# Transcriptome sequencing of three *Pseudo-nitzschia* species reveals comparable gene sets and the presence of Nitric Oxide Synthase genes in diatoms

**DOI:** 10.1038/srep12329

**Published:** 2015-07-20

**Authors:** Valeria Di Dato, Francesco Musacchia, Giuseppe Petrosino, Shrikant Patil, Marina Montresor, Remo Sanges, Maria Immacolata Ferrante

**Affiliations:** 1 Stazione Zoologica Anton Dohrn, Villa Comunale 1, 80121, Naples, Italy

## Abstract

Diatoms are among the most diverse eukaryotic microorganisms on Earth, they are responsible for a large fraction of primary production in the oceans and can be found in different habitats. *Pseudo-nitzschia* are marine planktonic diatoms responsible for blooms in coastal and oceanic waters. We analyzed the transcriptome of three species, *Pseudo-nitzschia arenysensis*, *Pseudo-nitzschia delicatissima* and *Pseudo-nitzschia multistriata,* with different levels of genetic relatedness. These species have a worldwide distribution and the last one produces the neurotoxin domoic acid. We were able to annotate about 80% of the sequences in each transcriptome and the analysis of the relative functional annotations allowed comparison of the main metabolic pathways, pathways involved in the biosynthesis of isoprenoids (MAV and MEP pathways), and pathways putatively involved in domoic acid synthesis. The search for homologous transcripts among the target species and other congeneric species resulted in the discovery of a sequence annotated as Nitric Oxide Synthase (NOS), found uniquely in *Pseudo-nitzschia multistriata*. The predicted protein product contained all the domains of the canonical metazoan sequence. Putative NOS sequences were found in other available diatom datasets, supporting a role for nitric oxide as signaling molecule in this group of microalgae.

Diatoms are a very diverse group of eukaryotic microalgae, estimated to include about 200,000 different species. They can be recorded in a broad range of environments (oceans, freshwaters, soil) where water and light are available and can thrive in a wide variety of temperature, light and nutrient conditions, indicating that a broad range of adaptive strategies evolved in this lineage^1^. As photosynthetic organisms, they have a huge ecological importance, being responsible for approximately 20% of the global photosynthetic carbon fixation, an amount comparable to that of all terrestrial rain forests^2^. Diatoms have complex signaling mechanisms that allow the perception of environmental cues^3^, produce signaling molecules such as sexual pheromones^4^ and can control their competitors and grazers by synthesizing specific anti-mitotic agents^5^,^6^.

The *de novo* sequencing of diatom genomes showed that this relatively recent eukaryotic lineage harbors a combination of genes and metabolic pathways first thought to be exclusive to plants and animals^7^,^8^. Diatoms have the urea cycle and the ability to generate chemical energy from the breakdown of lipids that were considered distinctive animal features, and also have the C4 photosynthetic pathway that was recorded only in some plants^8^.

Among diatoms, the genus *Pseudo-nitzschia* has attracted much attention because of its ability to synthesize the toxin domoic acid (DA), a neurotoxin causing Amnesic Shellfish Poisoning (ASP) in humans and reported as harmful also for marine vertebrates and sea birds^9^,^10^. The genus *Pseudo-nitzschia* is widely distributed around the world, with several species reported also in the Mediterranean Sea^11^.

In this study, we performed a comparative analysis of the *de novo* transcriptomes of three *Pseudo-nitzschia* species to obtain preliminary insights on their molecular toolkits and to identify physiological and metabolic differences amongst them. Two of the target species, *Pseudo-nitzschia arenysensis* and *Pseudo-nitzschia delicatissima*, are genetically closely related, and are cryptic species, i.e. they can only be distinguished with molecular markers^12^,^13^. *Pseudo-nitzschia multistriata* belongs to a different phylogenetic clade and produces DA^14^. The three species exhibit distinct species-specific patterns of the secondary metabolites oxylipins^15^, suggesting the presence of distinct functional traits also amongst morphologically and genetically closely related species. They regularly bloom in the Gulf of Naples^16^, have a broad global distribution^11^, and have different levels of genetic relatedness and different secondary metabolites production^15^. For two of these species, we recently optimized genetic transformation^17^.

The genome sequences of two other diatoms, *Phaeodactylum tricornutum* and *Thalassiosira pseudonana*^7^,^8^, and other transcriptome sequences produced within the Marine Microbial Eukaryote Transcriptome Sequencing Project (MMETSP)^18^, aided in the definition of shared and unique properties.

Whole transcriptome functional comparisons indicated that, in the exponential phase of growth, the three species express an overall comparable set of functions, however small differences could be found when looking in details at specific pathways, such as the pathways involved in the biosynthesis of isoprenoids (MAV and MEP pathways). This analysis highlighted the presence of a transcript encoding for a Nitric Oxide Synthase (NOS), present in *Pseudo-nitzschia multistriata* but not in the other two diatoms. We expanded the search for NOS sequences in other datasets available for diatoms and present the result of a phylogenetic analysis supporting, for the first time, the existence of such enzyme in this group of algae.

## Results

### Sequencing data and assembly quality

The total number of assembled reads was ~35 million for *Pseudo-nitzschia arenysensis*, ~34 million for *P. delicatissima* and ~118 million for *P. multistriata* (Table 1). The higher number of reads for *P. multistriata* is most likely due to the different sequencing methodology that resulted in deeper sequencing. The total contigs number, the N50 values of each transcriptome and the corresponding proteome sizes were comparable (Table 1).

The transcriptome size and sequencing statistics of other *Pseudo-nitzschia* species (retrieved from the publicly available transcriptomes sequenced within the MMETSP) were overall similar to those of the three species of interest (the only exception being one of four conditions for *Pseudo-nitzschia fraudulenta*), indicating homogeneity within the genus (Supplementary Table S1).

Completeness of the *P. arenysensis*, *P. delicatissima* and *P. multistriata* transcriptomes, as percentage of complete core proteins, was estimated to be higher than 85% (Table 1) using CEGMA analysis. The completeness resulted even higher (>88%) when considering the percentage of the partial core proteins (fragmented or truncated alignment) aligned against the reference dataset (Table 1) and resulted comparable to the completeness of datasets derived from the genomes of other diatom species (91.13% and 90.73% for *Phaeodactylum tricornutum* and *Thalassiosira pseudonana,* respectively).

### Functional annotations

Using the Annocript pipeline for annotation^19^, about 80% of the proteome sequences could be annotated: 15,818 (80%), 14,420 (82%) and 16,183 (80%) proteins annotated for *P. arenysensis*, *P. delicatissima* and *P. multistriata* respectively (Supplementary Tables S2, S3 and S4). Blastp results showed that, in terms of homology, the top ten matches mostly belonged to Heterokontophyta (Supplementary Fig. S1).

**Figure 1:**
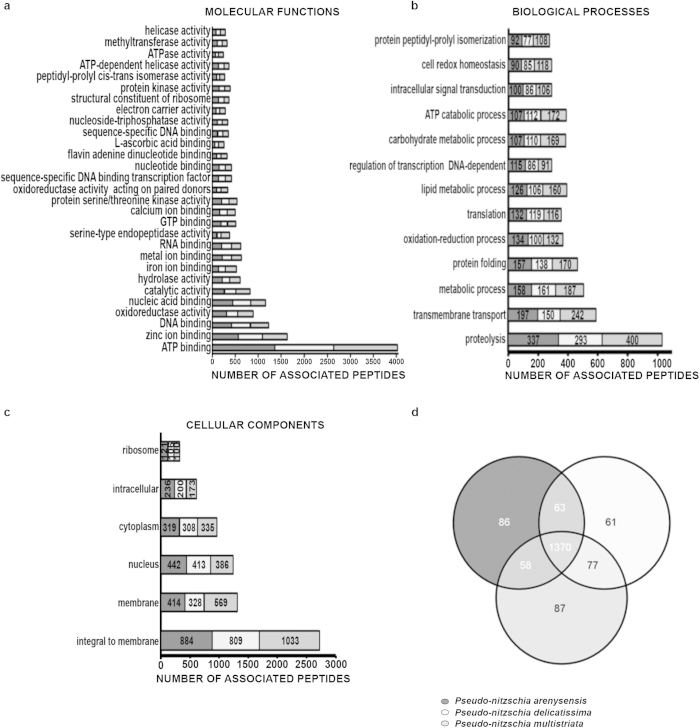
Breakdown of the GO terms describing the annotated proteomes. GO-terms belonging to Molecular Function (**a**), Biological Processes (**b**) and Cellular Components (**c**) associated to at least 100 peptides, for *Pseudo-nitzschia arenysensis* (dark grey), *Pseudo-nitzschia delicatissima* (light grey) and *Pseudo-nitzschia multistriata* (grey). On the y axis are the GO-terms, on the x axis are the number of sequences associated to each GO-term. (**d**) Venn diagram of shared/unique GO terms among the three *Pseudo-nitzschia*: external numbers correspond to the number of unique GO terms for each species. Numbers in the overlapping areas correspond to the shared GO terms. 1370 GO terms are shared among the three species.

The annotation process assigned a similar number of Gene Ontology (GO) terms to each proteome (1577, 1571 and 1592 in *P. arenysensis*, *P. delicatissima* and *P. multistriata,* respectively), the majority of which belonged to the Molecular Function category. The GO terms “ATP-binding” in the Molecular Function category, “Proteolysis” in the Biological Process category and “Integral to membrane” in the Cellular Components category were the most represented (Fig. 1a–c and Supplementary Table S5).

GO function enrichment analyses did not reveal any specific enrichments (Fisher test, data not shown), we were however able to identify uniquely annotated functions in each species of interest. The Venn diagram in Fig. 1d shows the unique mapping of 86, 61 and 87 GO terms in *P. arenysensis*, *P. delicatissima* and *P. multistriata,* respectively. Interestingly, while the unique *P. arenysensis* and *P. delicatissima* GO terms are mainly generic terms, the *P. multistriata* list of unique GO terms contains terms related to specific functions, such as ‘nitric oxide synthase’ (NOS) and ‘regulation of TOR signaling cascade’, absent in the other two species annotations.

The number of general pathways identified in *P. arenysensis* and in *P. delicatissima* was 62, whilst in *P. multistriata* it was 60 (Supplementary Table S6 and Supplementary Fig. S2). Essential and secondary metabolic pathways were present (Supplementary Table S7 and Supplementary Fig. S3).

The C4 metabolism and the urea pathway, both unexpectedly discovered in diatom genomes^8^, were present in all three *Pseudo-nitzschia* (Supplementary Tables S8 and S9). Notably, *P. multistriata* lacked the PEPCK transcript, differently from *P. arenysensis* and *P. delicatissima* (Supplementary Table S10). However, a blastp search in the *P. multistriata* genome (Ferrante, in preparation) retrieved a PEPCK sequence (data not shown), indicating that the enzyme is present in *P. multistriata*.

### Comparative transcriptomics

To identify novel and unique features of the target *Pseudo-nitzschia* species, we determined the homology level of the three transcriptomes among them as well as with respect to the pennate diatom *P. tricornutum* and the centric diatom *T. pseudonana.*

**Figure 2:**
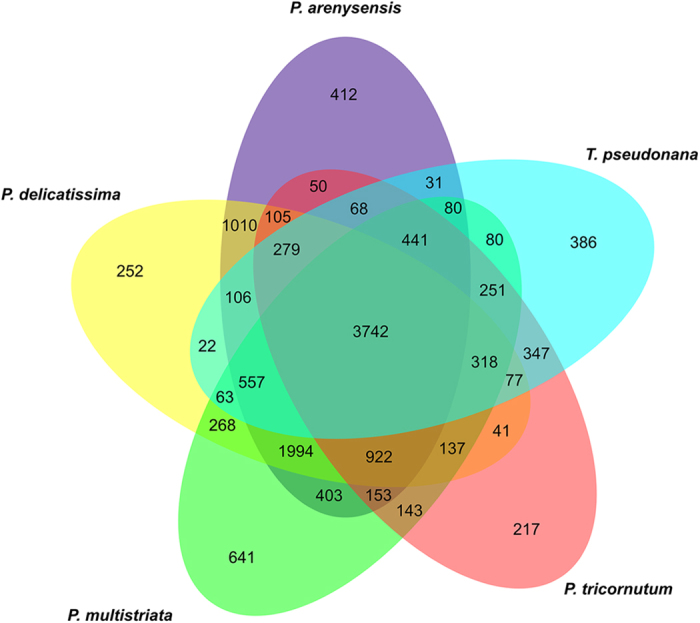
Venn diagram showing distribution of orthologous groups among five species of diatoms. External numbers correspond to groups unique to a given species (in-paralogs). Numbers in the overlapping areas correspond to groups of orthologs.

Table 2 reports the number of groups of homologous proteins found by OrthoMCL and their distribution in four classes of homology type. The species that had the highest percentage of similarity with all the other species was *P. delicatissima* and the one that shared less was *T. pseudonana* (Table 3). Specifically, we found that 10%, 14% and 22% of the proteome of the three *Pseudo-nitzschia* species was apparently not shared with other species, while 25% of the proteome of *P. tricornutum* and 39% of the proteome of *T. pseudonana* appeared to be missing from the other species (Table 3). The majority of the orthologous groups were common among all the species tested (Fig. 2): the central part of the Venn diagram, representing the overlapping among all the datasets used, showed 3,742 (27.52% of the total groups) groups of sequences common to all. The number of groups only shared between *P. arenysensis* and *P. delicatissima* was 1,010, highlighting, as expected, a closer relation between them with respect to *P. multistriata*. The groups shared only by *P. multistriata* and *P. arenysensis* were 403 whereas 268 groups were shared only by *P. multistriata* and *P. delicatissima*, suggesting that *P. multistriata* might be more similar to *P. arenysensis* than to *P. delicatissima*.

The four pennate diatoms together shared 922 groups of proteins, showing significant enrichments for functions related to the carbohydrate metabolic process, regulation of transcription and signal transduction (Supplementary Table S11). The species with the highest number of ‘in-paralogs’ (groups of proteins present uniquely in a single species) was *P. multistriata* followed by *P. arenysensis*, *T. pseudonana*, *P. delicatissima* and *P. tricornutum* (Fig. 2, external numbers).

Examining the annotations corresponding to the proteins included in the ‘in-paralogs’ group and to the unique proteins (proteins that were not classified into any homology group either in the same species or in other species) of *P. arenysensis* (Supplementary Table S12), *P. delicatissima* (Supplementary Table S13) and *P. multistriata* (Supplementary Table S14), we observed that around 55% of the species-specific proteins did not get any annotation, and around 33% were annotated as predicted or uncharacterized proteins. The majority of the remaining species-specific proteins corresponded to leucine-rich repeat containing proteins, zinc finger like proteins, trypsins, lipases, heat shock proteins, transporters and receptors, indicating that rapidly evolving proteins exist in these classes. In *P. arenysensis,* the most represented molecular function in the list of species-specific proteins was related to the serine-type endopeptidase activity, while in *P. delicatissima* terms related to energy metabolism were abundant. Furthermore, the *P. multistriata* species-specific protein list was enriched in functions related to lipid metabolism, proteolysis, nitric oxide synthase, serine type endopeptidase activity, hydrolase and lipase activity, iron and oxygen binding. Finally, the species-specific *P. multistriata* list included proteins that were annotated as antibiotic synthesizing proteins and acetylcholine subunit type receptors.

### Identification of a NOS sequence in diatoms

**Figure 3:**
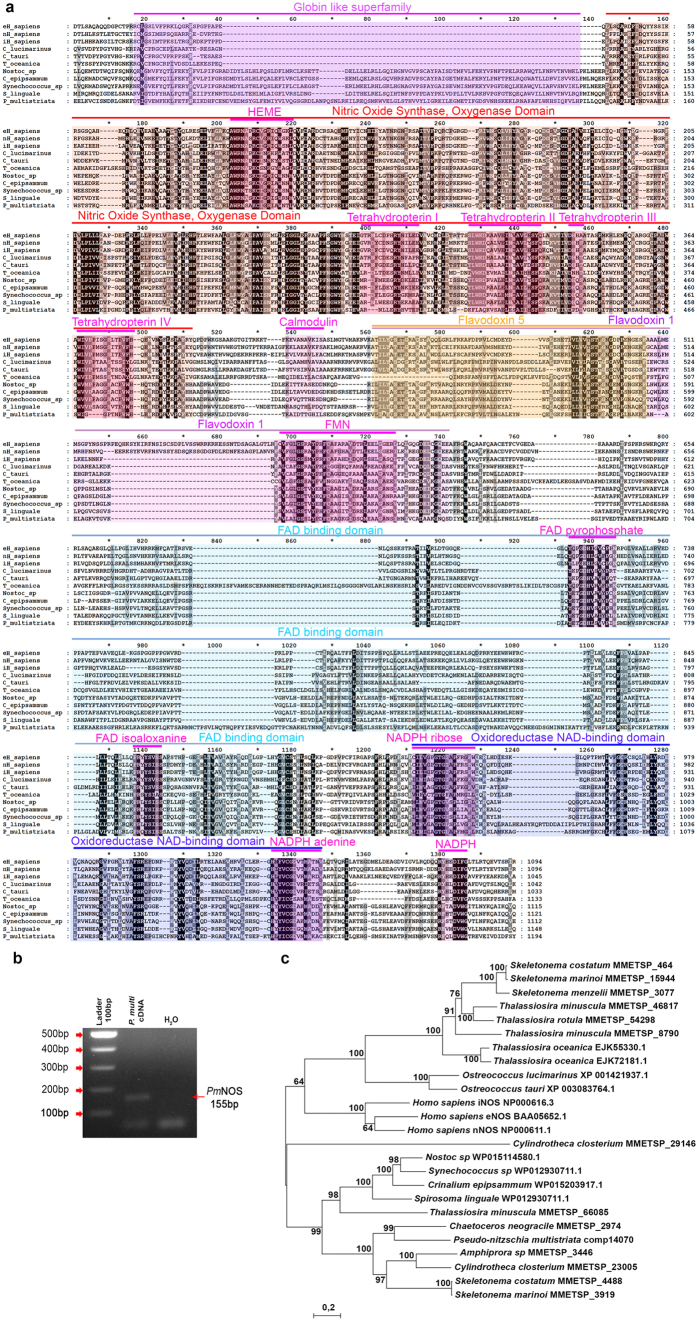
Analysis of the *Pseudo-nitzschia multistriata* nitric oxide synthase (*Pm*NOS). (**a**): Protein alignment of *P. multistriata Pm*NOS with the corresponding NOS of *Homo sapiens*, cyanobacteria (*Nostoc sp*, *Crinalium epipsammum*, *Synechococcus sp*), bacteria (*Spirosoma linguale*), Mamiellophyceae (*Ostreococcus tauri* and *O*. *lucimarinus*) and diatoms (*Thalassiosira oceanica*), showing domains and conservation; (**b**): RT-PCR amplification of a *PmNOS* fragment, lane 1, 100 bp ladder, lane 2, *P. multistriata* cDNA, lane 3, blank; (**c**): Neighbor joining three phylogeny of NOS protein sequences identified in diatoms, cyanobacteria, *Homo sapiens* and *Ostreococcus* species.

Because of the important role for nitric oxide as a signaling molecule in phytoplankton^20^, we focused on the ‘Nitric Oxide Synthase’ GO term uniquely annotated in *P. multistriata*. The presence of NOS sequences in photosynthetic organisms has been debated^21^. However, recently a NOS sequence was characterized in the green microalga *Ostreococcus tauri*^22^. A blastn search in the *P. multistriata* genome (Ferrante, in preparation) with the *PmNOS* transcript retrieved a locus with 99% identity (data not shown). The *PmNOS* transcript was analyzed for its sequence completeness, and the sequence was manually curated comparing the transcript isoforms and the genome sequence. The predicted protein had domain structure identical to the canonical metazoan NOS, with the two typical domains, the oxygenase (oxy) and the reductase (red) domains, fused in a unique sequence. Moreover, all the domains required for the enzymatic activity, Heme, Tetrahydropterin I-IV, Calmodulin, FMN, FAD pyrophosphate, FAD isoaloxanine, NADPH ribose, NADPH adenine, NADPH, were present (Fig. 3a). As an independent validation of the *in silico* data, we performed an RT-PCR amplifying *PmNOS* in three *P. multistriata* strains different from the ones used to produce the transcriptome or the genome (Fig. 3b and data not shown), confirming expression in standard growth conditions.

*Pm*NOS blastp searches in the NCBI database retrieved as top hits cyanobacterial sequences that displayed a putative canonical NOS (deposited in the database but not described in literature) followed by many vertebrates isoforms. The identity with the NOS sequence characterized in the green alga *Ostreococcus tauri*^22^ was lower (31%).

A *Pm*NOS blastp search against the available diatom genomes (*P. tricornutum*, *T. pseudonana*, *Fragilariopsis cylindrus*, *Pseudo-nitzschia multiseries*) did not retrieve any sequence, while in other diatom protein datasets produced within the MMETSP project we could find putative NOS sequences (Supplementary Table S15 online). Interestingly none of the other *Pseudo-nitzschia* species sequenced in the MMETSP displayed any canonical NOS sequence, only separated oxy and red domains could be found. On the contrary, many centric diatoms species displayed in most of the cases two transcripts (Supplementary Table S15 online), one coding for a canonical NOS sequence with similarity to the *Ostreococcus* NOS sequence and one that had similarity with the cyanobacteria NOS sequence, sometimes lacking the globin domain. Moreover, when different datasets for the same strain grown in different conditions were available, the NOS sequence was not retrieved in all growth conditions (see *Thalassiosira minuscula* strain CCMP1093 and *Thalassiosira rotula* strain GSO102, Supplementary Table S15 online), indicating a possible regulation of NOS expression.

A phylogenetic tree of all diatom NOS sequences, including two *Thalassiosira oceanica* sequences found in NCBI and deriving from the genome sequence of this species^23^, showed two well-supported clades clearly separating the sequences by their similarity with the cyanobacteria or with the green alga *Ostreococcus* and *Homo sapiens* sequences (Fig. 3c). *Pm*NOS confirmed its similarity with the cyanobacteria sequences together with more ancestral pennate diatoms such as *Amphiprora sp* and *Cylindrotheca closterium*. The two transcripts found in *Thalassiosira minuscula*, *Skeletonema costatum* or *S. marinoi* clustered each in one of the two clades.

### Isoprenoids and domoic acid production

Domoic Acid (DA) is a member of kainoids, a group of compounds resembling glutamate. The proposed route for its synthesis is the fusion of two hypothetical precursors, one deriving from the citric acid cycle (a glutamate-like compound) and the other deriving from the isoprenoid pathways (a geranyl type molecule)^24^.

Although toxicity of the strains used to produce the transcriptomes was not measured at the time of RNA extraction, the *P. multistriata* strain selected for the study had been shown to produce DA, while all *P. arenysensis* strains tested in the laboratory have never been found to produce DA (Ferrante, unpublished, A. Zingone, personal communication). We compared the expression levels of transcripts associated to pathways that have been associated to DA biosynthesis (isoprenoid biosynthesis, in Fig. S2 and Table S6, acetyl-CoA biosynthesis, geranyl diphosphate biosynthesis, isopentenyl diphosphate biosynthesis via DXP pathway, isopentenyl diphosphate biosynthesis via mevalonate pathway, L-glutamate biosynthesis via GLT pathway, L-proline degradation into L-glutamate, pyruvate metabolism, tricarboxylic acid cycle, in Fig. S3 and Table S7)^25^ in the toxic *P. multistriata* with respect to the non-toxic *P. arenysensis* and did not find substantial differences. In a study comparing *P. multiseries* cells in the stationary phase (high DA production) versus the exponential phase (low DA production) a number of genes resulted upregulated^26^. We searched for homologs of these genes and found similar FPKM (Fragments Per Kilobase of transcript per Million mapped reads) values for each given transcript in the three species except for *SLC6* (Sodium and Chloride-dependent amino acid transporter) for which the mean *P. multistriata* FPKM value was 7 and 11 fold higher than in *P. arenysensis* and *P. delicatissima*, respectively (35.16 FPKM versus 4.92 and 3.15 FPKM), indicating that this transcript might be more expressed in a toxin-producing species.

Finally, we looked at completeness of the isoprenoid pathway. Isoprenoids are a class of organic compounds including sterols, brassinosteroids, cytokinins, phytols, giberellins, plant hormones and carotenoids, and two routes are known for their production: the mevalonate (MVA) and methylerythritol phosphate (MEP) routes^27^.

The two isoprenoids routes were clearly identified in the three *Pseudo-nitzschia* species, with only CMK, one of the MEP route enzymes, missing in *P. delicatissima* (Table 4). The analysis was extended to other diatom datasets available in the MMETSP database at the time of the study, revealing that the MVA pathway appeared to be absent in two *Chaetoceros* species, and it was incomplete in *Nitzschia punctata, Coscinodiscus wailesii, Pseudo-nitzschia pungens fr. cingulata, Skeletonema marinoi* and *Thalassionema frauenfeldii*. On the other side, almost all enzymes of the MEP pathway were present in diatoms, except for *Nitzschia punctata*, which lacked a few of them (Table 4).

The geranyl diphosphate synthase (GPPS) enzyme, responsible of the synthesis of the geranyl diphosphate from the MEP pathway in the plastid, appeared to be absent in all analyzed species (Table 4). A *Nannochloropsis gaditana*^28^ GPPS protein blastp against the *P. arenysensis*, *P. delicatissima* and *P. multistriata* datasets demonstrated that the sequence was present, albeit annotated with a different name (Solanesyl-diphosphate synthase). Phylogenetic trees to look at the conservation of key enzymes in the isoprenoid pathways among diatoms always provided a well-resolved phylogeny of the enzymes, with two main clades grouping the pennate or centric lineages. For all the proteins examined the nodes in each phylogenetic tree were well-supported. In the clade containing the *Pseudo-nitzschia, P. arenysensis* and *P. delicatissima* grouped together, as expected, and resulted more divergent with respect to the other congeneric species (Supplementary Figure S4).

## Discussion

The comparative analysis of the transcriptomes of the three *Pseudo-nitzschia* species revealed an overall similarity among the datasets but also provided an inventory of species-specific transcripts. The two genetically closely related cryptic species*, Pseudo-nitzschia arenysensis* and *P. delicatissima* appeared to have very similar transcriptomes, while *P. multistriata* displayed a larger fraction of unique functions, including a nitric oxide synthase (*Pm*NOS), whose predicted protein structure had domains identical to the metazoan NOS. The transcriptomes of the three congeneric species analyzed in this study derived from strains grown at the same experimental conditions and collected in the exponential growth phase, thus providing a comparison between the genetic distance and their basic metabolic pathways. It is conceivable that a greater molecular diversity and larger differences in transcript levels might be detected when comparing responses to markedly different perturbations, which may highlight species-specific physiological capabilities/adaptations^29^. Even larger differences can be expected when comparing the response to the same experimental condition of species belonging to distinct genera. In fact, a study among two pennate diatoms, *Fragilariopsis cylindrus* and *Pseudo-nitzschia multiseries*, and one centric diatom, *Thalassiosira pseudonana*, grown in nitrogen-deprived condition, showed differences in non-orthologous gene subsets and in transcription levels, with less than 5% of the shared orthologous gene clusters similarly transcribed^30^.

For the general pathway content, our annotation retrieved sequences for all the major primary metabolic pathways, and complete C4, urea and isoprenoid pathways could be found, with little exceptions. A complete urea cycle, involved in nutrient sensing and contributing to the response of diatoms to episodic nitrogen availability, operates in *Phaeodactylum tricornutum*^31^ and our study indicated that in *Pseudo-nitzschia* species the same mechanisms could be used. In the *P. multistriata* transcriptome, *PEPCK* was absent but the gene was present in the genome, suggesting low levels of expression in the sample analyzed.

The presence of a canonical NOS sequence in *P. multistriata* was intriguing. It is important to note that we found the NOS sequence in the *P. multistriata* genome produced from an axenic strain, different from the strain chosen to produce the transcriptome, proving that this sequence is present in the species and does not derive from possible contaminations. The presence of a NOS enzyme in plants, green algae and diatoms is still a matter of debate^21^,^32^,^33^, however the product of its activity, nitric oxide, has been identified in diatoms and correlated with perception of stress cues^20^,^34^. Exploring different databases, we looked for the presence of NOS sequences in other diatom species. We could not find putative NOS sequences in any *Pseudo-nitzschia* transcriptome, or in the genome of *P. multiseries*. In these species, the oxygenase and reductase domains are present as separate genes. The absence of NOS in the *P. multiseries* genome suggests that absence of NOS in the transcriptomes of most *Pseudo-nitzschia* could be truly due to absence of the gene rather than to a bias in sequencing depth, sequence assembling or to suppressed expression. NOS sequences could not be found also in the genomes of the pennate diatoms *F. cylindrus* and *P. tricornutum* and of the centric diatom *T. pseudonana.* Two putative NOS sequences were instead present in the genome of *Thalassiosira oceanica*^23^ and one or more putative NOS sequences were found in the transcriptomes of species of the genera *Thalassiosira, Skeletonema* and *Cylindrotheca* sequenced in the MMETSP. We found a putative NOS in the *Skeletonema marinoi* strains FE7 and FE60, but not in the *S. marinoi* strain SkelA, where only oxygenase and reductase separated domains could be identified. In addition, for *Thalassiosira rotula* (strain GSO102) and *Thalassiosira minuscula* (strain CCMP1093), for which samples of different growth conditions were sequenced, putative NOS sequences could be found only in one or two but not in all conditions. These observations suggest that NOS expression could be regulated.

The presence of more than one NOS sequence in some species is puzzling. Without a reference genome for these species, any attempt to reconstruct the evolutionary history of these sequences proves difficult. Information about synteny or intron number and distribution might help in defining the relationships among the sequences. From our analyses, pennate diatoms, which are phylogenetically more recent than centrics^35^, seem to have only one gene, more similar to cyanobacterial NOS than to human or green algae, with the exception of *Cylindrotheca closterium* that has a second sequence with an ambiguous position in the phylogenetic tree.

Recently, it has been demonstrated that nitric oxide in marine phytoplankton is able to remove the oxidative damage due to different environmental stressors. NOS expression could be upregulated in response to stress conditions, as suggested by Vardi^34^,^36^, who demonstrated that nitric oxide is produced by diatoms exposed to sub lethal doses of the (2E,4E/Z)-decadienal aldehyde, a molecule that is synthesized when diatom cells are broken either by grazing or cell death. Functional studies on *PmNOS* will be needed to define the profile of expression and the possible role of this gene.

In *P. arenysensis* and *P. delicatissima,* leucine-rich repeat proteins were the majority in the list of unique proteins. Leucine-rich repeat proteins are required in various cellular processes and it seems that their major role would be protein-protein interaction. As they evolve rapidly, their repetitive structure can be useful in processes in which the rapid generation of new variants is needed, such as plant disease resistance and bacterial virulence^37^. The species-specific protein list contained mainly proteins associated to unknown functions, and proteins associated to known functions that were present in all the species considered. These unique proteins associated to common functions are likely divergent proteins belonging to related gene families. Unique and highly divergent proteins are likely responsible for the plasticity of the species and will deserve further studies.

Based on studies on *P. multiseries* in high DA producing versus low DA producing growth conditions^26^, we looked at genes hypothetically involved in DA synthesis. No major differences could be detected among our three target species, except for the SLC6-sodium and chloride-dependent amino acid transporter, very similar to kainoids molecules transporters (to which DA belongs) that could be involved in the transport of DA inside/outside the cell, which was, at least *in silico*, over represented only in the toxic *P. multistriata.*

The mevalonate (MVA) and methylerythritol phosphate (MEP) pathways were present in *Pseudo-nitzschia*, except for the CMK enzyme, which was absent in *P. delicatissima,* either due to technical issues or to a true loss during evolution. Compensatory routes in any case are known to overcome the lack of some enzymes^38^,^39^. Isoprenoids are considered to be the largest group of natural products, playing a wide variety of roles in physiological processes of plants and animals. Ancestral eukaryotes and animals generally have only the MVA pathway, while many photosynthetic organisms have acquired also the MEP pathway. The green alga *Chlamydomonas reinhardtii* and the red alga *Cyanidioschyzon merolae* have only the more recently acquired MEP pathway and eliminated the more ancestral MVA^28^. It was previously reported that Stramenopiles, as diatoms (*P. tricornutum*, *T. pseudonana, Nitzschia ovalis)* and brown algae (*Ectocarpus siliculosus*), had retained both^39^, however our comparative studies using several diatom species indicated variations and absence of some enzymes in different species, for instance in the genus *Chaetoceros.* Chemical searches for the ending products of the two pathways are needed to validate the possible different utilization of the pathways. The phylogenetic analysis of selected enzymes of the two isoprenoids pathways showed a good separation between the two groups of pennate and centric diatoms and confirmed the divergence between *P. multistriata* and the two cryptic species *P. arenysensis* and *P. delicatissima.* This divergence was strengthened also by the recovering of *P. multistriata* into the same clade with *P. australis* and *P. pungens,* two toxic *Pseudo-nitzschia* species belonging, together with *P. multistriata*, to the *‘seriata’* group^40^. *P. multistriata* was also more distantly related from the two other species in terms of percentages of shared proteins (Fig. 2).

When the analyses were extended to other diatom species, a broad diversity could be observed, suggesting caution in formulating general hypotheses based on information deriving from the study of a single diatom genome or transcriptome.

The representativeness of genes in whole transcriptome data with respect to genome data is not complete; however, transcriptome completeness CEGMA analysis for the three species indicated that the datasets were sufficiently representative of the potential gene repertoire. Despite the biases intrinsic to *de novo* transcriptome assemblies without a reference genome, we were able to describe and compare the basal physiological state of the species of interest. When comparing the three *Pseudo-nitzschia* to the two model diatoms *P. tricornutum* and *T. pseudonana*, we found that the pennate species had between 90 and 75% of peptides with at least one ortholog in another species, while *T. pseudonana* had 39% of peptides not shared with the four pennate diatoms, in agreement with what reported previously in similar comparisons including this species^7^,^30^.

Finally, we could find unique and still undiscovered functions, reporting for the first time the presence of a NOS sequences in diatoms.

## Materials and Methods

### Strains and RNA extraction

*Pseudo-nitzschia* strains were isolated at the Long Term Ecological Research Station MareChiara in the Gulf of Naples (40°48.5’N, 14°15’E). Clonal cultures were established by isolating single cells from phytoplankton net samples collected from the surface layer of the water column. Cultures were grown in sterile filtered oligotrophic seawater amended with f/2 nutrients^41^ at a temperature of 18 °C, at 12:12 h light:dark cycle, with a photon flux of 100 μmol photons m^−2^s^−1^.

Strains selected for RNA extraction for RNA-seq were *Pseudo-nitzschia multistriata* B857^42^, *Pseudo-nitzschia arenysensis* B593 and *Pseudo-nitzschia delicatissima* B653, both isolated on 27/04/2011. RNA was also extracted from the *Pseudo-nitzschia multistriata* strain B936, isolated on 24/05/2012, for RT-PCR validations.

Cultures with cell density of 2 × 10^5^ cells ml^−1^ were harvested by filtration onto 1.2 μm pore size filters (RAWP04700 Millipore) and frozen in High Pure RNA Isolation reagent (Roche Applied Science). Before proceeding to the total RNA extraction protocol according to the manufacturer’s instructions, cells were disrupted with glass beads (G1277, Sigma-Aldrich) on a thermo-shaker (Eppendorf) at 60 °C for 10 minutes at maximum rpm. RNA was further purified on Qiagen columns (74104, RNeasy Mini Kit, including a step with DNase digestion). RNA concentration was determined using a Qubit®2.0 Fluorometer (Invitrogen) and a quality check was performed by gel electrophoresis (1% agarose w/v) and an Agilent2100 bioanalyzer.

### Transcriptome sequencing, assembling and proteome annotation

*Pseudo-nitzschia delicatissima* and *Pseudo-nitzschia arenysensis* library preparation and sequencing were performed as part of the Marine Microbial Eukaryote Transcriptome Sequencing Project (MMETSP) (http://marinemicroeukaryotes.org/)^18^. RNA libraries with an insert size of about 200 bp were sequenced from both ends (paired-end reads 2 × 50-nt) on the Illumina Hi-Seq2000. Assembly was performed by the National Center for Genome Resources (NCGR, http://www.ncgr.org/) using ABySS and CAP3^43^. Transcriptome IDs are MMETSP0327 (*Pseudo-nitzschia delicatissima*) and MMETSP0329 (*Pseudo-nitzschia arenysensis*).

*Pseudo-nitzschia multistriata* RNA-seq was made in collaboration with the JGI (http://www.jgi.doe.gov/) within the project “A deep transcriptomic and genomic investigation of diatom life cycle regulation”. Raw reads are available at http://genomeportal.jgi.doe.gov/pages/dynamicOrganismDownload.jsf?organism=PsenittraphaseII, the library selected for transcriptome assembly, corresponding to sample B857, is HCUN. Poly-(A) RNA was isolated from 5 μg total RNA using Dynabeads mRNA isolation kit (Invitrogen). Purified RNA was then fragmented using RNA Fragmentation Reagents (Ambion) at 70 °C for 3 mins, targeting fragments range 200–300 bp. Fragmented RNA was purified using Ampure XP beads (Agencourt). Reverse transcription was performed using SuperScript II Reverse Transcription (Invitrogen). Double stranded cDNA fragments were purified and selected for targeted fragments (200–300 bp) using Ampure XP beads. The cDNA was blunt-ended, poly-adenylated, and ligated with library adaptors using Kapa Library Amplification Kit (Kapa Biosystems). Digestion of dUTP was performed using AmpErase UNG (Applied Biosystems) to remove second strand cDNA. Digested cDNA was cleaned up with Ampure XP beads. This was followed by amplification by 10 cycles PCR using Kapa Library Amplification Kit (Kapa Biosystems). The final library was cleaned up with Ampure XP beads. Sequencing was done on the Illumina platform generating paired end reads of 150 bp each. Raw reads were filtered and trimmed based on quality and adapter inclusion using Trimmomatic^44^ with the following parameters: threads = 20; phred = 64; ILLUMINACLIP:illumina_adapters.fa:2:40:15-LEADING:5-TRAILING:5-SLIDINGWINDOW:5:20-MINLEN:100. Reads were then normalized using the normalize_by_kmer_coverage.pl script from Trinity^44^ (ver. r2013_08_14) with the following parameters: seqType = fq; JM = 240G; max_cov = 30; SS_lib_type = RF; JELLY_CPU = 24.

Assembly was performed using Trinity on the trimmed, filtered and normalized reads using these parameters: seqType = fq; JM = 220G; inchworm_cpu = 22; bflyHeapSpaceInit = 22G; –bflyHeapSpaceMax = 220G; bflyCalculateCPU; CPU = 22; SS_lib_type = RF; min_kmer_cov = 2. Putative protein translations were extracted using ESTScan (v3.0.3, default parameters)^45^ based on the MMETSP protocol. Peptides shorter than 30 aa were filtered out. The contigs and peptide sequences for *Pseudo-nitzschia multistriata* can be found in the Supplementary Information. Peptide sequences, from all the species, were used for the subsequent analyses. To annotate the translated transcriptome we used the custom pipeline Annocript^19^ (See https://github.com/frankMusacchia/Annocript/tree/master/GUIDE). We used the Swiss-Prot (SP) and UniRef90^46^ (version: August 2013) for the blastp against proteins with the following parameters: word_size = 4; e-value = 10^−5^; num_descriptions = 5; num_alignments = 5; threshold=18. For each sequence the best hit, if any, was chosen. Rpsblast parameters, to identify domains composition of putative proteins in the Conserved Domains Database^47^, were: e-value = 10^−5^; num_descriptions = 20; num_alignments = 20.

The software returned GO functional classification^48^, the Enzyme Commission IDs^49^ and Pathways^50^ descriptions associated to the resulting best matches.

Finally, we removed from the transcriptomes sequences matching with bacterial ribosomal sequences. CEGMA v2.5^51^ was used to provide the completeness of the transcriptome data using the set of 248 most highly conserved Core Eukaryotic Genes (CEGs). For comparisons purpose, CEGMA was also used with the *Phaeodactylum tricornutum* (v2.19) and *Thalassiosira pseudonana* (v1.19) genome data downloaded from EnsemblProtists^52^,^53^.

Paired aligned reads were used to calculate the Counts Per Million mapped reads (CPM) and the Fragments Per Kilobase of transcript per Million fragments mapped (FPKM) of each sequence for each transcriptome as measure of expression levels. The CPM values were calculated as follows: reads paired aligned*1,000,000/total paired aligned reads. The FPKM were calculated as follows: FPKM = [mapped reads pairs]/([length of transcript]/1000)/([total reads pairs]/10^6^).

To avoid sequencing and assembly artifacts we selected only the contigs showing an expression level with CPM greater than 1. R scripts were used to perform further analyses (http://www.r-project.org/).

For comparisons among the proteomes, we used FPKM. Between species we used the t-test to compare the FPKM for groups of peptides associated to the same GO/pathway and the Fisher exact test to compare the corresponding proportions of transcripts. Inside species we performed a GO enrichment analysis of the homologous peptides using the Fisher exact test and correcting p-values with the Benjamini and Hochberg method^54^.

### Isoprenoid pathway identification

The list of the enzymes involved in the isoprenoid pathway was taken from Radakovits *et al.*^28^ describing the *Nannochloropsis gaditana* genome, and from Vranová *et al.*^55^.

To verify the presence of enzymes involved in the synthesis of isoprenoids in diatom species, the datasets available at the time of the analysis (see Supplementary Note) were downloaded from the MMETSP website (http://camera.calit2.net/mmetsp/list.php).

For each species, the corresponding isoprenoid pathway enzyme sequences were retrieved from the respective annotation tables generated in the MMETSP. The corresponding *Nannochloropsis gaditana* sequence IDs were retrieved from Radakovits *et al.*^28^, and the relative sequences were downloaded from the *Nannochloropsis* genome portal at the web address http://www.nannochloropsis.org/.

The *Phaeodactylum tricornutum* and *Thalassiosira pseudonana* protein sequences were identified by tblastn using *Pseudo-nitzschia* transcript sequences as query in the EnsemblGenomes portal^53^.

For each protein, the corresponding longest isoform, if any, was taken (see Supplementary Note). To verify the accuracy of annotations, the longest corresponding isoform for each protein was blasted in the NCBI database. For phylogenetic analyses, protein sequences were aligned using the ClustalX program v2.1^56^ with default parameters for the “complete alignment”. To generate phylogenetic trees we used the Bootstrap Neighbor Joining algorithm with random number generator seed = 111 and Number of bootstrap trials = 10000. Trees were exported to the NJPlot program and then converted in graphic format.

### Comparisons with other diatom species

To compare our transcriptomes statistics with the other diatoms sequenced through the MMETSP, the datasets corresponding to selected species (see Supplementary Table 1) were downloaded from the MMETSP website. The number of contigs was retrieved from the stats.txt file, relative to each species.

To predict orthologs and in-paralogs we used OrthoMCL (v2.0.8) with the 5 proteomes of *P. multistriata*, *P. arenysensis*, *P. delicatissima*, *P. tricornutum* and *T. pseudonana.* OrthoMCL is a tool to group proteins into ortholog/paralog groups by their sequence similarity exploiting blastp and a Markov clustering (MCL) algorithm (v12-135)^57^,^58^. The *P. tricornutum* (v2.19) and *T. pseudonana* (v1.19) proteomes were downloaded from EnsemblProtists release 19 (http://protists.ensembl.org/)^53^.

Orthologs from the five species were clustered using the following settings: E-value cutoff = 10E-5 and MCL inflation index = 1.5.

For each tested species, proteins into ortholog groups were counted by custom perl and R scripts and classified as follows: *i) one-to-one* orthologs: at most one corresponding ortholog for each species; *ii) one-to-many* and *many-to-many* orthologs: more than one ortholog in at least one of the species tested; *iii) in-paralogs*: two or more similar sequences in exclusively one species.

### Nitric oxide synthase analysis

Using the *Pseudo-nitzschia multistriata* sequence as a query for blastp searches against the NCBI database, the NOS sequences of *Nostoc sp* (WP_015114580.1), *Synechococcus sp* (WP_006458277), *Spirosoma linguale* (WP_012930711.1), *Crinalium epipsammum* (WP_015203917.1) were retrieved. NOS sequences of *Ostreococcus lucimarinus* (XP_001421937.1) and *Thalassiosira oceanica* (EJK55330.1 and EJK72181.1) were retrieved by blastp search against the NCBI database using the NOS sequence of *Ostreococcus tauri* (XP_003083764.1). The three *Homo sapiens* NOS sequences (iNOS-inducible NP_000616.3, eNOS-endothelial BAA05652.1 and nNOS-neuronal NP_000611.1) were downloaded from the NCBI protein database.

The NOS sequences from the other diatom species were obtained as follow: peptides files of each species were downloaded from the MMETSP website. Each peptides file was indexed (following NCBI instructions: http://www.ncbi.nlm.nih.gov/books/NBK1763/) and searched using the *Pseudo-nitzschia multistriata* NOS sequence as a query in a blastp search with default parameters. The sequences with highest similarity were blasted against the non-redundant (NR) NCBI database to verify the identity. Finally the NOS sequences selected were aligned and analyzed with the Mega6 software. Sequences alignments were performed by selecting the ClustalW alignment tool and the phylogenetic evolutionary analysis was performed selecting the Maximum likelihood Tree test with 10,000 bootstrap replications.

For RT-PCR amplification, the *Pseudo-nitzschia multistriata* strain B936 was used. Primers used to amplify a 155 bp long *PmNOS* fragment were: *Pm*NOS_Forward 5’-CCCAGTATCGTGCAAACAGG and *Pm*NOS_Reverse 5’-AGGTCTTTGTCCGCATAGCC. Taq DNA Polymerase was used (Roche Cat. No. 11146165001).

Refer to Supplementary Table S15 for IDs of the sequences used in the phylogenetic analysis.

## Additional Information

**How to cite this article**: Di Dato, V. *et al.* Transcriptome sequencing of three *Pseudo-nitzschia* species reveals comparable gene sets and the presence of Nitric Oxide Synthase genes in diatoms. *Sci. Rep.* **5**, 12329; doi: 10.1038/srep12329 (2015).

## Supplementary Material

Supplementary Figures and Tables

Supplementary Table S2

Supplementary Table S3

Supplementary Table S4

Supplementary Table S5

Supplementary Table S6

Supplementary Table S7

Supplementary Table S10

Supplementary Table S11

Supplementary Table S12

Supplementary Table S13

Supplementary Table S14

Supplementary Table S15

Pseudo-nitzschia multistriata contigs

Pseudo-nitzschia multistriata peptides

## Figures and Tables

**Table 1:** General statistics of transcriptomes and proteomes assemblies in the three *Pseudo-nitzschia*species.

**DATA TYPE**	***P. arenysensis***	***P. delicatissima***	***P. multistriata***
Contigs number	19,852	17,595	21,632
N50	2,224	2,560	2,340
Reads	35,278,872	34,418,919	118,619,362
G + C content(%)	46.9	48.1	48.8
Median Contigs length (bp)	1,568	1,717	1,730
Average Contigs length (bp)	1,865	2,092	1,984
Protein number	19,853	17,598	20,176
Median Proteins length (aa)	403	429	419
Average Proteins length (aa)	517	545	500
458 CEGs Complete (%)	85.48	89.11	87.50
458 CEGs Complete + Partial(%)	88.71	92.74	89.52

**Table 2:** Ortholog groups detected in the transcriptomes of the three *Pseudo-nitzschia*species (*P. multistriata, P. arenysensis, P. delicatissima*) and in those of the model pennate diatom *Phaeodactylum tricornutum* and the centric *Thalassiosira pseudonana*.

**Group types**	**Number of homologous groups**	**% on the total groups**
ONEtoONE	3752	27.6%
ONEtoMANY	7301	53.7%
MANYtoMANY	635	4.7%
PARALOGS	1908	14.0%
TOTAL GROUPS	13596	

**Table 3:** Total number of peptides and number of peptides with at least one ortholog in the three *Pseudo-nitzschia*species and in the model pennate diatom *Phaeodactylum tricornutum*and the centric *Thalassiosira pseudonana*, and percentage of peptides of each species finding homology with at least another species.

**Species**	**Total number of peptides**	**Number of orthologs**	**% with homologs**
*P. arenysensis*	19853	17156	86.4
*P. delicatissima*	17598	15908	90.4
*P. multistriata*	20176	15755	78.1
*Phaeodactylum tricornutum*	10402	7865	75.6
*Thalassiosira pseudonana*	11674	7135	61.1

**Table 4:**
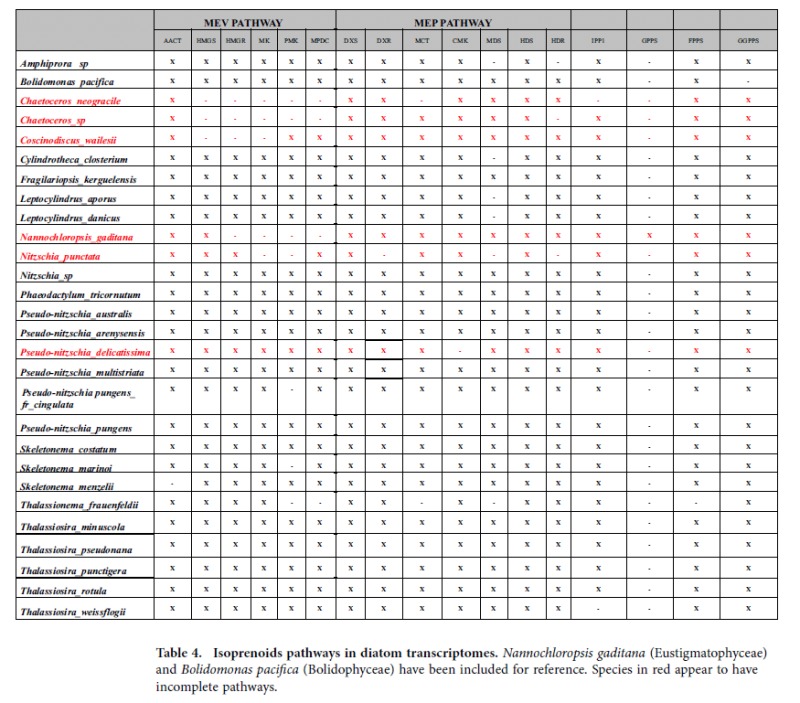
Isoprenoids pathways in diatom transcriptomes.

*Nannochloropsis gaditana* (Eustigmatophyceae) and *Bolidomonas pacifica* (Bolidophyceae) have been included for reference. Species in red appear to have incomplete pathways.
