# Supplementary material for: Transcriptome sequencing of three Pseudo-nitzschia species reveals comparable gene sets and the presence of Nitric Oxide Synthase genes in diatoms
Source: Sci Rep. 2015 Jul 20;5:12329. doi: 10.1038/srep12329 (PMC4648414; doi:10.1038/srep12329)
Supplement: Supplementary Figures and Tables [file srep12329-s1.pdf]

Title

Transcriptome sequencing of three *Pseudo-nitzschia* species reveals comparable gene sets and the presence of Nitric Oxide Synthase genes in diatoms

Author names and affiliations

Valeria Di Dato, Francesco Musacchia, Giuseppe Petrosino, Shrikant Patil, Marina Montresor, Remo Sanges, Maria Immacolata Ferrante*.

Affiliation and Address

Stazione Zoologica Anton Dohrn, Villa Comunale 1, 80121, Naples, Italy

Supplementary Material

This PDF file contains

Supplementary Figure S1
Supplementary Figure S2
Supplementary Figure S3
Supplementary Figure S4
Supplementary Table S1
Supplementary Table S8
Supplementary Table S9
Supplementary Note

Supplementary Figure S1

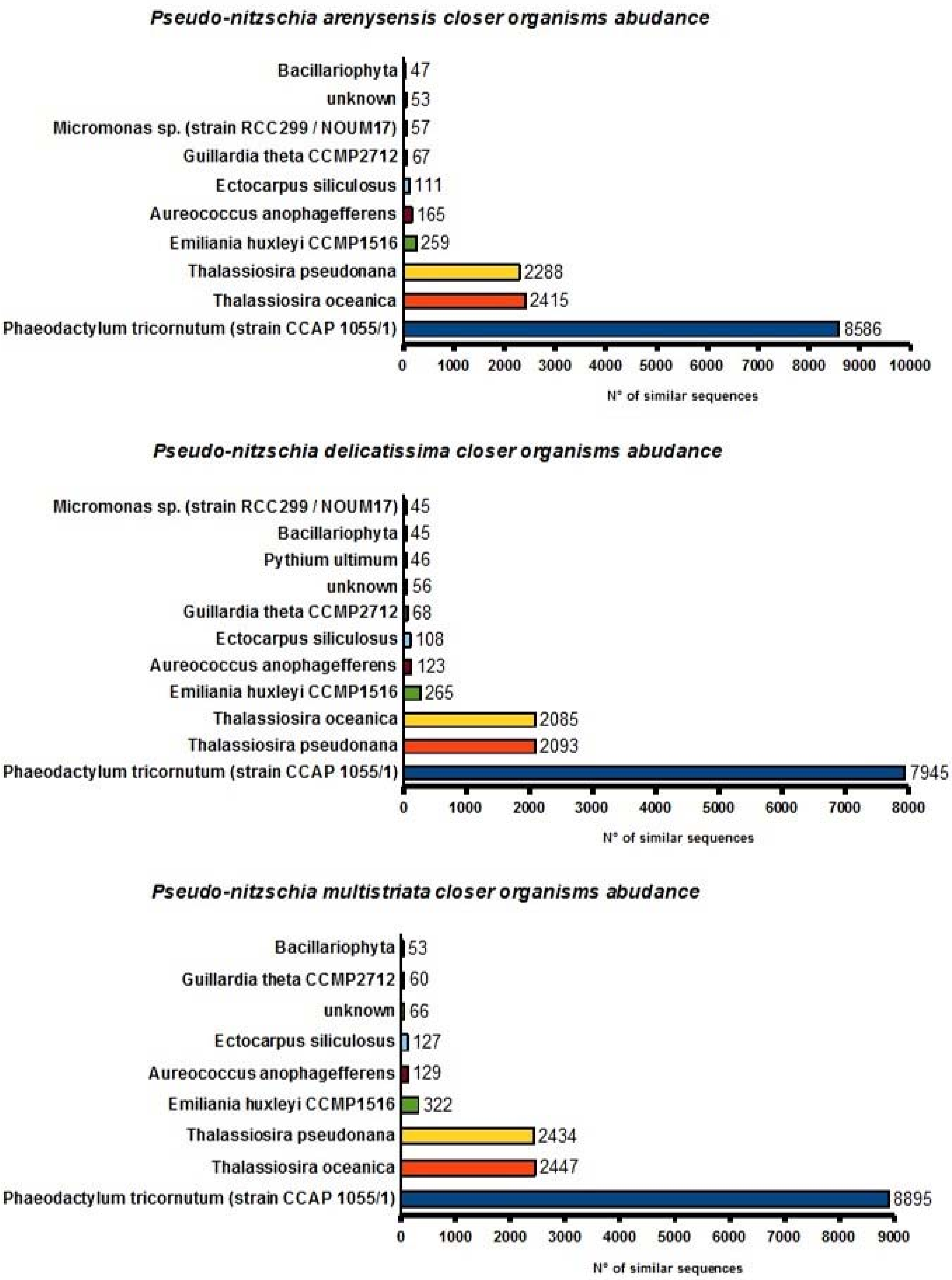

Supplementary Figure 1. *Pseudo-nitzschia* closer organisms abundance.

The graphs display the top ten organisms scoring the BlastP hits for each *Pseudo-nitzschia* proteome. Numbers on the side of each species-associated bar indicate the number of scoring sequences.

Supplementary Figure S2. Pathways expression based on the first level of annotation.

Each graph shows the expression of the general pathways for each species. The levels of expression are represented with box plots, each box represents the distribution of the expression values of the sequences associated to the relative pathway for each species. The black bar in each rectangle represents the median values of each sequence population. Points outside the main rectangle represent the value out-layers. Numbers in parenthesis indicate the number of sequences associated to the relative pathway for each species.

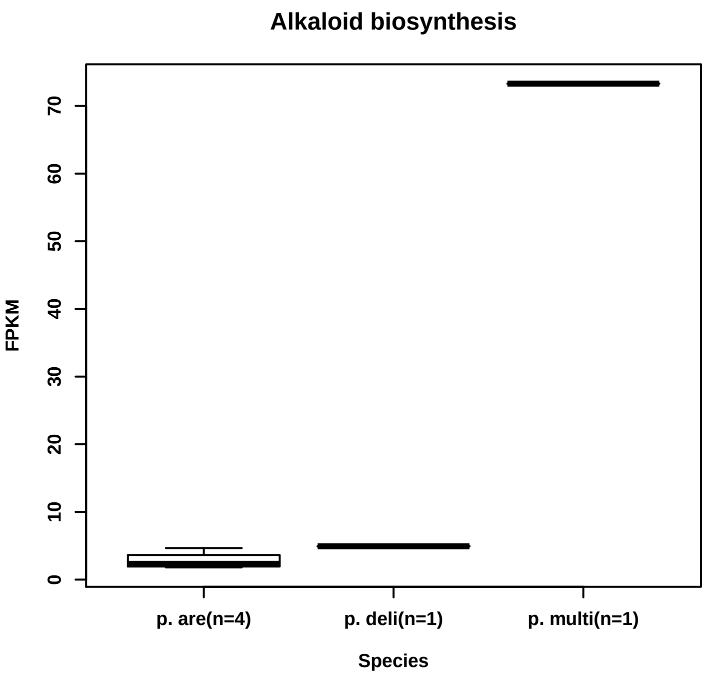
Alkaloid biosynthesis
FPKM
0
10
20
30
40
50
60
70
p. are(n=4)
p. deli(n=1)
p. multi(n=1)
Species

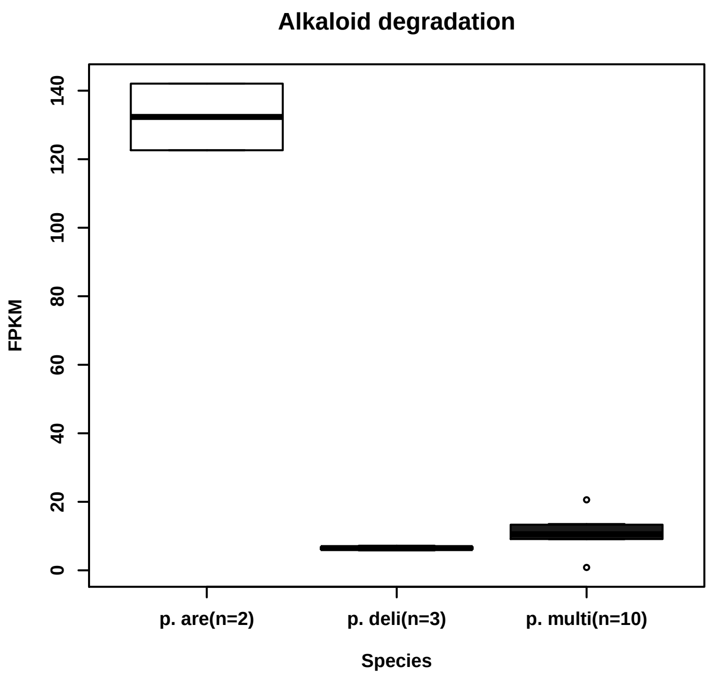
Alkaloid degradation
FPKM
0
20
40
60
80
100
120
140
p. are(n=2)
p. deli(n=3)
p. multi(n=10)
Species

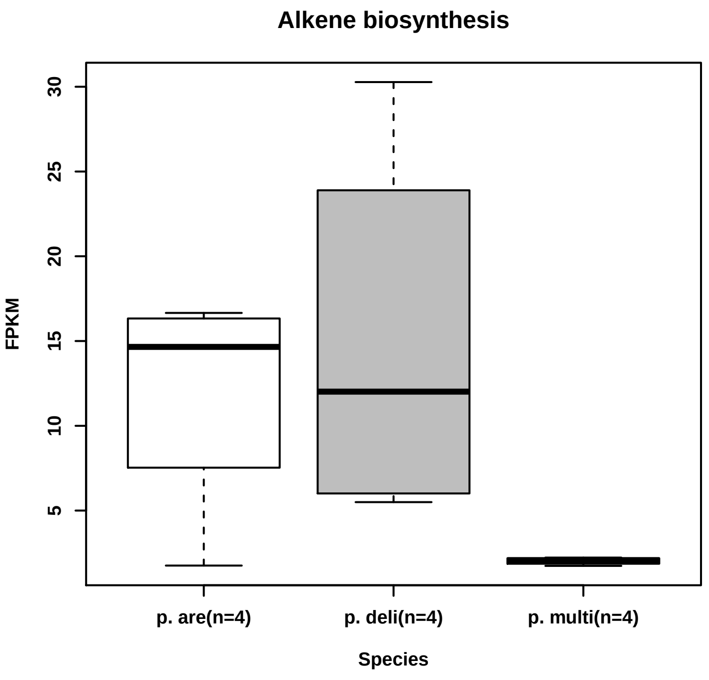
Alkene biosynthesis
FPKM
5
10
15
20
25
30
p. are(n=4)
p. deli(n=4)
p. multi(n=4)
Species

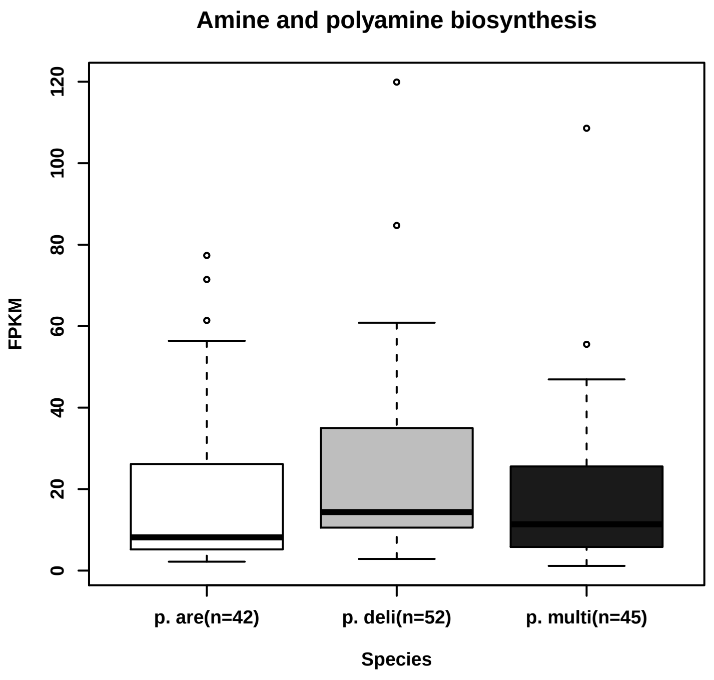
Amine and polyamine biosynthesis
FPKM
0
20
40
60
80
100
120
p. are(n=42)
p. deli(n=52)
p. multi(n=45)
Species

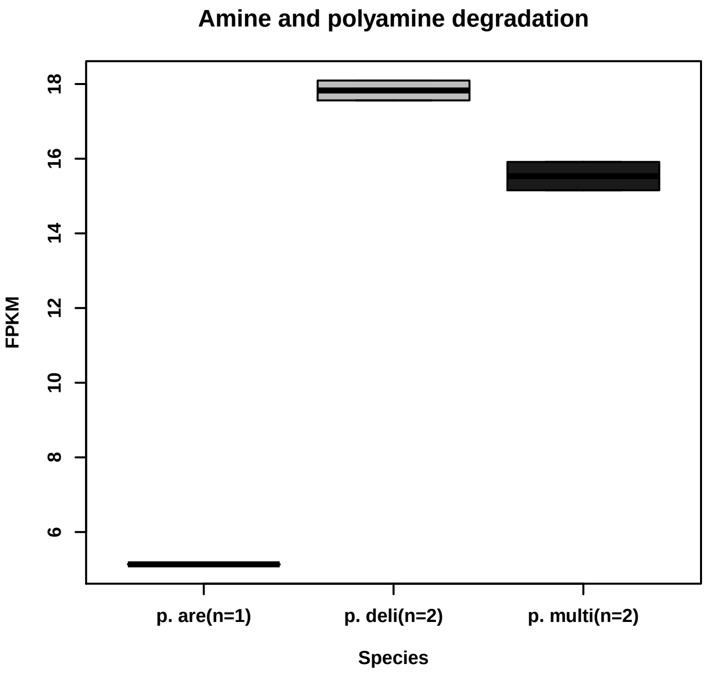
Amine and polyamine degradation
FPKM
6
8
10
12
14
16
18
p. are(n=1)
p. deli(n=2)
p. multi(n=2)
Species

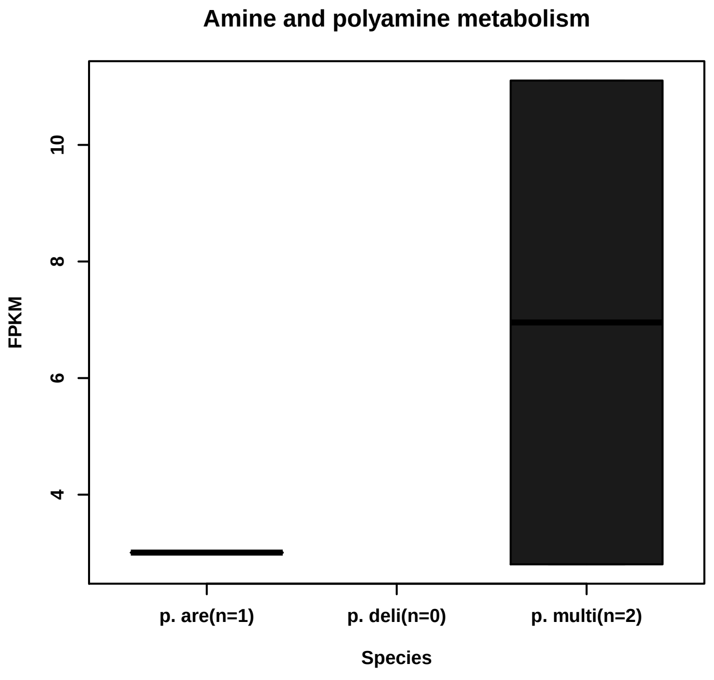
Amine and polyamine metabolism
FPKM
4
6
8
10
p. are(n=1)
p. deli(n=0)
p. multi(n=2)
Species

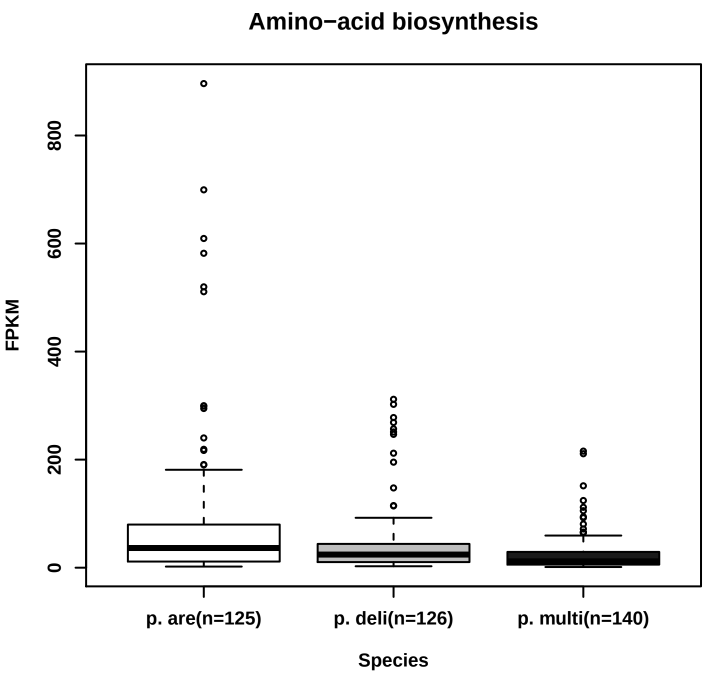
Amino−acid biosynthesis
FPKM
0
200
400
600
800
p. are(n=125)
p. deli(n=126)
p. multi(n=140)
Species

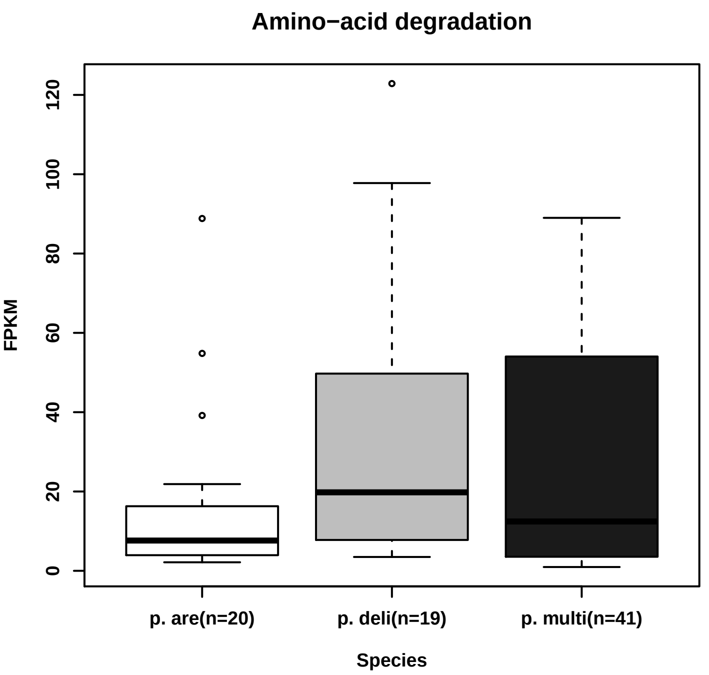
Amino−acid degradation
FPKM
0
20
40
60
80
100
120
p. are(n=20)
p. deli(n=19)
p. multi(n=41)
Species

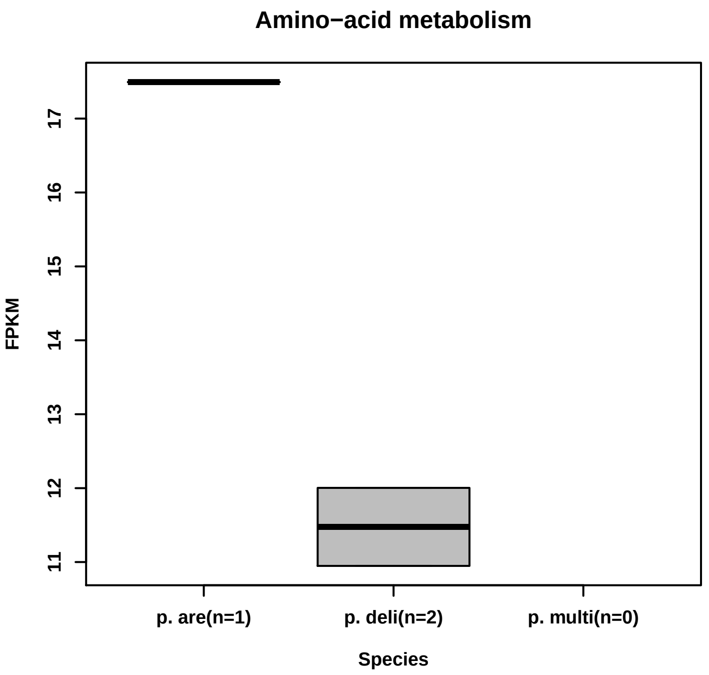
Amino−acid metabolism
FPKM
11
12
13
14
15
16
17
p. are(n=1)
p. deli(n=2)
p. multi(n=0)
Species

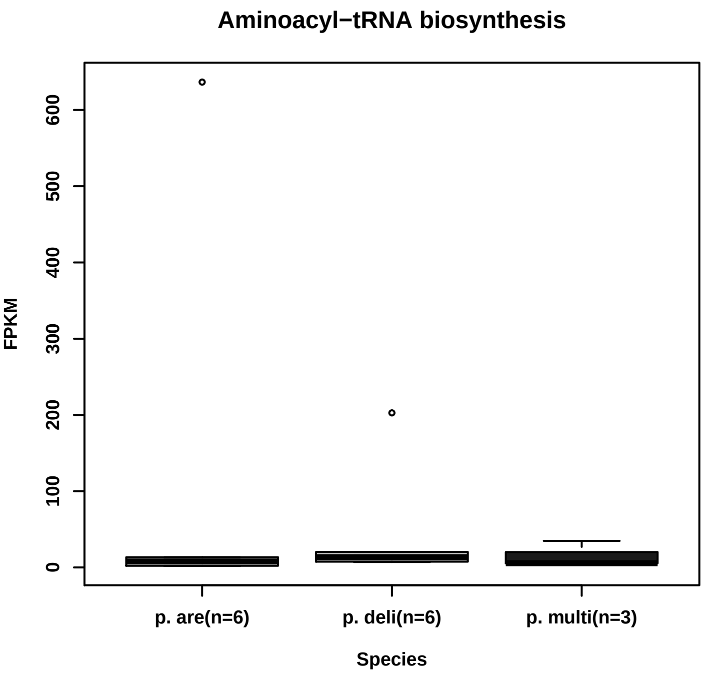
Aminoacyl−tRNA biosynthesis
FPKM
0
100
200
300
400
500
600
p. are(n=6)
p. deli(n=6)
p. multi(n=3)
Species

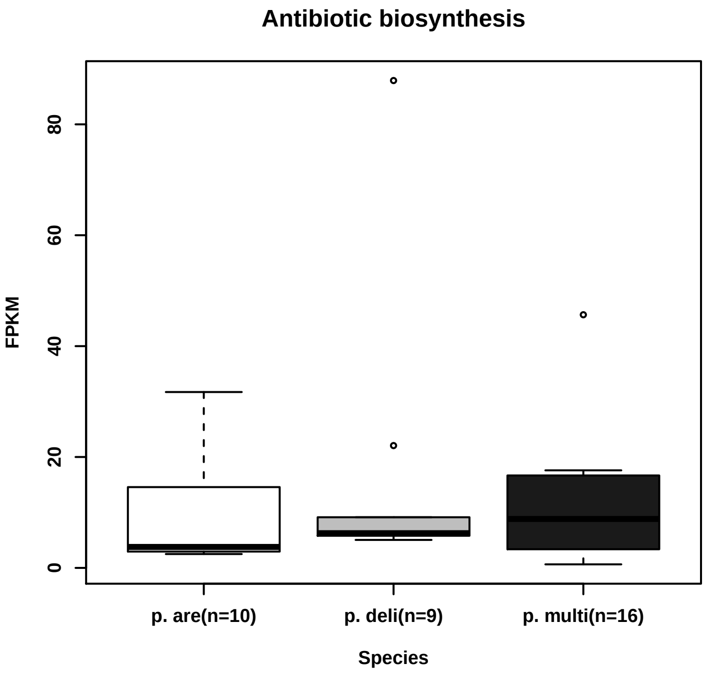
Antibiotic biosynthesis
FPKM
0
20
40
60
80
p. are(n=10)
p. deli(n=9)
p. multi(n=16)
Species

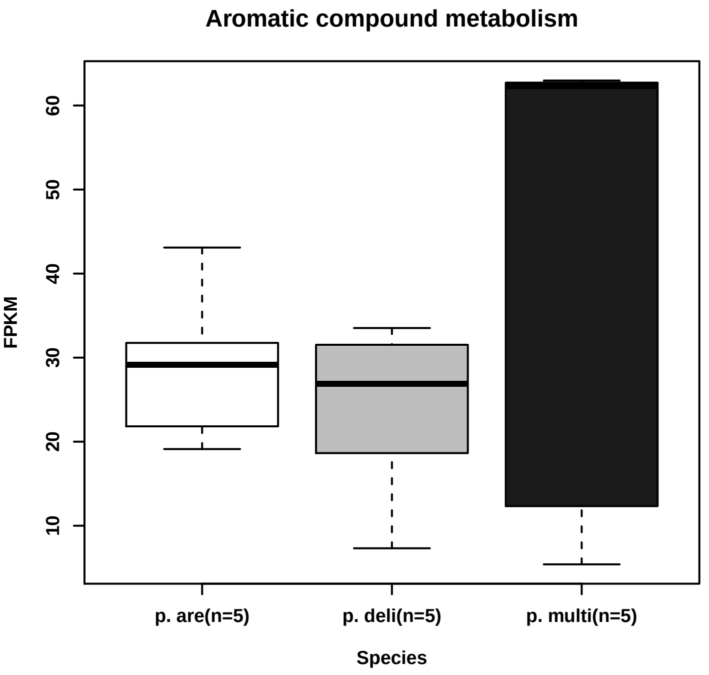
Aromatic compound metabolism
FPKM
10
20
30
40
50
60
p. are(n=5)
p. deli(n=5)
p. multi(n=5)
Species

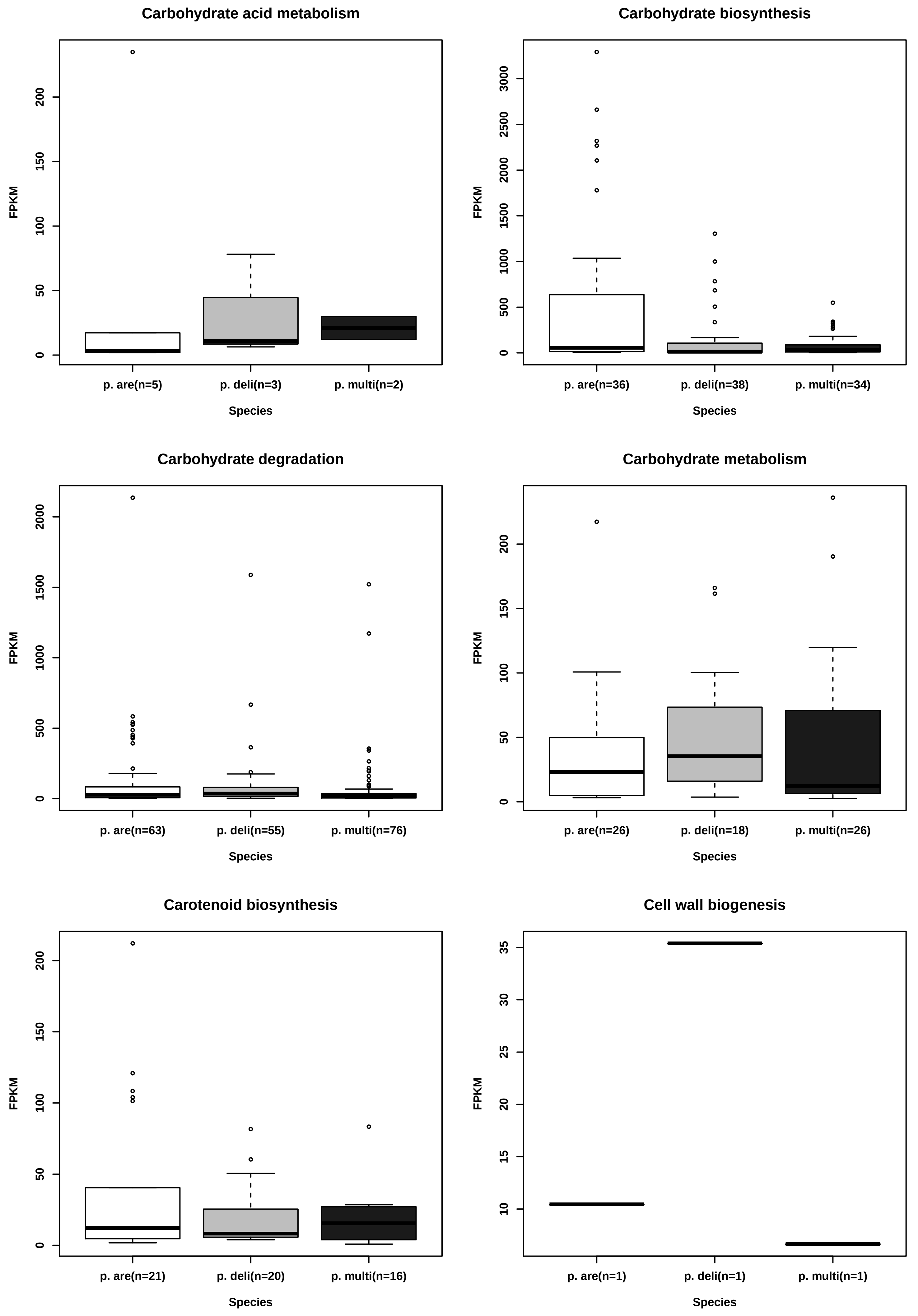
Carbohydrate acid metabolism
FPKM
0
50
100
150
200
p. are(n=5)
p. deli(n=3)
p. multi(n=2)
Species
Carbohydrate biosynthesis
FPKM
0
500
1000
1500
2000
2500
3000
p. are(n=36)
p. deli(n=38)
p. multi(n=34)
Species
Carbohydrate degradation
FPKM
0
500
1000
1500
2000
p. are(n=63)
p. deli(n=55)
p. multi(n=76)
Species
Carbohydrate metabolism
FPKM
0
50
100
150
200
p. are(n=26)
p. deli(n=18)
p. multi(n=26)
Species
Carotenoid biosynthesis
FPKM
0
50
100
150
200
p. are(n=21)
p. deli(n=20)
p. multi(n=16)
Species
Cell wall biogenesis
FPKM
10
15
20
25
30
35
p. are(n=1)
p. deli(n=1)
p. multi(n=1)
Species

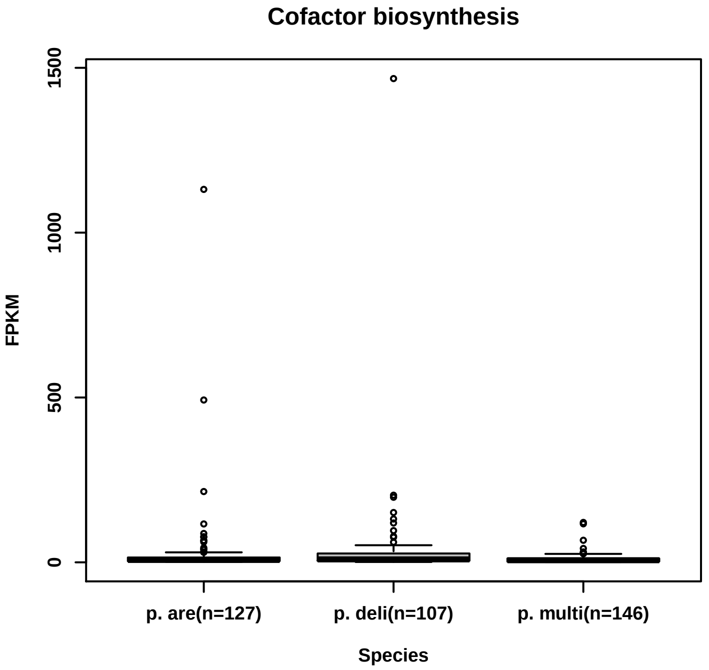
Cofactor biosynthesis
FPKM
0
500
1000
1500
p. are(n=127)
p. deli(n=107)
p. multi(n=146)
Species

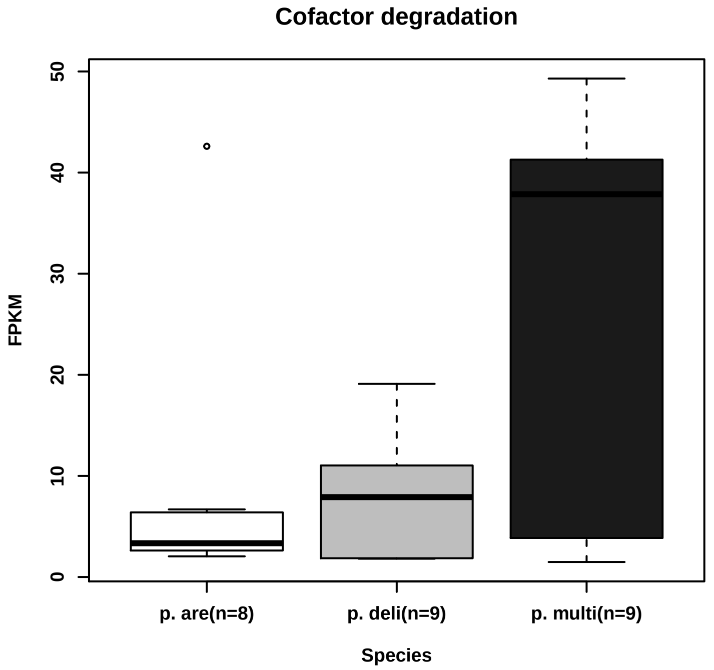
Cofactor degradation
FPKM
0
10
20
30
40
50
p. are(n=8)
p. deli(n=9)
p. multi(n=9)
Species

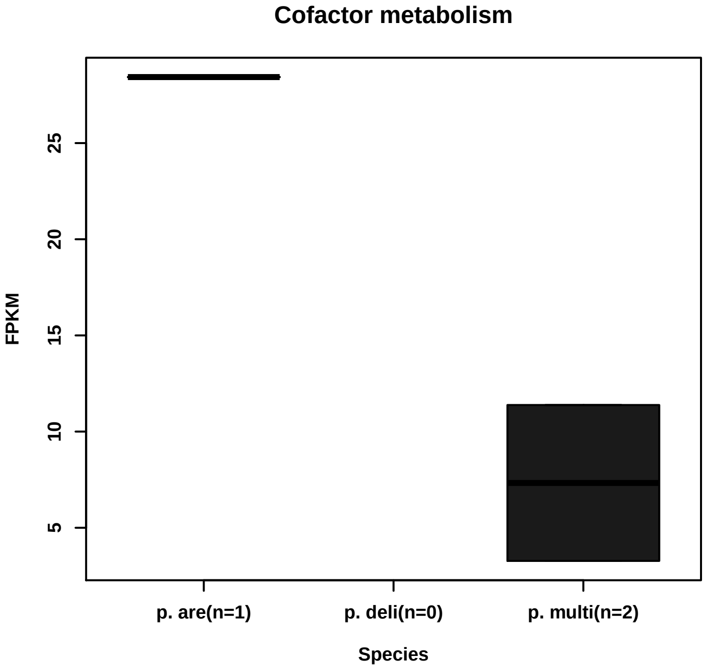
Cofactor metabolism
FPKM
5
10
15
20
25
p. are(n=1)
p. deli(n=0)
p. multi(n=2)
Species

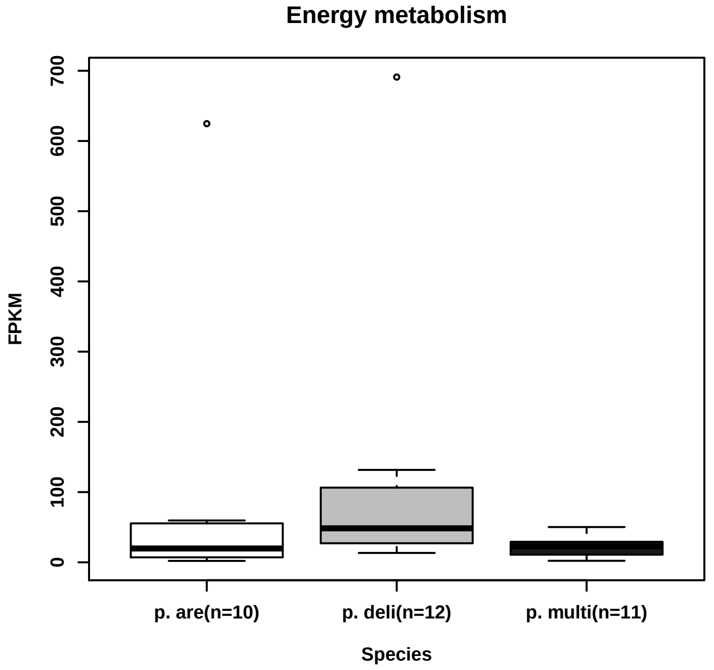
Energy metabolism
FPKM
0
100
200
300
400
500
600
700
p. are(n=10)
p. deli(n=12)
p. multi(n=11)
Species

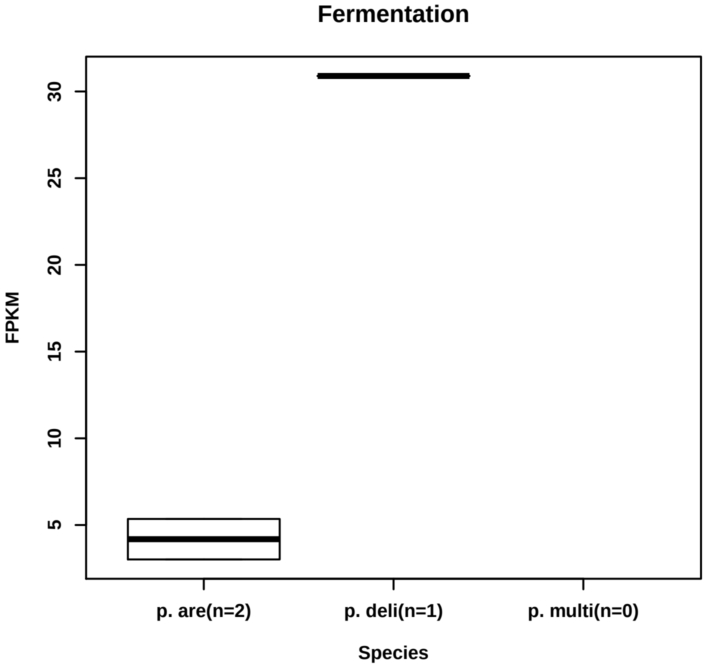
Fermentation
FPKM
5
10
15
20
25
30
p. are(n=2)
p. deli(n=1)
p. multi(n=0)
Species

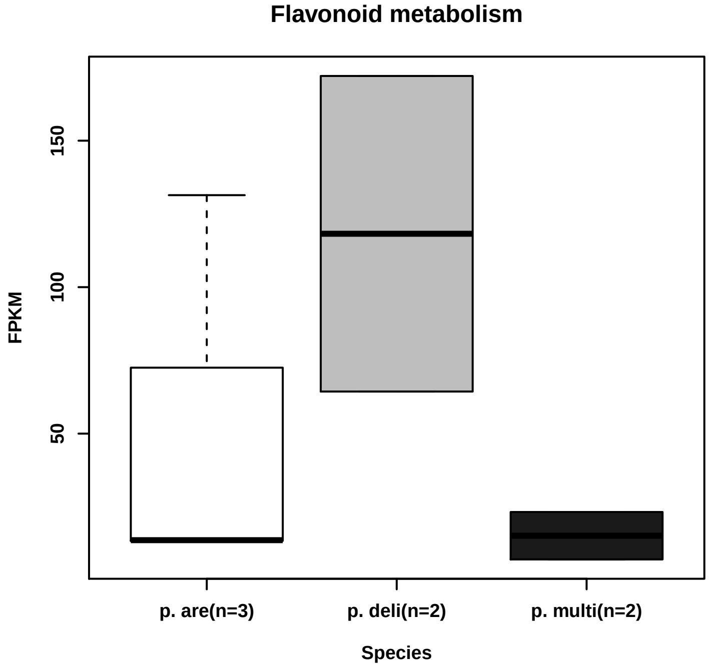
Flavonoid metabolism
FPKM
50
100
150
p. are(n=3)
p. deli(n=2)
p. multi(n=2)
Species

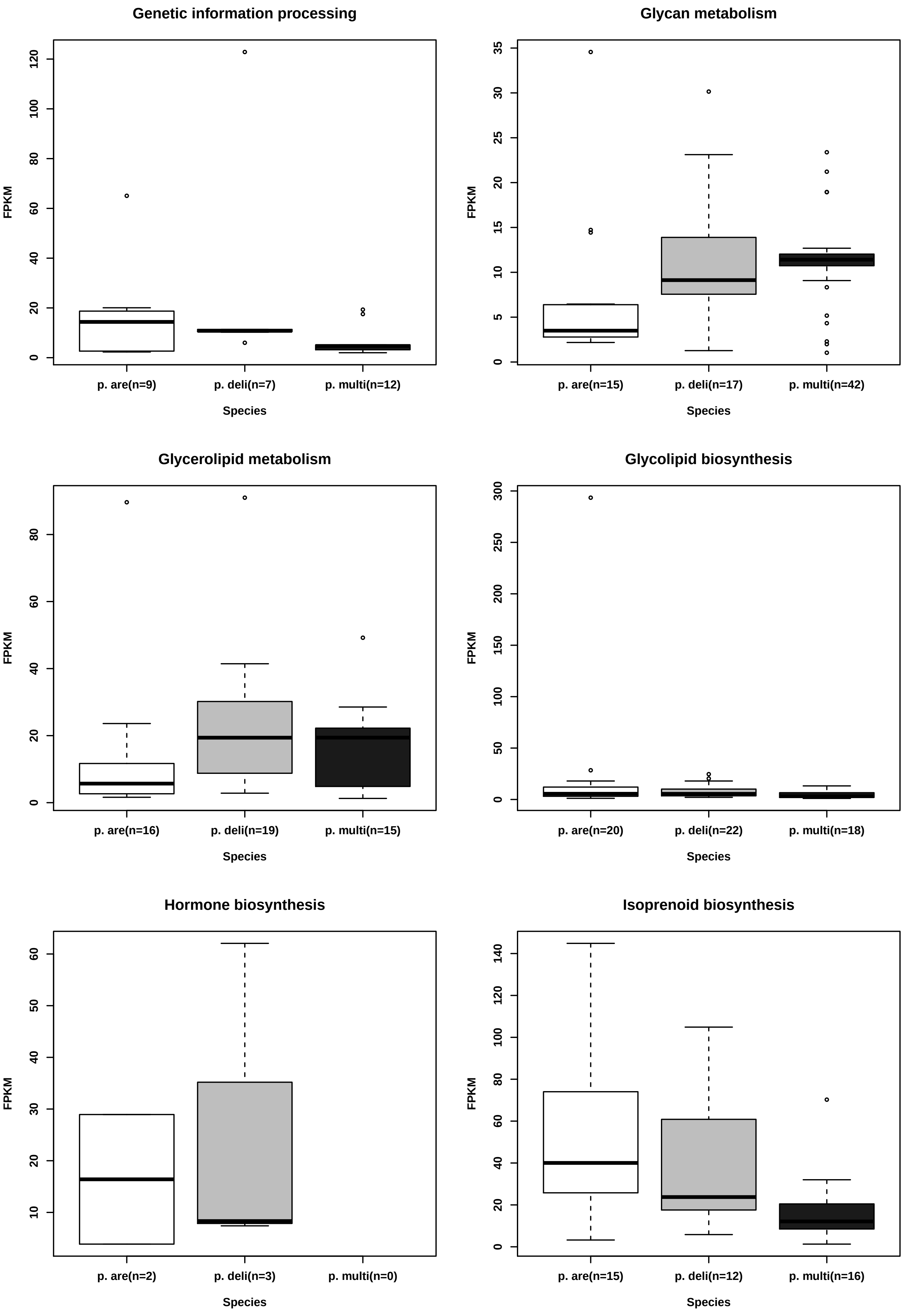
Genetic information processing
FPKM
0
20
40
60
80
100
120
p. are(n=9)
p. deli(n=7)
p. multi(n=12)
Species
Glycan metabolism
FPKM
0
5
10
15
20
25
30
35
p. are(n=15)
p. deli(n=17)
p. multi(n=42)
Species
Glycerolipid metabolism
FPKM
0
20
40
60
80
p. are(n=16)
p. deli(n=19)
p. multi(n=15)
Species
Glycolipid biosynthesis
FPKM
0
50
100
150
200
250
300
p. are(n=20)
p. deli(n=22)
p. multi(n=18)
Species
Hormone biosynthesis
FPKM
10
20
30
40
50
60
p. are(n=2)
p. deli(n=3)
p. multi(n=0)
Species
Isoprenoid biosynthesis
FPKM
0
20
40
60
80
100
120
140
p. are(n=15)
p. deli(n=12)
p. multi(n=16)
Species

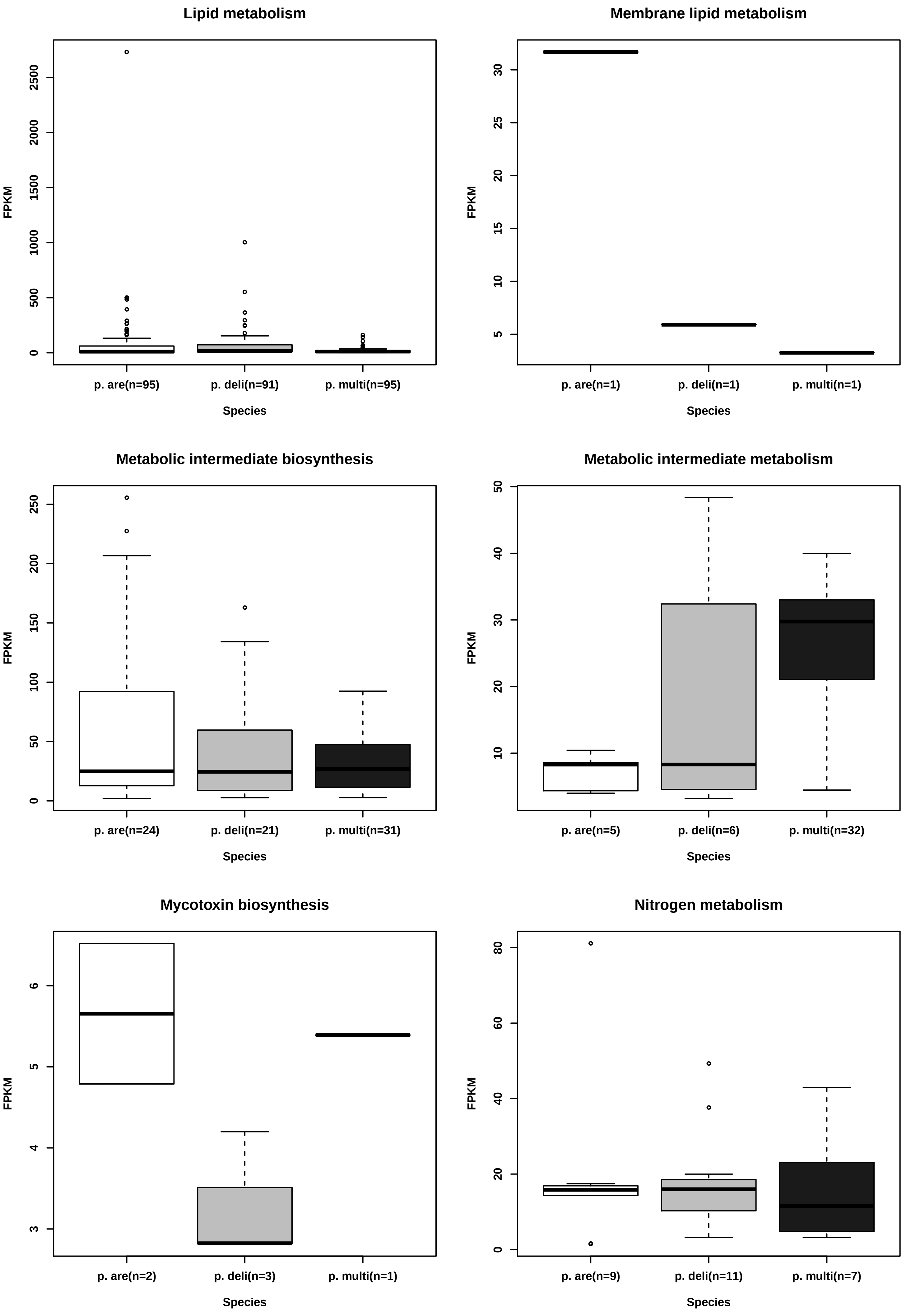
Lipid metabolism
FPKM
0
500
1000
1500
2000
2500
p. are(n=95)
p. deli(n=91)
p. multi(n=95)
Species
Membrane lipid metabolism
FPKM
5
10
15
20
25
30
p. are(n=1)
p. deli(n=1)
p. multi(n=1)
Species
Metabolic intermediate biosynthesis
FPKM
0
50
100
150
200
250
p. are(n=24)
p. deli(n=21)
p. multi(n=31)
Species
Metabolic intermediate metabolism
FPKM
10
20
30
40
50
p. are(n=5)
p. deli(n=6)
p. multi(n=32)
Species
Mycotoxin biosynthesis
FPKM
3
4
5
6
p. are(n=2)
p. deli(n=3)
p. multi(n=1)
Species
Nitrogen metabolism
FPKM
0
20
40
60
80
p. are(n=9)
p. deli(n=11)
p. multi(n=7)
Species

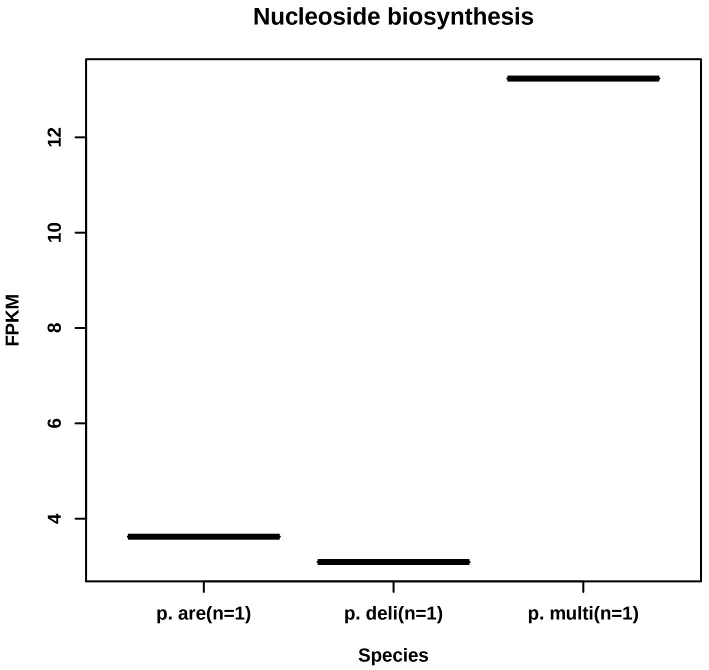
Nucleoside biosynthesis
FPKM
4
6
8
10
12
p. are(n=1)
p. deli(n=1)
p. multi(n=1)
Species

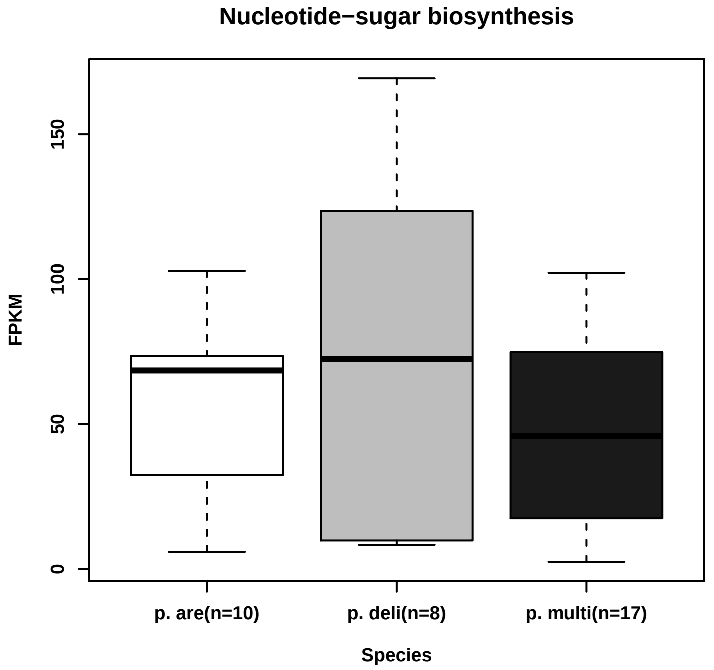
Nucleotide−sugar biosynthesis
FPKM
0
50
100
150
p. are(n=10)
p. deli(n=8)
p. multi(n=17)
Species

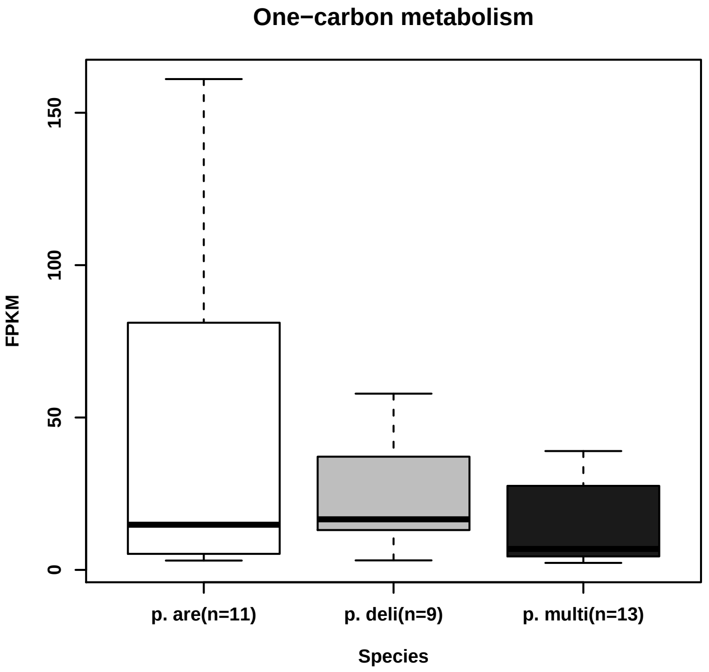
One−carbon metabolism
FPKM
0
50
100
150
p. are(n=11)
p. deli(n=9)
p. multi(n=13)
Species

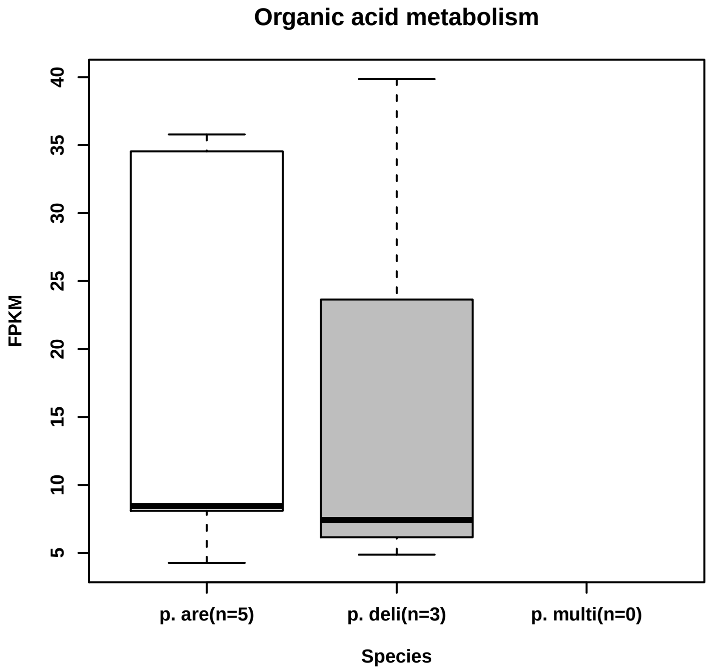
Organic acid metabolism
FPKM
5
10
15
20
25
30
35
40
p. are(n=5)
p. deli(n=3)
p. multi(n=0)
Species

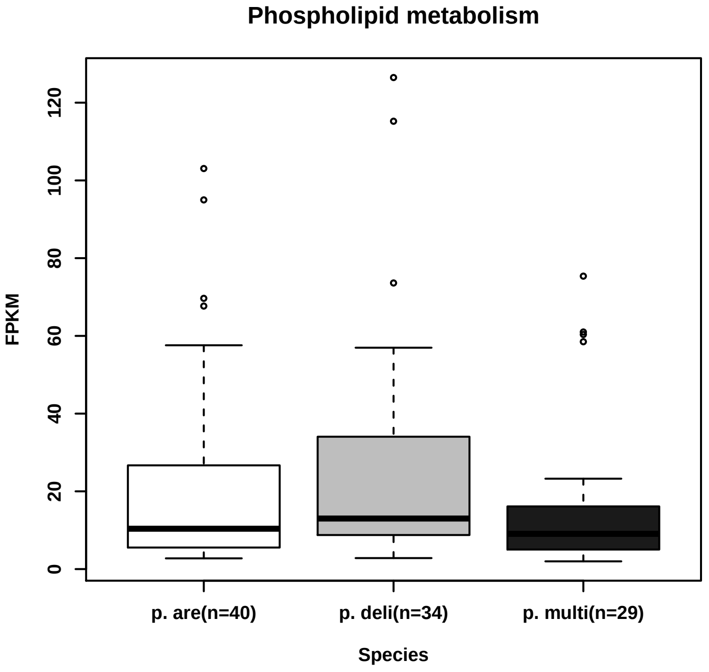
Phospholipid metabolism
FPKM
0
20
40
60
80
100
120
p. are(n=40)
p. deli(n=34)
p. multi(n=29)
Species

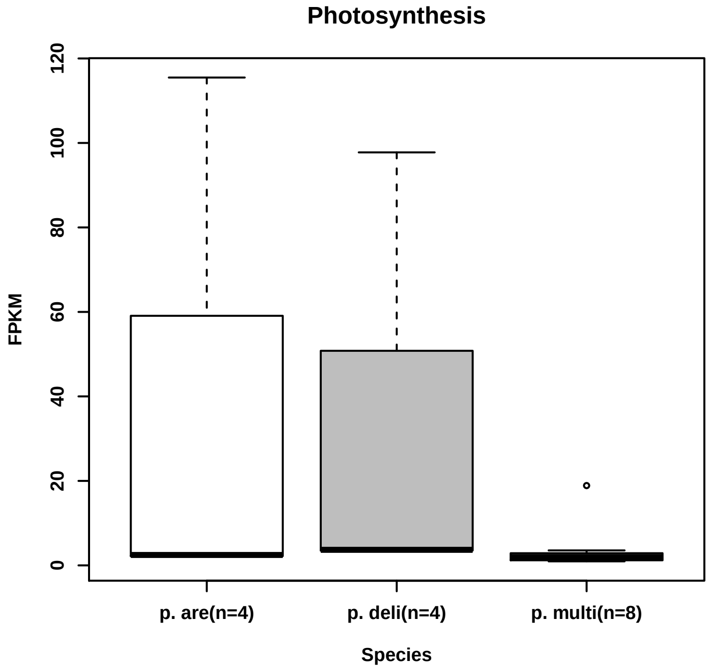
Photosynthesis
FPKM
0
20
40
60
80
100
120
p. are(n=4)
p. deli(n=4)
p. multi(n=8)
Species

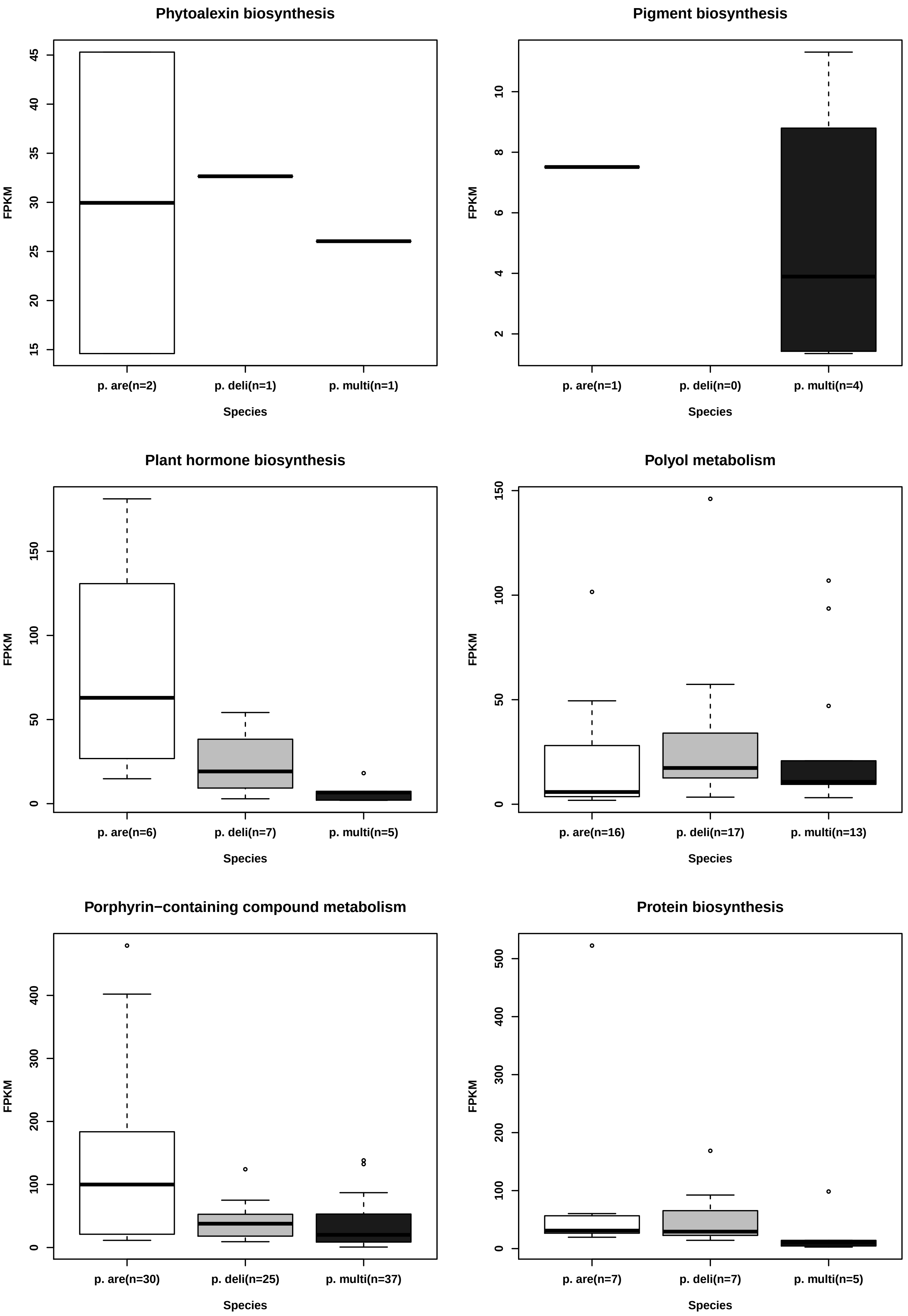

Phytoalexin biosynthesis
FPKM
15 20 25 30 35 40 45
p. are(n=2)
p. deli(n=1)
p. multi(n=1)
Species
Pigment biosynthesis
FPKM
2 4 6 8 10
p. are(n=1)
p. deli(n=0)
p. multi(n=4)
Species
Plant hormone biosynthesis
FPKM
0 50 100 150
p. are(n=6)
p. deli(n=7)
p. multi(n=5)
Species
Polyol metabolism
FPKM
0 50 100 150
p. are(n=16)
p. deli(n=17)
p. multi(n=13)
Species
Porphyrin−containing compound metabolism
FPKM
0 100 200 300 400
p. are(n=30)
p. deli(n=25)
p. multi(n=37)
Species
Protein biosynthesis
FPKM
0 100 200 300 400 500
p. are(n=7)
p. deli(n=7)
p. multi(n=5)
Species

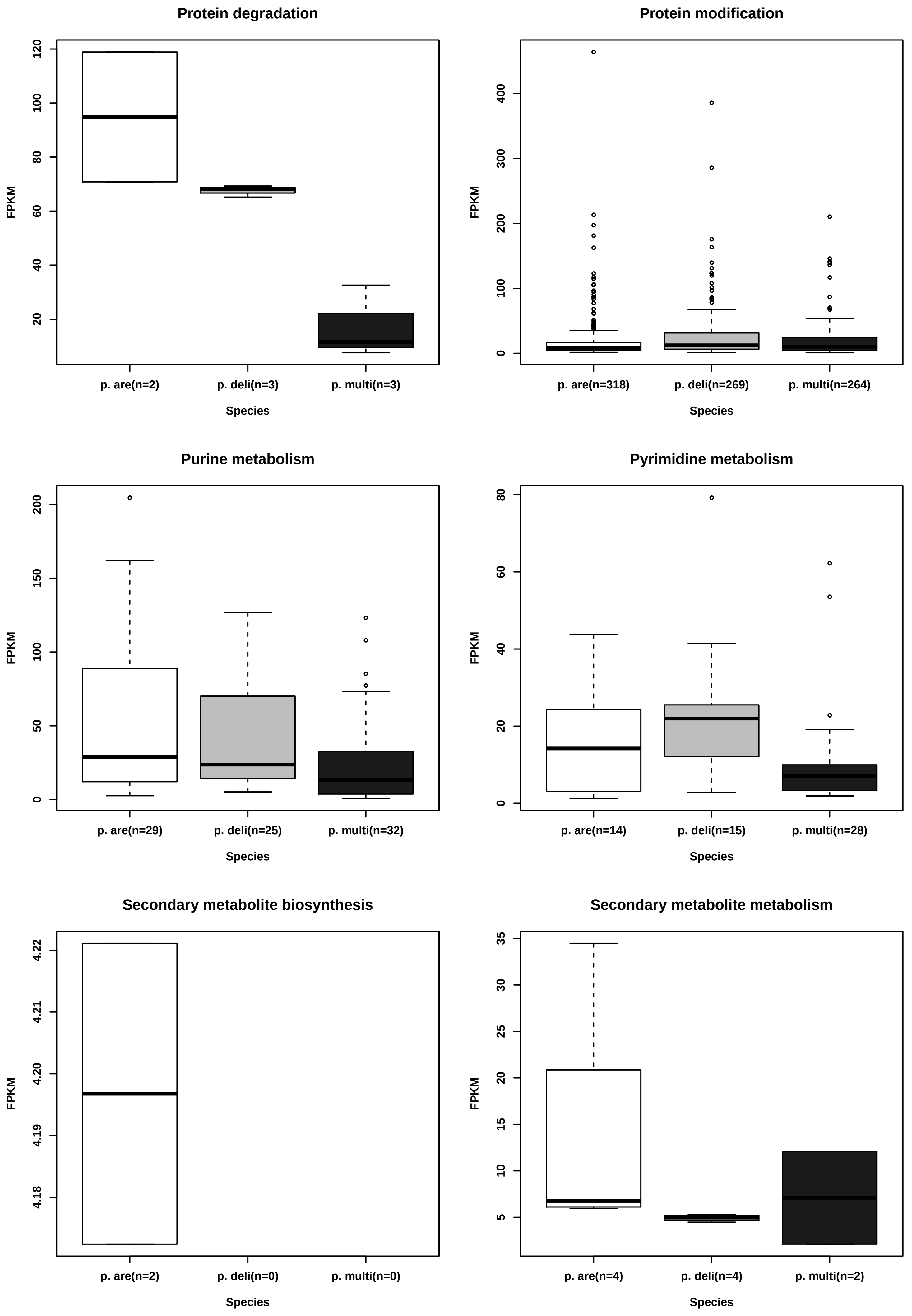
Protein degradation
FPKM
120
100
80
60
40
20
p. are(n=2)
p. deli(n=3)
p. multi(n=3)
Species
Protein modification
FPKM
400
300
200
100
0
p. are(n=318)
p. deli(n=269)
p. multi(n=264)
Species
Purine metabolism
FPKM
200
150
100
50
0
p. are(n=29)
p. deli(n=25)
p. multi(n=32)
Species
Pyrimidine metabolism
FPKM
80
60
40
20
0
p. are(n=14)
p. deli(n=15)
p. multi(n=28)
Species
Secondary metabolite biosynthesis
FPKM
4.22
4.21
4.20
4.19
4.18
p. are(n=2)
p. deli(n=0)
p. multi(n=0)
Species
Secondary metabolite metabolism
FPKM
35
30
25
20
15
10
5
p. are(n=4)
p. deli(n=4)
p. multi(n=2)
Species

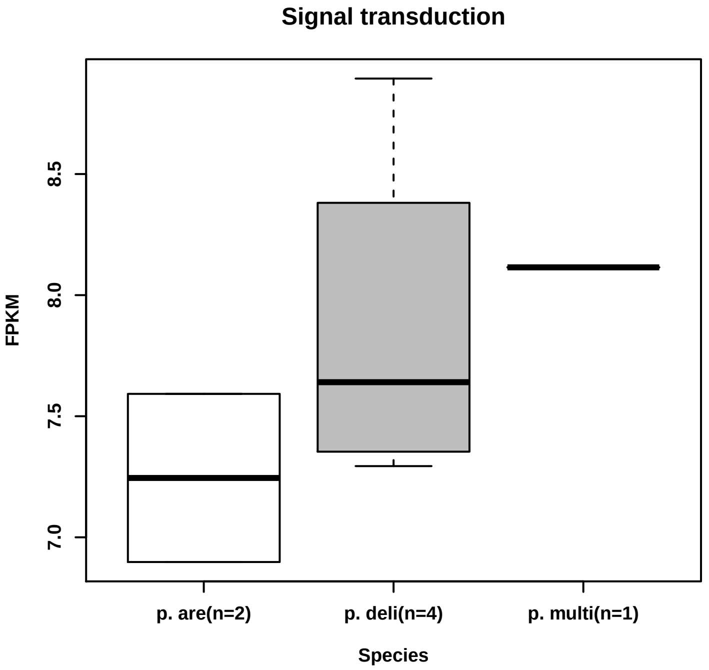
Signal transduction
FPKM
7.0
7.5
8.0
8.5
p. are(n=2)
p. deli(n=4)
p. multi(n=1)
Species

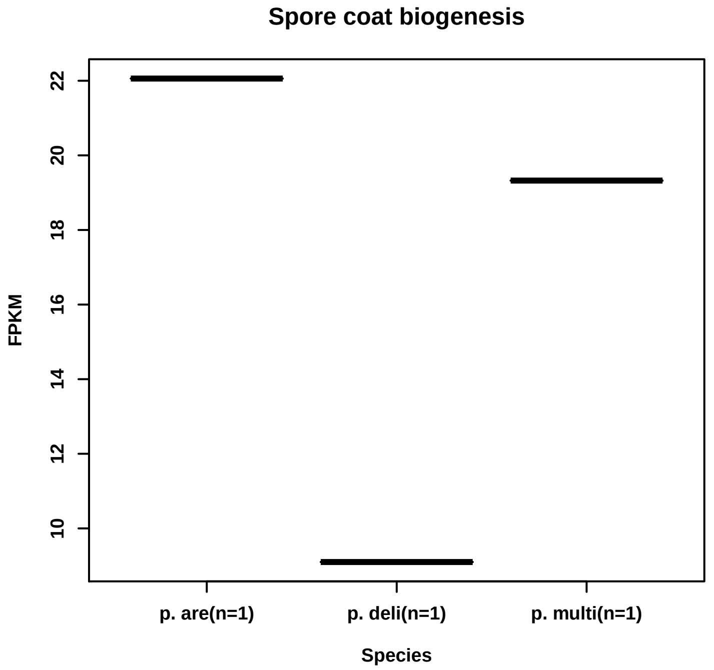
Spore coat biogenesis
FPKM
10
12
14
16
18
20
22
p. are(n=1)
p. deli(n=1)
p. multi(n=1)
Species

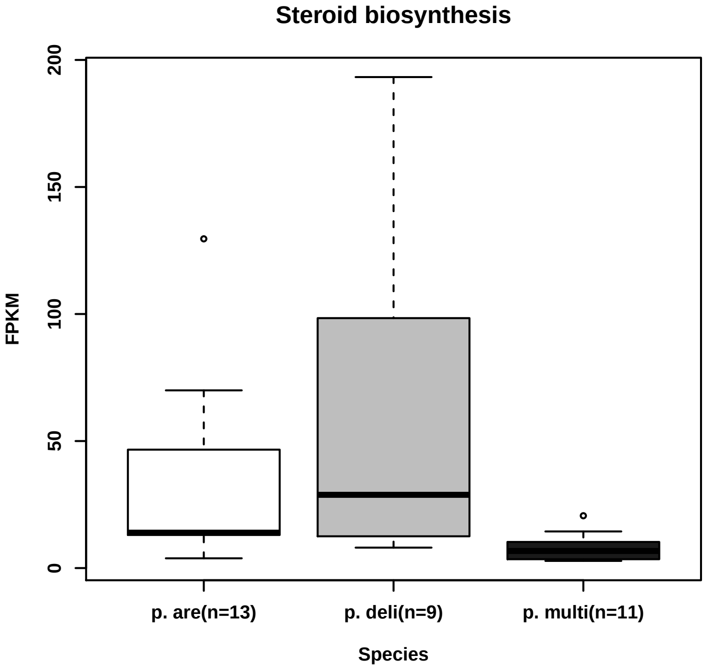
Steroid biosynthesis
FPKM
0
50
100
150
200
p. are(n=13)
p. deli(n=9)
p. multi(n=11)
Species

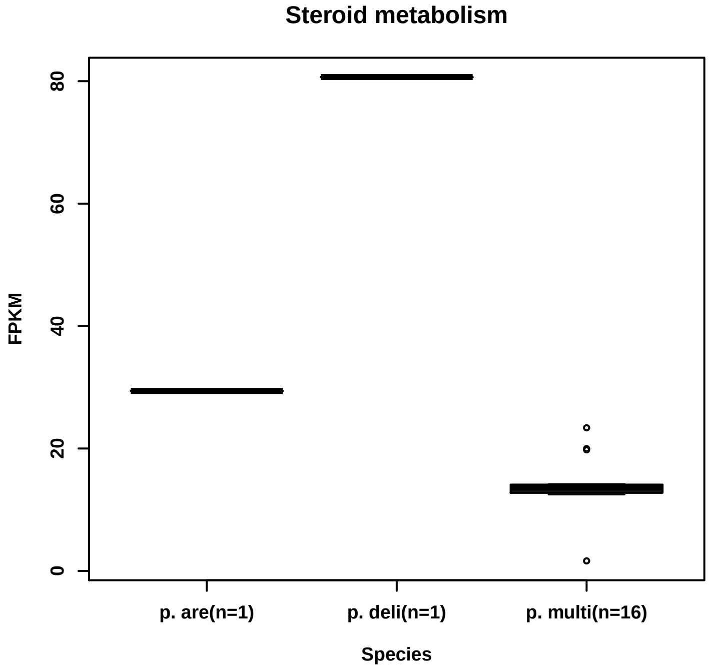
Steroid metabolism
FPKM
0
20
40
60
80
p. are(n=1)
p. deli(n=1)
p. multi(n=16)
Species

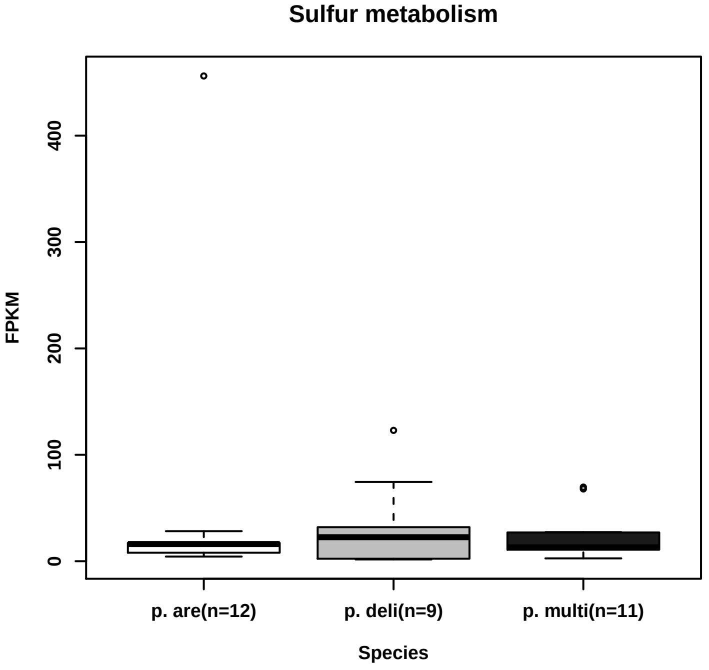
Sulfur metabolism
FPKM
0
100
200
300
400
p. are(n=12)
p. deli(n=9)
p. multi(n=11)
Species

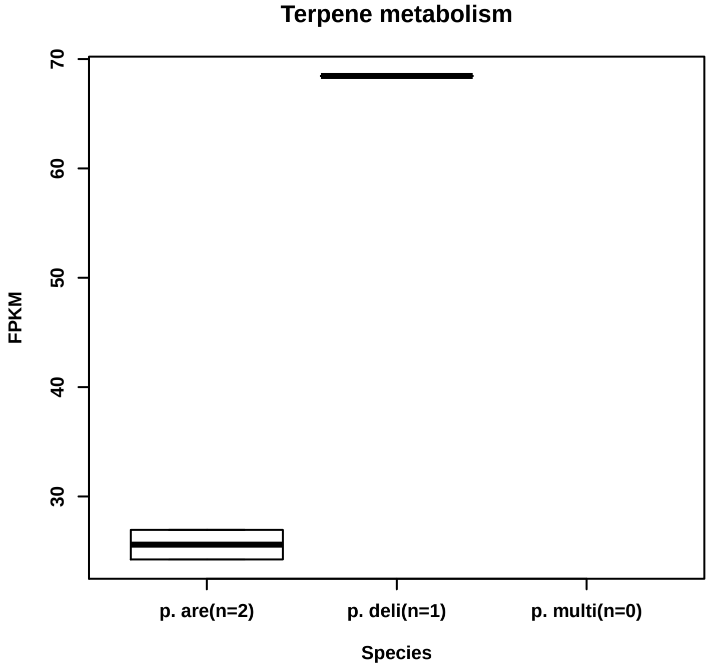
Terpene metabolism
FPKM
30
40
50
60
70
p. are(n=2)
p. deli(n=1)
p. multi(n=0)
Species

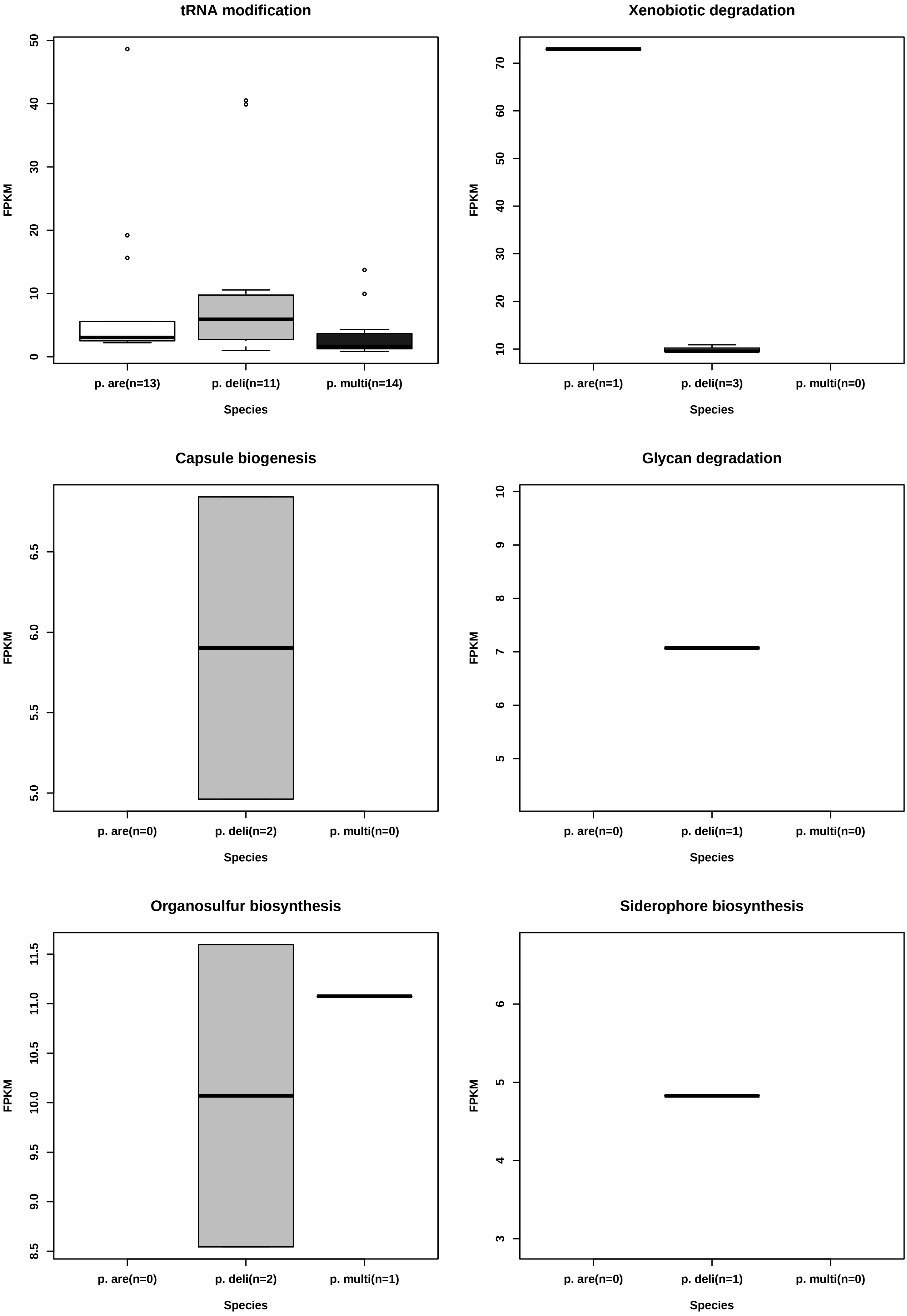
tRNA modification
FPKM
0
10
20
30
40
50
p. are(n=13)
p. deli(n=11)
p. multi(n=14)
Species
Xenobiotic degradation
FPKM
10
20
30
40
50
60
70
p. are(n=1)
p. deli(n=3)
p. multi(n=0)
Species
Capsule biogenesis
FPKM
5.0
5.5
6.0
6.5
p. are(n=0)
p. deli(n=2)
p. multi(n=0)
Species
Glycan degradation
FPKM
5
6
7
8
9
10
p. are(n=0)
p. deli(n=1)
p. multi(n=0)
Species
Organosulfur biosynthesis
FPKM
8.5
9.0
9.5
10.0
10.5
11.0
11.5
p. are(n=0)
p. deli(n=2)
p. multi(n=1)
Species
Siderophore biosynthesis
FPKM
3
4
5
6
p. are(n=0)
p. deli(n=1)
p. multi(n=0)
Species

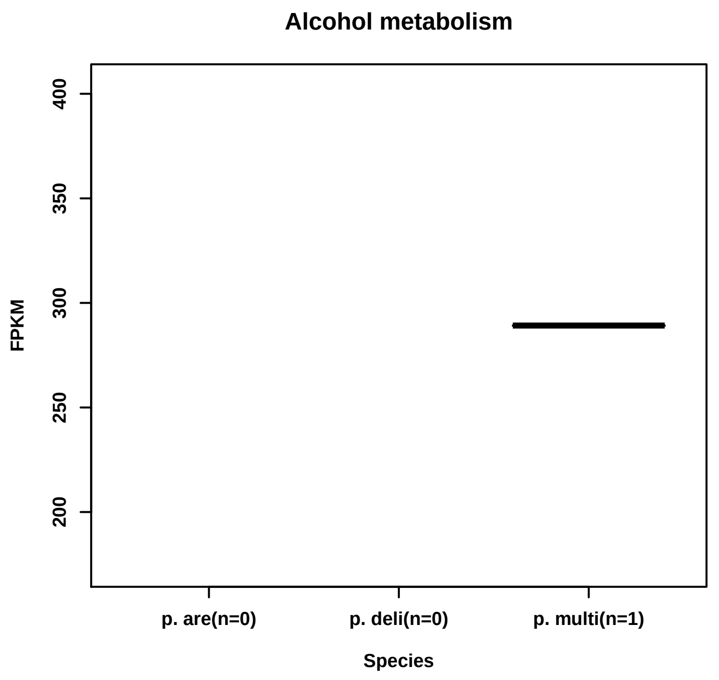

Alcohol metabolism
FPKM
200
250
300
350
400
p. are(n=0)
p. deli(n=0)
p. multi(n=1)
Species

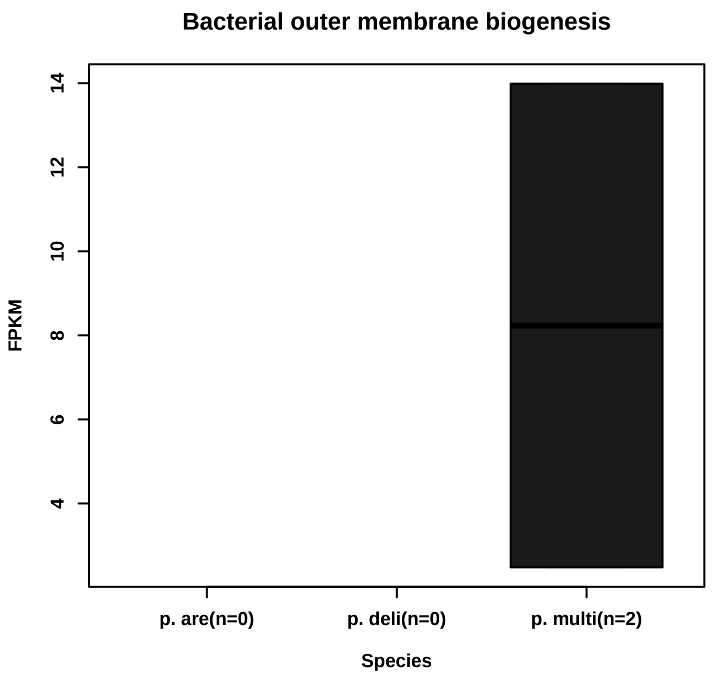

Bacterial outer membrane biogenesis
FPKM
4
6
8
10
12
14
p. are(n=0)
p. deli(n=0)
p. multi(n=2)
Species

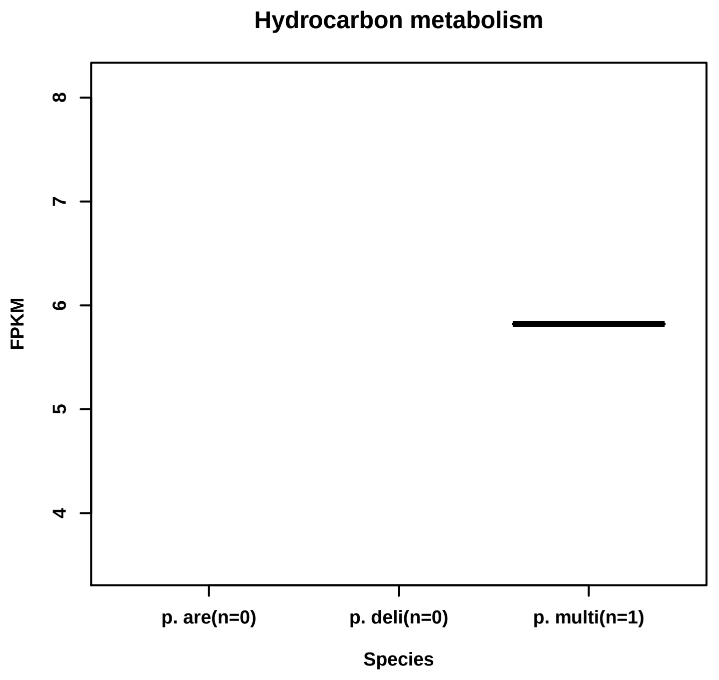

Hydrocarbon metabolism
FPKM
4
5
6
7
8
p. are(n=0)
p. deli(n=0)
p. multi(n=1)
Species

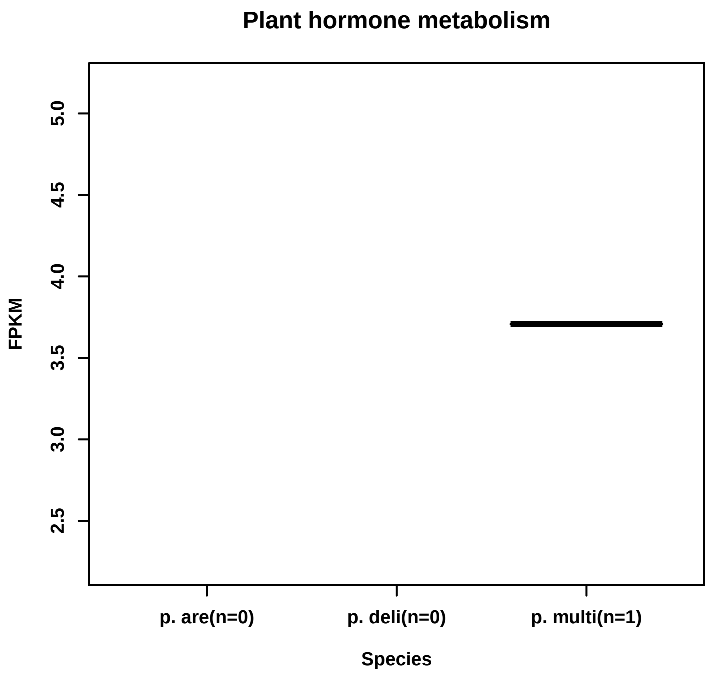

Plant hormone metabolism
FPKM
2.5
3.0
3.5
4.0
4.5
5.0
p. are(n=0)
p. deli(n=0)
p. multi(n=1)
Species

Supplementary Figure S3. Pathways expression based on the second level of annotation.

Each graph shows the expression of one of the specific pathways included in the more general pathways, showed in Figure S2, for each species. See Figure S2 legend for details.

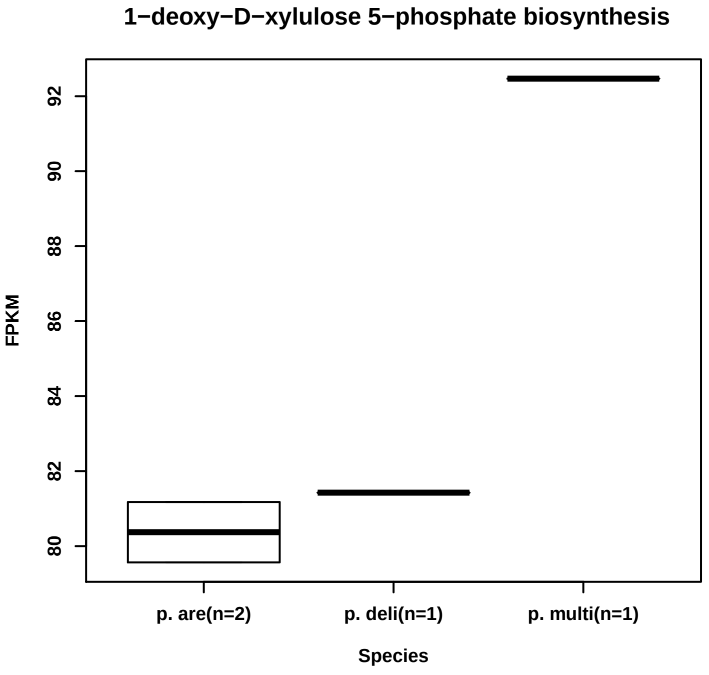
1−deoxy−D−xylulose 5−phosphate biosynthesis
FPKM
80
82
84
86
88
90
92
p. are(n=2)
p. deli(n=1)
p. multi(n=1)
Species

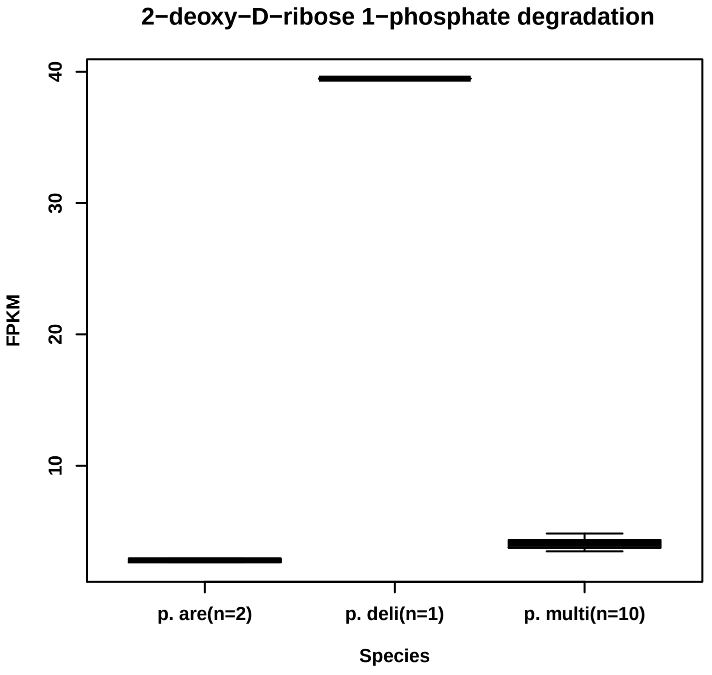
2−deoxy−D−ribose 1−phosphate degradation
FPKM
10
20
30
40
p. are(n=2)
p. deli(n=1)
p. multi(n=10)
Species

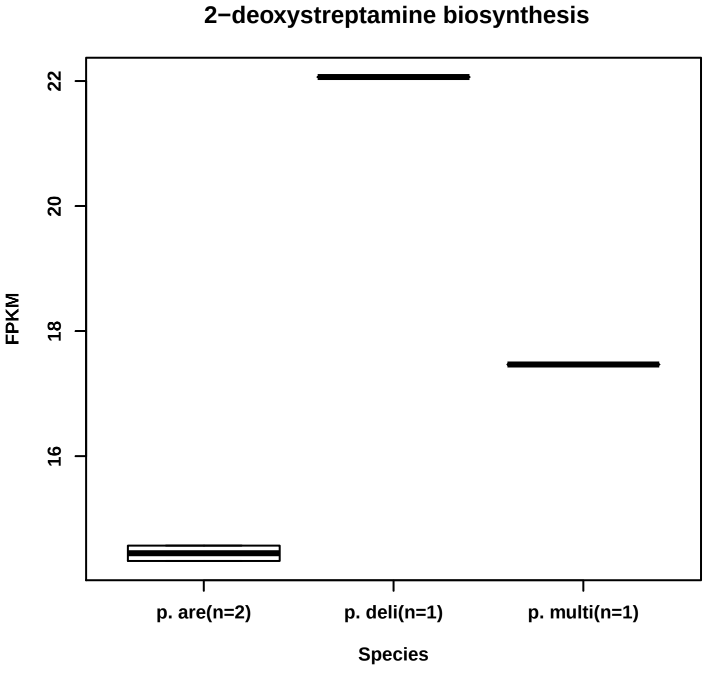
2−deoxystreptamine biosynthesis
FPKM
16
18
20
22
p. are(n=2)
p. deli(n=1)
p. multi(n=1)
Species

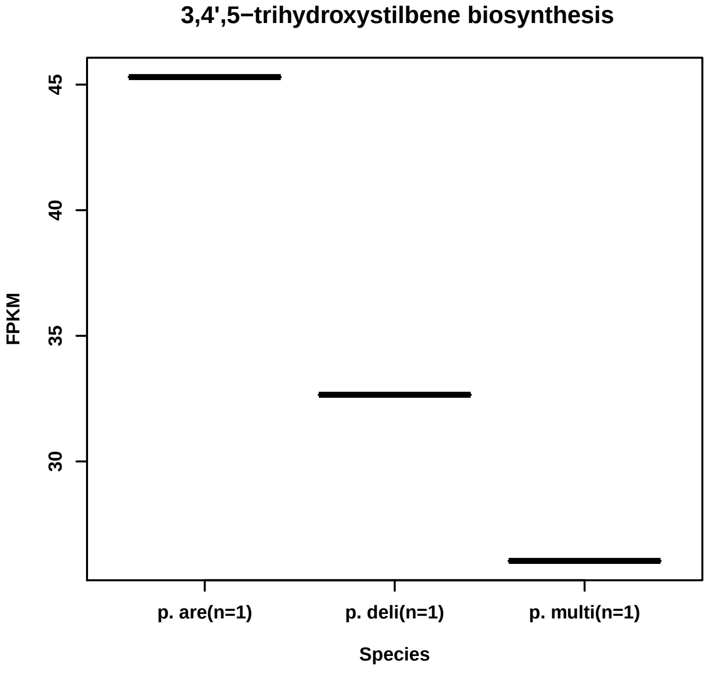
3,4',5−trihydroxystilbene biosynthesis
FPKM
30
35
40
45
p. are(n=1)
p. deli(n=1)
p. multi(n=1)
Species

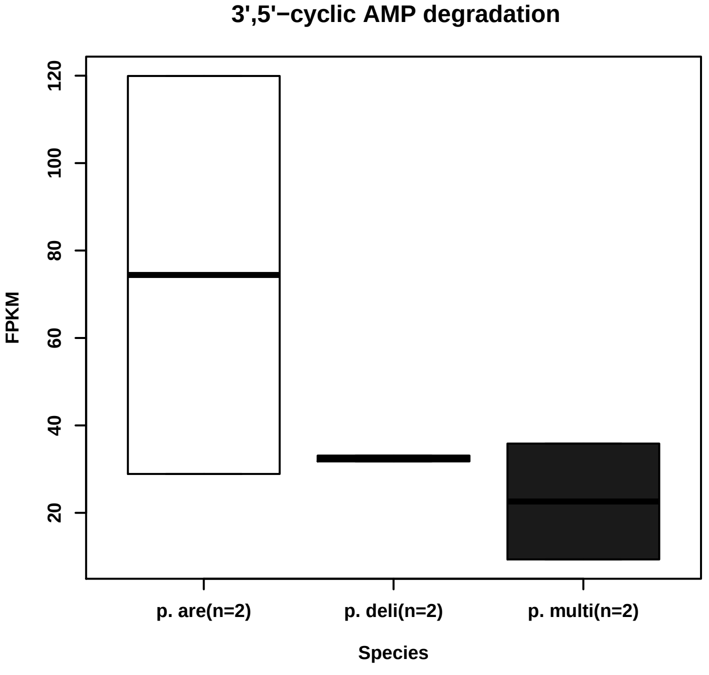
3',5'−cyclic AMP degradation
FPKM
20
40
60
80
100
120
p. are(n=2)
p. deli(n=2)
p. multi(n=2)
Species

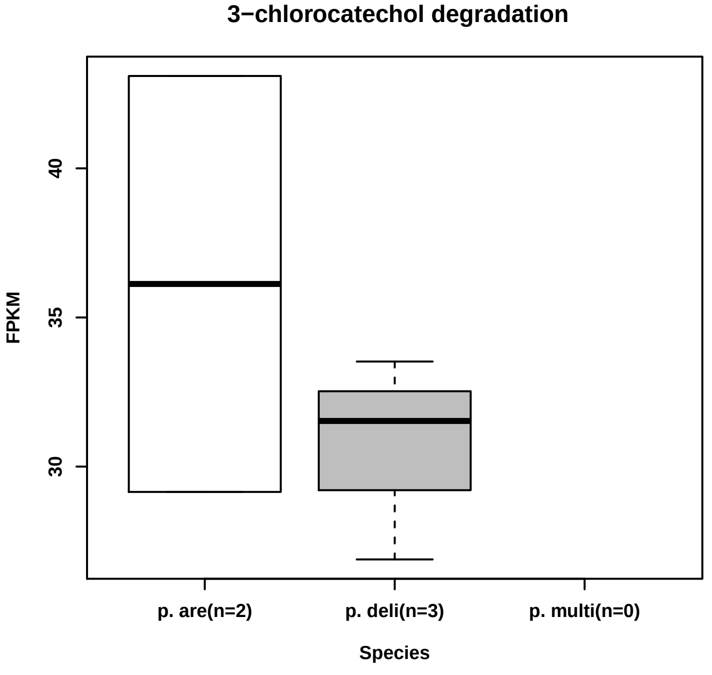
3−chlorocatechol degradation
FPKM
30
35
40
p. are(n=2)
p. deli(n=3)
p. multi(n=0)
Species

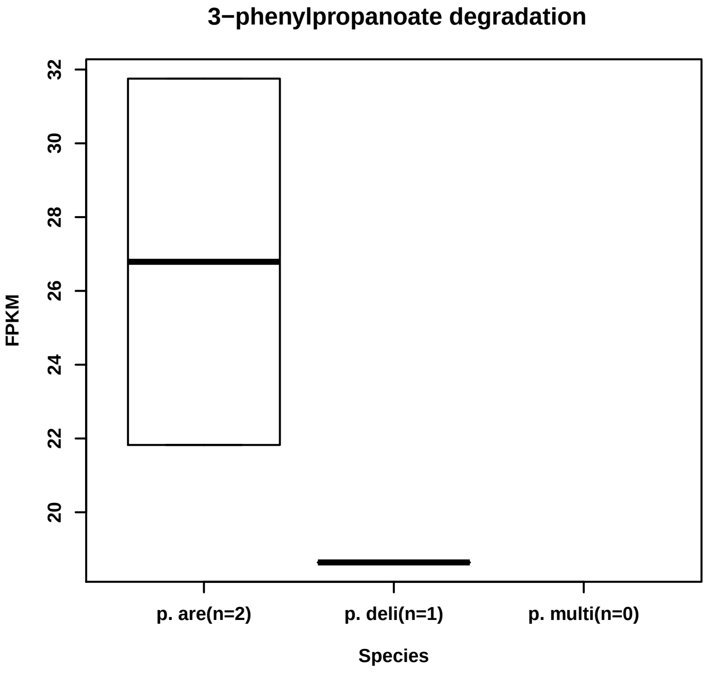
3−phenylpropanoate degradation
FPKM
20
22
24
26
28
30
32
p. are(n=2)
p. deli(n=1)
p. multi(n=0)
Species

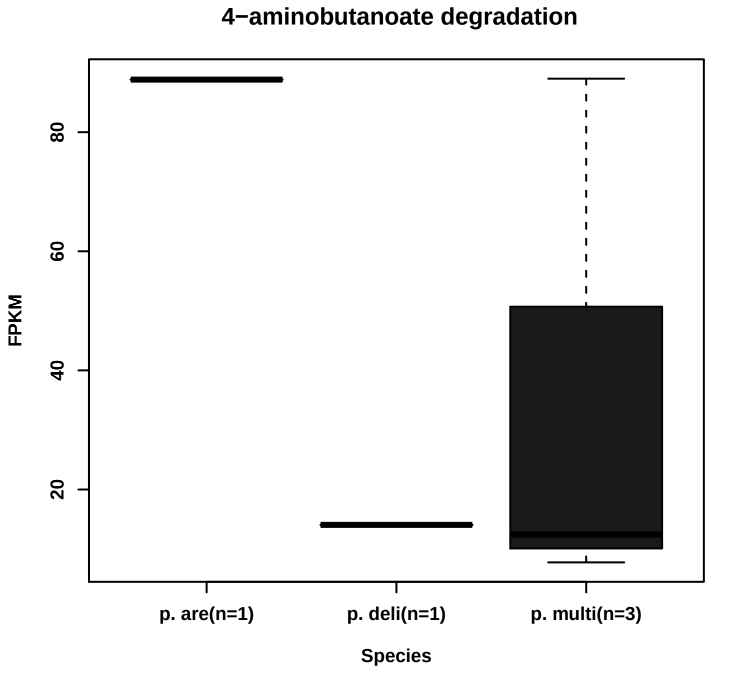
4−aminobutanoate degradation
FPKM
20
40
60
80
p. are(n=1)
p. deli(n=1)
p. multi(n=3)
Species

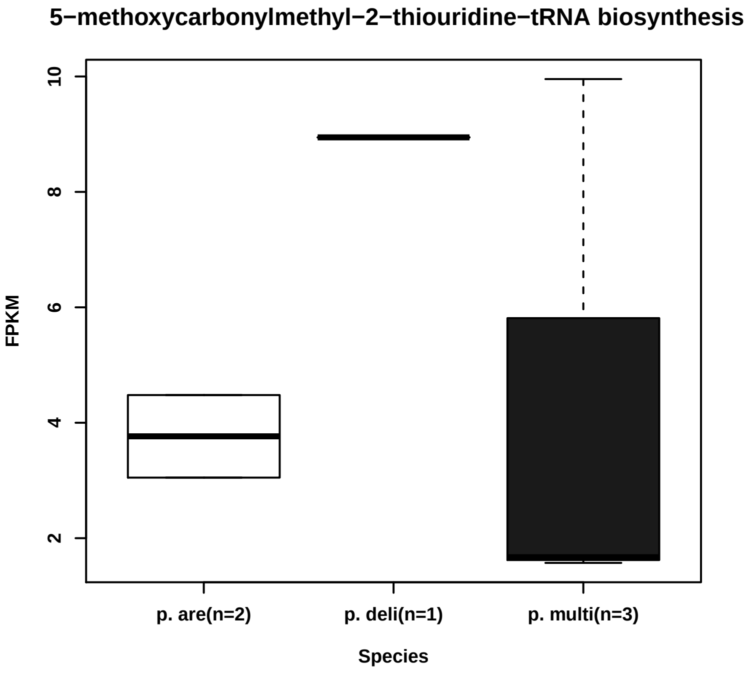
5−methoxycarbonylmethyl−2−thiouridine−tRNA biosynthesis
FPKM
2
4
6
8
10
p. are(n=2)
p. deli(n=1)
p. multi(n=3)
Species

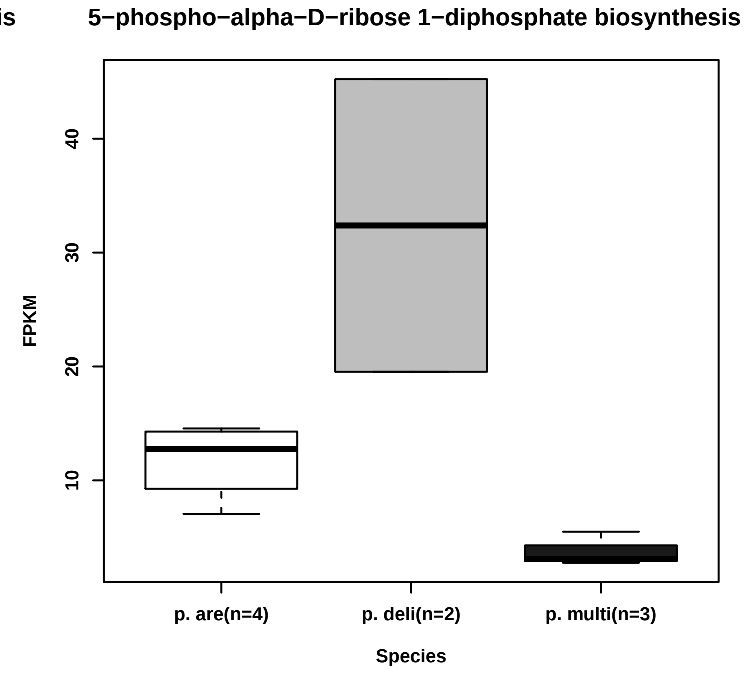
5−phospho−alpha−D−ribose 1−diphosphate biosynthesis
FPKM
10
20
30
40
p. are(n=4)
p. deli(n=2)
p. multi(n=3)
Species

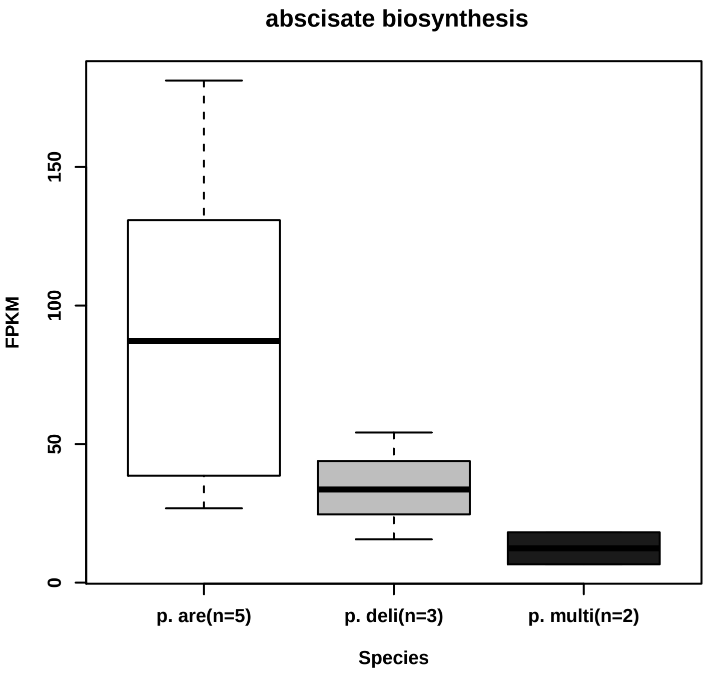
abscisate biosynthesis
FPKM
0
50
100
150
p. are(n=5)
p. deli(n=3)
p. multi(n=2)
Species

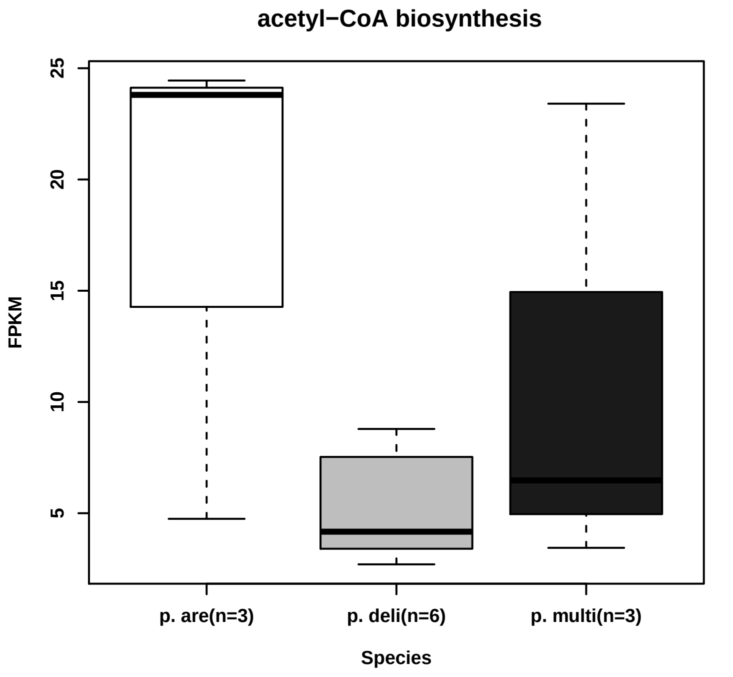
acetyl−CoA biosynthesis
FPKM
5
10
15
20
25
p. are(n=3)
p. deli(n=6)
p. multi(n=3)
Species

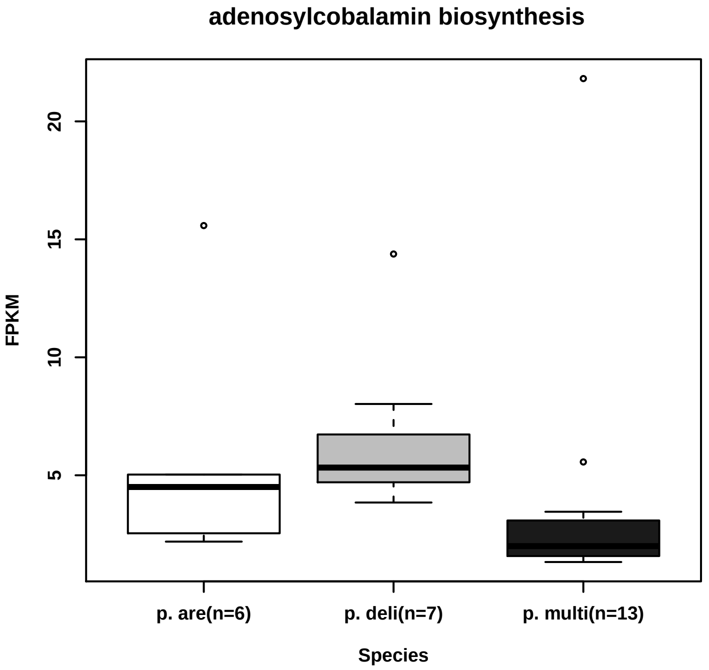
adenosylcobalamin biosynthesis
FPKM
5
10
15
20
p. are(n=6)
p. deli(n=7)
p. multi(n=13)
Species

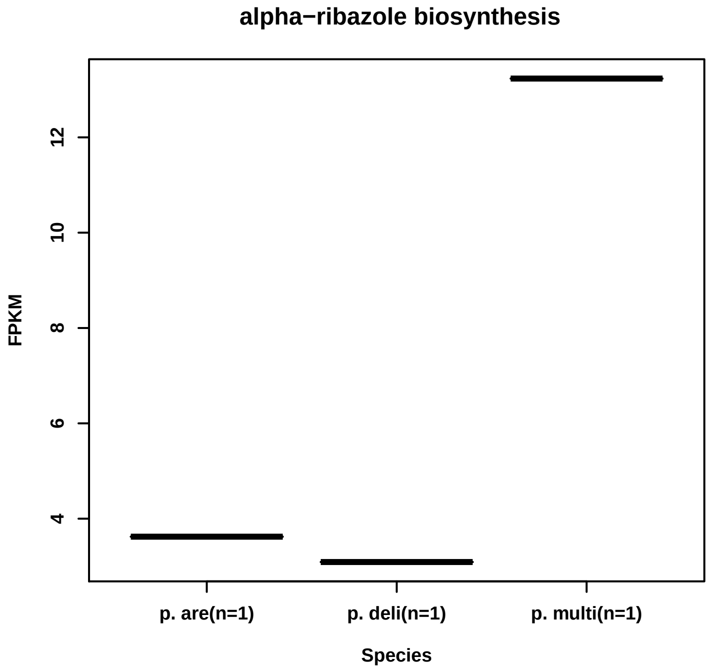
alpha−ribazole biosynthesis
FPKM
4
6
8
10
12
p. are(n=1)
p. deli(n=1)
p. multi(n=1)
Species

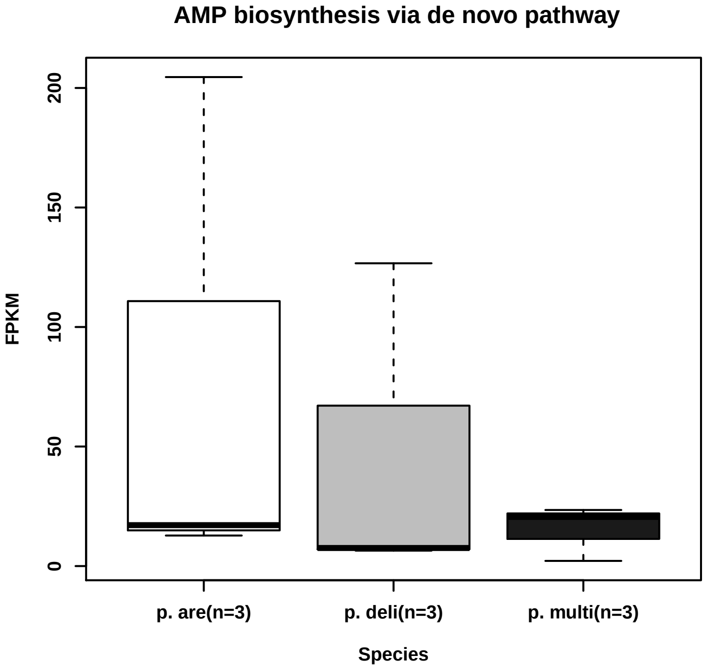
AMP biosynthesis via de novo pathway
FPKM
0
50
100
150
200
p. are(n=3)
p. deli(n=3)
p. multi(n=3)
Species

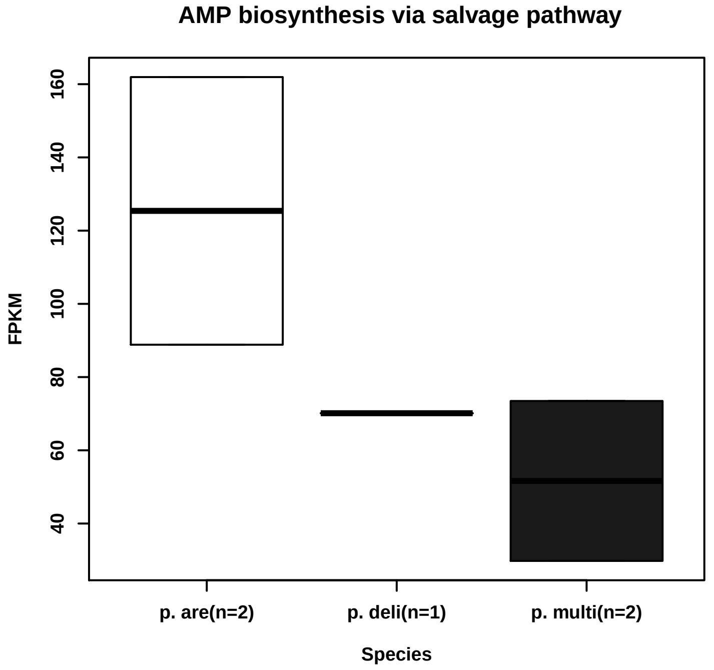
AMP biosynthesis via salvage pathway
FPKM
40
60
80
100
120
140
160
p. are(n=2)
p. deli(n=1)
p. multi(n=2)
Species

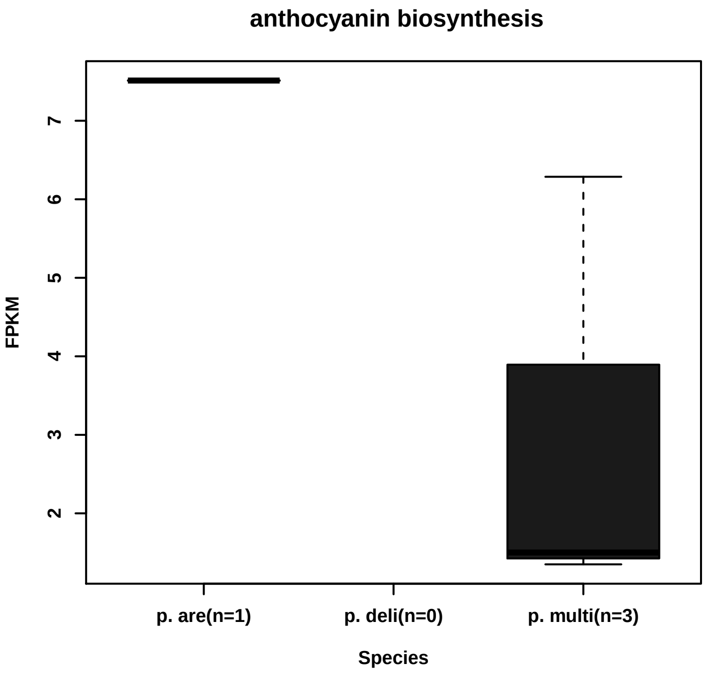
anthocyanin biosynthesis
FPKM
2
3
4
5
6
7
p. are(n=1)
p. deli(n=0)
p. multi(n=3)
Species

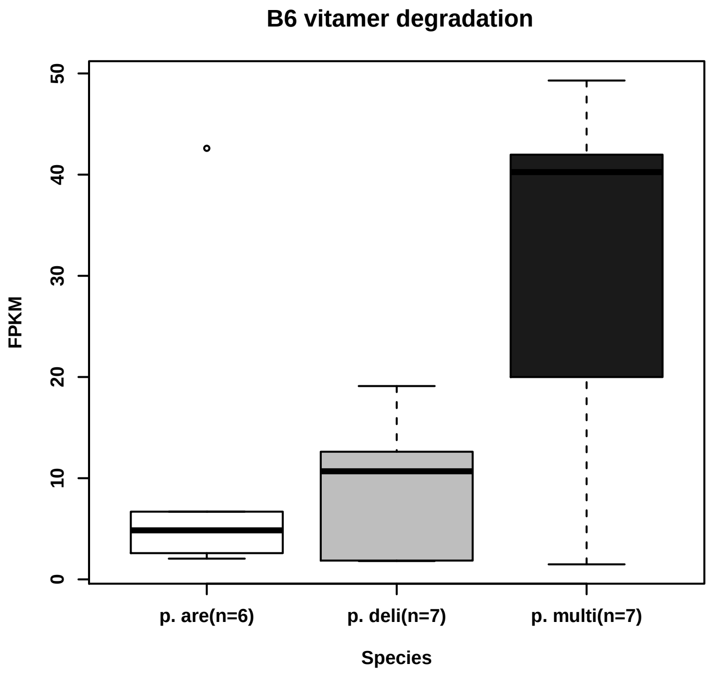
B6 vitamer degradation
FPKM
0
10
20
30
40
50
p. are(n=6)
p. deli(n=7)
p. multi(n=7)
Species

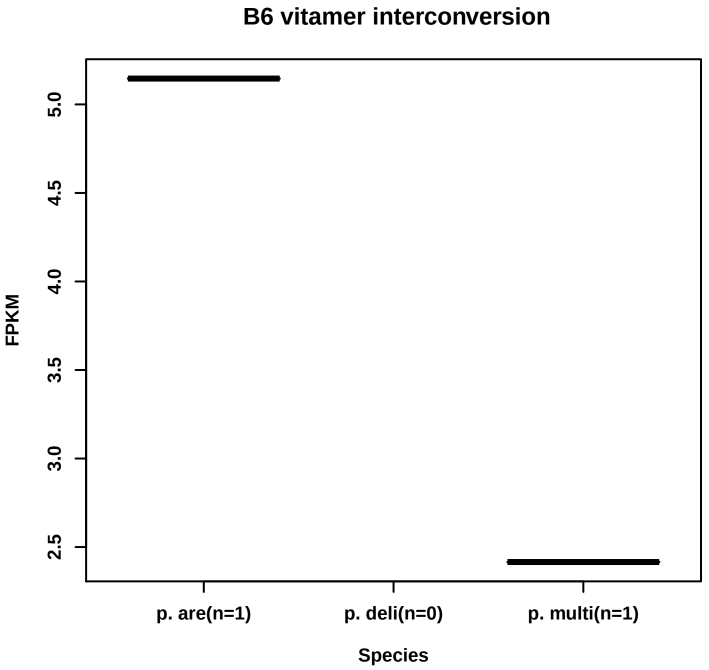
B6 vitamer interconversion
FPKM
5.0
4.5
4.0
3.5
3.0
2.5
p. are(n=1)
p. deli(n=0)
p. multi(n=1)
Species

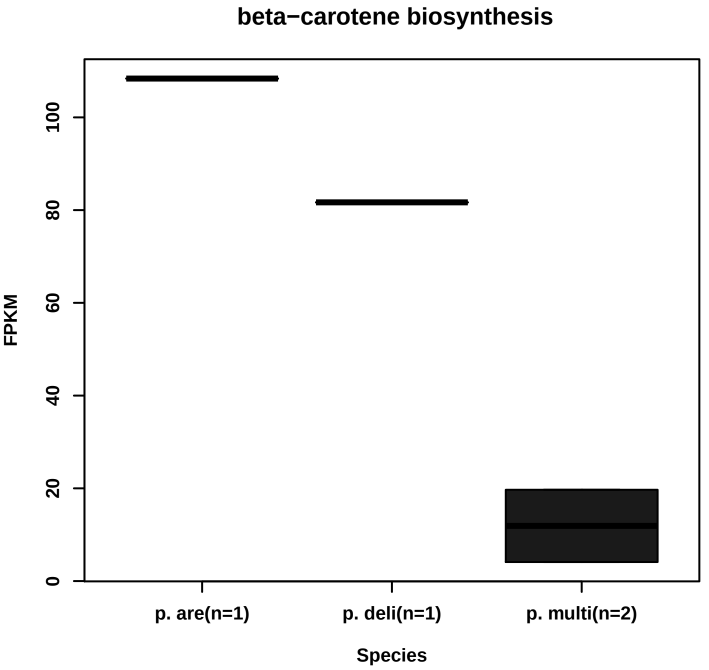
beta−carotene biosynthesis
FPKM
100
80
60
40
20
0
p. are(n=1)
p. deli(n=1)
p. multi(n=2)
Species

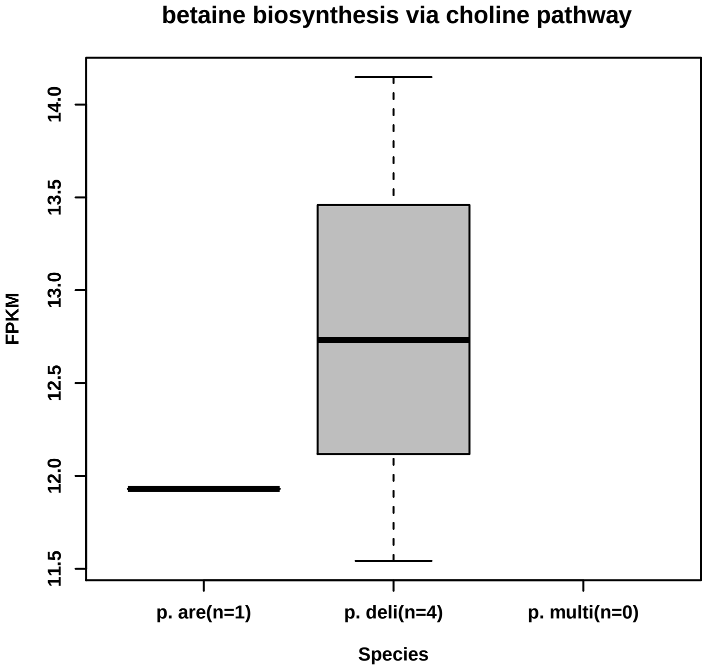
betaine biosynthesis via choline pathway
FPKM
14.0
13.5
13.0
12.5
12.0
11.5
p. are(n=1)
p. deli(n=4)
p. multi(n=0)
Species

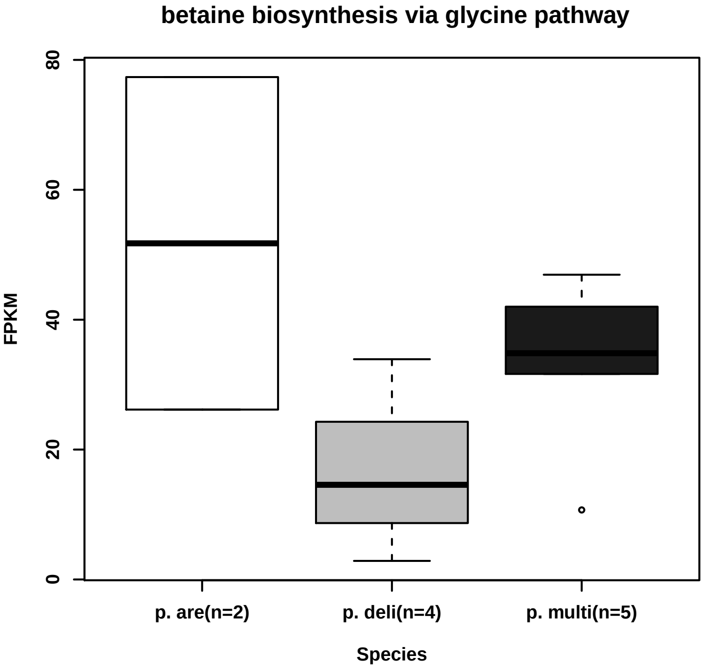
betaine biosynthesis via glycine pathway
FPKM
80
60
40
20
0
p. are(n=2)
p. deli(n=4)
p. multi(n=5)
Species

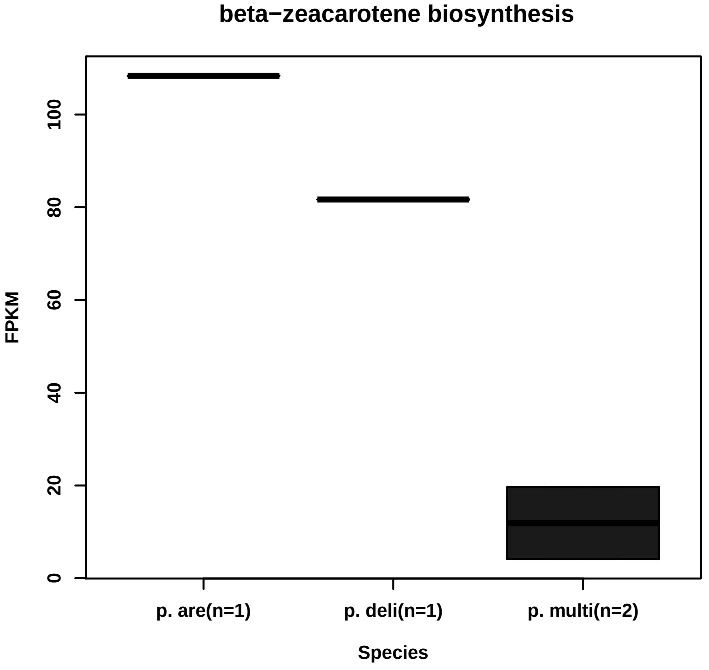
beta−zeacarotene biosynthesis
FPKM
100
80
60
40
20
0
p. are(n=1)
p. deli(n=1)
p. multi(n=2)
Species

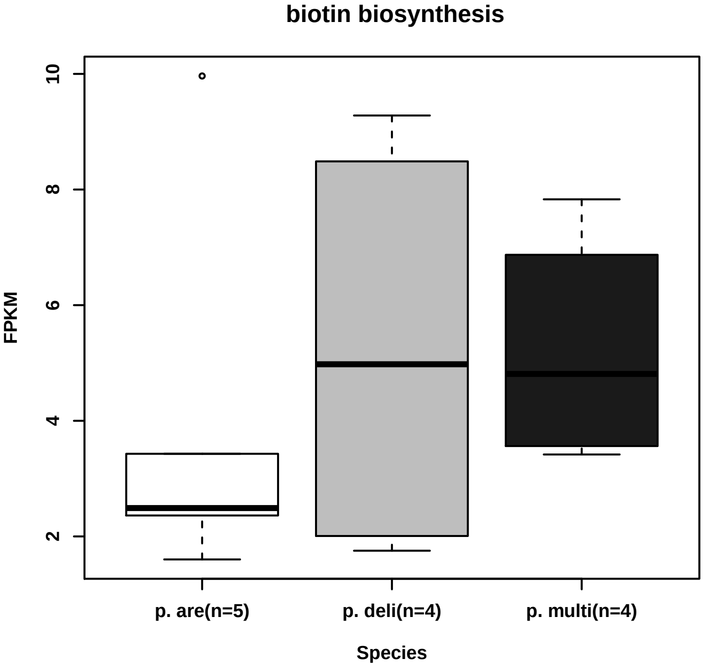
biotin biosynthesis
FPKM
10
8
6
4
2
p. are(n=5)
p. deli(n=4)
p. multi(n=4)
Species

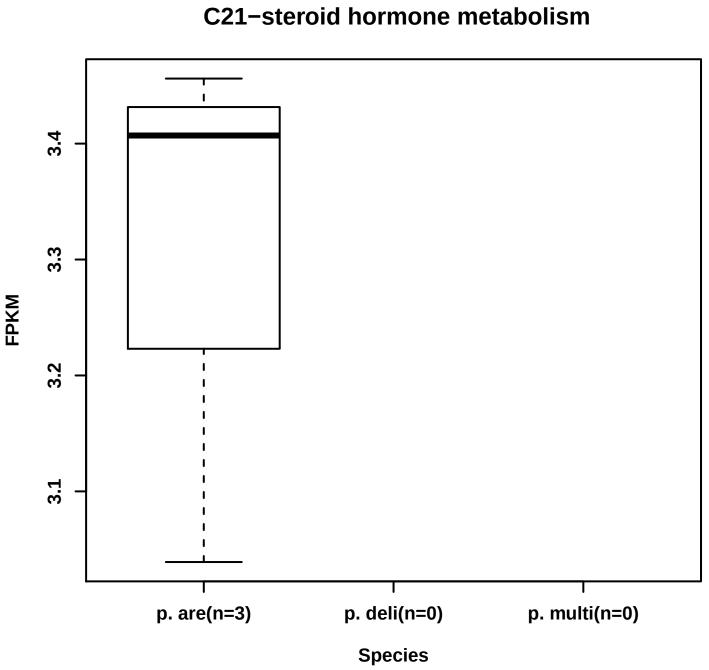
C21−steroid hormone metabolism
FPKM
3.1
3.2
3.3
3.4
p. are(n=3)
p. deli(n=0)
p. multi(n=0)
Species

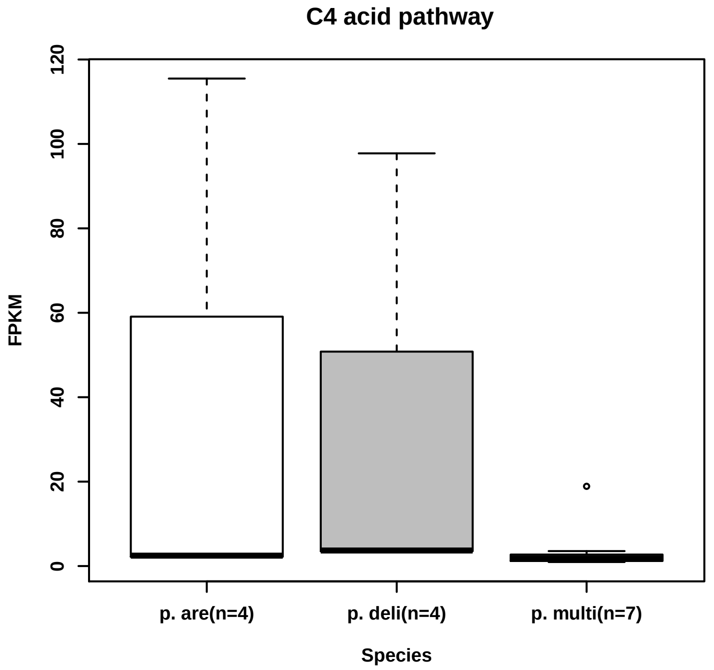
C4 acid pathway
FPKM
0
20
40
60
80
100
120
p. are(n=4)
p. deli(n=4)
p. multi(n=7)
Species

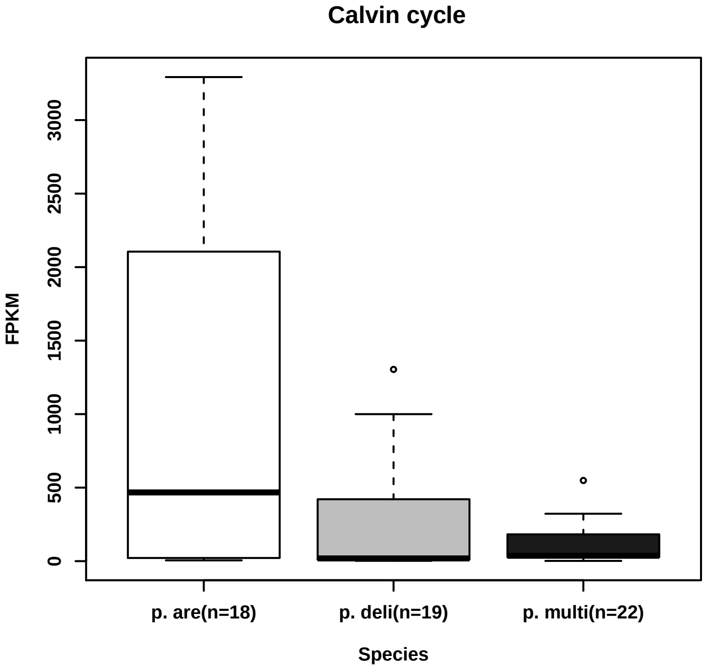
Calvin cycle
FPKM
0
500
1000
1500
2000
2500
3000
p. are(n=18)
p. deli(n=19)
p. multi(n=22)
Species

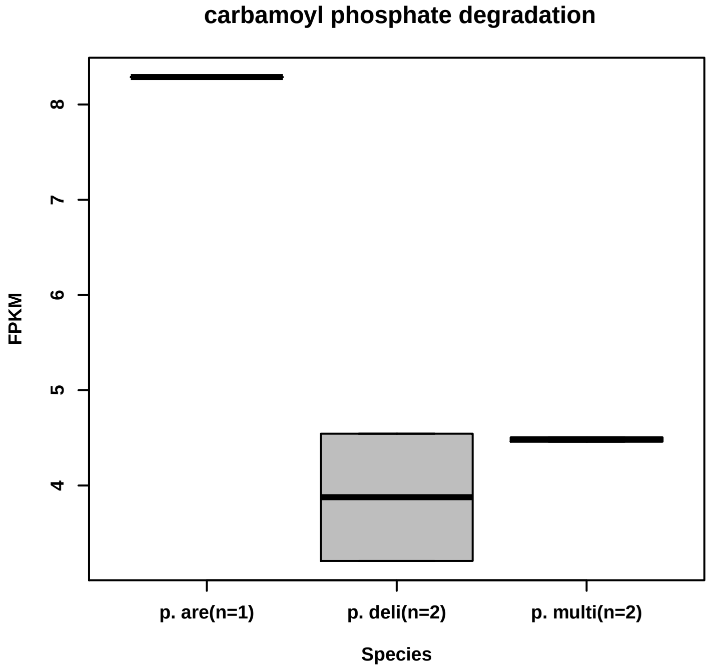
carbamoyl phosphate degradation
FPKM
4
5
6
7
8
p. are(n=1)
p. deli(n=2)
p. multi(n=2)
Species

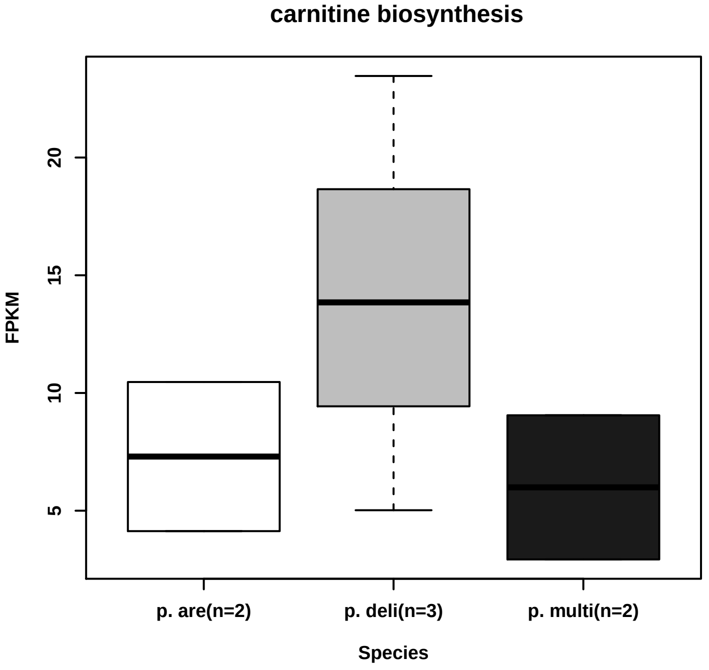
carnitine biosynthesis
FPKM
5
10
15
20
p. are(n=2)
p. deli(n=3)
p. multi(n=2)
Species

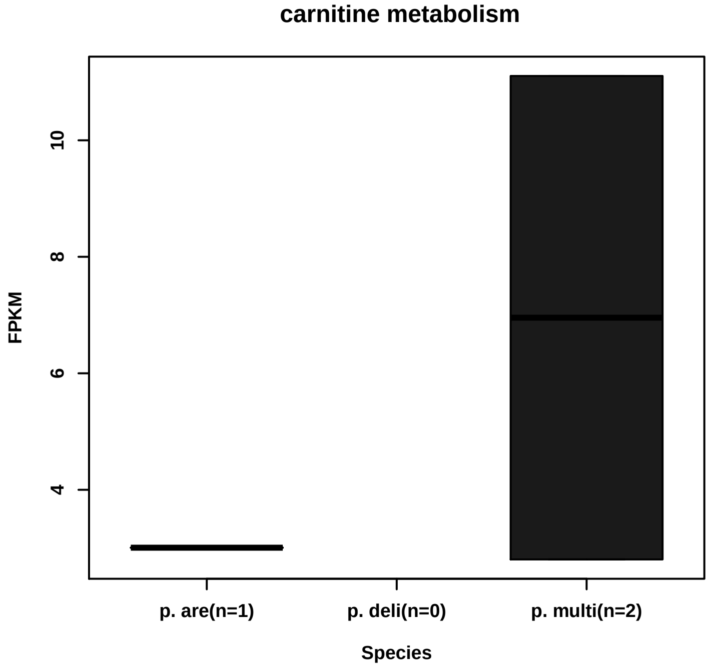
carnitine metabolism
FPKM
4
6
8
10
p. are(n=1)
p. deli(n=0)
p. multi(n=2)
Species

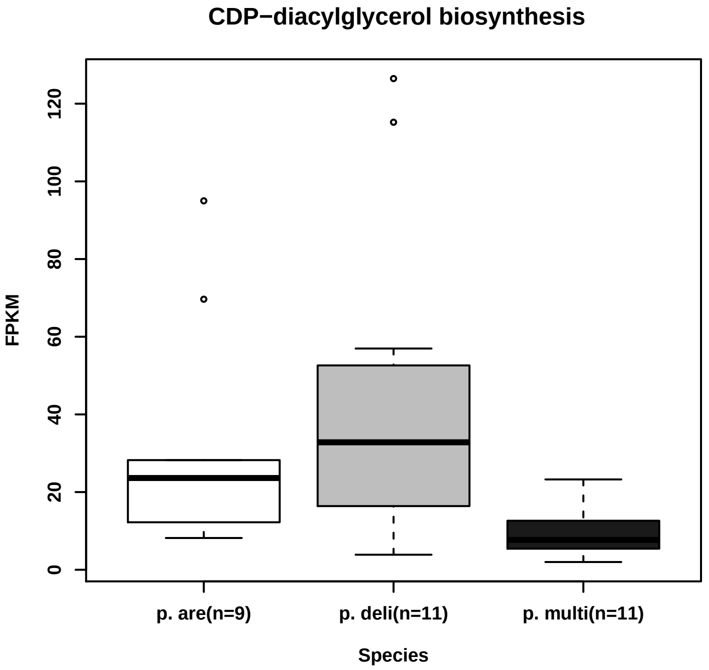
CDP−diacylglycerol biosynthesis
FPKM
0
20
40
60
80
100
120
p. are(n=9)
p. deli(n=11)
p. multi(n=11)
Species

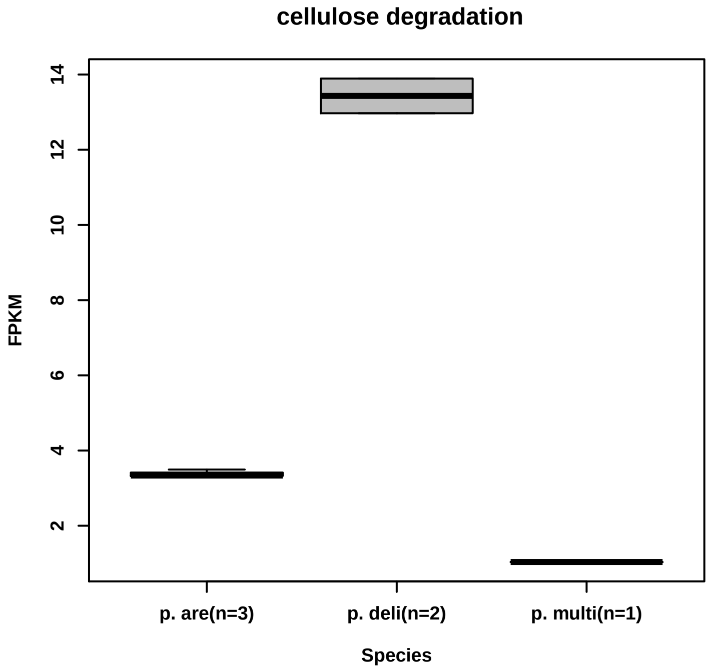
cellulose degradation
FPKM
2
4
6
8
10
12
14
p. are(n=3)
p. deli(n=2)
p. multi(n=1)
Species

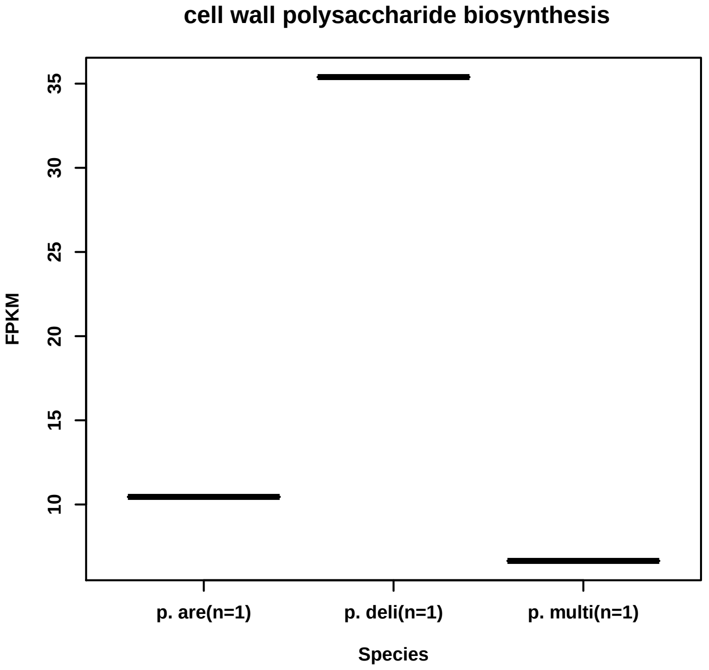
cell wall polysaccharide biosynthesis
FPKM
10
15
20
25
30
35
p. are(n=1)
p. deli(n=1)
p. multi(n=1)
Species

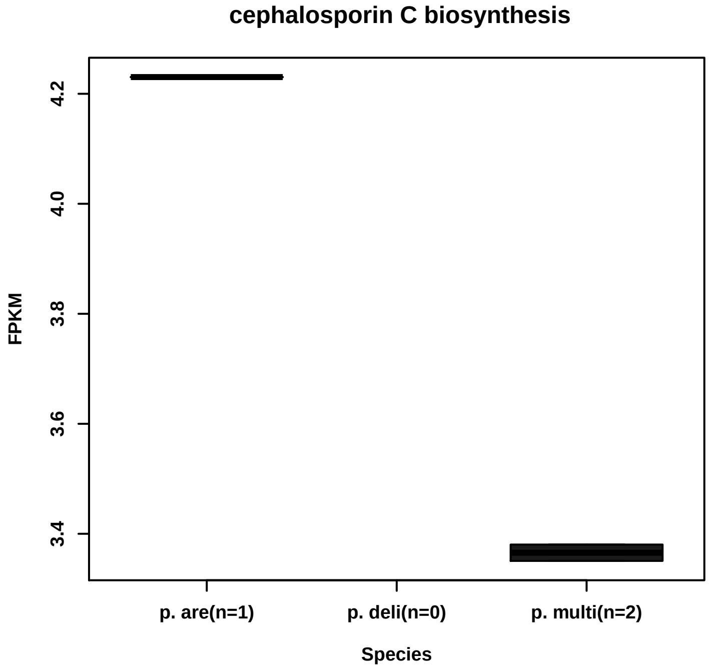
cephalosporin C biosynthesis
FPKM
3.4
3.6
3.8
4.0
4.2
p. are(n=1)
p. deli(n=0)
p. multi(n=2)
Species

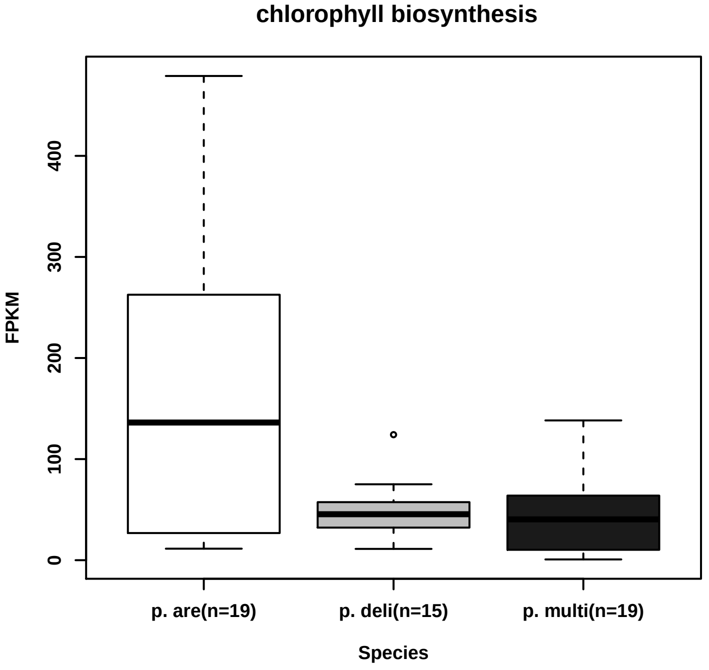
chlorophyll biosynthesis
FPKM
0
100
200
300
400
p. are(n=19)
p. deli(n=15)
p. multi(n=19)
Species

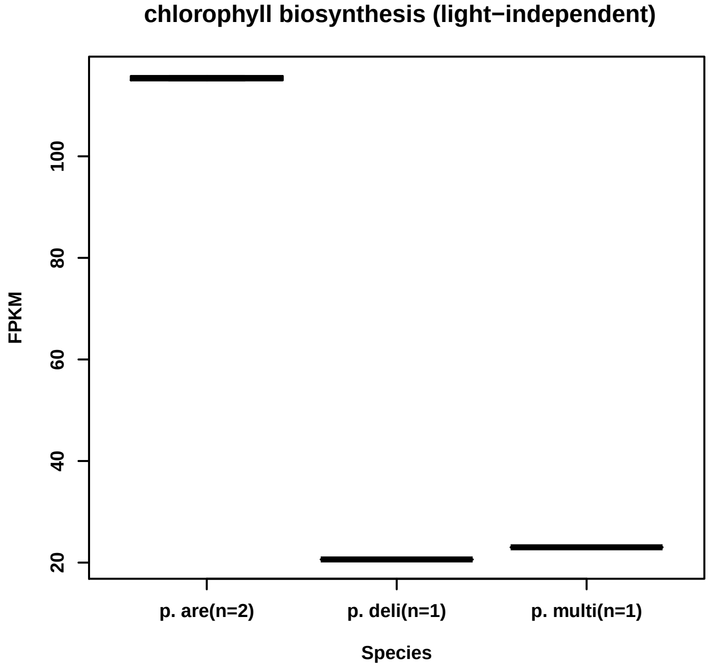
chlorophyll biosynthesis (light−independent)
FPKM
20
40
60
80
100
p. are(n=2)
p. deli(n=1)
p. multi(n=1)
Species

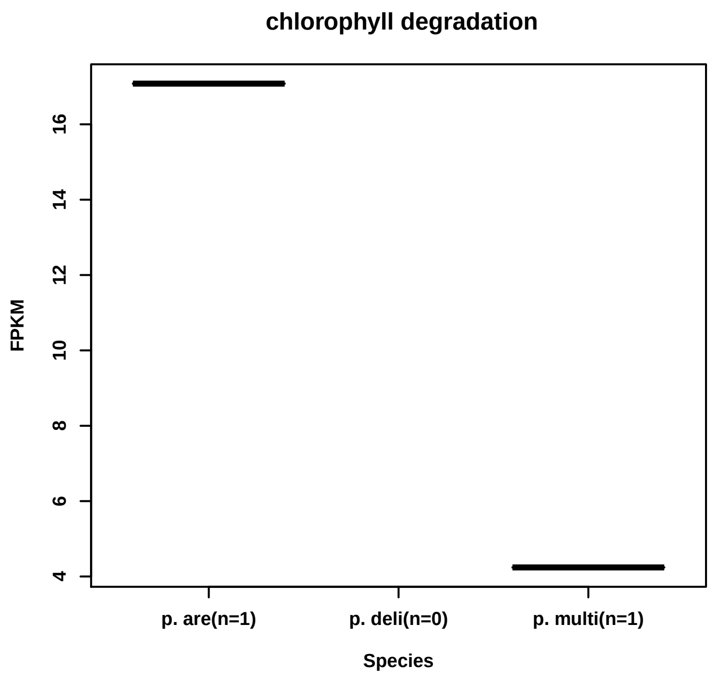

chlorophyll degradation
FPKM
4
6
8
10
12
14
16
p. are(n=1)
p. deli(n=0)
p. multi(n=1)
Species

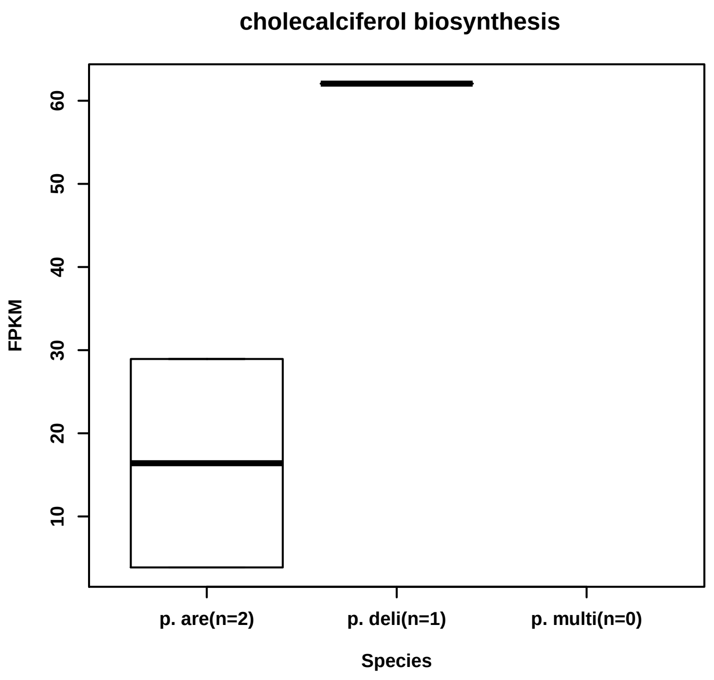

cholecalciferol biosynthesis
FPKM
10
20
30
40
50
60
p. are(n=2)
p. deli(n=1)
p. multi(n=0)
Species

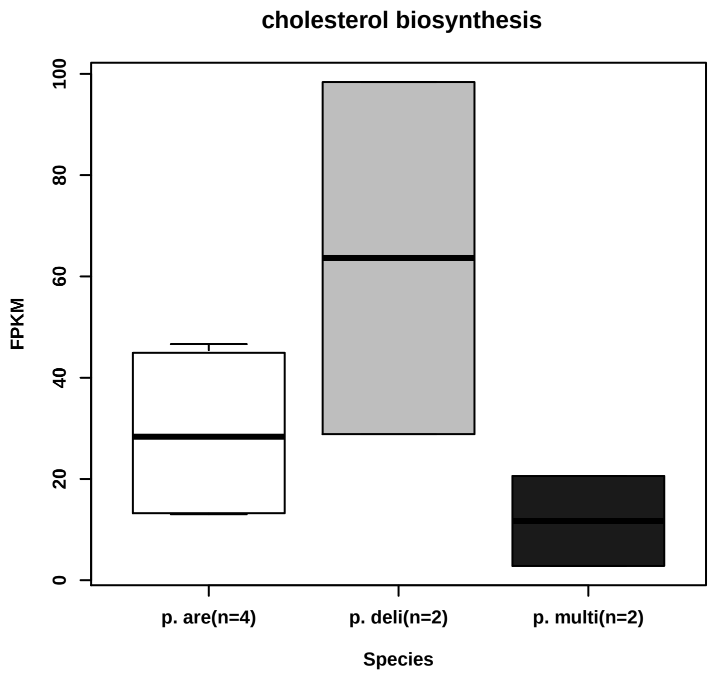

cholesterol biosynthesis
FPKM
0
20
40
60
80
100
p. are(n=4)
p. deli(n=2)
p. multi(n=2)
Species

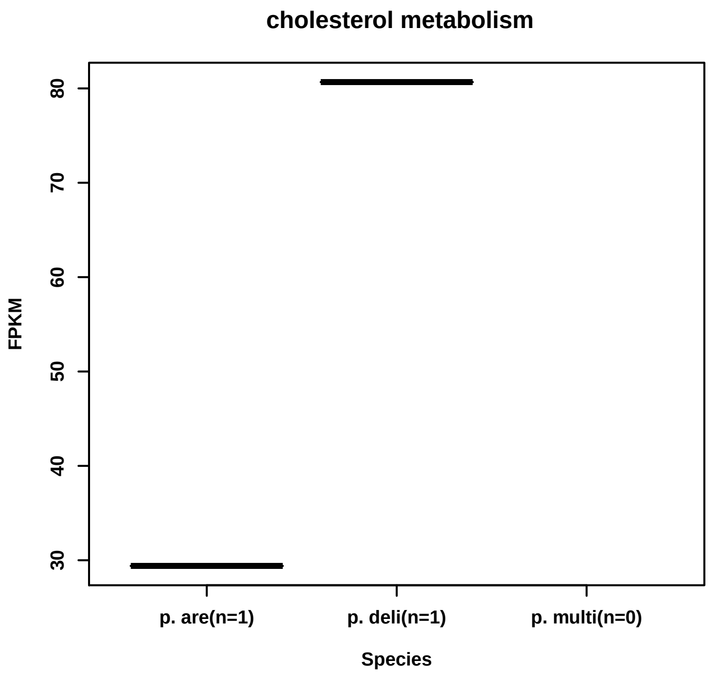

cholesterol metabolism
FPKM
30
40
50
60
70
80
p. are(n=1)
p. deli(n=1)
p. multi(n=0)
Species

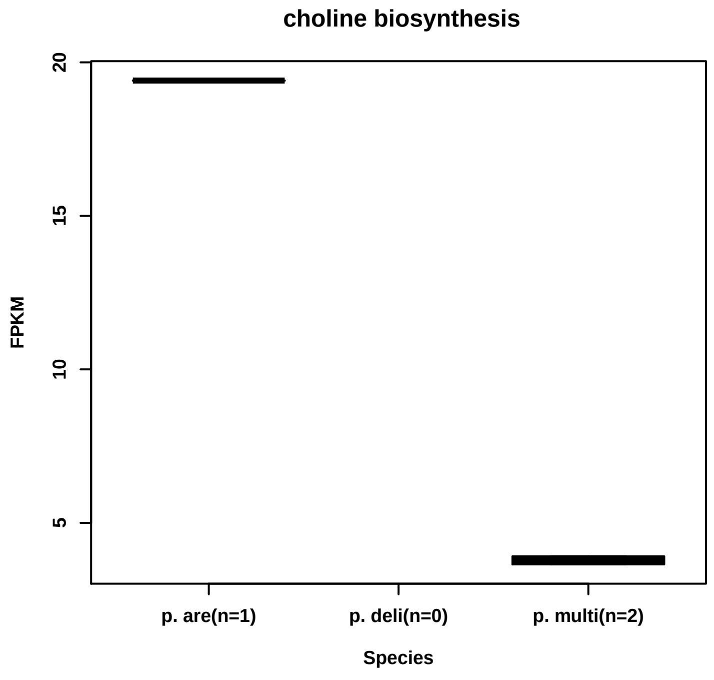

choline biosynthesis
FPKM
5
10
15
20
p. are(n=1)
p. deli(n=0)
p. multi(n=2)
Species

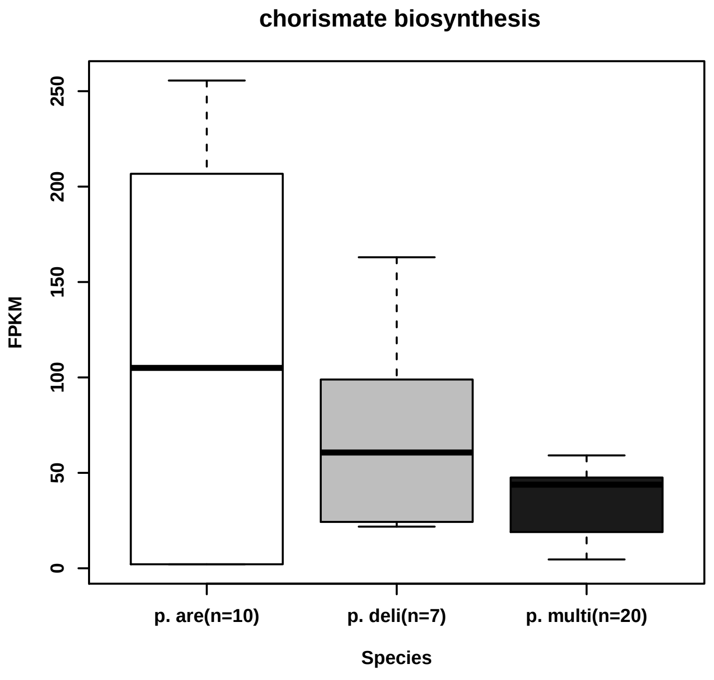

chorismate biosynthesis
FPKM
0
50
100
150
200
250
p. are(n=10)
p. deli(n=7)
p. multi(n=20)
Species

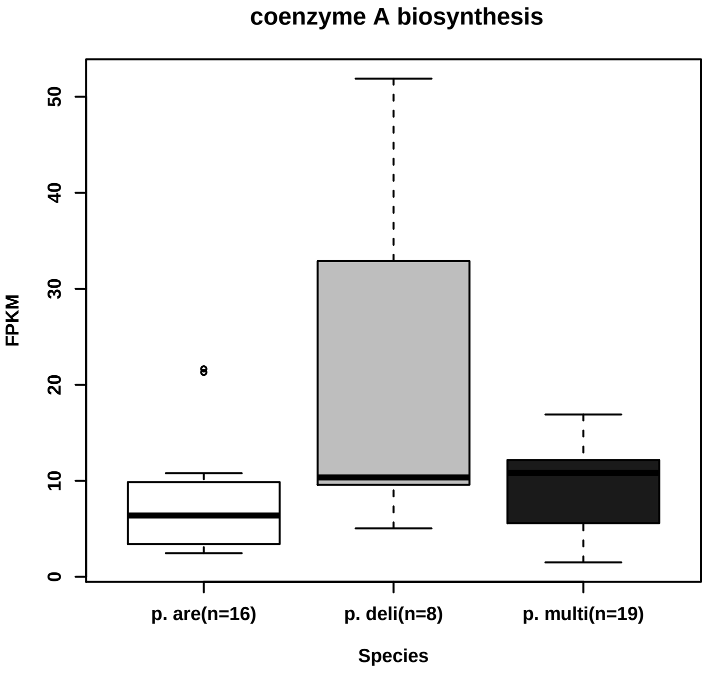
coenzyme A biosynthesis
FPKM
0
10
20
30
40
50
p. are(n=16)
p. deli(n=8)
p. multi(n=19)
Species

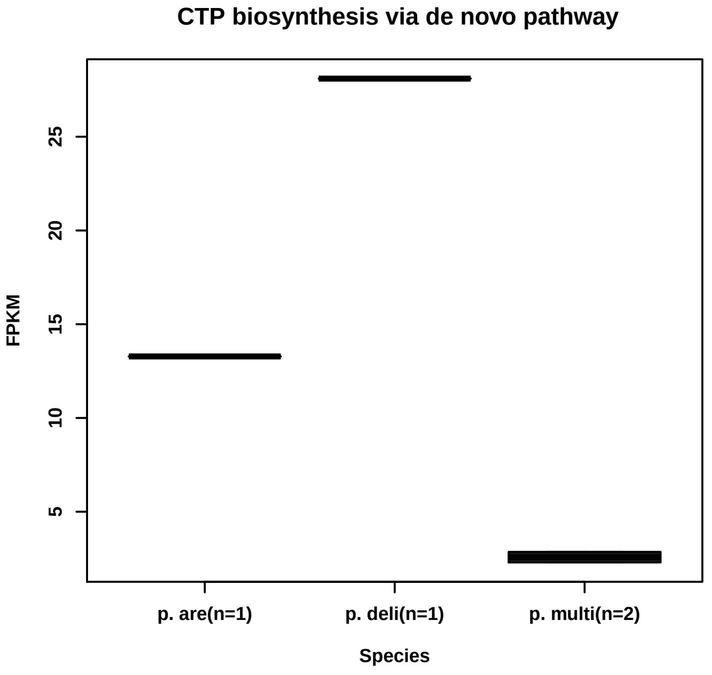
CTP biosynthesis via de novo pathway
FPKM
5
10
15
20
25
p. are(n=1)
p. deli(n=1)
p. multi(n=2)
Species

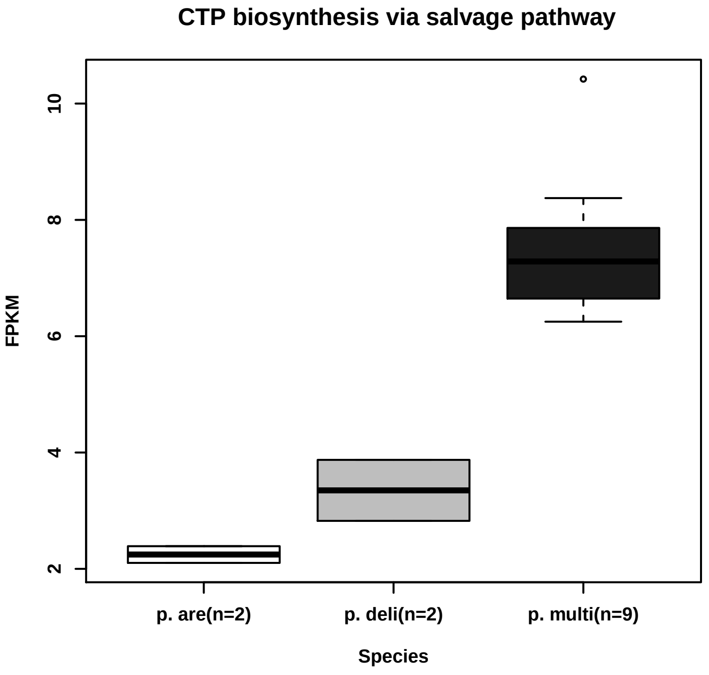
CTP biosynthesis via salvage pathway
FPKM
2
4
6
8
10
p. are(n=2)
p. deli(n=2)
p. multi(n=9)
Species

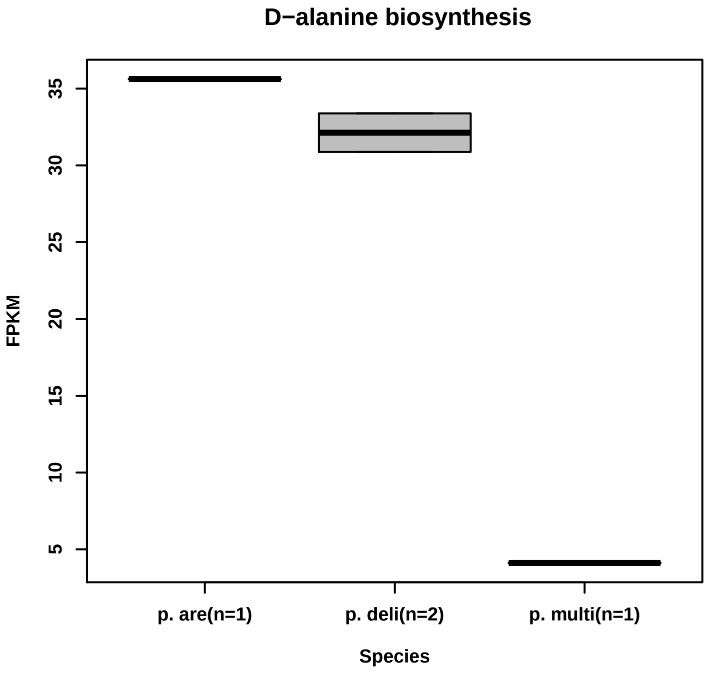
D−alanine biosynthesis
FPKM
5
10
15
20
25
30
35
p. are(n=1)
p. deli(n=2)
p. multi(n=1)
Species

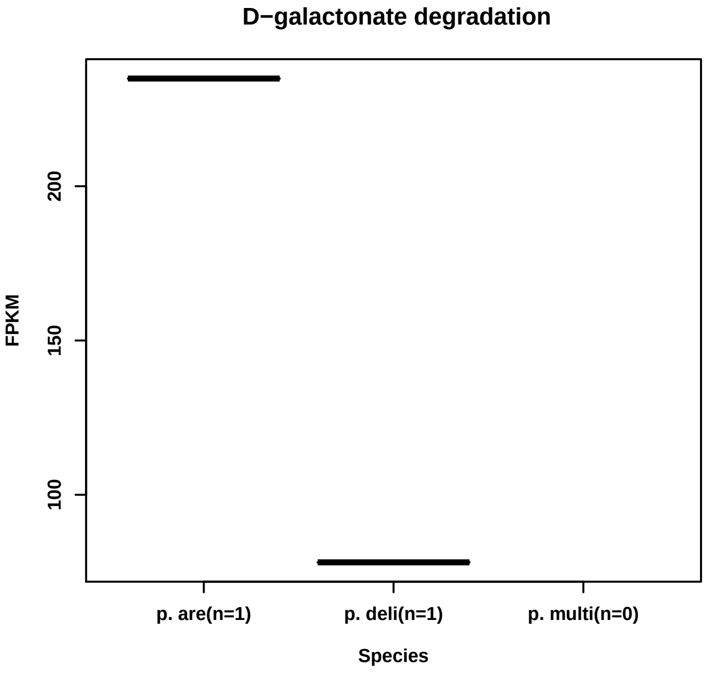
D−galactonate degradation
FPKM
100
150
200
p. are(n=1)
p. deli(n=1)
p. multi(n=0)
Species

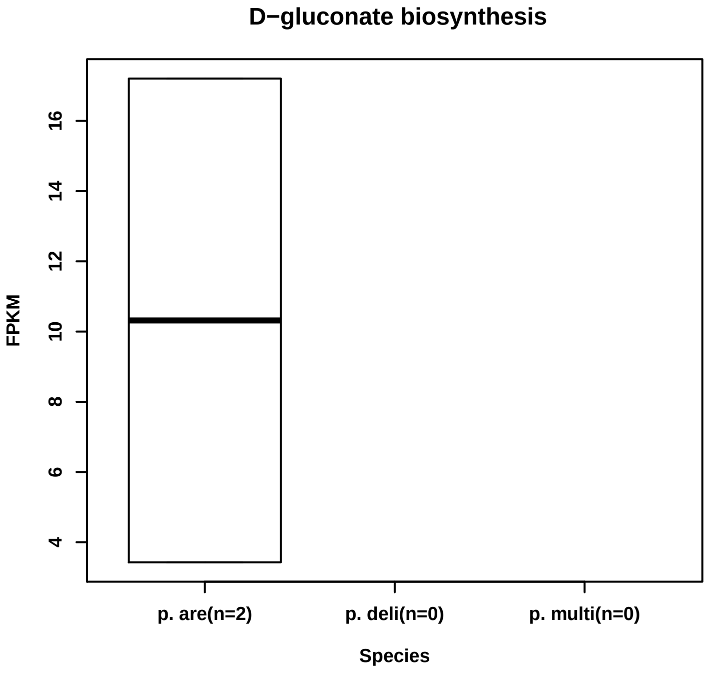
D−gluconate biosynthesis
FPKM
4
6
8
10
12
14
16
p. are(n=2)
p. deli(n=0)
p. multi(n=0)
Species

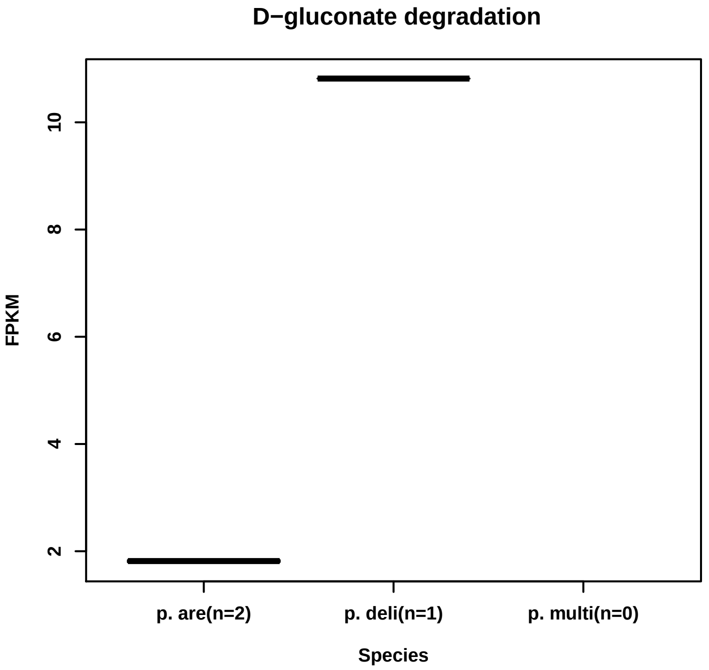
D−gluconate degradation
FPKM
2
4
6
8
10
p. are(n=2)
p. deli(n=1)
p. multi(n=0)
Species

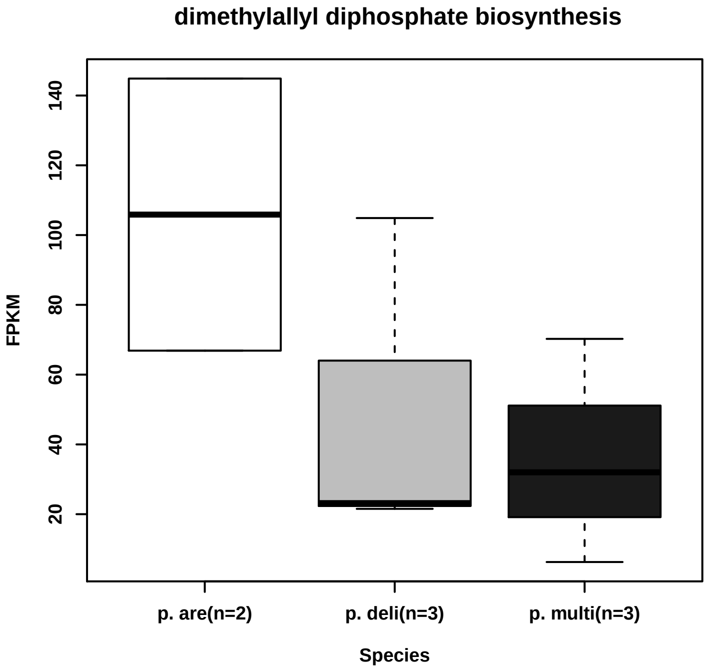
dimethylallyl diphosphate biosynthesis
FPKM
20
40
60
80
100
120
140
p. are(n=2)
p. deli(n=3)
p. multi(n=3)
Species

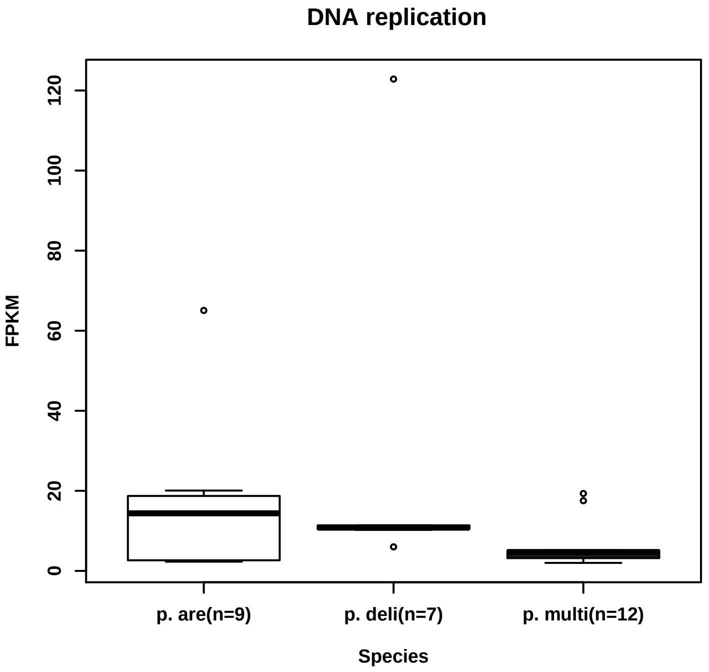
DNA replication
FPKM
0
20
40
60
80
100
120
p. are(n=9)
p. deli(n=7)
p. multi(n=12)
Species

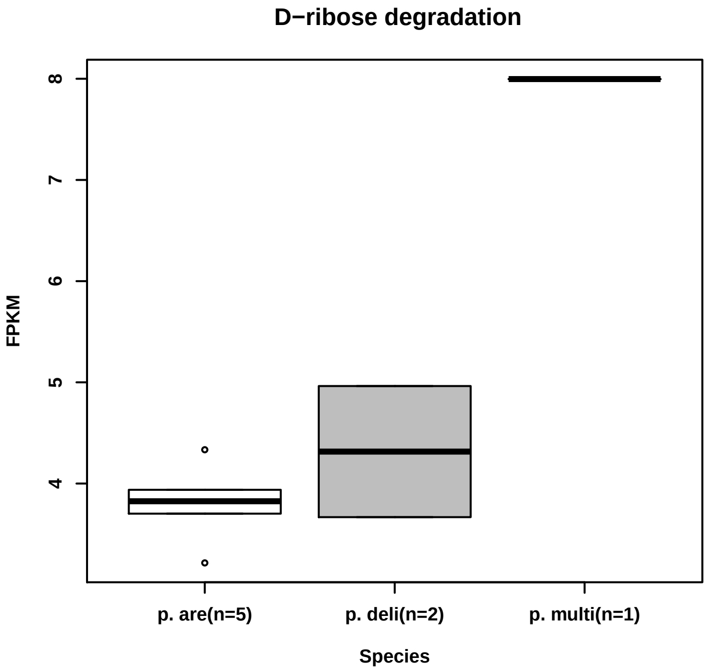
D−ribose degradation
FPKM
4
5
6
7
8
p. are(n=5)
p. deli(n=2)
p. multi(n=1)
Species

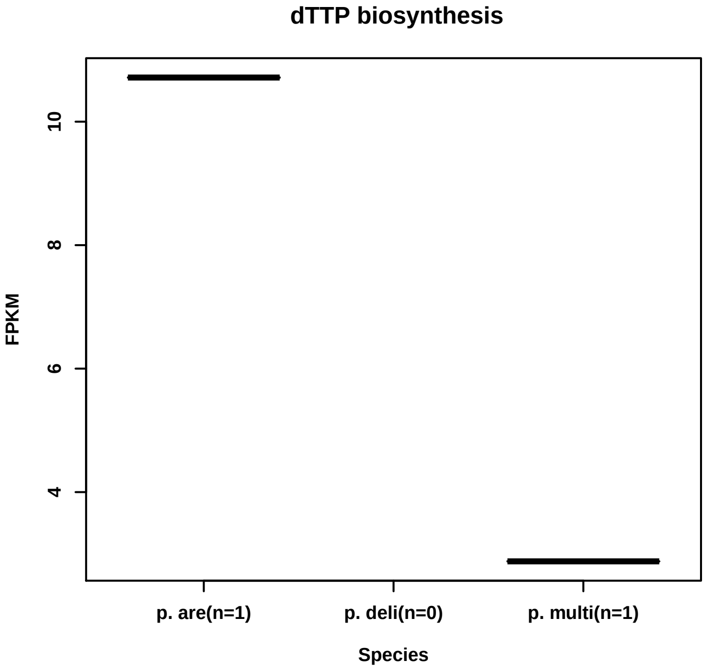
dTTP biosynthesis
FPKM
4
6
8
10
p. are(n=1)
p. deli(n=0)
p. multi(n=1)
Species

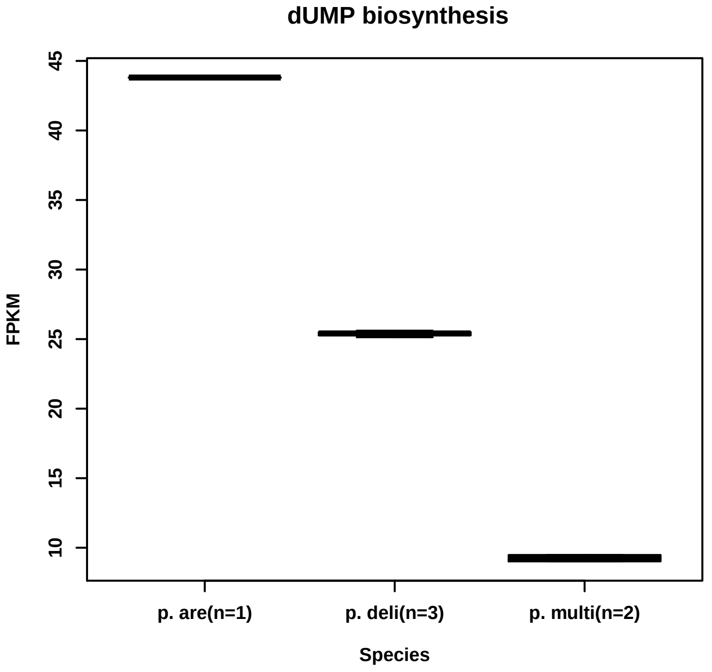
dUMP biosynthesis
FPKM
10
15
20
25
30
35
40
45
p. are(n=1)
p. deli(n=3)
p. multi(n=2)
Species

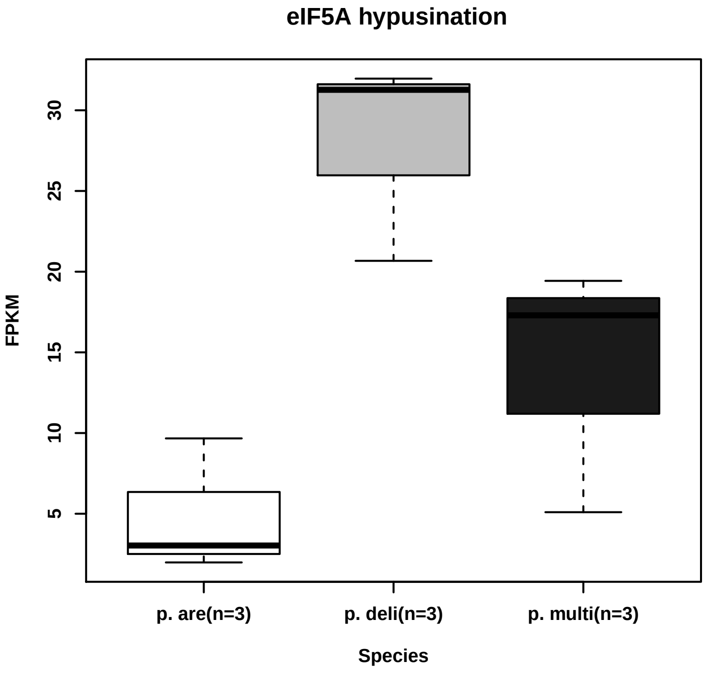
eIF5A hypusination
FPKM
30
25
20
15
10
5
p. are(n=3)
p. deli(n=3)
p. multi(n=3)
Species

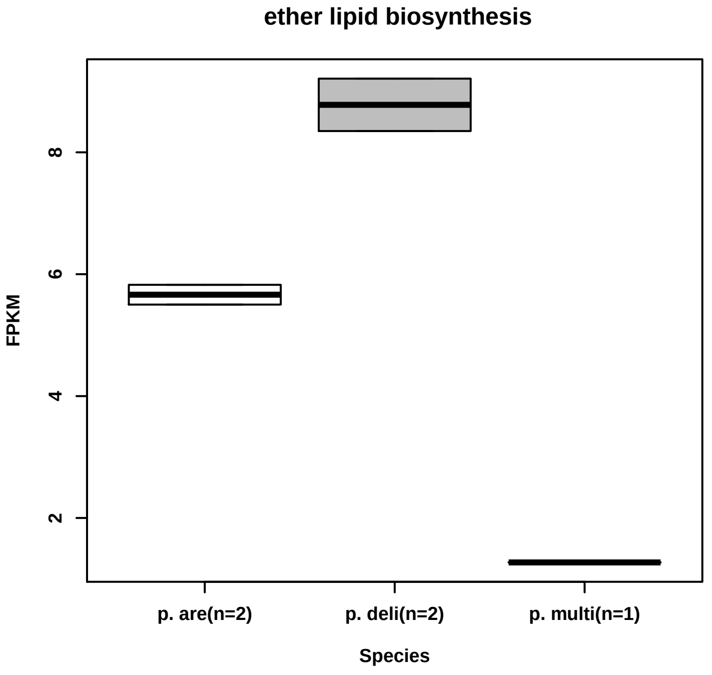
ether lipid biosynthesis
FPKM
8
6
4
2
p. are(n=2)
p. deli(n=2)
p. multi(n=1)
Species

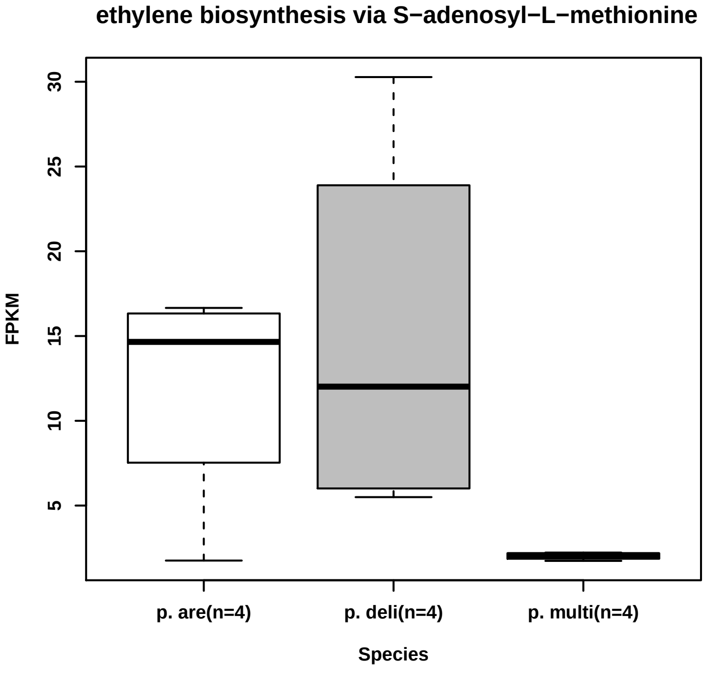
ethylene biosynthesis via S−adenosyl−L−methionine
FPKM
30
25
20
15
10
5
p. are(n=4)
p. deli(n=4)
p. multi(n=4)
Species

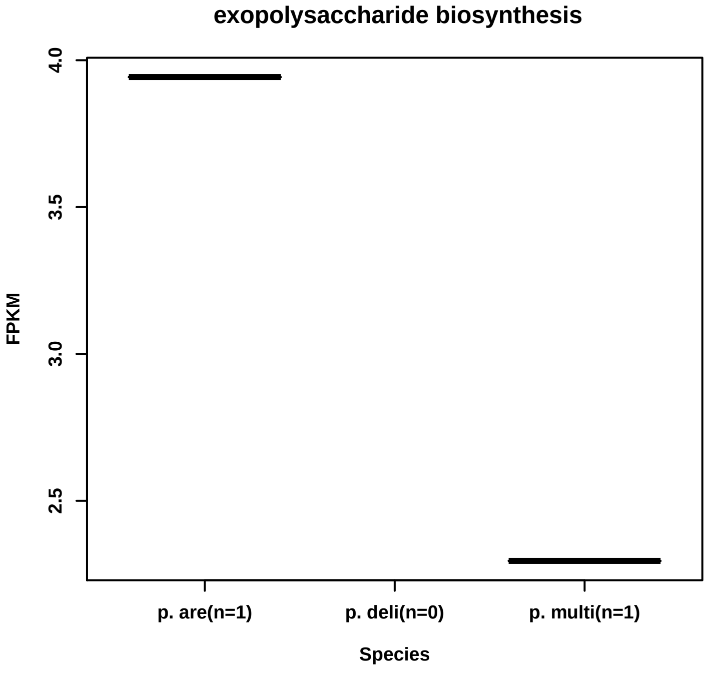
exopolysaccharide biosynthesis
FPKM
4.0
3.5
3.0
2.5
p. are(n=1)
p. deli(n=0)
p. multi(n=1)
Species

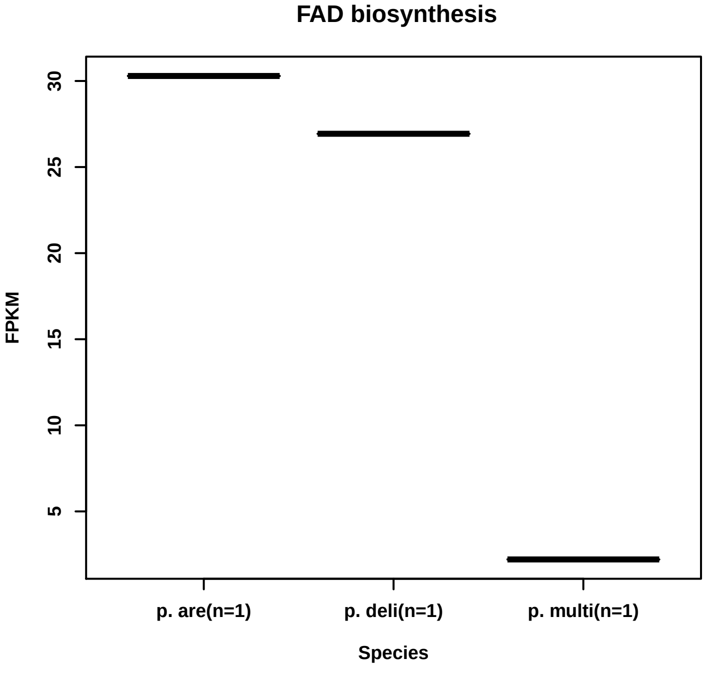
FAD biosynthesis
FPKM
30
25
20
15
10
5
p. are(n=1)
p. deli(n=1)
p. multi(n=1)
Species

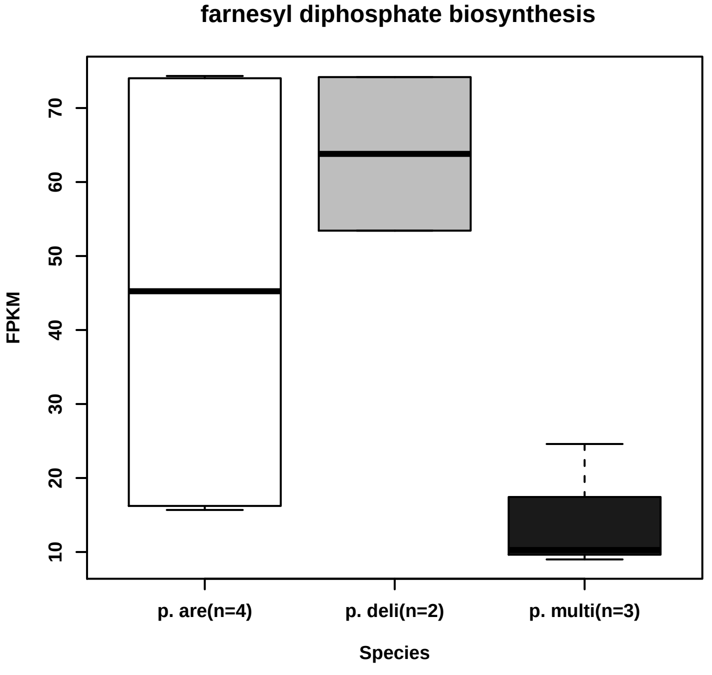
farnesyl diphosphate biosynthesis
FPKM
70
60
50
40
30
20
10
p. are(n=4)
p. deli(n=2)
p. multi(n=3)
Species

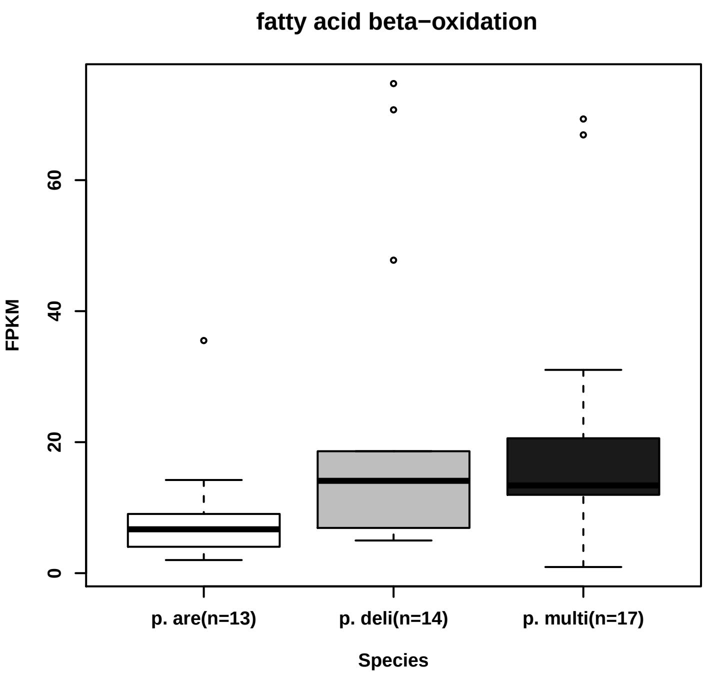
fatty acid beta−oxidation
FPKM
0
20
40
60
p. are(n=13)
p. deli(n=14)
p. multi(n=17)
Species

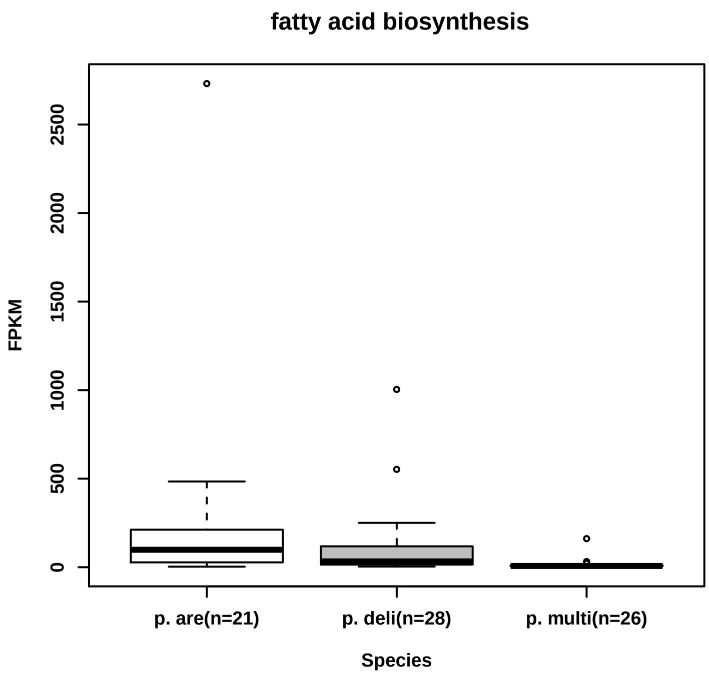
fatty acid biosynthesis
FPKM
0
500
1000
1500
2000
2500
p. are(n=21)
p. deli(n=28)
p. multi(n=26)
Species

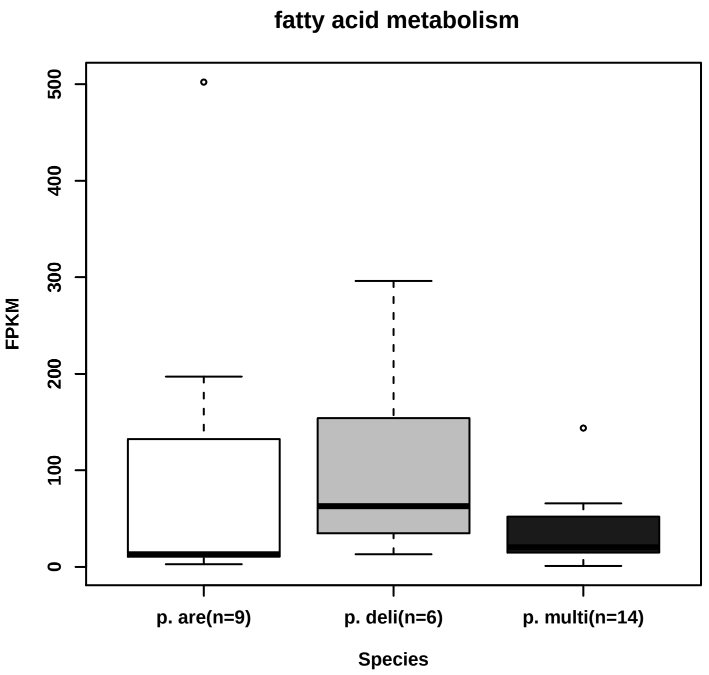
fatty acid metabolism
FPKM
0
100
200
300
400
500
p. are(n=9)
p. deli(n=6)
p. multi(n=14)
Species

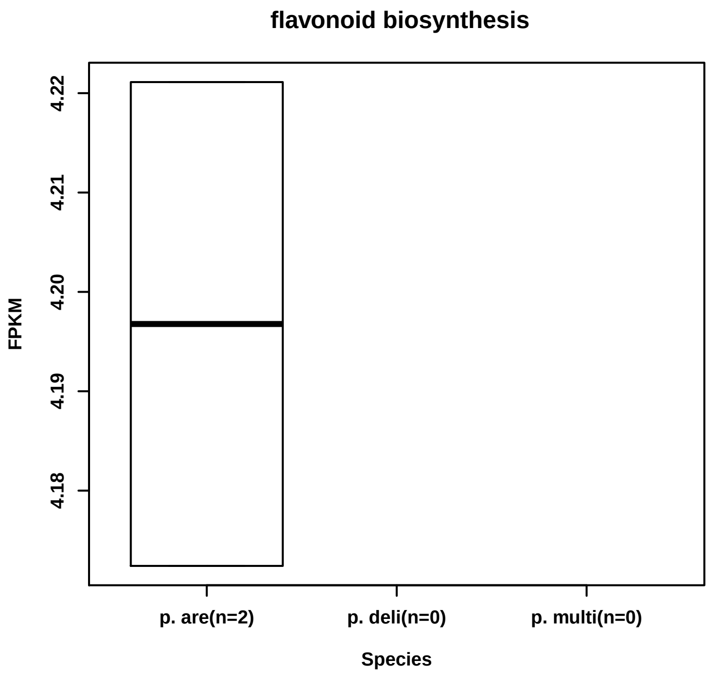
flavonoid biosynthesis
FPKM
4.18
4.19
4.20
4.21
4.22
p. are(n=2)
p. deli(n=0)
p. multi(n=0)
Species

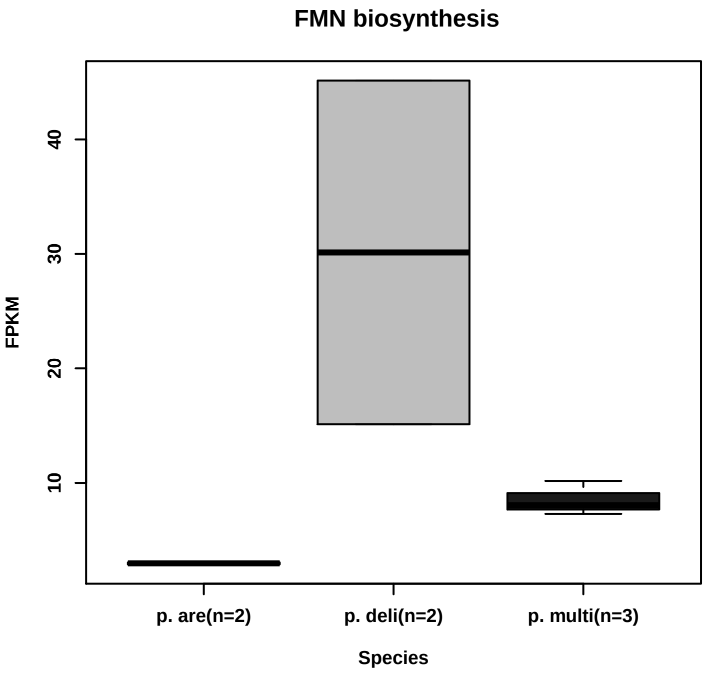
FMN biosynthesis
FPKM
10
20
30
40
p. are(n=2)
p. deli(n=2)
p. multi(n=3)
Species

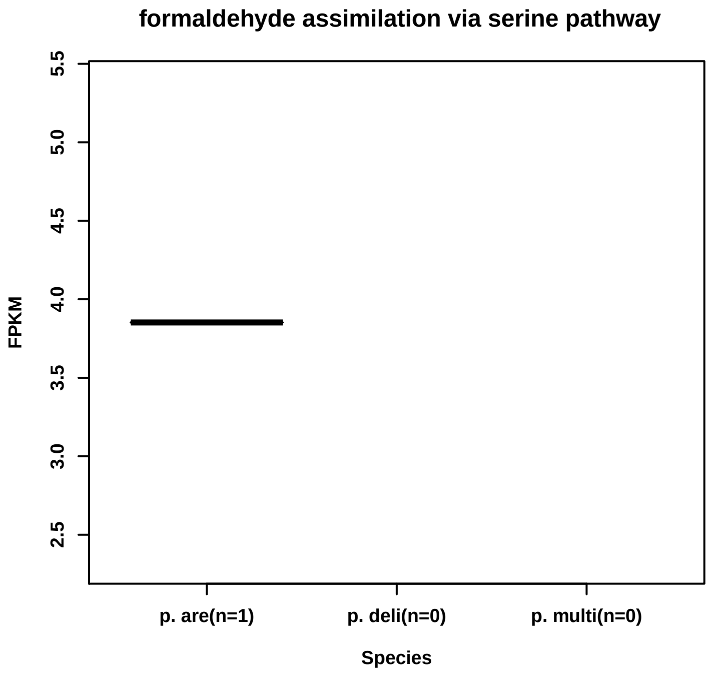
formaldehyde assimilation via serine pathway
FPKM
2.5
3.0
3.5
4.0
4.5
5.0
5.5
p. are(n=1)
p. deli(n=0)
p. multi(n=0)
Species

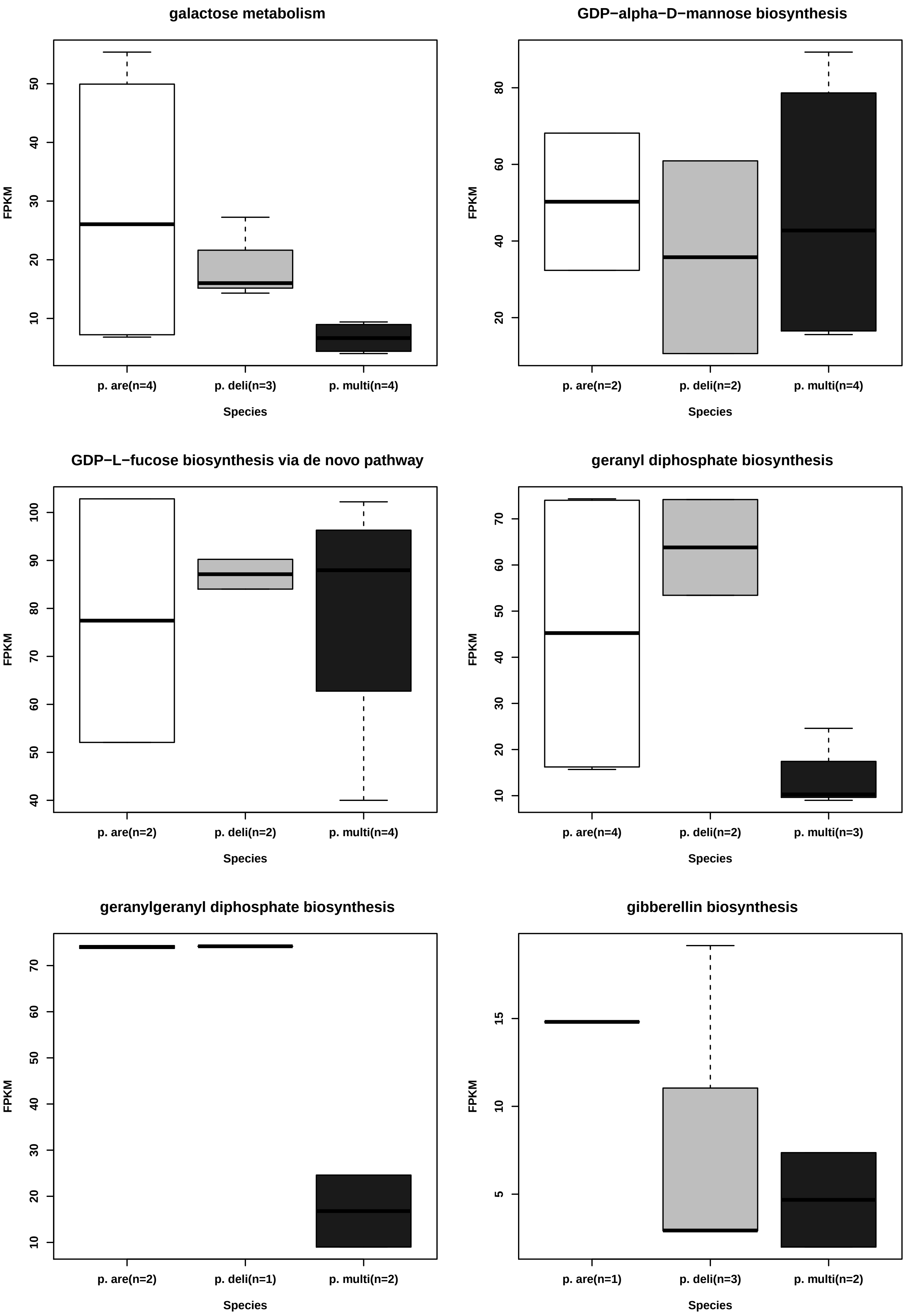
galactose metabolism
FPKM
p. are(n=4)
p. deli(n=3)
p. multi(n=4)
Species
GDP−alpha−D−mannose biosynthesis
FPKM
p. are(n=2)
p. deli(n=2)
p. multi(n=4)
Species
GDP−L−fucose biosynthesis via de novo pathway
FPKM
p. are(n=2)
p. deli(n=2)
p. multi(n=4)
Species
geranyl diphosphate biosynthesis
FPKM
p. are(n=4)
p. deli(n=2)
p. multi(n=3)
Species
geranylgeranyl diphosphate biosynthesis
FPKM
p. are(n=2)
p. deli(n=1)
p. multi(n=2)
Species
gibberellin biosynthesis
FPKM
p. are(n=1)
p. deli(n=3)
p. multi(n=2)
Species

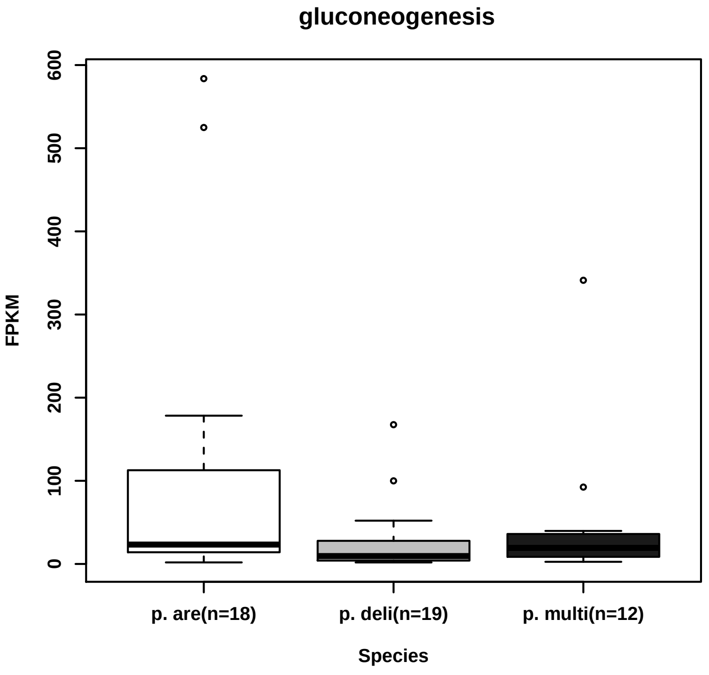
gluconeogenesis
FPKM
0
100
200
300
400
500
600
p. are(n=18)
p. deli(n=19)
p. multi(n=12)
Species

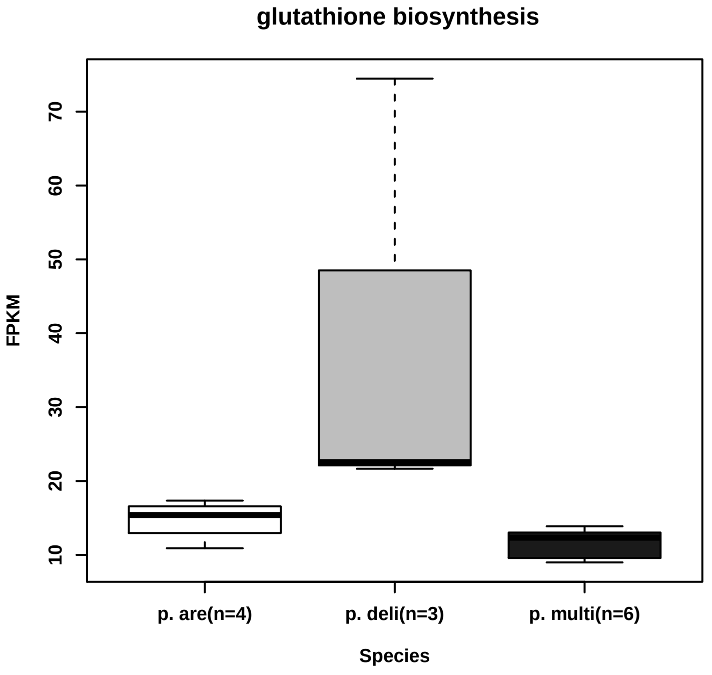
glutathione biosynthesis
FPKM
10
20
30
40
50
60
70
p. are(n=4)
p. deli(n=3)
p. multi(n=6)
Species

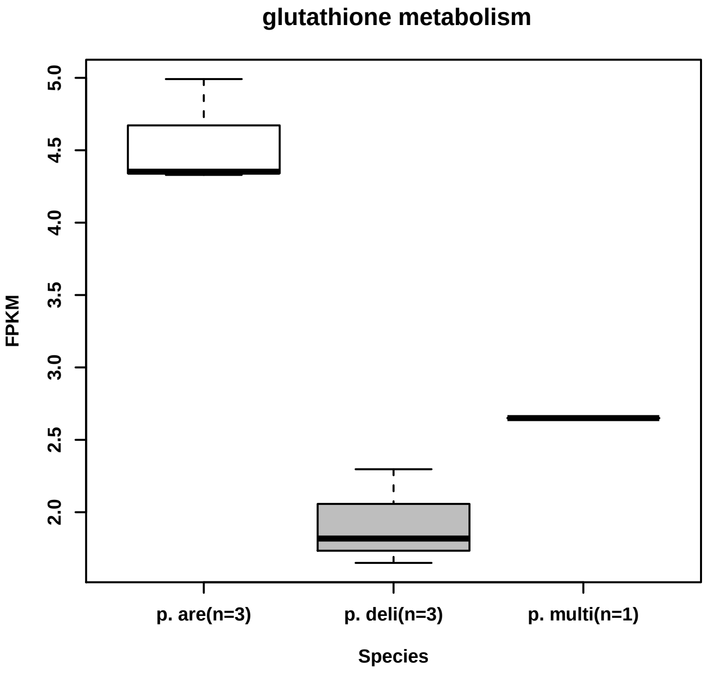
glutathione metabolism
FPKM
2.0
2.5
3.0
3.5
4.0
4.5
5.0
p. are(n=3)
p. deli(n=3)
p. multi(n=1)
Species

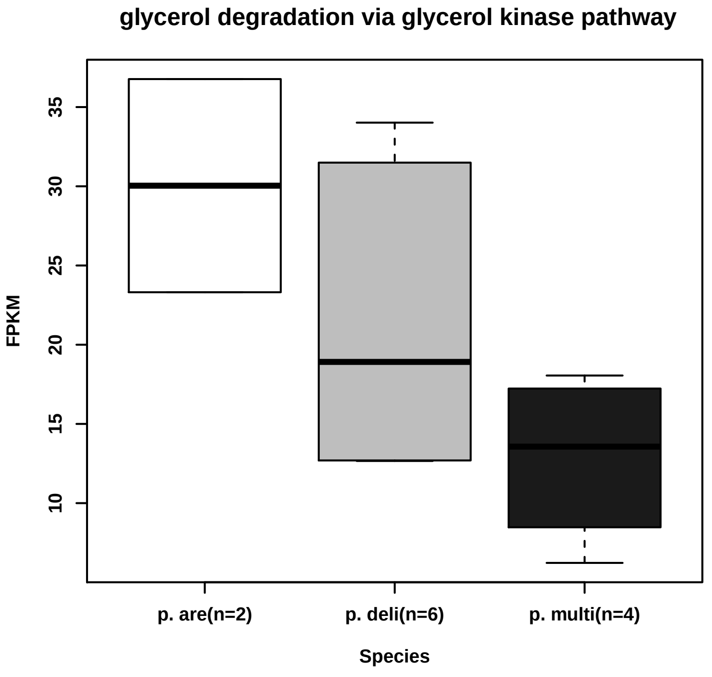
glycerol degradation via glycerol kinase pathway
FPKM
10
15
20
25
30
35
p. are(n=2)
p. deli(n=6)
p. multi(n=4)
Species

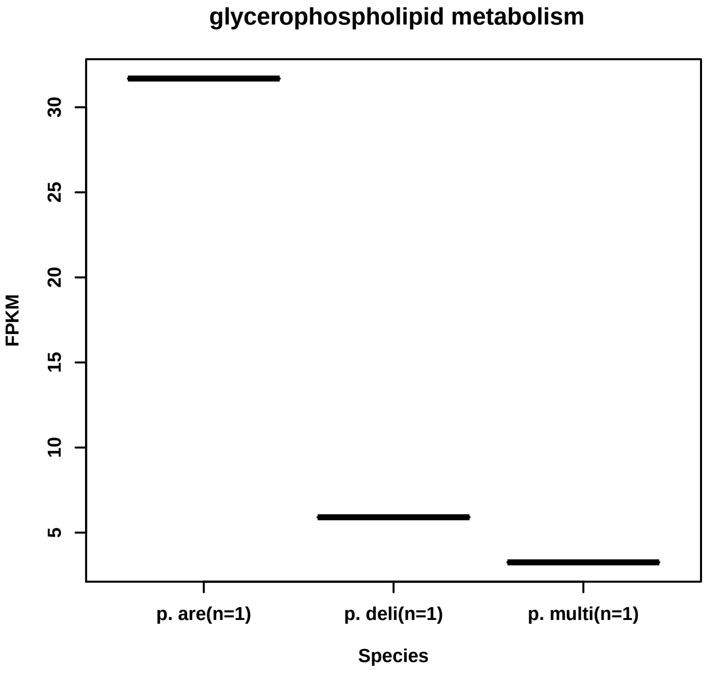
glycerophospholipid metabolism
FPKM
5
10
15
20
25
30
p. are(n=1)
p. deli(n=1)
p. multi(n=1)
Species

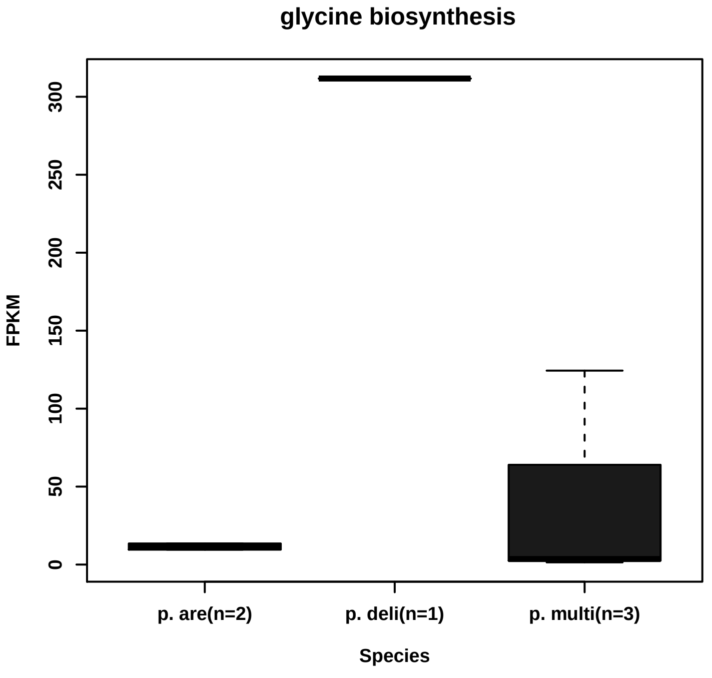
glycine biosynthesis
FPKM
0
50
100
150
200
250
300
p. are(n=2)
p. deli(n=1)
p. multi(n=3)
Species

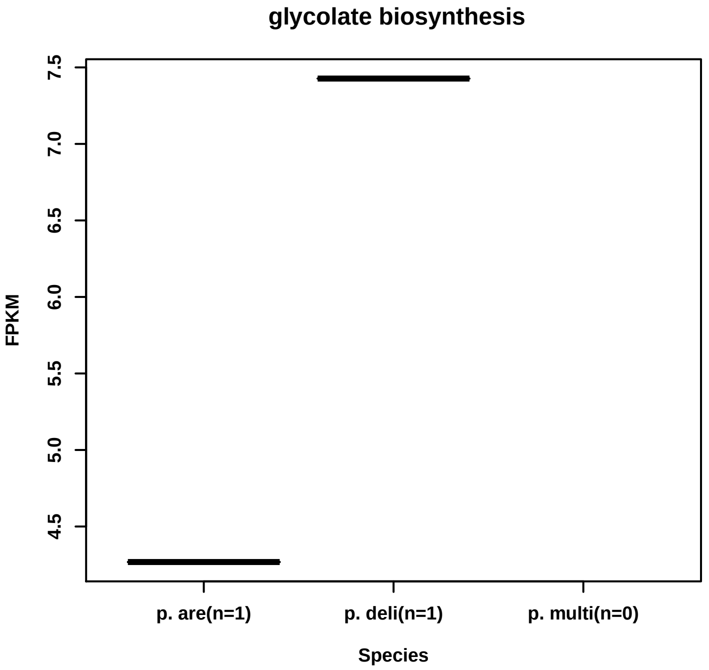
glycolate biosynthesis
FPKM
Species
p. are(n=1)
p. deli(n=1)
p. multi(n=0)

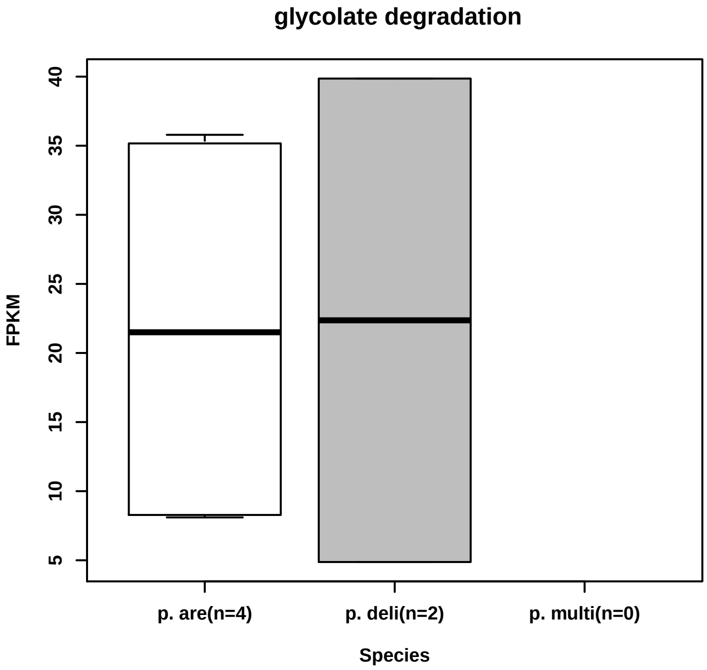
glycolate degradation
FPKM
Species
p. are(n=4)
p. deli(n=2)
p. multi(n=0)

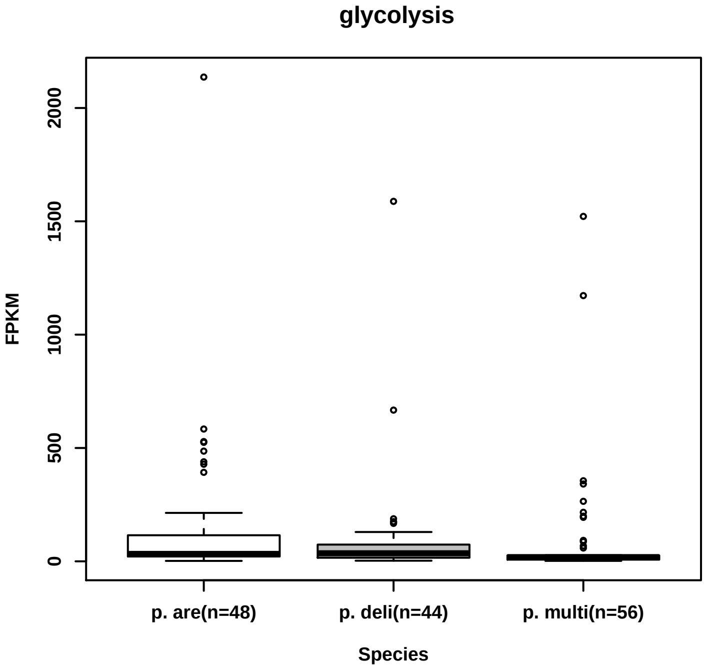
glycolysis
FPKM
Species
p. are(n=48)
p. deli(n=44)
p. multi(n=56)

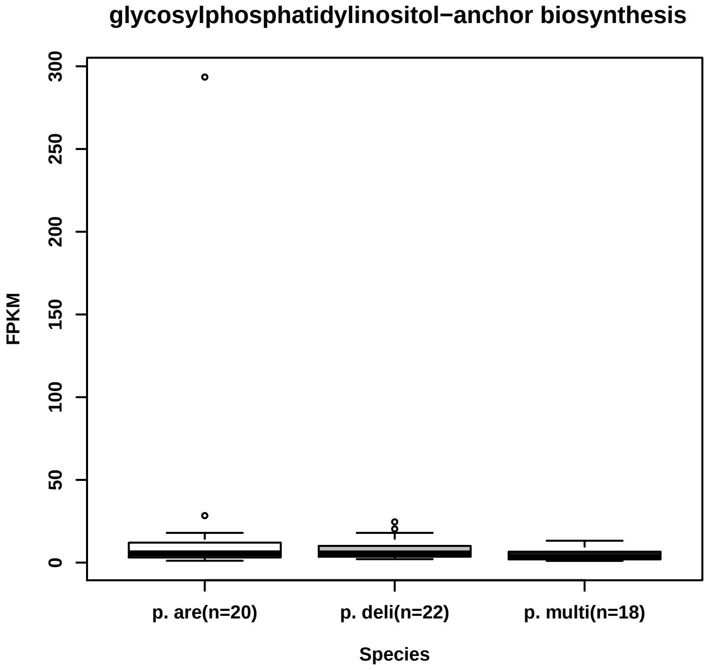
glycosylphosphatidylinositol−anchor biosynthesis
FPKM
Species
p. are(n=20)
p. deli(n=22)
p. multi(n=18)

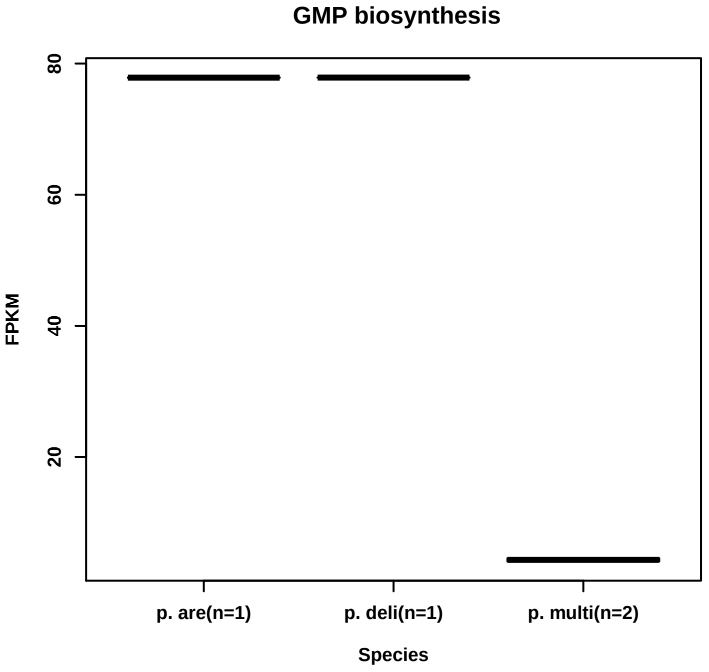
GMP biosynthesis
FPKM
Species
p. are(n=1)
p. deli(n=1)
p. multi(n=2)

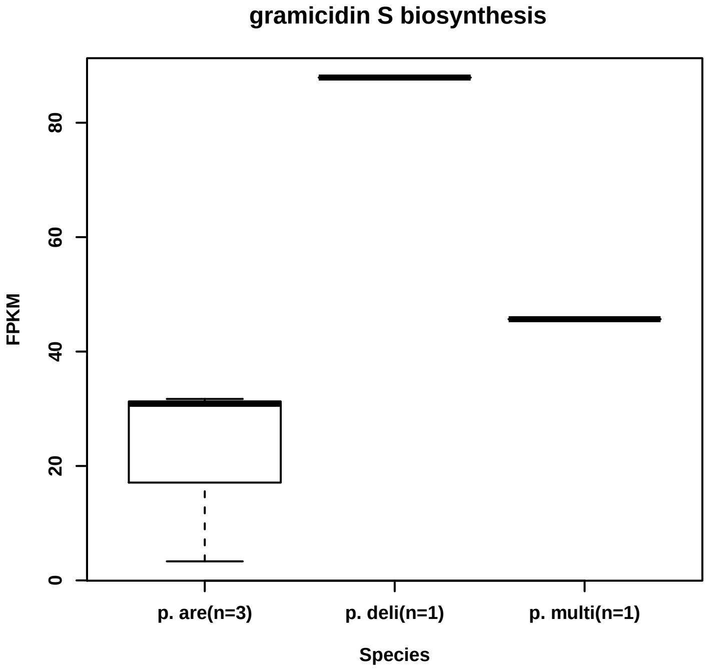
gramicidin S biosynthesis
FPKM
Species
p. are(n=3)
p. deli(n=1)
p. multi(n=1)

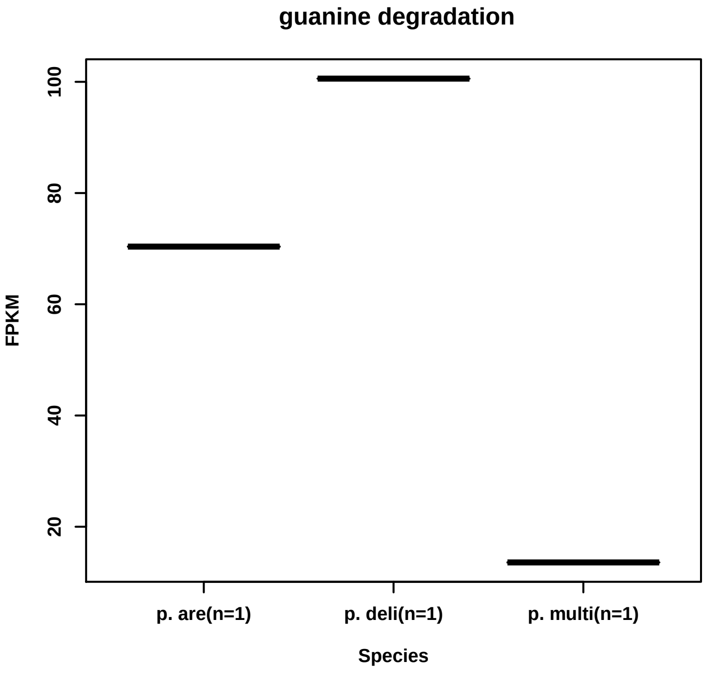
guanine degradation
FPKM
20
40
60
80
100
p. are(n=1)
p. deli(n=1)
p. multi(n=1)
Species

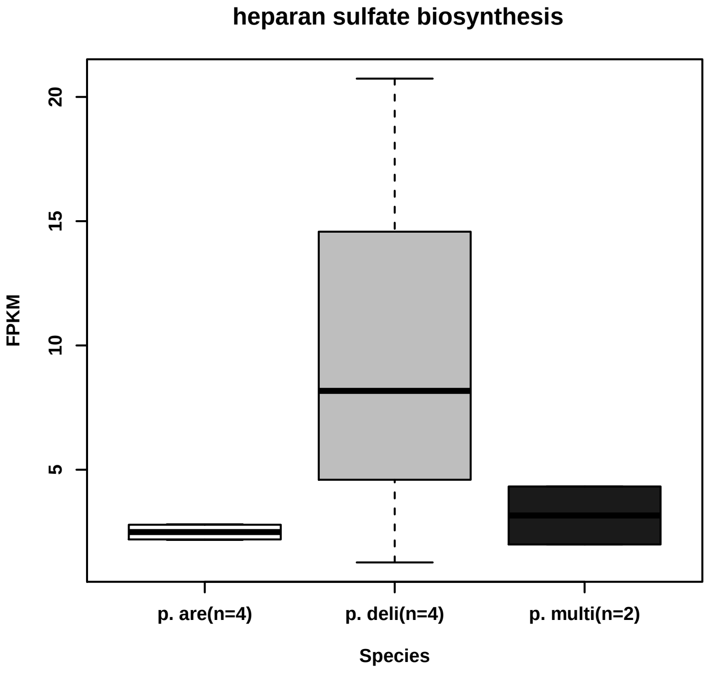
heparan sulfate biosynthesis
FPKM
5
10
15
20
p. are(n=4)
p. deli(n=4)
p. multi(n=2)
Species

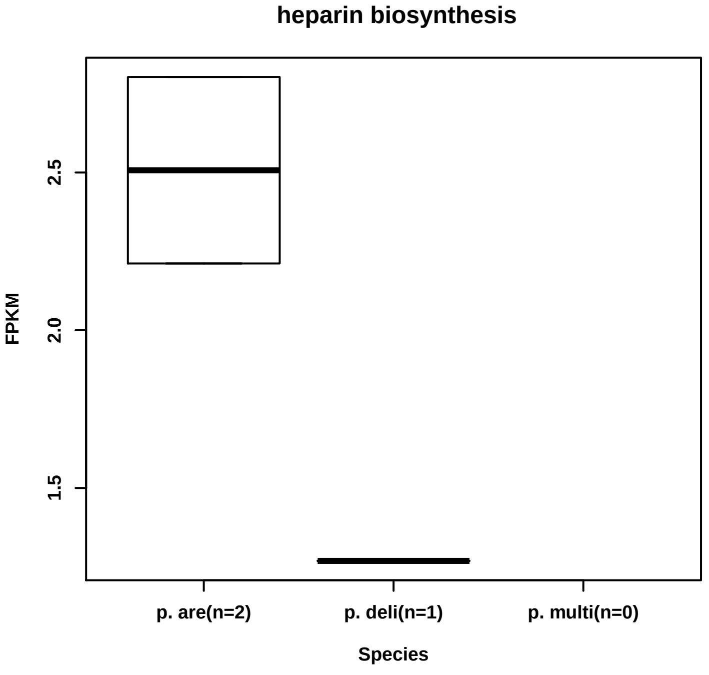
heparin biosynthesis
FPKM
1.5
2.0
2.5
p. are(n=2)
p. deli(n=1)
p. multi(n=0)
Species

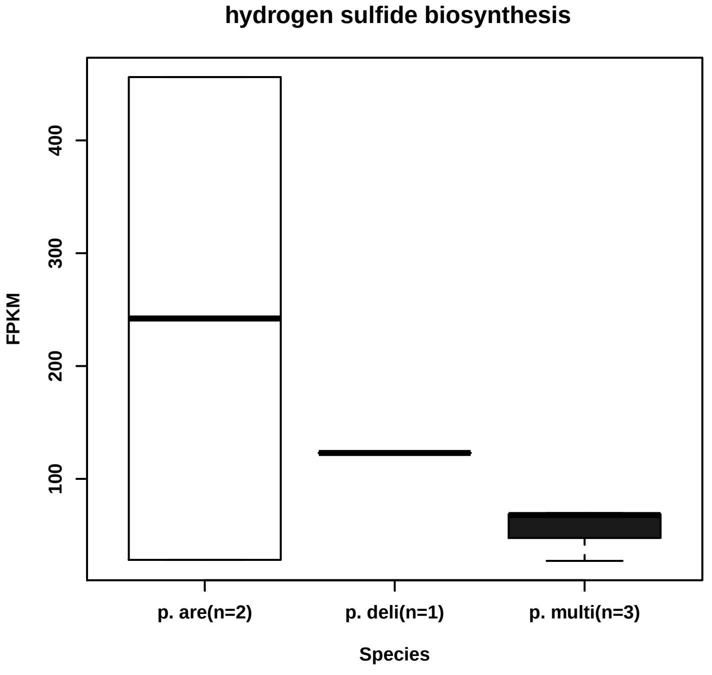
hydrogen sulfide biosynthesis
FPKM
100
200
300
400
p. are(n=2)
p. deli(n=1)
p. multi(n=3)
Species

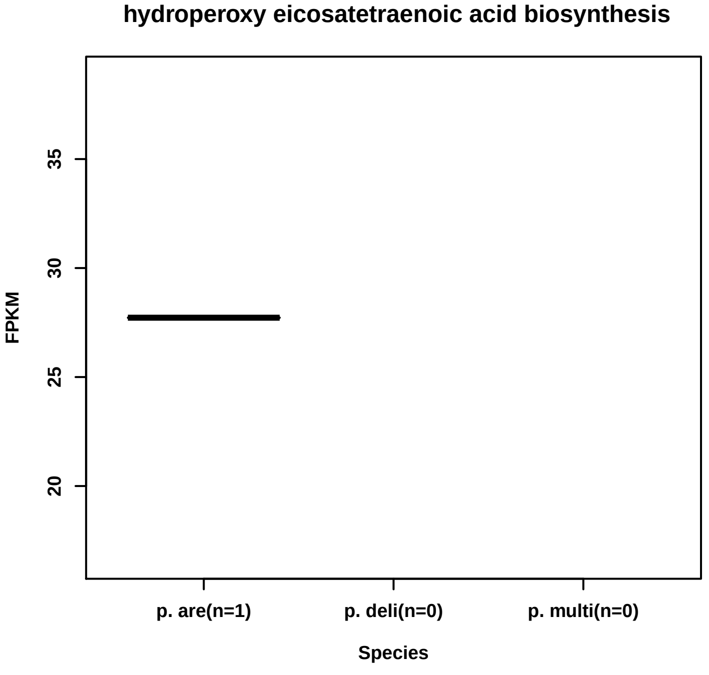
hydroperoxy eicosatetraenoic acid biosynthesis
FPKM
20
25
30
35
p. are(n=1)
p. deli(n=0)
p. multi(n=0)
Species

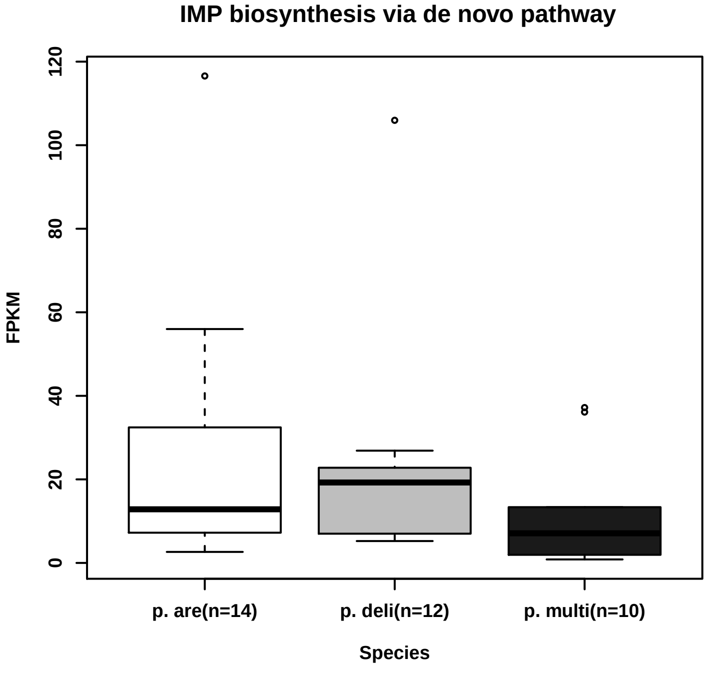
IMP biosynthesis via de novo pathway
FPKM
0
20
40
60
80
100
120
p. are(n=14)
p. deli(n=12)
p. multi(n=10)
Species

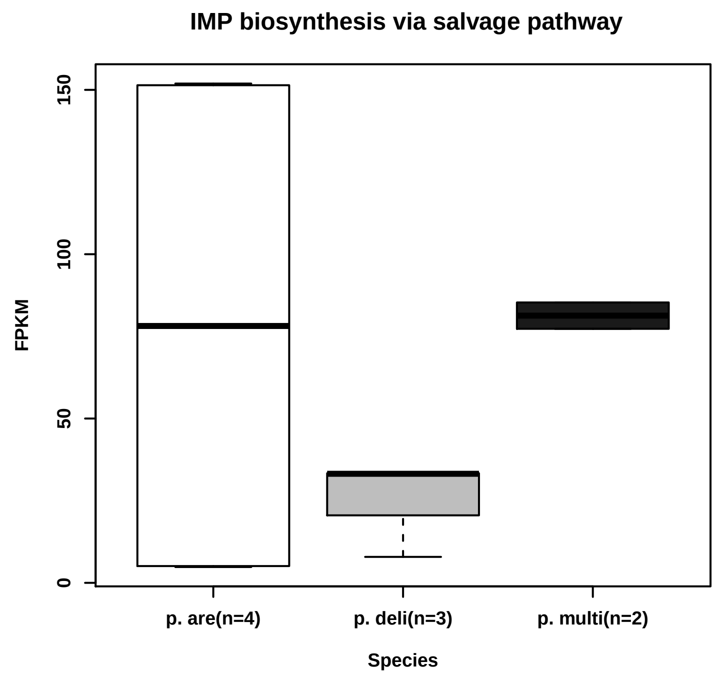
IMP biosynthesis via salvage pathway
FPKM
0
50
100
150
p. are(n=4)
p. deli(n=3)
p. multi(n=2)
Species

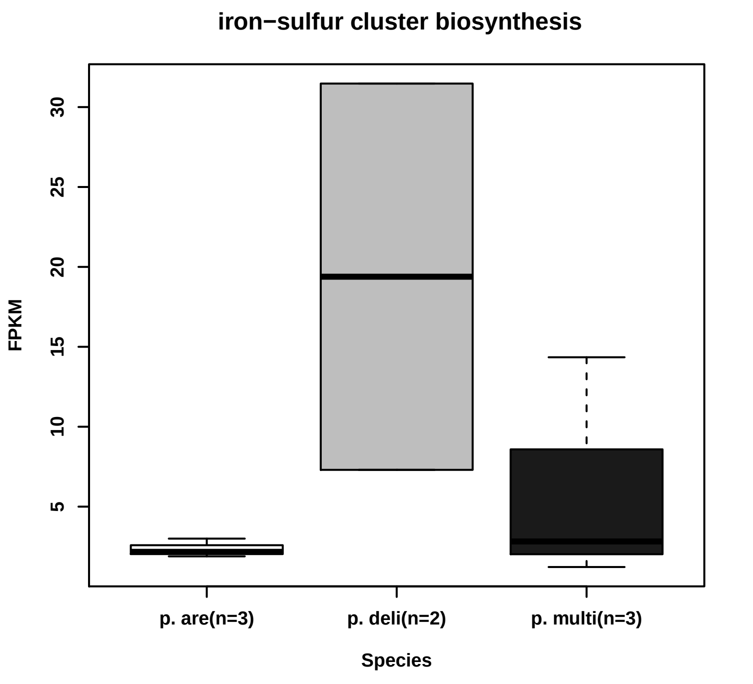
iron−sulfur cluster biosynthesis
FPKM
5
10
15
20
25
30
p. are(n=3)
p. deli(n=2)
p. multi(n=3)
Species

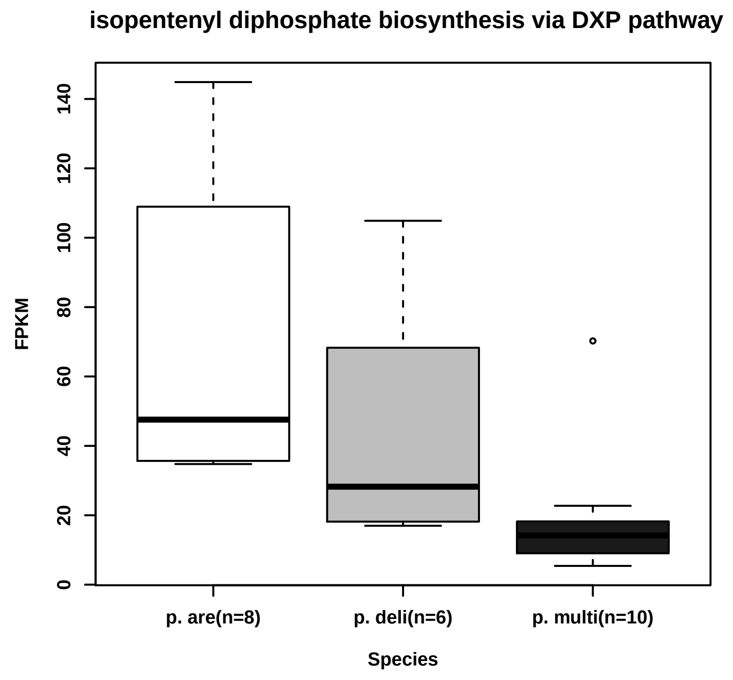
isopentenyl diphosphate biosynthesis via DXP pathway
FPKM
0
20
40
60
80
100
120
140
p. are(n=8)
p. deli(n=6)
p. multi(n=10)
Species

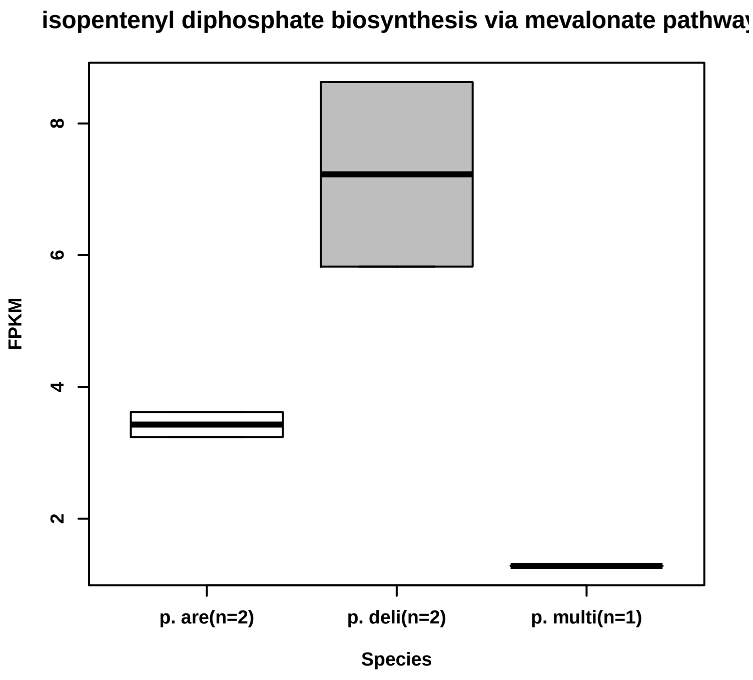
isopentenyl diphosphate biosynthesis via mevalonate pathwa
FPKM
2
4
6
8
p. are(n=2)
p. deli(n=2)
p. multi(n=1)
Species

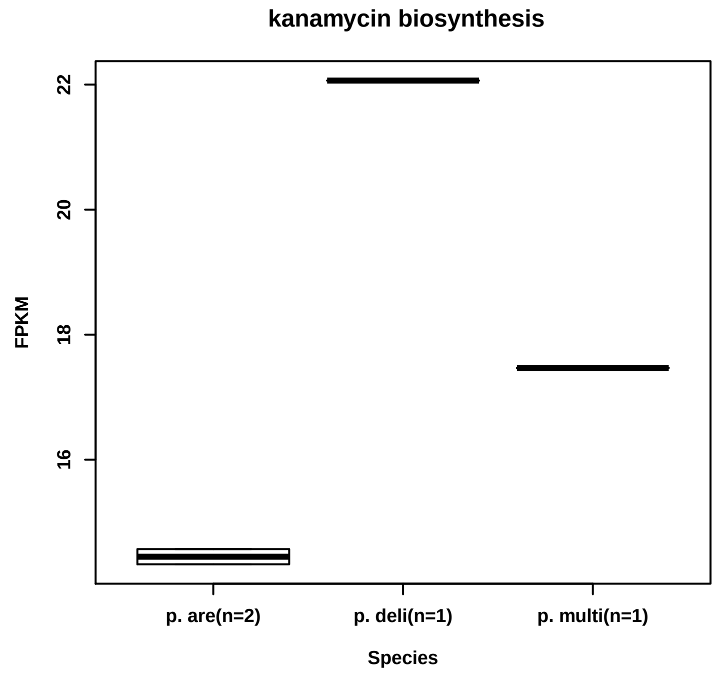
kanamycin biosynthesis
FPKM
16
18
20
22
p. are(n=2)
p. deli(n=1)
p. multi(n=1)
Species

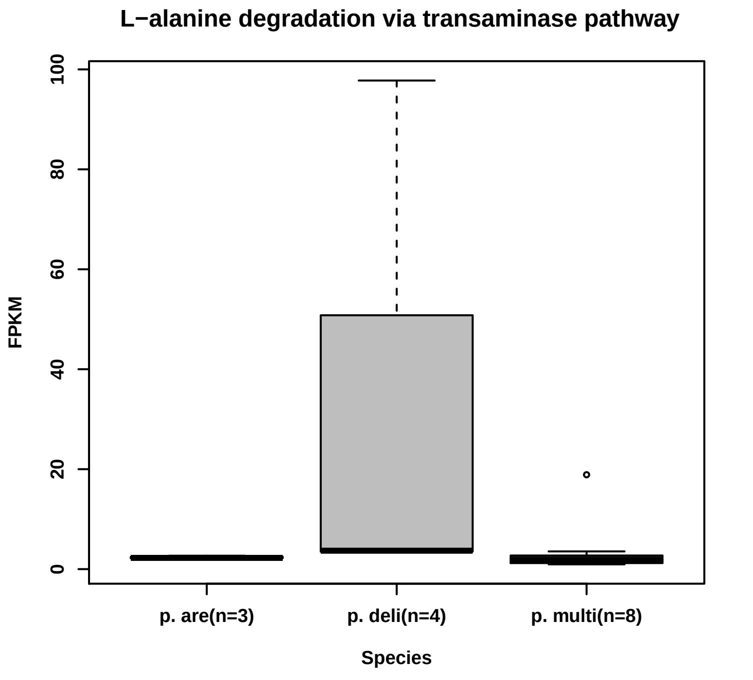
L−alanine degradation via transaminase pathway
FPKM
0
20
40
60
80
100
p. are(n=3)
p. deli(n=4)
p. multi(n=8)
Species

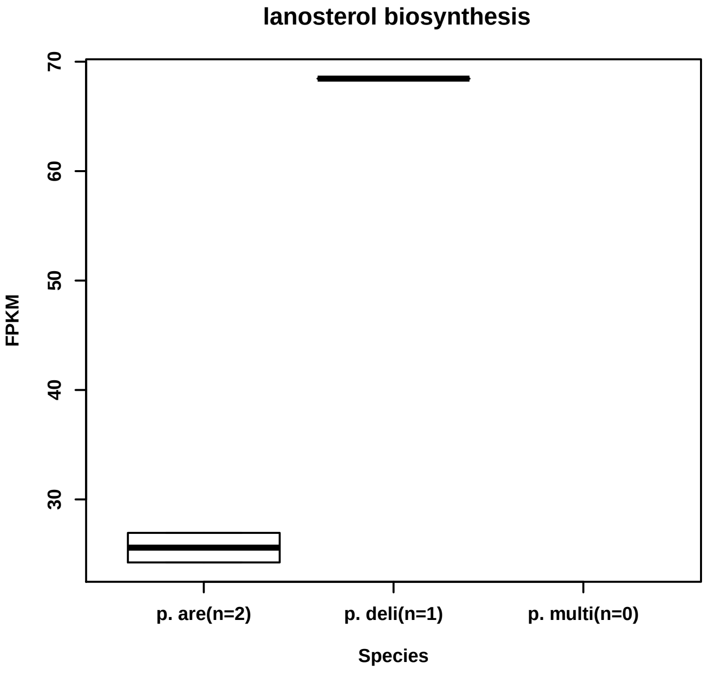
lanosterol biosynthesis
FPKM
30
40
50
60
70
p. are(n=2)
p. deli(n=1)
p. multi(n=0)
Species

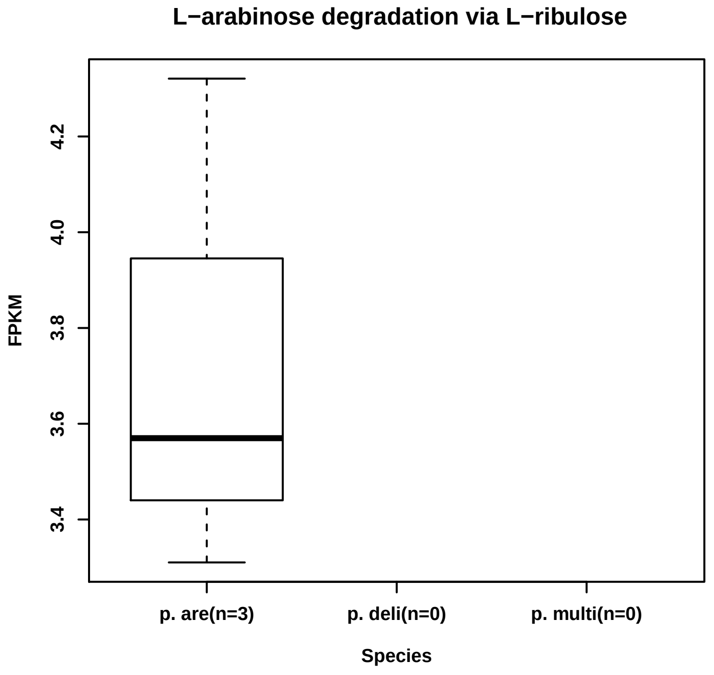
L−arabinose degradation via L−ribulose
FPKM
3.4
3.6
3.8
4.0
4.2
p. are(n=3)
p. deli(n=0)
p. multi(n=0)
Species

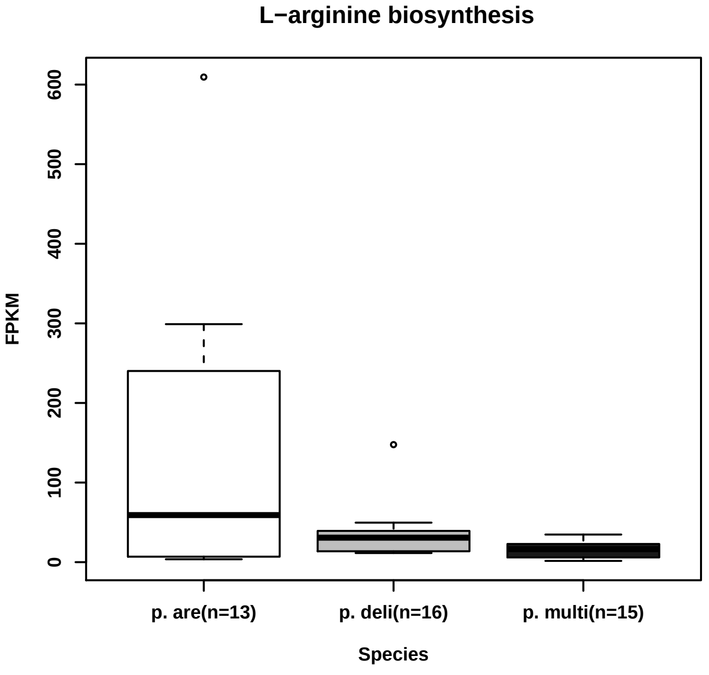
L−arginine biosynthesis
FPKM
0
100
200
300
400
500
600
p. are(n=13)
p. deli(n=16)
p. multi(n=15)
Species

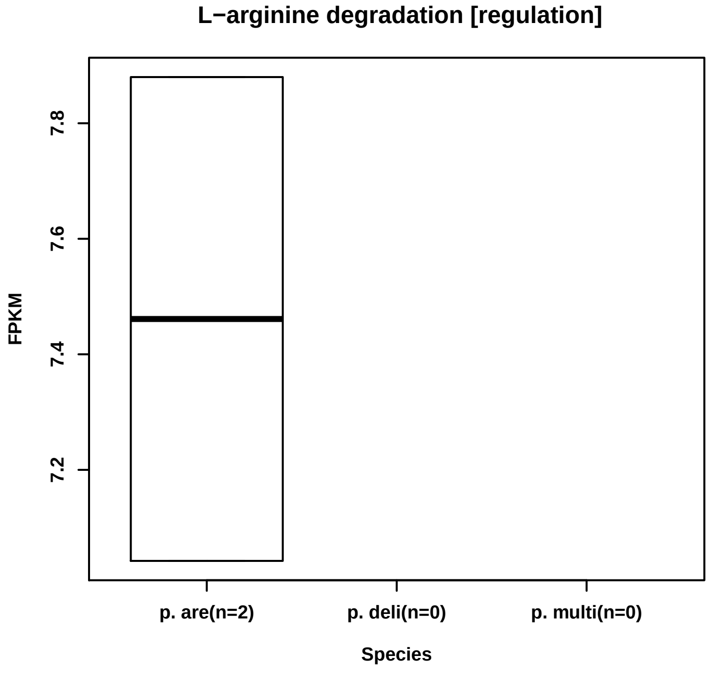
L−arginine degradation [regulation]
FPKM
7.2
7.4
7.6
7.8
p. are(n=2)
p. deli(n=0)
p. multi(n=0)
Species

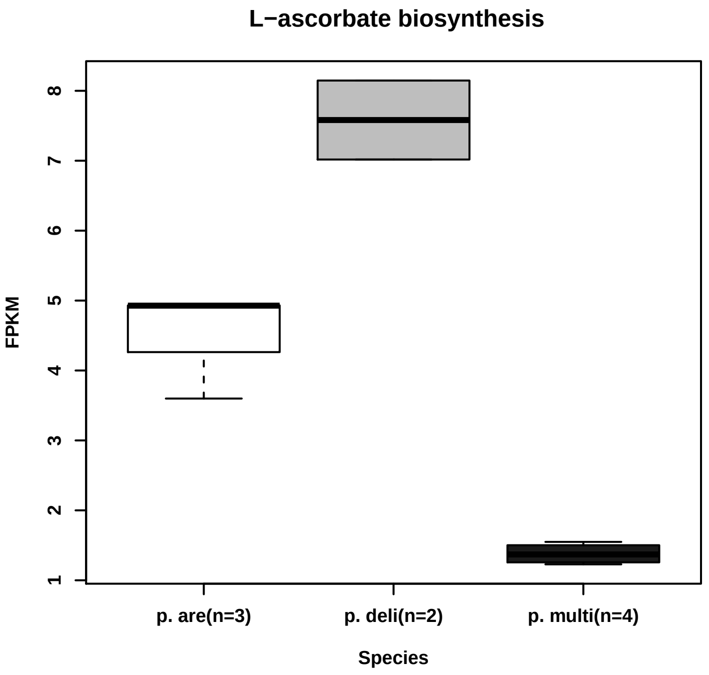
L−ascorbate biosynthesis
FPKM
1
2
3
4
5
6
7
8
p. are(n=3)
p. deli(n=2)
p. multi(n=4)
Species

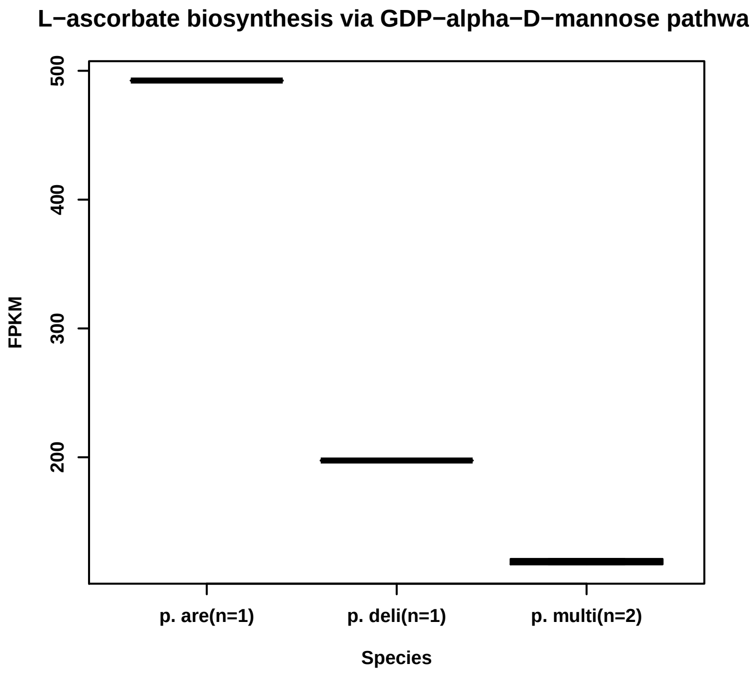
L−ascorbate biosynthesis via GDP−alpha−D−mannose pathwa
FPKM
200
300
400
500
p. are(n=1)
p. deli(n=1)
p. multi(n=2)
Species

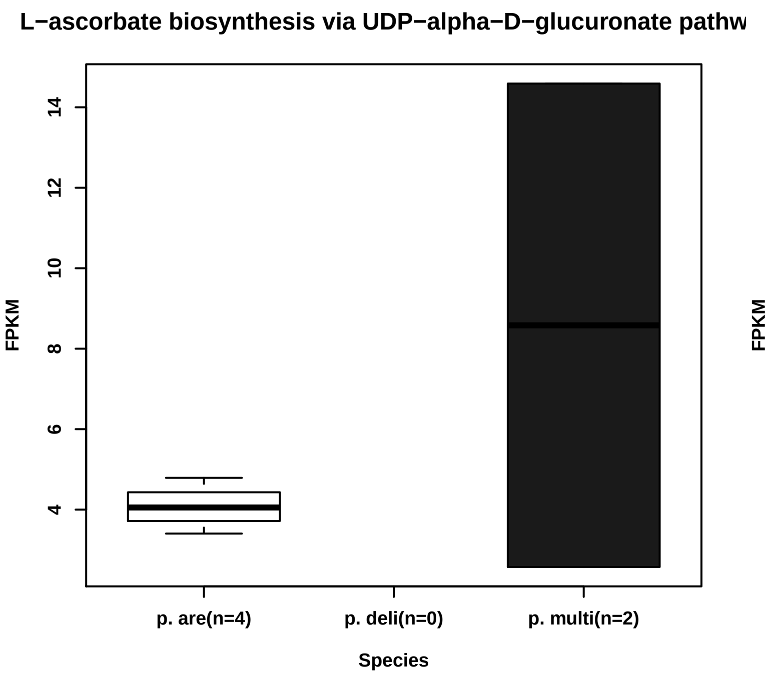

L−ascorbate biosynthesis via UDP−alpha−D−glucuronate pathw
FPKM
4
6
8
10
12
14
p. are(n=4)
p. deli(n=0)
p. multi(n=2)
Species

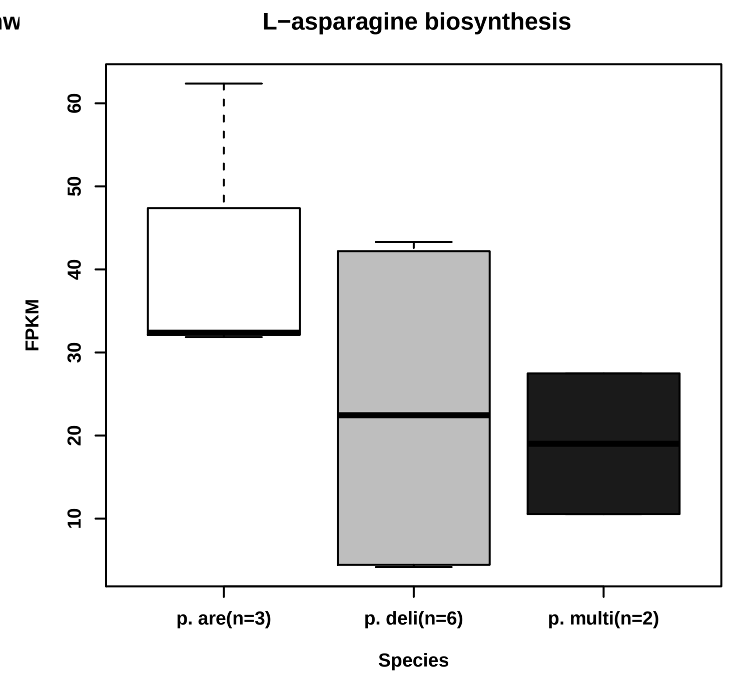

L−asparagine biosynthesis
FPKM
10
20
30
40
50
60
p. are(n=3)
p. deli(n=6)
p. multi(n=2)
Species

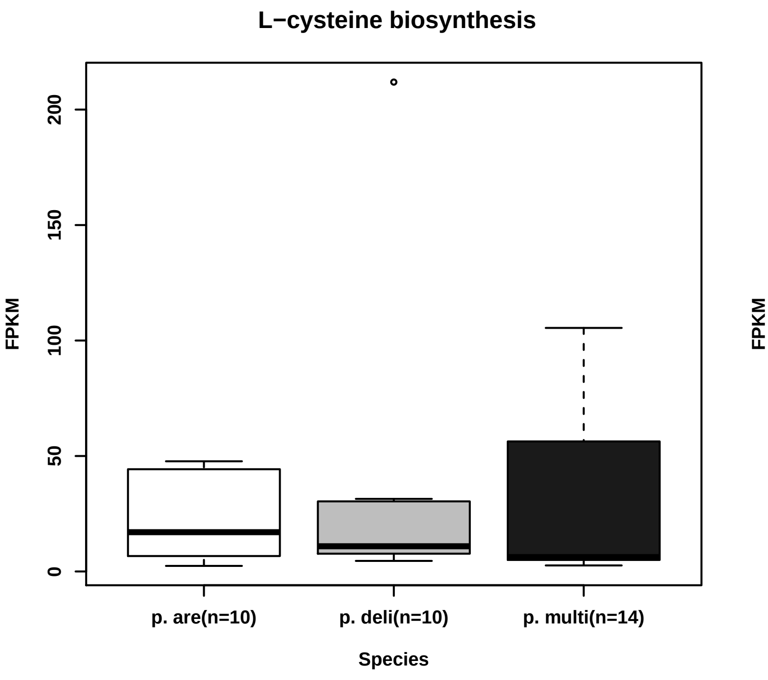

L−cysteine biosynthesis
FPKM
0
50
100
150
200
p. are(n=10)
p. deli(n=10)
p. multi(n=14)
Species

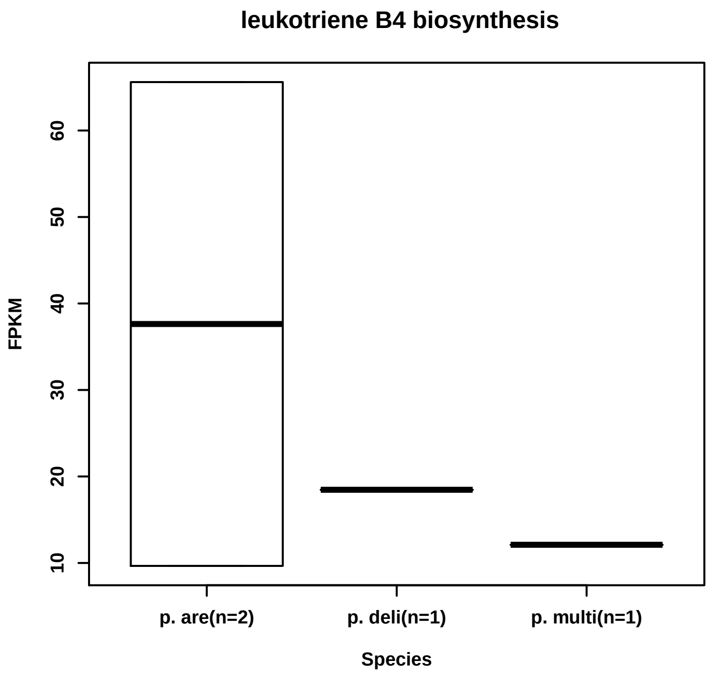

leukotriene B4 biosynthesis
FPKM
10
20
30
40
50
60
p. are(n=2)
p. deli(n=1)
p. multi(n=1)
Species

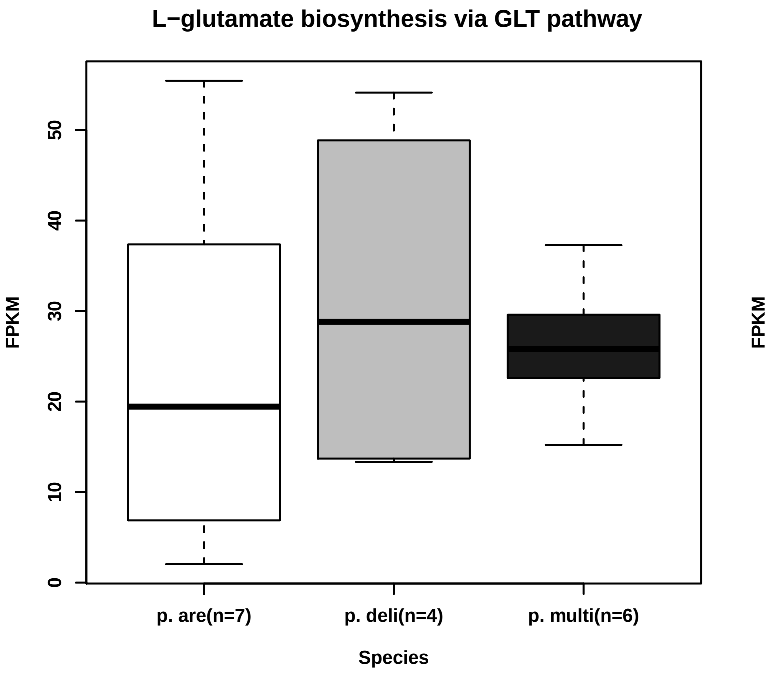

L−glutamate biosynthesis via GLT pathway
FPKM
0
10
20
30
40
50
p. are(n=7)
p. deli(n=4)
p. multi(n=6)
Species

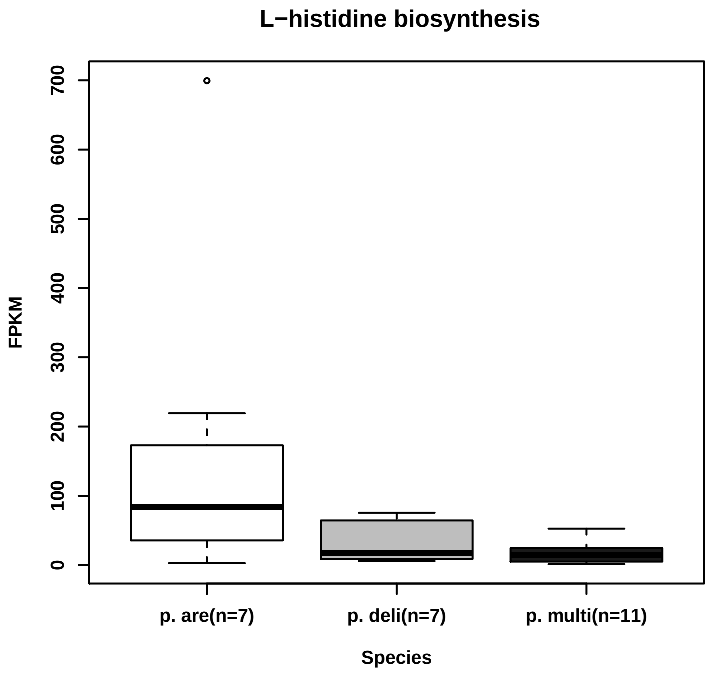

L−histidine biosynthesis
FPKM
0
100
200
300
400
500
600
700
p. are(n=7)
p. deli(n=7)
p. multi(n=11)
Species

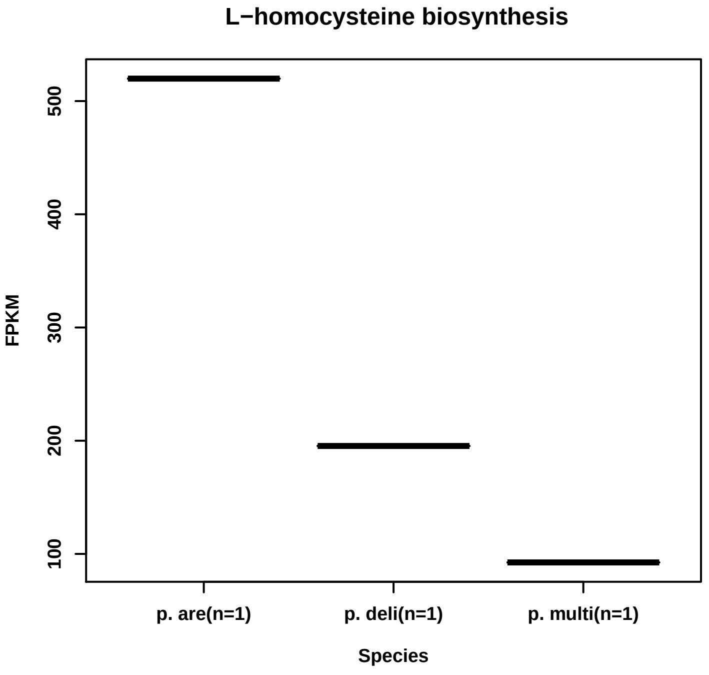
L−homocysteine biosynthesis
FPKM
100
200
300
400
500
p. are(n=1)
p. deli(n=1)
p. multi(n=1)
Species

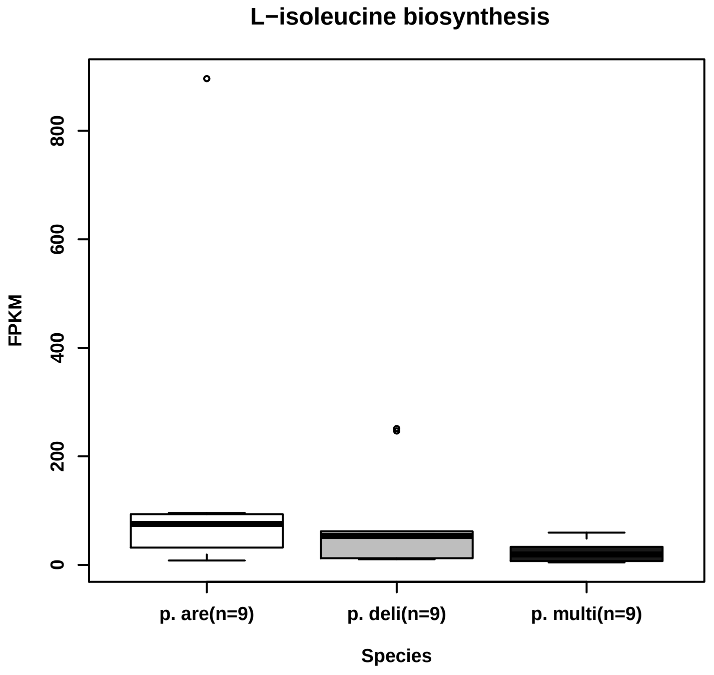
L−isoleucine biosynthesis
FPKM
0
200
400
600
800
p. are(n=9)
p. deli(n=9)
p. multi(n=9)
Species

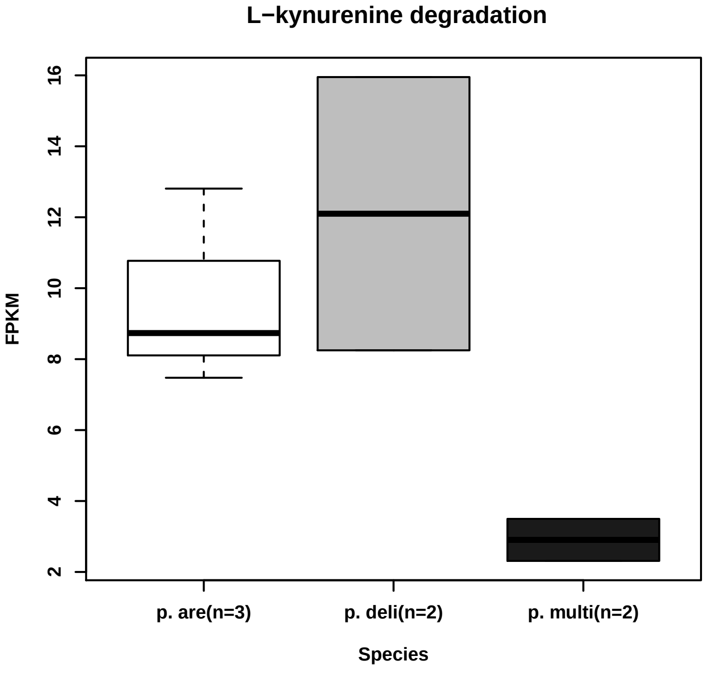
L−kynurenine degradation
FPKM
2
4
6
8
10
12
14
16
p. are(n=3)
p. deli(n=2)
p. multi(n=2)
Species

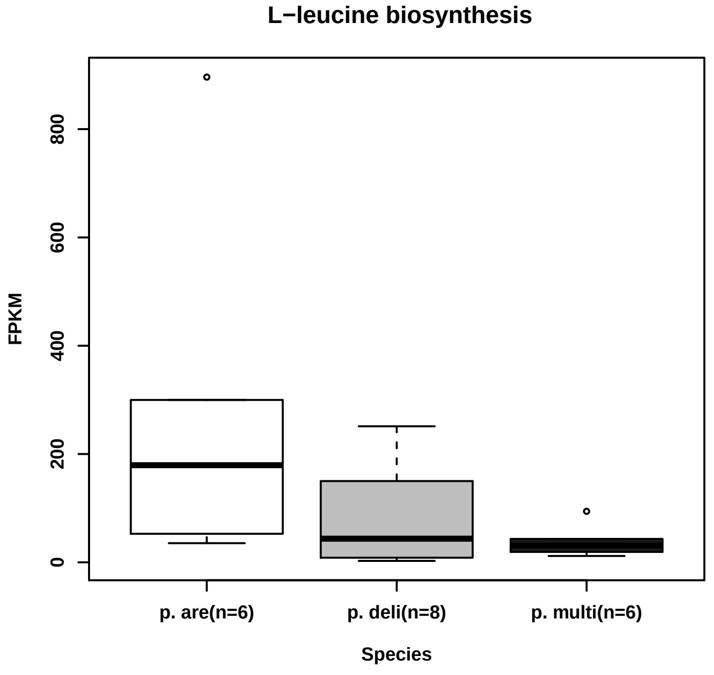
L−leucine biosynthesis
FPKM
0
200
400
600
800
p. are(n=6)
p. deli(n=8)
p. multi(n=6)
Species

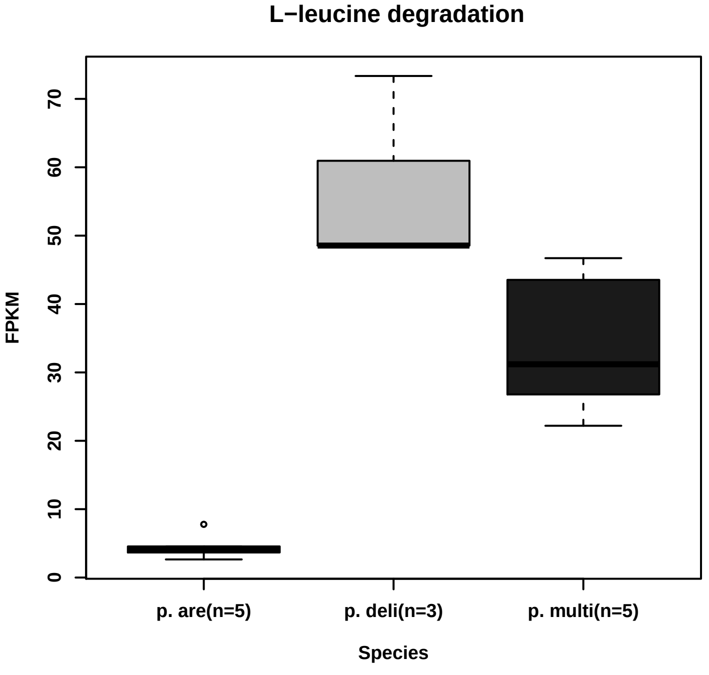
L−leucine degradation
FPKM
0
10
20
30
40
50
60
70
p. are(n=5)
p. deli(n=3)
p. multi(n=5)
Species

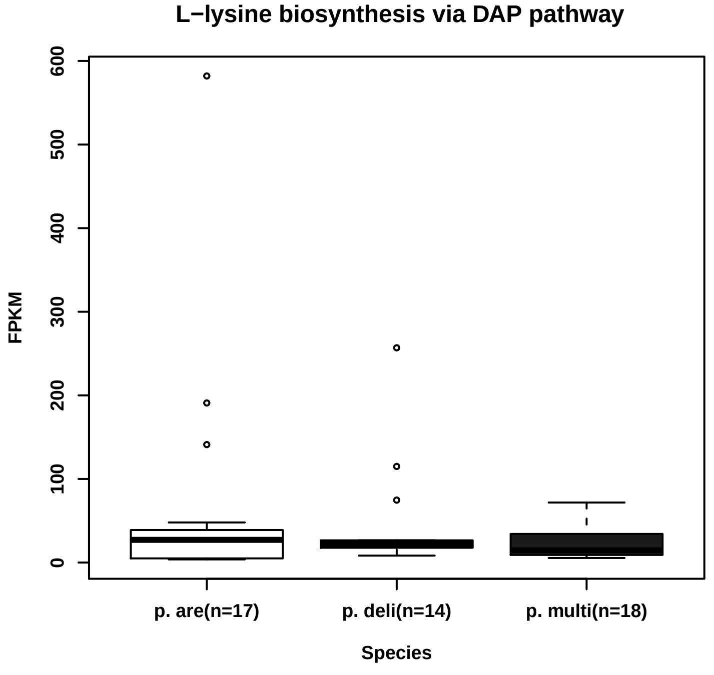
L−lysine biosynthesis via DAP pathway
FPKM
0
100
200
300
400
500
600
p. are(n=17)
p. deli(n=14)
p. multi(n=18)
Species

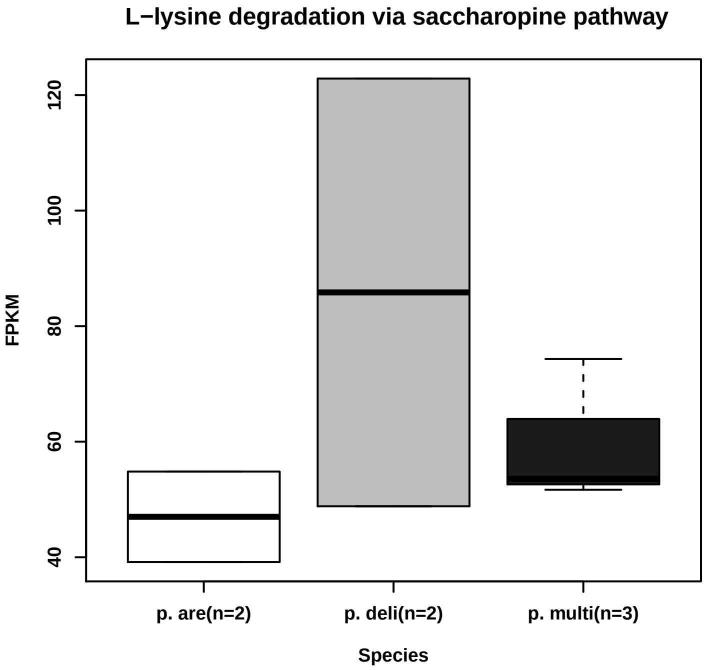
L−lysine degradation via saccharopine pathway
FPKM
40
60
80
100
120
p. are(n=2)
p. deli(n=2)
p. multi(n=3)
Species

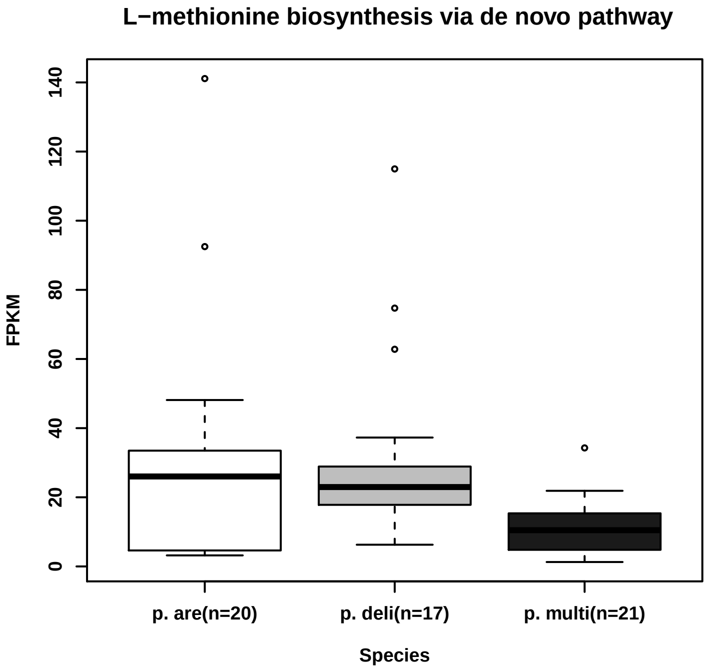
L−methionine biosynthesis via de novo pathway
FPKM
0
20
40
60
80
100
120
140
p. are(n=20)
p. deli(n=17)
p. multi(n=21)
Species

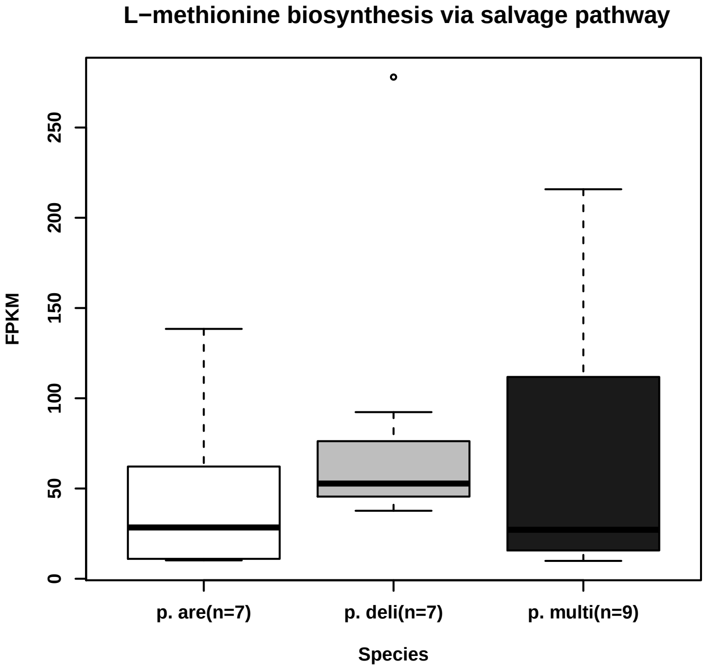
L−methionine biosynthesis via salvage pathway
FPKM
0
50
100
150
200
250
p. are(n=7)
p. deli(n=7)
p. multi(n=9)
Species

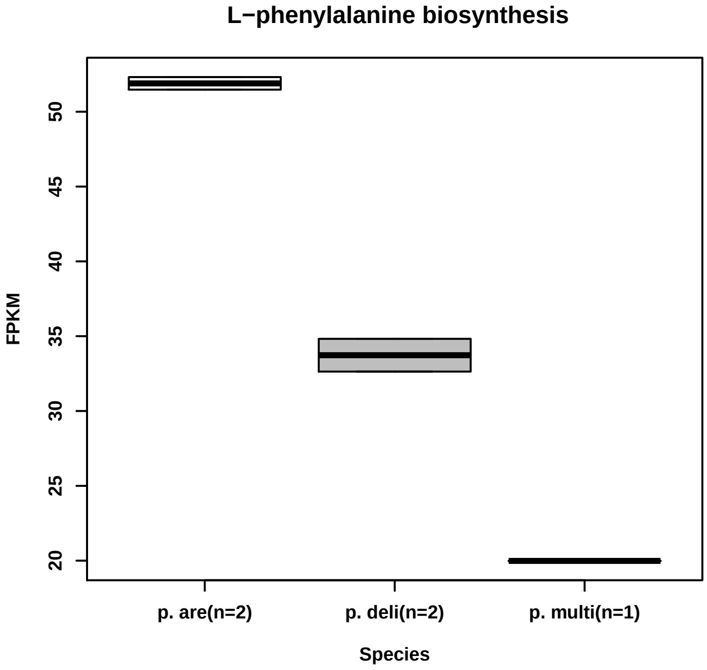
L−phenylalanine biosynthesis
FPKM
20
25
30
35
40
45
50
p. are(n=2)
p. deli(n=2)
p. multi(n=1)
Species

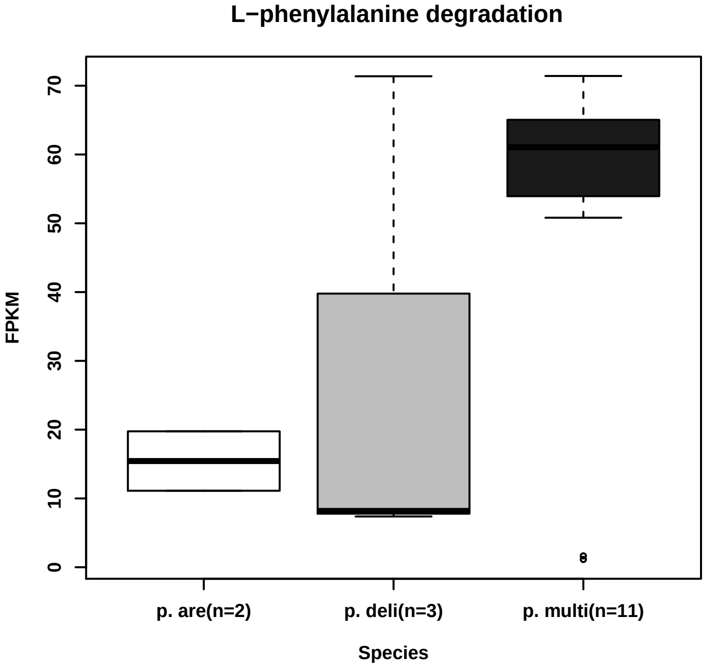
L−phenylalanine degradation
FPKM
0
10
20
30
40
50
60
70
p. are(n=2)
p. deli(n=3)
p. multi(n=11)
Species

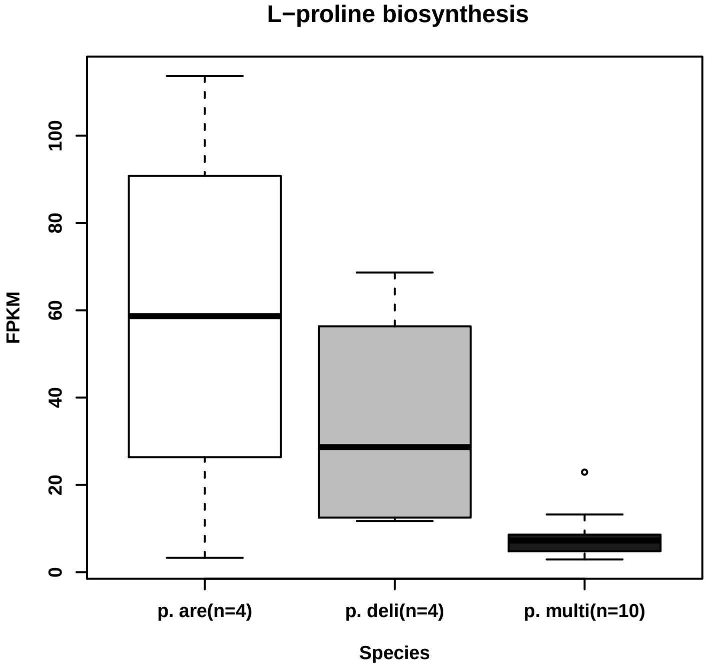
L−proline biosynthesis
FPKM
0
20
40
60
80
100
p. are(n=4)
p. deli(n=4)
p. multi(n=10)
Species

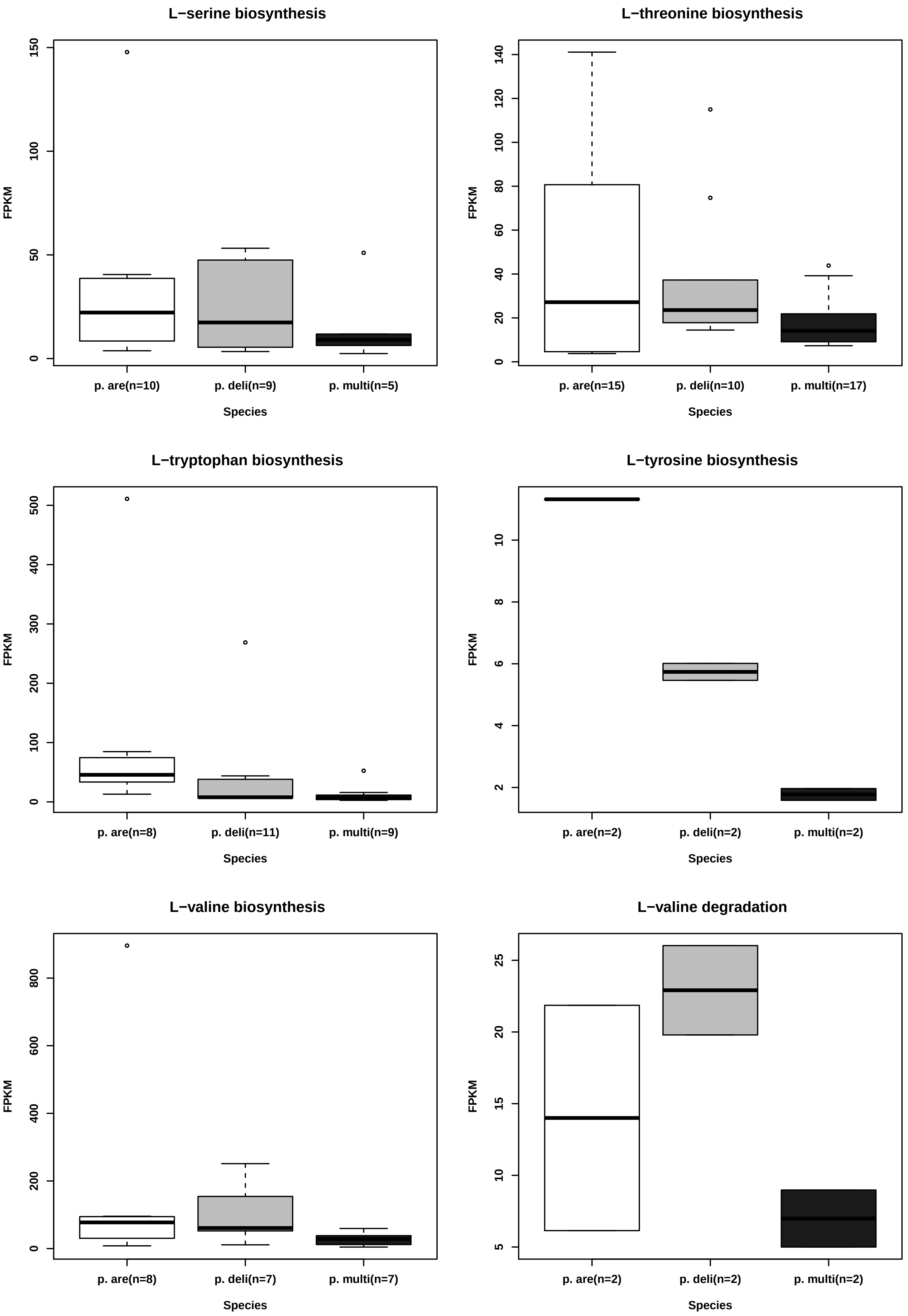
L−serine biosynthesis
FPKM
0
50
100
150
p. are(n=10)
p. deli(n=9)
p. multi(n=5)
Species
L−threonine biosynthesis
FPKM
0
20
40
60
80
100
120
140
p. are(n=15)
p. deli(n=10)
p. multi(n=17)
Species
L−tryptophan biosynthesis
FPKM
0
100
200
300
400
500
p. are(n=8)
p. deli(n=11)
p. multi(n=9)
Species
L−tyrosine biosynthesis
FPKM
2
4
6
8
10
p. are(n=2)
p. deli(n=2)
p. multi(n=2)
Species
L−valine biosynthesis
FPKM
0
200
400
600
800
p. are(n=8)
p. deli(n=7)
p. multi(n=7)
Species
L−valine degradation
FPKM
5
10
15
20
25
p. are(n=2)
p. deli(n=2)
p. multi(n=2)
Species

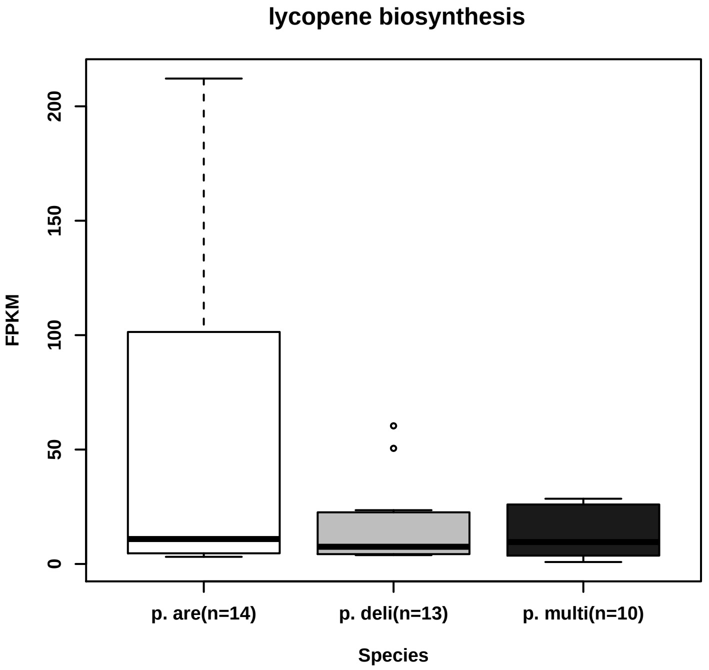
lycopene biosynthesis
FPKM
0
50
100
150
200
p. are(n=14)
p. deli(n=13)
p. multi(n=10)
Species

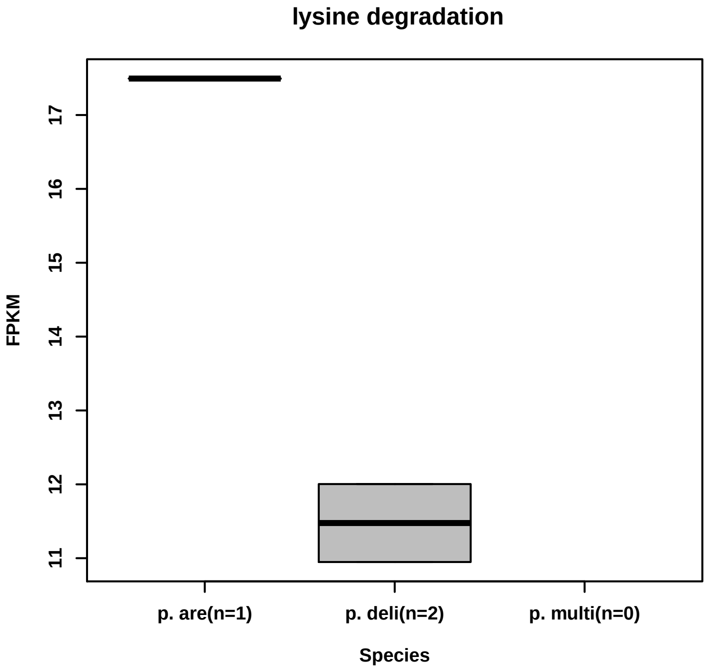
lysine degradation
FPKM
11
12
13
14
15
16
17
p. are(n=1)
p. deli(n=2)
p. multi(n=0)
Species

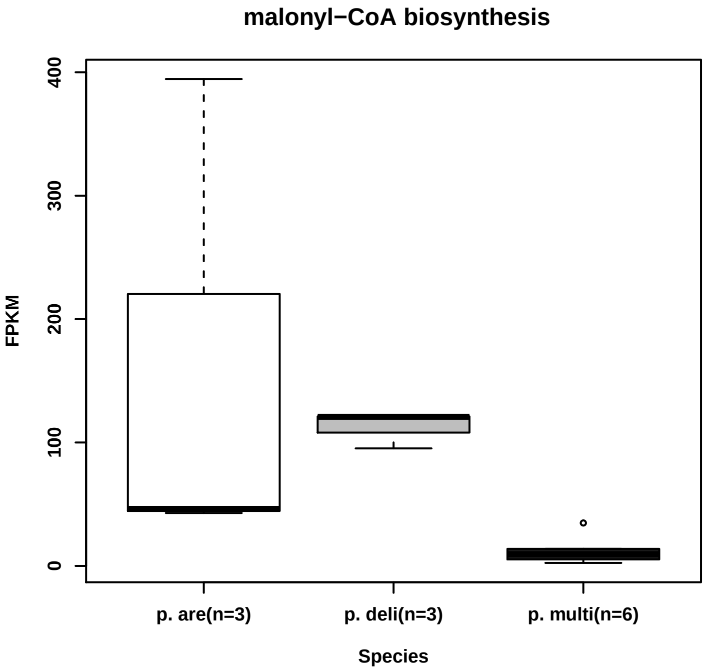
malonyl−CoA biosynthesis
FPKM
0
100
200
300
400
p. are(n=3)
p. deli(n=3)
p. multi(n=6)
Species

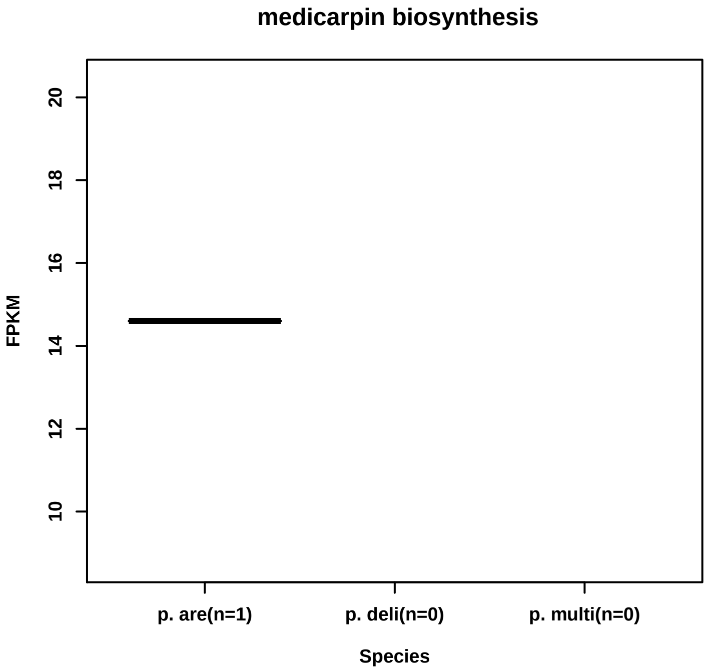
medicarpin biosynthesis
FPKM
10
12
14
16
18
20
p. are(n=1)
p. deli(n=0)
p. multi(n=0)
Species

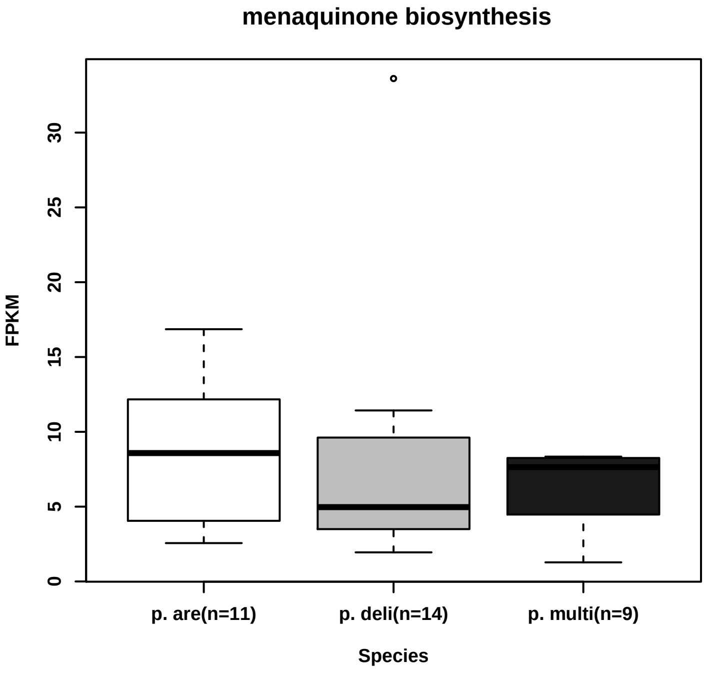
menaquinone biosynthesis
FPKM
0
5
10
15
20
25
30
p. are(n=11)
p. deli(n=14)
p. multi(n=9)
Species

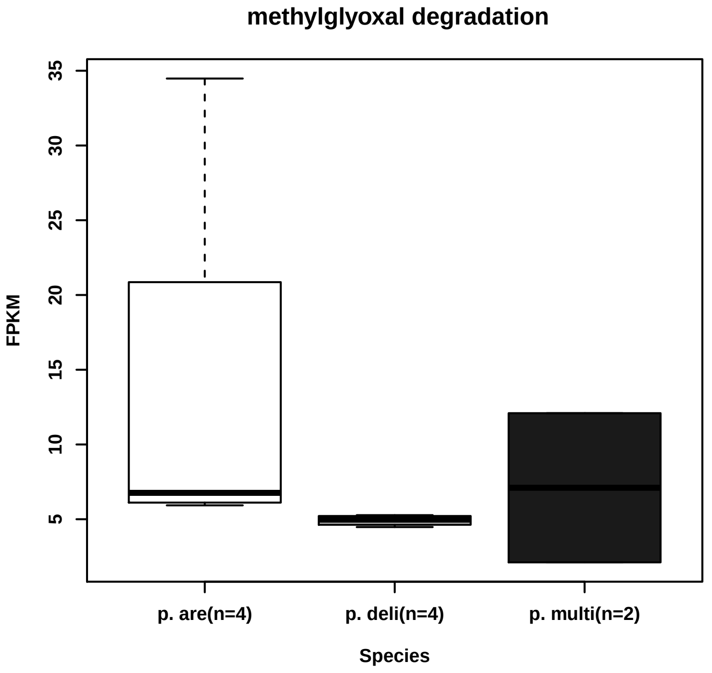
methylglyoxal degradation
FPKM
5
10
15
20
25
30
35
p. are(n=4)
p. deli(n=4)
p. multi(n=2)
Species

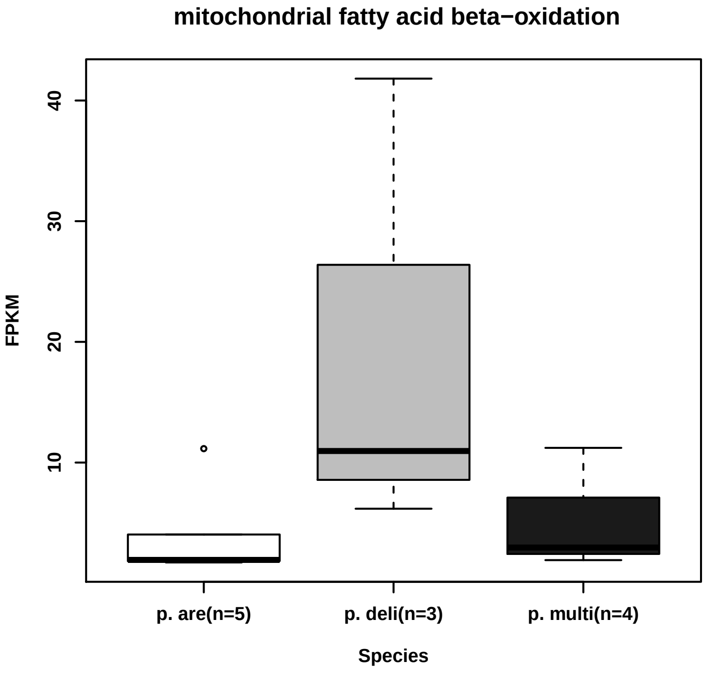
mitochondrial fatty acid beta−oxidation
FPKM
10
20
30
40
p. are(n=5)
p. deli(n=3)
p. multi(n=4)
Species

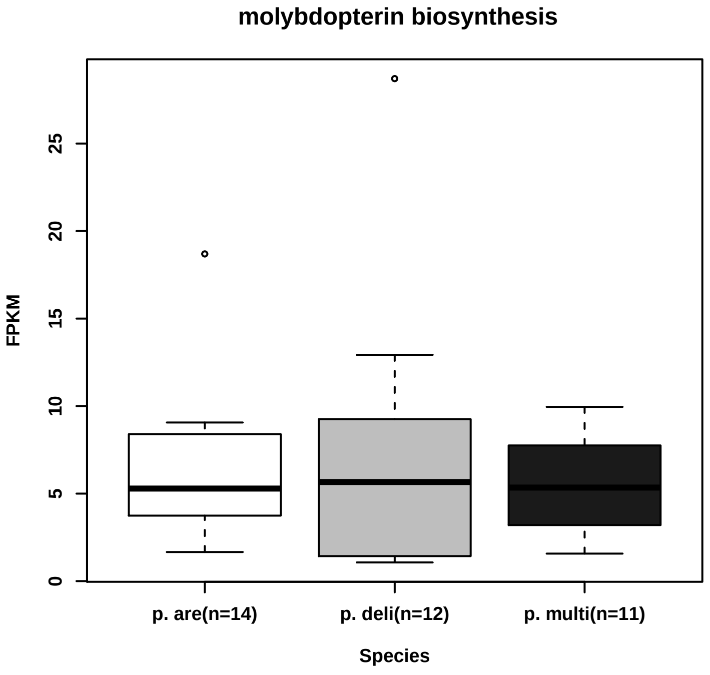
molybdopterin biosynthesis
FPKM
0
5
10
15
20
25
p. are(n=14)
p. deli(n=12)
p. multi(n=11)
Species

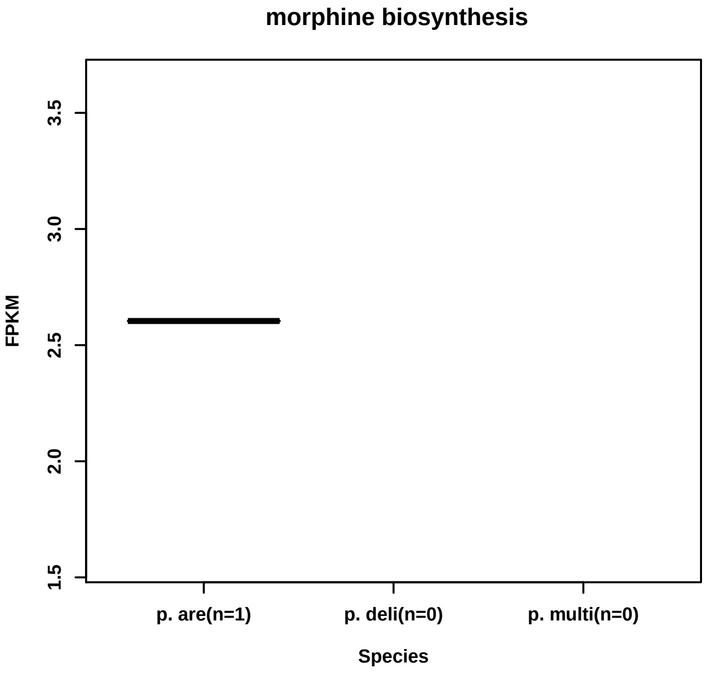
morphine biosynthesis
FPKM
1.5
2.0
2.5
3.0
3.5
p. are(n=1)
p. deli(n=0)
p. multi(n=0)
Species

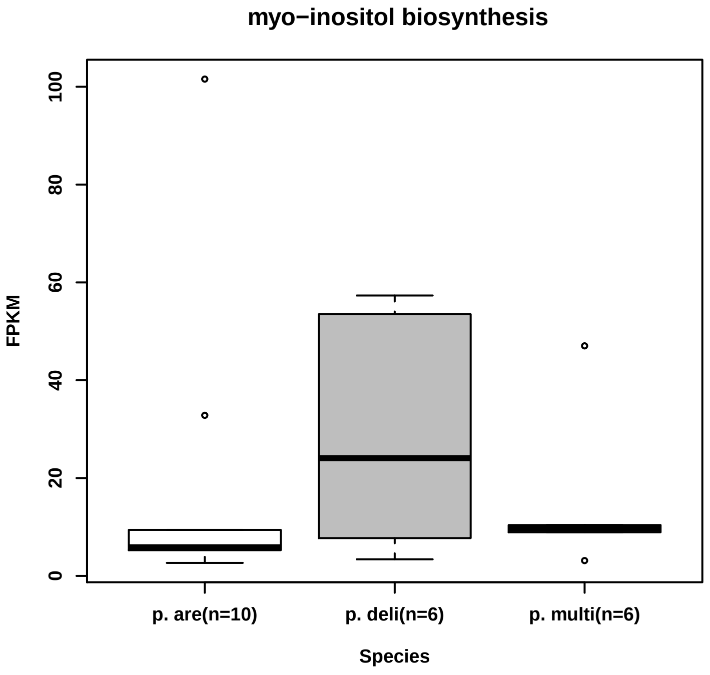
myo−inositol biosynthesis
FPKM
0
20
40
60
80
100
p. are(n=10)
p. deli(n=6)
p. multi(n=6)
Species

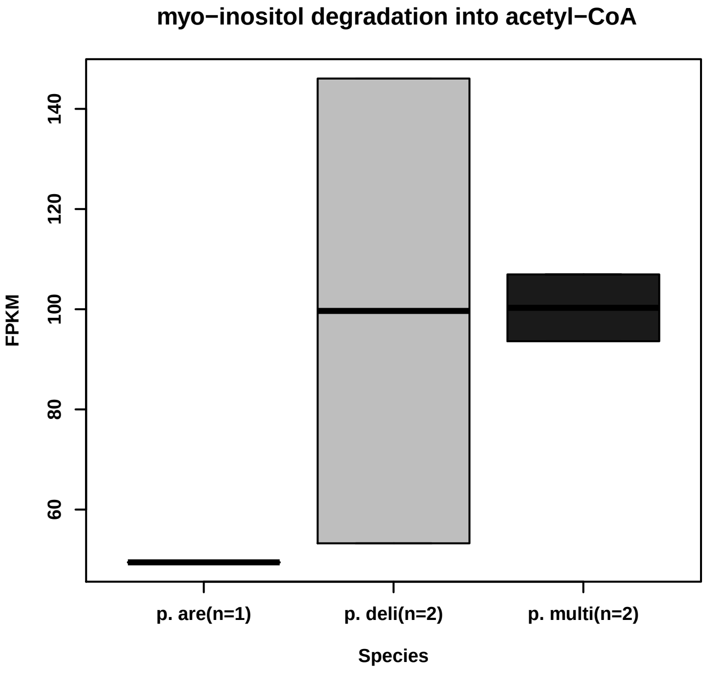
myo−inositol degradation into acetyl−CoA
FPKM
60
80
100
120
140
p. are(n=1)
p. deli(n=2)
p. multi(n=2)
Species

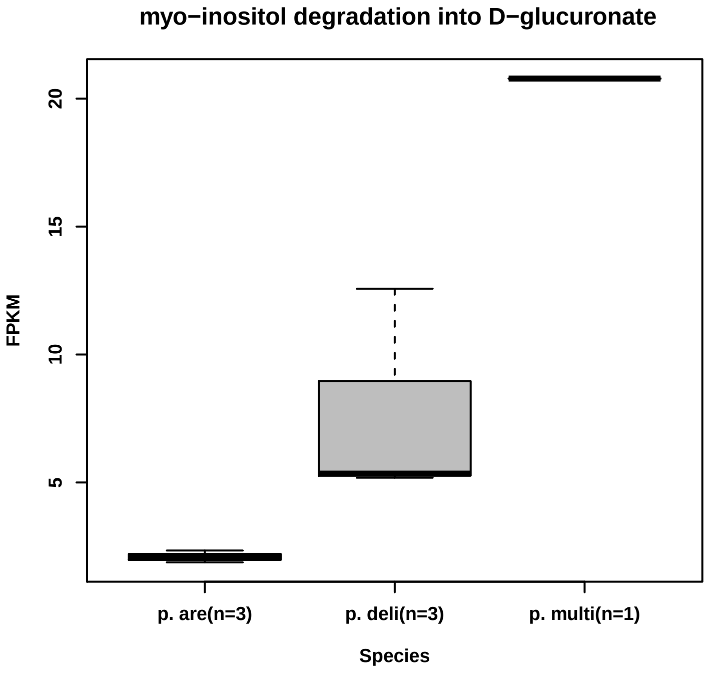
myo−inositol degradation into D−glucuronate
FPKM
5
10
15
20
p. are(n=3)
p. deli(n=3)
p. multi(n=1)
Species

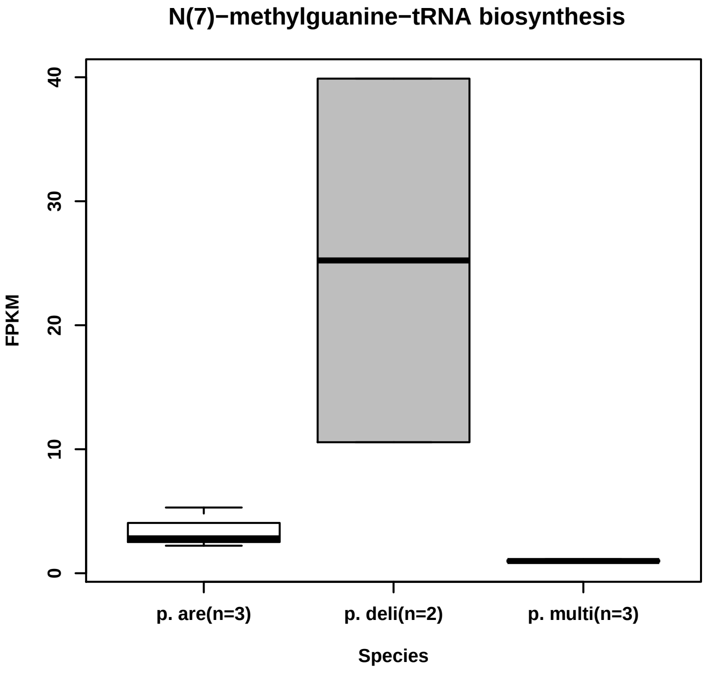
N(7)−methylguanine−tRNA biosynthesis
FPKM
0
10
20
30
40
p. are(n=3)
p. deli(n=2)
p. multi(n=3)
Species

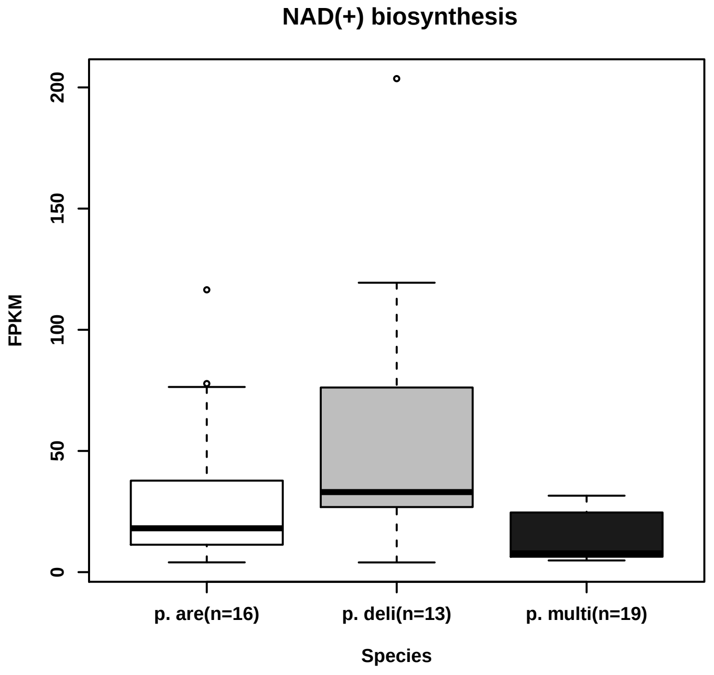
NAD(+) biosynthesis
FPKM
0
50
100
150
200
p. are(n=16)
p. deli(n=13)
p. multi(n=19)
Species

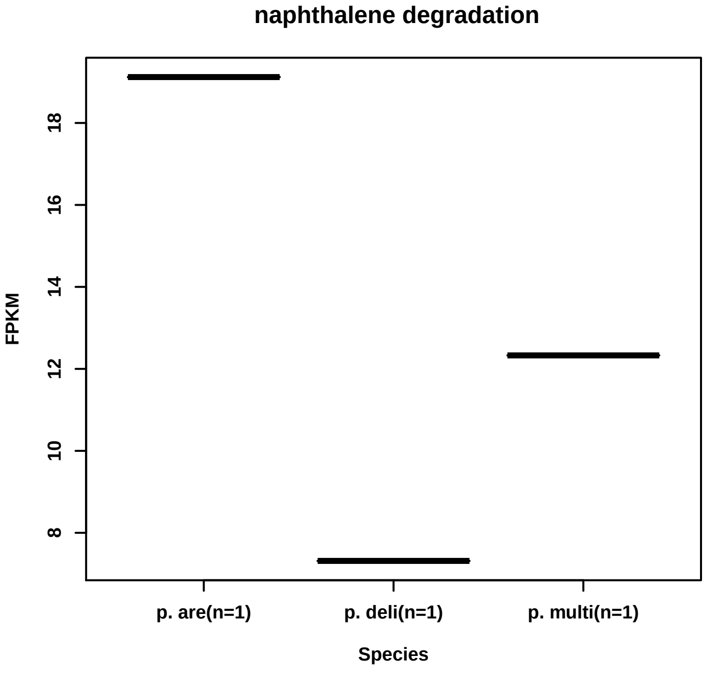
naphthalene degradation
FPKM
8
10
12
14
16
18
p. are(n=1)
p. deli(n=1)
p. multi(n=1)
Species

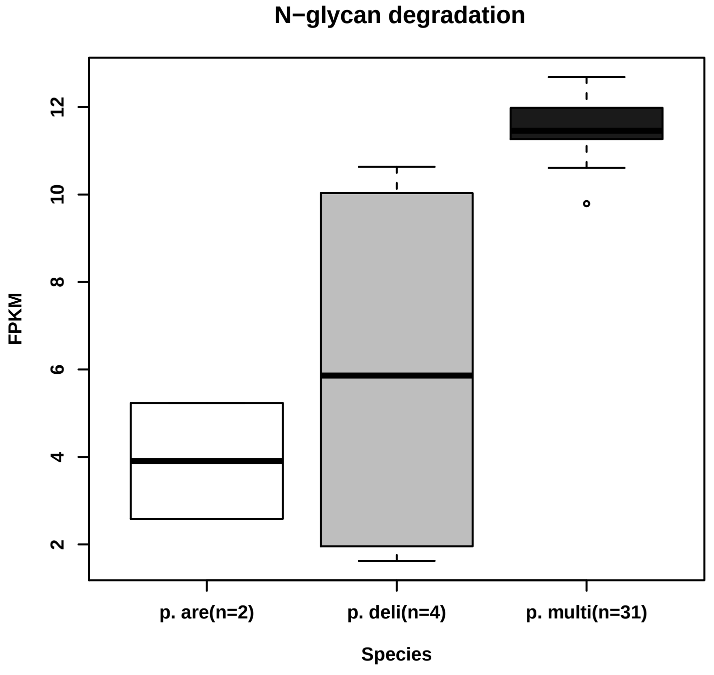
N−glycan degradation
FPKM
2
4
6
8
10
12
p. are(n=2)
p. deli(n=4)
p. multi(n=31)
Species

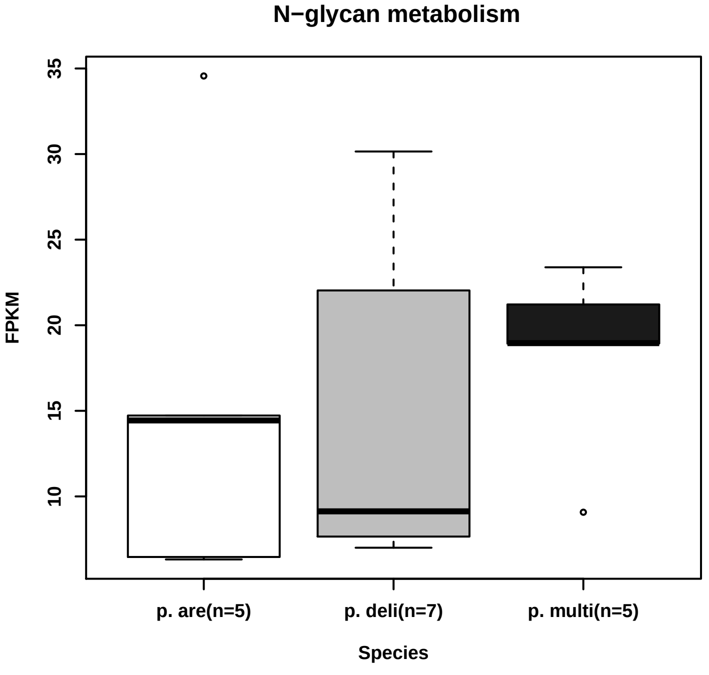
N−glycan metabolism
FPKM
10
15
20
25
30
35
p. are(n=5)
p. deli(n=7)
p. multi(n=5)
Species

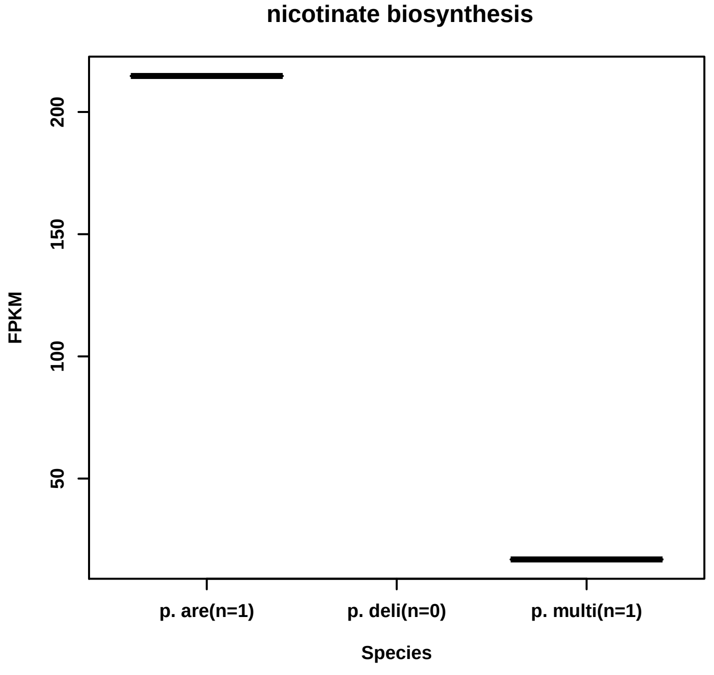
nicotinate biosynthesis
FPKM
50
100
150
200
p. are(n=1)
p. deli(n=0)
p. multi(n=1)
Species

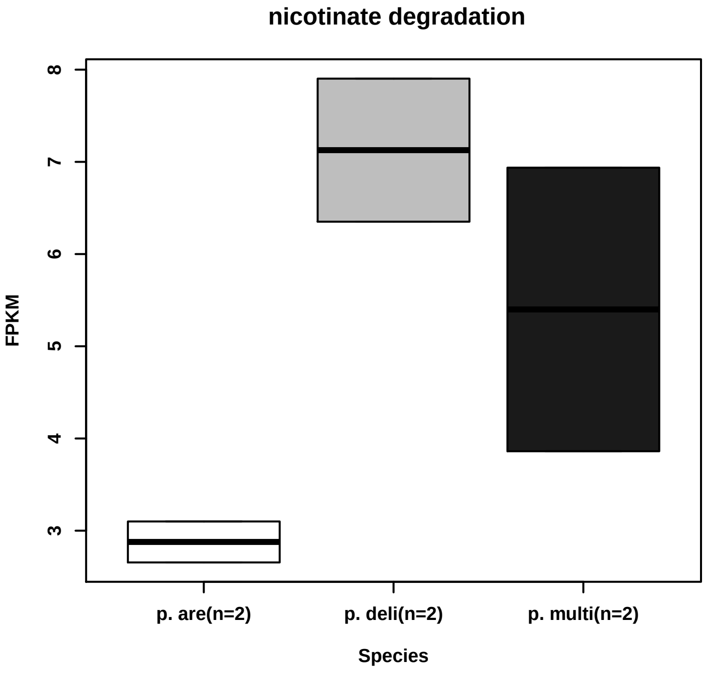
nicotinate degradation
FPKM
3
4
5
6
7
8
p. are(n=2)
p. deli(n=2)
p. multi(n=2)
Species

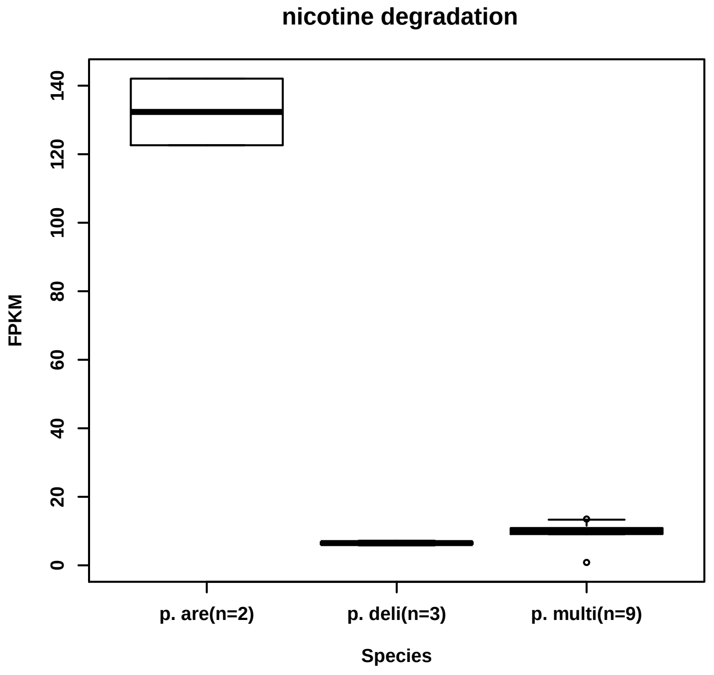
nicotine degradation
FPKM
0
20
40
60
80
100
120
140
p. are(n=2)
p. deli(n=3)
p. multi(n=9)
Species

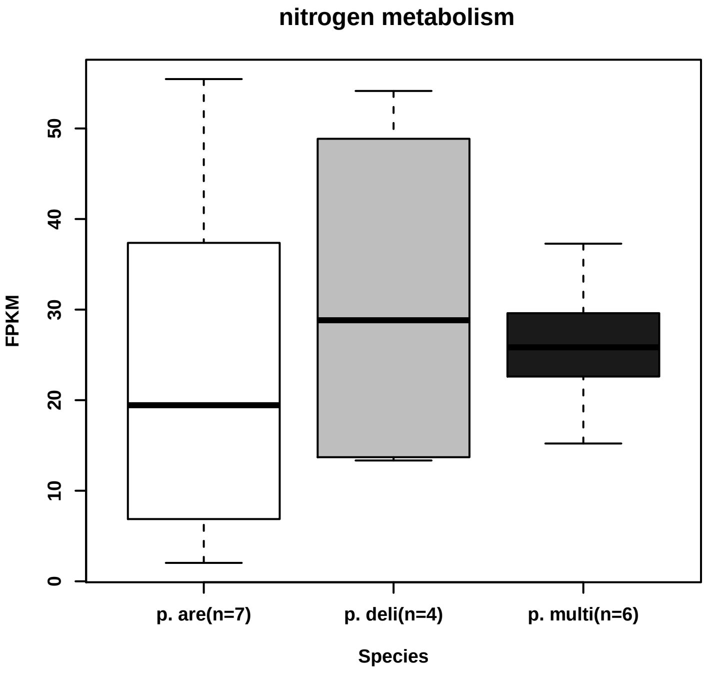
nitrogen metabolism
FPKM
0
10
20
30
40
50
p. are(n=7)
p. deli(n=4)
p. multi(n=6)
Species

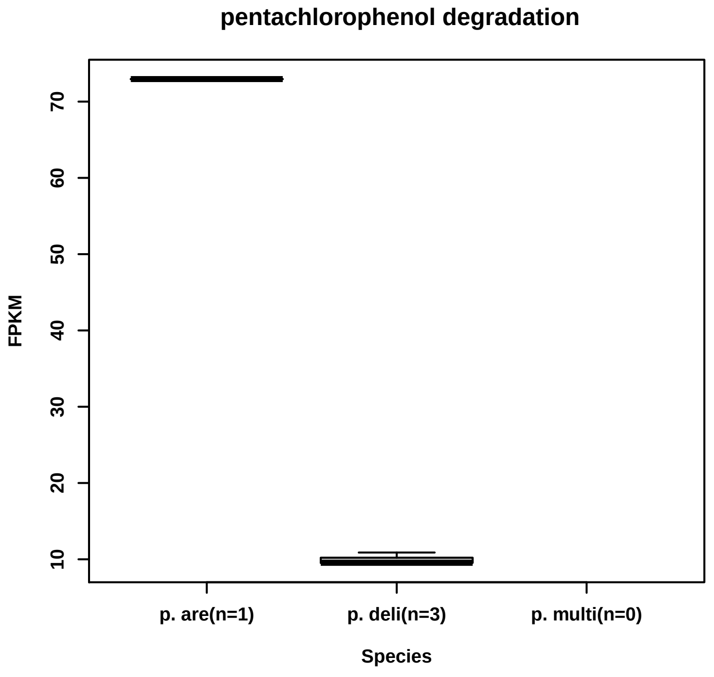
pentachlorophenol degradation
FPKM
10
20
30
40
50
60
70
p. are(n=1)
p. deli(n=3)
p. multi(n=0)
Species

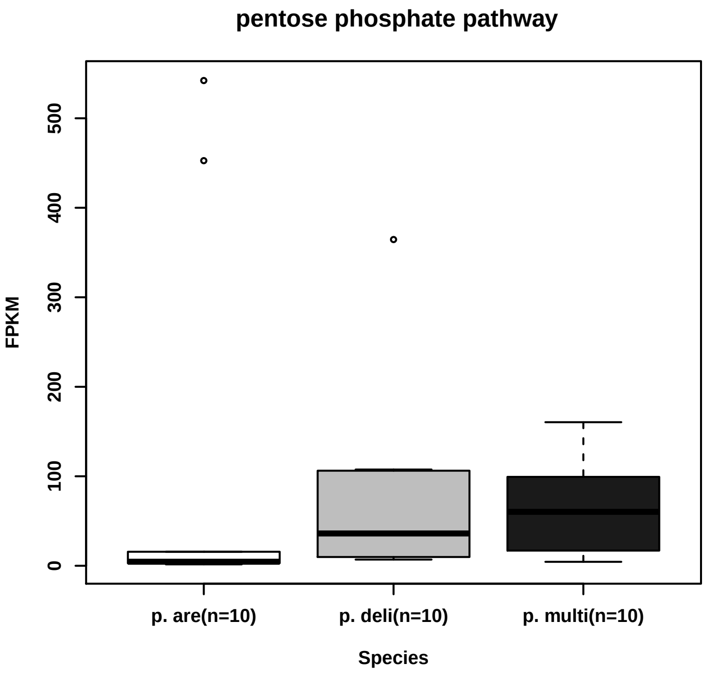
pentose phosphate pathway
FPKM
0
100
200
300
400
500
p. are(n=10)
p. deli(n=10)
p. multi(n=10)
Species

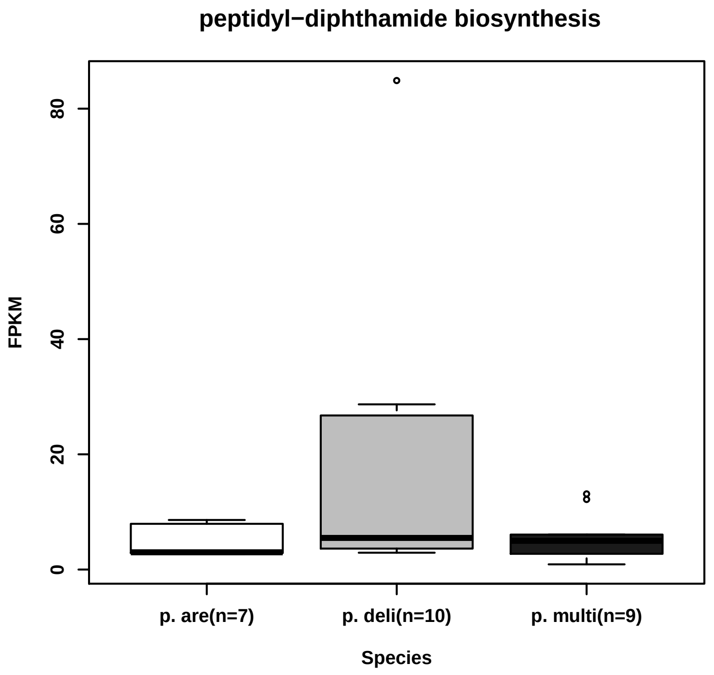
peptidyl−diphthamide biosynthesis
FPKM
0
20
40
60
80
p. are(n=7)
p. deli(n=10)
p. multi(n=9)
Species

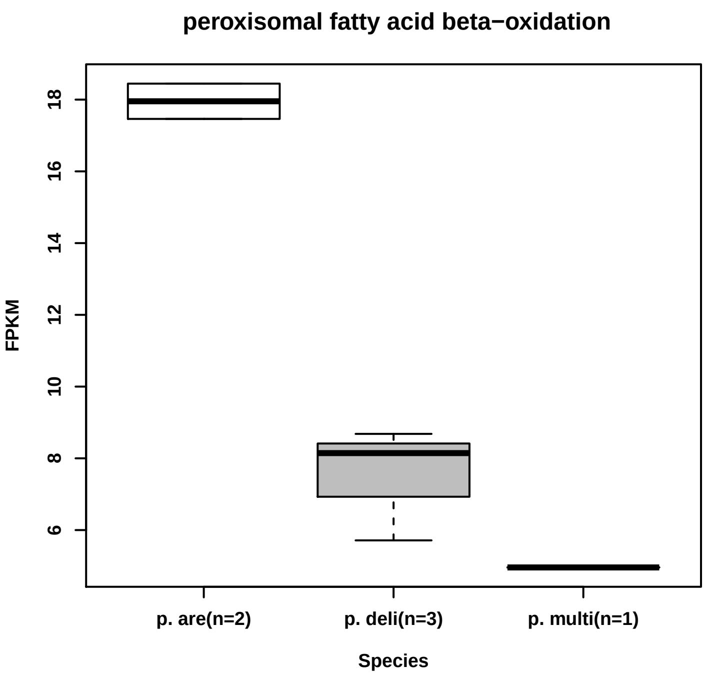
peroxisomal fatty acid beta−oxidation
FPKM
6
8
10
12
14
16
18
p. are(n=2)
p. deli(n=3)
p. multi(n=1)
Species

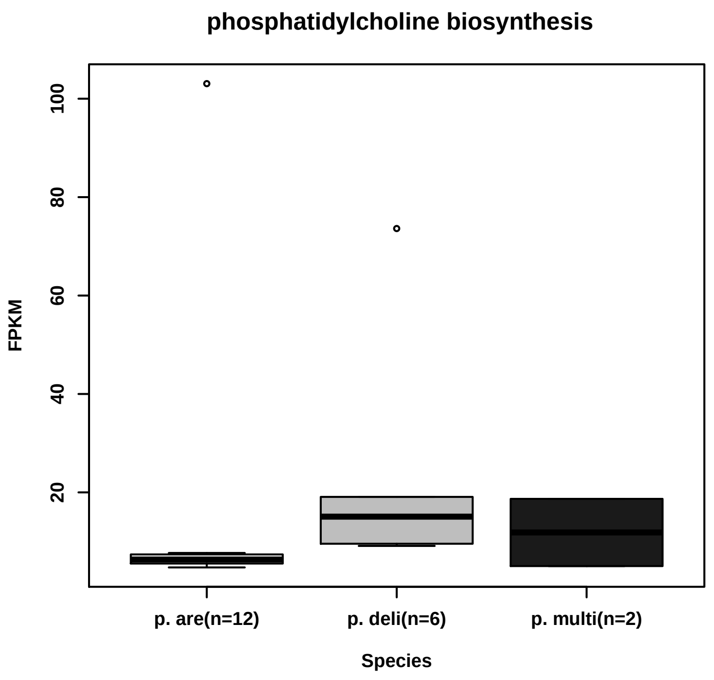
phosphatidylcholine biosynthesis
FPKM
20
40
60
80
100
p. are(n=12)
p. deli(n=6)
p. multi(n=2)
Species

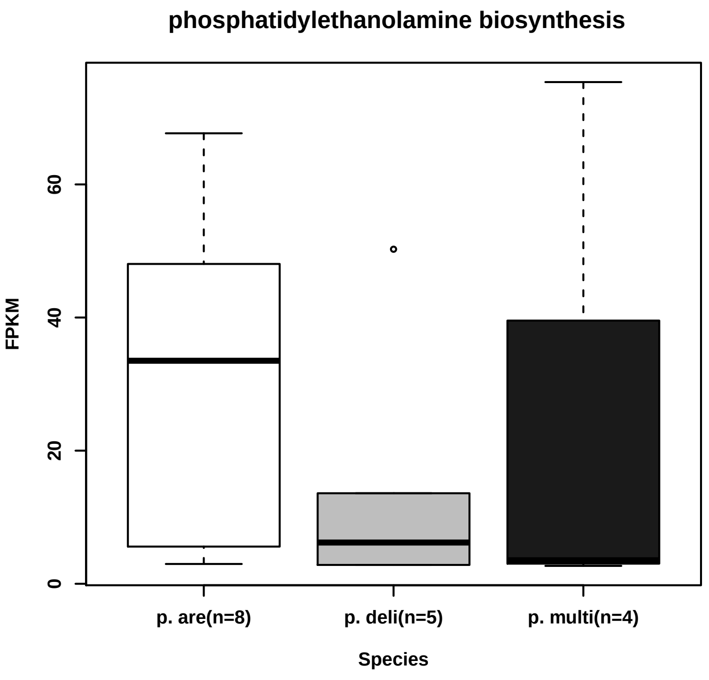
phosphatidylethanolamine biosynthesis
FPKM
0
20
40
60
p. are(n=8)
p. deli(n=5)
p. multi(n=4)
Species

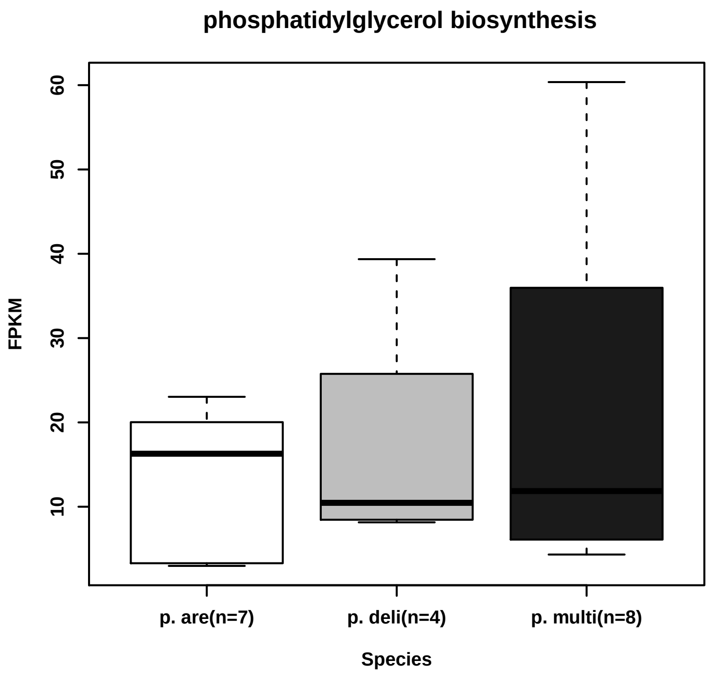
phosphatidylglycerol biosynthesis
FPKM
10
20
30
40
50
60
p. are(n=7)
p. deli(n=4)
p. multi(n=8)
Species

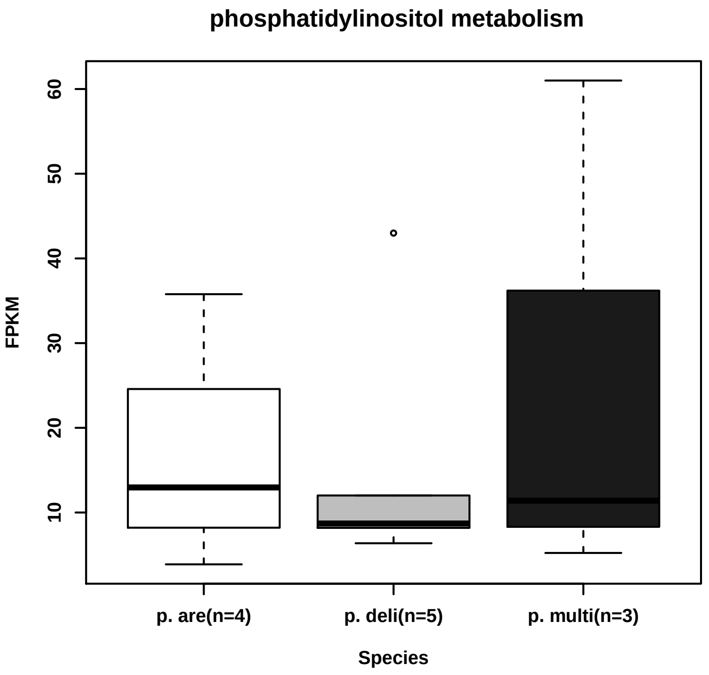
phosphatidylinositol metabolism
FPKM
10
20
30
40
50
60
p. are(n=4)
p. deli(n=5)
p. multi(n=3)
Species

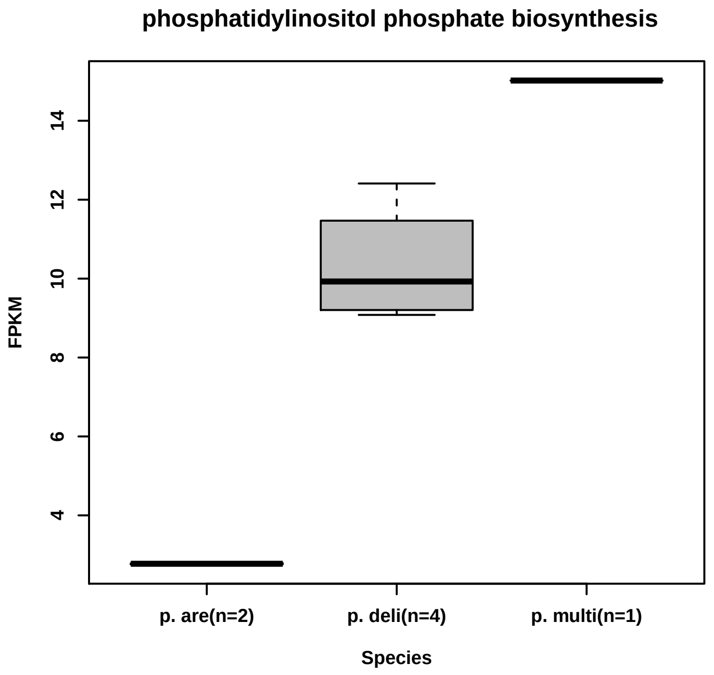
phosphatidylinositol phosphate biosynthesis
FPKM
4
6
8
10
12
14
p. are(n=2)
p. deli(n=4)
p. multi(n=1)
Species

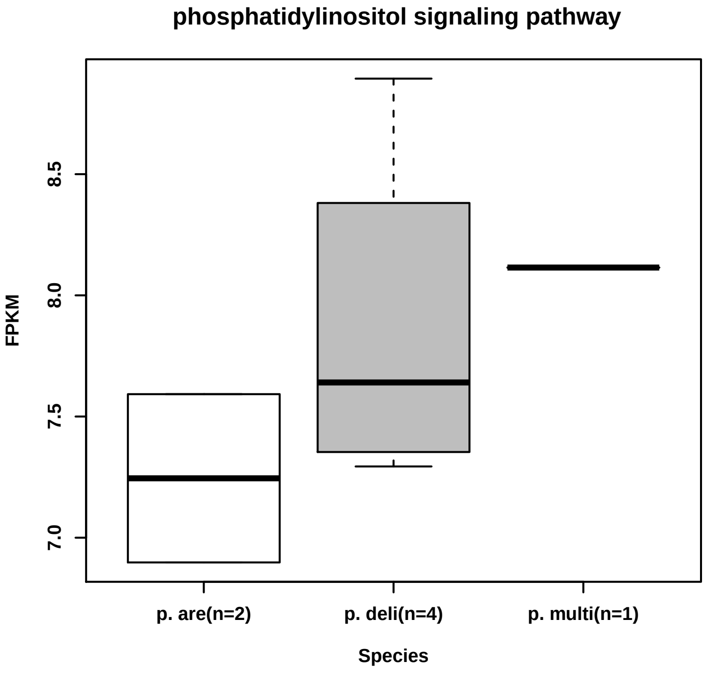
phosphatidylinositol signaling pathway
FPKM
7.0
7.5
8.0
8.5
p. are(n=2)
p. deli(n=4)
p. multi(n=1)
Species

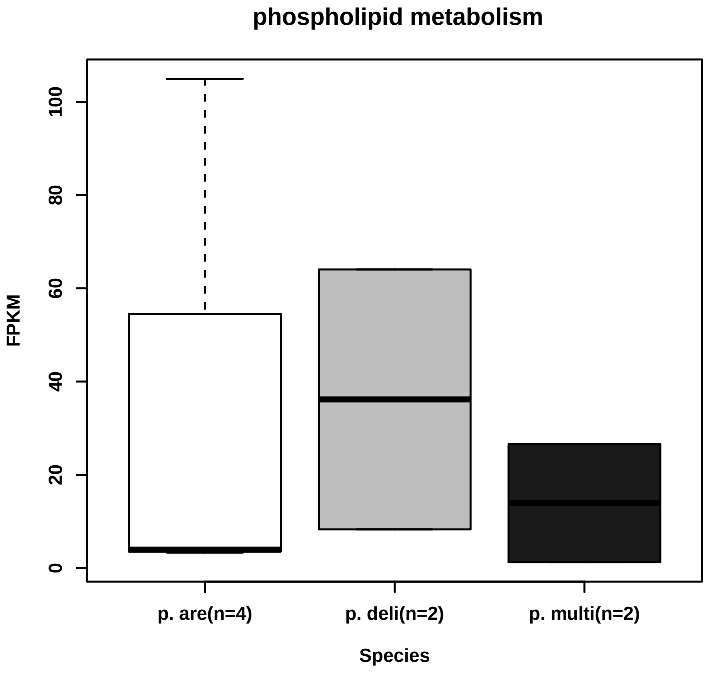
phospholipid metabolism
FPKM
0
20
40
60
80
100
p. are(n=4)
p. deli(n=2)
p. multi(n=2)
Species

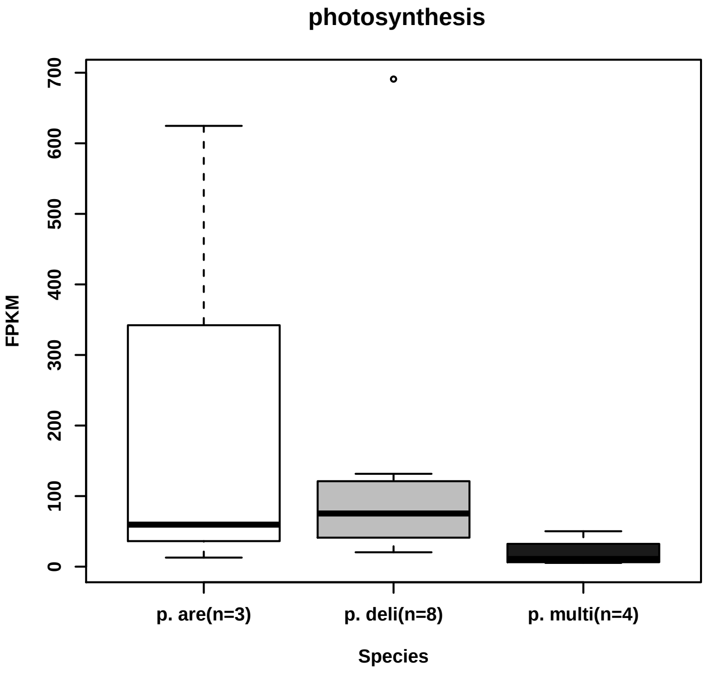
photosynthesis
FPKM
0
100
200
300
400
500
600
700
p. are(n=3)
p. deli(n=8)
p. multi(n=4)
Species

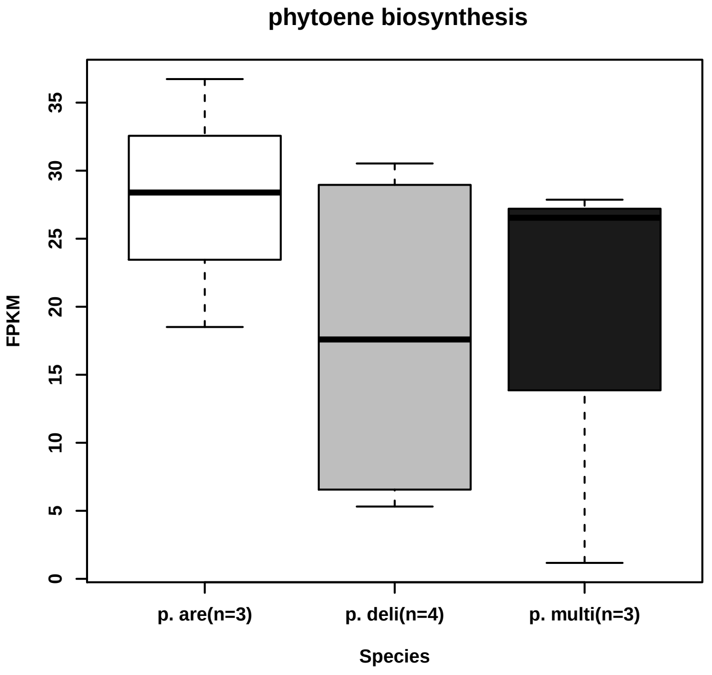
phytoene biosynthesis
FPKM
0
5
10
15
20
25
30
35
p. are(n=3)
p. deli(n=4)
p. multi(n=3)
Species

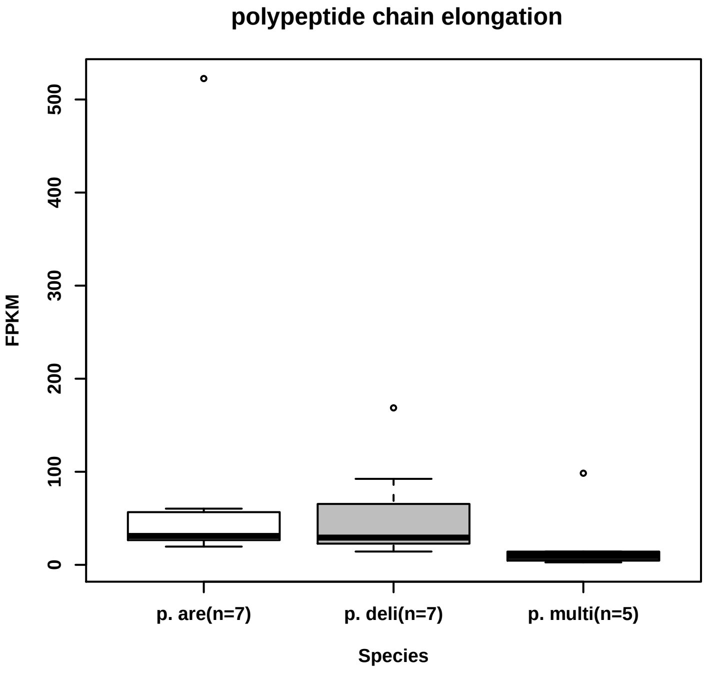
polypeptide chain elongation
FPKM
0
100
200
300
400
500
p. are(n=7)
p. deli(n=7)
p. multi(n=5)
Species

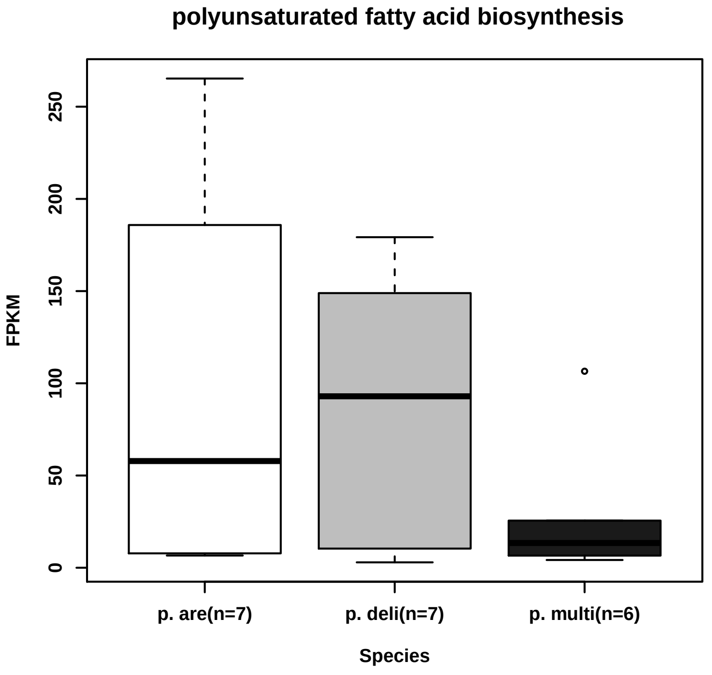
polyunsaturated fatty acid biosynthesis
FPKM
0
50
100
150
200
250
p. are(n=7)
p. deli(n=7)
p. multi(n=6)
Species

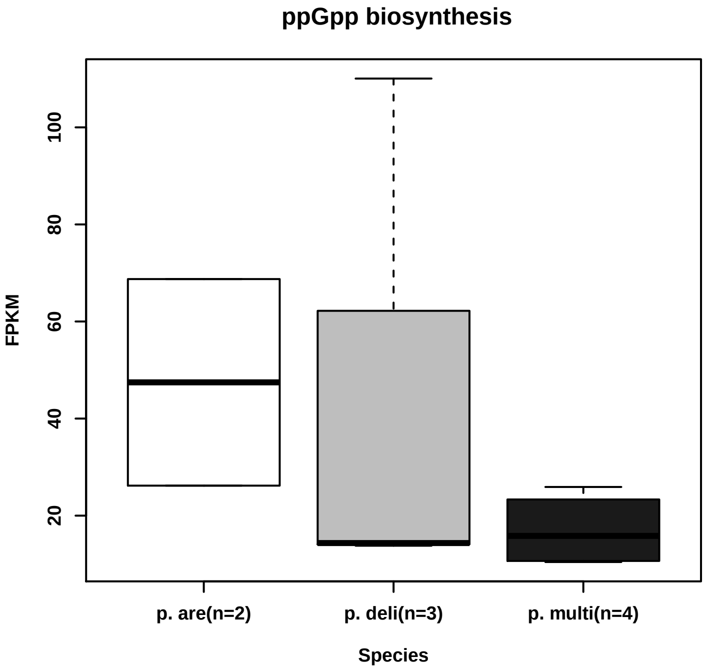
ppGpp biosynthesis
FPKM
20
40
60
80
100
p. are(n=2)
p. deli(n=3)
p. multi(n=4)
Species

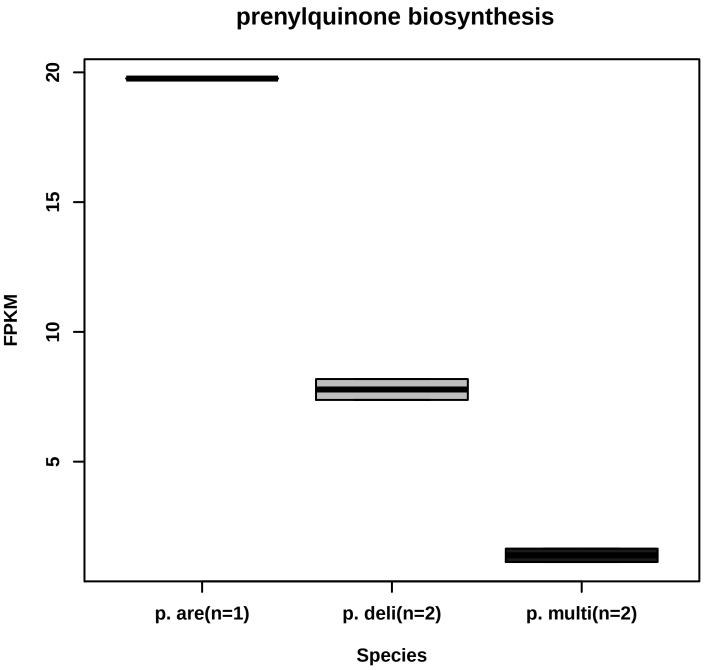
prenylquinone biosynthesis
FPKM
5
10
15
20
p. are(n=1)
p. deli(n=2)
p. multi(n=2)
Species

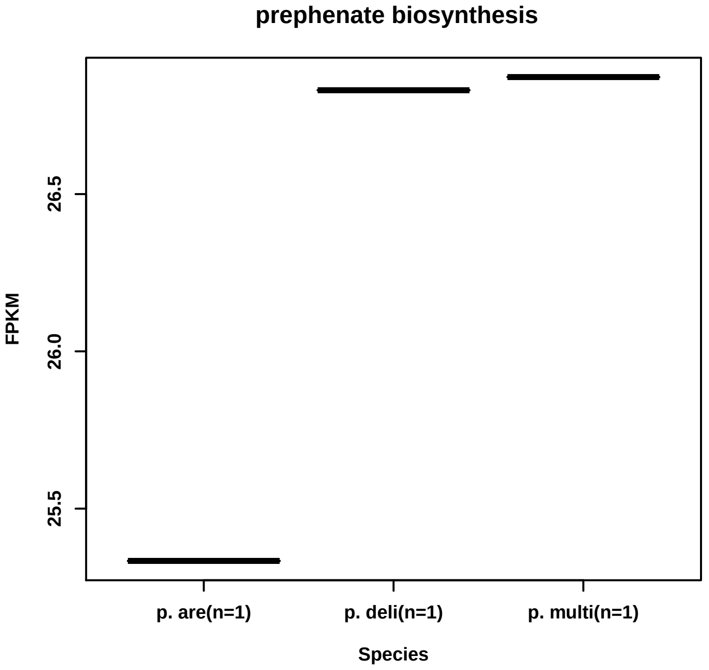
prephenate biosynthesis
FPKM
25.5
26.0
26.5
p. are(n=1)
p. deli(n=1)
p. multi(n=1)
Species

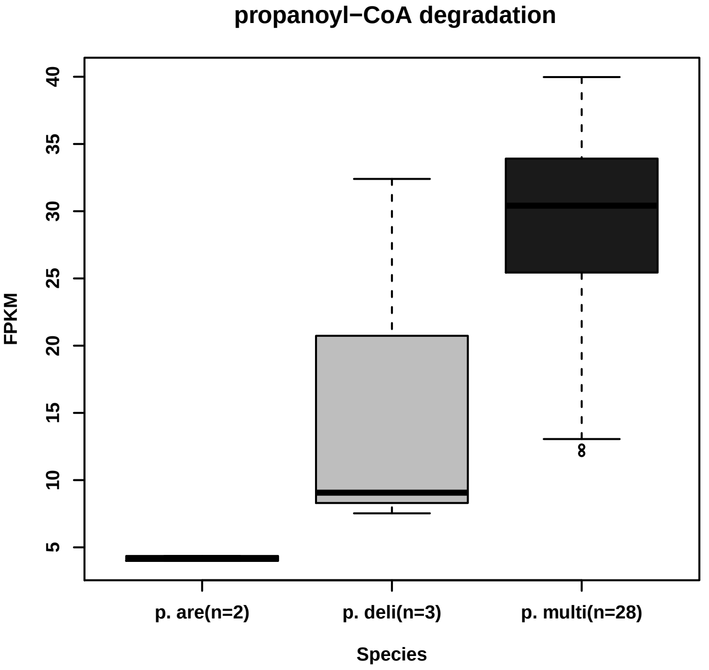
propanoyl−CoA degradation
FPKM
5
10
15
20
25
30
35
40
p. are(n=2)
p. deli(n=3)
p. multi(n=28)
Species

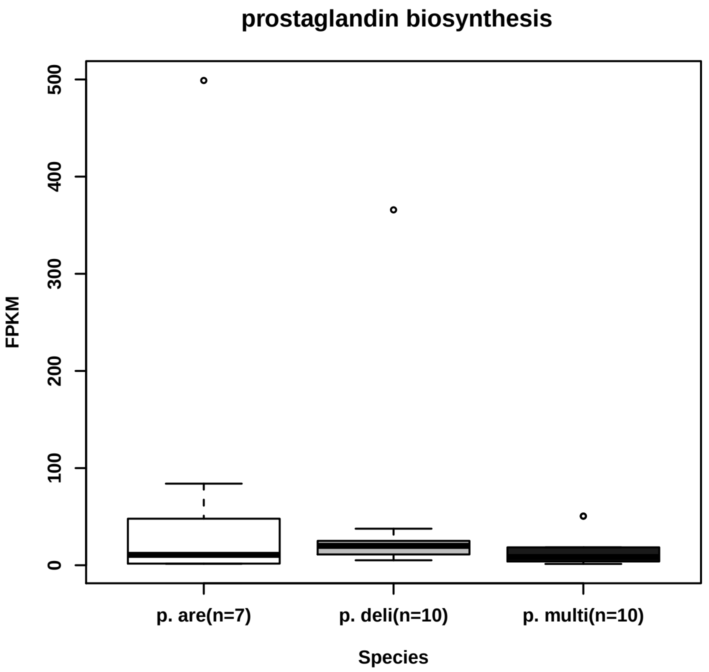
prostaglandin biosynthesis
FPKM
0
100
200
300
400
500
p. are(n=7)
p. deli(n=10)
p. multi(n=10)
Species

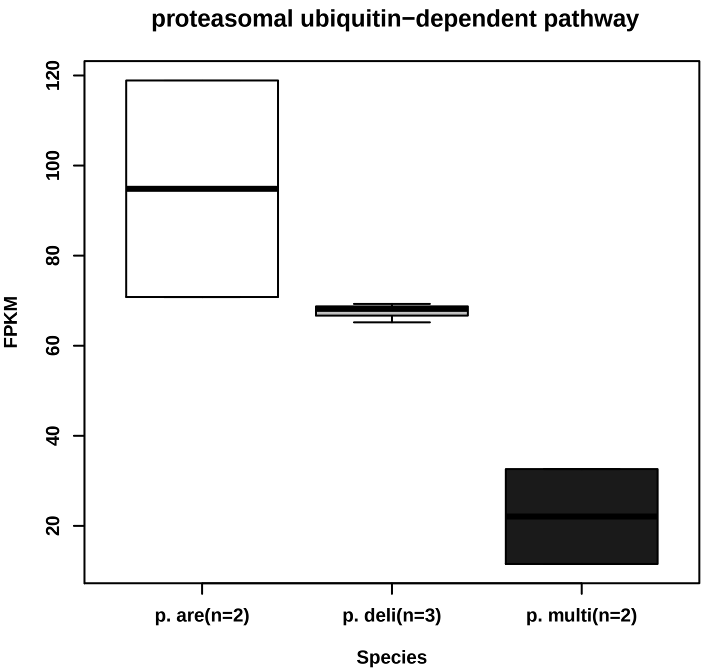
proteasomal ubiquitin−dependent pathway
FPKM
20
40
60
80
100
120
p. are(n=2)
p. deli(n=3)
p. multi(n=2)
Species

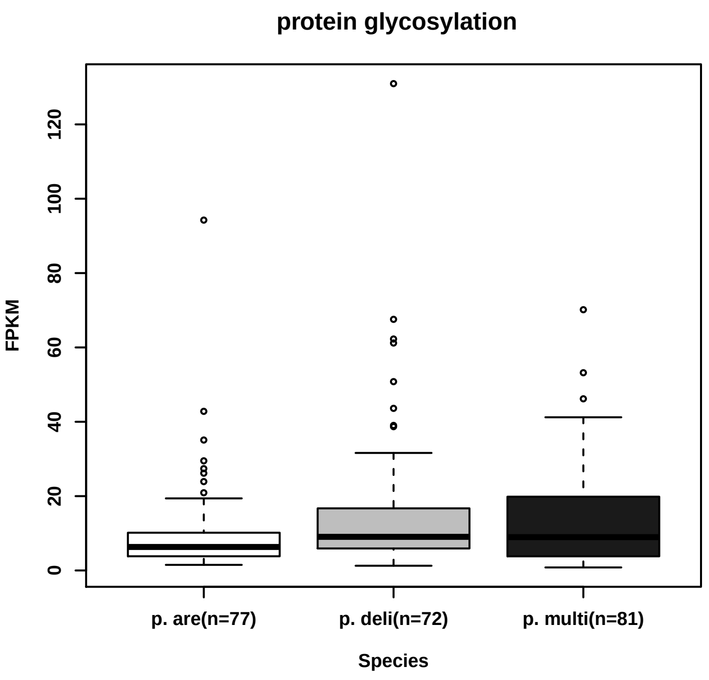
protein glycosylation
FPKM
0
20
40
60
80
100
120
p. are(n=77)
p. deli(n=72)
p. multi(n=81)
Species

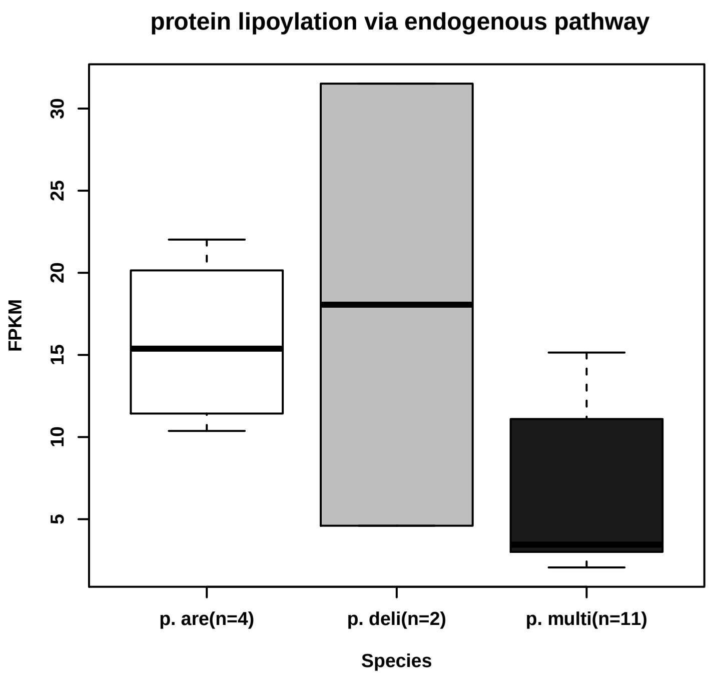
protein lipoylation via endogenous pathway
FPKM
5
10
15
20
25
30
p. are(n=4)
p. deli(n=2)
p. multi(n=11)
Species

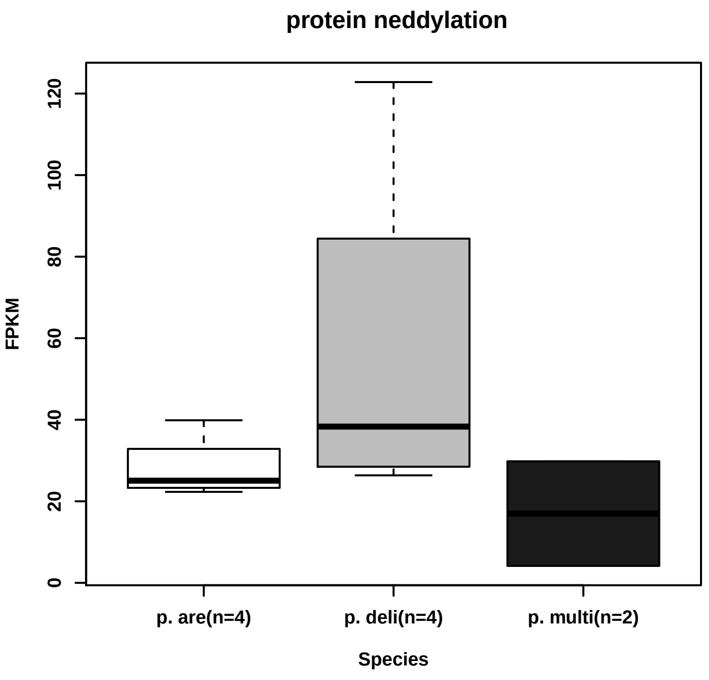
protein neddylation
FPKM
0
20
40
60
80
100
120
p. are(n=4)
p. deli(n=4)
p. multi(n=2)
Species

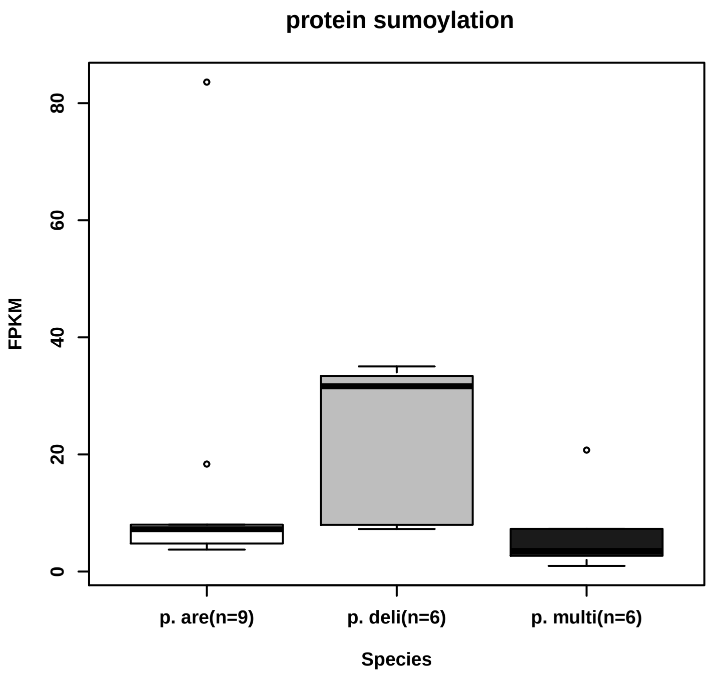
protein sumoylation
FPKM
0
20
40
60
80
p. are(n=9)
p. deli(n=6)
p. multi(n=6)
Species

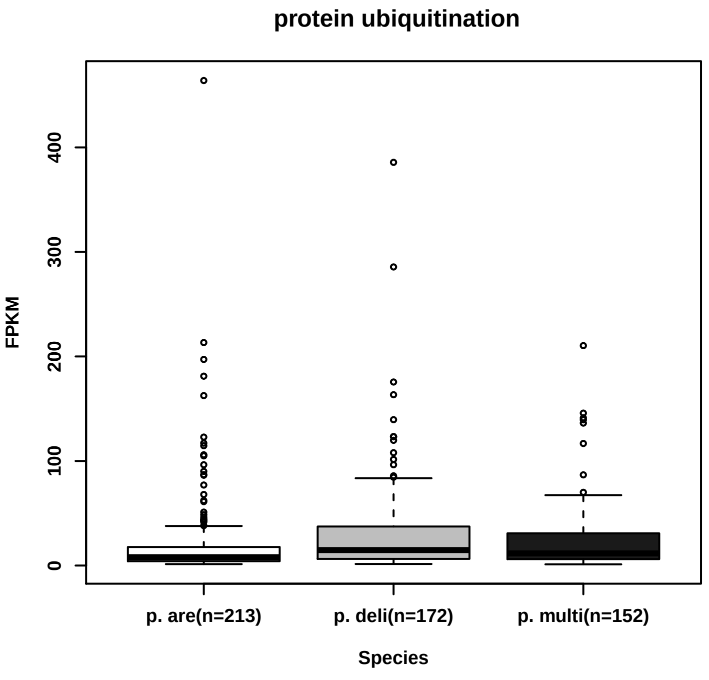
protein ubiquitination
FPKM
0
100
200
300
400
p. are(n=213)
p. deli(n=172)
p. multi(n=152)
Species

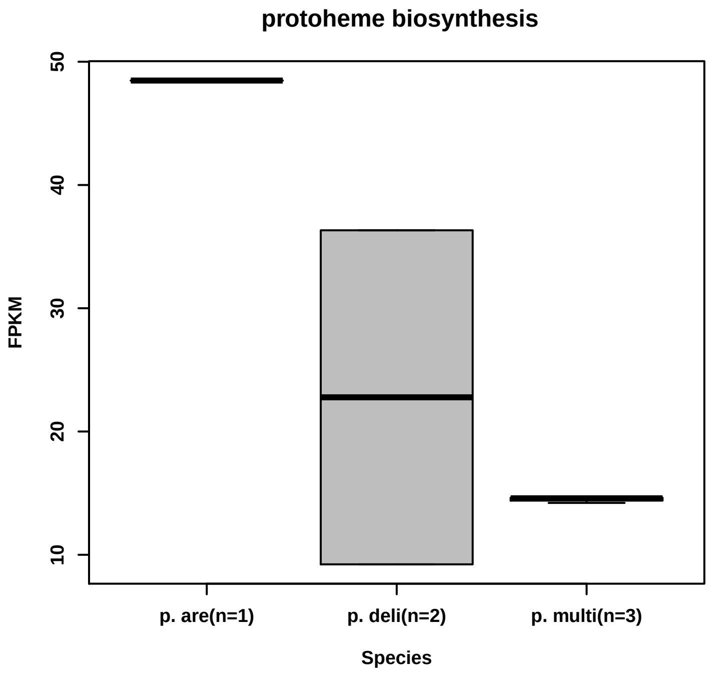
protoheme biosynthesis
FPKM
10
20
30
40
50
p. are(n=1)
p. deli(n=2)
p. multi(n=3)
Species

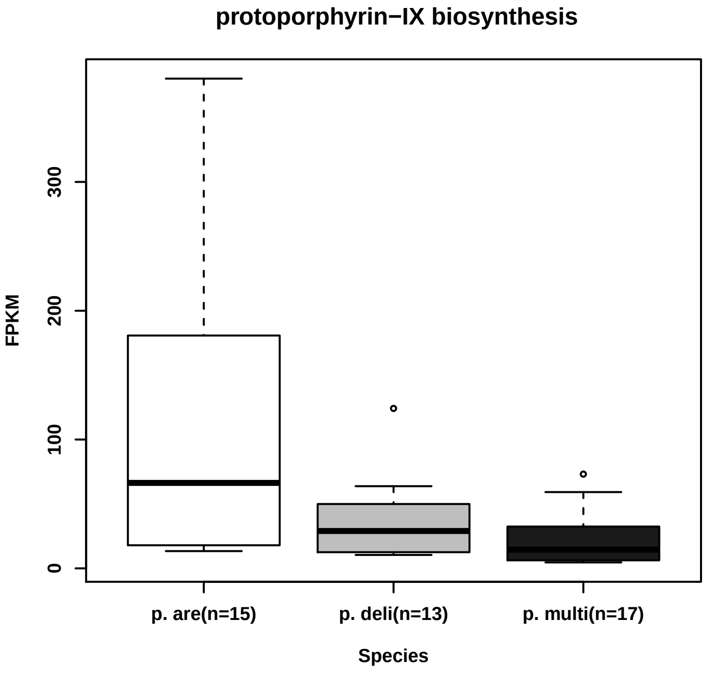
protoporphyrin−IX biosynthesis
FPKM
0
100
200
300
p. are(n=15)
p. deli(n=13)
p. multi(n=17)
Species

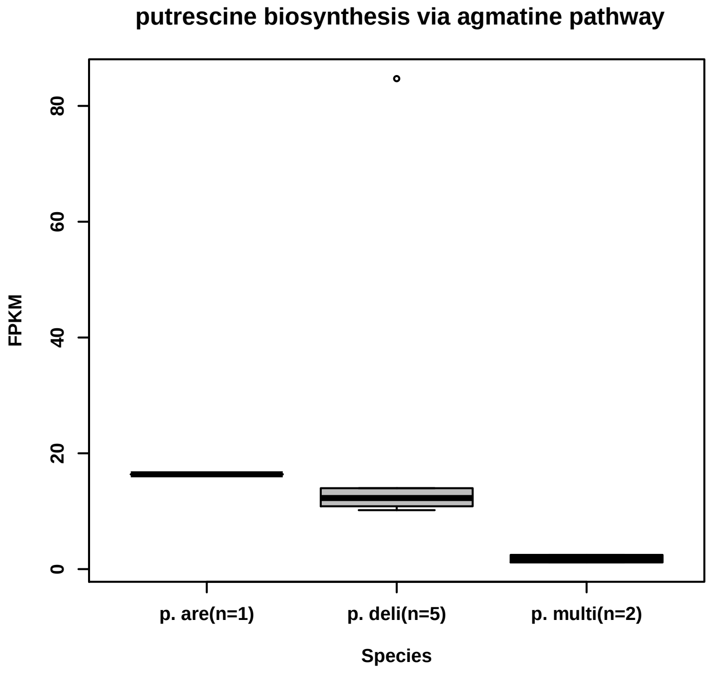
putrescine biosynthesis via agmatine pathway
FPKM
0
20
40
60
80
p. are(n=1)
p. deli(n=5)
p. multi(n=2)
Species

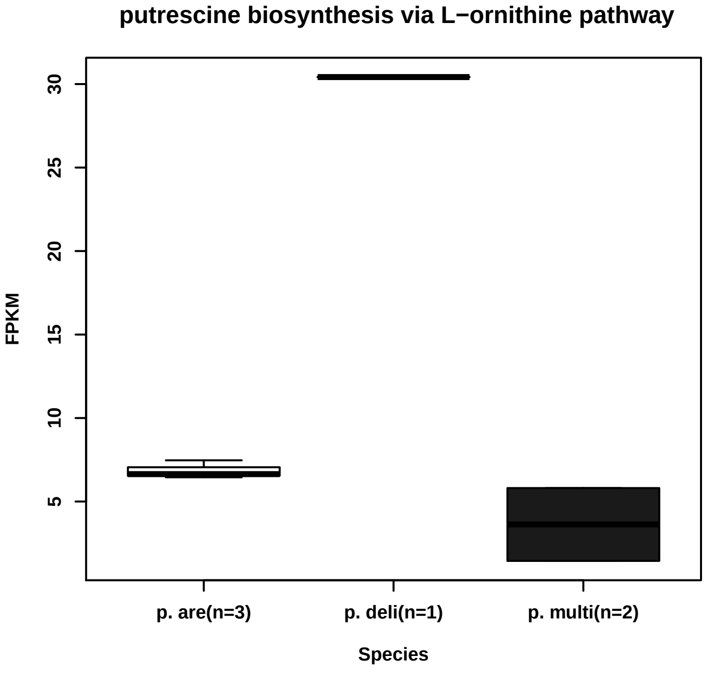
putrescine biosynthesis via L−ornithine pathway
FPKM
5
10
15
20
25
30
p. are(n=3)
p. deli(n=1)
p. multi(n=2)
Species

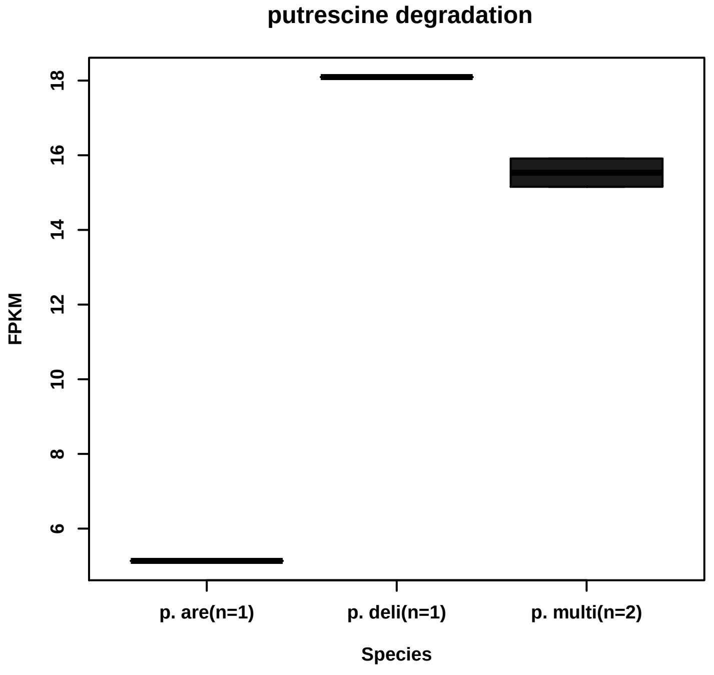
putrescine degradation
FPKM
6
8
10
12
14
16
18
p. are(n=1)
p. deli(n=1)
p. multi(n=2)
Species

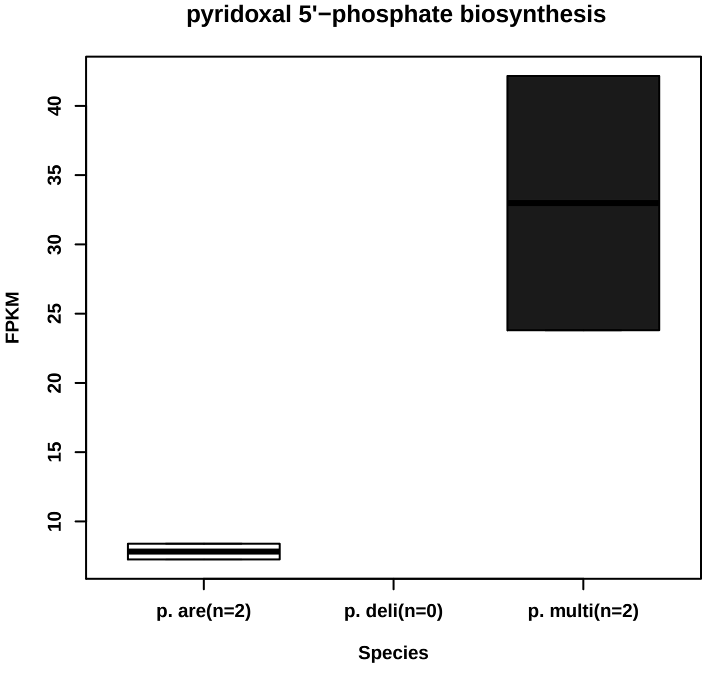
pyridoxal 5'−phosphate biosynthesis
FPKM
10
15
20
25
30
35
40
p. are(n=2)
p. deli(n=0)
p. multi(n=2)
Species

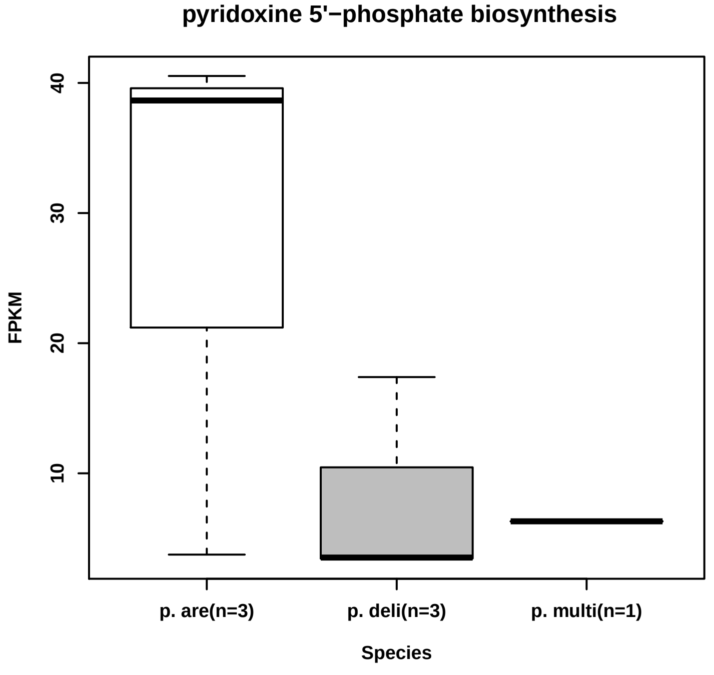
pyridoxine 5'−phosphate biosynthesis
FPKM
10
20
30
40
p. are(n=3)
p. deli(n=3)
p. multi(n=1)
Species

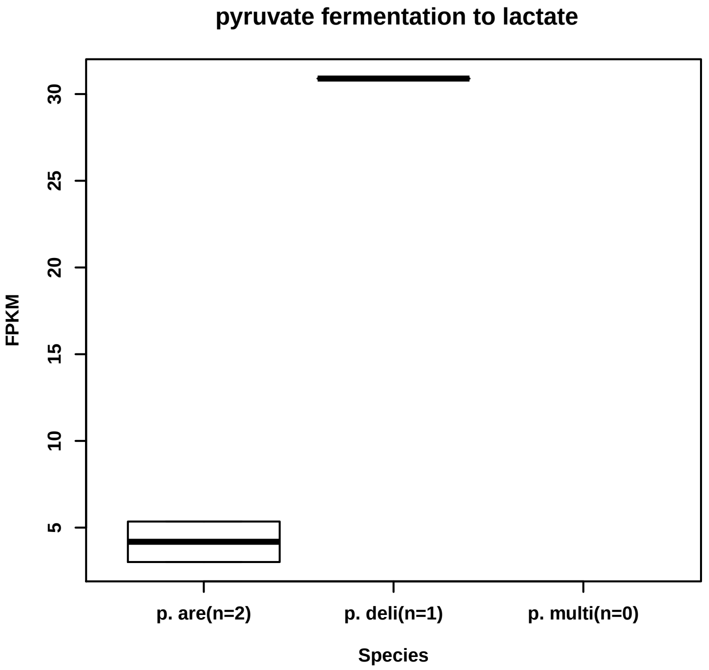
pyruvate fermentation to lactate
FPKM
5
10
15
20
25
30
p. are(n=2)
p. deli(n=1)
p. multi(n=0)
Species

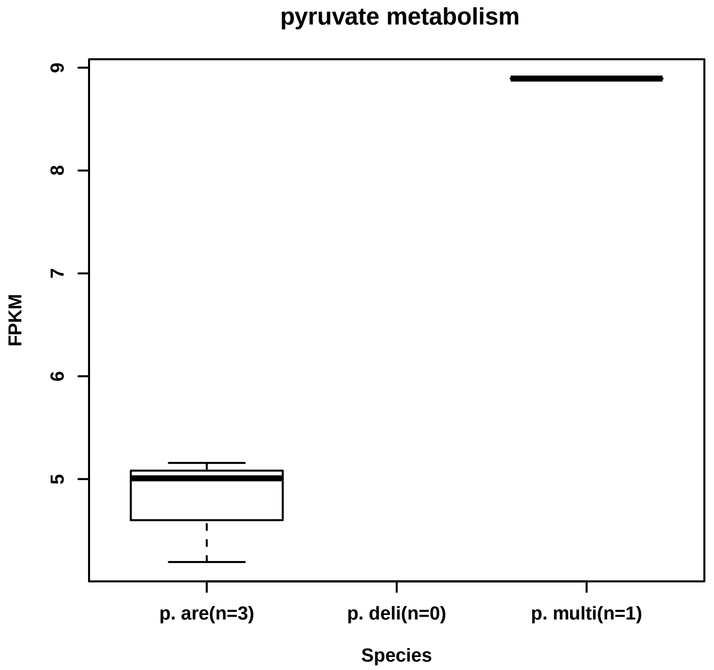
pyruvate metabolism
FPKM
5
6
7
8
9
p. are(n=3)
p. deli(n=0)
p. multi(n=1)
Species

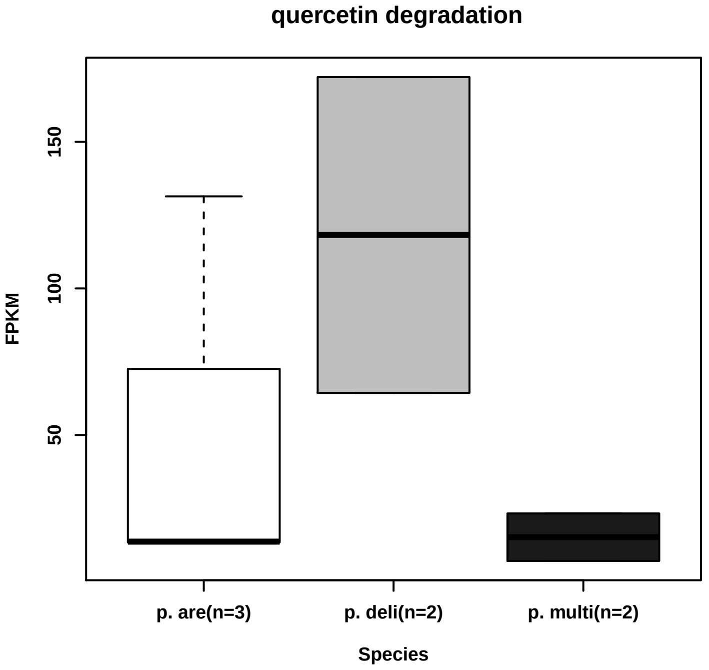
quercetin degradation
FPKM
50
100
150
p. are(n=3)
p. deli(n=2)
p. multi(n=2)
Species

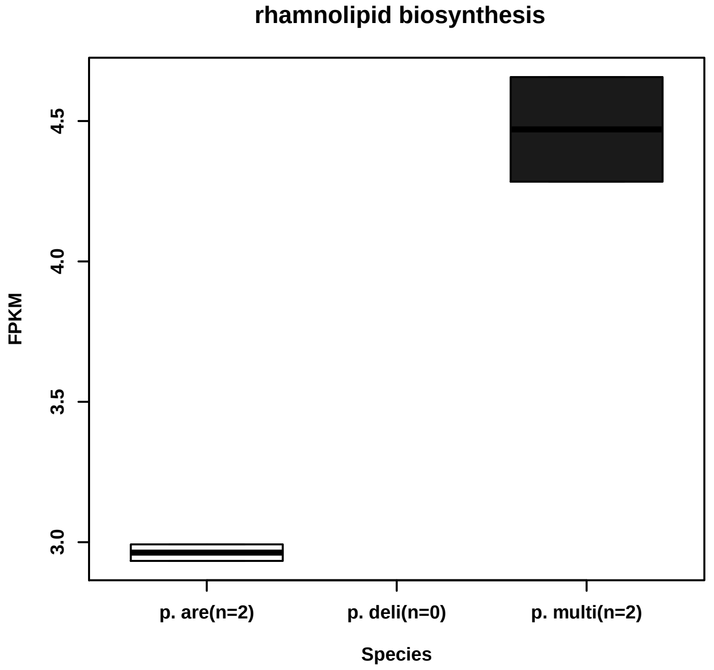
rhamnolipid biosynthesis
FPKM
3.0
3.5
4.0
4.5
p. are(n=2)
p. deli(n=0)
p. multi(n=2)
Species

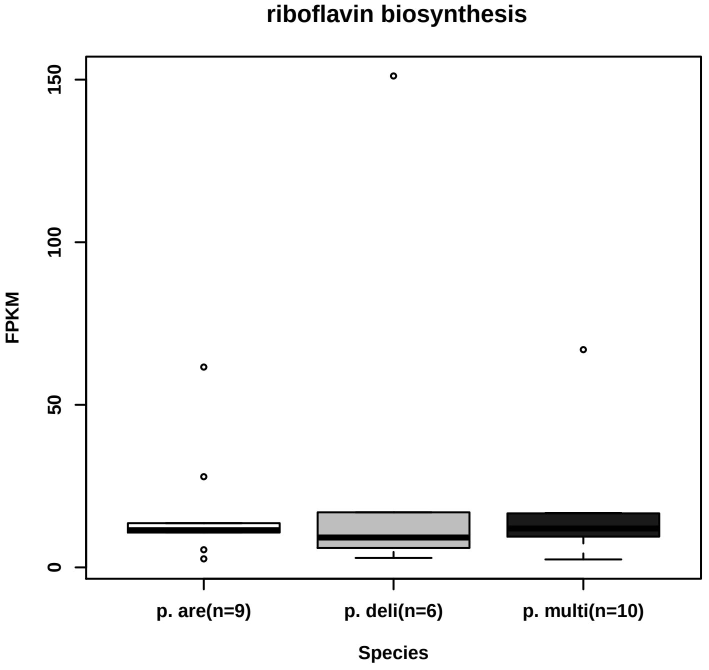
riboflavin biosynthesis
FPKM
0
50
100
150
p. are(n=9)
p. deli(n=6)
p. multi(n=10)
Species

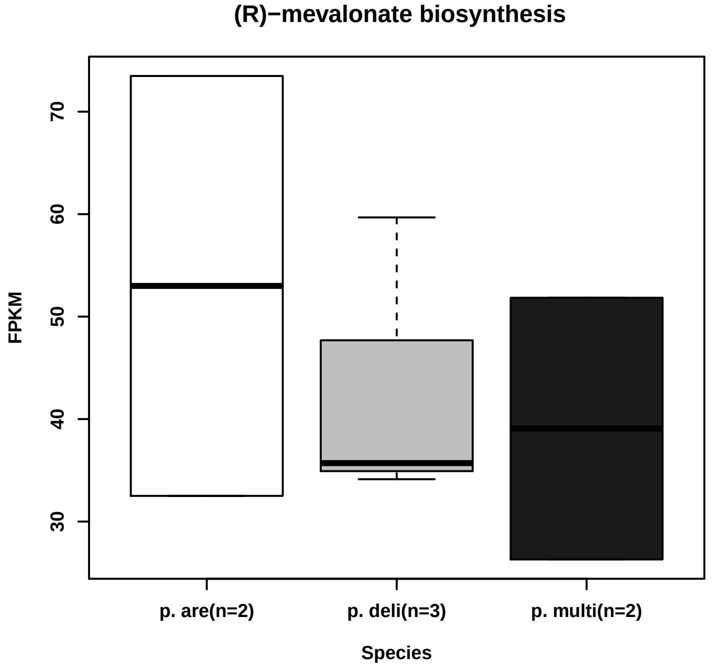
(R)−mevalonate biosynthesis
FPKM
30
40
50
60
70
p. are(n=2)
p. deli(n=3)
p. multi(n=2)
Species

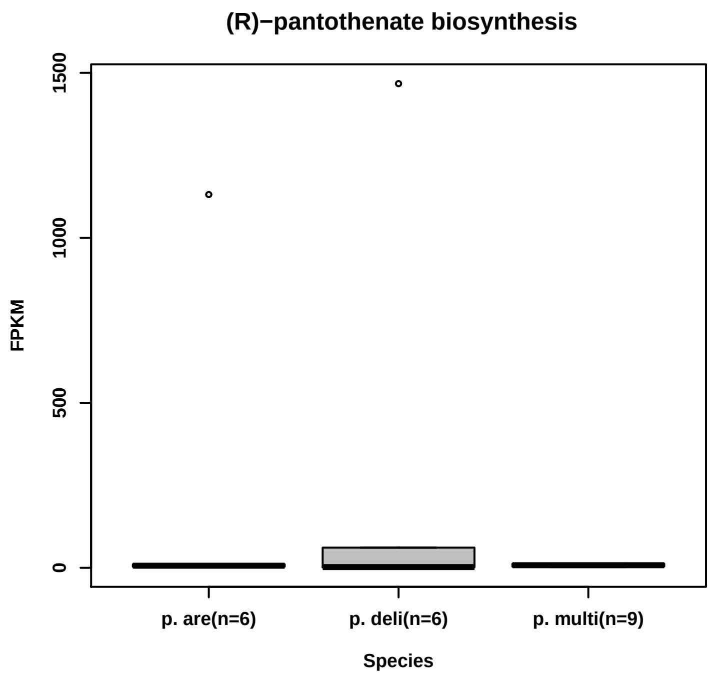
(R)−pantothenate biosynthesis
FPKM
0
500
1000
1500
p. are(n=6)
p. deli(n=6)
p. multi(n=9)
Species

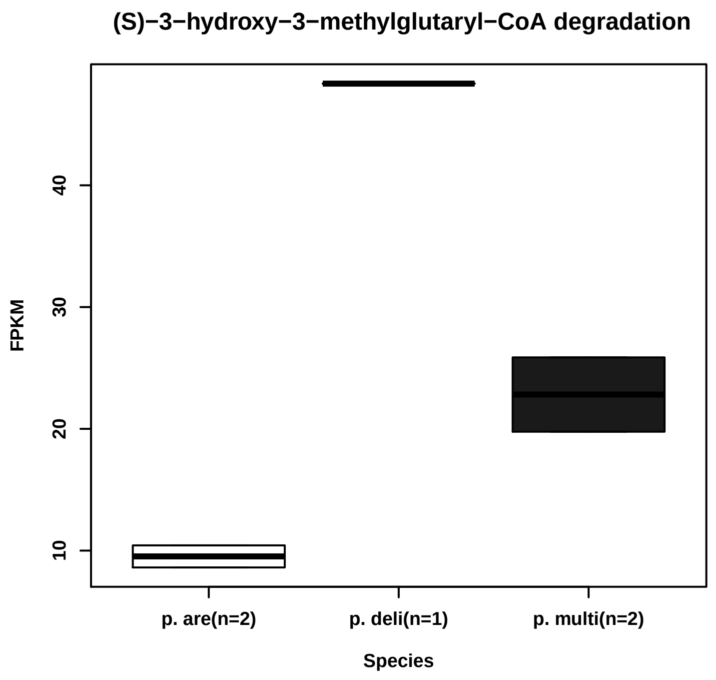
(S)−3−hydroxy−3−methylglutaryl−CoA degradation
FPKM
10
20
30
40
p. are(n=2)
p. deli(n=1)
p. multi(n=2)
Species

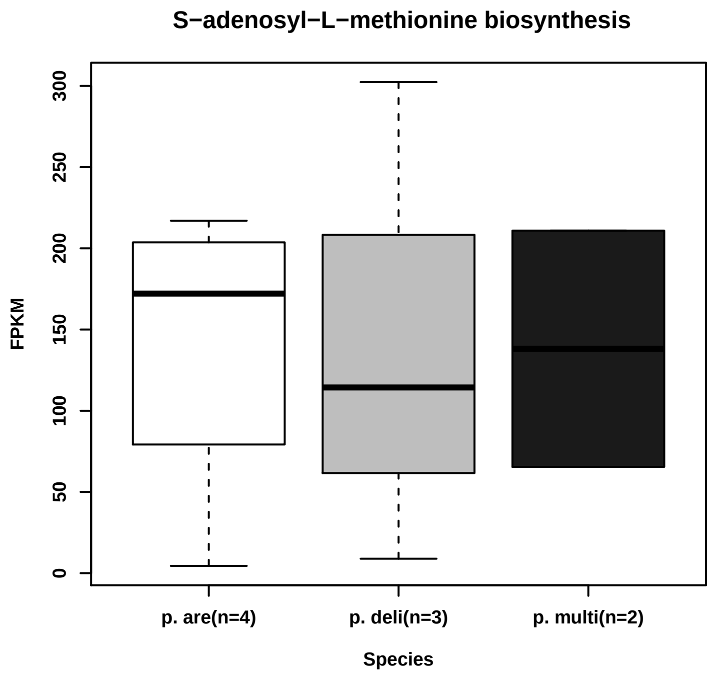
S−adenosyl−L−methionine biosynthesis
FPKM
0
50
100
150
200
250
300
p. are(n=4)
p. deli(n=3)
p. multi(n=2)
Species

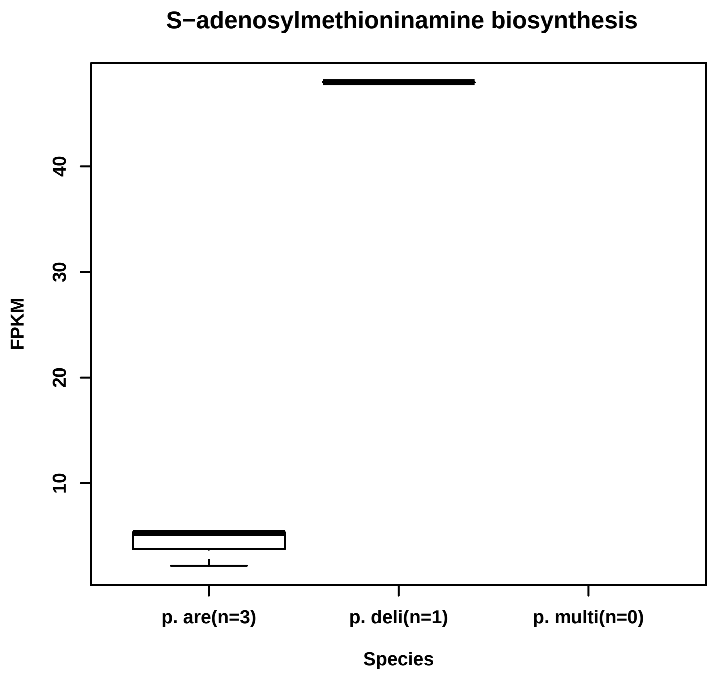
S−adenosylmethioninamine biosynthesis
FPKM
10
20
30
40
p. are(n=3)
p. deli(n=1)
p. multi(n=0)
Species

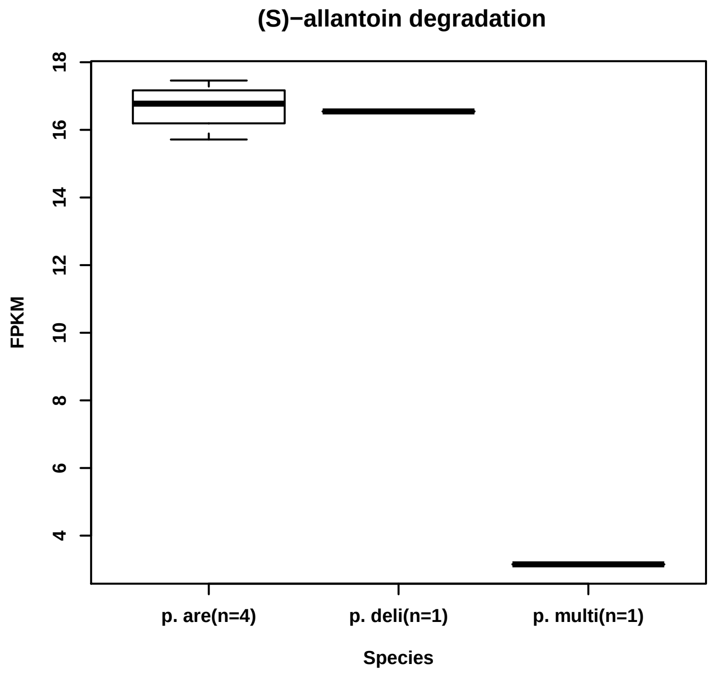
(S)−allantoin degradation
FPKM
4
6
8
10
12
14
16
18
p. are(n=4)
p. deli(n=1)
p. multi(n=1)
Species

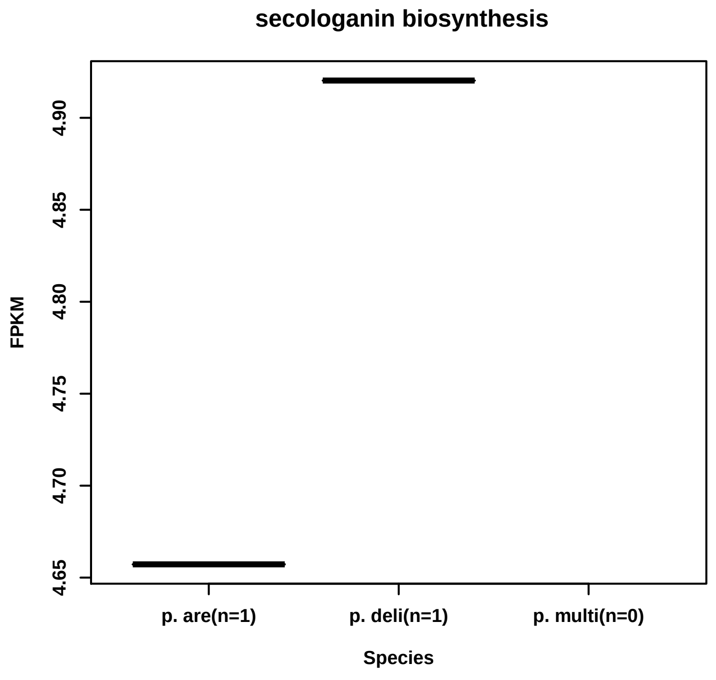
secologanin biosynthesis
FPKM
4.65
4.70
4.75
4.80
4.85
4.90
p. are(n=1)
p. deli(n=1)
p. multi(n=0)
Species

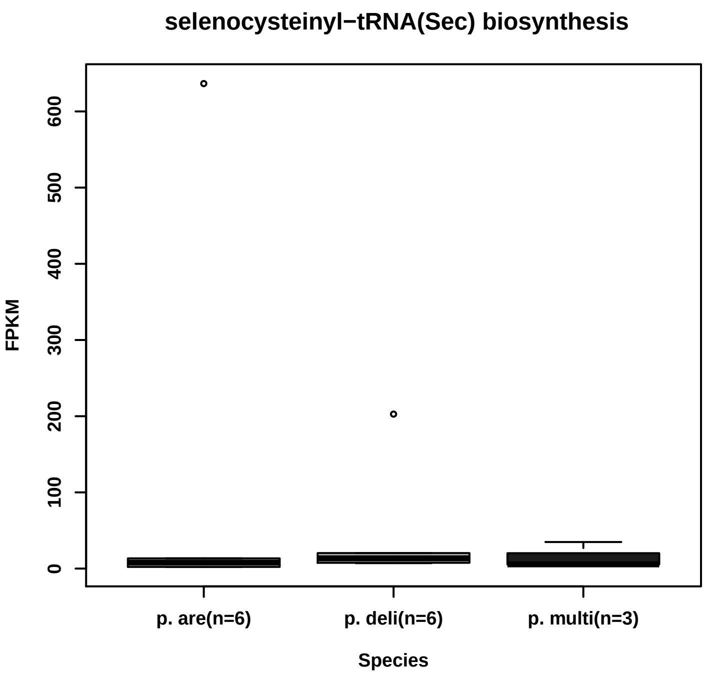
selenocysteinyl−tRNA(Sec) biosynthesis
FPKM
0
100
200
300
400
500
600
p. are(n=6)
p. deli(n=6)
p. multi(n=3)
Species

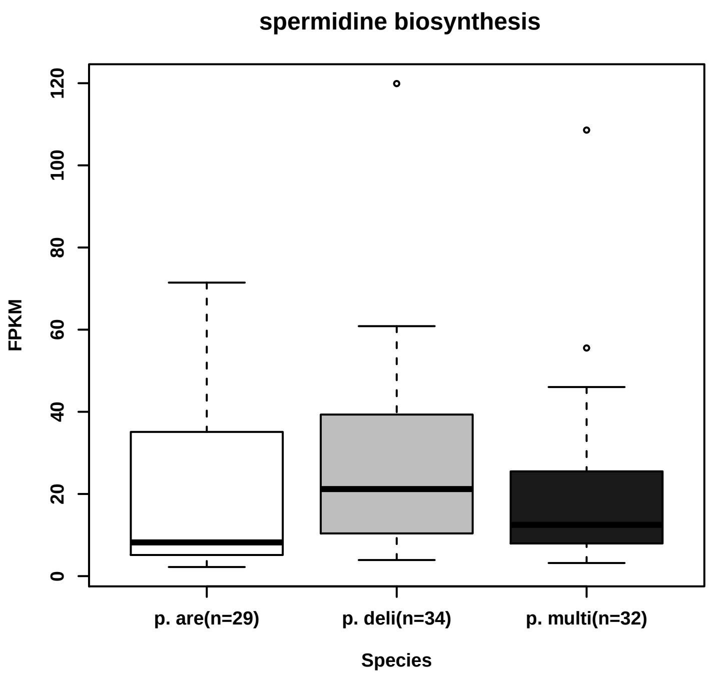
spermidine biosynthesis
FPKM
0
20
40
60
80
100
120
p. are(n=29)
p. deli(n=34)
p. multi(n=32)
Species

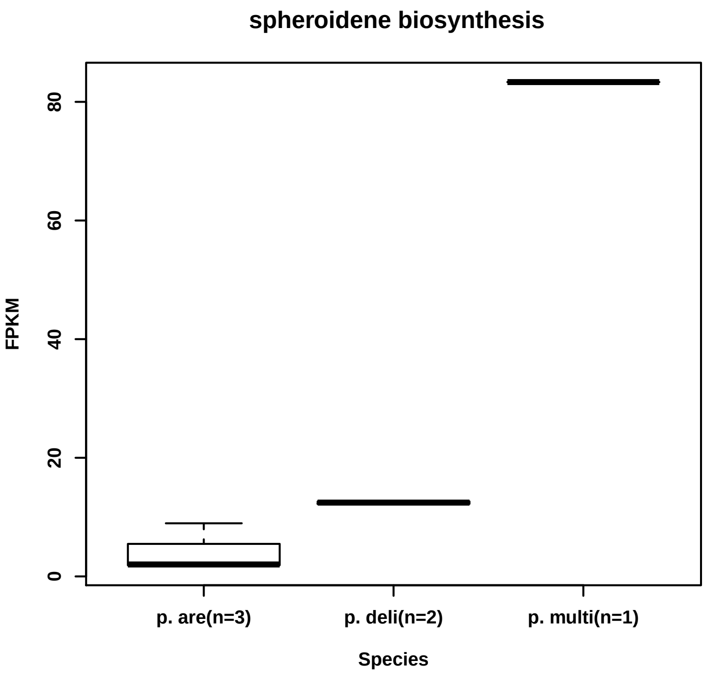
spheroidene biosynthesis
FPKM
0
20
40
60
80
p. are(n=3)
p. deli(n=2)
p. multi(n=1)
Species

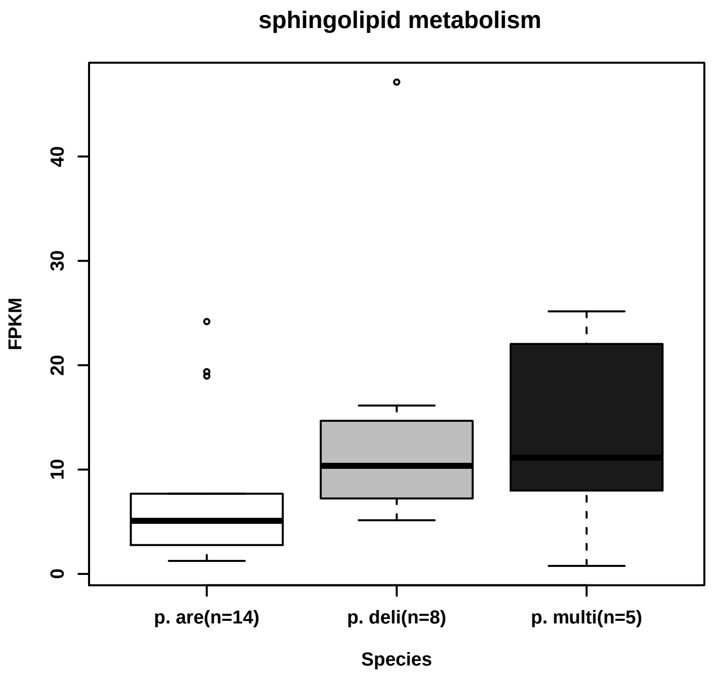
sphingolipid metabolism
FPKM
0
10
20
30
40
p. are(n=14)
p. deli(n=8)
p. multi(n=5)
Species

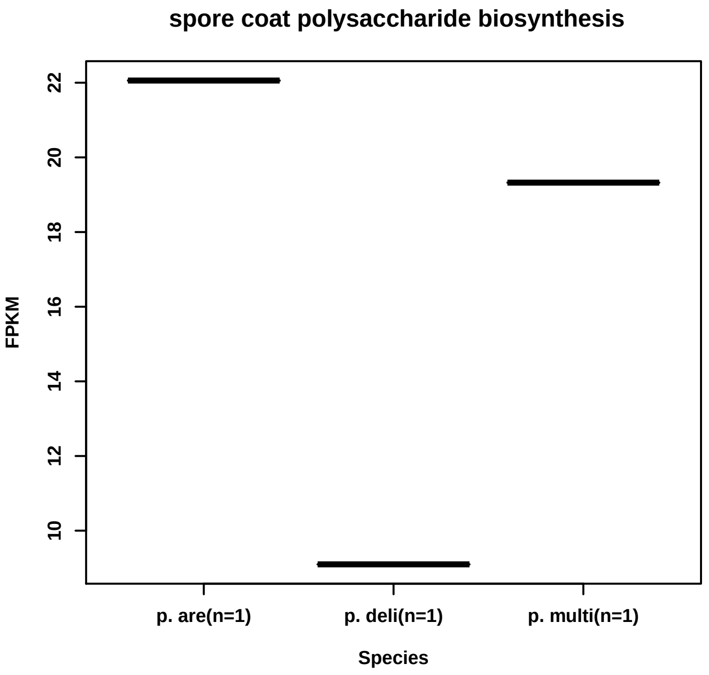
spore coat polysaccharide biosynthesis
FPKM
10
12
14
16
18
20
22
p. are(n=1)
p. deli(n=1)
p. multi(n=1)
Species

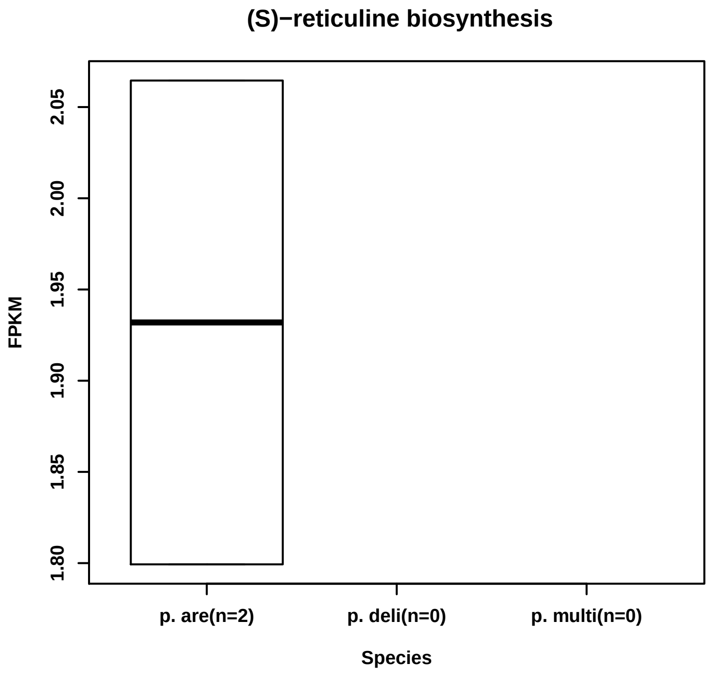
(S)−reticuline biosynthesis
FPKM
1.80
1.85
1.90
1.95
2.00
2.05
p. are(n=2)
p. deli(n=0)
p. multi(n=0)
Species

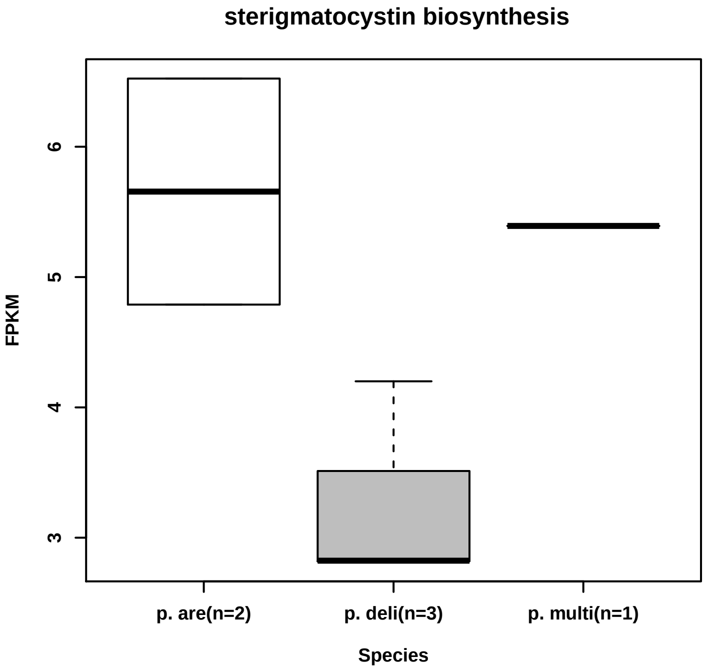
sterigmatocystin biosynthesis
FPKM
3
4
5
6
p. are(n=2)
p. deli(n=3)
p. multi(n=1)
Species

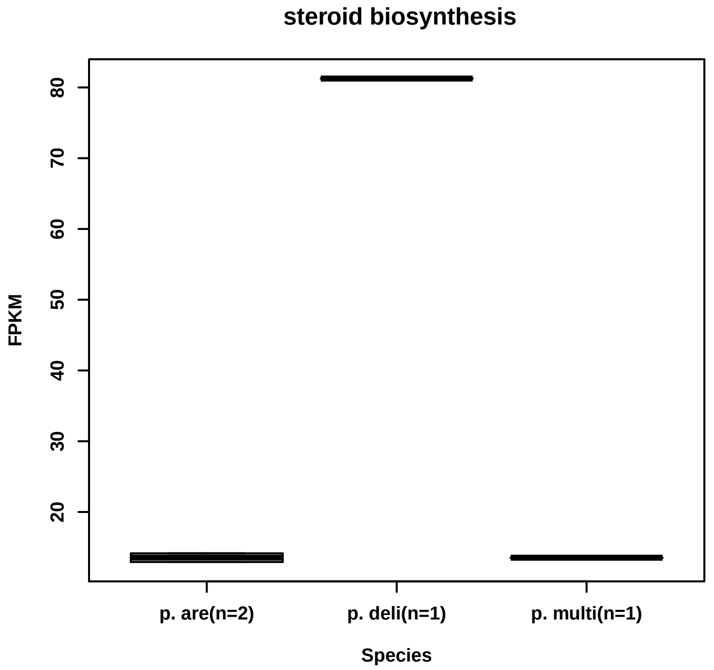
steroid biosynthesis
FPKM
20
30
40
50
60
70
80
p. are(n=2)
p. deli(n=1)
p. multi(n=1)
Species

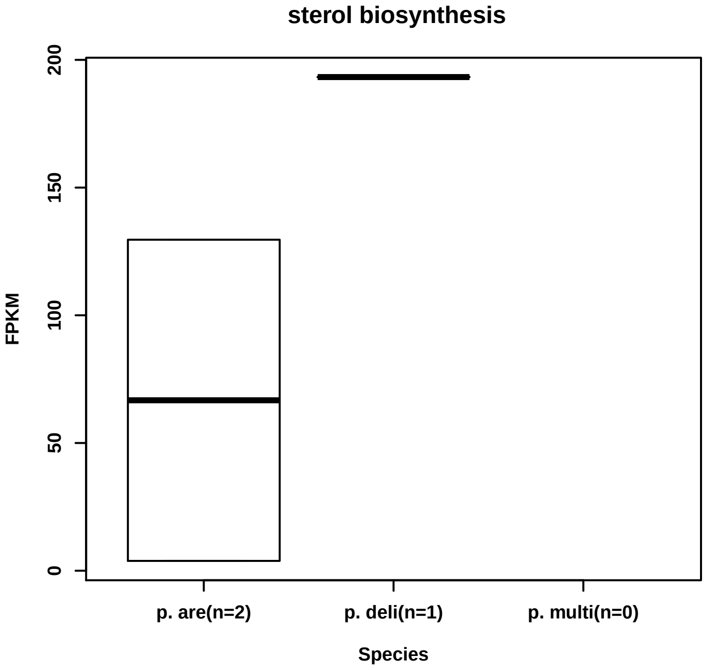
sterol biosynthesis
FPKM
0
50
100
150
200
p. are(n=2)
p. deli(n=1)
p. multi(n=0)
Species

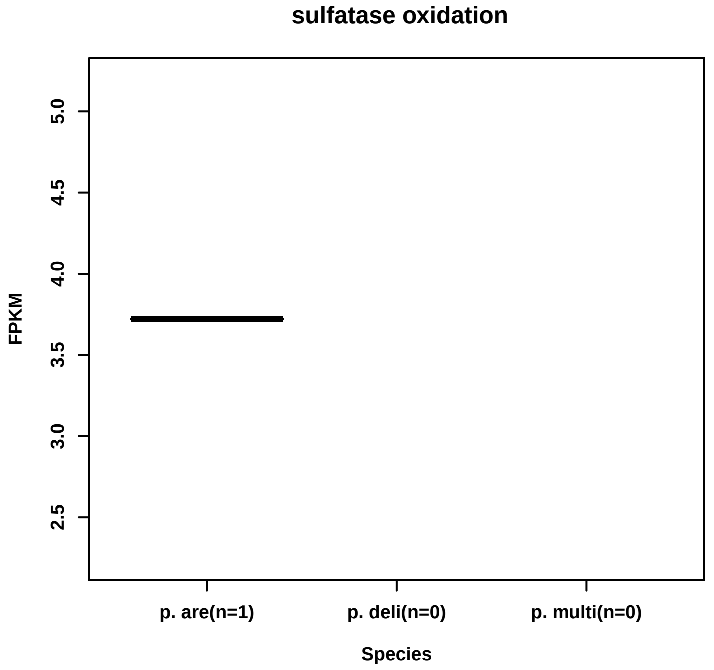
sulfatase oxidation
FPKM
2.5
3.0
3.5
4.0
4.5
5.0
p. are(n=1)
p. deli(n=0)
p. multi(n=0)
Species

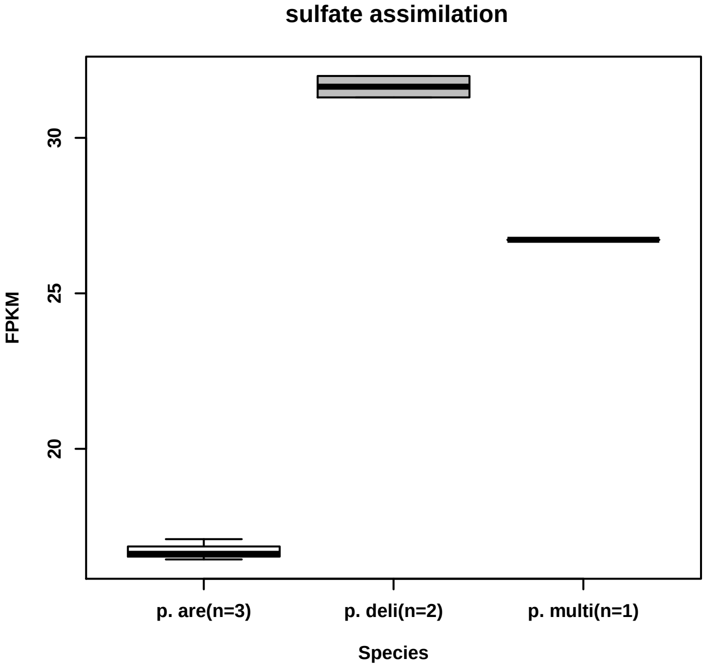
sulfate assimilation
FPKM
20
25
30
p. are(n=3)
p. deli(n=2)
p. multi(n=1)
Species

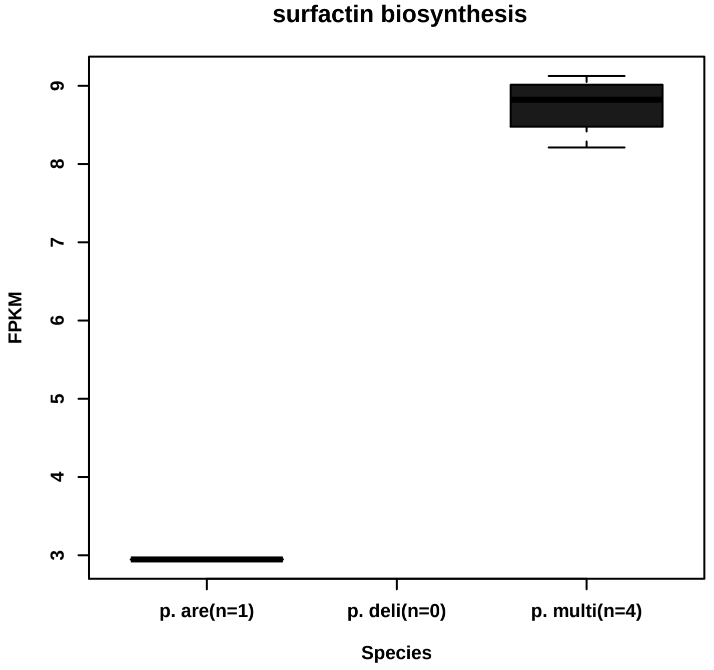
surfactin biosynthesis
FPKM
3
4
5
6
7
8
9
p. are(n=1)
p. deli(n=0)
p. multi(n=4)
Species

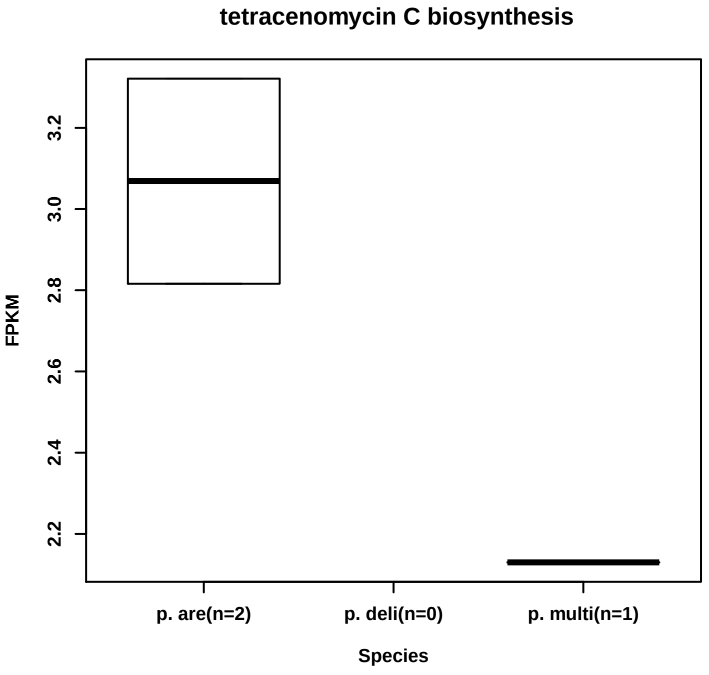
tetracenomycin C biosynthesis
FPKM
2.2
2.4
2.6
2.8
3.0
3.2
p. are(n=2)
p. deli(n=0)
p. multi(n=1)
Species

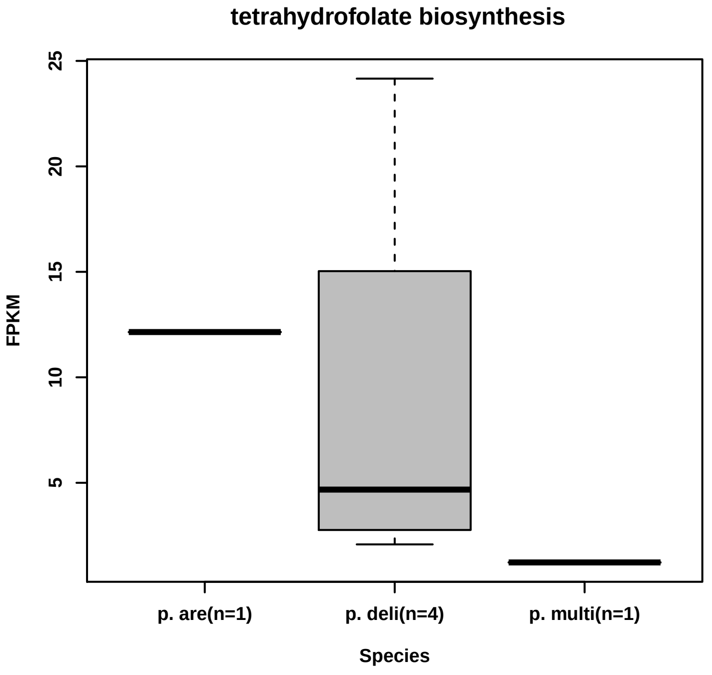
tetrahydrofolate biosynthesis
FPKM
5
10
15
20
25
p. are(n=1)
p. deli(n=4)
p. multi(n=1)
Species

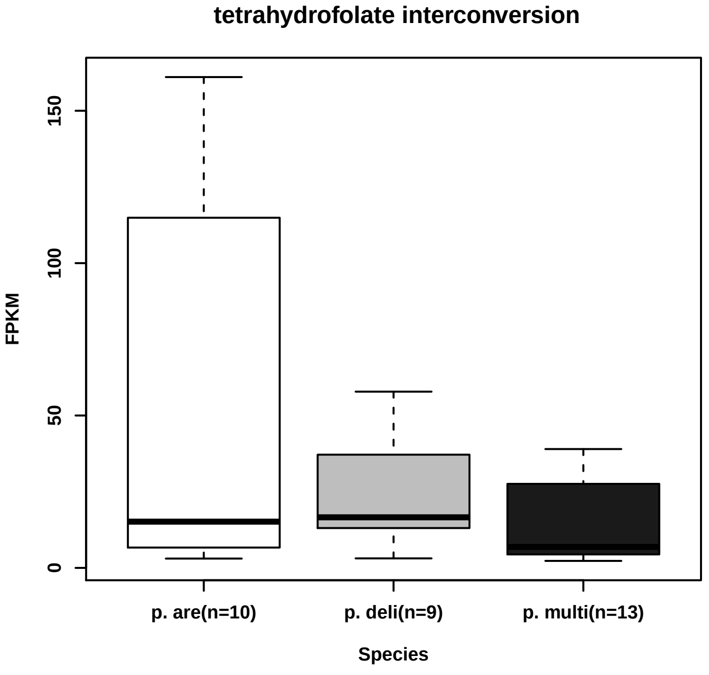
tetrahydrofolate interconversion
FPKM
0
50
100
150
p. are(n=10)
p. deli(n=9)
p. multi(n=13)
Species

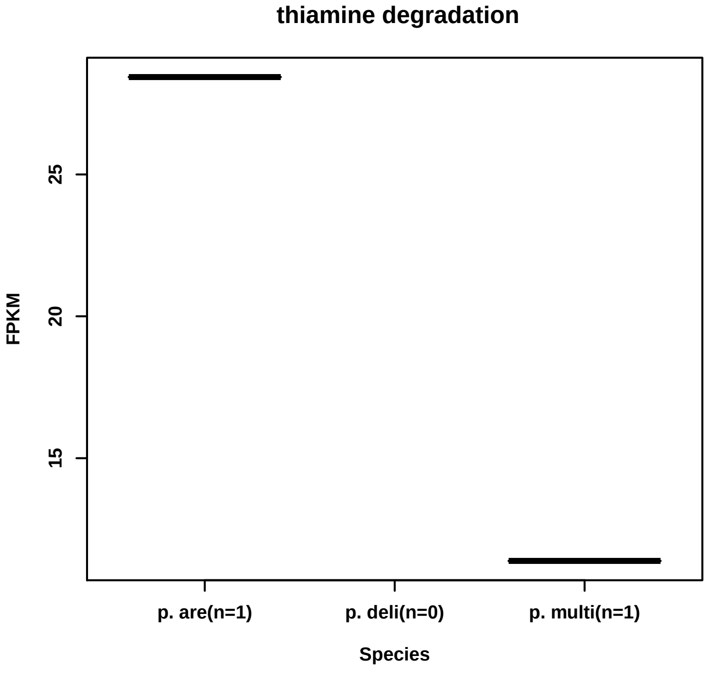
thiamine degradation
FPKM
15
20
25
p. are(n=1)
p. deli(n=0)
p. multi(n=1)
Species

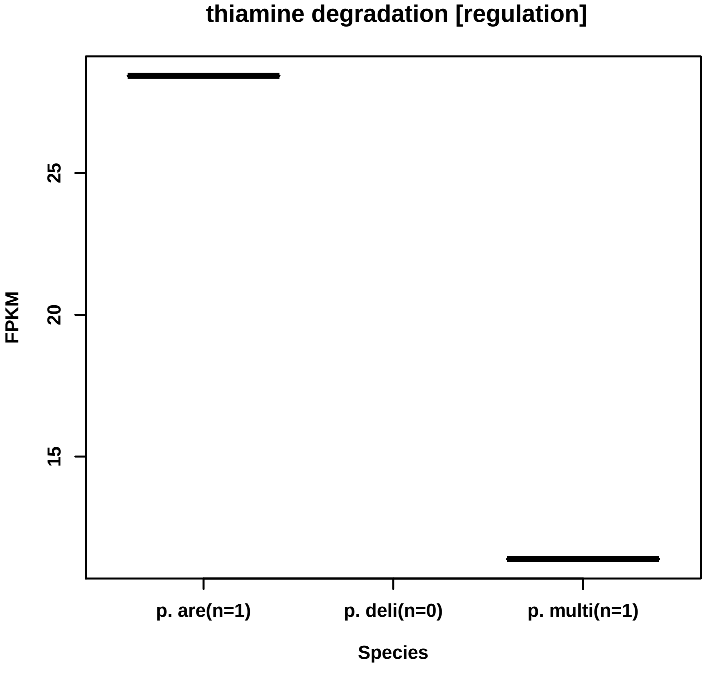
thiamine degradation [regulation]
FPKM
15
20
25
p. are(n=1)
p. deli(n=0)
p. multi(n=1)
Species

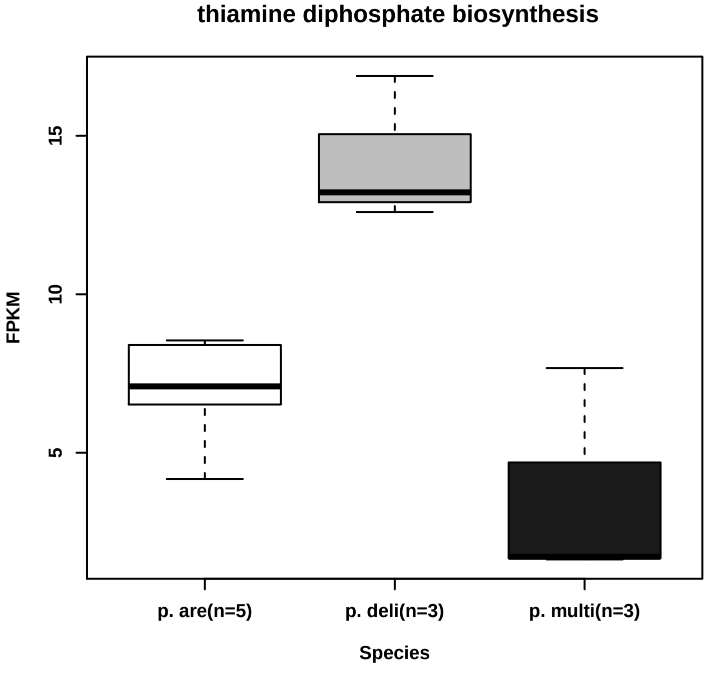
thiamine diphosphate biosynthesis
FPKM
5
10
15
p. are(n=5)
p. deli(n=3)
p. multi(n=3)
Species

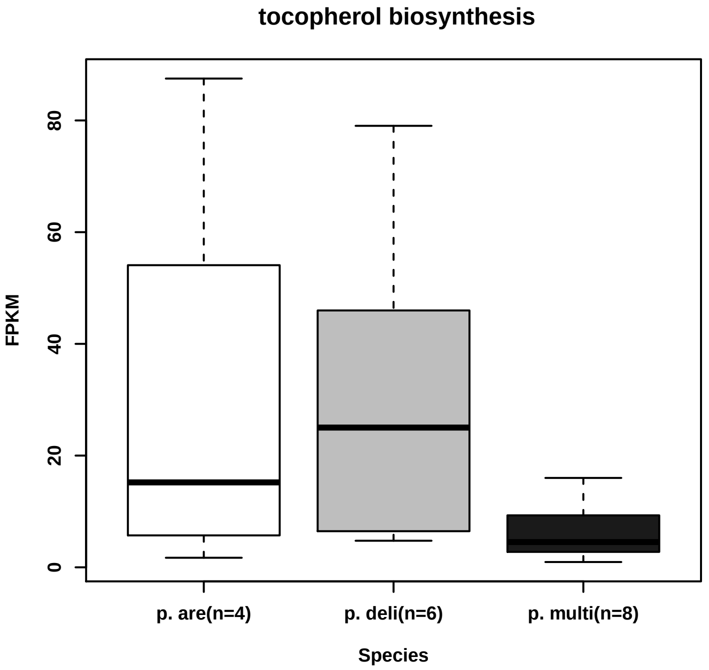
tocopherol biosynthesis
FPKM
0
20
40
60
80
p. are(n=4)
p. deli(n=6)
p. multi(n=8)
Species

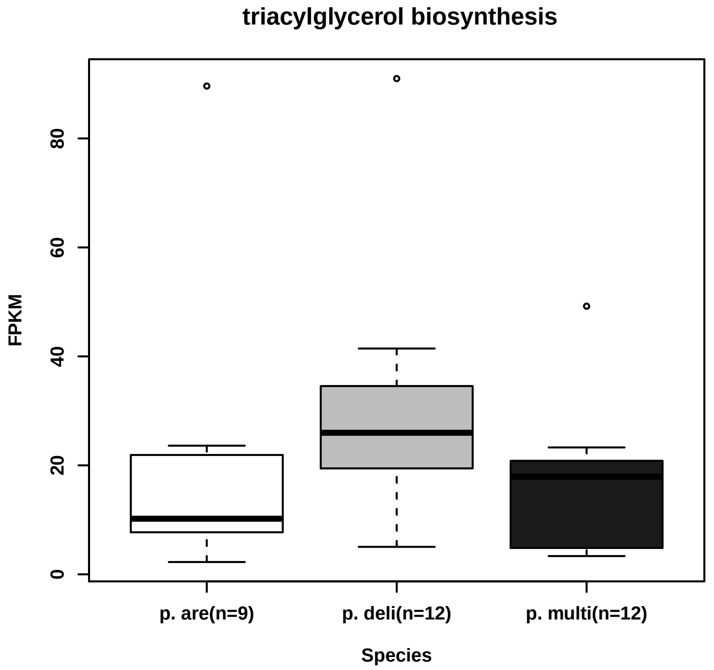
triacylglycerol biosynthesis
FPKM
0
20
40
60
80
p. are(n=9)
p. deli(n=12)
p. multi(n=12)
Species

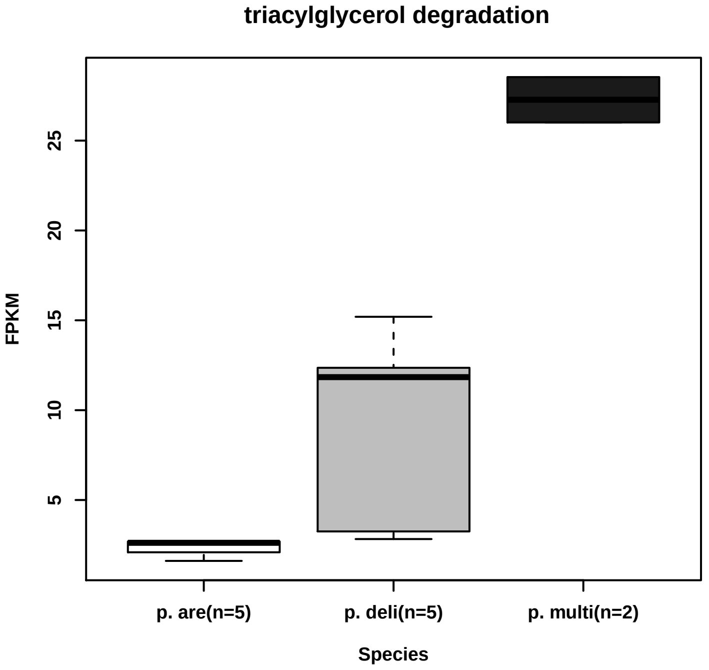
triacylglycerol degradation
FPKM
5
10
15
20
25
p. are(n=5)
p. deli(n=5)
p. multi(n=2)
Species

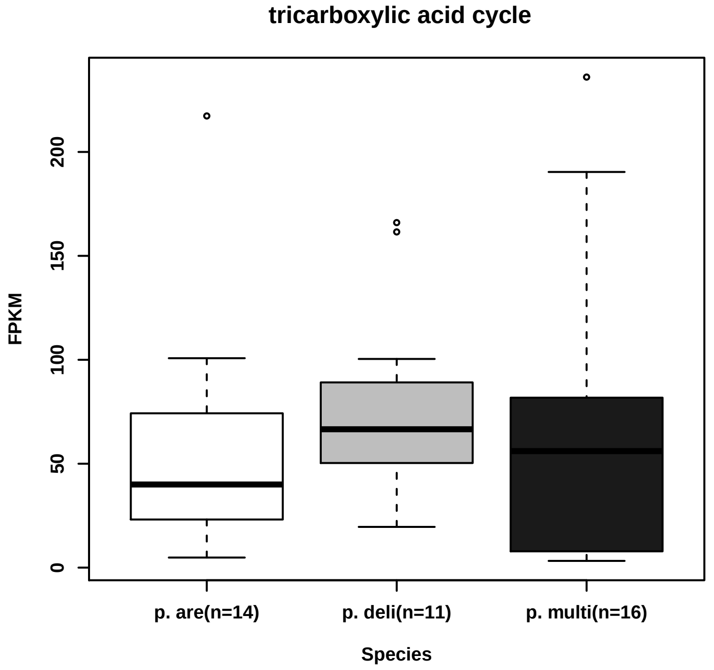
tricarboxylic acid cycle
FPKM
0
50
100
150
200
p. are(n=14)
p. deli(n=11)
p. multi(n=16)
Species

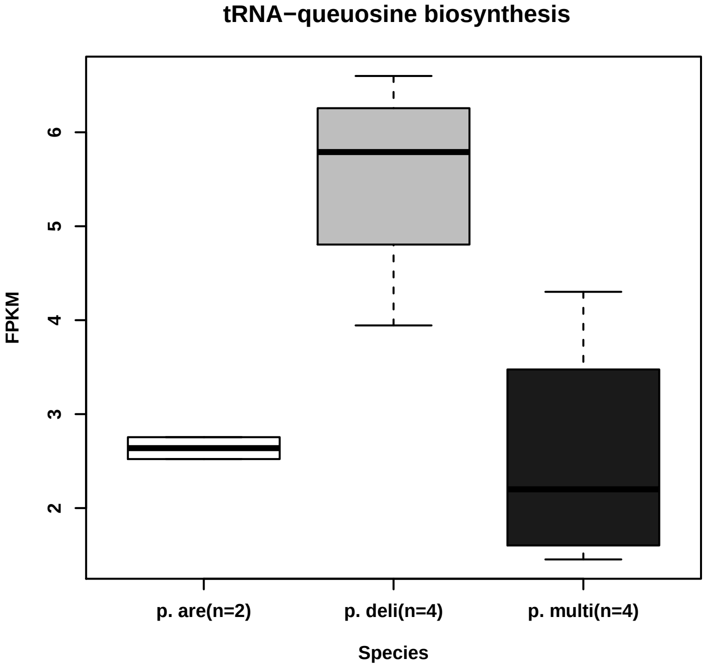
tRNA−queuosine biosynthesis
FPKM
2
3
4
5
6
p. are(n=2)
p. deli(n=4)
p. multi(n=4)
Species

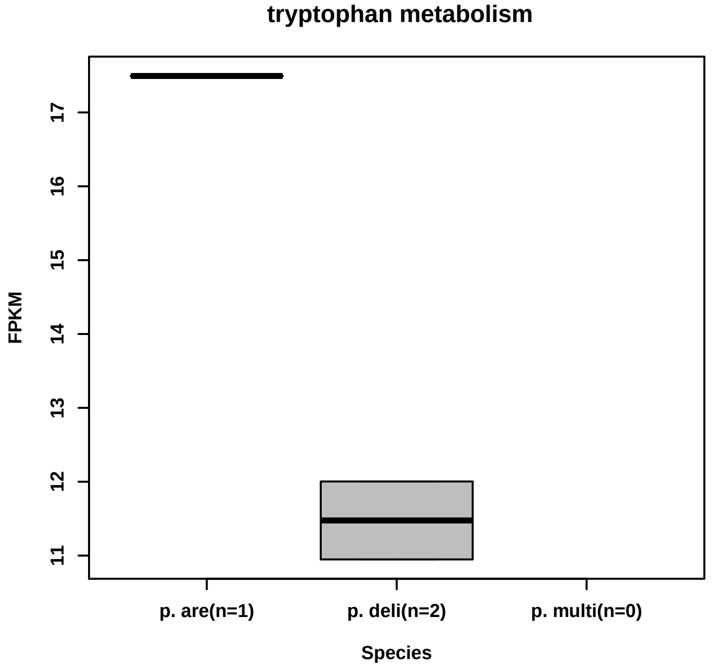
tryptophan metabolism
FPKM
11
12
13
14
15
16
17
p. are(n=1)
p. deli(n=2)
p. multi(n=0)
Species

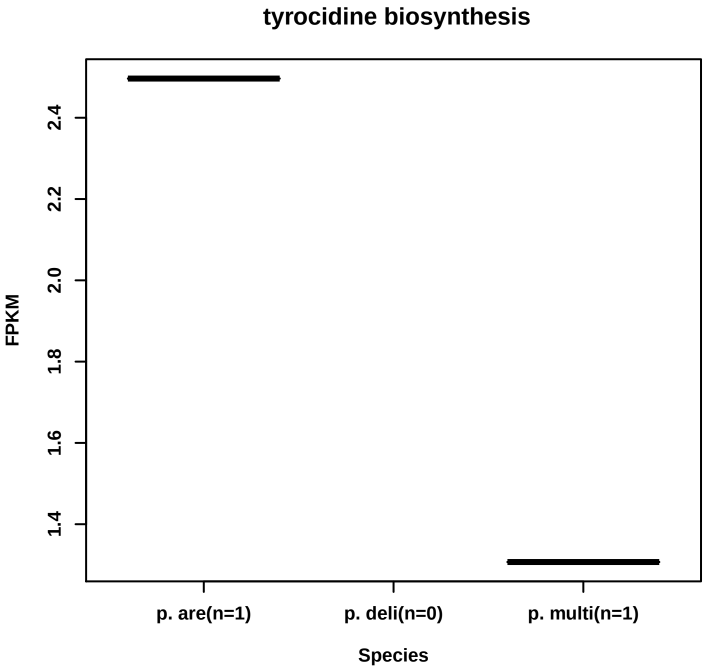
tyrocidine biosynthesis
FPKM
1.4
1.6
1.8
2.0
2.2
2.4
p. are(n=1)
p. deli(n=0)
p. multi(n=1)
Species

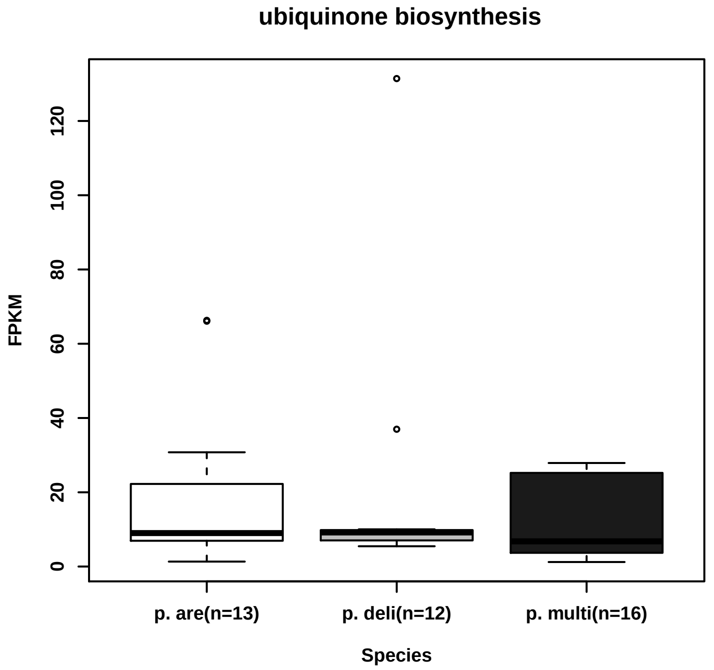
ubiquinone biosynthesis
FPKM
0
20
40
60
80
100
120
p. are(n=13)
p. deli(n=12)
p. multi(n=16)
Species

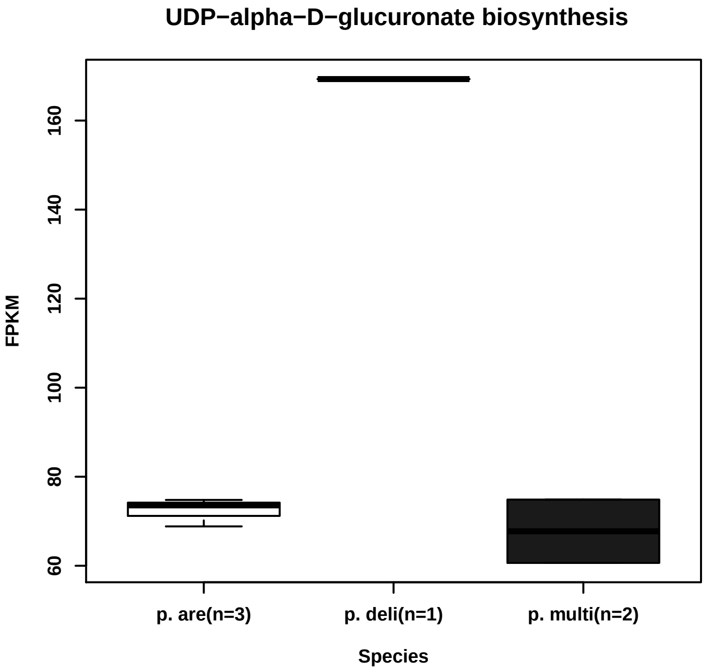
UDP−alpha−D−glucuronate biosynthesis
FPKM
60
80
100
120
140
160
p. are(n=3)
p. deli(n=1)
p. multi(n=2)
Species

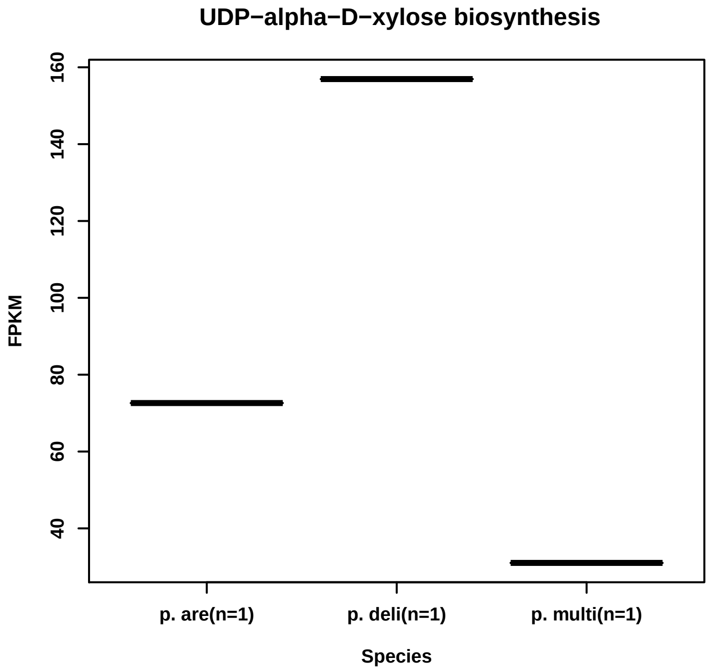
UDP−alpha−D−xylose biosynthesis
FPKM
40
60
80
100
120
140
160
p. are(n=1)
p. deli(n=1)
p. multi(n=1)
Species

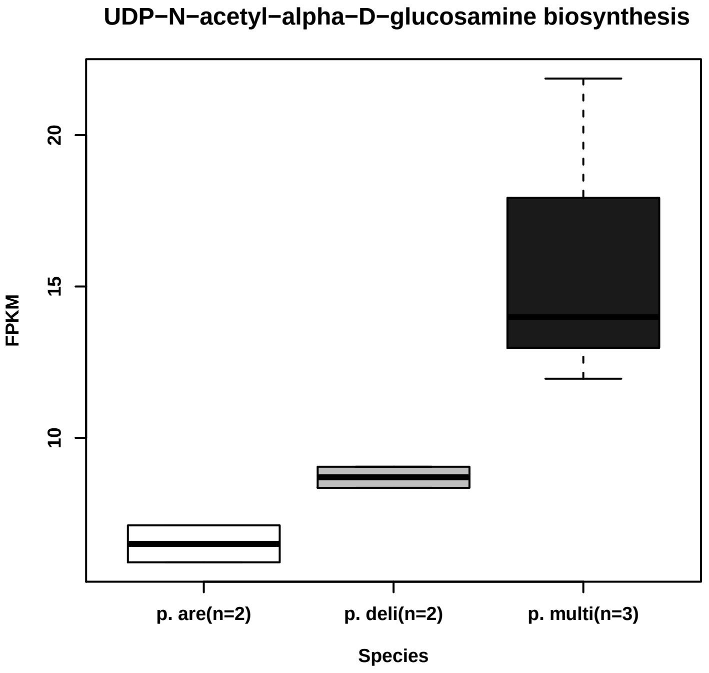
UDP−N−acetyl−alpha−D−glucosamine biosynthesis
FPKM
10
15
20
p. are(n=2)
p. deli(n=2)
p. multi(n=3)
Species

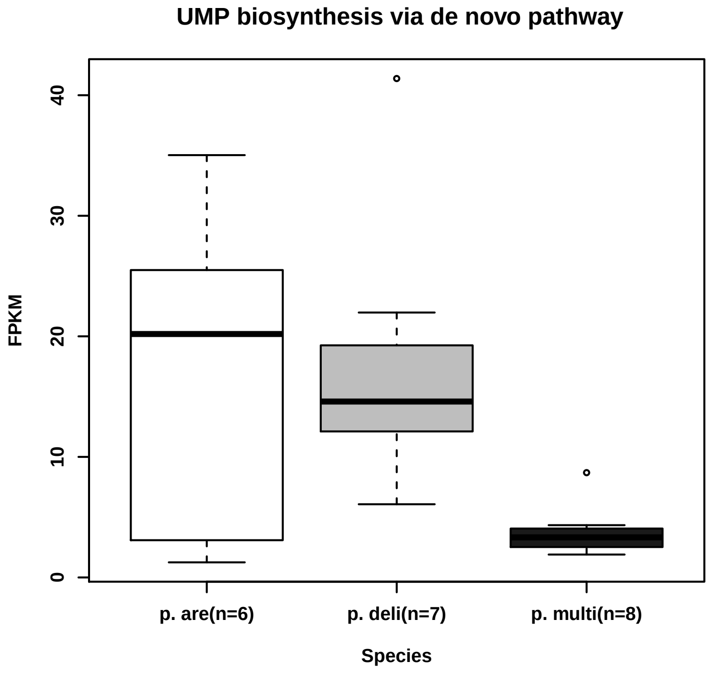
UMP biosynthesis via de novo pathway
FPKM
0
10
20
30
40
p. are(n=6)
p. deli(n=7)
p. multi(n=8)
Species

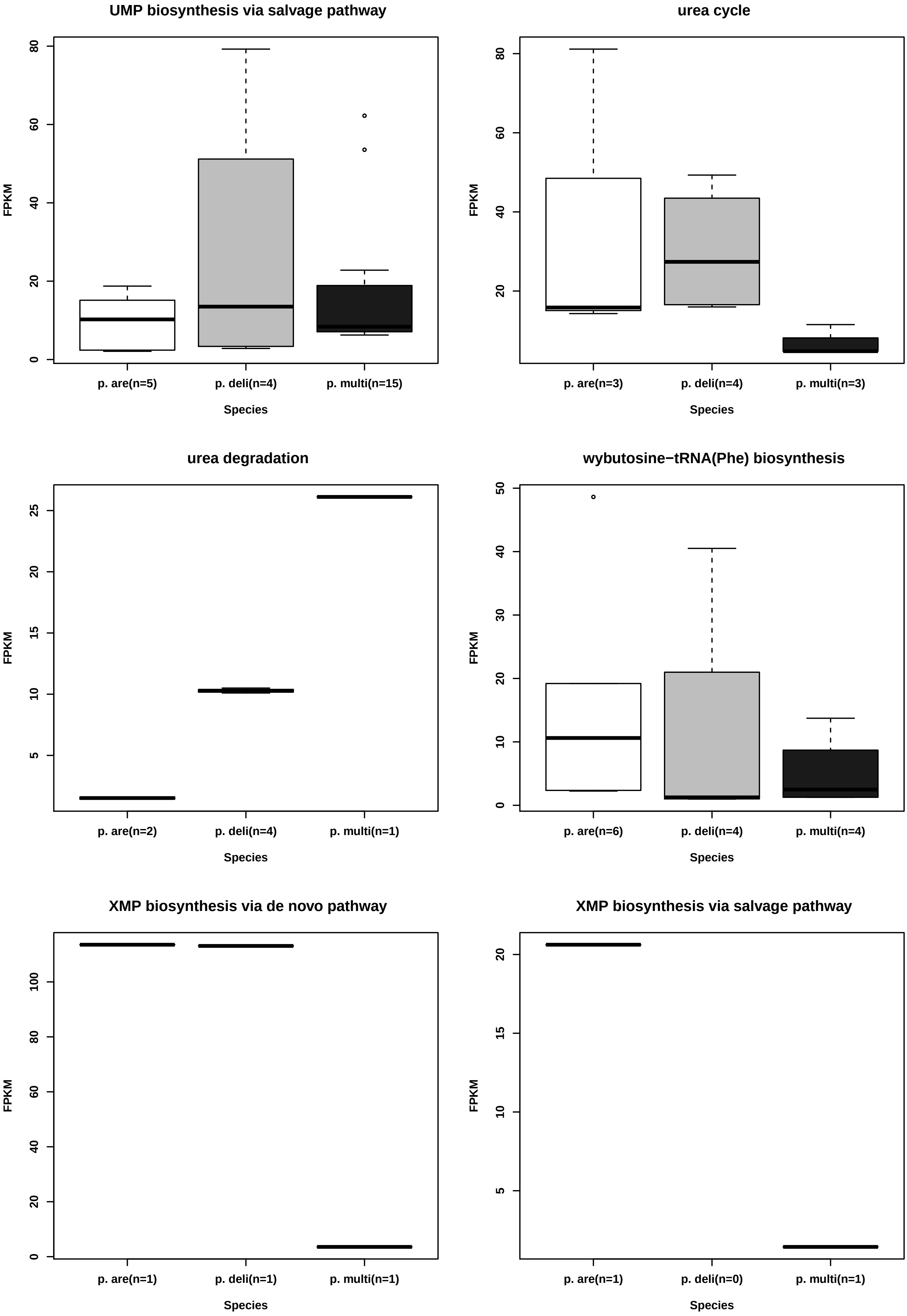

UMP biosynthesis via salvage pathway
FPKM
0
20
40
60
80
p. are(n=5)
p. deli(n=4)
p. multi(n=15)
Species
urea cycle
FPKM
20
40
60
80
p. are(n=3)
p. deli(n=4)
p. multi(n=3)
Species
urea degradation
FPKM
5
10
15
20
25
p. are(n=2)
p. deli(n=4)
p. multi(n=1)
Species
wybutosine−tRNA(Phe) biosynthesis
FPKM
0
10
20
30
40
50
p. are(n=6)
p. deli(n=4)
p. multi(n=4)
Species
XMP biosynthesis via de novo pathway
FPKM
0
20
40
60
80
100
p. are(n=1)
p. deli(n=1)
p. multi(n=1)
Species
XMP biosynthesis via salvage pathway
FPKM
5
10
15
20
p. are(n=1)
p. deli(n=0)
p. multi(n=1)
Species

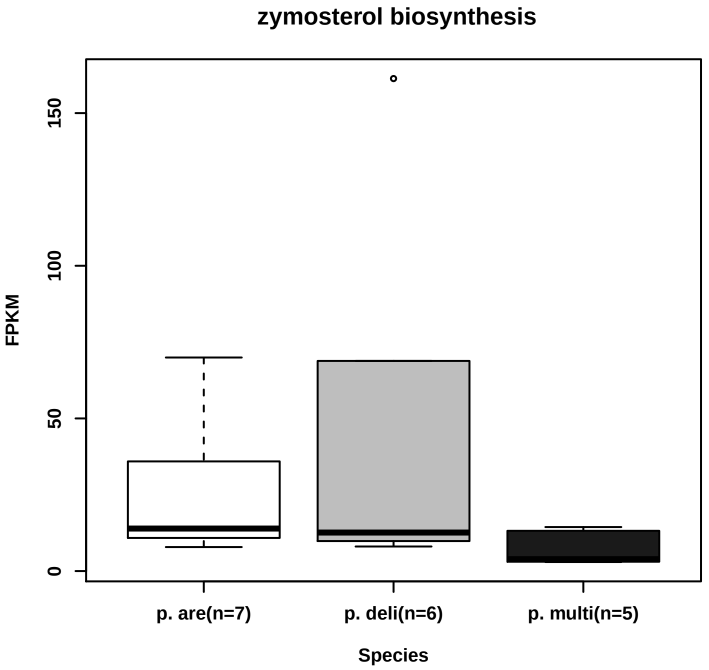
zymosterol biosynthesis
FPKM
0
50
100
150
p. are(n=7)
p. deli(n=6)
p. multi(n=5)
Species

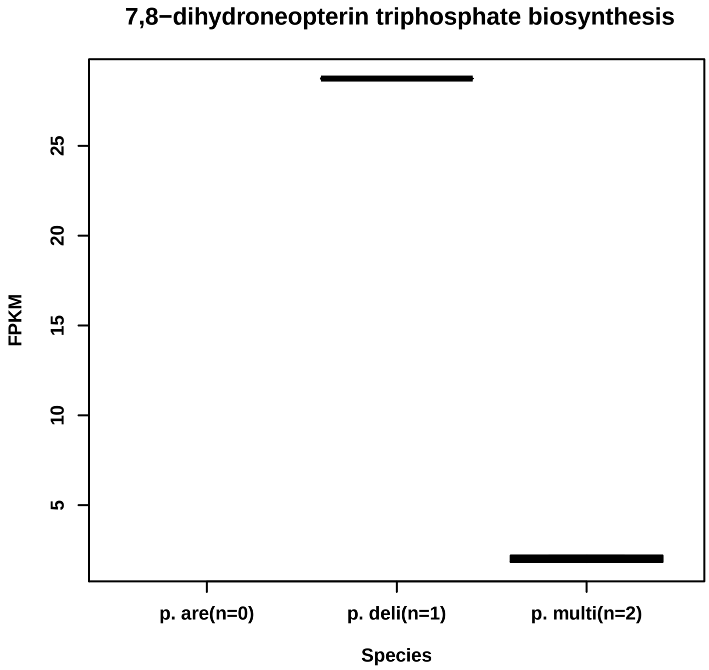
7,8−dihydroneopterin triphosphate biosynthesis
FPKM
5
10
15
20
25
p. are(n=0)
p. deli(n=1)
p. multi(n=2)
Species

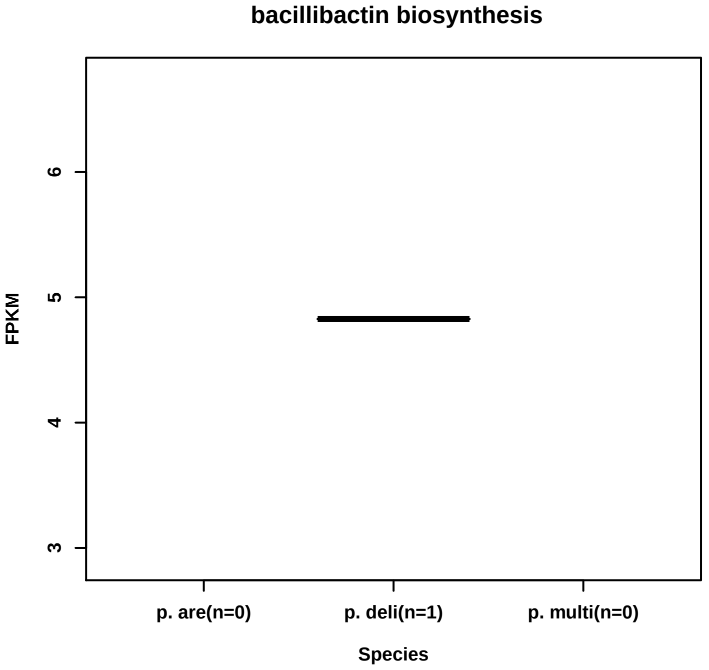
bacillibactin biosynthesis
FPKM
3
4
5
6
p. are(n=0)
p. deli(n=1)
p. multi(n=0)
Species

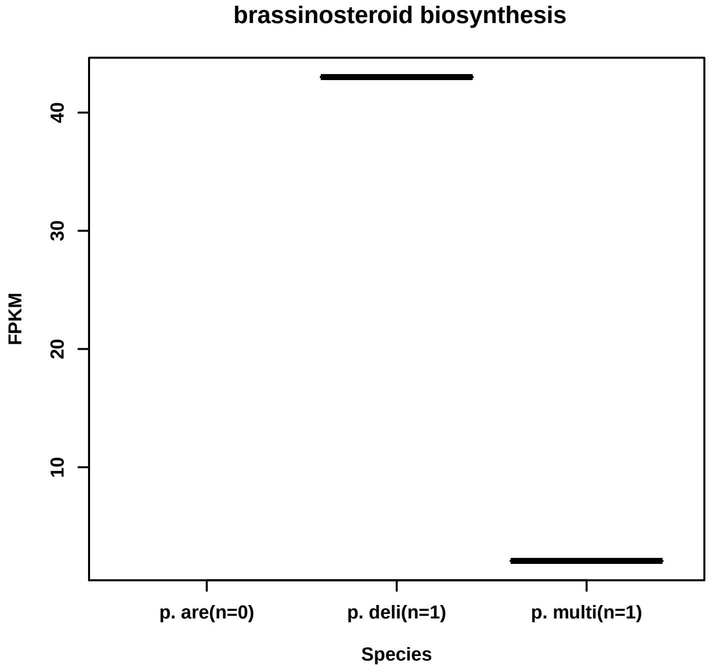
brassinosteroid biosynthesis
FPKM
10
20
30
40
p. are(n=0)
p. deli(n=1)
p. multi(n=1)
Species

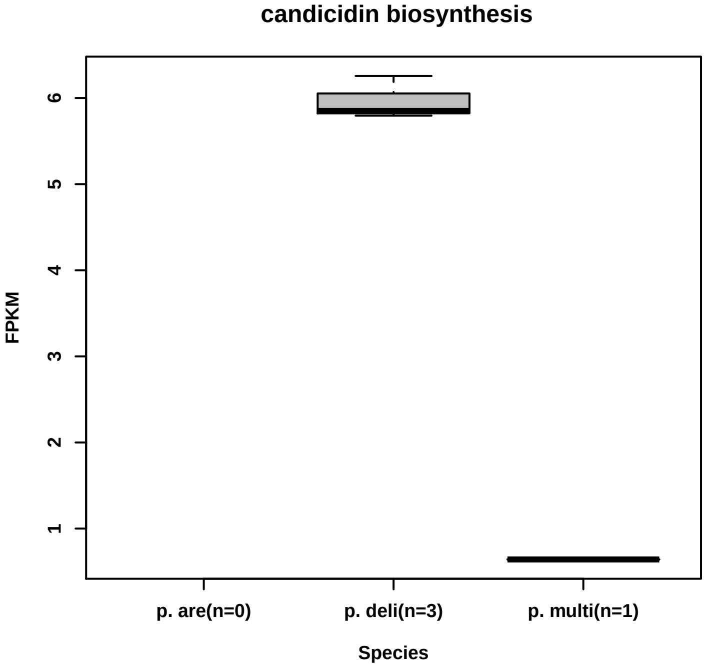
candicidin biosynthesis
FPKM
1
2
3
4
5
6
p. are(n=0)
p. deli(n=3)
p. multi(n=1)
Species

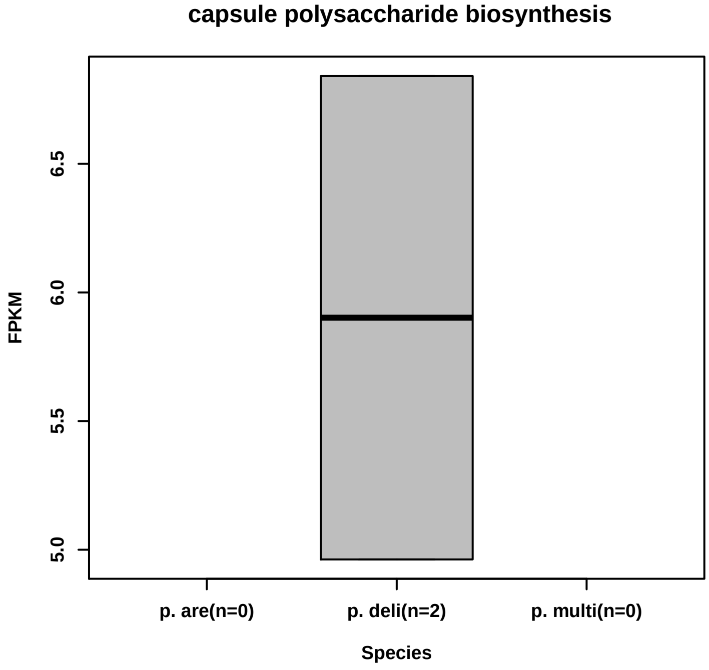
capsule polysaccharide biosynthesis
FPKM
5.0
5.5
6.0
6.5
p. are(n=0)
p. deli(n=2)
p. multi(n=0)
Species

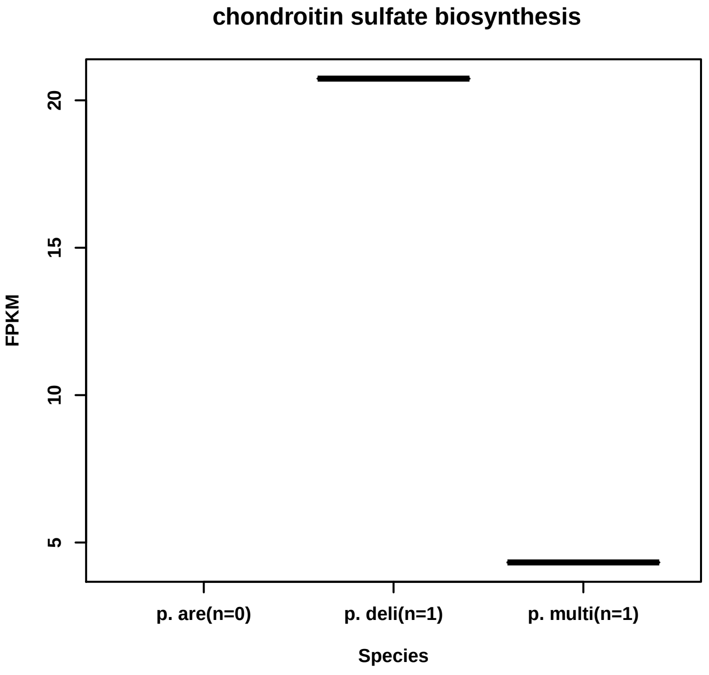
chondroitin sulfate biosynthesis
FPKM
20
15
10
5
p. are(n=0)
p. deli(n=1)
p. multi(n=1)
Species

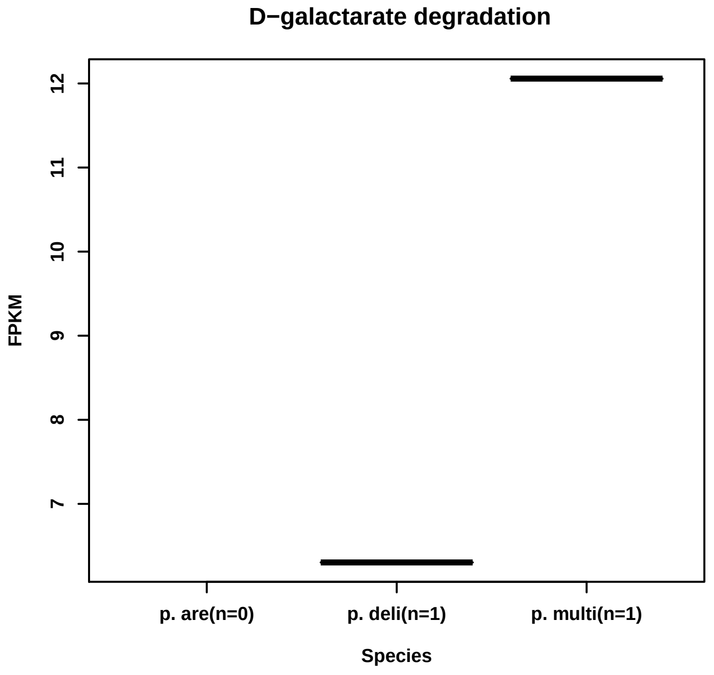
D−galactarate degradation
FPKM
12
11
10
9
8
7
p. are(n=0)
p. deli(n=1)
p. multi(n=1)
Species

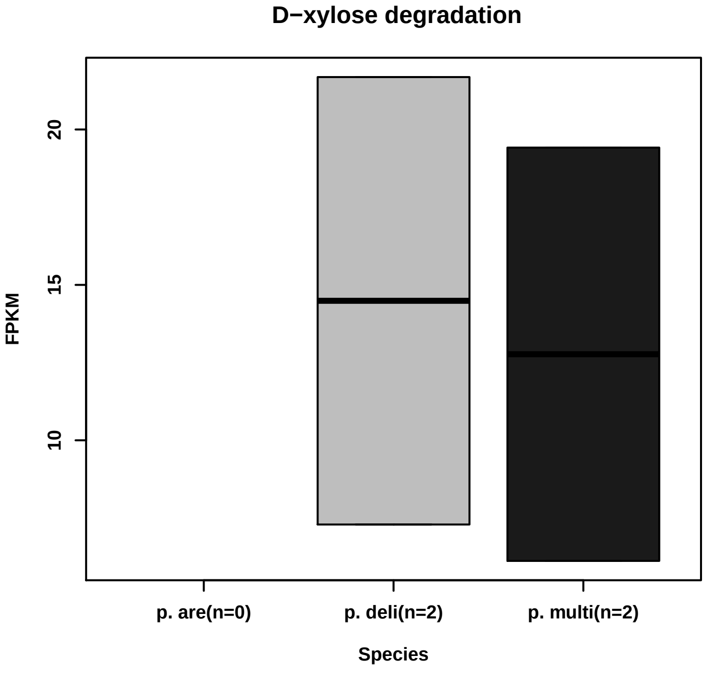
D−xylose degradation
FPKM
20
15
10
p. are(n=0)
p. deli(n=2)
p. multi(n=2)
Species

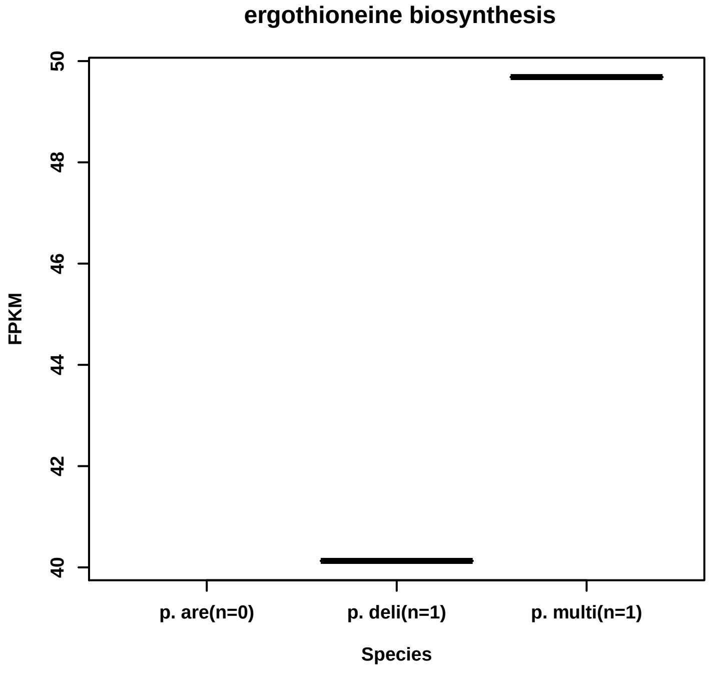
ergothioneine biosynthesis
FPKM
50
48
46
44
42
40
p. are(n=0)
p. deli(n=1)
p. multi(n=1)
Species

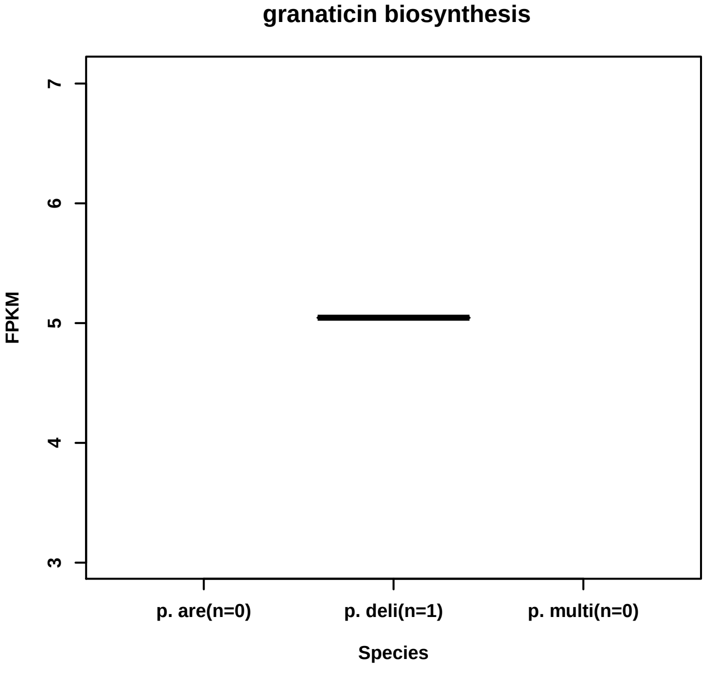
granaticin biosynthesis
FPKM
7
6
5
4
3
p. are(n=0)
p. deli(n=1)
p. multi(n=0)
Species

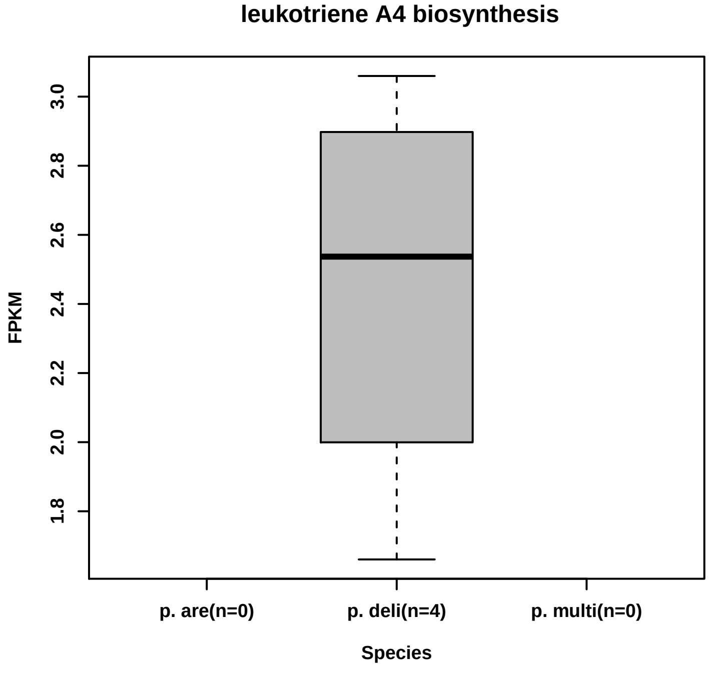
leukotriene A4 biosynthesis
FPKM
3.0
2.8
2.6
2.4
2.2
2.0
1.8
p. are(n=0)
p. deli(n=4)
p. multi(n=0)
Species

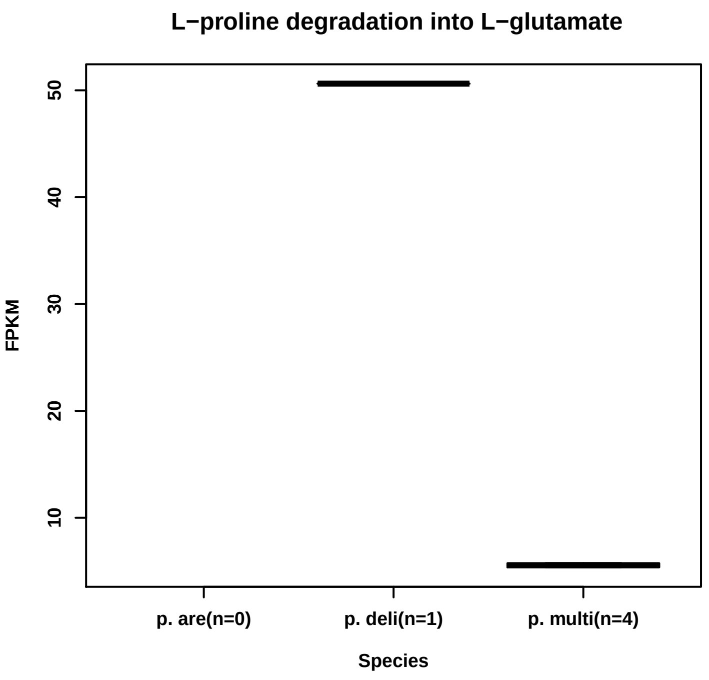
L−proline degradation into L−glutamate
FPKM
10
20
30
40
50
p. are(n=0)
p. deli(n=1)
p. multi(n=4)
Species

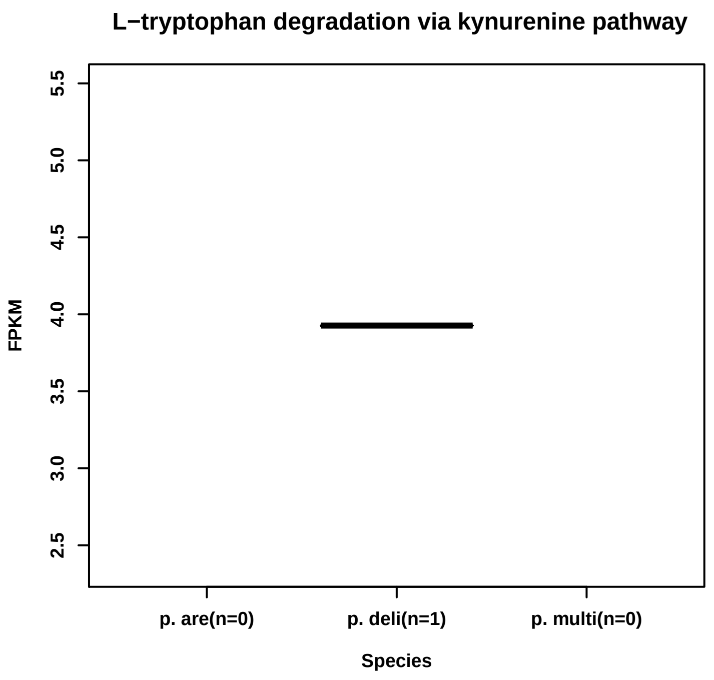
L−tryptophan degradation via kynurenine pathway
FPKM
2.5
3.0
3.5
4.0
4.5
5.0
5.5
p. are(n=0)
p. deli(n=1)
p. multi(n=0)
Species

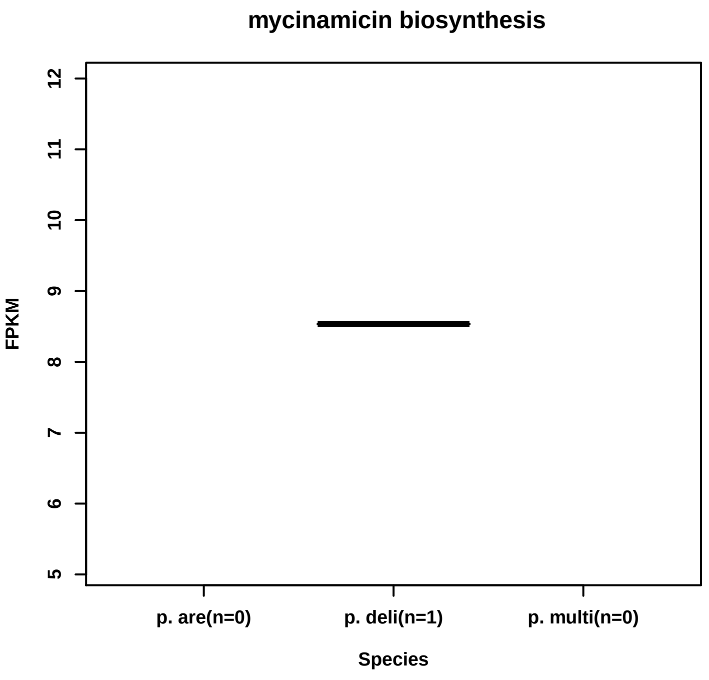
mycinamicin biosynthesis
FPKM
5
6
7
8
9
10
11
12
p. are(n=0)
p. deli(n=1)
p. multi(n=0)
Species

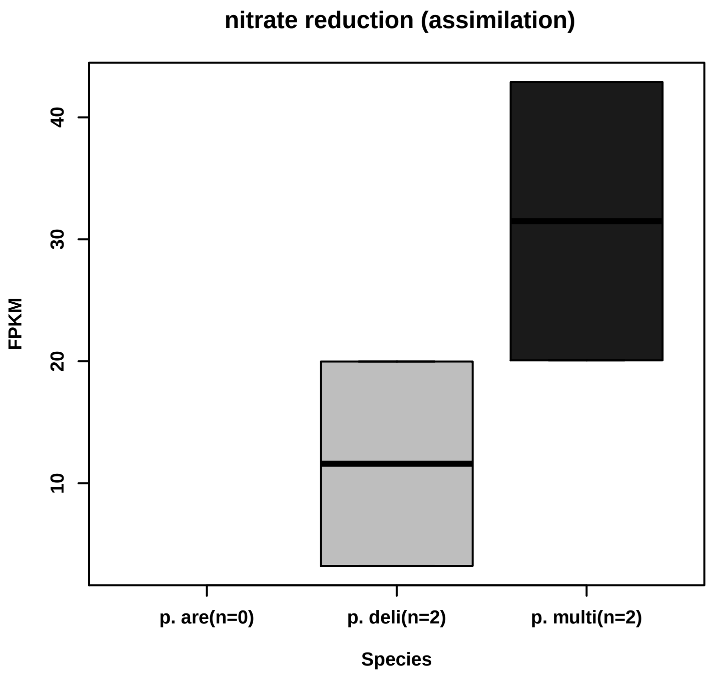
nitrate reduction (assimilation)
FPKM
10
20
30
40
p. are(n=0)
p. deli(n=2)
p. multi(n=2)
Species

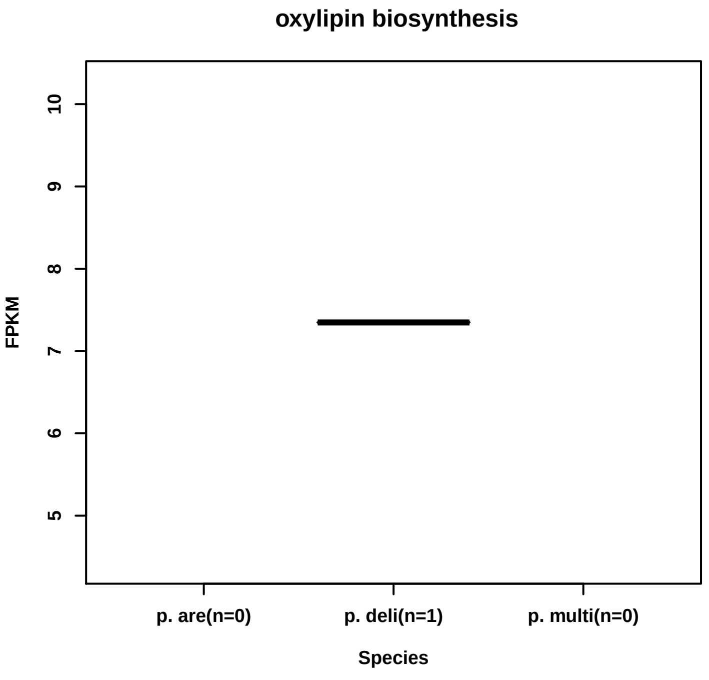
oxylipin biosynthesis
FPKM
5
6
7
8
9
10
p. are(n=0)
p. deli(n=1)
p. multi(n=0)
Species

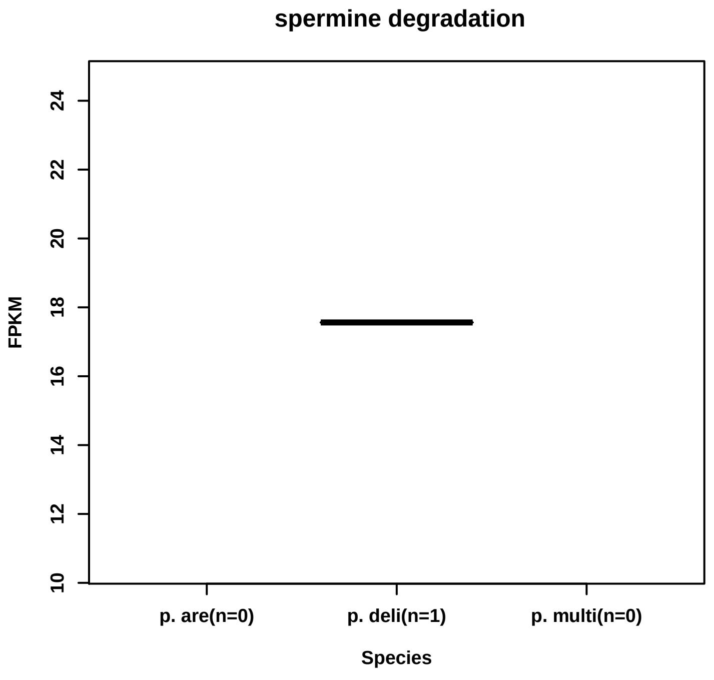
spermine degradation
FPKM
10
12
14
16
18
20
22
24
p. are(n=0)
p. deli(n=1)
p. multi(n=0)
Species

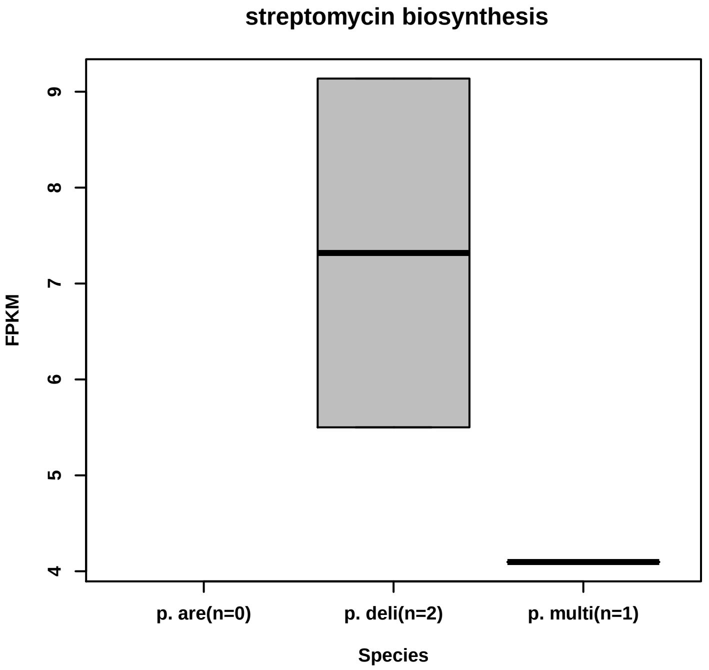
streptomycin biosynthesis
FPKM
9
8
7
6
5
4
p. are(n=0)
p. deli(n=2)
p. multi(n=1)
Species

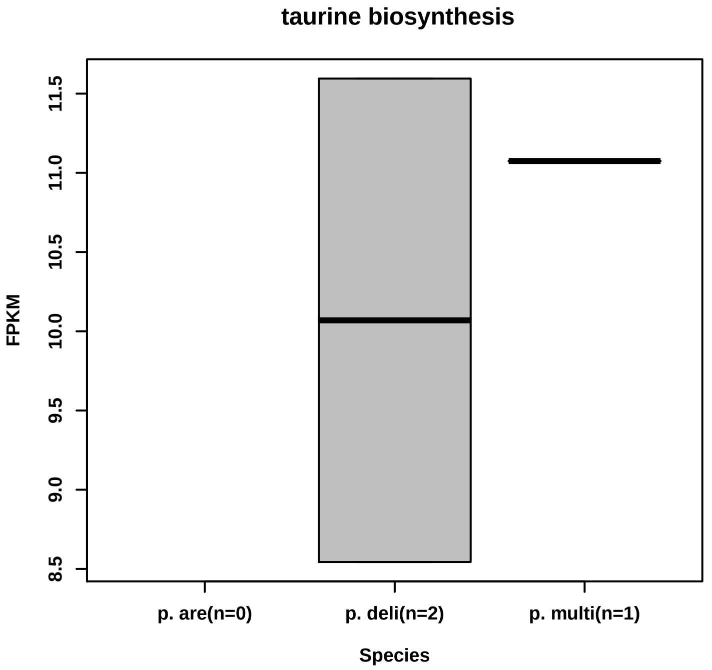
taurine biosynthesis
FPKM
11.5
11.0
10.5
10.0
9.5
9.0
8.5
p. are(n=0)
p. deli(n=2)
p. multi(n=1)
Species

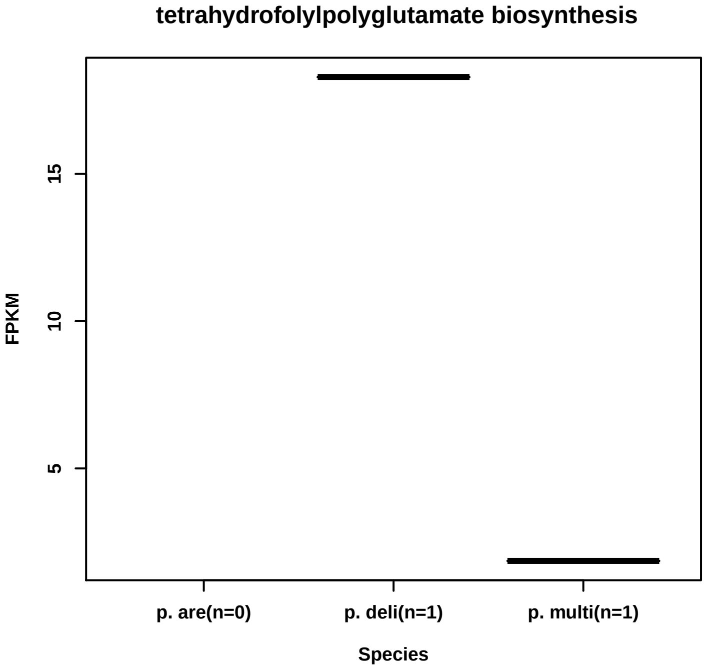
tetrahydrofolylpolyglutamate biosynthesis
FPKM
15
10
5
p. are(n=0)
p. deli(n=1)
p. multi(n=1)
Species

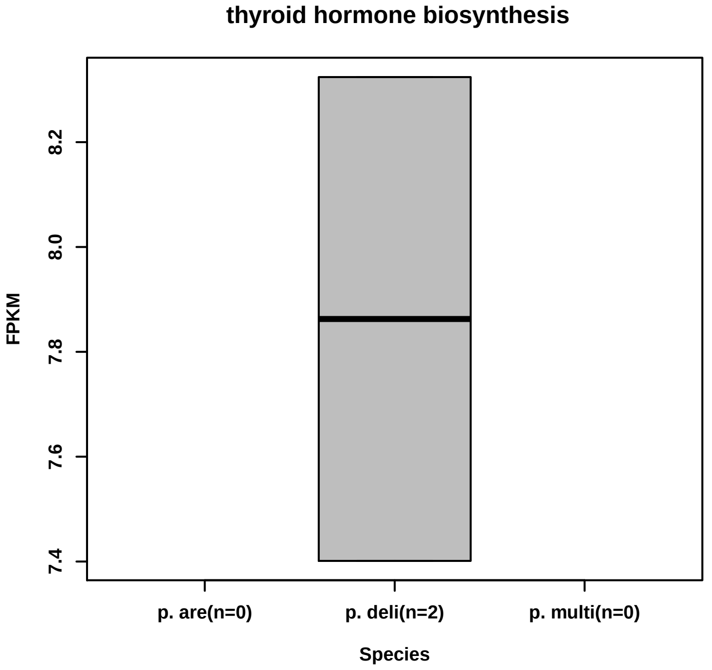
thyroid hormone biosynthesis
FPKM
8.2
8.0
7.8
7.6
7.4
p. are(n=0)
p. deli(n=2)
p. multi(n=0)
Species

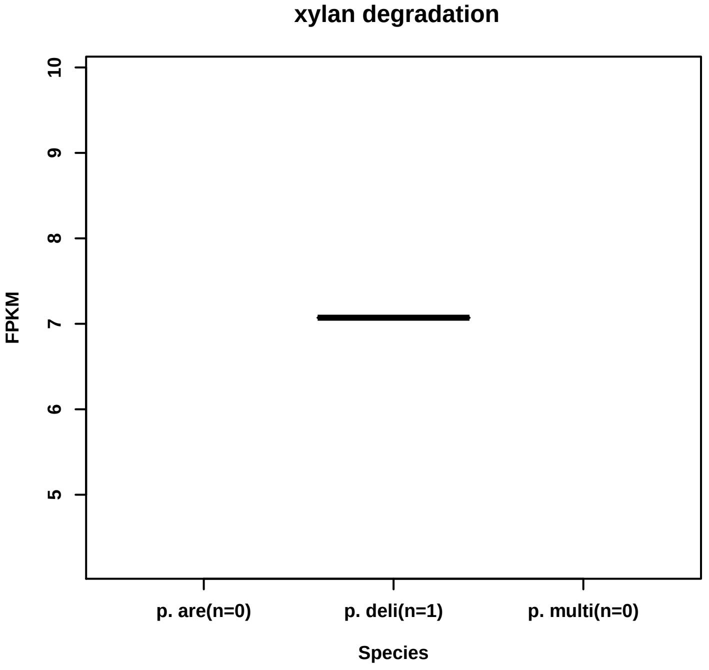
xylan degradation
FPKM
10
9
8
7
6
5
p. are(n=0)
p. deli(n=1)
p. multi(n=0)
Species

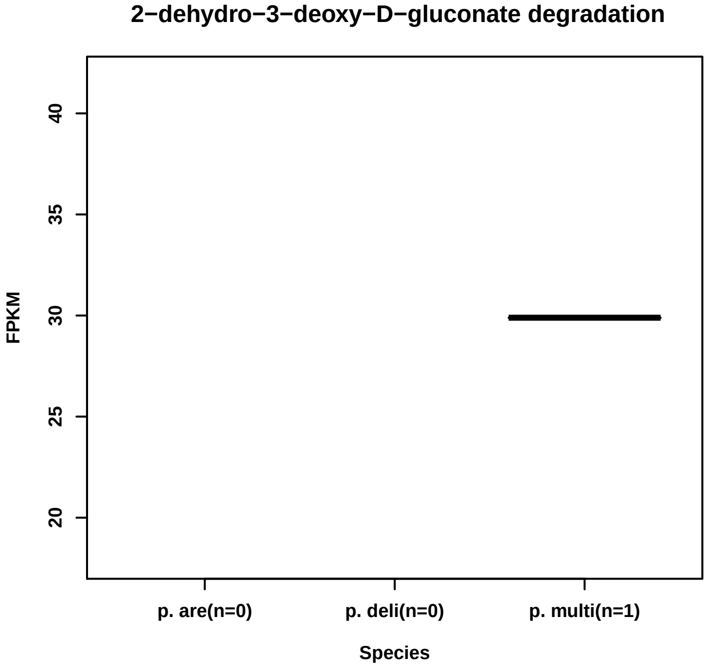
2−dehydro−3−deoxy−D−gluconate degradation
FPKM
40
35
30
25
20
p. are(n=0)
p. deli(n=0)
p. multi(n=1)
Species

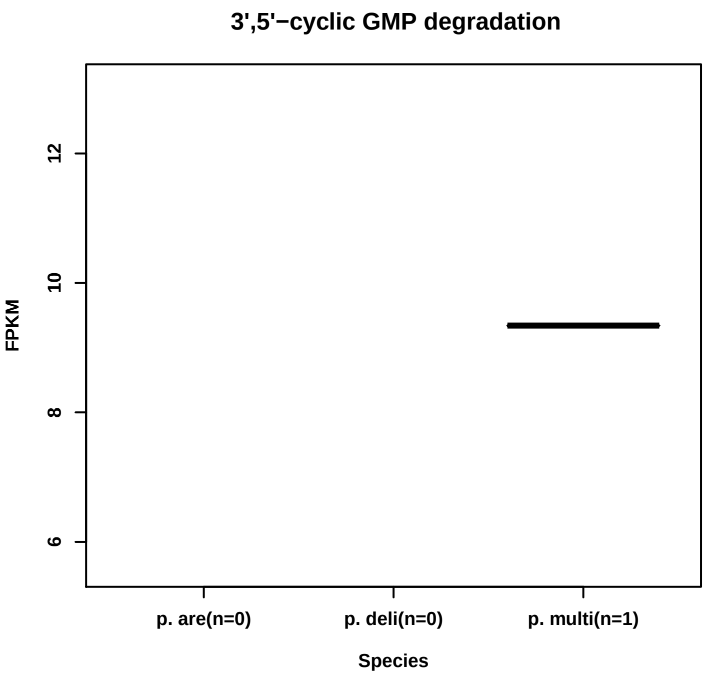
3',5'−cyclic GMP degradation
FPKM
6
8
10
12
p. are(n=0)
p. deli(n=0)
p. multi(n=1)
Species

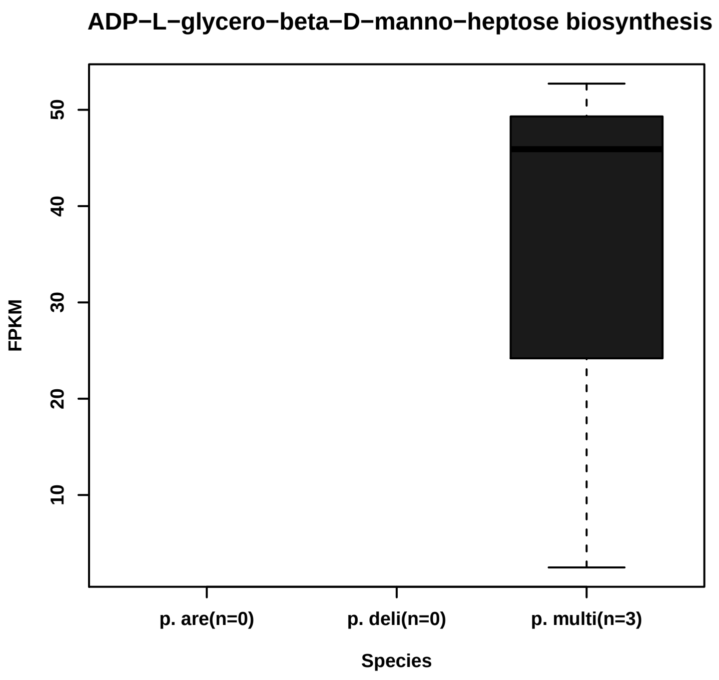
ADP−L−glycero−beta−D−manno−heptose biosynthesis
FPKM
10
20
30
40
50
p. are(n=0)
p. deli(n=0)
p. multi(n=3)
Species

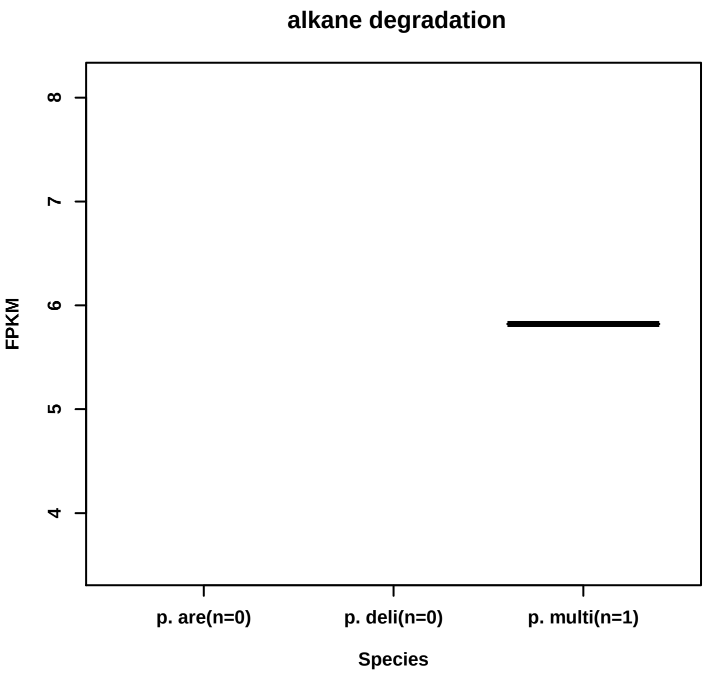
alkane degradation
FPKM
4
5
6
7
8
p. are(n=0)
p. deli(n=0)
p. multi(n=1)
Species

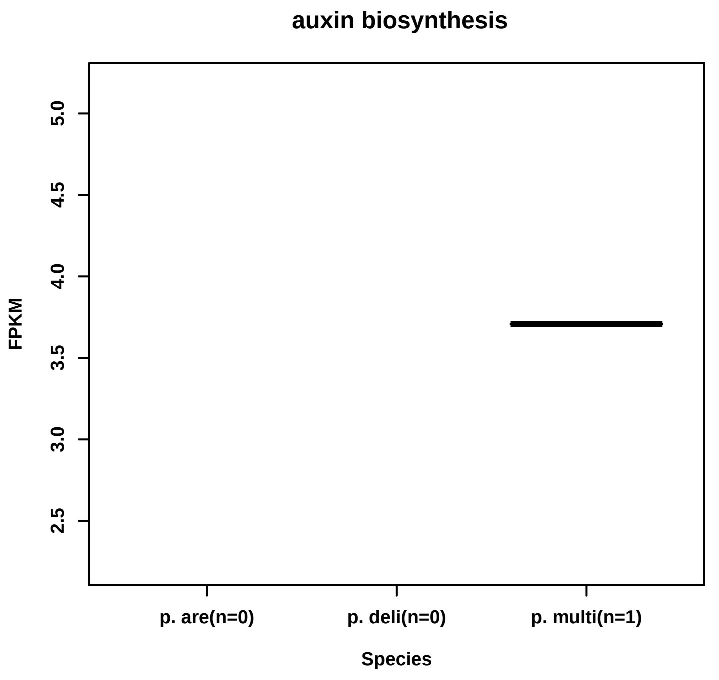
auxin biosynthesis
FPKM
2.5
3.0
3.5
4.0
4.5
5.0
p. are(n=0)
p. deli(n=0)
p. multi(n=1)
Species

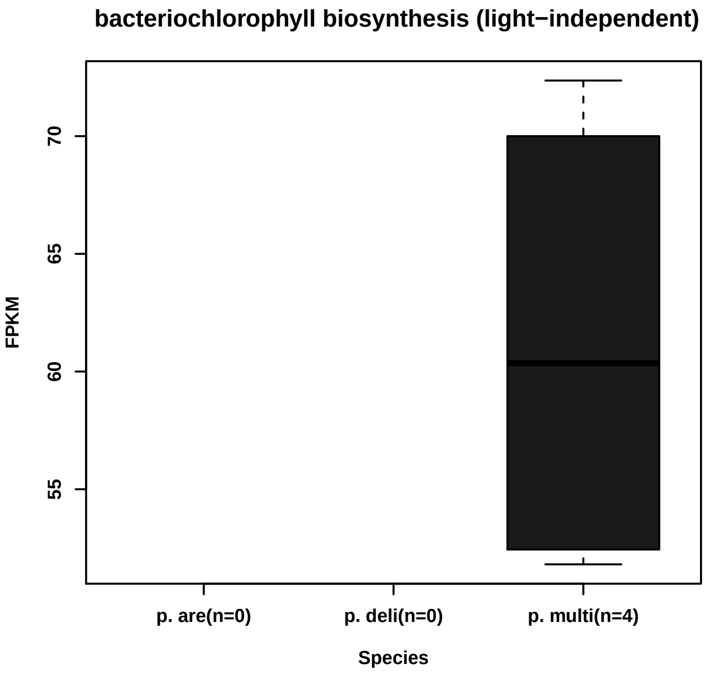
bacteriochlorophyll biosynthesis (light−independent)
FPKM
55
60
65
70
p. are(n=0)
p. deli(n=0)
p. multi(n=4)
Species

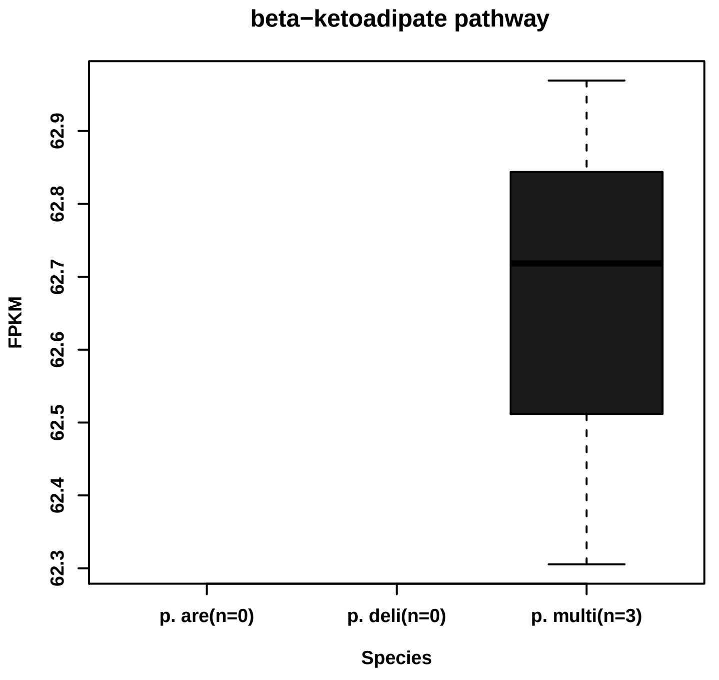
beta−ketoadipate pathway
FPKM
62.3
62.4
62.5
62.6
62.7
62.8
62.9
p. are(n=0)
p. deli(n=0)
p. multi(n=3)
Species

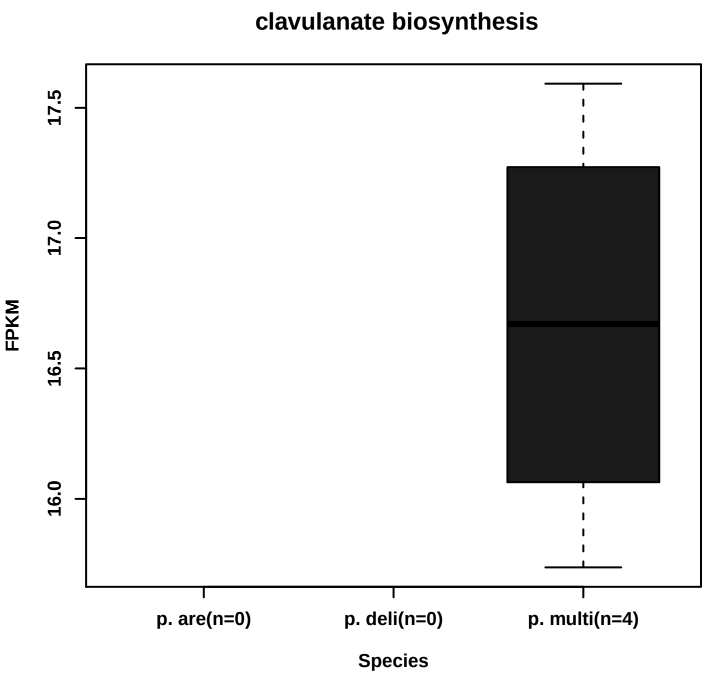
clavulanate biosynthesis
FPKM
16.0
16.5
17.0
17.5
p. are(n=0)
p. deli(n=0)
p. multi(n=4)
Species

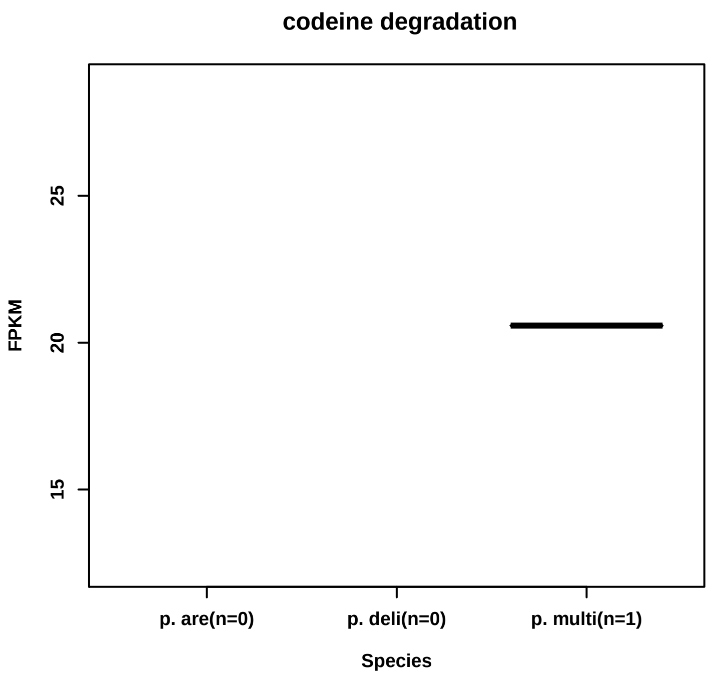
codeine degradation
FPKM
15
20
25
p. are(n=0)
p. deli(n=0)
p. multi(n=1)
Species

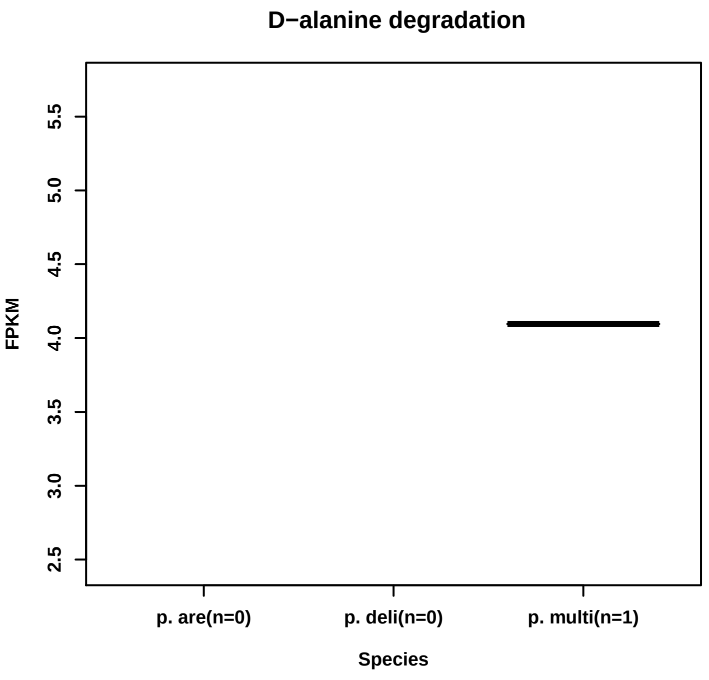
D−alanine degradation
FPKM
2.5
3.0
3.5
4.0
4.5
5.0
5.5
p. are(n=0)
p. deli(n=0)
p. multi(n=1)
Species

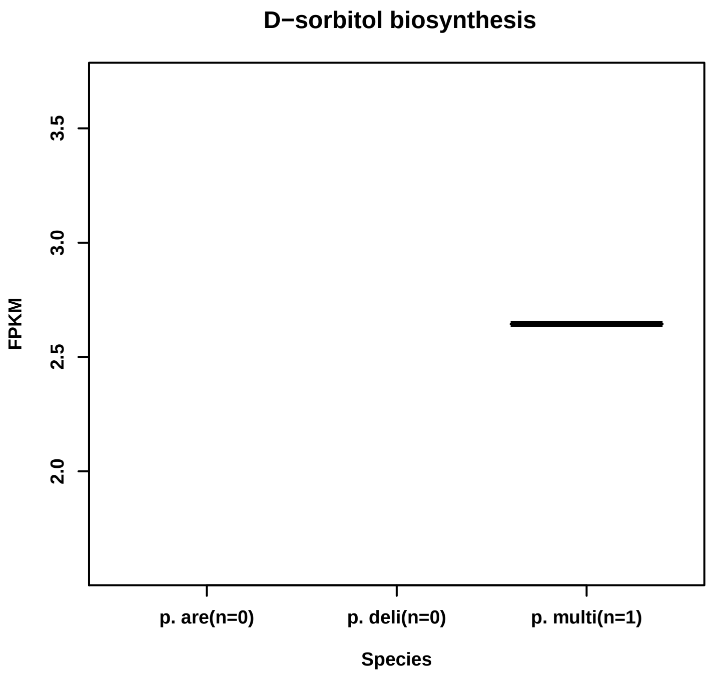
D−sorbitol biosynthesis
FPKM
2.0
2.5
3.0
3.5
p. are(n=0)
p. deli(n=0)
p. multi(n=1)
Species

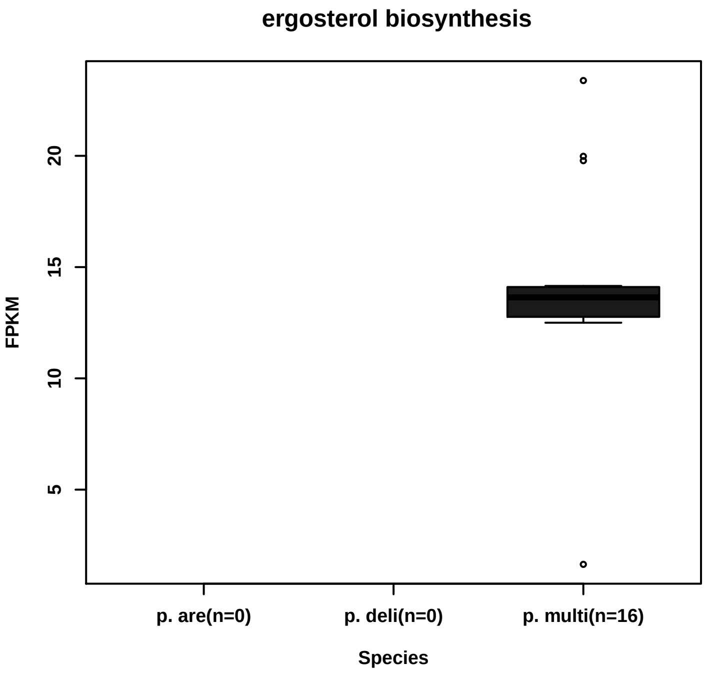
ergosterol biosynthesis
FPKM
5
10
15
20
p. are(n=0)
p. deli(n=0)
p. multi(n=16)
Species

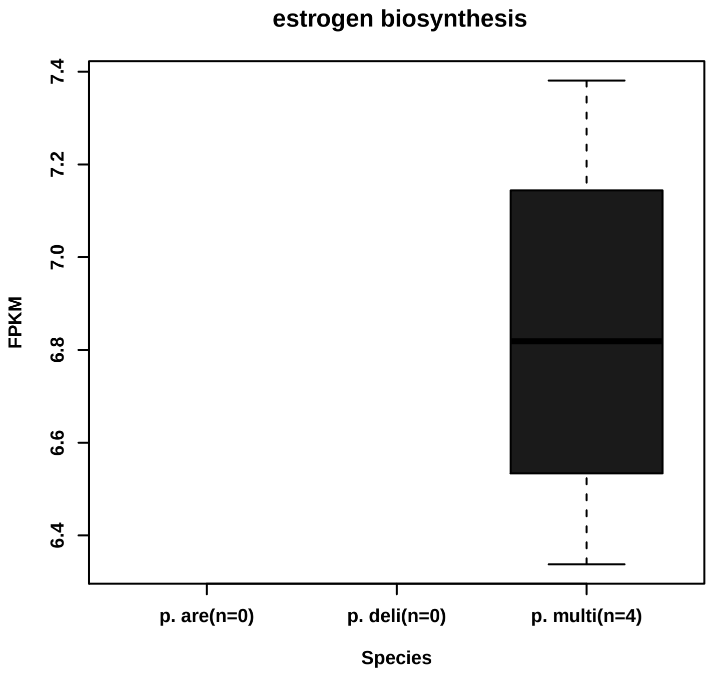
estrogen biosynthesis
FPKM
6.4
6.6
6.8
7.0
7.2
7.4
p. are(n=0)
p. deli(n=0)
p. multi(n=4)
Species

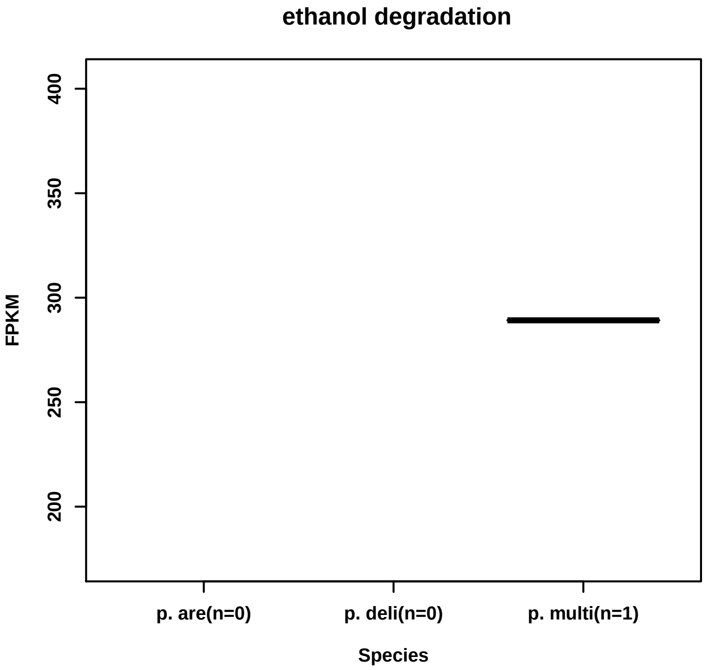
ethanol degradation
FPKM
200
250
300
350
400
p. are(n=0)
p. deli(n=0)
p. multi(n=1)
Species

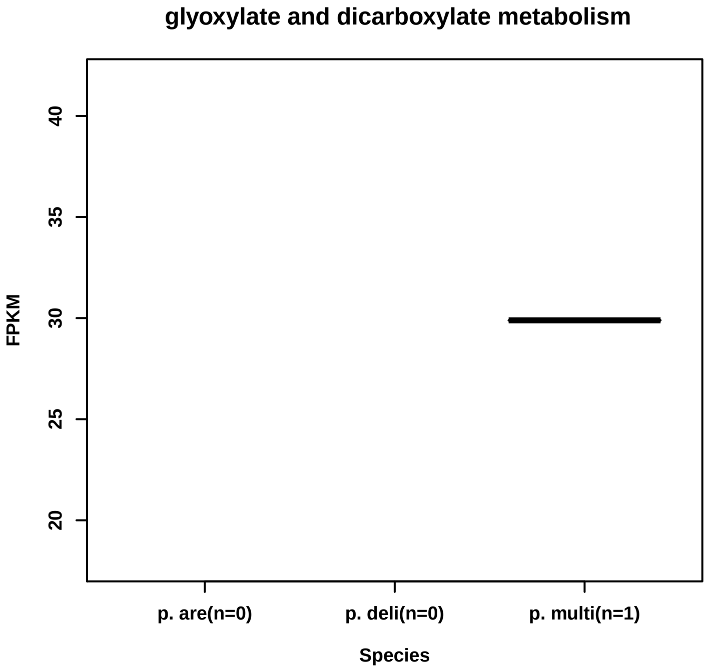
glyoxylate and dicarboxylate metabolism
FPKM
20
25
30
35
40
p. are(n=0)
p. deli(n=0)
p. multi(n=1)
Species

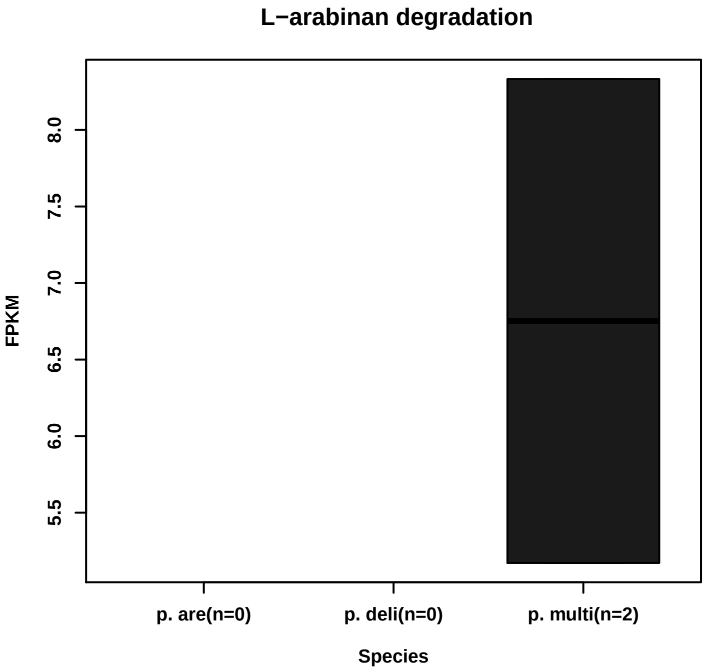
L−arabinan degradation
FPKM
5.5
6.0
6.5
7.0
7.5
8.0
p. are(n=0)
p. deli(n=0)
p. multi(n=2)
Species

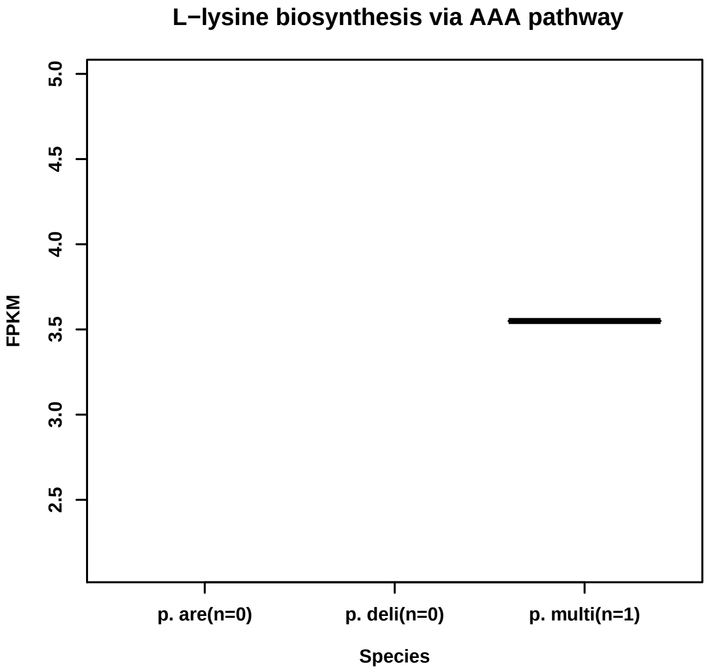
L−lysine biosynthesis via AAA pathway
FPKM
2.5
3.0
3.5
4.0
4.5
5.0
p. are(n=0)
p. deli(n=0)
p. multi(n=1)
Species

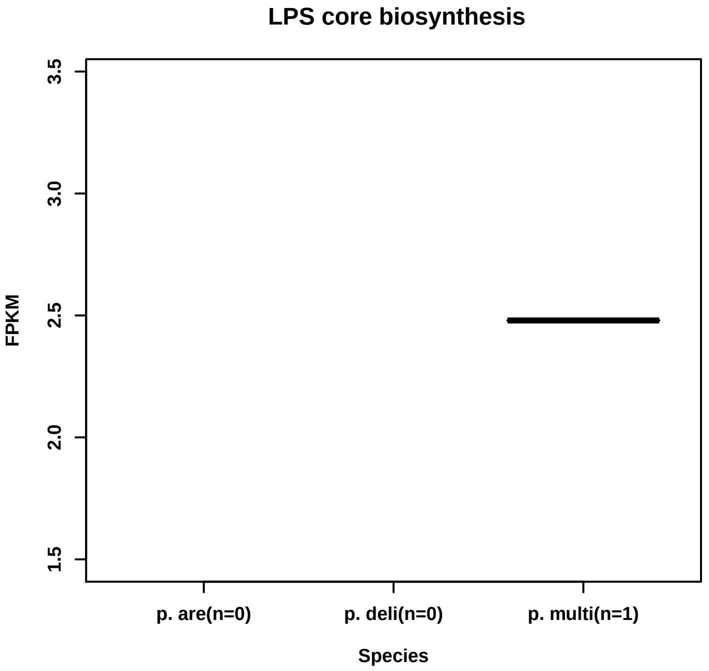
LPS core biosynthesis
FPKM
1.5
2.0
2.5
3.0
3.5
p. are(n=0)
p. deli(n=0)
p. multi(n=1)
Species

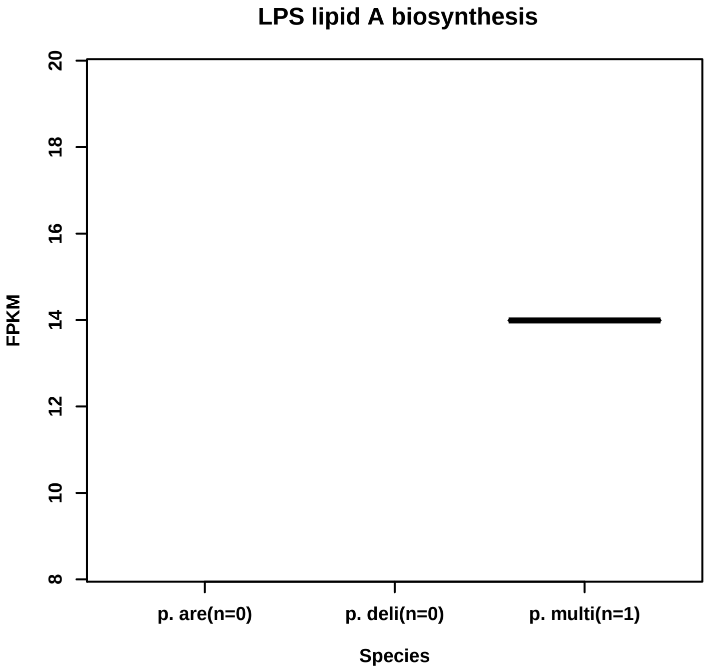
LPS lipid A biosynthesis
FPKM
8
10
12
14
16
18
20
p. are(n=0)
p. deli(n=0)
p. multi(n=1)
Species

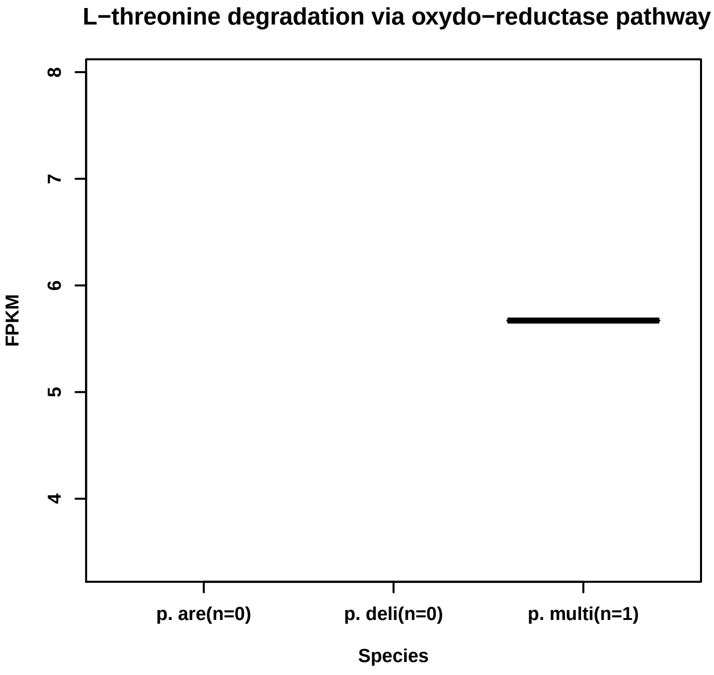
L−threonine degradation via oxydo−reductase pathway
FPKM
8
7
6
5
4
p. are(n=0)
p. deli(n=0)
p. multi(n=1)
Species

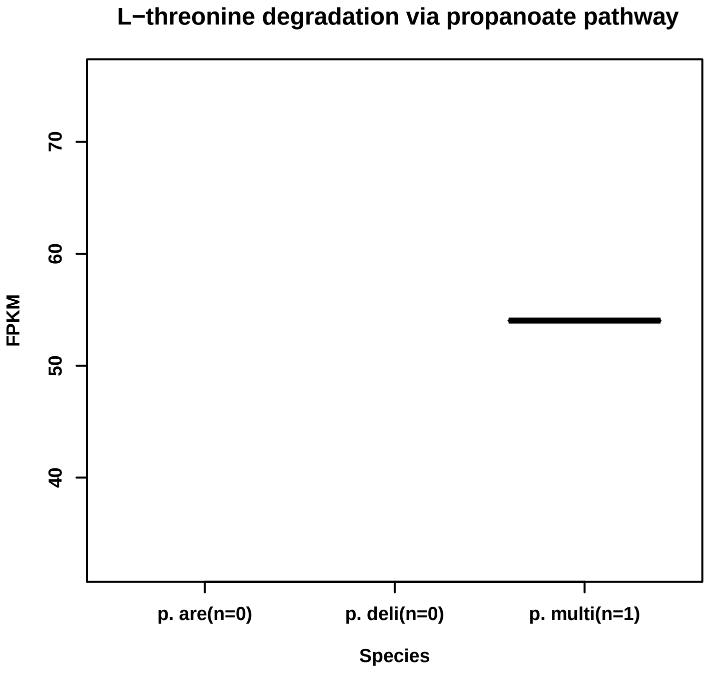
L−threonine degradation via propanoate pathway
FPKM
70
60
50
40
p. are(n=0)
p. deli(n=0)
p. multi(n=1)
Species

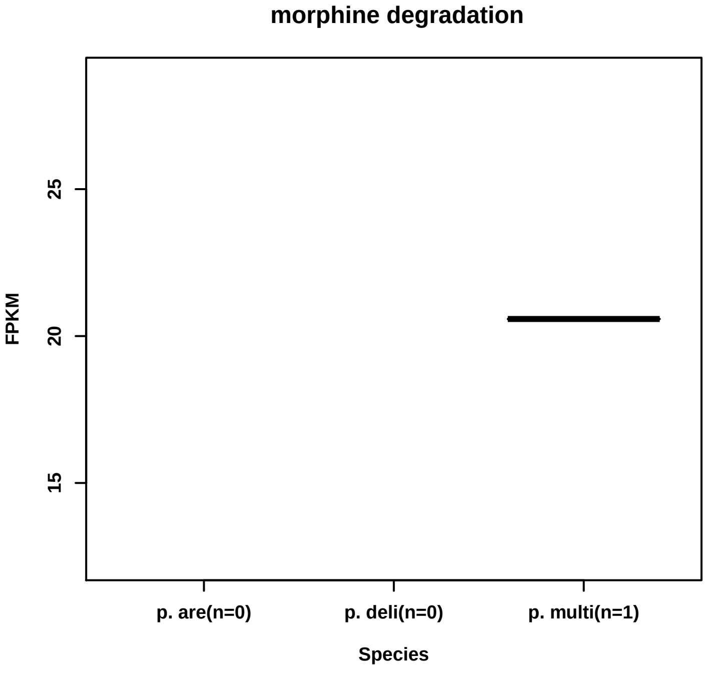
morphine degradation
FPKM
25
20
15
p. are(n=0)
p. deli(n=0)
p. multi(n=1)
Species

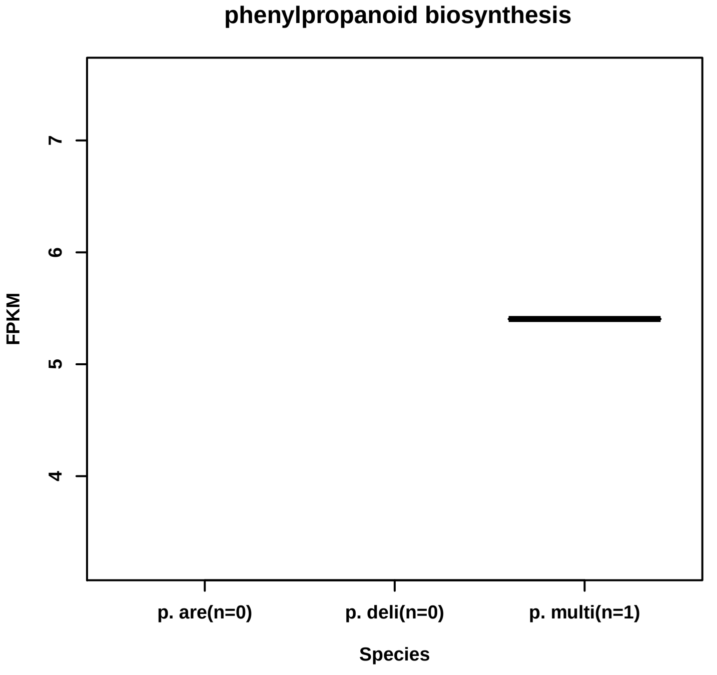
phenylpropanoid biosynthesis
FPKM
7
6
5
4
p. are(n=0)
p. deli(n=0)
p. multi(n=1)
Species

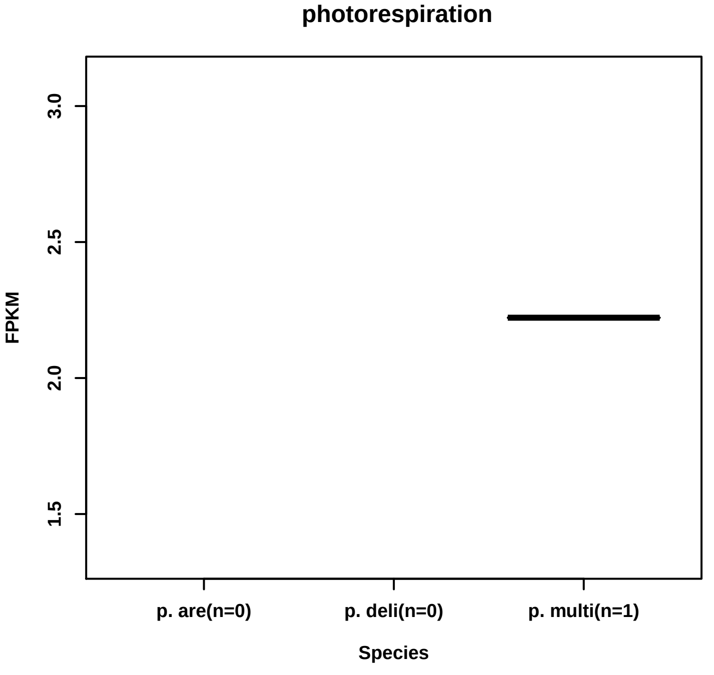
photorespiration
FPKM
3.0
2.5
2.0
1.5
p. are(n=0)
p. deli(n=0)
p. multi(n=1)
Species

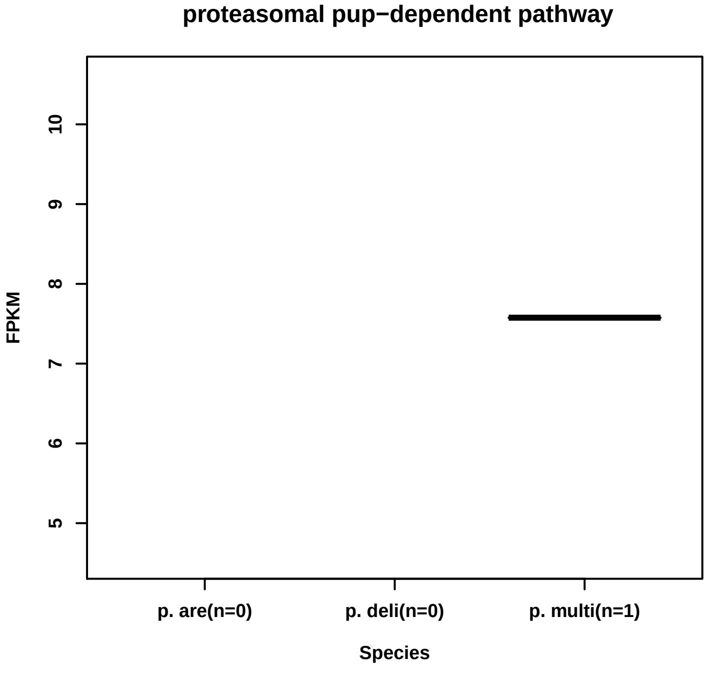
proteasomal pup−dependent pathway
FPKM
10
9
8
7
6
5
p. are(n=0)
p. deli(n=0)
p. multi(n=1)
Species

purine nucleoside salvage

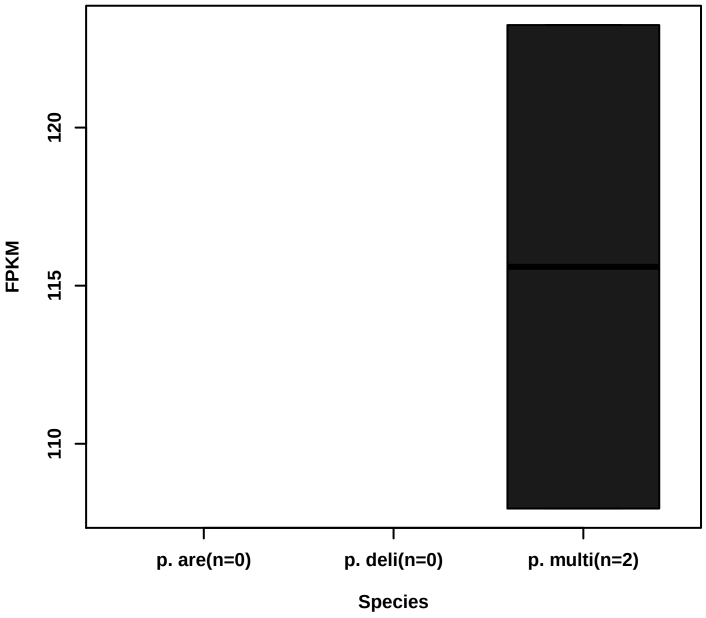

retinol metabolism

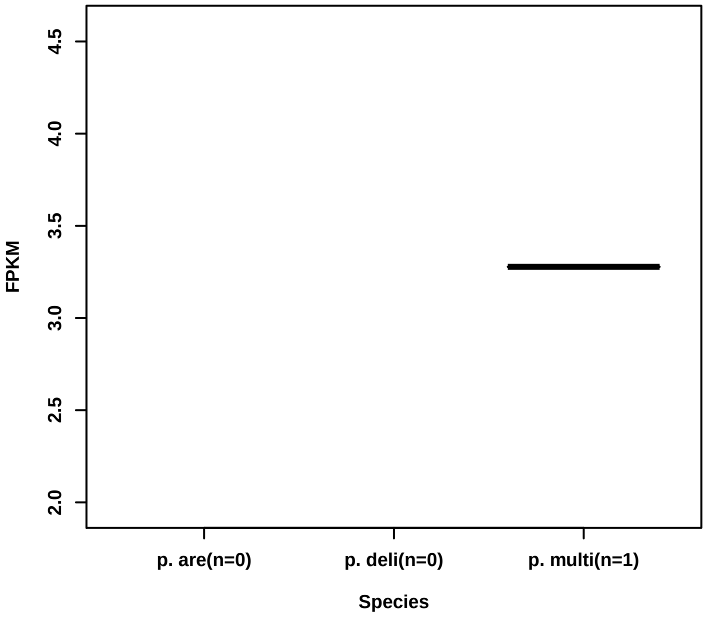

sulfur metabolism

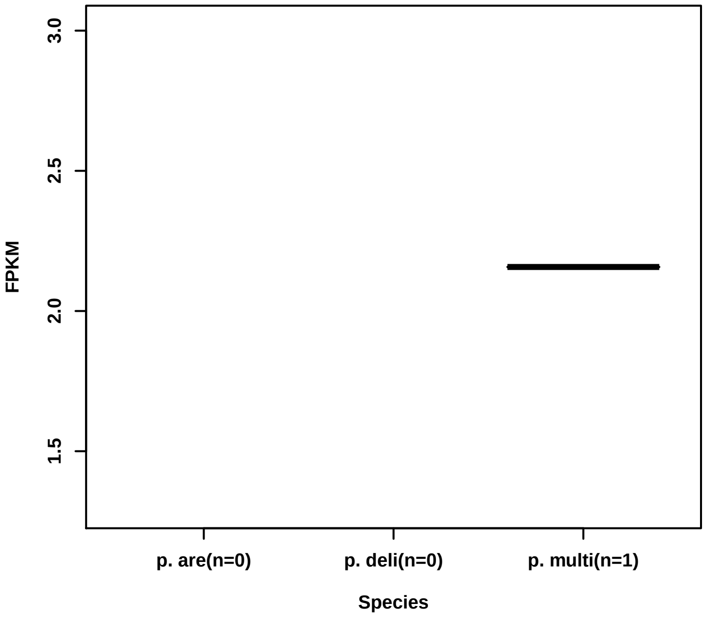

tropane alkaloid biosynthesis

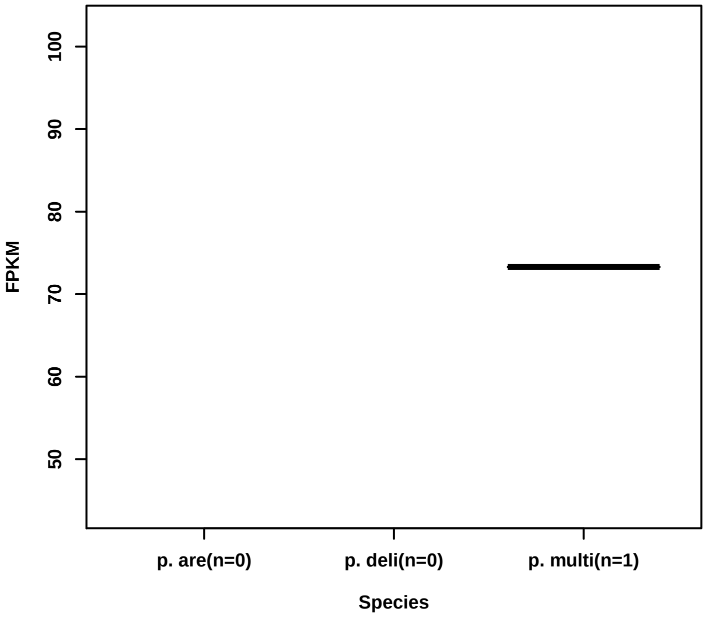

ubiquinone biosynthesis [regulation]

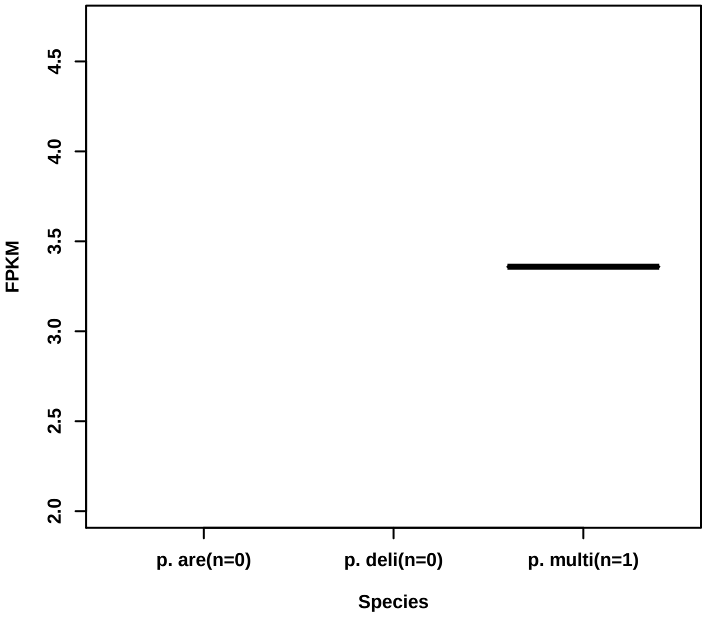

urate degradation

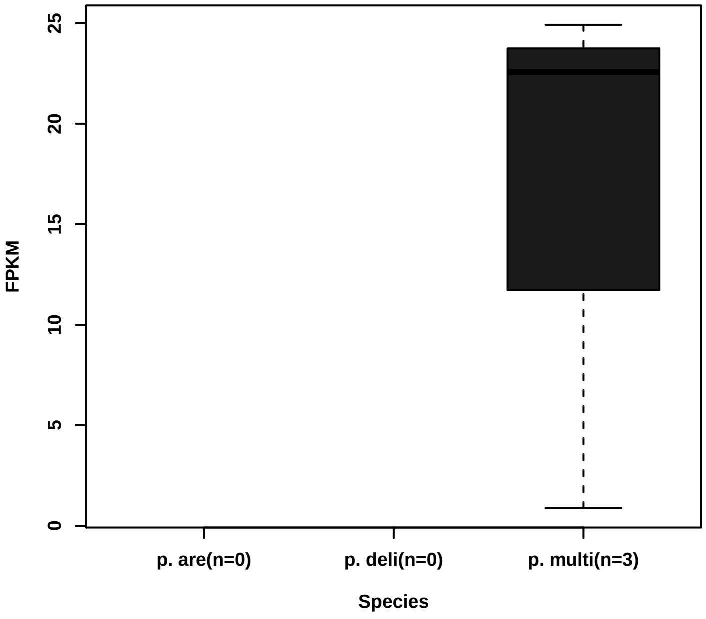

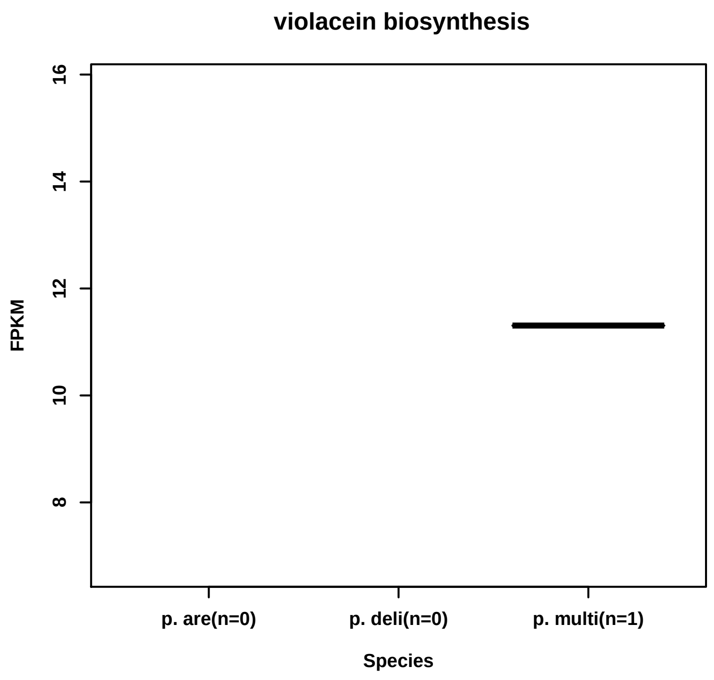

violacein biosynthesis
FPKM
16
14
12
10
8
p. are(n=0)
p. deli(n=0)
p. multi(n=1)
Species

Supplementary Figure S4. Neighbour joining tree of selected enzymes belonging to the isoprenoids pathways.

ClustalX neighbor joining tree phylogeny of isoprenoid enzymes including centric and pennate diatom sequences. HMGR = 3-Hydroxy-3-methylglutaryl-CoA reductase; MK = mevalonate kinase; MPDC = diphospho-MVA decarboxylase; DXS = 1-Deoxy-D-xylulose 5-phosphate synthase; DXR = 1-Deoxy-D-xylulose 5-phosphate reductoisomerase; CMK = 4-(Cytidine 5-diphospho)-2-C-methyl-D-erythritol kinase; HDR = 4-Hydroxy-3-methylbut-2-enyl diphosphate reductase; IPPI = isopentenyl diphosphate delta-isomerase.

The bootstrap values at each node represent the numbers of trees having the same node during the tree construction process.

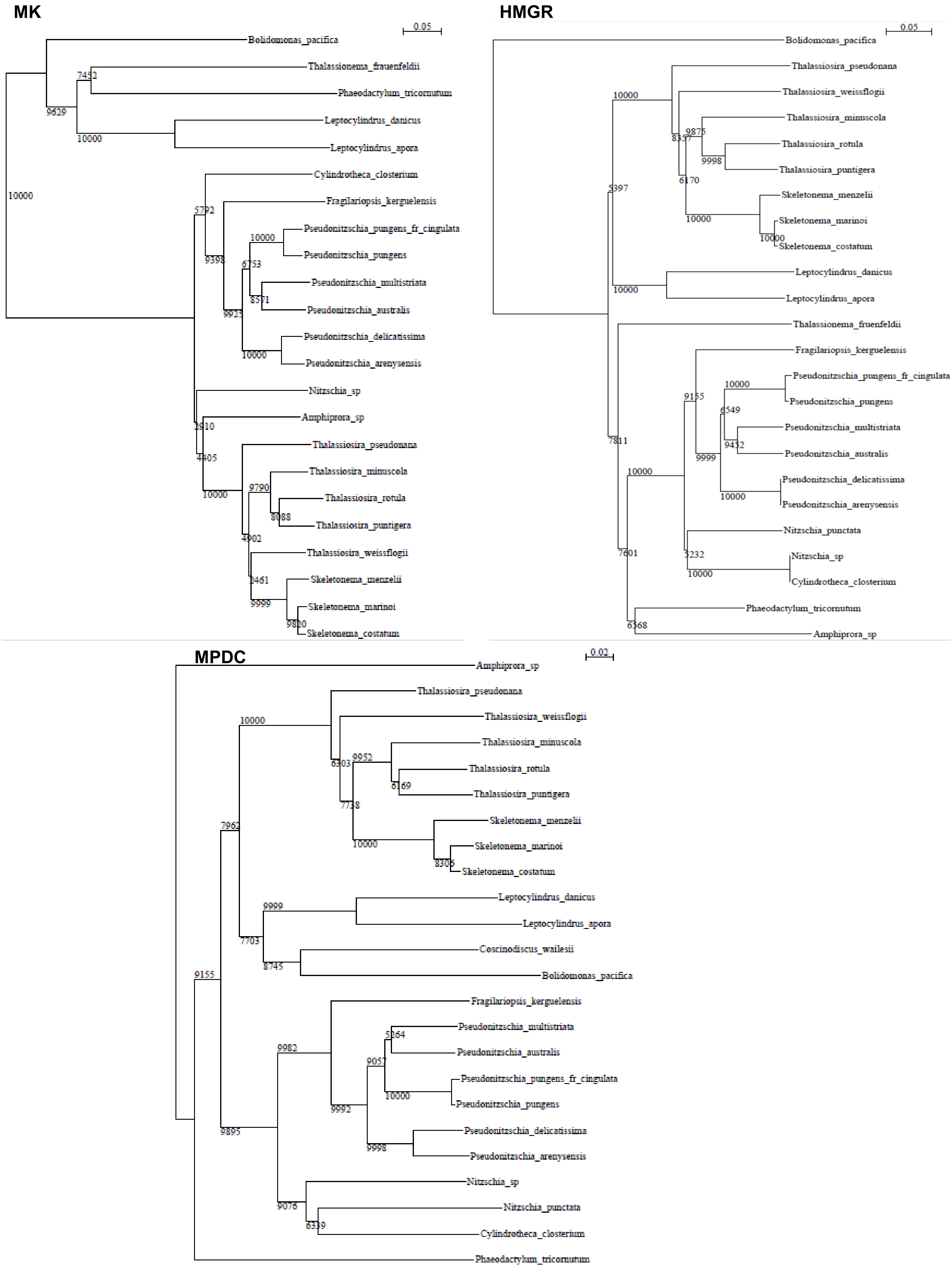
MK
0.05
Bolidomonas_pacifica
Thalassionema_frauenfeldii
Phaeodactylum_tricornutum
Leptocylindrus_danicus
Leptocylindrus_apora
Cylindrotheca_closterium
Fragilariopsis_kerguelensis
Pseudonitzschia_pungens_fr_cingulata
Pseudonitzschia_pungens
Pseudonitzschia_multistriata
Pseudonitzschia_australis
Pseudonitzschia_delicatissima
Pseudonitzschia_arenysensis
Nitzschia_sp
Amphiprora_sp
Thalassiosira_pseudonana
Thalassiosira_minuscola
Thalassiosira_rotula
Thalassiosira_puntigera
Thalassiosira_weissflogii
Skeletonema_menzelii
Skeletonema_marinoi
Skeletonema_costatum
7452
9629
10000
10000
5792
9398
10000
6753
8571
9925
10000
2910
4405
10000
9790
8088
4902
2461
9999
9800
HMGR
0.05
Bolidomonas_pacifica
Thalassiosira_pseudonana
Thalassiosira_weissflogii
Thalassiosira_minuscola
Thalassiosira_rotula
Thalassiosira_puntigera
Skeletonema_menzelii
Skeletonema_marinoi
Skeletonema_costatum
Leptocylindrus_danicus
Leptocylindrus_apora
Thalassionema_fruenfeldii
Fragilariopsis_kerguelensis
Pseudonitzschia_pungens_fr_cingulata
Pseudonitzschia_pungens
Pseudonitzschia_multistriata
Pseudonitzschia_australis
Pseudonitzschia_delicatissima
Pseudonitzschia_arenysensis
Nitzschia_punctata
Nitzschia_sp
Cylindrotheca_closterium
Phaeodactylum_tricornutum
Amphiprora_sp
10000
8357
9875
9998
6170
10000
10000
5397
10000
10000
9155
6549
9452
9999
10000
7811
10000
7601
5232
10000
6568
MPDC
0.02
Amphiprora_sp
Thalassiosira_pseudonana
Thalassiosira_weissflogii
Thalassiosira_minuscola
Thalassiosira_rotula
Thalassiosira_puntigera
Skeletonema_menzelii
Skeletonema_marinoi
Skeletonema_costatum
Leptocylindrus_danicus
Leptocylindrus_apora
Coscinodiscus_wailesii
Bolidomonas_pacifica
Fragilariopsis_kerguelensis
Pseudonitzschia_multistriata
Pseudonitzschia_australis
Pseudonitzschia_pungens_fr_cingulata
Pseudonitzschia_pungens
Pseudonitzschia_delicatissima
Pseudonitzschia_arenysensis
Nitzschia_sp
Nitzschia_punctata
Cylindrotheca_closterium
Phaeodactylum_tricornutum
10000
6303
9952
6169
7738
10000
8306
7962
9999
7703
8745
9155
5264
9982
9057
10000
9992
9998
9895
9076
6339

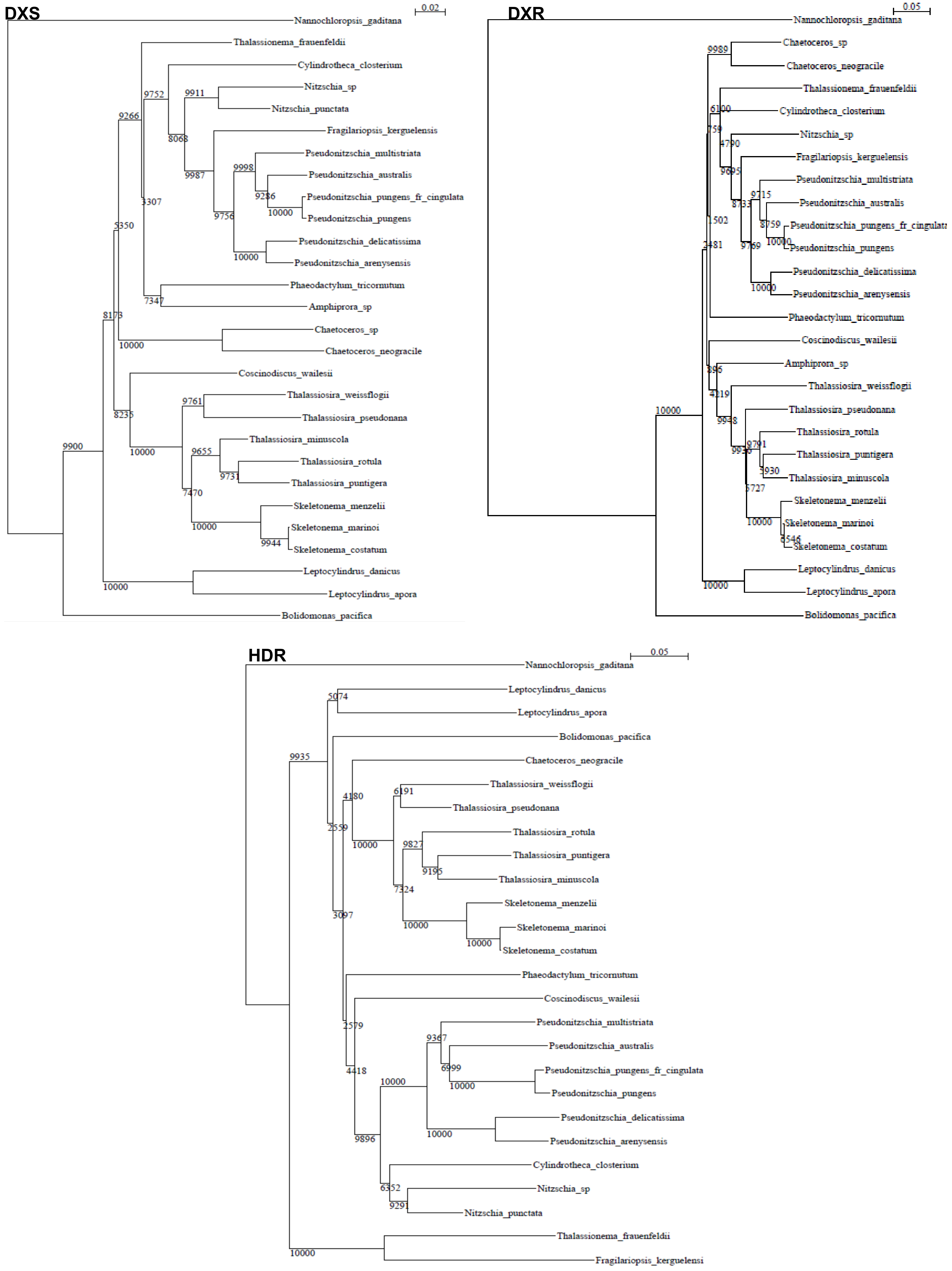

DXS
0.02
Nannochloropsis_gaditana
Thalassionema_frauenfeldii
Cylindrotheca_closterium
Nitzschia_sp
Nitzschia_punctata
Fragilariopsis_kerguelensis
Pseudonitzschia_multistriata
Pseudonitzschia_australis
Pseudonitzschia_pungens_fr_cingulata
Pseudonitzschia_pungens
Pseudonitzschia_delicatissima
Pseudonitzschia_arenysensis
Phaeodactylum_tricornutum
Amphiprora_sp
Chaetoceros_sp
Chaetoceros_neogracile
Coscinodiscus_wailesii
Thalassiosira_weissflogii
Thalassiosira_pseudonana
Thalassiosira_minuscola
Thalassiosira_rotula
Thalassiosira_puntigera
Skeletonema_menzelii
Skeletonema_marinoi
Skeletonema_costatum
Leptocylindrus_danicus
Leptocylindrus_apora
Bolidomonas_pacifica
DXR
0.05
Nannochloropsis_gaditana
Chaetoceros_sp
Chaetoceros_neogracile
Thalassionema_frauenfeldii
Cylindrotheca_closterium
Nitzschia_sp
Fragilariopsis_kerguelensis
Pseudonitzschia_multistriata
Pseudonitzschia_australis
Pseudonitzschia_pungens
Pseudonitzschia_delicatissima
Pseudonitzschia_arenysensis
Phaeodactylum_tricornutum
Coscinodiscus_wailesii
Amphiprora_sp
Thalassiosira_weissflogii
Thalassiosira_pseudonana
Thalassiosira_rotula
Thalassiosira_puntigera
Thalassiosira_minuscola
Skeletonema_menzelii
Skeletonema_marinoi
Skeletonema_costatum
Leptocylindrus_danicus
Leptocylindrus_apora
Bolidomonas_pacifica
HDR
0.05
Nannochloropsis_gaditana
Leptocylindrus_danicus
Leptocylindrus_apora
Bolidomonas_pacifica
Chaetoceros_neogracile
Thalassiosira_weissflogii
Thalassiosira_pseudonana
Thalassiosira_rotula
Thalassiosira_puntigera
Thalassiosira_minuscola
Skeletonema_menzelii
Skeletonema_marinoi
Skeletonema_costatum
Phaeodactylum_tricornutum
Coscinodiscus_wailesii
Pseudonitzschia_multistriata
Pseudonitzschia_australis
Pseudonitzschia_pungens_fr_cingulata
Pseudonitzschia_pungens
Pseudonitzschia_delicatissima
Pseudonitzschia_arenysensis
Cylindrotheca_closterium
Nitzschia_sp
Nitzschia_punctata
Thalassionema_frauenfeldii

CMK

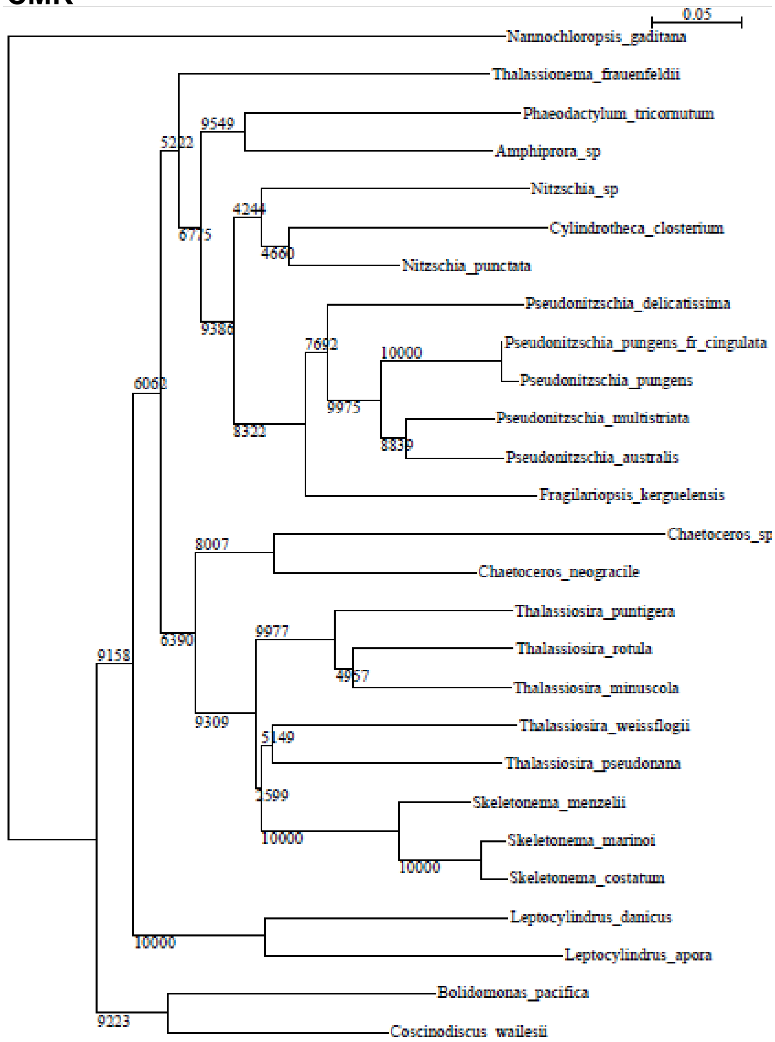

0.05
Nannochloropsis_gaditana
Thalassionema_frauenfeldii
Phaeodactylum_tricornutum
Amphiprora_sp
Nitzschia_sp
Cylindrotheca_closterium
Nitzschia_punctata
Pseudonitzschia_delicatissima
Pseudonitzschia_pungens_fr_cingulata
Pseudonitzschia_pungens
Pseudonitzschia_multistriata
Pseudonitzschia_australis
Fragilariopsis_kerguelensis
Chaetoceros_sp
Chaetoceros_neogracile
Thalassiosira_puntigera
Thalassiosira_rotula
Thalassiosira_minuscola
Thalassiosira_weissflogii
Thalassiosira_pseudonana
Skeletonema_menzelii
Skeletonema_marinoi
Skeletonema_costatum
Leptocylindrus_danicus
Leptocylindrus_apora
Bolidomonas_pacifica
Coscinodiscus_wailesii

IPPI

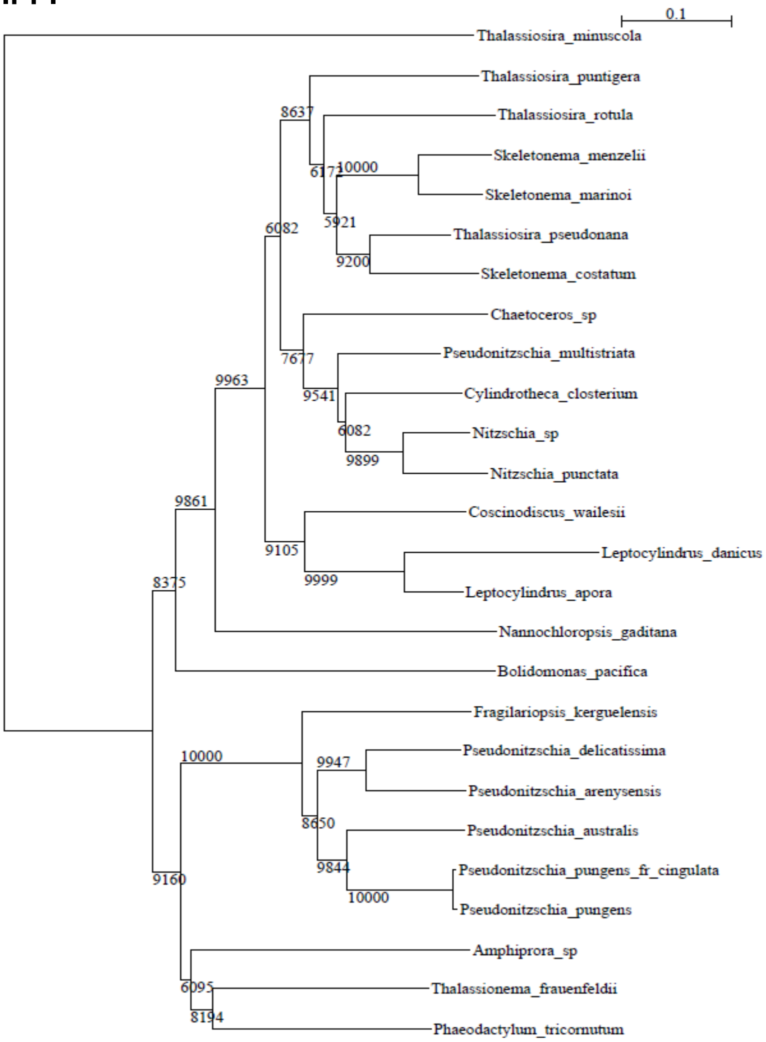

0.1
Thalassiosira_minuscola
Thalassiosira_puntigera
Thalassiosira_rotula
Skeletonema_menzelii
Skeletonema_marinoi
Thalassiosira_pseudonana
Skeletonema_costatum
Chaetoceros_sp
Pseudonitzschia_multistriata
Cylindrotheca_closterium
Nitzschia_sp
Nitzschia_punctata
Coscinodiscus_wailesii
Leptocylindrus_danicus
Leptocylindrus_apora
Nannochloropsis_gaditana
Bolidomonas_pacifica
Fragilariopsis_kerguelensis
Pseudonitzschia_delicatissima
Pseudonitzschia_arenysensis
Pseudonitzschia_australis
Pseudonitzschia_pungens_fr_cingulata
Pseudonitzschia_pungens
Amphiprora_sp
Thalassionema_frauenfeldii
Phaeodactylum_tricornutum

Supplementary Table S1. Transcriptome size and statistics of selected diatom species sequenced within the MMETSP.

SPECIES	Total Number of Sequences	N50	Average length	Median length	Maximum length	% GC
Fragilariopsis kerguelensis L26-C5 -Fe_LL	56173	1754	1035	680	16027	41
Fragilariopsis kerguelensis L26-C5 -Fe_SL	48729	1786	1078	744	16511	41
Fragilariopsis kerguelensis L26-C5 Fe_LL	51006	1818	1078	717	16160	41
Fragilariopsis kerguelensis L26-C5 Fe_SL	50930	1927	1109	709	23605	41
Leptocylindrus danicus var. apora B651	17776	1982	1478	1234	16580	40
Leptocylindrus danicus var. danicus B650	30346	1969	1245	893	29076	44
Nitzschia punctata CCMP561 Exponential	24774	1982	1456	1244	9581	46
Nitzschia punctata CCMP561 N-limited	24323	1799	1270	1005	10214	46
Nitzschia punctata CCMP561 Nocodazole	20545	1728	1265	1011	9835	46
Nitzschia punctata CCMP561 Si-limited	20590	1682	1248	1021	8377	46
Pseudo-nitzschia australis Glutammate as N source	18267	2219	1367	1140	8451	47
Pseudo-nitzschia australis NO3 as N source	25559	2213	1546	1371	9261	48
Pseudo-nitzschia australis Urea as N source	24075	2093	1515	1388	8730	47
Pseudo-nitzschia australis NH4 as N source	25365	2186	1524	1359	9246	47
Pseudo-nitzschia fraudulenta Silica limited 390ppm	54003	1697	924	503	10672	56
Pseudo-nitzschia fraudulenta 390ppm_Replete	15456	1940	1217	977	9952	56
Pseudo-nitzschia fraudulenta Silica limited 750ppm	16902	1800	1133	924	7371	49
Pseudo-nitzschia fraudulenta 750ppm_Replete	18640	1868	1056	705	11216	49
Pseudo-nitzschia heimii	16361	2163	1315	1029	13808	51
Pseudo-nitzschia pungens cf. cingulata	17200	2698	1510	1041	20706	49
Pseudo-nitzschia pungens cf. pungens	20080	2192	1587	1307	17488	49
Skeletonema marinoi FE60	19052	2029	1409	1107	13123	45
Skeletonema marinoi FE7	21683	1894	1343	1105	11734	45
Skeletonema marinoi SM1012Hels-07	30397	2475	1688	1311	15814	45
Skeletonema marinoi SM1012Den-03	31328	2519	1664	1269	14168	45
Thalassiosira puntigera C2	32778	1732	942	492	14941	55
Thalassiosira sp. NH16	35814	2192	1587	1307	17488	52
Thalassiosira sp. Th. Sp. FW	12781	2666	1695	1275	27914	47
Thalassiosira weissflogii CCMP1336 20nM Fe	18480	2208	1409	1111	12742	46
Thalassiosira weissflogii CCMP1336 20nM Fe	18662	2250	1428	1119	11100	46
Thalassiosira weissflogii CCMP1336 60 nMFe	18623	2242	1430	1133	13644	46
Thalassiosira weissflogii CCMP1336 60 nMFe	18624	2321	1474	1148	18032	46

Supplementary Table S8. List of the C4 photosynthetic carbon cycle genes[1] and their corresponding transcript IDs in the three *Pseudo-nitzschia*.

GENES	*Pseudo-nitzschia arenysensis*	*Pseudo-nitzschia delicatissima*	*Pseudo-nitzschia multistriata*
Carbonic anydrase	MMETSP0329-20121206|10<br>MMETSP0329-20121206|11309<br>MMETSP0329-20121206|11950<br>MMETSP0329-20121206|16679<br>MMETSP0329-20121206|19581<br>MMETSP0329-20121206|21379<br>MMETSP0329-20121206|21593<br>MMETSP0329-20121206|22047<br>MMETSP0329-20121206|2449<br>MMETSP0329-20121206|6511<br>MMETSP0329-20121206|7080<br>MMETSP0329-20121206|9814<br>MMETSP0329-20121206|9817	MMETSP0327-20121206|12916<br>MMETSP0327-20121206|13138<br>MMETSP0327-20121206|13750<br>MMETSP0327-20121206|19895<br>MMETSP0327-20121206|2135<br>MMETSP0327-20121206|7716<br>MMETSP0327-20121206|89<br>MMETSP0327-20121206|9960	Comp10350_c0_seq2<br>comp10602_c0_seq2<br>comp10604_c0_seq1<br>comp12090_c0_seq1<br>comp13851_c0_seq1/2<br>comp3866_c0_seq1<br>comp3951_c0_seq1<br>comp4639_c0_seq1<br>comp4674_c0_seq1<br>comp8539_c0_seq1
Malate dehydrogensase (MDH)	MMETSP0329-20121206|3423<br>MMETSP0329-20121206|8111	MMETSP0327-20121206|13132<br>MMETSP0327-20121206|2460<br>MMETSP0327-20121206|8089	Comp13442_c0_seq1/2/5<br>comp3147_c0_seq1
NAD Malic Enzyme	MMETSP0329-20121206|5779	MMETSP0327-20121206|7012	comp14698_c0_seq1-4
Phosphoenolpyruvate carboxylase (PEPC)	MMETSP0329-20121206|13428<br>MMETSP0329-20121206|4035<br>MMETSP0329-20121206|4516	MMETSP0327-20121206|10283<br>MMETSP0327-20121206|1569<br>MMETSP0327-20121206|18670<br>MMETSP0327-20121206|2331	Comp13217_c0_seq1<br>comp5966_c0_seq1
Phosphenolpyruvate carboxykinase (PEPCK)	MMETSP0329-20121206|3602<br>MMETSP0329-20121206|4400	MMETSP0327-20121206|12843<br>MMETSP0327-20121206|1562<br>MMETSP0327-20121206|19812	
Pyruvate kinase (PK)	MMETSP0329-20121206|12757<br>MMETSP0329-20121206|22082<br>MMETSP0329-20121206|6571<br>MMETSP0329-20121206|7148<br>MMETSP0329-20121206|8630	MMETSP0327-20121206|13875<br>MMETSP0327-20121206|10290<br>MMETSP0327-20121206|1193<br>MMETSP0327-20121206|12468<br>MMETSP0327-20121206|19729<br>MMETSP0327-20121206|7774<br>MMETSP0327-20121206|8994	Comp10607_c0_seq1-3<br>comp13968_co_seq1<br>Comp15562_c0_seq3<br>Comp7347_c0_seq1-3<br>Comp9010_c0_seq1<br>Comp9501_c0_seq1-8
Pyruvate-phosphate dikinase (PPDK)	MMETSP0329-20121206|17567<br>MMETSP0329-20121206|4315<br>MMETSP0329-20121206|13199	MMETSP0327-20121206|1616<br>MMETSP0327-20121206|9199	Comp4924_c0_seq1

Transcripts IDs were retrieved with a name search in the annotation tables.

[1] Kroth, P.G., Chiovitti, A., Gruber, A., Martin-Jezequel, V., Mock, T., Parker, M.S., Stanley, M.S., Kaplan, A., Caron, L., Weber, T., et al. (2008). A Model for Carbohydrate Metabolism in the Diatom *Phaeodactylum tricornutum* Deduced from Comparative Whole Genome Analysis. PLoS ONE *3*.

Supplementary Table S9. List of the Urea cycle genes and their corresponding transcript IDs in the three *Pseudo-nitzschia*.

GENES	*Pseudo-nitzschia arenysensis*	*Pseudo-nitzschia delicatissima*	*Pseudo-nitzschia multistriata*
Ornithine cyclodeaminase	MMETSP0329-20121206|16601<br>MMETSP0329-20121206|12770	MMETSP0327-20121206|9866	comp2508_c0_seq1<br>comp10073_c0_seq2
Agmatine ureohydrolase	MMETSP0329-20121206|21557<br>MMETSP0329-20121206|6021	MMETSP0327-20121206|14705<br>MMETSP0327-20121206|232	comp4538_c0_seq1<br>comp4538_c0_seq2
Urease	MMETSP0329-20121206|20423<br>MMETSP0329-20121206|3502<br>MMETSP0329-20121206|22452<br>MMETSP0329-20121206|4831	MMETSP0327-20121206|18997<br>MMETSP0327-20121206|10411<br>MMETSP0327-20121206|8851<br>MMETSP0327-20121206|1463	comp14288_c0_seq2
Aspartate aminotransferase	MMETSP0329-20121206|13842<br>MMETSP0329-20121206|14937<br>MMETSP0329-20121206|4801<br>MMETSP0329-20121206|7116	MMETSP0327-20121206|4731<br>MMETSP0327-20121206|5235<br>MMETSP0327-20121206|9520	comp11127_c0_seq1-5<br>comp12679_c0_seq1<br>comp12679_c0_seq3<br>comp14098_c0_seq1<br>comp7127_c0_seq1<br>comp8502_c0_seq1<br>comp9942_c0_seq2
ODC2 - ornithine decarboxylase 2	MMETSP0329-20121206|5745<br>MMETSP0329-20121206|19848<br>MMETSP0329-20121206|11971<br>MMETSP0329-20121206|6946	MMETSP0327-20121206|11700<br>MMETSP0327-20121206|12906<br>MMETSP0327-20121206|4293	comp12949_c0_seq1<br>comp8700_c0_seq1
Urea carrier	MMETSP0329-20121206|11970<br>MMETSP0329-20121206|4419<br>MMETSP0329-20121206|4956	MMETSP0327-20121206|13582<br>MMETSP0327-20121206|17340<br>MMETSP0327-20121206|596	comp11844_c0_seq1<br>comp15168_co_seq1
Arginase	MMETSP0329-20121206|21557<br>MMETSP0329-20121206|6021	MMETSP0327-20121206|14705<br>MMETSP0327-20121206|232	comp4538_c0_seq1<br>comp4538_c0_seq2
Fumarate lyase<br>Argininosuccinate lyase	MMETSP0329-20121206|21824<br>MMETSP0329-20121206|2578<br>MMETSP0329-20121206|14971<br>MMETSP0329-20121206|5583<br>MMETSP0329-20121206|6329	MMETSP0327-20121206|10118<br>MMETSP0327-20121206|10669<br>MMETSP0327-20121206|17196<br>MMETSP0327-20121206|17458<br>MMETSP0327-20121206|7445<br>MMETSP0327-20121206|7743	comp8093_c0_seq1<br>comp12477_c0_seq1<br>comp4933_c0_seq1<br>comp13109_c0_seq1
Allantoicase	MMETSP0329-20121206|23359<br>MMETSP0329-20121206|14584<br>MMETSP0329-20121206|23318<br>MMETSP0329-20121206|22834	MMETSP0327-20121206|2270<br>MMETSP0327-20121206|6752	comp7156_c0_seq1
Ornithine transcarbamylase	MMETSP0329-20121206|14902<br>MMETSP0329-20121206|15636	MMETSP0327-20121206|12919<br>MMETSP0327-20121206|19499<br>MMETSP0327-20121206|6375	comp8342_c0_seq1<br>comp5549_c0_seq1
Argininosuccinate synthase	MMETSP0329-20121206|14126<br>MMETSP0329-20121206|9021	MMETSP0327-20121206|15742<br>MMETSP0327-20121206|15160<br>MMETSP0327-20121206|11703	comp2518_c0_seq1
Urea-proton symporter DUR3	MMETSP0329-20121206|4419<br>MMETSP0329-20121206|4956<br>MMETSP0329-20121206|11970	MMETSP0327-20121206|13582	comp15168_c0_seq1
CPS III- carbamoyl-phosphate synthase (mitochondrial precursor)	MMETSP0329-20121206|10336<br>MMETSP0329-20121206|6833<br>MMETSP0329-20121206|7908<br>MMETSP0329-20121206|22067	MMETSP0327-20121206|6375<br>MMETSP0327-20121206|5850<br>MMETSP0327-20121206|11066<br>MMETSP0327-20121206|4753	comp15368_c0_seq1<br>comp12152_c0_seq1

Transcripts IDs were retrieved with a tblastn search in each *Pseudo-nitzscha* transcriptome using *Phaeodactylum tricornutum* proteins as a query. *P. tricornutum* protein IDs were taken from Levitan et al., 2015[1].

[1] Levitan, O., Dinamarca, J., Zelzion, E., Lun, D.S., Guerra, L.T., Kim, M.K., Kim, J., Van Mooy, B.A.S., Bhattacharya, D., and Falkowski, P.G. (2015). Remodeling of intermediate metabolism in the diatom *Phaeodactylum tricornutum* under nitrogen stress. Proc. Natl. Acad. Sci. U.S.A. *112*, 412–417.

Supplementary Note

```
>Pseudo-nitzschia_multistriata_NOS
MGIFSKHCCGGDTLNQVIHHQPEHKKDEADNEETCNRTDLWPTHTVTSRMERSESSLEFSRRVMTSNYRSQRD
ASTPQRSSSNSQSSIKSTCSSVESTESDLGHLKEKAIEEALTASYLDVRNVESRHGRSESSPPTEQSLNTSMH
SKRMILKRDSERGDFGDKKHFYESLVASTEDVLEKINEGWGIKGSFTMEPAMYGAPLSKVPLEQKHHAVLISQ
SWNKVLAFRSMFAEALISRWRLLASVEYIEEQALREKNPVVPNSRRHFPSFHQQLWQVGFSPDVQALNLIESN
NDPIAKCFETRTADLQVLVVSMIDTAVRGLCPHSQTIQRESYRPIDGSADSDPDISQVFFHRKDCTTFEDFCF
LFARYWVKPHHWLTLCRAFLWAMKSQNPYCIDDEKDDLDRDPADSVHARFFTGMIILPILEANLRRANYMRKG
VFEELKVCISNDRLGNKFDTIGVEMFKKLFETFPEIKDHFCENDVEDMSYGLFEMIYVLVQGSGRDLANPQSN
LRKILREQSMKWVELGLSTHVVYLIGREILEGMETIFGDSVNHSKEKLRNAFAFLWKHSIQFVLHPILLQQNL
QAEALKFYNDVAAELKWSTSTKQNRLFEVNMEILATGEYTHTQEELEIGARLAWRNSAKCIGRIAWNTLVVRD
RRHCTQPSEIFNEVCEHMKLATGGASIQSVMTVFRPQRHNELFGTRFWSSQFIRYCGYKDRDSEEIMGDPACV
DLTSYLIEKKLWTPPERRTAFDILPLVLKIPGNDIPFIYQLPKSVTHEVHLEHPEFPEVKDLGYKWAAVPAIS
NFMMNLGGIKYSAAPFNGWFVSIEVARNLLERYHVTEPLAIAMNMNLSDKFLAQKVSAELESAIIHSFEKSDF
TIVDPDTVGHSFITHCKRERSAGRECPAQWSWIggptnPVWHLEMRDFRVVPQFEYCCDPWRVLDIDGVSGTK
AIDTTGHILSEDDFSQKVSMPEVLIAYGSETGKAEAVANSLARTLKFCRPTVLTLNGALEYNQELGKFSHILI
ICSTFGAGEPPLNAKKFANAELAGKVTGVKYAVLALGSSLYPDFCKAGKGIDSQMEAAGANSIMNIVCADAAK
GNQSDIYNWSIFLKKLLLPNSLLVELESSGIPVEDTKAAEYRIRWLAKDEYDEEYSKHRLRTGTMKCRKNQDL
FEGSDPLRSTRHIELEQIDKTDIAYETGDHLSVVPINSMDMVSRFCNCFAPELEKAAESSGFHRLKLKASRKK
FLQKTSARMNCTPSVLWQTHQPFYIESVEDQGTTPCNAKHLTNNNLADVLQFSLDLSLSSESYLVEFLQMLNS
KLEEASLTLCCASDFQTMAKDAVQQCNEEGDSGMINNIKATYPTIVHFLEKFNDLFCKPLETRKNPLLGLADV
LVIMPRLGPRQYSISSSALTSPNKVSITVGVVKFETSRDVLVEGVCSHDLAGLEPGQMINASVVKSPFRAPRS
IKSPLIMIGAGTGLAPFMGFLRDRSLAMKKAGASRRGMFGKCHLFFGCRSEGEMLYKDQLLEWESSGVAKLHI
AYSREPGIHCPNRYVHDAMEDRGAELFALMTSEPKTHLYICGDVNMAKACSEKCISLLQEHGSMSKINATRFM
SNMRVSNRFQLDVWGTREVEIVDIDFSYHGNANTRLIKEASRRTIMREFNEKY
```

IDs of the transcriptomes in the MMETSP used for the Isoprenoids analysis.

SPECIES	MMETSP ID
Amphiprora sp	MMETSP 0724
Bolidomonas pacifica	MMETSP 0785
Chaetoceros neogracile	MMETSP 1336
Chaetoceros sp	MMETSP 0200
Coscinodiscus wailesii	MMETSP 1066
Cylindrotheca closterium	MMETSP 0017
Fragilariopsis kerguelensis	MMETSP 0733
Leptocylindrus apora	MMETSP 0322
Leptocylindrus danicus	MMETSP 0321
Nitzschia punctata	MMETSP 0744
Nitzschia sp	MMETSP 0014
Pseudo-nitzschia australis	MMETSP 0139
Pseudo-nitzschia pungens	MMETSP 1061
Pseudo-nitzschia pungens fr cingulata	MMETSP 1060
Skeletonema costatum	MMETSP 0013
Skeletonema marinoi	MMETSP 1039
Skeletonema menzelii	MMETSP 0603
Thalassionema frauenfeldii	MMETSP 0786
Thalassiosira minuscola	MMETSP 0737
Thalassiosira puntigera	MMETSP 1067
Thalassiosira rotula	MMETSP 0403
Thalassiosira weissflogii	MMETSP 0878

Isoprenoids protein sequences used for phylogenetic trees.

HMGR protein sequences

***>Amphiprora_sp/*HMGR**
PFHVHLKNVKRAMSSAMTLGMQIDAVINELSNLSSTQQYAIIVIGVGLVATLLLGSADH
PGLEMTTAPPKTAVSPSKPVKTDKNSQPEPRWYIFRMINYAMMVAFVASVADFVWNY
AAYAVDSAVLLRFLIGWSACLIYFFGFFGVSFVHDVVPPVDEGLAPSPPQPEKIPSAKM
VSSSRNQTVTIPKSMKKKAVEAPCPKDVPVCSDPSSFATSSKKAPPASNLTELSDDQIA
NLVLTGQMKDHELEKRLDCHRAVDVRRLVIREKLAKVGNDPSSLQELPSQPSLDYSRV
HGANCEIVVGYVPLPVGMVGPLRINDMTVYVPMATTEGCLVASANRGCKAISVTGAN
AMVIRDGITRAPCIRMPSAMEAARLKLWCDEPTNFAAMKAAFETTTSFGKLKSCSPTV
AGRNVYLRLVCFSGDAMGMNMVSKGSLAVIDLLRAEFPELKLIALSGNMCTDKKAAA
TNWLEGRGKSVVTECVIPKEVVNKTLKTTVEAMVETNINKNLIGSAMAGAIGGFNAH
AANMVTAVFLATGQDPAQNVESSNCITLLEETEDGDLHMSCTMPSLEVGTVGGGTTLP
AQAACLQAMGCKGGDRQNPGANAQRLALITATAVMAGELSLIAALAANTLVQAHMT
HNRKK
***>Bolidomonas_pacifica/*HMGR**
MGEGSPSLEPSTSATNLKGLDSLQLHDTQINNGVRPTMHHDNHMGNHEDKVDKLDKE
DNTSSARPVSSSISPSISPSISSSISSSISSSISSSIRPSTNSPTNSIAPKIDIEKLVEIAKTINSND
NKYTIRDVNALLGGGQETIETALLRRYVSGLQSPLSSLPYTGGGVDYDSISGRNCDNCV
GYVSVPVGIAGPVKYSFIDLTDGGVKEDKDLISHIPIATTEGGLIASVSRGCKALRDSCT
VTTCVTKDAMTRCPALEFKTVKDAVAFKVYVEGDGIGVVKESFEDTTKYGKLERVEC
KVVGRMVYFRVEAKTGEAMGMNMVSKGTENVLRVLKQDWEFNVITLSGNGCSDKK
PSATNFVKGRGKSVTAEAIIKREVVERTLKSNPENMKRTNIYKNQVGSAVAGNLGGM
NAHAANVVAGVFIATGQDPAHTVEGSMCTTVMDVTPEGDLYVSVTLPSICVGTIGGGT
ELPPQKASIKSMGCEGTGGSKRLAGLIG
***>Cylindrotheca_closterium/*HMGR**
MASAAYQPTTAPTLGMRLDAVLSQVDELSSTQLYGLIVAATVTLCVFLLGTGSNTDVE
FAATNSKMKTGTNDDGLKKPNASTNNTASTTRQPRWHIFKWVNYLASAAFLSSVGMF
CMNASQYLHHESKGVLVQFLVGWSVFLMYFFGFFGISLIHEDIPKDNQDDQGIEQPRN
TTTRYAAGSSSTTAAAKKQALHTAAPSAPVCSDPASFKASSSSSVPSNLKELPDEDIAN
LVLMNKLKDHELEKRLDPFRAVTVRRLVVNQKLSTLLTDGKTNQPVTNVLEKLPSTPS
LDYSRVFGANCEIVVGYVPLPVGLVGPLSLNDETVYVPMATTEGCLVASTNRGAKAIT
QGGGAQARIVRDGITRAPCVRMASAMEAADLKVWCEEPQNFAVLKQAFESTTSFGKL
QACNPTVAGKNVYLRLVCFSGDAMGMNMVSKGSLAVIETLQAKFPSLQLVALSGNM
CTDKKAAATNWLHGRGKSVVVEAIIPKDVVRGTLKTTVDALIFTNTNKNLIGSAMAGS
IGGFNAHASNIVTAVFLATGQDPAQXVXSSNCITXMEPTDDGDLWISCTMPSIEVGTVG
GGTSLPAQSACLEAIGCKGGGATPGENAQKLATVVAAATMAGELSLLAALAANTLVQ
AHMQHNRKPAPKK
***>Fragilariopsis_kerguelensis/*HMGR**
MASTEVMPTLGMRLDAMISHIDRLPSSQLYGVIVAATVGLCVVLLGTGNSNLELQQQQ
RQRQQQMMNVDDLKKPINNRIANVGGKQPKWHIFKWINYLAVGAFLWSVYTFCSNA
SQYLHHESQGVLVQFLVGWSVFLLYFFGFFGISLIHEDIPKEEDETTTMTVSKNIVSRKP
YNASTNSTTTMMKKKNSIHPPASCAPVCSDPTSFKSSGASLSSNSVNLEELADEEIANL
VLNNKVKDHELEKRLDPFRAVXVRRIAFNQKIASVLSDNTNKQNNINNTANVLDKLPS
TPSLDYSRVYGANCEIVVGYVPLPVGLVGPLTINDETVYIPMATTEGCLVASSNRGAKA
ITQGSGAKARIVRDGXTRAPCIRMRSAMEAADLKLWCEEPSNFLILKQAFESTTNFGKL
KECNPTVAGKNVYLRLVCFSGDAMGMNMVSKGSLAVIETLQNEFPSLQLVALSGNMC
TDKKAAATNWLNGRGKSVVVEAVIPREVVEKTLKTTVKALVHTNINKNLIGSAMAGA

IGGFNAHASNIVTAIFLATGQDPAQNVESSNCITLIEETDDNDLWISCTMPXIEVGTVGG
GTSLEAQSACLEAIGCKGGGATPGENARKLATVVAAATMAGELSLLAALAANTLVQA
HMVHNRKPASNK
>*Leptocylindrus_apora/*HMGR
MEGITSSPPTVGQQLDAFLEGLQNLDSTHKYLVAFATCLAVSFVVLNSGQGRYLDVKG
KDHQKAASQKKDLIVANDMPQKASATGKKSVVSGGREPRWYIFKIMNYVLATSFLVS
VVHFLLNSEEYIMSNDHIKTLGAVWTLFVLYFWGFFGVSFVDTDEHLEEEESPSSPSSSS
HKVAAPENVSTVLPPPXXPVHPPAPFAPVCSDPSTQKASSMKTPGDISSLSNEELANLV
LENKLKDHKLETELNPTRAVPVRRLVFEKKLGSLGKKGALNDLPYEHDLSYERVFGA
NCEIVVGYVPLPVGMVGPCTLNGESVYIPMATTEGCLVASTNRGCKAISAGGGAVSTL
LRDGITRAPCLRFESAAEAAALAIWVEETHNFLKLKNAFESTTSFGKLLSAKPTVAGKN
CYLRLKCFSGDAMGMNMVSKGSLAVVELLRQNFPTLKLVALSGNMCTDKKAAATN
WIEGRGKSVVVEATIPKRVVRTVLKTTVKAIVDTNVQKNLVGSAMSGTLGGFNAHAS
NIVTAVFLATGQDPAQNVESSNCLTLMEETKEGDLWISCTMPSIEVGTVGGGTSLPAQ
AACLKAIGVKGGGDIPGSNARKLAHVVAVATMAGELSLLAALAANTLVQAHMQHNR
KPATSK
>*Leptocylindrus_danicus/*HMGR
MAQTADPMTIGTRLDALIASGAEYLNNASQAQVCATLFVSSVAFSFALLNCGKGAHGS
SLDPTLKPLYKNTMEPIKTKVKSPDGSREPRWYIFKMLNYTAVATFSTSVLHFILYSDV
YMNNDAQMMKLMGAWTLFVLYFFGFFGVSFVDTDDHLEGSSPAEDEISEMTADQASP
SAVVAVAKQMPVKAAAPIHPPAPSHPVCSDKNLTKDALTSSSATPAVKKSSSLSLDLQS
MTNEELADLVLTNKMKDHQLETKLNPTRAVPIRRLVFEKKLASLGHAKSLDELPYEHS
LSYERVFGANCEIVVGYVPLPVGMVGPCTLNGESVYIPMATTEGCLVASTNRGCKAIT
AGGGAVSTLLRDGITRAPCLRFESAAEAAALALWAQEPHNFAKLKAAFESTTSFGKLL
SATPTVAGKNCYLRLKCFSGDAMGMNMVSKGSLAVVDLLRQHFPTLKLVALSGNMC
TDKKPAAINWIEGRGKSVVVEATIPKDIVRTVLKTTVKAIVDTNLQKNLIGSAMSGSVG
GYNAHASNIVTAVFLATGQDPAQNVESSNCITIMEETDDGDLWISCTMPSIEVGTVGGG
TSLPAQAACLKAIGVKGGGDIPGGNARKLAHVVAVATMAGELSLLAALAANTLVQAH
MQHNRKPATPAKK
>*Nitzschia_punctata/*HMGR
MAATFDTMPTTIGMKLDAMIAEIDNLSSTQLYGVIVAATVVLCVVLLGTGHSNLDLQH
SNNNDPLLKKQPAVAPSGNIKQPRWHIFKWINYLAVAAFLWSVCTFCLNASQYLHHEE
SQGVLVKFLLGWSVFLLYFFGFFGVSLIHEDIPKEEAGAASSAANRLSSVSQSNKTKTV
ETSSKNKALHGAAACTPVCSDPSSFKVSSSVPSNIKELADEDVADLVLKNKVKDHELE
KRLDPFRAVTVRRMTANRKLASVLPQNKPNVLDKLPATPSLDYSKVHGANCEIVVGY
VPLPVGLVGPLSLNNETVYVPMATTEGCLVASTNRGAKAITQGGGAQARIVRDGITRA
PCVRMESAMEAADLKVWCEQPENFARLKQAFEGTTSFGKLQACHPTVAGKNVYLRL
VCFSGDAMGMNMVSKGSLAVIETLQKEFPSLQLVALSGNMCTDKKAAATNWLQGRG
KSVVVEATIPKDVVRTTLKTTVAALVHTNMHKNLIGSAMAGSLGGFNAHASNIVTAV
FLATGQDPAQNVESSNCITLMEETDEGDLWISCTMPSIEVGTVGGGTSLEAQAACLEAI
GCKGGGATPGENAKKLATVVAAATMAGELSLLAALAANTLVQAHMTHNRKSNKK
>*Nitzschia_sp/*HMGR
MASAAYQPTTAPTLGMRLDAVLSQVDELSSTQLYGLIVAATVTLCVFLLGTGSNTDVE
FAATNSKMKTGTNDDGLKKPNASTNNTASTTRQPRWHIFKWVNYLASAAFLSSVGMF
CMNASQYLHHESKGVLVQFLVGWSVFLMYFFGFFGISLIHEDIPKDNQDDQGIEQPRN
TTTRYAAGSSSTTAAAKKQALHTAAPSAPVCSDPASFKASSSSSVPSNLKELPDEDIAN
LVLMNKLKDHELEKRLDPFRAVTVRRLVVNQKLSTLLTDGKTNQPVTNVLEKLPSTPS
LDYSRVFGANCEIVVGYVPLPVGLVGPLSLNDETVYVPMATTEGCLVASTNRGAKAIT
QGGGAQARIVRDGITRAPCVRMASAMEAADLKVWCEEPQNFAVLKQAFESTTSFGKL
QACNPTVAGKNVYLRLVCFSGDAMGMNMVSKGSLAVIETLQAKFPSLQLVALSGNM
CTDKKAAATNWLHGRGKSVVVEAIIPKDVVRGTLKTTVDALIFTNTNKNLIGSAMAGS

IGGFNAHASNIVTAVFLATGQDPAQXVXSSNCITXMEPTDDGDLWISCTMPSIEVGTVG
GGTSLPAQSACLEAIGCKGGGATPGENAQKLATVVAAATMAGELSLLAALAANTLVQ
AHMQHNRKPAPKK
>*Phaeodactylum_tricornutum/*HMGR
MRIHQYLYVPDRVSFVRSTSSRPTFRVPVTPSHPPCLLVRSDSISTKTSASNTLSARHPPA
PSAPVCSDPSSFTPIHTSKTNITTLDNAAICQLVLTNQIKDHELEKRLDAHRAVQVRRLV
VAHKLDTLEHINAHALDNLPSEPSLDYTRVHGANCEIVVGYVPLPVGLVGPLTVNGET
VYVPMATTEGCLVASTNRGAKAITAGGGATAVLLRDGITRAPCVRMPSAAQAAHLKL
WCETPQHFSTLKRAFESTTSFGKLLKCAPTVAGRNVYLRLTCFSGDAMGMNMVSKGS
LAVIETLQQEFPELVLVALSGNMCTDKKAAATNWLEGRGKSIVVEATIPKDVVTNTLK
TTVHSMVQTNLHKNLIGSAMAGALGGFNAHASNIVTAVFLATGQDPAQNVESSNCITL
LEETEEGDLWISCTMPSIEVGTVGGGTSLPAQAACLQAMGVRGGGATPGAHAQKLAQI
VASATLAGELSLLAALAANTLVQAHMQHNRKPAAK
>*Pseudonitzschia_arenysensis/*HMGR
MAETMTPMPTIGMQLDAMIAQIDALPSTQLYGLIVAATVGLCVVVLGTGNSNLEMEQ
HDLKKPKMVPVDGGKQPNWQIFKVINYIAVAAFLYSVFMFCSNASKYLHHESQGVLA
QFLVGWSVFLMYFFGFFGVSLIHEDIPSEVDSVPSQPKSVAPTKPVHPPAACAPVCSDPS
SFKAKKSCVPENIKELGDEEIADLVLANKVKDHMLEKLLDPFRAVTVRRIACNRKLNS
VHGNTSNVLDKLPSEHALDYSRVYGANCEIVVGYVPLPVGLVGPLTINDESFYVPMAT
TEGCLVASSNRGAKAICQGGGAKARIVRDGITRAPCLRMNSAMEAADLKIWCEKPAN
FAILKKAFESTTSFGKLIECNPTVAGKNVYLRLVCFSGDAMGMNMVSKGSLAVIEKLQ
EYFPSCQLVALSGNMCTDKKAAATNWLHGRGKSVVVECIIPKEVVRTTLKTTVAALV
HTNVNKNLIGSAMAGAIGGFNAHASNIVTAIFLATGQDPAQNVESSNCITLMEKQDNG
DLWMCCTMPSIEVGTVGGGTSLPAQAACLEAIGCKGGGATPGANAKQLATVVAAAT
MAGELSLLAALAANTLVQAHMAHNRKPAAKK
>*Pseudonitzschia_australis/*HMGR
MPTIGMRLDAMVAQVDKVPSTQLYGLIVAATVGLCVVLLGTGNSNLEIQLEQNCNND
LRKPKMVTVDAGKQPKWHIFKWINFVAVAAFLCSVFTFCSNASQYLHDESQGVLVQF
LVGWSVFLMYFFGFFGVSFIHEDIPREEVDVIPSKPQNKSLSTNNISHNKNKNNSISNKK
SVHPPAACAPVCSDPSSFRSSKVASLPDDLKELGDEEIANLVLSNKIKDHMLEKLLDPF
RAVTVRRIACNQKLASVHGRDQQNGNVLDKLPSEPSLDYSRVHGANCEIVVGYIPLPV
GLVGPLTINGESVYVPMATTEGCLVASSNRGAKAITQGGGAKARIVRDGITRAPCLRM
DSAMEAADLKIWCEQPSNFAVLKKAFESTTSFGKLKECNPTVAGKNVYLRLVCFSGD
AMGMNMISKGSLAVIETLQDKFPSCKLVALSGNMCTDKKAAATNWLLGRGKSVVVE
CVIPKEVVRTTLKTTVAALVHTNINKNLIGSAMAGAIGGFNAHASNIVTAVFLATGQDP
AQNVESSNCITLMEEQDNGDLWICCTMPSIEVGTVGGGTSLPAQAACLEAIGCKGGGS
TPGANAQKLATVVAAATMAGELSLLAALAANTLVQAHMVHNRKPAPKK
>*Pseudonitzschia_delicatissima/*HMGR
MAETMTPMPTIGMQLDAMIAQIDALPSTQLYGLIVAATVGLCVVVLGTGNSNLEMEQ
HDLKKPKMVPVDGGKQPNWQIFKVINYIAVAAFLYSVFMFCSNASKYLHHESQGVLA
QFLVGWSVFLMYFFGFFGVSLIHEDIPSEVDSVPSQPKSVAPTKPVHPPAACAPVCSDPS
SFKAKKSCVPENIKELGDEEIADLVLANKVKDHMLEKLLDPFRAVTVRRIACNRKLNS
VHGNTSNVLDKLPSEHALDYSRVYGANCEIVVGYVPLPVGLVGPLTINDESFYVPMAT
TEGCLVASSNRGAKAICQGGGAKARIVRDGITRAPCLRMNSAMEAADLKIWCEKPAN
FAILKKAFESTTSFGKLIECNPTVAGKNVYLRLVCFSGDAMGMNMVSKGSLAVIEKLQ
EYFPSCQLVALSGNMCTDKKAAATNWLHGRGKSVVVECIIPKEVVRTTLKTTVAALV
HTNVNKNLIGSAMAGAIGGFNAHASNIVTAIFLATGQDPAQNVESSNCITLMEKQDNG
DLWMCCTMPSIEVGTVGGGTSLPAQAACLEAIGCKGGGATPGANAKQLATVVAAAT
MAGELSLLAALAANTLVQAHMAHNRKPAAKK
>*Pseudonitzschia_multistriata/*HMGR

MAQTMGTVPTIGMRLDAMVAQIDQLPSTQLYGLIVAATVGLCTVLLGTGNSNLEKQL
QQNDDLKKPKASAAPVGVDRGKQPKWHLFKWINFVAVATFLYSVFLFCSNATQYLH
HESQGVLVQFLVGWSVFLMYFFGFFGVSFIHDDIPREAEEVIPSKPQSQPASGASKKTV
HPPAACAPVCSDPSSFKSKASPPDNLKELPDEEIADLVLSNKVKDHMLEKLLDPFRAVT
VRRIACNRKLASVLGRDQANSNVLEKLPSEHSLDYSRVYGANCEIVVGYVPLPVGLVG
PLTLNGESVYVPMATTEGCLVASSNRGAKAITQGSGAKARIVRDGITRAPCLRMGSAM
EAADLKIWCEQPENFAVLKKAFESTTSFGKLKECNPTVAGKNVYLRLVCFSGDAMGM
NMISKGSLAVIETLQSYFPSCQLIALSGNMCTDKKAAATNWLHGRGKSVIVECFIPKEV
VRTTLKTTVAALVHTNLNKNLIGSAMAGAIGGFNAHASNIVTAVFLATGQDPAQNVES
SNCITLMEENENGDLWMCCTMPSIEVGTVGGGTSLPAQAACLEAIGCKGGGSTPGANA
KKLATVVAAATMAGELSLLAALAANTLVQAHMVHNRKPAPKK
>*Pseudonitzschia_pungens*/HMGR
MAQTMGTMATIGTRIDNVIAQFDQLPSTQLYGLIVAATVGLCVVLLGTGNSNFELHHQ
RQKQLQQNNDDLKKPKMAAMPSGRQPRWHIFKWINFVAVGAFLCSVFIFCSNASRYL
HHESQGVLVQFLVGWSVFLMYFFGFFGVSLIHDDIPREEEESLPSKPQTKSVPTNTSAPK
KTVHPPAACTPVCSDPSSFKSSKPSVPENLKELSDDEIAALVLGNKVKDHMLEKLLDPF
RAVTVRRIACNQKLASVLGREDKSNVLEKLPSEPSLDYSRVFGANCEIVVGYVPLPVGL
VGPLTINDESVYVPMATTEGCLVASSNRGAKAITQGGGARAKIVRDGITRAPCLRMNT
AMEAADLKIWCEKPQNFAILKQAFESTTSFGKLKECNPTVAGKNVYLRLVCFSGDAM
GMNMVSKGSLAVIETLQKYFPTCQLVALSGNMCTDKKAAATNWLYGRGKSVVVECV
IPKEVVRTTLKTTVSALVHTNLNKNLIGSAMAGAIGGFNAHASNIVTAVFLATGQDPA
QNVESSNCITLMEEEVNGDLWMCCTMPSIEVGTVGGGTSLPAQAACLEAIGCKGGGV
TPGANAKKLATVVAAATMAGELSLLAALAANTLVQAHMAHNRKPASKK
>*Pseudonitzschia_pungens_fr_cingulata*/HMGR
MAQTMGTMATIGMRIDNVIAQFDQLPSTQLYGLIVAATVGLCVVLLGTGNSNFELDH
QRQKQLQQNNDDLKKPKMAAMPSGRQPRWHIFKWINFVTVGAFLCSVFIFCSNASRY
LHHESQGVLVQFLVGWCVFLMYFFGFFGVSLIHDDIPREEEESLPSKPQTKSVPTNTSAP
KKTVHPPAACTPVCSDPSSFKSSKPSVPENLKELSDDEIAALVLGNKVKDHMLEKLLDP
FRAVTVRRIACNQKLASVLGREDKSNVLEKLPSEPSLDYSRVFGANCEIVVGYVPLPVG
LVGPLTINDESVYVPMATTEGCLVASSNRGAKAITQGGGARAKIVRDGITRAPCLRMN
TAMEAADLKIWCEKPQNFAILKQAFESTTSFGKLKECNPTVAGKNVYLRLVCFSGDA
MGMNMVSKGSLAVIETLQKYFPTCQLVALSGNMCTDKKAAATNWLYGRGKSVVVE
CVIPKEVVRTTLKTTVSALVHTNLNKNLIGSAMAGAIGGFNAHASNIVTAVFLATGQD
PAQNVESSNCITLMEEEVNGDLWMCCTMPSIEVGTVGGGTSLPAQAACLEAIGCKGGG
VTPGANAKKLATVVAAATMAGELSLLSALAANTLVQAHMAHNRKPVSKK
>*Skeletonema_costatum*/HMGR
MTFSEPTTIGMRIDAVIASLESLPHTNPEILYASLIGASLMFSFLVLNSGGSSMALPDGGD
ADSLNKPKKISNAKPVSSSDEPQPKWHILRLTNYVVTFGFLLSVLKFASNATTYLDDST
SLLQFLIIWSISMCYFFGFFGISFIELDELESEPQNHQHQMLRKPMPTKESAEPPVKVVK
MHPPASSAPVCTDPTSFKKPSPSIPSNIKELSNGEIASLVLQDKVKDHQLEKLLDPHRAV
EVRRLKVDNQLDSLGRGGALAELPHKHDLDYKRVLGANCEIVVGYIPIPVGMAGPITL
NGESVYIPMATTEGCLVASTNRGCKAITQGSGAVSSILRDGITRAPCVRLPSAKEASEVF
LWIERPENFQKLKEVFESTTSFGKLLEARPTVAGKNVYIRLRCFAGDAMGMNMISKGS
LAVIECLRQVFPNISLVALSGNMCTDKKAAAINWIEGRGKSVVIEATIPKVVVRSTLKTS
VPAIVEANLNKNLIGSAMAGVVGGFNAHASNNVTAVFLATGQDPAQNVESANCITLM
EETPEGDLWISCTMPSIEVGTVGGGTGLPAQAACLKAIGVKGGGANPGDNAKQLAHV
VAAATMAGELSLMAALASNSLVAAHMTHNRKPASK
>*Skeletonema_marinoi*/HMGR
MTFSEPTTIGMRIDAVIASLESLPHTNPEILYASLIGASLMFSFLVLNSGGSSMALPDGGX
ADSLNKPKKISNAKPVSSSDEPQPKWHILRLTNYVVTFGFLLSVLKFASNATTYLDDST
SLLQFLIIWSISMCYFFGFFGISFIELDELESEPQNHQHQMLRKPMPTKESAEPPVKVVK

MHPPASSAPVCTDPTSFKKPSPSIPSNIKELSNGEIASLVLQDKVKDHQLEKLLDPHRAV
EVRRLKVDNQLDSLGRGGALAELPHKHDLDYKRVLGANCEIVVGYIPIPVGMAGPITL
NGESVYIPMATTEGCLVASTNRGCKAITQGSGAVSSILRDGITRAPCVRLPSAKEASEVF
LWIERPENFQKLKEVFESTTSFGKLLEARPTVAGKNVYIRLRCFAGDAMGMNMISKGS
LAVIECLRQVFPNISLVALSGNMCTDKKAAAINWIEGRGKSVVIEATIPKGVVRSTLKTS
VPAIVEANVNKNLIGSAMAGVVGGFNAHASNNVTAVFLATGQDPAQNVESANCITLM
EETPEGDLWISCTMPSIEVGTVGGGTGLPAQAACLKAIGVKGGGENPGDNAKQLAHV
VAAATMAGELSLMAALASNSLVAAHMTHNRKPASK
>*Skeletonema_menzelii/*HMGR
MTSEATTIGMRIDAVIASLESLPHTNPEILYASVIGASLLFSFLVLNSGGSSMALPDGGD
ADSLNKPKISKAKLVSPSDEPQPKWHILRLTNYIVTFGFLLSVLKFASNATTYLDDSTSL
LQFVIIWSISMCYFFGFFGISFIELDELENEPQNHQHQILRKPMPTKESADPPVKVVKMH
PPASSAPVCIDPTSFKKPSPSIPSNIKELSNSEIASLVLQDKVKDHQLEKLLDPHRAVEVR
RLKVDVQLDSLGRGGALAELPHKHDLDYKRVLGANCEIVVGYVPIPVGMAGPITLNG
ESIYIPMATTEGCLVASTNRGCKAISQGSGAVSSILRDGITRAPCVRLPSAKEASEVHLW
IESPENFQKLKEVFESTTSFGKLLEARPSVAGKNVYIRLRCFAGDAMGMNMISKGSLAV
IECLRQRFPNLSLVALSGNMCTDKKAAAINWIEGRGKSVVIEATIPKGVVRSTLKTSVP
AIVEANLNKNLIGSAMAGVVGGFNAHASNNVTAVFLATGQDPAQNVESANCITLMEE
TPEGDLWISCTMPSIEVGTVGGGTGLPAQAACLKAIGVKGGGENPGDNAKQLAHVVA
AATMAGELSLMAALASNSLVAAHMTHNRKPASK
>*Thalassionema_fruenfeldii/*HMGR
MSSADTAMTWGMRLDVFLSQMDSTMVSIDNLSSTQLYGLIVLVTGVISFSILGFGPKDE
LPAAVTNNNSNNNNXNNKKKIVGSNYNNKEPKWFVLRYLNYVAVAAFVASVAMFG
WNAQAILKDTSSLLKFLMGWCVFLCYFLGFFGISFVDPDDIVESSNTSPNATTTTTTPA
AAASKPKKVVLPPAPSAPVCSDPVSKTRSKLGDINHLSDEEVVALVLNNQLKDHQLES
KLNPDRAVVVRRQIVAQKLATLGCGGLDNLPCQPSLDYSKVHGANCEVVVGYIPIPVG
MVGPLTLNGESVYIPMATTEGCLVASTQRGAKAISSGAGATCTILKDGITRAPCLRLPS
AQQAAQLAKWCMDENNFKKLKKAFESTTSFGKLLKAQPTVAGRNVYLRLVCFSGDA
MGMNMVSKGSLAAIDLLRSHFPQVELIALSGNMCTDKKAAATNWLEGRGKSVVVEA
TISKQVVETTLKTTVAAIVDTNLQKNLVGSAMAGTLGGYNAHASNIVTAVFLATGQDP
AQNVESSQCITLLEETKEGDLWMSCTMPALEVGTVGGGTSLKAQRACLECMGVAGGS
REEPGKNAQKLATMVAAATLAGELSLLAALAANTLVAAHMKHNRK
>*Thalassiosira_minuscola/*HMGR
MPSSPTAPPPPPTLGMQIDAVIASLESISTPQLYGTVIAISLAFSFYLLNSGGGSSSPALQM
MTNDTKEQQDADADSLRKPARAAATSSSSSSESSSEDRPEPRWHILRFTNYVAALGFL
VSVLQFASNASAYLNDSNSLLQFLTVWSLFVVYFVGFFGISFVELDDFVDGAQISSSVA
RQRAQQQQQQQQQQQSQNMTVAASQAKTPGRKSHKTDGSDSPPIKVVNVHPPATSAP
VCSDPKSFKKPSAASIPPNLKELPNSEIASLVLQDKIKDHQLEKLLDPHRAVQVRRLKFD
ARLSALGNGGALDELPHRHDLEYKRVLGANCEIVVGYVPIPVGMAGPVTLNGESVYIP
MATTEGCLVASTNRGCKAISQGSGASSTILKDGITRAPCVRLPSAKEAAEVALWIETPE
NFATLKEAFESTTSFGKLLSARPTVAGKNVYVRLRCFSGDAMGMNMISKGSLAVIECL
RQIFPALSLVALSGNMCTDKKAAAMNWIEGRGKSVVIEATIPADVVRSTLKTSVTAIVE
ANLNKNLIGSAMAGTVGGFNAHAANNVTAVFLATGQDPAQNVESSNCITLMEETPEG
DLWMSCTMPSIEVGTVGGGTGLPAQAACLRAIGAKGGGENPGDNARRLAHVVAAAT
MAGELSLMAALASNSLVAAHMAHNRKPASK
>*Thalassiosira_pseudonana/*HMGR
MNHLLSNYQPSTSQIYSLVIVLSVAFSFRLLNSGDDLGSKLSSSTVEQAKRGEKGTSSK
YAKANNKNNKQSYDFPQPKWHILKLTNYVVTTLFLLSIITFLSNASVYLNDNTALMTF
LGVWSGLLCYFFGFFGISFVELDDLVVTDADGQQRQQGVVTQPQKQASCKSRKVESP
NDPPVKVISLHPPASSTPVCSDPIKSKSPTNTGIPTNVKDLSNEEIATLVLQDKIKDHQLE
KLLDPHRAVAVRRLKFDALLSSLGKTTGEDDKKGGVLSELPHEHDLDYKRVLGANCEI

VVGYIPIPVGIVGPVTLNGESVYIPMATTEGCLVASTNRGCKAITQGSGARSTILRDGIT
RAPCVRLPSAHEAAQVHLWIEEADNFAKLKEAFESTTSFGKLIEASPTVAGRNVYIRLR
CFSGDAMGMNMISKGSLAVIECLREQFPQLSLVALSGNMCTDKKAAAMNWIEGRGKS
VVIEATIPKDVVRSTLKTSVPAIVEANVNKNLIGSAMAGTVGGFNAHAANNVTAVFLA
TGQDPAQNVESSNCITLMEVSPEGDLWISCTMPSIEVGTVGGGTGLSAQSACLRAIGVK
GGGENPGDNARQLAHVVACATMAGELSLMAALASNSLVAAHMQHNRKPASK
>*Thalassiosira_puntigera/*HMGR
XDTSELKGSKSSNRSPIXPSQNSESMGDATSAPPTVGMQIDAAIAQLENVSTTQLYAIVI
AASLAFSFYLLNSGGGSSALTIMNEKDGDADSLRKPEARKRKDRPRASTSSDEPEPRW
HILRITNYVVAAGFLLSVLQFASNASTYLNDSTSLLQFLSVWSIFLCYFFGFFGISFIELD
DFVDQQPSQQQQQPSHKLKKAESISPDPPVKVVNVHPPSSSAPVCTDPTSFKKPPSNVP
GNLKDLPNSEIASFVLQDKIKDHQLEKLLDPHRAVEVRRLKFDAKLDSLGRGGALADL
PHKHDLDYKRVLGANCEIVVGYLPIPVGLAGPITLNGESVYIPMATTEGCLVASTNRGC
KAISQGSGASSTILKDGITRAPCVRLPSAKEAAKVALWIGTPENFLTLKKAFESTTSFGK
LLDATPTVAGRNVYIRIRCFSGDAMGMNMISKGSLAVIETLKKVFPDLSLLALSGNMC
TDKKAAALNWIEGRGKSVVIEATIPKDVVRSTLKTSVQAIVEANVNKNLIGSAMAGTV
GGFNAHAANNVTAVFIATGQDPAQNVESSNCITLMEETSEGDLWISCTMPSIEVGTVG
GGTGLPAQSACLKVIGVKGGGENPGDNARQLAHVVAAATMAGELSLMAALASNSLV
AAHMSHNRKPTSK
>*Thalassiosira_rotula/*HMGR
MVVATSAPPTVGMQIDAAIASLETLSTTQLXATVIAVSLXFSFYLLNSGGESSALTMMN
ESSSDSLRKPKXDRKRMKTQQQQSREETDEPEPRWHILRITNYVVATGFTLSVLQFASN
ASTYLNDSTSLLQFLMVWSLFLCYFFGFFGISFIELDDFVDQQQPSQQQPSQQTQVAPSR
QLKKAESSDPPVKVVNIHPPASSAPVCTDPTSFKKPSSKIPSNLKELPNSEIASLVLQDKI
KDHQLEKLLDPHRAVEVRRLKFDAKLDSLGCGGALTELPHKHALDYKRVLGANCEIV
VGYIPIPVGMAGPITLNGESVYIPMATTEGCLVASTNRGCKAISQGSGASSTILKDGITR
APCVRLPSAREAAEVALWIGKTXNFLSLKEAFESTTSFGKLLDATPTVAGKNVYIRLRC
FSGDAMGMNMISKGSLAVIDALRQIFPRLSLLALSGNMCTDKKAAAMNWIEGRGKSV
VIEATIPQDVVRSTLKTSVRAITEANVNKNLIGSAMAGTVGGFNAHAANNVTAVFLAT
GQDPAQNVESSNCITLMEETPEGDLWISCTMPSIEVGTVGGGTGLPAQSACLKAIGVKG
GGENPGDNAKQLAHVVAAATMAGELSLMAALASNSLVAAHMAHNRKPASK
>*Thalassiosira_weissflogii/*HMGR
MAHPSSYERVSSPPPTIGKHIDAVIAKIDSLPSSYVYTLAIAASLTFSFYLLNSGGGSNSM
QMEPHGDVDRASAIKKPKQKSRRSLDEPQPKWHILKLTNYVATSGFLLSVLKFVSNAS
MYLNDSTSLLQFLTIWSIFLCYFFGFFGISFIELDDFEQESHPQHQAPVSAINVSHAKHCN
AVKSGSLEPPVKVINVHPHSTSAPVCSDPASFRKPSSTIPGNLKDMSNEEIASLVLDDKI
KDHQLEKLLDPHRAVEVRRLKFDAQLQSLGRGGALAELPHKHDLDYKRVLGANCEIV
VGYVPIPVGMAGPITLNGESVYIPMATTEGCLVASTNRGCKAITQGSGAFSTIVRDGITR
APLVRLPSAREAAEVLLWIEDENNFKILKEAFESTTSFGKLIEARPTVAGRNVYIRLRCF
SGDAMGMNMISKGSLAVIECLRRKFPQLSLAALSGNMCTDKKAAAINWIEGRGKSVVI
EATIPKEVVRSTLKTSVPAIVEANTNKNLIGSAMAATVGGFNAHAANNVTAVFLATGQ
DPAQNVESSNCITLMEETSDGDLWISCTMPSIEVGTVGGGTGLPAQSACLKAIGVKGG
GDSPGDNSKKLAHVVAAATMAGELSLMAALASNSLVAAHMAHNRKPVSK

MK protein sequences

>*Amphiprora_sp/*MK
MSENQDSAAKRQRHERIGESFLKRTRAFGKLILFGEHFVVYKVPALVGAVSAYTDCEV
EFLEQANGDGDLQIIDNRPAVPNYKVKKAEEGKEAIGLVLKHFGLDPKESPMKLTFGG
DLCAVSGIGASAAQVVSLSRALSLALPKGMTEEEINAAGYEGEKGYHGTPSGIDNTAA
TFGGVLRFQRTDGDPIFVKKKIAEPIRIVYASTGITASTTTVVGDVKAKKEANEDWFDG

LLKKYVELVGKGEKALDEGDLTTLGKLMDENHALCQELTVSCKELDDLVDAARAAG
AIGAKMSGTGRGGLMLALTPTAELQDKVASALEKSAPQVWKTTFA
>*Bolidomonas_pacifica*/MK
XLGSEGESIIHGAPSGIDNSVSTFGGCLRMRRIEGEDGEIRMVKDLMKMEATEILITNTF
VPKSTKDLVAGVKRRWEEARKVYDPIFLAMSEISEAFLKGGLGLERVNELVRLAQGLL
RTIGVTHPAIEMVVEKSRAYGVETKLTGAGGGGCVITLLG
>*Cylindrotheca_closterium*/MK
MSDVSKTQAYGKLILFGEHFVVYHVPALVGAVAASTTCELEFVQGGEAVEIVDNRPA
VPNYKVKKAEEGRQAIDLVLKHFNVAEKQPKDTNLKLTFGGDLCCVSGIGASAAQTV
SLSRAIDQVLKLESTEEEINQGGYEGEKGYHGTPSGIDNTAATYGGLLKFQRTDTDPIFT
KKDIKEPIRIVYASTGITASTTEVVGDVRAKKEADPAWFDNLLEQYKTLVKKAEDAMD
QGDIPLLGSLMNENHALCQELTVSCDELDQLVIAARSAGAIGAKMSGTGRGGLMLCLT
PTEEIQNKVFEALEQRAPQVW
>*Fragilariopsis_kerguelensis*/MK
KLRTNNIMTEEIETSKRTSPPTNEKEDVDVTKKXKVDDEKNKELSTLAKTHAYGKLILF
GEHFVVYKVPALVGAVAAYTDCSIEVVDVDESDNVKTNEGDESKANNDTGIVEIVDN
RPAVPNYKTKKAEEGKKAVDLVLNHFKIFGKGKKAKLTFGGNLCCASGIGASAAQVV
ALARAIKQTFPQFATLTDDEINAAGYEGEKGYHGTPSGIDNTAATFGGLLKFQRTEGEP
IFEKKKLKEPIRIVYASTGITSSTTEVVGDVRAKKEADPAWFDALLKKYCDIVSKGEKV
LEDSNINELGKLMDENHKILQDLTVSCDELDTLVVAAKKAGAIGAKMSGTGRGGLMI
AIVGCEETQNAVADALDKIAPQVWKTSFQ
>*Leptocylindrus_apora*/MK
XVSCFGGAVYYQKEAATGSVKAFDQLTIAKMDIILTNTFVPRSTKALVAGVGQRKENF
KFAPYVITAIGNISDQFRDIITSNTQDPSVISDLIQTNQLLLQSLGVSHETLNEICRITQKM
DISSKLTGAGGGGCALTYIEPDCKYTKREVMDMIEQSGHGFKCLESSVGGFGVLFVDF
AX
>*Leptocylindrus_danicus*/MK
XTTTVAASAPGKVILFGEHAVVHSQPAIAASLDDLRIYAVIRHEEVTSKGMLTVNMPNF
KKGLVFRAPTASCLYETNVTRDVTQDVVSPSSDEVQKIHKFIQKAGASHTCNSALASD
QVEALTPLFFMMNGIIPALLNAKSSVSIYVRSEDLPLGAGLGSSAAFCVACSAALYRLR
EKTGIMGVAAVDRNIKDNNTRPEQASLDIINAWAFASEGVIHGNPSGLDNTVSCYGGA
VYYQKDAETGNLRAFHELVVPPLDMILTNTFIPRSTKALVAGVHKVKEDFEFTVDVFA
AIGRISDQFRKALDPGDRGYRDTAKISQLIRTNQNLLKAIGVSHKALDEICHITEQMGV
ASKLTGAGGGGCTFTYIPPDCEYTRDEIREKIEESPHGFKCLSSLVGGTGVRYMNPDTIP
IVDLAKMRNQTTDNSLPWDRTLQYILALGSLTALGFVAVGNMKKK
>*Nitzschia_sp*/MK
MPPSADDTTTTTKRPPEEETVTTDEGEETAKKAKTEGEETEATNADAAATATDTAEDD
CDTKGFGKLILFGEHFVVYKVPAYVGAVAAYTDCEMELLEDSKDKTDAPTLDIVDNR
PAVPNYKVKKADEAKEAISLVLKHFGIQDRSVKLVFGGNLTAVSGIGASAAQVVAMA
RAXYEGEKGYHGTPSGIDNTAATFGGLLKFQRTEGDPIFTKKSLKEPIKIVYASTGITSS
TTTVVGDVRAXNSNXTEGDPTFTKKSLKEPIKIVYASTGITSSTTTVVGDVRAKKEAEP
AWFDDLLAKYVALAEKGEKALEDGDITTLGQLMDENHGLLQQLTVSCKELDDLVDA
AKKAGAIGAKMSGTGRGGLMLALTPSDEVQNAVAXALEKAGAPQVWKTSFQ
>*Phaeodactylum_tricornutum*/MK
MAGTPLINSRSVCASAPGKAILFGEHAVVYGEPAVAAALDDLRIFVLFTPTSHNSTHAS
VIRVVMPDLPTPVDFALPVSLFTSMESTNTPPTPDDATVLAQLLHTADPTLDDFSVQAL
TPVLYLCNQILLKPLRFRSESNPSADGGYELWVRSQDLPVGAGLGSSAAFGVACAAALI
QYRQNLTATHAGTAAGTTSTNTPIQYGPPDSATLSEIDRYAYYSEILLHGTPSGIDNAVS
AHGGAIIFTKDVASTNGTVKMEHLTPSDDLTLSLVYTHVPRSTKTLVAGVRHFYQRHV
GFVTLILEAMGAIARDFEQAIRDRTNAHHGDHASDSHDFGERVLTMVRTNQYLLQAV
GVSHPSLDHVCAVVHEHFRDYGAAKLTGAGGGGCAFVLWKPGLAADVLATQRQRLQ

YALTTTLTPSPPYRYQCLASVVGGEGVLFLPAGDFPWDATTPSSATPSRSATNVGRLVS
ALTVSALVATTWMVWRSIGTKAR
>*Pseudonitzschia_arenysensis*/MK
MTESTSPTSVSKRIPEEEVADETAKKAKVETSADSDSTLEKTHAYGKLILFGEHFVVYK
VPALVGAVAAYTDCKIEVADVDEKDAANEKETGVVEIVDNRPAVPNYKTKKAEEGK
KAIDLVLNHFKIFGKGKKATLTFGGNLCCASGIGASAAQVVALARAVKETMPEFSKLT
DDEINAAGYEGEKGYHGTPSGIDNTAATFGGLLKFQRTDGEPIFEKKSMKEPIRIVYAS
TGITSSTTEVVGDVKSKKEADPAWFDDLMKKYCDVATSGEKAIEEGDMTKLGELLNE
NHKLLQDLTVSCKELDTLVDAARAAGAIGAKMSGTGRGGLMLALTPTEESQNAVAEA
LEKIAPQVWKTSFQ
>*Pseudonitzschia_australis*/MK
MAPSIEETTPPSADAETSEAPVTEDASKRPSEEEAVADTAKKAKVETETETNTDAKTDA
SADADVDAERLAKTHAYGKLILFGEHFVVYKVPALVGAVAAYTDCSIKVEDAGDSAD
DSETGIVEVIDNRPAVPDYKTKKAEEGKKAIDLVLNHFKIFGKGKKATLTFGGNLCCAS
GIGASAAQVVALSRAVKETLPEFSKLTNDEINTAGYEGEKGYHGTPSGIDNTAATFGGL
LKFERTDGDPIFEKKTIKEPIRIVYASTGITSSTTEVVGDVRAKKEADPAWFDDLLKKYC
ELAAKGEKALEDGDNTELGKLLNENHKLLQDLTVSCKELDTLVDAARAAGAIGAKMS
GTGRGGLMLALTPTEEIQNAVAEALEKVAPQVWKTSFQ
>*Pseudonitzschia_delicatissima*/MK
MTETSSPTSVQDTSKRAPEEEVAAETAKKAKVDTSAESESKLEKTHAYGKLILFGEHFV
VYKVPALVGAVAAYTDCSIEVAXVXEKDAANEKETGVVEIVDNRPAVPNYKTKKAEE
GKKAIDLVLNHFKIFGKGKKATLTFGGNLCCASGIGASAAQVVALARAVKETLPEFSK
LTEDEINAAGYEGEKGYHGTPSGIDNTAATFGGLLKFQRTDGEPIFEKKAIKEPIRIVYA
STGITSSTTEVVGDVRAKKEADPAWFDDLMKKYCEVATKGEKAIEEGDMTKLGELLN
ENHKLLQDLTVSCKELDTLVDAARKAGAIGAKMSGTGRGGLMLALTPTEESQNAVAE
ALEKIAPQVWKTSFQ
>*Pseudonitzschia_multistriata*/MK
MAPSTEDTTPEATVAETTPAAPAEETAAKRPSEEEAENSAKKAKVDDSAAPAVDADAE
RLAKTHAYGKLILFGEHFVVYKVPALVGAVAAYTDCSIKLEDAPADSKDESPTGIVEVI
DNRPAVPNYKTKKAEEGKKAIDLVLNHFKIFGKGKKATLTFGGNLTCASGIGASAAQV
VALARAVKETLPEFAKLTDDEVNAAGYEGEKGYHGTPSGIDNTAATFGGLLKFQRTD
ADPIFEKKTIKEPIRIVYASTGITSSTTEVVGDVKAKKEADPAWFDDLLKKYCEVATKG
EKALEDGDTALLGSLLNENHKLLQDLTVSCKELDTLVDAARAAGAVGAKMSGTGRG
GLMLALTPTEEIQNAVAEALEKIAPQVWKTSFQ
>*Pseudonitzschia_pungens*/MK
MAPSTDENPAEPAVAETPATDQIAKRPSEDEPDPETAKKAKVATTESDSSSTADAERLA
KTHAYGKLILFGEHFVVYKVPALVGAVAAYTDCSIEVEDAPSSDSTDDQETGIVEVIDN
RPAVPNYKTKKAEEGKKAIDLVLNHFKIFGKGKKATLTFGGTLCCASGIGASAAQVVA
LARAVKETLPERFGKLTEDEINTAGYEGEKGYHGTPSGIDNTAATFGGLLKFQRTDSDP
IFEKKTIQEPIRIVYASTGITSSTTEVVGDVRAKKEADPAWFDDLLQKYVELAKKGETAI
EKGDIPELGKLLDENHKLLQDLTVSCKELDDLVAAARAAGAVGAKMSGTGRGGLML
ALTPTEEIQNAVAEALEKIAPQVWKTSFQ
>*Pseudonitzschia_pungens_fr_cingulata*/MK
MAPSTDETPAEPAVAETPATDQTAKRPSEDEPDPETAKKAKVATESTAESTAEAKATA
KAKAERLAKTHAYGKLILFGEHFVVYKVPALVGAVAAYTDCSIEVEDAPADSTDDKE
TGIVEVIDNRPAVPNYKTKKAEEGKKAIDLVLNHFKIFGRGKKATLTFGGNLCCASGIG
ASAAQVVALARAVKETLPERFGKLTEDEINTAGYEGEKGYHGTPSGIDNTAATFGGLL
KFRRTDSDPIFEKKTIKEPIRIVYASTGITSSTTEVVGDVRAKKEADPAWFDDLLRKYVE
LAEKGETAIEKGDIPELGKLLDENHKLLQDLTVSCKELDDLVAAARAAGAIGAKMSGT
GRGGLMLALTPTEEIQNAVAEALEKIAPQVWKTSFQ
>*Skeletonema_costatum*/MK

XTRAFGKLILFGEHFVVYKVPALVGAVSAYTDCKLEYTTEPGLEVIDNRPAVPLYKEK
KMDEGIEAINLTLTHLGVDTKKSGVKLTFGGDLCCASGIGASAAQVVSLARAVNIADS
RSLSEDEINAAGYEGEKGXHGTPSGIDNTAATFGGLLRFQRTEGAPIFDKKTLVSPIRIV
YASTGITASTTEVVGDVREKKEADEAWFADLIGKYTKLCEEGQKAVEEGDMDKLGKL
MNLNHEYLQELTVSCKELDDLVVXARAAGAVGAKMSGTGRGGLMIALTPTEEIQNEV
GSALEKXAPQVWKTTFA
>*Skeletonema_marinoi*/MK
XFPVDQTRAFGKLILFGEHFVVYKVPALVGAVSAYTDCKLEYTTEPGLEVIDNRPAVPL
YKEKKMDEGIEAINLTLTHLGVDTKKSGVKLTFGGDLCCASGIGASAAQVVSLARAVN
IADSRSLSEDEINAAGYEGEKGYHGTPSGIDNTAATFGGLLRFQRTEGAPIFDKKTLVSP
IRIVYASTGITASTTEVVGDVREKKEAXEAWFADLIGKYTKLCEEGQKAVEEGDMDKL
GKLMNLNHEYLQELTVSCKELDDLVVAARAAGAVGAKMSGTGRGGLMIALTPTEEIQ
NEVYSALEKLAPQVWKTTFA
>*Skeletonema_menzelii*/MK
MTSFPVDQTHAFGKLILFGEHFVVYKVPALVGAVSAYTNCKLEYTAEPGLEVIDNRPA
VPFYKEKKMEEGIEAIDLTLTHLGVDTKKRGVKLTFGGDLCCASGIGASAAQVVSLAR
AVNIADSRSLSEDEINAAGYEGEKGYHGTPSGIDNTAATFGGLLRFQRTEGAPIFDKKT
LATPIRIVYASTGITASTTEVVSDVRAKKEADEAWFADLIGKYTDLCEQGQKAVEEGD
MDKLGKLMNLNHEYLQELTVSCKELDDLVVAARAAGAVGAKMSGTGRGGLMIALTP
TEEIQNEVASALEKLAPQVWKTTFA
>*Thalassionema_frauenfeldii*/MK
XLIYLLNILTSEYLHHGLEITVRSNFPMGAGLGSSAAFSVALTAALLRLKLALSEKPSLP
PLGRPAENHLDTINTYAFYAETLLHGNPSGIDNSVSTYGGGICYTKDLVNPGKGSMERF
RMPPLSVMLVNTNVPKSTKALVASVRTMYEEHPEVVGCILKCIGEITQEFRNLVEKKET
KNQKVLTLVNTNQHMLRSVGVSHESLDHVCHVTETISEGQAATKLTGAGGGGCAMIV
LQTNECNNSVSIEKLTQKIQKALEGGWHRWNWSSLGGS
>*Thalassiosira_minuscola*/MK
MVNNFPTQRTRAFGKLILFGEHFVVYKVPALVGAVSAYTDCELEFLDKPGLEVIDNRP
AVPLYKVKKKDEGDEAINLTLTHLGIDPKERGVKLTFGGDLCCASGIGASAAQVVSLA
RAVNIADSRSLSEEEINAAGYEGEKGYHGTPSGIDNTAATFGGLLRFQRTDGAPIFEKKS
FPSAIRIVYASTGITASTTEVVGDVRSKKEADEAWFASLLGKYTSLVGEAEQVVEEGDL
NKLGQLMDQNHALLQELTVSCKELDDLVDAARAAGAVGAKMSGTGRGGLMIALTPT
EEVQNSVAAALEKLAPQVWKTTFA
>*Thalassiosira_pseudonana*/MK
MADDFPIHQTHAYGKLILFGEHFVVYKVPALVGAVSAYTDCKVEYLNEPGLEVVDNR
PAVPLYKEQKMEEGMEAINLTLKHLGVDTQKRGMKLTFGGDLCCASGIGASAAQVVS
LARAFNVVDGRSMTEDEINAAGYEGEKGYHGTPSGIDNTAATFGGLLRFQRTDGAPIF
DKKSLKSPIRIVYASTGITASTTKVVGDVRAKKEADEAWFANLMEQYKVLVEDGQTA
VEAGDLTTLGKLMDQNHVLLQELTVSCKELDDLVAAAREAGALGAKMSGTGRGGLM
IALTPTEEIQSAVADALGELAPQVWKTTFA
>*Thalassiosira_puntigera*/MK
MSDFPTDQTRAFGKLILFGEHFVVYKVPALVGAVSAYTDCKLEYLDEPGLEVIDNRPA
VPLYKEKKKDEGEEAINLTLAHLGVDPKARGVRLTFGGDLCCASGIGASAAQVVSLAR
AVNFADSRSLSEDEINAAGYEGEKGYHGTPSGIDNTAATFGGLLRFQRTDGAPIFEKKS
FPSAIRIVYASTGITASTTKVVGDVRSKKEADEAWFADIMGKYTTLVEEAEQIVEAGNL
IKLGKLMNQNHSLLQELTVSCKELDDLVDAARAAGAVGAKMSGTGRGGLMIALTPTE
DIQNT
>*Thalassiosira_rotula*/MK
MDDEFPTDQTRAFGKLILFGEHFVVYKVPALVGAVSAYTDCKLEYLDKPGLEVIDXRP
AVPRYKETKKHECDKSINLTLTHLGVDPKARGVKLTFGGDLCCASGIGASAAQVVSLA
RAVNAADSRSLSEDEINAAGYEGEKGSHGTPSGIDNTAATFGGLLRFQRTDSAPVFEKK

SFPSAIRIVYASTGITASTTEVVGDVRSKKEADEAWFSGLMGKYTTLVEEAEQVVVGG
DLEKLGKLMDQNHALLQELTVSCKELDDLVDAARAAGAMGAKMSGTGRGGLMIALT
PTEDIQNNVATALEKIAPQVWKTTFA
>*Thalassiosira_weissflogii*/MK
XRAFGKLILFGEHFVVYKVPALVGAVSAYTDCKLEYVDDKPGLEVIDNRPAVPHYKET
KSAEGLEAINLTLSHLGVDYKNRGVKLTFGGDLCCASGIGASAAQVVSLARAVNIADS
KSLSEDEINAAGYEGEKGYHGTPSGIDNTAATFGGLLRFQRTDDAPIFEKKTLASPIRIV
YASTGITASTTKVVGDVRAKKEADEAWFANLIDQYKILVEEGQNAIEKGDLPGLGKLM
DKNHELLQELTVSCKELDDLVVAARHTGALGAKMSGTGRGGLMIALTPTEAIQNAVA
DALEKIAPQVWKTTFA

MPDC protein sequences

>*Amphiprora_sp*/MPDC
MTTPPQRMYMCTVTAPTNIAVIKYWGKASVEHNTPTNSSLSVTLDQSDLRAVTTVATS
PDFTQDCLWLNGKLEEGAQNSKRFRACVDGVKALATDKVDGETRAVVVSKAEWQE
WKVHVSSYNTFPTAAGLASSAAGYAALVAALTELYQAQETFDHQLSTIARQGSGSSCR
SLYGGFVAWRQGSAENDWKDSLAQPVAPVEHWPEIRAVILVTNAQKKHTSSTTGMQ
NSVRTSPFLKYRGEAVVPQRMHDMERAWLAKDFDAFAKLTMQDSNQFHSTCLDTYP
PIFYLNDTSRDIIRLIHAYNDFYATTMNDGGGSGSNGATSLRVAYTFDAGPNAVLYTTA
AHVQEVLQLVQFFFPEPSLKETNGQTTLPYVRGMLAVEETTTTPLDEQLVQYCQEQAL
LLPTSGKVQYMYVTQSGPGPQVLLPCSLASDVKTDGDDQKMEINLDPTTGLNTYVKS
>*Bolidomonas_pacifica*/MPDC
XLSVVFCAEEGYEGELSTIARQGSGSAAGAFREVSLMGSKEDGSDSMAEQICNENHWP
EMRAVILVISDLKKDTSSTEGMGTSVATSELLGFRARKGGIVDKRMEIIEKAYKERDFE
TFGRITMQDSNQFHATCLDTYPPIFYMNDTSKSVIKIVHAYNKWKGRVAAAYTFDAGP
NAVIYCLEDVMDEVLALMLQYYPGKMFX
>*Coscinodiscus_wailesii*/MPDC
XSTQDEHNGAPFPAVIHMETVSAPTNIACIKYWGKASIALNTPINSSVSITLSQSDLRAV
TTASASTLFSNDRLWLNGKEEDISKSRRFRACVVGVRALATDKICPTTGNVLVKKEDW
NGMHVHVSSYNNFPTAAGLASSAAGYAALVAALAKLFNAVETYPGELSTIARQGSGS
ACRSLYGGFVAWRMGGVKEDGTDSIAEQIADENHWPEIRALILVVSDKKKGTSSTAG
MGTSVATSELLTYRAKEIVPERMTIIEKAFLERDFETFGRVTMEDSNQFHATCLDTYPPI
FYMNDVSRSIIRIVHVYNDWSGRICAAYTFDAGPNAVLYTLKEYAVELCALMLKFYPG
EGQDYINNEEIATKARDYCLDEELVAAVLQNGLAPQTGDVKMIYYTRSGPGPMSLGFE
NALIDPKSGLNTYEP
>*Cylindrotheca_closterium*/MPDC
SGSACRSLYGGFVAWRKGGEKEDWSDSMAQQVADEHHWPELRALILVVSDAKKDTS
STAGMSTSVATSTLLQHRIHSVVPDRMKEIETAFSGKDFQAFGKVTMRDSNQFHAVCM
DTYPPIFYMNDXSRMVVRIVHAYNAWAGEIRAAYTFDAGPNAVLYTLDQYVVEVGA
XMKHYFPETGALSDYINNAEYTEKVQKFSLSXELLEATDKTGRVPASGDVKQFYYTKS
GPGPQTLSMEESNLDPKTGLNTYQP
>*Fragilariopsis_kerguelensis*/MPDC
XSTERCRYQVDLSKXNQSKIKSMSNTVENNNHAPVWMATVSAPTNIACIKYWGKADT
KWNTPTNDSVSVTLDQGDLRAVTTAAASSKFEKDRLWLNGKEEEGADDCTRFRACV
DGVKVLAADPDKWKDLKVHISSYNTFPTAAGLASSAAGYAALTAALVELMDAKETY
KGQLTTIARQGSGSACRSLYGGFVAWKAGSPEVSTEDGTPWADSIAVQIADENHWPEL
RAVIMVVSDAKKDTSSTAGMSTSVATSTLLQHRIAKVVPERMVAIEKAFLGKDFESFG
ELTMRDSNQFHAVCADTYPPIFYMNDVSRSIVRIAHAYNKWAGETRVAYTYDAGPNA
VLYTIEKYSVEVGALMTHYFPSIDADGFFNDSIYANDVTTFSLSPDLLAATDKTGRKSH
AGDVKMMYHTKSGPGPQVLSNEKDGNLDLKTGLNIYTPK

>*Leptocylindrus_apora*/MPDC
MSEIFSATVSAPTNIACIKYWGKASIPLNTPINDSVSVTLHQKDLKAVTTASCSASFKED
KLWLNGIPVSLEGNKRFYSCLNGVKALAKEKGKFDGMKVHVSSYNTFPTAAGLASSA
AGYAALTFAFKELFDAKEDYEGQLSTIARQGSGSACRSLYGGFVAWRKGNDPYNNGE
DSMAVQIAPESHWPEIRALILVVSDKKKDTSSTKGMSTSVATSKLLAHRADSIVAKRM
EDIEKAYLRKDFESFGKITMMDSNQFHATCLDTYPPIFYMNDISKGVIQLIHKYNEFCGE
IRAAYTFDAGPNAVLYTLEKYQIEILALVLHCYPCSDKSAFVSNSKLAEEARAFKLSDE
LVDLVAVNHASVGDVKVVYCTESGPGPLRLDEKESLIDLVTGDNLYNA
>*Leptocylindrus_danicus*/MPDC
XHVTVSAPTNIACVKYWGKASIPLNTPINDSVSITLQQRDLKAVTTASCSTAFETDELW
LNGAKVSLDGNRRFYNCLNGVRALGKKKLEGKVHVSSYNTFPTAAGLASSAAGYAA
LVYAFKELFDAVEEYEGQLSTIARQGSGSACRSLYGGFVAWRKGSDPYTEGKDSMAV
QIAPETHWPEIRALILVVSDAKKDTSSTKGMSTSVATSKLLAHRASQIVPERMEQIEKAF
LAKDFEAFGRITMMDSNQFHATCLDTYPPIFYMNDISKSIISLVHKYNDLYGEIRAAYTF
DAGPNAVLYTLEKYQVEILAFMLKCFPSSIGDDFVSNSELAKQASECELKPELLEFVQV
AEEGDVKKIYCTESGPGPQRLDDGLCLIDLETGLNKYSP
>*Nitzschia_punctata*/MPDC
AKRKDSAADAVPWSDSMAVQIADENHWPEIRALILVVSDAKKDTSSTAGMSTSVATS
TLLQHRIQTVVPERMEGIEKAFXKDSNQFHAVCMDTYPPIFYMNDISRMVVRIVHAYN
QWAGEIRAAYTFDAGPNAVLYTLDKYAIEVGALMLHYFPAPTAQNGTSYINKSDYAK
EIEEYKLDPELLAATEKTGRTPAAGDVKMMYYTKSGPAPQVLSMEEANLDPTTGLNT
YHP
>*Nitzschia_sp*/MPDC
MSSTSAADDGTPIYMATVTAPTNIAVIKYWGKASAEWNTPTNDSVSVTLDQSDLKAV
TTVAASSKFQQXPSLVXLNGVEEVDVYKNKRFRACVDGVKALAADKDKWSGLKVHV
SSYNTFPTAAGLASSAAGYAALVAALAQLMDAREVYPGQLTTIARQGSGSACRSLYG
GFVAWRKGTPEGGNLDSSRNTPWDNSLAEQVADEHHWPELRALIMVVSDAKKDTSST
AGMSTSVATSTLLQHRIPNVVPARMEAIEASFKAKDFDKFGEITMKDSNQFHAVCMDT
YPPIFYMNDVSRMIVRIVHAYNTWSNEIRAAYTFDAGPNAVLYTLDKYAVEVGALML
HYFPSSSSDDGGYINNPTYAESIKSFKLDPSLLEATDKTGRSPSSGDVKMFYYTKSGPGP
QVLGSEHSNLDPKTGLNIYQP
>*Phaeodactylum_tricornutum*/MPDC
MTTPATHPIFLATVSAPTNIAVVKYWGKADEHYNTPINSSCSVTLHQDDLRAVTTVAV
SKDFVQDRLWLNGVEVPHAATSRRFRACVDGVLALAPDKYHTDDDNNNKTVAIAQH
EWPTLHVHVSSYNTFPTAAGLASSAAGYAALVAALVQLTGATETFPGEFSTLARQGSG
SACRSLYGGLVAWHAGTADEQWRDSRAEQLADEASWPALRAVIAVVSDAQKDTAST
AGMQASVKTSPLLAFRAAHVVPQRMQELTQAWRRRDFPVFGKITMQDSNQFHATCL
DTYPPIFYMNDVSRQIIRIVTAYNDYAGEIRAAYTLDAGPNVVLYVLEPHRPVLAALLR
HFFPASGLEEQNDEVLDPALVHAAEATGRVPRDGDVRHYYVTRPGPGPRVLDNDADG
TIDPHTGLNR YQPSG
>*Pseudonitzschia_arenysensis*/MPDC
MSTADAPIYMATVSAPTNIACIKYWGKADAKWNTPTNDSVSVTLDQTDLRAVTTAAA
SSKFEKDRLWLNGEEEVGASESKRFRACVDGVKLLAADPAKWKDLKIHISSYNTFPTA
AGLASSAAGYAALVAALAELMDAKETYKGQLTTLARQGSGSACRSLYGGFVAWRAG
TPEGRSDDDIPWSDSMAVQIADEAHWPEIRAVIMVVSDAKKDTSSTAGMSTSVATSTL
LPHRISTVVPDRMKAIEXAFLAKDFAAFGELTMRDSNQFHSVCADTXPPIFYMNDVSR
QIVRIAHAYNKWAGETRVAYTYDAGPNAVLYTLDKFVIEVGALMTHYFPSGGVEGFC
NDAAFTXDIAAFELDVELLXATDKCGRTPASGDVKMVYHTKSGPGPQVLDNDKDGXL
DPVTGLNRYTR
>*Pseudonitzschia_australis*/MPDC

MTTEGEGAPIYMATVSAPTNIACIKYWGKADAKFNTPTNDSVSVTLDQSDLRAVTTAA
ASSVFEKDRLWLNGEEEEGASESKRFRACVDGVKLLAADPSKWKDLKIHISSYNTFPT
AAGLASSAAGYAALVAALAQLMDAKESYQGQLTTLARQGSGSACRSLYGGFVAWRA
GTPEGRXDDDIPWSDSMAVQIADENHWPELRAVIMVVSDAKKDTSSTAGMSTSVATS
TLLPHRIAKVVPERMVSIEKAFLAKDFEAFGELTMRDSNQFHSVCADTYPPIFYMNDVS
RQIVRIVHAYNKWAGETRAAYTYDAGPNAVLYTLEKHSVEVGALMAQYFPSGGVDG
FYNDASYASSVQDFALDPSLVEAADKCGRTPASGDVKMVYHTKSGPAPQVLDNEKDG
NLDPKTGLNRYTKPN
***>Pseudonitzschia_delicatissima*/MPDC**
MAQDAPIYMATVSAPTNIACIKYWGKADAKWNTPTNDSVSVTLDQTDLRAVTTAAAS
ARFEKDRLWLNGEEEEGASESRRFRACVDGVKKLAADPAKWKDLKIHVSSYNTFPTA
AGLASSAAGYAALVAALAELMDAKEEYKGQLTTLARQGSGSACRSLYGGFVAWRAG
TPEGRSEDDVAWSDSMAVQIADENHWPEIRAVIMVVSDAKKDTSSTAGMSTSVATST
LLPHRISTVVPDRMKAIEAAFLAKDFAAFGELTMRDSNQFHSVCADTYPPIFYMNDVS
RQIVRIAHAYNKWAGETRVAYTYDAGPNAVLYTLDKYSIEVGALMTHYFPSTGDAGF
FNDAAFAQEIAAFELDAVLLAATDKCGRTPASGDVKMVYHTKSGPGPQVLDNDKDG
NIDPTTGLNRYTRN
***>Pseudonitzschia_multistriata*/MPDC**
MTDAPVYMATVSAPTNIACIKYWGKADAKFNTPTNDSVSVTLDQSDLRAVTTAAASP
KFEKDRLWLNGEEEEGASTSKRFRACVDGVKLLAADPEKWKDLKVHVSSYNTFPTAA
GLASSAAGYAALVAALAELMDAKEAYPGQLTTLARQGSGSACRSLYGGFVAWRAGT
PEGRSDDKEPTPWSDSMAVQIADETHWPELRAVIMVVSDAKKDTSSTAGMSTSVATST
LLPHRIAKVVPERMVAIEKAFLARDFAAFGELTMRDSNQFHSVCADTYPPIFYMNDVS
RQIVRIVHAYNQWAGETRVAYTYDAGPNAVLYTLEKHSVEVGALMAHYFPPGEASVE
SFYNDASFASDLGGFSLDPSLLAATDKCGRTPASGDVKMVYHTKAGPGPQVLDNETD
GNLDPVTGLNRYTKPN
***>Pseudonitzschia_pungens*/MPDC**
MATEGEDSPIYMATVSAPTNIACIKYWGKADAKFNTPTNDSVSVTLDQSDLRAVTTAA
ASKRFESDRLWLNGTEEEGAASSKRFRACVDGVKLLAADPAKWKDLKVHVSSYNTFP
TAAGLASSAAGYAALVAALAELMDATEAYPGQLTTLARQGSGSACRSLYGGFVAWR
AGTPEGRSDDEIPWSDSLAVQIADETHWPELRAVILVVSDAKKDTSSTAGMSTSVATST
LLPHRISTVVPERMEAIEKAFLSKDFETFGELTMRDSNQFHSVCADTYPPIFYMNDVSR
QIVRIVHAYNKWAGETRAAYTYDAGPNAVLYTLEKHSVEVGALMAHYFPSGGADGF
YNDAGYEASVGGFVVDPSLLEATDKCGRTPASGDVKMVYHTKSGPGPQVLDNDKDG
NLDPVTGLNRYTKPN
***>Pseudonitzschia_pungens_fr_cingulata*/MPDC**
MATEGEDSPIYMATVSAPTNIACIKYWGKADAKFNTPTNDSVSVTLDQSDLRAVTTAA
ASKRFESDRLWLNGTEEEGAASSKRFRACVDGVKLLVADPAKWKDLKVHVSSYNTFP
TAAGLASSAAGYAALVAALAELMDATEAYPGQLTTLARQGSGSACRSLYGGFVAWR
AGTPEGRSDDEIPWSDSLAVQIADETHWPELRAVILVVSDAKKDTSSTAGMSTSVATST
LLPHRISTVVPERMEAIEKAFLSKDFETFGELTMRDSNQFHSVCADTYPPIFYMNDVSR
QIVRIVHAYNKWAGETRAAYTYDAGPNAVLYTLEKHSVEVGALMAHYFPSGGADGF
YNDAGYEASVGGFVVDPSLLEATDKCGRTPASGDVKMVYHTRSGPGPQVLDNDKDG
NIDPVTGLNRYTKPN
***>Skeletonema_costatum*/MPDC**
XPTNIACIKYWGKADSHYNTPINDSLSLTLDQSDLRAVTSASASLAFDKDRLWLNGSEE
XGAASSRRFRACVDGVRALATEKIXPDTKEVIVSKDDWKKMHVYIASYNTFPTAAGL
ASSAAGYAALVAALVELFGAKESYPGEFTAIARQGSGSACRSLYGGFVAWRAGGEKS
DWSDSIAEQVADEAHWGEVRAVICVVSDAKKETSSTAGMSTSVATSELLAFRAKEVV
SKRMKIIEEAXKAKDFEAFGKVTMMDSNQFHATCLDTYPPIFYMNDVSRSIVQLVHRY

NDWAGEIRAAYTFDAGPNAVLYTLDKHVIELLALVLKYYPCESEDLMGDYVNXKXM
VSKALAFELDSGLLQALEKSCXIRDIGDVKMVYCTKGGPGPQVLDAX
>*Skeletonema_marinoi*/MPDC
XSAPTNIACIKYWGKADSHYNTPINDSLSLTLDQSDLRAVTSASASLAFDKDRLWLNG
SEEVGAASSRRFRACVDGVRALATEKIDPDTKEVIVSKDDWKKMHVYIASYNTFPTAA
GLASSAAGYAALVAALVELFGAKESYPGEFTAIARQGSGSACRSLYGGFVAWRAGGE
KSDWSDSIAEQVADEAHWGEVRAVICVVSDAKKETSSTAGMSTSVATSELLAFRAKE
VVSKRMKIIEEAIKAKDFEAFGKVTMMDSNQFHATCLDTYPPIFYMNDVSRSIVQLVH
RYNDWAGEIRAAYTFDAGPNAVVYTLDKHVIELLALVLKYYPCESEDLMGDYVNNK
DMVSKALAFELDSGLLQALEKSCNIRDIGDVKMVYCTKGGPGPQVLDASSEVFEFKVE
>*Skeletonema_menzelii*/MPDC
PKADGDTNPTIHKATVSAPTNIACIKYWGKADSHYNTPINDSLSLTLDQSDLRAVTSAS
ASLAFNRDRLWLNGSEEVDAASSKRFRACVDGVRALATEKIDPDTKEIIVSKDDWKK
MHVYIASYNTFPTAAGLASSAAGYAALVAALVELFGAKESYPGEFTAIARQGSGSACR
SLYGGFVAWRAGGEKSDWSDSIAEQVADETHWKEVRAVICVVSDAKKETSSTAGMST
SVATSELLAFRAKEVVSKRMKLIEEAIKAKDFESFGKVTMMDSNQFHATCLDTYPPIFY
MNDVSRSIVQLIHRYNDWAGEIRAAYTFDAGPNAVLYTLDKHVIELLALVLKYYPCES
KDLMGDYVNNKDMVTKALAFELDSSLLLSLEKSCSVRDIGDVKMVYCTRGGPGPQAL
DASSEVFDFKVE
>*Thalassiosira_minuscola*/MPDC
XKSADNPEPIIHTATVSAPTNIACIKYWGKANSHYNTPINDSLSLTLDQSDLRAVTTASA
SLSFDRDRLWLNGDEVLDASKAKRFRACVDGVRALATDKLDPDTNQVIVSIDDWKN
MHVHIASYNTFPTAAGLASSAAGYAALVAALVQLFGAKESYPGEFTAIARQGSGSACR
SLYGGFVAWRAGGTKDDWTDSIADQVADELHWEEMRAVILVVSDAKKETSSTAGMS
NSVATSELLAHRAKEVVPKRMKIIEEAIKSKDFESFGKVTMMDSNQFHATCLDTYPPIF
YMNDVSRSVVQMIHKYNEWAGEIRAAYTFDAGPNAVLYTLDKYVVELLALVLKYYP
AQSADLVGEYVNNSAMSKAALEFELDPSLTEAIEKTCKVHEYGDVKMVYCTKGGPGP
KILDASCEIFDFKIE
>*Thalassiosira_pseudonana*/MPDC
IHTATVSAPTNIACIKYWGKASSKYNTPINSSLSLTLDQSDLRAVTTAAASTSFTKDRL
WLNGSEEANAFTSKRFRACIDGLRALATDKVDPTTNEVIVSKSQWQSMHVHVASYNT
FPTAAGLASSAAGYAALVASLVELYNAKESYPGEFTAIARQGSGSACRSLYGGFVAWR
AGGMKEDWSDSIAEQVADEMHWKEMRAVILVVSDAKKETSSTVGMETSVATSELLA
HRAKEIVPKRMKIIEDAIQAKDFEAFGKVTMMDSNQFHATCLDTYPPIFYMNDVSRSVI
QMVTRYNEWAGEIRAAYTFDAGPNAVLYTLDKYVVELLALVLKHYPAQSPELLGEYV
>*Thalassiosira_puntigera*/MPDC
XFEKDRLWLNGSEVLDASKAKRFRACVDGVRALATDKIDPDTNKVIVSKDDWKRMH
VHIASYNTFPTAAGLASSAAGYAALVAALVRLYGAEESYPGEFTAIARQGSGSACRSL
YGGFVAWRSGGAKDDWSDSVAEQVADESHWEEVRAVILVVSDAKKETSSTAGMSNS
VATSELLAFRAREVVPKRMGIIEKAIKAKDFEAFGKVTMMDSNQFHATCLDTYPPIFY
MNDVSRSVVQMVHRYNEWAGDIRAAYTFDAGPNAVLYTLDKHVVELLALVLKYYP
AQSEDLVGDYVNNSVLSKQALEFELEPSLTEAVEKTGGYASTVT
>*Thalassiosira_rotula*/MPDC
XNPAIHKATVSAPTNIACIKYWGKADSHYNTPINDSLSLTLDQSDLRAVTTASASLSFE
KDRLWLNGSEVLDAAKAKRFRACVDGVRALATDKIDPSTNQVIVSKDDWKHMHVHI
ASYNTFPTAAGLASSAAGYAALVAALVRLYGAKESYPGEFTAIARQGSGSACRSLYGG
FVAWRAGGAKEDWSDSIAEQVADESHWEEVRAVILVVSXAKKETSSTAGMSNSVATS
ELLGFRAKEVVPKRMKTIEAAIXAKDFESFGMVTMMDSNQFHATCLDTYPPIFYMND
VSRSVIQMVHKYNEWAGEIRAAYTFDAGPNAVLYTLDKHIVELLALVLKYYPAQSQD
LVGDYVNNEVLSKEALEFELDSSLTEAIEKTGRVHEYGDVKMVYCTKGGPGPMTLDA
SSXIFDFKIE

>*Thalassiosira_weissflogii*/MPDC
XKVVVALSKVDGDDPIIHTATVSAPTNIACIKYWGKANKHYNTPINSSLSLTLDQSDLR
AVTTATASPSFDSDRLWLNGQEVDSACTSKRFRACVDGVVALATDKVDSSSGKILVSK
DDWKKMHVHIASYNTFPTAAGLASSAAGYAALVAALVELYGAKESYPGEFTAIARQG
SGSACRSLYGGFVAWRQGGEKEDWSDSIAEQVADESHWPEMRAVILVVSDAKKETSS
TDGMSTSVATSELLAFRAKEIVSKRMKTIEEAIKAKDFEAFGKVTMMDSNQFHATCLD
TYPPIFYMNDVSRSIIQMVHKYNSWAGGIRAAYTFDAGPNAVLYTLEEYVVELLALVL
KYYPEVGEDYVNNVNLKNEALEFQLNPSLIECVEQSSRIHETGDVKMVYCTKGGTGPQ
VLDAVSSVIDFKIE

HDR protein sequences

>*Bolidomonas_pacifica*/HDR
MKLVSAALILLYASPLASSFAFSLSSTTSISTSSSLRRSPFISRSSLHANPLDVRDKGVETK
KDKRRSYMAKDTYYKNGFKGVREEVKVNMEQQFKSETVGELKSSDHVLFRDNVEVH
LAKDFGFCWGVERSIAMAYEARTHFPDRKLHITNELIHNPQVNGKLQEMDVQFVPKT
GGEDGGKDFSAVGDGDVVILPAFGASIEEMALFDSKNVEVVDTTCPWVSKVWNTVD
MHQRRGLTSVIHGKYAHEETVATASFCETYIIVKDMKEAEYVADYIVNGGDREEFLKK
FENAMSEGFDPDTDLKKLGLANQTTMYKKETRAIGQLFQRAQMSKYGPIDVKEHYME
FDTICDATQERQDAIHDLVNNKDELGLDFILVVGGWDSSNTQHLLEIPLHAGLRAFHIN
EGGCIGADNTITHRAMDGTIVKEEFMGKGLVKMGVTSGASTPDRAVQDALDRIFLLKK
VNPEYSSSQLQESILRADP
>*Chaetoceros_neogracile*/HDR
MKFTSAALLLSAVTTASAFAPNLNLSTRYTSISAPLQMSETTETEEEAKISKKQQRLLFM
KNDKFFRNGFKEVRKDVESTMEGQFKSNVVDEMKENKFVMEREGVKVHLASDFGFC
WGVERSIALAYEAVKHFPDRKVHITNELIHNPEVNDNLHAMDVNFIQKTDDGKDFTV
VGENDVVILPAFGASFEEMQFFDQKNVDVVDTTCPWVSKVWNTVDTHQKKGLTSIIH
GKYEHEETVATVSFCEDYICVKNMKEAEMVADYITNGGDKAAFMNHFKHAHSDGFD
PDTMLDCLGLANQTTMYKKETRAIGQMFQKTMMEKFGPVDAETHYMEFDTICDATQ
ERQDAVHDLVVKSDELGLDFILVVGGWDSSNTAHLLEIPQKAGVRSFHINRAECITAD
NTITHRTMSGEIVTESLILDTTKDVVMGVTSGASTPDAAVQDSLSSIFMLKKLSAKE
>*Coscinodiscus_wailesii*/HDR
ILFRELRSFGTTFANWTPPDVNTPVDTFAPSLVTASSNLLHLRHNTPTRIMKFNPSTLLL
LLLTSSPVTNAFVVITPSPIQLSSSSSPPTTTTRISMSTTADAADSSTTTRKSKKQDRLRW
MKEPSFHRRGFKDQRDGAEKRMNDSFKSDIVQEMRDSNFKIEREGVTVYLAKDFGFC
WGVERSIALSYEAVDHFPGKNIHLTNELIHNPEVNDELKNMNVQFIEKLEGGKKRFEA
VEDGDVVILPAFGASYEEMDYLDKKNVQVVDTTCPWVSKVWNTVDTHQKNGLTSIIH
GKYGHEETVATTSFCEDYICVKDYKEAEMVADYIVNGGDKEAFLEYFSKAVSKGFDP
DKMLNKVGLANQTTMYKKETRAIGQMLQKAMMKKYGPVEADKHYSEFDTICDATQ
ERQDAVAELVENASTLDLDFILVVGGWDSSNTAHLLEIPHKAGVRSFHIDRAERINADN
TITHRTVEGEIVTEPFLDLSKDVVMGVTSGASTPDSAVQASLGSIFLLKEVHDA
>*Cylindrotheca_closterium*/HDR
MKISGAAVFAIAIASVEAFSPVSVQNRNGATALFSTAEESKPRTKKEGRLGFFKSENFH
RKGFKEVRDNVEDTIKDQYQSSLVDDLKSSNYVIEKDNVKVYLAKDFGFCWGVERSI
ALAYEAVKHYPDKNLHITNELIHNPEVNDRLTEMNVKLIEKLGEGKKDFDVIEDGDVV
ILPAFGASYEEMDFLDKKNVEIVDTTCPWVSKVWNTVDAHQRKGLTSIIHGKYAHEET
VATTSFCEDYICVKDMKEAEMVADYMINGGNREEFLEYFKNAVSEGFDPDKMLEKVG
LANQTTMYKKETKSIGQLFQKTIMKKFGPSKVDEHYFEFDTICDATQERQDAVTELVE
NKNDYGLDFILVVGGWDSSNTAHLLEIPHHQGIRSFHINRAECISADNTITHRTVDGEIV
TEPLLLDLDKDVVLGVTSGASTPDAAVQDALSQIFLLKKMQGAAKEESASKSD
>*Fragilariopsis_kerguelensis*/HDR

SIMTTSHVVPVATSFQPIRRYGCSSLRSSASASSLSSSIIDKDTTTTDVVIEKKKQHENKK
KDDTTTTTTKEKKKKPVYIKNEREQIARKKDFYRGAGVFKDVKRDVTATMRAQFDSE
LMNAMKENPNYCIEKEGVEFFLAKTQFDSELMNAMKENPNYCIEKEGVEFFLAKDHG
FCWGVERSINLAYSAVETFPNAKVHITNELIHNPMVNDRLHLKNVNFIQKLERTPEEEE
NENEKSDCCSTKKKNNNAILDANPNAKDFSAIQEGDVVMLPAFGATLDEMKELDAKN
VKVVDTTCPWVAKVWNTVHTHQVRGLTSIIHGKYAHEETMATKSMAETYLCVKNID
EAQYVSDFILQELQQSDADKDRFESQEEQEAHQQQMTAAFMLKFRDACSAHFDPYVH
LRKIGLANQTTMYKKETRAIGQLLQKTMMHTFGPELLNDHYYEFDTICDATQVRQDA
VDELCTMTLDETHPITLDFILVVGGFDSSNTAHLLEIPHMRKVPSYHINTAECIKATNTIT
HRTVDGVVIEDEFPFLTPAMLWENEEQEQEGSTNTKKTKKLLRVGVTSGASTPDKEVQ
DALGRIMMINKLKEEEEEHKYE
>*Leptocylindrus_apora*/HDR
MKFSTFAILTLIQTTTVAAYTRSSTSLRMSSSTDSSSSPTTTKKIERRNIMKAPSFFRAGF
KEQREEVEGIMQDQFKSNLVDDLRSNEYVIERDGVKVHLAKDFGFCWGVERSIALAY
EAVKQFPEKNVHITNELIHNPEVNDHLKDMKVNFIDKTEEGGKDFEVVGDKDVVILPA
FGASLEEMKYFDKKDVTVVDTTCPWVSKVWNTVDTHXKKGLTSIIHGKYAHEETIAT
TSFCEDYICVKDMKEAEYLCDYMLNGGDKEEFMNKFAKAMSEGFDPDTMLEKVGIA
NQTTMYKKETRAIGQLLQKTMMEKHGPQNVNEHYMEFDTIXDATQERQDAVAELTE
NSEKLDLDFILVIGGWDSSNTAHLLEIPMNAGVRSFHINRADCITADNTITHRLMDGTIK
TEEFLLNKREDGSVKMGVTSGASTPDAAVQESLGQIFLLNKVMFGKDA
>*Leptocylindrus_danicus*/HDR
SNAFVVPGSRNGRAFSTKVGMSTTEKADEAKISKKKKRIDIMKNEGFFRNGFKEVRED
AEQTMNQQFKSELVDSLRAEEFKMERDGVTVYLAKDYGFCWGVERSIALAYEAVNH
YPDRKVYITNELIHNPEVNDNLSAMKVNFIEKKEDGSKKFEVVQDNDVVLLPAFGASL
EEMQYFDEKNVDIVDTTCPWVSKVWNTVDKHQQNGLTSVIHGKYAHEETIATTSFCE
DFICVKNMDEAEYVSNYILNGGDKEEFMKKFEKAVSEGFDPDIMLSKLGLANQTTMY
KKETRAIGKLLQRTMMEKFGPAELNERYMEFDTICDATQERQDAVSELTVNAESLGLD
FILVVGGWDSSNTAHLLEIPHMAGVRSFHINRADCIGADNTITHRSVEGEIVTEKFLIDA
DKKDVVMGVTSGASTPDAAVQEALSQIFVLNAVLKTKQ
>*Nannochloropsis_gaditana*/HDR
MSPRSMPVLLVAVLAVLCTASVTAFHTPSSPSAPSIWGAKRGAASAAALTTRRQAAAD
GEAVENVLSPKQERRRIIQSPNFHRQGFKAESEGVESQMVSEFTSQLINEMKDSTYTFQ
KGDLTIKLAKSFGFCWGVERAVAMAYEARAHFPEQRIHITNEIIHNPGVNERLADMNID
FLPVDEGKKQFEHIEKDDVVILPAFGATIQEMQLLDKKGVQIVDTTCPWVAKVWTAVE
KHRKLESTSVIHGKWNHEETIATASFADVYIIVKDIHEAEYVCNYIVNGGDKAEFMEKF
KNAVSEGFDPDVDLEKVGLANQTTMYKRETRAIGKLFEKTMMHKYGPAEVKNHYAE
FDTICDATQERQDAITDLMEEKLDFILIVGGFDSSNTAHLKEIPHMQHIPSFHIDRAERIS
ADGSIEHREIDGSIVKTANFLPDGPLTIGVTSGASTPDAYLQEAIERLFLLRTLKGQEAPQ
KDTVNA
>*Nitzschia_punctata*/HDR
KEERLNFMKSEQFHRRGFKEVRDRVEKDIQTQYQSELVNDLKTSNYMIENKDANVKV
YLAKDFGFCWGVERSIALAYEAVQHYPDRKLHITNELIHNPEVNDRLTEMKVNLIEKV
GEGQKDFSTIEEGDVVILPAFGASYEEMEMLDKKNVEVVDTTCPWVSKVWNTVDMH
QRKGLTSVIHGKXGHEETIATTSFCEDYICVKNMKEAEMVADYIVNGGDKEEFLKYFS
KAVSKGFDPDTMLKKVGLANQTTMYKKETKAIGQLFQKAMMKKYGPAEVDQHYME
FDTICDATQERQDAVSELVENAKEYGLDFILVVGGWDSSNTAHLLEIPHMAGIRSFHIN
RAECIGADNTITHRTVDGEIVTEPFLLDIEKDVVMGVTSGASTPDAAVQDSLSQIFLLKQ
LNKKKEEEGQKA
>*Nitzschia_sp*/HDR
MKFSQSVMLAVLTATSSVHAFTTTPAQQHQPLAFVGTKASPPTSSSVSVLHMSTTEEE
ATASTKKQTAGGMSKKKEERLQFMKSDQFHRRGFKEVRDKVESDIEEQFNAKLVEDL

KESNYVLDKTDEVGVKVYLAKDFGFCWGVERSIALAYEAVQHYPDRKLHITNELIHNP
EVNDRLTDMKVNLIEKSTKGEKNFEKIEEGDVVILPAFGASYEEMDYFDKKNVEVVDT
TCPWVSKVWNAVDTHQRKGLTSVIHGKYGHEETVATTSFCEDYICVKDMKEAEMVA
DYIVNGGDKEEFLKYFEKAVSKGFDPDTMLSKVGLANQTTMYKKETNAIGKLLQKTL
MKKFGPAKVDEHYYEFDTICDATQVRQDAVSELVDNVDEYGLDFILVVGGWDSSNTA
HLLEIPHMAGVRSFHINRADCIKADNTITHRTVGGEIVTEKFLLDTSKDVVMGVTSGAS
TPDSVVQDSLSQLFLLKQTQAKEP
>*Phaeodactylum_tricornutum*/HDR
MRFASSAVVFMAVASTVTAFQQTAFKPSRISIVVQQSTSATEAEEKRATKKEERLRMM
KSDRFHRRGFKEVREGVESNMEDQFQSPIVNSLRTSNFVMDRDGVKVYLAKDFGFCW
GVERSIALAYEAAEHFPDRKLHITNELIHNPEVNENLKAKNVQFIEKLGDGTKNFASVQ
DGDVVILPAFGASFEEMDYFDKKNVEIVDTTCPWVSKVWNTVDKHQKQGLTSVIHGK
YAHEETVATTSFCEDFICVKNFKEAEMVANYILNGGDKDAFMKHFENAVSEGFDPDK
HLEKVGLANQTTMYKKETRAIGQLFQKTMMKKFGPVKSKEHYMEFDTICDATQERQ
DAIHDMVESAQKDGLDFILVIGGWDSSNTAHLLEIPVHAGIRAFHINRAECIGADNTITH
RTVDGEIVTTQLLEDMDKEVVMGVTSGASTPDAAVQDSLSQIFLLKKMYDESKK
>*Pseudonitzschia_arenysensis*/HDR
MKFSSSSISALALLAANGSDAFAPAIQSPGLASTELFSTKSQSKKNDRLEFMKNPQYHR
RGFTDVRPKIEEKMEEDYTSELVEDLKSNNYLVEKDGVKMYLAKDFGFCWGVERSIA
LAYEAVEHYPDRKLHITNELIHNPEVNDSLTEMKVNLIEKESTTGAKDFSPVQEGDVVI
LPAFGASYEEMEMLDKKNVEVVDTTCPWVSKVWNAVDKHQKKGLTSVIHGKYAHEE
TLATTSFCEDYICVKNMDEAQMVVDYMEKGEDNTPEDKEAFLNFFKNAISEGFDPDT
MLTKLGLANQTTMYKKETKAIGQLFQKAMMKIYGPSKVDEHYMEFDTICDATQERQ
DAVSELVENAKDLELDFILVVGGWDSSNTAHLLEIPHNEGVRSFHINRADCIGADNTIT
HRSVEGEIVTETFLTSDPEKNVVMGVTSGASTPDGSVQDALGQIFLLKQMLSAKEE
>*Pseudonitzschia_australis*/HDR
MKFSSATLSAVLFLGSYANTCNAFSPAITSSQSRTIATSTTTALGATMSKKKEERLEFM
KNPQFHRRGFKDVRGKVEETMENEYKADLVEDLKSNNYLLEKDGVKMYLAKDFGFC
WGVERSIALAYEAVEHYPDRTLHITNELIHNPEVNDSLSAMKVNLIDKESSTGVKDFST
VEEGDVVILPAFGASYEEMDLFDKKNVEVVDTTCPWVSKVWNAVDKHQKGGSTSVI
HGKYAHEETLATVSFCDDYICVKDMKEAEMVIAYMEKGADNTAEDKEAFMKHFEKA
VSEGFDPDTMLDKIGLANQTTMYKKETKAIGQLFQKSIMKIHGPAEVDQHYMEFDTIC
DATQERQDAVAELVHSASGLGLDFILVVGGWDSSNTAHLLEIPHHAGVRSFHINRADC
IGADNTITHRTVEGEIVTEKFLTTLDDDKDVVLGVTSGASTPDGSVQDALSQIFLLKQM
ATNKEE
>*Pseudonitzschia_delicatissima*/HDR
MKFSSSSISALVLLASQGSEAFAPAPQGLGIASTTELFSSTSEKRTQSKKNDRLGFMKND
QYHRRGFADVRPKIEEKMEEDYTSELVEDLKSNNFLVEKDGVKIYLAKDFGFCWGVE
RSIALAYEAVEHYPDKKLHITNELIHNPEVNDSLTEMKVNLIEKESTTGSKDFSVVEEG
DVVILPAFGASYEEMEMFDKKNVEVVDTTCPWVSKVWNAVDKHQKKGLTSVIHGKY
XXEETLATTSFCEEYICVKNMDEAXMVVEYMQKGKENTPEEKEAFLKFFENATSEGFD
PDTMLNKVGLANQTTMYKKETKAIGQLFQKAMMKIYGPSAVDEHYMEFDTICDATQ
ERQDAVTELVVKADDLKLDFILVVGGWDSSNTAHLLEIPHHAGVRSFHINRADCIGAD
NTITHRTVDGEIVTEKFLTSDPEKNVAMGVTSGASTPDGSVQDALGQIFLLKQMLSAK
DE
>*Pseudonitzschia_multistriata*/HDR
MKFSSTALSVVLLLGAGCDAFSPLANANTNTGAKASATALQAAAQGSMSQKKGDRLE
FMKNPQYHRRGFKDVREKVEETMEEQYKSDLVEDLKSNNFLMEKEGVKMYLAKDFG
FCWGVERSIALAYEAVEHYPDKKLHITNELIHNPEVNDSLTSMNVNLIDKESTTGSKDF
SPVEEGDVVILPAFGASYEEMEMLDKKNVEVVDTTCPWVSKVWNAVDQHQRGGLTSI
IHGKYAHEETLATTSFCEDYICVKDMKEAQMVVDYIQKGKENTKEDKEAFMKYFEKA

VSEGFDPDTMLDKVGLANQTTMYKKETKAIGQLFQKAIMKTYGPAKVDEHYMEFDTI
CDATQERQDAVSELVDNASGLGLDFILVVGGWDSSNTAHLLEIPHKAGVRSFHINRAD
CIGADNTITHRTVDGEIVTEEFLTTLDDTDRDVVLGVTSGASTPDGSVQDALSQIFLLKK
MASEKAE
>*Pseudonitzschia_pungens*/HDR
MKFSSATVSAFVFLGSTCHAFSPVPRNTNVQTTVLRSVAEQAPEVNTGSMSAKKKDRL
NFMKNPQFHRRGFKEVRTSVEETMEQEYKSDLVEDLKSNNYLLQKDGVKTYLAKDFG
FCWGVERSIALAYEAVEQYPDRKLHITNELIHNPEVNDALTNMKVNLIAKESTTGTKD
FSPVEDGDVVILPAFGASYEEMEMFDKKNVEVVDTTCPWVSKVWNAVDKHQRKGLT
SIIHGKYAHEETLATTSFCEDYICVKDMKEAQMVADYMEKGADNTPEDKAAFLKYFE
KAVSEGFDPDTMLGKIGLANQTTMYKKETKAIGQLFQKSIMKIHGPAEVDQHYMEFD
TICDATQERQDAVSELVENASGLGLDFILVVGGWDSSNTAHLLEIPHKAGIRSFHINRA
DCIGADNTITHRTVDGEIVTEKFLLTLDEPDREVILGVTSGASTPDGSVQEALGQIFLLK
KLAAKEE
>*Pseudonitzschia_pungens_fr_cingulata*/HDR
MKFSSATVSAFVFLGSTCHAFSPVPRNTNVQTTVLRSTAEQAPEVNTGSMSAKKKDRL
SFMKNPQFHRRGFKEVRTSVEETMEEEYKSDLVEDLKSNNYLLQKDGVKTYLAKDFG
FCWGVERSIALAYEAVEHYPDRKLHITNELIHNPEVNDALTNMKVNLIAKESTTGTKD
FSPVEDGDVVILPAFGASYEEMEMFDKKNVEVVDTTCPWVSKVWNAVDKHQRKGLT
SIIHGKYGHEETLATTSFCEDYICVKDMKEAQMVVDYMEKGADNTPEDKAAFLKYFE
KAVSEGFDPDTMLGKIGLANQTTMYKKETKAIGQLFQKSIMKIHGPVGVDQHYMEFD
TICDATQERQDAVSELVENASGLGIDFILVVGGWDSSNTAHLLEIPHKAGIRSFHINRAD
CIGADNTITHRTVDGEIVTEKFLLTLDEPDREVILGVTSGASTPDGSV
>*Skeletonema_costatum*/HDR
MAALAGSAAAFAPSAQSLNRITSSLSMSTEVAEESKKRVTKKEKRLQFMKTETFHRRG
FKDVREGVEKSMEEQFKSQLVDELTSNDFVLERDGVKVFLAKDFGFCWGVERSIALA
YEAVEHFPGKTVHITNELIHNPEVNDKLHDMDVQFIEKLGEGKKDFSNVNDGDVVILP
AFGASFEEMSMMNDKNVDIIDTTCPWVSKVWNTVDQHQKKGLTSVIHGKYAHEETV
ATISFCEDYICVKDMKEAEMVVDYILNGGDKEAFLKYFENAVSKDFDPDTMLDKVGL
ANQTTMYKKETRAIGQMMQKAMMKKFGPVDAKDHYWEFDTICDATQERQDAIHEL
VEKSDDLGLDFILVVGGWDSSNTAHLLEIPQKAGVRSFHINKSDCIGADNTITHRTMAG
EIVTEKFIEDWGKDVVMGVTSGASTPDKAVQDSLSSIFLLKKVHAGED
>*Skeletonema_marinoi*/HDR
MKFSTSSVVAIAALAGSAAAFAPSAQSLNRITSSLSMSTEVAEESKKRVTKKEKRLQFM
KTETFHRRGFKDVREGVEKSMEEQFKSQLVDELTSNDFVLERDGVKVFLAKDFGFCW
GVERSIALAYEAVEHFPGKTVHITNELIHNPEVNDKLHDMDVQFIEKLGEGKKDFSNV
NDGDVVILPAFGASFEEMSMMNDKNVDIIDTTCPWVSKVWNTVDQHQKKGLTSVIHG
KYAHEETVATISFCEDYIXVKDMKEAEMVVDYILNGGDKEAFLKYFENAVSKDFDPD
TMLDKVGLANQTTMYKKETRAIGQMMQKAMXKKFGPVDXKDHYWEFDTICDATQE
RQDAIHELVEKSDDLGLDFILVVGGWDSSNTAHLLEIPQKAGVRSFHINKSDCIGADNT
ITHRTMAGEIVTEKFIEDWGKDVVMGVTSGASTPDKAVQDSXSSIFLLKXVHAGED
>*Skeletonema_menzelii*/HDR
MKFSSSSVLAIAALAGSATAFAPSARSLNRITSSLSMSTEVAEESKKASKKEQRLKFMK
TDSFHRRGFKDVREGVEKSMEEQFKSKLVDELTSNDFVLERDGVKVFLAKDFGFCWG
VERSIALAYEAVEHFPGKTVHITNELIHNPEVNDKLHDMNVQFIEKLGEGKKDFSNVN
DGDVVILPAFGASFEEMQMMNDKNVDIVDTTCPWVSKVWNTVDQHQRKGLTSVIHG
KYAHEETVATISFCEDYICVKDMKEAEMVVDYILNGGDKEAFLEHFKNAVSKDFDPDT
MLDKVGLANQTTMYKKETRAIGQMFQKAMMKKFGPVDAKDHYWEFDTICDATQER
QDAIHELVENAGDLGLDFILVVGGWDSSNTAHLLEIPQKAGIRSFHINKSDCIGADNTIT
HRTMSGEIVTEKFIEDWDKDVVMGVTSGASTPDKAVQDSLSSIFLLKNVHAADN
>*Thalassionema_frauenfeldii*/HDR

MLFRNLNLLLLLILNSESSHGFTASPPPSILSTQQRRTTVSTLSASVATDDDTKKKDDKM
KYLKFQRDAIAKKDNYLRGAGVFKDVKNDVTEXMAQQFDSELMNEMKKNPNYMLE
KDGIEFYLAKDHGFCWGVERSINLAYSAVETFPDNKLHITNELIHNPLVNKKLHDKNV
NFIAKDENNNKDFSQIEDGDVVMLPAFGATLDEMKLLDEKGVKVVDTTCPWVSKVW
NTVHKHQVKGLTSVIHGKYAHEETMATKSMCETYLCVKNLEEAEYLADFILNAKPGD
GRAEKFMEKFAKACSPDFDPHRDLLKIGLANQTTMYKKETRAIGQLLQKTIMKKFGPD
LINEHYFEFDTICDATQVRQDAIDELCDMHLSDDGPTLDFILVVGGFDSSNTAHLLEIPQ
MRGVRSYHINQADCISAENTIRHRNVKGDIVEGETLLLPKXPGSKLRIGVTSGASTPDK
EVQDALGRIMMLNNLRENGELKA
>*Thalassiosira_minuscola*/HDR
MKFTTTSIAAIAALVSTGSHAFTPSQHTTRATLRPLSMSSTEAVEEKKPTKKEKRLQFM
KTPTFHRRGFKEVRESVENNMQEQFESELVGELKSNDFVIEKDGVKVHLAKDFGFCW
GVERSIALAYEAVEHFPGKTVHITNELIHNPEVNDKLHDMDVQFIEKIGEGKKDFSKVG
DGDVVILPAFGASFEEMSLFDEKNVDVVDTTCPWVSKVWNTVDQHQRKGLTSVIHGK
YAHEETVATVSFCEDYICVKDMKEAQMLADYIVNGGDKEAFLKYFEKAVSKGFDPDT
MLDKVGLANQTTMYKKETRAIGQMMQKAMMKKFGPVDAKDHYLEFDTICDATQER
QDAIHELVENSDELGLDFILVVGGWDSSNTAHLLEIPQKAGVRSFHINKADCIGADNTI
THRTMSGEIVTEKFIEDVENKDVVMGVTSGASTPDKAVQDSLSSIFLLKKMNEASASA
>*Thalassiosira_pseudonana*/HDR
MKSPTFYRQGFKDVRPAVEKSMEDQFKSSLVDELKTNDFVIEKDGVKVYLAKDFGFC
WGVERSIALAYEAVEHFPGKTVHITNELIHNPEVNDKLHDMNVQFIEKLGEGKKDFSKI
GEGDVVILPAFGASFEEMTLMNNKNVEVVDTTCPWVSKVWNTVDQHQRKGLTSVIH
GKYGHEETVATVSFCEDYICVKDIKEAEMVADYIINGGDKEKFLKYFEKAVSKGFDPD
TMLDKVGLANQTTMYKKETRAIGQLMQKAMMKKFGPVNAKDHYLEFDTICDATQER
QDAISDLVENSDELGLDFILVVGGWDSSNTAHLLEIPEKAGVRSFHINKSECIGADNTIT
HRTVDGEIVTEKFIEDIENKDKKLVMGVTSGASTPDKAVQDSLDQIFMLKKVYSKEE
>*Thalassiosira_puntigera*/HDR
XTHQQADRLFLIVINSGPSTANSISNSNGRTIKMRFSSTSVMAVAALVSTGSHAFTPSPH
ARRAAARPPSMSTVEAEAPTEERSKPPTKKEQRLKFMKKDSFYRRGFKEVRDDVERT
MGEQFESELVGELKSNDFVIEKDGVKVHLAKDFGFCWGVERSIALAYEAVKQFPEKTV
HITNELIHNPEVNDHLHDMNVQFIEKVGEGQKDFSKVGDGDVVILPAFGASFEEMSLF
DAKNVDIVDTTCPWVSKVWNTVDQHQRKGLTSVIHGKYAHEETVATVSFCEDYICVK
DMKEAQMVADYIINGGDKEAFLKYFEKAVSEGFDPDTMLDKVGLANQTTMYKKETR
AIGQMMQKAMMKKFGPVDAKDHYLEFDTICDATQERQDAIHELVENSSTLGLDFILV
VGGWDSSNTAHLLEIPQKAGVRSFHINKADCIGADNTITHRTMEGEIVTEKFITDIEDGD
KEVVMGVTSGASTPDKAVQDSLSSIFLLKKMSSGGD
>*Thalassiosira_rotula*/HDR
MKFSTTSVMAVAALITTGTTAFTPSHHTLQRTTALRMSTVEETSVEESKPVRKKEKRL
QYMKTDTFHRRGFKEVREGVEKAMSEQFESELVDELKSNNFVINKDGVKVHLAKDFG
FCWGVERSIALAYEAVEHFPGKKVHITNELIHNPEVNDKLHDMDVQFIEKIGEGKKDFS
KVGDGDVVILPAFGASFEEMTLFDSKNVDIVDTTCPWVSKVWNTVDQHQRKGLTSVI
HGKYAHEETVATVSFCEDYICVKDMKEAQMVADYITNGGDKEAFLKYFENAISEGFD
PDTMLDKVGLANQTTMYKKETRAIGQLMQKTMITKFGPVNTKEHYLEFDTICDATQE
RQDAIHDLVENADELKLDFILVIGGWDSSNTAHLLEIPQKANVRSFHINKADCIGADNTI
THRTMSGEIVTDKFIFDIEDKDKEVVMGVTSGASTPDAAVQDSLSSIFLLKKMSSSD
>*Thalassiosira_weissflogii*/HDR
XSHHPHRTSLSLSQSTEVSQETTPATEKKPTKKEKRLQYMKDPNFYRKGFKDVRPNVE
KTMETQFKSPLVDELKTSNYVIERDGVRVHLAKDFGFCWGVERSIALAYEAVEHFPGR
TVHITNELIHNPEVNDNLHKMNVQFIEKLGEGKKDFSKVEDGDVVILPAFGASFEEMSL
MNEKNVEVVDTTCPWVSKVWNTVDQHQRKGLTSVIHGKYGHEETIATTSFCEDYICV
KDYKEAEMVADYIVNGGDKEAFLKYFEKAVSKGFDPDTMLDKVGLANQTTMYKKET

RAIGQLMQKAMMKKFGPVDAKDHYLEFDTICDATQERQDAIHELVENASELDLDFILV
VGGWDSSNTAHLLEIPQKAGIRSFHINKADCIGADNTITHRTMSGDIVTEKFIEDIEDAG
KTVVMGVTSGASTPDKAVQDSLSSIFMLKKLQSGKEE

DXR protein sequences

>*Amphiprora_sp*/DXR
XPLVLSSAAAFSCNQALAPNNKASSSLSAXFPGSKGTGEGWIDASSPVNTEESLQKTLE
KSLEGSNFQKRLSLLGSTGSIGTQTLEIVDACPDNFVVDALSAGNNAKLMAEQVMKYK
PKVASLATPEAAEELKQLLKDAGCSDMPSIVHGEEGILEAATVESADTVVTGIVGAAG
LKPTIEAIKLKKDIALANKETLISGGPVINPLVEEYGVNMLPADSEHSAIFQCLQGVPPG
GLRRVILTASGGAFRDMSKDELFQKCKDEPRLIQAKATTHPNWDMGAKITLDSATMM
NKGLEVIEAHYLFGASYDDIDIVIHPQSIVHSMVETQDTSCIAQLGWADMRLPLVYSVS
WPHRLKMPYKPLDLAELATLTFKAPDTEKYPCISLAYEAGRTGGTMTAVLNAANEAA
NEMFREDVGLGFLDIPKLIEGAMEEHKDDLKTSDITLDDILSCDAWAREYVVKMTDK
MKTEAVLL
>*Bolidomonas_pacifica*/DXR
MTYLLITLLLLITPTTLTFKYRAHRPTLPNQISTPLMTKRLHSTPPNKNWVNPCAPVSTP
DSLTLTLQKSLQGSTFKKNLSILGSTGSIGTQTLDIVRAKPDNFKVTSLSAGRNLDLLVE
QIVEFQPDIVSVMDVEDIKKLEGMLKGKFKPSKIVGGPSGIIEVSRDPSSDTLVTGIVGSA
GLQPTVEGIKHGKDIALANKETLIAGGPVVVPMVEEYGVNMLPADSEHSAVFQSLQGC
PPGGLSKVILTASGGAFRDFSKEELFDLCESDPSAVLAKATTHPNWDMGAKITLDSAT
MCNKGLEVIEAHYLFGAGYDDIDVVVHPQSIVHSMVEMRDTSVVAQLGWADMRLPL
VYALSWPHRLEMDYRRLNLAEIGSLTFKEPDQGKYPCIGLCYEAGRAGGTMTSVLNA
ANEMANEIYRKDVGMGFLDIPRLIEGAMEGAREQGLYKTVGVELNDILESDQWARDW
VGRKVEEGLGGKX
>*Chaetoceros_neogracile*/DXR
MKLTFGALVALLTVPATCAFTITAPSTKSTSLNAMKSGSKGTGEGWLERPAAPVNSEA
SARLTLQESLKGSSFQKRLSLLGSTGSIGTQTLEIVDACPDNFVVDALSAGNNAELMAE
QVMKYQPKIASLSTPEAAAELKDRLIAAGCKNMPEIMFGDDGILAAATYDTADTVVTG
IVGAAGLKPTIEAIKLGKDIALANKETLISGGPVINPLVREYGVNMLPADSEHSAIFQCL
QGVPEGGLRRVILTASGGAFRDFSKDDLFTACMEDPRAVQAKATTHPNWDMGAKITL
DSATMMNKGLEVIEAHYLFGASYDDIDIVIHPQSIVHSMIETQDTSCIAQLGWADMRLP
LVYSVSWPHRLKMPYRPLDLAELSTLTFTKPDMDKYPCIGLAYAAGRAGGTMTAVLN
AANEAANEMFRADIGLGFLDIPKLVEGAMEAHKSDLKIDDVVLDDILSCDAWAREYV
ESTAQSMRNGGKILSV
>*Chaetoceros_sp*/DXR
MPTSAWSFTVTTPAPSSRQTCRHAVMKSGSKGTGEGWLERPSAPVNSEAAARLTLQES
LKGSSFKKRLSLLGSTGSIGTQTLEIVDACPDNFEVDALSAGNNAKLMAEQVLKYQPK
VASLATAEAAAELKERLVAAGCQNMPEILYGDEGILAAATIDSADTVVTGIVGAAGLK
PTIEAIKLKKDIALANKETLISGGPVINPLVEEYGVNMLPADSEHSAIFQCLQGVPPGGL
RRIILTASGGAFRDFSKDELFRLCQEDPRAVQAKATTHPNWDMGAKITLDSATMMNK
GLEVIEAHYLFGASYDDIDIVIHPQSIVHSMVETQDTSCLAQLGWADMRLPLVYSVSWP
HRLRMPYKPLDLAELSKLTFARPDVEKYPCIRLAYEAGRAGGTMTAVLNAANEAANE
MFRADVGLGFLDIPKLIESAMEAHKNDLKLDDISLDDILSSDAWAREYVDSAAQKLLK
NGDKILSV
>*Coscinodiscus_wailesii*/DXR
MRINPTATLLLLVPLTTTSFHLSSPPHKVTTSLNTFQPPGSQGTGEGWISPASPVNTAAS
LQKTLSESLLGSTFQKRLSLLGSTGSIGTQTLEIVDARPENFVVDALSAGVNTELMMEQ
VLKYKPKIATMQTADAAEELKKRLTDAGCENMPEIMWGDEGIVAAACVDTADTVVT
GIVGAAGLKPTIEAIKLKKDIALANKETLISGGPVILPLVEEYGINMLPADSEHSAIFQCL

QGVPPGGLRRVILTASGGAFRDLSKDELFDMCKNDPRAIQAKATTHPNWDMGAKITL
DSATMMNKGLEVIEAHYLFGASYDDIDIVIHPQSIIHSLVETQDTSQIAQLGWPDMRLPL
VYSISWPHRLSLPYKPLDLAELSTMTFKKPDVEKYPCIRLAYEAGRTGGTMTAVLNAA
NEAVNEKFRDDVGLGFLDIPKLVEMAMEAHKDDLKTEGVTLDDILSCDAWAREFVNT
AKIEESQIFV
>*Cylindrotheca_closterium*/DXR
MKLSIAFSVLMAGQAVAFMPNAPKGVQTSMELVSGSKGTGEGWIDPSAPVNSAESLK
LTLEKSLEGSNFQKRLSLLGSTGSIGTQTLDIVDACPDNFVVDALSAGTNAELMAEQVL
KYKPKIATLATPEAATELKERLKAAGCDQMPEILHGDDGILAASTIDTADTVVTGIVGA
AGLKPTIEAIKLKKDIALANKETLISGGPVINPLVEEYGINMLPADSEHSAIFQCLQGVPE
GGLRRVILTASGGAFRDLSKDELFQMCKDDPRAVQAKATTHPNWDMGAKITLDSATM
MNKGLEVIEAHYLFGASYDDIDVVIHPQSIVHSMVETQDTSCIAQMGWADMRLPLVYS
VSWPHRLKMPYRPLDLAEVSKMTFMAPDYEKYPCIQLAYEAGRAGGTMTAVLNAAN
EAANEMFREDVGLGFLDIPKLIESAMEAHKDDHKTSDVTLDDILSCDEWARQHVVEKS
KTLMNQVLV
>*Fragilariopsis_kerguelensis*/DXR
MFMLFPNSFSSGVVSLLLRGVGILAVVVSLMASCNAFTIQSTSTASPTNLNLHSGSAGT
GDGWIEPSAPVNSEASLKLTLQKSLEGTSFQKRLSLLGSTGSIGTQTLDIVDACPDNFVV
DALSAGTNSKLMTDQVLKYKPKIVSMATAEAAQELKLNLEDAGCSYMPTIMHGDEGI
LAAATIDSADTVVTGIVGAAGLKPTIEAIKLKKDIALANKETLISGGPVINPLVKEHGIN
MLPADSEHSAIFQCLQGVPEGGLRRVILTASGGAFRDLSKDELFRMCEDDPRAVQAKA
TTHPNWDMGAKITLDSATMMNKGLEVIEAHYLFGASYDDIDVVIHPQSIVHSMIETKD
TSCIAQLGWADMRLPLVYSVSWPHRLTMPYKPLDLTEVSQMTFMKPDHEKYPCIGLA
YEAGRAGGTMTAVLNAANEVANELFRDDVGLSFLDIPKLIEGAMEAHKDDHKTKDVT
LEDILSCDAWARQHVMEKSXETKKTRFFDIGFNPNKEPITLNDLVER
>*Leptocylindrus_apora*/DXR
MKLTAAATFLFATEATHAFIVQRPSINADANVSLNMVFGKSGSKGTGEGWIDPCAPVN
SEASMRKTLDEALKGASFKKRLSLLGSTGSIGTQTLQIVDACPENFEIDALSAGTNSAL
MADQVMQYKPKVAAMATKEAADDLAERLKAYGFDEMPEILFGDEGTVAAATVDSA
DTVVTGIVGAAGLLPTIEAIKLGKDIALANKETLISGGPVINPLVEKHGVNMLAADSEH
SAIFQCLQGVPEGALRRIILTASGGAFRDFSKEELFSLCENEPELIQKKATTHPNWDMGA
KITLDSATMMNKGLEVIEAHYLFGASYDDIDIVIHPQSIIHSMVETKDTSCIAQLGWAD
MRLPIVYSISWPHRLTMPYKPLDLAEIGSLTFKKPDREKYPCIDLAYAAGNAGGTMTA
VLNAANEAANEMFRADVGLGFLDIPKLIEGAMEEHKADHKTTDVSLDDILACDAWAR
EYVAKASVKNESKIIV
>*Leptocylindrus_danicus*/DXR
MKLNIAAAFLLAQGASAFVPGTTQKSSANVLNVFGKSGSKGTGEGWIDPCAPVNTEA
AMKKTLNEALKGATFKKRLSLLGSTGSIGTQTLDIVDACPDNFQIDALSAGTNSALMA
EQVLKYKPQVASLATKEAADDLKERLKAAGCEEMPEIFFGDEGTVAAATVGTADTVV
TGIVGAAGLLPTIEAIKLGKDIALANKETLISGGPVINPLVEKHGINMLAADSEHSAIFQC
LQGVPEGGLRRIILTASGGAFRDYSKEDLFDLCKNNPEFVQQKATTHPNWDMGAKITL
DSATMMNKGLEVIEAHYLFGASYDDIDIVIHPQSIIHSMVETKDTSCIAQLGWADMRLP
IVYSVSWPHRLEMPYEPLDLAKIGSLTFDKPDREKYPCIDLAYAAGNAGGTMTAVLNA
ANEAANEKFRANMGLGFLDIPKLIEGAMEAHKADHKVGDVTLDDILSCDEWARNYVE
KESLNTATIFS
>*Nannochloropsis_gaditana*/DXR
PSFPPSLRRHILHFFRKLIHPHSGPRVHGHNVLQRGCLLPILLVALHRTLHDFGNVQVAN
LLLPEDFVRALVCGVEGGRHRSPRPARRVRQLDTGVLLVVGRLEGERSQLHQVEGLV
GHGDAVRPGDGVEDGQTHVRPAELSNHARILGLNQGVDDGLRMDDHIDVVVGGPEQ
VVCLDYFQPFIHHGGAVDSDLGPHVPVWMLEGVCHGDLLQILHGPVPEGATRGGEDD

AAEAPGGHALQGLKNGAVLTVGGDDVDAVFLQEGHNGWAAADQSLLVREGDGLAC
LDRFHRGQ
>*Nitzschia_sp*/DXR
MRFYAASILALAAMAAPEPASAFVGVNSFSLTQQSSSSQQSSMLHSSKMKSGSTGTGE
GWIDPSAPVDSEASLKLTLEKSLEGAEFKKRLSLLGSTGSIGTQTLDIVDVCPDNFVVD
ALSAGTNSKLMAEQVLKFKPKVASLATPQAAQELVDLLDDANMPKSERPEIMHGDEG
ILACATVDSADTVVTGIVGAAGLQPTIEAIKLKKDIALANKETLISGGPVINPLVKEHGI
NMLPADSEHSAIFQCLQGVPEGGLRRVILTASGGAFRDLTKDELFNMCRDDPRAVQAK
ATTHPNWDMGAKITLDSATMMNKGLEVIEAHYLFGASYDDIDVVIHPQSIVHSMVETQ
DTSCIAQLGWADMRLPLVYSVSWPHRLKMPYRPLDLAEVSQMTFMKPDFEKYPCIAL
AYEAGRAGGTMTAVLNAANEAANEMFREDIGLGFLDIPTLIESAMEAHKDDHKVNDV
TLDDILSCDAWARKFVAEKSETMKSQVFA
>*Phaeodactylum_tricornutum*/DXR
MRLTELSVSLIVGSAAAFAPTTGISATRTTSASTVRLQLVSGSQGTGEGWIDPSAPVNSK
ASLEKTLSKSLEGSNFQKRLSLLGSTGSIGTQTLEIVDACPDNFVVDALSAGTNVELMT
QQVLKYSPKVASMSTPEAAKELKRRLQDEGCKSMPLILSGQEGIVAAATVDTADTVVT
GIVGAAGLEPTIEAIKMKKDIALANKETLISGGPVINPLVEKYGINMLPADSEHSAIFQCL
QGVPPGGLRRVILTASGGAFRDFSKDELFRLCEDNPRAVQAKATTHPNWDMGAKITLD
SATMMNKGLEVIEAHYLFGASYDDIDIVIHPQSIVHSMVETQDTSCIAQLGWADMRLP
LVYSVSWPHRLKMPYKPLDLAELGSLTFSKPDHAKYPCIGLAYAAGRQGGTMTAVLN
AANEAANEMFRADVGLGFLDIPKLIEGAMEAHKEEFKKDGVTLEDILHVDQWAREHV
RQASEQLDKSKVIVV
>*Pseudonitzschia_arenysensis*/DXR
NMNFSLALALFLSAAPTASGFAPVSSSASATQLDVVSGSAGTGEGWIEPSAPVNSAESL
KLTLSKSLEGATFQKRLSLLGSTGSIGTQTLDIVDACPDNFVVDALSAGTNSKLMTEQV
LKYTPKVVSMATADAAAELKSNLESAGMSNMPTIMHGDEGILACSTIDSADTVVTGIV
GAAGLQPTIEAIKLKKDIALANKETLISGGPVINPLVKEHGINMLPADSEHSAIFQCLQG
VPEGGLRRVILTASGGAFRDLSKDELFNMCQDDPRAVQAKATTHPNWDMGAKITLDS
ATMMNKGLEVIEAHYLFGASYDDIDVVIHPQSIVHSMIETKDTSCIAQLGWADMRLPL
VYSVSWPHRLEMPYKPLDLAEVGQMTFMKPDHEKYPCIALAYEAGRAGGTMTAVLN
AANEAANELFRKDVGLGFLDIPKLIESAMEAHKDDYKKTDVTLDDILSCDQWARDFV
MEKSLTMRDRFIGDIGTNPNKEAIFLTDLSES
>*Pseudonitzschia_australis*/DXR
MNLSIALSLFLSAAPTAFGFAPISSSTPATTNLNVFSGSAGTGEGWIEPSAPVNTEASLKL
TLGKSLEGATFQKRLSLLGSTGSIGTQTLDIVDACPDNFVVDALSAGTNVKLMTDQVL
KYNPTIVSMATEKAAQEVRSNLEDAGISKMPTVLHGDEGILAAATVDTADTVVTGIVG
SAGLLPTIEAIKLKKDIALANKETLISGGPVINPLVKEHGINMLPADSEHSAIFQCLQGVP
EGGLRRVILTASGGAFRDLTKDELFNMCRDDPRAVQAKATTHPNWDMGAKITLDSAT
MMNKGLEVIEAHYLFGASYDDIDVVIHPQSIIHSMVETKDTSCIAQLGWADMRLPLVY
SVSWPHRLEMPYRPLDLAEVSQMTFMKPDHEKYPCIALAYAAGRAGGTMTAVLNAA
NEAANEIFRKDVGLGFLEIPKLVESAMEAHKDDHKINDVTLDDILSCDEWARAFVMEK
SESMKTELIL
>*Pseudonitzschia_delicatissima*/DXR
MNFSTALALFLAAAPTASGFAPVSSSGSSATHLNVVSGSKGTGEGWIEPSAPVNSEASL
KLTLEKSLEGATFQKRLSLLGSTGSIGTQTLDIVDACPENFVVDALSAGTNSKLMTEQV
LKYTPKVVSMATEAAAAELKSNLEDAGMANMPTIMHGDEGILACSTIDSADTVVTGIV
GAAGLQPTIEAIKLKKDIALANKETLISGGPVINPLVKEHGINMLPADSEHSAIFQCLQG
VPQGGLRRVILTASGGAFRDLSKDELFKMCQDDPRAVQAKATTHPNWDMGAKITLDS
ATMMNKGLEVIEAHYLFGASYDDIDVVIHPQSIVHSMIETKDTSCIAQMGWADMRLPL
VYSVSWPHRLEMPYKPLDLAEVSQMTFMKPDHEKYPCIALAYEAGRAGGTMTAVLN

AANEAANELFRKDVGLGFLDIPKLIESAMEAHKDDYKKNDVTLEDILSCDQWARDFV
MEKSETMKSPVLL
>*Pseudonitzschia_multistriata*/DXR
MKLSIALSLLLSAAPXTVAFAPVSSTAPSSTKLNVFSGSAGTGDGWIEPSAPVNSEESLK
LTLEKSLEGSSFQKXLSLLGSTGSIGTQTLDIVDACXDNFVVDALSAGTNSKLLTDQVL
KYTPKIVSMATEKAAKELKSNLEDAGMKNMPTIMHGEEGILAAATIDTADTVVTGIVG
SAGLLPTIEAIKLKKDIALANKETLISGGPVINPLVKEHGINMLPADSEHSAIFQCLQGVP
EGGLRRVILTASGGAFRDFSKEELFNMCRDDPRAVQAKATTHPNWDMGAKITLDSAT
MMNKGLEVIEAHYLFGASYDDIDVVIHPQSIVHSMVETKDTSCIAQLGWADMRLPLVY
SVSWPHRLEMPYKPLDLAEVSQMTFQKPDHDKYPCIALAYEAGRAGGTMTAVLNAA
NEAANEIFRKDVGLGFLDIPKLIESAMEAHKDDHKVNDVTLDDILSCDEWAREFVMEK
SETMKTELLLPR
>*Pseudonitzschia_pungens*/DXR
MNLSIALSLLLSAAPTAFGFAPISSSTPSTTTLNVVSGSAGTGEGWIEASAPVNSEASLKL
TLEKSLEGSSFQKRLSLLGSTGSIGTQTLDIVDACPDNFVVDALSAGTNSKLMTDQVLK
YNPKIVSMATEKAAQELKSNLEDAGLKNMPTILHGDEGILAAATIDSADTVVTGIVGSA
GLLPTIEAIKLKKDIALANKETLISGGPVINPLVKEHGINMLPADSEHSAIFQCLQGVPEG
GLRRVILTASGGAFRDLTKDELFNMCRDDPRAVQAKATTHPNWDMGAKITLDSATM
MNKGLEVIEAHYLFGASYDDIDVVIHPQSIVHSMIETKDTSCIAQLGWADMRLPLVYSV
SWPHRLEMPYKPLDLAEVSQMTFMKPDHEKYPCIALAYEAGRAGGTMTAVLNAANE
AANEIFRKDVGLGFLDIPKLIEIAMEAHKDDLKTEDVTLDDILSCDEWARQFVLEKSET
MKSELILPR
>*Pseudonitzschia_pungens_fr_cingulata*/DXR
MNLSVALSLLLSAAPTAFGFALISSSTPCTTTLNVVSGSAGTGEGWIEASAPVNSEASLK
LTLEKSLEGSSFQKRLSLLGSTGSIGTQTLDIVDACPENFVVDALSAGTNSKLMTDQVL
KYNPKIVSMATEKAAQELKSNLEDAGLKNMPTILHGDEGILAAATIDSADTVVTGIVG
AAGLLPTIEAIKLKKDIALANKETLISGGPVINPLVKEHGINMLPADSEHSAIFQCLQGVP
EGGLRRVILTASGGAFRDLTKDELFNMCRDDPRAVQAKATTHPNWDMGAKITLDSAT
MMNKGLEVIEAHYLFGASYDDIDVVIHPQSIVHSMIETKDTSCIAQLGWADMRLPLVY
SVSWPHRLEMPYKPLDLAEVSQMTFMKPDHEKYPCIALAYEAGRAGGTMTAVLNAA
NEAANEIFRKDVGLGFLDIPKLIEIAMEAHKDDLKTEDVTLDDILSCDEWARQFVLEKS
ETMKSELILPR
>*Skeletonema_costatum*/DXR
MKLPSIAVGFFLASHQASGFSTNLLLSSSSTKQSTSSLNVFSVSGSAGTGEGWIDPSAPV
NSAASLKKTLDEALKGSTFKKRISILGSTGSIGTQTLDIVDACPDNFVVDALSAGNNAK
LMADQVMKYSPKVASLSTPEAAAELRTLLKERGCTDMPEIVHGAEGIVEAATVGSAD
TVVTGIVGAAGLLPTXEAIKLGKDIALANKETLISGGPVINPLVEKHGINMLPADSEHSA
IFQCLQGVPPGGLRRVILTASGGAFRDFSKDELFEMCENDPRAVQKKATTHPNWDMG
AKITLDSATMMNKGLEVIEAHYLFGASYDDIDIVIHPQSIVHSMVETQDTSCIAQLGWA
DMRLPIVYSVSWPHRLKMPYKPLNLAEIGSLTFKAPDTEKYPCISLAYEAGKKGGTMT
AVLNAANEAANEMFREDVGLGFLDIPKLIEGAMEAHKEDFKVDDVTLDDILSCDAWA
REYVAENTKNVVKAPILFT
>*Skeletonema_marinoi*/DXR
XQASGFSTNLLSSSSTKQSTSSLNVFSVSGSAGTGEGWIDPSAPVNSAASLKKTLDEAL
KGSTFKKRISILGSTGSIGTQTLDIVDACPDNFVVDALSAGNNAKLMADQVMKYSPKV
ASLSTPEAAAELRTLLKERGCTDMPEIVHGAEGIVEAATVGSADTVVTGIVGAAGLLPT
IEAIKLGKDIALANKETLISGGPVINPLVEKHGINMLPADSEHSAIFQCLQGVPPGGLRR
VILTASGGAFRDFSKDELFEMCENDPRAVQKKATTHPNWDMGAKITLDSATMMNKGL
EVIEAHYLFGASYDDIDIVIHPQSIVHSMVETQDTSCIAQLGWADMRLPIVYSVSWPHR
LKMPYKPLNLAEIGSLTFKAPDTEKYPCISLAYEAGKKGGTMTAVLNAANEAANEMF

REDVGLGFLDIPKLIEGAMEAHKEDFKVNDVTLDDILSCDAWAREYVAENTKNVVKA
PILFT
>*Skeletonema_menzelii*/DXR
MKLPSIAVGFFLASHQASGFSTNLLSSSSITKQSSTSSLNVFSVSGSTGTGEGWIDPSAPV
NSAASLKKTLDEALKGSTFKKRISILGSTGSIGTQTLDIVDACPDNFVVDALSAGNNAK
LMADQVMKYSPKVASLSTPEAAAELKTLLKERGCTNMPEIVHGAEGIVEAATVGSAD
TVVTGIVGAAGLLPTIEAIKLGKDIALANKETLISGGPVINPLVEKHGINMLPADSEHSAI
FQCLQGVPPGGLRRVILTASGGAFRDFSKDELFKMCEEDPRAVQKKATTHPNWDMGA
KITLDSATMMNKGLEVIEAHYLFGASYDDIDIVIHPQSIVHSMVETQDTSCIAQLGWAD
MRLPIVYSVSWPHRLKMPYKPLNLAEIGSLTFKAPDTEKYPCIALAYEAGKKGGTMTA
VLNAANEAANEMFREDVGLGFLDIPKLIEGAMEAHKEDFKVDDVTLDDILNCDAWAR
EYVAENTKNVVKAPILST
>*Thalassionema_frauenfeldii*/DXR
MKLSIAAAVVCLCSSSSHAFAPTSTTTTTTTTTISKQQSTNSQLFNQKFSGSAGTGENWI
DPSAPVNSKESLELTLAKSLEGAQFQKRLSLLGSTGSIGTQTLDIVDACPDNFVIDALSA
GTNVELMTEQCLKYQPKVASMATPEAAAQLKANLDGKGFRGQILSGDEGILAAATVD
TADTVVTGIVGAAGLQPTIEAIKLKKDIALANKETLISGGPVINPLVKEYGINMLPADSE
HSAIFQCLQGVPPGGLRRVILTASGGAFRDLTKEELFHMCRDDPRAVQAKATTHPNWD
MGAKITLDSATMMNKGLEVIEAHYLFGASYDDIDVVIHPQSIVHSMVETQDTSCLAQL
GWADMRLPLVYSVSWPHRLKMPYQPLDLAKVSQMTFAKPDFEKYPCIALAYEAGRA
GGTMTAVLNAANEAANELFRQDRGLGFLEIPQLIEQTMEKHKEDLKTTDITLDDILECD
AWARQQVTDQSQSLLNKQYV
>*Thalassiosira_minuscola*/DXR
MKLPSLALSVLLLAHEAASFTPAASTRATTSLNVFSTPGSKGTGEGWIDPSAPVNTAES
LQKTLSESLKGSTFQKRISILGSTGSIGTQTLEIVDACPENYVVDALSAGNNAELMAEQV
MKYSPKVASLSTPEAAEKLKSILKEKGCADMPEIVYGDEGIVEAATVGSADTVVTGIV
GAAGLLPTIEAIKLGKDIALANKETLISGGPVINPLVEKHGINMLPADSEHSAIFQCLQG
VPPGGLRRVILTASGGAFRDFSKDELFEMCEKDPMAVQKKATTHPNWDMGAKITLDS
ATMMNKGLEVIEAHYLFGASYDDIDIVIHPQSIVHSMVETQDTSCIAQLGWADMRLPIV
YSVSWPHRLKMPYKPLDLAELGSLTFKAPDREKYPCIQLAYDAGKMGGTMTAVLNA
ANEAANEMFREDVGLGFLDIPKLIEGAMEAHKEDFKDKDVTLDDILSCDAWAREYVA
ENTKNVVKAPIPL
>*Thalassiosira_pseudonana*/DXR
MKLSLAFSTILLSQSSAFTTLTSTKSTTTTSSSLNVFSPPGSAGTGAGWIDPSAPVNTPE
SLQKTLSESLKGSTFQKRISILGSTGSIGTQTLDIVDACPDNFVVDALSAGNNAELMCEQ
VLKYKPKVASLGTPEAAKKLKELLVKAGCTDMPEIVHGAEGIVEAATVSTADTVVTGI
VGAAGLLPTIEAIKLGKDIALANKETLISGGPVINPLVEKHGINMLPADSEHSAIFQCLQ
GVPPGGLRRVILTASGGAFRDLSKDELFEMCERDPRAVQKKATTHPNWDMGAKITLD
SATMMNKGLEVIEAHYLFGASYDDIDIVIHPQSIVHSMVETQDTSCIAQLGWADMRLPI
VYSVSWPHRLKMPYKPLNLAEIGSLTFKAPDVEKYPCIRLAYEAGRQGGTMTAVLNA
ANEAANEMFREDVGLGFLDIPKLIEGAMEAHKEDFKVKDVTLDDILSCDAWAREYVA
EQTKNVAKSALIL
>*Thalassiosira_puntigera*/DXR
MKLPSLALSVLLLAHEASSFAPAVGGPTRAATSLNVFSTPGSLGTGAGWIDPSAPVNSA
ASLEKTLAESLKGSTFQKRISILGSTGSIGTQTLDIVDACPENFVVDALSAGNNAQLMAE
QVMKYSPKVASLATPEAADELRAILKELGCADMPEIVHGAEGIVEAATVGSADTVVTG
IVGAAGLLPTIEAIKLGKDIALANKETLISGGPVINPLVEKHGINMLPADSEHSAIFQCLQ
GVPPGGLRRIILTASGGAFRDLTKDELFEMCENDAMAVQKKATTHPNWDMGAKITLD
SATMMNKGLEVIEAHYLFGASYDDIDVVIHPQSIVHSMVETQDTSCIAQLGWADMRLP
IVYSVSWPHRLQMPYKPLNLAEIGSLTFKAPDREKYPCIQLAYDAGKKGGTMTAVLNA

ANEAANEMFREDVGLGFLDIPKLIEGAMEAHKEDFKDKDVTLDDILSCDAWAREYVA
ENTKNVAKQVLVL
>*Thalassiosira_rotula*/DXR
SGFLLAHDVSSFAPSIGSTRAESSLNVFSTPGSLGTGEGWIDPSSPVNSKESLEKTLSETL
KGSTFQKRISILGSTGSIGTQTLDIVDACPENFVVDALSAGNNAELMAEQVMKYSPKVA
SLSTPEAAEKLRTILKDRGCADMPDIVYGDEGILEAATVGSADTVVTGIVGAAGLLPTI
EAIKLGKDIALANKETLISGGPVINPLVEKHGINMLPADSEHSAIFQCLQGVPPGGLRRV
ILTASGGAFRDLSKDELFRMCEEDSAAVQKKATTHPNWDMGAKITLDSATMMNKGLE
VIEAHYLFGASYEDIDIVIHPQSIVHSMVETQDTSCIAQLGWADMRLPIVYSVSWPHRL
KMPYKPLNLAEIGSLTFKAPDREKYPCIQLAYDAGNKGGTMTAVLNAANEAANEMFR
ADVGLGFLDIPKLIXGAMEAHKEDFKDKDVTLDDILSCDAWAREYVAEETKTVTKQA
LIL
>*Thalassiosira_weissflogii*/DXR
MKVPSIALSGLLLLHSATAFSSLSSLPNASKQSTTLLHSSSSSKPIPGSLGTGAGWIDPSA
PVNTAASLKKTLAKALEGAAFQKRISILGSTGSIGTQTLDIVDACPDNFVVDALSAGTN
VRLMAEQCLKYRPKVASLSTPRAAEELKEILTKSGQFSKEDMPNIVYGNEGIVEAATVS
SADTVVTGIVGAAGLLPTIEAIKLGKDIALANKETLISGGPVINPLVEKHGVNMLPADSE
HSAIFQCLQGVPEGGLRRVILTASGGAFRDMTKEELFRMCEEEKEAVQKKATTHPNW
DMGAKITLDSATMMNKGLEVIEAHYLFGASYDDIDIVIHPQSIVHSMIETQDTSCIAQLG
WADMRLPIVYSVSWPHRLKMPYKPLDLAQIGSLTFQKPDTEKYPCISLAYEAGRMGGT
MTAVLNAANEAANEMFREDVGLGFLDIPKLIEGAMEGHRENGFKKEDVTLEDILSCDA
WAREYVKEKTKNVVKKPVVL

DXS protein sequences

>*Amphiprora_sp*/DXS
MMMIMKVAAGIVAIVGLQGVSSFTTTPLRTSTTTAAPRTSQLLAEQGSGTNASPPGPPY
SGPAVKPILDSISFPTDMKRLDMAQLKQLAYELRWETLEAVSKTGGHLSSSLGVNELT
VALHYVFDMPEDDIIWDVSHQCYPHKILTGRRFKMSGLRQSGGISGFCKRKESEYDSF
GAGHSSTSISAAQGMSIGKSMMGKRQNNCIAVIGDGAITGGMAYEAMNSAGYLKNRM
IVVLNDNGQVSLPTGTPSAGGVVPASQLSTYTSNLLVSKPFQDFRSFAKSFNKLLPEGIQ
GVNKRVDEYVRGLATGGTLFEELGFYYVGPVDGHDLDNLVPILEKLRDSPNNKPVLL
HLKTTKGYGYPPAEAASDRMHGVAKFNLGTGAQFKSKPTAPSLTSVFANALIDAATED
RRVVGITAAMPGGTGMDIFGRRFPKRTFDVGIAEQHAVTMAAGMACEGLKPFCAIYST
FMQRGYDQVVHDVAIQNLPVRMVLDRAGLVGNDGPTHHGVYDLSYMGCIPNLIIMAP
SDEIELKNMIMTQAAYDDGPTVCRYPRGTGYGPETLQDLFGYKLEDGQIPAKGEIIPMG
KGRVIRRPGASRGKSKKDRVCILTIGTRLHEALVAAKEVEEQDPTLGVTVADARFMKP
LDVDMVRQLADEHSVMITVEEGSIGGFGDHVLHFLTLDGLMDDGDLKFRPMVIPDAL
FEAGSQHEQYDLAGLNAEHIRGTILRLTKRKKAPQLEEVEEQVSRD
>*Bolidomonas_pacifica*/DXS
MKFVNALPTLLLTLLLTPTTSFLPYSPTITTTTTKTTPTTTKLNNNAGPPYGGPVRKPML
DRVKSPDDMKGMSIQDLKQLSEELRWEVIEQVSKTGGHLGSSLGVIEMTVALHHVFST
PEDKIVWDVAHQCYPHKILTGRRDRMNTLRQMGGLSGFCKMKESPYDAFGAGHSSTS
ISAIQGYSIAKSQLNPTGRNNCIAVIGDGAITGGMAYEAMNSAGYLKNRMIVILNDNGQ
VSLPTGTASAGGTGPASELSAATSRLLVSKQFQDFRSVAKGVSGLFPENVQEFNKKIDE
YARGIVSGGTLFEELGFYYVGPLDGHDLDNFVPILSKLRDSPSNKPVLLHVKTNKGNG
YPPAENASDKMHGVAKFDVATGIQKKGPKGPPSLTSVFANELCNIASTDKRVVGITAA
MPGGTGMDIFGRRFPDRTYDVGIAEQHAVTMAAAMAAEGLKPFVCIYSTFLQRGFDQ
VVHDVAIQNLPVRLILDRAGLVGNDGPTHHGAYDLSYLGCIPNLTICGPSDEVELKNMI
QTAASHDDGPIVVRYPRGAGYGEDKLKTLFGYDLPDGVPEKGETLEIGKGRVVRTSM
RSLPRSKSVAVLSFGTRLHESLVAAAEIEEAAPDLSVTVADARFMKPLDVDLVRSLAST

HSVIVTVEENSIGGFGDSVLHFLSLEGLLDNGELRFRPMVLPDAYFEAGPQFEQYEQAG
LNAKHIKGTILRLTEKIAVPIAV
***>Chaetoceros_neogracile*/DXS**
RKMKLFNAVAALSLAVPAANAFTTPVLSSKYVAKSALRMSEGPPYAGPLTKPLLDSVE
FPNDMKDFSIKDLKQLSNELRWEVLEAVSRTGGHLSSSLGVVELTVALHYVFDMPTDD
IIWDVAHQCYPHKMLTGRRHKFSTLRQLGGISGFCKRKESKYDSFGAGHSSTSISVAQG
MSIGKSMLNKRTNNCIAVIGDGAITGGMAYEAMNSAGYLQNRMIVVLNDNGQVSLPT
GTPSAGGTVPASQLSAYTSNLLVSKPFQDFRAFAKSFNNLLPENIKDVNKRFDEYARGII
SGGTLFEELGFYYVGPIDGHDLDNMIPILEKLRDGGSNKPVLLHVKTNKGQGYPPAESA
SDKMHGVGKFDLATGAQQKKTATAPSLTSIFADSLIQAATEDRAVVGITAAMPGGTG
MDIFGRRFPKRTFDVGIAEQHAVTMAAGMACEGLKPFVSIYSTFMQRAYDQVIHDVA
VQNLPVRMILDRAGVVGNDGPTHHGCYDLAYMACVPNLTIMAPSDEIELRNMMKTCL
DFNDGPTVLRYPRGNGYGAETLTELFGYELENGEIPTIGEPLPIGKGRIIRRPGGFGVNG
DKNTRGKSREDRIAILSIGTRLHDSLISASDIEDANPTVGVTVADARFMKPLDEDLIRSL
VDDHSILITIEEGSVGGFGDHVLHFLAKDGLLDDGNLKFRPMNLPDELFEAATQHEQYE
MAKLNHPYITELLQGLLKKNMKVPVLEEQTAEA
***>Chaetoceros_sp*/DXS**
MKLFHAAAAIAFAVPSAFAFTSPVLNTKYVAGSNTQLAMGPPYAGPTAKPLLDSVTYP
RDMKDFSIKDLKQLSHELRWEVLEAVSKTGGHLSSSLGVIELTVALHYVFDMPTDQII
WDVAHQCYPHKMLTGRRHLFGGLRQLGGISGFCKRKESEYDSFGAGHSSTSISVAQG
MSIAKSMLNKRTNNCIAVIGDGAITGGMAYEAMNSAGYLQNRMIVVLNDNGQVSLPT
GTPSAGGTVPASRLSAYTSNLLVSKPFQDFRDFAKSFNKLLPENIQDVNKRFDEYARGII
SGGTLFEELGFFYVGPIDGHDLDNMIPILEKLRDSDSNKPVLLHVKTNKGQGYPPAESA
SDKMHGVGKFDLATGVQYKKKATAPSLTSIFADSLIQAATDDRAIVGITAAMPGGTGM
DIFGRRFPKRTFDVGIAEQHAVTMAAGMVCEGLKPFVSIYSTFMQRAYDQVIHDVAIQ
NLPVRFILDRAGVVGNDGPTHHGCYDLAYMGCVPNLTIMAPSDEIELRNMVKTCADX
DEGPTVLRYPRGNGYGAEKLQEVFGYKLENGELPSKGEALEIGKGRIIRRPGGFGINGE
ANIRGKSRQSRVAILSLGTRLHDSIIAAAEIEAANPSVGVTVADARFMKPLDEDLIRDLV
DDNSILITIEEGSIGGFGDHVLHFLARNGLLDDGNLKVRPMVLPDELFEAATQQEQYDM
AKLNHPHITELVNNLLSKNMKVPVLEEKVTEQA
***>Coscinodiscus_wailesii*/DXS**
MDIKTLNQLAYELRWEVLESVSKTGGHLSSSLGVIELTIALHNVFSAPEDDIIWDVSHQ
CYPHKILTGRRDRFSTLRQLNGISGFCKRKESIYDSFGAGHSSTSISAAQGMSVGKSLLN
KRRNNCIAVIGDGAITGGMAYEAMNSAGYLRSRMIVVLNDNGQVSLPTGTQSAGGVV
PASQLSAYTSNLLVSKPFQNFRDFAKEINRLLPGDLQDINKRIDEYARGVVSGGTLFEEL
GFFYVGPIDGHDLENMVPILEKLRDSPSNKPVLLHIKTEKGKGYPPAEQASDKMHGVG
KFELGTGVQFKGKASAPSFTSIFANSLIDAATEDRAVVGITAAMPGGTGMDIFGRRFPK
RTFDVGIAEQHAVTFGAGMACEGLKPFVCIYSTFMQRGYDQVVHDVAIQNLPVRMIL
DRAGLVGNDGPTHHGCYDLAYMGAIPNLTIMAPSDEIELRNMVATCAAFDDGPTVLR
YPRGSGYGPDKLQNLFGYTLENDQVPTKGQVIEIGKGRIMRRPSASRGREKNDRIAILTI
GTRLHDALVAADDIESTDPSLGVTVADARFMKPLDVDLVRELVDQHGVLITVEEGSIG
GFGDHVLHFLALDGALDNGDLKFRPMVIPDQYIEAGTQFEQYDIAGLNAGHIKGTALR
LTSRIKVPVLQEE
***>Cylindrotheca_closterium*/DXS**
MRFSLAAVALAALSSVSAFAPSTGPQNVLGKSPLRMAEDNVQGPPYSGPASKPILDSVI
DAKGLSHTQPASNINSKMRFSLAAVALAALSSVSAFAPSTGPQNVLGKSPLRMAEDNV
QGPPYSGPASKPILDSVKFPSDMKKLSMSEVKQLAHELRWEVLESVSKTGGHLSSSLG
VIELTVALHYVFDMPEDDIIWDVAHQCYPHKILTGRRDKFSTLRQLDGISGFCKRKESE
FDSFGAGHSSTSISAAQGMSIGKSLLNKRKNNCVAVIGDGAITGGMAYEAMNSAGYLN
SRMIVVLNDNGQVSLPTGTPSAGGVVPASQLSTYTSNLLVSKPFQDFRSFAKSFNKLLP
EGIQDVNKRLDEYARGIVSGGTLFEELGFYYVGPVDGHDVDNLVPILEKLRDSPNDKP

VLLHIKTTKGYGYPPAETASDRMHGVGKFNLGTGVQVKSKSIAPSFTSLFANSLIDAAT
EDRAIVGITAAMPGGTGMDIFGRRFPKRTFDVGIAEQHAVCMGAGMACEGLKPFVCIY
STFMQRGYDQVVHDVAVQNLPVRMILDRAGFVGNDGPTHHGCYDLAYLGCIPNLTIM
SPSDEIELKNMVMTCAAFDDGPTVLRYPRGTGYGAEKLQSHFGYKLKDGEVPAKGEVI
EIGKGRIIRGPNAMIDNSSTTRGKERKDRVAILSIGTRLDEALVAAQEVEDSDENLSVTV
ADARFMKPLDVDLVRQLADDHGVLITXEEGSIGGFGDHVLHFLALDGLMDSGNLKFR
PMVMPDAYFEAATQNEQYDQAGLNSQHIKGTILRLTERMKVPVLEEA
>*Fragilariopsis_kerguelensis*/DXS
MRMRVSITVLLFSAAAITIPNAVLGFAPSSSSTTTTSRRKKIPFTQHAAVGDDGSGNPHT
VSGPPYSGPTVKPILDSVNYPSDMKGLDMRQLKQLANELRWEVLEQVSKTGGHLSSSL
GVTELTVALHYVFDMPDDDIIWDVAHQCYPHKILTGRRDQFPTLRQLGGISGFCKRKE
SKYDSFGAGHSSTSISAAQGMSIAKSVLNKRRNNCVAVIGDGAITGGMAYEAMNSAG
YLQSRMIVVLNDNGQVSLPTGTPSAGGTVPASQLSTYTSNLLVSQPFQDFRDFAKSFNK
LLPADLQGVNKRIDEYTRGLVSGGTLFEELGFYYVGPIDGHDLDNLIPILEKLRDSSNTK
PVLLHLKTVKGYGYPPAESASDRMHGVGKFNLGTGAQYKGKPTEPSFTSIFANALIDA
AVEDRSVVGITAAMPGGTGMDIFGRRFPKRTYDVGIAEQHAVCMGAGMACEGLKPFV
CIYSTFMQRGYDQVVHDVAVQNLPVRMMLDRGGFVGNDGPTHHGCYDLAYXGCIPN
LTXMAPSDEIELRNMVKTCADFDDGPTVCRYPRGVGYGADKLQSIFGYQLTNNEIPTK
GEAVPIGKGRIIRGTGGMVDTSSSSSTRGKKRKDRVAILSIGTRLHEALVAAQEVEDMY
DDLSVTVADARXMKPLDVDLVRQLADENGVLITIEEGSIGGXGDHVLHFLSLDGSLDE
GNIKFRPMVIPDAYFEAATQFQQYEQAGLNAQHIKGTILRLANKVEVPVLEQIEQEVL
>*Leptocylindrus_apora*/DXS
HKEEEAAAAATILISFPFQINNNIINILLLTMKYSSAALLSLACFTQTAYGFAPSSRHHVT
HKSNGNSNALQESPNDIDNGPPYSGPAYKPLLDSISSPADMKRLDVKQLNQLAHELRW
EVLENVSKTGGHLSSSLGVVEMTVALHYVFDMPEDDIVWDVSHQCYPHKILTGRRDR
FPTLRQAGGISGFCKRKESEYDSFGAGHSTTSISAVQGMSIAKSMLNKRRNNCIAVIGD
GAITGGMAYEAMNSAGYLKNRMIVILNDNGQVSLPTGTQSAGGTRPASQLSTYTSKLL
VSNEFQSVRDVLKNINQLLPSEALQDVNKRIDEYARGIISGGTLFEELGFYYVGPVDGH
DMENMVGILETLRDSDSNKPVLLHVKTEKGHGYPPAMAAADKMHGVGKFDIATGVQ
YKPKTKPVAPSLTSIFANALIEEAKEDPTVVAITAAMPGGTGLDIFGRRFPKRTFDVGIA
EQHAVTMAAGMACEGLKPFCCIYSTFLQRGYDSVVHDIAVQNLPVRIIMDRAGLVGN
DGPTHHGCYDLAYLGCIPQLIIMAPSDELELKNMIRTVRNYDDGPTCLRYPRGSGYGV
EKLKGLFGYSDEEINATEPTVLPMGKGRIVRKSARDAKDRANRVCILSLGTRLHESLVA
ANDIEATDSDVAVTVADARFMKPLDIDLIRELASENSVMVTIEEGSIGGFGDHVLHFLT
LDGLMDDGNLKFRPMVLPDQYFEAATQFEQYDLAGLNASHIKGTVLRLAQKIQVPVP
QES
>*Leptocylindrus_danicus*/DXS
TKMRFTSAALLALLPSAYAFVPAKNAQHNTALHMSGTESGPPYSGPKVKPLLDSVNFP
SDMKRFDIKELKQLAHELRWEVLENVSKTGGHLSSSLGVVEMTVALHHVFDMPEDDII
WDVSHQCYPHKMLTGRKDKFSTLRQAGGISGFCKRKESEYDSFGAGHSTTSISAAQG
MSIAKSMLNKRKNNCIAIIGDGAITGGMAYEAMNSAGYLKNRMIVILNDNGQVSLPTG
TQSAGGTRPASQLSTYTSQLLVSKPFQNVRDVLKNINQLLPSETLQDVNRRIDEYTRGLI
SGGTLFEELGFYYVGPVDGHDMENLVGILETLRDSDSTKPVLLHVKTEKGHGYPPALA
AADKMHGVGKFDLGTGVQFKPSKPPVAPSFTSIFANSLIDEATEDRSIVAITAAMPGGT
GLDIFGRRFPKRTFDVGIAEQHAVTMAAGMACEGLKPFCCIYSTFLQRGYDSVVHDIA
VQNLPVRIIMDRAGLVGNDGPTHHGCYDLAYLGCIPQLKIMSPSDEIELKNMIATCAKY
DEGPTCLRYPRGSGYGPDKLEKLFGYDMDAVPKHGEVLEMGKGRIVRKGGNKLLLG
AKQTDEGEKTTRGKDKKDRVCILSFGTRLHESLVAANEIEEADPDLAVTVADARFMKP
LDVDLVRELAAENSVIVTVEEGSIGGFGDHVLHFLTLDGLMDNGDLKFRPMVLPDQYF
EAASQYEQYETAGLNASHIKGTVLRLAQKIKVPVPQES
>*Nannochloropsis_gaditana*/DXS

MGVFSASSSSSLSFASFPSSSSTARSLPTAVLLLTTLTALTSSVTAFHAPSTSITSSAHMTF
PASSRTSSGGARSPLPIRARPRRTVFMETPLETGAGSSSTSNVPPPGTGPYQGPSSFPLLD
SVRYPHDMKRFDLKDLKQLAHELRWDTLHHVSKTGGHLGSSLGVIELTVALHYVFNT
PDDRIVWDVSHQVYPHKILTGRRDRMHSLRQTNGLSGFAKRSESEYDAFGAGHSSTSIS
AALGMSVGKLLTNKRVNNCVAVIGDGAITGGMAFEALNNAGYLRSRMVVILNDNGQ
VSLPTGTHSAGGVVPAGALSSYTSRLLSSKPFQDFRSFAKGLNRLMPSEIQDINAKLDE
YARGLIQGGTLFEELGFYYIGPVDGHDLDNLVPILENLRDSPSTKPVLLHVKTEKGYGY
PPAEVASDKYHGVAKFDVSTGRQFKGGNKGAPLSLTTTFANALCEIAAEDRTVVGITA
AMPGGTGMDIFGKRFPKRTFDVGIAEQHAVTFAAGMAIEGLKPFCCIYSTFMQRGYDQ
VIHDVVIQKLPVRMILDRAGLVGNDGPTHHGSFDLSYLGALPDIVIMAPSDELELMNLL
ETAYETNDLPSVVRYPRGAGYGLETLKSELGYEGLEELPKRGKAVARGKGRIIRRSAV
VPSTAAPGTCRVALLSVGTRLLDTVHAAKEIEENLPGVSVTVADARFVKPLDKEMVSS
LAMEHDVLITVEENSVGGFGSFVQQFLLNEGLLDGGKLRLRSMVLPDRFIEAGPQSDQ
YDQAGLAARHIVEKVEGLVRGREERMLQQEQQQPQPHQQQQQQTGAGVGTVAEMV
GVGAGKSSSSSSSSSRQGTSFQVLP

>*Nitzschia_punctata*/DXS

XGYLQSRMIVILNDNGQVSLPTGTPSAGGTVPASQLSTYTSNLLVSKPFQDFRDFAKSF
NKLLPGGIQDVNKRIDEYARGLVSGGTLFEELGFYYVGPVDGHDLDXLIPILEKLRDSP
NNKPVLLHLKTVKGYGYPPAEAASDKMHGVGKFNLGTGAQYKSKSTAPSFTSIFANSL
IDAAIDDRSIVGVTAAMPGGTGMDIFGRRFPKRTFDVGIAEQHAVCMGAGMACEGLKP
FVCIYSTFMQRGYDQVVHDVAIQNLPVRMILDRGGFVGNDGPTHHGCYDLSYMGCIP
NLTIMSPSDEIELKNMVMTCAAYDDGPSVLRYPRGTGYGAEKLQSLFGYKLENDEIPA
KGEVIPIGKGRIIRGAGGMVDTSSNNRGKERKNRVAILSLGTRLHEALVAAQEVEDMH
EDISVTVADARFMKPLDVDLVRQLADDNGVLITIEEGSIGGFGDHVLHFLSLDGLLDDG
DLKFRPMVIPDSYFEAATQFEQYDQAGLNARHIKGTILRLTKRMQVPVLEPVEQEA

>*Nitzschia_sp*/DXS

MARPGQFSVSLLWGLTYLSLQVANVAVLGFTAPSQHHRYRQPTTTTSLDAEETTKNG
HTISGPPYSGPASKPVLDSVQYPHDMNRLSMSELKQLSHELRWEVLESVSKTGGHLSSS
LGVTELTVALHYVFDMPEDDIIWDVAHQCYPHKILTGRRSKMSGLRQSGGISGFCKRK
ESEYDSFGAGHSSTSISAAQGMSIAKSTLNKRKNNCVAVIGDGAITGGMAYEAMNSAG
YLQSRMIVVLNDNGQVSLPTGTPSAGGTVPASQLSTYTSNLLVSKPFQDFRDFAKGFN
KLLPGGIQDVNKRIDEYARGLVSGGTLFEELGFYYVGPVDGHDLDNLVPILEKLRDSPN
NKPVLLHLKTVKGYGYPPAEAASDKMHGVGKFNLGTGAQFKSKATAPSFTSIFANALI
DAAVDDRSIVGITAAMPGGTGMDIFGRRFPKRTFDVGIAEQHAVCMGAGMACEGLKP
FVCIYSTFMQRGYDQVVHDVAIQNLPVRMMLDRGGFVGNDGPTHHGCYDLAYMGCI
PNLTIMSPSDEIELKNMVMTCAAFDDGPTVLRYPRGTGYGAEKLQSKFGYNLENDEIP
AKGEIIPIGKGRIIRGDGGMIDTASSTRGKQRKDRVAILSIGTRLSEALDAAKEVEEEYSD
ISVTVADARYMKPLDVDLVRELADDHGVLITIEEGSIGGFGDHVLHFLSLDGLLDDGNL
KFRPMVMPDAYFETGTQFEQYEEAGLNARHIKGTILRLCQKMKVPVLEQVEQEA

>*Phaeodactylum_tricornutum*/DXS

MRLSSALFLLITPTVAAFAPRASLLVRKTNTFVKAEGTNGGGSGPPFSGPAVKPILDSVK
YPSDMKNLDMRQLKQLANELRWEVLESVSKTGGHLSSSLGVNELTVALHYVFDMPE
DDIIWDVSHQCYPHKILTGRRDRMSTLRQSGGISGFCKRKESEYDSFGAGHSSTSISVAQ
GMSIGKSILNKRQNNCIAVIGDGAITGGMAYEAMNSAGYLKNRMIVILNDNGQVSLPT
GTPSAGGIVPASQLSTYTSNLLVSKPFQDFRMFAKNFNKLLPEGVQDVNKRIDEYARGI
VAGGTLFEELGFFYVGPVDGHDLDNLIPILEKLRDSPSTKPVLLHVKTTKGYGYPPAEQ
ASDRMHGVAKFNLGTGKQIKSKKPIAPSLTSIFANALIDAATEDRSIVGITAAMPGGTG
MDIFGRRFPKRTFDVGIAEQHAVTMGAGMACEGLKPFVCIYSTFMQRGYDQVVHDVA
IQNLPVRMVLDRAGLVGNDGPTHHGCYDLSYMGCIPNLIIMAPSDEVELRNMVATQA
AFNDGPTVLRYPRGTGYGLETLKNTFGYTFEGDEIPTKGEVLPIGKGRIVRRPGGFNQN
QSSTRGKSRKDRVAVLSLGTRLHEALVAAKEVEDIDPTLGVTVADGRFMKPLDVDLV

RELADEHSVLITIEEGSIGGFGDHVLHFLSLDGILDDGELKFRPMVIPDTLFEAGTQYEQ
YEQAGLNSEHIRGTILRLTKRLQVPQLQD
>*Pseudonitzschia_arenysensis*/DXS
MRLSISSLLLAAVIAPTFGFAPTSRHHASTKLYAEDSPKVSGPPYSGPAVKPILDSVNYP
QDMKGLDMRQLKQLANELRWEVLESVSKTGGHLSSSLGVTELTVALHYVFDMPTDDI
IWDVAHQCYPHKILTGRRDRFPTLRQLGGLSGFCKRKESEYDSFGAGHSSTSISAAQGM
SIAKSVLNKRKNNCVAVIGDGAITGGMAYEAMNSAGYLQSRMIVVLNDNGQVSLPTG
TPSAGGTVPASQLSTYTSNLLVSKPFQDFRDFAKSFNKLLPEDVQSVNKRIDEYTRGLV
SGGTLFEELGFYYVGPIDGHDLDNLIPILEKLRDSPSNKPVLLHIKTTKGYGYPPAETAS
DRMHGVGKFNLGTGAQYKGKPGEPSFTSIFANALIDAAIEDRSIVGITAAMPGGTGMDI
FGRRFPKRTFDVGIAEQHAVCMGAGMACEGLKPFVCIYSTFMQRGYDQVVHDVAVQ
NLPVRMMLDRGGFVGNDGPTHHGCYDLSYMGCIPNLTIMSPSDEMELRNMVMTCAA
FDDGPTVCRYPRGVGYGADKLQNIFGYKLDKNEIPQKGEVIPIGKGRIIRGAGGLIDTHS
SSRGKQRKDRVAILSIGTRLHEALVAAQEVEDEYPDLQVTVADARFMKPLDVDLVRQ
LADDHGVLITIEEGSIGGFGDHVLHFLALDGLLDEGNLKFRPMVMPDAYFEAGTQFQQ
YEQAGLNSRHIKGTILRLAKKIEIPILEDMEVDAGESTIA
>*Pseudonitzschia_australis*/DXS
MRLSITALLLAAYTTPNAGVFGFAPTSRHHASVLNSRTTTVLNAEGDSDKVSGPPYSGP
TVKPILDSVNYPHEMKGLDMRQLKQLANELRWEVLESVSKTGGHLSSSLGVTELTVA
LHYVFDMPDDDIIWDVAHQCYPHKILTGRRDRFPTLRQLNGISGFCKRKESEYDSFGA
GHSSTSISAAQGMSIAKSVLNKRKNNCVAVIGDGAITGGMAYEAMNSAGYLQSRMIV
VLNDNGQVSLPTGTPSAGGTVPASQLSTYTSNLLVSKPFQDFRDFAKSFNKLLPEDVQS
VNKRIDEYTRGLVSGGTLFEELGFYYVGPIDGHDLDNLIPILEKLRDSPNNKPVLLHIKT
VKGYGYPPAESASDRMHGVGKFNLGTGAQFKGKPGEPSFTSIFANALIDAAIEDRSVV
GITAAMPGGTGMDIFGRRFPKRTFDVGIAEQHAVTMGAGMACEGLKPFVCIYSTFMQR
GYDQVIHDVAVQNLPVRMMLDRGGFVGNDGPTHHGCYDFSYMGCIPNLTIMAPSDEI
ELRNMVMTCADFDEGPTVLRYPRGVGYGADKLKNLFGYKLDKGEIPAKGEIIPVGKG
RIIRGQGGMIENKSSTRGKERKDRVAILSIGTRLHEALVAAQEVEDEHEDLSITVADARF
MKPLDVDLVRQLADDHGVLITIEEGSIGGFGDHVLHFLSLDGLLDEGNLKFRPMVIPDN
YFEAATQFQQYEQAGLNSRHIKGTILRLAKKIEIPVLDEIAVDNSPGTIAGVYGSRLTGG
GFGGCTVTLVAQDNAEDLMAHMESEYKSKTGIDCPCFVTRPARGAHLLSIVDHKPLV
QE
>*Pseudonitzschia_delicatissima*/DXS
MRLSISALLLAAAAVPTLGFAPASRSHATTKLFAAEESKVSGPPYSGPAVKPILDSVNYP
QDMNGLDMRQLKQLANELRWEVLENVSKTGGHLSSSLGVTELTVALHYVFDMPTDD
IIWDVAHQCYPHKILTGRREKFPTLRQLGGISGFCKRKESEYDSFGAGHSSTSISAAQGM
SIAKSVLNKRKNNCVAVIGDGAITGGMAYEAMNSAGYLQSRMIVVLNDNGQVSLPTG
TPSAGGTVPASQLSTYTSNLLVSKPFQDFRDFAKSFNKLLPEDVQSVNKRIDEYTRGLV
SGGTLFEELGFYYVGPIDGHDLDNLIPILEKLRDSPSNKPVLLHIKTVKGYGYPPAETAS
DRMHGVGKFNLGTGAQYKTKGGEPSFTSIFANALIDAAIDDRSIVGITAAMPGGTGMDI
FGRRFPKRTFDVGIAEQHAVCMGAGMACEGLKPFVCIYSTFMQRGYDQVVHDVAVQ
NLPVRMMLDRGGFVGNDGPTHHGCYDLSYMGCIPNLTIMSPSDEMELRNMVMTCAA
FDDGPTVCRYPRGVGYGADKLQNTFGYKLENNEIPSKGEVIPIGKGRIIRGAGGLIDTHS
SSRGKQRKDRVAILSIGTRLHEALVAAQEVEDEYDDLQVTVADARFMKPLDVDLVRQ
LADDHGVLITIEEGSIGGFGDHVLHFLALDGLLDEGNLKFRPMVMPDAYFEAGTQFQQ
YEQAGLNSRHIKGTILRLAKKVEIPILEDMEVDAGESTIA
>*Pseudonitzschia_multistriata*/DXS
MRLSIAALLLAAYTSTNTGVHGFAPTSRYHARAPNTVLNAEGDSDKKVSGPPYSGPTV
KPILDSIKYPHDMNGLDMRQLKQLANEVRWEVLEQVSKTGGHLSSSLGVTELTVALH
YVFDMPTDDIIWDVAHQCYPHKMLTGRRDRFPTLRQMNGLSGFCKRKESEYDSFGAG
HSSTSISAAQGMSIAKSILNKRKNNCVAVIGDGAITGGMAYEAMNSAGYLQSRMIVVL

NDNGQVSLPTGTPSAGGTVPASQLSTYTSNLLVSKPFQDFRDFAKSFNKLLPEDVQSVN
KRIDEYTRGLVSGGTLFEELGFYYVGPIDGHDLDNLIPILEKLRDSPSNKPVLLHIKTTK
GYGYPPAEAASDRMHGVGKFNLGTGAQFKIKGGEPSFTSIFANALIDAAIEDRSIVGITA
AMPGGTGMDIFGRRFPKRTFDVGIAEQHAVTMGAGMACEGLKPFVCIYSTFMQRGYD
QVVHDVAVQNLPVRMMLDRGGFVGNDGPTHHGCYDLSYMGCIPNLTIMAPSDEIELR
NMVMTCADYDEGPTVLRYPRGVGYGAEKLQNLFGYKLEKGEIPTKGEKIPIGKGRIIR
GEGGLIDNKSSTRGKERKDRVAVLSIGTRLHEALVAAQEVEDEHEDLSVTVADARYM
KPLDVDLVRQLADDHSVLITIEEGSIGGFGDHVLHFLSLDGLLDEGNLKFRPMVIPDAY
FEAATQFQQYEQAGLNARHIKGTILRLAKKIEIPILDEIPVDNSESTIA

>*Pseudonitzschia_pungens*/DXS

AIXNSSEMRLSITALLLAAYTAPNAVFGFAPSSRRHVSVQSSRANTVLNAESESDKISGP
PYSGPAVKPILDSVNYPHEMKNLDMRQLKQLANELRWEVLEQVSKTGGHLSSSLGVT
ELTVALHYVFDMPDDDIIWDVAHQCYPHKILTGRRDRFPTLRQLGGLSGFCKRKESEY
DSFGAGHSSTSISAAQGMSIAKSILNKRKNNCVAVIGDGAITGGMAYEAMNSAGYLQS
RMIVVLNDNGQVSLPTGTPSAGGTVPASQLSTYTSNLLVSKPFQDFRDFAKSFNKLLPE
DVQSVNKRIDEYTRGLVSGGTLFEELGFYYVGPIDGHDLDNLIPILEKLRDSPNNKPVLL
HIKTVKGYGYPPAESASDRMHGVGKFNLGTGAQFKAKGGEPSFTSIFANALIDAAIEDR
SVVGITAAMPGGTGMDIFGRRFPKRTFDVGIAEQHAVTMGAGMACEGLKPFVCIYSTF
MQRGYDQVIHDVAVQNLPVRMMLDRGGFVGNDGPTHHGCYDFAYMGCIPNLTIMAP
SDEIELRNMVMTCADFNEGPTVLRYPRGVGYGADKLQNLFGYKLEKGEIPSKGEIIPIG
KGRIIRGEGGMIENTSSTRGKERKDRVAILSIGTRLHEALVAAQEVEDEYDDLSVTVAD
ARYMKPLDVDLVRQLADDHSVLITIEEGSIGGFGDHVLHFLSLDGLLDDGNLKFRPMV
MPDAYFEAATQFQQYEQAGLNSRHIKGTILRLAKKIEIPVLDEIPVDNSESTIA

>*Pseudonitzschia_pungens_fr_cingulata*/DXS

MRLSITALLLAAYTAPNAVFGFAPSSRRHVSVQNSRANTVLNAESESDKISGPPYSGPA
VKPILDSVNYPNEMKNLDMRQLKQLANELRWEVLEQVSKTGGHLSSSLGVTELTVAL
HYVFDMPDDDIIWDVAHQCYPHKILTGRRDRFPTLRQLGGLSGFCKRKESEYDSFGAG
HSSTSISAAQGMSIAKSILNKRKNNCVAVIGDGAITGGMAYEAMNSAGYLQSRMIVVL
NDNGQVSLPTGTPSAGGTVPASQLSTYTSNLLVSKPFQDFRDFAKSFNKLLPEDVQSVN
KRIDEYTRGLVSGGTLFEELGFYYVGPIDGHDLDNLIPILEKLRDSPNNKPVLLHIKTVK
GYGYPPAESASDRMHGVGKFNLGTGAQFKAKGGEPSFTSIFANALIDAAIEDRSVVGIT
AAMPGGTGMDIFGRRFPKRTFDVGIAEQHAVTMGAGMACEGLKPFVCIYSTFMQRGY
DQVIHDVAVQNLPVRMMLDRGGFVGNDGPTHHGCYDFAYMGCIPNLTIMAPSDEIEL
RNMVMTCADFNEGPTVLRYPRGVGYGADKLQNLFGYKLEKGEIPAKGEIIPIGKGRIIR
GEGGMIENTSSTRGKERKDRVAILSIGTRLHEALNAAQEVEDEYDDLSVTVADARYMK
PLDVDLVRQLADDHSVLITIEEGSIGGFGDHVLHFLSLDGLLDDGNLKFRPMVMPDAY
FEAATQFQQYEQAGLNSRHIKGTILRLAKKIEIPVLDEIPVDNSESTIA

>*Skeletonema_costatum*/DXS

MKFISAVVAAAIASAPVCNAFSPSSGRSPLRTIQQSTQSQKTSTSLLDTQTDSGPPYSGP
ASKPILDSVKFPSDMNRLNMSELKQLAHELRWEVIEAVSKTGGHFSSSLGVIELTTALH
YVFDMPEDDIIWDVAHQCYPHKMLTGRRDRFPTLRQLNGMSGFCKRKESEYDSFGAG
HSSTSISAAQGMSVAKSTLNKRKNNCIAVIGDGAITGGMAYEAMNNAGYLSSRMIVVL
NDNGQVSLPTGTQSAGGVKPASQLSAYTSNLLVSKPFQDFRDFAKSFNRLLPENVQDV
NKRIDEYARGVVTGGTLFEELGFFYVGPIDGHDLENMVPILEKLRDSDSNKPVLLHIKT
NKGQGYAPALAASDRMHGVGKFDX

>*Skeletonema_marinoi*/DXS

MKFISAVVAAAIASAPVCNAFSPSSGRSPLRTIQQSTQSQKTSTSLLDTQTDSGPPYSGP
ASKPILDSVKFPSDMNRLNMSELKQLAHELRWEVIEAVSKTGGHFSSSLGVIELTTALH
YVFDMPEDDIIWDVAHQCYPHKMLTGRRDRFPTLRQLNGMSGFCKRKESEYDSFGAG
HSSTSISAAQGMSVAKSTLNKRKNNCIAVIGDGAITGGMAYEAMNNAGYLSSRMIVVL
NDNGQVSLPTGTQSAGGVKPASQLSAYTSNLLVSKPFQDFRDFAKSFNRLLPENVQDV

NKRIDEYARGVVTGGTLFEELGFFYVGPIDGHDLENMVPILEKLRDSDSNKPVLLHIKT
NKGQGYAPALAASDRMHGVGKFDIATGIQQKGKAAAPSFTSIFANSLIDAATEDRAVV
GITAAMPGGTGMDIFGRRFPKRTFDVGIAEQHAVTFAAGMACEGLKPFCCIYSTFMQR
GYDQVVHDVAIQNLPVRMILDRAGVVGNDGPTHHGCYDLAYLGCIPNLTIMSPSDEIE
LRNMVATCAGFDDGPTVLRYPRGVGYGADKLQNLFGYDLEDGEIPTKGVPLEIGKGRI
IRRPGGVHALGEDIAANGSSDSKLGSGGRGAKREDRVAILSLGTRLSESLIAANEVEEM
DPDIAVTVADARFMKPLDVDLIRELAKDNGIIITVEEGSIGGFGDHVLHFLSLDGALDN
GDLKFRPMVLPDAYFEAATQYEQYEMAGLNSHHIRGTVLRLAEKVEVPELAELEK
>*Skeletonema_menzelii*/DXS
MKYISAVVAAAIASAPVCNAFSPSSGRSPLRTIQQSTESQITSTRLFDATADRPASKPILD
SVKFPSDMKRLNMSELKQLAHELRWEVIEAVSKTGGHFSSSLGVIELTTALHYVFDMP
EDDIIWDVAHQCYPHKMLTGRRDKFPTLRQLNGISGFCKRKESVYDSFGAGHSSTSISA
AQGMSVAKSTLNKRRNNCIAVIGDGAITGGMAYEAMNNAGYLSSRMIVVLNDNGQV
SLPTGTQSAGGVKPASQLSAYTSNLLVSKPFQDFRDFAKSFNRLLPENVQDVNKRIDEY
ARGVVTGGTLFEELGFFYVGPIDGHDLENMVPILEKLRDSDSNKPVLLHIKTNKGQGYP
PALAASDRMHGVGKFDIATGVQQKGKAAAPSFTSIFANSLIDAATEDRAVVGITAAMP
GGTGMDIFGRRFPKRTFDVGIAEQHAVTFAAGMACEGLKPFCCIYSTFMQRGYDQVV
HDVAIQNLPVRMILDRAGVVGNDGPTHHGCYDLAYLGCIPNLTIMSPSDEIELRNMVA
TCAGFDDGPTVLRYPRGVGYGAEKLQDLFGYDLEDGEIPTKGVALEIGKGRIIRRPGGV
RALGKEITANGSSDSKLGSGGRGAKREDRVAILSLGTRLSESLIAADEVEEMDPDIAVT
VADARFMKPLDVDLIRELAKDNGIIITVEEGSIGGFGDHVLHFLSLDGALDNGDLKFRP
MVLPDTYFEAATQYEQYEMAGLNAHHIRGTVMRLAQKVEVPELAELEK
>*Thalassionema_frauenfeldii*/DXS
MSGLRQSGGISGFCKRKESKYDSFGAGHSSTSISAAQGMSVAKSMLNKRRNNCIAVIG
DGAITGGMAYEAMNSAGYLRSRMIVVLNDNGQVSLPTGTPSAGGTVPASQLSTYTSN
LLVSKPFQDFRSFAKSFNKLLPEEIQGLNKRIDEYARGIVSGGTLFEELGFYYVGPVDGH
DLENMVPILEKLRDSPDDKPVLLHLKTIKGYGYPPAEEASDKMHGVAKFNLGTGAQIK
PKAGNPSMTSIFANSLIDCATEDRAIVGITAAMPGGTGMDIFGRRFPKRTYDVGIAEQH
AVTMAAGMACEGLKPFCCIYSTFMQRGYDQVVHDVAIQNLPVRMILDRAGLVGNDG
PTHHGCYDLAYMGCIPNLTIMAPSDEIELKNMVSTCAAFDEGPTVLRYPRGTGYGADK
LQSLFGYELEGGEIPAKGEVLPIGKGRIVRPAGGFSKNQINTRGKERKDRVAILSLGTRL
HESLKAANEIEEQDDKLGVTVADARFMKPLDVDLIRELVDDHSVLITVEEGSIGGFGDH
VLHFITLDGLMDDGDLKFRPMVLPDVLFEAATQTEQYEQAGLNAQHIKGTILRLTKRV
NVPVLEEA
>*Thalassiosira_minuscola*/DXS
MKELEQLAHELRWEVIDAVSQTGGHFSSSLGVIELTVALHYVFDMPEDDIIWDVAHQC
YPHKMLTGRRDKFPTLRQLGGISGFCKRKESEYDSFGAGHSSTSISAAQGMSVGKSML
NKRKNNCIAVIGDGAITGGMAYEAMNNAGYLSSRMIVVLNDNGQVSLPTGTQTAGGV
KPASQLSAYTSNLLVSKPFQDFRDFAKSLNRLFPENVQDVNKRLDEYARGLVTGGTVF
EELGFFYVGPIDGHDLENMVPILEKLRDSDSSKPVLLHIKTTKGYGYPPAMAASDRMH
GVAKFDIGTGVQVKGKAAAPSFTSILANSMIDAATEHREIVGITAAMPGGTGMDIFGRR
FPKRTFDVGIAEQHAVTFAAGMACEGLKPFCCIYSTFMQRGYDQVVHDVAIQNLPVR
MILDRAGVVGNDGPTHHGCYDLAYLGCIPNLTIMSPSDEIELRNMVMTCAEFDDGPTV
LRYPRGVGYGAEKLQDLFGYDLENGEIPAKGVPMEIGKGRLIRLPGGVRAMSKQPPAN
GSSDSKLSGAGSRGQKKEDRVAILSFGTRLSEALKAADEVEESDPDLAVTVADARFMK
PLDVDLIRDLAKDNGIIITVEEGSIGGFGDHVLHFLSLDGALDDGELKFRPMVLPDTYFE
AATQYEQYEMAGLNAHHIRGTVMRLAKKVEVPVLSE
>*Thalassiosira_pseudonana*/DXS
MKFLSSTIAAAILATSSAFTTTLPRRTSSIQPTSTRLHDTTDNGPPYSGPASKPILDTVNYP
HDMKRLDMKELKQLAHELRWELIEAVSKTGGHFSSSLGVIELTVALHYVFDMPEDDII
WDVAHQCYPHKMLTGRRDRFPTLRQLGGISGFCKRKESEYDSFGAGHSSTSISAAQGM

SVAKSLLNKRKNNCIAVIGDGAITGGMAYEAMNNAGYLSSRMIVVLNDNGQVSLPTG
TQTAGGVRPASQLSAYTSNLIVSKPFQDFRDFAKSLNRLFPENVQDVNKRLDEYARGL
VTGGTVFEELGFFYVGPIDGHDLDNMVPILEKLRDSDSTKPVLLHIKTNKGQGYPPALA
ASDKMHGVGKFDIATGIQVKGKAASPSLTSIFANAMIDAATEDRAIVGITAAMPGGTG
MDIFGRRFPKRTYDVGIAEQHAVTFAAGMACEGLKPFCCIYSTFMQRGYDQVVHDVAI
QNLPVRMILDRAGVVGNDGPTHHGCYDLAYLGCIPNLTIMSPSDEIELKNMVMTCAAF
DDGPTVLRYPRGVGYGAEKLQNLFGYELENGEVPAKGEVIKIGKGRIIRRPGGIHALKE
ELTANGSSDSRMGAGSRGLLKENRIAILTLGTRLSEALVAADEIEGANPDLAVTVADAR
FMKPLDEDLIRELSDNNGIIVTVEEGSIGGFGDHVLHFLSLDGALDDGELKFRPMVLPD
TYFEAATQYEQYEMAGLNSHHIRDTVLRLANVKVEAAPVLAEAE

>*Thalassiosira_puntigera*/DXS

MKCIEILSIAAAATTASAFTLPKTPVSKTSTTRLNEAINRVQPQGPPYSGPSSKPILDSVQ
FPRDMNRLTMQELEQLAHELRWEVIEAVSKTGGHFSSSLGVIELTVALHYVFDMPEDD
IIWDVAHQCYPHKMLTGRRDRFPTLRQLGGISGFCKRKESEYDSFGAGHSSTSISAAQG
MSIGKSLLNKRKNNCIAVIGDGAITGGMAYEAMNNAGYLSSRMIVVLNDNGQVSLPT
GTQSAGGVRPASQLSAYTSNLLVSKPFQDFRDFAKGMNRLFPEGVQDVNKRIDEYAR
GLVTGGTIFEELGFFYVGPIDGHDLENMVPILEKLRDSDSNKPVLLHIKTNKGYGYPPA
LAASDRMHGVAKFNVGTGVQVKGKAAAPSLTSIMANAMIDAATEHREIVGITAAMPG
GTGMDIFGRRFPKRTFDVGIAEQHAVTFAAGMACEGLKPFCCIYSTFMQRGYDQVVH
DVAIQNLPVRMILDRAGVVGNDGPTHHGCYDLAYLGCIPNLTIMSPSDEMELRNMVM
TCAEFDEGPSVLRYPRGVGYGPEKLQELFGYELENGEIPAKGTPIEIGKGRIIRLPGGVR
ALSSSEPAANGSTDSKLGASKSRGKKKTDRVAILSFGTRLSEALIAADEVEESDPDLAV
TVADARFMKPLDIDLVRDLAKDNGIVITVEEGSIGGFGDHVLHFLSLDGALDDGELKFR
PMVLPDTYFEAATQYEQYEMAGLNAHHIRGTVLRLAKKVEVPVLSE

>*Thalassiosira_rotula*/DXS

MKCILAAVLIAALATSATAFTIPTTPITKSSTSSTTQLNDAINRREPAGPPYAGPASKPILD
SVKFPRDMNRLDMKELEQLAHELRWEVIEAVSKTGGHFSSSLGVIELTVALHYVFDMP
EDDIIWDVAHQCYPHKMLTGRRDRFPTLRQLNGMSGFCKRKESEYDSFGAGHSSTSIS
AAQGMSIGKSLLNKRKNNCIAVIGDGAITGGMAYEAMNNAGYLSSRMIVVLNDNGQV
SLPTGTQTAGGVRPASQLSAYTSNLLVSKPFQDFRDFAKGLNRLFPENVQDVNKRLDE
YARGLVTGGTIFEELGFFYVGPIDGHDLENMVPILEKLRDSDSSKPVLLHIKTNKGYGY
PPALAASDRMHGVAKFDIGTGVQVKGKVAAPSFTSIMANAMIDAATEHREIVGITAAM
PGGTGMDIFGRRFPKRTFDVGIAEQHAVTFAAGMACEGLKPFCCIYSTFMQRGYDQVV
HDVAIQNLPVRMILDRAGVVGNDGPTHHGCYDLAYLGCIPNLTIMSPSDEMELRNMIM
TCADFDDGPTVLRYPRGVGYGPEKLQELFGYELENGEIPSKGTPIEIGKGRLIRLPGGVR
ALSEEPAANGSTDSKLGAGSRGKKKEDRVAILTFGTRLSEALIAADEVEEMDPDLAVT
VADARFMKPLDVDLIRELAEDNGIIITVEEGSIGGFGDHVLHFLSLDGALDKGDLKFRP
MVLPDSYFEAATQYEQYEMAGLNSHHIRGTVLRLAKKIEVPVLSD

>*Thalassiosira_weissflogii*/DXS

MKFIQSSIAALLLAAAAPTSAFTTFAPRTTPASTQLQSTRDAAASSTSGPPYSGPASKPIL
DSVNYPRDMKRLDMRELKQLAHELRWELIEAVSKTGGHFSSSLGVIELTVALHYVFD
MPEDDIIWDVAHQCYPHKMLTGRRDRFPTLRQLGGISGFCKRKESEYDSFGAGHSSTSI
SAAQGMSVAKSMLNKRRNNCIAVIGDGAITGGMAYEAMNNAGYLSSRMIVVLNDNG
QVSLPTGTQTAGGVRPASQLSAYTSNLIVSKPFQDFRDFAKNLNKLFPENVQDVNKRID
EYARGVVTGGTLFEELGFFYVGPIDGHDLDNLVPILEKLRDSDSNKPVLLHIKTEKGHG
YPPALAASDRMHGVGKFDIATGIQVKGKAAAPSLTSIFANSLIDAATEDRAIVGITAAM
PGGTGMDIFGRRFPKRTFDVGIAEQHAVTFAAGMACEGLKPFCCIYSTFMQRGYDQVV
HDVAIQNLPVRMILDRAGVVGNDGPTHHGCYDLAYMGCIPNLTIMSPSDEIELKNMV
MTCAGIDDGPSVLRYPRGVGYGAEKLQKLFGYSLENGEIPTKGTPVEIGKGRIIRRPGG
VRALSEDTEPANGSKDGKMGTGSRGLLKEHRVAILTLGTRLSEALVAADEVEELDPDL

AVTVADARFMKPLDVDMIRELAENNGILITVEEGSIGGFGDHVLHFLSLDGALDEGDL
KFRPMVLPDTYFEAATQYEQYEMAGLNAHHIRGTVMRLTKKVDVPVLVD

CMK protein sequences

>*Amphiprora_sp*/CMK
MRGLLTLLFVTLGAGASAFTVGSRDGTARYSATRILRQSTGSSATNTDVSTASSETDST
LSLFSPCKINLFLRIIRKREDGFHDLASLFQAIGFGDSLELTPIDGTADEFTCNMPGVPVD
SSNLVLRALELMRTKTGVEQYFKANLIKQVPAQAGLGGGSANAATAMWGANQLMG
DPASLEEMVEWSGDLGSDITFFLSRGTAYCTGRGEIMDSIDPPLPSGTRLCIVKPNVGLS
TPAVFKALEYDKLSTLDADEELLPTFLKGIDDVPDGYFINDLEFPAFKCLPELKELKDEL
LQEEGFDHVMMSGSGTSIFCLGEPSDKKAFEEKFAKR
>*Bolidomonas_pacifica*/CMK
LYYFVICHVSNTEGFHQRLPPQTPHTPRRSTSLNCAGSKDPSLASLTLFSPSKVNLFLRII
RKRDDGFHDLASLFCAIGLGDTLNLESADTDVFTCNMPGVPVDSSNLVVRALNLVREK
TGNEGKKFKINLHKECPAQAGLGGGSGNAATAMFAVNELLGRPATMDQLVEWSGEL
GSDITFFLSKGTAYCTGRGEVX
>*Chaetoceros_neogracile*/CMK
TLAFAPNKGYLNQFQSHHVSSRNAQMASRLFSTVDESEYDLSLFSPCKINLFLRIIRKRP
DGFHDLASLFQTIGFGDMLHLKYEESSTADIFECNMDGVPTDKSNLVIRALDLVRSKTG
NEDKFFKANLVKQVPAQAGLGGGSGNAAAAMWGANELLGRPATLEQLVEWSGALG
SDITFFLSQGTAYCTGRGEIMTPVDLLPDGTKVCIVKPDIGLSTPEVFRALDYDQLSTIDP
EELLKTFMEKGAVDAGDDAYVNDLEQPAFDCLPELGKLKEELKSVEGFDHVMMSGS
GTSIFCIGEPX
>*Chaetoceros_sp*/CMK
MRGDSVLLGLQTGVKWDDWDYYRAKVSDHDAYVNDLEQPAFDALPELKRLKDELK
SIEGFDHVMMSGSGTSIFCIGEPKDKDAFMKEFGQRKGINVFPAEFTSRKQGTWFQKPF
N
>*Coscinodiscus_wailesii*/CMK
MEDVPTDKSNLVLKALDLMRDKTGCQKFFKAKLVKQVPAQAGLGGGSANAATAMF
GANELMGRPATLDQLIEWSGDLGSDITFFLSQGTAYCTGRGEILDPITPSPFSSETKICIV
KPKNIGLSTPSVFRALDYDKLSSRDPQELLDTFLEKGVLGVEKNDYVNDLEDPAFRVV
PELLALKNEVEECGFDFVMMSGSGTSLFGLGGGDDAIEKTRERFTGRDDVSIFETNFLS
RGEGWFEGEIME
>*Cylindrotheca_closterium*/CMK
XGSSFGFAGPSATLQQYDTSTTLLRASRAAEKKASRTKWVKSRGFDDDIAGGSDAMTL
FSPXKINLFLRIIRKREDGFHDLASLFQAVGYGDTLELKKLDDATEDAFSCNMEGVPTD
MTNLVLRALELMREKTGVKQYFDANLIKQVPAQAGLGGGSANAATAMWGANELMG
NPASLEDMIEWSGDLGSDITFFLSRGTAFCTGRGEIMTPTDPLLEAGTKLCIVKPNVGLS
TPSVFKALDYDELSELDADKVLLPEFLNAESVEAVSGEYYINDLEPPAFRCVPELGELK
DKLTAVDGFKHVMMSGSGTSIFCIGEPSDPEQFQKDFGEDEELKVMFTEFINREEGTWF
ERP
>*Fragilariopsis_kerguelensis*/CMK
XSSHKEFSAEKRNKEFYTIPSDMLLRFEKIAKKRRMKLSTGVYFSFGFWCYMYXLCNW
ISVSSVRGFSVVKSNHIFSGSSTESSRKQYTTIKSTRVGQAKISTGHQAWRGSLHFDASL
KSSSTSIPELTQQENEQKTLVLFSPCKINLFLRIIGKREDGFHDLASLFQAIGFGDTLELAP
LKEEKSSIEKRNDEFTCNMPGVPVDSTNLVLRALSLMRQKTGVKMYFKTNLIKQVPAQ
AGLGGGSANAATAMWGANELMGRPASLEQMIEWSAELGSDITFFLSRGTAFCTGRGEI
LTPIDPPLPTGTKVCIVKPNIGLSTPSVFKALDYDELSDLDPDDVLLPAFLDSVGVENVA
REYYVNDLEPPAFRCVQKLGELKDSLLKVDGFQHVMMSGSGTSIFCIGEPEDTESFLKE
FGESEDIQVFFSEFIDRESNEWFQNPKNAA

>*Leptocylindrus_apora*/CMK
IASLLLLTSTTTAFSSWVPKRKSVSIHQNYRITTRQVTADAHSSVTTASSASDDASVEW
DMSLFSPAKINLFLRIIRKREDGFHDLASLFQTIGFGDYLHIRKLGSDAEDDVLTCNMDG
VPTDRTNLVMRAVDLVREKTGIKDQFFQMDLVKYVPHQAGLGGGSGNAAAALYGVN
ELLGNPATLEELVEWSGELGSDITFFLSEGTAYCTGRGEIMTPVPPLPNSAGTKLCIVKP
DIGLSTPSVFKALDYDKLSKADPENVLQIFQTEGMSPSEDEKFINDLERPAFKVLPDLEE
LKNDLISCGFETVMMSGSGTSIFCIGSTSDPEKFSNLIESRDDVNVYSTEFINRDGNGWY
TSPDDTSTKIQ
>*Leptocylindrus_danicus*/CMK
XSILLTTTQAFQIPSTFGTARRTALTVKSADAIDETLTKDENSETWDLSLFSPAKINIFLRI
IRKREDGFHDLASLFQTIGFGDYLHLKLLDDDEEAKEDILTCNMEGVPTDKSNLVMRAI
DLVRQNTGITDKFFKCDLVKHVPHQAGLGGGSGNAAAALYGANELLGRPATLEQLVE
WSGDLGSDITFFLSEGTAYCTGRGEIMTPVDPLPAGQTVYIVKPDIGLSTPSVFKALDYD
LLSTLDPEEILNVFMTEGVTTSNQSVFVNDLEPPAFKVVPELKELKDDLISCGFDTVMM
SGSGTSIFCLGKPGDEEKFEELIGSRDDVQVYPTEFINRVGCAADGGWYQDPNA
>*Nannochloropsis_gaditana*/CMK
MTHSFSVGGGGAGGGGGAGAMNRAHSSAVAGRSLVKSKEKVGAVEARELTAGKEG
GGVATARALAAPEVQDMTRRVSTHCGRPLFFLTRTPSINCLTCNVDLGMNLFLRIIRRR
EDGYHELASLFQTVAFGDTLLLKVLPAIAVQDEFSSSYTSLVTDQSNLVLRALDLFREK
TGKTQRFEVYLEKRTPVQAGLGGGSSNAATTLFAANQLLGTPATDEELIEWSKELGSDI
TFFLSKGTAYCTGRGELLEPVPPLPSSIKLYVVKPPVGLSTPAVFKALDLDALNGEDPR
DLLAAFTQHLSRGELGPASMYINDLEPPAFQCVPRLKAIKEALLARGFDCVMMSGSGT
AIFAMAPAPSSLPSSAGLSFDAAAFAREMEVDIWATTFTNRPSDDPKAWYPNPHA
>*Nitzschia_punctata*/CMK
XTTTESITEDEFTCNMPGVPVDSTNLVLRALELMRSKTGVQKYFKANLIXQVPAQALG
GGSANAATAMWAANTLMGNPATLEQMIEWSGELGSDITFFLSRGTAFCTGRGEIMTPI
EPPLPTGTKLCIVKPDIGLSTPSVFKALDYDELSDLNADEVLLPAFLNANGVENVPSEYY
INDLEPPAFRCVPELGELKKSLQKVTGFKHVMMSGSGTSIFCIGEPEDKEAFMKEFGET
EDLQVFFSEFIGREEGEWFRRPE
>*Nitzschia_sp*/CMK
XTNLLFILKDDDDDEREDERSYGLLEVSVKDFVRILLQLCRTFGDGDGVGLFQQFPNN
DDGFVPTITVSSSSSTIASHRHHRHRTGVFLDAVSSSTESSTTPSTSAQSQDDGSLTLFSP
CKINLFLRIVRKREDGFHDLASLFQAIGFGDTLELKLLQQDDSEEDGDEEEKTTTTTADS
FTCNMPGVPVDDTNLVLRALQLMRTKTGKTDQHFAANLIKECPAQAGLGGGSANAAT
AMWGANELMGRPASLEQMIEWSGELGSDITFFLSQGTAYCTGRGEIMTPTNPPLKSGT
KLCIVKPNVGLSTPSVFKALDYDELSTLDADKTLLPAFLETDGVENVSDEYYINDLEPP
AFRCVPELGELKSSLQKIKGFDHVMMSGSGTSIFCIGEPDDKDAFLKEFGNRQDELQVF
FSEFISREEGQW
>*Phaeodactylum_tricornutum*/CMK
MYFASAHVLFLAALSREAVAFTDNRFPIFNHPSAKVTTTSSLSAYTETNESSRGKISERT
LTLFSPCKINLFLRIIRKREDGFHDLASLFQAIGFGDTLELTSIDSDADEFTCNMPGVPVD
NSNLVLRAIQLMREKTGEKQSFRANLIKQVPAQAGLGGGSANAATAMWGVNELMGR
PASLDQMVEWSGALGSDITFFLSRGTAYCTGRGEIMSSIDPPLPSGTKICIVKIDIGLSTPS
VFKALDYDKLSNLNADDVLLPTFLRGIDQVPDSYFVNDLETPAFKCIPELRSLKEELLG
VAGFDHVMMSGSGTSIFCLGEPKDNKDFHDRFVSRKGLQVFFSEFISRPEGVWFEKP
>*Pseudonitzschia_australis*/CMK
MSNPFISFSFWCCYFGLFIATSSVEGFAVVEPCRVLRQSSLSASTQENQETLELFSPCKIN
LFLRIIRKREDGFHDLASLFQAIGFGDTLELTPLVETTEIGEGSDEFTCNMPGVPVDSTNL
VLRALDLMREKTGVDMFFKTNLIKQVPAQAGLGGGSANAATAMWGANELMGRPASI
EQLIEWSGALGSDITFFLSQGTAYCTGRGEILTPIDPPLPTGTKVCIVKPDIGLSTPSVFKA

LDYDELSDMDPDDVLLPAFLDTVGVENVSKHYYVNDLESPAFRCVPRLGELKESLLKV
AGFKHVMMSGSGTSIFCVGEPDDRESFVKEFGEMDDVQVFFSEFIDREKEIWFQKP
>*Pseudonitzschia_arenysensis*/CMK
MSSLHKISSLCIFYCFLCFFVATALVQGFASTXQNSALARSXKQTSLFGSSSEEPKATLK
LFSPCKINLFLRIIRKREDGFHDLASLFQAIGFGDTLELSPLDDDDADADEFTCNMPGVP
VDSTNLVLRALELMREKAGVDMYFKSNLIKQVPAQAGLGGGSANAATAMWGANEL
MGRPASVEQMIEWSGALGSDITFFLSQGTAYCTGRGEILDPIDPPLPTGTKVCIVKPNIG
LSTPSVFKALNYDELSDKDADKVLLPAFLDTEGVENVPKDCYVNDLEPPAFRCVPRLG
ELKESLLKVKGFEHVMMSGSGTSIFCIGEPEDKDAFLEEFEAMDDVKVFFSEFIDREKG
EW
>*Pseudonitzschia_multistriata*/CMK
MSNLISSFILWCCYFGLLTTTSSVQGFAVVEPNHVFRQSSLSASSQENQETLELFSPCKIN
LFLRIIRKREDGFHDLASLFQAIGFGDTLELTPLGETADEGEGSDEFTCNMPGVPVDSTN
LVLRALDLMREKTGVNMYFKTNLIKKCPAQAGLGGGSANAATAMWGANELMGRPA
SLEQMIEWSGALGSDITFFLSQGTAYCTGRGEILTPIDPPLPTGTKVCIVKPDVGLSTPSV
FKALDYDELSNMDPDDVLLPAFLDAEGVENVSAEYYVNDLEPPAFRCVPRLGELKESL
LKVSGFKHVMMSGSGTSIFCIGEPDDHESFMKKFGEMDDVQVFFSEFIDREKGEWFQK
P
>*Pseudonitzschia_pungens*/CMK
XSESPRVVSMVTSNSIRELIFYVETSKHSENTDHLVQELKLIYRVRPXDLYPFELILREK
QIASQRIGLQDKKMANASLSFFLWCCYFGIFAATSVVQGFAVVDSTRFARQSSLSASTQ
QDVATLELFSPCKINLFLRIIRKREDGFHDLASLFQAIGFGDSLELTPLEETSEDEEGSDE
FTCNMPGVPVDSTNLVLRALDLMREKTGVKMYFKTNLVKQVPAQAGLGGGSANAAT
AMWGANELMGRPASLEQMIEWSGALGSDITFFLSQGTAYCTGRGEILTPIDPPLPTGTK
ICIVKPDIGLSTPSVFKALDYDELSKMDPDDVLLPAFLDTEGVENVSEDCYVNDLEPPA
FRCVPRLGELKESLQKVAGFQHVMMSGSGTSIFCIGEPEDREAFLDEFGKMEDVQVFFS
EFIDREKGQWFQKP
>*Pseudonitzschia_pungens_fr_cingulata*/CMK
MANASLSFLLWCCYFGIFAATSVVQGFAVVDSTRFARQSSLSASTQQDVATLELFSPCK
INLFLRIIRKREDGFHDLASLFQAIGFGDSLELTPLEETSEDEEGSDEFTCNMPGVPVDST
NLVLRALDLMREKTGVKMYFKTNLVKQVPAQAGLGGGSANAATAMWGANELMGRP
ASLEQLIEWSGALGSDITFFLSQGTAYCTGRGEILTPIDPPLPTGTKICIVKPDIGLSTPSVF
KALDYDELSKMDPDDVLLPAFLDTKGVENVSEDCYVNDLEPPAFRCVPRLGELKESLQ
KVAGFQHVMMSGSGTSIFCIGEPEDREAFLDEFGKMEDVQVFFSEFIDREKGQWFQKP
>*Skeletonema_costatum*/CMK
MISTKVFALLXAAACGVXSVDGFVSSSTSRLGNXQKSNLHATTAESAPDSLALFSPCKI
NLFLRIIRKRPDGFHDLASLFQAVGFGDTLTLSLNNYGEXDEFTCNMEGVPTXSSNLVI
RALELMREKTGKTSVYFNADLFKRCPAQAGLGGGSAXAATAMWGANELMGRPAXLE
QLIEWSGALGSDITFFLSRGTAYCTGRGEXMTPIDPPLPSGAKLSIVKPNLGLSTPKVFK
ALDYDQLSTEDPQMLCDLFLKEGVVNTDDKYYINDLEQPAFDCLPELKALKDELLEVX
GFDHVMMSGSGTSIFCIGEPTDKDAFMKEFDERDGVSVFNAAFISREEXSWFQDPSQPL
AVVDESEEEVVAPIIEEGMTAEEAERVRESRLKAAEAMKESIPAEPDNSEIVVEDEGSSS
NFSKGLRSILNGXRKFEESRFGEKGASYPTPSKWLE
>*Skeletonema_marinoi*/CMK
XTMISTKVFALLLAAACGVVSVDGFVSSSTSRLGNIQKSNLHATTAESAPDSLALFSPC
KINLFLRXIRKRPXGFHDLASLFQAVGFGDTLTLSLNNYGEXDEFTCNMEGVPTXSSNL
VIRALELMREKTGKTSVYFNADLFKRCPAQAGLGGGSANAATAMWGANELMGRPAT
LEQLIEWSGALGSDITFFLSRGTAYCTGRGEIMTPIDPPLPSGAKLSIVKPNLGLSTPKVF
KALDYDQLSTXDPQMLCDLFLKEGVVNTDDKYYINDLEQPAFDCLPELKALKDELLE
VEGFDHVMMSGSGTSIFCIGEPTDKDAFMKEFDERDGVSVFNAAFISREEGSWFQDPSQ

PLAVVDESEEEVVAPIIEEGMTAEEAERVRESRLKAAEAMKESIPAEPDNSEIVVEDEGS
SSNFSKGLRSILNGQRKFEESRFGEKGASYPT
>*Skeletonema_menzelii*/CMK
MISTKMFALLFAAACGVSPVNGFVSSSTSRLGTIQKSHLHATTAESESLSLFSPCKINLFL
RIIRKRPDGFHDLASLFQAVGFGDTLTLSLNNHGDADEFTCNMEGVPTDSSNLVLRAL
QLMREKTGKTNVHFNADLFKRCPAQAGLGGGSANAATAMWGANELMGRPATLEQLI
EWSGALGSDITFFLSRGTAYCTGRGEIMTPIDPPLPSGAKLSIVKPNLGLSTPKVFKALD
YDQLSTEDPQMLCDVFLREGVVNTDDKYYINDLEQPAFDCLPELKALKDELLQVEGFD
HVMMSGSGTSIFCIGEPTDKDAFMKEFDERDGVSVFNAAFINREEGSWFQDPSQPLAIV
DEPEEEEEEEVAQIIEEGMTAEEAEKVRESRLRAAEAMKESSPSETDNNIATKDSGTNFS
KGLRSILNGERKFEESRFGEKGTAYPTPSKW
>*Thalassionema_frauenfeldii*/CMK
MEWRILMICVQFLSVVSSFGTQRPGKSSLFHPSTQLHAKPNVKLALFSPCKINLFLRIIRK
REDGFHDLASLFQAIGFGDTLELTILEGADADEFTCNMEGVPVDQTNLVLRALTLMRE
KTGNHNTYFYANLIKECPAQAGLGGGSANAATAMFGANELMGNPATLEEMVEWSAD
LGSDITFFLSRGTAYCTGRGEIMTPIDPPIEASTKLVIVKPNVGLSTPTVFKSLEYDKLSD
LDPENLLQQFMEESENVAMVDESAFVNDLEPPAFKNLPELMKLKEDLQKVKGFQHVM
MSGSGTSIFCIGTPADAEAFEKQFSYRDDLQVFFAEFIN
>*Thalassiosira_minuscola*/CMK
MTVSKALATLLLATIGALPTHGFAPSTHAHRMPTKNDVFSATSQKSAVYAATDSDVTS
KSSTDALTLFSPCKINLFLRIIRKRPDGFHDLASLFQAVGFGDTLHLSLTDKDADEFTCN
MEGVPTDSSNLVLRALELMRQKTDRKNVFFKADLVKRVPAQAGLGGGSANAATAM
WGANELMGNPATLDELIEWSGELGSDITFFLSRGTAYCTGRGEIMTAIDPPLPSGAKLSI
VKPDLGLSTPKVFGALDYDGLSEENPEGLRDTFLKEGVVNTDDKYYINDLEQPAFDC
>*Thalassiosira_pseudonana*/CMK
MNLLSHALTTILFATCSILPSDAFVSSPIGQQTTLTKNFSKNPFLRTQTRVAASDDSSSDT
LKLFSPCKINLFLRIIRKRPDGFHDLASLFQAVGFGDTLTLSLSEQPNDEFTCNMEGVPT
DSSNLVLRALELMRQKTGKTDVYFKADLFKRVPAQAGLGGGSANAATAMWGANEL
MGKPATLGELIEWSGELGSDITFFLSRGTAYCTGRGEVMTPIDPPLPSGSKLSIVKPDLG
LSTPKVFSALDYEQLSAEDPEELLNTFLEFGVVDTDDEYYINDLEQPAFDCLPELKALK
EDLLKVKGFDHVMMSGSGTSIFCIGEADDKDAFMKEFDSREGVSVFNAEFINREDGCW
FESP
>*Thalassiosira_puntigera*/CMK
MAISKALATVLLATFCASPVRGFVPPTHTHRSPSQGVYSATSEHSPSYATVDDADEATK
STSSESLTLFSPCKINLFLRIIRKRPDGFHDLASLFQAVGFGDTLRLSPTEGDADEFSCNM
EGVPTDSTNLVLRALELMRRRTGRDDAFFEADLVKRVPAQAGLGGGSANAATAMWG
ANELMGRPATLDQLIEWSGELGSDITFFLSRGTAYCTGRGEVMTPVDPPIPSGARLSIVK
PDLGLSTPRVFAALDYDRLSEEDPEGLRDAFLREGVVDAPDGCYVNDLEQPAFDCLPQ
LKALKEELLEVEGFDHVMMSGSGTSIFCIGEPADAEAFMKEFDGRDGVSVFNSEFINRE
EGCWFVDPEEN
>*Thalassiosira_rotula*/CMK
ALATVLLATSCALPTHGFIPPSSSHHRQPPSQGLLSSATSQKSLLHATDSAADEATKSAP
STESLTLFSPCKINLFLRIIRKRPDGFHDLASLFQAVGFGDTLHLSLATKSDDDEFSCNM
AGVPTDSSNLVLRALELMRQKTDRKDVFFNADLVKRVPAQAGLGGGSANAATAMW
GANELMGRPATLDQLIEWSGELGSDITFFLSRGTAYCTGRGEVMTPIDPPIPSGAKLSIV
KPDLGLSTPKVFQALDYDQLSTEDPQMLCDTFXKGVDTKDEYYINDLEQPAFDCLPQL
KALKEELLGVEGFDHVMMSGSGTSIFCIGEAADKEGFMKEFDSRKGISVFNSEFINRED
GCWFEDPENN
>*Thalassiosira_weissflogii*/CMK
MTSSGFCFLSKAILGFLIATSVILSAQCFAPSALESIAGRHLRASTTEDSSETLTLFSPCKI
NLFLRIIRKRPDGYHDLASLFQAVGFGDTLHLSLSDNDGKDEFTCNMEGVPTDSTNLVL

RALELMRQKTGKTDVFFHADLFKRVPAQAGLGGGSANAATAMWGANVLMGNPATL
EQIIDWSADLGSDITFFLSRGTAYCTGRGEIMTPIDPPLPAGAKLTIVKPDLGLSTPKVFG
ALDYDQLSVEDPETLLNIFLKDGVINTDDKYYINDLEQPAFDCLPELEALKDELLCVKG
FDHVMMSGSGTSIFCIGEPNDKDSFMKEFDEREGVSVFNTEFISREEGSWFVNPEK

IPP1 protein sequences

>*Amphiprora_sp*/IPP1
XSRLLVQAFQGTTPFRRRPTMMTTKATTATLAARIRSSNRMNHHPSRWFSSTTRITLSS
SSSSSYGDNMNQHDMMESDLLIAVNELDQAILPNNNNHHNKSDSRLTKRLGHTFGPAT
PRATLHRAFSLFLFDSSGRRMLLTQRAASKLTFPNVWTNACCSHPLQDMAVDEVDDGI
RDYPTFPGIKQAAWRKCRHELGLDLRALMATTEDGDDSDDDPTQQRQLQLQDKMQFI
TRFHYWAADSVTYGPSTEWGEHEVDYVLFLQLPPGIETSGSLLQPNPEEVGDYKWVTI
DELQAMLKSGNNKNLLWSPWFVGILERGGFEWWKDLSGALSGKYTNDQITFFDPPPE
HVADYNLPSHNRWTGVWKWNPGKNEPGSASSKTKDETMSTSS
>*Bolidomonas_pacifica*/IPP1
XLFSTFGIFFFLLLPILNAFTFTLRFHVPPMQFARGSKRFLPSTPSFLRYRASVPLLSSCGY
RVESRLFSAYGAGLSQEDMMKNDMLILTGNDDSIIGPESKKVAHTFNESSPRGGVHRA
FSVFLFNSKNEMLITKRAACKITFPNVWTNACCSHQLYNQSPEEVDHNSPEDVASRAG
TVESNGSKNAALRKLYHELGIRSIPHSSFRFLTRFWYWASDTGTYGEEAPWGEHEIDYI
LFARQDGVEVVMNEEEVDDIAWVSKDKLREMMNDPELKWSPWFVGIMNMKGFTYW
DNLDSIIDGDGNDFQDDTIX
>*Chaetoceros_sp*/IPP1
MSRNNWNGNSMSQQDFMEKDTVLVLDYDDKVIGSESKRASHEFNLEQPRGVLHRAFS
VFIFDESTNELLLQQRAKEKITFPSVWTNTCCSHPLHGMSPKEVDTPEDVAAGNVPGA
KYAAIRKLEHELGIPPSQLPLEKFKFLTRLHYWAADTVTHGPKSPWGEHEIDYVLFFVV
KSKNKITIVPNREEVEDVKWVTQQQLLDMMNDSSLLFSPWFRIIAKKWLLGANGWWI
DLEETIKTDKYCDYVTIHRXDPPKEHLGGLGNAACQFTSTGSTMTTGDVCKKQGAYG
KIKTHKESKLSQLSHLDEVWSAITLLYIKPLKSNLQSDHVVNTFDASDLEFCDDILCKVS
RSFAAVIRQLPATLLVDVLIFYLVLRALDTVEDDMTAFESNEIKIQYLLKFHETALVDPT
WSMDGVGEADEKRLLQEFPKCHSVYAALKSESKDVIANITKRMAEGMAEFVGKDLG
QGTVDVKEYNRYCHFVAGLVGEGLSRLFAASGLEDASLAKELYLSDQMGLFLQKTNII
RDYLEDYVDGRAFWPQSVWKKYSKTGDLGYFANQSDEDAKYHALCCLNELVTDALH
LVPDCLKYLSKLQCCEVFRFCAIPQVMAIATLDKCYHNKDVFTGVVKIRKGMSCKLIN
NTHDVXGVHAVFYEFAKSIANKARHAKASGFVDPTFESTIQICKKICDLTQGEVQKMK
KMRTVELSLALVAGW
>*Coscinodiscus_wailesii*/IPP1
MTTTWDGNAMTQTDMMIKDTVLVLDENDNVIGSASKKKSHDFSIENPRGILHRAFSVF
MFDEDSGELLLQKRASSKITFPNVWTNTCCSHPLHGMSPPEIDDPSTILTGQTTGVINGA
IRKLEHELGIPPSQLRADKFKFLTRFHYWAADTVTHGPDAPWGEHEIDYVLFYLCDKK
ELTVVPHPDEVDDVKWVSREALVKEMEVGLWSPWFRILANKWLLNEGGWWEDLAK
TMAMDPDESILRFDPPAEHMGGKGKAGAWLGTV
>*Cylindrotheca_closterium*/IPP1
KDTVVVLDDEDNIIGSASKKESHVFNTEQPRGILHRAFSVFLFDESTGELLLQQRASTKI
TFPNVWTNTCCSHPLHGMEPAEVDTPEDVANGTVMGAKYAAIRKLDHELGIPSNQLP
VEKFKFLTRLHYWAADTVTHGRDSPWGEHEIDYVLFFCVKNKSELTIKPHPDEVDDIK
WVSTQKLDEMMKDSSLLFSPWFRLICKKWLVTSWWKNLKQTMTTNDHCDYATIHEF
DPPKEHLGGNGKAGPLFEEKQGDESKKQGAYGKVKTHKEPMLKQLSHLDEVFAAFYF
LKVNPLKSNLETPFIEENFGADDLAFCDDILVKVSRSFAAVIRQLPSSMLVDILIFYLVLR
ALDTIEDDTTAFESNDIKIKHLANFRKTALGDPTWTMDGVGEGDEKRXKISTSVTXVY

AKLSEKSQSVIADITQRMADGMAEFVGKDLGQGTVDVEQYNRYCHFVAGLVGEGLSR
LFAASGLEKQSFAGELFLSDQMGLFLQKTNIIRDYLEDYVDHRAFWPQTIWKKYSKTG
ELGYFTRQEDEEVRERSLQCLNELVSDALELVPDCLAYLSKLQCKQVFRFCAIPQVMAI
ATLDKCYSNSNVFTGVVKIRKGTSCKLILRTNTLDEVHDTFYQFSQAIVGKAMAKRRM
GVVDPSYERTMKACETIQELTASGHRRQTSKRRMPFVLGGLLLAASLGSKHIPVDFPG
MQQSLLLLGIGMLSFGPYAVSQESDLKDANALQ

>*Fragilariopsis_kerguelensis*/IPP1

MKTSILLGILLRTNHCVTSFSSSLITPKSRSRHAIVTAKASTLLPALAISTPVDSTSEKGFI
ASSSLSHAQENIDKYDNDSAVYGADMNQEDMMESDLLVVVDHNDNVIELSDKQPISK
KAAHTFSEEQPRGVLHRAFSLFCFNEDGKLLLTRRAADKITFPAVWTNTACSHPLLKM
PLSEVDYWPDAFPQMPGIKRAAARKSKHELGLNIQPFLMDVQFVSRFHYWASDVQTH
GLEAPWGEHEVDYILFLQCPVQEFEIKVNPEEVADIKYVSIVELKDMLYNQPELKWSP
WFVGIMERGGFTWWEDLENTLQGKYTNRDIQFFDPLPNHVAKYNLPQHTRDKTGVW

>*Leptocylindrus_apora*/IPP1

MTQEQFMQKDTVLVLDEQDNVIGSASKKDSHVFHDEHPRGILHRAFSVFLFNEKDELL
LQQRASSKITFPYVWTNTCCSHPLHGLEPGEVDGPKDVADGSVMGVKHAAVRKLWH
ELGIPAEQLPVEKFKFLTRLHYWAADTVTHGKGSPWGEHEIDYVLFIKVPQDSLTINPR
PDEVEAVQWVSKKQLLHLLSVASDETTITEGNKNSLWSPWFRLIAYRWLLPDGTGWW
DNVDDVLHTDKFNDYENVHKFDPPKEHMGGGGNAGEWLGQLQ

>*Leptocylindrus_danicus*/IPP1

MTNNSTTNTATTAATWTGSGLTQSDLMTKDTVLVLDEAXNVIGSASKKESHVFDATT
NTQRGILHRAFSVFLFNERGELLLQQRAASKITFPNVWTNTCCSHPLHGLEPGEVDGPQ
DVQDGSVMGVKHAAVRKLWHELGIPAEQVPIEKFKFLTRLHYWAADTVTHGKESPW
GEHEIDYVLFIQVEQSKLTINPHPDEVDAIKWVSKEQLIEEMKVGLWSPWFRLIVNKW
VIPVWWNDLKMTLETDVHCDYENIHRFDPPEEHMGGGGNAGPWLGKLEIGSTGTISSS
SSTSNFLKLFATLGXAIAAVSSCVLHLYPELIESLPFEFSPVFEQTVALCITSLLVSTFYVV
SVQSQLKRQIMNMPEVPPEIPVLGSIKSFLTNVPWDLMENWHKQYGGIYRYTLLGRTC
VALADPAHLRIVLQSKIKHVKKDVDFAYKPFLPILGKGIVTSEGKSWMRQRLSVSSVLR
IDVLDEIPRITLGAVQRLCAKLDAAAESGEVIEMAEELRHLTLQVISETFFSLPAEESNTT
FAQMYLPIVEEGNKRVWHPERNFAFFLPFFWKHKAAVARLNSYVSKLVVERWEQRRS
EKTSNDPKRKEDVLDRIISAYDKEDAPKWGKAVVNQLRDELKTFMLAGHETSAAMM
TWALYELVSNEELMETVTKEGASVFGADYDWCKDGVNADYLPSREELGKLELSEGCL
RESLRKYSVVPTVTRKAVENIQVGSHTIPKGSTIMINIQAVHHDAKLWPDPMKFDPTRF
CDPSPQPKPYTFIPFIEGPRNCLGQYLALLESKMVLSMLLQRYSFTLQSGGEPRHPYMV
PVIPKTGVFVTVKRKF

>*Nannochloropsis_gaditana*/IPP1

HRYDHLNNMPRFKLSSIFSPSRKMTQEDFMRKDECLVVDDRDLLLRTENKYNCHRFTP
EQPRGLLHRAFSVFLFDKTGRLLLQQRAASKITFPQVWTNTCCSHPLSGFTPTEVDMPE
DLANGKVKGVKRAAIRKLQHELGIPSFQVPLRKFKFLTRLHYWAADAATHGPAAEWG
EHEIDYVLFIQVPTVTLRPNPEEVNAVKFVSLPELQEMMHPSSGLRWSPWFRIIAERFLP
LWWADLKLTLRTNTYFDPTTIHRFEPPTDPTGNISPSEFAQDTAASAPSGGLVPMGRAS
ASPTLPPAFGKGDEGKKQGAYGKIKSHSESLVSQLLRVDEVGAALRLKFARPFPETDV
GHAGDADVRFCADMLGRVSRSFAAVIRQLPKGLCLETVVFYLVLRALDTVEDDMEAF
QKDPGAKLRHLRGFYHVALEDPEFKMHGVGKGDEARLLENFDRVVRVFQGLTPGMR
TVIKDITRRMGAGMADFVDKDLGQGTISLQEYDLYCHYVAGLVGEGLTRMFAESGLE
APDLATSRQELANSMGLFLQKTNIIRDYLEDFVDGRAFWPQEVWKRHAPSAVPAQSLG
AFAEAEHRERALACLDELVTNALEHVPDCLAYMSRLHTPQIFRFCAIPQVMAIATLEKV
YHNPDVFTGVVKIRKGLACKLIMEANSMGDLYTCFSSFAGKIARRAAALEKRGDSVSA
QTLRVCEEIRVLCKEGEASSTNTKKYARALHAAFAALLLAIALVPVLALWGTESSRGG
FFARPIESPAGLSGADYVQMAFNVLVSVVATLALSYVVSKVWRPLGGQGNRSRSLAA

>*Nitzschia_punctata*/IPP1

AKELPIEKFKFLTRLHYWAADTVTHGNESPWGEHEIDYVLFFCVKSKDELTIKPHPDEV
DAYKWVTRQGLIDMMEDKSLLFSPWFRLICKKWLLXSWWKDLNTTMTTDKYCDYV
NIHCFDPPKEHLGGGGKAGPLFEKEAEGDESKKQGAYGKVKTHKESTLKQLSHIDEVF
X

>*Nitzschia_sp*/IPP1

MSTKTWDGNAMSQEDMMKKDTVLVLDDKDNVVGSASKRTSHEFEDGQPRGILHRAF
SVFLFDESTGELLLQQRASTKITFPNVWTNTCCSHPLHGMEPNEVDKPEAVADGSVLG
AKYAAIRKLDHELGIPASDLPVEKFKFLTRLHYWAADTVTHGDESPWGEHEIDYVLFF
CVKSKSELTVKPHPDEVDACKWVTRQGLIDMMQDKSLLFSPWFRLICKKWLLDSWW
ANLETTMTTDKYCDFVNIHRFDPPKEHLGGLGKAGPLFDAPTEGDASKKQGAYGKLK
THKESKLKQISHIDEVFAAVYFLYVKPLKSNLDTEYIRESFGADDLAFCDEILVKVSRSF
AAVIRQLPSTMLVDILIFYLVLRALDTIEDDTTSFPSQEVKIKHLLNFHKTALGDPTWTM
DGVGEADEKRLLQNFDKCHRVFAKLREPSRRVIADIAQRMATGMAEFVSKDLGQGTT
DVDQYNRYCHFVAGLVGEGLSRLFSASGLEKASFASELYLSDQMGLFLQKTNIIRDYL
EDYVDHRAFWPQTIWKKYSKSGDLGYFTNQDDPEVRKNSLACLNELVTDALELVPDC
LSYMSKLQCAEIFRFCAIPQVMAIATLEYCYANSNVFTGVVKIRKGSSCKLILNTNNLD
EVHDTFYHFANCLLKTANTNRAKGVDXPXYARTVKICETIMETTSPAKTRLDKARRM
PLIASSILVAGVGATMLKSDRPSSKTPLWQQGLLLLALGVATFGPSMIRKPSPSNLIEAK
DLEEKLMSSPQSRSKTNSSRNDETDSDEKKEAK

>*Phaeodactylum_tricornutum*/IPP1

MRRMPGKIGAMIILGAAWIGRVAGLAPSAKSRQFMVAQSWGLRAIKDRCATCLFSSN
AKSNDKAYGVNMDQEAMMESDRLIAVNENDRLVRNVNLSKRNGHTFNKETPRAALH
RAFSFFLFDDQDRMLLTQRAGTKITFPNVWTNTCCSHPLYDMTPNEVDVADDAYPMF
PGIKHAAVRKCKHELGIAPENLPKEDIQFLTRFHYWAADTVTYGDDTAWGEHEIDYIL
FLQVNGQVPVDANPDEVSDYKYVSMEEMKDMMEDPDLLWSPWFRGIMNRGGFEWW
ADLRGSLQGKYTNLDVTFFDPPKEHYASYNLPSHERKTGVLSSSTVEQT

>*Pseudonitzschia_australis*/IPP1/IPP1

MQLPLQISVAFLLQKHSLAFGLLSPHSKSSPLSPLSVAAPLTEIDEKNIEIFNIDSLDYGA
DMDQEDMMESDLLVIVDENDNVIELSEDQSVSKKAAHSFSNEQPRGILHRAFSLFCFNE
DGKLLLTQRALDKITFPGVWTNTACSHPLMEMPLSEVDVWPGAYPEMPGIKNAAVRK
AKHELGLDIRSLIPDVQFVSRFHYWASDVQTHGIEAPWGEHEVDYILFLKSSVKEFKLE
VNPEEVGDVMYVSISELKDMIYNQPDLTWSPWFIGIMERGGFDWWEDLENTLQGKNT
NEDIQFFDALPDHVATYNIPEHSRQTGVWKN

>*Pseudonitzschia_arenysensis*/IPP1

MRLHSRIPLSLLLLGGCSKGLLSRHNNPRTFSLGSAPAAESSYGADMNQEDMMESDFL
VIVDHNDNVIEVSEEVSKKAAHSFSSDQPRGILHRAFSLFCFNDQGKLLLTRRASDKITF
PGVWTNTACSHPLLQMAKSEVDVWPEAFPELSGIKNAATRKAQHELGLSLEQADMQF
VSRFHYWASDVETHGSETPWGEHEVDYILFLQAPLAEFELDVNAEEVAEVRYVSIDEL
KDMLYNQADLTWSPWFVGIMERGGFDWWQDLENTLAGKNTNEDIVFFDPLPDHVAS
YNKPDHTRQSTGIW

>*Pseudonitzschia_delicatissima*/IPP1

MRLPLRISLSLLLGGCSLTFGLIPHHTPRSLTTTSSLSSSAAAESSLEYGADMNQEDMME
SDLLVIVDHNDNVIELSEDXSVSKKAAHSFSSDQPRGILHRAFSLFCFNQDGKLLLTRR
ATDKITFPGVWTNTACSHPLLQMPNSEVDHWPAAFPEMPGIKNAAARKAKHELGLVV
EPKDMQFVSRFHYWASDVETHGPEAPWGEHEVDYILFLQAPVQEFPLNINSEEVGDAE
YVSIAELKDMLYNQPELTWSPWFVGIMERGGFDWWEDLENTLAGKNTNQDITFFDPL
PDHVASYNTPEHTRQTTGVWKNE

>*Pseudonitzschia_multistriata*/IPP1

MATQTWDGNAMSQEDMMMKDTVLVLDDDDNVIGSASKRKSHEFTPEQPRAILHRAF
SVFIFDESTGELLLQQRASSKITFPNVWTNTCCSHPLHGMEPPEVDGPKDVADGTVMG
VKNAAIRKLDHELGIPGSELDINKFKFLTRLHYWAADTITHGPDSPWGEHEIDYVLFYT

VSSKSDLTIKGHPDEVDDVKWVTRDVLIEMMEDKSLLFSPWFRLICKKWLLDHWWKD
LKRTMTTDDHVDLKSIHCFDPPKEHLGGGGKAGPLFGAEVEGDER
>*Pseudonitzschia_pungens*/IPP1
MRLTIKILVAFMLQTQSRGFGIVPRRHESSRSLPLSASAMTKDEKNIGSVNIDSADYGA
DMNQEDMMESDLLVIVDHNDNVVELSEDQTVSKKAAHTFSEEQPRGILHRAFSLFCFN
DDGKLLLTQRASDKITFPGVWTNTACSHPLMQMPASEVDIWPDAFPEMPGIKRAAAR
KAKHELGLDIQPLLPGVQFVSRFHYWASDVQTHGPDAPWGEHEVDYILFLKAPVKEFV
LDVNPEEVTAVKYVSISELKDMLYNRSDLTWSPWFVGIMERGGFDWWEDLENTLQG
KNTNTEIQFFDPLPSHVATYNSPEHTRQTGVWKNK
>*Pseudonitzschia_pungens_fr_cingulata*/IPP1
FSLTLKNADEYSIMRLTIKILVAFMLQTQSRGFGIVPRRHESSRSLPLSASAMTKDEKNI
GSVNIDSADYGADMNQEDMMESDLLVIVDHNDNVVELSEDQTVSKKAAHTFSEEQPR
GILHRAFSLFCFNDDGKLLLTQRASDKITFPGVWTNTACSHPLMQMPASEVDVWPDAF
PEMPGIKRAAARKAKHELGLDIQPLLPGVQFVSRFHYWASDVQTHGPDAPWGEHEVD
YILFLKAPVKEFVLDVNPEEVTAVKYVSISELKDMLYNRSDLTWSPWFVGIMERGGFD
WWEDLENTLQGKNTNTEIQFFDPLPYHVATYNSPEHTRQTGV
>*Skeletonema_costatum*/IPP1
MGNKQSSSSSSSTTNKKWDGNAMSQNDFMERDTVLVLNNDDIVIGSESKKASHVFST
DQSRGVLHRAFSVCLFDSSTGEMLLQKRASTKITFPNVWTNTCCSHPLHGMEVNEVDG
PEDVKDGSVKGVKAAAVRKLEHELGIPVGELPIENFKFLTRLHYWAADTVTHGEKSP
WGEHEIDYVLFITVPTKDSITLKPHPDEVDDVCWVTQSKLLEMFANDDLLFSPWFRLIA
NRWMIGGGENGKGGWWDDLDRTMNTDDFCDYENIHRFDPPTEHLGGGGDAGPWLD
TKEAPSANGTVEDTVGDASKKQGAYGKIKTHKESKLSQLMHIDEVCSAISLLYINPLKS
NLESDNIKKSFDTDDXAFCDDILCKVSRSFAAVIRQLPSELLVDVLIFYLVLRALDTIED
DMTFFASNDEKVKVLLAFHKTALVDPEWKMMGCGEGDERRLLEEFPKCHSVFAALRE
DSRRVISDITLRMATGMAEFVGKDLGQGTVDVQQYNRYCHFVAGLVGEGLSRLFSAS
GLEKPALATELYLSDQMGLFLQKTNIIRDYLEDYVDGRAFWPQTVWKKYSASGDLGY
FANPATVEHETAALNCIDELVTDALELVPDCLSYLSKLRCAEVFRFCAIPQVMAIATLD
KCYHNKDVFSGVVKIRKGMSCALINGTTDINGVHSIFHRFASSILRQAELSRKKGFEDPS
YERTVKACTTILELTETEAKRSGSSVVGSTLMLSTAAVGCLTYKYPNGSNTTALTTAIA
SXGMFSLFKTSFMKYVGKKGVSSLMPASKLTEKKEG
>*Skeletonema_marinoi*/IPP1
MSQSDFMERDTVLVLSDNDEVIGSASKKESHQFTPKNPRGILHRAFSVFLYDLSTQXAI
EDVMDGTVKGVKAAAVRKLEHELGIPVGQLPMDSFKFLTRLHYWAADTVTHGEKSP
WGENEVDYVLFVTVLNKDKITIEPHVDEVDDVCWVTKAKLFEMFADKDLLFSPWFRII
ANRWMISSGEETGKGGWWDDLDRTMNT
>*Skeletonema_menzelii*/IPP1
MSQSDFMERDTVLVLSDNDEVLGSASKKESHLFTPTNPRGILHRAFSVFLYDASTKELL
LQQRASTKITFPECMDEYLLFXXXXXXXXXXXHGMDVNEVDSAEDVKGGTVNGVKA
AAIRKLEHELGIPVGQLPMDSFKFLTRLHYWAADTVTHGEKSPWGENEIDYVLFATVP
CKDSITINPRPDEVDDVCWVTQSKLLEMFADKDLLFSPWFRIIANRWMISKGGWWDDL
DRTMTTNDFCDYESIHRFDPPQEHLGGGGNAGPLFVAEK
>*Thalassionema_frauenfeldii*/IPP1
MMESDLLIPVGSNDELISSMDVTKKKAHIFSKEGQPRGVLHRAFSFFLFDDQNRMLVT
KRASSKITFPGVWTNTCCSHPLQKMKPEEVDDPLEEKNSIEFRYDGVKHAARRKLRHE
LGIDPIHIPHEHIQFITKFHYWAADTLNYGTDNPPWGEHEVDYILFFKHKGSEPIPVLPCE
DEVEDYRYVTQDEFKDMLKDIPAKNWSPWFRGILDRGLWQWWDDLDHGTLTGKHS
NNDVIFFDPPKEYYASFNNDPSHNRQTGVLNAASV
>*Thalassiosira_minuscola*/IPP1
MPLSFSRSPLRRAPQRRNTAPLLLGLSILLLVIYYARRKIIDIDTPQLRKYEVPSSSNHAQ
GGELLNVYELPPSHATEPAHLTPVDPTTLVPKGTHPIDVAHRNGYLHTGHILYIMDDSG

SLLFLQRTPGVVTCPSTWSLLGEHANANESPRETVLRALEEELGFVGLNFDDFKNAGA
FTAELHPSHNLKNSLTVTINTVTDLPLYYIRHYGPRNDDRIDRQLTYLWVVNFPKKHAE
IDWKFDDEVADHKWVTLEEAASWLSGDGRNGKEDGEAKGGTGSDSAKEYGSDWKE
VKDDGPDEGEFCHHTIRSLYEVGLSHMI

>*Thalassiosira_pseudonana*/IPP1

MAPTTKKWDGTAMTQTDFMERDTVLVLSNDDTVIGSESKRSSHEFTPTQPRGVLHRA
FSVFLFDSSTNELLLQKRASTKITFPNVWTNTCCSHPLHGMDVNEVDGPEDVKDGSVM
GVKAAALRKLEHELGIPVGELPVEGFKFLTRLHYWAADTVTHGKKSPWGEHEIDYVL
FITIPSKSTLTLKPHPDEVDDTRWVTQSQLLEMFADESLLFSPWFRLITNRWMIGEGSEK
NVNGGKGGWWDDLKRTMETNDFCDYVNIHRFDPPSEHMGGGGDAGPMFDGAAVVG
DASKKQGAYGKVKTHKESTLKQLVHLDEVCSAITLLYLQPLKSNLESDVIKQTFDPSD
LAFCDEILCKVSRSFAAVIRQLPPTLLMDVLIFYLVLRALDTIEDDMTFFDSNEDKVKIL
QSFHKTALVDPNWKMMGCGEGDERRLLEEFPKCHSVYAALPEGSRRVISDITQRMAT
GMAEFVDKDLGQGTVDVQQY

>*Thalassiosira_puntigera*/IPP1

MRSRISLPAAMMMQRRHYRFFYGGISSTATGSRSNYHEAFASLALRKRQIASRTRRHV
TCSAIVSNWDGTSMSQAQFMERDTVLVTDENDILIDSASKKSSHEFTPSQPRGILHRAFS
VFLFDASTSELLLQKRASTKITFPNVWTNTCCSHPLHGMEKDEVDGPDDVRDGSVGGV
RHAAVRKLAHELGIPMEELWAMEREFKFLTRVHYWAADTVTHGEKSPWGEHEIDYIL
FATIPSKNKLTLKPHPEEVDDTRWVTQSQLLGMFDDGTLLFSPWFRLIAQRWMIGKTT
NVGQEGWWD

>*Thalassiosira_rotula*/IPP1

XASANEDEDDEDDDDASMATGGSRSKFCGAFGSPAAWNRQIIASTSRRNHFTSTAIAS
VSNTNINMNNNNQASSPSSPSTSWDGTSMSQAQFMERDTVLVTDHNDIVTSSATKKDS
HIFNPTNPRGILHRAFSVFLFDASTSELLLQKRASTKITFPNVWTNTCCSHPLHGMEPNE
VDGVEDVKDGSVGGVRNAAVRKLGHELGMDKGELETMSEEFKFLTRVHYWAADTV
THGEKSPWGEHEIDYILFATIPTKNSLALNPHPEEVDDIRWVTQSQLLQMFDDETLLFSP
WFRIIANKWMVGGGGWWDDLDRTMNTNAFCDYETIHRFDPPEEHMGGEGDAGPWL
CDPE
