# Supplementary material for: Transcriptome sequencing of three Pseudo-nitzschia species reveals comparable gene sets and the presence of Nitric Oxide Synthase genes in diatoms
Source: Sci Rep. 2015 Jul 20;5:12329. doi: 10.1038/srep12329 (PMC4648414; doi:10.1038/srep12329)
Supplement: Supplementary Table S15 [file srep12329-s13.doc]

| **Supplementary Table S15. List of diatom species present in the MMETSP transcriptomes database that were searched to identify the presence of a putative NOS sequence.** PmNOS was used as a query in blastp searches in the peptides file of each of the transcriptomes. The same was done in the genomes of *P. tricornutum* and *T. pseudonana*. The functional annotation of the top blastp hit is reported. A transcript ID is given only when the predicted protein product corresponded to a canonical NOS. | | | | | | |
| --- | --- | --- | --- | --- | --- | --- |
| SPECIES | MMETSP TRANSCRIPTOME ID | STRAIN | TREATMENT | Functional annotation of the top blastp hit | TRANSCRIPT ID | NOTE |
| *Amphiprora sp.* | MMETSP0724_20121128 | CCMP467 | Exponential | Cyanobacteria NOS, NADPH cytochrome reductase | 10308 | Sequences from all conditions are equal. For the phylogenetic tree, the sequence from the exponential phase was used. |
| *Amphiprora sp.* | MMETSP0726-20121227 | CCMP467 | N Limited | Cyanobacteria and vertebrate NOS, Nos Oxygenase domain, NADPH cytochrome reductase, Ferredoxin NADP oxidoreductase | 1139 |
| *Amphiprora sp.* | MMETSP0727-20121128 | CCMP467 | Nocodazole | Cyanobacteria and vertebrate NOS, Nos Oxygenase domain, NADPH cytochrome reductase, Ferredoxin NADP oxidoreductase | 3446 |
| *Amphiprora sp.* | MMETSP0725-20121227 | CCMP467 | Silica limited | Cyanobacteria and vertebrate NOS, NADPH cytochrome reductase, Ferredoxin NADP oxidoreductase | 2683 |
| *Chaetoceros cf. neogracile* | MMETSP1336-20130426 | RCC1993 |  | NADPH cytochrome reductase Ferredoxin NADP oxidoreductase | NF |  |
| *Chaetoceros cf. neogracile* | MMETSP0751-20121128 | CCMP1317 | Exponential | Cyanobacteria and vertebrate NOS, Nos Oxygenase domain, NADPH cytochrome reductase, Ferredoxin NADP oxidoreductase | 2974 | Sequences from all conditions are equal. For the phylogenetic tree, the sequence from the exponential phase was used. |
| *Chaetoceros cf. neogracile* | MMETSP0753-20130528 | CCMP1317 | N Limited | Cyanobacteria and vertebrate NOS, Nos Oxygenase domain, NADPH cytochrome reductase, Ferredoxin NADP oxidoreductase | 54073 |
| *Chaetoceros cf. neogracile* | MMETSP0752-20121128 | CCMP1317 | Silica Limited | Cyanobacteria and vertebrate NOS, Nos Oxygenase domain, NADPH cytochrome reductase, Ferredoxin NADP oxidoreductase | 5312 |
| *Chaetoceros cf. neogracile* | MMETSP0754-20121128 | CCMP1317 | Nocodazole | Cyanobacteria and vertebrate NOS, Nos Oxygenase domain, NADPH cytochrome reductase, Ferredoxin NADP oxidoreductase | 3199 |
| *Chaetoceros sp.* | MMETSP0200_2-20121128 | GSL56 |  | Ferredoxin NADP oxidoreductase, NAD reductase | NF |  |
| *Coscinodiscus wailesii* | MMETSP1066-20121228 | CCMP2513 |  | Nos Oxygenase domain, NADPH cytochrome reductase, Ferredoxin NADP oxidoreductase | NF |  |
| *Cylindrotheca closterium* | MMETSP0017_2-20120614 | KMMCC:B-181 |  | Cyanobacteria NOS | 23005  29146 |  |
| *Fragilariopsis kerguelensis* | MMETSP0733-20121128 | L26-C5 |  | Nos Oxygenase domain, NADPH cytochrome reductase, nitrate reductase | NF |  |
| *Leptocylindrus danicus var apora* | MMETSP0322-20121206 | B651 |  | NADPH cytochrome reductase, Ferredoxin NADP oxidoreductase | NF |  |

| *Leptocylindrus danicus var danicus* | MMETSP0321-20121206 | B650 |  | Nos Oxygenase domain, NADPH cytochrome reductase, FAD-binding and Oxidoreductase FAD NAD(P)-binding domain | NF |  |
| --- | --- | --- | --- | --- | --- | --- |
| *Leptocylindrus danicus var danicus* | MMETSP1362-20130617 | CCMP1856 |  | NADPH cytochrome reductase, Ferredoxin NADP oxidoreductase | NF |  |
| *Nitzschia punctata* | MMETSP0744-20121128 | CCMP561 | Exponential | Nos Oxygenase domain, NADPH cytochrome reductase, Ferredoxin NADP oxidoreductase | NF |  |
| *Nitzschia punctata* | MMETSP0746-20121128 | CCMP561 | N Limited | Nos Oxygenase domain, NADPH cytochrome reductase, Ferredoxin NADP oxidoreductase | NF |  |
| *Nitzschia punctata* | MMETSP0747-20121128 | CCMP561 | Nocodazole | Nos Oxygenase domain, NADPH cytochrome reductase, Ferredoxin NADP oxidoreductase | NF |  |
| *Nitzschia punctata* | MMETSP0745-20121128 | CCMP561 | Silica Limited | Nos Oxygenase domain, NADPH cytochrome reductase, Ferredoxin NADP oxidoreductase | NF |  |
| *Nitzschia sp* | MMETSP0014_2-20120614 | RCC80 |  | NADPH cytochrome reductase, Ferredoxin NADP oxidoreductase | NF |  |
| *Phaeodactylum tricornutum* | Phatr/ASM15095v1/JGI |  |  | NADPH cytochrome reductase, FAD-binding and Oxidoreductase FAD NAD(P)-binding domain | NF |  |
| *Pseudo-nitzschia arenysensis* | MMETSP0329-20121206 | B593 |  | NADPH cytochrome reductase, FAD-binding and Oxidoreductase FAD NAD(P)-binding domain | NF |  |
| *Pseudo-nitzschia australis* | MMETSP0142_2-20121227 | 10249 10 AB | Glutamate as N source | NADPH cytochrome reductase, FAD-binding and Oxidoreductase FAD NAD(P)-binding domain | NF |  |
| *Pseudo-nitzschia australis* | MMETSP0139_2-20121125 | 10249 10 AB | NO3 as N source | NADPH cytochrome reductase, FAD-binding and Oxidoreductase FAD NAD(P)-binding domain | NF |  |
| *Pseudo-nitzschia australis* | MMETSP0140_2-20121125 | 10249 10 AB | NH4 as N source | NADPH cytochrome reductase, | NF |  |
| *Pseudo-nitzschia australis* | MMETSP0141_2-20121125 | 10249 10 AB | UREA as N source | NADPH cytochrome reductase, FAD-binding and Oxidoreductase FAD NAD(P)-binding domain | NF |  |
| *Pseudo-nitzschia delicatissima* | MMETSP0327-20121206 | B596 |  | NADPH cytochrome reductase, FAD-binding and Oxidoreductase FAD NAD(P)-binding domain | NF |  |
| *Pseudo-nitzschia fraudulenta* | MMETSP0851-20130426 | WWA7 | Si-Limited 390ppm | NADPH cytochrome reductase, FAD-binding and Oxidoreductase FAD NAD(P)-binding domain | NF |  |
| *Pseudo-nitzschia fraudulenta* | MMETSP0850-20130426 | WWA7 | Si-Replete 390ppm | NADPH cytochrome reductase, FAD-binding and Oxidoreductase FAD NAD(P)-binding domain | NF |  |

| *Pseudo-nitzschia fraudulenta* | MMETSP0853-20130426 | WWA7 | Si-Limited 750ppm | NADPH cytochrome reductase, FAD-binding and Oxidoreductase FAD NAD(P)-binding domain | NF |  |
| --- | --- | --- | --- | --- | --- | --- |
| *Pseudo-nitzschia fraudulenta* | MMETSP0852-20130820 | WWA7 | Si-Replete 750ppm | NADPH cytochrome reductase, Ferredoxin NADP oxidoreductase | NF |  |
| *Pseudo-nitzschia heimii* | MMETSP1423-20130617 | UNC1101 |  | NADPH cytochrome reductase, Ferredoxin NADP oxidoreductase, FAD-binding and Oxidoreductase FAD NAD(P)-binding domain | NF |  |
| *Pseudo-nitzschia pungens cf pungens* | MMETSP1061-20121228 | pungens |  | NADPH cytochrome reductase, FAD-binding and Oxidoreductase FAD NAD(P)-binding domain | NF |  |
| *Pseudo-nitzschia pungens cf cingolata* | MMETSP1060-20121228 | cingolata |  | NADPH cytochrome reductase, FAD-binding and Oxidoreductase FAD NAD(P)-binding domain | NF |  |
| *Skeletonema costatum* | MMETSP0013_2-20120614 | 1716 |  | Cyanobacteria and *Ostreococcus* NOS, NADPH cytochrome reductase, FAD-binding and Oxidoreductase FAD NAD(P)-binding domain | 464  4488 |  |
| *Skeletonema marinoi* | MMETSP0920-20130426 | Skel A | Si-Limited | Nos Oxygenase domain, NADPH cytochrome reductase, FAD-binding and Oxidoreductase FAD NAD(P)-binding domain | NF |  |
| *Skeletonema marinoi* | MMETSP0918-20130131 | Skel A | Replete | Nos Oxygenase domain, NADPH cytochrome reductase, FAD-binding and Oxidoreductase FAD NAD(P)-binding domain | NF |  |
| *Skeletonema marinoi* | Skeletonema-marinoi | SkelA-20130924 | Combined Assembly | Nos Oxygenase domain, NADPH cytochrome reductase, FAD-binding and Oxidoreductase FAD NAD(P)-binding domain | NF |  |
| *Skeletonema marinoi* | MMETSP1039-20121108 | FE7 |  | Cyanobacteria and *Ostreococcus* NOS, NADPH cytochrome reductase, FAD-binding and Oxidoreductase FAD NAD(P)-binding domain | 3976  3419 | #3976 is equal to #3919;  #3419 is equal to #15944.  Sequences from both strains are equal. For the phylogenetic tree, the FE60 sequences were used. |
| *Skeletonema marinoi* | MMETSP1040-20121108 | FE60 |  | Cyanobacteria and *Ostreococcus* NOS, NADPH cytochrome reductase, FAD-binding and Oxidoreductase FAD NAD(P)-binding domain | 15944  3919 |

| *Skeletonema menzelii* | MMETSP0603-20130417 | CCMP793 | Replete | | *Ostreococcus* NOS, NADPH cytochrome reductase, FAD-binding and Oxidoreductase FAD NAD(P)-binding domain | 3077 | Sequences from both conditions are equal. For the phylogenetic tree, the sequence from the replete condition was used. |
| --- | --- | --- | --- | --- | --- | --- | --- |
| *Skeletonema menzelii* | MMETSP0604-20130417 | CCMP793 | N limited | | *Ostreococcus* NOS, NADPH cytochrome reductase, FAD-binding and Oxidoreductase FAD NAD(P)-binding domain | 1143 |
| *Thalassionema frauenfeldii* | MMETSP0786-20121207 | CCMP1798 |  | | Nos Oxygenase domain, NADPH cytochrome reductase, FAD-binding and Oxidoreductase FAD NAD(P)-binding domain | NF |  |
| *Thalassiosira minuscula* | MMETSP0737-20130205 | CCMP1093 | Exponential | Vertebrate, Cyanobacteria and *Ostreococcus* NOS, NADPH cytochrome reductase, FAD-binding and Oxidoreductase FAD NAD(P)-binding domain | | 46817  8790  66085 | #12094 is equal to #8790;  #67258 is equal to #66085;  #66346 is equal to #46817.  For the phylogenetic tree, the three sequences from the exponential condition were used. |
| *Thalassiosira minuscula* | MMETSP0739-20130205 | CCMP1093 | N Limited | Vertebrate and Cyanobacteria NOS, NADPH cytochrome reductase, FAD-binding and Oxidoreductase FAD NAD(P)-binding domain | | 12094  67258  66346 |
| *Thalassiosira minuscula* | MMETSP0740-20130409 | CCMP1093 | Nocodazole | Nos Oxygenase domain, NADPH cytochrome reductase, FAD-binding and Oxidoreductase FAD NAD(P)-binding domain | | NF |
| *Thalassiosira minuscula* | MMETSP0738-20130409 | CCMP1093 | Silica Limited | Nos Oxygenase domain, NADPH cytochrome reductase, FAD-binding and Oxidoreductase FAD NAD(P)-binding domain | | NF |
| *Thalassiosira pseudonana* | Thaps/ASM14940v1/JGI | CCMP1335 |  | Nos Oxygenase domain, NADPH cytochrome reductase, FAD-binding and Oxidoreductase FAD NAD(P)-binding domain | | NF |  |
| *Thalassiosira puntigera* | MMETSP1067-20121228 | C2 |  | NADPH cytochrome reductase, FAD-binding and Oxidoreductase FAD NAD(P)-binding domain | | NF |  |
| *Thalassiosira rotula* | MMETSP0403-20120918 | CCMP3096 | Light cycle | *Ostreococcus* NOS, NADPH cytochrome reductase, FAD-binding and Oxidoreductase FAD NAD(P)-binding domain | | 28511 | The three sequences are equal. For the phylogenetic tree, the combined assembly sequence was used. |
| *Thalassiosira rotula* | MMETSP0404_2-20130426 | CCMP3096 | Dark cycle | *Ostreococcus* NOS, NADPH cytochrome reductase, FAD-binding and Oxidoreductase FAD NAD(P)-binding domain | | 7832 |
| *Thalassiosira rotula* | Thalassiosira-rotula | CCMP3096-20130909 | Combined Assembly | *Ostreococcus* NOS, NADPH cytochrome reductase, FAD-binding and Oxidoreductase FAD NAD(P)-binding domain | | 54298 |

| *Thalassiosira rotula* | MMETSP0911-20130528 | GSO102 | Fe Limited | Nos Oxygenase domain, NADPH cytochrome reductase, FAD-binding and Oxidoreductase FAD NAD(P)-binding domain | NF | Sequences from all conditions are equal. For the phylogenetic tree, the sequence from the P limited condition was used. |
| --- | --- | --- | --- | --- | --- | --- |
| *Thalassiosira rotula* | MMETSP0912-20130531 | GSO102 | Fe Limited 2 | *Ostreococcus* NOS, NADPH cytochrome reductase, FAD-binding and Oxidoreductase FAD NAD(P)-binding domain | 49769 |
| *Thalassiosira rotula* | MMETSP0910-20130528 | GSO102 | P Limited | *Ostreococcus* NOS, NADPH cytochrome reductase, FAD-binding and Oxidoreductase FAD NAD(P)-binding domain | 46233 |
| *Thalassiosira rotula* | MMETSP0913-20130531 | GSO102 | Replete | Nos Oxygenase domain, NADPH cytochrome reductase, FAD-binding and Oxidoreductase FAD NAD(P)-binding domain | NF |
| *Thalassiosira rotula* | Thalassiosira-rotula-GSO102-20130909 | GSO102 | Combined Assembly | *Ostreococcus* NOS, NADPH cytochrome reductase, FAD-binding and Oxidoreductase FAD NAD(P)-binding domain | 7607 |
| *Thalassiosira weissflogii* | MMETSP0878-20121228 | CCMP1336 | Fe 20nM | Nos Oxygenase domain, NADPH cytochrome reductase, FAD-binding and Oxidoreductase FAD NAD(P)-binding domain | NF |  |
| *Thalassiosira weissflogii* | MMETSP0880-20121228 | CCMP1336 | Fe 20nM_2 | NADPH cytochrome reductase, FAD-binding and Oxidoreductase FAD NAD(P)-binding domain | NF |  |
| *Thalassiosira weissflogii* | MMETSP0879-20121228 | CCMP1336 | Fe 60nM | NADPH cytochrome reductase, FAD-binding and Oxidoreductase FAD NAD(P)-binding domain | NF |  |
| *Thalassiosira weissflogii* | MMETSP0881-20121228 | CCMP1336 | Fe 60nM_2 | NADPH cytochrome reductase, FAD-binding and Oxidoreductase FAD NAD(P)-binding domain | NF |  |
