# Supplementary material for: Transcriptome sequencing of three Pseudo-nitzschia species reveals comparable gene sets and the presence of Nitric Oxide Synthase genes in diatoms
Source: Sci Rep. 2015 Jul 20;5:12329. doi: 10.1038/srep12329 (PMC4648414; doi:10.1038/srep12329)
Supplement: Pseudo-nitzschia multistriata contigs [file srep12329-s14.pdf]

***Pseudo-nitzschia multistriata* contigs**

>comp162_c0_seq1
CATGTGGCCCTGGGAGTTGTGGATCTTTGCACTGGCACTAATTGTCTCGGCAGCGCTCCT
AATTTTACTCCACGCCCTCAAGGCCTGGAACGAGCGAATCCTGTTTGTCAAGCTAACGGA
AATGGGCCTGTGCATCCTCTTCTTTATCGCCCCGATGATCACCTCCAACTTTTTCCACAT
GCACCACTGGTATGCCGGCTGGCTGATGGGGATGCACTTTAACTACGATGTCTGGTGGAG
CCGGCTGGCCATGGCCTGGTGCTGGGGTATGTATGTCAACGGGGTCGCCGTGTACGGGCG
GGATCCCGTCATGACGTGCGAATACTCGTATTTTTTGTCCCTCGACAACCGGTGCCCCTA
CATTTCCTGCTACCTAGAGGGGCTTGCAGACGAGAACAATACGGAGGTGGTGGAAATGGT
TCCAGTCGACTGGAGAAATTGCTCGGCGACCACTGGTTTCATCCCCTGAGCAACTCGTCT
TGGAACGAGTCGATACCTGTAGAATGAATAATACAAAACCTTAAAAC
>comp235_c0_seq1
AAAGTCGACCGTCACAGCAAATGGAACACCAACTTCGTCGGAACGTGCGTAGCGGCGTCC
AAGAGCTGCGGTGGATTTATCAACACGAGTCGCAAGATCGTACTCCATGAGAGAAGCAGC
AATACCATCAACGATTGCATTGAATTCAGGACTGCTACTGATTGGGAGAACGGCACACTT
TTGCGGTGCAACTTGAGGTTTGAATTTCATGACACAACGTTGTTCGTCGTTCTCACGAAT
GTAGAAGGAGTGCTCCAAAAGAGAGTACAAAATACGTCCCATACCAAAAGAAGGCTCGAT
AACAGAGGGAGTAAATTTGAGTTCATGAACTTTTTTCGTTGACTGCTCCCAGCTAACCAT
ATCTTTCGTGACCTCAAATCCGTCAACGGTTACCTTTCCATCTGCCTCGAGAGCCTTCGC
AATGGGTTCAAAGTCCTTCCAACTCTCAGCCAATTTATCGAGTGCCCCAGATACTGTTCT
CGCATCCTTCTTGAAAACCATTCCGATCTTTTTCCGGTCGAATTTCAGTTTGGCGACCTC
GATTTCCTTAGGGGTGTCATACTTCACGGTAGCGACCATAGATGTGTTTGTCGCCTTAGC
ATGCACATCCAAATCGTAACAGGCACGATCGGCATGACCAACACATTCAACCCAACCGTA
GCTCGACTTGATTTCCAAATCCCAACAGTCAGCGGCATAGTGAGCCATTTCCGTGGCCAA
ATGCTGTCGAAAGCGAAGCCGTTCCGGGTCCATGCCGATCTTTTCCATATACAACTGGGT
TCGCGCCATAAAGTAACCCAATGTTTCGTTGTTGACCAAACCACCCTTGACGGCTTCTCC
GATCTTCAGCGTTTTGGTCTTACCCGTTCCCAACTGATCATCACGACCAAACAATACCAG
TTCCTTTTCAGCAACCGAAGAGAAATTGGGGTGCGATTTGTCGTCCGGGTTCACGAAATG
TTCAATTTCTCCCATGCAAAATTCTCGGACACGAAGAAGACCACTGCGAGGAGCAATTTC
TGTAGAGTTGGAATGAGAACAAAACAAATCCGCAAATTCGTTTTGTGAGTCGAAAACAAT
TTTTCGTTCCTTTCAATCGGTTTCCACTTCCTTCAAAATACTAACATACCGTTTCGGAAG
CCTAGTCCTATCTGAGCTGCTGCAAAAGGCATCTTTTGTGCGTTCAAGTCCAACAAACGA
CGGAAGTTCACAAACAGACCCTGGGCCGTTTCTGGTCGCAAATATCCAACTGATGTTCCC
TCCGGTCCGATGGAAGTTTTGAACATGAGGTTGAAAGGAAAGGATGGACCATAGGCTTCA
CCAGAGGGGCCC
>comp236_c0_seq1
TTTGCCTGGTTCCTACGGGCTTCGGAATTTTGGGAACGGTAGTAGTCGTGTTTGCTGATT
CCCTTTACGTAAAACGTGCAGTATCTCCTCCCGTAAACGTACGCATTGTCGAGCTGCCGA
TTGTTGAAGACCATCTCAAATGATTCAAAGACTTTGTCGGAGGCCTCGTAATATCGCTCG
ATTGGAAAGAAATTGCTCACGGTTGGAAC
>comp292_c0_seq1
GTGCCACATTTTGAGAATGTTTACCATGGTTTGTTCCGGGAATCTCTCGGGTACGTAGGG
GTTATTGGTTTTGTTTTCAGCCTTGTAGCGCTCCACGACTTCATAGAGATGGTCCTTCCG
GGCCTCTCGAAAGGATTCCTCGACATCGAAAGAGAAATAGACGCCGACCAATGGCATGTC
CAAATCTTTATGGAGCTTATGCACGGCGTCGCGAAGGAGAAACACCTCGGTGGAATTGAT
GTCCCCCC
>comp406_c0_seq1
TCCGCGCCGCCCTTGTGCGTTTCGATGAATTTTTTACAGCAGGTTGGTGCTCAACGGAAT
CGGCCCACGCCACGCCACGCCACGCCACGCGCACATCGACGATCGGAAACACACGGGCAA
CCCCGACGACCACCGCCACTTCCTACGGTTCCACAGGCGTTCCCTTGACCACCGCCACGT
GGGTTTCTTGGACCTTCCCATCGGGCCCCTGCGTGTAGGTCACCGTCTGCTCCTGGCGGG
GCCGCTCGTGGATCACGAGCGGCGGCGGCAACAAGAGGCAGCAGGCCCCGGCCCCGGC
>comp436_c0_seq1
CAGGACTTTCGCCATCTGGAGTTCCTCCAAACGCAGGCGTTCCTCCCAGGTTCTCTGAAT
TTCTCGCTGGCGGTTCACAAAGCAATTGCCGGCCCAGTATGCCGCGGATGCGACAAAGAG
AACCAATACTAGCAAGACTCGTTGCTGGGACGATAACAAGTACCTCCACCGGGAGACCTT
TGCCAGAGGCGAGGAGGAATCACTTTCGTCGTGCCCGTCGACCACGCTCACCCCGCC
>comp602_c0_seq1
CCCCAGCTCGTCGAAGAGTTCCCCTTTTGGACTGCAAAAGTGCAGATCTGAGTCTGTGTC

CTGTGGGATCCACAATGATACCCAATCTTGGCGACGGGTGCTTCCTTGTCGTTCGGAGAG
AAAAAGTAATGGAGAAAACATGAAACATATTCAGTGTTTTTCTTTGAGCGATTGTCACCC
CGTGGATAAACCTGAAGCTTCCAGCTGTATCCATGGGCGTGCAGGGTGGGAGAAATCTTG
AACTCTCC
>comp645_c0_seq1
GCGTATACATATCGTTCGATATAACGACATTGTGTCCGTTTCTCAAGTGGCCCTCCAGGG
CCTGCGAAAATTCTTCAAAATTGGATTGCATAGCCGATTCATGATTACTAGGAAAGGTGT
CGGCGTTGCGATAGACCTTAAACTCGGTGTCAACTCCCACGTTCACGTCCACTCTGGACG
ACTGTATCCCTCCCACAAAGTAAACGTTATCATCCAATGCCAACTTCTCGGCTACCTCGA
GCAATCCTTTCTTGATAGTCGTGGTGGCAGTTTTTGGTGGCCCAACATGTACGACCATCC
AGGGGCGAGAACCTGTGGGTGCTGTCATCGTTGTTGCTTCTGCTCGCTCTTGGGTTGCAC
CATGTAAAGACACGACGCTTGTCTCGTGCTCGGTAATGTTCGAGACCAGAACTTTGCACA
TTATCATGATGGCAACGTATGGAAAGAACAAAATCACCCTATACATCAGTTCTTTGACAC
TGTTTTTCTCAAATGCAACTCTTTGTTCTCGTCTGTGATGTCCAGGGATTTGTTTTTTTG
GAGAGATATTCGAAGGTGTTCCGGGCGGATCGCTGGGTGGATCCCTTGTCAGAACTCGAG
GCCTCAGAAGTGCCCGACTACTCATTGGTTCTTCAATGTCGGTTTTGTATTCGACGGTGG
GGTCTTGCTGACAAGTATTCGGCATTACAGAACAGAAACTTGAGTCAATTGTGATCTGGT
CTGTTACAACTCTGGTACAAGTAGTAAGAGCA
>comp689_c0_seq1
GTCGTCACTATTCACAGTCTCTGCCCGTTCTTTCCATTCTCTTTTGCCCCCTGCAGCCAG
GTTCCTCCATAGCTTCGCCATTATGCTGATCAGTGCCTTTGGGGGAAGGTCTGGGTTCTC
TATTTTGGCTTTTGGATAGGTTTCTTGCATGAAAAGTGTGTACCCCGTATGCTTTCGCTT
TTGCCGGCCCGCTTTCACCTTACGCTTTCT
>comp778_c0_seq1
TCGGGGTTATAGTATCCATCGGGTCGATCGTAAACGGGGTCGAACCGTCTGACCCGCTTC
CCTTCGTGGTTGTAAAAGGACCCAGAGGAATCGTACGGGTCATCGTAATAGTACTCGTCG
CCTCGTTGGTTGTCGTACTCGTTTCCGCCCCGCTGGTTGTAGTTGGGTCGATAGTTCATG
AGACCTCCGCCACGATCCATCCGCCGATTGGAATCCCGGTAATCGAGATTGTAGTAATTC
TGGTCCCCTCCCGGTGCTCCTCCGC
>comp882_c0_seq1
GGTGGACGTATTCGGGGGTGATCTCCATGACACGCTCCTTTCCGGTGGAAGAGATTCCTC
CGGCTTGTTCCATGATGAAGGACATGGGGGCTCCCTCGTACAACAATCGAAGCTTTCCGT
TGGGGTTTTTCGTGTCGCCGGGGTATCCAAAGACACCTCCGTAGAGGAGGGTACGGTGGA
CATCACCGACCATGGATCCGATGTATCGGCTGGAGAAACGCTTTCCGCTGGCTCCGGTTC
CCTCGCG
>comp889_c0_seq1
CCGTATTCATTAAGCTTTCATCCATTCATCCATCTTGTTTGGTATCCGAGTGCTGCTGTC
GTTGTTGTCCATCGTTCGGCCATTGTTTCCTTTTATCGCGTTTGGCCGTCGAGGTAGGCC
TCGACGGCTTCCGTCACGTCGTACCTGTGTCGCTTGAGAATGGCGATGCACACGTCTTCC
TCGGCCCCCGTGACGCTCCTCATCTTGGAGACGGTCGCATGCACCAGCTCCTCCCTCGAG
GTGGCCCCCCTCAGCACACATTCCCT
>comp936_c0_seq1
GCCACCACTTAGCAGGTTGGAGGAGACGTCCATGTATCTGAGAGAGTGCCCTTTCGCAAC
CACATCGTTGATGTGTCCATTGAGTGCGTTCTGGTGAAAGTCGATGACTTCTAGATTCGT
CAGAGTCGAAAAGAGGGCATCCGGGAGTGGTCCAGAGAGCAGATTGTTCTTCACGTCGAT
CACTTTGAGGTTCAGTAGCTTTCCAAAGGACCCATTGGACAGCTGGTCCTCGAACGAGTT
CGAAAAAAGGTACAATTCTTCAAGTTTCGACAGCGAGTAAATTTTTTCC
>comp1141_c0_seq1
CCGCTTCTTGCTGTTCGTGGTGACGAATCATGAGCATCCATTGAAGAAGGTACAATTGAG
GGAGGTATGACGATTTCATAGTCTTCCTCCACTGATAAGGGAATCGGAATTCTGATCTGC
TCCCATTCCTCGTCTGTGTGTGACATGCTGTCACTCACAATAGATCTGGTGGGGGAAGTA
TCGCTGTAGTCGTCGGAAGTTGTTTCGCCGGTATCGAAATAGGACAAAAGTGTGATCAGC
ATTATCTTATGAGCAATCCTTTGATTGGAACAAAAGGAATGATTAACTATGTTGATCGAT
TATGTCTCGTCTCACTTTGTCTCCCTGTCTCGTAATTTCAAAGCATGTGGCTGAAGCTTA
ATTTATCGTTGCTGTTTTGTTTCACGAAATCCAAGTCAAGCAAAAGGTGGATAGATTCCC
AATATCGAGCAGCGGGGTATTTCGCTGACCCTGAAGTATTACGAATATTTCAACGAAACC
AAAGAACAATCTCAATCGCGCCGTATACACGATGGGTCAATCGAGATGGAAACGAATAGA
TGGTCTTTGCTTTTGTGACGCTGAGCTTTTACTGACTCAAAAGTTATTAGATCTATGGCA
CGGAATCGACTTCCAGCTTCGTCTATTAGTGATTGTTGTCTATGAGCTAGGAATTTCGAC
AAACTTAGAAATCGTTTTTGTAACAGTTTTCATTCTCCGAATTTCGTTGTCACTATCGTA

CGGTAAAGTAAGTACGTACGGTGCAGTAATTCTAAATCAAAACTTGAGGTG
>comp1194_c0_seq1
CCCAGTTCTCTCATGCGTTTCAATTCTTCGCGCTTCACCATGCCTTGCGTAACAGCGCCG
ATAACCTTGAGTGTTGGGCAGGGACTTGCAATTTGTTTCAGGGTCGGCCATTCGGCATCT
GTGATTTTCGTAGACGTATCGAATAATTTCGCTGCGGTATCGATGTTGAGGACCTTTGAG
GATTGGTCATTTTCGAAAATGGAATCCCATCCGCCCCC
>comp1198_c0_seq1
CACCCTTGCAACGTTCCGTCGGGACTTGCGATCATCACATTGCTGATGACGTGAATGATA
CCGTTGGATGCCAAAATATCACGTTGGGTGAAGTACGGAATATTTCCACGTCCATTGCAA
GTTCCTAACTGTGCAGTTCGGCGGTTGCCTTCGCATCTTGTTCTGCTTGTGCCAAGTCGG
AGCATTCGAACGTATCGGTTGGGCCCATTGCAAAACAGATCTTGGGTTCTGTACACATTA
GGCGCAACGTGATAGAAAAGAAGAGCCC
>comp1252_c0_seq1
AGCGGCCGACTCAACGGGATTTTCTCGTGCGACGTGTCCTTTTGTGTTTTGCGCTGCGTT
GGCTTCGTTCATTGCGATCCACCCGAATCGTGGTTGCCTAGCTTGGTTTCGTTTGTTTAT
TGTATGACACATCGAATGGATCCATCTTCCCCAACCACTTCGCCGCACATGAGACTGGTA
TCGCACCGTGACACATTGGCGTGCTTCAGAGAGCCGTCCTCGGACACCTTCCGCTTGACG
TCGACTACATCGTCGTCGTTGTCTTCCAGCTCCATCTTTTTCTCGTCCGAGAACCGACCG
CTGCTTTTTTGTGCGGTCGCTTCGTTTTGACTCCCCG
>comp1480_c0_seq1
GTTTGGCGTCCGAATTCTTTTCATTAGATTGGGACCGTCTTGGTTTGCTCGGAATCTCGT
CGTGGCTCAAGCTGCCGTTGTCGCTACCGTGTGTGCTATTGTCCAATCTATCCAATGCTT
CCCTTGAAAATGGTCTCCCCCGAGTTCGTACTCGGCTATCATTAGACTTGTATGACAAAA
CGGAAGAAGCGCCACTATCTTCAGTAAGACTCCAATCACTGGGCACATTCCGCCGGCGAC
CGCCGCGTATCTTGCGGCCCAATCGCCCCTCCACCTTTTCCTCGACGGAGTGTCCTGTCG
TTGTTTCCGCGCAATTCTCGTAAGTATTTGTAGCGTTTGCTGTCGACTTTCTGCGTACCC
TTTTAATCTTGTTGCCTTTCTCGTCCACTCGCCACAGCTGCCCGTC
>comp1556_c0_seq1
CGGGGTAGCTTATTTGATCCAAGTCTGAATCCATCGAACAATCGTTGGTAGAGAAGCTAA
GATTTTCTTCAACAAGAAAAGCCTCTGTCTCTTGCCGTAATCTCTCTCGTCGAGCTCCAA
AAATGAAATAGTGGATGAGAAATTGAACAATGACGACACTTATCCAAGCAACCGCCGAAG
TAGATACCTCCAACATTGCTGTCCCTGGTCACTGAGCCCTCTAGTTATTTTCTATGCTTA
AGCTGTTG
>comp1578_c0_seq1
GTCCCTGGTCGCCGTCGTTATTGTCGTTGCGGCTGTTGTCTTCGGCGTCGTCGATCGGCT
GGTTATGTTGCTGAACCTGTTCATAGTCCCCAATGGCGTTTTCCACGGTCGACGGCAAGT
ATTCCTTGTAGGTGAAGGTAAAAGAGTGGGCAATGGCAAACATTACCATTTCGACACATA
TGCAATAATCTATCAGTCCGTTGGCAACTTCAT
>comp1593_c0_seq1
TTGTCATCTCTTGTTGTTTCCGCCCGTGCCGTCGATTGGTTCGTCCCCTTCCTCGGTGGC
TTTTCCTGAAACCGGTTTTGAAGAAGAACCTCTGCTTTCTGAAACAAATGAGCAGAGAAA
TTCCTACACAAAAGCCAATGACAATACCAGCAATCTCTCCCGCGCTGAGGAATTCTTTCA
TCTCGTGCACTATGTCTTCAGCGACTGTGCTGACGCTGGTTACTGTTTTTATCTCGCAGT
GGCGTCC
>comp1614_c0_seq1
CGAGCGCTCTCGAGGGATGCCTGAATGTTTGGAATCATACTCAACGATGCAACTCCTCCA
GACTGTTCCACCAAGGTAATTAATTCTCTGCGAAGCGAAAAGTTCTCTTTCGTAAGCACA
GCATTGTCGGTCGTCAGATTCTCTACGGAATCCTGGAGGTCGGTAAGAAGCTTCTTTCGT
CGTTCGCGGCTCTCCCTTGCCGACCTTCGATTTGCCATGATGCGTCTCATCTTGCGTGCC
TCGCGGCGTTTTTCCGGATCTAGTTCGTATTCGTTTTGATCGTCACCCCCAACAGCTCCA
CTCTTGTCTCCTTCAGTCTCCCCCGTT
>comp1640_c0_seq1
CGTGTATTTAGGAAGGTGACCTCGACCCCATAACCTAGATTTGAAGAATCTCCATAATGG
AAACAGCAAAAAGGGTTTCAAAATCTCCATAATGGATTCGGCCTTAAAAATGGCCTCGTC
GGGTGTTAGTGTCAGGTATCTACCGCCCTGCGATGCGGGTTTCACGATCGACTTCTCTTT
GTTCTTATAATCTTCCAAAAGCCGAAGCCAAGATCCCCC
>comp1664_c0_seq1
CTCGACGAGGCTTCCTCACTGCACTTGAGAACTTTGGACCGCCGAAACGATACTCAATAC
CATACGTGCTTGCTTGCACTTGGTCGAACCATGAAAAGCAAACTTATTTCGCGTTTTTCT
CCGATTGGTGTGATAATCATAGCAGGTTTGTTCTGCATCGAAGTCGATCTTTGTTACAGT

TTTGCGTTGGTTGGCGGCCAGTTTGAACTAAAGAACCATAACCAGCTTCTAATGGCAGCA
GACAAGAATCCTGTCACCGTATCAAACGAGAACAATGGCAGCGGTTCGGAAAATAGCAAG
GGAGGAAAGGTCTTTGACGAAGCATCCTACGAAAAAGAACGATTGGCCAAGGATGCGCAA
GCCATGGATGCCATGAAAGTCGTGGCTGAGAAAGAATTGAGCAAGGTTGACACGGGCAGT
GTTGGAGAGCTTCGATCGCCATGGAAGTGGGTGCTCCGGAAACGAATCTGGGACTTCATG
GAGGAAAACGATATAGCAAGGTTCCCTCGACCGGTGCACCATCGCATTCCAAACTTTGTT
GGTGCGGACCAGGCAGCGAGAAGATTGAGCGAACTCCCCGAGTTTCGATCGGCGAGCACG
ATAAAGGTCAACCCGGACACGCCACAGCGCCCCGTGCGACATTTCGTTTTGGAACAAAAG
AAAGTGTTGTTGACACCTCAACCCAGACTGCGAACGGGCTTCTTTTCGACCATTGAAATG
AAGGAGCTGCCTTCGCTGGTCGAAATAAAGGAATGCACAAACAGCAAGGGTGTTGTCAAA
TACGGAACTCCGGTCACCCTGAACGAGGAATATACGGTGGATTTGGTAGTGGTCGGCAGC
ACCGCTGTTTGCCCCCGAACGGGAGCCCGAGTTGGGAAAGGTGAGGGATTCGCCGAGCTA
GAGTGGGGAATTCTGTCTGCCCAGGGAAATCTAGAAGCCAAAACATGCCTGGTGGTGACA
ACTGTGCACGACTGTCAGGTCATCGAAGATGAGAACGACATGCCTGAGGGTTGGGCGCTA
ACTAAGCACGACGTACCCGTCGACATAATTGTTACCCCCACGAGGGTCATACATGTTTTC
GACCGAGCCCCAAAACCCTCTGGGATCTTGTGGGACCTCTTATCTCCCCAAAAATTGGCG
GCCATCCGTGTACTTCGGCAGCTGAAACAAGAGACGGAGGATAAGCTAGGAGTAACTTTG
CCGTCGGGACCGGACGAGTTGCTTCCGCCGGTTGCGTCTCGAAACAACAAAAATCGCAAG
AAGAAAAACAATAGAAGGCGAAAACCACGGCAACAGCAGCGCAAAGGTTCGGAAACACCC
CCCGCGAAGAAGTAATACCGTGCAAACTCGGTGTAAGACAAATACTGGCAACAGACTCTG
AACCAACCGACTATCCAAGGAGTTATAACCCTGAACTACGCCTAAGCTATCGACAAGAAC
ATTATGTAGTATACATTCGACTCTTCGGATCATTGAAACACTTGTCCAAAGATAATTGAG
TCC
>comp1667_c0_seq1
GAGATGCAATCTCATCTTCTGAAGTGCCTTGAAGTACAACAGCATTAAGGTTGTACGGTC
GTGATGACTCGGTGATGTCGATCATTTTCTCTGATTTGTTCATGGCTTCAGCTAGGACAT
CTATGACTTTCGATAAGAGATCTTGCCCGTTATCATCGACCTGAGTCACTGTATTGCCTG
AAAAGGCTTGATATGACACAGCGGCAAAAGGGTTGCGCGCTTTCGATGAGTCAGTTGCGT
CGATTACTTTTATGGATTTCTTATTGCCTTCTCGAGACATATCTTGCCCGTTATTTTCGA
CCTGAGCAACTACATCTTTTGAAAAACCTCGAGATAAAACAGCAGCGATGGGGTTGAGGG
GTCTCGGTGTGTCGGTGACATAATTTGTTCCCTTCGCATTGTCCATAATGCAAAGTGAAT
TATCCTGCATACTGCAATCTTCCTTAGAGGCCGTTCTAATTCCAGGTTCATCTTTCGATA
AAATACGAGATAAAGCACCATGTGATCCTGACGAATCGATCCAAGTTGCGGCCAAGAAAT
CATTTTCTGAAGATCTGACGAAAGAAAGATTCAAGTCTGGAGATGCAATTCTACTTCTAG
ATTCGTCCAAAGGCCTAAATGATGGCATAATGGAAATACTCTTCGTTTTTAATAGGGATG
CTTCGCCATTTGAATCCGCTTCACTCAAGTTTGACCAATAGTTTGACTGGGATGCAATCT
CCGACTTACTTGGTTTATAAAAATTGCTCTGTTCTAGAAGAGATCTTGCCGGCGAAGAAA
AGCCCGATTCATCATAACCAAATCGTGGGGAGGATTTCAGAGATGTGTTATCCGGAGAGG
AGCTATAATGATTCCTTGGTTCAGAATCATCCAACACGCTAGAAGAATCAATATACCCAG
TTCGAGATGAATTCAGTACCTGTGCATCATCATCAGTGAATTTATCATCATCCATGGATT
CCATTGTCTTTAAGAATTTACCAAGCATTTCGTCTATCATTTTTTTCTCGCTTTGAAGAG
GTGAACGAGGAATTTCTGCTCTGATCTCAGTTTTTTCAGAAATCTCTTCGATGACAGTTT
GCTCTGTTATCCCACTTATATTTTCTTCTTCCTTTTTGAAGGAAGAATATATTTCAGCTT
TTGTTCCAGTTTGTTCATAGTTTTCGCTGATCATTTCTTTCTCACTTTGAATCGGAGAAA
GCAGCTCTATTGTATCAATCTCTGTTTCAGTACGAAATTGTTCCATTTCTTCGGTAGTCC
TTTCTTTCTCGCTCTGAATGTTTAAAATTGCGATTCTACCTTCGATCTTTGTCTCATTCG
ATATTGTGAGCACCTCTTTTTTGGAACCGTGTCGACCCTCGTCTTTTGATTCTAGATTTG
GCTCCTTTTGGTAAATCGTGTTGTTTGATCCAATTGATTTGGTCGCCGACATTTTGTCAT
TAGATTCCATGGCATTCATAAAGTCAGTATGCTTCTCGAAC
>comp1667_c0_seq2
GAGATGCAATCTCATCTTCTGAAGTGCCTTGAAGTACAACAGCATTAAGGTTGTACGGTC
GTGATGACTCGGTGATGTCGATCATTTTCTCTGATTTGTTCATGGCTTCAGCTAGGACAT
CTATGACTTTCGATAAGAGATCTTGCCCGTTATCATCGACCTGAGTCACTGTATTGCCTG
AAAAGGCTTGATATGACACAGCGGCAAAAGGGTTGCGCGCTTTCGATGAGTCAGTTGCGT
CGATTACTTTTATGGATTTCTTATTGCCTTCTCGAGACATATCTTGCCCGTTATTTTCGA
CCTGAGCAACTACATCTTTTGAAAAACCTCGAGATAAAACAGCAGCGATGGGGTTGAGGG
GTCTCGGTGTGTCGGTGACATAATTTGTTCCCTTCGCATTGTCCATAATGCAAAGTGAAT
TATCCTGCATACTGCAATCTTCCTTAGAGGCCGTTCTAATTCCAGGTTCATCTTTCGATA
AAATACGAGATAAAGCACCATGTGATCCTGACGAATCGATCCAAGTTGCGGCCAAGAAAT
CATTTTCTGAAGATCTGACGAAAGAAAGATTCAAGTCTGGAGATGCAATTCTACTTCTAG

ATTCGTCCAAAGGCCTAAATGATGGCATAATGGAAATACTCTTCGTTTTTAATAGGGATG
CTTCGCCATTTGAATCCGCTTCACTCAAGTTTGACCAATAGTTTGACTGGGATGCAATCT
CCGACTTACTTGGTTTATAAAAATTGCTCTGTTCTAGAAGAGATCTTGCCGGCGAAGAAA
AGCCCGATTCATCATAACCAAATCGTGGGGAGGATTTCAGAGATGTGTTATCCGGAGAGG
AGCTATAATGATTCCTTGGTTCAGAATCATCCAACACGCTAGAAGAATCAATATACCCAG
TTCGAGATGAATTCAGTACCTGTGCATCATCATCAGTGAATTTATCATCATCCATGGATT
CCATTGTCTTTAAGAATTTACCAAGCATTTCGTCTATCATTTTTTTCTCGCTTTGAAGAG
GTGAACGAGGAATTTCTGCTCTGATCTCAGTTTTTCAGAAATCTCTTCGATGACAGTTT
GCTCTGTTATCCCACTTATATTTCTTCTTCCTTTTTGAAGGAAGAATATATTTCAGCTT
TTGTTCCAGTTTGTTCATAGTTTTCGCTGATCATTTCTTTCTCACTTTGAATCGGAGAAA
GCAGCTCTATTGTATCAATCTCTGTTTCAGTACGAAATTGTTCCATTTCTTCGGTAGTCC
TTTCTTTCTCGCTCTGAATGTTTAAAATTGCGATTCTACCTTCGATCTTTGTCTCATTCG
ATATTGTGAGCACCTCTTTTTTGGAACCGTGTCGACCCTCGTCTTTTGATTCTAGATTTG
GCTCCTTTTGGTAAATCGTGTTGTTTGATCCAATTGATTTGGTCGCCGACATTTTGTCAT
TAGATTCCATGGCATTCATAAACGCTGGTGGAACTATGTGTTCCATATGGACGTCAACTT
TGATACTATTTGTATCCAAGTTAGTATTATCGCTAGATAATTC
>comp1682_c0_seq1
CGGTCAGCAACCCATCGATCGTTTGGTTCAATACAAAGCGTGTGCATCCGCTTGGGCTAC
CACGAGATCTAATGGCGCTCGGAACGACGACAGTAACGCTGCCCCGCCCACACAAAGCAA
ACAGAAGAAAAAGAGGCCGTCGATGCCGCCAAAAACGACAATCCTTCCGTACGATCCACT
TTTCGGAGAACAAGGCATTTTGAGCAGTAGTGACGAGGAAGGTGACGATGACGCCAAGGA
AGAACTCAATCTTGACAAACCGGTGGTGAATTCGATCTGGGGATCATCGATTGGAACCAA
CACAAACCGGTACCTGTTATTGGACATACCGAAAAAGAAGAAAAAAAAAGGCAAAGACAA
AAAAAACAAAGGAAAACCCATTCAAAACGTACCGAAGAAAAGAAAGGGGGAAAAGTCTCC
AAATGATTGTGCGAAACTCTTTGTGCAATCCGTCTCTTCTCCGCAAGAGGGCTATGCGAT
CGCTGTCCGCTACTTAGTGTGGGGGGGCGTTGGCGGAGCTCCAGCCGAACGAGGAGTGTT
GATCCCCGGCTCAGAAGTTGCGTTCCCGGCTACACCGCAGGGTGTTTCCAACTGCTGGCA
GCGTTATTCTCCGCTGATTCACGTCATAACTCGTACCTGGCCGGGATTCGCGGCCTGCAT
ATTAACCCACCTCGTTGATTTTGTGTTATCGATCGAGGATAGCAGCGCAGTGTGCGAAAA
CGAGAGCCTGGATGCAGGATCCATTCGCAAACTCTATTTTCTATACGCATGGATACGCCT
CATATTGTCCCAGAGATTTATGGCAGCACTGGACCGAAATTTTTCGGTGAAAACGACCAC
CACAAAAAATTCCAACCCTCTCGACCTACCTTTGGCGCACCTAGATCACTTGGAAAGCCT
GGGATACCCCCTGAATAGTCTCTTGGATCGTTGCCGTCGTTGCAACAGTGAGAAGCACGA
TAACAGCAATGGTGAGAACGGTGATGCTTCCGGACGTTCCTTTGTCAATAACTCAGACTC
GATGGGGACTAGTCGAGATATAGTTCGCGATCTAGAGTCTATCCTTGGCGCCAAAGTGAG
CCCGAACTTTGGATATTCCGACAACACCTTTGTCCCGAGCAACACAAACAACACAGTCGA
ACCTAATCCGCTGAGCCGTTCAACAGAGAATGACGATAGCATTAACATCCAGGTCGAACA
TGCGAAAACTTCTGGTGAAATGTCTCTGGATGAAATGGAAGCAATGCTATTGTCGGAAGA
TAATAATAATAATGGTCAAGAAGGAGCGAAAGCCGAGCATCCGCCGACTGCTAGTCATCT
CGAAGACGCGATGAAAGCGACATATATGACAAGTGGAACTTCTGCAGGTGCTTGCATGGA
GATGGCAGAGCAACAAAGGCAGGTCGATTTAAACATGCGGCGACCTGCATGGATACGATG
CCAGCGTTGGGATGAATGTGCCATTGGAACTTTACCGGGATATCCTACGTAAGATGAATT
TGATATTATTCTGAATTTAGTCTAATTTGATGGTTTGGGCATCTGGAACGCCACTTGTGT
TCAAAAAGTAAAAACATTTGATGACACTGCGTCAGGTTGAAAAAAGCAGAGCTGAAGTAC
CGAGGAGTAGAGTTGTTTAAAGTATTTCACTACACACATGGAGGAACAAAGGAAGATAGT
AGAATTACAACTTCGTAAATGGTTTCCTGAAAAAAGAAATACATGTACGCAATCACACGG
AAATGAGA
>comp1697_c0_seq1
AAAGACTGTATGAACCATGAAAGTGATAGAAGGAGCCTCTCGGGTGGCGTTTCTGGCATT
CTTTCTGTCCCATATTCCTATCACCCTGTTCGTTGACGCGCAGGGACTCTTTGGTCCGCA
TTACCCGAAGCTCCTGACCGACTTGATCGCATGGTACTGCGGCCTCTTTGGCGATGTTCT
AATGAAAAACGCCCCGTCCGGTGAATACGCCTGGTTCTCGTCTCTGATCGGTTGTGAGAT
TTTGTTCCAGCTTCCCTTCTTCATTGTTGCGGCAAGGACGATCATGACGGCGGGAAAATG
CAACGGAAGCAGCACCACCAACCCAAGCGACGGCGGATCCACGCCACAGGAGAGCTACCC
GGAATGGTTTCGCATCGCATGCATCGTGTACGGTTCTCATGTCAGCACCACACTGGTTCC
AATCCTTTCGTGTTTTGTATGGAGCGAGGAAATGACGACAGGCCAGAAGTGCGCCACCAC
CGCCAGTAAGTACCGGATTGAGAGC
>comp1697_c0_seq2
AAAGACTGTATGAACCATGAAAGTGATAGAAGGAGCCTCTCGGGTGGCGTTTCTGGCATT
CTTTCTGTCCCATATTCCTATCACCCTGTTCGTTGACGCGCAGGGACTCTTTGGTCCGCA

TTACCCGAAGCTCCTGACCGACTTGATCGCATGGTACTGCGGCCTCTTTGGCGATGTTCT
AATGAAAAACGCCCCGTCCGGTGAATACGCCTGGTTCTCGTCTCTGATCGGTTGTGAGAT
TTTGTTCCAGCTTCCCTTCTTCATTGTTGCGGCAAGGACGATCATGACGGCGGGAAAATG
CAACGGAAGCAGCACCACCAACCCAAGCGACGGCGGATCCACGCCACAGGAGAGCTACCC
GGAATGGTTTCGCATCGCATGCATCGTGTACGGTTCTCATGTCAGCACCACACTGGTTCC
AATCCTTTCGTGTTTTGTATGGAGCGAGGAAATGACGACAGGCCAGAAGTGCGCCACCAC
CGCCATATACTCCCCGTATTTAATTTTTCCGTTGTGGCTATTGTGGCTTGCTGTGAAAGA
AACTTTCGTGGATGCAATGGATGAGAAAAGGTCGAGTGGAAAGAGGGACTAATGCATACC
GTGCGTTAGTGCAAATCGGATCGAACTGTAGGATCATTTATTTAGGTTACTGGCTGCTAG
ACAATATGACACTGAACTCTGACAGAGAAGGTATGAAAAGTTGCGATTACAAAGCGAC
>comp1702_c0_seq1
AATAAATCAATCAGCAATCATCAGAAGAAGTACGAATCAGTCTTTCTGTAGCGACTGCGA
CAATCAATAACATTGACTTGTCTAATCCGTTTGTGCAGCTAGTATTACAGCCGTTTTTGT
TGTCATTGTGACAAACGTACCGCGGATTTTGGTTCGTGTGCTCATCTGGTTGATGCCATT
GTTACTACTTGAATACCACCGACACCAGACATAATTCTTTGTAGCACTGCACAACCACTA
TTGCTCCCATTTTTCATTCGAGAATTCGAGATGGCTCGTCCAATACTCCCAACCCGAGAT
GGCATTCGTCTGTCTTCCTATGCCACCACTATTTGCTCGGCACACCTGCTCAGCCAGGCG
CAACCAAAGCCCAGCGCCAACAGGTTGAGGAGCGTCGAAACACCGTGGAGCATCCCAAAG
GTCTTGGAGAGCGCCCTGAGCTCCGGATCGTTTGCCTTCTTCGGATCGTCCGGGCGTATC
TTTCCGATTTCGTGCCCCGTTCCCAGCCGTTTTTCTACGGCGTGCCTCTGGTACATGACC
CTCGTCGTCGCCGGTTCGAAACAGAAGAGGTTGGCAAGAATGCACACCAGAACCACGACC
AAGGAATAGGGGGCGGCGGCCTCCTCCCCCCCCGGACTCGCCAGGGCGCCGCTCGTGGCG
ATTGCGACGAGGATGGACACCGCGGAGAACACAAAGTAGGCGGGAAAGAGCCTGGATTGG
AGCCTCCCAAAGGTGTGTCTCGGGAGGGTCTTGAACATGACGATCCCCGCAACGAAGCTG
ACCCACACGCTGCACCCGAACCAGGAGGAAAACGCCAGCAGGTGGAGGAGGACGAGCCCG
TGTCCGCCGCCGGCCGGGTTCGGAGCCGGGGAGAACCAGGTGCAGCCGGCCAGCACGGCG
ATCAGTGCCATCGCGCGAACCACCTGCTTGGTTCCGGTGGGTGCGGTTGCGGATGCGTCG
GACATGATTGTTGGCGGGAGGTAGTGCGTGTTCTAGGAACCGATGGAAGGGTAGGAATGG
GACGCGAGGCGGGAAATGGATTCGAAAAGCCTGCGGGCAAGGCAAGATGCGAGGTGTTTG
TTGTGCCGGATCCATCCGTCCGGGTCTTCTCGTTGCGACGGGAGACGAGAGGAAGGTCGT
TCGAAGTGTATCGCGAAGAGTTTTGGATCTTGGATTCGAGAAGGATGGATTCGATGATTA
GTGACTCAGTTTCTATGCCAAATGAAAATAAAGACTATTTCAGTACAGTCA
>comp1708_c0_seq1
CGGGTCTTCTTGTCGGTCTCGCACGAGGAGCACGCCAGATCAAAGACCTCCTGCACGGTC
TCCAGCACAGACGATCTGCGCTCGGGATCGGTTCCCCAGATGAGCTCCAATGCGTTCCAA
GCCGGAGTCGCGTGCAGCAACAGGTATTCGTTTCCGGGGTTCTGGGCATAGGAGTACGCC
CCCAGGGTCGCACTACATGACAATCGGCAGGCGATCAAGAGCGGCAGGTACCGGCGCTCT
TCCGGGCCAATGGGGCGAACCGAGTTGTATCCACGCAGCACGGCCGCAGCGGCAGCCAGC
GATCGTTGGTGTTTCCCAAATGCGTTGAGCATGGCATAGGCCATGGCTACAGACAAATCT
AAAATGTTCCAGCTGTTAGATGAATCACAATCATACCAGAAGAGAACAAAACTTGTGAGT
CAACAGTATGGCGCCTGGAGTTTTTGCTTGCGGAAGTTGTATCGAAAAAGCACAAAACTA
TCAATCTCTTTTTAAGAATGTTTGACCGCCGTCGTCATGACATTATCCACCTATCGATCT
TTTTCCATATCGAGGATGTCCATGATAGCGTTGACGATCCGGGGCTTTAATCCGTGGTTT
TTTGTAGCTCTAAGTTTTGAAGAATCCAACACGGTTGGAAAGAAAAGGATGCCTCCGACA
ACATTTTTGTCAATCGGTCGACATCCATATCTTCCAGATACGAGATCAAGAAGGATTCTC
TAGTTTACACGAGCTCGGTTGTACAGCCAACAGCGCGGCAATACACAACAAACGACGACA
AATAAAACTGAATTTCTTCTCACCTCTCTACAGAATCGCCAAAATCGATCACCCCCGAAA
CGCAGAAGTCGTCGCCGAGCAAAAAGTTTGCGTCGTTGAAGTCTGCGTGGATCAACGATT
TTTTGAAAGAAGTTTCCGCTACCTTGCTATCGATTAGGTCTTTTTGGAAGGCATCGATCA
CACTATTCACGATGGATCGTCTC
>comp1718_c0_seq1
ATTTTCATAAGCAACACGACGCTCTTCCTCTTTCATTTCAGATTGAATTAATCCAACTGT
TAAACCAAGGAATCGATGGACCTGCCCCATTATTTCCATGTCTCGTTTCGCTAGGTAATC
GTTCACAGTGACAACGAAGGACGTTTCACCGGTCAAAGCATTAAGATAAACTGGGAGAGT
GGATACAAGAGTTTTTCCTTCTCCTGTTGCCATTTCAGCCAGACGACCGTCATGAAGTAC
CAAACCACCCAAAATTTGAACGTCATAGTGACGAAGTTCTAGGACACGCCTGTCAAATAA
AGATCGAAGAACGCAAACGACAAAACAAGTAATGGTCCGTTGATGTCTTCCCCATTTTTC
AAACGTTCCTTGAAGGATTCAGTTTTTGCCTGAAGCTCATCGTCACCCAGTTCCTCAATA
GTTTCTTCCAGGCCGTTGATTCGAGTAACACGTTCTTGTAGTTTTGCTAGATACTTTTCG
TTCCTCGACTTCCTAGTCTTTTCGTCCGTGCTTGCGACGAAGTCGTCGACAAGTCCCATC

GAGAGGGGATGTCCAGGGGCCGAAGTCTTCTGTTGTCCAAACCAACCGATTTGAGTGTTA
ATTGTGTTGGGCGCTTTGGTCTGCTGCGGCAAGAACGCATGGCATTTACTGCAGCACAAT
ACGAGGACCAGTGACACCAGGATCGAGTACAAGGTGCCTGTTGCTTTTGACATCATGGTT
TGGTTCAGGACGACCGAAAAATTCAGATCGATAAACGGCTTATTCGCTGGGATGACAATG
TGCAGTGGATTTTAAGTCTAACCAGTTCTGATACAATTTGTGACACTTTCAGTATTGCGA
TACTTCTTTTCTGGAAATCGAAGGTGGTCTCTATGAAGATACAGAGGAAGCCCATCAAAT
TACGAGAGTACTCGACAAGTACACATGCGAGAACCAAAACCTACCGGTAATTAAATTAAA
ATCTGATCAGGATTCATTTTCCCACGAAATCTGTGTCAATCTGCGAAGTTCAGTTTGCGT
CAGTAAAACTGTGTTGTTCCGCTTTGACGTCTAGTGAGTCACGTTAGTTATGAACGTGGT
AGATGACATCAGTGCGTTCTACGCTACGTACGTAACTTTGTACTGTACAGTATTGAACCC
ACTTTTTGAGTGAAGGAAAAATAATCTGGGGGATATCCATACTCGAACTGGTACTACGGT
AGAACCACTTACAGAAAGAATGTCCGCTTTCGCAACTGTACGATGCATACCATCAGTACA
TACATTAACAACAAACAACAGATAACAAATACAACTGCACAAACAGTGGAAAATTCGAAC
AGCC
>comp1733_c0_seq1
CAACCATCTTTCGGTCGTCGCGTGCTGAACAACTGATTGAAAAGGAGTTGCTCCTATGTT
CCATGAGGTGAAAGGATTCATTGAATTTATTTGTGGTCCAAGTACGTGTATTGGCAAGTT
GTGTTGCAGAAGAGAATGTTATAGTGCAGTTGTGAAACAGAATGAGTTTTATTCGCTTTC
CGAGGCATACCCTTCACCTTTTTCTTGTTTGATTTCACTTCATCAGTCGTACAGCTATTG
TCATCCATAGCCGCGAACTTTGAATGTTTGTTCATGTACGTACCTTTCGAACATTGGGTG
TTTCAACCCCCGAGTCCGTCTTTGCTTCTCCGCCTTCTTCGGCGGGTGACTTTGCTTCTC
CACCTACGTCGTCGGGTGATGTCTCGCTTTTCTTCTCCTCCTCCTCGGCTGCCGTTTCTG
TGTTGTTTATCACAAACATCCCAGGAATGTAAGCAGGCTTTTCGGATTTCTCCTTGGGCT
CTGCCTGCATTTCCGCCTCTTGCCCGTGGTCGTCGTCACCGTTGGTAGTTCCGACTTCAA
TTTCTGTTTCGGGTTCTTTCTCGGCATCGGCATCGCACACGTTGTCGGCTTCGCTCCCTT
>comp1812_c0_seq1
GTCTTTTGATTCATACTGTGGTAGGATCATTTTAAAATGAAGTGTCTGGAGACATCAACG
AAAAGCGTTCATACCCCCTAACGAAGAGAAGGCATCGTCTTTCATAGTTTGTTGCTGTGA
ACTGCCACCCATGGGAGTTCCAAATCCACCGTCGTCACTTGATTGGGAAGATGCAATGTT
GTTTCCCATATTCATGTTTTGCATACCTTTGATAGCAGAGTTCATGTGGTTATTATTGTT
TGCCATTCTTCCCATGGAATTGTTTATTCCATTCGGAGAATTGAACATGTTTGTATTATT
CATTGCACCAGCATTATTCATTTGAAACTGATTTTGCATTTGTTGTTGCTGCTTCATCAT
CATCATCTGCCGTTGCATCATCATTTGCTGCTGCATCATTTGATTTGCCATTGCTTGGTT
GCTGTTTGGCATCATGTTGTTTACGTTGTTATTATTCTGCATCATCATCATTTGCTGGTG
CTGTTGTTGAGTCATCATATTTTGTCGATTACCAAAGGCGTTCATGTTGACATGTTGTTG
AGGCATCATTCCATTCATCGACGTCATCGAGTTAGCGTTGAAGCCGCCACCTGGACCCAT
TTGGTTCATGGCTTGCATGCCGAAACCTGATTGAGGTTGGTTTTGCTTCGGCTTTGGTGA
CCCAAACAATGCCAAAATGTCGTCATTGGATGTCGTAGTTTTGGTCGCTACGGGAGCAGG
ATTCTTCATGAGTTCTTCCGCTAGGGTCGCTGGCGTTGTTGCCTGTGGTGCAGACTTTGG
AGGTGGGGGGATTGCATTTTTAAGGGCCCCTTGTGTAACCTGGTTGGTAGGAGCGGCTGC
TGCCGGGACAAGAGGAGACGAGAATACATCCCCCCCCCCC
>comp1818_c0_seq1
CGAGTATTGCGGTGCCTTTTCGGCGAATGGTGGAAATTCTCTCGGTACGCTGATAGCTCA
CCAGCAAGTCGGAAAGGAGCAAACCAAACAAGCCGTTGGTTTCCGTCGACGTTCGTCGCT
TGCCCTCGGTGCAGAGAGGGATTGTCGAGTTGCGATTTCGACCAATAGCTCGGACAGCGT
TGCAGGCGAACCTTACAACGAGAATGACACGAGTCACAACGATTTGGAGGCAGTGAAATC
GTTGACGAGGACAGCAATGTATCGTTGTGGCACGGAAGAAGGATTGGATGCTCTGAAATC
CTTGCGCGAACTTTGCGACAATCGCCTTCCCTTTGACTTTGATTCCGTTCACCGTCGACA
AACATATGGGGGGAGATTTTCATCGCCAGACCCGAGAAACGCCGCCATCGATCAAGGCGA
AGAAACGATTTCTTTGCTGCCGGGTTTTTTGCCAACCCAAGCCGTCGGGGACTTTTTGGA
ATCCGTCCGGTGCATGGAAGAGCAGGGGTGGATGAGCACTAATCCAGATTCTGTGGACGG
TCTCCCGAGTCTTCACCTGAATCTCGTTAGCCAGGGAAAGCCCTTGTTCGACGAGACGCC
TCCATCCTGTGACGATTCTCGGGAGAGTCCGAGCGAGTATTGCGACGAAGATTCGTTCGA
AAGCCAAATCTTCCGGCTTTACGATATCGTTCGTCCTTACGTCTACGAAGAACTTCTTCC
CAAGGTCGATCAATTGCTGAACAACGCCAACAAATCCACTCCCAGAAAACGAAAACTCAG
GGTCTCGGATGTCTTTCTTCGGCGGTACGGGCAAGATATTTGCGGAGACGTCACACGGAA
CGGAATCTCGATCCACTACGATGTTTATTCGAGGGTCACGGCTGTCGTGGCTCTGGACGA
AGTCGCTGCCGAGGGCGACAATGGCCTCTTCACGCTGACCGTAGACGAGAACACGGGCGA
GACCTCCAACCACAAGGCCCTGCGCAGGTTCTTTCCCCTGCGGACGGGAGATTGTGTCGT
TCACACGTGGGACGTCTTGCACGGCGTCGACGTTCAACCCGGATTGGACCGAACGAGTTT

GATTGTGTGGTTCGATGAAGTAGCTGAAGGCGAAGGCCAAAACGAAGCAGAATCGGTATC
TCCGTGGTTGTCCTTGGAGAGCCAGAACGCACCACCAGGTGTCACCACGCTGCAAAACGG
AAACGACGTGAGGCAATTTGTGCTGGCAAGTGCTCTCTCGAGTCTCGAGGGCGACGAAGA
CACGGAACTCGAGGAAATCTTGTTGTATCTGCGCAGTGCCTCTAGGGGAAATACATTTGC
CCTGACTCGGATGGGCAGCATCTGTGACGAAGGGGCACTGTCGTCACCGGAATTGCAAAA
GGAAGCAGTCCAACTCCTGGATCGGCTTCGACCTTTCAAAAACCTCCCGGAGATCCTCCA
GGGACTTCTCGAGGGAAACGACGCCCAGAACCAACTGGCCTGCCGGTTTTGGTTCGAGGC
ATCCCTGAGTGGCAACCCACTGGCACAAAAGGCACTCGCAAACGAGATCATGTTCCGGGC
CTCGCAGTCCGAAAGGCCGGACCAGCGCTTGCTCGCTGCGGTGTTGTTTGCCCTCGCATC
GCAGCAAGAGGACGAGGACGGACCCTCCGATTCCCTTTCGCGGGTCATCGAGCACGACCT
TGCGGCGCGCAACGTACAAAGCCAGGAGGAATTCTTGGCCTCTCCGGTGGTACAGACGGC
CAAAGCCGCATTTGGCGCGGCATGAGCAAGGTTGCACCATTGCGCACCTATCATGGCGAG
ACTGCTTTTGGTGG
>comp1834_c0_seq1
AAAACTATGAAATTCAAATTCAGTAAGCGAACCAAAACCCCTCCAAGTATTGATTTGGAC
ACATTTACTCAGTCCAAGACTTGATCATCCTCACTAGGAGCCCCACTGAAAGTTTCTGGA
AATCTTCAAAGTAGCGAGGGCATGAGACTTTTTCAAGGAACATCTCACACGCAAACATAA
ATGTTTAGATCGCAGAAACAAATCAAAGTAGTCGCATCGGACTCCGCGCGGCTGCTTTCA
CGATGGGAGACCCAAAATGTAGTGGCGATTTAATCACACTGTATGCGTCAATATGGTGCA
GTGAGTGGTCAATTTCCTGAACATTCCCTGATTTAGGGCTTTTCGGGGTGGCGAGGATTG
CAGTTGACAAAAGATTATCCCTTTCATCGACCATTGCACCACGAATGCGTTCTCGACCAC
TACCAGTGACGACCCCAATACGACTTTTTGGACGACCCGGTGTCATTGTGAAATCGCTTT
GAAAGGGAGTTTCTTCGTTTGTATGCTTGACTTTTGTACCACTATCAAGCAGAATCGATT
GTTCTGTAAGGTCGTCTTTGAAGAAGACTCGAGGCGTGTTTGCCGACGAAGACTTCGAAG
ACGAAACATAACCTTTCGAAAAAGTGTCATGTAGCAATTTTGCATCGTCTCCACCCCAGC
TTGTACACATTCCCGGTGTACTTGATTTATTCGGTGTTGGGATGAGAAATGGGCTAAAAC
AATTGCTTTTCGATCCTTTTGCACATGGACTTCCAAGTAGAGCGTATTCATCTAGAGTGT
ACCCTAGATCCGACGATTCGCCCAACTCGAAATTGTCCTTCGATTTTGTATCTTTCTGGA
CTGTATTATCTTTCGTTCTCAATATCGGAGAATGCTTCAAACTTAACGAATTGGACAAGG
TTTCAATCTTACTTGAAGCTTGTTGAAGTTGAGTGGAATCCTTGGTGCTTGATGGGGTAG
GCAAATTGTTCGTAGGAAGTGGCGTTGTAGGTGGTGGACGTACTGGTGGGTACCCCGGGA
ACAACTGTGCCATCGGATAGAGGGGTGAAGGATGCCCCCAATGCATAGGATGTGGCGGAA
CCATGTACCCATTGCTATAACGATGGTGATGTGGATACAACTGCTGTTGCGGTGTTGCCC
ATTTGGGGCCATCTAGATGACGTCGACTCTGATTCATCGGCACTGCCTTTTGCAAACGAC
TATATTCAGACTGAGATAGACCCAGTGCATTCAGCTCGCCGATTGAACCACTTTTCACCG
CCTTTTCCACAATCCGTCGTCTCTCCAATGCGGAGTGATACACGCCTTTTATATATCCGC
CTTTGAGTTCACTCAAAAATTCTTCCAAGATCTTCTTGTTGACAGGAGACATCTCTGCAA
GCTTCGAGTTTTTGTTATATTCCGAACTAGACTTGACTCGTTTGACACTTTTTGGCGTAT
ACCCCATGCCGGGATAGGCACCAACCATCATCGCATCATATTGTCGTTTCATCATCGAAT
TGTAAGCAGCTGGGTTCTGGGCTGCGGACATCGGAGTTGCAAGAGGTACCATTCTATTTG
GATGCATGTGATTTAAACGAGTACTTCCGAGCGGTCTTCCAGCATAAGATTTCGCTTGAT
TTTTGATGCTTTTTGAATTAGAGCCTGATTGCTGTAGAGCCTGTAGGCATCCCTCAATAT
CCTCTCGTCTGAGTAAGAACCTGCCAGACTCATCGACTACGGGAGCTGTAGGATCGACCC
GTTTTCTCTCGAGATACTTTTCAATCTTCTTCTTCATGGATGAATTCCAATGGTTCTTGA
TGGCATTGTCAGTACGACCATTCAACATCTTCGAAATCTCCGCCCAGCGATTTCCAAAAC
GGATGTGACTGTCAATAATTATCCGATCTTCCTCCATCGTCCAGGTCTTTGACTTATTGA
TATTAGGGTTCAGATGATTGTGCCATCGCTCACGGCACTGTTTCCCACTTCTCCCTGCGA
TGCTATTTGCAATCGAACTCCATTTCTTGGCACCTGTTTGTATATCGATTAGCAGAGGTA
AAAAGAAATGAATAAGATATGATACGCCAAGCCGAACAAACGAATTGCTAGATCATCCCG
TAACGATAGGAGTTCTAATTTCAATCACTTACCGTGTTGTGCCACAAGAGCGAGTATCTG
CTCATCTTCCTGACGAGTCCACGGGACAGGTCCTCTACCCCCTTGAGATTGCCGCAAAAA
GTCATATCGCTCTTTGCATTTTTCACCGTCCCTCTCCATGGTTGAGCCGATCCCCTGCCA
GTTAATCATTAGATCTGAAGAATCTGCTGGCTGTCCGTTCTTTTTCTTTCGTAGAGCTTT
CTTTCGTGCAGACACAATCGACTTCAACTTTTTATCCTGAATGTCCAAAAAGAAAAAAGA
ATAGAATTTGGTATGAGTTTGTGCTGGATTTCAAGACTGTCGATCGCAACAACCATGATA
CGTCTTTTTATATTTAGAACTGAATCTTACATCTTCTTGACTCC
>comp1834_c0_seq2
AAAACTATGAAATTCAAATTCAGTAAGCGAACCAAAACCCCTCCAAGTATTGATTTGGAC
ACATTTACTCAGTCCAAGACTTGATCATCCTCACTAGGAGCCCCACTGAAAGTTTCTGGA
AATCTTCAAAGTAGCGAGGGCATGAGACTTTTTCAAGGAACATCTCACACGCAAACATAA

ATGTTTAGATCGCAGAAACAAATCAAAGTAGTCGCATCGGACTCCGCGCGGCTGCTTTCA
CGATGGGAGACCCAAAATGTAGTGGCGATTTAATCACACTGTATGCGTCAATATGGTGCA
GTGAGTGGTCAATTTCCTGAACATTCCCTGATTTAGGGCTTTTCGGGGTGGCGAGGATTG
CAGTTGACAAAAGATTATCCCTTTCATCGACCATTGCACCACGAATGCGTTCTCGACCAC
TACCAGTGACGACCCCAATACGACTTTTTGGACGACCCGGTGTCATTGTGAAATCGCTTT
GAAAGGGAGTTTCTTCGTTTGTATGCTTGACTTTTGTACCACTATCAAGCAGAATCGATT
GTTCTGTAAGGTCGTCTTTGAAGAAGACTCGAGGCGTGTTTGCCGACGAAGACTTCGAAG
ACGAAACATAACCTTTCGAAAAAGTGTCATGTAGCAATTTTGCATCGTCTCCACCCCAGC
TTGTACACATTCCCGGTGTACTTGATTTATTCGGTGTTGGGATGAGAAATGGGCTAAAAC
AATTGCTTTTCGATCCTTTTGCACATGGACTTCCAAGTAGAGCGTATTCATCTAGAGTGT
ACCCTAGATCCGACGATTCGCCCAACTCGAAATTGTCCTTCGATTTTGTATCTTTCTGGA
CTGTATTATCTTTCGTTCTCAATATCGGAGAATGCTTCAAACTTAACGAATTGGACAAGG
TTTCAATCTTACTTGAAGCTTGTTGAAGTTGAGTGGAATCCTTGGTGCTTGATGGGGTAG
GCAAATTGTTCGTAGGAAGTGGCGTTGTAGGTGGTGGACGTACTGGTGGGTACCCCGGGA
ACAACTGTGCCATCGGATAGAGGGGTGAAGGATGCCCCCAATGCATAGGATGTGGCGGAA
CCATGTACCCATTGCTATAACGATGGTGATGTGGATACAACTGCTGTTGCGGTGTTGCCC
ATTTGGGGCCATCTAGATGACGTCGACTCTGATTCATCGGCACTGCCTTTTGCAAACGAC
TATATTCAGACTGAGATAGACCCAGTGCATTCAGCTCGCCGATTGAACCACTTTTCACCG
CCTTTTCCACAATCCGTCGTCTCTCCAATGCGGAGTGATACACGCCTTTTATATATCCGC
CTTTGAGTTCACTCAAAAATTCTTCCAAGATCTTCTTGTTGACAGGAGACATCTCTGCAA
GCTTCGAGTTTTTGTTATATTCCGAACTAGACTTGACTCGTTTGACACTTTTTGGCGTAT
ACCCCATGCCGGGATAGGCACCAACCATCATCGCATCATATTGTCGTTTCATCATCGAAT
TGTAAGCAGCTGGGTTCTGGGCTGCGGACATCGGAGTTGCAAGAGGTACCATTCTATTTG
GATGCATGTGATTTAAACGAGTACTTCCGAGCGGTCTTCCAGCATAAGATTTCGCTTGAT
TTTTGATGCTTTTTGAATTAGAGCCTGATTGCTGTAGAGCCTGTAGGCATCCCTCAATAT
CCTCTCGTCTGAGTAAGAACCTGCCAGACTCATCGACTACGGGAGCTGTAGGATCGACCC
GTTTTCTCTCGAGATACTTTTCAATCTTCTTCTTCATGGATGAATTCCAATGGTTCTTGA
TGGCATTGTCAGTACGACCATTCAACATCTTCGAAATCTCCGCCCAGCGATTTCCAAAAC
GGATGTGACTGTCAATAATTATCCGATCTTCCTCCATCGTCCAGGTCTTTGACTTATTGA
TATTAGGGTTCAGATGATTGTGCCATCGCTCACGGCACTGTTTCCCACTTCTCCCTGCGA
TGCTATTTGCAATCGAACTCCATTTCTTGGCACCGTGTTGTGCCACAAGAGCGAGTATCT
GCTCATCTTCCTGACGAGTCCACGGGACAGGTCCTCTACCCCCTTGAGATTGCCGCAAAA
AGTCATATCGCTCTTTGCATTTTTCACCGTCCCTCTCCATGGTTGAGCCGATCCCCTGCC
AGTTAATCATTAGATCTGAAGAATCTGCTGGCTGTCCGTTCTTTTTCTTTCGTAGAGCTT
TCTTTCGTGCAGACACAATCGACTTCAACTTTTTATCCTGAATGTCCAAAAAGAAAAAAG
AATAGAATTTGGTATGAGTTTGTGCTGGATTTCAAGACTGTCGATCGCAACAACCATGAT
ACGTCTTTTTATATTTAGAACTGAATCTTACATCTTCTTGACTCC
>comp1835_c0_seq1
CGCGGTGCGGAATACGCCGTGAATTTCGTGCCCAAGATCAAGCTGGAAATCGCGGTTTCT
GCCGCGATGGCCGATCAGGTGGTCGAGACCATCACCAAGACCGCCCGCACCGGAAAGATC
GGTGACGGCAAGATCTTCGTGCTCGACATCCAGCAAGCCGTGCGCGTGCGCACCGGCGAA
ACCAACGATGACGCGCTTTGAGCGCGCAGGGGAAAGGATAAACACAATGAATTCCATGCT
AAAACTCGGCCTTGCCGCAACGCTGATTGCGCTGCCGCAGATGGGGCTGACCCAAGAGGC
GGCGGCTGAGGCCGTGCCCAGCTTTGATGAAATCGGCCCCTATATCATGACGACGCTGCT
GTTCTGCATGGCGGGCTTTTTGGTGTTCTTCATGGCGGCGGGTTTTGCCATGCTCGAAGG
CGGGCTGGTGCGGTCCAAGAACGTCACGATGCAGATGACCAAGAACATCGTCCTCTATTC
CTTTGCAACGATCATGTATTGGCTGGTCGGCTTTAACCTGATGTATCCCGGCGATGGCTG
GCTGGTGGCCGGTTGGGTCGGGCCGTTCTTTGCCATGACCGAGCTGGACCCGGTAGGGGT
TGCGGCGGCGGATGCGGCGCTTGATTATGCCTCGATCGGTTCTGACTTCTTCTTCCAACT
GGTGTTTGTCGCCGCCACAGCGTCGATCGTGTCGGGCGCACTGGCGGAGCGGATCAAGCT
TTGGCCTTTCCTTGTGTTTGTCATCGTGCTGACCGGGTTCATGTATCCGATTTCCGGGTC
CTGGCAGTGGGGCGGCGGCTGGTTGTCTGAGGCTGGGTTCTCTGACTTTGCTGGCTCGAC
CGTTGTGCATTCCGTGGGTGGCTGGGCTGCTCTGGCGGGGGCGATCATCCTTGGGCCGCG
TCTTGGCAAATATGGCAAAGATGGCAAGGTCACGCCGATGCCGGGTTCGAACCTTGCGCT
TGCCACTTTGGGCACATTCATCCTGTGGCTGGGTTGGTTCGGCTTTAACGGTGGTTCGCA
ATTGGCGATGGGCACTGTGGGCGATGTTTCGGACGTGTCGCGGATCTTTGCCAATACCAA
TATGGCGGCGGCTGCGGGTTCGATTGCTGCGCTGATCCTGACCCAAGTGCTCTACAAAAA
GCCTGACCTGACCATGGTTCTCAACGGCGCATTGGCGGGTCTGGTGTCGATCACCGCAGA
GCCGCTTGCACCGAGCCTGTTCGGCGCACTTTGGATCGGGGCTGTTGGCGGTGTGATCGT
GGTCTTGGCCGTGCCCATGCTTGACAAGTTCAAGATCGACGATGTGGTTGGTGCCATCCC

GGTTCACTTGCTCGCCGGTATCTGGGGCACGCTTGCCGTGGTCTTCTATGGCGAGGCCAG
CCTCGGCACGCAGCTTCTGGGGATCTTTGCCTACGGTGCCTTTACCTTTACCGTCAGCAT
CGTGATCTGGCTGATCATCAAGGGCACCATGGGTCTGCGGGTCGATCAAGAGACCGAGAT
CAACGGGCTCGACATGGCGGAAATGGGCATGGAAGCCTATCCTGAATTCTCCAAGAGCTG
ATCCTTCTCCTTCCTGAC
>comp1840_c0_seq1
GGCAAAAGTATGTCACCGGTTGCTATTAGCCAATTCAACCATTCCAGACGAGCCCGTAGT
CCATCGAGTGGGAGGAATGAATCAATTACATCTTCAGCTACAACCGCGGCAGCAACGGAA
CCGGAGAGAATGGTTTGGCGTGGGAGGGCACAGGACACAGTAGTCTCTTCTGGAACCAAC
CCAAACACTCTCACGCGAAAGCCCAAGACGATCAACAGGGACAACAGGGACAGCAACAAC
AACAGTCGCGTCCAGAAGGACAATGCTAACACCAATGATGGTGACAGCATTAGCAGGATC
AAAAACAACAACAACACGATTTGCGGGAACCGTAGACGGCAGTGCTGCCCTGGGTCCACT
GCCTGGATCGTGGCGTGGATTGTTCTAGTTCTCTGCCGGCAGCTGATGACCGAGACGGGC
CTCCTCCGAACTGCTCTGGCATCGTATGGTGCTCCTTGCCACTATTTTGACCGCGGCGGT
TGGACTGAGCAGGGTGACGAATGCGGATACGGACGTGGGAACGGAAGAAACCGACCCAGC
AGAAGCGCCCATGGATCGAGTGTAGATGCTTCTAGCAATCAAACTCTGGGGAGTGACAAT
GACAAAAATAGCAGCAGCATCAACATTACCGTCCCCAGCCTTCCGTCGGCTTTGGACCTG
CTGAACCCACCGTTCAAGAAAAACTACACAATTCGGGTCCTGGGCGGGGACAGCATCGGG
GATATTTACAACAGCAATTGCAATGGCAGCGATACTGCTGTGACTACAACCAGCAGGAAA
AACAAAAACAGCAACAACAGAAATATCATCGTGACGGCCTACTACAAGATCCCTTCCAAG
CATACCCCGGAGCGCTACAGGGACTGGATGAAGAATTTCCTAGGTCGATTCCGAGACCCG
GTGGTGGCATTTACGTCGCCCGACATGGTCGATTCCCTGGCGGCACTGCGATGTCCATTG
TCGAACTCGTCGTCAAGTAATTCGACTTCGGATAAGGATCTGAACCGTTCGTTGTCGCAA
TCACCGGCCTGCTTTCCCACGGTTCTGGTGGCCCTGCCCCTGGAGGAGCTGCCCCTCGCC
GAGCTTTATCCAAAATCTTTTTGGGTCGATCAGCTCGACCGGGATCCCGAGGGGAAGATC
CACGCTCCCCTTCGGGGGGATCCACGGGTCGGGGGGAGATTCGAGAGCGGGGAACTCTAC
TGGATCTGGCTTTCCAAGGCCTGGTTCGTGCACGAAGCCGTGGGGCTGTTAATGCAGGAA
CAACACCAGGGAGCGAACGAAGAAAAGAAATCGGTGGAGGAAGAAGTGGCAGCAACGACA
AGGTTAACATCAACAGCAACGGCAGCCGAAGGTGAGAAAGAGCATTGTCCCGAACGCGAC
AAACGCGGTGACGTCTTTGCGTGGATCG
>comp1841_c0_seq1
AACGACAACGACGACGACAACGACGAAACCAACGCGGCGCCGTCCTCCCCCCGGGTCAAG
TTCCGCGGGATCGATGCTTCCACCACCGGCGGCGGAAGCAGCGGCGGGGATTCCTTCGTG
GGGGGCAACCCGCTGGACCGGTTCCTGGCGGTTGTGGCCTCGGACTCCTTCTCGATCGCC
TCGGGGGCCATCGGGCTGCTGGCGGTGGTCGTCCACCGCTGGAACCTTGTTCTGGCGGCC
TCGGACGGGTCCACGGCCGCCTCCATCGCCGCCGCCGAGGCCCTGACCTACCAGACCCGG
ACCGACCTGCTGGGGGTCTTTGCGGCGGGGTCGGTCCTCCTCAACGGCGTCACCAAGCTC
GACGTCACCACGGCCCTGGCGGAGTCGGTGGTCCTGGAGGGGAAGGCGCTGCCTTCGCCG
GAATCGGTCGGTGGCGCCAGCGGGGAACGGCCCGGGCCGGAGGTCGCCTGGGGCCTGGAA
TCGGTCCTGGGGGCGACCCCGGCGAGGTCGGCCGTCCTGCTGGGAAGCGACAAGGATGGG
GGATGGGTCGTCCTGCACCGGGCGGGGGTGGTTCCGCCGGTTCCGGCCCCCGTCCCGGGC
AAGACCCCCATCCTGGACCGGGTGGGCTCCCCCGGGAACACCCGGGAGACCTACCTCCCC
ACGCTGCAGGCCCTGCCGGGGAGGACCGAGCTCAACTACCTGCCCCCCAACGCGCAGATG
GCGGTCATGCTCCCGATCGCGGCCGGCGGCGAAGCGCAGAGCGAAAACGAAAGCGAAAGC
GAAAGCGGAAGCGGAAACAGCGTCCTGGTGCTGGGGGGGAACGCCGCCAAGAGCTTCACG
CCGAGGGACATTGCCTGGAGCCGGATCGTTGCCGAGCGGATCGGGGAGAGCCTCTAGCGA
TCGGTTTTTCCGAGCAAGAGACGACGGTATCATGTCCGACACCGATGCATGCGACGGACG
CAACCGAGAGACGAACGAAATGTGGTTCCCGGCGTGGTGATCTTGCCTGTATTGCCCTAT
CGGTTGGGTAGGTCGAGTTGGGTAGGTCGAGTGTGTCCGGTTTGCAGTGTGGAAAGAGTC
AAGTACGCGTTGGTGGGATGGGGGAGGAGCACCGCCGACACACAAGGTGCCATCGGGGAT
GCCCGGGCGGCATGGCACTGCCGTACACAGCCAGCACAAAGCACCACACGCCATCTCTGC
CCGATGGCGGGCGACGCGATTCGATTCGTTTCGCGAAACACCTTTCTGTTGTTCGGTGAC
TTTCCCAGGCCGGACCTGGAGCACACCTTGCGAGTCGGGTCGTTGGCTTGCTGCCGCGCT
GGGCTTTTGTTGCGTGAAGCGCATGGTACGGGCACCACGCAATTTCTGTCGAGCGTGGTG
CTTACCGGAGCTCATCGCGGTGACAACCGACGATTCCGATTCCTACACTCGCCACGAACA
GCCGGCCATCCCTACAGCATAGCATAGGTATTAAAAATCATA
>comp1843_c0_seq1
CCGGAAGGTAGAGGTCGCCGCCGAGGAGGCCGCGGAGGACGAGACCGAGGCCCTCGAGGA
ACTGGAGGCCCTGCCGGACGGCGTCTTTGTGGAAGCCCCGGAGGAGGGAGACATGGCGGC
GGTGCACGGGTCGTCCTGGGGGAGCCTGGACGGCAGGCACAAGCGAAACCCGGGCCCCCC

GGGAAGCAAGAACGGAGGCAGGGCCTCGAAAGACCAGGACGGCGACGTGGAGATGGAACC
CGCTACCAAAAAGACCAAGCTCGGAACCGAGCAAGACGGAGCCCAGGGGGCGAAACCGGG
CGGACCGAAGGGAGGAAGCGGGGAGGGAGAGGAAAACGACGATGCCCTTTCGGACGAGTT
TGTCTACGTTCCCTCCTTTTATCCCCGGCCTCCCTCGACCAAGGTCGTGGTGGACGATCG
GCGGACGGTGGTGGACGAGACGGACGACGAGGAGCAGCGATTGCTCCAGCAGACGCGGAC
GCAGCTCCGGCAGGAACAGCAGGTCTCCTACGCGTCGTCCCTGGCCGCGAGGGCTTTCCC
CGGAGCGGCCCTCCGCGACACGATCGACATCGACTCGTCCCGCGGGGTCCGGACCTCCCT
GGTGCAGCTGGGCAGCGGGCACTCGCACTACTGGGGCTCGGGCTGGGACGAGGCGGCCCC
GAAGGGCCAGAGGCCGGCGTCCGCGTCGGCGATTGCGGTCCCGATGGGACGAAAGCCGGC
GGCCTCCGGAAACACGGTCCCCCTGGGAGAGCCGATCCTCCCCATGAGCCGCGCCAGTGG
ATCGAGGGTCAGCCGGGTCCTGGAGGGGTCGATGGACGCGGCCGCGATGCAATAAGAACG
ACAACACTAGTACGCACTGTACTTGGTAGCAACACACAAACACAAATACAAATACACGCA
CACTTTTTGCAACAACAACAACAACAACCAAAGACAAGCAAGGAAGCGTTTCAACAACGT
GAGCGGCTCCAACAGAAATTTTTGGCAATGCATTTCCATGCCGTTGTTTGCTGTTTTGGT
ATGCAAGATTCGCACAGTGCAGCGTTGTTGCGCTCCGAGCAGTGTTGCTCCTTGGCGTTG
CTTTCTAGCGTTGTATTTTAGATCTTTCGC
>comp1844_c0_seq1
GAAATTGTACCTCGCCATTTTTTGTTGAACGCCATCAAGCCAAGCAACAAACATTTTCCC
TCTGCCACATGGTATTCCATATTGCCAAAAAAGGACGGGTACAATGAAAACACCGCGAAG
CCTTCTTGCTGCTCTCTTTGCGACGGTGCATTTGCAACTGCCACCACCCTGTCACTCGTT
CAGCGCGAAACTCACAACAAATCCAAAGGCCGCTCCAGGTACCAACAACGTCAACTTCGA
GGACTGGATCACGGATGCTCCCGAGGAAGTCAGGGAATGGAAGGATCTGACCGTTAGAGG
GGATATCCCGGACTATGTCCGCGGTACCTGGATCCGCAACGGCGGAGGAATATGGTCTGG
CGGCGACAACGGCGACGGCGGTGAGTCCTTTTCCCACATTTTTGACGGCTTTGCCAAGGT
ATCGGCCTACCGGTTCGGAAACGGGAGGCTCCGCCACCAGGCGCGGTTTCTCGAGGGGAA
GTGGTACAAGAAGTTCCAATCGGACGGAAGTCTGCCTCCAAGCATCGGGACCGGGCCGAT
CCTCGACGCCTCCGGAACCCCCAAGGTCGGCGCCGCGAGAACGGTCGGGGCGATCCTCAA
TGTTGCCACGGCCTTTGACAACGCCCCGGTAAACATCTGGGATTTCGATCCGCTCTCGAG
GACAGCCGGTCCCAAAAGGGTGGAGGCCTGACGGATGCCCCGCCCCGGGTGAACCTGGAC
TACGACACGATGGACACCCTTTCGAGCAGCACCATCAACCCACTGGCCAAGGGAATCCGG
GGCTTCGAGCTCCTCATCACGGCCCACCCGCTGTACTCCCTCGAGAGCGACGACACCTAC
AACATAGCGGTGGAGATCGGCCTCGGCCTTCCCAACGCGAGGGTCAACCTGATCAAGGAA
ACACCGCTCGGGGACCGGAGCGTGGTCGGATCCTTCGCCACCGACGACGGGATCCCGTAT
TTTCACTCGTTTGGGTTGTCTCGAAACTACGCCGTCGTGGTGGTGCAACCCCTACGGATC
GACCCGGGCAATCTTTCCGGGTTGCTGGAGAAGGGTTTTATGCGGTCCATGAAGAGCGTG
GAGAAGACGAGGGTGGTGGTCCTTGATCTCAAGACGGGGGAGTGCGTCCTCGACCAGAGC
GTCCCCGAACCACTCTATTTTTATCACGCCGTCAGCACGGCCGAGGTCGACCACGGGCGC
TCGCTCTCGCTGCGCCTGTGCGCCTACAAGAGTTCCGACCAGCTCACGGGAGAGAACCAG
TTCATGCGGCTCGAGCGGTGCCGCAGGGGCAAGGAATGGAGGAACAAGCTCGACAAGGGC
GGCCGGTTCTGCGACGTCGTCTGCGACCTGGAGAAGGGTACTTTCTCGCTGCGGTGGGAC
GATCGCATCCGCCAGGGGTTCGAGCTCCCGGTCACGCGGTACTCGCGGGCCCACGGCGGA
AGCGTGGAAGAAAGGAGGGCGGCGGCCGATGCCCCGATCGATGGCCACCCGCGCTACGTG
TACTCCTTCGGGGCGTTCGCCCTCGGTGCGGAGGAGTACGACGCCTGGGGCCTCTTCAAG
TTCGACACGGAACGCCGGGAGGTCGCGGCCTCGTACCGTTGCGATTCCGTGTACGTGTCC
GAACCGGTGTTTGTCCCGGATCCGAGCGGTTCGCGGGAAGACGACGGCGTCCTGCTGACC
CAGGCGTATTTTGGTGCCGAGCGAGAAACCAGGCTCCTGGTCCTCGACGCCCGGACCATG
GAGGTGGTCGCCGAGGCCGGCACCGGCATGCGCACGCCGATGGATTTCCACGGGGGATGG
ATTCCGGAGGGCGGCGGCGGCCGCCCCTAGCTTCCACTGCTACCGATGCTGCCGCTATAC
CGCCATTGCTATTGCTACTGCCATTGCTACGCTTGGGTACCCGACACTCCTTTGCGCCTC
ATAAATTCCAATGTTGAGAGCGCAGATCGACGAAAGAGGAATGCAACCCACAACTTAAAA
TGTATCGAGTAGAAGTAGATTTTAGATTTTAGAAC
>comp1847_c0_seq1
GCCTGAGTCATGGTGACATCGGTCCCACTGGTTCGGCTGGAAATTTTTGTGCTGTAGGAA
CTTTCGGGCCAGGAATCGAGATCTGGAACCTGGATGTAATGAATGCCCTTGAGCCTTCGT
GTGTCCTTGGAGGCGAGGATACATCCATGGCCGACGACATTATGAGACTTAACATGATGG
GTGCCTCTGAGAAGAAACACAGCAATACACTTTCACCACAAGGTAGATCCCTAAAACCTG
GAAGTCATACTGATGCTGTAATGGCATTATCATGGAATAAAGTTCACCGACAAGTAATTG
CTAGTGGATCTGCAGATTGCACGGTGAAATTATGGGATGTTACACAGGCTCAGTCTGATA
AAGCCAACGCTGCAACTCTAAAACATCACGAAGACAAGGTACAATCCGTCTTGTGGCATC
CGAAGGAGGGGACACTCCTCGCCACTGGATCGTATGATCGAACTGTGGGTCTGGTTGATG

CTAGGAGTACAGAGAATGTGAAAACAGTAAAAATACCAGGAGACTGCGAATCTTTGGCAT
GGGACCCTTTCAACCCTCAATACTTATCTGCTGCTAGTGAGGATGGAACTGTTACGTGTT
GGGACGTTCGCAAATTCTCAACCAAAACTCCCCTCTGGTCCATTGTGGCAAGCGAATTCG
GAGGAGTCAGTGATCTGGCATACAATACCCATGTTCCTGGTATGATGGCAACTTCTTCTG
TCGATAAAACTGTAACAATTTGGGATACTGCTACTCAATCTCCAACAGCGAAAATGAACA
AAGATATGAAAGTTGGGAAGCTATTCACTTTGAAATTTTATCCCTCTTTACCATGGCTTA
TGGGTTGTGCAGGAGGTTCGAAAGAAATGGCGATATGGGATATGTCACAAGATGATACGA
TTCAAAAATGCTTTGGTGACCGGATAGACCATTCCTCTCTTGTGCAAACGGATGAACGTG
ACAGAAAGCCACAAAATGAAATAAGTTTTGATTCATTGATGAACTCGACATCAGTTGAAA
AAACTAATGGCAAGAGTAAAGGAAAATCATCAATTTCGAACAAAAAGAAGAAAAAGAAAA
AGGCACACCGAGCCGGCCGATGATTACTGCTTAAAAAAAACAGATGAAATGATAGTCAAC
GAATTTTTCAGAGATGACAATTTTCAGTACATGTGGAACAACCAAATGTAGGTGGCTAAT
TGGATGAATAGACCAAAATGCGAGTTAATTGAGCAGAATGACATTTCAAGG
>comp1848_c0_seq1
GAATAATAGTAATCACATCATACTACGACAATAGGATAATCTCCCTGCCAATACAAAAAG
TACTACCGCAGTATTATTTCGATAGGCTCTTCCGCCCCTACCTGCCTGCTGACATCCCTT
TGCTTAGCTTAGACGGCATATCTCCATTCCTCGCCGTAGTGCTTTGCAGTGTGCATACAG
ATACAGCCCAGCAAGATTGAATTCGGATAGTGGGGAAGATTCTGACAATTTGGCCACTCG
CTGGAACACGAGCAAAAACAAAAGCAACGCGGCAATGCAACAGAAACGAAGAAAACGGAG
GCGGTTCCTGGTTCCAATGGCAGCAGCAAATGCCACATTGGTTTTCCACAGCAACGCCGA
AGAGGGCGAGTGCAACAACGATAGCAAACGACTTGTCACCGAAAACAACATTCTCGTAAA
CACCGGCGCGAACCACACAATACGAATGACCACTACCACGAGAAGGATACGAACCAGAGC
GCGGAAACAACACATCTTGCTTTCCCCGAGCCTTTTTTTAGTGTTGGTCACTATTGCTTT
GTTTCAAACCGTTGACCCCCTGCTGGTCCGTGCATTTTGCCCTTCTCCTGGAATTGAATC
CATTTCCTATTCTAGATGGAAGCAAAGCATCGGGCGCCCGAGGTTTCTTTCCACATCCTT
CCTGAAGTTGTCAACGGTGACCCCACCAAAAACAGTCTTACCCAGTCGGGCCGAGCTCGA
ATCAATGAAGGTAGTTGATCTGAAAGAATTGATCAAGGATTCTGGTTTGAACGAACGAGG
ATTGTTTTCGAGGCTCAAGCGCAAGCAGGATCTGGTGGATTTTTTGGTTCAGGCGGGAAG
CAGGGAACAACAAACCACAACAGCGGCGGCGAAAGAGAAATCACAAATCATCAGCGACCA
ACCCAAAGAGCAGCAAACACAGCCCAAGACGCGACGCAAACCTCTGGGAATGCCCTCCAA
AAAAAGCGGGGCGCGACAAGATGCGGCAACAACAACAACAACAGCAAACAAACAACAAGA
TACTGGTACTGCAAGCCACAACGCTGTTGCCGACGAAGAATCACTGCCATTGCCAAAATC
GCCGCTGCAAGTGATCTACGATCGGGTACACGAACAATACCCTCCCCTGAAGTACCTTTC
GGAAGGAAACAACACCCTCCTTGGCGAACAGGACATCCGACAAAAGTACCACCCCATGCT
GCAACATGCAGCGAAAGCCATCACTTCGAAACGCGACAAGAACAACAAGGACCAAAACGA
CGACCAAGACGGCAAGAGTGACGATGGGGAATCCGAAACCGACGATGACACCTACACAAA
GCCGGCCCTCTCTGGAGACATGGACCTCATTTTTGTTGGGACGGCGTCGTGCACCCCGAG
CATTACACGAGGGGTCTCCTGCACGGCCCTTCGACTGCATTCCCTCGCCAAGCAACAGAC
GCAATCGGCCAACGTCAGGGTCGATGCGAGAAAGCCAAGCAAAGGCAACACCAAACAGAG
TGGCAAAAACTTTGATGTCGCCCAAAACTCCCAGTCGAACCTCGGAACCTGGCTCTTTGA
TTGCGGAGAAAGCACGCAGGTAAGGAAAGGAAAGGAATTGCGACGAAGCGAAAAAGTATC
G
>comp1848_c0_seq2
GAATAATAGTAATCACATCATACTACGACAATAGGATAATCTCCCTGCCAATACAAAAAG
TACTACCGCAGTATTATTTCGATAGGCTCTTCCGCCCCTACCTGCCTGCTGACATCCCTT
TGCTTAGCTTAGACGGCATATCTCCATTCCTCGCCGTAGTGCTTTGCAGTGTGCATACAG
ATACAGCCCAGCAAGATTGAATTCGGATAGTGGGGAAGATTCTGACAATTTGGCCACTCG
CTGGAACACGAGCAAAAACAAAAGCAACGCGGCAATGCAACAGAAACGAAGAAAACGGAG
GCGGTTCCTGGTTCCAATGGCAGCAGCAAATGCCACATTGGTTTTCCACAGCAACGCCGA
AGAGGGCGAGTGCAACAACGATAGCAAACGACTTGTCACCGAAAACAACATTCTCGTAAA
CACCGGCGCGAACCACACAATACGAATGACCACTACCACGAGAAGGATACGAACCAGAGC
GCGGAAACAACACATCTTGCTTTCCCCGAGCCTTTTTTTAGTGTTGGTCACTATTGCTTT
GTTTCAAACCGTTGACCCCCTGCTGGTCCGTGCATTTTGCCCTTCTCCTGGAATTGAATC
CATTTCCTATTCTAGATGGAAGCAAAGCATCGGGCGCCCGAGGTTTCTTTCCACATCCTT
CCTGAAGTTGTCAACGGTGACCCCACCAAAAACAGTCTTACCCAGTCGGGCCGAGCTCGA
ATCAATGAAGGTAGTTGATCTGAAAGAATTGATCAAGGATTCTGGTTTGAACGAACGAGG
ATTGTTTTCGAGGCTCAAGCGCAAGCAGGATCTGGTGGATTTTTTGGTTCAGGCGGGAAG
CAGGGAACAACAAACCACAACAGCGGCGGCGAAAGAGAAATCACAAATCATCAGCGACCA
ACCCAAAGAGCAGCAAACACAGCCCAAGACGCGACGCAAACCTCTGGGAATGCCCTCCAA
AAAAAGCGGGGCGCGACAAGATGCGGCAACAACAACAACAACAGCAAACAAACAACAAGA

TACTGGTACTGCAAGCCACAACGCTGTTGCCGACGAAGAATCACTGCCATTGCCAAAATC
GCCGCTGCAAGTGATCTACGATCGGGTACACGAACAATACCCTCCCCTGAAGTACCTTTC
GGAAGGAAACAACACCCTCCTTGGCGAACAGGACATCCGACAAAAGTACCACCCCATGCT
GCAACATGCAGCGAAAGCCATCACTTCGAAACGCGACAAGAACAACAAGGACCAAAACGA
CGACCAAGACGGCAAGAGTGACGATGGGGAATCCGAAACCGACGATGACACCTACACAAA
GCCGGCCCTCTCTGGAGACATGGACCTCATTTTTGTTGGGACGGCGTCGTGCACCCCGAG
CATTACACGAGGGGTCTCCTGCACGGCCCTTCGACTGCATTCCCTCGCCAAGCAACAGAC
GCAATCGGCCAACGTCAGGGTCGATGCGAGAAAGCCAAGCAAAGGCAACACCAAACAGAG
TGGCAAAAACTTTGATGTCGCCCAAAACTCCCAGTCGAACCTCGGAACCTGGCTCTTTGA
TTGCGGAGAAAGCACGCAGCTCCAGATCCAAAAGACTTCG
>comp1851_c0_seq1
GGCAGGTGCCGATGGACCGGCTGCCAACGGCCTTGTCGCTGCCGCCGTGGTAGAGGTAGT
TCCAGAACTCTTCGTCGTCGTCGCAGTCGCCCTGGTCCAGGACCGTGACGGTTCCGAGCG
TGCAGAGTTCCTCGCCCCGCTGGGTGCAAGCAATCTTGTCCATGATTTTCATCTGCGCGA
ACGGCATTTCGTGTTTGGACGATAGACGCGTTAGTTCGGGTTTTCCCGGGCACCAAAACA
AGATTGAAAGCAAAAACAGGCAAACGAGCATCCTTCGGTCGAGGAACGCATCGACAGCGC
TCTCCCAATCCACACTGCGCGCGCACACACATACATACACACACACACACACACAAAGGC
CACGACCGCACAAGTCTCGTTTCGTTGTACCCACCTCCTTCCCGTGCCACACCCACACGG
TGGCCTTGTCGGCCAACAGAATGAACCCGCTCCTCGAATCCATCGACGAGACGTCCATGG
GTACCTGGACCAGCTCCCCCTCCTTGCGGTTTTCCCGGGGCCCGTACCGGAAAAAGAGCG
GTTTCTCCTTTGTGGGCTCGACCTTGACAAAGCCGGACGAGATGCCCCCCTCCAGGTACT
CGAGGCGG
>comp1852_c0_seq1
CACCTTTGCGAAACGCCTGGCGGAGGGCTACGAGTACTACGCGGGGGTCCTCGGAGGGGA
GCTCCCGGCCCGCAAGCGGCCGAGGATCGCGCCCGTGGGAAACGCATTCCTTGTCCTCTG
GGAGGAAAACATTTCGTTCTGGAACCGGTTGTTCGGAGAAGACCTGTACCACCCGAGCCC
CCACGGGACCTACCTGGCGGCGTGCGTGGTGTACGCCACCATCTACGGCCGGATGCCGCC
GGCCTCGACCCGGTTCACTACCGAGCTCTTTGCGCGCTCCCGCAGTATGCAGCTCTCGGG
GGAGGAACAGCCGCTGCCCACCGAGGAGGAGGCGCTGTACCTGCGCTGGATCGCCAAGAG
GGTGGCCCTGGAGGGATTCGTCCCCAGGAGCCTAAGAACGAACCGCACCCGAACCTGATT
TTATCCCGGTGGGCAGTGCGATGTATGGGGGTTTGCTCTGCATTATTCGTTAGCGTCTGG
CGCTGCGGGATTCGGTGCGAGGCGTAAAGATCCTTCGATCGTGGGTGCCGGATGGGAATC
TATCTTCCGAGCCAAGCCAAGCCCGGCCGCGGGGAGGCCTACCCTTGAACGTCGTGGGTA
TGGGCCGGGGACAGCGGTCTCGAGCCGGCGAGGAGCCGCTGCCCGGGCAGCTTGCCCGTC
CGGGCGGCCTCTCCGAGCTCGTCCAGGCCGATCGTTTCGAGGCCGATGGCCCCGAGGAGG
TCGTTCTCGGCCGAGAAGTTCCGCCCGTACATGGCGTCGCAGATCCTCATGCCCGATTCG
TGGAGGACGGCGGGCCGGCCGACCAGCTTTCCGAGCGCCACGACGGGCGCCAGGCCGTAG
GGGACGTCCTCCGTGACGTACCTGGAATCGGCGGCGGTGGGGCCGTGGACGTCGTTGCCC
TTTTCGTAGATGTTCTGGCACATCTCCGAGAGCGATCCCGAAGCGGGGATCGGCACGTGG
AAGGACAGAGAAAAGTGGTCGTAGACCGTCTTGACGTCCAGCCCCAGTGCGGAGGCAATC
TCCAGCCGCTCTTTGTCGAGGGCCTCGAGGAAGCCGCCGATCTTTGGCGTGATGTTTTGG
AACTGGTACCATTCCTCTCCCTTGTCCATTCGGCTGATGTTGCCCATGGCGATGGCCAGG
TGGTTCTGCGGGTTCAGGTTCGACAGGGTGATGGCCAGCAGCCCCTCCCTCGGCCGGAAC
TCCGTGTCCGGGAAGAGCCGCTCGCAGAGCTCCAGGGACGAGGCCGAGTCCCCCTCCGGA
AGGCAGCTCGAGTCGACGGATTTCCGTATGGTTTTGATGTCGACGCTTGTGCCCGACGTG
CGTCTCGCGGTACAGACCGTGGTGCCCCAGGACGTGATGGTGTGGTGGTGGTTGCCGAGC
TGGTGGAGAAACTGGGAGAGATAGAGCGCACCAAAGGAGGCGTGGGACGAGACAATGATG
TGCTTCTTTTTCGGTGGAGCACCAGCGGCCGCAGGCGAGGAATCCGACGACGACGACGAC
AGGTGGGGCGCCAGGGCATCGAATATCTGTTTGTGTCCGTTGGCGGGGAGGGCAATGATC
AGCACGTCGGACTTCTTCACCAATTCCTCGGCGCTCGAAGCGATCTCTGGGTCGAATTCG
TGGCGCAGTGCCCCCGTGGAAGAAACCGTGGACGGCCCCTCGTCTCCGCTGCCGTTGTCC
CTGGATACTAGATCGGCCGTTCCTTTCCCGGACGGAGACCACAGAATGCTCTCGTGCCCG
TTCTTGGAGAGAATCGAGGCCGTCCCGAACGCAACGGCACCGGCACCTGCAATACCGACC
CGCAGCGGGCCCTGCGAGCATTCCGTGTGGCTTTCCCCCCGGTGTGTTGCCGAAAGGCGT
TCTTGTTCCCAGATGCGGCAGTTCGAACGCCTGTGGTCCGAGCCGGTGAACTCCCATCGA
ACATTCCGGGAACCGCCGCGAACCGCAAAGGAGGTCACGGCTGCGAAATTCCACGCCAGG
AGTGCCGCTGTTGCAACCCTATGGTGGTGCATCGTGAAATAGTGTCGATGAAATACGGAA
AACTGTCTTCTGTCGGCCTACACTACCGGTTCCGCCCGAACCCAACTTTTTTCATAAGCT
AGTATGCACTTATTCA
>comp1855_c0_seq1

CAACATACCGGAGGACCTCGAAACAACAGAGCGACGTCGGCCATTTGGCCATACACCTAC
CGACAAAAAATCAAATCAAAACAAAAGGAAACAACACGAAACGAAACGAAACGAAACCAA
GTCCGATCATTAGAACACAAGACATTGCAAAACAGCCATCGTAACGAACAGAACCGCAAA
CCAGCAACTTCTACCACTACCACCACCACACAACCGACCATGAGATACGACCTGGCCTTT
TTCCTGTGCTGCTGCCGATCGTTTCTCGGTGATGGCGAGAACGACGACGGCGAGGCCAGC
GGCTTTTACGGACTTCTGGGCGTCGAGTCCGACGCCACCCCCGACCAGATCAAGCGGGCC
TACAAGCGGCGGTCCCTGCAGATGCACCCCGACAAGCTGGCCCAGCGCGGAAAACCCGTC
ACGGACGAGGACCGGGCACGGTTCACCCGCATGAAGGACGCCTACGAGTGCCTCAGCGAT
CCGCACAAGCGGGAGACCTATGACGCGATCGGCGAGAAGGGCATGAAGTGGCTGGACGAA
CCCTTCTCGGTGGACCCGCAGGAGCTGGCGGGGAACTTTGCCAGGTCCTCGGTCCTGGAC
CGCTCCAAGATTTTTGCCATCTTTGTCGGCCTGGCGGTGGCCCTGCTGGCCCTTCCGGTG
GCGGTCTGCCTGCATCTGGACGGGGACCTGGGGGACGACGCCTCCTGGTCGGTGACCCTG
GTGCCGCTGTGGATCTGGAACGCCATGGCACTCTTCTACCACTCCCGAGTCCTCACGATG
CCCACCGTGTCGGCACCGGACGGTGTGCCGCCGGAAGAATGGATCGACCCCTTTCCGATG
AAGGACCGGATCCGGTCCCTGTTCCGGTTCCTGCTGCTGGTCTCGGCCGAGATCCTGCTC
GCTTTGAAGCTGGACGAGGTCCTGGAATGCGCGTGGGGCCTCGTGTTTCTCCCGGTCTAC
CTCTGGGAGGCCGCGAATGTCCACGAGCAGTGGCCGCTGTCGAGGCTCCGGACCCTCACA
GTAGAAGACCTGGAGGCGGCCCTCGGGAAGCCCCTCGGGGAGTGCACAGCCGACGAAACG
GAGGCGATCCGGAGGGCCTACGTGGTCGTCCCATCGGCCGAAAGCCCCGAGTTCGCCGAG
GCGGAGGAGCGGAAAGCCCGCGCCGCGCGGGAACTCGCTAAGAGCGCCGCTCGGGCCATC
GTTCTCTCCGCCCTGGCGGTCCGGCTCGATACCCGCGCCGACTGGAACTGGTGGCTGGTC
TTTCTGCCGGTCTGGATCCTCTCCCTTGGGGCCTGCCTGACCGACTACCTAAGTTTTGCG
GAAGCCTCGGCGAGTGCCGCCGAAAAAGACCCCGGGCTCTTTGGGGGGGACCGGGACGAT
CCCGAGGCAGCGGCAACGACACGGGCGTCTTTCGGAGCGAGCGGTAGTGCCGCGGCAGCG
GAAACCACCAGCGCCGTCCAGAGCGGGGAAAGCGGCGGCCCCGGAAGTGGCGGCACCGCC
ACCAACTACGGATCGGTAGGGGCCGACGGTGCCGCGACCCCTTCCGCCGCCGCGGCCGGG
TCGGACCTATCGGAGGAAGAAAAGGACCGGCTGCGGGAGCACGTGATGAACCAGGGGTCG
GAGTGCTGCACCCGTTGCTGCTGGCGGGGATGCTTTTTGCTCCTGATGGGCCTCGTGGCG
GCCAAGCTGCAGGGCGCTGCCTTTTCGGCCTTTTGGATTATCTCTCCTTTTCTGTTTGCG
GCCGGAATCATCCTCCTTTGCTTGGGGTTCGCGATTTTTGGCGTATCGGAAACGACCGAT
GGCGAAGAGCGCGGAGCCGAGTTTTATTCGCACCCCGCAGCGAGTGACATCACCACCAAT
GCACAGCCTTCGACAAACATTGTCTTGCCGGTGGCACCAGGCACTCCGGATACCCGAGGG
CTGCTGGCAACAGCTGGAGGGTCGAGCGCTAGTGAAAACGAGATGGAGATTCGTGGACTT
GATTAGCTATAATCACGCAGTGCAATACGCAAGGATTTGTTAGCTTTTAACTTGTTAGTA
TTTAACTACGAAGTAGTATAGGAC
>comp1856_c0_seq1
CAAAAAAATACTCCCGTTCAGAAGAACCGAAACAAAGCACTAGCCACCATGACTACCGAC
AAAGATCACACCAAATCCAACGAGATCTACCACTTTCCAACCCATGCGTCCTATCTTGTT
CCAGTGGTAATAAAAGAAACTCCACACCACGGAGAGGGTCAAAGGGGCGTCTTTGCGAAA
GAACCCATTGCGAAGGGAACCAAACTGTGGGTGTGGACCCATCGAGTGAAGTCGATTCAC
CGCGATGATCTCGAAGACTACATTCAAAAAACGTTTGGAAACGACCTAGAAGAAACAAGG
GTGTTTCTGAGACAGGGATTCGTGCTGCCCCCGAAGCATTCCTCCGAGACAATGTCGCCG
CCACAAAAACAGGGCGAAACCACACCACGAAACGCCAGCAACGACGACAATTTCTTCCAC
TCGAATCCCACCGACGCCGGGCGGCTCACAAACCACAGCTCCGATCCCAACACGGGTCCC
GATGGAGCTCTCAGAGACATCGATCCGGGAGAAGAAATGACGATGGACTATTCCTTCCAC
GGCAATCCGGAGTGGTACCAAGCGATTTGCGCTAGATTCGGGGTCATGACCGAGGCCCAG
ATTGCGGCATTGCACCACACGTAGGCAACGGTAGAATAGAATCACTCGGCCGAAAGGATC
AAAAGCTTTTTTCTAACAAACTAGAAAGTAGACCTCTTGGTTATCATGTCTATTTTTCGA
TAAACCATCAGCTGGAAGCGTTGATTCCGAACCTCTCCAGGCATTTT
>comp1858_c0_seq1
ATTGAATAAAACTGCGAACAAATTCAAACATCACCTTAGATAGTTCATCTCGTTGGCATA
TAATCGAAACGGAGCACACAGTGGTCACTAGAGGAAAGAAACATGGGCCCTTTGCCAACT
ACCTCCTTCTTATCCAAAAGCAAAAGAGAAGAAGAAAAAGACCAGGATGAAGTAGACGAA
TTTAAGAACAAAATCTTGCGCAATCAGAGCTGTCGACAATGGCTTGTTTCGGAATCTATT
AAAATCGCATTTTTACTATCATTCGGTGTTGTGCTCTTGCAATGGAAAATCATGAAACAA
GTCGATGTTACAGAGGATCTTAGCTTCCAGCCTTTTGTATCTGGCCACAGAGCTCTCAAA
ACTCTTAACGCCCCTCTAGGTGAAAAAAACGACAATCCGAAGTATCGCAATTGTCCTTTT
CGAGATTCGCCTATTGTAGAATCAATCTATGTTTATCCGCATCCAGGATCTCCCGAATGG
AAGGGAGATATTCTATCGAATTATACAAGAATCCAAACGAATTCAAGTAGCGAAGATTTT
ATCGTGTATCCGTGGATAGACAACGACCTACGTTGCAAAGCTGCTGGTGTTGGTCCCTAC

GATGGCACATCGCAGCTTGTTCAATACAATACAGAACTTTTGGTACGAGATATAATAACA
CACCCTGACTCATGTTTGAGGACGAATGACCCAGAAAAAGCAACCTTGTTTTACGTTCCA
TATCTTCCATCGGCCGAATACAAAAACGGGACGCTTTTTGGTAAGGATATGTCACAAAGT
AAATACGGAAAAGCCATCCAAAACATAATTACCAAACAAGAGTACGAATCGTGGGAAAAG
GAATTCGGGCTTACATCGGAATACTGGAAAAGACGGAACGGTTCCGATCACATTCTTGTC
TTCAGCGAACCCATGCACGGATTATGGCATCCAAAATCGAGACGCGGAAATTTCCATTTT
CTACATAGTCAATTCCAAACACATTCACCCATTGTTATTTCGAACGAACTCAGCAAAACA
TTCATTGATATGTATCCTTTGTGTGCTAGAAAGAATATTCTCGTGCCGTATCCAAATACG
AATGGAAGGTGGTTCAATGGTGCACTGGATAACGAGGCAGTCGCGATGATGACAGAGCAC
TCTGGGATTCGAAAACTATCCGAATCGCCGGGTGCGATTCAGAGTGAGATAGATTTGGCA
AAACAATCGGAACTAAAAGAAACATCCAACGGATCGAATTCAACGCCACCCCGCCTTTTA
GGATATTATTATAGTGGTGGGAATCATGGGACCTGTGTTGACCTTCGAAAAAGTATGAAA
GATGAATTCCGGTGTTCTCCATCCGGGAAGATGGTAAAGAAATACAAAAAAGCACTCAAC
AACTACGCCCATGGGTATCGCCAATCTACCTTTTGCCCTTGTCCGGGTGGTGATTCACCG
TCCGCGAAACGTAATTTTGATTCCGTGTTCGCCGGATGCATTCCTGTCATTCTGTCGGAA
GATTTTGTTTGGCCCTTTACATCTGAATTTGATCGGAGCAGTAATAGTGGAAGTGAAAGC
GGTATAGTTGCATTGAACCCGTATGACTTTTCTATTCGTTTAAACGCCAAAGACCATTGG
CATTCTAAGTTCGACTCCCCAAAAAATTGCAACCCAAAAATCAACACCGGAGAAGGGCAC
AATGAAACAGATCATCGGTCTCTGCAATCTGTGTTAGAGGCCGTTCCATCGAAAGAATTG
GCACGACTCCGACAAGGGTTGGCGCGAGCAGCCGAGGTCTACTCTTATTATAGAAAGCGA
CCAGACCTGCCCGACGAGCCCCTTCGAGAGGGGTTACTACCCGACGGTGGGGCTGCCCAC
ATGCTGGTTCGGGCATTGGCAGAACGAGCTTCAGGTGCATTATGGAATGCGTGTCGACAG
GAAATGAAGGGCAAGGACATTTCGAAGGACTCGGTCAAAAAATTCAAATGCTGAGAGCTG
ATACTGTGAAATAGGTCAAGAAAAATTGCGTATTAAAGAAATAGGCCGCAAACATACAAA
AAGATGCGATCATGCAATGAAATGCACACAAGATGAAATTGTTATTTGTCAACAAATGCC
GCTGCATACTTCGCAGAACCCACAACCAGAGAGTAGTACTTGATCAGGTATCGTTCAAAT
TGTAGAAGGCAATGGTTTTCGACGTAGTAATGGGAACTATCGATTTACGAACAAAACGTC
GGCCCAATAACTCATTTACAAGAAGTGTACATTATCGAACTGCTGAGACAAATCAGAGAA
CATGGAAGCAAGCGAAAAAGATATTACAACTGCTACAAAACTGCTGTACTGCGTTCACTA
TTCGTATCAATTCGCAAGAAACAACAGGCATTGACAAGAAACCTGTTGCCG
>comp1864_c0_seq1
TGCTATTTCGGAGTCCGACTGTTACCTCTGTCAAAAAATTTCGGGCTTCGGCTCCCTTCG
AAAACCCAGATCCGTTCTGGTTGTCACTGTAGAAATATTCATAATCAATGTCGATGCCAT
CTAGATTCATCTCATTCACAATGGTTGTTAGCCGATCGATAACCTTTGTTTCTCTTCCAA
AGCAGTAATCCCAACAGTCATTATTGTCACCAGCCCAGCTACCTCCCATGCCAGCACCTC
CAAAACTGAGAATCACCTTCTTTCCCATACCCTTCCATTTGGCGATAAGATCCGGCCGAT
TGACATTTTCGCAAACAGGAGGCATTGCGATTTCACATGTTTCGCTACAGATATTCTTTC
CTGGACTCCATTGGTAGCTAACAGCGAATGCAATTACAACATGTGTATACTGAGCGATCT
GCTCATCGGTAGGACACGCTTGCCAGTTCCCAACGTACGCAATAACTCTTGATTCGCTTT
CGTTGGTAATAGGAGTGGCGATAGGTGTTGGAGGTGATACATCCGGTACCGAGATGACTT
GAACAGGTGACTGTGTCGAGATTTCAGGAGTGGGTGTGGGTGTGGGAGCAGGTTCATCAA
TATGAATGTTGTTGTCGTTCGGCAAAATTGATATCTCGGCACAATTTACGAATCTTTCTG
GAGAGTCGCCGGTATAAAACTCGGGAGGAAATTGCTCGGGAGTGCAGGTCGAAAGTTGTC
GATTCCAAAACTCTTGTGGCAAGTGACTGTTTAGACTATAATACTCGGCATAGCCTTCTG
GGGAACACGAGTTGGCTGTTACCTAACAAGTCGTAGAAGTTTTCATATTGTCAGAGATGT
TTGGGGGATCATAGATCATGCAAACAGGTCATAAATCAGTTCGAAAAGTACGTACATACA
ACCACTGCAGTAAGACCTCTTCTCCAGTCAATCCTTGAGGCAATCGGAAGTTCATAGCGA
ATCGATTATTGTTAAAGGCTTGAGATCCGTAATAGTAACCACGTTCTGGATAATTTTCGT
CTAAAAAAGGAAAACGATAACAACCAATGACATCAGTTGACGGAACATTCTTGTCTTAAC
TTAGCTCTGTCAGCTGAATTGAATGCATACTTACCCTTCGGCATTTTGACATCGAAGTTA
TTGACATCCTCCACAAACTCAAGCACATGTTGGTCGAAGCATTCTTGAGTGGAGGCACGG
CCCATTGGACATGCACGAAGCTCCATGTGTCCAGTGTGATGGGCTGTAAGGAAGGTCTCG
ACCGTAATGATGCCGCCCTCTTGGTAAACGTTTCCATTTGAGTTCCAAGAAATTGGCTGC
TGCAATGAATCAAGCCAACTGTCGTAATTGACACCTTGTTCGGATGTGCCGCATACCGAT
CCAACTCCCTTTGTATTGAGACAATGAAAGCAATATTCTTTCATTGGAATGTTGGGCTCG
GTCCCCCAAGTCAAACCAAATTGGTTGGAGTAGTAATTTCGAGAAATCGGTTCGAACATG
TACCCATGCCCGTGGACTTCCTCAACAAGAAAGGAAAAACTCGAAATAGTTGCAACAGTG
ATTTGGAGAAACTTCATGGCAGTCTTTTGTTATAATTTAATGTTGGGG
>comp1865_c0_seq1
GGAGCGCAGAGAGCTTGACGACAAAATCGAATCAGTCCATCCGCCTTCTTCAGCTCAACT

AAGACTTCGGGAACAAAAACATGGATTTCTCCCTACCACACGTGCACGAAGACTCTCTGG
CAACCCTGTCGAAGATTCGGCATATCGATTTACCCGCCAAGGATGAGAGGGGGGGTTCTA
CTGGCAATGGGGAACCATCGTTGGACGAGCTGCTGATCGAAGAGGCGAACAACGCCGCAG
CAAGCTTATTCCTCGGAAAATGGAACAACGACAACAAGGAGAACAACAGTGGTGAGGATG
CGATGGACTGTGGGGACAACAGCAGCAAGAGCAACAACAGTAGCCTCACCGTGAATGAGC
GTCGAATGCTGATGAATTCAGCTTCCTATCCCGAACGATCCACCCCCCAAACCGAAAAAA
TCACGGAGGATTCTCTGGTGGTCATTTTCGAGAGTTTCGACAACCTGAAGTTTTGCTACC
CCAAAAAAGGAGCGATATTCTCTAACCGCAACGGGCACTTTCACCACGAGGATTTCATCG
GAAAACCTTTTGGGTGCAAGATCCGCTCGCGGAACAACCAGGGGTGGGGATTTGTCTACT
TGCTCAAACCGACTCCAGAATTGTGGAGCCGGTCCCTGAACCACCGGACTCAGATCATCC
ATTTCCTTGACGCCAGCATGATCGTCTTCTATCTAAATATCCGGCCGAACATGGTGGTTT
GCGAGAGTGGAACGGGGTCGGGGGCGCTCAGTCACTGCATCATGCGGAGCATCGCTCCCA
GGGGGATGCTGCACACGTTTGAGTTCAACCAGAAACGCGCCGAGGATGCCAAAAGGGAGT
TCGCCAGCAACGGGGTAGGCCATTTATCGAAAGTCCACCACAAGGACGTGTGCGGGAAGC
ACGGAGCGGGTGGATTCGACCAACCCCAGTCGTCGGTGGATGCGGTTGTGCTGGACCTGC
CGGAACCCTGGCTGGCAGTTCCCCACGCGGCATACTGCATCAAGCGAAACGCAAGGCTGG
TCTCGTATTCGCCGTGCATCGAACAGGCGCAGAAAACGATCGAAACACTCAAAAAGAATG
GATTTCATTCCATCAAAACTATGGAGTTTCGCCTAAAGGAAAACTACGTCGACGAGGTGG
AGTACTCGATGCCGCCCACAGAGAAGCGACAGCGGGTCGAGGACAACCGTGTGTATCCCA
GGGAAGACAAGGAGATTGCCAGTGGCATCGCTGCGGACGCTGCTGCCTCAAACCAAAGCG
AAAAACCGGTGGTAGAAACGAAGGAAGAAAGCGGAATTTGTAAGGATAGTCCAACCGAAG
AAAGCACCGAACAATTGAAGACGGGCGATGCAACAAAAATAGGAGAAACGCAACCGGCAC
CGAAGGGCGCGGAGAAAAATCCGGAGCCGAGTTCCTCAGAGGCCACGGCCCCGGCAGCAG
CTAAACGAAAAGCGCCAAAAACAAAACTAGTTGCGCGGCCCTTTGTCATCATGCGAGGCC
ACACGGCATTCTTAACCTTCGCAACCTCCGGAAATGGCCTTCGACCAAAGCCATAGTTTT
CTCAACACGC
>comp1867_c0_seq1
GGCATCGCAACCTATTCTCATTTGGGTATTCTGGTGATAATTGTATACTGGTATATTGTT
GTCATCGACGGGATGCCTTCCGTTACCGAAAGCACGATTTTTCTTTCTAGAGAGCCTTAC
ATCTTGCTCCTCGTGGGCGGTGCCGCATCGCTCGCCGCCGCCTCTTCTACCAGCAGCCTC
ATTTCGACGAGGGCGTCCTCGAGGTATTGACTCAGCCGTTCCGGCCATCCGCCGTGTTCT
TTCAGCGCCGTGATTCCGAAAATCACGCGGTCCTTGTGGACGGAATAGCTGAGACCCACC
CCGTCGGGAACGACCTCTCCGTATCCCCAGTTGTCCAGCTTGGGGTGTGTCAGATTGGAG
GTGGAAACCCTCCATTTCTTGGACCGGACAAACACCGGATCCGAGTACAGATCGGGCGCT
GCCTCGCCGTCTCGTACCAGCATGCTCAACCCCAAAAAGTGGCGGTCGACGCCCAGCCCC
TTGGCGGCGTTGCCGATGTACCGGGCGTGTGATTTGCAGGCCTTGTTCAGCAGCGCGATC
TTGTCGGTCGCCGAGAGCGGTTGGCCGCCGCCGTTCCGGCCCAGCAGGCTCTGGACAAAG
GCCGCGCTCGCCGGACTGACGGTCCTGGTCGTTTCGGTCCGCCCGTGCCGGAAGACACGC
GTTTGGGAGGCCTCGTAGGTTCCTACCTGCTTTCCAAAGAGCCGAGCGGTAGCCACCTGC
ATGGCCATCTGGACGTAGGCATCGGGAGAAAAGCCGGATTTCTTCATAAAGCCGGACCCG
TATCCCCAAAAGCTCTGTACCTCTAGGTCGTGCTTGCCGGTGGTCCATTCCCGAAAGTTT
CCTTTCGAGGTCTCGATCATCTCCAGAACCTTCGGGGTCATCTCGATGGATCCAAAAATG
TCCGCAACACCGCCGAAGGTTTCCAAACCAGAATTCCCAGTGGTGCTTTCGTCTCGGGCC
TGCTGGTAGGTCGTGCTCACGATGTGGTTGGCGAGCCCTACCACCGGCATGCCATCCATC
ATGCTGTGTTCGCCGAGCAATCCCGCCTTGCCGTTCGGACAGACCATGAGCTGCACCGAC
TTGTCGAACCAGCGGTTGGAGGCGTCCCCGTTTCCGCACAGGAGCTGTTTGCCACACTGG
GCCCGGCTGCTAGGGGAGGCGTCGTCCAGGTTTACCAAGACACAGCCGCTCTCCAAGACT
TCCATCGCCTCGGCCATTCCGGGGAGTTCCAGCAAGGCCGCGCGCGCGGTCGCCCACGCG
TCCCGGTGATCTCCCGTCAGGTATCCCAGGCCGGGTGCTTCTGTTTTCGAGTCTTCCTCG
TCCGCCAGTTCCAGAATCTGCGAAAGGTGGCCCTCCAGCGACTCTACCGAAAGGACCTCG
TTTGTCGACTCATCGATCAGCGGAATTTTGTAGAAACGCCCCTTGCGGGCCACGACGGCG
TGCTTGTAGTGGTGGGGATCGTAGATGCGGTAGGAGTCCTGTTGGGGCAGCGGAATCCGA
CAGGCGTGGAACATGTATTTGTAGGCCGTCATGCACAAGGGAATATTTTTGCGCCCCAGG
GTTTCGGGTGCCAGCCGGCCGGAAGCCACGATGTTCCGGTACTGACCAGTGGCGTAGAGA
ATGGAAGCGGCCCGCTGGACCTGGAGCGAGTTTCCCTTGCTCCCTTTGCCAGCCAGCGTT
CGGTCGTCGGCAAACTGGAAGTAATAACTGACGTTGATGCACACGCTGTCGCGAACCTGG
AGATATCCCAGGGTGTTCCACCAGTGCTGCAGCCAGGAAGAGTTCTTCTGGACCGAGGCC
GCTCTTTCTAGGAGCCGCTCCTGAAGTTTCTGTGCCTGGGCCGGGAATTTCTGGACGGCG
TTCTCGAGGGCTCGCTTCTCCTCCTCGGATTCCGCCAGGGGCAACGCCGTCGGCAAAAAC
CGCGAGAGGGTATCTTCGATCGCCGGGACCGGCAGCTGGGGGAGTGAGGACTGGTTTTTG

TAGGTGGTGTCCTTGTTGTGGGCGAGTTCCTCCAGCCAGGCTTCGTCGCGGTAATCTCCG
TGGGACTCGACCATTTTGGTCGGCCACGGGGCGATGGTGCTCGTGCTCGTGCTCATGCTT
GCTTTGATCGGTGTGGATGCTCTAGTCGTTTGTGTTCCCGCTCTTCCAAAGCCGGTCCTT
GCCGATGTTGCTGAGGCTGTTCGGATTCCGTGTCGAAGAATACGAAGATAGGGCGATCCA
ACAGTCGCCATTGTCGTCCTTCGATTGAGGTCAAAATTGCTTCTTCAATGATTCAAGGGA
CAGGTGAATTGTCGTGGATCGAGACGAAGACGGATACCATGGTCGTCGTGAGCTACGATA
CTATACTCGTGATTCCAACTGGAAAGTGAAGAGTTCATGATTATTTATGAATTTTTGAAT
TTTTTAATGTCAGTAAATTTTGTTACGAAAGAGGAAACAAATCTTATATAAAAATCCTCA
TAGCCCCC
>comp1868_c0_seq1
ACCATTGCATCGAAACTATCAATGATCGATACTATCCCATACCATAGCAGTAGAATTCAG
TTCTCACCTCCGTTGTGCAAATCACCATGCAGTGCGGAGCAAACGGTATCTGGGACGAAA
CCGGCTGTTTCGTGTACGAGATAACGGCCCCCCATCGAATTTGTGTTCGGAACAGTCCCT
GCAGCAGCCCCAAGCCTAGGCAGATTCAGGACTTCTACTATTTTGAATGCGGAGAACTCG
TTTCAATAGATCTCATCCGACACAATCGAGAGGATTACACCGATCCCATAGGCAGCAACC
ATTCCGTTGGACTCCATCCAGGAGCTCATGCATCGATAACCAGAAGACGGAGAAGACGAA
GCGACGACGGCCCGTTCCTCCGGCTTTCGGACGGTTCCGGTTGGATTTTGGCGAGAAGCG
ACGGACAGGATTTGGCCAAACGGGTGTATGTCGAAGACGCGAATCATGACTCGGACGAAA
AGCGATCGCGAACCCCCTTTTGGAACTTTCGGGCGGACAACTTACCCCTTGGGATAGAGC
TGCGCCGGCACCCCAACGAAGAGCTCCGGAATCCAAACGACACCAATCGATTTTCGTCTC
CTGGTAGCGGAGGTATTCGATACCTCCCCATGCAACGAATCGTCTGCGACCGGAAAATCA
CCACCGCAAGCGGTGTGAGTTTTTTCCGGGTTCAGGGGACTTGTGGCTGGGTCTTTGATC
GGCGCAGCAGCAACGGTAGCAACCACACGGGGCAAGAACCCTACCGCGAAATGATGATCC
GAGACGATCTCGTCGACACCGGTCTCTTTGCCTTTCGGGTCACTTCCACCGAACCAATTG
CCGTTCGGAGATCTTGCCACGTGGGGGATGGACCCAACAACCTAGGACCCACCGTGGTCC
AACACGGGGACACCCTTTTGGTCGATGTGGTCCGTCGATCACCCTTGCAAGATGCCAACG
GCAACGGTCCATTTCTTCGCTTGGTCGATGGATCCGGATGGTTGTTCCAGCACAGGCGCG
GTGTGCCCGGGTCACTTCTCGAAGAGGTGCCGATCGAAAACGGAGTTTTTCGTCTGAGAA
TCCTCCAGGTTCCGGACCACACAGCAACAAACCACGGTCAGCCCTTCTTGGATCGCTACC
AACACCGGGGTCTTTCTTCCAAAGTCACAAACAATTACAACAGCAACAATAACCTTTCTC
CAAGATTGAAACAGGGCGAAATTGTTTCGTGCGACCGCAAAGTAGTTGTGCCGAGTCCAA
CATCCTCTGCTGGGATTTGTTGGTGTCGGCAAGTAGGTACTGATCAATGGATTATGGTGA
AACAGCAGACGAATGAGGACGACGATGAAGACATGCATGATGGAGGACATATCGTTGCAG
AAATTGTTCCCGAACCAGAGCACCCCGCATCGTTCTTGCGGGAGAGAACGGCCGGGGACG
TCCGCAAGCGTGAAACATACGCCTCAAGCAACGGAGCCAAACGTCTTCAACAGCAACCTT
GGAGTCCGCGTCTTGTTCGGAGTTATGCATGCATGGTTCCTGGACTTGAGGAAATCGGAT
TCGATCGTCAGCACCAAATCCTCTCTTTTAAGAGCAGCAATGGCGTAATCTTTAATATTT
ACTGCGAAAACCGAGTGATCGGGATACTCTCGGTCAGAGAGAACACGATACAAGGCTGTG
TTCAGCGGTATCATCGAAACTGTAGTGATTTGGAACTGAGAGAAATCCTTCAAGATACCA
AAACCTGTCGAGGAGAGGGAGTTCATCGATGCGACAAGCGTGCTCGATCCTTTATGCCTC
CATCTCCGATCCGTACTCGATTTTTGGCTGATGAAGAGGACTGCAACAATAGCGAAGAGT
TATCGTTGAGAGAAAACCAAAACATCATCACCCCGGATACATCCTCTAGCAGCAGCGCGA
TTGGAACTGGGTTTGAAATGCACCAAGATTACAACAAAGACGACGATACCATCGATACCG
AACTCGAGTGTCGAATGGATCTAGTGGATTGCCAGAGGAAGATCCAAAATCTGTTGTACC
GACAGGGAAACATTTTGAGGGCCATTAAAGTGTACGAAGAAGAGCGGATGCGCGATGCCG
AGGCAATGAGAGCCAAGTCTGAACGAATCAGGATCGAACACAGGGAAAACCAACGACAAG
TCGAGCAGCTCCAACATCAGCGACGAGTCGAAGAAGATGACCGACAGGCACGATGGAGAC
GGACCTGTCGGCAATTGGTGTCCGAAAATGTATTTGTGGGGGCTTTCGTTGGGGGCGTTG
TTGGACTTGTACTGAGTCCATACTGCGGCCCCTTGCTGGTGCAGAAACTCGGATTGCAGC
ACCTATAGCGAATACTATGAGGCGATAAGGAGAATCAACTACCAACTTAGTTCTATTGTA
TTTTACTTGATCGCGCGATAATTTTTTGAAGATTCTGTGATGTTTGCATCCGAATGTCAT
CAACTGCTGGACGAAGCCCAAACAAGTTGTTTGGATTATCGCACGGCGATTCACGGTCAT
GATACGAAAAAAATCTCTAACCAATACTTTTCGAGATGTCGTACTGCCAAGTAAAATTCC
TTCATTTCATTCGCTGGAATCCCTGTCAGCAACTCATTCAACCTTCAATTTTTCATATCG
CGACGCTTGCCAAAATACCTTTTATGGTGCCTCCTTTTAGCTATAACAAGCGGCTGCATC
CAGTCACTCTTTGTCTATATTACAATGAAGCGCATCCTAGAGATCTTTGAAACTATTTCC
AACACAAGTTTTCATTGTTTGTATTCAAGCGCAGGTTGGAATCAGGGACAAAAAATTGCA
TGAGAGAGTCACGATAGGAAAAACATCGGAAAATGGTTCGGAAATAATCGGAATAAATGC
CATGTGTGGAATACCGGAAGTATACGTACGAGTAGGGTATGCAACGTACTTGTACTCCTA
CTAGAACCGCATGT

>comp1872_c0_seq1
CGAAAATTCTTTTTGGAGCTGGAAGTGAGTGATGCGGTGAGGAAGCACAACACAAACCGT
AACCACTTACAGAGAATGGAAGGAAAACAAGTACGGTACCAGTACTCTACGAAACCCCTT
GAGAACACATCGATCTCGACGCATCGATCGGCAATTTATCGGCAAACAAAGACTACAGCA
CATTGCAGCACATTGAAGAAAAGGTTACCTGAGCATTACGAAGTAAAGTAGAAGTACATT
CCTGCGGTCGGCATTCTTCTTCTGCGACAGCAATTTCCGGTTGCATCCTATCGACCGAAA
TCCATTGCATCGCACCGCATCCCATCGCATCGCATCGGAATTCACCGAACCAAATCTTCG
AACGGAACACGACAATCCGTACCTTCTTTTCGCACAACACAATACTACACAATACAATAC
ATTCCGTCACCAATCGCCATGATCACTAGGACAAGGACGACAAGCACCCTTTTCGCGGCC
GCAATCAAGGCGACCGCTCTCTGCTGGGCGGTCCTGTGCAGCAGCAGCAGCCACGACTCC
CATTCGATGGCCTTCGTCCAGTCCTTCTCGCCTACGCCTCTGGCGAATGGAGCGGAACCA
AGGGGCACGACGATCGCAAGCAGCAACAGCGGCCTTTCCTCCAAGACCGCCCTGTCGATG
GCCGGGGGGGTTCGAAGGCGTGGCCTCGAACAGCGAAGGGAAGGGGCTACCCCTACGGAG
GGTGGAATGACTCTGTACCTCAAAGCCGCCGAAGATGGGACCAGCGTCGGAGACTGCCCG
TTCGCTCACTACATCCGAATGATCCTCGAAGAAAAGAACCTCCCCTACACCGTCCAACCC
TGCGCGTCCAACGACGACAAGCCCGGCTGGTTGCTCGAATACTACGAGGGGAAAATGCCC
TGCCTGAGGCACAAGAAGGAAGCCTATGTCGAGTCGAATGTGATCGCTGCCTACCTCGAC
TATTTCTTCCCCTCCGGCGAAAGCGATGCCGACCACAAGGCCGCCCTGAAGGACGCCGAG
GACGCCATGGACGGCTTTTTCCCGGCCGTGGCGGGGTACCTCAAGGACAAAGAGAGCGAC
GCCGAGGCAGAGCTTTCGTGCGAACCACTGGCGGCCCTCCGGGAAAAACTCGGAGCCCTC
GAGGAGCACCTGGGAAACGCCGGCGGCGAAGACTGCAACCTCGACGGGACCTCCACGTCC
TTTGGAATCCTCGACTGTCGCCTGGTCCCCCAGTTGTACCACCTGAAGGTCGGCATCGAC
ACCTTCAAGGAGCACGGAAAGCCAGACCTGGAAAGCGAGTTTCCGACCGTGCACGCCTAC
CTTTCTCGATCCATGACACGCGAGAGTTTTGTCAACACAATGTACCCACCGGAAACCATC
GAGTGGGGCTGGACCAACGCACGGCAATAATGCCAACGCAGAAAAAGAAAGACCCAAGCA
AACAAACAAACAAACGAAACAAACAAACATACAATTCACCAATCGGTGCCGCCATACACG
CTGGCATTGTGCGACCAAAGAAAGAGGTTTTGCTGCTTGTTCACGTGGCGAGAAACACCA
CGAACGTACCTCCACGAGGCCGTCACGCTGTTCGGATAACGTCGAAATACCTAGTA
>comp1873_c0_seq1
CTCCCTGGGCGAAACCGAAAGTCGTAAGTGAATCAATAAGTGACAGATTGGGACTGTTCG
TTTAGTGGCTCGTTCAAATCGCGATTGCAAGGATATTTTGTTTTGGCTTCGGTTTCTTGT
CCTGAGCCTTGAGTTCTAATTCGCATCCTTTGACCATTCCTTGGCTTCCTTATGTCGCAT
CGCTGCCGTGCCGTGCGATGTCATGCCGCGTCGCGTGCCTGCTGCGAACCCTTCTCAATC
GCGTGCTGCCAAACATACTATTCAATCCACGTTGCCGATGGCGGCCGGATGTTTCGAAAA
ACGAATGCTACATCTACTCGACAACAACGACCAAAACAACGACAACCAACTATAGAACGC
TTAAAGGAGA
>comp1875_c0_seq1
AAAATTACGTCTAGAAGCAACGGCGGCATTCCGAAAGGAGCAAACAACCATTCCAACCAT
ACTTTGAAACGCAGTGTTCGAATCCCACAGGATTTCTTCCAGGATTGTTAATCAACCCAG
CCATCGTATTCCAATCGAAACCAAAGAAATCCATAGCGTTTGAAGCAGACCGTTCGCTCC
CGGAACGCAATATCCCACTACAATGAAGACGACCGATCGCATGGCGGTTTTCTTCCTTTC
GCTGCTGTCTCTGAACTCCGCGGCGGGATTTACCAGTTCGGCGATCCGAACGAGCACAAA
CCGAGTTTCTTCGCCCTTGGCACCCTTCCCACCGTCCGCAAACAAACAAGAGCGGCAAAC
ACCATTGATTCGCTGCAACGAAAAACCGGGCTGGTTGGACGATGCGATGGACGGCATACC
GTCGGGAGACTTCGACGAAGAAAGCTACAGAAACAGAATCGACCTGCGAGGGGGCATCGC
GGGGTTTTCGGTGGATCCGGAGCTCGGATTCGTGTGCATACTGGTGGCCGGGGGCGAAGA
CAGCAACCAGCAGCACAACAAGCACTGGATGCCAGTGGTCGTCTCCCCCGTCGACACGGA
TCGACCAAAATCCGCGGAGGCCCTGACGTGCGTGCAACTGGCGGGTGGGCTGGATCTCGG
GACGGCCATCCTGCCCCCGGATTCACTGGCCAGGCTGGTCGCCGAGCACGATGCCGAGGA
GCAATCCGGCGAGGGCACCGCCCGGGACAAGGGCGCTCCCAAGCGGCTCTCCCTGACCAA
AATCACGGCGGCGCCCAATCCCGATGCCAGCGAGAACGCCAAAGCCGGCATCGAGGAAGA
GGCGCAAGAGCTCGTCGCGACGTCTCCGGAACGAGAAAAAGCCATCTCGGACGCCCTTCC
GAAGGTAGAAACGGCGGTAAAAACCCTGCCCGGGTTGCAGGAGACGACCACGGAGAGCGT
CCGGGAGGCCATGCAGCGGTTCGCGGACGAGAAGGGTGCGGTCGACCGCGACGCCTTTTC
GTCTATTCTCGACTCGTTGCGGTCGCTGAACACCCCGGCGATATCGCTTGCACCCCCACT
CTTTCGCTTGGACGTGTCCGTCATCGATGGGAACGGGATTTCGCAGGTCACCGTCGACAC
TACTAATTCGATGATTGCTCTTGGCTTGGCAATGAGATACAAAGTGACTGTAGACATGGA
AGAAAAATACCGCCATCCCCGAGGCGGAAAGGGAGTCGATGAGTTGCTGGAGCGCTTTCC
TGTCTTTCGACCGATACAAGAATTAAACGAGGACTCGAGAATTGTTGATGGATTCATTCC
CTCCATGTTCGAAAAAGCAAGGAGTATTGACAATGACATGAAAGGCGAGTAGTCAGTTGC

TACTTGTCGGGATCGATTGACGCAAAGAGCTACGCAACAATCAACGCTGGGTGGAAGGAT
GATATCTAGACGTTCAAAACCGAGCGGTTGGTATACCTAGACTTTTTTTGTGATGTGTTC
GGACAAGACAAGACCTCTGATCCAATACGAAAAAAGGCAGTTCATCTTTTCCCACTGCTG
GACTTCCAGGCAGCAGTTGTCGACAACATTCCTTGTAGAAGAAGTGCTCCGG
>comp1876_c0_seq1
AGCTCAATGATGAATTGAGAATGATAATTAGAGACAGGAAAGCATGAGCCAAGTAGAGGA
AACGCCGGCTTCTCTGAAAGATACGAAAGATGCTGCATCGACAGTGCAAAAATCTCCGAA
AGCAGACTCTATACTAACTAATCTGGATGCAACAATCGCCGGTTTCGGTGATGGCCACAT
TTTTCCAGACACAGACACGTCGCCGGCTTCGAGCTGCTGCAGCAGCAGCAGCGACAAAAC
CGACCACCGAGCTAACGCGCTTGGAGATGGAATCGTCGAGCGCTTTTCGCCTCCCGGATT
AGAGGAGCCTTTCAAACAATTGCACTCGATTCTTTTTCCCGCCTTGCACCGGGGCCATGC
CAAGGACGGCATCGATCCTGGCCGTGCCGGTCCCGCCACACTGCCGCTGTCTTCTCCCTC
CTCCAAGTTCCAGAATCCCATATCATCTTCGTCCGCAGCGATTCTAATGGGAGTCAAAGG
AAGCGGCAAAACGCTCTTGCTGGAACGTGTCCTAGCGGCTTGTCGGGAACAGCTCCAATA
CAATCCTGGTGGTGGTCGTCGGCGTCTCCTGTATCGGATGGTCGAAATCAACGGTATTGT
ATGCAGAGGAGAAGACGTCGCATCGGTAGTATATGAAATAATCCGACAACTCTCTGATAT
TGCGTATAGATCTATCCAGACAACGAAGGCCGAGGAATCGCCCTTGAAGGCAACTAGGAA
AATTAGAAGAACCAGTGGTGACGATTCTGACACCACAGGCAAGCGCGGCTTTTCTTCGGA
TTCTATTCTTCGCCACAATGACGGGAAAACAGAAGAGGGAAATGAAACTGGCAGGTTAGG
GGGAAACGAGAAAGATGATAGTGGTGATGAGTCTATCTTTGTGCATGACGACGATGATGC
AAACACACGAAAAAGAAAAGCTCGGCGCCAGAGACGAAAGCAACGCAAGATAGACAAACA
CATGCTACGGCTTCGAAGGGCGACATTCAACTCGAACCTGTCTCTGTTGGAGTCCATTCT
CGAGTTTGCAGCCGCCGACGACATTCCGATCATATTGAATCTAGACGAACTGGATTCCTT
CACCGACGAAGGAGAGCGACAGATGCTTCTCTATCATCTATTGGATCGCGTAGCAACTCC
GGGATCCAACCTCGTTTTGCTTGGCACGACCTCGTCGTTCTCGGCACTGACGCTATTGGA
AAAACGAATTCGGTCCCGCGCCGAAGGGACGTCGAAAATAATCTATCTGCGCACTCCGCC
GACGTACCACGGACTCTTGGAGTTGTTGGATTACAAACTCAAAGGTTGCGTGGCACGAAA
AGACATATTGGACAGGTTGGCCCATCCAAAAGACATAGAAGCCAAGGCGCGCGAAATTGT
TTCCAAGAAGGGCCTGGGCGCGAGGAATGGTCATGCAAACCATACGGAGGAAAGCACGGA
TGAAATTGTGAAACTGTCTGTCGCAATGGAACGGGAATTCAGAGTCGGCAGAGATACAAG
GTGGTTTAGTCGCGTGATTGCCACATCTCTATCACTGTATCGTCACGATTGTATCATGGC
CATGTCTTTCAACGACAGCAGCGTTTCTTCAGGAAGCCCTTTGTCCGACGGCGCTCCACC
CAATTTCTGCGCGAAGTATATTCGGGACGCAATGGCTATGATGGGTGCTTCCGTCTTGGA
AACCATGGCGGGATCTGGTCAGCAACCCGATCTCTGCATAGTCGATGGCGTAGCTGCAAG
TCCTCGTTTGCAGGCACTTTTGGATTTGTCGACGCCACAGGTCGCACTGATGCTGTCTGC
CAGACGAATACTGAAGCGGGAGGAGCAAAGAGAACTGGTTGTGCTTGCTCCCCTCACCTT
GGAACGAATGCTGAAGGAATACGAATCGTTTCGACGGGGGTCGAAATCGATGGGGAATTC
AAAACTACTGAAAAGGGCCGCATCCCATCTCATGGAACAAGGTCTGTTAGTGCCCTCCAT
GGATCATTCGGGAGGTGGACCTCTTCAGTACGATGTGTCCAAAATGTATAGAACATTGGA
TAACTATACCTTGTTGCGACTTCCACTACACATTCCATACGACATTAATCGAGAGCTGGG
GAAAGCTCTGGAACAAAATCTTTTGGAATGTCCGACCGCACTGAAAGAATGGGCTAGATT
TATTTCGAAAGCTGCCTAATAAAATCGTCGACACGTATGGCATTAATGGTCTATTGTGCT
GTATCGTGTTTTCCATGCTTTATTCCTTTTTTGGACACAACATCCATTGTGTTTGAGAAC
AACATGGATCTATCTTCTAGGTTCGAACATCATACTCCCGTTTCCACCATGACTACTGGA
AGTTGTTGTAGGCATCTGGGGATTCATCATCTTGCCTCTTCGACTGTTAACAAGACACTT
TGCAAGGCTTTTTGGGAAACGAGCAAAATCGGAAAAGATTAAGTGAAAGTCAGAACGAAA
AATCGAATGCCCCA
>comp1881_c0_seq1
CATCTCTCCACCGACCGCCAGAACAGATTGCACCGCATCGGATCACCGGCACCTGCCGCA
CGCAAGAAACAGAGAGACCCAAACGGCCATGGTTGTCGGAGACGGGTCCCGCCGCCGTTC
CTCCCGCCTCGAGGCCGGGCAGAAACGGAAGCGTCCGGGCGGACTCGGCGCCGCCGCAGA
CGGCGCCACCTTCCACCGCCCGAGAGCCACCGGCTCGGCGGCTCAGCAGGACTGCGGGAT
CCTCCCCAGCGAGTGATACCTGATTTCCGCCAGCGTCACGGAGCGGGTCCCCTGGGGCTT
TGTCTGTGGCTACCCCGTCAAGACCGGAAGCGGCGATCTCTACGGGGGCGGCCACTACCT
CGAGGCGGCGTCTTCGAACGAGACGCCCCAGGGAAGGGCGGTCCTGGAGGCCGCGCTGCG
CTTTGGAACGGGCGGGACGCTCCCCTTTTGCCCCTGCAAGCGCGGGTACAAGCCGGTTTC
GGAAGGGCCGCCGCCGAGGCGCATGGTGGCCTGCTCCTTTCACGAAGCGGCCCGCCGGAA
GCGGTTGGCCCGGGACAAGGGAAAGCTGCGCGGGGGGGAAGCCGATCCCTCGGAGCTGTT
CTGGTCACCGAAGCTTTCGTCGGAAGAAAGCCGTGCGGAATTGCCTCCGCCGCCCGGCAG
CAGCAGAGAGAAAGCAAGGGAAAAGTCGTCCGAAAAAGGCGACGGCCAACACGACACCAA

AACGGCCACCTAAGGAAGATAGCACACAGTCTGCCAAACCGATGCGAATCGCTTTGCGTC
TATCGGTGTCTCTTGCAGCATCCGAGCATGAAACACACGGAATCTATGTTTCTCGCGGGC
AAGGTTTGGTGTTGCTGCCATTGTATTTATTTACCTTTGAAAGCATACCTTGGGAGGAAA
CACAAAAATGCTGGAGGGTCTTGGTTGCTTGTGTACAGTGTTGTGTGTGCTCTGATGCAT
TCCAATGCACTCCGGTGCAATTCAACACGTTTCCTTTGCGAAAACAAAGGAAGCTTCCCA
AACAACCACGACATGAATGAAACTTTTTCTGTTCTTGCAAAGACGGAAGAGGTGGAAGAT
GGATGGTTTTTGTGACGAGGGGCAAGGGCTGCTTGTTGCAGGGATACGCACTAACTCGAA
AACCAACAAGACCGAAACGGACCGAATCGGAACGAGCACCGAAGTAACCCCACGCGCTAT
GAACTGTTGTAATAGAGTCCAAATGGAAATTTTGTGATTAGGCTTAAATTATTCATGTTC
ACATTTATCTACGATTCATAAAGATTGGCTAGTGGTACAGCATCGGATACCAACAAACGA
>comp1883_c0_seq1
CAGGCATGTTCTTCTTTTGATTCAAAATGATAACGACTAGAAGTCCTTCGTATCATAATG
GGGTTAACCGGAAACGAACCGTCTACCGAATCTGCACAACGAGCTCGAGGACCACTCAGC
AGAAGGTTGAATCGCCTGTCATACGAGCACACAAGATTATTGTATGCTAGTCAGACAAAG
AAGCAGAGGATCTTATTCTTTTCATATAGAAAGTATGTTGGCGAAAAATAGAGCTCCTGT
CGCAAAAAGAAGACGCATGGTAGCTTACGACGCTCTTTCTTGGAACATAAAGAATGGGGT
TTCGGAAACAATCGAAAGAGGGAAGAGCTGTTGGAACATGTTTTAGGAGGAAGATAGGGG
TGTTAAACAAGGGGTTTATTTATTGTAATGATTGATATCTGGATATGTGCCGAAAACAAA
CCATGCTTGTATCACAAGTCCATTCACAGAGGAGCTTTCGCGGTGCTCCATCACTATATC
ACTATGCCATTTCTAAAATGAGGACGTTGTATTGACTGCAAGTGAAACAGCGAACGAACA
ACTCAAAGTAATTCTCTCAACGAAAGCCAAGGCACTGTTTGAACTATCGAAGGAATGCGC
GAAGGATTTTTCTTGTTCTACTGTAATTCCTTACAATCGCCCAGAATTCTACAGGATAGT
GTTGGATTTTGTATGCTGTGTGAACATTCGATAGGCATCATGAGAAGATTGCAAAGGAAG
TCACTGTCGCAGCGAATCGGTTGCATTGTAACTGTTAGTAACAAGCTCACTCTACGCAGA
ATTCGTCATTGTTCTTGGCAGTAACGAAGGCCGATCAACTTCTACTTCTTGGGGATCCGC
GTTCCTACGCATTCTTGAAAGAAGCGGCTGCACATCAATCCACGACAGATGCAAAAGGCG
CGCTGAGAGCATCCCAAAGCTGGACCAAAATCAAAGAGTCTCGTTAGAGGCTTATTTGTG
AAACTTCTTGAACACATTTCTTCTCGATACATAAGTATTGGCGCTGGTGATACTTCTCGA
CATTCATTGAGAGATCGATGGTAGTCGTGAATTTCTTTTGAAGGATGCAAAGGAGGATTA
CTGTAGTACAAAGCGGAAGAAAGAAAGACGAGAAATTTTTAACGACTCAGAATAAATCCG
ACCATTGACTTTGGCGTGCATCCTAATGATCTCGAAGTTTCCATTTCGTCGTTTCGACAT
GAAGAATGAAACCCGCGACGGCTGTTTAAGTTCTTCGGCTTCCATTGTTTCGAAGTACGT
GTAGAACTCTCTGAGATTTGGAACACTGGTGCACACAATCAAGGAATAAAGCAACAGCTA
AGTCCCATGTCCCGGATTGCAGCTGAGGCAAAGGGGTCCTAACGAGCAAAGCCCAACTAC
TATTGGGGATCATTGCCAAACAACACATGAACGAATCGAGCTATTGTTTGAAAAAATATG
CACTGGGGATCGTAAACATACCGTAGGGGGTATTGTGCTGTGAAAACATTTCTCGTAAGA
ACCAAAAGTCACATGGTATTA
>comp1884_c0_seq1
CTTTCGCATCCTCGATGCCTTCTCCTTGAATAATTGATATAGTTTTTGACTCGGGGAGAG
ATTTCAATGCTTTGCGACCGAATCCAGGAGGGGCTCTTAGAGGAAAGTCGTCCGCTTTTA
AGGTCAACGTTTGGAGGAGTTGATGGTGAACGACTACGTCTGCGTAACGCCGGATTGGAG
AAGTAAAATGAGTGTACTTTCCCAACCCGAGCCCATAGTGTGACAAACCCTGTTCACCCC
CTATTTGACCCGTCGATACGTATTGGGCTTCAGACATTGCTCTGAGTGCAAAAGGAGACA
GTAAGAATATAATGTGGGGGCCACGCGGTGTCGTATACGCTGCCATGTATATCGAGTGCT
TACCGAGTCGCAAGAGATTGAAATAGAGAATTGACTGCAGAATTTGTCTTACAAGACTTT
TCGGCCTTTTTGAGAGAGTCAGCTAATTTCATATTGCTACTGCCATCGAACGAAATCTTT
CCTGCATCAAGTATATCTTTGAGGTCTTCGAAACGACTTTCATCTACGCTTTGGTGAATT
CGAAGCAATGCGGATGTTGGGAATCTTTCGAAAATAGTTTTAGCGACCCAAGAATTTGCC
ATAATCTAAATAAGAAGATGGTGCACATCGGTTAGAAAATTGTTACCATTCAC
>comp1885_c0_seq1
GGAAGTCACAGCAACCGAACCCGTCGTGCTGTGATTATTAGTATTAAACCGCGCTCCCAC
TTGCGATTCTGGTGTACAATCCTGTCTAGGGCAGCATAGTGTTTGACAATGGCGTCGTCG
AGATGGAAGCGCTTCGCGTTCTTCGAAAAGAACGCCATCAGCCTCCCCTCGGAAGTCCTC
GAAGACCTCCTCCCCGCCGGCGAGTACGAATACTCGAACAGCGGTGGGCGAGGCCGCAGG
TCGGAGGAAGGTCCTTCCAATTCCAAGGACAAGGTGCGAATGGTAGTCACCACCGCTGCT
CTTCCCCTGGATTCAAAGCCGGGGCACGGAGCAACCGCCGGGATGCCGAACCAGGGAAGC
GGCGGCAATGCCAATGCTTCCGGGGGCGGGGAGTACACCGTGGCCCTCAACGAGATGTGG
TCCGGCTTGGCGGCGTGCAACCCCATGGAGAACATTCCCGGAGGCTACGCCGGTGGCACG
AACCAGACGATCACCCTGCCGAGCCAGGCACAGCTCTTCGAAGACGACAGCAACGTCGTG
TCGTCGTCCTCGGGAGCAGCGGTCGACGGACTCGTCCTGGCCTTTGTCACCTCCCGCGAC

ACCAACCGCGTGCACTGCTTCGACATCTCGGTCCGCTGCAACCACAGAAGCAGCGGAGGG
GCCCCGGGCATCGAGCCGAGCGCCGACGGCGACACCCAGGCCAGCGGGAAGAAGGCCAGC
GATCCGGAAGACCTGGACGGCTGGAGGGGGTACCTGGCGCCGATCCAGATGGGCACGCGA
GCGACCAGCGATTCGCGGGCGGCGGCGGGCCGGTCGTCGGATGGACGGGCTTCGGCCGAC
ACGGATGCGGCCCGACCGCAAGAAGAGCCCAGGGAAGGAATCGTGGGGATTGCGACGTGC
CGGGGGACCTCCGGGCACCGGCCGATGCACATGGCCTGCATCACGGCTACCAACCTCGTG
GTTTGCGTAGATCCCCACTTGTATCTGTCGTGTCGCCGTCCACTGTCTACTCCCGAAAAC
AACGAAGCTCCATCTTTCACCCTTGGTTCGAACTGGAACGAAGCCA
>comp1886_c0_seq1
ACTTTTCTTCGTACTTGCACTCGTTCTATTTGATTTCTCTCTGTTCTACCGTGCAATACA
ATATCGTTGTTAATCGTAACTCAAAGATTCGGAAGATCTCGTCGATGCCCTAAGTCGATT
CTACAGGAGTATTAGAAGTACCGACAGCTATGCGACCTCCAATTTCTAAAACGAAAGTCC
TCGTTCTGTTGGCGGTATTTGTTTTTGTTGTCGTGTCAACTCCTAGCGCAAGTGGTTTTT
GTTTCGTCCACCCCCATCGGAAAAGTCCCAGCGCACAGCCTCGGCAGTTGGTGTCTTCAC
TCCAATCCAGACCGGTTGATGTAAACCACAAGTTAGAGAAGGAGGGAGACGATGTGGATT
CTCTAAACCAAAGAGAGAAGCCGTCTAACAACACTACAAAACAACAGCGGGAAGCCGCCA
AGAACAAAACTCCTGTTCACTTGCTAGCTGGATTTCTGGGAAGTGGAAAAACTACGACCC
TAAAACATGTCCTGGAGAACAAAGAAGATGTCCGGGTCGGCGTCGTTGTCAATGACGTTG
CTAGCGTCAACATCGACGCCAAACTGATTGCCCAGAAGGATGCATCCTCCGCGGATGCTT
TTTCGTCGGAGGCAACCGCGACAAAGCCACCTATCAAAGCCAGCCGCAACGACGTCGTGG
AACTCCAGAACGGATGTGCCTGCTGCTCCCTCGCCGGCGAATTGTTTGACTCCATCGAGT
CGCTCCTACGGCCTAGAATCGGCAGCGGGGATTCCGAAGCGACTTCCCCGTACGAGGCCA
TCGTGGTCGAACTCTCAGGGGTGGCGGATCCCCTTGCGATCCGCAACCACTGGAGAGAAG
CCGTGGCAACCGATTCCCATCCCGGGGTTACCAACCAGGCCGAGCTGGGAAGGATTCTTA
CGATCGTGGATGCGACCACTTTTGGAACCGACTATATGACCTACGACGTCTTGAGGGACC
GAGACGGATGGCTAGGCGGCGAAAGTTCCACCACAATCAACACCAGCGAACGGCAGCGGC
TGCAACCTAACAACAACTGTTCCGGAAACAAGCAGGTTGCTTATTTGCTGGTGGAACAGG
TCGAAGCCGCCGACCTCGTGGTGGTCAACAAGCAAGACCTAGCGGGAGAGACGAAGACGG
ACGTCGCGAGACGCATGGTCGAGGGCCTGAACGAAGAGGCCGAGATTCTGACCACGAACC
ACGGGAAGATTTCGGCAACCAAGCTCCTGGGATTGCCATGGCCATCCGACTTGGAAGAAT
CCGATTCTCGGGAGCAAGAGGAGGAAAGCGCAGACGGATCCCATTCCCATTCGCACACAC
ACCAGCATTCCAGCAGAAAAGACGCGGATTCCCCTTCGCTTGACGCATACTCGCATGACC
ACGATGCAGCCTGCGACGACGCGGACTGCACTGATCCGTCGCATTCTCATTCTCATACGC
ATTCTCATACGCATTCTCACTCGCATTCGCATTCGCATTCC
>comp1888_c0_seq1
ACACCAAGGCACTTCCCCCCATTCTCTTCACCACCACGATCCGGCCGAAAGTGCCGCGGC
CTTTGCCGCTTCGATCGCTCTTGCGGGAAGCGTGGTCGGCGTCAACGGAATCGGAATCGG
GGCCCACGGGGACCTGGCTTCCCACCAGGTCACCGATTCGGCTGCCGTGGAAGCCGCGGT
GGCCGCGGCTCTTGCCGCTACCGATGCCAAGAGCAATGCCTACCACCACCACCACCACGC
CGGAGACCTGGTGGGTCCGAACCACATCCTCCACGGCACGGGCGATCCCATGGAAGCGGG
TGCTTCGGCAGCGAACGGAAGCATGCCCGTGGGGGCGGGCCTGCCCCCGGCGGCCGTCGG
ATCGCCCCGGGCGTCGGCGCCCGCGGCGGCCCCGGCCCGGGGGGACGTCGGGCTGGGACT
GGCCCTGGATGCCGCGGCAAAGCTGGCCGCCGCGGCGGCGGCCGGGGGCGACGTGGTCCT
GGCGGCCACGACGGCGGTTTCCACCGGTGGACCGGTCGGAAGCACCGCGAAGCAGGGGGC
CTGAACAGGAATGCGGGGTTTGGTGGGGCGCACGGCGAAGCAAAGCACGGCACGGGCCGT
ACGGCAGAAGCGCCGATCGATTCGGTATTGCGGGGCGGCGGTGTGGCAAGGATGGATTCT
TGGAGCCCAGAGGGATGGCGGAGCTGCAAGCCTTCTTGTTGGTCGGCGCACCGGGTTGCA
ACGTTTTGTTGCCTATTTATGGATGGATGGATGGATGGATCACGCCAGGACACATACCAT
TTTTCGTTTGCTTTGCAAGGGTTCCAAACACGCGCCAAAAAAAAAACAATCCTAGTACGG
TACGGTATAGGACCGAACGAGTTTGGTCTCGAAACGTGTTGGCACAGAAGGACGACGCCT
CGACTCGGGGTGCGGCCGCAATCGTTTCCCCGCCTTCTCGACCACCCGGTTTGGTTTGGT
TTGCGTTCGATCGATGGCACTCGGGTCGGAAGGTGTCGTTCCGGACTCGGAGATCGACCC
CGAAGCGTCCTACTGCGGTGCCCTCGAGGCTGGGTCCGTCTGCCATTCCGAATCAAAACG
CCACGACGTTTCGTAGGGTGACCATACTGGTCCGTACGGTACTTTACAAAGCAGACGGGA
>comp1890_c0_seq1
AAGGGATCCAAGGGAGGATACTACAGCATTGCCAAGCCCTCATACGGTCCTTCGTACCCA
ACCTACCCATCCTACCCATCGTACGGAGAAGTCAGCTACTCCTACTACGGAGGAAAGGGG
TCGAAGGGAGGAAAGGGATCCAAGGGGGGATACTACACTCCATCGTACAAACCATCCTAC
CCCAGTTACCCTAGCTACGGAGGCAACTGCTATGAAGTCTGCTACGGCTATGGTGGAAAG
GGAGGAAAGGGATCCAAGGGATACTACGGAGGGTACAAGTACGGTTACGGATCTGTCGGT

TACGG
>comp1891_c0_seq1
CGTTCATCATCTCAATCGAAAGTCGAACAAGAAGAATTGAGCTCCAAATTCTTAGTCTGG
TTTTGGTGAAGGAAATGCCACACAAGCAAAGTCGAGGTGCTTATTCCATGTTCGGAAACA
ACCGAAGCCGGACCACTTCTCACCAACACAGCGTGAATGGAAGATTTCATTCCGTTCTGC
ATCGCTGCCCCCTTGTCGCCGCTATTTGGTGTTTGCTCGCACTGGGTCAGAAGCAAGAAT
CGTTTGTAGTGTCACTGCACTTATTGCCACTGATCCATGCAAAGGCGGCAGCGATCATGA
CGCCTTCCGCAAGCGCTTCACCCAATGGCCACTCGACATCGTCTTTGCTCTTGGCAGCGT
CGTCGTTTCCGGAGAAAGAAGCAAAGAAGCTCCTCGAAGTGGAAGAAAAATTCTCCTTTT
CTGATCGATCAAAGCTCGAGGAGCGATTGAAAATGGAAGGATTCGCGCTTTGTCAAGAAG
TCATCATGGTGGATTGGTATTACGATCGATTGGTGGCGAACAGTGAATGCTTGGCATCGG
TGCTTCCCCTGGTCCGAAACGACCACTGGCTGAGATACCGTCAAATCACGAGTCGGAATT
GCATTGAATGCGTCGACGGCGAGGCAGAGTGGCAGCTCAAGCGAGGCACCTCCATCGGTG
GTGGCGACGGTGGTGCAACCGTCTATGAAGAAATTGAGGGAACTGAGGCCCTGAAAATTG
CCTGTTCGCTGGCGAACAGAGAGCTATCAAAGAGAGACGAGGCAAACCCAACCAAAAATG
AAACGGCGTTGCTCTTGTTATTTGAGGGGGAATCTTTCCCAGTGCCCCCGGTAGACAGTT
GCGACCTACAGCCATTTGCTAAAATCGTGACGCACCGAGCCAAATGGGAGCAGCGACAAG
GACAACAGCCAACGTCTGGAACAAAAACCACAAACGCCTCGTTGTTTTCGAAACTTGTCG
TGGACCTGGACACAACTCCCGATGGGTTTGCAGTCGGCGAGGTGGAAGCACTGGTGGAAG
CTTATGGCAAAACCGAGGGCACATCAACCGCCGTCTACCAGGAAGAAGCTGTTGCGATGG
CTCGGACAGACATCCGAGAGCTCCTGGAAAGGATTCTAAAAGGAGACGACAACAACGACG
ACCGTAGTACTCCAACCCGTAGTCCCTCTATGGGCAAACTGGAGCAGTTCCTCTTTCTTA
GGCAACCCCTGATCCATAAGGTGTGTGTCGAGTCTGGGGTCATTCCACAACCGCCGTTTT
CCGCCGAAGAAGAGCCGTCCTCGTGAGGCCATCCCCTGAAATTGGTCGTAACAGTTGCTA
CAGAACTGCTGTTTCGTAAATACGAATGATGACTACAATGTCAAGGCATGGGGAGGTGAA
TGACTTATTGGCAAGAAGGGTAGCAGAAATGCCGCTCTATGGCCAGCGTCGAAGCTCGAG
AAACAGCTTTCGTTTTCGAAAGCGAAGTTTCCTCCTTCCCCTTCTTTCCCGCGTCGTACG
ATCACTAAACATCGATGTTCGCCAGCTTCGGTACACTATTCTGCCATCGTGTTTGATTTA
AGTCTTCTTACTTAAGTGACCCGGAGTGATTCATTCGATGAAACAAAGGTTTAGACTAAA
AGCATAGCAAAACAATTGTTCACGCAGTGTCTGCTTGAAACTTCGTACAGAATGGTAGGA
AATTATTTGCTACATTCCTTCGCTAAAACACATGATCGGATCTATCGCTTATCTCTACCG
A
>comp1892_c0_seq1
GTCGGAATCATTCGCGTCGGTCAACTTTTATCAACCGAAGGTCTTCGTCTTGTCCAAAGT
GTATGGAACGGTGGTATAATTCACTAACCAAAGTTGGGTTAACTCGATCTCAGCCAATCC
AACCGCACCAATATGGCTCGCCGATTCAGTCGACCACTTGTACCCCATATCCTCCTAGCG
CTCGTCAGCTGCGCTTTCCTGGGTTCTACTAGGGTCAAATTGACAATCGATGCCTTAGTT
GTTCACTCTTCTGCGACGTCCTTTATTGGGAGGGGAGATGATGGAAATTTGGTTCAATTG
AGAGGTATTCGGTTTGACTGTGAAGGAGACTTCGAGAACGAACAACAGGCAACAGTAGTA
TCGAACATAGATAAAATTACTCCTATCATGAGTACATCTTCGACAAGACGAAATCTTTTC
GCCAACACCTGTTTTCTTGCAGCGGTGGCCACGACACTGGAACTCCCAGCCTCGGCAGCA
GAACCCGTTCGAGCTAAAGACACGGACAGTGTCCTCGCGATTGCCAAGAGAAAACTGCGT
CCTAAACCACCGAAGATATTGCGGCGGAAACTATCTCAGGATTTTGCAGTTCTTTTGATG
AGATCCTCTTACAATGCTCTGGATGAGATGGATTGCGTGGCCATGGATCAGTTCCAACGG
GATTTTTTCCTTATTCGAGTTGCTGAGTACGAAACATACACGAAAAATCTTGGGGCAGGA
ATGGTCACACAGGGTGACCTTACCGATCCCTTTTATTTTGATTTCATTTCTTTTGCGCAA
TATAAAACAATTAATCGGGAGGTCACACAGGATCCACCATACGTCTTTGAAGAACAACAG
GTCCCTCCTGAGGGTTCATCTGAACCGAGCATGAATCCTAATGGAACGGCAAGATTCGTC
CCTGTCATTGTGCAACGAGATCCAACTTTAACCAACAAAATGCTGATACCCACTTTCGAC
AAGAAGGTGGGAAGTAAAATCTTGGATAAACTCGAAGAAAATTTTGCCAATACAGAATCG
AAGATTCCCAACATCGGTTACAAACCCGACTCCGTCGCGCTATTGTCGGGAATTCGACAA
ATTGTGAATCTATTTCTCATTGGTGGTTACGCGTTCAAGGGAGACGTGAAAACAATTGCG
CCTGAATCAAATGGTGTACTAGCCACATATGTTTTATCGCTCAGCGCTCCTGCGACACTC
TGGAGTGGAAAGGTCTTGCAAGTCGAGGGTGACCCGCTTGATAACAACTTTCTACTAAAA
ACTTTGGCTGAATATATTCGGCGTAGTGGGTATATTAACATCAAATCTTCGATCAAGTAC
GAGGGGACGGGTGAGGAGATTACCATCACGGTTCAATAGCATAGTTATACCGAGTGTAAT
GGCATCTGATCGTGTGTAACAATAGAAAGTCAAATAAACAACAGCAAAAAAAA
>comp1894_c0_seq1
CGATAAAATGGTTCAACATCCAAAGTAAGTGCTTCACGGCACCGATCGAGATCGATGCGG
GTGGTATTTGAGGAAGGTCGTTGAGAGGAAGGATCGGAATCGTGGAGAATGGAGTCGAGT

CGAGTGGAGTGGAGTCGAATCAGATCGCTCTACTCTTATCTTGGGCAACATCAATGTAAC
CTTGGTCGGATCATCCCTTCATTATTGTCTCTAATCTTTTCTAATTTTGAATTATATGTA
TTTACCCTTTTCTAAAACAGAGGTCAGGAACACGAG
>comp1897_c0_seq1
ATTAGCGTTGCGACAGCAGCAACAACAACATGTTCTGTTTCAAGCACAGGAGGAACAATC
CATAGCAGTCACACTTAGTCCAGCACAATGGTTTGAGTCGAAGCTTTGCAAACGAAAGAA
TGGGTCGAACAGCTACTGGAGAGGTAGCCTATTCAATGAAAGAGATCACCGAAGGCACCA
AATCATCGCAAACAAAAAGTTGTCTAATCTTAATGGCAGATTTTTCCAACGCCATTGCCT
AGAATTTGTCAAAAGCCTTAGCATGTCGTCGCTGGATAGAATTGCAAGGAAAAGACGGTC
GATATTCCATATTGCTCTTGCTAGAGAGTATGGATTGTGCGGAAGAGAACAATTCGAACG
GGGCGTGTCTCGATTTTTCTACGTCAAGCAGTTATTGCAAGATCCAGATATTGTCTCACC
TCATCGAAAAAGTATATCGAGTCTTTCCGTAGACTTGACAGGAAGATTTCTTCTGGCGGG
TTCAGCAGATGCAACTATCACTATATATGATGTTTCAAAATGGGGTCGGAGCGAAAGCAT
CAGCAAAGACGACGCCAATAGTCAAAGGTATTCCTACTCCCCTGTCGCCAAGTCCATCAA
AGTTCCAGCAGTCCCTGATGTTCTCCAGCTCCCTGCAGGGCACTCAGCTTCTATCACATA
CACTCAGTGGTATCCTACAGACCCTGGAGTGTTCCTCTCGGGTTCGAGTGACGGAACGAT
CTTGGTGTGGGACACTCACAAGATGAAACCTGTCTTGCGGGTGCATCCCTTTCGAGACAA
CTCTACAGGTTGGGTATCGGCACATTTGCGCACGGGCGGAGATCATTCCATGATTGCTGC
AGGATCTTGGTATGAATCGGAAATCAAATTGGTCGATATACGTTCTGGGGCTTGCTCGCA
TCAATTAGTGGGTCACGGGAAGGGGATTTCGGCACTCAAGTGGTCGAGCAGTAATCCCAA
CGTTGTAGCATCGGGATCCCGCGACTCAACGGTGAGGCTTTGGGACATTCGCAAATCTGG
AAGCCGGGCGTGTGTCACAGTGCTAGATAGAGAACAGGTGTCATCATCGGCGAATTCGTT
TGGGTTGTCGTCCTCTGAATCGACTAGTGTGGGCTATGCCTCGGATTACTCTCATCTGCG
ACGAGAACCAACATGGGCACGCAAGCAGAGACGACAACAAATACCAGGCATAGCGCCCAA
TAACTATGACCACGTTCAACACAATGGGGCCAAATCCCATCGTGCCGGTCATGTTTCCGG
TCTGTCATTTTTCAGCAGTGGTCATTATCTCTGTAGCGTAAGTGGGCTTGACGGCGAACT
TTTGTTGTGGGATTTGCGCAGCGGATGCTTGTTACCAAGCAAGTTTGTAGCGCCAGGGAA
GTCATCGGCAGGGTCACCGAACCAACGACGAACGGCGATCATCGCAGAAGGATCGGGATA
CACGGACAGCTGTAACAAAAGCAGCACCATCTGGATCAGCCGTAGGGGGGTCCTACACGG
ATTTTCTACCGAGGGAGGGACACCAAAACAAACGCTGAAAGGTCACTTGACAAACATAAC
AAGCTTGGAAGCGATGGAACCAGGCGGAAAGTTGTTGTCAGGGAGTGCAGACGGCATGAT
TTTAGCTTGGGGCCAGCCACAGGGTGCCTTGGAAGTCGGAGATGCAACCATGAAAGAGAT
TGTGCAACAGGATATAGACGACTGGTGATGATGCGATTGCTGCTTTTAATTGTGAGTGTT
TA
>comp1898_c0_seq1
CAGCAGCAGCAGCAACGACAACGGCAGCACCCACCTCCCCACACCGACGACGCCACCCCA
CCGGATCGCTCTCGAAACGGCCCCGCTCTGGTGTCGTTCTCCTCCTCATCGCGATACTCC
TCGGCTCCACCACGCACACCATGCTCCCGGCGTCGGCCCGCGCCGCCGCCTCCGCGGCGG
GCAGGACCGCCCTCCGCCGGAGCAGCTCTTCGAGCGCCGGCGGCGACAAGGTAACGACGA
TCGTCTTTGACGTCGACGACACCCTCTACGACGTCGCCTCGGGCTTTACCGCGCACCGGA
ACGGGGAGGTCGTCTGGAGGTTCATGGTCGACCGCCTGGGCTTTCCGGACACCGCCACCG
CCCGGGCCGTCCGGGACGAGTACTTTGCGGTCTACCACAGCACCGCCAAGGCCCTGACGG
TGGCCCGGGCCGAGGGACGCTTCCCGGAGGGCGCCCCGGCCTTCGACCCGAAGGACCTCT
CCTCCTACTGGGTGGACCACCTGGACTACGGCAAGCTCTTCGGGACGCGGGCCCACCCCA
CCGAAGAGGCGGCGAAGAAGGCCAAGGCGGCCTTTGGGGCCGCCCTCGCCGAGTGCCCCT
GCGAGCTGGTGGCCTTTTCCAACGGGCCCCGGTCCTACGTCCTCAAGGTCCTCGACCGGC
TCAGCCTCCTGGAGCTCTTTGGGACCGGCCGGTCCAGCAGGGACGGGGCCCTCGTCTGGG
GGGTCGACGACGTCCTCCCCCACTGCAAGCCCGAGGCCGCCGCCTTTTCGGCGGTCTTTG
GCGAGCTGAACGCCAAGCGACGGGACGCGGGCCACCACGACGAAGTCGTCCCGGAGGAGT
GTGTCATGGTGGAGGACTCGATGAAGAACCTCCGGGCCGCCAAGGCCCTGGGGATGAAGA
CCGTCCTCATCACCGGGACCGGGGAGGAGGGGCGGATCCTCCCCGGGGACGCCCCCTCCT
CGGAGGACCCGGCGGTGGACGCGGCCTTTGCGACGGTCGAGGAAATGGTGGACGGCCTCC
CGGGCCTCTGGAGAGATCCCCCGGTCTTCGAGCCGCCGAAAGGCTAGGCTGGGCCAGGCT
AGGCCAGGCAGGCGCCTCGCACGGGCGATTGACGGCATCCAACCATACAAGCAGCGGAGC
CAAACGAATCGCGACGGCTGTTAAGGAAGTTAGAGAACGCCTTTTTCGAAAAACAGAAGT
TAGAAAACCAAATCAACACTACGTATTATACGGGCACGACGGTGAATAAAACCCGC
>comp1899_c0_seq1
CGAAGAAAGATTGTCGATGGCAGCAAACGCATCAAAGTGACAACGAGAATTAGAATTCGA
AAATCTATAAATATAAGACTGGGCATTAACGCCTTTTGAGCGATAGCAAAATGACACGTT
GTTCCACAAAACGAAAACGAAGTGCTGCCTTTTGCACCAACGGGAACAGCAACCATGGAA

AGGAGGGTCGCGCTATTGTTGGAAATATCTCATTTCCTCCAATTGCAACTACGAAAATAG
CCTCCGATAGCCCACTCGATGAAATCAAATTTTCTGATTGGCAATCAGCTAGAGTTTGCT
CTATTCGAGAAGACAACGAGCCAAAACACTGCCAGAGCTCTCTCCTGTCGTTCTTCGCCA
AAGCGAAGAGAATGAAAGCTGCACAATCTTCCCCCCAAAAAAGATTGCAAAAGAACGATA
ACGACAATTCGAAAAGTAACAGCAGAGGCTTTTGGTGTTCAAAAGAAAACAGAGCTTCGT
TGGTTTCGTCATTGCCAACTGCACCATCGTGTTCTTCCTCTTTCCGCCGCCAGCGCAACC
CAAGCATAGGAAAAACAGCAACAGAAAAACGATCACAAAAGTCTTCAAAACCCAAAGCCA
AATCCTTAACACAAGTCTATATCGATTGTGGTCAGTCGAGTTTTGGGCAGATTCTTTGCG
ACAAGTGCGGGATGCTATACATGCCCGGCATTGCGGAGGACGAACTCCAGCACAAGCAGT
TCTGCCAGGCCTACACACACGGGATCCCCTGCGTCAGAGGCAATGTAAAGGGCGGAAAAT
ATGTACCGGACACTGGATCAGTGAACGCATTGGCCGCAAGGGAGAAAGGGAGAAAGAGAA
ACGGTGCAGCAACCGTTGTTTTGTGGAGACCGGGCACCAAATGTCACAACCGAGCCAGGT
CGAAGCAACGACAGCAACATCCAGAAGGCTTTGCAGTCACCACGACTAAAGATGCCGAAA
TCAGCAAAAGCAATCACAACAAAAACCTACCGTCCCAATGGCCACTACTCGCTCAAATGA
TATCCAAAGACCTGGGCACCAATGAAGAGACCACACTGAATCATATTGCGAATCAGATGG
TTTTCTTGTACATTGAAAGTGACAACAGCAAATACCTAAGTACCAGTCAAATCCATAGAG
CATCGGCACCCGGTCGATCGCGCATTCTCGGAGTGGCCACCGTGCAGGTCTTGAGCCGCC
GAATTCCTGCCTACGAAATGATCAGCCTCCACGAACGATCCCTCACATCGGTGGAAGCCA
AATTGGGAATCGGCCTCCTCTGGACCCACCCCGTGGCTCGGCAGCGAGGAATCGCCACTC
GGCTGGTCGATGCCGCCCGCGGCCATGCCATCTTTGGAATGAAAGTGACCAAAACCGAGG
TTGCTTATTCCAGTCCCACACAAGCCGGTTATGACTTTGCTATACGGTATAGCTGTTGTC
GCAATGAGAAGAATGGCATTGTATCTTCCCGCAGCGATGAAAACGACGACTGCAAGGGTT
GCGATGGAGAACACGTCGCAATCCATCCGAAAGAGCAATCAGAGGCATCATTATGTCGCG
CCGTAAAAGGTCCGCTCGTGTATGAAATCGACTTGTAATCTAGATCTCGGAACTGCGCGA
AAGAAAAAATGTGTGGTTGCGTTTACGTTTGTTGCGTGTTCTCGATAAAGATGCAAAAGA
TGCAAATGATTTTTATGAAGTATTATATTTAGTGTGCT
>comp1900_c0_seq1
ATCGCCCGCTCCCAACAAACCAACGGCATCCTGATCATACCAACGAAAACGACTACCTTG
ACACTAATACAACTGCAACTATGAAGTTTTCTGTCGCCGCAACCGCCTTGGCTCTCTCTT
CTGCGACAACTGTTTCCTGTTTCTCTATTGCGCCGAACGGAAGGAGTAGTCTCGCATCTC
TCCGAGTCGCCACATCTTCCGAAGAAGCCGTCGATTCGAGCGTCAACGGGGATGACGACA
TAGTCCGAAAGAGCGACGAGGAAGTCTTCTCGAAACACAAGAGGCCGACATCCTTTGCCA
CCAAATGCGCTTCCTTGTATCACCCAAACCCCGGCGCCGAGCGTGGACTGGCACCCCCGG
TTTATTTCGGGTCCACTTTCTTGTTGGACGACGCCGCCCATGGCGCTCGTCTTCACGCAA
AACGCGAGGAACCTTACACCGACGACGACGGATTCGTTTATTCCAGATGGGGTGCCCCCA
CCAACGAGGCAGCTGCGGTCCAGATCGCTGCCCTCGAAGGCGTAGACGATCCAAACAAGG
GAGCCCTTGGAAAATGCTTGTTGTTCAACTCGGGAATGTCCGCCATCACATCGGCCCTCA
TGGCCGTCTTGAGGGCAGGAGATCACGCAATTTTCCCCTACACAGTCTACGGTGGAACCC
ATGAATTCGCCGAAAACTTTTGCAAAGAATGGGGCATCGAGGTGACCTACATCGACGCAT
CCGGTGTCGATGGACCAGAAAATTACAAAAAGGCCTTCCGGGAAAATACTAAGGTTGTAT
ACACCGAAACACCCGCCAACCCTACCATGCGATTGACCGACCTAGAAGGCGTCGGAAGAG
TCGTAACCGAACACTACGGAGCCGCCGAGGACACCCCCGGCAGCGATCCCAACGCACGAA
AGCGACCCTGGGTGATGGTCGACGGCACGTTCGCGACTCCGTACCACCAACGCGTCCTGG
ACATCCCCGGCGTCGATGTCTCCATCCATTCCGCCACCAAGTATCTGGGCGGCCACTCCG
ATATCTTGGCCGGATCCGTCACGTCTCGTTCGGAACCATTTTTGCACGGATGCGCCAAGG
TCCAAAAACTGATCACGGTCCCCCTCAATCCCATGGATTCCTTCCTGCTGGCCCGTGGGC
TCCGCACCCTGGACGTTCGAATGCAACGACACGGCGAAAACGCCATCAAGATCGCCCGAA
TGCTCGAAGAACATCCCGACGTGGAATCCGTCTTCTACCCCGGACTGGATTCCCACCCCG
ACCGCTCGATGTCCGATGGAATTTATTCGTCGGGAAGGGACGAAGACGACGAGGATGGAT
TCAACCAAACCTACGGTGGAATGGTCACCTTTATCATGGCCGGCGAGGGCGACATTGCCC
TCCAGAGAGCGCGAAACGTCTGCGAACACCTGCGGGTGATCAACCTGGCCGTGTCACTCG
GTGGAGTCGAATCGCTGGTCGAACACCCGGCATCGATGACGCACGGCATGATCCCGAGAG
AACAACGATTGGCCGGGGGTCTCGCGGATGGCTTGATCCGATTGAGCGTCGGATTGGAAA
AGGCAAAGGACCTGATCGACGACCTGAAGAGCGCTCTGGATCGATGCGACGAAGAAGGCT
GCGACCTCGACATCTAAAAAACAGGCCAACAGACCCAAACGCACGCACGCACGCACACGC
ACACACACAAACAAACAAACTGCAACTCGAACGAGGGTTTTTCGGCCGGGCGACGATGGT
TGCGTCTAGCTAGGCTTCTACTGCACTTGCACAGATGGTTTGGGCGGG
>comp1905_c0_seq1
TGGCACGAATACAAAATGGGAAAATGTATGTCGAAGCTCGCATCTAGGTCGGTTTCACCG
TGTATGATCACCAGTGCTTCGATTCGAAGCGCATCGGCGACATCGGCGATCCTTGCGTCA

GTTGCTCTAATTATTCATACATATATTAACAAACAATGGTTGATAGATTATTTCGATCGG
GCCCACAGGAGAAGAAAGCTGATGCGTCTTTGTGTCAATTGCGTGCCGGGTAGAATGGAG
TTTGCAGCAGCGCGTGAATTGGCTTCTACCTGGGCAGAAACTGGAGTTATAGATCCCCGG
GAGGAACGATTACTAGCACTTCTAGCTGGAATTCCTGAGGACCTTAGACAAACCATGATG
GAGGTTTCCATCAAGTGCATGCCGAGCAAAATGGATTTGGCAGATGGAGTTGCTTTGGTT
TCCGCCTTGGCAGCCATAGGAATCATCAATCTGCGAGACAATCTTAGGATCGACTTTGAA
AAACACTCAAAACACACCGCACCAGTTTGGTTCTATCAAATGGACGACGAGGGTCGAGTT
AAAAGGTTGCGTATAGGAGGCAGCATGATCTATTTCGATGGCAGAAACGATGAAGACAAT
TTTGATTTACCATCCGAGATAGCCCACCTCGATCAATTGGAGGAGCTTTCAGTGGTCAAC
TGTCGGTCTCTCCCTATAGACATTCAACATTTGACAAATTTAGAGATTTTAGTGTTTCTG
AGATGCGATTTCAATCAACAGCCTTTTTGGCAGCATGGCCGTGTCGACGAATATGGATTG
TTCGGAGTTGAACTCCAGCACGTAACAGGGATAGGTATAATCGAGTGCGAGTGTTTTTCT
TCCCGGCCCTTTTTCACATGGATGACAACTAAGCTGCCTCGGTTAAAAAATGTTCAATGC
GGATGTTTGCAAAATTCTGAAATAGACAGTTTTCTGGGAAGACTTCGCACAACAAATGTA
CGCTTTCGAGAGAAACTAAGATACCTCACAATAGATTCTAGTAAATTGAATGACCGTCAA
CTGGAAACTCTCTTGATTGACGTTTTGCCGAGCTTTTCGAGCGTGAATTATCTCAACCTC
TATCGCAACAAAATTCAGAGTTGCCAATCGGTTGTCAATCGAATTCACAAAGACGACGGG
ATAAGGTTTGTTCCTAATTCGATCGTTGCTGCGAGTTTTCGCGGTAATCCTATAGAGGAA
ACGATAAGAAGTGATCGCAGAGAAAAATCTGCAATGCTGAGCTTGTTGAAAACATTTAGC
ACAATTATTAATCTTGGAAAATTTCATGAAGGTGACTATGATCCGGATGTTGAATACGCA
TTGCGAATAAATCATGCCGGGCGGAGTATCATCGAAGGTGAAACTGATGTTCGGTGCCTA
CCTCTATCCATATGGCCCTTTCTGTTGGAAAAGGCATTTAGAAACTCTTATTGGATCTAT
GTGAAGGAATCGGAATTGTGTCTCCCGGAAAGAGCATCTTCTCGGAAAGCAAAGCGTAGT
GTGACAGGTGTATACTACTTACTGCGCCAACTACCATCAATAATTGGCGGGTCTGATCCT
ACCGAATCATAATGTTGTCAAGCGACAAAAAAATCATACAATGGAAATGATTGATTCTCT
TTCTAGATGGTTTACTTAAATTGTGCAATACGCACGGCACTTGGTAGTGAACACATTGTC
CTATAGAAATGCCGACTGGGATAATTCAAATCTATTGGCTTTTGTGGTATAGGTTGTAAT
TCATCGACAGATGCTACGG
>comp1906_c0_seq1
CGAGTCTTCAAGACGTCGAACAGAACATGATGATGCCGCATTGCATAAGGTAGCCTCTAG
AAAAATGTATTTACGCATAACTCTTTTCATCATCTGCATGTGCAGGGTTGTGGATGCGTT
TACGACAAGATCTTCATTATGCCACTACGCTGTCCCATCCAAATGCAGCGCAACTCCAAA
AGAGTCGTCTTCCGCATTTCCAAAGGTGAGATCGATACCGCCTTCCTCTCTGTTCCTTTC
CTCTGAGAACAATGAGAACGCCCTATATGTGCCAAAAGGGATTACTCTTAAAATTGCATT
CGATTCGCAGGGGGGAGTTGCTGATTTGTCTAGTGAAAAATCAGAACGTTTCACGTGTGG
AGAAAGTCTTGATATGGTACACCGGTTGAGGCGTGTTTCAGACGCTGTGTTGGTAGGACG
CTCCACCGTCGAGATCGATGATTGCACCTTGACGGTAAGGAGAGTTCCACTCCTACCACT
TGATAAGGAGCCATCCAACGGTACGGCACCAAAAGCGAAAAAAAAACAACCTTTTCGAGT
CGTTTTGGACCCAAATCTCGATCTCGAATTAGATCAGTTCAAAATTTTCCAAGACGGATT
GGAAACGATTGTTATACATGCTTTGTCAGATAGTGATCTGGTTGACGCAGGATCAAAATC
AACTAGTGCGGACGAAATGGGCAATAGTTACTTTGTCAAATCAACCAAAAAGGAATTTCC
CGCTGTAACATTTCTTGGACTTCAACCTATCATCGATCAAGATAAATTACGTCTCTGTAC
TCGGCAAGTACGCGATATTCTTGCAGAAGAATTTGGTATTCATCACATTATGGTAGAAGG
AGGCCCTAATACTGCTCTTCAGTTTCTAAGGGACAAAACGGTGGATCGTGCCATTTTGGT
TTATGCTCCCATATCATTTAAAGATCCTTTGCTATCCAATATTTCGACGTCGGTTCTCGA
GCAAGCGGGTCTAGAGCCTATTGAAGGCTATAAATTGGGAGTTGACTCACTGAAGTGCTA
CTCTCGACCCGAACTTTCGTGGCCGTCGACTCCCATTAAATCCTGGC
>comp1908_c0_seq2
AATACCTGTATCTAACCGAGGAACAGAAAAGATAGTGGTTGCATTCTTTTTTCCTATTCT
GAGTTTCAACCGAGCGAAGCTATTTTTTCTGATGAATGGAAGAGAACAATCGTTCGCGGT
AACAGCGATCCTTGTCGTCGGCTTGGTGCTCAACATGATAGGGGACAGGACCACTGCTTT
TCAGTCCATAGTTCTCGTATCGAGACCGGGACGACGTAATCGTGGCAAATTTCGAACGAA
TCAATGTCATAACCACAACTTTATTCACAATTGTCGTGATGCCAGTCTTTTTGCATCACC
TTCAGGAAAAGAGGACATTGATTTCAATGACGAACTCGACGATGAGAAGTTTAATGAAGT
CGTATCGGAGCAACCTATCGAATTTGAGTGGAGGGTTTTACAGCAAATGCTTGGTATCAA
TATTTTCACCGTCATATTGGCGGCACTGATAGTCTTTTTCCTGAGCATGAACATCATCCT
CGGCCCAGGTTGGCTTGGTTCAACCATTGGTATTCCAGGAACCGGCAATATTCAAGAAGT
ATCTCCAACGCTTCCAGGATCATTAGACTTGAACCGACCCGAATATCGTTTATGAGAAAC
GAACGATCGAAAAAGGTACAACCGCAATTCGAGTTTTCAGCGAGATCCAATCAGTCTTTC
ATGAAAAGGTTCCTTTGTTACGATACAGGAATGGAGAACCAAAGAGCAAATATCCAAACT

AGATTCTACTAGGATTTCAAAGCGAAATCTGCATTTGTATGTGTGGGCAGACACTTAGAT
TTACGATTGTAGCGTCTCCCTCAGCAATTTAAAGAGCTGGGCTAAGTGGAAGCGAAGAAA
TTTCATTTCTGTGCCAAAGTGTATCGAAAGAACGCCACTAGAATATTCCACGAAATTTCA
ACTTCTCTGTTTTCCAACTGCAAACTCCTAAGAAAAGAAACCATGCTTGTGAGTTAAATT
TTGAATTTAGAGAACTAATTACGGG
>comp1913_c0_seq1
TCCTTCATCCGGTCCTTCATCCGGCCCTTCATCCGGCCCTTCATCCGGTCATTTGGTCAG
CGAGGATGACGAAGATGAAGAAAATGACTCTGATTGCGAAGACGATTCCGCCTTTCTTTT
CAGAGGCAGGGAAGGAAAAAATTGCAACTGGGTGAAGAAGAAAGCGAGAAAATTTCGTCG
TTTTTGTCGGAAGAGGTCGAACGGCATAAGCGTGGCTGATTTCTGCCCTATGGCTTGTGG
TATGTGCAACGAGGACGATAATGACTCTGATTGCAAAGACGATTCCGCCTTTCTCTTCAG
AGGTAGGGAGGGGAAAAATTGCAACTGGGTCAAGAAGAAAGTGAGAGCTTTTTGTAATAA
GAAGTCAAATGGCATCAAAGTCAAAGTCTCGTGTCCAGTTGCTTGCAATGCATGCTAGTT
TTTCGACAACAATGCCGATGTTGAAGAAAATGATTCAACTGACTCCCAACTTCTTTATCT
ACAACTTAGACCTTTCACATATTTAAATATAATACAATAGATGCACAAATGAATCAAACA
TAGAAATCAC
>comp1914_c0_seq1
GAACTTTATCAAAAAACAACTTTAGACCACCAGACTAAGAACCCAGTCGAAGATGATATA
ATTACTTCAAGATTGGTGTCATTGGGAAGCCATATGATGCTCTACTGCTAGGACTTGGTA
GAACTAGTCTTCCAAAGTTACACTACTAGTTCGTGGCCTAGAATCCCGCGTTAATTTTGT
TTGTAATTTTACAATACTCCGATCAAACTCGTACCGGGAAATAAGGGTTCCGATTGCGGC
ATACCGGTAGTTCATCGAAAGCCAACGGCAGGTCGACTAGGTATGGGAAAAGAGAACAGG
ACAACCGGAATCGCACGACGAGGATTGCATTCATCACAGGCGTTGGTAGACTGAGATCGT
CTTTGTCGTCGCCTTTTCCCTCTGATTTTCTGAACGATAGAAAATGGCGAACCAGTTGAA
CCGTCACCGCGGTAACAATTATCGTGTCCATCAGTTTCAGACAGAACAACACAAGCCAAG
AAATCGTGGTTCACTTCTGTTTCTCTTTTTCATCCTGATTACACTTGGGTTGTACTCCAC
GCTGTCAATCGTTTACATACATCGCCTGACTCTGTATTCAAGGCCAGAAAATGCAATCTC
GGAGAAAACTACCCATGAAGATAGTTCTGTTGCTTCCTTGAAGAGGAAAGAAAATGACGT
TTCAAAGTCAAAGGGAGAGGGAGAGAAGATCGAGTCGTCGTCTCAGAAGTTTTCCTTTAA
AAGTCGCGATCGCGATCACGATAAGGTACTCCGGTCCTTGCAGGAATTGACGAGAAGCCC
TCCTCCGGGAAGTATAGTTTGTCCGTCTCCACATATGACCCCGATCTACGACAGAATTCT
TTTTTCAACATCGCCTAGATCTGAGAACACCACGGCAGTCTCATTGGGAGGAGCAGACCG
AAAAATACCGCGTATTGTTCACTTTAGTTTCAACAACCGCTGTGTACCAAACGACCTGGC
GACATCCATTAGACTCTGGAGTGATGCTTTGCCGGATCATTCGATATTCTTTCACGACGA
CGAAGCAGTGAAACGTTTGATGGAGGGAGACGACCGCAGCAACCAATCGCCACTATGGCA
TTCTTCAGAACACTTTCCAAACCTGCGTGATAGAATGCCATGCGTCACGTCCACTGGGGC
GATGCTCATTGATGTATGGAGGATGCTTGTTGTATGGACTTATGGTGGTATATACACAGA
CATAGACAATTGGCCAGGTCCAAAATTCAACTCTCAAACCATTCGAGAAGAAGATTCTTT
TTTCTCTCTCTCGGATGGCAAAAATCGTTGTTCGCAGTGGCTGTTTGGGATGGCACCTCA
ACACCCGATTGCAATATTCACTCTCCAGGATCTAACCCGACGCCTTGTGCTAATCAAAAA
CGTCGCTCGACCGAGGGTGGTCCGCATTACTGGTCCAATGACGTTGAAAACAGCCTTTCA
AAAGTTTAAGCAACTTTTGAAAGAGAATGCAACGATATTTGGAGAGTCGTCCTATTTTAC
TCAAATCCAAAAAGTCGATCTCGCAACATCTGATCAATACGCGAGAGGAAGCCTTGGTGG
CACCTTTGACGAAATCGTAGATCACTATTACGATGAGATTCACAACACTACCTACATAAA
TATAACAAAGAGGGAGAGAGCTGAGTTAATAAGCGGCATTGAACATTGGACAAAGCAAGT
GAACAGAACAAGCAAAGTTTCAGCGAACAAGACCGAGGGTGAAAGTTTGAACACAGTCGA
CAAAAATCTTCCTCTTCCCCCTTATGCTGGTGTTTCATGCTCGGATTATTTGGATGCCCT
CAATAAGTAAGATCGACGTGGAGTGTATAAGCAGAACAAAACGGAAGGCAAAATTAGTGA
CTTACCAATGGGATTTCTAGTTTTCTTTCGTTTCATGTGTGAAGTGTCGATTCCAATTTT
TTTAGCAAATACGACCATAGGTCAGATCAGGGATCACATAGATTATCCTGTAACAACCAA
GACAACTAAGCGTTCAATAGTTATAGAGGTATTGTAATGAGTTTTGAAATGGATGCGTTT
GGTCTAATTTCCAATTCTCATCCTATGAATACTAGATCAAAAAATGATCCTCGGTCCGGT
TGACCTGCTCGTTGTACCTTACCCTTGTTGTTCCCGCA
>comp1914_c0_seq2
GAACTTTATCAAAAAACAACTTTAGACCACCAGACTAAGAACCCAGTCGAAGATGATATA
ATTACTTCAAGATTGGTGTCATTGGGAAGCCATATGATGCTCTACTGCTAGGACTTGGTA
GAACTAGTCTTCCAAAGTTACACTACTAGTTCGTGGCCTAGAATCCCGCGTTAATTTTGT
TTGTAATTTTACAATACTCCGATCAAACTCGTACCGGGAAATAAGGGTTCCGATTGCGGC
ATACCGGTAGTTCATCGAAAGCCAACGGCAGGTCGACTAGGTATGGGAAAAGAGAACAGG
ACAACCGGAATCGCACGACGAGGATTGCATTCATCACAGGCGTTGGTAGACTGAGATCGT

CTTTGTCGTCGCCTTTTCCCTCTGATTTTCTGAACGATAGAAAATGGCGAACCAGTTGAA
CCGTCACCGCGGTAACAATTATCGTGTCCATCAGTTTCAGACAGAACAACACAAGCCAAG
AAATCGTGGTTCACTTCTGTTTCTCTTTTTCATCCTGATTACACTTGGGTTGTACTCCAC
GCTGTCAATCGTTTACATACATCGCCTGACTCTGTATTCAAGGCCAGAAAATGCAATCTC
GGAGAAAACTACCCATGAAGATAGTTCTGTTGCTTCCTTGAAGAGGAAAGAAAATGACGT
TTCAAAGTCAAAGGGAGAGGGAGAGAAGATCGAGTCGTCGTCTCAGAAGTTTTCCTTTAA
AAGTCGCGATCGCGATCACGATAAGGTACTCCGGTCCTTGCAGGAATTGACGAGAAGCCC
TCCTCCGGGAAGTATAGTTTGTCCGTCTCCACATATGACCCCGATCTACGACAGAATTCT
TTTTTCAACATCGCCTAGATCTGAGAACACCACGGCAGTCTCATTGGGAGGAGCAGACCG
AAAAATACCGCGTATTGTTCACTTTAGTTTCAACAACCGCTGTGTACCAAACGACCTGGC
GACATCCATTAGACTCTGGAGTGATGCTTTGCCGGATCATTCGATATTCTTTCACGACGA
CGAAGCAGTGAAACGTTTGATGGAGGGAGACGACCGCAGCAACCAATCGCCACTATGGCA
TTCTTCAGAACACTTTCCAAACCTGCGTGATAGAATGCCATGCGTCACGTCCACTGGGGC
GATGCTCATTGATGTATGGAGGATGCTTGTTGTATGGACTTATGGTGGTATATACACAGA
CATAGACAATTGGCCAGGTCCAAAATTCAACTCTCAAACCATTCGAGAAGAAGATTCTTT
TTTCTCTCTCTCGGATGGCAAAAATCGTTGTTCGCAGTGGCTGTTTGGGATGGCACCTCA
ACACCCGATTGCAATATTCACTCTCCAGGATCTAACCCGACGCCTTGTGCTAATCAAAAA
CGTCGCTCGACCGAGGGTGGTCCGCATTACTGGTCCAATGACGTTGAAAACAGCCTTTCA
AAAGTTTAAGCAACTTTTGAAAGAGAATGCAACGATATTTGGAGAGTCGTCCTATTTTAC
TCAAATCCAAAAAGTCGATCTCGCAACATCTGATCAATACGCGAGAGGAAGCCTTGGTGG
CACCTTTGACGAAATCGTAGATCACTATTACGATGAGATTCACAACACTACCTACATAAA
TATAACAAAGAGGGAGAGAGCTGAGTTAATAAGCGGCATTGAACATTGGACAAAGCAAGT
GAACAGAACAAGCAAAGTTTCAGCGAACAAGACCGAGGGTGAAAGTTTGAACACAGTCGA
CAAAAATCTTCCTCTTCCCCCTTATGCTGGTGTTTCATGCTCGGATTATTTGGATGCCCT
CAATAATTTTCTTTCGTTTCATGTGTGAAGTGTCGATTCCAATTTTTTTAGCAAATACGA
CCATAGGTCAGATCAGGGATCACATAGATTATCCTGTAACAACCAAGACAACTAAGCGTT
CAATAGTTATAGAGGTATTGTAATGAGTTTTGAAATGGATGCGTTTGGTCTAATTTCCAA
TTCTCATCCTATGAATACTAGATCAAAAAATGATCCTCGGTCCGGTTGACCTGCTCGTTG
TACCTTACCCTTGTTGTTCCCGCA
>comp1915_c0_seq1
ATCACTCAAAATTGATATGAGCATGTCTGCTCTCATCGGAATGAAGGTTCCGAACGGCTA
ATGATAGCTCTGCGTTGAAGGTTCCGAACGGTTGATAATAATTTTGCGTTCCTACCCCCG
CTTCTTAGTGGATTGGCTGGGATTGATTTGAGATTTATGGCTAACATATCTTCACATTTT
TTGCTTTCGGAAAATCTAACAAAACTAGAGATGTTTGAAACAAACCGCCAAACAGCAGAG
GAAAAGCTAATTCTGAGTCGCACCGAAAGGGTTCTCAACGCCACCTCCACTGCAATAGCA
CTCGCCTTCTTGAGGACGGCCCCCGTTGCGGTTGATTCGAGCTCCGGATTCCATTCCCTA
GATACCGACTACCTGTGCTGGATGTCATTAAGGGATCCCAATATCGCTACGTACCGTCGA
AACCAAAAACGCTATCGCTCCTTGCTTGACGACGAAGAGTCTACGATAAACATTACGTGG
AAAATATGAAAGCAATGTATCCAAAGATAATGAAGAAAAAGAAGCTAAGACTTCTACAGG
GGAAGACATTAAGGGAAGCGCCAAGAAATGCTTCCGACCGTCAGCACCAACAACCTTATC
GCTCCTTACTCGACGACGAAGAAACCACGATTAGCAACACATGGAAATCATCTGGAAGCA
ACACATCCGAAGATGTCGATGCCAACGTCGAGGGGGGAACGGAGGACATTCAAGAAGACT
GCGAGGAAGAGAATGAACCAAAGGTGTCGGAACAAATGCCTACCGAGAGAGAGACGAAGG
AAATTGAAAGTGTAAACAACATTGTGTTCACCTCGGACTCAATTGGGCAAATCGAAGTTT
CTTCTGCACAGGTCGCAGAAGAAGAAGAACACGAGACAAAGGTGGAGGAGTGCACTGACA
ATCGAAAAAGTGCCTCATGGACCCCGTTTCTGAAAAAGAAAATCCAGGCGACTGTGAAAA
AGTCTTATACTATTGTTGCTGGCCAATACTGCGAGAAAAACCACACACGTGCCATAGCCA
TGACCAAACACAGATCAAAAATGTGGACGGAGGCGATGAGTGGCATACGCGTTGGAATCA
AGGTCGATGAATCTTCGATGACAACATTGACCGCAGTAGAAGGAGATAGTGATTCCTCGA
CTGCGTCGAATGGAAATACTGCTCGTACAACTCCTTTTTTTTGCTAGAAGAATCAGCCGC
AGCATCACAAATAGAAAACTGGCTCGCCGACCACAAAGAAGGTAACGTTGTAAGTAGCCA
TGTCAGCAAGAATACAACTGGATTCGAGGTTCAACGATTAAGAGCTGGGCATATTATCAT
AACACCCGACGAAGCAACAGACCCAATGAATATTATCAAGCCGCAACCATACGTATCGGA
TACATCAGAAACAATTTTGACAAATGATGGTACCAATCTCCTAAAAACGGTGGAGTGTGG
AGCAACAACGATTCCAATACAATCATTTGCGAAACTGATAATTGTTTAATGTTCTACCAT
TGTCTTATGCCGAGAAACACGATGAAAAACTCGGGCTGTCAGCGATCGAGGTGATCGCAG
TGGTCTTCAGAAGACTTACTTGTATATATGCTAGGAATTTTCGCAAAGTACAATGCAACG
AATCACAATGGAAAGAATTACATATCCATGTTGTACCGACTACAGAATAAAAAAAATTGA
ATGATTCTATTGCTTTTGTTATGGTGTGATCAATGATTCGGAATTCCACTGGGATGAAAT
ATGAATGCACCGCGGAGGCAGTTATTCGTACAGAAATCACCAAGATTTGTTTGTTAGTCA

TCCGTCCAATTGATGCGAATGGCCAAGAGAGTCAAATCAATACAGAGACACAGACACAAC
AGCATGGTTGTACTTTTTTTTCACTTTTCCCAGATTGCCATAAAGCTATGCAGCTGTGTA
GACAAGCCTCC
>comp1917_c0_seq1
GAAGAACAAGGGTTTCGTTTAAGCCTACCAGGTATCCAACCTGTGGTATGAATATTACTG
TCAGCCCATCGAGCTGCTGACCGTATTTATGGTAGAGATCCTTTGCTGCTTCTGCCAGGG
TTTCTTCGAGACATTCGTATTGTTCCTTCCAGCCATCCAACTGCTCGTGAAAGCCATGAC
GAATCACGACCGAGGCTCCCTTTTCCTCCAAGGTTAGTTCACTATCGATGGTATTTGTGA
TGCGCTCCCTCAGATCGATCATAACTTCCACATTGCACTCGATACAAAGTTCCGAGAAGA
AGGAGTACTGGTGATCACACGAGAAACTGTTGGGGCCCTGTTGTGGCGGAGCTGTTGTAT
GATTCATTGACGCCAGAAGATCGTTCCGGAGCACGTTGCCAATCGAAATAGCTGCGGAGA
GTGTCGTGGTTAGGACCAGGAAATCAGTTGGCTTGACGGTGCATTTTTGGATTCGCATCA
AGATTTTGTCCACCGGACCGACCTTTTGCAAAAGTCCGATGAGAACACCAACCGATGCCT
GCATGCTTGGCTGCAGGAATAGTTCAATAGCGTCTTGGCGTGTCTGTATGTTTTCCAGAT
TGGTCAATGGCTTGAGCATCCACTCCCGCAACAATTGCTTCCCCCCTCGACCTGGTTGTT
TGTTTTAACAAAGCAAAAAGTTGAATCGCCGTTGTTAATGTTTGGACGGAGCGAAACGGA
AACGAAAAAACAAGGCAACCAAAAGGAACGTCGAGCTGTTAGTCAACGTGCGAGTTCGGA
ACAACGAAGTCTAAATCGGTCACTAAGATATGGGATTGCGTGTGGTCATTGGTCGATTGT
TGGAAATACCCTTGCGAACAGTTTTCCGTTTCGCTGTTCCCACTTACTCTTGGTACGATC
GAGCAAGGAGTATAGACTAAAACCTTCTTTGGAATGCCCGTGTCCCTTGGCAATGAGTGG
ATGGCGTTCGGTGGAAAAGATATTGAGCGCCGAAAACGTCGACGGAGAAAGGATCAAATA
CGACGATATTCCAGCCTCGATGATCCTGTTAACGGTGATGGTGTTCCCTTCCTCGAGTTG
GAAGACTGTTTTCTGCAGATACGACAAAAGAGCTCCCAGGGCTTGCACCAGAACCTTTGA
ATCGAAATCGATGACGGCTGCCAAATAATGATAACTGGAAGGGCGATAAAGGGTCTGTGT
GGATGACCCATCCCGGGGAAAATGCCTATCGTTGTCCGGGTGATTTCGATATCTACTGTC
GCTCCTTCCCATTGCTGCTGCGGTTGCTGATAGTCGTGCTTCGTCCATGGCTCTTTGTTT
GAGCATGGAGAGCACCTGCAATTTTTGCAAAATAATACTTCTGCAAGCACGAACGTCAAA
GATTCCTGATTTCAACAGACGATACGGAATGGAAGACCGAGGCGAGGGGTTTCCTCCTTC
TTGACGGTGATGGTGCCCCTGCTGCTCATGTTCGGTTTCCAGAAGAGGTTGAGGTTGTCG
CGTTACCATTTCAAGAAGAGGAATGTTGTTGACGATCCGATTTCTATTCGGTAGTCGGTT
TGGAGCCGACATTGAAGGGAAAATATATCGGGAACGAACCAAGTTGTGAATTGTAAAATT
GACAAAACAATACGACTGTTAGAGTGTAAAACAACTAATAATATCCTTCGGAATGGCT
>comp1918_c0_seq1
CCGTACCGTCCATAATATTTCGACGACAGCCTTGGCTAGCTGTACAGCATCGTCGGAAAC
ATACTGCACCATATTCATTATCACACAACAATGAAGCCGTTTGGAATTTCTTGCACTCGC
CGGAGCCAATTGCTGGTGATTCTGTCGACCTTGTTCGTGACTCTTGCAGGAGCTTTCCAC
ACCGAATCCTTGCTTCGCCACCCAAAGCTTGCTGCTTCGCGACAGGAAAAAATCATTCAA
TCGACAACGAGTGTGCGGCCCCATGTTCGCTCGAACGGAACCTGTGATATTTGCAAGTCG
TTTGACACCCTCGCCTTGAGGGCTGGTGGTGACGATGGTGCACTGGAGAAAAAGCCGAAC
AAAGTATCACAAATCATCCAATACTGCAACAAAAATTACTTTCTGGTGGGAATGTTCGTA
GCTGTTTCGTTGGCAAAAGCATTGCCGGCACTCGGGAAAAACGGCGGCATTCTTCGGCCC
GAAGTTTTGATTGGAAATTATGGAGTCGCACTGATATTTTTGCTTTCGGGATTTTCCTTG
CAAACTTCGGATTTGGCCAAGGCCATATCGAACGTCAAACTGAATGGTCTCGTGAACCTT
TTGCTTTTTGGCTTTTGGCCGTTTTGCATCGGTGTGCCGCTGCGCTTTCTGTTTACGGAC
ATCTTTCCGACCTTGATACCTCCGGCTCTGGCAGACGGCATACTCATCACGACGACCTTG
CCGACTACTGTGAACATGTGCATCATGCTGACATCGACGGCGGGGGAAACGCAGCATCG
GCCATATGCAACGCCGTCATCAGCAATCTTGCAGGAATCTTTTTGACGCCGTTGCTGCTC
ATGAAGTTCTTTGGAGCGTCGATCCATCTGCCATTTGGACAAATGGTGCTGAAACTCTGC
AAAAAGGTGCTGTTGCCCGTGACAATCGGCCAGCTTCTCAGGACCAACGGCCAAGCGAAG
GCTTTTTACAAGACCCATTCTAAAAAGTTCAAGCGCCTCCAGGAAACAGTGCTGTTGGGA
ATCGTTTGGAATGCCTTTTGCAATGCTTTCTCCGACGGCTTGGGCATTCAGCTGAAACAT
TCGTTGGTGTTGTTGGCACTCCTTCCAACCTTGCACATCGTGACTCTCGGAGCATTTTTC
AAGTTCTTCTCTCTTCCGTTCCTTGGGTTTTCAAAAGGAGAGACGATTGCCGGCATGTTT
TGTTCCTCCCAGAAGACCCTGGCTTTCGGTCTTCCGCTGATCAATACGATATTCCAGGGT
AATCCTAACCTGGCGTCGTTTTGTGCTCCCCTAATGTTTATTCATCCATTGCAAATGGCA
ATCGGATCGTTCCTACTTCCTACAATTCAAAAATATACGGGC
>comp1919_c0_seq1
AACAACAACAACAAGCAAGATATAGCAACGGAAGCAGCACTGCGGGAAGCAGCAGCGCCG
AGAACCATCGTGCCAGTGAATCCACCGACTGTGTCTGTCTAGGCCACACACGTAACAACA
GCAGCTGCCAGAAGAGCAGCAGCAACAACAACAACCACGACCACAACCACAACCACTACA

ACGAGAACTACAGCGACGAGGCCATCGAGGAGCTCTTTCACACCGAAAAAGACGCCGACG
GAGACAGCCCACGCCTGGTGATATGGCCGGAGCTCGACATCATGTGCCGCCACGACAAGG
GGAAAGCCTTTTTCTCGGATTCGCGAAACACCGAGTTCGAAAGCATCGCCGATTGCGGCC
ACCTGTTTCACTCGGACGGAAGGATCATACTCGAGATTGCCCGGGAGAGGCTGAGAGAAT
GCCTGCTCGATTTTTCGCCGTGCTAGCGGACGAGCGAGCGAGGAACGGTTGGAACGGTTC
GTGTTCGTCACTCGCACAAACGAACGATTCACCGAAACCGAGCCTCCGAAATGACAAATC
CATGGAACAAAACCAACAAAGCGCCGGGTACGTACGAGTGCTGAATTAATTTGAGAGTAC
CGGTACTTCTACTACAGTGAAACTAAGATTTGTCACGTTCGTAGAAGTACGGAATCGTAG
TCTAAGATAGCACATCAGCACTCGACATTGAGCGTCATAGACTATGGAACTGTTGTTTGC
TTTTACTACTAGCACGAAGTACAGTAATATTTATAGTTGACCAGTTCTCAAAAAACAGGA
TACGACAGTTTCTGTTTCCCTCCAGAAAAAAATTGCGGTAGATAACATGACGTCTCCAAA
AAACAATGAAACCCGCACGACGCAAAAAGGTGAACACAGTTCATCGTGATTCAAAACGCG
CCTGGAGGAGTTGTTTGTATCTAATCAGTACGAAAGAGTAGAACCACAATGAAAATAGGC
GACGAAGGGG
>comp1920_c0_seq1
AAAGTTTGACCGGTCCGTCTTCAAGACGGAAGACGAGGCCGCCGCCCTGGATCCCCTGGG
ATTGATGCAGATCGTCCGGCCGAACACGGGGACGAGCCTCACCGACAAGAGCACGAGGGA
CCTCCACCAAGCGTACAGGCTCGTCCTGGCCTGCCGGGACCTCCGTGACGGCCACGTGGC
ATCGGCCACCGAACCGGAGAAGAGCCACCTGGCCCGCTCCGTCCAGACCGTGAAAACCTC
CTGGAGGACCGCCTTTCGGGAATCGAACGGCTTTGGCCACGGCAAGGGCCTCGCCCCCCG
CCGCCAAGAGGTGTCGCTGGTGAACGGCCCCGTCCTCCACGTGCTGCCGGCCCTCGAGAA
GGCGGTCCGGATGCGGTCCGAGCCACAGCGGGCCCTGAAGATCGTCCGGGCCAGGGTCGG
GAGCCGGCGGATCGTCGGGGTCCGGTTTCCCTCGGACGACGAGGCCACGGGGGCGCTGGG
GGCCGAGCTCGATGCCCTGCGAAGGGCCCGGAAGGGTGTCGGCAGGGGCTTTGCCGACGA
GGACCTGGCGCCGGTCTGTCCCCGGACGATGGCGTGGGCAATTACCGAGCGCAAGACCAT
GAAAAGCTTTTTTGGGGTGGCGGGTCCGGGGCCTTGCCACCGCCCCCTGGATCCGAACCG
CGGGAGCGGGGGCACGAGCGCCAAAACCACGGCGCCGGGGGCTCGTTCCGGGAGGAAGCG
CGGCGGCGGCGGCTCCGACCGATGCCCGGCCGGGAAGAAGGCAAAACCCTCGACGCTCAT
GTCGTATTTTTCCAAAAAGAAAAAATGACTCCGTCCGTCCGACGCTATACCAACGATTGT
GTTAGAGCGAAAAAGTTTCGAAACCGATCTTTCGGATTGGCAATCACCGAATTACTAAAC
AAAACTACATAGAGTAGAATAAA
>comp1922_c0_seq1
GACGTTATATTGCCAATCTTTTGTCTATTACACGGCACCGTGCACTAGACCTATCGAAGG
AACTTGTGGCCATGGCAACAGAGGCAACGGCTGTTGTGCAAATAGCCCCGGAAGCAGCGA
TTTGGGTTGCGGAGATAGTGGTTGGTGCGGGACTAGCGATGAGCATAAACTTGGGGCTCG
ACCAACCCATCAAGAATGCGCAGATATGGGCGCAAGAGCGGGGTATTGAGGAGGACAGAA
TCTATACTTTGGTGCCGCAGTAGTTTGTATCTACTTGAAAAAGGAGCAGGGAGTACAACG
AACGACGAGGAGTCGTGTGGCTGGACGAATGGTGCCGTTGGTGGTGGTGCTGTGAGGAGT
TGAAAGAGTCGTGACCTTCGACCCAGGAATTTGGTGGAACCGCGGAACCAAGATCAACAA
AAACTATCACTTACTTTTAGTACTGTAAATAGTATTTGGAATCGTCCTATCAAAAATGGG
CATTTCCAAATAGTTATTTAAAGCGAACATCAAGCA
>comp1923_c0_seq1
AAGGGCCACAACTGCAATCCAAACCACGTTTCGTCCACTATGAGGCGCGGCACAAAAGTA
CGAGTCAAGGGATTAGTAAAGGCGGCAAAGCACAATGGAAAAATCGGGATTGTGACGAAG
GCTACGGCTCCGGGGGAGGGGCAGCGAGTAGGGGTAAAGCTGCAAGATGGAAGCGGCGTT
CTCGCTGTCAAGATTGAAAATTTGGAGGAAATCAAAAGTTCTCCGCGGACGACAGCGACA
GTGTCCAAACCAAAATCTGCCTCTGCGTCAACCCCTGCGTCCACCTTGCCAAAAGAACGC
ACCTTGAAAAGAGACAACGCCTTGCTCCGCGAAGTTGACGGAAGCCCGGACCCAAACGTT
CTCGCGCTGTACTATCATTTTGCCGACCGCGCGTTTGATTGTTTCAATGCCTCGGAGTAC
AACGTCCAGATGCTTCGTTACTACGAAAAGGGACTCTCGGTGCGATTGATTTGCCCAAGA
ATGATTGGTGGCAACGAGTACTTTTTGGTTGGATTGCAACACGCACAGCACGACAAGAAC
TCCCTGTGCGAAGTGGCGTTCAATTGCGGGAGGAGTTTTACGGGGATATCGATGCTGGTG
AAGAAACGATGTTTTGTGTGCCACAAGCCACTGCCCGGAAACAGCCAGTGTCCGAGCTGC
CTCTGTGCCTGTTTCTGTACCGATTCGAGCTGCATCAACAGAAATTCGACAATCCGTTCC
GATCACGAACGGTTGTGCAAGAAGATTGACTTGACGAAGGTAGTTGTAGAAAAAGAGAGT
CTCCAGTTGTTGCAGTAATTGCAATCAATAACTAGTGGAATGCACAAATTCCTTGGCATC
CTGGAAACATCATGTACCAGAAATTGCTATCATCACACTGTATCAGTAAATGCACGAAAA
GTCACAAGCCGACGAAAATGAGTTTCGGAAGAGAACTCGTACGAGA
>comp1924_c0_seq1
CAAGAATCTAACTTCGTCTATACCCGTTTTCAACTGTCCGACAGAGTTCAACTAGGGAAG

ATCGTCACGCACGTTCACATCCTTTATTTTGAGGTCACCCGCCAGACGCATATGCAATTA
GAAAAGGGCATCATTCTTCCGAATCGCCCATTTTACACATGAACAAATATTACATCCCAC
GACCTCTGCCTAACTATTGAATTCACTACTTCGATACATCACGGAACGAAAAAGGCATCT
CAACCGAACCCAAAAAAAAGGCGTACAAAACTTTGAATCCTACCTTACTTTCGGTCGCTG
GAGGTGATTGCTACAGGTAGAGTTTGGCGGAACGGAATTAAGTCGGATTGTCTACACAAG
AAAATTCATGGTGATTGAAAGCATAAACTTACTGCGTGAGCCGTCAAGTGTCGGATCGCT
ACGAATCAACAATCAAACCAAATTCTTTCATTCCTCTACTGGATTCGAAGAAAGCACTCT
GTTGGAAAAGTATGGTGAATAAGATGCCTGGAAGCAACCTTAACAGTAGCAGATCCACTG
GCGGCGCTGCTGCTATTCTATCTCGGAAGAGACTCCCTGCGTCGGGTGCAGGAGAAGAAA
GCAATGGTGTTCGAACGACAAATAATGAAAGTCGCACTACTACTATCGGGCGTGGTGATG
GAAATTCCATTCGGGATGCGAACACTGGAATTGAAAACAACAACAGTAGCAGCGCCCGAA
GCAGCACAAGCGCCAGCACAAATTCCAATAACGGAAGCACCCGCAAGATGCGCCGGGGAT
CCTTTCGCCGGACTCTGATGCTTCAAAGGAACAAACGGAGCAACAATGGTAGCTCTAGCA
GCCACTCAAAACGGGAAGGCAACGCTGTGGATGGGGGCGCCACACCAGCTACTGCCGTAA
CCAATCAGTTGCCGGTTTCGAAAAGAAATAGCACTTATCACGACCAATCGTCGGCACCAG
TTCCATTGAAGGGCTCGGTGGGTGCATCTGTTGCTTTCGCGTTGGAGGAACGAGAAACCG
GTGCGTGTTCTCCGACGTTGACATCTTCGAACGTGACGACGCCTGTTCGAGGCAGTCAGC
AGCGGCACGAACGTCTGCCAAGGGGACACATCGAAACCATTCGCAAGCACTACGGAAGCA
ACAGGGAAGGTGTTCTAGACATCTACGAGGATGTTCTCAAGATATCTCCCGATGCGTCCG
ACCGAGAAATCCGCATTGCCTACTTTCGTAGGGGCCGCGAAATCTTAGGTGACAATGCCA
GCAGCATCAAGATCAACAAGAAGAACCAGAAGGCGATCAAGCAGTTGGATCCCGCTATTA
AATCGCGATTTCAGGCCGTTAGTATGGCCTATGAAATTTTGTCAACGCCGGCCTGGAAGG
AGGCGTATCGAAAGCAGGGAGGGATACTAAACACGCCGGGAGCCAGGGCAAGTCCTCTCG
TTCCAAAGGTAGTAGTAGAGCAAGATGGTGTCGGCTTGAGCCATCCTAGAATTACCGTGG
GGAAAAAGAAGCAACAGGAACAAGAGCCTTTTGATCCTTTTGTGTCTTATCCATTGGCAT
CAAGGGCGTCATTTTGGGAGAATACAAACGCTAACACGGGCAATCCTTCAGCAATGTTCC
CAACAACGTTTTCATCCGATCCAGTGCTACCAGCACCATCCACTGCTTCTCGGTCACCAA
AACGCTTGGTTGGCGCTCTTAGAAAATCCAAGTTTCTCGGAAACAGCGGTGATGTAGACA
ACAGTAATTCTTCTCGAAGCAGACGACAGAGGCCCTCATCGGCCTCGGTCCGGTGGAAGG
ATCACGTCGAAGAACTCATCTTTGTAAACCATCCTGATGAACATGGTTCCAGTGACGAAG
AAAGCAGCGAAGAAGCTGACTACTATTCGTCCGACGACGAAAACCTACGTGGAGATGATA
GCACCGGTAACGACGCTGGCAATTCTAGAGCTAGTAGTCGCCCCGCATTGCCGGTAGCAG
TAGATCTTTATTCCGAACCTGCGGATCCTGGCTACAATGCGCAGGAGAACAATAGCAACA
ACAGCCTTAGCTACAGCAGTCGTGGCAGTGGCAGCACCAGAAGAAGAAAGAAGAACAAGG
CAAGGATTGTCATCGATTCGGAAGAATTGGAATCACATCTTAAACTCATGGACAGCGAAG
CCGAAAAACATTTTGTGCAAGATTTTTGGGACAACTTTGAAGAATCCATGGACGGGATAC
TGACCCTAGTAGATTCAATCGGGGGAGGCGGAGGTGCGGCTGCAGACATCTCGAAGGCAA
GCAACAGCAACA
>comp1926_c0_seq1
ATCATAATGAGTGCTGTTTGAACACATTACTAACGAATACAGATGAAAACAAAAAATCAT
TGATTATGCCAATCGATACCAGAATACTTTCCGCCTTCGGCTTGGTACAAATGCTTCTTC
TTGGTAATTCCAATGGATGGACTCATCCTGGAACAAATACGGCCAACATTAGTAGTTGTT
ATTGCGGTGCGATACGACGTCGATGGGGGCAATCAAACAAGATGTATAGCAGTTCCGCTG
AGAATCTCCAATCTATATGCAGCTATACGCATCAAAAACAACGACAAAGTCTGCGGGCGA
GTACGATAGCAGCTGCTGCGACAGTTCAGAACCAGCCCCTCGCACCTGGTCTTCCCAAAG
AGCTCGAGAAATTAAATCTGTTCGAACCTAAACCTCTGGAGTACAATAACGACGGCGACG
AAGTCCACGGACAAACAGTCGAGAGAGTGTCGATGGATCCTCCCATTTTCATTTTCAGAA
AAGCACTAACCCCCAAGCAGTGTCGCGGGATTCGGCAGACGGCATTTTCGATGGTCGAAA
TGGAACAGGGTAAAACTAACACCTGCAAGGACGACAATGACGAAATCCACGAAGAGGTTT
CCACTGCCTCTGATCAAGAACGAAAAAAGGAACAACATCGAAAACACCCTCGAAGGTTTT
CCAATGTGGGATGGCTCTCGAATGAGATGCCAATGGTGGAACAGGTTGCCCGCAACGGTC
ATGGTGTCTTTTTGAACGGGATGCCCTTTCACTCGACCCGAGGGATCGAACCTCTTCAGG
TTGTACACTATTACGATGTGGGCGGGGAATATATCAATCACCATGACTCGAACAATCGGT
TACTAACACTCTTGTATTATCTCAACGGTGTCGGAGAAACTTGGTTTCCTCTTGCAGACA
ACCACGATGTTGTTGTTCGGGATTCGAGAGAAGCAGACCTAGCCTGTCGCGACAAGTCGC
CCGACCGCGATGGCGTATTGGTCACGTGTCGCCGTAAATCTTCAATAATCGAAAGAGGAG
GTGATTGCGGGGATCTCCAGCTCGACTCCAAAGGTTGTAGCGGTCCAGTCGTTGCCCATG
TAGAAGAAGGAGATGCTGTTGCCTTCTACAATTACAACCTGGACGGGTCGATGAATTGGA
AGGCATTCCATTCTGGATTGCCGATTACCGAAACGGATTTGGAGTCCCCTGACGGCGAAA
ACAGCGCTAGCAAGGATGAGGATGGTGTTCATAAAAACGATGGAGGAAAGTGGATCGCAA

ATCACTTTTTCCATTTTGTTCCTTCTCATGTTCCCGAAACACCACCGCCTACTCCTGTCG
AAAAGTAGCCATCAATTCGCCAAGGGCAGCAGTATCGAGACCGAAACTGTTGACTCGCTT
ACAATCATTGATGCAATAATTTATCACTCAATTTTAGGCTTTGTCATTAAATTATGAATG
ATCTTTTTCT
>comp1927_c0_seq1
GGCCGGTACGTACGGTACGGTACGAAATGGAATTTTTCCGGTTTTCGATACACTTCTCTC
CCACTTTTTCACAACTTGTGGTAAGTGAAGGGAACGACATCAACGATGGAGGAGGATGGC
ATTGTCGTCCGCGAGATATCAACAATCGCTCGGGATGACATCTCTGGAGAGATCGATCAT
GAAGGATCTGTAACGAAATCATCGTTCCAACAAAAACCTAATTCGAATCCAACCACCGAA
GCAGAGAACACTAGGAAAAAGCCGTCCATACGACACGCCGAAAAGCGGCGGACCTCGACC
TGCCGATTCGCGGATCGTGTTGCGCAGCTCTCGGTCGATCACTACAAACGCACGGTGCCC
GCTGACAGGCGCCCGGCGCAGACCTGCATCGCTACGATTGTGGCCCATTGTCGGAAACAC
AACGGAGGCCGCGATGCCGGAGAGGAGCACGAGGACGGAGTCGATGTCAATGGCACCGGG
ACAGGTGAGGCCGACGTGTTGTGGGTTCTAGCAATGGGGATAGGAACCAAGTTCCTGCCG
GAATCGCGACTGCAACGAGAAAGGGAAAACGAAGGGCAGGAAGGCTACGGTTCACGGGTT
CGAGATATGCATGCCGAGGTTCTGGCTCGGCGAGCCTTTCGACGACAATTGACACTAGAA
ATCCAGGAGGATCTCAGGCTGCCAGCAGAAGAGCACGAACGACAGAAGGAAAGACCGTCC
AATCGTGTCTTGGTTCGATCGGAGGACAATTCTTCCACGGTGTGTTACAGGATACGACCG
GGTGTAACAGTACACATGTACTCCAGTTCGGCTCCATGTGGGAACGCTACACTGAAAAGG
TTTTGCAAAATGTCTAAGGAAAAATTTCGAGACGATCTAGGACCCGATGAATGGCCTAGC
GATGCACATGACCCACCATCTGGGCACAGTATCAAGCTCGGCGAATTTTCTTTGCTTGTG
AAACAAGATAGCAGCAGCAGCAGCAATCCGCAAACAAATGAAGCCACAAGCAACAGCACG
ACGAATAGTGAGGAAAAGAACAAGAATACTTCCTCGAAAATGGATGCAAATATGGATTAT
GGCAGCAAGCCAAAACGAACGAGACTCGCGGGAGATCGGCCGCCAGCCTTGCGGACCAAT
ATTTCCAAAGCATCAGCAACTTCACAGGGGCGAAAAACAGTCAGCCGAAGAGCAAAGCCA
TGGCCAGCAACTCTTTCGGACGACTGGACTCCCCCTGGAACCACCATTGTTGGGTTTCGA
CACAAGGGATCAATCCACACCTGCTCTGACAAAATTTGTCGCTGGAACTATTTGGGAATG
CAGGGATCACTCCTCGCTAGTCTTATGGAAGAACCCATCTATATGTCGACCCTGACGGTC
GGTAGGAAGCTCTCGGGACCTGTCTGCCGACGCGCTGTTTGTTGTCGACTCGATACACGT
TGCCGACAACCGAACCTCGCTATTCCCTTGAACAACAGCATCCCACTCACTGGCCGCACG
AACTCCAACAAAATTCAATACCGTGTGAACCATCCTGCTATTATGGGGACCTCGGTCTAT
TTGGACACGGGCGTGGTCGACACTAGCAACGTTGAAGAAAGAGGCCAGGATGTTCGATTC
CATTCCTCCTTTGTTTGGGCTTGGTGGGCTGGATCATCTCAGTGCGGTGTTAACAGCAAC
AAAATACCAAATACTGATTGCTATGGAATATTAGAATGTATCGAGAGTTCCAGTGGACTT
CTTGCGGACTTGTCGGCAGAAGTAACCGCGGAAAACGACATTGACAAAGGCATACGTAGC
ACTACTAGTGTCCCACAAGTGAGCACCAAATCACTGACATCTGCTTTCTTACAAGTGTTT
CAGGAGGCATCGGGGAATGGAAGTTATACCAAAAATACTGCTGGCTATGCTAGTAGTGCA
GCTAGCTACGAGAATAGCAACGACACGAAGTTGACGCCGGAAGGGGTCACTCCCGAATCA
TGGGAAAATGATACAAATACCCTCAACACACTTCGAAGCGTAAAGAAAAGGTTTTCACCC
ATCCACGAAGAAGTCAAGGATCGTTTGTTTGCAGAGCACAGAGTCCTTTCACAGTGGCGA
AGAAGGTGCTACCAGGATATATGCACTGAAGCAATCAATACTATGAGCTCTTGAACAAGA
CAAAAAGATTGATTCGGGGTTTCCGATACGCTTGCATAGATATTTTTCAGCAAAAGAATA
CTGTATTGGTCGTATTTTGTTGATGTTTGGGATAAAAAAGAAAGCATTGTGTTTTGTACG
AAAAGGTTGCTCCATTAGTTGATATTACTACCACAAGAAAGCCATCAGAGAC
>comp1934_c0_seq1
CAACGACAACGGCGATGCGGTCGTCCACCGGCACCGGCGGCGCGGGCGACGAACCCCCCG
TAACGCACGTGGTGGTCGCCGGCGCGGGGGTCATCGGCGTGTCGACGGCCTACTACCTCG
CCAAGAACTTTGGGGTCGCCACCACCCTGGTCGATCCGACGGGGACCATCGCCCCCGCGG
CCTCCGGAAAGGCGGGCGGCTTTCTGGCCCTGGACTGGAACGACAACTCCCCGGCCCTCG
GCCCCCTGGCCCGCCGGTCCTTTGCTTTGCACGAGGAGCTCGCGGACGACCTCGGGGCGG
ACACCATCTCGTACCGGAGGCTCGAGTGCGTCTCCGTCCCGGTGGGGGAAAACGGGGGCC
GGCGTCCGGCCGGCCGCAAGCTGGAGGGCATCGAGTGGGCGGAGGGAGCCAACGCGATGG
GGGACGCCTCGGTCCTCGGTGGGACCCGGACGATCGCCCAGGTCCACCCGAAGAAGCTCT
GCGATGCCATGTGGGAGCGGGTGCTGGAGCTGGCGCCGGAGAGCAAGCTGGTCCGCGGGA
GGGTCGTCGCTCCGGAGGACAAAGACGGAGACGCCGGTGCCGACGCGGCGGTGGCAGCCA
GGCTGGAGGACGGGAGCCTCCTGGAGGGGGACGCCCTCCTGTACTCGTGCGGGCCCTGGA
CGGCCACCAACATGTACGGAACCAAGTACCACTCCCTGGTCGTTCCCACCACCAGGGTCC
TCCACCAGTGCGTCTTCTTTTCGGGCCTGGGGGATCCCGAGGTCTACGTCCGGGGCGACT
CGACGGCCTACTGCACGGGCTTTCCGGACCCGCCGGTGCGGGTCGCGGAGCACCCGGGGG
AGGAAGAAGTCCGGCCGGAGGCCATCGACCGGATCCGTGAGTCGGTGCGGGCCGCGTCGT

CCTTTGCGGACGGCGCGAGCACCAGCTACCTGGTGTTGGGAGACGACGGCGACGACGGCC
GCCAAGCGCCCCTGGAACAGGCGTGCTACCTCCCGGGCACGGCCGACGGGATCCCCATGA
TGGGGAGGGTGCCGGGGAAGGAACGGACCTACGTCTGCGCCGGGCACACGTGCTGGGGGA
TCCTCCTGGGGCCCGCCTCCGGCGAGGCCATGGCCCACGAGATTGCCGCCGCGGGGGGGA
GAGGAAGCTCCCAGCCCTTCGTGGACCTGGGGATGTTTTCCCCCGGGCGGTTCGGAAGGC
CGCTCGAGCTGGTCCCGGCTTTCGGTCCGGAGCGGTGACCGCCGAGAGGGCACGCCGGCG
CACGACGAAGCGGGAACGCGGTGGTTTTGCCGGATCGCCCGTGAGGACGTCGTTTACAAG
CTGAATTCTTTCTGTTGTGTACCCAGCCCAAATTGGGTTGCTAAGCTTACAGTACTACAA
AACACAGATGTTAGCGG
>comp1937_c0_seq1
CCGTTGGCGAGTAAAACTGTGCGCTTCCGATGAAAGAGCTAAACGGGTATCCATTTCCCG
CGGATTGCAGAAGATGTCGATCGACTGTACGGTACAAGTATGGTCTAGTAGTACCCGCAG
CGGTCAACATGCTAGCCAGCTTGGGACCCAAACATTACAAATGCAAACAAATTCTGTGCA
ACGTCAACAGCTACGTGCGGGGGAGAGAGAGTTTCTGTGCTAGTTTTTGTTCTTCTGTCC
GACTAGGATTTTCCTGAAACCTGATTCGCTGATAGCACATGTCTCTTCTACGGACGCAAT
TTAAAAAAAGAAAAAGATGAGAAAACAGTCCAGTCCCGGAGGACTAACAACCCCCGAGCA
GGGGTTACCGTCCACGATTCCAGTTTCTTTGCCTTATATTTCGAAAATGAATGCCTATCT
TTTCGTGCCATTTTCAGGAACACTACTAGGGGGTGTCTGAGTTGCTGGCGTGGAATGACG
TTTTCACTGGAATACTGATTTAAAAAAATACTTATACAGCGCAACGGTTTCTTATGCTTG
TTCAAGTGCTTTTGATATGGATTAGAAAAATGAGGGTTGTTCGATTGGCCGCCGTGTGGC
CAAAAAAATTATGGAAGTTTCTTGTTGTTTATTATGGAAAGAAATTTTAGTCTCATTTAA
TTGGAGATTTCGATTTTTAGAACCTGACGACATCAACTAACATTCGAGGACCTTCCGTTC
CCTTGCCAAAGACTGCAGTTTTTCTTTGCTGAAGAAATCGAAGCAGTAGATCGATGTCTT
CGCATCGAAAAGGCCTAAACAATCACCATTGACTCGCACAAGCAGTACGAGCACTGGTCC
AGCTCTGCTCACATCCCAATATTTTTAGTTTTTAGACCCTTCAATTTCCTTCCCCG
>comp1942_c0_seq2
CAGAAAAGAACAAAAAAGGCGCTGCCATCGTACGCACCCGTTGCGGTTCGGCACGGCACA
CCCAATACCACCCATCCGGCCGGGACTCGCGGAAGCACCGCACCGGTTGGTCCCGTGCGA
TGGAAAGGATGCGAACCCGGTCTCGCGGTGCACCGCGGTGCTCTTCCTTCCTGCGAACCC
TTCGACACAACAAGAAAGCAACTCGGCGGCGACAGATTCACCATCGAGCAGGACCGAGCA
ACCAAGAGCGACAATATTTTGTGGCACAATTGTCCATGGAATGGTAAACTCTGAAATGTG
AGATTGAAGTTTGCTGTCATTTTCTTTGTAGGCAACACAGAGCAACATGACAAGTTGTGT
GGGCACTTCACTTCTGCTAATATCAAAATGATCTGTTGGCACTGTAACTGTCCCCATGCC
CTTGGAATCAACTCTAAAGTTCACATTCTGAATGCCTCCATTTCCAACAAACTCAATGCT
CTGCAAAGTGAAACCAAAAGTGAAGGGAACTCAATGCTCTGCAAAGTTAAACATGAGACA
ACCACAACAAATCTGGGGAGGCTCACAGGGCAAGAAGAGGCGCATGAAATGCACTGACAC
CAGCTTGCCATATTATTCTTCTCTCAGTTTATGGCACCCTTTAGATTTCACCCAGTCCAC
TGTTCCCATTGGTATTACCCTAGAACGGCATTGTCATAACAACCCACACGAACCTATCAT
TGGAGGCAATGCTTTCCACAATCTCAATTTTGAAGTCAACGTTCACGGTATCCACCTTGC
CTCTCCTGGGGAAAGGCTGCACATGCATCAACTCAGATGTGCAAAACGGGCAGCAGAAAC
ATTCCACAATGTCTTCCTGTCTAATTCAAAGAAGCTCCTTGATGATGGTATGGATTTGAT
AGCTGGTTATTATGGTGCTTCTCTCCAGAGGCAGTCCAACCGTGATTTCCCACAAACCAA
CTTTTCTGAGTCCATACATACTGCAAAAAAGGAGGGCAACCACTTTAGTGGTATGCTTCT
GTTACAGATCCTCTATCTATTGTTGAAGGATGGTGGTGCATTGTTGAGGCCTTATTACGA
TCGGCAAGAGATTGATGGTCGGATATATGATTTGGAACTAATTCTGGGGATGGAAGAATT
TCTAAAATGGTCTGGCACCCCATGCAAGATATTTCACTTGCAGCAAAATATCAATGGCAG
GAACCTCAACAATGCCTTCAAGTTCCCAAAAGGGAGAACGGGTGCAAATAACCTTGACAA
GATGGTTGTTCACTTTATTAACAACATCAATAATCACCTTGGCAGGAATGAAGGAAACAG
TAAGAATCTTATAAAGAATCAC
>comp1945_c0_seq1
ACAGAATTGGTCGTAAAGCGCAAGGCTTGGTGTGACGATAGGTTAAGCTCTCCCTTTCTT
TATCAAGCGGTCATTTTCCTGTTGGGAGTGCACGAAATAGGCAAAATGACGACTCCTATT
CACGGGAAACCAACCGAGGGAATGTGCTGCTTGTGCACTATGGAGGATATAACCGAAGAA
AATGGAAATTATGTCGAATATCAAACCTATCCCAGTCTCACGTGGCATCCCTGCGGCTAC
GAGCGATCGACGGTCGAGCACCTCCTGAAAACTCAGTTCGGAGCCTATCTAGAACGCGTC
AAGACAACGAATTGCCAGGCGGAACTCCGACGATTGCTCAGAGACGGTCCCCCCGTGTGG
TTGACGGATCCACATGCCCTGAAGCTTCCAGGAGCCGGCGAAGACAAAGAAACGACAACA
ATTACAGAAGCAACAACGACGACACCTGCTACCGACACGCACGTTTGTTCACTATGGTAC
GCGGGGGACAACACCGTAGTGTCGGCGAAGCTCAATGGAGCTGTCGAAGGGGGGGATCGT
GAGAATTTGTGGAATGAATTGAAAGAATTCTTGGTGGAAGAAGGAAAAGAGCCGGGAGAC

GATGACGACAACAATTGACTAGGCTTTAGGAAAAGGGTTTGAAACCGGTTTATAGAGTTT
TGCGGAGGGAATGGCTAACAGAGAGCAGATTTTCCTCCTACCGTAGTAATCCTGCAAACA
CGCATGCTGAAAGAGATTTTGTTGACTGCTGAGCTTTTGGGGAACTCAAAAAAGAAGATT
TACCTCTACTGCACTATTATGTTTAACACGAATACTGTAACCTGATAATTGTAGATTTTG
CGTTCTATGTTATGGTCGCAATAGGACGAAGCACTGAAGTAATATCCTTGACTAATGAAA
GTTATAAATATAGATAAATGTGGCAAGATCAAACACCCTTGAGTTTGGAAGGATCTGGAG
GAAAAGATTAAAAGTTCAGATACTTGTCAATATTAC
>comp1946_c0_seq1
CTCGTACGTTCCATAATACGTGAAGCACGGACTGGCACGATACCCGTTCGATACGATACG
ATACCGGCACGATACATTAAGATCCGATCCGACACGATCCAATCTGTGTTCCGTTCCTTC
CATCGATCGCCAACAGCCATGGGAAAGAAGAGCAAGCGGCCCAACCGGAGGCAGCCGCCG
CCGGCCACCGCCGGTGCCGTTTCCCCCGCAGCGGCAACCGCACCCGCACCCGCAACGGCA
ACCGCGTCCCCCCCGGTCTACTTCTACCCCTCCGACGGTCCCTCCGACGTGGAGCTCCTC
CAGACGACCTCCCGGTCGCTCCAGGCCAAGCTGGACCTCCTGACCCGGCTCGGGGGGGAG
AACGACCGGGCCGGCTTTGTGCGAGAGTTCGTCCCGCTGGACCTCCCGGGGCCCGACGCC
GCCGGCTACCTGGAGGACCTGACGACCGCCCCCGAGGCCGAGGGCCAGTGGAGGAACCTG
GTGGCGGAGATCGCCGCGATCCGGTGCGGCAGGGGGGTCGAGAAGATCGGGGGCGACCAG
GTGTCCCGGGCGGTCTTCCACTTCGAGCACCCGCTGCTGGAAAAGTGCGACCGGGAGGTC
GCCTTTTGCTGCGTCGGGGGCGAGTGGAGGGCCGAGGGGTGAGCGAGCGAGCGAGGGCGA
GAAACCGGTTTCTTACAGTGCGAGCAGTGCATTCTTTTAAACCACCTACGGGCG
>comp1950_c0_seq1
CATGTACTGGAGCCACGCCGGGAAACACGTCGGCATCGAGCCCGCCGGTCCCTTTTGGGC
GTCGGTCCCGAGGCCGTCCATGGAGGAGTTCTTTGCCAGGACGCAGAACCCCTCGGAGTG
CGCGCGGATCCTGCGGGAGGACTTTTGCACGGAGGAGTTCGGGGACCGGCGGCAGGAGGT
CGTCTTCATCGGGGTCGGCCTGCGGCGGTCGGAGATCGAGGAGGCCCTGGACGGCTGCCT
CCTCACCGACGGGGAAATGGACGACTACCGGGGGCACCTGGAAGAAAAGAACCGGCTGCT
TGCCAAGCCGGCGGCCTAGCAGCCCGGTTCGGTTCGACGCTCGCAACGGGGGAAGAAGAG
CGCCGCTCTCGTCGTTGGTGGGATTGCCGGTGCAAAACGAACCATGGGCGATCGATGCAT
TGCTCGAACTCGAACCATCCGGATGCGGTTCTGATTGCTGCTGTAGAGAAAGCGTGGTGG
TATTTGTACTATTGCAGCCTCGTTCGTTTGGTTGCGCTGGAAATGAAAACACCGGATTTG
TTTTTGGGCATTTCTTCTGACCACGCAAAAGCGCTGCCTGCACCGAACGACCGGATATAT
TTATTCCCCGGGGGGAAGACTAGCCGCATGGCTTTGTTTGCAAGCAGGCCCGTTGCGTTG
CCGCACCTCGAAAGAAAAGAGAAGAAACGAACTACGATACAAGGGTTGCAGGTCGATGCT
GATCCAATACAAAGGAATGGAATCATGGATTCCCTAAGAATATTG
>comp1951_c0_seq1
AACAATGCAAGCAACAGTAACACGTTTCGTGTTCGCCACCGCTGTCATTGCTGCTGGCTG
CAATGCCTTTGTTCCGATCACAACAACGTTATCGTCGGCGCGACAAGGCACCACGAAGGA
TGGGGATGATGTGCTCTTGCGGGCTTCGGAAGAAGATTCCTCTTGGTCGTCCACATCTAC
CGATGTCTCTGGGAATGCCGTTATTGAAAGAACTATTCTTCCCGACGGAGTCATCCTCAA
CGAGAAACTCGTCCTAAGAGACATTACGCCCGAGGAACGGGGAAAGGGTGGCGTCGAAGT
TGGCCAGGGTGGAAAGATCAAGGCACTGGAAGTATTGGCACGCATCCCCCGTGATTTGAT
CATCTCGACACTCGATGTTCCCGATCGTGCGATAGAAGCCGCTTCTGGAGCTAGAAATCT
GACCTGGGCAACCGACCTCACCGCCGCTTCACTATCGGCATTGCATCCGATCGAGGAAGA
GCTTGCTGCTTCTGCCTCTGTCCAAGCGAGAAAAGAATGGGTGCAGTCGTGGCAAGCCGG
GGGATGGGCTACGGACGGAGCTGATCTCGGGCCACCCGACGCCAACTTCGGATCGAAGGA
CGTGACCGGTTCATTGCTATCCACGGGAACGGACAACGACCACAATATCTACGCCAAATT
CCGCATGCCCTGTCATCCTGTTTTGTTGCGCGCTTCACTGGGATTGCAGGTTTTGACGAA
ATGCACCGAGGAAAAAGCCCGCGAGGCATTGACGGCACGTGGATTCACGTATCGAAGCAT
GAGGGATGCTCTGGAACCCCTGGTCCTCGAATCGACGGAACGAGCGAAGGGGTCCAAACG
GGAAAAACGGTGCTGGGATGTGGCGGATACCCTTAGCCGGGTCTTGTCGAGAGCGACCAC
GCTGCAGCTCGAAGAGACCAACGATAGCGAAGAACTTTCTCTCGCCTATGCTATTGTTCC
CCTCCACGAGCAATTGGAACACTCCGTGAAAGAAAACTCGAAGCTGGTATCCAACAACAA
TGGAGACGAAATCTTGCTTGTCGCGACCAGAGACATCGGCGAGGGAGAAGCCATCACCCG
GGATTACAATTCGAGCCCAAAGCTGCTCAACGACAAATCCGAGGGAGCCCTCCATCTCTT
GTTGCAGTTCGGTCTTCCACCCAAGGCATGGCCCAATTCCCCAAACTGAGCTTACGAGGG
TTTTCTCGATGCCCTCACGAAAATTGAAGCTATCCCGTTAATACGAGTTTGCGATCGATA
GAAAATAAATGAATAATTGAAG
>comp1952_c0_seq1
TGGATGCAACCATTCCTTCTCTCGCAGTAAAGAAAAGGCCACACAATGCCGATGTAGAGA
TTTCGACAAGTCCTTCCGGCAGCTCACTCGAATACTCGATGTCGTCTTCCTTTTCTTCGG

GCGAGGATCTCGGCGATCTCGAGGACGTTCCGATGCTTTCAGAGTTCGATACTTTGCAGT
CGAATGATAGAAGCAATCGTAATAGTAGAGATCTGGTCCAGCGAAGACGAGCATCGTTCG
CTCCCCGCTATAGACCGGCCATCGCATTCATTGCACTAGCTTTTTCGTCTTATATTTATA
TCGCCATCACCAGTCAGTCCGTGAAAGATCCGGTCAAAACTGCTGTATCCGAGAGCGTCG
TGGATCGACATAAAGACAACAAAATCCACGGAAACGCTACGACCAAGGAAGAGGAAAGTT
CCTCCCTAAGGAAATTTCACAGTCATTCCACATCTCCTGTGCTGGTCTCAACTCCTAGAC
GAAGTCCGAATCCATCGGCACTTCCTGCTCCGGAACCAACTCTGAAGCCCAGTCCGAATC
CATCGGTACCATCTGTCCCGGAACCAACACAGAAACCCAGTCCAAATCCATCAGAGCCTC
CTGTCCCAGAACCAACACAGAAACCTAGTCCGAATCCATCTTTAGGTCTGCCTACTGAAC
CACCCTCATCACATCCCATTTCAAAGCCCAACGTCGCATCTCCTTTAAACAATCGTGGTG
GGAAAAATGTAGAAGCGCAAAGAAGGAAGAAGAATCTCGTATTGCACGTTGGCCCTCAGA
AAACTGGGTCCACGACCCTTCAGGATGCATGGGCGCCTTATGGCATATTGGGTCAACCAT
TGAAGAACGACAATTATCTTTATGCATTCGTTCATAGGAACAGCGGTTACTTCAATTGTG
ATCAAAATAAATGGGGTGGTCTTGAAAACTGCAAATCTTCCCCCAGACTGAAACGGTTGC
TACAGACCGCAAAAAACAATGGTAGAAATCTTCTTTTATCGGATGAGAATCTTGGCGATC
GATTCTCCCCGGCATTGCGGGATGTCATTGATGATAATGACTGGGATGTCACTGTTATTG
TAGTATATAGACGCATACATCAGTGGCTTGTGAGTTGGTATAATCAGATCAATAAGACTA
CAAACAAGGATTCGAACGGCAACATACTCTTTGATAAAAATGGCAACGCCTATAGAAAAG
AGCACACAGTATGGCCCGACAAAGGAGGATCTCATATTCCCGCCTTTTCTACGTGGTACA
AAGAGTTTACTCAAAACTGGGATCCATCTGAATTCGTGGAGAAGCATCGAACCATTGAAT
TCATAAACGCATACAATTCATCTTTTAGCAACGTGGTCGTGCACAATATGCATGAAGACG
GCGATATGGTGAAAAATTTCATGTGCAATAGCGTTCCAGACGCCCACCATTGTTGCAACC
GCCTGAAGTACGACTCGAATAGATCACCAAGAGATAAAAACATGTCTGTAAATTTGAATA
TCGATATCCTTGCCGTTAAAGCGAGAGAAATTGGTGCAATGAAGAAGAGTATTTCAAGGA
AAAGGGCTGTTTCGGAGATCGAACGGTATACTAAGGTATTCAATAAGCGGATCCCGCTCA
AATGTGACAATGATGTAATCAGCGAAATCAAGAACTGGCTTTTAGAGAGTGAGAAAGCTG
TCTTTCATGAGACGTGGTCGGATCAACAGACATCGGATTTGGAAGATACGTTTGACGCGT
ATCTCCCAAGTGGAAAATTGTGCGATGTTGATTTCGAAAATATCTTCAATGATGAGCAAT
GGGTAGGCTTTTTCAAATCACTTGGATAATTTTTTTTCGCTAGCCAATATCTTTATTGCT
ACCAACAGTGTTCGAGAAAGCGCATGACGCTCAACACAGATTCAAGAGTGACACCTTTTC
TCTCCGGGTGATGATATCAGACGTTAGAAGCTATATTCAAGTGCCTGGCATGACATAAAC
CCGAAATGTGATAGATCGAACTCGTGGCTATTTCCTGATATATATTCCTTGAATACAATC
AATCTCGTGGAGAGATTATATGCTATACGATGTTGTCGTTGAAAATGTTATTGCTGGCTT
CGACACTTGGGCAAAGAAGAAGTAGTATTTCTCGCTTCTGGGAAAAACGGGAGTTCTTGC
CCTATTTGCTATTTGTAGACGACTACG
>comp1956_c0_seq1
CTATTCCATGATTGTCGAACGGGTTCTTGTTCTTGTTCGTGTTCTTGCTCTGTTGTAGGT
GGTTGCACCGAAAGACAGACGAGACCACCCATTGATATTCCATACCGTCTACCCGGCACG
GGAAAGGTGGTACCGTTCTCCCGTCCTCGAGGCAGTCCTAGATCGATCGATGCCTTCCCC
GAAGACCCGGAGAGGCTTGCCAGAAGAAACACAACAACAAAACCTGTGAGAAGAGAACCG
CATCGCTGTTCTGCTGCCCTAGTTGTCATTGTCATTGCCGTCGCCGCCGCTGTCGCTGTC
GCCATGCTGGTGACGATCGCGATCCTGGATTCCCTGCTGGACAGCGACCCGGAGTCCGTT
CTGTTCATGCTGTACATCATCTACTCCATTCTGGGCTCCTTTCTCAACTCGAGGGCCGTC
CCGGAGGCCTCCGCCCCGCACCTCCTGGACCAGGTCCGGGCCGCCGAAACGGGGATCGCC
GGCCTCCCGATCGAGGGGGTCCCCGTTCCCCCCTCGCCGTGCGGGAGGACCCTCGGCGAA
ACGGCGGCCACCTGCGGCTGGGCCGTGGCCTGGGGGGCCTACAAGAACAACCATTACCAC
ATCAATTACCACCACCATTACCACCACCACGGCGATCGACCCTTTTCCTCGCCCCGGACC
ACCCTCCGCCGCTACCTGGGAACCACCGTCCGGAGGGCGGCCTTCCTTGCCTGGTCCTCC
GCCTACGGCCGGTGGGTCGACCTCCGGGAGGAAGCGGCGGCCTTTCACCGGTCCGCGGTG
GAGAAGGCGGCGGGCGAGGGAGGCCTGTTCGGGCACTGGGGGGCGGCCAAGGGGCGCTTC
TGCGAGAGCCTCTGCCAAGGCATCGAGCCCCTGGCGGCGACGCAACGGTACGCGCTTTCC
CTGGCGCAAACGGCCGCCACCGCCGCCACCCTTGCCGCGAGCCTGGGCGCCGCGGGCATC
GAGCACTGCTTGAGGGTTGGCGCTGGTGCTGGTGCTGGCGAGAACCAGAACGAGCGCGAG
AACCAGAACCAGGACGAGCGCGGGTCCACGCCGCGGTCCGAGACCCAAACCGCGGATCTG
TGATCGCACGGAAACCGCGGGGAAAGCAGGCAGCCTGGTCCTTGCTTCTCTGCCCGATGT
GCGAAAGAGAAAACAATAGTGCGGATTTTGGAATCACTCTTCGCTTTTGACAATGTCTCT
GGAGGCTGCGCGAATCGAGCGATGCCACGTGATCGGACGGTGTGATGCAATGCGATGCGA
T
>comp1957_c0_seq1
CGGATTCCGACGGTGGTGGAAAGCCCTCGGCGGTGCCCGCAACCGCCGCAGCTGCACCGG

CACGGGGGCTTTCTCTCCCTCCGGCCGCGTCTCCCGCAGCAACAACGGCAACGACAACGG
CAACAGCAACGGCAACGACTGCGGCAAAGAAGGGGAGCACAAGGGCGGACCGAACCAAGC
GGGTGATCGGAAGAAAAGTCCTCAAGCTGTCGGCCGTGCTGCCCGAACACATCCTGAACC
AGCTCCAGCTCGCGGGCGGGGGGGGCAGCTCCGGCAGGAAGAACAGAAAGAGCGGCGACG
GTACTAACTCCGACTCCGACTCCGACAGCAGCGAGGACAACCCGGACACCGATAGCAGCG
ACGACGAAGACACTGGTGCCCAGAGTCGCAAAACACCTGGGTCCCGACGGAAGAAGGGTG
CGAATCCGGGAGCGAATCCGGGAAGCACGAATGCACCGCCCAGAGATTTTTCCATAGACA
AAGGCCTCATGGGGCTGCTGAAAGAGTTGTCCAAGTCCGGGTCCTCCTCTCAGCCGAAAA
ACAAAAAGATCCTGGGCGGCGGCGCCGAGGAAAGTTCCGGATCGGCCACCACGGGCGACG
AGAATCCCACGCCGGAGACGGAACCAAAGCCGACGCAAAAACCAAAACCGAAGAGCGAAC
CACTGGGCGCCGCGTTTCTTGCCACAACGGTGGAGACCACCACCCGAAAACGGAGGCAGC
CGGTGGCGGTGCGAAACATTCACGACGCAGAAGACTCGGCGGTGAAACCCGATGCTTCCG
TACACGCGTCGGCGTCCGCGTCGGCGTCCCCTCGGACACCTTCCGTCTCGGTCCCCCGAC
CATCCGTGGCACCACCACCACCCCAACCGCGGGCCCGGGCCGCGTCTTCCATGGGACGAT
CCGCGGCCCCGCAGGTGAGGGGATCGTCTTCCACATCCTACAACTCCGGCTCACACCCTG
CCAGCCACTATCGAATACAACAACA
>comp1960_c0_seq1
GGCTGTGATCAACAACAGACCAACTGCACGACACCCATCACAAGCGAATAGAAAGGCAAC
GCTGCAAGCACAACACACAAGCTACAGTACAATCAAGACCCAGTACAAGGCATCCAGATG
TCTCGACGGAACCAGGACCAGGGCGGGAGCAAACCCTCCATCGCCTTCCACATGATCCGG
CACGCCGAAAGCAACAACAACAAGGTTTACGGAGATGCCAAACGCCTGTATCGCTACGGA
TCCGACGACTACGACAGCGAGGGCTTTCGTCGGTACGTGGATGCCCACCGCAGGGCGGAT
CCCCACCTGAGCCCGCTCGGGGTCGAGCAGGCGGACGCCCTGGCAAGCGAGTATCTCAAC
CCGGCCCTGTTCGACTGCCAGGCGTCGAGCCCGGTTCGAATCCTGTGCAGCCCGATGCTG
CGGACGCTGCTGACCATCCGACCGGCCCTGCAGCGATTGGTGGTCTCCTCGAGCCAGACC
GCGGGCAGCGGGGGTTCCGGCTTTCGGCAAAAGGTACACATGGTGGTGGTTGCCCATTAC
CACGAGATCGAGGGATGCCACGACAAAGGGGTTGCCCAGCCCGGCATGAGCCCGAACGAA
ATCCGCGACTGTCTCTTCCGCGACCTGCTCTGTAGCAGCGGTGGTAAAAGCAGCAGCGAG
AGCAACGACACGATGCCAATCAGCCAGTCCGACATCGACGAAATTGTGACGCTGGAATTC
GTGGGGTTTGACCATCCCCACGAAGGATGGTACGGTGGCACGGGCCACGAGGCAAGAGTC
GATGCGGAAGCCAGAGCCGCGAAGTATTGTTTGTGGTTGACCGATTATCTCGATAGCCTC
ATGTACGGCAATGGCGACGGGGATCCCGACGAGACCGGCGACAGCAACAACAACGGCGAC
TTGTTCGACGTGGGCTTTCTGCTTCCGGGGGAGGAGCTCGAGGACCAGCACGACCGATTC
GCCAGGCCCCTGCGCCGGCGGCGCACCGTGCTGGCGATCGGGCACGGGGATTTCATGTCC
GTGGTGCTCAAGCGCTTGGTGGGGGGCTACGTCGGGTGCGGCAGGGGCGG
>comp1964_c0_seq1
CAGTCTGCAAAGTAATCGGTATTCAAAATACCGGACTTGTATTTTCCTTTTCTTCGCTTA
TGATAGAAGCATATCAAAGCAATCGATACTGACTGTTCTATGTCATTGGTCAAATTGATG
GTGGAAACAAGAAGACTATGGAGACCAGTTGCGAGTGGAATGGGAGAGTGAAGAATGTGG
TTAGAAGAAGTGGTTGCGATGGAGTCTTGAATAAATTGAAAGGATACGAAGATCATCTAT
CGTCAGTGGCTATGTCTATTGAAAGACATGAAAATTCACGCAGGAATGCGTCACAAAACA
AGCAGAATGAATCTGAAGGCTCAGGGAATTGGACAGAACGCTTTATCAGCAAGACAAATA
CGAACCCAACAACGACTTCTACGATAATCTCGCAACTAAATGATTACCATAGACATGGTG
CTTTAGCTGTGATAGATGCAAGAAGAGATACCAATTGGAATGGGAGAAATAAGGTCACAC
CACCTTCCGGTGCTCTCAAATCTCCTTGGGAAAAGCAAACGTGTGAATCCTTATCGGACA
CGAACCTTCAGAGAAAACACCCGGGAGATCCACCTCCTCTACAATTATCAGGAAGCAATC
CTGCTTTCACTGGCACAATTAGTAGATCATCGATGAGCGTTGCTACTACTCAGAATTCGC
TTCTTTCACTCCCCGGCAAGAAAGGAGTTCCGCACATCTATCACGATTATTCGAACGTCC
CAGATACAATAGGCGTAGTTCGAAAGAAGACAGGTGGAGTAACCCAGCCTTTTCCAGAAA
AGTTGCATTTGATGTTGGATAATGACGATGATCCTTCCATAGTAAGCTGGCTTCCTCACG
GAAGAGCCTTTTTGGTACGAAAGCCAGCAGAATTTACAGCTCAAATCATGCCAAAATACT
TCCGCCAAACCAAACTGACATCTTTTCAGCGACAGCTGAATTTGTACGGATTTCGGAGAC
TAACGCAAGGCGCTGACGCTGGAGCATATTATCATGAATTGTTCTTAAGAGGACGCCCTC
AACTGTGCTTACGAATGCAAAGACAGAAAATCAAAGGGACTGGGCACAAGCAGCCTGCTG
ATGTTCAAACCGAGCCAAATTTCTACACTATGCCCCTCCCAAAAAGATACGAACAGACAA
AAAAGAAAGAAAAAGAACAAACGGACGAAGTTAGCAAGGTTGCAAAAGTACGCACAGTAT
CTCATTCAGGAAATTCTATGCTAAAAGATTCTTCTTTCGGCAACTCTTCATTCGCACATT
TGAGTCCAGGTAGTAGAGGGGTACACGGCGCTGCGCATCTATTGAATGGCATGAAGAGTA
CAATCTGTTCTATGAAGGGTCGATCCTTGGTAGATCTAAAATCATTGTTGGGAAAATCTG
CCATAAGTTCCACTGTATCGGTAAGCGAAATGAATGTTCAACAAAGCAATAGGACATCGT

CTCCGACCTCGTCGCTTTCTACAACCCAATCATCGCATCACACAAGTTCGCCTATACTAA
CTGCAAACTACGAGTTGTACGTGCCCACACAACGATCGATGTCACTACTTGGTAGGGTAC
AACAATTCTCCAAGCCAAAGGAAAGAAAGCGTTCATCTGACATGGCTGAAGCACCTGGGG
AATATTTGTGGCCTGCTACTAGGAAGAGCACCATTGGGTCATCGGGTCATACTATAGCTA
CTTTTGGGTCGTCGTCGATCACGTACTCTTCCTCTCCTCCTCCTACGTACCTACATCTAG
ACTTTGAAGATTCAAGCAAACAAGGGGGGATAGATCAGCCAGAGAAACACCAAGTTTAGT
CGAGCCAAGTAGTTAAATCTATATATACACCACGAGCAATCAAAACCCCAAAACCTCGTG
TTACAACGGGAGCAGAAGCAGGGGAGATGACATGATTGAGGGATTTCAACCGAACAATGG
AGTGGCTACAAAATTTCATTTGAATAACACCGCAAGATCATACCGGTTAGATGCGCAGGC
TTTTGAAACGTT
>comp1967_c0_seq1
ACCAACGAAAAAAAGATGAATATAAATATAAACAATAACCGTGTCTAACCATTCAATGAC
ATTACTACGAGAATAGAAAAGTACAGTGATAATGCAAGATCGTGCTAACCGATAAAACTT
GCGCTGCTAGGGGTGATACCGGTAGGTATCAGACTCATAACCTGCAAAATGCACGTGTGC
ACTTCAGTTGCGTATGTTACGTGGTTTACAACAACAAGAGTTTACAATAAAGAGTGAGTG
GCTTATTGCTCACTCACTAGCATAACGGAATTATTGAAAAAAATGAAAAATTTCTCTCAT
GAATATGGCAGTTGAAGTCAAAATAATCAAATAGAGCGATGAGTACGTTGGGCTGAAACG
AAAGCTAAAAGGTGATTATCAGAATGCAATCACTGAAAAAATTCCGTTGCCGTTGCCCCT
TTATCAGTGCTTTGGTGTAGCCTCACCCTTCCTTGTGTGTAATAGCCATACACGACGAAG
CAAACACAGAGATATAAGGCTATGGATGAATTACGTGACTGGTGAGCAAGTAAAATATAG
TTTGAAGAGGCGTGATGCTGGTACTACGACCAGCAACGGAAAATCTTCCCG
>comp1969_c0_seq1
ACAATCCCCGCACGGAAAAATTCCACCAACTGATCTATTTTGCGAAAGTCAAACCGGTCG
ATCATGGCGATGAAAAAGCAAAAGGCCGGCAAAGGCAAGAAAACGAAACACGAAGAAATT
GTGGAGGAGGACGAAGACGAGAGCGACGACCAATCCGAGGCCGACGAGTCGGACAGCAAC
GACGACGACGATTCGGACAGCGAGTCTGGAAGCGATGGGGACGAGGATGTAGCAGCCTCG
TTGTTCAAGGGGGCGAATGAGGAGGAAGAAAGCGAGAATGACGACGACGAAGACGACACG
GACGACGAGGACCAACCCGCGTCGGCACTGCCATCGGAGCCATACACGTTTGATCTGCGC
AACATGCTCGCCATCAATACGGACCAGCTCGAGGTTGGTTCGTTGCACAAGAGTTCGAAG
AAGAAGAAGTCGGATGCGGGCGGCAACGAAGAAATTCCCCTCGCCAATGCAGCCTTCGAA
GTCAACGAGGAGTATTTGCTGTCGAAAGCTACCGATGGTTGCACCCAGCTCATCGAAGCC
CTGTGGAAGCTTCCAACGGAGGCAAGTGATGCCGGACCATTGGTGACTCTGCCTACCTAC
GACGAGGTTCGACTTCCGAGAGCGATGCCACCACCGCCCCCGAAACAAGAAACAAAATGG
GAGAAATTTGCCAAGGCCAAGGGAATCCCTCTCAACAAAGAGAAGCGATCGAGAAAGGTC
TGGGACGAAGGCACTGGTACGTGGATGTTCCGACACGGGTACCAAAAGGCCAACGCGAAA
GAAGGCGAGTGGCCGATCATGGAGGTCGGTGCAAACGACGATCCGTTCGACGATCCCTGG
GAGAAACAGCGGGAGGAGAAACGAGCCCGGCTCGATCGAAACATGGAGCGCCGGCTGAAG
AACGAGGAGCGGGCCGGAAACCTGACAAAGGGCGCGACCAATCGAACCATGAAGGCCCGG
GAACAGGCCTACAAGGCGGGCAAGGAGGCGGGCAGAAAGGATTCCAGGTCGACGCCCGTG
GGCCTGCCGATCGATCTGAAAAACAGCAACGACCCCCGAAAGGCCGCCGACAACAAGCTG
CGGGGGAAGTCTTCCACGCTTGCTGCGCTGTCCGCGGTTCAGAGGTCCACGGCGTCCCTC
GGGAAATTCGACAAGATGCGGGAGGGCGAACCCGAACGGAAAAAGGTCCTGTCGAAGACG
AAGAAGCGCAAGTACGAGAACCCCACCGACAAGAAGGTGCTGCAATCCGAGGGAGACTCG
TCGATGAAGATCCTGAACGCCGTCATGAACGGAGGCGGAGCTGCCAAGGCCAAGGCCATC
CGAAAGGGCCAGCTGGCCAAGGGAGAGACTGCGTACGATTACGAATTCAACGACGGGCTC
GGAGCATCTTCCTTCCGAAAGAAGAAGGGCCGTGCCGGGGTCGGCAAGATGAAGAAAATG
ACAAAGAAACGTGCGAAATAGATTGCCTGCTGGTGGCGCATGAGTTTTGGTTCTGTAGCA
ATCGTGATTCTTGGATGGGGTATACTACAATAACAGCCGTATGTGCCAAATACAACAGTC
AACGTCGCATAATATTGTACTTTTGTTGAATACTACTGCGTTGAACTGTACTGTACTGTC
CTTCAGTATCATGGTGCTGCTTCCTATCCACCAAATATCATTATTTCCAGATTCGATTGT
CACTGACAGCGTTTTGTACAATGACCAGGATGTACTTGTAAAAGAACGATTTCGAGGAGT
ACTACTTATGTTTCCTTCAGAAGTACTAATATACTGTACCGTATTATTTTGTACTAGTGC
ACG
>comp1972_c0_seq1
TGTTTCTTTTGTTCCATGGTGGCCTTCGCTCGTTTGTTCGTACATCTTTCATGCATGACC
CTGCTGCCCATGGTGTTATGGACTTTTGCCCATATGCACAGTATGGACTCAAGTCTAGAA
TTAGCGGGAATTCGACAAACTGAACACACTTTTCCATTCGATTCGTAAATGGATCATAGT
AGCGACGTTGGACGTGACAACACAGGATAGACGGGCTTCTTGTTAACAAAAGGCACTTCT
TTGCTTCAAATCGCTGTATTGATATGTTCTCATCGTGATCTGAAGAGTCAAATAAAATCA
TATTGTCGACTTCTGATACGAAAGGAGCTAGGGATGGATCGACACTATCGTCATCTGGAT

CCATTGTCTTGAGTCTGAAAAGTCTCTTCTCGACTTTGATTAGATCATCCCGCAAACACT
TGGTCTGCTCGAAAGGTTCTAGTTCACTTGTTCGTTTGTTACTTCCCCCCATCCTTTTGT
TTCTTCTTCTCTCGGTCGTTCCTACCGCTCCTTGAAGCATCAGTGCTTCTTCTTCTAGAT
TGGCAATTTCTTGTCGAATCGTACAGGACCGGCATTCAACATCTTTGACCCGTTCGACAC
GTGTAAAATTCTCGAAGCAATCTTCCAGCGAGCAAGAAGGGATGTGTGAGAGATTGGGAG
AAACAGGTTTTGCTGTGGATCCATATGCTTAGACAGATAGTTTGGTACGGAAGTTGGCAC
TAGGGGAATATCCAAAAAGGGTGCATTTTGGATCGGTCTGACGTGTTTGCATTTTGAACA
TCGAAGGGTAGAACCAAGCCAGCCAGACAACGGAGACGGCGTAATAGGAGACATGGTGCT
CCTCATCGCTTTCTTTCCATACCTGAAATTCGTTTGTGGGAAATTCGAGGGTAATCGATT
ACTTGTTCTGCACCACTTCTTTTCACCTTGGGGTTCATTTTTACTCCTTCGAGACGAGGC
CGTATTAGTTAATTCACTGCCCATGAATTCATTAAGTTCTTTTTTCTTCTCTTC
>comp1974_c0_seq1
ACTAGCTTGTAGTATTGGTTGTCGTTTTTCAACATATACGCTGTAGGTTGGTGGTGAGGA
TAGATCAAACAGCACATACTGTTGCTTCCCGTAGAATGGGTCGTCTATCGAGGGACAAGC
GGGATGTATTCTATCGACGAGCAAAGGAAAAGGGTTACCGAGCTCGATCGGCATTCAAAC
TGCTTCAGCTTGATTCCGAGTTCAATCTGTTCGATGGGGTAACGCGGGCTGTCGATCTCT
GTGCGGCCCCTGGATCATGGAGCCAGGTGCTGTCCGACAAGCTAGTGGTTTCCAAGCGAA
ATTCAGCGCCCGACGAAACCAAGACTGAAGAAACCTCCAACGCAACGATTGTGGCCGTGG
ACCTCCAGCCAATGGCTCCGATCGACGGCGTTCTTTGCATTCAGGGGGACATTACGAGCT
ATGAAACCGCGCAGGCGATCCTCAAGCCTTTTGGCTGCGGGAGCGAGGGCGGTACCAAGG
ACGGAGGCGAAACCCGTGCCGAACTAGTGATCTGCGATGGTGCCCCCGACGTGACGGGAT
TGCACGACCTCGACGAATACCTCCAGGCGCAGTTGCTGATCAGCGCGGTCAAGATTACAA
CTCATGTATTGGTGGACGGTGGAACCTTTGTCGCTAAAATCTTTCGGGGCCGCCATGCAA
GCCAATTGTATTCTCAGTTGCGTCTCTTGTTTGATCGTGTGAGCATTGCAAAGCCCACCA
GTTCGAGAAATTCCTCTCTGGAGTCGTTCGTGGTGTGCCAGCAATTCAACAAGCGCGGGT
CTGCCGACCTGCGAAACCTATCGCTGGACCGAGACACGGTTCTGGACGTGGTGGAAGGAT
CTCCCTCGTTGCCAAAGTGCATACCACCCTTTCTGACCTGTGGCGATCTGAGCGGGTGGG
GCGAAGCGCCCGGGACCATTATGGATGCGGACAAGAGCTATCCCATGGAAGAAGAGGACT
ACGTGCAGCCGATTCATCCACCCATTGCACCACCGCATATGCAAGCGATCGAAGAAAAGA
GATACAACGCCAGAAACAGGGATAGACGAATTTATCATCCAATTCTTTGTCCGGGATCTA
ACCTGGGATTACGAAAGATGCAGTGCCGCTTCATCGAACGGTTCGGGGGCAACTCGTGAG
ATGCTACACGGTACGGCAACTACCACTCATGAAATTTGGTACAAAAGATCGGAGTAAATT
AATGCCATTGTCAGATTTCAATTCTTTTCTCTGCAAGCTTTTGAGATCCACTACGCACTC
CGTTTCATTTTGCAACAAGGCTTAGGAACCGCGAGTCGCACCAAAAAGAAGGAAACAATT
TATACGACCATCTTAAAAGCTACGAAGTAGCACAAAATTCAGTCCGGTACGGACGTCCCT
TCGGAAGTTGGCAACTGGCTCAGACTAGCAATGCACACTTCCAAACATGAATCACA
>comp1975_c0_seq1
CCTGCTCTGTACAGTACAGTATTTGGCACGGAAGGGAAAGAGCTCCTCGGTACCGACAGA
CTGTAAAGAAACAACCAATCAGGTGCCATACACAGAGAATTGTCGAGCAAGACATTGGCA
AGAAGCAAATCGCAGGACACAGCTGATACGGTGTGAACGACACCGACATCGAATACGCCG
AGACCCTTTCCGAACACAACAACAACAGCAGCAACAACGATAAAGCCGACGACGGCAACG
ACTACGACGAAGACGACGACAACTTCGCCATGGCAAAGGCAAAGACCCTCGTGAGAATGG
TTTCGTGGATCGTTCTCGCGCTCGGAATCGCCACGGCTCCCCGCGGCGTCCGGTCCCTTT
CCCCCGGCAGCACCGACCCCCTTCCCTTGGGAAGCACGGGCACCGCGAGCGAGCCCACCG
CCGTGTCCCCCGAACCAATCCCCACCCGGAGGAGGCTCCTCCGGGCCGCCGCAATGGCCG
CCGCGGCCGGCCTTTCTGGCACCCGCAGCGCGGACGCGGCCTGCCTCACGGGGGACACCC
GGCCGGTCTGCATCGGCATCTACAAGGTCCCCCAGGACGCCGCCTTTTCCAACTACATCA
GCACGCCGGAGCAGCTCAAGGAGTTCGCCCCGGACCTGACCTACGTCCCCCCCACCCCGG
CCCCTTCCTCCTACAAGCAGGCCCTGGAAATCCTCGAGGGACAGAGGGTCGTGGCCGAGG
GCATCCGGCGGAACGTCGCCGCCGGCAGGCTGGAGGACGCCGGGGTGGGCGTCCTGGGCC
TCATCCCCCAGGTCACCGCCAGCGGCCGGTTCGTGGTGGACGAGCTGTCGGCCCGGGTCT
ACCGCAAGCAGCAGGCCGCAAAGAAGCTCGCGGCAGCCCCTCCCCCCCCGGCCGACGGCG
AGGAACCCGAGGCCGTCCGGGAGTACGACGAAAACGGGTACAAGCTCCCCACCACCGCCG
ACGAGCTCGCCGTCGACATGGCCTCGAACCAGATGGACTACGTCGCCGGGTACTTTGGCG
AGTGCGACATCTGCATCGGTCAGGGGATCCGGGGCGAGCTCGGCGTGTCGGCCGTCGCGC
AGCTCACCATCCTGGCCTCCCTCAAGGACGCGACGATCGCCCTGGACGACTTTGTGGCGA
CGGCCGCCTCGCTCGGTGCCCGGATGTAGCGGCGGAGCGAAGCCCGAGAGCGAGTCCCGC
TCCGAGCGAAACGAGACCGGGAGCCTCGGAGGCCAACGAGCCGTCCCAAAACGAAGCCGT
GTTGGGAACGAATGAATGGTGGTGAACCACGTGGTTTTGGTCACCCGTTTTTCGTTTTGG
CACGAGGCCCAAAAAATTCGAACACATCGAAACTCAGGGC

>comp1976_c0_seq1
TGAACGAACAGGATCGAACATCCATCCACGAAGCCATGGAACAGCAGTCGATTTCTGTCT
CCAAGGCCGGAATCGTAACCTCTCTGCAGGCCCGGTGCTCCGTGATTGCCGCTGCCAACC
CCATCGGTGGCCGCTACGACAGCTCCAACACGCTTTCCGACAACGTCGAACTGACCGCTC
CCATTTTGCAGCGATTCGACATTCTGTGCGTTCTCCAGGACACCGTGGATCCCGTCCTCG
ACGAACGACTGGCCAAGTTCGTGACCTCGAGCCACATGTCCGCGGTAGCGACACGGGATC
TCAACAACGGGGAGGCCACCCTGCCCCCGAGCACCAGTGTCGCCCAGCAACAGGAGGGCA
TCATCGACCAGGAGCTTCTGCGGAAGTACATTCAGTACGCCCGGACCAACGTCAAGCCCA
CGCTCCGGGGGAATGCCTTTGACCAGGAAAAGATTGCCTCCCTCTATGTGGCCCTCCGCA
GGGAATCGAAGAATTCGGGGGGGGTTCCCATCGCGGTGCGGCACGTCGAGAGCATCATGC
GCATGTCGGAGGCCCACGCCAAGATGCACCTGCGCGACTACGTCCGAGACGACGATGTGG
ACGCCGCCATCAAGATGATGCTGGAATCCTTCATCGTGGCGCAAAAATTCTCGATCCGGC
GGTCCCTCCGAAGGAGCTTTGCCAAGTACATCACCAGCGGGGAGGACCGGGCCCATTTGC
TGCTGCACATTCTGGGCGACATGATGCGCAAGGAGCAAATGTACCAGGTCATCCGGCTGC
GCCAGAAGGGACAATCGGAAGACCTCCTGGGAAGGCTCGAGGTGCCCGTGGACGAACTGG
AAGCCCGGGCCCGCGAGCGCCGCATCTACAACGTGACGGATTTCTGCAAGGGACCCAGCT
TTCAAGAAGCCGGTTATTTTCTGGACGAGAAGAGGGGCCTGATCAGCCGGTCGGCCGTCA
TTTCGACGTAGGAAAGAAACGATCCCATACCTGCCAATGGTATTGTTTTGGAAGAAAGCA
GGTACAGCAAAGCACTGCATTGAAGTGCCCTATACGCAGGAACCATTTCAATGGGCATCG
AATCCAACCCAACGGAAATAGTTTTATAATTTTATAACGTAGTAATGCGACATCTCC
>comp1978_c0_seq1
TACAAACACACGATTTTATAGTTACGACGTGCTAAGCACTACAGAGGACACAAGCAGGTA
TGATGCATTTATCATTGGCAGCGGTTACACTGACCTTCGTCTATAGTGCTTTATTTGGAA
ATTTCTGCCAGGCCTTTGTCATCGACACTAGAGCGAGCTGCCAAACGCAACGAGTCCGCA
ACTATGGGTCCGCTTCCTCTTCGACGACGAGGCATGTCATTCCTCCCGAAATAATGGAAG
AAGTCGGAACAGCTCGAGGCCAATTTTTTCTTTGGTTTTTTGGGTCATCAGGTGGCGGAG
GCATCGCCTTGTCCTCTTTTCCGAGGATGTACGAACAAGTGGCCTACATTCAATCCCTCA
AGGGGGTAGGTCCATCCTTGGGGGGCGACACTTTAGGAATTAGTCCACTCTGTGGATATC
CTCAAGATATTGCTGTCAAAGATGTAGAAAAGGTGGCAAACAGCAAACTTACGATAGACC
AAATGGTGGAGAAATTTCCACAGGAAGGCAATTACCTTGCTTCCAAAGGTTACATAACGT
TTAGTGCCTTCGAGCAAGCCAACGAAGGTTGTAATCCATTGGCGATTCGTGCCGTCTTCG
ACACCTTTTCCCAGTCCACCAATATATCCGATCCACGGGTTGCACAAGAGAAGCTTGATT
TGTACAAGCAAGATCCTTTCGCAATAAATGGTGCCCTTTTCAAAGCCAAGGTGACGGGAT
TTTCTGCCGTCTTTACGTTGTTGGTACTTTTGGGGTTGGCAGAATCCATTGCATTTGGAC
ATGCTAAAGATGGATGGTTTCATGACTGGACATGGTCCGATGGAATTCTGAATATTCCTG
ATTTTTGGATTTGAAGGGGATTGTCGAGCGAAC
>comp1981_c0_seq1
CTTGTTTTGTTTTCCCACTCTTGTTTATAGGATGAAAGCCGGCAGCTCTTCGTTCAACGT
TTCCGTGGAGGAGCGGCGCGTCAAGGCCAAGGAGCTCCTCCTGGATGTGGAAGCGAATCT
CGGAACGGAAACGATGCTCAAGGTCTTTGGCGTCTTGAAGAATGCGGACGAAACGACCCT
TGTGCAATGCAAAACGAAGCTCCTCGATATCTTTCGCGGCCACAAGGAGTTCCAGACGAG
GTTTCTGGAATTTTTGCCGCAGCAAATGCGTCTATAAATACTACTGGGAAGCGACTTCGA
AACTAATCCAGATCTGTTGTTGTCGAGATGAAGATGCTGTGGCAGCATCTGCCTATTTGA
GTGGTGTAGCGCGACAAAATTCATCACTAACATTTCAATGCGATTAGGGAGCTTTCAAAA
CGAAATTCAAGCGCTGATCATTTTATTGGTCTCGCTTCTAGAGGGAAGCTATATTCTGCT
CCGGTGGTAAATACACAATCATTTCAATGTTGGAAGAAAACTATAAATATCTACGGGAAG
CTCGGACGGGAATAATCGTCCTACTTCTCGATCATGTGGCGGATCTGAAAGTCTTCGAAG
GCGCGAGAAACGATCCGCTGAACTTGTTGTCTCTTTCTGACAGTCATGGTTCGAGACGAC
GACGAAGCTTCTTCTCGCAGCTCCCGAGTGATGACAGCAAATCCCTGTAGATACGAGAAG
ACGTCGCAAAAGGCTTTCCAGGACAGGACAGAAGCGAGATGCCCGTTGATGGCAAAGACC
GCTCCGAAATCTTGTGTCGTCCCAGGGGATTTTTCAAACCTGCATCGGATGGACCAAACT
GCGTATTGCGGGAGGCCACGGATGCATCTCACCACGTGGTCGACATTGTGGGTGATGTGG
CCCCGCAGGTACTCGGCGGTTTTGCAAGCAGCAAAAAGCATCATTTGGTAAAAGTTCCCG
ACGGAAGTTTCGAATGTGTTGATCGATGAATCGTATAATATAGGAAGACACCTGGGATAC
ACAAAGGTCCGTACTCGAAATTCCATTTTTCGACCCTCGTTCCCATCGTAGTCGATGGTG
ATACCATCCCGAAGCTTGCCTCCCTGGAATCGCTCGTAGTCGCAAAACACTTCGCCTGTC
TCTGTATCGAACAAAAAGCCGCACCAAGGAAAGAGCACGCTGCCGCTACGATTCAGTTTA
CTGCTGACGGAGAGAGAGATACTTTGAGTTCTTCCGTCATCTGCGTTTCTCTGTACAAAC
GACCTTACATTCACTAGGGTCTTCTTGGAATTCACCCGAGCCCCAAGCTCGGTTATACCT
TCATGAGTCCGGTCAAGGAAGCCCTGAAATGCCGCTTTGTCGGGACTTATAAAAAGGAAA

TCGTCTACGAAGCGAGAGATCAAGTGCATAGCTGGTTCGGTTGGTGTTGACTTGGCATTA
GCCGTAGAATTTGACCAAGGCAAATGCCCACCGTCCTCACTAAACATTAATTTTTCC
>comp1985_c0_seq2
GGCTTGTCAAGATCGATCGAGCAATTTTGAGGATTCCTACTCTGGCAATTCACTTGGAAA
CTGCCGAGGAACGAAAAGCCTTCAAGATCAATAAAGAAAACCACCTGAGTCCAATTTTGG
CGCAGGCGGCACAAAAAGCATTGTCTGGTGACACCGACGACAGCGAAAGTGGCGACAACG
GCAAGGTATCGTCGAAGAAGGATGATGAAGATGGTTGGACGGAATATCAAGAGCCGCTGT
TGCTACAACTTTTATCCAGCGAATTAGGCATTCCCATCGACGACATCGTAGATTTTGAAC
TAAGCTTGTTTGATATTCAAAAAGCTTCCTTAGGTGGTATTCACTCTGAGTTTGTCCATT
CGGCGCGGCTAGACAACCTCGCTAGTTGCTTTATTGCGGTCCAGGCACTGATGGATTGTG
CACAGCAGGATGGTTTTTTGGAGAATGATGAGGACGTAAGCATGGTGGTGTTGTACGATC
ACGAAGAAATCGGGAGTACTAGCGCTGTTGGAGCAGCAAGTCCTATTCTGGGAGAGGCTG
TTAAGAGAATTTCTTCAGTACTCAACGAGGAGGAACAAGCTGTAGCTCCCCATGATCTAT
ACGAAGCTACTGTACGACATTCCTTCGTTTTGAGTTCCGATCAAGCACATGCCCTGCATC
CCAATTATGCTTCGAAACACGAAAAAAACCACCAGCCACAGATGAATGCAGGAATGGTAA
TCAAACGAAATTCGAACCAAAGATATGCGACGACGGCAGCTACCGGTATTTTGATGCGAG
AAATTGCAAGGCGTTCAAATCTACCTCCGGTTCAAGAATTCATCGTCCGACAAGATTGTG
GTTGTGGATCTACGATTGGCCCTTTGATAAGTTCATCTACAGGTATTCGAGCGATCGATA
TGGGGTGCCCGCAGCTTTCTATGCATTCGATACGAGAAACTATGGGAGTTTGTGACTTAT
CTAATGGGTTGGAGCTTTTCAAAGCTTTCTTCATGAAATTTCGCGAGGTTGACAAATCAC
TAGAGAAGTAATTTGCAATAACTGTGACATTACTATTCATTTGGGC
>comp1993_c0_seq2
CCACGGAATCCTTGTCGGTCTTGTCCTTGTTGACGATGTTGAACGAGGTGATCCCGTGCT
CCTCGAACAACCTGATTCGGTCGACAAGGCCACGATCATCTTTGTTGGCGCGGACCGCGC
CGTTTGGTGGCTTCTTTTTCTTTTTGTTGGTGCGGTTTGGGAGAAAGAGCTCAACGCGCA
CCGGGATGGGCATTTGCGAAAGCAACATGGGTGATGGCGAGGAAAACAGTAGTTGCGGAT
GTGGATGCGAGTTTTTTCCGTCGCTCCCGCTGTGCATTCGCTGGGTGGACGAGAATGAAA
ACACCAGATGGCGAGAAGAAAGAACCAGGAACGCCGACAGAAAATATAGAATCGTGGATA
GGATCAAAAATGTCGAGTGTTGGATAGGAGAGAGCATTTGTTTCTTCGGTTTCTCTTATC
GTTTTGTCCCTCACTGCTGCTTATGCAGTTGTCTCATAATTCCACCTCCATACAACCATA
TCTACCCTGGAAGATGCGATGTGATAGAATTTGCCCAAATCTGTATTCCGTAGCACCTGG
AGCGATGGGCCACGGGGTACGAACGAACAGAAAATCATCATGTTTCGAATCATCAGCAGT
CCTACCGGACAGGAACGATTCGTCGCAATAGTTGTTCCTGTTCCTTACTGGTACTATCGT
ACGGTAATTCGCACTACCAACTCACAATCTCATCCCTTTTTCGTTAAAAATCACATCCAG
TTATGACTCAGAGAAAAAGATTAAACAATGGAAAAGTGAAAAGTATTACCGTACGGTATC
GTAGTCCACTTACGAATCAAGTAGAAATATCACAGAACG
>comp1995_c0_seq1
CGGGGTTGCTAGCCTCGGCACAAAAATCGAACGGATTCGAACGATCGACAACGAGCAGGA
CACTCGAGAGCACAAGAGAAAGGCAAACACGGAATCGAGAAAAACGCTGCAATCACAGAA
ACAGAGTTTTTGGCAAAGTGTTGCAGAAGTACCACTAGAAACCAAACCCTCCCCTCCATG
TGCGGACCAACCAATCGATTGCCCCCGGTGCAGGACGGCCCGGCGGTAGATGCTCGCAGC
TCGAGCGGCAAGCCACCCCTGCCCAACCCGGCAACGCCGACCACCGGACACCAAGCACCA
CCGGGCAGCAGCAAGAGAAGCTCCGCCGTGCCCAGCGGGATCCTCCACGTTTCCCCGGCG
GTCGCGGCCGCTGCCCTGGCCGCTTTCGTTTCCGTCGCCCCGTCGTACCTGTACGTCAAC
CGCGGGTTCACCGTCTGGTTCCACTGGGCCTCCCTCGCCTGCGTCCTGACCCGTTCGCAC
TTCCTGGCCCAGGCCCTGGTCCTCTGCGGGACCTCTATTTGCCTGGGCTGGTACGCCTGT
CTGGTCCACGAGTACGCGGTCCACGGCCGGGTCTTCGACGCCCTCCACAAGAACATGCCC
GGGGCGCTGGTGGACCGCATGCTGCTGCCGGGGGGCGGCGGCGGCGAGAGCGGCGGGTCC
CCGAACGGAGTTCTGGATTTCGAGTCGACCGGGTCGCTGCTGGCCATGGCGGTCTCGCAC
GTGCTGGATTTCTTGGGCCATCCGTGCCTGACCTATTATTTCTGGAGAAAGCACCGCCAG
CTCCAAAGCCAATCCAGTGTTGGAGGCAGCAACAGAAACAACAACAACAAGAGGAAGAGG
AAGTCGACCCCGAAGAAGAAGAAGAAAGCGACGACGGCGACGATGACGACGAGCTCAACG
GAAGTTATGACGACGATGATTATAATGAAGAATTCGAGGAAAAGGACGAGGATGGTAGTC
ACGAAGAAGACGAAGAGCGCTACAAAAGCGAGGAGGGCGGCGACGAAAACGTAGAACAAA
CAATACAAAACCAGGAATCACCGAAAACAGAACCACTGGAAGAAGACGAAGAAACAGAAA
TCGAAGAAGATGAGGACGATGACGATGACTATCTTGAAGACGAAGACGAACCCGACAATC
CTTGGGGGATCAAGCCCATTGACGATAAAGAGGAACTTCGTCTGCAACAACAACAACAGC
AACAA
>comp2005_c0_seq1
AATGCACGACGTTCTCGATGCGCAGTACGTGTACGATACTACCAAGGATGAATCCTATTT

GAGACGAGTTGTCATGCCGCTAGAAGTTTTGCTAACAAAGTACAAGCGAATTGTCGTGAA
GGATTCGGCCGTCAACGCAATTTGCTACGGTGCCAAGTTCATGATCCCAGGGTTGCTAAG
ATTTGCGGACGGAATTGAATTGAACGACGAAGTAGTCTTGATGACCACCAAGGGTGAAGC
GATTGCCGTAGCCATTGCACAAATGACTACTTCGGTTATGGCCACCGTTGATCACGGGGT
GGTTGCGAAGATCAAGCGTGTTATTATGGAAAGAGATGTCTATCCTCGTCGATGGGGATT
GGGACCCATGGCTCAACGAAAGAAGTCAATGATCAAGGAAGGAAAGCTTGACAAACACGG
AAAACCCAACGACAGGACTCCATCAGACTTTTTGACTTCCTACAAGGACTACAGCAAATC
CAAACCGCCACAACTCTCTGGCGAAGGATCGACTCCTGCAGCTGCGGGGGTACCATCGTC
CCCTAGTGGCGATGTTTCCATGGATGGCGGTGCTGGTAAGCGGGCCAAGGAAAGTGATGG
CAATAGCTCAGATGAACAGCCAGCCAAGAAGAAAGAGAAGAAGGAAAAGAAAGAGAAGAA
AGAAAAGAAAAAGAAAAAAAAGAAGGAGTAAGAATGAAAAGTAGCACGGCAAACTTGGGC
TACACAGTTTCCTATACGTCGAATGGATAATTCAAAACGAGCAAAAACTTTATCTCGCTC
TCAAATGATGCAAAAGTCGGCGTCAAATAAATCATAACTTCCCATTGGGAGTGATAATAT
CATACCTACC
>comp2006_c0_seq1
AATTTGAAGCTTGGAGCTGGTCGGAATTGTCTTCTTCATATTGCAATGAAGTTTGCGAAG
TATTTTCAAAATTTTGGGCTGTTGGAAGCAACGGTGCAGTCAGATCTTCATTTCCATCGA
CTGATTCTTGGTGCCTGGTCCTCTCTCTGCTCATTTCGCTTCTTTCACTATGTAATCTGG
TATTTTCTGCAGTTGCCACAGGCCAGAAACTGATCATCATGCCTAAACAAGCAGTGATAA
ACATCACAAAACCAATCAGAAAAAGACCAAACGTCGTTCGGCGTTGCACAGCCCCAACAA
TGGGGTCTCCATGGGCCTGGCTGTAAAATCCGACGTATTTCTTTTTATTCACTAAAACCG
CTTCGTTGCCTCTATCGTCATAGAGTGCACTGACAATCCATAATAATCCTGTTATGAGCT
GAATGGTGATTGACGTCCGAAGGTTCCATATTGCCAGTGCAGAGGAAGATCCTCTCGATA
TCAACGGGTACAACATCATAATACATCCAAGTCCCTGTGCCAAGATCACAGCTGCCATCA
TGTCGCATGCGCTGCCAATGGAATCTTCATGGTTCAAAGATCCTCGGATGAGTTCCAGAT
CCGATAAGTCAATGACGTGAGATTCGAGCCGATACGAATGGTTGTAGTATGAATTCTTGT
AGTCTAGAAGGACCGATAGAGCCTTTCGAGGAGAAAAGAAAATTGCACCTCCAACTGAGA
GCAAGAGGAGTGAATTCGAAAGAGAAAGGCATCCAGAAACCGATTGGTATATAGGTACAA
ATTCAGAGTTCATATCGTCGTCGTTAACCTTTCTTCTCGTCGCAGTACAGTTAGAGTTGT
TAAAATTAAATATGATGTTTTTTAACTGTTTCGATTGTGGTC
>comp2007_c0_seq1
AACAACAGCAACAACAACAACAACCACGACCACGACACGCAACACATCCGGATCTGGACG
AGCTTTGACGAGCAGGAGAAAAAGCACTGGCTGACCGTCCGCAAGGGGTTGTTCCGGCAG
CGGTTCCTCCTCCAGGACAACCTCCTGACGGCCTGGCAGGGCAATCGCGGGACGAGCCTC
CCGGAACGCATCCACGTGGCCGTCGACGAAATGATTCGGGCCGTCGACCGATTGGACCAG
CCGTCCAAAGTGCCGGTGAGCTCGCCAACGGGACACTTTCAGCAGTGGCACCAAAAGGGC
CAAAGGCGGTTTCGGAAGCGGTGACAACGCGAGGGATTCTGATGGCTGCAGTGGCAGTGT
TCCGGTGTACGGGTACCGCGCGGCGCATTGGTGTCGCTTTGCACCCACCAAAATCCACAC
GCAAAAACACACACAGGTACAAAAACACGCAACTAGAGAGGTTGCGAGAGGTTTAGAGAA
TTGGTTCGCTGGTCCTCAAACGAAATCACCATTGCATGAATCCTTCGTAAGAGATTAGTA
CAATACACGCGGTGCTAGTTACGGCTCGGTTGCGGTTGCTACACGCTACGAAAGTGCGCA
TTCGTGCGGTACGTGAACGAGTTCGTAGTCCGATCAACACCTAAATTCACAACGATAGAA
TAATGAACCCCAACAGTAATTTTTATTAGCGAAGCCA
>comp2008_c0_seq1
CTTTGTCGTACTACGTACCTCAAGGCAATAGACCAGCAAAGAAAGTCGTACAATAACACT
GTTGATGATGCTTTGCATGTCTCGCGATTGATTCATTCCTCCTTTTGAACACCGTACCCA
ATACATACGCACTTGGTTTGAATCAATTCAATCTACACAATGATTCGCCACACGCTATTT
CTTCTGTTGCTGGGCATACCGGCGATCTGTTATGCCTTCGTATCTCCCGTTCCCCAATCG
CATCGGTCCATAGCATCGGGTCTTGTAGGCCTAAACGTAGCTGCCCCCTTGGAACAAGAC
ACGCGAGAAGACGAGCAGCAGCTGCGAGAGCGGGAAGAGAAGCGACAAAAACGAAAGCTC
CGAAAGGAACGAGAGAAACAAGAACGCAATTCCCCCGTTGGCAAACTGAAATTCAAATTA
CTGAAACAGCTCAAGGCCGTCGCGAAAGACTACGAAATGCTACAGGACGGAGACCACATC
ATGGTGTGTGTGTCCGGAGGAAAAGATTCTGCCGTCCTGCTGGTCCTGCTGATGCACCTG
CAGGAGCAATTGAAACCGATCGGTGTCGACTTCAAGCTGACGGCCGTCCACGTGGACCAA
AAGCAGCCCGGGTACGACGGCGAACCTCTCGTAAAGTGGTTCGACGAGCTGGGGGTAGAC
TACGAAATTCTCGAGGAAGACACCTATTCGGTGGTCGTCGACAAGACCGCGGCCGGAAAA
TCGTTCTGCACCATGTGCTCGCGCCTGCGGCGGGGAATCCTCTATTCCAAGGCCCTGGAG
CTAGGTTGCAACAAGCTGGTCCTGGGCCACCACGCGGACGATTCCCTCGAGACCCTCGTC
CTGAACATGCTGCACGCCGGTGAGCTCAAAGCAATGCCGGCCCGGTACACGAGTCGGCGT
GGAAAGCTGGCGGTGATGCGGCCCCTCATAACCTGCATGGAGGACGACATCGAGGAGTAC

GCAATGCTGTCAAAGTTCCCCATCCTGCCCTGCAACCTCTGCAAGAACCAGAACGGCCTG
CACCGGCCCCAGGTCAAGCTGCTCCTGTCGACCCTGGATTCCTTTACTCCCGACGCCAAG
AAAAACTGGCTGACCTCCCTCAGCAACGTCCAGCCGAGCCACCTGCTGGACCAAAACCTT
CGAAAGGCCGTGGGAATGGACCCCATCACCGGGGAGGAAGAAGAAGAGACGGAGGAACAG
AACGTTCCTTGAACACGGTGAAAGGAATTGATAGATTAATTGGTTAGAACTAAGGGTAAC
GCA
>comp2009_c0_seq1
ACGATCTTCTCTGACCTAAATTGAAGGAAGCTTGCAACAATTAAAAATAGTCGAGATTCA
CATCTCCTAGTACGAGAAAGGACCTTTCTTACTACTGTGACGGATAACATTGTCATCAGT
ATCATTGCGACTAGTGATCAATCATCGGTTTCACTCGATCTATTGTTTACTCGAGTCAAC
AAACAACGGCTCTCTCGTTCTCTATTTGAAAAGCTACCCTAGAATCCGATCCTCCAATCG
GTAGCTTTCACTTTGTTCCGGTACATAGTACATTCGTACCGGATAGCAATCCGCATTGGC
GATGGTAACTAAAGAGGAGTTAATGATGACCACAATGGCGCAGTACTCAAAACCAAGTGT
CTCGGTGAGACGCAGGACTCGGCAGAATAGAATCATGCCAACTGTTCGCGGATTCTTTCT
GTTCACAATAGCTTTGATCTCTAGATTTGGATATTGCAGCAGTAAAAAAGACGACTTGCA
ACCGTACCACCAACTGGTTCCCAGAGTACATGCTGGCGTTGAACAAGATTTTCACGAACT
GATGTCCGAAATGCATCAAGCGGAGGACTCCGGTAGTTCCGTTGAACGAATGATGCAAAG
CAAAGAGGACCTTGCATTCTGCTACGATGCGTTGTACCTAGCCGATATGAACAATGATAA
CATGGTGAACAGGGACGAATTTGTTACGTTTGCACAGATCATGGGTCCAACTGGGTTTCT
TCCAAACGCAAACGTCTTTCAGGACTTACCCCTCATCTTGCAAAGTAATTTCATCATCCT
TTCGTGCCTTTGCTTGCAGATGCCGACATTTGACGGAGGAAACGTAGTTGATCCGCAGTG
TTGTGCCGTAAATCCTCACATCGACGTGGATGGGTCACAACCAGGAGAGATCCCTACCTC
CCAACAGTCACAATACCTCTATCAGGTTTGCTTTTTGACGGAGACTTCGATTGAACGATT
ATTGCTTAGTGAGACACCAACTACAGAACCCACTAAAAAGATAATTCCTACCAGTCCTCC
ATCAATGGCACCAACAAACACACCGACGAACGAACCGAGCGATGAGCCCACCAATACACC
AACAGTAGAGCCGACAGCGGAGCCCACTGAAATGCCCACCGACACACCGAGTGCACATCC
TACAGCTGAACCCACTGGAACACCAACTGCAGAGCCCACCAGCAAACCTACAGATGAACC
GAGCCCAGAACTTTCTGACCAACCTACCGACACACCGACGAAAGAACCTACGGAAAAGCC
TTCGGGAGAACCTAGCGGAGAGCCCACCAAAACACTGACGGATGAGCCCACAATCTCCCC
TACAGTTTCCCCTACACTGTTTCCTACAACGTCAGAACCTTCTATGCGACCGACTACGAG
CCCAACAGAGAAGGTTGAACCAACAGTCAGCCCCACTGTGCAAGATATAAACGTTACCCC
CGTGCCCACACAATCACCTACAGTGACAAGTGTACCGACACCGTCATCTGTAGTGGAGGT
TGCTATTGCCAAGTATGGAATTGCGATTGCCGACGGTAAGACCGAGGAGATCCCTCAATC
GTCATATGAATCCGACTTGATTGAAAGTATGAACATTGTGGCTGAAGATGTCGCAGACGA
CCTGAACTCATCATTAAACGAGGGTGATAGGCTTCTTCAGGAGCAACGTGAAAGTGGCAC
TGCTCTGCGACGATCGTTGCAACAAAATCATCGAAAGTTGGAGAGTAGTTTAGCCGTTAA
GCTTCCGACTGTTATTGATGACATTGCTGGATCGGAATGCCCTTCGTTTGTGCCACCGAA
TGACGAGTGCAAAGCGGTGACAGCTTCCGTGGAGCTAGATATCGCCGCGGTCGGAATCAA
TAGCTCAACTGATATCAAAAATTTGTTTGAAGAAAGATTGAATTCTGCTATCGAAAACGG
GGCTTTGCAGGCTGCCTTGGTCGAAGTAAATCCCAACTCGGTTGTGTCAATCATCAACGG
CGCCGCTCCTGGTCCACCGACGCCGACAGCGTCTCCCGTAACATTGACGCCAACTACAGT
AGCCACTGTAGAGCCTGTCCCTATCTCTCCAGGTGGAATAGCCGGCCTCGTTTTAGCTGG
CATTGGCGGTTTGATCATTGGGTTCCTCATCATAACTTATTTGCGTCGTCGAGGTGCAAC
GGATGGTGGAGTCAATGCTGTCGGTGCCTATGCCGTGCCTGCTGAAGAATCGGCAGATGT
CGAAGAAGCGCTTCTCAAGAACACAGAGGCACAGGAAATTGACTTGGGTTCACGAGAAGA
CAGCAGCTTTGACAAGGATGAAAAATATGAGACCCTGCATTCTCAAATGCCGACCATAGT
TGATTCGGGACCACCCATGGTTGACACTGGTACGGTTGCAACAGCAGCCTTCATTGGTAC
CGGGGGAGCGTTAGCAGCGGCAGCGGCTCTGGAAAGCAACGATAATCAAGCAAGCCTCGA
CACCAGCCCTGGAAAGAAGAAAAGAGCCTCTGGCAATCTATACGGTTCTGACGAGGCCTC
CTCCGCTGGAGAATCTGGCTGGTCGTCGAACCAAGATGGCTCTTCAGCAAATACTTCGAT
GGATGCTAGTCTAGACAGCATACCCCAATCAGCTATCTCTGGAGTCTCACCAGTGGTATT
AGGTGATATCGCTGCAGTGGGAAGGGATAGCGAAACTGTCGATCAAGCATCTCCGTCGCC
GGCGAGCCCAGAAAGGTTGCAAGTATCGACAGAGGAGGAGCAAGTCAATTATGTATCTCA
AAGCCTCAGTCCTACGAATACAATTAGTCCGGAAAATAGCAGGAGTGCTCAAAGCCAGAC
CAACAGTCAAGAAAATCAAAGCTATACGGGAACAACTGGAACGGAAGGAAGCATACAATC
AACCTATTCTGAGCTTGACGAAGCCATCCAGAAAGGTGACTGGGCCGCAGTAGGTGTTAC
GGCAGCTCTTTTGGCTTCTCAGGCTTACGGAGAGGAAAGTACGCAGGGCAGCAGCAAGAA
TAGCAGCGTAAGATTTATTAGAAAAGATGCGCCTTTGGATCCAGAACGCGCTGCAGAATT
GGACCTTCTCGTAGAGAGTGGTGACTGGGAAGGTGTTGTAGCAGCCGCAGCCAAGTTCGA
CGCTCAAGAAGCGTTGCGTGGAGACCCTCAATCGCCACAAAATTCTGCTGGATCTGCAGT

TTCTGTATCTTCTGCGGCTGGGTCGAACAATAGCTCAGCTCTCTCGGGATCAGTGGATGG
ATCCTCCAATGGCACCATTCCTTCCAATATGTCAGGATCGCAAACTATCGATACGACAAC
AAGTCCATCTACCTACACGGCTACCGGTCTAACTGCTACTAGCGATACCGCCTCCACGCG
GTCCAAGGCACGAAAATTGAATGAAATCCGAGACGAAGTAGAAGCTTTGGTAAATGCCGT
CGTTCCGGAAGAAGCTGAAAACATCGATGAAATGATGACACAGTTCCGTGGCCGCGAGGA
GGAATTGGTAGAAACACTTAGATCGATGCAAGAACGATTAGTAGCTCAAAAAGCGCGAAA
GGAGAGCCAAAAACAGGCCAAACGGCAGGCAAAGGAGTATGTACAAGACAAGAAGAAGCA
AGAATCAGCAGGAGACACTGATGTGGCTACTGATGCAGCAGACGACATGTGGATAAAAGA
AATCGAGGGATCGAGCGGTGCCGCCGATGCCTTAGCGGGCACTTCTGTTGCGAGAGGAGA
GGCACTCGAAACACCATTTGAAGTAGACGAAGATGCAGAGGCCAAGGCTATGAAGACTCA
GCTTAAAGAAGCCATCAGCAACGAGGATTGGGAGAACGTAGCAGAAGCCGCTGCAGGTCT
TTCTTCGCACGCTTTCAAAAAACCTGGAAGTTCGCAAGACAGCAGGTCGGAAGCATCGAA
CCGTAGCGTTGACATTAACGCTTTAGTTGACGAGGGTGATTGGGATGGCGTTGTTGCTGC
TGCAAGTAAGTACAGGGAGGCGGCAAGTACCCCGGGAAGTAGTTCGAATGCAGAAACAGA
TATACCGGATGATTCCACAATAGAAAGAAGAAGAAAGCGACGAGAAGAAAGGCTGAAGGA
AGAGGAAGAAGCCTTAGCACAAGCTCAGATATGGGATGCCATTGCCGACCAAACGAAGGT
AGATAACAAGGCAGAGGGAAGCGCCGCTGGACTGGCTGCAGATTGGGCCATCGATCAAAG
CCTTGCGGCGCTAAAGAAGGCAGAAGAGGAAGGCGATGAAGACAGCCAGGAATCTGATGA
AGACCAAAGAAATGACAATGAAAGTTTATAACAACTTTGATAAACATTAGCCCAAGTTTT
TGAATGTTACAATAATATTAATCACAACATCATTAGTGCCA
>comp2010_c0_seq1
CGAGTACGAGTACGTATTCATCCGAATCCCATACCGCATTGTTGTACAACTGCGGAACAA
AGCAACAGCAACGCAGCCTCCGATCGGGAACACCGCGAGAAGCGACACAAACGAAAAAAT
CTCGAAGCGTCTATAATGACCGGTCTCGCCGCCGCGGCCCCCTCCGTTGTCCTCGGCGGA
GCGATAACGGGCAGCGGGTCTTCCTTTGCCGCCGCCGGTGCGAGCCTGGCGCTGGCGGTC
TTTCGGGGCCATCGGGACCGGGGCGGCGGGGACGACCCCTTTCTGCACGGCCCGCCACAC
CGCCGCGGGAGGCAGGGGTGGCGCTCGGAAAATACCGAGCCTCGGGCGAGAGACACCGCG
GGCAGCAACGGCAACAACCACCGCAACCCGCTGGTGGGGGTCTACCACGCCTGGCTGCAC
CTGCCGCAGGTATTTCGGTTCTTTGTGGCCGGGAACCTGGGAAACCTCGGGTTTTTTTAC
CTCGAGAAGGTCTTTTTCCAGTGCCTGTCGCACATACTCATCGCCTCGACGGAGGTATAT
TCCTTCCTCCTGGACGGGATCGAGAAGTACCAGGACGGCCTGAGCTTCTTCTCGGCCTAC
CTGGTGCAGATCGTCACGACGCACCTGCTTTACGCCTTTCTGGTCTACGGAATGGACACC
ATCGACACGTACGAGAAATATTGTCTGACGCTGTGGGGGCAGTTCAAGGTCTACGGCTTT
GGTCTCGTAGGGGCGACCGGCCTCAACAGCTTTCTCATCGGCAGGGGGTTCGAGAAGTTC
GCGGCCTTTTGGATCACCACGGCTTCCTTCGCGGTGATCAACTACGTCCTGGTCAGCAGG
GTGGTCAGCGGGGTTGTGGAAGCGTCGGAGGCCGCTCCGCCGGGCGACGCCGGTGCGAGC
CCGGGACCGTGGGGGAAGAGGAGAAAGCAAAAGCAACGGCAACGCAAGTCCCTGGGCTTT
GCCGAGCGAGTGGGCAGGGGAGGAGGACGGCACCCGCAACCACCCGTTGCAGCTTCTGCC
CCGCTTGCTACTGCCAGCGGCCTGCTGGGATGCCCGGGGGAGGTGTGCAGCTATTGTTAT
TCGAGCTATAGAGAATAGATGGAAAGAAAGCTGTATGGATCATGCTACAAAAGCACGCAA
AAACCACGGATGCCATCCCAATTGAAGCACCTATGTCACGTCTGATGTTTGGTTTTTATC
AATAGTATTTTTTTGCTTTGAAACAATTAAGCGCATTACGGCACACCGTGTCCTTCTTAC
AAGCCGGATAAGGCATTCCTGAACGCTCTCTTTGAATCTCTTTTCGATTTGCCTCAACGG
GAAAGACGCTTTCTGCCAATGAACTGAGAAGAATGAACGAGGGGTCTGTAATGCACAGCA
TACTAACGGGGTGACAGTGGTAAAGACACCAGGATTAAGTAACCAGTCGCTTATTTTCTA
CAAAAATACGGGAGAAAAACTATCGTTTTGTAATGTTGTTTCAAATTATTTGTAATCCTG
GACGTATGTTGCGAATTCTGATGCGCTCTTCCAAATAGAAAAGCTCTAGACAGTTTAGAA
AAATACACAATATCAATTGATTTCCATCGACGGTGCCAGGATA
>comp2011_c0_seq1
CAAAAATATCATTCTCAACGGATAGAGAAAAAGCGGATATCACTTTTTCCGTTTTCACAA
CGAAGATTCATTGGAAAGACAACAACTCTTAAGTCTTCCATACCAAAATTCTAACGTGAT
ACCACATTGAGTGGTACGGTCTCTAACCAAAATCATCGAAACGAAAACTTCCCTGGCTCG
ACAAATACCCATTCAATATCTTACTACGAAGCTCGTGGTGGACAACGAGTTCTCCAAAAC
AAAATCTCTTTCATGGTCTCCTTTGTGCAACGATTCGTATTTGACAACGTTAAATTGGAA
ATGCCATCAGGTAAAGCAACGAAAATCAAGACACGATGACAGAAACAACAAAGACTACAT
TGTTGGCGGGACAACAAACAAAACTGACCAAAAATGGAGAACAAGTCCTCAGAGTTCTCG
AAAGTCGAAGCAAAGGAGATTTGCCCATGTTTGTTTGCGAGGAAATTCAAAACTCTATCG
TCGCACTGATGATAGCAAATGGGTTCCTCGATAAATGGAAGACTGCGTTCATGAGTGTTA
GTACAAGAACCAACTCCGCAATCCAAGACGAGGACGTGTTATCATTGCTTTCTTCGATTC
TCGAAGATGAACCAGAGTGTCGCGACAGCGAGATTGCGGCAACAATGACCCGGCTACTCA

TATCTAGAGAGACTGTGATCCAGGAAGGCATCAACGACATTGCTGAGTCTTTCCTCGCAA
ATCTAGAGAACACCGTTCACCAACTACTCTGCGACGAATTCAATCCAGAGCGAGCCGATC
GTGGTTTCTACTTAGATTGGTTCGATGAAGCAAAAATCACGGCAGCGATTCGTCTAGTTC
CTAGCGTTCTATCAACCAAGTGGAACGGAGCATATCCCATTCATTGGCAAACATGGAAAC
TGCAAGAAACCTCCGACGAGGATGCTACCTCTTATTCGTTGAAGTCCTTGTCCCTAGTTC
CAATGCTGGCAAAACTCGGGGCAGAACTCGGCTGTCGGTTTCGAGACGAAATAGAAGAGA
ACAACAATGATAGTTACAATAATGACAGTTATTTGGCTATGAGAGGGGGCCTTCTGTCAA
AAGATCTTGATGGGCTTAATGTATTGCAGAATCTGTGTCTCTATCCATGGGTTTGCTTCT
CCCACGACGAAGAACACCAAAAGTTGTGCGACGAGACCACTTCGAGAACACTCCAAACAC
TCAAAGAACAACAGCTATTGACAAAAGAAGACATTCGAGACCACGATCTTGTTTGGAACC
TCTTTTGCTTGTCGACAACAAGAGGGGAGACGGATCGGCGTAACCATTCATATTTTCCGG
AACAACGCTTCCGATTCTTTTCCGACTGGGATCCCACGACGCTAGCAACGCCGATTGGCG
GATCGGTTCCTCTATTCGGTGCGTGCACAAAAAGTTATGACGCGACCAAGCCCCGCCTGC
CAATACATTGGTCTATGGGATTTTCGAACGAAAGCATGCGTGCGTTTTCGATAGTCTTGA
AGGCCGGGATGAAGCACTATCCAGAAAAAATCGGGTTCGTCTTTGCGAACGAAGACGGTC
CATACAGTTGCGGCTACAGAAATAGTGACAACGGCACCAAAATGAACAGACGGCGTGCGA
AGCGAAGAAAACGACACAATGTTCCCTACTTCGAAGCTTGTGAAACATACGGTGCCCATC
GAGTCAAGACCGAGGTCCTGGATCGATTAACAACAACGACAGCAGCTACCGACACAAATC
GCTCCAACGACAAACAGCTAAGTCCCGAAGGATTTCTCCTGTCGGCAATGACTGATGAAA
CCATGGATCGTTCGGGATTGCATCTGCTCTTACAGCACTTCTCAGGGGCGTTGTCCCAGA
TATTGACGAAAAACGAACGCATTTCCAAGCGAGCGGCACTAGAAACGATCTCGAATACCA
AGGTGGGCCAGGTGCAATCGAAGTGAATGACAGTTTCTTTCTTGAATCATAAATATCATT
ATCTGTACAAAGATTTTCTTGTACTTCTTCAAACCTAGTGCAACGATTCGACCGCTACAG
CGTTCTGAGTTTCTTTATTTCTGTATCAATAATAGTAGCATGCAAAAGTACTAATCA
>comp2012_c0_seq1
TTCGTTCGATCAGTACTGGTCCTCATACTGTTACATGTACCGGTTTACAGCCAACGCAGT
CCACTATCATTACACGATCGATCGATACTACGGCAATACTCAATACTTGATACTGCATGG
TACCGGTGACCCACACCAGTACTCTTATTTCGATCACTCAGCAACCTCAAGAAGTACAAG
ACTGTTGAAAACTCGAAGAACTTCTGAATAAACACGCAGGACTATTAAATAAGTAACAAC
CATGGATGAAGAAGTCGTTTGCCCCCTGTGTTGTGAAGAGTTGGACATCTCGGACAGACA
ATTTTTCCCCTGTAAATGTGGTTATCAAGTATGTATGTGGTGCTGGCACCGAATTCGAGA
ATCGGAGTCCGGATTGTGTCCAGCGTGCCGTACACCGTATGGGGATGACCCTCACGAATT
TAGTGCTGTCGATGTAGAGGATGTCTTAAAAGCAAATAAGGAGAAGGAAGCAGCAGCGAA
AAAAGAAAGAGATCGCCAGAGGCATCAGTCCAACGAGGGATATATCTCGGAAGTGGAGGG
ACTTGCAGGCGGGAGGCAAGTTGGTCAAATGGAGATCCCTAAGGATCGAACTCAATTAGC
AAATATGAGGGTTATCCGTAGAAATCTGGTGTATGCGGTAGGTCTTCCACCGAATATTTC
TACAGAAGAACAACTCCGGAAACCCGAGTATTTCGGACAGTACGGAAAAATTGCGAAAAT
TGTTATCAACCGAAGCCAAACGACTTCAGGAGGAGACGCAAGACGAGCGAGTGCTTCTGC
CTACGTCACATTGGTGCACAAGGAGGATACGCTGGCTTGTATTTTGGCGTTAGATGGGTT
CTATATGGATGGCCGGAATGTACGATCGTCTTATGGGACCTCAAAATACTGCTCTGCATT
TATAAAGAACGTTCGCTGTAACAATCCAGAATGTACCTATCTTCATAATATGGGACTAAT
AGAAGACACGTTTACAAAACAGGAAATTCAAGCGGGATATGTGACCAGTGGAAGAGATGT
CTTGGCACGCCAGCAACAAATTGTTCAACAGGCATTGAGTGCCGCTAGCGGTGCCGCGGG
TACCACTCCTTCATATGACGAAGCTGTAAAAAATCCCACGACTTCCCTCGTTCCGACGCC
TCCAACAATAACACATGTGCGGTCTGCAGCGGCTGCTGCTGCATCTGTACAGACGATTGG
TTCTGGATTCCCTTCAATTTCTGCTGCCGGAGCCGGTTCATCGTCTTCGGCTTCTTCGAA
CCATATGAATGCGGCAAGCAGAGTAGCGTCTTCCTCTGTAATTTCTGCGTCGAAGAATAT
TGGACCTGCAGGCGCACTTTCCAGTGCATCAAAAAGCGCCGTCGCTTCCTTGCCGATGTC
CCAGACCGCTCGCAAAAATATGCCGGGTTCTATCAATGGCACCAACAGTGGATCTATGTC
TTTAGCACCAGCTGGTGCAACCGCAGCATCTGTCGTTGCCGGTGTTCATAGCGTCTCTAG
TCACTCAGAACCTCCGGCACCTCATACTACGCTCACACCGTTGACACCGTTGAAACGAAG
CACCACTGCATCGAAGGGTGCACCAAAGACAAATAGTGCATCCGATGTTCAAAAATATTC
AAATTCTCGAACAAGTACAAAGAAAACTAGTGGGCTCAAATCGGCATCGTCACAACCTGC
TGCTTCGAATCCCGAAGTAGGTATATCGTCGATCAGAAACAATGTCCCAGGTCAACTAGA
GGGTATCGGAGGGGATGTAATTTCTGCTCCGGTCTCGGTTCCCCCTCCTATCAACAGAGA
ACTAGCATCAACCAACGGTATTCCTCGTGGCCCGCAATTGAAGTTTGGTGGAGATGGACT
CGGTGGTCTTGGCGGAGAAGTATTTGATGGACCATTGTTGTCCTCTTCGAATACTAGGTC
AGCGATCGGTTCGGGCAAGGGCAAGTGGGACTCGCTCAACGATGCTGGTTTCCCAGGGAA
CACTAAAGCTCAATCATGGGGCAATGGTTCTCAGAATGCTCTGCCTGGTGCGAATAGAGA
CTCGATTGGAGGCGGAGTCATAGGAGGCGGGCCGATTGGTAACTTTCACGGATCGGGTAG

CTCTGCTTTAGCATCGATGCTGGGCATCAACTTGCCCACAGGTTCTGGATCCCTTCGCGA
GACATCTTCACAGCTTTGGTCATCGGAACCTGGGCGACAATCTTCTATGTCTTCATTTAA
TAATGATTCCTTGCCTTTGCAAGGTGTGATCGGAGGAGCAACGAAGGCAAGCAACAACCC
TATCGGCGGAGTCCCAATCGGCGGAATGAATAGTCAGATCCCAGCGAATCCAAATGTAAG
CAACAGCAGCGACATTGCTCTTCTTCAGAGTTTGTTGCCTGGGGTACATATTACGAGTGG
CGGCAATTATCAAGGGAGTGGCTTTGGATCCATCGGGAGTAGCAATAACAACCCTGGACG
GGGTCCTTCTCATCACAAAAACGGTGGTAGTGCATCGTTGGCATCTGGAGATACTCACCA
GGGCTTCGGTCTCCAACAATCTTTGGGTGGTCAACCAATAGGAACTATTGGGCAAAACCA
AGGCCATGGTAAACAGAGACAAGCACCTGGTAGTATCTGGTAGAAAAGCTGCATGTGATA
GGAAACAATTATCAGTGGATGAAACTGTCTCGATGCATATTCATACTCTCAACAAGAGAA
GTGGAACCAAAAAACTGAAATCAGAATGGAATAGGATGTGTGAAATGGAATGGATTGATA
TTTGAAAAAATACACGGCAATGGATTTTGATCGGAAAAATGTGCAATGGAAAAAAGTGGA
ATGGATTGGTAATGGAATAGTATGATTCTAGATTTCAGTTTATGGCGAAAAAA
>comp2012_c0_seq2
TTCGTTCGATCAGTACTGGTCCTCATACTGTTACATGTACCGGTTTACAGCCAACGCAGT
CCACTATCATTACACGATCGATCGATACTACGGCAATACTCAATACTTGATACTGCATGG
TACCGGTGACCCACACCAGTACTCTTATTTCGATCACTCAGCAACCTCAAGAAGTACAAG
ACTGTTGAAAACTCGAAGAACTTCTGAATAAACACGCAGGACTATTAAATAAGTAACAAC
CATGGATGAAGAAGTCGTTTGCCCCCTGTGTTGTGAAGAGTTGGACATCTCGGACAGACA
ATTTTTCCCCTGTAAATGTGGTTATCAAGTATGTATGTGGTGCTGGCACCGAATTCGAGA
ATCGGAGTCCGGATTGTGTCCAGCGTGCCGTACACCGTATGGGGATGACCCTCACGAATT
TAGTGCTGTCGATGTAGAGGATGTCTTAAAAGCAAATAAGGAGAAGGAAGCAGCAGCGAA
AAAAGAAAGAGATCGCCAGAGGCATCAGTCCAACGAGGGATATATCTCGGAAGTGGAGGG
ACTTGCAGGCGGGAGGCAAGTTGGTCAAATGGAGATCCCTAAGGATCGAACTCAATTAGC
AAATATGAGGGTTATCCGTAGAAATCTGGTGTATGCGGTAGGTCTTCCACCGAATATTTC
TACAGAAGAACAACTCCGGAAACCCGAGTATTTCGGACAGTACGGAAAAATTGCGAAAAT
TGTTATCAACCGAAGCCAAACGACTTCAGGAGGAGACGCAAGACGAGCGAGTGCTTCTGC
CTACGTCACATTGGTGCACAAGGAGGATACGCTGGCTTGTATTTTGGCGTTAGATGGGTT
CTATATGGATGGCCGGAATGTACGATCGTCTTATGGGACCTCAAAATACTGCTCTGCATT
TATAAAGAACGTTCGCTGTAACAATCCAGAATGTACCTATCTTCATAATATGGGACTAAT
AGAAGACACGTTTACAAAACAGGAAATTCAAGCGGGATATGTGACCAGTGGAAGAGATGT
CTTGGCACGCCAGCAACAAATTGTTCAACAGGCATTGAGTGCCGCTAGCGGTGCCGCGGG
TACCACTCCACGCAGAAGAACAGGAGGAGGAGGACCATCCGGAACAGGAAAAGCATCTTC
AAATCCTATCTTTCCTCCTCCTTCATATGACGAAGCTGTAAAAAATCCCACGACTTCCCT
CGTTCCGACGCCTCCAACAATAACACATGTGCGGTCTGCAGCGGCTGCTGCTGCATCTGT
ACAGACGATTGGTTCTGGATTCCCTTCAATTTCTGCTGCCGGAGCCGGTTCATCGTCTTC
GGCTTCTTCGAACCATATGAATGCGGCAAGCAGAGTAGCGTCTTCCTCTGTAATTTCTGC
GTCGAAGAATATTGGACCTGCAGGCGCACTTTCCAGTGCATCAAAAAGCGCCGTCGCTTC
CTTGCCGATGTCCCAGACCGCTCGCAAAAATATGCCGGGTTCTATCAATGGCACCAACAG
TGGATCTATGTCTTTAGCACCAGCTGGTGCAACCGCAGCATCTGTCGTTGCCGGTGTTCA
TAGCGTCTCTAGTCACTCAGAACCTCCGGCACCTCATACTACGCTCACACCGTTGACACC
GTTGAAACGAAGCACCACTGCATCGAAGGGTGCACCAAAGACAAATAGTGCATCCGATGT
TCAAAAATATTCAAATTCTCGAACAAGTACAAAGAAAACTAGTGGGCTCAAATCGGCATC
GTCACAACCTGCTGCTTCGAATCCCGAAGTAGGTATATCGTCGATCAGAAACAATGTCCC
AGGTCAACTAGAGGGTATCGGAGGGGATGTAATTTCTGCTCCGGTCTCGGTTCCCCCTCC
TATCAACAGAGAACTAGCATCAACCAACGGTATTCCTCGTGGCCCGCAATTGAAGTTTGG
TGGAGATGGACTCGGTGGTCTTGGCGGAGAAGTATTTGATGGACCATTGTTGTCCTCTTC
GAATACTAGGTCAGCGATCGGTTCGGGCAAGGGCAAGTGGGACTCGCTCAACGATGCTGG
TTTCCCAGGGAACACTAAAGCTCAATCATGGGGCAATGGTTCTCAGAATGCTCTGCCTGG
TGCGAATAGAGACTCGATTGGAGGCGGAGTCATAGGAGGCGGGCCGATTGGTAACTTTCA
CGGATCGGGTAGCTCTGCTTTAGCATCGATGCTGGGCATCAACTTGCCCACAGGTTCTGG
ATCCCTTCGCGAGACATCTTCACAGCTTTGGTCATCGGAACCTGGGCGACAATCTTCTAT
GTCTTCATTTAATAATGATTCCTTGCCTTTGCAAGGTGTGATCGGAGGAGCAACGAAGGC
AAGCAACAACCCTATCGGCGGAGTCCCAATCGGCGGAATGAATAGTCAGATCCCAGCGAA
TCCAAATGTAAGCAACAGCAGCGACATTGCTCTTCTTCAGAGTTTGTTGCCTGGGGTACA
TATTACGAGTGGCGGCAATTATCAAGGGAGTGGCTTTGGATCCATCGGGAGTAGCAATAA
CAACCCTGGACGGGGTCCTTCTCATCACAAAAACGGTGGTAGTGCATCGTTGGCATCTGG
AGATACTCACCAGGGCTTCGGTCTCCAACAATCTTTGGGTGGTCAACCAATAGGAACTAT
TGGGCAAAACCAAGGCCATGGTAAACAGAGACAAGCACCTGGTAGTATCTGGTAGAAAAG
CTGCATGTGATAGGAAACAATTATCAGTGGATGAAACTGTCTCGATGCATATTCATACTC

TCAACAAGAGAAGTGGAACCAAAAAACTGAAATCAGAATGGAATAGGATGTGTGAAATGG
AATGGATTGATATTTGAAAAAATACACGGCAATGGATTTTGATCGGAAAAATGTGCAATG
GAAAAAAGTGGAATGGATTGGTAATGGAATAGTATGATTCTAGATTTCAGTTTATGGCGA
AAAAA
>comp2013_c0_seq1
CCGCTATCTTTCTCGCGCAATTATTTTTAATACACAGGGAAAAAGATAACAACGTAATGC
GACAAAAAATCTTTTCCACGATGTCTACATCAACCAAAACCAAAACGAATACATTTCCAT
GGGTACTCCATGAAATGCTCGAGACGGCACACGAAAAAGGTTTCGATGGAATCGTTTCTT
GGCTGAAGGATGGAAAAAGTTTCAAAGTGCACGACCCTGCCTCATTTGTTGAAAAAATAA
TGCGAAAGTTCTTCCAGCAATCCAAGTACAAGAGCTTCCAACGGCAATTGAACCTATGGG
GCTTTCAACGGATAGCATATGGTCCAAACAAAGGGGGATATTATCATCCAGAGGGCTTCT
TTATTCGGGAAAAACGCTGCAAGATCCACATGATGCAACGACAGAGGATCAAAGGCTGTG
TGTTGAAAGACAAATCCAACAAGAACGCCAAAATAGATGATGATCATGTAGAGAAAGGAA
ACATCACTGAAATCGATAACACAGTTATAGAGGCATCAGGTGCTGCCTCTATCATAAGTC
AAGGGAGTCTCGTTGACATTAATAATGATGCATTAAATGTTGGCAGAGACTCTGCTGAGC
CTTTTTTACCACCTTTAAAATCACTTCAAAGTTCGATCTCTGATGAAGAGTCGTCGAGTC
TACTACAACTCGAACTGAAAGACGAATTCAGTATCGACCCTGAAGTAGGTGATTGCATCG
GCTTCGCAGGACGATCGTTCTTTTTCACAGAGGAGGAAGCAGTTGAAAACACACCATGTA
CGGTATCAAGCAGCATGACTCTTGCAAGAAGGCTTCACCCGCAACAACGGGTTGCTCTAC
CACCTGTTTCAGTTCTGCTCCCAAATACGTATCTATGTACTGCAAACATGATGTCACAGA
TTTCTCGACGTCGTGTTTCTATAGGGATATTTGGCGCCGCTTGCAAGCCAACGACTGGTT
GCTGAACAAATCAATCTATGACATTCACACGCTGGAGATACGAAGGCCGCCATAACATGA
TTTTTTACTTTGGTCCAAGTACACTGAATGATTGAGCACTTCAATTATCTAAGACCTCAT
TTTTTAGTACCATCTTTCGGGGTGAAAACACTACTTGTACAAAGACAACCCAAATTACGC
GTCCATGACTAGGTTGTGCTGCAGCGTTTATTAATGTACGAAACAAATGAATTAGTTCCA
TGCTATGCAACTTGGCTACTTTACTTCTACGAGACAAGTAAGATAGTGAATCACTTCTGC
TTTCTTTGATATCATCTTCTGGCCAACCGAGAAAGATTATTTCTTAATTGTCTACTTTAA
CGAATGGTAGAAGAAACATTGCTTGATTTCTTTTTGATCCGCAGAGTTTCCATCCCATAT
TATTCTGAACAAAGTTATAAAAAATAATACGAATC
>comp2013_c0_seq2
CCGCTATCTTTCTCGCGCAATTATTTTTAATACACAGGGAAAAAGATAACAACGTAATGC
GACAAAAAATCTTTTCCACGATGTCTACATCAACCAAAACCAAAACGAATACATTTCCAT
GGGTACTCCATGAAATGCTCGAGACGGCACACGAAAAAGGTTTCGATGGAATCGTTTCTT
GGCTGAAGGATGGAAAAAGTTTCAAAGTGCACGACCCTGCCTCATTTGTTGAAAAAATAA
TGCGAAAGTTCTTCCAGCAATCCAAGTACAAGAGCTTCCAACGGCAATGTAAGACCTCCT
CTAGGATAGTTCTATTGCTATTTGTTTCTGCTGCCCTAACAAACCGGTGTTTGTACTGCA
TATTTCTGCCTGGCTTTCGTTTCATTCTCAGTGAACCTATGGGGCTTTCAACGGATAGCA
TATGGTCCAAACAAAGGGGGATATTATCATCCAGAGGGCTTCTTTATTCGGGAAAAACGC
TGCAAGATCCACATGATGCAACGACAGAGGATCAAAGGCTGTGTGTTGAAAGACAAATCC
AACAAGAACGCCAAAATAGATGATGATCATGTAGAGAAAGGAAACATCACTGAAATCGAT
AACACAGTTATAGAGGCATCAGGTGCTGCCTCTATCATAAGTCAAGGGAGTCTCGTTGAC
ATTAATAATGATGCATTAAATGTTGGCAGAGACTCTGCTGAGCCTTTTTTACCACCTTTA
AAATCACTTCAAAGTTCGATCTCTGATGAAGAGTCGTCGAGTCTACTACAACTCGAACTG
AAAGACGAATTCAGTATCGACCCTGAAGTAGGTGATTGCATCGGCTTCGCAGGACGATCG
TTCTTTTTCACAGAGGAGGAAGCAGTTGAAAACACACCATGTACGGTATCAAGCAGCATG
ACTCTTGCAAGAAGGCTTCACCCGCAACAACGGGTTGCTCTACCACCTGTTTCAGTTCTG
CTCCCAAATACGTATCTATGTACTGCAAACATGATGTCACAGATTTCTCGACGTCGTGTT
TCTATAGGGATATTTGGCGCCGCTTGCAAGCCAACGACTGGTTGCTGAACAAATCAATCT
ATGACATTCACACGCTGGAGATACGAAGGCCGCCATAACATGATTTTTTACTTTGGTCCA
AGTACACTGAATGATTGAGCACTTCAATTATCTAAGACCTCATTTTTTAGTACCATCTTT
CGGGGTGAAAACACTACTTGTACAAAGACAACCCAAATTACGCGTCCATGACTAGGTTGT
GCTGCAGCGTTTATTAATGTACGAAACAAATGAATTAGTTCCATGCTATGCAACTTGGCT
ACTTTACTTCTACGAGACAAGTAAGATAGTGAATCACTTCTGCTTTCTTTGATATCATCT
TCTGGCCAACCGAGAAAGATTATTTCTTAATTGTCTACTTTAACGAATGGTAGAAGAAAC
ATTGCTTGATTTCTTTTTGATCCGCAGAGTTTCCATCCCATATTATTCTGAACAAAGTTA
TAAAAAATAATACGAATC
>comp2014_c0_seq1
TCCGATCTCAAAAAAATTCTTTTTGTGTACCTTAGGGAACTTGAGTATCGCATCGAGCTG
CAACGCCCACCGTATTGCAGTAAAGCTCTTATCATCCTCACAACTCCTCAGCAGAAGCCC
ACATTGCTAGTTTGCAAACACGTCTCCGTGAAATCTATATTTTGCGATTCCACTATCGTA

TTCAAAGCATCAAAACATACAAACAGAAAACATGGGATTGTTCGGAAAGAAAAAGAGCAA
GGCCTCGAAGGAACAGGCTTCTTCGTATTCTCCTCCGGATGTCGTCTCTTCCTCGACGGA
GGTAGAAGCCGTTGTGCCAGGTGAAGATGGTGTCATGGTTTCCGGTAATGGCAGCGTTAC
TAGTCTTGGAAGCGACGACAGCAAGAAAGAATACCTTATTGATGGCGAAGAAGAAATAGA
ATCAAACACAACGGATCAAATCATGGAATTCGTGAACAAGACCAAGGCCACCGCCATCTC
CTACAAGGACAAGGGAATGGAACAGATCACTGTTATTTCAGCCACTGTTCTTGCCTGGTG
GTTCAGTCTTTCCAAAGTTACACAGGTTGCGCTGCTAGCAGGAGCTGCTATTGGAGCAGT
AACCATCGTCGTCGCGGCGGCAGCTGGGGGACGATCCCCGGCGTTCCCATCACACATCAA
TGTTGCCTTTGCTGGAAACTCCTACTTTTACGCTAACGATCTTCCTCGGTTCGTCGAAAG
CATCGGAAACGGACACGTAACGCAAGATTCCGTAGTGCATAACTCCGCCAGCATCCTGGA
GTTAGTCATGACTGGAAACGGTATGTGGAACAAGTGGTCTACGCAAAACGGTATGATCAA
TGGTGCGAAGTTTGATACCTACCGAGGCACCACCGAATACCTGTACGACATGGGTGCCTG
CTCCGTTCCTCAACTGTTGACTGGAAGCGACCAAATGATCACGCCGGGCAACCAACTCGG
AGCTTTTGTGAACGATGGCCAAAATCCATGCTTCCAGCAAGACGCATACCGTGAATACAA
GGATAGCGTCACACTCAAACACGGATGGGATTTTGTCGTCATTACAGATCAACCCAAAGC
TATGGCCTTTGACGAGAGTAGACACGACGCGCTCATGGCCTTTAACTACACCTACGGACC
TATTCTCAAGAAGAAGCATATTTCTCCTATCATTGTGCAACCCCATGCCTACTCGTCGTC
GGGTGTAAACTCTGCAAGTACCAATGATTTAGCGACTTTCACTGCGCTTATCATGGAAGG
TGCGCAAATCTACCGAAAGTACCTTAACAAGCGCATCGGTTTGTTTGCCAAAGCCCATGT
CGCTCCCGTTGGAAACGCCTATTTGGCCATCTACGAGGAAAGTCCCAATGACCTCTGGCC
GAAGCTCTTTTTGGACGATGGTGTTCACCCTTCTGCCTATGGAACTTTCGTTTATGGATG
TGTTATTTATGCCACCATGACGGGATACATGCCGAAGTACAAACGTGTTGTTGTTGACGA
CATGGAAAACTCGGCCCTGTTTGCCTCCGCACGACGTGTTCAGTCGGCCAACTCCGCGGC
TGGTTTCCCCACTAAGGACGAGGCTGCTACCCTGTACAAGATTGTCAAGAAGGTCGCCAT
CAGCGGATACAAGCCTAAGGCTCTCAGGGGGTTCAAGATCCAAGAAGACGCCGCCGATTT
CCTCGATTCTACGTCTAGCAACGTTTACGACGGTAGCTACGCCTCGGGAAATTCTTACGC
CTCAAACAACGTCTACAACGAGTATGACAGCTACAACCAATACAATGGTTACCAAAACAA
TGCTGTCAACAACAATGATTACAACGTAAACAACCAATACGATAACTACCAATACGATAA
CTACCAAGAAAACAATGGCTACAACAATAACGCCAACGGTAACTACCAAGAAAACAACGG
CTATGATAACAATGAGAACTACAACGGCGATTACAACAATGGCAATAACTACCAAGTCAA
CAATAACTATGAAGACTATCAAGAGAATGCCAACGGTAACTACAACAACGCCAACCAACA
GAACATGGACGCCGATGCTGATGCCAATGAGAATAACAACAACGGCCAATATGAAGCTGC
TCAGCAATACTACAACGCCAGAAGTTATCAAAATCATAACAGTAATAACGTCAACGGCCA
GTACGGGGGAGGCAACCAGAACAAGCAGTTCTACTACAACGACCTGAAGGATAATGACGG
CAACTAAAAGGTGAAAAACCACACGTGACCAGACGATTGATTCGACATTTTTGATCGATG
ACAACCTGCAGTTTTGTGTAGTAATAATAATTGTGTTTGACAAAGGAACGTGCAGTGGCT
CGCTTTGGTCTCGAACGATAGAAAGAATCAATAATTTTCAATGAAATAATATATGTGTTT
TACATTA
>comp2015_c0_seq1
CAGCAGCAACCACATGTCTTGCACAAAATAATAATCATACAAGAGGAAAGACTCAGACGC
TGCAACGTGAATCATCCTGCTACACAAGTCTCATAGTCTCCATCAACAAAACTACTGCAT
TGACTGACCCTTGATATTTTTTTTTTGAAGCAAGGGCGAGAACAAGACCTGTTGCAACAG
GATTCAAATCTCAACTTCAATACACAACTATATTCGATCGTAATTTTAGGAAAAGACATT
CGCAGCGCAATAATATAATATAGCAGTGGCCCGTGCTGGAAACCATGGCGATTTCTACTG
AACTAAAAAGCGACGTCAAAATCACGGGAAAGGCTCCTTACAAGAAACGGTTGGAGGGGA
TCAATGCAAGCCCTGCATTATACGATTTATGGAAAAGTGCATATTCAACACACGTAACAA
CGAAAAAGGATTCCTCTGTTGAAGATTCGTTCCGAGCGTCTGCCACTCCCAGCACGAAAA
CCGAAGCAATCGATGTTCTTAGAAATGCCGGTGACCCTAGCAAGATCAAACGAAAGAAAA
ACAAGCCGGCAGTCGTGCCCATTCGGGTTCTTCAATCCATCAGATTGCTGTTGCCTCTGG
CGGGTGAAGAATCTTCGTCGCTGGACAGCGCCCTTTCGTCGTCCGCATCGCTACAACTGG
CTTTCACGCCCCCTCCCCCTGCACCAGAGCTGACCGAGGAGCGCAAGAAATACCTCGCAA
GGATGGAGAAACTCCGTCTGAAGGCGGAGGAGACCAAATACACCCGGATCACCACCAACA
TCAAAGACCAGCGCCAGGACGACGACAAAACGGCCAAATCCATGACCTACGCAGCGTCCG
TCGGGATCAATATGATTGTGGCACCCATATCCTTTGGAGCATTTATCTACTTCTTCTCTG
GTGGTGTCTTCGATTACTTGTTCCCCAGGGAAGAAGGCGACGACGAGTTCGATAAAAACC
CTACAGGAGTCGACATCAAGCGGGTCATCTTGGCCGTTATTTCGGGAGTGGGCATGATGA
TTATCGAAATGGTCCTCTTTGTGATAAGATCCCACGAGTTCGAAACGCATACAACCAAAA
AGAAGAAGAAGCGGGGAGTCCAGCCGTTTGGGTCCTATTCCGCAAACAGCGCCATGACCT
ACACGGACGGCAACCTCGATCGAAAAATTAACAAGACAATTGATCGAGAGGATCCCGCAG
TGAAGAAAAACAAGTGATGACCATCGATTGTTTGCAGTATGCGACGTGTCGAAAGAGAGC

TCGAAAGTCTATCGTAAACTAACGATATTAGTCACGACTAGTTAGATAAGGTGATGAAAA
AATATTATTAGAATAAAATTCTAATTGAATCCAATTTTTACG
>comp2016_c0_seq1
GTAAAATCACCATGTCGTTACTCGTAAGTTTTGAAGTTGTCAGATAGGATTCAGATACCG
GGAGTAACAGATATGGTCGAGCATGTCTTGGAATGGGAGCTTGCCACCAATGCTTTATTT
CAAAACGACTAGAGTTTTGTGTTGTTGACACGACGATGGAGGGTCCGATGTTGTGTTGAA
TGCAGTGCAATGGAGTGGCGGTAGTCACGAACCGAACGGCCGCCTTATTGGATAGGGCGC
GAAGTCGGATCCACGAAACCATCTTCTCACCGCTTTCATTTTCTTCACATGCGTTCAATT
CACTTGACATACGTACTGTTATCAAATAATACGCTTCGAAAACACAATTGATATAGACTG
GGGCATCGGGCCTCATTTTT
>comp2017_c0_seq1
GTCGGCTTTCCATTACCATCTTACTCTGTTTGATCCTCTTTGTCATCAAACCCGTTATCG
GCTACCATTCCCACGGCATGAAATTTCACTGATTCAAAGCATCGTCACAAATTAGAAACC
TGCAACCAACGAGGTAATACGGGAACACAACCATTGTCTCGTTGTAGAACGCAAGATAAC
TTCGATCTGTTTCAACTTATTGCAGGATTATAATCGTGCCTATTGGAAAACTTTCCGTCA
GATTTATTATCCAACAAGATGTCAGGAAAAGGAGCCTATCGTTCAATGGGAACAGGAAAC
GATGGAGGAAGGGTGACCAAAAAAGTTGGATTGCGATTGCGACTGATGGGTCTTTTTCGG
AAGAAAGGTGGCGTCAGCTCCGACGAGAAGAAGCTTATTCACCACGAAAGTGCATCAATA
GACGACAGTACAGCATGCGAGTCTCCAAGTAAAGAGAGCGATGATTCGTGTAGTGAACAG
TTCGATGTATATTTTGCATCATCTTTCCTTCCACACGTTGACACTAATGGGAAGAAAAAA
GCTCCCATTGGCACGAAGAAGAAATCAGTGCACTGGAAAGCGTTCGAAGATGCCAAGGAA
ATGGCAATCCCCCCATCTCCCCCCAAGTTAGTACGCACTCGCAAACAACCGCTGCCGAAT
GGCTATGCGACGAACAAAGCACACAAGACCGAAAGTACGCCAACAAGTCCAAGAAACTCT
TCCCAAAATGAGGTGCAAGTTCCATGCGTTCCGTTGGAAAATGATTACAACAATTTGACA
ACCGATGTCCAAGCACCACCCGAAGAAATCGTAGCTGAGAAAGCGAACGAAAGCCCTTCA
AACGCTCTGAACACTACCGCAGAGCTTCTCTCTTTCGATGATGATCTGATAGATACAGTG
GATAAATCCCAGGCTGACTTCAACAAGATGCTCGATAGGATGTCTTCAGAATCCAAATCC
ATCCGGATGATGAATCCTTTTTCGGATGAAACTGTGCTCATGTCGTTGCCCGAAGCAGGA
ACCGCTGAGAGCGAAAGCATGATCTCGAAACCTATGGGCGGGATTGTAAAATACTTTTCT
AAACTGAAGGCAGCTGTAGGCGAAACATCCAATCCATCGCATGATGGCGAAGGGGACACC
TGCACATATGGAACGACCGCTACCAGTGTATACCCAGAACTCCTCAAGACCAAAATGGAT
TCCATTGCAAAAGCATTTTGTTCAGTGGAGTTAGAGTCAACAGCGAAAAATTCTGAACTA
CCGACGAATCATCTGTGTACGTTCATAGAATGTACTGATCCAACAAACGATATCCCAATG
GGCACTCTCCCCGAGCATTTGCACGCCCCCTTGAACTTGAATTCCCCTCTGAACCGTTCG
TGGTTTTCTCTTAACGAAGATTCTGATTCCATCAGCAAGCTTTCAATGCCAATCGGAACG
AATGACATGGATTTGAGCGTCACTTCAGAGATGTCTCCAACTCCCCTCATGGAGAAAGAA
CCAATTGCCGGATTTTCACATGGTGAAACGCACCTGTAATTATGATCCTTGTTTCACCTT
TCTCCTCTCACTTAGAATCTGATAGAAATCCTATGGATTGGGCCAACAAAATAAATACTA
GTAGATGCAC
>comp2018_c0_seq1
CGTTGTCATAGACTCCAGAAAGGACGTATCGAATATTCAAGGCTCAAGCCCAACTGCTCA
CTGTATTGAATTGAACTGCTCCAAGAACGGTTAAGGTTTTGAAAGTATTCAAGCGATAAG
TGATTTGCAAAGAAACGGCACTATATCACAATGACAGTCACTTGTAACAGTTTCGCCTGC
CTCTTTCTCTTGTGTTTGGTGGCAACAGCGAAAGTGTCTTCGTCTTCGCAAAGAGCAACC
GATGCGGACAGCCACGATACGATGGACGTTCCTACAGGAGCTTTGCACAATGGAAAAAAA
ATACCATTGGTGGGTTTGGGCTGTGCTAGTGGAGTACGCCGAGAGCATGTCTCGAGGGCC
CTATCGATAGGTTACCGGTTTCTAGACACGGCTCAGAGCTACAATTGGGGTTACCACGAA
GACGAAGTCGGTACGGCAGCCAAGAAATTTATGGATGATAACATTGACGAAGAAATATTC
ATACAAACGAAGGTCCACCCGGAAGATCTTGGATACGAAGCAACGAAGAGAGCAGTGTAT
CAATCATTAGAAAGACTCCAGGTGCGAACAATTGATTCTGTCTTGATTCACAAACCACAC
TGCTGGACAGGTGCATGCACGAAAGAACCAGAAGGAACTTGGCAAGAATCATGGAAGGCT
CTTGAAGAGTTATACGATGATGGAATCATCGCTCATTCGATTGGAATCTGCGATGTTTCC
GATGACTTGCTGGATGAGCTTCTCTCCCAAAGAATCAAGCCTCACATTATACAGAACTGG
ATGGACCCCTTTCACCAAGACAAACGCATGAGAGACAGAATACAGAGCCATGGCATATTG
TACCAAGCGTATTCGAGCCTGGGGACTCAATGGCACCACCACAAGGGGCATTCAAAAAAT
CCTGTCATGAACCACCCCGTGCTCCAAGAAATTGCTGAGGCCCACGGTGATGTTGGCGCT
GATGTTGGTCAGGTCGTAGTGAATTGGGCAACTACCCGCCACGGGATCTCCGTTTTGCCA
GCGTCCACCAATCCGGGTCGCCAAGAGGGTAATCTCCGCAACAGTTTCCGGTTTTCGTTG
ACCGACGAAGAGCTCGATCGAATCGACGCACTCGACGGCAGAGTCCCGAAGGCCAAATCA
AGCAACGAAGTGTCGATTACGTTCACGAGCGACGGCGACACCGCCTCTGGCACGGTCGAC

GTCTATTGGATCGGCAACGATGGAATCGAGGAAGTGCCCGTCGGATCCGTGGAATCTGGA
AGATCCTTGTTTCTCAATTCATACCATGGTCACGTGTTCCGATTCAAAGACAAAGAAAAC
AATGTATACAGCGATCACACAATCGAGCGACAAGCCGGGATTCAGCAAGTCCACGTGATT
ACTCCGATCGACGACGAAGAGCTTTGATGAAGTTGGATTGGGCATGCAGCTTTAAATGCA
TTGTTCAGTCGACACGCAAATGTAGCACTATGCATTTTTATCAATAACATAAGAGATATG
TAGTTTTTCAGTGATTCAATCATCAAA
>comp2019_c0_seq1
AAAGAAACCCACTGACTCGATTCTTGGCGAAAATTTTCCACAGGAAGTACGAAAACGCCT
AAGGATTGTTCGGTCTGCCGCTTAGTATTCGTCTTTTTCTTCCTTTGTCGAGTCAAAATG
GCGACAGCGCCTCGGACCACGCAACAATATCCGATTGCGGGTCCAACCGCCGCGGGCGGA
ACAAATGAGGTCGCTACGGCTATTCCAGCCCTAAAAGTGATGCGGCTCCAAAAACCCGAA
CTCGATATGACAAAAGCTGGAACACTAAAACCTTCGGGCTGTTTGCTTGGTTCGAGTCTT
TGCCTACCGGACTCGTTTGGTATCATTCACGTGGGAGAGACGTTCACCGCATATCTCGGG
GCACTGAATGTTTCGAAAAGACTGCCTGTCACAAATCTAACGATAACGGCACAGTTGCAA
ACACCATCACAACGGTGGCACATGGCTTCGAAAACACTCGACGAATGCAACGCAGCGGGA
GGATGCGAACTTCTTCCTGGGCAAGGCATTGATTCTATCGTTTCCCATGCGCTGGAGGAA
GCCGGGCAGCACATTCTTCGTGTTGAAGTGGGATACGGAGGAAACGGAGGACCGATGAAG
TCGCTGCGAAAATTTTATCGGTTTCAGGTTTCGAATCCCGTCGTCATGTCGGAACTTACC
TACCGCACTGGCGATTCGTCGTGTTTTGTTTCAATTTCTCTCCAGAACAATGGCCACGAA
TCAAAGGGTGGGCTTACCGTATCGTCGGCAGAATTCGAGGCCGCTCCTGGTCTTGTTGTA
GAAAAAGCTATTGCTTGTGCGCCAAAAACTGACGACACAAATAAAATAGGTGCACCAACC
AATCATCTCGGCGAGGGATTAGATATGTTTGACAATTCTGGACGACTAGAGCCAGGAGAC
TCGAAGCGTTACATCTTTAAGGTCGGAGTATCGAAAGCAGACTCTGCTTCGGGTGCGTCC
GTCAAGGGAATAGCTGCGGGAGATCAGTTAGGGAAAGCCATTTTTACATGGCGTAAAGCT
TGTGGTGAAATGGGACGGATGGCAAGCTCACCTGTTGTATGTCCAATGTTACACCCAAAT
CTTGATCCAAATGATCCGGGAACGACAATGCAAGGGACTGGTACCGATTTCGTGGTCCAC
GTACAAGGGAGTAGTTTGTCCATTGATGTTGCGACTCATGCGGCAGGTAGAACGACAAAT
TCACCACGTGACGGAACGACACTCGACTTGCTATTGCCCCTCACAGTAGAACCCGTGAAT
CCATGCAAACAATTGCGTGTTGGGGTTCCTGTTGATTTTGCATTTTTGGTTGTAAATCAC
TCCCCAGAATTCGTAACCCCTCAGCTTCAATTTCGCTTGGAGCACATGAAAGGCATTGCG
GTCTGCGGTCCCAGCTTTAAGAACTTGGAAGAAATTCCCGGGAACGGAGGGAGTACCACG
GTGCTCGTGCGTTTTATTGCGTTGTCTACAGGGTTAGCACAGCTTACTGGGTGTTGTGTT
TTAAATTTAGCTACGGGGCATGTCATTGCTCAGCCACCCCTAATGAATATCATGGTCATA
GAAGAATAAACTTTATTAGCTTGGTCCATTACATAGCAAGGATCCGTTACCCGA
>comp2020_c0_seq1
ACGACACCTTGCGATGGCAATAATCGTCGCTCTTGAAAGAAATATCGAAAGGCAAGGGAC
AAACATAAACTGAAGACGATTGCGGCACCATGATTGCCACAAAACAGAAAAGGGGCCTCG
CCTCCCCGTTGAAAAAGGCAACAGCATGCATCCTATTCCTAGCTATTGCTGCTCAACCTT
CGTTCGCAACTCGGTTCGGAGTGTTGGAGAGTCTTCCGAGGACGCGCAAACAAGCCTTTC
TCTCGAAAAAGATAGCCGATGCCCCTTCTTGGAAGACCATTCCCCGCGGAGGTGATATCC
AGGAAGATGTTTCGTCGCCCACAGCTACGGATGACGACTGCGCGTTTGCCGTGGTGGCAT
CCGCCATCAAGGGTCTGGCATCGTTTATGAAGGGACCCAAGGCCGACACCATGCTGTTAT
TGTCCACCACGGCGCTGAATACGCCCCTATCAAAGATGATTGGCCTTTCTCCGATTCTTG
GATACCTAGCATTGGGAGTCTTGTTCGGTCCGAACGGATTGAGTCTCGTCAAGGATATTC
ACACCACGGAATTGTTGGCCGATATCGGAATCGTCTTTTTCCTCTTCGAAATGGGAATCC
ATCTCGACTTCAAGACGCTCATGAGCATGAAGCGTGATGTATTTGGGCTCGGTGGATGCC
AGTTCCTCGTAACGGGAGCTGTCGTTGCGGCCATCTGTAAGTTTTGTTTCGGGCTTTCCT
CCGCGGCACAGATTGTCTTGGGTGGAGGCTTGGCTCTGTCGAGTTCTGCCTTTGTCCTGC
AACTCCTAAAGGACAAGGACCAGATGGGTACCAAGTTCGGAAGGTCGTCCTTTGGTGTGT
TGCTCCTGCAAGACCTCGCCGTCGTCCCACTTTTGGTTGTCACCCCGATTCTGGCCGGAG
GCGGTGGTGGTTTGGCCGAAGCGTTTGGTGCTGCCGGAGTCAAGGCTGCCATCGCACTGG
GCACTATTGCCGTCGTTGGCAAGTTTTTCTTGAAGCCTTTCTTCGACAGCGTAGCCAAAA
CCAAGAACCAAGAAGCCTTTGTGGGTGCCATTCTAACGACTGTCTTGGGGATGTCCTTTC
TGACGGAGGGATTGGGTCTCTCCAATACTCTTGGAGCATTCCTTGCTGGTATGCTGTTGT
CCGAAACAGATTACCGCCATCAGATCGAACAAGAGATTGCTCCCTTCCGCGGAATCCTTG
TTGGTCTGTTCTTCTTCACGGTCGGCTTCGAAATTGATTTAGAATTGATTGGCTCTAATC
CGGGCAGAATAGTCTCCATCGTGCTTGGAATTGCTTCGATCAAGTGCGTGATAGCATCTG
TGCTGGCTTCGATGTTCGGATTGGACAAGGGAACCGCCCTTCGACTTGGCTTAATCTTGA
GCCAGGGCGGAGAGTTTGCTTTTGTTGCCTTCCGTCTCGCGCGAAGCTGTGGTATTCTTA
GTCCCGAATTGACCAAACTCATGCTAACCTGTGTGAGTCTAACGATGGGTCTCACCCCCT

TTCTGGATGACGTTGGGGCAAAGATGGCCATCGCTCTAGATGATGCCCCGTCGACTGGCA
AAGGAAAAGGAAAGAAAAATCGTTAATAAATAGATTCTTGAGGGCGAGAAAATGAAGTCC
AAACGGTGTTTTTCGGTACGCGAGATGTTCGCACACGTAGCAGTAAACCAATATATGTCT
ATGGTATTCAATTTCTTTGATTTGAATTTAGTATGATAGACTATACTACGGGTACACACA
TTTTAGAATAGTATTCTCGATTGAAAAAGTTGACAGTGTGAGACCGTTCGATTGGTGTGA
AATTTCAATTATCAGTTCCGATATTGTGTAGTGGTGAGTACATAAAAAAGCATTTGTTTA
TATAATTTAAATGCATTCTGACAAGACAATGATTGGAGTACAAATTGTTGAGTCAAAGTA
AGATACCGTTAGGAAC
>comp2021_c0_seq1
TTGGGCAAAAGAATTGTACGAAATAAATGTTCATGAGCGCGAAGTAATCAATGATGAACT
ACATGGTGTGTCGGTTTCGTTTCATCCCAATCCAGAACAAAATTGGACGGAAAAGCAATA
TGACTATTATATGCGTATGTTCCAGGCAGAAATCGACACCAACATCCCGACCGAGGACAA
AGAGGCCTATAAACTAGGATTAGATTTAGGGAGCTCATACATTGTGAGTCTTGAGTTTCG
ATTTCGGTTTATTCGGGCTGAACAATACGACATCCAAAATGCTGCAATACGGTATTGCAA
TTGCCTTTCTTTTTTGTTCGAGGTATTCGGGGAAAAATCACTCTTGAGACCTTTGCTAAT
AAACGACTTGACAAAAAACGAAATGAAATTTCTTAAGGAAGGTTACATACAAATTTTACC
TTCCCGAGATCGAATAGGGAGAAGAATTTTAGCTCAGTTGGGATCTTATGGCGGATCTAG
ATATACGGAATTCGAAAAGTTTCGAGTCAGTATCTACTTATGCTTCTCGGTACTATCGGA
AGATGTTACATCTCAACATCGTGGAGTAGTGTCACTGGGATCCTTCACGATTGAGGCGGA
GAGTTTTATGTGGAAAGAGTTACGGGTAATTTCAAAGCTCATCAAACGGTTCTTCGCAGC
AGTTCCTTTGCGTTGGTCGGCGACTCATCTCTGTATTCCTGACGACCCGGTGTACCATCT
GGTCAAATCCTGCACTCTTTTCTTTCTAGGGAAGAATGGAAGGAGAATACTGAGAATACA
TGTTGGCACACCTATGGAATGTGATTACAAACTTCGTTGCTTTGGAATTCCCACCGAAGA
CATTCCAAGAACTTGCACACATAAAATCAAATTAAAAAACCACAACAGGCTAATCGTTGT
ACGAAAAGCAATAGATCAATTTCGTGAGCAAAGCATGAGACCGAATAATGTTGTGAATAT
GTCTTTCCCAGGCGTCGAGTGCCCAGAAATCAATTGCGTTCTTTTTGGGAAATGCGCATA
CAGTTATCCGGGGAATATCGAATTCAGGGGCTTTCTTCGAGAAATACATACGAAACACAG
AAAAGTCGATTTAGAAAACCAAGGCATGATATATGACATTACACAGAGAGTTATTGAGGA
ACTACCCTCTCAAAATTTTCGATTCATGATGTATGACAGAAATACTCTATTCTATAATGA
AGTGACAAATTACGAGGAAATTTGGAAACTCGTAGAACTATCGGTAAGGGATTATCGAAA
GCGAAGCAGAGCCAGGGCGATGACAAACGAGGCAAGACTCTCGATATCAAAAATTATTGA
CAAAAGCGGGAGCGAGGACGAAACAGGCATGAATGTAACCCATCGTAGCAGTTGCGCGTG
TCTCAAGGGAGAATATCGAAGTACCGCGTGGGATGCATCGAGAAAAAAAAGATGAGCTCG
GACTTGCATGACAGCTCCTTTTGTGCATTCGCTGCTCTGCAAAGGTACAGTATCAAGGCA
AGAGTTCTTGAGAACACCTAAAAAAAATGTTATCCGTATACATCCGTGGGATCATTCGGA
TGATTGACGACATTTATTTTGAAACACTTGTCACGATGGTTTTGGCTGTTCCGTATGTTT
GTGCTCGGAACAGATCTAATATATGCAGAATTTTGGTAGATTTTTATTTTACGG
>comp2022_c0_seq1
GGAGAGAAGATGATGATGACTATTCTTTGCGGAAAAGTTCCTCTAGCGCAACTTCCTACC
CTCTCAGTTGTAGTGTTATCATTGCTGATTATTCAAAACTTTACTACTAGATGCGAAGCG
AATCTGAAACTACCGACATTTGTGGCGTCGCACATGGTGCTCCAGAGAGAACCTTTATCT
TCTAGAATTTGGGGTTGGGCTGACCCGGGAAGCTTGGTCCGTGTGTCATTGGATTCAAAG
ATATACACGGACACCAATGCACAACCGGATGGGTCTTGGATTATCGAGCTTCCGCCCCAA
CCAGCGGGTTCGGACCACAAAATTCTCATTGCGGATGGCGAGACTTCTATAACTCTACAA
GATGTTGCCTTTGGAGATGTTTACCTGTGTTCCGGTCAAAGCAACATGGAACTGAGCTTG
CCAGCAGTGTTCAACGCTAGTGCGGAAATCGAGGATAGTAGTAATTATCCAAATTTACGG
CTCGCAACCGTAGACAAGGTGACATCGGATACCCCACAGAAAGATGCTCCCTCAAAGTCT
CCCGATTATGCCTGGGCAAGATCTGGTCCTGACGCGATGAATCTGAATGATACCTTTAAA
TACTACTCTGCAACGTGCTACTTTTTCGGAAGAGAGCTCTACAAGGCACTGGATTCTGAA
ATTCCGATCGGCCTCGTCACGTCTTGTTGGGGCGGGCAAACCGTAGAAACCTTTTCGTCA
CCGGATGCCCTCGCGGACAAGAGCTGCGGCGGTACCCGTCCGGTGCATGGCACGGATGGC
GGTGGGGGGCGCAACGGAACCACCGTCGTCACCTCCGGCCCGGGTCCTCGGCCCACGCAA
TTGTGGAACGCAATGATTCATCCGCTGGTCAACATGCGCTTTGCAGGCTCGGTTTGGTAC
CAGGGAGAATCGAACGCGTTTGACCCAGAGTCCTACGCGTGTCGCTTTCCAGCAATGATT
CGCGACTGGAGGCTGAAGTTTGGCCTTTCGGACCTTTCGTTCTTCTTTGTTCAGCTGGCG
GGCTTTCACGGGACCAAAGACGACTGGCCCGGTCTGCGTGCCGCGCAAATGGCAGCCACC
CAGCTGCCCAGGGTCGGATACGCGGCGGCAATCGATCTCGGCGATCCATCCTCTCCCAGC
TCTGCAATCCATCCCCGGCGCAAGCTGGAGGTGGGCCGCCGCCTTTCCCTGAGCGCCCGC
ACCATTCAGTATGGGGAGCGCGGGGGGCTCGTTTCCACTGGTCCGATCCTGGCCGGTGTT
CAGCAACTCCCGACGTTTTCGACCACCCACACGATTCGGTTGTCCTTTTCTCCCGGCACG

GCGGATGGCCTACACGTGCACGGCTCTCCGGAGTGTTCTTTGTGCTGCGAGAAACTCCCG
TTTCGGGTTCTGGATGCAAACGGAACCTGGTCGCGGGTGGACGGGGCCGAGGTTCGAGAT
TCCGAAATCGATTTGTCCTCAAACGTTGGTCCGGTCTTTGGCGTCCGGTATGCCTGGGAA
CTCTATCCAGAGTGCTTGGTATACAACGGAGAGGGCGGACCGGACGATCATGCGGGCATT
GCTGGAGCTCCCTGGGAATGGTGTTCGGAACCATCCGGCGATCCTGCTTGGACGGGAACG
GCATGCAAAGTGCCGGCTTCGGCCAAAGGGTGAAATATTTGTTCGCTACGGACAACTGTG
ATCGGGCGATGAAACCGGTGGAAGGAAAGTCGACTCTGCAGCTCAATCAATATTCCAAAC
AATCGCTCGCAACTCGATCTACAAGCCTGCCAGGGTCCGCAGGCATTCTAATGTGCACCT
CCACTCTACACGCTCGGCAGTCGTGTTGGGAATTCGCATTTGTGCGTTGGTCGAATTTGT
CTTTGGTTTGCTCGATAGGGAGCAAACAAATCAGAAGAAACAGGACACATTGCCTACCAC
AATCTCCAGAGCGGCGACGGGACGGGACGAGTCCAAGCCTACCTCTCAGCCCGGGCTGCA
CCAATATCAAAAGAGAGCACTGCTACGGAACTAAAAGAATGTAGTGCAGTAGGAAGTTGC
AAATAATAAAAGGGTAAATCATTAACTATCGATCTTAGC
>comp2023_c0_seq1
AGTAGAGTGAAAGATTGACTGTTGCAAATAAAAATGTTGCTTGGTGTAAAACTGTTTATA
ATCGGCTTCATATGCTGTTCATTATCTGGTGTGTCGGCTTGGGTTGTACCAACGAGCAAT
CGGCTTTGTTCCACCCGGAGGACTCAGTCGAGAGCTACAGCAACAGTTCCATACCAATTG
CAACAGAAACACGGATCGCCTTCCACTCGTCGTGGGAATACGCGCCTCTACAATAAACTA
TTGGAGTTTCTCAGTCCGTATGATTCAAAAATCCCTGAAGAACTTCGAGATGAAATATAC
AAGGCAGAAGCCAATACTCCAGCGGCTAAAGATCGCGGAACACGGGTGGCAATCTACGCC
ACAATGGCTCTTTTCGGAATCATCCTGGCTTCTTTCAATGGATTTTTGACCGATATGCGG
GACAGAGAAACTGCCATGAATGCTGCTACGGTTGTTGCATCAGCAGCCGACCCGGGGACA
CCCAACAGCCTTTATGTTCTAGAAGCGAACGGTTTCGGGTGGGTGTTATCAAATCCCATT
TTCAAATTTTTGTTCACTAATAAAATTGGTGGTATCATTACTCTATTGTTTGGTGGAGGA
TCGGGATTGATGGCAGAAGCCGAACTTGATTCCCAGCGTATCAATGCTGAAAAAATTTAC
GAAGAGTTGGAACGCCGTCGCGAACAAAAACAGACGAAGAAGACCGGAGGAGGCGGCATG
AGTAAAAGTAAGAAAAAGAGACGATCTGGAAAGGAGAAAAAGCGTCTAGCAGCAATTTCC
GAAGTGGTAATGGATGATACTACCGAAGCAACTTCCGTGCTATCTAAGGAAAGTACACCA
GCAGAAGAAACTTCTTCTACAAATGATGTCGAGACGACAGGCATGGGAGGCGAAAGCGAC
GATAAGAAAGGGGTTTTTGGTAAAATGAAGGATCTCTACGAGAAGGCTGATTCTATGGCA
GCCTCTCAAGCCCTAATATTGAATAAAAATCTTGAAGAAGCCGGAGTGGTTGAGAAAATT
ACGGACGAAACAGGCCTGAAGGTGATCGGGCGGGAAGAAGGAAAGAAAATAGAAGAAAAG
AAAGCCTCCGACGACAATTAATTTCAAGACTAGGTATGCTACTAAAATATATATAACTTA
CCACAATAATCGACAAGACCCAAACGGAAAAAGAATCGATCATCGAAGAATCAGAACCGC
TATAGATATACATCATTTTGACAATAATTCTCTTCCTTTCCTTATTCGCATGGGTGCGAA
GAAACTTTGTGAAAATTAATATTACTAATTGACAGTATTACTTGCTAAAGTACTTGTCAT
GTTCGGTATCCAAGCATGCGAATGCCTTGATTTGGCAATCATTGAAATCCTGCACCAGTA
TTAC
>comp2024_c0_seq1
CTCGTACTATGGTATTCGTATGTACAGTACCTGGTACAGTATTTAACCAAAGTACGAATA
GTGATGATGATGTGTCGTCGAGCGTCATCTCATCTACTTCGTAGACTTTTAACGTATGGT
ATTACTGTGGCGTTCGGTACAGTACGTACGAGTACCTTCTGGTACGGGACTTAGTAATTT
CGAATTTCGAGAGAGATCACATCAGTAAAACAATCGAAAAAATTACTACACCAACACTCC
TCACTCTGTCGCCACAACATCTCCACGCAAGATAGAATAGAACACAGCACCCGCTACGCT
ACAGCAGATTAGACCAGAATATAGAAGAGGAAAATAAATACATCGACGATGAGCTTTCAG
CATCTAACACAAATCTTCCTTCTATCTTGCGCAATATTATGTGCGACAAGTTTCGTCTCA
GTGGATGCTTTCTCGCCGAAGGCCCCGACATCGTCGAGTGTGGAGCCCTCTTCACGTAGA
GAATGGTTGGAACAAGCGAGTTTTGGAGGAGTTGCTGCCATTGTTGGTGCTGTAGCGACC
ACAACTGCGTCGTTTCCTCAAGCAGCCAGGGCAGATTCGGGCGAATACGTGGCAGAGTTG
CAATCCTCGCTAGCGAAGTTGCAGCCCATACCGAAGATGCTGGAAGATCAAAAGTGGGAC
GAAGTTCGAACTATATTGAAAACTCCACCAGTGAACAAGTTGTGGAATTTGGGTGATTCG
CAAAATACCGTGCTGAAATTAGTAAAAGAAACAGGCAACGTTGAATTGTTCGAAATCAAG
GATGACCTAGCTTACCATTTGCAGATGTGCGACCAGCTGACATATGATAACAATTTCATT
TATTACCAGCCAGGAAATGGCAAACTCAAGATCAAAGAACCCATCGATGCCATCAACAAG
GCGTTGGTGTCGTTAAAGTCAATCATCGACGAGTCGTCCTAAAACCGATCAATCGCTTCA
TCTATTCATAATTTTTTTTTACGTTTCCCGACATTTCTGAAAAATACAAACAAAATCTTT
ATATTGTTCCTTTTATGACCTACAAGAATACATAAGGTAG
>comp2025_c0_seq1
TTCTCCGCTTTCGCTTTCGTTTGCGTTGGAGAGTTTACCTTCCGAGTATCGTGCTCTTTC
CTCAGTTTCACTGCGTCGCTCAGAACATCTCCTCTGTGTGCAATAGAATTTTTGCTATTA

CTTTTGCGCTGTTACTCAGAAGTGTGGGCCGTGGTTTGGTTATGTCCCTTTTGTTTGTTT
GATCGAAGTGAAATAGTGTAGAACCGTACACCGAAATCGGCATAACGACACGATAGGAAT
AACACTAAATCGAAGGAGCAGCACACTGCATCATACCAATCCACACCCAAACAAAGGGCA
AAACCACAAAATGGCATCGCCCCGATGTTCTTCTTTCGCGTTCGAAACTGGCTTCCCCGG
AAGCCCACACTCCATCACCAGTAACCACGGCAGCAATGGACCCTCCGAGATCACCGCTCG
ATCTCTAGCGCGGTCGTGCGGGCTGCTATCGGCTACGCTCTGGATCGGATACACGCTCGG
AGCTTCGAAAGCGCCACCCACTGCGCCGGTGCTTTCATCCGATTTGAACGTGCTAGCATC
TCTGCGCGTTCTAGCGGACCGGTTCGAGGAATGGATCCAGTGGATCGTTGTTCCCCTCTA
TCTGCTGGGAACCGTGACCTATCTCTCGGTGCGAACCTACAACGATACGGATTGGACTTT
GGTGCTATCGAGCACAAAAACGAGCGAAAAAAAATTCAGATATTCGCTCCTCCCCTCGCT
CAGAATGGCGAGCACGGAGACCATTCCCGAGGAACCCGAGGAAGAGGGCTTGAAAGAAGA
AACGACGACGCCCTTGTCCGTCACACCCCTGCCTTCACAGAAGTACGACAAGCCGGCCCC
GATCGACATGAACGGTACTTTCAAGGTGGTGGAGAACCACAACTTTGCAGAGTTTTTGAA
CGCCCAGGGCCTCCCTTGGTTTCTCTGCAAGGCAGCATCCAAGGCCCGACCCACACACAC
TTTTTCGCTGCCCACCTACTCGGATCTGACCATCACCATCAAGGGGATCATCGAATCAGA
GACCCACTACACGATCGACGGCCCCTGTACGGAAACGACCATCCGGGGGCGGGTCTTTCG
CGATTCGGTGAACTATCTGTACGAAGAAAGCGACGAAGGCGAAAACGACGGCGTCTGCGT
CGGCCTGCGCACCACTAAGGTGTCCGTCGGTGAGGGGTACGCGGTCCACGTTCAGCGGCG
GATCGTTCCCGCCGGGGCCACCTATGCTCCGCCCAAGGGGCAGCACGCGTACGACCTCGG
GACTCCCGCGGACGTCGACCGGCTGCTCATGACGAACACGCTCGTCTACGACGAACCGGC
CAAGGGGGGCAGCGGCGTCGTGGCCTCGCAGCTCTTCCACCGGCAATAACAGTGGCGTGT
GGCTAAGGCAGGCGAACGAAACGAAACAGAACGGAACGGGGTTTGGTTTTGCGTTGGTGC
TATAGTGTACCACGACGCAGTATAGAACAGGTAGCATGGTAAAATGACAAGAAATGAAAA
ATCATAGACCACCAACCATAGAGGAACAAACACCGTGGGTTCTTCGTTTGTTCCAATGAA
CCAGATGCATTGGGCAGTGTGCGGTACCGTTTGGTTTACACGTCGCATTCTCTGTTTCGA
CAAGATTGTTGTGATAACGACACGCCACGACGTTACTTGTACTATAGTACGATAGAAAGC
ATGATGCTTTAGTACATGTTTATGTTATGGATCCAAGGAAAAATGCACTAATTGATCTTT
GCCGC
>comp2026_c0_seq1
AACCATACCGTACCGGATGAATCTCCCGAAGGAAAGAACTACAAGAACAAGCAGCAAATT
AGGCTTCGCAGTCCAGCATCGATCACACGCATTGCACCATTAATAAATAACGACAAGGTA
ACTAACTGCCGACGCAATAGTTTGATTGGAAAAACCATCACCTGTGTGTGGGAAGATTTA
GATCCATCCATAATTCCCACCCGTCATGTTTTCAAGCAGCGTTTCAAGGATAATCAACGC
ATCTTCAAAAGCGGCAATAAAACGTCGAAGATGCATTCCTACATCATCACTGGCTTTGCA
GGCAATCTATCAACATGGTAGTTTACAACAGCCGGAATTCGAGCACAAACAGCAGGAGCA
TTTTTTCTCTACCAAATCGAAAATACCTTCGGTTCCGTCTGAGAACGCGTCACCGTCCTC
CACAACAAAAACAGAAGAAGACCAACCGCCATCCATTGCAACGACCGGAAATATTGCCTC
CCACCTAGCACCGATTCCGACCGAACGAGATCTTGCCATTGTTGGTGGCGTCTTTGCGCT
GACCTCTGCAAAACGCGACAAACTCGGAGAGCTCCGAAACGCCCTCGGCGAGGGCGACCT
TACACTAGACGACAAGGCAACCGACGAGGAGCTGATGGACCTGTACCTGGCGGATGGGCT
TAAGCTGATCACGAAGTGTACGGATGCCGCGTTAGATCTCTCGGGCACGAAGGATGAAAT
GAAACTCCTGAGCGACTTTTCTGCCTGGCTCGACAGCCAGCACGATCCTTCCGAAGACGA
CGAACTTTCTCGGACGGCAATGGAGATCAAGCTTGAGCTCATATCGGCTAAACTCGACAA
CCTGCTCGGCGAGGTTCCAGCGGACACCTGCCTAAGGCACGTACGCGACTTTCTCGATCC
TGAAACGATCCCCGAGGAGCTTGGGGAGGCCGGCGAGGACGAGAACCTTCTCTGCTCACT
CGGCGGCGCAGAGAGCGACGCCCTGGACGTGGGATCCCCCTTCTTTCGGAACGCGGTAGC
CCGCTACCGCCTGCTGCTCACCAAGGCCACCGTGGACAAGGTGTCTGCGTCCTGGGATGT
GCTCACGACGGTGTCGGACGGCGATATCGATCGCGCGGCCTCCGAGGGAATCGCACTGGT
GCCGGAACTCGAGACGGTGTCGATATCGGGAGTCCTCGGGTTCCTTCAAAAAACCGCCAC
TGGGAGCTGTTCCGAGCGCATCACAGCGGCATGGAACCTCATGGACCGGGACCGGGACGG
CTCCCTGGACGAGGCCGAGATGAACCACGTGGTGCACCTCTGCCTCGGCATCGAAACGGA
CGCCCTCCAGGTCCTCTTCAGGGAGGCCGTCGACGCCTTTCCGGTCCGTGCCCCCCTGTC
GGCAATCGGAGCGGGCAACGAAAAGGACGGCGGTGACGGCGACGTCGACAATGCGGGCGC
GGTGGGCCGCCAGGGATGGAAGGCCAGGAGAAGGGAGAAAAAGACCAGAAAGACGCTGGC
CAAGATGTTCGAGCGCGCGTGCCAGCGGCACTTTGACCGCGAGCTCGAGATCGACCACCG
GCTGCGGTGCGTTTATGCGTGGGCCAACAAGGCCGACCAGGGCAACCAGCTGAAGTCCGT
CCTGGTCGACGAACAAGCCGGCTGGAGCGGGCGCAAGCGGTACGTGGAGCTCTCGCCCAA
GATTTCCGAGGCCGAGTTCCGGGAGGTCCAGGAGATCCACTTCAGGCACCTGGACAAGCT
GGGAACGGAGCTCGTGACGAGCTTTCGCGAGGACCTCTGGGTGCTGCAGGGAAAGGGGCG
GGAGCGCAAGGACCTGGTCAAGAATAGCTTCCTCTTTCTGGGCGCGGTGAGCGCAATCGA

TACCGCGATTCTCTTGCTGTAAGCACTCGTCGGATCGGATTGCTCTAGAACGACTTTCAC
TGGAAGGGGTAAAGTCTGCTAGTAGTACTAGTAGCAATGGAATCTTCTAGTATAGTATAA
GTAATGATACACGACCACCGGTAGCATCAGAAGCAGTAGTAGTACTACCAGTGAGGGTGA
TTATGGATGTTTTGTTGCAAGTACTACTACTGGTGGTATTATTATAGTAGTAGTACTTCT
TGTTA
>comp2027_c0_seq1
GAGCACCCCGTCTTCAATTGGTTATATACCGTACTTACTACGGTACCATCTAACCAACCA
TTATTTGTGACAGTAGTCTTTGTTCTCATTTGTTCCTTGCGAGGACGAAACCAAAAGGCA
AGGCACCGAAAAAAAACACTAACACCATGGCCATTCGATACGAAGCTCCGCCCTACGCTC
CCCAGCCGGGCGGGGGAGGCAGCCTTTCGATCACCGTCAAGCCCGAACGATGGAGGCTCT
TCGCCTACGGTTTTTTCTGGAGCCTGATGATTTTTGCGAAACTGGTTTGCGCGACGTTGG
TGGAGCCAATCATCGAGGCCGGTGCACCTCCCGACACGCCTGCCGAGCGCCTGGGATGCG
GACCGTTCAACCGTGAGGGCGGACCCTTCGACCTCGAGTACGGCATGGGATTCACACTCG
ACGAAAGTCACCTGACACAAATCTTTGGATACGCCAACTCTTGCGTCGTGTGGGATTATA
CGCCCGCCCGCGAGATGATGGCAATGTACTTCCCGTTCTTCGAATACTCACTCGTCATCT
ATTTGTTCCTAGACTACACGAGCACCATGCTGTCCTACAGGAGGGGAGAGCTCCCGGAGT
GGTACTGGAAACTGATGCA
>comp2028_c0_seq1
AAAAAAGCACAAATCACTGCAGCTTTTGAAGCAGTGTCAAAATTGAATTTAACGAAGCAA
ACAATCCGCATTATCTCCAACATCGAGAAGTCGTGAATACCTGATTAGCTACCCCTCGCA
CCAACAGTCCGCGGCGCTACATCCGAGTCAAAGAGAAAAAAATTCGCTACGAATCCATTC
CATTCCTTAGAACAAATACGCACCCTTAAATACAAGCGAAATAAACAACAATGGCAACCC
GTTGCGCATTCGAAAACTCCCCCGAGGTTGGTGTCTTCAGTGCCCTCACAAATTCATATG
CCCTGGCCGGAATCGGGGGGTCGGAGAATTTCTACTCAACCCTGGAAGCCGAGCTGATTG
AGCACATCCCGGTCATCCACGCTACGATCGGTGGGGGACGGTATGTAGGGCGCACCACTG
CAGGAAACGTCCGGGGCCTGCTCGTCCCCAACACCACCACGGACCAGGAATTGCAGCACC
TCCGAAACTCCCTGCCCGACAAGGTTGTCGTCCAGCGCGTCGAGGAACGATTGTCGGCCC
TGGGAAACGTCATCTGCACCAACGACCATGTCGCACTCATACACCCCGACACGGACCGGG
AAACGGAGGAGATTATCTCCGATGTACTGGGCGTTGAGGTCTTTCGACAGACCGTCGGGG
GGCAGGCTCTGGTCGGGACATACATGAACATTTCCAACCAGGGAGGTATGGTCCACCCCC
GAACGACCGTCCAGGACATGGAGGAACTCTCGTCGCTGCTGCAGATCCCACTGGTAGCTG
GAACCGTCAACCGTGGATCCGACGTCTTGGGTGGGGGACTTGTGGTCAACGATTGGTCCG
CCTTTTGTGGGCTAGACACGACCGGGACGGAAATTTCTGTGGTCGAATCCATCTTTAAGC
TCCAGCGAAACCGAGGACAGGACCAGTCGATGCAACCGATGGACATTGTGCAGGATATGA
GGAGTTCCCTGATCGATCAACTGTCGTAAAATATAATTTTGTATACTGCAATAGTACTAT
TATTTCTACTACTAAACTATTGGTAGTGCAGTACGCTCTGATACCATGTTATACTTGCAG
ATACGAATAGCAGTACTTCTGGTAATCAAAATATGAAGCCGTCCTAGAAAAAGTAACTAA
CCCACTTTATAGAAGTAAAATAGTCAGCGAGCAACAAGTCAGAGTGTGCCATACAACCGG
TGACAAGAAAGAGTAGGATTGATGCCCAAAACAACATCACATGTTTCCATATAACTATCA
CAACACCAG
>comp2029_c0_seq1
CCCTCAGGTCATCACCATTCATCTGTTGGCGTTTCAGAGTTTCTGAATGGGGCGAGATCC
AATTCATAGCAAAATGATGATTAATATCAAAGGATCCATGGTCTCGGCAGCGTTTGTGCT
TCTTCTCCTCCTCGACCGCGCGAAAGAATCTTCGGCACTGAGCATTCGAGACAAAAGTCG
TGGCGGCAGCGGCAGAAGGGATTTTATTGAGGGAATTGCCACTGCCACGGCGACTGCTTT
GATCCTTCCCGAACAAGCCCACGCCACTCGAGCAGTTGGAGGTGCAGAAATCGAATGTCG
AGAAGCAGGAAACTGCTTGGAAAAATTTGAATTGGATGGAGCCTTAGGCTGGAATTGGGG
TGCGAAAGACCGCTGCGATGCTACGGATCCAAACTGTGGGTCAAATGGGAAGCTAAGAGA
TTCACCCCTTGTTGGAGATAGTGTACCAGATAGAATGGGATACGAAATCACACATGTCGT
TCAGATGTCTCTGGCAATTGGTCGGTCGGACGAAGATTGTACCGTTCGCATGGGTTTGTA
CGGAAATGATGTTGCTGTGAGTGTGGAGGAATTTCTGAACATAGTGAGCCCCTCCGGATT
GAGGACAACGAGCGAATTGATGTTTGAAAATGGCATGGGTGTCGCTGCGATACCAGTGAG
TCTCACAAGAGGAGGGATCTTGGGTCAGATTGTCCCGGGTCAAAGACTAGACTTCGGAAT
TCCATTGCAATCGGCTGCCTATGCTCGATCCAAAGGGATGGCCAACGCGGGCGACAACTT
TGTCCCGCAACCACGACCAAGGGAACTGAAAGATGTCCCTGTCCTTAGAAAGCACGACGT
GCCGGGTCTTGTTTCTATTCCGGCAAAAGGAATTGGATACGGGGGGACAGGCTTTGAGTC
GGAAGACGAATGTTTCGAGTCTGCCTTTCAAATCATTGCTTCTGCTAACCCTGCAATGGA
CAAAGAAAACAGACGAGTAATTGGGCAGATTGTGGACGACGAGTCTATGCAAAGCCTCGC
AAGACTGGCATCTCTTCCGACCAAGAAGGGCTTTCGAGGAGTGATACCTGGGCAAAATTC
GGGACCTCCACTGATCAAGGTTGTCCTCACAGATATCCAAGTTGCAAAAGTCCAGTCCCA

GCCCTCTCAAGGATCTCCTGTCGAAAGCTAATACTTTTATCCTAAACGAAAGTTCGGCAT
TGTACTAAACGCTAGTTTGTTTAGTTTTTAAACTCTTATCGACCCGGAGCACTTCTTCTA
CAAGGAATGTTGTCGACAACTGCTGCCTGGAAGTCCAGCAGTGGGAAAAGATGAACTGCC
TTTTTTCGTATTGGATCAGAGGTCTTGTCTTGTCCGAACACATCACAAAAAAAGTCTAGG
TATACCAACCGCTCGGTTTTGAACGTCTAGATATCATCCTTCCACCCAGCGTTGATTGTT
GCGTAGCTCTTTGCGTCAATCGATCCCGACAAGTAGCAACTGACTACTCGCCTTTCATGT
CATTGTCAATACTCCTTGCTTTTTCGAACATGGAGGGAATGAATCCATCAACAATTCTCG
AGTCCTCGTTTAATTCTTGTATCGGTCGAAAGACAGGAAAGCGCTCCAGCAACTCATCGA
CTCCCTTTCCGCCTCGGGGATGGCGGTATTTTTCTTCCATGTCTACAGTCACTTTGTATC
TCATTGCCAAGCCAAGAGCAATCATCGAATTAGTAGTGTCGACGGTGACCTGCGAAATCC
CGTTCCCATCGATGACGGACACGTCCAAGCGAAAGAGTGGGGGTGCAAGCGATATCGCCG
GGGTGTTCAGCGACCGCAACGAGTCGAGAATAGACGAAAAGGCGTCGCGGTCGACCGCAC
CCTTCTCGTCCGCGAACCGCTGCATGGCCTCCCGGACGCTCTCCGTGGTCGTCTCCTGCA
ACCCGGGCAGGGTTTTTACCGCCGTTTCTACCTTCGGAAGGGCGTCCGAGATGGCTTTTT
CTCGTTCCGGAGACGTCGCGACGAGCTCTTGCGCCTCTTCCTCGATGCCGGCTTTGGCGT
TCTCGCTGGCATCGGGATTGGGCGCCGCCGTGATTTTGGTCAGGGAGAGCCGCTTGGGAG
CGCCCTTGTCCCGGGCGGTGCCCTCGCCGGATTGCTCCTCGGCATCGTGCTCGGCGACCA
GCCTGGCCAGTGAATCCGGGGGCAGGATGGCCGTCCCGAGATCCAGCCCACCCGCCAGTT
GCACGCACGTCAGGGCCTCCGCGGATTTTGGTCGATCCGTGTCGACGGGGGAGACGACCA
CTGGCATCCAGTGCTTGTTGTGCTGCTGGTTGCTGTCTTCGCCCCCGGCCACCAGTATGC
ACACGAATCCGAGCTCCGGATCCACCGAAAACCCCGCGATGCCCCCTCGCAGGTCGATTC
TGTTTCTGTAGCTTTCTTCGTCGAAGTCTCCCGACGGTATGCCGTCCATCGCATCGTCCA
ACCAGCCCGGTTTTTCGTTGCAGCGAATCAATGGTGTTTGCCGCTCTTGTTTGTTTGCGG
ACGGTGGGAAGGGTGCCAAGGGCGAAGAAACTCGGTTTGTGCTCGTTCGGATCGCCGAAC
TGGTAAATCCCGCCGCGGAGTTCAGAGACAGCAGCGAAAGGAAGAAAACCGCCATGCGAT
CGGTCGTCTTCATTGTAGTGGGATATTGCGTTCCGGGAGCGAACGGTCTGCTTCAAACGC
TATGGATTTCTTTGGTTTCGATTGGAATACGATGGCTGGGTTGATTAACAATCCTGGAAG
AAATCCTGTGGGATTCGAACACTGCGTTTCAAAGTATGGTTGGAATGGTTGTTTGCTCCT
TTCGGAATGCCGCCGTTGCTTCTAGACGTAATTTTTCTTTCAAGAAACCCCGAGGAGTCA
AAAAGCCTTGAATTCTGCATCCCACTTTCAAACTTGAAGCTCGTGTATCCAGTTTCAGTT
CACGGTTACCATTCCATTTAGTATGATTACGAGTACGTACGAGTACGAGTAAGCAAGTCA
ACTAACTTGCTTGGGTGCGTGCAAGTTTTTATTAGTACGTACGTACCTATGTATTACTAC
TACGGAGGGGTATTTCCTAAAATTTCCTAAAATTCACGACGCCGCACATTTTTCAAATTT
CCTCTGAATTGTCGGAACGGAGAACGGAGAAGCTACCAACAACGTCCATACATACGTACC
TATAATACATCAAATGCCT
>comp2030_c0_seq1
ACGAAGACCATTCACAACTGGACTTTAACTCAGCGTCTGTGTCCATTAGTGATCACGACG
ACAACAGAAACAGCGAATAACAGATACCACAGTAAATCTTGGCTCGAGGTCGCAATAGCT
TGAAGATTTTTAACAGTAACGAAAACGAGATCAATTCTCAGATAACAACGACAATCAGAG
CACTGCCGTTGCAATGATTCTTCGATCGGTTTCGACAGCTGCTGTTTTGTGCCTTGTGGC
CAACTGCATGTTTTCTCACGGCTTGCCTATGCAAGTCACAATCAATCAGCGGCAATCGGA
ATGTCTGTATGAAACACTAAATGAAGGGGAAGCCGTGACCGCAAGTGTCTTCATTTTAGC
CGGGAGCCAGTTGATGGCGACCTATAGTTTGGAAGGTCCGGTCACAGATCTCAGCACTGA
TTCAGGAATAGCTTTATATGAATCGGCGTTGGCATTTGAAAGAACTGGTGGCTCGGAGGG
TAGCGAACTGCGGATTTCGGGGGAGGTCGATTTTGAACATTTGAACGATGAAATCAATGA
CGACGATGACGATGACGACGAC
>comp2031_c0_seq1
CCTCAGGAACAAAGAGACTTACCAGTCCCAAAAGTAAGAGACAGAGAGTACCTAACACTT
TTAGATTCTTGCTCAACCGTGGCAACGCTACCATTACCACCCAAACACACACACCATCCT
CAGAGGTCGGTAGATTGTTTCGATTCTATTCGAATAAAGCAAAAGATTCGATTATCTGTT
GACGGACTACTCGGACCCCATCTGCAATCTGCACCAGCACAAACCCCGTGCATACAATCG
AACAGACAGAAAATCAAGAATAAACCGATCGGCACTGCTACCATGGGAAGCAGTGGATCC
AAGCACACAACAACGAATACAACGAACACGATGCCCATTTCTCCCTCGCAGTTTTCCTTC
TTCCAGACACTGACGGCGCGATGCGACGATAGCAACCCCCCACCGCCACCACCTCCCCCC
GGAAGCGATTCCGCCCCCCCACCGCCCCCTCCGTCTTCTGCCAACGATGCCAACGACGAT
TCCATTGCCAAGGCCGCCGCCATGGCCTCTCAGATGATGAATCCCGGTCCTCTCGAACAG
GCCCCGGCAGACATGAAACGCCTGGTCCAGCTGGACACGCACGACGGCTTTCGGTGTGAC
ATCAACAAGCAGATCTCGCCCTACCTGGCGGTCGTCCACTCTTTCTGGCTGGGAACCAAC
ATGCTTCCGGACGGGCAGAAACGAACCTACACGTGGATGGCACAGGTTGCCGACGAATCC
TCCTTCTGTTTTTCCCGCCTTGACCCCTCCCAGTCCAGCGTTGACGGCCGAATCATCAAA

ACCCTGCTGGGGGGAATCGCAACGGCAAAGCTGCAGCTCGGCCTCAGTGCCGAGGGCCAG
GCCGACCAGTGCCTCTTGGAGGTCGACGTCAACGCCATGTCTTGGAACGGAAACCTGAAG
TACGGAAGCATGGGCGGCGGGGCCGTCTACGGGTGCTCTTTTCACCAGGGGATCACCCCA
AACCTCAGCATGGGAGGGGATGGAATGTTTGTCGCGGCAAACGGAAACATGATGAGCAAC
TACCTCCTCAAGTACTCCTTTGGCGCCAGCGGGGAAAGTACCAGTGCCAACGAGCCGCCC
TCCTCTGCGGTCTGCCTGCAGTTTGCCCCGAGCGGACCGTCCCCAATGCCCCTGACGCTT
AACTACAAACGAACCGTGACGCCCAAGCGCGTCACGCTGGGAGCCGAGCTCTCCTGTGAC
CCCCTATCGATGCAGTCCCACTGTGTGCTTGGGGGAGAGTTTAAGTTCAGCCGCAGCAAG
CTTCACGTAGCCGTCGACGGGAACCTCAAAATGCAGAGCCTCCTGGAAACCCCGCTCGGG
AAGGAACCCGGGCAGCCCCGGCTGTCCCTTTCGGCCGAGCTCGACCACCTCCGGGACGAG
ATGAAGTTTGGCTACGGGCTGGCCATTGACGGATAAGTGAAGGAGTGCGGTTTGTTGCGT
TCGCGTTTGTGTTTGCGTAGGAACGTACGGCACGAGGCATCAACGACACCGATACGGGCA
CATAATTGGGAGGATGAAAGTGGAATATAAAATACAGTATTATTGTACGATTGCAATTTC
ATCAAACTCTAATCGAATACACTGCCAAAGGAAAAGACATTAGAACGACCAATCCGGGTC
CCTCTGGCTGGGATTGTTCTAGTGCAGTATTGAAATACGGCAATGTGACAGAATTACTGA
CACTGTCGTGACGGAATGCAGCAGAATTATCTCGCACTCTGAATCCGAACTGACACACGA
ACTCATCTATTAATAAAATTATAAATTGAACAATATTAAATTA
>comp2032_c0_seq1
GTGTTGTCTTGAACAAAGCAGTGTTGGTACCGTAACTTCATGCTTCAGAGTTCAAAAGGA
GAAATGAAAAGGAACCATCATCTGGAACTATTCAGCAACTAATGCGATTCGCGAATTGAT
GAAGCTTGTAATCGCCACTCGTCTCCACCTGGGAAGAGCCTCCTCTCCGCCTACAGAGGA
ATCTCTTCACAGTGTCTTAGCCAACTTGAGAGGGGTTTGCGATGATAATGGCCTGCGCCA
GGATTACGAGACTGAAGTTTTGATTGCAGTAGACGCAACACCTAGGTTGGAGGGCTATGA
TTATGTCGAAGCTGTACGGAGTGCTCTACACTCCAACAGCGCCAATGAGGCGAACGGTAG
CAATCACATTCATATTCTACCTGTGACACCGTGGGGAAAATTTGTTCCTGCGTTGAATTC
ACTCGTAATCCACGCCAAAACACAGCAGGAAGCTGACCTAATTATGTTTGTATCAGCTGA
AGTAAATCTTTCTTGCAAGACTGTGCGAACGCTGTGCCAACAAGTCAGCGATAACAAAAA
TGTAATTGTAGCCGGAGCTGTCCTGAATGGTCACCAATATCACGAAATCGAGTCATCATC
GGACGAAAGGGGACGCGTCGTTCCGCTGACGGGCAGAACAACTCCATGGAACACTCTCTG
TGTTTGGGATCTCCACAAACTGTCCATGACAGGATTCTCAATTATCTCTGACTTGGGAAA
ATCAGCTGGTGTTGAGGAATGTGCGGCCATCGCATTATTGCAAAACCTATTTCCAGAGGC
TGAAGCTCGACTGGTGAATGTTGATGGAACCAAATGGGAGGAAGAATTCCAGGACGAAGA
GCGACGAAAGTGGCACGAGGAAAAAATGAAATCCAAGGTAGAAAGGCCACGGCGCCAACT
TGAGCTTCTCGGACTATCGGGGACTGTTCTCCACTGCTGACGGCTCATGTCGAGTAAAAA
ATATAAAAATAATTCGCTAACAGTTTACTCTTATACCGATGGC
>comp2033_c0_seq1
CGCGCAACTCGTACCAGTAGATACATGTACAGTTCTGTATCGTACTCGTGCAAGTGCCGC
TACTCGTAGTGTAGAAAATGTTGGCACAGTTATCTTGATTCTAGTAAAAACCCAGGGGAC
ATTAAAACATCCCAACTGCTCTCGAACCACAAGTCAACTCATCCCAACCCATATTTTGAG
AAAACAACTGCTCACGGAATACTGGTAGTACAAGCATGCGCTTCCCCAGAAACGAATCGA
TGTCGGCAACACCCATGCTGCTGATCCTGGTGTTGCTCGCCGCAGTGATTGAAGTCGATG
CAGGTGCAGTGATGGGCAAAATCCGCTCAGGCAATAGTAACCCGAACTCCCGCAGCCTCA
ACATCGAGAATCAATCCGGTCGAAAGGTGGACTTGTACTGGGTGAACATGTTTGAGAAAC
CCGAAAAAATTCGTTCCGCAATTCGTCGAGGACGGGGTCAGCGTCGGCTGTTCCTACGGAG
CCGACAAATCCATTAGCTCGTACGTGGGTCATACGTTCGAAATCCGAGAACTCCACAGCA
AAAAAACCGAAGCTTGCATCTTTGATGAATGTCGCAAGGTCAGATACACCGTCAGCAATC
GACAAGAACAAAAGATCGTCATCAATAAGGACTTTACACTCACCGTCATCGACGAGCGCC
AAAAAGCTTACCAGAAGGCGGACGAAATGTATTCGCGTTGCAAGGAGAAAGCCGATGCCG
TCGTCGATCCGCTCGGATCCATCGAGCTATTGACAAATTGCATGCAGGAAGAAATGACCC
AAAAAATTGATGTCAACAAAGAGGAGCGATCGTTTCAGTCCAAGATCCAGCGGAAGATGG
TCGCAGATCTTGTTCCGTTTGAGTGTGGCGACGTGAACAAAACAGACTCGAGAGAAATCA
TGAACACAACATGGTCCTACTATGATTCCAACGACGAATCCAACAAGGATTACACGGTGA
GAACCCTCCACGAGCTGCCCACTTCCAAGATTGTTGTCGTGGACGATTTCATTGCGGAAG
GAACCTGCAATATCTTGAAATCGTTGCGACAAGAAATTTCCGATGGCAAGATTGGAATTC
CGACCATGATTGCAACCAATGAATCAAAAGAGTCCAAAAGAATGCTAGAAGAGTTCTACA
AAATATACGATCTCATCAAGACTATTTTCCCGGATTGGGATGAGCTGGAATTTCTTAGTG
ATACGTTATTCGACTTCTTCAAGGATTCGGATGGATTCCAAACACCCTCGCAGCTTTGCG
TCGGTCCAGAGGAAGTTGCCGGGGCACATGACGAAATAAATGCCGGAAAACATCCAAAGT
GCAAGATTCCGGGCGGAGAGCCTGTCCGGGCTTCTAGCAAACGGTTCGTCGTCGAAGAAA
AAGGAAGCAAAAAGGGCCCTAAACGCCAGGTGGCGCAATTCTTCCTTTTTTGCGACGAAC

CAGAGCAAAAGCTAGGCGGCGTGCATTTTCCGTACGCCGGTGTCTACGTCGCACCCAAGG
CCGGAAAAGTGGTGGGGGCCATCCACCGGCATGCCAACGATCCTTCCGACGAACTGGATG
GCTACGTCGATGAATATCATTTTTGCCCGAACCACGATCTCTACACACATACAATAAACC
ACAACATTCCGGCGGAATAAGTAGTGATGGAAAGGAAGAGAGAACATCCATGAGGCATCA
TTCATGGGGCATCGAATAGTGTGCTTGCAGGCGCAACGACAAGAGTCGTTGAGCACGGAA
AATCTTGAAATGGGCACATATTTGGTGTACGATAAGAATAAGCCACCTAAGGAAAGAGTT
TCGTAGTAAATTAGATCGAAGAACTCCCCATTGCATTCGTGGTATAAGGTTTGTGTCGTA
AAATTTCTGAATTGACTAGTTTCGACGGAAATACTGTTTTTGCGTATCGACTTGTAATTC
CCTAAGATTGCAAGTACTACTAGTACAGTGGTCACAACAACAAAAAACTGAGATGCAACT
AATCGCTCATCAAACTGATTCCTGTTCAGTTCATCCATGTCATTGCTCAACTAGAGGCTT
TTGTGGCACGCTACTGCTTGAAAAAGGTTCTTAAGTCGTTTT
>comp2034_c0_seq1
CCAAAAAGCACAACCAAAAAGGATCCTCGAAGAGCCTCATCGACAACTCCGATAGTTTAA
GTCACAACCACACGAAAAATGAAATTTGGTGCCAACATTGCATCGTTTTTGATGATCTCG
CAAGGTAGTAGACAAGTTTTAGCTTTCACTTCTCGCAACATTATTTCGCACCGTAACCAA
GTTGCTGCCGCTGCATCCAGTACATTAATTAGAATGGCGTCGTCAACAGTAGCGACCAAC
CCCATGCTGGAACAAAGCGGTCTTCCGAAGTTTGAAACCATCGAACCCAAGCACTTGACT
CCTGCCGTGGAAGAGTTGTTGGAAAAGCTCGAAAATGATTTCGTCGAAATGGAAGATACT
CTGTCGAAGAAGGATGGCGCAGTTGAATTCGACGAGGTTTTGCCTGTAGTCGAACGCATG
CAATATCCAATTGGTTATGTATGGGGACTTGCCAGCCATCTCAATGGCGTGAAGAACGGA
GACGAACTGCGAGAGGCATACGAATCGAACCAAGGCAAGGTCATCGAATCTATGACCAAA
TTTTCGCAAAGCAAGGCATTATACGATGCGCTCTCCGCCATCGAAAAGCAATGGGCCGAA
GACGGAGACCAATGTGACTCGTTTTTGTCGAAACAAAAACGTCGTGCCGTCGAAAACTCC
CTCCTAGAGATGAAGTTGGGTGGAGTCGGACTCGAGGGAGCCGACAAGGAGCGTTTTAAC
GAAATCAAGCTGCAGCTTGGAAAACTTTCCAACAAATTTTCCAACAATGTTTTGGATGAT
ACTAAGACCTTCAGCTATACGGTTGAAGATCCCGCAATTATGGAGGGAGTTCCTGAGTCT
GCGAAGGCGTTGTGGGCTAATTCTCACGCACAATTCATCAAGTCCGAAGCGAAGGAAGGG
GAAGATGTTCCCGAGGTCGATCTCGATGCCGCAACAAAATCGGGGCCATGGAGGATTACG
CTGGACATTCCTAGCTACATTCCTGCCATGTCACACCTTGCTGATCGTGGAATCAGAGAA
AAAGTTTACAAAGCTTACATTTCTAAAGCGTCGGAGAAGAGTGATGGCAAGAACAACGTT
CCATTGATCTACGAAATCCTAACCTTGAAGCAAGAAATGGCAAAATTGCTTGGGTTTTCG
AATTATGCTGAGTTGTCTCTCGCGAAGAAGATGGCATCATCGGTTGACGAAGTCACTGAA
CTAACAAATCTCATGGCCGACAAAGCTGTTCCTGCCGCGAAAAAAGAATTGGAGGAAGTC
ACTGCTTTGGCTAGGGAGAAAGGCGGAGATGAATACTCCACTGAAAACCTTGAGAAGTTG
GAACCTTGGGATGTAACTTTCTGGAGTGAGAGACTGAAAGAATCCAAATTCGACATGACC
CAAGAAGAGTTGAGACCCTACTTTGCTTTGCCCGCAGTATTGGACGGTATGTTTGGCTTG
CTTAATCGTCTCTTTGACGTCGATGTCAAGGCAGCCGATGGCGAAGCCGAAGTTTGGAAC
GAAGACGTTCGATTTTTCAAAATTTTCGATTCCAAAACAGACAAGCACATTGCCAGTTTC
TTCTTGGATCCTTATTCTCGTCCCGAAAACAAGCGTGGGGGTGCCTGGATGGCGGACTGT
ATCGGCAAGAGTGAAGCAACAAACAACGATATTCCTGTCGCTTATTTGACTTGCAACGGA
TCACCTCCGGTCGGAGACAAGCCCTCATTGATGACTTTCCAAGAGGTCGAAACCCTGTTC
CATGAAACTGGACACGGGCTTCAACACATGCTCACAGACGCCCAAGTGGGTGACGTTGCA
GGAATCAATGGAATTGAATGGGATGCTGTAGAGCTTCCTTCTCAATTTATGGAGAACTGG
TGTTATGATCGTCCCACAGTTTTCGGTTTTGCAAAACATTGGGAAACAGGTGAACCTCTT
CCGGAAGAGAAATTCGAGAAGCTTTGTGCGCAAAAGATCTTCAACTCTGGTATGCAGACC
TGCCGCCAGCTATACTTCGGACAATTAGACCTGGCGCTTCATTCCGATTTCGATGCCAGC
GCCGGGGAAGAAGGAAAGGGAATTTTCGAACTCCAACGTGAGATCGCTAGCAAGTACACT
CCCCACAGCATGCCACTAGAGGAGGACAGATTCCTTTGCAGTTTCGGCCATATTTTTGCT
GGAGGATACGCTGCCGGTTATTATTCTTACAAGTGGGCCGAAGTCATGTCAGCCGATGCC
TTCGGTGCCTTTGAAGACGTTGGACTCGACAACGAAGAGGCCGTAAGAACCGTTGGAAAG
AAGTTCCGAGAGACTGTTTTGAGCTTGGGGGGAGGTGTGCCACCCGCGGATGTTTTCAAA
GAATTCCGTGGACGCGGTCCTAGCCCAGAGGCATTCATGCGCCACAACGGGCTTGAGTGA
ATCGGAAATGATTTACCAGATGCAACCGGTAAACTTTTTTTGAAATTATTGAATTGAAGC
CTTTTTCAGCCTGCTACTGAACTCGAGGAGTTTATAGGAAATTGCCTTTTTCCTTTTATT
ATGCTCCACGGTAGGGTTCAGTAAATATATTACTGTAATGAAGGTACTAAAATGAAGACA
TCGAGAACAATAATCCAAATTTATCATTGCA
>comp2035_c0_seq1
CGACGACGACGACGACGACGACAGTATCGAAGACGAAATCTGGGACGAGCACGGCAAAAA
GATTGCCTACCAACAGAAACAACTCGGTCGGAGACGGCGGCGCTTTGCTCCCGCGTACAA
TATCTGGGAAAAGCCCGCGCTCCGACCCATCCCGAAAAAGGTGGCCAAGCGGGCCGGCCA

ACAGGCCGTCCGTTCTATGATGAAACACGGGTCGGTCATGATCCCACAAATCCTGAGCGA
TGCGACCATGGAAGACCTGCGGGAATTCATTGTCGACAAAAATGAGCGTGTCTTGGGGAC
CCCCGACCAGTACCCCATGTCGGCATCGCACCACCGGATTTCGTACGGGATCGAGTCGAC
CGAGCATCCTTCCGTCGTGAGGGCATTAAAGGAGATCCACGACCACGGCGTCTTTGCAGA
GCTCATCCAAAACCTGGTCGGGGACGAAAACCCGGCGCTGTCGGAAATCACCGCCATTAC
GGCCTGGGGCGGTGCCGAAATGCAGTCGTGGCACCCGGACGTAAAGCCTGACGGAAACGG
GGTCATGTTTGGCCGGACATACAGCCACTCGTATTCACTCTTTCTGCCTCTCCAGGACAC
AGTAGGCGAGATGGGGCCTACAGATCTCTGTCCCGGGACGCACATGTGTGCCGACGAGGA
TTTGTGGGACATGTGCGAGGAAAACAAGATTGGCCTCCACGAGATTCGCAAGGCCAAGCC
GCGCCATTTATACGATCCCTCCGTCGAGGGAACGAAGGAATACCTAGAGGATCTTTCGAA
AGAGGGCACTTGGAGGGCTGGGGACGGGGCGCTCTTGAACCAGCAGGGATGGCACCGAGG
AACGGCACACACTGACGAAGACATGGACGATCGGGTGGTATTTATCGTGAGCTTTTTGAA
ACGCCCAAGTGCCGCCGATCCCCGGCAGCTAGCAAGAGGGACCTACTTCCACCAAAAGTG
GCTGAATTGGGGTTCAACCTGGCAGGACATGGCCGATGCGGCGGAATCGCTCAAGCGGCC
CTGGAATATTTTGCGGTGCCTGCATCTGTACAAAGCCTCCGATCGATCATGGGGATACGA
TTTGTTTACGGCGACGACGTTGCGGATTGCCAACGAACAAATGGGAGGCGAAGCCGGAGA
TCTGGAGGGCCTCATAGACAACGTGATGATTCCGTTGCGTTATCCGGAGTGGTTGCAGGG
GACCATTGACTACGATTCAGAGGAAGCCTGGCAGATGTATTTACGGGAAACGATCCAGAA
GACGCACGGGTTTTTGTATCGCGTGAACGCTATTGGTCTCACTGGATTTATGGTGGCTAT
GTTTATCATCGCAATCTTGGCACACTTGTACAATAGATTTTCCGGCCAAAAGAATCGAGA
AAGATCCGTTATTATTCCGGTTTTGAAAGCGAGTGCCCGGCGCCTTGTATGGACCCACGG
CTCTATTTTCGCTTTTGGGTTGTACGTTTGGCACGTGTTAATTCGATCGTCCCAGTGGGC
GATCGATATTGACTCGGGGACTACCCTTATGAGGCCGTTTCCACCCGATCCGATCTATCG
AGACGTGGATCCCGGTGTCCAGGGTGGCAACACCACACTTCCGAGGCGGTCAGATGTTTT
GATTGGTACCCGATACCATACGAATGCAATCGGGGCCTACCGGACGTGGCTAGATTATCA
TCCGGGGAACAGCGTCTTCAATGACTTTGTCGACACATATGGGGGCAATGGAAACTTTTA
CCACTCGCTCTTGCAACTGAACAAAGAAAGTCATGCTGTATCACAATTGCCCTTGAGCTT
GGCCGACCAGCTGGCATCGGTGGCCTTGAATACTATCCAAAGTCGACACGATGGTAGATT
CTTGATGCAGGATTATCGATCCGGCGATTGGAAGGTCCTGAGCGAGGCCGAAAGCAAAAA
ATACATCCACAAGAGACTCTTTGTCGGAAGTAAAGACACTCTGTTGGGGGCGCTGAATGA
CGAAACGGAGCTTATGCTGAATCGATATCGCTTCGGAGTCGAGCGGAAAACGTTTTCCAT
GGCTTGGAATTCTCAGCTTTTTGTTGCCGATTTGTCAGAAAGTCTTTTCTCCCCTGCAAA
TAAAAAATACTTGAAGGAGAATTTGAATCAAGAAATGGTTCGAGATTCTTCAACATCGTC
GCTTGCTTTTCGATCCATTCTTCCACATTCTAGTTTTCGATTGAGGAAGCGGAAAAAAGT
TCATGAAAACGCAAACATCTCTCACAACAATAGCAGAATTCGTAGATACATGGTGGGGGA
TAGCCTGCCATCGCTGTTTCCCGGGATGGAAATATATTACTCCATTGATAATTCTCGAGA
CGGCTCTTCAGATGAAATATTTTTCGCAACCTTGATTGGTATTTCGAGCAAGAAAACGTA
CGACGGGCGAGCTCGCTACCACATTGCCTTTGATGGTTCAGAGGTTCCTGAAATAGAGAC
CACTGAATTAGGTCGAGTCAAGAGGCAGTCGATCAGAACACGGCCGCCAATTACGGAAGG
ATCCAGAGTGATGGTCGGAGATGTCGCTGGGACCATCCTATTGGTGTTGGCCGATGGCTC
TATTGACGTTGTGTTCGACGAAGATGGCGAGATCGATACGGACATTCCGTACACAGATTA
CGAGTTATTTGTAGAATAAACGAATGGCATTTTAGCCCTATTCAATACTTCATAGAACTG
CAATCATA
>comp2036_c0_seq1
AATCGATCCAAAGATACTTTGTTCCACCGGTGCTGTGATTCGTAGGTTTCATCAGGTCGT
TCTTTAGAAAGTTTTAGTCGTGGATTTAGGAGATTCGGTGATCTACGTACTGTATTCGCT
TCGCCGACTTGAACGTTTCGGGATTTTCAAATTTCAAAATCCAATCGATGGCAATCGAAC
ATTCCATTTTTTTCGTTCGAATTGCCTTACTGTACCAGTCTCACGTACGAGTGTACCTAC
TCGTAGCACGTAGCAGTATTTTCTTTTATCTAATCGTACGTACCTCACATCACGAGTGAG
TGCCACAGTACGAGTACGCACGGTGCGATCGACCCTGCTGGATGCATCGCTCCAGTCTAT
CACAAACTTCTGTAGTATCACGATGGTCCAAGTCGTTCAAGTAGTCCTCAAGTGGTCGAA
AGAGGTCATCGAATTTGAGATCGAAGTACCGACCACACCCAACTCGTTCACGGGGAGATC
ATTCAAGGACAGGGTGCACTCCCTCACGAAGGTTCCGGTTGATCGTCAAAAGATTGTCGT
CGCAAAGACCAAGTCCAAACCGACTCCATCGGGAACCGGAAAAACCAAGAACCCAATAAA
ATGGTGGAAAGGTATTCTAAAAGACGACTTTGATTTTGCGTCTGCGATTGCAGCATCGAT
TAATACTGATAATTCGACAAGGATAGAATTGAGGTTAACGCTAATGGGAACAGCCGAAGT
GTTGGAATCCAACAGCAACAGGGAGAAAACAAAATTTATCGAAGACATGTCATTATCCGA
AAAGAAAGAAGCGGAGCAAAGGGAATGGATGAACTCTATGGAAAGCGTGGCGGCGATGAT
CCCAGCCCTTCAGGTCCCGCCACTGGACCGGCAGCCCCCGGGGAATACAAACGGCGATGG
CAATGGCAACAGCAGCGGGGATCCCATGCAATCGTCCCCCCCCACGATTGTCAGCGAATT

CACGGAATCCCGAGCCTACGATCGATTGGTGCACGGATATTCCCAGCTGCGCATCGACGC
CCTTCTTCGGGAGCAGACCCGAGGCGATCTACCAGGTGCCGACGCGGAAGAGCCGGGGAG
CCTGTCGACACCCCGATTGCACGGGCGCCCAGTCATGACGATGGGACTGGAACTTCAGCG
CGCCTACGTAAACGACCTTGCCGTATTGAAGGATGGAACCCTGTTGAGTGCCTTGGACGA
TGGCCACGTCCAATTGTGGAAACACTGCCGTCGAATCTGTGACATCGTGCACAAGCCGGG
CCAATTCGGTGGCTCCCAATTCTTTTCTGGAGTCGACAGCGTCCTGGCTCTGGACGACAG
CAACTCGCCATCATCGCCGTTTGCCTTTTGCACGGCAGGAATGGGCACCGTTCGGGTGTG
GGACAGCGATGGGGATGCCCTGTTGGGCCTGGTCCCGTCGCCGCTTCCCAATACGAGTCC
GACGGGTTTGGTGCGGATCCCTACCAACAGTCCGGACGCGCTCTGCCTAGCAGCTAGGTT
CCGGATCGCCTGGCCCGCCTCCCGCCGACCGAGACTCGTGCCCCAAGACGAAGCAGGCCG
CAGGCGCCTTATAGAGATCGAGCGGAGCGAGGCAGAGATGAACGATGCACTGACGGGCAT
GTCGAAAACGGTTCAGGTTTTCTTTAGCGACGGTGGCAGCAAAACACAAGGGGTCAAAAA
TTTCTTTTTGACCGCACCGGCTCCTGTTACGGCACTGGCATCATGGAAACAAGGAGATGA
TTCTATCCTAGCTGTAGGGGACACCCAGGGCGGGATTACGCTGCAAAAAATAGCCACGTC
GGAGCCGGGATCGAACGGGAGTCAAGCTAACATTTCAAACAACAAAAACAACAGCACACT
CCGGATTCAGGAAGTACGACGCATACAGTTTGCATCGTCCGAAAACGCATCCGAGACGTG
TTCTTCTATCGTCTACCTACAGTATATGGCCGAAACCAAACAGTTACTGGTATCGACCAA
GGAGATTCAGCTCACCGCTCTCACCAACCCATCGTCGAACATCCCAATCCCAATATTGAC
CTTGATTCCGTCTGCGGGTCCTCAAGCGGTGCACTCGATCGATATGGAGGCGGTGTTATC
GGATGCAACGCCCAGACCGCTCTCATCCAATCCTCTGCTGTTTTCCCTCGCTGGCCACAA
GGACGAGGTGCATTGCATAATGTCTCTTCCCAACGGAGATCTCTTGACGGCTGGCGGAAA
ACACGATGCTACCACGCAGATTTGGTCAAGGTCACAACTCCAGTCGGCAACGACAAGAAC
CGAAACACCAGCATCGGTACCCATTCTGGCGAAAGCAGCAACAGTAAATCTCTGCAAAGA
TGCTGGGTACATCTTTGCGGCAGAGGTGCTCGAGGATTTCAAGAGCAAGGGAGATGGTTC
GGGGGATGAACCGAACAATAAGAAAGAACAAAAGAGCAGTCCTTTCGCTATCGCAGTAGC
TCGTTATAATGTTGTGAAGATAATAGTATAGGAATCTTTTAGGTTGGAATTTGGAAACAA
CAATCTCTATGTTCAATGATTAAGACAAAGCAGGGTTTATCCTCTCGGAGGGTGTTTTCC
GAGCCAAATTACATTTCACTTGTTCATTTTGAGAAGCGTTCCTGTAGTTGCCAACAACTA
CTCCAGATTATCATATAATGAACTACTCTGCCAA
>comp2037_c0_seq1
AAAACTTTCATCGCAGTACCGATAATGCAGTCGTAACAACAACAATCTAGTCTAGCACTC
ACCCCGATCGAATAATTGGGCAGATACAGCTCTAGTAGGAAGGAAGTCCTTATTGTGCCA
TGCACGTGTCCATGGGAGGATACGAAAAGACTTAGATTACTCATCTTTCATAAAGAAGCA
AGAGCGACAGAAGATGGTCGTGAAAGGAAGATTATCCACTATGCGGCAGAACAAATCGAA
GAAAAAGACTTCGTTGCCTTCCAGAATATCAGCTGAGCACTGTTCCAAGAAAAATGACAA
GACTTCTTCGGCTCGAAGACGTTCGAGTCCAGAAAGACGATCTGCAAAGGAATCGAGGCG
ACAAACCGGTAGAGGTAGCTCGCGGCGGTCGAAAGCGATGTCTCCGAGCGGTGGTGGTCC
GAGGCTGCCATTGTCTCCGCGATCCTCCACAGCAGCAAAGTCTCCGACGTCAATAAAATC
TCAAACATCATCCTTCTCTCGAAGATCAAAAATTGAGCCGTTGTTATCAGATGACGAGCT
GAGGATTGCAGTAACGACAAGTTCGAAAATCGAAAAATCTACAGAAAATTACACACCTCC
GGTTGCAGGTAATCGCTCAGCTGTCGAAACTGAAAATCCCAAGTCTCTTGCTTCAGGTGC
TTCGAGTCCAAGAAAGCTCAAACCTCCGTCTGAAATTACTGCACGGGTTGCGCAAACAAA
ACAATACAAACCTCAGCCCGTTGCTGAGACTGCATCTATTGAAGACAGCGCAGAGAACGA
CAGGTATGAATTGGCCGAATCCAAGCTTGCGGCTGCAAGTCAAGGGATGTTTGAGGTGAA
AAATATTACAATCAACGAAACATCTCCAACGGGACAACACAGGGGATCGAGTGCTCTCGA
GGCGGGTTTGTTCACTGACGGTTCTGGAACCTTCGGTACCGATACCTATGCATCCGACAC
TTTTGCTGACGAGATGTCGTTTTCATTCGAATCTCACCAACGCCCGAAGAAGAAGTCTTA
TCGCAAGTACAGATTTAGCAGAAGTTGGGTTACGACGGATGCATCATCGTTCGACGATGT
TAGTGTCATCGTCAAGCAACGGGTAGCGTGGGGTTGCATTTTCCTGTCGGCAGTCCAATT
TGCAATTCTCACTACTCAAGTGCTGATGTGTGGAATCGCAAAATTGAATATTAACCCAAC
GATTGGTCCGTATCCTGATGCATTTTCTGAATGGGGAGGAAAAAATGCATATCTACTAGT
CGAAGGACACCAGTACTTTCGGCTAGTCACTCCTACCTTCTTGCATGTTGGATACCTTCA
TTTCCTTATGAACGCATTTTTCCAGCTCGAGACCTGTGCTTACTTGGAACGTGAATGGGG
ATTCAATGTATGGATCTTCATTTACCTTGCAAGTGGTTTGGGAAGTTGTTTGGCGGCTTC
CGCTATCGACCCAAACGTAATTGGTGTATGCAGTTCTGGAGCTCTGATGGGATTATTTGG
CGCCAGAATAGCGCAGGCTATTGTGTGGTATTCCTTCGAGCCTCGCCATGAATACATAGG
ACAGGGTGCCCTTCTTTTCGAACGACTGGGTGGAATAGTTTGCAGTGCTGCAGTCGTATT
TCTCTTAACGTTCTTGACATATATCGATTGGTCAGGACATTTGGGCGGTCTTGCGACTGG
ATTCTTAGTAGGGCTCGTGGTTTTCCCTTTTGCGTTGGAGGGTCGATCTACCAAAGCGGT
GCTAAGATTTACCGGAGTGTTAGGACTTTTGATTGGAGGAGTAGTGCTTGGAGTGCTCCT

CTTCCACTATGCAGAATTAGATGAAGAGCTCGCTAATGCATGCAATTACTTCCGAAGTCT
TTACACAGAAGGGTACAACTGCGAGTGCCAGGCCTTTGACTAAGTTTGGCCGAGCTAGCC
ACGGCACTGCGCTCAATTGAGCGAACGCGGTAGTACATCTCCCCTTCATTGATTTGCTTT
CAAAAGAATATATGTTCTAATTGTGGTCATTAATAGTGAAACATTTTATCTTACAAAAAA
AAAA
>comp2038_c0_seq1
TTCGATTCGATTCGATTCGATCGATCTAGATATTGGTGGGCCGATCAAACCAATCCAACA
ACCATATCATCATGACGTCCCAAACGAATTTTGGAACGAACGAAAATTCCGGAGCAGTCT
TGGCTCCCCTCATGTCGAGAGGTGACCCGGCAATGGGGGCGATAAAGTATGTGAAGCAGC
CCGGACCGGCACCCGCACCGGCGCTCCAGAACAACGGGGGGCAGTCGACAGCGAACGCGA
CCGTCGTCCAGATGCCTGCCCTCGCTCCCCAGCCGATCGCTCCTCCAGCACCTGTGAATG
CCGCAGGAGTCTCCCAGCCACCACCGCCAATGGTGATCAACATGCAGCAACAGCAGCAAA
TGCACAATATGCAGCAGCAATTACACACCATGCAGGCGCAACTCCAGCAAAAGATGCAGC
CCAAGCTAATCCCGATGAACACCCGCCCCAAGGTGCCCGCAGCCGCAGCTGCGGCAGCCA
CGACGGTGAACCACGTTCCGAGCCATGCGGCTCCGAACGGCACCTCCTCGGCCTCGGTGG
TGGGAACCGACCCGGGGACGCTTCGGACGGGGGGAGGGGGCGGCCAGCTCCTACAGTTTC
CCTGGAAACTCCACGAGATGCTCCACCTGGCAGAAAAAAACGGCAAAACGGACATTATCT
CGTGGCTTCCAGGGGGAAACGGCTTCAAGGTGCACAACAAAGAACTCTTTTGCGAGGAGA
TCATGCCGGGATATTTCGCCAGCCAGAAATACAAGACCTTTCAACGCAGCCTGAACTTGT
GGGGATTTGAGAGCGTGGCCAAGGGGCCGGACCGGGGGGCCTGCTACCACCAATACTTTG
TCAAGGGACATCCGGAGTTGTGCCACAGCATGTGAGTTCTCGCTTTGTTCTGTCCTTTCT
GTTCTCCTTCGCTTTCGTTCGATGTTGTTTGCATTCCGAAGGGAAGTTGTGCTTTTCTAG
CACTGACTTGTTTCCGAATTAATATCTATGTTTCCTTCCTATCTTGATTCGATTCCAGGA
CTCGTGTCAAGATCAAGGGGCAGAGCAATCCCCGAACGCCTGCAGCGGCGCAAGCTGCCG
CGGCAGCTGCCGCTAAGAGGGCCGCCTTATCGTCGTTTGCGAATCAGCAGCATCAGCAAC
AGGCTGTGACCGCCCCGGTTCCGCACCAACAGCAACAACAACACCAACAGCCAATCAACA
TGATGGCAATTAATCAGTTTATGCCAACCATGCCGGGGGTTGTGACTCCAATCGTTGGCA
TACCTAACTTTGGTATGCAAATGAATGGGGCGACAGCGGCATCTGCCACAACGAATCCCT
ATGCCTTTGCGGCAATGAACCAATTCGTCACTATGAACGCCCTCCAGCAAATGAATCCAA
CCCTGGCAGCAGCCGCCATGCAAAATCCGGCTATGAACGCGATGCTAACCGCACAAGTCG
CCGCGGCGGCGGCAATTCAAGCACAGGCGACACAATTGCAGCAGCAGCAAGCCCTGGGAG
CTGACATGGCAGCGGGACAGCAAGTGGCGCCCAGTATAGCACCCCCGACTACCCCATTCT
CTCCAGTCGGGCCGCAATATCCACCTCCAATGTCTTCGGCACCTACTTCTACTGCGGCGG
TGCACCCGGAACCAGCGCCGACAGCGACAGTTCCTACGAGCACCAGCACGACGAGTAAAA
GCAACAGCAATGGCCTATTGACTCTTTCGAATGCCAATGGCAATGACCACAGCTCGGAGG
CCAAGAACAAAACACAATCGAATGCCGACGCGAACTTCGCCGAATATTTGGCGCGAAAAT
CGGCAATGGACCGCCACATCGAACAACTAAGTGATCCCAGTGCACCCGTTCCTACTGTCG
CAGCTTCGAACAACAACAACAAGAAAACTGGTAGCACAAATTCAGCGGGTTCAGACGGAG
CTCCCGTGGCCGTATAGAAGGGGAACGGCCATGCGTCACAGCTTGACCAGTCGTACGTGT
CTTGTACCCTATATAATATTATAATTTATTATGTTGTATCAATGCTTCAAAA
>comp2038_c0_seq2
TTCGATTCGATTCGATTCGATCGATCTAGATATTGGTGGGCCGATCAAACCAATCCAACA
ACCATATCATCATGACGTCCCAAACGAATTTTGGAACGAACGAAAATTCCGGAGCAGTCT
TGGCTCCCCTCATGTCGAGAGGTGACCCGGCAATGGGGGCGATAAAGTATGTGAAGCAGC
CCGGACCGGCACCCGCACCGGCGCTCCAGAACAACGGGGGGCAGTCGACAGCGAACGCGA
CCGTCGTCCAGATGCCTGCCCTCGCTCCCCAGCCGATCGCTCCTCCAGCACCTGTGAATG
CCGCAGGAGTCTCCCAGCCACCACCGCCAATGGTGATCAACATGCAGCAACAGCAGCAAA
TGCACAATATGCAGCAGCAATTACACACCATGCAGGCGCAACTCCAGCAAAAGATGCAGC
CCAAGCTAATCCCGATGAACACCCGCCCCAAGGTGCCCGCAGCCGCAGCTGCGGCAGCCA
CGACGGTGAACCACGTTCCGAGCCATGCGGCTCCGAACGGCACCTCCTCGGCCTCGGTGG
TGGGAACCGACCCGGGGACGCTTCGGACGGGGGGAGGGGGCGGCCAGCTCCTACAGTTTC
CCTGGAAACTCCACGAGATGCTCCACCTGGCAGAAAAAAACGGCAAAACGGACATTATCT
CGTGGCTTCCAGGGGGAAACGGCTTCAAGGTGCACAACAAAGAACTCTTTTGCGAGGAGA
TCATGCCGGGATATTTCGCCAGCCAGAAATACAAGACCTTTCAACGCAGCCTGAACTTGT
GGGGATTTGAGAGCGTGGCCAAGGGGCCGGACCGGGGGGCCTGCTACCACCAATACTTTG
TCAAGGGACATCCGGAGTTGTGCCACAGCATGACTCGTGTCAAGATCAAGGGGCAGAGCA
ATCCCCGAACGCCTGCAGCGGCGCAAGCTGCCGCGGCAGCTGCCGCTAAGAGGGCCGCCT
TATCGTCGTTTGCGAATCAGCAGCATCAGCAACAGGCTGTGACCGCCCCGGTTCCGCACC
AACAGCAACAACAACACCAACAGCCAATCAACATGATGGCAATTAATCAGTTTATGCCAA
CCATGCCGGGGGTTGTGACTCCAATCGTTGGCATACCTAACTTTGGTATGCAAATGAATG

GGGCGACAGCGGCATCTGCCACAACGAATCCCTATGCCTTTGCGGCAATGAACCAATTCG
TCACTATGAACGCCCTCCAGCAAATGAATCCAACCCTGGCAGCAGCCGCCATGCAAAATC
CGGCTATGAACGCGATGCTAACCGCACAAGTCGCCGCGGCGGCGGCAATTCAAGCACAGG
CGACACAATTGCAGCAGCAGCAAGCCCTGGGAGCTGACATGGCAGCGGGACAGCAAGTGG
CGCCCAGTATAGCACCCCCGACTACCCCATTCTCTCCAGTCGGGCCGCAATATCCACCTC
CAATGTCTTCGGCACCTACTTCTACTGCGGCGGTGCACCCGGAACCAGCGCCGACAGCGA
CAGTTCCTACGAGCACCAGCACGACGAGTAAAAGCAACAGCAATGGCCTATTGACTCTTT
CGAATGCCAATGGCAATGACCACAGCTCGGAGGCCAAGAACAAAACACAATCGAATGCCG
ACGCGAACTTCGCCGAATATTTGGCGCGAAAATCGGCAATGGACCGCCACATCGAACAAC
TAAGTGATCCCAGTGCACCCGTTCCTACTGTCGCAGCTTCGAACAACAACAACAAGAAAA
CTGGTAGCACAAATTCAGCGGGTTCAGACGGAGCTCCCGTGGCCGTATAGAAGGGGAACG
GCCATGCGTCACAGCTTGACCAGTCGTACGTGTCTTGTACCCTATATAATATTATAATTT
ATTATGTTGTATCAATGCTTCAAAA
>comp2039_c0_seq1
CGTACTTTAATCACGTAGTTGGTGCAGGGTCTGATGCTGAATTCTGTAATTTATTTATTC
CTGTTCGAGAATTTTGTCGTAATGATACCGGGGTACGTACGTACGTACGTACCAACAGGG
GTCATTACCAACACATTAACAAGAATGGTATCGAAAAGTACAAATCCTACGGTACGAAGC
AAATAACAAGACTTCGTTTTGCGAACTTTTTTTGGGTGCGATAAGCTAAAGTTCAAACGT
ACTTAACACAACTGAAGCACTGCTACCAGCTGCAACAGAACTCTACTTTTTAAGATCTCT
AATAAAATGTTCTCTGAGCTTTA
>comp2040_c0_seq1
TCGATTTCTTGCTGGTGTGGAAGCAAACGAGAAAGGAATCGACACCTACCTATTGCGATT
CCCTGTTCGGAGAAAATCAATACTAAACGAACTTAGACTTACGAACAGCAAATATAAAAA
GCAACAATGTCAGCAGTATTCTTACCTCAATCCCGCATCAAGGAGGTGCACATTGAAGGC
CTTGCAATGATGAAAATCGTGAAGCATTGCCAAGAATCGCTGCCAAATATGGTCCAGGGA
GCGCTTCTGGGACTCGCTGTGGACGGGGATATTTTGGAAGTCACGCACGCCTTTCCAAAC
CCCGCCGGAAAGACCGACGACGACATCGACGAGGAATATCAGGTGGAAATGATGCGCATG
CTCCGCGAGGTCAACGTAGATAACAATTGCGTTGGATGGTACCAATCGATGTACCTGGGA
GTTTATTCGACTGCCAGTGTGTTGGAAAACCAATTTAGTTACCAAACAGAAGTTTCTCCC
AATGCCGTGGTTATTCTATACGACCCATTACAAACGGCGAACGGAAACTTTTGCCTCAAG
GCCTTTCGGTTGTCCGATGAATGCGTGGAAATGAAGAAATCGGGTAGCAATGATTTCCTC
AAGCCCTCCAATATATTCGAAGAGCTGCCCGTGAGATGCACAAACCCGAGCCTGGCAACC
GCCATGCTGCAGGACTGGACTCCCAAGTCGGGCATTGCCCTGGATCGTTTGGACATGAGC
ACGCAACCGTACCTTGAAAAGCATCTAGAATCACTGTGTGGATGGGTCGACGAACTAGCG
GGCGAGCAACAAAAATTCCAATACTACACACGGCACCTGGCGAGAGGCAACAATAAATCA
AAAGCGAGCTGGGCCGCCCCCGAAGCTCCGAAACGAATGGAGAGTCTCTTGGTCACAAAT
CAAATCGAAAACTATTGCGAACAGATGGACAAATACGTTGGCGGAGGCCTTGGAAAACTC
TTTTTGGCCGGAGGTTTGCACAAGGGAGAACGATCTACCAGTGCTTGAGCGAATTAACAG
GAGCCGAGCCGAGCTTTGTAAGCGATTGAATATGGCACAGAATACTATGCTTGCTCTTTG
GATCACTACACTTATCTAGCTAGATATGTACGGATGCACCAGAAAATACAGGAATCTAGT
TGGGATCCTTCATTTTGCGAAACATATCATATTCTTGTTCGAACTAATAGTAATATTGTT
ATGATTAATGATAAACAAGAGGAGTACCGG
>comp2041_c0_seq1
CAACTTTTCAGATCTCTTTTAAGAACCTATAACATCCTACAATGAAGTTCACCGCCTTTG
CCGCTCTTGCTCTTGCCTCCTCGACCGAGGCCTTCTCCCCCGTCACTCGCTCGAATGGTG
CCTCCGCCACCGCCCTCGAGGCCGTCCGCCAACCCATCATGGCCGGAAACTGGAAGATGA
ACCCCGCCACCGAGGACGATGCCATTTCTTTGTCAACCGGTCTGACCAAATTGTTGGGCG
ATGAGACCTGCCCCTTGGACGACGAAAATCCTCTCTGTACCGAGGTCGTTCTCTTCCCTC
CCTTCCCCTTCATCTCCAAGGTCAAGGATGCCGTCGAAGAAGTCGGAATCACCGTCGGAG
CCCAATCCATCTTCTTCGAAGACAAGGGTGCCTTCACCGGAAGTGTTTCCGCTTCCATGG
TTGACTCCATCGGATGCGAATACGTCCTTTGCGGTCACTCCGAACGCCGAACAATTTTCA
GCGATGACGACGACGCCATCAACCGCAAGGTCCTCAAGGTTCTCACTTCCGGAATGAAGC
CCATCTTGTGCATCGGAGAGACCAAGAACGAATACGACTTGAACATCAAGAACGAGGTCT
GCGCCATGCAGCTCTCCCGGGATCTGGCCGGAGTCACCAAGGAACAAATGGCCGATGTCG
TCATTGCCTACGAACCCGTTTGGGCCATTGGAACCGGTTTGGTTTGCGGATCCCAAGAAG
CCAACGACACCCATGCCTTCGTCCGAACCATCTTGGCCCGCATGTACGACGACGAAGTCG
CACAGGCCACCCGAATCCAATACGGTGGATCCGTGACACCGGACAGTGTTGATGAACTCA
TGGCCATGCCCGAGATCGACGGATGCCTTGTCGGAGGTGCCTCCCTCGATGCCGAAAAGT
TCGGACGCATCGTCAACTTCGAGAGCAAATAAATTTTTGGATCGTTTTGAAAGCACTTCT
TCCATGGGCGTTCCTCCGCGAAGAAAGTCTCACAGCGAATGATTAATGTAATAATAAAAA

TAAATTTGAGAAACGCAGA
>comp2042_c0_seq1
CAACAACAACAACAACAACAACATCATCATCACCACCAGCATCAACAACCACCAGCAACG
AAGAAACAAAAGAAACCTCTTTCTCGGAAACGCCAAATGGAACAAATGCTTCGCGCCGGA
AAACTGGACGAGGTCCGGGGCGACCACGAGGTTCGGGGCGTGGCCCACGTCTACCAGCAG
CAGGGGGACTACGCCGCCGCGGTGGCGGCTTCCTCGTCGGCCTCGTCGACCGTCCGGGTG
GTTCCCACCAGCGCCTACGACGCGGCGACCGGAACCACCGCCACCAGCACCAGCGTGACG
AGCCGCCAGAAGAACAGCAACCAGCTCAACAGCCTGTTGGCAAACGCCGCCTCGCTCGAG
AGCCAACGGGCGCAGAACCCCCGCTATGCGGGCGGGAGCCTCGGTGCGGGGCAGGGGTCC
CACAGGGCCACGGCAAAGCGCAGGTATGGATGGTAACCTACCGTGCGGGTCGTCGGAGAC
CGAGAAAGGGTGTAGAATGGAAAGCCGATCTATTGTATTATTGCATAAAACCATTTAGTT
ATATATTGGTA
>comp2043_c0_seq1
AAAAAATCGAACCGGATCCCGGACAGGGACACGCCTACACGAGCCGCCGCCTTCTCCAAA
TCGCATTCGAAGCGAATCGCATTTCGAATCGAACACTGCGGGATGCGAGTGTACGTACCA
TACTGCGGTGTAGGGAGGTATGCGCCGGGTGTCACCAAGAAACCGAAACGAGACGTGCAA
CAAATCGATACGAAAAAACACGAAACGAGACGAGACGTGCAAGGAACAAGCGACGACACC
GAAGACGAACACCTCGGGAAGATGCGATACCAAACGAGACGAGACGATGCGATGCGATGC
CGGCACCGGACCCCCCGTCGCGGGCAGCAGGGAATGCAACGAGTGAGTGAGTGACTGACT
GACTGAGTGAGTGACTGCAATACAATGCAATGCACATCCTCTTCTGTCGCACAGCACCGT
ATCGCACCCTGCCCAACACGGCCTCTCGGAGCCCCCACGGGGGTTGTTGGGCCGGAGGCC
GCCCACCGATGCGGCGAGAAACGGGACGGGCGGGCGGCCCCGTCGTTCCAAATTCCGGTG
CGATGGAATGGGATGGTTTGGTTTGGGATGGGATGGTTCCCGGACAACACAACACAGCGC
AGCACACCCGGCGGCGGTTCCACGGAACAATCGCCGCGAGAGGCCGGGGAGGCAAGCGAT
CGAACCCATCCCGCCGGAGGCGAGGGGGAACAAACAAGCACCCTGTTTGCGGGCCGCACG
CCGGTTCTGCCCCGGACGAAAGGGGGAAGAGGAGGTTCTTCCGCAGCGCAACGGAACGGA
AGGGAAGGGAACGGAGGAGGAATCAATGATTCGAAAGAAACACAAAGGCCAACACGATGC
CGCCACCGCACCAAAGCCGAGGCACAAAAGGCGATGTGCTTCCGCCACAAGGGTCGCTGC
GGGCACCAGAAACGATGTTCGATTCGTGGTCGAGAGGCCATACGCTACGGCACGATAGAA
CAGGGCAAAACAGAATAGAATGGAACAGAACAGAACAGAACAGAATAGAATACAATAGAA
TACAACCAAACGTCACAAAACAAAATAGAATACAATAAACCGGAACGGAACAGAATGTCC
GGCGGGCCTTGCGGTCCCAAACAGAACCAGCGACCGATCTCGAGGTGCTGCGCAATCGAC
GAGGAACGACGTACGCGACAGCAAGCAGCACAACACTAGAAGCCAAAATGGAAGGTCGGT
GTATACTAGTACTACTAGAACCGGCCGCTGCACCGGTTTTCCTTCCTGGACTCCACCACT
CGAGGGAGAAAGCTGTGGAATATCCTTTTGCTACTAATGCTAGTATGTTATTCAAACAAC
AG
>comp2044_c0_seq1
CCCGTATCATTCGAACACAATCTACATTCCTTTCCTTATGGACAACGGGCAATGATCGAC
CACATCGTGCAAAACGCGGACATACTTCAAAACGTTCTTAATTTCCTCGCGAACGATGGA
GATTCCGTCGCTCGATTTGCTCTCTCTTCGAAGGAGTTGTATGACAAAATCCTGCGAAGC
GAGACAATCATTAATCCGACTTTGTGGAAAGAACTCCTCCGCCAACGATGGAAACGAACG
ATGCGACGACCAATGGTAGCAAGCTCCAACGCCGACGACAACCAAAATGTCACGGTCGCA
GAACTTTCGTATCGAGAATTGTACGTCGCAAAGCGACAAGCCGACGCCGAGGCTATTCGA
CTGCTAGAAGAAATGACCTCCGACCTTCTGGTAGTGTTGCGATTGAACGAACCAAAAAGT
GTGCTGACCAAGAAAAATCCACACATCGGCAAAGCTTCGAACCATAATTGTTGGTACCAT
CTGCTGCGGGACAGAGCCGACTTCTACGATGTTATGAAAGCAACGGCAATGCGATACGCC
AGACCCACTGCGGCATCCTCTGCCCACGACAGGCTGATGGGATTTTGCGCGGCCCGTTGT
TTTCAAAGCATGCATTTCGCAGATTGCCTATGGGAATGGAGACGTCTTGCCGAGAAAGAG
GAGGAGGAGCGACTAGATCGCCAGCAGCAACGGGAGCTATCACCACAGCCGTCGCTATCG
CAAATGCGGGCCTGCCAACTGAGTGCGTCAAATTTCTTGGAACAATTTGCTTTGTTAGTA
TGCGAAATCCAAAAATCACCATTGCAACATCTGGAAGAACCCAATGGCTGCGCTTGTACC
GAGTATAACGCCAACGATGAAATTACGGGCATAGGGTTTAGGAATACCATGCGATACTCC
CATCAAGCAACCAAAAGTATGGACGCTATCGCCAAAGTTTGTCGAGAGCGAATCAAAGAG
AAATCAGAAGCAAATGCTAGCACGAGCACAAAGCTCGCAGTCATCAACGATGTTCTGATG
AATGACTATGGCTTTGCTGGAAATAGCGGAGATTATTACAACTTTCGGAACGTCCTGCTG
GACGAGGTAGTGGAGTCGAAAACAGGGATGCCCCTCACGCTTTGTGTCTTATATAGCTGC
ATTTGTCGAAGACTTGGTATACCCGTTCACTTGATAGGACTTCCAGGCCATGTTGTATTG
GGTTTTCATACAGATGACGAAAGCATTGACTTTGATGAGATTCATTCGTTCATTGACGTC
TTTCATGGAGGACGTATCCTATCGAGGGACGACTGTAGACGAATTGTTGAGAATTATAGA
ATGCCTTGGCACGACGAATTTCTGAACCCCATTCCAACCACCATGATCATTCAGCGAATC

TTCAACAACCTTAGAAATTGTCACGAAAGGGCGATGATGAATCCGAATCCTCCCATGTTT
TGTTCGGATCTAATGTTTCAACAACACATTCTGGGCATGATACATCGTTCTCCTCCCGAG
CTTGGTACCATTCTCTTGGAACGTCTTACGGAAGACTTGCCCAACGTTCTTAGTCCCGAG
TTGTTGCACGCCTATCACTTACTTTCCCCAGAAGAACTTGGCAACGGTCCAGTCGTTAAT
GCGACACACGCACGAATGCTTTTGGAGCTTTCTAGTTATGCTGGATTGTCGTACAACAAT
TACGCTTAACATAAATTACTTATTTTTTCTACCAAAAAAAA
>comp2045_c0_seq1
CAGCCGCGAAACAACGAAATAATATACCATGAAGTTCTCCATTGCCGCACTTACCACCTT
CGCTACGGCGACCAGTGCTTTCACTCACACCTCTTCCTTCACCGGAAAGACTTCTCTCAA
TGCCGCCTCCAACGGGTCCAACATGGACATGCGATACAAGGTTGCAGTTGTCGGAGGAGG
ACCTTCCGGAGCTTGTGCCGCCGAAATCTTCGCACAAGAGAAGGGAATTGATACTGTTTT
GTTCGAACGTAAGATGGACAACGCCAAGCCATGCGGAGGAGCCATCCCATTGTGCATGGT
TGGTGAATTCGACATCCCTGAAACCGTTGTCGACCGTAAGGTCCGAAGAATGAATCTTGT
CTCCCCTACCGGAGCTGAGGTTTCTATTGGAGATACTTTGCAGCCTGATGAATACATTGG
AATGTGCCGTCGTGAGATCATGGACACATACCTTCGCGACCGTGCCATTGAGTACGGAGC
CGAAGCTATCAACGGACTCGTCACCAGAATTGATGTTCCCGCTGACCACAAGACTTCTGA
GTCTCAATACACTCTTCACTACCAGAAGTTTGAACCCGGAAAGTCCTCTGGAACCGCTGC
CGAAATGAAGGTCGACATGGTCATCGGAGCTGATGGTGCGAACTCTCGTGTCGCGAAGGC
CATGGATGCAGGTGAATACAACTTCGCTATTGCCTTCCAGGAGAGAATTAAGATCCCTGA
TGACAAGATGGTTTTCTACGAAGAGATGGCTGAGATGTACGTCGGTGACGATGTCTCCCC
TGACTTCTACGGGTGGGTTTTCCCCAAGTACGACCACGTCGGTGTTGGAACAGGAACCGT
CGTAAACCGACCTGCCATCAAGCAATACCAGAAGGCCATCCGTGACCGCGCTGGAGACAA
GATTGCAGGAGGAAAGATCATCAAGGTCGAAGCGCACCCTATCCCAGAACATTACCGTCC
TCGTCGTGTCCAAGGACGTATGGTTCTCGTTGGTGATGCCGCAGGATACGTTACCAAGTG
CTCCGGTGAAGGTATTTACTTCGCTGCCAAGTCTGGTCGCATGGCAGCTGAAGAAATCGT
TGCCTGCATGGACGGAGGAAAGAAGCTCCCTACCCAAGAAGAAATCGAAAACACTTACCT
TAAGAAGTACGACAAGGCCTACGGACCAACTTACGCCGTGCTCGACATCCTTCAAAAGGT
TTTCTACTCCAACAATGGTGCCCGTGAGGCCTTCGTCGAACTGTGCGACTCCAAATATGT
CCAACAGGTCACATTCGATTCCTACTTGTACAAGAAGGTTCAAGGAAACAATCCTCTTGA
TGACCTCAAGCTACTTGGTGAAACCATCGGGTGCTTGATCAAGGGGTATTCCAACGCCAA
GCCTGACGCTGTCTTCTCCAACCCAGTTGAATCCCAGAAGAGACTTTAAATTTGTTTGAA
TGCAAACAATCACAAATGCAACAGAATTGCTATGGGATATCTCGACTAGAACTACCACAA
ATATCACCACAAAAAATCATGACGCACCCAGTATCTCAGTCGAGGAAAGAAATGATACTC
TACCGTCTAACTCTCGAGACGGCAGAACGAAATCAGGCTAACGCCGAAGGATAAACATTT
GTAAAATAGAGAGAAGATCATTTTTATTTTTAAAAAAAAAAA
>comp2046_c0_seq1
CGGTGCAACTGCTCTTCAGGATGCAGAGTTTAAGAACACAGCGTAGGGGTAGGCCCTTTT
CAAGAATAGCAGCTGTCGCCGTTGCAGCAATTGTGTCGATTGTGGCTACAGCACTGGCAC
CAGTATTTGGGTTTGCACCTCAGGCGTTCGTATCTTCGAACAAACCCACCAGATTCTTTG
AAAAATATGTGGATGAACCTATCTGTTATCGCACAGAGCGGTATTCCCGGTTGATTTCCT
TTTCCTCCAAGTCGGGATCTCTGGATGTGAAACTGTTGGAAGAAATTCGTTCCATGAGGG
TGAAGGAACTAAAGGCAGAATTGACAGAACGACGAATTTCCACCGCCGATGTCTTCGAAA
AAGAGGAGCTGGTTCAACGGCTCTACGAGGCAAAACTGAAATCGCCCGATCCGACAGAAA
CGGCTTCTGCATCAACAAAAAGAACTTCTAGCGATACCTCCTCCTCCTCTGGTACTAGCA
CTGGCGTCCTTTGTGCCGATCTGTTCTTCACTTCGGTCGACTCAGGTAGATCGATGTCGG
GCATGGATGGCAATTCCCAAAGTGTCACTATCAAGGTCGAGGACGTAAGCGATCCCTATC
CCACCATCGCCATTGATGTCTACGATAGTAGTGGAAGCAGCAGTGGTTCGTTCCCGCTCA
AACTCCTCGTGGACACGGCCTGTTCCGGACTCGTTCTACGTCCATCTGTAGTGCAAAGGC
ATTCCTCCTGCCTCAAATCATACTCGACCCCGGTCACCATGACTGGAGGAGCCGGCGCGG
CTGCAAACACTGGATTGACCCAGATCCAGAGCTACTCGCTCCGAGACTCGGCGGGTACAC
AACCCAAGCGACTGGGTCCGCTCCCCGCAGCCGTCCAGGACATTGGTGCCCTTCCACTAG
AACTGGACGGCATTCTTGGTCTTTCCTTGCTCAGCCAATTCGCGTGCACAGAACTCGATC
TGGAGAATGCTCGCCTCAATCTCTACGAGACCGAGTACAGGCCACCCCTTGCAAACGAAA
ACAAAGAAATCGTTGTTGCCGAAGCAGAACTGTCTCCAACGAAGCTTGGAATCTGGACGG
TAGATGTTTCTTTTGCAGTCGGAGGGAGAACGGGTCGTCCCATAAAAATGCTGGTCGATA
CCGGTGCTATGGCGACCTTTCTCACCTGGAAGGGTCTGGATGACATGGGACTTGGCCTCT
CTAGGTCGTCTCCGAGTGTACGCGAGTTGACCCAGCCCATGGGGGCCATGGGATCCGACA
ACGTTGCCATGAGTTTGTCCCACCGAATCGAGCTCGACGCGGATGCTCCCATTAGCTTTG
GAAGGAAGTCGGGTCGGCTGCAGCAAGAGCAATTGGGTCTTTCCATCGCAAGTAAGAGAA
AGGACGGCAAAGAACCATCTCTGCCCATTGATATTGGCAACATTGGAATATTGGAAAACC

AGCTCGCCTCGGACAACGTGGTTGGAATTCTGGGAATGGACGTCTTTTCCAAGGCCTCAA
GGATACGAATGACCTTTCGGGGTCCGGTTCCAAGAATCACTTTCTACCAAAAGAAAGAAA
CAACTCTGTCCAAGGATGAAATGAAGGACGCGACTGCTGCTGCCGACGTCGAACCCGAGA
AGCACACCGCAAAAACGGAGGATGCGGCTCCGGTGGATCCCGTAGAACCAGCAGCAGCAC
CACCGACAAAGGGCCTAGAAGCACGGGGGGAAGAGCCGGAGACCAAGAATGCTGTTCCGA
CGATGGTCGAAGCACCAACGCGTAAAAAGAAGAAAAAGAAGAAGAAGCGAAGGTATGACG
ACTAGTGTAGGTGTAATAGCAAATTTAGCCTACTTTTCCTGTTCAGCGCAAGGGTCGCTC
TTCTCGCTCAAACGGTTTGTCGAAGCACCAACCCTTTAAAATAGCATAGACACGGCGACT
ACTAGTGTAGGCGCAATAGCAAATTGTTGCAGTTCCCTGGCAACGGTTGGTTGTCTCACT
CAAAGGGCTTGCCGAAGCTCCAACGCTTAAAAGGAAGAAGCGTGGATGCGAC
>comp2047_c0_seq1
AAACTGTAGTCGTCATCCCGTTATCCTTTCTCATTCTCGACTGCAAATCAACTCAAACTA
TAAGTATTACTTGCACATCCCACAATGAGTCGCTGCATATTGGCATCAAACAAGTCTCTT
CGCCTTGCTCGCCATTTGGCAGCACCACTGCAATTACTCCCCGACGCCGTCGGAGCGACG
GCTGGTGTGGATAGTCCGAGTACTGTGAACAGCGAGGGCAATGACCTATACAACAAGAGC
TATTTCCATTTCCGTCGGCGGTTTTCCTCCGTTGTCAGGGACCAAGCCACAAGCACGGCA
CCAATGAAGACAGAGAATATACAGAACCAAGGGCTGGCCCGGACCGACAAAAGCATAGTC
CGGCGCTTGTTCGAGAAATATTCCATGTCCAAACAGAAGGATCGAATCCTTATGGCGGAA
TCCCTTTTTCAAGCCGCAACTACTCAAGCAAGTGATCCACGCTGGTACGGCCCAGGCCGC
ATCGGGCGTGACTTTCGTGCCTATCAGGCTCTATTGACGATGCATATTTGGTTCCTCCAC
CGACGACTCGTCACGGACGAGGCAGACCCTCATACCGCTGCGCTCATCCAGGAAGAGTTG
TTTGACATTCTCTGGATCGATACGTCGAACCGGATGAGGGCACACGGAGTCAATGAAATG
CTCATAAACAAGAATCTCAAGAAGGTTCAGCAATATTCATTCATGCACATGTTTCACTAC
GATCATTGCTACACAGGAGATTTGTTGGAAAATGCCGCCGATCGGCTGGAAGCCCTGAAA
ATGACGGTCAAGACCCACGTTTTGTTGCTCCCGTCGCTAACGGACAAAGACGCCGCTGAC
GGGAGTGGTGGCGATGCAGCCGTCAACGAGGAGGCGCTCCAGAAGCATGTCGAGCACGAC
GATCACGCCGAGCGCATCGCATGGTATATCGAAACCCAGTTTCAGAACATTTTGCACGAT
CTCCCCGAGTCGTTTTTCCAAAAGGCGCGTATCGCATGGGTCGACCTGCCGTCCTTCGAA
CATATGGTCGACGGAAACACGGGAAAAGAACTTACAATGCAACCAATTAACCAGGACGAC
TTGCTCCCAACGAACTGGACCAAATCGATTGCAAACGATGGAACCTACTACTTTTGGAAT
ATAGAAACGCGGGAGGTCCAGTGGGAGCGACCCGAATGAGTGCATCTTATTATCGTGTCT
ACTTTGTGTCTTGAAATAGAATGCAACACATAATTATCTGCCAATCTACTTCTTAGGGTG
TATCACAACGCTTTGTCAGATGCTGTCGGCGAATACACGCTTGATGGCTTCGAAATTCTT
GAGCTTGTCGGGAATGTAATGCTTTTCGAATACCAGTGGAAACGGCGTGTCGTACCCGCA
CACGCGCTGAATGGGAGCCTCCAGCGAGTAGAAGCATTCCTCCTGCAGCTTCGCAACGAC
CTCTGCCCCGAACCCGGACGATACCGGAGCCTCGTGGGAGACCACCAGCTTTCCCGTTTT
TCGCACGCTGTTCGCGACGCAGTCCACGTCCCACGGAACGATGGTTTGCAGGTCAATGAG
CTCACAGGAGATTCCCTCCTGTTTCTCCGCAAGATCACAGGCTTCTTCCAGTACTCGGAG
GTGACCGCCCCAACCCAGAACGGTAACGTCCGTTCCCGGGCGCATGACTTCTGCCTTTCC
TAGCTCAAGTTCGTAGTCGTCCACGGGTACCTCTTCCACCGATGCCCGGTAGAGGGCCTT
TGGTTCGAGAAAGACAACGGGGTCCTTCGATCGAATCGATGCCAACAGCAGTCCCTAAAT
ATGGATAGATATGCGTCAAAGTAGGGTTGGAATGGTTCTTGATTTGAAACGAGAAAGATC
ATGTAAAATTGGCGTTTGAATCAATTACAGTCAGCGAGGTCTCACCTTTGCAGCCCTTGG
TCCTCTCGGCATGACGACAGCAATCCCTGTTGAAGCACAAAAAGAACAGGAGAGGATTTC
CGTTCGAAAGAAATTCAAGGGTCGAGGATGAGGCTTTTGCAACTGACAAAACAAACTAAA
GCTGATTTTGTGTCGGCGATAGCGTACCTGGAATATGGGATAAAAAAGCCTCCGGAGATT
GGGAATGGTAGTGTCCGCCTTCGATGAAATCGTTTTATTGAATCATCGTGAGAAACTAAC
GATGAATTGTACAGGAAGTTTGAAAGTGTACAAATATAATTGAGAAACGTACCGTGACCA
ACAGCGCCACACGGGCTCCGCAATGTAACGCCTCCGCAGTTCCATTGATTGCCTGATCGA
TATCGAAATTTAGCCATTTCGTTGACAATCTTGGGGCATTCAGTTAGTTAATTCGTCATT
TTTAGCAGGAACAAGTTTACCGAAATGAAAAGGTATCTATTAAGTCGCACATTTTTGTGG
AAAATCCATTGGAACTGATTTCTGCGAATACTTACTTGATCGAACGCTGAATAAAAGTTA
AAATTGTATTACAGTACATGAAGAACCAATTTGGTACATGAGAAACTCTCAACGCTCTGA
TTATCGGGGCCGCGGCGCGGTTGACTTTGCACGTACCAGGAAATATATAATCGGCAAACT
GAATTTCCCCAACTGCCGTTCCACCCGACATGGTAGAGTAGCCAATAGCAAGCCCTTCAA
CGAGAGAGTAGGAAACAATGAGGTGCGAAGCAGCTCGTCAGCACCAAGCCATCAACAAGT
AGCAGCGAAAGATCAAGGAAAAGGACGCTCACCTGCAATTCCGTTCTCGGACAAAGGCGT
GTTGAAAACTCGGTCGGGCCCGAATTCGTCCTGCAGGCCTTGTGTGCACCGGAAAACACC
CCCGAACGCCACATCTTTTGCGGAGAAGGGAACGAACAAAGCGAACACGTGAGCAGGCAA
GATGTATATCTGGCAAACAACCAATCGATGGATAGCGACTTCTGAATTCGTCCAAGCATG

TGGTCTGTCTATATTGCTCACCTTGTCCAAAGACAATGGAACTCGGATCAGTCTCCAGAG
CAATGGAAAGTGCGTTGTTTATAGCTGTGAATAGATTCATGCTCTTTGTCTCCGAACCCG
GTGTGGTGGTTGCTGTTGTTGTTGTCGGTGATGCACTGAGAGCTACCGTCGAAAAGGAGG
CACCCGCGACAGAATTCTCGGTGGCCCCGAAACAGAAAC
>comp2047_c0_seq2
AAACTGTAGTCGTCATCCCGTTATCCTTTCTCATTCTCGACTGCAAATCAACTCAAACTA
TAAGTATTACTTGCACATCCCACAATGAGTCGCTGCATATTGGCATCAAACAAGTCTCTT
CGCCTTGCTCGCCATTTGGCAGCACCACTGCAATTACTCCCCGACGCCGTCGGAGCGACG
GCTGGTGTGGATAGTCCGAGTACTGTGAACAGCGAGGGCAATGACCTATACAACAAGAGC
TATTTCCATTTCCGTCGGCGGTTTTCCTCCGTTGTCAGGGACCAAGCCACAAGCACGGCA
CCAATGAAGACAGAGAATATACAGAACCAAGGGCTGGCCCGGACCGACAAAAGCATAGTC
CGGCGCTTGTTCGAGAAATATTCCATGTCCAAACAGAAGGATCGAATCCTTATGGCGGAA
TCCCTTTTTCAAGCCGCAACTACTCAAGCAAGTGATCCACGCTGGTACGGCCCAGGCCGC
ATCGGGCGTGACTTTCGTGCCTATCAGGCTCTATTGACGATGCATATTTGGTTCCTCCAC
CGACGACTCGTCACGGACGAGGCAGACCCTCATACCGCTGCGCTCATCCAGGAAGAGTTG
TTTGACATTCTCTGGATCGATACGTCGAACCGGATGAGGGCACACGGAGTCAATGAAATG
CTCATAAACAAGAATCTCAAGAAGGTTCAGCAATATTCATTCATGCACATGTTTCACTAC
GATCATTGCTACACAGGAGATTTGTTGGAAAATGCCGCCGATCGGCTGGAAGCCCTGAAA
ATGACGGTCAAGACCCACGTTTTGTTGCTCCCGTCGCTAACGGACAAAGACGCCGCTGAC
GGGAGTGGTGGCGATGCAGCCGTCAACGAGGAGGCGCTCCAGAAGCATGTCGAGCACGAC
GATCACGCCGAGCGCATCGCATGGTATATCGAAACCCAGTTTCAGAACATTTTGCACGAT
CTCCCCGAGTCGTTTTTCCAAAAGGCGCGTATCGCATGGGTCGACCTGCCGTCCTTCGAA
CATATGGTCGACGGAAACACGGGAAAAGAACTTACAATGCAACCAATTAACCAGGACGAC
TTGCTCCCAACGAACTGGACCAAATCGATTGCAAACGATGGAACCTACTACTTTTGGAAT
ATAGAAACGCGGGAGGTCCAGTGGGAGCGACCCGAATGAGTGCATCTTATTATCGTGTCT
ACTTTGTGTCTTGAAATAGAATGCAACACATAATTATCTGCCAATCTACTTCTTAGGGTG
TATCACAACGCTTTGTCAGATGCTGTCGGCGAATACACGCTTGATGGCTTCGAAATTCTT
GAGCTTGTCGGGAATGTAATGCTTTTCGAATACCAGTGGAAACGGCGTGTCGTACCCGCA
CACGCGCTGAATGGGAGCCTCCAGCGAGTAGAAGCATTCCTCCTGCAGCTTCGCAACGAC
CTCTGCCCCGAACCCGGACGATACCGGAGCCTCGTGGGAGACCACCAGCTTTCCCGTTTT
TCGCACGCTGTTCGCGACGCAGTCCACGTCCCACGGAACGATGGTTTGCAGGTCAATGAG
CTCACAGGAGATTCCCTCCTGTTTCTCCGCAAGATCACAGGCTTCTTCCAGTACTCGGAG
GTGACCGCCCCAACCCAGAACGGTAACGTCCGTTCCCGGGCGCATGACTTCTGCCTTTCC
TAGCTCAAGTTCGTAGTCGTCCACGGGTACCTCTTCCACCGATGCCCGGTAGAGGGCCTT
TGGTTCGAGAAAGACAACGGGGTCCTTCGATCGAATCGATGCCAACAGCAGTCCCTAAAT
ATGGATAGATATGCGTCAAAGTAGGGTTGGAATGGTTCTTGATTTGAAACGAGAAAGATC
ATGTAAAATTGGCGTTTGAATCAATTACAGTCAGCGAGGTCTCACCTTTGCAGCCCTTGG
TCCTCTCGGCATGACGACAGCAATCCCTGTTGAAGCACAAAAAGAACAGGAGAGGATTTC
CGTTCGAAAGAAATTCAAGGGTCGAGGATGAGGCTTTTGCAACTGACAAAACAAACTAAA
GCTGATTTTGTGTCGGCGATAGCGTACCTGGAATATGGGATAAAAAAGCCTCCGGAGATT
GGGAATGGTAGTGTCCGCCTTCGATGAAATCGTTTTATTGAATCATCGTGAGAAACTAAC
GATGAATTGTACAGGAAGTTTGAAAGTGTACAAATATAATTGAGAAACGTACCGTGACCA
ACAGCGCCACACGGGCTCCGCAATGTAACGCCTCCGCAGTTCCATTGATTGCCTGATCGA
TATCGAAATTTAGCCATTTCGTTGACAATCTTGGGGCATTCAGTTAGTTAATTCGTCATT
TTTAGCAGGAACAAGTTTACCGAAATGAAAAGGTATCTATTAAGTCGCACATTTTTGTGG
AAAATCCATTGGAACTGATTTCTGCGAATACTTACTTGATCGAACGCTGAATAAAAGTTA
AAATTGTATTACAGTACATGAAGAACCAATTTGGTACATGAGAAACTCTCAACGCTCTGA
TTATCGGGGCCGCGGCGCGGTTGACTTTGCACGTACCAGGAAATATATAATCGGCAAACT
GAATTTCCCCAACTGCCGTTCCACCCGACATGGTAGAGTAGCCAATAGCAAGCCCTTCAA
CGAGAGAGTAGGAAACAATGAGGTGCGAAGCAGCTCGTCAGCACCAAGCCATCAACAAGT
AGCAGCGAAAGATCAAGGAAAAGGACGCTCACCTGCAATTCCGTTCTCGGACAAAGGCGT
GTTGAAAACTCGGTCGGGCCCGAATTCGTCCTGCAGGCCTTGTGTGCACCGGAAAACACC
CCCGAACGCCACATCTTGTCCAAAGACAATGGAACTCGGATCAGTCTCCAGAGCAATGGA
AAGTGCGTTGTTTATAGCTGTGAATAGATTCATGCTCTTTGTCTCCGAACCCGGTGTGGT
GGTTGCTGTTGTTGTTGTCGGTGATGCACTGAGAGCTACCGTCGAAAAGGAGGCACCCGC
GACAGAATTCTCGGTGGCCCCGAAACAGAAAC
>comp2048_c0_seq1
AGTCCTACAAATAGTGAAAAGAAGGATTCTTGGGATGATACTGACGGCTTTCAGAAACAA
GCATCCTTCAGTCCTACAAATAGTGAAAAGAACAACCAAAACAAGAATCATACAAGTTTT
TCTGGTGACAATGCCTGGAATGTAAGCGTCTCATCTCCAGTGGGGCAATCTCCTTCAAAA

TTGAACAGCAGCAACGAACAATTTAGCAGGAATCGCGACACTGACCCCTTTCGCAATTCT
TCGGCGATTGGAGGTAGCAGCTATGTCTCGGCGGGCTCCTCTTCCACAGGACTTCCTGCA
GAAAATAGATCACTCACGGAGCTCTTGGAAGCTGCCAAGAGCAAGCGAAAGGACAGCCGT
CGTAGTTATGGAAATAGCGCCACCCGCCTCACACAGCACAATCAATCAACGAGCAGTCGA
ATGTCGTCTGCCTCAGTGAATTCGGCACCCGCCATTACGGCTTCCTACCTCCGACAGCAC
CACAACCTAGGCGGAAACAACGTAGCTGGCGACGACCCTTTGTCGCCTCGGGACGGTACC
TCAGTTTCGGACATTATTCAGAGCTTGGAAGACACCGATCGAATGAAGCGAGCTGAACAA
ATAACGTCCTCCCACCGTTCTATGGGAGACGCGGGCGTTGTGGCTACAGCCCGAGCCGCC
AAGGAGCGACTTCGGGAACGCCGGCGTCGGGAACGAGAATCAGCCGGCATCTATACAACT
GCAGGTCGCTCTGCTAATTCCCTTCGGCACTCATCTTCTCGGGATCATGGTTCCGATTCC
GATGAATCGGAGAACGGAAACGAAGCTTCTGAGTCCTGGTTGTTCGATCAGGTTACCGGA
GCTATGGGTCCAGTAGGCATTGCAGCCGATCTAGAATCACTAAGCGGTCGATCGAACCGG
TCAAAGAACTCCCACGGGGGAAAATCACACAAGTCGTCGGGTACATCGAACCGCCGTCGA
TCTTCTCGAAAGACAAGCAGCACGCGTCGGCACCGGTCTGATCGAAGTGTCGACAGCCAC
GGCAGTCGGACCAGTCGGTATTCCCATCGCTCCACGAAGTCCTTTTTGTCGCAAATGTCA
GAGCAGAGTCGGAGTGTTGCAAACGATTTGCTCCGTCTAGAGATGCAGTTGGCCATGGTA
GGGAACAACAATGTGAACGGCGGTGGAAAAGAGGCAACCATGCGTGAAATCGATAGCTTG
CCGGTGGTCCAATCGGCTCGCACTGGAATGTCCAGCAGCGCCAGCTTGGGTGGAACCTCG
AGGGCATCGGGTCGTAGCCGAAGATCAGTTACTTCAAGTCACCACAACTCATCGTCTTCC
AGTTCAGTCCTTCGCCGGACCAAGACCACAATTGTCGCTCCTCCCGGGAAGCTCGGAATC
ATTCTGGCAAACAAGGCAGATTCAAAGGGAACAGTTGTTTCGGGCGTGAGAACGTCTTCC
GTGCTAGTGCACAAAATTTCTCCTGGGGATCGAATTGTTGCCATCGACGGAGAGGACGTC
AGTCGCATGACCGTCAGCGAGATTACCACCATTATGTCCCGAAAGGCCGAATACGAACGA
AGGCTTACGGTAATGACCATTCCCCGAATTGGGTCTCCAAGCAATGGGAACACCAACAGT
GGCTATGCAGACCAGCAAAAGATGCGATCACCGAATAGGGTTCGATCTTCTCCGAGGGTG
GCATCCATCAGTGACAGCAAATTTGATTCCGCCGGCAGTGGAAGCAACTATCACAGTGTG
CGATAACTGCAACCGACACGAACGGCTAGAAATGAGTCGGCGAACCTACTGTGTCGCCAG
AAACAGCGAGTTCGTAATGGAGACCCTTCACTACGTACCTGACGCGCACGATAGTGTGTT
CGGGTACCAAAGCAAACATAACTCATACAAGAAGAAAATATTGGTTTAAAGATACCGTAA
TAACGAGACGTGTGATACACACAAAGTTTTTCTCTAGTGCGTCCACTATTCGATAAAGCA
ACC
>comp2048_c0_seq2
CCTTCCGTTTCCACTCTTCACCCTACAGTCGTCTTTGCAGCACAGACGACACAGTGCACG
GTTGCGAATACGAGCAACATTTGCTTCACACGAAAGCAAACAGCGAACACAACAGAATTG
TCACCATCAATCGATCAACCAGTAATTTGGAAGTCAAAACGCAATAAATCTCCAACAATA
CGGCGAACACGAATGGTCGGCTTGCAGTCGTTGTAGAAAAGTCGCTCTATTCATCTTTTG
TTCTTTCTTGCCGATCAATCGCATAGCAAAGTCACAGCGTGGTGATAGCTTTGTCACTGA
CAATACGCCATTCAAGTGTTGAAAATTGTTTAGAAGTGATTTAGAGGTACTCGTTCTTCT
TTGCACTTTCCATCGAATGCACCACAGCGAAAACAAAAGCGTTGGATGAACACATAAAAG
TCCGCACTGCACGTACAATATTCATACCAATACTACAGCAACTAATATTTCATAACTCGG
CTGTTCAGAATGGGAATCTTAAAGAAAATCTTCCGTGGGAACAAAAAGAAAACAAGGGAG
TTCCAAGATGGAGGCAGTAGGCCAATCTCAGATCCCTCCTTTCCTTCGCAGACTTCGTAT
TCGCAAACACAATCACAAGCAACGACCAGGGGTGGAGAATGGAGAGGAACGATGGTTGGG
GACAGCACGATCCCAAATGGCATGAAAATCGTTGGTTCCGCATCAACGTCCACCGCGAGT
TCGTATCCCAACAACAATCTTATGTTGAACACAAACAACAATAATATCAACAACACTAGC
AGTCATCCACAGCAGCAACTGATGGGTGGGTACCGTTCCCCACGGAGCACGGGGCCAGTC
GATACGGACGACGAAGGGATTATCGTACCCGAAAACGAGACGGAAGCCTTCTACGATAAC
GCACGGAATCAAACACAGGAGCAGTTGCGACTGGCTTACGAAATGTCGAAAGCAAACAAA
TCCAACAACAATAGTGCAAATATATCGAGCGATTCATCGAATTCATCCTTCGCTTTGACA
TCCAATCAACTCCAACAATGGGATCACCAACAACGGCATTCCAGTACACACCAATTCTCA
TCCTCTCCAACCACCAACAATAATAACCCAAAACCAGTTTACACCATGAGTTCAGACTTG
CCAAGCATGAATCCGCAGCAACCCAGTGGCTTGGACGACACGATGAGATCGGTATCCTCT
GCGTCCTCCTTCAATCTATCGACTGATGCCGAGGATTCTGAATACGAAAACCTCAAACGA
ACCTATTATCCTCCTGGCCAACTCACAAGCAATCTTGGCATCGGGTTGTCGGCCCTTACG
GCTTTGGACACTTCTGCTATTGACGCTGCCGGTTCAATAGCTTCATACACCACCGACGAG
GACCGCAGCAAGATCTTTCCCAATTTGCCAACGGACGACGATATGACGCTGGGAACAGAT
GCCTCTTCGAAGCGGAGTGAACCCCCTCCGAGCCTGCTCGACTTTCCGCAAAGTCGCCTC
TATGCCTCGACCAATGTTGGCAGTTCGCCTTATGGAGCACAAGTGACGGTTCCGATGCAT
GCCGCTCTCGCTATAAACACCAATCAACGCAACAGCGAAAACAATACCAACACTAACAGC
CGCAACAAACCCGGTGGAACGCCTCGTGACTTTACGAGTCCGAGATCACCGAAAGCTTCC

AAATTGGAGCACAAATTGGAGAAGAACCATTCCTCTTCTCCAAAGAAAAACATGAGCAGA
AACAGAAACAACGATAGCAGTGGCGACGATGGCTTTGGAAACATCTCAACAAACCGCAGT
CACGAAAGCAACGGCAGTGGTAATCATCGACAGAATAAAAGTCCTACGGCTGCTGCCATA
AGTCATTCTTCAGTTGAGGATTCCGGCTTTGCAGATTTTGGAAACTTCGCCGACTTCGCC
GACTTTCCTCGCGGTGAAGGTGTAGATGACAGCGGCTGGACAACGGTTAAGGAGGAAAAG
AAGGATTCTTGGGATGATACTGACGGCTTTCAGAAACAAGCATCCTTCAGTCCTACAAAT
AGTGAAAAGAACAACCAAAACAAGAATCATACAAGTTTTTCTGGTGACAATGCCTGGAAT
GTAAGCGTCTCATCTCCAGTGGGGCAATCTCCTTCAAAATTGAACAGCAGCAACGAACAA
TTTAGCAGGAATCGCGACACTGACCCCTTTCGCAATTCTTCGGCGATTGGAGGTAGCAGC
TATGTCTCGGCGGGCTCCTCTTCCACAGGACTTCCTGCAGAAAATAGATCACTCACGGAG
CTCTTGGAAGCTGCCAAGAGCAAGCGAAAGGACAGCCGTCGTAGTTATGGAAATAGCGCC
ACCCGCCTCACACAGCACAATCAATCAACGAGCAGTCGAATGTCGTCTGCCTCAGTGAAT
TCGGCACCCGCCATTACGGCTTCCTACCTCCGACAGCACCACAACCTAGGCGGAAACAAC
GTAGCTGGCGACGACCCTTTGTCGCCTCGGGACGGTACCTCAGTTTCGGACATTATTCAG
AGCTTGGAAGACACCGATCGAATGAAGCGAGCTGAACAAATAACGTCCTCCCACCGTTCT
ATGGGAGACGCGGGCGTTGTGGCTACAGCCCGAGCCGCCAAGGAGCGACTTCGGGAACGC
CGGCGTCGGGAACGAGAATCAGCCGGCATCTATACAACTGCAGGTCGCTCTGCTAATTCC
CTTCGGCACTCATCTTCTCGGGATCATGGTTCCGATTCCGATGAATCGGAGAACGGAAAC
GAAGCTTCTGAGTCCTGGTTGTTCGATCAGGTTACCGGAGCTATGGGTCCAGTAGGCATT
GCAGCCGATCTAGAATCACTAAGCGGTCGATCGAACCGGTCAAAGAACTCCCACGGGGGA
AAATCACACAAGTCGTCGGGTACATCGAACCGCCGTCGATCTTCTCGAAAGACAAGCAGC
ACGCGTCGGCACCGGTCTGATCGAAGTGTCGACAGCCACGGCAGTCGGACCAGTCGGTAT
TCCCATCGCTCCACGAAGTCCTTTTTGTCGCAAATGTCAGAGCAGAGTCGGAGTGTTGCA
AACGATTTGCTCCGTCTAGAGATGCAGTTGGCCATGGTAGGGAACAACAATGTGAACGGC
GGTGGAAAAGAGGCAACCATGCGTGAAATCGATAGCTTGCCGGTGGTCCAATCGGCTCGC
ACTGGAATGTCCAGCAGCGCCAGCTTGGGTGGAACCTCGAGGGCATCGGGTCGTAGCCGA
AGATCAGTTACTTCAAGTCACCACAACTCATCGTCTTCCAGTTCAGTCCTTCGCCGGACC
AAGACCACAATTGTCGCTCCTCCCGGGAAGCTCGGAATCATTCTGGCAAACAAGGCAGAT
TCAAAGGGAACAGTTGTTTCGGGCGTGAGAACGTCTTCCGTGCTAGTGCACAAAATTTCT
CCTGGGGATCGAATTGTTGCCATCGACGGAGAGGACGTCAGTCGCATGACCGTCAGCGAG
ATTACCACCATTATGTCCCGAAAGGCCGAATACGAACGAAGGCTTACGGTAATGACCATT
CCCCGAATTGGGTCTCCAAGCAATGGGAACACCAACAGTGGCTATGCAGACCAGCAAAAG
ATGCGATCACCGAATAGGGTTCGATCTTCTCCGAGGGTGGCATCCATCAGTGACAGCAAA
TTTGATTCCGCCGGCAGTGGAAGCAACTATCACAGTGTGCGATAACTGCAACCGACACGA
ACGGCTAGAAATGAGTCGGCGAACCTACTGTGTCGCCAGAAACAGCGAGTTCGTAATGGA
GACCCTTCACTACGTACCTGACGCGCACGATAGTGTGTTCGGGTACCAAAGCAAACATAA
CTCATACAAGAAGAAAATATTGGTTTAAAGATACCGTAATAACGAGACGTGTGATACACA
CAAAGTTTTTCTCTAGTGCGTCCACTATTCGATAAAGCAACC
>comp2049_c0_seq1
ATCGAATCTACACACCAATCGACAGCACCAAAATGGGAAAGAAGAAGGGAGCCAACGAAA
AGAAGGGAGCCAAGATCTTCAAGACGAAGTGCTCCCAGTGCCACACCGTCGAAGAAGGAG
GAGCCCACAAGCAGGGACCCAACCTCTACGGCTTTTTCGGAAAGCAATCCGGTGGACAGG
ACGGATACTCCTATTCCGCCGCCAACAAGAAGTCCGGGGTCATCTGGGAGGACCAGTCTC
TGTTCGACTACCTCGAAAACCCCAAGAAGTACATGAAGGGAACCAAAATGGTCTTCGCGG
GAATCAAGAAGCCAGCGGACCGCAAGGATCTCATTGCCTACCTCAAGAAAACCTGCACCG
CATGAGAAAAAAGGGAGACGCCGGTGTGGCTTGGAACCGCAACGTCGAAGGAACGTCGAT
AGGATGCATTGCTCATTCTCCGTTCCGGTGTTGCGCGGCACGGCTTGTCGCACATTTCTG
TTTGTTGTTGCTCCGGTGACGCGAGCCACGAAATGACCCCGTTGGAATCGACAATCGGCA
AAAACTAAAGAATAACCTAGAAAAATAAATGTTGCGATAACAAGAGCC
>comp2050_c0_seq1
GTTCAAAAAAATCTTTGAGCGACTGCCTAAGATGCATGATGGAATACCTCATAAGAAGTA
GAATCATATACGGTTGGTTTGCAATATTTTGTGTCGTAGTGCCTTTTCCCTCGTTCTCCC
TCCAACAAACGAAACATAGGATTCGAACGACGAGAAAACATCATGATCGGTTCGCTGATT
GGGCAAGAAGTAACCAACGAGGGCTACAAAACAATAATGACAAGATTAACCCCTTGCTCC
TATGGATTGATGGTAACAATGTTCGGGGAATCGGAAAATTCGAATGGAATGCAGTGGAAC
TTCAGCACAGAGTGGTCCGTTTTTGTCACAAATACAGAGTGCCGAACGCAATAGTTGTGT
GGGATCACGGTAGCCACCAGCTTTCGTGCTCAAAACAGTATCATTTCAACGATTCGGGAA
AGGAAATCGCCAGTTCCGAGGCTGATGTACCTTATAGCAAAGATGACAAGTCGTTTTGCG
TCAACCTAATCACCCTTTTTTCGGGCATTAGGCAACGAGCAGACGATGTCCTCGTGAAGG
AATCAAATCACCTGGTTTCACTGTCCTTTCGAACCAACGTCGAATCTGACTACAACACAA

AATTGAATTGGTCTTCGAATGCATTTGTTACAAACGATACCGAATTGAACTACAAATTAC
GGCGACAAGCTGTTCCGAACTCACCTTCGATACGTCTGAGTAGACGGAGACGAAGGGACG
CTGGCAAGATATACAATTCTGATAGCAGCAGCAACAACGAGGTGAAGGATACTTCAAATC
CACTCTTTTGTGATTCGACGGGATTTGTTGACCTGCTATGCGAAATCTCAAGGGAGTACG
ACATGGCAATGAACGACGTCGACCAGGAAGCCTCGATGTTGATTGAAAAAGCAAAGGATT
CTCTTCGCGAATGTTCCAAAGGCCAACGCCGTGGTTACAACCCCCGCAGGGAGAAAACAT
GGGAACGCTGCGTACAGGCTGAAACATTGCGCCGGTTTATTATTGACCAGGAAGAAAGAG
TCACCAAAGAAAAAGTAAATGAGAAAACAGAGAAGATAGTTACGAAACATTTCTGCCCTT
TCTAGCACCCTCTCGTCTTGACAAACGACAGCGGCGCCTGTTGGATCGCTACAACGCACT
CGTCAAAAACGGAGAAATAACGTCGTAAAGTGCACGAATAGCACCCCGAGGCCAAGAAAG
AAAGCAATTATCACATCACAGACCACGAGCCTAGATATTACATCATAGCCTAT
>comp2050_c0_seq2
GTTCAAAAAAATCTTTGAGCGACTGCCTAAGATGCATGATGGAATACCTCATAAGAAGTA
GAATCATATACGGTTGGTTTGCAATATTTTGTGTCGTAGTGCCTTTTCCCTCGTTCTCCC
TCCAACAAACGAAACATAGGATTCGAACGACGAGAAAACATCATGATCGGTTCGCTGATT
GGGCAAGAAGTAACCAACGAGGGCTACAAAACAATAATGACAAGATTAACCCCTTGCTCC
TATGGATTGATGGTAACAATGTTCGGGGAATCGGAAAATTCGAATGGAATGCAGTGGAAC
TTCAGCACAGAGTGGTCCGTTTTTGTCACAAATACAGAGTGCCGAACGCAATAGTTGTGT
GGGATCACGGTAGCCACCAGCTTTCGTGCTCAAAACAGTATCATTTCAACGATTCGGGAA
AGGAAATCGCCAGTTCCGAGGCTGATGTACCTTATAGCAAAGATGACAAGTCGTTTTGCG
TCAACCTAATCACCCTTTTTTCGGGCATTAGGCAACGAGCAGACGATGTCCTCGTGAAGG
AATCAAATCACCTGGTTTCACTGTCCTTTCGAACCAACGTCGAATCTGACTACAACACAA
AATTGAATTGGTCTTCGAATGCATTTGTTACAAACGATACCGAATTGAACTACAAATTAC
GGCGACAAGCTGTTCCGAACTCACCTTCGATACGTCTGAGTAGACGGAGACGAAGGGACG
CTGGCAAGATATACAATTCTGATAGCAGCAGCAACAACGAGGTGAAGGATACTTCAAATC
CACTCTTTTGTGATTCGACGGGATTTGTTGACCTGCTATGCGAAATCTCAAGGGAGTACG
ACATGGCAATGAACGACGTCGACCAGGAAGCCTCGATGTTGATTGAAAAAGCAAAGGATT
CTCTTCGCGAATGTTCCAAAGGCCAACGCCGTGGTTACAACCCCCGCAGGGAGAAAACAT
GGGAACGCTGCGTACAGGCTGAAACATTGCGCCGGTTTATTATTGACCAGGAAGAAAGAG
TCACCAAAGAAAAAGTAAATGAAACATCGCCGTCAGATACGAATGATGCGGTCTTCGTTG
CTAATTACCTGAATGAATTGCAAACCAAACGGGGATATATCAGTCCGTTGCATGAAGAAA
ACAGAGAAGATAGTTACGAAACATTTCTGCCCTTTCTAGCACCCTCTCGTCTTGACAAAC
GACAGCGGCGCCTGTTGGATCGCTACAACGCACTCGTCAAAAACGGAGAAATAACGTCGT
AAAGTGCACGAATAGCACCCCGAGGCCAAGAAAGAAAGCAATTATCACATCACAGACCAC
GAGCCTAGATATTACATCATAGCCTAT
>comp2051_c0_seq1
CCAAATACCAAAAACAGCACGAAACAACACTACGAGCATCACAACACTGTTCATAGTAAA
CTGACGGACGAGTTGCCACCACCGCTTTCACAAGTAATCCCAAAAAAAAACAGACCTAAT
ATACAATGAGTATGCACGTCATCAAACGGGATGGTCGCATGGAATCTGTCCATTTCGACA
AGATCACCAGTCGCATCGACAAGCTTGCGTTCGGCTTGGACACCAAGCACGTAGAACCTC
CGCTTGTTGCGCAGAAAGTAATTCAAGGTGTATATCCAGGCGTCACGACCTGTGAACTCG
ACGAACTTGCCGCTCAGACTGCCGCTTCGTTTGCAACACAGCATCCCGACTACTCCATCC
TCGCGGCACGAATCTCCGTGTCAAACCTACAAAAATCCACCTGTGAGGTCTTTTCGGAAC
TTACCGAAGCCCTCTACAACTACATCCATCCTACCACCGGAAAGGCGGGTCCGCTCGTCT
CGAAAGAATATTACGAGACGGTCATGAAACATAAGGATGTCTTGGACAATGCCATCAACC
ACGACAACGATTTCGAGTACGATTACTTTGGTTTTAAAACCCTCGAGAAGTCCTACTTGC
TCAAGCTCAATGGCAAGATCGCAGAACGACCACAGTGCATGTTGATGCGCGTCGCCGTTG
GAATTCACGGAGATGATATCGAGCGGGTCTTGGAGACCTATAAAGGCCTCTCGGAACGAT
ACTTCACCCATGCCACACCGACCCTCTTCAACGCCGGAACCAACAACCCTCAGATGTCCT
CTTGTTTTCTGTTGACAATGAAGGAAGACTCCATCGATGGCATCTACGACACACTCAAGA
GCTGCGCCGTGATTTCGAAGTACGCAGGTGGAATTGGTCTGGCAACCCACAACATTCGTG
CCAGTAAATCCTACATTCGTGGAACCAACGGTACATCCAATGGCATCGTCCCCATGTTGC
GTGTCTTCAACAACACCGCCAGATACGTCGATCAGGGTGGAGGCAAGCGCAAGGGAAGCA
TTGCCATTTACTTGGAGCCCTGGCACGCAGATATCTTTGCCTTTTTAGATCTTCGCAAAA
ACCACGGGAACGAAGCCGATCGCGCACGTGATTTGTTTTACTCTCTCTGGATCCCTGACC
TGTTCATGGAACGTGTCAAGGCCAACGGGACATGGACGTTGATGTGTCCCGACGAGTGCC
CCGGTCTCCCCGATTGCCACGGACAGGAGTTCAAGGAATTGTACGAAAAGTACGAGAAAG
AAGGCAAGGGTCGCCAAACAATTCAGGCGCAGCAGCTCTGGTTCGCAATCTTGGACTCGC
AGGTCGAAACAGGAACCCCTTACATGGTGTACAAGGATCACTGCAACAACAAGTCGAATC
AACAAAACTTGGGAACCATTCGTTGTTCGAACCTATGTACCGAGATTGTTCAGTACACTT

CTCCAGAGGAAACTGCCGTTTGCAACCTTGCCAGCATCAGTCTCTCCAAGTTGGTACGAC
CTCTCACCAACAATGGCCCACTCATTTTCGACTTCGAAAAGCTTCGACAGGTTGCGATGA
TTTTGACCAGAAACCTGAATAGAGTCATCGACCGCAATTTCTACCCTATTCCCGAAGCCA
AATACTCGAACATGAAGCACCGTCCAATTGGTCTTGGTGTTCAGGGATTGGCAGATGCTT
TTGCCATGCTCAAGCTCCCCTTCGACTCTCCCGAGGCAGCTCAATTAAACCGCGACATTT
TTGAGACCATTTACTTCGGTGCCTGTACTGCGAGTTGCGATCTTGCGAAAACTGAGGGAC
ACTACGAGAGTTATCCCGGGTCTCCCGTGAGCCAGGGAAAACTACAATTTGACTTGTGGG
GCGTTACACCCAGCGATCGTTGGAACTGGGCCAAATTGAAGGCAGAGATTGCCGAGCACG
GAATGAGGAACTCCCTGTTGGTAGCTCCAATGCCCACCGCATCCACCGCCCAGATATTGG
GTAACAACGAAAGTACCGAGCCATTTACCTCCAATATCTACAACCGACGTGTTTTGGCAG
GAGAGTTTACGATTGTCAACAAGCACTTGTTGCGCGATCTGACCTCGCTTGGTATTTGGA
ACGAGTCACTCCGCAACCGCCTCATCGCTGACCGTGGATCGGTGCAGAAGATCGAAGAAA
TTCCCAAGAAACTCCGAGATGTGTACAAGACTGTCTGGGAGGTTCCACAACGAATCATTC
TCGACTTGGCTGCCGATCGTTCCCCCTTTATCTGCCAGAGCCAGAGTCTGAATGTTCACA
TTGCCGATCCTTCGTCGAAGAAGTTGACCTCCATGCATTTCTATGCGTGGAAGAAGGGAC
TGAAGACCGGAATGTACTACCTACGATCTCGACCCAAGGCCGATGCAATTCAATTTACGG
TTGATCAAGAGAAGCTCGCAGCTGATACAAAGAAGAAGGCTAACATTTCGAGCGACAAGG
AAAATAAGAAAAAAGTTTCCAACATCCCCCCTATGGAATCTCTGAAGAAGAAGAAGCCCA
TGGTGGAAGCCAAGCCCTTGGTGGACAGTGCAACATTGGGTCAAGCCGAGGAAGAGGAAG
TCTGTCTGAACTGTGGTGCCTAAGTGGATCTTTTACTGTAAAGGATCGGGGGACTACAGT
CATTAACATCTCTGAGTACTTCGCTGGCGGCCATCATAAAGGGAATTGATGTAGGATTCG
AGTCAGCTTTTTGTGAATGAAATAAGACTTGTGTATTTAATATTAATAATTAAAAGTTTG
AA
>comp2052_c0_seq1
CTGGTTCACACCGTACACAATTCTGCAGAATTATCATTTCCTCATCTCTGGCTCCTTGTA
CAACCAGCATCTCCGAAAACAAAACTATCCCTGAAAATCTCGTTGCAAGATGGCGATTAA
GGAGGAGCTCTTGTTTTCCCTATCCATTACAACCGGGACTCTGTCTCTCGTTGGATCGTG
CTTGTTGATTGCCACGATCTTGCGCTCGAGAAAGAAGCTGAGCCTCCCATACAGGCGTAT
AATCTTCGGGACGAGTGTCTTCGATGTGTTCCAATCTTTGGGTTTGACTTCTTCTATCTT
CCTGAGCCCAAAGGATGTGGAGCCTCCAGTACCATACGCAATCGGAAACACCTCGACATG
TACTATTCAAGGATTCTTAACCACCATCGGAGCAATTGGAGTTCCGATCTACCTGTGCTC
TTTGAGTATCTACTACTTCTGCACCATCCCACTGAACATGAAGGCTAGGACTTTCAAGAA
AATCGAACCCTTTCTCCATGCTGCTCCATTGGTCTACAGTCTCTCTGTCGGCTTCTATGC
CCTGGCAAATCAATACTACAACAATGCCGGGTCAGTTTGTTACGTCGCCCCCTCACCGCT
AACGTGTGAAATCCTTCCAGGTGTTGAGTGCAATCGCGGGGAGGGAGCATCGAAATTCCG
CGTCAGGTTTTTGAGCTTTCCTAGCATGATCATCTTCGTCATCGTGTGCACCACCATGGC
GGTCATCACCTATTCGGTGAAGAAACAAGAGAGACGACAGAGGCGCCACTTGTTCCCCTT
GCACGGGGGAGGCGAGGTATCGGATGCCTCGTCTCCGGTATGGAGAAGACTGAAAAGGAT
CGGTTCAGTGTTCCTTGATGTCCTATTCTGCGTTTCGTTGTCGCGAAAGAACGAAAACGA
AAACGAGAATGACACCGGCATTGATTTGGATGGCACCGCATCGCGGGGAGAGAACGAATG
CCCATCGGAACCAAAAGCAGAATCGGCCAGAACGGAGATGTCGCCGGGTCCAAGCCAGAG
AATTTCTTCGTCAAGCAGAAGACGGTCCAGCACCCGAATCCGGGAAACAACGACACAAGC
GCTGCTTTATGTTGGTTGTTACGTCCTATCCTACGGTTTCGTCTGGGCTCAGAGCATCTA
TTCTCTGGCAACGAATCTACCCCCTCCCAAGGCTTTCACCCTGCTGTCATCTATATTCTT
CCCGCTTCAAGGTTTCCTAAACGTATTCATTTACTGTCGCCCTCACATTGTATCTCTGCG
AAGAAATTTTCCAGACGATTACTCCTGGTTTCAAGCTTTCGTAAAAGTTCTGAAGTCGGG
GGGAGACGATCCTTTGACAGTTCAAGAACGACGAATGATCAGAAGGATATCATCGAGCAT
AACGGTGTCCCACCCAAGCACAAGGAGACCATCGATGTCAGCGGGTATGGGGGACTATGA
GGCAGACTCACAGATGACTGTTACAATGACATCGAGAACCGAGATATTCGAAAGGAGATC
CTCCATAACTTCACGGACCACTGCGGACCTCGAAGAGGTTCCGGACAACATCCAGGACAG
CGAAAAAGCAGAGATGGGCTTTAGCAAGATCACCATCCCACAGGGAGATGCAGAACACGC
GTAGCATAATCACGATTGCAATCGCATTGAGTCGTCGGGGGAAAATCGCCAACAATCGGA
ATCCAGCGAACCCGAGGTGGACGATTCGGACCATTACAGAAGACCAAGCGCTCGCGACGA
GCCTGGAAACAAAACCAGCGTAACGCCCGAAAACAATTTGTCGGATGTGGAAATAATACC
AGAATCTCTCGAGGACTTTATACACGAAGCGGAGGAAGCACTCGATGATGCGTTGCACGA
GGTTCTGGAACTAGCCAGAGAAGCAGAAAACGAAGCGAACGGCGAAGTTTGACAACGCGC
AAACGAAAATGTCGCTGCTTCGAAACGTTAGATTGGTAGTAGTCAAGCGGAAAAGTGAAC
TAAACGGATGTTCCATCTGAATTACAAATTCTATTCAAGTCCTCGTATGATTGAAGAAGA
CACATTATTGTTAAAATCTCAAAAATCTTCTTCCGTGTCGTATCATGATGAAATATAATC
GCTGTTGAAGATTTGGTTTTTCATTTGAAGAGATACACCAATGCACTGCAGAAAGTGCTA

TTCGTAACAGTACCTGCAGTACCGTACAATGGTGTCAGTCTAGTACGAGTGGGTCAAATT
TGACTTGGTTGTTGCAGTCGAAAATCCTCGAAGCAGGAGGTTGCGTATCAAGGATAATAC
CTTATTACTACCGTACTACGCAGCACAGAATTATATTTTTATTAATTTTATTTTCGATGG
ATTTCTTTTTCAGCACGTATTCATTCTATTAGTTGATGCTGCAAGTGAGTCAGTCGGTTC
CTCAGTATCGTTATCAACACATCACGGTAAGGAGTGATTACCATACATGGGGATTTTCGA
C
>comp2053_c0_seq1
GCAGATTTTCGGGGAGTTTTTTACGACAAACTGAACGTTTACAGGCGAAGAATTTTCCCA
GCACATAAATTGGGATACCGGTACTAGAACGCGTACGAACGCTTTTTGTTCCGGCTTGGC
TAATCCCTCGAACACGTCCGGAGCTACCAATTTTTTTTTCAACGTGGTAACACAAGAAAA
CAACGGAGAAGTTTCCGTGCATTGATTGCGTTCTCTTTGATAAAGCATAGCAAGATGACA
GCTTCAGATCTTTTCTTCCCATTGGTTGCAACCTCAGCTGCTTCCGCTGTTGCTGGACTA
GTACTCTTGCAGACGGACATGGTGTCTCGCAAGATTCTTGACCCCGATCAACTCAATACA
AAACGACGATCGGAGAGTCGATACTCATGGAAAAAGTCCGTTGATAGAGCGAAGCTTCTA
ATGGACTACAGCTTGGCAGGTGGTCAACGAATTTTGACTGATGATCCAGAAATAGTCATG
AAACACACGGAGTATATTTCGAAGGAGGACGAACTAGATTCCCTTATTAAGAAAATTGTC
GATAGGAAGGATTCGGAATCTAGATTTTATGCCCTGAAAATCGATAAAGATCCCCTAGAT
GCCAGTGAAGAATTCTATTTATACATAATCAAAGAGCAAGAACAGAAGAATGTCAAAACT
CTGTATCAAATCCCGCTCGTGAAGTTTGGATTGAAACTTGCGCAGGCTTTCGAAGACCAA
CTGACCACAACAGCGCTCTGCTTTGTAGCAGATGCCAGTTCTGGAAAGGCGACAAGTATG
TTGGAATCTTTAGTAACAGAATCAAAATCTGGGGTGGCCGTCGTATCGGAGCCCCTCTGG
ATGATCCAAATAGCTCGACTGGCAGAAGCTTCCATCGTCGCTAAATCGAAAATCCAAAAA
CTTATCTTTGCTCTTTGTCGACTGGATGCATGGAGTGTTCGACATAAGGTAGGCGATGCC
AAAACGGTTATGTTCACTCTTCCCGGCCAAGCTGTAGTAGGCACACTCTTACCACTGGTT
CAGAGTGCCTTTCCGGAAGACCGCCATGTCTTTGCGTATGATGGGTGTGTGGAGTCCGTG
AAAAGAGGCATCTACGCAGAGCGCATGTACAAGCATAATAAGCTCATTCCTGATCTGAAA
TCAATAATCCAAGGCATGGGCAAGGATCCGTTGCGAATAACGACTCCGCTGCCGTCCAAT
TCTCCGCTGAGTCATGATGCTTCTCTCGGAGGTAGAGGGGGTGCTATGGAGCGAGCCTTC
TCACAGGTCCCTGTAAAACAAGCACGAATCGTGGAAGCCTGGCTTAGTAGCATCGACGCA
TATTTCAAACTGAAGAACGAAGAGACAACCAACGGATATGTGCCCTATTGCTTTAAGCTG
GGCTTCCTAACATCTCCGGTAGGAGATTTTGAATACGGAACGGATTCGTACTGGTCTCTG
AACTCTTTTCTGCAATATGTAACAGGTTGCCAAGGACGGAAAGTTCAAGAACATGTAATT
GATGCCGCCAGAGAATGGATGAAAGACTATAATTCAGAACAGAAAGCAGAAGATGGATCA
TTTGAAACGTCGGTGATTCTATCGGAACACGAACGAAAAATGATTGAAAATTGTTGTTTT
CAGCACAAGCAAATACTGATTGGTAACAAAACATTACAGGACACTGTTCTTCCCAAAGAG
CACTGGACAATGAAACAAGCATCTCGCAACGGATGCTCCTGCTGTGGACCAGATCCCTAT
GACCAAATGGAGGAGGAGGAATCAGATGTCATGGAAGAGGCCAAGTCGAACAATGCGATG
ACGGGTAATTTCGGTGCAGGAAAACCGAACATGACCATTGGTATGAATGCGAATTCATCC
AGACAAACCGGTTACGTCGATGCGACTCAGGTATTTGCTTTCGATCCAACGCGCTTCACA
ACATAAAACGTCGTTTTCTCTGATAGTGCGCTTCTGTAATCGTCATGTTTCTCACTTCGA
AACTCGTGCAATAGACATGCCTGTTACCCTTACACGGATTTTTTTACTTGAAATCGTACG
AAATCACTGTCTTCATCTCGAGTTCGTCGCCTTAAATACAGCTCGTATAGAGTGTAAGAA
AAAATTGATAGGCCAC
>comp2054_c0_seq1
CGTGCCGATCACTCGGCTTCCACTCGCCTTCGAAGCAACCAGTTTGGACCAGCTCTAGCA
CTCTCGTTGGCGGTACAAGAGCAAAGAAAGGTATCGCAGGCGATTTCCTGATCACGAAAG
CAGGTCGTCCACACACAGCGTCATCCGTAGCGGAATCGAATCCATCCCGGCCAGCTTCTG
TTTGTTGAGTTGTACAATGAACCATCCCACACTCCTACCGAGCAACCGGGACGGCACCGA
AAGCGCTGCCGCCACCAGCATCAGCACGACGGAAATGCTCGCGTCCACCAGAAGCCTGCT
GGGCGTCGACAAGAAGGGAAATACGCGCGCCTTGTTTTTGTACGACGGCGGCTGAGGCGT
GTGAAAATTCCTGGCCGGCTTTGTCCTCGAGCGTGATTCCCTGGATCACTTCCGGTTCGC
ACCGATACAGACAAGCCTGGCCCAGGAATTGTGCCACACCCATGGGATGCCCTCCGACGT
TTCGACCGCCGTTCTCTTCACGGAGGACTGCGCATACACCGAATCCGATTCCGTCCTCCG
CTTGTTTTATCCCCACCTTGGGTTTCCCTATTTCGTGCTGGGATTCGTCGCCCTGTATCT
CGTTCCGAAAATGCTTCGCGACGTTGGGTATCGACTCTTTTCCAGACACCGAGGGAAGAT
ATGGAAATTTGTCAAGAAAATTACCGGGATGGGGGATACCACCATGCGTCCGTACAGAGA
TAGTATCTTGGGTTGGAAGGACGAACAATTTTTGCCTGAAAGTTGGGGGTTGGAAGGATA
ATCCTTCGAAAGAACTGTCACGCTGACATTATTCCATGGTGAAGTTGGAATGACATTCGT
GTTTTCTTCGGACAAAGAACTAACAGCATATACTATTGTCAAGCACGAATCGACACAACG
CATCTAGTAAATGATGCCGCAAAAAAGTAGACTTCTAAAAATACGATACAATTTCGAACG

TAATGCCGCAATGACATGGGAAGTGAAAAAAAGAGAAGAGCAAAACATCATTTCTCAACG
ACGTTCCTAGATGAATATCAGGTTCCGGACAGTCGGCTGGGTGGAGCAGCGGACGCCTAT
CGCAATCAAATAATACGACGAAAAGTGCGATGTCCTTTGTAGTGATTGTTTTACTCGAAC
CAAAAAACATAGTGCAAGTGCCTTTTTAATGGGTCGATGGGATTAAAAAGTGAGGCATTC
ATGCTTGTAGACTGTATGTAATGTGTTGACGTGGTG
>comp2055_c0_seq1
GAAGAAAGCGTTCGTTTCCTCCCTTCGGATTTCTCTTGTTCGGCGCGAAGAACATTTGTA
TAATGGGGGAGAGATCATCGTGCTTTTCTATAATTCTACGCCGTACTACCCTGCCGTATC
GTATCGCCGTATAATTATTGGTCTCGATGACGCGTCGATTATAATGCAAGCTTCCAAGGC
CCAAGGCGGCAGGCATGAGTGCGATCTCGAACAAAGAACACTAGCAACTGCGACCCGCGG
CACGTTCCTCGATCGATACCGGAAACAAGCACACACCCTTTTGGAAGTGCGTACGTACGT
ACTTGTGTCACATTCTGAGGATCGCGACAGAAGCAGTTTGGGGAGATGCGAGATCTACGA
GTTACTTCGAGTCAATGAGATTCGTTCCTGCATTCCTTGTCTTCGATGTATGTATGAATG
AATCGAATGAAACCGAAGAGGGCAACGAAGAGCGAAGCTGTAACAACGATTCCAAAATTC
CTTCCCTCGAGCAGAACCCGATGCAAAGACCGAAGTTCAAGATTCCTTCGCTGTCGCACA
AAAACACACAGGCAAGCGCACGCGCATCGTTCGATGCACCGCTATCTCTTCTCTCTCTCT
CAGTCTGACTGTTAGCGGTAACAGGTTTTGTGGAACGGATAAACGAATCGTCGGTCCAGT
GTCATCGGCGTGAACAATAAAGAGGGGCAATAGCACTATTATACCAGAATCGTATGACGA
GTAGCATTACAGAGTATGTGTAGCGAGCGTGCCGTCTAGCTGCTTCTTTCCGGTTTTGCC
ACCGCCGTGACGAGACGCTCTATCAGTACAGCTTTTCGCCCGGATACCTTTTGGCCTCGT
TCCCTGAG
>comp2056_c0_seq1
TCACGTTGGTCATAGCGCTATCTATCAAATGAAGGAACAAATTACCACATAGTCTTTGAA
GTTTATGACTTGTAGTGGATAAGTAACGATAGAAGATCCTACTCGTTGATTCTTTCAAAA
ATCCAAGAAGAACGAAGCTCTACGAATTGGCAAACAATATTTCTGAATGCCACCATATTG
TACCTTGAGGGGGAAAAAGCAACGCGGTAGATTAGAATAGTCAAAGGAAATACGACCTGG
AACGTCAGCCAAGATGACTTCTGCGCGAGCACGCTTCACACCCTGGATGTGTACATTTTT
TGCCCTTCTCTTACCAATATTTCAGTCACTTCTGCTGCATACCAAGACGCTCGCTTTTTC
GCCATCGTTACCGGTGTCAGTCGGGTCTTCTAAATGTCGCGGAAGACAATATAAAGAATG
GTCTTGTCAAAGAGCGACTGCTTCGAACGAATCTAAAGTAAATGTGAGAAATTCTACAGA
AAAAAAAGAAGAGATCGCAACAACATCGCTTCCAATTCGCTACAACCCCTCTCTTCTGCG
ATTCCGATGTCGAATCTCGTACGATGGAACTAGTTTTTCTGGCTTTCAGCTGCAAGGAGG
GAGAGTAGGATCAGGGGGAAAACAAACCTATTTGAGTTCGAGAACCGAGAATTCAAACAA
AAAGAAAACGAAAAAGGGCAAAGGTAAAAGCAAAAGCAAAAACAACCCGAGGACGATTCA
GGGGGAACTCGAACGAGTCTTATCGACCCGTTTTCAGCGGGTGGTGAAGGTAATAGGAGC
AGGGAGAACGGATGGTGGGGTTCACGCTCGGGGCCAAGCCATTCACTTTGATTTATACCA
AAACGAAACACTCGGAAAACCAACGAATCGCAGCTCGGCCGATGGTTTCTCCTTCGAACG
CGACCTAGAATATACAATGAACCGCATGCTCCCCGTGGATATCCGAGCGTGGAACCTCGA
GCGGGCAAGTCCCGAAGAAGTGGCCGAAACTGGTAGCAACTTCACACTTCCTGGAGTCTA
CAACTGGAATGCTATGCGATCTTCGACTTCGAAATTGTACTCGTATCGGATCTGCGTGGG
AGATGCTATGGACCCATCCGAGCGTTTTCATCGCTGGCAATTGCCGTGGCGCGGGTTGGA
CGCTGCACGACTCCAACGCATCCTCAAGAAATTTGAAGGCCAGCACGATTTTGTTTGCTT
TGCGGGGGCACTAGAACAGCAACAAAAGAAGACCGGGATCGCTTTGGGTACGATTAGAAC
GATCCACGAGATTCGGCTGGTCAAAGAGGTGGATATCGATCGAAATCGATTCGACATCAA
TCTCAGCGACATGGGTGGGGATCCCTCCGAGCACTATTACCGCATTGATATTTATTTGGA
TGGGGCTCTGTACAAAATGGTACGAAACATCGTTGGAACGGCAATTGATGTCTGCAGGGA
ATCAGTGAGCGAGGAAGTACTTTCTGACCTGTTGGACCGACCAAAAGAATTAAAGTATTC
GAGAAAGAACAACCCCTGCGTGCCCGCACCACCAAATGGCTTGACCCTGGAGCGAGTCTT
CTATTCCGACCATGAACATTTCTGAGAACACGAGTGTACACGAAATTGAAAAAATAAATT
AATAGTGATGTACTAGACGCCGCGAAGACAAGTCGTCAGTAGCAGTCTTAGTAAGACGGA
ATTATAGAACTTGGT
>comp2057_c0_seq1
TTTTTATGAGAGACCTTTTGGCGCTCTGCGCTGTAGAAATTCTCAAGTGATGTTGGTCTT
CTAAAATTTAAAAAAAAATGTAGCCCAACTGCCTCCACCATCGTCTGATTTTTGACACTT
CATAATTTGAATAGTGCTACAATCAGAGATTACATCCAACATGGCTTGCCTTTGTCCATC
AGATTCCCACTTGATCGCACAGCGAATCAACAACAATGCCTCGCTTTGTATCGAAGCAGG
ACATTACAGAAACGCTATAATAATGCTCAAGAGAGCCCTCGAAATCAACGATAACACGAA
CAAGAATGTGAAGCGACCATATCGAGATGACGTCTGCAAATGCTTTCAATGCACTCTAGA
TGGATGTATCGCCTTCTCCGAAAAATCAACTTCTAGCACAAACAGTTGCCGACAATTCTA
CGATGCCTAGAGGTAGCAATTATGGTCGTTCTAGAAAGAGACGCAAGGTAGTTCCATCGC

CAGCAATAGCAAGAAGAAATATATTCCTTAAATCTCAACATGGAGAAGTCGACAGCGTAC
TTCCTACATCAAAACAAGAAGACGGAAACGAAGGATCCATCGAATATGGATATGTTTATC
AACAACCAATTCGAGTTGTACGTGAAGGCCATGCCATGGGATCAACACTCTTCCTTATAA
TAATTTTCAATCTGGCTCTTGCTCATCATCTTGACGCCATTGATAGTGGTCATTCGAAAA
GAGAGCGAGACAAGATCATAAAAAAAACTCTTTCTCTTTACCAATTGGCATATAATTGGC
AATTGAAACTTCTTTGCAAACACAACACCGTCGAAACAAAGAAATACAACCCCAAAACTC
ACTCGTTGTGCTCGTCAGTAACGAGCATTCGCTTCAATATGATCATTCGCAATAATCTGA
GCCAAATTCATCGCCTAGCCAATGACTACTCAAAGTATAAACGATGCCTGCAGCATCTGT
TGTCTACCGTGATGGTTGTCGTTGAATACAGGACTCGGAATTCAACCAGTAGCCATGAAT
GGAATGCGATTCCGAACGACCCGCGGATAATGGATCTGGATGGATTTTTGCAGAATGCCA
GCTCGGTAATGTTAGGAGATGAATGCTATGCTCAAGCTGCATGATATATTGATTGAAGCG
GAGGCACTAACGAAAGTTCATGATGCACGAAAGGGATAACCTCGTCGATTCTGAATGAAG
TTTTAAATGACTGTCTGATTTATGTTACATTCCAGACCAAAGACTACGCGTTTAGAAAAT
TTAGAAGATTTATAACAATATACTTCGTAATGAATATTTCTTATTGAGAAAAA
>comp2058_c0_seq1
CTCGTCTCAAGGGACCAGCAACAACGAAAGCAGTTAACAGAACGTGTACAATCAAATTTT
CAACTTCAATATCTGCAGGTATCAAATTAGGCCTTTCTCCTTTCTGCCAACCTCTTTTAC
ACAAAGTTACAACAGACAAACAATAATGTCGAGCGATTTCATCCATTTTATTACATCCTC
GGCCTTGTCGCTGACTATGGCCGGAAACACCGGCATTTCACCTTTCTTGACCCTATTGAT
TCTTGGATTGGTAGAGATAGTCAAGCCTGAACTACTGAATATGGGAAAAACAATTGAAAT
CATTTTAGCGAGCTGGTGGTCCATCGCTGTACTCGGAGTTTTGACTATCGTCGAGATGGT
CGGAAAGTGTATTCCAGCCGTCGATGAAATAATTGATTCAGTAGAAGTCTTCATTGTACC
GGTCATTAGCATTCTAGCATCTATGGCAACGATGGGTTTGCTTCCCGATGCTGGAACCCC
ACAATCTGATGCAGGACTAGGGCAAGACATCAATGTCATTAGCATACTTGATACAGAATA
TTCTGGTGATGGATATCGCAACCTTCAAGACCAAGTGGGAAAAGCCAACCTAGCTGGTAT
CGAGGATATTCCTTCCAACGAACTCGGAGAAGGCTTCCTAACCTTTATGAAGTGTTCTTT
GGTAGCATTTGGTATGCTCCTAGCGTTGCTGATCCACTTCTTCAAAATGTTGATCCGGCT
ATCTTCTATGGTTTGCACTGCAGGGTGTTGCCAACCTTGCATCACTGTCGTAGAGTTTCT
TATAGTTATTTCGGGGGTTATCCTTGCGATTCTTATACCGACCTTTGCTGTGATGGCGTG
CATAGCATTCTTGGTTGCAGCAGGGTACGTTGTTGTTGTCAAATGTTGCAAAACGAAAGA
CGGCAAAAACCACACAACTACCAGTGGTGTCGAAGTAGAAGTGGTGGGCAACAATCCTGA
AATAAACGACATTGAATGTCAACCAAGCGCCTGCAGGACATCAAATGCTACGATTCCAAC
GCATGTGGTACTCTATCCGGCAGAAACCAAGGTCGAATTCGTCAGACCCGTCAGTCCCAC
TACTCCGACGATAACTCCAAAAGAAATGATTCGGAATTCTAGTATAGGCGAACTCGTTGT
CGTACCTCTTTCGCCAATTCAAGCGAAAAAGGATTTCCAGGCGACAACTTATTAAGATCT
CTCTCCATAATCTCAATCCAAGTTCAAAACATCTAATAAAATGCTAGTAAAATTAAGCAT
ACTCTTCAGCAAACAAA
>comp2059_c0_seq1
GGCGTTTCTGAGCATTGCAGCAGCAATTCGAGCATAAGGACGGTAGCATTACTATCAGCA
ACAATGGCAACCCCCTTTTCAGTTCCGTTCGTAGTTTCCAATCCCGATTCATGGGGTCCT
CCGCCTCCCGGGGCCGACGAAGGCAATCCAAACGGTCCCCCTAGCTCCACCAAGTTTCCG
GACTTGCCGTACTCCCCGTTTGGACGAAGCGACCGCCTGGGACGAGCCGCTGATTTCACG
AACATCAGTCGCGGAGACCGAAGCCGATTCAATCGACACGGCCGCTACGACAAGAACGAT
CGCAACGACCGTCGCAGCAAAAACGACAACGACAGCGAGGATGAAGGCGAAGACTTTCAA
CTGGTCGATACGAGCAAAGCAACAACCCAGAAACGGTTCGTGCCTCCCGCAGCGAAGCGC
CGGCAACACACTGCCAGATTGCGACAGGTCAACGCCCGACGACAGAACCAAGGAGGACCG
TCATTCTCCCTCTCGAAACAAGAAGGTTTCGGTGGGCGAGGACGTGGTGCCGGAGGCCGC
GGAGGCAGAGGTGGACGCGGAGGAAGAGGCAGGGGCCGCGGAGGAAGAGGGTACAACAAC
AACCGCATCGATCGCCAACCGTCTGTTGCCGTACAGCCCGATTGGGTCAAGGTCGAAGAA
CTCGATCTCGCAAAAGTCACAAAGAATTTGTCTGGAACCAGCAGCTCCGATTCCAAAAAG
TCGAAGAAGGCCCTGGAGGAGTCGATTCCCACGCCACAGGATGTTTTGTGGTGTGGTTTC
CTCGATCAATACAACGACGCCTACGACAAGGTCACCGCTCGACAACCCGCACCCCTCAAG
CGCATGGCAACGAAGGAATTTTATCCCGTTACTACCACGGACGATCCCGTTCTGGAGAAG
TTGGCCGTCGATGGAATCGGTGGTGCCGGAGAGGGTGACAAGAGAAGCATCTTTGTTACC
GACAATATCTTGGCTCACCTGATGACGTGTAGCCGATCCGTCTATCCCTGGGACTTGGTC
ATCCAGAAAACCGCCGGTGGGCTTTTGTTCTTGGACAAGCGTGACAACAGTCAGCTCGAT
TACTTGACGGTATGGGAAACCGCATACAATGCTCCTCAACCCGCCAAAGAAGGCGAAGAT
GCTAGCGCCAACATCAACACCCCGGAGCGACTCGGTTTGGAAGCAACCACCATCAACCAG
AACTTTTCGCAACAGATTTTGAAGAAGTCTGGCCGAAAAAACATGGACTTGCCCAATCCC
TTCTTCGACGAAGACGACGCCGATGGAATGGAACCCGCCTCCGTCGCATACCGTTACCGT

AAATTTGCTCTGGACGAGAACACGACGCTCGTGTGTCGTACCGAACTCCACGGTCTCGTC
AAAAAGACGCAGTACATGACAGCATTCGCACTCAACGAGTACATTCCACCGCCCCAGAAT
GGAATGCCATCCGCCGCCCAAACGAAAACCTGGCGTGACACAATCGATTCGTCGCGTGGT
TCCGTCTTGGCGAACGAACTCAAGAACAATTCTTTCAAGCTTGCTAAGTGGACTGCCCAG
TCTCTCTTGGCCGGAGCCGATCAGATGAAGATTGGTTTCTGCTCTCGAGGCAACCCCAAG
AGCGCATACGAGCACGTCATTCTAGCTACTCAGTTTTATCGTCCGAAGGACTTTGCCCAA
CAAATCACTCTGAACGAATCACAAATGTGGGCTATGCTCCGCATGTTTGTGGCAATGTTC
GCCAAGCAAGAGGAAGGAAAGTACGTGCTTATGCGAGACCCAAACAAGGCTGTCTTGACC
ATGTACAAGGTTCCACAGGAGACCTTCGAAGAAGACGAGTAAACCCCCGTGTTTATATGC
ATCCGGGATCGCAGTTTGTCCCACTTAGTCGCCACCAGGAGTTAGAACTACCATTTTTAT
TGCTGGCATCTACTTTGGTCAAAAAAACACAATTGATTAAAAAAAAGTAGTAAAAGTTTA
GTAATTGATAGGAGATACACCAGCAAAAAAA
>comp2060_c0_seq1
AAGAAAATAGAAGCTTGCCATGCCTCGTATCGACACCCGAGAAGGCAATATCGTCAAGTA
TGTGCGACTGTTTGGTGTTCTTTTGTTGACTGTCTCTAAGTTGCACGCGCCTACAAGAAC
GACACCACCATTTGCAGTACCGACCCAATGAAAGCCGGAATCTGTTGAAACCTTTGAGAT
GATTCTCGTCCACTGTTCTTACTTGATCGCGATTGCTGCGCTGTCACATCTAGCAGACTA
CGCGAAGGGTTTCCCTTCTTTTCTTCAAACTCGAGGCAGCAGCAACCACGGAAGAACTCT
ATTCGTTTCGGGGTGTGCACCACTTGCTTTGATCAAGGACGACAATGGCCACCTAGACGA
TGAAGAGGGAACGGCTAATTGGACAATGGAAGAAGATTGGGCGTTGGTGGATAATCTGCC
CCTATTTACTGTGTCTACCACGACAGAGACGAAGACGTTTTGGACCCAACTCTGGTCCGC
GAATTCCGCCTTCTTTTCCGGAAAAGAGCCAGAGGATCTATACCAAAGAACTCTCGAACT
ACAGCAGGAAAGGCAAGCTACCGTCGCGATCATGGAGAAGAGCGAAAACAGGAGAGGACG
CAGATCATTGCTTGTGTTTGGGGACTCTCCACCAGTCTTGGAAAATTGGAAAATAGATGT
CGAGCAACAAGACAACAGAGCAGTTGGTCAAATTGGAAATGGAAATAGCGAAGGACGACG
CACGATCTGGTTTCATTACCACTCGATTGGGCGTTTGGAGGGAGATCCGTTTGCGGACGG
ATCGTCACGTGTGGTCTCTTTATTTCCCGGAGGATATATCGAAGCGGTAGGGGGTAGAAT
CTACGAACTAGGCCAACCAATGATGTTCGACAAATGGCATACAAAAGACGGAAATAAATT
AAGACCAAATACCCAGGAGATCAGCAGAGACAGTGTTAGAGAGGATGGTGAAAAGCCCTC
CATCCCACGCTGGTTGCTTCCTGGTGGCACAGCAGCGATGTCCGCTTTGGTTAGTGCGAC
CATTCTCTCGGCTTGCATTGGATACGGTGCGGGATTGTCCATGATTAGAGACCAGCCGTC
TCCATATAGCGCATGGCCAGTGCCGAACATGCTGACGGTCGAAACGGTTCTCGCAGGTCC
GTCGAACAGCAATGGAGTAAAAATGCTTCCACCTTCGTCGTCGTCCACAGAGGCGGCAGT
GACGAAATCTTCCGCCATCAAAACCTCCAACCTAAATCAAGCACCATCCACGGAGGAGCT
TCGAGCGAGGGCACAGTACAGAGTGTTGAGGGAGGAACGATTACTGGAAAAGATTTCTCA
GCGGTTGGAGATTGACAAACAAACTCTCAAACAGCTCGATGATCAACAATCCTCAGGATT
GCATCCTTGATGAGATTCTTCATCACAGGGAAATACCACATGAGATGTCGTCTAGAACTG
AAGCAATGATGCCATAGTCAAGGTTTGATTCACTCATGCTGGCAAGCCCAGGATATCTGA
AAGATTCTACTAGTATGCCTTTGGTGCAAGCAGCATATTTCGGTTGCAATGGTAAAGTGT
AGTAGCTGCATGGGCATTTCTGTCTTAGTACTAGTGTATTATCATACTAAAACTACTAGT
AGTACTTCTTCGTGTTACAAGTACCACCAGTACAAGCTACTATTACAACTATTATCATGT
TTGGGATGTACAAAAATTTAGTGCATTAGAGTACTACTACT
>comp2061_c0_seq1
AATGAACTCTTCATGACACAAAGTTCTTCAGCATCAAACAATCACCATCAATATCAGTGT
GAGCTAAGAAGCATTATCCACGATACCCTACCAAAATTAATTCGGGACAAAAGATCAGAA
AAGCACCAACTAGATTAAAATGATAAGCATTCGATCTGTATTTCGCCCGAAATTGCATCC
CGTTGTACATCGCGCTTTTCAACAGAATGGGGTGCGCTCCATGACCGTCCTAAGCAAAAG
CAGTGGAGAGGAATACAAGAAGCAGAATTACACGGAACGGATGCAGAAAACGGGTCGTCC
CGTGTCACCGCACGTGACGATCTACGCGTTGCCCGTTCCAGCCTTTGCGTCTATTACCAC
CCGTGTCACCGGTATTATGCTTTCCTTTGGGGCCTTTGGGGTTGGTGCGATTGATTTGAT
GGGCGGTCCCGGCGCATCCTTGTCTCTGATGGAATCGATCGGCTCGTCGGGATTCCTGGT
AGCTTCACCCGCTAAGTTTGCCGTGGCTTTCCCTCTTGTCTACCATTACCTGGGAGCCGT
CCGCCATTTTGTCTGGGATTACTTCCCCGATAAATACCTTAACAACGAAGATGTTCAAAA
ATCATCAATAGCACTTATTGGATCGGCTGGATTGATAAGTTTGGGCTTCGTGTTTGTGTA
AGCAGTCGGTAATGAACTGTACGGTTGGTATAGACTGCAATGTCATTCTCGACATTCGAG
ACAAAAAGCCGAATTTCTTACTGTAATTACTATTAACGAGTAATATCTTGCACAGAAATA
TGCGTTGAGGTACGCCGAAAATGAGAATTTCGGATTAATGCGGTTGGAATATTTGTTGAG
ATCACCAACTCTACTCAATGAAATAATTTAAGTACCCGATCATCTTCACTAATTCATAAG
TCGAAACTCAATTGA
>comp2061_c0_seq2

AATGAACTCTTCATGACACAAAGTTCTTCAGCATCAAACAATCACCATCAATATCAGTGT
GAGCTAAGAAGCATTATCCACGATACCCTACCAAAATTAATTCGGGACAAAAGATCAGAA
AAGCACCAACTAGATTAAAATGATAAGCATTCGATCTGTATTTCGCCCGAAATTGCATCC
CGTTGTACATCGCGCTTTTCAACAGAATGGGGTGCGCTCCATGACCGTCCTAAGCAAAAG
CAGTGGAGAGGAATACAAGAAGCAGGTGCGTCTATCAGTTGATTTGGGACGATTATCATT
CTACAGAGCTGGTTGAATGAAAGAAGTTGTGGCGGAGATGACGAAGACCCGATGCACAAG
TCGCTGTAACTTTAGGTCAATCGAAGCAGTTCTCAACTTTAATTTGCGTGAGGACAATGG
GAATGATGGACAAAATATGGATCGCTGAGTTGAGCCAATCGATCTGTCGTGTTGAAGAAG
TTTAATGAATTTTCAATCTAAAAAACTCATGTCATTCTCTCTTGAATTTCATGTTGAACT
GAACATTCCTTTGCATTCTAGAATTACACGGAACGGATG
>comp2062_c0_seq1
GTGGTACCGGTATACTCCTTTCTGTCGTATGAATAAACCGTTGAGAACGGCGACGAACAG
TGAGCTGCCCTGGAGATAAGATGTGATACTGTATAACTGTATTGCAAAACAACTGGTGTA
TCCACAACTGTAGTATCCACAACCTTAGTATCCACAACCTGGGCATCCAGAACTTGAACA
GCCACAACTTCCCCCCTCGCCATGAGATTACCAAAAGCGTCCCAGAGTACTTCCGAAGTG
TCGGACAGGAGTTCTCTCAGGTTTCATCTGAATGGTAACATATATCCTGCGTTGGTCATC
ACGTGCCTAGTCCTTCGTTTTGCGGTTCTAAACTTTGAGTCGGGGTTGTCGGAGTACTCG
AGAGCCGCCTCGAATGTAGTGAAGTTCGACGATCGAAACACCACCCACGGACCACCTGTC
TCTTCCCGGCATATTTACCGATCCGCCAATGACACCGCGTACACATGGCAAGGAAATATC
TGGATCCCCCCCCTCGGAGTTCCCCATTATACCGGGGCCGCAGTGCGGTGCCTGTTTCGT
TCCGAAAACACGATCTGGGTGGGAGATTCCACCATTCGGCAGGAGTACCACACCCTGCGG
TCGTTCCTCGAGGCCGCGAATCCAGGCAACATTGAAGCCTCGGAGTTTGTTGATGGTGCC
TTCAATCCCAACTTGGGCCACAAAATACCGTTCCTTTTCACCGCGCGGCACGGAGTGTCC
GGATCCGGGGACCGGTGCCTCCGTGTCCGACCAAAGGGCCCCTCGGAGGATCCGAACGCG
ACTCTGGCCATCGCAACCTACAACCAAGATCCTTCCAAACACTCCGAAGACTGCATCAGC
ACGGGACGGAACGACCTGTATCGCCTCAACTGCTACCGGGATGGAATCCAATGGATTGAC
CAGGATCTCCGCAAACCACACTACAAACACGGACTCCGGTCCTGGTTCTCCCTTGCGATC
ATTTCGCTCGGCGTGTGGGACACCGATCCGGGGAATAGGGACAAGTGCGCCTCGCTCGAC
CGCCTCGAGGAGCTCCTCGGTAAGATTGAAAAGGAGGTTTCTGGGCCGGACCTCTTTGTG
ATCTGGCAGGCCCACGCCCCCGCCTCGTACGAGGGCCCAACCAATGGCGCGGAGGCACTT
TCCCTGGGAAACCGGGCCCGCGCCTGGTTCTTAGAGCACCAGCCCGAGCACATGGATTTC
ATTGATCTCGGGAGCATCCTTGTAGGGGGCAACCGGAGTTTTTGGCCGAACCGGATCACC
GGGGACCACACCGCCCACTACGGGTTTGAGGCGCGGGCCCTGGGGGGCCAGTTGGTGGCT
GATGCGCTGCACCGGAGGCAACAACGGGATGGGATCGATTGCGTTGCGGACCCCGAACGC
TGATGGATCCACAAGGATGCCGGCAGGAGGGGCAAAGCGCAACAACCCCTAAGAGGGCAC
GCCACGGCGGGGCTCTGCGCCGCAATAGTTGCGATTGCGATCTAGGTTCAAGCACCTGGA
AACCTTGTCCCACTCCGACTTGAAGGCATTGCCTACGACGAAGGAAAAAATACTCTCGAG
CATGAGCCACCATTTTCTCCCAAGTACTTTCCCCCATACGCAGAGCCGACGCTTGTCACA
CAGCTGCTAGATCTCCGTTGCGTCTAATAAAACTGTACCCAGGCTTCGTAAGCAAGGCAG
CCCATTCGTGCCCGGGAATTTTCGACCTTGACGGATTCGGTATTTTGAAGAATGGCCCAC
TTGAATCGATTGTCGATATTTTGAGATTTTCTAGCTAGGTTTGCAATATAAATGTTTCCT
ACCACTATTTGTTCATACGAGGATGACAGAAGCAGTATCAATAACAGTGGTATTATACCA
CACAATTAATCTTCATGCTTATCTTTGAACCCATATAGATGCATCTTACATCTTGTTAGG
GTCGAAATCGGCCTGCTTTTTCAGCGTACCGGGACGGAACCATTCGGTGAGTTCAAATAC
GTCCTTCCACTCGAGCTTTTCCTTCGAT
>comp2063_c0_seq1
CGTCAAATCAATTTCTAGTGGAGTCATAATCTGTCGTTCCGGGGTATATTCATCTGATAA
TAATGCTGTCATCAAATTTTCGTCGCACCGTACTTTCGTCTCGAAACCTTTGGGCACGGT
CTTTTGCAACGGGCCCTGCTTCAGGCGGATCTGTTTCCGATGAAATGCTTCAAAAGCTAG
GAAGTCTCTCCACTCAAGCTCTCGTCGATGGTCTATGGGTAATGGGATGGCCGCCCGCCC
AGATTGATGGGGCTCGACCTCTTACGAGTGGTATGAAATGCGTAGGACGTGCTGTCACCC
TAAACTTTGTACCAGCTCGTCCTGACGTTGCACAAGACAAACCCCCTGGCGGTGAATCCC
CAGAGTACGAGGCATTTGAACTGTGCGGTCCGAATGAGGTCCTGGTTATGTCGAGTGTGG
GTCCTGCTGAAAGTGTTGGAGGAGACATCAAATTTCTTCGGCTAAAACAACGTGAAATTG
GTGGACTCGTTACAGACGGGAGTGTCCGTGATACGGACGAAATCATCAACTACGGTTTTC
CGTGCTTTTCGGATTCAACCACTGCTCGTCAAGGACCAGCTGCAATGCAGCCGTGGGAGT
GCAACGGCGTGATTAGCGTTAGCGGTGTGACCGTTAGACCTGGTGACGCTATCGTTGGTG
ATCAAGATGGTGTAGTTGTTGTCCCTGCTAAAGTTGCTGATACGGTTTACTCGATTGCTC
ATGGTCGAGAGGTCATTGAAGAGATTATCAAAGAGGAATTGATCAAAAACCCCGGTCCAC
CTGGAAAATTCTACCCATTTATGAGTGGAAAGATCAAGGTTGATTCTCCTCTTGGCGAAC

TCTTAGCCTCGAAAGGAATCACTCCAGAAAATTCCAATCAATTCGAGGGTACTGCTGACT
GGTAATATGTTACTTTCGTTCTGATATACGGTAGTTCTCATAATTTCCAACCTTCAATTT
GAAGCTTCTAGAAACGAACCACAAGGGCCGTGCCCTCTGTCACGAACCTTTGTCAGATTC
ACACAACGCATCGGGTTTTCCACAGATTATTTGGAGCTTAATTTAATCGAGATTGAAATG
TTTTGCTAGCTCGTCTCTAATTTTCTCTTTCCCTCTGATGATATCGCCATGGCAGGGAAT
AAGATTATCTGGTTCCCATTCATTGAGGACTATATCAATAACTTCTTCATAAAATTGTTT
GTCGCTTACCATCAAGTTCTTGTAGAATGGAAGGTATATTTTATCCATACCAAACTTCCA
TAAACGCGTTCCAAGGGGAATGGATTGCACGGGAGGCGCCGATCCTTCGTCGTCATTCCC
GCCCTCCAAGTGAGAGTTAGGGACACCGTCCTTTTGAGGGTAATTCCAGTATACATCAGT
GGTCAATAATGATTTCGACGG
>comp2063_c0_seq2
CGTCAAATCAATTTCTAGTGGAGTCATAATCTGTCGTTCCGGGGTATATTCATCTGATAA
TAATGCTGTCATCAAATTTTCGTCGCACCGTACTTTCGTCTCGAAACCTTTGGGCACGGT
CTTTTGCAACGGGCCCTGCTTCAGGCGGATCTGTTTCCGATGAAATGCTTCAAAAGCTAG
GAAGTCTCTCCACTCAAGCTCTCGTCGATGGTCTATGGGTAATGGGATGGCCGCCCGCCC
AGATTGATGGGGCTCGACCTCTTACGAGTGGTATGAAATGCGTAGGACGTGCTGTCACCC
TAAACTTTGTACCAGCTCGTCCTGACGTTGCACAAGACAAACCCCCTGGCGGTGAATCCC
CAGAGTACGAGGCATTTGAACTGTGCGGTCCGAATGAGGTCCTGGTTATGTCGAGTGTGG
GTCCTGCTGAAAGTGTTGGAGGAGACATCAAATTTCTTCGGCTAAAACAACGTGAAATTG
GTGGACTCGTTACAGACGGGAGTGTCCGTGATACGGACGAAATCATCAACTACGGTTTTC
CGGTAAGTTTTTCGTTGATACTCCGGACCTCTTAATGATTGTGGGATAC
>comp2064_c0_seq1
CTTTCCTTCTTCGAAGAAAAAACAAGATTACTAGTGTGACAGTGTAACACCGAATCCGCT
GGTCTCTCAAAACCCAAGATAAAAAGGAGAATGCGGTGCAATCGATTCATCCGAAAAAAG
AAGCACGGCGGCACCTGATGGACAAAGAACAAATATTCGGACAAGCAACAGTGATGGACA
ACGGCGCGAACTACCTTCCGGTGGAGTTGTGGCGGATCTGCCTCGGATTTCTCGGCGTGC
AGGATATTTGTCGATTCGAAATGGTCAGTCGGTGCTGCAAAAGGATCGCGGGCGACAAAG
ATTTCTGGAAACGATATTATTGCAGTGCATGGAGAAAGAATCTTCACCTCCCGTTCACCC
TCCCGACGCCGGAATGGAAAGCCATGTGTCTGGATCGTCGCAGTCACAGCCAGGGCCATT
TCCTGTCGACGGGGTTCCGCGGCAAGCGGGTCCGATGGGGCGGACCACACGTTGCGTACA
CCGGGTTCGAAGGCACCGAGGAGCTCGTGTACCGCGTTCGTTCCTCCATCCGGTCGGAGG
ATCCGCTCGTCCCGCTGGTGTCCGCCAGCGCGTTTCGCGGAGCCTGCACCGTGTACTACG
AAGCTACGATCCAGGGCGGAGGGAGCGTGGGCATCGCCGCGGCGGACGACGGCCTGCATG
CTTTTGTTCGGCACCGCCACCGGTACGGTTTCCTGTCGCGGTCGCGCCCGGTCGGATCGC
TGCCGGTCTCGTACGGGTTTCACGGCGGGGACGGGCGCATCCACTTCCACGAGGGCAGCG
CGGGGGCGACCGGCTCCCTCGCGTTCGATGAGCCGTTCGGCGCAGCAAGCGATGCCACGA
CCCTGGTCGGCTGTGGGTACGACGCGGCCCGCGGACTCTTGTTTTTCACCCGGAACGGGA
GGCTCCTCGGTGTTTGCCCCCGCGGCATCCGGTGCCTCGGGTACTGTGCCGCGGTGTCGC
TGACCCACCCCGGCGGCAGCGCCACGTTCAACTTTGGCTCCGCCCCCTTTGCCTTTGATC
TCGAACTATTCTGCGCGCAGCGGGCAAGGTTCCTCCAAAACTGCGAGAACAACAAAAACG
AGAACAACGACGAGAACAACG
>comp2065_c0_seq1
CATCTATTAATTCTCGCCCAGCACCATCCCTACAGCTCCACAGCTCCAATAATCTATTAC
AGTATTTTTATAATAAACACAGGAAATAGACCGAAGTGGTAGAATATCTATGACGACACT
GAGAATCGCTATTGCCTCAAAATAAAAAATCCACTATGCTTTTGACCCCAGCGATTTTCA
CAACAGGATTTTTCCTTTCCATTTTCGTTTCTCTCGGCCTCCTCACTGATGTAGTACGAG
CCGACTTGAAATACTATTTATACATTGAAGATGAAGGTGCTCTACACGATACAATAAACA
AAGACGGTGCGGCAGGCGAAGTCGTCGTTCTCGAGTCCGTTGGATCCTTACCATTCGAGA
AGAGTTCCTACTCAGCGGTTGGAGGGTCTTGCCCAACAGTTACCAAGGCAGGAGGGATGA
ATTCTCGATACTTATGCATTGGATCTCATGAGAAGTCAAATGAAAATGTTGTCTTCTTTC
CCATTGCCCCTGCTCCTGGACATGACTCAACAACAGCTTCGATACTTCCGTTGAGGTTTG
GATCGTTGGAAGAATCTCATGGAACTTCTTCAGGATTATTGTTGTTTCGGAATGCTTTAG
CTGTTGATTGGCTCGTGCCTACTACAACTTTAAATGAAGGAACTGTAAGCTTGAACGCCA
TATCATACGGTAGAGATAACTCTCGTGATGGATTTAACATGGAGTGGTTACGATCTGCAG
CTTCAGGCTACAAAGATGGTCAGATCGTAGCAGAGTGGAACTTGCTGACAGAGGCTGGTG
GCGATCCAATTTTGGACGGTGGTTCCATCCAATTTATCAAAGGTAAACCTCTACCAGCAA
TAGAACAAAAGCAATTGTCAGATGGTGGTAATAAGACTCTTGCTGCAGCAAATATTTCGA
ATTCCTCTCAACCTGAAACATCAGCTGTTACTTGCCAATCGATTACGCAAAACTTATGCG
ACGACAAGAACAACGATAAGTTTAGTACAATATGTTCCTTACTCATGCAGGTTGGATTGG
ATGATGTTCTTTCGTCCCCTAGTAGCAACAGCTATACTCTGTACGCACCGACAAACGAAG

CCTTCTCGAGAGCTTTTGGGAAAAATGCATCACCAGCACTCCTTTCTGGCCACGATCTTA
CTAAATTGCTCCTGAGCCACCTCTTTGTTGGCGACAACATTAAATCATCGCAAACGCAGT
CAATATCAGGACAAAATCCCCTTCGATATGAAGATATGGTATGTGGAGCGAAACAGATTA
TGGCTAGCCAACAAGTCACAAAAATTGGGTGTGACGCCAACGCCGAGGTATCGTACGTGA
CAGGTGCGGGAAACCAAAATATGTACAAGCCGTCGTTCTTAGCTGTTGATCAGGAAACTT
GTAATGGAATTATCCATACTCTTGATTATGTCATCCTTCCAGCTTCAACGGAAATTGGGG
ACGAACAGATACAGCATCATCATGACCATCACGTTGTAGATATTGACACAACAGGAGGGC
CAGGTGAAGGAAAATCCTCCACCCGGAAGAACAACCTAGATGAATCTGCGCTCATCTCAT
TTTTCAACGACAACATAGGACAGGTCGATGATGCAGAGGACAATATGAAGACTACATCAA
GCACCGCTGCAACGATTGAGGCAAATAATGAGGGAGGTCCTACTTTTGGGAACAATGATG
TCCATCCCGTCAGACGCAAAGATGTGAATCCAGTAAACACACCTCAGACCAACGAACAAC
CCCGAAAGATTGGGGACGGAGAACTCGGTCGCGATCACAGTGAACAAGATCTGGGCCAAG
GACAGCGAAGGTTAAAGAGGAGAGAAGCGTCAAATAAAAGCAGACAAGGGAACCTTCGTC
GTCCCAACTAACTTTCTCGAAGGATACTGTCTGACACGATGACAATTTCAGAACCAGTAT
GAAAAGAAAACAAACGAGGTTGGATGAGAAAAACGCTTCTTTTTACAAATCGTCTTCTAC
ACGTATTCTAGAAATATGCTTTTCCCTGTTTTTTTTTTTTTCTT
>comp2066_c0_seq1
CGAGGCCACTGGTGTTTGCACCGCTCTGATCCGCGAGGAATAGGGAGCATTCCGCCTCCC
GGAGATCATCCTTCGTTGATTGGTTCTTCGAACCCATTCCATTCCATGTGTGTGGATCCT
TCCGTTCTTTCCAACAATGCGCCGCAACACGATGGCAACAACGACAACGACAACATCAAC
CACAATCACCAACACCACGAAGATTCACAGGCGCCTTGTGCAAGAACCATACGAAACCAG
AGACAAGCAGCTGGTTCTGGTGGCCGGCGCAGAACAAACGGGGATCGGGGAGTTCTTTTA
CCGAAACGCGGCGGAACCGGACACCGCAAACGGCCTTGCCGGGTGGGAATGCCGACCCTC
CGGGAGGGAGGACTCGAAACGCTGCGGAACGGGGGACTCGCACCCTCGAATTCTTCCGGA
CACGGTGTTTTCGACCTCCTCTTCCGGGAAACCGCCGACGGCCCCGTCCAGCAGGTGCTG
ATGGACGGGATCCGCGACGCCTGGAAGAATTCCGGCCGCGGGATCGTTCTGGGGGCCGAG
AGCTTTCTCGGCGTCGGCGTCGGCCCGGAGGAATCCGGTGGTCGCGCCCCCAAGACGGTC
TACCGGCTCATGGAAAACCTGAGCCTCCGCTCCGGCGACGTCACCCTCGTCCTGGTCTAC
GGGACCCCCCGGATCCGGGAATGGGCCTCGGTCTGGTTGGGGCGTTCTTCCCCGGGCACC
TACGAGGACTTTCTCTGCGGGGACGCCTGGGCCGACCAGCGGCTCGAGCACTCGGAGACC
ACCATGAACCCCTTTCGGGTCGCGGAGGCCTACCTCTCGAGGGGCTGGAACGTCTTTGTC
CTGGACGAGGCTGCCGCGAGGGCCTCCGGCCTCGATCCCGCCCACGTTTTGGCGTGCGAG
GCCCTGGAGGGCGTCTCCTGCGAGGGGGGCTGGATCGCCGGCCTGGAGGGAGAGACCTCG
GGGATCCCCCCTTCCTACGAGATCGACGGGCTGGACCGGAGCGAGGAGAGCGACCTTGAG
AAGCTCTTTCTGGCCCGGGACTGCTGCTACCGGGCCGGCCTCGCTTCGACGAAGGGATCC
GGGACCCTCCGGACCTCCATCGGAGCCTCGGCGTGGAAGGACTGCCGGAGCCTCTCGTGC
GGTGCCGGTGCCGGGGAGAACCTCACGGACCCGTCCTTCTTTTTGCGGGCCCTCCGGTCG
CAGAAAGGCTGCGGGGGGGAGGCCCTCGACCTGTCCGGGCTGCTGACCGGCGGGAAGAGG
CCACCCGACGCTGCGGCCCCCCTGCTTCCGGTGGCCCCGGTGGTGGTCGTGGCAACAGCG
ACAGCGTTATCGACCCTGGCCGGCCTCCTGGTGTGGTGGGTTCGGCACCGATGGCGGAGC
GGGAAACCAGGACCGCTCCGGGGGGCCTCCGACGGCCTGTTCTCGGGGGAAACCCTGGGG
AGGCCGGGAGGCCCGGCTCCCCCGGCCTCGGCCTTTTCCGGCCGTCTCTGCAACGCCTGC
CGGTTCGTCCGGGCCGATCCGGCCTGCGTCTTTTGCGGGGAAGGAACCGTTGCCTCCCCC
CCGGGCGGCATGGGGAGGGAGATCGAGCGGAGGACGAGGGAATGCCTCGGAAGAGCAAAG
GGCCGGACCGACAGGGGCGGACGTTGTCCGGTCGACGCCTCCCACTTCGACACCAACCTC
CGAAAGACCCGCCACCAGAGCAGGAGCCACAAGAGGAAGCAAATGCTGCAGCAGCTGCAG
GTCCATCGGCAGCGAGACGAAGAGGCAGAGGAGGGAGCCGCCCGCGAGAGCCGGGTCGTG
AGAAAGCCGAAGGAACTCCTCGTGACGAAAACCGAGACCGGGGAGAAAATCTACGAGGTT
GCCGAGCGGGAAAGCTCCGCCCTTTGGAGCGGACCTTGCGAGGGAAACCCGCAAGCGATC
GTCTTTGAAAACGACGAGCGCACGCTCCTGTAGGATTCGCAACTACTGTACTTTGTACCG
TAGTAGTAGCAATGTTTTTGATTGTTCCGTGTAGGTAGTAGTATAAATAATGAAACACGT
ATGGACGAATGAAACTTGTGCGAGGAAACTCGTCTTCGGCTGCTGCTAGCTACAACCCAC
AGATGTGTACAGTAATCCAATATGAAATGCGAATTTCGAATTTCGCCATGGTTTTTTTCA
GTTTCAAAACTTTGTGCCAGAATTTTGTGATTGCCGAAGCAAAGAAAAAGACCGCGACGG
TCAAG
>comp2067_c0_seq1
ACGACCAACGCCAACGACCGGAGATGGCAATCGGGACTTGGGCACCGATACGAGTCCGCA
CCGGCAGCTCAACACAACCATCGAAGAAGGCGTCGTTCCGACACAACCAAGAATTGCGGC
GAGGTCCACCCACCCGCCCAAAATGCCCAGAATGACACGAATTCGCTCGCTGGTGGCATT
TCTCCTCCTCGGCGCTTGCGCCCTCCTTTCCAACGGGGTTTGCCACGCCTTTGTCTCGCA

CCGAACGGCGGCGCGCCCGGTTCCGAATAACCGTTTCCACCGCGGCCGCTCCCCTGCGGG
GAGCACCGACACCCGTCTCTGTGCGATTCCACCGGAAATCCTGGTCGACGTCGAGGGATC
CCGCGTCCAGTTCTTTGGCTGGTTCTTTGGTGCCTCCGGGGGGATGGGAATCGCTGTCGG
CGCCTTTCCCAGGATGTACGAGCAGTTTCGGGCCCTCCGGGCTCTAAGGGGATGCGAGCC
ATCTCTGGGGGGCGAAAGGGTGGGCCTGAGCCCGCTCTGCGGGTATCCCGAGGATCTGTG
CGTCAGGGACCTGGAAAAGGTTGTCAACAACAAGCTGAGCATCGAGCAAATAGTAGAGAA
ATTTCCGATCGAGAACAATTTCCTGTCGAGGAATGGCTACATTACCTTTCTTGCATACGA
ACGGGCCAACAAGGGGAACAACCCGCTGGTGGTCCGTGCCGTCTTTGACTCGTTTGCGCA
ATCCACGGACGCCTGCGATCCAAACGTGGCGCAGGACAAGATCGATTCCTACAAGGAGGA
CGTCTACCAAATCAATGGTACCCTGCTCCAATCCAAGCTCGTCGGGTATTCGGCAATCGG
TACGCTGCTCTTTCTCCTGGGATTGGCGCTCTCGGTTATGGTTGGACAGCTCAAGAATGG
TTGGTTTCCATCCTGGACACTGGCCGATGGCATTTTCAACATTCCGGACTTCTGGATTTG
AACAAAACACACCATTGTGGTCGACTGCTTGTTCCAAAGCATGGAAAACGGTACGATAGC
CATGATGATTATCACAGTAACTTTGCACTTGAGTTTTTCAGGTTGTAAGTTGATAGAAGT
ACATTCGTTGAATACTTTCAACTCGCAGGTGACAAGGTTTTCACCCTAGTATCTTAGTGG
CTGACAATGCGATCAGTAAGTACTAGTAAGATAGAAAGCACTAAATTCAATTATACAGTA
AGG
>comp2068_c0_seq1
CAGCTCCGTACTCCGTTACACCATAGCTCATCCCCAACCCGTTCTGCTACGTAACTAAAT
AAGTATACCACCAACAAGATGCCAGGACTAGGAAACAGACGCATGTCTACCAAGCGCAAG
TTCGTCGCCGACGGAGTCTTCTACGCCGAACTCAACGAATTGCTCATTGCCGAGCTCGCC
GAGGACGGTTACGCCGGTGTCGAGGTCCGAACGACCCCCCACCGCACGGAGATCATCATC
CGGGCCACCCGCACCCAGAACGTTTTGGGAGAGAACGGACGCCGCATCCGTGAGCTGACC
TCGGTCGTCCAGAAGCGATTCGGATTCGCCGATGGCGCCGTCGAGCTGTACGCCGAGCGC
GTCCAGAACCGCGGCCTCTGCGCCCAGGCCCAGGCCGAGTCCCTCAAGTTCAAGCTCCTC
GGAGGACTCGCCGTCCGACGTGCGTGCTACGGAGTTGTCCGATTCGTCATGGAGGCCTCC
GCCAAGGGTTGCGAGGTCATTGTCACCGGAAAGCTCCGGGGACAGCGTGCCAAGGGAATG
AAGTTCGGAGACGGTTACATGATCAAGACCGGACACGCCGGACAGGTATACACCGATACC
GCCGTTCGACACGTTATGATGCGACAGGGAGTCATCGGAATCCGTGTTTCCATCTTCTTG
CCCCACGACCCCAAGGGACAGCTCGGTCCCAAGATCCCTCTCGATGACGTCGTCACCATC
ATGGAGCCAAAGGAAGAGGTCGACCCCGCCGCCCTCTTGGCCGCACAAGAAGCCCAGCAA
CAGGCCGCTGCCCTTGCCGCCGCACAAGCACAGGCCGCCGCCGAGCTCGCTGCCCAGCAA
CAGGCACAGGTCGAGGTTGCCGCCGTGGGCGATGCCGGCATGGCCATGTAAGGGACGGGT
CCTGCCTGCCAGCCTTGCATCCTTTCGGTAGACTTCTACTGCGTACTACTATAGAATGGT
GCTTTCATTGACTGCGTTACTGCTTTTTCGTATTCGGTGTACTGACGGGTGCTATGTAGT
AAGTAACGTGCGGTGTACCGTGCTTCCTCTAAACCACATCCATTTATATATACCACACAC
ACAAAGTATCAGCATACCCAACGAGTAGCTTAGAATTGTTTCTCCGAACAAACGAGTCGA
TTTCTTTGCCGGACAAGCGCCGGAGGAATCATTGAACCACTCTGGTTCCTCAGGGGAGTG
GTTTAGTATGTCTTTCGGTTGGAACGTAGCCATGTAGTCACGAGGTGTGCCTTGCGACAC
CTGCTGAAACTCACTCATGGTGCAAGTACTTTTTCGTACCATGCTTCTTCGGTGCTCCGC
GGCAGCGGGAAAACACCTGACATCCACTCATGTCACCCAATGGACAATGGAAAATTGAAA
ATGGACAACAGACGCAAGCAATACGGTACAAGTTTTGCACGAAAGACGCAACATTTGTAT
GACCCAGCTACTGTAAGATGCATCGAAGAGAGCGACCAAACACCCTCGCTTTTCTAGAAT
TCTTTGTTCCATCCATTACTATTGCGATTGTTTCTACGAAAGGAACCGAACCACCCGAGA
ATACAGCATGCTACTAGTGCTAGTGCTGCGCCGATCCGTTGCTTGGACTCGGGCTCGTCT
TGATGTATAAAATAATACTACTAACACTATC
>comp2068_c1_seq1
CTGAGTTCCCATTTCATTCGTACAACGTTACCCGAACATTTTTCAATCAAACCAACCACT
GGACGGCTCATCCTGCAACACAAAGACAACACTAAATTTGTTGTCCACAAAAGAAAAAAG
GAAGAAACCAAAACAACAACAACAATGATTCTTGTTAGTTTCAAAAGCTTTCCGAGATGG
CTTGCGGCAACTGCGGCCATCTCTGCCTTTGCTCTCCCAACCACGGAAGCTTTCCATCCG
GTATCCTTGCAATCCACAACGGGAGTTTCTCACTCCTTGGGTGTCACTCCCGCTCCTTCC
GTCATGGTGGCCGGCGGGGCACGGCAACATGCACATAATCGGCCATTGACGGTGATGTAC
CGGATCCGCTGCGAAAACAAATACTATCAGCTGGAAGAACTGGAAGACGCCGATAACTGC
ACCACGGAGCTCTTCCTGAAGGAAGACGGGACGATGGACATAATGGAAACGGACGGGCCC
ATCTTCAAGAAAGCAGTAGGCCACTGGGAAATCAAGGAGAACCAATTCGCCATGACGATT
ACCAAAACCTTTGTGACCGGAACCGACAACAAGAGCGACCCCACCGGCATGGGTGAGTTT
TCCTTTGACGTCGAGCGCGTCTTCGAGGGCGAGATGACCGTGGTGGGCGGGACCGAGGTC
GCCATCAACGGCAAGATTCTGGCCGAGGACGTTGTCACCTCCAAGATGGAAAAGGAGGTC
GGCTTTTTCAACATGATCGACGGGACCGATCAGCGCCTGGAGCGCCGCCCGGACGCCCGG

CCCAAAAACATTGGCGTGATTCCCTCTCCGATCGATTCGAACGACGTAAAGGGCCACGAC
GGGATTCCAAAGGAGTGGCTGGAACCACAAGCACCACAGGTGCCACAAATGGACGCCTTT
GGAGGTGGAAGTTTTGGACAGGATGCCCAGGGATACGGTGGCGGTGGTGCTGGATACGGG
CAACAGCAGCAGCAGCAGGCTAGCGATTGGGGGAGTGGTAGTGCCGGATACGAACAGCAG
CAGCAACAAAGCCCGGGGATCTCCGAGTACGGAAACCCCGGCACCTTTGGACAGGAATCA
CAGGGCTATGGAGGTGGCGGTGGATACGGACAAGGAGACAACGGAACGAACGATCCCGGA
CCGTCCTTTGCGGATTATTACGGACAACAACAACAGCAGGAGCAGCAACAAGAAGGGTAC
TACGGCGATGGTTCCCAGCAGCAGCAAACAGAATACGAGCAAACCAATGGCGACGGCAAC
ACCGGTGGCAGCTGGGACGATTACTACAACTCCCAGGGCGGTGCCGGCGTATCGCCGATG
GGAGGCGGAGGGGGGCCCAACCCGTGGCCGTAAACCGAATCCATCCGACCCGGATCGCGG
TGTGGACGCAACGTGTTGTGCAGCGGGATCGGAGCCCGCCGAGCGGTGCGGCAATCGCAC
GCACAGCAATCCGACGAAACCAATAGCTACGACACAGAGCCATAGATAGTGTTAGTAGTA
TTATTTTATACATCAAGACGAGCCCGAGTCCAAGCAACGGATCGGCGCAGCACTAGCACT
AGTAGCATGCTGTATTCTCGGGTGGTTCGGTTCCTTTCGTAGAAACAATCGCAATAGTAA
TGGATGGAACAAAGAATTCTAGAAAAGCGAGGGTGTTTGGTCGCTC
>comp2069_c0_seq1
TTCAGCATCATGGTTGCGTCGGCTGTCTTCATCACGGATCTCCAAGGCAAGGCTATCATC
AGCCGAAACTACCGAGGCGATGTCGTAATGAATAAGGTCATCGAACGTTTTGCTACATAT
CTTGTCGACGTCGAGGAAGAGGCCAAGAAACCGGTTTTTCATGTAGACGTAAACGGCGAT
GTGCAATCTGGAGAGAATGTTGGGGAAACCGGTACGGGGGGAGAACACTATGTCTACGTA
TCACACCAAAATGTTTATCTTTGTGCTGTAACCACCAAAAACTCGAATGTGTTTTTGATC
TTTACATTCTTGTATCGTTTGGCACAAGTATTCAAGGATTATTTCGGGTCATTGGAAGAG
GAATCGATTCGGGACAACTTTGTGGTAATCTATGAATTGTTAGATGAAACGATGGATCAC
GGTATGCCACAATCACTAGATAGTAATATTCTTCGTTCCTTTATCACCCAGGACGCAAAT
CGAATGGCCGAAGATTCTAAGAGTAAACCTCCTGTGGCCTTGACGAATGCGGTTTCCTGG
AGAGCAGAGGGAATCAAACACAAAAAGAATGAGATTTTCCTGGATGTAGTAGAGAAATTG
AATCTGCTGGTTGCTGCGAATGGAACTGTTTTGCACTCGGAAATTAACGCGGCTGTCAAA
ATGAGATCCTTCTTGTCGGGAATGCCGGAATTGAAACTCGGATTGAATGATAAGCTCATG
TTCGAAGCAACCGGACGCGCAAACCAAGCCAAAGGGAAGTCAGTGGAGCTTGAAGATATC
AAATTCCATCAGTGTGTGCGATTGGCAAGATTTGAAAACGACAGAACAATTTCTTTCATT
CCCCCGGATGGTGAATTCGATCTGATGACGTACCGCCTCAATACGCATGTGAAGCCATTG
ATTTGGGTCGAAGCGGTTGTTGAACCGCACAAGGGATCACGTATCGAGTACATGATCAAG
ACCAGATCCCAATTTAAGTCGCGAAGTGTAGCCAACAATGTTGAGATTGTTATTCCAGTT
CCACCGGACGTCGACAGTCCTTCATTCAAATCTAGCATCGGAAACGTAACCTATCTCCCC
GACAAGGATGCTATTGTTTGGAACATAAAGCAATTCCACGGGGGAAGAGAATACCTGATG
AGAGCGCATTTTGGACTGCCTTCGATCAGTGCAGCAGATGTTGATCCAGATGGCCAGAAA
AAGGGAGAGAATTCGTGGAAGAAACCCATTAGCGTCAATTTTGAAATTCCATACTTTACC
GTAAGCGGTATTCAAGTGCGGTATCTGAAAATCATTGAAAAGAGCGGATACCAAGCATTG
CCGTGGGTACGGTACATAACGGCGAACGGAGATTACCAATTGAGAATGTCGTAACCAAGA
AAGTAAATGTTTAATCATAATATTGTACAACATCAACTTCGAAGTATCTTGAATCCAAAG
TTGCGACAATTCCGAAAAGTTTGAAGTTATCTTTATGAACTGGATACTTCAAAAGTCACT
GTGTTCTTCTGTATCACATGAATAAGGAACACTAGGTTACGAATAGAACATAACAGTCTC
AGTAATTAGCAATGATAGGTTGGGAA
>comp2070_c0_seq1
AATCGCATTCACACTATTGTGTCAAAGTGGTTACATAACTTCAGCGCTATCACGCTAATA
TCATATCTGTTTTGGAATTGCCGACGATGATGTTTGGTGCTAAACTTCCATCGAATAGAG
AAAATGGATCGCTGTCCTCGTTGTTATTTTTACAGACGTCGGCGCTATTAAAAAAAGCAC
CTATTTTTGTTCTGTTGATATGTCTACCTCTGGCTACATCATTCGTGTCGCCAAGATCGG
CCTTTTCCATCAATAATCATCTTTGCCACTACAGAAAGGTTGGAAGAAAGCTGTATGCTG
CGGATAGCGCAATCAGTGCTGATGATCCCATCCCTCCTGCTAGGGCTCGATTGCTTCCCA
GCGTGTTGCGCTTTCGTGGGGCAGTCGATGAGGGTTACGGTCGCGGCGGAAAGAAGCTGG
GGGTCCCCACTGCAAATCTTCCGAGCAGTCTCTTTCAAAACGCACTGGAAGATGTTGGGA
CAGGCGTCTACTTCGGATGGGTGGCCCTTGAGCGAAACTACGACAACGATATCGAATTCC
ACAAAGCAGTAGTAAACGTTGGGTTCTCTCCAACGTTTGAAGGGGAAGAAAACAAAGAAA
AAATTATCGAAGCGCACTTGATAATGGAAGATGGCGACGAAAAAGATACGGGCGACAATA
CAAATAACGCGGAGAAGAACGAAACTACCAGCGACAAGGCATTTATCGAAGACTTTTACG
GGGTTCCTATGAGGCTACAGTTGGTGGGATTCCTGAGAGAAGAAAAGAAGTTTGATAGCT
TTCCTGAGTTGATTGCTCAAATCCATGCAGATGTTAAAGATGCGAAACTCTCATTAGAAT
GTGAGCCCTACAAGGCCTGTCAAGCCGATCAGTTCTTGTGTATCAAAACATCGCCAGCTT
GGATTGGAAGCGGAGGAGGGGACAAAACGGCAAGCTGGGAGAATGCACCCCTCGAAGAAT

TTCTAGACACTCTGAAATAGAAAGAATCCAAAAGATAGTGAATGTATTTTAGGTAGAGGA
GCCCGCTGTAAATTGCAATCAATATTAGAAATATCTTTCTTTCTGTCTTTTGCAGAAAAG
CTTGATACTGCCAAGAATATGAACAAATCACATAAATTCCATTCTTCTC
>comp2071_c0_seq1
AAAAAACTAAACGACAAATACATTAACAAAGAAATCACAGCAACACAATGGGCGTACTCA
CAGTCAAGGTTGTAAAGGCTTCACATCTCGCCGACAAGGATTTAATGGGAAAGACGGATC
CTTACGTCAAGCTCGAACTTGAACAAGACAACCTTATTCGAGACAAGGACTATGGGTACC
AAAAGACCTCGGTCAAGCAGGGCGACGTCAACCCCGTCTGGAACGAATCCTTCAACTTCA
ACATCCCTACCCTCGACAACATGGTCCTGACCCTCCGCGTCTATGACTCGGACATTGGTT
CCAAGGACGACAAGGCGGGCTGGTGCAAGATCAAGCTCGAGCACGAGGGCATCTCGTCCT
CGCCCAAGCGCATCGAGAAGACCATCGACCGAAACCTGTTGCGGGCGAACGGAACGATCA
CGGTCGATATTTCGTACGAGGAGTAAGAACAAACAGGCAAACGTCGCGCCTGTGTATGTA
GATATGTGGATTGATTTTGATTGTGGTGCTGGCAGCGGAGACCGTCGAGGTGGAAAAACC
GTCTAGTTGGCTCCTTCCAGATACTGACCGCAACGGAACATACATTTATTATTTGAGCAG
TAACAAATTCTTGATAATAACCGTAAATGCATTTTGCACA
>comp2072_c0_seq1
CCAAACCAAACCAAACCAAACCACACCAAGCAAAGCAAAGCAAAGCAAACCAAACGCCAA
ACCAAACCAATCCGATCGCACCGCCACTACAGCAGCAATGATGCTCCTACAACGCGCAAT
CCTCGGTGCCTCCCTCCTCCTCGCTCCCGCACTGGCCTTTTCCGCCCGAAAGGGATCCGA
GCTGGTCCGGTCCGCGGCCTCGTCGCCAACGTCGTCGTCGTCGTCGCCTTTGTCCCGGTC
GGCCCTGTCCGTCGGGGCCGTCTGGACGGAGGACGAGGACTACTACGGGGACGACTCCTT
TCTACTGGGCCGCGCCGGCGCCTGCGCCGATTCCGAGTCCTGCTCCCTCGAGGAGGCCCG
GGCCTGCCTCGACGGCGTCCTCCGGTCCCAGGCGGACTGCGTCGGGGACGCCCTGGGCAG
CAGCCCGCTGTGCGGGAACGCCGACAAGGTCGCGGAGGTCGTCGCGGGCCTCCGCCAGAA
GATCGCCAGGGAAGAAAGGCGCCTCGCCCCCCTCAGGGCGGCTTTCCACCTGGCCAACCT
CTCGGTCGGGGCCTGGGTAATGTGGACCGTCCTGCACGGGGTGGCGGCGGTTCCCAACGT
TCCGGTCGACGGCCTCTCGATGGTGGGAACGCTTCCGGAAGGCCAGGCCCCCGAGACCTG
GGGCGTCGCCACGATCCTCCCGCAGGAGTGGTACTGGTCGGTCCGGGACGGGTACTTTCC
CTCGCTGCTTGCCGAGTGGGCGAAGCACGGCGGCCTCTCCGTCGATCTTTCGGCCTTTGA
CGAAAAGGTCGTTCCCTTTTCCCCGCAGGAGTGGGTCTGGGCGGCACAGAACGGGTCCCT
CGGAACGATGCTCGGGGAGCACCTCCGGTACGGGGGCTACCTCGTCGACTCGGGCTACGA
CACGGAGGGCCTCTCGCCGCTGACGCCGCAGGAGTGGGCCTGGTCCGTCCGGGGGGGCTA
CTTTGGGGACGCCCTGCGGCACTTTTACCGGAACGGCGGGATCTGAGCCGTTCGAGCCAA
CCCCGTTCACCCACCCACCCACCCACCGCCCGCACGGTTTCGATTGTACTGTACTCCACT
TGCACACTAGTAAGTAGCCAAAGCACACCCTCCGCTCGTTCGAGCCCACGATCCTTCCAC
CGGACCGGTCTGCACATACTCCCACACCCCACGCCACGCCACGCCACGCACCTTAGAAAC
GATGAAACAATAATAAAAAAATTACTGTACCCACTGCATGCAAA
>comp2073_c0_seq1
GATCTTTCGACATAGCATTGTTGCTGCTGAAAACAATGTTTTTGTGACCGACGGAAGAAA
TCAGAAGCGACTGTTCTCATAATAGCATTCAATTCTACTTCCGAAAAACTGTAATATCAA
TATCTCAAGATGGAACAAACTTACATCATGATCAAGCCCGATGGCGTCCAACGTGGACTC
ATTGGTGAAATCATCAAGCGATTCGAACAGAAGGGTTACAAGCTTGTGGCCATGAAGCTC
ACCAGCCCTACCAAGGAACACATGGAAAACCACTATGCTGACTTGAAGGAGAAGAAATTT
TTCCCCGGTCTCATCGAATACATGACCTCTGGACCTGTCTGCGCCATGGTTTGGGAAGGA
AAGAACGTCGTTTTGGAGGGACGCAAGATGCTTGGAGCTACCATGCCATCCGAAAGTGCC
ATGGGAACCATCCGTGGAGATTTCTGCATTGAAGTTGGCCGCAACATCTGCCACGGAAGT
GATGCTGTCGAGTCCGCCAAGCACGAGATTGCTATGTGGTTCCCTGAGGGAGTCATTAGC
TACGCTGCCACCGAAGCTCAGTGGATTTACGAGTAAACGCTAGGCTTTTGTCACCGCGAG
CCGTTATTTTTGAAACGGCCTTGGTATTATTGTAGAAGATGTAATAGAAGAATTTAAAAG
CTATGGATCGACC
>comp2074_c0_seq1
ACACGATGCTCGACATTGCCAAGCACGCCGCCACGGACGAGGTCCGCCTCAACCAGCTGG
ATCCAGACTTTGCCGCCCTGTCCTTCTACAAGCTCTTTGGCCATCCCACCGGGCTGGGGG
CCCTCCTGGTGCGTAGGCCGTCGGTGGAGGTCCTGCTGGAAGCATCGACCGTTGGTGGGG
GGCACTACCAGGGGGGTGGATCGGTCGACCTGGTGCTGCCCTTTCGGGGCTACACGGTGC
CAAAGCCGGGCCTCGCGTCCCTGGCCAGCGGGACCTGCCACTTTCGGGGAATTGCCGACC
TCTCGCACGGCTTTGACGCGCTCGAAAGGCTAGGGGGAATGCCCGCGGTCCACAGGCACG
CGGTGTCGCTGGCGGGGGAGCTGGCGAGGCGTCTCTCCGAGATGAGGCACGCCAACGGAA
GAATGGTGGTCCGAATCTACGGGGCCTGGGCTGGCCGGGGAGGGGAGCACCGCCAGAACA
ACGGCATCGGGCCGACCGTTGCCCTAAACGTGGTCCGCAGCGATGGAAGTCCGGTGGGAT

ACAGCGAGGTCTCCAAGCTGGCCTCCCTGCACAGGCCCCCCATCCAGCTCCGAACGGGGT
GCTTCTGCAACCCGGGGGCCTGCCAGCGGGCCCTGGGGGTGGGGGAAGAAACGGCCCTAG
AACACTACCGAACCACCGGCCACGTCTGCGGGGACCACATCGATCTCGACGGTGGAACCC
CCACGGGGGCCGTTCGTGTGAGCTTTGGGAAGGACAGCCTCTGGGAGGACATGGACGTCT
TTGTCCGGTTTCTGGAGACCACCTTTCGGAACCACAACCCAGAAACGAGCCCGGTGGTTA
CAGGTGGAAATGCTCCGGGCCACTGCGAACCAAAGTCCGGACCCGAGGAGAGCAAGGTGA
GCGTGTCCGAGCTCTACCTTTTCCCGATCAAGAGCTGCGCGGCCCAGAGGGCACCAAAAT
GGCCACTGGTCCTCCCCTCTGGAAAGCTGAGGTACGACCGGGAGTTTGCCCTCGTGGACA
GGTCGGGAACCGTCCTCCGCCTCCAGAAGCACCCGAAGCTCGGGCTCGTCTCCCCGGTCG
TGGACCTCGAGGCGGGAACCATGACCGTCTCCGCTCCGGGGTGCGAGGACCTCGTCGTCG
GCCTGTCCGAAACCCTGTACCACGGGGGCGAAAACGCCGTCCGGGTCTGCGGGGACACCT
GCGGTGGGAGGGTCTGGGGGGATGCCTCCGTTTCGGACTGGTTCGCCTCCTTCCTAGGAA
TCGAGTGCTGGCTCGCCCGCTACGACTCTTCTCTTGCCACCGACAGAGTCATCAAAGACC
GCCCCGGGTTTTCCAACGAGCAACCCATCCTGCTGGTCTCCGAGTCCGCGGTGGCGACGC
TGAACGGGGTGCTCTCGGAGCAGGACCAGCCGCCCTGTACCGCGAAGCGGTTCCGGGCCA
ACATTGTGGTCAAGGGTCACACCACCAGAGGTGGGCACCCGGCCTCGGACCGCTGCCACG
GCCACGCGGAGGACGAGTGGAAGACCGTCCGCCTGTCACGAAACCACCGCCTCGAGTTTT
CCGTCGAGGGCGACTGCCCGAGGTGTGCCATGGTGGACTACGATCCGGAGACGGGCCAGA
AGGGAAAGACCCTCCGGGCGCTGGCCTCGTATCGGAGACGGAACAACGGGCGCATCGTCT
TTGGAATCTTTCTGAGAGCATCCGAGGGGATCCTGCCCGACACGAGCAGCCTGGGCGCTG
GCAACGGGGATCGAGGAGAAGAAGGATCCGACGCCGGCACGATTTGGATTCGAGAGGGGG
ATACGTTAGAGTGCAAGCGATGATTTGCAAGTAATGCTATAGAAGAAAAAAATAGAGATA
CTAGCTACGGGA
>comp2075_c0_seq1
CGAAGACAGCCAACAAGCACGTATATTTTCATCAACGAATTTCCCATCGGCTCATCATAC
ACAGTGGTACTACGATACCAGAAGCATTGCCGCAGGAGAATCGAAACAAGCTTTTCAGAG
ACTCAAATCACGGTCAACGAAACATTCGGACCGAAGAAACGCCTTTATCAAGTGCAAGGG
AAGCAAAAGTAGAACACTTCCGAGATGATGAAAATAAACCAGATCACCTTCGCGGTATTT
TTGGCCTCGGTTGCATCGACCTCGGCGCTTCAAAACAATTATCTTAACCAGCTCTCGCAG
GGAGGACAAAATTTTCAACAAGAGCCAACAGAGTACTACCAACCTGCTGCCCCCGAGCCC
GGGTCGTATGCAGCAGATTCGTATGAAGCGGAGGTAGTTCCTACCGAGGAGAATAAAAGT
AATGAGTTCAATCCGGCTTATTACCAGCCCCCTCCGAGGATTCGCCTCGGATACGGATAT
GATCATCAAGACAACAGAAAGGGAGGAATGGCGCCTCTGAAACCCGGCGTCGACACGACG
CCCAATGGAGCTTCCGCCACCATGGGTCGTGGCAAAATGGAAATGCTTAGTCAGCTTCTT
AATGCCAAGGCGTCGGCAGAAGTAGCTTCCATGGGACGGGAGAAGATGCAAATGCTGAGC
CAGCTGCTGAACGCCTCTCCAAGCGGGGAATCCGCAACGATGGGCCGAGATAAGATGCAA
ATGCTCAATCAACTTTTGAGTTCGAAGGCTTCTGCAGAAGTTGCTTCCATGGGACGCGAG
AAGATGCAAATGCTTAGCCAGTTGCTGAACGCCTCTCCTAGCGGGGAATCCGCAACGATG
GGTCGCGAAAAGATGGATATGCTCAATCAACTATTGAATTCGAAGCCATCAGATGAATAC
GCATCGATGGGAACAGAGAAAATGGAAATGTTGAATCAACTTCTTAAT
>comp2076_c0_seq1
GCATGTCCTAGGTTGAACACTTTTTTTCCTGAAGAAATGAAGTTGCGGAAATGAGATGTT
GGTGGCAAGTGATGAGTTTGCTGATATGCTATTCAAAATTTGACTTCCACTTGCAGCGCA
CGTACGAGTACTACCGTACAGAGATGTCGACAGCGAGAAATTTATCGTCCATCTCAAGAT
TACCACTGATGCCATTGATTGTTCAGAACTTCCGTAGAGCGAGTGTAATAACATCGCCCA
CGAGAAAGGGTAGTCACTTTAAGGATTCAATTCTTTTCCAGGATGGAATGCGAACGCGAG
AAAATAGAATCTTCTGAGACAAGTTCCGAGAACGACGCATTTGACGACGATAAGAAATAC
AAGTCAAGTACGAAGAGAAAGAAAGAAAATCGAAAGTCAGAACGCAAGTCGGAAGATCGA
GCACGGAAGAAAGAACGACTAGGTAACAGTAGAGGAAAAGGAAACAACGAAGACGACTAT
AGCGATAGGAGTAGCAGTAGAGAAAGCGCTAGCGAAGACAATTATAGGAGGAGGGATCGG
AAGCGCAAACGACACGCGAAAAAACAGCGAGGCAGCAGCAGGAAAGCTTCTCGTTCCTAT
TTCAGTGACGACTCCTCCGATGATGACGAAAGTTCGATTAGCAATTCGAGTGAAACTGAT
TCCTACAGAAACGAGGCGCGACGGAAACAGAAAAAGGACGGAAGACAGAGGCGTCGGAAA
CGAAAAGAGAAGCGAAACTACCGTTATGATTATTCATCCTCCTCATCGTCCGATGACGAG
TCTTCAAATCATAAAAAACGCAAGCATAAAAAGGGAAAAAAAAAGAAAAAAAAGAAGAAA
TCGAGCAAATCCGAAGAACGTCGAAGTGTTATCTCAGGAAAGAAAATAAAGCTGCACATT
GAAAAGACAGACGAGGACTTGCTCGAGAAGAAGCCAGAAAGAATTATCTGGAGTTTATGA
ATTCTTCAGTTCGATAGACTATATAATAGGATAATGTAAAATCGGCAAAAATAGTTTCAA
AGTTATCTGCCA
>comp2077_c0_seq1

ACGAACAAAAACGAGTTCGTAATCAATGTGAATCACCATTTATAGATGCCACGACTTATA
CTTAAACTTTAACACAGCAAGAACGCCAGTGCTAGAAACAACGAGAACGAATTCTGGAAG
TATGAGCAACAGACTGGGGCGGGCAACCGGGACCGTCGTAAACGCGTCTCAGCTACGATT
GATCCTTATGGGAGCCATTGGTGCCTTGTTCCTCAATAACTTAATGATTTCCACATCGCA
TACAAACCGAATTATACATGAAAACTTGGATGGGTCTGATATGATACCACCTGAGTTTTA
CTCGTCGTCGCAACGACAAACGAAAGAAGAAACAGAGGAAAAATTCCCTTTGGAAGTTGG
ACGGAAACTTCTGACTGACGACGACGATGAAACCATTCGTCGTTTGAGAAATTCAAGAAC
TCTGATGGGTATTTTTTCTAGCGATAATATGTTTGATGCAAGTCATCGTAAATGGCACCG
TAATTTGTTCAATAATGTATGGAAGGACGACCGTGTGTGCACTTTGAATCAATTCCGTAA
CAGCGATGACATTGATTTTCAAAAAAAGTGCGAATTGATTTATACCTTTGTTACGGGAGC
CAATGAGGATCCAAACGCCCCGACCGAACGTCTAGAGGAATTCGACACCGTTGATACTCC
TATCGAATTGCCCGGTGGATACAAAAGTCCGATGAAGGAAGACATCAACTGGGACGACGT
TACGCATCTGAACATCCGAGAAAACATGAATGATGGGAAATCACCTACTTGGTTCTATTT
TGGTAGCAAAATGGCCAAACACTTTGAAAAGACAGAAACGCCTATAGACTACATTGTTAA
GTTCGACAGCGATTCAATGTTGAAGTTGCACGACTTTTTGGAATTTGCGCACAATCGATT
GCCTCCCGCACCTTACAACAAGAATATTTTTGCTGGTGCCCTCCGAGACAAGCGGCCGTG
GTATTTCAAGGACTCGAAACTAGGACACAACCAAGCAGACATGCCTAGGTACGAATCACA
CTGGGGAACCGAATATGATGGGGTACACCTTTATCTAGCAGGGCAGTGTTATTTGATGTC
TGCGGATTTGGCCGCCTTTGTTGCGGAAGAGGCGCCATTTTCAAAAATCAGAGTAGCCAA
AGGTGGGTATCTCGAAGGCCACGAGGACCATGACGTTGCTGCGATGGTGTATCACAATCC
TTCACCTATATCTTTGATTACCATCGGGAAAAGTCAGCGGTTTTGGGAACATCCTGTCAA
GGGTGCACCAAGATACAATAAGATCGTGGCGCGAGAAACGGCTCGGGTAAACAAGAAGCC
ATTCGAAGGAAAAATATTGAAACTCTATTGAGAAGTAGAAGTATTATATATCAAGGAGGA
AATAAAAACAGTCTACGGAGGTTCTTAGTCCAGTGAAGAATATATCACCCAAATGTTGCT
CAAGTTGTA
>comp2078_c0_seq1
AAAGCAAACATCCAAGAAATCCATCGGGGTATTTTATTCTTCTCATTAAAGAATCGATCT
TCCGAATCAAAAATTGTTCGGACTTTCACGTCACGCTTTTGTCTTCATAAAACAAAAACA
GATCTTTTTCCACACACTACAAGGTTCCCTGACAAGCACGTCTTGTGTTGTAACAACAAG
CACATATATGCTTATGATGTGCAGGTTCACAGAAAGAGAAGAACGGACGGGAGTAAAAAT
GAATCGCAGCTCGCAATATTTGCCAAACATCGGGGTACCTTGTCATCGGGGAGCGTCTTC
TTCTTCGTCATCTAAACAATTCAGTAAAGACCAAAAAAAGACAAAAACGCATTACATTCG
ACCGGAGGATCTCCGACCGTACGACATTCTTTGCGGACGAGACAAGGCAACTTTTAACAA
CATCGGCAACCGCAGGTTCAGGGTCTCGATTGGTATCAACATCCCGCGGTACGAAGCCGC
GAAGTCCAAGGCACAAAAGGCAGATGTTATAAAATCGGTTTGCAACGTACTTCAAAAAGA
CGTCGGCGTACGATTTCTGAAGAAAACGAAGTCCCCTTCTACAAAGGTTTCCGGGGACGA
TGAATGCGAAGACTACTATTACATCGAGTTGGACGAAACGGAAGCACGAAAAAAGGTAGG
GCATGCACTACGAGACATGTCGGTTGCTCGACAACAACTTCGACAAAAACGCGAAGTCGC
AAAACGACAGCAAGGCAGCCAAAAGAATATTGAGAATATTCATAATAAGAGGTATAAGCT
GACAACTGACAAACAGAATGATGGCGTTATAAATTTTCCTATGACGGAAAATTTTCAAGC
AGAAAGCTACACAGCCCAAAATAACATCGAAGACACGCTACTTGAGCCTTTGCCGATTAT
CGATAATCTATACAACGATCCATTGCTACCATCTCAAATTCAATTGGAATCGAGGGAGGG
ACCGGAAGAGCCTCGACGATTATCGTTGCAATTACCGCAGCAGCGAAGACCAGAAGAGTC
TCGGCGATTGTCGCTGCAATTGCCGCAGCAGCTAAGACTAGAAGAGCCTCGGCGATTGTC
GCTACAAATCTCTCAACAGCTGCAACCAATGCAAACTTCTGACGACGCATTCTCTCAAAT
TGAACAACAACGAATCCAATTTCAACAGCGACAGCAACAATTTCGGGAACAAATTCAAAT
GCAACTCCAGCAACAACGCAATCGACAACGAAATCAAAAGCAACATCGACAATTCGATCA
ACAACATCATCAGCCACATGCAATATCTATGCAAGACATACCCCAATTGGAGCAACAGCA
GTATGATAGACATCGGATGCTACTTCTATTACAACAGGAGCAACAGGAGCAACGGCATCA
ACAGCAGCAGCAGCAG
>comp2079_c0_seq1
CCGAGGTGCTGTTGATCTATTCTGGCATCGTTTGTTCATTGTTTCCGATGGAGCTTCTTC
TGCCCCTAAAGAAGCATTGCCACCCTCGCGTTATTCAATTGAACAGCATAAGTTCTATAT
TTCATCACCAATATTTCATCGCAGTCGATATGAGACGATACCCAACACAGCCGAATGAGC
CAATTGATTGGTCCCGAAAAATACAGGGTGCACTTTCGGGATTTGCCTGTGGATCCATGA
TTGGTGGAATTGGTTCTCTTCTCCAATCCAGAGGAAGGATCACCCCCCAAACCCTTCCAG
CAGCAATGTTCTTGGGAACGGTTTTAGGTGTTGGTTATGCCATTCGAATGTAACAAAGAG
TAGAAGTGAACAAGGAATAGAAATATTTGATTGAAATACGATAGAATTCGTGGTAATTGA
ATCCGGGGGGTTTTACAATGGGAACAAGAATAGAGGAGATACGTCGAGACTGTGAAATAT
ATTTTTTTCTATTTGGAAGGAGATCCTACGTACTGCATAAAGATTTTCGGGAGTTTTGGC

AGGGTCAAATAAACTCGTAAATGTAAACAGGTTCTCTGTCTTCTAAATAATTGACTTTTA
GGTGGGTTGTACAGGGGAAAAACATGGGATACAATGACGACCAAATACGATTCGGCAACT
TGCTCTTCTAACAAATCCAATTCGACATAAATGAGTACGGATTGTAAATGCAGTAATGGC
AAAATAGAAATCCTTCTGTTTTAGAAGCGTTCCTTCAAAATCAAATACAAGCCTATTCCT
TTTTGTTACGAAGCTACATCAAGAAAATATTGATTGCCAACAAAAAGGAACTGATTTTTC
TAATTCAATTCGATTTTATGTTATAAATTATGAGAAACTAATTGCAAAAAA
>comp2080_c0_seq1
GAACGCCCCAACAAAACTGAAAACTGAAAGCCAATCATTAACGAACAACACTTACCATCA
TAAAAATAGTCGGTGTCCAAAAGAATCTTAGATCGATGACTCCAATTTAGACAGTATCAA
AGGATATTCTTACCCATGGCGTGCAGAGGGAACCGCGCGATAAAGACAACATCAATCATA
TACTAGATGATCACCGAGATCCACTGTATTTTCGAATCTAAATTTCGATTCATCCGACAA
TGATCAAAAATTCTGTAACTGTGTCTGATCACAACCATGATGGTGACAACGACGATGAAT
ACTTTATGAGGAAATCGCTTGAAGTTGCAAAGAGAGCTCTAGATATCGGAGAAGTTCCCG
TGGGCTGCGTGATTGTTCTCGATAGACATCACCCGGCTGTTGTCAAGAAAAAGGATAAAA
TTATATGTAAGAACGAGACAAACGAATCTTGTTCTATTGGTGAAGAGCGTCGTGGAGTCG
TTATATCCCATGGTGCCAATCAGGTAAATGCCACAAGAGATGCAACTCGCCATGCTGAAG
TCGTCGCCATTGATCGTCTATTGACAGACGGTGTATCTAGCGATAAAATGCGGCTACCGC
CGGATTTGGCTCGAAAAGGCCAGTCATCACATGCAGGTGATAGAGTCCCTTATTCTGTTC
AAAAAGCTCGGAATGATCAATGGGAAGATCGATGGATAAATGAACCTTCAAATTCTGGTC
ATTGGACAAATTCATTTGGCTGGAGAAATAATTTTGATTGTAACGGTGCCAGCTTGGGTC
AAGACGAGGATAAAGAACACATAAGTATCGAGAGAGAGATTCGATCAGAAGACATTTTTC
GGCACTGTGACCTCTACGTCACGTGTGAGCCTTGCATAATGTGTGCTGCTGCTTTGGCAA
CTGTCGGGATTAGAAAAGTTATTTTTGGCTGTAAAAATGATAGATTTGGAGGCTGTGGGT
CCATTCTGCATCTACATCAAGTCGAAAAAGAGGATACGGCAACGCTACAAAAGATTGGTC
GTGGTGGCTGCGATAACAACCCGACTACAATGACGAACAAAAGCGTAGGATACGAAATCA
AAAGTGGAGTACTCGAGGAAGAGGCTATTACTTTGTTGCGTTCGTTCTATGATCGGGAAA
ATTTCCACGCCCCGGATGACAAGCGAAGGCGGAAAGACCAGGCATAGGAGAGAATATAAA
ATGATTCTCATATGACTACACACCATAGGAGGACAGTTTACTGGGTGATCTACGGTACTT
ATTTTCAGAATTTAGTGTATCCAATGACGAAGGAATGTT
>comp2080_c0_seq2
GAACGCCCCAACAAAACTGAAAACTGAAAGCCAATCATTAACGAACAACACTTACCATCA
TAAAAATAGTCGGTGTCCAAAAGAATCTTAGATCGATGACTCCAATTTAGACAGTATCAA
AGGATATTCTTACCCATGGCGTGCAGAGGGAACCGCGCGATAAAGACAACATCAATCATA
TACTAGATGATCACCGAGATCCACTGTATTTTCGAATCTAAATTTCGATTCATCCGACAA
TGATCAAAAATTCTGTAACTGTGTCTGATCACAACCATGATGGTGACAACGACGATGAAT
ACTTTATGAGGAAATCGCTTGAAGTTGCAAAGAGAGCTCTAGATATCGGAGAAGTTCCCG
TGGGCTGCGTGATTGTTCTCGATAGACATCACCCGGCTGTTGTCAAGAAAAAGGATAAAA
TTATATGTAAGAACGAGACAAACGAATCTTGTTCTATTGGTGAAGAGCGTCGTGGAGTCG
TTATATCCCATGGTGCCAATCAGGTAAATGCCACAAGAGATGCAACTCGCCATGCTGAAG
TCGTCGCCATTGATCGTCTATTGACAGACGGTGTATCTAGCGATAAAATGCGGCTACCGC
CGGATTTGGCTCGAAAAGGCCAGTCATCACATGCAGGTGATAGAGTCCCTTATTCTGTTC
AAAAAGCTCGGAATGATCAATGGGAAGATCGATGGATAAATGAACCTTCAAATTCTGGTC
ATTGGACAAATTCATTTGGCTGGAGAAATAATTTTGATTGTAACGGTGCCAGCTTGGGTC
AAGACGAGGATAAAGAACACATAAGTATCGAGAGAGAGATTCGATCAGAAGACATTTTTC
GGCACTGTGACCTCTACGTCACGTGTGAGGTAAGCATCGAGAATAACATTTTAAATCTGT
GTCTAAATGCAATTCGAATTCGAATTGGATATCCTGTGCTACAAAATTGCATTCCTCACT
CAAGCCTTAATTC
>comp2081_c0_seq1
AAAAACTTCGACTTGAAAAATATTCTTTCGAATAGCACGTACACTACAACTCTGAAATTT
CCTGTCCAATATACACACCCACCCGAGTTCTACGATGAAATTCACGTTGTCCATCCTTTG
TCTCGTTGTCGCTTATGTGTTTTCCGCCGAGGCAACGTCTCTTCGTGGAAAATCTATTCC
ACGAAAGGAAACATTGATGCAGGCCTTTCTATCAGCAGCACAATCTGGTGATCGACGTTT
GGATCAATACAACTACAACCAGTACGCCACTGATGACAATCAAAACCAGGGCGATGACCA
AGTCGACGATTCCTATGGCGACGACTACGTCTTTTACGACGATTATGCTCTCCAAAATTG
CGAAGAAGGCGATGAAGACTGCGAAACGGTAGCAAAATACACTGCGTATGATGATACGTA
CTTGGCAAACTGCGAAGAAGCAGACGAGGACTGCAATAATGCAGCCGCCTATATGACTGC
GAAGAAAGCCAAGGAGGAAGAAGAACTGAACAGCAACGAATGGAGTTTGAGCAACCTGAC
CGACAAATACAATTCCATGTCGAGAAGTTCGAAGACTTGGACGATTGTTCTGGCTGTGTG
GTTTACTGCTTTGTTGGTAACGACATCGTATTTCTGCTGTTGTCGGCAGAGTCTCTCGCA
GAGAGAGAAATCTCGTGGCAGGAGGTTACGAGAATCGTTCTTGGGAGGAAACAGAAGGGG

AGACTCCGACGGGGCCAAGACGTCGGACGAACGAAAACGTGGGAGATCGAAATTCAAATT
CTTTGGACGCAAGGGCAACAAAAGCAAGAGGGGCAATAGCAGGGGCCCCGTCTATTGAGT
TATTCAGTCGGATGTAGTTCGTCACTTCCCTAGTTGAAGATAACATTTTTTTCACTGCAA
GTACTTGCTTATCAATCAAACTATTTTCTATTTTTGT
>comp2082_c0_seq1
ACTAAATTCGATTCTTCAGTGCCTGAGGTATTGAATAAGACGATTATCGCATGCCATAGC
GACATGAAACAGAATAACAGAGACGTGGAACAATACAGTGCATCTAATCGAAAAAATAGC
GAAAACCCCTAAGTTTTTGGCAGAATCAAACAAACGTTCGTGGAAGGAACATACCCCTGA
ACTTAATCGAAGTCCGCATTCCGTTGAATTTCAATTTGTTTCATTTCCTTTCGTATCACT
CTTGGATACGAGTGCGTTTTTTGGGAACGACCAAAACCATTCGATTCAATTTTTCTTTGG
ACCAATCCGAAAAATAAAATAGCACAAGGGTGGTGATGAGGATAACGAATGGTACTCTGC
GATGTAGTAGCAGTAGGAGGGGTAATTTCATCCTCCTCTTTTTTGCGTTCTTGGTACAGA
GTTCCTCGGATGCGTTGTTTTGCAACGCAGAGGTTCCGGTTGGATCGTACGGTACCGCAT
CCGAATCTTCGGACAGAAATAGCGGTATAGATGGCGGGTTGTGGAAGGTTCCGACGTTGA
ACTCCGAGTCCGGCCCTCAAGCAAACGAAATCTACTTGAATTATAGATGTCCAGTGGGGG
ATCTTTCGCCAGTAATGCTAGAGGAAGATACTGGAACGTACGTTCTCTTTCGCCAGTCAC
CGCATTCCAATGAAACGGAAGAAAATCTCGTTTTGATTGATGGTGGTGAAAAGCTGAATC
GAAGCCGAGGCAACATCTATAGCGGTGACAATATTGACAGCAGTAAGGAAAAATTCGGGG
CCGATGATAATAGCGTTTTGGATCACATTAGGGCTGGAACTCGAAGATATCTTCGGGGAG
CTAAGATGTCTCGCGAAACCGAGAATCGTATCCTAGTCAGTACCCCTATATTTGTTGCAT
CTGGTTTCGTCGAAGAAGCACCCACCTCAGAGAATTCAATGCGTTTTCTCTCGACCTACA
GAAAAAACAGCCTAGTTGCCGATCGAAGGGATCTGAGAGATGCCAAAAATTCTAATGATG
AGACAAGTGCCGACCTCGAGAGCTACTCGACCTCCGAATTGCTCATGCTCCGCCCCTGTG
GGTGTTTTGACGAATTCGGCACGTTTGGGGGGGTTTCAGAAGGCAGCGACTCGGACATAA
ATCAAATCGGTGACCCCCTGCTCACCTTCTGTCCCGCTTCTTCCGACTGGTGCGCGTACA
CCACTAGTTACGATACCGATGCATCTTCACGTTCCCCGAAGCTCGAGTGCTTTTCCATGG
CTAAGGACGAGAGGGTCGCCCTCAACGCCTGGCCGCTGCTGTGGATCATGTACGCAGTCG
TTCTGTGCTCCTTCTGCTGCTCATTCCAGGGACGGATCTTGCTTGACTTTGTCCGTGGCA
CCATCTTGTATTACGTCTGCGGCTGTGGAGACCGCTACCGGAACCGACAGATGCGGAATA
TCAATCGTTACAATCAATTCGTGGTCCAGGAAATACTAGATGAGGAACGGGTGGACCGCG
AAAACGTTGTCCGCCATCGAACTAGGAACCAAAACGGGAACTGGATGAATTTCGACCCAC
CAAGAATTCCTCCCACGATCACGACATCTACCCATCATGAAGGATTGTCGCAAGACAATG
ACAACACGGAAGGAAGCAATCCCATCACGGAAGGAAGGGGGGACCACCCAGACGTTTCCA
TCGGCAACAACCGGTTGGTGACTTGGTGGAGGGGCGTGGTCTGGCCATGGCAAACCTCCT
GGAACCGGTATATCAAATACAATCTGTTACTCCGGGTCGAATGGATGGCCATCCGGGACG
AAATAGCCTTTATCGAATCTCGGCAAGAGCGGGGGTTGCCCCACGCCATTTACGAAATGA
AAACCCGCAGATGGAGTTCTACAGAGGAGGATACTTCTCGATCCAGCTGCTGCAGGACCG
GCAATGAATACGACCATTCGATCGATGCACCCCATTGCACGATTTGTTTCACAGAACTAG
AGGATGGAGACCGCGTGGGGGACCTTGGGTGCATTCATGTCTTCCATGTCGACTGCCTCA
AGATGTGGATCCAGAAACGCAATTCGTGCCCTCTCTGCAATATTCCCATAGCCAATCGGA
GGAGGCTATCTAGTGAGGAACTGCTGGTGAAGAACAACAACAACGACAACAGGGGGGCAC
AAAATGAATCACCACAGGAGGGTCACAGGAACAACCATGGAAGAACGATCGAGGAAGGTT
CGTTGGAAGCACAAGGGTCCAGCCTTTCTCCAACCATTCGCAACACGGACCACGGGTCAC
CTTCCAACCACCAGCGGCGATTATCGCACCTACCTGTAACGCCGTCTACCATCGCGGACG
ATGTTGATGCATATAGTACGTTTAGCGAGGAGATTGAAAGCGTTGTACAATTAGACGATG
ACGACGATGAATCAACCTTCGGCAGACGGACTCTTGAGATTGGTGAGGAAAGTGGGAACG
GTGAAGCAGAGCAGACGACCGTTGCTGTGGAAAGCTCACTGTTAGCCGCAACAACACGAT
TGAGTGCCTCCTCTGCTGTTACCGAGGAAAATGAAGACAAAAACTCGATTATGTCCATAG
AGGACGAATACTGCAACATTGACGATTTCCTCCTCTCGAAAGACAACCAGGGAAGAAGTG
TCGCGTCCGACCACAGCGAAGCGGCGTCTTTTGCCTCATCAGCACGCGCCGAAAATAGCG
TAGATGATGAAGTCGACCTTCCTTCCCCACCGCGCCACGTACGGAGACTGAGCTCGGAAG
CTCCATCCTCATCTGTCATGTTGCCAAACCACCAGCGCCAATACTAGATCTTTGCAAACC
TCCATGATTCAGTAAATTTACTTTGCAAACCTACCACCAGGGCGGAGGAGACAATATCAG
GGTAGAACCAGAAGAGGAAGATACCTGTTTTTTTGCTCGCTTTTGGAGGTATGCTTGCCC
TCTACGAATTATTTCTTCACCTGCTCAAGTTCACAAGGTGGTGCATAAAACGACAAATGG
ACTTTTGAGGATGAAAAAAAATGTAACTGTTATTTTATCACATGACTTAAAAAAATATGTA
TGTGTTTAATAGTCATTTTCCTCTGTATCTTATTATTGTTCAAAGAAGCGCTGCATTTCT
TGCGACAGAACTCTTCAACAGTCTTAGCTCTGCCTCTTTCTCTGACTGTTGTTTCAGTTT
ATTGAGGCCTTCTGATTTGTTTGCAGTTCCTGGTCTTCCTCCTCCCA
>comp2083_c0_seq1

ACTGGATCGATATCGTGAGTTTTCTATCCTCTCTTTTGTCTGTTGTTTCCAGCATTGGTC
GGAAACCCTTTGCGTTCGTTTAGCGTGGCGCGTCTTTCCGGACTTTTGAGTTGTCTCATT
CTTTCTTTGGGTTGCCTTTTGACGCGGTTTCTCAACAAACGATACACACACAACGCTACA
ACACACAACTATGTTCTTCCGATCTGCAGGAATTCAACGCGGCCACGATGATCCAGGCAG
TCTTTCGTCGGTACATGGTCTTGCAGTCTATGGAACGCGAGGGCGTAACGACCTCGTACA
TCCGAAACCGAAAGCGCCAGCGCAAAGCCAGCACCAAGATGTCCTACTTCCAGACGTCCA
CGGTCGACGAGACTGCTCCCGACCTTGGTTTTGGCTGCTGCGCCATGGGGTTTGACCTCT
GCGGGAACGACTACGATGCTGCAGATCTGGCCGCCTATCGAACCTTTCAGCGAAGGCAGT
ACGAGGAAAGGAAGAAGGCGCAGAAGGAGCGCGAAGAATTCCTCAGCCGATCCTACATGG
AGCAGAAAGGAATCGATACCTCCATCATGCTGCGTGGTCAAAGTTTGATCGATGCGTAGA
TTTTAGAGGCTGGGACGATGGAAGCGACGACGACGAATGTGTGTATCGTCGTTGTTTGTG
TTTTGCAATCTCGCTGTGATGGGGCCGACGAGGGTGGTGATTGTCGTGCTTCACCATTGC
CCTTGTTGCATGGAAGTATGTAGCAAGACCACGCTGCTAGAATCTGGTGGAAAAGAGCGT
ATTTTGTCATCGAAAAGGAGATTGTAGGGGTGGTCTCGTTCGCGATGGGTGGGTGTCAGT
GTTATTTATTATCATTATATACATTTGTTTTGATGAATGCATAAGTCGATTTCTTGGGCG
CCTTTGAGATGCTGTGTTTTCTCCAATATACGGATCGTAACGATTCGAGTTGTGCACAGC
ATGGTGAAGTCTTCTCCGCGGGATTTAAAAAAAATTATTGTATCCAGTGCTATGGGAGGT
CAATCTCAAAGACAGACAGACTTTTTATGTCCTTGATGTACTACGGCGTAACATACTATC
ATAACCAGCAGACTTCTTATTTTGCCCTAGATTGTTTACACGATTTACCCTCACTCTGTC
ACTTTAGTTTTTGGAGAGACATTTTAGTCTTCCGCTTGGAGCGATGTATCATAACGCGTG
AGAAAAAAACACTGTGAAGTCGGAATCCCATTCCTCTGTCCTGCGACACATGACCTTGCG
AAGAACGGGTCACAGCGAAC
>comp2083_c0_seq2
CAAATTTTCAAAACGACTCGTACAACTCTCACCGAAAGCTTTTCGCAAATTTGTGTAGAA
CAGAAGAAAACTGGACACGATACGAAGTGCACCATCCAGCAATTCTTTTGCTCGTATCGC
AGCGAGCGTTGACATCAACAAACACGTTAGGAGCACTTGCAACAATAAAGCTTTACCATC
TCTCATTGCGGAAAAACCGATTTATCACAACCGAACTTTTGTAGGAACAGGAAATCAAAG
GATCAATCAACGATGGGTCTTTTTCGGAAGAAGAATCGCGGAGCGGATGCTGACTTCGAC
ATTGACTTTGACGACTACGACAATTCAAGCATTCCGAGGGAAGTCAACACGAAGGGTGCT
CGTCACTCTCGCTCGCGCTCCCTTGGAAAAATCTTTGGTGGATCTTCCTCGAAGAAGACC
GACAAGAAGGGCAAGAGGAAGAGCGGAAAGGGCAACAACAACAGCGGCAGCATGGAAAAG
ATCAACTATTCCTCGTCCACATCGACCTCGAGCAACAGGGATGCCCGGGACGATCGCCCC
CAAACATTCCGCGCTAGCACCAGCAAGGCCAAATCCGAGACCAAGTCCCGACGTCACAAC
GAAGACTACGGTCAGGTGAACAGCCTCTGCAGTATCCCTCCCACGGCCGCCGACGCTGCC
TTTCATGGTCCTCCACGCTTTGACTGGATCGATATCGAATTCAACGCGGCCACGATGATC
CAGGCAGTCTTTCGTCGGTACATGGTCTTGCAGTCTATGGAACGCGAGGGCGTAACGACC
TCGTACATCCGAAACCGAAAGCGCCAGCGCAAAGCCAGCACCAAGATGTCCTACTTCCAG
ACGTCCACGGTCGACGAGACTGCTCCCGACCTTGGTTTTGGCTGCTGCGCCATGGGGTTT
GACCTCTGCGGGAACGACTACGATGCTGCAGATCTGGCCGCCTATCGAACCTTTCAGCGA
AGGCAGTACGAGGAAAGGAAGAAGGCGCAGAAGGAGCGCGAAGAATTCCTCAGCCGATCC
TACATGGAGCAGAAAGGAATCGATACCTCCATCATGCTGCGTGGTCAAAGTTTGATCGAT
GCGTAGATTTTAGAGGCTGGGACGATGGAAGCGACGACGACGAATGTGTGTATCGTCGTT
GTTTGTGTTTTGCAATCTCGCTGTGATGGGGCCGACGAGGGTGGTGATTGTCGTGCTTCA
CCATTGCCCTTGTTGCATGGAAGTATGTAGCAAGACCACGCTGCTAGAATCTGGTGGAAA
AGAGCGTATTTTGTCATCGAAAAGGAGATTGTAGGGGTGGTCTCGTTCGCGATGGGTGGG
TGTCAGTGTTATTTATTATCATTATATACATTTGTTTTGATGAATGCATAAGTCGATTTC
TTGGGCGCCTTTGAGATGCTGTGTTTTCTCCAATATACGGATCGTAACGATTCGAGTTGT
GCACAGCATGGTGAAGTCTTCTCCGCGGGATTTAAAAAAAATTATTGTATCCAGTGCTAT
GGGAGGTCAATCTCAAAGACAGACAGACTTTTTATGTCCTTGATGTACTACGGCGTAACA
TACTATCATAACCAGCAGACTTCTTATTTTGCCCTAGATTGTTTACACGATTTACCCTCA
CTCTGTCACTTTAGTTTTTGGAGAGACATTTTAGTCTTCCGCTTGGAGCGATGTATCATA
ACGCGTGAGAAAAAAACACTGTGAAGTCGGAATCCCATTCCTCTGTCCTGCGACACATGA
CCTTGCGAAGAACGGGTCACAGCGAAC
>comp2084_c0_seq1
GCCAAGACAAGAAGGATACAAGCAACGATACAACACCTTTTAAGACTGACTATCGCTCTG
CGACAGCCATTGCACTTGAAGAGGATTGATCGACAACTACACGGTAGTAAGGATCCCGTT
GCAACTTGTTTCGGAACGACAAAGAACTGGTTTGAATAGAACGGAAATCGTTGTTTGGTA
GTAGTACTTTATCCTCGACGAGTTGTTAAGACACGGACGCCTCTAAACCCAAACCGAAAG
CCTCTACGCCAAAGGCGCTCCAGTTCCCTAATTAGTCTATATCTGTAAAATATACATACG
CAACAAGATGACTCGACCGACGGGACCGCTTGGGATAGATCCCTTGGCATTTACTTTTTC

ACTGTTTGGCTTATTATTACTATTTGTACTGTATCTGATTTTGCCGAGAGGCCTGCGAAA
ACAATACTTTGGAGCCTACCCTAAACGACATGCATGGTCAGCTCGATCAAGAATGAGAAG
AAATCGAGGCTATGGAGATCATTCCTTGGGAGGTGGATCAATAACTGGAGCTTCGTCAGT
TGCCACTGGAACTACATTTGGAACACACGGGCATCCCGGATCTCACCGTCAGCTACAAGG
AATGGGGTTGGGAGCCAGTCACTCCGTTGCCGGATCTACAATTGGAGGAACCACAGCGTA
CTCAAACATGACCGGACGACGTGGATTCGACGAGGAACAGGTTGTGTTTGGTGGTGGCGC
AGCAGGTGCAGCTGGAACAAACCAAATGTTAGACATACAGCAGAACGAAGAAATTGTTAT
TTCGGCAGCTATGCAACAACTTCGAGATCCCGGTGTCCTGATCGTCGCCCACGGCAGTAG
GGGGAAACCCAAGACGGTTCGCCTACAACTTACAGAAAGTGCTGTAACCTGGAGAACAGA
GACACGAAAGAAGGGAGTTGCGAAGGATGCCAAGCCAAAAATGGGAAAACTGCATCAAGT
TCCACTCAGTCATATCATGTACGTCGATGTTGGAAAACAGACGACAGCATTGCGACGAGT
CGAGAACGCGAGTGTGAACGACGCACTTTGCTTTTCGCTTCTTACCAAAGAAGGGTCCCT
GGATCTAGAAGCGAATAGCCCTCGTGAACGTGATGCACTTGTTAGTTGCTTTTCATTGGT
ATTGGATGAGGTCCATGCCCAGAACTGGAGAGATATATACCGAGGTCCCAGTTCGGATAT
GCCGAGCTCGTTTGACGAGATCGATCAACAGCAGCAGCAGCAACAGCCCCAGGGGACGCA
AGGGCAAAGGCAACACGATCCGACAGCCGGCCGTGGCCACCCATAAATGGATGGCTATTG
ATGGAATCATCGGCTACATCGTTCTTTTCACCTTTGGTGAACGCACACATAACTACATAT
CAGTGACTTGGCAGGTCCTAAACTCACGGTTCAAACAAATAAACTCCAGTTACTGGCGCC
GTCACATCAAACCACAAATCAGTGCTCGATACATTGCGATACACTGACCCAATAACCAAA
CAGTTGTTTCGACTAGTCTGCAGTTGTTTCCATTTTGTTATTATAATATTATCATTGATT
ACAAAA
>comp2085_c0_seq1
CTCCTGTATCGCTACCGTAGTAAATCGCTACCGTAGTAAAGCAAACTACCGCACTTACAC
CAACGCTAAAGGTAGGTTCAGCAGCTTGCGTTTTGTTCTTCCGTGTTGACATAAAAAGTT
GCAATTGTTCGTGGTGACCCTGTTGGAATAATCATGTATTGAGTTTCGAAAAGCATAGAG
AAAGATATACAAACTCAACATACTACAACTACCGGAATAAAGCAGAAAGATGGGATTCAA
AAGGATAGGTAGAGGAAACAATCTTTCGGGGATTTTTCATCTATCGGTTGCGACAGTGGT
GGTTGCTATCCTTCGAAGCAATTCGAATGGTACACCCTCAGTTTTTTGCAGCGCGTGGGT
TTCGACATCGTCGTCCAGTACCAACGGTGGAACGTGCTCCTCTTCTACATTTTCTGCTTT
GAACAATGGTGCCACCGCTCGATCGCCAAGCGGTCGAATATCCGACAAAATAGGTGATAG
AAGATACTGGATGATATTGAACGCCGTGGTGGACGACAGCAGACAGGGAGGAAACGACAC
TTTGTCGACCTCTACAACCACAAAATCTCAAGGGGGTAGGAAGTCATCAACGATATCAAC
ATCGAAAATAAAGAAAACCAATCGGATATATATCAACGTAGATCGTTGGAATTCCATGTT
TGAACGATTAAAAGAATACAAGAAGACATACGGCAACACGAACGTCCCAAAGAAGTACAA
CGATGGCGGGACTCCCGCCCTCGGAAATTGGGTCAGCAAACAGCGCTTTGAAGCATTGGT
TTATCAAAATACAAAGGGAGAGGAGGGACAAATCACTCCGGAACGAATCTCGAAGCTGGA
ATCGATTGGATTTGTATGGGAACTTGGAAGACAAGATGAATGGGAAGAAAAGTGGCTAAG
CATGTTTTCAGAACTGGAGGAGTTTCAGAAAAAATACTGGAGCACAAAAGTCCCTATACT
CAACAACAAGGAATTGAATTTTAAAAGCGGCAGCGGCCTAGAGGACCCGATGTCCAGCCT
CGGTCGGTGGGTCAAGACGCAACGGATACAACGGAACAAGTTGTTGAGAAATGAAGCAGC
ACCTGCCATGACGAAGGAACGCCTGGAAATGCTTGAGTCAATCGATTTTGCCTGGAGTGG
AAAGCAAGCTTCAGATTACGATAAGTGGCTGAATATGTATTTCAAGCTTATGTTTTATTA
CCAGCGGCACGGCACGACGGTCGTTAGCGATTCGCAGGGCAGCAGTCGCTTGCTTAAGTG
GACGGAAGAGCAACGGCGCTTTTACCGAGACCTTATGACCACTAATAGTACAATGAGTGC
TTCTAAGACTGGTGCTGCGGCTACCATCTTGAACACGCTTACGGAGCATAGAATCAATTT
GCTGAACGATATTAATTTTGATTGGTCTCATTGCCACGAAGATAGGACCTGGGACGATAT
GTTCAACGAGCTCGTGGAATACTACGTAAATTTCAATTCCACATTGATTTCAACAAGTAT
CAATGAACGCCTTGGCAAATGGACCATCCTGCAGCGCGATAAATATAACGCGGGCTCAAA
AGATTTGGATCGCCAAAAAATTACAAAACTGAACACAATCAATTTCGATTGGAACGCAAC
CGAAGACGTTAACTGGAACGCTATGATGTACCGGTTAGAGGCATATAAACGCAAATATGA
TACCACCATTGTTCCAAACCATGACGGTGGAGACAGACCTCTGGCGAACTGGGTCAATAA
TCAGCGCGTCAAGCTGGGAAAATATGTCATCAACGAAGACGGTGAATTCTACGACGACCA
CGATTTGGTTGAACGCATCGCCCTCGACCTGGGATGCTCTCCCGAAACGGAGTTTGAACC
AAAAGGATTCATTCAAAAGTTGTCGCCCTCTACAAAGGCCACGCGAATACGACGGCTGAA
TGAGGTTGGCTTTGTATGGGACGCCCGAGAAGCCTCGTGGCAAGACATGTACCAACGTCT
GTTGGTGTATAAAGAACAAAACAACGGTTCGACAATGGTCCCTTTTGTACCCCAAGATCA
CAGAAGCAATAATAAAAGCAGCGGTTCAACGGAACAACAATCCGAGTCGCTGGGCGTTTG
GGTTAAGGTCCAACGCCACAAGAAGCAACTGGGGGAAATGTCCGAAAAGCGCCTGGTGCT
GCTGAACGAAATTGAATTTGTTTGGGACCCCCAAGAAGCCCAGTGGGCCGAAATGTTTGA
TCGCCTTTGCAAATACCAGAAAGAGAACAATGGCTGTACAAGGGTGCCTCGCGCATATTC

TCCCGATCCCGAACTCGGGGCATGGTTGAACACCAATCGGCGGGCCTACAAGCGAAAGAC
GCTTCCACAAGAACGAATCGATCGGTTGGATTCAATTGGATTTGTATGGGAAATACAATG
ATCAGTAACGGAGAGGATATATCTATCGATCTATAAGCCTCCTGATATTCCCAACAGATA
TAGTATAGGACACGACATGGAATATCCACAATAGACATAATATT
>comp2086_c0_seq1
TTCCAACTCTCATGACAATTATGTTGCCCCTTCTCACGCTATTGACCCACCATCACAAGA
GCCATCGCATTGATCAATTGTGATTCGGAGAGAACGCCCTGCTCCGAGTCGAGAAACAAG
ATCAAGAATGACGACAGGGAGCATGCTTCGTGGAAGAATGCTTGGAGCAGAGCTTGCTCA
CAGCGATGCTTTGCGATACTTGACAACCTACAATAACTCCACGGTTGCAGACAGCGAGAG
TGATACCTCGAATGTCATGAACACGGCAAAAGACTATGCTAGTAGGATCAAAACCGCAAC
TTCCGACCTGACCAGTACGCATCCCAGGGATTGGACGCATGACCAATACCTCGGTGTAGT
CACGGGCTTCGTGGCTATATGGACATTGCTCCTCTTGCTTTGTACCTGTCGAAAATGCTG
CGATAGACGACGCAAAATCCAGCGGAAAAGCGTAGACGACTCTTACATGAACATGAGCTT
TGCCGACAACAAACAAGCACTCCTTAACCAGCACCGATCTGGTCCCTACAAATACGACAT
GGTTCGGCCACGCGTCGTGCGTGGAACCGCAACCGATGCCGGTGGCATCGCGAGCGTCGA
ATCTCCTAGTGGCTATGATTATGATGCAATGGATCCCAATCGATTGCCGATGGGAGCTCG
CTTGCACGGAGCCAATAAGCTCCGCTCGGACGGATTGTCGGGAAGGGGGGTGCGGGTAGC
CATCATCGATTCCGGTGTCGACCACACCCACCCCGGACTCCACGACAAGGTTCGGACCAA
GCGTTGGTTTCGCCACGGGACATCTCTCCTGGAAGACGATCACGGAACCCACGTGGCCGG
AACCATTCACTACGTAGCTCCCGAGGCCGAGTTGTTCGATTACCGCGTCTTTGGCAAGGA
CGGAGACTTTACGGGAGACGTGGCCATTGCCAAGTCCATCCGATCAGCGGTCGACATCGA
CAAGTGTCACGTCATCAACCTTTCGTTGCGGTGCTCGTATCCGGTATTACCGGCAATCAC
CAAGGCCGTACAATACGCCAAACGCAAGGGTGTTCACATGGTATGCGCGGCCGGCAACGA
CGGAGACGGCAACGGCAACACCGACGAGCTCTATACATTCCCCGCCCAATTCGACGAGAC
CATCAGTGTCGCTGCCGTCCGCAAGGAGGCCGGTCTACCACCCGCAAACTTCAGCGAGTC
CAATGCAAAAGTAAGCTTTTCGGGCATTGGAAAAGATGTGATATCTCTCAAGCCCGGAGG
AGGTCACCAGATCATGTCGGGGACCTCTATGGCAGCCCCGCACGTGACAGGCTTGATTGC
CTGCCTCTTGTCCGACGAACCGACGAAATATACCGATTACCAGCTCCGCAAGTTGCTTCG
AGAGAAGCACACTGTTGACATAGCCGAAAAGGGATTCGACAAGCAGACGGGTGCGGGATT
TGTGACCTACCTGGTAGCAGAACAAGGAGAAGACGAAGAAAGCAATAGCCTCTTTGATTT
GTTAGCTCTGGTGTAGAAAAAAAGCTAAAAATATCTTCGGCATCGATTCGTGTTTCTATT
CAGTGCGGTTGCACGAAAAATTTTCCTTATTTTGGAAAGAAAGTGATTATCTATGCTGCC
ACACTCGATACTCGTGAAAGGATGAGGTCAAGAGCTTGACAAACGACTGCAATGTTTGAA
TTCTCGTGTTCTTTTGGGGGAGGGAAAATCCTCTTCAGCAATCATGACCATAGTAGATTT
ATTTTACTTTAAATTGTTTTGTTCTCTAAAAAA
>comp2087_c0_seq1
CGAGAAAGTATTCGATCCCTCAACCTTGTTGGTCTCTGTCGCACGGTACAATTCATCGAA
ACCATGGCTTCTATGCAGTTCAACACATTCGACGCCTTTGAGGACGCCGAGGACAAGGGA
GGCGGCGCCTCGGAAGAGAAAGTCCACGTTCGTGTCCAGCAGCGAAACGGAAGAAAATGT
ATCACGACCGTCGCCGGCCTTGCCGACGACCTCGACATCAAGCGAATTTGCAAGGCTTTC
AAAAAGAATTTCAATTGCAACGGTGCCGTACAGAAGGACGAAGACGCGGGAGAAGTCATC
CAGCTTTCTGGTGACCAAAGAACAAACGTTAAGCAATTCCTTGTGGATCAAGAAATCTGC
CACGCTCTTAGCATTGTGTTGCACGGTTTTTAAACGCAACAACGGCAACAATTGCAGAAT
CGATTGTTCGATCCGTCCATCTACTGCGGCGCGTTTTCGAAACGTTTCGTTTTATGGAAA
ATTTTTCAATCAAACTACGAGATGATTCTTGAATCATGGAGAAGCCGATGTGAACTGAAT
ATTTATTAGATCTCAAACTGGGAATAACGTTTTACTAATATATTCTAGATACATGGTTAG
ACAAAC
>comp2088_c0_seq1
GCAACAACAACAACAACAAGATGGACACCGTCCGGGTGAGAAGACGCGGAGAAAATCTAC
AACTCGTGTGGAGTCCGAGGCAATGTCGCCATCATCACGCAGGAGTTCCTCGCGTCGTTC
TAGTTCTAGAACGAATATCGGTGTTCCAGAAACACTGGAAGTGGTTCGTCGTAAACCATC
TCGACATGGTAGCAACAGCAGCAATCATTGCGGAGACGATAACTCCATTGTTAGTGCAAT
CACGATGGATGTTCATCTGTTTCATCCGTATTCCAGCAGAGACGACAATTTTGCTTTCAA
TACTAATTATTCCGAGGAGGAGGAAGAAGAAGAGCAACGCATTCCTGCGAGACCTACTCA
CCAACGCCGACCTAGACCGGAAAAATCCGGTCGTAGTAAACAGAAGGGAACTGGTAATAA
TGGAAGCAAAAGCATTGACAACAATATGAACAATATTGGAATTGGTCAGTTTCATACAGC
GTTGCGTCGTCGTGGTAGTCACAGCGATGAGAGCAGTCACAGAAGTGACAGCAGCCAACA
CAGTCGCCATCGGAAGTCGCATTCTATGCGTGGTGATTCAGAGCGTAATCTGTACCCATG
GGATTATGGAGAAGGACAAGAAATGGGAAGTGGCGCGATGCATCAGCAATTAAATCCATC
TACGAATATAACACCAGTTTTGGAGTGTGATGCGCTGATGAGATCGGTGTCGAGCAACGA

AGATACATATTTTTTCGACAGGAAATCTGTCTCCAACACGAAGACAAAAAAGATTGGAAG
CGAAGTAGGGATCGATTTTCAACCAGACGAGAATGCGAATCACAGTAGACGGAGTCGTCA
AACGGGAATGCAACAATCGATAGATTTTGCAACGGGGAGCACTGCAAATAACAGAAATAC
TGAGAATTCAGCGCATGCAGTACGGAGTGAATACCCGCCCAAACCTCATGACAAAACTGC
TATCGAGGATGATAACGATGGTTTTCCATCCTTCATAATGGACGACTACGACTACAACGA
CTACAACGACGACTATGACCACAATCAAAATATATTGCGACAGAAGCAGCAACAGCATTT
TCAACAGCTTCTGAAGAATGATACAAATCCCATACCTCACCCTCCGATCACATCGGTGTC
ATCTTCTTCGTCATCATCGGGCCGCATACATAACGGTACCAACGCCAGGCAAAACAGTTT
GTTTACACCAATTACGAAACAGGATATTACGGACATACAAGGCATTTTGCATTGTCTTAA
TTTAGAAGAATGCAATCCAGATGTTAGGGAAGACGCCTTGTGTAAGCTCTTCGACATGAT
TCAGAACATGAGCCGCGCTGGTGATAGACACACGCGTGAATTCATTTTGGAACATAACGT
GATTGAGGCTGCAACCAAAGCCATTTGGGCAGATATGCAAATCGTTGAAGTACAGGAAGC
TGCAATGCAGCTCCTCTTGCTTATATCTGCTACAACTACTCTTATAGGTGGTAGAGATGG
CGATCACGAACATGGGAAGAGCTCCGGTAGTGGTGGGAGCAACGACAGCAATCTACTCTC
CAAAAATGAGTCGGTTTGCGACTCCATCCTGTTTGCAATGCAAAACCATGCTACCGAACC
GGGTATCCAACTGAAAGGCTGTCTCATTTTCGCTTCTCTTGCTGGTAGCCTGCCCGATGA
AAGTGCCGCAGGAACCAATTCGGATGGTAGTCTCTCTGGGGCAATGACGATGGTTTTGAA
AGCCATGAGTAATCACGGAAACTCTCGATCCATTCGCAAGGCTGGCTTACAGGCCCTACA
CCACCAGTGCTTGCTTTCAATCCATGTAGAAAATAACAAAAGAAGCCTTGCAAGTGGTAA
ACTCGAGAATGGAATGGCAGCCGTTGATGTTCTGATCTACGCGATGGAGGAGTTGCAACA
AGATTTAGTTGCGATGGATTGGGCCTGCCAGTTGTGTTGGTGTTTGACTGCAAGAAAAGA
TTTATTGCAAGAGCTGGAAGAATCTTCGTTGCATGAAGCAATCCTTTCCGTATGCCAGCA
CTACATGACAAGTCCCGCTGGTATCAGCGTGGTCGAAGCAAGTATCGGAACCATTGGAAA
TCTAGCATACCTAGATAAAAAACGAAAAGAGCTGCTAAATATCGGGGCAGTTGAACTAGT
CCTTGACGGCTTGCGTTACCACAGCAATCACTTTGGGATAAGCTACGAAGCTGCCTCTGC
AATAGAAAACTTTGCTCTTCCCCCAGATATTCGAGGCTCATCCTCGATGGTTTCAAAAGC
CGATGCCGTGCAGATGCTCGTCCACGGACTCAAAATGTTCAAAAGCGAACACGAGTACGT
TGTTCAAGTATTTCGTGCTCTTGCTTGTATTGCCACCCAGTCTCAGTCAAGCAAAGAGAC
AATTTGCACAGAGGAAATTCTGGTTCTGGTCCTCGAATCGATTAGGAAGCACAAAACGCC
TGAAATCATTGCGACGTGCTCTACTTTAGTCGCTACTCTTGCCACGGGGGACTCGAACAC
GCAAAGCGAACTGATGATCAATAACGGAATAGTAGATATTTTGATCCAGTGTATGGAACT
AACCAACGGAGAGAAGGCCCAAGATGCCGCATGCTTAGCGTTGCGTAATTTATCATGCCA
AATTCATGATTCAGAACCACTTCTCAAGAACGCCAAGACCTTAAGCCTCATTATCAGTGC
CATGGAATCCCATCGAAGTTCATTATCAATTCAAAGTAGCGCCTGCTGTATCATTTGGAA
TTTATTGTCGAAGACAGAGGAGAAACAGCTCGTCTTCAACTCAAAAATTGTTCCCTCCAT
TACCTCGGCGATGCAGTCCCATTTCGAATCGGGCGATCTTCTCGAGCTAGCATGTGGAGC
TCTTTGGGTTTTCGTCGATACCTTCAATGATCAAAAGTTGTTTGTAGGGAAAGAAGTGAT
CGATATGGTAGCATGTGCGATGGTGATGCACCCAACGAAGACTCCGACCCTCGAAAATGC
GTGTGGTTTATTGTCGCACTTGAGTTCGGAGGAATCGCTAGCCGAAGCTATCAGCAAGGC
ACAGGGTGTCAACATCGTTGCCGAGGCCATGTGCAACAACGGAAGCTCGATTTCATTGTT
GGAAGCCGGCTGTTTGACGCTGAAGAACACTACGATAGCTTGTCCTAGCTTTGCTCGTGA
TGGATCTGTTGCCATTTCAACAGTAATAAATGCTTTGAAAGAAAACGTGAACCACACGAG
TTTTGTCGTAGAAGCATGTGACTTGCTGTGGGTATTGGCATCGGAAGACGACAACGCAAG
GTCGAAGATCTTGGCCCTAGACGGAATTTCTATTCTGATGCATTGCCTGGAAGAAAACAG
CAACATCCCTCAAGTCGAAACATCTGCCTTGGGAGCATTCAATCAGCTTGCGAAATCGAA
CACCGTGAATGTCGCATAGACGAAGCCTCATTTGTGTAGCATGCAACATAATAGGATAGA
TCTTTGTTTTATTTTACAAAAAA
>comp2089_c0_seq1
GACTTGCTGTATCTATTGTGCCACAAACAACAACAAGATATTCAGACTCCTCTCGAATCG
ACATAAAACAGTTACACATCAGACATAACACACCTCCACGATGAAATCCCATCTCTTTAG
TGCTCCATCACTCCTGCTTGCTACCGCCTTGTTCCTCGCAAAGGCGGCTTCTGTTTCAGG
CATGGGCTTTTTCAATTCCCTCATGGCACACATCCAGGAAAACGCGGTCGAAGAAATCGA
CCGGATGACGGATAGCAGCGGCAAGGGAATTTACTTTTCGTCGGGCGGTGCAAGCACCGG
AGGCGTCAACAGATATGCGCAGCCGGAACGTTTCACCGCCTTTCCTGGAAGTTCCAGCAA
GAATGACGAGGAGAGGATCCACCAGCGAAAGCAGCTGTTGGCGAAAGAACGACGCACCAA
GGGATACCTTCCCCCCATGGGTGTTACCCAATCCTATTCGTCACACGACGAAGAGCTCAT
AGAACGGTTGGTAGAGCTTCAGAATTGATGGTTCGATTCGTCCTTGTGTTTGACGCTTTC
ATTCATTCGGTCGATCGATCGGTCGATTAGCAAATCATTCCTTCGGTCTCTCGGTAATCA
CCGAGTCCGAAGGGAATAGATTTAGAGCAAAATACCCATAACTATGATCTCCCTACACTT
TGAATAGATTGTCGGGTGGTGGCATCTTCGCAGCATTCCCCCGCAATTAATTGCTATTGC

GATTGATTTCGGTCACAACCATATACAAGTACACACAAGTACACGAACGAAGACGCACGG
CAATATAGACGTACGAATTCATAATAAATTCAATTGTCAAGCAC
>comp2089_c0_seq2
CTCTTTAGTGCTCCATCACTCCTCTCGAATCGACATAAAACAGTTACACATCAGACATAA
CACACCTCCACGATGAAATCCCATCTCTTTAGTGCTCCATCACTCCTGCTTGCTACCGCC
TTGTTCCTCGCAAAGGCGGCTTCTGTTTCAGGCATGGGCTTTTTCAATTCCCTCATGGCA
CACATCCAGGAAAACGCGGTCGAAGAAATCGACCGGATGACGGATAGCAGCGGCAAGGGA
ATTTACTTTTCGTCGGGCGGTGCAAGCACCGGAGGCGTCAACAGATATGCGCAGCCGGAA
CGTTTCACCGCCTTTCCTGGAAGTTCCAGCAAGAATGACGAGGAGAGGATCCACCAGCGA
AAGCAGCTGTTGGCGAAAGAACGACGCACCAAGGGATACCTTCCCCCCATGGGTGTTACC
CAATCCTATTCGTCACACGACGAAGAGCTCATAGAACGGTTGGTAGAGCTTCAGAATTGA
TGGTTCGATTCGTCCTTGTGTTTGACGCTTTCATTCATTCGGTCGATCGATCGGTCGATT
AGCAAATCATTCCTTCGGTCTCTCGGTAATCACCGAGTCCGAAGGGAATAGATTTAGAGC
AAAATACCCATAACTATGATCTCCCTACACTTTGAATAGATTGTCGGGTGGTGGCATCTT
CGCAGCATTCCCCCGCAATTAATTGCTATTGCGATTGATTTCGGTCACAACCATATACAA
GTACACACAAGTACACGAACGAAGACGCACGGCAATATAGACGTACGAATTCATAATAAA
TTCAATTGTCAAGCAC
>comp2090_c0_seq1
CAAAAACAAAATACTATACCGCGATGATAGCAATCAATAATCTCATGCTAACCAAACAAA
CTGTTTTGAAGAACAAGGGGATAATGATGAATGTGAATTCATGCAAGAAGTCTCGAAATG
AGATAAATCCCAATGATGTATTATGCGGTCGCGGAGGACTGACGAATTCGAACATCGGTA
ACAAACGATTTCGATCTGTGGTCGCCAAGTACCAGCAAGAGTACCTTCATGCACGGAAGA
ATGACAAAAAGCTAATTGCTCGAAGAATCATTACTGAAATAAAAGAGAATGATGGACGTT
TTCTACAGCGAAGTTCTGATCCGAGTATCTGGTCTGAAATATCAATGAAGAAGGCATTAG
AGAAGACATCTCAAGCTTTAAGAGAGGGGCTGGATGTACGGCACAAAACGGTTCGTCCGG
AAAAATTAGTCCACAAAATAGATATAGATTCTAACAGGTGGAGAACACCGAAGAAGACGG
AAGCTAGAGTAGTTGAAGGAGTTGTTATCGAATCGCCAAGCAAAAACGAAATTGTAAGTG
GAGAGGACGTTCCTAATTTTGTACAAGAGTCTGAAACACACGAATTCGATCCTATATTCA
CGTTCTCCCATGCAATGATATCAGAGCAGAAAGAACATATTCAAGAATCAGAAAGCACGA
ACATTGAGGCAATCTGACGTCAGGACCACCATTGAGAAAACATGAGTACATAAATTTCTC
CTAGGGTTACAGGTTGAATAGATAAAATGAATCGTAATAGAGATTGGTTGTAGTCAAAGA
TTTGCTTTCGGTCTCTTATTTAAAAGATAACGTGATCTGTCTCGTA
>comp2091_c0_seq1
CAGGCGTAACACAGCAACAAACTCGCAGTCGTGATCTCCTATCTCGATTCCTCACGGCAT
CGACAATATTCATCTCCTTCTGTTTCGATAAGAACCAGGCGACTGCTGTTCTCAACCAGC
TCGTACAGAACACTGTTGTATAGAAGGAAAAGCCAAAGCCGAAGCCATCTATCGGAACAG
ATAACAATCATCTTACTCTCTATTTTCTTCTCCACAGGGAAATACAGACACACAAAATAA
ATTGCATCCCGATACAATATACGTTGACATATTAAGAATTATTCAAGATGGGTCAGGACG
AAAAAACACAAGAGGAGATGGACAAACAGATTGAGATCTTCAAGGTGAAGAAACTTATCA
AGAATCTGGAAGCCGCTCGCGGAAACGGTACATCAATGATTTCCCTGATCATCCCCCCTG
GTGATCAGATTTCTCGGGTCAACAAAATGCTGAGTGACGAATACGGAACCGCATCCAATA
TTAAGAGTCGAGTCAATCGTTTGTCTGTGCTCAGTGCCATTACCTCGACACAGCAACGAT
TGAAGTTGTACAACAAGTGTCCACCAAACGGACTAGTTATGTACTGCGGAACCGTGATCA
CGGAAGACGGAAAGGAACGCAAGGTAAACATTGATTTTGAACCCTTCCGGTCCATTAATA
CGTCGTTGTATTTGTGTGACAACAAGTTCCACACGGAGGATCTCAACGAGTTGCTTATTG
ACGATGAGGCCTTTGGATTTATCATCATGGATGGTAACGGATGTCTCTACGGAACCGTCC
AGGGATCCAGCCGAGAGATTCTCCACAAGTTCTCGGTCGATCTCCCCAAGAAGCACGGTC
GTGGTGGTCAATCCGCCCTTCGTTTTGCTCGATTGCGTTTGGAAAAGCGACACAACTACG
TCCGCAAGGTCGCCGAACTCGCCACCCAACTGTTTGTGCCAGATGGACAAAAACCAAACG
TACAGGGTATTGTTCTTGCCGGTTCGGCCGACTTCAAAAGCGAGCTGATGCGATCCGACC
TTTTCGATCAGCGTCTCCACAAAATCGTTGTTAAGATGGTTGACGTAAGTTATGGAGGAG
AGAATGGATTCAACCAGGCAATCGAGCTCTCCGCTGATTCTCTCGCCAACGTCAAGCTGA
TGAAGGAGAAGAAACTCCTCCAGAAATATTTTGACCAGATTGCCCAGGACACGGGACAGT
ATTGTTTTATGGTGGATGACACCCTAAGGGGACTCGAGATGGGTGCTGTGGAAGACCTGA
TAATCTGGGACAACCTTGAGATCCAGCGATACAAGGTACGAAACATCCAAACACAAGAAG
AAAAAGTTCTTCATCTTACGAAGGAACAGGAGGGCGACGACAAGTTCTTCCGAGACGCAG
AGACGGGGGCTGAACTCGAAACAATCGACAAGGAGCCCTTGGTGGAATGGTTTGCTGAAA
ACTACAAGGATTACGGGTGCAACCTCGAGTTTATTACGGATCGGTCCGCCGAGGGGACGC
AGTTCGTGAAAGGCTTCGGTGGAATTGGCGGTACTCTTCGGTGGAAGGTCGATTTGGCGG
AACTGAATTCTCTGGAGGAAGCCGCTAATATGGACCATGGTGTCGGCGGGGATGACTCCG

ATGACGATTCCGATGATGGCAGCGAATATGGTTTTGACGACGGCGACTTTGGATTTTAAA
CAGGAAAAAAATAAATGAATAAAAAATAAACACAAATTACGACT
>comp2092_c0_seq1
CGGTACTACTACTAATTTTTTATTTCGAAAGACCCCGAGTGAAAGTTTGGGAAACACAAG
GTACTTTTGGATCGAACGCAAACAACCATGAAGCTCACTAAGGCACAGAATGGGAACAAA
ACCACGCGCACTTATGCGTACAGCACGATCTTTCTTTTATGCGCCTCAATGCTTTACTTC
CGCGAAGGAATGAGCCTCGGAGAATACAATCTATACGACGACGCGGCAAAAATGTACAAC
AGTGCCGTTGTCGGCGTCGGATCGGACTCGTCGTCGGCAGCGGTTGCGGAAAAAAGCCAC
ATGCTTTCGCTGGAACCCTCCAAGAGCTCGCTCCACGCCGAATGGAAGCTCTGGGAAGAA
ATGAACCCGTCCGAGCAGGACGAAGCGATCCAAAAGGTTGGTGCGTACATGACCAAGTAC
GGCAAGCTGATCTACCCCAACGCGAAAAGGCGCCAAACCATCAAGCAGGGAACGTGTGAG
CTGGTGGAGTTCGCCTCGGGCCACGCCCTGTGCGGGCCCAAGCCGCCCAACGGTTGCTCC
TTTTTCAGCTTTGGAATCAACGACGACCCGAGCTTCGACCAGAAACTCGCAGAACTGTGG
GGCTGCCGGGGCTTTGCGGGCGACCCGACGGTCCCCCACCCCTCCAAGCTCCACCCGCTA
GTCACCTTTCACAACGTTGGGGCGTCGATGCTCCAAGACAACGAAGAGCGCCTCGCCAAC
AAGGGCGGGGCGGAGGAGTGGTGGTCGACCTCCATGCCCAAGCTGCGGTACTGGCTCGGC
CTCGAGCGGGTCGAGATCATCAAGATGGACTGCGAGGGCTGCGAGTTCGCCCTGGCGAGA
GACATTCTGCGGGAGGACCCCACCTTTTTGTACAACGTCGACCAAATCTCCATCGAAACC
CACGTCTCCAAGGCCTGGATGACCACCCGGGAGCACTTTTACTACTTTGCCCTGCACTTT
GCCCTGCTCGAGGAGGCCGGCTTCAAGATGGAGTGGTCGTCGATTTTTGGGTGCTCGAAA
CGGCACGAGGTCCCCGGGTGCATCCCCGAGCTCGAACAGTTTGGCTTTCCCTGCGGGTAC
GACCCCTGGCCGGGGAGACCTAATGTCGTTTTAGGCAGGAGCTGCCACGACTTTTTGTGG
AAACGGTACTAAGCATCAACCCACCGACCGACAAACCGACAAACCACTGCTAACCCATCT
CCATTGCACCGAAGTCCGGTGTTCTCATTGGCACCAGTAAAAACCCCGTTCTACCCTATG
GAAAGATCTTTTGTATTATTCAATATTGAACTAGTGTATGCTATCGCATCAAAATTACGA
TGGATTACTTCGAGCAGAAGCTTGTTCACCGGCTACCACCCGTAAAAAATAGAG
>comp2093_c0_seq1
CTCGAAATAGTGCGTCCTTTCGATCGGGAAACTGCCTTTGTTTATTTTGTTATTTTCTTT
TTTCACTTACATTACTGTATCAAAACGGAACACAACTTGTTCAAAAGAAATCAACAATGC
TCTCGATTCGAATTCCAGCTCTTCTCCTCTTTCTCGCAATGGCTGTCCAAGCGACCAATG
TTGTCTTAAAGGAAAACGAAAGAATCCCTGGAAAAGGAAGATACATTTACTCGTATGATG
ACGATGTTGCCAACAAGTGCGGAGTAGTTGTCATGATTGGCGTCGGAACAGCCATGCCAG
CTTCGGCCTACAGAATTCTTTCCAAGAAGACTTCCGCAGGTGCATCTATCGTTACCGTCT
TCTTCGACCACAATTCTGGGAGTTTAGACAAAACCGATGACAAGAAATACGCTGACTTCT
ACAATGCTTTCACTGAAAACCTCTCCGAATACGTTCCGGTTTGCGAGGGAAAATCACCAA
AAATTATCGCAGGAGGTCACAGTGCCGGAGGTCGTGCAGGACTCCAAGCAATGATGCATC
GGGGCATCGAACCGGATGGTTTCATCGGGCTTGACCCTTACCAGGCCTCTAAGGATAGAA
TTCGGGGTATCGGGGCGAAGGCAGCCCCAGCGACTGAAATCGCTTCAGACCTTCCCGTCT
TATCGTGGGGCTTTGAAAGGAGGACATGTGCTGTCGATCCAAAGATTGCTGGCAAATCTC
AGTACAATCTTTCTGGAGAAAACAATCGGGTATTCTTCCGAGCCAACAATGGTAGCGCTC
GTGGAGTTGGACACTGTGATTTCACAGATTCTGGATGTGGTCCTCTCCCCTTCTGTCCCA
ACCCACAAAACGACGATGTCAAGGACGTTGTGGCCGAGTCTATTAAAGTTTTTGCTGCTG
GTGTAGTGGCGGGAAGCATCAGTAAGAGCAACTTTGGATTTGATACTACGAATCCAAGTG
TACCTATCGACGTCTTCGTGAATTCCGAGGACGTATTCGCACCTTCGGAAGCAGAGCTGT
AAGCTACACTACAGTACATCTCATGAGCAACCAAGAAAAAAAAAACATCTCTCTAAATTA
CTACTACTACTAGTAAGTCCTTCTACTTAATTATTTTGTGTACCATAAAAAA
>comp2094_c0_seq1
ATTATTAACGATCAGCAGCTCGAAGCAACCTTAGTAGAGCCGATCGGTCATCGTACCGAT
ATACCTTGTCCTCGAAAACACCTCGGAAAATTCGAAATGATTGCCATCTGTCAAACGAGA
TCGCTACTAGCATCCTCTCTTCTGTTGTCTGTTGCCGTGTGTCTGAGTCAGGCATTTTTC
CTCCCGAGCGCAACTCACGGCTCCCATCGACAAACGCATAATGAACGAGGATTGTCAAAC
TTCAATTTGCCGCTAAGAGAATCCGATTTTCTGGCCTTGTCCGCGAAAAAGAAGAGACGT
CGCCGGCGAAAAGACAACACCGCTACGCCGTCAAACGAACAAACGGAGGACGTTGAAGAC
TTGTCTTCATTCGATGAAACACTCGGCCCGGGCGAACTTCCTGAATTTGATTTGGGCGAA
GAAGAAGAAGAAAAAGAGAAACCAAAGAAAAAAGTCAATCCCGATGAAATCACTGCGAAC
ATGATGGGAAGTGGCAGCTCCGGATCAAAAACAGTCGACGAGTTGATTTCCGATCGTGCT
CTGGAAAGCAAGCTTGAATTCGACGACAACGGAGATGCTAGCATCCCAGATTTTGTGGAT
TTGGCGCAGGCGTCTTCCACGACACCAACATATTCTCCGGATGATCCTTCGATGACTGCT
GGTGTTGGAAAGAAGAAACAAAGACGGGCCGAGCGTGTAGCTAATGCAATTGCCGCGAGG
GAGGCAGAGGAAGAAGAAAAATCCTTCCTTGAAAAATTTCCTCAGTTTCTGAACGAAAAG

GGTGAAGTGAGCGCTATTAAAATTCTAGAACAAGGAGCATGGATTGGCATTTTCTTACTC
ATCGGTTGGGAGTTATATATCAACTCACCTTTTTTCGAGCGTGCAGCACCTATGGCTCCA
ATTGTGTATGAGATTCTAATGTAAAATGACTTCATGTACTCGTATCCATCGTTATATGAT
GGGAAAAAATGCTAATCAAATTCAATCAGAATGAAGAAAAGAGGATAATATATCGGCCTA
ATGAGCTCATTACCCTACTTTGTGCAAAGTGTGCAATATATCTCCAAACTAATGTAGTGG
GTGTAGATCGAATGTCAGAAAAAAATCCTTTGCAAGTGCTTCAGTTCAGACGTCGACCGTT
CTATTTCAGTTAAAAAGGA
>comp2095_c0_seq1
CAGTGTTGTTGCATTTGCAAAGAACTAACTCAAGTACCATTTCTCGATAATTGTTAAGAA
GCACTTCAGTTTGGAATGCGTGGTCTTACTCGACAATTGGCTAGGGCAACAAAACTACGA
GTACTTCTTTTGCTTCCGTCGCTTTTGTCTCTTTGTTGCCGCTCGTTCATGTCTTCCCGG
GTGTGCTTTACAAGGGGACTATCGTCGTTGCAAATCGGAATACAAGGTCGTCGGAACCTC
CTTTTCGCTACCGAAGCATGTTCTTCCCGAAGATACACCAATGCCGATGATACATTCGTA
TCTGCCGCTTGTATTGGGAGAGACGCACAACGAGTGTCCGCCACCGAGGTCGAATTGCCT
CTTGCCGACGGAATGGTCGTGAGGGGCCAGCGATGGATCCACCTCGATCGTTTACAAAAC
AAAAACCAAGCGGCAACAACGGCACCTACGGTAGCAGTCGGTGGCGAAAATCCAGTGACG
ACTCGGATTTTGGCGCTTCACGGATGGCTGGACAATTGCCGGAGCTTTTACTGCCTCGCG
CCTCGATTGGTCGAGAAATTGGGAACCCGGGCCGAGCTCGTGGCGATCGACCTTCCCGGA
CACGGGAGATCGTCTCACCGGCCGATCGACGGACCCACCGCGGTCCTGAGCGAGGGTGCA
TACTACATTGCCGAGATCCTCGATGCACTGGAATGGGGGCAGGATGCAAACGCCAATAGC
AGCAGCAAATGCAATGGTGCGAAAGAAGATCAAACCAAGGTGACCCTGTTAGGTCACAGC
ATGGGAGGAGGCATGGCCGTTACGTATGCGGGAGTATTTCCGGAGCAGATTGGCAACATC
GTGAGCCTCGACATGTTTGGACCGGAACCGGGAGAACCCGAGGATGCAGCGGCCCACATT
CGGTCGCACGTGGTCCAGCGAAGGGGGCTGGGACCACTTGGACGGCCCCACGTCTTGTAC
CCATCCCTCGAACGGGTGATCGAACGGCGGATGAAATCGGCTCGCATGGCCCCGGGCGGG
CACCAGTATTTGTCGATAGAGGCCGCTACAGAGCTGGTCACCCGATCCACGGTGCCCGTC
TATGCCAGCGACGACGACGAAGCCGCGGAAGAAAGCTTTCCCAAGGGGTACAGGTTCCGA
CACGATACGAGAATGATGTGGCCTTCCCTCCAGTACCTCACTGCAGAACAGATAGCGTCG
ATCCTGGGGCGCGTAGACTGCCGGGTCTGTCTCCTCGCTGCAGAGGACGGGTACCCCTTC
CGGCAGGATCGCGTTGATCGTGCGCTGGCATGCCTTGAACCCGAGGTCTACAAAGTCCTT
CCGGGGAGCCACCACTTCCACGCCGATCCAGACACCGCTGAGGCCGTGGCGGATGCCATT
TGCGAGTTCCTGCGCGGGGGCAAAAACCGGGAATCGACGAACACCACGAGAGGCGCAAGA
AAGAACGCTTCGCCCTCCCAGGAGGCCACCGCGACGTATGGCATGCAGTAGTGTCTGCGC
ACGCTACTACCGCACCAAAACCAAACAGCAGTATATTGCGACACAACACCGACTTGCGAA
CCGAATCCCATATCTCCGGCCACGGGATAGAAGTCGATTGAGAGCAAACAATTCCTCCGT
CGTCTCATGACGATGAATGGAATATAGGGAAGTTTGACTGGATGTCTTGCTTCCTCATTG
AAAGGCAAGTCGTTCAAAGATTTGCGCTCTGAGAGAATGTAGGCTGGATAGGCCTAGCCG
TTGACGGTTTTGTGTAAAACTCAAATAACTCCGACTTAAAGGTATGGCTTCGGTTTTTCC
AGTACTGAACTTCACCTGAATTGTGGGGAACAACGAGAGTGGATGATTGCTTCTGGTCTG
ATTGTACGGGCTTTCGATCAGTATGAACAGTTTTTGGGTATGTTCAATCATACCAGTATT
TATGAATCATTAGACTTGCT
>comp2096_c0_seq1
CAGAATTCCATCAGAACACCGAGCCTACTTCATTGGAACTCACAAACTCTCAACAACGCA
CAAACACACAAAGTTCAGAGCACAAGAACGAATTCAACTACTATACTTACTTCCAACCAT
ACCGGAAGTATTGTCTACAAGCTGACATTGTCATTTGAGCAACGCACAACGCAAGGAAGA
ATCCGAATTCTTTGTCGACCAAAGCAGAATTTCAATCCCAGGCAAAAAAGAACAAGAATA
ATCGCATAGAAGAGGAACCGAAAGAAAACGAACCATGCCGATCCATACCGCTCGTCACCG
AACACCCATCTCCGTCGTCGCTTTTGCTTTGTTGCTTTCGATCGGCAGCACACGCAGCAA
CAGACAAGTCCTCGCCGCTCCAGCCCCAACGTGCCAAAGCTTCGGCGAGAGCTGGTATTT
CCCCAACAGTTGCGTGGCCGACTGCACCGCACAATCCGACGGAAGCAGCACGATGGACAC
GGATCGATCCCGAAACGTGAACGGAGATTTAAAGTGCTATTGTACCGGTCGGGAATCCCC
TCTCTGCGCCGACGATCCCATGTGTTCCGACCTAGAAATCCTCCCGGGATCCGCCAAGGA
GAGCTGCGAGGCGGTTTGCGGAAGCAGCGGCGGTTCGTCGGACGTAGTAGTAGTAGACGA
CGTGGAGTACGCGGGCGACGCTTCCGCGGCCAACAAGAACCAGACCCACTTTCGGGTGGG
ATGCTCGTGCGATGGGGGTGCCACACAGGTATGCGGCACCGACTACGTCCTCTTTAGCGA
CCTTACCTACCTCAAGTGGTGCACTAGAAAAGCCGACAACAACAGCCTACAGATCAAATC
GGCCGAAGCCTGCGACGCCTACTGCACAAAGACCAACCCCCTGTTCGACGGGGGAATCTG
GAACGACAGCGCCAAATCATGTGCCTGCAAAGAAGACTCGATGGAAGTTTCTGCCGTGGC
TTGCGACGACTCGTCCGCCAATACCAACGACGGGAGTGGCCTGGGCAATCCCTGCTATGA
AAATGTGGGTGTGTCGACCGCCGATTGCTCGTCGTCGTCGAACGCAAACGGGGAACAACC

ATCCACTACCCAGACACTGGTGTCATCGGCTGCGATCATGGGAATCTGGCTGCTGCCGTG
GTAAATGGGAAAAAGAAAAAAGGAATACTAAAACCAAGCTTGACAAGATTCAGTTGAGGA
CAGGACGTGTAAAATAAAATAGTAAGGCAGATGATAATTTGTCCAGA
>comp2097_c0_seq1
TCTGCGATCAATCAGTAATCGTACCTGGTACCGCAACATAAATCTCCTTCTATTTCAAGA
AACCTTGTCATTCGCTCGACCCCACACACCCATCCACACACCCAAAACAGAAAACATGCC
CTTCGTTCACATCACCTGGTTGCCCAAAACCTGCCGCAACGCCAAAGTCCGTAAGGAGGT
CGCGGCGGCCGTCATGAAAGCCATGACGAGCCACCCGGAGGCGGACGTGGCCCCGGAAAA
CCTGGTGGTTCGGTTCAGCGAAGCGGTGGACGGCTTTCCCCTCCCGAAGGGCCACAGCGA
AAACCCGGAACTGAGCGCTCCGCAAACGCCCGCGGGCGAGGCCTTCCTCAAGGACAGAGA
GTAGAAACGCAACGGGCGGGCTCGGCGCAAAGCGGGTTCGATAGGCATCGAAACCTAAAA
TAAGTGCTTCCGGCAAAATACGTTTGCACTCCACACAGCCAAAGAGTCTTCAGTCAGTAC
TCGTCTTGCTTTGTATCAGACACGAGGCATTTTCAATAGGGTAATTACCCAGGGGCACTC
TGTACTGAATGACTGATGCAACAACTAATATTTTAAACCTTTCATTTCCGCTATCTCTAA
CAAGGGCATCCGCCATTTCAATTCCTAATGCGTTTGGTTCGTCCCTCGCGAAGAGCACCG
AAAAAAGTAGTCTTTTGTTCCGATGATTTGTGGCACTCAATCGGAAACACCCCCAAGTGG
GAATTTTCAAGAAGGTGTGATTTTCATCCGAC
>comp2098_c0_seq1
ACCGTATCGCATTCCTCCTGTCCCTCTAGCGTTTGGCCAATCCGATAGGAAGAACCACCG
CCTCTTCCTTGCCACCATTCGTGCCAGAAACCGAAACATTGTACTACTAATAATGAGAAA
CCAGAATCTGCTCCTTTCGTTGGCGTCGCTGCTATTGACCGGGTCTTCCGTCGATGCGTT
TGCAACAATGAAGCCTAGAATTTCGCTTTCGAACACCGCCATCCGGGCCGAGGTCGACGG
CATGCCCGAAGCAGATATCGCCGCGGGCGTCCCCGATACGGCGGCTCCGCAGGCAGCCGA
TGCCGTTTCCGAAGCAGTGGGTGCCGCGACATCACCAGCGGCTCCTGCCACCGTGGACAC
CGCGGCCAATATCATCACTCCCAACCAATTCGCACCGCCCGTTGCCCCTAAAATCATCGA
TCCCGACGCCCCGATGCCCCTTCCCGTGGCACCTTTGACCGATCTGGACACGATAGCACT
GGTCGCTGGCCAAGAAAACTACGGCCTCGCGATCGTCCTGCTTGGAGAAGCGATCTGGTC
CTTTTCCAAGGCACCTTCGGTGGACCACGGCCTCAAGACGCTCGTTCCGGCGGTCCTCGC
TGCAGCCGCTCTATTCGTGGCCTCCGGGCCCATGATCACCAGCGGTGATGCCGGCAGCAT
CAGTACGGGGCTTTTCATTGCGACAGGGGTCAGCGTGTTTATGGGGGCCTGTTATGCGGC
CCGTTTGTCAGCGCCCTTTTCGCCCTCTCCAAAGGAAATTCCGGCTCTGGGGCTTGTGGT
GGCCTTTGCGGGGTTCTTCAGTTTCAGCCAGAATCTTGTCGTCGATGGGTTCGTTACGCT
ACCTTCGCTCCCGCACGTCGATCTTCCCTCTCTTCCTTCCATCAGCCTTCCGTTTTGAGC
TGCTTGATGAATAGAATAGTGCGTCGAGGAGTCGCATTTTGGTCAACATCCAATGCAAGT
AACACCAAAGCATCTGCATAATAATATTCTCAATGGGATAATGTAAATTCATCATTTAAC
GTGG
>comp2099_c0_seq1
CTGTATTCGTTCCACCGCGTTACAACCGAGCATTGATTTCAATCGTTCGTTTTCAAACAA
AATCAGGGATGATTGGCCACAAAATCATGCAGAACGATGATGGCAATCGTGGTGTTGTTG
ATGACAAGATTCCTACCGATATTGCAGCTGATTCTGGTGCCGTAACGGTACCAAGCACCC
AGACTGCTACCAGCGAAGACCGCATTGTGGAATCACTCTATCGCCATGTCATCGAATCGA
TGTGTCAAGACGTAGCGTCGAACATGCACGAGTTGATCAAAACTGGAGCGGGCGATGTAG
TTCCGTCGTCGTGGAAATTGTTCATGGGAACAAAAATACCAACACGAAGGGAATTGTATC
CAGAACTCTACAATGGAAGCGATTTTAACGCTGCGAGCGAAGGCAACAAAAATGAAGAAG
ATACCAGGGAAGGAGGTGATGAAGCGGGTGAGAATATGGACCCTAAAACCCTAAATAGAA
TAAGGATGACGGACGAAGAAATACAAGGCCTCCTCGACAAATATGCTGTGGATCTTCCAA
CTTCATCGTCATATTCGAATGTATCACCGACAGTGAGTAGAAAGAGGCGCACGCCACGGG
ATATGGCAGAGGAGGAGGAAGAGCGCCGTTTGTTGTCCAAAATTAAGAAGGGAAAGGAAA
GCAACTACAAGGACGATAGTGAAGACATCGAGGATGATGTTACGAATGGTGACGACGACG
ACGACGACGACGAAGACTTCAAAATGGATGATGCAGAGGGGGACGACGATGATGACGATG
ATCTAGGTGGGGCGCAGAAAAAGAAGCGAACGCTGTCGTCCTCTTCGGCTACACCTTCTA
CAGACTCTATAGCAGAACCAGCAATAACTACCGATCTACCAAGCAGCAGTTCTAACAGCA
TTAGCAATATCAACAGCCACCATCCAAAAACAGATATATGGGGGAAAACTCCGTCAAAGG
AACCCAAAAATCGTCTCTGCCTATGCCAGCTATGTGGTCGACTTGTTAGCACGTCTCGGT
TTGCGAGTCACCTCGATAAGTGCATGGGCTTGTCCACGAGCCGAGGAGGACCCTCGTCAA
TATCATCATCCACGGCCAGCGCGAAAAATATGTTATTGTCGTCAGGAATGCTCACGAAAA
AAGCAAAAAGGGGAAGCTCACTTACGAAGAAGTAATGCAAAAGGCACAAATAACTTCTAA
GGAGATCTGAACATTACGGTTGGTTCGAGTCAAGTTAAAGCTATGGAGTGCAGCAGTTCA
TAAACAATCGAGTGAGTAATGATCTTTAATTTATTTTACAGGGTTTTGGTGCAACGGCGT
CCATTGTTCGTCTAGCATGATAAATAGCGAG

>comp2100_c0_seq1
CCGTGTCAAGAGCCTCTGCATTCTTTGCGTTCCTCAAACCATGCACTAAGTGATCACAAC
CGTGAATCTCCTTTTCCCGCCCCGGCGTGATAACATGTCAACCCCGAGTTGACGTGTCTT
TTCTCTGATCATCGTCAAGGCATCATTGTCAATATCAAAAAATTGAAGTTAGACGTCATC
AGTGCAAAGTGCCAAGGTCTCGAGAAAAGCAGGGAAGGTCCGTGCCGCCTCTCAACTGCA
GTTACGAGTACACGAGTACGTTTGCGTTGTCCGCGATTCCAGCGTACAGAACAACGCGAT
CAGGTATTGACATCTATTCGGGCTTCGAAGCGAACTTCGAACGCGCAGTGTGGAAACAGA
TCGTTCCCCTACGATCGAACACGGGCTGCCCTCCCCTCCGTCCAAACGCACAAGACAGAC
CGTTGTAGACCAAAGAGTCACAATCCATCTCATTAGCAATGATGATGTTGACTATGACAG
CTCCGGAAGCGCCTACAACTACTGCCATTGGTGACGATACAAAACCTTTCCCTCCGGCGA
GTAGCTTAAAAGATGAAGAAACTACCAGCCTTAGAAGCAACGCTAGTAGTTGTACCAGCC
TCAATAGTAGTTGTAGCCACGACACTGACACTTCCTGTGCACGCGCACTCCCACCCTCAC
TCGATCCGAGCACGAAGAAACGCAAGCGCGTCTCCTTTAGCCTGCAGCAAACAGCCTATT
TCTCACCGGAGCAGATGCTGAGCAAACAAGAACGAAAGAGCCAGTGTTGGTACTCGGAAG
TAGAACTGAGCCTGTCGCGAGAAGAAGCCCGCGCAGCGATAGCGGCCCTGCACCACCAGC
TCCAGCTGGATGCCGCCGCCGCTGCTGCGGCATCCGCGGGAACCCACGGAAAAAGGCAAC
CATCGCCAGCGCAACCCACGGAACTCTCGAACGAGTACGGGAGCTGGGTGCTCCGGTGTC
CACACGACGAGACAAAGATTGTATGTCTGAGGGGAATCGAAAAATACGCGGACGCCGCGG
CCAAGTACGCTGGACAGAAACGCCTCGTGGACTCGGTGCTGCAGCAACAGAGCCTCAACA
GCGAAGACATACACGTGGCCCTGGTCTCGCGGACCCTGTCCCAGCCCTTTACGGAAGTGG
CCCGGTACTACGCCGTGAAGTCGGCCGAAGAACTGGACCTGTGCCGAAAGCACGAACAAG
AGAAGGAGCGAAACCAAACCGAAGAACGAAAGCAGCAGGAAGAAGTCGCAACCGTTCTCT
TGTTGATCATGGGACAGCAGGAGAACCGAGAGGGAACGCTCCACGAACCGCCGCACCACC
CACCGGGCACCCCGTCCCCTTCACCATCTTCCTCCAAGCTTCAAGAAGACAGTCCTATTG
TCACGCCCCGTGGATCTGCCTCGGGCAAGCGATCCTCCTCGTTCTCGAACTCACCTCTGT
GCGAACAACGAAACGTGAAGCCGTGCATACGAGCGGTTTCCGATCGATGAAGAGGCAAGA
GAGCCCGTGCGAGAGCAGAGCACCATGCAATGATTCATAGAAGTAGCACCAAAACCGCAC
AACACGACGCTACAAACAAGTATCCAACCAGCATCAAAGGAGTTGAATCGATCGATCGGT
TCATCCGCAATGTTCGGCTGCGCTTGTATCAAAGCCACACCACATTTTACCAATCCAACC
ATACAATCGATATCATACCTTGATTGATACAATAAAAGTTTTCAGCACTGCAATTCCACG
CAACAGAACGCAATAAAGCACAACGAAACGAACACGCATAACACACACACGAGACGTTTA
AGAGCAGCAATCGATAACAGTAAAAGCACGCACGAAGTATGCGGGCTCCTGCGCCACGTA
CGTCTCTATCTCAAAAGCAACTCTTACACCACCTTTTTCCACACCGACGGAAAGCCTGCA
AATTAGAGGAGAGCGACTTCTCTACTCTTACACCCGCAGCCTTTTCGGAGCACTACACCA
GCGTACATTGCAAACACCGCAATGGTACAACAACAAAGTATAACAACACATACTAGTTTT
TTTACACGCACATACCGAA
>comp2101_c0_seq1
CTTTTCTGTCATAAAAATCTATCTCAAATTACGCTCACTCTACAGGACGAAAGTGAAGGT
GCAACAAGTTAAAGTAATTTGATAGACAGAGTTTATCTCAAAAGAAGGACGAGAACCATG
ATCACTAATTCGCAGCGACATTCGGACGAACGAACTAGAACGCGGCTATTGAATCGCCTC
GGGTATTTTAAAACCCCAGGTGGAGGGACGGCAGGCCTCTCGTCTTCCGAGCTATCATCG
GTAATATCACAATCCACGATATCTTGTACAAACTTTTCATTCCCTTTGGCCAACCATCAC
CCCGATAATAGTCAAAATCTCACTCTTCTTGGCGATGCAGCTCCGGTCATGGTAAACCTG
AATGATAACTTGAACGAGGGAGATTGTCGTCATGACGACGACGTACCATATTTCAAGTCA
TCCTCTCCAACGAGCTGTATGATCGATATTTCTTCAACCATACGCAGCAGCAGTACGAGT
CACCAGAGTTACAACAAAAGTCAGAGAAGAAACTATAGACCGCCCGAGCCTCCTCCCACG
GACATAACCGCCATAACCACCGCTACTTATAATTCAACATCAGTTCAATTCAATGAAAAA
GCTGCTGTGTTGTTGATTCCATCAAGGTATCAGTATTCTGATCGCATCAAGAAATGCATG
TGGTCAAGTAAACAAGAAATGACAGAGATGACCAAGCGAAATGCCACCGAATTTGCTGCC
GAAGGCTGGGATTGGAAAAACGTTGTCATGGAGAGAGACATGCATTTCGACAAAGTCACC
GGGACACTGGTTCATCCTTGTCACTTGAATGATAAGCGACTATACATGCTCTATGACGAG
ATCGACAGAGACGCCTATGATAAAGATGATTATCAAGCTTATGACTGTGAACAAGAATAC
TGTTTTCAACCACTGGAACGGAAGTCTCATGTGATGGCTAGGGGAAATATGACTCGATGA
TTTCCGACTTTGTCTTTCACCCCCAAAACTTACTTGATTTTAAGAGCATAGGCACA
>comp2102_c0_seq1
GGAACGGAAATCGCAATTGCGATAAGGCGGCGTATTGCCACAGCCGTATCTGTCCGAGCA
AGCGGGGATCATGATCGGATGATGCCTCGGGAGTTTATGCTTCGTCGTTATCCGTTGCCA
CTAATCGAGTGCTAGTAATCTAAAAAATACGACGCTATCTTTCATTCGAACAAATATATT
GAACATATTGAACAATCATCTGTTCTGTATAGAACGACCATGTCGTCTATAATGGTGCGA
TCTAGCACCACCGATATCACGGAAACGGGTCATGATCGATACACCATTGCTGAAACTATG

GACGACAGTTTTGATGATGACGATGACGAATACGAAAGTCAATACAACTCTTCCCGTCAC
GAAAGAACTATAATCTCCGAGTCGACTTACTCTGGTACCTACGGAACCACTTCTTACATG
AGTGAGATGTCAGAAGTGCTCGATGAAGATGCTTCTTTCATAGATGACGATGACGACTAT
TTGTCGTTAGGATCAGAAGCAATGGTTGGTGATGGATGGGTTGATACAGCCTGTGGTTGC
CTAGATCGTCCAGTCTCGACTTACCAAAATATGTGTTCTAGGAAGCAGACTTCTTTGCTT
CGTCATAGCAGAGGCAAACCACGGCCCAATTTCAACCAAAGATCAGCGGTGATACCTACT
TCCAAACTGACCATGTCGGCCAAAAAGCGTCGAAATTTTCGTCTGAAAGATCGAAGCGTG
TGTGAACATGGCGAGAGAGATATCGAGGACGTCGAAGACAGCGACATTAAGACCGACGCC
CGAAGAAGTGACGAAATCAAAATGATGCGGATTCAACCAAGAGAATCTTACCAACTGGAG
GATGATTTTGACGTCAGGAAGGTTCGAGACACCGAAAAAACGAGAAACAAGAATTCTTCG
AAGTCAATGAAAGTAAAAAGAGAAGAAGCTGCAGTAAGTCACAATGATGAAGACGAACCC
AACGTGAAACAGGAGGATCGTCCCGAAAAAGCTAGTGAGAAGAAAATGGTTCCTGCGACA
GAGAGCAAAACCGAAGATGATCCAGTTATCAAAAATATTGAAGCTCCTCCACCCATGGAA
AGAGGAATCGAAGCAGAAGGCATAGAGATCAGTGCTCAGATGTCGGGCATTTCTACTAGA
GACCGAAGCGTGAAACGTGATCCTCCCACTGAATCAGAGGCAGTAGCCACTCGTGAAGAC
CAACCCGCCGATTACTATCCCGAGAACTATTCCAACCTAGAAGCACCTGCATCGCAAGTC
GAGGGAGAAGAACGTTCGAAGGACGAAGATGCATCCGAGGAAGATGTTACACTACCTTCA
CCACCTTCTTTTGGTGATAAAAAGACAAGCGGACTTGAGACAAATAGGAACGGTAACGTA
TCGCCAATTCCAGTCGAGGAGATATCCAGAAATCCTTCAGACATGGTGGTTTTGTCATCT
ACAAGTCAAAATGAGGACTTTGCGCGCACTCCCTCGAATGAAAACCAGAAGGAAGTTGGC
GATGGGGCATCGACGTCGAATAATATCGAAATAATTGATGTTTTGAATTTGAGTATAGAT
GATGAAGATTCTTGGACTCCTGTTGCTCGACATGATGTTGAGAAGGGAACAGCAACTGGG
AAAAATTCGAATGAATTTTCGCGGCGATCAATGAATAGCAATCGTGCCTTGCACCCAATG
AATAGTCCAAGTATGGAAGAGCGATGGATCGAAGAAGAAGCGAAATTGATGAAAAAAGTA
AAGGCACTTGGATTGACGAACTCTAGACCTCAAAACCATACGGTCATGGAATCAGCTGAA
GATGTCTGGAATGAAGAACGGATGAAATTGCAAAAGCCTTCCGATGACATTTTCGATGAC
CCGAAAGCACGAGAGGCTTTCTTGGATCGTCGGCAGGGACGTTTACGAGATGCCGTGATG
ATTTCTCGCAATAAAATGCTATCCAAAAATGGGACCAGTATCACGGACTCTCTTGATGGT
GTAGGCAAGCAGGTTTTTCCAAGATCACAGAGCACCGGACGAAACCCTCCACCATCTCCT
GAAATGCGGACGAAAATCATGCAGAGCCGAAGTTCGAGTCGTGACAGGATTACTAGAAAT
AGATCAATGGATCCTGGAAGTGCTTCACTTTTACCATCTCGATCGAAAGACCTCGTAGCC
GTTCGAAGCATCGAAGAAGGTCAAAGGATGCGTTCTTCCAATCGAGCCTCGAATAAGCAA
AGAACTTTTTCGAACAGTCACGACCCCATAGTTGTTCACAGCTACGTAGGGGATACATTG
TCGAGAGGTCCATACGATGAATTGCAAGGTCGAAACCAGCACAGCCGCACAACAGATCGT
CGAAACAGACCATCAACGCCATCAAGGTCAAGGTCGACGTCTATTCGAAGTGAACCAATT
GTTGCTGCTGATCTAACAAACGTCGATTATCCAATGAATAGCCACCGATTCAGCGATGAA
CGCGACGACCAAGAGTCACTAACTACTGCAAACCCAAATTCTATCGAGAGTAGTCAAACA
AATGTGTTCTCAGCTTCTGAAAAATTGTTGAAGCTGGAACAGAAAATCGAAAGGCAACTG
CGACAAGTAAGAGGCGGTGTGCAAGAAGACTTGGAACGGTCCAGAGAAATCAGGAGAATC
GAGTCGAAACTGTCCCAAAAGCTAAAATCATTTAGAACTGATGGCGCGGACTTGCAGACT
TTGTCGTCAAGAGATATCAAAAGACTCGAGAAGCAGCTGGCTCAAAAGTTGAGTGGCGAC
AACGAAAAACGAGCGTCGAGACTCAAGAAGATCAAACGCAGAGGTGTCTCTTCGGCACGA
TCGCATGCACATTCGTCGGGCATGCTCACTGACGACCAACAACAACTTTCTCTAGCATCT
TCCGCTGGACGAGATCGTGCTGGTTCAGCGGAAGTTGTGACGACTCCATCAGGGAAATCC
TTATCGCGAAGGAGTAGTAGGAGTCAAGAAAGTCGGTCGGAGAAGTCGAAGTACCAGACT
CTTCAAGGCTTGCGATCACGATACGTTCGCCGTGGATATTCACGAATGCCGGAGACTGAT
TAATTCACGCTGGTGGTGAACGTTTCACTCTCATATATGTGACCTCCCCGTGAATATACC
ATTTCACAAGGTATTAGTAGTGTTTGCACCAAATCAAGTTCGCGGGAAGGAAGTCTAAAT
TCAGGTTACCACAGCATGAAGCTTCGGATTTCGTGCTCTATATAAAGGTGATGCATAACA
GAAGATTAC
>comp2103_c0_seq1
GAAAAACACACATTTGTCCAACGCGAAGACAAAAAACACAAGCCAAAAAATCTTCCAACA
ATGAAGTTTGTTGCCGCCGCCGTTGCCTGCATTGCGGGCAGTACCGCCATGACCCTAGCG
ACCGCCGAACTCCGAGGAATGCGCAACCTCGAACAGGACCACAAGAAAATGCTCCGAAGA
GTCCCACCCGGATTCGACATTAACCAATTCACCCAGCCCCCAGAAGTCGCCGACGATTCG
TCGGGTACCGTTGTCGAGGTCACCAGCCCTCCCTCCGCAGCACCAGTTTTGCCCGACTGC
ACCCCCGTCGATGGAGCACCCTCCCGTACCGAGGAGTACGTTGTTGTCCAGTACGACAGC
TCTGCCAACGATAAGGTCATCAACTTTGACAAGAAGGAGGACCAGATCGTTCTCGACGTC
TTGTACGAGTTTGCCTACAAGAACGCGACGGGCCCCTGCACCCAAAAGGGAGCCGTGAAC
GAGGTCTTCGACGCCGAGTTCTTGGCCTTCAACGAAGACGACAGCACATTCTTGCTGCGC

ATCGGACGCATCACGAACTCCATCGGCGAAATGTTCTCTGGCACTTCCGAGAGCTGTGGA
CCCATCGTCTACAACTCTACCGATCCCGGATCCGTGGACCTCTCCGTTCGTCGTCTGGAA
GAGCAGTGCGAATGCCCTCTCCCCACCGCCGCCCAGCTCGTTGCGGGTTACAGCGCTGCC
CTCGCCAACGAGTACAAGGACGAAGGCCTCGGTGAGAACTTTATTGTCGGAACCCAACAG
TTGTGTGCCGAGGAGGGATGCAAGGCCGACAAGAAGGGAATTACCACCTACAGCTCCGCC
GGTGTCTGCCTGGGCGATATGGAAGCATTGACCAACGTCCCTTCCAGTTCACCCACTGAC
TTTCCCACGGACTTTCCCACCAAGTTTCCAACCGATAAACCCTCCAGGAAACCTACCGCA
AAACCTTCCAAGAGTCCAACAGAGGGTCCAACCAAGGAGCCAACCTTCGAGCCAACTCCT
TCGCCATCCGCGGAAGGGGACACTCCTTCGCCTACTGCCGAAGACACTTCTTCGCCTACT
GCCGATGACACTCCTTCCCCCACCATCACAAACACGACTTCTTCGCGATTCCTGGTAGAG
GAAGAACTCGTGGTCTCGCAAAACCAGGACGCATATGCGTACCTCACCGAGGCACAAGCC
AAGCAAGACGTGTGCTCACAGATCGATTGGAAGCTGGTCAATAACGAAGCCAATCGCCGA
ATGTGCAAGGACTACCTCGAATTGCCGTACGCCAAGACCGATGCAAAGAAGTGCGCCGAG
CAATCTTCCGAGACCAAGGTGCCCAAGAAATTCTTCTCCGTAGCCCGTACCTCACACGAA
TCCCTCGTCCACACCGGAGTCTCTGCTTCCAACCCCGATTTTGAACGCTATCACTTCAGC
GACTCCGAGGCCCTGGAATTTATCGAAGAAAATTGCGGAGCCGATGTCGCCCGCGCCTAC
CAATGCCTCGCCCCGCCAGCCTTCCGTGCGGATCTCTTCCGCTTCTGTGCGCTCTACTCG
AATGGAGGTGTCTACCTCGACTCCGATATTCTGCCTTTGGTCCGCCTGGAAGACCTATAC
GACCCCTGTGCGGTTGCCACCGTCGGACACGACTGGCCACAGGGGCGCCCCCAGAAGCAA
ATGAAGATCCTCGCCAGTCAGAAGAACGCTCCCATTTTCGGATGCATGCTCCAAAAAATT
GTTGGAAACGTGGCCAACGAATTCTACTCCGACAATGCCCTGGCCATCACCGGACCCATT
GCGCTCCAGGAATGCTACGAACTTCACTCCGAGGGTGTAGCCATCACCTACCACGACACA
CGCCAGGCCGCTTTCCCATTCACTGGAATGCGCGACGTGCACGGCCACTCCGTCTTGGCC
CTCGAGACACCTCCTACTGTCGAACGAGAAAACTACAAGACCGACGTGGATTCCCACGAG
GTCTACCGCCCAAACTGTCCACTCCACACGAACATCGCAAAGAAGGTTGCCGCAACCGTT
GTCGACAACATGTAAAGACTTGCAACTGCCCATCATCTTTAAATACAAAAATACCATAGC
AGGAATCGTTTTCACTCTTCCTATCGTCACGCATACGTAGCACTGTACCAAAACACTTAT
CTTTGCGACAAATCCACGCAAAATCACCCTAACCCCGATCTGCCAAGCTCTACCGCTGTA
GCGTTTAACACCCACTTCCATGTTGTATGGCGAAGGTGAGCTCCACGGAGCACCAGTCGA
AATAACAAAGAGCATCTTCCTTACGAGACAATATCTTAATATCGTACCGGAGGGAAGGAT
AAACGCGCGGTGGGATTTAAACCCAGCGTCATGGCCTGATTCTCTGATGTTTCTATCGTG
AAAGAGTCGTCTTGTGACAAGTTTGAGTTTACCTTG
>comp2104_c0_seq1
CGAAAAAGAACTTTTTGAGATCTCAGATCAAGATACCAATGAACGGTACACAATAATTCC
GGGCAATCCCAACGAAAAAAGACTTGACTTCGCATTGAAAAAGCATATGCAACCATGAAA
TTCTCCGCCATCTCTCTCCACACCTGCTTGGCAGCCACCGCTCTTTTTGCATCAAACTGT
AACGCCTACACCCCGGGAGTCCCAAAAGTAACTCCGGTAACACAGAGCAATGCCCTGGAC
AACAATGCCGATATTGCCATGTCGCAATACGAGGCTGCACTCAAGGCTGCTTTCGAACAA
GCTTCTTCCACTGCGGCCCCAGATGCACCCGAGGCAGAATCCTCTACTGCTTCGGCTACC
TTCGCCGAGCAAGTTGAGGAACAGGAACAGGCCATCGATCAATACGAGGCAGCCCTAAAG
GCTGCTCTCGAATCGACATCGGCAGCTGCGGGAGCTCCATCTTCGATCTCTTTTGGCGAG
GCAGATCAAAACGGACCCAACTGGGAGAGTATTTCCGAAAACATATCATCCCAAATTGAA
AAGGCTGAATCAAAGATCGGCGCACCGTTGTCAGCCGCTGCGAAGGGAGAAATTGTCGGA
ACCGTCTTGGCAGGATCCGCTGTTCTCGGAACGGCCGCCATCAACTCGCCGTTGCTGATG
GGTGCCGCTCTCGGATATGCCTCGACGCACGTCCTCAGCGGAAAGCGTGGAGAAGACCTC
GCCCGCGTAAGCAAGGATGCCTTCCTAAGCGCCGTCACATTTACGCAAACACAGCTCGAT
CAGGAAGAGGGAGACATCTCCAAGGCATCCGCCCGCATCATGCAGCACCTACAGCACGAG
GCCCAAAACAAGGCCATGGAAGTCGGAAAAGTTGCTCAGACACAGGCCCACCAAGTTACG
AGAGACATCACCAACGCCCCCACGCACTTAGTCGAGGAAGGAAAGAAGATTATCGCAAGC
GAGGATTTCAAGAAAATGCCCAACCGAACTTTCAAGGCTGTCCAGGCCTTTTTGGGCTCC
GACGAAGTGAAGCGGGCCAAGGAGAGTGTCGTAAAATCGATCAAGGACGGTTTGGAATCG
GACGAGCTGAAGGCCGTCAAAGACCGCGCGACCAGATCACTCAAGGAAATCTAATCAGGT
TATGATCGGTCGCTACAAACGAGAACACAGCAACATCGAGAACAATGAAAATTTCATTGA
AAGTGTTTCAAACTACCACAAGCGCTTTCTTTTTTCGCAAGAGAATTAGCAGCTCCTAGT
ATCTAAATTCAAGCATTCGTTTAGAATCCCAACATTGACTTTCTTAAATTTTTACTAGAA
AAGTACATAGTATTTCCGAAA
>comp2104_c0_seq2
CGAAAAAGAACTTTTTGAGATCTCAGATCAAGATACCAATGAACGGTACACAATAATTCC
GGGCAATCCCAACGAAAAAAGACTTGACTTCGCATTGAAAAAGCATATGCAACCATGAAA
TTCTCCGCCATCTCTCTCCACACCTGCTTGGCAGCCACCGCTCTTTTTGCATCAAACTGT

AACGCCTACACCCCGGGAGTCCCAAAAGTAACTCCGGTAACACAGAGCAATGCCCTGGAC
AACAATGCCGATATTGCCATGTCGCAATACGAGGCTGCACTCAAGGCTGCTTTCGAACAA
GCTTCTTCCACTGCGGCCCCAGATGCACCCGAGGCAGAATCCTCTACTGCTTCGGCTACC
TTCGCCGAGCAAGTTGAGGAACAGGAACAGGCCATCGATCAATACGAGGCAGCCCTAAAG
GCTGCTCTCGAATCGACATCGGCAGCTGCGGGAGCTCCATCTTCGATCTCTTTTGGCGAG
GCCGAGCCCGTCGCGAATCAAGACGATCAAAACGGACCCAACTGGGAGAGTATTTCCGAA
AACATATCATCCCAAATTGAAAAGGCTGAATCAAAGATCGGCGCACCGTTGTCAGCCGCT
GCGAAGGGAGAAATTGTCGGAACCGTCTTGGCAGGATCCGCTGTTCTCGGAACGGCCGCC
ATCAACTCGCCGTTGCTGATGGGTGCCGCTCTCGGATATGCCTCGACGCACGTCCTCAGC
GGAAAGCGTGGAGAAGACCTCGCCCGCGTAAGCAAGGATGCCTTCCTAAGCGCCGTCACA
TTTACGCAAACACAGCTCGATCAGGAAGAGGGAGACATCTCCAAGGCATCCGCCCGCATC
ATGCAGCACCTACAGCACGAGGCCCAAAACAAGGCCATGGAAGTCGGAAAAGTTGCTCAG
ACACAGGCCCACCAAGTTACGAGAGACATCACCAACGCCCCCACGCACTTAGTCGAGGAA
GGAAAGAAGATTATCGCAAGCGAGGATTTCAAGAAAATGCCCAACCGAACTTTCAAGGCT
GTCCAGGCCTTTTTGGGCTCCGACGAAGTGAAGCGGGCCAAGGAGAGTGTCGTAAAATCG
ATCAAGGACGGTTTGGAATCGGACGAGCTGAAGGCCGTCAAAGACCGCGCGACCAGATCA
CTCAAGGAAATCTAATCAGGTTATGATCGGTCGCTACAAACGAGAACACAGCAACATCGA
GAACAATGAAAATTTCATTGAAAGTGTTTCAAACTACCACAAGCGCTTTCTTTTTTCGCA
AGAGAATTAGCAGCTCCTAGTATCTAAATTCAAGCATTCGTTTAGAATCCCAACATTGAC
TTTCTTAAATTTTTACTAGAAAAGTACATAGTATTTCCGAAA
>comp2105_c0_seq1
AGAGCTTCACTTGCCATGAATTTCGTAATACTTTCAAGAGAAACAAGGCAGTAAATGATT
CTGCATTCAACGATCTCTGAGAACAGTTGAAGCGTCACTGTGGTACTGTATAAGTACGAG
GGAATCCTATGGCAAGAAAGTTATTCGCACCACTAGTATCAATATCACCACCTGTACTCT
TATGCAGGTCTGGAATTTCATTGGGAACATCCAACAATATTGCTCCTTCATCTAAATCTA
CGACACGCTTACTGTTGAACATACCAAAGTTATCACACCTGTCACTGACCACTACCGCTA
GGACGACGACGCAAAGCAGAAGAGTCTTGCCTAGAACAAATCACTTCTACACTGCGAAGA
AATGGTTTGGAAATATTCATTCAATCAGTGGAGAACCGATAATTAAGTCCACTGACTACA
AATCCAGCAGAGTGGAACAGGCATTTGATGACGGATCGTTGGAAACGACAAGGGCCCCTA
GCTTGAAAACAAATTCGATACCAATGATTCGAAAGGCGGGGAGTCAAAGATTACGGAACA
AAGACTGCTTTGCAGCGTCAGCAAGTCAGCAATTGGGCGAGATGTCTCCCAACATAAAGA
CCAAAGATGAAAATAGATATTCTGTCGTAATAGACAACGGTTTTATGAAAATATCTATGA
TACCTACTTTGACTCTGTCTGATGGGAAGACGAAAAGGATGGATGGGGATTTTTGCAATA
ATTGTGATTCTCCTAATAATTTTGAGAAAGTAATGCAGTCTCCTTCAAATAGAGATGAAA
ATGAATTCAAGAAGCAAAAGATTGAAATACCAGAGACAGTATTGCAATATCAACGGCAAC
TAAGAAAAACTCAAGCAGCGAAACGAGCATCTACACCACTACTTAAGTCTCAACTGCAAG
TCGTATATAACGACAAACACATTGTTGTTGTAAACAAACCGTCAGGAGTGCTTACTGTTC
CAGGAATCAATTCAAATCCAAGTATGTTGACACTACTTCACAAGAAATACGAGCACGAAA
TGGATCCACAAATGAAACCAGAACACATGATTGTCCATCGACTGGATATGGATACATCGG
GGATCGTAATGTATGCAAAAACGAAAAAAGCGTTGTTGAGCTTACAAGCCTTATTTCGTG
AACGAGATGGAGTAATGAAATATTATGAAGCTGTGGTATGTGGGCATTTAAATCCAGATG
TAGAGAGGGGGAGCATTGATCTTCCTCTACAACGAGACCATCGGTTTCCACCATTTATGC
GTGTTGCAACGCCGAAGAGTGAACATGAGGCCCAACAGGTTGTTAAGGATCTAAAAAACG
CTGGTTGGAAGAAAATTGTGAAGAAAAAGCCAAAACCCAGTAAAACATTATTCGAGGTCA
TCGAAAGAGAGTACGTATATTGTGATGGATATGCAGAAGTTGATGAAAACCTGTGTGGGG
CAAAAGAAAAGCAACGAGAAGTAAAGCGTTACCCAGTGACGAGACTAAAACTCACTCCAA
TAACAGGGAGAACACACCAACTTCGCGTTCACTGTGCATCGATTGGGCACCCAATACTTG
GCGATCCAGCCTACGGAATCTACGGCGAAGCTTCAGCTAATGGAGGATTTGAGGAGGATT
TGATGGATGAATTAATACGGGATAGGGCATCGATTAACTTGCAGTTATCTCTCAACGAAT
ACGCGAAGGTCAAAGGCCAAGTAATGTGTTTACATGCACGTGAATTGCATGTGAAACACC
CAGAAACGAGAGAATCGCTTGTCTTCAAACACCCACCTACATTTTGAACTTCCTCAGAAA
GCGTATCAAATAGACTACGGTTCAAGACAGTAACACTCTTAGATTATTCCTTTAGCGGAC
TTCATTGGCTGGCCCACAAACCCAAAAGAAAAAAGAAGCTTAGCACAGATCGCGCGAACT
CTCAATCCTCAGTGGCCGGCTGTATATAACGTTGAGCCTTGTTCAGATATGTTCTTGAAC
GTGCCGTGTAGATGCTATTACTACTACTACTACTGAAATCCTTTCTCTCGATTTGAAGGT
CCAAGGGGCT
>comp2106_c0_seq1
ACCCGCTCCTGTACCCGCTCCTGTGCCCCCGCAAGATGTGCCTACTCCCGTGACGTTTTC
TCCAACGGAAGCAACTACCTCGAGCAGCTGCGAGGACGACAGCACGTTTCGGTTCAAGAA
CAAAAAGAATAAGATGTGCGGCAGCAAAAGATGGTTGCAAAAATTCGCTGGCAACCTGAA

CAAAAAGAACACGCGGAAGCTCAAAAAACAGTGCAAGAAGAAGTCGGATGGAAAACGCGT
CTTTGACTTTTGCCGCGCTACCTGCGCCGTGGTTGGCCTGGGCCCGTGCTCCTAGATTTC
TGTTGTGTCGCAGGAATGCACGGTGCAACGGGTTGGCTTTGGTTTTCTTTGTGCCGAGCG
GTGGCGACCGGGAACGCTGGTGACGAAGGCAGTCGTCCCCCCCGGACGGGCAAATCCCTT
CGGTAGCGCTGCCATCTCAGAGAGAGAGAGAGAGAGGAAAAGCAGAAGAAGGCACGGTAC
GTACGTGTTTTGTTTCGCACACAATCGAAGATCGAATCATGTTTCGATACGACTAGCGCC
GCGCACTAGTTGCGGAAATGGTATGTAGTCTGTTTTTCAGTAGCATTATGTGTTAAAATA
TCTGTAACGGAGT
>comp2107_c0_seq1
GAAAGGAATTGATACGAGGACATACGTCCGAAGTACGCGTTCTTCACCTCCTCCGATCGG
CTTCAGGGCAGTGCCATATCGCATCGTCACGGTGTACAAAGAAATCAGCCTTCGGAGCAT
TGGTCACATCTCATACATCGATTCCTTCCAACCATTTCCGATGCGAAGAGGTTGGAAAGT
ACCGGCCCTATATCTGGCAACCACCTGCACTTCGAGTCACTTTGCTCTCGGATTTGTATC
GCACCAATTGTCGACGGCAGCGAAAGCGTCCGCCTGGAAACCCGCTTCGTCTCTGATTTA
CGGCAGCCCTAGCAGCGGCAGCACCTTCACCCGCAGGAACATGTCGTCCGAGGTTGTGCC
CGCCAAGGACTACGGACCGACGGACCAGTCCTGGAGATCCCTCCTCGAGGTGTCTATGGC
GCGATCGCGCAAGATCCGCGGATCGAACTATGTACAGATCTCGACCGCCACGAAAGACGG
CGAACCGCGGTGCCGGACGGTCGTCTTCCGCGGCTTTCTCAACAATAAGAACCTACCGGA
GGACCATCCGATGTCTGCCTCTGGTAATGTCGACGGAAAGCCGTGCGTCATGAAAATGTG
CACCGATCTTCGCTCGAACAAGGTCGCCGAGGTTGCCCACCAGCCGATTTCCGAAATGGT
ATGGTGGTTCCCAAAAACTAGCGAGCAATACCGAGTCCGGGGGTCGCTGTTGCTGGTTGG
GGACGATAGTGGGGACAGGGACCTACAGATTGCCCGGAAAGAACTCTGGGGGAACCTCAG
CGATCCGGCACGGGAATCCTTCCTAGACGACAAGCTTCCGGTCCCGGTCGGCGGGAGGGA
TGATGAAGGAAAGGTGGTCCCCGTGCCCGAGAACTTTTTGTTGATGCTCCTGGATCCGAT
TCACGTGGATTTTTTGCGGCTCACGGGAGCGCAATACCGGCAGATCGACGAGCGGCAATC
CGGACCAGATGGGTCGACATGGTCGGCCAAAAGCGTCAAGCCGTAACAGTACATAGCAGC
CAAGGAAGAAGGCTAAACGTTTACAGTACTATAGTAAAAATATTTGCTATCTAGG
>comp2108_c0_seq1
GAAAGCAATCTGGATACGAGTTCATCATCACACCGTTACGATAATCTAGACCAGCAAAGA
AGCAAACGCCAGTAGAATTTGCAATCATGAACGGGGAAACTTTTAATGTAGACCCGGATT
TTTCCGTTATTTTCGGGTGCAGATCGTCCCTTTCTCTGGTCGGACTGACGACAGCAGTGG
TCGGCTATTGGTTGATGGAGAGAAAATGGGACAACGAGGGAACAGGTGCACTGGAAAAGC
CAGACCAAAGAGTAGAGAACGAAGCCGGAAACGGAATTTACATCCACATGTACGATCCAA
ATAGGGTGGTGGCACAGGGTTTCCACCCCAGCGGAGAGCGAGATCTCGTTCGGGTTCGCA
CCGACGACAGCGAAGACAAGCGGTACCACCCGGGCCAAGCGGCGTCCAACAGCACAGAGC
CGATCCAATCTCTCGAGGCTGCGAAATACTACTCTTCTCCAGATATTTTATACGCCAAGC
TGGCACACGCTCTCCCCGTACCAAGAACGATGCTGGTCGGATTCGGCTTGTGGAGTCTGA
GTTTCTTTCTGGATCCATCCCTCGGAGGATTTCGGTTTTACGCCAACTTCTGGAACAGTT
GTTGCTTCATATTTTCCGGATTGCTGGGAGCAATCCTGGGTTTTCCGATGCGTACCGCTA
CACTTGAACGAGATGCCGAACAAAAGAAGAAAGCAATGGCAGCGTTCGTCGCATGTTCGG
TATTGCTTTGGATCGTGGCCATCGTTGACCCGGAGGTCGATGGACCTTGGTATTTTAACG
TTTTTGGAGTTTTGTTTATTCTTTTGTCCTATCCGGCATTCATGGCATCCCGCAAGATGG
GATATACGTGGTTTACCGAAGGGAAACCGAGAAGCACGGCTAGCATCACGATTCAAAACA
TGGGACCGTTGCTCTTGATTTTTGGTGTGTTTCTTCTTTGGGTGGGGACAAACGCAATTT
ACATGGTCGACTTGAACCAATCCTACATACCGTTTTGGACCACGGCAGTCCGCGGCTGGG
TCGTCTTCATCGCCGGCATGTTCCTCATTGTTCCTGCTCACATGGCACTGGATCGCGCAT
TTGATCAGGGCAGCCAGCCCGTCGTCCCGGGTTTCAGAGATACGTATGTGTACAAAATGG
ATGGAACTACTTTCGCGGCCCTGGCTCGGGAAAACATCACTCGTTTCGATCTTGCCTGGA
TGGCTAGGCTGCTCGAGACACCGCTGCTGGGATCCATTGGGTGGATTTTGATGGGGCTGT
GCAGCTTCATGCCATTTGGCATCATGGACCCGACGATCCAGAAATTCTTTACCATGTTTC
TCTGTTTCGCAGTACCACCCGTTCAGTTCGTGTTGCTAACTCCCGCCTTTTGGAGATCGG
ACAGGGCAGACTTCTTAAAGTGGATGCGTGTGTATTGCGCGCTGATGGTAGCATTGGTCG
TTGCCATTGGACATTCCAGCGGAATCGCCTTTCTATTTTCCTTCATCGGTATGGGGTTGA
TCTATGGGGGCAAGCGAAAAGATTTTCACGACGAGCGCAAACGAGGGAACCTTTGGTTGA
CCGAATCGCCGCCATCGGCGAATCCCAGCCCCCAGGTGTACGGGCTCGGCCAGCCGTTGC
ACGTTCTTGGATGGATTTTGTTCTGTACGGCCATGTCCGTACCGATGTAGGGTTTTCTCC
CGGAGCGTTCTCTTTTTTGTCCATTCGCAGTAGAAGTATTCGATAGGTACACGTATTTCA
ATGTATATTATCTCTTGACCTTAATACTAGTAGCACAA
>comp2108_c0_seq2
GAAAGCAATCTGGATACGAGTTCATCATCACACCGTTACGATAATCTAGACCAGCAAAGA

AGCAAACGCCAGTAGAATTTGCAATCATGAACGGGGAAACTTTTAATGTAGACCCGGATT
TTTCCGTTATTTTCGGGTGCAGATCGTCCCTTTCTCTGGTCGGACTGACGACAGCAGTGG
TCGGCTATTGGTTGATGGAGAGAAAATGGGACAACGAGGGAACAGGTGCACTGGAAAAGC
CAGACCAAAGAGTAGAGAACGAAGCCGGAAACGGAATTTACATCCACATGTACGATCCAA
ATAGGGTGGTGGCACAGGGTTTCCACCCCAGCGGAGAGCGAGATCTCGTTCGGGTTCGCA
CCGACGACAGCGAAGACAAGCGGTACCACCCGGGCCAAGCGGCGTCCAACAGCACAGAGC
CGATCCAATCTCTCGAGGCTGCGAAATACTACTCTTCTCCAGATATTTTATACGCCAAGC
TGGCACACGCTCTCCCCGTACCAAGAACGATGCTGGTCGGATTCGGCTTGTGGAGTCTGA
GTTTCTTTCTGGATCCATCCCTCGGAGGATTTCGGTTTTACGCCAACTTCTGGAACAGTT
GTTGCTTCATATTTTCCGGATTGCTGGGAGCAATCCTGGGTTTTCCGATGCGTACCGCTA
CACTTGAACGAGATGCCGAACAAAAGAAGAAAGCAATGGCAGCGTTCGTCGCATGTTCGG
TATTGCTTTGGATCGTGGCCATCGTTGACCCGGAGGTCGATGGACCTTGGTATTTTAACG
TTTTTGGAGGTAAGTGTTATTAGGCTACGAATGATGCTTGTTGCAGATTATGCTTTATCT
TGTGACATACGCTGGTTGTTGATAGCTGACAAGTAACCAACGAATCTTTGTTTTTTCTTT
AAAACCTAGTT
>comp2109_c0_seq1
ACGATCTGTCCAGACTGGTGCGAGTACTCAATAGTTGCCTCCCCTTTTCTCTTGCTGCAA
GATCGGAGAACATTTATTTTGGCAAACTGAATCGAATGTCACCGACGGAAGAGCAAAAGA
TGAAAATATGATATCGGAAATTGCGAATTCGTTGTTTTTGACTTACTTGGCGTCGGAGTA
CATCCTTTCCTCGCCATCCGCTGCTTCGTCGTCCTCAAAATCACTATCGACATCAAGAAC
ACTCAAAAGTGCAAATGCTAATAGTTACGAAAACCATTGCAAGAAAGCCTCACTCGAAAA
GGATAATTGTGTTGTCCTCGGATTTTTGCTTTCCATGGTGGGCATTGCGAACCTTTTGGA
TCATCATTACTACCTATCCGTGGTTTGGGAATCCGCAACAGAATTGTTTCCGAACATTCC
TTCCTACTACATTCAGATACCAGCCAAGCTATTGACCGCAGCAACCTCTTACGTACTAGC
GCTCTATGTTGATATTCATCGAATGAGAAAGAGCGACAATAGCAGACGGAGCAAGCACAG
GATCAAGAAAATACCCTTTCGAACGGTGTTCCTGCCCAGGGTTGGATGGGCCTTTTTGAA
AATCTTGCCCGCATATCCATTTCTGGCCGTCCTGATCTCCTTTGGCTTTTTGTTCGTTGT
AGATCTGTGGGACTTTGTTTTTCATTTGCCCCTGGACTGGTTGAACAATCCAATTTACTA
TGGAACCCTTTATGGTCCATTTGCCTACACTTACGTCTACGTCAAGCGGGAAATCGTCCA
TCGCAACAACGATGAGTGCACGGGCACCGATGGCGGGGAAAGCACGACCGAAAGTTCTTT
TGTGTAATAATCATTCCACCAACACCAAGACATCGTCGCATATGTATTTGGAACAATCCT
TGCAACTTGGCCAATATAATCCTTCTGCTACTAGTAAACAAATGCAACGACTAGTATGAA
TGCTGCACGATCCACCATAAATGAGGAAAAATCGCGTTGCATTTCCATGAAAGAAGAGTC
GCAAACGCGAAGATACATCGAATCGTATCAAGAAAAACTCAGAAGACCAACTGCACACGA
AAAACATCATTACTATTCAGTGTCTGGCAATAGAGAATGGAGTCGTCAACGTTTGGAAGG
AACACGATTTATTTTACCTTTTATTTTTCCGGCTATGCATATCGATGCATATCGAATAGC
CCGACCTTGATTCGATATACCATCGTTCAATTCCTCTATATCCTGAAGCTACTATTTATT
GTTGGTCACGCATGCAACTATGCGATTGCTTGAGTGGCAGCTGTGATAGGAACGAAACAT
CACAAAAACACACCGCTAAAAAAACGATGGGATGCATTGCGTCGCCTCGAGTAAGTCGTT
TGAAATGGATACAGAAATGACGAAATGTGTTTAATTTATTTGATTTGATAAAAATATACT
ACTGAAAC
>comp2110_c0_seq1
AATACTGTAACGATTGAACAATCCAACAACTAGCATTCCGTACCAAGAACATCCCAACAA
ACTTTTGAAAAATATATCGACCATGCCGTCAAAGAGCAGAGAAAACGCCCGCTCTACCGA
CGGTAGCAGCGATGTCGACAGAGAAAGGACGGGGGACCAGAGCAGCACGATCACGAGCCT
GCTGATCGTGATTCTCTCCCTCGTGTTTGTCGTGATTTTCGGTGGATCCCTCATCGGCGG
CATACTCAGCGGGGATCCCAGCAACGTCTTCTTCATCACGATCGTCGCGGCCTCGGCCTT
TGCCATTTCCGTTGCGCTGGGCGTCTCGGTTCGATACGTTCTCACGCAGCGCTCGAAAAG
ACAGCGGGCTGAACACGGCGACGACTATTTCGCCAAGGACAACCACGTGAGAGAAACCTT
CTCGGGCAGCAGCGACGAGGAGAACCACCTGTGCGCGAACAACCGACCCTCCGACTACGA
CCCGCGGGACGGCCACTATCCCCCCATCGAGTCCGAAATCAAGGAGATCGAGGCGAAGAG
CGTGTTTGGTGAGATGAGCGCACTGAGCCCCACGACCTACGACGGAGGCTCTCTCTCGAC
GTACCGCCACCACATCATCCGAGACAACCACCATCGGGGGTTCGACTTCTCCAGCATCAC
CTCGGCCAAACACGGAGACAACGCTAGCGGGCGCCAGGACCCTCCGGAGGGCACTGGTCT
GGCAACCATCCAGGCAGCCTGGAACACACGAGCCGTCTCTCGGGAGCCATCGGCTCCGAA
GTTTTCGACCGAGGGAGAAACCATTGTGGACAACGACTATTCCAAGGACGCCAACGACGA
CTGCGACGAGAAATCCAGTCGGTCGCGAAAGAGTGCAAGATCTCGGGGCGATCTCGAAGA
GAAATCGGTCAGAACGGAACGATCCAAATACAGCCAGCGCACCAAGAGTAGCAGCAAGTC
TCGTTCCAGGTCCAAAAGCCCATCGAATCTTCGCAACGGAAAGAGCCCCGCAAACTCCGA
AGCGAACTCATTCTTTAGCAGTGGCCGAAAAGAAAAGGAGGCTACAGTACATCCTGTGCC

TCCAATGAGCACAAGGCCTGGAGCGACGAGTTCAGTAAAACCTTCTGCAGTACCCTCAAC
GCCTGCAGCAAGTGAGGCCGGTAGCTTCGCCAGTAGCTTTATGGGATTGTTTGCAAAGGG
TAAAAAAACTGCTTCGGAAGCAGGTTCAACTAGTATGAGTAGCTCTGACAAGTCGAAGAC
GGAGCCAAAAGAAAAAGATCCTGATGGAAAAAAGAAAGATCGTAAACCACCGATGGTACG
ACCACCTATGATGCCTTCGAACAAAAAAGGAGGTGATGGCCTGCAAAGTGTTGGTCTCAC
TGGACCATCTGTTTCGACACCGTCAAACAACAGCACTTTTACCATTCCCCCACCACGAGT
GGTACCACGAACGCCAGCTGGTGGCGATGGTTTCTCGGAATTTCAATCAGTGGTTGAGAG
CACTTACAGCAATGGAGTTGCATCCTCAACCTTCGATCCCCAAGCTCGAGAAAGGGCATT
GCAAAAACTTCGAAGAAATCAAGCTGCGATAGGAGCAGCTCCAATAGGGGCTGCTCCGTT
AGGGGCAACTCCTGTAGATGCTGGTGACATGCCGATATACTACACTGATGACGATGATAA
GGCGTCTTCTGTGGGGGGTAACTCAGCCGCTCAATTAAATTCCTATGACGTCTTCGCACC
ACCGGGTGCTCTTGGCATCGTTGTAGATACAACTGATAAGGGTTGTATCGTGTATTCATT
GAAGAAATCCTCCCCGATGTTAGGGCTAATGCATAAGGGACATCTGATCATCGGCTTGGA
CAATTTTGACGTAAGAAATATGACTGCATCGTCTTTGACGAAGCTGATGGCCAAAAAGTC
GGATCAACAAGAGCGCAAATTCACGCTGGTACCCAACAATTAAATACGATTTAACTCGTC
CGATGCCAAGCCGACAATGGTATGCACGATATTTTACCGGTTCTGCATCAAGACTGAGAA
GTACAGAAATACTAATTCCAGA
>comp2111_c0_seq1
CAAACTTTTGCAAGGAAGTACCATTTACGTCTATTCCAATCATTGACGAGATCTGGCTGC
TGCCTCCTACCCTAGAACGATGTTACATTTGATGGATTGATTTGCAATCCATTCATAATT
GATTGGTTGACTGGTCGATTCGTTGCTTTGTTTCCTTCCTTCCTTCCTTCCTTCCAATCG
GTTCTTGAGGGAAAAGTCAGTGGCTAGTACACATGCCTATCATCGGCTTGCACGTGGCTA
AAAATAACTGACGAACTCGGTAGAAGCAAACCGACGACGATGACGGAGGAATCGGGCATC
TCAAGCTCGGTCAAGAAGCGATGCCTTGGTTTCCCTGCGATTTTTGGTGTTTCGGCGGTC
TTCTTGTTCCTTTGGCGAGGCACCACGGTTGCGTTATGTGATGCCTTCTTCTTTTCTCTG
CCGGTAGCGACACGGGAATTCGGTGCTCATCCATGCAACATCAATTTCGATTCCGAACAT
GGAAGATTGAGGATTCAAAGGGTGAAGGAACCAACCAAAAAGCACGATCAAAATACTTGC
TCAGGTAGGATCTATTCGTGTCATTCTTCTATAACCAGTCACCATAACGAGGGCGGAGAT
GACGAGGAGGAGGATAAAAACGAGGACAACATCAATACCAGTAGGGATTATTCGTTCCTT
ATGGTTCGTTCGAGCAGTAGACGGAAAATGTTGCGATCAACGCCTGGTACAATAGCAGCT
GCGGTATTTGGAACCACATCTCTGGCTGGAGTACCCTCGATCACGTTTGGGGAAGAGAGT
GATTCCTCGGAGAATATTTCCACAACTTACACCTCACAAAATGCCGACAGCAGCATTCGC
GATCGCAACAAAATCAACGACATTGCCACCGGAAGGAAAAACGAATCGTCGCTTCACAGA
GTGCTCGAATCATCAAGCGTTTACAGGGTCAAAACCGCTCCTACCGTACGACTCACAGGT
CGTCGGACCCCTGTAGTGCCGGCGTTAGATGCGAAACCGCCATCAGTTCACCGGATGGCG
AGCCAGAATCTCCTCCGGGATCTACAATCGAAACGAGCCTTGGTGCTGGGAGAACATCAC
CCCGACCCTAGGGATCATCTTCTCCAGGCTGCTCTGGTCCGGGACCTACACAAGGCTCGA
CGCCACAGCGGCAGCGGAAGCAGTGAAATGGCTGTGGGGCTTGAGGCAGTCCAGCAGCAG
TTTCAGCCGTTCCTGGACGCATACGTGGAAGGGATCCTGGACAATGACGAACTCAAGGTG
GCCACCGAGTGGGAATCACGATGGTATTGGTCCTTTGATGCCTACTTACCCGTGTTGAGG
ACCTGTCGGGACCTAGGAATACCACTGTTGGCGCTCGATTTGTCGTCAGAGGACCGCACT
ACCGTCGAGCGAGGGGGGCTTGAATCCCTCTCGCTAGATCGCTTGAACAATTACGTTCCT
GACCCCGACGCTTTCTGTTCGTTCGGAAGCACCGCCGCATATCGAGCCTACCTCGATTAC
ACCCTAAGACCACCCTACGAGCTCCTACAACGAATGGCCAAGCAAGGACCGGCATCAAGC
AATAGTAGCGGGATTCCCTTTTCAACCTTTGTCGAAAATGCTGAGAGACGAGGCGATGGC
CTCGGCCTCCACACGGTGGCTCCGCCACCACCCCGACGGATTGCTGGTGGCATGTGTGGG
GACCAATCACGCCTCATTTGGCTGTGGTGTCCCTGGTCGGATTGCACGAACGCTGGCCTC
GAACAGTGGGGAAAAGCGAGGCCGAGCAGCGGTGGCCTCGGTTCTGATCAATCCCGAGCC
GCTCAACACGGGGACCGAGCTCAGGATTTGCAACAAGGACGGAACGCTCGCTACAAACGA
CTACGATGACGAAGAAAGCCCAACCAGAATTGGGTTTCTACCAAACTCTGGGAACCAAGT
ATGTATCGAGAACAGCACAGATCTGCAAAACTATGCCCTTCAGTTCCAATTTGCATCATT
TTCGTTGTCCCCTAGCCAACAACAAGGAATTTTGCAAGAGGCCACCCTGGCCCAGCAGAC
CGCCGAGGGAGAAAATGTGTTGGGACTTGCGGATTATTTGGTGTTCTCGCCGAAGGAAAA
GACGTAATAGTGTGATTCATTCCTTCAGCCTAAGCAGAGTAACTGGCATCCGCAGGAGTC
ATTCGAGAAGAAATCGAAAGCAAAGTGACTCTCATTTGAACGAGACGAGAATAGCAACAA
CTGGCGACAACGAGATACAATGCAACATGAATCTATTACAGTTACAGGCGTACACGATCG
ACGACAGAATTGCTCGCAATTGCGCTAAGACTCCCTTCGAGACGAATAAATCAAAATTGG
AGGACAGGTCTCTACTTCCTGATTACCTCAAGTTGTACTAGAATCCGTTCTGTGCAGGGA
AATACTTCGGAGAGTAATACTAGGCACAGTAGTACTAAGAGAGGGATATGACTAATCATC
AATTTGTTGCCAAGTACATCCTCTGTCCGACAGTGACCGTCCCGTGACTTTTTTTTGCCA

TCCAATATAATGGGAACCAACATTTCGTCTTTATTTTCCAAGTTTCCACCCACCATTCCC
CACAGTGGAGTTTGCAGATGAGGGAGTCTGCCATTAGTTCAGGAACTCGGCATGGTGAAG
TGGGGTTAATTTGATTGGGGAATGATAAATTTTCTTCTTTGATTAATGAAACATTGGTTA
AAAGAGACTGCAATGCGTGTCTAAAGGACCTTACATAGATTACTTTGCCGGCAACTTTGT
TGCTTGAATGCCATTCAATAAGCTGTGGTACAAAAACATCACCACGGAGTTCAGGAGCTG
CGTTGTAGCATTCATCTAGAACAGATTGGAG
>comp2112_c0_seq1
GAATCTAGAGTAATAACATCGCAGCACATCGGGGCTGCAAGAAATATGTTTTTGGCGTTT
CATAGATCATCAGGATCACTTATCATCCGACGTCTGCTGATTTTTTTGCTCCTGTGCTGC
AACACAGCTATCGGTTTCTTAAAGGTCCCATATTCAAGATATCCCTCGTTTTGGAAAGCA
GCGCCATCGTCGTTGTTATTTCCGCAATCGAAAAAATTGATACCAGACAAATATTACTGC
AACGCCGGGCCTCACTCTGAGACTTTCATAAAAATGGCGGCATCAATCGCTGGAGAAAAT
TGCTCTTTCAAAGTGCAATCGACAAAGAATATAAAGGAAATCAAAGCAATCTTATTTGAT
ATGGACGGGACTCTTTTGGACACCGAAAGTCTAGCTGACAAAGCCATTTTAATGGCTTTA
TTTGGTTGCAGTGACAACGATGACGATAGTGACCAACCACGTTTGCCAGAATCGCTCCGG
TCGAAGCCCCCGACGTCGGAATGGCGGCTGCCCTGGGAATTAAAAAAGAAGATTCTTGGT
TTGCGTGGGAAAGAATGGGCACCGATATGCTTGGACTTTGCTAGGCAGCACTGCGAACGC
TCTCTCCCAACCGCTGAAGACTTAATCAATTTGTGGGAGCAAAATTTGAACGGCATGTGC
TCTGAGATCGAAGCTTGCGAAGGCGCGAAGGCGTTGGTCAAAAGTATCATCTCATCTTGC
AATTCTTCGAACTCGAAAATTCCTCTAGCTATCGCAACGTCAAGCCGAATGTCTGGCGTA
GATAAGAAACGGACCCGCCACGAAAAAGATCTGTTCGAATGCATCGATGAAATAGTCACG
GGGGATGATCCCGCCGTTAAAAATGGAAAACCAGCACCCGACATCTATCTAGAGGCTGCT
CGCCGTCTAGGTGTGGATCCAACAGAATGCTTGGTCTTCGAGGACGCGCTCTCCGGTGTG
AAATCGGGGAAGGCGGCAGGATGCCAGGTCGTTGCCGTTCCCGACCCACGCTTCTCCAAC
AAAGAACGAACTCGATTATTTCTCAACGAAGGCGGAGCCGATGTCGTGTTGACGAGTTTG
TGGGAATTTGACGGACGCGAGTTTGGAATGGATTTTGACATGAACGAAATTGCACAATCG
AGGGGACAACAATAACAGCTACTCCCAGCCAATGAGGAATGAATGTTAAGCTCAGCAAAT
AAATTCCAACAACTGGCCATCAACGCCTTTTCCATAGGGTAACATGTACTCGTACCATTG
TAGTAGTCGTTAGTGATGACAAAGCATACATTGCTTTCGTGAAAAGAATCACTGTCTAGG
GGTTACGACCTGTGATTGCCAGTTAAATTTCTTCAAATAGAGCGCAGCTCATATTGCCGT
CTCTCAGTCCAATGTTCAATCATGTCACCAACAGTTTTTCATAAGGAAACCAAAATTACC
TTGTGCTGTTTTCGGTTTCCGAAAATGGTAGGCGTGGTTGTATTGACGTATACGTGACGC
ATGGTTCGGTTTGATTGATCTAGTGTCATATTTAGATAATGATGTTAACGATGA
>comp2113_c0_seq1
CCTTTTCACATAACGAGCAACTATCTTCTACAAGGTGGCGACTTCGTTTCAATCAAAAGC
ACTAACAATCCTAACACAAAATCGATAAGAATGATTGAAAGGATAGAACGGCATTGCAGC
ATTGGTAGAGTATTATAAAACGCAAGGAGACAACGAAAAGAAGTTCATCAAGAAAAGTAT
TGGAATACAACAAACAACGGCAACGAAGCAGAAATCCCAAGTTCGAATTCCAGATTCACG
AATCTCAAATACCGCGGCATTTCATCAACAATTTCTCCGGTTATTTTCTGATTACCAAAA
CACAACAGCACTAACACCATGAAGTTCATGGAGAACGAGAAACTGAATCAAATTTCTTCC
GAGCTCTCGGAGGCGGTTCTCAAAGATTCGCACCGGATGATACAGGGCAGAATCGAAGCG
TACACAATGAAAAGAGCCGGCAACGACAAGAAATTTGCGCACGCACTGGGGCAGAAATAT
ATTGCCGAGATCGAAGACCTTCAGGAAGAACTGGCAGCGACCGTCGAGCGGAAACGAAGG
CGACGCAGCAATTCGGAATCTTTCCTCTTTACGACGACCAAAAAGTCGAAGCTCTCCCTG
CCCTCAACGCCGGAATCCACGAAGGCTCCCAGCAAGCGGGAACGCGCAAAGTCCTTTGAC
TGCACGCTGGATTCTACATCTAAGACGACACTCGGAGACTTTTCGGAGATTTCCACTCGA
AAACTCATGACGGATCTCATCCTGACGCTGAATGCATCCTTTCCGGACTACGACTTTTCG
ACTGTCAAGCCGAATCAATTCCAAAAAGTCAAGATGGCCGACGTCCGCAAGAGCATCCAC
GAGAATCTCTCAGAATTTGCGTCGCAGCGAAGCTCTCCGAATTTCTTGGAAGAACTCTGG
GATGCCCTGAACGACGTCATCGATTTGCAAGAGGCCAATGTGTACTCGTTCGATTACGAA
TTCGCAGACGACGACGAAAGCGGGAACAATTCCCTCTGGAATTTCCACTACCTTTTCGTC
AACAAGTCCGTCCGACGAATCGTCTTCTTTACTTGTTCAGAACGAATCAGTGATGACATG
ACGGGTGCCCAGGGAAGCGCCGACGAGGTCGACGACACCTCCGAACGGGTGGAATACTAT
GCCAGGGAGAATGTGTACACACCCGTGGACGACGTGGACTGGGATCCCGGAGACAACATT
GCCGGAGGAATGTCTGTGGAAGTCACGATTGGTGGGTAATACAACTTCAGAAGAAGAGGC
GAGGAGAGGAAGAGGATACATCGAGCAAAAGAAGACTCGGTCGGGGACGAGAAGGGACTA
CCAAAGTATCGAATGGGTTATTGCTTTACAGCAAAAGTCGATACGTGGATCCTTCCTTTG
TGGGTACTATGGAACGGAATTTTTACTCCATGTTGTATGCTTCGGATACATGGAATTTTA
GTATTTTTATATAAACTTTTATATAGATTCTGAGCGGGACAGCGCAAAACGTGGGGATAT
ACTAGGATTATGTTGTGTCCCTGTTGTCATGGCATTCGCCCTGCGGTTGTGCGGCATCGA

GTAGGCAAGCGATTTCATTACGGAGCGATGCAAGAGAGATTACAATGCGCATGCGACGAA
TGAATGAATTACATCCAACCGGATGGTGTTCTACGGAGGGCGTAACAAAATAAAATGCCT
AGATGATAATTCGATTTTATATTATTTTAGTATAATGGATTGATTCATTCAAAAAA
>comp2113_c0_seq2
CCTTTTCACATAACGAGCAACTATCTTCTACAAGGTGGCGACTTCGTTTCAATCAAAAGC
ACTAACAATCCTAACACAAAATCGATAAGAATGATTGAAAGGATAGAACGGCATTGCAGC
ATTGGTAGAGTATTATAAAACGCAAGGAGACAACGAAAAGAAGTTCATCAAGTAAGTAGC
GTAGGATGCAGTACTTTTTCTACGAGGTTGCTCCTGCAGCTCCAGAACAGTTCGGAATTG
TGCCTTTCGCTTCGATCAAAACACCGTTAAACAATCATTTTCTTCTTGCCTTTCCGGTTG
AACTTTGTTGCTGACATCGTTTGTATCAAACTTTCTCCTTTGATCATCAAATGCATTGCC
ACTATCAACACATATCCAAATTCGAAGGAAAAGTATTGGAATACAACAAACAACGGCAAC
GAAGCAGAAATCCCAAGTTCGAATTCCAGATTCACGAATCTCAAATACCGCGGCATTTCA
TCAACAATTTCTCCGGTTATTTTCTGATTACCAAAACACAACAGCACTAACACCATGAAG
TTCATGGAGAACGAGAAACTGAATCAAATTTCTTCCGAGCTCTCGGAGGCGGTTCTCAAA
GATTCGCACCGGATGATACAGGGCAGAATCGAAGCGTACACAATGAAAAGAGCCGGCAAC
GACAAGAAATTTGCGCACGCACTGGGGCAGAAATATATTGCCGAGATCGAAGACCTTCAG
GAAGAACTGGCAGCGACCGTCGAGCGGAAACGAAGGCGACGCAGCAATTCGGAATCTTTC
CTCTTTACGACGACCAAAAAGTCGAAGCTCTCCCTGCCCTCAACGCCGGAATCCACGAAG
GCTCCCAGCAAGCGGGAACGCGCAAAGTCCTTTGACTGCACGCTGGATTCTACATCTAAG
ACGACACTCGGAGACTTTTCGGAGATTTCCACTCGAAAACTCATGACGGATCTCATCCTG
ACGCTGAATGCATCCTTTCCGGACTACGACTTTTCGACTGTCAAGCCGAATCAATTCCAA
AAAGTCAAGATGGCCGACGTCCGCAAGAGCATCCACGAGAATCTCTCAGAATTTGCGTCG
CAGCGAAGCTCTCCGAATTTCTTGGAAGAACTCTGGGATGCCCTGAACGACGTCATCGAT
TTGCAAGAGGCCAATGTGTACTCGTTCGATTACGAATTCGCAGACGACGACGAAAGCGGG
AACAATTCCCTCTGGAATTTCCACTACCTTTTCGTCAACAAGTCCGTCCGACGAATCGTC
TTCTTTACTTGTTCAGAACGAATCAGTGATGACATGACGGGTGCCCAGGGAAGCGCCGAC
GAGGTCGACGACACCTCCGAACGGGTGGAATACTATGCCAGGGAGAATGTGTACACACCC
GTGGACGACGTGGACTGGGATCCCGGAGACAACATTGCCGGAGGAATGTCTGTGGAAGTC
ACGATTGGTGGGTAATACAACTTCAGAAGAAGAGGCGAGGAGAGGAAGAGGATACATCGA
GCAAAAGAAGACTCGGTCGGGGACGAGAAGGGACTACCAAAGTATCGAATGGGTTATTGC
TTTACAGCAAAAGTCGATACGTGGATCCTTCCTTTGTGGGTACTATGGAACGGAATTTTT
ACTCCATGTTGTATGCTTCGGATACATGGAATTTTAGTATTTTTATATAAACTTTTATAT
AGATTCTGAGCGGGACAGCGCAAAACGTGGGGATATACTAGGATTATGTTGTGTCCCTGT
TGTCATGGCATTCGCCCTGCGGTTGTGCGGCATCGAGTAGGCAAGCGATTTCATTACGGA
GCGATGCAAGAGAGATTACAATGCGCATGCGACGAATGAATGAATTACATCCAACCGGAT
GGTGTTCTACGGAGGGCGTAACAAAATAAAATGCCTAGATGATAATTCGATTTTATATTA
TTTTAGTATAATGGATTGATTCATTCAAAAAA
>comp2114_c0_seq1
CTGATTCTCTCCGGCAATGAAACTCCGAATCGCGTCGTTCCTTTCCGTGGCTGCCGCCAC
CCACGCCTTCGTCGTCGATACCAAAACCAGCCAGCGCCACAATGGACACGTGCTGTATTC
AACGACGGCAGCGACCCTCGGGTCTGCTCCGCCTACCGAAGCGGCAGAAACCTTCGGGAC
GGTGGTCGGAGACACGAGGGGCGCCGCCCTTCGACTGGAAAACGTGGCCATTTCACGGGG
AGCAACGCCGCTTCTCAACAACATCAATTGGAGCATCCAACCCAACGAGCGCTGGGGAAT
CGTTGGGATCAACGGCGCCGGAAAATCGACGCTTCTGGGAGCCATCACGGGAACGGTCCG
AATGGACGAGGGAAAGGCGCTCGTCCATTCCAACGTGAGGGTGGGATACCTGAGACAATC
CGCCGTGAGCGGATCGACCAAAACCGTGGCCGAGGAAGCCCGGAGTGAAATGACGCACAT
CGAGGAGGCCAAGATCGAGCTCGAGGCCGCCACCAAGGTCGTGGAGGACGGAGATTATTC
CGAAGAGGCGCTGGACAAGCTAGCAACCGCCCAGGAACACTTTGAAACGATCGGGGGCTA
TCAGCAGGAACAGATGGTCGACAGCGTCCTCAAGGGACTTGGATTCGAACCGGAGGACTC
GGATCGCTTGTGCAGCGATTTCAGCGGGGGTTGGCAGATGCGGATCGCCCTGGCCCGCTT
GCTTCTGTCCAAACCATCCTTGCTGCTCCTGGACGAACCTTCGAACCATCTGGATTCTTC
TGCTAAGGATTGGTTGGGAAAGTACATCGCCAGTTACGACGGAAGCGTCGTTTTGGTATC
GCACGATGTTTCTCTCTTGGATGTGAGCGTAAATTCCATTGCCGAAATCGCCGGAAACAC
GGTGGTCGAATACCGATCGTGTTCCTACAACAAGTATCTGGAGGAAAAGGAATTCCGCGC
CGCCAGTGCACAGGCCGAATACGAACGGAACCTAGAGGAGGCCGCCCGGCTCCAGGCTTT
TGTCGACAAGTTTGGGGCATCCGCTACCAAAGCCAAGAGCGCACAATCGAGAGTTAAAAT
GCTGGAGAAGATGAAAAAAGAGGGAAAGTTGGATCCGCCACCGGTAGCCGTTGTAGCCAG
GGAGCGAGATCCCGAACTCGTCTTGCCGCCTCCGCCGAAACCCAACGGCGAATACCTCAT
GAAGATGAAGGATGCCACGATCGGGTACGATCCAAGCGAGCCCCCGCTTCTTAAAAACAT
CAACCTAAAAATTCCGAGGGGAATGAAGCTCTTGCTTCGAGGGCCCAACGGAGCCGGAAA

ATCCACCCTTCTCAAGGCCCTTCGGGGGAACGTGCCGGAGATGATCCAATCGGGAGAACG
CGTCGAAAACGACCGACTCTTTCTAGGAACCTTCACCCAAGACCTGGCGCAGGAACTGGA
CCCGGATTCCCGCGCCGTTGATCTCGTCACGGACTACGCCCGTTCGGGGAGCGACGGAAA
CATCCGCGTGACGGACGAGGCCGCACGTGGCGTCCTCGGACGCCTTGGCTTGGGAGGAGA
AAAACCCCTCCGAAAGGTATCCGCCCTGAGCGGAGGAGAAAAGGCCCGTGTGGCCCTGGG
AATGTTCGCCCTGAAAGCCTCGAACCTGTTGATGCTGGACGAACCAAGCAATCATTTGGA
CGTTGGCTGCATCTCGGGCCTTGCCAAGGCACTGAGTGAATGGGGCGGCAAGGACGGAGC
CATTGTGGTGATCAGCCACGACCGCAAGTTTTGTGAAAAGGTCGGATTTACCCACGTTGG
AACTGTGATGGACGGAAAGCTTGTGTTGGAACAGCGATCCCTCCGAGACAGCGACTGGGA
CCAATACGATATCAACGCGGCTTCTAGGTCTCCAGCATAGTCAATGGAACGGAGCGAAGA
ATTTCGTAGTGGAGTAAAACTTTGTCACCACAAGAAGGAAGGCAACAATAAAATAGAGCA
AATTTTTGTTAGAAGGTATTTTTC
>comp2115_c0_seq1
TCGATACAGCATACGTGCAGAGTAACATACGGACTGTACGACACCGAGCACAGAGCAAAG
CTATCCATTCCCACAACCATAACTGTTGAATAGTTTGAAGCATCTGAACGTATATTATCT
GCATATTCAGGAACCAAGCCAAGCAAAGACCTTTATCGCAAAATGTCGCTAATCCAAGAA
ATCATGGCTCCTGGTGGTGGGGTTATGCTGATCCCGTTTGTTCGCGTCGTAATCAGCATA
TTGTTGGTGCTGACTATCTGTGGTTTTGTGGCGGATGTCGCGAGAATTCACATGGTGGTT
CTGGGATGTCTGAGTGGAGGCTTGCTCTTTTCCTTGTCCATGTTTGAGCGAGAATTTAAC
AAGGTTAAGAATCGAGGGCCACCATCAACGACTACTACCAAATTGGCGAATTCCAATAAG
ACAGATTAAGAAATCAGTAGTCAATCGACGAAGAGCGTACGAGTGCTACCGCGACAATCA
CCATTGGACCGGTAAATCAGTCGCCAGATTCAGTCATATTTGGTCCTTAATAGCAACATA
ATGGCACCGTCCGATGTCAGAGGAAAGTACGGAATGGAAGATCAGTATATCACAACATGT
GTGAGCATGTTGAGTAGGAGCAGTCGAAGGAATTTGAATTCGTTGACAGGGAAGGCAGTC
TCTACAGAATCATTCACACAGTCTAGATTATTCGAAGACGAGTACTGCAAAAAATTATGA
CAAAAATTATTATGTACAATTTAGGCACTTAATTTCAATTTTAGTCTTTTTAACGAAG
>comp2116_c0_seq1
CGATCTCTGGATGTGAATATCATCTTCACGGCAGTACCGGAAATTTTCTCCGCCAAGTTC
GAGTACATTTGGACATCGTTTCATCGTGTCGCTGGATTAGACCACAATTCATAGAGACAA
CTCATGTGGCTAACTCACGCAGGATTGGGGGTCGCGATCGCTGTTGCAGCGTTCGCCGTT
TGGACAAGAGGGTCGTCGTCGCCTTTGTCGCTGCCCTGGCAGGGGGGATTTGTCACCGTT
ATCGATCCGTGTCCATCGTTCGAAACCGAGCGAGTGGACCTTCCGTCTCCAGCCGACGAA
AACGATCCTCTTCTGGTAGATGCGGAGCACGTCCTGCTCGGCTTGGAGGCACCCGAAGAC
GGATTCCGCCTGGACGGCACCGGCAACGGCGACGAGTGGATTTGGAGCATGGGGAACGGC
GATCTTCTCCGAGTCAACCTTCGGACAATGGTGGCTCTGCCGTTTGCGCACACGGGGGAG
ATCCACGAAGAATGCGGTGCAATGCACATGGAATCCCGTTGCGGTCGCCCGCTGGGGCTG
CTCAAGCTGCCGCCGGAAGACTACGATCGTTACCAGCGATACTACGACTCGCCGGTTGCA
ACCAAGCAAGACAAGGGCAACGGTTCGGAACCGGCAGAAGCAGAAACCCCTCTCTGCCTG
GTGGCCGACGCATACAAAGGATTGATGCTGGTCTTTTCGCGGGGAACCATCGTCCCGTTG
CTGGAGGCCGTGGCCGGTCAGCCCCTGTTCTTTGCCAACGCCCTGGCCCGGGCCGGAGAT
GGGACCATTTTTCTGTCCGACAGCTCGTCCCGCTTTCGCCGGAGCGACGTGTTGCTCGAG
ACGCTGGAATCCCGCCCCACGGGACGGGTGGTGTCCTGGAATCCCGACACGGAAGTGAGC
CGGTTGGTAGCGAGCGATCTTCCCTTTCCGAACGGGCTTGTCCTGAGGGACAACGACAAG
AGCCTGCTGGTGGCCCTGACCACCCGGCACCAAATCATTCGCATCGACCTCTCGAACCAC
GGCGAGAGCGGGTGGGTCGCTCCGGAGCCGGTGTTGTTTGCTGCCCTGCCGGGAATTCCG
GACAACCTCCACGTTTTCCACAGCAACGAATGGAACCGATCGGTTCTTTGGGTGGGAACC
TCCACGAAATCGTCGCCGGTCGTTCGGTTCTTGAATCGTTGGCCACAGATTCGGAAGCTC
GTCGCGACTCTGCCTAGGGGAATGCTGCTCAAGTGCTTCAAGAAGTACGGGTTGCTGCTG
GCCCTAGACCCCGATACCGGGGAGCTTTTGCGCGCCTACCAAGACCCCACCGGTACAACG
GGTTATATTGCAGGAATCCATTTCGACGACTCGTACGCCTACCTCGGCAGCTGGAAAAAT
CGGTTCCTGGCCAGAATTCCCAGAGCCAAGTTGTTCTCGAGGTTGCCGGTGTCGGACTGA
CGCACGGATGGATGGATGCGTACGTATCTAGCAACAGTTACCACAGAACTGCGTTCAATG
ATTGGAAATCATGGCATCCAGAACATTGGGATGCGTTGCAACCGAACTCGACCACAAAGT
GCCAAAGCGCAAGATCTGGCATCATGCAATGCCTTGTACGAGTTCCTGGTGCTCTTTGGA
TTCGAAAACTACCCCGGGACCGCGACTGGGACGACGACGCTTTGTGCACTGTTGCTTTTG
TTTGCTCTTTGGTGCACACTTGGTATGCCTGTTGCGCCGAGGGTCTTTCAACAAAATCAA
TGTATTACTGCAAACTATGCCCTGAGAACGAATGCAATCGTATTGTTTAGGGACGCACTA
CTTCATCGTTAGAGCGAACGATTGTAACCTTGATAACGCTAAAATAAATAACATATCATT
TTGGAT
>comp2117_c0_seq1

GACTCCAGATTTTCCCCAAGTTAAGTCGAAAAATAGTTGTGGTGATAATAACCGGTCAGA
TCGTCTATCCAAACGTCGATAAATAGTCGAAAGGACCATCGGTACTCAGCAAACATGTAC
TTAACAAGATCAGAATACGATCGCGGTGTAAATACCTTTTCTCCCGAAGGTCGTCTCTTC
CAGGTTGAATACGCCATTGAGGCCATCAAATTGGGATCGACCGCTGTGGGGATTCAAACC
AAAGATGGATCCGTACTGGCTGTCGAAAAACGTTTGACATCTCCTCTACTAGATCCTGAC
AGTGTTGAAAAGATTGCAGAAATCGACAGCCATATTGGTGCCGCCATGAGTGGACTTGTC
GCCGATGCCAAGACTCTAGTAGATCACGCTCGAGTTGAGGCGCAAAACCACACCTTCACT
TACGACGAACCTATCGGTGTAGAGCCATTGACACAAGCAGTTTGCGATTTGGCCCTGAGT
TTTGGTGAAGGAAGTGATGGAGATTCTTCTAAACGAATGTCTAGACCCTTTGGTGTGGCT
CTTTTGTTGGCCGGATACGATGACTTAGAGGGATCCCAGTTGTACTTCTCGGACCCAAGC
GGAACCTTTGTCCGATACAAGGCAAAGGCAATTGGAGCTGGAAGCGAGGGAGCCCAATCC
AATCTTGAGGAGTCGTACTCTGACTCGATGACCTTAAAGGAAGCTGAAGAGCTTGCACTG
GGTACATTGAAACAAGTCATGGAAGAAAAGATATCCACAGACAATATCGAATTGGCACGG
GTCACGCCCGAGGGATTTCAGATTGTGAAGCACGAAGAAGTCGCAGCAATTCTAGAGCGG
TTGACTACGGGGTGATCTAGTTGATAAGGAAGCGCTTCTTGCGCCTCAAAATACAGTAAT
ATAAGAGAAATATACGATGCAGTCAAAGAAAGCATTGTTTTCTAGGCGAATATTAGAAGT
TAATCAGACGATTGCAACTTTGTGTTTCTATTCATAATTCCAACACACAAAATTTTTGCA
AGGAAATTTGCTTCGTAGAACACAGCGGTTTCTTTCAGACAAAACTTTGTCGAGAAGATT
GAATTTGATCCAGAATACAGATTTTTCGATTTCATCACCCTTGGGAAGATGTTTCTTTCT
CTTTTTTTTTCTTCTTCTTTTTCTTTTCACTATTTTTTTCGTCTTTCTTTTTCTTCGACT
TTTTCGACTTCTTCTCTCGACTCGGATGAGAGTATAGTTGTTGTTGCTGCTTTCGCACCA
TAGCTTCTGCCTCTTTTTGGGCGGCAGCAATTGCCGCCGCTGCCGCTTCGCTGATGCCGC
TAGAGGAAATAGGTTTCGCATATGGCGGCGGTTGCGACGGTGCTCGCCCATTGCGTTGTC
GTGCTTTTGTCGTAGAAGTAATCAATATAGATTGCGGGATTTTTTTATCCGTATTATTCC
TATCCATGGATACTGCGGTATCCGAGGATGACTTTTGAATGGAATCCTCTTGTCGATTAA
GGGTTCCGGTTAGAGGTG
>comp2118_c0_seq1
GCAATTTGCCGGTTGTGACCGGTTCGTTGACACATGGGAATACAAAATTGATCGACTAAA
CGCATCTCGAGACCAACACCAAAACAACCATGTCTTTCGAGAAGACCTCCCCAGGAACCA
CCGCTGCTTCTTGTTCTCCACCGAAGCCATTGCCCGAATACGTCAGCGACGAACTGGAAT
ATCTGCAGCCCTCCCAGGTCGACGAAGCCGCCCGCTTGCTGGCGGATTGCTTTCTGGACG
ACTCCAACCCTTCCCCGCAGTACAGCTGGATCTTCGAGGAATTGCTGCTCACGGAAGAGG
AGGAGGAACGCGCTCCCCGGGCAGCAGGCAATCACAAAGAAGACCCGCGGATTGGCAGCA
AGCGACGCTCGCGCCAGCGAGATGCCCTCCGTTGGATCTTCCGTAAAAATCTGGAAATCG
AGCTCGAGCACGACGCCGCACAGAACCGCACCACAGCGTGCCGGTGTTCCTTTCGTCGCA
AACCGAACCAGCCACCCGAGATGGTGTGTTTCTTCATCCTCAAGGACGTACAATCACCCA
CAACGAACAACAGCGGTAGCACCGACAAAAAAGACGGGGAAAGTGGTGATGGCACCAAGA
TATCGACATGGACGATGCTCCGGAATGGAATTCTTTGGTTTCCGATCCTATTTGGGTGGA
GGGTCTTTTACAGGTTGCTAGTGGTCAAGAATTATTTTGATACTGCAACCTTGGAGCTGT
TGTCCTCGAATCATGTAGTTCGTTCGTGCCGTGCTCTGGAACGAGTGGTGGTCCATCCCT
CGTTGCAGGGAAAGGGGGTAGGGACCTTTTTTTTGCGGAAGGCATTGTTCGGTGACGGGA
CGGACGATGGTTCCGCTGGCAATGGTAACAGCGGTGGCGGCAGCGTGATACTGCACACGC
AAGAAGAAAGGAACGTTGCGTTTTACTCGAGACTCGGTTTCGTCGTCGTGAAGAAAGATC
GACCATTCTCCGGTGGCACCAATTGGGCGATGGTGATGACAAGTTCTTGACCAGAAAGTC
GGCTTTGGGCATCGCCATTGGCTCCGGTACAACAAATAGGATTGCAACCACCGTCCGCTT
TTTCGTTTTGGTTCGTCGCATCGAGCGAGCGCACAACGCATATGCAACACGAATGCCTTC
CACACTGGGCCACTATTTCCCACGAGTGGCATTATTTTCTTTTGTACAGAAACGCAGCTT
TTTGTTTTGTGAGACGATCAATGAAGAGATGACAGCGCTCAGTCCCGACAGCATCTTATC
GTTCGGTGTTGTAACGGCTATTGAGCAGGGCTGACATTAGAAGCAAACTTCCACCAATAA
AATTAACTTCTTGCCAGATACCAGTGCCCAGAGTTTATTGGATCTTATTCCACGGTATCA
TTGGATAGGCAGTGATTCTTCTGTATTTTCAGATGCCACATCTTATTGCTTTGGTCAGGA
CTGTCCCAACTCATTTGGTGGCAAGTGTGAGCTACGACAAATTTTTGTGGAAGACAAGAC
TACGAGTAGTATCATTAGAATGTAGCAGTAACGCTAGATGAATTCTTTCC
>comp2119_c0_seq1
CATGGGCTCAGGAGCATCTTCAGCACTCACTCTTCAAAAGAACCATCGTCGGCGCATCAG
GTTCCAAACTAAATACGAAAACATCTTCGACATGCTAAAATCCACGATACAATCAAAACG
TTCTTCTACCGAAAAACTTTCCATGTTCATAGGCTCCACGACAATGCCTCGGAGGAGGAA
TCCTATCATTTTGTGGTACATGGCATTCCTTTTGGTGTTGTCGACCGCGAATTGTGTCGA
GAGTTTTTCAGTTTGTTTTGGCCTCGGAGCTCTTCTAACGCAACCAAAGGGATTGATCAC
CACGTCGACAAAAGGCGACATTCCAGCATTGTTGGAGGCGTCTGACTTTTTCGTCGATGC

CTTCTGGGTGGGCAAAGTAGGAGGAGGGGCCAATGAACTTAGCGAAAAACAACGAAGGAC
ACTTTCTGCTTCACAATTCTACGAGTTTCGATCGCGATATGCTGGTGCTGGACGCGGTCA
GTCAGAACTAGTTACATGTAGGTTGCCGTCGGGAGATTTGACCGGATGCGCTGGGATAGA
GGTTAGTCCAATACCGGACAAAGGTCTGAAAGGCCTGAATACAAAAAGTGGCCCGTTGAT
GTCGAATTTGGCGGTCGGTAGAGATTTTCGAAGAAAGGGAATTGCGGAAAAAATTAGTTAC
AGAAGTGGAACGCATCGTTCGTTACGAATGGGGTTACGATGACTGCTACCTCTATGTGGA
GGAACGGAACAAGCCTGCAGTACGACTATATCAAAAGCTTGGATACAAGAAGTTGTGGGT
CGACAAAGAGGCATCAACACTGCTTCCTTCATCTAATGGAAAGCTGACAAACTCTGCTAC
AAACATTGTATGCATGAAAAAGCGGTTGGATTTGGGAATAATTGGACGGCTATTACCCTT
CTAAGAACCAACTTAATAAAATGCCGTGGAAAACGATAATGATTGACGTGCTTCTGATTT
ATATCCATGCATCGAATAAGTCAAAACTATTTGAAAATATAGTTTAGATAATTGCTGTTT
CT
>comp2120_c0_seq1
TAAAAGTTTCGGTGCCTGTTCCACAAACCTTAGGTCCCATTGCCTCGAATCAAATCAAAT
CTATCGTGCGCCATTTCCATTCAAACACATTTTTTTTACTTTCTACACGATGGGTCGCTT
CTTCAAGAAATTTCGGCAGAGAATCGAAAGCCGCATCGATGCCGCTGTTGCCGAGGCTAC
TGGTGGCGGCGGATTTGGCATTGCCAACCAAACCGCTGCCCAGCTTTCCTTTGAGGAACA
GAAGCCCGATTCGATGCGACAGAAGGACGTTCGAATGATTTCGGGTTGCGAGGATTCTCA
AACCTCGGCGGATGTTTCCAACGTTGCACATTTTTCGCTACCCGATCCGGCAGGCCGTGC
GGGGGGTGCCTGTACCTCGACCCTCCTGAACATTTTGTACAAGGACGAAACCGTCCCGGA
AGACACCCTGAGTTTTACGCAGGTTCTCGAGCTCATGAGGTCCGATTTGCGCCGCGGGGG
CTATAGCCAGATCCCCCAACTGACCTCGCTCAACAAGATTGACGTCGAGACGGACTTTCA
GCTTGTGCCCAGCACCGCTACCGGAGTCCATCGGGCGGTCATGGTTGGAATCAATTATGT
CGGACACAGCCCTGGAGAGCTCCGAGGTTGTCACAACGATGTCTTGAATATGAAAAAGTA
CATCATGGAAGTACACGGGTTTGAAGAGGAAAACATTGTAATCCTGATGGACGATGGGAT
CCACACATCGCCGACTAAGGAGAACATGATTGCAGCTTACGAACAAATCGTTGCCGACAG
CGAAGAGGGCGATGCCATCTTTTTGCACTACTCGGGCCACGGAACTAAGCTTGTCGACCA
AAGTGGAGACGAAGACGATGGTTATGATGAAGCGCTAGTGCCCTTGGACTTCAGGGAAAG
TGGAATGATCCTGGACGACGATCTCTTTGATATTTTGATTGCGCGCCTCAGCGATGGGGT
TCACATGGTCTCGCTTATGGATTGCTGTCACTCTGGGACTATTTTAGATCTGCCCTACAT
TTTTAAGCCAATGCAAGACGGATCCATGCCCCAAGAGATGCAATTGGACGACTCAGTAAA
TTTAGATGGCATTCTCCAGCAATTTGGTGGCCAGGCATTGGGCATGTTGACTAATTTTTT
GAATTCAAAACTAGAGTAATCCGAGCGGCCATGTATTCAAAAAAAGGATGAAAAAATAAA
TTAATGCAATAAAACGGCGATAGAAACAATGTT
>comp2120_c0_seq2
TAAAAGTTTCGGTGCCTGTTCCACAAACCTTAGGTCCCATTGCCTCGAATCAAATCAAAT
CTATCGTGCGCCATTTCCATTCAAACACATTTTTTTTACTTTCTACACGATGGGTCGCTT
CTTCAAGAAATTTCGGCAGAGAATCGAAAGCCGCATCGATGCCGCTGTTGCCGAGGCTAC
TGGTGGCGGCGGATTTGGCATTGCCAACCAAACCGCTGCCCAGCTTTCCTTTGAGGAACA
GAAGCCCGATTCGATGCGACAGAAGGACGTTCGAATGATTTCGGGTTGCGAGGATTCTCA
AACCTCGGCGGATGTTTCCAACGTTGCACATTTTTCGCTACCCGATCCGGCAGGCCGTGC
GGGGGGTGCCTGTACCTCGACCCTCCTGAACATTTTGTACAAGGACGAAACCGTCCCGGA
AGACACCCTGAGTTTTACGCAGGTTCTCGAGCTCATGAGGTCCGATTTGCGCCGCGGGGG
CTATAGCCAGATCCCCCAACTGACCTCGCTCAACAAGATTGACGTCGAGACGGACTTTCA
GCTTGTGCCCAGCACCGCTACCGGAGTCCATCGGGCGGTCATGGTTGGAATCAATTATGT
CGGACACAGCCCTGGAGAGCTCCGAGGTTGTCACAACGATGTCTTGAATATGAAAAAGTA
CATCATGGAAGTACACGGGTTTGAAGAGGAAAACATTGTAATCCTGATGGACGATGGGAT
CCACACATCGCCGACTAAGGAGAACATGATTGCAGCTTACGAACAAATCGTTGCCGACAG
CGAAGAGGGCGATGCCATCTTTTTGCACTACTCGGGTAAGAGTCGAAACGTCATGCAAGA
ACTGTATTTTATCCAAGAGCTCGTACATTGCCACAGTGCAAGGCAAGAATGCCATACTCG
CATCATGCTTTTTAATTTTGTCTCTTGGGTGTATTGACATGCTAACCAACTTCTGTGCAT
TTCTTTCTTGCACCGGATGGCAACCTGCAGGCCACGGAACTAAGCTTGTCGACCAAAGTG
GAGACGAAGACGATGGTTATGATGAAGCGCTAGTGCCCTTGGACTTCAGGGAAAGTGGAA
TGATCCTGGACGACGATCTCTTTGATATTTTGATTGCGCGCCTCAGCGATGGGGTTCACA
TGGTCTCGCTTATGGATTGCTGTCACTCTGGGACTATTTTAGATCTGCCCTACATTTTTA
AGCCAATGCAAGACGGATCCATGCCCCAAGAGATGCAATTGGACGACTCAGTAAATTTAG
ATGGCATTCTCCAGCAATTTGGTGGCCAGGCATTGGGCATGTTGACTAATTTTTTGAATT
CAAAACTAGAGTAATCCGAGCGGCCATGTATTCAAAAAAAGGATGAAAAAATAAATTAAT
GCAATAAAACGGCGATAGAAACAATGTT
>comp2121_c0_seq1

ACTCCGGCATGAAGGGCAGCTGTGATAGAAATTGGAAACGAGGATCATTTCGGAGCAAGA
CAAAAAGAATAGATAATATCCAAATCCTGCACTCTCCATTGTATCGGTGCCCTTTTGTTT
TGAATTTTGAACCAAGAAAACGATAGTATGGCGAACGTGACGGATCCTCTCATCAAGTCC
ATACAGGGCTCGGACCCCCAGAACCTGATGGAGTACATCACCCGGCAAAAGATTTACGAC
AGCCGGTTTTGGAAGGAGCAATGTTTTGGGTTGACGGTAGCGGACGTCATGGAGCGAGCC
GTCGAGACCCTGACCTGCGTCGGCGGAACATACAGCGGGATGGGGAAACCAACGAAATTC
TTGTCGCTGGTCCTGAAGCTCTTGCAGCTCCAGCCCGATTATGAACTCATTCGAACCTTC
CTTGAACAAACCCACTTCAAGTATCTCAGGGCGCTGGGTGCCTTTTATCTGAGACTGACC
GGACGGCCTCAGGACATTTACGAACTGCTGGATCCCCTGTATGCCGATTATTCCAAGCTG
AAATACCGTGGTGTCAACGAGTGGAAATTGGTCCACCTCGATGAATTCGTAGACGAGCTC
CTGACGAACTCCTTTTGCTGCGGAATTGCCATGCCTAGGCTTCCTGCGAGAGAGACACTC
CAAGAGGCTGGATACCTTGAAGAAGGCCCGCGGCGGACGGCTCTGCACGATGCCTTGACA
CGGGCGGGAGGTATCAAGGAATATCTAAGGATCAAAGTCGACGAAGAAGCGAGCGCACGG
AAGAGCGGGATCAACGATGCCGTTAATGATAGCGCTTTCGGTGGGGCCATTTCGTTGTGG
GAAAGCGATATGGAAAACTCAAACCAATCGAGCGGGGGGAAGACGATGAAAATGGAAACA
CGAATGAGAACTTGGAAAGTACTAGAGATAATGAAGTCGTGGGGGGAGACAATCACGAAA
CAGAAAGCGAACGCGGCACGGGTCAAAACAACAGTGAGGGACGACTAGGAAAACGAAAAG
CCGCAGACACCGATGCAGATAGTGTGGAGGCAAAAAAGAAGAAAAAGAAAGAGAAGAAGA
AATCATCAAAGTACGGTTCCTTGTTCAAATCATCTGCGGTGGACAGTTCCAAAAACAGTA
ATTCTATGCTTCCCAAGGACGAAGAAGGAACGCAAAGCAGCAAGTCCGTCGATCAAAACA
GTGATGAATACTGGAATGAGCTGCGATCGTCTTTGGGCATGAATCCATTGAAGAAATAGA
TCTGTCTCGTACTTGAGTGCTCGATCGGTCAATTGTCGATTGACTATGAGTGCTAGCTGC
ACTGTACGTGTACGTGCAAATGCATTTGAACAGCGCATGAGAAAAACTATAGAAAAGACT
ATTAATATTTTTC
>comp2122_c0_seq1
ATTGAGGACGTCTACCATGCTTCAAGATGGTGCCACCACTTGCTAGAACATCAATTCAGA
CTCTTCAGATTTTATGTCAAAAGCATGGTTGGGGTTATCTTTTTGGAGCTGACGAAGCAA
CTGGGGATTGGTTTTTGGACATATTTTATGGACTAGGGGAATGGAAGCGATTCATGGTCC
CGATTCCAAAACAAGCACTACATGGAAAAACGCTTGTTTCGGAAGCTGCACTAGTGGGAT
TGCGAGAAATAATTGAACGCGAAGAATCGAAACGCATTTTGTCATTCAAAGATGTATTTC
CCAATCCAATCGTTATCTACGACTCGCATTCGAAAAGCACCTGGGAAAGATTCTGGAACG
ACTCTCCAACTGAAGTTGGAGTGGATTGTGAAGGAAATTCAATTTCACCACCAGTCCTCG
TCCAAATATCGACCGAAAATTACACAATTCTTGAGACTCCGTTGGACGGTCACTTATCTG
AAAATCTGCAGCGACTCTTGGGAATGAAATCCATTGTAAAGATATTTTGCGACAATTATT
CACACAGAGACAAGATATGTTTGGGATTGAATGTATATGGCGATTACAACGAAAGAGATC
AAGTTTCCAAAAAAACTCATATTGTAGATATTGAGATCCTCTCTAATAGGGTGATAGGAC
CTAGCAAGACACCCCGGGGATTATCGAAATTAGTTTGTCTGTGCTTCCCAGAATTGAACG
TGCGGATATCCAAGTCAGGAAAGTCAATGAAGCAACGGTTTAGAAACATTGGAAAATTTG
TCGAGATAGAACAAGGAAAGTAAGTCAACAAGCAAACATCAAAGATTATTTTGTTGGTCC
ACTGTAGCTACCGGTGTGCAATAAAGTCTGACAAATCAAAAAAAATTTGCCTTGAAATGA
TGATTTTTCTGCCCCTCATTGAACTGCCAACGCTACCTAGGGCAGAGCCTTTGAAAGGGA
TCTGTGATTTGAATGACACCCAACGACAGTATGCCGCTTTAGACTCTTGGTGTACTTTGA
AAATATATCAGCGAATGGTTTCGATAATGAAAAAGGAAATTGCAGGCCAAGTTAACTGAT
GATTTTGAAGTAGTGAAGATACAGTTGTTGTCTCTGAACCAACGCACTGATTTCTCACTT
CAAAATTCTGCGCTCCTGCTCTACGATGCAGTGGAAAGAGCAGCTGGAAGGTGGAATTCT
GTAAATTTATTCACAAGCTCAGCTAGCTATGCGTAACATTTCACTTTCTTTTCTACGACA
GGAAGAATGTAGATCCCCCAGATTATGGTCGTGCCTCCATCACATCTCCTCAGATGTAGG
TGCCGCCCCTCAGAGCGACTAGGAGCATTCTTCTTCCCAGAAGTTATGGTTCTGACCATG
ATAGTGCCTCTTCTAGTTCACGAAGTTATACATAAATTTAAGATTTTTTTAAAGATCGTC
CCCGATTAGGAGCACCAATCGGAAAGCCTAAAGAATTGAAGGCATTCATAATTTGGTTTT
AAGTGGTACTGAACGTTCCTGAAATATGCATGTGCCATCGAAGCAATCAGTTAATTTGTC
GTAACGAATGGGACACAGATGTCGAGTTTGTCAGAGCCTTC
>comp2122_c0_seq2
ATTGAGGACGTCTACCATGCTTCAAGATGGTGCCACCACTTGCTAGAACATCAATTCAGA
CTCTTCAGATTTTATGTCAAAAGCATGGTTGGGGTTATCTTTTTGGAGCTGACGAAGCAA
CTGGGGATTGGTTTTTGGACATATTTTATGGACTAGGGGAATGGAAGCGATTCATGGTCC
CGATTCCAAAACAAGCACTACATGGAAAAACGCTTGTTTCGGAAGCTGCACTAGTGGGAT
TGCGAGAAATAATTGAACGCGAAGAATCGAAACGCATTTTGTCATTCAAAGATGTATTTC
CCAATCCAATCGTTATCTACGACTCGCATTCGAAAAGCACCTGGGAAAGATTCTGGAACG
ACTCTCCAACTGAAGTTGGAGTGGATTGTGAAGGAAATTCAATTTCACCACCAGTCCTCG

TCCAAATATCGACCGAAAATTACACAATTCTTGAGACTCCGTTGGACGGTCACTTATCTG
AAAATCTGCAGCGACTCTTGGGAATGAAATCCATTGTAAAGATATTTTGCGACAATTATT
CACACAGAGACAAGATATGTTTGGGATTGAATGTATATGGCGATTACAACGAAAGAGATC
AAGTTTCCAAAAAAACTCATATTGTAGATATTGAGATCCTCTCTAATAGGGTGATAGGAC
CTAGCAAGACACCCCGGGGATTATCGAAATTAGTTTGTCTGTGCTTCCCAGAATTGAACG
TGCGGATATCCAAGTCAGGAAAGTCAATGAAGCAACGGTTTAGAAACATTGGAAAATTTG
TCGAGATAGAACAAGGAAAGGCAGAGCCTTTGAAAGGGATCTGTGATTTGAATGACACCC
AACGACAGTATGCCGCTTTAGACTCTTGGTGTACTTTGAAAATATATCAGCGAATGGTTT
CGATAATGAAAAAGGAAATTGCAGGCCAAGTTAACTGATGATTTTGAAGTAGTGAAGATA
CAGTTGTTGTCTCTGAACCAACGCACTGATTTCTCACTTCAAAATTCTGCGCTCCTGCTC
TACGATGCAGTGGAAAGAGCAGCTGGAAGGTGGAATTCTGTAAATTTATTCACAAGCTCA
GCTAGCTATGCGTAACATTTCACTTTCTTTTCTACGACAGGAAGAATGTAGATCCCCCAG
ATTATGGTCGTGCCTCCATCACATCTCCTCAGATGTAGGTGCCGCCCCTCAGAGCGACTA
GGAGCATTCTTCTTCCCAGAAGTTATGGTTCTGACCATGATAGTGCCTCTTCTAGTTCAC
GAAGTTATACATAAATTTAAGATTTTTTTAAAGATCGTCCCCGATTAGGAGCACCAATCG
GAAAGCCTAAAGAATTGAAGGCATTCATAATTTGGTTTTAAGTGGTACTGAACGTTCCTG
AAATATGCATGTGCCATCGAAGCAATCAGTTAATTTGTCGTAACGAATGGGACACAGATG
TCGAGTTTGTCAGAGCCTTC
>comp2123_c0_seq1
ATTAACAGTACCGTACTGTACTGTACGACGTCACAATTACAGTACGTACTCGTTCTTAGT
TCCGCTCACTTTCTGATTCTGAACCAGTACGTATCGTACGAACGAAAAATGGGATAGATA
AAAACGTCGTGAAAAAGCTTTTCGTCACACAATCTTCCAATATGCTTTCGTAGTATTCCA
TGGAGGCATGTCTGTTCTTTCCCGCAGTAGACCCATTCGATGGGGAATTTTAAGCGCAGG
AAAAATTTCCTCCGACTATGCAAAGGCAGTTGCAGTGACGGAGGGATCGAAAGTGGCAGC
GGTAGCTGCACGGTCGTTGGCTAACGCTGAGTCCTTTGCAGAAAAGCATGGCATTTCCAA
AGCGTACGGGACTTATGAAGAGTTGCTACGGGATGATTCTATCGACGTTGTTTACATTGG
ATCTATTGCCAATCAACATTATCCTCTGGCGATGCAATCTCTTCTGGCGAAGAAACCTAC
TGTCGTCGAAAAACCTCTGACTCTGAGCTATCAAGATACAAATTCACTCGTCTCGTTAGC
GAAACAGCAAAATACTTTTTTGATGGAAGGTTTGTGGACCCGCTGTTTTCCTGCTATGAG
AAAAGTTCAATCCTTGATCCAGCAAGGCACAATCGGTCAAATATCAACTATTCAAGCCGA
TTTTGGGTGGTCTACGAAAGATTGTGGGCCTGAAGATCGTATATGGGCCCCAGAAAGTGG
GGGGATGATACTGGATATCGGCATGTACATGGTTCAGCTTGGGCAGGTAGGATTTTCGGG
ATCTCCACCTTCCCGCGTTCAGGCTATGGGGAACTTTCGCAATGGTGTCGACCACACGGT
TTTGACAAATATTCAGTATGGTTCTTCTGGGTTTTTGCAGTTTATCGTGACAGGTGATGC
CAATACGGAGGAGCGAGTCGTCGTTCAAGGTACGAAAGGAAGAATTATTTTGGAACCTCC
TGCCCACGTTCCAACTGTTGTCAAGTTGCAGGCCGATGCCGGGCGTGGCGTGATCGAAGA
AGAGGTCTTCGAATTTCCCTTGCCAGACGACTCTTATACCACCTGGAACTATCCTGGCAG
CATTGGATTTACGCATCAAATCCAATCTGTTACAGATTCGCTGATCAACGGCGAAAAAGA
ATGTCAATACTTTACTCATCAAGATAGTCTGGAAGTGGCTTCTGTCGTGGACCAAATTTT
GCAACAGGTTCATAACCAACAAAAAGAGTAGCAGCAAGTCTCTGACCCGGACGAAGACGA
ATTCGCAATATCATTGTATTCGCAAAATATTTGTTAACGGAGCAAAGATGATTCAAGTCA
ACGTAATACTTTCACCATTGGCGGTTGGTATGTTGAACTTAAAATTCTACGCTCTTTTTA
AGTCTGTTGTTCAAATGTTTCATTTCTTTCGAAGAATATTCCAGCGACACGGATTACTAA
TGCTCAGCTCTAGTTTTGATGCATTATTCGTTATATAGTACGTCAAGTAC
>comp2124_c0_seq1
ACACTTTTTGTTTTCGATACCGTATCTCGCTGCCGCGACAGAAGACCGAAGCGCGAGAGC
GTTGCGGTACCGTTGCGGATCAGACACCGAACGGTCGTCCAGAAAAGAAAAAGAAAGAAG
AACCGCATCCATTACCGGGAAGGAAGGGTTCCGTTCCGGTCCCGAGAGAAGAACCAACAG
ATCCCACCGGCGAGAACAGAACAGAGCAGAGCAGAACAGAACAAGGATCACCCAGGGCGT
TTCGCGTTCCCGAAGACTGTCCACTATGGGCCAGTCCGCATCCACCGGCGGGGGAGCGGA
GGGACCGCCGGACCTTTCGTCGACGATTGTGATTGCGGAGACGGACGAGGAAGACGACGA
ACGCAGCGGCAGCGATAGGGATGCCTTTTTCTCGGATGCGAAAGCCGGCGGGGGTGTGGA
AGGCATCGGCGGCAAGAACGCCGACGAAACCGACAGCGGCAGCACGTGGAGCAGCAGCAG
CGACAGCGACAGCAGCAGCACACACAGTGGTTCCTCTTCCTCCTCCTCCTCTTCG
>comp2125_c0_seq1
CGTATTTGTTTCCCGAACTATCGAACAGGTGCGAGCGAAAAACGAGTAGGAACAGGCACT
ATTTCATCAGGCCAAGCCGAACCATCGATTCGCTTCCAAAGCCCGTTAGAGACCAACGGT
TGTACCTGTAGCATCGTTCTAGGAATTGAAAATGAGAAGGACCCTCAGTTGAAAGAACTT
ATCCATTATTGTAACCCTCTCTGTCATCGAACGAGACGAGATCTGAGTTCCAGACGAAGA
CCATCGGAAGAGCAGAACTTTTGTCGATAGGAACAGATCGACCTAGGTATCACTAGATCG

GAACCTTTCCTTGCTGAACCACATCGACACCTCCGTTGCCATTTCAAAATGATGTTCCGG
TTGATGTTCCGTCTCGACAAGGCGGTTCTAACCACGCTGCTATTATCTTGGACGATGTCT
TACTTTCTGTGTGGGAAGGACGCCGCTGTTCTGGTGGAGGCCTCTACCTCGTTTTCGATG
GAATCTATGATGGAGAACGGATCGACTACGATATCACAGGAATGTCTGCCATGGTGGCAA
CGGCAAATAGATGTCGCCGAGGTCTTTCTGGTGTGCGACCACCAGAGCGGCGATGGAGAG
GACGGCGATGACCAGTACTATTATACCAATCGGGCGTCGTGTCGAACGGGGGATTTGGGA
AAGGTTGCCCTAGGATTCACCGTAAAAAACCAATTGCCCTATGATTCGACGTTGTTCGTT
TCCATAAAAACCCATTACTACGGACGGAGTGCAACCATTCGAAATAGGACGGACGTTTGC
AGCTTTTCAAATCTAGGCTATATCAACGCAGACGGGGCCTTTTATTCGTCGGGCAGCAAC
AATGACGACAACGGCGACGCCGATGACGGCGGGGAGGGAGGTGATGGGAGCGGCAATCCC
CTGTGCCCGATACAGGCCGGCATGCACTACATGCTCATGGAATCCTTTACGCTCCAGGAC
TCGTCCGCCTCTCGCACCTCGAACTGGCAGGAATTCAAGCCCGACCTGTCCGTGTCCTTT
TATTTGTCCTCCGACGAGTCCAGCCCGCCCATTGGGTGCGTCCTGACGGGCACGAGGGCC
AAGCACGCCCTGGACCAGCGGCGGTCGAGGGACGGGGTGATAGCACTAATCGTGAGCGTG
TTTTCGTTCGTGATCGTCTTTGGCCTCTGCCTGCACAACCAGAAGCAACGAAAGAAGGCG
GTGGAACTCCTCGAGAACAACCGCGTGGCCTCGATGATTCGGCGGTACAACTATCGAAAG
AGCAACAGCGGTGGATCCTCTACGGGGCTGATGATGGGTTCGTCGAACCACGTGCCCAGG
CCACCGCCGGGGGACGGCCTGTTTCCCGGGGGGCCCTGCGGCAATCACCACGCGAACCGG
CAACCGGCTCCGCACCGCGATCTTCCGCCGCCGTGGCGATAACCACCGAATCGCTACTCG
CCACGGCACGAATTGTTTTCCCCGAAACACACAGTTGTCCGGATGGAGTCGGAACGGCGC
GCGCACAAGCATTGACCTCGAAGGCGCTAGAATAACAATTATTTTAGCATCGACAAAATG
CGTTTTATGTCAACTAAAAATAATGATACAACTTGCTATTCAAAAGGTGTCGATGCACCT
AGCGACTATCGAAAAATTTGCTAGCACTCGGAGCTAAGGTTAGCTTGCGTTGGCTAGCCA
CTGAATCTCGATTGTTCTGATTTGGCCCGGTCATTTCTCGGCGGGCAGCATCGCCAGAAG
CCTGGCCGCATCGCTGGCGGCTTGTATGTAGCCGTCGAGGGCCCGCTGCGCGTTGCGCTG
TGCCACCTTCAGCTCCCGGGCCGTGAAGTACCCCCCGAACAGCTTGAGGGGATCCGTCTG
GAGAATCCGTTCCTCCTCGTCGAGGTCCTCGTTCCGGCGTTCGTCGTCGCCGTCCGTGAG
GTCTTCCTCTTCCACCACCATCCACGACGAGGACGGGTTCGGATCCAAGGATTGCTCGTC
ATCGGTCTTTGGCTTTTTCCTCTCGAGTCGCACGTTGCAGAGTTTCCATTCGGGGGCGGA
CGAACGTGCCGAAGCCGCCCCGGAGTCCTTCGCGGGAGCAGCCCCCTCCTCCTCGTTCAC
CAGGTCCGCGATCCCGTCGTTGTCCCCG
>comp2126_c0_seq1
AGTATTCGTACTCGTGGTACTCGTACTGTAACATTCTGCTGTACTGACTGCTTCTGCCAT
CAGCATCCGCATCCGGATCTGTAAACACAAAAAGTTCCCAAAGCACGGAGAGGCAATTCG
CTCCGATTGAAGAATAGACCAGGATCCCAAACCAACCGCATCGCACACAATGAGGACTGC
TGCGCAAGCAGGAGGAACGATCCGACGCCGGACTCGCATCGCAACGAACGACGGTGGCAG
CGACAACAACGACGGTTTCGGCCTTCCGAATCCTTCTCGTGTGCGGCGGCAGCGCACCAG
AAACCGAAGGAGCAACGGCGCGCTGGGATTCGCCGGGGCGATCGTGGTGGCGATCGCGAT
TGCCGGCGGGTGGACCTTGCCGGGCGTCCCCTTTTCTGCGGCGGAAGCCCTTGCCGTTTC
GGGGACGGGTCTCCCCCCGGCCGCCGCGCTACGGAGCAGCGGCCGTTCCGAGAGGGGGGG
ACATCCCCGGGAACAAGCAAAAGAGCAACAACAGCCATCACCGCCACTCTTGGGTTCCTG
TCGACAGGGGTTCCCCGGGAGCCGCATCCACCAAAACAGGCTGGCCCGGACGCGCCTGCA
CCTTTTTTTCAACCAGAGGGACGAAAACGGAAACCTCGTCCAAAAGCCCGCGAAACGGGC
GAAGCCGTCCAGCGACCTCCTGGACGAGCTGCGCTCCCCCGGGATATCCTTCCCCAAACC
GATCCGGCTGACCGGCTCTTCTTCTCCGAAGGAACCATCCGCCGGCACGGATTCGCCGTC
CCTGGTCCTCCGCTACATGACGACCGAGGACCTCCGGGCACTGGTACCCATGTGCATCAA
CGAGTTTGGGAGCATCAGCCAGCAGCCACCGCCGGAAGACACCCACGCCAAGAAGGGATT
GCGGGGACTGGTCCCCCGCTGGATACAGGATCCGAAACGAATCCCGGAGCTGTGGGAGGG
GAAATCCTTCGAGCTCCTAATCTATTTGACCCTCCGTCTCAAGCTGATGCAGGGTGGGGG
AAACAGCGGCAAAAACAGCGGCATCAGCAGCGGCAACAGCAGCGGCAGCAACGGCAATCC
CGTTGCCAACAGCGGAAACCCCGGGTCGATACCGAACGACATGGTCATGCTCGTCCTCTG
CGAAGAAAACGAACCCAAACCACAAGAACAGTCCAAGAGCCTGTGGCGAAAGCCCAAGCC
GGCAGCAGCAATAGCAGCAGCAACAACAGCCACCACCGTGGAAGCAACCCAGTCGCAGCC
CCTCTCGCCCCTCTCGCCTCAACAGCGGGTCGTGGGCATGATCGAGCTCAGCCTGCAGCC
CCCGGACGCGGACCGGAATCCCCCCGCCCTCCCCCTTCCCAGGTGGTTCAAGGCCGCCCT
GGCCAGGCAGACCACGATCGACGGGCGTCTCCAGGGATGGATCACCAACCTCCTGGTCGA
CGACGACCGCCGGGGCCGGGGCTATTCCAAGCTGCTGGTCGCGGCCTGCGAGGGGATCGC
GAAACACTCCTGGGGCTGCTCGTCGGTCTACCTGCACGCCGATGCGGACATTTCCAGCGG
CCGGATCCCCCAGTCCCTCTACGAGGGAATGGGTTACGAGGCCGTCGTGGGAAGCACCCA
GAAGAATCCAAATGGCGCTCGCAGCGACGGCGATGGCGGCGAATACGCCTGGGCCGGGGT

CGCGGGGAAGGAACTGGAGCGGTTCACGGCCATCCGAATGGTCGACGGGGTGGCCCTCCT
GTGCTACTCCAAAAAGCTGTAAAGCAGCAGGCTTGGGACGGGCGGACCGAAACCATCGAG
AAAGTTTATCCAATCGTTGCGAATCCAATCGAATCCAAGCCAATCCAAGCCAAGCCTTGA
CGCGTGGCGGCAAGTCACAAATAGGATCTAGGCAGCAAGGGAGTTTGATGCGATGCTTTT
GATCGCAACCGATCCGGACTTTTGCCAGTGAAAATGACAGGCAGAACAAAGCACTTGGTG
TCGAAGCGGTGTCTACCAAATATGTTCCAGAGGGGAGCGAGCAAAACGAGAGGAATCAAT
TTGGGGAGTGGACGCATGAAACGGATGGAAGGCGAGCAGCTCGTTCGTGGCCCGACCGAA
GGACTTAGGCACCAATGGATGCATGCATCGCACTGTATGCATTGCTTTTGAGCTGCGTTT
CATCCACGTGCAAGCACCGCCCAACACCCAGACCAAAAGAGCCGACTCGACTCGACTCGA
CTCATGCGCATGCGTACCGTTTCACTGCCACTGCCCCTTGACCATTGCCACCGGTGCTGG
ATCTCGTTTTGTTCGGCTCGGGTTGCGTTGGACTCTGTCGACGGAAATGGCAACGATGCA
CACCAGCGCACACCCGCTTTGTGGGTGCATCGTTACACCGAGAATCACACCGAGGAAGAA
CAATTTCACGACATGCTTTTGCTGCGATGGCACCGCGCCTGGGCAGCTAGCAGTCTGTTT
TGCGCTGAGAAAGCAATCGATTCGTTCAAATTATATTACAAAAAAACTTCGTCGCACAAA
GTCAGAACTCGCATGTGGTGGGAGTTGATGGTCCGTTTCCATTCAAATAGTCTTAGGACG
AGCAGGAAACCAGGCAGGCATGATTTAATCCTCTAAAGTAGATACGGTATATTAACGTTT
ATTGAGAGG
>comp2127_c0_seq1
AAAGAATCCTATCCTTTTTGCAGCAAGTTTAGTATTGAAGAATCATGGCACTATGTGTAG
CGAATGACGACCGTAACAGAAGATTATGGCGACGATTACAACCGGGGCGATAGCTGCGAT
GGCTCAAGGAAAAGGAAGGGCTGTCGGTTTTCGTTTCGAGGTTGGTGCGACTTATTATTT
TGCACATGCATGAGCTATGAGGTCGAAGTTTGGATGAGACGCAAAGCAGCAAGATGGCCA
CGAGTAGCTCTCGGACGATGAAACTAAAATGAAATGCAGGAGTCTTAGCAGTGGGAAATG
GCTAGAACCGGAACATTTCGTCTCGAATACCTGCATTCGTGAGCTTTACTTTTTCTCGCA
CTGCTTTGAAATCCTTTATCAAACCAAACCATCTGCACTTGGTTGCGGTGATCATTCATA
ACAGTGGTTGGCTCCGACAATCATCGTCGGTGTTGCGATTGAAGGAACGAGCCCGAATGT
TTTGCAATCATTCGAACGTAACACCACACCTGAGGTTACACAACAACAAATCAAAAACTT
GCGATTCTTCAACAGCAATATTGTCATTTTTGTTCATACTGCGTGGAAGTCCTGGGTAGT
AGAATATGCAATCGGAACACAGAGTTCATATCTCATCGGCTCACACAATTTTTCCTTTGC
TTCCCCTTTCTCGAGACAGTTTAAAATAGATTTCTACCATGTCCAAGTTCACCTTTTGCC
TCCTTACCGTTTTGGTCGCTGCTTGCTCTGCCTTTGCGCCTACATCGCAATTGAGCAGTG
CCACCCGACAGGCCTCGATGACCTCGTCTCCCTTCGCCCTCAACGCCGTCGAAGACTATG
ACTCGGAAACCTACTGGGAAGACGAATACCCCCCATCCAAGGTCTTGGGACCTATCATGT
CCAAGATGCCCTCTGGTTTGCTCGCTGCCTTGTCGGTCGTCTTCTTGACCTCCCTTGTTC
TGTCGATCTCTGGAAGCGCTGCCCTCCAACAAGAACCCGGTGCCTTCGAGAACGGATCGT
GGGTCAAGTGGTACTATGTTTTGGGATCCTTCGGGGGACCCTTGGCATGGGGAACCCACG
TTGCTGCATGGATCCAGCGCAAGAACGGCATGTAAACGAATCTCTCTTAGCGATCGGATC
GGTGTAGTTCCTATCCTCCGGTAGCGAACAGTCTTAATTTCACTGCCGTAGAAGTGATCT
AGTAACAAGCACGCATGCATCGGATTCGATTGGATTTTACGATCCAGTCTATCTATCAAC
TACGAAACCAAACCAACTACGCTACGAGCCTTAACTATCGATGGGAAGTTGGTGACACGG
GAGACGACACTCGATGGCCGAATCTCGTGCTCCCTGTTTTTCTTGCGATGTAGAATTTAA
TTTACAAATAGATAATATTACTATATCGTA
>comp2128_c0_seq1
CCAACAGCAACAGCAGCGAGAACAACAAGACACAAAGTTCACTGCCCAAAAAGAAAGGCA
CCAACTTTGCTCACTGTTTCCAAGTACACCAGTTACCATAGAAACCACTTTTGTTGTACA
AGATGCCCATGCTAACACTCCAACAGGCCAGGTCCGATGCGGTAGCTAACGAAGCCGAAC
AACGAACCACGAGTCCCGAGCACAAGAGATTACTGTCCGAGCGAGGGGAACATGATACAA
CCATAGGAGAATCCAAGAAAAGGAGGAAGCTCTCCGAAAGTCTCGACACGGAGTTGGTAG
TCACTGGCAGCAGCGACGACAAAACGTCCAAAAAGATTCAAAATCAGAAGGAAACAATTG
ATGGTGTCACCATGCTCAAGCCGTCAGACAAGAAAAAGGTGGATCCTACAGAGTTGGCTT
CGGCATTTGCCTTAGCTTCCCTTGCGAGCCTAAGTCCGGGAGGCAGTAGCGTAGGTAGTG
CCTCTGGAATAAGTACTCCACCAAGGGAATCAAGGGAAGCCAATGTCATTCGAAAAACTG
ACTCAAAGCTTGATGACGAGGTACGAAAGGCTGCGTCTTTCGAGTCGGAAACACGTTCCC
CAAAAGCTTCGGAGCACCCCTTATCCCCGGAACAATTGTCTCTAAGAGAACAAGAAGACC
ACAGTGCCTCTTCTACGGTTGTGAACTCTCCCGCAGGAAACGCCAATCGAAGAGTGTCAT
TCGCACCAAACACGAAGTCAGCTGAAGAATCCAAAGTTCCGTCAACGCCTACAACGTCTG
CGAGTAGCTCAGGAGAACTCGCAACCTCAACATCACATCGATCGCTGGCAATGCCACAGG
AGTCTCCTGGCGGTAAAAATGGTCGTAGTGGTAGAAGTAGCAACAGCAACACTACCATAA
ATACAAGTCCGAACTCAATGATGCAAATGAGTCCAAGACCTCATCGAATGTACGGTGGAC
GAGGTCTCCCACCCTTTGTTCGAACCCCACCACAAGTGAACCAGATGAATCATCGTCTCG

ATTACGGAACACCACCTCCACCACCACATGCTCCTTATCATCATAGACTTCCTCCTCCAG
GAACAAATGGGTCGATGCCTCCAGGATCGTTTCGTCATGGACCTCCAGTTCTTCACCACC
GACAACAATTTGGATATCCAGGAGCACCACCAGGTCATCGTTTCAGTCCACATCAGCAGT
ACTTCCCCCCTCGTTACCTGATGCAACCTCCCCTCTTGCACAGAACCAATAGCTATGGCG
TGGGAATGGTGCCATCTGCTCCAACGCCACCGAACCAACAGGGTGCCCCGGCCACTACAA
AGTGCAATCAATGGGTTTGTGATTATTGCAACGTCGCATCTTTCTCTTCTTACGAGGAAG
CTTGTGCTCACGAAGAGATCTGTAAGAAAGCAGGCCCCAAGGCTGCAGCTCAACTCCTTT
CCGTAAGTAGAAGTTATAGTTCCGCTTCCATGGATGAAGATTCAGTCGTAAGCAGCAACA
TGTCAATGATGGTGGGTGGGCATCATCTTCCCGCCAACAATCCCCATCTTCATCCCGCTT
TTGGCAATGCAATGGAAGCAGCTCGACGTCATCATCATGCAAGTTTAGCATTGAAGATGG
GTCATCAACAGCATCGAATGCAGATTGATGGGAATAACAGCATGATCAGCGGCAGCAACA
ATGGGAGCAGCAGCAGCAATAACAATCGACCCGGAAAGGCAACACCTGTTTCTGCGGGCA
AAGGAAACTCACGCGAGGAGCAACAACCGATGACAGAAGAGGAACGTAGACGCCAAGAAA
TGCATTTGCATTCATTAGGCGGATACATAGTTTATCCAAACGACGTTGAGATGATTCCAC
CATACGTACATTTTTTGATGCGCCAAGTAGAACCGTGTTTGTTTACCGAAGCCGATCGTT
TCGTGGCACGCTCCAAGGGACCCGTGGGATACCCAGGATTTCAATGCCGCCATTGCCATG
GTCATGCTGGACTAGGAAAGTATTTCCCCGTGAGTTCGAAATCCCTCAGCACAAATTCGA
CCAGTCAAAACATTCATGCGCACATGCTGAAATGTCGCAAGTGTCCCGATAACGTGAAAG
AACAATTGGTTCAGCTAAAAATCGAGAAATCCCGTGCTGCACGTTTGGAACCTGGTTGGA
GAAAAGTTTTCTTTGATAAGGTTTGGAAACGTTTGCATCGTGAGGCTGGCGATAATGACG
AACAAGCAACATGCAAAACGCAATCGAACTGAAAACTTAAAAAGGAGTTTGAAGAGAAGA
AATGAAAACAAAGAAAAGATTAAGAGAACGAAAGGGGACTGATGATCGTTGGGAATGGAG
TAGAAGTGGAAATGTATTACTGTAAAAAAGAATGAAGGGGGTTCGTTGTGGCAAGAGATC
GATAAGAGACAAAACAAACGAGAGCAAGAGACGAAGTCAGAGCATATGTAAGAGTTCTCG
ACCAAGATCTCGAGACTTTTCTTGTTGCTAGTATCAATGAATGTCCCACCCAGGGTCTTC
TTGTGCATGCAGTGGAATACTAGCCTTTGAAAGCTAAACGGGATAAATTCAACGAAGATA
ATCTATCATTTTTTCTCTCTTTTGTAAGAATCTTAACGTAGCAGATGTTTCCATATGATC
TGTTGCAACAAGGCTGCAGTCGTACAATTCTATTCTCTCTGCCTCCTAGAATGATCCCGA
AAAAATTACTTCAATATTGGGAGGAACTGATATGACTAGATGTATTTGTCTTTGTGGTAA
TAATTACAAAAATGATATCATCATTTGTAATGCTGCAGTGAAGAATCATTATGCAAATCA
CAACAAGATACAAAAATGTCTCTGTGATAGTACTAGCTAGTACACTAATAGTGAGC
>comp2129_c0_seq1
CAAAACACAACACAACACAACACAATCCAGCAGCCGCGACACAAAATGTCACGCAAAGCG
CTTTCCGTCGACACCGGTGATGACCACCAGCAAGTCGAAGGAACGATGAGCGGCGTGGAC
ACGAATGCCAATACACCTGTTGCCGACAATAAATCGGGGTCCTCGCTGTTCCGTCGCTCA
CCCCAGTCGAAGACGCAAGCGCTTCGACCACCGTCGCCGCCAAAGACGTCGCTGGCAGCC
GAAAACGAGCGCTTGCGGAACGAACTCCGCGTCTTGAGACGGCAAATCCAGGAAATGGAA
GAATTCCAGGATTGGAAACAGCAAGACTCTAACGAGCAACATCGACACACACAACAATAT
CAACAACACCATTTGCTCCAATCATCTTTCTCCAGCCACGACTACCATGGACGGGCGTCC
AAAAACACCAGCAACAAAGTGATCGTGGAACTTCCGGAAATGTTCCAGAGCTCCCTCCCA
CCGCCCACCAGCGAAAGCGAGATCTGCAACCACCGCCGGGACGGTACCGTCAAGGAAAAG
CACAGCATCTCGACGGCGGACGATCAGGAAACGGTCGAGCCCATTCTCGACATCGAGGCC
CACCACCAGTCCGCGGCGGGCCTCCACCACCGGAACACGACAATGATAACGAGCAACCAT
ACCAAAAAGAAGAAGGAGGTACTTACGGAGGAAACTGCATCCTCCTCGGCGTCCTCGTGT
GGAAATTCCCTCGGCGAAACCGATGTGGAGGCGATCGCTCTGATCGAAGGCATTGACAGT
CACGGGAACAAAACCAACACCCACCACAACAAAACCTTCTGGAGCTCCCTCCTGGACCGG
GCAGGATGGCTGATCGGCCTGCTCATTTTTCAGTCGTTGAGCTCGTTCATCCTGGCCCGC
AACGAGTCCCTCCTGACGCATCACTCCGTCATTGTGCAGTTCCTGACCATGCTGGTGGGA
GCCGGCGGCAACGCGGGCAACCAGGCCAGCGTCGGTGTGGTCCGCGGTATTGCGGTCGGG
ACAATCCGCAGGTCCAACGCCAGGCGCGTCCTACTGCGGGAGTTTGCCATGGGCACTTGC
CTCAGCATAATTCTCGGCCTGGCGGGCTTTGTCCGGGCCAAGGTGTTCGCGGTACCCTGG
TTGGAAACCATGGCCATCACGACCAGCCTCTTCCTTATTGTCTTCATTAGCGTCGTGGTC
GGTGCGACGCTTCCGCTGTGCATGGAGGCAGTCGCCATCGATCCCGCGCACTCGAGCACC
ACCATCCAGGTCGTCATGGACATTACAGGGGTCATCATCACGGTGAAGGTGAGCAGCCTC
TTTTTGGATTCCGGGGGCGATTGGCTTGGGGCCGTTCTTTCGATGGACGGGGAAACGAGG
TGAGCGGCAAACAAAACACAAGTACAACAATTGCAGCACCAGCAACAACAATCACAACAG
ATCCATAATACAAGATTAGCCGTGATCGAGAAACGCCCAAGACATCGTATCATTGCATAC
GGAACGGACTGGTTTCGTTTCTTTGCTTCGGGATTCCGTCCTCGAGTCCACTCGTCTGCA
GAATTGATACCAATTCGAATAAATCTCAGCGGCGTGCGATATATGCACTAGATCAGTAAG
CAATAGAATTGATCTGCTTTCCCTCTGTCAAAA

>comp2130_c0_seq1
CTTAATACTCCCTTCGATTCTAGAACGCAATACACTAAGATGAAGATCGTTAGCAGCCTT
GCCTTTGCCGCCTTCTTGGCTACCACCGAGGCCTTTGGACCCGCCCAGCCATTGAAGGGA
GTTGTCTCAAACGGTGCCGCTAACGGCATGACCATGAGATACGGTATCAAGGACCAAGCT
CTTCGAGGACGAGTTTGTGCCATCGTCGATGCCAACCCTACCAAGGAAATTGTCGAAGAG
GAATTGCTCAGTGATTCCATGTCCGAGGCAATCAAGGGCGCCAACTGGAAGCTTCGAAAG
GCCCTCATCCGCAAGGTTACGAACCAGGCTGCACGATACGATGTTGCCGTCGACCCTGCC
TTCGGAAAAGCTGACACCCAGGCCGAACGTGAAGCAAAGGAAGCTGTAGCCGGAGCCGCC
AACAAGGCTGCCAAGGTCGCTGCCATCAAGGCCGCCGCCGACGAATTCGCCGCTAACCAG
GCCGCCCGAAAGGAACGCCGTGCCGCACACAAGGCCCGTGCCGCCGAAGCCCTCGCTGCC
CGCAAATCCGGAGCTCCCGCCCCAGCCGCTGAAGAAGCCGCCGCACCTGCCCCCGCTGCC
GGAGGTGAAATCACACCCAAGACCATCAAGGCCCTTCGTGACCTTACTGGTGCCGGTATG
ATGGACTGCAAGAAGGCCTTGACCGAATCCGGAGGTGACCAGGAAGCCGCTGCCGAATTC
CTGCGCAAGAAGGGATTGGCCCAAGCCGACAAGAAGGCCACCCGTGTTGCCGCCGAGGGT
AAGATTGCTGTCAGCACCGGAAGCGACGGAAAGGCTGTCATGGTCGAGGTCAACTGCGAA
ACCGATTTCGTAGGAAAGGATTCCACCTTCTTGGCATACTGCGACCGTGTTGCGGGAGCC
GCCTCTGCCTCCGGAACCGTTGTCGTCGATGACTTGATGAACGAAGAAGTTGACGGAGAG
AGCCTCGAGGCCACCAGACAGGCCCTTGTTTCCAAAATTGGAGAGAACATCCAGGTCCGA
CGATTGGAATCTAGGGGTGATGGATCCACCACCGTTGGTGGATACGTCCACATGGGAACC
ATCGGTGTCCTTGTCGAAATCGAAGGAGGATCGGAACAACTGTGTACTGATATTGCCATG
CACGTTGCCGCCATGAACCCTCCATTCGCCACTGCCGATGATGTCCCACAAGAGGTCCTC
GATAACGAAAAGCGAATTCTGACAGAGCAAGCCATTGAAAGCGGAAAGCCGGAAGCAATT
GTTGAAAAAAATGGTCGAAGGACGAATCCGCAAATTCCTCGAGGAGAAGTGCCTTGTGTCA
CAGAACTACGTCAAGGACAGTGATTTGACCGTTCAGCAACTCCTTGACAAGAATGATGCC
AAGATGATTGGATTCTCCCGATTGGTCGTTGGAGAAGGAATTGAAAAGAAGGTCGACGAT
TTCGCGGCCGAGGTTGCCTCTATGGCAGCTGGAAACTAAATTTCCACTAGCGCACACGGA
ATCGAAGCCCACGACCACCCTACAATCATCAATAAAACTCTAGCAATGACACATAATTGT
AAAAAAAGTATTTTATTT
>comp2131_c0_seq1
TGCACTCTATGTCCTAATGTTGTCATATCGTGACCATAATTCCGATGTATTGCCTATATC
GATACGACAATCTAGAATTCTTAAGACAATCATTGTCAGAAAGGGGAATTCAAAAAGTGA
GTTGAACTGACGTATGGGAACTGTCAGATGCATTATGATATTTTTGAAAACAATACAAAT
AACAAACACCTTTTTATAAGGAAGGGGTCATGTCATGCAACCTAATGACCCCTTGTTGAA
TCGAATGTCCATTGACACAACGATTCCCTCGACACGAACCTCCTGAGATAAGGTACAACG
AGTGCGAGTACGAGTACTCGTCGAGCACTTGTACCGGAAAGGGCACGAGTACGTGTGTTG
CTGCGTTTATGCTATGTTGCCATCCATTATTTCAATGTGCTTGTTGATTTGATTTTGGTA
TTTCAATAGATTTATTGGCCATCAATCATTGTTTCAAACCGGACAAAAGAGATCTTGAGA
TCACCGCACTATTTCTGAAAGAATTGAAGAAAATCTGCGAAAAAGAACGTTGAAAATGGC
CTCCGAAGATGATACAGATAATATGGCGTCCGCTGGATTGGAACTTAACCAGCTCCACCC
CAAAGCGGTGATGTGGATCCCTCGAATCGTGGGTGTGATTACGCTCGTTTGTGGCGCCTC
TATGATTTCGATGGCGTGGAAACGGAGTGACCGGTTGTTTCATCGATTGGTGCTAGCCAT
GGCGATCCACCTAGCGATCCTGGGAATAGCTCTTATCATTGGTGCCGCAGCCATTCCGGA
AAGCTTAAGCAACAGTGACGAGCCGAGCGAATCGATTCTCGGATACCACGGAACGGAGGC
CACTTGCACCGCCCAGGGCTTTGTCTTCTATGTTTGTTTCATGGCGTCCATGATGTACTA
TTGCAGTTTTTCCGCCTATAGCTACCTCGGAGTCCCCCGCAACTTTGTCAGTCGGGGCTT
TGAAGTGGCCGGAAAATACATCCACGCCTTCGTCAACTTTTTTGCCCTGGTTACGGCGGT
GGCGATCGTTCTCCTGGATGGATTCAACAACACCGGGCTCGGTTTTTGCGGCCCCGCCCG
CAGCGACATCAGCACCTACAAGCTCTTTCTCATGGTGCCGCTGATGCTGGAAATTGTGTT
TTCTTCGGTGGTCATGGTTCTGCTCTACGGGAAGGTAAAACGAAACCAGGCGATGATCTT
TATCAATGCCAGATCCGTGGCTCGCCAGGCTACCATCTACCTCGCGGTGCTGTACTGGGT
GGTGGTGCCCGTACTTCTTTCGCAGTGCATCTCCTGGGCGACGAATGGTGGGCACGGCCG
GAGCTTTATGACTTTTTCGGTGGTCAATTTCTCGTTGTTTGGCTTGTGGAGCATGCTATC
TTACTGGTATTTTTCCGTGGAGCAGAGGCACCATAAAAAGCGTCGACGCGGAGACGACGA
CACCGCCGACCCCACTAATACTTTTTTCGCGACGGAAAGGACGCAGATGGACAGCATGCG
CGGAAGATCGAGCAATTGCATCTTCTCCAGCAAAGAATTCAATATGTCACGGGCGAAAGG
CGAATCAGAGGCACCAAACTCTCAAACACGGACACAACGATTCTCCTTCAACATATTCGA
TGGAACTAATGCGAGCGGCAGGTTTGCGAGCTTTGTCTTCTCGGGGGACGACGAGGACGA
AGAAGCCGACAAAATAGAAAGCGACAAATGGAAGGATATACAGGACCACGTTTAATTGTA
CGTTGCAGCACAGATCATAAATGCGCAAAACACAACTTCACCGCACGAACAGAAGGTAAC
TTAGAAATCGCGAGCCAACAACTTAAGAACGAGATAGCAGAACTACATGTTTCCTTGTCG

ATAGAGGAGGAGTACGCAGGCATCAGTCCAGTACAAATGTGCAGCAAGACGCCACGGAAG
CGGAAGAATGGATCGTGATACAGAAAACGAGTGGTAGAATTCGCAGTGGGAAATTAGAAA
GTAATATTCTACACAACTCTACAACCTCAATGGGAATTTAGAAACTAGCAACTCTCAAAG
ATATAGAAAACGAGGCG
>comp2132_c0_seq1
ACAAGAACAATGAAGAGGAAGGTACAAGCAAGGAGGACGAAAAGAAAAACAATAATGGAA
ACGAGGATAATGAAGATCAAGATATCAGCAAAGAAAAAGACCAAAAAAACAATGATGGAA
ACAAGGATAATGAAGATCAAGGCATCAGCAATGAAGATGAGAATGTAAATGGAATAGTAG
ACGAAGAAAACAACGGTGGAAACAAGGAAAATGAAGACGAGGATATCAGCAATGAAGACG
AGATTTTTCATGATATAATAGATATGGAAAACGACGATGGAAACAAGGATAAGGATACCA
TCAGTGACGAAGAAGAGATGAAAGACAACAAAGAAGAAGAAATGATAAACAACAATGGAA
ACAAGGATAATAAAAACAAGGATATCACGAGTGACGAAGAAGATAAGAAGAACAAAAACA
AGAACAAAGACGAAAACAAAAACAAAGATAAAAACAAAAACAAAAACAAGGAATCTGAAG
ACAAAACGGATCCACCAACTACCGCTCCTTCCGCCAGTCCTTCTCATGCGTGGAGCAAGA
CACCCTCTATAGAACCCAGTTCCTTCGTCTATGGCGAGCGAGAAGCACCGATCACATCCC
CAGATATCATTTCCTCCACCGATTCCCCAACATTTTTCCTCGTAAATCAACTGGAACCGA
CAACATATCCAACGATGATTTATAAAAACACGATACCATCGTTCGTGGCCGTAAATATCG
GAGTGACACCAGTGCCGTCCTCCTTTCCATCCGATAAACCGTCGATTCATTTTTCCAATT
CTCCTTCTGACGGGCCAACAAGAGAACCATCATTGAATCCTTCGGATGCGCCATCGGTGT
CTCCTTCTGGGGGACCATCACAGGCGCCA
>comp2133_c0_seq1
CGTGGAATCAGAAAGAGAGGACATTATTGCTCATAGCATTCTCTCCAGATCTGCCACTGC
ACTACTTCGACGGCAGCAAGAAGAAAATTGAGAACAGGTATACGGAATTCCTTGTCTCGA
TTTTCTTCTCATCACTAGATCCCTTCGCTCCACCATCGGATGGGTGAATCTACTTTTATC
CATCCCGGATTTGACACGTCGAAGAATGTAGCCACGAAAGTGAAGAGTATCACGAACAAT
AAAAAATTCACGTCTGCGTTGAAGAAAACGAACCCAACGAAGCAAAATAAGTCACGCCTT
CGGAACGCTGTTGTACGCACTAGCGTTTTGCACGAAAAACATAGATTTTTCAAAAATGAG
TTCTTATTGCGTCTAAAGAAATTACCTTCCTCTCTTCTCGTCAATGCCGGAGAATCCTTT
GTTGCTTATTCGCATAATGTTAGCACCGTCTTCGACTGAAACATACTCATCTTCATTCAA
AGAAAAAAGAAGACTTATTTCTCGTTGATCGCATCCCAACTAATATTCTCACTGTTATCT
CCATAACTCCGTCTTTAACGATCTTTAGTCAATGCCTCCCAACAATAAAATGATCGTTAT
TGATTCTACCCATACTGAAAGCTCCCCACTCCTTATCAAGAAAGATGGTAACGATCAAAT
CTCGATCGGCACGGACAAGACATACAATTCATCCGAAGACTTTTCTGCCGTTGGCAGTGA
TGGCTTTCGCCCGGATCCTTCGTCCGTCGTGATTTTGCTACACCGCGGGAACGATAACGA
AAGCGAAAGGAAAGACCACAATGACGGTGATCACACCAAGCTTAAAAGCAACAACTGCGA
AGAACCTCTTCAAAAATCCCTCATTGAAGGCCTGGCAGAGGAATTCCACGACATTACCGA
AGCCGCACTCAATGAGGTCTACTATGTCCCCGACGATGAAAACTACATGATCGAAATGGG
ACTCACCCGAAATCTTAGCATTCTCCACGACGATGTGGTGGAAGCCAGCGTTTTGTTACA
GGGGAGCTTGCTACTTACATCCGAAGATAACTATGACACCGAGGCAGGAGATGACAATTG
CGCAGAACGATTTGTTCCGGGTGCTGTTGCTGCTGCCGTGATCGTTCCGTTGGGTGCTTA
CCTGCTACTCTTCAGCGCCATTGTCGCGATGAGCACTATTGGACCATTGCTCGAGCTTCA
GGGCGACACTACCGCAACCATGAAAGTCCTCTGGCGCATGATCGGGACGTCCCTGCTTCT
TGCACCTCGGGCTTTGATTGATATATACCGCCAGGCCGGCGTGCTGAGACTTACCCCACC
CCAATGGATCACATTTTTCCTGAGCACCTTTTGGTTCGTGGTCATGGCGATTGGCTTTTG
CTTGTCCTTGGATTATACATCGATCGGCAATGCCGTGATTCTGAGCAACAGTCAAGCGCT
GATGCTTCTGTCGGGCAGAATGTGCACCGGCGCCCCCGTGACGATGCTGGAATTCACCGG
CGCCATAGTAGCCTTTGGCGGGGCCGTGCTATGTTCTAGGGATGCCGCGGATACCATGGA
CGACGACAAGGACGAGAGCATAGGATTCGGAACGACCATCACGGGAGATCTGCTCGCTTT
AGCGAGCGCCATCGGCGGGGTCGGATACGTCACGCTGGCGAAAACATCGAGGGCTCAGAT
GTCATTGTTTGTCTTCATGTTTCTGACCATGGCTTTGGGTGCCGTCATTGTCGTGTTATT
CCAGGTGGCAGTGCTGAAAGAGATAGTCACCTGGGATCGCGACGTCGTTCACGGAGTATG
GGGCTTTCTGGTATGGGACCGATTCGATCGGCTTCCGTTGGAACTGGTGGTGGTGTTGAT
CTGCAACCTGGTTGGAACTATGGGCTACGTCCGGGCAATGCCCCACTTTGATGCCATCGT
GATTTGTTCGGTCCAGCTCCTCGAACCGACCATCGCCGAGTTTTTGAGCTACTTTGCGGG
CGTTGGGGTGTTGCCGGGGCTCTTGGGTTGGATCGGGAATGCGGCGGTCGCGGGTGGTAC
GCTTGTGGTGGTCAGCGAGAGCCAGAAATTCCATGACGCGTCGAAGAAAACGAGCGACGT
GCATTGAAAAGCGCTGTTCTAACTATTGCGATCGATGCCTAGCGAAGAATCAATTAAGAG
AAGCACGAAAACCGAAGATTAGATTGAATATACTTCGTAATGATAGATATCATGGAGCAG
GATGATCATAAAAAATTAAAATAGAAAG
>comp2134_c0_seq1

CACTGATTGATTCGTGATTGTTTGTACGGCACCACTATTGAGTGAGAACATCAAGAGAAA
CTGCCTCGGCGCACAATCGATCACTTCTGTCCTTGGTGGATTTTCATTGTCGTGTTAACG
AGATCAAAAATTGACTTTCTTCCTGCTGCAAAAACAAGACAGACTGTTATCAAAGACACG
ATTGCCTCTGGTTTTCGTTGTATTGTTTACAAAAAAATTTAATATACGGTAGATTCACAG
TAGCATCATAACCGTCTCAACTGATAACATGGCGACGCCTACGGAAGTTCTTTCTCAGGG
CGTTAGCGAAGAAAAAAACGAAGACAAAAATAGCGACGGCGAAAGCAACACGAAGCCGGG
TAGCGTACATCTCATGCCCTCAAACAACGCCATTCCTTTCCCTTGGAAGCTCCATCGGAT
TCTAGGCGATGCAGAGACTCAAAATTTCAGCGATATCATCTCATGGGTTCCAACAAATAA
CGGCTTTAAGGTCCACAAACCAAAATCCTTCGACACCGATATCATGCCCACATACTTCAA
CAATACCAAGTACAAGAGCTTCCAGCGCCAACTCAACATGTGGGGATTCGATCGCGTAGG
GAGCGGTCCCTTTAAAGGAGCCTACCTTCACCAATACTTTGTCCGTGGCAATCCAGACCT
CTGCGAAAGGATGCATCGCACCAAGATCAAGGGGATCCATTCCAAGAAGCTGCGCAAAAA
TAGATTCCAGGCGTCCCATGGTTCTGACGTCAGATCTTCGCATTCGTCTATGATGACTAT
GGACTCTGGCATGCCTTGTTTCCCGTCGCCTCTAGATCCCCATGAAAGCTTCAAGGCTGC
GGCACAAAAGGTGGCCGATCTGGAACGCAAAAAGGAGGAGATCCAGCGGAAGCTCGAGAT
GGTTTCGAACAGGGCAAACGCCGCCGTAGCATCGAACGCCCTCGCCAACATGTCGGGGAT
GATTCCGCGTGTATCTATAAACTCAGAGATGCAAATAGGGCAAGCGTCAACCAATAACTA
CAGGAGAAACGATGACTTTTTCCCACTTCCTGGTGCCGGCGAAGGAGATAGTCTAATGTT
TGGCGGGCAGACATTTGTCTTCGCAGACAATAATCAGAACCAGCAACAGCAGCAGCATCA
CCAATCACCCAGGCATCGTGTTGCTCGTCGGTATAGCCTCGAACCAAAGGGACCCGAGAG
TGATGAGTATGTTCTGACCGAACTTGAACAAGGAATGCACGGTAACAACATAGGCATGAA
AACTTCGGAAGAGGACTTTCTCATGCCAACGCCACTACCGCCAAACATGGACGTGATAAA
GGTTACCACCAAATTTCCCAACAGCACCCCCGCTCCTTTGGTAATCGGTCTCGACAAGCC
CAAGCGACGATTCAGTTTCCTTTCCACCCCCGTTGAAAATCCTTTGGACAGGCCCTACGA
GATACAGCCTCGCCCAAGTCTTGCGACGAACAACAGCATGATGAACAGGTCGATTATGAA
CAGTTTCGACAACCAGAATGGCATGATGATGATGAACTCGAATTATGAAAATGATTTCAA
TCGCAGCACGAGCAATGCCCGAGACATTCTTCTTTCAACGAACCCATCGAATCGAATATC
ATTCAACTAGCACCACATTTTGGGTCGGGAAGAACTATACATCATGTTCTATACTACTGT
GCTACATTTTATTTTTCTACATGCGCAAGCTTGGGGGGAGGGGAAGTGGAAGGGGTGTTC
CTTAGTCTAAAGTCCATAATACCGTTGTACTGTCTAAAATCAAATATTGTAACGTAAAAT
TACTAC
>comp2135_c0_seq1
TATTTACACAAGATACTATCAATAAATTCAAAACAACGACATCCAAAATGGTTATTAGTC
CATCTATTGTAAGCTATGGTGCCGCAGACGGCATTGGCACCATGATGACAGAGGGCGTTA
GGACTCTCGCCTCACAGCCCGCACCTTGGCTCCTCCAATCTCATCTGGAGACCATCATGT
TGGGTTCCGTCATTCTCGATGCCATGGCTGGAGGTATCATCTTCATCTTTTCCAACACAA
TCATGAAGACCTTTGCGGAATTTGATGACGAAATTGGGATCAATGTCATGGTTCTACTCA
ACAAAATCATTATCAATCCCCTCTTTGCGTTTGTCTTCTTCGGAGGACTCATCTCGGCAT
ACCCTGCGATTGTCATGATGACGAGACCTGACGAGTTCTCGAAGCCTGCTCGCTACTATG
CATTGGCCTCCACTTTGGTATTCTTTTTCGGAGAATTTCTGGTCACTGTGACGCAAAATG
TTCCTCGCAACGATGCTTTGATGGCCGCAGATCCTACATCGGACGAGGGAGTGGAGTATT
GGCGAAAGGATTTTCTCGGAGATTGGGTGGCCTGGAATACCGCTCGATGCATCGCTTCCG
TCGTTGCGTCCATCCTCGGAGGCCTTTCCCTCATGGCTATGAGAAGCTAACAATTCTCAG
TACCGCCTCTCTTGTTGCGAGAATCAATTTTGTGCGATCGTGCGCACTGTTGTTGTCACG
ATGCGACAAAATTGAAACCACCCATCATCATTTCTTCTCGTCGGCAAATCCTGGAAGCAA
CACGACACACGACGAAACACCATCTAAACGATGGACAGGTTGTCCATAAACAAGTGAATA
TTTTGCGCGCATATTTCTGCCGAATAAAACTTGTAAATGTATAATATTAGT
>comp2136_c0_seq1
TAGAAATGCCTGAGCAGATTTACGACACACAGAAATCAAAAATTAGCCAAGCCAACTACG
TCACGATTGGGATGGTATCCGCCTCATTAGGATTTGCATGCAAGGAAAACATGCAATATG
TATTCCAATCGCAATACGTGGGAGATGAACTATTGACCCTTTTCTTGCGTTCTCTTCTAG
CCTTACACCCGCTTTGTGCTGCAATCCAGTCCCTTGGTGTTATAAAACGTGACATTCTAG
GAGATCCTTCATCTTCCTTGGGGCGGATTCTTTTACCAGCAGTCATTTTGCATGGGGCGT
TTGATTTTGCGGAAATGTTGCTCGCTTACTTCCTTTATATTAATGACTTAGAAGAGCAAG
GGACGTTTGCCCCATCGTCGGCACAAGACACAGGTTCACAACCTTCGTCAGGGATTGGGA
TAGTGTCGCCGGTTGGAGTTGGAGGTATTTTTATGATCGTGGGATTGATGTATTACTTTT
TCCGTGCTCGTCTACAACAAAATCAGCTTGCACTGGCTCACATGAGCGAATCTACATCTG
CTCTCGAGCTGAGCTCTTTTGTGTAGACAACAAACGAAACGGGAACCAATAACATTCGGC
TCCGAAAATAGATGGAACCAATCGAACGATGACTTCATCCTTTCCTTCAAATCTCTCTTG
GTGTTTGCGCTTGTGCTTCTGCGATCGAAGAAAGGCAACACGTTGTCATAATGCTCCATG

AAATGTAGCCGTACCTTGCAGCTGAGGTCCCGTCCCCGATATGACCAGAATGATTGTCTC
CATGTCTAGTTCCAGCTTTTGATTTTGTGACGTTCCCGTGAATGGGAATGCTATATTTGA
ACGGATGTGTTCAATTGGTGATAAAGCTATCGCATCCTTTATCAGAAAATAAATTTATTA
CTACTTCGAATTTCATAATCTAAGCGCAGTTGCTCCTAACTGCTCTTAGCCCGAGTTATT
GTAGCCTTGTCTTTCCGTCTTCCGTTATTGGGTAGTTGGTTGGTGCAATAGAGGTCTAGC
AGGGAGGATGGGATGTGTCCCAGGAGTCTCTCGTTTTTTGCTTTTGCAGTTGTATGGGGT
TTGAGGAAGGGGATTAAGTTTTTGTTTAGGGCTTGTTCTGTTCCGTTCTGTTGAGGCCAG
TGATTTGTGGGCACTTCCTGTCATTGGGGTCAAGTGCTTCATGGCATAGGATCGTGAGGA
CCTTGCAGTTGGCTGGGTGTAGATCAATGACATGCAGTACATTTTGACTGTGTCCTTTAG
GATGGTGACAATGGCGTTGCCAACTACAGGGGCAAGGATGAGGGCTGGCATGATGGCGGT
GTGTAAACTTTGGGAGAATTTGGTTTGTACAAATTTTACCTTGTGAGCATGTGATGCGGT
TGATTGTTGGTGTTGTTCTGCAAATTTTTGCCTTGTTTATTTCCGGGGTGCCGTGGTGTT
GGAGTGTACTTTTTCTGGGGTTACGTTGCCTAAATTTGTCTTCTGTTGGTGGTGGTTGCT
GAATTTAGTAGTTAGTCGTCAGTCTTGTTGTTGTCATTTTTTGGCATTTCTTTGCTGTTT
GTTCATTTTTGTTGGGTGTCATTTTGTGACATGGGGTGTCTAAGTGGGCAAATGTGATTA
GATCTACATGTAAAATGTGTAACAGTCTGGGATTCAAG
>comp2137_c0_seq1
ATCTCATTCTCCAAGAGAACAGAAACAGTAGTAGTAGAACTGTACAGCAAATCGCAAGCA
TGGGGCAGAGTGCGTCGAAGGGATTGAGCAAAGCGGCGGAAAAGGCAGCGAGACAGGCAC
CTGCGAAACTGAAACGACCACCGATTCCCAGCCGGGTGCTCCGGACCCAGGAAGCCTCGG
CCCAGAATCCAGAGAGTTTTCTGCGGGGACAAGGGATCGGTGCCCAGGACCTACGGGACA
GGGGCCAGGAAATGTACCTGCAGCACGCGCAGCAACAGCGACAGCAGCAGCAACAACAGC
AAC
>comp2138_c0_seq1
GCCGGACCGCCGCCGCCGCCAATTCCCTGCTGCGGCGCATGTCGGTCCGGGCCCGCCTGT
CCGGAAGCGTCGACGGGGCCCTCTTCATGAGCAAGTACGAACGGCTCAACCAGGTGATCG
ACTCGCCCCGCCACAAGCACCGGAGGGCCAGGGGCTCACGCCACGAGCGGGGCCAGCCCT
ACACCGGAAACGACAACACCCAGGGGCACAACCACGACCTCTTCCTCGACGAGGAGACGG
TCGAGGACGAGGTGGGGGGGAACGCCGACTCGTCCTCCTCCTACCGGGAGCACATTCCCT
CCCCGACCAACAACGTCGTCATGGACCCGACCCTCTTCCGGGCAAACCGGTCCGGCCAGC
GGGTCCAGGTCCGGTCCAGCATGTTCTTCGAGCAGGACGGGAGGATGCTCATGCGGCTCC
CCCGGGACCAGGTCCGGCTGGTGATGGACTACAACCTCGAGCCGGGGATCCTCTCGGTCG
AGCAGTGGAGGAGCAAGGAGGAGGCGGACCGGACCATCGAGCGAATGGCGGTTTCGGCCG
ACGCCGCAACACCCCGGACGAAGGGCGGCCCCAACCATGGCTCGCAAACCGAGCGGCGGC
GGCGAGACCGCGAAAAGCGGCGCCTGCGGCGAGAAAAGGAACGGGAACGACGCTTGCGCA
GGAGGCAAAAACGAAAGGAAAAGAAACAGAAAAAGAAGCTGCTGCGGGAGAAAGCAGCGG
CAGCAACAGCAAAGAACGACACGGACGGATCGGCAACGGAGGACCGTGACGCCAGCGGTC
ACGATCCGGGGGATTCGTCGTCCTCGTCGTCCTCGTCCTGGTCCTCCAGCGACTCGGACG
CAAGCAGCAGCTGCGACACGTACCTGGACTACGACCTGGAGGAGGGACGCCCCGGCCTCG
CCGAGCTCCCCGAGCTCAAGTACGTCATCACGGTCCCCGACGACCTCTACCGCCGGATGG
TGGGGGAAATGAGCGCCAAGATCTTTCCCCCCGCCTGGGGATTCTTCAAGTGCTGCCACC
AGGAATCGGAGAGGGCCGACATCAAGCTTGCCCTGGTGATCCTGGCGGTCGTCATGGCCC
TGCTGTTTGTCGGGTCCCTCGAGTGGCGGACGACCTAGGGGTTGGCCTCGGGTCCGGGGC
TTGCGCCGTGTCTTGGTAGGGCCGGGCAAAGCACAGCGCCGCGTAGCGTAGCCAAGCGCA
GATGCGTGGCAGAAAATAGCGCCAAAGAAAAAAGAAATCA
>comp2139_c0_seq1
AAGATAAATTAGGTAACCATCACTGCTCTCCGCACCCTCAACACATACGTGACCGTATCG
ATCGCAATGTACGAAACGATTCTTTGCGTTTCACTTCTGAACTGCATAAAAGATTGGATC
CAGATCATATGATAAATTCATCAAAAACGACTGACGGTGTTTAACTTTCATACAAATCAT
GCTGGGCGAGAATTTTACCGGTGCGACCAAAGTGTCGTCGCTTGCGACTTCTCTCTTTAA
TGAATCATTGAGTGACTACATTGACTATCACAAGCCGGACCCAATTGCTCGACGGCAACT
AGAATCGTGGAAAATGAGCTGGTCAGAGGATACTAACGATAGTAGGAAGAGCAATGACAA
TGCAAACGACAAGAAGAAGCATCTCGATTGGAAACCCACGAATGCTATCGAGGGGAAGCT
TGAGATTTGTGGTGAATATGTTGCCGCTACGATAAAAATGGAGTGTTTCTGGACCGATTG
GAATCCGGCAGTAAATGACGACAAAATAGAGGACGGCAGGCTCTGCTTCTCGTGCAAATT
GATCTCCGGCCGGTTGATAGACAAGAAAGACGGCGAAGAAAGAACCGAGCGGAAAGTTCG
CAAAAAGATGCTCACTAGGCTACGAGGGGACTCATACATTTCAAAGATGCTCGCAATGAC
CGAAGGAGGGAAGTCTGATACCAAAGATATTCAAACGCATCATCAACTCTGTCTAGCCCA
AGCTAGTATTTATGTGAACGCTACAAAGTTCGAAATGGAGGAACGTGTGGATGTGTCTGA
ATCCGTGGCAGAGACGCTTCGGAGGTCGCTGTGGCCATCCATCCACTCGTCTCTGGATGT

GGTGGAAGTTATTTTGGCCCTTCCGTCTTTGCCGTGCCGCACTCCTGGCAGCAAAACGAG
GGCTACAACTCGGTTAGCCAATCGTGCAAAGTTACGGATGCTGGAAGATGCCATGCTGGA
TGAATGCGAAAAAGAGGGAGACGATCAAATAATTGAAGACTTGAACATCTCAAATGAACA
AGCGTCTTCAAAAGAAGACATTATAGAGCACGAAAGTGCTGACTCTTCGTCACGCAAGAA
GAAAAAATCTAAGAGATAGTGCGAGAGATTATCAAAGCATTGGATTTCTAAAATGAGTAG
CATTGCTGCTGCTGCTAGAATTTTGATACGAAGTATTTCACCGGTAACTCCCATTGGCGG
TTGCTTACTGTTTGTAATAACACATATATTTGGTCGTGCA
>comp2140_c0_seq1
CACCGGTACCTATCCGGTGTTCCAAAAGTACGAGTGCTTGCAATCTCGTCTACAGACTGT
TTGGCCTCCCTCGATGCTTAAACTCATTCCTTATCGTTGTTCTTTAGTTAAAGCCGAAAA
AAGGAAATCAAGTTATCGTCTGTTCAACGCAGATACATAATAGAAATCAGAGTTCAATAC
TCAACTTGATACTTTTTTTCAGAATCAGCAGATGTCGCGTATATCGTCTCCGTCACCAAC
CCTACGACGTTTCTTATTGGTAGCGCTAGCGTTGTCGTTGTCGTCCAATGCTCCTGTACA
CGGGTTCTCGACAACTTCTCGTCCATTCGAAGGACGAAGGCGATCCATGATAGCGGCGAA
AGGCTTTGGAGCAGCCTCAGCCGAGAAAAAAACAGAACCGCCCAGGAATGCAGCCGATAG
CAACACACAAAACGACAACAATAGCGGAGAAATATATTCGAAGCCTTCCATGTACGACAT
GGCTTTTGGATATCGAAATTTCGAAGAGGAAGTTGACTTTTTGATGGACCAGCACCGGCA
ACTCAATCCGGACAATGCTCTGCCTAAGCGGGTTTTGGAATTGGCAGCAGGACCGGCGCG
TCACTGCATCGAGGCACTCGGGGCCTCCTACATTGAATCGGCTACAGCGATCGACAATTC
TGCGGACATGATAAAATATGCCAGAGAGATTGCTGCCGAGGAACTCAAGAGCGACGATTA
TACTTTTGAAGAAATCAATGGAGCCGACGATGTGACAAAAGATTCCGCTTACGAACACCG
GGTCAATTCCTTGAACTACGTCCAAGCCGACATGACCAACTTTTCGTTTCCTGGTGATGA
TGAAGCGCAACTCTTTGACAGCGCATGGATTTTGCTCGGATCCCTTCAACACTTAACGAC
CAACGAGCAAGTGATATCCTGCTTCAAATGCATTAACCGTGTCCTTCGACCGAATGGAAC
CTTCATTCTCGAGCTGCCTCATCCCCGGGAGACCTTTTCCATGGTGGAATGCACCCGAAA
CGGCTGGGAAATTCCCTTGGAGGACGAAAGTGGAGGTACCTGTGGTGAACTAAAGATTGT
CTGGGGGGACGACGACGACGACTTTGATCCAATAACGCAGGTACGACAATTTACTGTCTC
TATGGAATTAACTGGAGAGAATCCAAATGATGCACCCATTCAGAGTGTTAGAGAGGTGGT
ACCTATGCGGCATTTCACATCGCAAGAAATCGATATGCTTGCTCGAATAGCAGGTTTTGA
GGTCATGTCGATGCATGGCGCGCTGCAGTTCGATGTCGATGTGAATGATGATGACCAAGC
CTTTCGACTCGTTTGTGTCCTTCAAAAAAAGTAGATCATCATTTGTTGATTAATAACAGT
TATATCTAAAAAAAAAAAA
>comp2141_c0_seq1
GACCTGCATCCTCCGTGTGATCAGCATCCTTAGTAGCAACACCGCCTCGATATAATCTCA
GCAAACTCAAGACAAGATGACTGTTGCCGGTATCATGCGCCAATACCAAGTGGTCGGCCG
AAAGGCCCCCACCGATGACGTTCCTAACCCAGAAGCGTACAGAATGATCATCTTCGCCCC
CAACGCCGTTATCGCAAAGTCGAAGTTCTGGTATTTCATGCATCAATTTAGAAAGATGAA
GAAGACCACCGGTGAAATCTTGGACTGCGTTGAGATTGTCGAAAAGAACAACAGAATCGT
CAAGAACTACGGTATCTGGCTTCGTTACAACTCCCGTTCTGGTACCCACAATATGTACCG
AGAGTTCCGAGATGTTTCCTTGACCGGAGCCGTTTCACAATTGTACGATGAAATGGCCGG
ACGTCACCGAGCTCGCCCTCGATCCATCCAGATTATCAGAACAGCGGTTGTCGGTGCCAA
GGACTTGAAGCGTGTCAACGGTATGCAATTTGCCCAGGCCAATGTTAAATTCCCTCTTCC
CCACCGTGTTGCCAGCGGAAAGCGAGGACAAAAAGCTTTGTCTTCGTCCACTTTCACCAC
TGTCCGACCTACAACCTTCCTTGGTTAAGCAATAAACGAAATTCCAATACCCCAAAATTT
TGGTGAGATTAAAAGCTACAACCGCGATCATCGCCTGCACTGTGACATAAGTGCCTTGAT
ATACAATCGTATTGTTAGAAGTAGACGTATGAGCGAGAAGAGGAATCAAATGTTCCTTGG
CAATAATTCCTCGAACTTTATCTGTAGATTAGGAACTTTTCTGCTTAGTTCCAATCCCAA
ATTAAGCACGGAGTCAGTTATTGAGATACTTTAAAAGAAGTGGGAACATCTACGATGTCT
TTTCGAAGAAAGGATTGGGAATAGCGAGAGCATAAATTTCATCAAAGGCGTTATGTTTTT
TGATACAGAGAAATGACGAAACCCCATTCGCTTCACGAGCCCTTCAATCCCGCTAAGAGT
ATCGGCGTGAAGACATTATCGGTGTATAATTCAGTCTAGTTTCTAATTACGGTAGTTCTC
GTAGCTTGCACGGGTCCATTTTTCATCCGGCATCCTCTTCCCGTGCTACCACTTGAAACC
GTGGTAACCCGAAAACGTGGATTCGTCGTAGTCCAGGTGTCCGATGTCGCCCATCATCCG
TGCGGACACCATGCCGTTCGGGCGAACGACCAGATAGGTAATGGAGCAGTTGAACGTGCA
CAGTCTCAGCCACGCCTCGGGCGGCAGCTGCAGCGCGCGGCAAAAGAAGTACCGGATAAC
GTTGCCGTGGCAGACAATCACCTCGAAGGCGTCACTCTCGCCCGCTTCCTCCGGAGATGG
ATCGTCGCGTTCCCGTTCTCCCTCGCCGTCGTCTCTGTAAAAGTACTTCCGAAAGGCCGC
CTCGATTCTGTCGTGGTTCTCGTCGATTTCTTCCTCTGCACCCTTAATGTCCGGTCGAAT
GGGCACCATGGGAGCCGGTAGGGCCTCGTTGAGCATCGGATCCGGTTCCCGCAGGAGTGC
TGTTTCCCCGTGGAATTGTTCGGCGATGAGTGCCGCCGTCTCCTTCGCCCGTGTCATATC

GGAGACGTGGATCGCCTTGACGGCGCATTTCCCGTTGAAGCGATGGTCTTCGAACGATGC
GGAACCCCTGGCCATCTCGACCAGTCGCTTTCCCGTTTGAATGGCCTGCCGGCGGCCCAG
AGGCGTGAGCTTGCGGTGTTTGTCTTCGTCCGACCGCTCGTCGTACTGACCGTGTCTCAC
CAGTATCAGGTGCCTCGATCGCTTGTTTCTCGTGCCGTCGTTGCCATTATTCTGTTCGGC
TCTGCTGGATGCATTTGCTTTGGCTTCGCCGTTTCGAAAGGCTTCC
>comp2141_c0_seq2
GACCTGCATCCTCCGTGTGATCAGCATCCTTAGTAGCAACACCGCCTCGATATAATCTCA
GCAAACTCAAGACAAGATGACTGTTGCCGGTATCATGCGCCAATACCAAGTGGTCGGCCG
AAAGGCCCCCACCGATGACGTTCCTAACCCAGAAGCGTACAGAATGATCATCTTCGCCCC
CAACGCCGTTATCGCAAAGTCGAAGTTCTGGTATTTCATGCATCAATTTAGAAAGATGAA
GAAGACCACCGGTGAAATCTTGGACTGCGTTGAGATTGTCGAAAAGAACAACAGAATCGT
CAAGAACTACGGTATCTGGCTTCGTTACAACTCCCGTTCTGGTACCCACAATATGTACCG
AGAGTTCCGAGATGTTTCCTTGACCGGAGCCGTTTCACAATTGTACGATGAAATGGCCGG
ACGTCACCGAGCTCGCCCTCGATCCATCCAGATTATCAGAACAGCGGTTGTCGGTGCCAA
GGACTTGAAGCGTGTCAACGGTATGCAATTTGCCCAGGCCAATGTTAAATTCCCTCTTCC
CCACCGTGTTGCCAGCGGAAAGCGAGGACAAAAAGCTTTGTCTTCGTCCACTTTCACCAC
TGTCCGACCTACAACCTTCCTTGGTTAAGCAATAAACGAAATTCCAATACCCCAAAATTT
TGGTGAGATTAAAAGCTACAACCGCGATCATCGCCTGCACTGTGACATAAGTGCCTTGAT
ATACAATCGTATTGTTAGAAGTAGACGTATGAGCGAGAAGAGGAATCAAATGTTCCTTGG
CAATAATTCCTCGAACTTTATCTGTAGATTAGGAACTTTTCTGCTTAGTTCCAATCCCAA
ATTAAGCACGGAGTCAGTTATTGAGATACTTTAAAAGGTGAGGCAACTTTAGTAGTCACT
CGATGGATAATACCAAGCGTGGCTCTCGTTATCAATGAAGTGCTTTCTCGATATGAGGAA
ACTCTTGTCTTCATAGGTACCTGCAGCAAACCCGTCTTAACGTCAATCTCTTTCTGCATC
ATCAGAAGTGGGAACATCTACGATGTCTTTTCGAAGAAAGGATTGGGAATAGCGAGAGCA
TAAATTTCATCAAAGGCGTTATGTTTTTTGATACAGAGAAATGACGAAACCCCATTCGCT
TCACGAGCCCTTCAATCCCGCTAAGAGTATCGGCGTGAAGACATTATCGGTGTATAATTC
AGTCTAGTTTCTAATTACGGTAGTTCTCGTAGCTTGCACGGGTCCATTTTTCATCCGGCA
TCCTCTTCCCGTGCTACCACTTGAAACCGTGGTAACCCGAAAACGTGGATTCGTCGTAGT
CCAGGTGTCCGATGTCGCCCATCATCCGTGCGGACACCATGCCGTTCGGGCGAACGACCA
GATAGGTAATGGAGCAGTTGAACGTGCACAGTCTCAGCCACGCCTCGGGCGGCAGCTGCA
GCGCGCGGCAAAAGAAGTACCGGATAACGTTGCCGTGGCAGACAATCACCTCGAAGGCGT
CACTCTCGCCCGCTTCCTCCGGAGATGGATCGTCGCGTTCCCGTTCTCCCTCGCCGTCGT
CTCTGTAAAAGTACTTCCGAAAGGCCGCCTCGATTCTGTCGTGGTTCTCGTCGATTTCTT
CCTCTGCACCCTTAATGTCCGGTCGAATGGGCACCATGGGAGCCGGTAGGGCCTCGTTGA
GCATCGGATCCGGTTCCCGCAGGAGTGCTGTTTCCCCGTGGAATTGTTCGGCGATGAGTG
CCGCCGTCTCCTTCGCCCGTGTCATATCGGAGACGTGGATCGCCTTGACGGCGCATTTCC
CGTTGAAGCGATGGTCTTCGAACGATGCGGAACCCCTGGCCATCTCGACCAGTCGCTTTC
CCGTTTGAATGGCCTGCCGGCGGCCCAGAGGCGTGAGCTTGCGGTGTTTGTCTTCGTCCG
ACCGCTCGTCGTACTGACCGTGTCTCACCAGTATCAGGTGCCTCGATCGCTTGTTTCTCG
TGCCGTCGTTGCCATTATTCTGTTCGGCTCTGCTGGATGCATTTGCTTTGGCTTCGCCGT
TTCGAAAGGCTTCC
>comp2142_c0_seq1
GAACGACACAACGAAGACATCGATCAAGATCGCACGGATACTTTTAGACATCGCTGGTTG
ATCACCACCAACGTCGTCCCGCTCTAGCTAAATTAAACTTTATTCCTTAAAGTTAGTTGG
TGTTACAATCAATCGATGGATCCCGCTTGTACAACCCGGGGTTTGTGACAGTTTTCCCCC
CGTTCATTACACCACAAAGAAGCAGTGCAGCGAATCAAATCGAGCAAAGTACAATCGAAA
CGATTTTTGGTCTGTTCCGTTTCGTTCCGGATAGTGACATGGACCGTCGCTCTTCTCACA
GTAACAGCAACCAGAAAAGAGGAACATTCGGTTTCTGTTTCATCGAGTCACTGTTCTTGC
TGCTGCTGCTGCTCCTGTTGCTCCGTACCACCATTGCAGCACAGTGCGCAATGCCGCTGT
CGTTTGTTTGCAACCGCACACACACCGCCGCAATCAGAACCCTGCGATCGTTTTCCACCA
CCGCCGTAAACGCAAACCCCAAAGCCAGAACAAACAATATGCCGAAGCGAAAAGCCGGTC
CGAAGTTCTACGCCGTCGCTTCCGGTCGGATCCCAGGAATTTATACGACCTGGGCCGAGT
GTCAGACCCAGGTCAAGGGATTTTCCAACGCAAAGTTCAAGAGCTTTGGCACCGTTGAGG
AGGCCGAGTCCTTTGTGAAAAAAGAAAACCCGAACGCCGGTGAAAAAACGCATCGAAAAC
CTCAGGGATCCGTTGGTGCTTTGGTGTCAGAGAAGGCGACCTCCGTAAAGAAGCAAAAGC
ACAACCTCGGCAAGCCCTCGTCGTCCTTTGCCGGATACGTTGCTCGAAACATGGAGCGCT
CGGAAGTCCGAGCCGATCCCACCCTCAAGAGATTCGTCGAAGCCGAGATCGATAAGATTC
TTTTCTCGGAGCAGCACACCGACGCCGCCGGTACGGACTGGAGCATCGTGCCCCTGGTGC
CCCTCCCCGCGACCGACCCTCCCGCGACCGAATTCGAGAAAGGATCACGAACCGTCGGCT
CCAGCTGCAACTCCGACCCCGACAGCGACGGCGACAACCTCGCCCGGACTCCTCCACCCC

ACAAGCTGTCCTTCCACGTCATGTTCGACGGTGGATCTCGCGGGAATCCCCACGGGCACG
CCGGAGCCGGAACCTACGTTGTCACGCGATCCTATTCCCGCTCCGGACCACAAAGCGAGG
AACCCGACAACGCTGCAGCAAAGGGGGCACGCAAAACACCAAAGACGACGAGACCAAAGG
AAAGCCTTGCCGAGGAACGGGCAAACATCCGGACGTATCTGGGGATGGGAGAGCTCACCA
ACAACCAAGCGGAATACCAGGGTCTCTTGACCGGGCTGGAATACGTTTCGGAAGCGCTGA
AGCGGGTCGACCGGCTCGCCTCCGTCGAGCTCCTCGTCCAGGGCGATTCGGACCTGATCC
TCAAGCAGGTCAGGGGCGAGTACGCGTGCAAGAGCCCAAAGCTCAGGCCCTATCATTCCC
GGGCGGTTGCGCTGATCGAGGACCTTTCGAGGGACTGCCGAAGACGGCTCGGGTGCGCCC
CCGAGATCGCCCTGGAGCACGTCTACCGCAACCACAACGAGGTCGCGGACGCGCTCGCCA
ACGACGCCATGGACGCCCGGAGGAGCTGGACGACCACTACCACGAAGAAGACCGGGGCGA
CCGCTCCCGGAGAGAAGACGACGAACGACCAAACCGTCGATGGAAGGGGGCCGGGGAACG
ACGACGGGGCCATTGAGGTGTAAACTATAGAATGCATTGCAACAACAAAAAAGAAAGTAC
TGTACTAGACTGCAACGCAGGAATGCAATGCCGTGATGCGGCCGCAATGCAACAAGAGTA
CAACAAGTGTGCGACACGATGGCATGGCGCTTCTGTATTTGTGTTGCGAACACCGAAAAG
ACGAATGCCTTTCCACAGTTGGAAGAAATTGCCTCCGGG
>comp2143_c0_seq1
GCCAACTAGTTTTGTCTTAGTCATCAAACTCCCTTCCATACATCATGCGTAACTCCAACG
ATAACAATAAACACAATGGCAACTCCCATTCTACTTCGACTTTGGAAGATCGGCATTTTC
TTTCGGATCCACGCCCGGTACGAGATCTCAAGCTGCCGACATGGGAAGTTGATATACACC
GCGAGGGAGTTGACATGCCGATCATGACGCTCAGGGTCGACTCGACAGTGCTGCCTCCGC
GAAAGAATAAAAAGAAAAAGGTCCTGTGGAAACTCCCCGACAACATCGACACCGCCCCAA
AATTTGGGGAGCAGGACAAGCGCCGGGTCCTGGAATTGTTCCGAGAAAAGAAGAAAGAGC
TCAAAAAGCTCAAAAACAAACAGCGAGCCTATCGAAGAGCTTCATCCATGGAGTCAATGA
ATAGCAGTGGGGCCGGATCAATAGATTTGAGCACTCATGCGGGAGGCGGTGGTAAAGACG
ATTTAAATTCGAATGCGGAGGGTCTTCCTCCCGAAGCGAATGGAAAGGACAAGCAACACG
AAAATCAAAAACAATGTCAGCCGACTGGAACGAATGAAAAGAACAACGGAAATAATTCCA
ACCCAGATGTTGCAATTGTTGCCAGTGACGGTTTGAATCCTTCGTCGTCAAAAACTATCG
AGGAAAGGGGCGGCAGAAGGGGTGCTCTAAGTCCACCCGGATTTGGGAATCCGCCGCCTA
GGACGTCGCCACTGAAACTGGCCCCGCCAGGTTTGCCATCTAAAAACAACGAACACTTGG
CAGAGAATAATTGCAAAGCGGCAAATGGGCACTCATCATCGCTATCACCGGCAACGTCAA
ACTCGACATCTTCACAGCCGCCCCTTCCTTCTCAAAATGGACACCATCCTCAACAACATT
TGCACCACGGTGCACAGCATTCTCTTACACCACCACCACCACCACCACCAGGATTGCAGA
ATATTCACGATCTCAACCAGGACGGTATCAGGGCTCCAAAAGCTATCCCACCGCAACAAC
CACCGATGGCTATCGGGCCGTGCTTCGTTCTTCCACCCAACGCTCCACCAGGAATGTCCA
TTGGAAAATTTGTAACCGAAACATACTATTTGTCGCTCCGGAACGGCTTGGTTCGAGAAC
TCGATGCGTATTATTATCAGAATGGTGGTGAAAGGAGTGGTGCTGACGTAGCGAAAGTAT
GTAAGGCGGTGACGGTGGGGGGTGCCCATGCCTTTTGCACAAACCAGCAAGATCGCTTGA
TGCAGCTCAATACCTTTTCCGGAACCGAGATGCGCATCAAAGGAGTCCAGGAACAACTGA
CGGTAGGGAATGGCGTCCTTGTTCTCATTACTGGTGTGAGCATCCGCTTCAGTGCTCCAA
ATGGATCGTCACCACAGCAATTACCCTTTTGTCACACGCTGATTCTAGCTCCCGTATCAA
CAAGAACACCAACAGCCGATTCCTCCACCATCGGATATCAGATACTAAACGACAATCTCG
TGATTTTGACGGCAGACGAATAAATCATAAAGCAGGGACAATCTCTCGTACTCTTTTTGT
TCCATGAGTACATTCGCAACAAGTCATCAATTCCTCTCTTTTTTTCTCAATCATTCTGTT
GTCACTGATCCCACTCATTGCTCGAGGAAAGAAAAAACGAATCATCATGGTCGGAATCAA
GAACGAGTCACTTGTTTCTCGTACATGGAGGTCTATGTTGGAAGATTGATATATTATTGG
AACATGCTCTCACCTTTCTTGCGAGAAACAGAGATGAACGGGTCTGTCTTAGTATCCATA
CAAATTGAGAAGCGCTATGCCCCGCCGAAAGGCGATGCTTATGTGAGGTTGGAAGTCTCT
GTAACCAGAAGTGATAAGACGATCATATCTGACAACGAGGGAGTCATAATGTGCGTTGGC
ATGGAATTGAGGTATATGTTCTTAGTTGGGGGAATCCTACGACACAAAAGAAAACAAGGT
CTACACTAGCTGACACTACAAAGGCTTTCTGCAAATCGTTGGAGATACTAGGAGGTTGGC
AATGGATTCCTTGACTTGATCAAACCGGTCGTCGGAAGACCTCTCATCTCTCGAGAGAGC
TAAAGGCAAAGAATTATCGTTATCCGTCGTTGGCGGTATCCCGCATTTGCTTCGCATCGC
CTTCACATAGTCCTGCAATAAATGTGCCAAAAATGATGCGGCGAGAGGCATCCGAAAAAT
AAGCAGCGATCCTCTTACAAGAGTGTTATCTTCGGTAGTCATCCGTGACAAACTGTGTTG
TCACAGAGTTGGTCTGGATGCTGGCTAATAGATACCATAGAAAAGAAGGAATACGTGACT
AAGAAAAAAAGACGATGGTAACGATTGCCCTTTTCGCTTTTCGATAGCACTTCTACCAGAG
TTGGCTTCCAACCTTCTTCTCACCCCTATTCGAACGTACGGTACACTTTTCTTTTCAGTC
AAAATCTGTGCATCGGTATGAGTCGTACCTGTACTCGTACGGTTCTTTTTCGTACCTGTA
CGTCTTGACGATTTTTTACACCTTCGACTATTTCGACTCATCATTTTTTTGTCGAATGGT
TCCTTTTGGTCACTGCGTCAGATTTTTTCTTACGTTTTGATTGGCTAAAATACGTCAGAT

GTATTATGGTTCAGATCGTAATTCTTTGGTTCAAATTTTTTAAAACATTGGAACTGGCCA
ATCACTACGTCAGTGAATATAAGTGTATTCTTATTGGCTCAAAGACGTCAGAACTCGTCA
TTTCTTTTTGATTGGTTCTCTCTGATTCAAAACAAGAATCCTACATTCTGTCTCGTGTCG
AGTGCGAAACACTAAAAACTCCAATCCTTTCGTCACCCATCAAG
>comp2144_c0_seq1
CCATCCCTTGATTCCTCAATGCTTTTCTTGCTTGCTACCGTTACACCGAAGGTAACCTTG
CCGTACACGAATTGCCAGACGTGTGTCATTCCAAAAGACAGCCGGCGGCAATCCAACCCC
TAACACCGCTGTGGGCATCCAAAAGCGCTTTGTACCCATCCACCGTTTTCCCAGAATGGC
GTATTTGTTGCCGCACCTGCACAGCGGGTGGGAGGTCGACCAGGCCATCGTCACGGCGGA
AGACCGGGTGGTCGTCATCCGATTCGGCCACGACAACGACGCGACGTGCATGGTGCAGGA
CGAGATACTGATGAGCATCGCCGACAAGGTCAAGAACTTTGCCGTGATCTACCTCGTCGA
CATCACGGAGGTCCCCGACTTCAACAACATGTACGAGCTCTTCGATGATTGCACGACGAT
GTTTTTCTACCGAAACAAGCACATCATGGTCGACCTCGGAACCGGAAACAACAACAAGAT
CAATTGGGCCCTGGCGGACAAGCAGGAAATGATCGACATCATCGAAATGGTGTATCGCGG
GGCCAGAAAGGGACGGGGTCTGGTGGTCTCGCCGAAGGACTACTCGACCAAGTACCAATA
CTAGTGGAAGCGGTACCGATGGTGCGGCGGGGAGGGAGGGAACGGGCGCGCGAGAACGAA
CGAAGCGCCGGCGGGTTGGTTCGCTGCAAACCATCGCACCGGAGGGACTTTTTTGTGGGG
AGGAGGTCGGGAGCGACGTGGCGGGTTGCTTGTGTCTCGCAAAGTGTGCCACAAAGAACA
GAACCGAGGAAGGCTCTTGCGCATTGCGATTTCCGATGGCCGTTGTGGTTGTGAGGTCTG
GCTAGTCTTGGCTCCTTGCCGTATGAATTCCAGTGACAAGAATTGGAGGGAACACTTTCG
CTCGAGCGGGAGGCTCTGGAGCATCGCTGCGAGACTGCATGTGTACTTTCGCAAAGCGGG
CTCGACACAACACCGCATTCCTATGCCTAAAAAGTAATACTACTGTACTACTGGTTTTTG
CAAAACGATGGAATAGTCTCGAATTTTGAATTTGCAAGCTGCC
>comp2144_c0_seq2
CTGCTTGTTTGGAGACCACAGGGCGGGAGGGATTTGTGTGTGTGTGTGGTTGTGTGTGCA
ACAGTGTGCATTTGTCTGTGTGCTTGTGGTTGGCGTGTCAAAGTGGGCGCGGTTGGGTGC
TGCACCGAGAACACCGCTGTACCACCAAAGAACTAACGAAAGTCATTCTCGTTCCAATGC
AATTCCACACGAACCCACCGCGACGAACGATGCGTGTTGTGTTTCGATGGTTGGTTTGTT
GCGCCGGCGCAATCGTGTGTGCATCGATGCATTCGTGGCTCCGAACCATTGTCCCGGGGA
ACGCGACAACGCCCGCCAACAGTTGCACGACGATGTTTTTCTACCGAAACAAGCACATCA
TGGTCGACCTCGGAACCGGAAACAACAACAAGATCAATTGGGCCCTGGCGGACAAGCAGG
AAATGATCGACATCATCGAAATGGTGTATCGCGGGGCCAGAAAGGGACGGGGTCTGGTGG
TCTCGCCGAAGGACTACTCGACCAAGTACCAATACTAGTGGAAGCGGTACCGATGGTGCG
GCGGGGAGGGAGGGAACGGGCGCGCGAGAACGAACGAAGCGCCGGCGGGTTGGTTCGCTG
CAAACCATCGCACCGGAGGGACTTTTTTGTGGGGAGGAGGTCGGGAGCGACGTGGCGGGT
TGCTTGTGTCTCGCAAAGTGTGCCACAAAGAACAGAACCGAGGAAGGCTCTTGCGCATTG
CGATTTCCGATGGCCGTTGTGGTTGTGAGGTCTGGCTAGTCTTGGCTCCTTGCCGTATGA
ATTCCAGTGACAAGAATTGGAGGGAACACTTTCGCTCGAGCGGGAGGCTCTGGAGCATCG
CTGCGAGACTGCATGTGTACTTTCGCAAAGCGGGCTCGACACAACACCGCATTCCTATGC
CTAAAAAGTAATACTACTGTACTACTGGTTTTTGCAAAACGATGGAATAGTCTCGAATTT
TGAATTTGCAAGCTGCC
>comp2145_c0_seq1
AGATATCGTAGGTGTCCAAACCTGTCGATCCTCTAATGCAATAGGGCAGGGGAGATGAGA
CATGACAAGACAAGACAAGACAAGTCAAGTCAAGTCAAGTCAAGTCAAATAAGTACGAGT
TACTCAGAAACGAACAGAGCTAAGCTAATAATAATAGATAGCCCAAAAGTGGTCAATGCT
TTTGGATTTGCTCCTCCTGTATTGGCTTGAGGGAATATTGCAATCGCATCCTTGTACCTG
CCAGCGTTAGGTCGCACGCCGAACTGTTTGTTGCTTGTCTGAAACACGCAGAGGCTAGAG
TATCCTCGATTGTTTACGAAACGAAGCATCGCTGGTGATAATTTGTTCGCATCACGGAAA
CCGAAAAGATTATGACGGCACTGTCAACTGCTATCAGGACCATACACGACCTTAAACCAA
ACCATTTTTTTTCGGAACTACCGGGGCCGTTGCCATCTCCCCAGGGAAGGCGTCGCGGCC
AAGGTCAATTTCATCACTATCAATCACCCCAAAGCACGGAGCGATCACACAGGATCATGG
GCAGCGACAGCGACAACGACCCGAACCTACTAGGCGATGACGCGGAAAAGGACGCGAGCA
ACGGCAGAAAACGCGAGAAGACTCGCTCGCTATCACCATGCCCGTCGTTTTTGCCGTTTG
ACGTAGAAGACAAAAAATGTGCGGTTTACGTTGGTGACATTCCATTCAATGGACGAAATC
GATGCAGGATTTGTTTTGGAAACGGCGACCGAAGCATAGATAGGGGCAGCATACAGGATT
GTGACAACGATACCGTCGATAGCAACATCGCTAGCGATGATGACATTCTTGTTTCAAGTG
ACCGAAGCAATCCCAAAAATAAAGATGGGGCATGCGCATGCGAAACCGAATTGAAGGCAT
ACATCCGAGGGCGAATATTTGCTGCATACAAGACCCCCATCCGATACATCGGGATTCGTT
CCTGTCGATTCTTTGCACTTCCAGCTCGTTCTACCAGCTCATCTACCAGCTCATCTACAA
GCACGGAGAAAAACGACGCTGAAGACGACGAAGATACGACCAAACCATGCGACACAAACA

GCAACCTCCACCGTCGTTCTGACCGCCGCCTTCGAATAGCGGCCTGTATCGAATTTGAAA
ACCCAAGGCTGGCAGAGTATTCATTGGGACTCAAGTACACGACCTTTTCCTTCCGAAGCC
ATCCGACCGTACGAGCGGCCTTAAAATTCCGTCCCTGGGCGATAGAATCCCGTCTTCCAC
AACAACTTACCCCAAGGATGCCTCCAAAACACCAGAATTCAAAGGAACAGCAATTGGATC
CTCCAAAGAATGTCCTACCCAGGCCAATATCCAGCCCACCGCATCGGAGAAATCTTGTTT
GCGACATTGATTGCAACTCCAGCGACGAACAATCGCCATCGTTGAGCATAAGTTTTTCGC
CGTCACCTCCAACCTCCTCGTGCCTTGTCCTCATCACAAACATTCCCTCCGATGTGACGT
GCGACGAACTCGTGGCATTTCTTGTCGAACGAATGAACAGGGCCTTCCGCGGAAAACCTT
CACCGACAATTCTCCGCCTCGCCATTCGGAAACCCAAGGCTATGGATGCCGATAGAAGCG
ATCATGCTACCGATAGAAATGACCAATCCCGACCAATCCGAAACCACGCATGCGTGGAAT
TTCAAGATGCAAGGGCTGCCTTTCGGGCAACAACGTTGAAACACCGACGCTGGCACGCGA
AGCCACAGGAATGCGAGACCGAAAACAACGTAGGCAATGGCTTTGGGGCTGGGGGCTGCA
GTGACACAAAAGGCAGCGACAAGACGAGAGATTCGGCAAGAGCGCCCTGCCTTGGAATCG
AAATCTGGGATCCCACGAAACACGACGTAAATGATTTTTTCATTGGAAACGAGAGTAGTA
TTCCTGAAGCAATCAATACCGATGACGGCACGGAAGACGATATATCTGAAAGCTCCAATT
TGGTCGCCGAAAAGGATGAAAAGAGCGATGGTGTGCGCACAGCATCTGAGCTGACCGAAA
ACGAGATCGAACCAAAATTAAAAACCCTTTTCGAAGAGCTGAAAGAGGATGGCTTTCTAG
TGTGTAAGCACGCGAGCATAAACCAGAAGCTTGATCACCTCTTTGCGATACCCGGAAGTG
GAAAGAAGGCATCCATGTGTCAAGGATACGCATTTCGTGGTAGAATATGTCGGGCTTACA
GGCGATCTCTAGGCATCGATAATAACAACAATAAAAGGGGATCTGCCAGCAGCAATCACT
CATGTCCATACGTACACATCGATTCACTCGAGGATTTGGAAGATCCCCTCGACGTTCTAG
CTATATTATACTTTGTCCATAACAACGACGACATCAATTTTGTTCCTGGACACGGCTGGA
CACCCCAAGAATACCTCCAACAACAGCAATTTAAGCTTTCTGCACTACCTCCATCTCAAC
ACCAACAACCTACCCAGTCAAAATCTATGGAAGAAGTAGTTGAAAAATTGCAGAATGATC
TGTCGGAAGTTCGAACCCAATTGGCCATCACCCAACAAGAGCTTCTCAAGTCTGAAAATG
CAAACGAATACCTACTCGAAAAACAATCCAAGGCTGCTCTGATCGAACCATCCAATACAT
CCTCAAAACAAGAAGTACAAGAAAAGGATACAATCCGGTCGCTGCAAGACGAAATTGCCC
GATTGAAAATTCATCTCGACGAAAAAGACCAAAATTTGAAAGCATCAGAACGAGATGCAG
CAGAAATCAGGCGCGAGTGCTTTGCCTTGATAGAACAATCCAGAAATTCCACGAAACAGG
TAGCAAACAACCATATATCCGATGATAACTTGAAATCGGATATCATGAAGCTCAAGGAGG
AACTTCAGCAAACTCGATCAGATATTCAAAAACTTGATCAAACCCGCTATTTCAATCCCA
ATTCACACGTCGGGCACGTCTGGGCCCAAGGGAAAATGGACAATTTCATTCAAACTAGCG
TACCCGCACTAATGGAATTAGCGGGCGGAACCCAAAACGAGCAACAACAGCAGGATGCCA
TGTATCATTCTTTGAATAATTCCCTCTCTTCATTGACCCGTAATGGAACCAATGAATCGA
AAACAACGGAATCCGTAAAATCTGTCGCTCTGCAAGCCGAAATAGCCGCTGTTCAATCTT
TATACGGCAGAGAAGCGGTAGTCACGAGCAGTGGGTCGTCAACTACAGTGACGCGATACA
TCATACTCCCCGTTCTCAATTCTCAAGCGGGAGACATCACGGTGACCCTAATCATCACTC
TTCCAAAGGGCTACCCATCGCAAGGCGTTGTTGAAATTGATGCCAATGCCCTACCTCACA
GTTTTCCAAGGATCAAAGAATGTATATCGAGCTTGTTGCTCGTTTGTCAATGGGAAGCAG
AAGCTTGCACCGGGAGTCCAGCAGTGATCAGAATCATGAAAAAGGTCGAGCAATGGGTAC
AAAACGATTGGGAGATCCTCCAAAGGAAACTCACTTCAATGGGCCATGGTATGAATGAAG
AAACGCGAAAAACATTTTTCCATTCGATGTGACGAGGAAGAGGCGCTAGTTAGAAGCAAT
GTTTTTCTCGCCTTCCACCTTGCCTCACAAATTCCTTAGCCTACTTCATACTGCAAGGTT
CAGAACGATTGAAACAATTGAATAAACCCATAGTTTCTGGAAACCCTGTTTCTGATAGCT
CGATAAATCACTATAGGCTACGAAGATCGAGGAAGTCTGTGACTTGCGACATTATTCTAT
TTCTGTGCATATGAAACGATCGAGGAGTCATGCCTACTTTAATACTAGAAACGGAATGAT
CCTGCG
>comp2146_c0_seq1
CAACTGTAAGACTAGCAATAGATCTAGCAATAGAACTAGCAATAGCACTAGCATTAAGAT
GTCCGGGGGGCACAGGCAGGAACTCAAATCAAGCAGTCAACAGTCGAACTCGATTGCTAC
GCTACGCTAGGGAACAGAGTTAACAGAGGCAACAAACCGTACCGGCAGGCAGCAAGCACA
AACCACAAGAAAACAAAAACAGCGCACGCAACGAAACGAAACGCACGCTCCATGGGCAAG
TTTGTTCGGCGGTCGTACAAGACCGCCGGCAAAAGGTCGTCTGGAGGTTTTGGTTGTCGC
TTCAACCCGCACCACACAGCCTTTGTGCTGCTTGGTCTGGCAATGATTTTGCTTCGCCAC
AACCAGAGCAAGGAAATCTTCTCGAGATTCTCGGACCTGGACGTTGGGACACTAGCAAAT
CCATTGCCAAATACAGCGACAGCAACAGCGACAGCGCCACACCAAGGGTCGATTCGGGCC
ACCACAGGAGCACCGCCACCGCAAGCTGCGGCTTCCAACGCAACACCCACCACAGAGTGC
ACCCGAGAAGAATTGCTCACGATCCGAAAACACCTCAACCCGGAGTTTTGTCCCACGGCC
CGCGAATTCGCCTACTTCCAGTTCTGCAGCCTCACCCAGGCCACCAAGTGCGTCGACGCG
AGCAACTGGCTGGAAGACTACTATCGGGATCTCCGAAAGGAAGGCAGCCTTCCCGACGCC

TTTTTGGGACTGTCGGTGGGCTGCAACAAGGGCTTTGATGCCCTCAACACGCTAAGGATG
GGAACCCACGACACGGCCTTTACCAAAAAGGCCTGGAAGCAGTCCATGGAACTGGACGGG
GCAAAACTCTCCGAATCCGTCTGCAACCAGGACATTGTCGACGAATTCCAGGTGTCTCTC
GAGGATAACAGCAATCCTGCCGATGCAGCTGCCGATGCATTCGCAACCGGCGAAATGCAC
TGCTTCGAGCCCATGCCACCAACGGTGAAAAAGCTCCAGCATGCCGCAAAGCAACTCGGG
TACGACCAAAAGGGATTCAAGGTCGTCCACGCCGGGGTCTCCAAAACACCCGGAACCGCA
TGGTTCGCAACGGTAGGCTCCGAATCAGGCGTAGAAAACGTCGGCATGGACAACTGCGAG
GCCATGGGTCCGATACGAAAGGCAAAATACTGCACGCAGGTTCCCGTTGTCTCCCTCAAG
GACTATGTCAAACAACACATCGACGGTGACAACGACAACGACAATGCCAACGGCAAAAAG
CGAACCATCCACCACCTGTCCATCGACGTCGAGGGTTTCGACGCAGACGTTCTGTCCGGA
ACGGGTTCGGATGTCCTGAAACGGGTCGAGTACCTAGAATTCGAATACAACTGGATGGGA
TCCTGGAAGGACCAACACCTCTACGACGTGGTAACCGCCCTCGACCAGATCTCCATGACC
TGCTACTGGGCCGGAACGAGGCGCCTGTGGCGCATCACCGGCTGCTGGATGCGCTACTAC
GACATCCACACGTGGTCCAACGTGGCCTGCGTCAACCGGAACCTCGTTCCGAAACTAGCG
GCAAAGATGGAGGACGTCTTTCGTGCCACCCTCGGCGACTCTGCCATTACGGCACACACC
TTTCGGAACCCGGTCTGGCGGCGGGCCAACCACTGGATGAGAGGCTTAAAAAACGATCCC
ATGCTCGCCACCGAGCCCCACGAAGCAATGGTGTCGACGGAGTACCTCTAGGAGTATCTC
CAGGAGTATCTGCAGGAGGATCTCCAGGAGTATCTCCAGGAGTATCTCTAGTCCTCCCAC
GCCCGCTGTTGGTTCCGTCCGTGACGCAGACGCCCACTCGTGTAGTTCATAAGTGTGAAG
TGGGCCGAGACTCACTACGGACTTACTAAACAATCTTACCAGCGGTACACCAGAATCGAA
TCTACAGTATCTAGTACGAAGTAGTAGTAGTAGTAGC
>comp2147_c0_seq1
GTTCGGGTAGATTCCATACAACCAACCGCACACACCGGAGAGCTGCACGACTGGATGCCA
AGGGCATGAAAATCAAAAGAAGCAATGGAACGAGCACGAACTGCAAGTGTATCATTGTTG
TTCTTCGATAACAAATCTTCGTGTTCAGAGCAGTAGTGCGATTCGCGTCCCTCGCAATGA
GGCGTTCGTTTTTCACATTGGCGAGATGCAGCCCTAGCCGTTTGTCATCCGTTTCTGCTT
CTACAAACATGCTTTCGGGTGCTGCGAGGAAAATGGCGGGTTTATCGGCAACGAATCCAG
CACTCGTTCGGATTCGAACGAACACGAGTGGCATGTGTAACAGAACGTTTAACAGCACGT
GCAACAGCATGTTGCGCCTTAGATCGATCCTTGACTTGGAGGGTCGAGGCAAGGGCCCTT
GCGTTCGCCGATTTTCAACAGCTCCGGCTATCGTTGGTGACGATGATAGCCTCGGCCACC
GCCAAGGCCACAGCCACAGCCACAGCAACAGCAACAGCCCGATCCAAGTCAAGGCCAAAC
AACCAAGCTACAAGGATGCCATCCGTGGCGGTCCCAAAGAAATTCTGCATCCAACAGAAC
AGACGACCAAGCACAACCACCAACCGCCAACCACCAAGGCCGGAGATGACTGCAAGCAGG
AACAAAAACAAAAACACCCGGGCCAGAACAGCCGCAAAGCAAGAACGAACACCACGCCAG
ATCGCCTGGTCGACTTTGGAGACATCTCGCGGGCGATGGTTGCCATCCGAAGTGGAATCC
GGCGCACGAAATGCACCCAGTCCTTTTTCCTCTCGGAACTCTGCCGGGCCAACATTTTCC
TCAAACCCGAGGTACACCAGTTCACGGGAAGCTTCAAGGAACGAGGGGCACGAAACGCCA
TTCTTTCTCTGCTCAAGGAGCAAGAGTCTCCCAGAGTGATTGCTGCCTCGGCGGGAAACC
ACGCTCTCGCGCTGGCCTACCACGGAAAGCAGCTCGGGGTTCCGGTCACGGTGGTCATGC
CAACGGTGGCACCCCTCCTCAAGGTAGACCGGTGCCGGCAGTTCGGTGCCACCGTTCTAG
TGGAGGGATTGCACATCGGGGAGTCCAAGACGGTTGCCGAGGAACTGGCGGCGAGGGAAG
GCCTCACCTATGTCAACGGCTACGACGACCCTCCGATCGTGGCGGGCGCCGGCACGATCG
GAATGGAAATCGTCGAGGACGTTCCGGACGCCGACGTCGTGGTGGTCCCGGTGGGCGGCG
CCGGGTTGATTGCGGGGGTATCGTGTGCGGTCAAGACCCTCAAGCCCTGTACCAGGGTGG
TTGGCGTGGAACCGGAGCGCTGTGCGTCGTACACGGAAGCCCTGCGGAAGGGCCACCCCT
TTGACGTTTCGGCGTCCGGCCGAAGCAGACACAAGAACCAAAACCAAACGACGCTGGCGG
ACGGGCTCGCGGTCCCGGAGGTCGGCTCCCACGCCTTTGCTTTGGCGAGGCAGCACGTGG
ACGAATGCGTCCTGGTCGACGAGCGGGCCATCAGCCTCGCGGTCCTGCGCCTGATCGAGA
ACGAAAAGATCGTCGTCGAGGGCGGGGGGGCCACCGGGCTGGCGGCGCTGCTGCCGGGAG
GACCCCTCTCCGGGAGGCCGGAGATTGTCGGAAAGAACGTCGTGGTCCCCCTCTGCGGCG
GAAACATCGACACGGCGGTCCTGGGCCGGGTCCTGGAGCGGGGCCTGGCTTCCGACGCCC
GGCTGTGCAGCTTTACCGCGGAGGTCTCGGACCGGCCGGGGGGCATCGAGGGCCTGACGA
AGGTCCTCCGCGGGCAGGGGGCCTCCATCAAGGACCTGTACCACGAGCGCGCCTGGCTGC
ACAGCAGCGTCGACCGGGTGAGGGTCCGTTGCGTGGTGGAGCTCCACGGGAGGGACCACG
CCCGGCGCCTCCAGCGGTCCCTCGTGGAGGCCGGGCATTCGATCGAGTGGAACCAGCCGA
CGCACTACTAGCGAGTGCATGTGGGCGGTGCCAGGGTTGTGTTGGAACCTTGTGGGAATA
CTACGTAGTAAGTTCGGCACGCATTCTAGTCGAACTCGAAACCGGCAGAGACACCATGCG
CCGGACAATCGTAGC
>comp2148_c0_seq1
CATTGATTCAACTGCTGATTACCGTTGAGACAGCAAGCGAGCGAGTTGGAAGAGTCCTTT

TTTATTCGTGTTTCATGAGTCGTTTCGATCAATCAAGGTGGAGGATTTTGTTCTTACCGT
ACCGTACGGTAGAATGGTACAAACTATTGTTTTTTATTTCAAGTGTTTTAGTTTAGTTGT
CGCGAGGCTTTACGAGCAGGGTACGTACGATACTACTAAGAACTACTATTGCGTGTAGAA
AAATTGTAGAAGGAATTTTTTGTGTTGAAGGCCTTGTAACAGTGGATAAATCTTTTACGA
AATTCGTATCAGAAATACGAATCCACACCGGCAAAAGTATCGGTACGACAATAGAAAGAC
TTGAATCAATCAAATCTGTAGCAACTTCCTTTGACTCTCTTCATCATGAGCACCGAAGAT
GATTTTACCGTTCCCTCATCGGAAGAGGAAGAAGCCGGCCTAGCGGACATGAAAAAGGTG
TTCGAGACGGAAATGACCGACGACCAAACCGGAAAAAAGAAGAGTTATTGGTCGTTTGCT
ATGGAAACCATGGGAATGGCTTCCGGAAAGGACGGAGATGAAGATTTATGGTTGGAAACC
GTGGAGCAGGGTGTGCCTATTTTGAAGAAGTACATAAGAGAAAAAGATGCAAAGTTGGCC
GAGGAACAGGAACAGGCCAAAAAGAAACTCCCATTTACCTCTTACATGGATCTGGTCCCC
CTCGACGAATTTCACACCACCCAAACAAACTTTTTGAAGTCGTTTTTGAAGTGGTCCATC
AAGGACAAAGAAGATATTGTCGAAGGAAGCGAGGGTGCAAATCTCGTCGTGAATGCCTCC
AAGGCCCGGCGTCGATTGGACTCTTACTTCGAATGGATGAAAGACAACATGGCCGAAGAT
ATGGCCGAAAACCCCCTCACGTTTGAATCTGTGAAAGAGGTACATAAGGCTTGGGGCCTC
GAGGCAACTCACTCCAAGGATGATCAACTCATCTGGTGGTTTGATCTCGGAACAATGCAT
ACGAAGAGCATCAAGTCTCTTGCTCCTCAGGATCAAATGAGATATATTGTATGGTTTAGT
CACCTGATGATGTTCGACCACAGAGGCCAGGACAATGGTTTGCTGCTAGTTGAAGCTCTC
GATAATCTTGGATTCTGGAAAAGTATGACTCTTCTTCCCATGGAATTGGGTGCTAAGATG
GACCGATTCACGATTGGCGTCTTGCCCATTAAAATGAAAGGAATCTACATGTTTGGTTGC
GCCCGTTGGGTACATCTCATGGTGGGGTTGATGAAGCCTTTTATGAGCAAGAAAATGCGG
AGTCGTATGATCATTGTGACTGAGAAGATGGCTCCAGAACCGCAAAAGTATTGCGACGAT
AACTTCGGTCAAGGAAGCATTCCCGACGGCTTTATGTCTTTGAACGGAGGATCCAAGAAA
GACGCATTGATCCACCGATTGAACAAGAAGGGAAAGCAAAAGGAGAAGAAAGCCGCGACG
AAAGCGAAGGAGGAGGAATAAGGAAAAGGGAGCAACGAGGAGCAATGTTCAGTTCTTAGT
GTGGTGTTTGACGACGTTTTCATTTCGAACGAAAAATTGTGCCGTGGATGCTCCTTCGAC
AGAGTAGCAGCGTCACTAAGAACATCTCTCAATTGTAGGATCCCTTGACCATGACTGTTT
TGTAGTTCGAAATAACGCGCTGTTGTGTCAAGCATTACTAATAGTTTTAGCAGAC
>comp2149_c0_seq1
GAAACAAATAATAGAATACCACGAACAGGAGTCAGGTCAGATGGAACACAAAGTGAGATG
AACCCACAACATCGTTACGTTCGTCCGTACGGTCTGCCAATTCAATAGCGCACAGCTTCT
AGCTTATCTTTCTGTTCGCCTTGCACTCTGAAAACAACATATTGCCAAAGCCTCTGAGCC
GTTGACCATACTGTAAAACAGTTGAACATCATAGATCGAAACCATGCCTAACAACAACGG
TGACGATAACGACTCCTCTGAGGAGCTAACTGCAAAGAAAATGGACTCGGTTTTGAACAG
GGTCTCCTTCTTTGAGAAGTCACAGGAAATTATGCAAAGCAAAATAGCTAGAACGCCACC
CCGAAAGAAAGAGACAAAAAAGAAAGAAGATCCGTCTCCGACACCCGAACCGGAAAAGGT
GCCCGAGAAAAGCAGAGAAAGTAGTACAATGTCTAGGGTGCAGCAATGGGAAAGCAGAGC
TTCTGGAAACAACGAAACGAAGAAAGGATCCTTTTCCGGGACCGAAACTTCGTCCAGTGC
CAATCGGGACATATCACTCGGATTTTCAGGAACTTCTGCATCGAGCTCCTCCGAGGATAC
CGACGAGCAGGAAGTCTTGATGCATTCTATGACCAACGACGATGATATTCCACTCGAGGA
ACTTTTTTCGAATGCAGCATCCCTCAATGTTAGACTGGAAACCGCAAACTCTGCCAATTC
TCCGCCGCAAAAACTGTTCTCGAAAGACGGCGAGGAAATCGTCGAGAACACTAAAAAGCA
AGACGAAAACAAAGAATCACCTATTGCTGAACCTACAGCTGTTCGGACAGTCTTATATGA
CAGCGATGATGATGAGATGGACGTCGAGAGCGGTCAAGGACTAGCTAGATATATGCCCGC
TGCCGGATTCATGGCAATCCCGTATTCCAATGGCAAACGGAAAAAGGATGCAGATGGAAT
GAGTGTTGTCAGCGATGACATTAGCATTGCGGCCAACGTCGGGATTCTGAACACCAAGTT
TGAGATGCTACGATACACAGTCCCTAAACCTGATTACAAAGAATATCCACCAGATAACTC
TACCGAGAGAAAATACAAGGTGATAATAATGCTGTGTTGTGTGTTATTGATAGGTGTGAT
CTCAGTCATCATTGGGCTTTTGGCCTCGAGAAGGCCTGAGATTTTACCTTCTCCCGTGGT
TACGACTGCTCCAATGACTGCACCTAGCTCGCCGATAACACAGAAACCTTCTTTAGCACC
CGTTGACATGGTACTAGTGGAAGAGGGACCTTCTTTGCCACCCGTTAGCGTTATACCGGT
GGCAAAGGAACCTTCTTTGGCACCTGTTGGCATGATACCAGTGGCAAAAGAACCACCTGT
TGAGATCGGATTGCCACCAATAAAGTACAAGTTTAAAAATACTGCCACGATTGTTGGTCA
AGCAGATGGAGATCGATTGGGATCGGTAATCTCAATGAGCTCTGATGGAACCTTCCTAGC
AGCGTTGAGCAATACCCCAAATGCCCCAGTCCAGACTTTCCAGAGGGACGAAAATTCAGG
CGACTGGGTTCCCACCATGGCTACCCCGGTAGATAATGCAACTATTTCGACGAATACTTC
TTTAGCTTTTGGTTCCGACATTTCCTCCGCGACGATACCAGAAGATGGAACTCCAGTGTT
AGCCATTTCATCGCAAACTCAAGTACAAGTCTACCAGCTGGTGGGTCGGTCTTGGGTCAA
TCGCGGGCAGCCGATTTTGTGGGACGTTGATACCGTTGCATACTCTGCAATGACTCTTTC
CTCTGACGCATCCACGCTAGCTATGGGCTGTGTGGACGACAATGGCGTCATTATGTCAGT

CCAAATCTACAGATATGATTTATCATCAAAAGAATGGACTCCCATCGGTGGAAATCCGCT
TCTTTACGATGCCCAGAACCGTCACGTAGGTGGCAACCAACGGAGAACCCTGCTCTCAAT
TTCTCTCGCTCTTTCAAGAAATGGGAGTGTACTCTCGGTAAAAGAATGGGCTGTGGCAGA
TCCACAGGTTGTCGTTCAGACTTTCGGGTTCAAGGACCTGAGTAGTGGTAGAGATGATCA
TAGTGGAAGCTGGGAGGCCATGGGAGGTCAATTGATGCATCAATTTGGTCCAGCTTCTGT
AGCTCTCTCTGAGGATGGATATACCTTGGCCGTGGTAGCCACTCATCCTGGTGAAGCCGT
AGTTTATCGATGGATCGAAGGTTATTGGAAAAAGATCAGTGGCGATGCGAATCATGGTTA
TCTCCCTAGCGGGGGTTCATCTGTTTCCCTCGCCCGGAACGGCTCCAGAATATTGATTGG
AAACCTACAGAAGGACGAGGCACGCATATACGACTACAGCAACAATGAAACATGGCTGCG
GACGACAACTTTATGGGGTTCTGGTTCAAGTGGCTTCGGAACTTCAGTGGCAATGGATGA
AAGTGGTAGTACACTAGGAATCGGTGCACCTTCTTTATCCAGCCGTAACGGAAACAATCT
CGGCCAGGTTCGGATATATTCATAAGAACTCATTAATTTTGTACAACATTTCACGCGGAT
GTACCAGTTGTTAAGAAAAGACTAGTAAAGTACACTTATGTAATTGAAAGACCGCACTAC
TAGTAGG
>comp2150_c0_seq1
GAGGAACTAGAATAGAATTGTCAAGCACATCCTACGATAGGATACGTATTCGAAAACGAG
AGGTCCCATTTGAAATCCCATACAGTATTTGTGGTCTTGCGATCATGAACATCTACCGTT
CCTGCCCATACCGTACGGTAGTATTCGAAGACAAAAGTCGTTGTCGTTGCTTTGCTAGAC
ATACTGTTCCACACCTGTTCTCAACGAACGTACCGTACGACGTTTTTAACTTCACCGAGA
TTTAGAAAGAACATCCCCGACACATTTTTTCACATCTACTGTTCTGAAAAAGACTGTGAG
GATCGGGCGAGGACAGGATATCTACACCCTCTTCGTAGATAGAGTTGTGTTGTCCACGCT
CTTAATTTCTTTTCTTCCCAACCACGAGCGTTGGTTTGTTGAGTGCGATAATGCCCAACA
TGACCACATAGAAACGAAATGATCCGTCCGGATTGCCATCGATCGCATCCATCAATCGCA
CACGCGCAGGTATCACCACCAGCGCAAGGCAGGACCGCGCCTTCGAGATCTTCGCGATGC
CCCTTTCGATGCCATGGCAGCACCCTTCCTGGTTGAGATTGGGTTGCCACGAAACGCCCT
TTCTTCGGTACCAAAACGTCGTTTCCGAACACAATCACTCTCTCGGTGAAGGATGAACGG
AGAAGAATGAGAAAAAATCTTCGCCTTCAGTACAAAAAGTTAAACATCTTACCTAATGTT
TACTGCACAGAAACATTGCAATGGCCAAAACAGCGACTCAGCTACATCCTATGCACGAAA
ACATACCGTGATTCAGAGCGCAAGAAGGAAATACTTTTTGATCGCAATGCAAGTCTGAAA
GACAGAGAATCTGCATGATCAACCAGCAGCTTGTTGCGACCTCTGCAGAAGAGAATGCAG
TCAGAGGATACTCATAGAGGTGCAAGAAGAATAAGCCATGGGAGGTTTTGACCTGTACTA
TGCATTACATGACGGTTTGCAACAACAATGCGAGTATGTTCCATAGCTACTACTGGCTAC
TGCCACAGTAGATTTGGTTCCACGCCCCCTCTCTCACGAATGTTCTACAGCTATCTTGTT
GAAACTGTGATTCGAGTCCGGAAACACAGCGGTGATCCATTTTTTCAAAAATATTGAAGA
AAATGATGGTATTCATACGACTCGTACGACTACAGCGTCGTATCATGTTTCTTTTGTTGA
TCGCTGCTGTAATGTGCCTTCAAAAGGCCAAGGTGCCACAATGTTTTGGGATTGATTGAC
AGCTGACACGGTTCGGAGTCGACGGACCATTATAGTTTTACATATGCCGAAAACGGAGAA
GATTTTATGCGCCTCCGAGCGCGATCAAAAGGTTGTCATGAAATCTGTCAATTTTGATTG
GTTTAGAGTTTTCCCGTTTGACCAATCAGCGACCAATACTTCAATTTCTGCATCTGTTAA
CGTCTCTTCAAAATTCTTCAAACCCAGTTCGAGAGAACTTCCTTCCTTCCTTGACACCCT
TTCTACATGGCGGAAGATTGATTGAAGGTTTTTGGTACTTTCGAAAACTTCACGGAAACT
TACGATTCCTATCCGTTTCGGCTCCTTTCGCCGCAGGCGAACATCAACATCACGAGCAGC
TCTGACACAAATAAAATCACAGACGAGAAAGGTTCATCGTCACTGACAGCAGTATTGACA
TCATCACAAGTATTTGACAACACACTCACCATCAAACAAAGTGCAGGAGTCGTCAAGAGT
GTTTCTCTCCTCATAAAATAGAATAAACGATAGAAAATCATCAAAGAATACAGCGAAGAG
AAAAACGAATCAATATTAACCAGAAGACAAAACAATAAAGGCCAGCATCCATCGTCATAG
ACACAAACACTCATATAGCAACAAGAAAACATCAAGATACTATGAAAATCTACCGAAGAG
CCAATACTGAATTGTCCACCGAAACAACATTCCAACAAGTAGAGAGCGAGGAGGACGATC
ATCAACTATTTTTTGTCACTTCCTATTCCGACTTTGAGAAACTAGCCCATGGCCCTCGTG
GTAGCGAAGCCAAGCACTCTGTGCACGTATATCGCTTTTCCCCATCCGACGGTTCGCTTG
TCCTCTTAAACATCCAGGGAGACGCGAACGTCATTAAAAACCCGGCCTTTTCTCGGCACC
ACCCCCGTTTGAATGTGGTCTACACTTGTACCGAAGACTGTCACGAGAACGGTCAAATTA
TTGCTTACAAACTGAAGCAGGATGGCTCATTGGAACAATTCGGAGAAGCCGTGGATGCCG
GGGGGACCTCCACCTGCTACTTGACTATCGACAAAGCCCACCGCAATCTACTGGCAGTGA
ATTATTGGAACTCGACCCTGGTGACGATCCCGATGGACCCCGAAACCGGAGCTCTGGTCG
GTGGTGTCAAGAACGTTTACGATCCAAACATGGGAAAGAGCATGGTAGCCTGCGCCAAAA
AGGATGGCGGCGTGAACCACTCCTGTAACGACGCATCCACTATCTCGGCCCGCCAGGCCG
ATCCTCACTCGCACGCACTGGTCCTGGACCCGTTCGTGGGTCGGGTGGCGTACGTTCCCG
ATTTGGGAAAGGATCTCATCCGGGAATTCTATTACGACGCCGCGGATGGCAAAATTGCCA
TCGAGCTCAACGTGATGCCGTCCGGGCTTTGTACCGGCAAACCGGACGGACCGCGCTACT

TTGATTTTCATCCGGAGTACAACATTGGATATGTTGTCAATGAATTGAGCTCCACCGTAA
GTCACTGTAGAAACAGTGTGGCTTTCGAGTTTTGTTTCATTCTGTCTCGACATCCACTTA
TGCATTCTTGTTTGCTCTTTCTTTCTTTTTTGTATTAA
>comp2150_c0_seq2
GAGGAACTAGAATAGAATTGTCAAGCACATCCTACGATAGGATACGTATTCGAAAACGAG
AGGTCCCATTTGAAATCCCATACAGTATTTGTGGTCTTGCGATCATGAACATCTACCGTT
CCTGCCCATACCGTACGGTAGTATTCGAAGACAAAAGTCGTTGTCGTTGCTTTGCTAGAC
ATACTGTTCCACACCTGTTCTCAACGAACGTACCGTACGACGTTTTTAACTTCACCGAGA
TTTAGAAAGAACATCCCCGACACATTTTTTCACATCTACTGTTCTGAAAAAGACTGTGAG
GATCGGGCGAGGACAGGATATCTACACCCTCTTCGTAGATAGAGTTGTGTTGTCCACGCT
CTTAATTTCTTTTCTTCCCAACCACGAGCGTTGGTTTGTTGAGTGCGATAATGCCCAACA
TGACCACATAGAAACGAAATGATCCGTCCGGATTGCCATCGATCGCATCCATCAATCGCA
CACGCGCAGGTATCACCACCAGCGCAAGGCAGGACCGCGCCTTCGAGATCTTCGCGATGC
CCCTTTCGATGCCATGGCAGCACCCTTCCTGGTTGAGATTGGGTTGCCACGAAACGCCCT
TTCTTCGGTACCAAAACGTCGTTTCCGAACACAATCACTCTCTCGGTGAAGGATGAACGG
AGAAGAATGAGAAAAAAATCTTCGCCTTCAGTACAAAAAGTTAAACATCTTACCTAATGTT
TACTGCACAGAAACATTGCAATGGCCAAAACAGCGACTCAGCTACATCCTATGCACGAAA
ACATACCGTGATTCAGAGCGCAAGAAGGAAATACTTTTTGATCGCAATGCAAGTCTGAAA
GACAGAGAATCTGCATGATCAACCAGCAGCTTGTTGCGACCTCTGCAGAAGAGAATGCAG
TCAGAGGATACTCATAGAGGTGCAAGAAGAATAAGCCATGGGAGGTTTTGACCTGTACTA
TGCATTACATGACGGTTTGCAACAACAATGCGAGTATGTTCCATAGCTACTACTGGCTAC
TGCCACAGTAGATTTGGTTCCACGCCCCCTCTCTCACGAATGTTCTACAGCTATCTTGTT
GAAACTGTGATTCGAGTCCGGAAACACAGCGGTGATCCATTTTTTCAAAAATATTGAAGA
AAATGATGGTATTCATACGACTCGTACGACTACAGCGTCGTATCATGTTTCTTTTGTTGA
TCGCTGCTGTAATGTGCCTTCAAAAGGCCAAGGTGCCACAATGTTTTGGGATTGATTGAC
AGCTGACACGGTTCGGAGTCGACGGACCATTATAGTTTTACATATGCCGAAAACGGAGAA
GATTTTATGCGCCTCCGAGCGCGATCAAAAGGTTGTCATGAAATCTGTCAATTTTGATTG
GTTTAGAGTTTTCCCGTTTGACCAATCAGCGACCAATACTTCAATTTCTGCATCTGTTAA
CGTCTCTTCAAAATTCTTCAAACCCAGTTCGAGAGAACTTCCTTCCTTCCTTGACACCCT
TTCTACATGGCGGAAGATTGATTGAAGGTTTTTGGTACTTTCGAAAACTTCACGGAAACT
TACGATTCCTATCCGTTTCGGCTCCTTTCGCCGCAGGCGAACATCAACATCACGAGCAGC
TCTGACACAAATAAAATCACAGACGAGAAAGGTTCATCGTCACTGACAGCAGTATTGACA
TCATCACAAGTATTTGACAACACACTCACCATCAAACAAAGTGCAGGAGTCGTCAAGAGT
GTTTCTCTCCTCATAAAATAGAATAAACGATAGAAAATCATCAAAGAATACAGCGAAGAG
AAAAACGAATCAATATTAACCAGAAGACAAAACAATAAAGGCCAGCATCCATCGTCATAG
ACACAAACACTCATATAGCAACAAGAAAACATCAAGATACTATGAAAATCTACCGAAGAG
CCAATACTGAATTGTCCACCGAAACAACATTCCAACAAGTAGAGAGCGAGGAGGACGATC
ATCAACTATTTTTTGTCACTTCCTATTCCGACTTTGAGAAACTAGCCCATGGCCCTCGTG
GTAGCGAAGCCAAGCACTCTGTGCACGTATATCGCTTTTCCCCATCCGACGGTTCGCTTG
TCCTCTTAAACATCCAGGGAGACGCGAACGTCATTAAAAACCCGGCCTTTTCTCGGCACC
ACCCCCGTTTGAATGTGGTCTACACTTGTACCGAAGACTGTCACGAGAACGGTCAAATTA
TTGCTTACAAACTGAAGCAGGATGGCTCATTGGAACAATTCGGAGAAGCCGTGGATGCCG
GGGGGACCTCCACCTGCTACTTGACTATCGACAAAGCCCACCGCAATCTACTGGCAGTGA
ATTATTGGAACTCGACCCTGGTGACGATCCCGATGGACCCCGAAACCGGAGCTCTGGTCG
GTGGTGTCAAGAACGTTTACGATCCAAACATGGGAAAGAGCATGGTAGCCTGCGCCAAAA
AGGATGGCGGCGTGAACCACTCCTGTAACGACGCATCCACTATCTCGGCCCGCCAGGCCG
ATCCTCACTCGCACGCACTGGTCCTGGACCCGTTCGTGGGTCGGGTGGCGTACGTTCCCG
ATTTGGGAAAGGATCTCATCCGGGAATTCTATTACGACGCCGCGGATGGCAAAATTGCCA
TCGAGCTCAACGTGATGCCGTCCGGGCTTTGTACCGGCAAACCGGACGGACCGCGCTACT
TTGATTTTCATCCGGAGTACAACATTGGATATGTTGTCAATGAATTGAGCTCCACCGTTG
CGGTGTTTGAAATCAACCGTCAGCTTTTGAACGAAATCTACGCAGCATCCAAAAATGGGG
AGGACATGAATCGGTTTCGAGGCCAATCGACCCTTAGGTTGGTCCAATCGATTAAAACCA
TCCCGCAAGCGTTTCCCACAACAATGAACACCTGCGGTCGCATGTGCGTGCACAAATCCG
GTAGATTCGTGATTGTATCGAATCGGGGACACCAGTCAATCACCGTGTTTCGCGTCAAGA
GGAAAGGGTCCAAACGGGGTGAGCTCCAAACCGTCGGATGTTTTCACACCCGGGGTGAAA
CTCCGCGGCACTTCCAGTTCGACAATTCCGGCCAATACTTACTGGTTGCAAACCAAGATA
CCGACTCGATTGCGGTCTTCAACTTCAACCTTAGCAACGGGGAGCTGAAGTATTCTGGCA
ACGAGTACCGTGTGCCATCGCCTAACTTTGTCTGCTGTTGCCCAACGTATGCCGAGGACG
ACAGCGACTTGTATCACGAAAACATGGGTTCTTCCATCAGTGCGGTGACCTCCATCCAAG
CAGACGAGAACAGCAGTCCCGCTTGCTCCGACAGCGAAGACAGCACGCTTCCTACCTGGA

CAGATAAATCCTCCGACGATGACGTCAAAGCCGAGCTTGCCAAGGCTAGAGAAGAAATCG
AAGCGTTAAAAAAACTCTTGATGGAGCGGACTCAATAGCTAACCGCCGCTTGCTTGCATG
CAGTAAGTCTCTCTAGACTGCCATCTCGAAGAAGAAAGAATATTTGTTACCTCCTCATCA
GTTTTTACTTTCCTATTTTCATACCTGCGTGTCATCGGACCAATACGTGTGGTATGACGA
CTCTACGTGCGCGTTGTATGCTCTGTTTGCACATCGTGATTTAACCTCAATCATGACAGT
TTTCCTGTATCATTGCATTGATAGAAACATAAAATATCACAGCGATAGAATCATTTAGTG
CTAGAACAATACTAATATTATAATAATGCATTTTTCAG
>comp2151_c0_seq1
ACTTTTTCTTCGACGCCACACGTCAAAAAGTTGTCTAGTAAAGCCTATTGAAAAATACTC
TAGTGGGATAATGAGAAGACCTACTTCCAAACCACCGGTGACACGCCTGAGGTCACCCTC
AAGATCATCAACATACAAGACGAGTTCAAAGCAAAACAATGGCGCTGCTGCCTTGGAGGA
TGAACGTTTCAAACAATTTCTCAAAAATGCCGACGAGATCGAGTCGATCGAGCATTTTAT
GTATTGGAAAGACGAGTTCTTTACGACGTTTCGTCCATATCTGGATCCGCTGGGAACGGC
GACAGCGAGAGAAGCTGACGAGGAACTCTATTTCGAGTTGGAGAAACTCGCAAATGCGTG
CGTCCACACCAATGAACTAGTAAAGGAAGGCAAGATCAGCCCCGAAAAGTCGAGCGTGAA
GGGGCAAAAGAGCATAAACGAAATGATAGCCTCTACGTGCACCTGTGAGCAACATATTGA
CAGATACTTTCCGAAGACACAAGAAGAGGAGCTAACTTGTGGTTACAGCAAGTTTGAATT
AGCTGCGGTTCTGGTGAGAGATGGCTTCAGGGTGTATGGAATGATGGTGGCAACTCGGAA
TTACGTCCAAGGACTTGTGATCGAAGGGGGTGCCCTCCAGAAAATTCTCAAGTCCAATCA
AATGTCCATCCTTCAATACTACTTTCGGTGCCTTGACACATTTGCACAAACCATGGCCGA
CCTAGGCATGTTCAAACTAATGGTGAGATGCGAAGAACTCTACAGCGTGCGACCGCGACG
CAAAAAGAACAAGACGAAGAAATTCAACAAACACGGTGCAAAGGATGCTCTAAGCGACAC
AAGCTGTGAGGGTGAAGCTATTAACGGCGGACGCATGTTTCAGAAATCTAAACTGAATTA
TCGAGCTATCATGGACAAGGGATGGTCAAATGGATTGTGTTTGGGACAAGAAATCTTAGG
ACCTTTAAAATTTTCAAAGAAGAATGATAAAAAGAAAAAAAAGGGGGGGAAGAAGAAAAA
GAGACTTATCAAAGAAAATAACAAAGATGCCAATTGCAAATCCGGAGAGGACAACGAAGT
AGCGAGTAACGACGAGACTAACTTCTCACCAGAAGCTGGATCGTCGTCCTCCATCGAAGA
CGATTCTGTCGACGAAACCGCCGAAACCGTTGACGAACTAATGTCGATGGCGTCCTCTTC
GGAGGACGAAGAAGAAGACGAGGAGGACGTGATATATTACTTTGACCCGGTCCGGAAAAT
CGTTGGCAAGGTTTCACGAAAAAAATGCTTGGCGGAAATCACGATTGTGAGTGTAGACAG
TGAGACTGGTACCGAACGAGCAGAAGGAGTTGTGGAAGAGTGGGATTCTAGGGATGACGA
AATTGGGAAACAGGATTTGATACTGAACCTCAAAGAAATCTTGAGGAGCAAGGCATAGAA
CAATTTAGAGAATAAAACTAGCAATTGCAATTAGCTTTAGACCCCT
>comp2152_c0_seq1
ACTTCCCATGGCTGTGCACCGCAAAAGACCTAGTCGAAGTACTGCGGGAACAGTACCCCA
CGTAGCTTGTACCTGCAGGATCAGAGAGTTCCACAAGAACTTTTCTCAAGAACTTTCATG
AGCTAACTTGAGTGATTTGCACTTCACTCCATGTGACACAAATTCCGAACCACAGGTATA
GTACTGTATTTTCATTTTGAAAAAAGTATATCACAGATAGAATCATTAGCTGCAAGAGCA
ACTTGCGCAGATGTCAAAGCATGCTGACACCCATGTCGATGTCGACAACGATCAAGAGAA
AGATAGCGACGATACAGACATTGGAAACAGCCCGAACGCGCGTCGGAGTCTTTCTCAAAG
GTACAATGACCGTTGCTTGAGTTGCAAAATGCTTCTAGGCATTGTACCGTTTGTAGTATT
GGCAATTGTATCGCCGGTGGACGAGGAACTGTCTGTTTGGCTTGCCCTGGCTTTATCTCT
TTCTCTTAACTTGCGTTTACTCTACCGTGGATGTTACGATCCAAAATACCCGAAGTGGTA
CTGGATGGAAATGGCGCTTTGCCTTAGCCTCCTTGGAATTGGCATCTTCAATGGTATCAC
GGGGGTTGCAGTTTCCCTCCTCCAAATCATCCCTTCCACCGCCATGTTTCTTGCGATCCC
TATTTCTATGATGTTCGAAGATCCCTTTTTGTATCGTGAGTTGGCTCCAACCGTGACACC
AGACGTTTCATCTAGCCCTGGTTTTATGCAACTGTGTCAATACCTCTCCGCATATTGGAT
GTTTGTCATGTTTTTGCAATCAGTGTCTATGTGGGTCAGTTTATTGTTTCTTACTTCTTT
GACATCGGGCGAGCCTTCGGAACCCATCCCGTACCTTATTCTATCCGCTGTGCTTCCTAT
TGGTTTCATCATTCTTGGCTTCCTTACGACGAAACCTCTGGCCGATTATCTGAAATCAAA
GGGCGAGGAGAAGGATGGTGACGAAGAGAACGGTGATGTCACCTGTGATGTCTAATTTTG
GCTCGAACCGGAAGCAAAACAAGAAAGATTGTCATCCGCTTTAGATACTCAGCGGCATGA
TCGATTGGAAATGTACTAAGGCTAGTGTACACTGGAAGTTTGAGACAAAGTCTTGAATGC
ATCACTATCAAATAGCAATACTAAAATTCTTTATAAGATACGTGCATTCTTTATAAGGTA
CCGGCA
>comp2152_c0_seq2
ACTTCCCATGGCTGTGCACCGCAAAAGACCTAGTCGAAGTACTGCGGGAACAGTACCCCA
CGTAGCTTGTACCTGCAGGATCAGAGAGTTCCACAAGAACTTTTCTCAAGAACTTTCATG
AGCTAACTTGAGTGATTTGCACTTCACTCCATGTGACACAAATTCCGAACCACAGGTATA
GTACTGTATTTTCATTTTGAAAAAAGTATATCACAGATAGAATCATTAGCTGCAAGAGCA

ACTTGCGCAGATGTCAAAGCATGCTGACACCCATGTCGATGTCGACAACGATCAAGAGAA
AGATAGCGACGATACAGACATTGGAAACAGCCCGAACGCGCGTCGGAGTCTTTCTCAAAG
TGGTACTGGATGGAAATGGCGCTTTGCCTTAGCCTCCTTGGAATTGGCATCTTCAATGGT
ATCACGGGGGTTGCAGTTTCCCTCCTCCAAATCATCCCTTCCACCGCCATGTTTCTTGCG
ATCCCTATTTCTATGATGTTCGAAGATCCCTTTTTGTATCGTGAGTTGGCTCCAACCGTG
ACACCAGACGTTTCATCTAGCCCTGGTTTTATGCAACTGTGTCAATACCTCTCCGCATAT
TGGATGTTTGTCATGTTTTTGCAATCAGTGTCTATGTGGGTCAGTTTATTGTTTCTTACT
TCTTTGACATCGGGCGAGCCTTCGGAACCCATCCCGTACCTTATTCTATCCGCTGTGCTT
CCTATTGGTTTCATCATTCTTGGCTTCCTTACGACGAAACCTCTGGCCGATTATCTGAAA
TCAAAGGGCGAGGAGAAGGATGGTGACGAAGAGAACGGTGATGTCACCTGTGATGTCTAA
TTTTGGCTCGAACCGGAAGCAAAACAAGAAAGATTGTCATCCGCTTTAGATACTCAGCGG
CATGATCGATTGGAAATGTACTAAGGCTAGTGTACACTGGAAGTTTGAGACAAAGTCTTG
AATGCATCACTATCAAATAGCAATACTAAAATTCTTTATAAGATACGTGCATTCTTTATA
AGGTACCGGCA
>comp2153_c0_seq1
ACCGATCACGCATCATTCAATAGGAACAGGCGTCGCTTTGCCTTTGCATCCGATAGACGA
TTGCAAGAGGGAGGGGCGATTGCATCAAGACGGAACCAGCCGTGGAGACTTATTTCTCGG
GTCGATTGATTGTTGAGTTTCAGTCTCCGTGGATCGCCGATAGTTGCCTTCGGTTCGGCA
TTGTGCTCGAAGGAAGAACAGAACCAGACTGAAAGGCCCATTCCATTGCTCTCGTTGCTA
GTCCACTTCATACGAGATGGCGACCCCGGTATCCACCGAAGCTCCTCTAAACGGGCTCGT
GAGGTCGGGATCCGATCAAGAACGCGATCTGCAAGCAAAGATGCAACACATGATCAGACT
CCAATACGCGGAGCAAACCATCTCGAACACGAACGCGAACGCGAATCAAGATCTGGCCGG
CGGCGATGGCACCAAAGGGCACCAAACAGGTGCACCTCTCGAAAACGATCGGGAGGACGA
TGGCAGCAGAGGCGGAAGAGGCTTGACCATGACGAAACGGGGAAAAACCAAGACCCATGC
GAACCTGGAAAATCTCGGGAACGACACAGACACAAACAGTGTTGACAACAATGCCGAGGT
AGGGAAAGGAACCATTGTATTTTCTGGAAGCCTGCGCGGTTCGATGCTTCCCCCAGGTAT
TGTAGATGACAGGCTTGACCAGAATGCTACGGCGACCAAGGGCCTCGAACGGGAGCACCA
GCGATACCAGATGCCTTCGCATTCGACAACCCTGGAATCACAGCAGCAGCGTCTTCTTTC
GTCCGCACCGCCCCAACAGATTTACTCCGGGTACGGAAGTGTCCACGAACAAAACCACGA
ATTCCATTACGGCAATCCGGATTACAACAACATCGACGACGAAGACTATTACTTCGACGA
GCACGGAGGGTTCCGGCGGGACGGGGACCACCGCACGACCTACTGCGGGCAGCTGTTTGG
ATGCCTGGTGTCTCCCATCGCGAACCTCGTCGGCCAGGAAAACCTCCGCCGGTCCTTTTG
CTACGGTGCCATCGACGGCTTGCTGACCGGCTCGGGCATCGCGTCGGCCCTTTGGGGGCT
CGGTCTCCTGTCGGTCCGGACCAAAACCGAGCTCCGCGTCGCCTTTGTCGTGGTCACCCT
TGCGGCCTGCACCGCCGACGCCCTCTGCATGGCCATGGGCCACGTGTGGACCACCTACAT
CGTGACCTCGAACCACGCATGGGAGCGCTCGCACGAGCGGCGGCTCCTGGAGCACGACAA
GGCCGACTCGAAGGGCCGGCTCGTGGACATGCTGCTGGAGCGCGGGATGCTCAAGATCGA
TGCCATGAGCCTGGCCGACACCCTCGAGGGCTACCCCGACCTCTTTGTCAGCGCGCTGGT
CGGGGATTCCCTCCTCTCGTCCGGGATCCAGGACGCGCTGCTCGACGAGCGCACCGACGA
CGGGCGCAACTACCCGCGGGAGCACTACAGCCCCGGCACCTATTCCGACCCCGCGACCGA
TTTCGACGGGGGCGGGGCCTTTTTCGGGAGCTTTGGGTCATGGAAGTTTCCCATGCACTA
CGACTCCGACGAGCGGGGCGAGTTTCGCAACATAGAGGACCGACACGCCCGGGTCGTGTA
CAGCGAGAGCCAGAAGGAGGCGACTTTTATGATGGTGGGTTTTGCCTTCTTTGCCCTAAT
TCCGAGCCTCCTCTGGTTGTTCCTGCCCCGGTGGTTCAACAATCCCGCCAACAACGGCGA
TTTCGGGCAGACGCACGCGTATTCCTCTCTGTCGTCGCAGGAAGCTTACTCGGGTGTTGG
AGATGGCGAAACCGTGAACGTACCGAGCCTAATCATACTGATTCTGAGTTCCATCATCTG
GTGCCTGGGAGTCTGGAAAAGCAGCTTTGTGGACTCCAACTGGGTCGTCTTTGGCTTGGA
GACCGTTGCTGTATTGATTTTGTGCATATCTTCCGCTTACTGGATCGCCGTCCTACTCGT
TCATTTTCTCGGTTTGGAGTACCTTATATTAAGCGGCGAAACATCTCTCGGTAGTCCCGA
TTTGTAATCAACCTGACCATGTACAAACAACGAGGCGGTTAACGACTACGAAATTACAAC
GTGGTAAAGAATATCAGGTGATTCAATACTCGAGCAATACGTGATGGCATGAATTCTGAA
ACACAGTCATTTCTGAAATTGGCAGATCATGGTGTTAAAAAGCTCATGGCACAAATATTA
TAGTATTATAATATC
>comp2154_c0_seq1
GGTCACAAACAATAACATCATTGAACCATGAAAGTATTGAGCACGTTATACCTAGCTTCT
GTGGCATCTGCCTTCACGACGCAGAGCTCGCACCGATCCCCCCTTGCTGTCGCGAGTCGA
AAAGTAAAGCAATCCACTGTTGCTCGTTCTCTCCTCCCAAACAAGCAGCAACAATCGGAT
GCATCCCCTTTTGCCATTGACGTCGAAAGTTCTACCGTCCTCGATGCCAAGGGAACAAGT
AAAACCGCTGAGGACGGAAGTGCAGGAGTCCGACAACTTTTGGGTGTGCGTGGCGCAGAC
AAATCGGACGACATCTGGAAAATTCGTCTCCAGCTCACCAAGCCTGTGACATGGATTCCA

CTTTTGTGGGGCGTGATGTGCGGAGCCGCAGCCTCTGGAAACTACCATTGGATTTGGAAT
CCGTTCGACCCTAACGACAGAAATGTTTTGCTTGGATTGGAAGACACAGCTAAAGCTATC
ACCGCCATGGTCCTAGCAGGTCCTTTTTTGACGGGTTACACTCAAACCATTAACGATTGG
TATGACAGAGAAATTGATGCTATCAATGAGCCTGATAGACCAATCCCTAGCGGTGCCATT
GCCGAAGGGGATGTAATCGCTCAAATTTGGATTCTCCTCCTTGGAGGATTGGGACTGTCT
TACGGTCTTGACGTTTGGTGCGGCCACGAATACCCCATCAATCTTTACTTGGCAATTTTC
GGATCTTTTATTTCCTACATTTATTCTGCTCCTCCACTCAAGTTGAAACAAAACGGATGG
GCCGGAAACTTTGCCTTGGGTGCCTCCTACATTTCTCTTCCGTGGTGGTGCGGACAAGCC
GTCTTCGGCGAGCTTGATCGCCCCGTCTTTTGGATCTTGCCCGTGCTATACTCGATTGCG
GGACTTGGAATTGCTATTGTGAACGATTTCAAGTCGATCGAGGGTGACAGAGCGATGGGA
CTGAACTCTCTTCCTGTTGCATTCGGAGTGGACGGAGCTGCAAAGATCTGTGCAGGATCG
GTCACGATCACCCAATTCGGTGTTGCCGCGTACCTCTACTCTATTAACGAACCAATTTAC
GCGGCAGTCTTGTTGGCGTTGGTGTTGCCCCAAGCTTACTTCCAAGCCACACTTTTGCTA
CCCGACCCAATCGAGAACGACGTCCAGTACCAAGCTTCTGCCCAGCCATTCTTGGTCTTT
GGTATCCTAGCAACGTGTTTGTGCCTTGGACACCACGAATTCCCGGTATAAGATGGGGAA
AGCAAAAGATGTTTACTGCGCTGATCTATTCGATTCACTCGATTTTCGTAGACAGCAAGT
GAGTAGCCATCGGTATAAGAGTAAACTGTTAGCGAATTATTTTTATATTTTTTACTCGAC
ATGAGCCGTCAGCAGTGGAGAACAGTCCCCGATAGTCCGAGAAGCTCAAGTTGGCGCCGT
GGCCTTTCTACCTTGGATTTCATTTTTT
>comp2155_c0_seq1
ACGGTGATGATGATGATTCATGATGGTGGGTGATTGAGAAGTCGTACTCTTTTTACTGCA
CTTCTTCTGTAATCCGTTTAGATCCACTTAACCACAACTCGCACAAACCAATTTTCACCA
CCGTGACATCTCCTTGATTGAAAGATTAACTACGATGAAGTCCACCATCCTCTTTTGTCT
CCTTTCTGTTATCCTCGGAATGTTCTTTGCGCCACAATCAGCGTCGGCGTTTGCCCCCGT
GAGTGTCGCTCCAGTCGCGTTGACGCCGACCACTAGCACACAGCTTTTCTTTTTCGGAAA
GCCAAAAGATGACGGAAGCCCCGGAGACTACGTGTGCAAGGACTGCGGTTATGTTTTCAC
GAAGGGTCCCAAGGCCTGGGCCGCCCTCGACGACAGCAAGTACAAGTGTCCCCCTTGTGG
CGCGCCCAAGTTCCGGTTCAAGAAGGTTCCCAAGGGATCCGAATCGGGGAAGGTCGAGGT
GAAGAAAAGCTGGTTTTGAAGAGATACAATATGTAAACGGAACAAGAACAAATACTCCAC
GATGCTACTCAAACAACACACTTGAGCCGACCGCATTCACACGATCACAAACACAAAATG
ATTTCACTTCGCTATCCGAGAAAGCAAGATGTCACACGAAGTACTTAAGAGGCATGACGT
CATGGATTTGATTCCCTGGGTCAACTGCTGGTTTCCCACTCCATCTGTAGCTTCAAAAAT
AGCCACGCACACTCACTCCTGCTGTAGAATAATAATAAAAACGCACAAAATTCATTGATC
AAGCGTTCACAAACATGGCGGAATCGAATGGTTCGAATAGCTAGTGCTACAGCT
>comp2156_c0_seq1
CACGGTTTAAAAGTCTCCTTTTTTGAATGTCGGTTTGAGTACGAGTACAAGGTACTCGTA
CTAGTACGTACTCAAATTCGTACAGGGATTGAAAATTGTACTGTACTGTAAATTCGTTCC
GTCCCATATTTTCGATGTTCTATTGCAACACATCCCTGGCGTCAGATTACTTTTCATTGA
TACCCTTAACTTCTATCTTAAAAATTAGGAAAATTAGTATCGAGGAGCTCTGAATATCCG
TGCAAACGACAACCATTCGAAGACTATTCAATATTGCTACGACATAAATCTGAATACGAA
ACAATCAAGCATGTCCCGATCTCCCCTTGCCGCCCCAATAGCACCACGTCCAGCTCGAGC
CAACTGGCGGACAGCAACCAAGCGCCAAATCCTGGGTAACTTCCAACAAGGTAGCCCGTC
GATGTCCTCGTTATCATCCGCCAAGATTGATGGAAGCAACACTGATCATATCCAAATGTT
GATCGGTGGATTGCAAGAAAAGCACGACCAGATGATCGCTTTTCTCAAATCAGATTTGGA
CGACTGCAAACTCGAACAGGAAGAGGTTTTATCCACTGGCCTGATCAAGCTTCCCAAGGC
CGTCCGAAACATGTCTGTCAGAGATTTCAATCGGCAGCACGGCTGCGATTTGTTGGCACT
GCTCAAGAGCAAGGACGGGGTGATCTTATCGAAGAACAGCGTCAACGGCATCAACAACAT
CCCCAAACACCCCAATAATACGACGGCCTGTATGATAGATGCAAACAAGAAACGATGCTA
TGAAACTCCTGCACCACGAATGAGACGTCCTGGTGCTCCCATGGGCACCATCCTTCGAAC
CGCTCGACGAGGAGAGGAACTATATTCCCAAAACGGATCCCCCGTCGCGGCCACCGAGCC
AGGTACCGTCATTGCCACTGTATGTAAGAAGCGACGAGGCAATGAATCAGCCAACGTCGA
GATCAACATCGGGGAGGGCCAATACATTTCTCTCAACGACCCTAGTGGCATATCAGAACT
CGATGCCCAAATGAAACAGACGGCTGCCAGCCAACTCAAGGTCCTTCAGGATCAAATGGC
AAGTTTGATGGCCCAGCTACAGCACTAAAAAAATGTGGCGAAGGATTATGGCCTACTCAC
GACTGGATAGCAGTCAATCTAAAGCAAAACAATTGATAATGAATACTTATCTGTAAACAT
ATGGCTATCAAATAGCTTGCGAAAAGAAACGATTTATTCGGAGTACAGCATTTGTAATCG
TGTAGATTGTTGCGATGCAAACAAACGAACAAGATTCAATGGAGCCATTTCTTGTAAGCA
TGTTGTTTGAATGAGGAAAAAATGTGATATTTGAACGCTGTGACGTTGAAGAAGAAGTAA
CTCCCCGGATTCAAACACGCAATTGACTTTCAACTCGAAAACTGTACAATGCTGTCAGAG
TTCGGTTTGCTCTCATCCATCTCGTAAGAGAAGATCTTATCTTACTATTCCAAGCACTTT

TTTGTTGATGATCTTGTTGTTTTACCATGTTATTGTTGTTGTTGCTTTTATTCTTGTTGA
AAAATAACAACAACAAGAAAAAGGAGAACAAGAACAACAACAATGAGG
>comp2157_c0_seq1
GGTTTTACCGCCCTCGAGCTGTCTGACTTGCGTGCAACTGTTCTCTTCTTGATAGAAGGG
GCCCGACTCATGGAACAAAGTTTTCGCCCGCGACGGTGTCTGACTTGCGTGCAGCTCTGT
TCTATTCTTGAGAACAACAGGCATCACCGTCATTTTCGGCTCTCGCAGTTTGGCGCTGCA
CACCCTTGTTGACACAACATCCGAAAACGGCAAAAAGACTGTTCAGCACGGAGCCCATCT
ACGGCACGGCAACAATGGTTTCTAGACGGCTAAGAGGCTCAAACCCCACCCGCTACCAAG
ACAGGCGGGAAGAAGAGGAGGCGATCGATCCCAGGAGCGGCCCGGGAGACGAGGAGGCTG
CCTCGAGGCTGCGGGAGCTGCGGCTGCTGGCCTCGGAGGCGATCCGGCTGGCCAGGGAAG
CCCGCGAGGCCGCTCTGCGCCTGCAGGAGTACCGGACGGCGGTCTCCATGCCCTACCGCC
AGGGCGGGATGGTGGGCGTGGACGACGCCTCGACCCTGCGAGAGGACGGAAGCTGCGCCA
GGAGCTGAGCCCGGCGGAAGCCTTGGCGGCGTTGGCCGGCCCGCTTTGCCCGACCGGGGA
GGCGAGGGGTGTTTTGCGGAGGCATCGCTCGGTACGGACCGGTGTCGAAGCCGAACCTTC
TTGCGGTCGCTCTGCTTTGCGTCCCCTTGCTCGTGCTGCGAAGACAAGGAAGCCGAATTG
CGGTGGAAAAAGGAACGGCGAATCCAAGCAAAACCATTGCCGGCCAAAAACCGACAGGGG
ACCCTTGTGTCGGTGGCGATGGCCTTGGAGTGCAACACGGTCGGGCGTGGATCGCACATT
GCTGTCTGCACTCGTGCGCAGGCGGAACGGATCGTTGCGAAGACGATCGATGTGCTTCCG
ACGACCCGGCCGGAAGGAGGCAGAGCATTGCCGTCTGCACTCGTGCGCAGACGCAACAGA
TCGTTGCGAAGACAGAGAGGGCAGATGTGTACGTGTTATCGAGCACTAGGGCCCAACGAA
AGCCTTCCAAGGATTTCGCGTCCATTCTCGGGTTGATTCCACGGTTTGCAAGCAGTCATA
GTAGTGTACAGTACTAGTAAACAATGTATCGTTAAAACA
>comp2158_c0_seq1
CAGAGACGAGGGACGCACAATTAAATAACTTCCAAATTAGTTGGTGCTGAGCTTGCGTCA
AATTGCAACAAGCAACTACTACAATGCTTCGTCAAAGAGCCCAGTTTCTAATATCCAGGG
CATCAACTTCTGTTTCCGGGAGAAATGGATCCCAAAACTCTGCCAGAAGTCTTTTTTCCC
GCCGATCTACGGGGTCATCGGAACGATTCAAACTATCGACGCGTGCGACGTCCACGCTAT
CTTCTTCCGCCCATTCAGAGGATGCGGGTTCTTCTTTGTGGAACACAGCAAGCGCTGCTG
CCCTGATTGCAGCCGCTGCAGCAACGACCACATTCGTTTCATCTGATTCCACGACGGACT
GCTGCGGAATTGTTGGCGTGATCGGTACCGACAAGAACGCGGATGCCCGCGAATTCCTAC
TGGACGGACTGACGATATTGAAAAATCGCGGATACGACAGTGCAGGAATCGCTACCGTTC
CTTCGGATGGGAGCGCTTTATCGATTACCAAGTATGCCTCGGACGGGGACAATGCTGATT
CGATCGAACTCGTTGCGAAACACTCCCAAAACCACGGACATTTTACGGGAATTGCCCACA
CTCGATGGGCAACTCATGGAGGCAAAACCGACGAAAATGCGCACCCCCACTTGGATTCCT
CGGGGAAAATCGCGTTGTGTCACAACGGAATCCTAAACAACGCAAACGACTTGAGACGAG
AGCTGCAGAGTGAAGGCCATACCTTTACCTCGCAAACCGATACTGAGGTCATTGTCAAGC
TGGTAGGCAAATACTACGCAGAGGGCAGGAGCGAAAACATTACATTGAAGGATGCTACTC
AAAAGGCTCTTGATCGATGCGATGGAACGTGGGGATTAGCCATTGTATGTACCGACACAC
CAGACGAGATGGTAGTAGCCTGCAATGGATCGCCGCTGGTTATTGGACTTGGCGCCGATC
GTACATTTATTGCATCAGAAACATCCGCGTTCAATAGGTATACAAAAAATTTCATCAGCA
TGAAGGATGGAGAAATTGGTGTTTTGCATGCCGATGGGCGAAATCTCGATCTGAGTCGGA
AGCAGATAGCACCCGATCAACAGGTAAAACTATCGCCCGACCCTTATCCTCACTGGACTT
TGAAAGAACTTAATGAGCAACCCGAGGCCATCGCGCAAGCACTAAACTTTGGAGGCCGAA
TGTCAACAGACAGAGTGACATTGGCAGGATTAGATCGAAACGAAGAAAAAATGTCGAAGG
TCAAGCATATGACTCTGAGTGCCTGTGGGACGTCTCTGAACGCGGCAAAATACGGAGAGC
GAATCATGAAGCATTTGGGATCCTTTGATAGTGTTGCTTCTATAGATGCTGCAGAAACCG
AATCGAAAGACTTTCCTTGCATGGATCGACCAGAGACTACAGGACTGATTGTGGTTTCAC
AATCTGGTGAAACAAAGGACGTCCACAGAGTTGTCAAGAACGCCATGGAGAAGGGCATAG
TATCAATGAGCGTGGTCAACGCCGTTGGTAGTTTGATTGCGCGCACCACGAAGTTGGGAG
TATATTGCAACGCAGGAAGAGAAAATGCAGTTGCGAGTACCAAGGCGTTCACGACTCAAG
TCACGGTCTTAGCTTTGATTGCCTGCTGGTTCCGCGAATGTCGTGACCGGCTATCGGGTC
GACCCGCAAATATGGAAGCGCGATTGGTCAAGGATTCGCTCATGAGATTACCAATCACGA
TTGGGATGACTATGAAAACATGCCAAGAACAATGCAAAAAAGTAGCTGAGCGACTCATGG
ACAAAGAGCATTGCTTCGTTCTTGGAAAAGGATACGCCGAACCAGTGGCTTACGAGGGTG
CGCTCAAAATCAAAGAAATGTGTTACCTCCATGCGGAGGGCTACAGTGGTGGTGCTCTGA
AACACGGGCCATTTGCTCTCATTGAAGGTGAAAAGGGAAAGTTTGGGGCAACACCTATCA
TTATGTTCATTTTGGACGATCAACATGCCCACCATATGAGATCAGCGGCCGAAGAAGTGA
AAGCACGCGGAGCTGAACTCATCATTATTACAGACAAGCCGTCTTTGGCACGGGACCTGG
ATGATAATCCTATTGTTATCCCGCAAAATGGTCCAATGACTGCTCTAACAGGCGTCATTC
CGCTCCAGCTCATTGCATACGAGCTTGCATTACTGCGTGGAATCAACCCAGATACACCCA

GGAATCTTGCAAAGGCGGTTACGGTTGATTAGAAAACTCTCCATTGTTCACAACTCCCTT
TCCGGTGATATTATGATTAAAATTCCTTTCATCCATTCTCTTTGGTGCTACACGAGCTAC
CTCTAGAATGACTGTAATAAGATACTCATTATAATGATATATAACAAATAAATTTTGATG
A
>comp2158_c0_seq2
CAGAGACGAGGGACGCACAATTAAATAACTTCCAAATTAGTTGGTGCTGAGCTTGCGTCA
AATTGCAACAAGCAACTACTACAATGCTTCGTCAAAGAGCCCAGTTTCTAATATCCAGGG
CATCAACTTCTGTTTCCGGGAGAAATGGATCCCAAAACTCTGCCAGAAGTCTTTTTTCCC
GCCGATCTACGGGGTCATCGGAACGATTCAAACTATCGACGCGTGCGACGTCCACGCTAT
CTTCTTCCGCCCATTCAGAGGATGCGGGTTCTTCTTTGTGGAACACAGCAAGCGCTGCTG
CCCTGATTGCAGCCGCTGCAGCAACGACCACATTCGTTTCATCTGATTCCACGACGGACT
GCTGCGGAATTGTTGGCGTGATCGGTACCGACAAGAACGCGGATGCCCGCGAATTCCTAC
TGGACGGACTGACGATATTGAAAAATCGCGGATACGACAGTGCAGGAATCGCTACCGTTC
CTTCGGATGGGAGCGCTTTATCGATTACCAAGTATGCCTCGGACGGGGACAATGCTGATT
CGATCGAACTCGTTGCGAAACACTCCCAAAACCACGGACATTTTACGGGAATTGCCCACA
CTCGATGGGCAACTCATGGAGGCAAAACCGACGAAAATGCGCACCCCCACTTGGATTCCT
CGGGGAAAATCGCGTTGTGTCACAACGGAATCCTAAACAACGCAAACGACTTGAGACGAG
AGCTGCAGAGTGAAGGCCATACCTTTACCTCGCAAACCGATACTGAGGTCATTGTCAAGC
TGGTAGGCAAATACTACGCAGAGGGCAGGAGCGAAAACATTACATTGAAGGATGCTACTC
AAAAGGCTCTTGATCGATGCGATGGAACGTGGGGATTAGCCATTGTATGTACCGACACAC
CAGACGAGATGGTAGTAGCCTGCAATGGATCGCCGCTGGTTATTGGACTTGGCGCCGATC
GTACATTTATTGCATCAGAAACATCCGCGTTCAATAGGTATACAAAAAATTTCATCAGCA
TGAAGGATGGAGAAATTGGTGTTTTGCATGCCGATGGGCGAAATCTCGATCTGAGTCGGA
AGCAGATAGCACCCGATCAACAGGTAAAACTATCGCCCGACCCTTATCCTCACTGGACTT
TGAAAGAACTTAATGAGCAACCCGAGGCCATCGCGCAAGCACTAAACTTTGGAGGCCGAA
TGTCAACAGACAGAGTGACATTGGCAGGATTAGATCGAAACGAAGAAAAAATGTCGAAGG
TCAAGCATATGACTCTGAGTGCCTGTGGGACGTCTCTGAACGCGGCAAAATACGGAGAGC
GAATCATGAAGCATTTGGGATCCTTTGATAGTGTTGCTTCTATAGATGCTGCAGAAACCG
AATCGAAAGACTTTCCTTGCATGGATCGACCAGAGACTACAGGACTGATTGTGGTTTCAC
AATCTGGTGAAACAAAGGACGTCCACAGAGTTGTCAAGAACGCCATGGAGAAGGGCATAG
TATCAATGAGCGTGGTCAACGCCGTTGGTAGTTTGATTGCGCGCACCACGAAGTTGGGAG
TATATTGCAACGCAGGAAGAGAAAATGCAGTTGCGAGTACCAAGGCGTTCACGACTCAAG
TCACGGTCTTAGCTTTGATTGCCTGCTGGTTCCGCGAATGTCGTGACCGGCTATCGGGTC
GACCCGCAAATATGGAAGCGCGATTGGTCAAGGATTCGCTCATGAGATTACCAATCACGA
TTGGGATGACTATGAAAACATGCCAAGAACAATGCAAAAAAGTAGCTGAGCGACTCATGG
ACAAAGAGCATTGCTTCGTTCTTGGAAAAGGATACGCCGAACCAGTGGCTTACGAGGGTG
CGCTCAAAATCAAAGAAATGTGTTACCTCCATGCGGAGGGCTACAGTGGTGGTGCTCTGA
AACACGGGCCATTTGCTCTCATTGAAGGTGAAAAGGGAAAGTTTGGGGCAACACCTATCA
TTATGTTCATTTTGGACGATCAACATGCCCACCATATGAGATCAGCGGCCGAAGAAGTGA
AAGCACGCGGAGCTGAACTCATCATTATTACAGACAAGCCGTCTTTGGCACGGGACCTGG
ATGATAATCCTATTGTTATCCCGCAAAATGGTCCAATGACTGCTCTAACAGGCGTCATTC
CGCTCCAGCTCATTGCATACGAGCTTGCATTACTGCGGTAAGTCTGCAAGAATGTTACAT
ACGGCGAAAATTAAATGTGCGCCAATTGCCTCTGTCCTCATCATTATTTTCCTCACTTCT
TGTTCGCTTTGTCCGTACGCAGTGGAATCAACCCAGATACACCCAGGAATCTTGCAAAGG
CGGTTACGGTTGATTAGAAAACTCTCCATTGTTCACAACTCCCTTTCCGGTGATATTATG
ATTAAAATTCCTTTCATCCATTCTCTTTGGTGCTACACGAGCTACCTCTAGAATGACTGT
AATAAGATACTCATTATAATGATATATAACAAATAAATTTTGATGA
>comp2159_c0_seq1
ATCATTCTAGCGGCACAGTACGAATTGCTTTTTGCAATGCTTGAACGGTAGAAGTTTCAT
TCCGAGCCCTTTGAGTAGTTGGATAGAAAGAGATATCGAACATGCACTGACGTGCAGTAC
CGTAGCAAGAGATCAGCAGAAAGAGCACGATGGCAACATTCGATACGAAACATTGTTGGG
TTCGAGATTTGAAAACAGGAACCTTCCTCGCGAATTCAAGAGAATCGGTTCGTGTGATGG
GTGTTGTTACTCATATCAGTGATTTTTCCATGGAAGATAAAGCACTGCTACACCCTTTGA
TGAAATGTCCGAACTGGTCGTCTCCCATCGAAAGGCCAATCACATCTGTAAACAATCGAG
GGGAAGCATTGTCACAGACGCAAAATAGTGGCACCGACGGCGGAAACGAGGCTGTCTTTT
TGTCTGTTACAATTGATGATGGGACAGATTCCATTGTCGTTTGTGCTCCTCGACAAATGA
TAGAGTCCCCATCGTCGGAGATAGTGATAGATATTGGGACGACCTACGACTGGGTACTGA
AATTACGTCAAAATTCCTCAACAAAACAATGGTTCGCCGATACCCTGATCAAGATCGAGA
ATCCCATCGACGAGCATTATCGGTGGATGGAACTCAGTCACCACGATCAAATGTCTTGTT
CTCAGTCATCGCCGTCGTCATCATCATTACGGGTTCAATATTCTAATCTTTGTCACAGAG

CTGGGTTTCCAACTCGCCGAAAGAATGCACCCGAGGTATATCGCTTGATTCGACTCAATG
CTCAATTGCAACGTGAACAACAACAGCAGGAGCAGACAGGGAAACCAATAGCGAAAAGAT
TCCGCAACAGTATTCCACAAAAACGTAGTTGTTTAAGATTCAATAGCGACAGGCTGCCGC
TGCGTCTACAAAACCGCCCGAAAGGACTACAAAGGAGCTTTGTCAATACCAACAGGATTA
TCCCTGCCCGACGGAGTTTGAATTCAAAATTGCAACCCACCAAATTGCACACTGGAACAT
ATTGTTTGCAGACACAGTCATCAGCGAAGACGAAAGAGCAACCGCCGCAGGTTCAGTCGC
TGAATGGCCTTTTGCTAAAAGATTTGGCTGCGGTGTTGCAGAAAACACAACGCGACATTC
AAGAGATGACCGAAGACCTGCAAATGGAGGGAAAAATTTACCAGAATGAGAGGGGGGAAT
ATCTACCGCTGTAGTGAAAAGCTAGTGAACCGAAAGAAGCTTCCATGCGATTAGTGAAAA
ATTAAAAATGGGCAAACAAAGAAAGTAGCGCTCGTAGATCTCAAGTCTACAACAGTTTCG
TCTATGATCTCGATACAGTGTGCATGATTGCGTCTTGTCTTGAAATATACGTGCTACTGT
AACCATACTCTATGCTGGACTCACAATGAGTTTATCTATCAGCTTTACGC
>comp2160_c0_seq1
ATTTCTACTATCCACTATCTTGTGCTATGCATAATCGCTGTAGAACGAACTAATCAGTAC
AGTTGGAACAAAATGTCGTCTCCCGCCACCCAAAGTGCAATCATTCCACCAGGCAGTAGA
AGCGATGAAAACCGTCTCGCCTCGTGCCTGGTCTTACTCAACGAGGTTGACGAATGGATT
TCCGGTCCGATGTTCCGCTTGGGACTTCCCAAATGGTTCGAATTTTGGTACTCGATTCCC
GCATGTTTCTTTGGTACCACATTCACCAGTGTCGCGGGTCCAGTGTGGATAGCCTTGTTG
GCATTAGTGTGGGGACAGCATGACGAAAAAGAAACTTGCGGAGCAGAGCTGGAAGACAAC
ATTTTTCTTTTGAAAATCATAACTTTTTCCGTAACTGTTGGTTACGTTGTGATGTGGGGA
TTATGGCAAATTGGTGGACACGAGTATCTTGCAGGTATTGCTTTCTGGAATGGTAATGTT
TATGTTCTTGGGGTCCCTTTCAATATCGCAGTACTAGCCTATATTTTAAATGGCTTTCCA
TATAGTAGCTACCAGGGCAGTGAAACAAACTGTAGTGCTGAATACAAGAAAAACATTTTT
TCGATGGCTATTTATCCGTTGTTTTTATGGCCAATCTTAATCACGTTGTTAAATTGGATG
AAATCCACCTCCAAGCGCAGCCGCCCCGCCTTGAAGGATATCGAACGAAAAAATGCAGAA
GGCTGCGGCAGAGGAACAAGCGAAAACTGGACACAAAAGAAAAAATTTCCAGCAATGACC
CAATTGCTGGCCAAATACAACGGAGATGCATCTTTCCCCTCTGGTGACGTTTTGTGTGCG
ACAATGGTTGCCTTGTCTATCTTCAATATTTCTTCTGATGTCAGCCACGCTTATCGTCCT
TCCCACCTCGCATCCACTATTGTGATCCTTGCGGGAACTGGTCGCATGTATGTTCTAGCG
CATCACTTTTTCGACGTGATGGTGGGAGCAATTCTCCCTTTCTTGGCACACAGATTGGCC
ACCAAACTGGGATGCGGAATGTTTGACATGGAATGGTGGCATCCTCTCCTGGTGTTGGGA
ATCTTTTTTGTCATAAACTCGATCACGCGACGAAAAAGTGACGAAAAGAAATCGACCAAA
AACCCTCTGGCTGAAAAAGAAGCATGATGTGGTGGGTGGTGTAGGATGCTCTAATCCCAA
GACCGTAGGGAAATTGTAGACACAACGCAGGAGTGAAAAAATTGATGCAGATCAATTGCA
TGAATACAATTTCTTCAGACGAATACCAGTTTCATTTTCAACATGGCAATCCAAAATATA
CAGGGAAAAATTCTATAGATTAATGTGATTTATAACTTCCTTTTGCTTGCGTT
>comp2161_c0_seq1
CCCGATCCCAAATATCACTTCCCATTCAACCACTGTTGCTCTGAAAGTGAACTTCGGAAC
GAACCAAACGGAAGCACACGATACAGTTGTATTCAAATCGATACAAAAAAAAGACCAACC
AAAACAAAACAAAACAAAACAGAGCAGAGCAGAGCAGAACAGCACAGCACAGAACAGAAC
AGAACACAACAGAAACAACAAAGATTCATCGATGCAGTACCGAGAGGAAAAGTCTTTTCC
ACACTACTACAACGTCGTCATCGAATCTCCGCTCCCTCAAACCGATCGACAGTGCTTTTG
GTACAACAGCAACGACATTTCCGACATCCAGAGTCCAAGAAGCAGCAAGAATCACAATGA
GAGCCTCCTCCTCGGTCTCCACTCCTCCCTCATCTGTTTCAAGATTGCCCTGTGCGTCCT
TTTGTGATTTCGAGGCGTCTTTTAAGGACATGTTTCGATGCCAAGGCGTCGAGTTTGCGA
TCGGCGACGGCAACGACATCCTCGCGGGAAGAGACTCCGACACCATATCAGCCACCAGCT
CGGCGAGCACCGTCTCCCAGCGATCCATCCTTGTGATTGGTTCCGACGATGCGATTGAGG
TCGACGTCGACGTCGACGTCGACGCGGACTGCGAATCGGCCAGGGACTACCTGGAGCGTG
TTTCGAACAACAGGAGCAAGAACACCGACACCTCAAGTGCCAGCCAACAAGAAGAACAAC
GGGACGAGACATCGTCGTCTGGTTCCTCCTCCCCACGGCCGTCCCCCCGGGGCAAGAGCA
AGGGCAACAAGAAGCACCACAAGCTCTACCACATTATTCGATCCCGGAGCAGCAGCAACA
GCAACAGCGAGGTCCCTTCCTCCCCCAGGCGACGGCGCAGGCCGTCCTTTGGGTCCTATC
TGAAACGGAACGATTCCGATTCCACCGACAGCACCCGCTCTCTCAGCGAGGACGAGAGCG
AGCGACAGCCCCGAGAGCAAGAAAAACAGCCCCACGACCTCGAGCCCAAGGACTCGGACG
ACACCCGGGACAGCTGCGAACCCGACAGCGGTCGAGAGGAAAGGCAAGCACAGGCACAAC
CGAAACCAACGGCGTCTCCGCTCCCGCGAAACGGCGCTCTGGACGGCCGTCCGGTCTCGA
TTCTCCGGCGCAAGGAACGGTCTGCGGACCTGACGATTGCAGGCAGCGGCAGGATCCTCA
CCTTTGCCCCGTGCACGGTCTTTGCGGACCCGGCCGAGCCCCTCCGGGCCCGCCGGAAAA
TCCCCCGGCTGCCGAGGCAGAGGGTGAGCATCCTGGTGGTGGAGGGAACGACCGGAGCCT
GCTCGAAGCGGGAGGTTCTCCTGAGCCTGCCCCGGTCCTCGTCGAATTCGAATCCGCGTT

TGCGCTCCTCGGCGTCGTCGTCTTGCTACTCTTCCGGTTCCGGATCGACCGATCGCTCCA
GCGACCGCGAAAGGAGGCGGCTCTTGCGGAGGGCCCAGCAAAAGGAGGCGCTGCTGATCG
AGCAATCGGCCAGCAGCGAGCTATTGCTCCCGGCCTCAAACGGGTGCTACGTCTTTCGGT
GAGGAACGAAACTGCTGCGATACGGTACGATACGATACAGCACCACATCGCACGACATCG
CACCACGAAAGAGCACACACAGCATTAGAACACACCGCGACACCCAAACGAGGCACGCCT
CACACACAGCATACATCACACTGCGCACCGCGACGCTCGGCGAGGCACGGATGCAATTCA
ACACGACGCTATTCACGGGACGACCAATTTTTGGCACCATTGTTACAACAGCTTTTCGAA
TAATACAAGAGTTTAGAATACAGTTTTATGAAACAACCTCTCCTAGCGACCGCGTCATTG
TTTTATTCATGAACGTGTCGGCATCGTTGTGTTGTCGTAGTTTGCAAAGCTGGTCGCCGT
CCGTGCCATTTTCGGTGTGTCCAGCTCAATGTTTTGTTGCAATAAGAAGGACACGCACCA
TACAGTGACGTTGGATTAAGTTGTACTAGTCTTTCGTCAGCACTTTAAATTTGAAATCAA
GTACAGCGATATGCTTCCTACTTAGTTCTCATCGCTCACCCCTCAAAATAATGACGCCAG
CTTGTGTGCCTCCGAGAACGAACCGCACCGTACGTGATGTTGAAGAGTTCTCTTTCTACC
TGCCCGAACGGGTAATGCCGTATCTCCGGTAGAACAGAAGACTTTCTTCCCAAACTCCTA
ATGCACCTTACGCATAGTTCGTTCACATAATGCGGAAAATGCAACCCCAAATGCACTTTC
AATAGCCTCAACGGCAATGATAGTTCTCAGACTGTCTAACTTTTTCAAATGCTTCGGATT
AAATCCCATTAACGGGCATAGTTGCTGTCATGTGTGTTAAGACCTTCAATACGGAACGCC
TCCAAGATACCGTCGGGAAGATAGATGGTTCGAGTACCGTACTGTAGCAGCTCACCAGCA
ATTCATAGAAAACTCCGTCCCAAAATAAAAGTACCCTAGGCATCTCTTCTGGTGAAACGA
GCTCAATAATATTTGGGCGATTATTGCAAAGTATAAAATTATAAAGATCATATACAG
>comp2162_c0_seq1
CTAGCACACCTTTCTCATCAATTCGTTTGAAAGCAGGCTCTCACGAGGATCTCATGAAGG
TCGTTATAGATTTCAGAAATTGGCTGCGGATGATTTTGCACCATTGACTGATTCATCCGT
GTCTACATGAACATTTCTTCTCACAATCGGCGGCGTCGGTCCTCCCTCCACCAATACCAT
TCTCTCCGGGATTCCCGTCGAATCTATCTCGCATTAGCATTTTTGATTGCATTGGTGGGC
TTGAACAATATGTGCCAGTCATTTCGGTCTCTTCGATTGCTCAATAGGGGTATGATCCTT
AAAAGTGACTTGCTGGGACGACAAATCTTTGACGAAGCGATGTCAGGACAGCAAATCAAA
TCACGATTGTTGGCCAGCACGGGGGCATGCTCAATGCGTAGTTATGCGATGACTCCCACT
AGACTTTTTGGCAGCAAAAAGGGGGTCCCCGGAAATCCACTAGGAACTGTATCGATTTAC
AACGACCAGGAGGCACTCAAAGAAATCGACGAGGACGCCCTCCAAAGGACTGTGCACCGA
ATTTCGAAGATAATAGGCTACGAAACATACGATGTAACGTTGCTCTTGGTCGATGATGAC
GAGATGAGAGAAACGAATCTTGAAACACGGGGCATCAACTCTCCTACTGACATTTTATCA
TTTCCCTTTCATTCTCGCGAGGACAACAAAGCTGGCCTGTTGAAAGAGCCCGAATTCGAT
ATACCTGACTATTATACCCTTGGCGACATGGTTGTTTGCGTCCCCTACGTTATCCGACGA
TGCAAAGAAGACATTCTCTTTCGAAAGGAACAAGCGGAGGGTGCTACGAACGACGAAAAA
AAATCAGAATATGGGGATGATGATAGTGATCTTATTGACGACGATGATCGTGGAGTGTCT
GGGGCAATGGCTAACATTGATGATCCCGAGAAAAGAATCAGAATGCTCTTGGTGCACGGA
ATGCTTCATTTGGTTGGATACGATCACATCGAAGATGATGACTACGTGGAGATGGTCGAA
AGAGAAGAAGAATTGCTCAAAGAGCTTGGAGATTTATTGTAATCGGAGGTGCTGTATTAA
GCTATTGTAAATGAAATCAAGTTTTCTTCCGCCTAGATAAACAATCCACAGTTCTCATTG
GAATGTTTCATATGCCTTGCTTTTGCCGTTGTTGATTTTCTTGCATTAATCAGGTATGGC
ATCGTACAACGAATAAAATAATATTAGAC
>comp2163_c0_seq1
AACAATTCAAACACCTGTTCATCAACTCGTATCCCGTGGAAGCAACGACGGCAGCACCTT
ATCGACCGCCGTCGATACAATAAGCAATCAAACCCAGTTGTTCGACCTACCTCTTCCCAT
TTTTGACGAGAAAAATACTAAAGTTTGCACGTTCGATAGCTTCCATTGCATCGGTTTTGC
AGCTCTGTACTGCGTTTCGCCCCAGCAAGCAATCGAGATTTCGTGTGTGCTTCGCATTGG
ATGCAAAACAGGAATAAAAACAAATCTGCCGAAATTTTTCAGGTACAATTCACTACTGCT
TCTACGACGAAGAATTTAGCACGATCGCGCGTTTCACGTACAATTTTTAAATTTGTTGCG
AGATTCCATCCTTCTTCGTCGGCAATATTAAGTACACAAGAATATTAAGTACACAAGAAC
CCACAGCACTGTACCTCCGCAGCGAAAGCGAGGATTATCATTATCATCGCAGGAGTCACA
AACTGATTTGTATTACATCTTGGCGATAGGACTACTTCCAACATGATCACTGAGGTACCC
ACAAGCTCATTTCCCGAAACGGAAGGTAGCAGCACAAGAAAGCCTCGAGCAGCGCAACAA
CAACAACACAGTATTAATATCACATCGATAGGACACCTTAACAGCTATCGTTCACCGTCA
CCTTTCGGGAGTGCGTTAAAGACACCTTTGAAAAGTGGAAGTATAATGAGGACGAGAAGT
CTTGATGATCATCACCAGGAGCAGCAAAAGCAGATAAATTGCAATCCCCCCTTATCGCAA
GACATTGAAATGACCGGAAATAGCTTTTGCGAGGGAGGATCCGCATCTTCGTGTTGCAAC
GACGACAAGGACGAAGGCACAACTGTAGCAACCGCGTTGCATTCTTCCTTTGACATTTCT
TTGCTGAGGGGGGGTGGAGCAAGTTGCAGCAGCGGAGATCTTACTCTTTTTGAAAACGGA
CTCGATGAGGAGGAAGGAGACAGCCAAGTTATAATACATACGAGGAACTCGTGTTTTGAT

CCAGGAGATCTTACACCGTCGGCCAAAAGCGATAGCATTCATCACCATTACCAGAAACTG
CGGCAACCGAAGCTATCGTCCGCAAAGGAAGTGAGCTACATCGATCTGAAACCACCAGCT
ACCGTTCGAAAGCCCAAGATCTCGGCAAAAAGTCGGAAGGACAGACGAAGGAGCAGCGAC
ACCGCAACAACCGTCGGAACGCATCTATCGACAGGATCGTTTGCCTTTCCCATGTCGAAG
ACTCCCGCTCGCACCCCCGGGAGACCGCTGAGGCACGGAAAGAACACCAATGCCAGAACT
CCAGGAAAACCAGCGGGTTTGCACCGAAGTCCGGCTCCCTCTTCTACGGCGAAACGGCGC
AGAGATCCACTCGCCCATCCCGCACGGCTCTCGCCCATGAGGCGGTATGGAACATTATCT
CCCTCTTCGTGTGCCGCTACTCCTGCTGCGAACACTCCCACGAAATCTCAACCTCCCCCG
CCCTTCAGTCTATCACACCCGGCTGCCCTGGCCGGGAAGGGTGGAGCCGACAAGATTCGT
TCGCTATCTACACAAACACCGATACACCAAAACCTATCGAGCGATCCTCATGGGGCATTT
TTGAAGAAGCTTCCTTACCACACGGGAACGGCAGCTGGAGCAACACCCCTCGAAACAACG
TTTGAAGTAGATGGAGACAGTGTAGATACGAATTCTCCGGCTCGATTCCGTTTTACTTCC
TTTCCAGCCTCTCTGCCTCGAGTGCATCACGCGAACCATTATCCTAGGTCAACGCCACCG
GTTTGCCCGGGATCGGTTCGAAAACGAATGTCATTTGATACCGAAGAAGAGAATGCGAAT
ACGGAAGATGCCAAAAATACAGCGACAGAACATATCGACGATGAGATCGAGAACGATCCA
GTTGGACTTAATTACTCCATTCACGATGCTGTCAATCAATCGAAAGATGATGACGGGACG
CACAACACCAGCCTCAGTAGTTTGTCGGTGGACGGTGGTCACCACCAGCACTTGCCTCAC
CTTCCGCATCAACACCCACCTCATTTTGCATCGGTTCCAATGAAAAATTTATTGCTCTTC
CCCGGAGAGCAAGATGACGAGAATCGTCAAAACGAGCTTGGAACCAATATGTTTGGATAT
AGTGACGACGAGGAAGACGATGTATCCTCTAAGTTCGTTGGTGAAGATATTCGAACAAGT
GCTTCCCCCGACCAGGAGGGTACTGTGAGAAGGACTCGATTGAATTTTAATGTGTTTTTG
AGTCCGTCGAGCGACAAGAAAAACCTTGAAAACTCACAAGAAAATGGATTCGGTGGCGAC
AGGCAAATCCAACTGATGTCAAATGCTGATCTCTCTCGACCCTACGATCCTCAGAACCCA
ATCCACAGCTTTCATTCGTCGCTTTCTTCGCATACGCAAATCAACGCCTCGGCGCACACG
AACGAAGAGAACCTCATGGTCGGAACGTCATCCTCTTCCATGAGCCCACCGAGCATCGGT
TGTGAAGAATGTCAACAGCAATGCCAATAGCCTTCCAAGCACACCAAGGGAGGTTCACTT
GCACTTTCCCCCCGAATCGGAATGTTCACCAATTCTTGGTGTTCCGAAGCATGATTTTGT
TGAGCCTCCATCGTGTTCCAGAGCACTGATGCTGAACCGCCAGCAGCTCGAGACAATATC
TTCCGTTCGTAAGAATGGCACTTCCTCTCCCATTCATTCTGCGTCAAAGCATCTTTCACA
CCAAAAAGAAGATAGCACTTCTTCCATCTCCTCGTCCTCCGAGTCAACCACAACAACACA
AAAGAGGCGTCTCAGACCGATGCCCGACATGTCCGCGTTCGAGAATGTAAAAGCGTCCAG
CACACGGCCAGCCGATCGGTCGATTGATGACAACAATACCGCGACCGATAGTCGTGGGGC
CGGTTCGCTAACTCGAAGAGAACGGGCGTGCCCGCCAACTCCACAGCGCACGCCCGCATG
GGCCAACGATGGAGGAGGCCGCCCTATCTTCCCTACACGCAAACAAAACTCGCTCATCAT
GACAAAGGTTCTTTTGTCGTGCCCCTCGCAAGTTTTGGAAGGAAGATCTTCTCTGGAAAC
ATCTGTTTTAGACGAGGACAGCAAGCATAGTGGCAGCAGAAATTCCTTTTCGAACAGTTG
CGATTCTATAAACTATCCGAATGGAGAAGATAACCAAAATCTTCCTGAGAAGTCAACAAA
CGAAGATGGAACCACGAATATGGACGTCGAGAAATGTGAACAGCCACTAGGATTATCTAC
CTTATCGACAGGCAATGCCCACGAAGAACGCAAAGTAACGCACGATCAGATCGCTGCGCC
GCCCAAGCTCATGCGTCGACTTCCGTCTAATCCAGATATCAATGCTGTGATTTCCTTTTC
AGACGATTTTGAAATTCTTGGTAGTCTCGGAAGTGGTACATTTGCCGACGTGTACAAGGT
CCGCCAGAAAAGTGACGACCATCTCTATGCTGTCAAGCGCAATCGAAGACAATTTCGAGG
GAAACGCGACCGGGATGTGGCCTTGGCGGAAGTCCAATCCATGCAACGTCTCCAAAGCGT
GTTTGCAACAAGCAATGCTTCTGGAGAAAGAAACCACTCGGAAAATTCTAACAACTCCTC
GTCCGATAGCAAGAGAAACTGCTACAGTTTATATCTTCTTTTTTTCTATCGAGCTTGGCA
AGAGGATGGATACTTTTACTCGCAGACTGAATTGTGTTGCCGAGACACATGTCGTGAATT
GTTGGATTCACTCCGCTTTCTCTGGAATTCTGCTAAGAAGAAATACCCAAGACTTGTGAA
AACACTCCCTCCACCTTCAGGTGTCGAAGCCGGCAGCAGTATGGATGCCTTTGGCCGTCT
AGTACCAAACTTGACGGTATGGAAGATATGCCATGACATTTCGGCTGGATTATCACACAT
TCATTCTCACGGAATCGTACACCAAGACATCAAACCCTCCAATATATTCTTTGTGACAAA
CGCTCGTTTTGGAGCGATGTGCAAAATCGGAGACTTTGGCCTGGCGGGAAGCATTGGAAG
CTCTGGTGAGGGCCAAGAGGGTGACACGAGGTACATGCCTCCGGAGTTGTTGGCTTCTGC
AACCAGGCACAGCAGTTCGGATATTTTCAGCCTTGGGCTCACTCTCTACGAAATTGCAAT
GGACGATCATGTCGAGATGCCATCGGAAGGGCCGCGATGGCACCAGCTGCGATCCTCTGA
TGAACCAAAGCTACCCGCTTGTCGCGGTGACAACCTCCAGCGTTTGTTGCAATGCATGAC
GAATCCCGACGAACGAAAGCGTCCAACCGCCGATACTATCCTAGAGAATACAAACGTCAA
AATTGCTGGGCATGAGGTGGACACGTTTTTGCAAAACTACATTAGAGACGTCGAATCGTT
CGATAGGCAAGAAGAAGAGCGTCTTGCCATGGAGAGGTCAGACGATCAGACCCCACGAAA
TGACCACCGATCCGCTGCCGTGAGAAGCCCGAGTTTGTCCATGCTTCTCCCCGTAGCACC
ATGCATGTTTTCACCACCGTCCTCGAAAGTCATTCATTCATAAAGCCAAAAATACATAAT

TCATAATGATGCATGCAGCAGGAGCTCGCGTGCAGAACCAGAACTGAAAAGATTCCACGT
AGAGAACCTGTCTGCATTTGTGACAGAGATACAATTTTGTCCTTCTTGTTTCACAACTCA
ATACACTTACACCTTTAGTTAGTAGAAGTAACTAATAGAAACGATTACAGCAC
>comp2164_c0_seq1
TGTCGCAAAGAGGAAAGATACCGATCAGAGATCAGAAACATGGAACGAAGTGGATTGAAA
TACACCAAACCCAGCAAATTATCTGGTTTAGCATTAGCGGAAAGCAATCATGTCAGCTCC
CAACAAAGATGACATGGAAACTACTGTTAGCAGGAGCATCGATGGTTACAGCGATGAAAA
CGATGGCAATAGTAGCAGTACGAACGGCATCAATAGGGATCGATATTACGATGGTGTATA
TTATGGTGATAGCGACGATGATCCTGCCGCTTTGCTTTTGGCACTAGACGAAGCCGAACG
GAATGAATCTTCATTTGATATTCTTGCCGACCACGGCGAATTTTCGTATAGCGAGCACGA
TCAGCGCATGTACACGCCTGCACTGGACATAGATGCTCTTTTACCACCGGATAACAATAT
GCTGCCTATTTCGGAGGAGGAAGGAGAAGTAGGAGGTGGCACAGCATCTTCTTCCCTGTA
CGAAATTCCCAGCGGATACCGCAACAATCGCAGTCTCGCCGAAACGCAAGGAGAAGACGA
ACTGGATGACGTCGTTGACGACGAGGATATGTCTTCTCAAACCTCGTCTCTTTCCGGTTC
TGAGCCATCGAGATCCATTCAGGAGAAAGTTGTCGAAATTAACACTTTCTTGGACGATGC
CATGCAACAACAACCAAGCAACAAAGCCAACAGAGGTTTTGCCAAAACCGGATCCGGTGC
CAACCTTTGGAATCGCTTTAGCGCCAAAGGAAGTAGCTTCGATGAGAATGCTAGTGCCGT
TAGCGATGCTAGCTTTTTGAAAAAAGGCACCGTGTCGCTGGTGCAGGAGCTCGTAGCTGC
CGATGCATCATATTTGAACAGTAAGAGAAAAAGTGGCGGTAGCCCTACGGAGGATCCCGA
AGCCCTGGCCACTGCCCTCCTGCCGTCGGCTCCAGTTGGGGAAGAAGTTGACGCAGGCGC
AGATACAATACAGCAAAAGCGGTATTTCGTCCGTAGGGCTATCTGGTGTGCTATCCTTAT
TATTGTCTTGATTTCTGCGGCTGGGACTACTGCGGGTTTATTGATAGCTAAGAACCGCAG
AAACAACAATGAGACGACAACAGAAGTAGTTGTTTCCCCCGGGGAGACCACAGAAAAGAA
AAATGAAACATCTTTTGATTCGACAAATAATAGTTCTGCACTTGATGAAGTGAATAACAG
CAGTATTGGCTTTAGCAACAGCAGCTCCAACAGCAGTGATGCTAACGATCAGAACGGTCC
TTCTTCATCGTCCTTGTTGAATCCTTCCATGCGACCTTCGACTGCTCCATGGATAACAAG
CACAAATTTCCCCTCCACAATGCCTACTAATACAATCTCGTTATCTCCGGTTCAAACCGC
CTCCGTAGAGCCATCAGTTCGCGACGACAATGACACCAACGAACCAAGCAAAGAAGACAA
TGCAGAAATCACAGGAGATGATGTTATCGACAAGAGCGATCGACCATGGGACGACGACGA
TTTTCAGCCGATCGAGATTCAGGGCGACGACGATTTTTACAAAAATGGAGAAGAAGAAGA
CAACGATGGAATCAAAGGAGGGGGATTCGAGGATGACAAACCTGTAGATCAACCCTCGGG
AGACGACGACTTTCAACCAATCGAAATGTTGGGCGAGGATGATTTTTACACCGGTATTGA
ACCTCAAGACCCGTTGCATGACAACACGATGGGTGTACCCAGTGCTGTTGTTCACGACAA
GAAACAGAAGCAAACGAAAGAATCAAAGATTAATAGCACCAAAAAGAAAAAGTCAAAGAG
CACTAAAACCAGAACGAAAAAGCCTAAGAGCACTAGAACCAAGGAGCAAAAGAACGAGAT
TCCACACAGCGAGGATTCAGTGGATGGATCTCTCCTTTACGGATCAGCCGAAGCGGTAAA
TATCAAGCAGCGGTCCTTTTTCTCGAAGGGTAGAAACATCGATCCCCAACCGTCGCGTTA
CAGAACTTACGATCGTCGCTATCCCCAGCCGCAACTACATAATCAGTATTATGGTAAGCG
TCCTCAAAAATTCTGGTACCATAAATACGACAGCAACCCGTATTTGTCGGTGAAGACGTT
TCGAAAACAACCACATAAGTGGCGACAGCAACAGAAGCAGTGGCGAAGAATTCCACGGGG
GTATTAGAATAGAGGGATGAAAAACATGATGCGTTTGCATGTTCTTCAAACGGTTACTCG
TTGGGATGCTGATTGCACAATGTTAATGTTTTAACAGATGGAGAGCGATTTCAGGTTTGA
ATGGGGACAAAAATTGCCGATGCTGATTTCGCAATCGATGATGGAAACATTCGCACAAAG
GTGGAATTTTATGCAAACAGCGCATATGGCGCTTAGTTACGAGACAAAGAAGCGATTATA
TTCACCGCCATAATATCGTGAAATCTTCAAATCATTTCAACCATTTCATCTTCCATCATC
AGCCTGAAAAAAAAACTCTGAGCAAGCCTCTTCCTACAGCTATGAGCAAAATGATAGTAG
TATTCCTTTGTCAGAACATAAGCCACACTCGGTATTACTCGTCAACAAGCAGAATGTTAT
TCCAAGATATTCCGACGGCAACGCTTGGAACTGATTTGAAAATGACATTGTGTATTCCAT
GGCAATGGACAAGAATCTTTTGGCATCAACCAACCTACGTATCGTTTAATTTAGAATTTA
GAATCATACACGGTG
>comp2165_c0_seq1
GCATTGAGGTAGTACTTCTAGTAATTCCGAGCTCTTTTGTAAGTTCCGTTTTGGCTTCCA
AAGGCTACTTACGTACCGTACTACCGTACGGTACTGCAAATAGTGAAACCTCGGACATCG
GAGAAAAGAATCTTCTAGCGAAACAAAACCCATCGTACGGTACTTGGTATGCTGTTCACA
AGAAGAACATCCAAAAATCCACATCATCCATCATGATGGAGAACCTCGTCCCCTTTGTCG
CCGGAGCTTCCTACGGGTTGACCACGGTCGTGGTTGGCCAGCCACTCGACACCGTCAAGG
TCCGGATGCAGGGCCTGCCGAGCGCGGCGAATTCCAGCGGCGTTGCGGTCGCGGGCGACC
TGTTTGCAAAGGAGGGCGTCCGGGGGCTCTACCGGGGGGGTCTTCCCCTGTTCGTCGGCG
GTGCCTTTATGCGATCGGCCCAGTTCGGCGTTTCCGGTGCGGCCAAGGTCTGGCTCGAGG
AGAACACCAACGGGGAAAGCCGGCACCGCGCTTGTTTGGCATTCTGGACTGGCAGGTCA

TGATTGCCGGGGCGGCGGGCGGTGTGTCGAGGGGCCTGGTAGAAATTCCCACCGACTTTT
TCAAAACGAGGCGGCAGGTGGAGCACGGCTGGAGCATCCGGGAGGTCATGGACGGGACCG
GCGTGACGCTCGCGCGGAACACGGTGCTCTACACCGCGTTCATGTTCTACCTCGACATGG
CGAAGCTGCTGTGCCGCCAGGGGTACGTTCCGTCTCTCCTCATGGCGGACGACCGGGAAA
ACCTGTCCCCGTTCGTCAAGGGTGCGGTCTGCGCGAACCTCGCGTGGCTCACCTGCTGGC
CCGCCGATGTCGTCAAGACCCAGAGGCAGTCTGGGAACTACGCCTCCCGGAGCAACACCG
AGCTCCTGATGGAAAACCTCCGAAACGGCCGGCTGTTCCGCGGCATCGTACCGGGTCTGG
TGAGATCGACCATTGCCAACGGCAGCAGCATGGTCGTCTACGAATACGTTCTCACTACCC
TCACCAAGAAGTTCGATCTCGAACGGAGAGATCTGGCATAGGCGCGAGCACTGTGGTCTT
TTTTGTTCTTGTAACAAGAAAGGGAACGAGAAGAAAAACCAGGTGACCACCGCAAACTCA
TGGACACGCGAAAACAATCCAAATACGCCTCCCGAGCACGATCCCGTGGGTGGGTTATTT
TGTTGGTCCAAAACGAGAGCTTTCGTTCCAAAAATCAGCATTCCACAAGAGCCGATGGGA
AAAAGAAGTCTAGAACAGTTCCACGATTGTCGGTTGGAGAAGCGCAGGCGATCACAAAGT
AGGAACCACGCATCCTTTTCGTTCTGGGCATCCGTACAAGTAGAACAGAATCGCGAAAAG
GCACAACGACGCTGCGCTGGTGAGATTTGTTCGGGGAGCAAGAAAAAGAAGAACCTGGGA
AGATAGAGGTCGTCGCGCCAACGGAACGCGTCGGACGGGATGTAACTGGGTTTGTAGTGC
GGCACAGCAGAAACATTTTGCGACGGTGCCATCTACAAAAAGTAGACGAACAGTAGCTGT
GTGA
>comp2166_c0_seq1
CAGTTCTTATTCTCCTCGAACATGTGTTTCTTTCACGGTCCCTTAGTTACAAGTCAACAC
TGCATAGAGAATTTTATCAAGAGCGATCGCACAATATATCAAACAAGATGGGTCACATTT
TTCTTTGTTCTTCGAGGAATATTCCCCGCGACGATTTTCGCTGTCCTTTTTTGAGACAGA
TGACGAAGTTTTCGTTCGGTGTAATTTTGGCTTGACCGAAGACATTGTGGTCGTGAATGT
TATTGCATCGTCATAGCGCAGCACACACATTATGAATCGTGCGTCGAGTTCCCTTCTCCT
GATGCATGATGGATGCCGAAGCCTATTCTACCGGTCGTTCTCTGCCCATTTATCCGAAGA
TCTCATAAATTGATGCACAAAGTTTTTGTCAAGTTGGAGCATCAGCAGGAAGAACAAGCC
AAAAACAAGGAAGGATTGATCAAGATGATCAGAATGAATTCGATTCTATTTGTGCTCGCC
TCTACTATATTGGTCTTCGGATGTGAAAGTCATGCATTCATCCCTCTCTTGCGCAGTGCT
TCTACCCTACGACCAGCACCCACATCGGAAATCAGCTATGGTAGAAAAACTGTGGTAGTA
TCTTATTCGTTTTATCCGGTTGTGGAAGCTGTGGAGCACATCGGTTATGCGGCGAAGGTA
CTCATTGAGCATGATGTGGGCAAACCACTGGGTTCAATACAAAGAATTATATGCGTCGTC
GACCCAGACGTCGAGGCACGATTTCTAGTGGATATCACCCATCTGTTGCTCGACTTTACG
GTCATTTTCAATTTTGATGGGGCGCTCTTGAACTTTGCGCAAGTAGTTGGTAGAATTGCT
TTCATCGCGATCGACTTCCTCCCTGGGCACGAGTTTCACTATGAAGAAATGGCAGTGCAG
CTCGTGCTGCTGGGAGTAAGTATAAAGAGAATGGCATTCGCTGAGACAAAAGATTCAACT
CCAAGTGCCTTGGAGAAATTCGACCAGTTGGATCACGTCGGACAAGAAACACAATGCGCT
ACCGATACTGATTTCAGCACTGTCGATGAAAGCATGTTGGTTGAAGCTTGCGAAGAGTGA
ACCAAAGTACGAAGACGATTGTCTTCTCGAGGGTTGTCTCCTCCGCGAAGTCTTTCTACG
TTCACCCAAACCCAGTCCACCGTGCCGATCTTTCACGTCTGGAACCACAAAGGTAATCGC
CAAGCCAAATAATAATCAAGATAGAAGAAGGAAAGATATAGCTTTCATACCAGTCTCCTC
GTGATTTATTGGACCCAAGAAGCTTAGATTCTCAATCGTGGAACCTGCCGACACTTACTG
TATTGGAGAATCTGCAGGCGTTTGTTGCTACATGATAGTGTCCGACAGATGAATAGATTG
GGGAATAAAGGCATTTTTTTCTGATCCGCAAACAATTGCAACGCATTTGTAGTTGCTTCAA
ACTACTGCCCAAAATAGATAATCGATAGTAATACAGCATGAAACACC
>comp2167_c0_seq1
CCTCCGACAAACCCTCCGACAAATCCTACGGCAGCACCTATACCAGGAGATTCGCCAGAT
GACGATTCGTCGGCTCGGCCTACCTGCGAGCACGAGGAACCAACGGGGGCTCCAACCACC
GCCCCAGTCCAATTCGTACCGGTGCCAATCGATCCGCAACATACCATTACTCAGCTCCCC
ACGTGTGAAAAAGATGGTCTTCCCGGTGTTCCGGAACCCGCGGTCACATCAGAGGAGAAT
GTCTGCAGCGAGTACGGCTCGAGATCAATTAACGATTACATCTGCTCAATGAACAACTTC
AGCAGATTCTGCGAGCTCGTCAGCGACGTCGATCTGTCGAACTTCTTATCCGGAAACGAG
GGCGGTAGGGTCACACTCTTTGCCCCCGTGAACTCGGCCTTTAACAGCGTGGACCTGGAG
GGACTCGACGTTGACGTCCTGCGCAACATCGTGTACACACACCTCCTCGCCGGAGAAACC
ACCGCCGAGTCACTCCAATGTGGAGAAGACTCCGAGATGCTCCTGGGCATGTCGGTGAGC
ACAACGCAGTGCTACGAATCGACCCTCGGGAAGGCCCAGGTCGGATTCTTCAACGACGAG
ACCTGCGTTCCTATGATCCTCTCGCCGAACGACATCACCCTCTGTAATGGGCTTGTCCAG
CCCATAAACCACCTGATCCGGGTGATCAACTTCTAGCAAATAGACTAGACGAAAAAATAG
AAGAAAAACAAAACAAATCAAAAGCAAAACCAACAAGATGGGAAACAAAGGAAAGATGGA
AGCGTTTTGTCTTTCATAGGAATGAATTTCCAGGCTAACGAATGCAGCGGCGAACGATGA
CATGACCTTACACATTAGAGAGCGACAAAATTGAATGCGAGTCAATGCAAGACAACGCGA

GGCATTGTGCTGCGTTGTGCTGCGTTGT
>comp2168_c0_seq1
GCCATCCGCGATGCCCTCTGCCTCTCCCTCTGCGGCTCCATCCGCGGCACCCTCCGCGTC
GCCAACTGCAACACCCTCCGCGTCGCCCTCCGCAGCACCCTCTGCGTCGCCCTCTGAGTC
ACCATCCGGTGCACCAACCGTGTCCTTGGCACCCTCCTCGGCGCCATCCGCGGCGCCATC
CGCTGCGCCATCCGCGTCGCCATCCGCTGCGCCATCCTCGGAACCATCCGCGGCGCCATC
CGATGCGCCATCCGCGTCGCCATCCGTGGCACCATCCTCGGAACCATCCGCGGCACCGTC
CGACGCACCATCCGACGCACCATCCAGTAGCCCCTCTTCACCCCCAAGCTTGAGCCCTAC
CATCGACCTGACCAGGTTTTGCACCAGAGACAAGGTGAACTACTTGTTCGAAGGGGAGGA
AGGAAAGGACTGTCAATGGGCCGGGAACAACCCAGAAATTCGTTGCAACCGCAAGGACAG
CACACGTAGGAACAGAAAGGTGAAAAAATTCTGCAAATCATCCTGTGCGCTCAAGTGCGT
TTGCAAGAACTCGAGACGAGTGTTTAGACTTGATGGATTCCTAACCAAGTGCAGGAGAGT
CAACGCAAGCAGATGCAACGATCCCGCAAGACGACAAGGCAGAATGAAAACTGTCTCTCA
CTTTTGTCCTAAGAAGTGCGGTACGTGCTGGCCTGGTGCACTCTAACTGTAGAATCGAAA
GGAGGGTCTTCTTGCCGATTGTCCAAGACACGATAAGCCAATTCCATCGACAACTGCAAG
ACGCTTCTCGACGTGATCCAAAACACGGCGTGTCTCTCCCTTTGTTGCATGTTCCTCGCC
GGTTTTCTTCTGGATGAAGTATCTAGTGAAATAATTTAGATGAACAACTAAAAAATAAGT
AAATTTTTATCCCGTAAA
>comp2169_c0_seq1
CAAGCTGTCGGCGGATCGACTGGACGATTTCCTTCCAACGGTGGTCTTGTCCAACCTGCG
GGAGCTTACCCTGACCAACTTTGAGGGTCTGACCGACACCCACGTCCACGTGCTTCTGCT
TAGCACGACAACAATGCTCCTGGGTGCCGGTGCCGCCAACTGCAAACCCGGGAGGAGGAA
TCTTCCCCTGGGTTCTCCGAACCAAAACCAGAGACAACCGCATTCCACCGGGAGGCAAGG
GAACCAGTGGACGAGGCTGGTTCTCGATGGCTGCCACGGGCTAACGAGCCACGCGCTGAC
CTCGATTGCCAAATTGTGCAGCCAGGACCACCTGAGGGAGCTGTCGCTGCGGGATTGCCC
GGGAATCGATACGATCGCCCCGCTCTCGGAGCTGCTGGTGACCAAGCTGGGGGCGAAGAA
GGCAACCACGGCGACAACTATGGCGACGACAGCAGCGACAACAGCTACAAACACAAACAC
AAACACGCATTCCCACCGAGACGAATTACTGCGTCCATCGCCGGTACCACTCTCTGCGCT
TGTCCCTGCAGAAGCCCTCGGTCGCAAACTCCGGGACTTCAGCGATGGCGAATGTTCCAC
GCGTGCTGCTAGAGTGCCGGCCCCGATGGCCCGGCTGTTCGAAACGCCCCCCGCGGCTTC
CGTGGAGACCACCCATCGACGGGGGAGGGGGTCACCGCCTTTGTCGTCCTCCACACTGGC
ATCGCTGTTTGATCTCCCCGGGACGTCCCCCACCCGACGGAAGACCGCCGAGTCTCTGGT
CCTTCTCCCGCCCCCGCCACCGGTTCTGGCCCTCGGAGAGAGTGGTGCCATCACTTCGGC
CGGTTCCTCGCCGCTGCGGTTGCGGCCACCGCCGCGCTTGCCGTCGGACCCGCTGTCGCA
CCGACGTTCCTCCCCGGGCTTGGAAACGCAACCCCTTTTGCTGTCCTGTGCCGACGGCGT
GACACCAAAACCCTCTCCCCCACGAAAGCACCAGCTTGTCCCAGAAAAAGAAACTGCCGC
CGCTGCTGCTCCTTCCATGGCACATGCATCGCGGAGATACCACCGACACAAGCAGTCCTT
GTCCAAGGAAGCCGCTTTGCCGTTTCCACCCGGTTTGCTCCCAAGCAAAAACAGAGACGA
ATGGGAAAGGGTGTGCCGGCTGCATTCTTTCGCCCCGACGTTCGAATGCCGCGACCATCG
CTCCGATAACCGCGCAAACGGCAATCCCGGCCCAAGCACGGCGTTGAATGACGTCCGACC
GCCGTCGTTCTTCGGGGAGGGCACGACGACGGTGGGAAGCCTGGAAGCCCTGGATCTACG
CGGAACCGCCGTCCGACCCAGGGAAATAGTCGGTGTTCTCTGGTCTCTGACCCACCATCG
ACCGAGGAAGAAACCTTTGATGAGCGTCGGGGCGAGAACCTGTTTTCCAAACCAGAGCGA
CAGCAACACTGCTTCTGCCTGGTGCCGTGTTTGGTTCGAGGAATTGCACCTGAACGGTGG
CCTTGACTACTACTCCTGCCGCCGCCGGAACGAAATCCGGGTCAATCTCCGCACAGGCGC
AGGGGAAGACGCAACACACGCAACGATCGACGCAGCAGGTGCATGTTTTCTCCCGGCAAC
GCGCTGGTGGGACGGTGCTATGGCCGAGTCCGATAGCTGCCCCGGTGGCACGGGCTGGAC
CGGGGAGGACCGGAGAGATCTCGAGGCTTCGCTGGCCATGGACGAAATGGAGCTGAGGAT
TGGTGGCGAAAAAGCAAAGAAAAAGAAAATAACACGCAATTGCTACCAATTACGAATGCA
ACGCAACGCAATACAATAACGCAAATCGAGCGAAACGCAAGGAGCCTAGCACTTGTGGAA
AGTCAATCGTGCTCCGTTTTGTGCACTACACTTAAATTTAATTTACGACACACTTCAGAA
CTTCGTACGGCACTAAAACAATCATAAAGACTGCTATGAACAAATACATAAA
>comp2170_c0_seq1
AAAAGTAGTTAGCGTAGGTAAGTGAGATGAGGTCGATGTGAAGATGATGTGTCTAACCAT
GATGGAACAAGAGAGTTGCACGTCCCGCTTTTTAGCGGATGCGTCGACAGGGGTCATCTG
TACTTTTCAATTGGATTCTCCTTTACTTTGATCGAAAACACCGAACTTTTGCCCTGAGAC
AAGTCTTGACCCCGAACGAAATCCTTCTGATTCATCGCAACCACTTTTCTTTCCCGATTA
TTTGATAGATTTAACTTAGTACAAGTCGACAATATGCAAAACGAAGCAGGAGAAAACGTT
GACCTCTACGTCCCAAGAAAGTGTAACTGGACAAACAGATTGGTCGAAGCAAAGGATCAC
TCTAGCGTGCAGGTAAATGTTGCCAATGTTGATCCCGTGACAGGATTGGCTACCGGAGAT

GCTACACCATACTGTTTGGTTGGTTACATTCGAAAGATTTCTGAGGGAGATATGGCCTTG
ACGGCGTTGGTTGAGAAGAATGATGCCGCCATGTTTGCATCAGAGGAGTAAATGAAACAC
GATCACATCAGCCGGATGAAAGCGGTGGAAGCAATATTAGTGTAACGCTCTAGAGATCCA
TTATTTCGAC
>comp2170_c0_seq2
AAAGTAGTTAGCGTAGATTTAACTTAGTACAAGTCGACAATATGCAAAACGAAGCAGGAG
AAAACGTTGACCTCTACGTCCCAAGAAAGTGTAACTGGACAAACAGATTGGTCGAAGCAA
AGGATCACTCTAGCGTGCAGGTAAATGTTGCCAATGTTGATCCCGTGACAGGATTGGCTA
CCGGAGATGCTACACCATACTGTTTGGTTGGTTACATTCGAAAGATTTCTGAGGGAGATA
TGGCCTTGACGGCGTTGGTTGAGAAGAATGATGCCGCCATGTTTGCATCAGAGGAGTAAA
TGAAACACGATCACATCAGCCGGATGAAAGCGGTGGAAGCAATATTAGTGTAACGCTCTA
GAGATCCATTATTTCGAC
>comp2171_c0_seq1
CTTCCCGATAGGTGTTGTTGGTTGTACAGGTGTCTCTCACAAGGCAAGGTCTCTTAGATT
CGTTGCTTGTCCACCCAATAAGATCAAATTGGCTACTATCGCTGTTTCGTTACGGTAGAG
ACCAGCAGTTGACAAAACGAAGGATACTCGAATACAGCAGTCTTCTCCAGAAACAACAAA
ATGAGTGACGCTCTGCCTCCGTGCCGTCTTTTGTTCCGCTCGAAGGAGGGGATCGAAATC
TTCTCCGTGCGATCAGAACGCGACGCAGAACCTTTGCCACCAGCGACCGATCGGAAATTT
CTTTTGAAGGGAGCTTCCTCCTTTCAAACTATGCTGCCCGATGGATCGGCTGTCTTGGTC
CATCGTCCCGACAAGGGGATTGTGAAAATCGACTTGGACGGAAACGACACCCAAAACTCC
GATCAGCCGTTTTTTCCCAACACTTCGAAGGTTCAAATAATGGACGTGTCCCCAAAAGGA
AGCTATCTGTTGACATGGGAACGCGGCAGCGCCAACGAGAGTGAGAACAACCTCAAAGTT
TGGAATACATCCACCGGGGAATGTGTAACGGGCTTTCGACGGAAAATGATCAACCGAAAT
TGCTGGCCGTACTTGCAATGGACGCACGACGAAAAATTCGCCTTTTTGTTGACCACCAAT
GAGGTTCGCATCTATCCGGGCACCTTTCCTCAAAAAGCAGAAACCAGATTTGTGGATAAA
ATTCGCATTCCACAGATCACTCAAATGAGTTTGCCGTCGTCTACTGCAACCGATAGTGTA
GGGAAAATCTTGTTTGCAGCCTTCATTGGGAAGGACAAGAACAAACCAGCGCGGGCCGCT
TTGTATGAATACCCTCCTGCTAATGAGTCTGCTTCACCGTATCCCGCGATTGCTAGCAAA
TCTCTTTTCAATGCGGAGGAGATGACAGTTCATTGGAGTCCCAGAGGAGACGTCGCTCTC
ATCGCCTTGCAGAGCTCTGTAGACAACACCGGAGAGTCCTATTACGGATCCTCATCCTTG
TTTTTGATGTCCCAACAGACCTCGAACGACGCCATTGCCGTGCCACTTCCTCAGGAAGGA
CCCGTGCTAGATGTCGGATGGATGCCCAATGCCGAAAAGCCACCTTGTTTTGCCGTAGCA
GCTGGACGGATGCCGTCGATGGCATCCTTGCACAACGGTCGGGACGGAAAAGCTACGTTC
TTGTTCGGAAACCGCCATCAAAACACGGTGTCGTGGGCGCCCCATGGACGGTTCGTGAAT
TTGGCTGGATTCGGAAACTTGGCTGGAGGCATGACATTTTGGGATAAGAACAAAGGAAAA
CAAATTCCTCCTGGAACGCCTGTCACTGCGAGTGAGACAACACAGTTTGGATGGTCACCG
GATTCACGACTGTTCGTCGTTGCAACCACGTCCCCGCGCATGAACGTGTCGAATGGCGTC
AATCTATACCGGTACAATGGAGAAAAGATCAACAACAATGTACTACCCTGGAACAATGAG
AATTATCTTCCAGATCGACTCTTGCAAGCAACCTTTGTGCCGGCTGCACCCGAAGTTGTA
TATCCAGATCGACCGCAATCTCCGTCTTTGAAAGACCTAGGGACCGGACCCGACGCCGCT
GCCGCGTCAGCGGCTGCCGCTCCCAAACCAGCTGGGAGATACATTCCACCGTCCCAACGA
AATCGTGCTGGACGAGGAGGGGGAAACAGCCTTGCGGATCGTATGCGAGCGGAAAAAGAA
GGAAAGATGCTTGGTGCACAGAAAGTCACTAAGACTACCCCTGGAATGAAACGCGGTGTT
GTTGGAGCAACTGGGAAGGTTGTGGTTGGTATGGCACCACCAGAGCAAAAGGAAGGAAAA
AGCAAGTCGGCGCTGCGAAGGGAAAAACAGCAACAGAAGAAGAAAGAAGAAGCCGCACGA
AAAGCCCTAGAAGAGAAGCTGGCAGCGGAACAAAAAGCACAGGAAGAGGCAAACAAACCG
GTGGACCCGGTCAAACGTGCCAAAAAGATCAACAAAGTTCTTCGACAAATCGATGAATTG
AAACAAAAAGACCCATCCGAATTGAACGAAGACCAAGCCAAAAAGGTTGCCTCCGAACCA
AGTCTTCGGGAAGAGCTTGCCAAATTGAGCGTGTAGGTACACAGATTTGTGGCTGTACTT
TGCTAACCGATAAAGTTCTATTCCTTTAGAAAAAAA
>comp2172_c0_seq1
AAAGTACCGGTAGTACACAAAGTTCGAAGTACTGTAATCCCTATTATCTATGGTTCGTAC
GTACATAGGTACTCGTAGTATCCGAAATCCGAACGGAAATAATACGATTACCGTACAGTA
CCGTACTACTACTACCAGCCGGTATGTCTGACAAATTCAAAAATTTTTACTCCAAAACTC
CAATAGAATATTTTACTTTCCCTTGCCCCCTTCAACAAACAACTCAAGGACGCGCAACCG
ATGTAAAGCCAAATCGTAATTATCGAAAGCTAGCAAGGGGCAATTGTATTAAGGATACAT
TAAGTTTGTAGAAAAACATGCTAGACAAAAATGCGACCATAAATCAGTGAAGCTCGGGCC
CTGGGAATCTAAATTCTGCAGCGAAATAATTGACCTCGAACCTCAGTAGCCTACGGTATG
AATAGCGCTAGCCGCGATTGAGACAACGGCACTACTCCTGATTGTACAACTACTTCTCAT
TTCAAAATACAACTCCGAGAAAGAAACGAAGAGCACTTGCACAGTACGACTACACAATAC

AATAAGAACCACGCTCGGAGGACAAAGAAAGGAAAAATTGGAGATACGACAATTAACGAT
GGGAGCTGTTCTTGCGAAACTTTTGCAGGTCTTCTGGACAAAGAAACTCGACATCGTCGT
CATTGGCCTTGAAAATAGCGGAAAGACTACACTCCTGTCGGTGCTAGCACATGGCGAACC
TGTAGAAACTGTGCCCACGATCGGACTCAATGTTAAAGTATTTAAAAAAGGACAAGTCAA
CATGAAGTGTTGGGACATTGGGGGCCAGGAGCAGTATAGAAGTGAGTGGTCCCGGTATAC
CAAGGGATGCGACGTTGTTCTTTATGTAGTGGATGCCGCCGCTCCGCAAAAGTTAGGGGC
CGCAAAGAAGGAGCTTCACAAATTATTGGATGACGGAAGTATTGGTAGCACGCCCATGCT
GGTCTGTGCCAACAAAATTGATTTGTCGCCTCACGTTGGAGAGGCGGAGCTCATTGACAA
GCTGCAGCTCAATTACGTCATGGAGACTCCCTGGATGGTACTGCCCATTAGTGCTCTAAA
ATGTACCAATATTGAACAAGTGGTGGAATGGTTGACCTCACAGGGAAAATGATCATAGTT
ACTGTTGATTTCTCTGCTAACTTGCGGTTTAGCACACATCACTTACCATAGAATGAACGC
ATGAAAAAAACGTTAAAAAATCCTGATGCAACGACGAACAAATCCCCTTCCCGAATTCAT
GTGATCATTGTTCGCAATCAATGACACATTATTAATTGTTATCTAAAAAGTATGAGTATT
CCCATCAAATATCTCATTTCTTCACTCTTCATAGTACACGATTGTTGGTTTGCAAGAAAA
CGGAAGAATATCAGAAGCGTCTGCCACATACGATAGCAACAGTGCCATTACTCATATCAT
TACTCACATGGAAACAACACTCCGCAGGTTTCGTGTAATATATCTAAAATACAACTCTAT
GTCTCGTACTTACGGATAAATTTCTCATTCCCTTGATATTGTTCATGACGAATGCTGTAA
ATTGCTATTGGGTTATCAAAGAAGGGGCGAGTCACTCATATCGCGCACAAAATGTAAGCA
AAGTATGACGAATGAAGTCACAAGGGCTTGTTGGAATTTAATTATCGGAAGTGTCAATCT
AATGTCCAGAATGAGTACTTACAGTACACGTACTTCCACTATACAAACAAACACCTGAGG
CATTGAATGAAAATTTGAGATGTTCAAGAAGGTGATTAATTCTTTGGGTTTGACATTAAT
TTTTTGAGTTCTTGTTCACGATGAAATGGGGGCGATCGTCTAGAGAAACCACGGAGCTTC
CTCTTCGATAACCTT
>comp2173_c0_seq1
ACACTAAAGAAAATCACCAGCTAGAACCAAGCCTTCAAAAGAAGAAAAACTATGGACATC
CTTCAAAGAGAAATGAAACGTAAGAAGGAGGCTCTCCTCAAAGCAAAGAAAGAGGCAGAT
GCTTCTTCTGAAACTTCCAGCGCCGGTGATGGGAGGAAGAAGCGCAAATACTTGAAAGTT
TCCGATCTTCGTAGGATCGAGGACGAAGAGCAAGAAGAGAAAGAGAAAATGCGCTGGGAC
CGGAACACCAATTCAAATAGGAAGGCTGTGTCGATATCATCGGCATCGAAGGAAACAGAC
GATGACGATGCAATCAGTCAAGCACGAATGAAATTTAAGAATGCAAAGAAAGGCAAGTCA
TCGACAACCTCTTCGCCATCTTCATCACCTGCGGCGACGGTCAGAGACAAGATCGATTCT
TCACCAGCTTCTATAACGCAGAAACTTCGCGAAATGGGTCTTATCGTACGATACTTTGGA
GAAGGAAACACGGCTCGTTTGGAACGACTGAAAAATGCCCTTGAGTATCAATCCAAAACT
TTAGAGGGTCTATCGGAACTGGAGGAATTTCGACTTGGAAAGGGTCATGGAATTCGTAAT
CCCTTCTTGGAAAATGAGAAGCAGTTTAAGAATAATCAGCATCAAATGCCGGGCATGGGG
ATCAATGACAAAGGATCACTCGAAAACTCGAGACTGGAAAGAAAAAAACAAGGCAAGGGG
AAACATGCGACAAAATCCGATGTAGATGGCGAGAACGAGGAAGATCAATCAGATCCTCCA
AAGTTCATATACAAGTACCTGAAAGGTTTATTGAAAGAATGGGAGGTCGAGTTGGCAGAA
AGAGCCGAGTCTGTAGCGAGATCTGTGGCGGGTAGGAACGAATCAAAGACTCTGAAGCAA
TGCAAGGACTATATCCGACCACTTTTCAAACTATTGAAGAGCAGAAAACTGGAAGAAGGA
CTGCAGTTACATTTGCTGAAAATTGTCAAGTTTGCCAAACAAGGGGAATTTGTCAAGGCG
CACGATTCCTATATTGATGTTGCGATTGGGAGAGCTGCCTGGCCTATCGGAGTTACCATG
GTCGGTATTCACGCTCGCAGTGGACGTGCTAAAATCGAGTCTGCGAATGTTGCACACGTC
ATGAACAGTGAGCTCCAACGCAAATATCTCACGTCTGTAAAGCGTCTTTTGACTTTTGAT
CAAAACAGACGAACGGACGTTGATCCCTCCAAAAAGGTGAGGTGATAAAAATCATCACCT
CCGGATTTACGAGGCAAAACATCTGGTCATATTACCGTCATATTACCCTTTCGTCTTCAT
CTCATCGCACGAGATAGGTAGTTTTGAGCTTACAATCCAAAAAAAGGCAGATTAATTGTT
CAAGCATTTGTTTTTGTCTTCTGAAATATTGTTGTGGAGAGAGAAAGTTTACTATGAATA
ATGCGAATCTGTTTTGCCATCTACGTTCTAAGCATGTCTTGTCAAGTATCAAATCATCGT
GAAGTAAAATTTCGTCATACCTGAAGTTCAATTGTACGAAGTTTGTTGAAGTTGAAACTG
CTACAGCCAGTACAAAAGCTTGACTTATGGTATCCGTACATAAATATCATGATTATACTA
CTGCTCAACGCCATTTACTAGCAAC
>comp2174_c0_seq1
GTACGATGAGTACTAGTACTCGAACCTCCTCGTAAATACAATCTATCACAACGGACGATG
ATGGACGGTCCGAATGAAAGCACACCGTTCCTTCCTTTCAGAGTTGTTCCACATCGTGTG
TGACCATGAGATTCCCCTCCCTGAAGCAGCAAGAAAGATTATCAATCTATATCTAGTGCA
CTCAATCGAAAAGAAGTGGAGCAACCTAGTCACTATTGTATCCAATAACGATAGCCCATT
GACGAGCAAACGCAACACAATACAACACTTAAAAGAAACATCCATCATGGCCAAAACAAA
AACAAAAGCCGCCACCAAGGGAGGCTCCAAGCGCGAAAAGCCCCCCGCTCTTGATCAGTA
CAAAATGCCCCTTATCGCCCTTTGTCTGAGCATGCTAGGATACCATATGTTCCGGGGCAT

GATGGCCAAGGAAATCAAGAGAGTCGACATGACCGCCACCGCCAACGAGCTCGAGCTGAG
AGAGGTCTTTTTCGGAGAGGACGTGAACACAGGATCCACGAACTACGCCGTCTTGTGCTA
CCCCGAGAACGCCGTCTTTCCCGTCTCTTCAGTCTTCCAGGATGCCGCCAATGACGGGAG
CGCACCCTGCCAGTTCCGTCTCATGGATTGCGACGCGGCCATGCCCTTTTCCGAGGAAGG
AAAGTCCGTCGTCAAGAGGTTCGGCCTCAACAAAAAGACGCGCCCGGTTGTCTTTGTCAG
CGGTGCCGTCGGCCAGCCCAAGCAGGTCCCCGCGAAGTACCTCAAAACCGGAGCCATGCT
TACTAAGGCCCTGAAAAACCTCCTCGAACCCAGGGCTGAAAAAATCGAGACGACGCAGGA
CCTCCGATCCAAGTGTCTGGACAAGGACATTTGTGGTTTGATGCTCAAGGGCGGCAAGGT
TTCGCCGACCTATGCGAAGGAAGCCATGCAAAAATTGGTGGTGGAATACCCCAAGGTCAC
CTTTGCGGCGGTAGATTCCAGTGTCTTGTACGTGAAGGGCATCGAGGCCGAATTTTTGCC
GGAACTCACACCGGGGCTTCCCCGCTTCATTGTCTTTCAAAAGACCAGCGGGGGTGTCAA
GTCGGACAGCTCTCGTCTCAAGTCGACGGCAATCCCCCTGGATGTGGATGTGGCATACGG
ACCCATGTCCAATCTCGTAGCTTCCGTGGTCAGCGGAAAGGCCGAGATGACCAAGATTCC
GGCATCGCCCACCATCAAGACGCGCACCAAAAAACTCGAAAAATCCGAAAACGAAAAACG
ACAACGTCGCAAGAACAAGACCAACGGTAGCGGCGACTCTAGTTCCGACAGTGGGACGCC
GCATTCGGGTGCAAACGATGGTTCGAGAGAGGGACGAAGAGCAGAACGAGAACGCCGCAG
GGCAGAGCACCGCAAGAACAATCCCAACTACAGGGAAAAGACGCCGGAAGAAATCAAGGA
AATGGAACGGAAGCGCCGCATCCGCATGGAGGAAGAGGCCAAGAAATGGAACATGGATGC
CGAGGATCCTTCAGAGGATAGTTCTTCGGGTGAATACATGGTGGAGGACGAGGGCTTTGA
CGAGGACGAAGAAAATCTTGACGAAGAGGACGAAGACGTCATGGACCTCGACTAAATGCG
AACGAACGAAAAAGTGTCATATGCGCATGCATCTCTCTGATCTTGACCACGGTAGATCAA
TAATATGCTAGAATGCAATCATTCAC
>comp2175_c0_seq1
ACGAAGTACTCCTTCACAACTAATCGATTCATTCATCGGAACGGTGAGCATGCACGGGTA
CTGCGTGTACTATCAAACCAGCAGCGTAGAAACCATCTACACCGAATGTGCAAACCAATC
GAATTCTGAATAGAGCAGGCACAGCATTTTCGGTTTTGTTTCGCGCAAAACCAAAGAAAC
CAAAGGCAACGAAACCAAGCCGACACTCGGCCCGATTGAGGATTGTCTCGACTCCGATCC
AAAACACCGCGGCTTGCGCATCGAATAGTGGGAACAAAACGAGGCGAGACAAAAAGAAAC
AGTCGTGAACCCAATCCGTACATTGCATGATATTTCGGTCGAATATATCCATCGTTTTAT
TATTCCACACGATCTATCCACCTACTGGTCAGCTGCATATAGTCCGATCGATAAATTCTT
TTGCTCCTGAGGCAGCGTTATTCGTTGATCGATTGATCCCAGGCCTTGGTTTCCGAAAAA
GTTTACCGAACAACGTTGCAGAACCTCACATCAAGATATCAAGCAAAGCGAAAGGAGCAA
AAATAGGAAACCAAGCCATACCAAGCTGCGAGAGCTATGGGCCTTTTTGGGAGAAAGAGC
AACATTGGCAGGGACGAGAAGCAACAACAGCTGAAACCGCCCTCGATGTACGAGCAGGGA
GGTCCCAATGACAATGCCGACGATGTGGACTTGTCCGAGAGCATGAACGACGATTACGAC
GACTTTCACGAATACAACCACGACGACGACGACGCATCGGTTGCGTCCCACATGGAGGAA
ATGACGACCGATCCCCACGACGATTACTTTGATTTCATGGATCCGGAAGCAATGGCAAAT
GCGGCATCGGCATCGGCATCGGGTCCGCAACAGCAGCTACAAGAAGTTTCCAGGTCGTCG
TTTCCGCAGCGCCACACGAACGGCGACAAGTATTACAAAAACGCGTCCCCCTCGTTCCAC
AGCAGCCACGATGCCCTCAAGAGAAGCAACAGCAGCAGCAGCAAAAACTGCGCTGCGGAC
CACGAGAATGACACCAACGCCATCCCGGAAAGGACCAACTCTGCGACGGCGCTAGTTCAC
AACAGCAACGAGAGAAGCAACGGCCACAGCCGCAAGAGGGAAGACCCGCTCGTGCCGGTC
ACTTCGTCCCTCTCCCCGGCGGCCACGGCCCGCTCCGTGTCCTCCAGGAGCACGGCCACC
GGCAGAGTCTCCCGGTCCCACAACTCGCGTTCGACGTCCAAGACGCACTCGAGGTCCCGG
TCGTCCCGGACCCAACTCTCGCACTCGCACTCGCACTCGAGGAAATCCTCCCGGTCCGCC
GGCGGTGGGTCGAGGAGCAAGATGAAACTCGGCGAGAAGCTGAGAAATGGCGGGGACCGG
AAAAGCCGACCTTCGGCAAGCGGCAAGGCCTCTTCCACCACAGCTCTGGAAAAGCAGGAA
CAAGTGGCTCCCTCCCCGGCGGCGAAAGCAAGGGCGTCTGCCAAGAGCAGCCCCTCCGGA
GACCTCGTCGAGCAGCAGCAGCACGAAATCCTCGCCATGCAAATCCAACAGCTCTCGCTT
CTGCAGCAGCAACGCGAGCAAGAACAACAACATCCCAGCCCCGCCCACCCCATCGACGAG
AGCGAGACCACCCGGGATCTCGTCAAGACCTTTATCGGAGAAATATGGAACCGCGGCGAA
ATCAGCAGCATCTCCAAGGTCTGCTCGCCCCGGCTGCGCTTCAACAGCGAATCCGGTCTC
GAAAAGGTGGGCCACGAGGGATTCTCGCAGATGGTCCTGTCAGTCCGCTCCTCCCTGGAG
GACTATCACTGCGAGATCCATTCGATGGTCGTAGAGGGACGCAAGTGCTTTTGCCGCCTC
AAGTTTACCGGCAAGCACGTCGGTACGCTGCTGGGCTGCCCGGCGACCCACAAGCTGGTC
TCCTGGAGCGGGGCATCGGAGTTTACCGTTTGCCCCAAGCGCCACCAGATCCTCAAGGTT
TGGGAACTAGGGGACATAAAGACACTTGAAAAGCAGCTGAAGATAGACTGACACGGGACG
AATCTTGCCACACACTATAGCTCCAGAAACAGAAACGGTACGACATGGCACAGCATAGCA
CACAACCCAATGAAAACGCTGTTTGTGTTTCGGCGAAATGAAACATCAAATGGAATTAAA
ACGATAAGGAGAAAACCAAGAGGGGGTTGGGACCAACCACAGAACCAATACACGGCGGGC

GCTCCGAATCTGAGAGGCAACTGCTGGGAGCGAAGCGAAGCGGAAGGGAACAATTATCAC
GCAAGTTTCGAAGGGAGGGAACTTCACTCCCTGCGTGCCGATACAGCATCCTCTGCTGAC
CCTTGGAATGGAATGGATTTGAATCCGATGGAAGTATTGCATTGGATCGTGCAAAAGTTG
CCGTACATCATGCCTGACGTCCTTATACACAGGAGGAACATCAATAGTAGATGGAACAAC
ATAACATAAACAGCAAACCGCGAGATTAATTCGCAATCAAACAC
>comp2176_c0_seq1
CCAAGGCACAACAACAGCATTCTCAATCGCTGTCACCCAGGCATCATCCAACCATTCTGC
TCTTCAAAACCTCCCTGATTGTACCGCTCCTCGCCGCTGTCTTCTTCGCCATGTCCCTCA
ACCCAACGCCCGCCGATGCAGCTGCCAAGCGAGACGCCTTCGCCGCAACGCACACAACAA
CAACAGGAGAAGCAAAGGAGGGCGACGATTCGTACGGCGCGTCCCACACGTCTTTTTACA
CGACGGAAGAAAAAAAAGATTCGTACTCTACTCTTTCCAAAACTTTGGAAACAAAGTGCA
GCGACAAATTGGTGCGGCAGGTGATCTTGGATATGATGGGCGTCTGTGCGGATATTACAG
ATGCGCTCCGTGTGAATTTGGTGACAGTAGAAGGAAGTGCCAACACGTTTGGAGATTCTC
AATTGAGCGTTGATGTTATATCGGACAAGCTGATGTGGGATGCCTGCAAGAAATCGTCTG
TCATCAGGGAAGGGGCGTCGGAGGAGGATCCCATCGTCCACAACGTCGATATTGAGGGCG
AGAGTGGGGGGGAGTTTACGGTGTGCTGGGATCCGCTGGACGGATCTTCGATCATCGACA
ACAATTGGGCGGTCGGAACGATGATAGGGATCTGGCCAAAGAAAACCGGACTGATCGGAG
CCACCGGGCGGGACCAGGTTACCTCATTGATTGCTCTGTACGGTCCCCGAACGACCGTGC
TGGTGGCACTGGACGATGGCGTATACGAGTTTACCTACGGCGCTGACGTCCCCGATGAGG
CCACCACCGATGGAGGAGACCCGACCTGGATCTGCACCCGATCGCGGATTCGGCTGAAGG
ACGACTCGAAAATTTTTTCGCCTGCAAACCTCCGGAGCGCCCAGGACCTACCGGGATACG
CCAGGCTTGTCAATCACTTCATGACAAACCGCTATACCCTCCGGTACACGGGTGGGCTCG
TCCCCGACGTCTATCAGCAATTCACCAAGAACATGGGGGTCTTTTGCAACCCCACGTCGG
AAAAAAGCCCCGCCAAGCTTCGGCTGGCCTTCGAAGCAGCTCCGTTCGGACTGATTGTCG
AAAAGGCGGGGGGACTAACGTCAGACGGCGTCACCGGTGGTTCCATCCTGGACGTAAAGA
TCACCGCCGTGGACCAGCGAACCGCCCTCTGTTGCGGATCGAAGAACGAAGTATTGAGGT
TTAACGAGTTCCTGGAGCTATCAAAATAGGTTAAGAGCATATACGATAGCGACATGAGAA
GTTCGACACTCTCAAAGTGTATTATGCCATAATATTAGATTTTGTTCACGGTGCCACGTA
CTGTAGAAATAGGATATAATCCAGCTGATAATACTTTCGAATACAATCATTAGCATGTGG
GATTTCAAAGGATATCAATTTGTTTAGTTCATGATTGATTCAATCTAATCG
>comp2176_c0_seq2
CCAAGGCACAACAACAGCATTCTCAATCGCTGTCACCCAGGCATCATCCAACCATTCTGC
TCTTCAAAACCTCCCTGATTGTACCGCTCCTCGCCGCTGTCTTCTTCGCCATGTCCCTCA
ACCCAACGCCCGCCGATGCAGCTGCCAAGCGAGACGCCTTCGCCGCAACGCACACAACAA
CAACAGGAGAAGCAAAGGAGGGCGACGATTCGTACGGCGCGTCCCACACGTCTTTTTACA
CGACGGAAGAAAAAAAAGATTCGTACTCTACTCTTTCCAAAACTTTGGAAACAAAGTGCA
GCGACAAATTGGTGCGGCAGGTGATCTTGGATATGATGGGCGTCTGTGCGGATATTACAG
ATGCGCTCCGTGTGAATTTGGTGACAGTAGAAGGAAGTGCCAACACGTTTGGAGATTCTC
AATTGAGCGTTGATGTTATATCGGACAAGCTGATGTGGGATGCCTGCAAGAAATCGTCTG
TCATCAGGGAAGGGGCGTCGGAGGAGGATCCCATCGTCCACAACGTCGATATTGAGGGCG
AGAGTGGGGGGGAGTTTACGGTGTGCTGGGATCCGCTGGACGGATCTTCGATCATCGACA
ACAATTGGGCGGTCGGAACGATGATAGGGATCTGGCCAAAGAAAACCGGACTGATCGGAG
CCACCGGGCGGGACCAGGTTACCTCATTGATTGCTCTGTACGGTCCCCGAACGACCGTGC
TGGTGGCACTGGACGATGGCGTATACGAGTTTACCTACGGCGCTGACGTCCCCGATGAGG
CCACCACCGATGGAGGAGACCCGACCTGGATCTGCACCCGATCGCGGATTCGGCTGAAGG
ACGACTCGAAAATTTTTTCGCCTGCAAACCTCCGGAGCGCCCAGGACCTACCGGGATACG
CCAGGCTTGTCAATCACTTCATGACAAACCGCTATACCCTCCGGTACACGGGTGGGCTCG
TCCCCGACGTCTATCAGCAATTCACCAAGAACATGGGGGTCTTTTGCAACCCCACGTCGG
AAAAAAGCCCCGCCAAGCTTCGGCTGGCCTTCGAAGCAGCTCCGTTCGGACTGATTGTCG
AAAAGGCGGGGGGACTAACGTCAGACGGCGTCACCGGTGGTTCCATCCTGGACGTAAAGA
TCACCGCCGTGGACCAGCGAACCGCCCTCTGTTGCGGATCGAAGAACGAAGTATTGAGGT
GAGTGAATTTTTTGCTGTTGCGCATGGGTTTTGGTTTGACTGGACGTCGTTTCTTCTGCC
AAAATCGTTTTGGGATTGTGTTTGCCGCCCACTGACTCAGAACTTTTACATCGTCGGCTA
CTGTACTTGTACAATGCGTATTTCGTACAATGTGATTCGAATCTTTCCTGTGCATTATTG
ATCGGCTTGGGCTGAATCAGGTTTAACGAGTTCCTGGAGCTATCAAAATAGGTTAAGAGC
ATATACGATAGCGACATGAGAAGTTCGACACTCTCAAAGTGTATTATGCCATAATATTAG
ATTTTGTTCACGGTGCCACGTACTGTAGAAATAGGATATAATCCAGCTGATAATACTTTC
GAATACAATCATTAGCATGTGGGATTTCAAAGGATATCAATTTGTTTAGTTCATGATTGA
TTCAATCTAATCG
>comp2177_c0_seq1

GCACAACACAACACATCACCTGATGTCCTGCGGTACCACAACAGTCTTCTCCCCCACGAC
ACCCAGGAAAACGATTGCAAGGCACGCCACATCGCACCACATCGCACCGCACCGGTCCAA
AGAGCAACCATGGTCCGCACCGGCTTGTTGAGACAGGGGACTCTCCGCTGCAGGAACTCC
TTCGCCGCGAAAGCAGCCGCCCACTCCCTGCCGGGAACGGGAACAACGGGCCCTTCTGCC
TCCTCGTTCGGGGGTGCCCGCCGGTCCTTTCTGTGCCAGCATCGGCCGGGAAGACCGATA
GCGATAAAGGCATCGGCATCGGCATCGGCAACAATCCACCCAAACCCCTTGTGCGCTTCC
TACCGCCGCTCCTTTGCGGAAGGCTTTGCCAGCGAATCGGACTACCACACGGCGGCCGAC
GAAACCCTGGAGGACCTCCTGGACGCGTTGGAGGCCGCCATCGAGGACCACTGCGACGAC
ATGGTGGCCGCGGGCGGAGGCAGCGAAAACGAAAACGACGACGAGCCCGAGGCGACCCTC
GCCAGCGGCGTCCTCACCGTCGGCCTCCCGCCCCACGGGACGTGGGTCATCAACAAGCAG
ACGCCCAACCGGCAGATCTGGTGGTCCTCCCCACTCAGCGGCCCCCGGCGGTACGAGCAC
GACGGGGAGGACTGGGTCTGCACGCGTGCCGAGGGGGAGGGACCCAAGACGCTGGCCGAG
GCCCTGGAGGGGGAGTTCGAGGAGCTGTACGGGATTTCCCTGGGCCTGTAGCCACGGGGG
TTCGCGGAAGAGCAAAATGATTCTCGAGACGAAGACCGTCGAGAGGCCTACACCGGTGGC
AACCGCTGCGTGCAAAGGCAATCGCATCGCAGCCACTAGCATCGCAGCCACTGGCATCCC
ACGCTTGCTGCCGTGGAAAATCCTCGTTTGCCACGAGTTGCAACTATAACA
>comp2178_c0_seq1
CATTATCGTTCTGTAGCGAGAACTTTTTAATTCCGTTCTTGACCACTGTAACGAACATAC
TCGTAGCTGAACACTAGTTCGAGAACAACGTATGTTTTGACAACTGTTATCGCTGCCCCA
TGACACATCTTGGTTGAAGAAGAAACATCAACATAATATATTGTTGTTAATACAATATAC
ACTTCACGCTCCCAATTACGACGTAACGTATTACAGGAAGGAACTATCAAGTACGTAGGT
TATCGTTAACCACTCTTTTACCAACTTCGCACAAAACAGCAACACGTAGTCACGACATCT
CTATCGACACGAGGCAACGCAAGACACTGCACAATACCACGCGGAAACAATGACCTCCAG
AATCCTTTCCCTCGCGAGAATGATTCAATCCAGGACGGCGGCAGCGACGAGATCCGTTCC
CGCCGCGCTCTCCGAACCCCTCAAGGTTCTTTTCGGGACCACTCCATGCGAGGACGCAGC
CGAGGACAACGAAGCGAACCACGCCGATCGGCGCGAGCACGAAGAGCCCGCGGCGGAGCA
AGCCCCGGGCCTCTTCGCGGCACTGGTGCGCGGTCCGGCAGCGGCTGGTCCGGCTGCGGC
TGCGAACACCCCGTGGAACGCAAGGGAGGAGCGGAAGGCCTCCATCCGGGACTACTACCA
CAGGGCCATTGCGGAAGGCCGGGAAGCTGCCGCGAACAACGACCGTGTCCTGCGAAATCC
GGACCTGTCCGTCCCCGACGGCGAGCAGTACCTGTACATTGCACAGTAAACGGGACGCGA
AATCCGGTTGCGGCCCTGCCGAGGAGGAGTTTTTTCCGTGCGATTGCCTGCCGGTTCCGA
AACGAAAGAAACCTTCCAGTGAACACTGTTGGAGACGTTGCACGGGCTGTGAGATAGTAG
GCGCTATACAGTGAATCCTAAGCCTATCTAAACAACAACCCCAACAAACACACCATGCTG
ATGATATGCAGCTGCATAGTTTTAGTGTTCGACCGGAAGAATGGGTCACCAGAAAAAATA
GCAACGAGCAGATATGCTAGTCAGATTCGAATTCGCGAGAACAATTCTATTGAAAATTCT
ATTGTGTAGGAGTGTAAAAGACTGGTTCAGGTTTTGTGTTATCACTTGGCATCCACAAAT
GTAGAAAACCATTAATTTCTGCACGTTGAATTCTGAAATAATATTTCTATTGCATACTCT
CGGTCGATACTTTTTTTGAAATGAAGCACG
>comp2179_c0_seq1
CAGAGCACAGATAATGCCTACTGTATTTTCAAAGATCGAGGACCTTCGTTGAACGACAGG
CATTAGTTCATTATTACCATCATAAACCAGTCTAATCTGTACTTTAGTAGACTCGAATCA
AGAATTTAATTCATTATTTGCGTTGTGTTGGGTTAACCAGATTGACGTACAAAGCTTTTC
ATCATGATGCCCGTGCAAAAGATCCTGTCGACTGTGGGCTGCATCTTGCTCTTGGCCTGC
AGCGGTTCTAACGCTGCTGCCCCTGCACCGAAGCGAACGAAAAAGGGAGTTCCCAAGGGA
GCCGTCTCTCCACGTGGTGCCCTGGCCCACAAGGATCAACTCCGAAAGAAGACAGTCGAA
AAGATTGAGGCAGCAAAGCGATTGAACCGCCGCGAAAAGAAGGAAGTTGCCGATGCCGAA
ATGCAGACCTACAAGACTTTGCGCCGCGAAGGTGCCGACGAGGTAACCAACTCTGCTGAA
GCACAAATGTTGCGAAACTACCGTCTTCGCAACAAAAAGCGAAAGGGGCTTCTTCGTAAA
AAGCAAACCTCCTTGGCCCAAATCTTGTTCCCTGGAGTTTCTCCCGAAGAGTATAATTTT
CAAGAACAAGTTTGGATTTATTCCGACTTAGTGGAATCGAAGAAGACCCAAGTTCCTTAT
GAATATTACGATTTGCCTGGATGCCCATATCAGGTCGAATCTGGTATTAAGAGAAGACGA
CGCGAGAGAAAGAACCTTGGTGCAAGATTGCAGGGGCACGACACGAAGCCTGCTCCATTC
AAACTCTTTGCCAAACAAGACAAGGAATGTACTCCCATGTGTCTTGTTACTTTGGAAGGA
AAAAAGCTCCGTTGGATGAGAAATCTGATTCAGCGTCAGTACCGAGTTCAACTTCAGTTG
GATGGCCTACCAGTATTGATGAGAAGCTCGGAACTGAATTATGCAGTAAGGGGATACCCC
ATCGGATTTGTTTCTGCCGCTGGTGGGACAGTAGCAAAGAGCAAGCGTAACAATGCAGAT
GTATACCTATACAATCACTTAAAGTTTGCCATTACCTACCGCACGGGCGAAGATTTTGAT
GGTATTCGCATCACTGGATTCGATGTCCACCCTGTCAGTATTGCCCACGATATTCCCGAA
AACAAAGCTGTGACCGAGACCGCTTCGAGTTTATCCACTTGTAAAGGAATGGGTGTCGAA
AACGATCCATCTAGCTACCTATCTCTATCTTTTGACAAAGGAAACTCTGTGGATGTCATC

TATAGTTATGATGTTACGTGGATTGAAAACGATAGCTTGTTGTGGGCAGACCGTTGGGAT
GTTTATTTGGTCGGTGCACCCGATGACGATATTCACTACTACTCCATCATCAACAGTCTC
ATGATTGTCTTGTTCATGACTGGAGCTGTCGCAACCATCGTCATCCGCACTCTTCGCAAG
GATATTGCTGGATACAATGAGTTGCAGACTTTAGAAGAAGCTCAAGAAGAGACTGGCTGG
AAGTTGGTGCACGGGGATGTCTTCCGCCCTCCTCAATTCTCTCCTATGTTGCTTTCGGCA
ATGGTTGGTACAGGTGCACAGATCGGTTGTGCTTTCGGCTTTGCTATGCTTCTTGCCATC
TTTAATGTGACAAACTCGATGAGAAAGGGACAGATTTTGACGTCTATGATCTTGCTCTAC
GTCCTTTGCGGGTCAATTGCAGGATTTGTGTCTTCTCGCATTTACAAGTTCTGCGAGGCC
ACAAACTGGAAGATGAATTCCATCGCGACTGCGACTGGTTTACCAGCTGTATTTGTCGGC
ATGTTTACCCTTTTGAACATCTTTTTGAGTTTTGCCGGGGCTGCCACCGCCGTTAGCTTT
TTGACCATCCTGGTTCTCTTCTTGCTTTGGGTCACCATCTCTGCGCCTATGGTCTTTGTT
GGAGCATTTATTGGACTCAAGGCACCAACCATTGAAGTTCCTACCAAAACAAAACAAATT
GCTCGTGTCATCCCTGAGGCTCCTATCCACATGAATAGTGGTGTTATGATGTTGATGGGA
GGTGTCCTTCCCTTCGGATCCGTTTGCATCGAACTGGCATTCATCATGTCGGCGCTCTGG
CTTCACCAAATTTACTACGTCATGGGATTTCTTTTAGCCGTTTTGGGGATTTTGGCAGCC
ACTTGTGCTCAAGTAAGCATTGTATTGTGCTACTTGCAGCTCTGCGCCGAAGATCACCGT
TGGTGGTGGAAGTCCTTTAACAACTGCGCTATGGCTGGGATTTACTTGTTCCTCTACAGT
CTCTGGTTCTTGTCGAGCCGATTGGACTTGGTAGGAATGTTGCCCGTTATTGTTTACTTG
ACATACATGTCAATGATATCCATTTGCTTCGGACTTTTCTGCGGAGCTGTTGGCTTCATG
TCCAGTTTCTGGTTCACCCGTACGATTTACGGACAGGTCAAGGTGGATTAGACGTGCCGC
CGTTTGTGAGTGCTTGGCCGCTATGCGACGACGAAGCTCTAGCTGTTGCCGCCGGCACGC
TAATTTCACTTTTCGAGAGAGCAAATGTCTCAAAAAATAGTAACGAATCAATAAAATACG
ATCTGCTTCAAATTACTCTTTGTTTTGTGTCATGTCTGGTCCTACGAGGCTGGTGATAGA
ATTCAATATTGACCAATTAGCGACCCTTTCAGAGAGGTTCTAGCGGCCCCGTATGCAAAG
AACTGTCTCGTTTTGGGCCATCGTTAGAGTGGCATCAAACTTTATAAAAGAAACGGC
>comp2180_c0_seq1
TATCTCTCCATCAGCAAAAGAACACCATTTCTGCAACTGCGCCTTCTTTTCGTCTCAGAA
TACTCTCTGTTCAAAACAAATAGGAAAAGATACCATACTTGTTGCATAGTATCTCCCAAC
TACCGTAGGTGCACCATCAGCTTAGAGCAATTCACCAAATCCTCCTTTCAAAAAGAGTGG
ACCTGGACATAATGGTTCCCATACAATTTCCGCATCGTTCCCAGTCGCAGCCTCCAATGT
ATTCGCATTCGAACCGTCAAGTGGGATCCTCCCCTGACGAGATGCATTATAGCATGGAGA
TGAAAGATATTGAACGCTCGAGTCAGCTCGAGGGATCCCGCAACAACAATGCCCAAAAGC
TCCTAGACCAAGAATGCCCGATCGTGTGGCGATGGGTTCCCTTCCATGCAAATCACGGCG
ACTCTGGCATTTCTTCCTTGTCGCAACCGCGGGTTATTTATGCTACTTCCAAGAAAGTAG
GAAAGCCCGAGCCCTCTCCATTGCTAACAGGAGGAGAGCTGAGAGAGGCTCGAGAACTCC
TAATTCGAAGCAAGGTGAACTCTCTTATCGAAGCAACGTCTGCCACGGAACCTTCCCCAT
TGTCGCTTCGCATCCCCCATGTTCCAAGAAATATTCCTCCACACGTTAATCTTGTGCGTC
ACACTCAACACCAAGTGCTTTTGGAGTCGCAAGGTGCAAGCGGCAATGCCAGCAACGGCA
TGAACTCGTCGTCCAAAAGAGCAGAAACAGCAGGAGGCGAACTTGAAGTTGCGAACTTGC
TGCTCAACATGGGTGGGGGAAGTTCTCCAAAGTCCGGAGAGACAGCCCAAAAGAAGAAAC
AAACCACCAAAAAAACCGCTTTGCCCTCAAGACCGAAAGGACAGACGGGAAAATCTGCTG
CGGATAAGAAGCCGTCCTTGCTGGAGGATACTTACCTTGACAATTGGGTAGCGGGTTCTA
TTTCATTACACACTCCCGAAGACGACGATGTTTTGTCTCCTCTCCATTGCTTCATGAGAC
GATACTGTGTCGAGGCCTTTTCCGCTACTCCCGAAGATGTTGCTACACCAAGATATGGAA
AATCGCACGGTTTCAAGGTTGAAGTTGGGCAAGTGGGGATCCGTTGTCTACACTGCAAGG
ACTTGCCTCCCAGCAAACGAGCCGAAAGGGCAGTTTGCTATCCCAGTTCATTGCGAAATA
TTTACCACTCGATCGAAACCTGGCAACGCCGCCATAGTCTGGTCTGCAAGTGCATCAATC
CTTGGGTTCGCAAAAGTATTTTGGAGCTCATGGAATCCTCCAAAACCCGAGCGGGTGGGC
GACGCCAGTATTGGGAAGATTCAGCACGCCGCCTCGGTATGGTGGATACATCTCAGGGTG
TTCGATTCTGTCGCAAACCTGGTGATCTAGGTCCTATTCCATCTCCGGGGAGTGGTCGCT
TGCCAGGAGAGCCGGACGTGCCACAGCACGAACCAGAAAAAATTGTACGAAAAGAAGACA
AGGACCTCGTTACAGATTATCTCTTTTTGCTCATGGATCAAATGCAAACCTGTCAATTCA
CAGAAGAAGACAGGACGGGAGGTCGTAGCAAAATCAAGGACAACGAAGTTGGCTTTCCTG
GGATGGAATGCCGTCATTGCCAAGGTCGTGCGGGATTTGGACGGTACTTTCCTTCGTCAG
TAGCTGCCTTGAGTTTGGCCAATTCTGATCGGAATGTCTACAACCATTTGCAAAAATGTC
GAAAATGTCCCGAGAGCGTTAAGACAGAGCTAATTCGCCTTCAGAAGACCCAAGCGCAGT
CCAAGAATCGTAGGGGTTTGCGAAAGCTCTTTTTCAATCGGATTTGGAAAAGAATGCACA
AGGTTGACAAGGACGACAGCGAAAACTCCCGTCCATCGGAGTCGGGTTTAGTCGGGAGAC
AACAACTACCTCAAGGACGTCCTGGTGAATTCGTACAGTACAGCTTTTCTCAGCTACCCC
CTGCAATCATGAATCATTCCTACCAACATACTATGCATCCTCAGGTGGCGGTCCACAATT

TTAGGCGATATTGATCTGTTTTTCGAATTCAGCGCGCTAGTACTCCATCGTACTGAGCTC
ATGGAATAATATTGGCGTTCGCCACAAAAGGGGATGAGTCTATATCCATATACTTGCATC
AGAAAATTGAATTCTGCATATCTGTATATATAGAGCCGACAATAGTCACAAGATACAAAT
TCAAATACAAAGAGACACCGCATTATCTCAGCTTACACAACTAAATAAAAAACTGCATAA
TATACAAAATTGAATCGGTACTTCTCTGATTCATAGATACTCTAAAACACTGAAGTTCG
>comp2180_c0_seq2
TATCTCTCCATCAGCAAAAGAACACCATTTCTGCAACTGCGCCTTCTTTTCGTCTCAGAA
TACTCTCTGTTCAAAACAAATAGGAAAAGATACCATACTTGTTGCATAGTATCTCCCAAC
TACCGTAGGTGCACCATCAGCTTAGAGCAATTCACCAAATCCTCCTTTCAAAAAGAGTGG
ACCTGGACATAATGGTTCCCATACAATTTCCGCATCGTTCCCAGTCGCAGCCTCCAATGT
ATTCGCATTCGAACCGTCAAGTGGGATCCTCCCCTGACGAGATGCATTATAGCATGGAGA
TGAAAGATATTGAACGCTCGAGTCAGCTCGAGGGATCCCGCAACAACAATGCCCAAAAGC
TCCTAGACCAAGAATGCCCGATCGTGTGGCGATGGGTTCCCTTCCATGCAAATCACGGCG
ACTCTGGCATTTCTTCCTTGTCGCAACCGCGGGTTATTTATGCTACTTCCAAGAAAGTAG
GAAAGCCCGAGCCCTCTCCATTGCTAACAGGAGGAGAGCTGAGAGAGGCTCGAGAACTCC
TAATTCGAAGCAAGGTGAACTCTCTTATCGAAGCAACGTCTGCCACGGAACCTTCCCCAT
TGTCGCTTCGCATCCCCCATGTTCCAAGAAATATTCCTCCACACGTTAATCTTGTGCGTC
ACACTCAACACCAAGTGCTTTTGGAGTCGCAAGGTGCAAGCGGCAATGCCAGCAACGGCA
TGAACTCGTCGTCCAAAAGAGCAGAAACAGCAGGAGGCGAACTTGAAGTTGCGAACTTGC
TGCTCAACATGGGTGGGGGAAGTTCTCCAAAGTCCGGAGAGACAGCCCAAAAGAAGAAAC
AAACCACCAAAAAAACCGCTTTGCCCTCAAGACCGAAAGGACAGACGGGAAAATCTGCTG
CGGATAAGAAGCCGTCCTTGCTGGAGGATACTTACCTTGACAATTGGGTAGCGGGTTCTA
TTTCATTACACACTCCCGAAGACGACGATGTTTTGTCTCCTCTCCATTGCTTCATGAGAC
GATACTGTGTCGAGGCCTTTTCCGCTACTCCCGAAGATGTTGCTACACCAAGATATGGAA
AATCGCACGGTTTCAAGGTTGAAGTTGGGCAAGTGGGGATCCGTTGTCTACACTGCAAGG
ACTTGCCTCCCAGCAAACGAGCCGAAAGGGCAGTTTGCTATCCCAGTTCATTGCGAAATA
TTTACCACTCGATCGAAACCTGGCAACGCCGCCATAGTCTGGTCTGCAAGTGCATCAATC
CTTGGGTTCGCAAAAGTATTTTGGAGCTCATGGAATCCTCCAAAACCCGAGCGGGTGGGC
GACGCCAGTATTGGGAAGATTCAGCACGGTATGTTACGGTGTGATGGGTATGCTTTGCGA
TTTCGTGAACTACTTTTAGATATATATGGTCTCTAATTTGCTTGGTCGTCGGTTATTCTT
CGTTTCTGCTACTTTTTAATTGACAGCCGCCTCGGTATGGTGGATACATCTCAGGGTGTT
CGATTCTGTCGCAAACCTGGTGATCTAGGTCCTATTCCATCTCCGGGGAGTGGTCGCTTG
CCAGGAGAGCCGGACGTGCCACAGCACGAACCAGAAAAAAATTGTACGAAAAGAAGACAAG
GACCTCGTTACAGATTATCTCTTTTTGCTCATGGATCAAATGCAAACCTGTCAATTCACA
GAAGAAGACAGGACGGGAGGTCGTAGCAAAATCAAGGACAACGAAGTTGGCTTTCCTGGG
ATGGAATGCCGTCATTGCCAAGGTCGTGCGGGATTTGGACGGTACTTTCCTTCGTCAGTA
GCTGCCTTGAGTTTGGCCAATTCTGATCGGAATGTCTACAACCATTTGCAAAAATGTCGA
AAATGTCCCGAGAGCGTTAAGACAGAGCTAATTCGCCTTCAGAAGACCCAAGCGCAGTCC
AAGAATCGTAGGGGTTTGCGAAAGCTCTTTTTCAATCGGATTTGGAAAAGAATGCACAAG
GTTGACAAGGACGACAGCGAAAACTCCCGTCCATCGGAGTCGGGTTTAGTCGGGAGACAA
CAACTACCTCAAGGACGTCCTGGTGAATTCGTACAGTACAGCTTTTCTCAGCTACCCCCT
GCAATCATGAATCATTCCTACCAACATACTATGCATCCTCAGGTGGCGGTCCACAATTTT
AGGCGATATTGATCTGTTTTTCGAATTCAGCGCGCTAGTACTCCATCGTACTGAGCTCAT
GGAATAATATTGGCGTTCGCCACAAAAGGGGATGAGTCTATATCCATATACTTGCATCAG
AAAATTGAATTCTGCATATCTGTATATATAGAGCCGACAATAGTCACAAGATACAAATTC
AAATACAAAGAGACACCGCATTATCTCAGCTTACACAACTAAATAAAAAACTGCATAATA
TACAAAATTGAATCGGTACTTCTCTGATTCATAGATACTCTAAAACACTGAAGTTCG
>comp2181_c0_seq1
TTCTTATTTAAACAAAGCGAATTTTAATTTTCTTCTTTCCAAACATATCGAAGAACGATG
TCAGACCTCTCGCTCCAATCGAAAGAAACTGCCTGTGTCTTCATTGAGTACCAAAATGAA
TTCACCACCGAGGGTGGAAAACTCTATCCTGCAACGAAAGAGGTTATGGAGAAGACTAAC
ATGCTCGAAAACTCAACTGAGCTCCTGAAGTTCTGCCGCGAGAAGGGTATCCTCGTTGTT
CATGTCCCCATCAGCTTTCGTCCCGGTCACAACGAAATCGCAAAGGATCCCTATGGCATC
CTGGCCGGAATTAAGGATGGAAGTGCCTTTGAAAGTGGAGACTCTGGCTCCGAAATTTAC
GAGCCAATGAAACCCATCGAAGGTGAACTGATTGTCCAGGGCAAGTCTGGATTGTGCAGC
TTTGCGTCGACTAACTTAGAATTCCTCCTTCGGCAACACAATGTGAAGAATGTTGTTTTG
GGCGGTTTTTTGACAAATTGCTGTGTCGAAAGCACCATGCGAACTGCCTATGAGAAGGGC
TTCAAGGTATACACCCTGAATGATTGCACGGCTGCCACTAGTTTGGCTGCCCACGAAGCA
TCGTTCGAACATAACTTTGGCATGTTCAGCGTTCCAACCAGCAGCGAGGACATCAAGAAA
GCTATTCAGTAACTCTCTTGTGATTGTGACTCTCATTTGTTACTCTTCAGGAGTTGTGAC

TGCTGTTCGCAAAATTTGGGAAATAAATGGGAAAGACCAAATAAAATTTAGCACTTGGTA
TTCGTTTAAAAAAACACAAAGCAATGCTACTTGAAAGCTATGTTTCTTTTTGGTATGATT
AGGAATGTCCCAAGATATGTCCGAAGACAGAGTCGTTTCCAAGCAGGAAGGGGACAACTA
CGGGTATTCGTAATGCTGCCTCCTCACAACCGTATACTCATAGTACATAATGCTTTTCAC
GGACTACTGCCCTTCCTTCTGTGTTCATTCACTCGGTTCTTTGAAGATTGAGCTCTTCAA
AGATTGTTCAGTCTCAGCGGAAAACGGAGTTGAGTGCGAGAAACTAATTGCACAACATCA
AAATCAAATATCTCTTTTGGAAGAAACATCCGAAATTGAATGTTTGGTTACTCGTTACCA
AAGAACATTAGTACGAGTAGTTCGACCATGGTCTCAAGTCATGCCATTAGTTATTTTGCT
AATTTCTGGAAAACTGGATCTTGGGAAAAAGTCTCAGACAAGCTATCAATGCAATTGTAT
CCGCAACCAACATCAGCAAAAAAACAATCTGTTTCCTTCTGGAACTCGCCTCCCGATGAG
CAGGGGAATCGAGCACCACGACACTCCTCCTCAATGCGTTTTTGAGCCCACTTGAGTTGC
CGCACTGTCGTT
>comp2182_c0_seq1
GACTCGCATTTCTCTTTACTCTTCACCTATCGCAGCATCCGCCGCATCCTCCTTCCTTTG
CAGGTACCCCACCAAAGACATCAGTCTAACGCAACAACATCCATACTCTAGTAATACAAC
CTAGCCGTAAAAGAATTAATTGCAATATACTTGAATCATGCCTACCAACGCTATTCACCG
AAACTACCGCAAAACCTTCACGACTCCTCGTCGACCTTACGAAAAGGAAACCCTTGATGC
CGAGTTGAAATTGGTTGGAGAGTACGGTCTCCGCTGTAAGCGTGAGATCTGGCGTGTTCA
GTACGCACTCGCCAAACTGAGAAAGTCTGCCCGTACCTTGTTGACCTTGGACCCTAAGGA
CCCCAAGCGTATTTTCGAGGGACAGGCCCTTCTCCGTCGAATGAGCCGTTACGGTTTGTT
GGCCGACGATGAGTTGGAACTCGATGCCGTCCTTCAGTTGACGACACAGAAGTTGTTGGA
ACGCCGACTTCAGACCAAGGTCTACAAGCAAGGCCTTGCCAAGTCCATCCACCACGCCCG
TGTCTTGATCCGACAGCGTCACATCCGTGTCGGTTCTCAATTGGTTACCGTTCCTTCTTT
CAACGTTCGAACTTCCTCCGAAAAGCACATTGATTTCTCCGTCAACTCTCCTTACGGACA
AGGACCTCCCGGACGTGTTGCCAAGAAGCGTGCTGCCTCCCGAGCGGCCGCCGGAGACGA
TGAAGAGTAAACATCCGCCAGCCTATCAATGATAAGGTATAAGTTGCTGTGTAGCTATAG
TTTATCTCTCGTTTCTTTTTTTGACAAAGCTTGGCATTTGAAATCAACACAAGGCAGTCA
AATACGTTCGCACAAGCTGATGAGGGTAGATCATTAATCAATCTAAATCAACCCGAAGAG
ACAAGCGGCATCCCCATTCATTATTGCTATCAACATTAAAGTATTTAGTGAATACCATTA
TAGATCTAC
>comp2183_c0_seq1
TAGAAAAGGACCCAGTAGAAGTCCTTATTTAGTTTGATTAGTGTTGCTTGTTTTCTTTTC
ACGACCACTGCTTCTTCTGACGTCGGCAACCAGCAGTATGAGCACCGATCCAGAAGATAG
GATGAATACACAGGAGGATAGAGAAGCCAGGGATGGTCTCCTACAACTGAATACATATCT
AGGAGGCCATGCTGATGGATTAGTCTCAGATGATTCATCTGAAGAGGATTTTCATCAAGA
GGAAGCTGTTGTCCCTCCTCCCCCTCCAGCCGCGCAACCACCAGTGCCACCTGCTCCACC
AACACTACCAGCAGCATCCCATCCTTTGTTCAAGACAGCACAGGCTACACTGGCCAAGAG
GAAGTTTGTTCCACTCAAAGAAAAGCAAGATTTGCTGCTTAGAATCAGGGAGAAGCTCAG
CCATGATCTCATCAAAGCTGTCTTTCCTGGGGGGGACGCCCTGTCCCTAGAGATGCAGGG
GGTGTATCACAATATAACTGCAACAGGGAACACGCATCACAAGAGGATTGGAATGGAGTC
AGGCCTTTACAGCCACAATGCTTGCCAAATTCTTTACAGGAATGGAGATCAGGAAGGGGA
GGAGCGACCAACAACAAAAAAGGGAGAGCAGAAGGACAGCTGCCGCTGTAGCTGAGCTCG
AGACTGTTTCCAGAGCAGTAAAGAAGGCAATGAAAGCAGAAGAAACCAGCAACAGACTGA
AAGGATCCAAGCACAAGAAAGGGAAGGTCTATTGGCAGCAGGGAGCAGCCTCGCGTTTAC
AGGATCAGTGTTCTTATGATCCAGCCAAGCTGGATAGGTTTGGTTGTCCTATCTGTGACC
ACCATTCCATCATGCCTGTAGATGATTTAGATAAGATTCAGAAGCACAATCAAGGGAAGA
GGAAGGAGCATGATATAGCTGTAGCAGAACATGATGCAGCGAATAGAGATAAGAAGGGGC
ATCCTAAGCTCAAGGGTATGCTGGATGTAAGGATGGCGTGCTACTGTTGCAAGTTCAACT
GTAAGCTGGATCCAAGGGGGGGGACGTGCCCTGAGTGCAGCAAGCCTGGTGTCGATTATC
TAGAGTATGAGAACGGCGTGCCAGTGGCTTGTAACTGTCCCCTTTGCAACTGCCAGTGTT
CCATCTATTTCTATCTAACTGACAGATTCAACATCAACAAGAATGAAGAAAGGCTTAGAA
GTGGTCCCCAGCCAGAGGAAGAACAGTACTCAGGTAATCCAATTTTTGCTCGGTCGAGCA
CGTGTCGGAGACCCTAAGTAAATGAAAAAATGAGGTATTTAAATAGGCTCAACGGACAAA
AATGAAAGTGGTACTTTGGTACTCTATAAATTACTTTGGTACCACCACATG
>comp2184_c0_seq1
CGTTGCCGAACGAAAAAAAACATAGGGAGGAAACGAAGATAAATACCCTTCAACCAAACC
ACGAAGCACGACATTGAGAATACCTTAGCAGCATCTCGCGTTTCGTTCCAGCTTCTTACT
ATCGATAAGGCGTTGGAAATAGCAGTCTACAACGTAGCAGTCGATATACCAGCAAGTGAA
GCTTAACGAGTGTTTCGTGTGACGAAAGAAAAATCGCAACAACAACAACGATAACGATAC
AGCACTGTGCATCCATACCGACGGTAGTTATACTTGGAACGCAACGACGACGTACTGAAA

GCTGGCAAAGCACAGGGCGGTCCAGTACATAACGATAATTCGAAATATTTTGCATTGAAG
GTGTTGCAGAAGATCTACCTTCAATTTCAGTAAATTAGATCCTATCCGATCAACGCATTG
CAAGATAGCACGAAGCAAAACAACAAAATGGCCTCCCAATACAACAACGGCAACAATAAC
GGGAACATGTCCACCATGGACGCCACCCGCTCGTACGTGACCGCCCGTACGGCCGCATCC
CAATCGGTCGCCAACCACACCACGAGTGGTGATACCTGCTCCCTGGCTCCCCCCCCAGGC
GCCATGGCCCAGCCCCAGCCGGCCCCGTACCCCTCCTTTGTCAAGGTGTGCGGCTGCGTG
GAGATCTACAAGCCCGGCCGTGAGGTTTTAGAGTACGACTACACGTACGACGACGTCTCG
GGGAAGAAACCCCCGCAGTCCTACCCGAGGATTCGTGAGCGGGGGGAGTTCTACATCGAT
TCCTATAACGGGGAGCCCTGGTCGTGTTCCTTTGGGACGGCCGAGAAGAACGGAACCTGG
TTTAATTCCTCGGATCAGGCCGGATCGATCATGTCGTGTCTGGTGTGGGTGCTCATGCTG
TACTCGTCGGTGACCGTCACCCTCCTCACCATTACGGAAGGAATTCCCGTTACCTGCGGA
ATGGTGTACAATGTGTTGTGTTTCATGGCGTTGGCCTGTCACGCAAAAACCAGTCTCACC
GATCCCGGAAGCGTTCCACTGGCTGCAATCCCCAGTGAGCGACAACGCCTAATGAAAGAT
TCCCACTCCATGTGCGGGCAGTGCCAAACCTTCAAGCCACCCATGTCCCATCACTGCAGA
ATCTGCAACAGGTGTATCAGCAGAATGGATCACCACTGTCCCTGGATGAACAACTGCGTC
GGTGGGAACAATCTGAAGCACTTTTTGCTCTTTTTGATCTACACGTGGACCTGTGCGGCC
TACGCACTAGCGCTCTTTGGATGGAACTACTTTTTCTGCGCCGATGAAGACTGCACCTTC
CACCCGGTGGTCGTCCACCTGGTCCGAGTTATGACCATGCTCTGCGTGGGAGCATTTTTA
TTTACCTCCAGCATGCTCATGAACGTTTGCTACGGACTCATGACGGGGATCGGGACCATT
GATCGGCTCAAAAAGAAGGCCACCAACACCATGAACCAGTCGGACGAGGAACCCATCCCT
CTCAAGGACGTCTTTGGCATTCAGGGATGGATCACCTGGCCCTTTCCCGTCGATCCCATC
TTTGAGGACTTCGACCAAGTCATGGGATACTGTCTGCCTCAGCGCCTGGACCGAGAACGG
CAGTTTATGGAATACAAGCGGCCCATTCCAGAGATCCAGCAGGTCACCTCGCACGAAATG
GAGCTCCCCAAGTGCATCAACGATCTGCTGCCCGTGTAGATCAGATCGAAAGCAACCGGA
CGGAATCGAATCATCGCCTCGATTCGATAACGAGCGGTACGATAAAACACATCTTGGGTT
CACAAAGCACTCACTCGCACACACCTCACCACCTCTATACACACAGACACAACGTCCTTT
TTTCCTGCGCGAAACGACAACGATACCTTTTGGAGCGTTGCGTGTTTGCAGCGACCATCA
AATAATAATATACTAAGAAAAACAACAAAACCCATC
>comp2185_c0_seq1
AATAATTTGTTCTATCCGAGGTGTGAGGTGTAGTAGTGCTTCCTGTCCAAGAATAACACT
TGTAATATTATAGAGAAATTTCAATCATCCACTATTATGAAACCGTCTGGATCAAAGGGT
AGGAATGTCTCCCTCTTCACAACTTGGCTACTCATTGTAGGCAATCAATGCACGGGATTG
ATCGGAGGAGAAGCGTTTGTTATCACTAGTCATGTCAAGATTTCAGCTTATTGTTCTGGA
TTGGGAAGAAGGCTAGAAAATACTTTTTTGTGCGGCAGCACTGGTGACAATGACGATTTT
TCTTTCAGAAGGACAGATGGCGACGCAAGGTATTGGAACTTACCAAACGATTTTACGATG
TTCCTGACACAAAGGGCCATTCAGTCCTTCATATTTTTGACGAGCCAACTGCGAGATCCC
GAAACATGCTACTGGGTTGAGCAATTTACCGAGCCAAATTTGGTCCGCCTCGATACCAAA
CAATCCTTCAGTCTGGGTGACACTTTGCCGAATCTGAGTGACATTTCTAGCGGCAAATCC
GATTGGCGGCCGAACGCCGACGGCGATGATCCGAAAGACGACGAGGACGAGGAGGAACGA
CGGACCTTGCTTCTCTTAAAGTACCACGGCCTAGCTGCTATGGACACTGTTCGGTTTCCT
ACATGGGAGTCCTATTTCGAAGAAATGTTGGAACAACCGACCGATAAGTGGATCATTGCA
AGTGACCAATCTCATATTCCAGATTACACATGGGAGATAGATCCGTCCTCCCTATGCAGT
AGAATTTTAAGTGTCAGAGAACAGCTTTCGAGAGAATTTGCTAGCGATCTTCAAGCCGTT
TCCAATATGGGTGGACAGACGTTAGATTGGTATTGGGATCGACTTCGTCAATCTCGTGAA
GATGAACGACGAGAAGAAGGACAACAAACGGAAGGGAAAAATAGTGGCGAAAACGAGGAC
AATCCCTTCGTAGACCCGACTTCGCTTAGAGGTTCAAGGCAAAACTTACTATTTCTTGAA
ATAAACGTCGATAGGCCCGAATTGGATCACAAACCCAGTCCCTTGCGAAGGGGGAACTTC
GATCTTTTGGTGTTGTTGGCAACCGAAGAAGCTATTCAGCGGGTTCTCAATCGATGGCAA
TCGAAAAAGTCTGTGGGGGATTTGGATGGTTCGGAATTTGTAAGCTACGAATTTCTAGAA
GACTTTTACAAAGAGAGACAGCACTTGTTTCAGGGTCCGTTGCCGTCCTACGGAAAAGCC
GATGACATTTTGGAAGAATTGTTGTCTTCTGCACTCAGTTTCAAGGGCGACGGAGAGGTC
ATCAATCCTACCAAAATAGCCGAAGTGGTCTTGCGGGAACGAGAACGAGTGGCACTGGAT
TGGAAAGATATCGCATTGGATTCCCCCCAGGACCACATGAAAATCCAAAAATTGCGATTG
AGTCGAATGATGATGGGCAATGATAACAACGGCAATGGCCAACAAGGCGAGAGCAGTCGG
GGTGAATCATCCAGCAATGTTGGTTATCACGACCCTGGTCCTGGTGCTTTCGAGTAAAGT
TCTTACCCGGACGAACGAACAAAATCCATTCTTTTGAATCTAAAAGACAACGTCGAAACT
CTCCCAATCTTGCAAAGGCATAAATCAACATTCTACATATCGAGCGCTCTCACGTACGCA
AGTAAATTTGAGCTTGCACTTTAAAGTATACTCTAGTTGGGTATCTTTTCTTATGTTGCA
TGAACCACATCGAAGAAAAGGAATGGAGGTTTTATCAAGCTTGAGAGATCATTTATCTTT
TTTTACGTTCCCTCTGACTTATTGCGTACCGCCGTCTATCATGGATCTCTAAGTTGGTAG

AAGTTAATAGAATGGAACGCATTAAGCTTCTAGTAGCGGGTTCCTTCCATTCAGTGAACT
ATTCTTTGAATGCGAATGATCGCTTCTAGTTATTTCCGAAGTAAAGTGGTGGATAGGAAG
ACAATTTCACGGCGCCTTTCGTGTCTTAAAGGACAGCGTCCTCTCAGAATGGACGCACGG
CATATACGGATGCATGGCGTCTAGTAATTTATGGCATGGACTTTCTTCAATTTCCTTACA
CCCCCA
>comp2186_c0_seq1
TGGCACCATATCGGATAATACCTCTTGTCATACCAAAACCGAAGAGATGATTCATATTAA
TACCGTCGTGTAAATGGAAGTGAAGTTATTAATTTCGTCCATGTCACTTCAACCGAATTG
ATAAAGGCACAAAACCTGTATTCTTTTTTCGAAAACCACAAAACGCAACATAGAAAAGCA
CGTTACGAAGATGGAACAAATAAACAAGAAAAAACCTCCGACGTCGATCCGTGGACTATGG
AACGAAGACCAACAGATCAAAGCTTGCTGACGATTCCAAGCGTCTGCGTCTTTTTAACCA
AAGTAACCCGAACAAAACACAACTGACGGGTGATTCTCGATTTGTTGTTGCCGCCAAGAT
CGCAACCCTGAATAACTCGGCCGTCGATCTATACATCAAGAGAGACTTTGAGAATGCCTC
CGGATTCTTCGAGGAAGCCACGCTCCTTCGACTTATATCTGATTCCCTCTTTCCGCCAAA
AGTCTTGTTTGATCTATCGAGACCACCAAACAACAAGATGACTTGGGACAAAGGTACGGA
TGCTGCGAAAACGTACACAATGGGCGACTCGGCCGTGCCTTTGACATCAAATTACATGTA
CCAGCGGACGGAATTCGATGAAGGCATCTCGTTGTATTGCGATACCAAGGTCATCCACGA
CGGAGATCATCATCTATCGGCGAAGGCGACGCTTCTATTCAATGCGGGACAGGCGCGAAG
AGAACTCGGCGACCTTGTTGGCGCCGTTGCCTTCTACGGGGATGCACTTTCTGTGCTCCT
ACCAAACCTTGAAACGGACGAGGCCACGCATTTTTCACCCCACCGCATTGTTGTGCCCGT
CCTTCACAACCTCGGACAAATCGCCTATCGAATGGGAGCTGCTTCGAAGGCAATCTGGTT
GTATGAGATAGCACTGGTTCATTGCCGGGTGCTCAACGGCGAACGAAGCTTGTCCGTAGC
TTCGACTCTCAATTGTTTGGGCGTAATGCATTACCACTCACCAGCAAATGACGACTCGGA
CCAGGCCATTCGATACTTTTTGAGGGCCCTGGATGTACTACACGAGGTCATGGGGTCGGC
CTCAGAGTACGAAGCAACCATTCTGAACAATCTAGGAAGGGCATACGTACATTATAACGA
GTTTGATCTCGCCTTCGAGAGTTACGATCAATCCCTTGCGATTCGAAAACATTACCTCGG
GCCGGACCACGTCGACTACGCCGCAACCGCCTTTAATGCTGGTCAAAGCCTTCACCATAA
GGGTGCGCTCTATTTCGACCGAGCCATGGAACTTTATGGAGATTTTCTTCGCGTTGCATT
GGTTCGATTCTCGAAGAAACATAGGGATGTCGCTGTCGTTTTAAGTGGAATGGCACAAAT
CCATCAAGAACGGAAGGAGTATCCAAAAGCATTGAAACTATACGAGGAAGCCTTGGAGGT
CGCAAAGGAAGCACTTGGCAATTTTCATTTGGAAGTGGCGGTCATTTGCAATCGGATTGG
AAATTTTCACTTCGAACGAGGGCATTGCGACAAAGCCTTGGAAGCTTACAAAGAAGGCTT
GCACATAGAATTGCGGCTTTTCGAAAGAACCCATCCCAATATCATCGTTACGATGAGTAA
CATCGGTGGAATTCTGCGCCAAAAGGGAAGCCTTGAATGTGCCATTCGTATTTACAAGGA
CGTAGTGAAACTGCAAACCATGCGGTATGGTAAGAATAGTGCCGAGGTAGCGACAACGCT
CAGCGTCATTGGCATGATATACGATCAAAATGGAAACTCCGATAAGGCCCTTGTGACGCT
TCAGAGAGCATTGGCAATAAGGCGAAGCGCCACTGGAGTCAATGACGTCGATATCTGTAC
CACGCTTACACTTATCGGAACAATTTTGTATCGAAAGTCGATGGTATCAACGGCTATAGA
ATTCTTTTCGGAATCACTCAGTATCCGCCGCATAAAACTTGGAAAGAACCATCGATCCAT
TGCTTACACATTATACAACATAGGGCTTTGCCACCAATCCCTGGGTAACCTGAACCAAGC
AGTTGATTGCTACAAAGAGACACTAAGAATAGAAAAAATGGTCCTTGGAGAAGACCACAA
GGATATTTCTATGACTTTATTGAAATTGGGGGAAGCGTGCAAGGCCAAAGGTGACTTGGA
TGAAGCACTTGATGTTTTAAATTATGCCCTTGCAATTGAACGCAGCACGAAAGGACAAGA
TAATCCTGCAGCTGTCGTAAGAATAATCAACGAAATAGGAACTATCCATCTCGAAAAAGG
TGATTTTGTCCCCATGATGGAAGCTTTTTGCGAGGCATCAACATTATTTCAACAAGCAGG
ACTTACTGCCGAAAGCATTGCGATGCCTGGGGCCTTGTATCACATCGGAATCATTTTTCC
AAGTGGTGCTCCTGCGGCATGAATGAATATAAAACATTAAAAACTCAAACTTACTAGTAC
ATCAAATCTTTCAGGTCCATTGCAATGTCCTCAAGTGGATCTACACGCTCTTCATCATCC
GAGGAATCACTGATGTGTGACTTATTGAAGTTGTCTTTTAGGTGCAATACCGACGAAACC
GATTGAAAGGCTCGTTTATCGCTTTTCCTAAGGCCCAAAAGTGCATTGGATCCATCAGCT
TCTGTATTTCGAAAAAATGCCAAACGAGATGAGCTACTACTCATGGCCACGGATGCTGTA
GCTCTTGCTGTGCCTGCTTTTGCAAGATAAGATTTTCTCCCGAGAACATCAAGAAAAATA
ATTCTTTTGAACTCAATCCGATGAAGAACGCTCAACTGCTTTGTTTGCCGTTGGGTCTTT
ATTTCTTCTTGCGATGAACTCCGACTGGCATGCCAACCTCCATCATTATTCGACTTGTTT
AATATTGCGT
>comp2187_c0_seq1
AAAAGAACTTCAGAACACTTGTGACAGCACGCCAAGACACACAACTCGTTACAGCGAGAA
TATCCCCTCTAACCACACTACAACACCGCAATTCACATATCTCACAACCTCAAGTCACCA
GCTGCAACATACCAAGTAACATACGTTGTTCCTTCACTGAAAATTGATCGATTCATATTC
CATAAAGCTGCCAACAAAGAAAGAGAAGATCAGAGTTCGAGTGGTGCATCAGAAGAAACA

GAAGCAGAAGCATGTCGTATTCCTCGGCCGCCAGAACCAACCTGTCCTCGCAGGACGTCC
GAATCAGTAACTACAAGCTCGGTGAGGGAGCCTTCCGCGTGTGCCACGAGGGAACCTACG
TGGGAGGGAATCGGAACGGCCAATACGCGGCCTGCAAGCGTTTCAAGCCGCAGTTTCGAT
CGATGGAAACGGAATACTTCGAGAAGGAATTTATGATCGCCGATACCGCCATCCACTACG
CGGAGCAGTGGAACGACATTTGCGACCCCGAAGATAAAATCCTCATCACCAAGGGACACA
TCATTTACTCCAACAGTGGCATTCCGTACATGGCGGAGCCGGTGATCCGGGATTTCAAAA
AGTTCACATCGAACTCCGGTTGGATCGATGAGAGCGATATCTATACGGAAGCTATGGAGG
CCTTTAGCCACATGACCTACCATAACTCCGGGGGCAACCACCTGGTTTGCGACCTCCAGG
GGCGCTACCGGAACAACTACCGCAAGTCTCGCTTCGAGCTGACGGACGTTGCGATTTGTT
CGGTCGCCAGGGAGTTCGGTCCCGCCGATCTCGGCGAAAAGGGGATCGAAAACTTTTTCG
CAAACCACCAATGCAACAATTTCTGTTTCCCCCACTGGCGTCGTCCCAACCTCGCGCGCA
GATGGTTTCCGAGCCAACCCGGCACCTCCATGTTTCCCGCAGGCGATGTTCGCTTGCAAC
GACCGAGCCCCGGGACCCACTTCCAGCTGGAAGGTATCAGTGTTCGACCAGGCGCCTACC
AATTAACTACAACAGAAGATGGGCTGATTCGAGGGCTGTTTCCAGTTCCACAAACTTAAC
AAGTCACTCCAGTTCAAGGTGGTTCTAATGGCCCAGTTCGGTTCCAAGGTCCCCCAATAC
CATGCGGCCAAGAAAGCACTGTCTCCCATTAATTGTTTCTTGCAGTATATTTTCTCTTTT
TGGGGCTATGGCTGCGGATTGGAGGAAGTCGCCCGGGTCCTGATAAAATAATGGCTAATT
ACCAATAAGAACGAATCATTTTTTGGTTTCTAATTTTTGTTGTCCTTTCTGCTCACCCTG
GCGTCATCATCGTCAACGAACGCAGTATGGGGCAAGCAACCGCCAAGGTTTGTTCATTCC
TCGAGAGGAACCATACTTATCCCGATGCAACAATAACTCGAAACGGACGCAATGAAATTA
CTAAGTCAGCACTCCCACTAGCTATGATTATTGTTGATATTATTATCTGTGAGCCTTGCA
ATCCAATGGCACCAAATGAAGTGTGTATGTCGGCTTTCTGATATGTGCCTTGAAGCGAAC
CGCCGAAAGCAACAAGTCTCGAAAACATGTCGATTGTCTGCATGCCAAGAGCCACGATGA
ATATGACTGGATTGGGCAAACAGCCACGGGCAATGCCATGGCCAGCGCCGTGTTTTCCTC
ATGCTCTCATTTCCGCTGTAATTTCGTGGTCCTCATGATACTCTATCTCATTCTAGATCA
TGGGTTCTCCCCGTTCCAACCACCTCATACGTATCGCGTTCTCTGACCCTCTCTTTTATG
GATTCATCTGCCGATTCACGTGTTCCTCCTGTTTCCATAGCCACAAAACCCAACATCTCC
CTTGGCCTTTCATTATTCCTATACGCACAAGGATACCAATCTAACTCGAAGGATAAAGAT
GTGTAATAAACGCATGACTCGTAAAGACACATACTGCAAACGATTGATGACAAAATTTCT
ATACCATAAACACAACATCGGCTTTTGCTTGGAATCCTCCTGAAACGTTGAATGTATGAT
CGTGTCATACCATACGAAGCAAAGCTCGATCATGTCTGCCATGCACTTTGTCC
>comp2188_c0_seq1
CGTTCCATCCGATACCGCTACGCGCCACACAAACCAAACAACTGCAACACAATGGTGTCC
CTCTCGTCTGCTCTATACTCCCACTACACACTTTCTGCCCTGGCGATTGCTCTTCTTGTC
GTGCTCCCTTTCCCTTTGCAGGCGGGTGGTGCCAGTGCCAGGAGAAAACTTACCAAGAAA
GCCACGCAAGAACCGATCCCCACCCCTGACCCCACCCCCGCCCCCGGTCCGAAAACGCTC
AAACTCGCCTTCGACGCCCTCCGCCTGGTCTCGGAGGATGTTGCCAGCATCGGCTTCGAG
TCCAGCTCCCTCTCCACCGGCAACCCCCAGCCCACCTTTGTGGGTGCGACCACGGTCTCC
TTCCTGAATCTCTTTCCGTTTGACACAGTGACGGCGGAAAACGTCGAGAGCCTCCACGCC
GACCCCTCTTCTGGCGGCGCCATTGGCACTATCGAGTTCCCCTGCATCATCACCAACAAC
GGCTTCTCGAAAGGCCTGTGCCAGGGAGTTGTTGACTTTTTGCTCGACGGCGAGCCAGCC
GGTGCAATGGTCTCCTACGGAGGGCACCTGATCAACCAGGAGCACTACCAGGGCATGATC
ATTGGCGGGACCGGGGCCCTCGGGAACGCCATCGGCGGGACCTACCAATCGGTGAAAACC
AACATCCCCACTGCCGACGGCACGAGTGTCTACGTCATCGAAAATTTTGTCGCGACCTTC
GTTCTTGCCTAAGCCCGGTTACAGTTGGAACAGCGACGCTATTTGTATTTTAGATTGTGA
GTGTTTCATTCGCAATCCAAAAGTGCTGCATTGCAGCATTCTGTCTGCAATAATCCAGTC
TTAGCAGTGTTCACTATTTTGGTAGCCACGATTCAATATCGTCGATCATATTGAGGACTT
AAACCGATAAGTTTTGTTACTTTCTAG
>comp2189_c0_seq1
CTCCTCGAAAAAAAAACCAAAACAGACGACTGACATAGAAATGGATCAAGACACCAAGAA
AAATACGAGCAAGGGCGAAAAGAAAAACATGAAAAGCAAAAAGAGCAATCAGAACAAAAA
AAACAAAACGAAGAAAGACAAGAAAAACGATAGAAAAAGGGACGATGTCGCGGACGCAAT
CCCGAATGTCCTTGATGTGACTCCTATTGTGGCTGTTGCTCCTATTGCGGCTGTTGCGAT
CCCACTTCCAGACGATATACACCCCGACCAGCACTACACACCGCCAGTTTCCGCCCAATC
CAACACTACGGATCACCAATTTCCAAATGAGGGAAGTATCAACCCTGTGATCTATCCAGT
GGTGAGCGAAATCGCCGCTGTCGCAGTCCCTCTCCCGGACGACATCCATCCCAACCAACA
CTACATACCGCCTGTTTCCAACCAGCCCAGTGCTATGTTTCCCAACCAGGAAGGCAGAAA
CCAACCAAACATTGGTTCGCCGATAAGTGGCAACGGAAATGTAAAACTGAAAGACCAGGG
CTACAGTTCAGGACTGATTTATTCAATTGGCTTAAACAACGTGATATGCCCACTACGGAT
ATGGGTCGTGGACAACAGCGGATCCATGAAGAAGAGGGATGGTCACCGTATTTCTGAGAA

TAAAAGAACCAAGCAACCTTTTCTTCTCGAATGCACGCGATGGGCTGAGATGCAGGATAC
TGTGGAATACCATGCACGAATGGCAGCACAACTCGAGTCCCCGACAGTTTTTCGTCTCCT
GAACAAAGCTTCTACAGGCCAGCAGCAGTTTAGTATTGGTGAAAGAGGCCATCAATTCAT
CCAAGAAGATCTGAATATCGCCTTGGAAACTATATGGCGTACAGTTCCCAATGGTAAAAC
ACCCCTGGTGGAGCATATATACCAAATCCGTTCAGTCATCATGCCGATCAGAAACAGCCT
CTGCCAGGATGGACAGAAAGTGGTGGTGGTCATTGCAACCGACGGGCTCCCAACTGACAG
CTCTGGAACTATTTGTTTAGAGCGCTTCAAGGTTGCACTCCGATCACTGCTTCAGCTACC
TATTTTGCTTGTGATCCGTCTCTGTACAGATATGAAGTCAGTGGTAAAATTCTACCACAA
GCTTGACGCTGATCTTGAATATAAGCTAGAAGTTTTAGATGATTTTTTTGGGTGAAGCCC
GCAAGGTTTATCGTTTCAACCCTTGGCTCAATTATGCCCTGCCCTTGCACCGGATGCGGG
AGATGGGCTTCCACCACAAAACCTTTGATTTGCTGGATACACGGCTGCTGACTAAGCATG
AGCTGTTGAACTTCTTTGTCCTTCTCTTTGGAGCTGATCAAATGGACGGCATTCGGGATC
CATGTACAGACTGGAAGGCATTTCTAGACCATGTCTCTGGGGTGATCAACCAAGAGAAAT
TGCAGTGGCACCCTCATTTTGGCGCAAAAAAACCATGGGTGAACATTAAGAAACTTAACC
AATGCTATGTGGACGGGAATATGACACAGTGGCCACAATCCAATTGCCGGTGTACCATCA
TGTAGTGTAAAGCTATTCATTCTGAACCAATATTTGTTAGTTTTGAATGTTGAAAGCTCT
ATTTGTTGAATTGATGTGCTCCATGTTTTTGTGTTTTATCACAAGGCCTTCTCCAGTCAT
GAAGCAGAAAACAACACAATTTCCTATCGACACAAACCATTTATAATATTGTTTAACCTG
AC
>comp2190_c0_seq1
CAAAGAAATCAAAGCAATCAAGGTCGGTTTGCGAAGCAAAAATCCAGAGGAGTATGCGTG
GATTGTAGTATCGCTCGGAGAATAGAGGATCAATCGCTCTGATAGAGCAGAAAAAAAAAC
AACAGAAAAGAACTAGGAAGAGTACCATTGAGAACAATTGATATTTCACCCACTCACCCG
CTGGCTCGCTCACTTTGGTGGAGCCCAATGATCCATTCCAGCCTTCAACCAGTCGGCACG
CCACGGTGCGACACAGGAAGAAGGTGTCTTTCCACTTGCGATAGTCGAACTTTCGATGGA
ACACGACGAGTACTGTATCGTATTCAAAATTTTGATTGCCGCCGAACATGATTGTGCCCT
TTCCTCCCATCGTCGTAGAGGCCGCCCTGACCGCATCGTTGCTCCTGAACCCTATGGCGG
CGGATCCGATACCACCACCAATGGTTCAAACGCAAACCAAGAACTACGAGTTCGGAAACG
GTTCCGTTCGCATCGACAATCGCATGAAATTTCCGGGGATGACCCTCGAGGAACCGAGGT
TTTTGGGTGCCGGTGGGGGGGGATCCGTGTGGGGCATGAAAAGAATCTTAGACCCGGGTT
TGGATCGAAGCGGGGATGGCAACGGCAATCCCGGTGGCGTTCCCAAAAACGTCGTCGTAA
AAATAAGCTGGTTGCGCAGCACCGACAGCGTCCGGAACGAATGCGATGTCTTGTCGGTGA
TGGAGCAGCGAGGGGTCACGGGCGTCGAGCGCTGTCTCGGGAGCGTGGAGTATCCGTACG
ATTCCCGCCGTGTGGCCATCGCCATGGAGCCACTGATGGAAGACGACGGCGATGCTACCA
CCTCGAGCCTGGACGACCTTACACCCGAACTAGCCCTCCGGAGTGCCCTTCAGCTCGGCA
AGACAATGGCGCAGATGATCGCGGCCGGTGTGGTGACAACGGACCTCCAGCCACTCGTCT
CTAGATCGACGGGCGAGGTGCTGCTGATCGACATGACGGAGGCCAGGGTGCTGGCCCTTG
CCGAATCCGGCGACATTGCGGAGGGCGACAAGCTGCTGATCGACGCCTTTTGCACGGAAG
TGCTGGGGTTGATCCCCGACTCGCTTCTCGACGATGCCTCCGAGTCGTTCGTCGCGGAAT
TGCACCGCCTCGAAGCCAAGCCAGGTGTGCGGATCGATCGCAGGGTCAAGGACAGACTAA
GGGATCTGCCCATCACAGGGGTTGAGAAGCTTTAGTGGCGGGCTTTCGACGGGGATCAGA
AATAAGAAAGACGGGTAGGGGTGGGGCTCGAGTCGGCAGTATATTGCAAAAGATTGTAGT
CATTGTCGGGTTTGCATTGGAGAATTCTTTTCATGGTACGGTTTCGTATCGAGAAGCAGC
ATCACTGCAGTGGTATTGGATAATAGTTAGCAGTGTAATTTCAATGATCAAGCTTTCTAG
TACCAACTTGAGCAGAATACAGTAGAACAACATATAGAACAACAAATAGTGATGCATCAT
TATCTG
>comp2191_c0_seq1
TGACGATTGTCGAAAATAAATATGGATTCTGACAACAGAAGGCTATGACAATCAATCTTC
AAATCTTGAAAATTATTCCTTGCTAAGATGAACACTGCCCGGAACAGCTTCTCGGCAGCT
ACTGGTTTTGGCAGAGTCGAAGCATCCATTTTCGGTGAATCTGCCCTAATCGATCGAGAG
ACGGGACGGGTATATTGGGAATGTGGTGATTCGAACACGTTGTATGAAAGCATAACGCCG
AAATTGGATCAATACGGAGTTGAACATGCCCAAGCAACCTTCTTGACCATCGTAATCACG
CTAGCGTTTCTGAGTGTCTTGTCGGGATGGTTACAGCGAGTGATTGCCTATTATAACTCT
TCGGTGTCAACTCGATCACTATCATTATTGATACTACTAGCGGAATGGACAATACTGAAA
CTTCCAAAGATCGAAAACAAGTGGTTGGTCTTAGCCTCCTTGATCTTTTACTTTTATGAA
TCGTACAACTGCAGCACGCGACATTTCCTTGCCAATGCGATGTCGAGTTCGAGTGAATTG
GACACTTATATCGATAGTTTACGGAATGAACAGCCAATTGTGACGTGGAAGGTGAAAACA
TTTCACTATGAGCTTCGAAGAATATTTGCAATCACGACCATTATCCGATCGCTATTCCGC
AGTGTCAAGAAAACCGGTGCTGTGCAAGAGTTATCGACATCGACGTCTGCGTTGCCCTCG
TCAAGACCCCTCGACATGGGATTACCGATAAAAAGCGCGAGAAACACCCCGCCCATGTTT

TTGATTACTCGCAAGGTAGTTACGAACGAGGCTTCCGCAAATTATATGTACACCAGTTGC
AATGACAATACGATTGCAGGTGTCTGGACCAGGGCACAAACAGATTCGGATGACGGTAGT
ATTGTTCCCTTCACCAAAATCTCTTTATCGAAAGTACTTGTATTGGCAGACAAACGATCG
AGGGAACACTACTTTCAGCAGCAATCCGATTTCGTTACCAAGTATGGTCGAGAGGATGAG
TTCGCCGAATTTTCAACCCACATTGATGTGACTGGATATCGCCCTCGGCTTTTGGCACAC
AACGATTGTACACAATGGAAAACAAGAATGTTTCGAACATCTATCTTTTGGATGTTTACG
TGCCTTGGTTTAACTGTCCCCTACCGCGTCTGGTTCAAGCGCCACTGTGACTTTATTCGA
GTCACAATAGTGAAAGAAACCAAAGCAATGGGTGCAACGGATAGTTACTTAAGAAGTTGG
TTTCCATCCCAGGGATCGCTTCGGTTCAGCAAAAAGAAAAGGGATTGAACCGAGTCTTGC
CGTGTTGTGCCAGCAAACTGTATGAAAGTAGTGCAAATATTGGGTGATTCCTGTTATTCT
ACAGGCGCTTTCTAAATTTTCTACAATGTGATACTTTGACACACGTACATTTAGATTGAC
TCAATCTAAATCTAAGTTAATCGAACCATACCCCTGACCCCGTTCTAAATAAGAGATTTA
GATTGGAACTCCCAGCTTAAAAAGTATGAATGCCTCGACTACCATGGGGCAAGCATTGTT
CGTCTCAGCAGATTCCAAATTCCTACTATTACAAGTCTGGTAGTTCATAAGAAGGTTGTC
TACTGGAAATCAATGCCCCTTTTTCTAAACCTGGTTCTTCTCATTTTCAGAATAAAGAGA
TTAAGAAAAAACTTGAAGAGGAATGGCCCAATATTTTTTGGCACAAACTTAGGATTCATG
AATATTTACACTCCTTTAAGTGCTTGCTTTGAGCTGTGACGAGAGAATACATACTGAACA
CAGAGAACACACTGAATCCTGAATATCCAATGAATTCTGAATATCTACTGAATAC
>comp2192_c0_seq1
CGCAGCAGCGGAGTCGTAAAATCGCAAGCAGTTCTTACCGTCGCCACCGATTATCATTAA
TATCGGCAGCCCCATCTAGCCGAAGCGTTTCAAAACCCCAAAATTCGATACAGTCTTCCT
CCACTATGAGGTTCTCTGGCAGGCAGATTTTCTCGGCGGCTCTCTTTTGCACCGTATCGG
ACGCCTTTGTCCCCCGTCGAAACAATTTCCGAGTGGTCGCGCCACAGACGGACGACCATG
CCGCACCACTGCGGGTCCCTTTTCATCCCGATTTTCAAGTGGGAGGCAGGGAACCCATCG
TTGCGCTACATGCGAAGAAGAAGAAAGGAAACGCTAAAGCGGCAGCCCTTGACGCGCTCG
ATGCCCTGGACGATGACTTGAATGCCCCCATGTCGAAAAAAGAACTAAAAGAATTAGAGA
AAAAGAAAAAGAAAGAAGAGGCCGCCAAAGCAGCAGCAGCTGTTCGAGAGGAGAAAGCAG
CCCCGCCTGCTAAACCAATGAGCAAGAAGGAAAAAATGCTTGCTAAAGCTTTGGAGCTTG
AGATGATGGATGAAGCGGCGGCTGCTGCTAAGGGAGAAGAGGACGACAAACCAAAGCTCT
CGAAGAAAGAGCTCAAAGCCCTGAAAAAGAAGGAAGAAAAAAATGGCAGCCAAAGCGAAGA
AGAAAGCAGAAAAAAATGGCAGCAAAAAACGCGGGCAGTGAAGGAGAAGAAGGAACGAACG
GTGCCGTCAATGGAGACGCAGCGCCAGCCGAGCCTGCTGTAGCCGTTCCCGAGAAGAAAG
TAAAACTTACTCTCGAAGATAAGATTCGCAAGGAACGACCTCCTCCTCGAATCCGAGTGA
TGGAAGGTGCTCAGCCTGGATTTGTGTCGCTTCGAATGGAAGATATTGGAATTACCTTCA
GAAACCAAGAAGTTTTGAAAGACGTGACTTGGGGCGTCCAGTCCGGTGAAAGAATCGGCC
TTGTCGGTAAAAACGGGGCGGGAAAAACAACGCAGCTGCGTATTATGGCGGGAGAACTTG
AACCGACAACAGGTGACGTAGTCAAATCTTCTGGTGATCTTCGAACGGCAATGCTTCGTC
AGGAATTCGTCGATGAATTGGTCAAAGAACGCACCCTTAAGGAAGAATTCATGAGTGTAT
TCGCAGAAGAAAACCAAATTGTTCAAGACCTCAAAGATGCCGAAAACCGACTCGAAACCA
TGAGCGACGCTGATCCTGAAGAGATGCAAGAAATTCTAGATAGAATGCAAGATTTGCAAC
AGAAAGCTGAAGACAAGGATGTCTATGTTCTGGAGAGTAAAGTAAAGAAGACTATGGACT
TGATGGGTTTCAATGATGATGAAGCAGATGATCTCGTTGCGAGCTTTTCTGGAGGCTGGA
AAATGAGGATCGGACTCGGTAAAGTTTTACTGAAGGATCCAAATGTCCTACTACTTGATG
AACCCACAAATCACTTGGATTTAGAGAGTGTTGAATGGTTAGAAGCATTCTTGCGGGAAC
AAAGCATTCCTATGATTATTGTGAGCCACGATCGTGAATTTTTAGATCAAGTTTGCACGA
AAATTGTTGACGCAGAAGGGGGTATTTGCACAGAGTATGACGGGAACTATTCTCGTTTCC
TTCAGTTGAAAAAGGCTCGCATGGACTCCTGGCAATCAGCATACGATGCCCAAGAGAAAA
AAATTAAGTCAGAACGTCAATGGATCAATAAATTTCAGGCAAAACAACCTCAGGTTGTAA
AACAGAGAAAAGACAAGCTAGAGAAGTTCATGAAAAGTGATGAGTATGTGCAAAAACCTC
CATTTACGGGCAAGCCATTTAGATTCCGTTTTCCTCCTGCTCCTCGATTGAGTCCTGAAG
TCGCGCAAGTTGATGAGATGAGTCATTCTTACGAAAACGAAATTTCGAACAACCGTCTGT
TCGACAAGGTTGATTTATCTATCGAGAAAGGGGATCGAATTGCAGTCGTAGGACCGAACG
GAAGTGGAAAGAGCACATTTCTTCGTCTTTTGATCGGAAGAGAAGAGGCTGACGAAGGCT
CTGCTCAGATTGTTGGTCAAAATGTAGAAATGGCTTATTTCGAACAAAATCAGGCTGATG
CATTGGACTTAGATAAGACTGTGATTCAAGTGATTCAGGGTGCAAGCAACGGACAATCAT
ACAACGAGCTCCGTGCCCTCCTGGGACAATTTCTTTTCAAAGGCGATGACGTAGAAAAGA
AAGTGGAATTTTTGAGTGGTGGTGAAAAGGCGAGACTTTCTCTCTGTTGCATGATGCTTC
GATCAGCAAACCTTCTCATCTTAGATGAACCGACGAATCACTTGGATATCCCTGCCAAGG
AGATGCTTGAAGAAGCACTTCAACACTTCGATGGGTCAATCATGGTTGTTTCGCACGATC
GATACTTCATTTCCAAAGTCGCCACAACAATTGTCGCCATCGAAGACAAAAAATTAACCA

AATATCAAGGGGATTACAAGTTTTACATGGAGAAAAGTAAAGCTATCAGAGAAAAAATAG
AAGCCAGGTACGTAAAGGGTCTTGATCGGATCGGCAGCGCACCGGAAATTAATCTCGAGG
AGCTCATCAAGCCGAAGAAGAAGAATTTTGGAGGAGCAAAAACCGCAAATCAGGTCACTC
GAAAAAATAAGGGTGTAAAAAATGCTAAAAGACAACAGCAGAGATAATTCAAGTAGTAAC
AACGAAAATGTAATCAGATCGCAGTTTCTACCCAACCGTTTGATTCCCATAAGTATATTA
AGGGTCGGGAGATCGATATCCTCCAAGACAAAAACAAACCATGGGATTGGCATAACTAGT
GAACCATGGAAGCCAAGAAATCTCCTACACTGAAGAAATATGAGATATCAACGTCTCATA
GAGAGTAGTAGAAAGCATATCCATTGCGCACTGCTGGTATCTCCAATAGACAATCTTCTT
CATAGATCATCTGTGACGTATTGTATGTCAGAGATTGATTTGGGGTCACCCACAATCCAG
TATACAAAATATATTGTTTATGCATAGAAATACCATACATTATGTAACAATAACG
>comp2193_c0_seq1
GACACATACAATCGAACAATTATTTATTACAATACCATGGGATCTACCAAAGCAGCACGA
CCTTCCGTATCCAAGCCGGCCCACCAGAAGTCGTACAGAAACGTCGACGAATCAGGTGAA
GAGGTGAAAGGGTTCTCGGAATACATGTTCGAAGCTCTCCATCACTTCATGATGTTCTCA
TCATACATTCCTCTCTTCCTCGGGCAGTTCATCGAAGACTTTCAAATCGCAGCGGCAGCT
TCCTTGGGTGTGGTTGTTTTCAATGCCTTGCTCTCCTTTATGATGTACAAGCTGGGAAAA
ACCCAGAGCTGGCCAAAACCACTTGATATCTGTTTTTTCGTTCTCTTCGGCATCATGGTA
ATTGTCGTGTTTGCCTATCCGGCTTCAGCCGACTTCTGGAGATTCTGGCAGGGTGTATAC
TTCAACTTTGGTTCTTTCCTGTTTCTCTCTGTGATGTGGTGCTTCGGGATGCCCTTCACC
GAAACTTACGTCGAGGACGAGTACGGCAAGATCGGCGTCACTCATCCCGTCTGCCAATGG
GGTGTTAAAATTAACTGTGGCGTTTGGGTTGCCTCGTTCTTCATTTTGGGTTGCTTGAGT
TTGCCGGCTGGTATCGCACAACAACTAGGAAAACCGAGCACAGACCTGTTGGCCATTTCC
ACCTACGGAACCATTGTTGTGATGGTTTCGGCGCTTTCATTTTCCTTCATGTTGTTCCCT
TGGTATATGGAAAAATACGAAGACCCGATTTTCGACAAGTTCATGGATCAAATTCTTGAA
TGGAACGCTAAGCATCCGGACGAAGACTGGGCTATTGAATTTGAAAAAGCCATGGCAGAA
GAAAAAGCAGCAGCCCAAGCGCAACTCAGTCAGAATGTTTGAGGATCCCAACGGTTTTTA
CGTCAGTTTGGGGCGGACCTCGAAGACGATCTTCACCAACGAATCAAACCTCATCTAGTC
AAATAATGTATTGATAAAGCATCT
>comp2194_c0_seq1
CAACCTGAACATACTTGGACCAAAAACACCCACGACGCAGAGATCCCCTTTTCAAGTACA
ACATGCCCTCCGACAGCAACTACTCCGCGGTGGCGAGATACACCCGCCAGCCCCTCTCGA
AGGCCGCGAGGCCGGTGGCCCTTCACGTGGTGGACGAGAGCTGGGCTTCCGACACGCTGA
GCGACGACGAGGTCGAGATCACGCGCCACGAAGCGGCGGTTGCCCCCCTGCTGGAGGAAG
GCGAGCTCGACGCGGACGCCATTGTCGTTTCGTCGACGGGGAATTCCGCCTCGGCCGCAA
ACAACGCGGCAGAGAGACGAAGAAGAGAGGAAGGCTGGAATGACCTGGGACTGGATCAGA
TTGACGCAAATGGAGTGGGTGTTAGTACCTCTGTACCTTCCCGAATGTCTACGCAACAAG
AAGGATAAATCTCATATATGTTGCGGCCTCTCATTGTTCACTTGTTTACACAAAGTGTTG
AACAGCTATGCACGATCTGCTTCGAATTGCTTGGCTTTCCGCTCTATTTTGCCTACTCCT
CAATGGCTTTGCCATGCACCTAACAATTTAAACGAGAAACAAAATGGCATTTCTGTGATG
CCTCTCTAGGCGAGCGGCGACCAGGATAATACAATGTGCTAATTTATGAACTAATTCAGT
GTCATGACGATGACACATGGCACCAGAATGAAATGCAACATCGTTGATTTACATTAAATT
TACAGTAGATCTTGAACACGGTTGGAGAGAACAGCATCGTGAATATGTCGGTTGGAGAGA
CCTTGTTTCTACTTTCTTTTTTGTTGTGTTTAGCTAGAAACGTTCAAGTAAATGCGCTCC
CCTGACAGAAACCTATCCATACGATTTATCTCGTTCAGAACCCAATTCCTTCTCGATACA
GTCAGTATGGCATGAATCAGGATAGCGATCCCTGTGGTCAAAATATTATGCTGCACGATT
GAAGTGGATGGTAGAAATATCTGATGATTCCCATTTCATAATTATTTGTGCATTCCAATT
CATGGACTTTGTTCTCAACGGTGTGCTTTACCCACCAAACAAGCATTTACAAGATTCCTT
TAAGGACCAAAAAAGTTCTTTCAGCAGTATCTCCAGGCAACCTGAAACAAGATTTTTTAT
ACAGGGATAACCAGAATATGTTACCTTGCGAGTATTATGATAACTGAATGGTTAACGACG
ACAACAAGAACGAGGTCTCACTTTTTACTTGTTACAAGAACGAGGTCTCACTTTCTACTT
GTTACATAACAGCATTAAGCAACGATTGACTTGGGA
>comp2195_c0_seq1
CAGCAACAGCAAAAACAACAACAACAACAAGGAAATCCCCTGGCCGTGGACATCGAAACG
GAAACCAAGCTCACAAAGGCACAGCAGGAACTCATCGAGGAGCAAGTCGAAGCCGAAGCC
GAAGCGGAATACGGTCCCGACCCTAGCGGCGATGGCGACCACCACCACCACAAGAGCATT
CGCTACCTCTCGAAATCCCCGGGGCAGCAGCACTTTGAATACGACGAGGCCTACCTGGCA
CGCGACTACGACGCCAACTCGGAAATCGTCGACGTAGACCCCTCGCAGCCCCTGTGGGAC
GCCAACGGCCGGCGCCTCAGCCCTCCCCCCTACGACCCCACGGGCATTGCCTGCATCGAC
GCCGGAAAGATCCGGAGGTTCCGGACGGCGCCCGAGGAAGGCAACAAGGACTCGCTCTAC
CCCACGACCTTTGGGGTCATTGGAGACATTGGGCAGTTCGAGCACTCCCAGCAGGTCCTC

AACCACATCCGGGACCACCTCCAGGGGATCCGGGCCGTGGTGCTGGTGGGGGACATTGCC
TATCCCGACTACGACGGCCGGAAGTGGGACACCTTTTTCGACTTTTTGGACGACCAGAGC
AACTTTGACGAGGTACCCCTCATGGTCGCCGCGGGGAACCACGACATTGACAAGCAATAC
AGCGGAAACGAGATCTTTCTGGGATACGAGCACCGGTTCCGGATGCCCCGGGTGCATCCC
CCGGAACTCGGAGTGTTTGACGGCGACTGCGAGGACGGAGCAATGAACATGGACGCTCCT
CCCTATCCCCTGCCCTACGAATGGGGGAATTCCTACTATGCGTTCACCTACGGACCGGCC
CGGCACGTTGTTGTCAACGCCTACGCGGACATGTCCCCCGGATCGACCCAGTACGAATGG
CTCGAGAAGGAACTCGGTTCGATCGACCGGGAGGCGACGCCGTGGGTCTTTTTGACGATG
CACCCCCCGATCTACAACACCTTTTCCCTCCACCACAAGGACCCGCAGATCTTTGCCGCC
AGGGAACACCTGGAGCCGCTCTTTGTCGAGCACCACGTCAACCTCGTCCTGACGGGGCAC
ATCCACGCCTACCAAAGGACTGCCTACGTCGCCTTTGGAAACCTCACGGACACGGGCCCG
ATGCACGTCACGGTCGGCGCCGGCGGCCGCAAGGCCGACGCCCCCTTTTCCAGCAGGGAG
CCGGAGGAGTGGCTGGAGGTCCGGGACGCCTCCATGTACGGGTACGGCCGTCTCACCGTC
CGCAACGAGACCCACGCCGACTGGAGGTGGATCCCGATCGCCCCGTCGGATCTCCACGAC
TACAACCAGGTCCGGGGGGAGGACGACCTGCACCTGCCGTCCCTGGACCACGACCGGGAG
GTCATTGTCAACCAGTACTACAGGCACCTCGAAGGGACGGGCTGAGGGAGGGGGAAACGC
ACACGCAGGCCGGCGAGCGGCGCAGAGGACGCCACACGAATGCCCTGCTGCTCCCGCCAT
CGCAGCTGCAGCTGCACGTGCGATAGATGGGGTACGCAGTGTTTGTACCCATACCAAAAG
ATACAGTATAGTGTTATTATTATTATAGAATTCTTCTCTAGACGG
>comp2196_c0_seq1
ACCAAACCAAACCAAACCGAAGCAAAACAAGCGACACCGACACCGACACCATGCCCGACG
CCGCCCCCACCGCCATGACCACCCCCCTTCCCTACTCCATCTTCACGAACGGATCCGCCT
GCGGCCCGGGGGGCGTCGAGGCCGTCGGGGTCATCCAGTCCCTGGTGGTCGTGGACGACA
AGAGCCAAGGCGCAAAGGCCCAAGCCCCTGCCGAAACCCAACCCCCCGGGCCCGCGTACT
CCTACTTTTGCGAGACGACCACCCACACCCACACCGACCCCGATAATGGCGGAACCATCG
AAACCACGGTCTACACCAAGGTCGGGGTGACCTGCTCGCCCCTCGACCAGGGGGACCTCG
CCCTGGACGCGGTGACCTGCGAAGAGGCCGGCTGCGGGAGCTGCGAGACCGCCTACCCCG
TGGCGGCGACCCTTGCGGTCCCGGTCTTTGCCCCGCTCCCGGCCCCCGATGCCTGCTACG
GGATCACCGCGGACGCGACGGGGGTCACCGTCCTCAACCGCTTCGACGCCGGAGCCGACC
GAGAAGGCATCGAGACCTACTGGGAGGTCTACAAGGCGAACTCCTGCCTCGGGGACCTGG
TGGAGGCCGCGAGTGCGAGTGCTTCCAGCGCAGGCGAAGGTCGCTCCGGTTCCGTTGGAT
CCCTGGTTGTGGCCGCCGCGCTGGCGGCCTCCCCCTTTCTTCTCAACTAACTAGCGGGGT
CCACCCACCTGCCCACCCACCCACCCACCGATGGATCGGACGAGCGAAATGCACCGATGG
AATGATATGGAATGGAATGATATGGAATGCCCACCCTCGGCCACGGGCAAAGCGGACAGC
GGTGGTGTCGGCGGACGAGAAACCAAAACCAAACACCACGGCAGGACCGGAACGAAACGA
TCGCTCGATGGCGTCGAATCGAATCGAATCGAAAAAAATTGACTCGAAAAAAATCGAATC
GAATCGAAT
>comp2197_c0_seq1
GTTCTACAAAACCTGAGTTTCACGATCGACGCAATCGGTGTTGGATGTAACGGCTCAGTA
GGAGAAAGGTGTCAAGCATCGCACAGTGACTAGAGATATGCCGACTGAGATTTCTATGTT
GACGCAGTCAAAGCAAGAGTTGCCAGACCGCGTCATACTCAAGCCAAGAACATTCCCAAA
CGAGTGAAAGATCATTGGGTGTTGCATTGCATGGCATCGCTTCGTCCTCTCATGCGATTC
ACAAATTAAAACTCAACTAGAGAAAAAACAACTCCATCAGAAGCAAACATATACAACTCC
TTGAACCGCACCACCCCAACATGAAGTTCTCTACACTCGTTCTCGTCACTTTCGGTTCCA
ACGCATTGGCGTTTTCCCCAAGAAGCAACGACATCAAGGCAACGAAGACACAAGCGACTT
CTGTCGCGGACAACGAGCCGATTCGGCAACCTATTTTCGACCCCTTGGGCCTCTATCCCA
CCAACTCGCACGAGCGCCGTTTCGGCATGATCTCGGCGCTCGAACAGCCGCTCGAAAAGG
ACGAGACCGTGATCGACCCCCTCTCTCTCTACAAAGACGAATCACAGGTGACCAGGGACG
TCGAGATGTCCCCTTCCTTACCGTTCCTGAAACGCCCGGAGCACCTCGACGGCTCCCTGC
CGGGGGATCGAGGCTTCGACCCCTTCAACTTTGCGTCGGACCCCTCGGCCATGGCTTGGT
ACAGAGATTCCGAGATCAAGCACGCACGCCTCGCCATGCTGGCAGCCGTCGGATGGCCCC
TCGCCGAGCTCGCCCACAAATCGATTGCTACGCGGTTCGACCTGGATCCGGTCCTGGGGA
TCGGAGGAAGGGTCCCAACCGTCTTGAACGGAGGCCTGGGCCTTACGAACCCGCTCTTCT
GGGTGTCCGCCATTAGTGCCGCGGCTGCCCTGGAATTTGTTGCGACAAAGAACGGAGACA
GCAGAGAGCCTGGCGACTTTGGATTCGACCCGCTGTCACTTGCCGGAACCAACGGCCAAC
AGAGTGTTTTCGTGCGAGAAGCAGAGATCTTTAACGGGAGACTGGCCATGCTTGCTATTA
CGGGCTTTGTTGCTCAAGAGTTTTACACGAACACGGCAGTGGTGAACCAAACACCAATTT
TTTTCAAACCGTTCAGCGACGTGGTTGCTCATTTCCTAGGAGCCGGAGGTGGCAGTATTT
GAAAAGGAGCTCTCAGCAGAGTGTGGCAATGTTGTATGGTAATAATAGAGAAAATTAATG
ACGAAATAGACGAAAAATCGAAT

>comp2198_c0_seq1
CGGCGATAGATTAAGTCCTCACGTTCGACTCCTAACTAGCAAATATCACAAAACAATATC
ATCCTACCATTGTACATTTACCATGAGACGCGTTTCTTCTCGCCAACGTTTGGTATCTTG
GGATGCTTTCGATGCAACCGCTGTTTCTGATTCCGGAAGCGGCAATGACTCTTCGGTCGG
GGGATTTGAGGAAGATGATCGATCGTGTGCTTCTGGATACGAAACTGCGTATTCACGATC
CTATCACTCATCGATGGCTCTGAATATGCTCTGCGAAGGAAATCTTTACATCAGCAACAG
CATTGATAATACAAATAGCATGGAAGAAATAACGGGAATCGACGCGACGACAGAGCTAAG
AAGGCGAAAAAGAGACCGGAAATCGTCGCGTTCTTCACCTACTGAGCGTGCTAATAGTTC
CAAGATGGTCGAGACACCAAAGACTTTTGTATTCACAACTCTCTCCACGTCAAAATCCCC
AAAACTCGTTCCATCGCACTCCTGGGAGACTGGCAAACCGCCGCCGCCGTCGTCCGTTGG
ATTCGATGACATGATTAGCGATTTACACCTCAAGATATTCTCGTTCCTAGATTTGTCATC
ACTGCGATCGGCAATGTATTCCAGTCGCCACTATCGAAATTTGATCATCTCCAATGATGC
AAGGAACAGTCTCTGGATGGATCATTGTGAAAAAATTTGGCACATTTCAGAATTTATGGA
AGAAAGTGGACAGAATCAATACCATTTTCAACCCCATCCAAAATTCACGGACAACTTCCA
TCTACCAATAGCTGCTCATACTAGCACTGTGACCAATTCCACTGGATTTGAAACTGATCC
AGTAGACACCACAAATATTTCCTTGCTACTCAGCTTGACACCAAAAGATTTTCCTACCTG
TGTCGACAAAGATGCTTTAAAGTCACGAATGCAGCTCAGCGGTACCATTCGGCAAACTAT
TCCAGTTGATCGACAGGACGACGAGGACCAACAGGTTCAGTATTATAAAGAACAATCGAC
GGGTCGTTCTCTCATTCGCTACACCGGATACGTTGGCCAAGGTGACCGATGCATTCGTTC
CAATCGTCCTATTCCAAGACCATCACAACGAGCTCGCTTAAACGAGACCTTTGAAAGTGC
CAAAGATATAAACGAAATCAATAGCAGTCGGAGCCTAATGAATTCCATTGGTCCGTCGTT
TTTGCACATTGACGACTCGCACCACCCATTGTTGTACAGTCTTCTACAGAAATGCAGTTC
CAAGGCCATGAAGGGTGATGGCATGAAATCTTCCAAGTCGTCGTCATCGTCAAACTCATC
GGTGCTTTCGCCACCATCCCCATCGCTCGCATGGACCCCCTTTGTCGTGCCGTTTGTCGA
CAAGAGCAGCAGCGACGATAATAGCACGTTCCTGAACGTAACTCCTCGTTTCATTTCGTA
TTACGAAGTGAGCATTTTGAAACAGGATGGAGGTGAAAACCAGGCCGACTTTCCAGCAGA
ACGAATGGTGCCACCACCTCTCCATCGAAGGAGCAACATGGATTGTGTAGCAGTTGGTCT
CGGAACGAAAGACTTTAACAATCGAACCAGAATGCCCGGGTGGGATCGACAATCCTTTGG
GTATCACGGTGACGATGGTGGGATTTTTCACGCTTCTGGAGGAATGCTGAAACAATACGG
ACCCAAGTTTGGTCCTGGAGATACGGTGGGATGCGGTGTCGATTACATTTCAAAGGGAAT
ATTTTTTACATACAACGGCAAGTTTCTAGGCTATGCATGGAACAACGTCAGCGATGATAT
CTTAAATAATGACCTATACCCGGTGGTGGGACTCGACACCAATTTTGCGGTCCATTTGAA
TTGGGGAACAAGCGGCCCGTTTGAATTTGACCTATCCAAATTCATTAAAAAACATGAGGT
AGCGGTCATGGCAACATATTCCCTGGATGGGTGCTCGTGCCTTTGTCCCCGTGATACTAC
TGTCACTGGCGCAACCGCAGCAGAAACAGATTCTTCTACGAAGAAGGTTGCCCGATCGTT
TTCCGGCCGTTCGACGTCGAGACGACATCGAAAGGGTTTCATTGGGAAACGCAACCAATC
AAGATAGTGTGAATGGAATGCCTAAATCATTTGTTTCTCTGAAGAACAGTCGTTGTCAGT
CCGTAGTATAGTATGTGTGGTAGTATTGGAAAAAGAAATGTAAAACAAACAATTTCAGTA
GTGTGAAAAAAA
>comp2199_c0_seq1
CAAAGACTTGACACAATTTCATTCCCTTTGACTACCAGACAGTAGATACCCAATTTTGAA
ATCATGCGCATCATTTCTGCCGCCCTTCTTTCGCTTGCAAGTGTCAACGCATTTACACCT
GCCGTGCTCCGAACACACACCGGATTCCAATCTACCGCTCTTTCAATGTCTACTGTTTCG
CAAGACGAACTCAAAAAGCAAGTCGGCTACAAGGCCGTTGATGACTATGTCAAGAGTGGA
ATGGTTGTCGGTCTCGGAACTGGTTCCACCGCCTACTTCGCCGTCGAACGTGTGGGAAAT
CTTTTGAAATCCGGAGAGCTCAAAGACATTGTTGCCATTCCCACCTCGGAACGCACCAAG
GAGCAAGCATTGAGCCTCGACATCCCCCTTGTTACCCTCGACACCCACTCCAAGTTGGAT
GTCGCGATTGACGGAGCCGATGAAGTCGACCCCGATTTGAACCTCGTCAAGGGAGGTGGC
GGTGCCCTTCTCCGAGAAAAAATGGTCGAAGTCATGGCGGACAAGTTTATCGTCATCGTC
GATGAATCCAAGCTCTGCGATGGATTGGGACCCGGATTCCCCGTTCCCGTCGAGATCACC
CCCTTCTGCCACGAGCACACCATGAGACAGGTCGCTGCCCTCGATTCCTGCGCCGGCTGC
AAGCCCGTCCTCCGAATGGGATCCTCCAGCAGCAACCAGCCCGACGGGGACGAAATTGCC
GTCACCGACAACGGAAACTACATTGTCGATCTTCACTTCGAGGAACCCATCAAGGATGCC
CGAAAGATGGCCGCCGATATCAAGAACGTGTGCGGTGTCGTCGACCACGGACTCTTCTGT
GACATGAGCACGGCCGTGATCATCGCCGGAGCCGATGGAATTTCCGTCAAGGAATAAACA
GTCTGTACTGTATACTTACTACGAGGAATCGCCCTGCCGCTCAATATTTTTTGGAAGAGC
TCTTGCATTTTACCTCGAATCTTCTGGACCACGAGCTACTAGATAGGCGCAACGAATTCT
TTCGGGCGAATCGGTATGCGAACCAATCGCAGACACACCTGATCTCGGGCGCAATATTTT
GAAACTGAATTTTAACAAACTATTTCTGAGAAATCCAGCAAGCTAGGTCGTAGATGCGAT
TTCAATACTAATAAATGACACGTG

>comp2200_c0_seq1
GCCTGTTTCATAGTATTGCTCCAATCCATATCACCCCTTTGGAAAGTATCATGTCCTCCC
CGTCGTCGTCTTCGTCGACTTGCCACGGACGCCTGGCCTACGGTGTCCGGCGCCTTCGCC
CGGGTGCCGAACCTTCCTTCCAAAAGGATTTCGAGGCCTGGCTTGCCACACATAACGAAT
CGCGCCTCGCCGCGGCACTCGCCTTCCCGGACCCCTCCGATGCCCATCTCTTGCATCTCT
TGGTGTGGGACGACAACACCACGGAGGAGGGTGCAGACGATGTTCTACTAGGGCCCGAGA
GCGACGCCTTTGAGGAGGAGGACGCCGATGGGGTCCTTCATGTCTACGGAAGCGGAAGTG
CCACCGGCGGTGCATCAAATAGCACGTCGACGAACCGGAAACTGCCCCCCGTGCACATGC
AAACCCCCTTGGCGGGGTACATGCGACGGAGATTGCGCACCACGGCAGCCTCCAAAGATA
CTGGGAGTCTCCTCTTGATTGGCGTGTTTAAGCGTGCCATTCATTCCGGGAAACGCGACG
ATCTGGCACAAGCCTTTCAGCGCGTTTGCGATATTTGGTACAAGACGGTGCCGGGCATTC
TCGCGGCCATGGTCTTTCCCGACCCGACAGATACTGGCTTTGTCCACGACATTCGCATTT
TTGACGATAAAGCAAGCTACGACGCGCATGTGGACAAGTCCAACCCGGAGCTCACCCGTG
CCATGGAGGAATGGTTCTCCCACTACGATACTTCGATTCCGCACAAGGGGATCATGGTGG
CGGAGGACACCTCGGATCCGGCCATGCACACGAGTTCCATCAAGAAAAGACCGGTCAAAG
TGGACTTCAATGTCTTCCACTACGGACAAGGGGGTTGTATGGGACGATGCCTGCATGTCA
CATTTTGACAGAATATAGCAGCTGCAGATGTGTAGTGCGAAGCATTTCCAGCAAAACGAC
AAGTGCAATACTCAATTTATTTTGCAAAGAACCCTCTTTCATTCTTGTTGGACACTCCGC
ACTCCAAGCTCAATCTCTGGTGAAAATAACGCGAGTGGAATCAACAGGAATACCAGCAAC
ACGCATATCAATTTTGCAATTGCTTTTGTTGATGGATCTTCTTCTTGTCCAAGAACCATT
GAATTTACGCAATGAAGAGCGGACGGATAAAAAAATAAGATGGGCCGTAAATGTTTTGGA
ATATTCTTGCGTTGCAGTACGTGGTCATAAGCTAC
>comp2201_c0_seq1
CGGCAGCTTTTTCTCGCAGAACACCACCCTCAAGCTCTTCAACCTGGAGGGGAATTTCGT
CCACGACGAGGGCGTCCAGACCATCGCCGACGGGCTGCAGGCCAACCCGGGGGGGACCAA
GCTCGAGGAGCTCTACCTGGGCTGGAACGGGATCGGCGACGAGGGGTGCCTGCACCTCGC
GCGGATGATCGAAACGAACTCCACCGTCACCAAGATGGGGCTGGGAGAGAACGACATCAC
CAGCATCGGGGCACGCGCCCTCCTCAACGCCCTGGCCCAGAACCAGACCCTGCAGAGGAT
CACCGGCCTGTACCACAACATGATCGACCGCAAGTTTGTCATCGAGTGCATCAACCGGAT
CCTCTATTCCCACCTGGAGACGCTTCCGGCCGAGCTCGAACAGGTCCGCCGGGCCGAGTT
CGAGCGCCTGAAGCTGGCCTCCATGAGTGCCACCGATACGCTCCTGGAGCCCCTCGGTTC
GCACCTGCCCATCCCGGACGAGATTTCGGAAGGGGAGCTGGACTGGGCCGATCGCCTCTA
CAACGACGAAACCGATCTGGTGGAAAGCGGGGTGACCATCGGAGAGGTCCCGAGGGAAGA
GACGCCGTCGGCACTGGGGCAAGTAGACACCGAAGAGGGAGACATCGACGAAGCCGCCGA
GCCGAAGACCATCGCGATGATGAGCCAGTTCGGCAAGATGCCCGAGCCCAAGTCCCTCCT
GGATCGTCTCGTGGTCTTCCAGGCCGCACCGCTGGTCTACCTACACCGGGAAACCGCTTT
CCGCCAGGCGATCCCCCTCCACGATTTCGATCAGGAGACCGCCATCATCCAGAACTCTCT
GAACGGCTGTTCCAGGCTGGACGCGACCATCGACATGGACGTGCAGCCGGCCACCGCGGA
GTCCTTTGCTTCCTTTCTGGCATCCGGCCGCGGCCGCGTGCTGCACTTTACGGGGTATGG
CCACCCCAACCACCTCCTTGCCCTGGAAGACGAGGCAGGCTACCTCGACGACACCTTTTC
TCTGGACACGCTGCCCGACCTGATCAAGTCCGCCAATCCCCCCCTCGACCTGGTCGTCGT
GAACTCCTTCCACTCCGGAAGGATCGGAAAGGCCTTTCTGGATGCCGGCGTTCCTCACGT
CGTGTGCTGCCACCACCCCGAAACCTTCCGCGACAAGGCGGCGAACAGCTTCCTCAAGAA
CTTTTACCGTGCGCTGGCCACCAACAAGTCCCTCAAGCAGGCCTTTTACCACTCCCAGGA
GGCGGTCCGTGTCGAAGAAATTTCCAAGGACGTGGATCGGTACGTCCTGCTGCCCCGCAA
GCCCGAGGACGATCCGTACCACGACGTGCCGATTTTCTACACGCAACCGGTTCCCGCCAA
CACGCTGGACCAGGACACGGACAATCTCCTGCTCGAAGACGGACGAAAGCTCCTGCCGAA
GCTGCCGAGGCACTTTACCGGACGGGAGGTCGACATGTGCAAGGTGCTGGATGCCCTCCG
CAACCAAAAGCTCGTCAGGATTGGTGGAATCCGGGGGATCGGAAAGAGTTCCCTGGCGGC
AGCGGTGTGTCGATACGTCCAGAAACGCTCCGGGTTTTTTGAATTCGACAACGTGTTCTG
GCTGCCCGCGGCCCGCGGTGTGGTTCCGGATGAGGACACGCTCTTCGCGGACCTGGTGGA
ATACACGAGCCTCATGGTGAAGGCCGATCACAACATCTCCGAGGACGAGGATGCGCTGGA
ATGCCGAGAAAGAATCGAGATCGAGCTGGAAGGCCAGAGCACCCTGCTCGCGATCGATTC
TCGAAAGTTCGACAGCGAGGTGTCCGCGGAGTGCCTCGAGACCTTCCTGAGCGAGCTGCT
GAACACGGACCTCGAGGTCAAGGTCATCTTGATCAATTCCAACGAGGAGGACGAGGAAGC
AACCCTCCGGCTTGCTCCCATCGACTTCAAGTCGACCTCCCTCTTGTTTGGAATGGTGTC
GCGCTTTATTACGGCCAACGGCTGTCCGGCCGCGCAATCTCCGGACGAATACGCGGATCT
GATCGTTCCCCCGTCGATCGCCATCGGCGAGCCACCAAAGTCGGCGATCTCGGTTCGGCA
GGAGCGTCTGCTTTCCGTCATGGGCGACGGGATTCCGTTGCAAGTCATCCGGAGCGCGAA
ATCGATGCAAGCCAACATCTTTATTCGGTTGATCAGCATGGCGAACACGCCGGAAGTCCA

TGTGGATTCACTGGATAGTCTCGAAGTATCCATCTCCAAATGGACATCTCGCATGGAACA
CGCCATCGAGAACAAGAATTTTTTCCGTGCTCTCGATCTCCAGCACGTTCTGAAGGAGCT
CGAGGGACAGCGGTCCAAGTTCCCGAGCGTCGACGATCTGGTGGCCAGGGAGAAGGAGCT
CCACCGAAAGCACACGCTTTGTTTCAAGACGAGACGGTACGAAGAAGGCAACCGCATCAA
GAGGGAAATCCTGGTGCTGAAAAAAGAGATCTTGCGACAAAAGAGGAGGCTGGCCACCAA
TCGCCACATCACGGACGCGGCTCTCACGCAGTCCCAGACCAACAAAATCGCTGACATCCA
GGCGCAGATGAAGTCCATCATGAAGCTCGCAAACTCGTCATTCAGCTCGCTCACGGACCT
GCAGGAATCCCCGGACAAGAACGAAGCCGTGTTCCGGCTCGGTTCTGCCTACCACAGCTG
CCAGGTCCGGATCTACCCCGGAAACGTGGAAGACTTCGATCCCGGCGACGATCTCGGCGC
GTCGATCTGCTGGACGAACGAGTGCTGCGACATCAACCTCGACGAGCAGGGGGCATCGCT
GCTGAGCTACGGCGGCCAGAACCTCGCGGACGACGCTGAGACCCTGCCCGCCATTGCGGA
AACACCCTGGGGAACCACCAAGTGCGGGACCGGAAACGCCGTGATCGTCGGGCCCGGGAA
CTACGACGACCTGCGGGTCCACTGCGTGATCCTCGCGGTCGGTCCGATCTCTCCCACCCG
CGACGAAGTCCTCGATGAGAGCGACGAGGACTCCCTGCACTACATCACCGTCATGATGCG
GTCGTGCCTGCGCTCCAGTTTCATCCTGGCGAAGCATTCTCAGGTGCAATCGATCGCGGT
CCCGACGCTCACGACCAAGGTGGGGAGCCCCACGTACGAGTCGACGCTGCTAACGAACCT
GAAACTCCTCGTCGACGAGGCCAAATTCTCGGACCTCAACACGCTGCACATTGTTGCGAG
ATCGGACGAGGAGTCTACAAAGCTGATCGACATGGCGCTGACGATGGGCCTGACCATGTC
GTAGTGAGAAACACGGGACGAGACACACTGCCACAACCACTAGGGGAATTACGGTTAAAC
ATTTAACATTACACACAACAGTATTGCGACG
>comp2202_c0_seq1
GTTTACGTATCTGTTTCATTTTCGGCTCGATGATAATATCGGCACCTCAAACATTGGCTT
ACGATATTTTTATGCTTCATCGCTTTGTCATTTTCTCAAAGCTACGTCCGAAATATTTCG
AAAAATGAGTGGTGGAAGATGGACGATTCTCGCATTTCATTGGTATCCACCGAAGAAGTT
TTGAGGTCCGAAGCTTACATGCTTTTTTACAGAGTTGTGGACCATCCGTACAGCAAAAAG
CTGGCGAATCAGGTTAAAGCATTGAACGATTCTTACGATGTATCGGATTCCTTGAAAGCC
AAAGAAGAGAAAGAAGCTGCGGTTGCCTCTCGAAAGAGAAAGGAGAAGGAAACGAAGGAC
GCTTCAATCGAAATAGAAAAAAATTCTGCCGACGCAAGTTCGAAAAACTCTTCTCGCCTG
TCCGATCGTTCTATCGGATCTGGCCCCGCCGCCAGTGTTAAGAGAAATCACAGGAAGCGA
AAGGCTCCTGAATTCTTCTGCGGTGAAGAATGGGCACGATCGAAAACTGTCATTGCAGAG
AAAAACATCGCCAGATTTCGCGATGTCGAAGGCAAGGTCAGCAGGCACATTACGTTTACC
CCTGAATTCGAAAAGCTGCTTTCGGAACACGCGTTGAAGACTAACGCTAAAATTGACCAG
ATTCCTAGTTCAGGAAGCAGTCTCCTGGATAATTGCGGCGACAGTTCAACGGAGGACATT
AATATCACTCTGCTCAGATGCTTCCTGGCAGTGAGTAAGAACGATGGTATCGTTGACTTC
GCCCCAACTCCAACTCCCACCATGACTACGAGATCATCAGCTGCATCTCGTGGAAAGTTG
CAGTCGTCGGCGATTCATGTCGTGGACCCCACTGACGATCTTCTTTGACCGGAGGGAAGA
AAAAACTCATAGCAGAAGGACGGGTACAACTGATGACAACCACTTCTTGTTTGCACAAAC
GAAAGCGAAGTACAGAATCATTCTAGCTGTGGCTACCTCTCTTGGACAGGCGTAGAAACC
ACCAGCTTAAGTGCATTCCTCTCTTTGTTTGACATTCTGTACCAATCTGCTGTGTTTATC
AAATCAAATGCTATTTACAACAAAAGGACACTTGTTTCGTTGGCGTACACGAGTTGCCTT
AAATTTCATAAATTCTACAAGAAAATAAAATAAAGCTATCATTACTAACAATG
>comp2202_c0_seq2
AAACATCCCCGATTGGCCAGGTCTCCTGTTTGACGCTAAAAGGGATCAGCTGGGCCCTAG
CTTTCGATAGCATTCAGATCAAATTCAACACGTGACGCCATATTCGAATAGAATTGGTAG
GTTGTACCTAACTCTCCTATTTGTAACTTGACCCTCGTGTTGTCATACTAATAGGAAGAT
CGGTCATGGTCCTCTTCGAGTAATTCTCTCGCACCCACTGTCCAGATTTTCTTGTTTCTT
TTTCCGATCGGTGCTGTTTGAATTTCGGTGAAAAAGAGATCACGCATACCTCTTACCCGG
CTCGCAACTGATGCGGCACAAGAACCTGCATTGCATGTCGGAAATTTTAGAGAAGCTACG
CTACACGTTTTAGTTTGTTACCTTGTTTGTCTGTGTTTATCGTATCATCGTCGGGTAGAA
TCGAAAAGAGCTTCCAAGTTGTGAAAAAATCTGTTGAAGCTGCTATTCAGTTGGAAGTCG
AAAAAGACTGGAAACATGCCCCAATTAATGGAAGATAAAAACGATGGAATTGGAAACGAC
AATGATTTGATGATCCTCCCTCCTCGTCCTCCTCAAAGAGCAGTTGCAAGAGCCGTTTCC
AACCTTGGAAACACATGCTACATGAATGCTGTCTTGCAAGCTCTAGCACATGCCCCCGAG
CTATGCATGGCCATGGATGTCCATGGTCACCGCATGCGATGTCCTATCTACAAAGAAAAC
GCCGAAAAGATGGCTGCACTATCAGCGGCATCGTCGGCTCCTTCCTCTCGCTCGTCTTCT
CCAGACAACACCTTGAATCAAAACTTCGATAGCAACGGAGAACTGAAAAGTGTGTACACT
TATTCTTCGACTGGGGGTGCGGGAACCAGAAAGTCAACGAGACGCTCGGCTCGTACCAAT
AGCCCGACCAGCAATGAAAATAGCGAAGACAAGAGCGTTGATGATCCCAACGTAACCAAG
TTTTGCGCCCTTTGCGAAACGGAACACCACATGGACCGTGTTCACCGAAACACTCACGGG
AGCAACATGACTTCGAACAGCAAGGCCGTATCTCCCACGAACTTTGTCCATGGTTTTATC

GACCATGTGGCCCCCGCGAGTTTCCGTGTCGGCCAGCAAGAAGACTCGCACGAGTTTCTT
CGTCTCTTGATCGAAGCGATGCAAAAATCATGCAAGCAAGCCAGAAGGCGCAAGGTCACC
ACAATAGACCAGGGGAATCCACAAGATACTCCTACAGAATCTCTTCCTTCCGAAGCAGAA
TCAGAAAGTGCAAATTCTCTGGTCAAGAAACAGGAGGAATCACCCGATACAGAGTATCCT
TTTCAGCTTTTTAGTGGAAAGGTGGAGTCAAAGGTGACCTGTGGGTATTGCCACACAACC
AGTTCTACCCTCGACCCCATCGAAGACCTTGGGTTAGAAGTTACACCTTTGGCAGGAACA
TCTCTCAACGCGGTTGGAAGAAGAAATGCTCCCAGTCCTGTTCCTACATCCTCCTTGGCG
GATGTTTCGCACGCTCTTCAACGGTTTGCGTGTGCGGAGGATCTAGACGCCGGATACAAG
TGCGAGAAGTGTGGCAAGGTCGGCAAAGCAACGAAACAAATGCGCCTGGCTTCGATCCCT
CCGATTCTCACGCTTCATCTCAAGCGATTTCGTTATGGATCCTCGGATCCATCCATGGCC
AACGCTGGCGGTGGAGCCGGTCTCCTTGGAAACGGTGGCAGTGGCTATCTTCCCACACGC
CGAAGCCAACGCACGTCCGAAGTGAGTCAACTCATAGCATCCTCCGGAGGTGTAACTTCA
AGTGGCAGCGGAGTTGATTGGTTCAGCGGCGGGAAGAGTGGATCGGCGAAAATCGAAGGT
CCGTCGGAATTCAAAGCAGTGATGGATCTGAAACCCTATCTGACTGAAGAATTGCAAAAG
AAACATGGAAATATGCTTTGTCGCCTATTCGCTGTGGTTGTCCACGTCGGAAAGAATTCA
CACAGTGGACATTATTTGGCCTACGTCCGAAATATTTCGAAAAATGAGTGGTGGAAGATG
GACGATTCTCGCATTTCATTGGTATCCACCGAAGAAGTTTTGAGGTCCGAAGCTTACATG
CTTTTTTACAGAGTTGTGGACCATCCGTACAGCAAAAAGCTGGCGAATCAGGTTAAAGCA
TTGAACGATTCTTACGATGTATCGGATTCCTTGAAAGCCAAAGAAGAGAAAGAAGCTGCG
GTTGCCTCTCGAAAGAGAAAGGAGAAGGAAACGAAGGACGCTTCAATCGAAATAGAAAAA
AATTCTGCCGACGCAAGTTCGAAAAACTCTTCTCGCCTGTCCGATCGTTCTATCGGATCT
GGCCCCGCCGCCAGTGTTAAGAGAAATCACAGGAAGCGAAAGGCTCCTGAATTCTTCTGC
GGTGAAGAATGGGCACGATCGAAAACTGTCATTGCAGAGAAAAACATCGCCAGATTTCGC
GATGTCGAAGGCAAGGTCAGCAGGCACATTACGTTTACCCCTGAATTCGAAAAGCTGCTT
TCGGAACACGCGTTGAAGACTAACGCTAAAATTGACCAGATTCCTAGTTCAGGAAGCAGT
CTCCTGGATAATTGCGGCGACAGTTCAACGGAGGACATTAATATCACTCTGCTCAGATGC
TTCCTGGCAGTGAGTAAGAACGATGGTATCGTTGACTTCGCCCCAACTCCAACTCCCACC
ATGACTACGAGATCATCAGCTGCATCTCGTGGAAAGTTGCAGTCGTCGGCGATTCATGTC
GTGGACCCCACTGACGATCTTCTTTGACCGGAGGGAAGAAAAAACTCATAGCAGAAGGAC
GGGTACAACTGATGACAACCACTTCTTGTTTGCACAAACGAAAGCGAAGTACAGAATCAT
TCTAGCTGTGGCTACCTCTCTTGGACAGGCGTAGAAACCACCAGCTTAAGTGCATTCCTC
TCTTTGTTTGACATTCTGTACCAATCTGCTGTGTTTATCAAATCAAATGCTATTTACAAC
AAAAGGACACTTGTTTCGTTGGCGTACACGAGTTGCCTTAAATTTCATAAATTCTACAAG
AAAATAAAATAAAGCTATCATTACTAACAATG
>comp2203_c0_seq1
GACCACTGAAAAAAAACAATAATCTACGGTGCCGAAAACCGAATGCGGTGCAATTTATTT
ATCCCCTGTGCCAGCGAATTATTCTACCGTATCTTGAAGGAAGGCTGCCTACAACTAGCC
CCCTGAAAAAATAAAAGTATGATTGCTCCCATCGATGTGGCTGAACAGATCGAAGGTCGA
CCAAAGATCGACGTCGACCTGCTTACAGAGGCCTCTGTAAGTGGCGAAGTCTTTTCAAGG
AGGAAGAGCAAGGTGCAATTTCATGCAGTCGAAGTACGTGAGTATGCTCGTTCGATTGGT
GACAATCCAACCGCGATGGATGGACCTCCTCTTGGATTGGGTTGGACTTATCAGAACCTG
AAAAGAAGCAGTTTTTCAGAAAAATCGCAGCGCACCGAAAGCAACATTGTTGTAACTACG
GGCAGTGAGACTGACACCACTGTTGACGACCACGATGAATCTATCACCATTCCCATTGAC
GAGTACGAAACCGCGACAAATGAACGGCGTCGCCGCCGAATCATGAAGCTATTGAAACTC
AGAGAAAACGCGACATTTCAATTGAAGAAACAGAGAAGTCGCGCCACTCAGCGGCAGGCA
AGCACTGGGAACTACTACTATAAAAGCAAAGAAAAGGACGACAAGTTGGATCCCAACGAG
CTTACGGATGAGCAGATCAACCAACTGAGTGCAACGTGGCTAAAGATCCAGCCAGTCCCT
GGAAAAACCCGCCGAAAGATTTTGCTGGAAGCAACCGATATGTCCCAGAATCAAATTGAA
GACACAGTTCGTGAGCTAGCGAAACTCCGAAATCAGCGTCGATCCACGCAAGCAATGGCG
GAGACTGGTCTTGACGACTTTCAAGCAGCAGTGGAGTTCTGTGTCCGACGCTTGCGGAGA
ATACGATTGGGAATGAGCAAAAAACGAGAGCAGGAACTCCTATGGGAAAAGGCTGAGGAA
TACTGGAAGAGTATTGAAACGCCTCATTTCAATAGAGACTCCGCGCCCCGTAGCGCTCTA
AAAGTGTGTGAAGTTGACGACAAAACAGAGGACTAAACGATTCCGACAACCCAGAAATAG
ATAAGTTTGCACTTTCGTTGAACGTGGCATAGGGAGCGCGAAGATTACATTCCGAATGTT
GATTTCATATCCCAAAAGTTCACACCGTAGGCCTGTGTAGAAATGAATTCTATAGTTTGG
CTTGAATTTAGCAAACATTAACATTTGAAATGTTAGCAGGCAATGGTATCCTTCTTCCTC
AATTTTAGATCCCTTCTTCCTCTTTTTAAGGTATGTGCAAGACCCAAATAATTGTTTTGG
GAAGGTAGTGCACTACCACATTGAAGAATGGGGACTACAGTGATATGCTAACGCTCCAAA
GGTCAACTTTAAAATAACATTTTGAACATTATTTCTAACA
>comp2204_c0_seq1

CTCCCGACAACTACAGTACAGTACAGTACAAGAGAACGACTTCGTGCCCACGGTACTAAA
TTTTGTCCACTATGGCGGATAAAGGACAACGACGGCTGGTCCTCAGCGAAGAAGATTACA
CCTCCAAGCTATCGTTGATTGTTGCACGCGACTATTTCCCGGAAATCTCGAGGCTCGAGC
GAGAGAACAGCCTCTTGGACTGCCGCTTGGCGGGAGACGCGATCGGAGCCGTCGCCGTCC
GCCGAGCAACGCGCAATTCTTTAGAAGAGGAAGAAGCAGCCGCTGCTCGGCGGGAAGGCG
ATGATCGTGATGTATTGGAGTCCGAAAGCAATGAAAACACGCTAGCTCGTCGAAACCCAC
CGGGTGCAAACATACAGATACGGAAACGCATGCGTCCGTTAGAAGAAGAAACCGTGACCG
GGTTCGTGATGAGAGCAACAAACGAGGACGATGACGAGTTTGACTCGAATCTAAAACAAA
ACATAAGAGAAAATAGACAGAGACTAGAAGCCATTTATCAAACAAAAAGCAATCCAACCA
GCAAAACCAACGAAAACGAAAGGCCAGGAGGAGCCATCAGCAACCTTTGCTTGGAGATGG
CATCCGATGACTTTGCTCCGGAATCTAATCGAACAGCATGGAAACACAAACCTCTAATGC
GGAACGGCTTGTTCTTTAATCCGACGCCGATGAGAAATTCGTCTGAGACAGACAGTGACG
CAATTGGAAATGTAAGAGAACAGGCCCTCCTGAAAAATTCAACTGCATTGATGGTGTTAT
CCGATGGTTCCGATGACATGCCACCCCCTTCGAAGCAGCATACTTCCGGATTGCTATCTA
ACCGAAACGCCGGAAAAGATAAATTGACGCAAAATTTGCTCAAGAGCCAGCTGGTGGAGT
ACATACCGAAGCACAAACTCGAAAAGAAAATCGAACCGAGCGCGACGAGATTTCCGTCGA
ATCCATCGTTGATTCCGATTCCAGGCAGTGCTCTGCATGGGAGTCTTGACGAGGAAACAG
ATTCTGATTCCCACACGGACTACATGTCTTCGACGGATGCTTCCACGGATCTCGACGCCC
CTCTTAGATCGCTTTCGGAGGAACGCCGACGTTTCAAACGCAAGAAAGAATCTTATCGCC
AGTCGTACCCATACGTGGCAATGTCGTCCCCGAGAGCCAAACAACAATTGCCAATCAGCA
CCCATGGAGCCATCGATGGCCCACCATTGGTTTTGTCACACCAGGAAGAACAACTAGAAA
AACCGGAATCGACTTCGTCCTTCCGAATGCCGTCGAAAAACGAACGGGAGTTCGCAGCGA
GCAACGCAGAAAAACTGATGGCACGCAGGGCCAGGCTGGCGTCGCCCTCGTCCTTGCGTC
GGTCTTCTCTCAAATCGGGGAAACACAGTTCTATGTGCCAGGCACTGGGACCCGGTTCGC
TGACCCCGGCTGCCCTCTCCTTGATGAAGGCAAAATCGATCAAGTTACGAAACCGAGACG
CATTCGGGTCTGCACTTAGAAGATCCTATACACCTCAAGTTGTCCACCGCTCGTCCTCGT
CCAAGAGTTCTCGGAGAAGGAGAAGCGCAAATCGACCCGGGGATCATGCCTACAATGCGA
CGCCCCAAAGATAGAAATACGCAAAACGCTGGGTTAAAGTAATAATA
>comp2205_c0_seq1
GAAACCCAATTTTACTCTCACCTGTTATCACAATTTGACCTGTCATTTCTAGAGTACTCT
CTGACCATACTATTACTATCACCATTATCACACTATGAAGTTCACTGTTGCTGCTACTCT
CCTCGCCACATCTGTTACAGCCACATCTGGATCTGACCAAAGTGTTGCCAATGATGCAGC
ATCCGCGAGTCATCGTGGACTTGCTGTTTCTTTGGAAGATCGATTCGAGGTGACTTCTGT
ATTGAAAGAGCGCCGTGCATCCAAAAATAAAGGCAAGGTCATCCGCAATCTACAAAGAAA
ACTTCAGCAGCGGCAGCTTGGCAATGAGGTCGAGGAAGATTCACAAGTGCATGAAGAAGA
CCTTGATCTTGGTATATTTTCTCGTGGATTGCAAGCCAATGCTACAGAACCCGAGGAAGA
GCTATCCGTGATGGAACAGCTAATCGAACTTTGTGGGGACACAGAAGAAACAGACGAAAG
CTTTTCGTGCACATGTGCTGATCTTAATGTCGAAGCATACACTGCAAGTGTAACTTGTGC
ATACGCAGAAAATTGCCTCAGTCCCACCGAGGACATTTGTGGAGGAAATACCACTTTCTG
CTTCCTAGAAACCTACAACCTCGAAGTTACGGCACCAGGAACTGGATCCTCTGCGATCTG
CTACGAGCTAACTTCTCCTCTTTCGTTCAATTACTGCTACGGGCTCACATACACGGACGA
GAGTACCACTCCTTCTGGTTGTGTGTTGGAGGTAGAAGGAAACCAGTGCAACTCGTGCGA
ATTTTACTTTTACGATTCACAACCGAACGTTACATGCAATAATTTTGATTGCAGCAACGC
CGATGAAACCATTGGAAGTGGTATCATCTGCGGAGATGATACCATCGTCTCGGCGAAGAT
CGAGGATCACCTTACGTATGCTCCCTTGCCATGTGAAGGTGGATGCAACATTTGTCCCTC
GAATGGTGAGATGTCATTGGTAGATAACAATGTAACCATGATCACAGGTGATGTCTATCA
GTGTTGGCAGTTAAATCTTGCAGCACAAGCCGGATATCTCCAAGATATGCCGGGTGATTT
ATGCAATAGTTTGCTGGAAGTCGTGAACGAACCTTGTGGGTGCGTTGTTCCTCCTGCTAC
CGAAGTCCCGGAAGTTACCCCCACTTCAGACGAAGACACAAATCCCGAAGCAGCACCCAC
TGACGCACCTATGTCGTCAACCGAGCCCAACGAAGCTTCTGATGGCGCAACAGTAAAATC
AAGATTTTCTTTTGCTGTCACTGCGTTGGCTGCAACCTTTTCATGGATGATAATTTAAGA
TATGAATTATCCATTCATTGCTCAATACTTCTACATTGTATTGAACTTCCGGGTCGTCTG
TTTACTCAATTTCACTATCGATTTGGTCGGGCGCTGACGACAGCTCATTGACACGTCGTA
CCATGTTTAAAATCGTATCGTTAAACTCCAATACATAATTTATTGTTATATTTTGTACTA
AAAAA
>comp2206_c0_seq1
CTTGAAGCGAAGACCAAACAGATGACTCGCCTTGTACGGGAGGTAACTTTCCGTCCCCAA
TACAGTAGATGGCTCCGTTTCTGATTCACTCGCTGGTGACATCTCATTTTGAACGACCGA
AGACCTCCGAAACGAACACAAAACCACAATAAAGTCACAGCAAACCATACCAGCAAAGAA
GCCACAAAAAGCACCCACCATGATGAAACATTCCACTGCTATGAATTCCGGCAAGAAGAC

GATCGTCGCATGTCTATGGATGGCCGTCCTGTCGATCGCATGGAGCAGCAACAACGCTGG
TGTGTTTGTGCAGGGCATGGGCGAGAAGACTAGCAGTTTGCCGCTCACCGCAAACACGCC
GATTTCTTGCGACGAAATGATCATCCTGCATCGCTGGGAAGGATCTTGCTGCAGTCTGAA
CGTCACCAACGGTGGCGGGTGCATTCTCAACGTCTTCGACGGATACTGCAACGTGAAGGG
ACAGGAATGGACGCTGAAATACAATTCGACGTACGACGAAAAGCCCTGCCCACCGTCGGA
ATTCTCTAACACACAGCTTGGCATGAAAGGAGAACCGACTCTGCCACCCAGTGGTAGCAG
CAGCAGATTCACTACCAAAGCAATGGTTGTCTTGGGAGCATCGATTTTCGCGGCACTGTT
GTAGTCATTTCCTGTAATGTTCTTCGCCTTCGTCAGTAGGAATACAAAGGAAGGAAATTG
TAAAGGATGGCTATTGTTTGGTTCGATGTAGATCTTGCGTGAAGGAAGATGAATACATCG
AACGGACTTCCAAAAGAGATGCAGTTTGCTGTAGATACTGTCGTGTTAGATTGGAATTCT
TGATTTAATTAGATAGAGGAAGATCTTCTATGGAAAGATGATGCGGTTGTGATTTTGGGG
CGATTTCTGTGAGGCTAATGATCTTTTTTATCAATCATTGGCAACAAGTTATCACGAGCT
AGGAGTCGAATAGCGGAAAAGGCCGACGTCCTGTGGTTCTCTTCGGAGCAAGTTTGAGTG
GAAAAAGTGCAGGAACAAAAATGCAGACAGGATCACTTTATTTCAGAATAAAAAGATCAG
GATGGATTCAAGATTTTGTTTCAAAATCGCGAAAGCGATGTGATGTTTGGTCGATAGCAG
GAGTACTAGCACTATTCTTGTCCAAGTTGTTGTAGTAGTGTGTGGATACTAGTAGTAGTA
CTACTAGTAGT
>comp2207_c0_seq1
GCATTTTTATCAGACGTCTATTAAATACATTTATCCCATAAAATTTCCAATTAGAATCGA
CAACATCGCACGATTCATACCACAATAGTAGACGAAGGAAGAAGATGAACAACGAGACCT
TGGCAACAATCGCCCGCGAAAACACTTCATTGGCACTTAGCACGCTTGCGATTGCAGCGG
CCGGAGCTGCAGTCGTTTTTGCGTCAACCGCAATCTCTCGAAAACCGGATAATTCCCAAA
ACGACAAGCCGATACCGCTTCCTGTTGCACCAATTGGTTCGAAATTCGCGATACTCCCCA
AATCGGGAGTCACCTTGCGCTACACTGATACCCAAGAGGAACAAGTCACCGAGAAAGACT
CTGGTGAAAGCAGCTCTGAGCAAGTAACTTTCGTATTGCTCCACGGCTTTGCTGGTTGTG
TCGAAACTTGGGAGTTATTGACTCCATATCTCGTTAAAAGCAACAGCACAACTAATGAAA
GCGAGGTTGCGGTACGAGTTGTAGCCATAGATATGGTTGGTAGCGGATTCTCGGACAAAC
CTATTGGAGACGACTTTGATTATGCCTACCGAAACCAGGGTAAGGTTGTTTCAGAATTCA
TCTCCGTTCTAAGCTTGTCGAACGTTGTGATTGTAGGGCACAGCTCAGGAACAGTGGTCG
GCGCCGCAACCGCGAGCCAACAACAAAGTAATGAGAGCGGGAAAACCGCGGTCGTGGGGG
CTGTGTTTATGGCATGTGCGCTGTTCCGTCCAAAATCAGATTTTTTTTCCAAAAGCTGGC
TCAAGCCTTTGTTTCGCTGGATGACCAAGAAAATGATGGCCAATCGCAAGCAGAGTTTGG
AAAGAATGCACCTCCCAGCGCATGCTGAGCGAGTTCTGACCGACGACTTCGTCGAAAAAT
TTGCGGCACCCACTAGACTTCCAAATTTTCACGATGCGCTGGTAGAAACAGTGATGGCCA
AAGAAGCTCCATATGAAGAATTAGTAGATGAATTGATAAACACGCCGTCAGCTCCGTTCC
CAATGCTATTTGTTTGGGGGAAACAGGATACTTATACACCTCTTCCTGCTGAACAGAAAG
ACTCAATTCAGCAAAAACTTGATACTATGAACAAGACATCAGAGCAAGAACGACAACCCA
TTGTCGAGACGACAGAACTAGATGAATGCCATCATTATCCTCAACACGAGCAGCCCGAGG
CCTTAGCGAACGAAATTTTGACATTTGTCCAAAAGAATGTAAAACTCCATTAAGTTTTGA
AATTTATACTCACTTACGATGGCTTTGGTCGTTTGAATCTTCATGTGCGAGACCTGCTAT
TTTATCTAGTAAAGTACTTGGTTGCCAAGGGTTCCTCCGATTGCAATGGCGAGTGCTGTG
GTGATAGTACAGTTCTTTATCTTCGTAAATTTCAATGACAACCTTAAAAAATAAGGAATT
TGGTTTTGTTCAAAAGATTTCAGTCAATTTTTCTACACAAACACAATAGCATGATGCTAA
ATTGGATTCCTGTATACTGAAAGTGGGCCAAAGTGAAGAATATCAAATACCAATCTTTTT
AGCCTATTGTATTTTATGCTGT
>comp2208_c0_seq1
GTGGGAATTTCTCAGTATCATTGCATACCAAAACACCTGTCCAGAGGCAAGCACATCCAT
CCTCGCCAGCTTGGTATAGATTGTTTAGAAAGACCGCGTTCAATATGACTCAATCCATGA
GGGCAATTGCGCTGCTGCTCGTGTTCGGGATCGTCGCCACGATCGTCCAGGGCTACCGGG
CACCCTCGTCATTGTTGTCGCAATCGGCGGCGACGAATGCGAATGCGAACGCCGTTGTCG
GTGGTGCGTCGCCGAAGCTCTCGCCGCTCATGCGCTCGGTCATGAACATTCGTGGCGGAG
ACGAGGAGGAGGAAGAGAGCGATGGCGAAGAAGAAGAAGAGAGCGACGACGACGAGGAAA
CCGGCTTGTCGTCGTCGGGATCGGGGATTGACTACGTGGCCGTCTTGGAAAAAGCACTGG
ACGTGACCAAGACCAAAATCGTGCCGACCGTCATCGATTATTCCAAGAAGGGCTTTCTGG
CAGCCAAGAAAACATCCATCGTCGTTTACGGAGCCCTACACAGAGCCATTCAAGCGGGAT
TGGAAGGAGAGGAGTCGGAAGAAAGCGACGACGAGGATGACAGCGACAGCGACGACGAAG
GAGAAGAAGCGACGCTGGTGGAGAAGATCTTGACCATGACGAAGAAGACAGTGAAGACCG
TGCAACGCATGGTGAAAGCAGCACTGACGGTGCCCGAGGGCGACGAAACCAGCGAGGACG
AGGACGAGGAAACTATCGATCTGGAGGAAGGCGAGGACGATGCGATCGACACCACGGAGG
AAGGAACCGAAGAGGGCGCAGCGACCGGACCCGAAGAAACACCGGCTGCCAAAGACGGCA

ACGACGACGAAGCGGCGGTGAATGACAATGACTTTGGATCGTATCTCTCGGAAGCATACG
AGGTAGACGACCTGCGCAACAGCGGAAAATCGCCGGGAACCGTCATTCTGGACGGTTCCC
TCCAAGATGCACTGGAAACGGCTCGGAAGCAGGCGCGCATGCTTCTGGTATTCATTCCCT
CGGAAAAGCCCAAGTCGGAACAGGGAGGACTGTCTTTGTTTGGTGGTGGCCGAAAGGGCA
CCGAGGAATCCGAGCAAAACAATCTCGTGGCGATCGAGAGTTTGTTGAGCAAAGAGGTCG
CAAAGGCTTCCAACAGAAAAGCACGCAAAATGCAAGCCGAAGACTTTGGTTCGTTTGCCA
TTTGGGGCACCAAGGCGGGTTCCTCCGAAGCCGCGGTCGCCATCAAGCGACTAAAAGTCA
AGGAAATCTCGGCCAAGGGAGAGAAACGAGCTATTCTGTGCGTCGTCTATCCTGCCAGCA
GCGGTGGTGTTATTGCCCCCAAGGTTCTGGCACAACACCATTGCAATCCACCTCTCAAGG
CAGAGTCCATGGCATCCTGGATGAATGCACTCCGCAAGCGACACGGAAAGCAATACGAAG
TTATGCAAACAGAGTTTACAGAACTTCAACTGTACAAGGAACGGAAGGAAGGCTACATAG
ACAGCGTCCAGAGTGACAACCAACGCAAGGCCAAGGAAGCGCAGGAAGAAGCAGAGCGAA
AGGCCAAGGAAGTCAAGGAAGCGGCGCGACAAGCCGAAATCGACGCCCGGCGCCAGGAAC
TCGAACAATCACTGCCCGAGGACATCAAGAGCGGGAGCAATGTCAAGAAGATCGCGCTGC
GATTCCCCGACGGGCGAACAGGACAGCGCGGCTTTGCGTCGGATCAGCCCCTGACCGTCC
TCTTCAATTGGGTAGACGCAATGTTCGAGATCGAGCGCGAAACGGTGGTCCTGACGACAC
TGAACGGCAAGCAAACATTTTCGTGGGAAGGCGACGAGAACACCACGACGTTAGAGGATG
CCGGATTGAACAAAATGACGGCGTTCCGGGTTTCTCAGGCTGCCAAAGCCGACTAAACGA
GTGGATAGCAAAAATTCTCGATGTTAAATGGATATTAAATGGGTTACTGGAAGATGATCT
CGTCTCCTGAAAGCCCTCTTTTTTTGGTTTGTCGTTTTGTTGTATATCGTGTGTCACACA
TATTGTCAATATTAGTTTCAGGGACTGTTTTTATACTCTTATGGTGTGGTCCGAAATAAT
ATTTACAAACAGGGTGTTTGGGGTATGTGTTCGCGCGTTGAATCAAGCGCTGAGATAGAT
GTAGACCAGAGCACTTGAAGACAGGTTTTTCATAGTTCTTCAACCAATGCAAGTAATTTT
GATTCTAGCTTTATTTTATAAAATCTGATTGCCCAC
>comp2208_c0_seq2
CTGCCAGCAGCGGTGGTGTTGTGAGTATCGATTCGAGTTCGCGCCCGTTGGTGTCGTGAG
AACGATGCCAAAGTGTCGTTGTTTGTATTTCACTCGTTCTTTGCGCGTTTGCAACCGCAC
GTACGGTTCTTTCGAATGTATCGTTTGCTCACCGAGTTCTGTGGTTCCTTTCTTTTTCAG
ATTGCCCCCAAGGTTCTGGCACAACACCATTGCAATCCACCTCTCAAGGCAGAGTCCATG
GCATCCTGGATGAATGCACTCCGCAAGCGACACGGAAAGCAATACGAAGTTATGCAAACA
GAGTTTACAGAACTTCAACTGTACAAGGAACGGAAGGAAGGCTACATAGACAGCGTCCAG
AGTGACAACCAACGCAAGGCCAAGGAAGCGCAGGAAGAAGCAGAGCGAAAGGCCAAGGAA
GTCAAGGAAGCGGCGCGACAAGCCGAAATCGACGCCCGGCGCCAGGAACTCGAACAATCA
CTGCCCGAGGACATCAAGAGCGGGAGCAATGTCAAGAAGATCGCGCTGCGATTCCCCGAC
GGGCGAACAGGACAGCGCGGCTTTGCGTCGGATCAGCCCCTGACCGTCCTCTTCAATTGG
GTAGACGCAATGTTCGAGATCGAGCGCGAAACGGTGGTCCTGACGACACTGAACGGCAAG
CAAACATTTTCGTGGGAAGGCGACGAGAACACCACGACGTTAGAGGATGCCGGATTGAAC
AAAATGACGGCGTTCCGGGTTTCTCAGGCTGCCAAAGCCGACTAAACGAGTGGATAGCAA
AAATTCTCGATGTTAAATGGATATTAAATGGGTTACTGGAAGATGATCTCGTCTCCTGAA
AGCCCTCTTTTTTTGGTTTGTCGTTTTGTTGTATATCGTGTGTCACACATATTGTCAATA
TTAGTTTCAGGGACTGTTTTTATACTCTTATGGTGTGGTCCGAAATAATATTTACAAACA
GGGTGTTTGGGGTATGTGTTCGCGCGTTGAATCAAGCGCTGAGATAGATGTAGACCAGAG
CACTTGAAGACAGGTTTTTCATAGTTCTTCAACCAATGCAAGTAATTTTGATTCTAGCTT
TATTTTATAAAATCTGATTGCCCAC
>comp2209_c0_seq1
AAAGGACTACCCGTACTTTTGCTTCGTAGTATGGTACGTACCGTTTGCGCTATCAGTAGG
CTACAGTAATACGCAACAATCCGAAGGGAAGGAAATGGAACAATACTGTAACCCCTCGTG
CACATAGCCGACATCAAGCCGACGCTTAGATCATCCCTCCATCGCAAACATATCGAATAG
ACGTGAAGAAGTGAAAACGAGATAATTTCAGAGGTCACAATGCCCGATTATAGAGAAGCA
TCGAATTTCTGCGCCCGCTATTCAATTGTTGGCATGATTTTTATGGTCCTGATTTCCATG
ATGCTGACATACCAACCTTTTTACATCGGAGGGATCGAGGACTTAGATCAGGCCCGAAGC
AATGCATACGGTGGCGCCGGAACATTTTTATTTGTTTTCATCTTATCGGTTGTATATCTG
GTGTTTGATGCTCTAAGGGGAGGAGGCAACAACAGAAATCGAAGGGAATCAAGAATTAGT
CGTGGGAATGAGTACGATGCGGTATCGTTGAGTGGTCTCGATGGAACGGAACAGATGATG
GAAAATATGGCAAATCTGGAACTTCCTCCGAGTGTACAGGAAGCTCGCTTTACGTAGTGA
ATGAATCCTGAGGAAGCTATTGCTTTGGTTCGGTTTGAAGTTGAAACAAAGATCATCGAA
CAATCAATGTCTGAGTCGGGTATGTGGTATTTACTACACTCGTTGAAAACAACGGGTCTT
ACACTGTACCTCTTTCGATTGGTCTTGAATATGCTGACTTTCAAGTGGAGTTCGACACCT
ATATCTGGCTGCATCAAACAAAACCTGCGAGATCGCGATCTTTCCCCCAGTAAGAAGCAA
CGAAAATCTCATTGGTTTACAAACCCTTACACGATTCACCTCGCTCACAATCGACCAGCT

ACATAACTTCACGATTTGTGATAACATGTATTCATATAGAAACACGAGTTTATTCCTCTA
ATTATCTAATGCAACTTGCGGG
>comp2210_c0_seq1
CTGACGACGATTGAGAAAGATCCCACCCGTTCTGTCCTGTTTCTTCCCTTCGACAGTGAC
GACTTCGAGATTGCCATACTTCGAGTGAATGCACGAACGAGAAGACCTAAGTCCATTTTC
CACCAAAATCTTCAAGAACGCCATGAGACAATCCACTTTGTATTGCTGTAGTGTCCTTCT
GTCTGCGGTTGGGACAGGCCCGGCGATGGTCGATGCCTTTGCGCCAGCCTTGCAAGAACA
GTCGAACCCAATACGGCTACAGGCCACAACAAAGGACAGCGATTCGATCGATATCGACCG
ACGAGGAGTTTTCCGGACAGCAGCGACGGCAGCGGTCGCAGCCATAGCCGGTACGTCGAT
ATCCCTGCCCGCCAATGCGAACCAGGCCGAGGGCGTCAACCTACAGCAATACGAGGATTT
CACGAAGGCCCCAGAGGGGTTTTCCTACCGCGACGTCAATGTCGGAACCGGGGTTTCCCC
CTCCATTGGAGATCGGGTGGTGTACGAATGGAGCGGATACACTATTGGTTATTTTGGTCG
GCCCTTCGAGGCCAAGGGTGGTCCCCAGGGTGGGGCCTTCGACAAAAACCAGGATTATTC
CCGAACCGTTCTGGGGAAAGGAGAGGTTGTCAAGGGATTGGAGCTAGCTATGCAGAGCAT
GAAGGTTGGAGGCATCCGGCAGATTATTGTTCCTTATGGAGAATTGGGCTATCCGAGCAC
CAAGGAGGATGCGTCCCACGACAAGATTGGGCCAAAACCGACTACCTTCTCGGGGGAACG
TGCACTTAATTTCGTCCTCGACAACCCACGAGTGGATCGAACTCTCCTATTCAACGTCAA
GATGATCCGGGTAGACAAGGCTGATGGGAAAGGTGGATTCACACGTGGTGAAAAGGCATA
GGTAGCTTCGCGCATATGCACTGTCACTCCTACGTCTTTTTTGTGGTGGTGATTTGCGTT
GTATGAATGGCTTGGCTCTTACTGCTACCAGCAGCAGCTGGCTCCAACAAGTGATGGGCA
CTCATACTACTAGAAGTACTCCCAGCATAAGTAGCTAATAATTATAGTATGGCAATACTT
GTTGTTGGTACTGCGCAGAACACTAGTATTGTACTACTAATATGTTTATATTCAAAAACA
TTGGCGGTTGCTACTGCTAGTTTGGTTTAGTCTGGAAACATTGAAGGTCTGCTTCTATTG
GTGTGGTCTGCTTCTAGCCATTGTGGGG
>comp2212_c0_seq1
CAACGAATCAAAGGATTGCACAGCACCCTTCGATAGAACCAAACCCAAAGAATACCGCAT
GGAGAGCGACGAGGTAGAGACAAACCGAACTACAAAGGGACAGTTCGACTAAGATTAAAA
GCAGTACCGTAGGCCAGTTGCACTGACTACGCTGTACTACGGAAAGAAGTGACTCACATT
TTGCTCGTTAGCTACAAACCAAAGAAGAAAAAGAACCAAAAGCAACACACGAAGAAACCA
TGAAAAGTACCAGGGAGGATTCAGGGAGAGACAAGAATGACCGGGGCGGTCGTGGTAAAA
ACGGAAGAAAACAGAGCGGGAACCAGGGGCGCGGCGGCGGAGGAGGGAGCAAGGGCGGAA
GCAGGCGGTCGAAAAACAAGGGCCAAAACAACGGCAACAGAAGTTCCAATAGGTCCAATC
GCTCGTCTTTCTCTTCGTCCTCGTCCCCTTTGGTCTACCCGCAATACGCTACGCTGGAAG
AGTGCCTCACCAGGTACAATAGGGGGGATCGCAACCTTATCAGGGGAACCATTCGCTGCT
TGCCAGTGCGGGACGGACCCGCCTTTTGCACTTGCGACCGCGGGTCGCAGTCCAAGGACG
TTGTCCTGGATGGGCCGCTCGAGCGCAATCGGGCCCTAGACGGCGACGTTGTTTTTGTAG
AAATCATTTCCAAGCCACCGGAAGAGAAGGAAAAGGGGACAGCACCTAGTGATCGCACAC
CCAGAGAGGAGCCGGGCAGCGAAACCAAAGAACAAGAATCAGAGGACGAGTGGTGGCAAG
ACGACATCGATCAGGTGAATCTCTGGGATCCGGTGGTGCCAATTGCGCACAAGAAGCTGA
TTCCAAAGTCAGATTCGTCATCGCCTAGTGTCGCATTCGATAAGGAACAAAAGCACGGCA
GGGTGGTCTTTGTCGTTCATCCGAAAGAATGGTCTAGCGAGATCAAGTCTAAACAAAACA
GTCATTCCAGGGATCGCTCGGCCCCCACAAAGCGTATAGTTGGAACCCTGAAACGGCTCA
AGGGGGGAACGGCCCTGCTGACATCGTCCAACAACCGAAGCATGCAGCAGTTTCGCCTAT
CGAACGCCGACGCCGAAAAATATAAGGACGCCCCAGAGGGGTCGATTTTTGCCGGAAAGT
ACACCTACGGAACATGGCAGGAAACCCACAAATGGCCCGCCTGCTCGAACCTCGAACAGT
TTGGGGCCACCGGGAACATCGAGGACGAGACAGCGGCTCTCCTGATAGAATTCGGGGTTG
ACCATGGAGAGTTCCCTTCCCACGTTCTAGAGGAAAGTCAGACCGTTGTCGCCTCGGGAC
AGTATTCCAACGGGACGGAATCCGGATGGAGACCCACTGAAGACATGTATGAGGGACGAA
GGGACTATCGAAACCAGCGGATTTTCACCATTGATCCAACCACTGCAAAGGATTTGGACG
ACGCCCTCCATATAACTGACCTAGGAAATGGGCAGATTGAAATAGGAGTGCACATTGCTG
ACGTTTCGTTTTTCGTGACTCCGGATTGCGCAATCGATGTCGAGGCACAAAGGCGATGCA
CCACCGTGTACCTCGTGGATCGAGTTATCCCAATGCTCCCAAGGCCACTCTGCGAGGTTG
CCTGTAGTCTCAACGAAAACGTAGAGCGTCTCGCTTTTTCTTGTGTATGGAGGATGAACC
GTGACGGAAGCCTCGTCAAGGGACAGGACGTATGGTACGGGAGAACCGTTATCAAATCGT
GTGCCCGACTGGATTATTCCACCGCTCAAAACATCATTGAAAACAAGGTAGCCTATCCAG
GGGAGAACGAAATCGACGAAGAGCTCTGGCCGGTCTCTCGGAGGCCAACAGGGGGGCACA
CCATCGGACAGGTAGCCGCAGACGTCCGGCTGATGAACGAAATAGCCCAGGCCCGCCGCA
GGCTGCGCTTCCGAAACGGAGCGGTGGCCCTCGATGCGGTCAAGCTTACCTTTCAGCTGG
ACGACGACCGGGAAACACCTCTGTTGTGCGAGCCATACAAGATCCGGGACTCCAACAAGC
TCGTCGAGGAGTATATGCTTCTTGCCAACTTTTTGGTTGCCCAGCGACTCATCACCAACA

CTGGGCCCCGGGCCTGCCTGCGCAACCACGAGCCACCGGACTACGAGGGGCTAGAAAAGG
CCGCTGCCCTGGCGATGGAGGCCATTGGCTTTCGCATCGACATTGAGAGTTCCGAGGCTG
TCCAGAAATCGCTCAACCGCCTCAGCCAGGAATGTCAGGATCCAATCCTGCTTAAGTGCA
TCACCGAGCTGCTCAAGAATCCGATGAAACCGGCCACCTACATTGCAGCCGGCACACTCT
CGCAACCCGAATGGGCCCATTTTGCATTGCACATCCCCTATTATACACACTTTACCAGCC
CCATTCGACGGTACGCGGACGTTATCGTGCACCGGCTGCTCCAGGCCAGCATCGACGGAG
AGGACGCGGTCGAAGACTTTCCGATGGAGGTCAAGGAAATCAGCGCCATCTGTGGTACTT
GTAACGAGAAGAAAGAAGGATCTCGCAAGGCCCAGGAGCGCAGTGATGCCGTCTTTCTGG
CCCTTTACCTCCGCAAATTTCCCAAAAAGAGACAGCTGGCGGTCGTCACGTCAATCGGGA
TGAAAGCCTTTACAGTCTTTCTCCCCCACCTGGGGATATCTGCCATGGTCTATCTAGATG
AGCACAAACACTGGATCGAGGCAGAAGCTTACGAGGACCCAAAATTTGGCCGCAGGATCA
AACTAAAGCGGACCCAGACCCACAAGGGAGAGCGATGGAAAGAACTCCTCGTCAAAAACT
TTTTGCGGCTCCGGGTGACCTGCGGCTGCAGCGACAAACCCCCGTTGAACGTGAATCTGA
AGCTTGAGGGGCCGTGGGAGGGAGATGACGAAACAAGGTAGTAGAGGACAGATACGAAGG
TGAAACGATCGAAAAGCGAAAAGACGGAATTGCATCATAACGTAGTATGGTACCCAATTT
AAATCAAGTGCTCAATAGTTCTCATCACCTATGGAGGATTCCATATCGCAGCTTAGAATA
TAGTGCTAGATTACGATATTCTACAAACGTTATTTTCAGATATTCTTGTACCACCAAGCC
CAAGGTAAGTGAGCAATCGTATACGCCAATTGATCAGACATTTGTAGCAGTGAAATAATA
GAATGGTACTATTCC
>comp2213_c0_seq1
TGCTTGTGCTTGTGCTTGTGCTTGGCCAAGATTGAATTTGATCTGCATTCCTTTTTCTTG
TCCGACCAGCAGTACTGGGGAAGGATACGGCAATAGCAATGCATCGACAAGCCAAAGAAC
TTACCAACCAACAGGCCCATGGGAACGCTCTTTGGATCTCAACGACGGTCTCTCATTAAA
GTTATAGTATGTAACCATGTGCCAATGTCCCAATCTATCACTGATGAAGTGTACAATGTT
ACCGAGCTATTGATTACTTTGTTGGTACCGCAATCTGTAACATTAAAACGATTTTTGTTT
CCGTAAAATAATTCCCTGACCTGGACTGACCGAGCACCAACGCATGAATGCACGATCTAC
TTCAAAATCCACGCAATAGTAAAGGGGGGAAAGTAGCTATTGGTGCGAGTACAATCGAAG
CAGGATTGGTTGCATAATTTGGTACCATTCTCGCAAAGACAAATCGAACGAATCGTCGCT
GTTTTTGATTGCACCACCAAAGGTGCAACTCTTGGTTTACGCTTTCTCTGCAGAGTCAGT
CGCAAAGCATGCGCCATACATGGCAGTGGCACTCGGCTGCAGCTAGGCCCGTAAAGATTC
CCGGGCATGTATAGAAACAAATATTGAATCGTCGTAGTAATCATAATAGCAAACTATG
>comp2214_c0_seq1
ACAACAACAACGCTACAACCCTAGTTTCGAGGACGACGTTTTCAACGAGGAGCGCTATTA
TTCCATCGAAAACGGATATGCCATTAGCTGTTACGAACTGGCAATGGCCCTCTACCAAGA
CCTGATTCTCACGGAAAACGAGGAAATACGCAAGGCCAACGACAAGGCCATGGAACTCGC
GGAGTCCCAGCTCGGACTGAACGAGGACGCCACGGAGGACGAGATCATGGACGCTCTGCT
GAGCCAAACCACGAGCGGAGGAATGGGCGGTGCGGGGGCGACTTCTTCCTTTGTGTCGAT
CCCCCTCGACATCGGAGACGACGAGCTGCAGGACCTCTTCGGGGCAGGCACCGGCCGCTT
TTCCGAGGCAAAGGAGGCCTCCCTCCTCGAAATAGAGGGCTTTCTGGACACGGCCATTCT
GACCTTTCTGCAGCACGCCGATTATCCACCGTCCGGGGACGGAGCCAGGTCCGGCGGGTT
CTTCCACGGGCAAACGCCGCCCAAGAAAAAGAAGATTGTCGTTGTCAACGGCAAGAGGAA
AAAATCGCAAGAGGAACTCCGAGCGGAAGAGCGACTCTTTCACTGGCGGGCCTCCCTGGC
CACCGCCTACCAAAACTCCGCCAGGATCGCTACGGTTCGGAACCACCACATCCGGGCCAG
GGAGCTGACGAACAAGGCACTCGAGCTCTATACAAAGGTCGTGGTACCCTACTACGAAGA
AACGGAGAGGGGGTCTGGCAAGGGCAGCAAGGCCCGGGAGAACGCGAGGCAGTTTGTATC
GTCGAGCATGGCTTCCACCATGACCAAAGAGTACGCCAAGACGTCGATCGGCGAACTCAA
AAAGTCTCTGGCCGCCACCGCGCTGTACCTCGGAAACTACAGCGAGTCCATGGGGCAATA
CAACAAGGCCATGACGTACTTCCAGAGGAACCATCTCACTCCTTCTCCCGACGGCATGTA
CAACACACTCCAGGGCGACGACGTGGGCATGCACCAATACAGAGAAATCGTAGCGACCTA
CCAAAAGGAGCTGGAGGATTACCGGGGGAACGTAAGGGCGATGTACGTCTACAAGGACGA
CTCCTACGAGGCGGGGGTCCTCCTGGCCCTGGCCAGCATGACCATGGCCATGGGAGACCT
GCCGGTGACGATCCGATCCTACGAGGAGGCCATCGAGATCTACGACCGCATCCAGAGCAA
CCCCGATCGGAAAACCAGGCTGGGCCTCGCGAGCGCCCACAGCGGTCTGTCCTCGGCGCT
CTTCTTCTCCCGGCGGTTCGCCCACAGCGGGCACCACTACGGAAAGGCCCTCGGGCACTT
CCGATCCGTCTACGGGGAGGGGACGCCGCCCCAGGAAGAGGCCGAGCTGGCGATCGAGGA
CATGAAGGACGAGCTGATCCAGGCCTACGGCGAAGAGTACTACGAATCGCTCTATGCCCA
GTACACCGCGATGGGCGGCGGGAAGGGGGGGGGCGGGCCGAGCGGAAGCGGCTCCGGTGC
GGACGGCCCCGGGGTATCGGGGAATTTCGACGTGGTCGGGTACCACAACGCGACCTTCGC
CGCGGAAGAAAGCGCGGCCGCCACCGCCGGCGGCGACGATAGCGACGACGACGACGACCA
CGCCCATCGCGCGCACAGCGGCGAGCTGTGATGCGATGCGATGCGATGCGGGCAGGGGTG

GAGTCCTTTCCGGGCCAAGCGCACTGCCCGGTGGTGGAGGATTCCCGGAAAAAAATTGTG
CATCGGATTTCCTATCGGATGGTAGTACGTAGCACCAAATGGATTTGGAGACGTGTTTTG
GCGCGCTGTATATGCTCGGGAATGCGACGCAGTGTATTGCCGATACGATTTTGGTTAGAA
TAAAATAGAGCCATCGC
>comp2215_c0_seq1
CAATCTGTTGGGATGCAAGATTTGATCTCATTGTGTATTCCGCGATCGGCAACACCTCGT
TCGACTGAAGGCTCACACTCCCATTATTGTGCTCTAAGCATCAAAACTCCTTGATAATGA
AGTATTCCGTTGGTCTTGTTCTTGCATCACTTATCCCCGCTGCTTCCCTCACGAGGGAGG
GAATTGGCAGGAGGGATGCGATCTCGGCGGCCGCCTCTGGTGTGGTCGGAGGCTTTCTGT
CCCAAGTACAGCCATCCTTTGCGGCTGGAACCCCCCCAACCAAAGAGGAGTTGGGAAGAA
TCGTTCTCGGCTACAAACAGATCACTGCATTGCTGGAAAACTTTGAAGAGTCAACAACAG
TCTGTAGAGAGAACGGAGGAGAATGCAAGCGTGACGCAGAACCAATCCGACGGGTCCTCG
GCTTGCGCTCGACTACAGACCCTTTGTTTCAAATTGAAAAGGTTTTCAATAAAGTGAAAT
ACATGGATGATGATATTGATTTGGAAGTATTCTTCGAGGCAACTGAAGATTGGAATTCTG
CCATGACCTTGAGTAACAGTATGGCTTTCATCAGTCAATTTGGGGAGTACAACCCTGGTG
GTGGTGTAGAGGAAGTATTGAAGTACCTCAACGAGTCCAAGAAGGCGGTTCTTGTAGCTC
AAAGTGCGTTGAAGCGAATCATTGACACTTTGCATCTGGAAGGGGTCTAGATAGAGGTCG
TTTTCTATAATTCCTCGAGTAAGAGTACCGAAACCTTGCACCATTCTCTATACAACGAAT
ACTGCTCGAGGGGAAAAATATTTTCCCTGCTACCTCAACTTACGACTAACTAACAACTTC
TTACGCCCAAGAATGACAGTGTGTCGTAGACATAGTAATGCCCAACTTAGTTTCGTTAGC
AATTGATTATTTATTTTTTCTT
>comp2215_c0_seq2
ATCAGTCAATTTGGGGAGGTACGTTTAAAATCAGTTGTAGTGAAGCATAGTTTTTGGGGG
CCGATTGATAATCTAATCATTGTAACGTTCAATATTTTCTATTTACCTGTTTCGACTGTG
TTTGCACCACAAACGAATCGCGAAATTAAAGTACAACCCTGGTGGTGGTGTAGAGGAAGT
ATTGAAGTACCTCAACGAGTCCAAGAAGGCGGTTCTTGTAGCTCAAAGTGCGTTGAAGCG
AATCATTGACACTTTGCATCTGGAAGGGGTCTAGATAGAGGTCGTTTTCTATAATTCCTC
GAGTAAGAGTACCGAAACCTTGCACCATTCTCTATACAACGAATACTGCTCGAGGGGAAA
AATATTTTCCCTGCTACCTCAACTTACGACTAACTAACAACTTCTTACGCCCAAGAATGA
CAGTGTGTCGTAGACATAGTAATGCCCAACTTAGTTTCGTTAGCAATTGATTATTTATTT
TTTCTT
>comp2216_c0_seq1
CCGTGACCTCACCGAGGTCGAGATCTTTGGCTCGAGATCAAAGTGAGGTTGGCGTTACAG
CAGAGCTCTTCCACGCATAGTTATTGTTATCATTTACGTAATCATGTGCGTTGTGCGGAT
TATTTGCTAAGTATCCATGTGGGCTGGGTTACAAGAGCACCTGTTAATGGCAAACGCAGG
CGTCGATCGGTTCCTCCAGCATACTCTATGTCACTCTAGGGGGACTGTGTAGTAGGGATG
ACGCTGCTGGTTACTGGGACTAAAGGAGGAGTTGCCGATACAATGCGAGTTCCTGGTGCG
GAGGACCCTGATACGAGTCGCTCTTACGGTCTGTGATTACGGGACACTGATACAAGTTGC
TGATACTACTAGGAGTTGATACGAGTGATAGTTGCTGAGAGGGATGGATGGGACGCTGCT
GAATCAAGAGTGCACCGAATGGCACGGAGGGGACAAAGTTGCGGATGCGAGTTGCTGATG
CGACTGGCTACGAGCCAAAAGGAGTGGTCAAGGATTAGGGTCGACGATTATGGTCCAAGA
TTACATTCGACGATACGTTTTGCCGATAAAATTCGAGTTGATGGTAGATACGGAGGACGA
TGATACGAAGGATGCTAATACGGAGGATGCTGATGATTGAGTTAATGGACAGATTATCGA
GGCAAATAAAGTGTTCTCTATCTTATTGTCTATCCCTTGAAATCTGAGCTTTCCTCCTAG
CTCTAATAACCATGGCTCTTGTGCCAGATTTCAATCCTCTTGCTCCAATTCAATCCCTAC
AACTTGTTCTACTCACACTCACTGGTACTCAGGCTGGATTCTGCTCTTCCATCTCACCCG
ACTCGAACTGCAGTAGTGTCACTGGAAGATCTCTTTTCCAACAACCGATACGGAGGACGC
GGGGGATGCGAAGACGAGTTTCTGATACAGAGGACGCCGACGCGGGTCGCTGGTGCATGC
CTCTGACACAAATCGCTGATACGGAGGACGTTCACTGATACGAATTGCGATTGCAGCCTA
CGATTAAGTCGGTGTTGGTGCGGATAGGATGCGAACATGAGTTGGCAATACGAGTTGGTA
ATATTTTTTTGTTAGTAATACAAGTTGGTAATATGATTACGGTTAACTATTACGGTTTAC
GATTACGGTCTGCGATTACGGTCCAATTATTTCGGTGTTGGTGTAGATTCATAAAGAATG
TGAAGGGCACGCTGCTGAATACGAAGGACGCTTAATACGGAGAGGAAGCTGAACAAAGAA
AATCATCGCCTAGTGTCA
>comp2217_c0_seq1
AGATGACCTCCATGAAAATCAGCAAACGAAATCAAAAGTCACTAGAAGAACGCATTCGAG
CATGGGGAAACACGCTCTCGTCCTGACGGTGGTTTTAGCTCTGGCCCAAACAGAGTGTTG
CCTGGGCTTTCAGTTGGCACGAGAGACGCATTTGCCCTTGTCTTTGGCTTCTGGGTTATC
TAAAAATGACGAACCCTCCAATGACAACGGTTGTGTCTCAGGATTGTGCAACTCGGAATT
GTCGAGACGGGATGCATGTCGTTCTGTAGTTGGTACTGCTTTAACGATGTCTAGTGCCCT

GTTGGGTGGAGCCGATCCGGTGTTTGCTACAAAAGAGGACGAGAATGGCTTCGCTCCTGC
TGTTCGACCAACGGCGTATCGCGTCGACTCCACGATACCTCCAACACTGCTCCCACTTTC
TAGTGCAAGAAAAGAAAAGGCGACTCTGAAAGCATTGGGAGGTGGGTCGGGAACGGATAA
GGAAGAGATAAAGATCGATACACTTAACCTCAACAACTTCATGAACAAACTCGTTTTTGG
TTCGATTGACTTTGTTTCTCAGACGGTGAGCCAATCGAAAGACGAAAGTAAAGTAGGTCC
GGGATACGCATCATTCGTCTGCATGGGAATGCCTTCTAAGACGACGTCAAATGATATCGA
TTTGGCTACGAGCCTCATGGGCTCAATCCTCCAAAGCCGGCGGGAGGAATTAGCCACTGG
GTTGGGTCTTTCCTTTTGTCCCATGTCGTCACAGCCTGCTTTGGATGCGTTCACAAAGTC
AGGGGATGAGTCAACGTTACAATCTGCCTTGAAGGGAGCTGGAGTGGGAAGCGAAACCAT
TGACTTGTACATGCCCTTGATCCGTTATGCCAAAAGCAAGTCTGTAGATTTCTTGGCCCT
GTCGCCCGAATTTCAGGACATTGAAGCTGCAAGGACCAAAGGTTTGGCATCTGTAGACTT
TGAACGACGCAAATCGTATGTTTTGGATCCAGAGGGTTTCATTTCTATGACCCAGGATCC
TAGGTATAAGCTCTACACGGACAGATCTCTGTTGAAAGACTTTGAACCAACAAATAGTGA
AGACAACATCAAGGGTTTCTTCGCCGAGCGAATTTTCGTCCACGAAGCAGGAGCTTCTGC
CGTAGCCAAATATACCGTGAACAAACCTGAATCAATGGTGGTGATGATATCGCCAATATC
GGATGTGCGCTACTTGCAGGGTATTAACGGACGAATCCCAAGAGTCTTCGGATCCCTCGT
GGAAAAGAACAATGCAAATGGTGTGAACTCTCAAACTGTTACTAACAAGGTATCAGACAA
TGCCGTGACAACGATACTCCTCAACCCAACAGCCCTTGATACACTATCTGCGGGCGAAAA
ATTGCGCCTAGAGATTGGCACTGGACCAGACACTATAGATTATCAATCCAAGATTGCAGA
TTACATTTGGTTCTCATCATCTCCCAAGGTGAGCCTAATTCCGAGACTGATGAATTAATC
ATTAACACATGTATTATGCCGAATGCGATCTTGACTATTCCAGGTGGCTCATGAATATAA
GTAAAAGCATG
>comp2218_c0_seq1
GCTATCGGCGTGAATTACTCGTATTGCTCGTCAAACTATTACTTCCAATTCTAGCTTCGA
TGCTTTCAAACTTCAGATAGGAACGGAATAAGTTGCAATAGGATGAAATTCAAATTGTTT
GACAAATTGAAGAAAGCTTTAAAGCCTAAGGAGTATGCTGCTGCTGTCAAGCTCCAAACG
TTTTTTCGCGGCATTTTAGCCCGTGCCCGTGTGAGCAGAATGGTGAACAAATTGATCACA
GATATTCTGATCAGAAGGGGTGAATTGCCACCAGATTTTTACGAGCAGGAAGAGAAAAGA
AAAGAAGAGGAGAAGAAGAAAGAAGAGGAGAAAAAGAAAAGTGCGACAAAGGGTCGAGGC
GCAATGCTGGAGATACAAAAGGGATCAGTGAAAGATTTGACGTTGAAATTTGACAAGAAT
CCTCCAAAACCACGCGAGACAGCATCTTCTGAAGCATGTCCTGTGGAAGAAGAACCGCCA
TCAGAGCCAAAAAATTCTATCTATAAATCATTTCGCTGTGAGCACAATGCGACGAAACAC
GTCATTTCCTCTGAAGATATTCTCAATGAGCAAGCGCGACGATTGAACGAAGAGATAGAA
CATCTATTGCTGGACATCAAACGGATAGGAGAAAAAGGGAAGAAATCCGTCAAATTTGGT
GAACTGTTTGACGATGACGACGTAGCAAATTACTACGAAGCTTTGGTTGGAACCTTGAAG
GCCGCAAAAAAGAAAGGACTGATCAGTTACGAGGGTCAAATTCTACTCAAAGGAGTCCAC
GATGATCATGAAATTTGTATTCTTTGATGTTGCAGAGCTCAAAATTATTTGAGAGGAGTT
CTCTCCTCTAGTCTGTCGAATTAATGGTGACAACGTGTACAGTATTTCTTATTAAGCTAG
TCGATGTAACTCAACACAAGATTACCTGAACATACATGATAGATTCTGGTTACCTGTCTT
AGTATACCATGCGGCTTCCATACTTATCTGGAAGTATCCACCAACAGAGCTCGTGCAGGC
TTATCACCCTCAAGTGATATAGTACCGTCGCATTCATGGATTGTTGTCACGACAAAGTTG
ATTTTGACTCCACTGGCGATTGAGTCACCTCCTTTTGTCCACGTCTGTAAATCAACGTCA
AATGTGTACCCGGCTTCACGAATTGTTTCTGCAGATACCATGGCATTGATGTAACCAAAC
ACTAACATTCCCACATGGGAAGGAAAACACTCGTTAACGACCCCTTTCAACTTTTGGATT
ATAACAGACAAAAAATAAATAATTTGAGATCACTCTCTTCAAAAGATTCATTGCTTCCAT
AAAATGAGAACATGATCCGGCGGTCAGGTTCTTCTTACCTGGCATCCGATCGAAGGATAG
AACACGAGAACATCACAAGAAACATCGTAATGAACGTGAGGAAGTTCGTTGAGTATCCAT
CCCTTTCCCAAGGACGATTTGTCTTCATGGTCTTGAAGTTTAACGTTGTCGTACGCCATT
AATATTCCTCCCAGACCATTGGAGAATTTTAGCATCATCTTCCGGAGAGAATTTTCAACT
GATTGCTTACAATCCTTGAGAGACCAGGG
>comp2219_c0_seq1
AGAGACGCACTTCAACACAGATGTTTTAGGTGGGGGAACTTGTAGAGGTGAGTAAAATTT
GTTGCGAAAAGCGCAGAATGGAACAGTCGAAATCCCTCTAGACGATTATTTTATCAAAAC
AAATCAACTCAAACCTCAACTTGACGGCTGATCATTGAAATTGGAATTGAATAATAAAAT
CTTGTCTATTACAAGACAATGGTTAGACACCTTGGCGATACTTATGGGGTGCTTAAAGTG
GCGACATTAATTGTTTGCTGCTTTCAAGAATCCTCCTCCTTCAACCTTCACAACAATCGT
TTGACTCACTGCCATAGCACTGTAACAAATATCAATCAAAATCCTTCGACTGAACTTTCA
AATCCCATTCGACAACAACGTTTGTTGGCGACCTCGTCGGGTGTTGGAAATAATTTCGAA
GAAGCGTTGCAACATTCACAAAACCATGCGTCGTCCTTGAATGCAGCAGGAGGAGCAACA
TGGGACGATGTACTTATCCCGTCATTAGAGCTTCCAGTCAACGACCATACAAAAATAACA

GCTATGGATAAACTCGGAATAGCTATTTTCGGAGGGTGCTCAATTGGCGTATTCTTGTCA
ATTGTCTCGAAGTCTGCTGGATCATGGAGATTTTTCTTGGCTGGAGGACTATGCGCAGCA
ATTTCTCATACGATTCCTACACCAGTTGATGTCATCAAGGTGAGATACAGCGGAAGGTTT
TTACCTTGTAATAGAAGATGGTACTTCATATTTTGTCTCACTCGGCATTCTGACGACTTT
GACTGCTGGCGACAATTTGGTCCAGACTCGCAAACAAGTAGATACTAGATTTGTAGATAA
GTCTTTTGTCGAGGCCGGTCGCGTAATTTTGAAAGAAGATGGGTTCAGCGCTCTTTGGGC
TGGGTTGGGACCAACGACCTTTGGCTATTTGTTAGAGGGAGCCGTCAAATTCGGGGTTTA
TGAGGTACTAAAGCCAGCCATCAAGTCGTGGCTTTCTGTTATAGCTTCGATGTCGTCTTT
TTCTTTTTTGAATTCGCATTTCTTTTCCTTTGTCTTGAGTGGAATTGCATCGGGCCTTGC
AGCTTCGTTCATTTTATGCCCTATGGAAGTGATAAGAATACGGTTGGTGGCGGAACCAAA
TTATACATCTGGTAACTGGATACGGGGAGGCTATAAAATATTGAAGAAGGAAGGTGTGCA
AGGATTTAGCAAAGGATTGAACCCCATGATATTGAAGCAGGTTCCGTACACTGTAACAAA
AAATGTTTCGTTCGATGTTTTCACGAAATTTTTCTACTCCTGTCTCAGAAACAGTGGAGT
TGCGACAGTATCGCATGCAATGAAATTTACGATTCCCTTGGTGTCCGCTGCGATTGCTAG
TGTTTTATCATCTTTAACGAGTCAGCCTGGCGACACCATTCTAAGTCTATCTTGTGCTCA
TGGTGGTGATTACAGAACACGTGATATAAGCCGGAATATCTTGCGCAGTGACCGTGGCAT
CGCGGGTTTCTTCGTTGGCACAAGAACGAGACTCCTCCATGTCGGAATGATTGTCACGAT
CCAGCTCTTGATTTATGATTATGTAAAGAGGATATGTGGGATAGGTGCAACAGGTTTGGC
ATAAATCGAGAACTATGTACACTCTCCATTCATCTTCACTTTTTAAATTTGTTCGCACAG
ATTTACTCTAACCTTCAATTTCGACTTTAAAAATATCAGCAAGTCTACC
>comp2219_c0_seq2
AGAGACGCACTTCAACACAGATGTTTTAGGTGGGGGAACTTGTAGAGGTGAGTAAAATTT
GTTGCGAAAAGCGCAGAATGGAACAGTCGAAATCCCTCTAGACGATTATTTTATCAAAAC
AAATCAACTCAAACCTCAACTTGACGGCTGATCATTGAAATTGGAATTGAATAATAAAAT
CTTGTCTATTACAAGACAATGGTTAGACACCTTGGCGATACTTATGGGGTGCTTAAAGTG
GCGACATTAATTGTTTGCTGCTTTCAAGAATCCTCCTCCTTCAACCTTCACAACAATCGT
TTGACTCACTGCCATAGCACTGTAACAAATATCAATCAAAATCCTTCGACTGAACTTTCA
AATCCCATTCGACAACAACGTTTGTTGGCGACCTCGTCGGGTGTTGGAAATAATTTCGAA
GAAGCGTTGCAACATTCACAAAACCATGCGTCGTCCTTGAATGCAGCAGGAGGAGCAACA
TGGGACGATGTACTTATCCCGTCATTAGAGCTTCCAGTCAACGACCATACAAAAATAACA
GCTATGGATAAACTCGGAATAGCTATTTTCGGAGGGTGCTCAATTGGCGTATTCTTGTCA
ATTGTCTCGAAGTCTGCTGGATCATGGAGATTTTTCTTGGCTGGAGGACTATGCGCAGCA
ATTTCTCATACGATTCCTACACCAGTTGATGTCATCAAGACTCGCAAACAAGTAGATACT
AGATTTGTAGATAAGTCTTTTGTCGAGGCCGGTCGCGTAATTTTGAAAGAAGATGGGTTC
AGCGCTCTTTGGGCTGGGTTGGGACCAACGACCTTTGGCTATTTGTTAGAGGGAGCCGTC
AAATTCGGGGTTTATGAGGTACTAAAGCCAGCCATCAAGTCGTGGCTTTCTGTTATAGCT
TCGATGTCGTCTTTTTCTTTTTTGAATTCGCATTTCTTTTCCTTTGTCTTGAGTGGAATT
GCATCGGGCCTTGCAGCTTCGTTCATTTTATGCCCTATGGAAGTGATAAGAATACGGTTG
GTGGCGGAACCAAATTATACATCTGGTAACTGGATACGGGGAGGCTATAAAATATTGAAG
AAGGAAGGTGTGCAAGGATTTAGCAAAGGATTGAACCCCATGATATTGAAGCAGGTTCCG
TACACTGTAACAAAAAATGTTTCGTTCGATGTTTTCACGAAATTTTTCTACTCCTGTCTC
AGAAACAGTGGAGTTGCGACAGTATCGCATGCAATGAAATTTACGATTCCCTTGGTGTCC
GCTGCGATTGCTAGTGTTTTATCATCTTTAACGAGTCAGCCTGGCGACACCATTCTAAGT
CTATCTTGTGCTCATGGTGGTGATTACAGAACACGTGATATAAGCCGGAATATCTTGCGC
AGTGACCGTGGCATCGCGGGTTTCTTCGTTGGCACAAGAACGAGACTCCTCCATGTCGGA
ATGATTGTCACGATCCAGCTCTTGATTTATGATTATGTAAAGAGGATATGTGGGATAGGT
GCAACAGGTTTGGCATAAATCGAGAACTATGTACACTCTCCATTCATCTTCACTTTTTAA
ATTTGTTCGCACAGATTTACTCTAACCTTCAATTTCGACTTTAAAAATATCAGCAAGTCT
ACC
>comp2220_c0_seq1
GAATGTGTGAGACGCGTGTCTCCTGTTATTGTACAACTGACCTGTACGCCACTGGATTCC
TCTTTCATCCATCTTTTACTGTTTTGTATCTGAAATCTGAAATCTACCTGAAGTAAGAAC
AGCAACCGTAATCACACTGAATCGCTGCGAAGGGATTAAAAGCATCAATACTACGTACGG
CACTCTATTACGGCTGCAATGCCAATATCGATGTCCATAGTTGGAAGTTTGCGTTTCAAG
TGGATCGTTTGATTGGGAAAAGGACAAACGATACGAAGGCATACCGCGCACAATCTGTTG
AGATACGAAATCGAAATCAAAACATCAATTCCATCATAGCATGACTCCGCACCACATCTT
TCGCCACGGTAGATTCCACAGCACGCTGCGGACATTGTCCTTGGCAGGCGGATCCCTACT
CGCCGGCTGCCAGATAGCTTCCTCTCCGCTTTTGTTCGAGAACGAGGATACGTGTCACTG
CAGCCTCAAGGTCAACAACAATGCGGATGACACCATCTGTACGGTGCGCAATCGACTACG
GGAGATCCACTCTGTTTTCTCGAGATTTTCCCGTTTGCTGGAAGCAAACCGTTCGTCGCT

GGAGCCGGCTTCGAAGCCTGCCTCCAGGATCGCATCCGAAAACAACACGATTTTGGCGAC
ATCGGTACGGCAGAGAAAAACCTGCCTTGAACCCGGGAAGGCAATCGGTAGCGTCGGAAA
CACTGCTCTGTACGACTTCGTTATCGTTGGATACGGCAATGCGGGCCAGAGTGCCCTGCG
CACGCTTCAGGAACAATGCCCGGGGGCCAGGGTGGCCCTCGTGGATCCATTGCGTTCCCC
AAACAAAGGAACCAAAGGCAAAGGCGTCGACACCGATTATTTCCGCGATACCGTGGTCGA
ATTCGACCCGTCCTCGAAAACCCTTCGGCTGCTGGCCGCGGATCCAAGCCGCAGAGCGAT
TCGCTACAAGCACGGCGTTCTGATCGCGACGGGGGCACGGGGGGCACCCCCACCGCTGGA
GCTCTTCCAGGAAGAGTCGCTGCCCCGAGTCCTCGAACTGCGGCCTACCGAGCTATTCCG
AAACACAAAACGCCCGGCAATGGCCCCCGAAAAGGTCAGGAACGCGGTTGCGGAAGCGGC
GGCGAAGGGGGCGAAAATCGCAGTCCTGGGGAGCGGGTGGGAGGCCCTCGATCTCCTCTT
GGTGGCGGAGCGGGCGTGCAAATCCACCAAGAGAAGGCCAACGATGTGCTTCGCCAGCTC
GGGCATTGCGTGGCACACCCTGCCCCCCTATCTGAGCGCGGAGCTGCGAAAGAAACTTGG
CAAACGCGAGGTTGATGTGCAGGATCGTTCGTTCGTCAGGTACGTCGCAGACTACCAGCA
ATCGAACCGCCAGCAAATCGAGCTCCACACCGCAAAGACCTACGACTTGATGGATACTCG
CAGAACTGCTTTGGACCTGTTGGTTGTTGCCCCCGATTCGTTTGGTGACAAGGGAACCGC
GGCGCTTCCAACCATGGACGTTCCGGAAAGCATGAAGGAGTCCAGCGATGGTCGTCCGTG
GTACAAAACTTGGTCCCAGATGGCCAGACGGAGAAACGCGGAGCCATCAGCGCTTGTTTG
TTTCGAAGACGACGGGAGAATATCGGTCAATGCCGAGCTACACGTAGCCTCACGGATCTA
CGCCGCGGGGGGCTGCGCCAAGTACCCGAACAGCTCCACGGGCCATTCGTGCATTGCCGG
GGAGGGATCTATCGACGGGTCCGAAGCGGGTCGTCTGGCCGCCCTGAACATGTCCCGGGA
CTATCGCCTGGCAACACGGGCATCCTCGAGCTATGGGTTTTCCTCCTCCGGAGATGATAT
CGAAGAAGCGCACTCGTTTGCGACCAATTCACTCCCGATATGGCGCTCCGATATTACGTC
TTTTCCGGCCTGTAACGGGGACCGGATCTCGTCTCTCTCTAGCATTGGTGTGCAAGCACT
GTGTGTGGGGAAGTGTGACTCCGAGCGACAATCGACGAGAGCGTTTTGGTGGACCAACTC
TTCCGCACGGCGGAAGATGAATCGCTTCATCAAGGACGAATCAGAAAGTTCGCAAGACGA
CGATGGGGACGATGGCAAAGACGAGGATGTTGACGAAGGGTCGATCAGGCGTCGGAAAAG
TCTTGTTATCAGCCGCCACAAGACCGGCCGAGTCAACTCCCGCGGTCTCGTGAAGCCGGT
GTACGGGGTCGGGGTTGTCTTTTACCTCGATCACTACGGAAGAATCCAGGGGATCATGAC
CTGGGGCTTGCCGTTTGCGGATCGACCGGGGGGAAAGATCAACCCGGAACTTCTGGAACA
CATGAAGCACATGATTGCCTCAAACGCAGGGGTTTCCGCTTTGGATGCGGAAGACAACCA
CCAACTCGTGAATCTTGCACTGGGGAAAGCGAGTCAAAAGTTGGTAGCCCTTTCCGTAAA
AGACCAAGTCTCTCACACCACTAGAACATGGCACGGCCTGGACGGACCTATCCAAGGGTT
TGCAACGCCACTTTACCGATACACCGAGGTGTCCAACTCACGAAACAAAACCGTCAACGT
CTTGAAGCGAAAAGACGGGAGCGGTCTTGGTGTACTAGGTGAAGGGCTGTACGTGCGGGA
CGATTTTGTATTGGAAGAAGTGGCCGACAATGGACCCGGCGATCACCTCTCGGAAGAAGA
AAACGAGGATGCTTCCCCCGCGAACATTCCTACCACCATGTACCCGATCACGGTGGCCCC
GGCGTCTCACCACGACGACGCCTACGGCAAGAAGGAAATCTCTCTCGAAACGATGAAGGA
ACTCAATCGATACCTGGCGGTTCAGCTCCGGTGGGAAGCAAACGAAAACAGAGCGCGTCC
CGGCAAGGAAGATCCACTTTGGTTGCGCCCCGGAGACGAGAGAAAAAACACGAGCGGAAA
ACAGAACGTCATTGACGCGTACCGACGAATCATGTTTCCCCATCAATCGAGCTAAGAGCG
ATAGGCATGGCATGGTGCATCGTAATCTGATTGCCTTCGTTGTGAGCTAAAACATTATTC
TCTGGTCTCTGTTGCTAAACTAAATTAATATTTGCGACATCCCATC
>comp2221_c0_seq1
GGAACATCGATGAGAATTAGATTGCTGAGGCTTTGTGCAGAACATAGTTTGCAGAATGAA
ATATTCTTTCGTTGGATTCTTACTAGCCTTTCCAAGATATCGAGGCAAGTTGAACTCGAC
AACTTCCTTGGTCGCCGGGTTCTCACCTTCCCCTGTTACCAGAAGGAACTACAGAAATGC
CATTGGGTATCTGACTGGTTGCCGTTCGTATGTGGAAAAAACTGCCAGAAGCAGAAGATC
TTCTCTTTCGGCACAAAGTGGTCGCGATTCAAGAGATAACTACGGTAGTAACCCTGATTC
TATAGAAAACAAGGCAGGTACAAACAACAACACAAAGAACTGTAATTGGCTTGTGGTGGG
AGATGGAGATCTATCGTACAGTGCATCCATTGCAGAATATCTAGCCAACAAAGGCATTCG
ACTCTTTGCAACCGTATTGGAAGAGGAAGAAGTTCACGATTCGGTCTACAAACGGTCCTC
GCAAAACAAAGCAGCCATACTGTCTTACCACAACCTGCACGATAGCCTCGTACGCTCGGA
TACTACGGGAGGCGATGATACGGCAGCAACAAACTTGCAATCGCAACACATAGTTTTATT
TGGCGTCGACGCTACCCATCTGATAGACTATTTTCCAAGGACCAAATTCAGCACCATCGT
ATTCAATTTTCCTCACTGGAGGGGTAAGACGAATGCAAAACGCAATCGAATACTTCTAGA
CAGTTTCATGGGTAGTGCCGCTGAAGTACTCGCCGAAGAGGGCAAGATAATCATTTCACT
CTGTGAAGGACAGGGTGGCTTTCCTGCATCAACCGCCGAGGAATGGAAACGGAGCTGGCT
TGTACCTCAATACGCAGCAGGACATGGATTGCTGCTGCACAAGTTGAAGCAGTACGACCC
GATGTATCAACAGACTTCTCACAGAGGCAAGGATCGACCCTGGATCAAGGATGGCACGGA
CCAACGATACATATTTGGGTTTCCAAATGAGGGATGCATAGACGAAGATTTACAGATAAG

CTGTCGTCACGAGCTTAGAGTTGTGTTACCAAAAGATTTTGTAGAAGGCTCCGCAGTTGG
TCGACAAGAGATCATAGAAGGCGACGCAGTTTTTCAACTAACCAAAGGATTTGTCCCAGA
TGGAATTCGAATGGAAGTAGTGACCCGCGAAATATTGTATCCTTATTTCTTTGACAATGA
AGAGGTCACTCTTGCTATTTTTCTTCTAAATTATAGTGGGGAAAAGGCCCCACTTACCAG
AGAATCTGCCGATACCATTCGATCTAAAGTAGAAGCCTCTGTGCAACAAGATTGGAATCT
GAAAGTGGCCAAAAGCGGGCGGCTGGTTTCTCGTCCTTATCCTACGCACTTGCTACCAGC
TCTAATACGGGAATCGAGTGAAAGGAACTTAACACTGACCAATTCAAGTCCTTAGACGTT
ATCACAACCATGTTTGAACTATACTAGTCTTTTATTATGCAAC
>comp2222_c0_seq1
CAATGCCGGAGGTAAGTTTTCCAATAGCTTGGAAACCAAAGAATTTCTTTTAAAATCCTT
TTTCGCTAACGCATTCCTAAACCATGTGACTGTATTTCTTTTGAAAAAAAAATTTCAGGTA
TTGCTGAAATGGCCGAACTTCACCCAAGTGTTCGCGAAAAGACCGATGCTTTGGACGCTG
CCGGAAATACAACTGCCGCCATCGGAAAGGGATTCGCAATTGGTTCTGCCGCGTTGGTTT
CTCTTGCGCTTTTCGGAGCCTTTGTTACCCGCATCCGAGATTCTTCCGGTCAAACTCTGT
TCACCGACGGTGTCAACATGCTCGAACCCGTTACCTTTTCTTTCTTGATTATTGGAGGAA
TGATTCCTTTTGCCTTTGCTGCTCTCACCATGAAGTCTGTCGGTATGGCTGCCATGGAGA
TGGTTATGGAAGTCCAGCGTCAATTCGACGAAAAGCCTCACTTGTTGGACGAAAACCCAT
CCGAACGTCCCGACTACGATGCCTGTATCGCTATTTCCACCCAGGCTTCCCTCAAGGAGA
TGGTTGCTCCTGGTGCTATGGTCATCTTCACTCCGCTTGTGACGGGTATCTTGTTCGGAG
TCACCGCCGTTTCTGGACTTCTGGTAGGAGCCTTGGTCGCCTCGATCCAGCTTGCCATTT
CCATGTCCAACTCTGGAGGTGCTTGGGATAACGCCAAGAAATACATCGAAAAGCAATCTG
CCGACTCCGAGCTCAAGGGCAAGGGATCTGATATCCACAAGGCCGCTGTCGTCGGAGATA
CTGTTGGGGATCCGTTCAAGGATACCTCTGGACCTGCCCTGAACATTGTCATGAAGCTCA
TGGCTGTCCTTTCCCTTGTATTTGCCGATACTTTCTATGCTATCAACAATGGAACCGGAT
TGCTCCAGCTTTCCTAAACAGTTTGTACTCGGTTTATTGATCGGTTCGTGTTGCATCCAT
CTCAATGGCTTTCAAACTTTTCAAATCATTGACTCTTTGTTCTATTTAGTTAGGTGAAAT
TAATGCATAACATTGATTAGCTCTAAA
>comp2222_c0_seq2
GTTTGTCTGAAATAAATTAAGAAGTAAAATCACCATGTCGTTACTCACTGGGGCATCGGG
CCTCATTTTTGGCTCGGCCGTTGTTGCCTTGCTCTGGGGTTACCTCCAATTTAAGATCAT
TGCTGCCATCCCCGTAAAATCCTCTTCTGGATCCGGAGAAGAAGAGGGGCTCGCAGATCA
AACCGACCACGTCACAGCGACCCAGCGCTTGAACGAAATTTACGAGGCCATTTATGAGGG
TGCCGAGTCCTTCCTCAAGGCTGAGTACTCGGTCTGCTTCTGGTTCGTTGTCAGTTTCTC
CGTGCTCATCTTCATCTTGGTTTCCTGGGGAACCGGATGGGACATGTCGCGCGGTAGCTT
TACCGCCATTTCCTTCTTGCTCGGTGCCTTTACTTCGATGGCATCCGGATACCTAGGAAT
GAAGGTTGCCGTTTTCTCAAACGTTCGCACCACCGTCTCGGCGCAAAAAGCCGGATGGAC
CGAATGCTTTAACACCGCATTCCGAGCTGGAGCCGTCATGGGATTCGCCCTCTGCGGTAT
GGGAATCTTTATGCTCTACGGAACCGCGCTACTCTTCAAATCGCACTACCCCGATGCCAA
AGAATGGATCTTGCTCACCGAATCTCTCACCGGATACGGATTGGGAGGATCCGTCATTGC
CATGTTCGGGCGTGTTGGAGGAGGAATCTACACCAAGGCCGCCGACGTTGGTGCCGATTT
GGTTGGAAAGGTTGTTCACGGAATCCCCGAGGACGATCCGCGCAACCCCGCCACCATTGC
CGATAATGTTGGTGACAACGTCGGAGATGTCGCTGGTATGGGATCCGATTTGTTTGGATC
CTTCGCTGAATCCACCTGTGCTGCCTTGGTCATTGGAAGCAGCATTGGTTTGTCTGGAGG
ATGGGATGCCATGGTCTTCCCCATCATTGTCTCGGCTGTCGGAATCTTTGTCTGCTTGGT
GTGCTCCTTTATCGCTACTCACATCTGGACCGTCAAGCAAGAATCCGATGTCGAACGCGC
CCTCAAGGTCCAGCTCATCAGTACCACCATTGTTATGGTTCCCGCTGTTTACTACGCCGC
CATGAATTACCTTCCTGCCTCCTTTGAATTGCATGCCACTGTTGGAGATGCCGTCTTGAC
CCTCACTCCTTTGGATGCTACAATCTGTGTTGCTATGGGAGCCGTCGGAGGACTGACCAT
TGGTTTGATCACCGAATACTACACGTCTCACTCGTACGCACCCGTCCGTGAATTGGCCAA
CTCGTGCAAGACAGGAGCTGCTACCAATATGATCTACGGTATTGCCTTGGGATTCAAGTC
CGCCATCATCCCCGTTGTTGTCTTGGCCCTTGTTGTCTACTTCTCGTTCGACATTTGTGA
CATGTATGGTGTCGCCTTGGCCGCCATCGGGTTCTTGTCCAATCTTGCAACCGGTTTGAC
CATTGATGTCTACGGACCCGTTTGTGACAATGCCGGAGGTATTGCTGAAATGGCCGAACT
TCACCCAAGTGTTCGCGAAAAGACCGATGCTTTGGACGCTGCCGGAAATACAACTGCCGC
CATCGGAAAGGGATTCGCAATTGGTTCTGCCGCGTTGGTTTCTCTTGCGCTTTTCGGAGC
CTTTGTTACCCGCATCCGAGATTCTTCCGGTCAAACTCTGTTCACCGACGGTGTCAACAT
GCTCGAACCCGTTACCTTTTCTTTCTTGATTATTGGAGGAATGATTCCTTTTGCCTTTGC
TGCTCTCACCATGAAGTCTGTCGGTATGGCTGCCATGGAGATGGTTATGGAAGTCCAGCG
TCAATTCGACGAAAAGCCTCACTTGTTGGACGAAAACCCATCCGAACGTCCCGACTACGA
TGCCTGTATCGCTATTTCCACCCAGGCTTCCCTCAAGGAGATGGTTGCTCCTGGTGCTAT

GGTCATCTTCACTCCGCTTGTGACGGGTATCTTGTTCGGAGTCACCGCCGTTTCTGGACT
TCTGGTAGGAGCCTTGGTCGCCTCGATCCAGCTTGCCATTTCCATGTCCAACTCTGGAGG
TGCTTGGGATAACGCCAAGAAATACATCGAAAAGCAATCTGCCGACTCCGAGCTCAAGGG
CAAGGGATCTGATATCCACAAGGCCGCTGTCGTCGGAGATACTGTTGGGGATCCGTTCAA
GGATACCTCTGGACCTGCCCTGAACATTGTCATGAAGCTCATGGCTGTCCTTTCCCTTGT
ATTTGCCGATACTTTCTATGCTATCAACAATGGAACCGGATTGCTCCAGCTTTCCTAAAC
AGTTTGTACTCGGTTTATTGATCGGTTCGTGTTGCATCCATCTCAATGGCTTTCAAACTT
TTCAAATCATTGACTCTTTGTTCTATTTAGTTAGGTGAAATTAATGCATAACATTGATTA
GCTCTAAA
>comp2223_c0_seq1
AGAGCTATGAAAAAACAACTCCTCTATTTAATCCTTTCCCTGGCACTGGTGATCTTCTAC
GGATCCTGTGATAATGGAAATGCAGACCCCGTCATCGTTGACGGTCCTCCGGTTATTGAA
TCATTAACCGTTAATGCTGGAACTATAGAAAATACCGACTTTATGGCTACTGCGGTAGTC
AATGATGGTATAGCCGGTAGTACCATTAGTACACTAGCCACAGCGAACTGGACAATCACC
AATGGTGGTACTACAGTGGCCTCTGGAACAGAATCACTGAGTGGTGATTTAGCCACTATA
AACATTGCTGTAGCTGGTGGTTTTGGTGTAGGTGACTACAATCTCGATCTGGTTGTGTCA
GACTCCAATGGTAACTCGTCTACTGAGAATGTGAGTTTCACTGTTGAATTGCCTCTTCCT
GATTTTGACATTTCAGGTGATTGGAAATTGGAGCCAGTAGCAGGAGCCCTGAAAGTAGGT
CCGGCACAAGGTGACGGCTCTTGGTGGTCAAGCGGTGCTGGAGACGTAGCTGGTAGAGCT
TGCTTGTTTGATGATGTATATACATTTACTGGTAATGCTACTGGTACATTTGGGGTAAAC
GTAGGTGATATGACCTGGTTAGAGCCTTGGCAAGGAATGGACCCAGAAGGATGTGGTACA
CCTGTTGCCCCTTTTGTGAGCTCTACTGACTTCACTTATACTTACCAGAACAACCAACTT
ACCCTCGAGGGTGTAGGTGCTGCACTTGGCCTTGTGAAGGCTCACAATGGCGTTGAACTT
GGAAATCCTGCAGATGCTCCAGATCAGGTAACTTACGAAATTGCCTCTCAAACTCAAGAA
GGTTCTGTGCGTAGAATGACTCTTCAGATTTTTTCTGGTGGAGAAACTTGGTGGGAATTC
CTGTTGATTTCAGGAGAGCCTGATGCAGTTCCAATTGATGTTGTTGGAGACTGGACACTT
GAGCCTGTAGCTGGTGCTTTGGGCGTAGGCCCCGGACAAGGAAACGTGTCATGGTGGGCA
AGTGGTGATGGTGACGTAACAGGTCGCGCATGCTTGTTTGACGATGTTTATACTTTTGAT
GCCAATGGTAACTTCTCTATGACGCTGGGTTCTGAAACCTGGTTAGAGCCATGGCAGGGA
ACTGATCCTGAGGCTTGTGGTACTCCTCTGGCTCCTCACGATGGAATGGGTTCTTATACT
TACACTTTGTCTGGTACTGACCTTCAGATCGTAGGAACCGGTGGACATGTTGGTTTAGCT
AAAGTGACGAATGGATCTGAACTAGGAAATCCAGCGGATGCTCCTGGTGATATTACTTAT
ATCGTTTCTGATTTCTCAGTGGATGGTACTACCAAAAGAATGACACTGCAAATTTTTGCT
GGTGGAGAAACCTGGTGGCAGTTCCTGCTGATCTCGCAATAATACCTTTAGTTCATAGTT
TTTATGGCCCGCTCCCATGCGCGATGGGGGCGGGCTCATAAAACCTCAATTACTTAAGAG
CATGAAAAAAATCAACAGCATTTTTGCAGCAATACTGGTAGTATGCTTCTATGCCTGCGG
TTCTGATGACGGTGGTGAAACACCGGGAGGCGGCAATACAGAAACCATTACAATTCCAAC
CACGGGATTTACTTCTCCCACGTCATATCCCAATATGACCCTTGCCTGGGAGGATGATTT
TGACGGTGCGGTTCTTAACTCAGCGCACTGGACTCACGAAACCGGTACCGGTTCTAATGG
CTGGGGTAATGCCGAGCTTCAATACTATCGCTCAGAAAACACTACGCT
>comp2224_c0_seq1
GGCCTTTGTACTCGTTCTCTTTCCGCCGATGAAAGATCAAAACAGGTTTACCAAGAAAAC
CCTGACGAAACCCAAGCTAAAAATGAGTGCCGCTGCCGCCCGTCGCAAGAAACAACTCCT
GGCTCGCAAGAAGCAACAAGAGGAGGCTGCCGCCGCGGGCCACGATGCCCTCGACCCGGT
TTCGGCCCAGCTGCAGAAGCTCCTAGAGGATCCCGAGAAACTTGCCGAGGAAGCCACCGC
GTACGAAGCCTTGCAACTCGCCCAGTCGCAGATCCGAAAGAAGGTCCACGCCGGAAGTTA
CGCCGAGGCAATCGAATTGGCTTGTTCCGCGAGTCTGATCATCCTGGAGCAGGGCCGAGT
CTCGGTGGCCTCGCAGCTCACCGCTTTGTTGGCGAATGTCTTGCGCGAAACCCACACCGA
AGAGACCCCGGCATTGCTGGAAAAACTCAAGGGCCTGCACGCCGCGCAGGAAAAGGCTAT
GGACGGAAAGACGGGAGCGGAAGCAGTTCGTTTGAATAGACTCCAGCGCGACTGGCTTCG
CAAATGTGTCCAATGGAGCTCCGAACTGGGAAAGACTCGATACGGAAACCTGGAGCTCCA
AAAGCTCTTGGCGCAGCAGTCCTGGAAATTATCCCAGCAACTTGTAGCTGAGGGTGCTGA
TGAAAACGTCGACGAGGAGGACAACGAGGTCATGGAACTCAAGGCCGACGCGGTGACCCA
CATGGCACTCGCAGAGGAGCCGATGGGCATTGTCGAGATGCTCAAGACCATGCCAAAACC
CACGGCCGCAGAAACCAAGTCGGGGCACACCTGCCCCCCCGCGGATCGTGACGCTCTCCT
GACGCGAGCCGTCTTGTGCCTCTGTGCGGTCGAGAACCTGAGGGATGCCTCAGCCCTCTG
CCGCGCCTTTTTGGAACAGGTCGAGGACCGCGATGCCGACGCCCTGACCGCCTCGTATGT
CAGCAAAGACGATGGAAAGGCCCCCTCCCACATTATCTTTTGCTGCATGCTCATTCGCAT
CTGCGAGAAAGACCCAAGAACGGGACCTCTTTTCTCGTGGCTGCTTCGCTCTTTCAAGAG
AGAACTCGATGGATTCTACAAGGTGCAAATCATTCAGTCCTACACTTCCAAAATCGGCAA

AATCTATTTCAACATTCAACCTCCACCCAGCATGATGAACATGTTGGAAAACATGATGGG
CTCCATGGGAGGAGGAGGCGGTATGGGAGGATTAAACCCAGCGATGATGCAAGCAATGAT
GCAAAACATGCAGATGTAGAGACGCCCGTGGAGTACTCCCGCTGTATCACGTCCCATGCA
CATTTGTGAACAATCCAATTTTAGTTTCACCAAGTTATGGTTGAATTGCATTTTGATCGA
GGTTCATAATGAAGTGCAATTAATAGTATTCATCATCCTCATTTTAAACTACAGGATTCA
AATAATGGAACTGGAAAGGAATCAGTACCATATTTTTGCAATACAATAGTAATAGCACTC
ATTACGTTCACCGATAAATAGAGAAAAATGTATTTCTATTAATCGCTTTTCCAT
>comp2225_c0_seq1
ACTAAAAGCAAACAATTTACTGCATTGTATTTTAAAATAAAAACTTCTACATCAAGAAGA
AAGAAGCACATCAATAATAACACAACAATTGGTGAAAAGGATTATATCAGAAAACAGCAA
CGACAAACTCTCGCTCGGTCGAATTATCTTCTCTATCGATAGAAGGCAGCAATAACCACG
CAAGTAATTGACATCGAGGGCCGGATGGGTGGAATCGCAGAGGATGGTAACACTAGTGTT
GTCGACATCGCTGTCGGCGGCATGACGTGTTCGATGTGCTCCAGTGCGATACACAAGGCT
TTGACGGGGATGACAGGGATTAACAACGTCACAGTGTCACTTGCCACCAATATTGCTAGC
GTGGAATACGTCGAAAGCGAGACTCTTACCGCAGAAATTATTGCGGAAGAAATCGAAGAT
ATTGGATACGACGTGACCGATATTATAAGAAAAGTCCCGGCATACAGAAGAACTCGAGTG
CTCAAATCAGTGGAATATGCCGTTGGTGGAATGACATGTTCTATGTGTTCGAGTGCAGTA
ACTAAAGCCTTGACGGGGATGCCGGGAGTCCAAACTGTTGACGTCTCTCTCTCAACCAAC
ACTGCAAGAATATCTTATTACGACGATGATCAAGTAGAGAACGATACAAACGAACCCTTG
GACTATCCCGGTGATTTCCAAGAAATGATAGAAGACATTGGATATGATGTAAACGGCGTA
GTCGATGATTGCGGAGAAGAAAGCTTGTGCGAAAACAATGACGACAACGATGTCGACGAG
TTGGTTCCCAGCGAAGCTGAAAATCGACTGGAACGTATTCTGCGGCAACAAAATTCCGAA
CTCAAGGCCAGGAGAAATGCTTTTTTGTGGAGCTTTGTAGGAACCTTCCCCATTTTGATT
ATAACGATGGTGATGCCCCGAGTTTTGCACAAGGACAATTTCGTTTGTCAATTTATGAGA
CGGCACGTAGATGTCTTCGGCTGCAAACTATTGATAGAAGCGATTATCCTGTGGATGTTG
GCCACGCCGGTTCAATTCTTGTGCGGGCATTCCTTCTACAAGACTGCATTCCACGGGATT
CGAAGAGGCGTTTTGGGGATGGATGTACTGGTGTCGATAGGGACGACATCGAGCTACGTA
TATGCTTTCCTGGCCATGTGGAAAAATGATTCCGGGTACCGCTTTTTTGAGACTTCTGCG
GTACTGATTTCATTCGTTCTTCTCGGGAAATGGATGAACGCAATGGCTGTTCGAAAAACT
AGCCAGGCGTTGACACAATTGATGAAGCTACAAGCCAAGACTGCTATTAAAATTACCATC
CCTCCCTCCACCAATTCATCGACCTGGAATCCACTGACAGACCCGTATTCAGAAGAGATC
GTACCCATCCAAAAAATCAAACCGGGAAATATTGTAAAAATTTTACGCGGTGCTAATATT
CCTGCCGATGGCACTGTTATCCATGGCGAATTAACGGTTGACCAGAGCATGATTACAGGA
GAATCCATTCCGGTCTTGAAATCACCAGACGACGCTGTAATAGGTGGAACAATTTGTTCC
GAAACAGGAACCGTGGAGGGAGATAACAATGCTCTAACAGCGGCTGCCTTTGTGAGGGTG
ACGGGAGTGGGAGCTGACTCTGCTCTTTCGAAGATTGTTCAGATGGTTCAGGACGCCCAG
TCACGGCAGGTTCCCATACAAAAACTAGCCGATGGAATTGCATCCATATTTGTTCCAGTG
GTTGTCGTCTTTTCAATATTGACATTTCTGACATGGTGGGTGTGCTGCAGCACGGGTCGC
GTCCCTACCGGATGGTACGACAAAGAGAATCCAATTGCCTTTTCGCTTATGTTTGGAATC
GCGGCCCTGGTCATTAGTTGCCCGTGTGCCCTCGGCTTGGCGACACCAACAGCCGTCATG
GTAGGCACTGGTGTCGGAGCCAGTCATGGGATTCTAATGAAGGGCGGCGAAGCGCTTGAA
GTAGCCTCGAAAGTTAATGCTGTGATTTTTGACAAAACCGGGACCCTCACAAGAGGGAAA
CCAGCTGTAACTGACTTTACTCGGGTGGTCTCGGACGAATATTTGAGGAGCCTGATGAGT
AACGATGTGGCACAGATCTCAGCAGATTACTTTTTGCTTTGGTTGTTGGGTTCTTTGGAA
CGCAATTCGGAACATGTTTTGGCCACAGCCATCGTCAAATATGCGGAACTCAATATCGAA
AGTTTACTCAGATCTTTGCCATTTGCTCAGCCTTCTGACTTCGTACCAGTTACTGGTCGA
GGGGCATCAGGGGTCATCAACGGCAAAACCGTTGCTATCGGTAACCGGGCCTTTGCTGAA
CTTCACACAGGCAGTATGAAGATATCTTCAGAGGCTGAAGAATGCATGCAGAAGTTGGAA
GGCGATGGGAAAACAGCTATCCTTGCGGCAGTCAATGGCGTCGTTTGCGCTGTTCTCGGC
ATCGCCGACGAAATCAAGACCGAGTCCCCCGACGCTATCCGATATCTTCATGACCGAGGG
ATGGATGTATGGATGGTAACAGGAGATAGTTGGCGAACTGCCAAAGCGATAGCAAATGAG
TTACAAATACCACGAGCCCGCGTTATTGCCGAGGCATTGCCTGACTCAAAGATAAACCAA
GTAAGAAAGCTACAGAAAGAAGGGAAAATCGTTTGTATGGTAGGAGATGGTGTTAACGAC
GCTCCAGCTCTTGCAGAGGCTAACGTAGGAATAAGCATGGGAACAGGATCTGACATTGCA
GCCGAGGCTTCCGATATGGTTCTGGTCGGTGGCAATATTTCCGGTACATGCACTGCGTTG
CATCTCAGTCGAGCTATCTTTCGCCGGATTCAATGGAACTTCGTCTTTTCTATGGGTTAC
AATATAATTTGTATCCCGCTGGCCGCAGGTATTTTTTATCCAGCTACCCACGTGCGCCTT
CCTCCTACGATGGCAGCAATCGCTATGGCACTCAGTTCCGTGAGTGTTGTTTGTAGTAGC
CTGGCCTTGCGGCTGTACAAGCCACCGGATATTGTTGCAAGATCATCGAGTTCGCTATGC
TGCAAACCGCTGGGACAGCTGCGACTACTTGGGCGACGGACCACTGAACAAGAATATGAA

ATGATTGCTCAACAGCCTCTTGCTTGGTCGGATGAGAATTCCGTAGGGTCGGACTTATTT
GGGGATGAGATGGTCTAATGCCTCGTCATAATCATTATACAGCAAACTAATTTTATTGCA
CTCTACTCCAGTAACACAAGTATTCAATCCGCCTATCATTGTCGTATCTAAAGGTGACCC
GACTATCAGCAAATTTTCAATGGCTCAAGAATTTGTTTTCTTTTCCTTGATTTTCTAGCG
TCGATGGTCTCCACGATTCGATCGAATGCAATTTATGTACGGTACGGTACGTACGGGGGG
TACCTGTACGTTAAGATCGTACTTCTAACAGTACGTACGGTAGCCTAATGCAGTACGTAG
CAGTGCAGTAACGTGA
>comp2226_c0_seq1
CAAAAACTTTGTCAAACAAAGTTCAGAAAGAGACAGCAATTACCTCTGTTATCTCATTAA
GAAAATGCAAAAGGACGGAGGGGACCCAGATTTCAGGAGTTGCGGTTTGGATCGATCGTG
CCCTAGAAGTGTGACAACGCCTCAAGGCCAGGGTGTACGACTCGATCAATACCAAAGTAG
CATCACCGGGAATCAAAACAAACGACAGAGACGACAGAGTCGACAGAGTTACTCTGGTCC
AACGACCGTTGCCATTGTTGCACAGACTTGTACCACATTTTGTCCTGCTTCAACGAGGAC
ATCTCTTGCCGTGATTCCGGACCATGATTTTGAATGGTACGATCTCGAAGCTATTCGTAA
AGTAGACAACAAGGAAAACACGATCATCGTCGGCCATCATGACTACAATGCAGGTCTGCT
CTTCACTACTGAGAGACAGGTCTTGGGAAAAGGAGCAGAGATGAACATGTTTAATACATC
TATCACCATCGAAAGTGATGACAGTGAAGGAAAAAGCAAGGATGGGAAAAACAGCAGGGG
AAAGAAAGGAAAGAATAACAAAGGAAATGAAGGGAAAAACAGACAAAAGAGGTTCTTTTG
GGAGAAGAACGATAACGACATAATCGAAGAAACGCCAGCCGAAAAAGAAGAACGAGAATT
TCGAGTACAAATGCAAGAATTGGAGCAAGAAATGAGTATTGATTTATCGTCTCAGTCGTC
TGGATCGAAACACAATCGAAACAAGAATGACAGCGACAATGCTTTTTTTGCCACTTGGCG
AGTTGGAGATCCTGTCATTCGATACTAGTATCATCGAAATAAGATGTGCGTGTTGTAAAC
AATTCATTTGACGCGATCCGCTCGGGAGCATATCTGGATAGAATTGAATGAAGTTGCGAT
TTCAGAAAAACTATGAGTGGAGCCGTGTCCAACCACAACGAAAATCCCGATTTCGACGCA
GGTACATCGTTTTACCTGCATGTTTCATGATTATATCAAATGCAGCAGTATACGGCGGTA
TGCTGTTTGACTTGAACTGCCATCATTTAGAAAGAGTACGTAGGCGTAGTTAGTTGACAT
TAGTTCCC
>comp2227_c0_seq1
TCCGATCTCCTGCCTGGCCCATCATCGAGTCACCCATCGGTTCTCACGAGCAGTCTTCCT
TTTCCATCCCTACAGAGGACCACAATAAATTTTTTACCAAATTTTCAACAATGAAGGTCT
CTGCCGCAGCTATTCTTCTTTCCCTCAGCGGTGCTTCCGCCTGGTCCCAACCTAGCCGTC
GGGACCTCCGATCGCTCGGACAAAAGACGGTTTCTATGCAAACCACTTCCCGCACTAATG
GAAACACCATGAAGATGGAAGATTTCGGTTTCCTCAAGGGAACCGGAATTGGATTCGAAG
ACCTTTGGGAAGGAGAGGAAGTCATCTCCGAGTCCGCCCTCGAAGCCCGTCTCAACAAGG
ACGGACTCCGATTCCGCATGAACCGAACTCCCGAGGAAGCCGACGAAGTCGGACGCCTCG
AGGGACTTCCCGGAGTCACCGTAAACCTCCCCATCATTGGTGAGACTTTCTTGGGACCCC
CCAAGGTCGAATCCATCTGGGAGGCCATTGGATTCACCGCCACCTCCAACAACGAGGCCC
GACAGCTCGAAAAGAAGAAGGCCATCGAAAAGTGCAAGATCGCCAAGGAGGGTGTAAGCG
AGAACCAAACCATGTTCGGACCAGGAGGCGGTGGTGCCGATACCCGAAAGGACTGGCTTG
ACAAGTACGGATACCCCCGACTTGTCGGAACAGGAGGAATCTTCTACGCTGACCAATTGT
CCAGCGACAAGGAACCATCGGGTGGATTCAACATGGGAAAATCCGGATCCATCTGGCCTG
TTCCTGATGTTGTCAAGGAAGGACAATATGGAGGCAGCATGGGATGGGGTATGAAGAAGA
AGGGACCCGCCGTTGACGGTCTCGCCAAGGAAAAGCTTTAAACAGAAAGCTACTGCCCAA
AACTCTACTTACATCCCATTAGCCTAGCACAGCACGGCAACCCATACTGTGTCATGGTGC
CTACTTTTCGATGCAGTGATGACACTCGTTCTGAATACAACAAACGTACTACGATAGCAT
CATTCATAGCCTCGTTGTGATATAGATTTCATTATTACTTTTAATATCACGACATGTATC
ATTGCCTTACAAGAACGAAGCCTTGGACGCGTTTCTCCAGTCGTTACTCGCTGTGATAAA
ATGTACATTGTGATAGCCCCGCCGTATAACAACCACAGCGAAGCAAATCCTATGTCACAA
CGCC
>comp2228_c0_seq1
CGACGACGCGTACGAGGTTCCAACAAACCCTCTACTTTGTTTCAAAACTAAATTTTGCAA
GGCACCGATTTCTGCTTCAACAGGGAGCTTGTATTCAGACAAACTAGACAGTTGAAGAAA
CTCAGATACACCATGGCATCCGAAGAGAGCAAAGAAATCCCAACGAAAAAAATGGGTGAT
TTAAAAATCGACGCCGGAGGCAAGAGCAAATCAGGCGGGGGCGGCAAGAAAAAGGAAGCC
AAACCCCAACGTGAATGGACGCCGGAAATGCAGGCAGACTACGACCGAATTTTGTCCGTA
GGCGAGGAATGCCAAAATCCAGACGAACTTAGAGGCCTGATCTTGGCAAAGGGCCGTGGA
TCTGAGCACCCGACAGGGTTCAACTTGTACGACGGATTCGAACCAAGCGGTCGTATGCAC
ATTGCACAAGGTGTCTTCAAGGCTATGAATGTTAACAAATGTACTTTTCCTGGAACGAAT
TCTACCTTCATTTTTTGGGTAGCTGATTGGTTTGCCCTGATGAACGACAAAATGGGAGGG
GATTTAGACAAAATCAAAACAGTTGGAAAATATTTGCTCGAGGTTTGGAAAGCAGCTGGA

ATGGATTTGTCCAACGTCAAATTTAAGTGGTGTTCGGAAGAATTTACAACCCATGCAGAC
AAGTACTGGCCAAATATGCTTGATATTGCCCGACGCTTTAACGTCACCAGAATCAAAAAG
TGCTGTCAAATTATGGGTCGGATGGAGGGCAGTTTAACTTCGGCACAAGTATTGTACCCT
CTCATGCAATGTACTGATATCTTCTTCTTAAAGGCTGACATCTGTCAATTGGGTGTCGAT
CAGCGAAAGGTTAACATGCTTGCACGTGATTACTGCGTTGCTGCCAAAATAAAGAACAAA
CCCATTATTTTGAGTCATCACATGCTCTTTGGACTCAAGGCTGGCCAGGAAAAGATGTCG
AAATCAGATCCTGATTCGGCTGTTTTTATGGAGGATACTGCCGAGGATGTTGAGCGAAAG
ATTATGAGTGCGTACTGTCCGACAACGGAAGACGAGAAGACCGAGACCGACACAGGCGGC
GAGGAAGATGCCGGCAAGGCCTCCATGCAATTGAAAGAAATTAAGATTAAAAATCCATGC
TTGGATTATATTGAAAACATCATTTTCAGCCCTCCTGGTGCTACATTTACAGCCGGTGGA
ACGAATTACACCGACTTCGAAACGGTGCGTGATAAGTTCCTCGCAGGGGACATATCCGAG
GAAGAATTGAAACGAGGATTGATTGATGAGTTGAACAAACTACTTGAGCCAGTTCGACAT
CACTTCACGACAGATGAAAATGCAAAGGATCTTTTGAGCAAAGTTCGACAGTACAAAAAA
GAAGCTCCACCCAAGGTAACCAATGTAAGACGATTGAATTTAGTCGAGCTCTCAAAGGTT
CCAGCAGGTAGCCATTTGGTTTTCGCACCTATGCCTACTGCGACTCCCACGCTGCAAGAA
GCTATCGACGTCTTGACCTTGCTTCGACGAGCAGAAGAAGGTCAACCATGTGTCTTGATG
CTACCAGACTGGTCCGCCCGGGTGACAAATGCATGCGATGGTGATACCAAGAGTATCACC
GCTTATTTCACTATTCTTGCCACCGCTCTCAAGGCTCTGTGTGCCGAAACGATGAGCAAG
GTTCAAATAATTTTGCAAAGTGAAGCTATTCTGTCCGATCCTTCCAACTATTGGATAAGT
GTGATTAATGCCGGACGTCATTTCACCTTGAAGGACGTCATGGGTGAAATGAGTGATTCA
GACCCTGTCGGTTGGGTGATCGCAAGGCTTATGTTGGTTGGCGATGTTGCTGGTGTAGAA
CCGAAATCAGTAGCACTTTTGGAAGGTGGAAATAACATTCACGCGGATGGTGCGTCTATC
GGATCGAAGATGATCACCGAATTCTTTCAAGAAAAGCTTGTTTCTCTTGTTCAACCTAGC
GTAACTACAGGAGCAGGGCCAAACATGCTTCTCCAAAGACGTGAATTGGAAGCTCACAAG
ACTGAAAACGACGAGTACTACTTGTTGGACGATCCGAAGGTCAATGGAAAATCCAAGATG
AAGAAAGCATTTTGCGAGCCCAAAAATATTGAATTTTGTCCTCCTATCAATGTTGCTGCT
GCGTTTGCATTTGGTCTTGAACCGGACAGTAACGGCGAGATGATCATTTCTCGAACCCCC
GAAAACGGGGGCGATGCAGTGTTTAAGAATCGTTCTGAGCTGGAAGCTGCGTTTGCAGAC
GAGTCCCTACATCCCGGAGATTTGAAAGCGTTTGCCAGCTCGACCATGGTTGCTACGTTG
ACTAAGTTGTCGACAGCTATCAAGGCTGATGGTGATTCGGCGAAAGCAGGAAAAACACTG
AAAGCTATGGCAAAGAAACTCGCCAAACAGAAGAAAAAGTAACAATTTTATCGATTGTTT
GGTATCATTATTTGTTGGTGGCTCAAACG
>comp2229_c0_seq1
GCAGCAGCAGCAGCTATTCGGGGTCCGGTGCGCATTCTTTCCAGACAAAAAGTGCGCTGA
AGGTCTGGACGACAACGGCCACCGAGGAAGAGGAAGCAGACCGCCAGCCCGATCCATCCT
TCGATCGGCGCTCCCTTCTCTTGGCGGGAGCGTCCTCCGTCGCGACCGGATTCGTTCTGA
ACGAAAACAAGGATGCACTTCGCCGGGCACCCGCTATCATGACTTCCATCGAAGCCCCCT
CGGAGGCCGGCGGCGTGGTCGCTCCTGCTACCACGAGCGTGTCATCCGTCGAGGAAGCCC
TGACGATCATCGAATCCCTCGGCGACAAGCGATTCCTTCACGCCGTGGTGGCGTCGGACT
ACAAATTTCTGTACGAGCAAACCCCTCGGCAAAATCGATCCGGGGCGCCGAAATCGGTGC
TTTCCGACATTGATGCCGAGGCCATATTTTCCAAGAAAAAAAATGTGTCCTCGTCGCCAC
GATCGATTGTTCTGGCAGCGAGCGGAAACCTGGCTCCCGGCAAGGCGCCGTCGCTGTGGC
CACTCGAGACTGCCCGTTGTAGTGTGGCCGGTAGCTGCGACAGCATCCATTACGCCTGGC
CGGAGCAGGGCGGGGCGATTCCCAGGAGGAACAATGATGAATTTGCGGCGAGGAGCGCCA
TGATCGTCGATGGAATAGATTGCGGCAAGATGTCGCTGGAAGATGCGCTGGAGGGGGACA
TGCAGGTGCTGGTGCAGGCACCGACGTATCTTTTGGTTCCGAGCTACATGGAATCTAATC
TGAGGAGGGGCCTTCAGGGAGCATTCTTGATATGAGACTACGTGAACCAACCCAACCTAT
GTTGTGGAGCCAAAGTGGACGGTGATCGATCGGGTAACAGTTGTAGGATCGACGCACCAA
TTTTCGGAGGATAGGACTCTTCACAAACAGTGTTTTTGTAGTATTCACCATGTTCGAATA
CCTGTCGCTGATAAAAGTTTCATATCCCAAGTGAGAATGATGTAGAATCGTAAAGTGATT
AGGTTCCCAAGGTATTCATAATCAATTTAGGCAACTGTCATTGGGCAGCATGACATGTTG
TCGTATGCTTCTAAAAATAATCCTACTCAGAAATTTCTTACATTCATCGTGATGTACTGA
ATGAACACTTTTGTAACTAATATTAAACATAAACCATTC
>comp2230_c0_seq1
CCGCAATGGCGGGGATGGGCCCGCGCAACCAAAAATTCTCAGTCGTTCCCCTTTTCGAAT
GATTACATTTTGATTGATTTTTGACGTTTCTGTGATTGATTCCTGACCGAGATGCTATAT
GCCACATGCCGCAACATTTTGAAAGTGGCTACGATATTTTCATTCTTGATAGTTACTGGG
GAAAATGCCATATTTGAGTCGCGCTTGTGGAGCCAAGATTTTCTATGAGACGAAAGGACC
AATACATGGAATCCCAGTCTTGTTGTTGGCGCCGGGAGGCATGCGATCCTCTATTGGAAA
ATGGGAAATGTCTCCGTACAACCCATGGATGAGGCTTTCGTCAAAAAATGAGAAGCACGG

TAAATTTCTTCTGATTGGAATGGATCAACGCTTTGCGGGCCGATCAACCGGGACGGTGAA
CAAAGATGATGGATGGCACACATTCCTTGGGGACCAACTGGCTCTCCTGGACCACCTCCG
CGTCCCTAAATGCCATATCTTGGGAAGCTGCATTGGTCCATCCTATGCCCTTCAGCTAAT
GAAAATGGCACCCGAGCGGTTTGGACGATGCGTCATGCTTCAACCAATCGGGCTGGCACA
TCACACAACAGAACCGGGAGAAGCATGGGAAGGACTGAATTCGGATGCATCGTGGGATTG
GGTGAACAGCTGGGCTGATGAAATGATTGGAACGAAAAAAAGCTCGGACCGAAGCATCCT
GAACGATCTATACTACAATATGTTTGTGGCTCAACCACATAGAGAATTTGTGTTCAGTAT
TACTCGTCAAGATGCTGCCAGAATACAACAACCCTTGCTGATTTTCATGGGGAAAGACAT
CTATCACCCTTCCGAAACTTCTAGACAAATATCTCGGATTGTGAAAAATACCGAGCTGGT
CGAAGTATGGCGTGATGCTGGTCCTGAGAAGATAAACGCGGCTGCTTCTAAGATTTCATC
TTTTTTGCTTACTGATACGTTTAGTTCGACTCCCCCAAGGTAGCAATAAGAACTAGGGAA
AAAGAGAAGAAGCTCAGAAAAGCTATTTTAATCACATGCATCACATCAAAATTTCAATTT
CTAGAAGAATTGTAGATCTCGTAGAACAGAGAACAGGCACATTCATATTTTGATTGTGAA
TCTGGTTTTAAGCTCTGCTTTGATCGATTCTAGTTCCAAAGTGGCTCCGCCCACAATAGC
GCTGTTTTCTTTCGCCCGACGCAAGAGATAGGGCATCACCTCATTAACTTTCCCATATGG
TAC
>comp2231_c0_seq1
CGTACTCGTACCAGTACGAACCAGCAAAAAACTATGTTTTCCTGCTCACAGAATCGCAGC
AAGGGCAGCAGAATCTGCAGAATCGACCGGTGCCCGCCCCCCAGCAAAGAGGGATCGAAA
AAACGACAAAACAGCAAAATGAGAACAGCTCGCAACGGACCCGAGCAAAGGACCAGCGGC
AGCTGCTGCCTTGCGGTGGTGCCAAGACTCGTCCTGGTCGCCGTGTGCCTCCCCCTCTCG
AACGGATTGGTTCCTAGCACTTCCCCCGCCACGGGTCGTCCTCCACAACGGTCTTCCGCC
GCGGCGCTGCGGGAGAAGAGCGGAAGCGGCGGTTTCCTGGAGGGTCTCTTCGGAAGCACC
GCCCCCGCTAAGAGCCCGCCGAGGGTCCGAACACCGGAGGGCTTTGTCACGCCCGAGCCG
AAACCCCTGCAGATCTCCGAGTCCGCCGATCCGGCCAGGTTTGCGAGGAACACCCTGGCC
TTTGTGATCCGGTTGGGGGTGGGCGCCTTCGTCCTCGGGTGGAAGATCGACACGGTCTTT
TACAAGCAGCCGGAGGACGACGGCAATGACAATGACAAAGACAACGGCGAACCTAGCGGC
GACGGCCGCAAAACAAGGTACAGCCTGGACCTCGGGCCCTTTTCGATCCGGGATTCCAGC
TCGGTCGTCCTCCCAAAGCCCGAGCAACCGCTGGTCCTCTACGAGTACGAGGCCAGCCCG
TTCTGCAAGCGGGTCCGGGAAACCATCAACCTGCTGGACCTCACGGTAGAATACAGGCCG
TGCCCGGGCGCCCGGCAGGGAAAATTTTCCGACGAGCTCTTCGAAATGACCGGGAGGCGG
ACGGTGCCCTACCTCTACGATCCCAACACGGGCGAGGGGCTCTTCGAATCGAGCGACCAG
ATCGAGTACCTTTTGGCCAACTACGGGCCCGCCGACGCCTCGACCTACGACCGAAAGGCC
CTGTGGCCCGTCACCCTCGAGCCCTTTTCGGTGGCCACGTCGACCCTCTGCGCGATATTG
CTGGGCATGCCCGGAAAGGCCCGCCGGCCCGACGCCCGCCCCGACAACGAGGAAATGCTT
CCGCTGGAGCTCTGGGGGAACGAGGCCTCCCCCTTTGTCCGCAACGTGCGGGAAACCCTC
GGAGGCCTGTGCCTCCCGCACGCGATGGTCAGCTGCCCGAGGGGATCCTCCAACCGCGAC
CGGATGGTGGAACGGACCGGGCGCTTTCAGGTACCCTACCTCGTGGATCCCAACACCGGG
ATCGAGATGTTCGAGTCCGAGGAAATCGTCAAGTACCTCGAAGAGGTCTACACGGTGTCG
TGAAACCAAAGAAAACTAAAGCAGGAG
>comp2231_c0_seq2
CGTACTCGTACCAGTACGAACCAGCAAAAAACTATGTTTTCCTGCTCACAGAATCGCAGC
AAGGGCAGCAGAATCTGCAGAATCGACCGGTGCCCGCCCCCCAGCAAAGAGGGATCGAAA
AAACGACAAAACAGCAAAATGAGAACAGCTCGCAACGGACCCGAGCAAAGGACCAGCGGC
AGCTGCTGCCTTGCGGTGGTGCCAAGACTCGTCCTGGTCGCCGTGTGCCTCCCCCTCTCG
AACGGATTGGTTCCTAGCACTTCCCCCGCCACGGGTCGTCCTCCACAACGGTCTTCCGCC
GCGGCGCTGCGGGAGAAGAGCGGAAGCGGCGGTTTCCTGGAGGGTCTCTTCGGAAGCACC
GCCCCCGCTAAGAGCCCGCCGAGGGTCCGAACACCGGAGGGCTTTGTCACGCCCGAGCCG
AAACCCCTGCAGATCTCCGAGTCCGCCGATCCGGCCAGGTTTGCGAGGAACACCCTGGCC
TTTGTGATCCGGTTGGGGGTGGGCGCCTTCGTCCTCGGGTGGAAGATCGACACGGTCTTT
TACAAGCAGCCGGAGGACGACGGCAATGACAATGACAAAGACAACGGCGAACCTAGCGGC
GACGGCCGCAAAACAAGGTACAGCCTGGACCTCGGGCCCTTTTCGATCCGGGATTCCAGC
TCGGTCGTCCTCCCAAAGCCCGAGCAACCGCTGGTCCTCTACGAGTACGAGGCCAGCCCG
TTCTGCAAGCGGGTCCGGGAAACCATCAACCTGCTGGACCTCACGGTAGAATACAGGCCG
TGCCCGGGCGCCCGGCAGGGAAAATTTTCCGACGAGCTCTTCGAAATGACCGGGAGGCGG
ACGGTAGGTGGTTTGCGCGTGCCCAGTTTCGTGCCGCGATGGTTTGCCCGTGTTCATCGT
GCTCCCTCTCGCTGTGCCCTACCTCTACGATCCCAACACGGGCGAGGGGCTCTTCGAATC
GAGCGACCAGATCGAGTACCTTTTGGCCAACTACGGGCCCGCCGACGCCTCGACCTACGA
CCGAAAGGCCCTGTGGCCCGTCACCCTCGAGCCCTTTTCGGTGGCCACGTCGACCCTCTG
CGCGATATTGCTGGGCATGCCCGGAAAGGCCCGCCGGCCCGACGCCCGCCCCGACAACGA

GGAAATGCTTCCGCTGGAGCTCTGGGGGAACGAGGCCTCCCCCTTTGTCCGCAACGTGCG
GGAAACCCTCGGAGGCCTGTGCCTCCCGCACGCGATGGTCAGCTGCCCGAGGGGATCCTC
CAACCGCGACCGGATGGTGGAACGGACCGGGCGCTTTCAGGTACCCTACCTCGTGGATCC
CAACACCGGGATCGAGATGTTCGAGTCCGAGGAAATCGTCAAGTACCTCGAAGAGGTCTA
CACGGTGTCGTGAAACCAAAGAAAACTAAAGCAGGAG
>comp2232_c0_seq1
GTGTGCTCTTCCGATCTGGAAAAATCGCAATCAAAGATCCATGACACATCAAAAGTCAAT
AGCAGCAACAGTACAAGTAATAACATAAGCTCGTTGTCGTCGTCATTATCGGTGATAGAG
GACGCAGCGCAAACTCATTTGACCGGACGAAAACGATATCGGACCGTTGTTCCGAGACGA
GTCTATCTCGACTCAATAGCTCAACAATGTGACGAAGAGCAAGACAGCTTCTATGTAGTT
CAGTCCGATCCTGAGACATCAAGAACAGGGAAGCGTTTGCTCGAATCGTTTGAGGAAGCT
ACGACATTGAACACTTTTTAAAAGTGGACAGATTTACGCACTTTTTCCTATACCAACAAG
GATCTCTAGATGCTTGGTTAAAAAGTGGAAAGTGAATACAGAACGTAATCTAGTGAAAAT
CGAGGCAGGATTTTTCCAAATAATTCATTCATACTAAAAGTCACTCCTGGGGGAGATAAG
GGCCACACTTTCGGG
>comp2232_c0_seq2
TTGAGAGATGCCGAGGAGATCGTTCGCGCCGGAAAGAGTCCATGTCATAACACAAATACT
TCTACGATACTACCGTATACTTGTGGAATGGCGGAATCAAACTAATGTGTGAAAATTTCT
TACTGCGATAGAGTGCAAACGGTATTAAATTGATCAAATACAGCAGGAACAAAATCCGAT
GTCTAAGGATGCTCCGCCGTGCTTCTCCGATGGGAGGAAATTTCCTCTGATCTCAATTCC
AAAACCAGCAGATCGGGGTGTCACGAACTCGAATATAAATGATCGAGCGCCTCCCAAGTC
TACTTCAAAAAGAATAAGAATACGAAGGAGAAACCCTTTCTCCGATGGAAGGAGGAACAA
CTTTCCTGACCTCGATAATTTCTTCAAATCGACATCGGAATCACCGTCACCTGGACGTCT
TCGCGTAGGACATGATAGTTTCCATTCACATAGTAGACACGACGCATACGACGATGAAGA
TGAATGCTACCACTTATCCGACGTAGGTGAGACGTCTGTACTCAACATGTCCATCGGAGC
AAGAGCGGCACTGAATGAGCACGCTAGACACGTGGCAAAGTATGGGATTGACGGTGAAGT
AGCTGAAAAACAGGAGAGCGATGAAAGTGAGAATCAGAAAAATAACAGTTACGAGATATC
GAGTAATGCTGGAACCAATGTATTTCTTGACCATCAATGGGAAAGACTCTTTGACCTATC
ACAAGTGAATTACAGTAGTGAAGGTTCCGATTCTGGAGGGATCCCACCAACGCCAAACTT
TGGGACAATAAATTCGAAGTTCGATGTTGTAAATGCGAGTACAGATAGATCTGGATACGA
AGAATCATCAATCGAGGTATTAACAGATGTAAGCTACGACTACTTCAATAGCAGCAGAAT
TAATTTGCTGGTAACACCAGAAAGAAACAAAAACTTTCGAACGGCAATGATTGTGACGCG
TGACGGCAATGGCGTGGAAGACGATCTTTTTGGAAATCACCAAAACTTTTTATTTGATGG
TGCCGAAGCCGATGGCCTTCCTTATCATGACGAAATTGAAACTAGTCGGGTTGGTTCCTG
TCGCTTTACTCATAGTTCACAGGAAGGAGCATTTGTCCAGGATCCTTGGGTGTCCTCATT
CAATATAGGGGATGTTTCACGAATAAGTGCCCACTCAGTTTCGAACGCAAATTGTACCCC
AAATACATCGTTTCAGAATTATCATAGCCATGGCAAAAAGGGAAACAAGAGCAACGATAA
CTTCAACGGCCCATTGAGTGCACACTTCGGAGCAGATTCTCCATCAATGATTCTGAATGA
CTCATCCTTTTCGTCCATCGGAGGAATGGGCCCGTCGCAATTGCCTTTCGATAATAACGG
CTATACATCTCCAGAAATTCCTTTCAGTCCTTCTAATGATCAAAAAAGTGGCATTCGAAC
GAAGGAGAAACCTTCTCCAGATAGAAGTGCTAATTCCTCGTCTTCCATTCAAAGCACGGT
CTCGAGTTTTATAGCAGAAGCGAGGACGATGGCAAAACAGGTGGTCAGCGATGTAGAAAA
GTCGATAGGAGCGATGGAATCACTCCCAGTGGATTTTCTTCGAGCTCTAGAAATCGGCCG
GGCTGACACGCTGAGTCCAATTTCTCGGGCCTTGAGCACCCCCCCAAGCTCTCGTCGAGA
TGGCAGCAAGAAGGAAAAATCGCAATCAAAGATCCATGACACATCAAAAGTCAATAGCAG
CAACAGTACAAGTAATAACATAAGCTCGTTGTCGTCGTCATTATCGGTGATAGAGGACGC
AGCGCAAACTCATTTGACCGGACGAAAACGATATCGGACCGTTGTTCCGAGACGAGTCTA
TCTCGACTCAATAGCTCAACAATGTGACGAAGAGCAAGACAGCTTCTATGTAGTTCAGTC
CGATCCTGAGACATCAAGAACAGGGAAGCGTTTGCTCGAATCGTTTGAGGAAGCTACGAC
ATTGAACACTTTTTAAAAGTGGACAGATTTACGCACTTTTTCCTATACCAACAAGGATCT
CTAGATGCTTGGTTAAAAAGTGGAAAGTGAATACAGAACGTAATCTAGTGAAAATCGAGG
CAGGATTTTTCCAAATAATTCATTCATACTAAAAGTCACTCCTGGGGGAGATAAGGGCCA
CACTTTCGGG
>comp2233_c0_seq1
CTCGACAAAGACCGTCAAGAACGCAACTTAGGAATCTCCCTGCCAATTGTTTGGAATAGA
AGAGCATAGACAAACAAAACGAAAAGAAAACAAAAATAAAAAAGGGTCCTTACTTTTCGG
AAAGGCCTGGAATACGCACACACATACACCAATAAGTCTCCGGATTGCACAAAAATACTA
CCATGAATCGAGGAGGAATGCAACCCGGGATCATTATCCTTCGGGAGGGTACGGATACTT
CGCAGGGCCTGCCCCAACTCGTCAGCAACATCAACGCCTGCCAGGCGGTGGCGGACGCGG
TCCGAACCACGCTGGGGCCCCGCGGCATGGACAAGCTGGTCGCCGCGGGATCCAAGGCCA

CCATCAGCAACGACGGGGCCACGATCATGAAGCTCCTGGAAATCTCCCACCCCGCCGCAC
GAACCCTGGTGGACATCTCCATGAGCCAGGACGCCTCGGTCGGGGACGGAACCACCTCGG
TGGTCCTGCTGGCGGCCGAGATCCTCACCAAGCTCAAGGCCCTCGTCGAGGAGGGGATCT
CCCCGACGCGCATGATCCAGACCATCCGCAAGACGGGGAGGTTTGCCGTGGAACAGGTCG
GGGCGCTCGCCGTGGAGACGGACCGGGAGGAAATGCTCCTCAAGTGCGCCGGAACGGCGC
TCAACAGCAAGCTCATTTCCTCCCACCGCGACCTCTTTGCGCCCATGGTCGTTTCGGCGG
TGACCAAGCTCCACGAGGCCGGGAACGTCGACGAAATCATGGACTGGGTCTCCATCAAGC
AGATCCCCGGGGGAGACGTCCGCCAGTCCTTTTTGGTAGAGGGCGTGGCCTTTAAGAAAA
CCTTTTCCTATGCCGGGTTCGAGCAGATGACCAAGAAGTTCGAGAACCCCAAAATACTCC
TCCTCAACGTCGAGCTCGAACTCAAGTCGGAAAAGGAAAACGCCGAGGTTCGGATTTCTA
GTCCGAAGGACTACCAATCCATCGTAGAAGCGGAGTGGCAGGTCATCTACGACAAGCTCG
ATGCCTGCATCGACTGCGGTGCCAACATTGTGCTGTCGAAGCTGCCGATCGGAGATTTGG
CGACCCAGTACTTTGCCGACCGCGGCCTGTTTTGTGCGGGCCGCGTCGCCGACGGCGATA
TGAAGCGGGTCGCCAAGGCCACCAACGGATCCATCCAGACGACCACCAACGGACTGACCG
AGTCGGTCCTCGGAACCTGCGGGTGCTTCGAGGAGACACAGGTCGGCGACGAGCGATTCA
ATTTGTTCACGGAATGCCCGTCGTCCCTGACGTGCACCATTGTTCTGAGGGGAGGCACCG
AGCAGTTCATCGCCGAGAGCGAGCGCAGCGTCCACGACGCCCTCATGGTCGTCAAGCGAA
CCATCACCTCGGGGTCCGTGGTCGCCGGTGGAGGCGCCGTAGAGATGTGCGTCGCCAATT
CCCTGAGACGCCGGGCCCTGGAAATCGAGGGAAAGGAGCAAATGATCGTGGCGGCCTATG
CCAAGGCCCTGGAGGTCATCCCGAGGCAGCTGTGCGAGAACGCGGGCCTGGACGCCACGG
ACGTCCTCGCGGCCCTGCGCCGCAAGCACTTTGTGGAGGAGGGGGACGAGAACGCCTGTT
GGTACGGTGTCAACATCGAGGAGGGCGGCATCACCAACACCTTTGACCTGGGCGTCTGGG
AACCCAGCGACAACAAGATGAATTCCTTCCAGGCGGCGACGGAAGCGGCCTGCGTGATTC
TTTCCATCGACGAAACCGTCATCAGCCCGAGGTCACAAGACCCCGCGGCCCAGGCAACGG
GGCAGATGGGCGGTGGCGCCGGTGCCATGATGGGCAACGCCATGGACATTGCCAACAGCG
GAGGTCCGAGGACGGGGCAGATGGGACCGGGAGTCTCCTACATGAAAGGAAGAGGAGGAG
GTTAGATCGATAAAATATTTGAAAACCAAACCCCATGCTTTCTGTCCACACGACAGTCAA
CACAAGTGAGATAGCTAGATAGTACAATGGTCTGATAGGTAATAGTGA
>comp2234_c0_seq1
AACGAGTGCTTGTAAAACGAGTCAACAAGCAGCTATCGAAAACTTTTTCAGCCGGTGACA
AAAACTTATCACGATGAAGTTTGTTGCTGCTTGTCTCCTTGCATCGGCAGCATGCCCTGG
AGTGTCGGCATTTGCCTTCCACAGTACACCATCTAGCAATGTGGCTGCCAGCACTGAAGT
TTCCATGAAACGCGCATCTTTGAAGCCATCTTTCCAACTGAACGCTGAAACTGTAGATTC
CGATACGGTTGCTGGTCTTCCGGATACAATCGAAACTGAATATGAACTTTTGGGACTTCC
AGCAAGGCCTGGACGCCCTTTGAAAGTGGCTATTGCAGGTGGTGGTGTTGGCGGTTTGAC
GGCCGCATACTACATGCTTAAGAAAGGCTTCGACGTGACTGTATACGAGAAAACATCGGA
ATTCGCCCGATTTGGTGGACCCATTCAGTTCGCCTCAAATGCTCTGTCAGTTCTAAAAGA
AATTGATGATCGTCTTTTCGATCGAGTTATGGAAAAATTCACTTTTACGGGCATTCGCAC
ATGTGGAATTAAGGACGGACTTCGATCTGACGGGTCGTTCCGTATGACAGATGATTCTCT
TGATTACCTCGTGAACCCCGATGCTCCTGCCGATTGGTTTGTTAAATTCCCTTTGAAAGA
GTGTGCTGATTTGTTCGGCCTTCCTTACACTGGAGTAATTGATCGACCAGATCTACAGGA
AATTTTGTTGGACGAGTGCAGACAAATTAAGCCTGATTTCATTCAAAACGGAAATCCGGT
TGTTGGATATGAGAATCTTCCGAAGGGAAGTGGAGTTTCCATCAATTTGGACGACGGAAG
TACCGCTGAAGCTGACGTTTTGGTGGGGTGTGATGGTATCTGGAGTGCAGTACGAAATCA
GATGTACAACGAGGGCGGAGTGAAATCTTTGAGCAAAAACAAAAAGAAGCGCCAAGGATG
TGACTATTCGGGATACACTGTGTTCGCAGGAGAGACAGTTCTCAAAACTCCCGATTACTA
CGAAACTGGATACAAGGTTTACATTGGCCCACAGCGTTATTTGTAACCTCTGATGTTGG
AGATGGGCGAATTCAATGGTACGCCTTTTTTGCGTTACCACCGGGCACCAAAAAAGCGCC
TTCCGGATGGGGTGGTACAGAGAGAGAAGATCAGGCGGATCCCGAGGAAAATTTGGTTGA
GTACATCAAATCTCTGCACGAAGGGTGGTCTGACGAAATTATGACTGTTCTCGACGCAAC
GCCACCTGATTCTGTGAGCAACGTGACCTTTACGACAGAGCACCTGAATTCTTCCGAAGT
TGGGCCGATGGTAACGTCGTCCTCATGGGGGATGCAATTCACGCTATGATGCCTAATTTG
GGACAAGGAGGATGCCAAGCCATTGAAGATGCGTATGTCCTTACTGAAAATCTCGCAAAT
ACGAAATCTACCGACAAGATCGAAGCCTCCCTTCAGGATTTCTACAAGAAGCGCATCTTG
AGAGTCAGCGCAGTTCAATTTTTGAGTCGATTAGCGTCCGATTTGATTGTCAACGCATTT
GATACGCCATGGAGTCCTCACGACAACCTTGGGAAGTCGTGGAAGAGTTACTTGACTTTC
TTCTGGAAGCCAATTTTGCAGTATGCTATTTTCCCTGCGCAGTTTGCGTTCTTGTATTCG
TACTACCCATCTGGAAGACTGGACGATGTCGCCGAAAAACTAAGCGATGAGTGGAAGTCT
AGACATTTTGAAGCGAGCGAAGAAGCATTCAAGAAAGCCGAGGAAGGTTTTGTAAGGGAG
TCGGGACCATCGTTCTTCCAAAAAGCTGAGGTCGAAGAATCAAAACCATTGGTCGCTGGC

TTGAAGAGCAAGTTCAGTATCTTTTAAATTGCAGCCCTAGATCTTTGATATTTCATTAAT
TACTAAAGATATTTTCCCAATGGC
>comp2235_c0_seq1
CGGCCGGGTACATCGGAAAAACAAGTCGTACAATAATACCACGGTTGTCTCCCATCTCAA
TCTTTTTCACAAACTAACAAGCCAACGAACGAACGAACGCAGGGCAGGCCAGGCCAGGAT
CAAAGTCGTTCTCCGTTCTAAAGCCAGCCCGGGAGAGTCTATCGGGAACTAGTCCAGTGC
ATCCAGCGACACCTCGTTGCGTGTGCCAGAATTGCTTCTGCGGAGCTAGCTCTCGAACAG
CGGAGATAACTTTCACCCTTTCTCCTTTTCGTTGCATCGCCGTACAACAGCCGCCGCCTG
TCGTTGCATTTGTGTTGTACCAACTACGAACGAGACCCGAGGAAAGCGAAGGGCTTTGCA
ACGAGAGTCGCTCGAAGCACGATGACGAAGCAAATCTTGCTCAAGACACCGCGTGGGAAC
ACTGCGGTGATCCCCGTCGATCACTTCGTCTCCAAAGCCAAGGCGGGAGCCACCGCAACA
TCGGGAGCCAAACCGACACTCGCTTTTTCCTCGCCCGGGGAAAGACGCACGGCTTCGGTG
TTATCCAGCAGCAACTACTGCGACTACCCACACGGTCACCCCGCCACTACGGAAGCAGAG
GAAGAAGCCGCCGCCATTGCGGCGGCCCTCGACACGAGCCGAATATTCGAGGACGAGCAC
GAATACGACGTGGATCGGGAGAGCAAAGAAGAGCTCCTCTTCCACCTGATCGGCGCCTCG
AAGCGAAGAGATGTGGGACGCAAACGAAACCTCCGGTCTCTCTACAACCTCGAGGTCGAA
GGCGAGGACGACGGAATGGAGATCGAAACGGAGTACGCCCACCAGCATCCGCACCAGAAC
GAATGTCAAACTCACAACAACGGGTGGTCGAGCAGCGACGACGAAAAGGAGCAAACGGTA
GTGGCCACTCGCTTGCACGAGATGGACATCAATCGAGACGACTACTACTACTACTCCGGG
GCTGGCAACAGGAGATTGTTCAAGAGCCCCCGGCGATTCTACTCTCACTCCGGGGTCCGC
AGCTCCAGTGCCGCGTTGGTTCCGACGTCACCCACTCCAGCAGTTAGAAGACGCCAGCGC
GAGGATCCAGGACTTGCTCGGCAAGCGGATCGACCGAGCCAGCAGGGGTCTTCTCCTCCG
CTGATGGTCGCCTTTTATCACCAGGGAAGAAAGGTGTCACTGGATAGGATCTGGGATTGG
ATAACCGGATCGTCTGCTGCGAAATCCGAGTCGGGAACAACTGATGATGTTGGTGTTGCA
GGGCGCAGCAACAGTGAAACCGCATCTTCTTCCTCTAGCACCCCGCTGTTTCCCCTTTAT
CTCACCCTCTCGACGTCCGCTCGATTGCTGGGTGGTATCGACCGCCAGAACCGTGTCGGG
TCCAAGTTTGGGGGAGGTGGTGTCTCTTCGTCCCAGAATTCCGAGCGGGAGCGGAAGGAG
CGCCTCAAGCAGCTGGCTTTGGAATCGATTGATTTGGCCAAGGACCCCTACCTGATGCGG
AATCACTTGGGAACCTACGAGTGCAAGCTCTGTCTTACGTTGCACACGAACGAGGCCAAT
TACCTGGCCCACACCCAGGGAAAGAAGCACCAGGCGGGGCTCGCGAAACGTGCCCACATG
GAAAAACTGCGAAAAGAGCGGGAGGAACAGCTCGCCGACCGGAACAACCTGAAGCGGCCC
CCTCCCGGCGGACGATTTGTGTGCCAGCCGGCAACCACTCGCAAGGTCCCCAGGATTGGA
CGACCGGCCTACCAGGTATTCAAGTCGAAGGACCCCGATACGTCCCAGCGATGCCTGACC
TTCGAGCTGACCTACAAGGACATTGAGACCGATCTGCAGCCCCGCCACCGCTTCATGTCT
GCCTTTGAACAACGGGTCGATCTGCCTAGGGACAAGCGCTACCAGTACGTCTTGTTTGCA
GCGGAACCCTACGAAACCATCGCGTTCAAGATTCCGAACGAGCCTCTCGACAGAGATCCG
GGACGCTTAGTCACCCACTGGGACGAGGACGACAAGAAATTCACCGTGACCCTCTATTTT
GCAGACGGAAAGGACGGGGGCCCGAGCAACGAGAACGTCACTGGTGGCAGCCACAAGGAA
GCGGCCAGCATGGAAATAGATGGCTCGGCTGCCGGAACGTCCGTGTTTGCCCCGGCCCTC
GGGAGCCACTGACGACCGGACCGATTCAAATTTTCTGCCACGAAGAAGAACAACAAAATA
ATATATTCAGTTAATTACTGATTGATTGAAATAGGAATTGTCACTAGCAGTAGCCAAACA
AAATTCAAATCTGCTCGAATACACTTTACAATAATCTATTCAAAGAAGAACGACCCATGT
TTTTCCTCTGCGCACCCTGCTTCAAACGTTAACGACAGATACTCGTGGAATCAAATAATG
TAGATTTTCTTCTCCATCACGGCTTTACAAGCAAAGCTTTTTGTGCAATAAAAAACACAC
CACGAACTCGCGGAGGGATAATCTATTCATGGCAGTTTACGACGTTGTTAGTGAAATTCA
ACGACTGAATCCACATCTTCTCAAAGAATGTTGTTTCCAATAGGTCTAGATCCAACTGCT
GGACGAAAGACATTGTACTCGTATTCCGTTGAATTTGCCCTCAGAGACTCACAACGCACT
CCAACGTTTTTCCAGGAAAAGAAGCCATTGAGAATGTCTTTTTGTGGGCCCAGCATGCAG
TCAATGGAACTAAAACTATTTTTACTAGCGGATTAATTATGCTGGGTTTCTCAATAAGCT
ACAAGTAGTTTTCTTTCTATGTGCTACAAGTACCGTATGAAGATTAAGATGCTCCA
>comp2236_c0_seq1
GGATGTTGCTCATAACTTGCGAAATACCCCTTATTGAGGGATGTTACGTGGTTCTACCGG
TACACTAAAGAGTGAGCTCATTCGCCATCTACTCTTGATTGGGGTTCGTATTGAGCGCCA
CACCAAAAGTGCAAATTTACATCGAACATTCTCAAATCGTGGAACTCAAAACGACACAAT
TCGAACAAAGAATACTGTAATCACCACAATCGACTTCGACCATGACACGGGCAGCACCCG
ATACAATTTGCATACTTCAAGCCATATGAATCGACCTATAGATAACTCTTCTATCACCGA
ACGCAATAGTAATATATACTCACACTCTGTCATGACGCCACCTCAATGTTCAACCAATGA
CGATGGCGGCGGCTTTCGCCTCCGCATTCCGCTGCTGGATGACGACAGCTACCATGATGA
TTCCGGGAACAGGGTACTTTCACAGAAAACGATCGATAAGTTTCGCAGCGATGGCTTTGC
GGTTTTTCCTAACGTTCTATCCCCGGACGCCGTTGACGCCCTGAACGATCGCTTGGAAGA

CGTCCTGCGGGGGGTCTACGACCGGGGGCAAAAACCCGACAAGACTCCGCGCCTTCTTAA
AAGTCATAAGCCACAGTCACTGCCTCGTGACGAATCCACGTATCCATCGAACGTCGAAAC
CGAAAACCCAGAAATAGCGGCAGCAGCAGAAATAGAACCGAAAGGCAGTGGCAAGAAGAA
AAAACACAGAAAAAAGAAGGGATCCCCTTCCGTCGGGCCCATCGGGTTCAGCGGAAACCT
TGAAAACGTTAAGGTGCTACAAATCATCAACATCCACAAGGCGGATTCCCTTTTTCGGAG
GCTCGAAACCAGCGCCAGCCTGGCGAAAATCGTTGCCGAGCTTGCCGGATGGGAGGAAGG
TGCCCGTCTCGCCCAGGACCAGGTCTGGGCCAAGCCGCCGGGGGCACCCCCGCTGGTCTT
CCATCGAGACTCGCCCTATTTCATGTTCGATCCCCCGGACGTCGTCACCGTCTGGCTGGC
CCTGGACGACATGGACGGAGCCCTGGGCCCGCTGGAATACGTGAAGGGGAGCCACCTGTG
GGGGGACGGCCGCGTTGGATCGGCCAAGCAGTTCTTCCAGGCCGACACCAGGAGCCTCCT
TCGCTCGGCTGCGGAACGAGAGGGAATTCCCGAGGCAGACCTGGAATTCGTCAGCATGGA
GGGACTGGAGCGTGGCGGCCTCTCGGTCCACCACGGGAGGATCTGGCACGGGTCGGCCGG
GAATTCCAGCACCTCGTTGCCACGGCGGGGCCTCGGCCTCCACTTTGTCCCCTCCACCGT
GAGATTTACGGCGGATACAGCGAAGAGCAAGCTGTGGAAATCTTACGTGGAGGACTTGGA
GGGCGATCCATCGGAAGTGGAGCTGCCCGAGGAAGATTTTCCGTTGTCGACAGCTCTTTA
GATTGGAATTTTGAGAGAAGAGTAGTATGGTAGAAAGCAAGCACAGTAGACAGTGGGATT
GGAAACTCAATGTGGTAGAAGGTATTATTAATTGTAGAACAATGAGGGAATCTACAATTT
GCACAGTTGCTACCTCATTATTCTTTGCTCCGAACCTTCGTAACAATTATTTTCGGCTTT
TTGAAGAAATCGACTGGAGTTGGAACATGGAGTAAGCTTAGCTTGTGTCTTCGATGGCCA
CGGGTTTTCGTTCTAGGTTGCCTGTACTGTGGCAAAAGAATCCAACATCCGAACAAGAAT
AGGATGTCATCCGATAAACACTTTTGCGCGGGATGGTGGTATGTGTTGACAGGCACTACT
TGAACGATGTGTCTCGGATGTCTGGGAAGAAATGAGCCTTGGTTTCTGTTGTTTGTAACC
ATCTACGTCTGTTAGAACTTGCAAAGCCACTCTCTCCCAAAAATGCACCATCCATAATCA
ACCGCATTTTAATATTTTTTGCCTGCCGGTGTCACGAAATAAGAATTTAAATTATTTTAC
CAATAGTATCAAAA
>comp2237_c0_seq1
CACTGCCATTACGGACGGGACGACACGCAATCCACGAGTATCATACAGCTTCTTTGTATC
AACCTGAATAAGTCCATAAATCCAGAGATAGAGAGATGACTGAACAAACGTCCGGCGCCT
CGCAACCTCCGGCGTTGAGTGATTTGAGTTCAGAAACGCTTCTGAGCCTGGGCGATTCGC
ACTTCGTTGACGAGAACTACGAAGAAGCCGCGGACGCATATGCCGCGGCACTATCGCTTT
TTCGTGAAACCGAAGTTTCTTCGCAAGTCCGGGCCCTTTCTCATCGATCATCGGCATTTT
TCAAGCTCGGCCGCTACGAAGATGCCCTGGAGGACTCACAAAAAGCACTGGAGCTGTTGT
CAAACAACAAACCGTCGGGGCTGCGAATGGGGGAGGGAGAGATTTGTCACCGCCGAGCAG
GCCTAGCAGCCCTAGAGTTGAAGCAGTACCAAGAGGCAAAAGAAGCCTTGCAAGCGGCCG
CGAAACTTGCGTCATTGAACCAAAAGAAAACCAACAAAGAACTATACGAAGACCTGCTCC
AAAGATGTGATGCGGGATTGTCTGCTTCCAAGAAGAAAACGGCGACTTCCGCAGCTCCGT
TGCCAGACTTTCCAAAGAACGAAGACGTTCCAGAAGCAACTTCAATGCCATCACGGGTGA
GTTCTCCGTCGAAAGCCAAAACCACTACCCGCCCAGCTTTGGCGGGAGTCAAAGGCAAGA
CACCAAAATACCAGTACTACCAGTCAGATAAGGTCATGACTATTTCTGTCCTCGAGGCTG
GTGTAAAAGAACAAGATTTGACCGTTCGATTCCAACCCAAGCATTTGGTGGTGATCCTCC
GAAAGGACAATACCGATTTCACCGTCATTGCGGGAAATTTGTATTCGGAAATTGATGTGG
AGAAGTCCAAGGCTGTTTTCAAGGATGAAAAGGTCCTCGTAAAGCTTCGAAAAGTGGAAC
ACTATGAATGGCCCGAGTTGCTTGGAAAAGGAGACGACAACAACAGTAGCAAGGCAACAA
AAAAGAAGGCGTCGGAAGAAGACTCGAGCAGTGATACCGCAACCAATCCGAAAATGATCC
CAAGCGTTCCCCAAGATTCGAAGAAAAATCGTCCGTACGCAAGTCACCGCGACTGGGACG
CGATCGAGAAGGCTATCGAGGAAGACGAAAAGAACGAGAAACCCGAGGGCGACGAAGCTA
TGAACAAATTGTTCAAACAAATATATGGGAATGCCGATGAAGACACGCGTCGTGCGATGG
TCAAATCTTTCCAGACTTCGGGTGGAACAGTACTGTCAACTAATTGGAATGAGGTAAAGG
ACAAAGATTACGAAAAGGAACGTACAGCTCCGAAGGGTGTCGAATGGAAAACTTGGGATG
GAGAAAAGGTTCCAATGAAGGAAGATGATTGAATGATTGGGCAAAGATAGAATTGCCATG
AGTGAACGAGATATTGATAATACGAACTACCGTGCTGAATAGCGCCAAGAAAAATGAATA
AATCTCTATTGTAATTAAAAGATTTTTTCATGCATGAAAAA
>comp2238_c0_seq1
CTTTTTCTCGCACGGAGCGATTTATTTCGAAGGTTCTCGTCGCAACGGAATCGCAGTGGA
TCCGAAGAGCACACACACATTACATAGCATACTACGTAGTAGAAAACGCTACGAGTTACT
GTGCTTGCTGTGCGAACTCCAAGATACATACCGTCCTCCTACGCTGCGAGGGAGTGTCAC
CTACCCCCTCCATCCAGCAAGTGTTCTCACTGCGTACCGGTACCCACCGCCGCGCTTCGC
CATTTCACACCGGTAGGTAGGCACCACCAAGACCAATACAGTATCGCACGTCGCACGGTA
GGATATCTTTCTCGCGTTTCCTTGCATTCCGTCACCAAACCGCAACAATAGCCGTTGCTT
CTGTGTTGATTTATTTAGCTCCCACAATACTTCTACATATACTACTAGAAGTACATACTA

AAGTCGGTACAGGGCATGGACCGAGACCCAGATTGATTTTATTTTATTCCCACGGTTTCT
CGGCAGTTGGAAAACAAACCCTGCGCACGCGTTAGCACGATCGAATCGAATCGAATAGTA
CGAACGACGTCATTCTTTGCGCCATTTTGTGGACCGTTCTTCTAGCAGTCCAACACACCG
TTCCAATCAGCGACGAAGGCGTTCGATCCGTCACGGACCGGAGCGCGAAGCACAATCCAG
ACGAAACCATCGTTTCGATCCATCCGCGAGCACTGCCTTTCCTTGCATTGCATTCGATTC
GATTCGAT
>comp2239_c0_seq1
AACCAATCCAATTCAGCCTTCGCTAGAGCTTGTTCTTTGGGAACGATGGCGGACCAAAAG
AATGCGGTGTGGCTCCATTACTTGCGTTCCTTCCTACGAAGCGACCAAACCGACGCGTCT
GCCGAGAAGAAACGCAAGAGCGAAGCGGGATTGGAAGAAGCAACCAAGACAAACCCAACG
TCGAGCGATTTCTACTGCGAACCAAATGGCCCTGGGCGGATTCCGGTAGCGGTACACCAC
CCGTTTGTTGCGATGGATCAGTCGTCATCGTCGTCACCACAGCCCTCTAACAAGCGTGTG
TGTAGGAGTCGTTCATAGGAAAAGAAAAGGAGAGGCAGAAACCAAGAATATATTTATTCT
GTCACGGAATGTCCGAAATCGAATCGCTGTTACGCATAGGTTTGCATAGTTTGCTTTTTC
TGCGAATGGGGCAGTTAGGGAATGGGGAACGATCGAATCAATCGAAAGCAAACGAAACCA
TGGATTAGGACTTGGGCGGGATTGGGAAGGGATACAACGAGGGACGGGATGGGAAATCGT
GTTTTGATGCTGTCGGGCAATCGAATCTGTCGAAAGATCGAGACAACGCAATGCACTTGG
GAATATCATAGTACTTAAGAAAGAACATTTTCATTATTTTATTACAACGAATACTACATT
CACATTC
>comp2240_c0_seq1
ATTTGCAAATCGGGTCATCGTCATAACTCTGCTTTCAAGAAATTTCAAAACGAAAAACAA
TGGACATCGAAAAACAATTCTCCGATCTCTTGCAATCTGTCGAATCTCTAAAGGCAGATA
GTGTCAATGAGATTCGCAAATTGCGCGTAGAAAATGAGAAGCTTAAGGAGCAGCTAAAAG
TCTTCTCTGGTGGAAGGAAGCAGAAGAGGATGACGACAGCGAACGCAGGCTTTGCGGAAT
GGGCACAGGCAGATTTTGTCCGCATGGCCAAAAACACGGACGCACTTCATGGAATGGACA
ACGTCGAAGTAGCTGATGGCGAAGATGGAATGGTTCTAACAGACAGTGTCGCCTTCGATC
CTGAGAAGAGCTCGTATGCCAGCGGTTATGACTACAAGTATCTTTCGGCGAAAGAAGTTC
TAATCACTTTGCATGATGTAGGAGATAACAATGTAAACATCGATGCTGGCCCTGGGATGT
GGGCGAACTACATTCTTTCCAAGGAACGGAACAGTACTTTGAAATGCGTGGACAAAGCGA
AGCTTCTAAAGGAAGGTAAGTATTACCACGTGAAGAGTATCAGAGAGGGCCTCGATATTG
ATACACTCGTGGCTTATTGCAAGGAGAGGAATATCAAAGCGATCTGTCCTACCAGTGTTT
CCGACGAAATCTACCTCGCGACGCACAACAAGGTTTTGCGTGGACAGGGTATCTACCCTT
TCTGCTGCGAAGATCCCCAGAGCTATAAAACGCTCGACCACAAATGGCTCTCATACCTAT
TCTGCGAAGAGAATGGAATTCCACAGGCAAAAACTCTGCCCCTTCGTGTGGACACCGTTG
ATGCCTGTCGTGAGCTTGTTGCAGAGACTTGCGCCGCCGGCAAACCCGTTTTCGTCAAGG
AATGCTTTGACACGTGTGCTGGAGACGGCGTCATCAAGGTTGATAAAATTGAAGAGTACG
ACGATGCTGTTGAGCGCATCACCAAGGGAAGAGGACCGCAACCAGCTGATACATCTGGTA
TTGACCAACATATCATTGTTCAGGCTGGTCATCCGGGAAGGATTGCTTGCTGTCATAATG
TTTTCTACAAAGGCAACTTGGTGTCGCTTTACATTACCAAGGAGAATATGAAAATCATGG
ACAAGCTTGGAGAGCTTCGTTGGGATATGACTATTGGAACTTGGAGTACCAAAACGGATG
ATTTGTCGACATCACTTAAGCTCGAGCTCGACGATCCATGTGACAGACTTATCCGAGACC
AAGCAATCACGATCATGAAAAAGGTAGGCAAAGCCCTAAACTACACCGGTATGTGCGAAG
TTGAATTTATTTGCGAACATTCTCCAGATGCGATCTTGAATGTGCTAGAGTTCAACCCTC
GCTTCAGCGGAGCATGCCATTCATTTGTTGGTGCAGGAATGGTACAAGACTACATGCATG
TTATTGGGCTGATGGTCAACACCAACGACGATACAGACAAGGTTATTGCCGAGAAAGCAG
GTACCTTCCATGCGCGATCTGACACGCATTTGCCGAAGTCGAACTTCAGAGATTACAACA
CTCTCAAGTTTTACATGCCACAGCTTGCAACTATTGCCAAGTTGGGCAAGATTTAAACGT
TCCATGCGTAGTATTTCTGCGAGGTCATAATATATTTGTTCCGATCAAGATATTCCAAGT
TTCTGCAACATTCTTTCAAATGTGTCGTACGGCATGCCTTCAACTATCGCTGTCTATTCG
TTCATAAGTATATGAATCATAGTTTTTTAAAAGTAATCAATATATTTTTCCGTTGAAAAA
AAA
>comp2241_c0_seq1
GTCTATTTCTTGAATTGATACTAATACGCCATGAACTTGTTCGTCACTCTTCTCGTCGCT
CTCTCTGTATTGGCAACTGTGCCATCAACTTCGGCCAACGCGTCCTTCTCTCGTGGAGTT
GGAGCTCCTCTTTTCGGGATTTCTCGAGGCGGAGGTCTATTCGGAGGAAAGGATGATTCC
AAGAATAACGAAGCTAGCGTTGCTGCCTCTGGGGAAAAGGTCATGTACCCCGCCATGACT
CAGCAAGAAGTCGAGGATTGGTTGGAACACATTCCTGTCTTTGCAGTCACGGACTCAAAC
GGTGCAGGAGTTGTTCTCAAACCCGACGAGGATTCGAGTGTTTTCTACTTTTTTCTCAAT
CCTTTGGCTGCAAATGCCACGTTGACCCAGTTGAAAAGTGTGAACGAAGACATGGACTTG
AAGGTGTCTGCTTTTTCTCTTGGAAAGATCTGGTTCAAGCTCTTGCAGAGTGATGCTGAT

CGTGAAGTTTTGCTGAAGAAGCCTGGTGACGATGATGACATGACCCAAGCCACCAAGGGT
GTAGAATACCGCCTCGTGCCAGACACAAAGGATCTCCTAGGTGCCCGTATGTTGTTGACG
ATGAACCCTGAGGACGGCGAAGCGTTGAAGAATGGCCAAGAAATGACACCAGAAATGGCC
CAAAAAGCTGTTGAGAAGGCTATGAACGAATCTCCTAAGTTCAAGGAAACCTTCAATGAG
ATCCCTGTCTTCACCATTGCTCAAATGAGAATGCAAAAGCAACCTGCCGAAGGCGAATCT
TCTGCTGAACCTGTTACCCTTCTTCCGATGTACTTTTCTTTGCAAAACATGGTTGGAACA
TGGCAGCAATTTATGGCACAGTCGGCACCAGAATTGCAAGGAACCGAACCTGCTATCAAT
TTGATGGCTCTCCACGATTTGGTCGAGATGATGCAGAAAGAATCCGAAATTGATTTCCGA
TCTGTTGTGCTGGTCCCTTCCATTCCCGAAGGCCCAGGCGGTGCGGCACCACAGGGCCCA
CCTGTCGCAAGTGCGGCAGATCCTATGGCTAGCATGGGTGGTGCTACTCTAGGTGACATT
TAAAAACAGTAATAGAAATACTAAGGGAAAATCAACTCCA
>comp2242_c0_seq1
CGGAGTGATTACAATATCGAAACGGGAGCTGAAACTGATCTCCTCTACACCGACTTACAA
AGCGACAAAATCTTTGAACAAAAAAAAACATGTCCGAAACAAACCCAGAAGAAGGTGACC
CGTCGGCGGTAAGTAGCAAAGTCGAACCCACTCAAGCCGGAGATGAACAAAAGGTGAAAA
AGGATGACGGCACTAACAAGGACAAGGGAAAGAAAAAGAATCCAGTTCAAATGACATATG
TGTCGGCCACTCAGATTGAACGAATGGTTACAGTCAGCTTATATATGCACAACCTCTCCT
GGAAGCACCTTTCGTGTCGGCAGTTCTATTGGTTCACCATGCCACAAGCTGTTCTAACGG
CGGTGGCGAGTGTGCTGGCGTTTGTGGCAACATCGGACGCTTTGAGCGACGACGAAGCGT
CAAAAGTTATACTTAACTTAATTGTCGGTTCAACGGCGAGCTTAGTTGTATTTCTACAAA
CCATGAGTGGGCTTAAGAATTATGGCACAAGAGCAGCGATGCACCAAGGAGCTGCGGTTG
ATTTGAAAGATCTACTATGTGATCTACATATCTTGAAAATGAAAATGAGTCAAGTTGAGA
ATATAGACCAGGAATATCTCGACATGATGCGAGGCATTCAACCTAAGGATCCCGAGGCAG
ACGCAGAAGCAGACAAAGAGGCACTAAAGTATAGTGAAACCTTTGGGACCATTCAAAGTC
GTTTGACTCAGACACTCAATGCATGCAAATCGAACATCCCTATGGAGATAAGCGCAGCAT
TCAATGGACTTCGAACCACGGCACATCTTGCAATGACAAAAGCAAATTTTGAATCCTTCG
AAAAAATCTACGGACACGAAAACTTTCTGAATACCTACTATTCCAAGGCTGATGACATCC
TACAAGGAGAAATCCTTGGCTCCAGATTCTTTCCGATGAAACTTCCTTACTCCAAGGTAA
TGGTGAAGAAAACTATGGAAATTCTTTACACGCAATTCAAGGATTACGACCGTCATGATT
TCGGCTTCGACTCGTCTACCGACGACGCGACAAACGATGTTGAAAAACAGGGTGAATCAA
AATAATTGTCGGCTCATCATGTTTGATGTTTTTTTACCGATCCACCACCGGGTGGTCTTA
CATATTCTTTCTGTGCTTTGTCCTCACTCAGCAAGCTCGGAGTCAATCTTTCAACCCTCT
TAAGCTGCTGTATTATAAAATTGAATTCTTGATTTGGCTGTAGTATTACTAATACTAACA
TATCAACATTT
>comp2243_c0_seq1
GAACAGAACAGAACAGCACACGGTTGCCAACTGCGATCCGAGTTTCCCCGATCTCCTCCG
ACAGAATCCAGTCCGGTCTCCCGATCCAATCCAACCCAACCCAATCCACACCATGTCAGC
GGCAAAGACAATGGCAGCCCTCGCGGCAGCGGTGATCGCTTCGTCGATGCCGGCAATGGC
CTTCAACTCGGTCGCGGCCCCCCGTTCCGCGGCGCGAAGGGCAGCGGCAACGGCAACGGC
CACCACCACGAGGCTGTACGATAGCTACGACGACCAGTACGCGAAATTCCAAAACCGGGG
TCCCCCCCCGTCGCAGCAGCCGGCAGAGGCCGGAGCGGTGGAAGAAGAAGCACCCTCCGC
CCCGGTCCCCTCCGCCTACGGCTCGTACCTGGACAACCTGGGCAGCGCTCCCGTCGCGGA
AGAAACGGAGGAGGTCTTTTACGAGGCCACCAACTCGTACGAGGCGCCCCCGGTTTTCGA
AGCGGAGCCCGAGCCGGAGCCCGAACCCCTTCCGGAACCCGTGTTTGCTGCTGCCCCCGC
CGTTCCGCAGCCCGTGGTAGCCGCCGCTCCCCCCGCGGCGGCAAAGCCTCCCCCGGCGGT
TTCCAGCCGCCCCCCACCGATCGAGTACCAGTCCTCCCCGACCCTGTCGGCCGAGTCCTC
CACCCTATCGGTCACGCTCCCGGTCGGACTCCGTCCCCCCAAGCTCGGAAAGTCCAAGGA
CATTTCCTACGGAGAGGGATCCCGAAAGTTCCGCCGGACCGTCTACTCCCACGACGACTG
GGTCCGCCACCGGGACCCCGATCGCTTCTTCCGGTACATTGCCAGCGTCCCAACCTCGGG
GATCTACCGCAACCTGGCCCGCGAGGTCTGGGCCGTCGTGGCCGTCACGATGCTGGTCCT
GGTCTACAACGGACTCATCACGGGCTACCAGGACGTCTCGGGGGCCCTGCACGAACCGGT
CCTCTCGAGTCCCTTTTTGCCTCCGTTGACGCTTCCGGCGACGCCCTTTACGCTGGCCAG
TCCTTCCCTGGGATTGCTTCTCGTCTTCCGAT
>comp2244_c0_seq1
CATTCATGTCCGGACCGCAACTACCGGCGAAGAGCACTATTCGCAAGAGAAGCGTGGCAG
CTTTGGAGAGCGCCAGCAACCGCAAGGCAACCAGCAACGAAAGAGGAATCTCAAACATCC
CGAGAAAAGGTTCCCCAATGGCCATGACCGCGAGACCGGTCCGGTCTTCTAACAAAGAGG
ATAATGCCACGGCAAACGAGGCGAAATCTTCCAGCGATGCAATGGCATCGAAATCCCCAA
AGCGACAGCACTACGGTTTCCAGCCGAGTGCTAACTGGATGATTCGTTCCCCTCTGCTGT
GGGCAGTTGCAGTGGTTCTACCGCTCTTTTATTGGGTTTACTCGAAACAAGGGCACGGAA

CGTCACTTGCGGACGCAACGAAGACATCTGATATTCCCACCGGGGATCGAGACACAACCA
CCACCACCATTTTGGAGGAATACCGAACCCTGATTGAACTCTCCATTCCACCCGGGGCCC
TCACTCCAGGTGAACACCGCAAAGGTCGCTACGGAAAAGTCCACGCCACCTTTTGCGCCA
TTGACTGGGACCTTCAATCCGCTAACCCATCAACGGTCCCCATGTTCAAAGACCTCAAGG
GGCGGTCCTCCTCTTGCAAGGCCACCAAGACAGTGGTGACCGATTTCTACAAGCTGGCCA
GGGCCGCCAGGGACTACGACGCCGAGCAAAGCGACCGGAACGAGACAGACGAAGCCCCCT
ACCCGTCGGGAGTGGTCTTTCACGAGACGCGGTGCGGTTCTACCCTCGTGGCCAACCTGC
TGGCGGGATCCTCTCCCACCATGGGCACCTCGCGGGTCTACTCCGAGTCGCCTCCCCCGA
TCGCGGCCCTCCAGGCCTGCGCCAGGAACGGTGGGAATGCCTGCGACCCGGACCTACATT
CGTCCCTGATCCGTGACGTCTTTTACGTGATGGGACGACGGAGGCCAGCGAGCCCCAAGG
CCGAGCAACACCGCGTCTTTTACAAGATCCAGAGCATCGGTTCTATGTCCATAGACAAGT
GAGTCGTCTTTGATTTGTTGTTCATGCTTTCGAATCCATTTTCTTGCTCACCAGTAATTT
ATTCTCGTTGACTGCCAAAGATTCACCGCGGCCTTTCCAACCGTCCCCTGGATCTTTGTC
TACCGCGACACGGTAGAGATCCTGCAGTCCCACCTACAGCAGGGGCGAACTAACCCGACC
AACTCACAGGGGCAGCAGAAATCGACCGTCATTCCCAAAGTCTGCACCCGCAACTACAAC
CTCGGCCTCAATCGGCAGCCAGCCACCACCCTCGAGGCCATGAAGGACGCAGGTGTGGCG
ATAGACAAACCGCCCAGTGCAAGCCTGGATCCCATTGAGTACTGCGCGGCCCACCTCGCG
GGCCTATCCCTAGCGGCACTCCACGAGCACGATCGGTGGCAATCCAACCACAAACAAGCG
GCCAGCGGGGGCGGACGCTTTGTCGAGTACTCGCAGCTCCCGGATCGGCTCTGGAACGAG
ATTCTCCCGGACCACTTCGGGCTAGGCCGACCGCTGCCCGAGCAGGCAATCTCCAACATG
GAAACCATCGCGGGGGTCTACAGCAAGGGCCGGGGAAAGAAGGCCAACCAGGAATGGGCC
AGCGACTCGGAGGCTAAACAGGAAACGGCCTCGAAACAGGTCACAGAGGCCGCGAAGCGC
TTTGCCTCGGGAGCCTATGAGCGGATGAAGGCACTGTCATCGTGACGAAGCACGGCATAG
CAAGAAATAAAGCAATTGTAGTATGTACGCCACATGTTACCATGCTCTACGGCATCGAGA
TGACAGATATTTGCAACCCACGATCAAAGCGGCTTGTTTGTTATTGATCTCGGGTGTGAA
AAAGTGCCAGCGTTCCTTCTCGATGCTCGAATTTTCTCTGCTGTATCCGCTGTGTCCATG
TTGGTTTCTGCCCATCGAACCTTTGCCAATCGAGCTGGATTGGAATGGACAACAGCAGTT
TGCAGCTAGTTGCCCTCTTTATTGCCGTTCGTTCTTGCTCCGGGAAGCCACAAGATAATT
TTCCATGTTGAAACAGTGGTTTCCCTGTTCTCTTACATGGGAAGACTCATGTGTATCTGG
AGCAACGGATTCCGTCATGGTTTTGTTTGCCGATCCTTCGGGTTGTCTACAGACCCGAAC
CCCGTTGTGGTTTGGAATAGCGCAAGTCGTAATAGTAACTTGGTGTCAACATAGCAGGGG
TTCGCTCGGTAGAACGATCTGTGTGTTCGAGGAAATTTTCCAATATTGCAAGACAAGCCT
CGCAAAACACCCACGATCCAGTCGAAGCGTGATTGGCTCGCAGGAAAGGAGGGTTCAACT
CGAAAAGAACAGCGGCTTGCGTGATTCGGAACCATGAAAGTTTGTTCTGGGATGTGCAAT
AGATTTTGTGGTAGTGTTTGCGATTTTTCCTCCATGAGCAGAAGCCAACGCAGAAGCTTT
TCAATAGCAGTTGTGAACACAAATACGTACTCTAGTTTAGCTTTGTATTCAAACGAAGAG
CACTAATACAATACGATTGGTGCGAGTGCTTGTATCCTTGAATCAACGAAAAAACTGGGC
GACATAGTATTTTCCTTTGTCAATCCAATCTCAATCTGTTCCCCACACCACCTTGTCCTA
CTGCTACTTCAGAGGATGAGTTTGACTACACGGATCGGTTTTTCGAGATTAGGTCGTATG
CTACTGTAGCCGGTGCAATGGCAGTATTGATTGCAAGAGCATTAAAAAATGAAAATAGAG
CAG
>comp2244_c0_seq2
CATTCATGTCCGGACCGCAACTACCGGCGAAGAGCACTATTCGCAAGAGAAGCGTGGCAG
CTTTGGAGAGCGCCAGCAACCGCAAGGCAACCAGCAACGAAAGAGGAATCTCAAACATCC
CGAGAAAAGGTTCCCCAATGGCCATGACCGCGAGACCGGTCCGGTCTTCTAACAAAGAGG
ATAATGCCACGGCAAACGAGGCGAAATCTTCCAGCGATGCAATGGCATCGAAATCCCCAA
AGCGACAGCACTACGGTTTCCAGCCGAGTGCTAACTGGATGATTCGTTCCCCTCTGCTGT
GGGCAGTTGCAGTGGTTCTACCGCTCTTTTATTGGGTTTACTCGAAACAAGGGCACGGAA
CGTCACTTGCGGACGCAACGAAGACATCTGATATTCCCACCGGGGATCGAGACACAACCA
CCACCACCATTTTGGAGGAATACCGAACCCTGATTGAACTCTCCATTCCACCCGGGGCCC
TCACTCCAGGTGAACACCGCAAAGGTCGCTACGGAAAAGTCCACGCCACCTTTTGCGCCA
TTGACTGGGACCTTCAATCCGCTAACCCATCAACGGTCCCCATGTTCAAAGACCTCAAGG
GGCGGTCCTCCTCTTGCAAGGCCACCAAGACAGTGGTGACCGATTTCTACAAGCTGGCCA
GGGCCGCCAGGGACTACGACGCCGAGCAAAGCGACCGGAACGAGACAGACGAAGCCCCCT
ACCCGTCGGGAGTGGTCTTTCACGAGACGCGGTGCGGTTCTACCCTCGTGGCCAACCTGC
TGGCGGGATCCTCTCCCACCATGGGCACCTCGCGGGTCTACTCCGAGTCGCCTCCCCCGA
TCGCGGCCCTCCAGGCCTGCGCCAGGAACGGTGGGAATGCCTGCGACCCGGACCTACATT
CGTCCCTGATCCGTGACGTCTTTTACGTGATGGGACGACGGAGGCCAGCGAGCCCCAAGG
CCGAGCAACACCGCGTCTTTTACAAGATCCAGAGCATCGGTTCTATGTCCATAGACAAAT
TCACCGCGGCCTTTCCAACCGTCCCCTGGATCTTTGTCTACCGCGACACGGTAGAGATCC

TGCAGTCCCACCTACAGCAGGGGCGAACTAACCCGACCAACTCACAGGGGCAGCAGAAAT
CGACCGTCATTCCCAAAGTCTGCACCCGCAACTACAACCTCGGCCTCAATCGGCAGCCAG
CCACCACCCTCGAGGCCATGAAGGACGCAGGTGTGGCGATAGACAAACCGCCCAGTGCAA
GCCTGGATCCCATTGAGTACTGCGCGGCCCACCTCGCGGGCCTATCCCTAGCGGCACTCC
ACGAGCACGATCGGTGGCAATCCAACCACAAACAAGCGGCCAGCGGGGGCGGACGCTTTG
TCGAGTACTCGCAGCTCCCGGATCGGCTCTGGAACGAGATTCTCCCGGACCACTTCGGGC
TAGGCCGACCGCTGCCCGAGCAGGCAATCTCCAACATGGAAACCATCGCGGGGGTCTACA
GCAAGGGCCGGGGAAAGAAGGCCAACCAGGAATGGGCCAGCGACTCGGAGGCTAAACAGG
AAACGGCCTCGAAACAGGTCACAGAGGCCGCGAAGCGCTTTGCCTCGGGAGCCTATGAGC
GGATGAAGGCACTGTCATCGTGACGAAGCACGGCATAGCAAGAAATAAAGCAATTGTAGT
ATGTACGCCACATGTTACCATGCTCTACGGCATCGAGATGACAGATATTTGCAACCCACG
ATCAAAGCGGCTTGTTTGTTATTGATCTCGGGTGTGAAAAAGTGCCAGCGTTCCTTCTCG
ATGCTCGAATTTTCTCTGCTGTATCCGCTGTGTCCATGTTGGTTTCTGCCCATCGAACCT
TTGCCAATCGAGCTGGATTGGAATGGACAACAGCAGTTTGCAGCTAGTTGCCCTCTTTAT
TGCCGTTCGTTCTTGCTCCGGGAAGCCACAAGATAATTTTCCATGTTGAAACAGTGGTTT
CCCTGTTCTCTTACATGGGAAGACTCATGTGTATCTGGAGCAACGGATTCCGTCATGGTT
TTGTTTGCCGATCCTTCGGGTTGTCTACAGACCCGAACCCCGTTGTGGTTTGGAATAGCG
CAAGTCGTAATAGTAACTTGGTGTCAACATAGCAGGGGTTCGCTCGGTAGAACGATCTGT
GTGTTCGAGGAAATTTTCCAATATTGCAAGACAAGCCTCGCAAAACACCCACGATCCAGT
CGAAGCGTGATTGGCTCGCAGGAAAGGAGGGTTCAACTCGAAAAGAACAGCGGCTTGCGT
GATTCGGAACCATGAAAGTTTGTTCTGGGATGTGCAATAGATTTTGTGGTAGTGTTTGCG
ATTTTTCCTCCATGAGCAGAAGCCAACGCAGAAGCTTTTCAATAGCAGTTGTGAACACAA
ATACGTACTCTAGTTTAGCTTTGTATTCAAACGAAGAGCACTAATACAATACGATTGGTG
CGAGTGCTTGTATCCTTGAATCAACGAAAAAACTGGGCGACATAGTATTTTCCTTTGTCA
ATCCAATCTCAATCTGTTCCCCACACCACCTTGTCCTACTGCTACTTCAGAGGATGAGTT
TGACTACACGGATCGGTTTTTCGAGATTAGGTCGTATGCTACTGTAGCCGGTGCAATGGC
AGTATTGATTGCAAGAGCATTAAAAAATGAAAATAGAGCAG
>comp2245_c0_seq1
CTTTGATTTCATCAAAGGAAAAAACGCAACGAAATACCATATCCATACCTTGGTACATCA
TAGAACATTGGAGACTGAGTGAATCTAGTACTAGAATCGAACCAAGAAAGAGCTTAGACA
ATCATCATGACTGTATCCTCGGCATATCAAAAGAGTCCGATGTCTTGGACCGAAAGACCC
ATCCCGGGGCTGACGATTACAACGGCCTTCGCAGTTTGGCTTGGAATTACGACGGTGCTC
GTTCCGGCTTTGATTCAGAATTCATATTACAGAACGGAGTCCGGAAAATCCCCTCTTCTT
CCACCCATCACCATTCTTGGGTGGTCGATAATGTTTTTCAACAACCTGAATGTATTGATC
GCCTTTTGCGAGATTGCATTAGGAAATCACATTGCATTCATCCAGAACCATTACCAAAAA
CTGCTGAAGGAATATCGCGACGCGAAAACAGGAAAACCCAAGAAAGCAGAATATCTTGCG
GGATTTGCCTACCTGAACATGCCCTTGTCGCTTCGAGACCTATACGACGGGGGGCAAAAA
TGGTCCCAGATGTGGTCGACATACAGTTTGTGGGATCCATCCTATTCCAATCCAGAGAGC
TTCGGATTTTTCATTGACGTTGGAAATGGTTGGTCGACGATAGTGCCATGCTGGACGTGG
AATTTTGCAATTTTGTTTCCCCATTATTTTGCACGGAGTAACACCGATGGAACGGCAGTC
GTTCTTCTTGGATTTATTGGCGCTTGCTCGTATTGGCAGGTCTTATACGGTACAATCATA
TACTTTTTGAGTTTCTTGTGGAACAAACGATACCAGTACAAGCCATTGGTGGAAGTACTT
GGATTTGTCGGTTTTGCGAACGGAATCTGGTTCTTTTTTCCCATCGCGTGTTTGTGGGCA
AGTTACAAATTATTGCTCGATCGAGATCCGGTATCTCTCTTCTGGTACTAGTATCGTTAT
GGGATGGGCTACAAGCAAGATTTGGGCTCTCATAGTTAGACGATTGAAGCGGATCAATCT
ACGGCACGTTACTTTAAATTAAAGTTCTGACGAAATAGAAACGAGCCTCTTTCCGAGGAA
CTCATCATCTATACCAAAAACAAGTCCATTTCGAGCTACTTCACTATAATTTACAGTGTA
AAGTTTATCGTTCGGACTTTAGTTTTCTCTGATGCCATCGGCTAGATGTGAGTTATCAAG
GACGGTTACTGTTGTTGCCACTTACAACTACCGAACAACATCCGCTCGTCACTAGCATTC
ATACTAGTACCGGTACAAGTACGAGGACGACTACAAGTACTAGTACATAACATATTTTTT
AGTACGAGTACCTGGTACGAGTACTAGTTCTCGACCTCGTGCCAAACGCGCC
>comp2246_c0_seq1
TGATAACATGCCGCTAAGATAAGACTTCTAAAAAAATCACTTTGACGGTCCAGTCGTATC
ACGAAAGAACAGCACAGAAAGCCAACACAAACAAGAGAGGAAGAGAAACGGAATCACTCA
TTCGATCAGTTACCGACCGAACCAATCGCCGACGAAATCAATTTACCTCCCGACAACCTA
CGGCAAACCGCAAACAGTCAGCACCACACAACACCGGAACCACAATGACGAACGAGAGCG
AGTCCGTAGTGAAATTCATCCCCTTTTCTTCCACGGTCGAATCGCCCTTTTGGGTGCGCT
ACTGCCACCTCAAGCTGGAAACGATTCGGCTCAACGAAGACCCGATCGACATTCGGGCTT
CTTACGGGGCAGCAGCAGCAGCCACGTCGGCGGCGACCGGCGGGACCAACCGCATCCAGT
GCCAGGAATCGGGCCTGCTCCTTCCGGGGGCGGACGACGCCGGCGGACACGATGCCGCAC

GGGAAAGCCCGATCCCCAACGACCGCGTGGCGTGTGCCGGGGTCCTCCTCGGATACAACA
CGAAGGAATCCTTTCAAAAGGTCGACAAGAACAAGCTGCTGAAGGACGTCTTCTGCGACC
GCTTTTTTGGTGCCTCGTCGTCCTCCCTGGCGGCCCCGTCGCTGGTCTTGCTCACCTTTC
CCGACCTCAAGCAGCACAAGGTCCTGTACTGGTTTGGCTTTCCGACGATCATGGCCAGGG
CCCCCATCCTGGCGAGCACGGGGGGTTCGCCCGGGCAATCCTGGTGGGCGGGCCTCGCGG
AGGGACTGTACGCGTACCGCGTCAGGGAGGACGCCCTGCCGCCGTATTTTGCGGTGCGGG
CCGGCGGTTTCACCGCGGGGGACGATCCCGGCCCGCTGGACATTGCGGCGCCCCTCGATC
GGGACCACCGCGAGGCCCTGGATCTTTCCGAGGGACAAGAAGCGGTCTTTGGATTCGTGG
ATCCCACGGCGATCGGTGTTGCCAGCTCCAGCAGCAGCGGCAGCGGCACAAAGACAAACG
ACATTTTGATGATGGGCTGGCCGATGCGGAATCTTGTTGCCTACCTCGTCCTGCATTTGG
GTTTGGGAGGGAAAAAGGTTCGGATTCTAAGTCTCCGACCCGGGGGCTGCCCCGGCCTGA
GGGGAAGGATCGACGCGACCGACAGCAACGAGTTTGGTTCGATGCTGTCGGCGGTGGATG
GCGAGCGACTGGCCGAGAATTCTGTCCTGATGACCGTCACGCTCCCCCATTCCCGAGACT
ACGACTACGGAAAAAGCGACGGCAAGGAGGACACGAAAGAACCCACCTATAAGGTTGTCG
GGTGGGAGCTCAATGCCCGCCAAAAACCCGGTCCCAGGTGGGTCAACCTCAAGCCGCTCC
TGGACCAAAACCACCTGGCCATTCAGGCGGCCGATCTCAACCTCAAGCTCATGAAGTGGA
GAATGATCCCCGAGCTGGACGTCGATCTCTTGCAAAAGAACTGCAAGGTTCTCTTGCTGG
GCGCGGGAACGCTGGGCTGCTCCGTAGCTCGAACCCTCCTCGGGTGGGGGATCCGCCACT
TTACTTTTGTGGACTACGGGACGGTCAGCTACTCCAATCCCGTCCGACAGGTCCTCTTCA
ACCTGGAAGACTGCCACTTTGACCACGGCCAGGGAAAGCCCAAGGCGGAGGCTGCCGCCG
CCGCGCTCAAGGAGATTGCCGCCGACGTGGTCAGTGAGGGCATACGGCTGAGCATTCCCA
TGCCCGGCCACGCTTCCGATAACGACGAATCCCTCGCCGAGACGGTGGATCGGCTGGATG
CACTGGTCAAGAACGCCGATGTGGTCTTTCTCTTGACCGACACGAGGGAATCGAGGTGGC
TCCCGACCGTGATGGCCGCGCACCACGACAAGATTCTCATCAATGCCGCCCTCGGATTGG
ATTCGTGGCTGGTCATGCGACACGGCCACGGTGGTGCCGATCTCGGTGGCGACGACAACA
GCAGCCAAAGCAATCGGCTCGGGTGCTACTTTTGCAACGACGTCGTCGCCCCGGAGAATT
CCACCAAAAACCGGACCCTGGATCAGCAGTGCACGGTGACACGGCCGGGACTGGCCCCCA
TCGCCAGCAGCATGGCCGTGGAGTTGCTCGTCTCCCTGCTCCACCATCCCTCGAAACAGC
GGGCCCACGCACCGGCGCCCGGAGGCAACAACAGCGGCAATTTTGCCCCGACCGAACGTT
CCGGTAGCGACAGCAGCCCGCTGGGGCTCATTCCACACCAGATCCGGGGCTCGATCGTTA
CCTACACCATGATGACACCCTCCGTTCCGGCCTTTCGGTGCTGCACGGGTTGCTCGGCGC
CCGTCCTGGCGGCGTATGACGTCGACAGGTTCGGACTGGTGTCTCGTGCCTGCGCCTCCA
TGGACGGTTCCTACCTGGAAAATTTGTCGGGACTCACGGCCTTTCGTGCCGAAGCGGCCG
AAAAACTCGCGGATCTGGACGAGCTGGATTGGGACGACGACGACGACGA
>comp2247_c0_seq1
CGGTACTTTTTTCTTTGTCGCGCCTCATGACATAACCATAATCTAAAATATCAGTATGGT
TTTTGGAATTACAATTGTAACGCGAGTGCCTCTGGCGTTGATTATATATATGGGGGTCGT
CTCTTTGCTCCTACTTCGAAGTATATTGACGAATTACAAAACAGCCTTCGATGCCATCTA
TCGGGACCAGACACCCGGAATCCAAGCACTCGATTACCGCACCTCTGTCCGCGCAAATGC
CTTTTCCCAGTATCTTTATCGAGACCTAAATGCCACCGCGTATACCTGGCAAGGAAACAT
CTGGATTCCCCCCATTGGGGTTCCCCATTATTCTGGAGTTGCCACCCGATGCCTGTTTCA
ATCCGAGAACACGGTCATGGTTGGGGATTCTACTATTCGGCAGCAGTACCACACTTTGCG
ATCGTTCCTCGAATCAGAAAACCCCGACAACATCCCCGTGGCGCAGTTTGGGGAGGGGCG
CGACTTTTATCCAAACCTGGGTTCGAAAAATGCATTCGAAACAACCATGGAACGCAAGGT
GCTTGGGTCAAAGGGCAACTGCTTCCGAGTCCGATCCACGGGCACGGATGGGAATAGTAC
ATCCTACAGCCATCACAAAGAAAAGCACGGCCCTGATTCCACAAGCAACAGCACCCCCGG
TAGAAACTACGGGTACCATTCCGAGGAATGCCTCGAGACCGGAAAATACGATTTGTTCCG
ATTGAATTGCTTCAAGGAAGCCATTGACTGGCTCCAGAGAAACAGCACCATACCCGACAT
CCGACACCGCTACAGCTTTGTGGTCCTGTCCCTGGGAGTATGGGAACTCGATGAATACCA
AAAGGCGAGTTGTGCATCGCTCGACCGCCTCGAAGAGCTCCTCGGCCTCGTTGCCCGGGA
GCTTTCCGGACCGGACCTCTTTGTCGTCTGGCAGGCCCACGCTCCCGATTCGAGCCACAT
CGGCGACGTCGGGGCCGACACGCTGGCCCTGGGGGACCGGGCGAGTGCCTGGTTCGTAGA
GAACAGGCCCCGTTACATGGATTTCATCGACGTCGGCAACATGCTCGTCCGGGCCAATCG
TGCGTTTGCCGACCAGCGGATCGGTGGCGACCACCACGCCCACTGGGGATTCGAGGCGCG
GGCACTGCAGGGACAGCTCGTGGCCCAGGCGCTGCACCGGAAGCAACAGCGAGAGGGAAT
GGATTGCGTCGTCCCGTATACCCACAGCGCCTGACCAACTGCCGGTTGGTTGGTACCCGG
ATTGGATTGAATCGAATCGAATTAAATTAAATCAAATCGAATCGAATCCAATC
>comp2248_c0_seq1
GAACGACAAACGATGAAGTGATGAGACGACATACCCGTACCTGATACCTTCATCGGCAGT
TTCGGCATCATTCCAATTTTTTAACACCACAATCGCAAATTAGGTTTGCTGCTCTTTAC

TTGTTTCTTTAGACTACGATGAAGTCCGCAAGAGGTCACACCGGTAGCTCTAGTTCTCCA
ATCCTGATAAGAGGAGCTATGTTGCTACACAGGGTCGCAGAATTTGCGATTTTTGCGGTG
CTCGCTATCGCCACGACCACGATTTTTTCGAACGTTGTGGCGGGAAGAGGAGGAGTTGGT
CCGGTGGCAGCGGAATCATCGACCGCGGGGCCGTTTGCGGATGCCTTTGCTCCGACGTAC
ATCGCTCCGGCCATTTCGAGACACGCTCGGCACTATGTCTCCATCCACACAAACACCAGA
CCAGAGTCTCCCGGAGGTTCTTCCCTTGCCGCCTTAGCGGCGGCAAGCGATGTCCCGAGA
CTCGGCCCGGACGATCTGGAAACCCTGAAGTCACAGGGATACGTCGTTGTCGAAGACTTT
CTGCCGAGCGACTCGGAATGGAAGAATTCCCTCCGTTCCGATGTTTTGCAGCTGAGAGAG
AAGAACAAATTCAAGATAGCCAGAATCGGGCAGGACTCGACCAACACGCTCAACGAGGAG
ATTCGGGTCGCCGAGACCTGCTTCCTGGGCCGCAACAAGGCCGAGTTGAGGGACGTTCCC
AGCGCCGACCGAGAACGCCTCTACGATGTCCTGGACGCCCTGCGACAGGACCTGGAGGAC
CACGGCGCAAAGGGAAACAAGCTCGACGAGGGCCTGTCGGAATTCCTGTACGCCTACTAC
CCCACCGGTGGCTTTTACCGCCGGCACCGTGACGCCATCAAGGGATCCGCCAGCTTTCTC
CGGGAGTATTCGCTGCTCCTGTACCTGAACGACGATTCCTACGACGCCGAGACCGACGGG
GGCCGGCTGCGGATCCACATGGATTCCGGGGGCGACTTTTTGCCGGCCGGGGAAGCCCCT
CTTTTCCAGGACGTGGACGCCTGCGGGGGGACGCTGGTCCTCTTCGAGTCCGACCGGTTC
CCCCACGAGGTTCTGGACACGGTCCGGGAACGCTTCGCGGTGGTGGGGTGGTACAACCGT
CCCATGTCGATCGGGGACCTGGCCTCCATCAACAGCGAAAGCGGCGGCGTCGGGGACAGC
GCCTCGGATCCCATTCGGCTGGCCGGCCTAGCGGTGGCAGCTGGTCTGGTGACGGTCGGC
CTAATCAACCTGTTGGGATCGTAATGCGTGGCTTGGCATGGCATGGCATCGCAACAGAAC
AGAACAGAACAGAACCACCGCCAGGGTTGTGTATTTGTGTTCTCTGTATGCCTATTTTCT
TCCGCGAGGCAAAGAATCGCAGTGCCGAAGGCTATTGCGTACGGAAGTGCAACCCAAGAA
CTAACACACGGTCGTTACAGACCGAATGCCATAATGCAATAGGATAGACAACGGTGCAAT
GCTATACAACATATTTCAAGGGAATGGTGCAAAACCAACCGACGCGAAACAAACGGAACG
GAACCGAACCAATCAGACCAGCAGCGCCCATCCGAACGCATCCATTAATCATGTGTGAGG
AGAGAATGCCCGGTCGCTCTGGGTTTCTCCCCACTCCTAGACGAAAGCTTAATACTGTGA
AGAATCTATGAACTCCTGGCCAGGCGAGGTTGAAGCGCAACATACCTTGTATGTACTAGC
TCTTTTAAATCATGATACTATAAAATAATCGTGTGTTTCTAGAT
>comp2249_c0_seq1
TATCAACTACGAGTGACACTTACAACTTCAGGTTTGATCCACCATCATCTCACAACCGAC
ATCGAATGTACCGTAGAAATAAGATTTGTACCTAGTGTCTTCGCTAAGTGCCGTGCCAAA
TTCCTAGGTACTGGAGCATTCACACACTCGAAGGAATGATTCGCAAACGACTGCCCCGTA
TTATTTTCCAAGCATTGCTACACTGGTCGGCATTGCTAGGAACGATTCGATGGTATAGTC
TTGGAAAGATCGGTGTATCAATGACCCATTTCGCCCTCTCTCTCTCTACGTCACCCTCGG
CAGCTACGATGACTGCCGGACTAAACTCTATTCGGTCAAGTTGTCGTTGCGGGAGTCTTG
TGATCGATGTATTTGGGAATCCAAGCGCCAAAGGAGGGGGCGATAATGGTAACTGTACAT
CACGGAATCGCGCAATAAACTGCCACTGTCGGGGCTGCCGACGGTATCATACGGGTGCGT
ATGCAAGCTTTCTCGAGACAGATTGGAGCAATATTTTCGTTCGTCAAGGCAGAGACAAGA
TTGGAAAATTTTCCTCGGATTGCAACGAGTTTGGTCCGGTCGAGCGTTGGTTTTGCAAAA
ACTGCTCTAGCAAATTATTGCTGGTTCCGCAGCATCTTTTTTCGTCTGAGGAGACTGCTC
ATGGTGATGGCGGGGACAAGGTCGATGACGATGACGATAAGGAAGAGAAAAAATATGCAG
AACGAAAAATAGCACCAGACACAAAGTGCCTACTCAATTTAGGCCCGGTGATAGACGATA
CAATACACCCAGACACTGCAAATTGTTGGAAGGAACAACTGGATCGATCCGAAAATAATC
TCCACTTCGACAAAACGAAATCTGTTTGGGCACGCGCTCTCCCCGAAAATGAAGACCGTG
ACTATATCTATGATGACGATCATTGCCAAACGTTACCAACCCCGTTGAAGTGGTTCGGTG
GATGTGCTTGCGGAGCATGTCGTTATGAATTTATTCTCACGCATCCGACCGAGATGCAAC
ATTGCTATTGTCGGCTGTGCCGAGAACTATCAGGGAGTCCGTTCATGACTTGGATTCCGG
TAGAGAAGCGAAATTTTCGATGGATTGTCTGTACGAAAGGAGTAGAAACAGAAACATTGA
CATCGTCTTCTGCGTTACAACTCGTCCGAACGACGCCTTTCGGTAGCCGCCACGTTTGTA
ATATGTGCCAAAGTGTGATGACTATTGTCTACGATGAACAACCCGATACTATATGGCCTT
GTGCAGGTAGCTTAGATGACTCGGCTTTGCCAAAGAGCACCGAAGAAATGGGGGAAACAC
TCAGTAGGGTTTGTCACATCTGCTGTCGCTACAAACCTACCTGGTTGAGAATCAGAAGAG
ATGGAATGGAGCGGATTGAAGAGGCGTGTTGATAGTAACGAAATTGTTATTTAACAAC
>comp2249_c0_seq2
TATCAACTACGAGTGACACTTACAACTTCAGGTTTGATCCACCATCATCTCACAACCGAC
ATCGAATGTACCGTAGAAATAAGATTTGTACCTAGTGTCTTCGCTAAGTGCCGTGCCAAA
TTCCTAGGTACTGGAGCATTCACACACTCGAAGGAATGATTCGCAAACGACTGCCCCGTA
TTATTTTCCAAGCATTGCTACACTGGTCGGCATTGCTAGGAACGATTCGATGGTATAGTC
TTGGAAAGATCGGTGTATCAATGACCCATTTCGCCCTCTCTCTCTCTACGTCACCCTCGG
CAGCTACGATGACTGCCGGACTAAACTCTATTCGGTCAAGTTGTCGTTGCGGGAGTCTTG

TGATCGATGTATTTGGGAATCCAAGCGCCAAAGGAGGGGGCGATAATGGTAACTGTACAT
CACGGAATCGCGCAATAAACTGCCACTGTCGGGGCTGCCGACGGTATCATACGGGTGCGT
ATGCAAGCTTTCTCGAGACAGATTGGAGCAATATTTTCGTTCGTCAAGGCAGAGACAAGA
TTGGAAAATTTTCCTCGGATTGCAACGAGTTTGGTCCGGTCGAGCGTTGGTTTTGCAAAA
ACTGCTCTAGCAAATTATTGCTGGTTCCGCAGCATCTTTTTTCGTCTGAGGAGACTGCTC
ATGGTGATGGCGGGGACAAGGTCGATGACGATGACGATAAGGAAGAGAAAAAATATGCAG
AACGAAAAATAGCACCAGACACAAAGTGCCTACTCAATTTAGGCCCGGTGATAGACGATA
CAATACACCCAGACACTGCAAGTTGTTCCTTCCAACAATTTGCA
>comp2250_c0_seq2
CGACAACAAACCGATGACTGTTGTATCCGACGCCACATCTATCTAATCATCAAATCATCC
AGATCTCACAAAAGAGCTAGTACACGTCAGTTCCAGTCAGGCTGTACGGCAAATCCGGGA
CGCTGTGTTGCGTCTCGCAAAAAGAATGGAGGGCGGAGAGATATATAGGGACAACCAACC
CGAAACTGACCCCAGTACGGTCACAAACCCGTACACGAAGACGAAACACCAGCTGGAACC
TTCCCACCGGCCCAAAGGAAAGTGCGATCCGGACATCGAGGAAGGATTGTTGTACTCGTA
CAGCAGGAACGGGAGCCGAAATAGCGTTTTCTTTGGAAATGGAAATGGGGTTAATCCTGT
TCCGTCCCGGAATCTTGGACCCATTCCGGCCGCTCCGGTTTCCGTGTCTGTAGAAAAATC
TGTAGAAAAACCAGAACCAAACGATACAGATGCCACAAGCATTCACGGCTGCAACAACTT
TGATAACGGCATACAAGCATGCACGGCATCAACCATCATCAACGGCAGCAACAGCAAAAG
CAACATCAAAAGCAACAGCAACAACGACACGATATCCTTTCGAGAGATCCTTCCGAGGGA
CCGCACCCGGATCCAAGAATTGTTCGAGGAATGGTTTCCGGTCGATTACAAGGACGAGTT
CTACGACGATCTCTGCCAGAACCAGTCCATGGGCTCACAGCGGTTGTACACGCTGGTGGC
AACCACCACCACGACAGCAGCAAAAGCCCCTTTGTCTCCCGCCCCCGAGCCGCAGCGGGA
ACGGCTGATCGCCTGCCTGCTGGGCTGCAAGCTCACCGCCAGCAAGCTCAACCGAACGAG
CCGGAGGCTGCTCATCCCGGGGTACGAGTCGCGAAAGACGGACCGCCCGGCTGGCCGCGG
CGCGGGGGGCGACACAGATCCCGCAAGCGACGACGAAAACGAAAACGACTACCACTGCGA
CGGCGAGGAGGACCGCAGGTTTCTGGATCGGACCGAGGTCTTCTACATCATGACGCTCGG
TGTCGTCGGCGAATACCGCAAGCGGGGCCTGGCCTCGTATCTGGTGGAACGTGCCCTGGA
AGACCAGATCGGTGCCGCCGCCGCCACGGAACACAGCGACGGGAAGCAGCCCGACGGAAC
CGCGCTTTCCAATGGCACCAAGGGCACCGATCCGACCGAAGCAACGCGGCCCCCCTGCGA
GACGGCCTACCTGCACGTCATCGTCGACAACGAAGCCGCCATCAAGTTCTACGAGAAACT
CGGTTTTGTCCGGCTCCGGGAGATCGCCGGATACTACACCATCCGGGACGAGAAGCACGC
TTCCTTTCTGTACGCCAAGTTCTTCGACCGCGCCAGCCTCGAGAAACGCAGGGCCCGGCG
CGCCCGGGCCGCGTGGAAGCGGTGGGTCCTGGCGGTCCCGGGCCGGCTGCTGGAGGCCCT
CGCGGTGGGCCCCTGGCTGTGGTCGATCTGGTCGTCCGTTCTGGTCCGGCACGGCCCGCC
GCACGAGGGCCGCCAGGGCGGCGGTGGGGGGGGAATGAAGCACGATTGACGGGGACCGTG
TGTGCGGGCGCTGGTGCGGCAAGCGAGGCGGAGGGAGGGGACTGCGGCATCGGAACCGAT
CGACCGGCAGGCACCGCAACTCGCAACGCAACAACTCGGGGGGGAACACGTTTTGGCACA
TCGGTCTCGATTGCAAGCATATGTAGTGTAATTTAGTATGCATGTATACATTTC
>comp2251_c0_seq1
GTGCATTCCCGCGCCGGCTGTTCAAACACGTGTGTGGATTATCCATCTACGACGCACAAC
CAAATCTCTCAGACCTCTTTCAGGATTCCAACAAACCCGCAAACAAAATAGCAGCTTCAA
ATTCTCCAACTTGTTCATATTACAATGATACAGGGATATCTCTTCTTCCTTTTGCTTTTG
TGTGTGTTGAGTTATGACGCCATGGCATTCGTGGGACACGCGACATCCAGACAGCAGAGT
ACTACTTCGTTGGATGCCCTAGGACGCAGAGAGGCTTTCTTTGGCATTACATCCGCCCTG
GTAGCCGGCACCACCCTGGTATATAATCCCGAAGCAGCTTCCGCCAAGTATTCGGATTAT
GCCCGACGCGAGAAGGATTGGCACGATCGAGGAGAGGCTGGGCAAATCAAGGTGTCGACA
GCGAAAGACCTCCGAAGGCAACTAGCAGAAATCGTTCCCCAAAACTCGGAGGGGTCCAAG
ATCTTTTGCCCGAACGGCGCTTCGGCAGCCGTGAGTCCCCTGATGGAAAATAAGTGCAGC
GATACCCTCATGGCGCTTCCCTCGGTCTACGGGCGAACACAAGACTCCATGGGAAACTCG
ATTCCCGGGTTTCAAAAGGGATACGACTGGTCTGCTAGCCAATCTTCCAGCATGTCAGCT
GCCGTTGGAGGGTTCCCCAAATATTAAATCAGCGAGCAAAACGTCGTTAAGCCCGTGTTG
TGCGTGCGTGATTGGAGAGAACGGCGGAGTTTCCCAATGGGGAAGTGAGATGAGAGCGGG
ATGATACACGCAATCACTTTCACAGGCTGGAGGTGACAGCCAGCTGAGAACTCTGCTTTT
ATGGGAGTAATCGGATGTCTCTGTTGCTGCAACAATTCGATTCTGATTACTTATCAATTT
ATATTGACTGGCTAATTCTGGTCGACATTTTTTGATTAAATGCAGAACAAAAATCCAATA
TAATTCTTTATTGTAGGTTGACTGATTGAATGACGCAAAAAAATGAATTTCATGATGGTA
TGCCAAGCGATTTTCTTCGTGTCTTTCCTAATTTTATAGATTCGATTATTACACATGAA
>comp2251_c0_seq2
GTGCATTCCCGCGCCGGCTGTTCAAACACGTGTGGTACGTGGTCGTAAACCTGAACAAGC
GGCAAGAACACGCAACGGCCTCAAGCCACAAGAACTCTCGTTCATTCCTTCTCCGCAGGA

GAAATGTTCTGTTTGCTCATGAACAGGTGCTTCGTTCCTTATCCGCTGCTAGTGGATTAT
CCATCTACGACGCACAACCAAATCTCTCAGACCTCTTTCAGGATTCCAACAAACCCGCAA
ACAAAATAGCAGCTTCAAATTCTCCAACTTGTTCATATTACAATGATACAGGGATATCTC
TTCTTCCTTTTGCTTTTGTGTGTGTTGAGTTATGACGCCATGGCATTCGTGGGACACGCG
ACATCCAGACAGCAGAGTACTACTTCGTTGGATGCCCTAGGACGCAGAGAGGCTTTCTTT
GGCATTACATCCGCCCTGGTAGCCGGCACCACCCTGGTATATAATCCCGAAGCAGCTTCC
GCCAAGTATTCGGATTATGCCCGACGCGAGAAGGATTGGCACGATCGAGGAGAGGCTGGG
CAAATCAAGGTGTCGACAGCGAAAGACCTCCGAAGGCAACTAGCAGAAATCGTTCCCCAA
AACTCGGAGGGGTCCAAGATCTTTTGCCCGAACGGCGCTTCGGCAGCCGTGAGTCCCCTG
ATGGAAAATAAGTGCAGCGATACCCTCATGGCGCTTCCCTCGGTCTACGGGCGAACACAA
GACTCCATGGGAAACTCGATTCCCGGGTTTCAAAAGGGATACGACTGGTCTGCTAGCCAA
TCTTCCAGCATGTCAGCTGCCGTTGGAGGGTTCCCCAAATATTAAATCAGCGAGCAAAAC
GTCGTTAAGCCCGTGTTGTGCGTGCGTGATTGGAGAGAACGGCGGAGTTTCCCAATGGGG
AAGTGAGATGAGAGCGGGATGATACACGCAATCACTTTCACAGGCTGGAGGTGACAGCCA
GCTGAGAACTCTGCTTTTATGGGAGTAATCGGATGTCTCTGTTGCTGCAACAATTCGATT
CTGATTACTTATCAATTTATATTGACTGGCTAATTCTGGTCGACATTTTTTGATTAAATG
CAGAACAAAAATCCAATATAATTCTTTATTGTAGGTTGACTGATTGAATGACGCAAAAAA
ATGAATTTCATGATGGTATGCCAAGCGATTTTCTTCGTGTCTTTCCTAATTTTATAGATT
CGATTATTACACATGAA
>comp2252_c0_seq1
CCACATCTTCGAGAGAAGAAATCGACTGAATCCGTAGTACTGGAATAGAATGAAATTGAG
GAGCCACTCCATACCATCATCTAGTTCTACATTAAGTATGCAAGAAGTTCTAAGTCCGAC
TAAGATAGGGGAATCGTTCCTCCGAACCAGTCGCAACCTTTTCGACGTGGCCGCCGAAAC
CAGCGAAAGGACTCTCTTTGTGGTTCCTGCCGGAAGCATTCCGGAAACGGAAAGTAGAAA
GGAACAAATTATTCAGGTGGTCGGTATGACCGTATTTTTTGTGGTCCTCCCTTTTGTGAT
CACCATCCTGTTTTGCTGCGTTGCTTATAAGCACTTCAATGTCACTGTCGAGAGGGACGA
TATCCGAAATGCGACGCCAAGGGTTCCACAGCGCCGATCTACGACTAGGAGAGAGCTTCC
AACGGTGGAATTATCTTTGGGAGACAACGATGGCGATGGCGAAGAATCGTGCTCGGAAAC
GTGTACCGTAATGAGCCAGGATATGGACCTGAAAAGCGACATCGATGCCAAACAACCCGA
CTCAGTCCGATGACAATATGCTGAAATGTTTTATTCCTGGATACAAAAGTAGCAATAAGA
CAGATTTGATTCGAACTCTATCTCTCGGTAAAAGATTACTTTCTTTCCTTCACCCCACTT
TTGATTCGATCACTGTTTCATCGGAATCATGAAATAGTGAGGGAAATAAGCTATGGCATT
CCGGCGTAGAACACAACAAAGTAAGTGGTTTCCAACAACAAAAAGAAAACCCGATGGATA
TCATCGTCCGACAGAGAACGATCCGGGGTAGTTTGGTGCGGCTTCTGGTATGGAAAGCAA
CAACCTCGGATTATATCGGTGAAATGACATCGGCGAGATAGCATCGGCGAGATACGAGTC
GGACACAGCACTATAGCCTCTCTCGTGCTCGGGGCGCTGCAGGCACAACCCGCAGAGGCT
ACCTTCCGGCATGGACCTATCGTTGATGCAGTAAATAATATTAAAAAACACTTAATCCAT
>comp2253_c0_seq1
GGAATTCATGGAAAGCATGGTCACGTTTTAATGCTATTCCATCACCCTTTGCCAGGAATA
AATAATTTGCTCTTCATCGGATCGTTCCGTAAGAATAAGAAGTTGATACTCCAGAAAGAG
CCTCATTTTAGTACGGTGTGCAACTTGCACTCGGGTGCAAAATCATTTTATGGTTTTCAA
TTGAGCGGGGCTACACAATCAAGAAGCTGAGCACAAAACGAACTTGAATACAAAACTAGT
TACGTTCTCGTGGATTATAATAATCACCAGAGGAGGAGATGTTTTAATCGCTGGCTAAGG
TTAAGTTAAGTTTTGTTCTGTATAACTTATGAATAAATTATGCATAGTTGCATTACGTTT
TTATTATGATACATATTATTGTTTCTTTGGCACGTTTACTGATGAAATCAGTTCGCACAA
GATGGATCTTTCTTGGAAAAACCCAATAATTTACAACGATTGTAAGTAAGTAATAGCTCT
TGAGTCAATCGCCAAGTAACCAATAATCGAAGCCCCTTCCTCCCCTTTTTGTTTCAAGTC
TTTCCCATTGATATTAGGCAAATGCCCGACTCCATAAGGATGTTGATGATGACGATGTCG
TCGACGTGGCTCTGGTATCGGGTGTAACGGGTATTG
>comp2254_c0_seq1
CGGGCTTCTCCAAGCCCCTGAGCCCGTCGGAGTTTGCCGCCCTAAGGGCCTATTTGAGGG
AGGAGGACGGCGCCGAGATCGGCGAGGACGAGATCCCCGTGGAACCGGCGAACGAGGCCT
CGGTCCTGGGCGGCTACGCCGACACGGAGGAGCTCAACGCGAGGTGGAGGTCCTCGAGCG
CGATGAGGTTCATGGACGACACCCTGGACGACGATCCCTTCTCGGACCTCCTCCCGTCCG
ACCTCAGCCCGACCCTCCTGGTCAACCGCCGGGAGGCCAAGCCGATCCCGAAGCACCTCC
TCCACCACAACAACCTGGCGCTCCTCCGGAGGTACATCACCCCGTCGGGCCAGATCATGA
ACCGGGTCCAGTCCCGCTTGGGCGCGAAGGACCAGCGGAAGATCGCCAGGCTCATCAAGC
GGGCCCGGTACCTGGGCCTCATCCCCAACAGCGGGCAGTTCGTGGTGGAGGACCACGGGA
GCGTCCACGAGGACGACCTGGGCGAGGACAAGGAGTGGGAGGCCGAGCTGAAGCGGAGGG
GCCTGGTGGTCGGGAAGGAGCAGCAGCGCACAAGCACGAACGGGCCGCGCCGGTAGGAAG

CGGGACCCTGGAGCGAGGCCACCGCCGAGGCGCTATGGCACAGCGGGACGAACTAGACTA
AACAAACATCGAACCTTGTATCCC
>comp2255_c0_seq1
AACAGCAACAGCAACAACAGCAAGAAGAAACCCAGTGGCAGCTCTGGTTCGTCTGCTCCC
ACTGGCAGAGGCAACCATGCGAGGCAACCGAACCACGGCAACAGCAACGGCAAAAGCAAA
AGCAGCCACCAAAAACCCCTGTCGGACAAAAGGCTGTCCCATTCGCTGTCGTGGGCGCTC
CGGCACGCTGCCCTCGACCTAGGCCTGAGCATCCGACCCGACGGCTTTGTCCCGGTGCGG
GAACTCCTCGACTCCACCCACCCGAAACTAAGGGGGGCCACGCTGGAAGCCATCCGAAGG
GTGGTCGAACACAGCGACAAGCAGCGCTTCCGGCTCGAGGACCGGCCGGGGGACCTGTAC
CCGTCCGCCGGCGAAGGGCAAGGGGAGACGGTCCTCTGCATACGGGCGAACCAGGGCCAC
AGCATTTCCACCATCGACCCGGAGCTCCTGCTGACCAGGATCCCTCCCGAAACCCTGCGG
TCGCTCCCCCGCATCGTGCACGGGACGAACCCTTGGGCGTGGGAGTCCATCTTGAGGGAC
GGCGGCCTCCGGAAGATGAACCGGACGCACATCCACTTTGCTCCGGGCCTCCCATCGGAG
ACAAGCGTGGTGTCGGGCATGCGCAAGACCGCGTCGGTGTACATCTACGTGGACGCCGAG
AAATGCGCGAAGGAAAGCCGGATCGACTTCTTCCGGAGCGACAACGGCGTGCTGCTGACC
GACGGCGTTGGGGACAGCGGGGTGCTGCCGGTCGAATTCTTTTCCCACGCCACGGATTCG
TCGGGCGAGATACTCCTGGACAATCGGACAAAGCCGGCCACTTGACGATCGCAAGCGACC
AGCACAGCATTTTTCAATCCACCATCGAACGCATCGCATTCGCCAACAACAAACACACCG
ATCGATCGATACTGTACAGTCAAACAAACAAAAAATGCCGATGCGGCAGGGAGCCGAAAA
ATATATCACTGTCCATAATTAATGTCGTAGGC
>comp2256_c0_seq1
AACGAAACTGAAAACAACATCACCGAATGCTACCGCTACTCAGCATCGTAATCGATGACC
TTCATCCTCTTTTGTCGATTTAAAATACGAAATAAAGGGTTGACGAGTGGGTATCTTTCT
ATCTCCCGAGACACACCCAACATTGAGAATAGAAAATTAGGCATCGACCATGGCGCGAAA
ATGGGCTGCCAAGCTTGACATGTACAGGAAAGTCCCTTTGGATCTGGCTGAAGGGACTAG
GCGAGGTAATATTGTCTCCTTGGTAGCAATATTTGTGATGTCGTACCTCTTTTATAAGGA
AACAAGAGATTATTTCACGACAAAATTAGTACCCAGCTTGTACCTGGATCGACGGCATTC
GGAACAAGAGCAAATTAATGTCACCTTCAAAATTACGATGATGGATCTGAGATGCGATTT
CGTGGAAGTCGACGTTGTGTCGGTGTTGGGAATCAATCAAAATGCTACGAAATTCGTGAA
AAAAGTTCCATTGGACGCAAACGGAGTTTTGAATACGATGGCGGAAAGAAACTTGGATCA
AAAGGATATTCAAGATATAGCTCTACATGACGCCGCCGTCACAAAATCGATTGAAGATCT
TCACGTAAACGGCGAGGATGCTATCCGACTCGATTCGAAAACTCTTGGCTATGCACTCCA
TGAAAACGATCTTGTTTTTGTCGACTTTTTTGCTTCATGGTGTTCGCACTGTCGTGTATT
GGCACCTACGTGGGAAACTTTGGCCAAGGTCATGTTAGATGCAAACGAAGAATCGGAAGA
AGAGGGTGGGGATGAATACGGGGAACAGGAATTAAGAACGGCAGAGGCATTAGACGTACC
AGTTGTCATTGCGAAGGTGGATTGCGTCGAACATGATAAACTTTGTCGCGAACAAAACAT
TCAAGCATACCCTACACTGCGTTTGTTCGTCAATGGTAAACCATTTGATAGTGGGGATTA
TGAAGGCCATCGTACAGTGCTTGACATGGTTCAATACCTAAAATTGGCGGAGAATAAATT
GGGCAAAGAAAAAAAGCTTTCAGCCGACGGCTTGCACAGTGCATTGGAAAGACACTTGGA
AATGTCGGTGGAAGAACGACACTGGGCCGAAGCCTTCCAGCGACTACAGAAACGTGACCA
AGATGTCTCTTGGAACCCAATGGAGCACCCCGGGTGCCAGATATCTGGTTCGCTTCTTCT
CAACAGGGTCCCGGGTAACTTTTATATCGAGGCACATTCGCCCTTTCATAATTTGGCTCC
ACACATGACGAACTGTTCACATGAAATCCACTATCTTTCGTTTGAACCCACAGGCGTGGA
TCTGAAGGAAGGAGACCCGCTCCCTCCTAACTTCTATAAATCGAATTCGCCTCTCAATGG
TAACGTTTATGTGACACAAAAACTGCATGAATCGTATCATCATTACATAAAATTGATCTC
TACAAATGGAAACAAGTTCCAGGTTCTTCAAAGCAGTCAACTAGCATCGTATCCGCACGA
TGATACTCCAGAAGCTAAGTTCATTCTTGACTTGTCACCGATTGCGGTCTCATACGAAAA
GATCTCGCGAAAGTGGTATGACTATATCACTTCCCTAATGGCCATCATTGGGGGCACCTT
TACAGTCGTTGGGTTTTTGGATTCTGGTATCCGAGTTGCTACCAGGAAGAGAATAAAGTT
ATCTCACAACAACAAGCCACGGACCATCTATTAGCTTCACAAACATGCTTGTCTGGAAAG
ACATGTTCCTTCTCGAAGGTAAACGAAGCTCTTTATACTATAAAACCTCGACTTTTCATC
TACTTTCAATCAGAAACGGTGGTGTAATAATACTAAATGAGTTATGTACCTCTTTGGAAC
AAAGAAGACATAACCTTGAAATTGAACTGTTCAAGTTGTGTTTTCTGACCTTTGCCTAAA
ATAACCACGACTTGTTTCCAAATGTTAAACTTAATTATGTACGTTTACCG
>comp2256_c0_seq2
AACGAAACTGAAAACAACATCACCGAATGCTACCGCTACTCAGCATCGTAATCGATGACC
TTCATCCTCTTTTGTCGATTTAAAATACGAAATAAAGGGTTGACGAGTGGGTATCTTTCT
ATCTCCCGAGACACACCCAACATTGAGAATAGAAAATTAGGCATCGACCATGGCGCGAAA
ATGGGCTGCCAAGCTTGACATGTACAGGAAAGTCCCTTTGGATCTGGCTGAAGGGACTAG
GCGAGGTAATATTGTCTCCTTGGTAGCAATATTTGTGATGTCGTACCTCTTTTATAAGGA

AACAAGAGATTATTTCACGACAAAATTAGTACCCAGCTTGTACCTGGATCGACGGCATTC
GGAACAAGAGCAAATTAATGTCACCTTCAAAATTACGATGATGGATCTGAGATGCGATTT
CGTGGAAGTCGACGTTGTGTCGGTGTTGGGAATCAATCAAAATGCTACGAAATTCGTGAA
AAAAGTTCCATTGGACGCAAACGGAGTTTTGAATACGATGGCGGAAAGAAACTTGGATCA
AAAGGATATTCAAGATATAGCTCTACATGACGCCGCCGTCACAAAATCGATTGAAGATCT
TCACGTAAACGGCGAGGATGCTATCCGACTCGATTCGAAAACTCTTGGCTATGCACTCCA
TGAAAACGATCTTGTTTTTGTCGACTTTTTTGCTTCATGGTGTTCGCACTGTCGTGTATT
GGCACCTACGTGGGAAACTTTGGCCAAGGTCATGTTAGATGCAAACGAAGAATCGGAAGA
AGAGGGTGGGGATGAATACGGGGAACAGGAATTAAGAACGGCAGAGGCATTAGACGTACC
AGTTGTCATTGCGAAGGTGAGAGATATTTCTACACTAAGCCATAATGAACGGCAGGTAGT
CCCATTTTCCTAACCGATTACTTGTTTGTTTAATATTCATAGGTGGATTGCGTCGAACAT
GATAAACTTTGTCGCGAACAAAACATTCAAGCATACCCTACACTGCGTTTGTTCGTCAAT
GGTAAACCATTTGATAGTGGGGATTATGAAGGCCATCGTACAGTGCTTGACATGGTTCAA
TACCTAAAATTGGCGGAGAATAAATTGGGCAAAGAAAAAAAGCTTTCAGCCGACGGCTTG
CACAGTGCATTGGAAAGACACTTGGAAATGTCGGTGGAAGAACGACACTGGGCCGAAGCC
TTCCAGCGACTACAGAAACGTGACCAAGATGTCTCTTGGAACCCAATGGAGCACCCCGGG
TGCCAGATATCTGGTTCGCTTCTTCTCAACAGGGTCCCGGGTAACTTTTATATCGAGGCA
CATTCGCCCTTTCATAATTTGGCTCCACACATGACGAACTGTTCACATGAAATCCACTAT
CTTTCGTTTGAACCCACAGGCGTGGATCTGAAGGAAGGAGACCCGCTCCCTCCTAACTTC
TATAAATCGAATTCGCCTCTCAATGGTAACGTTTATGTGACACAAAAACTGCATGAATCG
TATCATCATTACATAAAATTGATCTCTACAAATGGAAACAAGTTCCAGGTTCTTCAAAGC
AGTCAACTAGCATCGTATCCGCACGATGATACTCCAGAAGCTAAGTTCATTCTTGACTTG
TCACCGATTGCGGTCTCATACGAAAAGATCTCGCGAAAGTGGTATGACTATATCACTTCC
CTAATGGCCATCATTGGGGGCACCTTTACAGTCGTTGGGTTTTTGGATTCTGGTATCCGA
GTTGCTACCAGGAAGAGAATAAAGTTATCTCACAACAACAAGCCACGGACCATCTATTAG
CTTCACAAACATGCTTGTCTGGAAAGACATGTTCCTTCTCGAAGGTAAACGAAGCTCTTT
ATACTATAAAACCTCGACTTTTCATCTACTTTCAATCAGAAACGGTGGTGTAATAATACT
AAATGAGTTATGTACCTCTTTGGAACAAAGAAGACATAACCTTGAAATTGAACTGTTCAA
GTTGTGTTTTCTGACCTTTGCCTAAAATAACCACGACTTGTTTCCAAATGTTAAACTTAA
TTATGTACGTTTACCG
>comp2257_c0_seq1
TCAAAAAGAGCAATACCGGTACATACTAGATATATCATCAGAGAGGAAGACAAACAAGGA
ACGATCTAGCATTCACACACAAAATGCCTTTGTTAGGAGTTGGTTTTAGCATGGGAAGCT
CCGGTGACGGAGACTCACCGGCCTTTGGAAAGTCCCTCGACGAAATAATCTCCGATCGGC
GCAAGGAGCAAGCCAAGGCCTCTTCGGGCAAGAGTGTCGCCGCGAAAAAGAACGGCAAGA
TCGAAGCAAAAAACAAGAAAACAGCGATGGCCGATCGCTCGATCGCAACGGGCCGGGCCA
AGAGGGCTGCGGCCATAAAGGCGAGACGTGGATTAGCTGGCAACGGAGCGGGCAACGGAG
CGAGCAACACGGCGGGAAATGGAGCAGCCAAAAACAACGCTAGCAGTGGGAAAAAGAAAA
AGGCTAAGGCGTCGGCCATGGAGATCGAAAAGGAGGTCTACCGCCAGTCCCGAAAGACGT
CAGAGAGCAAGAAGAGGGCCGAGCAAAAGAAAACTGGCGGCAGGCTGCCACCCAACTCGT
CCCTGCGGGCGAAGAATAATACCAACAAGAAAAGCGTGGAAATTTCGCCCGAGGCCCCGG
CCGTTGTCACCATAAAGCAGCCAAGGAAAACTCAGCTGATGGCCGCCATCAAGGGCATGG
AGCAAGCCGGGTGCCCCGTTCCGGACGGTTTCCAGGTCGCGATGCAATTCATTCCCTCCC
CTGTGACCAAAGCAACGAAAGGAATGGGGAAGAAATCGCAGGCCGGAAGTAATAAAAACA
ACAACAGCACTAACAAAAGGAACACTAGTGGACGAGGTCGGGGTGGAAAGAAATGATCGG
CCAATTTGTGTTTCTGTGTTGTCATGATAATTTGTATTTAGATTGGGTGTCTAGGCTTTC
CGGATTTGAAATTACTCTTGTGGATGGATGCGAGGGAGCATGTGTGACAGGCATCATTAT
TGTCGTTCTGTTCTTCTCACTGCAATGCTAATGATTTCATTTTGGTGGCGAGGAAAAGTA
ACTCGCTAGGTGTTACCTACCGCTTGAACAAAACGAGTGGATGTCGATTATCAAATTTAA
GAAGAGAAAGGAAGTCAACTAGAATTAAATTTGAACCTGCCGTAAGATTGAAATGCATCA
CATTTGGAAGCAAACGCAGCAAGTTAACGCAAGCGCATCAATTTTGTATTGACCATGACT
ATAAAGCAACAAGAAACGATGCAATGCTACAATAACCAGGGAAAAAAGGAGAAAGGCATG
CTAAAATGCCGGGGAAGGAAAAGATCGTAAAAGAATAATAATCATCAAATGTGTAATGTA
TGCATTGTAAACATCAAGTCAACCTTTCCCGAACATTGTTACGTAAGTTGAAACGATAAA
GGACACTATATGCAGTGAACGTAAAAAGTGTTCGTCGCATAGTGTTCCAGTCAAGCAAGC
CGGGATAACAGAGGTTACTATACTCTTATACCCTACTCGTAGTAACTTGACCACTACCCT
TGAAACATGGTGTTGTCGTAGCGGAGGGCCTGCCTATTTGGCTTTTCTACAAAATTCCGT
TGCTGTCGTGCACCGAAAGGTTTGGTACTTTTCGTGAAAGCATTATAGAAGTCAGTTACT
TTCATTTCGCTTATTTCTTTATCTGTTTTTTT
>comp2258_c0_seq1

AGTTGACAGAAATCACCAAAGCAGTCGCCTTACGGCCATCATATCATTGGAATCACTACG
AAGAAACATGAGTGCCAATGAAAATAATAACATGATGATGGGGAAGATCGATGATGAGGT
TGTCGAGTTGCGCTTTTCCAACATAAATGTTGATACGGAAGAACTAATCTCGGGCATCAT
CTCCGACGAATTTGAAGACATCGTAGAAGAATACGCCGATGCTACCGCTGAACGTAACAC
AAATAGAGGTACCAAAACCGCGAACAGGTTGTCGGCGATTAGCTTTTCGAAAGGTACCGA
TGGAAGACCTTCCATCTTGAGCGTTCGGCCATCCCTGATGTCACTGGGAATGTCCGATAG
GTTGCTTGAGAAAGTAATGGAAGATCTATCGGACCCATCGCTAGACCCGTGCGACTTACC
TCCACAAGAGCAAAACTCAAGTGATAATGCTAGTGACACCTCTTGGGCTATGAATGTTGC
CGCTCCGGAAGGTACCAATGGAGACCTCTACAACGACGATCGTCATGATTCCTTTGGTGC
ATCCCCATCTGTGTCTGCACCGACATTGGCGGATTCTCCCTCATCGAATCCCTTTACGGG
GGTTGCGATGGAGTCACACAGCGAGCAATTTCACGCTGGTGGTAATTACGCGAAGGGGAG
AATATCTATGACGGCAGACCCGCTTGTTTTTGGTGCTGGAGGCATACCGATTCCAATAGA
TGCACACAACGGCGAAAACCACTATGAATCGACGCAACCGTTCGCTGATCCAGCCATTTC
GCAGAACATAGGAGATCAGACTTATAATGATAGCATATCGCCTCCCCCTCTTAAGAGACC
ATTTGCGATGCGATTTTCGGTATCCCGCCGCACACTCAATATGCTCGACGATGATTTTTT
CCACGATTTTTCGCCGCATTCCTCGGGGCACGAGAATGACGAACATCCGAAACCCGTCAA
CGTCGTTTCGAGTGAAATGCAACAAACATATCTGAACGAGACAGAAGAAGTTTTCAAAGC
CCAAGAACTCGCCCTGACGAGGGAAATGGAAAAAATCGCAGTTCACGACCAGGATAAGGT
CAACTTCGAAGTTCATGGAATCCCTCACATGGGAACTCTCAACGTTGAACCTGTAAACAC
TGATTGGTACTTGCATCAACTCGAAATGGAGTTGCAGAGCACCATCCAAGACAACTCCGC
CTTTCAAGAGGCACAAAGGATGAACCCAAGCTACGTCAACAGCAAGACTTTTCGACTCAT
GTTCCTTCGGACGTTTTGGCAGGAAAGTACCTTCGATGTTAAACAAGCAGCGGCAAAAAT
CGCCCTGTATTTCTCAACAAAGAAACTTATTTTTGGCGGTGGAGAAATCCTTGGACGGGA
CGTTCGGCTCTCGGATCTTTCCGTCGACGATCGAGCTGCAATGGATTGCGGAGCACTGCA
AACCATGCCCGATCGGGATGCGGCCGGGAGGGTAGTCATTCTTTATGCTCCAGGGCAACG
CGTCTTCAACACGGTAGAGAATTGGTTGAGGGCGATGTGGTACATGCTTTCTGTTACTGC
CAAAGATGAAGACAATCAAAAATCTGGGATGGTCATGGTTGTGTACCTCAGAGGATTTAC
CAACCGACGCGATACCTTTGAACAGGCCAAAAAGATCACCATGGTTCGAGATTCCATACC
ACTAAAGATCGTCGGGTTGCACTACTGCTACAATGATGAGTCGATGAGAGCTTTGGTGAC
GGCCCAAAAGGTGCATTTCTTGACCCGAAACCAACGAAGTCATACCCGAGAACATTTGGC
AAGACACGAGACATATGCTTTCGACTCGAGACCTACGGAATTCACGTTGATAAATGCATT
TTATTGGAAAACGGGCACCTAGGAATGGCGTGGTACAATCAATGGATTCGATTGCGAGGG
GTGGCTGAAGTGGAAATGGTGGCGAGCAGCGGCACCGCCACAACGCCCCGGACAATCGAC
ACCACGATAGTTCCAAAGAAGTTCGATGTCTTGTTCGGTCGGGGTCGAAACACTCGTGAA
CACTGTGGCAATCTCCGATGTGCGCATCTTGTCGAGATGCACCAACTGGAATACGAAAAC
TGCAGCAAACCACAAAAGACAGCTCTTGCCCTCAAGATCGTAGATATGGTTAAGGAATGC
GGGGGAAAATTTCTCAAGAAAGATAGAAAGTATGGATGGCAGGAAGTGCCCGACACCGCT
GCCCGAGAGAAAGTTTCCCATTTTTTCCGCCATTTACGATCAACAAAATCTTCTGCCGAT
GATGACAAACAACCGGGAGTCACCGAGTCCCAAAAGAGATCTGCACCTTGCTAGTCCGCA
TACAGTATCACGAAGTCAAAAAACTTACGCAGACCCGTTTCACCGATCGTTTTCAGTCTT
AAATTAAAGAAGTGAAAGTTCTTCAGTTTACGCTGTGTGGGGAACAATATCACTGAAAAG
TCAAAACTAAGGTTTAGGCATTCTTGATATTGACTCGTCTAATTATTTTCTAAGTAATGT
TTTTCCAACATGGTCATGCACTGCATAGTTAAGTGAAACCAAATTGTAAGCAATGAAATC
TCTCAGTACACCTTTCTCCAGCAATGAGTTTCCCAACAAAAAATCTCACAATTTAGGTTG
AGAATGGCTTTTGATGGAAGCAATTTTCAGACGCATTTGCTCTCGC
>comp2259_c0_seq1
AAATCGGAAGCAATCACCATGAGCGGAATCAACGAAACTTTGACTTTGAGAGGAACCCTT
AAGGGACACGGAGACTGGGTTACTAGCCTTGCCACCACCCCCGAAGACCCCAACTTGTTG
TTGAGTTCTTCTCGTGACAAGTCTGTTGTTGTCTGGCAACTCTTCAACGGATCCACCCCT
ACCACCGTCAACGTTGAGGACCACGAATATGGATTCGCCCGACGTGCCCTTCGTGGACAC
TCCCACTTCGTCTCTGATGTCGTCATCTCCTCCGATGGTGCCTTTGCTCTTTCCGCTTCT
TGGGATAGTGAGCTGCGATTGTGGGACATTGCCACCGGAAAGACCACCCGTCGATTCGTT
GGACACGAAAAGGATGTCCTCTCCGTAGCCTTCTCGGTTGACAACCGTCAGATTGTTTCT
GGATCCCGTGACGGAACCATCCGTTTGTGGAACACCCTCGGAGAGTGCAAGTTCTCCATC
AGCGGAAACGGCGAAGGTCAACAAGGCCAGGGCCACACTGAATGGGTCTCTTGCGTCCGA
TTCTCTCCTTCCCAATCCGTTCCTCTCATCGTCAGTTCCGGATGGGACAGACTTGTCAAG
GTCTGGAACTTGAACAACTGCCAACTCCGAAACGACCTTTTCGGACACACCGGATACTTG
AACACTGTCTGTGTCAGTCCCGATGGTTCTTTGGCCGCTTCCGGAGGAAAGGACGGAACT
GCCATGCTCTGGGACCTCAACGAAGGCAAGAGACTTTACAGTTTGGATGCCGGTACCATC
ATCAACGGATTGGTCTTCAGCCCCAACCGATACTGGTTGTGCGCAGCAACCGAAGATTCC

ATCAAGATCTGGGATTTGGAATCCAAGGTTGTTGTCGACACTCTTCGACCTACCGAGATC
GAAGGTGCCAAGATCCCTGCATGCACTTGCTTGGCATGGTCGGCCGATGGATCCACTCTC
TTCGCTGGATACTCCGACAATGTTATCCGTGTCTACACTGTCACCCCTAGCTAAACTGTT
TGATTATCAACGAGTAACGATAATTAATTGTTTTACCGGAC
>comp2260_c0_seq1
AGTGACCGTTCCAACAATCGAGAACGACCATATCAGCTGAAAAATAACTGCACACGATAG
CTACATTTCAGGGCAAGCGAAGAGCATCCGATCAACGTGAGCATCACTATAATCACGGTA
TCACGCCACCATCCAACAACAGGAGATGCGATTTTTTCGAAGCCTTATTTTCGTCGCCGT
ACCCACCCTGATTCTGCTGTTCACGATGCCCTCCTCGTCGAATGGACTCAGCAATGAATC
AAAAGAAAAAAACCCATTACGAGGTTCTCGGCATCGAAGATCCCGACAGGTGCGAGCTTTC
TGACATAAAAAAAGCCTACAGGAAGCTGGCTCTGAAGTATCATCCGGACCGCGTTCCGGT
CGGGGAAAAAGAATCGGCGACCGTGAAGTTCCGAGAGGTCTCGGAGGCGTACGAAGTGCT
GTCCGACAAAAAGAGCCGAGCCGAGTACGACGATTCTCTGAGATACGGTGCCAACGGAAA
CCGCAACACGGGGCAAGGTCGTCGTCCAAACTACCACGGCGGATACAACACGAACCATCG
CCACCGCTACCGCGATCCCTTCGCCCAGTTCGACGATCTCTTCCGCAACGATCCCTTCTT
TTCGGAATCATTCCGAGGCATGGACGACCTGTTCTCCAAGACTTTTCAGCAGAGAAGGAA
TGGCGGCCATCCGAACACGAAATCAAAGGGCTGGGGCGAATGGATGCTCGACAAACTCGG
AGTCCAGATGACTACTAGCAGCACCAGCACCCGTACGAATAGGGATGGAACCTTTTCATC
GTCGTCGTCGACCTACAGCAATTCAAGGAGCTCGTACACATCCAAAAGCACGAGAACCAT
TATAGAAAATGGTCGAAGAATAACGATTCAATCCATGGAGAAGGACGGGAATCGCATAGA
AGAAAAATACGAAGGGAATAAGCTAGTGCAAAGGTTGATCAATGGCCGCCCCCAAGACAT
TGGGAGAATCGCTGGTGGCGATCTTTGAATTGGTCGCTCCAAACAAAAGTAGAACCGATT
CGTGTACTGGGTAGCCTATGTGGCAATTGGCGGGAAGCATTTTGCGCTTTCGATTCAATA
ACGAAAGGATGGTCTCCCATGGAAAGTCCGTCTGAAATACATCGAAAGCGTCGAAGGCAT
TTGCTGAATCGAATAGCTAAAGAATGAACAAACAATCTATCCTATCCTACCAGAGCCCCA
ACATATCTGGTTTAAATCTAGTTCTTCTGATACCATCTTGTGTGTGTCCATCGAGTCGTG
TTAGGAACGATGAATTATTACAAACTAGCTGTATTCTTTCAAAAATCTCTTCTTTTATTT
TAAAT
>comp2261_c0_seq1
CTTTTTTGGTTTTCGGTGATCTTTGGCTTTGTTGAACAGAAGAAGAGTGAACCCAGATGT
CACAGTAGGAAACTCATGATATTATCTCATTGGGATCGATATTATCCTGTACAGCACTAC
AGGTCTACCGAGTTCTCAAAAATCCGCATTTGAAGTCTATTTCTATTCGCTCTTGTGTCG
GGAGCCAGAATAGCACGGAAGATCCAATCGGCGAAGCCTTCGAGGAGCGGAAAGGATATT
TCTCATATTTTGTTTGTATTTAATTTTTTCAATTGATCGGTGATCTTGGATTTGATAAGA
CTATGTTGAGGCGCGTGACATCCACTGCGACAGCGAGTAGAACTGCCAGTAGTCTGCTGC
GTCGTAGCAATCCTTCGATGGCCATCGGTGGAAACACTAGAGCTAAGCATCCTTCTAGTC
GTGCCTTTGCCGCTGCGACATCGGCCGAAAAACGTGCTTTGAACGCACAACAACTCAAGG
GTTCTAGTGGAAAATCTTCGTCGACAGCTGCGAAAGAAAGTGCGACCGGAGCTGCCCCGA
CGGCGGAGAGCGGCGGCGGCGGTATCGGACCGGTTCTAGCGTTATTGGTAGCTACCGGAG
GTGCCGGTGCTGCCTACTATAACGGTCTTCTTGACGACTTTTTGGGAGCCGACTCTTCTC
CGCTGCCACAGAAATTGGAGGACGTCAAAAAACCCAAAAAGAAGAAGAAAGCGGCAGAAG
CAACTGACTCTCCAACTCCCATAACTAACATAGAGAAAAAGAACGCAAATGCTGCCGCAG
CAGCAGATGAGAGTCTGTCTACCAAGGAACTCTTAGAGGTAAATGAAATCATAGCCGATG
TTATTGGCGAAGTGAATGAGGAGGTAGCTGCAGTCACCGAAGCCATAGAATCCATTGCCA
AAGAGAATGAGGCAGCTTTAGCCTCCGTTGTGGCTACCCCTGTTGATATCACCGCGAAGA
TTGAAGAACGTCCTCCAGTTCCAGCAGAGGCAGTTCCAAAAGAAGTAAAAAAAAAAACTC
CAGAGCCCGAGCCCGTGCCTGAACCTCAACCTGAACCTGAACCTGCACCCGTTCTGATGG
CTTACTCAACGCCAGCTTCCACTCTGGATGAAGCCAAAATTATGTCGGAAATCGCCGAAC
TAAAGAAGGCGCTTCACGAGAAATCCGATAAGGCCCTCACGGAAGCCCACTCGGAGCTCG
CCAAGATTTCTTCTATGGATGTTATGAGTCTCGACCTAGATCAAATGACCAGCTCACAAC
TCAAAGTCAGATTGGTTCAAATGGCAAAAGACCTAGAGGAGCGAACAAAGTGGGAGGCGG
TGCGCTTGCAGGAATTCTTGTCCATGAAAGAAAAGGAAGTCGAAGATAGATATGTCCTGC
TCATTAAGAAGCTTAGAATTGAGGCGGAGCACCTCCAAGAAGAAAAACTTTCGGAGCAAA
GAAAGGCTCTCGTAGCCCAAGCGGAAGAAGCTTTGAAAGAACAGCAAGCGAGATCGGAGG
CTTTTCTAGAAAATTCGATGCAAATTGAGGCAAAGGCTCACGCCGAAGACAAGGCTGCAT
TTGAGCAAAAAAACGGATGAGGCGATGAGCGCCAAGTACGAGGAATTGTACGGAAAGGAAC
TGGCAAAGATCAAGGACGACTTTGCTACAAAAATGAATCAACGAGTCCAACAAATGGAAC
GGCTGACCAAGAAGCTATCGGATCTGGAAACCTCGCTGAAAGACTCCCAAGACTTCCAGG
TGGGTTCGCTTGAAGCCCACCGCATCACAGCCGCGGCCATTGCCCTAATGGAAAAGCTCG
AATCTGGAGAACCAGCGGGTGCGGCAGTCAACGCATTGGAAGCAGTCGCCTCGGAAAATG

CTGTTGTCAGCGCGGCCGTCAAATCTCTCCCCAATTCTGTGACCCGGTCAGGCATTATGA
CGGTCCAGGAACTCCAGGCGGGATTCGAAGAAAACATATACCCACAGTGCCGCCGTGCTG
CTAACATCCCCGAAGGGCAGGATGGCTTGGAAGGGCAACTTTTGGGATCAATTTTTGCCA
CGCTACGGCCTGCACCAGGCCCTGAAGATGTGGCGCCAGAATCTGAAAAGAATTCCTCTG
AATACGTCTTGGCGAGAATTCGGCGCTATGTACGTTTGGGCGACCTGGAAAAGGCCATTG
GCGAAACGAAGAAGCTGAGCGGCCAAGTGGCTTACACGGCAAAAGATTGGAATAGCCACG
CGAGGGATCGGGTGGCCGTAGAAAAGGCCCTGAAGGTCATCCGAATGGAATGCGCCCTTG
CCAATGACAATCTTAGCAAGGCTGCGGCATAAAGACCCTCGTCATTAATTTTTCTATCG
ATTGTGTATTGCAGGTCACGATAGATCACAATGCAAGGTACTCTTGTTGCCTTCTAGCTT
AAGCATAAGTAGTTGATGCGCTTCAATAT
>comp2262_c0_seq1
TGCAGCAACAGGAAGCTGATTGTATTGTCTTGAAACGTAGGACATCGCTCTCAGACATGA
GAGAAATCACAACAATACAATTATCATGTACAGCGCTAATAATTTCGAGGACGAGCCTGG
TTTAGAAGGCATCGATAAGTTCGAGGATATCCCTGTCCCGTCGGAAAAAGTCAGATTTGA
TGGACTTGATGAGGCAGCGGCCAAAGCGGAGCGTCGGCGCAAACGTCGTCGTCGCCGGAA
CCAACGAACTGGAATGATTGCCGCGTGCATTATTCTCGTTTGTATTTTGATTGGTGTTAT
AATTTCCGCGACCGTGGTGGAGGAAACAGAACATATGCTGTATCAAGATGCGTATGTGCC
CGAAACAGAAATGCCTTCAACAAGTCCGACCACTGCAAAACCCACAATGCACACGACGCC
CTCACCGACGGTTGCGAGAAAGCCCACATTTTCCGTTAAGACGCCATCGCCAACCATCCG
TCACACGCCGTCGCCAAACCTTGACTTGCAAACAGACTCCCCTACCGAAACGCCGGGTCC
CACGTCCATCATGACCGACGTGTATACTTTCGAGCCAGTTGGGGACACTTACATGTATTT
AGACGGTAATCACAAAGTTCAGGCATATGGCCACGAGGATTCGCTCTGGGTGCAACGCGG
GAACAAAGAGTCAACATTACCCGGTCACGAAGTAACAATTCCAACCATCGTGGGCCTCTT
AGAATTCGACACCACCCAAGAAACTCCCACCAACATGGCTCTGCCGAAACGATCACGGTG
GCCTGCCGAAGCTGAAAACAATGTGGTAGTGACGCTTCGACTCCACCATGTAGCCAAGAG
TGATTCGGACGAACTTGCGGTGGAAGATATACTACCAGTAAACATCGAAGTGTATAGACT
TCCCAATAATCACGATTGGGTAGTTGAATCTTTGACAGGGGATGACTTTCAGAATTCTCC
GAAAGCAGCCAGGGAGGGAATTTTGGTTGCCCAGCATGTTATTGAGGCAACCGATACCTT
AGTAGATATCAATGTGGAGTCGGCGATATTCTTGCCCGAAGACATCAATGCACGGATGTA
CAAAGACGATCAAGTACTCCTGCTGCTGAAGGTGTACTGGGAAGAAGCCCCTAACGAGGG
CGATTCATTTGAAACACGCGAGTCAGATGGTCGGTCACCGCAGCTTATTTTCTCAAACAT
GATGTAGGAAAGCAGTGTTCAAATCCACTCTTTCTTTGCAAAGAAAAACGAATAATTACA
CGTCACATTGTAACCCGGCACAATCAAGGAGGTGTAGATTGACTGGATTGTAATTTTCAA
AACTGTACAATATTTGCGCAAGGGCTCTACGAATTAGGGACAGTAGATGCAAAAGGAAAA
TAGCATTCCATAAGCAATATAAGTCTCCGTACGTTCTTTTAGGCGTGCTCCTTCAAGGGT
ATTTAGTGTGTGTTGTGTGTTCAAGAGGAGAGAATATAACACTTCTATTCGACGTAGCGA
TATATTATGCCAGGTGAAAACGACAGAAATTGTGTCATAATGGGTCCATGTCCATAAGAA
TGGCACTTAAATATCCCTCA
>comp2262_c0_seq2
GGGAGTTTCTTGGGTGGTGTCGAATTCTAAGAGGCCCACGATGGTTGGAATTGTTACTTC
GTGACCGGGTAATGTTGACTCTTTGTTCCCGCGTTGCACCCAGAGCGAATCCTCGTGGCC
ATATGCCTGAACTTTGTGATTACCGTCTAAATACATGTAAGTGTCCCCTACCGAAACGCC
GGGTCCCACGTCCATCATGACCGACGTGTATACTTTCGAGCCAGTTGGGGACACTTACAT
GTATTTAGACGGTAATCACAAAGTTCAGGCATATGGCCACGAGGATTCGCTCTGGGTGCA
ACGCGGGAACAAAGAGTCAACATTACCCGGTCACGAAGTAACAATTCCAACCATCGTGGG
CCTCTTAGAATTCGACACCACCCAAGAAACTCCCACCAACATGGCTCTGCCGAAACGATC
ACGGTGGCCTGCCGAAGCTGAAAACAATGTGGTAGTGACGCTTCGACTCCACCATGTAGC
CAAGAGTGATTCGGACGAACTTGCGGTGGAAGATATACTACCAGTAAACATCGAAGTGTA
TAGACTTCCCAATAATCACGATTGGGTAGTTGAATCTTTGACAGGGGATGACTTTCAGAA
TTCTCCGAAAGCAGCCAGGGAGGGAATTTTGGTTGCCCAGCATGTTATTGAGGCAACCGA
TACCTTAGTAGATATCAATGTGGAGTCGGCGATATTCTTGCCCGAAGACATCAATGCACG
GATGTACAAAGACGATCAAGTACTCCTGCTGCTGAAGGTGTACTGGGAAGAAGCCCCTAA
CGAGGGCGATTCATTTGAAACACGCGAGTCAGATGGTCGGTCACCGCAGCTTATTTTCTC
AAACATGATGTAGGAAAGCAGTGTTCAAATCCACTCTTTCTTTGCAAAGAAAAACGAATA
ATTACACGTCACATTGTAACCCGGCACAATCAAGGAGGTGTAGATTGACTGGATTGTAAT
TTTCAAAACTGTACAATATTTGCGCAAGGGCTCTACGAATTAGGGACAGTAGATGCAAAA
GGAAAATAGCATTCCATAAGCAATATAAGTCTCCGTACGTTCTTTTAGGCGTGCTCCTTC
AAGGGTATTTAGTGTGTGTTGTGTGTTCAAGAGGAGAGAATATAACACTTCTATTCGACG
TAGCGATATATTATGCCAGGTGAAAACGACAGAAATTGTGTCATAATGGGTCCATGTCCA
TAAGAATGGCACTTAAATATCCCTCA

>comp2263_c0_seq1
TGAACACAAGGGATGACTCGTGGCTCCCTATCATAGCGATACGGAACGATAACCGGACTG
CACATCTCGTGTTCCTGCAGTCTGTTTCTTTCAATAGTGCCCGTACGGTACCGTACCGTG
CTCTTGTCGTCCGGCGTCTCTCTTGCCAAACACCATGATTCCATCCATGTTTTCTTGCTT
GAATTTCCTACTTACGTGTGGCACATGCCGTGCAGTACTTGGTACGGTATGGCGTCGCAT
TACGAGCTCTGTGATCGCCAACGCACGAACCATCCATCAACGGATACAAATCCATCAAGA
ATGCTTTTTAAAAATTCTTTCGAAAGAGTACGAGTACGAGTACAAGTTACTGTTTAATAC
TACTCGTATCATCCGATGGATGAGAACCGCCCTAGGTGTTTTCTCGCCGATGCCTCGATT
TTCTGCAAACATCCAAAAACATAATTCGAACCCAATCGTCAACACCCCAGTTGAGGAAAT
AAAATACAAGAACATTCTGCGCCGTGCAGCTAACAACATTCGCGACCATGAGACTCATTC
AATCGTTGGCCTGCTGCCTTGCGTTCCTTTCCTCGGGTCCGCCGCACACACTCTTGTGGT
GGACCGTTTCGCTGGCGGTGTCTGCTTCGTTTGCTTCCTCCACGCCTCATCTCTGGACGA
GTGAACACAGAAGGCACGCTAGCTCTTCCAAGATGGTTCCAGGTGGGCAGGGGGCGTCGA
CGAGAGCGATTGATGCTCTTCGGACAAGAAAAAGGTGTCCACTGAGAGACATTAACGACA
ACGACAACGACTGTGAATGCGAATGTGAACCCGTTTGGGATAGAGACGATGGAAATTACG
ATGGCGATGGCATCAGCATCGGCATCAACACCAACGACGAAGATAATTTCGATGCAAGCT
CGTCCTGCTGGTACCCGATCAATCGGTTCGCTCTGGAGAAGGACCACGAGCGACGATCTG
TGCGGGCCGCCGAATATGCACACGGCACGGCAGATGCAAAGATCGGGGGAGAGTCCACCG
ACCAAATTCCTTCGAGTACTACCGGAGAGGGAATCTTTGTGTAGCACGGCGGCATCGCGT
CTCTTTTTCAACAAGGCACGAGTCGAAGCAATCTGTCGTACGTAGCATCTGCTGAGCCTC
GTTGCGCTGCACAGGGAACTAGCCACATCGTGTTAGGTAGCTGGCCAGCCAGTCATCGAA
TCAGACCGCCATAGCTCCAGAGTTCACGATTCAATTCTCTCTGGAAACTTGGGGAAACAA
AACTTCTCCGACGTACCATTCCGCCCAACCAACACAACTATATGCGCAAGACTACAGGCG
AAGAAACACACACAGCCATTCAAGATAGAGGGACTCGAGTTCTGACGGATGAATTCTTGT
CTCCTAGTTTGATTCACTTTTCTCGACAGCATCGCAAAACATAGCTGTAGAAGCAAGTGA
ATACGGAATCGAGTGAATCGAATCGACGTCGTCGCAGTTGGGAATATTTCCGCAATGCTA
TCGCTTTGGTGTTGGATGCATGGCCTGCAACCCATCACCGGAAAGCAACTCATTTTAACT
CAACGCATAAGAGAATGAGTTGAACACGAAAAGAAAAACAAATTGCTGCCGACAAACACA
TAGCCTGTCTGCACACTAGACAATAAGCTATGATCAACCGATCTAGAAAGATGGAATAGA
GTCGAGTCCAGTTGATTCCATGCGATTCCAATGACCTATTTGGAGGGGGGAATCTAACCG
AATTTCGAATTGACAACTATGTTTATAGTACATTCCAAGAGCCAATTTCGGGTTTTGGTT
ATACTAATGATACAATTAATTACATAGCAACACTTTAATAACCCAG
>comp2264_c0_seq1
CCAATAAATACCATTGTCTCCAACCGGGTTACTGCAGCTCACAGGAAGCGAAGATATTCC
GACCATTATTGATTGGAACAAAAGAGTGTCAAAATGGAATTCGGCACTCTCCACGAGACG
AAAGATGTGATTGACCACAACGGGAAATCAATCGAAGTTAATCATGTTGACGTCCGTGAT
AAATCACGCTGGGACGTCGGGCTAGACTGTATCAATTCTTTGCTAAGACCAGGAAGCGCA
ACACTAGAAGCAATGACAGACTACCAACGCCTCGGTTTAAAAAATTTGAAGGCGATGATT
ATGCTTTCTGGAGATATGGAAGATGACACAAAGAATCATATCCCACAAGCACTTCTCGAA
TCGAGCGAATCTGCAAGCAGCGACTTCTTGTTGGCAGAATATGCAGGTTATGACCGCCGT
TCGCAAGCAAAAACTCTGACTAATAGCATCCATAAGGTTCTCTCGGCTAAAAAATGCGCT
ATAAAAATGAAAAAAATAGCGAGTTCAAACGTGAACGCTCGCGAAAGTGCCGAGTCCTTG
TTCTTGGAATCCGTCGATTTCGAAAGCAAGGAAAGAACAAATTTGCCACCACATTGGTTC
GATCTAGACAATACATCAAAGCTGAAGTTGAGGGATCTTTTGTCGTGGGAGAATCTTTCT
CGTTGGGATTTCAATGTGTTCGAAGTCGATGACACCTTGAAGGGGAAAAACACCCTCATC
TTCGTCGCCTGGGCAATCATTGGATCCCCTCATTCACAATATGCAATGGATATGGCTTGC
TCAAAGATCGAAGGGGACACGAATACAGAAGTAGAGGGGCTAGCAGAAAGAAAAGGCTAC
GACTTTGTATCTGCACTGAAAATCAGGGAGAAGACGCTTCTCAATTTCTTGCGCGAAATT
GAAAATACATATAGAGACGTGATTTATCACAACAAAGTTCATGCAGCAGATGTGACACAA
AATCTAAACGCTCTGTTTCAAATGGGGGGGGAATTTTTTTCGGACGGGAAACTAGAAACA
TTTTCCATGTTGATTGCGGCCGTCGTTCATGATGTTGGACATCCTGGCGTCAATAACGCT
TTTCTTATCCATTCGAAATCCGATCTAGCGATAGTATACAACGATGTCTCTGTGCTTGAA
AATATGCATCTGTCCACAATGTTTCGAATAATAACCGGGAACTCTCGAGATCCGAAAATA
GATGTTTTCGAGAACTTCTTGGAGGAGGAAACGGAAGTGGCTAGGAAACTTATCATCAAA
GCAGTACTTTCAACCGATATGACGAAACACTTCAAAAAATTGAATACTATCAGAGGTGTA
ATACTATCTCAAGAAGATGGTGAAGAGGTGACTGAGTCACTTCCGATGGATATCTGTTTC
ACGAGCGAGATACTATCCTTTATGTTGCACGCAGCCGACATCTCAAACCCCTCCAGAGAT
ACAGCAACTGCGATAAAATGGGCCGATAAAGTTCTCGAAGAGAGTTTCCACCAAGGAGAT
CTGGAACAAAGTATGGGTCTTCCAATAACACCACTGAGCGATCGAAACTCGACGCATAGA
GAGAAATCTCAGATTGATTTTATCAAATTCATCATTTTGCCTACCTTTGAGCTGCTTGGA

CAGTTGATACCACGAGTTACAAGTGAAGTTATACCAAAGATATTCGAGAACCTTAAATAC
TGGCAGGAACAGGATCTACTTCGTGAAAATGAAGTGCGAAGCCGTAGAAGATCAAAAACT
TGAACAAACCAGCAAAACAATTTTCAAAAACTAGCAACTTTTAGAATTATAACAGCAAGT
TACTCCTCGTAAGAACAGAACAATAATTCAAATCCATCAAATCTCGCTACAAGGA
>comp2265_c0_seq1
ATTGAAATGAATAGATTCCGGAAGGGTCGAACAAGTAATGAAGGCAACGATACAACAAGA
TTGTAATTGCCTTTCAGCTTCTCTTCAACCTACAATGGAAAGGCTGCGGGAAAGATTACT
GAAATAGCTACGGAACTATTCAACCGGAGACTTGCATTGACTCCAGATACGTTGCGTGTT
CGACGCAACGGTACGGTACCATTGGCCTTTATCGTGCTTCTACCGTCAGATCATCGTACA
AGACATGACCCCGTTCGGAATGGTTCATCAATTTACGAGGCGCAAAGGCAACTCTAGTCA
CGTATAAGAATGCAGGACTGCTACTATCGTATCAGCTTTTGCGAACTTGGGTAGGAAGAA
GTGCGAAAAATTTCGTGTCTTTCATTTGTTGTGATGCCAAAAGGAGTCGTACTGTACGGT
ACTATCCATACCGTACCTTAAAGGTATCTTGAAAGTGGAGAGCACTGTCGTGAGAGAGTG
TATAGCTGGCCTGAACCTCCATACCAACAGCGAAGTAAGCCGAATTAGCTCAGTTGGCAG
AGCGCACGACTCTTAATCGTGT
>comp2266_c0_seq1
AGAAGTCTTACTCGTGCCGAGTCCGAGGAGAGACGTCATGGGCATTAGCAGAGAAAACGA
AGAGATCGATTCTACAATCTTTGCACTCGGAACCGAGTTGAACCTGGCCTATACTTCGCA
TTCTACAATCACCACCCGAGCACACAAGAGCAAACCTTAATTCCTCGAGCAGTTCCTCAT
CTGTAGCTGCTATCCCGGCACGGGCGGAGAATCATTTTTCGTACGACCCGGGAGCCCTAG
GTGGTTTCGGGCAGTCTCTGATCGATCGAAACCCGCTTCGTCGAGGCTTCGCTGATCGAA
ACGGTGACACAGGCACTGGTTGTAGGCATTGGTTGCAAGCTCTCCTCGTTAAAGAATACT
AGAGCACCATGACAGATACTCCCCAGCTGGCTGAGATTGCGATAATCTCGCCGGAGCTGG
GTCTGAAAACCGACGACGTTTCGGATCGAAGAGCCCGCCAGGCAGCGGCGGAAATTCTTG
CGTCCGACCACAACAACACAAATGGCGACGAAACCGCTGGTTTGTCGTCGCAGCCGCAGC
AATCGTCGGTGCTGGAACAAGACCAAGGTCTTCTCATGGACGTTCTCAAAGAGCTCGACA
GGGAACGACAAAAGCGTGCCGAGCTGGAAGCCCGGGTACGGGTTTTGGAGCAGGAGGTCC
AGTTCCATCGAAAAAAGAGCACCACCAAATCGAACAAATCGTCCAAATCGTCCGAAGCGT
CCGTGAGGGACTACACGATTGTCAAAACAGAGCGAGACGGATACAAAGAAATCCTAGAGG
CCATCACGCAAGACCGACCCGCTTTTTCCACCTACGCCCCCGACGACAACATACAAAAGA
GAAGGTTTGGCCGCCGAAACAAACAGCAGCCACCGAGCCTTCCCATCCACATCGTGCGGC
TCCTCGAGGTGATGCCGTGGGATTCCCGTGCACAGCAGTACGTTTTTGGAGAAGAGCACG
TTTACGAATGGCAGTTTTATTCCGCCGGCAAGAAGTGGCAGAAAGAACTCCGCTACTTTC
CCACTTTTTTCAAAACCCTGCCCATCGTTGTGCCTTCACCAGGAAAGATTGTCTCCGACG
ATGCCGCGACGAAAAAACACCGTCGTTTTGGCAGCGCCAACATCGCTCCACCGAAGCAGT
GCGTCTTGACCGATCTAGAGGGGAAAACAATTTTGAACATCGACAAGGGATACCCGCTTC
CCGAAGATGGAGGAGGATGGGCTTGGGTTGGGGCCTGGAAGGTCGAAAAGAACAACGACA
CGGACGAAGACGGATGGACCTATTCAAATGAAACCAGCATCATGTCGGATCCTTCCACAT
ACTACGGCGAGTTCCGGGTTCCGGAACAGGGCACGCCAAACATTGCAAAACGCCGGCGCA
AATGGAGCCGGTCACGCGTCCTTACGGACTACCCATATGCGTCCCAGATGACCCGGGAGT
ATCTGAAACTGATCACGGAAAAATCGATTATGGACTCCAACATTGTGAAACTCTCGGGGC
AACTCGTCGACACCAAAATGAATCTAACGACACTCGAAGCAGAGAAAGAACAAACCGACG
AACGAATTCGTACGCTTGAGCGCGATCTAGAGGACAAAAACAAGATTTTGGGCCTGGTAG
AAGTTGGAGCCGGATTGCAATTGACTAAGGCAACATCAGCAGCAGCAGATACCTCCGATG
CTTCCATAAAGTCAACGGATTCCGCAAATAAGGTAGACGAAATCCGATCCGCTGTGACCC
AGTGGGTTTCGAATACGGTCCAGAAAGCCGATGGTGACAACAACACTGTCGTTTCCAATG
GTTCCGGTGAAGAAGCAGCTCTGATCACAAAGTTGGTTCAGCAGCAGCAACAACAACTGC
GGCTGCATGATCCCAAGCAGCAATTGTTTGATTCCATTCGTGACAAGGGCACGGGCCTGT
TCGAAATCTTGAAACAAAAAGGCGAACAGCTGGACAAGATCAAACAGGGCGGCGGCGCAG
GTGGAGTCGGACTTCCCTGGCAAAAAAAGGAGGAAGACGCCGACACCGACAGCAAGGAAA
CCGAAGACCCCGAATCTTCCACTTCCTCTGCTGCTTCGGCGCCGACGAAATCCAATGTGT
ATACCCCCTCGGCGCGAATGAGATTCTTGAGCAGTGGTAGCAGCAAAGACTGATTAAAAT
TTGTTTCCCTCCCTTTTGACGAGGAGTATTATATCAAGCAATGATCATTATTTTTATTTC
AACCGATATTCATGGTAAGCCCCCATATAGGCGTGTGTAAATATAATCATGATCTAGGGA
CATCAATGAATGGAGAGAAATACAAACCTGGATTCTAGATGCACGTAGCTGCGTGCTTCA
TCATGTTTTACCGGCTACGATCCTTTGGAAACGCCAACGCAATCTGCATATTGAATTTTT
ACATCTGAAATACTTAGCAGAAAATCGCGATCAAACATGCGAGCTCGATAGTTATGGCTA
CGGTTACGGTGATGATAATGTGGAACTATAATGGTCATAAAGTGATCGAATCACTCAAAA
CATATAGGATTGTCCTAGCCGAAAAAAAGATCTGAAACTAAGGAGGTTTCGAAAAGAGGG
AGGAAGATGACGCCATCGAGGCGTACGTTGTAGCCATCGGTACAAGTGCTACTCATAGTA

AGAATAGCACATGAACACAAAAGATGTGGTACAAGGTATGGTCGTCTGTCTTCTGAATGT
ATAATTTTGTTTATAATATTGTCATACAATATTTACTATGCATTGTAGTCCTCGGTTTTG
TTTGGATCACTGCCTTCAGCTACCGCTTGCAATGTGACCATATCGGTAGATTCTAAAGAG
CAG
>comp2267_c0_seq1
CGTTGTTGCCACAAGGGCACTGATGTACCCGTAGTCGTACCTGTTGCCGGTATAAATTTT
TTCGTCAACAAAAAAATTGTTGATCGGCGAAGATCGGTATCTTCAGCGAAGACTTTCCGG
CCGCAGGCTCTTTGGCAAAAAGACATCAGTCGGAGACCGCATCTAAGTATTGACACGGCA
GTGCGAACTACGAGACGATGGTGATTCGAGCGTCTTCCGTAGTGCGCCTCTCGAGGCACT
GGAGGGCGACGTCCGCGAGGATTTCTTCGGGGGCTTCCTCGAGGAGGCTTGCGTCTGCCG
CTGCTGGCAGCAGCTGCACCGGGGATTCGTCCACCGTGAACAATCTCTTGCTGGCCGCGG
GTGCGGCATCGATTCTCGCTGCCGGCGTTGCACTGGGATCGGCAAACGCCACTGCCACCA
ACACCACCGACTGCGAACAAGACTGGGGCAATCTTCCAGCATTTGGAAGCAGCTCGGATT
CTATTCCCGGGAGTGAATATTCCGAAAAAGCCAATGGTAGCAACAACAACACTATCTTGC
TTTCGAAGATAAAGCGCAAACGCACACAACAGGAGATCGACGATCTGGAAAAAGAAAGGC
AAAAATTTGAAGAGCTTCTGGGACAGAACATTCTCTCTACATCGGCTGCCTTGCCTGGCG
GTTCGGATTCAAAAAGCACTGAGAGTGTTACCACCAAAAAAATGTATTTTTACAGGACGT
CTCAGATCGAAACGGAAAAAAAGAGTAAATTTATTCTTTTGGCTGGTCCAAGCTCGGACC
AACTGGGCAGCGACATTGCCCACCTTCTCGGCAAGGAACTCAACAACATGGATGTTGGAG
CATTTGCCGATGGCGAAACGAAAGTGGAACTCGAAGATTCTGTTCGGGGAAAACACGTCT
ACCTTGTGTGCTCTACCTCCAGTAACGACGCCGTTATGGAATTGACCTTCATGCTTTCGG
CTTTGCGCCGGGCGAGTGCCAAATCCATCACGGCAGTCATTCCATACTTTGGTTATTCCC
GCCAAGACCAGCAATTCGGAAGAGAACCAGTAGCGGCTTCCGATGTAGCTATTATGTACG
AAGAGATGGGAGTAGATCACGTCATGTGCCTGGACCTCCACAATGATTCCCTTCGAGGCT
TTTTCAGTCCGAAAATACCAGTCGAGAATCTGGTTCCGGTTCCTGTCGCAGCAGCATACT
TCAATGAAGAATTAGTTGGCGACGGTGAGAAGATCACCGTTGTTGCATCGCACGAAGGAC
AGGTGAGCCGAGCCACTATCTTTCGAAACGTCCTCCAACGACTGAGCGGTGAAGATATCG
AACTTGCATTCATTACCAAGGCAAGGCAGAGAAGGGGCGAAAAGAAATACACACCACAGG
TGGTTGGGAATGTGAAGGGTCGCAAATGCGTCATTGTTGACGATTTGGTAAACACTGGAA
CCACGCTCGAGAGCAACGTCGAAAAACTAGCCGAAATGGGAGCAAAATCAATTCATGCCT
GGGCGACGCACGGGGTGTTTGGTCCAAAAGAATTGTGTCAGGCAAGGGAAAAGATTGGAA
GCCTGAAAGATTTGGAATACCTGCTGATATCGAACTCTGTCAAGTACGACGGTGCCCTTC
CTGATAAGATCCGGCAGCTGAATGTCGCTCCCCTTCTAGCGGAAGCCATTGCTCGCTCCT
TTCACCACGGATCGGTTTCAGGTATTCTCAACCTGAATGAAACCATTCAAGAACGGTACG
ACAGTCGGTAAACTCAGCAGCCGAGTCGAGTGACAACAAATGAACGAAATCATAAGTTAT
ACGTGCCAGCGATTGATCTTTGGTCGTTGTCCTCCTGCTGGTCAACAATCTTTGGTTTTC
AGTTTCAACGAATTAGTACATAATTACATAACATCAATTCTGTTGAGTAATATGATGTTG
GATATGAATACCCCCGTGCCTATCATACACTTCAAGTTTCAACTTTGGCACTATTTTCTG
TTGATCAATATGTTTCACCCTTTTGTATCAATACTTAATGTTATATATTTTAGGC
>comp2268_c0_seq1
GGCCGAAGGAAGCCGAAGTCTATTGCTGATGAGACTACATACAGTGAGTGATCGGTTCAA
CGAGCCTGTGGCGTACAGATGGAATTCTCTCGTAAAAGGATTGCTCTGCGAACAGTCACC
GCGGTACTTTTCTTGGGTTTTTTAACGGTAAAGCAATGGAGATACATAGTCCTATTTCAT
ACGAGTAGTCATCCAAATGATTGGCAGGCGCAAGGGCGCACAGAAATCGATGAAGCATAT
CTAATTCAGGGGAAAAACCAATCGCTAGAATATAAGACAACAATAAGATCGGAGAATGAA
GAACTCCGTCACCATCGCCCAGGTGTTGTCGTGGTCAATGAGATTATTCACAGAGCTTTC
TTTGGACTTGGCCACAGACTTCATCGATCAGCTGCTTCGTATCATTTAGCCCGATCGTTA
TCATCGCCTGGAGCATCCCGATCACCCGCCGGTGAGCTTGATCCAGTCAATACGGACCAT
TATAATCAGCAGCCAACTATTACGCATCTACGGTTTCATTGGGAAAGTTGTCTTGCTGAT
GGCGACCAAGGCAATTCGAGTCTCCATGACGAAGCAGGAGAGAAAAAAGAGTACAATGTG
TTTCGCTTTTTGTTTGGGGAAGATTTGTGGGAACTAAAGGATCAGCCAGAAAATTCTTTG
ATGGCATCGACAGATAGCGAAGGCGACAGTGACAAAAACGTGGGTGTCCAAAATATAAGG
AACATGATCGTTTTGCGAAACGACATTCCAGGTTATATTGCTGGTCAATTGTACAAAGAT
TTGAAACTTCCCGTCAAAATGAAACCAAAAGAAGCACCCTCGCGAAATAACAAGGCAGAT
GCATTGTCCTCGGCAACAGTTGCTCTGACTGATAAGCTCTATGAATATGACACCATCCTT
GATAAAGTAATGCACAGCGATGTCCATTTTTATCGCCGCCTTGTTGACAACTATCGATTT
GAACGCGAACTTCAAGACTTTCAAGTTCAACATAAATGGGGCCAACGTCCACTAGTTGTA
GGCTTGCATCTACGCGCTGGGAACGGAGAAGATGCTCATTTTGTAAAGTCCGGACGCGGG
GCATCAATAGAGTACAACGAAAGTACGATGATCACCCGACTAATGATGTTGATAAACGAA
GTGGCAGCCCGTGAAATCCTGAAAGCAGAGCACAATGAGCAAGACGATGCTCTTTCGCCT

CTTTTATTTGTTGCAACCGACACAGCGCATCTGATTCCATTGGTGGAAAAAGTAGCAAAA
CTCAACGCGCATAACGACGCTGATTCAATGCATGGCTTGGAAGGATTACGTTCTCTAAAT
GCCACAGCTACAAATACTCGAACAAAGACTCTACCTCTACAAATTATCACCTGGCCGCAA
AGTTATTTACCAAAAAATGCCGGCGTTGGATTTGACGCTCTGAAAGGAAAGGGTGATCGC
TGTTTAGAAGGTTGGAAATCTGCTATGAGTGATATATTGCTGCTGAGTAAAGTTGATGTT
TTGGTTGCAGCAAGACGGTCAACGTTTACACAAAGTTTACCAATATCGCTTGGTTTCGAT
CGGAGTCGGAAATACACTGAGATAGCTAGAGCTAGCGATATTAGTTCTGGCATTTCAAAC
GATCCTGAAAGCGATCGGAGACGATTTTCTTTCTGCGAAGTATCAGAATCTGACACAGCC
CGGATTACATGCTTTGGAAGTATGCAGAAGTGGTTATTTCGAGGCAAAGATGATGAAAGT
ATGACTTCTAAAACATACCAAGAGGAAGACGGGAAAGGAACTCAGGCTAGTGTAGATCCA
GAAATTTGGACGTTATCTATTCCTGCGACAACTTCAAGTGCTTCTGATCAGCCAGAAAAG
AGAGAGCAAGTTGAGCACAAGGTCACTGTCCTTTTGCCAGATGTAGTTCCACCAAACGAG
ATGAAGGAAGCGAGAGATTTTCTTCGACGTGAAGGTTCACATGGCAGAAAGGAGGCTGTT
AATGAAGGTAAATCACGAGAAGCTATTTTTCGCTACGGACGATCGAAAATATACAAAAAG
TATCGCGCAGCCCACAAAACAAATGACAAGACAACAAGCGGTTGGAATCTCGTTTTGTCA
CACGTAGGGAACAAACGGATAGAGAAGTAGGGTCTAAGGAAATAAGAAGTAGGCTGACGA
TCTTGTACCGTGTTTTGTATTATGTCAGTTAGATTTATCGGATGAAAACCAGTAAATTTT
CCGCTTATCGCATCTTTTTCTAGTTTTCATCTGTGACTTGTTGTGACATTCTCCTAACCA
TTCTGCCGAATCCAGATTCAGAGTCGCTAACACGACGTGAGCTTTCACGAAGCATAGACG
AACG
>comp2269_c0_seq1
GAAATCGCTTTCTAAGATTTCGCGAGATAGAAATTGCGATTGAGGACAAGGCTCCAAAGA
CAACACCAGCACTCTAAAAAGAAACAAAGAAGAGTTTGATGCGACGAAGAAGAGCATGGC
CTACCACCAGACGAGAAGACATTGCACTATTTCTATCTGTGGAGATAGGTATCGAATCTA
TGCTCTGTTCCTTGTCGTGGCAACAGTTACATTGTCTTGTTCGGGTTTCACCTCTACGGC
CTTTATTGTCAACAATGCTCCTAAATTGACGATGCTCGGGCATACAAGCAGTTTTACGAA
GACAATTCAACCAAAACGTTTCTCTAGGCCATCCGAAAAATCCAACCTTGTGTTGCCGAC
TACGACATCAGTAGCCTCTGCCTCCTCTTCGTTCGAACCTCTCTTTCGGGGAGTTAAACG
CGACTTCGAAAATCGATGTCCTGAATTCAAATCTGACATTGTCGATGGTCTGAATGCTCA
GTCCTTGGCGACAGTATTCTTTTTGTTCTTCGCCTGCCTAGCACCCGCAGTAGGCTTTGG
TAGCGTTCTAGGAGGGCTCACGGGAAACCAAATGGGGGTGATCGAGATGGTTGCCTCCAC
AAGTTTGAGCGGGATATTGTATGCGACTTTCTCCGCACAGCCTGTACAGCTGATTGGACC
CCAGGGACCAGTTGTAGCATTTGTGGCAGCCCTCTTTGGCTTGGCTTCTTCTCTCGGCCT
TGATTTTTTGTCGCTCTATGCAGCTACTGGATCGGTAGCGGCTGTATGCTTGGCGATCTT
TGCTTTCACCTCAGCTTCAAACTTGGTGCAAAAGCTCTCCCGTTGGACCGACGAAACATT
CTCGGTTTTGGTCTCAGTGTTATTTCTAGCCCAGGCGGTTGGGGATGTCGGTGCCACATT
TGCACTCCCGGCTTCGGTGGTACCCTCAGTACTCACCAAGGCAATGGCCACCGCTTTAAT
GACACTCGTAACATGTACTTCAACGTTCGGAACTGCTGTTGCACTTAAGGGATTGCCCAA
GACGCGATATGGAACAGCGGCAATTCGAAAGCAGCTCTCCAATTTTGCTCCCGCAATTGG
CGTTGTTGTGGGTTCTGTGGTGGCTCGAGCCGCACGTATCCGTTGGGGGGTTTCTTTGCT
GGCCTTGCAGCTTCCAGCGAAGTTTGTAACGTCCACGGGTAGGACCTGGTCACTTCTTTC
CACCCTGGCATCAAATTCTTGTTCCAAGCTTTGGCTGGCAGCGGTTCCGGCCGGGGTAGC
AGCATCGATCCTGCTCTACCTCGACCAAAACATCACGGCTCGCCTCGTGAACCATCCACG
GTTCAAGCAAACAAAGGGAAATAGAACATCGATCACCGATGGAATGCACGGGGACATGCT
GGTGCTGGCCGGACTCACGGCTCTGTCCTCCTTCCTAGGCCTGCCCTGGATGTGTGGCGC
ACCAACGCGGAGCGCCGCCCATGTCCGCGCCCTGGCTACTTCGGACGAAGATGGAACAAT
CTCTGGAACACTCGAGAACCGTGTCAGCGGCTTTTCGATTCATGCCCTGATCGGGTTGTG
CGCGGTGGCCGCTGCCCCGCGTAACCTTCTGGCGACGGTTCCCAAGGCAGCTCTGTCGGG
GATCTTTTTGTACCTTGGATTCACTTCGTTACAGGGTCTAGAATTTTGGGACCGGATCCG
TGGTCTGTTTCAGGACGATGTGGTCTTGGATAAGTTTGATTCTGTAAAGCGTTCTGCCAT
TACGGTCTTCACAATGGCACAGATATTGTGTCTGTGGACCATGATGCAGGTGACTCAATC
CAAGTATGGAGTTGTATCACCCCTCCTCATTGCACTATTGCCCTTTGCACGATGGGGGTT
GCTAAGGATGGGAGCCGTGACGAAGAACGACATGAAAATATTGGACGACTAATTTCCAAG
AGCCTAAATACAATTTCAACTGACAGGAACTACTTTTTCTACAAAATACTAGTAGTAAGG
AGTGCGTGCAGATACACTGCTTTTGCTCTATAATGCTAATACACTAAGTTAAAGTTTGCT
CTTTGACTCAGAAATCATGTCGATTTCATTTTTTTCAGAAAAAAT
>comp2270_c0_seq1
AGACCCCAAAAAAATACAATATAATTGTCCTCTATCTCCGTCCCTCCACAGTCGGCAAGA
ATATCATGCATTCATCCCTGATCCTGATTATGCTATTAGTTGCCTCCGCGAGACTCCCTT
GCGTGTACGGCAACACTATAAGAGCAGCCGACATGGAAACCGCTAAGCGCCTACAAGGCT

ACCAAAAACGAGGCTACCAGTGGCCTGTCCTAGAGTTCGTTCCAAACACCCCCGGCTGGA
AGAAGCTAATGGAAGACCGGCTTGAGCAGATTTCCAACATCGAAGACCCCGCAGAGCGCT
TCAAGGGCTACACTGCCACAATGTATTCCGGCATAGTAATTCAAAACTACACCGAGCATG
GCTTTGCCCGGACCCGCATCAGCGAATCCCTCCTGAAGGAATTGCAGCAGGGCATTCGTG
ACGGATTCGAAGACCGCGTCAACGAGGGGTACACGCCAAGCATCACCGGCAACCAGGCCT
GGCACATCCAACGGGACGATCTCACCCAAAAAGTTGAGGACGAACTCCACGACAAGCTCG
AGGAGTGGGGAAGGGTGGATCTCGATCTGACCTACGTGTATGGCCTGCGCCTGTTTCGCA
ACACGACCACCATTAAGATGCACTTGGACAAGAAGGGGTCGCATTCCATGGGATACGTGT
TGCACGTCGATTCCAGCGACGACTACGACTCGGATGGCGACCCATGGCCATTTCTCATCG
AGGATTTCCATGGAAGGACCCACGAAATAACCATGGTCCCAGGCGACCTGATTCTCTTTG
AAGCCGCAAAACTAGTTCACGGTCGTCCTCATCAGCTTAACGCAACCTGGTATTGTAACG
TTGTCGGTCACTACTATCCTCGGGACGAGACGTGGGCCTCTATGAACCACGTAGCGGAAG
CGGAATACGCCGTCCCACCGAAGTGGGCAAACGAGCCTTCTGGTAGCAGCAAAGCCGACG
ATGCCATGGTGAGAGAAATCCAGTTCCGGGGTGGATTAAGAGAGCCCGGTTGCATGGATG
GGTGGTGCCGAAGCACATCTCTCGAGCGGATTCAATGGAAGGGACCCTCCGGACCCTCCA
CCCACTGGATCGACGCCAACGGAGAATCGCACGAATTCATGGGACGACTAAAGCAAAAAG
AAGAACAAAAAGCGGTAGAACTATGAGAATATATGCTCCGTAGAAATGGCATACGAAAAA
TTATTTGCAAAAATGGAACATCCGTTTTAAGTTCTCTGTAAACAACATTCATATTAAATT
TCTTTAATGTATTAAAAAA
>comp2271_c0_seq1
ACTGGAAGCAACCGGAATGGGAAAAAGGTTTTCTCGCAACAACAATTGAGAGCGAATGCC
TTGCTGTCTTCTTCTGGTTATCTAAAGGATAAAACGGGAATCTCACTAATCACCGAAAAT
ATGGCATCCATTACAGCTTCCTCTGCCCTCGTCAATGTTGTTGACGGCGCAGAACTTTGT
CTGGTTAGACTCCTCGACGAATCCGCGGCGCCAGGATCCATCCGTCCAACTTTTGTCCAA
GACTGTGAAGCCTGTATCGCACACAGCGATGCGTCGCAACTGCTCAAGACGATTGTATCC
GACCAAGGCGCCATTGCTGCCCTTGTATCGCTCGACGAAGAGGCCATCTCGGCAATCTCC
CTTCTCGCGGCTTGTTTAGACAGAGTCGAAGACAGCAGCGCTGTGATCTTGAACAATCTT
GCCGATTCGATCATTGCGATCAGTACTGCAACTGGTGCAAGCTCGAAGGCAATCTCTTTG
TTGGCGACTTTGTATAATATGAGATCGGATCCGACCGAAAAGGTTGGACTGTTGGTCAAA
ATGATTAGACTAGCAGTTGAGTCCGGAGATGTTTTGGAGTCCGAAAACTCGGTCTTGGGC
AAATGGATGGACCCTGCTCAAGTCGAAACAATGCTGAACGAATGGAATGTAGAACCAACC
GGTCGCCGTGAATTGTACCAGGTTGCCGCCCAATCTTCCCAGAAGTCTCCCGCTGCCACA
CAGCAATTTACCCTGTTGGTAGTAGAAACCTACTCCAAATCGGATGTCGATGCCGCCGGA
TTGGAAGCAGCTAAGCAAGCAGCCATTGGAGCTATTCGTGATCCCGTGACCTTGTTTGCT
CAACAACGAAAGATCCTTTCTCTACCTGCTATACAGGCATTAGAAAAAAGCGATGCACCA
TTGTTTGCTCTGTTGAAGGTCCTCCAAGAGGGGAAATTAGAAGATTATAATTCGTATATC
AAGTCGAATGGTGGCGATTCCGTCCTTGCCCAGTGGCAGTTATCGGCCGAGGATTGTTCT
CGAAACATGCGCATTTTGAGCCTCTGTAGTCTTGCTGCCGACCACGAGGAAATTCCCTAC
TCCGTCGTTGCAGAAACGCTGCAGGCCGATGCCGGAGATGTCGAAAAGTGGGTGATTGCC
GCCGTTTCCTCCGGTCTCTTGTCAGCCAAAATGGATCAATTGCAACAAAAGGTTATGGTA
GAACGCAGTGTTGTGAGGAAATTCGATATGGATCAGTGGAAGGCACTCCAGTCGCGGCTT
CATCTATGGAAGCAAAACGTCGGTGGAATTCTTGAGGCTTATAAGCAAAGCCTGAATAAG
TAAGGTGGGCCAGTTGTACAATAGTGTAATAAGCACAATCGCAAAACAACTTAGATATAA
ACTTAATATTAACAAGCAAG
>comp2272_c0_seq1
GAATCGTAAGCGAGGAAAAGGGCAAACAAACCTCGATTCAAATCCTGCCAAAAGTCTTGC
ACATTCCAACCCCTCTCCAACCCAAACCAAGCTACAGTACAGAAATATATCATATCCAAA
ATGGCTCCCTCAACCGAAGACACTACCCCAGAGGCCACCGTCGCCGAGACCACCCCCGCT
GCCCCGGCCGAGGAGACCGCTGCGAAGCGACCCTCGGAGGAAGAAGCCGAAAATTCCGCC
AAGAAAGCCAAGGTTGACGACAGCGCCGCTCCTGCTGTGGACGCCGATGCCGAACGACTG
GCCAAGACCCATGCGTACGGAAAACTCATCCTCTTCGGGGAGCACTTTGTCGTCTACAAG
GTCCCCGCCCTTGTCGGCGCCGTGGCAGCGTACACGGACTGCTCCATCAAGCTCGAGGAC
GCTCCCGCTGATTCTAAGGACGAGAGCCCGACCGGTATCGTCGAGGTGATCGACAACCGC
CCCGCCGTTCCCAACTACAAAACGAAGAAGGCCGAGGAGGGCAAGAAGGCTATCGATCTT
GTTTTGAACCACTTCAAAATCTTTGGAAAAGGCAAGAAGGCGACCCTTACCTTTGGCGGA
AACCTCACCTGCGCCAGCGGCATCGGTGCCAGCGCCGCCCAGGTTGTAGCCCTGGCCCGT
GCAGTCAAGGAAACCCTCCCAGAATTCGCCAAGCTGACCGACGATGAGGTAAATGCCGCC
GGATACGAGGGTGAGAAGGGATACCACGGAACCCCCTCTGGAATTGACAACACGGCCGCA
ACCTTTGGGGGCCTCCTCAAGTTCCAACGGACCGATGCCGACCCCATCTTTGAGAAGAAG
ACGATAAAGGAACCCATTCGCATCGTCTACGCATCTACCGGCATTACCTCGTCGACCACC

GAGGTTGTCGGAGACGTCAAGGCCAAGAAGGAAGCCGACCCTGCCTGGTTCGATGACCTT
TTGAAGAAGTACTGCGAGGTCGCTACCAAGGGAGAAAAGGCGCTCGAGGACGGCGACACG
GCGCTGCTCGGATCCCTCCTCAATGAAAACCACAAGCTTCTCCAGGACCTGACCGTGAGT
TGCAAGGAGCTCGACACCCTCGTCGATGCTGCCCGCGCCGCCGGGGCCGTCGGTGCGAAG
ATGAGCGGAACCGGCCGCGGGGGACTCATGCTCGCTCTGACGCCCACCGAGGAGATCCAG
AACGCCGTGGCGGAGGCTCTTGAGAAGATCGCCCCACAAGTCTGGAAGACGTCCTTCCAG
TAATCACTAGGTAGCACTCTTTCGTGCGAGTGACCGTGACTGCAACTGCTACTGCTACTG
CATTGAGCTGCTCTGTACAAAGCAATGCGGAGCAGAACGCAACGCAACGAGTGAAAATCA
AACGAGAGAACAGAAACGACTCTCACCTACCATCAAGGTGGGCAATCAAGAAATAAACCA
CGAAGTACTAGCTATGGTAATGCGGTAGGGACTCATACTGTAATACTAACAAAAAAGGTA
TCATCAAG
>comp2273_c0_seq1
TTTACCAAGGAAACAAAACAAAATAACTCAAGTTCATCGCACCATGCCTCTCCTCACTGC
TACTGTTGTCAAGGAAGAAACGAGCTCAAAGCTTGGAATCGCTTTCGAGCGCCTCGCTGA
GGATAAGGCCATGACCATCAAATTGATCCGAGATGATAGTCTTTTTGCCGGAAGCGATTT
ATGCGAGGGCTTGATTGTTGCCAGCGCTGTCGGTAAGGACATGGAGGGTATGTCTCCTAA
GGACTGCGCTGATGCCTTGCGAGAAGCCCCTGGAGGTGAGGAAATCTCTTTGGTTTGCAA
GGGAAGTAAGGCCACCATCAAAAAGGAAAAGAAAAAGCTTCTGAAGAAGGAACCCAAGAT
TGGTATTTCCTTCAAGAGCACCACCAGTGCTCCAGGAAAGATCTTCATTTCTAACATTAA
GGAAGACAGCAAGTTCGCTGGAACTGATTTGACTGTTGGACACCAAGTCCTCACCATCAA
CGGACAGAAGTGTCCTGGCCGAGTGACCGAGGCTGTTGAAATGTTGACCAAGAAGGGTGT
TTCGGAGATCACTCTTGTCACCATCGACCCCGAAGACGAAATTGTTGTTCCCAAGGTCAA
GGAAGAGCCTGTCGAAGAAGTTGTTGAAGAGAAGAAGGAAGACACAGAAGCGGCTGCCGC
CGAAGAGCCAAAAGAAGCTGAAGAAGACTTGACACCCGAGGGCAACAAGACCATCCTCGA
CACTATGTTCGGTGTATGCATGTGCTAAACGCCAAGCGCATATTCTGGCTATCTATCAAC
CAACAAAAGTAGTACTCGAAGCAATGCCTATTCATGGGTTCGACGAAGACTGTGGTGACA
CATCCAATACTGGGGTGCCCGGTATGACCCTTGTTTTGAGATGTCAGGGACATGCTTTTC
CAGTGCCCGGCACACAGTGCTATTGGAGCACTTGCGTGTGCCTATTATGTTGAGACATAT
ACGTGTTAATATTAAAAAATAAAAGTAAAATTCTACTTTGCCATGTG
>comp2275_c0_seq1
AGATATCGCATGACACTGAAGGGCACGCGGCAGCAAATCCCACAACATCGAGCAACGGCC
TCTGCAACCAACAGAGACATTGAACATCAACAGGCGGAACAGTGCACGCAATCAGCCGAA
GATCGAACAAGAACAACAATCATCAAAGCGTAGTGCAACAGTTCGAACGACGCTTGGCAA
TAATGCTGTTTCTATTGTCGAGATCTCGTTCTACCACTATCCCGGTCTTCCTTTCGTTCC
TGTTGGTTGGATCGTGCATCTTTGGGGTCGGTTCTGCATCGGAAACGGAGCATGCACCGT
ACCTTCGGCGCGATTCCGGGTCGAAAGAAGGGTCTCCAATCTCCGTGGTAGGGGACGATG
ACCCGGACATCAAAGAAATGTACATCACACAGCAACTGGATCACTTCGACAGGACCAACG
AAGCGACCTACCAGCAACGGTATTTTGTGGACTACAGCCGCCACGGCAGCAAACAAAGTG
GTTCGCAAGAGACATTGCCCATCATTTCCTTGCTGTGCGTGGGGGGCGAGGGGCCCGGCT
TTGACGAATCGGTCCTGATCGACTCGGTGCACTGCAGCGGAGACATGCTGGAACTGGCGA
AACGAATCGCCCAAACCAAATCGCACCGCGTCCATTTGTACGCCCTTGAGCACCGCTACT
ACGGCAAGTCCTACCCGGCTTTTGTGGACTCCAAAACGGGGAACAAAACATCGCCGCTCA
CGGTCCAAAACCTGCGCTTCCTGAGCAGCCACCAGGCCCTGGAAGACCTGGCCCATTTTG
TGCACACCATGAATAAAGAGACAAGCACGGACGGCGATCCCTCCGCGAACACATGGGTAA
CCTTTGGGGGGAGTTATCCCGGCCTGATGTCGATGTACGCGCGGTATAAATACCCACACC
TGGTCTTTGCCAGCGTGAGCTCGTCGGCTCCGCTGCAGCTCAGAGTGGACTTCCCGGGAT
ACAAGGCCAAGCAGGGGTGGGGCCTAAAATACGACAAAGTCGGCGGAAGCGAAGAGTGCC
ACCGCATCGTCCAGGCCGGTCACGAGCAGGCTGTCGCGGTTTTGCACCAGCCCGGGAACA
AGACGAACGCCACCGAGGCCGGCCCGGTAGTCCTGGCGAAGAAATTCGGGGTCTGTGATC
CGGGCAAGGCTCTTTCAATGCGGCGAAACCAGGAATTGCTCCTGGGAGACGGGCTCGTCG
TCATTCCATCCCAGGGCAACGATCCGAAATGCACCGGGGGGGAGCTTTGTAACATTGCAG
GATTGTGTCGGTTCATGATCCAAGAAACGGAAAAGATGAGGAACGCAACCGCACACAACA
GCACGGGGAAAAGCAATATCGAACTGGAAGTCTTGGCCAAAGTATCGCAACAACAACGAT
CAGAGCGAACCCGGGGAAACGAAAAGACATACAAACACAACAAACGGCAACAAATGATGC
AGAGAATGAGGTTGCCGAGACGGCTCGCAGGCGGTGGTGACAATGACGATTGTGTCGACG
TCGATTTCGAGGGGATGGTCAAGTACTACTCCGATTTGGAGGTGAGACCGTTTGGGGATC
GCTCGTGGCTGTGGCAAACGTGCACCGAGTTCGGATTCTACCAGACCTGCGGCGACGACT
GCCCCTACGCGGCCCACTTTCACCAGGTCGACATGGATCTCGAGATCTGTGCCCGCCTCT
TTAACATTTCGAGCGATAGGGTGTACGAGAATGTGCAGTCCTCCCTCGATGCCTATGGCG
GAAAATCCTTCCTCGAGTCGGGGGCCACCAGCCGCATCCTCACGGTCAACGGCAACGTCG

ATCCGTGGTCCGCACTGGGGCTGGAGGAAAACGACTCGACGAGCTTTTTGCTGCCGGTCA
AGATGGTCGACGGTGCCAGTCACCACTTCTGGACGCACGCCGTCAAGGATACCGACGCGC
TCGAGATCATCAAGATACGGGAGTACATTTATTCGTGCGTGATGAGCTGGCTTGGAATCA
CCGTCCCCCACAATCCATTGCTGTCGCTGCATGCGAGTAATGTAGCTCGCTAGCTAACAG
CAACAACATTGGCACGAGTCTCAACAACTAAACCAAAGAGACACTATAACAATAGACCTA
GA
>comp2276_c0_seq1
AACTATTGAACGAAGTCGTATTATATTGAAATTGAAATGAAGCAAAGTCAAGAGCTCAAA
TAGGAAGGATTGTTGGAGACATCGTGGCATTACTTGGGAGGTGCCGTTGGAAAGGGTACT
GTTTTTTTTTTTTTTCGTAATCATTTGGATGGACAAGAGACGGCATTGGCTTGGAGACTA
GTTTAGTAGTACTTGGATCGGATATTTATTTGTGCTTGTTCGTTCATCTATCAAATCATA
ATTGGTTTGAGTTGAAGTGTTCTATCAAGATCGATTGTATAGAAATCTTTTCTTCCCGGA
GTTCCATCCTCGGTCTTCTGTCTATTGTCTGTTGTCTTTTGTCTGGATTGAATATATCTT
GCGTCCAATCGCTCATTAGATTGGACAAAAGACCTGCAATGGCCAGCAATCGGAGCAGAA
ACCGGACCCAAGGTAGATCGCACACCATTAAGGTACATGATCACGACGTACTGAGCGGAA
GGGGTGTAAATATTGCGCAGCATCCTGGGAATGAAAGATTCCGATCATTGATCAACGCAA
GCCGCGATGAAAAGTATTGTTCCGACTTTACAACCGAGGAGAAGAAAGCCCTCGCGCAGG
AAGTGATTGATCATATTGAGTCACTTGATCCACCCGGTCGCTTCCTCAAAAGAGAAGGAG
TCTCGCAGAGCTCCCGTGGATTGGAAGGACCATGGGAAGAACTCACGAAGAAGGAAGCTT
CTAAGAAAGCAACACAGGCGCTTCGTGACTGCAACCGCCCCGATCGAACCGGATACGCGG
CCCAGGTGAAGGCTCCACCAGATGTAAAAGTGACAGCCGAAGAGCGGAGGAGATCAGGAA
TATCTCTGAAGGATCACGCGGCTGCAAAAGTAGAAGAGCGATCGAAGCAACCAAAAAGAA
ACAAGAATAGCAGTAGCAGTGACAACAGGAAGAATCCCCCGTCGTCTGTGACTGCTGCGT
CTCGAAAGAGCACCAAGCGATCTATCCGGGGATCATCACCTGCCAACAATTATAAGAAGC
ATCGACTAGAAGAAACCCCATTGTCGTTCCTACAAGAGGGCCCATTTGGGGCCCAACCAG
AACTCTCTGATTCTGTTCCTTTCAATGAGGCAACGTCGTCTACTGCCTTTGCCGAGACAT
CCGTCGGAGCCACTCCAGGTCCCCCTCACGAAACTTCTGTGCCGGTAACCGAAACTCCCG
TTCAATACCGTCCAACATTCAATCAACGAGAACCCCAAGACTATGGATTTCATCACCAGA
GTCACTACCAGAACCAACAACAGCAGCATCTTCATCCATATTCGAGATCATTACCTGTAC
CTATAATGCCTAACTCATCTGCCTCGTATCTCCCTGTTGATACCGGCCACCATCAGTTTC
AATCTCAAGAGTCCAACGAAGGGGATTCTGTCCCCTACAGGGCGCCTTACTCTCCATTGG
TGCGCAACAGTCCTCCACGACACGGCAATGGCCATAGCGAAGACGTATTGCGTCTTCCGA
TGGATTTCCGACAAACTCTTTTTGATGGGGAAGAAGACGATTTTGAATCCATGGCCGCAA
ATGCAGCTGCTGGGCTTCCTCATGGACACAACGAAGGCGGGTTTGATATGATGCACCACG
ATGCCCATCACCTGGACGAAACCAACGAGAACATATCAAACGGGAACGTCGCGAACGCGA
GCAACCAGCTGATCCTACCCTGGCCTAGTCCAGAACAAAAATAAACAAAATTGCACACTT
AGAAGGCATGAAAAAATAAGAGATACTTGATCTGTGAGGACTATTTACTTGGGGAAAAAA
CACAAGGTCCACACCGAAGCATCCTACCAACATCACCACATTACCACGGCTCAGAATCAA
TTTTACGCAAACTTGCAACAGTCCGAGCGAGGACCAAGTCGTAGATACTTTCACTCGCCA
TCTCTGTGCTTTCAAGGCGCTGTATGGACTACGCACCAAATGTGGATTTCAACACGAAAT
ACTTCTATTCTAGGCCGTCTGTTTCGCATCGTTTTTGGAAAGCGGTGCCAAACGGCGAAA
ATAATACAGGAATATACTTCATTGAAAGGATAACACTAGAATAAACATGAAACTATATTA
TTGTTCGCCTATCGCATAATGCTGGCGTGAAACCGGTTCCGGGAGGTTGATGATACAGGA
TAGAGTTCGATATTGATTGAAAACTGTGAAAAATATTTTTTTTCACGAAAATGAGAGAAT
TCTGCTCATCTTGTTGCGCGCGCCGCAGAGCTTCTTCGCACCGCACGCAGGCCTTTCGCG
CAATTTTTTTGTGAGGGCGGCCATTTCCTGAATTTAGTTGTCTCGTCTGCCTCGTTTTCG
CGAGAGGGCAATAATGATAATAGGGTAGTAGAAATATCAAAGGAAAGTCTGGACATCACC
CGGCACGAAGAAGAAGGCTTTAATAATGCGCGCAGGCCGCTCCCATCGATGAATTTGAAT
CTCGGCATGCGAGATTTTTAGCCGCCGGCTCTTCGGTTGGCAAATCCCGACCGATGTTGC
GAGGCCTTTCCTTCTCGTTCGTCGTTGCTTGGAGAGAAAAGATTATTACAGTAGTTTTTG
TCACGGTTTAAA
>comp2277_c0_seq1
ACCAAACCAAACCAAATCTACCCATCCTAACAAACATAAAAAGCAGCAAACCCAAACAAA
CAAACAAACAAACAACACCAACACCAACACAAACACAAACACCAACACCAACACCACCAT
GCTTTCCCCGAGATCCACCATCGCCGCCGCCCTGCTCGTCCTCCTGCTCGCCGTCGATCC
CTCGCACGGACTGGCGGGCGTCGTGAACCGCTTCCGCAGGAGCGCCCCCTGGGGCCGGGC
CGTTGCCCGGCAGAGGCGAACCAGCACGAGCACCACCAGCGGAGTTGGCCTCCCCGTCCG
GGCGGCCGTGGCCAAGCAGGGACTGCTCACCGAGCCAGAGCCAGAGCTCTCCCCGCACCC
GCCCGGTTCCACTCCCTTTGACGCCGACGCCTACCGCCAGCAGATGACCGATCTGGTCTA
TCAGCGAAGCATGGAGCGAATCTTCCGCGACTGAACGCGAGCCGGCACACCGAAACACAC

CACACCACAAACTACAGAACGATCCCAGCGCGCAACGACTCGACTCGAGGAGCTCCCTAC
GCACTATCGGAAGGGAATAGTGTACCCCAACCATGGGCTCCTTTCTTTTTGAAACGCTAC
GAGCAACGTTGTTGTTATTTTTAGAGAGACTAGAACCATAGAAGCATATGACATAAATAT
AAAACATAGAATCAAAACCAAC
>comp2278_c0_seq1
TGCGGTCCTCACTTTATCACTCCAATCAAGCCATATTCTTCTAACCACCATTCTATCATT
GAAATATCTGGCACCGATTGCCCAAAACAACGAATCCAAAACCAGAAACACACGAGCGAA
CGAGCAAGATGTCCCCCCGTTCCACCCTGAAACTCGCGAGCATCCTGCTCCTGGCCCTGT
CGTCCTCGTGCGCCGGGGTCCGGTCCGGCGCCCCGTACCGGGCCGAGAGGTCCCAGCGCC
GGGGGCTGAACAAGCGGACCCGGGGCATCGGCGGTGTCTCGGGCCGGGCGGAGTACGGCT
CCAAGTCCAAGTCGGGGGGCAAGTCCGGCAGCAAGTCCGGCAGCAAGGCCGGAAAGGCGG
GCGCGAGCGCCAGCGCTAGCACCGGAAGCAGCAGCAGCAGCCCCAATACGGGCGGTGATG
CCGTGCCCGCGTCGATATCCCCACCCACGGCGGCCCCCGGTGCCTCCGAACCCGAGGCCA
GGGCCCCCACGCCCGTCTACCGCCCGGTGCCGGCGCCGACCGAGAGGCGGCCCTCCTTCT
GGTACCCCGGCTCCGATCGCGAGCCCACCGAGGGACGGATCGACCCGTCGGGGGGCAAAC
TCCCCAACCAGAACGAGGTCGTCGCCGTCGTCGACGAGGAAGACGGAGGTTCCCCTTTCG
GACCCGGGAGTGGCGAAGGGAAACAGGACGGCAGCCCGTCGGATGCCTTTCAGGACCTCT
TCGACCTGGTGACCGGGGACGGCGGCGGAAGCGGCACGTCCATCGGTACCGAGACCGGGG
ACGACCAGGCGCAGGGAGGCGGTCCTTCCGGCGGATCCGGAAACGACACGGATGGCTCGC
CCGGCGACAATAGCGATGGCCGAGACCCTTCCACCGGAGGCAACGACAGCCGGGGAAGCG
GGCTCGACGGAGACCAGGCAGGGGACTCCTCCCAGGGAACGCAAACCGGAATTTCGACAG
GAGTCTCGACAGGAGGTTCCAAAGAAGACTCCAAAGAAAGTTCGACGGGAAGTTCGACTG
GAGACTCTACAGGAAGCTCTACGGGAGGTTCCACTGAAGTCTCCACAGGAAGTTCCACCG
GAGGTGCTACAGGAGGTTCCACTGAAGGCTCCACTGAAGGC
>comp2279_c0_seq1
CTAAACCCGAAAAACACCAAAACCAAGCCTTTCCAACCAAGATCTACCAAACATGAATAC
CATGAATCTTTCATTTGTTGCCATCCTTTCCGTTATTGCCTTGCAAAGCACACAAGCCAT
GATCTCTCCTGCCGTGAGTTGCTGTGAAGACAATGGAGGAAAGTACGAAATTGTGGACGG
CAGTGATGGCCAGTCGGGAGTTTGCAGGAAGGATGGAGTTAACACAGAAGCCTTTTACTT
CATGGACCAGAACTGCCCTCCTCCGCCAACAGTTTCTCCATCCAATGCACCCACCATGAA
ATACTCTGTCTATGCCGTCAATAATTGCGACGCCACTGTCACTGCCAATTTTGTTCTCAG
ATCAAGTAGGAGATTAGGTGGAAACCGGAACAGCCAGGTTGAGGTCCAGCAGGGACAATG
CACATATGTAGGTCCTACCAATCTGGATGTTGTGACTTACACAAGCCAGGGAAGCTATGT
CAGCAGCGGAAATGATTCCTGCTTGAACATAGGAGATTCCAACAACTCAGATTGCAACT
>comp2280_c0_seq1
CACAAACGCCAGTATGATACGGTGCATCTGATTTCACTGCATTCTTCTCGCTGGATCAGA
GAAGGCTTTTCACATCGTAACGGGACTTCACGTCACGTCACGTCGCATCCTGCCCTCTCA
ATACCATTGCGATCGCACCAGATCACCAAGCGAAGCACCTCTGCTTGCCGACGATCCTGG
CAGTTTCCTGACGCTGGGTGCCTGCACTCTTGGTTTTATGGACGCAATTCGTTTTTCCCT
GTCATTCATACACAGGCAAGCAAAACTCCAGAGGACCGAAACGAAACACAACAAGGTTGC
TACGGCACGGCAACCCCAACGCAACCGGGCACTCCCGTTCCTTACCTACGACTCAGACGG
CCAACCTGTATAGAATCCGCATACAGTACAAAAGGCACACGACAATATACAGAACACAAC
ACAACACAACACAA
>comp2281_c0_seq1
TTTCGCTTTTCGTTGAGCAGGCCCCAAGAAACTATCATCGAACATCAAGATGCACGATCA
CTTGAAAGAATAGTAAGAACCAACCTTCACGAGTTAATCAGTCACACAATAATTATTTTC
ACAGAGTGTGCCAGCTGCTCCCACATCTCTGCTATTCAATCAACAAAGCAAAACTTTTTG
ACATCCAAAGAAGAATTGAAAATCACTTCTTGTTCCGTGTCACAAGAAATTCAGCTATTC
GAATAGGTTACAGTATTGATAGTGATCAAGTCACTTGTGTTTTGCTACTCTCGGCGTGCC
CATTGAAACTACGGTGCAAATATCTAAACGACAGGAAAGAAAAAGGATGCCACACTATTT
TGCTTCCAGCTGTCGATCACTCATCATCTCCTCCTCTCGCTGGGCAGCGACGGCCCAGCA
CCATGTGCAGCAACGGGTGTGTTTGCCCCCCAACCGCCTCGTGGCGAGGCGAGCTCTCTC
GGCTGCCACCGCTACGTCGAGCCACCAATCGGCCTGGCGACGAACCGCTCTGGTGGGTGT
GATGGGGGTCGTTGCGGTAACAATTGATTACAACAATCGAACCAGCACCGGCGTCTTGTC
CCTTGGTGCCCTGTCTTGCGACCACTGCGAATGCGAAGCCGTGCGGAGGAGATCCAATGG
AATTCCAACGTCCGGAGATGTCTTTTCGGTGGGAAAAATCACCCACGAGCCAGCGACGAA
GATTTCGTTTCCTGCTCTCTGTAATGGCATGTCTCTGGGTGGTGTTGGCGTTCGAATCAA
ATACGTCTTTGTGAAGGTTTACGCCGTGGGAGCCTATTTTGATCCTATTGCCATGGCGGC
CGTAAAGAAGGGCACGCAAGCAGACATAGAAGGGGCTCTGGTAGATCCAACCTATCCTCG
AACCATCCGCATCGTCATGAATCGCGGCTTGTCCGTGGACAAATTCATTGCTGCCTTGAA

CGAGGCAATCGAACCACGCCTTAACGGGAAGAACCTGGAGACATTGGACGAGTTCAAGGA
ACTTTTTCCAAAAATCGACTTGATCGAAGGAGACGAAATCGAGCTCACTATCCGAGGCGA
CACACTCCTCCTCAAAACCGGTCTCGGAGTGGGAACCATTCGTTCCCAGCCTTTTACGGA
AGCAATGTGTGATGTTTATTTTGGAAAGGAATCTGTAAGCCCGACTCTCAAAGAGGACGT
TCTCAAAGGGATCCCAGCGTTGTAACAATAATTTGATAAAGTGAATTTCAACAACTGAAT
ACGATAAAGCCCGTGGTGAATTTCCAAAGGAAGGAACCTGTGTGTCTCCGTGATTCGAGT
GTTAACAAGCGAAGAAACAACGTGGTGACAATGATTCATCGACGAACAACGAGTTTTCAA
GCAATACAAAAACAATAGGTTGAATCGATCGATCGATCAATCAATCAACGAACGTTCGAA
AGCAATAATTGCATGTAGAATAGAAAGATACAACAACGATTTATTCAACTTTTCGACTAA
CATTTAGTTTCGAAAAGCACTACTCGTGCTTGCGTTCATGAAAAGCCGAGACCTAGTAGT
CAGAATACAGTCTGTTGATCATTCAATTTTTTCCGTTCAAACCTGTACG
>comp2281_c0_seq2
TTTCGCTTTTCGTTGAGCAGGCCCCAAGAAACTATCATCGAACATCAAGATGCACGATCA
CTTGAAAGAATAGTAAGAACCAACCTTCACGAGTTAATCAGTCACACAATAATTATTTTC
ACAGAGTGTGCCAGCTGCTCCCACATCTCTGCTATTCAATCAACAAAGCAAAACTTTTTG
ACATCCAAAGAAGAATTGAAAATCACTTCTTGTTCCGTGTCACAAGAAATTCAGCTATTC
GAATAGGTTACAGTATTGATAGTGATCAAGTCACTTGTGTTTTGCTACTCTCGGCGTGCC
CATTGAAACTACGGTGCAAATATCTAAACGACAGGAAAGAAAAAGGATGCCACACTATTT
TGCTTCCAGCTGTCGATCACTCATCATCTCCTCCTCTCGCTGGGCAGCGACGGCCCAGCA
CCATGTGCAGCAACGGGTGTGTTTGCCCCCCAACCGCCTCGTGGCGAGGCGAGCTCTCTC
GGCTGCCACCGCTACGTCGAGCCACCAATCGGCCTGGCGACGAACCGCTCTGGTGGGTGT
GATGGGGGTCGTTGCGGTAACAATTGATTACAACAATCGAACCAGCACCGGCGTCTTGTC
CCTTGGTGCCCTGTCTTGCGACCACTGCGAATGCGAAGCCGTGCGGAGGAGATCCAATGG
AATTCCAACGTCCGGAGATGTCTTTTCGGTGGGAAAAATCACCCACGAGCCAGCGACGAA
GATTTCGTTTCCTGCTCTCTGTAATGGCATGTCTCTGGGTGGTGTTGGCGTTCGAATCAA
ATACGTCTTTGTGAAGGTTTACGCCGTGGGAGCCTATTTTGATCCTATTGCCATGGCGGC
CGTAAAGAAGGGCACGCAAGCAGACATAGAAGGGGCTCTGGTAGATCCAACCTATCCTCG
AACCATCCGCATCGTCATGAATCGCGGCTTGTCCGGTGAGTTTTTGTTCTTTTCGTCTGT
CGACCTCCATGTTTTATTTTCCGATCCTTTCAAACTGACAGAAGCCTGTCCGTCCGCCCG
CCGGCCCCTTTGGTTGATGTTTTCATGACTATTGTTTTTGTTGAATACTGTGCGGTAGTG
GACAAATTCATTGCTGCCTTGAACGAGGCAATCGAACCACGCCTTAACGGGAAGAACCTG
GAGACATTGGACGAGTTCAAGGAACTTTTTCCAAAAATCGACTTGATCGAAGGAGACGAA
ATCGAGCTCACTATCCGAGGCGACACACTCCTCCTCAAAACCGGTCTCGGAGTGGGAACC
ATTCGTTCCCAGCCTTTTACGGAAGCAATGTGTGATGTTTATTTTGGAAAGGAATCTGTA
AGCCCGACTCTCAAAGAGGACGTTCTCAAAGGGATCCCAGCGTTGTAACAATAATTTGAT
AAAGTGAATTTCAACAACTGAATACGATAAAGCCCGTGGTGAATTTCCAAAGGAAGGAAC
CTGTGTGTCTCCGTGATTCGAGTGTTAACAAGCGAAGAAACAACGTGGTGACAATGATTC
ATCGACGAACAACGAGTTTTCAAGCAATACAAAAACAATAGGTTGAATCGATCGATCGAT
CAATCAATCAACGAACGTTCGAAAGCAATAATTGCATGTAGAATAGAAAGATACAACAAC
GATTTATTCAACTTTTCGACTAACATTTAGTTTCGAAAAGCACTACTCGTGCTTGCGTTC
ATGAAAAGCCGAGACCTAGTAGTCAGAATACAGTCTGTTGATCATTCAATTTTTTCCGTT
CAAACCTGTACG
>comp2282_c0_seq1
GGAACGAGAATCGAATGCCAAGAAACACTTTCCTCTCTCCAAAGCGAAACACGAAGCGTC
CCCACGAACACGCCGAAACAGGCATGTTGTGATGCTATTTATTAATGTCATATCGTGCTG
GTCATATCACCATGAATACTATGCTTCGTGATAAGCTATCAAAAGAATGGCATTAGTGGT
GATAGACTTTTAACACTGAATGGATTTTCACATTTTGAGAGACGATACACTGGAACTCAA
TAGAAGACGTCAACAGCAATCAACAATCTGCAGGACCACCACATCAAACCAAGAAACTTC
CATAGAAGAACCACCCCCGACCAAAGCGAATCGGTTATGGTAGAATTCCGGGATGGAACA
AAGCCGGCACAAATAGCAGGAACTGCTGGAACTTCCCCCGAAACAGCCCACCACGACGAC
AGCGACGCTGGATCGGCGTCGTCGTCCCGGAACACTCTTCTTTGCCTGAAATGCCACAAG
AAGCAGATAGTGTACGAGTGCGACCCATGTGGTTGTCCCTCGTTCTGCACGAGCTGCGCC
ATGAAACTCGCCTCCGGCGGACGGTGCAAGACCTGCCACCAGGCTTACGGAGGCCTGCGC
CGGCTGCGGGTATGAACAATCCAAAGCGGGCCAAATGAAACCACTCGGATCGAACGGGCA
GGTACAATTCACAGAAATCTGAGAGGCAAATGTCTAATGCGATCAAGCCAAATCGATTTG
CACTAGCACGTGGAAGGCGTTTTTAGGACGGTGCCATGCCCTGGAGCTCGTGAGACTCGT
CCT
>comp2283_c0_seq1
TATCCCTCCACCATTCCTCTCTTCTGAAGAACGATACTTAGCGAAGACAACGCCTATACT
GTATTCCTTCCTCCCAATTCAATATCCACTTATCTGTTGATTACAAGAGACAGAAATTCT

TGGACAATCACCATAGCAGCAACTCGGATCAACTCGGAGGGAAACAAGAGTAGAGAACGA
TACCACCATATACAAGCGACAATCCACAAAGTAGACCAAATGCAAGCAACCATGGCATCG
TATTCTTCTGCGCTGGCGCTGCTCCTCGTGCTATCCAATGCCTCGTTCTTCGCGAACGCC
CTTGACATCGAATTCGATCGGATCCAATGCGACGAAACCCTCAGTGCATACTCCTCATTT
TCGGAGATCGAAGTGACGTGCAACGATGGAAAAGACTCCCGGTGCTCGTTCGGTAACGAT
GTGAACATCAAGGGCATGTGTAAGTGTTGAAGTGCAACTTCTGCATCAACATCCCACCAA
CAAAACGATTGTGATGCGGGCTGGTGCAAGCGAGTGCAAGTTACTCTCTCTAACAAAAAA
GGTTATTCTATTGGTGGATCTGTTGCTTTCTATTGAACGTTTTCTTTGTTGTATCGCGCA
GTGCACTACCACAACCTCTATCCGTACCTTACGAACAACACGGGGTACGCCTCTGCCAAT
CTCAGGCTGCTGAGCGTCGAATACAATCTCTTTGACCTCTTCCCCGTGGATTTCTGTGGG
GATTGGGCCGTACCCTACAACTCCACCTACGGCCACGATTGCCCCGACTGGGACAGCTAC
TACGCATTCGACATTGACTACAAGCTGCCGTGGGACGACGACGACATTACGACCTGGTTC
GCCACCGGATGGCAGGGCGTCTCCAACCTGGCGATTTACTCCAACCAAACGGTCGACAGC
GAGCTGATTACATACTGTACGATGCACTGGCACACGTATGTGACCCCCTCAGAAGACGAG
GATTTTCGAACGCTGCCCTCGGCGGCCGTCACCGGGATCGCCCTGGCCTCCTTTCTGGTT
GCTTTGTTTTGCTGCTGCTGCTACGTGAACTGCTGCCGAAGGCGACAAAAACACGTGACG
GACCGCGACTACTACGACGACGAGGTGTCCCGCAAGAGCAGGTACGAAAGGTACGAGGAG
CATGCCTGTTAGACGGTATTCGAAAGTTCTCGAAAGAAGTTGTGACATCGATAACAGTAT
CAACCAAAGGAACCAAGACTGTTTTAGTTGTTTAAATGCTACGTATTTGAAGTAAGCAAC
GATCCATCCAATAGATTAACCTATAAAGAGACCAAATGATGCTGGACAAAGGATTCATCG
TCCCGTTAATGGATATAAAGACCGGCTGCATTCTATGGAATCCGAGAGGATCGGTTCCGA
AGGAGGAGATGGTCGGCAAAACGTTCCTATCACTCTATATGTAACGTAATGTAATGTAAA
ATTTTGTGATGCATGGA
>comp2283_c0_seq2
TATCCCTCCACCATTCCTCTCTTCTGAAGAACGATACTTAGCGAAGACAACGCCTATACT
GTATTCCTTCCTCCCAATTCAATATCCACTTATCTGTTGATTACAAGAGACAGAAATTCT
TGGACAATCACCATAGCAGCAACTCGGATCAACTCGGAGGGAAACAAGAGTAGAGAACGA
TACCACCATATACAAGCGACAATCCACAAAGTAGACCAAATGCAAGCAACCATGGCATCG
TATTCTTCTGCGCTGGCGCTGCTCCTCGTGCTATCCAATGCCTCGTTCTTCGCGAACGCC
CTTGACATCGAATTCGATCGGATCCAATGCGACGAAACCCTCAGTGCATACTCCTCATTT
TCGGAGATCGAAGTGACGTGCAACGATGGAAAAGACTCCCGGTGCTCGTTCGGTAACGAT
GTGAACATCAAGGGCATGTTGCACTACCACAACCTCTATCCGTACCTTACGAACAACACG
GGGTACGCCTCTGCCAATCTCAGGCTGCTGAGCGTCGAATACAATCTCTTTGACCTCTTC
CCCGTGGATTTCTGTGGGGATTGGGCCGTACCCTACAACTCCACCTACGGCCACGATTGC
CCCGACTGGGACAGCTACTACGCATTCGACATTGACTACAAGCTGCCGTGGGACGACGAC
GACATTACGACCTGGTTCGCCACCGGATGGCAGGGCGTCTCCAACCTGGCGATTTACTCC
AACCAAACGGTCGACAGCGAGCTGATTACATACTGTACGATGCACTGGCACACGTATGTG
ACCCCCTCAGAAGACGAGGATTTTCGAACGCTGCCCTCGGCGGCCGTCACCGGGATCGCC
CTGGCCTCCTTTCTGGTTGCTTTGTTTTGCTGCTGCTGCTACGTGAACTGCTGCCGAAGG
CGACAAAAACACGTGACGGACCGCGACTACTACGACGACGAGGTGTCCCGCAAGAGCAGG
TACGAAAGGTACGAGGAGCATGCCTGTTAGACGGTATTCGAAAGTTCTCGAAAGAAGTTG
TGACATCGATAACAGTATCAACCAAAGGAACCAAGACTGTTTTAGTTGTTTAAATGCTAC
GTATTTGAAGTAAGCAACGATCCATCCAATAGATTAACCTATAAAGAGACCAAATGATGC
TGGACAAAGGATTCATCGTCCCGTTAATGGATATAAAGACCGGCTGCATTCTATGGAATC
CGAGAGGATCGGTTCCGAAGGAGGAGATGGTCGGCAAAACGTTCCTATCACTCTATATGT
AACGTAATGTAATGTAAAATTTTGTGATGCATGGA
>comp2284_c0_seq1
GTTTCTTCTGAACGCTCAATTATAGGCATTACAACATCACCAACAACTACCAAGCACTCG
TTTTTTTAATCATAGGACAACCCCGATGAACGAAATCTGTGGTTTCTCGTCGTCTCGTCG
TCGGCGACAAAGGAAAAATCTACTGTGGAATTCGATAGCAGTTCTAATTTCTGTTGGATC
GATGGGCTCGACCTACGCAGAGGATTTCGCGACGCAATATCCCACAACAGAGTCAACACG
GTTTGATGACGATGACAACAGCTACGATGATATATTATTTTTTGATCTTCCTTCCATGGC
AATAACACTCGATGTTGATACGGAAGATAACGTCCTCATTCCCTTCGAAGACAAGTTAAC
GGAAAGCATTGAACACCATCTCGAGCACTTCTATCAAAAGAAACTTCTCACATCAGAAAT
TGGTAACCCAAAGTTCATACATGTTGACCTCGAGTCAAACCTTTTGTGGACAGAGGTCTC
GATGGCACCAAACATGCGGCAAACGGACGATGTTACCTTTGTCAAAAAGTATCAGGTTCA
AGGGATTTTCAATAGCAAAATTAAGCTCCAAGTCGAACCTATTGTAACGGAAGAGGGAGA
GAAAGAAAAGCTTTCCTTGCTCAATCAAGTGCTCATGGATTTATTTTTCCTCGAAGCCTT
CGAAGACGATTATCAATGGGACTTGCTTCATGGTTTTCTACTGGAGCCTCTTTTGCACGA
CATAATTAATGTCGAAATCAATATCATGGACTCGGGTTTCGTACATCCGCATGATGCAAA

CGGCAACCTTGTTTTTGAACAAGGTGGTGACGATTTCTATTCCAAATCGATTTTCTCCTT
GCAAACCAAAGACGTTCATTGGATTATGGTTGGCGTTATTCTTGCCACAAGTTTTTTGGT
TGGTCTGATCGCACTGTGGATCTGCTTGTGTATTGCAACGAAAGGGGGAAAACGGCATTA
CTGCTTCCGCTGGCTGCGAGGAAAGACTACGACCTATTATGACGACAAAAGTCTTGAGGG
AAGTTCGGTAACGGGTAGTACAAGCTCGCACGACTCGGTATCGGCGGAATCGGAAGCCAC
CAGTCTGAGTTACAATCTAGACGCATGGGCCAATGCCATCACGAGCATCACGCTCCGCAA
TCCGGCGAGGAGAAAAAAGCGCGGAGCTAAAGTCGTCAAACGACCCTATTTCCGTCCCAG
TGACGAGCACTCGTCCGATCTTAGTTGCATAGCAGAAGGAGATAACGAAAGCTGCACTTC
CACTGTCAAGTCGGCCGGAACATCCCGAAGCGAAAGGAGCCGAAGGAAAAGGCGAAACTC
GAAATCTTCCTGGAAGTCTTCGACAGCTTACGATTTGCCATCCTTGGATTCTTCATCCGT
ATCGTCGGCATTATCAAGTGTGATATACGAAGAGGAGCCAGAAAAAGAAACAGCTGACAG
CGACGAAAATGCTAATGGCATCACTGAGGATGATGATGGCACAAAACAATCTGCTCCACT
TTTGTCAGAACGAGGCCTGATAAGGTTGATGCCCAGCGACGACGAAGATGATTTTAAGAT
GTGATGTTTGAAACATCGCATCGACCAAAAATCTAGGCCAACAAACAGCCACCGCAATCT
TTTCAATATGTAGAAATAATTCAAACTTGTAATTTAACATCACAATAACGCA
>comp2285_c0_seq1
CGGGAATATCATGTTACCCTTGTTATTCGATATGCTCCCTCACCCCTTGGTTTGTCTCCA
TGATCTATCCCATCACCAGCCTTCCCGCATACGAATCCTACAGGTCGAACAAGCACAGCA
TGCCAATGCCAACAGAAGCGGCAGCAAGAGGGTACCCCAGAACCGCCAAGAGCCACCGCC
CCGCTGTTACAAATGCTGTCTTCGTCGCGTCACGTAGCGTCGCTATCAAATATGAGCACG
TCTTCAATTGGGCTCCCGAGATTCGTTTCCGGCGACGAATGGCTGCGTCGCGAAGTCTTG
CAGGGGGCTAGTGAACCCGCGTATCGAAACCAATCATACCAGTGATTGCAATTATGGATG
CGGGCGACCGTGAATCAATCAATCAATGACGCGAGCGACCGTGAATGAGTGAATAAACCA
ATCGCCCGAGAGGATATGGGGTGTTGCCGCGCAGGATGTCAACAGAAGCTGCTCCCGATC
GATAGGAAAGCGTGGCTCTATGGGGGTACAAGAAAACCTCAATTTTCCGTTGTAGGACTA
CGGAGAATAGCGGTTTTCGTGAGAACCTCAAAGCAACCAAATCCTGGCAGGCGGAGACGG
CTGGGCGAAAGAACAAATATTTCCTGTCCTATGTTGAACATTTCATAACTGATAGGCCCG
TGGAAAATTTCAAGAGCGTAAAATGAACCAATGAACCAATCAATCTCCAGCTGGTCGAAT
TACCATATTGCACGGCTTCTCGACACTCGCTTATCCGACTGTCGATTGACCTTGCATTCC
TTTCCAAGCTTCTTGTCATAATTTCCATCAGGTAGAATTTAGTTCCAACTTGATAGAAAC
TTGCTCTGCAGAAAGCAACATTTTCTCTAACATAACTGATGAATCAACAAGTAATAGCAA
CGATGAAAACACACGAGTTCGATATTCGAGTAGCACTCGTTGGATATGTGAGCGTCGGAA
AATCCACGGTCCTCAATGCTTTGTTGGGCGACAGGTTCAGCGAAGTTTCTTTGAAGCGAA
CTACCGCGGGTGTCAATCTCTTTCGCATCTCTCAACCTTCGCAAGTCGAAGGAAAGAAAG
ACCTCGAAGGAAGCGCGACACAAGAATGGTCCACTCTTGATGATTGCAAGGTGATGAAAG
GAGCGAACGCGACGCACGAAGAGATCTCCGAAGACAACAAAAAGTTGCGATCCAGCGACG
TTGTGGAGGAAAAGACCTTTGATATCCAGGTCGATTACCAAATCTGTGAAATGCGTCCGG
ATACGAATTTGATTCTCATCGATATTCCTGGTATTAATGAAGCAGAGGCTAGCAAGAAGT
ATAAGGACTACGTAGAGTCAAACTGGGATACGTTCGATTGTGTCGTGGTTGTCATGGATG
CCATCAAGGGTGTCAACACAGAAGAACAAGTCGAACTTCTCAAGTTCGTCCAACAGAACA
ATCAGAATACCAAGGATATTCCTACCATTATTCTCGGGAACAAAATTGATGATCCGGACG
ACGAAGATGCCATTGCTCTCATGCACGAGACCCATACCAAAACAATGGAAATATTTGGCC
ATGTTGACTCCAAGTACATGCCCCACGAAAGAAAGACGAAGGATAACGAAAACAATTACT
TTGGTTCGAATGAAACTGACACTGCATTCATTCCAATCTCAGCGAAAAATGCCTTCACGT
ATCGAAAGGCCGGGAGAATCGACACCGATCATCTGAAGGATGCCAAACACCGAGATCTTC
TCAACAAGATAGGGAATGATGAACTCGGACGTAAATGGTCCAAGATGGATATGGATAAAA
AAGTGGAGGCCGTAACAGAAATCCTTCGAGATCCCGCAGAACTCGATGAGCGACTTGCTG
ATACCAACTTCAATACGTTCCTTGCCGTCCTTTCATCGTTTGTAGGCAGCGACGAAAGGC
AACAAGACATTCTTAGAAAGCAGACAGAGCTGGAATTAGCGCGCATTTCACACGGAGTTT
TAGGAGAGAAGGCCATTGCTGCATCAATCTTGGAAGTCTTCAAGAGGATGAAACCGATCG
GTTGGGTCGACACGGGCATCCTGCACACTGCTTTTTGGGGCGCCTTTGAAAATTTCAAAT
GCGGAGCCTTGATTGACGCATTTGAAACCGATGTGAGTCCGAAGGCGTTCGGACGCGCAT
TTACGGAGCTCGAGAAATATCATGATGTGGCTTCCAAGCTTGGGTGGACGAAGGAATCCG
AGCGTGCCATCTTCGAGATGAAAAAGATGTTGCGATCGCAACTTGTTTTGTTATTGCAAA
AAGTGAAGAATTGGAGTTTTCATTCGTTCTGTATGGAAGCCGGTGTGCGGCTTCACTCTG
ATTGGGGTGAGCATGGTTGCGGACTTTGCAGTGGGTATGGTTGCAATACTGTTTATCGGT
ATCCTCAAAGCAGTAGTCGGTATGCCCAAAGCAGTAGTACCGAGCGGAGCTATGGTGTAT
GCCGTCGAAGTAAATTTGCTTCCTGGCCTGACACATTGACAATCCCAAGTGCGACCACTT
GGGCAAATCTCTCACCACAGGACTGGATCACCATTTTGGCATCTCTTTCATTGGTCTGGA
ATCAAAGTTGGTTTATTGAAGATTTTGGTCACGAAAAACTAGAGCTAGAGGCGGCGCTTC

TCAGGTACCGTAGCTTCTTTTATTCTTCCCCTGTCGGGACGCTTTCTTCCGACAAATTTA
CGGGAAATCAGGATGACGATACAGCATTAAACGAGTTCATTCTTTCACATTCATACAAGG
ATGCTATCATCAATATATATGGCACAAAGAAATCAAAGAAAAGTGAATCCCAATCGAAGA
ATCGATTTGACTTGCTATTAAGCGATGTGATCGATACCAATGGAACAGACAACGCTGCCA
TTACCAAAGTGAGAATGCCGATGTCGTTGGCGGATCCTTCTCATTGGGGTAACCTGGCTT
GGAAGTACATCAAATTTTGCAACAAACAGGAAGATATGATTGACTCCCGTGAGAAGAAGA
AGCCTCGACTGCTTTAAAACGAAGAAAATCAAGTTGCTGGCGATGCTGTAACGCACTTCC
AATAGAAAAGAATCGTACAGCAAGGTATGGTATAGGACGTTGCAAAAACAAAATGTTCTT
TTTCCGACGCGTTGCGAAGAATGTATGAAATATTTGAAATAAAATACTTTTTTTACAA
>comp2286_c0_seq1
CGAATACACTATTTCGATTGTTGGATCTTTCGAGACATTGCACAATACTTCCTAGAATTC
AAACCAGCGGAAAAGTTCTATAGTGCTCAATCACGTTAACAAGTACAAGTACCCAGTAAA
CACCATCTCCTACGAGAAAAACACTGAAAGCACAGCGACAATGCCGAAAGATTCTTCCAG
CAACATGGCCATGACTAACGAACGGTGGTCTGCCCCGGGAGGAACAAACGAAAATGATGC
CCTTGGCGCCTTTGTTTCCAACAGTACGACGCAGACAGATCGATCCAACAAGAGCGATAG
TCTTCGACAGATGCTTTGTCAGGATATCGGAACAAGAACGCTGGTCTTGACGAAAGGACC
GTCCTCCAAGGCTCGGTCTCTGATACGGTCCATGCCGATTCCATCGAACAACAGCCTCGG
TAGCATGAGCACGATCAGCAGCACGGAAAGTCTCTGGCTTTATGGAGAGAGCCAAAAATT
GGGGTCGGTCACCAACATTTCCAAGAACAGCCTGCTTCGGTACGGCAACATGGACAGCAG
GAACAGCCTCCTGAGCCTGAACAACCGATGGAGGGCCACCCCCCCAAACGCGGTGAACTC
CAACCCCCAAAAAATCTTCTCGTCGTCGTTCAATGCGCCGATCTCTACTGGGAACCAGCA
GAGCCGGTGGAAGGCAACACAGGGCGGGGACCAACCGATCGGCATGGTAACCCGAAAGAC
GTCGGCAACGTATCTGTTTCCTGCAAAATCGGCCTAAAGCCGAACGTTGAGAAACCGACA
CGAGCCAACCGACTACCCGACAGTATCGCACTATACTATTATGCATCACCACACCGCATC
GCAGCAGCACGCATAAGCCAAAAAGGAGGAAACCAGAATCTATGGCAAACTCCGAAACAA
GCTATTTTACATGTACATTACTAGCAAATAGATTATAATCATTCGTTTCTGACTAAGAGC
TAATTAGACATTTTACACACGCTA
>comp2287_c0_seq1
TGGATGTTCATCTAGAGCACAAACCTTTTTGAATTTCTAAATCCTTTTCCGGTGTGCCAC
TTACTCTTGGCTCTGGTTGCATTCAGAAGCACAAGTACCGTATGGCCGAAGGAGAAAAGA
ATTTTCTTAACAGCCGACGGAGGAGGCGATGAATTTCAATGTGCACTGTATGTATCCTTT
CTTAGCGGCGCTCTTTTTCGCTCCGCCATGTCCTTGCTATGGTTTTGCTTCACATCATGC
CCTCGTCAATTTGCAGCAATCGCAGAAAACTTTAAGCATCTTTGAAAAGAATGGTGGTTT
CTCGCCGACTTGGAAAAGAAGGAAAAGATCCGACAAAACTAAAGTAGCACTTTTGGGCGG
AACGAGTATTGCAACATCGAACGGCGATCCTACTTTTGGTGGACTGGCAACAGGCATCCT
ATTCATAATTCTTTGCACTGCTGTGGGCACCTTTGTTTTTGCAAATCAAGTGTATACACC
TGAAATTATTGAAGGAGCTCAGATGATTCGAAAGTCGAATAGAGAAAACGAAATACGAAA
ATTGCTTGAGGCTGTTCGCAGTCACCAGGAAGACGGGAAGGAATTAATTGAATTGAGGCT
GCCTTTGGAAACCGCATTGGGATCTACTTTGGAGGAGTACGTCGCAGCTGTTGAAGGGTC
TGAACAAAAGTCTGACACAATCTTCACCATGGCAGATAAAGATTTGGTAAAGATTTTGAA
ACAAAACGTTTGAGGATGATGAGACGAGAGCCACAACATCCGCAGATCCCAACGTCCAGA
ATGCAAATCATCTTCCAATCACTTGTACCATGGGCTGTTACTGTACTTAAGCTTTTTGTC
TCCAATCTTTAGGAAGAATTTAGTATCGTAAAAGATTGCAAATAGTGGTAGTGGCTTGAT
AAAAGTCAAAAGTCATTGTAAAAATCAATTCAATTACCACCAGTATAGGCTTTATTGAAC
AGTTTGATAACAAGGAGGAGATGCCAAGTCCCTTCCGCCAGAACCATACTGCTAAAAGTA
GTGCTGGCAGTGAGAGAAGGGAAAGAGACTGATTGGAAAACTTTGGATATGAGGACAATA
TAGGACAATATTCCCCTCCCTGAAGGAGTGATGCCACCAACGAAAATATATTGAGGAAGC
TCAGCAAGGAAGTTGGGAAAAAAATTGAGAGAAAGCCTTGGGCAGAGAGATGCACTCTGCA
GACATACTTAGACTACTGAATGTTACTGGGGGAA
>comp2288_c0_seq1
CCTTCACCAAACCCGCCCCCGGTTCGAGGGAAAGGATGCGATTCGACGTCTACTACTCCA
CCGGGACGGTCGGGACACGGCTCCCCCTGGTCCTCCCCCCGGGGGAGGAACCCCCCAGGG
ACAAGATCGGCCGGGTCAAGAAAATGACCCACCTCATCCGCCGGGACGTCTCCTCCCCGG
AGATCCTCGAGGCCATCTTTTCGGACCCCTACGCACACGGCGGGGCCAGGCCGAGGGAGG
GGACCTCCAACCGCGCCAGGAACGACCTGCGGCTCGCCCAGCAGAACGACCGCTTCCATA
GGGCCTTCCACGACGCCCTGCACGGCATCATCGACACGACGGGGGACCACCCCAGGTCCC
ACTTTCTCAACTGGGGGACCAGCCGCTTCCTCGAGCAGCTCGCCAGGAGCGTCTTTTCCT
TTCGGCGGATCAAGAGCGGGTCCAACCAGATCAACGTTCCGCCCCCGCACGGGGGGGACG
AAGGCGAGCCTCCTCGTGAGGGAACCGAGTACATCGACATGGAGAACGGCATCCTCGAGG
TGGTCCAGAAGATGATCCAGAAATCGCCCCGCCTGAGCGAGCGGTGGGAGCTCAAAGAAA

AGAAACAAGCGGCCTCCAAAAGCTCCGGAGGCAGCAGCAGCAAAACCGAGAGGCACGCCG
GGGACCACCTTGCCTGCGGGGGGGGCCGCCACGGGGAGAGCAGCGACGAGCACGACGGCA
ACGAACACGAACACGAACACGACGACGGGGAGGGAGAACGCATGGACCTCGCGGACGAAC
ACGAACACGACGACAACGACGACGAAGAATTTGGCCACCGGATGCTGGGATTGTAGAAAC
CCAACAAAACGGGGGACGGAACCATCCGACGGATCGGGGCTCGGAACCGAAAAAGCACCG
CGGCGATGCAGACCACGCCACGCCACACACGCACACACACACGCACGCACGCACGCACAC
CCGGCGCGAAAAAAACAAAACAGGCGGATCGAAGCAAGGAACGGGAACGAAACCCTTGTC
TCCGTCGAAAGCACAGCTAGAGAGCAAGTGCGTCGCAACAGGCATGAGCTCGTACGAGAT
ACCAGGGTGCAAAGAGCATTGTGTTGCGATATACTATACTGTAATGTAAAAATGTACTAT
AGATCAATCGATCGATCGATCGTGGAATATCGCT
>comp2289_c0_seq1
TTGACAACCCCGAAGTGACACAGGATTATTCCGAGCAACCATCATCCGTATTGGAAGAAA
TCTGTTCTTGGAAGACAAAATTGGAGGACACTACTTCAACCAGATCGAAAAGCGAACTCG
AAGGCAGCAGCAGCAAAGAAAGCGCCATCGTAGCATAATGTCAACGCTTCGTCAAGTTAG
AATATCGCAATTTGCGCTGATTCTCCTCTCTGTGTGGATTCCAAGAATCGTCGTCGACGG
TTTTTCATCACCCACCACTCAACATCGAATATGCGGAAGGAAAGCTCCATTGTTTTCTTC
TGTGGAAGACAAGCCACAGACTTCTTCGGTTAATGAAGATGAAGAAGATGGTCCAGCAGG
AATCGGCGGAGCCGAATTTTTTGGAGGAAACAAGCAAAAAGAAGAATTATACGACCCTGT
AGCCGAACGAGAGGCGGGAAAGGGCATTGAACTAAAGGCGACGTCATACAACCGCTTCTC
TCTGGGTGTTGGTGCCTTTGACTCGGTTGAGGTCGCTTCCATGGCCCAATCTCTGCAAGC
ACAAATAAACGAGGTCTTGTACAGCGACAACAAAAAGTTAAACAGGCTTCTAAAAAGGAA
CCAAAAGGACTCGGAAAGCGACCAGCAGAAGTTGGATTTTCCTGATGTTAACTTTGGAAG
GTCCTTGTCATGGGACACACCTATGACAGTCTCGTCAACAAATCCATTCGAAGAAATCAG
GGCTGCCAAGAACTTTTATCAAAACATTGATTTGGCCATTGTTGGCGGAAGGAGAGTATC
AGAAGGTGTCTCAGAATTGTCTTGGGAGCTCTCCGTAGTATGGCCCACATTCTGGTCACC
GCGAGTTGTTCTTTCTGGTTCCTCAACGGTCACAACCGCCTCCGATAAAGTAACAATCAC
GAAGCAAGTAGATCGCATTTTCGGAGCGGACAACAATGCTGCGTTGCTCTCGCTGCTTGG
AGGTCAAATCACTCCACGGTTCTGGGACTGGTACCACATTGGTATGACTCCATCGGCAGA
GCAAGTGCCTAGGAAGGTTATATCTCAAAAAGGAGGAATGGCGGTGTACCAAATTCCTCC
TCAATTGGTGACTGCACCGTCTCTCATCGAGACGGGGACACGAGACAATCGCAACGCACA
AATGGTACCCAATCACTCCTTTACATGCGTTATCCGAACCATGGGTCCAAAAGATGATAG
GGACATTTACGTACCAACGACACCGATTGAAGTCAAGATCGGAAGGAACGACAGGAGTCA
AAACGACAATGCTGAAGATGGCAAGGAATCAGATGATAGATTGCTCCTATCATGGTCCAT
TCCTCTCTCAACCCAGTTCCAAGCTCTCAACAAAAACCTTCTCTTACCTGCGGAGGATGA
CGAGGATGCCGAAGAGGGTGCCTTTCCTGAGTGTAATTATGTTTGGCAACCGCTTCGTCA
AGTTGCCACCATTAAGTACGGAGGCAATGTCCAAGACGAGGAAATTTCAGGAATTCGCAA
AAAGCTGTACGAGCAAGTGATGAAAGAAGGATTGAAGCCGAAATTGGATGAAGCCGGAAA
GCCCAAGTTCTTTTTCTGGCAAAACGATGTGAAAGCATGCTACATAGAAGATGGATTGGG
GATGGTTGTTTACGACTGGAGACCCGAATTTGCAAATTCAAACGAAGTAGGGATCGAGCT
AATGGTATAATAGATTTACATTACAGGA
>comp2290_c0_seq1
AAGTGTTCTCATATTCAATTCCTTCACACGAAAGTAACAACTCCGCTATCAAAATACGAC
AAAAATGAGGCTGGTAACCTCCGGTTTCTTACGGTTGCCCTCTGGTGGCAACAGAGGTGC
CCGAACCGCAGCAATGGTCGTCGCACGGGCATGGATGGTTCTTTTCGTGTTCTTTCTTTC
GACAACCACTACGACGAACCGATTCGCGTCGGCCTCAACTCGCAATCCGTTGTTTTCCTC
CAATGTCGTGACGCTCGATTCCTCGAATTGGAAAGAAACCGTGGTGGACAATCCGCACCT
TGTGTTGGTCAACATCTGCCGAAAGGGGTGAGGATACTGCCAGCTTCTCCAGCCGTAAGT
TTTGGATGCGTGTTGCTGTTGCTGTTTCTGCCAATGAGTTCCTTTTGCTGGTTTCGATCC
ATTCACCTTTGTTCTTGCATGCAATTCACACAAATAATACAATGTATTGCGTTCCTGTAT
TTTATGATCCTCCATGTTCATCTTTCCGCCCCGATCCCTCGTTTCGTCTTTGTATTCGAA
AACAAAAAGAGAATGGGAGAAGCTGGCGGCTTCGGTCAAGGGTATGGTCACCATTGCCTA
TTGGGACACGGAGGGCGGATCCCGGCCTCCCAGGCTGTTGGGCGAGTACAAGGGTACGCC
GACCATTAAGCTTTTCAAACCCAAGCGCAGACAGCGCAAGAAGGGTTCCAGTGCGGAAAA
GGACGTAGTGGAATATCAACACGGGGAACGCAACGCAAAAGACATGCGCAAGTTCCTCGA
GTACCAGCTTCCCAGCTTTATCGAGCGGGTCAAGTTCGGTCCCGACGACCTCGCAAAGGC
CCGATCCAAGGCTGACAAGTACGGGCTCCCCGTGGCAGTGCTCTTCACCAGCAAAGCAAG
CACTTCCACCACGGTGAAGTGGCTTTCTGTGGAGTTCCGCCGCCGGATGCTGCTGGTGGA
GGTCCCACCCACGGAGAAGAACAAGGGTATTCGTGAGGCACTCCTAGAAGAATTTTCTGC
AGGAACCTCTGCCGACGATAGCAACAGCCTTCCGGCCCTCTACGTCATTCCACCCCAGGA
GGGACCACTCTCGTCCGAGACCGACAAGGGCACGCTACAAATTTACGAAGGCGAAAGCTT

CAACCGCCGAAAGTTGCAAAGTTTCTTCGACAAAACCGTCCTGAAGAAGCCTGTCTTGGA
ACCCCTTGTCAAACCTGAAGAAGACCCGCAACCAAAAAAGAAAGAGAGTGTTGGAGGGGA
CGAATTCTAAATCACCCATAAAGCCCATAATCCCCAAGACAATTGCGTGACGGCTCTTTT
TTGTTTGCAAAGTTGTGCTCGCTTGTTATTGCTCGCAAACGTTTTCCTACCTATGCCTTG
ATTGAGGAATGACGATCCTGCAACCATCGTTCTGTTCACATCCTTTACAATACGAGGCGG
ATCTGAGACACATCGAGGCAGTGTTAGGTAAAGCCTACGCGAGCAATGGTTCAACGGATA
GGCTAGCAAACGATCAGTGTAAGACGGAATTGTACTAAGGAATGATGGATATCATTAGCG
AGGGATCAATGGAATGAGCGATCCATTCTGATGGTTCCAGTGAAATGATGTAAGAAAGAG
GAACTTAGCGATCAAAATTTAAATATCAATGATTTCGTTCATTTTAGATTTATCTGGATA
ACAGCAGTTCAAT
>comp2290_c0_seq2
AAGTGTTCTCATATTCAATTCCTTCACACGAAAGTAACAACTCCGCTATCAAAATACGAC
AAAAATGAGGCTGGTAACCTCCGGTTTCTTACGGTTGCCCTCTGGTGGCAACAGAGGTGC
CCGAACCGCAGCAATGGTCGTCGCACGGGCATGGATGGTTCTTTTCGTGTTCTTTCTTTC
GACAACCACTACGACGAACCGATTCGCGTCGGCCTCAACTCGCAATCCGTTGTTTTCCTC
CAATGTCGTGACGCTCGATTCCTCGAATTGGAAAGAAACCGTGGTGGACAATCCGCACCT
TGTGTTGGTCAACATCTGCCGAAAGGGGTGAGGATACTGCCAGCTTCTCCAGCCAGAATG
GGAGAAGCTGGCGGCTTCGGTCAAGGGTATGGTCACCATTGCCTATTGGGACACGGAGGG
CGGATCCCGGCCTCCCAGGCTGTTGGGCGAGTACAAGGGTACGCCGACCATTAAGCTTTT
CAAACCCAAGCGCAGACAGCGCAAGAAGGGTTCCAGTGCGGAAAAGGACGTAGTGGAATA
TCAACACGGGGAACGCAACGCAAAAGACATGCGCAAGTTCCTCGAGTACCAGCTTCCCAG
CTTTATCGAGCGGGTCAAGTTCGGTCCCGACGACCTCGCAAAGGCCCGATCCAAGGCTGA
CAAGTACGGGCTCCCCGTGGCAGTGCTCTTCACCAGCAAAGCAAGCACTTCCACCACGGT
GAAGTGGCTTTCTGTGGAGTTCCGCCGCCGGATGCTGCTGGTGGAGGTCCCACCCACGGA
GAAGAACAAGGGTATTCGTGAGGCACTCCTAGAAGAATTTTCTGCAGGAACCTCTGCCGA
CGATAGCAACAGCCTTCCGGCCCTCTACGTCATTCCACCCCAGGAGGGACCACTCTCGTC
CGAGACCGACAAGGGCACGCTACAAATTTACGAAGGCGAAAGCTTCAACCGCCGAAAGTT
GCAAAGTTTCTTCGACAAAACCGTCCTGAAGAAGCCTGTCTTGGAACCCCTTGTCAAACC
TGAAGAAGACCCGCAACCAAAAAAGAAAGAGAGTGTTGGAGGGGACGAATTCTAAATCAC
CCATAAAGCCCATAATCCCCAAGACAATTGCGTGACGGCTCTTTTTTGTTTGCAAAGTTG
TGCTCGCTTGTTATTGCTCGCAAACGTTTTCCTACCTATGCCTTGATTGAGGAATGACGA
TCCTGCAACCATCGTTCTGTTCACATCCTTTACAATACGAGGCGGATCTGAGACACATCG
AGGCAGTGTTAGGTAAAGCCTACGCGAGCAATGGTTCAACGGATAGGCTAGCAAACGATC
AGTGTAAGACGGAATTGTACTAAGGAATGATGGATATCATTAGCGAGGGATCAATGGAAT
GAGCGATCCATTCTGATGGTTCCAGTGAAATGATGTAAGAAAGAGGAACTTAGCGATCAA
AATTTAAATATCAATGATTTCGTTCATTTTAGATTTATCTGGATAACAGCAGTTCAAT
>comp2291_c0_seq1
CGCCTCTTTCTCATTTTGACTGGAATCGCTTCGTGCCTCCAGGACTGCGTTTGCCATGGC
GACCATCAACGGACTTTACGAGCTAGCTAGGAGGCCTATCAAGAACGGCAATGGCTGGTT
CGATTCGCTCCCGTCCCTGCCCGACTTTAGCGACGAGTACGAGCTCGTGAGAGACACGGC
GGGCACTTTGATCAGCAACACATACGACGAGATCGAGCAACGGTTGCCCGATGATCTGAA
GAACAATTATTTTGTCTTTGAAGACCAGGTCGGCGAGTCGTTGGCCGTGATTGGCTTGGG
CGATGTGCGGCCGCGAACCGTGATCAAGTGGGCTGCATACCTTTTGGTTCTGTCCATTGT
TGTGCTGAAATATGTGTCCAAAATGAAACGACGCCGCCGGCACAAGATGCTCTACGGAAA
CAATGCGTTCCCGCCATTCACCAAAGCTAGCATGGCGAAGACCGTCTCCGCACTAGTTAC
CAACTCACAATCCCCCTGGTTTTTCAAGAAGTGTTCCGAAGATATCAACTCATCGGTCTT
TCGCTTGAGGTTGCCCTTTGTCAAGGCACCCATGGTCGTCGCTGTTGGAGATTTGGTTAC
CGCAAAGGAGATTCTGCAGGATCCCTTGACGATGAAATCCGAATCACACTTTTCTTCCGT
CAGCTCTATTGCCGGAGGTCCAAACATCATCACGTCGGAAGGACCCCAGTGGAAGAAATC
TCGAAAGGCTGTGTCTCCTGCCTTTATGAAGAAGCACCTTGACAGAATGCATCACGTATG
TAGGGAGGAAACGGAGGACTGGATCAACAACAAACTGAAACGATACATCGATAAGGACAA
AGACTTCGATATCGGCAAGGAAATGATGCGCCTGACGCTGTCGATTTTGTGCAAGGCTGC
ATTTGATTACGATACCGAAGTGGACGAGAGAGCCGCCATAGCATCAGAGCTTGAGATTGT
GACGAAGGAGTTTGGTTACGACGACATGAACCGTCCTCTCAGGGTGAGATTTGGCATTCT
TCGCTCATCGACGCGTCGCGCGAGACTGGCCCGGACCAGGATTCAGGACTTTGCCAAGAA
AATCCTGCACTCGCACCGAAAGAAGAAACAAACGGAAAACAGCACCGCGGTCGATGGCAA
CGAAACTATTTTGAGTTGCATCGTCAAGAGCAGAAAGTACGAAGACGATTCGCGAAGAAT
TTCCGACATTGTGATGTTCCTGTTCTCGGGCGTGGAGAACACGGCATACTCTCTGGCATG
GACTCTGTTCGAATTGGCACGAAATCCCAGGGTGGCATCGGAGCTGAACAGTGCTCTGAA
CGGAAACAACGACAAGCGCGCCCAAGAAATGCTCAAGGACGTCCTTCGCGAGGGAATGAG

GCTTCGTCCACCCCTCCCCAGCATCGGGCTCCGCACCGTTGGCAGGGATTTCTACATGGA
AGACAGGTCCATCGTCATCCCCAAGGGTTCGCAGATCATTGTTCCCTCTCTCGTGACAAC
ACGGTTCGACGTGGAGGACGCCGAGTCCTTCCGCCCGTCGCGCTGGAGGGACCACCCGGA
CAAGTCGTTCCTGTTGTTTTCGACGGGCCGACGAAACTGCGTCGGCCAGTCCCTCGCGCT
CGCAGAAATCACGTGGGTCTTGTCGCGTCTCTGCGCCAAGTTCGAGTTCCACGTTGTCGA
CGACGGCGCTATCGAATACTGCGGGGCCACCATGAAGTGCCTCGGCACGCGCCTGAAGGC
CAAGGTGGTCCCGAAACACCTGTAGGCGCGCGTCTTCTGCGACGACCGATTCCAGCTTAT
TATCAGGCCAAACACAAGCGAACAAACGGGGTGCACAACATAATAAGTTTTCAATAAAAA
GAGAAAAGCCGAACGAAACACTTTAGTCGGCTGCATTGGACAATGAATAACAGCTAAAAC
AGAATGATTACAAACAACCGAGGCCAAAGCTCTCTTGATCTTCTCGTATAAGGTACATTC
GAGAAAAACATCGGCCTTCATTGGCAAGAGAAGAGAATGGTGTATTCCCTACTACTCAAA
>comp2292_c0_seq1
TGTCACACAGCCAAGCCACAAATTAACGATGGTTACATCATCATTCCTACGGGTATCGGC
ATACGTATTTTGGATTTGCTTTAGTATTTCCCAAACGATCTGCTGCAGAGCTTTTCTAAC
CAGCCACTTGGTACATCGAACTCAAAACAGGCAACTTCGAAAAGAGGTACCGAGTCTGTT
ACAAAAGTACGCATCCGAAAGGACAACGACAAACGGAAAAACAACTCGAGCGAGAAGAAG
AACGTCCTCTGCCATGGCGACCAGTTCCGACAACCAAACAGACCTTAGCAGGAACCATCT
CTTTAAAATAAGAACGATCACAGCATTTGTGAGCTTGAAGGATTCCGATTTTCATCACGA
TGCGGTGGCACTGAAATCAAAACTCGAAACTACGTCGAGAGCCCTTGAGATGGCCCGACA
GCAATTAGAGGGAAAGAGCAGTGGCAGCGGCTACGAAATCCAAACACTGAGGGTTGCCAC
GAATCCATTTCCCGAATATCTTCTTGGAAGTGATAGTAGTAGCAAGACCGCATGCGATGA
AGCTGTCGTTCGAGAGCGATTGAAAGTGTTGGACGACTGCTTTGAGAAACACGGAATCGC
ATTTGTATCCCTGGGCCCTGCCTGCAGCCAAGAAACCGTCGAGGTCTGCACGAGAATCGT
CGATTTTTCGGGGCGCTTTTCTTGCTCGGTCCAGCTGGCATCCAACGACGTTGCCCTGGC
CAGGGTTTCGGCCGAAACCATCCTGCGCAACTCGAAACTGGGAAATCCCGAGGACGGTGG
CCTGGCAAATTTCCGATTTTGTGTGGCCAACGTCAAGCCGTACATTCCCTTTTTTCCTGC
CGCCAAGGCAAGGACGGAGGACGACGAAACTTTTACGATCCGTTTCGCAATCGGTTTGGA
AAACGGGGCACTGTTGAAGGATCTCTTGGAGAAGGACGCAGGTCGAACCATTGCAAATAT
TTTGCCCCCGAAAACCTTGTCGCCCGCCAGCGGCAGCAGTTTCGCGGCGGGATTCGCCGA
ATCGGTGCGTCCGATCCAAAGCATCTGCGACAACCTTGTGAGGGAATGGGATGGTTCGTC
CGCTGCTCTGGAATACATTGGATTGGATGCCTCCTTCAACCCGTCCCTGGACGAAGAAGG
CAGCGTCGCTTCCGCGGTCGAAGCACTGGACGAGGTCTCGAGGGGATTTGGCGGAGTCGG
GACCATCGCCGCTACGGCCGCCATGACCACGGCAATCCAGACCAAGCTGCCGAGCGAGGA
GGAGGGTTTCGGCATCAAGATCACCGGGTACAACGGCCTGATGCTTCCTCTCTGCGAAGA
CAAACGACTGGCCCAGCTGGCCACCGAAGGCAAGCTGCGGATGACCGATCTCCTGAACGT
TAGCAGCGTCTGTGGAGTCGGCATCGACACGGTCCCCATACCGGGCGATGCCAGTGTCGA
GGAACTAACTTCGTTGTTGCTGGACGTGGCAGGACTGGCCGAGCGCTGGAACAAGCCCCT
GAGCTGCCGGGTGTTTCCCGTTCCAAACAAAACAGTCGGGGAGATGACCACCTTTGATTC
TCCGTTTCTGGTGAACTCAAAGATCTTGCCTGTCTCGTCGTAGGAACTCGATACATTCAT
AAAAAAGTTTAAAATTACGCACGAGCACGACACTTTGCAAAGG
>comp2293_c0_seq1
TACAAAACTTTGAAATCAATACTGCACCCAACAAATGCCCCCACAGAGAGGTACATCCAA
CGATAGAGGAGTCGATAGCCTCTCTGAACCACTAGTGAGTGAGAGCATCGACGATACTCT
TGGAGCTGCTGCTGAATCGCCCGAAATTGCGAGTATCGCCAATAGCAAGGATATTGTTGT
GCGGAAGTGCTGCAAACGTGGTGGCGAAAACGAAAACAATGTTGTCATCCGCGTAAACCA
CAACGTCTTTTTGAATTTATTGCTGTCAGTCCTCTACGGAATCTCGAACTCGTTGTGGAG
CGGGACGGCATATTTGGCCTTTCTGAAAAAGATGGGCCACGGTGGTAACGGCCCAGTGGG
AAACATCGAGGCAGTTTCGGGGCTTGCCTCGCTGGTCTGTGCGCTGCCCATAGGTTATTT
TGCCGATAAGATGGGGAAATCGATCGTGATTCGTTCAGGCGGAATGTTGCTCTTCACAAC
CTCGATCCTCCAGATTGGGATTTTACAATGGGTGGGTACAGATTTCGATAAAAATGAAGA
CGATGAAGACTCAAACAGCAATAGGAACAATACAGCATTGTGGCTCATGGGAGCAATAAT
GGTGCTTTGGGGACTAGGAGATGGAGTCGTAAACGGGCCATGTCAGGCACTATATGCCGA
TTCGACACCTGAAGGCCAGCGATCAACGTATTTTACCTATCAATTTGCATGCTACCTTTC
GGCCTCTGCGGTGGGTCCTCTCGTATCCATTGTTCTCTTCCAGGTCCTTGGAGATGAATG
GGATTTATACCACCTGAGACTTGTCATCTATACCGGATTGGGAATGGAAGTATTCAATTC
TTGGTTGATGATGCTCTTTGACGACAGCAAAGCATTGGACGAAGACCAAGATACAGTCGA
AAGTACCGATGAGAACGATGGTGATGATGCTTCGTTTGACTGGTCGAACGAAGGGATAGA
ACAGAGATTGCCCTCCGAACGTGATACCACCCCAACGACCCTCCAAAAGCGGCAAGAGTG
GATCCCCTATCTAGTGTTCTTTTCCGGACTCGTGTCCTCCCTCGGCAGCGGAATGACTGT
GAAATTCTTCCCACTTTTTTTCAAGGACGAGATCGGTATGTCACCCTCACAGGTGCAAGT

AACATATGTTCTGGTCCCGCTGGTGATGATTGTCATGGGATCGCTCTGCACGAAGATTGC
CAACGCCGGTGTGGGCCGAGTCCAGACCACCGCACTTTACGAGGTCGGCGGGATTTCCCT
CCTTCTAGCCATGGTGGTTTTCAAAAACTACCTGGACGTCCATCCGTTTCTATTGGTGCC
AATTTACGTGATGCGAACCAGTCTAATGAACGCAACGTATCCACTCCAACAATCAATCCT
GATGGATTTCGTTCCGAAAGACAAACGAGCTCGGTGGAAGTCCCTAGACTCCGTGGCATC
CTTTGGTTGGTGCGGATCGGCGGCCTTTGGCGGCTGGCTTGCGGATAAGTATGACTACAC
TCACACGTTTTTGCTGACAGCCGTTTTTCAGAGTGTGTCGCTTGGGATCTGGTGCTGCCT
GCTGCCTCTTGTTCCCCGCGTGGAAGGTAGTGAGTTGGAGGAAGACGGTGAGGAAGGACC
TCTGCCTGACCAAGCCGAAGCGGCAGATGATCACAGTCCGGGATTTAGCACAACAGAAGA
GAATGGCCTAACGGAGCTGTCAGAACCCCTTCTATAGCTATAATAAATTAGAGTACTGAT
AGCCATTTACAG
>comp2294_c0_seq1
TCGTTCGTTCGTTCGTTTCAATCGCCAGCCAGCCACACGTTACGAGCGAATCGGAAGCTG
GTCCCGGGATGCCTGCACGGTGCAAAGGAACGGACCGGCCCTGGTCTGTATCCTGTATTC
GATTGGAAGAACCCATCGGGTCCCGGAACGTGGAACGGATTGCGTGTGTCTGTTTGCAAC
GTGCTGCCGGAAGAACTGACTGGAACCACTGGTTCCAAGCCCACGAAGCGGGGAACCCAA
AGCGGTTGTTGGACAATTCGGGTCGTGCCCCTTTAGGAAGGGTTCCCGAAAACCAGCAGC
GGAGGGCTCTGGTTTGGAATTGTCCTAGATTTCCATTCCGGTTTTGTTTGCACTAGCCAG
TGGTTGGACTGCACTCAAACCTCATAGCACGAATGAGGATGCTGCGGAAACGAAGCACAC
CCACGTCGGCCAAAGATTGATAATAGTACTCCAACCTCTAACCCCCAGGTGCGACTATGA
TACATACGATATTGCTTCCAGGACGTACGTATCGTACGTACGACTTGTATGGTATGGGAT
CGAGTTGATGAAACTTCTTACAAACAGTTGGGAAGAACTTGTACGGCCTGTTTCTACGAT
TGATGAACCAAGAGTTTCCGTACCGTACCGTACCGTACTAACCTTAAGTAATAGAACACG
TACTACTGCGA
>comp2295_c0_seq1
GAGACACAAAATCAACGATATCGAACGGGATACCAACACAAAGTGCGTCTGGATCGACTG
TACACTATTCGAAGACTGGTTCCTCCAATGCTAAAGGCAACCGCGAACCCAAAAAAATTG
TTGAAAAAATGAAACACCGAAATTTCCCCCCTTCATCGTCGTTGATGCTAGTGTTTTTCG
CACCATTGATTGCAAATCTGGCGAACAGCAACTACCATCATGCAGTAGAAGGATTCTCGT
CACCGCTACTGATAAAAAATGCCTCCAAGCCGAGAATAGCAACACCAATTTTTGCCGCCA
AGGGCTTTGGCGGAGGCGGTGGGTTCGGGGGTGGTGGCACCAAAAAGAAAACCAAGAAAC
AGAGCGGAAAAAAGAAGGGTAATCGGTTGGCCGATTCCCTCGCAGACCGGCCCAGCAAAC
CCGTCGCGAAAAAAGGACAGACCTATGTCAAGTCCGAACAAGACGCCATGCTCGAGGAGT
TGGCGGCGAAATCCTCCCGAACGCCCATCGGGAAGGCCGTCAACGAGGCGAAGCGTGCCC
TGCACGCCGCTGGTATTGACACCGATGTCGACCCGTTTTGGGATCTCATGCCGTCCCTGA
TATCGTCGAAGTTTCCGAGCTTGCAAGATTCTCAGCTGGAACGCGTCGCGGGCATGGTGA
AGCACGCACTTCATCCAGACCGCCAATCTTTGTTGGACCAATCCATCGTAGCAGACGAAT
GGCGGCCACACGAGGAAATCCACGCATACATGCCAGACTTGGGAGAGACGAAACCCTTTT
GGGATTCCAGCGAGCTTTCTATTTGCCAAAAGCTGAGCGAGAACTATGACACAATTTGCA
GTGAATACCAGGCTCTTCTGCAGGACCAAAAAGATCGGTTTCAATCTGTGACCAGCATGA
ACTACGAGAGTGGTTGGAAGACATTGGTTCTGTTTTACAATGGTCATCGAATCCCCGATT
TTCCTTACCATTTGTGTCCCAAAACTACGGAACTCATGGAAAGCCTGCCGTTGGCCGGAA
GAATTGCCGGTTTCAACAGGCAACAACCCCAGAGTGGAATCCCACTCCATTCGGATGGCA
ACAACATGTGGCTCACCTTGCAGATGGGGATCAAGGTTCCGGAAGGAGAAAAGGCCTGGA
TCCGGGTAGGGCCCGAAACCAAGCGATGGAAGGAGGGAGAATGTATGCTCTACGATACCA
CATACGAGCACGAAACCTTCAACGAATCGGAAACGGAAGAGAGGGTGGTATTGCACATTG
ACTTTTTCAACACACTGAAGATGACTCCGGTGGAGATCGAAGTAATGCGCTACATTTACA
GCATGAGAGAAGAATTTATGAAAGCAGAGGGAGTTTCCAAGGTGGGCAATCAGATTTTAT
AAAATAAAGCTAGAATCAAAATTACTTGCATTGGTTGAAGAACTATGAAAAACCTGTCTT
CAAGTGCTCTGGTCTACATCTATCTCAGCGCTTGATTCAACGCGCGAACACATACCCCAA
ACACCCTGTTTGTAAATATTATTTCGGACCACACCATAAGAGTATAAAAACAGTCCCTGA
AACTAATATTGACAATATGTGTGACACACGATATACAACAAAACGACAAACCAAAAAAAG
AG
>comp2296_c0_seq1
CGAAAATCATGTTCAATGTTGTGCAAGAGATGTAGAGGACAAGACGCACCTATCCTTCAC
AGAAGTGCAAGAGCAACGAAAGAAAACACGAATATCCCTTCTAGTAATTCGCTTATATAG
CTTGCGTAACCTTTTGCAGCAGTAAAATCAGTGGGATCGCCATCAGAAGTGATAGGACGT
AAGTCGACGATATATATCTGCGTTCATATTAAGGCTCGAAAGGGAACTGAGGAGCCCGTT
TGAGTCACTACAAATTCTCGGGCCAGTGTCTCTGAATCGGAAGCACACGAAACTTCAACT
TTTGACTGAAAACTGAGCCAAACATCACATGATGCTTCGTTCCGGGGGAAAGAAATTTAA

GGCGGCCCTGACCTCGGGTCCTTGCCGAAGATATCTTTCATCGTCGCCACCGGTGGCTGC
AGCGGCCGCCGCATCGCCCGGCGACCACGCTTCGTCGTCGTCATCTTCTTCGATTCGTTA
TGCAATGCCTCTTTCGATGCTTTCTCTGGCGGTTGGATTTGCTGCAGGCCGCCTTTCCTT
CCGATCGCCGGAAGAAGAAGAGCGGCTCAAGCAGTTGCCCAAGGGAAACCCGTTGGCCTG
CTGTGGGGACAACCACACAACGGACGATGGTTCGCAGGCACACTCTCACTCGCACTCCCA
CTCGCACACGCACTCTTCCAATTTTTGGAAAACGACGAGCTGTGATGCTCCTCAGTTGTC
CGAGGAGCACCAACAACTTGTCAAAACGCTCCGGAGACTTGTCGGGAAAGATTGCTTTCT
GGACGGCCGGGAGATAAACACCGAAACCACGGCCTATCTCAAGGGAGCACGCATGGGAAA
GGGAACCGCCCTGGCAATTGTCCGGCCCACCAAGCTGAAACAGATTCAGGACATCGTGCA
GGCCTCGGTCGATGCCGGGTGTGCCGTGCTGGTGCAGGGATCCAACACCGGCCTGACGGG
TGCCTCCGTCCCAAGGCCACAATCGGACGGACGGCCGACGGTGGTGATCTCCATGAAGAA
CTTTGACACGATCTTTCCGATTGATGGCGGAGAAAAGGTGGTCTGTCTCGCGGGGGTAGG
ACTGGCCTCCCTAAAACATTTCGTGGCGGACAACTTCCCCGATCGTGAATCCCATTCCAT
CTTGGGGTCGACGTTTCTGAATCCAACCACCGCAGCTGGTGTTGCGTTTGGTTCTGGGGG
TACCCAGAGCCGCAAGGGCCCAGCATTTACCGATCGGGCTTTGTATTTAAAGGTGGTACC
AGATAAATATGGTAAGCACATCGTTAAGGTGGTCAATACATTAGGAATCGAAGACCTAGA
TTCGGAAGAGGGCGAATTTGTTGCCAACAAAGCGTTGGACGGAGTCATTCCCACACTCGA
TTCCTACATCGATGTTGTAAAGAACGAACAGGACAACACTATGAAGAAATCCAACAACAC
GTATGGGAAAGACCAATGCCACGACAAAAAATACAAGGAGAAACTATGCTCCCTGGACAA
TAAGATGAGTCGATTCAATGCAGATACCAGTGGCCGTGACTGCAACCGGTCGGAAGGCAA
GGTCCTCATTTTGGCCACGGTGCACGATACCTTCAAAGCACCCACCAAAGCAAAATCCCT
ATGGATTTCTTTCGATAGCTTGGAGCTGGCCACAAAGTTCAAAACGGAGGTCTGCCTCGA
CAGCCCAGAAGATCTGCCTGTGAGTTGTGAGTACATGGATCGTGATTCCTTCGATGTCAT
CGACGAGGCGGGTAGATTCATGGGGCACCTGATCAAGTTTTTTGGTTCGGCCAGCCCGGT
GGTAAGCGCTGGCTGGGCAATCAAGCTGAAGATCGAAGCACTCGATATTCGGGGAGCTTC
GACCTTTCCGGACACTGTCCAATACTATTTGAATAACTTTCTTCCCGCCGTTCTTCCCAA
GGAAATAATGAAAATGGGTTACGCCCGGGATCATCACATTGCCACTACGGTCGGCGACTT
CAATGGAAGTTTGGGTCGTTTCGAAGAACGTTTCGAAAAGTTCTGCAAAGAAAACGAGGG
AAAGATCGATGTCTACGAATGCAAGACAAACTCCGAAAAAAATGGTGTCACCGCCTTTCG
ATTCATCGCGGCTGCTGCCTTCAAAACCTATTGCGTCGGGACCGGCCTCCAGGGGTACAG
CGTTGATTATGCCCTGCCAAAGAAGGATTCGTCGTCGCCAAAACTGCCAGAATCCAACCA
ACCGGTCAAGCGGATGAGATACAGCCATTTCCTGTGCAACGTTGTGCACGAGGATCTTGC
ATACGAACAGGGAGTGGACACCCACGCTGCCAAGATGGAGCTGAAGCACGTGGTAGACGA
AACGTATCACGGACGATTGCCGGCCGAGCACGGCCACGGGACGGAATACCACGCCCCAAA
GGACACGCAGAAACGCTGGATGAAAATGGACCCCCTGAATGTCATGAATCCTGGCGTTGG
TGGATTGTCGACAAAATATCGATACGAAAAGGAATAAAGCAATTCAAATCGAGCGCTTTT
CGAACTCCCGAGGTCTCAATCATTATGATAGAATATCCTGGCACCGTCGATGGAAATCAA
TTGATATTGTGTATTTTTCTAAACTGTCTAGAGCTTTTCTATTTGGAAGAGCGCATCAGA
ATTCGCAACATACGTCCAGGATTACAAATAATTTGAAACAACATTACAAAAC
>comp2297_c0_seq1
CGCCCACGTTCGGATCTGTCCCATGCCTTGTCGGTGCCACCGCAATCGATGAACCCATCT
AATCAGCAGTTTCTACAAACAACAGCTTGATTTGTCAGGAGCTAACTATCCTACTGAAGC
TTTTCAACCGCAGCTATAGCATCAAAACAGGAAACGGGAACAACAACTAGAGGAAGCGCT
AGCAACAAAACATCCAACGAAATTCTTGATTCTGTTTTTGCCAGTGATCGAAACCAATAC
GAATAACCAATAATCATGAAATTGACTACAATCGCAACAAACGCATTCCTGCTAGTGGCA
GCGGGATCCTTCCAAGGGTCTCAGGCATTTGCCCCTACTTCACATACACAACACAAAACT
AGCAGCCAACTATCTATGGCCCAATCGGGAACGCTTCCATGCCGTCCCATTGGAGTCGGG
CACGCTGCCCCCAAAACGGTGGTAACGAACAAGGATTTGGAGGCCGTAGTTGAAACAGAC
GACGAATGGATCCGAACCCGCACTGGAATTGCCCAGCGCCGGCTCCTGGTAAGTGGCGAG
TCCCTTCGGGAGCTAGGTGTCAAGGCCTCCAAGGATGCCTTGGAAATGGCAGGCGTTTCG
GCCGAAGATATCGATATTGTCATCTGCGCCACCAGCAGTCCCGAAGACATGTTTGGAGAT
GCCACCGCGATTGCTGCCGAGATTGGATGCTCGAAAGCAACCATGGCATTTGATTTGACA
GCCGCATGCAGCGGTTTTTTGTTTGGAACCGTCACGGCGGGGATGTATTTGAGCAGCCCT
AACACGAAACTGAAAAACGCTTTGGTGATTGGTGCCGATGCCCTTTCCAGAGTTGTCGAC
TGGGAGGATCGAAACCAATGTATCCTTTTCGGGGACGGAGCCGGCGCAATGGTCGTCACC
AACCAAAATGACAGCGATGACGAAAGCGATGAAATAACGCCGGGATTGCTTGGATACGCC
GCGCATTCCAACGGAGAAGGATACGAAGATTTGAATTGCGTATACAAGGGAACTCCGGTT
GAAGTATCAACACCAGATCCAATCACATTAGAAAACTCCGAGTACAACAACCTGGCTATG
ACTGGCCAAAACGTATACAAATTCGCCACGAGAGAAGTCCCCCGGGTCGTCACAGAAGCT
CTCGAAGAAGCCAACATGACCGCCGATGACATTGATTGGTTACTACTTCACCAGGCCAAC

ATTCGAATTATGGAAGTGGTCGCCAAACGCCTAAAAATTCCGAAGGAAAAGATCATCACA
AACTTGGCTGATTACGGGAATACCAGTGCGGGATCCATTCCCATCGCTCTTAGTGAGGCG
GTCCAGTCGGGACAAGTGAAGAAGGGGGATGTCATTGCGTGCGCCGGATTTGGTGCTGGT
TTATCGTGGGGATCCGCCATTCTGAAGTGGGGCTAGAAAAGAAATGACAAAGCAAACAGT
ATCATCAAGGCATTTGAATTGATGACGACATTTCCATACCACCGAATATACCGTACTGCC
ACAGCCCTCTCCGACCATGAAGCATATGCCATGACTTTTCTAAAAAAGGCATACTTCGAA
TGCCATTGCTAATGTACTTTTCTAGAGATTATCTACCGTTCTCCCATACCAATATTTTGT
ATTCTGCTAATTCTTCGTTTATTATTCT
>comp2298_c0_seq1
CAAGAACCGTCAAGAACACGACAGATCAACGAAGACAAACAAAACCAAGAGGACGTTGCA
TCCAAAAGAGAGAATCTTTGGAAGCATCGTCTCCCGTCAAACGCACCAGCCACACAAAAT
CCAACGGCACGGCATACGGAACCAAAATATAACACAACACAACACAACACCAGCACGAAC
GAAGAAGGGCAACGCGACATGACCGAGACCGACGAACCCATGGGCGAGAGCGAGGGAATG
AGCAACCCCACGACGCGTCGGAAGAAACCTGTCAAGCCGGCCCTGAAATCCAGCAGCAGC
AGGAGCAGCGGCAACAATGGGACCACGGACCAGGATTACGACCACCACACCACCACGGAC
GACGACAGCGAAACGGACAGCAGGAAGACCAGCAGCACCGGCAAGTTTGCGAAGAAGCGC
ACCAAGAAAGCCAACCGCACCCTCAAGTGGGACGAGGCCATGATCCAGGAACACGACCAG
CTGCGGGGAACCCGAATGAAAATCGAGGAGGCCAACACGCCCTTTGCGTATTACGACAGC
GGAAGCGAAACAGACGGCTCCTTTGGCAGCGCCCGCGGGATGAGCGTGGCGAGCAACGAC
AGCGGAGGCAGCCACCGGGAGCGTGGCAAGGGGGGGCCGCCGAACCGCGCGGGAGGGATC
TGCTGGGACGCCCTCACGAACAAGCTGGAGGCCCACGCGGCCGTTGCGGCGTCGTCGTCG
TCGGCGGCGTCGGCCCGGTTCGCCCCGGGGAGCGGCGGCGAGTCGGAGGGCGACGGGGAC
GGCCACCTCCGCCGGCGCGACGAGCAGCGGGCGGAGCTCAAGCGCCTCGAGTTCCAGGAA
CACCGCAAGCGGCACTACAACGAGATGGAGGTCCTCCGGAGGTTCCGGCGGGAACACCCC
GACGGGGAACCCACTGCGAACGGGCACGGAGAGGACGAGGGCGAGGACGAGGACGACTAC
GACGAGCAAAACGACGGAGACGACGAGAACGATTGAACGAAACGGAATGGAAGGAAACGG
AAGGAAAGGAAAGGAAATGAAATGAAACCAAAGGGAAGGAAAGGAAAGTTAGTAAAGCAA
AGGAAGGAACACAGGATCAATCAAAGCAGAGCATATCGCAACACAAATCAAAACAGAACA
GAACAGAACAGA
>comp2299_c0_seq1
GAAGTACTTCGCAAGTACGATAGCATGCCATCAACCATTCACTGATTCTTTTGTTACCGC
TGAACAATAAAAGCTGTAAGCAACAACCATCGCAATCCACCTGCACCTATTCCAGGTAGA
TCGGGAGGAAAGCAACGCAACAAGACCGGAAGAGCAGCCAACAACAGCAGCTAACAACAC
GATAGCATCATGGCGCGGCCGGCCGAGAAAGCCCGGGCGATGCTCAACAAGTGGGTCAAG
ATGCGGGAGGAAGGGAACGCCACGCCGAGCGTTCGCGGAGCCCGGCCGAAGCTGGCCTCG
GAGTGCGAGCACCTGGGGGACGCCGAGTTCTACCGCACCCAGATCAACAAGGAGATCACG
GGGATGATCGCCAAGATCCAGAACCCGGCCCTGGGCGAGCACACGATCCGGGACCTCAAC
GACGAGATCAACCGCAAGTTCCGCGAGAAGCACCACTGGAACAAGCGAATATTTCAGCTC
AGCAACGGGACGATCGATTACAACAAGCGCGAGAGATCCGCCCGGATCGAGGAGGGAGAC
GTCCAGTTCCTGGCGGTGACGAACAACGGAAGAAAACACGGGCCGACGTACCGGTATTTT
GGTGCCGCCAAGGACCTGCCGGGGGTCAAGGAGCTCCTCGAACAGCAGCAGGAGAAGATG
CGAGCGATGAACCGCAAGAAACGAGGGCCGCACGAAATCTATAAGCACGTGGGGGTCGGG
TATTTTGGGTGGAGGGACGAGGAAGACGGGGTGCTTTTGGAGCTAGAGGAAGACGCTTTT
CGGAACCACAAGAGGAACAAACAGCAGAAGACCGACGCGAATGGTACCGGTGGGCCCGCC
GTGCAAGTCACGGAAGTGGATCTGGACAGGCTTCTCGAAAAGGACTACCTGGCGGACGTG
CCGTCACAGGAAGAAATGAAACAAATCATACTTGCCGAGAAGAAAAAGGCCTTGCTGGCA
AGATTTTCGGTGTAATCCATCGGCGAACGATGCAATAGGCGTGTTCCTCCTAACTACGAC
TGTTGTACAGGG
>comp2300_c0_seq1
CAGCAATCTTTCTTCCAATGCAAGATAGTCACAGTCGAAAATATCGAATGAATACGATAC
AAGGATACTGAATAATACAAAATAATACTTCATCATGATTCACCCCGCGAAACGGATCAA
TCAAATCAACCTGCAAATAGCAAACGAAGAATACGACGAAGCAATCGTATCCCTTGTCGG
AATCTTGAGAACGATAAAACTCGTCCTGTCCGGTGATGCAAGGATCAGTATAGAATCAGA
GCGCGTTGAGATAGACAGAGACAACGACCAACGCGATGATACCACACCCTCGTCAGGCAC
AAGTCTTGAATACGATTTTTTCTCGACCCCCAAGAGTCCTTCGATTCTGGAAACAGCAGA
CGTCGGCAACGAATGTTGTCACAGTAGCTACTATCAACCCAACCCCCAACGGACCCGAAG
CGATCTGGCTTTGGCGTCTCACAACAAGAGCGACGAGAGCACTACTTATTGTAGCGCACA
GAAGCACCATCCGCAAATATTTCGTGATCCCATGATGGTGAGGGGCAATTTCTTTCGGGC
ATCCCTCGACAACAAAGTGTGCCAACAACTCTCCTATGTCGCCCTCTACAACCTTGCGCT
ATCTCACCACCTGAAATCGATGCAACTGTCCAGGAAGAAGTCGGAAAAGCAAACGGAATA

CCTGCAAAAAGCTCTCTCCCTCTACGAGCACTTCCACGAGGCACTCATGGCACAGACGGC
CAGCAACAGCATTCCGATCACCCATGGGATGGCACTCCTGGGAAACCTGGGGCACATTCA
TCGAGAGCTGGGCAACCAGCGCAGCTCGGAGACGTGTCTTCAGTGCCTGTTGTCAAGAAC
GATGTACCTGGTCGTCACCGGACAGGTTGACAAAATGGGGGATCTAATGGACACTACGTT
CGACATTGTCTTCCCCTGCCTCTCCATAGGAAGCCCCGCACCAGCGGCGTAGAGAACTAG
TACCTACACGCTTTCAAAAGGAGCAAACACACACGTGCTGATACACGCCATACTGGCGTC
GAACTAAAGCAATCCCAACATCCATAGACTATAGATGCACCATATTACTTCGATCAAATA
GTGTTTTATTAATTGCGTCCAATTAATGTCCTTGGTTAATGCTTCTCCCAGTAAAAGAAC
GGTTGGTTGCCTTGACTGGTCCCAGTCTTTTGGGTGTCCATAATCCCGATGCACTCGGTG
GACGAAGAAACCTTATTCGCTCCTGTGTTAGTTCGCATCAAAGCATAACATATCTAGTGG
CAGTTTCGAGCAAAAGAAATTCCTTGAGTTCTTTTTGTTTATCATCCTGAGACAATACCA
GCGAAACTCGAAACCAGCCCTAGGTCTTTCTTCAACCATTCGCCGCAGTATTCAGCTGTA
GCAACCCTCGACTTCAACACATATAGTTGTATAATTTGCAAAGAACGCGAACGTTGGAAC
AAAATATTTTTCGATGATATAATTTCTAGACCATGAGTAGGCATTCTGCAAAATACTT
>comp2301_c0_seq1
GAAATATTTCTTCGTACAATTACAGAATTGAATCGTCTCATATTCATTCAACACAACATC
TTACAGACAATTTTTCATCATAATTAATTTCAGATTGTTCCCGTTATGACAAGCTATGGC
AACCTATGTAGTTGCGAACGACACAAATAGCCGGCGACGACAATCGTCTCAAGTGATTTT
GCTCTCTCCAGAGGAAATTCTTGGTTTGGTCGATGCAGTTGACGAGGATGAGGACAACGA
GACGGAGGCCAAGGTCGCTATTATAGAATTAGCAGCAACTTCCACTACCATGTCGGTTCG
GGTCACCTCGCAACAACGACATCGACGTTCTGGGCCATCACCAACGGATCTGCATGAATC
ACCACAACAACCGCGAAGAGTGTCCTGGAATGGAGAGGCAATGATTTCAAGTGATGATCA
AGGCATCATCAGCGATAACATCGGCCTTTACGACGACGGCATCGTTGATTCTAAAGATGA
CAAATACGGTGACGACGTATCCGATGACGATTCAATGACAACCAATGCACCAAATCCACC
GTCAAGAGAGACAAAAAATGATCTCAAGACTCGTAGCCATTACTGGAACTCATTAAACGA
TCAGCCCCGAGATATATTACAAGCTACGAGAAAGAATACTAACACAAACACATACCACAG
CACTGACTATCCGATGAATGCTCGAGCATTGCCGCCTGCTGGGATCATAGACAATCCGGT
CTTTCGGCCCATCGATGCTGATCGCGAGCGCGGGAAACAATACCAAGAAACTACGAATCG
CAGCTGCGAAAACAGCAAATGCTACTACGACTACCACTCAAATAGCCGCACTCTTTTTGA
TTCTACCCGCACAAGTTCACGACACAGCAATAGCAACGCCAACAATCACAAACAGCGAGC
TACATCATCGCGGTTTGAAATTTTTCAGCAAACTACAGGACTAATATTTTGAGACACCCA
CATAATGATACGCACCGAACTGGATGCTGTAGTAATTTTCGATTAAAATAGCAATTACAA
ATGCGCAGATAATTCAAATCCAAATGGAGTAAACACAAGACGA
>comp2302_c0_seq1
CGAAAGAGAAAACAGCGGATTTGGCACGATCGAAGCAATGAGCTCGCCCATCAAGGAGTA
AACAAGGCATAATTGATTCCAATCTGAAATGCTGGCAATAGAGCGACCGTTGGAGGCGAG
ATCCGACGAAACAGAAAACAACAGATTTGGCACGATCGAACCAATGAGTTCGCCCATCAC
CAAATGGACAGGGGATATTGGATTCAAATCTGAAATAGTCCTAAAATAAAGGCAGAGTAA
TGGGAATTTTGCAACAGAGTGACCGTAGGAGACGGCTGTGACAAGGACATCGGCATCTGC
CCATACTACTCTGCTGCCCAGTGAGTTCGACAACTGAAGGCAACCCGGTCCTGAGGATGG
ACAATGTGGGCAGTTGCTTCATCAGTCAGACCCGTCGCTCTTGTAGACTGTGCTATCTTT
GGCGACATCGCAGTCTCGAGCTAAAGCTCTCGACTGCGCTTACTATAATTGTCACACTAT
AGTACTACTTCAACATGAGCTCCCCCGATACAAATTCTGAGTACACTCGCTGTACGAGCA
ACGCCACAGATATGACATATTGCAACACTCGCTCTTTTTTAATCCAGCAAGACAGCTTGC
AACGAAATCTAATTCGCAACTACAGCGATTTGTGTCTCGATCCCAATCAAGGACATTGCT
TGTAACTAGGAAACTGGAGCAAGACGGCAGAGTTCTCCCCCATTCGGAGCTGCTACTACT
GGTTGAAGAAAGGTTACTCAAATTTTACATCTCTAGCATCAGAGCACTCTTACAATTGAT
CGCTATTAAAGTTCTGACTTTTTGTCATTTCCGTGTACACGGGTCATTAGCTTTCTCGTT
CCTGTTCATCACGTGCTGAACGCATCTATCTCTTTGAACTATTGAATCACTTACGAATCA
ACACCGCCTCACTGGCCACAATGATGCCTTCATCAGAATCTTGTGCCTGCACCGCCTCAG
TGGCCATAATCACTCTTGGTGGAGCCGCACCAACATTGGTTGGGGGGTGAATGATCGTGT
GATCCAAAATGAAGTGAGCCATCTTGTAAATATCTGTTTGCTTTGGCGATATATTAACAG
AGGAGATTAGGAGATTACAAACACGTGGTTTGTCCGTTTGGGTGCATCTTGCACTAGTGG
TGTATAGGGAGATACTAGCCTTGGGGTCGAAACGAGCAGCTGTCTCGAAGAAAAAGCCAT
GGATCGACTTATAGGGTACGCTGACGAAGTCCATTCGTCCAGTGACAATGATTAGTCGGA
GATTTGTGTACAGAAAAAATTTTGTGCCCTGTTCCAAAGCCTTAAGAACAGTTTCTTCTT
CCAAAAGCAACGGATACACGTGGTGAAATTGGACGTTCAATTGTTCTGGGTTAATTTCGA
TTGCCATCGGAAGAAAACGATTGAGCCCAAATGGATTTTTCTCGTATCCAATGCAAGTTT
TCGGTCTGAAGACTACTTTTGGATCAGTATGGCATCCGACGATGAAACTGCTCAATATCT
CTTGAAGTTGCAAGACATCAGCCGTCCCGGCGAGGAATGAGAATCCTATAGAGGCAAGAT

TCCATCGATTGCGAGTATAAATGCAAACAGATGATTTCATCCCCACAGATGCAGGTCCCA
GATAAACGCTGTAATAACGAATCGAGCGGTACGGCAGAGACGTGTACAGAAACCTCATAT
TCGATAGACCAGATACCAGAATAGCCATTACACGAAGATTGGTGCACAGAAATATATGTG
ACACTGACCTCAAAGCCAGAAAGATCTTTTCTTCATCTAGGAGGATTTTTGACTTCGTAT
GGAACTCTCTTTCCAATTCGATGGGATCCAAGCCTCGTTGAGGATCGCCAAGCCATTGGA
AAGTACGACCATAGTCACGTTGTTCTTCTGTCAAGATAGGCATTGCAACAGGAGCGCCCA
GCTTCAAATCGTCAACCTCAATAATTCTTTTTGACAAGTAATATTTGAGCCTGATTGCAT
CAACACAATTCTTGTCAAACTCCAAGCACCTGGTAGAAAAACCATCGTTGTAAATTAATG
TAAGAAACTGACAAGCACAGC
>comp2303_c0_seq1
TTCAGATCTTTCGTCGAGCTAAAGTTGAAAGAGCCTTCTTTGCCAGAATTACGATGAAAG
ATTCAATATCTGCCACAATTATCATCGATAATACACAAATTTGTTGGATGTACCAGGAAG
ATGCGTCTTAACGAAACCGAAGAATTCAGACGCCCGTCACCCCGTTAATTTGAGATGGCA
TGTTCCGAAATTCTTACGAAAAACTTGATGACCTCATGACATTTGTTGTCTTTTCGATCG
TTCTCTGCTGTGGTGTCGAAATCCCCATAGAATGAGGATATTTATCTACTCAACATTCGG
ATTGAGAAATACACAAACGATAGTTGGAAACGGCATTTGAATTTTTAATCCGAGTTGCCA
AATCTTTCAAAGTTGCGAACATATTTTCCGATTCTCTGCTTCAATGATTGTCGAAAATAG
CTGGTTACATGCCGATGATCCGGTACGTTCAGTTACCTCGAAAAAAACTATTTTATCTTC
TCGCAATCAAACATCGGCAAATGAAGGACGTAGAGAAAGTGCTCCTGGTAGGACGCTGCG
GTTCCGCAAGACTACCTCCATACTTTGGATAGAAAGTGCTCGAGATATGTCAGATGAAGA
AATTGACGCCTGCTATTACCAAAATCGAGACTACGAGTCTTTCCGCTCTCGCGAAAAGCG
CATGTCCCGGGGTTTTTCTGCCTGGAGCTTCATGAACGGGACGAAGAGCGAAGATTTGCT
AGGGGTTGAAACGCGAATGCAGCGTTTTCACAGACGTCAAAGAAGCAAAAATGCTGTCTT
CGCTGTAATTTTTGAGCAAGAACTTCGTCAGGAAAATGGTCGTGACGCACATTCTTCGAC
TGGAGATGATGATATTATCCTTGCACAGGTTTATCAGCAGTATACGATGGAGAGCACACG
ACTGGCCAAGGAAAGAGCCGTGTTGAATGCCTCTCAAGTCGAGAGACTAACATTGCGCAC
CAATGGATCGTTGATGAACGACGTTGAAACGGAGGAAATGAACAAAGAGACAAAGTCCGA
CAGAACAGCAAATGTTGGGTCCCCGTGGCAGATTCCAGTTTCCAACCGAAACAAAACTGA
CAGAAACCGCTATTCGGAGAAAGTGAGATATGTTTCATGCTCTCGCCCCATTCTTCCTCA
ACAGTATTCTGGAATGTCAACTCCGGATATGAAATTGGACGACCAAAAAGAATCCCAAGA
TTTTCGGTACGAAATGCAAACTGAAATGCAGGGTCGAGGATCAAATTATGGAAGCAGACG
ACAAGAAGCACCAGTGCAACAACAGATTAACCGAGCTGAAGAAAAATTCCACTACAGGAG
ACCTACAGGTGTACTCCGTTTGAAGGCCCAAGATGAATGTCAATCAATGCCGAATTATGC
AACTGCCAAGAACAATTATGCGGGTACTCTCGCCCAGAAGTGGATGTGGAACCCAAATTA
TTGTGATCTAATTTCACCATCTTCTCCGGTCCCTAGAGTTTTCAGTTGGGATCTTCATTG
AATAATCCGTGACAAAACATGCCCGACAACTAGATTTATAGCATCCACGGACAAACGGTG
GTTGTAAGTCTGTGTATCGCTTCGATAACTTGACGGATGATCACATCAGGCAAATAGTAC
CCAGAAATTCGTAGAATTAGATGATGCGGAGTGAAACACCATTAAAGTGCTTGATAATCA
GGAT
>comp2304_c0_seq1
ACGATGGATATCATTAGTATCAAAGTGTTGAACCATCCCCTGCATTGTCGTTATCCGTAG
CTGCTTCCGTATACTGAGCATTAAATCAACCAGACGAATATAGCGGCATCGGAAAACATT
CCCATTCCGTTTCGAAACTCGATACCATTGAACTGAAGCGAAAACACCAAAAGGCAGCGA
ATAGCATTACACTATGCGGTCTGTGAAGTATCTTGTTCCGTGCTATCTAACGGCATCGAT
CGTTAGCATTGTTGCTGGATTCCAGCAACAGCAACCGAACCAACAGTTTCGTCTTCAACG
GCGTGTCGTCGACCCGTCGACGCTTTCGAGGCAAAAAATGCCAGCGAAAACTACTCTCTC
GAAGAAATCTAACGATGCCTATTCGAGAACATTCGGGCGGACAACTCCTGGTTCGACTGC
TACACAACTGAATGCCCTCTCGTCGTCCCCTTTGGGTGCACTTGCAGTGCTGGCAGGAAT
TGTTGTGGTCCACGAGAGTGGCCATTACCTAGCAGCTCGTGCATTTAACATAACGGTCGA
AGAATTTTCCATCGGATTCGGTCCCAAGCTGGCCGGATTCGAGGCCCTTGGGAATGAATT
CAATCTGCGAGCGTTGCCGTTGGGTGGCTTTGTCCGATTTCCGGAGAACTACGATTCGGA
GGCGGTTTTCGAGAACGAGGAGCTCAAGCGCAAGGCCAAGCGAACGCTGCGCCGGGAGGA
AAAACGAAATCCAGAGGACGGAAACAATTGGAACTGGAAGGACGAAGCATTGGATGCAGC
CTCGCTGGGGTACTGGGGAGAGAAGAAATTCGAGGAGGCGCGCATCGAAAAGAGGATCGA
ACGCGAAAATAAAGTAGCTTCGACAAAACCCTTTTGGGCAGGTTTGTTTGGTGCTTCAAA
TGTTGCTGCCAAAACCAATGGTGGAGAGAATAACAATAGCAAGGAAGACGGTGAACTAGT
TGACATTGACAAAATCTATGAAGACCTGGACGAGCTTGAAGAATTTGAGGTCGAATATTT
TGAGGACCCGCAACTTCTACAGAACCGACCCTGGCAGGAACGAGCTGTTGTTCTTAGTGG
TGGTGTGATCTTCAACCTTATCCTGGCCCTGAGCATTTATTTTGCAGCGATTGGACCCCT
GCCCGTCGGGAGCAGCGAAGGATTGCCCCGTGCTATCTTCGACAATGGCGTCGTAGTTTC

CCAGGTTCCTAGGTCCGATGGTCCATCGAGTGGTTTGCTCCAGCGTGGGGATGTTATTAC
GGAAATCAATGGTCGACCTGTACAAGTTCTTGGTTCTAAAAAGACCTCCGTCGCGGCGGG
GCAGCAACAGGTCACGGATGTCATTTCCGCGATTCGAGCAACACCCGAAGGTGAAAGTGT
GAGTATCAGCGTGTTGAGAGGAGCAACGAAGCCAGTGGCTACAACAGAAGCGTCATCCTT
GGATGCAGTCGCCACAAAGCAGACAACAACGGAAACAAAAACATCAAAAACAACAACAAC
GGTTCGCATCAAACCCTCAACTGCCGATGGGAGCAATGTGCAATCGATCGGAGTCATGCT
CAGTCCCAGAATCGAACGATTGGAACGATTGAAGAGCGACAACCCTATAGTGGCATTTCA
ATTGGCCTGGGGATATCTCTCTGATATTTTCACGCAGACACTCCTCGGAACACTCTCTGT
CCTGACCTCGTCTCTGTCGCCGAGCGGACCACCACCTGGTCAAAAGATATCGGGACCCAT
TGGTCTCATTTCGCAGGGATCAAATGTGGTTGCGACTAAGGACTGGACGGCAGTTTTGCT
ATTTGCCGCAGGGCTATCAGTCAACCTTGGTGTCATCAATGCCCTCCCTCTGCCGGCATT
GGATGGGGGGCAGCTGGTTTTCGTGGTGGCGGAAGCGATTACTGGAAAGAAGGTGGATCA
AAAATTGCAAGAGAAGGTAACATCTGTTGCCGTCTTGTTCCTGCTATGGCTGAGTTTTTC
GGCTGCCATTGGTGACGTTGGTGGTTTGTTTCAATAAAATATATTCACGAATAAGAAAAT
CCAGGCAAGCATGATAAACGAAC
>comp2305_c0_seq1
GCGAACGACAAATGTGTGATTTGTGAACACTTCAGCATCTTCACAAACAGCTCCCAGTTA
CAGTACAGGACCATTGCCTTTGCTTTGCGCCCTCCAACACTCAAAGAATCAGCTTGGTAT
TTAGTTCCGACCCCGGATGATAAGGGAAGGAGATAACAACGATGACACCGCCGTTGCAAA
ACGACAAGACCTTTGATGTTCCGAAACGGCTTCGAGGCATGTTTAGTCCTAAAAGAGTGC
AGGTGCTGAAGAAAATGGGAGCCGGCGCTATCATTTCTTCCCTTCCTGTACTTGTGTGGT
GGAAATGGGCTATTGATCAGCGCAACAAACGTGCCGAAGACGTCCGAACGCGTGTCCGTG
TTCCCAACGTCCAAACAATCGATGATTTAATGATAGAGAGGTGCAGGCCGGGAGATATCC
TTTTGTTTGACCGACGATGGGAGCATTGTGCCGCTGGTCCTTTGGCGGCTTTTGTCTGTG
CCTTGGGACGGACTGTTTTGTGCTTTGCCGACAACAGTAAAGCCATTGGGGAAGGAAAAT
TTGACCATTGTGGTATTGTTGTCCCGGGGTATGTGAAACATGAAAGTGATAGATACGATC
CAACCAATTTACTACTGTTGGAAGCCACTGCTGCGGAAGGTATCGTCGCCCGTCCGTTGT
TGACCAGGTTGGAAATGTCCGAATCTCGCTCGGTTGTCTTGCTACCACTTGCATTGCCTG
GGGAAAGAAGAAATGATCAATTTTATGAGTCGTCGTCAAGCGTTATCGCTACCGAACAAA
TTGTCAATAAAAAATTAGAGGAATTCCGAGACAATTGGGTGAAATTGTCGAAAACGATGA
ACTATTCCACAGCCCATTCCACCCTGGGACTGATTGGTGCAATAGCCTACCTGTTAGGAT
ACCATACTGCGAATCCCGGCCCGACCTCTCCTTCGGCATGGTTCGTTACGAAAGCGTTAG
TCGACGCGAAAGTCGCCCAGAATGTTTCTGAACGCTCCGCCAAGGAAGCAAAAGTCGAAG
ACTTTTTGAGAGACTACAGATTTCAGGATCAGGATGTGATTCGTCTGCGGCCGGGCTGGC
GGTTTCTTGCACCCGTTTCGTTCCGAGAAACATAACTGATGAAACCTTTTTGTATTTTCT
TTTTATTTCAACCAACAAAAGGATTAACAAGGAAGTACCAAGACACAGAAATTGCTTGGT
TATTTGTTGCAAAATACTACTACGTTGTACATTCCTATCTTTGTCAAAAAGTATCTGAGA
CACCATTCGTCTTTCTCAACACCAGCGTTGACTAGTCAACAACAACGCAGCTTTGGTAAA
AGAAGTGTTAGTAGTGCTGCTTCTTGCAGTAGGGCAATGTCTGTTGCAATACAACACTTA
GCTTCACTGGTGCACCATTACTTTTGAACGATAGCGGAGAATGAGACGGTTTGTTGTGAG
TTACCATATCCTCTTGCAAAACAATTATGAAGAAAAACCAAATTGCTGATGATCATGAAG
AAAAGCAGCAATATGTTACTGCATGGTAGATAAAAGTAGGGATCCTTCATATGGATTACC
ATGTGGCTATTACAAAACAAACAATGAAATAGATGCCACTATTAGCAGTGAAAGCTTGCG
CATTTAATATATAATCTTGTTGTGTGCTGCACATACATGCAGAAGGG
>comp2306_c0_seq1
CTCGGCTTTCAAAGAATGCTATCTGGTACGATACGAGCCCGGAGAATAGAACACAACAAA
AATACAATGACCAGGGGGGAAATAGGAAAGGAAAGCACCAGTAGCATCATTCGTTGCCAT
GGTTCGACGGCGATGCTGATAGCGCTTCTGCTGTCGTTGACATCGGCATGGACATCGGAG
GCATTCGTTGCTGTGTTGAAATCGCCGGCGGAATCAATGACCATGTGTAGAACGATAGGG
GTAGCTGCGATGAGATGCTCGGGCTCGGGAAACATCTCCCCCATGCAATCGGGGTTCTCG
ACAACTCTATTCTCCTCCAACGAGGACGAAGACGATGGATGGGGAACGAGTCCAACAGAA
ACAGAAGGGTCAGGAACCACACCGTCGACGTCGCTCGAGACGGACCGGAAACTGAGCGAG
CTCCGGTCTCTCCAAACGCAAGCGTCCAACAGATCACAGGCAACCACCACGAATTCTTCG
TCTTCGGGAGAGGAGCCGGAACGAGACATGTTCATACCCATCATGGCCGTCGTTTCGTTG
GCGGGATTGTTTGGCGCCTACGGATACGAAACGCTCCGCCTAGCAAGCCGTGGGGAGCTG
TATCTACCGTTTTGATTTCATTCTTCGGAGACGATAGGCAAAGACGACGTCCAACCGACA
ATTCCTGTGCACACACGGACACAAAGCGCGCGGGGGACCCGCAAATCGATTGATTCCTAT
GAATGGGAATGTGAAAACTAGACGTGACGACGACAATAGTGTTGTTTCATAATGGCTACA
TTTAAAACATTT
>comp2307_c0_seq1

CTTTTGGCGCAACATCTCCAGTTGATTCCACATCGGTGAGAATTGTACAGTCCGAATCTA
TCTCCGATTCGAATCGAAATCTTTCCGGTTGCATCGTTTTTCATTGAGCAATTCATATCT
CCTAAAATAACTATATTTGAAAACCAAAGATGGAAGCCGACAATGATAAGGACGACCCCT
CGGTTGAAACAACAACTAGGAGCCAAGACGAACAGCAGCTAGATCATGAACTGAAAGATG
TTGAAGGAAACAAAAACATAATGCCACGGTCAAAGGACATAATTTCTGGTAACTACGGAC
ACAGGAAACTCGCGCCCGAAAACCATGGAAGTATATTCTGGGATTGTTTCGTCCGCTCCT
TGTCTCCTCTCCACGCGGGAGGCCAGTTGACGAAGTACGAGAAAAGCTTACTCGCTGATA
TGATGATGGCGTGTGTTAAAAAAATGGGAATGCGTTTCGTAAAAAGAATAAGAGACGAAT
CGCAGAGCACAGGGAAGGAAAAATTGCAGAGTCAGGAAGGTCATAGAAGCGTTTGGTGTG
AGTTAAGAACTTTTAACGCGAGATCCAAGACCTTGAACGACCTTCGTCGTTACCCAGATT
ATAAATGGAAAACGCTACATTTAGAAAGAGGGGATAGCTCCAACATTGTAGCCTCCGCCA
AAGCTACAGCTTCAATTGTTGCTGAAACGATACTCTCAAGCAAAGCAAAACGAATAAACG
AAGCAATATCCGTCCATGAAAAGAAAGACTTCAGGAAAAGCGTTTTGGACCTTGATGAGA
AGAAGGAATATTATCAATACCGTTTCGCTATGAATGCGGTGGAAAAACACATACCAGAAA
ACGGGATTTTTGATGCCTTCGACGAGAAGAGCAATCTTAGGAGTAACAACTGTACCAATT
ACGTTCGTGCAGAGAGCGACAATGAAACAGATATCTTTGTCGCTCCTGGTATCGCTACTG
ACATGGTTCTCGGACAGTCGCTCATAATGACGCAAGATCATCGCTGTCAACAGCGTTGGC
TTCGGGAAGAACACGCCAAAGCTAGGAGAGAAAAGTTGCAACACGACAACGATCTCTACT
GCGCACTCTTTAAAAATGTTTTCGCTGTTCCAAACCAGGAGTTGAAACCCTATTGGTGTC
GGGATGCGTGACGCCTTAAAATTTCCTAATCGAACTCCAACGCGCTGTAACACTGGAAGT
TTTTTGGCAGCATTCCTTGACCATCGGATTCTTGTTCTCTTTCCCCTACTGTATCTCTCA
ACTAGAAAGTGTACCAAACACGCGGAACTACTAGGAGATTCATCAACCGCTAGCCTCTCA
ATAAACGTTAATATACCGTATCTACTTTAGAGGATTAAATCATGCCTGC
>comp2308_c0_seq1
ACGCATGGATTGCAACCATAACCACCGTGTCAATATCCACCAAAACTGATCTCAAACGAT
TTCAAATACATTGCAAGACATCTTTGTACAACATATCAGCATCGAAGCAATAATTAAACA
GAAGAGATTAGACCATAGATTGAATTCCGGAACAGTCCATATCCAATCATTCAGACCGCA
CGGCGGGCAGCCTATTTTCTATTTTATCAAACCAGTCAACCCAAAAAAAAACACAAGCAT
ATAACCCCAGAAACATACATATTCCTATCCACAGGACAATGAAAATTGTGAAGAAGAACA
TCAGCGCAAAGGACGGTAGCGGTACCGTTATGCTTCCGCCGGAAACCCCCGAGGATCTCT
GGCATTCTTACAACCTCCTAAAGAAGGAGGATCTGGTTCGATGCACGACCGTCCGCAAGG
TGATCAAGGAATCCAGCACCGGGTCGACGACCAGCTCCAAGAAGCGCCTGATGCTAACGA
TCTTGTTGGGGAAGATCGACTTCGATCCCGATGCCCTGCAGGTCCGCCTGAGCGGGCAGG
TCCGCAACCAGAACGAGCTGGTCCGCCTTGGTGCCCACCACACCCTCATGCTTGAGCTCC
ACCAGAACTTTACAATCGAAAAGGACTGCTGGGACCAGATCTACCTGGAGATCCTCGAGG
AAGCCTGCAACCCGACCGCCAGGCCAAGCTGGCCGCAATAGTGATGCACAGCGGGTTGGC
CCATCTGTGTTTGGTCACCGGATCGCTGACCATCACCAAGGCCAGGATCAAGACCAACAT
TTCCAAGAAGCGGACGGGATCCTCTCAGAACAGCAAGGCCATTACCAAGTTTTTTGAGGC
CATCTACCAGGCGATCTTGAGGCACGTCGATTTCACCAAAGTTAAGGCGCTCCTGATCGG
AAGCCCCGGATACACCAAGGACGACTTTTTTGCCTACCTCAAGGAAACCTGCGTCAAGCG
GGACGACAAGCCACTGCGGGATGCCGTCCACCAGGACAAGTTTGTCCTGGTGAGAGCCTC
AAACGGACACAAGTATGCCCTGGAGGAGGTCTTTTCCGATCCTGTCATTGCAAAAAAAAT
CGAAGACACCAAGGTCGTCAAGGAGGTTGCAATCCTCGGAAAGTTCATGCGGATGATGGA
CACGGACCCCGACAAGGCCTACTACGGGTATTCCCACGTCAAATCGGCGGCGGACCAGTG
TGCCATCGATTCCCTCCTAATCACGGACGAGCTCTTCCGGGCGCCGACCCCGCAGGTTCG
TTGCATGTACGTGGATCTGGTGGAGAACGTCCGGTCCAACGGCGGGACGGTCTACATCTT
TTCCGCCCTGCACATTTCTGGACAGCAGCTGGGACAGGTCAGCGGCGTCGCGGCCATCTT
GCGGTATCCCCTCCCGGACCTCGAGGAGTTGGAGCTAGAGTACGAGGCAGCGATGATGGG
CAACAACAACGACGAGAGCGAGGACGAGGACGAGGGCATCGATGAGGTCCAGGAGGACCT
CGACGACATGCACTTTTAGCGGTCTCTTCGAGTTGGTGAAGTGCACGCAGCATCCGTGCG
TACTGGGAACACCAATGTTCTCAAGCCTGCTCCACAAATCGACTGCCCTATAAATTTGAA
GGCTATTGCTTGGCCTTGTGCGGTCCTGGAACTCGGCATGGGATGGAACAAAACCAAGAT
CGGCGATAGTTTGTGCCGCGGCAAAGTAATGAGCGATGTGCGCTGAAGAAAGCAAACAAA
ACGGATCGACGCTTGTTTGTTTGTTCGACGTCGTCCAATGCATCAAGAACGCTTGGCTGA
TTCTCATAGCCAGACCAGCCGTCGGGAACGGTGTCTCTGTTTCCGCGTGAACGAACGGAA
ACAGCAACATATTGCGTTCCATTCACAACCAAAGCGTCACAAGAATCTCGTAGGATTCGT
ACGGAGTGATGGTGATATAGCCATAACCTTGCGACTGTCTTACCACCGGATCAAAGGTTG
TTCCCGCTAATAAAGATGGTTGATAATTTATATTCCAAGATGGAAATTTTGTAGTATTTC
TTCGAGAGAATTTTCTGATATCACGTGCTTTTGACACGCCTAACAAACACTCCCAGCCAC
GTTGCTGGAGATATTGAATCGCTGTGTCATTTTACCCGTAC

>comp2309_c0_seq1
TGCGTCTGCACGAAGTTCTAAAAAATCCCCCTTGTGCCGTAGCGCTGTGCACTTCGAGGG
AAAAGTTACTTACTGTGATTTCCAACGAGGAAATATTACTGTAGATTACTGTAGATCAAT
AGATCATCAAACTGCACGGTTGAGATCTCAGAGCTACGAATCGTGTTTCTTTTCACGACT
TGACGCAGCGATCTTGCATTTGTTGTTTCCATTCGACCCATTCGACTTGAAGGAATAAAC
TGTAAATAACGGTTGTTCAATTCAAAAATTTCAAAACTAAGTTCATTTGCAGGAAACAGA
ACCAGAAACAATAGAACTCTCACGATACCGTACGGTACTGTAATACTTCAAGCAGCTTTA
TCTCAGCATCTATGTCAATGAAGCAGTACGAGTACTGGTGCCAGAAGAAAGCGAAACGAA
AAGAGCAATCCTTTACTTCGAAGTACTACGAATTTCAATACCAACAAAGAAACATCTCAT
CATGCCTCCACTTTCTGAATCAGGTCATGGCGGCGACGACGTTGATGGATTCCAGAATGG
GATTGAGCGAAAATCAGGGGCCACCGATCTCGGGTTCACTTCCTCTCTCATGGAGTCGAT
TTCCGCATTTGAATCGAAGATAGAGGCTTGGGCAGAGCATGAAAAATCGAAAACCGATCT
CAGAGCTAAGTCGTACAACAATACACTGATGCAACAGCAAGCTGCAATTGACGCTCAGGT
AACAGAACTAGCAAGTGTTCAACGAGAACGCGGAGCAAATAATTTAATATCAGGAGTAAA
GGAAGGAAAGGGCGACAACAGCGAAAGCAATAAAGAAAACATCGCTTCTAGAAGGATATC
ATTGGAAGAGGAGTCTACAAAAGTACAGGTCGAGATTTTGAGATTGAAGACAGAACGAGA
TAATAGGGAAAAAAGAGTGAAGGATATCGAGCTTGAGGAATCAAAACAGAGTATTCGAGC
AAGTGATGCAGCTGTATTGAAACGAAGGGCGGAAGAAGCGAAAAACACAACCATCGATGA
TCTAACGAGAGGCGTTGTCAACTATAAAAAGCTTGGTTTGGATTTCGAGCAAACCGGAGA
TGGAGAATTGGAGTTCAAATTCGACCAGCTTGACCCTCATGATCCGTCGAGACTATTTTC
ATTTACTCTTTTTATCAACGATGAAGATAACTATGATATTTCTGGTTGCAATCCGAACAT
CATTGCTGAGAATTTGACAGAAATTCTGGAAGAGACCAATGCATCGTCAGATCGAGATAA
TATGTCAACACTAGCTCGTCGTATGCGTCGAGCCTTCAAAGATATTTGTGAACCTGAGCG
TTACTGATTCGGTCGTGCGGTATACAGTTTAACAGCTCGAAAGAAAAGATGTCTGATACT
TTAAGTTGAAGGGTGAAAATAATACATCCAAACGAGCAGTCTATTTCATCTACTTTAGTA
CAACAACGAAATGCAATTGAAAATTCGATGTATTAAACGTGTAATCAAATAATATTTTGA
TATTTCTTTCACAATTAGAACTGAC
>comp2310_c0_seq1
GAGCAGGACAACCTAGAAATCTCATTGAAATCCATCGATCAACACAATCGGAGACAAAAA
ACAACAAGCGAACCAGGAAGACGACGAAACAACAGCAAGCGGCAGCAAACGACAACTAAC
ACCACACAACACCACAAACAACCCAAACGCACACACACCACACAGCATGTCCGATGCCAT
TTCCGTCCTTAGGGGCTGGTCGAACTCCGGCAAGCTGTCGGAGGTGGCCTTTTCCGACGA
GGGAAAACTGCGGCTGGGGGAAACCGCGAGCCTAACGGCCTCGGAAACGGTGGAGGTGGC
GGTCGGCGGGGGCAAGACCTGCCGCTACACGGTGGGGTCGATCTTCCTGCAGCTCGTGGA
CGTGAACCAGTCCCTGCTGGCCTACCGAAAGGCCTGCAAGAAGCACAAGGTGAAGGACGC
GGTCAAGGCCATCGACAAGGCCGAGCTCATTGCCTTTTTCAAGGGAGGGGCCGGGCCAAA
GCCGGCGGTCCCCGAGCACGCACGCGAGGGAGCAGCCCCGGTGTCTTCCTCCAAGCCGGC
GCCCCCTCCCCCCCGGCCGGAGGGGGACCGGGGCCGGGACCGCCATTCCAACCGCGAGGA
CAAGAAGCGGCACCGATCGAGCAGCGAGCGGGAACGGGAACGGGACAGGCACCGGAGCAG
CAAGAAGCGGGAGCTCTCGTCGTCGAGGTCGTCCTCCCGCCAGCCCGAGCGCAAGGAAAA
GGCCCACAAGCCGATCGATCCCGAGATGCTCTTTTCCAACCTCTCGGTGGTGGTCGGCAA
GCGGGAGATCAAGGCCAAGAGGGACGCCGAGCAGGCCAAGCTGCACGCGGCGCTCTCCAC
CGAGGGCTTTGGGACGGTCGCCAAGGAGCTGATCGCCGAGTTCCGCAGCAAGCACTCGAT
GGCCCACGAAATCCCCGTGGGAGACTCGGCCTCGATCCTCAAGGCGGGGGCCGGGAAGGA
CCTCTCGCGCATCCTCAAGCTGTACTCGGAGACCATGGAGAAGAAGAGCGCCCCCGGCCG
GAAGCCTACCTCCGGGAGCTCCAGGGCGAAGGGCTTCTCGGACGGCCAGAAGCGGTCCTG
GCGTCCGTACCTGGTGGACAAGAAGCCCATCATCGTGTTGCCCAAGGGAATGACCGCCCC
CATCACCCTGGTCAACGCCCACAAGTTCTTTGCCGAGTCCCAGTTCGTCAGCCGGACCGT
TATGCAGAAGGAGCTCCGCACCAAGCGGCAGGCCCCCAAGACGCACTTCGAGCGGCAGGT
CAACGCCCGCTTCGGGGGCGGGACCGTCCAGTACGAGCTCATGGACAACCCCCGGTCGAA
GCTCCACCGGCCCGAGGACTGGGAGCGCGTCGTGGCCGTTCTGGCCCTGGGCCAGTCCTG
GCAGATGAAGGACTGGCCGGGGCCCTACTCGAACCCCGTCCAGCTCTTCCACCGGGTCTT
TGGCTTTTACGTCGGGATGGAGGGAGACAACGTCCCCAAGGAGCTCCAGGGGTGGTCGGT
CACGCAGACCCGGGTGAGCCGCGACAAGCGCGGCCTGGACAGCGTCACCTACGCGAGCTT
CTGGAACGCCCTCGACGAGTGGATGGCCATCCACAAGCCGGAGCTGCTCCCGCAGCCGAA
CGAGTAAGCGGCGACGAGCTTTGCGGTGCCCTTCGTTGCGAATACTACTATACTAGAATA
CTATACGAATTGCCACATCCTAAACCAAACCATAAACCAGACACGG
>comp2311_c0_seq1
CTCCAACTGGAGGTTCGAACGAGTTCCATCCAGCAAGAAAACACAAGATTTTGTTGTCGA
TTCCGATGTTCAAGTGAAGAAAACGAAGTCGTCAAAGATCTTTGACTCCTCTCCGAGCGA

GAGTCAAGAAGGTGGAATATTTTACTTCCAGCGCATTTGTTCTCGAAACGAATCTCACAA
GACAAAAACTGCACATGATTCCTATACCCGGGTCTCTGAATCCTCATCGGATTGTGCTGT
AACTACCAATGGCGATCAACAACTGCCAAATTTCTTCGAAGTGAATGTCGACGGCGACGA
TCTATTTGCGGACTTGGAAGCCGTTGCTGATTCGAAAGACGATAGCGAAATATCCTTCGA
CGATTTGATGGACATTGATGTCGAAGACGGCGCCTCCACAGAACACATACAGAACTACAA
CGGCCACCCATATTCTGATTCCATCTACGACGTCGATGGATTCCTCAACGCGACTGTAGA
TAACTTGAGAAATGCTTCCCTGGAAGATGACGAAGACATCAATATGATGAGCTGCGACGA
CACTGCCAGCAACAATATGAATACCTACTTGTCGCCCGTTACAAGCAAGGCGAGTCTTTC
GTCTACTCTTCCTTTTGGATCGCTGGATTCCCCCACGTGTGTCGCACAAGACATGGCATT
GTCTCCCATATTCAGCAGCAGTCTCTCCATGCCGTCGGCATCCACTAGGCCCGCTTTTCA
ACAAATGCGCAAGATTATGACGAAACAGCTCATGGACATAAACAACGGCAGCATGCCCCG
TTCCCTCCACAAAGATTCTCTGCTTCCGTGTGCCGAAGGCATGGACCAGATGTATCTAGA
TACACTACAGAAATTGTCCATTAGTATGGCACGTAGCAATCGAACCAGAAAGTCTCTCAG
CATCCCCCTGCAAACAATGCAAGACGGCAATGATTCACACGGACTACAGCGGCAACAGGA
CTACAACAACCGAGAATATACCCGTGTCAATCAAGTTTTGAAATCCGTTCAAACCTCCAG
TCGACATGTCGATACATGTCTGCAAAGTGTTCGCATGATGAGTCGATAGGATAGCTTACA
AACTACTGTACAATAGCAATATCTAATTCCTTTTTCGTTTTAAACTAAGCACCTCCTCGA
AATCCCAATCTGCGAACGAAACGTCTTCGTCAACTGATATACACGAGCACAAACAGTATC
ACAGGAACGCTTACGAAATGCTTCCCTTCCTATTCATCTTGGCAGCAATGCCAAGAAAAC
ATGCTGTTCCATGCACTTTCGTCCTCGCCGAATTCAGGCAAGCGAATTTTTCAACACGCT
TCAAATATCTGATACGGCGCGAATTCTCGTTCAACAGGTTAAACGAAAGCGGATAAACTG
ATAGCGTAACAAAGAATAACATTCTTGCAATAAAGATCATTTGGACCTGTTTGATAAGTT
CAGAAAAATATTCAACGGAACAGAAAATGGAAGATTTTTCTTTTGATATTTTCCCGATAT
CAAAATCCATTAATGTATCTGGTGGGCAATTTGCAAGAACGTTCTTGTGAGACCACTAAA
CATTGAATTATTGGAAACGACACGAAGTAAAAAGTAGTTGAGCAC
>comp2312_c0_seq1
TAATGCGAGAGTACCCAAGTACCCTACCGCAGTTACCGTTAATATTGTTCGGTCCAGTAT
CACAATCGGGGAAGACATGACAGGCTCAACCAACCTTACCGTGGGTTACGCAATTTATTG
CAATGATAATACCGACACATTCAGAAAAGAGTCAGAGGCATGGAAAACAACTTCGGGATG
ATTTGTCGCAATGATGGGATCGGAACTCAGAGGGCAATGCTGGCATTCAATCGAGGGCGG
GTCTACGACTGTGGACGAGTGCCACCGATTCCGAGACGATCATCTGACACAACCTCGGCA
GGGGCAATCACGCTGATCCGCTTTTTGATGACCTCGCACACAAACCACCAAAGGCACAGG
TTTTCGGAATCCGGATGGCGCAGAAGAATCGAGAAGGGACAAATCCTTATGGACGGAATC
GTTGTAAATGATGCCGAAATGGAACTTACAAAGGCAAACGTAAAGCAGCGGGTCGAATAC
GTCAGGGGTCCATGGAGAGAACCGATTGTTGCCGTGGTTGTACATGATGATACAATAGAT
ACTGGTCCCGAACCTTCGTGCCAGCTCAAGATCTTGTACATCGATGACCACCTGATGGTG
GTTCACAAACCTAGTAGATTGCCCACCATGCCATCGCAAACATACTTTGAATTTTCTGTC
CTCAATGCTCTGAGACAACTCCATTGCATGGAAAAAGAACTGTCCAAAGACAAAGAAGAT
GAAGACAATTGTGAAGTAGAAAGTTATTCGAATACGAATGCGAACACCGATTCAATTTCA
AAAAATCACTGTGTTGTCGCCACATCTACGGTGAGAAGATTGATTCGATCCCATTGCTCG
GCACTGCCCCAGCCTGTCCACCGACTGGGGGTGGGAACCTCCGGTATATTACTGATAGCT
ACATCTCTTTTAGGAAGGCAGCGTCTGACCGATGCGATGCGACTCAAAAGCCACAACGTT
AACAACAACAAGCTTCGAAAAACATACCGAGCACTGGTCTATGCATATCGCCGTGACGAA
AGGCTCTCTATCGCCACCGATGACGGAAGTCCCGAGGTAGATGGAAGTAGCAAGGAACGC
TCGACAAGGGAAGGACCGAAAATGCTAATTCCTGATTTCATGATGATTGATTGTCCGATC
GGGCCGGTGCCGTTTCCAATCGGAGGGGATTCCATTCACGCAGCTTGTCCAATTAGCGGC
GACAGCAATGACAAAATTGACCCTTTGTATAGTCATGTTCACCTTCACAATCAAGTTTCC
GATGTTCAAGAGAACCAGCCTCAGGAGCGGTCAACTACTGCAAGCAATAACAACAAGAAG
ATCAAGCATGCCCTATCGCTGGTAAAGGTCATCCGTAGGAATATTCCCCGCCATGAGAGG
AATGTTGACAAGGAAAATGGCGGCGAGACTGATAGTGACACCTATAGCGGTCACAATTTT
GATACAACGGCGACTGCAGTGGTAGAGATCGAAATACCTACGGGCCGTCCCCATCAGATA
CGGATCCACATGGCCTACGCTGGATATCCCTTGGTGGATGATCCATTGTATCTACCGGGT
GGAATTCCCGACGTTCGGCCAAGACCATTCAAAACTCGCAAACGAGAAGAAGAGGACTTG
GACCAATCGGACGAAACAGAAGATGGTGGTATTGACTGTGATGAAGACAACGGGACGCAC
TCGACTACGATGCGCGTAGCTTTGCCGAGGGATTGCGGTTACCATCTCCACGCCTACCAA
ATAACTCTCGAGCATCCGTGCTCTTCTCGGCACTCCAGCGTTGCAACTATTAGCACTGCC
AACGCCGATGTCAGCGACGCTGCGCCCAGTGCCTATGCCGAAGAATGTGCGGTGAGCTCG
AGTTCGGCGATGATGACATTCACCGCAACTCCTCCTCGGATTCTGCGTTGATAGTCAATC
AATCAATTGGTCTCTCTAAAGCAGTCGCTTGCGTTCTTGCTTTAAATGATAGAATAGAAT
TGCTCATCCTTCTAGTCGGTCAGGTGATAAAACTAGGTGATTTTTTCCCCACTATCATAA

TAATACGCCATGCAGATACAACATCATCATAACAATTCTGTGCGTACGGTAGACCAAAAG
GTGCTATGCTCAATTAGTGTTGTGACACTCTTGCTCGATGAAGAAGATACAATGCAGTCC
CAAAAAAACAATCGATTTACTAG
>comp2313_c0_seq1
AACACACACACCAGTGCCATAAGTAGGAGTTGTAGTGCGTACTCCACCTCTTGTGTGACG
AAACAGAGTGCACGTTCTGTTCATCGAGTTTCTTCCAATTTCCATTTCTGCTCTTTTCCT
GTACGGCAAGCGAAGTCGTGCTCGTGGTCGTAGTCGTGGTCGTAATTGCTAACAGTACTC
GTCTCGACGCATGGAGCGGGAAGCAAGGACAAAGTGTAAGCACCACCGGCAGCACAAGCC
CCAGAGAAAGCTCGCAGACGCCCTCCGGTGTGCCGGCTTCGATATCTTCCACGGGCCCTT
CTCCCCGAAAAAGTACAACGATCTCCTCGAGTGCGAGGGTCTGGAAGGTCTCTCGAGACT
GCCGGAGGAGCGAAGGTGCACGGGCAGCAGCGGAAGCAACAGCAGCGGAAGCAACAGCGA
CTACGAGGCCTACCTGATTGGGAACACTAAACACCTTTGGCCCGTGTTTCTGGATTGGCT
GCAGCTGCGGCGTCAATCAGCAGCAAGCACTGCCACTGCCACCGCCACTACCACCGCCAG
CAGCATCGAAGCACATCCCCTCGACACCTACGAACGAACCGCGATCGAAAGGGTCGTCCG
GGAAAGCCTCGGCCCGGACGAAGAGAAAGGCAAAGGCAAAGACGCCAACTGCAACACTAA
CTGCGACACGAACGCCTGCCGCTGCTACGAGCTTTTCTTCTCGTCCGACCTAGGGACCGG
CCTGGTGTCCATGGCGAGGGTGGCCTCCTGCAGCGGCTTCTCCTACCTGGATCCCCACAC
GCACCTCAGCATCCACCCCCGCTACGGAACCTGGCATTCCTACCGGGCGGTGCTCCTGCT
GGCTTCGGAAGGCCTTCCGCCGGGGCTTTTCGAAACCGGGACCAAGACGGAGACACCGAT
CCTGACACCGATCCAGAACCCCCTTTCTCCTATCGACGCGCGGAAATCGAGAAAGGCCTT
TGACCGGGCCCTAGCGATCACCACGGGCACCACAACGGACGAGCTAATGGACGAGGCCAT
GGAAGAGCTGTGCGAGATGCTCGGAGCCGGTTTGTACAGGGACGTACGCGACGAACGCCA
GGAGGAAAACGACAACGACAACAACAACAAAACCAAGTCCAAAAACGAAACCGTCGCGGC
GGCGTGGATTGCGCTGCGGGACTCCATATCGATCGGCCGGGAGGACTACCGCTTCGGAAC
AAACCAATTGCTCTACCACTACACCAAGGATCCGAGGCACCTGCTGCCGTGACGGTGGTG
CGATGCCACGTCTCGTTTCGTTTCGTTTCGTGTCACTTCAAATCACTTAACTTAACTTAA
CTTGATCTAATAGAACAGAAT
>comp2314_c0_seq1
GTCGACACTATTCTTTACTTTTCATTTACTGTACCTTCTTCAACAATACCATTACCTTGA
TTATTAGACAGAAGATCCTTTGCTCCTGCCAATGCACCGACAAGAATCTGTTTCGGAAAT
TCATTACGAGCAATTTCTCGTCTTTTTCGTAATACGATGCCGTCATCACCAGATCACGAT
GATTCCTGCATCACAGTGAAAACAACACAGGGACGAAACTACAGCGTCGAGCGGTATTAT
ATGCTAAGCGAAATCGAGCAGCACTCGATACATCCTGTCTACCTGAATAACCACCATTCA
AGCTCAGAAGTGCACGATAGTAATGACATTTCTCACGGCGTCAACGAGAATGAAAATTCT
AGTTGCAGTAGAGGAAGCAAAAAAGGACATGACATCAGTCGCAAGAACAACATCAAGTTC
CCCCCTTTCGTAATTCTTTATCTACTGTGGAATGCACATATAGATGAGAAGACCACAGCG
ACCACAGCATCCTTTCAGAAAGGTCCCTTTCGAGAAGCGATTCAAAAATTGAAATGCCTC
TTGGAAATGAAACGAACATCGACAGTTTTGAATCAAAGCGAAGATCCACCAGACACACCA
AGAGTTTACGTTGTCGTAGATATGTTGGTGCAACCATCGTCGGTAGCCAAAAGAAGCAAC
GCGCTTCGGTCTGATGAATTTCAGAAACACGTTTCAAACGCAGAAGAATTGGCACGCTGC
ATACTACTATCGGAAAGCGTTGGAAACAACAAACAGCAAGACGGCGACTGCCTTGGTGAT
ACTACGCCATACATGCTTTCTGCCATTGATCTGGCCGGTGTGACTGTTGGACTTACCGAT
CATCCACGCGCGGCACCCGGTTTAGAATCCTGCCTCGATGCCATTTTAATCGGGTCTAGG
GATCGGAGGAAATACAGCGACAAGACTGGCTCACATCGTGACGGTGTCGACCTGAAGAAA
AGCTGCATTGGGATCGTTTGCAATTCTATTCAGGATTTAATTGGTTTCGATGAAGAACGC
GAAACAGATGCCGTTCAGGGTGTGATGCAGTCACGAACGGATGGCGTGCGAATGGATTGT
CACAAGGAAACCGACGGGGAGGAGGAAAGAAAGGAAGAAGCACTGGAGTCACCGCTCTCG
AGTTTCGCACACGAGTCACACCGATATTGGCGAGTACACAACGCGGGATTGCCACCGGAA
CCTACTCTCGAAGAAAAGAAATTCGATGCCGAGTATGGTCCCATCGAGAACAACCGCATT
ACGTGGATCTTGGGAACTGTGATCGCTTTGGCTGCCTATTGCTGGAAGGCTTCATAAAGG
AGATGAATGCATGTAGTTTGGGACAAGTCTTTCGGACAACCGTTACAAGAAAAGGCAATG
ATGATCCGGTTGCTTTAACAATCTCAAAAAATTAATCTACTGCTTAAGATCTAACCCCGA
ATTTAGACCATAAA
>comp2315_c0_seq1
ACAACAACAACAACAACAATACCAAAAAGGGCCAAGGAGGAAACTCAAACAGCAACAACA
ACAGTAACGGTGGAAATGGCGGTGCCGGTTCGAGTGGAGCAGGGAACAATAACAATAACA
AAAAGAAACCACGCACCAAACCAAAATCAGACAATCGTGTCAATCGACAACGAGACCGTA
AGATGAATAAGCAGGGAGCTTTCGGAGTGCAATGATGATAACGAAACAAGTTTTGAAACA
TCAGCCTAAGCTACACAACGTTCATCCATTCAGACCGAGGTACGATGCTGTTGCATCCTA
GATGACCTCGTGGATACTTTATCGGCTTGAAGCAATTCGATCACCAGGGGAAAAACCCTC

GGCTGAGGAAAGAACAGCCGCCACGCCATGTCCGTCTCGACACGAACACATGGGTGCAAT
TTTTATAGAACGTGCGCCGTTGCGGTCAGTCGCCAAATGCATCATCTTTCGACGAAATCT
TACCAAAAGAAGACGAAAAACACAAACGCCAAATCCGAAAAGCTGAACAACAGCACAATC
AACAGCACAGTAGCCTTAAGTAGGTTGTTGTAGCTCGAATTCATTCCTTATTTTTAATCT
AGCTTTGAATAGTTGTCCGTTC
>comp2316_c0_seq1
GTAGAAATTCGAAGCGAAACTAGTAGAAAAAATCTATTACGGCGCACCCATAGATAACCG
TTAGTAATCCAGGTGTGATTGTAACATACCAAAAATTCAATGCAGACGATACAATACTAT
TTAAAATTAATTCGAGAATCATCTGTTTGGTTATAAACCATGATGATTTCAAGGTTGGCA
CCAGCAACCTTACAATTGCTATCCGCCTTCTGGGCGCTGGCTCCAGTAATCTCTACTCAG
AGTAGAGACCTACAGAATGGCATGGGCCTTGGCTGGAATGAGAATCCAGGATCTTGCCAC
GGGACGCTGTTTTACTTTCAGAGCGAAGTTTTCGTTGATTTCGGTGGATTCGATACCGAT
GTTAATGATCTTACACGTATCAGGTGGGAAGAACTTGAAAGGTCGTTTCGGATGTCATAC
AATGCCCTCTCTGAAGATCTCTGCGACAATTCATACCGTCGCGTTGTCGACGTGTCAATC
AACGAGCAACCAGATGGTGATATTTTGATAAAAAGAGGCGGGAATCAATACAGCTTGAGT
TATACCGTCGAAGCAGTCTGCAGAGGCTGTGACCCTAGCTCCGTGACTCTCTTCTCCCCT
CCTAATCCTGACTACTTCAGAAAGCTCATTTCTTCAGACGGAGCAACCATTGACTCGATG
GATGAAAATCCGTTGAATGCGGTTCTGCAAAAAAGGCGTCGTAATTCCGAAAAGTCATCG
AAGTCTGAGAAAGGATCGAAAGGTTCCAAGTCAAAAGCCGAAAAATCGAAAAAGAGCCAC
TACTACAGAAGTGGCACAGGTAGTGTCGATGAAGGCACTATCAACGGCTCTGAGAACGGA
AAAGGGACTGTGGCTGGAAAAGGGGTCGAGGCTGGAAAAGGTAATGATGCTGGAAAAGGC
GATGGAGGTGAAGGAGATGCTGTTGACGGTCGCGAGGACGAATATGATTGTGGGTGCAAA
TCGCAATACCCAAAATACCGTGCACCAACTGAAGAAGAGTTTGCTACAGCATATGGTTTT
TCAATTCGAGAGATTGAAGAAAAGCCACGTGCCCAGCGACATGCTGGAGTTATAGTTGAC
AACGTTCTAAAAGTCACAGAAATCCAAGATGTATCTTGTTCGATTGTGACAGAGACGTTT
GAATCAACCGTTGCAATCGAATTCTTCGGAAATTATGAAGCTGTAACAGATCAGGAGAGA
AGTATTCTAGAACAGAACTTCGTGTGGGCTTACAACGGTCTCCAGGATCACAGATGCGAT
AGCCCTGAATTCCGCAAAGCGACGTCAGCTGATATTTTCTTCATGATCGACCCCATTGCA
AAAGGTGAAACAACTTTTGTTTATTTGCTAAATGTGAAAGGAACTTGTCGCGGGGAAGGT
TGCTCGAAGAGTGACGATTTACTGCTTTTTTCGGCATCTGAAACAGGCGGCTTTTTCAGA
AACCTTCAATTCGTTGACGGAGCGGAATGTACATGCCCGCTTTGGGGTACTGCATTTGGG
CCACCATCAACTTCCGAAATGGAAGTTAGGTATTTTCAAGTCATACAAGATCTCAGATTT
TTGGACGGTCCATTTACAAGACTCAAGAGTATTGATGCTAGTGGCTTCGTCAAGGAAATT
GGAGAGGGTGCAAGCACACCTTTTCCAACATTTCGGCCGACTCTTCCGCCGGCTGAAGTC
CCGGCAATACCGGGTGTTCCAACACTGAAACCTGTCACCCTCAGTCCGACCAGAGAAGGA
GAGACTCGTTCACCCACCCGTGAACCCACAGATTTCCCAACACGCAAACCTGTCACGCTC
AGTCCAACGAGAGAAGGA
>comp2317_c0_seq1
CGAAAATAAGCCGATCCAACAAGAGCGTCAAAACGTTCGACATTTGCGTGCGACGTCGTG
AGTCTGTGTCTTCCATCTTACCAAAGGCAATCGAGGTCCAAAGGGATGGCAAATTTTCCT
CGGAGCTTCGGTTGCCATCTTTTGGGGTTTCTCGAGGTTGGGCGCCGGAAACAAAAAGCG
CGCAGAACAACAATTGTTTGAGCGCCAGGAACGCTACACTCTCGTATCCCTCTTGCAAAA
TGAAGCCGATCGTGAATATCTCATGAGAGAAAAGAAACTACAGAAGCAAGAAGCAACCAT
TATGTCAGAAGTTCCAGGATGGGAGGTAGGAAAGTCGCAATATTACAACGCATCACGATG
GACGCCTGATCATATTATGGATTCGATGAAGAGTAACATGAAGAAGTAAAAGAATTCCGA
TTAGATCGGTAAATTTGGCGTGAAGGTCGATGAGTGTAGAGAGTTTTTAATCTGTACAAA
TTTGAATGGATCCCTTCTAACAGACACCATGATGCTGAAAAGTAACGATAGTTCAACAGA
TTGCGATGGAAATACTTCTCCAATTAGAAGGAAATATAGTACAAACAGTTGTATTGACAA
ATAAAATGATGCAACTAATGCATTGATTGGTCATACGTCGTGTAACCGTGTGGTGTCTGT
TCTTCACAAAAGTTCAGTACCCACGCACCAGACGTTTCGACGTACTAGGGGTGGTGTCGA
TAGGATACCGATTTAAACAGTTTCTGTAGTCTACGCATCTATTTGCCGGTTTGTTCATTC
GTCCAAATGAAGATTTATATGGGGTTAGCCTGAATACTAGAAAAGTTCTGAACGATATAT
AACTAGAATTTGATTTGTCTAAAAA
>comp2317_c0_seq2
CGAAAATAAGCCGATCCAACAAGAGCGTCAAAACGTTCGACATTTGCGTGCGACGTCGTG
AGTCTGTGTAAGTATTCCTTGTTGTCAAACCATCATGAGCGTACGACCAGACCCAAACTT
TATTCCGAAACAGGAAAGTGGTCCTTTGGGTGGGTGGGCCCATACAAAGGTCTTCCATCT
TACCAAAGGCAATCGAGGTCCAAAGGGATGGCAAATTTTCCTCGGAGCTTCGGTTGCCAT
CTTTTGGGGTTTCTCGAGGTTGGGCGCCGGAAACAAAAAGCGCGCAGAACAACAATTGTT
TGAGCGCCAGGAACGCTACACTCTCGTATCCCTCTTGCAAAATGAAGCCGATCGTGAATA

TCTCATGAGAGAAAAGAAACTACAGAAGCAAGAAGCAACCATTATGTCAGAAGTTCCAGG
ATGGGAGGTAGGAAAGTCGCAATATTACAACGCATCACGATGGACGCCTGATCATATTAT
GGATTCGATGAAGAGTAACATGAAGAAGTAAAAGAATTCCGATTAGATCGGTAAATTTGG
CGTGAAGGTCGATGAGTGTAGAGAGTTTTTAATCTGTACAAATTTGAATGGATCCCTTCT
AACAGACACCATGATGCTGAAAAGTAACGATAGTTCAACAGATTGCGATGGAAATACTTC
TCCAATTAGAAGGAAATATAGTACAAACAGTTGTATTGACAAATAAAATGATGCAACTAA
TGCATTGATTGGTCATACGTCGTGTAACCGTGTGGTGTCTGTTCTTCACAAAAGTTCAGT
ACCCACGCACCAGACGTTTCGACGTACTAGGGGTGGTGTCGATAGGATACCGATTTAAAC
AGTTTCTGTAGTCTACGCATCTATTTGCCGGTTTGTTCATTCGTCCAAATGAAGATTTAT
ATGGGGTTAGCCTGAATACTAGAAAAGTTCTGAACGATATATAACTAGAATTTGATTTGT
CTAAAAA
>comp2318_c0_seq1
CACCGTCAAAATAAAATTATACACAACCATGAAGATCGCTGCCGTCCTTGCCCTTGTTGT
CTCCTCCGTTGCCGCTTTCGCGCCTAACGGACAGGTCCGACAAACCACAGAACTCGCCGC
CGAGCGCCGTGAATTCATGTCCTCGGCTGCCGCTGCCTTGGGTGTTGCCGGACTCACCGC
TTTCGCCCCTGAAGCCAAGGCCGACCGTGATTATGCCGGTGTTGGTCTTTTGGGAGGTGG
TAACGTTGTCGATGTTAACAACGCTAACGTCCGAGTGTACTTGAAAATGCCTGGTCTTTA
CCCTGGTGTCGCCGGAAAAATTGCCTCCAACGGACCTTACAAGACCGTAGGTGACTTGTA
CGGCATCCCGGGACTTTCCGGTGCCGAAAAGGACTTGATCAAGAAGTACGAAAGCCGATT
CACCGTCACTCCTCCTTCCGCTGACTACGTTATCGACAGAATCAACAACGGACTTTACCG
TTAAATCATGCAGCGCTCCAATTTGCCGCAACAACTGACACGTTGACGCCTCCCGGTCCC
GGACATTACTTCAGGCACGAAGGATGTCGATGACACCGGTAGAATTTTTACAGCGAGATG
TGTCTTAAAGGATGAGATAAATGTATTTATGCTACACTAGATAAATATTAGATCATTTAT
TGAACTGCATCAATTGTGTGTTTCAGCATTCAACACCAAATGTCAAGGGGAATGAAGATT
ATCCCGTTAGAACGAAACTTTTGAATAAGGTACGAAGATTGATTCTGTACCAATACTCAT
GAGAGTCAACGAGTGATACAATACCTCGCACTTTTATTTAGTAGCATCTTTTTGATTGGA
CGAAGACTGTTGTACACCAGCAGTACTATCTGATATCATTGTCCGATGCCACTGTTGATA
GTAGTAGTACTATGAAACAACCATCAG
>comp2319_c0_seq1
CAAAGACAAGCGTTGTTTGAATACAAAAAAAAATCATCATCATTGCCATTGCAATCATCT
ACGACCGAAGACTTTTCCAAATAACCGTGGAGGCACAAAGAAGAGCACATTGTCTGAAAT
TGAGCGCAACTCTTATTCTATTGTTGATTGAGGGAAGGTCGTTCGAATCCAGAAGTAAAC
CCAATTATCTTTCGGATATTACTTGTACTACGTTACTAACTTTGTATCATGGAAGGCATC
ACCCATCGACAGTCCTCGTCTTCGGCCATCGATGTCACCACAACTGATACCATAGAAGAA
TCCGTCTTTTCTCCGTTGGAAATAGAAGAATCAAGCGCAATATCGGACATCGATGGCTAC
GACGACGATGGCGTATCCCCCCTCTTGGGAAACAACGATGGCAACGGGACCAGTAGAACG
AGCTCTAACACCAACGGAAAAGAATTGGCCGACCACAGCCACGGTGCAGGTCCTCCAGGA
TCGGCAGGGACACCACAAGTCATAATCAACATAATTATATCCTTTGTTGGAGCCGGTTTG
TTAGGAGTGCCAAACGCCTTTTCCAAATCCGGTTGGCTTTTGGGCTCTATCACTCTTGTA
TTGGTCTCCACTCTGAATGTGTACGCCATGCTCTGCCTACCGAGGGTGCAAAGGGTCGTC
CAGGCGAAGTATCCAAACGAAATTGTGCAATCTTACGGTGATGTGGGCCGACTGATTCTT
GGAGAGAAGGGCGAGAAGGTCATTTTTCTTTGCCTAGGAATATCGCAGGCGGGTTTTGCC
ACGGCCTACATTATCTTCATTGCCGCCAACCTCAACAGCATATATGGATGGTCAAGAGGA
CTTATTTGCGCAACATGTGTTCCAGGGCTCTTGGGATTGGTCCAATTTCGAGAGCTGGCG
TCGTTGTCACCCTTCTCGCTCCTGGCCAACACGGCCAACTTCTGCGCCCTCAGCGCGGTC
TTGTTTCAGGACTATGAGCACTACACGCCCCATAATGATGCGATTCACAAGGCCGACTTT
GGGGGTGTCATCTATGTCATGGCCGTTACCATTTACAGCATGGAAGGCGTCGGTTTGATT
CTGTCGCTTAAAGCGAGCTGCAAGCAGCCCCAACAGTTTTCTTACTTGCTTATTGCAACG
CTGGCAGCGATCACGACGTTCATGGCCATCTTTGGCAGTGCGGGTTACTGGGCCTTTGGG
GATGCAACACTAGCTCCTATCACCCTCAACCTGGCCAACCACTGGTCGGCAACCTTTGTC
AAGTGTTCTCTGTGCCTGGGGCTGTACCTGACCTATCCCATCATGATGTTTCCCATATGG
GCCATTATCGAAGATGCGAACACCACACAGCTGTCCCGCATTTCTGTCCGATCCGCTATT
GTGCTGGTATCAGCGCTCGTAGCCTATTCGGTTCCGAACTTTGGAAAATTCCTAAGCCTG
GTGGGGTCCTCCATCTGCACCCTCCTGGGGTTCGTCTTTCCATCCTACTTTCACTTGCAC
ACGTTCGGAAAGGAACTGCCGGTCTGGCAGAGAGGTTTGGATACATTTCTGCTGGTCGGA
GGCTTTTCCTTTGGCTGCATGGGGACCTATCAATCCGTGGTGGCTATGATGGAGGGGGAG
CTGGAGGGCCATCACTAGATCGACGCAAAACACCGCATCAAAACCAAGAATAGACTGACG
TAAAAAGGGACCAAATGAGATAGATTTGCGCTAGAATATCTTATAGTTCTTCCCAAGGAA
TTTTTGAAATACTGATTTTTGAAGCTTTGGCTGGATTCCAGATGCGAATCCACTAGCATC
AGTAAATAAAAAGGAAAAACTAGTATCTGTTCATCCATAG

>comp2320_c0_seq1
GAACTATTCTAATCTTCCCCCAACTAGCGACGGCATTCGAAGCCGCTCTATCGAAGTAT
CAAAGAAGAAACGCTACGATCCAACACAATGTCAACGCCATCAAAAACACCGACAAGAAT
ACCAATTTCTCCGATTGCTTCCGAGGTGAGTCTGTGTCAGTCTCGTTGTGTGCACTTTTT
TTAGAATGGACGGAGTGTTTGTCGGGGAGAAGCTGTTGCTGAATCCGCAACGAGAACGAT
GGGCACGACGGTATTGTGCAGCACAACAATACAGAGACAGGGTGGGCGGGTCTTATGAAA
CGCCGTGACCCTGCGAACAGCGGGATTCTGCGACAAGACGAACGCCAACGACGGCAACAT
GCCTGCCTCGTTACAAATTGCCAACCGGAGAGAACCGATGGTCGACACCGGAAAGAATTC
TTGATCCGTTTGAATTCTGTGGGGTTGCGGCGGAGCGATCGAGGCGCCCCGATATAAAGA
AAATGCGATGAATCGTCCCAGTCCAAATCGCGAGAAGCGGAACGATCGAAACCGGATCAA
GTCGGTTCGAAGGGCACTGTCCTTTGACCTGGGTGCTCCCAGCGGCAATTCCAGCCACCA
GTTCGTAGAGCGAATCCTGCACGACTGTTTCTCGCCCAGGCGGTACCATCGCGTCGCCGG
GGACGGGGGCAGCGAAAGCAACGGCAAGAACAACACGAACAAGAACATCTACGCAACCGT
AGCCGGCCGCGAATCCGAACGCAGCAACCGAAACGATATTTTTCGTCCCCGCACCAACCG
GCACCATCCCGGTTTCCGCCGCGGCGCCCACGAACGCGTCTCTCATTCCTTTCCCTCCCG
TTTTACCCTACTGTCCCTCCAGGAAGAACGCGAGCAAGCCCTCCGCGACTACAAGGTCAC
GATCGAATACAAGCACCTGAAATCCCATGCACCGGGGGGGGTGTACCTGATCCCCTCGAT
GAATTCCCTGCGGGATTTCCACGGAATCATCTTTGTCCGGCGGGGGCCCTTCACCAACGG
GATTTTCAAGTTCCGGCTGAACCTGCCGGCCAAGTACAACGACACGAACATGTGGCCGAG
AATCACCTTTTCGAGTCACGTCTTTAATCCGTACGTGAACGAAGCCACGGGAGAGCTCGA
CGTCCAGAGCGCGTATCCGACGTGGGATCCCTCCCGGCACTACCTGGTGACCGTCCTGAC
CTACCTTAAGAAAATCTTTTACGCCAAGAACTTTGCCGACGCACGGGCCAACACCGCTGC
CAAGGAGCTGGCCGAATCGGACCCACAGGCCTACAAGAAAAAGGTGGAGGAGTGCGTACA
CGAATCCCAAAAGAACGTCTACGTGAACGAGGAAGGATCCACGGCGAAATTCACCGAAGA
AGCTGTCGCCCACCGCGTCCTGCGGGACCTACTCAAGCACCACATCAAAAACGAAAACCA
GGTCTCGAAACAGGCAGTCATGACACAGATCGAGAAGGCGCGGAAAGTATAGTCATCACA
GCAACAATACCATTACAATGGATTAAGTGTTTTTTAATATTATTTACTGCATCAACGATA
GGTC
>comp2320_c0_seq2
GAACTATTCTAATCTTCCCCCAACTAGCGACGGCATTCGAAGCCGCTCTATCGAAGTAT
CAAAGAAGAAACGCTACGATCCAACACAATGTCAACGCCATCAAAAACACCGACAAGAAT
ACCAATTTCTCCGATTGCTTCCGAGGAAGAACGCGAGCAAGCCCTCCGCGACTACAAGGT
CACGATCGAATACAAGCACCTGAAATCCCATGCACCGGGGGGGGTGTACCTGATCCCCTC
GATGAATTCCCTGCGGGATTTCCACGGAATCATCTTTGTCCGGCGGGGGCCCTTCACCAA
CGGGATTTTCAAGTTCCGGCTGAACCTGCCGGCCAAGTACAACGACACGAACATGTGGCC
GAGAATCACCTTTTCGAGTCACGTCTTTAATCCGTACGTGAACGAAGCCACGGGAGAGCT
CGACGTCCAGAGCGCGTATCCGACGTGGGATCCCTCCCGGCACTACCTGGTGACCGTCCT
GACCTACCTTAAGAAAATCTTTTACGCCAAGAACTTTGCCGACGCACGGGCCAACACCGC
TGCCAAGGAGCTGGCCGAATCGGACCCACAGGCCTACAAGAAAAAGGTGGAGGAGTGCGT
ACACGAATCCCAAAAGAACGTCTACGTGAACGAGGAAGGATCCACGGCGAAATTCACCGA
AGAAGCTGTCGCCCACCGCGTCCTGCGGGACCTACTCAAGCACCACATCAAAAACGAAAA
CCAGGTCTCGAAACAGGCAGTCATGACACAGATCGAGAAGGCGCGGAAAGTATAGTCATC
ACAGCAACAATACCATTACAATGGATTAAGTGTTTTTTAATATTATTTACTGCATCAACG
ATAGGTC
>comp2321_c0_seq1
CTGGAATCAAATCCAGTCAAACCCAATCCGATCTACCTAACCAACACTCCATTTCATTTG
AACATGCCGCAGTTGCAGGCGCAGCAGCCTAGGATACTGGCAGCGTCGTCGTCGTCGTGG
ATGACGAAGGGCAACGCGTCTTGGCTCGCGACAGCGGTCGGTGGAACTGCATTCGTTGCA
GCATCGACCGCTTCTGCTTGGATGGTGTATACGCATAGAACCAAGAGACTGGGGGGTTCT
ATCGTCTCGGGCCTTTTGTCGCCTTTGTTTCGTCGGAGTCGAAGCCTGCGGTCGTCGTCG
TCGTCGTCCAAGCCACAGATAGCAACAGCCCCCAAATCCTACTACTTGGATTACAACGGA
ACGACTCCCATCTATCCAGAAGTCTTCGACGCCATGGAACCTTACCTGAAAGAACACTTT
GGGAATCCGTCGTCTTCGCACCTATTTGGAACAGAGCCCCGCATTGCCATTGAAAACGCG
AGGAGGCAAATACTGACGCAGCTGTTGGGGGTGAAAGATACCGCCGAAAGCAATCCGGGC
AAGAACGGCCTGGATCTCTCGGCGGTTTGGTTCACGGGCTGCGGGACGGAAAGCGACAAC
CTCGCCGTTCGGTTGGCTTTGATGTCTGCTCCGTCTCGCTCGTCCGCCAGTGGTGGACGA
CAGAAACATATCGTCACGTGCAACGTCGAGCACCCCGCCATTGAAAACTACCTCCGTTGG
CTTGAAGAAGACCAAAGCGACGAAATCGATGGCGCGGTTCGTTCGTCCGGAAGCATCAGG
GTCACGCGTGTTCCGGTCAACAACGAGGGAAGGGTTTCGGCGGAAGATGTCCTGAAAGCC
ATCACGCCCGATACGGTCTTGGTGACCCTGATGCTCGCCAACAACGAATCGGGGGCCCTC

CAGCCCGTCAAAGAAGTAGCGGAAGAGTGCCGGAGAAGGGGTGTCTTGTGCCACACAGAT
GCAGCGCAAGCGGCTGGCAAGGTTTCAATCCACTTGAGTGATATGGGCAATCCCGACATG
GTGACAATCGTCGGCCACAAAATCGGGGCACCAAAGGGAATCGCGGCGCTCTACGTCCGG
CCGGGTTGCCTGAACGAACACGGGCGCGCCTTCCGGGCCAACCAGAGCGGGGTCCTGCTG
ATCGGCGGCGGACAGGAATTTGGGAGGCGGGGCGGACCGAGAACACCCCGTACATCGTT
GGCCTGGGCCGGGCGGCCGAGCTGGCCCAGGAGCACCTGGAGCGCAACGCGGCCCACACG
GAGGAGCTCCGGGGCCGGCTGCTCGACAACCTGGAGCGCAGGCTGGGGGCCGCCAGGGTC
CGGGCCAACGGCCCGGGGGATCCTTGCCAGCGCTTGCCCAACACCCTGAGCGTCGGGCTG
GACGGGATCCGGTCCAGCGACCTGCTGTCGAGCGTGGGGCACGTGGTAGCCGCCTCCGCG
GGCGCGGCCTGCCACAGCTCCGGGGGGTCGATCTCCTCCGTTCTGGTGGCCATGGAGGTA
CCCGAGTCCTTTGCCCGGGGAACCCTTCGGCTGAGCGTCGGGCCGGGAACCACCCTCGGG
GACGTCGACGAGGCCTCGGAGATTATTGCCCGGGCCGCCCTGTCGCAGTGGGGGGAGGAA
GGCCGCGCGTAGCAGCCTCTGTTTTGCCCGGGGGGTGGACGGGGCGCCGCAGCACGCATA
CAAGACGAAACGAATACCCCGATGCACGACGCACTATTCCGTGTGTACGAACCACACAAA
ACGCCTAGGCCTTTTGGAAAGCCGGCCGAGAAAACAAAATGCGCTATCGAAAATCATTTC
CATTGTAGTACGTTGTAGAAGTTTTGTACATTATTCTTGGTTGCTTAGCCATAATACACA
GTGCCGCCGATGGTTTCTGGTGCAGCTTACGGCCCCGCCAGGACGAGTCCGACGA
>comp2322_c0_seq1
AGAAGCAGCACGTCTACAACGGGCGGCCGAAGAAGCGGAAGCCGCGAGAATCAAATTGGC
AGAAGAAGCGGAAGCAACCCGTTTGAAGAAGGCAGCTGAAGAAGCCGAAGCAGCGCGGCT
CAGGCAGGAGGCTCAAGAAGCCGAAGAAGCCCAGCAGAGACAGGCAGCGGAAGAAGCAAG
AATTGCAAGAGAAAGGGAAGCAGAGAGAATTCGTGAGTTAGAAGAACAAGAAGCTAAAGA
TAAGGCGAGACAAGAAGCCATAGCTCGGGAGCAAAGGGCCGTTGAGATTCTCCGTCAGAG
AGAAGAAGAGGAGGCTGCACAGTTAAAAGCAGAAGAATTGCGACTCGCATCTGGTGGTAA
AGCTACGGTTGCAGGTGTGAATATTTTGCGCGATTATGTGGCCAAGTTCGGTGAAGCATT
TAATCCTGCCCAGCAGCTTGAGCAAAATCCTAATTTAGAGCAATTACGGACTTCGTTGAA
TCAACTCGGAATATCGCCAGGAGTTGCAGGTGGACTGGCCGCTGCTACACTTATTGTCGG
TGGCGGGGCGACGGCGATCAAAGTATTGGCAGATCAAGCGGAATCCAACATTGACGATGC
GCGAGAGGAGGACGGAGCGATGGGCAAAGGAGACGAGTCAAACAGCGGCGGAAAAGATCT
ACTGGACTCTCGAATCGCAGACCTCGAAAATAAGAAAGAGGAGAAGACAACACAGCAGGC
ACCGACCGTAAGAAGAACCCTAAGGATTGTTGATGACGACATCAATGGGAATAGTCCCAA
CGGACAATCGGAGACGTCACGACCGAATGCCGACCCCCCTGCGTCTATAAAGTTTGGGGA
CAGTCCATCGCAACCCCAGCCTAAAGTATCTCCACCTCCAGTGAAGAAGAGCTCGAACCC
GATGAATACACAAGGACCATTGTCATCATCCTCGAGTTCGCAAAAGTTTGATACCCAATC
GGATAAAAACGGTAACAAGGTATCACCTGTAAAGGCGAACGTGTCTGATAGTTCATTAAA
TAGTTCTTCCCCAACTTCCACCAGTTCCTCTGGTTTTGGCGTACCTCCTCAAAAAACTGT
TGGTCCATCAACATTATCGCCCCCCCCAGATACTTTCGGATCCCCGCCTTCCAAAACAAG
CTCTCCATTTGGTGTTCCA
>comp2323_c0_seq1
CAGCAAGTTATCGAGGGTAACAACCGAAACACATTAGAAAGCACATGCAACATCTTCCTA
TTCCATAGCCTGTCACCGTGGCCACACCCATGTCAATATTCCGCTCATTACTATTGTCTA
CTCTATTGTGTCCACGCTGCCGTACATACACTCCGTATCACTGGAACAGATAGAACCAAA
TCTTCTGTGTTCTATCGCTGGCTCTCGCCTGTTCTTCGATGAGTTGTCAGTTCTGGGACC
GCTGTCTCCGTTAGAGTTTCATTCAGCCGGCACTAATTAAGGCTTCTGCAGGTCGCAACA
GTACCCTTAGGCCTGTTTGGCAATGAAAACGGCAGTGAGAACAACTTCGTCTTCGAGCGA
TGATCCAAATCACAGAAGCAACAACAGAAGTGAAGCCACAACATCGAACAATAATACCCA
TACTACTCCGACGATTTGGCGGTCGACTTACCGCTACGTGTGGTTGTCCGGGATTATTGG
TGTTGTCGGCCTCCAATCGATCACTCTCTTCAGATTGACGAGTGGGTTTACGCTCGACGA
AACCGGGACTCTTTCGAATGCCACCAATAACGCGTGGTCAGACCTTGGAATCCCCGAAGC
CGTTTTTCTTACCGGTGCCGCTTTGGACCGCAACAGAAGCAATGCTGAATCCAAGGCGTT
CGCGGAGGCCCTGGACAAGAAGAGATGGGACTTTCAACGGAGGCAAAAACTGCGAAAACG
ACTAGCTTTTCCTGGAAGCAATAGAACGGCTCGTTTCAAAGAACGCAGCAGAAAATTTCC
TATAATTTCTTCGAGATCCGATGCTACGAATGAATCATTGTCATTACACCCTGTTGCCGC
CGTTTATAATAACGAAAGCACCAAAACATCCAAAGCAAAGGAAACATGAAGATTGCTTCC
AACAATGAAAGGCCTCTGAGCGGAAGCACGTGTCAAGGAATTCAAACAATTACAGGAGTC
TAACTCCTGGACCGACAATCCATTCGAAGGGTGAGTTTTAGGTACGATTGACAACAAAAT
GGTGATGCAGAAGAACAAATTCATATATGTACAAGGGCAAGCAAAACGATTTGACGATAA
AACACTATTCGTCACGGGATCTTTAACAGTTTTCGGAAATGTAGTCAAAATCTCTATGAG
CATATCTTGTACATATATTATTATAACCTTTGGTAAAGGCGTTTATTAATTTCATTACGT
AATTTGCAGGTGTC

>comp2324_c0_seq1
AACAACAACAACAACAACAACATAATCATCGCCAGCAGCAACAACAACCGCTCGAGGGAT
CCTTCACCACCGCTCGAGAAACGAGGGCCAGGGTGCCGGGGCTGGCTTTTTTCGCTCCGG
ATGGCGAAGGTGCACCGAGAACCACCGCGCCGGCGAACCGAAAGCCATTCAAGCAAAGGT
GCTTTCACTGCGGAGAAAAGGGCCACAGCAAAAAGTTCTGTCCGAAGCGAGAAGTCTCCT
CTCTTCGGAACGACCACGCGCCCGAGATCATTGAGCTCTGAGAGGAATGGGAAGCCACGC
ACGATCCTAACCTTCCGACAGGGCTTCCACTGAAGGAAACGTCCGGCGCAAGAGAATGTA
ATATGGCTTTTGGTCGGACGAAGGATTTTAGTGTGTACCACAACCCACCGCACTGTTGGT
CGGCTGCACGATAGGATGCTACTCAATAACACTGGAGAAAATAGATGTAGAACTATATTG
CTATAGGCTTTTTCGATACAATCGTCCCGTT
>comp2325_c0_seq1
AATGCTTTCACGCACCGTCGTTTACAAAAGCCAACGGCGACTTTAGTATTCGGTGGCCCG
ATAACAACGCAAAAACAACAAACAAAATGAGCGCTGCACCGTCTCCATTGCTCGGACTGC
GAATGAGCAACTTTCCCCGAACCCCCGCCGCGGTGTCGGATAGATGCGGTAACACCGACC
GTGTCAAGGCAATGCTGAACCAGAGCGAATCTACGTACAGGTGCAATGATTACATCCGGC
GGCGGTCGAGAAAAGACAAGAGTGGTTGCCACGCCACGGTCGGGGAAGGAGCGTCGGACT
CCGGCTGCCCCGAGCTCTCTGTCGAGCAGGAACAGCTCGACACCGTTTGCAGGGAAAAGA
TGTGCGAGTGGAGCTACCGGGTCTGCGACCACTTCCAGACCGGCCGCGAGATCGTAGCGG
TCTCGTTTTCCCTCCTCGACCGGTTCGTGGACAAGTGCAGCTGCGAGCGAAGTGCCTTCA
AGCTGGCGGCCATGACCACTCTGTACATGGCGAACAAGATATACGGTGGGCCACAGGCCA
TCAGCATCGGCGCCCTCGCGGAACTCTCCCGGGGCGAGTTCGAGCCGTCGCACATTTCTG
AGATGGAGGTCATCATCCTGAAGACCCTGGAGTGGAGGCTCCACCCTCCCACCGTCCAGT
GCTTCATCGATGCCTTTTTCCACTACCTCTCCCTCCCGTCCCACGGGGCCTGGTCCGTTG
CCATTTACGAGCGGGCGACGTTCTTTGCGGAGCTGGCGGTGTACGACTACGCCTTTGTGC
CCAAGCGGAAGAGCCTGATCGCCCTCGGCTGCCTGATGAACGCGATGGAGGGGATGGACA
ATAGCCTATCGGCGGAGCAGCAGAGGGGCTTTGTCGACGCCGTCAACGCGACCTTCGACC
TGGAGTTCACGACCGATTCCATCGAGTCGGTCCGGAACCGGCTGTGGTACGTCTACAGCA
TGAGCGCCCAGTACAAGGAGGACGACGCGGTGGCGGGAGCGGAGCCGGGCGACGTCGTAA
AAAAGTCTGTGTCCCAGAGGACCTACGGGGTGTCGTCCACCGCCGCCACCGACCTCTCGT
CGTCGCCGTCCACGTCGCCGGTGTCCGTCGCTGCGGCGTCGGGGCCCCACCACATTGCTA
GCTAGCCAGCCCACCCACCAACCAACGCAGCCCAGAGCGGACAAACCAGACCAGACCACG
CCGAGGCACGACATGGCACGCCACAGCCACCCAATACAAAAACAGGGCATGCATCAATTT
TTTACAGGAATCCATGCTTTCACCCCCACATACATCCCCATCCATACATCCACCTACACA
TACAAGAGAAGAATCCGATACGTAGAATAGTGTATCGATATCGACGAAGAAGAGTACCAT
AATGTATAATAAGTACCATTAGAAAAATG
>comp2326_c0_seq1
CACTGTACTTCGCAACGCCGCCTGGCTTCCGACTCGATTGTAGCGACGACCGTGGCCCGC
TACCATTCCCGAAGAGCGCAGCAAGGCTCAGTCCCATCCACTGCCCGGTGCACGCGGGTC
GGACGATCAAAGAAAAAGAAGAGAGAGAGAGACAGAGATACCGTCCCGCAAAATAGTATG
AAGTCGCTTTCGTTGGTCCTTGCGGTCTTCTTCTTGGCAATCCTTGCAGTGTTTGTGATG
AACTTCGTGGTTCTCTTTTGGTTCTTTCCGGTTCCCGAATCCGCTTCTCGCCCGTCATCG
GCATTCGCGGCGGCGAAGGGTGGTGGTTGGTTTCGAACCCCCGGGGAGAGCACCGAGCAA
CAGAAACACCGGAAAAAGCAACAGCGCCCTCCCCTCAGACCCAGCAACAAGCGGCACCCC
CTCCACCACCAGACGGAACAGGCAACGGAGCCACCAATAATAAAACCGCCAGATCCAACC
GAGTCGGCGACGAGCGATCCCATCCCGGCGAAGAACGATAACAGCACAAGAAAACCAAGC
GTCGGTGAGTGTCGATCGTTCGGCTGTCCGATCTATCCCCCGGAACTGACGAATCCCTTG
CAGAACGCGACCATCCGGGACGCCCTGGAATCGTCGCGTCTCGGGGGGTCGCCCGGTTCG
AAATCCTTGTTGCCGGATCGAGAAAAGGCGGCTTCCATCGGGTTCGCCGCGGCGTCGTTC
GCGACGCTTTCCCAGAAGGGCGAATCCCACACCGTCAACCAGGATCGGGCGGTCTACGTC
CCGTCTTTTCTGTCGGACCTGCTGGTCTCGTCGGCGCGGAAGCCTTCGGGCGCGGCAGCG
GAACTCCCGGCACCCGAGTCCTTTCTGGTGGGCTTGTTCGACGGCCACGGAGAACTGGGC
CACGATGTCGCGGAAGAAGTAGTGGAAAAGCTCCCCGTCCTCTTGGCCGAGAAACTGACC
AAAGGGCTCGGCGGCGGCAGCAGCAGCAGCAACGGCGAGTTTGACGACTTCAAGAGAGCC
GTTGCAACCGATAGCTACAGAGACAATAGCAACAACAGCACCAATACCGTCAACAACGAA
GCCATCGTGGTACACGCCAAGGAGTACGACAAAACGGATTTTATCATACGAAAGGCCCTG
AACGAAACGTTTCTAGAGGTAAACAAGAAAGGGACGTCACCTACGTTTTATCTAGGCGGC
TGTACCGCTAGTGTTGCGCTCCGTTGGGGATCAAAACTCTATGTCGCGAACGCCGGGGAC
AGCACCACTATCCTTGTATCGGCTGCCGAGAAGAAGGCACCAAAGAAGAAGGAGACTCCG
TCCCTCGATGTGAAGGTGGAATACCAAACCCGCAGGGACAAGGCCAACCAGCCGGGAGAG
CGGGAACGGATCGAGAAACTAGGGGGGAAGGTGCACGTCAACGCACAGGGCTTTGATCCG

CGGGTCATCGTGTACAGCCATGTCGCAAAAGACACCATCGGTCTGGCAATGTCGAGGAGC
CTCGGCGACTGGGAATGGAAGGTCGCGGGTGTCACCGCCGAACCCACCATCGACGTGATC
GACCTTTCCGAGCTCTCGGAGCCACCGGCACAGGAGCGAAGCCTCTTTCTCCTCGCCGCG
AGCGATGGCTTCTGGGACATGCGCCAGCAACACTTTCTGGCCACCCTGTCGGCTTCGTCC
TTTGTGGTCCCAAGCCGCGACACCGGCAACGAGAGCGAGGAAGAGAAACTTCTTCGGCCC
CTCTTTCACCTGTACGATATCATCCGGCGAATAACTCCAAAAGAACAGACGGGCTACAGA
GACGACATCACCGCGACGATCGTCAGATTATAACGTAAAAAGCACCAGGCCGGCACTCGG
CTCCTTGGATCACACGAATGGTGCTGCGTCAAGTAGAGCTGCATAGAATATTTATTTATC
AAATGGACGATCATGCCTCAAATTTTTCATTAAGTAGAAACACGATGATGTTATTTATCG
GACAGCAGAGTAATGAACCACCAAGAAAGACTACATCATGCTGCAGAGTATGTTCATGCT
CATCGAATCGATCAATTCCTCGTTCAGAATTTCTAACGATTTT
>comp2327_c0_seq1
CTTTTGCCCAAAATGGGGACTGTGCGATAAAAGGCTCATCCTGCACTCTCCGGTTCATTT
ATGACAAGTATCATGACGTTCCATTCCTGTAGCAGGAGACCGACGAAAATTCCCACAAAG
ATGGTGCCATGTGCAAGGGAACTTTTGTTTCTCGTTTGCGCCACGCTTGTGATCCTTCTC
GGTTGCCTCGGCTCCACCGACGCACTGGCTGGTCCCTCCCAGCCCCGAAACAACGGGCCC
GTCACCATCAAGGACGAGCGAATTGTTCTTCCGAACGGCGTTCGGGCACAGGTGGTCTCG
GCGCTTCCCGAGGGCTGGCCGAGGCAACAAAACCAAGACGAAATTTTGAGCAAGAAGGGT
GTGGACGAGGAGAAGCCAAAGCCACCCATACTCTTTTTGCACGGGTCCTTTCACGGAGCC
TGGTGCTGGCAGGAACACTACATTCCTTTCTTTGTCGAGCGGGGGCACCCGTGCGTGGCC
TTTAGCTGGAAGGGAACCGGTGGGACCCCCGCCGGGGAGGGCGTCACCAAGGTTCGTGTC
GTGGAAGACCACTGCGCCGATCTGGAAGCCCTGCTCGATTGTCTGCCGTCCATCCTTGGC
GACACGGGCAGCGTTGGCGACGAGAGCCTTCGACCGATCGTGGTGTCCCACTCCATGGGG
GGTGTCGTCGTCATGAAGTTTCTCGAGAAAATGGCGCGATCGAAAGACCTACAACCCAGC
GAGCTCTTTCGCGGCATCGTCTCCATGTGCTCGGTCCCGCCGAGCGGCAACAGCAAGACG
ACGATGCGGTACCTGCGCAGATCCCTGGTCGACACGTACAAGATCACCGTGGGTTTTGTC
CTCAAGAAGGTGATCACGGACGACGCGATCTGCCGGGAGTGCTTTTTCGGTGGCACCGGG
AAGGGTCCGGAACCGGGCGTCTCGGATGAGGACCTGGCGCGCTACCAGGGCTATTTCGAG
CGGGACACCCGGGCGATCCTCGACGTGACCGACCTCAACCAGATCGTCCCCTCCCGGTCC
GCGGACTCCGGGGGAAGGGCGCCCTTTGTTGCGGACCTGCCCCCGTGCCTCGTGGTGGGT
GCTAGGGACGACTTCATCGTCGACGCCGTGGCGAACGCGGAGACCGCGACGTACTACGGC
ACAAGCGCACCGGTCTACGTCGATTCCCCGCACGACGTGATGCTGGGGGGGCAGTGGAGA
AACGGAGCCGACGCGCTCCACGACTGGATACAGGAAACCTTTCTGGCTTGAAAGAGACGA
ACGGGAACAAAACCGTTGGTTGGGGCGCATAGGTTCAAGGGAAGAATCAAATTGATAGCG
ATGAAGTTGACTTTGTAGTTTGTTACTGTAGAAAGAAAATGCCTGGAGAGGTTCGGAATC
AACGCTTCCAGCTGATGGTTTATCGAAAAATAGACATGATAACCAAGAGGTCTACTTTCT
AGTTTGTTAGAAAAAAGCTTTTGATCCTTTCGGCCGAGTGATTCTATTCTACCGTTGCCT
ACGTGTGGTGCAATGCCGCAATCTGGGCCTCGGTCATGACCCCGAATCTAGCGCAAATCG
CTTGGTACCACTCCGGATTGCCGTGGAAGGAATAGTCCATCGTCATTTCTTCTCCCGGAT
CGATGTCTCTGAGAGCTCCATCGGGACCCGTGTTGGGATCGGAGCTGTGGTTTGTGAGCC
GCCCGGCGTCGGTGGGATTCGAGTGGAAGAAATTGTCGTCGTTGCTGGCGTTTCGTGGTG
TGGTTTCGCCCTGTTTTTGTGGCGGCGACATTGTCTCGGAGGAATGCTTCGGGGGCAGCA
CGAATCCCTGTCTCAGAAACACCCTTGTTTCTTCTAGGTCGTTTCCAAACGTTTTTTGAA
TGTAGTCTTCGAGATCATCGCGGTGAATCGACTTCACTCGATGGGTCCACACCCACAGTT
TGGTTCCCTTCGCAATGGGTTCTTTCGCAAAGACGCCCCTTTGACCCTCTCCGTGGTGTG
GAGTTTCTTTTATTACCACTGGAACAAGATAGGACGCATGGGTTGGAAAGTGGTAGATCT
CGTTGGATTTGGTGTGATCTTTGTCGGTAGTCATGGTGGCTAGTGCTTTGTTTCGGTTCT
TCTGAACGGGAGTATTTTTTTGGGTTCGGATTTTTATGCAAAAGTGATTGCTATGAGAGG
TCTCGTCTTCACCTAGAAGTAACTTCTACGAGTATGCGTAGTGCCACTTTCTTGGTACTC
GTGCTCGTATCTTACAGTGCTCAGGCAATTTTTTCAAGGCGGATGGATGACACCGTACGG
TCTTCAGTCTTTACTTGTAACTGTACATACGGTACAATAACTACGGTACCATACTATTGG
GATCTGTTCTCTGATGTCTTGAAACTGCTCAGTTTTCGTTTGGGTTTGGAAGGAGGGAAC
GGG
>comp2328_c0_seq1
CCGCAAAGTTTGCGCACCGCTCCTCGCTGAGCTGCATACATACCGTACCGTGCTTCGACA
TCCCCCACACGCCGCACTCCGAGCCCAAGTCCCATCGACGAAAAGCAACACCAGCATCAC
CACCACCGAACAGAAAAGAAAAGAACAGAACAGTACAAACCCATCGCGCAACAAACATGG
CCTCCGACGGACCACCCCAGCCCATGCCCATGAACCCCGGCGTCCCCACGGCCTCGCTCT
CGAACCCCATGGCACAGCAGCAGCAGGCGCCGCCGCAGGCCATGGCCATGGCCATGCCCC
CTCCCAGCATGATGGTCCAGCCGACCCCCATGGCCATGAACGTTTCCATGACCCAGGGCA

TGGTCCAGGTCCAGCAGCAGCAGCAACAGCAGCAA
>comp2329_c0_seq1
CAAATATTTTTGAAAATATTATTTGTTCACTTCGTACGAAATACGTCGGAAGCAATCAAG
ATACCGAAGTTATGCAACTGACGATCGATTCATAATCATTACTGTATTCGTCATATTTGT
TGATCAGATTGGTAAAAATAGAATAAGGCACTCTTCACTTCCGTTGTCACAGATTTCTAC
AATAGATCATAGTAAGATTTTCCGGACCAAAACCCAGTTGGTATGTCCATGTGCCAATCT
GAAACATTCTTAATATCATAGGAGTAGATATCCATCAAATGGTGCAGCAGGTGACGCCTC
TTCGAAGCCCTGGAGTCGAAAAGGATGGTCAAGAATTTGGGCTTGTTCCTAGATACGTGC
TGAGGAAAAAGAAAGCAGTGAACAAGGAAGGACGAGGACCCACAGGCGACTCCAACCATG
TAAAAAACAACCAGAATGGCCACCACGACATGATGAAGGATTGCGACGAAGAAGACACCC
CCCAAAAGACTCCACAAGAAGTGGACAAGCTGTTGCACGAGACAAACTCGCTGCTAGCTC
GCCGATGGAGATTGTCATCCCTTATAGATTCGGAGCGCGACAAGAAAGTCTTACTGGCTA
CCGAGGCCTACCTACCGTCAAAGGCCGCCGACCGCATCGAAAAAAATTGCAAAACCAAGA
TGGAACTTTTTGGGTCTGAATTGATGGATAAAAAAATAACTCTGTTCGAAGATTTGTCCT
CTGAAAAGTCCGAAGTTGGCGTCGAAGGCGGTTATGTCCAGCTTCTTCCTACCCGTGATC
TCGCAGGCCGCGCAGTTCTGTTTTTTCAATACGACCAAGACCTGTATTCAGAGCAACCAA
TCAATTTCCGCAAGATTTTGTTTTATATGATGGCAAGTGCACTGGAAGACGTAGATACGG
CCCGGAAAGGAATCAGCATGGTAGTTTGGAATGGTGCTCCCACGAAATGGTTCGATCCCT
TTTGGCGAGTCTTTGAAAAGATCAGTGCTCCATTCAAAATATCAAAACTGCATTACTGCC
AAGTGTCACAGTACGAGTCATCGTACCAAGTCCTTGAAAAACGAGAGCGAGGAGAGACGG
ATGTTCGGATCCATAAGGGGTCGTTGCAGGAATGCATGCATGGTCTTGTAACATATGGTG
TTCCGAGCCACTTTATCCCACTGAATCCTCTTGATGGAAGCATAAATTTGAAAGAGCACT
TAAAATGGATCGAGCTCCGCCGACGGATCGAATCGTTTCCAGAATCTCAACAGGCATGCA
TTGTGCTGGTGCCCGGGCAAGACGACGTTCTTCTGGGCAAGCGGAAGATGTCTATGAATG
GCAACGTGCTTTATCACCAGATGATTATGAAAAACATCAAAGCCTACCACGAGGCAACAT
CTGAAGACGCGCGCGATGATATTCTGATGGCTGTATGGGATTCCATACAGTGTCGCGGAG
GGCGGTTTTTGTTGCAGGTCGAGGACGGTCAATTGTGGGAGAGGCTGGAGTTGCAGTCGG
CTCTGTACAAAATCAGCCAGGCTTTTGACTTTTTATGCATGCAGCTTTATGCACCAAAGC
CAACGAGCTCGAAAGATTCCAGCAGCAAGAATTCGAAGGACGACGAGGACATATTTTTTG
GTTGGAACGATTTGCTCGATGGGAAAAAATGCTATGAATGTTTCGACCTGATACACTGCA
AGGGCGATGGTTTTGGGAAGCCAGCCGCGTGTTGGTAGGGGCAATTGCTCTCTGCTGTGG
CAAGCGCTACAGCAAATTAATTTGGGACTCTTTGTGCAGTATGGAATGTAGAGCAGATTG
GATCATGGTTGTATTTCTTGCATCATTATTGTTTTATGTGATAATCACTGCCTGTAAAAA
GTTGTAGTTACAATGCTTATGCATGCCTTACCCCTGCTGTATTGGTGTAGATTTGCTGCG
TTTGCTACGAGTCCTTTGTCATACCATATGAGTTCGTGGAAGATGCAATCACAGGGCAGT
CATGACGTCGTCGCAGCTTTGCGAACAATGAACCAATGTCATCTGTTTTTCCCTTTTTCC
GGATGACATCCCCGCGCTTGTTGAAAGTGAGGCAGTAGGTATATCAGTCTGTGGCGTGAT
GAAAGTCTGGCCTTTGCGAATTTTTCCTGACTGCTCACATACCCGTCCCGGCGGAATGTA
TTGGGGTGCCTTCGCCGTTTCGTTGCCGGTCTCGAAAGCATCCCTAAATACGACCTTAGT
CATGTCGATCTAATCAAGAATTTC
>comp2331_c0_seq1
CTATGAAGCAAACCACAAACGATCCAGGTCGAAGGAAGAAAGAAGAAAGCATATAATATA
AGCAGTACGGCATAGCATAGATACGATCAGATCCATAGATCGGCACCAAACACACCGCCG
GGAGTAGAAAAGAAGCAAGAAATGCCATAGAGCAACGACAACAGCAGCAACAACAAGATG
AACAACAGCAACAACAACAAGATGAACGACAACGAGACGAACGACAACGACACTGTGAGC
ACCAGCACCAGCAACGACAACGACAACGAAAAGGGCCACGGCTTTGCCCGCCACCACCAA
TGGCTCCCTTCCGGCCCGGCCGTCCTCCCGCTGGCCCAAGCAGCGGGCGTTTTCCTCGCT
GCCGGCCCCCGAACAGGGGGAGCCGCCCCCGCTGGCCCTGGCGGCAGCGGCGTCCGCCTT
TCTGATCGGGGGGACGCTGCTCTCGGAGCGCCGGAAC
>comp2332_c0_seq1
CGACAGCAATCTATCATTCCCTCACCGAAATTCAACTCCGGTCCATGTAGGAACGATCCT
GTTTCCATGATTCGTGTTGCTATCGACGAACACGGAGCAATGGAATCGTAGGTACTCAAT
GACAGGCACAAGGTTCATCGATTGTAGAACTATCGTTTACCACTCCAAAAGCACAATTCT
CTTTTCGATCGGATAGAAAAATACTCATCTCAAAATACTGTAGGAACAACAACTTCTAGA
GACAACTCAATCTAACATACACCAAAACCGATCCAATCCAATCCATCGACCTCCTTCATT
GTTGAAAAAAATGGTTTCCTTCCTTCGTTCCACGGCCTCTAAATTCACTGTATTGGCCGT
GGTGTTTCTCGGCTCTGGTGCCGCCCCGATCCATGCAATGGAACCCGAACTCGAGGATAC
AGAAATAGGCAATGATGCGCCTCTAGATACCATCTGCAAAGACAAGCCAAATTGGCGCTA
CAACGATAAAGAGAGAAAAACCTGCGAGTGGATCGGGAAAAATCCAGACAAAAGAGCAAG
GCTGTGCGAGAAGAAAAGGGTCAAAACAAACTGCCCCGAAACTTGTGGGGTGGACTGCAA

CGACTTGATCAATCGCAACGGACCAACGGACCCTATGCCACCGTTGGAACAAACTCCGCC
ACGAGACCCCTGCAAAAACGACCCCAGTTGGCGCTGGAACGACAAAGAGAGAAAAAGCTG
CGACTGGATTTCAAGGAAGCCACAGAACAAAGAGAGGCTCTGTGTGAAGAGAAGGGTCCG
AGAGGCCTGCCCCGAAACATGCGGGGTGGTCTGTGAAGACGTCAGTCGCAACGTACCAAC
AGACCCAATCCCACCACCATCGAGTCCAATTACGAAATGCCCTAAATTCTTGGGTGCCAA
AGTAGATTCGGCTGTCCCAGCTTTCGACAGAATACCGAGTGCAGAGAGCTATTTCGAAGA
ACCAACTATGGACTGCTCCGAGTACCTGGATGGCCTCACATGCTACACTGACTACCGCTA
CAATGGATGCAGCGATGATACAATGCAGTGTACTCCTTCGAAAGCCAGTGTTTGTAGCAA
CAACGTCTGGCAAACCAGTGTTGCAGTCAATCCAGCGTTATGTGGGTCTGATTTTGGTGA
GATGCCCTATCTCTCGGTGTTTGGAGAATACTGTGATCCAAAGGAATGCCCCGTATCGCA
ACCCAAGAACGGTTCGGATTGCAGTAGCTACGAAGGCGAGTTTAGTTGCATGTATAACTA
CAAAACCTTTGGGTGTGACGGCTTCGACGATAGTCACGGGTGCTTCCCCACAACCTCATT
GAAATGTACTGATGACAACAAATGGCAAATGTCTATTTTCTCGATTGCGAAATGCCCCGA
CGGGTCGCCACAGGGCCAGGTAGCCTGCGACCCCGAAGAATGTCCCTTGAAAGAGCCCAT
GCATGGTACAGATTGCTCAAGCTACGAGGGCAAGGACGAGTGTCACTACGACTATAAAAC
TATAGGATGCGATGCCGACAGTCTGACATGCGCTCCCACAAAATACTACACCTGTATGGA
AGAGCCCGATGGCAAGTCGGACCGATGGCAGCTTCAATACACGATGCGTCCGGCATGTTA
TTATGAGGGCGCTGATAAATCGTGCGATCCGAGCGGTTGTCCTCTTTCTGCTCCCATTCC
TGGGACGGATTGCTCGAGCTACAATGGGGGCAAACAGGACTGCATGTACAACTTCAAATT
CGATGGATGTAGCTTTGACGACCTTCGGTGTTCGCCCACTAGTTACTTCTCCTGCAACTC
TGATTTCAATACGTGGGAAGTAGCAGAGACCAAGCCCTTGGACTGCGAGGTTATCATTCA
GGACTTCGAGGGCATATCAGAGGATTTGCCGGTCGGAGAGACCTGTAAGTTGTGCCCTAC
CGTTGAACCAGCCGATCTGTGCCCCGCCAAGCAACCGAGAGCTGGAACTAGCTGTACCTC
AGACAAATACGATTACGAAGTGGGTCTTGTTTGCTCTTACGACTTCAAGTACGAGGGATG
CTCCACAAACAAACTCAAGTGCAAACCTCATGAATTTTTCGAGTGTACCGAACAAGATGG
ATGGGTAGTGAAAATGACGGAACCTGAACCTTGCAATTAAGAAGAGAGAGCTGGTATAAA
TCGTCAACAAATGCGTTCTTGACAGCCTGTGGAAAACAACGTGGTAGATTATCATGACTT
CAAGAGCCTGATAGATTCGGTTGACTGTGTCGAGTGAAAAATCTTCCAAATGTAGTATTT
TTAATTACTGCCATAACAGTAAACAATGAGTGTGAATTGATT
>comp2333_c0_seq1
CGCAGCACAAACGACGTTCATCGGCACTTGTTTCATTTCCATCTTCCTGAGGATCAGATT
TTCGCATACATTATGTTCTCGGCCTTGGCACGCAAATCCACCTCGAAATCGGCTGCTGCG
CTTACGAGCACGGTCCGCCGAACGGTAGGTGTCCGAACCTTTGCCTCGGAGGATTCCCAC
CAACCCCCTGTCTCGGTTTACGGAACCGCAGCCCGTTACGCCAACGCCACATACGTTGCC
GCCTCCAAGGCTGGAGCACTATCCCAGGTAGAAGGCGAACTCAACGGACTCGCACAGGCC
GCAAGCGACAGTCCCGCCTTTGCTGGATTCCTTGACAATCCCTTGATTTCCCGGGACGAA
AAATCTGCCCAGATTGAGGCGCTCCTCAAGGAAAAGAATGTTTCGGAAATCACTACGAAT
CTCTGCACGACCATGGCCGGAAATGCCCGTCTCAACGAGCTTCCCAAGGTAGCTGATACG
TTTGCCACACTCATGAAGGCCAAGCGGGGCGAGGTTGAGGCTACCATCATTAGTGCCGAT
CCCTTGACCAAGAAAGAAACCGCTGACGTAGCGGCTGCGATCAAGGCTACCTCCCAGGGA
GCATCGGAGGTTGTTATCAGCTCCAAGGTCGATCCTTCTATTATTGGCGGAATCCAGGTT
CAAATCGGAGATCAATTCTTGGATTTGTCGATCAAATCTCGCATCGAAGAAATCTCTCGA
ACTTCCATCTAAGTAAACCATATTTTTATTACTTGGTACGAACGAGTGTGAGAATCTACC
CATCATCAAAAAGTAAACATAGGAACGAGCATATGTAGGAGAAATGAAATTCGATTGTGG
ACGAGCGGCGCAATAATTGCAATCCAAACTTGCATCTTCACTACGAATCGAATCGACGGT
AGCCCAACCACATAATACTGCAATAGTAGTTATCGACAAAAACAATTCAGGCAACAGTTT
GAAACAAAAGACACGACCAAGTAGTTGTATTGTTATAGCTAGAGTGGCCCAGTAAAAGTC
GACGGACGCCAAACCTCTTACTCTGGTATTCTTTTTTGCTTCAAGCTTCCAACAATATAA
ATGAAGCGAAGCAATTTTGAGATGTACCGTACTACTGAACAATGCTTTCCATTCACAAAC
TACCTAGTGCTTTCTGTTGTTCACGGGAGAAGTTTTTGTAGTAAGGAGCAAACTGCAGAA
AAACGAAATGCCAAAGCACAACTCCACTCTGTTGTCCTCAGGTGCATGATGAGTTCAAAT
TAGAAGTACGGTACTTCATGGGGGAAGTCCCTGTGAAATCAAAGGGACCTGGTTGAGGTG
TTTGTGGGGGTTTGTTTGACAAGGGGGGGGGCGGAAACTTGCGACATGCGCATAATTTCC
TAATAAAAAATTATAGAGAAACTTGAGCCTAGTGGCCCAAGTAGTACGGCATCACGTACA
GCCTGGCAGTGCATCACAAGGATGCATGCACGTGTATTGACCAGCTCCACCGTAAGGCCA
CGGACCTTCATGATCGTCCGCAAACCTCTCTAAGGTAAGTATGGGCGTCATCACGCACGA
TGCGGCCAAGCGCTACTACCCATTGATATCAAATAAATTTTAAT
>comp2334_c0_seq1
GATTTGATTTGATTTGATTTGATTGGTTCGCCGGACGGGCACCGCACGTCTTTCCGATTC
AAGGGATCTGTTGCGAGCACCGTTGCGGTGCGGGTGGTGGAGAAACCAATCCGTGTGTTG

CTGCGGCCCGCAACAAACGGTGCGTGTTCGAAACGATGGTGTGCGTGTATGTGCAGGTGG
TTCGTCGTTTTTCGTTCGGGTAGGACGGGACCATCTCTCTCGGAAAGGTATGCGGCGTCG
AAGTAAGAAAAATACCACGGCGTGTTTCAAACAGGCAGGACGTCGATTAGAATAAACAAT
AAATGGATCATGGCCGTGGG
>comp2335_c0_seq1
CTCTTCTGCCAAACAGACCGGATGATCGATATGTCCTATCCTGCCTATTCCCGTGCCGAA
CGTCTTGCTGACGGGGCGGTGCATATTGCTGGTCTGCTGGCGGCGCTTACCGGTGTTGCC
GTGCTATTCGCCATGTGGGCGCTGCGCATGGACGGGGTGACGCTGGCCTCGACCATCGTC
TATTCCGGTGCGCTTTTGATGATGCTGGGGGCCTCGGCGGCCTATCACCTTTATGCCCAT
ACCGCCGCGCGGCCCATTTTGCGCCGTCTGGATCATGCTGCGATCTATGTGAAAATCGCG
GGCACCTTCACGCCGCTCAGCGTGCTTTTGGGCACTGCCTTTGGCTATGTGGTGCTTGCG
TTGGTCTGGGTGCTGGCCATCGTTGGGGCCGCGACCAAGATCCGGGCCAAGCGCGGGCAG
ATGCCAACGGGATGGTTGCCCTATGTGGCGCTGGGATGGATCGGCGTGGCGCTGTTTATC
CCGCTTACGCCGATTCTTCCGGCACATAGCCTTGCGCTGTTGCTGGCGGGAGGGCTGCTT
TATACGTTTGGGGTGGTGTTTTACAGGTGGGAGAGCCTGCGGTTCGCCAATGCCATCTGG
CACCTCTTCGTGGCCTTTGCCAGCGGGTGCTTTTTCCTTGGCATCGCATCGGCCCTCGCG
ACCCGCCTCTGACGGGGCGCAAGGGCGTTTTGGATCCGGGCCAGAGCGCGCGCGCTCAGG
CGTGCTTGCCCGCCATCAGCAGGGAAAAGACATCAGTGTTCCGGCAAAAATCCGGCGTTG
CCGTCGCGGTCTTGTTCTGCTGGAGCCATTTTTCGCAGGTCTCGGGATTGACGCAGCCGG
TGCAGGCCAAGACCACATCGCCCAAGGTGTCAAAGGTCATCTGACCTTCCATGATTTTCT
GTTCCAGATCGACGCCCAAGCTATTGGCCATC
>comp2336_c0_seq1
CTCCTGGATAAAAACAATACAAATACATCGACAATCGGATAGAATTAAAGATTGCCCTCA
CTCATACTATCACAATAGAAAACATACTTGTCTTCTTCTCAGGACGGCACAACTATTTGT
TACCATGAACGTTGAACAGAGGACCATGCGGTGGACGAACGAGCCAGAGAAATGGAAGCG
GAAAGAAGATGGCATTAAAGTGACTTGTCCAATGGAAACGGATTTTTGGAGAGGAACAAT
GCATGGTTATAACAAAGATGATGGTCCTTTTTATTGGATGATTGCCGAGAACAATTTTGA
GGCAAGACTGTCGTTAAAAGTGAAATTGAAAGAATTGTACGATCAGGCGGGAATGATGAT
ACGCATAGACTCGGAAAATTGGATTAAACTAGGAATCTGCTGTTATCGCGATCAGCCCCA
CGTAAGCTGCTGCTTTACGCGAGGTGTGTCTGACTGGTCAACTCATCGTTTGTCACAGAC
TAAGGTTGATCACTTTCACGTATGGGTGAAGAGAAAAGAAGAAGTGATTGAGTGTTTCTA
TTCTTTGGATAACGAGAATTGGACTCGTATCCGACAGGGATACTTCTCGGACAGGCCGAG
ACTCAAGGTTGGTATGATGTGCGCCGCCCCCGAATCAGCAGGATTTAAAGTGACGTTTTC
AGATCTTTTGATCAAGAATCTTGACGATGATGGCGATGGCGACAGCGACAGCAACAACTA
AAGTTTGGACAACCAGAGGTCTCGAGGACGAAGGTCTTCGCGGTACTGAAGACTGATCAC
TACTCACATCAGTGAGCTTGCATCATAACATACCTAACAGTTTTGAAACAGACGTGGGTT
TACGAAAGTCATTGTCACATTCTACTACTACGTAATGCTTTGCCTTTCGAAGCAATATCT
AACTTTTATTCTTAATTATTATTGTGCCGTTAGCACGAATTAACAATTGAGTGGAAGACT
GGATTGCAACCGTAATACCAAGCAAAAAAGGACTATCCAA
>comp2337_c1_seq1
GGCCACGAGCGAGCGCGAACGCCAAAGCACGGTCCCGGGCAACTACCGCGCCGAGGCGGG
GTACGGGGCCGGGCGAATGCTCCCGGGCGACCCCCACGAGCGAACCGAAAACCGCAAGCG
AGCGCACAAACGACAAGAGGCCCGGCACGGTCGCCGGCCCAAAGCTACCCCCGCCCACCA
CCGTTCGGAACGAAGCGGAGGCAGAGCGCGTTTTAGAGGAATTCTGGTCGGTCCTGGGAT
CGGCCGCTTCGGGTGAGGATCCAACGGAGGCATCGGAAGGAACGGGAAAGCCAAAACGAG
GGTCGAGCAAAGGGACAAACTTGGAAGCCACGAGCAAGAGCCACAAACCACATCCATCAG
CAGCAGCTACAAAGACAACACCACCCACAGCAAATGAAAAGACAACAGAAATGGTAGATA
CAACAACAATAACGGACCCAAAACCAACGACGTCGACGGAGGTCTCGTCGGCTCTGGGAG
GACTCAAAACGATCCATCGCTCAAACGATCCGGGGGAAACGAATCATTCGCTCTCTCTGC
CGTCGCTACCGATTGTAGGGAACGAAGTGGTGTCCTTTGTGGCGGAAGAAATGCCGCAAA
TCTGCAGGTTTCAGCTCACGATGACACTGAGGCAGCCGTCGTCCTCTCCGCCGCACGAAA
GGAGCAGCGGTGGTTTGGATGCGGACTCGATCGTTGTCTCCACAACCCCACTGGGCAGGG
CTGCTTCCCGCACGATAGTGATTATTCGACGGAGGGAAGCAAACAAACCCGGCGATGTCC
GGGCTTCCCAGCCAGCGACAGACCACACCGCGGCTCTGTTTGTGGGAGAATTCCCACGCA
AGATAGAGGCTTCCGACGTCACGTGGAGGGTTTATCCCAGCAATACGCCCGGCAACGAAA
GCAGCGCAGCGGTGCCTATTTTTTCTGCGGTGAAAATCGTCTTTCGGCTCCCCTATCCGT
ACGATCCTTCCGTTGCCCCGGGGTCTCTCGGTGGCAGTGCGATCGGCAATGCTTCGTCGT
CTTCTACCCTGGATGAAATCAACAGCGTGCTGTGCGGATCGTGTCACACGCCCCTTATTC
TCCGGAGGCGCCACGACTGCGGAGCACACGACAACGATGTCGAGGCCAGTAGCAACAACG
GCCGCAGCAACTCTCACAACAAAACCGCAATCCATTCCTCCTCGAGCGATACCGAGCCGT

CCCAATCTGACGTGGGTACGATTCGTCGGGTATTTCCTCTCCCGGTGGGACACTGGGACG
AAATCGCGGAGTATCTGATTTGCTATGACGGTCAACCAGTTGTGGATTTTTCTGCTGGAT
CAGAGTGTGCAGAACCATCCATGGCGTTGCAAGATGCCAGCTTGCTTTGCTTTCACAGAA
GGGATGTGGAACATGCGGTTTGCGCTCTCGCCGTCGACGGATACGGTGAACCGACGGAAC
GAAGGAAAAACGATGCCAATAGAGGCGAAATCGATGGTTTCGGTGACCTGGGAACCTTCG
GAACCAAACCGGAGTCCTCGTTGTTTGTGCTCCAGGAGCCACAGGATAGCGGTGGTCCCA
ACCGCAACGACCCACAGGGAGCCCCGGGAGACGCAAAAGAGGAAGAGCACCGGCCCAATA
GCGAGGACGAGGTTCTTCCCTCCTCAGCGGTGGTGCGTGGAAATCGATCTTGGCAGGACG
CGGCGGACGGGGATAGCGTCAGCCTCTGCTGCGGTCGGTGCTGTGCTCCGATCGGGTTTG
CTTCCCTGGGTAGCCCCGAAACCTGGAGATTTTGGAAGCACCGCTTGTGCCTACCAATGC
CGAAACTGCCTCAGGAAAACGAACAAACGCGAGAAAGTGAATACAGGATTGGAGAGAATC
CCGTTGAAGCGAACCACATCCCATTCCAAAACGCTTCGTTGTTGCAGCCCACAAAGCCTT
TCGCATCGTGTTCTTCCTTCCTTGCTCGAGAACTGGTCCGATACGCCGAATCCAAGGCAA
TTTTCACGTTTATCGTTCGGTGTGAAGAAACAGAAGATGACGACGACGATGCGGAGGGCG
GAGAGAGCACAAAATCGAACAGCGAAATCAAGTGCCTCCTCCTGAGGCTACTCAGCTGGG
AAACCGCGATTGCGACATCCTTTGAAATAAACGATTCTTCTCTCTCATCGCGCAGGCGAC
TCCGGTTTCAAAGGGTGGCCAAGATTGTGTTCGAAGAAACCATCGACCCAACCGCAAGCG
AAGCAGCGGCAGGTGCCTCGGCGGGGCACAACGGCAACGGCTCGTCGGCCACAACCCAAT
GGTTTTGGGGGGGAGTCGACCTCTGCTGCCCACCTCCGTCTGCGGCAGCGACCAAAACCT
CTGCGCCGAAGGCTTCGTCCGGAGAACGAGGAAACGAGGGAGACGATCCGGTTTCTACCG
TGCGGCTGCAATTGCCGAGATTGGAATACGATCGAGTGCTTGACGACCTCGTGTCCGGAC
GCTCCCTCTTCCAGAAGGAGGTTGCCGACGCCACGATCATGCTGAAAATGGGTGGGATGT
CGGAAGGACTGGGACTCACCGCGGTGGCCCTGGAGTAATGGGTGCGTGCGAATTTGGCTT
CTCTTTCGAAAACGCGTCCGGTTCAACGAGGGAAAGAAATCTGTTGCATTCGGTCAAGGC
CAAAGCACACTGTGGAAAGAACGGAACGTCTACTTGGGTTTGGACGGAACGCAAATTGCT
TGATTTTTTTTCGCTAAAGAGTCTTTCAAAATGCAATTGCTAGCTACAATTACCTTGCCG
CAACCATCATATGTATTGTGTTAATTAATAATGGATAAAAAGATTGATTTGTCAACCTTC
TTACTCCTTGCCATCCTCAACCACTCTTTGTTGGTCCTCCGGAGGTATTT
>comp2338_c0_seq1
CTGTAATCGTAGGTTGCTAGCAACACGCAGTTGGGATCATTCAACGGAACAAAGCCGGGA
AATATTGTTTAATATGAGACATTCGTACCTGCCACTTCTGCTATCTAGGATACGGCCGCA
AGCAGAACTTGCTTCTGCTTCTGCCATCAGAAGCCACCTATCTCGCTCTTCTACGAGGCT
GTATTCGACCGCAGATTATCTGAGTCGATTGTCAAACACGCCGCCCTCCGCTCTCTCTTA
TTCCACAACAACAAACAAGCCATCCTCCGACAAAATAAATGCCTTCGATGGAAAACATAC
GACGTTTGAATCCTATTCGAATGCGCACGCGGCACCTCAAAATCACCGTGAGCCCGTTCG
GCCCTTCCCTCTGTCGTCGAGGAAATCGATTTCGGGCTACAAGAACATCGTCCCGCCCCC
CTATGCCGAGTCCGGAATCCCACCACAAGTCGACGATACCGTTCTGATATTTTCTGAGGA
CGAGTATCTCGCAAACATGAGGCACGCAGCAAAACTGGCACGGAGGGTCCTCGACCTCGC
CTGTTCGGTGGCCAAGCCCGGCGTCACCACGGACGAAGTGGATACGGTCGTGCACGAGGC
GCTCCTGGAGGCGGGGGCCTACCCATCCCCCCTGAACTATGTCGGATTCCCGAAATCTCT
TTGTTCCAGCATCAACGAGGTCATTTGCCACGGAATTCCGGACACGCGCCCCCTCGAATT
CGGTGATATTGTCAGCTTCGATGTGAGTTGTTTCATCAATGGAGTTCACGGAGACAATTG
CGCGACCATCATAGTAGGGGACGAGCAAGAGAACGACGAGATCGGTACCGACTGGAGGGG
CGTTCCCTACCTCGGCAGCGAGTACTTTGAGAACCGACCCGAAGCGGATGAGCAAATGAT
TCGATCGGCACGGCGGCTGGTGCACGCGGCGAGAGAATCCCTTTACGAGGCCATAAATCG
GATCGGCCCCGGTTCTTGCCTCACGGACATTGGAGCCGCAGTTCAAGACGTCGCGGATTC
CTACGGATATAGCACCGTCGCAAAGTACCGGGGGCACGGCATCGGTACGGACTTTCACCG
CCCGCCCTTCGTCAAACACTACCGCAACTACGACAAGCTTGAGCTGCTTCCCGGCATGGT
GTTTACGATTGAGCCGATGATCGTGGAAGGCGCCTCGGATTGTTTCGAATGGAGCGACCA
CTGGACAGTGGCAACGTCCGATGGAGGGATGTCTGCACAATTCGAGCACACTGTTTTGAT
TAAGGAAGACGGTGTAGAAATTTTGACGGTTCCGGAATGAGTTGAGTTTGCCCAAGCTTG
ATTGAATAGGATTGAAGTTGGTGTTGTGAAGAAATAAACGCGGCCCCTAGAGTATTTTGA
AATCACCCAGAGCATTTGTGACCTCAAAAACAACATTATTATATACCTTGTCCCGACGTT
CACATTTACCGCAAGAATGAACGCAATGCGTTCACCAAAGTATTGTTGAATTCTGCCCTT
AATTTCAATTGCTGATTGAAAGACTTTACGATGTCGAACAAGGAGCATAGTAGGATAGAT
ATATCTTCGTTTGTGATACTGAACTCTATTATAACTTAGCCTTTTTCATTTCTACGCTGT
AACATTCTATTTCGTCTCCCTCCTCGAAATCCGAAAAGCCCGAGAGACCCAGACCGCATT
CTTCGCCTAGCTTGACGCTTTCCACCGTTTCTTTAAATTTACGAAGACTCGAGGCAGTCA
CACTCTCGTCATCGGGGGAAATCAACTCACCATTGCGGTATACTCGAAAGTGGGAAGGGA
GGTCAATCGTATCCGAGCCGCCAGCTGGTCCCTTTGCTCTATACATATTGCCATCCAATA

CGTTTAGCCCGGCTATTCGCTCTTCGCCGTCCTCGGTATCTATTGAGAATATGGCCTTCA
CTTTAGC
>comp2339_c0_seq1
ACCACCACCACCAACAACAACAACGAGAAGAATCGTCCGTCGGACATGGTGGCTCGAGAA
CAGGAATCGCTGTTGCAAGAGGTTTGGGAATCCTGCGATTCGATTGCTGCCAAGCTTGGT
GATGGCACTGTTTTTTTTCTGGCTGCATCTCTGCTGATAGGGGCAGCCGCGTTTGCGTTC
CACCCGCAAACGACGATTCCATCGAGACCTCTGTTTGCTCCCGCGATTGCTTCCGTTTCT
ACCAATAGCTTGCTACTGCCACTCCACGCAACAACCACTGATGCCTCCGACAACGGCAGA
GACGACGACGAGGAGGAGGAATTCTTCGACGCCTCGTTCTCATTCTCCACGGAACGCCTC
GTTTCGAAGTCGGGGTTGCCCGTCTGCCGTTACGCCCTGGGGGGTGCGGCGCGGTCCACC
CAGCCCAAGAGCATCGTCAACGAGTACCGGGAAAGGTTGGAAAACGCAATTGTTGGCGCC
AACGCAACTCCCGCTGCCCCCTTTTTGTTTTACTACAATCCGCACCGGTATCCGGACTTT
ATGCGGGGGATTCGTGAACTGGCCAACAAGGACAGCGGGAACAATCCTATTCGGCGGGAG
GACCTATTCCTGGCCAGCGGTGGGACTGATCGGTCCCAGAAGGGCATGGAGCAGCGCTTA
CGGGACGCCCTCGCTTATTCCGGGGGGGACTACCTGGACCTTTTCGTCCTTGAATACGTC
TGCCCGGAGGAAGCCGGAGACGACAGCGGCGACCTGGTGAAAGCCCTGGAACTGGCGCGG
TCCTGGGTTCGGGACGGTTCCGTCCGGTACGTAGGGGCCTCGACCCACTCCCACTCGGTG
GGGGCCTGGCTGGGGTCCCTGGACCGTGGGGAGGAACGGGACAACGACAACGACAATGAC
GGCGCGTCCTCTCCGCTCCCACTGCTGGACGCCATGATGCTCCGGTACAACATGGGGCAC
AAGGCCGCCGCCGAGGCCCTCTCCCTTCCGGTGTGTGCGGAACGGGGGATCCCGGTTCTG
GCATTTACCACAACGAGGTGGAACCGCCTCCAGGACGGCCATCCCGATTGGGGCCAACGG
GCCCCAACGACCCCAGAGTGCCTGTCCTTTGCCCTGTCCGCGTCGTCGGACGAAAGAGGC
GCAAGCGCGGGACCAGACGCACCCACGACGACGGAAGACGGCGCGGGCCGGCCGATCGAA
ATCGTCCTGCACTCGGCGCGCGACGCCGAGGAACTGGGGGAGGGCATGTCGTGCCTGGAT
TCGTCGTTCGTCCCTCCGACCATCGACGCCGGAAATCGTGCCGCCGTGGCTATCTGGAGG
GACTACGGAGACCTGGAGTGGAACGACATGGATGGATTCGATGAGTATCCGGAAGAACAG
CAGGGAACCAGCATTCTGTGACGCGGTGAGACGGCAACGGCTGTGCTCTGTGCCGCAAGA
CAAACTGGTTGCCGCGCATGACTATTCTACCTTTGGTCCCTCTGAAGAGATATGTCGTTT
CAGTAGTATTTTTTTAGCAATTATAATACAATATTACTGCATGCTTGTGTTTATGAAGAT
CCGATGGGATCCAGTTTGGTTCAAAATGATCCATAGCAAACGTTTGTGCCGCGGGACCAA
AGCAGCAGTTCAATGATCTACCATCTGTTGTTTACAAATTGAGTCAATCCAC
>comp2340_c0_seq1
TCCGTCTCATTTCTAAGAACAGGGCATGCTGAAAACGCGATCGATTTCCCCATTCGTCGT
CATCTCACAATCATGTCGAGCAGTGAAAGTATTTTGAAACTGGATGGTCGTAACGACGCA
TCAAAATCGGATTGTGGTGATGACAGCGTCTTAAATTACCTCGAGACTCCTCTGAATGCA
TGCAGCCTTGAGAGTTTTTGCAACACCATCCATTCAGAACTTTGCGGAGAACAACAATTC
ATTCCGCTTTGTTTGGGCTGTTATCAACTCAACGAAATCACAGGAAATAGGGAGGGATTG
TTGGAACTTTATGCGAGCACAACCGAAAGTTTCGGAACCGGAATGCATCCACCATTGACA
ATTCTAGGAGACGGAGATGGTGCCCCAGGCGTATTAGATGGAAAGTGGTCAAGTCGACTG
CCGTCATCTTCTACTTCAATTGACTTCCATAACTATTATGCAACGGCCCATGCCAATGGC
GAGATTTGGGTTCACAACATCACGAACAAAGCGGTCGAGATTGCCAATGATGGCGTGGAG
AAGAACACGAATCCTTTCCGAGCAACACTTGCCGGGAAATCGAGGTCGCCGAGTGAGAAT
GAAGGACTCTGTCTTGCTCTGGCTTGGGAGGACCCGCTCTATCGGAATGGCGACAGCAAT
TGTAGCACTGCTGTTGATACTCGCATCATATCATCGTACAGCGATGGACGTACTGCTATT
CATAGAGTGAGGGAACATCCACCCACGTCGGCGGAAGATGCCACGCAAACGGTGGAATTG
TCTCTAGAGCACCACTGGGATTCTCATACTATATTCAAGTGCCCTGCTGAAGTTTGGTGC
GCTAATTTTATCGATTCCAATACTGTCGTCACGGGGGGGGACGAAGGAAGTTGGAAAGTA
TGGGATTTGCGACAGCCACTCATCAAACCAGTTTATCATGGCAAAAATGACTTTGAGGCC
GGTGTGACGGTGCTATCTCCCCACCCACGACACAAAAATCTACTGGCTATTGGGAGTTAT
GACGAGACCATCGCCTTATTTGATCTCCGTCGCATGACGACAGATTCAACCAGTGCGAAC
AAGTGTCCTGAGGAACTGTACCATTCTGAGCCTCTCGGAGGCGGCATTTGGAGGTGCAAA
TGGCATCCAAGGGATGATAGCCGGATATTAGTGGCAGCTATGCATGGGGGTTGTGTTTCG
GGAAAATTCGGTGGGTTGGATAGCGCGAATTTTGAAGGTCAAGAAGCTGTTATCCCCACT
TCAATTAGTTTTAGTATACAAAAGCAATTTAACTTACACCAGAGCATGGCATATGGAATT
GATTGGTTGGTATACCCAGAAGGCACGACAGAAGATACGAGCACTGAAGCTGCCGCCAGT
TGCTCATTCTATGACAAAGCTATGTACTTGTGGAAGACAGGCCCCGAAAATATTTAACTA
ACTAGCAGATCTATTTTCCCA
>comp2341_c0_seq1
TTCCGAGGTACGTACTCAACATGCGTACGAAGGTATTCCCGATTCTATTCGATGGATATT
TCTTCGAGGCCATTTGTTTCTCAGTGTTGAAGTCTCACCTTGTTTTCGTGCTCTCGAACT

TCAATCTGTCTTCAGGAAACTGCGATGAAGACCAACAGCAACAGCCCGAAACTTTCAACA
ACACCACAAGATACCTCGTTTATGACGGAAGAAAAGATGAAGAGCATCTATATGCCGAAA
CGAATCGATTTTTAAAGTGATCGAGGCCGGGTTGACCGAAGCAGCTGTTGCTACGTTTAT
AAAATTTTGATTCGGAATCAGGCTTTCATTTCATAGAAGTTTGAATTGATTTTGAAATCA
ATAGCTTTCTTTCGGTTGGTAGAACTCGGAGGGAGTTTCGGCATTGCTCAAAGATCGTCG
ACAAATGCTAACGTGTGAACCGAGTCCGTGGATGAATCACTCCTTCAATAAAGATTACTG
AGGCGATGACCATTGCTAATGTTGCCGGTTTGTGGTCTTTAATTCTTGCGAGTGTTCATT
TGGGATTCCTTGCGCATGTATCAGATGTATCTTGGTTCATGCCATAAAGATATGCTTCTG
CACCTGAGGTCAACCGCTCTTGTGTTCAAAAAGATCGCTTGTTTTTTCTTTTGTATTTGC
CAGCAGGCATCCTGTCTGTTTTGATTTATTTTGTAGTTCTTAAATTTAAAAACGATTGTA
TTTTATAACGCTGAAAAAAA
>comp2341_c0_seq2
CCAGTGGCAACAAGAACACACCAAAGTAACTTCACGAGTTTCGTAACAATGAATCGACAA
TCTACTAGCAACGATTACGCGCACGACACCACATGTCGCTTGAGTGCGGAAGACAAGGGA
ATGATGGCTCAAGTATCGATGACGAGCTGCAACCGAATCGCTGGACATTCCGAGGAAACT
GCGATGAAGACCAACAGCAACAGCCCGAAACTTTCAACAACACCACAAGATACCTCGTTT
ATGACGGAAGAAAAGATGAAGAGCATCTATATGCCGAAACGAATCGATTTTTAAAGTGAT
CGAGGCCGGGTTGACCGAAGCAGCTGTTGCTACGTTTATAAAATTTTGATTCGGAATCAG
GCTTTCATTTCATAGAAGTTTGAATTGATTTTGAAATCAATAGCTTTCTTTCGGTTGGTA
GAACTCGGAGGGAGTTTCGGCATTGCTCAAAGATCGTCGACAAATGCTAACGTGTGAACC
GAGTCCGTGGATGAATCACTCCTTCAATAAAGATTACTGAGGCGATGACCATTGCTAATG
TTGCCGGTTTGTGGTCTTTAATTCTTGCGAGTGTTCATTTGGGATTCCTTGCGCATGTAT
CAGATGTATCTTGGTTCATGCCATAAAGATATGCTTCTGCACCTGAGGTCAACCGCTCTT
GTGTTCAAAAAGATCGCTTGTTTTTTCTTTTGTATTTGCCAGCAGGCATCCTGTCTGTTT
TGATTTATTTTGTAGTTCTTAAATTTAAAAACGATTGTATTTTATAACGCTGAAAAAAA
>comp2342_c0_seq1
AAAGAAAGGAAAACTGGTGAAGGAGGTATGTCCCAAGCAGTGTGGCTTTTGTTTGTGAGG
AGGCCAGCAGTGTGGAACTACGTGTACACGTGAACCAACCAATTTATGAAAGTGAAACAA
TTGGAATTTATCATTGTGTTTTGTGAGATCGGTGCGTTGCGTTTTCGTCTTTGTCGTAGT
GATTGGCAACATTGTGGGTTCGTAGTCTGTGGTGGTGGTGCGGGCCTTCGTTATACCGAC
GGCGACGACGGGGTTTGTGCTTGCGTTTGTGTCTGCCCGGATCGTTCCTCCAATGGACGG
TTCTTCCGCGGACGGATCGATCCGGTGGGGGAGGACGTCCCTCCGACGCGGCGGCTCGGC
CAGAGTTCTTCCCCGGTGGTTGGGGCTGCTGCGGCGGCGGCCCGGGTCCGGCTCCGAGTG
GCACCCGCAACCGCCGAGGCGAGAAATCGATGCAGCGGTCCTCGTGGCGGTTCGCGTCCG
GGGGATCGTCGTCCTGCATTGCATTCCCTCCCAAAACCCTGCCATCGATCGATCATCGGT
CGGACACTGTCACTCGTCCAAAAATTACGCGTACGAAGCAGAAAACAAAAACACTTTTGG
GTATTCCAGCAAAGCAATACTATGCGCACGGCACGTGGCGTAACTTTGCATACCGTATAG
GGCAATGCTTTCTTCCGCAAGGCAAAGAAAAATATTTCTGTAACAAGCGATGGAAAGAAT
GTTCCTACCATTCATCCAAGCGGATCGGACGAAACCAAGCTTTCGAAAACTACGATACCT
CTGTTTTGTGAAGTGTTGCGCATGGGCTGTGATAGTGTGCTACCACCTTCTCCGAGTGTA
ACAAAGAAACATCGGAACAACCGATCGAAAGAATGCTACAAAATCAATCGTTCCGAAAAC
TGGTGTTGCTTTTCGGTCCACTGTCACGTGCGGGCTGCAATGGTGTGCTATGTTATCTGT
TTGCTTCGAACGAAAGAAAAAATCGGAACCAACGAAAGAAAATGTTTGGATTTTCCGAAG
AGTTTTTCGATTCGTACCGGTATACCACAAAATATGGTCACCGAAAACGGAGGCTGGGGT
TATAATTTTATATAACCCCCGAACGGAGGTATGCCATAACGGTACATCAGCAGAACGTGA
AGTACGTACCTCGTAGAAGTAATTTTAAAATACCGTACGTTCTACAGTAAAGTAGAAAGT
ACTGTACTTCGTACGAAGTACAATTCTTATGAAGTACCGTCCG
>comp2343_c0_seq1
AAAATACAAGAGAACACAAGACAACGAATGACCTTGCCAGCAAAACAATCAATAAGTAAG
AAATATAAACGCAACTTAATTCTTCAATTATGACGTTTCTCGACACACCAGTCTGCAAGT
GCTGTGGCGGGAACAACAAGACCACGATCGGCGACGTCTTCAAGCTTATCATACCAGTGG
CATGGAGCACGGTTGCTTTTATGGCATGCCTCGCCTATCTGATCTCACCCCTTTCGCGGG
GCGATACTCCTCGGTGGGGAAGCGCCGCTCTCGTGGCTCTGAGTGGAGCGGCCGGAACCG
GTTTGTCGATCGCTATCTATAGTTTCCACAGAACCACATCTTCCTTTGCTATCGACGAAA
ACTATGTGGAAAGTAGCAACGACGGAGATGGGGAAAGAGAACAAGACGAAGATCAGCAAG
GGACAACCAAATTGGAACCACCGATGAAGAAGATACAAAGACAAAAGATGTTACAGATCC
ATGCCGTCTTGGCGCTTGGCCTTTCGATTTTATCGATAATTCTGGCTATATTTTTTGGTG
TGTTTGCCTCTAATCTCAACAAGTGCGCGCACGAAACCTGTGGAGCCGACGTGAGCTGGA
GTTGCATCACGTTGGTAGTTTCTGTTTTGTGGATGGGTATCACCTACCTGGGATTCAAGC
ATCTACGGCAGCAGCTGTTGCCCGAAAAGGACGCAACAAACAATGAGAACGACCAAGGAC

TGGAGATTCCCTGACGGCGAAATCCTCTGCGAACTTTCAGCGACACACCAGTCTTCTAAG
TGGCTAAAATTTAGGTATTCTAGCTATATGCGCGTGTTGCTGCAAAATGTTCTTGATTTG
ATGTGGGAACGAAACCGACCGAATGCTGGAATGCTGACTGTATGAATGTTATCACGCTTG
AGTATTGCATGTGCCATAATGTTACTGCTACTACTAGTATGATTGTTTTGGATCATGTAA
CAGCTGATAGCAGCGTTGCTAGCAAAATCATGGAAGTCGAAACATAAAAAGAAGTCCAAA
ACAGTTTTGACTGTAATTGGTTCTTGAAGAGGAAGAGGGGTCTAGGGGGTGGATTTTCAA
GAAACTACTCAGTTGGCTGTTTTTAGTTGTAGAGCTGGTATGGTTTTGAACTGTAAGAAA
CATTGTATCAATAAAGTTATGGGAATGAGATGCAAGCTTAAATGCTGGTTGCATGCGACT
ACATTGAAAATACAGTGCCGGCACCTTCGACTTCCACTGCCACCCAAGCAGACCAATCAT
CAGTAATTACTTCCCTACCGTAGTAAAATACAATATTATTTCCATCTTTCACCGTG
>comp2343_c0_seq2
AAAATACAAGAGAACACAAGACAACGAATGACCTTGCCAGCAAAACAATCAATAAGTAAG
AAATATAAACGCAACTTAATTCTTCAATTATGACGTTTCTCGACACACCAGTCTGCAAGT
GCTGTGGCGGGAACAACAAGACCACGATCGGCGACGTCTTCAAGCTTATCATACCAGTGG
CATGGAGCACGGTTGCTTTTATGGCATGCCTCGCCTATCTGATCTCACCCCTTTCGCGGG
GCGATACTCCTCGGTGGGGAAGCGCCGCTCTCGTGGCTCTGAGTGGAGCGGCCGGAACCG
GTTTGTCGATCGCTATCTATAGTTTCCACAGAACCACATCTTCCTTTGCTATCGACGAAA
ACTATGTGGAAAGTAGCAACGACGGAGATGGGGAAAGAGAACAAGACGAAGATCAGCAAG
GGACAACCAAATTGGAACCACCGATGAAGAAGATACAAAGACAAAAGATGTTACAGATCC
ATGCCGTCTTGGCGCTTGGCCTTTCGATTTTATCGATAATTCTGGCTATATTTTTTGGTG
TGTTTGCCTCTAATCTCAACAAGTGCGCGCACGAAACCTGTGGAGCCGACGTGAGCTGGA
GTTGCATCACGTTGGTAGTTTCTGTTTTGTGGATGGGTATCACCTACCTGGGATTCAAGC
ATCTACGGCAGCAGCTGTTGCCCGAAAAGGACGCAACAAACAATGAGAACGACCAAGGAC
TGGAGATTCCCTGACGGCGAAATCCTCTGCGAACTTTCAGCGACACACCAGTCTTCTAAG
TGGCTAAAATTTAGGTATTCTAGCTATATGCGCGTGTTGCTGCAAAATGTTCTTGATTTG
ATGTGGGAACGAAACCGACCGAATGCTGGAATGCTGACTGTATGAATGTTATCACGCTTG
AGTATTGCATGTGCCATAATGTTACTGCTACTACTAGTATGATTGTTTTGGATCATGTAA
CAGCTGATAGCAGCGTTGCTAGTAAGAATGCTTTTGATTTACTGTAGGAAGCAAGATGGA
GTTTGAAATGCAATTTGTAATCTTCCTACTAACAGGCCTAAGTGTTTTCTTTCATACATC
ATTTTCTTCGTACTTGTACTAGGCAAAATCATGGAAGTCGAAACATAAAAAGAAGTCCAA
AACAGTTTTGACTGTAATTGGTTCTTGAAGAGGAAGAGGGGTCTAGGGGGTGGATTTTCA
AGAAACTACTCAGTTGGCTGTTTTTAGTTGTAGAGCTGGTATGGTTTTGAACTGTAAGAA
ACATTGTATCAATAAAGTTATGGGAATGAGATGCAAGCTTAAATGCTGGTTGCATGCGAC
TACATTGAAAATACAGTGCCGGCACCTTCGACTTCCACTGCCACCCAAGCAGACCAATCA
TCAGTAATTACTTCCCTACCGTAGTAAAATACAATATTATTTCCATCTTTCACCGTG
>comp2344_c0_seq1
CTTCTCGTCAAAGAACTGCGACTGCTCTGCCACCACAATTGAAATCTCGAACAAAGATCA
TTGCATAGCATAGCATCACACCACACCGCACCTTACTTTACCGATTGCCTCCGAGGATGA
ACCGGCTCCGACCACTCGTGGCACGAGGCTTGCCATCGGCGGCAGCAACTGCAACTGCAA
CCACAACAAGAATGGCGGCTTGGAGGAGACAACGGCTGGCAAGGAGCCATCGAAGCTTCC
AGCTGCGTCTGGGGTCCTCCTCGCACAAGCACAAGGACAAGCACAAACCAAGCAGCAGCA
GCGCACCCAGGCCCGGCGGCACCTGGATCTACCTCGGCTGGACGGTCCTCGGCCTGGTCG
GCGTCGACCAGGCCCTGCAGCACAAGCAGGCCTCGGCCGACGCCAAGGCCCGGAAGGCCC
TTTCGGTCCTCCAGAAGGACGCCGAGGCTTCCGCAAAGGCTGACTGGGAGCGGTCGGTCG
ACCAGCGAGCAAACACAGCCCCCGTCGGTGCCTCGAGAATCGCCAGGGTCGATCCCTGCC
TCGACGGGACCAAGATGCTCACCCGCCACAGGGGCGTCCAGAGGGGGGACGCCGTCGACG
TTCTGGAAACCGGCGTCGGGCCTGCCGGCCGGTACCACCTGTGCCGGTGGACCAAGGGCG
GTGCGAATGAAAATGCCGGCGATGGCAGCGCCGTGGTGGTGGTGGGCTGGTACCCGATCG
ATTACCTCGAGCCGCTCGAACGTGGAAAGCACGCCTAGCCGGTGGTTGCACCCCTGGGTG
GCACGAACGCAAATTTCTACTAGCCACCGGTAACTCGGTAATTCAATAGACACGGACTCA
TGCAACAATTATCTCTACCGTGGAAGACACACATTACACTACACATTACATTACACTACA
TTACCATTCGCGTGCGTGCTTAACCTCCGTGGGTGGAACCGCACGGACCCACTCGCTTCG
CTTTGGTGCTTCCCACCCACCGTTCCCGGTGCCGGTGCGTTGCGGGAGGGGGTTCGCCCG
GGCCTTCCTCGTCGCTCGGCCAGTCCCCGCCCATGCTTTCGGAGCAAGGGGGGTCGCGGT
GGGACGAGGAGGAGGAGGAGGAGGGCACCCCGGTTCCCTTCGGCACGGGGTTGGGATCCC
CCAAAAGGTCGCCGCCGGCTCCTTTTTCGGTCGGGTTCCGGTCCCCGCACGGTGCGCGTT
CGCGTTCTCCCCACGCCGGGCACCACCCGTCCAGGGACAGGTCGGACTCCTCCGAGCGGT
TGGGGGCCACCCCCAGCTCGACCACCACCGGGACGCCCTGGGCGTCGGTTCCGAGGGTGG
CGGAAGGCTCCCGCTGCTCCGGGGAGCCCTTCCCGGGAAGGTGCCTGCGCGAGCCCCACC
CCTGGGGGGGGGGACAGCTGCTCGAAGAGGGCCCGGATCTCCGGGAGCTCGTCGCACGGG

CCGATCTTGTGCAAGAGCTCGCTGCGCCCGCACCGGTGGGCCCTTCCGCTTAGACGGCGC
CAGGGGCCCTTCCCCGTTGCGTTCCGATCGAGGGGCCCGGCCCGCCGGGGATGGGCGGTT
CTAGATACCATGGAATGGTGTGTGTGTGTGCGGATAGTGGTGGTGGTGGTGTCGTGCCTG
GACGCCTCGTGTCGTGTCGTGTCGCCGAGGGGGTGGTGTCGTGCTTGGTTTGGTTTAATG
GGACAAACTCGCCGCAAGAAAGGATTTTTCTTTCGTTCGAAGAGCAACAGCGAACAACAG
AAGCGAGGGTTTGCGGCGGAGTGACGGAATTCCGGGGGGAGGGGG
>comp2345_c0_seq1
CCCATAGTTCCTGGATTGATTCAATAGTCAGGAAGCATAAAATGTCGTTTGTATCCAGAT
GGATGCAAAGAATATCCCAAAGGAGTCGGGATGGATTGGCGTTGTCACGAGCTATGTACC
TTGCCGTTCCACGAGCAGCTCCTGCGAATACTACTATTCCGGAACGAATCAAAGCGGTGT
TGTTAGAAGAATCTCGACAACCTTCGAACAACAGCGTTGCAATATCGCTTCTCAAAATTG
ATAAAGAGATGGGTTTGGTGAAGTATTGGGGCTTGGCGGAATATATGCGGCAAAAACAAG
ACTCGTTTGTTTTGCCCTCGATTCTTTCATCGAACTCAAATTCCTCTTTACCAGAAAATC
CCGACAGCAAAGTCTCCGTCACGGATTCTCCTGGAACTGGTGCTGCGATCAGCACCCATT
CAGCAAGCTCTGGTGCAAAGGTAGCCTTTATGATAACATCTCCCATGAAACATGATTTGA
TCAATAACTTGGGATATGAAACAAATATCATCAAGGGCATGACTCCACAACAGGCATCGC
TCGTATTGCATCATCGGGTCCTTCCAGAATCCTATGATGATTGCATCTCAAAATTAGAGG
AACAATTCCAAGAGGAACAACAAAAATTACAGGAGCAAAAAACTTGCCTCCAGGAAAAGG
AACAATTCGAAATGGAGAAACAACCTAGGGAAGTCGATGTCGAAGATAATTTGACTACAG
CAGAGATCGCTGCAATTCCTTCTGATAATGCTAGTAATAGCTTGTACGTTGCTTCGCATG
ACAGAGATGAAGGATATGATCGAGATAACAGAGTCAACATTGATCAAAAGCAGTTGATTT
TGTCCTCTTCTCAGAATGAAGAATTATCATCACTGTCTTCCGACAGTCGCCTTCCGAATG
ACCTTGCAACAGAATCACCCACACTACCATCAACAGAGGATCGATTTATCCCAGAGAGTA
GTGTTCCTAAAGAAAATTCTACTGATACGATTGTTAGAAAAGAAGAAGAGGATATGAATC
TTTGGTACGAGGTGGTAGAAATCAAAGGTGAGAAAGCATCCTCCGCTGCTAGTGATGCGG
GTGTATATTCAAACGAAGTACGACATGGATTGTACCGCAATCGAGATGAAGCAATGTTGA
GTTTGGAGACCCGCCAAATGATTAAAAATCGTCGAGAACTCGATACCAATGATAATGGTG
CCAAAAATTCTACAGAAACGTCGTCATCATTTGTCCTGCGTCCCATCTCCGACGAAGACC
TCCGAGTGCAATCATGAAGAAAATAGATAGAGGCTGCCTGGGACGAATAATCAAGGTGCA
TGGTTGTGTTTCCATCAAACCTAAGGATTGGCAAGTATAGATTTATAGACCGATGTCGAG
CAAGGGGTAAATTTCTAGGGTTCGAATAAAGCAACAGATTGTGTGTAATAATTTGGTAAC
TTCGTAGACGGCTTAGTGCGATCCGTGGTGCTATGGTGCCTCTTCCGAC
>comp2346_c0_seq1
CAATAGTTTCAGCATAATAGAAGATAACTGTTCACGATGCCTTCCCAAAAGACTTTCCGA
ACAAAGCGCACGCTTGCCAAGAAGCAAAGGCAGAACCGCCCTTTGCCACACTGGGTTCGT
CTTCGAACTGATAACACTATCAAGTGGAACGCCAAGCGTCGTCACTGGCGCCGCACTAAG
TTGGGTCTTTAAAGTCACTGTTTGCTTTTCTCAGTAAAATATGGGGGCAATGAATTCGTG
GACGAGCTTGTAAAATACAACGTCCGACGGAACATAATAACTCTTACGCCAAAAGTATAA
CGAACCTCTAGATCACAACACACCATCGAAACAATTGTCTCTGTTAATACGCACATGTAC
TTTCTATTATTTGTAGAATGAAAGCTAGATGCTATCGCATTTCAGAACGGGAGAGCGATG
GATGATGTTCTTCTAGACAGGAATACAATGTTGAAAAGCAAAATAAATATCGCTAGACTC
TGTTGCACCGCCATGCGGACTTCCATTTGGCTAGTCCGTTATAACTATTTAACGTATCCA
TCTTGGCAACAACGTATGACTTTCCTGCCTGGAGAACGAAGTGATAACTATCTTCTCGA
>comp2347_c0_seq1
GAACAATTTAAACTAAATTAAATTTCCACCATGGGTCGCCGTCCTGCTCGCTGTTATCGC
TACCAAAAGAACAAGCCGTACATCAAGTCTCGGTACTGCCGTGGTGTGCCAGAATCGAAG
ATCAAGATCTTCGACGTCGGTGCCAAGAAGGCCCCCGTCGATCTCTTCCCCTTCGTGGCC
CACCTCGTCTGCGACGAAAAGCAGCAGATCTCGTCCGAGGCCCTCGAGGCCTGCCGTGTT
GCCATCAACAAGCACTTGGTCAAGACCATTGGAAAGGAAGGATACCACATCCGCATGCGC
GCCCACCCCTTCCACGTCCTCCGTGCCAACAAGATGCTTTCGTGCGCCGGTGCCGATCGT
CTGTCCTCCGGTATGAGGCACTCGTACGGAAAGCCCATCGGCGTCGCCGCCCGTGTCGAC
ATCGGTCAGATCCTCCTCAGCGTCCGCTCCAAGGACGCCAGTGAGGCCCACGTCATCGAG
GCCATCCGCCGGGGAAAATTCAAGTTTGCCGGACGCCAAAAGATCCTCAAGTCCGCCAAG
TGGGGCTTCACCAAGTACCCCCGCGAAGAATACGTCGAGAAGCGCCGAAGCGGAGAGCTC
TCGTGCGACGGAAACATCGTCAAGGTCCACAACCGACGCGGTCTCCTGGTCAACTCTGCC
CTTTACCAGGCCGGACAGGAGTAGGCAGCCGAGCCTAGCCTGCGCACCAACACCGATCGT
GGCAACGGAACGGAGCCGCGTACTGCGTCTTGTGTGGCCCGTTGGGTTGTTCTAGATTGT
GTACCACCCCATACAAAAAAAGAATTATTATACACCGA
>comp2348_c0_seq1
GTTCCTTCTGTTTCTTCACGAATGGATACAGAAGGAAAAGATTTTTTTCATGAGAGGTCG

CATTATGTCAACGCTGGTTCTGTTCGAACCAGAGAGAGATTTATTCTCAGAACCACTTTT
CGATCAAGTGCCGCTTAATATACTCATCTGATGATTGTTATCTTTGTCCACACAATAAAA
ACGACAGTATCTTCAAAAGTTTTCAACTGCAGAAGCATTCTACACTTTGATTTCATATCA
TTCAATATGTTTAGTCTAATCATTTGGCTTTCATTTGCAGGCGCGACACGTTGCACTGCA
TTCGTTCCTTCTTCCACCCTGAGTTCTCTTCACGGAGCCCATGATCTCCTCCTCCTTAAT
GTTGCTACAGATGACTCTGCTGATACTATTACTGGCGAAAATGTAATCCGTGAAGTGTTT
CCCGGTGTCCACTTACCCAAGGATAACGTACCCCTGGCTCTCAACAACGACGGCGTGGAA
GCAAAAACAAAAAAATTTGTTTTCAAAGCTGCCCCACTCCTTTTTGCTCTGAATGGGAAG
CCAAAAGCGGACGGGTTGCTTCCCTTGCAAAATGCTTACCTCGAGGAAACCCTCGATGAT
TACACCATCCCGGACGATCCAAACTACGATTTTTTCCCAAACAACTGTAACGCGAAGACC
CTTAGGTATCTTCTTAAGGTAGACCCATTTTTTGCTTCAGCCTTGACCAAAACCCGCTGG
GGGTTTGAACTCAGATCCTTCGATCGAAGAGATCCCGATTGCAAGAATAAACCATCACTA
TACCGTGAAATGACACGCACTTTGACCGGCGTCGGCCGTAACGTAAACTTTTTCTTTGAC
AAGCGCATGAAATTGACAGGTTACAAGGTTTACGACGATTCCAAAGGATCGAAAATCTTC
GAATTCAAGAGTCAACAAGACGAGGACTACTGGGCAAGTGCTGCGATTTACAACCTATTC
TTTTACGCCTCATCCATTCACGCGACGATTCATGTCCTTCACTACCTAATAACTTCTGCA
TTTCAATATGTTTCAAAAGACTTCGAACCAATGCACCAATGGGCAAAGTTTTACGCGAAT
AACATTCAACAAAAGTATTCGCAGGTTGGGACACTTTTGATCCTTCCTGCACCAGAAATT
CTTGACACAGACCAATCCAAGATCACTGCTTTGATTACGGGAAAATTGGGTTTTGGTTCC
ACCGCAGCGATCACACCTATTCTATCTCGTCTATTGAATACATGGGGCCAGAATCCCTCT
GCATCGTCCTGGATCGATTCCATGGTTGGCATCTCATCCAAAGATATGGATAGAGCAGGG
ATCCTGACCGAGTTCAGGAAGCATGTCAATCTTATCTCACCATTTGCAAAGGAAGCATCT
GATGCCTTTCGCAATATAAACCCAGAAAAGGCTGCGGCCGTCGAGGAACGCTTGAAGCAG
TATCTAAAGGAGTGTGGGAGCTTCACTTCAGAAATCAATAGCCTCGAAGACTGGATCGAG
CTTATGAGCATCACGGCTGATGTTCATGGTTCAACTCTTGGCTACACTCGTTTGGCTACT
TCATCGGATGTCATGCGATGGCGTGACATTCGCGATCCAAACTGGGATTCCCTCGGACTC
AATATGATCACTGTTTTGTTAGGAACGGTTTGTGGTATGGAAGAAGGTCGTCATACAATG
ACTTCGACGGTCGATGCTCCGTACGACCCCGCACTCCAAGCCGTCCTCGACAAATACGAC
GAGAAGGCAACTCGGTTGAAGGAAGAATACCAGGAGAATATCCAGAAGGATCCCGATTTT
AGTGATTATGGATGGATTCTTTCTGGCTTTTGTACCGATGGTTTTGATGGCAAGCAGCTT
ACCAGTGCCACTTACATTTAATCGGCTGCAATGACTCAGGATCAATTTGCATCGATAAGT
TTGGATAATTGGTTTGTCGGCCTGTGATCAATGAAAATAACGAATCAGTTTTAGATTGAT
TTTATGGATGAAAATTCAATACGATTTGTATGACCTCAAATGATATCCATAGAATAGGAG
ATACTGCATATTAATTCGAGTAGTTAACTGTGAAG
>comp2349_c0_seq1
AAAGTATCTGTTGCACGCATCCCGAGAATAACCGACACAACACTTATTCACTCAACTTTT
AAACACCCAAAGATGAAGTTCACTATCCTAGCATCTCTCGGCTTCTTGGCTACTGCATCA
GCCTTTGCGCAACAAAGTTGCAACCGACCGTCTTTCACTGCTCTCAATATGGCATCCCCC
GCTGAATTCGTTGACTCAGAGATTGAATCAAACGATGTTGTTGTTTTCTCGAAGTCTTAC
TGCCCTTTCTGCACGAAGACCAAGGAGTTGTTCGATAGCTTGAACGTCGAATATGCCGTC
CACGAACTCGACCAGATGGGAGACGAAGGTCCCGAATTACAAATGGCTCTCTTCAAGAAG
ACGGAACAAAAGTCTGTGCCCAATGTCTTCGTCAAGCAACAACATATTGGAGGCAACGAT
GATACGCAAGCCGCAGCTGCGGCCGGAAAGTTGCAGGAATTGCTTGGATTGTAGATAATG
AATTACGGTAAAGAAATGAAAATCCATTTATAATTCTCCTCTATGAAAAATGTTCTGCAC
TTATATCATGGATGCGGCGTATTTTCAACAACTTCAAACGTTGTTTCTTCGAATCGTACG
AGTAAGTACCGGTTGGAATACTGTTTTTCGATCTCCAATCCTGAGGCACAGAGCCGATCA
TAACAAAAATCAATTATTGCTGGCAACTAAACGATTCTAGAACACATGCAATCAAATCAT
TTAGGGTCAGTTTAGTCGAAACCACTCGAAAAACGATATTTTATATCTGAGGCCTTCATG
TTACGTG
>comp2350_c0_seq1
GGCTCACATAGTTCGCAAAGAAGAACAACGAAGTGGACAGCCAAAATTCCTCCCCATCGA
AACCAACACAAGCATCCGTCGATCGTTTCTGCCTATTTCCCCCCATTGATCCGAGCATGT
CGCCGAGAAGGTCGTTGTCGAAGAAATCGACCGTGACGGTCGTCCACCCAAAGAGCGGTC
GGTCGATGGATCTATCCGGAGAGGAACTCGATGAGTTCTGCAGGGGGCACGGTCTCTGTC
GTCGTTGCGCCAGGGTAATTACCCACAAACGAGTGTTCAAGTTGTTTGGAACGAACCATC
CCAAATGGGAGCCCCTCACGGTACTCAAACCGACGACCGCCGCAGATGAGTGGAACAACA
ACGGGGACAACAAAGGGGGTAATCAGGGGGGAAACCACGGGAACGTCGAGCAATACAAAA
AGAACGATGAAATCGACAACGACGAGGATGATTCCTATCTTGTCTACAAGGGTTATTGCT
TGGGCGAGGGCTGTTACACCATGAAAGAAGCCAGAAGGCTACTGGGAGAAACGGGGGACA
AAAATTCAAGGTACAACAAAGAGAACAGCGGCTGGAGATCCAGAATGACCAAATCGACCC

GTTTTAAAAAGAAGAACCCGAAGCGACCAGGGAAGCGCCGCACCAGGACGAATCCGGAAA
CCGGTTCCGTGGTCACCAATGCGGCCTCGTTGTCTTCTCTGAGGTCTTCGTCGTCGTCGA
GTATAGTAACCACGAGCAGTGCCAGCACTAGACACAGCATCATTAGCGGAATGTCGGGCC
TTTCTGGATTTTCGGGGATGAGCGGCAGTGTGAAGAGCACCAGGCGCAGCGGCCGATCAC
GTCGATCAACACAAAGGTCAAGCAGTAGATCTTCCTCCGATGACGACGACGACGAACACA
ACCGCAAGGAGTTCTCCTCGGAGAAAAATGCTGTCTCTCACATTGTCGTTGAACGGTTGG
AGAAGTTGGTTTCCTACGATTATTTTACTGTGTTGGACCTCAGCAACATCGATATGAGCA
ATTCGTCCAAAAGTACCAAAAACGAGAGCAATGCAAACCTCGATGCTCTCGTCAAAGCAA
TCGGAATCGCAAAAACACTCAAGGCGGTTGTTTTGGAAAAAATGCGGACTCGGAGACGAGG
GCCTCGAGAAACTTGCTGGTGGACTCGAGCAACAAATCGTGACGACGTCGTTGCACAAAA
GCAAGAGTGCCGATGCCGGCGACGTGCTGCTTCCGAGCATCCTGCGGTTGCGGGGAAACC
GCATCGGAAACAGGGGTGTCCAGGCACTTGAATTTTTGTTCCGCTCCTCGTCGACCCTGA
AGGAAGTGGACCTGAGCGACAATCACATCGGGAGCAAGGGTGCCTGCTCGTTGTTCGAAT
CCCTCGCGCAAAATCCAAACGGAATCCCCCTGTCAGTGCTCAACCTTTCCCAAAACGAGA
TCTGGGAGCTCTCGTCCCCCATGGAGAACAACAATGCGTCTACTTTTTTGTACACCAACT
CGACACTGAAGGAACTCAACCTGGATGGGAATTTGTTGCACGACGGGGGTGCCGAATTCA
TTGCCCAATCCATACGATCAAATCGCAGATGCGCTCTCAAGAGACTCCATCTGGGCTGGA
ATGCAATCGGTGACGACGGCGTCCGGGCACTGGGCAAAGCTCTCGAATCGAATACGAGTC
TCGAGGTCCTTGGACTGGCAGAAAACGATATCACAAACACCGGGGCAAGGGCCCTGCTAG
CGGCATTAGCTGTCAACACAAGCGTCAAAGAAATTAGCGGCCTCTATCACAACCCCATCG
AGCGCAAATTCATCATCGTGGCCATCAAACGATTGTTGCACCGAAAGAGCAGCGTCAGGG
GGGACAAGAACAATACCGATCCCGCCAGATCATTGGCAAATTCTGAACACTCTGCGGCGG
CCGCAGCTTCGACCAGAACGGGGGCTACGACGGCAGCCGACAACGAAGATTACGAGGACA
GCGAAGAAATGATTGTTCCACTCAAGCCAGCCGCGTCCAACAAAGCTGTTGTCGTCGTTG
ACGATGGTTTTTCCCGTTTGCAACGGTCATACGAAGAGACCGCCAGATCATCCCAGGGTG
GAGACCATCACACACATGCGAGCACCGCATCGCATTCCGGTGGCAGTCTAGCATTGGAGG
CGATCGAAAACTGGGACTGGGGTACGTTTGGAATCGAGGAAATCGAACGCAAAATATCGG
GGGATCTAGATGACAATATCGAAGTCCAGGTGGACGGTTATACCGATCCGTCATCGGCAA
AGGATTCCGCTCTGAGTAACTCAAATACAAAGAGAGGACTTTCGGCCGACACCAAAAAGT
TGGTTGATCGAATGTTCGTATTCCAATCTGCTCCTCTAGCATACTTTGACCGGAAAAAAA
TGCAACACGTTGGAGTTCCTCTCCTGGACTTTGATCTCGAAGCACGAAGCCTCAGGGACG
TTTTGCCCGAGGACAAGAACATCGATGTCGTGTTCCAAAATGCCACCCAGAACAATTTCA
AGCTGTATTTCTCCAAGTCCTTGAGTCCGGTGATGCATTTCTCGTGCCATGGAAATGATT
CCGATTGTCTCATACTCGAAAACGGATTCGGATATATGCAAGCCCTGCCGCTGGACTCTC
TGAAGAAAATGGTCGCCGCCAGCAAGATAAAGCTGGTGGTGGTTTCTTCCTGCAATTCTC
GTCATCTTGGCGAAGTCTTTTTGAACGCAGGCGTACCCCACGTGGTGGCGTTGCAGCGAG
AAACCACATACTCTGACGAAGGGCCTATCGATTTCGCCAGGGCGTTTTACGGGGCGCTCG
GGCAGAGAAAAACGCTTCGAGAGGCTTTCGACATTGGAATCAACGAGTGCAAAAAATTTT
CCAAACCGCCCCGGCTTTTGGACGCTTATAAATTGCTTCCCGAGCATTGTTCGAACCACG
ACGTGGAAATTTTTTTCCAACAATCTCCACCACCGATAACTGAATCTGTTTTGGCAAAAG
ACCTGTCCGAAGATACGAGCATTTTACCAGAGGTCCCGAAACAATTCGTGGGAAGAGAGG
TTGATCTTTACGAAATTCTTGAATCCCTGAGGGTAGATGATGTGGTTCGAGTCGGAGGAG
CCCCGGGATCGGGAAAGAAGAGTATTCTGGCTGTATTGAGTCGCTATATTTTAGAGCGAT
CCCGTTCGTTGGAGATTGATTCCGTTTATTGGTTTCCTACCCCTCCCGGTGTCACACCGG
ACCCAGACTCTCTATATGGAGATTTATGCAATGTTATGAAATGGATTGTTGAAGCCGAAG
ATGATGTTTGGGACGAGGAAGACTACGCTGAAAGTCGGGAACGCATTGTTATCGAGATGG
AGGGAAAAAACAGCATTCTCGTCATCGATGGTCGTATCTTTGCAAACGAAATAGCAGGCG
AAATGCTCGAACGATTCCTCACCCACATTCTGAACGAGGTCAACGTGAAAATTCTATTGA
TCACGGCCACCGACGAGTCCCGAGTCAAATCGAAGCGATCGAAATCGGAAGAAGCTACCA
TTCATCTGGGTCCACTCGATTACACATCAACGGTCCGAGTTTTTGGCTCTGCGTGCCCGC
TTCTATCTTTGAATGAAAAGGATTGCAGTTGCACCGTTGATACCGTTGAAGAATTTGAGA
GTTGTTTGCTACCTAACTCAAAAGCTGCTGGACCGACCGTCTTCGTGGATCCAAAGCAAG
ACGTAGTTCGATCTCCGCACCAGAGCGAGCTTTACAATTTAATGGGCAGCGGCAATCCCC
GAGATATTGCGAGAAAAGCCGCTTCATGCCAACAGGAGGATCTATATAGATTGCTGCGAT
TGGCGCAGAGGCCAGTGGTTTCCGTAGCGTCCTCGACAGAGCTCGAACGACAAGTTGCCA
AGTGGATGGCACGAAAGGAACAGGCTGTGCAAAACCACTATTATCTCCGTGCCAGCGACA
TTGACAAAACATTGCAGGAATTGGAAGATCTCAGAAAAACATATCCTAGTTTGACAGACA
TGAAAACGAAAGAGAGAGATCTCAAGGAAAAGTTTAGAGCTCTGTTGAAAGCAAAGCGCT
ACGACGAAGCAAACTCTGTCAAGCGGCAACTTTTAACCCTGAAGCGCTCCATGGTAAAGG
AGCAGTCTTCCTCCAGTTCAATAAGTTCCCAATCAACGGCTGCCCAGGAGTCCATTCTAG

AAATCCAAGAACGGATGAAGACCATGATAGCACTTGCCGAAACGCTTGGGCAGTCTATGT
CAAGCGTTGGGAGTCCTACTAGCCACTTGGTTCAGGAGTCGCCCCCGGCCGAGGCCGAGT
TTTGCTTATCAGACACTTGCACACTCAAGATATCACCTGGTTGTTTGTGGACAGATTTTC
CATGTTCGAAAACCGGGGGCACTAGCGGAAAAGATAATTCCACTGTTTCTGCTATGATTG
TTTTTACCAATGAGGCATGCGACCTTACGAGCAACAGCAAGGCCATATCGGAATCGGGCG
ACACTGATCGTAAATTGAAACTCTCCGAGAAAATTCATGATCTCGAAACACTGACCGAAA
CGCAATGGGGGACCGTAAAGTGCGCAACAGGGGAATCTGTGGCACTTGACGACATTGGCG
CCGGTGGCAATCGCAATCTCTGCATCGTCTTAGCAGTCCCTCCTCTCGTCACCAATGTTA
ACGAGGATGCGAAGGATTTTGACAGCGACCAACACTACGTAGCAACACAGCTTAGGAGTG
CCATTCGATCTTCGTTTCGAACGGTTCGAAGAACCAACAACAAGGTGACAGCCATTGGTA
TTTCTACCAGCCCATCGTTGTCATTGAATGGGAAAGGGAATCGCTATCGCACCAACGACG
AATTAGATTCAGATGAAAACACAAGTTCCTTCGTCACCCTGAAAGCAATCGTCGATGAAA
TCTGTCGTGGTGGTGGCGGCAAAAACAACGAATGCCTTCGGACGGTCCACTTGTTTCCCT
CCACAGGCATCGTAGAATCCAAGATGCTGATTCGGACTGCTTTCGAAATGGGATTAAAAT
GCAGATAGCAAAGCGATACGAGTGGTTACCGCACGGGAGTTTGAGTTTCTGTTTCTATCT
TGCGGGATGAACAACGAGAACGACAGCAACAAGTCACTAAAGATAATCACTGCTACTAAT
TATTTTTCTACCGGAGTAAAAATGTTTGAAAAGTCGTAGCCGCACCGAGCACTATTGTTC
TGAACCAAAAACTTCAGATCCGATGGGAGGCATTGCTTTGCGGGTTCGATGTGATAGCAC
AGGTCTTTCATGCGATCTGTACACAAAAAGTTTTACCCATTCCTCAAGTATTAAGTACAT
TATTTAAATTTCCCAAA
>comp2351_c0_seq1
CGGCATTGCACCTTGATGTCTTCTAGACCACGCATACTGTGAGCTCTCTCGGAGGCAAGC
GACATCACATACGTTATCCCGTGTTGTTCACGTATAGTGCGTGGTGTACTGGATGCCAGA
ATTACGACTGCGATCGCCAGCAATTTGATTATTTCGTACGTTTCGACATGTTCTCGACAA
ATACGCAGAAATAAGCGACATAAATTGGTATCAACCCATCACCTACTATACGTTTTCGCT
CCTATCGTAACCACAGCATCCGTAAAATTTTGGACACGCGAATGATGCATACATCTATGT
GCCAGGTCGATACACTTCAAAAGAACAAATACCACGGGAGCACTGCTCTTTGTTTGAACA
CAAGCTCAATCCATTGGCAGTAGAAAAGAACAAATAACATCACGTCATTCGAATACATCA
CGATCAGTCATTCAATCAAAATTCATTCGAAAGATGACATTAGCACCGAAGCTAATGCCT
TATACCGGCAACAATCTTGTCCCCATAACATCAAAAGTTCCTGCGCAACAACCCAGGGTT
GTCCACACGAAAGCTAAACCCAAAACTAACAAAGCAACAACATCAATAGCTATGCAAACA
CCATCGGATTCCTCAGCGGCAACCGTCAAGCCTAAGGTCACGAAACGTAAACCTCTTGAT
GTGCTTCAAACAATACTTTCATCTGCTGATGCTGTTAACGGCAGCAGCAGTGAAAACTCC
TCGGCGGCAGTACAACAACAGATGACAACACCAAAATCAACATCACTAACAACGCCAAAA
GCACTCACCTTTCAGAAACCTTCAGAGGTAGACATCGCTGCATACGATCTTGAGACAGTC
AAGGCGATTCGATCGAACAACCTAGACTTACTTCGGCAGTTATGGCAAAGTGGGAAAAGC
ATGGACGCATGCAATCAATTTGGTGAATCTGTCTTGCACATGGCATGCAGAAGAGGTTAC
CGCAAGGTTGTCGAATTTCTTTTAGTGGAAGTGAAGGTGAGAACAGATCGGTGCGATGAC
TTTGGGCGAAATCCCTTTCACGATGCACTGTGGACGTCGACTCCAAATTTCGACGTTGTG
GACCTGCTGATAGACCACGCCGATCCGGCCTTGCTGCTTACAGAAGATGTTCGCGGAAAC
ACTCCTTTTGCGTATGCTCGCAATGATCACCGCGATCAATGGAATGAGTTCCTCGAGAAA
CGCAAAGGCAAACTGATCAATCGAATGCGGGGTAGTGTTGATTCGAATTCTGATGGGGGA
GTTGATGCGGCAAAGAAGGTCGAGGTTGTCGGATAGTCAACACGACGGAATTTGTACATA
AGTTTACTGCTACTGCTACCAATAGATTTTCATTTGCGTACTCGCTACCTGATATGCAAT
GCAATAAGATGCAACAAAGCTGTGGGA
>comp2352_c0_seq1
CCTTTTTAGAACCCGAAACATGTCTCTGTCCAACCTCAAGTGCATAGTAACTGGAGCCTC
GTCAGGAATTGGCAAGGCCACTTCGATTTCATTGAGTGCTGCAGGCGCCAAGGTAGTCGG
CGTTGGACGGAACGAAGAGTCGTTGAAGGAACTCAAGAACAACGGACAGATTCATGACTA
TGTTGTGGCGGATTTGGTTGAGCCTGGAGCCTGTGAGCGCATCGTTCAAGAGGCCGTATC
AATGTTGGATGGCGAATTGACAACTCTCATTAACAATGCTGGCGTATTAAAGGGAGGAGC
AGCTGGAGCACCTGGTACGGATCTATCCAACTATAAATATAACATGACACTCAACACACA
GATTCCATTTGAGCTTATGCACTACTCGGTTCCTTACTTGGAAAAACATCCGAACGTTTC
CTCGATTATCAACGTAAGTTCTGTAAATGGCAAACAGGCATTTGCTGGATGCCTTTCGTA
TTGCATGTCGAAAGCGGCATTGGACATGATGGCACGATGCGCTTCGGTCGATTTGGCTCC
AAAGGGCATTCGGGTCAACAACGTCAATCCAGGTGTTGTTACAACGAACATACACACTGC
TGGGGGACGAATGGACGAAACCCAGTATGCTGCATTCATCAAACGTAGTGTCGAAACGAC
ACATCCATTTGCTCAAAGTCTAGGACGAATCGGCGAAGCTACGGAGGTCGCTGAGCTGAT
TGTATTCCTGGCAGGAGAGAAGGCGAAATTTCTTACTGGAGAATGTATCGCCATTGATGG
CGCACGACAGAACCTAGGTTCAAGATAAACAGCTTTTACGGACAATACAAGCGGTGCATG

GATTGGAATTGACGTGGATAAAGCTAAGTTGTGGAAGGAACTTATGGTGAATGGTACGGT
AGATAAAGGCAATGATATTGCCTTGCTCTCTTTATTCGTTGCTAGATGATGAATGCACTT
TTGTTGAAGTGCGCTTGGCAAGCATACCGGTAGCCCGAATAAAATACCGCCCATTTGATC
GACACAGAATATCATTTTTTAATTTATTCAATTTGTTCCTGGACTTCTCTTCCTCTAAAT
CCAAAGCTAGCCTTGCTGAGATCTTCAAACAAACATTGAAAGGGCTATTCTACAGAGTGC
TTTCCCAATAGGAACATTCGAATATAAAAACCATTTTTTTCAGCACAAGTTGTTATCCAT
ATGACCTGTTGACACTGTATACGTGAGATACAACAAACTTCTTACTCTTGGGAAAGAAAA
TTATTTGCTTCTCTGTTGCATGAATTATCTCCATGAATATTTTCCTAGTAGTTCCATGGT
GTTCATCATGCATTACCATGAATGACTCTTCTCTCAACTTTCAAAAATGGCATCTAGTAC
TCTCTATTCCTCCCAGCAGTTGATTTGCACTGTCCCTTCATTACACATATGAAGCTTACT
GCTAGTGATACTACTATGCCCAGTCTAAGAAGAATGTATAAATGGA
>comp2353_c0_seq1
GAGCTTCGATCATAACAAAAAGGAGGGAACTTAACGTCTTGCAACTATGAAGGATAAGAT
TCTTGCCGCTGTATACATTCTAGGATGGGCATTAGCCACTGGAGACTGTTTCTCCCCCAA
TCCTGCCACACCGATCTCTGCGAGAAGAAGACAGGCCGAGGCTTCGGTCGTTGTGAGGTC
CTCCAACGAGGATGGCGACAGCGACGAACCCCTTGTAATCCCGACCAGTGATGTGGTGAA
AAAGGTAGCTGTGACGGGAGCTACGGGGAAAACAGGAAAACTGGTGGTGGAAGAGCTGTT
GAGTCGGAACGTCCAGGTAGTTGGTCTGGTGAGGAACGAAACAAAGGCAGAGGAACTGTA
CGGCGGTTCCCCTTCGGACATGCTGGACATTCAGGTATGCAACCTTTCAGACCCGTCCTC
CATCGAAGAGGCCCTCGAGGGATGCGATGCCACCATATGGTGTGCTACCGGCTTCTCAGA
TGCCCCCGGTGGCCCTCCTACCACGACCGTTATGTCGTCCGAGCAGTCGATCGACGTCGT
TGGAGTTCCGGTCGTTGCCAAAACGATGCTTCGAAACAGTGGTTCGGAAGAACGAGAACA
AGCGTTCCCTAACGTGGTCATGCTCAGCAGCGCGGGGACCACACGACCGGGCTGGGACGA
GGAAAAGAAGGATCGACTCCGGGGGTGCGCCGACATTCCCATCGTCCGGCTCAACCCGTT
TGGAATCCTTGGCCTCAAGGCCGAGAGCGAGGCGAAGCTCCGGGATACCGGTGCCGACTA
CTGCATCGTCCGGCCATGCGGGCTGAACGACAACTGGCCCGAGGGGGCCCGGCCCGTCGT
GTCGCAGGGTGACGTGGCCGTCGGAAGGATCAATCGCAGGGACGTTTCCAGGATCCTGTG
CGACGTCCTGTCGCTCCCCGAGGCCCTACACAAGACCTTCGAGGTCCTCGGGCTGGGGGG
CTACCCGCCCCCTTCGAGCATCGGGCCCGCCCTGGAACGGCTCCGGACGGATTCTGAGCC
ACCCTCCGAGGCCGAGGTGGAGGCGACGTACCTGGCCCTCCAGCAGCTGCTCCCGGGAGA
GCGCCAGGACGCCGCAGCCCTTGCAATGGGACAGACCTACGAGCAGCTGGACCGGGGAGA
GACGGGACGGCTCGGTGAACGGGGCACAGAGAACGTTGACGCCGCAGGAATCAAGCCCAC
CACGGCGGCCGCGTAAAACTCAGCGGGTCGACTTATTCGCACAATATGCCATGAAATATA
TCAACGTAGCGGATGTTGTCACACCGTTTGTTTTTACACGAAAGTTCCAATACGGTGAA
CGACGTTTCGACAAGAAAGTTTGTAATTTAGTTTTGTCTAAGTTTTTACACAG
>comp2354_c0_seq1
CGTTTCACAAAATAAAAATATACCAATTTCAAAACGCTGAAATAATCAAGTATTCACAAC
ATCATGCCGATACGAAATACAAGCACTGGAAGTTCGTCCTCCGGCAAGAAAAGACCGCCG
GTTTTTCCGAAACGTCTCTACGACATGCTACAGAGTGCAGAACGCGACGGATACGCCCAT
CTAATTTCCTGGATGCCGGATGGAAACTCATTCAAGATACACGTCGATGGCAGCCCAGAC
CCAGAAGACGAAAAAGCGATTGTTCGACTACTCAAACAAGTATTCAATCAAACGCGATTC
AAATCGTTTGTAAGACAGCTCCAACTCTATGGTTTCGAACGCACATACAAGGGCCCACGT
CGTGGGGAATGCAGGCATCCGTTATTCATCAGAGGTCGACGAGACCTTTTGGACAAGAAA
TCGATTGACAACTTTCAACAAAAGTCGAACGACAAGACGAGTCGCAGATCCAAACACGTT
CGGCAATTGTTGCAAAAATCTCCTTCCCGAGCAGAAGTTATTTCCTTCATGAGGAAGAAC
GACTCGAGAATCGAGACATACAGCATGCAATCATTGGAAAATACTGCATCTAGTCCCTGC
CAATATCTTAACACGTCGGTGATTCCTACGAAGCTTCATAATCTTGTTCTTTCGGATAGT
GACTGCAGCACGAAAGGATCTGACTCTTGCAATGAAAACTATGATGACGAGGACTTGTCG
GTAATTGTTTCGAATCCATTGCTTGACGATCCGCCGTTGAATAACTATCAACAAACTGTA
GACAATTTATACGGCGGCGATCTCATTTCGATTCACGACAGCGAAAGTGTTTTCCCCGAT
TGGACTGGAATAGAACTTGAAATACTGCGCAGTGCGCTTTAAGCACTAGAAAAGGGTTTC
ACTCCCGCCATCAATTATATATCAGTAGAATAGATCATAAAATAACCAAACTATTGTTTC
T
>comp2355_c0_seq1
CGCAACGGGAAACAATGAGGTTCTCCAAAATCCGCTACTGCTTCCGCTGACCCCCGACTC
TGTCTATGAGTTTTACGTCCACGAATTATTTCCGGCCGTTGTCTCCTCCCTTCCGATTCT
CTTTTACCGCATCTGGAACGATGCGCTGTCCTGGGCTTGGCTGGCGGGCCATAAGCTCTA
CGTGTTTGTCCAGCCGACTTGGATGACCAATGGTGGAACCATTGAGGCCAACAATGAAAC
TACCGCAAATATCCTTATGCACCTCCACCTGGCCGAAAAGGACCTAGGCGGCATGATCCA
AAACATTTGGGAGCAGGCCGTACAGTCCTCGAAGCAGCATGTCAACGCGACCTTTTTTTT

CGAATTGCTCCGGGAACACAGCCTCCACTCAAACTCGACCACCGGTCCCCTTGAGCTGCT
GGCCAGGCTGCCGGCGACAGCCAACATTATCATCCAGCAGGAAGCCGCCGCGGTCGAAAA
ATCCTTCTGGACCTGGTTTTCCCGGGAGTGGCCCCTCATGGACTGGAAGATCGGGGTTTT
CCTGTGGCAGACCTTTGGCGGGGTCCTCCTAGTGGTGGCACTCCTGTCGATCATGCCGGG
TAGACTGCACACCTGGTCGGGGAAACTCCTTCGGTGGCCGATCCTGGGCCTCACGTACCT
CCTGATCAACGTAGAGCTAGTGGTGTACGTCTGTATCCGCCTCGGCATTCGCCTCATCGA
GGCGATCGTGGCAACCCCCAAACACCGGGCCCTCCGCGCCAAGATGGCGAACGAGTCTTC
GACCTACGAGGAGTGGTACGGGTACGCGTCCGAGCTCGACGCCTCGCAAAAGCGGACCCT
CTGGCAATCGACCACGACGGACGAGACGAGCTACCAGTACAACTGGCCCCTCATCCAACA
GCTCATCGCGGACATGAGAGTGGCTCGCTGCGACAAGAAGGACCCTATACTTGCACTGGC
GGTCCTCCAGCAGTGCACCCGCAAGAACGTCGGCGGCATCATGAACGAGGACCTCTTTAC
GAAAACCTACACGGGGGAACCCAAGTTCATCGTCATGGAGTTCATCGGGGAGGTGACCAA
AACACTGTACTGGGTGACGGAGCAAACGCTGACGCTGGAAGAGCGAGAGGAGCTGCGGAA
CAAAACAGCGGGGAGATCGTCGACGTCGAGGGGCGGGCCGAAAACATACGAGCAAAACCT
CCAGCAGAAATCCAAGGCGGAACACCACAAGCTCTGGAGGAACCTTGTGGCCTTTGCCAC
GCTGGATTTTAGCACTCGAACGATGTCACAAAACAACGAACAGGAAAAGGAACCCTTGCC
AAGCGAATCGGAAGCGAACGACCCCGGTTCTCCGAAATCGACCGGCTCTGGTCCCCAGCA
GCCCCCGGTCAACCGGGCCCAGCTCCTCAAGTTCCTCAAGCGGGCCAAGCACGCCTACGG
GAAAACGGCTCTCTGCCTGTCGGGAGGGTCCATGATGGGCCTGTACCACCTAGGCCATCT
CAAGGGACTCATGGAGACGGACTGCCTCCCCGAGATTGTCAGCGGGTGCAGCGCGGGGAG
TGCCATCGGAGCCGTCCTGTGCACCCGGACCAACGACGAGCTCCACAGAGACCTCGACCC
GACGATTATCGGGCCCCGTCTCAAGTGCTTCGACCGGACCTGGTCGGAGTGCCTCCGGTC
GGTGTGGAAGACGGGAAATCTCTTTGACGGGGACGACTGGCTAGAGATGATCAAGTGGTA
GGTCGCTATATGAGATGGTTGTGTAGCATCCGTTTGCCTGTAAGCAGATTTGTTTCTTGG
ATGCCGTGCATGGCATTCTAACTCTTTGGGTGCTGTACACATTTTTATATTCTTCGATGA
TTGCTCGCTTTGGTTCTTGCTTTTGTTTTTGTTTTTTCCTTGGTTTGGCACGTTTCCAAA
CACTTTCCATGGCACCTTCTCCCCCATCCACCCAACCCAAACAACAGGTTCACGCTCGGC
GACACTACTTTCGAGGAAGCCTACCAAAAGACGGGCCGGGTCTTGTGCATCGCGCTGGCG
GCCACCGCCAAGAAGACCCCGCCGATCCTCCTGACCTATATCAGCTCCCCGAACGTGACC
ATCGCCAGCGCTGTGGTAGCGAGCGCCGCGGTCCCCGGCTTTGTGAGCCCCCGGCGGCTC
CAGATCAAGGACCCGGTGGACGGGACCATCCGGAGCGCGGGCCCCGAGACCTACTTCGAC
GGGTCGATCAAGCACGACATTCCCACGGCGGGCTTGTCGGAGATGCTGAACTGCCACTTC
TTTGTGGCCTGCCAGTGCAATCCCCATATCGTGCCGTTCTTTTTCAATGCCAAGGGCGGC
GTCGGCCGGCCCACCCGCTGGTCGGGGGGAGAGAAGGAAACGAGCTGGAGGGGGGGCTTC
CTGCTGGCGGCCCTGGAAATGTACATCAAGAACGACATGCGGGCCAAGTTTGTCTTTCTG
AGGGACCTGGACGCGGCCGTGAGCTTCACGGGGACCATGATGACGCAGTCCTTCGAGGGG
ACCACCACGATTGTCCCGCAGGTGGAGCTCCGCGACTTTTTCACGCTCTTCCGCGACCCC
AGCGTGCACCGGCTGTACCACTACCTGAAGGTGGGCTCGGTGGCGGCCTACCAGCACGCC
GCGATGATCCGGTGCCACTACCGCATCGCCGACGCCCTGGAGGAATGCATCGAGCGCCTC
GAGGCCGGGGGCGAAAAGGGGCAGCAGCAGCATCACGACCACGCGGCCCGGAGAGAGGCC
TTTGGCCAGTACGAGTCGCCTTTGAAACGGAGGGCCAGCCGGAGGGCCTGCTTCGAGACC
GGAAGCATGCGGTTCGAACCGGGCCGGTGCGATCTTCACACAGAGAGCCAGAGCCACAGC
CACCGCAGCCAGCGGAGCGTCCCGGAAATCGTTTCGCAGAGGACCGACAGCGACGACAGC
GTGTCCACGGCCTCCGAGACCGGGACCCTCCCTCCCGGCGGGGGAATGGTGCACCTTGCC
CGGTAAGGCAAGGCAGCGCAGTGCCACGGTGCGGAGCGGAGCGCGGGACCAGGACCGGAG
GCTGCCTCCGTGTCGTGGTCGCGTCGTGTGTCTGTGTGTGTGTGTGTGAAACGCGCAGGC
ACCGGCGTTTCTTGCCCCGGCACGGCAGATCGAGACCGCAACGAACTATAGCAAAGCAGG
GCATAGCGCAGTAGCAGTACGTACTCTGCACAGCAACCCAGCGGAGTTCGAGGCCACTCG
ACCCAATACCGCAGAACCATAGTACACAGAACTACGAAACGAAACGAGAGGCACGATGCT
ACTGCGCTGCACTTGTAGGCACAGCAGCAGCATCCCAAACGCACGCTTTTCCTAAGAGAG
GCATGTCTTAATACCGAACACTGCGCACAGGAACTAGGACTACAACT
>comp2355_c0_seq2
CGCAACGGGAAACAATGAGGTTCTCCAAAATCCGCTACTGCTTCCGCTGACCCCCGACTC
TGTCTATGAGTTTTACGTCCACGAATTATTTCCGGCCGTTGTCTCCTCCCTTCCGATTCT
CTTTTACCGCATCTGGAACGATGCGCTGTCCTGGGCTTGGCTGGCGGGCCATAAGCTCTA
CGTGTTTGTCCAGCCGACTTGGATGACCAATGGTGGAACCATTGAGGCCAACAATGAAAC
TACCGCAAATATCCTTATGCACCTCCACCTGGCCGAAAAGGACCTAGGCGGCATGATCCA
AAACATTTGGGAGCAGGCCGTACAGTCCTCGAAGCAGCATGTCAACGCGACCTTTTTTTT
CGAATTGCTCCGGGAACACAGCCTCCACTCAAACTCGACCACCGGTCCCCTTGAGCTGCT
GGCCAGGCTGCCGGCGACAGCCAACATTATCATCCAGCAGGAAGCCGCCGCGGTCGAAAA

ATCCTTCTGGACCTGGTTTTCCCGGGAGTGGCCCCTCATGGACTGGAAGATCGGGGTTTT
CCTGTGGCAGACCTTTGGCGGGGTCCTCCTAGTGGTGGCACTCCTGTCGATCATGCCGGG
TAGACTGCACACCTGGTCGGGGAAACTCCTTCGGTGGCCGATCCTGGGCCTCACGTACCT
CCTGATCAACGTAGAGCTAGTGGTGTACGTCTGTATCCGCCTCGGCATTCGCCTCATCGA
GGCGATCGTGGCAACCCCCAAACACCGGGCCCTCCGCGCCAAGATGGCGAACGAGTCTTC
GACCTACGAGGAGTGGTACGGGTACGCGTCCGAGCTCGACGCCTCGCAAAAGCGGACCCT
CTGGCAATCGACCACGACGGACGAGACGAGCTACCAGTACAACTGGCCCCTCATCCAACA
GCTCATCGCGGACATGAGAGTGGCTCGCTGCGACAAGAAGGACCCTATACTTGCACTGGC
GGTCCTCCAGCAGTGCACCCGCAAGAACGTCGGCGGCATCATGAACGAGGACCTCTTTAC
GAAAACCTACACGGGGGAACCCAAGTTCATCGTCATGGAGTTCATCGGGGAGGTGACCAA
AACACTGTACTGGGTGACGGAGCAAACGCTGACGCTGGAAGAGCGAGAGGAGCTGCGGAA
CAAAACAGCGGGGAGATCGTCGACGTCGAGGGGCGGGCCGAAAACATACGAGCAAAACCT
CCAGCAGAAATCCAAGGCGGAACACCACAAGCTCTGGAGGAACCTTGTGGCCTTTGCCAC
GCTGGATTTTAGCACTCGAACGATGTCACAAAACAACGAACAGGAAAAGGAACCCTTGCC
AAGCGAATCGGAAGCGAACGACCCCGGTTCTCCGAAATCGACCGGCTCTGGTCCCCAGCA
GCCCCCGGTCAACCGGGCCCAGCTCCTCAAGTTCCTCAAGCGGGCCAAGCACGCCTACGG
GAAAACGGCTCTCTGCCTGTCGGGAGGGTCCATGATGGGCCTGTACCACCTAGGCCATCT
CAAGGGACTCATGGAGACGGACTGCCTCCCCGAGATTGTCAGCGGGTGCAGCGCGGGGAG
TGCCATCGGAGCCGTCCTGTGCACCCGGACCAACGACGAGCTCCACAGAGACCTCGACCC
GACGATTATCGGGCCCCGTCTCAAGTGCTTCGACCGGACCTGGTCGGAGTGCCTCCGGTC
GGTGTGGAAGACGGGAAATCTCTTTGACGGGGACGACTGGCTAGAGATGATCAAGTGGTT
CACGCTCGGCGACACTACTTTCGAGGAAGCCTACCAAAAGACGGGCCGGGTCTTGTGCAT
CGCGCTGGCGGCCACCGCCAAGAAGACCCCGCCGATCCTCCTGACCTATATCAGCTCCCC
GAACGTGACCATCGCCAGCGCTGTGGTAGCGAGCGCCGCGGTCCCCGGCTTTGTGAGCCC
CCGGCGGCTCCAGATCAAGGACCCGGTGGACGGGACCATCCGGAGCGCGGGCCCCGAGAC
CTACTTCGACGGGTCGATCAAGCACGACATTCCCACGGCGGGCTTGTCGGAGATGCTGAA
CTGCCACTTCTTTGTGGCCTGCCAGTGCAATCCCCATATCGTGCCGTTCTTTTTCAATGC
CAAGGGCGGCGTCGGCCGGCCCACCCGCTGGTCGGGGGGAGAGAAGGAAACGAGCTGGAG
GGGGGGCTTCCTGCTGGCGGCCCTGGAAATGTACATCAAGAACGACATGCGGGCCAAGTT
TGTCTTTCTGAGGGACCTGGACGCGGCCGTGAGCTTCACGGGGACCATGATGACGCAGTC
CTTCGAGGGGACCACCACGATTGTCCCGCAGGTGGAGCTCCGCGACTTTTTCACGCTCTT
CCGCGACCCCAGCGTGCACCGGCTGTACCACTACCTGAAGGTGGGCTCGGTGGCGGCCTA
CCAGCACGCCGCGATGATCCGGTGCCACTACCGCATCGCCGACGCCCTGGAGGAATGCAT
CGAGCGCCTCGAGGCCGGGGGCGAAAAGGGGCAGCAGCAGCATCACGACCACGCGGCCCG
GAGAGAGGCCTTTGGCCAGTACGAGTCGCCTTTGAAACGGAGGGCCAGCCGGAGGGCCTG
CTTCGAGACCGGAAGCATGCGGTTCGAACCGGGCCGGTGCGATCTTCACACAGAGAGCCA
GAGCCACAGCCACCGCAGCCAGCGGAGCGTCCCGGAAATCGTTTCGCAGAGGACCGACAG
CGACGACAGCGTGTCCACGGCCTCCGAGACCGGGACCCTCCCTCCCGGCGGGGGAATGGT
GCACCTTGCCCGGTAAGGCAAGGCAGCGCAGTGCCACGGTGCGGAGCGGAGCGCGGGACC
AGGACCGGAGGCTGCCTCCGTGTCGTGGTCGCGTCGTGTGTCTGTGTGTGTGTGTGTGAA
ACGCGCAGGCACCGGCGTTTCTTGCCCCGGCACGGCAGATCGAGACCGCAACGAACTATA
GCAAAGCAGGGCATAGCGCAGTAGCAGTACGTACTCTGCACAGCAACCCAGCGGAGTTCG
AGGCCACTCGACCCAATACCGCAGAACCATAGTACACAGAACTACGAAACGAAACGAGAG
GCACGATGCTACTGCGCTGCACTTGTAGGCACAGCAGCAGCATCCCAAACGCACGCTTTT
CCTAAGAGAGGCATGTCTTAATACCGAACACTGCGCACAGGAACTAGGACTACAACT
>comp2356_c0_seq1
CTTAAATTCGTTTTTGGATCCAAAACACTCTAATGAATCAAATTGACTTGAATTCTTGAT
TGCCATCGCTATCGGTTTCCCCCATTGTATGCTATGATCATTGGTTGGATTTCATCCCAA
AACGATTCATCAGCAGCGCAACAAAAACAACAACAACCGTGGTCGTTCGTCCATGAAGCA
AGACCCCATGGATGCGTATCGTCGTGAGAGAGCTCGCGATCGCAGCAATAGTTTTGATCG
GCACAACAACAACAACAATCGAAGGTCTCGCAACCAAAACGGTCCCGGCGGCGGTGGTGT
CTCTCATTACGGGCCCTCGCCATACGGAAACAACAATAGTAGCAACAACCAAAACAAAAA
CCACCAATATCCTCCGAATTCAAAGTATGCACCCAGCAGTGGTTCGGGTGGACCACCTCC
GCCCCCACCCCACAATGGCATGGGAGGGCTCATGGGAAACAACGGAAATCTTCCACCCCC
TCCTCCCCCGCCACCCCCGCGACAAGCAGCGCATGGTGGGGGTCCGCCGCCGCCCTCGCA
GTATGGGGCCCCACCCCCTCAACAGCAGATGCAGCAACAGCAGCAGCAACTACCACCCCC
ACACACCCAGCATTACGGGTCGCAGCAGCCTCAGCAACAACAGCAACCAGCAAACAACAT
GTATGGGGGTCAACAGCAGCCTTCCTACGCTGCTCAGTCGCAGCAGCAGCCGCATCATCA
AATGATGACCCCCGGATCGGCCTCGGTAGGGGTGCCTCCTCCTGCAACTGGAGTCCCTTC
CAACAATTCGTACTACGGCGCTCAGAATCCGCAGCAGCAGCAGCAGTATGGGGGCAATGT

TGCAGCTTCGTACTCCAATCCTCCTGCAACCAATGCCATGGCTGCACAGAAACAGCCATA
CATGCAGCATCAGCAGCAAGCAGCGCGAGTCCCTCCTGCCACAAATGGCTATGCTCCCCA
GCACCAACAATCACAGCAGCAGCAACAAACGGGATGGCAGCAGCAGACCCAAAACCAGTC
CGTTGCCGGAGCCGCGGGATTGGTGGACATTTTGGGCATAGCTGACAAGGCTGCTTCCGC
AGTGCAGGCACTCCAAAACCAGAATCGTCTCAAACAGCCACAGCAGCAACAGCCATACCA
GCAACAGCAGCAGCAACATCAACAGCCGTACCAACAAGGTACACAACAGCAGCCGGCTCA
ACAGCACGCTCCTCCCGTGCAACATCATCAGCAGCAATCGTATCAAGCGCAACAGCACCA
ACAATACCAGCAGCAACCACAGATTCAGCAGCCACAGCAATACCAACAAGCGCAGGTTCA
GCAGCCTGCGGCCAACAATCAGTATGGTGCATATGGTCCTCCAGCGCAAACATCCACGCC
GTATTCGACCGGAGCTCCTCCATACCAGCAACAGGCGCAGCAACAGCAGCAACAATATCC
ACCACAACAAGGTCATTATGGCAATCATATGCAGCAGCAGCAGC
>comp2357_c0_seq1
CTTGGCATCATGACAGGATCCATTATCGGCACCACAATCGCGGTTGCAATAACAAAGGGT
AACGTTTTGGTCAGAACTCGAAAAAAATATCATTTTCTACACCTTTTCTGTACTACCAGT
GTACTACTTAAAATCTACCAAGTGGTTTATCAAAGATAAAAAATGTCTTCGCATTTCCGA
AGAACTGTCTACGTGACCGAGAGAAGAGATGCATGTCATAGTTGCACCACTGTACATTTT
GCGATACTTTCTCTCATGATTGCTCGCGTTTTACTCAAAACGCACTTTTTTCACATACAA
ATCCGTGCCCTGTAGCTTTGAATGCCATTCAACCGTCACGCATTAATGTCCCAATCTCAG
GCTTAAAAAAGTAAGAGACAACAAGCAAACGAGAGATACAAGTGTTCAACGGCCCTATCG
ATCCCACCGCACCCCATCGGGCCTTTCAGCAACTTCAAACACAATTTCTATGCAATCCTC
GACAGACAGTACGAGTACTATGGTAGAGCAGCTACAGCTGTTGCCACCGGACTCTGTGTC
CTTATCTCCCACTGAACCCCAAGTACGAGTAGTCTCCCCCCGATCCCCGAAGAACGGTGT
GGAAGGAACACGGTGGGCCGACCACCGGTTCCGCCCGGTACACCTGAACCCCGAAGACTG
GATAGCGCTGGGATCTACCCTCTCAATGGCAGAGCTAGTGGACCCCAACATGAGCTACGA
CTTTTCAGGCAACATCGGAGCAAAGTTCTCGGGCCGGTGGCACCTCGAGGAGGACGAAGA
TGGCCGCGTTGTCAAGCTGTTCATCAACCCCTTCGGCTGGGGTGGCATCTACAACGACGT
CTTTGCCAGCATCGACGACGTGGTTGGACATATCCACCGCAATGATCCCTTCCATCTCTC
GGAGGACATCTCCCGCCTGACTAAGCTCACCTTTTTGGGCCTCTGGAACTGCAGGTCTCT
TCCTTCGAGAGGACTCTCGAGCCTTCCGTGCCTCCAGAAGCTGCACCTTGACAACTACAT
GTGCCCGGAGCTCCTCTCCAACTTTCCTTCTAGCATGAAGCTCAAACACCTTACCGAGCT
CGTGGTAGATTTTAACCTAACGGAACCTCCTGCGCAGTTCCTCGAGTGGATCACAAACAA
AGATATGGTGCCGATGCTGACCGAAATTGAGTTCGTCCAGACGAAGGAGGAGCACCTCCC
CCTCTTGTTGGATGCACTCCTCGGCGCAGAAATCGCCTTCCGAGGACAGCTCACCAGCAT
TTCTTTTCTCGAATCGCAATTCAACGGAGATAAGCTCGAAACGCTGCTCTTTGACTTTCT
GCCGAGATTTCCCAATGTGGCGACCCTCAATGTTGAACATCTGAAGATCGAGAGCTTCCG
GCCCATCCTCGAGCGGATCGAGCAAGGCGAGGCGAAGTCTCCCGTATCGGGATCCCTCCG
GGACCTTTCCATCGACGTGAGCCATATCCTCGAGCGAATCAAAACGGTCGCGGAGGAAAA
GGAGGCCGTGCTGCGCTTTCTCAAACTCTTTGGGAGGTTATCTCGCCTCGGGAACGGGCA
CCCGAGGTGGGACTCCGATATCAGGTACGCCCTCATGAAGAACGCGGCCGGGCGGAGGCT
CCTGGAATACGGGACCATAGGCGCTCCAGCCGGTCTCCCGCTTGCGCTGTGGGCCAACGC
GATAGAGAGGGCCTCGAGGCGCGAGTGGTGGGAAGAACGGAACGCACCCGCCACGAAGGT
CTTTTACCTGGTCCAGAACGCGCGGGAGATCGCCATGGAGGCATCAGAGCGTTATACAAA
AGAGGGAAAAGCAAGGGAGAGGGAACAGGCGGAAGGGGAAGCCCATGCGAAACGAAGCCG
ATTGTTTTACTGAAGAAGGAAGTCAGTCGCCCGTCACCATTTTTCTTATCTTGTCATAAC
ATCTGTACATCGGGAATTGAACACAATCAATAGCTAATAATTCATAACACTACCGGACGG
TTGGTACTAATTTAATTCATATATTGTATGCGA
>comp2358_c0_seq1
CGCAACCAATCCAGACAAAAAAAAAGTCCGAGCCCGGATACCAATCCACACCGGACCACA
ACCACCACAACCACCACAACAAGCTGCCATGAAGTTCTCGCTCGTTGCGCTGCTGGCGGC
CGCCCCCAGGGCGTCTGCCTTTGCCCCCCAGGGGGCTCCCTCCCACCTTCTGTCCCCCTC
GCTCTCGGCCTCGGCGGCCCCCGTCGAGGACTACGTCGACGCCAAGCTCACCGAGCACTC
CCACTGCACCGAGCCCGGGGACCGCGACATTCTGGTCCGGGCCGCCCGCGGGGAGGTGAC
CGAGCGGACCCCCGTCTGGCTCTGCCGCCAGGCCGGCCGGTACATGGAGGCCTTCCGGGC
CTACTCGACCAGATACCCCTTCCGGGAGCGGTCCGAGACGCCGGAAATGGCCGTCGAGCT
GAGCCTCCAGTGCCACCGCAAGTACGGGATGGACGGGATCATCATGTTCTCGGACATTCT
GACGCCCCTGCCGACCCTCGGGATCGACTTCGACGTGGTCCCGGGCATCGGGCCCGTCAT
TTCCACGGAGGTTGCCAGCGAGTCCGACGTGGAGGCCCTCGGGTCGGTGGAGGGCGTGGA
GGCCTTTGACGAGAAGCTCCCCTTCATTCGGGAGATCCTGGGCGACCTCTCGAGGGAGGC
CGAGGAGAAGAACACGGCCCTCATCGGCTTTGTCGGGTCCCCCTTCACCCTGGCGGCCTA
CACGATCGAGGGCAAGTCCTCCAAGCACTGCCTGGCCACCAAGAAGCTCATGATGGAGGC

GGCCCGCGGGGAGACCGCCACGATGGACCTCTTCCTGGACAAGATCGCCACCATGATCGG
CCACTACGCCTGCCACCAGATCGAGTGCGGGGCCCAGGTGATCCAGTTCTTCGAGAGCTG
GGCCCACCAGCTCTCCCCCAAGCACTTTAACGACTTTGCCAAGCCGGCCGCCCAGAAGGC
CATTGCCATTGTCAAGGAGAAGCACCCGGACGTCCCGGTCATCTACTTTGCCAACGGGGG
ATCCTCCTACCTGGAGCTCCAGAGGGACATGGGGGCCGACATGATCGCCGTCGACTGGTC
CGTCGACATGGCCGAGGCCCGGAGGATCCTGGGCCCGGACGTTGCCGTCAGCGGGAACAT
CGACCCGACGGTCCTCTTTGGGTCGAAGGAACAGATCGAGCAGGCCGTCCGGGACTGCAT
CGACAAGGCCGGGGGGCCCGGTAACAGGCACCTCCTCAACCTCGGCCACGGCGTCATGCA
GGGAACGCCGGAGGAAGCCGTCGGCTGGCTGGTCGACGAGGCCAAGCGGTATACCGGCAA
GCAAGCAGAGTAAAAGCGCAAAGCGAGCGACGGGCCAAAGACAAACCACCGTCCAAACCA
CCGAACACCACGATCGGTTTGGAGTAAACTAGACGATAGTAGTTACTGTAGTACGTAC
>comp2359_c0_seq1
CTCGTATTCGACTAGGGGTCCAGTACGTACGTACCTGTAAACCATCAACAGTACCGGTAA
TAGAGGAAAAGCAATTTGTAATCCCCTTCTTCTGATACCCTACGCTAACTTTTGAATGCG
AGACCGAAGAGATATTCTTTGAAGAGTTCTCAAACTGCCTCTCAACCGACTTACGTCCAG
CTCATTGCTATATTTTCAATACAAAAAAGGAACAAGGAGAGGGTAAAGAAACGATGACGA
CAAGCCTCGTTGCTAGCGTTACGGCTGTTGGAATTCTTAGTTGTCTGAGTCCCCTTTGTC
AATGTTTTACCAATGACATGCCGTTACGGGCTTCCGTTGGAAGCGTTATTGAAAGGGACC
GACGTCATGGAATGCGAGAGCACTTTCTTCTTGCGTTGTCTGCTCATGACGAAGCTTTCC
AAGATTGGTCATCAGATAGCAGAATCGATGTTCGAAACTTATTGACACAACGAGCGATAC
AATCGTTCATGTTTCTTTGTGAAGAATGCCGCGATCCTCACTCGGGGAAATGGATCGAAG
ACTTCCTAAAGACACAGAATCTACTCAATTACCACGGTACAGGCGCCAGCTTTATGGCGG
ACGTCCCTTGGGACGGGCCACTGTTGGAACTAATGGATCAACCAGAAGATGTAATGATAG
TGAGCGCGAAACGTCGCGGAAGAGGCCACGGGGGCTGGAGCAAAAACAACCCTTATCTAC
AGGACCGATACGTGGAATTTAAAATTACTATTGACCCCGCTTCGTTGACTGATCGCATTC
TTGCTGTTAGAGAGCAACTTGCCGCGGAGTGGGTGAATGATCTAGAAATCTTGGGGAGAG
CGAACCAACAAATCCTGGATTCCTACTTTCAACTAGCAAAAGACGAGCGCAACCAAGATC
CTGCGATGTCACCAGCTGTTGCGTTTGAACGTACAGCTGTGGACGCTCTGAACTCTCATA
CATCGTTTGGTGCCTCGGGCTCGCCGTTTCGCATGGGTAGTTTTGATTTGCTTTACAACC
TTTGCACCCAAGCTAGCATTCATCGTCTGCTTCGGTCCCTTGGCGACAAGGGCGACGATG
CAAACCTCGAGTTGCTTCGTGATTTCTACGTAGATCGCCTCGAAGATTTTTTTGACGGCG
ATCTTCCGTTTGGAAAAGCAGACGATTTTATTGAAGAACTGTTGCTTCTGTCGCCGTCAA
TAATTCATCGTGACAACGGCAATGTCGCACTCACCGATCCATTTGGAATTTCCGAGCAGA
TAATAGAATTCAGGAATGACATCTTTTCGGAATGGATTGAAGCCATGAAACTAGTTCCTA
CCGCACACCAGGACGGAGTCAGAAAACGGCTTCTCAACAAAAGGATGGAAGCCTGGAACG
AATACACGAGAAAGTCCAAAGGTAGTGGTAATACTGGTGCAGGATTTCAATGATTGCCAA
ATCAAGGCATTCGCATGCTTGGATACCGAACATGACAAGTACTTTAGCAAGTAATACTGT
CAATTAGTAATATTAATTTTCACAAAGTTTCTTCGCACCCATGCGAAT
>comp2360_c0_seq1
AAACCAGATTGGGAGAGAAGATACTGTATGCCAGGATCGAGCAGCATGCTTGTGACGATA
CAGAGGCCGACGAGAGTCTTTTTCCCAGCTAGATTCGTTTCCCTCGCGCTTTCATCGGTC
CTTGTTGTTCTGGCTCTGTTTGTGTCTCAAGAGTCAACGGCGCTTTCCACTGCAACGAAC
AACAGCATGTTCCGGAACAACCCGGAAACCATCCGCAGGGTCCTGCTCAACGCCAAGACC
ATCGCCGTGGTGGGGGCGTCCCCAAAACCAGAGCGCCCCTCCAACTTTGTCATGAAGTTC
CTCCTGGACCACGGATACGAGGTGGTCCCGGTAAACCCCGGGCTCGCGGGCAAGCAGATC
CACGGGCAAACCGTTTACGCCAGCCTGTCGGAGATCCCCGTTTCCGGGAACAAGGGCATC
GACATCGTCGACGTCTTTCGGAACAGCGCGGCGGTCCCCCCGATCGTCGACGAGGCCATC
TCCGTTGGCGCGGGGTGCGTGTGGATGCAGTCGGGGATCGTGAACGAGGAGGCCGCTGCA
ACCGCGACAGAAGCCGGCCTCGATGTCGTCATGGACGCCTGTCCAAAGATCGAGCTGCCG
AGGTTGCTTCCGACCGGGAGCGAGCTGTGAAAAGCAGTAGGGCCGTGGCGCATGTCGCTA
AATTGCAAAACTAGTAGTACGATAGTAACCCGTCTAATATTCACCAATCTGCATTTGC
>comp2361_c0_seq1
GACGAAATAAGGTACGGCATTGTATCGTATCGCACCAAGAACGGCAAACGACAAACGAAG
ACACTCGGTGCAGTCGCCTTAACGCACAGCACAGCACATCGCAGTCACATCGCAGTCACA
TTCGTAAAGCGCTCCAAAAGATCCGATAGAAAAGAATGTCGAACGGGTCAGGCGGTGTTC
AGTGTCATTATGACGTGCTTGGTTGCTCCCAAGACGCTGACACATCTGCGATTAAGAAAT
GCCACCGCAAGCTCGTCTTGAAATATCATCCCGACAAAAATGCAGGCGACGAAGATGCCG
CGGAGAAATTCATACTGGTTCAGAGGGCCTACGAAGTCTTGACTGATCCACAGGAGCGAA
AATGGTACGATGACCATCGCGATGCGATTTTGGCGGGTTGGTCGGCTTCGGAAGCAGCAA
ATAGCAACACGGCGGATATTATGCTCTTCAACGTCGCTCCGTATATGCATCCGGGATGCT

ATTCTTCGTACACCGACAGCACGGGGGGATTCTATCAGGTTTATCGAGGCGTCTTTGAAC
ATATTGTAGCATGTGAGAGGAAACAGTCGGAAACGATTGTGGAGCTTCCCACAACTTTCG
GAAACAGCGAAACAAGCTGGTCGGAACTTTCGTTCTTTTACAAATCCTGGGAATGTTTTT
CCTCGGCCCTGAATTTTGCATGGGAGGACACGTATAACGCGCGGGAAGATGCCCCTAGCC
GTAGGGTACGAAGGCTGATGGAAGAAGAAAACAACAAAGCGAGACGAACCGCCAAAAAAG
CGTACAAGAATGATATACTGCAACTCGTGGCGTTTGTAAAGAAGAGGGATCCCAGATATA
AGGCGCACACCGAAGAACAGGAACGGCTGAAAAAAGAAAGAGAGGAGAGGCAAAAGCAAG
AAAAGATCGACCGGAAGAAAGAACAGCAAAAAGCGAAGGAAGAATGGAGAGAAGCTTCCC
TCCGTGAAATGGAGAAGGCGGAAGAAGAGGATCGGTTGAGAGGAAGGATTAGGCTAGCAG
ATTTAGAAGATGATTACGATTACGGAGGCGGAAAGAAAAAGAAGAGAGGGAAGAAGAAAA
ATAGAAAATCAATGATCAAGGAAGAAGAAAAAGAGGAAGAA
>comp2362_c0_seq1
CGAAAAAAAGTGAAGCTTCAAGAACATACGAAACATCTATTACACAACAAATGACTACAA
TGCTACCAACGACGCCATTTTCAATTGACAACAAGTATAAATCGACGAAGAATACAAAGA
ACAAAAACAATACCCGTCGCCCACACGACGGGCCAAGCAACGGCACGAGTGGCAGCGTCG
GCAGGCCACCGATGAGGAGAGCAGCGAAAACAGGAAGATGGCAACACAAGCAACGACATT
ACACACAGGTGCAGCAGGCCTTTCGCCAAAAGGTCGTTTCCGCAAACCAAAACTCGATAT
GTAACACAACCAACCGTCGCGTAGGCCGACGCACGCTTCGGCAACTCCTGCTCCCCGTGG
ATGCCAGTCGAACACATGGGAGTGCCAACGAAGACGACAGCGAAGCCTACATTGTCCCTA
TATTTCAGCGAAGGTACTGCTGGACGACGACCCAATGGGAAACACTGTGGATGGACGTGT
GGAAGCGAAAGTCGGTCAAGCATTCCCTCGGGCGGTTGACCTGCACAACCGTTCCCGAGG
TGGAACCAATGACGACCGGTACCCAAATAAGATATGCGCATCAGCAACAAACCAATAGTA
ACGGCAGCAACGTCGGTTTCAATGACAACATCGGTGCTTCCCCGCATGATGACGACGTTC
CCGTTCGCCCTCGATCGGTCATCATTGACGGACAACAGAGATTTACTACCGTCACGTTGC
TGCTGGCAGCGATTCGCGACGAACTTTTGAGCACGGGTGCGATCACAGAGGATTGCGGTT
GTAATGACTCCCCTGGTCCTTCCACTTTCGAACTCAAGCCAGAGGAGGCTGCGTTGGTGC
AATCGATCCACCAGATGCTCTTTTTGGACGTTCCGGCCATGAGAGAGTGGGTCGAAACCG
AGTCTACCAATCATCGTGCGCTTGAAGACGGCACGGTCCTCGATTTTTGCCGTCTGGTTC
CTACCTTTTGCGATCGGAGTGCATACCTGGCCGCCATTTTGCCCCCTAGTGCACCTCAGG
TCCAGAGATTTCTAGCCAAGTCCTACAATCCTACGTGGCACCGTCCGTTGCTGGCCAAGA
AGTACTTTGAAACAAAGATTCGTGACCAGCTATTGGAGAATGTATCAAACCAGCAAAACG
AAACAACAAAGGCGGCCTTCCTCGGGCAGCTCACCCGGTCGTTGCTAGACGGGATCGACA
TGCTTTACTTTCCCGTGGACGTCTCGGGTGGATACAAGGACGGAACCGAAGACACACAGG
TCATCTATGAGCGGCTGGCCATCCGAGACGCCACATGGTGCAAACCGAAGCGCCATTCGG
AGTACCACTCGATGGACGGGACCGACATGATCCGCAACCTCTTTTTGGGATCCATGGATA
CATCCCAGTCCAAGGTGGACTTTTATCAGAACTATTGGCTACCACTAGAGCGCAAATGCT
ATCCAAACCTAGGCGAGGACGAGGACGGCGACGAGAGCAAGAGCATAAGCACTTTCTTCC
GTGCCTTCTTGGATCACCAAGCCTCGATCCTCGATGGGGTTCACCAAAAGCGGCTCACAG
AAGCGGTAGCCTCTCCGAACCCTGGGCTTATTGGCGGGCGCATCTACCGAGACTTTGAGC
TCTGGATGGGACTCGACTTGGAGCTCTGGCAACAGAAGCAGGGAGCCACGAAACCCGAAC
CAGCAACCACCGAGGACCACACACGAGACGTAGGTCGAAGGCTCTTGGAATTCGCCAGGG
ACAGCATCACGGAAGACTCACTAGGATAAGTGATATATATATATAGAGGCATCGATAGAA
AAATATATAGATTAAACATAATAATTCACTAACCACAATAGATTAGCG
>comp2363_c0_seq1
CCAGCATCCATCCATTGCACGAGTCAGATCAGATCGCATCGCATCGCATCGTTGTGTTCT
CCTGCCGTACCTATCCGACAAAAGACATCGCATCACATCACATCGCACCACATAGCACCT
CTCTGCACCCCAAGAATCCAAGCCAACTTTCACCCCAAGCCAGCCAAAACATGATCTACC
AAAACGTCTTCCTCGCCACCCTCGCCCTCGCTTCGACCGCCTCCAACTTCGGTCTTGTTT
CCGCGGTTCGGGGGGAGCGCAAATACCTTCGCAGCAATGACCAGCCATTGGAACAGGAAC
AGGACGAACGATCCCTTCTGTTCAAGCGTGGCCGCATGGGCAAGAACAAGAAGGACAAGG
ACAAAAAAAATAACAAAAAACCCGGTAGTAGTGTTAACTTAAACCCTCAATTCCAGATCA
TTGCGAACCCCCCCACTGCCGCCCCTGCCAAGACGACGACCGATCTCGAAGACTGGAAGA
AGGCCTGCCAGGGAACTCCGATCAACAACATCGACCTGTGCACCTTGCCCATGATCACCG
CCGACGCCACCCCCGAAAACTGCATGAAATGCCTCTTCGCCTTTGGCTCGTTGTCAACCT
CCACCGCCCAGGGATCCGCCAGCAGCTGCAGGAACAACGAGGCCTGTGGAACCAGGTGCT
CAACCGACCTTCTCAAGCCCTTTTTCGAGTGCGGCAAGAGGGTCGACAAGGCCTACGTGG
ACGCCGGGAGACCCGCTGTCTGGGACGCGAGCACCCTCAACACCTCGCAACTCGCCGAAG
ATGCCCCCGGGGCACAAGAACCACAAGAACCACAAGAACCACAGGGACAAGGACCCGCAA
GCGAAGTGGTGATCGACACGGTCAATTGCCCCGCCACCCTGCCCGAGAGCGGAACAAATT
GCACCTTTGTGGGCGATGCCAACATCAAGAGCTGCCCCTACTACGAATACAACCCCGTTC

CCATCTGCACCTGCGTCAGGGGCGAGGACACGTGGGTCTGCACCAACCAGGGATCCGGGA
CCCTCGACGTCGAGAAGGAAGACCTCACCACGATCGTCACCGTCACCAACCCGCTGGCAC
CCCCCCTGGCCCCGGTCGCGGACGTCGGCATCGGCGTCGACCTTTCCGTCAACAACCTGG
CCATCAACCCCAACTTTTGCGGGGCGGAGGCCCCCATGTCGGGAGACGCCTGCAACCCTT
CGGGATTCGAGGAATTCACCTGCTGCTACGACCACGACACCTTCGGGACCGTCGAGTGCA
CGTGCTCGGGTTCCACCAACACCTTCTCGTGCGCCGGGGGGTCCCTCTCCTCCTGCCCCT
AGATGCGTTCCACAGGGGTTGCGAATGCGAATGCGAATGCGAATTCCAATTCCGTGGCGT
GCCACCACACCCGCCGCGAATAACAACCGCGAGGCCCTTCGTTCCGCCTCCTCCCGAAGC
TTCCTGGTTCTCGGAACGGAACCGATGACACACCACGACCACGGCGCTGGTGTCTTGCTC
GATATCGAACCGAATCGAATCGACCCATCCACGGAAGAAATGATATGATATACTTGCCCA
TGATTTATGATTTTTACTGTACGACTAGACAATTTATAATTTAAAGAAAAGAAATCAATT
CTTAAAAAA
>comp2364_c0_seq1
GTGACAACTCCCATCCATCGCAGCACAATCAAAAAGTTCGAGTAACTCGTTTGATCGCAC
TGAAATCAATTTGAAAATTCACCAAAACACTTCAGCAATGGAAGGTGCCAACAGGGACGG
CAGCTTCTTCGAGGATGTTATTTCCTCTGATCTTGCGCTGCGAATCATTCAGTGGCTGAA
CGTCGCGGACAGCGGGCGGTTGGCGTGTACGTCGCAGCGTTATTACTATCTCGTCCACCA
ATATCGACGATGGAGGGGGCCAGAAATTGCAATTACAACGGATCTTCTCACTCTCGCCGG
GATCGAGCAACCAACAAAAACCGAAAGAGTATTGTCGTATTCTTCGGATGAAATTTCGAG
TGCCATGGCGAAGGATTGGAAAAAAAAGCTTCAGTCGAAACCCAATCTGATCCTGGGATT
TTCCACTCCTTCCAACCTAGTTGATGCCATCAAGAAAGACACGTGCTTTCCCGTCTCGCC
AAGAACATGTGAGGCAATAAGCCTCAATGTCGTTGCCCATCAACGCATTCAGGTCTACCA
ACCAGAGCTGGGCCAGAGTGGTGTACAATCGAGACAACCAAAGCTGGATTACAAAAGCAG
AGCATCTTTGATGGCTATGAGTTTTCCAGGCGCCACAATAATGCCGTATCTCATTCGGGG
TCAGCCCCAAGTCGAAGACCTCGAGTTTATGGAGCATAGGCTGCGCTTTCACAACAAATC
CAACGACGAAAACTTTTGGAAGGCTATGATTCTATACGCAGTCGGAAGCGGCGTGCAGGC
GTCCGACCAAATTACAACCCGAATCCAAGCGCTTATGCCGAGCGCCGTGATCGTTGGCGG
TGCCTGCCGAGAGGGATTCGTGTCCATGCCCACTTATACAAAGGAGGAGCTTAGTTCCAT
GTCCATAAAGAACATCAAATATGTGTTGAGTCACTGCTGTTCCAATGCAATTCACGAAGG
CGTGGTTGACCGAGACGCAGCAAAGAATTTTTTAGAAAAGTCGGAACTAGTGGACTGCAT
TTATGCCGCACTAGAAAAGGAGCGAGCAGTGACGAGCGATAGGTACAGCTCGGTGTACAC
GATTTCCGGGGCCGGGATACTCGGAGTCTTTCTGGGAGGCAACGTCCCAATCAAGAGCGT
GGTATCGCGGGGTGTCCACAGTGTCTTGAACAACAATGGTCCGCCGCAACCATTCTCAGA
TCTAGTTGTTCACGAGACAGCCCTGAGCAGACCCGGAGACGACGACTATTTGTTTGGAGG
CCACGATCAGCCGCCTATTCACTTGATTAGAAAGATACGAGACAAAGCTACGGGGGTGCT
CTATTCACCAATCGAGCTGATGGTGAAGTACTTTCCGGCTGCCGAGAGCCAAATCGACTT
TTTGGGACTCAAGAGAAAGGGATGGGATGGATTCCAGCTGTCGAGTTTGAATGAAATGAC
GCAGGCGCTTAACGCATTCGCAATTTTGACCGATGGATCCGAGCCGGAATTGGAAACCCT
AGAAGAAGCCGAAATGGACTTCTTTTGTTTGAACGGAGAGGCATGCATGGAAGACATGGA
TCGGACTATGCTGAAACTCAAGGAGCAGACGAAAGGAGAAGAGATATTGGGTGCCTTGAT
GTATACTTGCAATGGACGTGGTCCCGAAATAGGTTACCTAATTCCCGAGGAAATGAGCGA
TGCGAAACGATTTGCTCGTTCATTCCCCGCGGTTCCGTGCCTGGGGTTTTATGCCGCCGG
AGAATATGGTCCTATTGCGCTAGCAGGAAACGAAAACGTCTTTCAAAAGGGCAGGGCATC
TCTCCAAGGGTTCACCGCTGTGTTTGCTTTGTTTATCGTTCCCGTTCGGGATTTGCCTTC
TACATATGATTGGGACGATTCGATCCAAAACGTTGAACAGTTTGTTCGACACCGGTTGCA
CTAGAAAGCAACACGACGATACCAGAGAAAAATCTTGTTGGGACGATTGTTATATGGTTG
TATAGTAAGCTGTAGATTAGAAACATGACGTTAGCGATTCAGTTGGCTTACGGGTTGGAA
ACAATCTCTTCATGATTCCTCGAGTTGCACTGTTTCGATTTTTTTAAACAATATACCGCA
CTGCAATTGTACAGATTGTTTCATCGCTCGACCGCAATATTTCTCTGGCCTTTGTTTCCC
CGGGTGGCGCTGCAAACGCAGTTGGTACAGCTTTCTTAAATTCAATGCTATCGCTCTTTT
ATTCGGCCGTGTTGAGTTGTGTGTTCATGGCTGTGCTCTGCCCACGGAGTTTCATTGTTG
TCATTTCCATATAGAGAAAGGCAAAATGTGTATCAATAGTGGTCGAATGGTACGAATGCG
CAGATGATGGGAAGTGAGCAAGAGCGATTTGGTTTGCACCTTCTCATTCCAACGAACGAG
ACGAGAGAGCCGAGGGCGAATGCATCACAACGGCCAGCAAGACGACAACGAAAATATACA
CCGGGTACTATGCTCAGCAGGCTATGGGCGAAGACCTGCCAAGTTGTTGGATCGGAAAAT
CTCACTCTGCTCCCAGGTTGTGTACCGCGAACCGACCATGGAGATGCATCGGACGAGGCT
TAGATTGGGAGAATGCCACTTCGTGGGCAACGGCAATAGCAGCAGCAACAACAACAAGAA
AAGGGAAACCCCTATCGAGACATCAGTGCTCAAACGCAAAAATAGTGCACGTACCAGTTT
AACGACTCCGTGTTCCACTGGGTCTCGAAACGCAGAGCGGCCAGAAGTGTGCAACACCTC
TATTCGTAGTAGCTGAGCCGATGGCAGAGAAAGCCAAGCAACAAGGCTTTTCGTCACAGA

TGACTGGCTACATAAGATGCCATAGAACTTTAACACTAAATCCGAG
>comp2365_c0_seq1
CTAAGATACATTCTAATCGTGCTTATGTAGTAGTATTGGTCGTGATTGAACATTGATGAA
CATCGACGAACGAAACGAAGCTGATATGATGAACTAGTCAAGTCAGTTTCGACACGAACA
GCAGCGCTGTGCAGTTTAGCAATCTCTGTCCAGATTTCCATCGAGGCCTTCGGGACCATT
CTCAAGTGGTGAACACGGCGATAAATGGGGCATTTACTACATATATTTAGGGGGGTGGGA
CCATCCTTCCGTTCGATTTTTGAATTTGTCGTTCTAACCCCTTGCGAATGATATCTGAGT
GGAACAATCGCTGGCTCCTGTTTCCCAAGTGGTGATGATTCAGACATTATTCAATATTAC
TCTAAAAGCACATCTGAGTAGATGCACAACTTAACCGACCAATTGTCGACAACATTGGAC
TCGCTGGACCTCATTACGCCGAAATTTCTCGCCTCATACAACAGTTTCAGCTTGGAATGT
AAACGCAGCAATGAGATGGAAATAAATAGTTCCTGTCACGAAAGGTATATTGCGGACAGT
GGCAACCTCTGGCAATTCAAGCAAAACTGCTTCCGATTTTCCGTCTCAGTATTCCCAATG
ACGTTGCACAAAAATAATTAAAGACACTCTTTGAGTATGGGTCTCGTTTGTTCCCCAAAA
AACGGAGCACATGTACACACGATCAGATTAGTCGGACTGACAACCAACAGTTGAAATGAT
CAATCAGCTGTCAATTTTCCATTTCGTGATTCTCTACACGACCAGAATCGACAAAGCTTG
TAAACTGGGAATAGGAAAGGACATTCCCCTTCGGACTTAGTTTTATACTATAGTTGCCAT
AGTTATTGCCATA
>comp2366_c0_seq1
GACATTTTGAAAGCCAAACCTAACCGATTTCAATCCCCAACCAAATACTTCGCGATGACT
TCCTTTAAGATTCTATTTTCTTTCCTCTTGCTTGCCGCCTCGGCACGAGCCGCCGCGAGC
GACACGATCATCCGCGATTTGCAAAAGGAGCTCAAACCGCAAAAATTCATCCGGCGCTGC
ATGAAGACCGGGGTCAGTGAGGATGAGTGCGAGGAACTCGTTGAGGAGTGCATCAAGGAC
ACCGAGACATTCAGGATGTGCAAGGCCAAATTCAAGGAATGCAAGACGTCCACCGACAAG
AAAGGGTGCCGAAAAAGCTTGGTCGAGGAGTTTCCCGAGTGCGAGAGGGAGAAGAAGGAC
AAGGATTGCAAACAAAAAATCAAACAGTGCAAGGAGCAGGGCATTCCCAAAAAGGACTGC
CTAGCCAACCTTCCAGACTGCAAGCCGTCGACCGTCAAGAACGCCTGCAAGGCACTCTTC
CCCAAGGTTAAATGAGGAAGTGCAATCCAGTGATCCATTCATCATTACATGATAATAGTT
AACAACGTCTTGGTTGGCGCAACAGAAACATGTTTTGTCTTGCGGTTTTGCAAGGCTAGC
AGCAGAATTTCAGAAGATATTCAACCTCATTGTAACTCTCATTCATAATAAAATCAATAA
AAAATAAAGAATATGCCGTACCGGTACGTAGCAAGTGCAA
>comp2367_c0_seq1
AAAGGCCGAGAAGAGTGACAAGGCTTCAAAGGCCGAGAAGAAGGAAGGAAAGTCCGATAA
GAAGGAAAAGAAGAAAAAAGGCGATGACGAGTCCGCCGCACCCACTTCCAGTGCGTCCCC
GACATCTAGTGCGTCCCCGACATCCCCGACGGTTCAGCCATCAGGTATCAGTCCAACTAA
GGCACCTGTCGTAACGACCAAACCTCCCACCACTTCTCCCACTACTTCTCCTACCACTCG
TCCTACAACTTCTCCCACTGTGACCTCATCCTCCGGGGGAACCAGTAGCACCACTTCAGA
CGGTTCAGGCGGTGGTACCGCTGGCAACAGCGATGAAAACACTTCCGAAACATCTGACTC
TGGCAATGCCGCTATAGCGGAAATGCC
>comp2368_c0_seq1
AACGGATCGACCCCGAGATAAATGTTTGAGAGACGGAAATTTCCTGCGATAACCAACGGG
TTGGCGGTATGGAGTTGAAGCAGCGCATAGAGTACTCGTACTCCTACTTATTGACAAAAA
AGACGAGCCGGAACAAGAAGGATTGCGTACCCATCTCTTCGTGTGTGCCTGTGAGAGTGC
GTGTGGGTGTGTAAAAATATTGCCTCGTTATGGGGACAAGAGCAAGCGGAGGCAATGAAT
TCATAATGAATCGCAACCGCGGACAACGACTCACAACCCACGAGGAACCTCAACGGCAAT
CAAATCCTTCGAATCCTCCTCGGCTCCAACAACAAAACCAGTATCCTAGCTGCGCTTCCC
AGACCCTAGAGGAAGAAATCATCCCATTTCTATTGATCTGCCACATCGCCATTGCAGCGG
TAGCGATTTTTTCTTTTCTTGGATAGAACGGAAAGAACGAAACGAACACACACAATGTAC
CAGGTCTTGGACGTTCTGCCAGGCTGGATGGCCACCTACCGCCATTGTACCGAGCCCCTT
GGGGTCGGCTGTGTTGGGGCCTTTGCCTACAACGTCGAACAAGACGGCAACAACGAAGAC
GGCACCAGCGACGACAGCAACACGATCGTGGAAAATCGTTGCCCGGTCGAGGCACGGCAG
GACGGCGGCAGCAGCTGCGATGAAAGGCTCCTGGAAACCACTCTAGAAGAATCGGGGATC
CGCGACGGCAACGCCTGGAGAGACCTGTGCTTTGCATGCACCAGAGTGGGGGAGGTGGCC
GGTATCCACGACGAGAATGGAGCGTCGAACGGCCCCAGCAACAGCAACGATGAACACTCA
CTCTTGGTCCAGACCATCAAGGTTCGCGAAAATGACATTTCGGCGGGTAGCACACCCTCC
CAAAACTCTGCCAACCGCAGTGGGGACAACCCCCGAAGCGATGCCATTGGAGACAACAGT
TCCGACAGTAGCACCACCAAGAACAAAAAAGAGAAGATCAAACGTGAGATCGATAGTAAT
GACAAAGAGCAAACTATTGTGGTCGAGACCATCAAGATCCGCACGGCAGACTTTCTCGAG
GACCGAGAACCATTCTCCACCACTCCCGACCACGGTGATGCCAAAAACACCACCATCCAC
GGCAAGACTGGCACGAAACAGAACCTCTGGCCAATCGATCCCCCCGGGCACGTCGGCGAC
AAAAAGGAAAAACAGAACGGTTTCCACAAGCTCTTGCTCTTCCACCACAAGCTCAATATC

AATCTGCAAAACCACATGCGCCAGCACAAGGAGCGCCAGCGTATCACCAGGGGCAGGATC
AGACGCAAAGACAAAGAGAGCTGGACCAGAAACGAGCGGGACCAGGCAGAGGAACAAATG
CTGTTGCTAACCCCACGAGCCCGAACCGAACCTAGTTGCCGGTTGTCCAGCAACCAAACC
CATCCCTGCCGGCTAGAATCCACCGCAGCGGATGTTGACTTGCACATGGCTACCGAACCA
CCACTCAAGCGTTCCCACTCGTCCCCCCAACCAACGCTTCAGAGGTCCTTCCGTGGCAGC
AAGAGCCACAAGCTGTCGCCTTTCCGAGTGGCTCGGTCGCTGTCCTTTTCCGTGATGACA
TCGCCGTTTGGAAAGAGCAAGAGCCACAAGACTGGCAAGACCCCGCGCTTCGAGTATTTG
AAGGATACCGAGTAGCGCAGTGAAGGAGTGAATCAATCTTTGAGCAACAAGGCGCTGCCC
TGATCGACTTTGCGCAACAAACTATTGAGGATCGTCATTTCCTTCGGGGTCATGTCCAAG
TGTCTCTCTTGTCGTGGAGAGGGATAGAATCAAATCCAATCGAACTCAACCCAGTGCAAT
TCCTATCGTTTAGACGTGTGGAAAGTTGATGCAATCCATGATTGGGACATCGCCCTTTTC
TATTCCGCTTTCTTCTGCAACTGCGAATTGTAGAATAGAATCGTTCGTTTCCCGGGTACT
ATGGTATGGGCACAAAACACAAGACAGCTACTGAACGTGGAAGAATCCAATTCTATACTT
TTATGCGACAGAATATGCTCTGGTATGGTTCAATATGGCAAAATGTGTCTTTCGGGGATG
TTCGCTAGCAGTTGTACAACACACAGCAAAACACACACAACTAATTTAAAGCATCAAGTT
TTTAATGGTAATGTCTTGATTAAACGTGCGAAGCACCACTAAACGTCATTTGCCATG
>comp2369_c0_seq1
CCGTCTAGAACGAAGCATACGTCAAAGAGTTCAACTGATTTCATAAATTCCATTTGCACA
TTGTCACTCACTATCAAAACAAAGATGAAAACTTCCACTCCAAGTAGCATTTGCCGAAGC
TGTCTTCGCACATCATTGTCCATCTTGATTCTCGTCGTTCTCGCCTCGTGTGTGAATGCA
GAAAGCGAATGGCAGCTCCGCAAAAGAGACCTCGGATTTCGCGGTGGCAGCAACCCTGAA
AAAACTTCATTCGGGGTTATTGGATCTCTCGAGAGCAAGGCAGGTAAATCCGATAAATCC
AAGGAAGGTAAATCAAGCAAATCGATTGCAGAGAAGACCAGCGATTCCAAGGCAGGTAAA
TCGTACGCATCCAAGTCCGACAAATCTGACAAATCGAAGGTACCCAAATCAGACAAATCG
AAGGGAGAAAAGTTTAGCAAGTCCAAGGCAGAAAAGTCTCTTCCCGATAAAAAGTCAAAA
GTACCCGCTGTTGAAAAGTTGTCGAAATCGAGCAAATCTAAAACTGCAGACTCGTCAACT
GACCATGTACCAGAAGGAGGAGCTGTAGACGAGGGAGCTGTAGACGAGGGAGCTCTCGGC
GGCACGGTCACCCAGTCGCCTACTGCCGTACCTACCGAAGCGGTTTCTTCCAAGGCATCG
AAATCAGTTGGCGTGAAGAAGTCCAAAGCGCCCACCGAAACAAAGGCATCGAAATCACTC
AGTTCGAAGAAGACAAAGGCGCCGACCGGAGAGGAATCAGGAGGACTCATCGATGATGGG
GCTATCGACAGCGCAGCCACCACGGTGCCTTCTTCCATGACCTCTTTTGGCTCCAGTAAT
ACGAGCTTCCCTCCGAGCCTCTATCCTACTACTCGCGTAACCACCAAATTACCCTCTGGT
TTTCCTACCAGTTCTCCTTCTGTTTCTCCTTCGACTTCTCCGTCTACAGGCGCACCAACT
ATTGGCGGTGATGAGGGAAAAAAGACGAAAGCTCCCAAGGGAGACAAGAAGCTGAAGTCC
GCTGCTTGAGACAGCGATCCGAGTGTCAATTTCTACTGTAGCAACAGCCCCGGCTTGCAG
AGCGGGGAAGGTTGCAGAACAATCTATGAATTGCAAAAGAATCCTTACTTTGCAATATCC
GTGGGACAAAAATGAGAAACGTATAGGTGTCGGTCCTCTAGATCTTTTCGTGCCTGAAAC
AGGTTTTTAGTCAACAGATTTTAAATTGAATGAATCGTGTTATTTTCTA
>comp2370_c0_seq1
CTCGGGTCGACCAAAAAAATCTCGACACGCGGCTCGCATCATCCGCCACACCACCAACGC
CAGAGAACGCTTTCGGGGAACGATTCTGAACTCTTTTCCAACAACAAAACACCCATTCAT
CCTTCTTGCTCCTCGTTCTTTCTGTATTCACCCGACCCAACATTGCCCGAAACGAAACGA
ACTTGTTGCCATGGCACCCAAGACGGACACTCCTGTTCCGACCCTCCTCGCCCTGCTCGT
TGCCTGCACTGTGGTTGCGCTCGCAGCGGTGCGAACCGCGGACGCCTTTGCGGCCACCGC
TCCTGCGCTCCGGACCGCGGTTTCTTCCCGGTCCGCCCTGCCCGCCCGGCCGCGGTCCAA
GTGGGACGACCTGGTCGACGAGGACGACGAAAAAGGCAGCGGCTACGAAGGGGGGACCTT
CGAGAGCGGTATCCCCGTCCCGCCCGACATGACCTACGTCGAGCGGAACGTCAAGCGGTG
CCACGAAAACTTTCTGAACCTGCGGAAGGTCGGCGGCAAGGACCTCTGCCACGACGTCTA
CGCCAAGTCCCCGCTCAAGAAGGAAGAGGTCTGGTACGTCGGAAAGGTCGCCAAGATCAC
CGACGTGAGCCTGGAGGACTGCATCGCCCGCCAGTGGAACATCATCGAGACGCACGCGGC
GAACCTGCGCCCGATCGAGCTCTACCCGCAGCGGGGGAACCTGGAGATCTGGACGGCCCC
CGGGGACACCGAGCTGGACGTGGCGTACAACCGGCCCGAGCTGGTCATGGCCAGGATGGA
AAAGTACGAGGTTCTCGCGAAGGACCTGAGAAACAACCTGATCGGCTTCCAGGGCGAGGT
CTACCAGGAGGGCGAGGAGGGCTTCCGGACGTGGCGGAAGGAGGACGGCTCCCCGGCGCG
CCCGGAGATCAACCCCGGCGGAGAAACCAGACCACCAACGGAGGAAGAGATGGAGCAGCT
CAAAGCCGAGGCGCAGCGCCAGAACATCAGCTTCCAGGACGTCTACAAGGAGTCCCAAAA
GCGAATGGATTGATTGTGCGTGGGTTTGGATTTGGTGGTCGAACATACCATCAAAAATGC
CAGTCACAAAACGCACCGTAAAGCATTCGCTTCGGTGGATCCCGTTTTTAGCACGAGAG
GAGAAACCAGTTACACATAACCTCATCTATTTGACGCTGCAAAGGTTCATTATTTGCAAG
CGAATTAAACGCTCAC

>comp2371_c0_seq1
AACGATTGCCATACCTTGCTTACGGAAAAGCGAAACAACCCGAGCTGTTTCTTCGGTTCT
TTTTATCAGCATGGCGGATTAATAATCGTACCGTAGTGTCATGCGTGATCCAGATAAGCG
TCTTGGTACGATCGAAATCATCCATCGGCTGAGAAAAGCTCGAAGCTCACAACGAATAAA
TTTCCATAAAGAATAATTTGCCATAAAGAACACCACAAAATATTGATTGCAACAAGACGA
ACGAAACCAACCAAGAGATGACACTGGCGATTCAAGTGAGTACGAAAACCGAAACCCCGG
GAGATAGCTATGACGAAAGCAAACTCTTTAATAGCTCGTGTCCTTCGAAGAGTGGTCAAG
AAGAGAACAACAACGAAGTCTGCAAGTATACGAAAGCAGAATCATCCATTTCGCGTCTTC
GCAATACCACAGCTTCTGTCGCCGACGCCCTGCTAAAGCTCGGGGGCTCCCAAGACGATG
TCGAACCGACTCTTTCTAATCCTCTCAAGAGAAAGCGCTCTTTATTTGTCCTCGGGGAGA
GGAAGTTTACTACCTACGTCAAAGATGCTGCCAGCACCGTGTCCGAAACGGATACCGAAT
CCACTGATCACTTCTCGAAACGCTTCAGGAACGACGGGTGCGAACAGCGGCGTGCCCCAT
CCGATTTCAAGCTGAATCTTCCTCTTTCACTATCGATTCCAAGAACAAATGGAACCGGCA
ACGAAGTAGACGATGCGCTTACTTTTTCAACATCAGAATTCCAGCTTCCGCTCCAAGATT
TCAATGAACGAAAATGTCCGGGAGAACTCGGGCCAATCTCGTCTACCCTTCAGGATTTTC
GGCCCCTACGAGCCCGCCCACACATGCCAACTTTTATTGTCCCCGAACATCCTCCTCCGC
CACAGCAAAACTACTACTCGCCGCAACTATTACCAAGTGATAGTCCTATGATGACTTCCG
TTGTTGCGGCAGACGAGGGATGTTTCGTCTTGCATTCCTACACCATAGTTCCAAGGCAAA
ACCTACATCCGACCCCACTTCCACTAATGACGAATGCTCCTTATCCTTGTTATAGGTAGC
GATAGAAAGTCGTTGTACATAAGCAGCAGGACGAATCATCGAGGAAAGAAGAATTTCCCC
TTATTCTTTGCGCTTTCTTCTAAGCAGACATCGAACAAACAAGCTATAACAAGCGAAGCT
AAAAGCGCGAACGCATCGTCCGAGAAGCAACTATTCAAGTATCCAAAAGAAGTTTTTTTT
GCATAGCAGATTTTGAAAACATTTTCACACAATCTTTTTGATGAACAAGAAACAATAGTT
TTTCACGAACTCCATTTTCACAATAATATATAATTTATTAACGATTTTCACTATCTTCTC
C
>comp2372_c0_seq1
ACAAAACGAAAACGCTCTTCATACACCGACGATAAAGTTTACCGATAAAAAAGCAAAATC
ACCATCAAATAAAATGACTAAGATTCTTTTCCCACTGGCACTCATCGGTTCTCTTGTTGC
TAGCCTGCCCCAACTTGCAATGGCTCAGAGCGATCTGCCGGAAGAGTCGACAGGATCCTT
TTCCATCGATCAGTTTCACGAAAGCTACGATGGAGAACTCCAGAGCATTGCACAAAAATT
TCTCAGCGAATACGTCCCACCAGAATCCGCAAACGAGCTTTCACCATGCCAGGAAGAGGG
CGCGAAGACGATCGCTGACTACTGTGGTGAAATCACGGAAGACAGCCATCCTATTTATGT
GTGGTGCAAGGCTGCATTCGAAACGGGCCTCATGAACAAGTACGCAAACCCCCTCCGAAA
GTACACGATCTTCGCGCCCAGCAAGGCCGGCTTTGTGAACTACTACACCGAGCTCGCCAG
AACCCAAAACGTCCCCCACGAAACGCTCATGCCGATTCTGGAATACATGGAACTCGAGGG
CAACATCTACGGGCCCAACCAGCTCTGGTGCAAGAAAAAGTACATCACCACCACCGATCG
CATCGCGGGGCACGTCAAGCCCCCCAAGATCATCTGCCTGAACGACATCAACGGCGATCC
CGTGACGTTTCTCCGTGGGTCGGACAAGGAATCCCAAAAGGTGTTCACCCCCCAGTTCGA
AAACCCGGGAGACCCCATCAAGGTTTGCAACGCCAACATCTACCCGATGAACAACATGGT
CCTCTACAAGATAAAGGGGTCCAACGAGGGCAAGGGGAGAAAGAGGAGGAACAGAAGAAA
CAAGAACAACGGCGTCACCACCGCTCCCGTCTCCACTCCGATCGTCGGAGTCGCCGAGGT
TGCCGCTGCAATCCCCGCCCCGCTGGCCCCCGCCGCACCGGTTCCCGCCCCCGTCGCCGT
TGTTGCACCGATCCCCTCCCCGATTGCCATCGTCGCTCCGGTTCCTGCCCCGGTTGCCGT
CGAAGCACCGGTCCCGGCCCCGGTTGCCGTCGTCGCTCCGGTTCCCTCTCCGATTGCCGT
CGAAGAAGCACCGGTTGCCGCTACCGATCCCATCCAGGTATAAGAACACGGTTCGGTCCC
TTTTGCTCTTTCCGATGATATAGAATCAAGGAGCGAAGTCAACTTGAAGCATACCAAAAC
AAAACAAAACGAAACCAAACCAAAGTGAACGAGAACAACGACTGCACGATCGTTGTGAAA
GTGAATACGGAAAGTATTTACTATATGCATGTTTTAGAGAAAATAACGAAATCATTAACA
AATATTTATAGAATAAAAATCTTACCCA
>comp2373_c0_seq1
TCGTTCATCAAAGGTGAAGTACAACGTACAACAGACGAAGCAAGAAACAATTCTTCTATT
GACTACCAGTGAAGGCATATTCAGCATACCTTTTGTTTCACATCACTACCGCAAAAAACT
AAGCCAACCTACCGGAATCAATGACATGAAAGGTCTCTATCACCCGATCACCGCCATCGG
CGTCTGTATTGCGGCGTATTGCTGCGTTGCCAAGGTGCACGAAATCCTGACGTTCCTCGA
CTTTTTTGAGAAACCCTCTGTGCACCACGACGAAGCTTGCGTCAAGGTCATGTCTATTGG
AGCCTCAGAAGACCAGACCCAATTCAACAGCAATTCCATCGTTATGACAGAATGGAGAGC
TACGGGATACCACCCGGGAGAAGAGAAACCACCCGGAGCATTATACGTGTCGAGTGGATT
CAAGCAAGGTACTACGGCAAAGAGTGCCCAAGAATTCGTCGAGAACAACCTCAAAATCCA
AGAGCTTCCGATTTTTAATTATCCAGAGAATATCGATTTTCATCCCCATGGGATGTACAT
TCGGAAAGAGGATAGCACGCTCTATGTCATCAATCACGCGTACGAAAATGGCGGAGAAAG

AATCGATGTGTTTGGAGTTGGAACCGCGGATGATGGTGACGATGACACAGAGAACGACAT
TCCGAATCGCCTAGATTATCAATATAGCATTACATCCGACTGGATGAACAACGATATGCA
TGGTATTCTCAATTCTTTGATTGTAGTCGAGAAGAACAAATTTTACGTGACACAATACCT
CCCTATACCAGAGCGCCACGACGGGTGGTTGAGTTTTGAAAGGAACATTGACATTGAAAG
ATTCAAAACACTGGTGTTTGGGAAAAGGGGTACCTATGTGTGGTATTGCGAATACGACGA
TGAGTCGGGGTCTCTCGACTGTCGGAAGGTCGCCGACCAATTCATCGGTGCGAATGGAAT
CACCCACAACGAAGATTACTCAAAGATTTTCGTTGGGGACTATTTCACGATCAACGTGTT
TGACAGGGATGCGTCCTCCAACGAGCTGCAAAATCGGACGACCATCGACGTTCCGAATTT
TGTGGACAATCTCAAGTTCGATAAGGTGTCGGGGAATGTTTACGGCGGGACCATCAACAA
CCTGTGGTACACCGTGTTCGTGCACCCTTTCCCGGCCGAAAGCGAACAGTCGACCTCTGG
CATAATGGAATTGGCTTTCGATCCACAGACATCATCGTGGCAAGCGAGGGAAATAATATC
GAGCTCGAAACTCAATGCTATTTCGAACGGGTTGAAAATGGGTGAGTTTTATGTAATGGG
AGCGGGGGGTCTTGGGTATCCAGGCTTATTGGTTTGTCCAGTAATAAAAGAAGCAGGACA
GAGCAATACTGGAGGGGGCAAGGCGTACGATTCTGAGCTCTGAGATCAAACGCTCGAAGC
AGAAAATGCTTCAAACAGAAACAAATGTCTATCTAAATCTACAGTCTAGTTTTATCTATT
CGTTTGGGGCCGTCCATTCCGTTCCTTTGTTATCGACAAAGCGGGGGCGTATTAAAC
>comp2374_c0_seq1
AGAGCTTTGAGTGTTCTCGAGATGTCTCATAATTTTTTACGAAGCAAATCAAGCACCACC
AACCAATCAATCAAACTATCGATCAGTCAATCAATCAATCATCATGGGAGACAGTGGACG
ATGCATGTATAGTGGCCTTTGTTGTGCCTACACGGCCTGTGATTGCAAGCACGTGGAGTT
TTGCTACAAAGAATCTTGCGATTGCCTTTGTATCCGCCATGCATGCTGCCTTTCGGTGAA
CTCCAAGTCTCGGGGCTGCTGCATGACAACCGACAAGGAGAAAGGAGAGTGCTGCAAGAT
TGCCTGCCTTTGCTGCGACTGCGGAATCATCAAACCCGAAACTCTTTGCGCCGGTGCTTC
CCAGTGCTTGTGCTGTTACACGGTGGAGAGTCTCCCCTGCAGCGATGAATACGTGGAGGA
ATGCGTCTGCGCCTACTTGTTCATCCAGTGCTGCCCAAAGTGTGGTTGTTGCGCAGCGCC
GCCCTCCTGTCCCGCTTTGGATAAGATCCGAAATGACGAGTTGGCTCCCATGGCCATGGA
GCGTTCTTAAGCTATATTTGTAACAATCAAATCGATTTGAATCAAGTGCAACAAGATCTC
CTGGATGAATTCCAAGCTCTTCAGAACTAGAATGGACAATTAGATTTGAATTGCAATCTT
TGTCAAAAAGAAGATGCGTAAGTATATAAATTGAGGTGGAGAATTAAGAAACTGTGCTTT
GTGTCGAATTCAAGAATAAATGTATAAGTGGATAAGAGAAAAAATGTTAGTCCCCTCCTG
AAGTTACACGTACATTCA
>comp2375_c0_seq1
AAAACGACAACGCAACAACATCGATTCCCAACAAAACTAGCAACCAAAATCGCATTATGG
GATCTAAATTTACAAAGTTCGACGCAGCTCTTCCGAGTGTCGATGGCAAGGTCTTTGCTA
TCACAGGTACGTCACAAGCCAAGGCATAACGAGTGAAGCAATGCCATTCTTTCCTCTCGG
AATGGAAAGTTTCCTTTTATAACGGGTATGGAGATCGAAGTGAAGTAAATCAACCAACCA
TCCGCTTGGATCTTGCACATCCGCCTTGCGTGTAGGATTCATCGTTCTAATTAAAAGCCA
TTGCCTCGCACCGCAACCTAAAATAACGTGTACAAACAGGAACGACCTCAGGTACGGGCT
ATGTCGCAGCCGAAACCGCGGCAAAACACGGGGGTGAAGTTCTCTTGCTGAACCGCCCTT
CGGAACGGTCGGTCTCTTCCCTGGAAAAGCTCCGGGCCGCGGTTCCTGACGGAAAGTTTG
TGCCCATCGACTGCGATCTCCAGGACTTTTCTTCCGTCCGCGCGGCTGCCCAGACAATCA
AGGACAAGGGCTACAAGTCCATCTATTGCCTCTCGAACAATGCCGGAATCATGGCGGTTC
CTGACGAAATTACCAAGGCAGACGGTTTTGATAAGCAGATGCAAACAAACCATCTCTCTC
ATTTTTTGCTCACCAAGGAGCTCTTTCCCCTCATCGTTGCTGCTTCTAAGGAACACGGTG
ATGCTCGGATTGTCCAGCACAGCTCGGGCGCCCGGCACATGACCCCAAACAACGGGTTGG
AAGAAAAGTACTTTCTCAAACAAGAGAAGGACGGCATGCTTGGAGGAGACACCGAAAACG
GATTTATGAAGGGTGGCAAATGGTACCGATACCATCAAACCAAACTCGCCAATTCTGTCT
TTACGCAATGCCTGCATGACAAGATCCAGGCCAGCGAGAATGCTGACTGCAAAAACATTC
TGAGCTTGTGCGCCCATCCCGGTGGTTCTCGTACCAATCTTGCTGATCATCTCCCGATCG
ATGGAATCTTCATGAAAGCATTGCTAAACGTGGTAGCCTATACTTTTATGCAATCCGCAG
AAGATGGTTCCATGGGGCTTCTCAAGGGAATGATGGATTCCAGAGAGAGCGTTCGGGGTG
GAACTCTGTACGGGCCAGAAAGGTTTGCGGGATGGGCCGAGCCGAATCCGCCGAAACCCT
ACGAAGTAGATCCAAAAGCAAAGGAAATGCTTTGGAGTACAAGTGAGAAAGCGACCGGTG
TAACGTTTGAGATCTGAAGAAACTTGTGTTAGTACTTCGGATGGATTTGTCATTATTCAT
GAGAGTTTGAGCAATGGAGTCGTCTTGCTCATTAAGATTGCAAAATGCATGAAATCTTGT
ACCATGTTAAAAACTGTACTATATTCCAACTGATCAAATATTGTCACAATTAGCGGATTT
AAATAGCATGCAATAACATGATGGTTGGCATGGAATAATAAGTAGCAGTTCCAGGAATTT
CACTCTGTAACCTTTCATGAGCAACAATAAAACTGGAAATATAATCAGTGAACAATAAGC
AGTAGGAATGATAACACATGGTATTTCCTCCTCCTTTTCAACAAGTGTGGCATCATCACT
CCTCCTACTTAATGCTATTGCTATCAGTGCTTCTTTCCTTAACAAAGATTTGTGTTCTGA

AGCAGTTCCTGGTATGCAAAGAATCCTTCATCTCTCTCTATACATACATTAAAGATCTGG
TCAACATTACTACCCATATTGCCAACAGCGCTTCTTGTCTGGTAATTCTGCAGTACTAGA
ACAACAATCAGACTTATTACTACTAGGC
>comp2375_c0_seq2
AAAACGACAACGCAACAACATCGATTCCCAACAAAACTAGCAACCAAAATCGCATTATGG
GATCTAAATTTACAAAGTTCGACGCAGCTCTTCCGAGTGTCGATGGCAAGGTCTTTGCTA
TCACAGGAACGACCTCAGGTACGGGCTATGTCGCAGCCGAAACCGCGGCAAAACACGGGG
GTGAAGTTCTCTTGCTGAACCGCCCTTCGGAACGGTCGGTCTCTTCCCTGGAAAAGCTCC
GGGCCGCGGTTCCTGACGGAAAGTTTGTGCCCATCGACTGCGATCTCCAGGACTTTTCTT
CCGTCCGCGCGGCTGCCCAGACAATCAAGGACAAGGGCTACAAGTCCATCTATTGCCTCT
CGAACAATGCCGGAATCATGGCGGTTCCTGACGAAATTACCAAGGCAGACGGTTTTGATA
AGCAGATGCAAACAAACCATCTCTCTCATTTTTTGCTCACCAAGGAGCTCTTTCCCCTCA
TCGTTGCTGCTTCTAAGGAACACGGTGATGCTCGGATTGTCCAGCACAGCTCGGGCGCCC
GGCACATGACCCCAAACAACGGGTTGGAAGAAAAGTACTTTCTCAAACAAGAGAAGGACG
GCATGCTTGGAGGAGACACCGAAAACGGATTTATGAAGGGTGGCAAATGGTACCGATACC
ATCAAACCAAACTCGCCAATTCTGTCTTTACGCAATGCCTGCATGACAAGATCCAGGCCA
GCGAGAATGCTGACTGCAAAAACATTCTGAGCTTGTGCGCCCATCCCGGTGGTTCTCGTA
CCAATCTTGCTGATCATCTCCCGATCGATGGAATCTTCATGAAAGCATTGCTAAACGTGG
TAGCCTATACTTTTATGCAATCCGCAGAAGATGGTTCCATGGGGCTTCTCAAGGGAATGA
TGGATTCCAGAGAGAGCGTTCGGGGTGGAACTCTGTACGGGCCAGAAAGGTTTGCGGGAT
GGGCCGAGCCGAATCCGCCGAAACCCTACGAAGTAGATCCAAAAGCAAAGGAAATGCTTT
GGAGTACAAGTGAGAAAGCGACCGGTGTAACGTTTGAGATCTGAAGAAACTTGTGTTAGT
ACTTCGGATGGATTTGTCATTATTCATGAGAGTTTGAGCAATGGAGTCGTCTTGCTCATT
AAGATTGCAAAATGCATGAAATCTTGTACCATGTTAAAAACTGTACTATATTCCAACTGA
TCAAATATTGTCACAATTAGCGGATTTAAATAGCATGCAATAACATGATGGTTGGCATGG
AATAATAAGTAGCAGTTCCAGGAATTTCACTCTGTAACCTTTCATGAGCAACAATAAAAC
TGGAAATATAATCAGTGAACAATAAGCAGTAGGAATGATAACACATGGTATTTCCTCCTC
CTTTTCAACAAGTGTGGCATCATCACTCCTCCTACTTAATGCTATTGCTATCAGTGCTTC
TTTCCTTAACAAAGATTTGTGTTCTGAAGCAGTTCCTGGTATGCAAAGAATCCTTCATCT
CTCTCTATACATACATTAAAGATCTGGTCAACATTACTACCCATATTGCCAACAGCGCTT
CTTGTCTGGTAATTCTGCAGTACTAGAACAACAATCAGACTTATTACTACTAGGC
>comp2376_c0_seq1
AAAACTACGAACGAATTTTGAATCTTTTGAATCATTCGAATTCTCTCGGGACGGTCGAAC
CGGAACAGGAGATGCCCAACGGCTTCTTTGGTCTGAGACAGAGTTCGAAGGACGACAGAG
ACAGAGACAGAGACGCCGACTATGGACGAATCCGAGCCCCCGACACCGAAAAAGACCACC
AGGGTCTTTGTCCTCCTCGGCCAGTCCAACATGGTGGGAATGGGCACCGTCCAAGGAGCG
GACACCGAGGGAACCCTCCAGCACGCCGTCCAGCAAAAGAAGCTGTACCGGTACCTGGTG
GACGCGGCCACCGGCGAGTGGAAGGAGGTCGTCGATCCCTCTGTTCGGAACATCTTCACC
ATGGGAAGCGGGAACGACCCCCGGGACGGCGTCCGCCAGAACGAATTGCTGACGGTGAAA
CACTCCCGGATGATCGGGCCCGAGATCGGGATCGGCTGCGTCCTCGGCCGGTGGAGGAGG
TCGGAAGCGGCCGCACGAACCCCAACCCCGAACGGCAGCAGCAGCAGCAGTAATAGAAGC
AGCCACCCCAACGACGAAATCGAAACCGTCCTCCTCAAGAGCTGCATCGGCAACCGGAGC
CTGGGCTGGGACCTCCTGCCCCCGGGGAGCCCGTCCTTCGAGCACACCGAGCCTTCCTCG
GGGAAGAAGTGGACCTACGCGGGGTACGGCCAGTCCCCCAGCCGATGGGAGGCCGGGACC
GATCCCGTCCCCGACCCTTCGTGGCACGCGGGGGAGCAGTACGACGGGGACGTCCGCAGG
GCCCGAAGGGTCCTGGAGGGCCTTTCCGGAGACGGCGAAAACGACGGTGCCGTGTCCGTC
GCGGGCTTTTTCTTCTGGCAGGGCGACAAGGACCGGTACGACCTCGCGTACGCGGGGAGG
TACAAGGAAAACCTGGCCCGGCTGGTCCGGGCGCTGCGGAAGGACTTTGGGGCCCCTGGG
GCGCCCTTTGTCCTGGCCACCCTGGGCCAGACGGCGAGAGACCAAGCCGGGACCGGTGCC
GACGGGCTCATCTTCGAGGCGCAGACCGGGCTGCAGGAACTGCCCGAATTTTCCGGGAAC
GCGTTCTGCGTCTACTCCAAGCCCTTTTGCCACGGCGGGGCGTCAAACGGCCACTACAAC
CACAACGCCGAGACCTACATGGACGTGGGGCTGGCGATGGGCCGTGCCATGGTGGACCTC
CTGCGGACGATGCCACCCAGAGCCCGGGCCCGACGGGCATCTTGTAAACTAATACTCTAT
CTAAATTATTGTACGATTGGCA
>comp2377_c0_seq1
TGTCCTGCCGTGTGCAACGCTAAAGTAACACGGACGAACGAATCGAACGGAACGGGGTTG
CCTGTCGACAGGAGCTAGTGGTGACACGCTAGAATTTGTCTTGCTCTTGGTTTCTCGTTA
TCTCGGGCATTTCTTTCTCTCCCCGCGTGAATTTTCAATTTTCAAACACGCACCCAGCCT
CGCCCCCTCCAGGACCGCATTGGAACCGCATCGCCGTTTTTCGTGTCAACACTATTCTTT
CTCCTGGCGCAGCACGCCACCCCTCATTTTTTCGTCTTCAACCTGGACCGGTAGCGCATG

GAAAACCCGAAATTCGCGTGCACAGCCCAAGGAAATATATGCGGTGCAATGCATGGAGCG
CGCGAAAGGTCGGCATGGGGTTTTCTTTCTCCGGTTCGCGATCTCGATGATGATGATGAT
TCATTCTTTCTTCCTTCCATGACTAACGGTTGTTCTGTTCTGAAAGCCCAACGCATTTTC
ATTGGGTTTCCTCCCTAAATTATCTAGAAGAAGAATTTAATCGCTTGTGTTGGGTTTGGG
GGGCGAAAAAGAAGCATTTTGATTGGTCATCTTCATTTCCCGAGAAGAAGTTTGATTGGT
TAGAAAGTTGACATTCTTCTTCTTTCTCTTTGACTCGCGGCGGCACGGGAGAGGTGATCG
GCTTTGACGCGGTATGTTGTCGGTTTGTTGCCACCGGAAGAAGTTGCTTCCGATCGCTTC
CAGTCGCAGTCACGGGGAATACGGTAGAAATTAGAAATTCGAATCGGTTTTAGGAGGAAA
AATAAATTATCATACCGATACCAGCAGTAAAGAAGTACTAGTAGCAGTAGACGTATGCAA
GAGACTTGCACACACCAGAACGTGATGTCGGAGATGATATTCTTGCAGTGCACAGTTTCT
GGAAACCACCACTTCTGTCTTTGAAATTTGTGTTAATTCTCCTCATCTTAGTCAAAGAGA
AAGCAGTATTGAAATCCCATGACAAGGAGGCAACCCAAAAAGAGTGAACAAGAAGCAACA
GTACTACTACTAGCTACTACAAGAACAATAAGAACACAGA
>comp2377_c0_seq2
TGTCCTGCCGTGTGCAACGCTAAAGTAACACGGACGAACGAATCGAACGGAACGGGGTTG
CCTGTCGACAGGAGCTAGTGGTGACACGCTAGAATTTGTCTTGCTCTTGGTTTCTCGTTA
TCTCGGGCATTTCTTTCTCTCCCCGCGTGAATTTTCAATTTTCAAACACGCACCCAGCCT
CGCCCCCTCCAGGACCGCATTGGAACCGCATCGCCGTTTTTCGTGTCAACACTATTCTTT
CTCCTGGCGCAGCACGCCACCCCTCATTTTTTCGTCTTCAACCTGGACCGGTAGCGCATG
GAAAACCCGAAATTCGCGTGCACAGCCCAAGGAAATATATGCGGTGCAATGCATGGAGCG
CGCGAAAGGTCGGCATGGGGTTTTCTTTCTCCGGTTCGCGATCTCGATGATGATGATGAT
TCATTCTTTCTTCCTTCCATGACTAACGGTTGTTCTGTTCTGAAAGCCCAACGCATTTTC
ATTGGGTTTCCTCCCTAAATTATCTAGAAGAAGAATTTAATCGCTTGTGTTGGGTTTGGG
GGGCGAAAAAGAAGCATTTTGATTGGTCATCTTCATTTCCCGAGAAGAAGTTTGATTGGT
TAGAAAGTTGACATTCTTCTTCTGTAGGTTCGAATGTGCAAGCTGCTCATGATGATTCGT
TAGTTTCGTCATTGTTGACTGTTTCTCAAGCTTTTACCGTATGTTGTTCGTGTCTTTTCG
AGCAATGCCATGGAATACTGGCATACCGTACAGTTCTCTTTGACTCGCGGCGGCACGGGA
GAGGTGATCGGCTTTGACGCGGTATGTTGTCGGTTTGTTGCCACCGGAAGAAGTTGCTTC
CGATCGCTTCCAGTCGCAGTCACGGGGAATACGGTAGAAATTAGAAATTCGAATCGGTTT
TAGGAGGAAAAATAAATTATCATACCGATACCAGCAGTAAAGAAGTACTAGTAGCAGTAG
ACGTATGCAAGAGACTTGCACACACCAGAACGTGATGTCGGAGATGATATTCTTGCAGTG
CACAGTTTCTGGAAACCACCACTTCTGTCTTTGAAATTTGTGTTAATTCTCCTCATCTTA
GTCAAAGAGAAAGCAGTATTGAAATCCCATGACAAGGAGGCAACCCAAAAAGAGTGAACA
AGAAGCAACAGTACTACTACTAGCTACTACAAGAACAATAAGAACACAGA
>comp2378_c0_seq1
CGAAGTTAGAAAAACCTCGCCACGAAGGCTATCCAGCTGAAGCCGCATCACAACAACTTG
CAACTTGCTCCCGACAATAATCCAGTCGCCCTATCAAACCATCTATTGCCATCGGCACTG
TTACAAACATCTGTGTATTACTCGTTGTCCAAACGCTGCTGTCTAGCTTTCCGATTTTGA
CCATTATTGCAAACCAAGCTTTTAATAAAAGAAGAGATATACAACATGAGATTAGCAGCT
GGCACTGTCGCTTCTCTAGTTCTGACCATCGGAGCGCTTCTACTCGTGGTCGCAGTGGAT
TATTCTTCGAAAACGGTTTTGAACGGTGGAAGAAGACCACCCAAAAGTGCTTTCCTTCGA
TCTTCAAAACGCCGCAGACTGTACAACTATTCGAACAATGACGACGGCAACAATGCG
>comp2378_c0_seq2
AACAATGACGACGGCAACAATGCGTACGGCGGTGACTATGGGTACGCCGAAAACAATCAA
GAGAACTCTTACGAAGGCTCGTATGGTAACAACGACGACGGCAACTATCAAGACGATGGA
GGCTACAACAACGGAGACGATGCCGCAACCTATGATGGAGACTACGGCAACAGCAACTCT
GCCTATTCAAACGACGACAACACCTATTACAACTCGAACTCTGCTTACCAGGCCAATGTT
TACAACGGTAGAGATCAATACTGGGATGGGCAATCCGTCCAGATGTACACCGACGACGAA
GAACCCGTGATTGAAGAAGAGGGAGAAGGCGGAATCAAGATTTTTGGAAAGTACGCCGGA
CTCTCCATTGCGTCAACGTTTTCTTTGGTATTTCTCTTGCTGATGGCAGCCATTAGTACA
TATGTCTTCCTTCTGCTCGCCAGAGGCTTCAACCTTATCGATTTCTATCACAAGTATCTT
TGCAAAAGACGCCCTGCGAATGAACCCAAGGATGACAGTTTCGTGAAGCTCGAGGACCCC
GTGTTGTCATAAATGGACAGGCGAACGAACAAACGATCGAACGGAACGAATTGAGGCCAT
CCATCCAATGAAAGGCGAGCAATGCTTTCGGAATCATCGGGGGGATCAGTCTTTACTACA
CACCCATGCAACATCATCTCTTTGCATGATTGGTATAGAGGTGGAAAACAGCAGAACGTG
AAGGAGCAAGCTACTTCGCGATACATGACGACCCATGTTAGTGCCTTTACCACCCTCTGT
GTTTTTTATTAAATCTATTTAGTTGTACAGAATTTGACATGTTTCTGAACGTATTTAACA
AAACAAAAGGATTGCACGAACATCGTGCCAAGTGGAAGTACCAGTAGAGGAAAGCAATGA
CTATTGGTGATGGCGTATGCCAACATTGAAATCACTCCTTCGTGCTTCTTTACGTACTGA
TCCTTTCATCACTTTGCGGAGGGACAATCAAATACATGAAATGGAACGGCCTGCGGCAGT

TCTATGAAGTTCTACTCCAAGAAGTCAGCATGCTGCCTCCTCACATTCATGCTAAACAGC
AGACACATCGTTGGCATGGTGTTCCCTACTAACCGCCGAGTTTGGTCTAGTGGCATGAAA
CTCTTGCTGTCCTTTCGCCTTCCTTGTGTGTTTTTGTAAATTTCACCCAGGCTTTTGAAG
TGTTTCAGAGTTTTGCAAGAAACTACAACACCATATTGTACTTAGTGTTTTGGATTTCAA
TCAGGAG
>comp2379_c0_seq1
GTGTGCTCTTTAGTGCCGTATAACTACGTGTTTACAATAAGAGTGAGCTTATTCACTAGC
TATTCGTGATTTAGGCTCACACGTACTACTGTATTGGGAACGACATAGTACAATACGGTA
CAGCCGTAGGAGTTAACTAGACAAATAGCTACGTTTAACGTGTACCTTAAATTGCGATTT
GTTGAGTCTTGGGAGCAATTTGGTTCTCGTAAATACTGGAACGGCTGATCCTCAATGGGG
GGAAATAAAGATATGTAATTTAGCCTGTCTGTCAAGCCTTTAGTACCACTGGAAGTGATT
CCCGATTACATACCTCGATATGTTTTCGGGAATGACTCGAATGACCCGAATGACCCAAAT
GACCATCGGCTATTACGAACGTCCCCGGTGAGGTGCGCGACAAAGGACAAAAAAATAAAA
ACTAGTTTTTGTAAGCGCGCGGTACGGGAGCCGTAGGTTATAGAGCAACTATTGCGCGGT
TGCGCGCATCTTGAATAACTGACGCATGTGATCCGCCATTACCAATTTTGATTGGCTGAT
TATATTTAATAGATATCATGATGACGTCTAAAAAATAGATGATTAACAAATCGAGACTAC
TTCTGAAATAGATTTCACGATTTATTCTCGATCTATTAAATCGGGAATAAAACGGGAATA
AGTAGAGAATAAATAGAGAATTGAATTATTAATTAATACGAAAGAATGAAAACCGA
>comp2380_c0_seq1
GTAGCACTGAACGGTACCGCATGCAAAGCGAAGAGAAAGAAATTCATATTGCCTCTTCTA
CTCAGTAGACTCCAAACACCACGTTGCACATCACACGACACTATTGACTAACCATGAACA
ACTTCAATCGATTGTGGACGATTCTGTGGCTTCTGGGAGCAGCAAGCATCCCAACAACCT
GCACTGCCAGAATCAGTCACGAGAAACAAGCGGAGCGAGCGGACTTTGTTCGGGCCATGG
ATCGAGCCCTAGGCCAAAAGAAAAAGGACGACCTCTTTCGCTCTCGACTCGCGCAGTTGG
CAGTACCCGCAAGTTCCAATCCACGATACGTACGATCCCAAGATTTCAATGCCGAAGAGC
CCCGTCGCTTGGAAGAATACCAGGATTACGCAATCGACCTTTCTGAGTATGCCTTGAAAT
ACATTGGCTGCTCAAATATCCGAACCTGGAGCGACGACCTTGCAGCAGAAAATGACGGTG
GTAGCGACTCCGTCCTAAAGATGAACAAGTTTGTCGTCCTTCGTCTTTGTCCCCGAGACT
CGTGTTCGAATTACAACCAGTACGGTTGCCTCGAACAATTTGGTGACTACTTAATTCCAA
TGGAAACATACATGCAGACCATGGCCGAGACTTTCTTTTCTCAGTACCAGGAATATTGCC
AGACCTGTTACGAATGCATGAACACAAATTACAACAACGATGATGGAGGTAACTACAACA
ACGATGACGGAAACAACTATAACAAT
>comp2381_c0_seq1
CGCATTCAAAGATGGAGCACGGTGTGAAGCCTTTTACGATGGATGACCGGCATCTCTTAA
ACAAGTGGTACTTCTTCAAATCGACCAGTCACAGTGGTTACCGTATTCAATTGTTGTGGA
AGGTGTAGTGTTTGTTAAACAAGGAGAAACAAAGAAAGCACAAGTTGAGCGTTTTAGAAT
TGGTGACCGGTCAGATGTTTATGGGAATGACCCAATATTTCTGTTCATGAGAAAGATGTG
GAAGACCCGTGCCTTATTCCTACCCAACTATGGTATCACTATCTTTCTGATACCAAACTG
TATGTAACAATGGATCTGACAATCCGACGGAGAGCTAGGAAAGTTGTGGCTCGCGGATGC
CCAACTCGAAGCGCTCATAATTCGAAAGCAGGACCTCTCCTAACGGCAAGCATTCTATTG
AACAGTGGCAACGATTTCTCCCGTGATGACAATGACAACTCGCATCTTCCTCTTCGCGCT
CGCAGCCGTGGCAACCACATTGCCTTCTTCCGTGGCTACCCCAATCGTTGATTTTGATTC
CTCAAGGATCGTCAACGGAGTCGACGCGGAGCCCTGCCAGTTTCCCTATTTTGCACAATT
CGAATCAGGCTGTGGTGGCTCGCTCATCGCGCCCGACATCGTCCTAACCTCAGCGACTTG
CAAACCAGTGAATGACGAGGGGGTTGTAGTAGGCGCTTACCGCCGGGGTCGGGACAATGA
GTTTGGAGCCAAAAAACGCTTTTGCAAGGAATTCGTCGTGCATCCCAATTCTATGCAGAG
GCTTAAATACGATTTTGCCTTGTGCAAGCTCAACAAACCTGTATTCATCGACGAATCATT
TGTCAGGCTGGAATTGAGCAAAAATGTTTCTGCTCCAGTTGGTGGAACTAGAACAATTTC
CATCGGAATGGGCTGGACGAACAACAAGGTAGATGTTCTGCGAAACGTCACTATTCCGAC
AAGAAGCAACGGTGACTGCAAGAGAGATTATTCATGGTTTGACCGCCGCACCCAACTTTG
CATGGAATACTACCCCACAATCACCCTACCGCCCTGTAACGGTGTCTCGGGAGCTCCAGT
TGTAGAACGAGTCTATCAGGGCGACGGCTTTTTTGTGGACGTACAGCACGGGCTGACCTC
CGATGGAACGTATTGTACGCATGGTTTTAATGCCCGCATCAGCGGGGAGATCGAATGGAT
CCGGTCCACTTCGTGCCTGACGCTCCACAGCATTGGTTCCATGTGCGACACCATCACTTC
GCCATCCACTTCGCCATCCATTTCGA
>comp2382_c0_seq1
GCCGGGAGGAGTGTTCTAGTAGGCTCAATAGCGAATACACGCGTACCCAATACACACGCA
GCAGCACTTCGGATGATAGTAAGAGTATGAAAGCTTGGAGAGCTTCTCCAAATTCTAACC
GTTTACCCATGAAAAGTTCCTGCCATTCCTCCCGTGATTCCCTCGTCTTTGAGTTTCTCC
CCGAAGCCACTAGAGAACTGTTTCACCGGAATTCGATCGACGCCATGGACGAGGACATAA

TGAGTTCCGACGGTGTCCACCTCGCCCATGTCTATCTTTCCCAGGCCACGAGCGAGACCG
AAGACGCGAAAAACAAAAGCGAGCCCGATCAGTGTAGCGAGGGGGAAAGCAAACAGAGGC
AAACACAAACGCCGGACGCAGCAAAACCGATCGATGGAAAAATCGCGAGCAACCACCTCG
ACGAAAACCTGTTGCAGGTTTATCGAAACAAACTAACGATTTTAACGGAAGCAAACACGG
AAAAAGAGCGTCGGTTGGAGGAGTTGGAGAAAGAACACAACGACCACAATGTACAGCACA
AGGAAGGCATATACTGGCTTCGATTGCAACTGGACACTACCCGGCGACAAAAGAAAAACG
CCGAAAAGCGCCTGGCAGAGATGAAGAAAGAATTTCGAAAGCTCGGCGCAGGAGATGCAT
CAACGAGCGATGAAGACGATCTGACCATTGGTAGCGATTGGGACGAGAAAGACATCTTGA
TTGCCGAGTTGCGCGAGAGACTGCGAAAGAGCGAAGCCGGTTTCGGGGTGATGGAACGGC
AAATGGCTACGATCGAAAGCTCGTCCGCACTGGTCATCAAGTCCCTAAAGGAAGAAATAG
CCAACATCATGGAAGACCGAACCAGGGTGGAGCTCGATCTGCTCAACAAGCTCTCGGATC
TCGACACCGAACGGCAACGAAGACAACTGGAATTCGCGCTCGAGCTCCAGGACAGGGACG
AAACCATCGACGCCCTGCGGAGGGGCCAGCAGGCCTCGCTGTGCACGGCGGACGCGACGA
GTTTCTCGGGCACCTACAGCGATGCGTCCTGGTGCGTGCACGGGGTCTCGTCCAAGAGCC
GGTCGGAGAGCCACGCGGACGAATCGTTTTCGAACCACCAGCACCACCGGCCCGGGGGGG
CCGAGCTGCACCGGATCCTCGAGGTCGCCTCGCACGACTCGGAGGGTGCGTGCGCGGGAT
ACGGCGGCGGAAACGACTCGGGCAGGTCGGGCGAGCTCGTGTGGAAAGAGAGGCACCGAA
TCGACTCGTCGATCTGCAAGATGAAAACCGTCCTGGAGTCCACGAATGCCTCCATATCCA
ACATGGAAGGGATTGTGAAGAGCTTCGATCCGAACGACGACGCCGGCGGTGAAAACAAGG
AGAGAGAAACCATGATGTCGATCCTCCAATCCGCCTCGCTCATAAGCGAGCAGGTCAAGC
TTTCGATCTCGCTAATCGAGCTGAAGCTCAGAAACAAGTCCGAGAGCCTTAAATCGGGTG
TCACAGTGAACGAAGGCCATCAGGAGTGTTCCTCGGTCGACGAAAAGACGATAGACACTA
TAGAAGAAATCGAAGACAATGCGCTTATCGAACTTGGAAAGGTGGAGGAAACATTGACCA
ACCAACTGAAAGAGTTGGAGGAACGAGCAAAGCAAGAACTGATGAACCAAAACCCCGATA
TAAAGAACGGGTCTTTGCCAAGTTTAGGAGACCATTGCCACAAGGATACCAGATCAAACA
CAGACGACACGCTGGTGATAAGCCGCGGGGTGCTACACCGCCTGGAAGAAGAACTCGTCC
AGTTCGCCGAGTGCATGCAATCGAAGAACGACAAGATCGATGAACTGACGGCGGAAATCC
GCAAGCAAAAAAAACGCGAACGCGATCTAAAAAAGGAATTGAAAGCAGCATCGAGAGGGA
CCAGCAGCGAACGCAGCAGCGGATCCAAAAGCACCCGAGAACACTACAGATCCAGGAGCA
AGTCCGGAGATACCAAATCAACGAGGAGCAAGAGAATGCCCGAGGATAGAACCAAGGACC
AGAAGGTCGAACCGAAGAATCTAACCCGCACGCGGACCTTTAGCCACCAGCCGATCGAGC
TTCCCGCCGAGCTGCAGTCGAAGAAAGAGGTTCTGCAGATCACGTCTATCAAGCTCGCTC
CCGATTTGTTGGTTCCCCGGGACCAATCCGCGACGATGAGACGAAAGGGCATGCCGGAAG
TTTCTCTCCGGGGGGGCACGGATGCGCTCTCTCCGTGCAAGCTGACTTTCCCGAGGCACA
ACAAGAACCTGTCGGAATTTTTCTCGGCCACCAACCAGAAGCTCGCGACCGTCGCAGAAA
CGCCCTTGGACACCCTCGAGGGACCATCGAGGCACCTCTAGCAAGGCGCGACAGGGTGAT
GGTCTGTGGGGGTGAGGGAGGGCGGAGCAGACTCTGTCAATCGCCACGCGACTAAGGGGA
CACAGCGGGGAAGGGGAGGGGGATAGATCAACCAATTCGTTTCGATCATATTAGGTATTC
GGCAAGTCTCGAATTGCAAGCGTCGCAATGCAATACTATAGTATACCATTTTCGACTCCA
ATGGATTATGAGTAAAATCAATAACACAGAAATCAATTTACACCGCCAGATCGGAAG
>comp2383_c0_seq1
TCCAACCAAAGAAAGCCTCGAGTCGCTTTATGTGCAAGCGAAGTCTTCACAAGACCACAG
TTCCTATCGAGTGGAAGATTAATTTCCAACACGGCGAAGACTAAGCAAGGTCGCGATGAA
GATGAGGACCAGCTGTAGATTTCCGATTCTACACCATGGTTTCTTTGCGGCGTTGGCTTT
CGTTGCGTTGACCACGAAGATTCTACCGGGTGGCGTTGGAAGCGTCTGTGGATTCCTTCT
CTCCGGCGACAGGGTTAAAAACGACAGCAGCAGCAACTTATCTTCTCGACATTATGTCCC
CGTATATGATAATCAGCAATGTCGGATGCATGCAGTCCATTCGTCCTCTGAATTTAATGG
GAATGATAAGCAGAAACAAGAATATCCCCAATCGCCACAGCCACTCCAGTCGAGGGAGGC
TTTTTTTGGGACTCTCCGGCTTGCTAGCCTCGGAGCGATCGTATTATCACCGATCGAAAA
TGCCTTCGCATTCGACGGTGGTGTCGGTGGCTTAGGAAAGACAAAACCCGAAACAGGTGT
CGAGCTGTTCGACGAGACATCCGCCCCCATTCAAAATGAGAAGGGGATCGTCTCATCGGA
GATCAAGGGAAAAGATGGATCTCCTATACTGGTCGCCTTTCAGACGCCGTGGCCACTCCT
TCCGACATCAGGAGCCCTCGAGGCGCGCGACATCCGGTCGACGGAGTCCGCCTTCGTTCA
GGTCACCGGTCTGTCTGCCGCACGAAGCAGTGACCAAAAATGGAAGTCGAAGAAGGAGTT
TGAAAAGGTCCTAATGGAATCCGTCTTTGCAGCGCAGGGGAAATTTGGAGCCTATGGCCA
GCCGTACGACATCCAGGCAAAGAAGGTAGCCCGGGCAGATGGCGACGAGAGCGACGCAAA
GCCGACCCTGTTTTCCGTGGGGTTTACGACGCTGACCCCCGCCATGCGAGAATCGGAGCG
TCGGGCACTCGTTGCGGCGACCCAGGTGGGGGCCGATACCCTGGTCATCTTGGTGGTAAC
GACTACCAGCGCACAATATGCTTCGAATGAGAAGACCTTCAAGGCGATCGCCTCGAGTTT
CGAAGCGATTGCGGCTCCCGAATCCGGTCTAAAAAACAAACGATCCTCGAAATAACAACG

AACCCGATGAGAAATGACAAGACAGAACTACCACACAGCACCCATACTTTGGAACGAAGA
CTTTTGCGCCTGGTCGATCCTTAACTTCAATTCAGATCTATGACGATTTTCCCAGATTTG
AGAACACAGCGTAGAACCAAAAGGTGTTCCGTGATGTACATCATAATCGTTCAACTTTTT
ACTTTTTTCATAATTTATTCACTATATATAGATTAAGCATGCAATACGTAC
>comp2384_c0_seq1
CACATACACGTAGTCACAATCTTCGTCACCCTCTCCACTCCTGCTTTCCGAATTCATGTT
TTAAGTAATCTTGCGGGTATTTTGTCTCGCATCTAGCCGCACCGTTTCCTGAGATGGTGA
CCATAACAAGATACCATAAAGTCCTTCTGGCTCCTCTGGCGTTCCTCTCGTCTCGTTTTG
CGCCGACAGGAAAGAGCAAGATGAGAAATGAGGTTCCTGATGACGGTGCTACCACATCCA
CGATTGCCAGTGACGTCTTCCGCAAAGAGACAAAACGCATGACACAAGCTACAACTGCAT
CAATGTCTAAGCCTTAAAAGAAACTGCCGGACCAATTGCAGCTAATTTCGAGAGAACTTG
GCAATGACAAGACAAGACTAGAAAATAACACCCAAACAAATAGGCGTCGATGACGTCAAC
ACAGGACATAAATCACCATGGCACAATACATTATGACAAACCGGGAGATGAACCATCACA
TGACATGCAAGGCGTAAATACAGTACATACTATCGAAAGCCAATCAAACCAACGGTTGCT
ATGGAGTGAAATGGACGAAAATGTTTTGATTCGTATGCGAGTCGTCCACACGTTCCATCC
ACTCCGTCGTTCGCAGAGAATGCAACGCGAAAGGTTGAAGGTAGAGTACGGTATAAACGC
AAGAAACCACAAATCATATCAGACTTATTGTTGAGCGCCTGTATCCGAGATAGAATAGAC
ACGCAACAGCAGAAAGACGGCTGATCAAAACACTGATCCAATTACACAGGACATTATTCC
ACACACCACAAATCAAACACGGATACAAATTTCCGATCATGGAAAAAACTTAATTTGCTA
ATTTCCCTCAGATATTTCGTCACAAACCATCTTTATCAGTTGGAACAAAGTACAGCAGAG
ATGAATTTACTACATTCGTGCTGCATTGTACGGTACGAACTGTTGGAATATCTAGGTGGG
TAGGTGGGCAATTGCCGAAGATTAGAAAGCATTGAATTTTTACTTCTTCTGTACTTGTTT
GCATTCGATCATCTGCTGCAGTCGACACCATTTCAGGCTCCACCAAAACGTGTCAGCTCA
AGCTACGTCGATCGTACTAAATGCGAACGTATCTGGCAACAGACGAAACCTTTCAGATCA
AGATACTTCTGGAAAAACTTTATCTTAGAATTGTGCGCTTCACTTCACCCATCTAGATGA
AACATTGCTCAAATTAGAGACACCTTCGCTTTTGTTTCGCTCATGATATTCATCATCTCG
TCATTTTCCGTGAAAGAGATGATTCCTGTACTTAACGTAGATCTTCTACTACCAGTAGGT
TACTGCCGAAAAATCTTTCGTCAACACTGTATTGAACCGTACACATCGTAGCTAAATAGC
TAAATACCGGTAGTACGAGTATAGCTGACATCAAAAACGTCAAAAAAAATATAGTTCTTC
GACCGGGGGTAGGTACGTAGGTGGTCATCACGAACTTCAGAATTTTACCGTACCGGCCGG
TACGTGGAGCGAAGAACACAATTTCACGACACAAATTCAATCACCGAACTGGCGTGGACC
TGTGGACTGGTAACTACGGAACCGATTTTTGCTTCCGACCAACACGCCTGAAACGGTTCG
TGCCCCTTCGTGCGAAGGCACTCCGCGCACAGTATCTCCACGTTCCAGGGAGCGCTTTGC
AAGCGAAACGAAAACCAAAGAACATCGTCTAGATAGGTGGACTGAATTAACTCTTTTGCC
GACGTAGACATCCATGGAGTGCTATTCAAACCTAGTACTGTATTTTAACGGCGTCACGCT
TAAGATCTACGATCGTTTTTTAAACTGTAGAACACTTAATGGGAAACTGCTGTCGGTCGC
CGTTGGCCCAACAGATTTTTTCGGC
>comp2385_c0_seq1
CTTTTGAACCGTTTGGTGACGTTCGTATCTTCGCATGTCTTGCTTCTAGTATTGAGCCAT
TGATTGCGCAAATACAAGGTATCAATAATTTAATACAATCCCCAAGGCACTGTTGGAGAC
ATTGAAAAACACTTCACTGGCGACGAGAGGAGAAGGACGACAGGAACAACGCATGTACTT
TTCAAAGCACACGAGCAAGAACAAAAAAGTACGATCGCAAACGCAAACACAAACGCAAAC
GCCGGCGACGTGAAGTCGTGTACTGTATACGAAGAATCAGTCACAAAAGAACAATCCGAT
TCACGCAAAAAAACGATTGCTTCGATTCATCGCATCACATTTACTTCATAGCAGCTAGAA
AAGGTAATCGGTGCCCCTTTCGTTATCTGATTGTAGTGGAATCGAATCTATGAGATCATC
CGCAATCAGTATTAATTTCAAGAACATCAAAAAGCAGATAGTCTTTTTGCAATACTCGGA
TACACCAGAACATAACTCATAACATATACCAGGCAACACTACTCAAACCATCCAACAATG
GCAAAACCCGTTAGAACATCAACACAGCGTCGTCGTCCGGTTGTGGAAACTTCCGCAGAC
GGTGCTCCTTCTATTCCAAACAAAAAAAGCAACAAGGTGAGAAGCAAGAGGAAGCAAAAG
AAAGCGTTGAAGCCAATGTATGTTGTATCATATAGCGATGGTGGCGATGACGAAGCCAAC
GAAGCCAAAAGGGAACTCTACGCACCATTTCGTCGCGAATTTCCAGATACTGCGGTACGA
AGCTCTCCCAAACAGAAGCCTAAACACAAAAAGGACGCCACTGCCAGCGACCACAATCGC
AGTCACAAGAATAAAGGTAGCAAACATTCTTCTCTCTTGTGCAAGCACCGAAATGGTGTT
GTCTCGTCGCACGCAAGAATGTCTTTCGCCGGGGAAGTACTAGAAGGCGATCGCCACGGA
GACAACTACTTTGCTTCGAAGCAAGGAAAGACATCATCATCTGCACATCCACGCCAACTA
GGACTCCCACTTATGCCTACAGAAAGCTCAAGCAGCGAAACAATCAGTATTCTTAGTGAC
ACTGTATTTTGCGTGGGGCAAAACTCGGCCGATGCCAATGCGGCTACAGCGGCGGCAGCG
GCGACAGCATCTCCCGAAGAAATCCAGGCCTTCAGTGCTATCTTAGACCACATGATGAAG
GAAGGAGGAGCGGAGGATGCCAAGAAGAAGAAGAACAATAATAACAACAAGAAGAAGAAA
GACAAGAAGAAGAAGAA

>comp2386_c0_seq1
AGTTCCAAACGTATTCATACGGAATTCGCACCTGTACCTTATGTTGTTGTCACAGTCGCA
ACCTCTTTCTTGTATCGAGAAACAAATACCTACGGTATTTTCTTTGGTCCGATACGGTGA
AAGTGTTATAGAAAGCATATTTCTTCTCGCCTGCTGAGTACCTGGTAGTACCTTTCAAGA
AACAGTTTAGCGGGGATTTCAGAAACTCTCGACCCAGTTACAGTACGAGTACCATTGTTT
CATAAAGATGAATATTTTTCTTTTGTGTGGTCTCATTCTTCTTTCTCGACAGATCTTCGC
GTGCGCTGTCGAGTTAGAAAGATCACGGCGAACTTCTGCAATGCTGCCAACGTGTGACTC
GGATCGGCCGAATTCGTGGGCAACGCACAGGACGCTTACGTTGCCACAGTTGTGTCTCAC
AGGTTCGGTTGCGGAGACAGCTATGGCGAATGCCCTTTGCTGTGTGTATACGCACGTAGT
TTCCGGTAGCGGAATCACAGATGTGAATTCTGCATTCTTCGAAATCTACGACGGGAGCAC
AATCGAGATTGCAGGCTGCGGGACTAGCAAGACATCCATCCAGTTGATGCAAGATGTAAT
GGACAGCATATGCGACATGTCCATGGGGAGCGAAGACTGCTACAGTCAAAGGGTTTGCAT
CGAGAACGCCAAGAGAACTTCTGTGCCATCGACAGTTCCAACTCCTGTACCTTCTGCAGT
ACCTACGCCACCAAGAAACAATGAATCCGAGTGTGAGGATGATGAGGCTTTCCTCTTTCG
TGGAAAAGCCGGAAGGAATTGCACTTGGATCAAGAAAAAGCGAGAAACACACTGTAGCAA
AACCTCCAGAGGGAAACGAGTGTCCGAATCCTGCCCTTTGGCGTGTGACCAGTGTTCGAA
CAACGACAACAACAGCGGTTGCAAAAACGATTCCACATTTTTATTCAAGGGCAAAGCCAA
GAAGGATTGTAACTGGGTTAAGAAAAAAGCTCGCAAGAGGCCCCGACTTTGCGATAAGAA
TTGGAACGGCACCGACGTTCGAATTTCGTGTCCGGTGGCATGTAATGCTTGCTAAGAAAA
TAGTAGGCACACAGTGTGTTGTTGTGCAGTGAAGTGCAGGCTTTTTAAATAGCTTCAAAA
TGATGTATTTCGTTCTCAGGAAGTATCGTACAGAATAATATCATGGTACTAGTATTTCTC
CGTTAGGAATCAATTTGACTTGATAGTTTCTTGAGTACTGAGCACTCACTTTGGTGGCAA
ACGATCGGTACATGTTGCGTGTAGAAATAAGACCTATATGGACAGGCATGGCTTCTGCCA
GGTACTTCGATCCTAACTATACTATTTAGAAATTTTCGACCTACAATAACTAGTAGAGCG
ACATCCATG
>comp2387_c0_seq1
CGTGATTTCTCAGTCAAATATCGAATACAGCATATTGATACCGGTACTATCCTCACATGA
CCACCTCTTTTTCTGCATTCAGACAAGAAGTACCGGTATATCAAAAAGCAAAATTTGTCT
CCACAGCATTTTGCTGCCCGACACAGGCGTGACAAAGCTTTCAGTGTAAAAATTGACTCA
TACCCCTATCAAGCGTATCTCTGATGTCGAGAGTGGGAAGAGGTGTACCGACAATTTTCT
TACCCGCCCTTTGCGTCTACCCAATATGGTTGACAATCGGGATACGGGAGGATGCCTTGG
TCTCTGCCGTTGTAAACTACTATCCTATGATGGTAGCAATGGTCCTAGGCTCCATGATCG
CTGGGTCAACTCCACTTGGAGGAGGAGTCGTTGCCTTTCCGGTATCTGTTCTTGTGCTTG
GATTTAGCCCGGACCAAGGCAGAGATTTTAGCCTCTTGATACAATCTGTTGGGATGACAG
CAGCTTCATTTCTCATTTTCTATAAGAAACGATGCCTATTGAGTGGATGCGAAGATGCCA
TGGTGATATTTAGTATCTTGAGTGTCATTGGAATGATCGTTGGCTTCGAATGCTTTTCTG
GTCTTTCTCCGTACGTTGTGAACATTATGTACACAACCACAGTAGCGTGTATTGCTTTGG
TCTTGGCGTATTTCGATGTTGTTGCTAGAAGACGAAAGGCTGTGGTCAATGACAGAAGCG
CGGGGGGGAATGATGCCGGCAAGACTCACCAACGCAGTATGACAAAGGTATCAGATGACA
TCAGCGACAGTGACATCGAGTCGGAAATCATTCGAGAAGGGCCCCACGTTGATTCCTCAT
GTGTACCACTAGAGACAACAATATCGAATGTGTCAGATTCTGCCGGAAGCGATAGAAGGT
GGATGGCGACAACATTTACCATGTACATATGCCTCCCAGTATTCGCAACTGCAGGAGGTA
TCCTTTCTTCTCAAATCGGATCAGGGGCTGACATCGCATTCTACTTTTACGGTTGCGTCT
ACAATGCTACTCAGTCTCGACATGGATATTCCCGCAGTGGTAATACAGAAGGGAAAATAG
GTGGAAATACCTTGACGGCTGGATCCGTCATAATAATGGCTAATACCAGTATATTCGGCT
CCATACTACGCGTGACAACACAGAAAGATTCGCAAGCAATAGTTGATGATCGAGTATACC
AGGCATTGATGGCATGTATACCGATTGTGGTACTCGGTGCACCTGTCGGCAGTCTCTTTC
TCACACAGTCAAATCAACAACGGTTGAAATATTTATTTTATGTCCTTGGTATCCTCCAGC
TTGCAGTGTTCGGGATCATCAAAATTGGAAATGATTACTTGACATGGAGTGCGATTCTTG
GACTGCTATCCTTTGTGATGATTTGTCTCGTAACTCATCATACGCTATCCTCTCGTTATG
GTGAAAAGGGATCTGTAACATTTGAATCTTGACAGAGAAATTTTGAAGCGACTTCCGTAA
TATAGATGATTTTTGTCGGTATTGATATGACTAATCCTTCAAAAATATGATTCTGATCGG
AGAACTCACTGTCTTTCATTGAGAGAAACGTGCAGGTGATCAATAGAATTTGAATACATT
AATTTCTTTGCTGTGACAGCTATGGAGTTTCTTTCAGAATGGTTGAAAAAGAATAACTTC
ATCCGGAAGCCAAAAAGATGGCTTCGTCATTTAAAGACTTCATATTTGTGGATCGCTACT
CATCATCGTTTTCTTCTTCTGGTTTTGGTGCAAGGAGAACGCCCTGAATGATTAGACCCA
GAGAAAAAAGTGAAATAATGGAATTCATAATAGGAAAGAGCAGCGGGCCGTCTACTGTTC
CATAGTTTGCAAATTCAATCGCTGCTCGGACCGACGATGTTGCACTGCTAATGCTTGTAA
GCACTAGCAGCAGAATTACCAATGGTAGCAAGACTGTTGTCGACAAGCCCGTCACTACTA
GTATTTGCTGGACGATTGACACTACCGTAGGCCGAAACAGAATGGATCGCAAATCGTCCA

ATTGTGGATTGACCAAACTCTTTGTCGCAGGAGCCCTGACGAACGCCGTAGGATTCAATG
CATATCGTGAGTCGCCAAACAAGACCGCCATTTTATCTGGGATGGACAAAGTATAACCAT
TTTGGTCATCCTCCTCTATGATTCTTTCCATTGCTCTCTTGTTGATGATATAGGCTGTGG
TACCAAGTGCACTTTGGCTATTCGACGTGGATAATTTCACTATTCTCTCCTCGTTAGTTT
TGGACACCTTTAGGTCTGGAACGTATGGAATGTATGCCAGATGAATCACATCCCATTTGC
TGTTTTTGTTTTCGATGAAATCAGACACAGCGTCTATTGTGTCCTGTGTCAATATTCGGT
TACCAAGGCTCAAATTGTCTTCAAAAATCAACACGTTAAGATCCTTGCCATCATTTTTGC
TGTCAATGGAACGAGCATTTACCTTTTGAAAGACATTAAAAAAATCAAAACCACCCGGGG
ATACCGATGAACCCCTAGAATTTGCTCCGAGAACATCCCTGAAAACACTGACATGCGAAG
TATAACATCCTCGAACTCTATCTTCATCGTCGGTCTCAAATTCTTTGATTGTAGTGCTGT
CGAGAAGATTTATGGTGTTCAAGATATTCCGTGTGGATTGTATTCTGTTTCCGTCGACGT
CGCCGTTCGGACAATGAATGAGGCAAACAGAGTCGAGGAAGGACCAGTCCAAAGAAGATG
CTGTGGCACTGCTAGAGGTATCATCGTTGCTGGTCTCGTCAACGAAACTTTCACCAATAC
TTGCCCATACAGCAAAATGTCTGCTAAATGTAACACTTTGATCAGGTTTTGTCGCGAATG
CCTGCGAAGTCGTTGAGAAAGCTTGCACGTGGTTCCTAGGGGCAATGAAGAGCACCAGTA
ACAAAACAGTCAAACATATCTGCAAGAAACTCAAAATCTTCCTTTGTCCGTAGAAGCTTC
TTGTCTGTGAAGATGTCACCGTCATGGTCCCCGTTGAATGCAGTTACAATATATGGAATG
GTAGTACCGAATGCTAGAGATTGTTTGATGGTGTTCGATGAAAAACAGAATCGGTGTCGT
CGACTTTTTGATGGATGTGTTGTCGAGGTGAAGAAAACATGAGGTCTTCTTTTCTTGATG
TGTACTGGTAGGTACCGGTACGTACGATATTCGTACAAGAAACGTACTCGTACGAGTAAA
GTACTTAATCGGAGG
>comp2388_c0_seq1
GCCCGGAACGTCCAAGGTCCCAAAGTGAGCCTTTTGGCCATGGCGTTGCTGTGTGTTTTG
TTGCTGTTTCGTACATTTTCCGATCCCTTTCCTGAGACCCAGGAACAGCCGATGCTTCGC
GTTCCGGTTGCCAATCGGCTTTCCCATGATTATGCGATTACAGTGTCCAAGACGGACCCT
TCCATTGCCGTGTCCGTGTACAAACAGGGTGACATTGTGTCGAACAACATTGTAAAACAC
GGATACTGGGACGGAGGTCTGACCGAGGTCGTCTCGGCCATTCTTGATGCCCGACGACAA
CATCATCCTTCCACTGGCAACACGCAACCACAGACCATCGTCGACTTTGGGGCCAACGTG
GGATGGTTCAGCCTCCTGGCTGCCTCCAAGGGCCATCGATCGATTGCCGTCGAGCCCATG
TCGTCCAATCGCGGGGCCTTGTTACAGTCGGTGGCACTGAACAGCTTTGACGACCTGATT
CAAGTCGTGGCGGCAGCACTCGGCGACGGTTCTTCGGATTCGCCGAGCAGCCTGTGCATC
CAACCGAGGGTGGCGGGGCACAACATTGGTAACGGTCAAACGACGACCAAAGTGGACGAC
TGCGGCGAGGTGGTTCAGGTGAGGACCCTCGGTTCGATCATCGGAAACAGAAAGGTGGAT
TTCGTCAAGGCCGACTGCGAGGGATGCGAAGCCAATGCCATCATGGGCATGAAGGACATC
ATAGCGGGAAAAAGTCCACCGTGTTCCTTTGCAATCGAGTGGAGGGTAGAGACCATGAAG
GAACTCGGTGGGAACCCAACGGAGGTCACAAAGCTCCTTTTTCAATCCGGGTACCTCTTC
TTTCGGCAACAGTGCGACTTGGTGACTGGAGAGGGTTTGAAACTGACCGAAATTCCTTCC
TCACTGGTGCTGGAGAAGCTTCCTACCGGCTCCATGGATCTCGTCTTGCTTCACAATTCC
AAGCGCTGCTTCGAACGAGACGGGAAAGCCTTTCGTTACGTGATGGATCGCGTGGCTTCG
TTTCAACCCGTCCTTCTCTGATACCCACAACAAGAGTGATATCTCAAGTTTATTCCCAAT
TGACAACGAGATTGTTATACTTATCGTGTTACAGCGCTATCTGCAACTTCACTGAGATAT
CGTTGATTATATTGATGTATATCAGAAGATGATAATTTTGATGTAAATTGTTAAGGTATG
AGC
>comp2389_c0_seq1
TACAGTAGTCCCTACGGTACGATAGTTCCTGTATTTCCTACCGGTAGGATAGAACCTTAA
GCACATATGCTTTCAAGCACCGAGAGACAAGACAAACTTTTTTTCAGTCGTACATTTTTG
CTGTGATGTTGCGAACAGAAGAAAACAAAACAAAAACAAAAACGAAACGAAACGATCGAG
CAGCAACCATGTCGGCCGAAAAAGTCGGAAAACGTGCTGGCATTGGATTCACCTATCTTG
TTATATTGGTCATACCTCTACTTTTCTCTCTTCGCGATCATGCGATAATATCGGATTGGA
CCAGAACCTTGGAGTTAAATTCGAATGTCCTTCCGAGCGGAGTTTCTCCTTGGAACAGGC
ACGGTCGACCGCAGACACAGGCCACCGCATTGCTGGAGTTTGACAAATTTTCTCGAAATA
GCTCCTGTGTCTACGAGACGGAAGTCGTGGATGAATTCTGTAAGAACACAACGTGCGACG
AACACGAATTCTGCGCGGAGGGTGAGGATCCGGGCCCGTGCGACGAACACGAGTGGGACA
CGGAAGACGAAGACGGCGAAGAGGGATGCAAAATGCTGTGGTTTGCGGCGATCTACGACG
GCGGTGACGAAGTTGCGTGCAACGAAAAAGGGACCGGGTACAAAAACTTTTACTCGGTCG
CCCTGAACCCGCAACGGTGAACGCAGGAGAATCTCTCCAGCCCGTCCTATTGCTTGGGCG
ACTCGAACTAGAAGACGACAGCGAGACAACAACAACGACAACGTCAACGACGACAACGGC
CACCAAGACTGCGTCGTTGTCAAAGGTCGGGCGATGGGCGGAGCAGAAAGGAGCGTGGGT
GTTCAACGTGCCCAGGCTTTCCTTTCAAGAAATCGTGAACAGACACTACCCAGAGATGCC
CCCCTCCCATCGACAGGGCCCGTGGTTGCGCCTGGAAATCCCACGGCTGATCAAGGAGCA

CGGACTCATGGACAAGGCACCCAATATGTGCAAGCGTCGTTTTCTCTACACCGACACGGA
CGTGATGTTTCCCAACAAGCTGAAGAAGGCCAACCTCAAAAGACTCTCGGACACCCTGTC
CAACGACGATGCAGCAATCATGTACGGTCGCGAATCCTCAAAGGAAGCCGAAGCCGTCAA
TACGGGGATCATGTTAATAGACGTTGAAAAATTTGGCAAAGAAATCCCTGGAATGCTTGC
GTACTTTAACGAAACCGGACAGCACGTCGCATTCGACCAAGGCCTCGTGAACGACTATTT
CAACGCAACCGATCTCGGGCGTTCCTTGCTGCCGATTCATTACAACTGGAAATCCTACTG
GAAACTGCATCCAAGCACCTTTGAAGAAGTGAAAATCATCCACACCCATGGACCAAAGCC
GGGGAAGGGCCTGGCGGAAATCGGATCGTGCGACATTTCGGGTCTCCCCAACATACCGTA
CTACTTTGGATTTTTTCGGACGGGAATCTGCTGCGACCGAGGGAAGACCGCGGCGTGGTT
TATGGACTCCTACGAGAAGCTGTTGGCCCCGTCCGAAGATATTTGCGGAAGCGCCTAGGA
ACGACGCCGCGCTAGAAGTCTCCCGGACCTGTTGTATGTATACTACGAAACCGTTATAAA
TTGCTGTATTGTTTAGCTACGTA
>comp2391_c0_seq1
GAAGAAGAAGAAAAAGAAGAAGACAGATCCGACAGCAATGAGACGCCCCTGCTGGAAACC
GTAGCAACCGCGACCAAGCCATCCAACGACGACGCGGCTCGAAGCTTTCTCGAAGAGTTC
GACGTGGCGTCCAAAACGGACGAAATCTCTGCGTTGCTCGAAGAAAACGTGGCACTAAAG
ACAACTTTTTCCATGGTAGCCACCGACCAGAGCGAGGTAACCTATTCCGACTTCTGGACC
CGGTATTACTTCCGTATCGCCGATCCCGACCGCGTGGCCGAAACCTACGGAGTCTATTAC
CGAAAGGAACTGGAGGCATTTGAACAACGACGGGAAGAAGCCGAAGCCGCTGCTCGGGAC
GCCGCTGCCAACGACAACCTTGCCAGGGGAATTTCCAATGTGACCAACTTTTTCGGTGGT
GTCGTGAACCGCCTGACCCAAGAAGACGACGAGGGTGGTGCCGACGAGGCCGGCGTCGAC
GAAAGCATGGATGTCGACGACGAAAGTGCTCCACAGACGTCCGGCCTCGGAGGGTTCTTT
AACAGCGTCACGGGTGGAGGTGTCGGCCGCCCACCCTTTGTTTTGAATACCGCCGTAAGC
GACGACGAATATGATGATTATCAAGCACCAGAAGAGGAAGACGACGATGATTCTGAGGTG
GAACTAGGATGGGACGACGACGACGAGGATCTCGAGTCTGACGATGACGACGAACAAAAT
ACCAGTGCCACCATGCAATTCAAGGACGGCGACGATCTCAGCGAAACAGTAGATTTTAAG
GACGCCGAAAAGGAAGGCCTGCTGGATCAGTTGGAACAGGCCAGGGCCGAACGGGATGCC
CTCCAGAAGACTGTCGCCATGCAAACCGAGGAACTAAAGAAAGCGAGAACGGCCGACATT
GCCGCCATTTCTCCTCCCGCTGCTGTTCTGGAGGGTGGAGACAAAGACGAGGAGGTCCAG
AAACTGTCTATACAGCTCTTTGAAAAGGACTCGGAACTGGCTGCTCTCAGAGCCAAGCTG
GAGGACGACAAAAACGAAGCGAAGGATCAACAGCAGCAGCAAGAAACGCTCGAGAAGTTG
CTTCAATCAATCGCGGAAAAGGACGCGGAACTCGAAGCCCTCCGCAGGAGTACTGATGAG
CGTATTGAAATGCTGCAAAATACAGTGGATCAAAAAACCACCGAGCAGGAGCGATTGCGA
ACAGAGTGGGACTCCGAGAAGGAGGCCCTTTTGTTGGAGAAAGAGGCGAACCGGCAAGAC
ACTTCCGTGGTCACCGCTGAGAAGGCTGACGACGCGGAGATGTTGGCACTGCGTTCTAAG
ATCGCCGAGCAGGAACAGAAGATCGAAGAGCTCGTTGCGTCCAAGGATGCTGCCGAGAAG
GCAAACAGCGTGGAAATAGTGGCACTTCGGTCCAAGGTTGCCGAGCAGGAACAAAAAATC
GAAGAACTTGTCGAATCGACAACCGCCCAGGAGGCAGACGGCGTGGAAATGGCGTCACTG
CGTTCCAAGGTCGCCGAGCAGGAACAGAAAATCGAAGATCTCGTTATGTCCAAGGCGACC
GCCCAGGAGGCAGACGGCGCGGAGATGGCGGCACTACGGTCCAAAGTAGACGAGCAGGAA
AAGAAAATCGAAGAACTCGCTGCCTCCAAGGCGGCCGCCACCGAAGAGCTCCAACACCAA
ACCGAGCAGAGTGCCCAAGAGCAGGAAGCCTTGCGTACCACCCTCCAGTTGAAAATCGAA
GGCTTGGAACAAAGACTGTTGGTTGCTACGAATCAGGAAGAAAGTGATGTCGCCGCCATG
GAAGCTCTGATTTCCGAAAAGGAAGCCACTAATGTCAAACTCCAGACTGCCCTAGCGGAA
TGCCAGAGCAAGCTGGCAAAGGTCACAGAAAGAAACTCCCAAGACCAAGCGGCACTCCGC
GCTGACCTCGAGGCGCAGATCGACACCCTGATTGATCAATCCTCAAAGCGGGACGATGAA
TACAAGCAGACCAAAAAACAGCACACATTGGAGGTGTCCGAGCTGGGGGAACAATTGGCG
TCCTCCCGGGCCGTCCTTGATCTCCTGAAGGTTTCGAACATTGCCGAGGCCACCAATCTC
CTTGCTTCATTGGAAGCCAATCGATCGGAACTCGAACGGAGCAAGGCCGAGCTTTCGGCT
GCGCTGACGGCTCCGGCCTCCCCGGGGTCGAGCTCTACTGGGGTGCAGGTGCGGCTTCCG
AGCGGCGACGACGGTGAACCAGTCTCTATGCCTTCGGCATCTCCCAACGAACCAGGTAAC
AACGCTGATGCCGACGACGATGACGACGACGACGGCTGGGGCGACGACTGGTAAACAGCA
CAAACAGTTTCGATATAACTCAGCCTGGTGCGTTGTCCCCATCTTTTGTTTTCAAACAAA
TTATACGCACGTGCAAACATACAGCACACACCACTATGAATGGAATGCACCTAGTTTCAA
ATTTATGTCATTAGCATTGCACAGGAGTCCTACAATAATGTTATTAATTACATAATCATA
CAGCATGCC
>comp2392_c0_seq1
GGGAGGAGCTCCGCCGGAAGCGCGAGAGCGAGGACAACCTGCAGAAGCTCCTGAAGGACG
CCCAGTCGCGCTTCAGCACCCTGCACGACCAGAACGAGGACATCTCGGCCCGGAACCGCG
AGCTCGAGGGGCAGATCCGCAAGCAGGCCGGATCGGACCACGGGCAGCAGAGCGACGCGG

ACACGACCCGGACGACGCAGCTCACCGAGACCATTTCCCTGCAGGACGAGGAGATCCGGA
GGCTCCGGTCGGAGCTGGACCGGGCGAGGCCGCTTTCGTCGCCGTCGTCGCACGCGGGCT
CCTCCCGGGCGGCGTCCTCGCTCGAGCGGGAGCTGGAGGCCGCGAGGGGGGACCTGGCCC
AGAAGGAACACGCCAACACCGTCCTCAACAAGTCGCTCAAGGAGGCCCTGGGCCTGCTCA
AGCCCCTCCAGATGCACCTGGAGGCCGCCGAGATGGAGAAGAACGAAACCTCCAAGGAGC
TCCGGAACCTCCGGAAGCGCTTCCGCCAGCTGCAGATGGGAGAGATGGTGGACGACCAGT
CGAGGTCGACGGTCGGGGGCAGGGCGATCGACGGCGGCGTGGAGCTCGCCAAGATCAAGG
AGGAGCTGGAGGAGACCGTCCGGCAGCTGGAGCTCGAGAACTCGGAGCTCCACGACGCAC
TGGACGACCTCACCGAGGGCGGAAACAAGCAAAACAACGAGGCGAAGACGAGGCACCGGC
TCGTGGAGCTCAACAGCCTGTACGAGGTGACGCAGAACCAGCTGGAGGACGCCCGGGTCG
AGAACCACGCGCTCCTCAAGGCGCTCCGGCAGAAGGAGGCCGAGGAGATCGAGCGGAGGG
CCGAGGTCGTCCGGCTGGGCGAGCAGCTGCACAAGACCGAGTCGGAGCTCACGAACGCCA
AGAAGATCGCGCAGTCGGCCCTGGTCAAGGTCGAGGAGCTGACCATGTCCAACATCGAGC
AGCTCTCGATCCTCCGGGACTCGCCCGGGAGTGTACACCGGACCGACCGCTCGAACCAGC
ACGGGGGGTTCTCCTACTAGCCGCCGGACCGGGGTTTCCCGGGAACCAATTCGCGACGCA
CGAGAGACAGGCCGAGAAGGCACGGCACACGGGGACGGGGAAGAATGCAAACTTTTTTGG
ATGGAATTCGTTGGTAGTGGTCTGCCACGCGAACCAAACCTAGGAATCCACACAAGAAAA
TAATATGATAGTATTGTCGTTGCAGAACGAATTCAAAGATTGTCGCAGCGATGGGTTACT
ATGCGAATGCTTTTTGAAAGAATGCGTCCTCTGGCTCTAGGCGCAGACCCCGGGACGCAA
TTTTCAATTATTGGTAGTTTCAGTTCTTTGCCGTTGTTGTTTGTTTCCTGGATTTTGCCA
AATAGGGGTG
>comp2393_c0_seq1
GAAATGTTTGTCAATCTTTTTGCGATGGGAAGGTGGAAGTAACAGTTCTTAGCAAAGATG
GAGAAAGCAACTGCTACGAAGATCAACACCATTTCAGTGGCTGAGGATGGTGAAAATAAG
GAAATAGAAGTTTTTTCGTTGAATCTTCGTGGAGCTACAGGTAAGGATTGAGCTAGCCGG
ATGGAAGTGGCATATTTCGCTCGGAGGGCACAGAACTTTTTGACTCCAGGAAGACTATTC
GAAATGCAATGTCCCTGATTCTACGTTCATCACCACAGTCCATCTCTCTTCACTAGGAGC
TAGCATCGTAAATTTTTTGTCTAATGCAGACGAAAACGACGAAGTTGTGGACATCGTAGC
AGGTTACAAGGATGCAAAAACGTTTCACGATACCGGCAATCCACCCTTCTTCGGTGGCAT
CGTAGGACGCGTGGCCAACCGAATCGGCGGGGGCAAGTTTTCTCTGGGTAAGAAATCTTA
TTCGATTTTCCCAAACAACCCTCCGAATGCTTTGCACGGCGGGAAAGTCGGGACATCCCG
AAAAATCTGGGCCCCAGAAATCATTCGCGAAGGGTCTGCATTACAGTTTTCGTTGCAGAG
CCCTGATGGTGATCAGGGATTTCCTGGAGATATCACCATCAAAGCTACCTATATATTGAG
GCCGTCCTTTTCTCCTTCTGGAGTTCTCTTGCAATTGGATATGCACGCCGAACTGAATAA
TCCAGGAGAAGGCACAGTGAAGGAAACTCCGATCAATTTGGCAAACCACTCCTATTTCAA
CCTCGGAGACCAAGAGAACGGGATACTCGACCATTACTTGCAGGTGGAATCAGACTCTTA
CACACCAGTAGACAAGAATTTCATACCAACACGAGAAGTTCGATCACTGGATGACGATGG
TGTTATGGATTTTCGTCAAGGAAAACGTTTGCGGGACGCGTTGGAGAAATATGCTATCGA
AAAAATGGATCTTTCGGCACAGCAATCCCAAGAACACTTGGCAGCTCGTAATTATTCCTC
GCTTCCAATACCGTACGGCTTTGATCACAATTATGTTGTTCGCAAGCAAAGAGGAACCTC
ACTACCAAAGATTGGGTTCTTGACGTATGGGTTGCGTAGGTTGACGATATATTCCGATGC
TCCGGGCGTCCAAATTTACACCGCCAACAATTTGGGCGATAGCGATCAGTCTTCTTCGGT
AGATGTGTGCAAAGCAGAATATGGCCCTTGGAGTGCTGTTTGCCTGGAGACGCAGCACTT
TCCCGATTCCATTTCTTCTGCAAAGACAGCATCCTCGAGCACTCGTGACTTTTGGGCCGG
AAAGTGCCCAATATTGACAAACGCCGAACCCACTTACAACCAGACGATGGTCATGCACCT
CGAGATGGACCATTCAAATGCGGAAGCATCTTACTTTGGATCCGACACGAACGGGAAAAA
GTATTCCTCGATCGAAGAGATGTGGCGAAACCAAGATTCATCCACGTGGTATACCCGTGC
GAAGGGTTGGTACGAAGACAATTGCCCATCCACCATTGATGGAGTTCTGGGCGGTATAGG
TCACATATCGTGCAAAGATCTGGAGGGATCCCGTGAATTTTTAAAGCAATTGAAACTCCC
CTCCAGGGCTGACGGAAAAGCCTCTGTAGCTTGTGAATGCGGTGCAGGAATAGGTCGGGT
CACCAAGGGGCTTCTTCTTGACTTTGCAGATCGATGCGATCTGGTGGAGTCATCTTCTCG
GTTACTTTTCTCGGCTCCCGATCATCTCGGCGACAGCTCATCCAACAAATGCCGCTTCTT
TTGTGCCGAGCTGCAAGACTGGAAGCCCGGAGCTAACAAATATTCCATAGTATGGATTCA
GTGGGCCTTGTGCTACTTAACCGATTTCGACATTGTTCAATTTTTGCGTCGTTGCTCCTT
GAGTTTGGTCGAAGGAGGATGGATAGTATTGAAGGAAAACACCTGCGAGGACCAGGCATT
TGTGGTAGATATGGACGATGCAAGTATCACGAGATCACTGGAATATTGGTTGGATCTCAT
TGCCAAAAGTGGGCTGCAGGTGAAACGATTGACCTGGCAAAACAATTTTCCGGATGAAAT
CTTTCCAGTTCCAATGATTGCACTACAAAAGTGATCTCAAAAGTTGCGACAGCTCTGAAC
TGATTTGTAAGCATTTATTGACGACTTTCAGTTGTAGCGAAAAAACTTCCTTTCTTCAGT
ACGAAAACGCTCCTCATACGTTGCATGATACTAGTCAAAGCATTAGTGATTCTACTAGTA

TTGCATGTGAGTCAGTTGGTAATATTAGAGTCAAGAAACAAT
>comp2393_c0_seq2
GAAATGTTTGTCAATCTTTTTGCGATGGGAAGGTGGAAGTAACAGTTCTTAGCAAAGATG
GAGAAAGCAACTGCTACGAAGATCAACACCATTTCAGTGGCTGAGGATGGTGAAAATAAG
GAAATAGAAGTTTTTTCGTTGAATCTTCGTGGAGCTACAGTCCATCTCTCTTCACTAGGA
GCTAGCATCGTAAATTTTTTGTCTAATGCAGACGAAAACGACGAAGTTGTGGACATCGTA
GCAGGTTACAAGGATGCAAAAACGTTTCACGATACCGGCAATCCACCCTTCTTCGGTGGC
ATCGTAGGACGCGTGGCCAACCGAATCGGCGGGGGCAAGTTTTCTCTGGGTAAGAAATCT
TATTCGATTTTCCCAAACAACCCTCCGAATGCTTTGCACGGCGGGAAAGTCGGGACATCC
CGAAAAATCTGGGCCCCAGAAATCATTCGCGAAGGGTCTGCATTACAGTTTTCGTTGCAG
AGCCCTGATGGTGATCAGGGATTTCCTGGAGATATCACCATCAAAGCTACCTATATATTG
AGGCCGTCCTTTTCTCCTTCTGGAGTTCTCTTGCAATTGGATATGCACGCCGAACTGAAT
AATCCAGGAGAAGGCACAGTGAAGGAAACTCCGATCAATTTGGCAAACCACTCCTATTTC
AACCTCGGAGACCAAGAGAACGGGATACTCGACCATTACTTGCAGGTGGAATCAGACTCT
TACACACCAGTAGACAAGAATTTCATACCAACACGAGAAGTTCGATCACTGGATGACGAT
GGTGTTATGGATTTTCGTCAAGGAAAACGTTTGCGGGACGCGTTGGAGAAATATGCTATC
GAAAAAATGGATCTTTCGGCACAGCAATCCCAAGAACACTTGGCAGCTCGTAATTATTCC
TCGCTTCCAATACCGTACGGCTTTGATCACAATTATGTTGTTCGCAAGCAAAGAGGAACC
TCACTACCAAAGATTGGGTTCTTGACGTATGGGTTGCGTAGGTTGACGATATATTCCGAT
GCTCCGGGCGTCCAAATTTACACCGCCAACAATTTGGGCGATAGCGATCAGTCTTCTTCG
GTAGATGTGTGCAAAGCAGAATATGGCCCTTGGAGTGCTGTTTGCCTGGAGACGCAGCAC
TTTCCCGATTCCATTTCTTCTGCAAAGACAGCATCCTCGAGCACTCGTGACTTTTGGGCC
GGAAAGTGCCCAATATTGACAAACGCCGAACCCACTTACAACCAGACGATGGTCATGCAC
CTCGAGATGGACCATTCAAATGCGGAAGCATCTTACTTTGGATCCGACACGAACGGGAAA
AAGTATTCCTCGATCGAAGAGATGTGGCGAAACCAAGATTCATCCACGTGGTATACCCGT
GCGAAGGGTTGGTACGAAGACAATTGCCCATCCACCATTGATGGAGTTCTGGGCGGTATA
GGTCACATATCGTGCAAAGATCTGGAGGGATCCCGTGAATTTTTAAAGCAATTGAAACTC
CCCTCCAGGGCTGACGGAAAAGCCTCTGTAGCTTGTGAATGCGGTGCAGGAATAGGTCGG
GTCACCAAGGGGCTTCTTCTTGACTTTGCAGATCGATGCGATCTGGTGGAGTCATCTTCT
CGGTTACTTTTCTCGGCTCCCGATCATCTCGGCGACAGCTCATCCAACAAATGCCGCTTC
TTTTGTGCCGAGCTGCAAGACTGGAAGCCCGGAGCTAACAAATATTCCATAGTATGGATT
CAGTGGGCCTTGTGCTACTTAACCGATTTCGACATTGTTCAATTTTTGCGTCGTTGCTCC
TTGAGTTTGGTCGAAGGAGGATGGATAGTATTGAAGGAAAACACCTGCGAGGACCAGGCA
TTTGTGGTAGATATGGACGATGCAAGTATCACGAGATCACTGGAATATTGGTTGGATCTC
ATTGCCAAAAGTGGGCTGCAGGTGAAACGATTGACCTGGCAAAACAATTTTCCGGATGAA
ATCTTTCCAGTTCCAATGATTGCACTACAAAAGTGATCTCAAAAGTTGCGACAGCTCTGA
ACTGATTTGTAAGCATTTATTGACGACTTTCAGTTGTAGCGAAAAAACTTCCTTTCTTCA
GTACGAAAACGCTCCTCATACGTTGCATGATACTAGTCAAAGCATTAGTGATTCTACTAG
TATTGCATGTGAGTCAGTTGGTAATATTAGAGTCAAGAAACAAT
>comp2394_c0_seq1
ATTGTATTGCAGAGACGAATGGTGAACAAAGCACAAAGTACGAGCACTGGGGCAAGAGGG
GCCTAAAATCGAAGAAGTGCAGGACTGCTGCGACTACGGAGGAAGAGGGTGTTTGTTGTT
TTCGAAGACGTACTAATTGTTCTGTACCATACCGTACGTACCGTACTTCTATCCTCGAGC
AATCGTTAACCGGTGAATAATACTGTAATATCGAATACGAATGAGAGGGAAAAACAAAAA
CACAGATACGTACCGGTAGAAGTAGTACGAGTGAAGTCCGTACTCTCCTTCTTCATTCTG
TGTGTTGTGCATACTACTATGTTACCCTACGGTAGGTACGTACGAGTAATCATATTCAGG
GAAGGATGTTCTCCGGCTCCTCCGCAGGCAGAAAATTCTACGATACTAGTAGTACCGTAC
GTACCAAACCGGTACCTCGTACCGTATGTAGTCGATGAACAACCGTGATATGTTTCATTC
GTTTGAGCCCTCTTGTACCCCCTGTACGTTTAAAGGTACGGATAAACCGAAACATGATCC
TTTCTAGGGGTTGTAAAATGGCCCTTTCGCAACTAAAGGATTCCCAAAAAGACTGAGTAT
TGCTTAAACGTAAAGTACTCAACACTCTTTGGGTCTGTGCATACAACTACTGTTACTCCC
AATCGACGACTTCAATATGTCAGGCGTCGATGTCGGTAAGGCCGCTGATGAAAAACACGA
CGCCACAAACGTCCTATTGAACGCCATCGAGGGCCTTGCATTTGAAGAGACGGCATCGGT
GAACACGCACGGAGCACTCGCCAAAAGCGACAGCGTAACCGTGGGAAATACAGGAGAAAT
CCGATATGAAAACGTGTGGCAAAAGATGGATGGCAAAGCCCGCATCGGAGCAAAGAAGCT
GTGGGACGATACATTTGGGGACGCCATGCCATGTAAAGTGAAGAACGAACGCCTCGGTGC
CCTCTGTGTCGTCGCTTTCAGCGGAGACGAAGTGGTGGCCGTCTCTACCGTTGTCATCGA
AATGAACAGTTCTTTATATTGCAAGGTTGGGTGGCTTCGATGTCTTGTCAAGGAAGAATT
TCGACGTAGAGGTATTTTCACTGAAATTATGAGTCGATGCAAAATAGCCTTAGAGGAGTG
GGCTATAGAAAACCCCCTTGCGGGCCTCTTGGCTATGGGTGTTATGTTGGAATTGAAATT

TGACGGGAAAAATTTGAACAACAAAGATCCCGTGTGGCCCAAAACTGGTTTGACCCTGGT
AGGCTACACCCAGCGAGGTGTACAGATTCGGATCGCCTGGTTAAAGCAAGCTCGCGTGGA
GTTTTAGATTGCTATACATGGAGGCTCTACATAAAGGAAGATCGAAAACGTTGAAAGAAC
ACAAATTCATGGGAAAATCCACTTGGTTCAAATGAAATTTACACATTGTAGAGATTGTGT
GCAATTCATTGCTGGAGTAGCGCTAAAAGCATGAAAACCAAGAAATCAATATGTCACTAG
TCATCTTGAATAAAAGAATCCA
>comp2395_c0_seq1
CTAGGATAAAGAGGCACGACCATACGGTTACCCATTTTATTCATAGGGAGGCTTGCTCGA
GCAAAAGGACACCAAGGAACGCCCGACGATGAGATAAGAGAGAACGACGACTCTTTTCCC
ATACACGAGATAACAACGAGGAACTGAACGAAGATTTTGATTCCGAGTACTCTTTGGTTG
TCCGTTTTGCGCTTTTTTGGTTAGGATTAAAAGTGCACATCCATCACCACTGCGGATGGA
CACGCCTTTGCATCATACTGGAATTCAGCCGAGTGCTTTGTTTGTCTTCATTGAAAACGG
ATTCAGCGTCCATATTTGTTTCAGCATTGTTTTAGAAGGAAAGGATCAATGGAAGCTTCA
GAATCCGAAGCAAATATCGTATCCAAAAGAGTCGAATCAAAATGAACCAAGGAAGATTGA
CAATAAACATGTTCAGCAATAGCGTTGAACTATCTCCAGAGTCATTGGAACGGAAGACGT
CGAAGAAAATAGCTTCAAACTGTCTTCCACCCAAATCTCCGGCATCGATGAATGGGAAAA
AACTTTTGTCCGTTGTCACCAATGCTTCATCTTCCTCTAGTCGCAGGCGAGGACCATCTA
GACGAAGATCTTCCAGTCGACGAAGAAGTCGTTCTAAATCTCGCAATTGTAGAGGAAATA
GCAGTAGCGATGCATCGTGTTCTTCCGAAAACAGTTCGTCTTCGGATACAGATTCTCTCA
AGCTACTTCCTTTGTCGCCTCGCATTACAGATCCTTCTCCCATTCACTTAACTGTTGATA
CTGATTCAGATGATTTAGTAACGCAGAACAATTTTCTCTTCTCTCCTTTGAGAAATCCA
TGAAGAGTAATTCGACTTTCAGCTACCTAGATAAAAACTCGAGTGAGTTGGCAGACCGGA
GAAACAGTGGTTGTCAAGATAATCATACACAGAATCGTCGGCGTCAACACCGCCGATCAC
ATCGTCGGGAACATAGTTGGAACTCGAGCACTCCCATTAATTTGAACACGGTAGACACTG
GAACCAGCTCGAAATCATGTCTGACCGTACCCAAAATCTTGCGAAGGCAACGTGTCATAG
TTGCCGACGTCGCGTCGGTCGGAAATGCAAGTGACACTCTTGTGTCTATTGTTGAAACAA
GCAACGGGAACGGACGCGGTCGCATCACCGGACGAGAACTGCACGAAACAGCCAAAGTCT
TTATGAACGAAGGTGAATACCAAGAAGCCTTGCAGATGTTCGAGGCGATATTACGGGCCC
AGCTTGCAAGATTCGGTTGCGAGGAGCATTCCAGCGTTGGGGCAGCATTACACAACGTCG
GTGTTGTCCGATTGCGTATTGGAGAGCCCTTGAAAGCAGAAGAAGCGTTTCTGCGGGCGT
TAACGATTCGTAGGAATGTGTTGAAATCAGACCATCTAGATTTAGCGGCAACTTTGGCGA
AGCTGGGCTCCGCAAGGACCTCTCTCGGAAAGTTCGATGAGGGACTTTTCGATCTTCGAG
AGGCATTACGAATTTCCCGCAAGGTTCTTGGACGAAACCATCGAAACGTTGCGCAGATAT
TATGTCATATTGCCTGTTTGTACTTTGAAGCAGGCGAGCTGTTCTCGGCGCAGGCTACAT
TCCAGGACGCACTAGAGATTTATCGAGGAGTATTTCAGACCGAGTCTGACCGAGACGCTT
GCATGGCGCAGCTTACTGAAACATTATGTAATATTGGGTCCATACAAAATAAGAGGAAGA
ACTTTTCAGGGGCAATCGAATCATTTCGAGAGGCTCTATATCTGCAACGAGGAATCTTAG
GTCACGACCATCCAAAAGTCATTGCCACCCTTGATAATCTTGCCTATTCATTTTCAAAGA
GTAAATCTTACAATCAAGCCCTCATTTGCTACAGGGAAATGCTAAGCGCAGAAATATCTC
GGTACGGTTCATTCACATTGGAATGTTGCGACACATTGGCAAAGCAAGTGCTAATTTTAG
GAAAACTGAAGAAAGTTGATGATGCACTGCAAGTTGTAAACAAATACCTGGAATCAATCC
AGGCCAAGTCCGAACAGGAGGTTTCAGCTGCAAATGACGAAGTTGCCGAAGAAGTGAACC
AAATGCTATTGGATCTGAAAACGGCAAAAAAGAAGTCTTCTAAACCAAAAACCAGAAGGC
GGGAACGATCCCTAGATTGTTTCGATAGAATACTCTTTGAAAAAGGAAAAGATAAGAGAG
CATCAGAGCTCTAAATACATATCATGGTTTTTCGAACGTGGCCTCTGTGAAGTAAAGAAC
AGCTATCCCCATAACGAAGAACTACGAAGGCCCAAAAGCTCCAGATACTTGCCTTAAATG
GCTCGTATATTGGATTGATGCCAGAGAATACAAACTATTAAATCAAGAAGTTAAAGCATT
GATTGCTTATTACTACCACTAATCTCATATAACGTCACACTAAATGACATCATTTTTCCA
TGTA
>comp2396_c0_seq1
CTTTGTCGTCATCTTCTCCTTCAGACTAGAGCAGCTGTATCACCCATCCAAACAACTTCC
TCTCGAAGACTGTAGCGAAGCAACGATAACTGCAGCAAAATTCAAGCGACGCGAGAAAAA
ATTCACGGTGTCTGAACTATTTTAAGAATGGAAGGGCCAGAAGCCAATCATGGAGGGCGC
CCCAAAACCCCTCAAGAGTTTCTGGGGTGCGTCCCCATTGGCCATCCGGACTCGATGATG
TCCGTGGAGTCCGTGAGAAAGGCAGTCGAAGAATTTCGAACGAAACCCACAGACGTCATC
GTCGCAACCTTTCCAAAGACTGGTACCACACTTGTAACCTGGATCTGCCACCTGCTGCGA
ACCGGAGCATGTCCTAAAAAATCACTTGGGTTTGAAACCCCTTTACGAGGTCGTTCCGTGG
CCTACTCTATCGTGGGACATTGGATATGACCCCAACGTGCAGGGCAGCGAATTCAATCCG
CGGGTCTTCAAGAGCCACCTGCGCATGGCGAGCATCTATCCTAGCTGCAAGTACGTGGTC
ACCATACGGGATCCGGCCAAGACGGCTCTTTCGTTTTACAACTTTTTTATTGCAAAAAAG

GTCCCCTTCTTGGTGTCGCCGGAGGACGGCAAGCTCCTTATGGACGTTTCGACATTTTTA
CTGGACACCCCATTTGTCAAGGGAAATCATCCCAAGCGCGCTAGCATCTGGGACTATTAC
TCCGAATACCACGCTCTAATGGAGTGTCCCTCTGTGCTGATGATTATCTACGAAGATTTT
GTGCAAGAGATGTCCTCGCAAACGCGAATCCTTGCCAAGTTTATGGACATTCCAGAGATG
AATAAGGACGATGCAGATAACGAAAATCTTATTGCTCGGACTGTTTCCATGAGCAGCAAG
GAATACATGGCCCAGCACATGTCGAAATTCGACGAGCCCTACGAGCGGGCCAAATCCCTC
AGAAGAGCTGCCGATATTTCACAGCTTGCACCGGGGGCCAAGATTGCCGTGAAGAAACAC
GTTCAGAAGTTCGACGAGCGAGCGAACTCGTTTCTAGAGGACCAATGGAAGGCCACTATG
GGAACACTGGGGTACGACGATTACGACTCTTTTTGTTCCACCGTTCGGACAAGGAACAAG
AGATTGTTTGGAAATAGAAACATACAATAGAAGAATAAAGAACTCAGAATGAACTAGATC
GAATTATAAATATTGATAGCACTGAAAACAAAATTCAATGGAGAATGGCA
>comp2397_c0_seq1
GTGCCGCCACTGCCCCGACGACGATGCCATGGGAGGTGCTGCTTCTGCCACCAGTGTCTT
TGAGGTGAAGACCCCTGTCGGTTTCTTGTGCCGCCACTGCCCCGATGATGATGCGATGGG
CACCGATTCTCTCTTGATGACCGCCATCTCCCAGGACTGTGCTGGGTTGTGCCAGAAGGA
AGCTGCCGCCGAACTCGAGGTTGACTTCTGCAACAAGATTCGATCCGCCCAAAACTCCAA
GTACCTCAGCTCTGCCAAGTCGTGCGCCATTCGATTTGACATTGAAGAGAAATCTAGTAA
CAACAAGCGTGTTTCTTCTGCTACCAATAAAGTTGCAACCGTTGACTCCACTATTATCCT
GAAGGGTGTCGGTACCGGCGATGCTACCAAAGAGGAACAAGCTATCCTCGCAAAGGCCTT
TGTCTCTGCCTATAACGATGTTCACTGGGGAGCCAACCACTACTTGACCGATGCCGAGAT
CCCATTTACCGCTGCTACTGGAAGTCCTGAGGGATTCTTGTGCCGTCACTGCCCTGATGA
CGACTCCATGGGTGGAGCCCAGCAGACCAACACCTTGGTATTGGATATCGTCAGCCCCGT
CGAAGCTTTCTTGTGCCGCCACTGTCCCGATGACGATGCCATGGGAAGTCAATTCGATAT
CCAATCTCTCAACTCGAACCCCTTTGACAAAAAGGCCATCGAAGTTGCCTTTTGCAAGAA
GATCCAGAACTCCGTCAGCGACAAGTTGGCGGCTACCAAGTCTTGCTCTATTGCGGTCGA
ATCCGTCATTGCTGCAGCCTCCAACACCAAGAAATGGTAAATTGCAAACTAAAGTGAATG
GTGCAAATGAAAACAAACGACACGAACGAATCTAATCAAAATACATGAAAAATCGTCATG
GTTATGTGACTGAGAACAACAAGGACAACAATTAGAACAAGAGGAACTAAAAGGCACCAT
CAAGCTAGATTCGTTCCGAAAATACATATTACAAAATCTTTAAATTAGATAATCAGAACT
TACATATCATCAAAA
>comp2398_c0_seq1
CTTTGTACCGATCCAAGAATGCCATACGGTACAAGCCCATACGGCAGAGGCCCAATCTTT
ATAGCAATCACCATGGAAGCTCACGGCAACTACAAGCCTGGTTACGACTCTGAATATGAC
ACAGCCAAAAACGAAACGACCTCCGATGACCACCACCATGGGAGTGGAAGTGTTGAAAAA
GAGGGAAAAAACGATGACTACAAGATTAGCTCTTATACCAAGATGAATTGCCCACCGTCA
AAGCAAATGCGACAGCCGCTGTCTTCATCGTCTTCGCAGCGATCAACAAGAAGATTGCCT
TCTTACAACAACAAGATTGTACAAGAATCTGCTAATAACTGCATTAACAGTCGTTTCGAC
CATGACACTTTTTCCACGGCCAAGTCGATGGCGTTGAAGTTTGCAAATCCATGTGGCCTG
GACATGAACCAATACAACAACAATTATTGCCCACCGCCAAGGACACCATCTACCGTTGGA
AGTAGTTTCAGTGACGATGAATCAGTTGAAACTTCTTCTTCACTTGCACAGCACTCCTCA
CACCCGAAAAGAAACAATCTCAAATCCAGATGCAGCGACGACAACCGCAGTGGCCATCCA
TTTTTGAGGAGAACTGTGATTCAACGTCGGTCGTCGAGAAGCAGTGAGGCATACTCACAA
TTTTCTACCGAAAAAGATGAAGGCGGCTACCCACCGCTCACACGAGGGATAGCTCCGAAT
TTTGATAAGAATGACGGCGAAGATGTGGTTATTGTAGTAGGCGATCAGGAGTATCCACTT
CCACGGACAAATGAGAAGTTCGTTGTCAAGTATTTACTTAGTCGACAAGAGCGAGTAGAC
GATCACTTCTCGTATGTCGATCTATCCACACACTCGCCCAATGAATTCGAAGAAGTAATG
GAATTCTTTGAAAACAACCGAAACACAGAGACATGGGACGAGATCCATTGGAAAAACATC
CATATAATTCTGCCCTGGTTCGTCGAGTTCCAGTCATTGCCACTGATGACAGCGGTCGAC
TCCTTTTTGTGTCACAACGGACTGGGATCAGGTGGTCAAGGATTTGGATGCAATGACAAT
GGGAATACCAAACGTAATAAGCACATCAGCCTCTCAAATCTGCTCATCTTGACGCAAATT
GCCTTTCGCTGTGGCTTGGAAAGCACGAAAAGTCATGCTCGGAGACTTCTGAAGCAATGT
TTGCTAGAACCTCGCAAGGAATACGGTGGCTCAGACGTACTAGATGACAGTAACCACACC
AACCGACCACCGATGGAAGCTGCAACAGAGGATATTGAATTGGAATGGACTTTGCATGAT
TTACAAATCTTAGCACAAACTTTGCAGGCATATTCCGATCTAAGGGAATACCTGTGGGAT
ACATCAGTCATTGTATATTTGCCTCACGATCTCGATATTTCGAATTCAACTGAATTGGTT
TCGAATGCGCTTTTCCCATATCTATTGCGCGAAGGAATGATGCAGATGATGATTGTCGAA
GGAATCCGCATCGACTCATCCGCTGATCTATCATATTCACAAGCCAATGAGAGCAGTTTC
AATAGCTCTGTAAGCATAGGGTTCTCGTATTCGGAATCCACCTCGTGCAGTGACACTACA
ATTCCTACGAATCCTGCTGCCCAGCGGAAGAATCTCACGCAGGAGGAGATGCGGGTCCAC
TTAAATGAGATAGTAATGTATTTGGACAAATTTCGCGCCGAAAAAGTTGCCAGGATAGAA

ATGCACAAGGAGCAGTTGGAGAATTCCCTTCTTGCAAACGAATCCTCATTTGACGACATC
GGAGAATTATCTGTGGACGGTGTTACAAATAGTCGTTCAAGTCAGGATTATGGTAGTCGA
AGCGACAAGTATGACTCTCATCAAGTGGAAAGAAGATCTTCAACGTCCTCTCGGGGGAGA
GTGAGGGGGGTCTCAACGTTTGCATGCTGAAAAATATATTGAAAGCTCATACCACTATTT
GGGATGACTTTGTGCAGAAAGAATTAAGACGTTAGTTATAGAAAATGATACGTTTTGAGC
TTCTTTGAGCGTTGTTGCTCGCTTCACTTGCCTGTAAAGTGATACAGTCCAGTCAACAAA
ATCACTCGGTACTTAATTCGCATAAATGAATCACCATTTCACTCCGGGACTCGCAGTGCC
AGTAGCATCTGAACTGGTTTTCGATGCTTTAGGATATAGCTAGTACGAGATCCAACTCTG
ATCTCATAATGGTTTTTGGTGTGTATAAATTGTACTTCTACGAGTGCAAGCCTTATCAAC
ATGTGTAGATGAAATGACACGCACGCCAATGTAGATCTTAGATATT
>comp2399_c0_seq1
AGCATTTTTACCTACCGGTAGTTTGATTTGACAATTTCCAAACACCAAACTGGCGGAAGG
AGCCACCATACCGTACCCGATACGCATCACCTCCTGGTTCGATACCATCGGTCGACGCAA
CGAATAATCGCTTTCCGCAAAAGGATTCACAACAAGGAGCGCCATGGCCGGATACGTTAC
CAAGCCCGCGGCGGCGGTGAACAAAGCCCTCGTAGAATTTCTGCCAGAGCTCGACTCAGA
CTACGCCACATATCCCATGAAGCATGATCGGTGGTACGAACCCGACGAGACCGGTCCGAA
GGGGGAACCTTGTTTCATTGCCAAGGGGGGAACCTCGTTGGCAACCGGCAGCCCTCCAAT
CAAGAAGGACTATGTGTACTGCACTACGGGCGGGAACGGGGAAGGCTATTACTCGCTCAT
GTGCCGGACCTCGTACATAAACCTCTACTACAAGCTCAAAAGCTGCGCTCCCCAGGGGAC
CTGCACGGCGTGTCCCTGTTTTGCAAGCAAGGCCACCCGAGACGCCCTGGATCGGTACGA
CGACTGCAAGCGAGTGGTCTACATGCGACAGAAATGCTTCCCAAAGCCGGACGACAAGCT
GGCCTCCGACATCGTCATGGGCATAGCCAAGGACACCGCCAACATGGTCTACCACGGCAC
CCAAAACGAGCAGCTGATAGTCAACGCTGTCTTTTGAGAACCTTGTCGTTTTCTCGAGCG
AACGAGTAAGATATTTTGTCAATATAAGGAACGAGTGATGGAATCGAAGCACAGAATAAT
TCTCCCATTTGTGGTATGCTATTGTATCCCATCTTATGGTGGTGAAAGAGTGATCTTCTC
CTGTCCTGGTCGGCTATTTTGTACGCATATTAGCTTGGCAATAGAACCTGCTTCATACTG
GCAATGCGTCGTAGATTCCTATCTTCTTTTGACTTTCTATTGGACCGCCCTAAGGTTGTG
CGCCTCAACTGTTTTTTTTTTTTTAAACTAAAAAGGAATAGATGTAGAGTAGGATTGTAG
ACCCCGTTACCTCTTTCGCTACTACAATTACTGTCCGACACAGCCGAAATTGCATTTGTG
TATTATATCACCACCAG
>comp2400_c0_seq1
CGTTCTGTACTAGATAGCATCCTTCGCATCTCGTTCTCCAAAAGAAAATGGAATAAGGCC
CTGGGCCAACCGCCGCATCAATCACATAAAAAAGACAACAAATGGAACCAATAGAGGACC
AACACTGTTACACACACGACAATATTTCGACTATGCAGACCAAGGGCGATGATCTTGACA
AAATCAATATTCGTATGAAAGATGATGGTACAAAAAGTGGCATTTGCACCTGTGGTGGAA
TGTCTTCGTCTGACATGATGACGACGGTGGCGATGGACGAAAAATTGAAAACCGCCAATA
GCAGTGGCAGTGGAATCGGCGAAAACAATGAAGAATTAGGAGCTATACATTTTGAAGGCG
CGAAAAAGAGCCCAGTTTGGCTCGAATCATTCTTCGTGAATCTCATGACACGCCGGAACA
TCCGAACCTCCAAAGATTACAAAGAACGTCACCATCAACAGCTTTCGCAACACGGTTTCT
TAGAGCGACACCAACACCTATTGAAACTATTAGAAGTTGCATATAATGCCAATTCGGATG
ACATTGATCATCACAAAAATGAAAACCATGGCAAATGCGATGCGATGGTCGAACTGTGCG
TTGAGTGTATCGATCGTGTTGAGGCAGCACTGGAAGCTGACACGCAACGTATGTATGCTG
AAATTGAGGTCTATCAGAAAGCCGTTCACGTTGCAAAGAATAAGACTAAATCGTTACAGA
AAACGACAGAAGGTGTTGTTGGCGCTGGCATCGCCAAGGCAACTATGGGAGAGGATCCAA
CTGAAGATCAGGCGCACGGGAAAAATAACATAGCTATTGAAGAAGCCTATCGACATGAAA
TTTCCATGCTGGAACAAGAGGCTCAAACTCGATGCGACGAACTACTCCATTACAAAAATA
TTCATAAGGAGCAGACAACCATCACTCGTGAACTTGATTTATTTGAAGAGGCAATTCAAT
TGGAACAAAATTCCCTTGAGTTGCAATCGCAAGCATTTGATAACCGTCGGCAGATCCTTA
CAAAAACGCTTTCGGAAGTCCAAAATGAGGTCGATAGACTTACATGTGTATCGCTACCTC
GAGTCTTGTTCGACTTACAAGTAGATCAAGTACGAGGACTACACTATCCTTTGATAAATC
AACTTCGACTCGCTTTTCGACCAAAAGGAGATGTGCCCGCACAAGAAATCCAAGTGGCTT
GGTCACAAGCAACGCAGCTGCTCTTGGTGTTGGGAAATCTCCTTGACTATCCCAGCTCGG
ATTGGAAATTGGTACCTCTCGCAGATTGCGCCAAACTCATTTATCGCAAAGAAATTTTCA
ATCTGGAGCCGGGTGATTGTCGTAGCCTGATGGCTTGGAACGCACTTTTGGACCAAATCG
TGAAACATGCTTTATCAATAACGGTCGGAGGTGGAAACAAGGTAAATCTCAATTCTGTTT
CTACAGATATTTATGACCAAGACAGCACGAGTACGAGCACGAATGCAAAGCCTCCCTACA
TTAGCTCGCCCACGATGATCGGAAATACAGAATTAGCCCGACTAGATCCGATTGACCACG
TTAGTTGGTCGCAGGTTATACACCGGATGGCTTCCAACCTCCTTTGGTTGTCCAATCGAG
CAAGCGCTTTGGCAGCTACACAAGTCGGTAGCATAGTGCATTGTGTCGTCTAGAGAGTGG
CGCTCAAGAGAAGACAGAGTTGTGGTACGCGAGGCTTTGAGCAAAATAAAATGGCATATG

AATGTATTATAACATTCTATTCAAGTTCTGACTATTGGAAGAGTTCAAAGAGCTAACCTG
TATAGGAAAGTTCTCCGATCCAGCTAACGCTACTTTTACATTTCCAATTCGTGGTCACAT
GCTGTAGTGTATCCCCTTCCTTGGAATCTGATCAACTATGAAATGTCAATCTTTTTGAGG
GAGGGTTAATTTTATGCTTCTTCCTACTAATGTCTGTATGAGAAGAAGAGCTACTGCGAA
GAATCACCAGTTAGGGCAACCTTAAAGAATAAATAATTACAAAATAACTAGTCACCCT
>comp2400_c0_seq2
CGTTCTGTACTAGATAGCATCCTTCGCATCTCGTTCTCCAAAAGAAAATGGAATAAGGCC
CTGGGCCAACCGCCGCATCAATCACATAAAAAAGACAACAAATGGAACCAATAGAGGACC
AACACTGTTACACACACGACAATATTTCGACTATGCAGACCAAGGGCGATGATCTTGACA
AAATCAATATTCGTATGAAAGATGATGGTACAAAAAGTGGCATTTGCACCTGTGGTGGAA
TGTCTTCGTCTGACATGATGACGACGGTGGCGATGGACGAAAAATTGAAAACCGCCAATA
GCAGTGGCAGTGGAATCGGCGAAAACAATGAAGAATTAGGAGCTATACATTTTGAAGGCG
CGAAAAAGAGCCCAGTTTGGCTCGAATCATTCTTCGTGAATCTCATGACACGCCGGAACA
TCCGAACCTCCAAAGATTACAAAGAACGTCACCATCAACAGCTTTCGCAACACGGTTTCT
TAGAGCGACACCAACACCTATTGAAACTATTAGAAGTTGCATATAATGCCAATTCGGATG
ACATTGATCATCACAAAAATGAAAACCATGGCAAATGCGATGCGATGGTCGAACTGTGCG
TTGAGTGTATCGATCGTGTTGAGGCAGCACTGGAAGCTGACACGCAACGTATGTATGCTG
AAATTGAGGTCTATCAGAAAGCCGTTCACGTTGCAAAGAATAAGACTAAATCGTTACAGA
AAACGACAGAAGGTGTTGTTGGCGCTGGCATCGCCAAGGCAACTATGGGAGAGGATCCAA
CTGAAGATCAGGCGCACGGGAAAAATAACATAGCTATTGAAGAAGCCTATCGACATGAAA
TTTCCATGCTGGAACAAGAGGCTCAAACTCGATGCGACGAACTACTCCATTACAAAAATA
TTCATAAGGAGCAGACAACCATCACTCGTGAACTTGATTTATTTGAAGAGGCAATTCAAT
TGGAACAAAATTCCCTTGAGTTGCAATCGCAAGCATTTGATAACCGTCGGCAGATCCTTA
CAAAAACGCTTTCGGAAGTCCAAAATGAGGTCGATAGACTTACATGTGTATCGCTACCTC
GAGTCTTGTTCGACTTACAAGTAGATCAAGTACGAGGACTACACTATCCTTTGATAAATC
AACTTCGACTCGCTTTTCGACCAAAAGGAGATGTGCCCGCACAAGAAATCCAAGTGGCTT
GGTCACAAGCAACGCAGCTGCTCTTGGTGTTGGGAAATCTCCTTGACTATCCCAGCTCGG
ATTGGAAATTGGTACCTCTCGCAGATTGCGCCAAACTCATTTATCGCAAAGAAATTTTCA
ATCTGGAGCCGGGTGATTGTCGTAGCCTGATGGCTTGGAACGCACTTTTGGACCAAATCG
TGAAACATGCTTTATCAATAACGGTCGGAGGTGGAAACAAGTGTATCCCCTTCCTTGGAA
TCTGATCAACTATGAAATGTCAATCTTTTTGAGGGAGGGTTAATTTTATGCTTCTTCCTA
CTAATGTCTGTATGAGAAGAAGAGCTACTGCGAAGAATCACCAGTTAGGGCAACCTTAAA
GAATAAATAATTACAAAATAACTAGTCACCCT
>comp2401_c0_seq1
CTCAAAAGGTATCTAAAGTATCATACAGCAATAAATTAACAGCAACTAGAATGGATCTCT
TTCAAGAAGAGGACTGGCCAAAAGAGGCCGACAAGATGTACGAACGAATCGAGGTCTTGG
GGAAGGGTAGCTTCGGATTAGTATGGATGTGCGAGCGCAAAACAAAGGCTGATGACGAAT
TTGACGACAAATATGTCGCTGTAAAGAATATCGAAATCAAGGAAGAGAAGGGAAAAGTAT
ATGCCCAACGAGAAATTTCTATTCTTTCGGAACTAAAACACCCCAACGTTATTCGGCTTA
TTCGAGAGTTTCCGATTTACAATAACATCAGCCGATTGGTTGCTCTTCAGCTTGCTCGGG
GACCGAACCTTCAACGGGTGGTCTCGAAACGTGGTGCTCTTGGATTGCCTCTCTGTCGAC
TGATATCAAGAGAGCTAGTGGCAGCCGTGTCCTACCTGCATGGCAGGGGTGTTTTGCACC
GGGATATCAAGCCAACGAATCTCATTCTAGAAAACACCGAGATTCGTCCCGTGGATTATT
ACGATTATAGCCGCGACAGCTCCATTTGGAGTGACGGGCGGGATGCCGAAGAAATGGTCG
AACGTAACAAATGGAAGGTCATGCTGGTCGATTTTGGTTTCGCCCGAGCCTTGACGGCAA
ATGAAATCGATTTTTATAGAAGCGAAGCCGATGATAAAACTCCAGATTTGGCCAAAAAAT
CAAGGCGCCATATGCGTAATAGTATTACTTTGGAGTCGAAAGGTCCTCCCACTCAAACCA
AGCCGCCGTCTGCTGCGGTTAATGTAGACGACTCCGACGAAGAGCTAGCTTTAATTACCA
TGGCGGCAAACTCGATGAGAACAAATATGATGGACAACGCAGCACACCAGCCTTCGAAAC
GACGAACTTCGAACGTGTCAAACCTGGAAGAAATAAAGGAGGCTACGAACGCCACCAACG
GGACTCCAACGCCCACAGAAGATAGGGTCGCACCAATCAAATCCAAAAACCGTCGTGAAT
CGATTACACAACAGAAGGTTCGGTCGATGAGTGCGTTGGGGACCAAAGCATACGCGGCGC
CGGAGATCAAGCACGATCTTCGCAACAAAACTATTGAAGACATCAACAAGACAAATGCCG
CACTGACAGAATGCGTTGCTGATTATGGAATGATCGTGGACGCATACTCGGTCGGATGGA
CACTACGGGTCGTCCTGACGGGGGTTCCTCCCAATTCCACCATTTCCAAGTACATGCGCA
AATACAACGGGCAGAAAGCGTCGGCCGAATTCACGGACGAGGTTGTGTGCTGTTGTTCGT
TCTTGGGATCGCCTACCGATATCGAGGAAGAAAACAAGACGCAGACAAAGCCAGACTTCA
GGGTTCGTGACACGGACGAAATACCGAGAGCAGCAACTATATTCATTTCACAGCTGACCA
AGAAAGATCCCGAGGAGCGCATGAGTATTCGACAGGCTCAGCTTCACCCATGGATCAAGG
GAGACGAACAAAAAGGGGAGCCACTGTATAAAGTACCACAGGGCGACTACCCTTCCCATC

ATGGAGACCCGGTTGTTCCTTTGAAATGTGCGGCCAATCTTACAAATATTGTCGAGATTC
ACAACATCAATAAAGACCAATAGTCGACAATGCATTTATGGAATACTGTTTTTAGAGATA
GAGCTTTTCAAGAACCAATATTTTCCATTTTGAACCGCGGAGAGAGGATCGACATATCCG
AGAACTCTATTTTGCACCATTCTAACCACTTCTACTACAGCATTTCATTTAGGTTCAAAT
AGAAATCAGAGTAGGAGAATCGTGGTGATACTGTAACGATGGGTTATGAAACAACTCTTG
GCGTTTTTATTGACTGCGAACACCACTACTACTATCGGTAGTAAGTTCTGTAATTGCTTC
GAAACGAAGCGACAAACTAGGACGAGTAAAATAATAATAATATTGATATGCG
>comp2402_c0_seq1
TGCCGCCATTATTATTTTTATTATTGTCAAACCACCATGAGCGAAGTACAAGTAAATGCC
GTGCTCTCGACACAGAGCGCAGACACAGTCGATAGCACACAATCTAGGGATGCCAATGGA
AACCCGATAGTTTCAAAAAGCAAGGCTCGCTCTCTGCGAAGAAAGCGTGCGAAACAACGA
AAGAAGGCGGCAATGCAGAATGGCGGACAAGGAGTGGAAGAAAAGAAAACAGAACCTCAG
GATAAAGAGAATAGTGGTGCCCAGCAACCAAAGGGTCAAGCGGGCGCGAAGAAGAAAAAA
AGGCACAGGAAAAAGAAAAACAAGGCACAAGCAGGCGATGGAGCAAAACCTGATGCTATC
AAGAACAATAATGGTAACAAAGTGCGATCCAGCTCGGCGGCAACTTCAAAGAACGTGGAA
CCCAAGGTTACTGCGTCAACAAAGCTTTCAGAGAGTGCTAAACCCAAAGTTACTGCGTCG
ACATCAGAGATCACAAAAGGAACAGAAGAAACGAGCCCTGCAAAAATTGATGATTTGCCT
GTCAGAGAAGAAACCATTGCTCCAGTTGCAACTTCGGTGGAGATAAAATCGGATACCACA
CAAGCAACTATTTACGACGATGATAATGAAAACAACATTGGAGAGAAAGAGGATTGTGCG
TGCGCTTGCACTCTCATGTAAAATTGAAGCTTTGCATTGCCACATTTCCACAATGATACT
TTCAAGATATCGATTATTACATCATTTTGTGTCGTACTGCAGTGTTTGCCAGTATCACTC
TTTGATTGTAGAGTCCGATGAAGACAAAACTCATCAAATGGAATCAAGTTCAAAATGTCT
GTTCCCAGAGTCCGTGAATTTCGTGAACCTTAAGACATGCGATGATACCACTTTCGATTT
CTGTAACAGGATGTAACATAAAACTACTTAACATGTGAATATTTTC
>comp2403_c0_seq1
CGAATCAGTTGAGCAAGGAAGCAAACCTAGCGACTTTGACTCTAGCGGTAACTCCATCAT
ATCAGCGCGACGATGGCGTACTTTGCTGTAGCAGCAAGAAGAATAGCGACGGCCTCAAGC
CGTCGCGCCGTCCAATCAACGGCCAACCGATCCTTCTCTTCCAATGGTGCCGATGTCCTC
GCGGGTAGCGGCGTCTACGGCGGACTTCAGGACCAGGACCGCATCTTTACCAACCTCTAC
GGAGAACAAGACTGGCGTCTCAAGGACGCCTTGAAGCGTGGCGACTATCACATGACTAAG
GAAATCATGACCATGGGACCCGATTGGATCGTTCAGGAGATCAAGGACTCTGGGCTCCGT
GGTCGCGGTGGTGCTGGCTTTCCTTCGGGTCTGAAGTGGTCATTTATGCCCAAGGAGACG
GATGGTAGGCCCTCCTTTTTGGTTGTGAATGCCGACGAATCCGAACCGGGAACCTGCAAG
GACCGAGAAATCATGCGGAAGGACCCGCACAAGCTCGTCGAGGGATGCTTGCTAGCTGGA
TATGCCATGCGCGCTAGAGCCGCTTACATCTACATCCGTGGTGAGTACTTCAACGAGGCG
GTCGTCTTGGACGAGGCGATCCACGAGGCCTACTCTGCAGGCCTTATTGGAAAGAACGCC
TGCGGCAGCGGTTACGACTTTGACATTTTCCTGCACCGTGGAGCCGGCGCCTATATTTGT
GGAGAAGAAACCGCCCTAATTGAGTCTCTCGAGGGACGGCAGGGAAAGCCCCGCCTCAAG
CCCCCATTTCCAGCCGGAGTTGGCGTTTTTGGGTGCCCATCCACAGTCACGAATGTCGAG
ACGATCGCTGTCTCGCCAACCATTCTTCGGCGTGGTGCCTCGTGGTTTGCCGGGTTTGGA
AATGAAAACAACCGCGGCACCAAGCTTTTCGGTATTTCCGGCCACGTCAAGAATCCAACG
GTCGTGGAAGAATCCATGAGCATTCCCTTGCAGGAACTTATCGAGAAGCACTGTGGTGGC
ATGCGGAACGGGTGGGATTCCCTGCAGGCCTGTATCCCCGGTGGATCCTCTACGCCTGCA
ATGAACAAGGAGCTTTGTGACGAGGCGTTGATGGAATTCGACCACCTCAAGTCCCTTGGA
ACCTCGCTCGGAACTGCGGCCATTACCATGTTTGACGATTCCGTCGACATGGTCGCCGCG
ATTCGACGGCTCGCGCACTTTTACAAGCACGAATCCTGCGGTCAGTGTACCCCTTGCCGC
GAGGGAACCTCTTGGCTGGAGGACGTTCTCATCCGGATGGAGAAGGGAGACGCAGACAAG
CGCGAGATCCCGATGCTCGAGGAAATTGCTCGGCAAATCGAGGGACAGACCATTTGCGCC
CTTGGAGATGCCGCCGCATGGCCCATCCAGGGATTGCTGCGCCACTTCAAGGAGGACATC
GAAAAGCGAATCGACGACCCAGCTAGCTTCGACCATGACGCTGCATTTCAGACATCCTGG
AGTGGGGATCCTTTCGCGAACGACGAGTGGGTCGACGAGTTTGGAGATGGTTTAACCTAC
AAGAAAGCGACCGCTTAAACCAAAGTCGGGATGGAAGTAGAGTACACATGACAAATCGAA
TGATTGAAAATCGAAACAAGAGAGAAAAGAGGGTTTTGTCGAAATAAAACAACGCAAAAT
ACAACCCAAGCATTCGTATTTGCGATTCCCTAAGTCAGAACAAAACCAGCTCTGGTTTCA
AAAAAGGTAGACCGAAGTAAATTTCTGCACCACTGAGGTTCCATTATTAAAACATTAGTC
AACTTGCAACTTGAAGCGGCT
>comp2404_c0_seq1
CATAGGGTATCAACAGTGACCGCACCAGGGACTATTGGAACGACCACCTCTCTCTTTCAC
CTGACCTCAGATTTTCTAGTGAAGACAACGATGGCAGACGGAGAGGAACCTATTACTAGT
GATGATGTTGCCAATGGCACCGCCGGAGACAGCAACGCGAGCCCGAATGAAACAGAGGCA

TCGGCCCTCGAAGACAACATTGCCACAAAGGGGAGAAATTCGTACTACTTCGCACACAAG
CACAAGGCCAAGGGACCAAAATGGGACGGGAAGGCCGAGCCTAAATCACTATCCAAAGAA
GACATGAAAGCACTGAGTCTAGAAGATCCAACAAAACTATTGGAACAATCGGGAAAGGCA
TCATCCTTCGCCTACCACAAGAGCAACATCACATCGTATGCATTCTTGAACGAAGAAAAG
CAGGTCAAGCTTTATATCACTTTGGAAGGAATTGGCGAACAGTGCAAAGACGAAGATATC
CAATTGGATTGGGACGAATCATCTCTTTCGTTACTGATCAAGAACTATTCCGGCACCGAT
TCGACGGGGACAGAGGATCGTACGTTGTCGTTTGGAAGACTTACGGCAAAGATATCGAAT
GCGAAATTCAAACTAAAACCGGATAAGATTATTCTCACGCTTAAAAAAGAGAAAGCAGGC
ATGGAATGGCACACAATCAACGACAAGGGATCACCAGATCACGAACTCGTATAGCAATCG
AAATTTTAAAAAGTGTGAAATAGATGGTAGACTATCGTGCACTGATTTTCGCAGGACGTT
TGGTTTATCCAGAAAGTTCTGAATCTAAAAATAGGGAAGGAAAATATTTTGACGTATAA
>comp2405_c0_seq1
CAAACATCAAAATTTGGAAGATTCCCAGGAATCACACAAATTTAACACAACCTTTCTTCC
ATCTAGGAAGAAATAGCTTGTTCATTTAATAACACTATTTCATTTCCCTACCATGTTGTT
CCGTAGCATCGCGTTATTTGCAGCTTTGGCTTCACCTGCTTTCGGCTTTGCGCCTGTCTC
CACACCTCGTCCATCGGTCATGGCTCTTCGCGTTGCCACTGATCCCATCAAATCTGACGA
TTTGGTTTCGAAGGAGGCAAATGTCAAGTCCGTACGCAGGACCAAACCCACCAAGAACCC
CATGAGTCCTGAATTTGAACAGATTAGATCTGTTCCATACAACGAGGCTTTCCCGAACTC
TACTAAGGAGCACAAGACCGTCACTCACGAGGAAACTGGACATGTCCTTAATGTCCCATT
CCGTCGTGTTCATCTAGAAGATCCCGACCAAAAGTATTTGGATCTTTACGATACATCGGG
ACCTCAAGGACATGACCCTAAGATTGGTATCCCCAAGCTTCGAAAGGATTGGGTCGCTGC
TCGCGAAGGAAAGTTTGAAAGGTACACTCAGATGCATTTCGCTAGACAGGGCATGATTAC
CGAAGAAATGCTGTTCGTTGCCGAGCGCGAAGGTGTCGAGCCCGAGTTTGTTCGCTCTGA
AATTGCCCGAGGAAGAGCCATTATCTGCTCCAACAAGAAGCACCTCGAATTGGAGCCTCA
AATCATCGGACGCATGTTCAAGGTCAAGATCAATGCCAACATCGGAAACTCTGCTATCAC
CTCCTCTATTGAGGAGGAGGTGGAAAAGCTCCAGTGGAGTACTCTATGGGGAGGAGATAC
CCTGATGGATTTGTCTACAGGAAAACACATTCATGAAACGAGAGAGTGGATCATGCGAAA
TAGTCCCATTCCTGTCGGAACAGTTCCCATCTATCAAGCACTTGAAAAGGTCGACGGAAT
TGCTGAGGATTTGAACTGGGAGGTTTTCCGCGAAACCTTGATCGAACAGGCAGAACAAGG
TGTCGATTATTTCACAATTCATGCAGGAGTGTTGCTCCCTTACGTTCCAATGACCGTTAA
GCGAATGACTGGTATTGTGAGCCGAGGAGGATCCATCCACGCGAAGTGGAACATTTTTCA
CCACAAGCAAAATTTTGCTTATGAGCATTGGGATGACATCCTTGACATTTGCGCCGAGTA
TGACATTGCGCTTTCTATCGGAGACGGTCTCCGTCCCGGTTCGATTTACGACGCGAATGA
CGAAGCCCAGTTTGCTGAACTGAAGACTCAGGGTGATCTGACCCGTCGAGCTTGGGAGAA
GGATGTCCAAGTCATGAATGAGGGACCAGGCCATGTTCCTCTTCACAAGATTCCGGAGAA
CATGCGCAACCAACTTGACTGGTGTAACGAGGCTCCATTTTACACTCTGGGTCCTCTTAC
GACTGATATCGCACCAGGGTACGATCACATTACCTCTGCCATCGGTGCTGCGAACATTGG
AGCTCTTGGAACGGCCATGCTCTGCTACGTCACACCTAAGGAACACCTTGGTCTCCCCAA
TCGTGATGATGTCAAGGCTGGAATCATTGCCTATAAGATTGCGGCTCATGCCGCGGATCT
AGCCAAGGGATTGCCTGGTGCTCAGGATCGCGACGATGCTCTTTCCAAAGCTCGCTTTGA
GTTCCGCTGGAATGATCAGTTCAATATTTCTTTGGACCCTGTTACCGCAAGAGCATTCCA
CGACGAAACCTTGGATAGCGACGCATCCAAGAGTTCCCACTTTTGCTCCATGTGTGGACC
TAAATTCTGCTCTATGAAGATTACAGACGATGTACGTCAATACGCAGCCGAGAACGGTTA
TGGAGTAGAGGAAGAAGCCATTGCAAAGGGCATGGAAGAAATGAGTGACATGTACAAGGA
AATGGGCAACAAATTGTATCTGGAAGACATGGACAATGTTGTCAACCCTTTGGAAGACTT
GACCCCTTAGAGGAATTTATATTACAGAACATCCGTTATGACGTCTACAAGATATTTTAT
CTTGTCGATATTTCAATCATCATTTTTGTTTGCGGGAGCAACCGAAGAAACAGCTGAGAT
TCATTATCGAATACCGCTTGAACCTGATCATGGCAATGCATGCGTGAGGGAACAACAAAA
CGATTGATGTAGAAACGTGATTTCTAAACGAATGAAGAATATAAGTTCTTCTATCAATAT
ATAGGATTATTTTTTAGGAACATC
>comp2406_c0_seq1
AGGACAACGACAACGACAACGACGTCGGTTTTCGACATACTGTGTCGTTTTCGGTGTCGT
TTTCGTCGTCCTTGCTCGCCGGTGCCGTCGATGTACTCCACCCGGGAGGTGCTTTCGGAT
CCTCCCCCGTTTCCGGCGCCGGTGTCCTTTGTTCCGCCCGGCACAACCTCGGCGTCGCCG
AGAGCAAGAACAAAACCATGGCCGATCGCCAATGCCACGCCCAGAGCCGCAACGGCAACA
ACGAGCCCGAGCCCGAGCCCGCCACTGCCAGCACCATCCAAGAATCCAAAGAATAGCCCA
ACGACAAGGCAGAGAACAACCAATGGCGGAGACGCACGCGAGGATCGGCA
>comp2407_c0_seq1
CCTTCGACCCATACACAAGGAAAAGCAAGCAAAAACAAGCAATCGAATGAACCATGGTCT
TTGGCAGAAAGAAGAAAGAAACCACCCCAGCGGAGGGAGAAAACGACCTGGCCCTCGCAG

CCGGTGCCCCGGACGACGCTTCCTCAACGGGAGACTCGGTACCCCCGCCCCCGCCGGAGG
CCTTTGCGGGAGGCAAACAGAACACTGAGTCCATCGAAGCCGACCAGTACGTCGTCACGA
TGCCCCAGGCGTCCGGTTTGGCCGACGAAGCGAAGGAGGGAAAGGACGACCCGGACTTTT
ACAAAAAGCTCGGGGTGATTCTTCTCTGCATCAGCACCTTTTTCGTGCTGCTGGGGGTGT
CCATCAAGTACGGGGTCAGCCGCAACCAGCGCGCCGAGAACAGCATCGGGGATCCGCTGT
TGGTGGAGTCCGGGCCGGCGGAAGAAGGCACTTCCGAGCCCCCACGCACCGTGGACCAGC
AGAGCGTCCCCTGGGAAGACACCCCCAGCGACCCCACGGGCACCAACACCACCGGCGTCG
GCGTCGACGCAGAAACGGCGCCCGGAGCCGGCGAGAAGACGGTAGTGCCCTCCCAGGTCG
GGACCACGGGCGGAACCCTGCCCGCGCCGAACCCCTTTGCGGGCTGCACCGCCGATGGCA
TTGTTCTCGAGGCCACCTGCGTGGACGGGGCGTCCCTCGTGAGCATGGACTTTTGCCTGG
CGAACGGCGGCGGGGCCACCGACGAGTTCTGGGCGTGGGTCGAGACGCCCGACGCGTACG
CCCGCTTCGTCGAGACCGACTGGGGCTGGCTGCGGGACGAGGCCTCGAGGGAGGTCGAGG
GCCTCCCCGGTGGCACCTACGTGATCGGCCTGTTTTCCGACGGCCGGCAGCTGCTGCCCG
AGTACCCCCTCCTGGTGTCGCAAGAATTTACCGTGTCCTGCCCCGAGCTGGAAGTGAGAA
ACTAAGGGAGCGGAGACACCACGGCACGGCCGCCGTTTTGCGCATCGAACCCGGAAGTCT
TTGGAGTGGTCCACGCTGTGGGCACGCTGTGGTTGTGAACGGGTCTACCATACTATTCCA
ATAGTATGGTATAGTATAGTATAGTATAGTTGAGTACAGTACAGTACAGTATAATAT
>comp2407_c0_seq2
CCTTCGACCCATACACAAGGAAAAGCAAGCAAAAACAAGCAATCGAATGAACCATGGTCT
TTGGCAGAAAGAAGAAAGAAACCACCCCAGCGGAGGGAGAAAACGACCTGGCCCTCGCAG
CCGGTGCCCCGGACGACGCTTCCTCAACGGGAGACTCGGTACCCCCGCCCCCGCCGGAGG
CCTTTGCGGGAGGCAAACAGAACACTGAGTCCATCGCCGTCGCCGACGACGACGACGGCG
ACGGTGAGTCCATCGAAGCCGACCAGTACGTCGTCACGATGCCCCAGGCGTCCGGTTTGG
CCGACGAAGCGAAGGAGGGAAAGGACGACCCGGACTTTTACAAAAAGCTCGGGGTGATTC
TTCTCTGCATCAGCACCTTTTTCGTGCTGCTGGGGGTGTCCATCAAGTACGGGGTCAGCC
GCAACCAGCGCGCCGAGAACAGCATCGGGGATCCGCTGTTGGTGGAGTCCGGGCCGGCGG
AAGAAGGCACTTCCGAGCCCCCACGCACCGTGGACCAGCAGAGCGTCCCCTGGGAAGACA
CCCCCAGCGACCCCACGGGCACCAACACCACCGGCGTCGGCGTCGACGCAGAAACGGCGC
CCGGAGCCGGCGAGAAGACGGTAGTGCCCTCCCAGGTCGGGACCACGGGCGGAACCCTGC
CCGCGCCGAACCCCTTTGCGGGCTGCACCGCCGATGGCATTGTTCTCGAGGCCACCTGCG
TGGACGGGGCGTCCCTCGTGAGCATGGACTTTTGCCTGGCGAACGGCGGCGGGGCCACCG
ACGAGTTCTGGGCGTGGGTCGAGACGCCCGACGCGTACGCCCGCTTCGTCGAGACCGACT
GGGGCTGGCTGCGGGACGAGGCCTCGAGGGAGGTCGAGGGCCTCCCCGGTGGCACCTACG
TGATCGGCCTGTTTTCCGACGGCCGGCAGCTGCTGCCCGAGTACCCCCTCCTGGTGTCGC
AAGAATTTACCGTGTCCTGCCCCGAGCTGGAAGTGAGAAACTAAGGGAGCGGAGACACCA
CGGCACGGCCGCCGTTTTGCGCATCGAACCCGGAAGTCTTTGGAGTGGTCCACGCTGTGG
GCACGCTGTGGTTGTGAACGGGTCTACCATACTATTCCAATAGTATGGTATAGTATAGTA
TAGTATAGTTGAGTACAGTACAGTACAGTATAATAT
>comp2408_c0_seq1
ACCTCAGCTGTGAAAAGGTGCACCCCTCGCATCGAACGACTGGAACCAAATAAAGCAACC
CATTGCTTTTTATTCCATCGCCTATTCCATCTATCTTCGATCGGTTCCACCAGAGTCCTT
TTTTGTTTCTTCGACCGTTGCAGAACACAATATCCGATATCCAAAAAATCCGTTCTACAA
GCAGTGCTTCGTCTTGCAATCGGCCACCATGGCGACGAAGAGACTGAGGCAGTTGCTTTC
GATACAGATCTCGCGGTCTATTGTATGTAGGAGAGCAGCGGAGTTTCAGCCGGCTGAGTT
CGCCAACCAAACCTTTTCCCTTTCACACAGAGCGTTTAGCGACGACAGGTCCGGTTCGTC
GTCGGAGAAGCCACCCCCAAGGCCGCAGTCGCTGGGGAACGAGTGGCGGAAGCAGCAGCT
CGACAAGCTCGAGAACCGTTTCCAGGGAAAGCTACTGGAAACCCGCACGATTGAGTCCGA
GGAGGACCTGCAACCCATGTGGAAGGAAATGGAGGGGAGGGTGACAAGAAGGAAGGCTCG
GACACTGGCGGACACGGGCGGCAAGAGCGGGAGGAGAAACATCAAGAAAACCGACGAGGA
TGTTTGGCTGCAAGAGGGGATGTACGGTACGGACGACAACAAAGACGCCTCAAAAAAGCA
ATAGGTGGATCGAAACCGATTCGATTGCGTTCAAGCCGATCTTCTCTGTGTGCGTTTGGG
GTTACTTCGATTTGGGGCAACAATTCGTTTCTGAGGGACGGGAATACAATTCCATATTTC
TGATCCTACTGTGGATTTCATCCAAACTAGAAGTATTGATATAGCTCCAATGATAGTTTT
GTGTAAATTGGAAAACCGTCAGCTGAAACCATTTTTTCGGTAAAAATTCAAATGAATTAG
AATAAAATCAAACCTCACGCCTTTCCGCCACAATGGAAGCATTATCGGCCTCCCAGAATG
GATCTTCTCGATAATCGGTTGGTTCTTCCTCATTGAACCTCTCCGAATACTCGTTTCG
>comp2409_c0_seq1
CATTTAAATCGGTTCTGTTAGTTGTTGGATTCCATCATACTTCCTCGACACAATTTAAGC
ATTAATTTGGTGCCGTAGTGTGCAGTTTGTAGAAAAGCAGTAGCCATGGCGCCGAGCAGA
AAAGGTCGAGTTTCAGCCCCCCTGCTAACGGCGGGATTATTCCTGGCGTTCGTCGCCGGG

GCATTTTCGTTCGTCCCTCAACGACGATGGGCCCAGGGAACGTCGACCCGATCATCCCTC
TGCATGTCTGCGGAGTCTAATGCGTCGAGCAATACCAAAGGAAAGCTGCTGGTGCTCGGA
GGGACGGGCTTCCTCGGACAAACGATTTGCCGGAGAGCGCTGCTGGAAGGTTACCAGGTG
ACGAGTGTGTCGAGGCGGGGCCTCCCCCCAGCGACGAAGGACAACAAATCCGAGCAGCAG
CAAGCCGGTCCCATCGGCAGCAACAGCATCGATTATCGAGCCGGGGATGCACGAAAAGAA
AACACCATATCGTCGATTCTTCGGGAAGGTGGCTACGTCGGCGTCGTCCACTGCATCGGT
CTGTTGCTGGACGACGCCTCGGGACTCGGGACCTACAACAGGTTCGTGTCTGGATCGGGG
TCGGTTCCGGACGAGAACTCCTCCTACGACAGCATCACGCGGGTCACGGCCTTCAATGCC
ATCGATTCGACGATCCAGTACCTGAACGATCAAGAAGGCGGCCCTCCCGGGGGTCGGCTT
CCTTTTGTCTTCACGTCGGCCGCGGAAGCCGGCTGGCCCGACGTTGCCGGGGGAAGCCAA
ATAGAAAAATTCCTGGCACCGGAATGGCTGCAGCGGTATTTGGTGGCCAAGCGGGCGGTG
GAATCGAAACTCTTGGGAACGGATGCCCAAAAATTGCTGCGTCCGATCATCGTGCGGCCC
TCCCTCATTTACAGCTTGGACAAGCCAGCCAGCTATGTTCCGGTGGGAGCGTTCTTTGTG
GGCAACAGGCTTGGATTGCCCTTTGTGGACAGGCCCGTGACGGTGCAGTCGCTGGCCAAT
GCCGTGGTCAAATCCATGTCCAAAGACTCCACGGTGGAAGGCGTCCTGCGTTATCCCGAA
ATCGATGCCCTAAACTAGAATGCTAGAGAACACACGAATCAATCTCAAAAACCCTTGTCC
TTCGCCAATTTACAAACAAAAACAGTCGTACTGTTACTAAACGAGATTTTAATCTTATGC
ATGAAGTCATCAAGAATTCTAATCAAAAACTGCATTCATTCCGTACAATACAGCGTGCAA
GCTTTACGAAGTATCCCTGCACAGAGTGCAACAGTCGGGCTCGCAATCAAAGCCGAATCC
GGTCCGTCTGTTGTAGCAATCTACGACGAACTGACCAAGCTTGTCATTCAATAAGTCGCA
AACCTCAATAGGGACCGTACCCGACAATTGGTTGCCCT
>comp2410_c0_seq1
AAGAGAACGAGTACGATTACAAGAGCCCTGACATCGACTTTTAATGCGAACGGGATCATT
CTTGTTCAGAATTTCATCTGACCATCCACCATCAACGCAATCCAGAACGTCAGTCATCGC
GATCGGTCGTTCGGTGAAATTGACCGAGCAATAGAACACGAACCGGCAACGCATCCACTC
TGTTGGACCAGAAAAACAACGATGAGCACTACCCGTAGTCGGACCCGTGCTAGGGCCAGT
ATTTCGATGGCGTTGGCAGTGACCTTGTTCCTTTCTCTAGAGACGTCGGTGGTAGCCATG
ATTCGTTCCGTGGAGTCTGCATCTCCGTTGATTTCAAAGGCGCCCACCACCAAGAACATT
GATGTCGACGTTGATGTCGACGTGGACTCTGATGGCATTAACGACATCGAAGTGAACAAC
TCCAATTGCAAGCAGCGGGGGAAGATGCTCAAGGAACGATTCGATGAGCACGATTTGCTG
CGTGCCTACCAAGAAATCAAGGCCGAGTACCAGGAAAAGGCCTTCGGAGGCAGCTGTGAT
GCAACGGACGGAAGCCAAAGCATTGGCAAAGGACAAGAAGAACCACCGCAATGGAGGGTG
CTCGCCACAGTGAACCCAAACAAGGGGAAAAACACTAACACCGAACACGACGACGAACTC
ATCGTTGTGTCGATGCTGGAGCACCCCTCCGACCCTCTGTGTCCCTACGTAAAGATGGAA
ACGGTGATCCCTGTGCCCGTCGAGAAATGCTGGAACTTTTTGTCCCTGGATCGGTGGGAC
GAGACCATGCCCAGGATGGACCCCTTTTACGAGGGCCTCGACGTCTACGGAGAGTACGCG
GTGGCGAGCGGAAGCGGCGGAGAGGGCGATAAAAGCGACCACGACGCCGCGCGATGCCAG
CTCGTCCGCATGATCCTCGCACGGAAGCGCACAAAACGGATACTGGCCTTTGGAAAGCGG
GAGTTCGTCTTTGTGAGCGTCGAGGATACCCCACTGCAGGACGGAACATGGGTCTCGGGA
ACCGTCAGCGTCCAGGTGGACGAATCGTCGCTGAAATCGCCGCTGAAACGGAACAAGTCC
TATACGAGGGCCTTCCAGGACTCGATCGCCTTCTACAAGCCCCTTCCGAGCAGGGCGGCC
ACGGAGGGGAAGGCAGGCGGGGGCGATGCCGACCACCGGTCGGCCGGACCGGCCTCACC
ATCGTGTGCCGGATCGACCTCAATGACTCCAACGGAAGGGGGGGATGCATACCCATGTGG
CTTTACGTGAAAACGATCGGGGCGACGGGGGCGAAATCCGTTTGGAACATGCGCAGGTGC
CTGCTGGAAGAGTATGCCTAATGCTGCAGCAATCGGTCGAAGCCATAGCCGTGCACCTCA
CACGTGCCGCGGAACGGCAATCAAATCCTCCCACAGTTTGTATCGAAGAATACTACACAT
GGAAAATGTGCGTGCGACACAATCGTACCAATGCAAAGAAACTCTAAGGAAAAGTGTCTG
CTATCACATAAACATATTTAAGTAGAGGATTTATCATAGCAGTTAGATAGATCAGGAAA
>comp2411_c0_seq1
GGCTTTTCGTGCCTTGTACTCTACGTTTGTTTCGTTGCCTGACATCACCATCCTCGGTGA
AGATAATGGAGAAAAATCAGGAACAATTCTTTGAGCTTTGAAAGCATACTTTTGTTGCTT
TTGCGTCTTCGATATTTTCGTCTCCATAATAATTGATTATTCAATCTGTTCCTCTCATTT
CTATATTTTCTTTCCATTTTCACTCAATCGGTGCTAAACCTGGACTATGATCGATGACAT
TGATTCTTTTAAAACTATGGCAATTCGATGTGAACTCAGGGTGGTGTTCTTTATTTAATG
CCGAGTTGGCGCTGAGCGTGACGATGACCGGTATATCGACTTTGTTGTCTGTAGTCATGC
TTCCATTGAATCTAGTGATTTACACAACCGGGAGCTATTCCGACGAGGTTGTCAAATCTT
TGGACTGGTTCGCCTTATTTCTTTCACTTATCGTTGTCCTCGGAGGAATTACCTCCGGTG
TTCTTATGTCGGCTTGGTATAATTCGACTCGTTTTAATTTGATGGCCAATAAACTTGGAA
ACCTGGCCGGGGTCTCGTTGGTCATCTTTTCGGCAATCGTCTCCTCGACAGGTCAAGACA
CGAGCATATGGAACCAGAGTGCCGTATTTTATATCGGATGTGCGATACCGGCCATAGTAG

GTGTAATACTGGCTACTTATATGGCCAGCAAGCTCGGTTTGGAAAAACCAGAACGAGTTG
CTGTGGCTGTTGAAGGATGTTATCAAAACACCGGGATTGCAACTTCTGTAGCTGCATCCA
TGTTCACCGGGACACAACTAGCGACAGCAATTGGAGTTCCTTTGTATTATGGACTTGTTG
AAATGATTGTACTGGCAATTTTTTGCCTGGGATGCTGGAAATCTGGGTGGACTAAGGCGC
CTCCTGATGAGAATTTTTTCACAATGATTGGAAAATCGTACGAAGTGGAACAGGCAAGAT
TAGAATCTCCTAACGCAATCGAGGTTGTGCACAACAATAATCCTAAAGATGATGGCGACA
TTGAAGATTTGGTTTTCAACCAAACGATCGAGGGTTATCAGGTAGATGAGAGGTCGCTAC
AAGAAAAGACCAATGCAGGTTTGATGGAGCAAAGACAAATGAATGGGTCTCCCCTAAGGG
CTCGCTCGTCAGCCGAAAGTTCTCGAAGGATAGATGATGACACACTCAAAGCCGAACAAG
GAGAATTCACTTAAAGGGAAAATTCAATGTTCCTTTCCATCACCAGCATTTGCGACGACT
AGAAACTGTGGAAGTCCCACTGTTATTCAATCATCGATCCCCACACGATTTTTTGTTAGA
ACATGATTGACAAGCGCATCAAATTAATAACACTACGATAAACCGATTTTTTTTCACAAG
GTGTCGAAATCTAAAAAGTATGTGCACTAGATTTATGTTACTATCAAAATCCCCTAAAAG
AAAAGGTTCGTGCCCGATTTTTCCCTTCCTCTCACAATGACTTACAGTAATAATTTCGAA
TTTTGGTCATTTTTCCCTGTAGTGAAGTCATGCTAGCGAAAAATTTCAATGCAAATAGTT
TTCAGT
>comp2411_c0_seq2
GAGTATTTCTTTCTGTCTTGCGAAAAAGATTGCTTTCCGACAACCCGTGATTCTTCACTC
GTGAGGACCTTCTTCACACACAGTTGTTACGCACAAATACAACTTTTAAAGACCTAATAA
CCTTTAAACATGAGTCGAGCTACTGAAATCGGAGCCCACTTCCTCCTCTTTTTCCTGGTT
TTCGGAATGTCCGCAACTGTGGACATCGGTCATATGCGGAAGCAGTTGCGGAATTGGAAA
GCATTAATGATCGGATTGTGCCTTCAGTTTGTCATTCTGCCGTTCGTGGGATTTTGCATC
GTGAAAATACTGAATATGGACCCAGCAATTGGAGTATCTTTGTTGGTTGTTACATCCTCT
CCGGGAGGATCCTATTCGAATTGGTGGTGTTCTTTATTTAATGCCGAGTTGGCGCTGAGC
GTGACGATGACCGGTATATCGACTTTGTTGTCTGTAGTCATGCTTCCATTGAATCTAGTG
ATTTACACAACCGGGAGCTATTCCGACGAGGTTGTCAAATCTTTGGACTGGTTCGCCTTA
TTTCTTTCACTTATCGTTGTCCTCGGAGGAATTACCTCCGGTGTTCTTATGTCGGCTTGG
TATAATTCGACTCGTTTTAATTTGATGGCCAATAAACTTGGAAACCTGGCCGGGGTCTCG
TTGGTCATCTTTTCGGCAATCGTCTCCTCGACAGGTCAAGACACGAGCATATGGAACCAG
AGTGCCGTATTTTATATCGGATGTGCGATACCGGCCATAGTAGGTGTAATACTGGCTACT
TATATGGCCAGCAAGCTCGGTTTGGAAAAACCAGAACGAGTTGCTGTGGCTGTTGAAGGA
TGTTATCAAAACACCGGGATTGCAACTTCTGTAGCTGCATCCATGTTCACCGGGACACAA
CTAGCGACAGCAATTGGAGTTCCTTTGTATTATGGACTTGTTGAAATGATTGTACTGGCA
ATTTTTTGCCTGGGATGCTGGAAATCTGGGTGGACTAAGGCGCCTCCTGATGAGAATTTT
TTCACAATGATTGGAAAATCGTACGAAGTGGAACAGGCAAGATTAGAATCTCCTAACGCA
ATCGAGGTTGTGCACAACAATAATCCTAAAGATGATGGCGACATTGAAGATTTGGTTTTC
AACCAAACGATCGAGGGTTATCAGGTAGATGAGAGGTCGCTACAAGAAAAGACCAATGCA
GGTTTGATGGAGCAAAGACAAATGAATGGGTCTCCCCTAAGGGCTCGCTCGTCAGCCGAA
AGTTCTCGAAGGATAGATGATGACACACTCAAAGCCGAACAAGGAGAATTCACTTAAAGG
GAAAATTCAATGTTCCTTTCCATCACCAGCATTTGCGACGACTAGAAACTGTGGAAGTCC
CACTGTTATTCAATCATCGATCCCCACACGATTTTTTGTTAGAACATGATTGACAAGCGC
ATCAAATTAATAACACTACGATAAACCGATTTTTTTTCACAAGGTGTCGAAATCTAAAAA
GTATGTGCACTAGATTTATGTTACTATCAAAATCCCCTAAAAGAAAAGGTTCGTGCCCGA
TTTTTCCCTTCCTCTCACAATGACTTACAGTAATAATTTCGAATTTTGGTCATTTTTCCC
TGTAGTGAAGTCATGCTAGCGAAAAATTTCAATGCAAATAGTTTTCAGT
>comp2412_c0_seq1
CGTAGGTGCATCGTCCATCGTGCATCGTGCATCGTGCATACCACACTCGTGCCCAATAGT
CTCTTTTGCCATCGAATGCATGACCCGTCGTGACTGCAACTGTTTCACAACTCCCAACCT
TGTTTTCTAAAAGAGCAACCAGCCGATTCAATCAAGGAAGGATGCTCCGCAAATGCCTCG
CGGGCCCCTGCTTGCTCCTCATCGCTGTTGCGGCATGCGCGATATCGACTGGAAGTAGTT
CGTTCCCGGCCACCGAAGCCCTGTCGTCGTCCGGAAAACCAAAGCGGCCGATCGTCAGTC
TCACCGGAAGCGGGGACGTTTCCAAGTTTCTCTACGGCAAGCTCCAGCGGGCAACCTCCC
TGTACGGATCGGGGTTAGTGGGGCCGGCCACCGCCGTGGTGCACGGAGACAAAGACACGA
GGGAGCTCCTCCCGAAGCCCGTCGTGGCGAGCAGCTCCAGAGACCAGCGAAAGGACCTCG
ACGACATGCTGTGGAAGCAGTTTGGAATGGCCACGTGCGAGGGAATCGTCGAGCGGGAGG
ACCTGCTTACGAAAATGTCCTATCTTAAACCGCGGCTGGCCGACTTGCAGGACACGGTAC
TGTTTCTGGACGCCACCACCAGTGGCATGGGAAAGAACAGCGCCGGAAGCGGAGAGACCG
GTCTGGGGGGATTGCTTTCCGGTTTCCTGGGGGGAGGATCCAAGCCCAAGCCAAACAGCA
CACCCAAAAACAAGCCGCGATCGAACAAGAAGAAGAAGAGCATGCAGTCGGATTGCTGGG
TGGACGTCGACCTCGTTCGATCCGCCATGTCCCGCGGTTCCAGGGTATTCGTCGTCTGCG

AAAGCAGCGACACGACCCGTTGTGCGAAGGCGCTGAACAAATGTATATCGGCGATGGACG
GGGACAACAGCATCGTCACCCTCGTGTCGCCGGAGGACGGAACGATCCTCGGGGGCAACG
AGGGAGAGGATACGGCCTGGAAATCCCTCCGGCCCCAGGACCTGGAGGGGGAGCTCCTGC
AGGCAGTATCCGTCCGGAGGGATCCCGTCGCGGAACGACGCGGCGGCGGGCCCTCGGGGG
ACAACGGGAGCCGGGCCCGGTTAGCCGCGGCGTCCGCCGACTACATTGCCACCGACTCGG
CCAAGGGCCTCCCGCTGTCGCGGCAGGACCTGGCGGAGGTCTGCGTCCAGTGCGCCCTCC
GGCTGCCCACCACACCACCTGCACCGACGGAGGAGCCCGGTACGGACGTCGACGCCGACA
CCGGCAGCGCACCCACCGGTACCCCCACGCTCCGGGTGGTGCGGGTGATCCCTCGCGGAA
CGGAACGGGCCGCGAGGGACGGGATTGACTGGACCGAGCGACCCAACAAGAACTACTTCG
CGATGATGGGTGGAAAAAAGGCCAAGGCCCGGGAAGGCACCGTCGCGAGCGCGGATTGGG
CTAGAACGCTCGAGCCCCTGGTCGACGGAGGAAGCATCAGGGTCTTCCCAAAGCGTCCGA
GCGAGATGTGAAGGAGGGCTGCGCAAGGAATTATTACTTGTACTTAACAAGCTACATACT
TTTGATTAACAAACTATTGCAGTATGTTTTATCGAAGGAATTTCATCATTTCCGTTCTAT
AGAGCATTTCAGTCTTCGGGCGATGACAGAACTACCACGAATACGTTTGAAGTTCGTACT
TCGTTCTGTACGTTGTATTTTCACTTCATAACAAACTTTTACTAAGAACTTAATAATTAC
GGTAA
>comp2413_c0_seq1
GCCGATTTCAATTGTTATGAAACGCATAGTATTGCGGAAAAGATCGAGCGATCAAACCAC
CCCCAACAAAATAAGGACAGAACGAAGCAAAGACCACGGCATAGGCATTCGTCATGGACC
AAGATAACGACGACAACGAAAAGGAATCGAAGATCAACCGGCAATCCAGGGGCTTTCTCC
AAAACGCCTTGATCTTAGTGGCAACCCTAACTGTTGTTGGCCTGATCATGGGATACATCC
ACACGCACGACCCGCCGCAACTCAGCAAGAACATTCAGCACCTGGTGGAGAAATGGGAAG
ACAACCTGGTAGCACACGAAAGCGCGGCCCAGGGGCAACTAAGGACCCCGCCGATCGCAA
CGAAAGAAACCCCGCCGGAGAAGACCTCCAAACAGACCGAATTCGACCTGACCTTCCAGG
TAGAAGAACTGGACCAGAAAGTCCGGGCCCGAAAGAGCACCGAGGGCGTCCTCATGGAAA
CCGATCCCGAGGGACTGAGACTGACCAAGAAATTGCAAGCCGCGACGCTGAAACTACTTA
AGCACCGGTATGGCTACGGTACTAAACACGAACGATTCCGCGTACGCGTTGACCTCGTCT
ATCCGGAGGTCATTGTAGGTGAGGGAGAATCGAAGAAGGCATTCTTCGTGATCGAAATGG
CGCCGCCATCACTCATCCCTTGCAGCGTTTTCTACTTCATGGAAATGGTCCGGACCTACA
AGTCGGGATCCTTTCACCGGAATGCGGACCACGTGCTGCAGATCAACGCCCAGACCGAAG
CCACTGCGGGCCACAAGTCGATGCCCTTCCAGGAATACAGCCCCGAGCACCCCCATAAGA
AATACACTACAGGATACGCCGGGCGGCCCAGCGGCCCGGGTTGGTACGTTTCTATCAAAG
ACAACACTAAGAACCACGGGCCTGGCAGCCAGCAAAAACACAACCCCTACGAGGCCGATG
CCAATTTTGGGAGGGTGGTGGAGGGAATCAAGGAGGGAGTTATCGACAAGATGCATTCCG
TTCCCCAGAAGGGTTGGCTGGACAAGGACGACTGTATCATCATTCCCAAGATGACTATAC
TGGTCCAGGCACCCGACGACGAGTTGGAATGGATCGAATGGACACCCGACTTGTAATACT
ATACTAATTGAAACAAACGATGTGCTCCTTGGCAAACCATAGTTGTACACTCCGCCTGTC
TTTATGTTTCTGTCTACGGCAGTGTTTATTTTGCCATGGGAAGGAAGAAGAGTAAAAGCA
GAGTAATCGATTGGTTGTCTCATAGATTATATCATATCCTTGGTCATGC
>comp2414_c0_seq1
AAACAACAAAAGCAAACGATTGGCAACATAATTGCCTGTCCCATTATTTGGCGTCCTCAT
TACACAACATAAACGAAAAACAACTCATATCTTTGTCTCTTCCGTTATTGCTATGCAATC
AATTGCTTCGGCATCAAACAAATCCCATCGAAAATTAATTGACCACAGACCGACAGAGCA
AAAGAATTCTGCAATGAAATGCAGCTTCGAAGAATGAAGATCCCGTTCCAAAAACTACCG
GAAGAGAGAGAGCCAATCACCGAATCCGTGCATGTGTCAAAGATTCGGATTGTGCACAAG
AGGCCGTCAACTACATCACGAAGAAACTCCGACACTGGACCGAAAAGAGCTTCGCATCGG
TCGTACGCAAGAACGAAGGATACAGAGGTAACCACAACGCCGAAAAGAGCCTCTCACCTG
TTGTGTGAAAGAACAAAGGATGTCGAGGCAGCCAGTGTAGAGACGAAGAGCACTTTGCAT
CGTTCACAAGGAAATACCAAAACAATCCAGCCACCTTCGCCGACTTGTGTCAAAGAATTC
GCACGTCAAAGAATCGTTGTCGAGGAAGAAAAGCAGGGCGAAATCTTCGAGGAAGAAAAA
CATGATGAAATCTGCGAGGAAGAGGAGGCCAATGTCTCGGATGGCTGGGCCAGTAGTCAC
TGCACTCGATATGCCTGTGTTGAATCGTTTCCCGATTTTTTCTCCGACACTGAGTTCCTT
AGTTCCGTGCAGGGTAACTCAAGAATGAAAGGATTCAAAGACTTCGCTCGCGTTGTGAAT
ATGAAAGTGGAAGGTTTCCAAGAACAATATCACGTCAACCATCATGCTCTCGAAAAAATA
TTTACCCTTCGGCATTTTTGTCACAACCTTCCCTGTACCGATTTGAATGCCTGTTCCCCG
TCAGCTGTCGACGATGCCACTGATGCAAAGGACATATCCATGACGGTTGAGTTTCGGAGT
AAGAACACACCAACAATCGAGCGAATAGAAATATAGATTGTACGACGGCATATGTACCAT
TCAAATACACTAGTAAATCACACAAATACGTTGTATACTTTTGTAATGTTCCAAGAACAC
TCAAATTTCATTG
>comp2414_c0_seq2

ACTGAAACCGTTCACCACTGTCTGAACAGATTATTTACTGTAATATCTTCGAATTCAATA
CTCTTTCTTCCTGTGCAATCCGTTGCGGTCACAATGCGATTCTCTTTTCGACAAGCTACC
GACAGAGCAAAAGAATTCTGCAATGAAATGCAGCTTCGAAGAATGAAGATCCCGTTCCAA
AAACTACCGGAAGAGAGAGAGCCAATCACCGAATCCGTGCATGTGTCAAAGATTCGGATT
GTGCACAAGAGGCCGTCAACTACATCACGAAGAAACTCCGACACTGGACCGAAAAGAGCT
TCGCATCGGTCGTACGCAAGAACGAAGGATACAGAGGTAACCACAACGCCGAAAAGAGCC
TCTCACCTGTTGTGTGAAAGAACAAAGGATGTCGAGGCAGCCAGTGTAGAGACGAAGAGC
ACTTTGCATCGTTCACAAGGAAATACCAAAACAATCCAGCCACCTTCGCCGACTTGTGTC
AAAGAATTCGCACGTCAAAGAATCGTTGTCGAGGAAGAAAAGCAGGGCGAAATCTTCGAG
GAAGAAAAACATGATGAAATCTGCGAGGAAGAGGAGGCCAATGTCTCGGATGGCTGGGCC
AGTAGTCACTGCACTCGATATGCCTGTGTTGAATCGTTTCCCGATTTTTTCTCCGACACT
GAGTTCCTTAGTTCCGTGCAGGGTAACTCAAGAATGAAAGGATTCAAAGACTTCGCTCGC
GTTGTGAATATGAAAGTGGAAGGTTTCCAAGAACAATATCACGTCAACCATCATGCTCTC
GAAAAAATATTTACCCTTCGGCATTTTTGTCACAACCTTCCCTGTACCGATTTGAATGCC
TGTTCCCCGTCAGCTGTCGACGATGCCACTGATGCAAAGGACATATCCATGACGGTTGAG
TTTCGGAGTAAGAACACACCAACAATCGAGCGAATAGAAATATAGATTGTACGACGGCAT
ATGTACCATTCAAATACACTAGTAAATCACACAAATACGTTGTATACTTTTGTAATGTTC
CAAGAACACTCAAATTTCATTG
>comp2415_c0_seq1
GAACGAACGAACGAACGAACCGGTGGAGGGAGGAGAAACGGGAGCGGAGAGGAATCCGGA
GCCCAAGGATTTCGCTGGTATTGTGTTGTGTTGTGTTGTGGCGACGAGTTTCCGAGCTGA
CTTCTGTCTTTTGCATCCGAAGGTGTCGACGAAGCCGAGACGAATCTCGTGTCCGAAACG
ACGGGAGCATGGATCTGATCTGATCTGATCTTGTCGCGTTTCGGTTGCACATCGCAACTC
GCGTGCCGTACGGTACAGTACTACGAGAGGAATGGATCAAACGTCGATCGTTTTACTAGA
GTTTTGGTTTCGACTACTGTAAAAAAA
>comp2416_c0_seq1
ACGAGTACGAGTACCTGTTGCGAGGTGATGCGATGCCCAAACGGGAACTTCCCGAAGCGA
CGTCGAGTGGAATTGTTGAATGAAATAAAAGCTGCTCGAATCCAAACTCCCAAAACATTC
CCATCCGAAACGAGCTGGTGCATGTCAGTGCTGCACAGTAGCAGGTACTGTACCCTACAC
AGAGTATCCTCGTGTCAGCCTTGACAACACACTCTCTCACGACAGAAACGTAACACTTGA
CATTCCCAATACTATGGTGCAAACGATCTACAGCAATGCCAAGGTCTCCCGGAAATGTCC
TCACCGACGAGACCCAAGAAATACCAATGCTGCAATGGAACCGAACAACAAAATTGGTAG
CAGACGGATATCGAAACGGAGGAGATCGGCAATGGCGAGGAGGGCATTCTCCTTGAGCGA
GTACGCAAATGGAATAGGGACAGCGGTTGCAAACCTATCGGTTGCGTTTTTTCTCGTGAT
TCTGTTGCTGGCAGCTCCCTCTTTCGTCCGTGCCACCACTCTTCCCTTTGGACGAATAAC
AAAAAACGCACCGCAGCAGGCGAGCCAATCGCCGCTGTCTTCCCTCAAGCAACCCGCCTT
TCACATGCGTGCCGGGAGCACCGCCGCTGCCGCTGGATTCGTCACCCCGACGCTGGAGCT
GATGCCCCTCTCATATTACCTGAGGATCGCCCTGGCGGGTGGACTCGCCGGGGCCACCGG
GAGTGCCGTTTTGTATCCAATCGATTCGGCCAAGACCCTGCGGCAATCGAATCCGACTGC
CTACAGCTCGGTGAGGGATGCCCTCTGGACCATGCTCAAATCCAAAAACGCGCTCCGGCG
TGTCTACCGGGGGATTCTCCCGGCGGCCCTCGGAGCGATTCCCTCGTCGGCCCTCTATTT
TGGGGCCTACGAATCCTCCAAGGTCACCCTGAAGCGATTGGTCTTTCAAAAGAACCACAA
AAACGTTGACAGCGACGACTACCGAGCACGCCTCTTCGTCCACGGCCTTGCGGCGGCCAG
TGGTAACGTGATTTCCTCCTTTGTTTTTGTCCCCAAGGAAGTTATCAAGCAGCAGCTCCA
GATACGAGCGTCGGAAGCAACCACGGGGACGGTTCAGGCCATTGGCAGCATCGTGCGGCA
AAACGGTATCTCCGGCCTGTATGCGGGATACGGAGCCACCCTTCTGCGCAACATTCCCTC
GGCCACGATTCGTTTTGTCTTGTACGAGGAACTGAAACGAAAGTGGGCCACGGCCCCTTC
CGGAGAGAGCGGAAGCAGCCCCCAAACCCAAAAATTGCGGGCCGCCGGCACCTTTCTGGC
CGGCGCCGTCGCCGGAGCGACTTCCAGTTTCGCCATGACGCCAATCGACGTGGTCAAAAC
GCGAATGGCAACCGGGAGTTGTCCCCTCGGCGTTCGATCCTGCATGCTCCACGTCGTCGG
GGAGGTGGGCTGGAAGGGCCTGTACGCCGGTGCCGGATCGCGGGTCTTGTGGAGCGGGGC
CTTTTCGGCCATTGGCTTTGGGACCTTTGAATTGGTCAAGGGGTATCTGGGGGTATCCTC
GACCTCTTCCAAACTGTCGCCTTCTACATCCACGAAGCCGACACCATTCACGACGGCAAA
AACGTCATCCTCGCTCTAGAAGGCAATGGACCATCTGCTCACCTCGAGATGCATTCTTTT
GGTTGTTGCTTGCCTACCACCGGCACGCATTCTCTTGCTTGTCTTCCATTGCAAAAGCGG
AATGGGATCCCGATGAAACCGCTTTCGACGATTGAATGTTTGGCGACAACCGGCTACCAA
TCCACACCACGCCGAGCCATAGCCCAAACAAAACTGCAATTGGCGTTGTGATACGTTCAA
ATCCAAAATTCTGACAAAACACCCTGCTATCGAAACGCAATTTTATATGCTTCTGCTATC
AAATGATCAAAACGATTGAAGAGAGAACTATTCACTGATGTTATGAAAAATCCAGATTAA
AAGTATTATCTCAATAGGAC

>comp2417_c0_seq1
GCGACACGCCGGTCGGAATTTCCATCCAACGACACCGAGCGCGAGATCCAGCAACGATAC
GGAAAGGTAATGGGCTCCGCCGTCAACCCGGTGCTGCGGGAAGGAAATTCCGACCGGCGT
GTCGCTCCCCCCGTCAAGGCCTACGCACAGAAAAACCCGCACCGCATGGGCATCTGGTCG
AAGGCCAGCCGAACGCACGTGGCGCACATGACACAGGGCGACTTTTACGGGTCGGAACAG
TCGACCATCATCGGAGACAAGCCGACCAGCGTTCGGATCGAGTTCATACCCAAGAACGGA
AGTGCGTCCCGCATTCTCAAGGAGGACATCCCCCTCGAGGCGAGCGAGGTCATCGACGCT
TCCTGCATGAGCGCAGAAGCCCTGACTGCGTTTTTGGCAGACGAAATCGAGGACGCCAAG
GACTCGGACATTCTTTTCAGCATCCACCTCAAAGCCACCATGATGAAGGTCTCGGACCCA
ATCATGTTTGGCCACTGCATCAAGGTCTTTTTCAAGGATGTCTTTGACAAGCACGGCGAC
ATCCTGAAGGAGATTGGTGCCGACCCCAAGCAGGGCCTAGCCAGCATTTTGGAAGCCGTG
GAGAACAAGGTCGACGACAAGAAGAAAAAGAAAGAAATCATGCTGGATTTCAAGGGATCC
TACGGAACAGAGGACCGTCCTTGGCTCGCCATGGTCAATTCCGATAAGGGGATCACCAAC
TTGCACGCTCCCAACGACATTATCATCGACGCATCCATGCCCGTCGTCATTCGAGATTCG
GGAATGATGTGGAACGAGGATGGGGAATTGGAGGATTGCAAGTGCGTCATTCCCGATAGA
TGCTACGCAACCCTGTACCAAGAAGTGATCAATTACGTGAAGACCCACGGGCAGTTCGAT
GTTGCGACCATGGGAAATGTCAGCAATGTTGGACTCATGGCGCGCAAGGCCGAAGAATAC
GGATCCCACGACAAGACGTTCGAGCTCGAGGCCGGCCACGTTGCCGTGACTGACAACAAC
ACCGGTGAGGTCTACTTTCAACACATCGTCGGCGACGGTGACATCTGGAGAATGTGTCAA
ACCAAGGACGAGCCCGTCCGCGACTGGGTTCGATTGGCAATTGAACGAGGCCGGGCCACG
AACGACCGGGTGATTTTCTGGCTCGATAAGAAGCGCGCCCACGATCGTTCTTTGATTGAC
AAGGTAAACACGTACCTCAAAGACCACGACCTAAGCGGCTTGGACATATCCATCATGAAG
CCCGTCGATGCCATTCGCGTCACGATGGAACGAGCCACCCTCGGAAAGAACACCATCAGT
GTCACCGGAAACGTTCTCCGTGACTACCTAACGGATCTTTTTCCCATCTTGGAACTGGGA
ACCTCGGCAAAAATGTTGTCCATTGTCCCGCTGTTGGCCGGTGGTGGCTTGTACGAAACC
GGCGCCGGAGGCTCTGCTCCAAAACACGTGCAGCAATTCGTTGAGGAGGGTCACCTGCGA
TGGGATAGCCTGGGCGAGTACCTTGCGCTTGCGGTGGCATTCGAGCAGCTCGGTTCGACG
ACCGACAATGGCAAGGCCGTCCTACTCGGCGAATGCCTCAACAAGGCCGTCGGACGCATC
CTGAACAACCGTAAGAGTCCGTCTCGAAAGGTCAACCAAATCGACAACCGAGCGACCAAT
TTCTATGTCGCCCTCTACTGGGCGGATTACCTCGCACAGGAGGATCCGACCTACCAGCCC
ATGTTCGATGCCCTGTCGAATGCTCGCGCTACGATTGTCGAGGAATTCAAGGAGTGCCAG
GGAGATCCGGTGGAACTCGGCGGGTACTACCTTGTCGACCCCAAGAAGGTTAGCAAGGCA
ATGAATCCTTCTCCAACTCTCAACAAGATCCTGGCCTCGATCGGAGAAACGCTTTAACAA
CAGTACTAATGCACGATAGGTCGTTTTTGTATACAATGTGATTGCTTTGGCAATGTGCCA
TCCGCATATGATTATTTCTTCATCTTCTGTGTTCCAAACCTCATCGTAGTGAAGAGTAGG
TCCGGAAGCCATGAATGGGATGTCTTGGTTTTTACGCTGCTTGATTGTTTCGTTCTTTGC
GAGTTCCAAACATCGGAAATTAGTGTACGAGATTCACAGTTGTTTTCGCCGAGCATCCAT
GGTTTGCTTGCATGAGATACTGGAGTTACATTTGTAATTTTATGGTAATAATTCGTATAT
TTTACTCGTTAAAAAAAAAA
>comp2417_c0_seq2
GACGAGTACGGGACGTACGTACTGACTACACGGGTACCAGTACTACTATTCTCCTTGATC
TGAGCTGAGGAGAAATTAATGAGAAACTCGGATCTAGCCTTACAGGAAGGAAGATATTCA
TCCATCATTCCCTCGATCGTACGATATGCTACGGTACGCAACACGATTGCCTAATGGCGC
CGTGCAGTACACAACGACAGCGAGCAAGGGGCCAATGGCGTTCTGCCATACGGTGCGTCC
AAGGCATTCCTACGGTACGACGGCACAGATCCATTGTACAGAATCCGCTCCGAAGAAACT
TTTTCTTCGCTCGATCACATCGTTGAACCATTTCCAACAAATCCACCGATAGCAAACGAC
CCAACCAGTCATGATGATGCATCGCGCGGCCAGAGCAGCCCGTTCTTTGTCGCTGGCAAA
CACCCGACGCCCCCACACATTTGCTGCTTCCAAGACTGCGGCTCTCTCTCGAATGGCGAC
CAAATCGTTTTCGACCACTCGCTTCGCTGCTTCCGATGCCGCTTCTGCTTCCGATCTCGA
CATTCCTAGCGCCCCCACCTCGAACCATTCCCACACCGCTCTTCCCGGTGCCCAGGCCTC
CATCATCTACACGGAAACCGACGAGGCCCCGGCCCTGGCTACCTACAGCCTCTTTCCGGT
GGTGAACAAGATCGGGGCACTGGCGGACATCGACATTGTCCCCTGCGACATTTCCCTTTC
TGGACGCGTCCTGGCCCTCTTTCCCGAGAAGCTCGAACCCAACCAGCGCGTTCCGGACAA
CCTCGCGTACCTCGGCGAACTCGCGAAGGTGCCGGAAGCCAACATTATCAAGCTTCCCAA
CATTTCCGCTCCCCTGAACCAGCTCGAGGACTGCATCAGCGAGTTGCGCTCCAAGGGCTA
CGATGTTCCGATTTATCCCCGCGAGCCATCCAACGACACCGAGCGCGAGATCCAGCAACG
ATACGGAAAGGTAATGGGCTCCGCCGTCAACCCGGTGCTGCGGGAAGGAAATTCCGACCG
GCGTGTCGCTCCCCCCGTCAAGGCCTACGCACAGAAAAACCCGCACCGCATGGGCATCTG
GTCGAAGGCCAGCCGAACGCACGTGGCGCACATGACACAGGGCGACTTTTACGGGTCGGA
ACAGTCGACCATCATCGGAGACAAGCCGACCAGCGTTCGGATCGAGTTCATACCCAAGAA

CGGAAGTGCGTCCCGCATTCTCAAGGAGGACATCCCCCTCGAGGCGAGCGAGGTCATCGA
CGCTTCCTGCATGAGCGCAGAAGCCCTGACTGCGTTTTTGGCAGACGAAATCGAGGACGC
CAAGGACTCGGACATTCTTTTCAGCATCCACCTCAAAGCCACCATGATGAAGGTCTCGGA
CCCAATCATGTTTGGCCACTGCATCAAGGTCTTTTTCAAGGATGTCTTTGACAAGCACGG
CGACATCCTGAAGGAGATTGGTGCCGACCCCAAGCAGGGCCTAGCCAGCATTTTGGAAGC
CGTGGAGAACAAGGTCGACGACAAGAAGAAAAAGAAAGAAATCATGCTGGATTTCAAGGG
ATCCTACGGAACAGAGGACCGTCCTTGGCTCGCCATGGTCAATTCCGATAAGGGGATCAC
CAACTTGCACGCTCCCAACGACATTATCATCGACGCATCCATGCCCGTCGTCATTCGAGA
TTCGGGAATGATGTGGAACGAGGATGGGGAATTGGAGGATTGCAAGTGCGTCATTCCCGA
TAGATGCTACGCAACCCTGTACCAAGAAGTGATCAATTACGTGAAGACCCACGGGCAGTT
CGATGTTGCGACCATGGGAAATGTCAGCAATGTTGGACTCATGGCGCGCAAGGCCGAAGA
ATACGGATCCCACGACAAGACGTTCGAGCTCGAGGCCGGCCACGTTGCCGTGACTGACAA
CAACACCGGTGAGGTCTACTTTCAACACATCGTCGGCGACGGTGACATCTGGAGAATGTG
TCAAACCAAGGACGAGCCCGTCCGCGACTGGGTTCGATTGGCAATTGAACGAGGCCGGGC
CACGAACGACCGGGTGATTTTCTGGCTCGATAAGAAGCGCGCCCACGATCGTTCTTTGAT
TGACAAGGTAAACACGTACCTCAAAGACCACGACCTAAGCGGCTTGGACATATCCATCAT
GAAGCCCGTCGATGCCATTCGCGTCACGATGGAACGAGCCACCCTCGGAAAGAACACCAT
CAGTGTCACCGGAAACGTTCTCCGTGACTACCTAACGGATCTTTTTCCCATCTTGGAACT
GGGAACCTCGGCAAAAATGTTGTCCATTGTCCCGCTGTTGGCCGGTGGTGGCTTGTACGA
AACCGGCGCCGGAGGCTCTGCTCCAAAACACGTGCAGCAATTCGTTGAGGAGGGTCACCT
GCGATGGGATAGCCTGGGCGAGTACCTTGCGCTTGCGGTGGCATTCGAGCAGCTCGGTTC
GACGACCGACAATGGCAAGGCCGTCCTACTCGGCGAATGCCTCAACAAGGCCGTCGGACG
CATCCTGAACAACCGTAAGAGTCCGTCTCGAAAGGTCAACCAAATCGACAACCGAGCGAC
CAATTTCTATGTCGCCCTCTACTGGGCGGATTACCTCGCACAGGAGGATCCGACCTACCA
GCCCATGTTCGATGCCCTGTCGAATGCTCGCGCTACGATTGTCGAGGAATTCAAGGAGTG
CCAGGGAGATCCGGTGGAACTCGGCGGGTACTACCTTGTCGACCCCAAGAAGGTTAGCAA
GGCAATGAATCCTTCTCCAACTCTCAACAAGATCCTGGCCTCGATCGGAGAAACGCTTTA
ACAACAGTACTAATGCACGATAGGTCGTTTTTGTATACAATGTGATTGCTTTGGCAATGT
GCCATCCGCATATGATTATTTCTTCATCTTCTGTGTTCCAAACCTCATCGTAGTGAAGAG
TAGGTCCGGAAGCCATGAATGGGATGTCTTGGTTTTTACGCTGCTTGATTGTTTCGTTCT
TTGCGAGTTCCAAACATCGGAAATTAGTGTACGAGATTCACAGTTGTTTTCGCCGAGCAT
CCATGGTTTGCTTGCATGAGATACTGGAGTTACATTTGTAATTTTATGGTAATAATTCGT
ATATTTTACTCGTTAAAAAAAAAA
>comp2418_c0_seq1
CGAGAACATCCAAACAATAACCAAAACAAACAAAACAAGCACCAATCTTTGTCCACGGCA
TATCGAACACTCGCTCGATTCCCCAAACAATCACTAGCGCAACAACGACTAGATTCCACA
ACGACCTCCGAGTAATCCATTGTGTATTGTAGCAAAGCAAGCGAGCAAGCATTCCACGAA
CTGTTGACATTATTAAAGCACCATGACGATTCTATACGCTCTCGTATCTCGACAACAGAC
GGTCCTGGCCGAAAACACCAATGCGTCGACCACGGGCAACTTTCCCACCGTCACGCGGGT
CCTGCTCGAAAAAATCCCCCCGCAGGACGGCAGGATGACCTACGTGTACGACGAGTACGT
CTTTCACTACGTCGTCGAGAACGGGATCTGCTACCTCTGCATGTCGGACGAACCGAGCAA
GCACCGGATCCCCTTTGCCTTTCTGGAGGACGTGAAGCAGCGATTCCTGCAGCAGTTCGG
CCTGGAGCAGGCGCAGCAGGCCATTGCCTTTTCCATGAACGAGGGCTTCAAGGCGGTCCT
GGGCGAGCGGATGGACTTTTACGGGACGGAGGAGGCCGACCGGTCCATCGACAACATCGG
GACGGTCAAGTCCCAGATCGACGAGGTGAGGGACACGATGGTCCAGAACATCGAGCGGGT
CCTGGAGCGGGGAGAAAAGATCGAGCTGCTGGTCGACAAGACGGACCGGCTCAACCAGCA
GGCCTTTCGGTTCGAGTCCAACAGCCGGTCGCTCCGGCGGACCCTGTACTGGCGGCAGAT
GCGGACGCGGGCCTTTCTGGGGGCGGCCGTCCTGGCCGTCCTGTGCCTGGCGACGGTGTC
CGCGTGCGGGTTTGGCCTGCAACGCTGCCGATCGTCGTCGTAGGCGGGCAAGGGTGCGGA
GCGCGGCATCCACAACGAGTGTAGTATAATCATCGACCAATGCAGTATAGGTTTTTTTAA
AAGTAAAATTGTGGTAAGAA
>comp2419_c0_seq1
TAGTGGTTGTAGACAACGATGGTATGTATCGTTCAGTTGCGGGAACTCCACTTTTAATGA
CCGCATTCGATTGATTTTATCTGGCAAAAAAAATCAAAAAATCACGACGATAACTTTTTT
TGAGTTCCCTCTTTGTCGTCAACCGAGCTGGATCTTCCAAAGAACGGGACCGCTTGAACT
TTTGCTCATGTTCCTGTAAATATCTAACGTTGCAATTTTGTCGTTTTGACTTTTGTCGTA
CGATATTTTCGTCCAGGTGGGTTGGCTGGAG
>comp2420_c0_seq1
GCAAGAGAAAAAAAGAGCGGTCGAGGACGTGATTGATGCCTGTCAAGAAGAATAGTAATT
TGAAATTCAACCAGGACAAAAGAGACTCAAATACAAGAAAGAGAGAAAGAACGAAAAGAG

ATAATTGGAAGAGCAAAAGAGAAAGAATTCAGACCTTCAGGATAATAGTCAAAGAACGAT
CTCGCAACAACGAATCCCTCATCATCGCCGTTGTCGCATAGCAGCTGAAACGTGCCATTA
CCAGCAAAGATACGATGGCATCCAACAACGGTCAAACTCCGGGAACAACCTTCCCCGTGA
TGCCGTTGTCTATTCCCAAAGATATTGCCCAAAACGAAGCAGCGTTGTCGTCCTCTCTTC
CGTCGTCCTCGTCGTCGACGACAACGATATCAAAGCCCGGTGTCCCGGGAACACCAAAAA
GCATATACGAAATGATCATGTCCAATCCACCTCCGACGTCGGCTAACTACACCCCACAGG
CTGCAATGCATCGTCCCGTTGAATATTATAGCGAAACCAACAGGGTGGATCGAGGACGCG
GGTACAACAAAGACTACGCCCTCGACAAACTCACACGAAAATCCCTCCCGTACTTCATGC
CGTGGAAGACTTCCGGCGGACCCACCGAAATACCCCCCAACTATGCCTGGTTGATGGACG
GCTGCGCAGGAAAGGCTGCCATCGGTGTCTTTGGTGGCGGGGTGATGGGGCTGTTGATGG
GGGTATTTCTAGGAGCCCTCTCGGATTCGCAGCCTCCCATCCAGGTCGCCGGTGGAAAGG
ACGTGCCGCAGGCACCCTTCAAGGAGCAGGCCCGGTACACGATGCGGGCCACCGCCGAGA
AGTCCATGTACTGGTGCCGTAATTTCGCCTTCATCACGGGGGTCTTTGGTGGGTCCGAGT
GCCTTGTGGAAAAGTTCCGTGGCAAGCACGACATGTGGAATCCGGTCGTCAGCGGATGCA
TCACCGGCGCCGCCCTCCAGGCACACGCGGGGCCGCAGGCGGCTGCGGTTGGGTGTGGTG
GATTTGCGGCATTTTCCATCGTGATCGACAAGATTATGGGAAACTATGACTAGCGAACAA
TGAGCACAAATCAATGGAAGAAAGACAGAGACTGGTAAAGTTCGACCGAGTCAAGAACGC
TATTTTATGCCGTGTCGTACGATCCGTGTCAGTTTCATTAGTAGAAACTACATTGATTTG
AAGTTCACACAATATGCACTAACTTTTTCGTATCATAGACTGAAAACAGTAGCTACGGAG
CAAACCAGTAAAATCTTTATTCTTATTGAGTTCTTTGAGTTGATATCGATGTGGAATGTA
ATTCATCGGCATGAACTTGCTGCTTTTCCTATTATTTAAATCGAATCGACTGTCCCTG
>comp2421_c0_seq1
CTTCACCAGTTCGTGCGATAGAACATAGTTCGGATTGAAGCAAAGTACGTCAAATTATAC
GATGGATTCGGAAGCGGTATCGTCCCATGAAGATAACCCTCCATCTCGGGTGAAAAATCA
TAATAAATACAGGAAAGACAAGCCTTGGGATAATGACCAAATCGATCACTGGAAGAATCA
ACCGTGGAATGAAGAAGATGGTGACCAGTTGGTCGGTGGTCGTTTACTAGAAGAATCGTG
CTTTGCGACGCTCTTTCCAAAGTATCGAGAAAACTACCTTCGCCAGGTATGGCCAGTTGT
AACCAAAGCATTAGATGGGTATAGAATTGCCTGTGAGCTGAATCTCGTCGAAGGATCGAT
GACCGTTCGTACAACGAGCAAAACATCCGACCCCTACATTATTCTAAAGGCCAGAGACCT
CCTCAAGCTTCTGTCGAGGAGTATCCCAGTGGCACAAGCTTTGAAAATTCTAAACGATGA
ATGGGTCTGCGACATTGTCAAAATCGGTGGTTTGGTTCGAAACAAGGAGCGCTTTGTGAA
AAGAAGGCAACGGTTGTTGGGGCAGGAAGGTTCCACTTTGAAAGCCTTAGAATTGGTAAC
AGGGTGCTTCATTCTTGTTCAAGGAAATACAGTCTCGATTATGGGTCAGACTTACCACGG
ACTGAAACAAGCTCGAAATGTTGTTGTTGATTGTATGAACAATATTCATCCAATTTACCA
CATTAAGAGATTGATGATCATGAAAGAACTTGCCAAAGACGAAAAGTTGAAGAACGAGGA
TTGGGAACGATTTCTACCAAAATTTGCGAAGAAGAATGTGAAAAGAAAGAAGCCTACGAA
AAAGAAAACACCCAAGGCTTACACACCGTTTCCACCTGCACAGCAACCTCGAAAGGTTGA
TTTACAGCTCGAAACTGGAGAGTATTTTGTCTCTGAAGAACAGCGAAAGTCACAGAAATT
GGCTGCAAAGAAAGAACAGGCGAAACTCATTTCTTTGGAGAAACGAAAGCGGCGAATGGA
AGAAGACGAAATCGCACCAGCGAGGAAGACTGACAACAAACGAAAGAAAATGGCCTCCAC
GGAGAACGCAAATGTTGATGTACAAGCTTTTAAGAGCAAAATTAAGAGTCGATCAAAGGA
AGTAAACCCCAAGGATTCGAAGATATCTGATTTTGTTAAGGACTAAGCGAGCAAATCATT
TTGTTACGAAAGAGAAAGATAAATGATCTACTTGAATCATTTTGGCTCAGCACAGATTTG
TGCAAACACAAATATTTAATGCTATCAACGTATTGTATTTCTTTGCCCA
>comp2422_c0_seq1
TGAAAAATTCGGCGCGATCACACATCACAATCAAATAATCACTCTTTCTTCGTACTATTT
CATTGAATTCTTTACCCGGCCCTGTATCCTTTGTTTACACAGTGCAGTTAGCCACAGAAA
CACTTGTCGAAGCGCAGCTACTACATAGATTCATTCAATTGCGTTAAATTTAATAAAATC
GAATTGAATTCAATTTAATTTAACCGTGTTCAAGATGGAGGACAGCGATGATGCCCGGGA
TATGGCCATCAAAGAGGCCAGGCTAGAGCAAATGCTGAGTGAAAAATTGATGCAGGGATA
CGTTCTAATGGAGTCAACATGTCCGAAGTGCTCAACTCCACTCGTTAAAAACCACCAGAT
GGTCCCGCGATCGCTCCAGTGTCCGTCCAGCGACCAAAACGATACAAGACGAGTCGATAA
GTGCGTGCTGCTCCCTCAGGAATCCTTCGATCAACCCTTCAAGCCCGTCGAAGGGGTTCC
AGTGTGCGTGCAGTGCAATTCTCACGTGATCACACAAGAGACGGAGGTATCCATTCTGGA
ACAATGCGATTCCATGAAAGATAAGGGATCCATTTATGTGGCTCTAGAGGAAATCACAGA
CGACAAACCGGAAATTATACACCTAGATGGTGCCTTCGAAGTGAGTGTAAGTCCGAGTAA
GGAACGATCGAACAACACCTTCCGAGATGGTGACAATGTCACCGAGCAGAAACATGAATC
ACCAACAAGGCAAATGGTTGACATCGAGACAACCTTTGACGACGGTACTACCAACTACGA
GATTACCTTATCTCCCCGAGAAGGAGAGGAAGGCAAACCAATCGTCCTTGATGGTGACGA
AACCAAACCAATCGACCTCGTGGAAGAAGATCCACTAGGATCGTATGCTGAAAGGCGTGA

ACTCGCAACGAAGGTGTTAGGTGCGAAAATGTTGCAGGGTTACACTCTCCAGGAGACGAC
TTGTGAAAAATGCTCCATGCCTGTCATGAGCTACAAAGGAGTCGTAAAATGCGTGGTATG
CCCCGCTCTCGCGAAAAGGGCGAAGAAGAAATTGCGACAGAAGCAAAAGCATCAAGAGGC
TGCACTCGGCGGGCAGCAGCACGAGCATGAAGAACCAAGTGACGTCTCGGAAGATCTTCA
ACACCGGGACGAGCAATTGGACCGTATCAAGACCGTGCGCAAAGAGATCGAAGAAAAAAC
GCATCGCGAAGAAGAAGCGGATGCCAAACGGGCGGAAGAACAACAAAAAGCGATTAACCA
CGAAAAGGCTCGTATTTTGGATAATATGGAAGACGATTCAAAAGATCATGCAGTTTCGCA
ATCAGGACCAACAGAAGACCCTCAGATACCAATGTCGGATGAACAAGCCGCGGAGTTGGC
AAGATTGGGAGCAATTGCCAAAGAAGAGCAAAGAAAGCGCAAATTGGCAAAAGAACAACG
TCAAAAGGAAGAGGAAGCTGTCGAACTCGAAGCACAAAAGAAAGCTCTTCAAGAAATTGA
GACGAAGGCGTTGGCAGAGGAGTTGCAGCGTATTGAGGGTATGGACAAAAATACCCTGCA
AAACGATAAGGCTTTGGCAATGAAGAAGGACTTGATGGTTGAGGAGTACCGCAAACGAAT
GCTTGGAAAGGCGGCTTTGGACAATGAAGTTGCTCGACTCGAAGAAGAACGTCTCAACGA
AGAAATGGAAGCTCGGCGATTAGCAGATGAACAGCGAGCAGAATCCGAAAGCCGTATGAT
CGCTGCACTCGAAGCTGATGCCGCAATGAAGGCACTAGCTGCCGAGGATGCCATCCGCAA
AGCAAAAGAAGCCTTGGCGGAGGTTCAATCCACTAAGAAGCAAATCATTTCGCAAACGAT
CGAGATCGCAGAAAAAGAAGTCATTGCAGAGGCGGAACAAACGATCATGGCAAACCGCGA
GGACTACACTGAACCTGTCATTCTTCCTACCGATTCAGAAATCGAATCAGAGCGCTGGGA
AACTCTCAGAATGGAGGGTCGAGCGATTATGACAAGACGCATTTTGCAAGGGTGGGAAAT
TCTCCCTGAGGCGTGCAAGGGATCCGAATGTTTCATGTCGCCTCTTATCGGTCGTCTAGG
ACGCAAGGAGTGTTGCGTTTGTGGTGGGAATGGTTCTGGGACCGATGGAGCTTACTCCAA
ACAAGAAGTAGCAGAACAACAACAATCTGAGGCCGAACTAGCTCCTCCACCATCTGTCAA
GCTTGAGACTTCTGATGACGAATGCAAAGGAAAGGATTGGTATGATATGATGGACTTCGA
AAAGAAACGGGCAATCTACAGCAAAGAAATCGGAAAGAGAATGCTTCTAGGCTGGGTTCT
TGTTGACGCGACCTGTCCCAAGTGCGGAATGCCTATGATGATGGATGATCTGGGAAACAC
CGATATATGTGTTGTACACGGTGAGATGAACAAGCACTTCGATGCCTCTACAATTGCAAC
GAAGGATATGGCAGCTCTTGAAACTGCTGGGGAAGTAAGTGTCGAAGAAGTTCCCGAGGA
GACTGGAGAACAAAAGAAGCATGTTGAAAGTGGATCTGACGGAACAAGCCCTGAACTTGT
AGTGAAGAGAACGGACAGCGATCTATGTTCTACAATCCAAAAACAAGCTAAGAAGCAACA
AACGAAGAGGAAACCTGTTACGACCAGTGACCCACCGGCATTCAAGCCGCAGGCACGTGT
TTCGGTGGAAACCGCCAGGCAAATCTCTCAGGCAAGTGCTTTAGTTGAGAGCGCTAGGCA
AATCGCTGGTCAACCATCCCAAAAGGTCAACGTCAAAGACGTTATCCCAATCGATACAAA
TGACTTTGCAGATTCTTCCGCTTTTGAAAAAGTGACTGGAGCCCAAGGTCGCGAAGAGAC
CAAACCGATAGACATATCGATTGAAATGGTTGCAAACATGTTCTTGAAGAGCCCACATGG
ATACGATTTTCAGGATTTTGGACACTCGATGAACGTTGACGAAATTAAAGAGCTCGTTGA
CATTTTCCTAGTGACAAACGTCGACAACAGTGTTTCAGATGATTTCAAGCTCTCGGTGGC
CCAAAGAATTCTTAGCAAGATGAACTTGGACACTGCGAAGTCTCGAGATGACTCTTTGCC
GCTTCGTATCGAAGTAGATGAACAACAATTCAACTTCAACGACATAGAGAGTGACTATAC
GGCGACAAAATCGACGGGCAAGTTTGGAAAACGGGCCAAACCAAGACCCGAGGACCAAGC
ACGAGGCTTACCTCCCGTTTCACCAACCAAAAGAACAACGCCTCGGGGCGGTTTGAACTA
CGTTGGTAGTCCGACTTATCGCCGTTCACGAAGCGACGACATGTCCGTAGCTAGCCGTGC
TAGTACTGTGGCATCCGATGCCCTGGAATCTATTTACGACAGAATTGACGCTTGCAAGGA
AAAGCTCTTGGATCCCAATAACACATTGGATGAGCAAATTGCTACAGCTTCACTTCTCGA
AAGGTTAGCGCAAGCCGCTGTAGCAGTGAAGGAAATGGAATACGTCGAGTAAAGCTTTTG
AAGAGATGACTGGCCGGGAGCATTCAAGTTCAATGTGGAGCTAAGAAAGGGACCAACTTT
AATTATTTGTTTCATACGAAGTACTTCCCATGGTGAAAATACAAGAGTATTTTTAGATTG
TGGTAATTTTAAGTCACGTTTGTTTTATAGTAGTTGACACAAGTTTTCG
>comp2423_c0_seq1
CTCCCAAAATCCTGAAAGCAGAACGCTTTTCTATTGAAATTTCGTACCAGTGAAGAAAGC
TGCTTTCAAGTCGAATGCCGAAGAGTCATACAAGCAGTTCTTGTGAGAATTTAATGTTGT
AAGCTACTCGTATCCTACATGTTGCAACTGTCACGTCTATATTCTAAATCCTTTATTCCG
AAGCAATTCCAACAACCATGACGATCGCTGGAAGATTCGTTCCGTTCGTTCGATCAACAA
AGGCCTTCAAATCCTTGTCGAGTTCGGTGACTTCAAGATTCAATTCGAACACAGGAGGAA
GAAACAGACTCTTCGGTACTGTTGCGAGAAAATCCGGCACGTCTGTGTTCCACCAACTGG
TATCATTTTCTAGGCGGGCTTTATCCTCCTCCGCAACAGTCATCCATCCGTTGGATCCCT
TGTTGCCAGACGAAATTACACGAGCGTCGGATGCTGTTCGAAAACATCTCGGGTTGACTC
CCGACAATGCCGTTCAGAATCTTCGATTTGTATCTATTTCGTTGCTAGAGCCACCCAAAG
CCGATTACATTCAGGGAGTGAAAACTCCTCGTAAGGCTGAATGTATCACATTGAATCCGC
AAACGGGAATTGCTTCCAATTTTACCGTTACGTTGGAAAACAACGAAGCAAAGATTGTGG
AATCAACAGAATATCCAAAAGGAACCCAGCCCCTGTTGACGCCAGAAGATTGCGACTTGG

CGGAAGCGATTGTTCAGTCATCACCAGAGGTTGCCAGAGCATTACGCGATCGATATGGCA
TTATAGACTCATCTCGTGTTGCGTGTGATCCATGGTCAGTGCATTTGGCTTCTCCTGCCG
ACCAGGATCTCGTCAATTGGAGAGACGACGGAGTTCCCGGTCGATTGGTGCAAACATTTC
TTTATCATCGACAGTACGGTGATGGATTGGAAGACAATCACTACGCACATCCAATCGATA
TTGTACCGGTCGTGGATCTGAACGCACGAAAAGTCGTGACGATCCACGGAATGGAGCGTT
CCCCAGCCCCCGAAATTCCAACGTCATCGGTGCAGTATCACAGAGATTTAGTCTCGATAA
ACTCCTACCTCGAGACGGAATGGCGGAAGGATAAACTCGCACCGCTGGATGTTCTCCAAC
CCGAAGGCCCTAGCTTCACCGTGACGGGAAATCAAGTCGAGTGGAGCAAATGGAAATTCC
GTGTTGGTTTTAATAGTAGGGAAGGATTGGTTCTTCACGAAGTCGAATACGACGGACGGC
CAATTATGCATCGTGGCTCCTTGGTGGAAATGGCGGTCCCATACGCCGACCCAAACGAAC
ATTTCGCGCGAAAGTGTGCCTTTGATGTTGGAGACTACGGATTGGGTTTTTGTGCAAATT
CCCTCAGCCTGGGTTGTGACTGTCTCGGTCACATCCACTACTTTGACGCAATATTGAACG
ACTCCCAGGGGAATCCTGTTGAGTTGAAAAATGCCGTTTGTATGCACGAAGAAGATACCG
GCGTCTTGTGGAAGCACGTCGAATATCGAAATGGTCACAATGAGTCGAGGCGCTCGCGGG
AGCTCATTATTAGTTCCATCGCAACTGTCGTAAACTACGAATATCTTTTTTACTGGCATT
TGCGTCAGGATGGTTGTATCGATTTCAAAATCAAGCTTTCCGGGGAATTGTCGACAAATT
TGCTTTCCGATGGTGAAAGTGTGCCTTCCCATGGTGTCATGGTCGCACCGAATGTCAACG
CACAAATTCACCAACACATGTTTTGTGCTAGATTGGACATGTCCGTCGATTCCCATAAAA
ATAGCATCAGCGAGGTCGACATGTATTCGGAACCATTGAGTGACACCAATCCCTACGGCA
ACTGTTTCAACGTCAAGGAAACCATTCTCGAGGACGAAGAGAGCGCCAAACGGGTCTATG
ATGCGAACAAGGCACGAAGTTGGAAAATATTTAACGCGGAAGGAAAAACCAATGGCATGA
CGGGCAAACCCGTTGCGTACAAATTGCTTCCTTTCAGCATGGGACCATCTCAACCGATTC
TATTGACAGATCCATCGTGTGCAGTAACAAAGAAGGGAGAATTCGCCACAAAACATCTAT
GGGTTACACCACATAGCGACAAAGAGCGGTATCCAGCGGGAGAGTGCACGGTATTGGGAG
ATGGAAGCGACGGTCTTCCTGACTGGACAAAGGCAAATCGAAATTTACAGGGCGAGGATC
TCGTTCTATGGCATGCCTTTGGAGTGGCCCACGTGCCAAGAACTGAAGATTTCCCGGTCA
TGCCATGCGAAACAACAGGATTTACGTTGAAGCCCGACGGGTTCTTTGACGGAAATCCAG
GAATCGATTTGGAGCCAACTGTCAACAAAGCCAGCGAACTCGCAGAGGGTTGTTGCCCAA
ACACTAAGTAAAGAAATAAGAATGAGTAGCTCATGACTGTGTAAACCGTA
>comp2424_c0_seq1
TCTCGTTCTGTATCCTGAGAAACGAAGTTCATTTGACGCAGGGTAATCGTACAACAGCAC
TCGTTACAGTGACACTGCTTACCTAAAATCGAATAGTACTTCTGTGAATAACATACCACA
CCAAAGAACGAAACTGCAGTGAGAATCCACACGAAACAGGGCAATGGATAGAATTTTTGG
CAAGAAGAAACAGGCGGCGGCGGCCCCTAGCCTCTCTGATACCTCGACCAATTTGGGAGG
CCGCGTCGATGAAATGGACAAAAAGATTGCCGGTCTCGAGCAAGAATTGAGGGTCTACAA
GGACAAGATCAAGAAGGCAAGAACGCCGGCGGCGAAAAACGCCGTTCAGAAACGGGCGAT
GGAGGTTCTAAAGCGAAAAAAGATGTACGAAGGCCAGCGAGACATGGTGGCAGGACAACA
ATTTAACATCGACCAGGCGAGTTTCGGTATCGAGAGTGCCAAGGCAAACGTTCAAACGAT
TGCGGCGATGAAGGGGGCTAACCAGGAACTAAAGAGAACAATGAAGAAGGATCTGAATAT
CGACGACGTTGACGATCTAGCGGACGATATGGCGGAAATGATGGACGAATTCAACGAGAT
CAACGAGGCCCTCGGGCGGAATTTCGCAACTCCGGAAGATATCGACGAAGCCGACCTTGA
CGCCGAGCTGGAGATGCTGGAAGACGAAATGTTCGAGGAGGAAGCCGAGGCTGCCGAGGC
CACTCCGGCCTACCTGCAAACGAACGAAATGCCAGATACGCCAACTACGGAGTTGCCGCA
AGCCGAGCTGGGACTGCCGGCCGTTCCCAACTAGTTTTTTCTCTTCTTTCTGAAGAAACG
GATCGATTCAAGTTGCAAAAACTTGTTGATGTTTCTAAAATATTCTTGTCAAGACAATGT
AGCGACTGGAGTCTTTCGTTTGCGTTGTGTTGTTTTTTCCTACTGGGTTTCCAAAGCAAT
GGAACAAGAATTATGTTATAGGAGATGTCGCATTACTACGTTATAAAATTATAAAACTAT
TTCCGTTGGGTTG
>comp2425_c0_seq1
CGAACAATTTCACAGAGTGTCTCGAGTCTCAAATTAAAAAGATTGTGAATTAAGTACACC
GTTTACCAGCCTCCCACACCGCGGCAGACCAAAACACACCGAACGGCCAAAATGATCGAC
GCCATCCACGCCGGTCTGGACTACCTCATCGCAAACAAGGACTACCTTCTCCTCTTTTGT
TTCTCCGTCGATGCCGTCAACACCCTGATCAGCTTCCCCCTCATCGTCTGGAAGGGTCCC
AAGTGGCTGCTGAGCTCGACGGAACCGGGCGCCAAAGAAAAGCTAGAGGACGGCGACGCC
GGCACCGAGGTCGTCGATCTCTCGGACACGGACCGCAAGTCCTTCCGGGTCCTCTGGGAA
ATCTTTACCATCTGCTACGAGGGGTATTTCGGGTTCACCGCCAGCACCCTGGCGTGCCTC
TACACGGCCCCGGAAACGAGGCCCCTCTTTGCCTACCCGCTCTTTGCCCTCTACGTGTAC
AAGGCGAAGGCGTACATGACGACCCTTTCCACGGACTCCGCCCAGGCCAGGGCAAAGCTG
GGCTCGATCCTCTTCTTCTACTGGCCCTGCTACGGCGGCTACTGCCTCCTGCACTCGATC
GAGTGGGCCCGGGGCCGTGCCTGAGGGCCGGCAGCGGTGCAAGGGCACCCCGGCGGTTCG

GCAACACTACCACGTACAATAGCGATGGTGCCAAGCAAGGAATGCACAAAAGGGCCCGCG
GTTAGATAGCTATGCGGAACAGCAACACCTAAATAGACGTGTCTTATCTAGAAA
>comp2426_c0_seq1
GCCCAACGAAGTAGCAAATGATCAAGCCACGTGTTGCCATCCTTGTCTCTATGATATTGC
ATCTTGGCCATTCCTTCGCAACGAAATCGCCTATTGCGCATCAAAGAAATGAGATGAGTC
TCTCGGCAACTATTGGCACCGTATCGTCATCGGCATCCCCTCGCAAGGTTGCCATTATTG
GTGGCGGAGCGGCCGGACTCGTGACAGCTCGAGTACTGAAACGAAATGGGATTGAGTCGA
CAATTTTCGAGAAAGACTCAAATGACACCGGGGTTGGCGGCGTCTGGTCATACAAGCAAA
ACTCCGATACTCGCCCCATGTATCGCGGTCTAAGAACCAATCTACCAAGGGAACTTATGG
CTTATCGCGAATTCCCATGGGGGGGGGATGGAAAGACGAGGAGCTTTGTGACGCACAGAG
AAGTCAAGGATTATTTAAGTAGATATTCTAAGGTTATGGGTGTGGAAGACACAATTCGAT
ACGATTCCATTGTGACCCAATTGGAAATCTTAGAGATCGACGAAAAGAAGATGGAAAATG
ATGAGGGAAATGAACAATATTCCTGGCCTCAAATCAAAGTGAGGTGGGAATCGGTGCTGG
ATGGAATAAATGCTGGTGACAGAAATTCTCAGTCAGAAGTTTTCGACGCTGTTTGTGTTT
GCAATGGCCATTATTCTGCACCTGCCAACCCAATCATTTCCGGCATGGAAAATTTCGAGG
GAAATATTATTCACAGTATTACTTATGACGATCCCTCAGTTTTCAAAGATCAAACTGTAC
TTTGCGTAGGGGGAAGGGCAAGCGGGTCAGACCTTGCACGGGAGATATCCCACTTCGCGA
AGCACGTGTACCTTTCAGATACGACATGTCCTGCCTTGGTCGACGGAAAACCCATCATCG
AGGAAAATGTTTCGTGGGTACCGAGAACAACTGCAGTCCTCCCGAACTCTAGAATATCCT
TTGGACCTACATGTGGAGAAACCCCGAAAGTTGACACTATAATTCTCTGCTCAGGCTATG
ATTATGAATTTCCCTTTATCAATGATAATTCGAACTTAGATCTTTCGGTTGTGCCCGGTG
AACGGAGGGTGAAACCACTCTACGAACAGTTGTGGCACGCACAATACCCTTCGCTTGCCT
TCATTGGACTGCAGCATAGTGTGGTGCCCTTTCCTTTCTTTGAACTTCAAGCAGAAGCTA
TTGCTTCGCAGCTGGTCGGTGATTCGTCTTCCAGATCTTGGACTCTTCCTCCGATGAAAG
AAAGAATGGAGTGCGCTGAGAAGGATGCAACTAGTGGTGGACCGAAAGCACCTGGACGGG
TCCAAGATACGCACTACCTCGGATCTTATCAGTGGGAAGAATGCTTGAAGTATGCAAGAT
TTGCAGGTGTTTGTGACGAAGATCTGAAGAAGTACATAATGACCAACAAGGCAATATATG
ATCACTCAGGCGAAGCGAGAAAAAATCTTTTCCCTGGCGGTCCAGATACGTATAGGTATA
TCAGTTATGTACGAGATGACAATAACGAAACTTTTACTGCAGTTTCGTTACATCATGAAA
TGGATTTAGAAGAAAACCTTCATCCAGCCCATTCGGATAGTTCAGTTAAGGCAAGTTAAC
TACAAAATTCGGGACTTTATTTTATCCTTTGGTATACCGCGACACATCTGTTTTAACTCG
ACTTCCATCAATAGTGGTAGGATAGCACTGTCGTATTCGTTGTTGTTTTTGTAAAGGATT
CCTTTTCAACTGAAAGTGAACGATGTTGGTGGGAAAAGATGTTGACTGTGACATAATTGC
AATCCTCAAGATCGGGTTGGGTAGCCCATCAACCGGTATAGATAGAACTCGCAGAGAGTG
CTACGCACCTCTAAACGCTCGAGTGAAGTTATATCACAAAGATAATCAAACCTCTCAAAA
CATAACACTGCGAACCGATGGTTTCTTTTTTCGATCGACCTGTATTTACCAAATCAGACA
TTTTGGAGGGTTCAGGAAAATCTAATTCCTGGTACGACCAACTGGAGAAGTACCATATTG
AACGCAAGGTGCAACTGAATGAAGCGAAAGTTGATGCAAAGAACTCAAGTAAAGTCCATG
CGTCATTTGGCGGCAATAAATTTCATAAAGAA
>comp2427_c0_seq1
AGAAAATTTAAAACACTCCAAACCAGAATATAAACTATCACCCAAACCATCATATTTACC
TACTAGTACACCATAAAATAATCATATCACCACCACAATGGATGATGATGCTGATTCTTT
TTCTGCTGGTGATGGTTGTTGGCAATTGTTCACCATACTATGGTCATACTACTAGTGATG
CAGACCTATCTGACAACAGTGGTGGTGGCCTATACCCTCACAGTAGGAGCCCCTCCCAAG
ATGACAGCAGTGTTTTCAGTGGTGATACTACTAGTACTACTAAGTCCTCACATGATGTGA
CTTAGTACTAGTAGTAAGAACAGTTTGTCTCTTGGAACAAGTCTTTCTGATGATGCCCTT
ACTACTACAACAATGCTCCCTTTGACAACAAAATTCAAGAACTCCTCCTTGATCTTGACC
TCTCTGATTCATCTGATCTGGAGCAGGATGAATTGGAACATCTTCTCCATGACTCCTCAG
TTCTCATGAGTCCAACTACTAGTACTAGTAGTAGTAAAATTCTTCCTGCTGGTACACACA
TGTCTTTAGATGGTGATGAAAAAGCACACAGCAACAATGACCATGAAGAAGAATGCAACA
ACAATGTCACTCAGCATCAATCTCCTTGTGTCAGGAATTTTGTGCAATTCTTTGAACAAG
TACAGGACACTACTAGTCCACCCAAGAGGAGAGGAGATCAGCTCTGTATAGGAGTACTAC
TGATTCTGCTGGAAGTATCATTCATGATTATCTCCAACAATGAAGAGTTGTTTGGTGGGT
TTCATTTGAGGATGTCAAGACTGGTAACAGTCAAATACTGTCAATAAAGCTTGCATTAGT
GGTGTCATGGTGACATGGAATGGCAAGAAGTCATTGGGTGATGATGCAGAGGAGTGGTCT
CCTCCCCCCCTGTCCAGTATCCATCACTTGGAGGAGGTGCAACCCCAGGTCCCACCCATG
CTGTAAGGAACACATTGGATGTTTACTAAGATGGAAGGGTTGGTGAATTTGTTC
>comp2428_c0_seq1
CCAAAAGTGGACGAAGCAACAAAGGAACCGAATCCCGATGACGACGACAACGCAGTGAAA
CTTGATAAAGTTGGGAAGAATTCCACGGAGGACGACGATCAATACACCAAAAATGATACG

GATAACAATAAGACCGACGACGATGAAACTAAACCTCAGATCTTGGTTGAAACCTACGGC
AGCTCATCGAGAAATAAAAACGAGACGTTGCCGTTTGCAGCAAGAAATACTACAAATGGC
GCGGAGGTATCGAATGTTACCGATGTTGCCATAGAGAACTCCACAATGACATCGCTGTAC
TCCCTGAATGGTACCGATTCCATCCTGTCGCTTTCGGCTGGTACAAACAACAGCTCGATC
GGCAATACAAGCACCCTAGACAGTGGTATTCTCCCCACTAACACAACGAAACCTTCAACC
GATAGTAGCGACAAAGATATACCAACGAACTCTTCGGCGGTGGCCAACACAACCGCACCA
TCGACTTTTGCTGGAGAAAATGTCGCCATCCTCGCCGAAGACTTTAACGCTACGGCTTCC
AACGCCTCATTTCTCGCTAACGACACAGTTACAACCGAGGGTACCACAGAAACGATCGAT
GCCAATGCAAATACCACCCTGAAGCGCATGAATGCGCCCGTGATTGTGATTCCTGACAGC
GACGGTAACGACGGGCACTCTCTTTCGAACAATACAAACACGTCCAAAACTGTCAGTGGT
TCCTTCGAGTCGTCTAATAATGTAACCATCGGTGGCGGCTCGTCCGGGTCGTTCAGCAAT
TCAACAGTACCATTGGAAATTCCAACCAACGATATTCGTACCGTGGCTACGGAGGGCAAG
ATGGCAACTGAGAAAAAGAAGAAGGGAAAGTCGAGAGCTAGGGAGAGTGACAGCACGAGC
GATACTAGTGCTATGACAAGCTCAACTGAAGGCGGCGGTGCAAGCGAGGCAAAGGAGACA
TCGGAGTCTACGGAGAGTGAGAGCAAGGGCGAGACCACCGGGCAAACGAATCTATCCGGA
AAGACCACAAGTAGCAGCAAGAAAAAGAAGAAGTCGAAGTCGGGTACGATGAAGAATGAT
GGCAGTGAGAGCAGATCTGCAAACCCAGTCACAAGTGCAGTAGGTACAGGTGGCGGCGAT
AAGAAGAAGAAGAGCAAGAGCAGAAAAGGGTCGAAAACCCAATCGATAAAAGCTGAAGAG
CTCGGAGGATCAATAAAGAAGTAAACGAACTATTTTTCGC
>comp2429_c0_seq1
ACGGTACAACTGGTGTACTGTACGGTAACGTAGTTTTGCGATGAAGGTGCAGATAGACGT
CGAATCAAGGAAATCGGCAATCGGAATGAAGTCGGAAGTGACACAGAGGGTTTCGACATT
CCTTCCATACATGTCCCTCGTATGCCAGAGTGATTTCTGTTGATCTCATTTGACAACAGG
TACAAGGATCAGAAGAAGATAAATGGTCCGGATCGGATACTTTCGGAGCTTTTTGTTCGT
GCTCATAAATACTTTCAATCTTACAACTTTAGCCATGAGAATAGTTGTCCAAAAGGTGAA
GTCTGCAAGTGTCACAGTCAATGGATCCATCGTTTCTTCCATTGGACCTGGCGCTATGGC
TCTGGTAGGCATACACGAGAATGACACAGACGAGGATTTAGAATACTGCGCGAAAAAACT
GCTCGGTTGTAAACTTTGGGAAAATGATGCAGGGGCCTTATGGCGGCATGGTGTCAAACA
ACGCAACCTTGAATTGTTGTGTGTCTCTCAATTTACTCTATACGGCAAGTTAACAAAAAA
GAATCAACCCGACTACAAAGCATCTATGAAGGCAATTCCTGCTAAAGCAATGTACGAAGA
CTTTCTTGGAAAGCTGAAATCGAGCTACGAACCAGAGAAAATTCAGGATGGAGTGTTTGG
CGCTATGATGGATGTTGAATTGGTAAACGATGGTCCTGTAACGATTGTGATTGACAGTGA
TGCTGTCTCGTCATCCAAAACACCACCAAGTGATTAATGTGATTCCGTAGCAGCAGTTCA
GTTGTTGTGCCTGACTTGGACACATTTGGAGATTTATAAATTATAGCACATTTTTCTTCC
ATTGACTTTTTCAGAGGAAAATCAAATGGTGAAATGTATCATGAAGCGTCCTTAGAGCAA
ATTGTAGCATTTAGGCGAGAAAGGGGTAAGCGGGACGAATCTATCACAGATTCAAGACGA
CTTGATCATAAGGAAATGGCTCAGATCATCATTATTTCATCCATGATAGACTTTGTATAA
CAAGGCACCCAATGCGTGTTCTTTGCTGGTGTTTGTGATATTGATAGAATTTAATTTTCG
ATGACAACTAAATTGTTTCGATCTGTACTACGCGGTATACCTTGATTCAGCTTAGAGTAC
CATTACTTGCAATTTATAAATGAGCGTC
>comp2430_c0_seq1
CGCATCAAAAACGGAACAGCACGGACCAGCACAGAATTGAATCGATGAACAAAAACCGCT
CACTCCCTCTATTCCTCTCGACAGCCACCGCGTACGCCTTCGGCTTCCTGCTGTGGATGA
CAGCCACCACCATCAAGGAATGTGGCGGTACCGTTCTTGCCCTTGCCCTTGCCACTCCAC
CTACCACCATTCCCGTCGCCGTTTTGGGGGCCACCGGAAGGCTCGGGAAAGAAACCGTCG
GGGCCCTGCTAGAGAGGGGCATCCCCGTCCGGGCCCTGGTCCGGCCCTCGTCGGTCGAGA
AGCTGAAGCAGCAGCAGAATCAGAATGAGCTACCAGGCGACGGAATCGGTATCCTGGACC
ACCCCCAAGTGGAAATCATCGAGGGGGAGCTTCTCGATACGCCCGCTTCCGGAACCTACA
GCGACGACACGGTCTTGCCTTCCGAGGAGCTCGTGGAATGCCTGACCGGGTGCTCCGTCT
GCATTGCCTGCTACGGTGCCACCCGCAAAACGAAACCCTCGGAGCTGCTGTCCTGGGTGT
TGTCGACGAAGAGCAACGATGACGAACCCGACCCCTCGGAGGACACCGATCCGATCCACC
CCAAGCAAATCAACTACCGGAGCATGCTTGCTCTGGGGAAGGCCTGCGAACGGATCAACA
GCAGCAACAACAACAACAAGGACACCACCAGGATCGAGCACATCGTCCGCATTACGGGAA
AGGGAGAAGACCCTACCGGAATCTTCTCCGTCCTGCTCAACGGTTTGGGTTCCTTCTGCA
AGCTCTGGAACTACCAGGGCGAGACCGTGCTGCGCTCAAAGGCCTTTGGGGGCCCCAACT
CCGGCACCAACGAAAACGAGGGCAGCGTCGGATACACCATCGTCCGGCCGGGGGTCATGG
TGGAAGACCTAAAGGAAGACGATCTCAAGCCCGGCCAGCGCCGTTACCTGGAGCTGAAGG
GCAACGGGGGGAACGATTTACCCGTGACGCCCGTTACCCGCCCGCAGATCGCGGGTCTCC
TAGCGGAACTGGCGGTGGGGGAGGCTGCTGGTGCGGGCCGCCGGCGAAGGGTGACGCTTG
CGGCCATGAACGTCGTTGCGGAGGACGAGGCCGGAGCCAGCGGCAGCGATCTGCGGGACC

GGATCCGGGGCCTCGCCAGCGACTCGAGGAGCTTTCCGGACTCGCTGGTGCACGAGCACA
AGTCGGCCACGAAGCGGTTTTTCCGGAGGCTGGCGGCGGCCGTGGCCGCGGTTGTGGTGG
CTGCTGTGTCGCTTCTGGGCAAGGTGGTCCTGGGGTAGTTTCACACTTTGGCAATCATAC
CTTACCGTACAACACGGACGACCACCGACCAAAACCCGGCGGAGGCAGCTCCACCGTTCT
CGAGTTTAGAATGTTAGCTTTGTATAGGGTTCAATTTTGTTGCATTCGGCTGAGAGGGCT
AACCTGATCACCACACGTCGACTTCCTGGGGATTCCATCTCCGGTATATTTGCTATACGA
ACCAGCAGTACGAAAGAGCTGGTTGCTTGAACTGCATTTGAAACTGAAAGCGTGATTTTC
TGGAGAACTTGAGAATATTCTACTGCTTCATTTGTATTTAGATAATAGTAGTATTTTTTA
CAATATTGAAGGTTCAGCAAGTATACTCTCTAAAGTCTGCTGTAAGGAGTAGAAGAGATA
ACAGATACAGAGCAACAGAAACTCTAGCAAT
>comp2431_c0_seq1
ATTGCATCATGGAACTGCTCTTTCTCACAATCAGCACTTTATGTAATCCCTCTCAGCAAT
GCTCAATGTGCAAGAAGTTCGTCTGGTAACCTTCACTGACACGTTCGATATATGCAAAAG
AATCAGATTCAATAAGTAATAATTGGAACGAGCAAAACACAAGAAATGAACGAGTCTTCT
CCTGATCCTTCGCCCGACACCACGGACGACGCCGTGCTGCTCGAGAGAGCCGAAGAATTT
TACGGCGAAGAACGTCTCTTGAAGGCAGCTGAGGTGCTGAAGCAAGTGAAAGACAAAGAC
AATTGCTTGACGGATCGCCACAAAAGAATTCTTCGGTGGGCGGATCTTATCGAGAGCGGT
ATCGACAACTTGTTGCAAGATCCAGAACGAGATGGAAGTCCCTGGATCAAACAGAGGGAA
CAACACGGACATCGGGATTTTCTTGTCTTTTATCAGTTGACAGACAACAACAAACTGATA
GCACGCATCGACTGTGCCTTTGAGAGCAGCCTGTTGGTTCCTTTGCTATCGGTTTTCAAC
GAGTCGGAACTTTTCTCGTCGTGGATGCCGTGCTGGGAAAGACCCGTACGGCTCGGGATA
TCACGCTCCGAAAAACTCAAGGACGAGGGGCGAGGGAAACAAATCATCCGAGTAACAACC
TCCCTGGCGTGGCCCTTTGCCGACCGGGAAGTGGTGTTTCGCGTAATGGCAGCCGATGCC
ATCGAGGAGGACCGCAGCTGCATTGCCATCCAGGCCGTGAGCCAAACGTCGGACGACGAT
CCGGTGATACCGAAGGCGACGAGGGAAGCGGTCCGCGTGGATTTTTCGAACTTTATGTTG
ATTCAGGCTTGTCCGCCGGACCATCCGTGCGTCGCTCGATCCAAGCACCACAGCAACGAC
AAAGACCACGAGCCACTGATCTTGATGAACCTGACGCTGGAGGTCGACCCGCACCTGGGC
GTCGTGCCACAGTCGGTGCAAAATTTTGTCACGAGGACGGTAATCGGTCGGCTCTGGCTG
GCGCTCTTGCACGTCGCGGAAGACGTTCGGGACGGAAAACGTCCGAGGCACAAGGAAGCC
ATCGACGCAAAGCCCGAGCTCTACGACTGGATCAAAGAACGCGTCGGCGTGATGATCGAG
AACCTGGGGAAAGAGAAAGAGAAGTTAGGGTGCGAACCAAGTATGTAAGATATGTTTGGG
AACGTATTTAAGTGACTGGTTTAATACTTTTAATT
>comp2432_c0_seq1
ACAACAACAACAACAAAAAGCAAGCAACAACCATGGACCGAAGAAGGCAGCAGAAAATCC
AGAGCGTCCTGCTCGATTCCTGTGCGGCGGGCGCCGTCGCAATGCTCTACCACAAGCAGC
CCGAATTCTTCAATCCGTGGAACCTGAGCCCGGCGCACTGGGTGCTGGGCGGCCTCGCCA
TACGGCTGGCCATCAACTTCTACGACCACCTGGTCGTCTACGCCGTGGACTCCCTCTTTG
GACACAAGTACCTGCCGACCCGGACCACGGGAACCCCGGTCCGATACGTCGAGCTCGACG
CCAAGTCCGTCGTCTTTCTCTCCATCAACGCGGTCCACGAGTGGATCTTCGTCCAGCGGC
TGTGCCACTACCTCTGGCACTCCCAAGAGGTCTCCCTCGACCCGGCGGACCTCGGCCTCC
TCAACACGGTCGGCGCCCTCTGGGTCATGTTCGTGGTCCTGGACCTCTGCTACGCGCCCT
GCCACCACCTCCTCCACACGCCGGCGCTCTACCCCCTGGTCCACAAGCACCACCACCGGC
AGCACTACCCGACCCGGGGATACCTCGACGCCGGCAACGAGCACCCCATCGAGCACGTCA
TCGGGGTCCTCTGCACCTGGACCGCAGTCCTGGCGGCCGTCCGCCTCTGCGGGGCCCACT
CCCTCGTGGTCTTTTTGTTCTTCAACGTCCACGCGGCCCTGGCCATGCTGAACCACTCGC
CCTACGACGTCGAGTTCGACATAATACCCTTTGGGATACTCACCTACTCGGTGGCAAACC
ACGAGATGCACCACCGCAAGTTCACCGTGAACTACGCCCAGTATTCCATGTGGTACGACC
ACCTGGTAAAGACCTTTGCGGGATACGAGGGGCCGGCGGTGGCCAAGGCCGTCACCAAGC
AGCTCTAGCACCGCAGGGACCGAAGCGGGGCAGCGGGTGCGGGTGTGAGCAAGAGAGAGA
GAAAAAAAAAAAGAAAAAAAAAACGGCCATCGAACCGCTCTGGAATCCACCGTGGCCGGT
TGCAAAGACCGCTTGCTTGCCTCGCCTCTTGCCCCCCTCCGCGGGCGGTTCGGTTCGGTT
GCGATTCGTTGGTCGCTGCGAACGCCTCGTTGCCGCCGGATCGGAAACCGAACCACAGTG
TGCATTGTACAATAATAGTAGGAGAATGTGATAAAACGAAATGCGAGTTGTCGTAGCCGA
TTAAGCAAATAGCACCGTGTTCCGTCTTTTTCGTTGCAGCGTTGTGCTCGATACTACGTC
TTGTTCGTGCCATTCCTCTCACGGCAACCAGC
>comp2433_c0_seq1
CATTGAGTTACCGTACTAAAACGAAAGTATTAAATATTCCACGATGTTTTCCAAGAGAAC
TTTTTGCGCCGTCGCCATGGCCGTTGCCATGGCTTGCTCTACCGAAGCCTTTATCCAAAA
GGGAGGTGTTGCCAGCAACGTCATGATGCTCGGCAAAAAAAGCGGTGGAAAGATCGCCAC
CGCCCCAAAGGGTGCCGTCAAGAAGAGCAGGGGAGTCAAGCTCTCTGCCTCCAAGGCTGC

TGATTCCGAAGGAAAGTCAGGACTGGGAGAAACCCTTCAAGTTGGTACCTACTTCGCTCT
TTGGTACCTGTTTAACATCGCCTACAATATCTACAATAAGCAAGCCCTCAACGTCCTTGA
TTACCCTTGGACCATCGCCACCATTCAAATGGCCACCGGTATCTTCTACTTCGTCCCGAT
GTGGCTGACCGGTTTGCGCAAGGCTCCTAAGCTCTCTTTCTCTGAGCTCAAGACTCTTTT
CCCCATTGCCCTTTGCCACACCGGTGTTCACGTTGGTGCTGTTATTGCCCTCGGAGCTGG
AGCCGTTTCCTTCGCTCACATTGTCAAGGCGTCTGAACCGGTCGTCACTTGCCTCATGAA
CTACCTCTTCTTGGGACAAGTTCTTCCTTTGCCTGTTTACATGACCCTTCTCCCTATCAT
TGGAGGAGTTGCCATTGCCTCCATGAAGGAACTCTCCTTCACTGTCCTTGCCCTCGCTTC
CGCTATGCTTTCCAACGTTTCCTCGTCGGCCCGTGGTGTCCTTTCCAAGAAAACCATGTC
CGGAAAGTCCATCGGCGAAAATCTCGATGCCCAGAATCTTTACGCTGTCTTGACTGCCAT
GTCCACTTTGGTCTTGATCCCTATGATGTTGGCTATTGAAGGAACTGGATTCTTCTCTGC
CTTCAAGAACGTTGTCTCCGCCGGAGACTTCACCAACAAGTCCTTGGCTGCTCTCATGGC
TCTTGGTGGTGCTAGCTACTACGCATACAACGAGGTTGCCTTCTTGGCTCTCGGAAAGGT
CAACCCTGTCACACACGCTGTCGGAAACACAATCAAGCGTGTTGTCATCATTGTCGCCTC
GGTTGTCGCCTTCAAGACACCAATGAGCACCGGATCGATCATTGGATCCTCCATTGCCAT
TGCCGGAACCCTTTTGTATTCCTTGGCCATGAACGCCTCTAAGAAATAAATATCTTCTCA
AGACAAAAATTTTACAGCGCTACGCAAGCTACAGTATATAGTTAATCTTCATTCAGACAG
ATTCTTTTGATATGTGGAATAAATCTTTTTACCTGGTGGTTGTATCAGAAAGCCAATAAA
TTATGCATTCGTCATGCTTGGAGTGTTTGCTCTTACGAATATATTGGAAAACGATCGTTG
ATCGATGAAACATTTACACAACACTTAAATAAAAGCCAGAGTTGAGATACCTTGAAAACT
CGTTGAAGACGTCGA
>comp2434_c0_seq1
CATGCTCGTACCAAAGATGACCCCAATGGAAGCTTGTCACAAAAAGAGATTTGAATAACC
TAGTGGCTGTGTATAATGAGTCCAAAGGATGAAACAACAATACCGGTTGTCATAGTCGAA
AGCCATCAGCATGTGTTGGAACATGTCCACGATGTCTTGCGAAAGAAGAAGTTATTCGAC
CAACCCTGGTCGATGGTCCATTTCGATGCTCATCCCGACCTGGCGTGTACGAAGAGTGCA
CCCGCAATCGCATGCTTTGCTCCACGACAATACTATGGTATGGGTAACAGGAACAACAAA
GAACCCGGCGACAACGCCACTGGCAACGATCTTGACGGAGAAGCCGACGGAGAGGAGGAA
AGGAATCTATACGAACTGCTAGACTTAACACCCTCGGGCATAGCGGAGTGGATAATGCCC
TTGGTGCTGGCTGCAAACCTGAATAGGATTGAGTGGATCAAACCATTTTTTTCCAAGCAG
ATTGCAGAGGGATTTTACCGGTTCGAGGTGGGTGCCGAATACCAACGCAACAACAATGTT
GATGGCAATGTCGACGACGGCGGCACCGGCGGCGATGATTCTTCTCATCGAGACGAAATC
AATAGTTTCTTGGATCTTCCGAGGGATGCGAAATTAAAGGTCAATTTTGATCATCCCTAT
TATCTTGATGATCTTTCGGTCGTTCCGTCTGAGGATCTTGTTCTCAAACAAACACTCGAA
CTCAAAGTTTCCGAACTTCAGCGACGCCTACCGATAGAGCAGCAAAATGACACATCGGTG
TTTTGTGGCAGTGACAAAAGTGGAACGAGGAGCAGCACGAATGGTTCTGCCACCAAGGAT
GACCCACGACTCTGGATTCTAGATATTTGTCTCGATTATTTTGCATGCCAAAATCCTTTC
CTATCCGATCTAGAAACCAAAAACGAATCGGCGACCCGCGCCTTTTTGGACGTGATGGAG
GGATCAATCTTTAATCACTGCACAGACGAGGACAAAGACGGGAACAGCGTTGTGGAAATA
AGCAACAACCAGCCTGCAACCAACGCCAGCTTTGTTTACCAGACAGAACTTGTTTGCTTT
TATAAGCTCCTGAAAGAGCTGCTTTTGGACTTGTGTGGCCAGGAATGTACGGAACGAAAA
TATCAGGAAATATCGCATTCGAAGAGTGGCAATAGTCTTGTGTTCGATCAAATCTCCAAA
TACTTTGGTGGCAAAGAAAGAGCCAAAGTTTTGATCGGAACACTCGTCGACGAAATCAAT
GCGAACGACGATGGACGCGACGATGGACG
>comp2435_c0_seq1
GAGAAAGGCATTCTCCATTCCAGCAAGGATCCAAACCTGGAAGTGCTCGTGGGCTACGCA
AGATATCCAAATTTCACCATGTTGTTATCGTCGAATAGTGTCTATGTGGACGGTGCGATT
CTCACGTTTGGGATGCAGTTCACCGGTTTCGTCGTTGCCGCCGTTTTGCAAACAGAAACC
TTTTACGATGTTCTGGGAGGGATCAACTTCTTAGCCCTGGCAGTCCTTGCGTTTGTATCA
GGACAGGGGACAATCGACGCCGGCAGAAACAATGTGTTCGTTGTCTTGTTCGCGGTCAGC
CGGGGTTGGCTGCTGCTGTTTCTGGCCTGGAGGGCCCACCACCGAAAGGGCGATTCACGA
TTCGACGGAGTCCGGAACGTTCCCTCCACCTTCTTTGTCTATTGGATGGTCCAGGCGATC
TGGGTATATTGTATTTCCCTCCCGTTGCTAGCGGTGGCCGAGCATGCGCACGACCACCAA
CCCGATGCGGCAGCAGATACCGGCGGCGTCGACGGTTACTCCCTGCTGCTCGTTGCTATG
GCGATGTCCATTGTAGCGGAAATCCATAGCGATGTTGCCAAGGCGGGATGGGTGGCGAGG
GGCCGGCCCGGAGGATTCTGTCGGGAGGGCCTCTGGAAACACTCGAGGCACCCGAACTAT
GCCGGCGAGATCCTGACGTGGGTGTTTGCGGCGGTGTACGCAACCTGTGTCTGTGGCTGG
TCCTGGAAGGTGGCCACCGTTGCATCGGTCTCGCCGCTCTTTACGGTGCAGATCCTGCTG
AATACCTCGGGGACGGGCGTCTGGAACGCCGAGGGGAAAAACCTGCGGCGCTATTACGAA
GACGAAACGATCGCCGGGAACTACGAGGAGTACCGAAAGACGACACCCCCACTGTTTCCG

ACACTGTTCGTCCCCTACGAGAGTCTCCCGCGGGGGTTCCAGCGCCGGATTTGCTTCGAA
TGGAATCGCTTCGAGTACAGGAAACCGTCGAAAGAGGGAAAAGAGCAATGAGGTGAACCA
GAAACCCTGCCGATGCTCGGGGGAAAGAGGGACGAATGATTTGGAGTTTATTTGGATCAA
TGATGGGAAAGCCCTGTACAAAATGGATTCCCACAGAAAATACAACACAAGAAGTACCAA
TGATTGTTTTATCGTTCAAGTATGTGAGAATTTAAGAAATGAGGAAGGGGAACGACTAGT
TTACAGTATCATTTATAATCTGAATTCGATCACTCGAATCGCGTGTTTGGTGCACGAGAT
GTCGTTAAGGTGAAATGTGTGTTGTGTTTGCAGGATCGTAAATCGAACCGAGGAATAGAC
CTAGGTTGGACTAGCGATCACCGCTGAACAAAACTAGATCGAACCTCACACGGAATCCGA
CGTGGCCATCGTTGTTCGTGGCGGGGACGAGTTCGTCGGTGGGCGCCGCGGCGATGGATT
GGAATCGGACGGGGATTCCTCTTCGCCGTCGATTAGCCCGGCGTCGTGCATCAGACCGTT
GATGCCTAGCGGCGCAATGGCACGGAAAGCAAAGGTGGTACCGGAAAAACGCGGGGCGGA
ACACATGCAGAAACGAGTGCAAGGCTGTGTGAGCAGCGTTTGGCAATGGCTTGCTGTCTG
CTCCCATTCACTATTATATTTAATTGAGAACAATGCAAACCACA
>comp2436_c0_seq1
CGGATCAACGTTCATAACGATTATTTTTGCGAACTGCTGCAATTGACTTGTGTCATCGGC
ACGAACAGAGGAACTGATTGATTACCACATTACAGAACAGAACAGCAACTGAAATACCTG
GAGTCATTGTACTGTAGACTGCATTCGTATCCTGAAAATAAGCGCACTACGTACTACTCG
CACGAAATGTCGTCGAATCGATCCTTGTCCGAAAACAATGGCGGTGCGATCCCAATCACC
CGCGACGACGATCCCGAAGCGCAAGGAAGGGTAATTGACACAATGGAAGAAGGTCGGATG
TCTGCGTCCTTGGAACTCGAACCGCGTCGCGAATCGCTGCTGCGAGAGGACTCGGTGGAC
AGGTCCTCCTCCAATAGAAGCAGTGCCAATAGCAGCCGCGACGACGAAGGAAGGGCTCGA
GCAGTCACGATGATCGGCAGCGTCGCCAATTTATGTTCCGCTACGCTGGGGGCCGGAATC
CTGGCTCTGCCGTTTGCCTTTTATCAAGCCGGGATTATATGTGGCGTGTTGCTATTATTG
ATAAGTGCCCTCGCCACCTCGTCCTCCATCGGACTGCTGGTGGAAGCCTGTGATCGCTAC
AACCTCTCGACCTACGAAAAAATCGTCGAAAAGGGGCTCGGCCGAAACGCGAGAAAGCTT
GTCGAAATCTCTATACTGGTATTTTGCTGCGGAACCGCCGTCGGCTACGTTATCGCGGTA
GGGGACATCATGGAGCGGGTGGTGCCGTCGCTGACGACCTCCCAGAAGCAGCTTTCCATG
TCACTGGTGTGGTGTGTTGCGATGCTTCCCCTGTCGTGCCTGAAAACAATGAGGAGTTTG
GAATGCGCCTCCTCCGTCGGAATCCTGAGCATCGGAACCCTGCTGGCGGCGGCCGTGGTA
CACTTGGTGCGTGGGGACGGTAGCGGCGGCCGAGGCAATGATTATGGCTACAGCGACGAC
GAAACGCTGCTGGAGAGTTTTTCGTCCGAGCCGGCGTCGATCGCGTCCCTGCTGGGTCCC
GCGGGTGGGAGCTGGGTGTCGGTGCTGCAGGCCTGCCCCATTTTCTTTTACGCCTTCAGC
TGCCAGGTGAACGTGGCCCAGATTTTCGACGAGCTGCCCGCCGTCCGCGGCGGGGAAAAA
CTCCCCACCATGAACCGAGTCACCTCCACGGGGGTCCTGGTGTGCGGCCTGCTGTATGCG
AGCGTGTCGCTCGTGACGCTCGTCGACTTTGGCGCCTCCGTCGAGCCCAACGTGTTGTCG
AACTACGACCTCGGTCGGGGCGCTCAGCCCTTGCTGCACGCCGCCTTTCTAGGAATGGCA
CTGGCGGTGGTCATGGCGTTTCCTCTCAATATTTTTCCGGCACGTGTCAGCCTGATCCAG
ATGTGGGAGGAAACCACCAAAGAGAACAACAATGCGTCGTTGTCGTCGTCTCTGCCACCC
GAATTCGAGGCGGTCCGACAACCATTGCTATCGGAGGAACACCGGGCGAACGGCGGTGGC
CTGGACTACAATTCCGACGGAGAGAGCTCGGGTGCGGCGGGCAGCGGAGAAGACCCATTG
CACGACGAAACAGGAAGCCACGACGACGTCGACGAACCCGGGGGCTTCCGACCCGTCCGG
CACGCGTTTGTCACCCTGCTGCTGGCATCCCTGGCCCTCGGCCTGGCGCTGGTGGTCCCC
AACATATCGGTGGTCTTTGGACTGCTCGGGGGATCCACGAGCTCCCTGCTGGGCTTTGTG
GTGCCGGGACTCCTGGGGCTGAAGCTGGACCATACTCGGGTGGGCGCCTGGGTCCTGGTG
GTTTGCGGGAGCATGATCGGGATCCTGACCACCGGCGTGACGATCTACTCCATGGTCCAT
GCTTCCTAAACAGAGCGGAATGGAATGGAGCGGTGCGCACTCTACCAAATCGACAACCAC
TTGCCGTACGAACAATCCTTCACTGACTAATAATAAATAACATAGAGCGATACAATAGAA
CAAAAAACAAACGGGA
>comp2436_c0_seq2
CGGATCAACGTTCATAACGATTATTTTTGCGAACTGCTGCAATTGACTTGTGTCATCGGC
ACGAACAGAGGAACTGATTGATTACCACATTACAGAACAGAACAGCAACTGAAATACCTG
GAGTCATTGTACTGTAGACTGCATTCGTATCCTGAAAATAAGCGCACTACGTACTACTCG
CACGAAATGTCGTCGAATCGATCCTTGTCCGAAAACAATGGCGGTGCGATCCCAATCACC
CGCGACGACGATCCCGAAGCGCAAGGAAGGGTAATTGACACAATGGAAGAAGGTCGGATG
TCTGCGTCCTTGGAACTCGAACCGCGTCGCGAATCGCTGCTGCGAGAGGACTCGGTGGAC
AGGTCCTCCTCCAATAGAAGCAGTGCCAATAGCAGCCGCGACGACGAAGGAAGGGCTCGA
GCAGTCACGATGATCGGCAGCGTCGCCAATTTATGTTCCGCTACGCTGGGGGCCGGAATG
TGAGTCGAGGAAAAGAGAACAAGGGAACCAAAAAAGGAAGCTTCTACTGTGCTGCAAAAT
GATACACG
>comp2437_c0_seq1

CTCCTTCCGAACGACATCGAAGCATCATCGAAAGGGAATGAAAACCTGTGCCTCTGCATC
GCAAGTGGAGAATCCCTAACTACCGCAGCTGTTCGAAACCGCATCCAAAGCCACTAATCC
AATTCAATTCAATCCAATCCAAGGCAAAGGAAAACAAAACACAAACACAAACACCCAACA
CCCAACACAACACCAATCGCAAGCAAGCAAGCATGGGGCTGATGGTGTCCCTGATGGCCA
AGGCCGTCGCCACCGGCGCGGCGGCCGGATTTGCCTCCCACAAGCTGGTGGAAGGGCTCT
CCCCCGGGCTGCCCCCCCCGGCGAGGGCAGCCCTCGCCCTCTTGTCCTCCCTGGCCGGAA
CGGCCGGCTACCTGCGGGCCTGGATCCAGGACTACTGGGTGGACCGAAGCTACCGCGGGG
ACAAGGACCTCACCGGCACGACCGCGGTGGTGACCGGCGGAACGGTGGGGGGCCTGGGCT
TTGCCGCGGCGGAGCTCCTCTACGAGCTGGGCGCCACGGTGGTGGTGACGGTCCGGACCG
CCGAAAAGGGAAGGGCCGCGGTGGAGGGGCTGCAGGGGAAGGCTTGCGACACCGAAACGG
AAGGCAGGGCCTTTTACGTCCTGTGCGATTTCCTGTCCGAGGACTCGGTGAGGGCCTGCG
CTGCCGAGCTAAGGGCCAAGTGCGGAGGGGGGGGGATCGACTTTTTGGTCCTCAACGCCG
GGATCAGCGGCGGCGGGACCGGAGGATCCCCACAAGCCAAGCCAGAGGCCGAGCGCGACG
CCGACGCCGCCAGGGTCTGGATGACCAACCACGTCGGCCCCTTTGTCTTTGCCAACGAGC
TCATGCCGCTGCTGGTGCAAGCCGCGAGAAAGGACCCGGCTCGCCACTCCCCCCGCGCCG
TCTGGGTCTCCTCGGGGGCGCACAAGAGGGCCTCCATCGACTGGGAGGACCCCTTCCACC
CGTCCTTTGCGGCGTCGGGAATGAGGGTCTACGCCCAGTCCAAGCTCGCCAACATACTGC
ACGCGAGGGAGTACCAGAAGCGCGTCCGGGAGTGCCTCAACGAGGGAAGCAAGAAGGAAG
GCGGCCCCGACGTCAGGTGCTTTGCCCTCACGCCGGGGGCCGTGTGGACGGGAATCCTGG
GCGCAACCAACCGTCCCCTGCTCTACCCGCTCCTCTGGGCCACCATGCGGACGCCGAGGA
CCGGCGCCCAGGTGATCAAGATGGCCTGCCTCGACGAGGGCCTGGAGGGGGGGAGTACCT
CTCCAACTGCTACGCGAAGGAAACCGAGGGCAAGAACGGCTGCTCCAACGACGAGGCCCA
GTGGAAGAAGCTCTGGGAGCTGACGGAGGGCCAGGTCGCCGCGAAGGCCTACGCGGACCC
CTCGTTTTTCGGCGCCCGCGAGAAGTCGGAGTGAGCGACGCGCAGTGCGTTGCGTTGCCG
TGCGGTGCTGTGCGGTGCACGCGCCAGCGTACACGAAACGGGCTGGGACGCCGGGAACAA
CAGTAGGTAGTACGACTACGGTGACAGAACCAAGCGAGTGACACGACGACAAACGCACGG
AAAAGAGGAAGGACCTTCCTTGGAAACGATGTATTGTTCTATTTCAAGCAGTAATTAGGA
TTTTTTAAAAGTTTTGAAATGCATACATACCAATTGGATTGCTGTAATTATGA
>comp2438_c0_seq1
TTTCCACGTTGCACAAGCCACCACATTTTTGTGTTCGTATCTTCTTCCTTCGAACGTTTG
ACGAATATTCCACACGAGCCTACACGGTTTTTCCCAACTACTAACACCCAACTCCGTCCC
CGTATCGCCGAGAGACACGCTACGAAAAGAACAACCTTTTTTTGACACACAACATGAAAT
TCTCATCCTCCCAAACATTTATTGCTACAGCCACCACCATGTTTTCCATCGCCACCCGAA
GAACCGCAGGATTCGTCCACAAAAATGCCGTCGCCAAGACCGCCACCCGTGCCTATTCCG
GTAGCGCGGCAAATCTCGCCAAGGACGGCCAGGCCGAGGTGATCCTCGTTGGATGCGGAG
CCCCTAACCGCGGGATGGGATGGTACCACGCCGTTCAG
>comp2439_c0_seq1
ATGGCAATGGCAATGGCAATGGCAGCGTCGGCCTTCCCAACGACGCGCAGACGCACCAGA
AAGCGAGGAGGCGGTCGTCGTTTTCGTCCGTCATGTCCGCGCTCGGAAGCGAAACCAAGA
GGCTGTCCTGCATGAAGAAACGGAGGGAGAAGCAAGCGAAACATCACCAGGAAGGCAAGG
ACGAGGACAGGGGCATTTGCAATGGCAATGTAGACAACGACAACGACAAGAAGGAGAAGA
AGAAGACGCCGACGAAGGACAAGGACAAGGACAAGGACAAGAGGAAAAAGAAGAGGGAGA
AGAAGACGGAAGAAAACAAGCCAAGGATCTCCGAGAACGAGGGTGTGTCGTGCTCCTCCT
CCTGTTCTTCTTCGTCCACTGCCGAATTGCCTCAGGATGCCCGGGTCCCGAATCAGCAGC
CGACCTTTGTGGTGTCGGATCCCCACGGATTGGTCTTTCACGACAAGGACAGGGACAAAG
AGAAGGACAGGGACGATCACGAAACCAAGAACAAGAGCAAGAAGAAGAAGAAGGAAAAGA
AGAAAAAGAAAAAGACACCCAAGAAAAAGGACGGGAAGAGAACCCAACAGGGCACGAACG
AGAAGACCGGACCGCCAGAGGAAGCGTCGGCGTGCGAGTCCCACAAGCATTCCTGCTCGC
ATTCCTGCTCGCATTCTTGCTCTCACTCGCAACGGCGTTTGCCCTCGGACCCGAGCAGCC
TCGGACTGGACGGGGCCGTCTCGCCGGCGGATCTCGTGAGGCGCCTCCGCAGCATCCGAT
CGATCCATTCGACCATGAGCGGATCCCTGGCCCTCCACTCGCACCACGAGGGTTCCTTGT
CCGTGTCCAGGACGGATTCCAGGACGGGCCACAGCTACAACTACGGCTACAACCACAGCG
GGACCGAAAGCAGAAGCAGCTGCAGCAGCAGTTGCGACAGCAGTTGCGACAGCAGCACCC
AGAGCACGACCAGCCACCACAGCTTGGAGAACCGCGAGAACCACAACCTTTTCCCGGTCG
CAACGAAGGAAGGAATCTCGCAAGGAACCTCGGAAGGAACCTCCTCCTTGTCGTCTTTTC
GCTCGCACCAGCAGCCCCTGCAAATGTCCCAGAGCTCTCTCTTCTCGTCGCCCCCGCCCC
TGCGCTTCCGGAGGGCCACGCGGCGTTCCTCCCTGGGCAGCAAGAGCAGCGCGACGAGAA
TGACGGTGTGCTCTACCATTGAGGAAACCTTCTCGCACGGACAGGAGGAGCGGCTGCCGA
GGCCCGGCTCGCGTCGGTCCCTCACGCCCCCCAGGAGGCGTCGTTTCTCCCATCCCCAGC
AGCAACAGCAGCAACAACAA

>comp2440_c0_seq1
GAAACATTAAAACCGTGAAAACGGGTTCTATTTTCGAGATCGTACGGTACGTATGGTAGA
ATCCTACGTACGATACGAGTACGGTACGTAGTACGTACTCGTATAGGTACAAGTAGTAGG
TTTGTTTCGTATCGATTAATGGGTACGATCATACTTTTGTCGCATGGACCAAGTTTTCTT
GGAAATTACCATCTTCTACCGGTATGAGGATCAAGGAATCTCTTAGATTCCTTGGGACCT
GAGAACGGACAGACAGGTCGTTTTTCTTCTCATTCTATGAAACTGAGTGAATCGGTTCAG
TACAGTACAATACGTTACGATACGATACGATACGGTACTCGTACTATTGTGTGGCTTCAC
GATACGAGAAAGTACGTGCGTGGGACTGAACGGTACATACGTACTGGTAATCGTTTGTAC
TGGTACAGGATGGCTTGTAGAACCTGTCCGCACGACACAAACATCATCTCTGATGGGTTC
CAACAGCTGCTTACCATAGCGTTCTATTCGCATGGTGTCATCGCTCAATATGGAACAGGT
ACTAGGCTGCGTAGAAACGTACTGTAGGTAGATCGAGTACGAGTAGCAAATTTTTGAATC
GTTGATGTGATGTCGTTTGTTTGTTGTTGTTCGATTGCATTAAAAAACTTACCCGTTGCA
GAACTGAATTGAAAACAATAATACTGTAACTTCTTGTAAATATAAATACTTCTATTCGTA
CAACTCCTACGCTACGGTACGTACTCGTATGTAATTCAAAAGAAGGCACAAGAACACGTA
ATTGGAAACTCTGTTCAAAATCCTTGTCGCCGCAAACAATCCATCTCCGTACCGTATCGG
ATACTGCTGCGGAGTTTCTGCGAAGTAGACAGTGCACACTGCGACAGGAACAGCAATTAC
AGCACTTCTGCTTACTCGTACTGGGACTCATAGACAACACAAACACAGTAGAATAGCAGT
GCATTCGTTCAAGGAAGAGCAATAATTACGCTCGGAGTGACATCTTTCTGCATTCCGTTT
TCCATTTTCCATCCATCACCATGTCTTCTCATTCGGCTGGCAGCTCCAGCAGCGGCGGTG
GCGGCGATCCCCGGCAGGCCCAGAGCACAAGCAGCAACAATGGCAACGGCAACGGCAACC
CAAGAGATCCCTCGGGCTTTCTGAACACGGACGAATACGGAAGACCCTCCGAATCTTCCG
CTCCCACCGTCGTCAAGATTGTCGGGACGGCCAGGGACGACCTGAACGGTCTGCTGGGAT
TCTGCACTGCCTTCAACCGCGATCGGGAACGATACATGGTGCGGATGGCGTCGGTCGATC
CCGGGAATTCTCCCAACGCCACCGTCATGGCGCTCAAGCCGGCCAACCTCACCAAGGCTT
CGGGGCTGGAGACCTACCGAGCAATGTTCCAGCAGCTCAAAACGGATCCCAGGGTCAAAA
GAAAGCTCTCGGAATACTACCAGCAGGCCGACACTCTTTGCCGGGGGCTGCCCCTTCCGG
CCGGGACGAAACCGGAGCACGCTGCCGGGGCACTGCTGTGTGTCGTGGTGGGCCTCGTGT
ACCTTCTGGGATTGACCAGAGTGATCATGCTGTCGGCCTGCCTCGTCATGGTTGGCTGCA
TCCTGGCCGACGACGTCCTTCGCCACCGAAAGCCATGGAGGGTGGTCCTTCAGAACCTGC
CCTCGAGGTCCAACGCGGTCCTCTCGAGACAGTTTCCCTTTTTGCGCGGGAGGCTCAGCG
ATCCGGTGGCTGCCGGTCTCGTGGGCTTGCTTGTGCTGTGGACACTGCAATCGGTGTTGT
CGCGGAGTCCGGCCCCGGCACCCCGTAGCGGGACAACGGGAGGCGTCGGGTCCCGGTCCG
GCCGTGACCTTTCGGCGTCCTACGCTTCCGTGAACCGGGACGTGCTGGAAGAATTTTACA
ACCTCGGCTTCAAGGATTCTGCAGAGGGGAGGGAACGCGGGCACTCGTTTGCCGAAGAGG
TGGAGCAACTCCTCGGTAGCAGCAGCAGCAGCAGCAACATCGGTGACGACGGTGACATTC
CGTACTCGTATTCGGAAACACCGCACCTGCCGCTGACTCCACCGTCCGCCCTGGGGTCCC
CTCCAAAAACCTTTTTCCAGAAACTGGTGAGCATGCGCACGATGGGGTCTTTGTTTTATC
TGTACCGCATGGCCATGCAGATCGGAGTCGATCACTCGACGGGGATTTTTTCCGTTGCCC
AGCTCTTTTCGAACATTCAGCACTCGATGCCACCGTGGCAAAAGGGGATGCTAGCTTTTT
CGTGCTACAACCTGGTCAGCAACCTTCTGTTCTAGAGGGATCAATGGAAAAAATGGAATT
TCTTCAAATGGTATTGTTGCGATGCATGGAATCGATTTGTGATTCAAGGGACGATCCGAA
GTGAATGGATTGAAGCGAAGTCATTTGAGTAACGAAACTAATTCCAATCGAAAAGCAGCT
AGAACCATTGGCTCTAGCTAGGCAAGCACGTTGCACAGGAGGGAAAGCAACAAAATCAAT
CTTGCACTGTACCGTGCAAACGAAACTGGAAAAATAGGGATCGAACGAAGGAAGAACGGC
CCGCAGTCCTGTCTTCCAAACCAACTAAAAACATAAAAATCATGCATTTACAAACAACAG
AAACATAAGACATCCAACGAAACCAA
>comp2441_c0_seq1
AGTACAACGAATACATACCCTAGCGAAAGTCGACGTTGTTCGCTTCTCAAGACACAAGTT
AAATTTATATTCAACTTCTAATCAACAACAACGTCAATCATGTCCGACGACGAGTACAGC
GCCGCCTCCGAAGAAGTCGAGGAAGAAGAAGTCACAGATCTTTCCAACAGTGATGTGTGC
ACAAAATACCAAGAGGCATCGAAGATCGTGAACCTCACACTAGAGGGTCTCGTTGCTCAG
ACTGTCGTTGGTGCTAAAGTATTGGACCTTTGCAAATTCGGTACTACCGTTATGGAAACC
GCGGCATCGAAGCTTTATACTAAGAAAGTTAACGGAGTAGCGATCGACCGTGGTGTTGCA
TTTCCCGTTTGCGTTAGTGTTAATGATATCGTTTGCAACTGCAGTCCCCTCAACGATCCC
GAACCAGAATCAATTGCGATGACACCTTTGAAAGCCGGTGATGTTGTTAAATTCGATTTG
GGATGCCACCTCGATGGGTACATCGCGGTCGCAGCCCATACCGTTGTCGTGAAGGAAACC
GCCGACAGCCCAGCCCCACCGGAAGACGAGGAACTCGCCAAATGTGCCGTAGCTACTTAC
AACGCTATGTTGGTAGCTGCTGCTACCATTGCTGCCGGAAAGACCAACGCTGACGTCACG
AAAGCCGTTGACAAGGTCGCCAAGGCGTACGGCGTCACCCCAATTTCGTCCGTCCGAATG
CACCAAATGAAGCGATACGTACTGGATGGAGTTAAGGAAGTTGCCCTCAAAGAACCGCTA

CCAGATGATCCCGAGGAAGAAAAGGTCCCAGACTGCACCTTTGAACAAGCTGAGGTCTAC
GCAGTTGATGTCGCCATGTCTACCGGAGATGGTAAGGCCCGACCAGGCACCATGAGAACC
ACCGTTTTCAAGCGCAACGTTGAACAGCAGTACTTGCTCAAGGTCAAGGCTGCTCGCCAA
ACCCTTGCTGAGGTTGATAAGAAGTACCCAACCATGCCTTTCACAATGCGCGATTTGACC
GATGTTCGTGCCGCCAAGCTTGGAATCAACGAATGCGTTGCACACGGACTATTAACTCCT
TACCCTTCACTGCACGATTACTCCGGAAAGGTCTGCCACTTTAAATGCACCGTTCTTTTG
TTGCCATCTGGAACAGCAAGAGTAACTGGATTGAAGCTTCCTGAGTACTTCAAAACCGAT
GTACAGGCCGACGAAGAAACGCAAAAGATCTTGGCAGAGATCGCCGAAGCGGAAGCAAAG
AAGGCCGCAAAGAAAGCTGCAAAGAAGAGAAACAAGAAAAAGAAGAAATAAACAGAAAGC
CGCAAGTACGTGCTATCGAATTTCTGGAAACGGCGAAAGGAGACGGCACTAGTTTCGTTC
GTTCGATGTGGGGTTTCAAATGTCTTTGTCAACGACTCTCTCAACTCCAGTGGGGCGAGA
AGGAATTATCACTTAAATTCTACTAAAATTTAAATTGAAGATGTCTAGCTACGA
>comp2442_c0_seq1
CAGACACTCCCAAAACAACATTATCTCACTTGACAAACACAAAACACACAGTACAATACT
TGACTCCAACCAGCAAACGAAGAAACAGATAGAGCAGAGCAGAGCAAACCACAACACAAC
ACAACACGACACAATCGGCCACCCCCGCCACACCGAACGCACAAACGCACCGTCCAAAAC
CAACGCGTGCAACACAAGATGCTCCCGAAGATCTCCTTTCTGCTCGCCACCGCGGCGTTT
CTGCGCTTCGCCGCCGATCGGCTCGGCACAGCGGACGCCTACAACGTCGTCCGGGTGTGC
TCCAACAAGCACTGCTCCAAGAGGGCAAGGGGGGACGTCCTCCAAACGATCCACAACCTC
TTGGGAACCACCGGCGGGGAAACAGAAACCAGGGTCGTCGTGGAAGCATCGGGGTGCCTT
TCCCACTGCGACAAGGGCCCGAACCTGGAGGTGAAAACGAAGGACGGGACCACGATCCTC
CAGGGAATGATCGACGCCCAGATGTGCGCCTACCAGCTGGGAGAAATATCCTCGGCGTCG
CCCTCCTTCCCCCCCGTCCCCAAGCTGCTGGTGGCCGCCAGCAAGGTCATCGAGCAGAGC
GAGCGGTTCGCTTCCGAGGGCAACCACCGGGAGCAGATCCGGTACCTCGGGTCCGTCATC
GACAAGGTCGGGGCCTCCCCCTCCCTCTCCCCCCTCACGGCCGCCAACGCCCATGCCCAC
GCGCTGCGCGCCCGGGCCCACCTGAAGCTCGCGTGCAGCGAGGGGGAGGGGCTTCCCCCC
AGGAGCGAGTCGTTCGCGGCCTCGGTCCGCGACGCCCGGAGGGTCGTCCAGGACTTTTCG
GAGGTTTCGACCCCCTCGAGCCTGTCCATTGCCTACCGGACGTGGGCCGACGCTCTCCTG
GAGCAGCAGAAGGAAACGGAGGACGAACCGTCCAAACAAAGGGACCTCGGCCAGGTGGTC
GCCGTCCTGACGCAGTGGCACCGCGTGCAACCCGTCTACCGCACCAAGCTCCAGGGAGAA
ATACGGGGGTTGCTCGCCAACACGTGACTCTGGTCCGCAACAGACAAAGGACGGAGAGAG
GGTTCACCACAGCAGTAGTGTGTCGTCTCCGGAATATAAATACAGTATGTGTTTTTTACT
GTACTTCATAGAGTGTCAGGCATGGAACACGAAACAATTTCTTAATTTGGCAAC
>comp2443_c0_seq1
GGTCTTTCGCAGTATGACTAATCAATGGCATTAGATGTACCTCTCAAATATATCTTTGAA
TATAGTTTTGTATCTCTTCAATCAAACAATTTATACAAGCCATTCCTTTAACTTCGAAAG
CCTGCCGCAAATTGCACGATACGTACCGCACTCGTAACGCAGCGAAGGTCTGGTATGGAT
TGCGTTTGTAGTCGAAGCTTGTTTGGTGTATCAACGAATGTTTTTTTTTTAAGACAGAAA
TCCTCCGTTTTTGACTCTCGATAATACTAACACCGAAGTTGATTGGAATAGCCAACGGGA
AGCATCGAGCAAAACCAGTGTATCGCGGCGTTGGAGACTGACACGCAAGCTAACGGACGA
ACAAACATACTAAACTTCAACAAATTATGTCCGTGAAACAGAAAGCACAGCCACAAAGAG
TGACACTATTAACGGGGAGTGGACACAGGGTTGACGCGGATCCGCAAGAACAAAATCATG
TTACAGCTGTTGCCAAGCAAAAGCAGCAGCAACAACAACCGCCGTCGAGGAAGATTCGGC
GTTTTGCTTCGTCTACATCTACGTCGTCACTGGTTCGGGTTGTGTTCTTTGTAGTTTCTG
TACTTATGCCAATGGTGTACGTAAAATACCACTCCGAGTTGCTTCCAACGTTTGCAGTTA
TGATCGTGGAACCCAAACAGCAGCCCAACGTGTCGGTATCTCCACTCCTCAACACTAGCA
CGAGGGTTTCCACCCCGGGTGCCTACGCTGCTGCTACAGGCTCACAGCAAAAGGTGAATT
GGTCCATCGGAGTAGGAAAAAACGATAGCCAAACAGAAATCCATTCGCATACAACAACAA
CAGCAGAATCGATTGAAACTGTTTCCAAGGTTGCGTTGTTGCCCGACAACAGAGGAGGGA
ACGCGAAACGCAACAACAAGAACGATGATGGCAAGCAGAAACGAAAGAAGAGGAGGAGCA
CAAATCTCTTCAAGCTAGAGTCCAAAAGTGTGAGCGAACTCTTAGAAAATGACCAGGGGA
GGGCCATCGTGGTCTTGAGCATGGGTAAAAACAAAACCCATGAAGAAATCGCAGGACAGC
AACCGCAACCGCAACAAAAAGGACACCCTGCCATAGGCAATCAGACACAATTATCCCCCT
TAGAGCGCTTCGTGTTCTCAGCCCGTAACATTGCCAAGTTCCGTGGTTGGATTGTTGTTG
TAACAGACGCACCATCCCTCGATGATCTAGAGGAAAGCATCAACGCGGCTCTTGACGAAA
ACGATGCCAATACCCTCCTTGTACGGCCCCGCCCCGGAGACCTACTAGAAGACCGATTCC
ACGACGAGCAGATGGCGTATTTCCGGCTGAAAACCTTTGCCTTGGACTACATAGACATGG
ATCCACGGCTAGACGGCGTCCGGATTGTTTATTATATGGACGAGGCGGTGTTATTCGGGA
GCATGATCCATGAAACGATGCAGGAACTGGAGTTTATGTACGGAATCGGGAGACCTACAA
CAGAGGCGACGAAATCCGACAAAACTATTGGAACAGCCGCCCTTCAAAGCAGTGGGGATG

GGACCGAAGCTCTGGAAGATGCGGTGACAAAAGTCCAAGCTTCTTCCGCCCGTGGTAAGA
TGTGGATGTTCCGAGCCAACCACAACTCAAGCGATCACGACCATGCGATGGAGAGTGGTC
ACATCGTTCTAGATCGAAACATTTCGAGACCCTGCCTCGATCGGTGGAGACGAGAATTCG
ACGAAAAGGCGGCTGGCGAAAGAATAGTTATGGACCAGCGCCTCTTGAGCTTTCTGTTGG
AGGAAGAGGAAGAGTACAAAACCACCACGGTGCACAAAACGTCGAAACCACTGGAATGCT
CCATCCAGCTCATGGAACAGGCCCCGTATGCGGATTATCCGTCGGTCGACTCGATCCGCA
ACCAGACCTATGCGCTCATCGAGCACAAGAAAGTGAGGCATCCCCCCATGGTTCACACAG
GCAATGTCATGAGGATGGAAGCAAGAAGCCCGGAGTTCATCCGGCCTTACCTACAGGACC
TATTGCGCCTGAGAGGGAACGAAAAGGATGAAAAGGATGAAGGGCTTATTGGAACTGACA
CTTTTGCGAGGCCCGATACGCAGGAAGGAATAGAGGAAACGATATCCTCTCCTACCGCGC
GAGTTGTGGCTTGGTTGCGAACAACGCAACAAAACACATAATACTAGCAGAGATAACAGA
CCACGAATTACCCGGGCTTTTATTTATGTTGGTATGAAAAGTTGTATTGGGTCGGGTTGA
GCTGCATTGGAGTTAGCATTTCTATCGATTCAATTTTTAACACCACTCTCTCGCCTCAAT
AAAAATGAATACGTAGTTCAAACTTTCTGAGTTCCCTTCTGAAACCGATAGATACCGAAA
CACGGAGCCTGGCAGTTGATACAAATCGGTTTGAATCGGATCGAGTACTGCAGTATGCTG
GATTATTGTCTTCGACTATCAATTTCAACCAAATGTACTCGGACGGTTTCTTTTAGAACT
GCTCCTTGTTGTTCGCCACCCCATTACAGGCGAAATTAATGCACTATACCCCACAAAAGT
TTTGTTCGTTCTGCACTATGCTGGATTCAATTCTCCGGATCTCCGTTCACAAAAACTTGG
GCAAAAGTACTTTCCGTAGTGTCTTGTTTACTGCTGAGCCAGTGTGGATCCTGAGACGTC
CAGAGATCAGTGCCTCGCACCGTAGCGTACATCCCTATGAGGAAGGCAGTGTAAGTCAAG
AACGAAAACACGAGGCAACTTATACGCAGTAGGAAAATTCATCGAATAAACAAAAAGAGA
AAGATTTTTGTTGTAGTAATACCAGTGATTGAGAAGAGCAGCCAAAAATAGCACCAAAGA
ACAAACCGAGAAGAAAGACCAAATTCTAGCCCTTTTGTCATCTTACCCATAGTCGAATTT
TCGCAAGGAAATGGAACGCAAACACCTGAGGTATGAATAAGGGCTACGCGATCGAGGCTT
CTTGGCTGTGGTGGTTTCTAAGATTGATGCATCGAATCGAACTGTTCGTTCGCTACTATT
GCCTACGCCTTCCTGTCCGGGTCCATCTAGTGATGCAATCGCTTCCTTGCTAGATAAAGC
ATCATCGTTCTGAAGCGTCAGCGGCAAGCATTCATCTG
>comp2444_c0_seq1
ATCTCCGGACTGTAAAGTATAGCCGGAGACTCAGATTTTTCTTTGTACTACTATTCAGTT
CTTTGCTCACAAAATGAAGCTCCTAATAAGCAAATCAGCAAGCAACTAGCAAGCAAATGT
ACCATGGTAGCAGCAGCAAGAGGATCAAGGCCGCGCAAAGCAGCTCGCAAACATCAGGGG
CGGATTGTGACCCCCCAACAAGGCGTGACAAATCATCAGCAATGGTGTCGGTACCAAAGA
GGGAGTTTGTGCAAGCACTTGATCTAATAGGAGGAGGGATACAAGAACTAGCAACATACT
GATTAATCATTTGAGATCTTTATGGGGATCCGTCGAAATTTAGTATCATTGTCGATGAGC
AGCGTCAATGACCGACGTCCGTGTCTTAATTTCCTTACTACTTGTCCCCCCTTAATGCAA
TCGAAAAAATCATAAAAATAGTACTGTGTATATGTTAAA
>comp2445_c0_seq1
GAATGATCCCTCAGAAGTCTACAATCCATCGCAACCAACCCTGGAGCACAAGAAGCCAGC
TAGTAAACCATCTCTTCAAAAGAGGAGGCGCAAAATCTCACAGCAGCTGTAGTATCCCGA
GAGAGCTAGTTTACACAAGGAAAATTCGTCGTCGGCCTTTGGTATGAAAATTCGGAGCAA
AAACTCTCCGTCCCCTTTGTCGTTGATAATTCCAATTGCTATCATGTCGGTGCTGGCAAT
GACAAGGAATGGCCTCAAAGGACGTATTCTCGTCGACGGTTTCGCTTTGAGTCGCCAATC
ACGAGCTCGGTTCTCGAGAAGTGCTGCAGATAAGGTATCCTCGACTTCATCATCATTATC
TTCATCTTCGGATACAATCAATGGTGGCAGCTCCAAGGTAGAGAGTGACGCATCGGTTTC
TACGAGGTTGTTCTCGGAAGAGATCAATGTCTTGTACGATTCGAAATGTAACGTTTGCAA
ACTCGAAATGGATTGGTTGGTATCCCGCGACATTCGCTTAAACGGTCCGGACAACAGAAA
ATTGAGACTAACGGACCTGGAAGACAATTTCGACGAAACCGATCCCGCTAACGGTGGAAT
AGACTATGATACGGGCATGGCGGCGATATATGCCATCAAACACGACGGAACAATCTACAA
GGGCGTACCTGTGTTTGAAATTTCCTATGACCAAGTAGGACTGGGATGGATTTGGAAAAT
AAACAAAGTGCCAGCAGTCAATCGATTTTTGAATTGGGGATACGAGATTTTTGCACGGAA
CCGGACGAAGATTACCAGGGGGACGCCTCTCGAGGAATTGCTGCGATTGAATGCCGAAAA
GAAAAAGGCAAAGGCAATGGAGGATTGTGAATCTTGCAATAACAAGATCATCTAGAATAC
AAGTAACTGTTTCGTAATCAATGTCAAATGTGAAAGAACAACTTGAATAATACATTTTAA
ACCGACG
>comp2446_c0_seq1
CAAGGAATGAATGATCCGCAATCTCGCATCTTTCTTCTGAAGTAACATTCAACATAGGTG
AAAGCAAAAGAAGTACAATGAAGGCAGCGATATTCCTGTGGCAGCTAATCGTTTCAGCTG
GAGCGTTTTCTAGCTCGCCAGTCACAGGTGTTGGGCGAACACACTCGACACCACTGCGAT
CTACAGAAACAGATCTGTCATCTCCAACGGGCACCTCTTACACACTAGATGGAGAGGAAA
TTCGTGGGCCCATAACGCCGCTTCGAAACTTTATCCTTGTCAAAGTCAAGGATACTCTCA

CAGCCACGTCCGGAGGAATTCTTCTGCCCGATCAATCCAAGGAGCGTCCCACCGAAGGTT
TAGTTGTCGCTGCTGGCAAAGGAAAGATCCATCCATTCACTGGAATTCGTATTACCAACC
CAATCAAGGAGGGTATGTCTGTACTCTACGGAAAGTACGACGGAAAGGCCATTATTTACA
ATGATGATTCCTGTCAAATGATTCGGGACGACGATTGTTTGCTGTATTACAGTGGCGTTA
CGATGAAGCTGGATGCCGTCTTCCCAGTTAGAGATTATGTTCTCATAGCGCTGGATGAGA
ATCCTGAGTCTCTATCAACCAGCAGTGGTGTTGTGATCGCCAGCCAGGTAGTTGCGGACG
ATGTGCCGTGTGAGGGCATTGTTGTCAAAGTGGGTGAAGGTCGTTTGAACAGCAGTGGAG
AACTTTCACCTTCGCCGGTCGAGGAGGGAGAACGTGTAAAATTTAAGGACTATGCTGGAA
ATGATGTTACCATCGAGGGGAAGCAATACTCTGTTGTAAAAATGGTTGACATTCTTTGTT
CTCTTAGCAAGGAAGTTCAATCAATTCGAGAGACTGGTGGTGATGAAGAGGCTCCATAAA
CCATTCGGATAATTAGGTTGATAATCTGCTGACTAAAGAACTAATTTCTCTATCAAAAAA
A
>comp2447_c0_seq1
CAGGCGAGAGGAACAATTGTAAAGGAGGATGGACCTGTTGCAATCGACGAAGTGATACTG
ATCAGTCAAATCGGGTGGCATTCTTTGTCTCTCTGCGACCGAGAGGGCGCTATCAATGCA
GCATCAAAACACTGCTGATCGAACAATAAAACACTACGTTAGGTTTCCACTTCGATTCCA
TTGTATTCAATCCTGTCGAGTCGGCTACTCGAGTCGATTCTACAGTCTAGCACAACACCA
ATCCGGCTTACACCAAATCAGCGATTGATATCAATATAAATTCGAATTCTTCGTTGCAGA
TTCAGCTAGGATTGCTACCATGGCGAAGGATAAGGACAAAGACGCGGCCGCCGTGAAGAT
TCAGGCAATTGCCCGTGGCCGCCGAGACCGCAAGAAAGTCGATGGGCTCCTCCAAGGGCT
TATCGAAAGCCTAATGGCATCCAAGGAAGGAAAAAAGAAATCAGGAAAGAAATCGAAATC
CAAACCAAAGACCAAGAAAAAGGAAAAGAAGGAGAAAAAGAAGTCGGCTTGGTGCTGCAA
AGGCGACGTAAGCGCCGTCGAAGAAGAAATTATCGAGGTAGTTGAAATTGTTACCGTCGT
GGATAGCAAGCCAGAAACAACACCCAAGGAATCCACAGCAAAGACCTCTGTACCGGCGAA
AGGTGCAGCCGCCCCAAAGGCGCGCACGCCAAAGGTTTCCGAATTCGATCGCTACAACCA
ACAACAAAAACAAGAACAGGCCGCCAGAGCGATGGCGAGGCCACCGGGGCTAAAGCCCAC
AAAATCGTGGAGGGAACCACCTTCGGGGGGAAAAGCTGCCCGGCCCGAATGGATGGAAAA
GGCAAAGTCCTTTCGGGACGCATCGGAGAAATCGGCCGCGTCGTCGACGGCGGCGGCCTC
CAAGGGTCCGGTCGACCCCAGGAAGCCGCCTCCCCCGAAACCTCAGGGAAAGCACAAGAG
CGCCCTCGTGAACCGCTACCTCGAGGCCGCGGCGAGGCAAAACGAGGGCGAGAAAAAGAA
TGAGAACGAGGCATCCAAAATAATCGAGACGCGAAACCTGGAAGAAGAGAAGGCCAAAAA
GGAAGAAGAGGACGCCAAAAGGGAAGAAGAAGAGAAGGCCAAGGCGGCCGAAATCGAGGC
GGATCGGGCAAAGAACGCGGGCAAGAAAAAATGGAAGGGCAATACGACTGCCTACCAGAA
CCACCCTCCGAACGTCCCCAAGCCGTGGGAAAAGAAATCTAAGCAACCGCCGGGTTCGGA
AGTCCCGTCCGGGTCCTCCGACGCGGATGTAGCCGCAAAGCCCCGGGAGCCGGTGCCAGA
AGGCGGCGACGCCAAAGACAAGGTGGCTTCTCTCAACGACAATCTCATGAACAAAACATC
GGAAACGACCACCGGAAAAATGACGATGTCGACGCTGTCTCCACCCTCTGGATATGACGA
GAGCCAGGGGCTACCGCTGTGGTGGATGCAACTCGTTCCGCACAACACAAACCACAAGGG
GAACACTGGGTCCGCCAAGGGAAAGAACAAGGTCATATACAAAATCGACAAGAATCATCC
CCCGGCAAAGAGGGCGAAACTTCAGGCCATTTCGGAGGGCGACGAACCCGAAGCACCCAA
AACGACGTCGAACCGAAGTGCTCCGGCGTCTTCGTCTCGTAAGAAAAAGTCCGGGATTTT
TGCCAAGTGGTTCTCTCGCCCCCGCAGGCGCTCGATGGGGGAATTCTAACCACATCGACA
CCGACCATCGTCCGGGGATCTATTAGGCGCCGAAAGATGTGGTAGTGTTTTCCTCTCCGG
AAACCGTGATGCCGGCGAGGGCGATCACACATGCAGTGCCGTCTGTGAGCAGCAGTAGAA
TCAATGCCACTGAAAAATTGATACTAATAGTAGTGTCAATCAATCCATACGTGCTTTTCA
AACTTCTATTGTTCGGGGT
>comp2448_c0_seq1
TTTGACAAAGTCGTTTGCCAAAGTACTGGGACATATATAGCAAATGACTGACATCTATCT
AGATGGAAAGGCAACGGGACAAGGTGTCGTTTTGAATTCATTCAGTGCACCGAACCATTG
CACGCGACGGACGTACGGTTGGGAGGCATGAAAAAGGATGGAATAGCGGAACCTCCGGGC
AGAAGCGGGTATGCGCACGTTTCGCAAAGGCCCGGACACGCAAAGCATTGCCGAGAGGCG
AGGGGTGCGGTGTGGAAGGACTTGTTGGCGGGAGGGATAGCGGGAACGGTCGGCATCGGG
ATCGGACACCCGTTCGATACCATCAAGGTTCGTTTCCAGCAAGGGCACACACCACCGGAA
GCAAAGCGTGCCGGGATTCCCACGGCTCGGACCCCGCAGCCGTCTCCGTATCGCGGGCTG
TACCGGGGGCTCGGTGCGCCGCTGGCGACCGCCGCACTCGTCAACGCGAGTGTCTTTTGC
GTCTACGGGGCAACCGCGAGGCAGTGGGACGAGCTCTTTGGGACCACCACCACGTGGGAG
GAATCCTCGACCGCATCGGCAATCGGCAGGGAGGCCTTTTGCGGTGGGGTCACGGGATTC
GCGACCTCTCTGCTGATCTGTCCCATAGAACTTGTCAAGGTCCAGCTCCAGACGCTGCGC
CCACCGCGGCAGGGTTCCTTCCAGCCATCCCCGCCGGGAACCCTTTCCGTCGCACGGCAG
ATTTTGGGGAGCGAGCACGGCCTCCGCGGATTGTACCGGGGGCTGCTCGCTACCGCGTTG

CGCCAGACGCCTTCGCTCGCGATCTATTTTCCGGTGTATCGGTCCCTAAAGGACCGATTG
GGCGGGACGGGGGGGACCCCAACCCGAGAGCCGTCCTGGTACTCATCGGCACTGGCGGGC
GGGCTGGCGGGGTGTGTATCGTGGGCGGTGGTATACCCGATCGACGTTTTGAAGAGCCGA
ATCCAATCGCTACCGATGGAAGCCCCCGCCCGGGAGCGGTCGCTCCGCCGTGTTGCCCGG
CAGACCCTGCGAAACGAGGGCCTGGTGCGATTGATGTGCTCGGGCGGACTCGCCGTTACG
CTCTTGCGGGCGTTTCCGGTGAACGGAGCGATATTCTTCGTCTACGAACTGGCAAGCGAG
CGATTGGCTCCGTTGGGCAACCCTCTGGGCAGTAATGGCAAATCCGTGATCGGTGCGGAG
ACCAGCCTGGCAGCGGCTGGGATTGGAGGTGCGAGCTCGAGGCTCCGGGGCAGAATTACC
AGCCAGGTAGAATGCAGTGAATTCAATGCTACCGCCGATGTATGACACTAAGTGCAACAA
AAAAGGTGTGGCATATATTGTTGTAAACTATTGATAAAAAACTTGTAATCATATGTTGG
>comp2449_c0_seq1
CCTCTTTCGACAATACCCAGACACAAACACAATCTTAACGAAAGAATGATTGCGCTACCG
TGCAAATCAGAGAGTAAAAAGTAATCACATCGATTAAATCCACGGCGACGTTAAAGAATC
CGTCTGTGAGAAGTAGGAAAAGGAACATAGCGTCATGGGCCAGAAAAAACAAGACGATGA
CGCTGGCGTCGCATCGTCACTACCGAAATTGTCTTTCATTGACAGCATGAAAGCTATGCG
CTCCAATTTCAAATACACCACAGTGCTTTGTTGCTTCAAAGCAAAGAAGCAGACCAAAGT
TTCCTCGTTGGAATTCAAGATTTTGCAACTGAAAAAGAAGTTTGGGGTTGACTATCTAAC
GTTAGTGAGCGAGAATGCACCAGTCGAGGACCTGAAGGATTGCTTGAAGACAGCTCTTGA
AGAACTTTCCAATCTACAAGAAGAGATAGATGATACCCTCGATGAGATCGATGAGAAAGA
GCATAAAGTGAATGAAAAAATAAGTAGGACGAAATCCGCCGACGAATTATTTGGTCCGAA
CAAGAACAAAACAGTTCGATCGAAACCGAAGCAAAAGAAAAGTAGAAGAGTCGATAACTC
CGAAAAAGAAAGTGACTGGAGAGATGATGATGGATCCTTCAAGTGACCGGGTAGAGTGTT
CTACAACGCAATTGTTATTTGTAGATGTCAAGAGGGGAATCCAAAAAAAAATTAAATGGA
GAAGAAACGAAAAGAGCCGCAGAGAACACAAAATCAGATATGAACTAGCGAAGGAAAAAG
GCACTACAACATCCAACCTACCGTATCATTACAGGCGCAGCGGTGTGTTCACCATTTTGT
AAAACACGTGATACAATAGTCACAGAAACACCCATTTATTGCAGTCTCTATCGTAATAAT
GAACAACAACATTGACCTACTACAGACGAGTTACATAGCTTGTGTATTGCCATGACATGA
TAGGTTAAAATAGTCAATCGTCA
>comp2450_c0_seq1
CTTCTTCATTTGTTTCCATCGCATTGTCGTTCTACCGCAAGGAACAATAGCTCCACTAGC
AACGACTAGCAACGCGAAGAAGAGAGATTGCTAGCTCTAACGCAACGCAACGCAACTCAA
CGCAACACAACACGAATCGACGGAACACTCTTCACACGACCAAACGAATCCAAATTCCAT
CCCAACCCGAAACAAACAAGTTTGCACCAACGCCAAACACAGTCCGGTTACGATGAGGGC
CCTGTCCGTCTTTCGCTACGCCCTGTCGTGGTCGCTGCTGCGGTTCCTGGTGGCTGCTTC
GGCTTCGGCTTCTGCTTCCGCCTCCGCCCCTGCCCTTTCCCGACGGTTCCTCCGATCCCT
CCAGCAGGAGCAGGACGCTGCCCCCCCCGTGGTCCGGACCGATCTGCTGCGGCTGGTCCC
CTTCCAGATCCAGATCGCCATTCGCGACGAGACGACCGGGGAGGAAGCCGAATTGGTCTC
GGAGATCCTGTACGAGGGAGGCGCCCGGACGCTCCTGACGGACACGATCACGGACTGGCT
GGTTTCCTCTTTTGCCAGCAAGTCGACGGGGGCCGCCGGGGAGCTCCTGGGGATAGGAAA
CGCGACCAGCTTTGACTCGATCGCCCTCGAGATGGTCGGCGACTCGGCATCCACCGGGAC
CATCGACGGCCAGGAACTCAGCCTGGTGCAGGTCTCCTTCGAGGGTGTCTCGCTCTGGGA
GCGAACGGGGACCGGCACCGCCCCGATGGAGGCCGAGAACGTCGAGCTCATCCAGCGGGC
GACCTTTCTGGAGGACAAGCGCCTCCGGGACGACCTGCAGGCGGCCATTGCGAGCCTCCT
CTACGACCTGGGCCTCGAAACCACCGAGTCGATCACCATCCTCGACGTCCGGGCCTACAT
CACGCCGCCGGGATCGATTCTCGACGGCAGCGGCGGAGGCAGCGACGCCCAACCCACCGA
TTCGGCGGGAACCCAGGCCCAGGCAGGCGATAGCGGCGCGGACCAAAACAACAACCTGGA
GATCATCATCATCGTCGCCATCGTCGTGGCCTGTCTGGCGTTTGCGCTCCTTATTTTCGC
CGTCGTCTGGGCCTGGAGGGCCGACCGAAACGACCGGGGAAGCAGCAAGTCGTCGTCGGC
CTCGGGCGGCAGGAAGCGCACCAAGAAAAAGAACGCCTACGACAGCGTCCGCGACGACGA
CGCGGAGGCAGCCGCCGCGGCGCCCGCTGCCGGCGGGAAGGGAGGCAGGCGCGGCGGGAA
GAAAAGCACGCAGCAAACCGCCAAGCAGCAGCAGCGAGAGCGCGAGCAGCCCCCACCGCA
GACCCGCGGAAGCCGGGGGATCTTTTCCAAGAAGAATCGAGAGTCCCCCGAACCGGCCTC
CTCGCCCAGCCGCGGGGGAATGGTCATCGGGGACACAACCGGCGGCCGGGGCGTCAGCGT
CGACATCAGCGACAACGAGACGGACGACGACGACACGGCCGGAAACGCCACCAACGACTC
CTACCCAATCCTCCAGCCCAACGACTCGGTGGTCTCCTCCGACATTTCCTCCAGTCTCAC
GGCCTACTACAAATCGGGGATGGGCTACGGCGGTGGCAGCGGGAGCAAGAACAGCGGCCG
GAGCGGCGCCAGGGACTTTAACGACGCCGCCAGCATGTCCAGCATGGATTCCTACGGGTA
CTCTCTGGACGGGTACGCCCCCAGCCTGGGGCCGGCCCAGGGGGGCTACCCCGTGGGGCC
CATGCAGGCGGCCGCAAACGCGCCGATCCCCATCGGGACCTCCCAGGACGAGGAGCTCGC
GAACCACGACGATTTTGAGGCCATCCGGCAGCTCCAGGAAGACGAGTCGATCGTGGACTA

CCAGGCCGAGGCGTAGTAGTAGTCCCGCAGAGGTGTCTGTCTGTGGGCGAGCCGGCACCA
CACCACACTACTCTACACTATCCACGACGATACGTCCATACCACATCACACGATAGGTGC
ACAGCACAGCACCGCACCACACAAAACCACAACTATACACCGACCGGTTTCTCTCTGCAT
ACAAAAAACACGCACAGTATTCCTATTATACACATATAACTACCA
>comp2451_c0_seq1
CAGAGACGATGCCGACCCGACTAAACTAAACCGAGCATAACCAAGCGACAAAAGTCAGTC
CTTTTGTTCCTACACAATGATAGCGTATCGAAAGCATCTGTTGGCTCGAAACAGAGGCCA
CACGGGCGAAAAGATAACTCTGCAGCCACCAACACCTGGAGATGCAAAAGGAAAGCACAC
GCGTACAGCGGGTTTGTCCTCCTGCGTTCGGAGATTAGCGATGAAATTCCGATTCTACTC
CAATAAGACCATGTTTTCCATAAAAACAATTGCTCTGGTTGTTCTAGCGTTGGTTTGTGT
TGGGATTTGGGACCGGTTCTTCTATCGAACTGGCTGCGTTCGGCTCGGAGGAGACCAACC
ATGGCTCCCTTCTCTTTCTTCACCGTTGTTACCACCGCCGCACCCTTCCACTTTTGCCGT
TGTGATCAACACCTACCGGCGACCAGAACGGCTGCGAAAGACCGTTCGACACTACGCCGA
AACCTGCGGAAGGCGATACGGCGTGGGCCGTGTATTCGTGGTATGGGCGGACCAACACAT
CGAGCCGCCCACGGACGACTTCTTTTGGGGTACCGGTGGCCCTCCCCTGCGGGCCCTGCA
GGGTTACAACAACGGCGACGACACCGCCACTGGTTCCGACGCGAACCGGGTCCCAGTGGA
GGTGCTGAAAAAACACAAGAACTCCCTGAACGTACGGTTCGAACCCATCCCGCAGCTCCA
GACGCGGGCGGGGACCACTTCGGTCTTTATGGTGGACGACGACGTACGGGTGGCCTGTCC
CTCGCTGGTCGAGGCCTTTTCGGTCTGGAGGGAGAACCCCGACACGATGGTTGGCTTTTA
CCCACGGCTCGCACTTCCGCCCATCCGGAAGCATGATACCAACCCTTACGATGCGCCGGC
AGGGCACGGAGACCTCTTGTACCACCTCTGGCCGAAGGTGTACCTGGCACAAAAATTCAA
CATCATCCTCACCAAGGCTAGTTTCCTGCACTCAAGGTATTTGGAGCTATACACGAGCGA
CGACTCCTTTCCGAGAGAAATCAAGGACTACGTCGACGAACACATGAACTGCGAAGACAT
TGCCATGTCCGTGCTGGTCGCAAACTATACCAACCAATACCGCACCACTAGCAAAGACCA
CACCGACGGTTCCACGCAAAACCCCTCTCGTCCAGTTCTGTACGTCGAGGGAAGCGTTTC
CGATGTGGGCATGTTTGGCGGGATCAGCTCTGGACCGGGACACTTTGCGACGCGCACAAA
TTGCCTGAACGAGATCACGAGCATCGTTCGGGCCCGGGGATGGGGATCGCCGCTCGAGAG
GGACTTTGACCTGGTGGAGAATTCCTGTGGGCAGCACTCGCCCGGATTTTGGTGGCAATC
TGCCCCCAGCAACTCCCTAGAGTGGCTGGGGCTGGTCAATGTGTTGTCGTAATGGTGAGC
GCCATGCGACAAAAATGAATGGGGTAAACTATTATTTTGGATTGTTTTGATCTACACTAC
GAGCAAGCAGTAGCAAAGAGATGCTGTACTACACTTCAAGCTACTCAATAAAATATACCG
ATAGTTGTTGTTTTTGGATACGCTACCGATCTCGCTTGCTCCATCCCTTCGTGTTTCATT
GTGCTAGATTTGCTTCCTATACTAAGTTATCGATTAGATGAAATTGCAGTTTAT
>comp2452_c0_seq1
ACTGGTCGATTACTCTGGATACTGAATGCAGATCGATCTGAGATCAATTGGAATAAAAAG
GAATATTTGATTCGTTCATCCAATCACATGGATGAAGAGACTATCGACGTTGTGTTCCGG
CCCTCGCATTTCGAATCATAGCAGCCAATTAAAGCAGCTACTTCTGCCATCACAATGAAG
GATGTCAAAGAGCTAGCCGATAACGTAGAAGATCTGAAGAACGATGGCATGATGAACATG
GACCTAGCGTTTGATGGCATGTGGTCGTTTTCAGGGTATTGTGGAATGATGGTACAACTT
GCACATTTTTTTGTCGAGTCAAAACAAGGACGGGAGGAAAACAAAGGTTTCGGAGAAAAT
GATGAAGACGACGAAGACGAATTTTTGTTCGTGGGAAGCATGGATATCTCTGCAGAAGAA
ACAATAATCCGATGCCAAGAAAATTTGGAAGAACGAAATTTGCATATGGCTGACGGTGTA
TTGCGGCGATTGGTGGAGGGATGCTGTCCACCAAAGATTCGTATTACAAGGCTCGATGAA
GATACATTGAAGAAACTTATCGACTATAAAGTACAAGGAAATCACTTTTTCGGCCGGCAC
GAATTCCGAGAGGCCATTGATTTCTACGACTTAGCGTTGTCTTCCATTCCCGAAACCCAG
AGGAGACTGTATATTGCGCCAAGAAACCAGATCCAAGAGATTGTGAACGTGTTATCTAAC
AAAGCGGAATGTTTGTTGCGGAAGGTAAAATACGAAGAAGCGGCCGAAACTGCCACAGAT
GCGCTGATCTTCATGGCCGACCACGAAAAGTCACGCATACGGAGGGCCAAAGCTGGATTG
GAAATTGGAAAATACGACAGATACGAAGCGACGTCGCGAGGAGACGGAAGCATGACAGGT
GTAGCATTTTTGGTTCAATCGAAATACGATTTGGAGGAAGTTTTGAATGCACCTGATGCC
ACATTGGTAGGGAGGCAAACCGCGGAAGAAATTTTACATCAAGTGGATAAACTGCTGAAG
GGCGCGAAGAAAAAAGTCTTGAGCAAGGACCCAAATCCAGAATGGGATTTAAATGTTTTA
AAAATTCAATCGAGGTGTTGGTAGAACCGGTCTTCAAGTTACATGCCTGACATGCATACT
ACTAGACATTTGATACACTGATGCTTGCACATACTAGCTAACTACTTATCTGTTTTAGTC
TTGAACTGACAGTTTGATCTCTATCTTGGTCACCAACCAGCCTTCAATAGAAACAACCAC
AGCAATGTGTTCCTAATAAAGAGACAATTCTAGCTGTATTGGTGGGGTTTATGGGACATT
ATTGACCATGTTAAGACGTACAGCAGTGCTCCATGTCTAATCCAAGGTTCTAACATTGTT
TTGTTTTTTAAAAGGCCAACACTACTAGTATGATATTCAGTGGAAATAAAAATTGTGATG
AATTTACCTAAACTGAGGACTTTTTTTTTGTTTTTGGGGGA
>comp2453_c0_seq1

GCCATTGCCCTGGATTCCAACATCGACGACAACGACAACGACGGCGAAGCCAACGACGAC
GGGGAGGGAACGTCTTCCTCCCCGGAAGGAGGACGAAACGTCACCACGATGCTCCAGAAG
ATTCTTTCCCTGGGGGGAAGCGAAGAGGAGGGCGAAAATGGGCAAGCAGAAGCGGAGCCA
GCTCCCACGCCCACCCCCACTCCCCCCGTCGAACCGGAGGAGGCGGTCACGGATCCCGAA
TACCCGGACGCGGCGGTGGTCAGCGGGGCCTCCTCCCGGATCCCCTCGGTCCAGCACAAC
GGCCGGCTGGTCTGCAACCTCCCGGATCACCCCATCCACGCCGGTGCCACGGCGGTGGTC
GCCGTTCTCACCGGCCGGATCCTTACGGTTTCCAACGCGGGAGACTCCCGGGCCGTCCTC
TGCCGCCGCAGAGCGGGCGCGAACCCCGAAGCCAACGCCGGCAGCGACTGCTACGCCTAT
CCCCTGAGCTACGACCACAAGCCCTCCCAGATGCACGAGAGAAACCGCATCCTGCGATCG
GGCGGCTTTATCAACCACTTTGGACGAATCAACGGCAACCTCAACCTTTCCCGGTCCATT
GGAGACTTGAAGTACAAGCAGGTCCCCGGGATCCCCCCCGCCCACCAGATGATCACGGCC
GAGCCGGACATTATGCAGATCGCCCTCGATCCGTCGGTGGACGAGTTTTTTGTGCTGGGC
TGCGACGGGATCTGGGACTGCCTGACCAACCAGCAGGCCGTGGACTTTGTGGCCAAGCGC
ATCGACACGATGTCGCCGACCGAAATCGGGAGGGAGATGCTCGACGAGATCATCAGCGAC
GACCCCCGGGTCTCCCAGGGCATCGGGGGGGACAACATGACCGTGATGATCGTCGATCTG
CAGAGCAAGAAGCGGTCGCGGAAAAGCGCAGCGGCGCCGGAAGCACCGCAACAGGGCGAA
CCCCCCTCGGCGGGAGAGCCGGCGGGGGCCGAAGCGGACCAAGCACCGGAGGCCGCCACC
GAGTGAGCACCCACCCACCAAAAAAACAAAAGACGACAGACACCGACACGATAAGCACT
>comp2454_c0_seq1
TGATAATCCTCGACCGGTTTTGCCATCTAAGTTGCAACAGATTTAGATCGACTACAGGAA
ATGCTTCGACATCTAGGACATTTTAACTTATGTCGCATTCAGATATGATTCCTGTTGCTC
TATCATGAATCATACCGATGGACAGCCCCACCGCAGGAATAAGGGATACCGAGGAGAAAA
AACGAACACAAATAAAAACAATGACGAGGAAATTGGGATCAATCGGAAGCAACATGATCG
CGGGACACATTGCCACACTCATCAATTTCAACATTTCCGGATGAAACGAAATATTTTGTA
CTCGTCGGCAACGGCGTTGTTGTTCATCGTTCTAACGATATACCAATTTCAAGATTATTT
TGATGAACGATTGCAAGACAAGTATTGGAGAGCGATGCTTCTCTTGCCACTGCGATATCT
TCGTGTCAAATTCATGGACCAACGACCAACCCGTCCAAAAACTTTCACGCTGAAAGCAAC
CAGATCGTTTGTGATCAATCTTGATGCAGACAAAAAGCGATGGAGATTTTTTCAAGAATC
GAATCAGCAGCAATTACAAAGTATTACTCGATTTCCTGCGACTTCGCTTCAAACCATTCA
CGACGACGAAAGAGAACACTACTTTCTAGAAAAATATCAGTTTCTTCGCAATCTCATCAA
AAATGAAGGCAAAGGTTCGGCAGCATGCGCCATATCACACTTGAAGTTGTTATTAGAATT
TGTCTCCAATACCGCAAACGATGATGACTACATTTTTGTTTTTGAGGACGACGCACTGCT
AACATCCCATTTGCTTCAATACGGATTCGTAGTGGGGCCAGACAATGCGGATTTCATTCT
GCTATCGGAGTATTCATCGGCACATATCTATGTTCCATTTGACAAAACAGATGTAAACAC
GAATGTGAACGATCTTTCAAAAAAATCGTTGGAAGAAAATTTGTTACAAAATTACGCAGA
GAAAATCAATACGGCAACACGTGTGGTGAGTGGTTACACGGCAATTGGATACATTGTAAC
AAGACGCGGTGCAAAAGTCGTATTGGATGGAATGAAGAAGGAAGAAGAGCTTCCAGTAGA
CCTATACTTTTTCGCAAATCCGCAAATAAAGGCCTATCTACCGTTGCTTGGTTGGCCATT
GGTACAGCACCACCAGCCCTCCCAATCTGTTCGAAGAGACAAGAACAAAGAATGACGAAG
AGTAGAATTTCGCACATCAAATTCAAGCATTTCATTTTATGATAAATAAGGACAGATAAT
CATTTTTGGCTTGCAGATAAAATTGCATAAGCTTCGAAAGGCGATATTAAATAGACGTGT
TTGCACAAAGGAAGATGTAAGATGCCCTTCATTCATGTGCACTTGCAACAGATATTATGC
AGATACTACTAGGAAGAGAGAACGTTATAAAAAATGCAAAAAAATGTAAAATACTTCGAA
GATGATACTCATGACATCAAAGTAAAAGAATATAGAACATCATACTAGTAGAGGA
>comp2455_c0_seq1
CGTGTTGCAATGAATATCGGTAGAAGACCTGCACTCGAACGGAAGTACCCCGAAATCACG
ACAACGCAGCTACAGCAAAACTAGAAACAAATTTAATGCCACTTTGAAAGAAGGTTTTAT
TTACAACATGGAACACCAAGAAGGGGATGATGTCTCGTCAACCGCAGAAATAATTCTTGC
GGATCTTTGCAAGAAAGAAACCGAGGCCGCCGAATTGAAAGAGGCACTGAAAAACTTGCT
GGACAGCCATGTCCGTACAAAGAGAAGGCATGCCAAAGAATTATCCGAGGTCCACAAACA
ATATTCAAAACGTTTGGTCCAATTGGAAATCGATTTGGAAACACACGACATACATTTTAC
AAACTATGCAAAGGATCAGATGGGATTGGAAACAAAAGCGATTCTCGAGGACAAAAAAGA
ATGTGCCTGGAAGAAACCGAAATCGGATATTTTGGCGCAGCAGAACCTGATAGCGAAGCT
GATTGCAGAGAATGCCGACCTGGAACACCGATACAAAAGAGATTGCACGGGGCATAACGA
TAGGATACTAGAATTGGAAAACGAGAACCGATGGCTCCGTCGATCCCTGCGACGCAGGGT
CGATTCCAGCAGCAGCACCGGTGATGCCGAATCCGCCGGGGGGCAGCTGGCACTGGAAAC
CGATAGTAATTGCAGCCTTGTTGTCGAAAAATACGAAAAACAGAACACGGAATTGTTGCG
CAGTGTAGATGAACTGAAGGAGAAAATCCGTAGCCTGGAACTGAAGCACGTTCTCGCTGG
CGGTCCTACCAACACAAAGGAGGAGAACGGCAAAGAATTGTCGTGCAATGACAAAGATGA
AAAATTGAAGGATTGCAAGATAGCATTGTTGAAATCGAAAGCCATTGCGTACCAAGCGCG

AAACATTTGGCCTCCGGATACGGAGTGCAGCGCGCCGCCAGAATCGCTATCGTCGTCTTC
GACGGCAATCAAAGATTTGAAGGAGCAAATACAAGAGCTCGAGAACATTCTCATTAGGAA
AGACAAACAGTTGAGATCGCAATCAAAAAGATATCGGAAAACAAGAG
>comp2456_c0_seq1
AGATTCTTTCTATCGGTGGACACATTGAAAGTTCTCGGTACTCGTACTCTTATTCATCCA
AGCATAATTCGACAACAGCATAGCGACAAACTCCATTCCATTCTTCTGTCCGCTTGTTCG
ATAACCTCAAGGCGGACTCAACACGCACACGGAAGATATTGCTGTCTTGCGTCGCTCCAG
AATTCTTTGAAAGACATGACTCCTTTAAACCCTCGAAGGACACTTGCAATTCTTGCGATA
CTGCTGCAAGGTGCAGAGTCATATTTTAATCCCAATGTATACGCTCTCCCTAGGTCGCTA
TCGGGTGAACTCTTTGCGAAAAGTGCAATTTGCTACAACAATGCATACGGTTCGTTTAGA
ATACGACAGTCTTCACCTATAACTTCGACGTGTACGAAACATACCGGAAGCGATTTCATG
AAATTTCCACAACCGACGACATCTACATCCTCATCGCTGGCAGTGATGAATCCAGTGAGG
AGAAGAAGCGAGACAGGAAATGGGGGACGAGGAACTTCGCCGGCTTTCCTTAATACATTT
CTAAATAACGTCGGCACTCTCACTGAGTCATTAGTTTCAGCAACAATGAAATCTCTTCGA
AAGCACTGGTGGTCATTTCCAATGATTATGGCCTTAATTCCACCTTATTGCGTTCTATTC
AGAGGATCATATGCTTCGATGCCTCATTGGTGGGGTGTTGTGAACATGGATTATGTTGCT
GCTGCGAAGAACGCGAATTTGATCATTGCTCACTTCTTGGGATCCAATATCGCGTATGCG
GCAAGTGGCGCCTACGTCCTTCACAAGTTCCACTTCTTTGGACGATCGAAGAGTGGTGGC
TTGGAGTTTCGGCTCAGTCGATTCTCAATGTTGGGAATTTGGATTTTGATGGCTGGTATG
GTTAGTACAATTTTCCACTCTGTTCAGGCATTGGGATCATATGCTCTCGCAGAAAGCATG
TGCTATTTGGATCATGCGGTGGCCGGCTCTGCTACTTTTTATTTCGCTGATACATGTGGT
GCTCCATCCAAAACAACCTCAATAATAGGTGCTTTAGCACTCATGAGCCTTGTGATGTCA
TCGCCTGCGTATGCTTGGATTCATTCTTGTTGGCACTATCTATCAGCGCTGGTAGCAACA
CGATGGGCCATAGATGGTAGTGCTAGAATGCACTAAATGTCTTCCGAGCCATGATGGGGT
AATTAAGAATCAATATACTTTTTATGTTCAAAAAA
>comp2458_c0_seq1
CAACAACAACAACAACAACAGCCGTTTCGCACCGGAACGAATTCGCCGGGTATCCTCCGC
TGGTGTTACGGATCGGTTTGTGCCCGGTCGGGAAAACACCGATCACAAACACGACGCCCG
CCATACAGTTGTCCCTCCCCGCACGGGTTTGGCGCTGGCGGTAGCACCGACAGCATCGCC
ACTGCCGTCTGTTCGTGACGTCTTGCTGGGCCGGGGCCGGCCCCTCCAGAAACACCCCGG
TAACCTCCGCCTGCGGATGTTAATCTCTGATCGCTTGGACGAATACGAATCGTCCACCAA
GCCGGAGAAAACGTGGCTCGCTGAGGACATTGTAAAGGAAGTCAGGGCAGGCGGGGGCAG
GTTTTTGCAAAAGAGAAAGCCGACTGAAAACGCGAAAGCAAAAGAAGCGGGGCCGGGCAT
TTGCGTTGGCGTTGGCGTTGGTATTTCCGGTGAACGCAAGAATGCTGCTGCCGATGACGA
TACTGTCAACGATGGCACCGTCGAGTGCGGCTGGGAGGAGGTTGACGATCACACGGCCCG
CCTCAAGGTCGCGTACACCTTTCGAACTCTGCGCAAAACGAAAACAAAAGCAAAAACGAG
AGAACCAAGGCAACAGCAGAGTACAAGGGCAAAACCATAACAGGCAAGACCAGACTGGTC
GATCTTGCACTCCCAGTACCCGCATCTCATCGATGGGTGGCCTCGTGGTTTCTCTGGCAA
AGCAAACAACGACGACACTACTTTTCTAAAAAACCGCAACTCGCAGACGACAGCTCTGCT
CTCGGTGGAATGCATGGTGATTCGTTCGTAGAACCAGAACGAAAATGTCTTTGGCTTGAG
GCGCGGTCCTCTGTACGAGTGCTTTCTTTTTCAACAACATCTGTTATATATTATAGAAAC
ATTATTTTTTCTTAAAAAA
>comp2459_c0_seq1
CGAGAAATTGTCTCGCAAGGAAACGAACGGGGCACAGAGAGAAAAAGGCGAAGCTTTGGA
TCGGAGACAACGATCAATATTTTGACCCAAGAAAATTGCGGGCGGAGCGAAGATGGTTGC
CATACTAACACGGGGTGCTTTTCTGAAAGCGGCGGCACTAGCGTCATCGCTCACAATCTC
CCCATCGTCCCCGGCAGTCGATTTTGATTTGGCCAGCGGCACCCAAACCGGAGGTGTCGT
AGGAACCGCACAGCCCGGACCGTTTCCATCGGTGTCTTCCACCGTGGTTGGAACAATTCC
ATCGCCGCTCGAAGACCGCCAATACGCCGCGGCTGCTGCCTTTGTGCGACGGGAAATCAC
TCGCATCGTTAGGGAGGAGAACCCCTCCATGGCGGGGTCCATCCTCAGGCTTTCGTTTCA
CGACGCGACGGTGCGGAGTGTCGCCAGCGATCCGAGCATTGGCGGGGCGGATGGTTCCAT
TCGCTACGAGCTCGAGTGGCCCGAAAACCGGGGCCTAGCGAAGCCCCTGGCAGTGGTGAC
AAGCGTCTACGAAGAGCAGCTGGTTCGATTCTCGGTACCGGGAAGTTCTCCAATAGAAGA
AGAGAACGCACCACTTCCCTCGTGTCCCCTGTCCTTTGCCGACACGCTCGCCCTATCGGG
TGCCGCTGCGGTGGAGGCCGCGGAGGGCCCACAGATCCCGGTCCGGCTGGGCCGGAGGGA
CAAGGAAGTTGCGGACAATCGGTTCCTGGATCGGCCGATCCCCGGATTTACAACTATGGA
TCAAGGCCCCGGCGATCGGGCCTCGATCACCTCGAGCCTTCCCTCGGCCGGCCTGGACTC
GCTCGGTCTCCGAAACTACTTTCGGAGGCTCGGGCTGACGGAGAGCGAGCTGGTGGCCCT
GAGCGGGGCCCACGACCTGGGCCGCCACGTAACGCTATTGGGAATGCGCAAGGACTGCCT
CCGGGACCTTACCCGCGCCTGTCTGGAAGATGCTCCCGTGCTGTGGCCGTTTGTCACGAA

GGATCCCGACACCCTCTCGAATCGTTACTTTGCGACTCTCCTCCGCTGGAACAAGCGGGA
GATCGAGAGAGGGGAGGCCTCGTTTATTCCCACAGATGTCGCCCTGGTGGTCGACGACGG
ATTGAAGAGGCACGTTGTGGCTTTTGCCAAAGACGAGAAGCTGTTCTTTCGGCGTTTCCG
GACCGCCTACCAAAAGTTGGTCGATTCCACGGCGAGCTCGCAGTCGAGGTTTTGAGTTTA
CTCATGCTCGTGTTGTTGTCGGTGCCTGGTCGAAAGATTGATTAGTTGTTCACGCAAACA
TCGTTCCCTGCATTCTGCGTACTAGGCCAGAGCCACAATCACCGGAACACAAACTTTTGA
TGCTCTTGCATGTAGTGTCGACCGACGATGTGTAGACACCAAGGTTCCTTCGGGGTACGA
AAAATACCTATTCGATTCGAAGGGTATTGTCGGCCATGCCCTTCGCATAGACCTGGACGA
GTGAGCTCGAACCCACTAGTCGGTTTGGTTTGAAGTGCTTCAGTTTCGGTTGAATCATGG
TACATCCTCTGGGTCGAATATTTTTGAACGATGCTACAGGGAGGGAGAGAAAGGGAAATT
CCGTTAGCGGATGAAAAACTACTATTGTTACCTCCTCTTCCCGAAATATAGGCGTTGGAA
CTTGCAAGGGAAAGTAGTAGGGAAGAATTAAAAATTGCCGGTGCTGTCTTCAAATCGGAA
GCGACTACAACAAGCAGTATTGAATGCCAAAGCAACGACTGCACTACTACTGCTGTTGAT
AGTGGTACTGAGGTGAACATCATAATAGTACTCGTACTAGTACTACTACTAGTATGGAAA
ATATGTTCATTCTCTAATGATAAAATAGTACCACAAATATCAATTTAATATCCAAGAAAA
TGGTACCTCCAGAATCAGCATAAAAAACTGGACTAGACTGGCT
>comp2460_c0_seq1
TCCTCGCGGGTAGCGGGTAAGTCAAGGAATCGGTGTTCTATGGAATAGTACAAAACAAAT
GAAGCAGTTTAACAATGGATTGCTTCACTGCGTACAAAGTTGGATCGATGCAAAGCATAT
CAACCAAAGAGAATAACTTGAAGAATTCCACACGTAACCGTAACGGTGTGCACCAGAGTG
ATTGTGAAGGAACGAAAGTCAACCTGACTCTCTCTTGAATAATAGTAGATACGGGACACA
AAACGAGACACTGCAGAACGGCAACAGAGGGCGGAGTTATATGAGGAGAAACCGGAGCGA
ACAATACAACAATACGGAGACCAGTATCTCTCACAAACACATTACATATATCATAAATAT
TGATTACAAATTAGTTTTTCGTTGGTGATTTTTTCTAACTTTTTTTCTCTTTTGCAAATG
GTTTCACGTTGCTCCGATGGAACATGATGCAACGTGAATGTAACCTACTGATGAACACAA
CTATTTCGCAGCGTCTACGGCGGACTTCAGGACC
>comp2461_c0_seq1
ATCCCATTCCAAATCACAATCATCGAACACAGGTGAAGAGACATTAGTATTCAAACTCAA
TAGAAAGAGCAAGAAGCGATCAATCAAACCTTGTGACTAAGTAAACATAGGAACAAAAGA
ACAAAAGAACGAAAGAACAAAGAACAAGCAAGCAAACAAGCTCACAATAATAACTCAAAA
GAACAGCAGGATAACATTCTACTAACCAGCTAACCAGCCAACCATGAGTGAGACTACCAG
TACTGAGCCTAAACCCACAGCCGCGATGGCAAAGCGGGGGACCAAGAGCGAGGAGAAGGT
CAAGCCCGACACCGATCAAAATCTGTGCACTGCCACAAACAACACAAACGACCAGCCATC
GCTCAACAAAAAGAGCGTCCGCTTCGGAAGCGTCTTTGTCCGGCTGTATGGGATAACGAT
CGGGTGCAACCCGGCTACCTCTCTCGGGGCCTCCGTCTGCCTTACCTGGGACTACGACGA
ACTCCTCCCGGAGCCGCTGGACACCAGGGAAGGGAAAAAGCCCAAGAGGAAAAAGAACAC
GTCCCCATCCACTTTCTACCTGTCCTACTACCGCCGCGAGGGCATCCTGAAAAGGGCTGG
ATTTTCGAAGGAAGACTTTGATCGGCACGAAAAGCTGATCCAGAGAGATCGCTTGAAGCG
CAAGATATCGACCTACCAGTGCTATCCGATTCTCTTCGCCAAGAGCTTTGGTTTGAGCAG
GGCCCGCCGTAAATCGAGGACATTTGTCAAAAAGTACAGAAAGGAGAAGAGAAACAAGAA
GAAGCAAACTATCAACAAACCAAAGGAACAAGCACGGCAATAATCGGAAACATGACGTAT
TGTGTGATGTACACCCCTCTATTTATTCCTCTATTGCATTCGTAAAGTGTCGAATAGCGC
CGAACCGAACCGAGCGAAACCAAACAATAAAACACAAACGGAACAATTTTGGGAATGAAT
CATTGCTCTCTTTTGCATCACTACTGCATTGTTTGCTGGTTTTGCTTTGTCTGAAGCCAT
AAACCAACGCAAGCAAGCATGCACGACGGTTGACACAGAGAACACGGTATGTAGCAATGG
CATCGATCCGTGTCTCGAACGGCAACCGAGATTCATTTTGTTTTCCATCATCCCGTATCT
GCGAGGAAAAGCGACGAAACGAAACACTCGATCCGTCAAAATCCATCTTCCTAAGCGGAT
TGGGTTGAAATGCATTGTTTTTCATTGCACAACAGCCGCTTCACTCCAAGAGCGAGCGGA
ACCCAAAGATTTGAGCGAGAGCAAACAAAGTGTGCCGATCCATACTTCCGTCCAATAGCG
GCAAGTGGCAAGTAAGTCCATGGTGCTACTTTTACTTTTGAGTGGTATTGAAGCCTGTGC
TATCTGGCGACGAATCTCTATGGATCGCCAAGAGGAATGAATCATCCTTTGCATACCACG
CAATAAATAGAAAATAAGTGTTGTATTTTTTATTAGTGGTAA
>comp2462_c0_seq1
CGCATACTCGTACTTCTTTTATGCAATCCCGATCCGAGCCCACCAAGCGCTGCCGCCTGT
TGTCTTTGCTGATCTGCGTTGCACTATTGATTCATTGCAGCGAGAACAGCACAACCATCC
ATCCGTTAGCGTTAGCGTTCGCGTTCCCACAATTGCGCCTGGGAGTTCGCGTTCTCCGCA
CGAGAAAGACACCCCCGAAAAAGATCCACGCCAACCCAATCGTCCCGAACCGAAAGCCGT
ATTCTTTTGTTGGTAGAACCACATCCCGCACCATGTCCACCGAAGAAACCAACGGCGGCA
GCGGCGCCAGCGATCTCCCCCGCATCGACGGATCCGAAACCATCGGAAGCACCAGGTGGC
TCAAGCTAGAAACCCTCTCGTACACCGACGCCACCGGCAGCCAGCGCAAGTGGGACTACG

CCACCCGCACCACGAAGGCGAGCAAGTCCTCCACGGACGCCGTGGTCATCATCCCCCTCC
TGAAGCGGCCCTACAAGAAGGAGGGCGAAACCGGCAACGAAAAAGACGAGGAAGACAAAG
ACGACGTTCTCGACACGCTTCTGGTCGAGCAGTTCCGGCCCCCGGTCGGGAGGGCCACCC
TCGAGTTCCCGGCCGGGCTCATCGACGCCGGGGAGACACCCGAGCAGGCCGCCCTCCGGG
AGCTCCGCGAGGAAACCGGCTACGTCGGGGAAGCCTGTAGGATCCCCCCGGTGGTCTCGA
GGCAGGTCTGCATGTCCCCGGGCCTCTGCGACGAGACGGTCCAGTGCGTGGTGGTGGTGG
TGGACCTGGACAATCCCTACAACGATCCTTCGGGGGCCCTCTTCGAGGCAACCCCCGACG
AGGGAGAGCACATCACCGTCCACCGGGTGAGCCTCAAGGACGGCTTCCAGACCCTCCTGG
ACAAGGGATCCTCGCCCATGGCCATCATGGGGCTGTACCTCTTTGCGATGGGGCTGGAGG
TCGGCAAGACCATGGGCAAGTGAATCGACGACCGCATCCGTGAACGGTGCGCACGAATCG
TTGGACATGGCTTTGGTCGACCACGGCATTGTCGTCGTCCGTATCGAGATGTGAGTAGCC
ACATACAGAACAAGTACTGTGCCAGGGTGATACAGAACGACTCTTTGCAATCTTTGTGCA
CTGTTTTACCAGGACGGAGAAAAACGAATCGAGATTCGCACAAGCTTGCATACCCACGAA
ACACCAATCGATGGATCCATGGCATTCGAGGAAGTGACAAAGAGGGAGTTGTGATTTACT
ACTAGTTGCTCTATATGGATACTATAATTCAGGAAATAACTGTAACCGTCTAAACAATGA
AGAGTCAAGAGCA
>comp2463_c0_seq1
ATCGAAACTATCAATAGATTTGATTGTTCCTCTCATTTCCCAAGAGAAAGACGAATACTA
TTACAGCATGAAGAAGAAATCCAAGAGCAAAACCAACCGAAAGGAAAGTTCCGAAGTGGA
TACAATGGATTCTCCCGTGGCCGAAGAATCAACACCCATAGAAGACAAGCTCGAACGAGG
AAATCCAAACGTTGAAGATGGCAACGCCGAAACTTCTATTTTCTCCACGAGCGATGGAAA
GTATCGGAACAAACAGCGATGCTTAGTCCTTTGTCTTCGAGGTGCTACTGCTCGTTACCG
TCATCTATTAGAGGACTTGCGTACACTGTTACCGCATCACAAGAAGGAATCTAAACTTGA
CTCGGGAAAAGGCGGCGCTGCGACGGCTGTCAACGATATCGCACAGATCAGGGGCTGCAA
TACCGTTTTATTCATGGAGTGCCGCAAACGCCAGGATGCTTACCTGTGGCTTGGTCGAAC
TGGTGATAATCATGGGCCATCGGCCAAGTTTCATCTTACAAACGTTCATACGATGGACGA
ACTTCGCCTCACGGGGAACTGCATGAAGGGAAGTCGCCCGATCTTGACATTTGATGAGTC
CTTCGAGAAACATGGTCATCTTAAACTTTTGAAGTCGCTCTTCACTGATGTCTTTGGAAC
ACCAAGGGGTCATCCGAAGAGTAAACCTTTTGTGGACAGAGTTATGGCGTTTTATTATGC
TGACAAAAAGATATGGGTACGAAACTACCAAATAGTGGAAGAACAACCCACGAATGCACT
GGAGGCTCACCAAGCAAAGAAACTGTCGGGACAAGAAGAAAGCACGTCCCTAGTCGAAAT
TGGACCTAGATTTGTTCTCAATCCTATTCGAATCTTCAGGGGTTCATTTGGTGGCCAAAC
ATTATTCCAAAATCCAGACTTCGTTTCGCCCAACGAAATTCGCGCGGTTGAGAAAAAACG
GAAAGGGCGTTCCTATGAACTAAGGAAAGAAAGCCAGAAAATGCGCAAAAGTCGACAGGA
AGACTATGTTCTGCCTGAGGATCCTTTGAATTCTGTTTTCCGATAAAATTAGAATTCAGT
TAACG
>comp2464_c0_seq1
TGAAGCAATACTGATCTTTGTATTTAATCAGCATCACAGGTCCTACAAACCACGCTTTTT
AGAAAAATGAGATACGATGCTGCAACACCGAGTACAGTCTTCGGTGCCGACGACGAAGAA
AAAGAAAACCCCGATACCGTCGATTTACTACTCACCAAAGAACTTCTCGGACTTTCCTTC
AAAGAACGAAATGCGTTTCAGGAAGAAATCCATGGCGTGGTATGTTTGGCTCGAGAAGAG
ACTCCTTCACTTCTTAAAAAATCACTCGAGAGATTACAGCAAGAAATCGACGTGATGCCT
GATTGCCGAAAAGAAGCTTACTTGAAGAGCCAAACACTAGCGAATGTCATCAAGAACAAC
AGTCCTTCTTGTACCACGTATGTCAACACAGATGGTTTCCGGTTAAGATTCCTTCGGTTC
GAGTTGTTCGATGCTACCAAGGCCGCTGTCCGCATGACAAAATTTCTTGATGTGATCCTG
GATATATTTGGAGAATATGCACTGCAGAGGCCAATCCGCTTGAGTGACTTTTCCAAGATG
GAGCTTCGGAACTTACGGAAGGGGCGATACCAATTTCTCCCTTACCGTGACCGTGCTGCT
ACCTGCGGGAGACGGGTGCTTTGCGTCTTTCCCGACCAGGAATGGGAGGCCATACCACCT
CGTGTGCGCAACAAGATATTGATTTATCTCGTCTGGGTAGCAGGAGAGGACGTTAACACG
CAGAAAGAGGGATTCGTTGTTGTATGCTGGATCGACCATACTTTCAAGGTATCCAAGTCG
ATAAAGGTGCACACCAAAGACCATCAAACGCTTACTGTAAGGATCAGTGCCCTCCATATC
TGCTCATCAGATACACCTTTGCATCGTTTCCGGCGGGCAATCATAGCCATGAGGATAGGG
CACAATCGATCCAGACTGAAGCAGCATGTTGGAGAATCGATGGAGCTTCGTTACGCACTG
CAGTCTTACGGCATCCCTGTCGATCAAATTCCGATAACGTATACCGGAAAAGTGAAGGTG
ACGTACTTCAGACAATGGCTGCGATTACGGGACTTTGTCGAAGGCAACCGATGCGACAAA
ATAAATCATAGCATTGAGCTCAGTCGAACCAATCAAGTCGATGATAATAATCAGATGATT
TTTTGTTGCGATTGGAACAGTTGCTGTGAAGATAAAAGCAACGGCAATAGTTGCATTTGT
TATACCGCAATTGAATCTCCGTATTTGAATGATATAATATTCCGAAAAGGTGCAAATACA
TCATCCCATCCTGGAAACGCGGTAATGAGTCGCTTGATTGCAGACAAAACCAGGCAAGAA
CTCTATGCACAACACGAACAGCAATTGGTCGGCGAAAACAACAAAAGGACCAAAATGAAA

TTGTTTGTCTCAGAAATAATTCAGGAAATCCGAGAGAAGAATAGCACAGAACGAAGAATG
TGCCGCTTTCTGGTGTGGGACGAGAGAGGTTGGTGGACAGAAATTTTGAACGAGTATGAC
ATTCAGACACGCGTTGAATATATCGCAAGGGGTATACGTAATACTGTTACGAAAAACTAT
CGATCATCCACACCATTGATGCAATCACCAATAAACATGGGATATAAATGCGCGTACATA
GTGAAGGAAGACGAAAGAATGATGCTGGGAGCGTTTGCGACGCAGCGTCAACTCAAAGAC
GATGCAATGTTTAGATACCAAGATTTTTGCAGCGGTTTGGATAAACGCAAACAAAAGAGA
TTTAAAGCCATGACCAACAGCAGTGGTGATGGAGATGGGATCATGATGGCATCTGAAACT
AGATTTTGCGGAAGCTACTGTTTTTGAAATTAGATTGTGTACTAGATAGATCAACGGGTC
TGACTCAGATTGGCTCTAAGGTATCAACACCCAAAAGCCAACGCCAACAAAAGGTAGAGA
TTCTCAAACAGCAGTTCGAACCCAAAATAATTGTACCCCATTTCATTGCCTTGTGTTTCG
GTGATCTCCAATTATTAGGAAGGAACGCATTCCTGAACAAACATGGTAACGGTTTAACTC
GTGATACATGTTCAAAAGATCCCATCTATAATCCATCAAATTGCATGGCACAAAGAAAAC
ATTCAGCATTGATTTTGAATGCATAGAATCATTTTGGAGAATTTGATGATTATGGTTTTT
CTACATACGAATAAATCATGACTCCTAACGTGAA
>comp2465_c0_seq1
AGAATATATCCATCAGCTTCTTCTCCGGTTTCTGCCGGCAATATCGCTAGGAGGAGAAGG
AGCCTTGTTCCGGAAGAATAAATGGAACAGGTCCAACAGATGGAAATAGGATTCTATTGT
GGGATGTCTATGAGATCTCGATGGAAATCACTGGTTGCAGCCGTCGCTTGTGATGTGCGG
AACAAGACCCGGACCAAACAATCGTCACACCAAGGGGCGAAATACTTAAAAACACAAGGA
ACAAAGTACAATACTTCGGTAAGCCCCGGCCTTCCGCTCCTTTCCCGTTCTCGTGCCGCA
ACCGAATGGACAAAGGCCAGCACCACGACGAACCGCAACGAACCTTCCAATGGCCGGGCA
TCGAACTGTTCTCTTCTCCTTTCCCAGATCGTCAATATTTCGCGAGCGAACAAAGAACAA
CCAGTGGCTGGCTATGAGAGAATGGAATTCTCCTTGAGGATTGCGGCGATGGACGAATGA
TCCGCCTCGCGTTCCCTGCCCTTCGATGGTGCGATGGACCTCCAACCATTCCGACGATGA
ATGGATGGATTCGGTCGCTCGCTGTCGAATGTAAGGCAAGGTAAGGAAGAATGCCTTGGG
TGGCAAACCGAGACTTGTTCGCGCGGAGGCCTTATGACGCCTTGACGATCCAACCCACGG
GTGCCGTCGTTCTCGACAAGCCGACACAGATGGTGGGCGATCGATCCCGTCCCCCCGAGT
CTCCCGGAAGATTCGTGGCGGTCAGTGCCACACAATACGATGCGATGCGATACAATTCCT
AGTCTGAATGACGCGAGAACGGAACGAAATCGCGCCAGGCACCCATTGATTAGGGGTGGG
GAGCGGGAGGACCGCGGGCCCTTGCCCGCCGAGAGGCATCCCTCCACGATATGCGACCCG
CCAAGTACAACAATGTGAGGTGCACTGGCAGCAAGACCTGTGGGGGGACCGAAATAGTGC
TGCCACGGTCCCCCGAAAGTCTTGCAGCTGCTAGCGCTTCGAGAACTCGACGAATTCGAA
CCTGATCCCTTGTACTGTATCGCGAAGCTATCTTTTTCAAAATAGCTATGCATTGATAGA
GGAAGACTGCCTGGACAGGTGACAGGGTGGGAAGCACTCGCACAAACCAAGATTCTAGGC
CATGAGAGCACACCTTGTGATCCCAAGGAAAGATAGCACAACGAATTTACTCCCATTGTT
GAGAAGCTTCGACGAAAATTTCCCCAAGACTCAGCCATTCTTCTGGTTGACGACGTTTCC
AAAGGAGGAATCTCCCTCAACTACAGCCATTCGATTCTACCAGGAAAAATAATGCAGAGG
ATACCCATC
>comp2465_c0_seq2
AGAATATATCCATCAGCTTCTTCTCCGGTTTCTGCCGGCAATATCGCTAGGAGGAGAAGG
AGCCTTGTTCCGGAAGAATAAATGGAACAGGTCCAACAGATGGAAATAGGATTCTATTGT
GGGATGTCTATGAGATCTCGATGGAAATCACTGGTTGCAGCCGTCGCTTGTGATGTGCGG
AACAAGACCCGGACCAAACAATCGTCACACCAAGGGGCGAAATACTTAAAAACACAAGGA
ACAAAGTACAATACTTCGGTAAGGTACGTTGCTCGTATGCCTGTGGTATCAGTTGGTTCA
CAACCACACGCACAAATCATCTGCCGCCGCTCTTCTCTGGGGATACCAATACGCGTATCG
CACTCGTAGTATCGCGTCGAGTCGCGTCGCATTGTGCGGAACTACACTTTACCCTTCTCT
GAATCGTACCGTACAAATCGATCCAGAAAAGGGTAGTGTGTGGTGCCGAAGTTCCCTCCC
CCCCCTAACTTCCATCAAAACCTCTTTGTTCGGCACAAAGCCCCGGCCTTCCGCTCCTTT
CCCGTTCTCGTGCCGCAACCGAATGGACAAAGGCCAGCACCACGACGAACCGCAACGAAC
CTTCCAATGGCCGGGCATCGAACTGTTCTCTTCTCCTTTCCCAGATCGTCAATATTTCGC
GAGCGAACAAAGAACAACCAGTGGCTGGCTATGAGAGAATGGAATTCTCCTTGAGGATTG
CGGCGATGGACGAATGATCCGCCTCGCGTTCCCTGCCCTTCGATGGTGCGATGGACCTCC
AACCATTCCGACGATGAATGGATGGATTCGGTCGCTCGCTGTCGAATGTAAGGCAAGGTA
AGGAAGAATGCCTTGGGTGGCAAACCGAGACTTGTTCGCGCGGAGGCCTTATGACGCCTT
GACGATCCAACCCACGGGTGCCGTCGTTCTCGACAAGCCGACACAGATGGTGGGCGATCG
ATCCCGTCCCCCCGAGTCTCCCGGAAGATTCGTGGCGGTCAGTGCCACACAATACGATGC
GATGCGATACAATTCCTAGTCTGAATGACGCGAGAACGGAACGAAATCGCGCCAGGCACC
CATTGATTAGGGGTGGGGAGCGGGAGGACCGCGGGCCCTTGCCCGCCGAGAGGCATCCCT
CCACGATATGCGACCCGCCAAGTACAACAATGTGAGGTGCACTGGCAGCAAGACCTGTGG
GGGGACCGAAATAGTGCTGCCACGGTCCCCCGAAAGTCTTGCAGCTGCTAGCGCTTCGAG

AACTCGACGAATTCGAACCTGATCCCTTGTACTGTATCGCGAAGCTATCTTTTTCAAAAT
AGCTATGCATTGATAGAGGAAGACTGCCTGGACAGGTGACAGGGTGGGAAGCACTCGCAC
AAACCAAGATTCTAGGCCATGAGAGCACACCTTGTGATCCCAAGGAAAGATAGCACAACG
AATTTACTCCCATTGTTGAGAAGCTTCGACGAAAATTTCCCCAAGACTCAGCCATTCTTC
TGGTTGACGACGTTTCCAAAGGAGGAATCTCCCTCAACTACAGCCATTCGATTCTACCAG
GAAAAATAATGCAGAGGATACCCATC
>comp2466_c0_seq1
TTTTGAACAAGTTCGGGTACACCATCACACCAGTTTGTGATTTGTGATTGCTCCACAGTT
CGCAACGCCATAAAGTCTCTGACGATAACGAACCACGCAACCAGAAAGCTTGAATGGCAA
CGGCAACTTTATTATCGATCGTTCTCCCGGGAGACGATGTCACGGACAAGATTGTGCCAA
CCTCACTAAAGAAAAGCATTCCAAAACTCGGCACCGGCCTTCGAATCGAACGCGACGGCA
AAGAAGATGGCCACGAGAGAGTATTTGCAACCTGTGCGGGTCGGTTGGTACACCGCTCCA
ATACCTACTACATAAAACAGAACGTCCAGCGATATCGTCCCCTCCAGGAAGACCGGGTGA
TCGGCATCGTCGAGGATCGAGTGGCAAGCGATGGCGCCGGGGGCGATATCTACCGAGTCA
ACATCGGTGGGCCCCACCCGGCCATGCTCAACAACCTAAGGTTCGAGGGTGCCACGAAAC
GCAACAAACCAAATTTTACGGCGGGAAAGCTCGTTTATGCGCGGATCGATACGATGCCTC
CCTCCATGGATCCTACTCTTTCGTGCCAGCTGGGTCCCCACGACGCCGGGGTTCCCCGAA
AGGATTGGATGACGAACGAGGGGACGTACGGAGAATTGAAAGGAGGAACGTGCCGAAAAA
TTCCCCTCGGATTGGCCCGTGAGCTCTTGTATCCGAAGAATCTAGTCCTGAACGAGCTCA
GCAAATCCATAGCATTTGAGGTCTGCATAGGAGTCAATGGTTTTTTGTGGGTGCACAGCC
CACGGCCCGAATACACGATTCTGATTCTGAACGCGATCATGAATAGTCAGGTACTAACGG
AAGCACAGGTCAGGGGCATGGTCAAGTCGCTGGTAGACACAGTCAACCGCCAAATTGAGG
ACGAGGAAGACTAACTATTCGTCTTGGTTGTAGGGTGAACAATTTAGGACATGTGCATTA
AATAAATTGCGTGACACTGATTAGCTTAGCGCTTTGTATTTAATTGATTGCAAAACATGT
ATCCTCTAGTCGATTGCCTCGGATCTCCGATGTGCTGAAGATAATTCTTGACCGATGATC
GGCCTCTTTTGACAAAGCAATCATACTTTTTATTCTACTTCCTGGCTGAATATCCAGCCT
CAAATATATTTAC
>comp2467_c0_seq1
CAACGATTCCTAATACAGTTGTCCACTTTAGAATTGGCCAAGAGAGAAAGTAAAGAATTG
CAACGTTCAGTGTCAACCAGCCAGACCATTCAGATGGCGCTAATAACAAGAGCCTTGCGC
TTCATAATAATTTCTTCTATCTTGTCATCAGCCTACTCATCTTCTATCAATACACCGGGA
TCGACGTCGAGACGAACGAGACCTCCGAACGAGACCTCTCAAACTGCACAGGTACAACAA
TCCTTGCCCTGTGATGGTAGGGCCATAGTGACTAGCAACCGACGAGCGTGTGGATTTTTG
TTTGGGACTTGCCCTCCGCTATTCTCTACAAGTGGTGCCACAGATTGCTGAAGGATCGCA
TTTACGAAGGCAAAGGAGGCTATCTATTTGTGAGGTACGAGAGCAACTCCATATTGAAGC
TTCTATATCCTTGTAGTAGATATAGTCCCGACAAGTGCATGATTTGTGGGGGAAGCTTTC
ATGATCATCTGGAACTATGGTGGTCAAAACATTTGTACTCACCCTACGATCAGTACCTGG
ACAAGACATATAACGACGCATTAACAATAATTGAAGAGGATGACGGGCGAGGGCAGCGTC
GTCATGGTGTATCTGCTGATAATATTGGGAATAAAATAATTCGAAATCGAGTGGATCGAA
ACAGCAGACCGTCGCGAAGAAGCTCTTCCTCAATCCATTCTCTAACGGATCCACCTGCGT
TTAATTTTCACAAACGCTACGTTGAGGATGTCACCGACCTTGTTAAAAAAATTGCGAATC
TCAAAAGCAAACAACAAGTGAAGCTGATAAAGAAGAGAATAAAGCACCTGGCGAAAAATA
GACGCCACGATGTTTGTAGCGTCCCAGCAACCGATGTGACTTACGTTGCTCCTGGTACTG
CTGCTGGCTTTATCGACAACGAATTATACACAAAAAGGGATTTTCTCGTCGATTTCTTGA
CTCTCATGGCCAGACAACCTGTCAATGAAATGATTCAATTGCAAGTTGAGGAGTATACGG
AAAGGCGACTTAAAAACTCTAGGATCATCAATCGACTGGGTCAACAATCAACTTCAAACT
AATAGGAACTTCGTGAACTACAAGATTTATACGTGGGATTAAGCTCCGATCCATACAAGA
TTTTTTGTAAAATTTCTAGTTTTTCTTGATTCGGTAGTGCAATGCGCCATTCCAGACCCT
GCTCTGGAAAATAGAACCAAACTGAGATGCAACACCGACTCCCTCAAAAATCGTAAGATT
GTCATCGCTACGGACGGGATTGCCGGTTCCGTGACCTTCAGTGTAACGCAGACCCCTCCC
ATTTTCGATGAAAATATAGAATGCCTGGAGTTTGTCCTTCTTCAACGTAATAACATCGGA
GTGATCGAGCAGCACCTTGGTCGCAACATTGATTCCTTTTCCCTTTACGTTCATTTTTGC
TAGCAATATCCAATCATCGCGTTTGTTCTCTGAACCGGTAAAAGGCCCTTCTTTGGTATA
TACGTGAACACACACCGTGTCGCTATCAATGAGGTGAAGATCGAATCCATCCATATCAAG
ATCGTGTTTAGGCAAAACGTCAAACATTACACCGTCTTGACCGGACCCTCCATCGAAAGT
AGTCTCTAGGCGACGTCCACCACTCCGTTCGCCGTCTTCTGAAAGGACGTCGTAAAAAAT
ACCCCCATTCCAGATTCGATTTTGGTATACTTTACCAAATGGCCGTGTTAATCCCACACC
TTCGTATACGGTCAGATCCTCGTTTGACACAGATACGCTCCCAGCTGCAATCCCATTCGT
GTATCTAAATCTACCTTTGGTCAAAACGAGATAAAAACCATAGCGAGTGTTGGCATTTAG
AGGTAAATTTTCGATGTCCATACAGCTCTTTTTCCCTCTCCCATTACCATCAACAGATAC

AACAACACTTCTTTGCCACAGCCACGGTTTGTTTTCAAACGAGATGTGAGTGCCGGTCCT
GGTCCAAAGCTCGACGTTGACGGTTTCATCCACGGCAAGATTCAAGTAAATTTTTCGTAT
GTTGATGTCTTTCACGGGGAGTATATCAAACATAGTGCCTGACGCCCCATTTCCACCAAG
AAATGCAGATGACAGTTCATTCATTAAATTTACAGAAAATGACATGGAAGGAGCGGAATG
ACTGATTTGATGAATCAATATTCCTGTTCGGGCTGGTATCTTCTGATAAGAGTCCGTGCT
TGGATAGGGACCTTCCATAAAATTTACTGATGGAGCCAAGAAATCAACTCCTTCTTTATG
GAAGAGATCAAATTCATCACCGTTGTTGCGTCTATTTTCGAGATCGTATG
>comp2468_c0_seq1
GATACGTTCAAGGAGTCTCGATACAATACGGTGTTACCTTACGAACTTCATAATGAAGAG
GTTGCCCACAATGGGGTACGAGTCAGCTCTACGGTCACAGATTTCAAAAGGTGCGACTTG
TTTGCCGTAAAAATAATGGGAATTCCGATACCGGGTGACAAGTTTCCAGGAGATCAGAGA
CCTCTAATTCGTGGAGGGGATATTGATGTTCGTCATGCCGCGTCGCGAATTCTTTCCTCC
GTCAGCCATACAACAACTATCAAGCTAGAAAGAAGCAATGATAAGGACGAAAACGACTAC
CACCACTACTGCTGCTACTACACAGCCCAGGAGTACTGGTTACTGGCACTTATTGTCGCT
AGTATGGTTTGCATTATTGTTCATCTCAGGAACGGTGCATTATACGGTGATTCCCATGTG
CAGATAATGTCAAAGAAATATGCTAGAATCCGGTATGTTCTATTGGGAATGTCTACGATT
GCTGCCCCGATCCTACCATTTGAACACGGAGTTCTTGTGATTGCGATGTTGGGCAATGGA
TTCCATTTGTTCGAAGTAATTCGATCGCACCATGATACGACTACAGTTTACCAAAAGCTA
GAGACGACAAAACGACACGAAAAACGAATGCCGCTGGAGATTGACACGTCAAAATATGTC
GATGACTGCTCTGGTGATTTAAAAATAGAACTTGCTGTCAAATGGGATAGTACTGCATGA
ATAGGAATGACTTTACTTTCGATTGCGTGACCCTTGAACTCCAAAATATTGCTCCCGGGA
GTCATCATTACGACAAAATCATGTTCTTCCGCCACTGATACACTAGGCTCGATGAAATTT
TTCGAATTGCATCCCATTTAATGCTGACAACCTTCGAGTATTCTTTTGTAACCAGGTGCA
GCTTTTTGGAAACATCGAAGGAGAACTCAAAAACTCCAAAAACTGCATTTAGATAGGTAA
CCAATTTTTATCATTCCTACGAAAAGTGTTCTGTTCAATACTTCTCACTCAAGCTCCCAT
TCTTTGGGACTCGTGTAGATCGTGTTGCCTTTAACTATAATGAGATTTACGTTCCATGCA
AAACGAGATTACTCAAAAGTTCAACCTGGTCCTTCTTTTTGCAACGTTGACATGAAGTTG
CCGCCCAGCATTCAAGTTGGGGGTCCATTGATTAGATTTGTGGACACGAGTAGTATTGTC
CTGAATTTTTTTATACGCAAGTCACTGCTTATTTTCATTCAAGCTCCTTCGGCGTCTCAA
ACATATCCAAGAGGTTCAAAATATTGACGAAATCCTGCTTTTTGCAATGTGGGTACTTAT
TATGAAAAAGCAGTGTCCCCTGTTTTTTTTCTTTTTTGCGAAGTAGGTCCTGCATTATGA
CCGCTGCCTAATGCAACGTTCAAATGATTACAATTATCAAATCCATCTCTTTTCGTGCGA
AGTAATAGTGATACTTTGATAGCTTTGGTAAAACACTTTACTAGTAAGTATGAAATTCGT
TTGTAAGAGAGCTATCCTTGGAAAACGAGAAATATGAAAATCAAAATCGTTTTCGTCTTG
AAAATCAATCCG
>comp2469_c0_seq1
CGAAACAGGAACCATTGCCATTAATTAGAAGTACTTCAATTACCCAGCGGAGAAGGAAAC
AACAACGAATTAAAACATGAGCCAACATGAAAGCACCATCGACGAGCGCAAGAGTGATTA
CAAAGTGACCACAGAGCAGCCAAAAAACGACCAAGTTTTATCAGTCGAAGACTGGAATGA
TGCGTATTTGGCACATCAATATCGCCTATCTCTCCCATCCTCTCCAACTCAATCTAATTT
TCGAGTGCTTGCACTTTTGTTTTACGAAGAGAAATACGATGGGAACGGATCTCCGATGGC
AACCTCGCACCTTCCCCCATGGGTCAAACAGATGGTAGGGGATCGCAACTTCATTGTAGG
AACGAACGACGAGCCAGGATATATTGGAGGGGCAATTTGTGCGGAACGGGCAGCAATGGT
CCAATTGAGATTCGTCCCGGCCTTTCGTATTACTAAGCTGGTCATCGCAACGGATTGCGA
AACCGTGCCAATTTCTCCTGGCATGCTATGCAGAGAATTCTTAGCGGGACACGGAGAAAG
CGTGCCGTGGGACCTAAAGGTCATCAGCACCGGTAGTCAGTGTACACGATGTAAACGAAA
AGACGAAGACATCATTCGGCCATTGAAGGGATTACAATCAGCGAAAGAAGATGGCGATAA
ATCGTATTGCTGCATTGAAGGGCATGAAGGGCACTCGCTGCCTACACTTAGGACGACGAT
TGCCGAATTGTATCCGTTTCCATCACCGTATACCCGGCTGACCGCGTCGCAATCTGTCGC
GTTGGGAACCAAATTTGGCGAGAGCTCGCATCGCAACCGCCACTTAGATTATCTAGATTC
TACAGCCAAACGGTTGCTCGAATTAGCAATTTCGGAGGCTCAACAAAGTGTTTTGAACGA
GGCCGGACGAGAAAACCACCCGATTCATTTTGGTGCAGCCGTCGCCTTTGAAGATCAGAG
CATATCCACGAGCAATCAGCGGAGCGCGTTGGAATATGGATGCAGCCTGGATGCTGTGTC
GCAGCTAGTGCCATACATAGAGGACTCGGATTGTCCCCCCGTTTTGATAGTGCAGGTAGA
TCAGTATGGTGTCGCGCATTCTCCATTTGCCCCGGCGCGAGCGTATCTTAGCGAACACGG
TTACGAAGACTGCCGCATCATCGTCCATTCTACAGATATCAACGACGTAACTTGTTTAGA
CAAGTGGGACCTTATTAAGGTACGAGTGGCCAACCTGGCTCCTACTCCACCGGCTTGGAC
GATGAGCGAGAGCACGGATAAAATCAGGACGCCTTCGGACATCGACGATCCTTGCGATTC
GTGACATTTCGGAACGCGATGGATAATGCAACTATTTTTAAAAAAAGTTTCTTTGCCTA
CAATAGTATAGAGGATGTATGTTTAGCCCGAAAACACGAACTATTAAAAATCGGCA

>comp2470_c0_seq1
GCAGAGCAATCTGAAAAGCACCAAGCAAACAGACAAACACTTGCAACACTCATCTTCAAA
TCAATTCACAATGTCTGATACCGAAGTCAAAACCGGAGAGCCCACTGAGTGGAAGGCCTA
CTCTGAGTACGCCATCGAGGTTGATCCTGACCAAGATGACAAGGCCAAGACCATCAAGCT
TTGTTCTTTGGCCCGCCCTCACATGAGAGCCTTCCACTTCTCCTGGTGGGGATTTTTCGT
CGCCTTTTTCATCTGGTTCGCCATTGCCCCCCTTCTTTCCGAGATCAAAGTCGATCTCGG
ATTGACCAAGCAAGAAATTTGGACTTCCTCTATTGTCGGTGTCGGAGGAACCATCTTGAT
GCGATTCATTCTTGGACCCGCCTGTGACAAATGGGGTGCCCGTATTCCCATGGCTGTCGT
CCTTTGTGCCGCTTCTATCCCAACTGCCTGCACCGGTCTCGTCAAGGATGCCACCGGACT
TGCCATTCTTCGCCTTTTCATTGGTTCCGCCGGAGGTACCTTCGTCATGTGCCAATACTG
GTCCACCAGTATGTTCTGCAAGGAAGTCGTTGGAACTTCTAATGCCCTTTGTGGAGGATG
GGGTAACCTTGGA
>comp2471_c0_seq1
ACAACACCGAAACCCTCATTTGCTCAAAGCCACGAGACCAAAAGGGATCATTGTCAATCA
ATTGCGAGGGAAAGCGTACGAGAAACAATGGTTGATTCCGGAAACCAGGACAGCCCTGTG
ATGGCCAAAACGGAAAGGAAACGACGGAAGATCACCGTGGTTCAAAAAGCGGTCTTTGCG
GCAGCGACAGTCACGACGATGGTGACAGCGGGACGCCTCTGTTCCTCGTCCTCGTCTGCT
TGGTCGTCGCTGTTCCAAAGATACAGCAGAAACAACAGCATGGACTCTGCGCACGGGCAA
ACACACACGTACCTTCAAACCAACGAGCGTCGGCGGCGGCTCGGAGAAATCGAAGCCAAC
ATGGAAAACGACGACTTTGTGGCGGAGAGCCAAAAGAAACACCACCCCATGTTTTCTGTA
TCGGACACCTTCACCATTCTTGGACCGACGGTCTTTCCCAAGCCCACCTATGTTTACGGG
GATGACGTCCCGAGCGACAAGGACCTCGTGACGTACCTGCAGCCTACCTCCGGGAGTCAT
CGGCCCGACCGAGACTCGGTCTTTGTCTTTGCCGCCGAGTACAAGTTCGACACATACGTA
CTCTTTCTGATGAGCCTCTACGACACGGGATTCAGGGGGGATGTGGTCTTTGGCATTTCG
AAAATGGACTGGGAAAACAAAGAGGTCCGGAAGTTCCTGGAGCACTGGCAAAACGCCGCC
GGGAAGGACGAGATGACCGTGGTCGTGTATGTGGTTCCCTACCACTGCTACAACCTCGAG
GGGGAAGTGGTACCCAGCCACAGGGGCGGGATGCGCGTCTGCAAGTGCAACGACATGTTC
GGCCTGAGAGCGAAAGGCGGTGATCCCGATGCACCCACCATCCCCATCGCGGACAAGCGG
CACGGGCGAACCGCACAAACGATCCGCTACGAATTGTACTGGATATGGTCCGAGCACTAC
GACCCCGACAGCTGGATACTCCTCATCGACGCGAGAGACACCATCTTTCAGCAAACTCCC
TTTGGAAGAGTGCCCCGGAGGGAGAACCACGGAGACGACGGCGGGGTCCTCATTTTTTTC
GGAGAAAACGCGGACGCCACCAGCCTGGGACAAAGCAAGTACAACCGCAAGTGGCTGACC
CTTGCCTACGGGGAGAGCGTCGCCGACGCCTTTTACGACAAGCCCACCATTTGCAGCGGG
TCGACCATGGGCGAGCAGAAGGCCATCGAGACCTACCTGCGGGCCGAGGTCGCCGAATCC
GACGAATCAAAGACGGTCATCTTCGGGGCCGACCAGGGCTTCCACAACTACCTTTACTAC
AGCAACAAGATGGCCAACGCAGAAGCGATAAGCCAGATCCTAGTGCAGGACCAGGGCCTC
GGACTCGTCAACAACATGGGGGCTCTCCGCGACAAACCCCTCTCGGAGTGGGGCAACGGA
AGGATGCTCCAGGAAGAAGCGTCGGAAGCGCACCCCCAAAACACGGTCTTCAACTGGGAC
GGGACCGTCTCTCCTATCGTGCACCAGTACGATCGGCACAAGGTGCTGTCCAAGTGGTGG
TGCAAGACCAAGTACAAAGAGTACAAGAACCGGTGGAACGAGATGAAAAAATGAACCCGT
CCCAGCGAACGCGACAACAAAGGCGATCAACCTTCCGGGGCAAAGAACTAAAGAAACATA
TGTAAATCAATCAATCAAACAAACAATCAATCAAACAAACAAACAAACAGTGCGTTACCC
TTTTTTGAAAAGCATGGTGTTTGTCGCTGGTGACCGGTGTAGTGCAGTTAGTTGGTTGGT
TCGTTAGAGTGCAGGATAGAATTGTTCCTTGAACCGATCCGTAGGAGTATGTACCTATTG
ATTGCAAACATGTATTTCGTAATGGTTGTGTTGGTCAAGTAGATCGGATTCATTCATTCG
CGGAGAAAGCAGAAAGTGGACACCAGTCGTTGTGCTCCAAAGAATGAGACGAGAGTCCAT
TCATCCACAAGAACAAGGAGGATGGTTT
>comp2472_c0_seq1
CGGATTTGAGAAAATATACTTATTGATCGGAAAAACAGAATGAATCAATCGGGTCACGCA
TTCGTCAGGTACAGTTTTAGTTTTTCAAAGAGCGCGCGCAAACTTTCAGAAACCCTGCTC
ATCCCAAAACCGCCGATTTGATGATCAACCAGATATTTCTCCGGAGTTGTCGATCGATCA
TGGCCGCAATGGAAGCATCTTGAAATACAACTCATGATACAGTAGTGTATTGTCTTCCAG
ATTTTTTAAGCATTTCATTGTCAACCTTTCGAAAACATTGACTGCGATCGGTACGGTCAC
CCACTCATATAGGAAGCGAATACCAAGGCAGTCCTGGATCGAAAAGAAAATGGTGCTGTA
TGGTCAGGTCGTAGCAGGCCCTCCTGGTTCAGGGAAGACAACCTACTGCACAGGCATGCA
GGAGTATCTACGATTGCTGGGAAGAAACGCATGGGTCATCAACCTTGATCCAGCGAACGA
AGTTCCGTCAGCGACAAAGAAGTCTACCGTAGAAAAAAATGGTGCGGACGTAGAGGGTGA
CGAAAAGGATCAAGACGAGTTCGAAAATGATCTGCCCTACGAATGTCTATTGGACGTGTG
CAAGAGCGTGGTCAATTTATCGTCGGTGATGAAACAGCTCGGACTGGGTCCCAACGGCGG
GTTGGTCTACTGCATGGAGTACCTCGAGGCCCACGTCGACGAGGTGGTAGACATGATCGA

AGAACAGATACGGGAACAAGGGGACAACAACGCGTACCTGATTTTCGATTTGCCGGGGCA
GATCGAATTGTACACGCACGGGACCGCCGTCCCGCGTCTCCTTGCGAAGCTTGTCAAGGT
GTTTGATTTGCGGTTGGTGGCGATCCAGCTCATCGATGCACACTATTGCACAGAGCCCGC
CAATTTTCTTTCCGCGGCTTTGCTAGGAACCACCGTCATGCTCAAGCTGGAACTGCCGAC
CGTCAATGTTCTCAGCAAAATCGATCTGCTTGCCAACTATGGACCACTGCCATTCTCGTT
GGAATTCTTCACCGATTGCCAGGATCTAGAACGACTGGTTCCATACCTCCAGCAGCAGGG
AGATTTTGATTTGAACGAGAACGAAGACGATTTTACAATATCTACACAGCAATACTTTGA
CGAGGACAATTATGCCGACGATCCAGAGTATCAACGCGCGCGCAAGATCAAGAGCTCCTC
GCCATTCTTCCGAAAGCACACACGACTCCACAAAGTAATGGCCGAGATCGTGGAAGAATT
TGGTTTGATTTCGTTTTTGCCGCTTGATATATCCAGTGCTGAATCGGTGGGGAGAGTCGT
GGCAAAGATTGACAAATGCAACGGTTATATATTCACTCAACAGGTCAGAGGAGGTTCCGG
TTCTTCCAGTAGCTCCGCCAATGGTTCCAATACAACAACTGCTGCTGAAGACCTCTTTCA
GTGCGCACTTGAATCCGAACCATCCAACTACGAAGCGATCGCCGATATTCAAGAACGGCT
TTCAATAGGGAGAGCAAAGGGCCAACAGAGTGGGCGGCGGGATACATGAACATTCTGAAG
ACTCGGTATTATTTACGACCGTTGTTTGATTGTCGATTGCTAGATCGTTCCGATTGATTC
TTTTCTCCTCTCTATGAAATATATTAATAATCTAACCTAATGGGATGACCTATGTTGTTG
ATACAAG
>comp2473_c0_seq2
TGTGGTGGAATGGATCGGAAGACACGATCTGATCGGAAGATTGGTCTTTGGTCGCCCTAC
AACGCGATACGATGAGTGTTCTGTATTCTTGTACGAGAAGGATGCTAGTTCTTACTTTTA
TTACGGTTGCAACCGATTCAATGAACGCGTGATTGTTAGTGTATCACTCTAGATCAAACG
ACGTGAAAGATATTCTTGTTTTATGCGTTTCGAAAAGGAAACAGAAACAACCACTCGGCA
GAAAATAAGTGTTTCGGAAACAGTGAGCGGAATAAAAACTGCTTGGTTAAAAAAATAGCT
GAATGCTTTACCAGTCCGACGCCGTATCCATCCAATTCCTACTAGCAATTGCTTCTGTTT
GCGTTCCGGAAGAGGAGGAAACGAGCGATGCATTCTTCTAACAAGAGCAGCTGCGTTGCG
CAAGGGGACTCCGAGCTGTAGAAACAAGAAAAGCAAATAAGATCACTTTACCAATGTACG
GTTTCAACCGCTATATAGAAAAGCGAATAACAATCATTCATTCTTCCTTTTCCGGCAAGG
AACAAGAATGAGACCAAAAAAGAAGAACGACAAATATAGCGAGGGCGATTCTCATTGGCT
ATTTTTATCGATGGGGCACAAATTCCGTTCCTTTTCTCCTTGCTGCGTCAACCCCCAAGC
AATGAGTACGATTCCTTAAGTTGCGTACCGTAGAGAAGACGAGAGAACAACAAGCGATTG
TTTCTTCGATGCCATGCATTCGGCGAGGAACAATATTCCCGAATGGAATTGTTCCCGAAG
AATAAATATGATGTCATCATTTCTACAAAGAACGATTGGTCGCTAGAAAAGAATACCGTT
GATACCGAGCACCCCCGCATGTAGAAACAAAACGTACAAAAGTCAGACATTCCTTTCTCT
CTCCAGAAGAGATCTCAGTCGTGCTGCATCGACAGATGTATTCGACCCGCCGTA
>comp2474_c0_seq1
AGTCATCCCCCCCCTTAAGCTACTGTATGTGTACGTAGTCGCACCAATGAAATCTGTCGA
GTCAGAATGCTTTCTTAGAATTTGCCATCTCAGTGAATCTGAGATCGAGAGGTCTGATTA
GAAAAAGGTATCGAAAACATCACCAATGCTTGCTCAATTGCCATGCAGGGATGAAATGTT
TGAAAAAAGGATGATGATTCTTCTAGTCAGACGATTTGTCCTTTTTTGGATAAGAAAGAA
GAAGGAGTCACTTTATTTTTGCACATCACTCAATCGCACACTCTAGATTGTCAATTTTTC
AAAACTAAGCCAATTTACCATGAATACCTCAAACATTACCAAGAACCAATCTGTTGGGGA
CATGAGCGATGTTACAACTGTTGCATCATCTCTTGGAGGATCGTTCCCGCCTTCGCCCGC
TTCCTCTGTTGCATCCGAGTCGGTTCGATCCTGGATGGACACCGAGCTTGTTGAACCTCA
TGTTGGATACAAGAAGGTGCTTGTCACCGGTGGAGCTGGCTTCATCGGCAGCCATGTGTC
GGAATACCTTCTCGCTCGTGGAGATGATGTTGTGATCATTGACGAAATGAATCACTACTA
CGACGTGAACATAAAAGAATCGAACCTCAAGCTTCTTAAAGAATCCTATCCAGAAGAGGG
TCGAGTAACGATTTACCGCGGTGACATATGCGACAGAGATCTTTTAGAAAGAATATTCGA
AACAGAAAACATCAAATGGGTTTGCCACATGGCTGCACGTGCGGGTGTCCGTCCTTCGAT
TCAAGATCCTTTTGTCTACATTCACTCGAACATCAACGCTACAACCCATCTAATGGAACT
GTCGCACAAGCATGGCATACAAAATTTTGTCTTCGCCTCTTCCTCATCTGTATACGGGGG
CTCGAAATCCACCTTTTTCTCCGAAGACGAGGTTGTCGATCATCCGGTAAGCCCTTATGC
CGCCACCAAAAAAGCATGCGAGCTTTTGGGTTATACCTACCACCACTTATACAATCTGAA
GGTAACTGGCTTACGATTTTTCACCGTGTACGGTCCGCGCGGACGACCGGATATGGCCCC
ATTCAAGTTCACAGATCGTATCTATCGTGGCGTCGAGATGCAACAATTCGGCGACGGCTC
CTCGAGTCGTGACTACACATACATTTCCGACATTGTCGATGGTGTAGTCCGTGCGATCGA
TCGCCCTTACGACTACCAGATCTTCAACCTCGGAAAGGGCGATGGTACATCACTGAAGAA
ATTCATTGGCTTGGTACAAAAATACGTCGGCAAGAAGGCGATCATCAAGGTCCTCCCCGA
TCAACCCGGTGACGTCCCGTACACGTGTGCCGATGTCGCGAAAGCCAAACACTTGCTTGG
CTACAAGTCAAAGGTGTCGTTTGAAGATGGAATCAAACGAACCGTGGATTGGTACAAACG
CAGTTATGCCACACAAAATAACGAACAAAAGAATGAATGCCAACAGACAATACCTGTGGT

GCAAATGGCACAACTTGCTGTCTAGTGGGAATGGTATCGACTCGGTAGATACAACTGTAT
ACTAGCTTAATTAAACGAAGAAAAAGGATTGCGTTACCGG
>comp2475_c0_seq1
AGAATTTTTTTTCTGGATTAACACGGCAAGGATGTCATCAAATACCTTGAAAACCTTTTT
TTGCGTTCGGTGTATCAGATAGCGATTGATGTCCCAGTATATACAAGGACCCAATTGCGA
TAAAAATTTCAAAGATGCCGCCAAAGAAGCGACCCGCTGGGAGCAGTATTATTGTACCAG
GAACGAAGAGGAGAAAGGGAGTTTCCGTGAGAATAAAGGAGGAATCAAATACGCGTTCCG
ACGAAAGGAGGAAATCTCCGAAGGATGGTGTCATGCAGCGCTTCCTGGATTTACTGGGCC
AACCGAGGCACAAGCTAGGGGCATCGAACAAAATAATACAAGGTGCATTCCCAAAGCAGA
AGAGAAGCCATTGCTCGTACAAGTAATCAATGAACTCTCGAAGGCATCCAAACTAAAAAT
GGAAAAGTCAGGAGTAGACAACGATTTATACTATACCCTAGTTTCGGACGAAGTCGCTCA
AAAATACATCGGTCTTGATGTTTCTGCTAAAATGGTTTTACAGTGCATTGAAAAAGCTGG
TAATAATGGGATATGGACCAAGGAAATTCGAATGCAGACCGGATTGCAACTACAGGGCCT
GAACAAGACCTTCAAACAGCTAGAACAACGTTGTCTTATCAAGCCAGTCAAATCAGTCAA
TGCGAAAGCGAAAAAGCTATACATGTTATATGACTTGACTCCTTCAAAAGAGATCACTGG
AGGTGTTTGGTATTCAGGCCTCGAGTTTGACCATGAGTTTATCTCAGAACTCCGTACCTT
CGTCATGCACTGTGTGAGAAAACTCAATGGTGGGAAAGGGGTAACACTGTCTGAAATCAA
GAAAAAGACGGAGCAAGCAAAAGTCAGTAGGGTTGAATTAGGTGTGGACGATATCAAGCA
ACTGGTGCAAACTCTCGTGTTTGATAATTATATCGAAGAGGGATATAGAAACGATAGCGA
CGAGGTATGCTATGTTTATGCAAGACCTGTTTCCACAACGTGCGATTTCAAATGGTGGGA
TGCTCTTTCTCCCGACTTCCATTTCCGAGCAATCAAATTCGAAGATGGCGTAACTCTTCT
CCCTCACGAACCCCACCACCACACTGCGTAATAAACACAACATATTTTTTGATTTCTTCC
TGTAATAATAACTGC
>comp2476_c0_seq1
CTGCTGCTCGCGACAGAAACATTCATGAGACTAGGATCAAACAATTCAGCTTACAAATTT
TTGATTCGTTAGTTCTGTAGCGTCGACTTTTGAATACTGTGAAACGATAATTACTGATTC
GATGCAAGCGACGAATAGACAATGACGACACAATCCGATGGGTTGGCTTTGTCTGGGAAG
ATCATCACGAGTGAGAACGGTAAATCGACTGTGAATTCCGACAACGAAAACATTTCGGAA
AAGGACGCTAGTGCTGCAAGTGGGAAAGGATCAGCCGTGAAAAAAAGAAATGACAACATA
TGCAACCAAAATGCCGTCCAAGATGACGACGAAGACGATCAAATGATGGATATTGAAGTG
CCTTCTGGAATGGTCTTCTTGGAAGACAGTGCCGGCGACGATGGTAGAATCAACCTAATT
CAGCAAAAGCACCATACAAAAAATCTGCCACAGAAGCCAGCCGTAAATCTGCAAGTTGAC
GACCGAGCGATTTTGGATTGTTGGAATCTGACCGTGGCTTCCCACGAGGCTGCTATGGTT
GTCACTGCAACGAACCCCACAAATACTTCTGCATCATTCGATTCGTCGCCGTCGACAACA
ACAACCTCTCTGACATCGCCCTTTGTGACTCTTTTGGAAAATGAATATCGTTGGAACGCA
AAAGACAAGGCTTCTACAGCGCCAGACGCAACGGATGTCTTTGGGAGTTGGGAACCAAAG
CCGTTGGCCCTGCCGATATGGGCTATCGTTCCTTGAAAATGACAACAAATGAATAAAGTG
CACAAGGCTTCCTTTGGCCAAGGGTAGAGAAGAATGTAGCTGTTGTGTTTTAGCATTGTG
CTGCAAGCAACTACGACTTTTGACTTAGTAACCAGAATATAATAGTTTATTGACCGACAA
AAGCCCTACTGAAACCGCGTGTAAGATGTACAGGACCGCAAGGAGTTCCATCATTTGCCG
GTGTGTCTGCATCAAAAGCTTTATCTCATTAGCTTTTGACGTATGTAAAATTAAGATACA
AG
>comp2477_c0_seq1
AGGAAACAATAATCGGTGTCGTAGCTCGAACAGCACCATCTCAATAGAACAATCTCCGCA
GCAGTAAAACATGCTCAAATTATCGACCACAGTCTTGCGACGCAAGCAACGTGAAATTTT
GCGTCGCCTGCAATCTACTTCCGCGAAATCTGCTTCAGAATGCATGGAATTGGAAGACAA
ATACGGGGCACACAACTACCACCCTCTTCCAGTTGTCCTATCGAAGGGTGCTGGTACAAA
AGTTTGGGATACTGATGGCAAGGAGTATTTTGACTTTCTTTCTGCGTATTCTGCAGTCAA
CCAGGGACACTGTCACCCGAAAATTATAGGCGCCCTTGTCGATCAAGCGCAAAACTTAAC
ATTGACATCACGTGCCTTTCATAACGACGCCCTCGGTGAATATTGTGAATTTGTCACAGA
CTATTTTGGAATGGATCGTGTTTTGCCAATGAATACTGGAGTTGAAGGAGGCGAGACTGC
GATCAAGTTGGCCCGAAAATGGGGTTACCAAGTCAAAGGCATTGCTCCCGACAAGGCACG
ATGTTTGTTTGCCAACAACAACTTCTGGGGACGAACTTTGGCAGCAGTCAGTAGTAGTAA
TGACCCCACTGCTTACAACGAGTTTGGTCCTTATATGCCAGGATTTTCTCTCATTCCCTA
CAACGATGTGGAGAGCCTCGAGAAAGAATTCGAAAAGGATGCGGAGAACATCGCTGCGTT
CATGGTCGAACCCATTCAGGGAGAAGCGGGAGTTATGGTTCCAGATGATGGTTACATGAG
GAAGGTGAAGGAGCTATGCGAAAAATACAATGTTCTCTTGATCGCTGATGAAGTTCAGAC
GGGATTATGCAGAACTGGATATCGATTGGCAGTGGATCACGATGGAATTCTTCCCGATAT
TCTTGTACTAGGAAAAGCTTTGTCGGGAGGTGTGCTGCCGGTATCGGCTGTCTTAGCTAA
GGAGGAAGTAATGCTTACACTAAAGCCAGGTGAGCACGGATCAACTTATGGTGGTAATCC

ACTTGCATGTAAGGTTGCGATGACTGCTCTTGAAGTTTTGAGGGACGAGAAATTGGCAGA
GAATTCACAGGTCCGCGGGGAGCAACTAAGAAGTGGTTTGGAAGGATTGATAGGAAATTC
CTCGGTTGAACACGTACGTGGCCGTGGTCTCTTGAACGCTGTCATTATTAATCAACCCAA
AGGTGGGTATGGCGACACCGGGAAGGCTTGGGACCTTTGTGTTAAAATGCAGAACCACGG
ACTCTTGGCCAAACCAACACATGGGAATATCATTCGCCTAGCTCCCCCTTTGACTATTAC
TGAAGAAGAAATCGATCAGTGCTTGGATATCTTTGAGAGGTCTGTTCGTGAGCTTGATGC
CTAGATCTAACGGCATTAGATTATCTAACAATTACATTTAATTGAATTTCATTGACGTCT
GGA
>comp2478_c0_seq1
GATTACTGCATGTCGCTCTCACAATTGAAGATTCAACATCTTAGAAAACACTCCTTAACC
ACACAAAACTCTAAGGCCACTTTTCTAGAAAAAGAAACTATGTCTGAAACGAAAACCCCT
GTCAAGGAGGAACCCAAGGGAGCCGCCTTCACCGAGGAAGACATTCCTGAAGATGCTACA
TGGGGAGAGGTATTGCAATCCTGCTGTGTCCACAGTCCCAAGGAATGGGGACTTATCTTC
ATTGGAGTTTGTGTTGTGGTTTTCTTCCTCTACTTCTTCTTGCTTGGTCTTGATCTTCTC
GGATCTGGGGCCAAGGTCATGGGAGGATGTGCCGCCGGATCACTTTTCGGCGACGACACA
AACCCCGTTGCTGGACTCATGATCGGTATTCTGGCCACTGTTTTCCTTCAATCTTCTTCC
ACTACGACGTCCATTGTTGTCTCCCTCGTTGGAGCTGGTTCAGTTTCCGTCAACCAGGGT
ATCTACATGATCATGGGAGCCAACATCGGTACTTCCGTCACCAATACTATTGTCGCAATG
GGACAAATGGGAGATGGAGATCAACTTGAACGCGCCTTCGCTGGAGCCACCGTTCATGAC
ATGTTCAACTTCTTGACCGTCGCGATTTTCTTCCCTGTCGAACTTATCACTGGATACCTG
AACTGGTTGACCGGTCTCTGTGTCAGAAATTTCACCGCCCGTGATGGAGAAAAATGGGTC
GGTCCTATTAAGAAATTTGTCGGACCACTAACCAAGAAGATCATCATTGCGAACAAGAAC
GTCATCAAGGATGTCGCCAAGGGAGGAAATTGCGAAGAATACTACCCTATTGAATGTGAG
GACATGAGTGCTCCTACTAAGAAAACTTGCTCGCGTGTTGGAATCATCGCTTGTGACAAG
GGAACTGGAAAATGCCCTGCATTCTTCGATGCCAACGCAACTCTCGCCGACGATCAAGTT
TCCGGAATTGCTGCCTTTGTTCTTGGATTGATCATTCTTTTCGTATGTCTGTTCGGAATG
GTCACCATGCTCCAAAAAATGCTCTTGGGAATTTCTTCACGCATCATCTACAAGGCCACA
AATATCAACGGATACCTCGCAATGATTATTGGTGTAGGTGTAACCATTCTTGTCCAGTCT
TCTTCCATTACCACATCCACATTCACTCCTCTCGTTGGATTGGGAGTCATTCGGCTGGAA
CAGATGTACCCCATCACTCTCGGAGCGAATATTGGAACCACCATTACCGCACTTCTTGCC
GCCCTTCTTTCCACAACTGAAGCCCTGCAGGTTGCGCTTGCACACTTGTTTTTCAACATC
TCTGGTATCATTGTCTGGTATCCTGTTCCATTTATGCGAAATATTCCGCTGAACGCGGCA
CGACACTTGGGTAAAGCTACACGAATGTGGCGTGGTGTCCCCATTGTGTACATCCTTGTC
GCCTTTTTCTTGATTCCTTTGGCCTTGTTGGGAATTTCCCTCCTCTTCACCCAAGGATCA
ACTGGACTGACCACCTTGGGATCACTTGTCACTATTTTCTTGTTGGGAGGAATCATCTAT
TTCATTTACTGGTTCCAATTCACTGGAGGAAAGAATAGCATTGCCGAATGCATGGCCAAG
CGCCAGACCCGCCGCATTGCCATTGAAGACCTCCCATCGGATATGGAAGCGTTGAAAGCG
AAGATTCAATCGTTGGAGGAGCACACTGGTCTTCTCACAGAAGAACCCGAAGACGAAAAG
GAAGCCCCTGAGGAAGCTCCCAATTCCGTTAGTGCCGAAGAGCAAGTTTAAGTGCTACGG
CATCACTGCAGATAAAACTATTTATGTACTACAGTATGAAAAATTAAAGCTCTATAAGAA
TTTGTTTTTATTATTTCACTACAAAC
>comp2479_c0_seq1
CATAAAGAGGTAAGACATTCCGTCCCCCGTCATATGAAAAGAGGAGGATATTGGAAAGTA
GATGCATGTGAAGCATGCTGTTGTGGAATCCGTGGTAGAAGATAGCCAGAACCGAAAACG
AAGCCGACATGATCCGATGATGTTCTCAGTGTTCCCTCCGTTCAGCGGATGGCATCCGGA
ATTTGATTTCCCGGTCATTTTGAACCATCCTCGGGCTGTCATGGTCGCAATAGCAACGAA
CAAAGCGCTGGCCTCAATGCGACTAGCTTCATTGTCGGTGACGCCCTCCTGCGACCCAAC
CGCTACTATTCGATCTCAATCACCATCTTCGGTCATTTCTTGAAGAAAGCTACCTTAGTT
CCTCACAGAGCACGCGCGGGTACTTAACGGATACCGCAGAACGGCGGCGATACTAAACTT
CAGATAGTGAAATCCAACAAATGCTGTTTTCATCGTTCATCTTTTCTCCCAACTCTTCTT
GTCTTTATGTCATCTACCGATACACATAACAACATCACACAACAACACCTATTTATATCG
CTCTACCTCAT
>comp2480_c0_seq1
TGACGATCATTCATCTTCCGAGAGGAGCAGTAACAACTGAGAGATAGAAGTAAAAATCTT
GCATCGGGTCAAATTCAGAATAGGGTCACAGAATTTGAAGAAATCATTTTTTCACCATGC
TTGGTCTTATCTCAGAGCAAGCAATTAAGTTCTGGTACAATCATTACAACGTCGAGACGC
AGTGGTGGTCATTCTGGGTCCCCATGGGCTTTTGGACAGCATGTTGGTTGTACGCGAAAT
TGATAACAAAAAAGGATTTTTCGCGATGGTGGGAAGTGCACACTTTCCACCATGTTGTAG
CAATGACATACGGTTCGCTAAGTTTATACTACTGTGACGATTCGATATTTAACGAACGAA
ACGGCATTTTGTGGACGTTGTCGTACTTCATCATCGACGTCATCGAAAGTATACTCGTGG

GGCACATCCTATATGTCGGACATGGTTTAGCTTGCGTGGTGCTCGGATTGGCAAATTACA
ATTTACCGCTGCTGCGCTCTTTGAGGATGAACTCAAAAGCGACGTACATCGAAGCATCAA
GTATTGTACTGCATCAGGTGAAACGACACCGAAAACAATGGCTGTTTGGCATTTTTGCAG
CCGTGTACACAATTTGTCGCATTATTTGGATACCATTAATGGCAAAATCTCTCTTGAATA
ATGAGATGGATTTTACACACCCCGTAATTCTTGTTCTGGCTGTGTTCTATTGTTTGAATA
TTCATTGGTACATCAAAATCATCAAAATCGCGATAGAAGGAGATGGCAGAAAGAACTCGA
AGAACGAAACAAAAATAAATCCAAAAGAAAATTGAAATAACAGAAACTATGTTGGTCAGG
ATGATCAAACAAATGCTTCTCAATTAGTACAATTCGTACTGCCACAATCTGTTATTTTCT
CTGGGGTATTCTACAAGATAATAATATATTTACATGATTTTTTCTTCGTATCGTC
>comp2481_c0_seq1
ACCACAACTGCGAACACTATTGCAAAAGAAAAAATACAAATACGACCACAAACTCGAGCT
GAACCGTACCGGACTCGGAAACAAGGAACAAGACCAAAGAAAAGCACGCAAAGAGACGGA
CACAGAGACAGACGAACGACAGCTACTGTTACACCATGCCGGCAGCAACCAACAGCGGCG
GAGGAGGAGGCGAGCAGTGGGCCTGGCTGGGCCTCCTGAAATGGACGCTGGCCCACACCG
ATGGCACGCGGCCATCCAGCGAGAGCCCGACCATGATGTCGGACGAGGACAAGGCCTTTC
TGGAAAAAGTCATGGCGGAGGGTATCATCGACGAGTCCGAGCGGATGAAGTTTGTCCTGC
AGGAGTTTTCGAAGGCCATGGAGTACTACAAGAGCCAGAGCCAGAGCGATGGACAGGAAG
CACCAACAACATCGACAACGACGGAAGAAAAAGAGCAAGAGCCACCGCTACAGCCCCCGG
CGCCGCCGAACGAGGACGATCTCGAGGAGCTGCTGCAGGAACTCCGGGACATTGTGGAAC
AGGTCGACTACGCGAGGGGATTCGTGAACCTGAAGGGGTGCCGGTACCTGCTGGGGGCGA
TCGCTGCCCCAGAGGTTCCGACAACGATCCGGGACGCCTGCCTGGGGATCCTATCGACGC
TGGCGCAGAACAATCCGCCGGTCCAGAAGGAGCTGGTGGAGCTCGGATCGATCAAGGTCC
TGAGCGACCTTTTTCTGTCCGAAACACAAACCGACGCAGACGCGGACACCGCAACAGAAT
CGGGCACGAGAGCGGTCCCGCTATCCACCAAGACCAGGACCATGCAGGCGCTGAGCGCAA
TTGTTCGGTGCTACGACCTGACGGAGGGCGTCTTCGAGGGCCTCCCGCAGGCCCCGGCGA
TGATGGTGCAGGGGCTAAGCACCGATCCGAACGTTTCCAGCACCTCCCTCCGGACCAAGA
CACTCTTTTTCCTGCGGGCCTTTCTGACCTCCGACCTGTCGACCCCGGCCCGTGCCCAGG
CCTTTCGGTACGCAATCGCAGCGGTGGCCGACACGGACTCGCTGTACCTGAAGGCTTCCG
ACGAAAGCGGCAACGACTGGGCGTCGATCCAGCTCCGGGAGTCGGCCGTCTCGCTGCTCG
GGCAGCTGCTGGAGCGGAGGCTCGCGGTCGGCCTCCTCCTGCAGCGCAAGCAGCACCTGG
CCTCCAGGGGGGTCCAGCGGATACGGGAGCTCCGGGCCTGCACCGGGGAAGACGCGGAGG
CCACCCGAACGGAGCTGCAGCAGTGGGAGGCCTTTTTGCTCCTGCTGGCGCGGACGGAAC
CGGAAGCACCAGAGGAAGGGGCTTCGGAAGCCCCGAAGCTGCTGCTCTAGGTCGGTGCGA
AGCATCAAAAACAAATAGTTTATAACACCCTAGTCTACGATACAATATAC
>comp2482_c0_seq1
CAAATTCTCATAACAATACAGTACCAGACCAAGAGAAAGTAAGAGAGCAACAAAAAGACA
ACAAGAATGATTCTTCCACGTCACCAGGTCCTCTTGATTATCTCGTCGCTCCTACCTCCT
GTCATACTTCTCTGTTTTCATTTTCCCTACTTGCTCACCCTTTCGGCTTCGACTGGAGGC
TTCATGGTGTCTTGGAGTCGTTTGGGGAGTCTCCGTACAGCTGCGACAAATAAAATTGTG
CAGAAATACGAATCTGAGATAGGGCCCGTTGTTCTAAATGTTCTCGATTTCGGAGCCATA
GGGAATGGTATCGACGACGACACAGATGCGGTACGACGAGCGTTGCAAATCGCGTCGAAC
CTCGCTGGAGGGATAGTCGCTACCATTCTTTTGCCGGAGAACCATACATTCTCTACCGGT
CCGCTCAACCTAACGAGTCACGTAACTCTCCAAGTGGATGGTACCTTGCGAGCACTTAAT
TGGACCGAAACAACCTGGCCACAAATCCCTCCTCTTGTTAACTATGGAAGAGCTGAAGAC
GGCGGTTTTCATTTACAGTATCAGGCCTTTTTGTATGCTGTAAATGCTCACGACATACGA
ATAATAGGAAAGGGAATGATTGACGGACAAGGACAACCATGGTGGGATGCTGCCCAAAAC
CACTCTTCATTATTGAAAGCGGGACGTCCCAACCTCATCCAGTTGGTTCGATGTCGCAAT
GTGGAGGTAACCGGGGTCATGCTGAAAGACTCCCCCTTTTGGTGCCTTCATCCTGTTCTC
TGTAAAAACGTTCACATTCACCATATTAAGATTCGATCGCACATGTACGCATTCAATAGT
GATGGAATAGACCCTGATAGTTCCAAAAATGTGCTTATCGAGGATAACGATATTTCTTGT
GGTGATGATCATATTGCAATCAAGGCGGGACTGTGTGGCAAGACTGCGGCATTGGACTGC
GGCAAAACAAAAGAATTCAATGATGGTACCTACGAAACAGTCAATGTTACTATTCGGCGA
AACATGTTTCGCATCGGTATGGGGATTTCCCTGGGCAGCGAATGTAGCGGAGGAATTCGA
AATGTTCGGGTTGAGGACAATGTGATCGGTTTTTGTCACCCTGGCCATTGCGAAGTAGGT
TGCTGCGGATGGAGTCCTGCCTTGCATCTGAAAACCACGGGCACTCGTGGCGGTCACATG
AAAAATATTTTGTTTGCAAACAATACTATACACAACACAACGTCTGTGATTCTTGTGGAA
ACCGATTATCAGGACGATGGTAAGGATAGGAACGATAGGCCAACATTGATTCAGAACCTT
TCATTTCAAGGAAACAGTGGTGTTGGAACTGCAAAGGGCATACGCTTTGCTTGCAGCAAG
TACATGCCTTGCAGGAATGTTACGTTTTTGAACAACAAGTTTGATCCTGAAACATCAGTA
GAGTGCGAAAATGTCGAAGTTATCGATCAGAATCAAAACAATATTTGTTCTATAAAAACC

CTTAGATGACTTTCTAACCGTAAACAACAATAAATTG
>comp2484_c0_seq1
ACAGTTTGGATGGATGAGAGCCATGTCATGGTTCTTTCCAAGAAGACTGTCAAGCCAATT
AATACAAGCTGTTCCACAGAAAATCAATAAAGAAAAGTTAATTGCAACAAACAGACTTGG
TCCTGGTGTTGGGGATTGGTCATGATGATAGTGATGACAACCAGTGCATTAAGCAGCAGT
CTTAATGTTGGAGTTACTCTCTGTAATTTTTCTTCTTAGAGAAGGGGTCATGGACAAGCT
TATCAATGGCAATTCAAGCAGGGTGTTGCTTAGATGGATGGGTACTGGTGGGAGTATAAT
GACTGAACAGTTATACGTGACATCTCTTCTTGGTAGATACTACTATAGAGACATGGTGAT
GGTTGTAGAGTTGTGGTTGCTTGAGAGAAAATACTGAAAGAACTTGTGCTTAGAGATACT
AGTGCAAAGAAAATATGAAACTAAGTATGGGGGGCATGAAATCTAGGCATGGTGGTTTCT
CTTCAGGAAGATCTGAAAGAATAGGCTTCAGTCTTTGTCTTCCAACACCACAGATACCAG
AGGCCACTGCATTATAGAAACTGAATTTGATTGATGGGGGCTTGGACTGTTGTTATTGTT
GTCTACTAGTAGCACAAACTTATGTTGTTATATTGATGTAAATCAAGTGCTAGAAGAAAA
GTGAGCCAATCCTCTTATTGAAAATCAACAAACTAGTATGACATCTAAGATGGATTGAAG
TGAGAGTGTATATGAAATTTTTTGCGTTAGCGACCCAGTGTGAGCCTA
>comp2485_c0_seq1
GATGAACCATCTGTTCATCTTGAAGTATTCTTCTCTAATGTAATATTTCAGGGAAGTAGA
TTGAAAAATGCGATTCGCATCTCGATGAAACCCAATTTGTTCTTACGGTTAATGGAAAAA
ATGAATGAGTAATTTTGTACAGGTACCCGTGTCTCTCACCCGAGTTAGGTTCTTCTTCGA
GTATTACGTACTAATTGTTCATTCTTCATTTCCCCGGTACATATCAAATCAAGGTGATTT
CATCCGCGAGAAGAGTGAGATTGCCTTCTAGATAGTTGTTTGGGGACCATAGAAAAATCG
ATGGGGACCATAGAAAAATCGAAGACGAATAATGAGACTGCGAGACAAGCCACGAATAAG
GTTTATCTGAATCCATCGCTTACTCCTTCAGCTTTTTGCGAAATGAGCTGAGTTACGCAC
TCGTTATTTACTACGTGGTATTGCCTCACACAACTGAGTCGCAAACTAAACTCAGCCTCT
CCACGGCTAGAAGGAAAAATAATGAATAGCATTGCTAGCTCCCGAAAGAATTTGTGAAGA
AACTGCGGTTTCAAATAATGAATGAACAAAATTATTATTTACAACT
>comp2486_c0_seq1
AATCCTTTATTGAACTGAATGCTGAATACCGGGAAACTGATGTTACTTTGTTACGAGTGT
TTTGGATCGCATATTCTCAACTCCAAAGACTATTTCCAACCAAACAAAAAGTCCGAGACG
AACCCAACATGCTTTCGAAAGCTACCGGAGACAAGGCTTCGAGAGCAGTGATTTTTCTGT
CTCGCCTGCACCCGACTAGACGTGCCGCCGCCTTCGCAACGTTCGGTTACGGCTACGACC
GCGAATCTCGGCTGTCGTTCTCTTCGCCTCCGTCGTCCATTCGGCGCCAGGACGAAAACG
ATTCTCGCAACCTTCCCGCGAGTCCCGCGCAGAACGATGTTCCGATCGATTTCGCGGTGT
CGTCAAAAATCGAGGGCGAGGAATCACAGATCGCTACCGTCACAGTGCGCCCGGGCGAGA
GGCTTCGGGCGGAATCGGGATCGATGATATACATGACACAAGGCGTGGAAATGGCGACGA
CCCTAGAAGGTTCATCTTCTGCATTTAAACGCATGATGACGGGCCAGAATATCTTCCTGA
CCGACTTTACCTACAGAGATACAAACAACGAGAACGGTGAGGGAAAAGGGACTGTAGCGC
TGGGAACGGACTTTCCAAGCAAAATCATCCGCTTTTCTCTCGAGGAGCACGGAGGAAGCC
TAATTGCCCAGAGAGGTGCATTTCTTGCAAGCAATACGACGGTAGACATACAAATGGAGT
TCACGAAAACGATGACCGCTGGGTTTTTTGGAGGACAGGGTTTTATTTTGCAGCGCTTGG
TGGGAGCAGGCGATGTATTGATCAAAGCAGGAGGAACTCTTATCGAACGGGAATTAGAAG
AGGGAGAGGTTTTGCGCGCAACATCTGGATCCATTGTTGCTTTTGAGCCGACCGTGTCGT
ACGATATTCAAATGATGCAAGGAGTGAAAAACGTCGTGTTCGGAGGCGAGGGACTGTTCG
TTACGACACTGACGGGTCCCGGACGTTGTTGGCTGCAGGGCATGCCACCGGATCGAATGA
TTGCAGAGATCGCGAGTCGTATTCCATCCGGTGGTGGTTTCGGTCCTGTGATACCTGTTG
GCGGTATGGGGGGGACCGGGGGAGGAGCGGAAGGTGCCGAGGGTGGTGCCGGGGCACCAA
TCGATGTCCCGGGCACCACCGATGTCGATTCCGGGGAAGAAATAGTTGCAGCAGCGAACG
AAGGTTTGCCAATTGACCAAGAAGCAACCACCACGATGGACGATGCTTCGACAACGTTCT
CAAACGCTGTCAGCAGCGAAATTGATGATGCATTTCAAGAAACTACTTTCCAGGACGACG
GAACGGACTTTTCGGGACCTTCGTTTTCGGACGATACTTCTTTTTCTACCGGCAATACCT
TTCGAGACGAAAATTTTGAACAGCAAGAAGGCGAGTTTTTTGGTGGCGATGACGCTGAAA
TTTTCGACTCGGGCGCGACGGAAGAGGCTGCCGAAGCTGGCAGTAGCATACTGACGACGT
TGTGGGATATATTTAACGGCGATTAGATATTGCGAGATGTTTGTACGTATTGAAGATTCG
TTCCACTTCCGTGAGAAGCTGGGTTTGAATCATCTCAATTTGCTCGGGGGTTGGGTCCTC
TCCTTTCACGTGATGTGTGTGTACTGGCTTTCCCATCACAGCAAGAATGGGCTTACGGAA
GGGAATGGGCAAGAAAAAACGCCCATAAATCAAAATAAGGGCAAACCCCAATTTCCTAGA
GAATTTCTCCAAAACAGTTGTCGCTCCTGGAATTCCCTC
>comp2486_c0_seq2
AATCCTTTATTGAACTGAATGCTGAATACCGGGAAACTGATGTTACTTTGTTACGAGTGT
TTTGGATCGCATATTCTCAACTCCAAAGACTATTTCCAACCAAACAAAAAGTCCGAGACG

AACCCAACATGCTTTCGAAAGCTACCGGAGACAAGGCTTCGAGAGCAGTGATTTTTCTGT
CTCGCCTGCACCCGACTAGACGTGCCGCCGCCTTCGCAACGTTCGGTTACGGCTACGACC
GCGAATCTCGGCTGTCGTTCTCTTCGCCTCCGTCGTCCATTCGGCGCCAGGACGAAAACG
ATTCTCGCAACCTTCCCGCGAGTCCCGCGCAGAACGATGTTCCGATCGATTTCGCGGTGT
CGTCAAAAATCGAGGGCGAGGAATCACAGATCGCTACCGTCACAGTGCGCCCGGGCGAGA
GGCTTCGGGCGGAATCGGGATCGATGATATACATGACACAAGGCGTGGAAATGGCGACGA
CCCTAGAAGGTTCATCTTCTGCATTTAAACGCATGATGACGGGCCAGAATATCTTCCTGA
CCGACTTTACCTACAGAGATACAAACAACGAGAACGGTGAGGGAAAAGGGACTGTAGCGC
TGGGAACGGACTTTCCAAGCAAAATCATCCGCTTTTCTCTCGAGGAGCACGGAGGAAGCC
TAATTGCCCAGAGAGGTGCATTTCTTGCAAGCAATACGACGGTAGACATACAAATGGAGT
TCACGAAAACGATGACCGCTGGGTTTTTTGGAGGACAGGGTTTTATTTTGCAGCGCTTGG
TGGGAGCAGGCGATGTATTGATCAAAGCAGGAGGAACTCTTATCGAACGGGAATTAGAAG
AGGGAGAGGTTTTGCGCGCAACATCTGGATCCATTGTTGCTTTTGAGCCGACCGTGTCGT
ACGATATTCAAATGATGCAAGGAGTGAAAAACGTCGTGTTCGGAGGCGAGGGACTGTTCG
TTACGACACTGACGGGTCCCGGACGTTGTTGGCTGCAGGGCATGCCACCGGATCGAATGA
TTGCAGAGATCGCGAGTCGTATTCCATCCGGTGGTGGTTTCGGTCCTGTGATACCTGTTG
GCGGTATGGGGGGGACCGGGGGAGGAGCGGAAGGTGCCGAGGGTGGTGCCGGGGCACCAA
TCGATGTCCCGGGCACCACCGATGTCGATTCCGGGGAAGAAATAGTTGCAGCAGCGAACG
AAGGTTTGCCAATTGACCAAGAAGCAACCACCACGATGGACGATGCTTCGACAACGTTCT
CAAACGCTGTCAGCAGCGAAATTGATGATGCATTTCAAGAAACTACTTTCCAGGACGACG
GAACGGACTTTTCGGGACCTTCGTTTTCGGACGATACTTCTTTTTCTACCGGCAATACCT
TTCGAGACGAAAATTTTGAACAGCAAGAAGGCGAGTTTTTTGGTGGCGATGACGCTGAAA
TTTTCGACTCGGGCGCGACGGAAGAGGCTGCCGAAGCTGGCAGTAGCATACTGACGACGT
TGTGGGATATATTTAACGGCGATTAGATATTGCGGCAAGTATAATTATGACAAGCTCGAG
CAATGGGGACGGAAAGGGCTGTCAATCACATCATAAACAAGTGATATTGTATTATTTATT
GTTTCTCTACTTTTTGAAAGAAAGAACTCATCTCTGCTATTTGATGATAATCTTTTTCTC
GTCCCATCCGTAGAGATGTTTGTACGTATTGAAGATTCGTTCCACTTCCGTGAGAAGCTG
GGTTTGAATCATCTCAATTTGCTCGGGGGTTGGGTCCTCTCCTTTCACGTGATGTGTGTG
TACTGGCTTTCCCATCACAGCAAGAATGGGCTTACGGAAGGGAATGGGCAAGAAAAAACG
CCCATAAATCAAAATAAGGGCAAACCCCAATTTCCTAGAGAATTTCTCCAAAACAGTTGT
CGCTCCTGGAATTCCCTC
>comp2487_c0_seq1
GCTGTAGGTGCTGCTGTAGGTGCTACTGCTGGCTCCGTCCGTTCCCATAGCCTTCGGTGG
ATGGTGGCGGGAACCTCCGAGAAGACCACCGAGAACAATGCCGCACTGCGAGACCAAACC
ACGGAACAGAAACGCGGGCACGGGAGCAACAGCACCGGCCGCCACCGCTCGATGCCGGCG
TCTCCCGACCGCCACAACCGCTGGAACGAGATGTACCGCCGGCTCTTCCGGTACAAGGAC
CGCGTCGGGCACGCCAACGTCCCCCAGCACTATCGGACCAGCGAAAAAGACCACCACCAA
AAGCTCGGGAAGTGGCTGGACAAACAGCGGCAGCTCAAGGCGCAGGGGACGCTGGACGCC
GGCAGGGAAGAACTCCTGGAAGACCTGGGGGTGGCCTGGGACGTCCAGTCGTGGAAGTGG
GACAAGATGTTTCGCTTGCTGGCCCGCTACCAAGCGGAGCACGGCCACGCCAACGTTCCG
CAGAACTACACCGTAGAGGTGCGCACCGACAAGGCCGACGGTGGCGACGGCGACGATGAT
AGGCACGGCGAGGCATCTCCGTCGTCCGCCAACCTCGGCGGGTGGCTCAAGGACCTCCGC
CAGCTCAAGCTCGAGGGAAGGCTCGATCCGACTCGGCAGGAAAAGCTGGAGGGCCTCGGC
GTCGTCTGGGACATGCATTCCTGGCGGTGGAACCACATGTTCCGGCTGCTCGTCCGTTAC
CGGGAGCGGCGGGGTCCCTCGTCCGTGAACACGATGAAGCAAAGCCACAAGGAAAACGGA
GAAAACCTGGGGCGGTGGTGTTCCAGGCAGCGAGAATCCTTCAAAAAAGGCGACATGGAT
CCCGAGCGGATTGCCGCGCTCGAAGAGATTGGCTTTGCGTGGACGGTGGCGGCCTCAAAC
AAGGCGAGAAAAAAGAAAAAGAACTCTGCAAAGAGGACAAAAAAGTAACCAAGCAGTGCA
TCGCAATAACCAAAAGAGGGCAGGGGGGG
>comp2488_c0_seq1
TGAATAATTACGACTGTACTTCGGAAATCGATTTGCTGAAGCAGTGTGAGGCTACATCCT
TTTCCACGAACACTTTCAAATACAGCTACTCGTACTTGCTCTGTTCGGACGGCGTTATCA
ATTTGTATTGCGTGAAACATGAGATCGACATCGAGAATATTCTACATCACCGTTGCTGTT
ATTTGCTTAGTCACTACAACGGTTGATGGATTTGCTGCTCTTCGACCGGGCGTTACGTCG
ACGACAAAAATAACAAGCCAGTTGAAATCTGAATCTTCGACCACGGCTAACAATCTAGCG
ATTGTCAGACAAACACAACTTTTTGCTGGATCGAACGCCGGAGTCGTTCCCGGCATTGGC
GAGGAAGGATGCAATTTGCCATCGGTCTCGGGAATCAACACAAAGGACGATCTCACGCAA
GCCTTTGTCGTGAAGGTGGTCTTTCTTTCTCTGGCATTGGGAACCTTTTTGTTTTCTTCG
GGGCTTGAGCAATGTTCGGGGTTCCTCGATAGCAACTTTCCGTCGCAGTACGAATTTTTC
CGCAACACATGGCCTATAAGCTTTGGATTGATCTTTTCGCTTGCTGGAGTCACGCACTTC

ACGCTGAAGGAAGAATATGAAAACATTTATCCAACCAGGGGAGCTTGGGGCGTCTGGTAC
CTACCAGGTTCAAGGGCCTTCCACGTCGCCTGGACGGGTGCCGCTGAGCTGGCAGGGGGT
CTCGGACTCTTGGCAGCCGGGGTCTCTTCTTTCGCCGGAAATCCGAATCTGGCCCCCCTG
ACGAGCGCTGGATTGGAATCCGATTCTGCCGCGGCACTTTTCCTCCTAGTCCTTGCGGTA
ACGCCTGCTAATATTTTCATGTTCACGCACGGTGCCAAACTTCCGATGGACTCCGAACCC
CTCCCCGTTTCCTTTCATGGAGTCCGATGGATCATGCAGGTCGTTCTATTGGGATTCTTG
TATCAAATGGGCCAAGAAACATTTCAGGCTCTGTTGTCGTAGAGCAGAGGAAAAGAGAGC
CTCTCATATGAGTTTCGCAACAATTATGCAATGCCCACTTTCCCTGCTTTGGTTTAGAAA
TGGCCCTCGACCACTTGATCATGATGTCAACCAATGTTGGACGGGACTTTGGCACATGAT
GGTTCTGTCGATGCGTCATCTCCTGATAGTAAACAATGACTAGTGAACTATACCTAGTTT
CACGTGTAAATATAGATGGGAAATCGCTCAGGACAGACGCAGAAATCGCCGGAGTGGCTG
TTCCGTATTTTCTAACGTTATGTGGAGCGATCGCTTGAATTTACTACATTCAATAGTAGT
AGAAGAATAAAAGAATTACGAACGC
>comp2489_c0_seq1
AAAATACTTTCTACTTGTTCTATCGGTATTATTTCGCTGCCTTCAACAATTTTTGCGTTA
GTTGAGATTTCGATAATATTCACAAGCACACCACCACCACCACCACCACCATCATGATCC
AGCAACACCGGCAATTGTCACAGCGCTTGGTATCAATGCCGGCCGCATTGATGGTAGCTG
CATCCTATTTCCTTCTACTTACGCCACCAATTTCTGCCTTTCTTGTCGGGGGTACAAGCA
CCGTGCTTTCACGTGGCTCACTCAGCGTTGCGACTTCTTCGCTTCAGATGACAGCGACCA
ATGGCGCCAGAATCGACCAACACGAAGAGACAAAAGATGACGACAACTATGACGTTGTGA
AGGTAGATTTGACAGATGGACGGGACTACCCCATTTATATTGGAACGTCCTTCACCGACG
AACAAATCAGCGAAACCCTGACTTCTCACATCCACGGCAAACAAGCTCTGATAATCACCA
ATGACAAGATCGCCCCTATGGGCTTCCTGGAAAAATACGAGAAGCTGTTGGCCCCGCATA
AAGAAGTGCACACCCTTGTCTTGCCCGACGGTGAATCAAACAAAAATCTAGAAATCTTGC
AATTGATCCTCGACAAAGCCCTCGAGGAAAAATTGGATCGGAAATGCACACTCATTGCGT
TGGGCGGTGGGGTCATCGGTGACATGGTTGGATTTGCAGCAGCCATATATCAACGGGGCG
TTGACTTCGTTCAAATTCCCACCACCGTTATGGCCATGACGGATAGCAGCGTGGGTGGTA
AAACGGGCGTGAACCATCCACTTGGTAAGAACATGGTTGGTGCCTTCCATCAACCGCAGT
GTGTTTTCATCGATACCGACTCCCTATCAACCCTTCCAGATCGGGAACTGCAAAGTGGTA
TCGCCGAAATTATTAAGTACGGGTTGATTCGCGATAGAGATCTTTTTGAGTGGTTGGAAG
TGAACATGGAAAAACTGTTGAACCGAGACCCGGAAGCAACTCGCCATGCCGTAAAACGCT
CATGCGAAAACAAGGCCGAAGTTGTCAAGGCAGACGAAAAGGAGGCAGGTGTTCGCGCCA
CGCTGAATCTAGGTCATACATTTGGACATGCCATTGAAAGTGGATCTGGTTACGGAACAT
GGCTGCACGGAGAAGCTGTTGCGATTGGTACGGCCATGGCGACGACGATGAGCGCCCAAA
TGGGATGGATCGATAATGAATTGGTAAAGCGAACCTACAAATTGCTAGAAAAAGCTAAAC
TTCCCACCGACTTACCTCTTGATTCCCCCATGAATCGAGAAATCTTCTTGAAATATATGG
CGGCCGACAAAAAGGTAGCCAACGGTCAGTTGCGATTGATTCTTCTGAAGGGAGCTCTGG
GAAATTGTGTCTTCACAGGAGATTTTGACCAAGAAGCCATGATTGATACCATTGATGAGT
TTGTTGCAGAATGTTCCGGCGCACAGTGAGTAACTGAAATAGCTCGATTGTCAAAACAAA
AGCATAAATAACACATTGATCAAAGCTCTTCATAACAGTTTTGCTACTGTGGCATATTCA
GTTTTATCCGGGCAAGGTCTCGAGAGTGGAAAACAGTTGATAGAGATTATCATTTTTGAA
TAGGTCGTGAGAGTTGCGAAAGATTACAATTGTATGAGTGCTTGGTGCGGTTAGTTTCCA
TCGAGGGATCATCCTTCTGATAACAATTTCTAAATGGTGCTATGTAACATTTAATAGCAT
TTTCTGGC
>comp2490_c0_seq1
CATCACTGTGGTATCGCAAGATACGAGGTCCCATTCAGTGCCCTACGAACTCTCCCCAAG
TAAAAAATCGACAACAATTCGACGTTCATAATCAATAACCGGGTAACAAAGAGTGGTAAA
ATAAACGAAACACATCTGATTGAACGTTAAAACGAAGCAGGAAGTCCCGGAAGAGACCAT
GTCATCTACGTCAAATAATTATACGCCACCCCCGAGGGATACCGCTGCGATGAAGGATAC
GGGGAGAGAAGGTCGCCTTTCTTTTCGGAATTTTGCCGAGAACCAATTAAAGAAAGAGTT
CAAGGCCGAAGCTATGAAAAAATGCGATTTGCAGATCGGTGCCCTGGCCGAATGCATCAA
GGACGAAGGCCTGTTTGCGCCCTTCAATTGCCGGCCAATACAAAAGGATGTAGACGAGTG
CATGATGGTATACAACTCCGCCGAGCGTTTTAAGTTATACGTGAAAGAGCACGAGGACGA
ACTACAGAGCAAGCCGTTCGTATAGCTGCTAGGCCATTGTCGCACCAACAAAGTTCCGGG
TACACATACCTCGGACAATCACAATCTACTTTCGCCAATTCGAGAACTAGATCTTTCCGG
AAGTGACGAATCAGCTTCTCCCTTTAATTATTTATTGTGCTTTTAAGTATTATTGCAATG
CATCCGTAGGCTACAAAAAGCTCTTCAATTATCGAAGAAGAGGAAAATATACTTCTGAAA
GAACCACGAATACCAATCTGAATATGGCGTTGAAATCGGAGCCAGT
>comp2491_c0_seq1
TGGCAACGGCAACGGCAACGGCAATGGCGAAAGCGAAAGCGAAAGCGGACCCCCGGGTGA

GTGCAAGCTTGCCTACCGCAAGTTCTTGCTGTCGTCGTCGGATTCCCTTTCCCAGGGATG
GAAGGACCTGTACATCGACGATTACTCCCTCTTTGCCCCCGTGACCTCCAGGGAAGACGA
CCAAATGGCCGACTACTTCAACCGACCCGACGTCCGCTCCGCCCTTCACGTCACGGACAC
CATGGAGCAAAGCTGGCCCAACCCGGACGTTGGCTTCTCGTATACGAAGGAATACAATGC
CTGCAACTGGAAACCGGCCGAGTTCATTCGGTACCCCAACACGACCATGGTCGACGTCTA
CCGGGAGATCATCCCCATGCTGGACCGGGTCTGGATTTACAACGGCGACACGGACCCCTG
CGTGAGCTACGAGGGGACCCGGGAGGCCGTCAAGCAGATTTTGGTGGACGAGCTGGACGG
TGGGTCCTACCGGCCCTGGTTCTACCAGCAGGAGGCGGCGTCGCTGGATGTCCTGGCGGA
AAAATCCGTCCTCTTCGGACCCGACCTCGTCGCCCAGACCCTGGAGCAGAACCAGTTCGC
CGGCGAGGTCACCAGCTACCAGAACGGCCTGTCCTTTGTCACATTCCACGGGAGCGGGCA
CATGGTCCCGCAGTTCCGCCCCCAGTCCAGCCTGCACTTTTTGAAGCGCTTTCTCTACGC
GGCCGACAACGGGTCGGCACTCTCGCCCCTCCTGCCGACCAACGCCACCCTGCTGGCCAT
GAGCGACGACGACTTTGAGCAGGCCATGGACGATTGGACGGAATCCGCCCGGGACGAGCT
CTAGTTCGGGAACGTCGCGGGCGGGCCACGGGGCGAACCGGGCGGCAGGCACACCGGAAG
GACGGAAGGAACGAACCAACCCGGATCGCGGGAACGAACTCGCAGCGATCGGCGGAGCCG
ACTCTTTGGAATCGTTGCCAGCATCGACCAACGACTCTTCTACGATAAATGTGTGCGTGG
GCTAATTGCTTGCTTCATACCTGGCTCGTCGGTTAATTAATAAAGGAATGAATCAATGCC
GA
>comp2492_c0_seq1
TGAAGCAAGAAACCAGAAACCAGAAACAGTAAAAGCGACTCCGGTAGCGCGCTCTTGTCT
TATTGAGCATTTGAGTATTTAATTCGCGTCTATTGCATGCTGTTTCTCTTTTTTCTCCGA
TTGCATCAGACTGGCGCACGATACGTGTTGGAGATAATTCTTCGATAGTAATGAATGACG
ACGAAACAGAAACGATTGCGTTGGCAATTAAGACTCTCGAGAAAGGTGTCAGTCGGATAT
TGAAGCGCCGTGGTGAAACTTTCAACGACAACTCCCGCCATCGCATTCAGATGCTGATAG
AGCACGCAAGGCATGTACTGCTTGAACTTTTCAAGAAGCGTCGGAGTCTGAAAGCTTTTC
TTCGTTGGTTCCACAAGGAATGGGTCCGAATCGAGATCGATCTTGCAAAACACCCAGTAC
AGATTTTGCAGCATTCAATGCAGCATTTTCTACGGTGCCAAGACGCTTTCATATTGCTCA
CTAGTGGAGAGTGCAGACATGAATACGATATGAAACTCGCGAAAAAAACAAAGTTTCGTT
CTAGATTGTCAACGCTACAAGAGCGAGACGATGGCTACACTGCCCTATACAAGGGCACCG
GAGTTTATTCAGGTTATGGTTTTGATGAGTTCAACAACGAGATCGTTGGTATTCGGGCTC
TGCTCGTGAAAGAGCTGAAGAAATATCACAAAGCTCCTTGGGATTACCTGGAGCGACAAG
GGGGGACGGAGCTTCTAGAAAACCTGCTTGATCAGCACGAGCGCGAACAGAAAGAACTAC
AACATGCGCTGCAATCATTGAAACGACTAATGATCGAGAATATGACAAAAAGGCTGAAGC
CGAAAAATTTAAAGTTTGCCACGCAAGACAAGTTGGAGCGACTGAGTCTATCTACAAAGG
GCCAAACGATAGACTTGGAACGAAGGGGAGATGCGTTCCAAATAGACGGTCCTTTGCTTC
CCAATGAGAACAAAAATCCAGATGATTGGCATTCTTTTTCACTCGAGATTAAAGAGGAAA
TGGATCAATCATCTATCCCTCCTTCCAAAATTCAAAAGCGAAGGAGGGTCATTATAGACA
GTGACGATGGCAGTGACAACGACGATGAAAGAGAGGACGCGCCTAAAAAGAAAGGCAAGA
GACAGAACAATAAAACATCTCAAGCATCCGGTTTGGTGGTCCGAGTTGACTCCTCTCGAA
CCAATGCAGAAAAAGAAGATTCTTTAGCTGCAATCAAGTCTCAAGTAGGTATCAGCACAC
AGGACTTGGAGCAAGCACGAGAAATTTTGGAAGAAGAGAATGCAAGCACAAAGAAAGTTT
TCGAGGATGAGAAGGTTTTGCGCTTAGAAAAGATTCTGAGGCGAGTTCTAGCACGAGATG
AGATAGACGAAAATGAAGTTTGGGACGCACGTGAATGTCTACGGTATGCTTGTATGGAAG
CAGGAAACAAATATTTGTGGGACTCAAGTGTTGGATGCGTTGAAAAAGCGATTGAGAACT
TTGAAAAAGCTAAAGAGATTGTGGAGTTGCAGCAAAAGTCACAACAAAAGGTGACCAGTA
GTGAGGCATCGCTTTTTGTACAATTAAATCTGTTTTATCTTCATGGACAGGCTCTTGTCA
ATATCGGCATCTCCTTGGTAGATACCTCCCACCATAGGTTAAGCATTCCAAGGGTTAAGG
TAATGCGGGCAATAGAAGAGTTTGAAGGAGCGAAGAAACAAATGGCAAAGTTACGGAGTT
TAGCAGGACATACTAGTCAGTCAACTTCGAGGGCAAGTGTTAAGGAAGCAAACTCCTACA
TTCTCAAGTCAAAACAATTAGAATCACTAGCTTGTCGTTGGATGGGACGGGGATTATGGT
TAATATCAGAAGAAATGAAAGCAGTCACGTCATTTGAGGAGGCATCGCAATTTTTAAACG
ATGACATACTGCGGAAGTGGCAACGAGACGTATACTTTGAAATTGATGTTTTCGATATAA
CAGCCGAAGCTATTTATGCAACTTGTGATTTGGCTGATCGCTGTTACTCGATGATGGAAG
CGCTGGATCACAAATCGCCATCATCGCGGAAAAAGGGGCATGATTTGTTGGAGATCATCA
CTCGTGCTCTAAATCGTCATATTGGAATAATCGAATCAATCGAGCTATGTTCTTCTCCAG
TTCGGACAAAACATTTTCGCGATGAATATGATATTTCATCGATCGAGGACGTGTTGTTAT
ACCGCAAGAACGTCATGAAGTGGTGGAAAGAACAATGTGAAAATGAAATGAACGATGTGA
AGCCTAACCAAAATTGCAACAGTCGTCTCCCAGAATCGAGATCAGATAGCTCTTCGTTGA
AGACGAAGAAGATACTGACATCTGACGGCAGTAGGAGGAAACGTTACGAAAAACGTCGGA
AAGCATCTTCACACATGCATGGCAACAGATCAAATAATTCACAAGATTTTGGACAAGAGA

ACGATGCCGTGTACGCTCTTTCCGGGCATTATCAACCACGCCCACCAGTCAAGTTTCGGA
GATGGGGAGATGAATATCTTATTGCCCAGCAGCAACTTGCGAAAAGTGAATGTGATGACC
CACATGAAGAAACCAATTCATCCTTCTTAACTTATCCGTCTATTGCTCCGCCAAAGCCGG
TCGAATTTCAAGTTTAGGCAAAAGCTGGAACCGGACAAGAAAAAATTCATCTGATGTCCA
TCAATTATGACATCCATGGTCCACTTATACACTTGGACACATGTCCTCGAATCGACGAAA
TAGGGATCCTGATTTCTTCTTCTTGTCTGACGGTACTTTTACACCCAAGGTTACAAAGTA
GCATAATATATTTTTGAAGCCAAATTAAGTAAGCAGATTCTTTCTTGTTTTTTAAGAACA
GAAACTCTGCAAATGACCGTATATATTTATGAAATCTAAAAATGGAAAAAAAAAA
>comp2493_c0_seq1
GATCCATCATACAGACAGAAAAATTCCAATAACAACACAACACAACACAACACTTGCACG
ATGTCAACGATATTGAACGAATCCACCCAAACAAGAATGCTCGAGTGGGCACTTGCACTC
TTCTCACTCACGGTATTGGGTTGTGTCATGTACAAAAAATCGAGATTCGGGGCAGAAAGC
GAGGACATCGATGAATCAATCCACCCGAACCCAACCGCGAACACCACCACGGCCAATCGA
AAAAATGTTCCGAAATGGGAAGAACAGAAGAGGAGAGCGGTTATTTTCAAACTCTTCCAA
ACCGAGCACATCCAAGAGAGAGTAACGTGCAGTCGGATCCGACGCAGCCGTTCCGTAAGC
ATGGGATCCGCTACGACAAAAGACAGCGAGTCGCTCGATGGCGAAGAAGACCGCGAGGAC
GTATGCTCTCTGGACGGAGAGGAGGACCACGGGATAGTGATGGTAGGCGGCGCGGACGGC
GTCCACAGCGACGGCGGCACCGAACCCGACGACACCGACATTGAAACCGGTGGAGACCCA
GGCGGCACCGGCGGAAAGCCGGTGGCCAATCTCTGCGCCATCTGCCTAGAGGAATACCAC
GAGGGAGACACCATCGTGTGGTCCTCGAACAAGAACTGCCGCCACGCCTTCCACCGGGAT
TGCCTGACGAGCTACCTCGTAAAGATAAACGCGAAGGACACGTACGAGTCCTACCCGTGC
CCGTGCTGCCGGCAAAACTTTTTCTACGAAGACCGCGGCGACGGCGGGAAGCAGAACGCG
AGTGCTTCGACCGGCAGTGAAGAAGAAGAGGTGTGACGGAACACCGGTGACCCGATGCTC
TCGAGTGGTGATCGCATCGGCAATGGTTGAACCGCGACGTGTCAATGCGAATCGGGTGCT
GGCAAGGACACACCGCGTCCTTCAGCGAGAACGAAATGCTCTTGGTCTCTTGGGCTCTTG
AGTCATTATTGATTTTGGTTGCGAGACCAGCACCACCATCCATGGGCTATCATGCTTTGC
AGGGTAGAATTTGTTTGTTCCCCAACGTTATGGTATTGGTCAAACAAAAACAAACCAACA
CTCAGTTAACAGAAGAAAATGAACCATTGTGATTTTGCAAGAAAATGCGAATTTTGTTCC
TTCAAGAACACGGGTTGGCTCTATCGATCCGACCAACACACCCGAGTAGCTAGTAGTAGT
AGTAGTACC
>comp2493_c0_seq2
GATCCATCATACAGACAGAAAAATTCCAATAACAACACAACACAACACAACACTTGCACG
ATGTCAACGATATTGAACGAATCCACCCAAACAAGAATGCTCGAGTGGGCACTTGCACTC
TTCTCACTCACGGTATTGGGTTGTGTCATGTACAAAAAATCGAGATTCGGGGCAGAAAGC
GAGGACATCGATGAATCAATCCACCCGAACCCAACCGCGAACACCACCACGGCCAATCGA
AAAAATGTTCCGAAATGGGAAGAACAGAAGAGGAGAGCGGTTATTTTCAAACTCTTCCAA
ACCGAGCACATCCAAGAGGTAAGTTCGAACCAACGGAAGCAACGAAAAACACTAAAGCCA
CACAAATCCATTCGCGCCACTTCCGTTTTCTCACGCGCTCACGGCAATTCCTGTAAATTA
TGGCTCCTGCATGATCACAGAGAGTAACGTGCAGTCGGATCCGACGCAGCCGTTCCGTAA
GCATGGGATCCGCTACGACAAAAGACAGCGAGTCGCTCGATGGCGAAGAAGACCGCGAGG
ACGTATGCTCTCTGGACGGAGAGGAGGACCACGGGATAGTGATGGTAGGCGGCGCGGACG
GCGTCCACAGCGACGGCGGCACCGAACCCGACGACACCGACATTGAAACCGGTGGAGACC
CAGGCGGCACCGGCGGAAAGCCGGTGGCCAATCTCTGCGCCATCTGCCTAGAGGAATACC
ACGAGGGAGACACCATCGTGTGGTCCTCGAACAAGAACTGCCGCCACGCCTTCCACCGGG
ATTGCCTGACGAGCTACCTCGTAAAGATAAACGCGAAGGACACGTACGAGTCCTACCCGT
GCCCGTGCTGCCGGCAAAACTTTTTCTACGAAGACCGCGGCGACGGCGGGAAGCAGAACG
CGAGTGCTTCGACCGGCAGTGAAGAAGAAGAGGTGTGACGGAACACCGGTGACCCGATGC
TCTCGAGTGGTGATCGCATCGGCAATGGTTGAACCGCGACGTGTCAATGCGAATCGGGTG
CTGGCAAGGACACACCGCGTCCTTCAGCGAGAACGAAATGCTCTTGGTCTCTTGGGCTCT
TGAGTCATTATTGATTTTGGTTGCGAGACCAGCACCACCATCCATGGGCTATCATGCTTT
GCAGGGTAGAATTTGTTTGTTCCCCAACGTTATGGTATTGGTCAAACAAAAACAAACCAA
CACTCAGTTAACAGAAGAAAATGAACCATTGTGATTTTGCAAGAAAATGCGAATTTTGTT
CCTTCAAGAACACGGGTTGGCTCTATCGATCCGACCAACACACCCGAGTAGCTAGTAGTA
GTAGTAGTACC
>comp2494_c0_seq1
CGATTATGTCCGAGTGACCTGAGAAAAGATCCTTGCAAGCCATCGCAAAGAACACAGTGC
CCGACCCTTCTGCGCTGCACCACAGCAAGCACCGACCGACACCGAATCGCACCACCATGG
CAGTGAAAAACATCGACAGCAGCAGCGGCATCGGCGATTACCGCCCCCACGAGGGGACCG
GACGACTCATGATGTTCTTCATGGGCTTTGTCGCGATCATGGGCCTGACCTTTGTGAGCC
AGCTTCGGGACTACCACCGCACCGACCACGGCGGGGGCGGTCGTTCCAATGCCGTGCACC

TGTCGCAGCCCTCTTTCCCGGCGGCCGATTCCAAGATCGCCGCGGGGGCGGAACAGATGG
TCGAGCACCTGCAGCGACAGGCGGAGCAGATTGCGGCGGAGACCGCAATGTTGTTGGTGC
CCAAAGGATTGAGCGGGGCGGCGGAAGCAAAGCCCGAGGCGAAGCCGGAAGTAAAGCCGG
AAGCAGAGCCAGAAGCAAAGCCCGAGGTGAAACCGGAAGCAAAGCCGGAAGATCCACCGA
AACCAGCGGAGCAAAAGAAACCGAGACGCCTGAACGTCGCTCTGTTCTACGCCGACGACT
GGACCATGAAGGTTCTTGGAAAGCTCGACAAGAACGTAAAGACGCCCAACATCGACCGGA
TGGCCGACAACGGGATGATCTTTACCAACAACTGTGTGACCACCAGCGTGTGCTGGTCGA
GCCGTTCGACGCTGGCCACCGGGACTTACTCGGCCGTGCACAAGCACACCTTTCCCTTCC
AGGAGGCTGACTTTGAGACGGAGCACTGGACGGAAACACTCTACCCGCTGATGAAGCGAA
GGGAGCGTGGGAACTACTACACCGGGCTGTTCGGCAAGTGGCACAAGCTGGAGAAGGAAA
ATGAACTCAATGAGGCCTTTGATGAGAGCATCCGTTACTACGGTAGCCACTGGGAGGAAC
GGAACGGAAAAATGCGCCATGTCACGGAACTCAATGCGGCCGACTCACTCTGGTTCCTGG
ACAAGTGGGACTCTCGGCGGAGGCAAGTCCCGGCCACCCCAGAAGAAGGGGACGACCTCA
CTAAGCCCTTTTTCTTGACGACGTCGTTTTTTGCGACCCACGCCCGGGACGGGGAGTTTC
CGAGCTACCAGCCGCTCAACGCCACACGCGAGATCGAGTACCCCGACTCGGTGATCATCC
CAAAGCCCAAGACGGCCACCAAGAAGCACTGGAACGACCTCCCGCACTTTCTCAGGCACG
ACGGAAACGAGGGCCGGACGAGGTGGAAGAAACGCTTCGACCCGGAAAGCTTCCAGGCCA
ACATCAAGGACATTTACGCAATGGCCACAGATGTCGATGCGGCCATTGGAACCATTGTGG
ACAAAATCACCCAGCTAGGCGTCTTAGGTGAAACCGTGCTGATTTTTACAACGGACAACG
GAAACATGCACGGGGAGCACGGCCTTGCCGAAAAATGGTACCCGTTCGAGGAATCTCTCC
GGGTCCCACTCGTGATTCAGGATCCCCGCATGCCCAAGGAGCGCAGGGGGACCATCAGTG
AGGCCTGGACCCTGAACGTGGACCTCACACCGACGATCCTTGGGGTCGCGAACCTGCCAC
CTAGCGATTTTATGCAGGGCCGTGACATTTCCGACCTCTACCTCTACAAGGACGAGGATA
AGGAAAAGGCCCTTTCCAACACCGAGGTGGCCGATAAGGTAGCTTGGCGCAAGGATTGGT
TTTACGAGTTCAACCTGGGCATGAAAGACGATGCCAGTGACCATCCCTGGACAAACTTTA
TCGATGCCTCTTTCGCACTGGTGACAGACGAGTGGAAGTACGTTGTGTGGCCGCAGCACG
ATGACTACGAGCAGCTCTTTCACCGGAGTGTAGACCCCTTTGACGAATGGGACCTGTTAA
ACAAGGTCCTCCGCGGGAAAAACGAGGAGGTTGAGGTGGAAGGAAACGGAGACACCGTCA
ACGGGCCGTTCAAGGACACGGTGCAAACCACAAGGAAAATGTACGAGGAAATGAAGCAGC
GCTACGCCGAACTGAAGAAAAAGGCCCAGGCTGGAGAACGGATATAGGCAGGCGCGGAAT
GACGCGATTCGACGCGATGCTCACCGCTGTAGAAGATTGTTAAATCAAAATTGAATTAGA
ATTGGGATGTTCTAAACGCAACCACAGAAATGACACGGAGCGTTTTGTTTCATTTCATTT
TATTTCGTTTTTTTTCTGTTCAATGTGTTTCATTGCATTGGGTTGCTACATCGTTCGGAT
CCAATGTTTTGTCATTGCATTTTTGTTTCTATTTGGTGCAAGTGCGACAACTTCGAGCAA
GCTCAGAAGTCGTCAGCAGCTTTCAAAAACGGTGCTTGATTGCCGCCTTGCCATAAAGTA
GAATCAACAATCTCATGGTGCGGGGCGGACGCAAAGTTCCGTACATATAGAGACACAAGC
AACTCGGCCGGCATCAATCCCAGCGATTCGGGAATCGTACAACGACTTCATCCAGATTTG
TCCTCATTCGCCATAGTCCTTTTGATGTCCATGTCATTGTCAACGCCTCC
>comp2495_c0_seq1
GGAGGAGAAAGGCCCGGCACAACAGCTCACGTTCCGTCATAAAATTATGGTCACCTTCTA
CATAACGATGTTCGATGAGGGCTACGAGATAGAATTCCAGCCAGTAGAAGAGACCGAATC
ATCGACAACCCACAATTGCGGTGTGCGACAAAGAATTCTTCGAAAATGATTTCAAAAGAA
GAAGAAAATAAAAACAATGCGTTTGAGGTTATTACCTATTTTGATAGCGCTACTAGCACA
TCGGCTGTTGAGAATCTTTTGACACCTATCCAGGATGCAGCCACCCGTGCGACCATGGCG
TTTTCATCGGCTGGTGAAGCAATCGTTAATGCAGGCGAATATATTTTATCGCCGCTAAAG
GAGTCATCGCCGAGCAGCAGAAGGAACCGCGTGAATTTCTTCAAAGAAGAGGACGACACA
GAGAAAGAACCCCCACAGGTAGATATTCGGCCTCGTGTCGATGGAACAGGAATAGAACTT
GTTGCCAATAACGACCACTGCGCCGCTAGAAAAGAAAACACCAACAACAAATTGGGAACT
TCTTATCTACGCGGTTCCATGCTGATGTTTGATCCGATTGCGGCAGAAGCTGAGAACGGT
GAACTGGAAGCAATGAATCTGAGAGTCTTGCGCGGAGAGAGCCTTCCTATTGATCGCACA
AGAGTGCGCATCGGCGTGCTCAATCGCGAAGGTGCAGCAGTGGATCAAATCATAGGATCG
ACCCCTTCGAGTGAGGGGACTCGCGATCCAATATGGGGCTCTAGGACATGTTCATGGACA
CTGAATGCAAAGGAATCGACAAAGTTGCTATTCACTCTAGTCGCAGCAGATTCTGAGAAA
GGATCGAAGGTAGAGACCAAAACAAACGGAGCTATAAAATTTTGCGCTGAGATTTCGGTA
GCCGACCTTCTATCAAAACGCAACCCGAATTTTTGGATAAACCTAAAGAATGAGGACGAT
CCCTGTAGCAGCAACAACAATGGCCGTATTCAGATCCGTCTGACCCGACCCACACAAGCA
AAGCTGCACGAACAAGAAACCTGTTTGAGAATTTCGCCCGGCCCAGGACTCCCTCGAGTT
CAGAGCTATGCCATTCGAAAGATGGCCTTATATTCATGCTCGCCCGTTATGTTGAATGTG
TATGATGTATCCAACAACCCGCGAATACAAACAGTCAACAACACAACGAAAGCACTCGGG
TACGGTGGAGTCTTTCATGCCGCGATCGAGATCCAGGGAAAGGAATATTCATTTGGAGGA

ACGGTCGACAAGAAATCAAAGGTGTCGGGGGTTTTTCAATGCGCTCCTAAGCAATGCCCT
ATGCACAGTTATCGAGAATCGGTGTTCCTTGGCGATAGCGAAGTCAATGGGCAGCAGATT
CAGAGTATATTGGCGGAACTTCGTCCGAAGTGGTTGGCAAGATCCTATAATCTATTCCGG
AAAAACTGTGCATTCTTCAGTCGCGAATTTGCAATCGAACTAGGCGTCGGTGACATTCCA
GAATGGGTGTTTTCCCTGGCTACGACTGCGGAAACGATAGAGCCATATTTGCAAAAGCTC
AATGCGTACCTCAAAAGTAGAACAAACGCTGCCTCGCCCGACAAACGAATCACCAAATCA
ACAACTACGACGTCGACAAAACAGAAGTCCAGCAAGCGTGTTTCCATTGATACGGACGAA
GACATAAAAGCACACGAGGTTGCGAAGAATACACAGGAAGCCCTTCTAGATCATGCCATG
GCTGCCAGAATCCAGCGAAGAGTCAGGGCTTCGAACGTCAGGCGCGCGAGCATGTATCGT
GCACAGACCCAGCTCAAAATTGAGCCGGTAGGGTTTGCGTCGGCATCGGCGTAGGCAGAA
AAACGACTGTTGGTTTCATCAGAAACTATTCATTGCTCCCAACTGTAGTGGAAGCATCTA
GAGTAAGGGCGGATTGAAAAATAAGGAAGTCAGAACTGAACAATGAGCTAAAGACGTACT
TGTTACTATTCTTTTTATGAGTTCAAAATAAAAGTAATTAAAACTAAACAATGAATAAAA
AACAAAGTAGTG
>comp2496_c0_seq1
AATCCCCTCGTCGGTACTGTTCCCTGGTCAATAAGTATCTTTCGTCGGCCCATGGCTTCT
GACTGCCCGTCCTGCATTATCGAGAGATAGACACTGTATGGCACCGTGCATTCCAATCAT
TGCACTGGAGCCATCACGAAAATTTCGCCATGCCAAATCAATTTTTCATAACAAGCCCGG
AGATCGCAACCGTTGGTCAGAGTGGTCAAGCTTACTTGCAGAGAACTGTGGTAATTGTTC
TGGCCGTCGCCTGCATCGGGATCAATATTTTCAATTTGCGCGCGGACTGTAGATTGGTGC
TCTTCACTTGGACTGTTGATCTGAATGCTTATGAGCTGGAACGAACCGCCAATGTGACGA
ACACGGTGTCTGATGTTCTTGGCGTCGTTGGAATTCAGTTGGATAACTCTAATCGGAACT
TCGATATATTAGATTATTACGTTGAGGGGGACGATGGACTTTACTTTGGACTCAATGGCT
TTGCCTTTGTCAACAGCAAAAGTTCAGAATATTGGAACAAGAAATATAAGTGTGTTCCGT
TCGACTCGCTACCCGAGACAAAAATTACGATGACGGAGGAAGACGTCAAGTTCTTCGACT
CGATAAAAACTGTTGCCATCCTGTCTTCTTATTTTGGTTTTGCAGGAGTGCTCTGCGCAT
TTGTGGAGTTTCTCTTTTGTGTCTTTTATCCGAGCGTCATCGCAGGCTCCGTTTTCTTGG
CGCTAGCCGCGTTTATGAACTGTGTGTATTTGTTGTTATATTATCTTGGAAGCGTAGAAT
GTTTCACTGCAGGGATGTGTAAACCGGACCTGAATTTTTTTTCCTATTTATTTACTACGG
GAGGGTTTTTCTTTGCGAGCTGTCTATTGGTCTTCGGATTTAGAACCTACCCCTGGTTCT
TGCGAAAGAATCGAGATCCCGCAAATTGTAAGGTTGTTGAGGCAGACATTGTCGATGAAG
AGGGGTACGATCGGTCGGAGCATCAAGAAGAGCAAGGTGGTGGCGTCCCAATCGAGGCCT
ACCGGGAACTGGAAGAGGAGCTCGCTGAAACAAAGCGGAGAATGCTCCGAGCCGAACGAG
AACTCCAGGATTATCTTGCCCAGAAGGCCGGGAAAACATCGCCTCACACCGGCAGAGGCC
ACACTGAAAGTAATGCTTCGAACGACAAACGCATTGCATTCAGACACTACGGCTCGCAAC
CCGCGGAATAAGAATCACAAGAGAAAATCGATGGATGAACCAATGGGAAGCCACCAGGTT
TCGACGAAAGAAAAGGATGAACGAATCGAACCATGAGAAAACGTTGTCACGACGGATTCA
GAGACGCAACAATATGTGGAAAATAGCCGTGTTCCAAACAGCACCAAGAGCCGCAAAATC
CTCAGAGCGCTTGCGAATTGGTCGCAAAAAGATGTTCATTGCGCTTTGCAACACCAGATA
CCGCCCCCTGTCTGGTTGATCTAGTTCGGCCTTGATTGCTGAAAAGCATCGCATGCGCCC
TCAAATTTGATTGGTTGCAAAAAAGATACTTTTTTTAAACAACACTGTTTCACTCTATCA
TAGTACTTCTTTTTTTAATTGCAAATTAATTTCGTC
>comp2497_c0_seq1
AAAAACTAGAAGGCGCCTTCTAACAATGCGAGTACTATGTACCGTACTGCACCCCTTTTA
TTGCATGGCATCCTCGCCGTATCGTACCGGTAGGCTCTACCAGGCGAGGCTGGTGATTGA
TTTTCTTTTGAGAAGGAGGAATCTTCTAAACGACCCGAATAGTCGTTGATTGCCCGGCAA
GCTGGGCGTCGAATAAAGATATACCGATATGTCTTGTGACACGAACGAGTGATCGATTGG
ATGTTTGGCGCGATGCGTATGGCACGCCGGTAACTGCACCGAAACTGGAATTCAGATGTT
GATTTTGAAATTTCAACGAACGGCTCGAGTTTCCGATGGATCCTGTGAACGCCTGGAGGG
AGTCCGAAAGGATCCGCCAAATTTTGTTGCAAAAGTAGAAAGGAAAAACCAACCCATCAA
TTTTCGCTTCAATCCCTGAAAAGGATCGTTCATTTTGCATTCTGCGAAAAAATCATAGAA
TTTCATTCGATGCCATTGTCTCGCAACCCTCCCATCCCTCAAGAGAAAATTACCAAGCAA
TACAATAACTATTACAGAACTAAAATTTGCACCATGTTGGGAGAAGAAAGCGCAAAGCCT
ACTGACGGCACCCCCTCCTCGAAGAAGGGGATCTTCATGTGGTTCAAGGAGATTTATTCG
ACGTGCCTGTTGATTTTCTGCTCGATCATTGTCCTCACGGTGATCTTTGAAAAGAATACC
AAGATCTCGGAAGCAACAGCATGGGGTGCCTTTTTTTCTCTTTTGGATCGCCCTCTACTGG
CTATCCATGGTTGAGGGAGGACAGGCGTCTCTCGTTGGTCTCCCCCCCGTGGATATGGAG
CTCTACAAGGAAAGCCACCCCACCACCCACAAGATCATGGCCATCATCAACAAAGGCGAA
ACACTCGATCGGTACCTGATGGGACGCCAGTTTATGGTGCTGGCCCTGGTCTTTGTAGAG
AACCTCTGCGTCCACCCCATCGATGCGTCCTTGTCGATTCTGGGCATGCCTATCGCCATC

AACAAGATTTTCCTGGACACCGGATTGGCCGTCTTTTTCATGACGGCCATGATCGGAAAG
ATCTCGGCCCAAGTGAACGCCTCGAGGTGCATGTTGGACTACGTCAATAACTTCTTTGCC
TACTTTACCATGCAAACCGCCCGGTTGATCGAGGTCTCCGGTCTCTTGCACTGTTGCTAC
CCCGTCCAGATATTTTTCGCGTGGGCCGCTGGCCAGCCTCTCGAATCCAAGGAACCACCA
CGAAGTCTGGTACAGAACATCTTCTTCTGGGCACGCGTCCTCGTGTCGACTGCCATTCTG
TGCTTCGCCTTTGCTGTCACCCTGTACGCCATTCTCAACGGCCAGACCACCATGTGGGAA
GGCGTCCCCAGCTGGCTGACGATCTTGCTCTTTTTCCTCTTCATGTCCGTGGTCGGTATG
CTCGAGGGCATGCAGATCGCTTTCTTTGCCGTGGCCCGTATGACCGAGGCAGAACGTGCC
AGGTCTACCTGGGCCAAGCGAACCTGCGACGTCCTTTTCGAGGGCGACGGGCGCAACCTT
CCCGGATTCATGATCGGCCGTCAGATGTGCGTGACGCTCTGTTTTTTCATCATTGCCCGC
GTCACCACCGTCCAGCTCCAGGCCGGGGACGAAAATATCTTTGGGGTCTCCGATGCGACC
CAGGCCTTTTTCGCCACAGGGCTGCTCGGGGCACTCATTACCACCATCGTGGCTTCCATC
GCATGGCAGCTCGTGGCGTCGGCCTTTCCGATGGCCTTTTTGTCCACTCCCATCACCTAC
GTTCTGCTCAGGTTCTGCCTCGCACTCGAGTGGACCGGCATCTGCCAGGGCTCCTGGGTG
GTGGCCCGCATCCACCGAAAGATCGTCAAGTTCAAGCGCGACGAGGTCTACATCGGAACC
GCCGAGGAACGCGCTGCAAGGGCCAAGGCCAATCCCAGCAAGTTCGAGGAAGACAAGGGA
GAGAAATACGACGTCGTGCCAGGGCACATGTATCCCGGGGTCCCCACCCTTCCCCCCGAC
TTCAAAGGGGTCCACCGCTCCCTGGAAGAGATCGAGGACCTAGAGAGGGACCTCCTGTCG
CGCCAGCAGGAAATCGAGGGCCGCCTGGCAGAACTCAAGGCCCAGAAGGCAATCCTAATC
GGGAACGGAGTTGAATCGACCCGATCGGTGGATGTGGAGTGCTAATTCATAACCAACACC
GCAGCAGAGCGATGCGCACTCCGTCGCAAGTCAAAATCCATCACTACACTGCGGTTTGCC
GCAACGATGATTCGTTTCTTCATGGATGGAAGGCGCTCCAATCTGTACGCACTCTCCACA
TCCACGTATTGGATTTCCCTTCTTCATTTAAATAATTGCAGTACGAGTACTATTATTAGG
TTTGTGTATTTTGAAACAAATTTAATGTCTTTTACAGCGAG
>comp2498_c0_seq1
CAGATTTCCTTCCTCGAAGATTTCTGATCTCGGATACCCAGAGAGCATTTCTTTCGGAGT
GATTTTCGATGCAACGACGATGAAATACTATTTCAGACCACAGCAATTAGTAGGCCTTTC
TACCATATTGGCTTTTCTCGCCAGTACCTGCCGCGCCTCTTTCCCGGCAAACGAAACGAC
ATATCTAAACTTTCACATTCCCACCAGCTTGCGACACAATAAAACATGGCCCCATGCTGC
TTCCATGTTTGGACGCATGGGTCACGGGCCCGAGGGGTCTCTGGTCTTGCCGGTGAAAAT
GTACGTGGATTCGCGTTACTTGTGCAACGATAATTTCGATTTCACCGAGGTGAACAAACA
ACTGAAGATCCCCTCAGACGGCGGCGCGCCATTTATCTTGTTGGTCGAATCTGGTATTTG
CACCTTTGTCAAGAAGGTCCGCAACGCCCAGCACCTCGGAGCTGCCGCCGTCTTGATTGC
CGACATGGAACACGAGCACTTGCCCGACAACCTGGAACTGAGTGGCGACATTCTCCACAG
AGCCCATCCACATCACGACACAAGCAACCACACGGAGCACCGGAATGCATTCCGTATGGC
CGACGATGGTTCGGGTCGGGATATTTCTATTCCCAGCATGATGATTGCCAAGAACGAATA
TGACGAACTCAAAAAAGTCATTGACTCGAAGCCCAATTCCACTGGGATTGTTGTCGCCGA
AATGTCCTGGCACGTTCCCAAGTTCGTCAACAAGGTTATTTTGGATCTTTGGCATTCGCC
TGCCGATATGCACTCCAAGCAATTTCTGGCCTCAAATTTCTCCGTCATCGCCAGGACGTT
TGATTTGAACGAAATGCACGGAAAAACCCACGACAACGACAGTTATAATGAGAACATGAA
CCTATTGCAATTTAGGGAATGGCCTGTTTTGCTGGACGGAAAAGCCTTGGGATGTGTCGG
AAATACAGATGCCCCGGACGAACCCTGCTACTTGCTCTGCACGAACGGGGGGCGTTACTG
CCACGCCTCGCATCACCTCACCGATGGAAAATACATCGTCAAAGAAGCCCTGAGGAGAAT
GTGCATCGACAAACACTACAAGAGTCCGAAGTTTCACTGGGACTACATCGATCACTTTTC
CAAGTTCTGTTGGGATTCCGATTACTTTGCCAACGAGAAATGCATCGAGGATGCCTACAA
GCATTCGAAAATCGACAAGAAGGTTATCGAATCGTGCTTTGACGATAGCGGGGATCCCGA
ATCAGATCATTCGAATGCACTCCTGCAGCACGCCCTCGATATGCAGCTAAAGTGGGGAGT
CCACAAGAGCCCGTCCGTGACCATCAACCACGAAATCAGCCCGCTCGTTGAGTTCCAAGG
TTTAACCCCGGCAACCATCCTGTCTGGCCTCTGCGACGCCTTTGCGTACGGCGATAAACC
CCATGTTTGTTATGCCTGCCAGCACTGTGGGGATCCCGTGAAATGTGCCCAAAGATCCCC
CATGAAGTGCCTGAGCACCGACGGTGTAGAAAAGGAAGACACCAGCGCTCACAAGGAAGT
AAATGGGGGCGGCAAGAAACATAAGAAGAAACGTTCCTTTTTCTGGCGCCATTTTTTCAT
TGGTGTTTTTGTGTTGGGAGGACTTGCAGGCGGATATGTTTACTACCAGAAAATGGAAGA
GAATGGTAGTGGAGGGCTCGGATCTTACACGCTGCAGGATGCATTTTTATCTGACTCCAT
GTAACTTACATATTTGGCAATTATAGCGACATCGAACGTCGTAGCTCATTTCATGAGATT
ATTTTGCAAGGAAACCAAAAACAAACGAAGGAGAACAGTCGACGTGCACTGTAGTAATGA
TTGATTGATTCATATTATTGTAATTTATACGATGCCTGCCGTAACGGTACAGTGCACGCG
ATTGTCAACATCAAGAAGCAATTACACATATTTTAAAGATTTTATAACATAATATGATAA
ATGTCCGA
>comp2499_c0_seq1

CTTCCGAAGTCGATTCGAAGCAACGTAAATAAAAATCATCTTGCAAGCGTATTCCAATAT
TCCAATACTTCGCCTGCAACAAACTCGGATCGACTTTATTGTCTTCGACATTCTACATCC
AGCGTCACCTCCTATACTCTAAACAGTAAAACAAAATGTATCGTTTCTCCTTGGTTATCT
GCTGGGCGATGATGGCCGCAACGGCCATGAGTGGGGTCTCGGCCTTCGTCGTCCCAACGT
CTCCGGTAGCATCCGCGGCCGCGAGATCATCCTCGAGCACCCTGTCTATGGTAGACCCAA
CGATAGCTTCTTCCGTCGCCGAAAGCGTCGGTGTTGCGATTTCATCCGGTAGCGGAATGT
TGTTGGCGGAAACCGAGGCCTGGGTAAAACCCACCGCGACGGTATTGGGACCATTCTTGA
ACTTTATGTCCTTCGCTATGTTGTGCCGCGTGGTTGCATCGTGGTACCCCGATACCAACC
TGAATGAATTCCCCTGGGCTGTCGTGGCCTGGCCATCGGAACCCTTGTTGAGACTGGCAA
AGGGTAAGATTCCTCCCGCCTTCGGCGTTGATATCACACCCGTATTCTGGCTCGCTATTT
TCACATTCATCAACGAAATCTTGCTAGGACAACAGGGGCTATTGGTCATGAAGATGAAGT
ACGGGATCTAAGTTGTCGAGACAATACTTGATGATTTTATCGAACGAAGAACGGAAGCAC
GGACGACTCGACAAATATACTCGGAACACAATAATACTACAAATGCTACGTCAGACTTGG
ATCGATCCGATTGTTTTACCAAAGGAGCGGAAGCTCGCCATCATTGCATTGCTAATCAAA
CTACAGTATACGCCAAACCACACCACCAGGCTTTGCTTGAATTTATATTGACTAGGTTTT
CTATGAATAGCCAAGATTTATTCTTGAAGTGAAGATTAGCCCTGTGAATGCTGATGCACG
ATAGCAAGCAATTGGCATTGGTGCGATCTGATGACTGCACT
>comp2500_c0_seq1
CCCACACCCCGCGACAAACATACAGGAATCATTGAACCGACCCACGGTCTCAAAAGAAAA
CCACTCCGTTCACCCGTCGAAGACACTACTATGGTTCGAGCGGCGAACGAAAAGTCGGCG
GGCTTCTACTTCGTGCTCTTCCTGTGCTTTTTGCTGGGTGCTGTCTGTATCTACCACGAC
CTCGGCGTGATCGCGTTCGTCCTGGCGGTCTTTGTCGGGATCTTTGGCTTTGGGCTGCGG
GAAAAGTTCTCAAATGAGACCACGGCGAGCGCCTACAGCGTTTTCAACGAGGACGGGAGG
GCCATCGTCGGGGGGTTCACGTCGAGCCAGTTCGAACGCCAGCTCCGCGGATCGGTGGCG
AGCCAAATGGCGTCCGGCGCAGGCGGAAACCCGGACGCAAGCGCCGCATCCGGACCGATC
GCGAGAGCCCGAACGGAGGGGCCGGCGAGGAGGACAAACGTCGCTCTCTCGGATGCAGAA
CGAACAGCGAGACGAAGGGCCGCGGCCGAGGCAGCAGAGCGACGATTCCAAGCCGCGAAA
GAAGAATAGAATGAAATGGAATGGAACCAAACTAGACCAAACCAAACCAAACCGAAGATT
AAATTCGCACGGAATATCTTGGTTTTGTTGTTCTTGGAAACCATGGTCACTTTCATGGAA
TTTCCGAGTGATCGTCTGAGACCCTTCTCTGCGTGAGCGATAATATTGGGATGCTATGCT
ATGCAATACCAGGTGCCTTGCCTTCTCCCTGCTGTCAAGACACGATTTACCCATGCCGAG
ATCGAAAACAAGGCTTTAGCTCCTCTTTCATCAAACCAAAAGCAGACAGAACTTGATTGC
GATCTCAAGTCAATGTACTATCAAGTTTCATATGGCTGCAGCGACATATAATTGACTCTC
TCGGTGCTAAAACAATCTAAACGCCTTTCTTGTTTGATTGATTGCTGTTGTTGAATCATT
ACTGGCTATCGTAATGTAGGTTTCTGCGTTATTAAGCATTAATATTTTTGGTACAGG
>comp2501_c0_seq1
AGGATTTTGATGAAGAAACAAACGGATCGCAGAAAGAGTGTCTGAGGGAATCATTCGTGC
GCCACGGAAATCCAAGGAAAAAAAGGACCATGTACCGGTAGTATTTATTTCGTATTTTGA
GCATATAATCATAACACAAAGCGCGATGTCCCGAAATTGTGAACGAATTTTTCGTCTTGC
TGCGAAGTATTTTCGTCGGGAACTGCACAACCCCCCAAAAACTCTGCTACAGGACCACAG
GCATGAACAGTTGAAAACAATGGTGCCAACACCGAATCAATAGCTCGATACAACATAACA
AATACATTCATCGTTTTGCACTTGGAACACTCCCATCATCATCATACACAACAGAAACAC
TGCACTTGTTGAAACGATTGCTTACAGGGCATCGAAAAAGAACTATTGTGAAAATAACAG
CAAGACTAAATAATACAGAAGACGACACAAACGATTCACAAGGATATTGAGAAAGAAAAC
AGGCGCATACACTTAAACTACAATCGCAAGCCTAGCCGAGCAATGTCGACACCGGGGTAC
CGTTCGATTAAGGCTCGAAAGTGCAGCCGACGAGTGCTCTTCGAATCCTCGAACGGGAGC
GTTCAGCCGTCCGTTGACGACAGCGACAGCGACGATTGGGAGGATGATGCATCCTCATCT
AGTTCCGATTCCGAGATGAGTCGCCGACATCGGTGGAGTAGGCAGGTGACAAGTCGGAGC
TTGTCAGCGAAAAATCTTAAATCATCATCTTGGGGCATTTGGGGCGTGTATGATAGCAGC
CGTCAAATGAACTACGACGGTAGCCTCGACGGAAGCAACAGCATGAGCAACAGTAACAAG
CGAAGCGATGATAGCAACAAACTCGACGACGATGACATCGATATCGAAATCGAAGGCAGT
GACAGTGACGACGAATCCTTTCTCGAACAGAAGCAACAGGACGAAAAGGTGAAACTAAAA
ATGGTTGCCATACTGTCAACAGTGGTCTGTCTACTCTTGCTGATGACGGCCGTGGGTCTG
GGAATAGCGCTGAGCAAAAAGAAGAGACGGCCACCCGCGATACCTTTGAATGGTTTGTCC
GATAACAATAGCTCCGTCTTCGACCCGTTCGCTCCGTCTTCGACCCCTAGCAGCAGTTTG
GCTCCATCCGTTGCTATGAATACAAGCAGCAACACAACAACGATAGGCACAATGAACGTG
ACACTCGACGACTTTGACCCGATCTTTCTGAACGAGACGGATGACAATGTCGGAGCGAAT
GAAACATCATCCCCCACGCAAAGCACCACGATATTCACCACTGATAGCCCCACGCTGAGT
CCAACGACGGCAAGCCCCACTAAAAGCCCTTCGAACAGCCCAACGCAAAGCCCTACGAGG
GATCCAGAAATGATTATTCCCGACGCAGTGGATCTGACCCCCGCTGGTGATACCTACGTT

GTGATCCTCCCGGACGGAGAGGTGTACGAGAAGGAGAGTGCAACCCCCACCCTCTTGGTG
CGAAGCCGCAACAGCGCGGGAAACAATGGTGCTTCGTTGATCACCCAGCAGGTCAACGAG
GATGAAATCCTCGCGGGCGATGGCCTGCCCCTGGATGCCCTGTCATACGCCCTCCTCCAC
TTCGACATGGCAGACTACAAGTGGTACGAGGACGGCAATGCCGAGTTGCTCTCCACAAAG
GCGAACGCCTATTTGTGCCTAGAGCATGTTCCGAACACCCGCGAAAACGATGCGTGGGGG
AATCCGAATGACCAAAAGATTTTTTCCGCGTGCCGCGTCGAGAATCCGATCCTGGAAGAG
GACTGGCTCGCAAATCCAGCATTCCAGGCAGAACTCACCGGGTACAAGATGCCGGAGGAC
TGTTTTGGCGGAGAGGTCAAAAAGACCTTTGTGAGCCCTGATACGGGGAACGTTTGCATC
GACGTCTCGATGTTTGTGGACAACTATCCACCCTTTGCGGAGGTTGACCCGAGCGATGAT
TTTACATTGCCAGATCCCGACGATGAAGACCTGGTAGAGACGCTCGGAGTTCGCCACCGG
CTCCTTCGCCCTAACATCTTCAAGAATATGGAATCTACCAACGAGACGGAAGCCTTCTCG
GAGTCAGCGACGCCGCGGGTCGGTGGAAACTACAAGAACATGCTGTTCATGGTGGCCCAC
ATCGAGCCGGGCGTGGACGCCAGCGCGGAATTCTACAGCCGACAGGACACCGACAACGCA
CCAAAGCTCTATGTGACGCTGGAGACGCTGACGGAGGCCCCCACGGTGGCTCCCACGGGT
TCGGAGTACCCGTCGCAGCATCCAACGGCGGACGCTACGAACGATGTCACTCTTGGTTCG
AGCGGTGGCGTCGGCACCATCGGGGCGGTAGACGGGAATGCCACCATGGTACCGACGTCT
GGTTCGACCACCGCCTCCAGCGAGAGCGCTTCGTGAGACGAAATTGGAGCCGTAGCACCG
ACGTCTCGTGCGACCACCGAAACTGGCAACGGCAAGGCGGGAGACGAAACGGCGGCGGCC
CTGGCAGCGGCAAAGGACGCTATCGCCGTGTGAGGAAGTGGCCACGATGGAGCGATAACG
ACACGGTCGAAGACGGCAGAACAATACGTGAAGTCTTTTTAATTGTATTGACTAGCAAAA
AGTGTTCGTCGCTGATCGGAGGTGCCTACTTGCTAATTTTTTGAATGGCTGGTTATGGTA
CTTGTAGGATGCAAGAGGTTGCACTGGGACTACGGCGACGAAACTATATGCCACACGGAA
GTATTTTGTATTGTACACTAATAAATAAAGAAGAAAAAATCCAAAA
>comp2502_c0_seq1
GTTCCGCTAAAATAGAAATGTCAGAAGAGAACGACTGTCCATACTTTAGCGGTACGGACA
ATTTGATACGATTTCAGTCAAGATACCTCTGTACAATAACTTCATGCTATTGAGGTGACT
GCAGCATCAGAACTTTCATCAGAAAGACAAAAGAGCGCCGAGTCTTCCCGCCAACACCAG
TGTCAGTCTCGGATAGAATAAGAGAGAGAGAGAAATTGGAACAAACATGATCATGGTGAA
ACCGCGACGGCGACTAGAAAGAGTTTTCATTCATGTTCCAGCATTGCTATCGCTCTTATC
CTTCTCATTACCGTTGGTATGCGGGTTTGCACTGACAACGATGACAATGACGACGAAAGG
AATGAATAATCTTTCTTCCGTTGAGGACTTCCTTCTTCGTTCTCGACTTGAAGGGATAAA
GAAAGGTATCAAGAAGGACTACGCAAACTCCCCGGGAGTAAAACTTCTCTCGGGGGAGGT
CGATGAGAAGTCAACAATGCCGACATTTATTTTTCCAGGTGCTGGTGGGCTAGATGAATT
GGTTTTGGAACTCCAAGCAACCATTCCAAACTCGGAAGTAATCGACTGGAAGGAATACCG
AGGGACGCTCCTAACCGCAGGTTACGATAGCGAGGCGGTGGGCGAGGCTATCGCCGAACT
AGTTCTAGAACAAATTGGTGGCGATGGCGAAGAAATCAATAACGGTGGCTACGAGAACGA
GGAACTGGAATCTCCATCGATTTCCTCGGATTCATGTTCTGGCGTTCGATTCGTTGGAAT
ATCAGTTGGTGGTTTTGCTGCGAATGCAGCTGCAACTATTGTTCATCGACAAATAACTGA
AAGATCCCAGCCAAAGAATGTGGAGAAAAATAAAGAAAACGACATTTTGAACGTCCATTT
GGTTTTGCTTGACCCATTCTGTGGCCGTGGCGTCTTCGGACAGAACTACGGGAGAAACAA
TTTTGGAAAGTACGCCACATCGGCTATTCAAATCCTTAACACAGACGATCCGGTCCCAAC
GACAAACGACCCCTTATCCAATTGCTACTGCCTCGATGTGACGGAGGCTCCTGAGAAACA
AGATTTTGTAGTGCTTCCGGGAGACTCGATGCATTCCTGGCCTGTTGCCTATTTTGCACG
CTATTATTACAAGAAATGCCAGGACCCAAGTTTAACTCCGATGTTGTTTCCTCGGGGCGT
GGTAGAAGAAGTATCGTGATGAAATAGCTAATGTATGCCT
>comp2503_c0_seq1
ATCCGAACCATTCGCGTCACCGACCAAGAGAGAACGCACTGGGAAAAGAGAGGCACAGAG
CTGCTCGATTCAATAGATAGGTATTTTTCAAAGCGATGAGCGGAAGGTGTTTGAAGCTGC
TTCGGCAGGGAAGAGGCGGTCGACAGATCCGTTCCGCGGCGATTTCGTCGTTTTCGACGT
TTTCGTCGTCGCCGGTGGCAACGGCGACGACATCGAGCGGCAACGACGGGGGATTCCGGC
GTCTTTGTTCTCTGCCCTTGCCGTACGGTGGCAATCTCCGGGGCCACCCACAGTACACGC
ACCAACCACCACTGCCGCTTTGCCAGAAGCGGTGGAGGTCCGGCAAAATGGGCCACCAGG
TCGAGTCGCTGAAGGAAATGGCGCACCGGCCGGAACGAGAAGCGGCGCAGGAACGGCGCA
AAAAGAAAAAGGACCGAAAGGCGGCCAAGAAGGCCGGTGGAAAGAAGCAGCAGGCTTCCG
AAACGGACGGCCCGGGCGGCGGCGCGGACACGGCCGGTGGGGACGAAGACGAGGACGAGG
ACGACATCTTCTTTGGCGACGAGGACGAGGACGAAGAGGCCGGGGACGGCGACGGAGAGC
CCACTCTGCCGGATCCGGTGGCCGTCCGGAGCAAGATGGACGCCGTCGTGGACCGCTTCT
CGGAATCGCTCCGGTCGGTCCGCGGCGCCGAACCGACCGCCGACATGTTCGACGACGTGA
TGGTCCAGGCCTACGGTGCCCCCACGCCGCTGAACGCCGTCGCGCAGGTCGTGATCGTTT
CCCCGACCCTTGCGCAGGTGACCTGCTTCGATCCGAGCGTGGCGAGAGACGTCGTCAAGG

CCGTCCAGCTCACTCTGGAGCTCAACCCGCAGCTGGAAGAGGGCGGGGCGGGGAACATCA
AGGTCCCCCTGCCCAGGATTTCGAAAGAGGTCCGGGAGAAGCTATCGAGGAGCGTCAAGA
AGCGCGCTGAGTCCTGCAAGAAGCGGCTGCGCGGGGTCCGCCGCAAGGCCATGGACGCGG
TCAAAAAGGGCAAGGACGGAAAACTCGCGGGGGTTTCCAAGGACGACGCCTTTGCGTCGG
GCAAGGAGATCGAGGCTGCGTCGGAAGCCGCCACGGAACGGCTGAAGGCCGTCGTCGCCG
AAAAGATGGACTCGATCATGGCGGTGTAGGACCAGCGCCACAAGCAACACCCCGATCCCA
AACAGCGGAGCAAGCGAACGAGCGGTTGGTTGGTTGCAAGCAAAGCCCGGGAGTTTCGAC
TCATCCCAGTGGCACAGCTATACCCAAGCATAAAGAGAACAAACGCGAATAAATAGCGCG
ATAATAACAATATTGAAACATAGCCC
>comp2504_c0_seq1
GAAGATTTATAATAAATAAAATTTTTTATGACAGCAACTTTAGAAAGACGTGAAAGCGTT
TCTGTATGGGAACGTTTCTCTGCTTGGATTACTAGTACTGAAAACCGTTTATACATCGGT
TGGTTTGGTTGTTTAATGTTCCCTACATTATTAACAGCTACATCTTGTTACATCATCGCG
TTCATCGCGGCACCTCCAGTTGATATCGATGGTATCCGTGAGCCTGTTGCAGGTTCTTTA
TTATACGGTAACAACATCATTACTGGTTCAGTAATTCCAAGTTCTAACGCAATCGGTATG
CACTTCTACCCAATCTGGGAAGCAGCTTCAATCGATGAGTGGTTATACAACGGTGGTCCT
TACCAATTAGTAGTTATGCACTTCTTATTAGGTGTAGCTTCTTACATGGGTCGTGAGTGG
GAATTATCTTACCGTTTAGGTATGCGTCCTTGGATTTTCGTAGCATTCTCAGCTCCAGTA
GCTGCAGCATCTGCTGTATTCTTAGTATACCCAATCGGACAAGGTTCATTCTCTGATGGT
ATGCCTTTAGGTATCTCTGGTACTTTCAACTTCATGCTTGTATTCCAAGCTGAGCACAAC
ATTTTAATGCACCCATTCCACATGGCTGGTGTTGCTGGTGTATTTGGTGGTTCTTTATTC
TCAGCTATGCACGGTTCATTAGTAACTTCTTCTTTAATTCGTGAAACAACTGAAAACGAA
TCTACTAACAACGGTTACAAATTCGGTCAAGAAGAAGAAACTTACAACATCGTTGCAGCA
CACGGTTACTTTGGTCGTTTAATCTTCCAATACGCTTCATTCAACAACTCTCGTGCTTTA
CACTTCTTCTTAGCTTTATGGCCAGTTTTAGGTATCTGGTTAACTGCTATGGGTATTAGT
ACTATGGCGTTCAACTTAAACGGTTTCAACTTCAACCAATCAGTTGTAGATTCTCAAGGT
CGTGTAATTAACACATGGGCTGATATCATCAACCGTGCTGATTTAGGTATGGAAGTAATG
CACGAACGTAACGCGCACAACTTCCCATTAGATTTAGCTTCAGGTGAAGTATTACCAGTA
GCTTTAACTGCTCCAGCTGTTAACGGTTAATTTTTAATCGAAGTCTAC
>comp2505_c0_seq1
ACCATACCATAGGTGGAATCGAGTCCAACCATCCATCGGTCTGCCATCTCCAACCAACCC
CGAGGACGGCCAAAAGATGGAACACGCTGCAAGCCCCGGCACGGGGGCGGCCGGAAACGA
ACCGTCTGCGTGCGAGAAAGAATGCTCGAAGCAGCAAGACGCCCTCACGGCCTGCATGAA
CGCCATTCGCGACGCCCGGGAGGTTGTTGCCTCGGGTGCCGGTGCGGGGGAGACCAACGA
CGACGACGGCGACGTTTCCAGGGTCGATACGTCCTGCCTGGCCCCCGCCGTGGCCTCCTG
GACCGAGTGCTGCGCGTTGGCCAACAACGGTGCCGTAGAATCGAACGAGGGCCACATCGG
CATCGGGTAGCTCGCAGCCGGTTCCGTTCGGTATGTACCGTGGAAGGTGCACCGAACCCG
>comp2506_c0_seq1
AACAACAGAAGCAACAACAAAAGCAAAAACAGAAGCAACAGCAGCAGCAACAACTAGTAC
TACAACAACAACAACAACAGCAGAAGCACCCAGAGCTGGTCGGTATTGGCATCCCGCCTC
GATGAGCAACACGATGCCTTCCACGGGAAGGGGCATTCCCGATACGGATGCCACCAGAAA
CAACGGCAACGGTTGCACCAGTAGCATGCCAACCGCACAGGCAATGCCATAGACAAGCCT
CGTTCCAAATGCAATCACCACACCAACGACAAGAACGAAAAGAGCAAGAACGAAGGAGAA
AAACATGCCACGGGAAGAACAACCGATGCTCTTTGGATGTGGTGTGTGCAACGATCGAGG
CAGGCCCACGGCAATCTCTCGATGCGCCAATTCGACACGCGCTATGCTGTGAGTACAGTA
CATCGCTGTGACAGTTAGTAGATATATATATTCTTGATTCTTATCATACGTAGAAAATCC
TGTACTACTTTTAGGATGGTGTCTACTACTAGTAATGGTGCTCCTGGAGAAATAATAGTA
CAATG
>comp2507_c0_seq1
CAAAAAACGAGCGAAACGAATACCGTGTAAGCGATCCATACTCTTAACGGATTCAAAAAT
ACTGTATTGTGATTCCACACACATCATCAATATAACGGACGGGACGCAATCCTTCCCGGG
GATAACACAACCGCTACTATCAAGCAGCTTGGGTATTCGAAGCAGCGCATTGTTCCACTT
CCCGTCTTGTACCCAGCTTGCACGGAGGCATCCAAACACCACCGCATCACCGATCCCGCT
GCATAACAAGCACGTCGCATCGTATCTGGCATTCCGTGCTCTTTCGGTCGACAAACACAA
CTTAGCGTTCACATCAATACATTTCAAGACAATAGTATCACAATATACAACACGACACAC
AAGCAGCAAGCATGGACGATCTAACCTCGATCCCCTGGTGGCAGAAAAACGGCAACGACG
AAGACCTCATGCGCGGGGACTACGACGACGCAGGCGACGACGACATTTTGAGCACGGAGC
AGCCTGTCTACGCGAGAGAGGGTGATTTCCACCGGGAGCGAAGAAAGCAGAACCAGAACG
AATACGAATACGACTACGAATACAAGAACCAGCGCCATTCGAAAGCAGGAGCGATCCACA
CCACCGCTGAGACGAACCCGCGGCGCACCACTGGATCCAGAAACGAGCGATTCGTTTCGG

AGGATCCCAGTGCCAACCGCAGGTCCGATGCCAACGCCAGTTCTACGAAGAACGCTTCCA
CGAGACGGAAGCAATCCGTTTCGAGAACAAAGAGCGGAAACGCGTTGCGTGGCGGGAACG
CCTCCCCGGAACAAGCCCCGGAACCGGAGCGACCCCCTCCGGCGAAACGAACGTCTTCTT
CCTCCTCTGCTCGGCGGCATTCCGGCGACAAGAGCGACAAACGCAGCAGCAACGGCGACA
AGATCCGCAAGCAGGTCCGGTGGATCTGTAGCTCGTGCACCTTTGTCAACGCCAGGAACT
GCAGGGTCTCCTGCACCAAATCCACGATCCTCACGTGCGAGGTGTGCTCGGTCCCGTACG
TGCTTCCCGCGGCCGCGGCCAGGAGCAGCGGCGACGTCCACGATGCCCGGTCGATCACGG
AACAGCAGGCACCGCAGCACGCCGAGGACCGAACTCGCCGGAACAGCAGCAGCAGCCTGC
CACAAGCAAACACCAAGAACGACACCGCGAGGCGCCAAAAAGACAGCCAATCGCGCCACT
CAAAGGGAGACAACTCGGACAGCACGTCCTCGACCGGGGCATCCACCAGCAAGAGCAATG
GGAACAACAGAACCTACTCGATCTCGGAACTCGTGCGGTGTCTCGGGGCCACGGGGGAAG
ACGGATCATCCTCGGTCTCGAACCTCCCGCCGTCGCTGGAGCGGAGGGTCCAGGACTTTC
GTTTTGCGCAGCAAAAGCGACGAGAAAAACACGGCGATCAGAAGGCATGGGGGATTTACG
GCCTCTATGCTCACCTGTCGGACATTCGGGCCGATCTGGAATGGGCCGAGGACGCCGCCT
GGCGCCGCCGTAGGAGCAAGCCCTATCTGTCCTGGAAGGACTTTGACGCGGCCCGCGACA
AGGGCGCCCACAGCCGGCCCTACTTCACCTATGGGGTCATGGTTTTGTGTACCGTGATGA
TGCTCGTGACCCTTGGCGTCAACGGGTGGAAGTTCGAGCCACTCACCGTCAATCCACTGA
TCGGGCCCTCCTCGGAAACCCTCATTGCCTGCGGGGCCCGGGACACGAACCTGATTGTGA
ACGAGAACCAGTGGTTTCGTCTTTTTACCCCCATGGTCCTGCACGCTGGACTGGTGCACT
ACTTTGTCAACATGCTGGCGATGTATTTCATTGGCGGGGCCGTCGAAAAGGCGCACGGGG
CCGCCTTTACCTCGGTCTTGTTTGTGATTCCGGCTGTGGGGGGGAATATTCTGAGCGCCA
TTTGCCTCCCGGGGTTTATCAGCGTGGGGGCATCGGGCGGAATCTTTGGCCTGATCGGGG
GTTGCATGGCCGACATTTGCCTCAACTGGAACCTGCTCTTTTTGAAAACCACCACGGACG
AAGATTCCCGGCTGAGGCACCTTTGCGTCCTGCTGTGGCTTGCAGTGGACATTATCGTCA
ATTGCATCCTGGGCTTTACCCCATTTGTCGACAACTTTACCCACCTGGGCGGGTTTCTGT
ACGGCTTTTGCATCGGCTATGCCTCGATCGAGCGGCTGGCCACGGGGTTTTTTGGGATCC
GGATCGGGGACTCGCACTGGTCCAGGCTCAAGAGCGTGGGTCTTCGCTATTTCGGGCTGG
TCCTGAGCGTGGTGACGATTTTCGCCACGACCATTGTCCTGGCACAGTCGGACGGACTGA
CGAGTCCCTGCCACCGGTGCCGGTACATCTCCTGTGTTCCCTTTCCGTTTGGTGCCGAGC
GGGACAAGTGGTGGCACTGCGACGACTGCGACATTGTGACGGCGGACCTCTTTGTGGCCA
CGGACGGCTCGGGCCTCTACCAGCAGGTCGACATCCACTGCCCGAACGGCGAGACGGTGG
AGGTCCCGATCTGGGACGAGGGCATTTACGACACGGAGGTCCTCCGCAGGAAGCTCCCGT
CGTACTGCCGGGGATTTTGTGCCGAGGTCTTTGCGCACTGAGATCAGCGGAGGACGGGCG
GGCGGGGACTTGCCAGGGGTTCTGCAACACATACGCACGCACGCACACACACACACAAAC
ACACACACACAAGACACCCAGAACCCCACACGCATCCCACACCACGCCCCATAGAGATAA
TGCATACTGTACATACTATTATACACACATACATCACGAACACCTGCAGCGATGCACGGT
TCCTACACGAGAGCAACACCCGGCCGTATGGTATGGCACAAACACTAGGACAAACAAACA
GTAATGATAGGAC
>comp2508_c0_seq1
CGAACTCCTAAAGCTTTTGGCTACTGTAACAAATGCACGGTAAGATCGCATCGACAAAGA
GTCCCTTCGATTAATTAACGATATCGCGATGTTGTCGAGAAAAGTTCTATCCAAGCCTTT
CTTGGCCAAGCGTCTTTTGGATGTGGCCTCTCCGTCAACTGCAGGGGCCAAAAGTATAAG
ATTGGTGTCTACATCCCAAACCAAAAAAGGGTTTCCTCCTCCCATTCTGTTCGACTACGA
AACGATTCGGAACAATCTGACGGTGGCCGATGCTATTCCTTCAGTCAAAGAGGCATTTTG
TGCTCTTGCTGCCGGAAAGGTCGATGTCCCGATGCCGATGCACATCGGCATTGAAGAATC
ATTGGCTGCTGGCCCAGGTGATTGTCACATTAAGGGCGGCTATGTCTACGGTGAAAAAAC
GTTTACCGTGAAATTGGCGTGTGTCAGCTTCTACAAAAATGTTACGGAGCGCAACCTTCC
TGCTGGATCTGGGGTTTTTGTGGTCGTTGATGCCCAAACTGGAGCCCCGTTGGCTTTCTT
CCAGGAAAATCGCATCATGACCGACTTGCGAACTGGTGCAGCTGGGGCAGTTGCTATTGA
ATACACAACTCCCAACGACGATAGCAATGTAATCGGCTTTATTGGCACGGGCGCAATTGC
TCGAAACATGGCCCGTGCTGCTGCAGCTGTTCGACCAAACTTTACTGGTGTGGCATATGG
ACTGGACTTCCCTGGAGCTGAAGCTTTTGCCAAAGAAATGTCGTCGGAGTTAGGTTTAAA
CTTTTCAGTAGCTTCCTCCTCGGAAGAACTTTGCCACCGGTCGAACGTCATTTTCACTCA
AACCACGGGCTCGGAACATGTTCTTGAGCTCGAACATCTCCGGAAAGGAACAACCATAAT
TGCTTCTGGGAGTGATCAACCAACGAAGCAGGAACTTCCGAACGATGTTTTGGCATCCGC
AGATAAGTACATCGCAGATCTGATTAATCAAACCAGCCGAGTAGGCGAACTCCGTGGAGC
AATTGAAGCGAATGTCATGACGACCGAAGAAGTCTATGCCGAACTCGGTGAAATTGTGAA
CGGGAGTAAACCAGGGCGAGAAGATCACGAAGAGCTCATAGTGGTAGATTTGACAGGCAC
AGGAGCCCAAGATGCAGCGATTGGGCAGGTCGCATGGGATAAATTGAGTAGGATTGAGTA
GGCTGCAGAGTATGGTGTCGGGAGATCGACTGAGCACTGCTCTGAGCAAAATTGTTCCAT

GCGTTCTCTTTTTTCAGAAGTCGCTTTTCTGAAACAGTTATACTGCTTCCTCCGTTTTAA
TCCAATCAAGCAAGCGTATCAAGTCTATGTCATAATAGAGCTATTTTAAAAC
>comp2508_c0_seq2
CAGCGATTGGGCAGGTATGTATCGATAGTATGACGTCATATTTCAAAGGTTTCATTGTCC
CAAACTCAATGTTAACAATTCTAATCATTGCTTCGTCACTCTTTACACCCACACCCCGGC
ATTCATATTGATGACAAAAAGGTCGCATGGGATAAATTGAGTAGGATTGAGTAGGCTGCA
GAGTATGGTGTCGGGAGATCGACTGAGCACTGCTCTGAGCAAAATTGTTCCATGCGTTCT
CTTTTTTCAGAAGTCGCTTTTCTGAAACAGTTATACTGCTTCCTCCGTTTTAATCCAATC
AAGCAAGCGTATCAAGTCTATGTCATAATAGAGCTATTTTAAAAC
>comp2509_c0_seq1
AGCGGACAACAAACTCTGAACATCGAACAATACGTTATCGGTAGAGTTAGCTTACGTCGA
TTCTTTCTAGGGCAAAAGCGCAATGGTGTCCTCTGTCCTTTCCATATTGGCGCTCTTGTC
GGCGGTGCAGCTGTCACCGGTCGAGGTTCTCGCCGCCGAAGATGTTGGGCTCTCCTGGAC
GATTTATCACTCGTGGGATGCCAACCAACAATTTGTCCGTCGTGGGAGTCTCACGTGGGG
CCCCGCCCCCGTCGACGAAAGCAAAGACGACGACGGAAAAAAGGAGTTCCAGATCCTCAA
CGATGATGCCAGCGCCAATCTGACGGCGGGCGATATTCGATCCATGCTCGAGTACGGGTG
GTACCACATCAAGATCCAGAGCAAGGACGACGAGAACAGCTTTGTCTTCCAGACGGTTCC
GGCCTGTAACCTGCGGCGGGCCAATTTTAAGGATCAGTTCGACGTGACGCTACCACGCAG
CAGCCTGGATGATCGCCAGGATCCCATGACCAGTTTTGCCTACACGCCACTGGTCAGTCC
CCTGGCACCCAAGAACTGCGACGATTACGACGACGAGAGCGACGGCACCACCAGGTCCTT
TTCGAGCAGGGTATCGGTCCAGCTGGACACCCCCGCCATGACCATCAAAAACATCCTGCC
GCAGTCGAAACCCCCGCCGGGGATTGCCTTTGTGAAACAGCCACCGGGGGGAAGTGGGGA
GCAGCGAGGCGGGACCGACGACGGTGAATCCTACCAGGATCCCGAGGCACCCACCACCCC
TCCCGGTCCTTTTGGGTTTTTCTCGAAGTACTGGTACATCATTCTGCCGATGATGATTTT
GCAAGTCCTTCAGGTGCCAGAAGAGCCTCCCAAGGAACAACAGGGACAGCAGCAGCAACA
ACAAGGGGGCGGTGGTGGCACGATAGCTCCCCAGGCAGGAGCATCGCCCCAAAAGGTGCG
CCGCGGAAAGACCAGCAAAAAGAATTGAGAGAACTAGCACGTCTTGTATCGAACTGCTGC
TTTAGACGAGAAGTGATAGCAAGCATCGGAGCCCTACCCTTGCGCATCGAGTCGAATACT
TGTTTTTGTAGTAGCGATTATTAGTTACTTCTAATTTTTATTTGCAACGGAATGCGACTT
CAGCTTAGTACAGCGTAGTCCAAAACTGAATCTTTCACTTCATCGAGCGAGC
>comp2510_c0_seq1
CATTATTGTCACCATGAGCAACTGGGTTCGAAAGAAAAATGACCAACATACCAGCGACAT
AGAACCAGAGTCAACGGAAACATTTGAATCAGACGATGAGGAGGAGTACGACGAAGACGA
AGACATTGAAGACATCTCGTTTTGGAGGGAACACGGACTGGCAATTTTAGTCGCCTTTGC
TGCATCAATCATTGTTCATTTTCACAATCAAAACTTGATATCTTTATCGGGATTTTTAAA
CCGGCTCCCGTTCGGAACAAATAGTGAGAGACACAGTCATCTGGACGGGTTTTCGAGAAT
GGCAAATGTCAGCTTTTGTCGGCAACGAATTCAACCACCGGCCTCGGAAAGATTGAATTC
GATGGATTTTTACATCCCTCGTGATCATTTCGAATCGTTGCTGACATTTTACGTCGCAGA
TCTGGATGAAGACGATTACTACGCCAATACGGATATCGCCGTTTCAAGTAGCAGGAAGGA
AGACCTTGGCGCCCGTTTGAAGGATAACAAGGAATTCGAATGCCTTTCTGAACAACATCA
CATCAATCCTACTGACAAAAAATTGAAGGGCACAACTTACTTTTACAAAGACCCAACGCT
CGAAGAAATCTATCCTGAACTAATGAAGGAAAATACTACACCTAACTTGAGTATGGTGAA
GAAATCATCGAACGAAAGAAAAAGACAACTGGAACAGCCTCCACTTAGATTTACTGGATT
TGCCGCAAAATTTGTGAATCTTGACACCGATCCCGTACTCTTATATTGGGATGGAAAAGG
TGGACACGAAAGTTCTAGAAAGCTTGTAGGTGAGATTCCCCCAATGGAGTCGATTGGCAC
GGCAACAATGCCAGGACATTCATTCCATGTTACTCCAATATATGATCCTTCTACAGTCCT
TAACCATTGGGTTGTGACACTAGATACAGCTCTTGTGTTTTATGAACCAAAAAGTGCAAC
GGAAATGAAACATACCCTGAAAGCAAAAGACCCTAAGTCTTATGCAATGTATCAGAGACA
GATGGTCAACAAAGCCTTTTCCAGAGATTATACGATTATCAGTGGACGCACATGGCTTGG
ACATTTTCCCAGACCATTTCCCTTGCATTATATGCACGAAGCTTCTTTTATTGGCCAAGA
GCATCAAGTTGGCGGTATGACGTTGAAGGTGGCATCGGTAAGACCGCGCGTCTTCACGAT
AGAAAACTTTTTGACATCGGAGGAATGTGAAGAAGTAGTTCGACTTAGCTTAGAGCAAGG
CCTTACGGAATCGACATTGCATTCAAGCCCACAAGCAACACAGAATCGCGATGTTTCAAC
TCGTAGCTCGAGTAACGCTTGGTTGTCTAGAGAAACTAGCTCGCTAACAAAAATGATTTT
CGAGAAAACAGCCGAGTTGACGAAGATCGACAGACACCTATTTCAGAAATTCCATGAATC
TAATGCCAAGCGTCATTCGATCGCTGAATCGTTGCAGGTTGTACGATACAAAAAAGGAGA
GGAATATACGCCACACCATGATTTTGTATCTCCTTCTATCAACAGTCGCCATCAACCGTC
GAGGTTTGCAACCCTATTAATTTATCTTAATGATGTGGACGAAGGAGGAGAAACACGATT
TCCTCGAGCTGTTAACAATTACAATGCGGAGGGCTTGGAAATCGTGCCGAAGACTGGGAA
AGCTGTTCTTTTTTACAACACATTAGAGGACGGAAATCTGGATGATTTATCTCAACATGG

AGGAAACAAAGTCCTTGCCGGCAACAAATGGTTGGCGAATTTATGGATCTGGGACCCCGT
GATCGGATGATTTTGATGCAAGAATTCTTCGCGATCATCGTAATTTGAGGGCAAAAAAAT
ATGAAAAACATCTACCTTTATTAACGTGAGACGAAGAGGACCAATTAGTTCATACGTAAA
AAATCCATTGGAAAAGCAAAACCATCATTATTTCTAT
>comp2511_c0_seq1
CATTTAGTTACAGTAATGGAACGTGCCAATCGAAGCAACATTTTACTTAGTGTTCATGGT
TTCATGGTCGCAATTTCAATATTGATTCTTCTAATATTCATTTTTGATATTTTGGGATCC
CTGCATTTCTATAATCCTGATACTCCATTCAAAACGTCAAGACAATTATTTTTCGAGCTG
GCAAGGATGTCCAATTGAAATCGAAGTAAAATAAACGTGAACATTGTCATGGCGTTCGTC
AAGAGTATTAAAACTCAGCAATCGTGTTCTATCCACCGGAGAAAACTCGCGAGGACTTGC
CAAGAGGAAACTTTTACATCATTGTGACGAGTTTGCGTTATTTTGTCCCACTGAATTTAT
GGAAATTATGGGACGTGATGTGTATTTTTGTGTATTTCTACTTATCGTTCTTAATTTTCA
TTACGGGGTCCCATCAAACACCGTAAAATCATTGTACCGTGGGACAAGAGACTTGCAAAA
TATGTTTGTTTCGGATTTCTCTTTGAGGACTCCCATTGCTATTAGTAATATCGTTGTCGT
CGCAGCTTTCACGAACGAACTCTGCGATCCGAAACCCGAAAATCATCGCAATCGTTTATC
ACCATTCCACAAAATAACCCTTGTATTCAATCGAAACAGTTTTTGGTAGCTTTCGTTCGA
CTTCAGAGAACAAGTACTAAAGATGGATAGACACATCGTGACCGAATTCTTGAGAAAGGT
CAGAACCATTAGCGATGGTCACAACGCCATTGGCGCTCCTCATCATTCGTGGTCCTCAAT
GAATAACCCTCGTGGCATACCGAGGAACAAGAGCAACTGCAGCAACGACTGCCATTCCCA
TACTCAGAATGTCAACGCTACGCAACGGGAACAGTCAAGCCAAGACAGCCATATCCTGCA
TGGACGTCCGCAGGAGAGAATGCACCATCAACCTTTCTTCATGGAAGAACGCCTTCAATC
AGATCTGGCCGTGAATCTTTCGTCCCTGAGCAGCAACGGTAGCAATGGCAGCAAGAATAC
CACAAGCAGCAAAACTAGCTTCAGCAACCATATCCACATGCAACCGTTGACACTCCACCG
AGGCCGGCAACAGCAACAGCAACAGCAGCAG
>comp2512_c0_seq1
CAATACATGGTAATTGTGTTCGCTCATTATCTTGAAATAAGAAATAACAACAAAAACAAT
CAAAATGGATCGCGATTCTCTAGTATACAAGGCAAAGCTCGCCGAGCAAGCCGAACGATT
CGATGAAATGGTTCAAGACATGAAGGAGGTTGCCAAGCAACCCCAGGAACTTACCGTCGA
GGAACGCAACTTGTTGAGCGTTGCTTACAAGAATGTTATCGGATCCCGACGTGCATCGTG
GCGTGTTGTTACGAGCATCGAACAAAAGGGCGATAGCGACAAGATGGAAATCATCAAGGA
CTACAAGGCCAAGATCGAGAGCGAGCTTGTTGAAATCTGCAACGACATCCTTGGAATCAT
TGAAGAATCATTGATCCCCAACTCGACGTCGGAGGAAGCCAAAGTTTTCTACTACAAGAT
GAAGGGAGATTACCACCGATACCTCTCCGAATTCCAAACAGGAGACACAAGAAAGGAATC
TGCTGGAGCCGCCTTGGATGCCTACCAAGCCGCCAGCGGAATTGCCCAATCCGACCTTCC
TCCTACGCATCCTATCCGTCTCGGATTGGCACTTAACTTCTCTGTCTTCTACTACGAGAT
CCTCAACGCGCCCGACAAGGCCTGCTCGATCGCCAAGCAAGCTTTCGACGACGCCATCGC
CGAGCTCGATACTCTTAACGAAGAATCCTACAAGGACTCCACTCTTATCATGCAGCTCCT
TCGCGACAACCTCACTCTGTGGACGAGTGACCAGGCCGACTCCGAAGTTGTTGACAAGGG
AGACGCAGAAGGGGAATAAGCATTCAACTGTCCGAGGCTTTGAGGACACGAAAGGAACTA
TTGCGTGTGAGGTAATCCTGCCCCGAATTTGTGACCATGACCTTATCCCACAATGATAAA
ACACCGCCTAGGAATAACTTTTTTTTATTGGAACAATCATCTATCAGCCGATGCGCTTAG
CATCACTTTTTTAGAATTGCATAAGTGCACTAAAATTAGCATGTGGAAATAATTCACACA
CAAATGGAGCAACGGCTTTGAAAAGATACTTGAGAAAAACAGATGCCTCTCATGACGAAC
AACATTCATTGTATTTGGATTAACATTTTGTAGTATTCGAACAACAGGTTGCTCCTTGAT
ATGAATTTGATACACAGCCCCTGTTGTGGATTGGGCTAGTCTGGTCGTGACGGTCGACAA
CAGAGCACGAAGTATGGTCTTTGCCTTGCACCTCAATCAAATTGTGGAACACA
>comp2513_c0_seq1
GCCTCGTTTCTGTTCAACAAAAAATGAGGCCTGATTCTGTTCTACCCCAGGAACAGGAAC
CGTCGTAACTCTGGCTCCGACTTACATACTCTGGCGCCAACAGTCTAATTGAATAGAAGG
AGTGGAAAGTAGCAAACAATACCAACCTGTAAGAACAAAACCAACACCAATTCACCAGCA
TGGAAGGAGCCGAAGGCGGCGAAGCCGTTTCCCGAAAAGTAATTGTCGTGGGGATGAACC
GCCCGCGCCTTTTAAAAATCATATCGATGGTAGACGACGAGGTCGCTGGGGGCAACGCTC
CAACCGGGGTGGAATACGTTCCGTGCCTCGCTGCCATGTCGTCGTACGAAGCAGAAAACG
GGGATGCAGTCCGGTACATGTCCGACTTTGTCTTTCGCGACGGCTCCCCGATGAGCAGCT
TTTTCGACGACGAAGCCTTCCGCGAGTCGCTCGACACGGTGGTGATGGTCGGCTACGAAT
GGCAGGAGAACGACAAACAGCTCGTCGAGAAGTACTTCGAATCTAATCTGCTCTCGGTCA
CGATTGTATGCGTCTCGCCAAATCCGGGTTTTTCCTCGCTAGGCGAGGAAATGAGCCATT
TCAAGAATCTTTCCGACGACGAGAAAGAACGGCACACTACAAATCATACCATGGGTCCCA
AGAAGATGAAACGATTCATACTCGACACTACAACACCGTTGCAAACAACCACGGAGAAAA
AAGATTCGGCGGAAAAAGACAACGCCGTTGAATCCCTCGACGGAGAAGAAAACGAGATTG

AAATAGATTCGCCACCCGACAAGCCAAAGGAGGAGGAGGAAGTTCCACCAAAGAAAAAGA
TTCCGGTAGCCATCGACCATAACGCGACATCTTTTGCGTGCCGAATGTGCCGAACGGTGC
TCTTTGGAGAGAGTCATCTCGCCGACGATCACAAAAAAGCTCTGCACTCGCTCAAGCGGA
CACCCAACCGTGCTTCCGGAACCGTGGCTTCCTGCCAGTCGTTCTTTTGCGACGAGTCCG
TCCTGGAGTGGCTCGCCCCGGACGGTGCGGACGTCGAGGGGAAACTCGCGTGCCCGCACT
GCAACTTCAAGCTCGGTCACTGGAACTGGTCCGGTGCGCAGTGCTCTTGTGGGACATGGG
TGGTTCCGGCCATCCAGATCCCCATGGGCAAGGTAGACGTGATCCTGCCGCCCTCCGAGA
GGGCAAAGGTTTCTCCCATCATGATTGTCGCTCCAAAAGCATCCTATGCATAGAAAAGAG
GCCGACGAATTCTTGTTATTTCCTCTGGGATTGCAAGCAATAGAAAACTTTCCTTATTCT
TCTCAGCCACTTGTTTGTTTGACGATTCCCACTCAACAGTGGCTAAGTTTTTAATCGCAA
ATATGCAGGGATTGTTCGCATTATCATTACTTATTTCAATGTTTGAATTGAGTCAGATTC
TTCTTCACTCACACATCATAACAGTCACTGTGGAATACTAACACTGCCAGGCGAAACCAG
GAGGAGGAGAAAGTGCCTGCTGATAGCACCAATAATACCGCTGCTAGTAGTACTAGTAGT
AGTACTATTACTACTGTAGCATTTGGACTATTGGACTGAAATCAATAAAGGTCAGCAGCA
AGCATTCAATGGTGGAAAAGAAAGGATGTGAGGCAGAAATATTCCGATCGTGAAGAATAA
CAAAGATAAGTATTAGATTCAAAATAATGATGATGATACTAATAATGTGACAGTTGGCAG
TAACAATATTACTCCTCGTCGTACCATACTTG
>comp2514_c0_seq1
CTGTTGAAACCCATGCCTTCGCAATAAAATAGGATACGGGATCTCAACAAACGCTTTATT
CAATTCAAACACAAACAATACAGATAATTTGCTTCGAGACAAGAAAAATCAGCAAGGAGA
TGGCAACGAATGGATTGTCCCGTCGTCGCGGAACCAGGAGGCGAAAGCGCAATTGGGGTC
CTCTGCTCTTAGGTGGCGCGGTGTTGTTTTTGTTCGGAATCATTGGAGGTAGCATTTTAT
GTTTTGTAATGTTAGATGTTGGACACACTGGAGATAGCACGGTTGCTGTTGATGCTACGG
CTGATGTAACGGGTGATGCAAAGGCTGATGCAAAGGCTGATGCAACGGCTGATGCAACGG
CTGATGCCGGGAGAAGTTCCTTTTCGCGACAGAAGAGACATGTCAGAAAACGTAATATTT
TGGAATACGACCCCGACGACTCGAAAAAGATACCACCCAAGTCTCTCGACGATTTCATGA
AGGAGGCGAAAAAGAAGTTTATGGATCATTTAGTCCAAGAAGGGACTTCTGGGAGCTTTC
CATATTATCCCAGAGATGAAGTTGCAAAGGATCACGATTTTACAACGTGGACGGCTCCTG
GTGGAAAGCGCTTCGAGGAATACAAACACGGCGATTCTCCCTACACGTACGCTCCGAACG
AATCCGATGCTTTGGCTAGAAGTCGGCGATACCACGTGAAAAAAGCGATGGAACACGCCT
GGCGCGGATACGAAAAATATGCTTTTGGTATGGACGAATTAGCACCCGTAAGCGAGAGAG
GTAAAATACGCTGGGGCGGGCTTGGGACGACGCTAGTGGATAGTTTAGATACCCTTTGGT
TGATGAACATGACAGACGAATTTTATCGTGCGAGAGACTGGTGCAAGACCAGCTTGTCTC
ACAACAAAGATGTTCAAGTCAGTGTATTTGAGACTACAATTCGATCCCTCGGAGGTCTTT
TGTCTGCGTACGATTGGTCCCAAGACGAAGTCTTTCTCGACCAAGCTAAGGATTTGGGGG
CGCGCCTCTTTAAAGCCTTTGATACGGAGTCTGGACTACCGGCTAGTCAAGTGAACCTTA
GAACGGGGAATGCACAAAAGGCGCGATGGATGTCGACAGGTGCTGTCATTCTGTCCGAGT
TTACCACGTTGCAGATCGAAAATCGTGCCCTGGCACGTTTCTCTGGCATTCGAGATTACA
AGTTGAAAAGCGAAGAGGTCTTTCAAAGGATGAATAGAGTAGAAAACGTAAAAGATGGCC
TGTATCCTTGGTCGATTCATGGCACGAAAGACGGAGCGACGATCTACAACCAAAAAATTA
CTTTTGGGGCTATGTCTGATTCTTTCTACGAATACATGCTAAAAATTTGGATTCAGGGCG
GACAAACGGAACCAATCTACAGAGAGATGTACGACAAATCTATTCAAGGCATGCACGACC
GGCTTCTTTCGGTTTCCCGTCCTTCTGAATTAGTTTTCATTGCCGATGAAAATAACGGAC
GTATGGATCACAAAATGGATCATTTGGTTTGCTTCATGGGTGGTCTGCTTGCTCTCGGAG
CCTACACCGACCCACAAGGCCTGGACTCGAAACGCGCGCAGCGAGATTTAAAAACCGCCA
AGGCTCTGACATACACGTGCTATCAAAGTTATGCCAGAATGCCGACAGGAATCAGTCCAG
AATTTATTCAATTTCACATGGATGCAGACGACTTCGGACCCGGAAGAGGCGCACCCCACT
ACTTGCTGCGGCCCGAAACTGTAGAATCCTTCTTTGTTCTTTACCATTTGACCGGAGATC
CTGTCTATAGAGAGTGGGGCTGGGAGGTTTTTCAGGCCATAGAACGGTATTGCAAAACCA
AAGTCGGGTACGGTGAATTGTCAGATGTACAAAATTTAAAAATCAAACCACGAGACACTA
TGGAGAGCTTTTTCCTCGCCGAGACACTGAAGTATTTGTATTTGCTCTTCGATCCCGAGA
CACCAATTGACTTGCTCCATAAACACGTATTCAACACTGAGGCACATCCTATGAGAATCT
TTCCTGTCATGGATAGCGATGGAATAATGGATTTGATAAAATAACAAAAAGTAGCTGCCC
GGATGAATATAAACCGAAAGCGG
>comp2515_c0_seq1
TTCAGAAAGAAACCCGAGAAAGAGGGCCACCGCACACGCCGTTTCCGTGTATCTCTGTGC
AGTGAAAGTTTTCATACCATACGTACGATACCATGAGGATAGTCTGTGTTGCCTTCGTGC
TGCTGCACCCCGCGGCAATCGGCCACGCACTACTGCTTCCACCACCCGCAACGCGAAAGC
TTGCCGTGATTGGCAGTATCGAGCCAGCAACCCGACCATCCCGTTCCCACACCCGGAATC
GATTCGGGCCGTCATCCACATGTGACACCGCCCTCTCCTCCAAAGCTCCACCCGAAGAAT

ACGACGACGGGATTGGCGATCTACAAAACCAAAACAACAGGAACGACGGCGACGACAACA
ACTGGCAGGAGCGGAGCGACGATGCCGCGACGAATTATGCCGAGGGCGAGTCTCTCTCTG
CGGAATTCTTTCAAAACCTACGGGATCGACAGGAAGGCGGGGCCGTGGACGCGAATTCTT
CGCTGTCGTCACTCTCCCGGTCCCCTGTGTCACGAGAGCAGTTCGACAGCGGCTTGTTCG
AACCAAGTGAATACGACGACAACTATACGGACGCCCGAGCCAATGCCCCGCCAACTCCGG
GGACCAAAAAATTTACCGGTCGACGGAGCGAGGGGAGCTTGGACTATTTCGGAGCCTCCT
CTAGCGGGAACAACCAGAGGAGTGGCGGCAGCAACAATAGCAACAACAACAACGCGGTGC
GGCAGCAAATGATGAGGAGGGAGTACGACTTGGTCAGCGGGGCGACGGGGAGGACCGCCC
TCGGCCTGCAGGCGGGGCTCGCGTTGACCATGCTGGTGTTCTTTCTCTACGTTGGGTTCT
CCGGGGGCATCGTGAGCGGAGAAGGAGCAGTCGACGCGGACTTTGGTGGGGACGACCTCA
TCCAGTTCGAACAGATAATACCTTTGCCGCGGGATTCCGACAAGAGCGTTTGGATATAAC
ACGAGAAAACACACGAAGGATAACACTGAGTTACTTGCAATCGCACAAGTTCATGCCATC
AAAAAATCACGAAACAGAGAGGATCAATCTCCACCTCACGAAGTCATAGTCGAATACTAC
CGCTACCGGTACTTCTCGTTAATGTGTGTTTCTTCTTTTCTGGGCCTCCTTGTGCATTGT
GTATTCAAGTATGCTACAGTTTCCGTACATAGTACCGTGTTTCTATTCTAGTGTGCTCAG
GAATGGTTTGGAAAAAGCAGTAAACTTTCGGAAGGTGTTGCATGTGTAGAGGAAGGTATG
GATAAAGGGGATTTT
>comp2516_c0_seq1
CTGTAATGCCCATCAAGAAGTACGAAATTCCTTTTGGAAAATGAGAACAAATCGAATCGA
TTCCTCTTTCCAAATTTGGAGAAGCATGCTTGTCGCATCACGCTTTTTTTACACCGATGA
TGTGACAAAAGAAAAAAGCTCGTGTCAGCATTGGGTGGTGTGCAGTGGGCGGTGTGCTAC
AATAGAACAGTAGAACGGTAATGCAGACCTAATCTAGAACACGACGCCGAGGAAAACATC
AAAGGTGACCACTTCGGTTCTAGGCGAAGGAACTGCGCTGAGCCATGCCGCTAAGGCAGG
AAATTTCACCACAATGCTTCAGCGGAAGAGAGTACGAACGGATGAACCGTGCCGTAAAGC
CAGCCCTCAGTTGTTCTGGTAGGGCTTCTTTCCCCAGAAGCAGAATAAGAACTGCAAAAA
CTGGATTATCCATGTTAGTAGTCAAGTAGAAAATACGAGAATGGTACCTAAATACTAGGA
TCCTAACAGTAGTAATACAACCGTTTCTCCAAGTGATGTAGCGGTAGGGTTATGGAAAAA
GATGTGAAATTTAGTGGAATGGAAGTACGTACTTCAGAGCGGAACATACCAAATTGGAGA
ATAGCTCAGCTCTGA
>comp2517_c0_seq1
TGAAGAAATGGTACGCATCTTAAACATCTTTCGTTGCCTCCACAAAGATCGCGTGTAGAA
AGGATCGAACCCACTGGCCTCGAACAAAACTTTCCTGTTGATTCGTCTATAGCCAAAGAA
GGAGCGACTCAATTCATCATTCACCCGTCCATCCATGTCTCTTACCTTCGACATTTGTTT
CATTCATGCTATTGCCGACCTACAGAACCCCGGAAATCTTGCTTTGTGCACCACCATCGA
CGACATTGATGCTGTTGGTGGAGACCTGCACTGCCTAGGATGGAATGGAAGTCGAACGTT
CGAAGACATTGACGAGAAAGCCTCGCGAAACCCCGGTCTCGACTTTCTGCCTGCGGACCT
TGACGACAAAGACGAACCACTCCCGAACTGGCGGTACGAGAATGGCTTGCTTCGAGCGCC
GTTCCAGGGTTCCGAAGATGGTGTCGGCGGGAGCCGAGATCTCTGCTTGGGGCCGAGTGA
TGTTGCCCTTTTCGAAGCACCCTGCAAGCCTTACGAACTGAAACTGCAAGAATGTCCGTC
CGAAAGCAACGTTCGCAACAGCATCGTTCTCGACGAGGGATACGACAATGCTGTCCCTGC
CGATTCTGCTGCCAGCTCGGAAGAAATGGCAGCTAGCCGTCACATGCGTTTTCTCGTTAC
GGGGCAGGGGCGTGTGCAGTCCCTGGCTCCCGTGCTCGAACGGAGAACCGGCAGGGATGT
GATCGAAACGAAACCCTTCTGTCTGACCATGCTAAATGGCAACGATGACGGCCTTGGTAA
GAAGCCATATCTCGTTCCATGTCAACCAGAAAACAAGGATGGCAAAGACACCAATCACGA
CGGCCGGCACGGACTCGCCTCCCCGGTCCAGGTGTTTGGCGTATACGGGACCTACTCGGA
TTCCTTTGTGATGGTGCTGCAGACTGTCTGGGGATACAACGAGGTCGTGAAACTGGCATA
CCAGGTCGGTATTCCAACGGGATCTATGTCGTCTTTTACTTCTTCCGCCACGATTGTCAT
TGAGCAGCAAGTAGGCTCGAGCAACAGCCATGTAGACAACAGCACACCGATGTTGGTGAA
GGAGATTCCATTAGTTGAAGATCATCATGTTGCGAATAGTATCAGCGGCATTCTTTCCTT
CTCTTCCTGCCAATACGAGATGGTTGGTCCCATGACTGCAACGCTCCACTCAATTCGTGA
CGACGTGAATGCTTCGAATTTCGAGGCCTCTACAGCATCAACAGCAGCTTCCCGCGCCTT
CTTTGTGGTCGACGATCCGCAAAAGGAGAGCCTCAACGGGTTTTTCGAAATGTTCGTTAT
CATCTTTTGTTCCATTGCGGGATGCTTTACTTTGTCGATGGTGGCCGATTGGTGCCTCAA
GGACAAGATTCGAGCATTCGGAACTCTCTGGGGGGAAAACAGCGACGGGAAGACCGTCTG
TCTCGACGACGACCTGGACGACAACTTTGACTTGGAAAAGGACGAGGGCACCGAGATCAC
CTCCGAGCCAGAAAAGGAAAACGAACGGACAGCCGACGAGGACAACAACACGGAACATGC
CAGTGCCGATGATAGTAGCAGGAATATCGACGGCAGCGACGACTACAGCATGTGTAGCAA
CGAAAACCATGGCAAAGCCGACAACGAGGAAACTCCTCCTCTCTTTCCGAATGATGTTTG
AAACCGATACGCAAGATTATGGAAGATGCGCATGTCCTGTTTCTGAAGAAAAATGGAATG
CAACAAAGGATCGTTCGGTGCGGCAAGATCGAGAAAGACATGGATGCTTGCACAGACTGA

CGGAGGAATCGACTTTTGGTAGCTTGTGGACACTGGGTTTACTGCTCTTCCAAAGTTGGA
AATTTTCTGCTCTGTGTCTGTCTACGGGTATTCTTTACATCTTTGGATCGAGGTTTTGCA
TATCTCTCTGCCCGAATAGGTTTTCACTCGATGATGCACGGTGTTATACTGTAAGAGATG
AACGTTTATGATACTATTCGCAGATAAGAGAGGACGTCCTCCCTTTGGCATGCATTGGGA
CTGCGGTACGCTCTGTTCTTGCACGATATCTAAGTATCAAATATCACTTTGCTGCGCTTG
CAGATTGAACGAATGCATGGCTCGACCGCATATCTTTGAGGTTGGGAGCTCAAGAGACGA
ACTTATGCGGTCTGGTGGACATACTACAAGCGGTAGACTTAGAATCCTGTTACGTGTCAG
TGCAGTATTATACCAATCGTTTGTGTGCTGGATCCCCAATTATAAAGTAGTTAACATCAT
ACAGTAAGTCT
>comp2517_c0_seq2
GCTTCGTCAGCGATCGAACAGGAAGAAAGCCAATGTCATCCATCGAACACGAACCTCGGC
ATCGATCATGTCCGGTGGATGATATGGAATCATGGATTCAATTCTTCTTAGGTTGGAGCG
TGAACACTGAATCTTGGCGATTAGTACATCCTACTTTTGCTTCTCTGGCATTCTGCGAAA
CAGGCTGTGGCACATCCAAGCGATTAACAGATCAACAAAACCATCAACATCAACATCAAA
ATACTCCTTGCAATATACAACAGCAGCAGCACCATGCTACTTTCGAAATCTCCCATCCCA
TGGTTGCCGGCCTCGATGTCTCTGCTGTCGCCATCATCCATCACATGCCTTCTCCTTTTC
CCCTCGATTTCCTCAATGCCAGTTCCTACGAGTCTTGAAGAAATGAACCCCGGAAATCTT
GCTTTGTGCACCACCATCGACGACATTGATGCTGTTGGTGGAGACCTGCACTGCCTAGGA
TGGAATGGAAGTCGAACGTTCGAAGACATTGACGAGAAAGCCTCGCGAAACCCCGGTCTC
GACTTTCTGCCTGCGGACCTTGACGACAAAGACGAACCACTCCCGAACTGGCGGTACGAG
AATGGCTTGCTTCGAGCGCCGTTCCAGGGTTCCGAAGATGGTGTCGGCGGGAGCCGAGAT
CTCTGCTTGGGGCCGAGTGATGTTGCCCTTTTCGAAGCACCCTGCAAGCCTTACGAACTG
AAACTGCAAGAATGTCCGTCCGAAAGCAACGTTCGCAACAGCATCGTTCTCGACGAGGGA
TACGACAATGCTGTCCCTGCCGATTCTGCTGCCAGCTCGGAAGAAATGGCAGCTAGCCGT
CACATGCGTTTTCTCGTTACGGGGCAGGGGCGTGTGCAGTCCCTGGCTCCCGTGCTCGAA
CGGAGAACCGGCAGGGATGTGATCGAAACGAAACCCTTCTGTCTGACCATGCTAAATGGC
AACGATGACGGCCTTGGTAAGAAGCCATATCTCGTTCCATGTCAACCAGAAAACAAGGAT
GGCAAAGACACCAATCACGACGGCCGGCACGGACTCGCCTCCCCGGTCCAGGTGTTTGGC
GTATACGGGACCTACTCGGATTCCTTTGTGATGGTGCTGCAGACTGTCTGGGGATACAAC
GAGGTCGTGAAACTGGCATACCAGGTCGGTATTCCAACGGGATCTATGTCGTCTTTTACT
TCTTCCGCCACGATTGTCATTGAGCAGCAAGTAGGCTCGAGCAACAGCCATGTAGACAAC
AGCACACCGATGTTGGTGAAGGAGATTCCATTAGTTGAAGATCATCATGTTGCGAATAGT
ATCAGCGGCATTCTTTCCTTCTCTTCCTGCCAATACGAGATGGTTGGTCCCATGACTGCA
ACGCTCCACTCAATTCGTGACGACGTGAATGCTTCGAATTTCGAGGCCTCTACAGCATCA
ACAGCAGCTTCCCGCGCCTTCTTTGTGGTCGACGATCCGCAAAAGGAGAGCCTCAACGGG
TTTTTCGAAATGTTCGTTATCATCTTTTGTTCCATTGCGGGATGCTTTACTTTGTCGATG
GTGGCCGATTGGTGCCTCAAGGACAAGATTCGAGCATTCGGAACTCTCTGGGGGGAAAAC
AGCGACGGGAAGACCGTCTGTCTCGACGACGACCTGGACGACAACTTTGACTTGGAAAAG
GACGAGGGCACCGAGATCACCTCCGAGCCAGAAAAGGAAAACGAACGGACAGCCGACGAG
GACAACAACACGGAACATGCCAGTGCCGATGATAGTAGCAGGAATATCGACGGCAGCGAC
GACTACAGCATGTGTAGCAACGAAAACCATGGCAAAGCCGACAACGAGGAAACTCCTCCT
CTCTTTCCGAATGATGTTTGAAACCGATACGCAAGATTATGGAAGATGCGCATGTCCTGT
TTCTGAAGAAAAATGGAATGCAACAAAGGATCGTTCGGTGCGGCAAGATCGAGAAAGACA
TGGATGCTTGCACAGACTGACGGAGGAATCGACTTTTGGTAGCTTGTGGACACTGGGTTT
ACTGCTCTTCCAAAGTTGGAAATTTTCTGCTCTGTGTCTGTCTACGGGTATTCTTTACAT
CTTTGGATCGAGGTTTTGCATATCTCTCTGCCCGAATAGGTTTTCACTCGATGATGCACG
GTGTTATACTGTAAGAGATGAACGTTTATGATACTATTCGCAGATAAGAGAGGACGTCCT
CCCTTTGGCATGCATTGGGACTGCGGTACGCTCTGTTCTTGCACGATATCTAAGTATCAA
ATATCACTTTGCTGCGCTTGCAGATTGAACGAATGCATGGCTCGACCGCATATCTTTGAG
GTTGGGAGCTCAAGAGACGAACTTATGCGGTCTGGTGGACATACTACAAGCGGTAGACTT
AGAATCCTGTTACGTGTCAGTGCAGTATTATACCAATCGTTTGTGTGCTGGATCCCCAAT
TATAAAGTAGTTAACATCATACAGTAAGTCT
>comp2518_c0_seq1
CTCTTCCGATCTAGAATCGCGAAGACCGAACTCCCCGCTCTTTTCTTCGAACTTCGTCTT
GCACCAAACCGTTCACAACTGCAAAAGAAAGAATTCTCAGACAGCAACAACCATGGCCGA
ATCCAAACCCAAACTAGTCCTTGCCTACTCCGGTGGTCTCGACACCTCTGTCATTCTAAA
GTGGTTGTGCGAGAAGGGATTTGATGTCATCGCCTTCTGCGCCAACGTCGGACAATTCGA
GGAAGATTTTTCCGCCGTCGAAGCCAAGGCACTCGCTGTCGGTGCCAGCAAGTGCGTCAT
CACCGACTTGCGAAAGGAATTCGTAACCGATTATGTCTTTGACTGCATCAAATCGAATGC
CATCTACGAAGGTCGATACCTTCTCGGTACCTCGATCGCCCGACCCTGCATCTCCAAGGA

AATGATCCGAATCGCCAACGAAGAAGGCGCTACGTACATTGCCCACGGAGCCACCGGAAA
GGGAAACGATCAGGTCCGTTTCGAGATGTGTGCCCAAGCTCTTTCCCCTACCATCAAATG
CATTGCCCCATGGCGCGATGCCGAATTCATCGAATCCTTCAAGGGACGAAAGGATTTGTT
GGCCTACTGCGAAAAACACTCCATCCCCGTCGATGCCAAGCCCAAGTCGAACTACTCGAT
CGACGAGAATCTTTTCCACACCTCCTACGAATCTGGATGTCTCGAGGACGCCGCGTGTGG
TCCCGAAGAATCCATGTTCAAGATGACCGTTTCGCCACAAGCCGCCCCCGAGACCGGGGA
AATCGTTGTCATTGAATTCGACAAGGGAATCCCGGTCAAGATCACCAACAAGTCGGACGG
AACCGTCAAGACCGATCCCCTCGATATTTTCTTGTACTGCAACATGATCGCCGGAAAGCA
CGGAATTGGCCGCATCGACATTGTTGAAAACCGATTCGTCGGAATCAAGTCTCGTGGGGT
TTACGAGACTCCCGGAGGCACCCTGCTTCGAGCGGCGCACATGGACTTGGAAGGCATTTG
CATGGACCGCGAGGTCAAGCGCATCACCGAGGGCATGTCGAACGAATTCGCCCGATTGTG
CTACAACGGATTCTGGTTCGCGCCAGAGATGGAATTGATCCGCCACTCGCTCGACTACAG
CCAGCGCGACGTCGTCGGAGAGGTCGAGATCGAGCTCTACAAGGGCAACTGCACCATCAA
GGGACGAACCAGCCCAAACGCCTTGTACAACGCCGATCTTGCCTCTATGGACATCGAGGG
TGGAGGTGACGCCTTTGATTACAACCCCGCCGATGCCCAGGGATTCATCCGCATCAATGC
CGTCCGTCTCAAGACCTACGCCGCCCTCCGAGCAAAGGATTCTGCTTAGAAAATGTAATC
TATACAAAAGGACAACGAGTTTAAGCATTTGTGA
>comp2519_c0_seq1
TCTACCCATCCATTTTCTTCTTTATCTGCCCATCCATCGAAGACGAGAAGAACCGCACGG
TAGAAAGTGAGCGCAGATTTGGCAAATCTTTATCGTCATCGTCGTCGTCGTCATCATCAT
AATCGCCGTCGCCAACCACCATCGATCGGCACGCGGACCAGCAACACATCGAATTGTAAC
ACAGAGACAAACGCAGATCCCATCGTGTCGACAGCCGCTACGAGTAGGCACATCTCGTCA
CGGCACGGCTCGGCTCGGCTCATCGTTTTGCAGCATCACGACAGGCATGGCCGGTCGAAA
ACTCATCGACACGGCGAGGTCGCGCGCCTTTCGGACCCTCTGGATGCTCGAGGAGATCGG
GATTCCCTACCAGCACGTCGCCGCCCTTCCCCAGTCCGAGCAAGCCAGGCGGTACAACCC
CCTCGGAAAGATTCCCGTTCTGGTCGAGGACGACGGCTTTTCCCTCTACGAAAGTGGGGC
CATCCTCTCGTACCTCGGCGACCGCTACCGGAGCAACAGCGGCGGCGGTCTGGTTCCGCG
GGCCGGCACGAGGGAGCGGGCGATCTACGAGCAGACCATGTCGGTCCTGCTCACGGAACT
GGACGCCCAGGGCTTGTGGATCCATCGCAAGCACGAGGTGATGGGCAAGTTCTTCACCTA
CGTGCCCGATGCGGTGGAGCACGCTAGAAAGTATTTCCACAAGACCAATCGGGTGCTGAT
CCAGCAGCTGAAAGACAACAACAACAACAGCAACAAGAACAAGAACAAGAACAACAAGGG
GAACGTCAAGAACGACATGAACGAAAGGATCGGGCAAAGAAAAAAGAAGCCCGGGAGGAA
ACCCAAAAAGGTCGGCAAAAAGGTAGGCAAGAAGAACAGAAACGAGGACGAGGAGGACGA
GGAGG
>comp2520_c0_seq1
CAAAGATTGTCCCAAATCACAATTTGGAAATCCTCTGGATTCGGAGCATCACATAGGTAC
AAACAGATGCTGTTTAACTCTTCTCCAGTTGGCCTGTTTCTTTTGGCGACAGCATGGTCG
CAACCATGCTACGGATTCCACGTTCGTCAGGAACAACCGAGATTGCCATGCTCAGCAATA
TCCTTGTTTCAAGAATCCTCGTGCGGTTTCATGGAGGAGAAAACCGACTCGTTTGACGCC
AGCGTGTCTTCTAGGAGAGATCTTTTTCGAAACTCCGCAGCAGTGGCATTCGCTCTTTTG
CTCAACGAAGGTTGTTCGAACCAGCCAGCATTCGCCTTTGACGGTTCTGGGGCATCGGCT
TCCGCCGGATACTCTCCGGCTACCAAGGCCGAAAAGAAAAAGAAATTCCAGGAACGCGTC
GCCGCGGATGTCAAAGATTTCAAGGCATTGGGAAAAGCTATAGACAAGGGAGAAACCGAT
GGTCCGGAATGGGTCAATTTTTTCATCCTCTTTCAGCGACGAGAACCCGACGAGGTCGGA
CGCACGTACGCCGCCCTCGCCGACCTGAGGGGCTTGCCGGGAAAAAAGAAAGGCGAATAC
GAGGGTGGCGACGGCCTTTTGCTGGCTAACACTTTCACCAAACCGGGAAAGCCACCAGAC
AACACACCAGCCGTGAAATCGTTCTTGAAGCTCTCCAAGACGTTTGATGCGATCGAAGCT
GCCGGAAAAGCAAAGGATGCGGCAAAAGCCAAAAAGGAATGGCAAAAAACAAGTGAATTG
TTTTCACAGTATCTTTCTGATGTAGAGCTACCGGGCGATTTGAATGATTCGTTGTACAAA
TAAATAAGTGCGAAGCAGTCTCAAGGGCAGACATTTCCACACTCTGAACACTGCACAACT
CAAATTAAAAAGTAAACTGCCACGACTATATACGAACGAAATACAATGAGTACTCTGTTG
CCTCGGGATTTTGTTCATCGTAAAGTAGCTTTTAGACCAG
>comp2521_c0_seq1
GACGTATTTTTCTTTTGAAATGACGTTTGCATCACATTGTTTCTTATGGCACGTAATGTT
TGCTTGGGCCTTTCGCTGACACTTCACAAAGCTTGATAAGAATCTCCTCCCAGCATCCTA
CCGAGAGACGGGATCCACCGATTATTTCGTACAAAACAAAGTGCAATGCCAAGATCCTGT
GTTATTACCGTTTTGAAAAAAGAGCAAAAACAATCTTTTGCTATCTTCTCATTCTTTTGA
TAGAAAGGTCTTCAAATCATTGAACCGTTTTTACACCGTACGTATCATGAAAGTTCTTTG
CTTTCCATCCTTTTTCTTCGTTTTCCATGCTGCTATTGCTCCGGTGGCATTTTGTGTCAA
CTCCGAAGATCGTTCTGCATTGTCTTTGGGGAAGGAAGAAGTCCCCGTGTCATCTCCTTG

GATTGACTTGTCTCAACTCAACTCCAACCCTCCTCAAGATAATGGGCAAGAAGGTTCTGT
GCAAGGAAGAAAGGCCCCTAAGAAATCAAAATCAAAAAGCAGCAAGAAGGGGTCAAAATC
CCCAACTGATTCGCCCATGTCCTCCCCGACTAGTCCCCCCGTGTCTTCCCCTATAGATTT
AGCATGCGTCGATGTATTTACGGGCAACCGCACTTACATTCCAAGTTGCGCGGGTGCAGT
CAACGAGATTATAGTTCAATGCAACAGTGAAGGGAACTGCAACTATAACCAATACGTCAT
TGAGTTTGATAGCGACGGAAATGTGATACTGGACAATGACACTTGCCTATTGCTTGCAGA
ATTTTCTTACAACAAAGTCATGGTCAAGTCGTCTCAATGCGAACTGCAACTTGAACCAAT
CCAACTCAGTCACGTTGGAACATGCAACTTTCCTAACAAAGGACTCAATTATGGCGCACC
AAATATGTTAAAAGCCAACATCCGTCCTGATGGGACCATGGACCTATTTTACAGTAAAGA
TGACGGGGAAACCTTTTTTGATGACGTTCGAGTTGCTACTATGAAAAATGAAGAAGGAAT
TCGCAAAGTAGACCCCGCCATATTTCGATCCGCAGCTCAGTGCGAAACATTGAACCCAGC
TCCCTATCAAATTGATGAATTATCATCAGGGTATGGACCCTGTAGAAACGATGCTGATTG
CAAACAAGTGAATACGGACACTTGGTGTTCACTCGATACTGGTGACAATTTTCCCACTGC
ATACTATTGCTATGATTGTTTGAATAATAATCATTGCATCGGAGGTCAAACTTGTGAGAC
CAACAGAAACAGTCGTTCATGTGTCAACCCCGACATTAACCAAGTGAGAGGCATTGCAGG
GATTACAACGGGGTACGATGTTTTCGACGCTGATCCTCTATTCACTGCTTATGCTTCTGG
GCTAGCGGACCAGGCAGAGGATGCTGGTGCCGTCAATAGGCGTAGATTTTTTGAGCTTGA
GTGGTCGTCAGATCCAAAATACAAAGTCGATCTACCATCAAATAAAACGTATTATGAGCC
CATTGGATTCGAAGTCACTGCCGAGCTTGTAATGACAAGCCAAACATCAGTCAAAGCATT
CGCCTCACTTGATCAATTCTCTGAGGAACAGAATAAGGCACTCGGTCTTGGAGCGTCGGG
GACCATCAAAGGTGTCCAACTTGGAGGTTCGGTTGGCATTAAGTCAAAGAAAAGAATTGC
CGAAGACGCACATAAGAACCTGGTGACCGTAACTGCAAAATCTGCAAAACAAATATACAG
ATTCCAAATCGCATCAAACAAAAAACTCAAATTGACGCTCAGCGCTGAAGCAGACTTGAA
GAAAGCAAGTAGTAGTAAGGATTGGAATGACTTTTTCCGCATGTATGGTACCCACTTGAC
CCTAGGAGGCAAGGTGGGTGCATGGCAGAGGCAAATTTACTCATTCGATGAAAATCAAAG
ATCTCAACTTACAACTACTGAGAGAACATTCTCACAATCTGTAAATATTGGTATTCCAGG
CATTTTTGGGTTCGACCAAGACAGTGAAAGCAGTGAAAGCAAAGAAATCGGAAAAAAAAT
TCAACAATTGCAGGTGAAACAGAAAACTTATTCGAAGGGTAACGTCGACAACGTTTACGA
TGAGGCAGCTATCATCCAAAGGCGGTTGGAACCAATTTGTGACCACGTTGGTGAGGTGGA
GGGGGTGACTAAAGACGAATGCCAGGAGGCGTTGATTGAGTATTGTGTCGAAAGAATCAA
AGATACCGGTCAAATCACAACTACGAAATGCGAACTTTCAGGAGATGAATTTAACTTTAC
TTGCTTGTACGATAATCAATGCCCGAGACAAGAAACATGCAACAGAGGCATCTGTACGCC
TCGATGCAAGGAAGATAGCGACTGCGGTAAATTTGAAGAATGCATTGATGTGGATAACGT
ACCGTCCAAAGAATGTCACCCTATGAAAGGCGAAAGCCAACTGCACAAACACCAATGTTC
ATATGAAAATAATGACACTCTGTGGGTTGGTTGTCAATATAAAAATGTACATTGTCCATC
GGGTTATCAAGAAGTTGAAAGAAAAGGTTTTCCTACCTGCTTCTGGGTGCCCATGAAAAA
AGTCAAATGTGTGAGAAGAGGGCACCATAAACCACCATGCGCTAGACCTCTGAAAGGAAG
TTGCGGTCAAGGAAACACAGGTAATGGATGTTGTGCAAATGGCCAAAACTGCTCTATTTG
GGGTCATTGCGGGACAGACCCACAAGTTTTTTGGCCCAGCAAACAGCAATGCGAGGAAAT
GGGCGCAAGAGCGTAGTATTGGCGCAAACACTACGAACAGGAGAGGACAGGCTCTAATTG
AACCAGATCAGAACCGGACCGAGATCGAGAGCATTCGATATCACTCAGTATTGTAAATAG
TGCTTGGAATCCTCCTATCAATAAAGAGGGATTTCCAAGTAGTAATAATTCTATTTAAAG
CGAACATCAAGCAACA
>comp2522_c0_seq1
CTTCACGGTACGAGAAGGTATTGTATCACACGGCAGTCATTGCTCTGGTTTCTTAACATA
GTTTGTTGGAACTAACGAAAGCAAAATACACATTTGTGCTGAGTTGAGAAAAAACAACAA
CACGGAAAGTTGCTGTAAACCAGCAGTTAATTCCTCTCCGCGTTGACTTCCTCTCTGAAC
ACCATCCATCGCACGATAGAGAAACGCTCTAAACAAATCATCATGTCGCAACCCTACAAG
GCCTCTGCCGAGGACGTCTTGAAGGAATATGGGGTTTCCCTCGACCAAGGTCTTTCTGAA
GCCCAGGTTTTGGAGCTCCGAAAGAAATACGGAACCAACGAACTCGAACACGAGGAGAAG
ACCCCGCTTTGGCAATTGGTCTTGGAACAATTCGATGACAAGCTCGTCCAGATTCTCCTG
GGAGCTGCCTTGCTTTCGTTCGTCTTGGCTTTCTTCGAAGAGGTCGATCACTGGGTAGAA
GCCTTTGTCGAACCGGCAGTGATTTTGTTGATTCTCATCATCAATGCCATTGTCGGTGTC
TGGCAAGAGTCCAACGCCGAGAACGCCCTCGAGGCCCTCAAAAAGATGCAGTCCCTGCAC
ACCCCCTGCCTGCGGGACGGAGTCTGGCAGGACGAACTTCCCACCGAAGAATTGGTCCCT
GGAGACATTGTCAAGATCAGCGTGGGAGACCGGGCTCCGGCAGACGTCCGTCTCTGCAAG
CTCCTTACGGCCACTCTGCGCAGCGATGAAGGTGCTCTGACCGGCGAGTCCGAGACGGTC
CTCAAGACCACTGATTCCATTTCCAGTGGAGAAAAGATCTCTGACCAAAAGAACATGGTC
TTTGCGGGAACTACCATTAGCGGAGGAAAGGCAATTGGAGTCGTTACCTCCACGGGAATG
ACCACGGAAATCGGAAAGATCCAGTCTGCCGTCACGGAGGCTTCGGAAGATGCCGAGAAG

ACCCCCCTCAACCAGAAACTCGACGAGTTTGGAGATTTGCTCACGCAAGTCATCACCGGC
ATTTGCGTCTTGGTATGGATTATGAATTTCAACCAGTTTTTCGATCCTGTCTTTGGTGGT
GTCTTCAAGGGATGCATCTATTATCTCAAGATTGCCGTTGCTTTGGGAGTAGCTGCCATT
CCCGAGGGACTTCCCGCCGTCATCACTCTGTGTCTGGCGCTAGGTACCAAGAAGATGGTC
AAGCGCAACGCGATTGTAAGAAAACTGCCATCCGTTGAAACTCTTGGTTGCTGTACCGTC
ATCTGTTCGGACAAGACTGGTACCCTTACCACCAACCAAATGGTTGTAGAGGCATTGGTC
GTTCCCGACGCCCAGGAGGGAGACCTCCAGGAATTCTCCGTGACGGGAACCGGATACGCC
CCCACGGACGGAGTTGTCCAAAACTACACTTCCACCACTGGAAACTTGGAGCAAATGGCC
AACGTTTGCAGTCTCTGTAACGAAGCGGTCATTCGTTACTCGGAAGGAAAGTACGTTCGC
GTCGGAGAACCCACCGAAGCCGCACTCAAGTGCCTAGTGGAGAAGATGGGCCTTCCTGGT
CAATCCGCCCCTTCCAACGAGGTCGAAGTCGCCGATTACTTCGGAAAGTCTTTCCAGGGA
CAGTTCGACAAGGAAGCCACTCTCGAATTCAGCAGAGACCGCAAGTCGATGTCCGTCCTG
TGCAAGAACAAGGCGACGGGAGACCGTGTTTTGTTCGTCAAGGGAGCCGGCGAGCTTCTA
TTGGAGCGATGCACCAGCATCATGCGCCCCGATGGATCTGTTCAACCTATGTCGGCCAAG
GATCGACAGACCTTGGCAGATCGCGTCACAGACATGTCCAAGAGACCGCTTCGTGTTCTC
GGTATGGCCATCAAGACCAACATGCCTCCAAAGGAGCAATTGCCTTCTCAGCTCTTGAAC
CTCGACGAGTACGCTCAAATCGAATCCGGATTGACCTTTGTGGGATTGACCGGCATCAAG
GATCCACCCCGTCCCGAAGTTCGATCGGCCATTCAGCGTTGCCAGGACGCCGGAATCCGG
GTGATCATGATTACGGGAGACAACAAGACTACCGCCGAAGCCATTGCCCGCGAGGTCGGA
ATCTTTGAACACGACGAAAAAGTCACCGGCAAATCGTTCACGGGTGCCGAATTCTTCCAC
GGTTCGATCAGTGACGACCAGCGTGCGCACTTGATTATGCACGGAGGTGGCTCCAGAGTG
TTCTCCCGAACGGAACCCCGAGACAAGCAATTACTGGTCAAGCTTCTTCGATCTTGCGGA
GAAGTTCCTGCAATGACCGGGGATGGAGTCAATGATGCACCAGCCCTCAAGCAGGCCTCC
ATCGGTATTGCCATGGGAATCGCTGGAACGGAGGTCGCCAAGGAGGCTTCTGATATGATT
TTGGCCGACGACAACTTTGCCACAATTGTTTCAGCGGTCGAAGAGGGCAGATCCATCTAT
TCCAACATGAAGGCATTCATTCGGTACATGATTTCTTCCAACATTGGAGAGGTTGTTTCT
ATCTTTTTGACATCGACCATTGGATGTCCCGAGGGACTCATTCCTGTTCAGCTTCTTTGG
GTTAACCTTGTGACAGACGGTCCTCCCGCTACCGCTCTTGGATTCAACCCCCCTGACCCG
GACGTCATGCTCAAGCCACCCCGTGGAAAGGACGATTCTCTCATTACGAATTGGGTATTC
TTCCGATACATGGTGATTGGCTTGTACGTTGGATTTGCAACGGTTGGAATCTTTGTCTTT
TGGTACACTGAATACGAGGGATTTGTCAACTGGTTGGGACCTTCCATCATGGAATTACTC
GGAAGCAGCTACGACGGACATTCTATGGTCAGTTACGACCAGTTAACACGTTGGGGACAC
TGCCGCACCGGTGAAGAAGCCGTCGGGACCATCTTCGAAGGATTTTCCGTCAACGACTTC
GATAACACTGGGAATGGCCTCATTGAGGACTTTAGCACTGATCCTTGCTCTTACTTTACC
AAGGGCAAGATGAAGGCCAGTACTCTCTCGTTGAGTGTTCTAGTGACAATCGAGATGTTC
AATGCTCTCAATGCCGTTTCTGAAGACGGATCATTGCTGACTATCCCCCCATGGGTCAAC
CCGTACTTGTTGCTGGCTATGGCCTCGTCGTTCATTCTGCACTTCATGATTCTCTACGTC
CCTTGGATGGCCACTGTCTTTTCGATCAGCCCCCACGACGCGGTAGACTGGATTTTGGTC
TTCTTGTTCTCTTTCCCCGTCATTATTATCGATGAGATCCTAAAGTATGTTGGCCGTCAA
ATGAACGAGACCGAACGAAAGGAGCGCGAACGACTCAGGAAAGAAAAGGAGGCCTAAGGG
AAGAAGGAATATAATACGATTCCAAAAAAGAAAAATGCTCAAAAAATATGGAACTTAGTA
GAATAGTAGTCATGATAAAAGCTTAACTTCATAATGACATGATTCATGCCGAGA
>comp2523_c0_seq1
CAAAGGATTCGATACATGTGCATGTATTCCACTGAAGAGGCTCTTCTTAGAGAACTCAAG
AACGCTGGCAATTGCGGAGGGATCAACGGATAGACAACCGAGATCTTAATTCTATCGAAG
CCAAGACAGAAACGGGCGGAAAGCAAGAGTGAGCACTGGCTCCAAGGAATAGAAGCAGGC
TGTCTCGATCGAAGCAACTCGAAATCGCGTCGAGACGATGGCTTTGGGTTCCGAACCAGA
GGGACCACCGAGGTCGCCCGAAGCGACCATTCGCGTGGCAGGTGGCGACGAAGCAATCAA
ATCGACGGTGAAAAGCCTGCGCAAACTCATGGGTGGAATCTGCGACCCGCGCAAAAACAA
TGCCGACGGAAGCGGCGAGGAGGAGGAAGACAATCAATACGACTTGTATCTTCACGAGCA
GTACCAGCATTATCTTCACGATCAGGATCGGCATCAGGATCGGCATCAGGATCAGCATCA
GCACCAGGATCAGCTTCAGGATCAGCTTCAGGATCAGCATCGCCCCACCCACGATCCGAC
TTGGAGTCCGCCCGTACGACCGTCTAGTCCTAGTGCTACCGATGGAGCCACCACCGTTGT
CGCAAGTAGCACCAGCAAGAATGACGACAGCAACAATCCTCCTCCGCTGGTAGACGGGAA
TGGAGCGACAGCGACACCTTCCGAAGAGAAGGATTCTCTCGATAGCATGGACAGTGCCAA
AAAAGTGTTGTTCCCCAACGAAGAAAACGACGAACCGCTCGTGGACGAAACAACACCAAT
CTTCGAAGAATCATGCGACAACAGCATCATTAGCAACAACAGCAACACCAACTTGGAAAT
TGATCCGATTCCAGAACAGCGGCATCCTCGTGATCATTTGCACGTCTGGACCCGCACTTT
GTTGTCTATCGCAGCCCTCGTTTTTCTGGCATTGGGAACTACGATTCTATGTATGCAAAC
GACCGATACAGAAGGTCTCAACATCAACGAAGTCTTAGACCGCATACGGAAGTATCGTCG

CACAACCCATCTCCAGGAAAGGTCGACCATACACCCTGCTTCTGTCACCAATGACGTCGA
CAGCACCATTCGTGAGGCATCGCTGCCGCCAAAAACTCGGCGAACAACAGAAGAAATCAA
CGACCATGCCAAGGAACCCAATAGTTGGCTCGACACTATGTACGACGACGCAGAGGAGAT
TCTTCGATTGACTTACGATCGAACCCAGTTGTATCTACGAGAGGCAGTGGGGGAGATCCT
GGAGACTCTAGGTCATCAGAAAAATAAGGTGCTAGAAAAAATCTATGAACACCACAAAAC
CATTCAATCAGAGCAATTCAACGAGCCCGAGCTATCCCTCAACGGCATCACGAAGTATTA
CTCCGAACTCGTAGATATTCGAAACGTAACGGTCGCTCTGGAGCTCTTTCTAAAATATTT
TAGCGGCAACAGCACGTGGGAACAGATGGGGATCAATGTGATATCCAAAGAGTATTTTAC
GCAGTACATTCCGAAGACCCTGGAGCTTGTTCGAGGAGAGGCAAACCTCGAACACGTCCA
CAAAATATGGACGTCCGACCTCACGAAACGCATCCGGAATATGAGGGTCTTTGACGAAGA
CGACATCTACAACAAACTGAACGAGATAGGTGATACTATAGCCAGGTTCTACCACGAGAG
TGGATTGCACCAATACGTCGAACGGCTCGAACAAAAGCTAGCGGACGCCGTAGGCGAAAC
GAACATGACTATCGTGGATGCGATGCGGGCAGAGATCCTGGACCGGCTCGCTGAGTACTA
CCAAAACAATCCGTTGAGCGATTATTTGCAGTTGCGACCACTGCTTTCCTCGGACATCGC
GGGTGTCGACGAAGCGACCGCGTCTGGAGCTTCCTCGGATGAATGCGCGACGACCGGCGC
CGATTCCACGCCAGAGCGGGAAACAGACGAGCTCGACCAAGCCGAGACGGGCCTCGACAC
GGACGAACGAGGCGCCGAAGAAGAAGAGGTTCAAATCGTCGACCGCGATCCATCAGGCTC
CGACGAGGAATCCGAACTCCTCGTTGATACTGTCCAAGACAACCCTATAGAAACGAACGA
CTCTTCATCTATAGAAGAAGAAGAAGGAGGAGAAGGAGAAGAGTCCAGCCCAACGACGGA
ATCGAACCCGTCGATTTCTGATGTGGATGTTGTCGATGCAGAGCTCGACGACACGTTGGA
ACCAGAGGATGACTCGGACGCAACCACCACTGAAGACGAAACTATCGGCAAACACGCAGA
CGAGGCTGCCGACTTGATTGAGTCTGACCAAGAACCCAAGCTCCCTGCAGACCCTACCAT
AGCCGACTCGTCGAGTGGGACTGATAACTCCCTACTGACAGAAATGGAGGGTTCCATCCC
AAAGATGGAATCAAAGCCTTCGCTTTCTGATGTGGGTGTTATCGATGCAGAATTTCACGC
AACACTAGAACCAGATATGAATATTGAGGGAAGCGAAACCAATGACAAAAATTCAGACGA
AACCGTCAACTTGAACATCGCAGACCAAGAGCCAGATGTGCCTGCCGAGACTGCCACAAC
TGACTCTGTTGACACGGAGCAATCCTTGCGAACAGAAATAGAGGAATACAACACAACGAC
GGAATCGAATCCGATGGATTTCGACGAGGGTGTCATCGGCGTAGAGCTCGATGAAACGAT
TGAGCAAGAGAAAGACCCAAAGATATCTATACCTGCGGAGGAATACGACATCATCAACAA
GAAAAAACCTCTGGTATACGATGAAGTGGATCTTCAAAACCCAAACGACAGCGACCAGGA
CATGCTTGTACCAAAAAAAGCATTAGATGGAGATAGCGACGGCTTGTCTTCGGGAGAGGA
GCATTCGATCAAGTCCGACGTCAAAGAATCACTCGGCAAAACAACTACAAAAGAACAAGC
TGGAGAGGAACACGATGATCAACCGGAAGGCACCGATATACTTGACGAAGCATCGATAGC
CTCTGAGAATCCCGAATTGACCACTGACGAATCTACTCCTGATGACTCTCTCGCCGATGA
ATCATCGCAAGGTGATGAAAGAATACCCAGCGAGCTGCACAATAGCATTCCAATGGGAAC
GTTCCCCTCGGAAAACAAAGAAAAAGGTGGCGAGGCAAAAGATGCGGACAGTGATATTCA
AACCGTGTCTTCTGAGGACCACGGCACCGGCGACGAAATTGGTGAAGATTTGGAAGCATC
TTCATCGCAATCAATTCCACCATCGGATGTGTCGGACGCTGACATCAAAGAAATCGCTGT
CGAAAGCCCAATTCAAAAGCCGGCTGAGTTGGGCGCACACGACTCTAAAGATGAAGAGGA
TGGTACCGATGGTGAAAACCTACTCGTAGTCTCAGCTGAAACACTAGATTCTCAGCACTC
TATCATCGAAGAAGCTACGCCAGATAGCGATAAAGAACGAAACTCGGTTCAGGGACACGA
TGAAACAGATGTACACGACCAGAAAGATTCTGGTAATCCTGAAATTCTAAATGAGGGGAA
TTTGATCAGCTCCGATAATGAACGTGATTCGACTAATCAATCTCCACAAACGCCCACCTC
GGCCCAGCCAGCACGAGATGTTCCCGATAATATTCCAGATATGATCAAGGGTATAGAATA
TCACATGACTAAGATGGAGCAATCAGTAGCACCCGATCTCGATAAAAAATGCTCATCGGT
CGAATGTATGATTGAAGAAATAGAGGCAATTGTAGACAGAACAAGAACGGAAAGCTACGC
CGCTAGAAAATACGATGCAGCGGACAATTTCGAGGAGAGGAATCCAGTTGTTTCAAAGCC
CTTGTCGGTGAGACGTCGCTGGAACTCTCTTCAGGGATGGATGCAGAGAAGGTAATAGTG
GATGATAACATCATGAAAACTCCCTCTATTTTATCGGAAATCGTTATTCTGGCA
>comp2524_c0_seq1
GATCGATAGAGCAACACAACCCTCTTGTGACAGATCTCAGGATCCGATTGGAAACTTTTG
GTCCCCTTTTTGCCGATTTTCGATTTCCTGAACAAAAAGTTGTCGTTCAGTTTTCGGGAA
TGACCCATAAACCATACCGGTAAGTAAAGAAGCTATTAGCTATCCTACTGTAGATACAGC
ATCATCATTTTTCACTTTGAGGGAAAACCGAATCAAGTCGTGACCCCCATTTCAAAACAG
GCGCAAAAAAAGTACCTTAACTACAGTACTTGGGTACGTACGTACATCATAACCTTTAAA
ATTTTCTCACGTGCTTCTGAGAAAAATTTAAAATGGTGACCGAAATCAATCAACGAAAAC
GTTACAGTGATAACACAACCCACTTTGCAGCAAAGACAGTGGTGCAGCTCTAATTGTTTA
ATCCAGACCATAAAAGGCAATGAGCTGGTGATGATTGTACACATAACGAATACAGTGGCA
GAAATAGATGAGGACTGATCTCGGTGACGGCGTCAAAAATCGTTCACATCATGGAAACGA
GCGATGGGGATGACGGTTGCAAAATTGGAAGCTGTCGATTGAGCGAAATTGAAACGAAAC

CAATAACCAAAGCACCTTCGACTGTCTTTCCCGCAGTGGTAGCTTGTGGATATCGACGGG
TCGGAATCGCAAAATCTGGGAATTATTTTCCTGCGTTTTTGGTCGCTTTCCCGAATCACA
GAGACAGCAGCAACCCGGAAACAGTGCAGAGGAAGAGTGCAATCTGTTTTGCGTGGAGAT
CAAAACGAGATTTTATGCTACTTGCACGGTCTTCTGACTCTCTAGTCCTTCCTAAAGCAG
CTTTGAAAAAAGTTGTTGATAGCAACAGTGTTCCATGGAGACGATTTGACATGAACCTTG
TTGGAAACAACAACACCCCATACTTTCTAAGCGATCCCAAACTACCAAATCACTTTTCGG
TACGAATGAAAAAATCTCTGTGGCTCCTTGATCATTTTTTACATCGGGCAGAAACATCTG
CAACGGAAACGGCGAAGGCTGCCTGGGAAACATTTTGCCGCCCATCCGATACCGAAGATT
TGATCACTCCAAGAGAATTTACTCCGGGAGCTGATTCGACTACCAACCAAAGGGATGGAA
ACATTGCAAAACCAAAGGGCGAGGACACACAGGCACATTCGACCGAAACAAAAATCAACG
ATCCGAATACTTCGTTTAGCAGCAGTCACAACGAAAACGTAACGACACTAGCAGATTCAT
CCCGTTTAGGACAGTACTTTGCTTCCGAAGAAAATTCTCGAATGGTGGTGGAGTGTGCCT
TAGAAAAGATTCTTCCTCTGTTCCACGATGGCAAGAATAGTGACATCCTATTCCTTGAAC
CTAGTTGCGGACATGGTGATATCGTTGCCTCTTTGGTAGAGGCTTTGGAAGAGCATGAAA
TATCACCACGATCGGTCTTTATCCAGGGTTACGACATCGACCCTTGCGTCATTAAGACTT
GCCAGCAATCTACGACATTCCATAACAGTAGGTCTGCCAATTCTTGTAAGGACTCCAACG
ATTACCGGGTTCTCTGGGAATGTTCAAGTTTTTTTGAAACTACCCGTCAAAAATGTTTGT
GCAGTTTCGAGTCGGCTTACGGCAAGAGAGACGAAACAAAACGATTGCTGGTGTGTTGCT
TAGGAGGCCCCCCGTACACAAGTGGCCAGGGAAGCGGAAGCAGTATACAACGCGACTTGC
CCACGAGATTTGTTAAACATTGCCGAAACGAATGGAAGGCGGATGTTGTCTCATTCTTGC
TGCCTGCTCGATACAGAGAAGGAATGAAACACAGCATTACGGATTGCACTTTTACAAAAA
GAGCGGCAGCAAACGCTGAGTCAGTCACACACAAAAATACAATCAATCATTCTATCGGAG
GTGCTCAACAGATTTGGCGGTGCGAAACCGAAGAACTCAAGGAGTCGACATTTTTCTTTC
GTGGGAAAACCAAAGTGAAGCAGCCTTCAATCATCCAAACATTTTACAGCTGTAAAATAT
AACGTCCGTTGATTATCCATGAACAAGATGCTCCGTTGTCTTCAGACTGCAGAGTACGAT
ACGAAACGAACGATATTCCTAACTTATCATTTTGAGAATAAAATATTACTGGTACTACTG
GCAGTATTGGCTACGGTGCACTGGACTACAACTATCTTTAGTGCACAGTAGCCAGTAACC
TTGACGAATTCTCACAGCCCTGGACTTGCTGAGGAAACAAAAAGAAATTTGTTTTTAGTG
TGGATTTGTGTAAACCCCGGTATATTAATTTCTGCAAATAAATTAGTACCTTTACC
>comp2525_c0_seq1
CGGATCGATAGCCGGGTAAGAATTCCAAAAATGAGGTCGCCTTGCAGTGTTATTTTATAT
TGTGTTGTTCCGTATAAGATCGCTCCCGTCGATTTCACAACTTGACCGCAGCACAGTGTC
AAATCGTAGTGAAAGAGAATGCCTGTTTCTGAAAATCTGCGTTCCCGATCTCATATTCAT
GCGTGCATAGCAACCCTTTCGACGTGTTGAAGACCCTGAGCCAGACCAACAAGGGCAAAT
CTGTTCCCCTCTCCACACTCGTCGGTCAGATGTACAAAGACCAGGGAGTGGCGGGTTTTT
ACCGCGGTGTCGGCGTCAACGTAATGAGAGCCATTGTGCTAAACGCAACCAAGATGGGTG
TGTATGACGTCGCCAAGGGACAGGTAGTAAAGAGCACCGGGTGGACCCGCAAGGATCCCC
GAACGGCGTTTTGCTCTAGTTTCCTCGCCGGTTTCTTCATGACGTGCACCGTGGCTCCCT
TTGATCGGGTGCGAACCAAGCTCATGAGTCAACCAACGAACGAGAAACTCTACACCGGTT
TGTCCGACTGTTTCGTCAAGACCGTAAGCCAGGAAGGGCCACTGAGTCTATGGAGAGGTT
TTGTACCAATCTGGGCGCGTTTCGCACCGATGGCAACCCTGCAGTTGTTGACGATTGAAT
CACTCTATGCTTTCTTTGGGTTTGACAGTATTTAAATTAATCACATGCATTTACCATTGT
TA
>comp2525_c0_seq2
CGAAGACGAGAACAGGTTTTGTTAATTCACGTTTTCACAACCTTTAGACCACAGGCAATA
GAAAAACAAAAAAAGCAAGATGTCTACCTCAAATAAGAACGTCCCATCGATTCTGCAGTC
CACTGTCATGGGCGGAACAGCCGCTGTCTTTGCTGTGAATTTCACCCACCCAATTGAACT
CGTAAAATCTCGGGTGCAAGTCAACAACATGGGAGTCATTCAAACAATTTCGGATACAGC
TAAGAACGAAGGTATTGTGGCATTCTGGAAGGGACTTCCATGGGCGTATTGCAGAGAGGG
AAGTTACACTGCTATCCGACTTGGAGCCTATGCTCCCGTAAGAGATGCAATCGGTGCCGG
TGCACCCGATTCGCCAGTCTACATGAAGTTTTTGGCGGGCGCCATGACCGGGGCGATCGG
ATCGATAGCCGGCAACCCTTTCGACGTGTTGAAGACCCTGAGCCAGACCAACAAGGGCAA
ATCTGTTCCCCTCTCCACACTCGTCGGTCAGATGTACAAAGACCAGGGAGTGGCGGGTTT
TTACCGCGGTGTCGGCGTCAACGTAATGAGAGCCATTGTGCTAAACGCAACCAAGATGGG
TGTGTATGACGTCGCCAAGGGACAGGTAGTAAAGAGCACCGGGTGGACCCGCAAGGATCC
CCGAACGGCGTTTTGCTCTAGTTTCCTCGCCGGTTTCTTCATGACGTGCACCGTGGCTCC
CTTTGATCGGGTGCGAACCAAGCTCATGAGTCAACCAACGAACGAGAAACTCTACACCGG
TTTGTCCGACTGTTTCGTCAAGACCGTAAGCCAGGAAGGGCCACTGAGTCTATGGAGAGG
TTTTGTACCAATCTGGGCGCGTTTCGCACCGATGGCAACCCTGCAGTTGTTGACGATTGA
ATCACTCTATGCTTTCTTTGGGTTTGACAGTATTTAAATTAATCACATGCATTTACCATT

GTTA
>comp2526_c0_seq1
CATGTCCCAGGAAAGTTACAAGTACACCACAAACGACCCAACCCAACCGAATTCCGACGT
CTCCATACCCCTACAGTATTTTACAGCCGAAACAAGCGAACCAAGCCAGTTATTATCGAA
CATAATGACCACCAAACGCGCAGCATCTGTCGAGAAACCGCCGTTGTCACCAGTCGCCAG
TGTCTCGAGACCGATTTCGTGGTGGACAATCTGCTGCTCCCTCCTCCTTCTTCTTCCTGC
GGTATTGGAAGCCAATAAAATCGATGACGACGAACAATGGGGAGCCAATCTTGAGGACCA
TCCCGATCCCAACGACGAACACTACGAAGGAATGATCTACGTCGGCAAGCACGACGCCAA
CGGGAAACGGATCGGTTTCGACCAAGACAACGGAATTCGGTACATATTTGGGACCTCATT
CGACGAGACCCACAAGGACATCGAAGAAAAAGACTTGCTCCACCACCCCCTGCGGCCGGT
CCAAGACATTCCCGACCACGAGCGCGAAGCTGTCCTCAGGGAACGGCGGCATGGATCCCT
CAAACAAGACGGCGAGAGCGGTCGCTATGAAATGCCAAAGGCGTTTGGTCACGTAGATGA
GCACCCGCTGGATGGTGGGGTCGTCCCGCCCAAGGTTGTCCACGTGGATCCCTTCTTTCT
GGACGAAACCCTCGTCACCAACAAGGAGTTTGGAAAGTTCGTCAGGGCGACCTATTATGA
GACGGAAGCGGAGAAATACGGATGGTCCTTTGTCTTGGAGTCGATGGTGGACAAAGCCGC
GCTGATGCAACACAGGAGAGACCAGCAACAAGACGAAGACCTGGAGCTAGAAAATGATCC
AGAAGCCGAGGACTGGATGGCCGTCGACGGAGCTTACTGGAGGCGTCCCGAGGGACCCAA
ATCGAGTTATAAGTACCGAGAAGATCACCCTGTTGTCCACGTATCTCACCGAGATGCCGC
CGAATATTGCAAATGGGTAGGCAAAAGGTTACCAGGCGAATGGGAATACGAAGCCGCCGT
GAGAGGCAAGAACACTGGACCCTCCAATAGAACAATGTTCGCGTGGGGCGACGATGACAG
TGATTGGGAATTGGCGTCCAAGCACGCTAATCTGTGGGGCCCGGGCGCTTTTCCGAACGA
AAACGGCGCCCTGGATGGATACCGGGCAACCTCTCCTGTCAAGCACTACCCGCCTAACGA
TCTCGGATTTTACGACACAACCGGAAACGTCTGGGAATGGATGCGTGGCGGCAAACACAA
GGCACGGATCGTCCGCGGGGCTTCCTTCGTGGACTCTTTGGACGGATCGGTCAACCATGC
CGCTACGCTGGGGGCCCGGTCCACCGTTCATGCCACTACCACCACGACCAACGTTGGGTT
TCGATGTGCCAAGGCTCCAAAACGGCGGACGGAATATCACTTCGTGACGCACGACGAGGC
AACCCATGGGCACTTGGCAGTCGAGGACCAGTTTGGAAAGCGGGACATGATCCCGCAAAA
AGGCTGGGAAGATCAGTATCATCACGTAGACGAAGAGGAAAACGAGAACGACGAAGATCC
TGACAACCAGTACAACAATCAGTTGAAGAAACGACGGGTAATCAAGAGACGAGAACGTTA
TTCGAACGAATTGTAAACCAATCAAGGAAAACGTAGACTAATTTTGAGTCTATGACATTT
TTTATTGCGTTACCACAAAATGGATATCTCA
>comp2527_c0_seq1
CCGATCTAATGAGTGGCGATGGCCTTTTTCCTCTCTTGTTTTCAACAGACTATCGTGTTT
GGTATCCTCAATTTGATTAGTAGCGTCTGGGGAGTCGTTCTTGCGATCGGGTTGTTGACC
GGTGGAGCCCTGATTGGGTCAGACGGAGTTGAAGAGTGGGAAGATGAGGCCGGATCCGCC
AGCTTGTATATTCTTGGTGGCATTTTGATGTTTGTTTCGGTCTGCGGCGTCGGCTTTTAC
AGCTTCCAGATTCATGCCGCCAAAAAGTTCAACGTTTGTGGACTCAAGTTGGTCATCGCC
TGGGATATCTTCACGATCTGTTTCAGAATCGCTGAGTTGATTATCCAGCAGCAACACTCT
GGCGATGTAGGAGTTATGATCAACAACCTCTTCTGGACTGTCGCAATTTACGGACTTAGC
CTTTATCCCATTGTAGGCTTGGTCTTTGAAATCCAGAAAGGCATTATGAGCGAAGAAACC
TACCCACGCGAAGCCTACAGCTGCTGCTGCGAGCCCAAGGTCTAATGCGACTGGAACGCG
TCAACGTCGTCAACGACGCAAAGCGATCAAAACAACGTCGACTCGGATGACGCAAAAATT
TCTGAAGGCATCGGTTGTGCTTCTTTCATGTTATTTGAGGGTTAACGAACAAGGCGAATT
GTCGCTATTTTATGTATTTAAGTAATGAATTATTGTGACTCGAA
>comp2527_c0_seq2
CTTGAATCAAAAGAAAATTTGAGCGTACAAGAATCCTACCTTATTAGAGGATATGCCGTT
CGAGTACTATTCGCATGCGAAGTATGAACGCTTCTTTTTTGGCATCGATGGACACGCATC
GCTTTCTCAGTTTTTTCCTTTTTCCAAGCACACAATTTCCACGAGTTGAAAAGCAAAAAC
AACGTGCTTTGCAACTATTCTGAAAATCAAACAGAAAATATCTCATCGTCAAACCATTCA
TCCACCATGTCTTCTGAAGAGGTCAAAGAGGTCCCCGTCGCAACGCCTGAAGCTGAGGTT
CCAATGAAAGATCAGGAGAAGATCGGAGCCAAGTGCTGTGGATGTTGCTGTGACTACCGC
CGTGCTACTATCGTGTTTGGTATCCTCAATTTGATTAGTAGCGTCTGGGGAGTCGTTCTT
GCGATCGGGTTGTTGACCGGTGGAGCCCTGATTGGGTCAGACGGAGTTGAAGAGTGGGAA
GATGAGGCCGGATCCGCCAGCTTGTATATTCTTGGTGGCATTTTGATGTTTGTTTCGGTC
TGCGGCGTCGGCTTTTACAGCTTCCAGATTCATGCCGCCAAAAAGTTCAACGTTTGTGGA
CTCAAGTTGGTCATCGCCTGGGATATCTTCACGATCTGTTTCAGAATCGCTGAGTTGATT
ATCCAGCAGCAACACTCTGGCGATGTAGGAGTTATGATCAACAACCTCTTCTGGACTGTC
GCAATTTACGGACTTAGCCTTTATCCCATTGTAGGCTTGGTCTTTGAAATCCAGAAAGGC
ATTATGAGCGAAGAAACCTACCCACGCGAAGCCTACAGCTGCTGCTGCGAGCCCAAGGTC
TAATGCGACTGGAACGCGTCAACGTCGTCAACGACGCAAAGCGATCAAAACAACGTCGAC

TCGGATGACGCAAAAATTTCTGAAGGCATCGGTTGTGCTTCTTTCATGTTATTTGAGGGT
TAACGAACAAGGCGAATTGTCGCTATTTTATGTATTTAAGTAATGAATTATTGTGACTCG
AA
>comp2528_c0_seq1
TCGATAACAACTACACCGACTCTTACAAAGATTGCGTCTTCACGATGGCGATTATGTTCA
ACCACTCCTTCCCCCGGCTGCTAGTATGGGTGGTAGCCGTGCTGGCGACGCTTCACGGAA
CTGCAGGGGAGGATGCGACCAAAGCAGAGGCAGAGCCAAAGGTCGTGGCGTCGATCGAAG
TCCAGCTAGATCCAAACAGGCCAATGGATGGAGTCGATAAGGAATTCGGCGACGATGGGG
AAAAATTCGAGTGCAAACAATGGGCAAGCGAGGGAGAATGTGAAAAGAATCCCGGCTTTA
TGCTCCAAAATTGTGCTGCTAGCTGCAAAGCTGCAAACACAGTCATTGTCAGTGTCTATG
ACGGAGAGGATGTCGGAGTGGCAGCCTTTCGTTTTGGAGAGGAACATTCCTCCGCTTTTG
GTTCGCCCAGCGAGACCCAGGAATCAGTTTTGAAAGTGGCACAGGAATTGCAAGGAGTTC
TGGATAAGGGGAGTTACGAACCTCCGAAGGAATTAACTCACTGCGGCAAACGCCAGTGCA
GTGCCGGAAAGCTTTGGAAGCGTGCCGAAGAAATGCGCAAGGCAGATATGCACGACGATG
CGGGAGCAGATTTGATCCGAGCGTTGCTGAAAACAGGTATCGAGGTTGACTTTGTTGAAC
GGTGCAAGCGTTCGTTGCAATGGGCGTTTGGAAGCATCCAACGGCAGCGAGAGAGAGAGA
AGAGAGATGCGATAGAAGAGGCCAAACTTGAAGCCCGGCAAGCGGAAGAACGTGTGGCTA
TGGAAGAAGCCCTCCAACGGAAGAAAGAATACGAGGCAGATTTTGTGAAATTTGGAGTCA
AGGTCCAGGAAATGGTTGCCGATGGATCAAAAGTGTCGTCTGCTTCGGTTGGAGCGGATG
GTACGGTCGAAACAGAAACAAACAATGGTCTGATTTTGGAAATCATGAAATCATTTGTTG
CGGAGGGTCCAGAGGGTGGAAACTGGAGCAACGTGCTTCAGCTCATCAAGAAGGTGCGTC
CATCAGAAAAGACCACAGAGATACTTCTTATTGAGGCTCGGTGTCACGAGATGATGGGAG
ACCACAAAAATGCTCTGTCGGCCGCTGGTCGCCTAATCCAAAAAGCGGCATCATACACTC
CTTGGGTTGACGACAGCCCTAGAATGATTGCTGTTACATTAGGAGCGAATGCTGCGATGC
AACTAGGTCTATCAGAAAATGCAATCTCCTTCTACCAAACCGTTCTGAAGTTCGACCCCG
AACAAAAAAAGACGAGGAAGCAGTACAGAGGCCTAAAGAAAGTCGTGAAGCTTATGGCCA
AGGCAGACGAACAGATTGAAAAAGGATACAACAAGGCCGCTTCCACATTTGTTGACGACT
GCCTTTCAGCCATGCGCGGTCTTGACGTAGACTCGCCACTGTTTAGAAGCAAGATTCAAC
TGAAACAATGTACCATTTTGAGTGGCATGGGCAAACACGAGGAAGCCCTGGCAAACTGCG
ATTCTGCCGTAGAACAAAGAGCCGGGAACGAATCGGTGTCGGACGAATCGAAAAAGGAAG
CTTATTTGGTGCGTGCCGAAGCTCTCCTTTTGGATATGGACTACGACGAGGCGGTGGGAG
ATTTTAGGATGGCCTTCGACCTCGTCCCCGAAACGCAGGAAACCATGCAAGAAAAACATG
AGCTCAACCAGAGACTGAAACAGACCCAGCGCCAACAGGAATTGTGGAATGGTGGGGTGA
AGGATTATCAGTTCAACGAAAACACCGGCTACCCAGATGGTAGACCGCCACAACGGGATC
ATGCAAAGATCCTACAATTGCCAATCGACCTCGATCAACAAAGCAAGGAGGTGAAATGCG
CGTGGCTGAAGAAGCAGTTCAAGACGCTTGTCAGAAAATATCACCCAGACAAGTACAAGG
GAAACAAGAAGAGAGCTGCGAGAAAGTTCAAGGAGGTGAAGGAAGCAAAAGAAATCATTT
CGAAGGCTTGGCAGTGTTAATTTGAGTACCCTATTTGATATGGCGGACTCGGTTGCTAAC
ATAATTCTATTCAATAATTAATAGGAATGCTAATTACCTTCTAAACTAAGTGCTACGTCT
GAACATATCCAAGTACAAGTATAATGGAAAATTTTTTAGTCAGAAATTAGTGCGAG
>comp2529_c0_seq1
AACAAAATCATTTCATTCGATACTTGCGATACACCGGATGGAGTCTGGGGCACTCGCATA
AGCTATCAATTAAAGCATTGCAATGTACAGCGCATACAATAGAATGAGGCGTAACTTAAA
ATGTTCGTCAATCTTCCAGCTGGTCGTGATTGTTGCGCCTGTGATAGCGCTGCAACTTAT
TCTCGTGACCCAGCGTCTCGACGGAGTCGTTGGTGTCGGCGTCGACGATCACCAACATTT
GTACACGGATAGCTTTCCTAGGAACGATGAGTACAACTTAATGGCCCTGGAACCGGAAAC
ACCCTTTGGAACCCCATACGGCGGCTTCAGCAGAGCTACGGCCAGCCGACTGGCCGCTTT
GCCCTTGAAACTTCCTTCTATATCCAAAGTAGAGAAAGGGGAGAACGACCAGGACGACGA
GGATGACGGCGAGTTTGATCCTATGTATACAACTGTGAGAGATAATGCCGGCCGTGCCTT
TGCCTGCCGGATATTTCTGGAAGACGAACTGAAAGCAAGCAGTCTCGGAGATAGTATGTT
CGACACTCCAATACTGAAAGAGAAACCCAACAGCAGCAGCAAAGAATCATCGCCGTCGGA
AGAACGCAACGAGGAAACCAGTGGATCGGAGCAAGCTGACCCTTCCGCTTCTCAAAGTCT
CTCCGAGATTGGGTTCGACCCTGTAATGGTAGGGCATACAATTCACCAGCGGTTGAAAAA
ATTAACTGGTTTGTGCGCCCAGTTACATCCCCCTGGTTGGTGGAGTTATGAGTGGTGCCA
CCAAGAAAACATTAAGCAATTCCACGTCAAAGTTCTCCAATCAAACGGCAAGGCACAAAA
ATTTGAACTCGAAGGCATAACCAATCTCGGAAGCTTATCTGAAAGGAGAATCCAAGTCGT
AAATGAGGACGACGATGATGATACGAACGACAATGAAGATGAAACTGACCTTGCACAAGG
ACGAAAGGAGATTGGACGCGTAATCGATACCTTTGTCGGAGGTGAAACCTGCCCAGAGAC
TGGTGAGCCGCGTGTGACAGAAGCTACATTTCGATGTTGCTCTGAACGATTCTTGAGGAA
AACCAAGGGCGGAGTGTTGAAAAACGGGAACCAGGTGGACACAAACATTATCAGTATTTT

CGAAGCAAAGGAAAATAGAAACGCCGTCTGTCGTTACAATATCACGCTCTGTACACCTCT
TCTGTGCGATGATTTTGATGACGGGACTGGCGAAAAAGACTCAAAAGCGGGAGACACAAA
AACAGAATCCCCCTTGGAGAAAGCATACAAGAAGGCTACGAACGGTAAGGGCAAAAATCA
AATCAATAACGAGTTGGATCCTTCCACAGCGGAGAATATGTCCGTCGCCGAGATTTTGGA
AGAGACGTTTGGGCAAGCGGGAAACTTTTGCATTCGGCAGTACACTGGGGGCTGGTGGGC
GTATGAGTTTTGTCCGGGAAGTTACGTGCGCCAATTTCACGAAGACAAATCGTTCAAGCA
GGATCAATTTAAAGGAATAGTAACAGCACAAAAGGAGGAGACAGCAGGGCCAGGCGTTAC
GGAATATTTCTTAGGACGATTTATTTCAGAAAATCACAAGGGAGTTACAAAAGAGAACGA
ATGGGAACAAGTTGTGAACGCAACATCATCGTCGCAAGGAGGGTCGAAGCCAAAACCCTC
TGCGGATGACAAATCACCAACGGTTCCGTTCGCTAATGCTGGCGGAAATGGTGCATACTA
CTTCCAAGAGTACACTGGGGGCGACATTTGCGACGATGTGGATGTCACAGATTCTGCGAT
GAAGGCAGGCGCATTTGGGGAAGGCGGAATCGAACGCGCCATCACTGTTCGATACTCCTG
TAATGAAGACCTATCAGTTGCGGTGAAAGAGGATTCCACCTGTCATTATATTATTGATAT
TACTGTCCCAACTCTTTGTCATCATCCTCTGTTTAGAGCACCCGTATCGAAGAGACAAAT
AGTAAAATGCCTTCCGATCTCGTCGTAAAAAGGCGACCATGCATGACGTCGTAGAATTTC
AGTTTTCACCAATTCCATGAACGCATCAAGCTTAAGCGCTGCAAAAAACAGATTGTAATA
TCAATGGCGGTTACTTTAGCAGGCATCATTCCAAAATTATGTTATAGCTTACAAATGCGA
CGCAAAAAATAAAGGAGGATTTCAAAAAATAATAATAGTATTTGAATGTTAAAGTTCA
>comp2530_c0_seq1
GTTCGCTATCGATGCAAATCAACCCCCGTGTACTTATTTCAACGAATCTAGGCGACACAT
TAGAGTTGAAGACTGCGCAGTCAACAACTACATTCATGGTGAATAGAATCGATACGATGA
TGATAATGATGGCAGCTGCTTTGTTTGCGGTCGGCCGCCAAGCAACCCACGCCTTTCACG
CTGCTCACTCCCAAACAGGGTTGGTAGGACGAACCCTTTCACGGACGGGATCGTTCGCTG
CCGAAACAAGCCCGGGGCAGAGCTTCTACAACAACAAATCTATAACCCGCATGTTCAGTG
CAGCAACGGAGCTTTCCGACGAACAAAAAAGTGTGCTAGAGTCAAAAATCAAAGACACGG
GTGACAGAATTCGGGCTCTCAAGGACGAGGGAGCCGAAAAGGAGACGATAGCACCGATCG
TTCAGGAACTCCTCGCCCTAAAGGCAGAGCTGGATCCCACATTCAATAAGAAACCGAAGA
AACAACAGAAAAAGCAGAAACAGCAAAAGAAACAAAAACAAAAACAAAAACAAACCGACG
ACGATAGCTCCGATTTCATTACGGCGCGAGCAGTAGACTATTCGAAATGGTACAACGACG
TCATTCGTGTGACGGGATTGGCGGAAACATCTCCCGTCCGAGGATGTATGGTCATCAAGC
CATGGGGAATGGCCTTGTGGGACAAGGTTCGAACCGAACTAGACGATCGAATCAAAGAAC
ACGGAGCCGAGAATGCCTACTTTCCGTTGCTCATCCCGAAATCATTCTTGTCAAAAGAAG
CCGAACACGTCGACGGTTTTGCCAAAGAGTGTGCCGTTGTCACTCATCACCGGCTAAAAT
CATCGGATGAAGAGGACGGCGGACTGGTGGTCGATCCCGAGGCAGAACTTGAGGATCCGT
TGATCGTACGTCCTACTTCAGAAACCATGATTTGGTACATGTTCCGAAAGTGGATTAATA
GCCACCGTGACTTGCCATTAAAGGTCAATCAGTGGGCAAACGTTATGCGCTGGGAGATGC
GAACACGCCCTTTTCTTCGCACTTCCGAATTTTTGTGGCAGGAAGGCCACACGGCGCATG
CGACAGCTGAGGGAGCACGTGATGATGCCAAGACCATGATGGAGGAATACGCCTCTCTAT
GCGAGAATCTGTTGGCCATGCCCGTGGTCCGAGGTGTCAAGTCTCCTTCGGAGCGCTTTG
CGGGAGCCGAAGACACCTTCACCATCGAGGCCCTGATGCAAAACGGATGGGCGCTGCAGA
GCGGAACATCCCACTTTTTGGGCCAGTCGTTCGGCAAGGCGTTCGATGTCACGTTCCAGG
ACGCGGATGGGGAGCAAAAGGATGTGTGGGGAACCTCGTGGGGTGTCTCAACCCGGCTCT
TGGGGGCACTGATCATGACGCACTCGGACGACGAAGGACTCGTGCTTCCACCTCGTGTAG
CCCCCATCCAGGCCGTCATTGTTCCTATCGCGCCAAAGAAGAACGACGAGGAAGGGAAAG
CCGCCATGAACAAGGCACTCGACGATTTGGTAGCCGGTCTCAAGGCGAAGGGTATCCGTG
TCAAGGTAGATGATCGCGATTTCATGCGTAACGGAGCTAAGTACTTCGAGTGGGAACGAA
AGGGTGTGCCTCTGCGCATTGAGCTTGGGCCTCGCGATATTAAGAACAACAGCTGCGTGT
TCAAGTACCGAGCTGGAGACAATTCTGACGAGAAGATCAACGTCGATCTCACCGACGCCG
CATCCAAGGCATCGGAAGGCTTGGAAGCCATGCAGCAGAGCCTGCTCGACCAGGCCCAAA
GCCGATTGGATAATGGAATTACCGAGGATGTCTCGTATGAAGAAATGAAGACGGCCCTTG
AAAGTGACGAGGCTTCGTCGTATCCAGGAAACGGTCTGTACCTAGTTCCCTGGAAGTGCG
ACGCCGCAAACGAGGACGCAATCAAAACGGAATGCAAGGCCACAATTCGGTGCTACCCCC
TCGCCTCCAACAACGACGGGCAGCACGAGGGAAAGAAGTGTTTCTACAGTGGAGAAGACG
CAACTCACATGGCACTGTTTGGCAGAGCTTTCTAACTAATTGCACACTGTAGTTCTACAA
CTATTTCGTAATCCGACATAAACCGGCACATCAATCCATGCC
>comp2531_c0_seq1
GGCTTCTTCAAACAAAACAATTTGAGAGATCGAAAATGCTCTTTTTTTCAAAATTCGATT
GTGTCTTCGCTGCCGCTGTTGTCGTGCTGGGATCAACCCCGTTTGCGAATGCGGAGTCGT
GTGTTGACAAACATGAGAAAATAGCGGATTGCATGGAATGGGCTTGGCATGGAGAATGCG
ACAAAAACCCTGGATTCATGCACCTTCAATGCAGAAAGTCGTGCGGATTGTGCAAGCCGT

CGAAATCCGAAACAGATAATGAAGATACCTGCGTCAACTGGCACGCCAAATGTGATGACT
GGGCCACCAAGGGAAATTCGCAGGTAGACAATTTGTGCAAAGGGAACTGGAATAAATGGA
AGAATGGCAAAAAGCTTACCGGAGCATACATAGTTGAGATGTGTCCCAAGGCTTGTAAGG
TCTGTGACATTCATTTGGACGACCGTGACATTGATCTTGGAATTGGTCTTCCACAGAGCT
TTCCAGGCATGGAGGACGACAAGGAATTATTCAATCTGCTCTTAGGAAAGGTCGCAGAAA
CCCGAGCGTACGTCGAATCTATCGAAGACGAAGAAATTCGCGAGGTCTGTAAGATGTCCC
ATCCAAATTGCGCACGATACGCTCTGAGCTCGGACTGTCAAACCCACTTTGACCACCCCA
TTATGAAATATGGGTGTGCCGCTGCCTGTCAAACCTGTGAAAAGCTGGTGGAGGATAACG
GAATTTTCGAGGCTCGATACATGTGGGGGGATGCTCTCAGAGAATTCAATGAAAAGAAGT
TGGCCAAGGAAACCATCAAGGCAACCGTATAAACCCAACAAAAGCGGTGAAGGGAAGATG
ATCTCTATGCGCATTAGCAACATCAATGAAAAATAAAATATAACCAGTGGACAGATCCAT
AAAAAAAAATTATGACGATTGTTACGGTATGATCAGTTTTGATGCGTTCAACTATTATTC
GGAAACTGATGCATTGTATTCCATTAAATACAACTAAGTATTGACTGAACTTTGTTCACC
AATATTCAATAGGTTCGTGAAATCGAATCAGGATTGCCCAAAAATTTATTGAACCCAGAA
TATTCGTTCAATAATGGAGGAGGTGGTGACATTGGTTCTGAATAATGACAAATTGACAAG
GACAAATATTCTTATAGATTCTCCTCAGATGACGT
>comp2532_c0_seq1
TAAAGCCGTAAATTTCTGTTCACCGAATTACTTTTCACATCAAAATGAGCGTCAAGAACC
AAAACATGCCTCTTGCTACTTCTGCTGCTGCCGCACCAGGCCTCCTTACCCAAGTGGGTA
TGGCAGGAACCTCTGCTGTGATCACGGTGTCCTTCATCCATCCTATTGATGTTGTCAAAA
CCCGTATCCAAGTTTCGGCGGAATACTCTGCGCTTGGTATGGGTGGAACAGTGAAAAAGG
TCATGTCCGACGAGGGCGTCATTGCACTGTGGAAGGGAGTGAACGCAGCATGGCTTCGTG
AAGCTTCTTATACTTCTCTTCGTCTTGGTCTCTACGAACCTACCAAGGTACTGTTCGGGG
CCAACGGATCTGACGCTCCTTTTTACAAGAAGTTCGCAGCTGGTAGTGCTGCCGGTGCTA
TCGGAAGTCTGGCCGGAAACCCTTTCGACGTTCTCAAAACGAAGATGATGACGGCTGGCG
GAAAAGAAGTTCCATCGATCACAAAAACTGCAGGAGATCTCATGGCCAACCAAGGAATTG
GAGGATTCTACCGTGGAATTGATTCGAACATCGCCCGTGCCATGGTTTTGAATGGAACTA
AGATGGCGTGCTACGATCAAACCAAGGAAATTGTAGCTTCAACTACCGGTATGTCCAAGA
AGGCTCTCCCTACTCAGTTTTTGTCTGCTGTTGCCGCCGGATTTTTCATGACATGTACGG
TGTCTCCCTTCGACATGGTCCGCACGCGATTAATGAACCAACCGGCTGATGCCAAAATTT
ACAGCAACGCCTTTGACTGTGCTATGAAGATTGCCACACAGGAGGGACCTCTTACTTTCT
GGCGTGGATTCATGCCTATCTGGTCTCGATTCGCTCCTACCACTACTCTTCAACTTGTGA
TTTTCGAACAATTGCGTGGAATTATGGGAATGAAAGCTCTTTAAAAATATTCATGATGCA
GATATATTTGATAGACTGAGGCGAGAATCATTTCAACGGAAATGGCTAGAAAAATAAATT
GTTTTAATGTATCAACTGTTACTATTACCAAAAAA
>comp2533_c0_seq1
CAGGATTTAAGAAACTTTACCCATAATTCCCAATGATTCCTGAGTACTGTACATACATAC
CTGATCTGCACCTTCTGGCACCAAAACCACCATGGTGACATCCCCTCCTGAAGAAAAAAA
TCTGCACTGCTTAGTCCCTGGTCTCCATTCAAGCAACCTAACAACAAAGAAGAAGCCTAT
TGAGTATGATAAGAGTGATTGGTACTACTACTAGTACTAGTAGTTCTTGTATCCCTCCTC
CTCCTCGTCCTCAGTCCCCCCCCCCTCCGGTTGATAGTAGTAGTACTGTACCACTACTAC
TAGTACT
>comp2534_c0_seq1
AATCAAACGATCGACTGAATAGAGAAGAGCGAAACGACCAACAACTGAAGAGGTGCAACT
AATATTGTTACTAGTTGCCGTATTCAAAACCATGACGGCGGATAGCATTGAAAAGAGTTT
AAAAGAAGAATTCCCAGACGCAACCGAGGCGGAGTGCTTTCGTTTTGTTCAGGCAGCCAA
AGCAGTCCATAAGAAAGCGAACGCAAAACTTGATAAAGACAAACTCATAAAAGATACTGC
AAGCGAGAAGCTGGAAGAATATCTCGAATGGAGAAGTTTGTACGGGCTTGACTACGATCG
ACCCGCAAAGGACGCAGACGATAGTTCAATATGGGAGTGGGCCGCTCGAAAAGCTTTGGG
TGCAGCCGAAACAAAGAAGCGCGAGGCGGAAGCCCTTCGGGCGGCGGTAGAAGCCAAACG
AGCCGCCGACCGAGAAGCCAGGTTGAAAGCGAAAAGAGGCGACGTCGATGACAAACCACC
GAAGGTGGACTACGACGCGATGAGAGAACAAGTAATGCGTGAAGAAGAACTAGCGAGAAC
CGGAGGCGGAGACGACGGCCAAACTGATGATGAGGAGAACAACGACAGCAAGTGCATTGG
AGAAGGAAAGGCTGAGTCCAAAACAACACTTCCCCAGGTTTTCTTTCGACGTAAAGACCC
GAAGACGGGAAAGTTACTATGTGATAAAAAAGGGACGGAGCTTATTCACGTGTTGGCAGC
AAGGATTGATCGTTTCGCCACCGACAACGAGACCTGGGCACTGGCCGTCGCCCTTTATAT
CGATGCGAGTGTGGATAGAAACTCCAAACACTCTTTTGCAATTTTAGTCGATGCAAGATC
TGGCGAGGGCTGGCCTAATCCTGTGATACTCATGATTGTCTCCTTGATAGGTCAAATCGT
CACGGAGATCGAGAAGCGTCAGCCAGAACGTTGCAAATCTTTGATCATCTGTCCCTTGCC
CCGAGCTCTGATGATGGTTTGGGGAACCGTGAGGGGTTTTTACAGTGCCGAAATGAATGA

ACTGATTATAGCGTTTTCCGGTCCATCGGGCATCGGCTCTCCCCTCCCGAAAAAGAAGTT
GGATCCTCACGTCGAAGATGCCACAATCGAATTTTTTGAACAATGCAGAAGAGATCTTTT
TGTGTGTGAAGTTGAGGATGAGAAAGTATGAGAATTTAGCCGAAGATTCCTTTTGTTGGA
ACTAGAATTTCAATGGTCAAGTGGGGGTGTGTCCAATAGAAATGGTGCAGTTTTACCGCA
ATC
>comp2535_c0_seq1
ACATACTTACTAGACAAAATTGAGATGCAACAAATTTTGCAGTCGGCGATGGTTTGCACC
GCTTGTCGTAATCGTGTAAAAAATTGAGATTATGTCTTGAATGTGCCTTTTTTGTCGAAA
AGACTCCGACAATTACAACAAATTTCCCCAAATACTTTGAACAACAGTGCCCGATGACAC
CAGGGTTGCCTGTATAATTAGAAGGATTAGACTAAAAGCAACCGTATTATAACAGCTAGC
GAAGTATCGTTGTTCGGCGAGAGGAATCCCAGGGAGTCAACAGCAACTAGTTAAGAACTG
AATAGAAACGTATCCACACGATATTCCGCTTTGAATATTACAAATTGTTAATCGACCATG
AAAGGGAAGTTGAAAAAGTTCTTCAGCGGTAGGAACCTGTCGTCTGAGATCTCCGGATCC
TTCGATGGTGATGCTGCTGCGCACGACTCGTTGCTTGGCAGCAACAAGGAGGAGGATGAC
TGGGATGCATGGAGCATTGACAAGAAAAATGACGATGCGAGTGATCCGGGCCGTGGTCGT
CTAGACCGTCGGGCGGGAACGCGACGATCGCATTCGGAACGTCCGTCGAGGAGAAAGCGC
TCGAAAAGCGAGGTGAGCAAGAGGCTCAAACGATTGGAAGGTGATTTCATGACCGCTCTG
AACTCATCGAAGAAGAAATTCAGCAGCAAAAGTGTGGGTGGCGTCTTTAAAGGAAAAAGT
TCGGACGTTCGTTCGAGAATTGATTCTCTAGCGCTGGAGCGCTCTACATCTGATTCTGCA
TTCGATACCAGCAACATCGACAGATCCAAATCGGAATCGAACTTTGGGCCGAAATCCACT
AAAAAACTGCGCGGAGGACGACGCAAATCAATGCTTGACGAACCTACTACGAATAACGTC
TCTACTCGGACCCGAACTAGGTCATTGTCCAGAGGGAGACGCTCTCTCAAGGCCGACGAC
CGAGAAGGAAGTTCCTCCCAGCGTTCTTTCTCGAAAGCCAGGAGATCGGGCTCCAAGAAA
CCATCGAGCAAACGATCGATCTCGAAGCCACGAAAATCAAAATCCACGCAGGACTTTCCC
GAATTGATCCAACGAGACTCATCCGAAGGACAGAACAGTGTCTCGACCATGACCAAGGAA
TCGAAGCAGAAAAAGAAGAAGAAGAAGGACAAAAACATGGACAAGGACAAGGACGAGAGA
AAGCAAGCTGGTGATGATTTTTCCGTACCATCCAAGGTGGAAATGAATGGCGACTGTGAA
AGCCAAGGAAGTATTACCATGCACGATAGCATTAAGCTGGAAGAACCAACCCGTTCCAAA
AAGGGAAAGGACAAAGAAAAGAAGGAAAAAAAGGAAAAGAAGAAAAAGAAGCGAAGCAAA
TCGAAAAGCAGTTTTCCAGATCTTCCTCCAGACGATTTTGGCGATGGTCTGGAAGGACTT
GCAGAAGAGTTTAGAAGATCTAAGCAAGCTTATAGCAAAACTGGCTCTGAAGGAATGCCT
TCCGGGGAACTCCCTCACGGGGAAGACTCGAACTACACGAAAGTTAATAGCATGTTCGAG
AGCGCTTGGACTGGAATGGAAAGCGGCAATGCAAATGGCGATTCGAGGAATCCTTTCGAA
GACGAAAAGAAACACAACACGGAAGATGATATTTTGCGACAAGCGGTCGACTTTGAGGTG
AAACAAGCCGACACCACGAATGAAAAGAACGAAGAGATATTGAGACTTCAACAGGAGCTT
TCGGCTGCGTTACAAAAACAGTTGAAAATGTCGGAAGAGCATATTGAAGAAAGAAATGAA
TTCATGCAAGTTTCGAGAGACCTCGACCGTTTGAAAGTTGAACTTGCAGAATCACAAGCC
GAGCGCGGTGAAGTTATGGAAGAATTGAAACAGCGCGATGAGGTGATCGAAAACGATAGG
AACCGGATTGCTGATCTGGAACAAGCAATCGACGATCAACTTGGAAAGGAGGAAGAACTA
GTGAAAAATCTTCACCACTCAGAAGAAGAAATCGAACATTTATTGGAAGAAATACAGAAG
TTCGAAAAGAAGTTGGACAACGGCGAAACGGATGGCGGAGGAGCTTCTTTCGTCGAGCTG
CGCAATACGAAGAAAGAATTGACGGACAGGGAAGAGGAAGTCGTTGCGTTAACGGCAAAG
ATTGGGCTGCTTGAGAACGAGTTGAAAGAATCACTGACTGTCCCTCAACTCCAAATTGAA
GAGCTGGATCAAGAAAACAAAGCATTGCAAGGGAGGTTGAAAGGAGAACGACTAGAATAC
ACATCCAAGCTGTCAGCCAAGGATGACATCATTTCAAATCTCCGGTCAGAGCTCAACACT
TTTACTTCCACTCCCGACGCCCAAGACCTTCAATCCGCACGACAAAAACTGACCGAGGCT
CGAGAAGACGCTACTCAGGTCAGAGAAGATCTTGCCAGGACCAGGAAATATGTTGAAGAG
ATCCAGGGAGATCGAGAAGATTTAGTTGAGAAATACAACTTATTGAAAGACAAGTCTGTC
TTTATGGAAAAGACTGTGAAGGATTTGAGCGAAAAGTCGGATAATCTGGCTAAAAAACTG
CTCGTGTGGACCGAAAAAACTTATGACTGGAAGAAGAAGGCAGAGGCAGCAGAACGAAAA
TTACAGGAGCAGAAGGCTAGCAAAGACAAAGGTGGAAGCGAAACGAAAAGCGCGAATGCA
CCCGGAGATCCCCAAACTATGTTTTTGCAGGCCGCGATGGAGAAAGGCAAACCCGAAAAG
AAATCTGGCTCGTGGGGAATATTCAAGAATGGCTCCGGAGACCAAGACTCGTCTCCCGAA
GAAGGCGAACTTCGTGCACTTCGAGACAAGAACCTAGAGTTCGAAGCAAAGATTGCACAA
CTGAACAGCGACCTCGTCAAGATGCAAACGGCGCACAAAAACGAGCTGTACAGCACCAAG
AAAAAGATTGCGCAGTTGGAGGGCGAGAACGACGCACTCGTCCTGCAAAATGCCACCCTC
TCGCAGCTAAGCCAGAGGTGATCGCTCCCAGGCACGAACTGTATTTATATTATTGTATTC
ACAATCAAACCTATACCAAGCTCGCGCAATAACATATTTAAAGAAACTGTCTGTTGTG
>comp2536_c0_seq1
CTCGTACCAAAACGATTCAATTCTACCGTACACCTATCATTCAATCAAAGCATTTCTGGT

CGTATCATAGGATTGAAGTCATCGACACCGAGGGAAACGAAGATAGAAAAATTTATTGCA
ATAGAAAGTGAAAATGGCATCTGTTACGTACTCATCTACCCGTGGAGGGCAATCCAATCT
TGATTTCCGGACAGTCGTCATGACGGGTCTGGCACACGATCGCGGACTCTTTGTTCCAGA
CACCTTTCCTACCGTCGAGGCAGAAGAACTGGAGAGTTGGAGAGGGCTTGACTACCCCAG
CGTAGCCGTTAATGTCATATCAAAATTTGTTAAAGAAGATCAAGTGCCCGCCGATAAACT
GGAGGACATTGTCAGGCGATCCTGCGCCGCCTTTCGTCACGAAGAAGTAACGCCCGTTAT
AAATGTAGCGGGACACAAAGTTTTGGAACTCTTCCATGGACCCACTTTTGCCTTCAAGGA
CGTGGCTCTTCAAATGCTCGGTAACTTCTTCGAGTATTTTTTGGAGACTGGGTCCAATGA
TGGGCGACTTGCTGTTCTTGGCGCAACATCCGGAGACACCGGTTCCGCTGCTATCTATGG
TCTCCGTGGGAAGAAGGGGGTAGATTGCGTTATTCTCTTTCCCAAAGGTAGAGTTTCCGC
GATTCAGGAACGCCAAATGACTACGGTTCAGGATGACAATATTCACTGTGTTGCCATCGA
AGGGACTTTCGATGATTGCCAAGATATTGTTAAGGCCAGTTTCAACTCGCCTGAATTTAG
AGATCGTGTCAAGCTTGGAGCAGTTAACTCCATCAACTGGTGCCGTGTATTGGCGCAAAT
TACTTACTACTTTTGGAGTTATCTTAGAGTTACTGACGAAGATGAACATAAAGATGCTAA
GGTTGGCTACTCTGTTCCCACAGGAAACTTCGGAGATATTCTGGCAGGTTTCTATGCCAA
GCAAATTGGTCTCCCCGTTGGAAAACTCATCGTAGCCACCAACGAGAATGATATCCTTCA
TCGATTCTTTACGAAAGGAGAGTACCACCGTGAAGCTATCAATAAGACTATCTCACCTTC
CATGGATATTTGCGTCTCTAGTAACTTTGAGCGATACCTTTTCTTCTTGGCCGGAAATGA
TTCCGCGAAACTTGCGGAGTGGATGACAACGTTTGAGGAAACGAAGAAACTCACAATTGA
AGGGGAATTGCTCGCTAAGGCACAAAGCGACTTCAGCTCGGCCAGGGCCGATACAAGCAT
GACTTTAGATATTATTCGAGAATACAATGAAAATCACAACTATGTTATGTGCCCGCACAC
TGCCGTGGGTGTCAGTGCCATACATCAAACGAACGATGTCGTCGAGACAATGGTGTGCCT
GGCTACTGCTCACGAAGGAAAATTCCCAGCTGCTGTGAACCAGGCAATAGATCCCCTTCC
GACACCACCAGAACAGCTTGCGTGTCTTCTCGATCTCCCTACCCGTCTTTCAGAATGTCC
CAACGACGTGCAACATGTCCACGATTTCATGGAGAAAAAGATCGCTGAGAGAATGGCGAA
AGCTTGAAGAAACCAAATGCGTTTCGAGTAAAAGACTCGCAAACAGAATATGAAATGGAA
GACAAATTGTGTAATTTCTCATCCATAGAAGCATCAAACAACGTGAAGGCTTTTCTATCA
CGATACAACATGAATTATTAAAAAGGTCGAATCGCTTCATACTTAAATTGAGCTCAGATT
AGTATGACCGAAATAGCTAAATATGAATAGAAGC
>comp2536_c0_seq2
CTCGTACCAAAACGATTCAATTCTACCGTACACCTATCATTCAATCAAAGCATTTCTGGT
CGTATCATAGGATTGAAGTCATCGACACCGAGGGAAACGAAGATAGAAAAATTTATTGCA
ATAGAAAGTGAAAATGGCATCTGTTACGTACTCATCTACCCGTGGAGGGCAATCCAATCT
TGATTTCCGGACAGTCGTCATGACGGGTCTGGCACACGATCGCGGACTCTTTGTTCCAGA
CACCTTTCCTACCGTCGAGGCAGAAGAACTGGAGAGTTGGAGAGGGCTTGACTACCCCAG
CGTAGCCGTTAATGTCATATCAAAATTTGTTAAAGAAGATCAAGTGCCCGCCGATAAACT
GGAGGACATTGTCAGGCGATCCTGCGCCGCCTTTCGTCACGAAGAAGTAACGCCCGTTAT
AAATGTAGCGGGACACAAAGTTTTGGTAAGTAGCTAATTTGTTGCAAAATATTTAAAGAA
CAAATCTGAGTGGTCTGCATCGCCGGAAAAAGCAGACACGAGACCCTTACTGATGCGACT
CTGCGATACTTTCTCGTTGCAGGAACTCTTCCATGGACCCACTTTTGCCTTCAAGGACGT
GGCTCTTCAAATGCTCGGTAACTTCTTCGAGTATTTTTTGGAGACTGGGTCCAATGATGG
GCGACTTGCTGTTCTTGGCGCAACATCCGGAGACACCGGTTCCGCTGCTATCTATGGTCT
CCGTGGGAAGAAGGGGGTAGATTGCGTTATTCTCTTTCCCAAAGGTAGAGTTTCCGCGAT
TCAGGAACGCCAAATGACTACGGTTCAGGATGACAATATTCACTGTGTTGCCATCGAAGG
GACTTTCGATGATTGCCAAGATATTGTTAAGGCCAGTTTCAACTCGCCTGAATTTAGAGA
TCGTGTCAAGCTTGGAGCAGTTAACTCCATCAACTGGTGCCGTGTATTGGCGCAAATTAC
TTACTACTTTTGGAGTTATCTTAGAGTTACTGACGAAGATGAACATAAAGATGCTAAGGT
TGGCTACTCTGTTCCCACAGGAAACTTCGGAGATATTCTGGCAGGTTTCTATGCCAAGCA
AATTGGTCTCCCCGTTGGAAAACTCATCGTAGCCACCAACGAGAATGATATCCTTCATCG
ATTCTTTACGAAAGGAGAGTACCACCGTGAAGCTATCAATAAGACTATCTCACCTTCCAT
GGATATTTGCGTCTCTAGTAACTTTGAGCGATACCTTTTCTTCTTGGCCGGAAATGATTC
CGCGAAACTTGCGGAGTGGATGACAACGTTTGAGGAAACGAAGAAACTCACAATTGAAGG
GGAATTGCTCGCTAAGGCACAAAGCGACTTCAGCTCGGCCAGGGCCGATACAAGCATGAC
TTTAGATATTATTCGAGAATACAATGAAAATCACAACTATGTTATGTGCCCGCACACTGC
CGTGGGTGTCAGTGCCATACATCAAACGAACGATGTCGTCGAGACAATGGTGTGCCTGGC
TACTGCTCACGAAGGAAAATTCCCAGCTGCTGTGAACCAGGCAATAGATCCCCTTCCGAC
ACCACCAGAACAGCTTGCGTGTCTTCTCGATCTCCCTACCCGTCTTTCAGAATGTCCCAA
CGACGTGCAACATGTCCACGATTTCATGGAGAAAAAGATCGCTGAGAGAATGGCGAAAGC
TTGAAGAAACCAAATGCGTTTCGAGTAAAAGACTCGCAAACAGAATATGAAATGGAAGAC
AAATTGTGTAATTTCTCATCCATAGAAGCATCAAACAACGTGAAGGCTTTTCTATCACGA

TACAACATGAATTATTAAAAAGGTCGAATCGCTTCATACTTAAATTGAGCTCAGATTAGT
ATGACCGAAATAGCTAAATATGAATAGAAGC
>comp2537_c0_seq1
CGAAAAGGAAGCCAAAGAAAAGGCGGAAAGAGAAGCAAAAGAGAAGGCTAAAAAAGAAGC
CAAAGAAAAAGCAGAAAGGGAGGCCAAAGAGAAGGCTGAAAAGGAAGCCAACGAGAAAGC
CGAGATAGAAGCGACAGCGGAAGCGGGAGAGGAAGCAAATGAAAATACGGAAGGAAATGC
TGCTGCATCGGCGACCACTATATCATCCCCCACTAAATCAACAACAGAACCTGCCACAAC
GAGTACCGTAGATGAATCGAAAGCAGTTGCTGAAAGTGGAAACAATGATGGATTTTTGAA
GCAAGTTCAGGAAGATGGATTCGTCAATGCTTTCTTGAAGCTAGATGTGACGACGCAAGC
GACGGCTGCTGCAGGAGTGCTGGCGTCAGGAGCAGCTGCGGCACTCTCCATTGTAAAGAG
GGACGACGACAACGGCGACGATAATGGCTCAGGCTTTGGTGGGAACTCCTCGTTTGGAAA
TCAAGGACGTCAAAACAACCAAGGGGGGCCTTCATCGTTTGGAAATCAAGGGGGAAATCC
CTCGTTCGGAAATCAAAGGAACCAAGCTGGACCTCAATCTTTTGGAAATCGAAATAACCA
AGGTGGGACGCCTCCGTTTGGGAGTGCACCTGCTGATGCTTCTCAGAACAATAATAAAGA
CGGAAAATCCCTGTTTTCTTCTTTTTCTTTTGGCAATCCACCTTCTGCCAAAAATTCCAA
TTCCAATAATGCAGGAGGTTTGGGTGGTCCCCCAAAGAAAAATGGAGGCTTTGGGGGTGC
GTTTGGATCTAGTCAAACAAGTTCGAAGAAGCAAGGAGGATTTGGTGGTGGTGGTGGATT
TGGG
>comp2538_c0_seq1
CTTACTTCTCTAAATGTCTTCTCAAAACTATCCAATCATGGTATCCGCTACGTTAAAAAA
ATATCTGAAATTCCTCCTATTCCAGTGCTTAATCCTTTCTGTGTTGGGGGATGACTTCGA
CAACGCTATATCTGCCAAAATCAAGAGTCTCGCAATTCGGGGAGCTTCGATCGCATACTA
TGATAGTAACAAAATGCCATCACCTGTAACCCGAGGATATGGTCAGATTTCTTCGGATTC
ATCCTCAGCCTCCGTGACGCCCGATACAGCCTTCATGATAGCGTCTGTCTCAAAGCCATT
TACCGCATCGGCTGTTGCTGTTCTCGTTGACAAGGGGATTATATCGCTCGACGATGATAT
CTGTGATGTCATACCGGCAGGATACTCCGACACAATGTGCAGGAACCAAAACCACCCAAA
CGTTAAAATTACGTGGAGAATGATGATCACACATCGGTCGTCTATGAAAGGGAACATCCC
CGATGCGAAGGATCGATGGGGAGATCCTATCAGTCCAAGTTATGGCCCTTCAGGTGGCTA
TACTTCCGATGCACCCGCCGTAGGGAATCCAACCTGTCCCCTTGGAGAAATCGGAGATTT
TTACCACGCCTTGCTTACCGACGATCCTAATGCCACTACCGACGTTGGAGCTGGTGTGAT
CCTACAGGGTGGAAAAGAACTTGATTGGTTTGACTTGGCAATATCAAACGGGAGCATGTG
GAGTCGATCAAAACCGGGGCAGCGAAGAGACTATTCGAATGCTGCGTACGGTTTTGTTTC
TGCTCTCATAGAATTCGCAACGGGGCAGTCCTTTCCTGATTTTTGTCGAGAACATTTGTT
CGAGCCCCTTGGCATGAGCCACACGGAATGGTTTCTCGACGACCTACCTGATGGTTCTCA
AGTGGCCGTACCGGTTGAGAAAAAAAGGAACGGTGGCTACATGGACGTTGGACATTACTG
CTTTATTGATTATGCAAGTGGGCAGCTTCGTACGTCGGCGAACGATATAGCACGGTGGAG
CAATGCTATGCTAGACTATGGCTCGCCCATGTTATGGTCAACAGCTGTTGGTAAAGAGAT
TGTGAAATGCCAAGAGAAAAACGTAAATGGCGAACCGATCGGAAATAACAACTGTGATTT
TGGCTATGGATGGATCTTGTTAAACAATTCCATGAAACAGAGCACTCAAGAAAAGTGGCT
GAAACAAGGATTCAGTGAGTATGACTGGACCGATGGTGTATGGCACGATGGATCAGAAGC
TGGCAGTCAGACAAACATTGTCATCCTTCCGAAAGCAGGGATATTTGTTGCCGTCTTGAC
AAACACAGACTTGAACTCGGAAACCGCAGCCCAACAGCTGACACAGGCCGTTGTCGAAGC
ACCCCTTCCTTCTTTGCCACCAACTCCAACCCCGCTATCGAATCCCACTCTTGCTCCACA
GCCCACTAGAGCCCCAGCAAGTCCAACAAGAGCACCAACAAATCCTACCAAAGCACCGAC
AAACCCAACAAGAGCACCAACAAACCCT
>comp2539_c0_seq1
TGTACCGAATCTATCTCAAAAGAAACTGTGGGAGGAAAGCTTTCCGTTTGAGCAAGTACT
CGTACCCTTCGAACCACCACGTTCAAAGACATATCGTTCACCACCACTTCGTTCATTAAC
AACATACTAGCTCATACCGATTCAAGTCCCAATCGGTCGCATCTGCATTCCTCGTTAGAA
GCACTTTTACTTTTTATCACCTAAAAGTATTTCTTTTCATCCAACGATATTTATTGACCA
CAAAAAAACAAAAACACGGCCGGTGACAATGCGATAGAGAGAAGGAACTGAGTGCAATCG
GTCGGAAAACACATAGAAACCTCGGAACAATAAACAATTTTTCCGGGAGGAAAAATCGTG
AAGTGAAAGATGATTCAACATGTCTTGTTCTATCCTTTTCGGGTTTTGTTTTCTCCATAT
TTCAGCGACGACCAGCTGACGTCGTTTCTTACCGGAGCTTTCACGGTAGGTCTATTGTGG
CTTTTAGACGGACTATATCACTCAGTCCCCGACCACTTCACGAAAGATAGAACAAGGAGC
GACGGCAGTATAGATGATGACGACGACGACGACGATCTTACTTTCATCGACCCATGGAAT
GATCCGTTGGCCTACGTCTCCACCCAGCAGCGACGAATGATGAGAACGTCAATATCTACC
ATGGTAGATATCCAAAAGAATATTGGAGAGCTTCCGAAGCGTCTGAAGAGAGAGCATTGG
CCTTGGGAAACAATCCGGCGGAAGGTGATGGGAGAAGAAAGCGACAGAGACGAAGGAAAA
ACCGAAGAAGAGAGAGATTCCCAAACAACAGAAAACCAAGGCGAGGAAAAAAGAATGAAA

AATTTGATGTCTGGTGAATCTGAGGAAGAAGCTAGTTACCAGGAAAACAAAAAAGAAGAA
AAACCGCTTTGTATTGGCAGCATTTTTGGATTGGACGTCGGCGGAACGCTAGCAAAACTC
GTATATTTTGAGAAAAAGGCGCTCGATGTGGATCCTCTTTACACCAGGAGCCTCAGAGAA
AAGCATTATGCGAAGGCACTTTCTGCGCAGACAGTTCTCATGGCTCGGATGGATGACAAT
ACCGATGGCCGTGCGAAGCGATCGGCATCCATGTCACCGATGAGAAAGATTCCAGCGCCT
CTGGGAAATCGATTCAGGAGCAGCACCGCCGAGGAATACGAATTCAAGGAAGAAGATAGC
TTTGAAAAAAGGATGCTGATACCGCCTAGCAACGAGATACCGGGTGTCATGCGTCGAAGC
CAGACTATGACGGGACATATAGCACATCCAGTGAAAATCAAGGAGAGCCAAAACTACCCC
ACCAAAATAGAAAGTTCTCTGTTCGTTGAAGAAGACGGAGAAGAAACGAATATAATGCCG
GAGCATGCAACTAAAGAAAGAAATTCCACAAACGATTTGCAAAGCCTTTATGCGATTCGA
CAAGAATCTCTTCCCGACGACGTTCGACAATTTCGGCATGTGGTAGACGATAGCGACGAC
GAAAACGACAGTGTATTCAAACAAACGACATCCTTAGAATCTTCTAGTAGTGGCCTCCCG
CCATCATTGAATCTGAAAAAAAGATCTCTGTCCATGCTCGACATGACGACGGCGCACGAC
CAAGAAAAGGTCGAAGCTCTCAATCGGTTTTATGACTTTGCTCGCAGGCTCGACACAAAC
GAAGATTCTATCAGCGACATCCAGCTAAGTTTTTATTGCCGGGAACTAGGAGGAGAATTC
CACTTCATATCCTTCGAAACACGCAAGATGAAAAACGCCATGGATCTGATTCGATTCAAC
AAGCTGCACATGAACATTCGCAACATGGGCGCCACCGGCGGAGGAGCCCATAAATTTGCA
GACAAATGGGAAAGAGAACTGGCGATTGGAATGATCAAACAAGACGAACTGGACAGCTTG
GTAGCGGGGATGCAATTTGTATTATCCACGGTGGTCGGGGAGTGTTACACATTTCGACCG
AAACAAAATCGAACGGCGGAAACAACGGGCGATGGGAAAAACGAAACCGCCGATGGCGGC
AAAAACCCAAAGCTGGAGTGGTGGACGCGGAAAGTGCAGAGAGATACCATATCCTACTCG
TCTGCCTATCCGTACATGCTGGTTACCATCGGCACGGGTGTTTCGGTGTTGCGGGTGGAC
GGTCCAAGGAAACACGAACGTGTTTCCGGATCGACCATCGGGGGAGGAACCTTCTGGGGC
CTCATGCGGCTGTTGACGGACGTCGAAAACTTCGAGGATGCCATGAAACTCGCCGAAAAG
GGCGATCCAACCAAGGTAGACATGATGGTGGGCGATATCTACGGAGAGGACAGCGACGCC
CTGGAGAAACTGGGCCTTACCGCGGACATTGTTGCCTCGTCCTTTGGCAAACTGGTCTCC
AAGGAAGACCCCGCAAAGGGGCTGACCCAGGAAGACCTGGCCCGGGCTCTGTTGTCGCTG
GTCACCAACAACATTGGTCAGGTTGCCTATCTGAACGCGAAGCTCCACAAGACGCCCCGG
ATCTATTTTGTGGGGAGCTTTTTGCGGCACAACATGATTTCCCAGCGACGTCTTTCGTAC
GCCATTGATTACTGGAGCAAGGGGGAAATGGAGGCGCTCTTTCTCGAGCACGAGGGATAC
TTTGGAGCCCTGGGAGCCTTTCTCATGACGCAAAACATCGAAAAGAAAAGCGGAGGGTTC
TTTCAGTGGGAGAACAATCCCTAAAGCACCACCAATAGAGTCGTGCCGTACAACTGCGGC
AGGCAAAACCGACGCCCTGAAATCTGTCACGAAGAAAGTTTGCGGTGCAGACCCTCGATC
GTACAGCACACTACCGCGATTCCAATCGCAGCGAAAGAATTGGGAATAGAGAACACACGG
TCACTTCAAAGAATCGTGAAGTTCTTGGGTTTGAAACAAAGGCATCTACTATTCATTATT
CATACTTCTAGAAGAGTAGCGTTGATGTACTAAAATTTACTAGTGCCATGCGCCACAAAT
TGAATTTTA
>comp2539_c0_seq2
TGTACCGAATCTATCTCAAAAGAAACTGTGGGAGGAAAGCTTTCCGTTTGAGCAAGTACT
CGTACCCTTCGAACCACCACGTTCAAAGACATATCGTTCACCACCACTTCGTTCATTAAC
AACATACTAGCTCATACCGATTCAAGTCCCAATCGGTCGCATCTGCATTCCTCGTTAGAA
GCACTTTTACTTTTTATCACCTAAAAGTATTTCTTTTCATCCAACGATATTTATTGACCA
CAAAAAAACAAAAACACGGCCGGTGACAATGCGATAGAGAGAAGGAACTGAGTGCAATCG
GTCGGAAAACACATAGAAACCTCGGAACAATAAACAATTTTTCCGGGAGGAAAAATCGTG
AAGTGAAAGATGATTCAACATGTCTTGTTCTATCCTTTTCGGGTTTTGTTTTCTCCATAT
TTCAGCGACGACCAGCTGACGTCGTTTCTTACCGGAGCTTTCACGGTAGGTCTATTGTGG
CTTTTAGACGGACTATATCACTCAGTCCCCGACCACTTCACGAAAGATAGAACAAGGAGC
GACGGCAGTATAGATGATGACGACGACGACGACGATCTTACTTTCATCGACCCATGGAAT
GATCCGTTGGCCTACGTCTCCACCCAGCAGCGACGAATGATGAGAACGTCAATATCTACC
ATGGTAGATATCCAAAAGAATATTGGAGAGCTTCCGAAGCGTCTGAAGAGAGAGCATTGG
CCTTGGGAAACAATCCGGCGGAAGGTGATGGGAGAAGAAAGCGACAGAGACGAAGGAAAA
ACCGAAGAAGAGAGAGATTCCCAAACAACAGAAAACCAAGGCGAGGAAAAAAGAATGAAA
AATTTGATGTCTGGTGAATCTGAGGAAGAAGCTAGTTACCAGGAAAACAAAAAAGAAGAA
AAACCGCTTTGTATTGGCAGCATTTTTGGATTGGACGTCGGCGGAACGCTAGCAAAACTC
GTATATTTTGAGAAAAAGGCGCTCGATGTGGATCCTCTTTACACCAGGAGCCTCAGAGAA
AAGCATTATGCGAAGGCACTTTCTGCGCAGACAGTTCTCATGGCTCGGATGGATGACAAT
ACCGATGGCCGTGCGAAGCGATCGGCATCCATGTCACCGATGAGAAAGATTCCAGCGCCT
CTGGGAAATCGATTCAGGAGCAGCACCGCCGAGGAATACGAATTCAAGGAAGAAGATAGC
TTTGAAAAAAGGATGCTGATACCGCCTAGCAACGAGATACCGGGTGTCATGCGTCGAAGC
CAGACTATGACGGGACATATAGCACATCCAGTGAAAATCAAGGAGAGCCAAAACTACCCC

ACCAAAATAGAAAGTTCTCTGTTCGTTGAAGAAGACGGAGAAGAAACGAATATAATGCCG
GAGCATGCAACTAAAGAAAGAAATTCCACAAACGATTTGCAAAGCCTTTATGCGATTCGA
CAAGAATCTCTTCCCGACGACGTTCGACAATTTCGGCATGTGGTAGACGATAGCGACGAC
GAAAACGACAGTGTATTCAAACAAACGACATCCTTAGAATCTTCTAGTAGTGGCCTCCCG
CCATCATTGAATCTGAAAAAAAGATCTCTGTCCATGCTCGACATGACGACGGCGCACGAC
CAAGAAAAGGTCGAAGCTCTCAATCGGTTTTATGACTTTGCTCGCAGGCTCGACACAAAC
GAAGATTCTATCAGCGACATCCAGCTAAGTTTTTATTGCCGGGAACTAGGAGGAGAATTC
CACTTCATATCCTTCGAAACACGCAAGATGAAAAACGCCATGGATCTGATTCGATTCAAC
AAGCTGCACATGAACATTCGCAACATGGGGTAAGTAGCACTGAAGTTTTGGTGTTTTTTG
TTCTTGTTCGTGTGTCACGGTTCTCGTGTGCCATGCTTGAGATCTCCGACCGAAACCGAC
TGATGCTTATTACCTCCAATGTTTCCGACGCAGCGCCACCGGCGGAGGAGCCCATAAATT
TGCAGACAAATGGGAAAGAGAACTGGCGATTGGAATGATCAAACAAGACGAACTGGACAG
CTTGGTAGCGGGGATGCAATTTGTATTATCCACGGTGGTCGGGGAGTGTTACACATTTCG
ACCGAAACAAAATCGAACGGCGGAAACAACGGGCGATGGGAAAAACGAAACCGCCGATGG
CGGCAAAAACCCAAAGCTGGAGTGGTGGACGCGGAAAGTGCAGAGAGATACCATATCCTA
CTCGTCTGCCTATCCGTACATGCTGGTTACCATCGGCACGGGTGTTTCGGTGTTGCGGGT
GGACGGTCCAAGGAAACACGAACGTGTTTCCGGATCGACCATCGGGGGAGGAACCTTCTG
GGGCCTCATGCGGCTGTTGACGGACGTCGAAAACTTCGAGGATGCCATGAAACTCGCCGA
AAAGGGCGATCCAACCAAGGTAGACATGATGGTGGGCGATATCTACGGAGAGGACAGCGA
CGCCCTGGAGAAACTGGGCCTTACCGCGGACATTGTTGCCTCGTCCTTTGGCAAACTGGT
CTCCAAGGAAGACCCCGCAAAGGGGCTGACCCAGGAAGACCTGGCCCGGGCTCTGTTGTC
GCTGGTCACCAACAACATTGGTCAGGTTGCCTATCTGAACGCGAAGCTCCACAAGACGCC
CCGGATCTATTTTGTGGGGAGCTTTTTGCGGCACAACATGATTTCCCAGCGACGTCTTTC
GTACGCCATTGATTACTGGAGCAAGGGGGAAATGGAGGCGCTCTTTCTCGAGCACGAGGG
ATACTTTGGAGCCCTGGGAGCCTTTCTCATGACGCAAAACATCGAAAAGAAAAGCGGAGG
GTTCTTTCAGTGGGAGAACAATCCCTAAAGCACCACCAATAGAGTCGTGCCGTACAACTG
CGGCAGGCAAAACCGACGCCCTGAAATCTGTCACGAAGAAAGTTTGCGGTGCAGACCCTC
GATCGTACAGCACACTACCGCGATTCCAATCGCAGCGAAAGAATTGGGAATAGAGAACAC
ACGGTCACTTCAAAGAATCGTGAAGTTCTTGGGTTTGAAACAAAGGCATCTACTATTCAT
TATTCATACTTCTAGAAGAGTAGCGTTGATGTACTAAAATTTACTAGTGCCATGCGCCAC
AAATTGAATTTTA
>comp2540_c0_seq1
CAGAAAAGTGTCTTGCAAAACAACAATTCAAACAAACTTGAACATTCTGTTTCATCGTAA
CGCCTTCCTATCATGAAATATTGTCAAGCTGCTGTGCTCATGCTAGCCGCAGCTAGCGGA
CCAGTGCCAGGATCGAGCCAACTACGAATGCGAACGAAAACACAACCGAAGAGGGACGAA
GAATTGCAAAATAGAATCAACGATGAGGAAGAAGTTCCATTTTGGACGAGTGCACTGCTC
AACAATCTTGATAGTATGGTTGCGCCTACACCAACACAGGCACCTCCCGCTGGTGCATCG
ATACATCCGAGCCTGGGAGACACATTTTTGGAAACAGGTTTTCCAACCCCAACCCCAACT
ATCATTCCGGGAAGCATTTCTGAAACAGATTCTCCATCCAGCACACCAAAGTCAATCAAT
ATCGTGGACATAGACAATTCTTCTTTCTTCTTTTCAGACATTCTAAATGTTGCGTCCTAT
TCGACGTTCTGTCGAGCAGGGGAGTACACCAACGTCCAGATCGTGAGCGCCAGGGAAGAC
GTTGTATATTGTATAACAAATTTTGAGGGTGGCGAGGTTGAATTTGGGTACGAACAAGAC
TATCCCGATCGTCCAATAATGAAACTAGATATTACTCCACCCCTTGTTTGCGGTGACGAC
GAAGTCGAGGTGGAAGATTCGATAAGCATCGATACTCCGTATAAGAACTACTGTGGCTTC
TTCGGGAAGGCAAATTTCCAGCCCATGGCAATAACAGTGACTGATATAGATGGAGAAAAC
ATTGGTTTCATTGATGCTAATATCACGGAAACGGCGTCGTACACAACTTACTGTGTGGAC
GAAGAATACTCAAATGTCGAAATCGTTGATATCAACGGAGACATATACTGTGTTACCGTT
GCTCCAGGAAATCCCACAATTGAATTTGTTGATAACTCTGGATTTATGGCACTGATTGAT
GTAGAACCGACCATGTCTTGCAACGGCATTGATGGAAGTATATCGGTTCCATGCGGTTTT
GAAGGCGTCGCTTTGACCGTGTGGCCAAATTTCATCTCAAGCGATAGTAATATCACTGAC
AGCAACAGTACTGTGTTTTAATATGTTTCCAATTAAGTTTGGGCAACAGCCATAAACCAA
GAAGCAATGTGCCAGCAAAGAAAGAGTATAAATAGATAGAAATCTTGTTTGATGAAGGCA
AAATGGTCACAGTAATACTAGTAATAGGGCAGTAATAATATTAGTAATACGTTGAAGGAG
T
>comp2541_c0_seq1
CACAACACAACACGACAAGTTACGATACGACACGGCGCGGCCCAAGCACTGCTGCTTCCC
TACAGAGCACGAACCCCGACCCGAGAACGCAACGCATCGAAACCCCACCCCCGCAATGGA
CAAGTTTTCCCCCGCCCACGTCGGCGCGAACGAGCTCCCGGACGGCTTCACGCAGGTCAA
CCCGAACGGCGGGGGCGGTCCCGGAGGCAGCGAGGCCGACTTCAAGAAAGCGGCGATCCA
GGAGCAGAAGCAGATGATCCTGGAACAGGCCATGGATCTGCAGGCCCTGGCCCGGCTGGG

GAGGATCAGGCTGGTCAAGCCGGAAAAGGCCGCCGAGATCGAGAACACGATCGTCTCCAT
GGCGATGCAGGGCAAGCTCCCCGGGAAGATCAACGAGGCGAAATTGGTGGAGATTCTCGA
GCGGAAGAACCGACAGGACGCGAGGTCGTCGGAGGGTGCCTCCCACGGAATCCAGATCCA
GAGGAAGCGATACGCGCTGGACTCGGACGACGAGGATGACGACGACGACGACATTTGATG
CACGATGCCTAGCGACAACTCCGTGTCCGTATTGCACAGCCATTCTGCCCGTGCAAACGC
ACGTCGCCGTATCGTCTGCCTGCTAGAATCTCTGGCGGTCGACTTCGGATTGTTGTTGTG
CAAGCGTTCTTCGCTTCGGGCAGGAAAACAAGCATCAAATCCTGTTTTTGTCACTTCAAC
TATCTCTCGTCACAAAACAAAAGGGAAATAGGTACACACGACGAACGTTAATTTATAGAA
AGGCAGA
>comp2542_c0_seq1
AATGTATTCCTCGAAAACAAAACACTGGACGGTTATCGATAAGTTGCGATTCATCCGTGT
GCTAGCATGATCCATTCCGTCTTTTGTTTAGGAGCTAGTGAGCCAAAATCCATTTCACTG
CATCGTGGCGAACTGGGCAAAAATAAAGCAAACTATATCTCCGCGGAGCTGAACGGAAGG
CAATGGTCAGACGCAACTTCTACGACGAGGAGTCATCACCAACAGCAGGAGCTATCACTA
CAGCAAGTCTCCCAATCCCAGGACGAAGCTTCACTTTGTTAAAAGCGTCTACCTCCGCAT
CGTCTCTGAATGTTAGTTCCAGCAGCGCATCTTCTAGGAAAGCCGAAGCACACAGCGTTC
ATACTAATCAATCCGGCGGGAACAAGTTTGAAGGCTCAATGATTGCGCCATCGTCTCATT
GTGGGACGCCGCCTCGGAAAAAAATGGGAGCGTTCCTTCAATTCGTTTTTGAAAGGCAAGA
ACCCAAACCTTTCACCAACGATGTCATCCTCTCCGGGGAGCAGCATTAACTCTATGCTAC
CTGCAGAAGACATGTCCACCGGCACGAATGGAAGTGGAGCGAGTCAATCCACGACGGGGG
GAGGAATGAGAGGGAGCAAGAAAAAATCGTCGTTTTCTATAATAAAGGGATCGAAGCGAT
TGGTCTCTCGGAATTTATCGGGGAAGAGCGGTGGGCTCCCGTCCATGTTTTCTAAAGGTG
GTGCGAGCAGTGGGCGAATCCAACGAGGGCACCAATCAATGGATGCCTTGGACGTTCAAC
TGCGAAAGGGCCAACAAATTGCCAAAAACGATTCAACCACTCTGGCAAGGCGGTCGTTGC
CTTCGACGTCACTGTTGGCAGTAGTAGCACCATCAGCAACGACCCATACAAGAATGCACT
TCCCGTCTTCCGTAGCAGAATACACTCGGACGGGCCCTTTGCACAGTTCCGGGTTGCAAA
AAGAAGTAGACTGTCACGGAGACAATAGATCGGCGAATACGGAAACGACGGTATCCACAA
CTCCACCAACATTTACGAGCGCTCTGGACGAACAATTGAGAGGTTCAACTTCTTACAATA
ACATGAATGCGACTTCCACCGCGTGTCATTCGCCGTCTTTCCAGATCAATCCAGTCGTCT
CCGAAGGGGAGGAGCAACCCAACAGTGCCTCATCGGCTCCACAACTGACACAGCTTCACC
GTACACGGTCCAATTCAAGAACCCACATATACAGTTCCAGTTCCAACTCAAATTCCCGTA
CGAACACACCAGTGAGACAATACAATCATAATTACCACGATGAGTTGGATTTTCGTAATG
TTCCCGAAATGCCGACTTTGAAAGGACTGAGGATACAGAACAAACATCCATCTTCCGAAT
TTCACGATGTTGGTCTTAATCTAGATGACTCGGACAATAGCCTGGTCCCAGAACCGAACA
ACGGTGACACGAATCTGATGAGCCAGAATAACAGTAGCCATTACAGCTTTAGCAACACAA
CTCCAACGATCATCACCGATGGCAGAATCACAGAACCGCCCGAAAACCTCGACGGAGCTT
TGGAGACTATCGGTGGGATGGTTACGGTGCATACCTCGTCTTCTTCCCATCTCCTTCATA
AACTGGGTCAGACAGAAAGAACTGTTTTTCCGGCCTCGCCTGAAACGAAGAAGGCGTTTA
CGAAAATTCATAATTCTTCCACAGATTCCGAAAGTCCCTTTCTCGGTGGAAACAAGGCTT
CAAACGTTGATTTGGCGGGCATGTACAATTCTCAAGAGAGACCCGTTTCCTCTTCGAATC
TACTAAGTAAATCTCTCCCGACGACGACATTTGCCAAAGACAATATTATTCATAGCAACA
GTTCGCGCTTGTTGAAACCTATTCAGAGCGCCGACTCGTGGGCGCGGGGTCGACGTTACC
TAATTGCACCTGCTGCCTTGGCAGCTTGTCCATCAAGCACGATACAGGGCCTGAGTGGAT
TGTGCACTGAATCTGTCAAAGAGGCGGCGACTTCAACGTGCCCTTCGAAAGCCTCAGGCG
CGATTGAATTAGGAGATGCCGTGATGACCTACATTGGCGAAAAATATCACTTATCCTTTG
GCAAATTTTCGTCGTGCAAATTGGTTTTACGACAAAATTATCTATTCGAATACGATTTGT
CCGCTCCTTTGACAAACCTTCCACGCGGGATTGCTCACCTCCAGCACGCTACTGCACACA
TCCACAAAGACTTTGAAGATGGCCTGGAATTGCACTTTTACGCGAGTCCATGTGCCAAGG
TAGATCATAGAGTTCTGTTGATCCGAGTGAAAAACAAAGACGAACGGGACCATTGGATCA
CATGCCTAAACAAAGCTGCCAGACTTCAGATTGAGGATCTATGGGAAATAGACGAAGACC
ATCCGCTAGGAAGTGGTCGGTACGCTTCTATTTTTGCCGCCCGGCGGAAAAAATTCGAAGA
CCGCGAACAACAGCGACGAAGAGGATCGATGCGACAAAGAACGCTCTCCTATTGATGATG
GTAAATATAACTGCGCTCTCAAAGTCATTAACAAAAACGAGTTCTGGCGAATGGTTATCA
AAGGAAGAGAAAGACCCGATACGATAGTGCGTGAATTGGCCGTGCAGTCAACGCTGACTG
CGAAGTTTGGGACGAAACAGAACTTTTTACAAATTCAGGGATTTTTCGAAACTTCGGACA
AGGTTGTCATCGAATTGGAACTTCTCGACGGAAGGGACTTATTTGAGTACATTTCGTCGA
AGGGGGTTTTGGAAGAGGGAGAGGCAGGATGCATCATTCGCGACATTTTAGTTGCATTGG
ATGCCATGAATCGGGCAGGTATTGCTCACCGTGATATAAAGCCAGCTAATATATTGATGA
CCACGTCAAACGAATCCAAGTACGGAGTCTCGGTCAAGATTTGTGATTTCGGTATGTCGA
CATTGGTCGGTGCGGATGGATTTGTGAGAGGCCGTTGTGGATCGCCTGGATATGTAGCTC

CTGAGATCTTCAAGGCCGGGCCAGGGGAAGGTTATCGAAACAAAGTAGATGTCTTCTCTG
CCGGCGTGACCTTTTATTTGATGTTGTGTGGGTACGAACCGTTTTACGGTGAAACTGAGA
AGGCACTCATCGAGGCAAACAAAACTGCAGAACTCGACTTTCCCGAGAGTGAGTGGGGTA
AAATATCACAGGACGTCAAAGATTTGGTTGAATTGATGTTGGATAAAAACCCGACAGACA
GACTCACCGCTAAAGAAGCCCTTGAATATCCTTGGATACAAGGACTCGAAAAGAATGAGA
AACACAACACAAACCGCTGCCCTGGAGGTACTAATGATGACACGAACAATCCACGTCTCC
AAGGAGGCATTTGTGCCTTGATGTAAGCTCATATTAGGTACTATGTAGGTTAATGCTTAG
CAATTATCCAATATAAAGAAATAATTTGCTACTTTACGTCAACTTGTATCTTGCACGAAA
TAATGAATCGATCTTTCCGAATCCTTGGAGGCGTTCTACTTATTGCCAATTATCCATGTT
TCTCTCTGTTGGCAAAACCCTCCTGCCGGGTCCAAATCGATTGCCTGGGAAGAAAAGAAC
CATATCGTTCGCATCGAATCAAAATCTGACATTTTGTGTTTTCTCTTTCAATATTAACTT
TTTGGCATCGTTGTCAAAGTCCGTGC
>comp2543_c0_seq1
CATTTATTGACTGCCATCGTCTGGTATCACACGATACCCTTCCACAACACAGCGATAAGT
TTGAAATCTTCCTGTACTGTATATCTCAAAAGCACAGCCGAAAGGGTCACAGCAAAACAC
GGAAGATCGAAGTAAAACGGGGAGAAGGAAAGTTAACCGAAGCACATAATCACAAGTCAA
GAAGGGTATCATTGTACGCAGGGGGGGATCAAGCAACCAACACCGGACCCGAAACATACC
GTACAGTAGCTTTGGGCTATCTGCTTTTGCTCCTGTGAACTTTTTAAATTCCATTTATCG
AGAGCAATAGCAACTCCTGTCACAACCAATAACATACCACACCAAACAAAACGACATTCT
TGATTGCCGGCCACAATGTCCAGCAGCGAAAAAGCCGATGGCGGCTGGAGCATCTCCGCA
ACGGGGAAACGAGCGGCGAGCTTCATCAGCGGATTGTTCTTTGGGGCGCCCGGTGGGAAG
GACGGCGACGAAAACCCGCAGCCTAGCAACAAGAGGCGCAAGACGACGACCACCACCAGC
AGCGCGGAAATGGACCTGGTCCTGGAAACCGTCCGGGAGCACACCGCCAGGGCCGAGGCG
CGGGAGAAGGCCGCCGCGGCCCGGGCCGAAACGATCGAGGCCCTGCTGGAGGCCGTCCTG
GAGCGTCTAGAGCAGCGCAAAAGGCCGGCGGCCCGCGAAACCTTCTCGACCCCCCGCAAG
CCGGCCAGGCCGCTGCCGCTGAACCCCAATCCCCCGTCCGCCTCCGGGCAGAAGCTCTGG
ACGCAGCAGGAGCGGTCCATCGTGGTAAAGGGGCTTGCGGCGTACGGGATGGGTTCCGCC
AAGCGGGTGGCGGAGCTCCTGAAGCACCGGAACTACAGCCAAACGAGGGGCTTTATGCGG
AGGAACGCCGATTCGATCCGGAGGGAGATCGAGGCCACCACCGGGTACGCCACCACCGAG
GCCGAAAAGCAGGAGATCCTGGATTCCATTGCTGCCGAGAACGAGGAAGACGGCGAGGAC
GACA
>comp2544_c0_seq1
ACCACAAGCACGAACCAGGTACGAGTAAGAGTACGCTATGTACGTACGTAGATTGAAAGG
TAGCATACGTATGCGTATTTGGAGCGAGTAGTACGATACGATTACCGTACAATCCGGTAC
TAGGCACCTGTACCTGTACTGTTCCCACGACCGATGATGTCAGAAAGGAAAAACCAAAAA
CACCCAACACAAACACAACTGGCCAGCGGTCCGTTGCGGAACAGAACCCCCACTCCCTTG
GAATGCCGAGCAGCAGTTCGGATTCTAGATCCCCCCAGACGATTGCCTTGCCGATAGTTC
CATAGATCCACAGATCCGTGCACGGAACCGAAGCGAACCCACTCATCCACTCTTCTTTTT
GGAAACAAGACGAACAAAGATGCCGTTGCAAGAGCAACCATTGCTCCGTCGGGCATCCGG
CACGGGCAAAAGCACGAGCACGAGCACGAGCATCGGCACCGGCATCAGCATCCACCGCCG
AGCGTTCTTCTGGATGCTGTATGCAGCGGTTTCGGCATCGAGCCTCGTGTTGGAAAGCCG
GGCATGGACCACGACGGCAATGGCGGGGGCGTCCTTTTCGAACAGCATTGGAGCACCTGG
TTCCTTGTCCAAGTCTTTGTCCTTGTTTGCGTCCACTTC
>comp2545_c0_seq1
GCACAAACGAGCACGGGCAATCTCGACCAATACAGTACAGTACTAACACCCGACGCCAGG
CCCAAACGACACACCCAAACCGAACCAACGCAAACGAAACGCAAGCGCAACCCAACGAGC
AGAATGGCGGACGAGGGAACCAAGATCGAGAGCGTCGGCCTCGCGCCCGAGGTGTTCGTC
CTGGTGGCGGCCCACGCCGCGGTCCACCCGGCGTCCCCCGTCCACGGCGTCCTGGTCGGG
AGCCGGGAGGGGGGAAGGATTTCCGTCCACGGGGCCTACCCGGTCTGCCACGAGACGCCC
ACGGGGGTCCTGGTGGAGACGGCCCTCTCCCTGGTGCAGTCCTCCCTCGAGGAGGACACC
AGCAACGAGACAACCACCGAGATCGTCGGCTGGTTCACGGCGCCCGAGCTGCTGCACGAG
AAGGCCCCCGGCCCGGTGGCCCTCCGGATCGTGGCGAGCCTGGCGGCGGCAACCAGCGGC
GGGGGGACGGACCCGGTCCTGCTGGTGGTCGACAACGAGGCCCTCCTGGCCCTGGCCGGG
GGGGGAGGGGCGGTGCTGGCATCCGAGGCCGTCCGGGCGTTCGGGAAGGACCTCGGCCGG
CAGTGGAAGGAGCCCGTCGGCTCCCTGGCGGTCGAGAGCGACGCAGGGGCCGCGGAGGCC
GCGGCATCCGCGATCCGGGAGGCAACCGGGGCGGCGGCGGGTCCCTTCGTCAGGGACCTC
GTGGACCACTGGAAGGCGGGCGCGGCGTCGGAATGGACCACGGCGGCGTCCCTGGCGGCG
TTTACCAAGAAGCACACGTAGGCAGGCGTCGTGTGCGCTTGGAGCACCGAGGCAATCGAA
TCCCCAAAGAATCCCATCCGCGGCCGCCAAACCGCGGATCCTACTACTAGAATGCCGTTC
ACTAGTCTAACGCCA

>comp2546_c0_seq1
TTCTCTCGCAAACGGTTAATACTATTGCTACCTGCACTGATTAAGTCAACATTGATTGAT
TAATTCCTCCTCGATCCAAACAGTGAACTCGAACCGATTTTAATTTTGCGTATACGATAC
AAAACCAACAAAAATGCTGCCAAGGTTTCAACTGTTCACGTCCTCCAAGCTTTTTGGACT
TTCCAGCAGTCTCCTAGCATCGTCTGTACGGAGCTTCTCTTCCACTCCGACCACTGCCAG
CGAGCTCCTGAGGTCGTCCCTGAAGGACCCTAGCCTGACCGAGTGGCCGGAAGATCCTTC
TTCTCCTACAGATGAACCCAGATTCCCCGTGTTCGACCCTTCCTCCCCAACCACGCTCCT
CGCCGAGATTTCCACCCGAGACCCTAAGGCTGCCATCGAAAAATGTTCCGATGCCCTGCC
GGCATGGAGAGACGAAACCACGGCGCCACACCGGGCGTCTCTCCTCACCAAATGGAGCAG
CCTCATGACGGAAAACGCCGACGATCTCGCCACCATCATGACGCTCGAGTCGGGAAAACC
CCTGGCGGAATCCCACGGCGAGATCAACTACGCCAAGGGCTTTCTCGACTACTACGCAGC
CGAGGCCATTCGACCCACGGGGGCCGGGGGCGGATTCTTGGTGCCCTCCCCGTTTCCGGG
GCCGAGCGGTGGCCCAAAGGGCCAGGTAATGGCGGTGAACCAAGCCGTCGGAGTTGCCGC
CATGGTGGCGCCCTGGAATTTCCCCCTGGCGATGATCACTCGTAAGGTCGGTCCGGCACT
TGCGGCCGGGTGCACGACGGTCGCAAAGCCCTCCGAGCTGACCCCCCTGTCGGCGATTGC
CCTCAAGCACCTGGCCGATCGAGCAGGTATTCCGGAGGGTGTATTTGAGATTGTTACAGC
AAGTACGGAAGGCACCCCGGCAGTAGGCACCGAGTTCTGTACCAACCCAATCGTTAAGAA
GATCAGTTTTACTGGGTCCACTCGGGTAGGAAAAATTCTCATGGCGCAGTCTAGCGATAC
AGTCAAGCGTGTCTCTATGGAACTCGGTGGAAATGCCTGCTTTGTGGTCTTTGCCGACGC
CAATGTCGACGATGCGGTGAACGGTGCCATGACCGCCAAGTTTCGGAACGCGGGCCAAAC
ATGTGTGAGCGCCGATCGCTTTCTGATCCACTCGTCGATCCACGACGAGTTTGTGTCCAA
GTTTTCTGAACGAGTGAAAACCTTGAAGGTCGGTCCGGGGATGCACCCCGGCACCGAAAT
GGGGCCACTCATCAACGCATCAGGAGTTCAGACCATCACGGAGAAGGTCAAAGCGGCAGT
GAAAGAGGGGGCAACAATCTACGAGGAGGTAGCGTTGCCGGAAAAAGGATCGGGCCCCCA
GTTCTATCCCCCGACCGTCTTGACAAATGTTTCTGCCGACAGCGAAATTTGGAAGACCGA
AACCTTCGGTCCCGTTGCCGCCATTCGCAGTTTCGAGACGGAAGAAGAGGCCCTTGCGAT
TGCAAACGACTGCAACGTGGGGCTGGCCTCGTACATGTACACGAACGACCTCTCGCGAGC
CTTTAATTTTGCTTCCAAGATGGAGGTGGGCATGGTCGGCGTCAACGAAGGAATGATCAG
CTCGACCGCGACGCCATTCGGAGGTGTGAAAGAAAGCGGCATCGGAACCGAGGGCAGCCC
CCTCGGAATTAAGGAGTACCTCGAGACGAAATACATATTTATGAAGACAGCAAACTAACA
GACATAGCAGGAAGAAAGACGCGCCCGATCAGACGTTCGCTGATGCTAAAGAACGAGACC
CCGGTACCAATTGATAATATTAACAAAAAAGATCCAAGCTTTGAAGGAAAGCAAAGTAGC
GAGGAATGTGTGAAAACTGCTACAACGATTCATAATAGCATTCTCGTGTATGCAAAAGGA
CTACCCCGAACCAGCCCAGCCCTGGTCTTCTTGATTTTTTCATACACGGAATACGATATC
GCCAAACATCATTCGAAGCTACGACAATGAATTATTGCATCCATCTATCGAATAAGTCGA
AAAAAGTACACATTGCAGTAGCAAACAAATATGAAATCACCCCTTCTTTTCTACCAGAGA
CCAGGAATGACGTTAATAATGCTGTTTTTATCTGAAACTGTTGGTCAGTTTTCCGCGATC
GATTTTTTTTCCTCGCTTTTTTCTTCCTTATATTCATTTCTCATGTACCGGGA
>comp2547_c0_seq1
CGGAACCAACCAGCAAACAAAACCATCGCACCGGCAGACATCTCTCTGTCAAACCCACGC
AAACGCAAACGCACCGAAACGAAACGAATCGAAACGAGCGAAGCCTGCATTCCAACCGGG
TCCGTCCACACAAAACGCCCGATCCACCTCCGTGCGCGAAAGCCTTCCTTACCATCCAAC
AACCATGTCTTGTTCTGCCATCCGAAAGAGTCCCGATTCGATGCACGCGGCCGCGGCTCC
CGTTCGCGTTGCTGCTCCACCGCCACCCCACTCGCACTACTTCGAAGAGTCCGGGCCGCC
GATCGGAGCGGGCTCCGAGAAGCTCCACACGAAACGCCAGGTCAAGGGCGCCGCGGTCGC
GGGAGGGGTCACCGGCCTCGTCCTGCTCGGGCCGGCCGCCGGATTGCTCGCGGCGGGCGG
CGCCGCCCTGGCGACAACCAAGGGCCGGGGCAAGCTCGGAAGGGCCGCGAGGGCGACCGG
GGATTCCGTGTCGGACCTCGGGAGGAGCGCCAAGAAGTTCGACCAAAAGCACGGCGTCCG
GGAGAAGACCTCCAGGGGAATCGCAAAGGGGTGCGACTGGATCTCGAAAAAACTGGACAA
GGGCAAGGAAGCAACACGGAAGCCATACTAGCTAGTAATGCCCCGAGGAGTTGGCTGCGC
AGGCCGGTGGTTTGGCCTTTTCCCATCGCACGATTTGAAACCGTTGGGAGAAGGTGTTTT
GCTTTGAGTCGGGTGTTTTGCTTTGAGTCGGCATGGTACACTTCGTGATTTAATAATATT
TTTTTTAATGTCAAAAAA
>comp2548_c0_seq1
CTTTGTGTCAATATCACACTAATAATACCTTCAACGAATCCAGCATTTTAAATTCGAAGC
AGAAACCTATTTGATTTACTCAACAGTTCCGTTGTAACACCATCGAAGCATGAATCATTC
GGCCTCGTTTCCGTCAACAGGGATAAATAACGTCGAGATGAGGCGGGGAAAACGGCTTCT
GAGCCGAATGGTGGTCGACGTTCCGTTCTTCCTATTACTGTTATTTGGTGGTGTCAACGG
GCTTGCGCCCAGTAGAACGAGTATTAGACAACCAAGAGCACCGAATTTTCGCCAGCAGCG
CTACAATTCTTTAACGTCTTTGCCCCTGCATTATGCCATCGATTCCACGAGACTGGATAC

TATAGGTGAGGTATCGCAAAACATGACGTTTCCGCTCCTTCTTTCCGACGATGGGCACTC
CATACAATCTCTTATCACCACCACAACAAAACAAAAACGAAGTAGTGTTTTAACAAGAGT
TTGGGGGAAAATAACCGAGTCAATGAAATTCGATAGAAAGGAAATCGCGAAAATGGGCAT
TGATTTTGGATTGACTTACAACATGATAAGTAACATCAATGGCAGCATCACGCTATCGAC
TGCTTGGTACATAGCATCAATGAAAGTACGTGTTATAGATCGATTCCTTGTTTGTTGCTG
CTTCGTGATTGCTTCAAAGAAAACCCGTGAAGATTTGATTATTTACATAAGACGGAAATT
CCTTTTTGTTTTTTCTGTTGTTGTTCATCCGTTTGTTTATAACTCATACGTTGCACACCT
GATAAACTAATGAACAACAGAGTGGTCTTTCACCTTTAGCTCCCGGAAATTGGAGAAGCC
TCTTGGCGGCATATGCGAGTTTGTACGTAGTTGCGGCGCTGGCGCGTCCGTTTCGGATCG
CGTTTGCCCTCGGGGTAACCCACAAAATGGAACATTTTGTTCAGCACGTTCAGAAACGAA
TAGGTTGTACACGAACGAATGCCATCGGGTTGATTTTTGGCTTTGGAGTTGTGATGTGGA
TTACGTGCTGCGCCGCAGGGGTGACGCTGGCGAGCACCCTTGCGGGAGTTCCGGTATGGA
AATACGATTGAATAAAAACGCAACACAACAAAGCATAAGTAAGCAACGCTCGAATAAAAC
GAAGAACAATACAAGCAAGCGGTAAGAATCGCAGCAAAGACATTAATCGTATGCAAAATA
ATATCACTTCAAAGGTCAACCGGTGGCGTTCCCTTTTTCTTTCGAGATGGTGTCGAGTAG
AGTTTTCAGTCTTTGAAACGATTCGATACAATTCAAAAAATATGAAAAAAGTAAATCATT
TACAGCAAAATAGAAAATCTGAAATGGAACGTGGTAAACGAATATGTTCCGTGCCCACAA
GTAGACCGATTGTACACATCAATTGGAGCAATCTCAAGAGCTATTCCGCATCTTCTATTT
GCCTTGCTTGACTCGTGTAAGTAGTCTACTTTCGCTTCATGAAGCTAATCGTCCTCCATA
AATTACAACATTATAAATTATACTAGTGAAATAGTAATGCGGAC
>comp2548_c0_seq2
CTTTGTGTCAATATCACACTAATAATACCTTCAACGAATCCAGCATTTTAAATTCGAAGC
AGAAACCTATTTGATTTACTCAACAGTTCCGTTGTAACACCATCGAAGCATGAATCATTC
GGCCTCGTTTCCGTCAACAGGGATAAATAACGTCGAGATGAGGCGGGGAAAACGGCTTCT
GAGCCGAATGGTGGTCGACGTTCCGTTCTTCCTATTACTGTTATTTGGTGGTGTCAACGG
GCTTGCGCCCAGTAGAACGAGTATTAGACAACCAAGAGCACCGAATTTTCGCCAGCAGCG
CTACAATTCTTTAACGTCTTTGCCCCTGCATTATGCCATCGATTCCACGAGACTGGATAC
TATAGGTGAGGTATCGCAAAACATGACGTTTCCGCTCCTTCTTTCCGACGATGGGCACTC
CATACAATCTCTTATCACCACCACAACAAAACAAAAACGAAGTAGTGTTTTAACAAGAGT
TTGGGGGAAAATAACCGAGTCAATGAAATTCGATAGAAAGGAAATCGCGAAAATGGGCAT
TGATTTTGGATTGACTTACAACATGATAAGTAACATCAATGGCAGCATCACGCTATCGAC
TGCTTGGTACATAGCATCAATGAAAAGTGGTCTTTCACCTTTAGCTCCCGGAAATTGGAG
AAGCCTCTTGGCGGCATATGCGAGTTTGTACGTAGTTGCGGCGCTGGCGCGTCCGTTTCG
GATCGCGTTTGCCCTCGGGGTAACCCACAAAATGGAACATTTTGTTCAGCACGTTCAGAA
ACGAATAGGTTGTACACGAACGAATGCCATCGGGTTGATTTTTGGCTTTGGAGTTGTGAT
GTGGATTACGTGCTGCGCCGCAGGGGTGACGCTGGCGAGCACCCTTGCGGGAGTTCCGGT
ATGGAAATACGATTGAATAAAAACGCAACACAACAAAGCATAAGTAAGCAACGCTCGAAT
AAAACGAAGAACAATACAAGCAAGCGGTAAGAATCGCAGCAAAGACATTAATCGTATGCA
AAATAATATCACTTCAAAGGTCAACCGGTGGCGTTCCCTTTTTCTTTCGAGATGGTGTCG
AGTAGAGTTTTCAGTCTTTGAAACGATTCGATACAATTCAAAAAATATGAAAAAAGTAAA
TCATTTACAGCAAAATAGAAAATCTGAAATGGAACGTGGTAAACGAATATGTTCCGTGCC
CACAAGTAGACCGATTGTACACATCAATTGGAGCAATCTCAAGAGCTATTCCGCATCTTC
TATTTGCCTTGCTTGACTCGTGTAAGTAGTCTACTTTCGCTTCATGAAGCTAATCGTCCT
CCATAAATTACAACATTATAAATTATACTAGTGAAATAGTAATGCGGAC
>comp2549_c0_seq1
GTGACCTTTTACGAAAGAGGAATCCGATTCGATGTATTCTTAAATGGTGATTTCGATTTT
GACTCTAGATTCTTCAGGACACGAAGAAATCGAAGAGTAAGAATCTCAAGAGACTTCCAA
GGAAGAATCAATCGAGTTGGTGATGTTCGAATTCGATATGATTTCCAAGGAAACGTAAGA
AGAATTGGAAGAGTTACCATGAGCTACCGAAGAGGATTGTTAAGAAGAGTTGGAAACTTG
AGAATCTCTTATAACAGTTGGGGAAATCCAAGATTCTTTGGAAATGTAGGCTTCGATGAT
TTTTACAATGATTATTACTATGGAAATAGTTGGAACGTAAACTTCAACTGGAATATAGGG
GCTATCTGTGTGTACAATGATCCTTTCTTCTATGGAAATGAGTTCAGAAATAGTTACAGA
AGAGTCAGAGAGGATGCTAACTACATCTACTACAGAGCCAATGATGGAACCAATGTATCT
AGAGACCGAATGATCAGACGAAGGAAAGCTTCTTCTAACAATGTAGCTAATGGACAGACG
GTGAGAGAAGGTAGAAGAGGAAATTCACAGGCGAGAACAAATCAACGAAGATCTGTTCAA
AACGAGTCTAACAACACTAGAAGAAGAACTGAGTTTAGTACAAATAACAATAGAAAGAGA
AGATCGGTTCAAAATGAATCTAACAACAGGAGAAAATCCGAGTTCAATACAAATAATAAC
AGGAAAAAAAGAGAGGTTAGAAACTCTTCTACCCAAAGAAAAGTAGAATCAAAGAGATCC
ATAAAATCGGATCGAAAGGTTCAAACAAAAAGAAAAACAGAAACAAAAAGGAAAACAAAT
ACAAAGAGAAGGAAAGCCGTGAACGGTGAACGAAGAGGAAG

>comp2550_c0_seq1
AATCGCGAGTATTCTGTACTTCATGCTTCTCATATGGTACTTCGTACTGCATTATGGTAT
GACACATGGTATCGGATACAATACTCGAGTATTCTTTTCGTCTAACCACAGAAACAGCAA
TCAAGAGCAGCTAGTTGCAGAAGATATTGCTTTGTCTAACTGAGGGTAGACTGGAGTCGA
AAGCTTCAAAGTAAAGCTACGGTGAAAGTAAAATGATGGTCGATACATCCGCAGCTTCTG
AATGTTTGGAACTAATTTATTTATCCATCTTCGCATATATCGAATCCGTGTTAATATCTT
TTGGCATTCAGCACGACACAGCAATCATCGAAAAAAATCCACGTGAAGAGTTGGAATGCC
GGAACAAGACCGATTGTATTGATAGATGTTTAGGAACGAAACGTCCTGTGGACTTGTGGG
AGTTACGAGAGCTCGCTCTTTCCGAAGGTGGATTGCTTCGAGCTGGATACCGGCAAAAGG
CATGGCCGAAGCTCGTAGGTTTGACAGAGGAAGATTATATTCCAGAATTAGAACTTTCTT
CGACATACAGCGAATCACCGTCATCTGTAAGATCACGAAATGAAAGTGCATTATGGAGTC
CATCAACTACTGCTACTACTTCTACTAGTTGCTTGATAATTGAAGAAGAAAAGAAATCAG
AGATAGAGGCCAATGTAGACTTGATTCGTAGAGATGCAGGTAGATCGGTGATATTCCGAT
ACCACCTTGGAACCTCAGACGCAAATCCAATTGTTCCGCCGTGCGCGAGCGAACGATTGG
CACAAGTGTTAGAGAACACAATAAGACAAGGCAATTCTTCGCTGCATTACTATCAGGGGT
TGCATGATATTGCAGGTGTCGTTCTTCATAACATGGATTATCAAGTGGATATCACCTCAC
AGATACTACAAAGAATCAGTCACAGTCACCTAAGGGATGCCATGCGGGAGAATTTTTCCA
ATATCAATTGGTTCTTAAACGTCCTTCTACCGCCTCTGGTGGAGCGAGTAGACCCATCCG
TACATTACTCGCTACAGATGGCTAACGTAGAACTGTCAACAGTCTGTCTACCCTGGATGA
TAACGTGGTTCACCCATGATATACACGATCCAGAGACTGCGGGGCGATTGGTGGATGCTT
TTTTATCCGGACATCCACTCATGCCGATGTATTTTGCGATGGCAATCATTACACATCCAT
TGCTGAAGCAAGAACTTTTGAATGCTGATCCTGAGGATACATCGTCGATCTTCATGGCGA
TCAAGAGCATGCCGTCAAGACTCAAGACAAAGGTCGAAGAAGACGAGCAACTTAGTGAAC
AACAGCATGTATCCTTGCAAGAAGTTCTGGAGGATTGCATTACAATAATGTAAGTAGCGA
TGTGAGGAGACAGAAGTATGTCCGTTGATTGAGTATGGAGGAGTTTTTTGCGCAATTGGT
CGTATGTTCTAACCCTTTTGGTTCGGACTTCCGGTGTTTAGGAATAGAATGCCCCCGCGA
TCTTTATTGGATCTAATTGACTCGAATATCTACTCTCGATCCGAGCTTTTACAAAGGATG
TCGTCCATATCACTTTTCAAGGCCCCAGCCTCGTGGAGTATGGCGTCGGGTGGGTTGCCT
CGCTCTCGGAATGAACTCAATGACATAGAAGGGACGACATACTGCGTTCGAGCAAAACTT
GCAAGTGGCGTTCCGATGCTAATGAACACCGTTTCTGAATCTACTGTGATCCGAAGAAAA
TCGTGGGGTAGTCGCAAATACTTGCGAAAGAAGAAGACGCAGAGGATGAAGAAACTAACA
CGCATCATCCTCGGAAACACAACGAACACCAAGATGTCGTGAAATCCCGTCTACTGATTT
GATCCTTAACGAAGAGATGAAAAAATAATATTCGGTCGAAGTCTGGTGCACAAAACCAAT
TAAAGGCCACTAGAAGAGCTGTGTTTGCAGTCTTCAAGAAGACGCCAGCACCAACATTTA
TATGTTAACTTTGCTCTGCGTCAATACATTCCTTCGTAGAAGGAGGTTATTGCAACCTGA
CATCAAGGAAAACTAAGCCAGTACAAAGTTCTCGCAAAAGTGACGGTCTTAAAGGCCGCT
ATCGTTTGCTTTCAAATACATTCGTGCTGAATAGGGATTGAAGCCTGAATTCTTTTGCAC
CTTGCAAATCGTTGCCCTTGTGTTTTAAACAAATTTCTTCAGTTGTCAGAGATGAATCTC
GCATCCTACAACAGCGATATTGGTTTTTCCCTTTACTATTGAATGCATGCCCAAACGGAT
GTAGTACGTGTAGTGCATATTCAACATGTATTTATGTGACCAGCAAATAAGATACAATAA
TAAAGACAGTATTTGAGATTCT
>comp2550_c0_seq2
AATCGCGAGTATTCTGTACTTCATGCTTCTCATATGGTACTTCGTACTGCATTATGGTAT
GACACATGGTATCGGATACAATACTCGAGTATTCTTTTCGTCTAACCACAGAAACAGCAA
TCAAGAGCAGCTAGTTGCAGAAGATATTGCTTTGTCTAACTGAGGGTAGACTGGAGTCGA
AAGCTTCAAAGTAAAGCTACGGTGAAAGTAAAATGATGGTCGATACATCCGCAGCTTCTG
AATGTTTGGAACTAATTTATTTATCCATCTTCGCATATATCGAATCCGTGTTAATATCTT
TTGGCATTCAGCACGACACAGCAATCATCGAAAAAAATCCACGTGAAGAGTTGGAATGCC
GGAACAAGACCGATTGTATTGATAGATGTTTAGGAACGAAACGTCCTGTGGACTTGTGGG
AGTTACGAGAGCTCGCTCTTTCCGAAGGTGGATTGCTTCGAGCTGGATACCGGCAAAAGG
CATGGCCGAAGCTCGTAGGTTTGACAGAGGAAGATTATATTCCAGAATTAGAACTTTCTT
CGACATACAGCGAATCACCGTCATCTGTAAGATCACGAAATGAAAGTGCATTATGGAGTC
CATCAACTACTGCTACTACTTCTACTAGTTGCTTGATAATTGAAGAAGAAAAGAAATCAG
AGATAGAGGCCAATGTAGACTTGATTCGTAGAGATGCAGGTAGATCGGTGATATTCCGAT
ACCACCTTGGAACCTCAGACGCAAATCCAATTGTTCCGCCGTGCGCGAGCGAACGATTGG
CACAAGTGTTAGAGAACACAATAAGACAAGGCAATTCTTCGCTGCATTACTATCAGGGGT
TGCATGATATTGCAGGTGTCGTTCTTCATAACATGGATTATCAAGTGGATATCACCTCAC
AGATACTACAAAGAATCAGTCACAGTCACCTAAGGGATGCCATGCGGGAGAATTTTTCCA
ATATCAATTGGTTCTTAAACGTCCTTCTACCGCCTCTGGTGGAGCGAGTAGACCCATCCG
TACATTACTCGCTACAGATGGCTAACGTAGAACTGTCAACAGTCTGTCTACCCTGGATGA

TAACGTGGTTCACCCATGATATACACGATCCAGAGACTGCGGGGCGATTGGTGGATGCTT
TTTTATCCGGACATCCACTCATGCCGATGTATTTTGCGATGGCAATCATTACACATCCAT
TGCTGAAGCAAGAACTTTTGAATGCTGATCCTGAGGATACATCGTCGATCTTCATGGCGA
TCAAGAGCATGCCGTCAAGACTCAAGACAAAGGTCGAAGAAGACGAGCAACTTAGTGAAC
AACAGCATGTATCCTTGCAAGAAGTTCTGGAGGATTGCATTACAATAATGAATAGAATGC
CCCCGCGATCTTTATTGGATCTAATTGACTCGAATATCTACTCTCGATCCGAGCTTTTAC
AAAGGATGTCGTCCATATCACTTTTCAAGGCCCCAGCCTCGTGGAGTATGGCGTCGGGTG
GGTTGCCTCGCTCTCGGAATGAACTCAATGACATAGAAGGGACGACATACTGCGTTCGAG
CAAAACTTGCAAGTGGCGTTCCGATGCTAATGAACACCGTTTCTGAATCTACTGTGATCC
GAAGAAAATCGTGGGGTAGTCGCAAATACTTGCGAAAGAAGAAGACGCAGAGGATGAAGA
AACTAACACGCATCATCCTCGGAAACACAACGAACACCAAGATGTCGTGAAATCCCGTCT
ACTGATTTGATCCTTAACGAAGAGATGAAAAAATAATATTCGGTCGAAGTCTGGTGCACA
AAACCAATTAAAGGCCACTAGAAGAGCTGTGTTTGCAGTCTTCAAGAAGACGCCAGCACC
AACATTTATATGTTAACTTTGCTCTGCGTCAATACATTCCTTCGTAGAAGGAGGTTATTG
CAACCTGACATCAAGGAAAACTAAGCCAGTACAAAGTTCTCGCAAAAGTGACGGTCTTAA
AGGCCGCTATCGTTTGCTTTCAAATACATTCGTGCTGAATAGGGATTGAAGCCTGAATTC
TTTTGCACCTTGCAAATCGTTGCCCTTGTGTTTTAAACAAATTTCTTCAGTTGTCAGAGA
TGAATCTCGCATCCTACAACAGCGATATTGGTTTTTCCCTTTACTATTGAATGCATGCCC
AAACGGATGTAGTACGTGTAGTGCATATTCAACATGTATTTATGTGACCAGCAAATAAGA
TACAATAATAAAGACAGTATTTGAGATTCT
>comp2551_c0_seq1
AGCATTGCCAATCTAATAAAAAAAATCACCGAACACTTCAAGACAAAGGAATCCTACCAA
AATGATAGCTTTGAAACCTTTCTTTCTTGTTGCCCTTTTGGCAGGTACCAGCTCAGCCTT
TGCTGTCATCCAGCCTGCATCTTCCATCGCGAAGGCGACGACGACGTCGACCATTCTGAA
ATTGAGCGGAGGCGCTGTCGGAGCAGGAACTGCTCCTGAGCTCAAGCCTCCACCATCTTT
ATACCAAGGAGCTGTGGCCGCCGGAGTTGGCAAAGCCTCGAAGACATGGGGAGATATTTT
CAAACTTGGTATTGTTAGCGGTTGCCACATTGCTTTCGGGGCATACCTCGCAATCACTGT
CGGAGGAGCATGCTCGGGAATCGCAGCTGAAAATCCTGGCCTTCAGAAGATCATTTTCGG
TTCCTTTGGGTTGCCATTTGGTCTCATCATGACATTAGTGACAGGAGGTGAACTCTTCAC
AGGAAACACGGCTCTTGTAACTGCTGCGTATAAAGAAGGTAAAATTAGCGGAAAGGACTT
GATCAAGAATTGGGTTTCTTCTTATACTGGGAACCTCGTGGGATCTTTGCTTTTAGCTTA
CTTGGCTTACAAGAGCGGAACTTTAGCCGCTAGCCCAGCGTCGGCTGCCATTGCTACAGC
GAAGTGCTCTCTTCCATGGGATGTTGCATTTGTTCGTGGTATTCTGTGCAACTGGCTGGT
ATGCATGGCTGTCTATATGGCATCTGGTTGTGCCTCGATGGCAGGAAAGATGACTGCCGT
TTACTTCCCCATTTCCGCCTTCGTCGCGCTTGGATTGGACCACAGTGTTGCGAATATGTT
CATCATTCCTCTTGGGATGCTTCGTGGTGCAGACATCACTATCAAAGCATTTTTGATGAA
GAATCTAATTCCAGTTACTTTAGGTAACATCGTTGGTGGGGCACTTTGTGTGATGGCTCC
CTTTGGTTCGGTCTTCGGTAGCTGGGGAAAGAAGTCCGACTGATGCGGTATAGGCAATTA
GTGCCAAGATACTTGTTCAGTGCCAACTCCTATCTCATCAAGCAGCATGCGGTAGATTCA
TGCCCTTCAACTAATATGAAATCCAGTGAATTGTCTATGTCATCATTTTGATCACTTGTT
TAGCAGGGTAGAATATTCATTCGAACATTTACTTTAACTAGCGTCGTCTTTCTTCGATTT
CGTGTTTTCTATTTTGGCTCTTTTTCGTTTTTTTTCTTCCCATTCGTGAATTGACAAAGT
GATAGGCACATATGCTCGATCTACTG
>comp2551_c0_seq2
AGCATTGCCAATCTAATAAAAAAAATCACCGAACACTTCAAGACAAAGGAATCCTACCAA
AATGATAGCTTTGAAACCTTTCTTTCTTGTTGCCCTTTTGGCAGGTACCAGCTCAGCCTT
TGCTGTCATCCAGCCTGCATCTTCCATCGCGAAGGCGACGACGACGTCGACCATTCTGAA
ATTGAGCGGAGGCGCTGTCGGAGCAGGAACTGCTCCTGAGCTCAAGCCTCCACCATCTTT
ATACCAAGGAGCTGTGGCCGCCGGAGTTGGCAAAGCCTCGAAGACATGGGGAGATATTTT
CAAACTTGGTATTGTTAGCGGTTGCCACATTGCTTTCGGGGCATACCTCGCAATCACTGT
CGGAGGAGCATGCTCGGGAATCGCAGCTGAAAATCCTGGCCTTCAGAAGGTACGTATCGG
AGTGGTAGAATATGGAACAAATCATTTTTTCCTCTGGTTTGCTTAATAGAGATCAAAGTT
TTCATCGAAGCCTAAATGAATTTGACTACCGTATTCAATCGGTGTTTCAGGATCTTTTGA
AATCGACACTCTTCCGTTTCGATTTACAATCAGGAGTTGTTCCGAGTTCTAACTGTTGTC
AATAATTTATCTTCTCGTTTAACATTTGCATTTACTTTTCATAAAAAGATCATTTTCGGT
TCCTTTGGGTTGCCATTTGGTCTCATCATGACATTAGTGACAGGAGGTGAACTCTTCACA
GGAAACACGGCTCTTGTAACTGCTGCGTATAAAGAAGGTAAAATTAGCGGAAAGGACTTG
ATCAAGAATTGGGTTTCTTCTTATACTGGGAACCTCGTGGGATCTTTGCTTTTAGCTTAC
TTGGCTTACAAGAGCGGAACTTTAGCCGCTAGCCCAGCGTCGGCTGCCATTGCTACAGCG
AAGTGCTCTCTTCCATGGGATGTTGCATTTGTTCGTGGTATTCTGTGCAACTGGCTGGTA

TGCATGGCTGTCTATATGGCATCTGGTTGTGCCTCGATGGCAGGAAAGATGACTGCCGTT
TACTTCCCCATTTCCGCCTTCGTCGCGCTTGGATTGGACCACAGTGTTGCGAATATGTTC
ATCATTCCTCTTGGGATGCTTCGTGGTGCAGACATCACTATCAAAGCATTTTTGATGAAG
AATCTAATTCCAGTTACTTTAGGTAACATCGTTGGTGGGGCACTTTGTGTGATGGCTCCC
TTTGGTTCGGTCTTCGGTAGCTGGGGAAAGAAGTCCGACTGATGCGGTATAGGCAATTAG
TGCCAAGATACTTGTTCAGTGCCAACTCCTATCTCATCAAGCAGCATGCGGTAGATTCAT
GCCCTTCAACTAATATGAAATCCAGTGAATTGTCTATGTCATCATTTTGATCACTTGTTT
AGCAGGGTAGAATATTCATTCGAACATTTACTTTAACTAGCGTCGTCTTTCTTCGATTTC
GTGTTTTCTATTTTGGCTCTTTTTCGTTTTTTTTCTTCCCATTCGTGAATTGACAAAGTG
ATAGGCACATATGCTCGATCTACTG
>comp2552_c0_seq1
CTCAAGGTTCAGCGGAACTTCAATGAAAACCAGTCACTTGCAAGCGATAATCGGGAAGAT
TCGCACGTTCTTTCCAGTCTTTCTTCACATTAGTCTTCTGATTCTCTAGGTCACGTACAA
TTCACTATCAGAAATGAACAGAACCTACAACGGCGAGCGAGGCAACCACCCGGGCATTGA
GCAGAAAAAGGACACCACAGCATCACCCCAGGCAGACCATGCTGCATTGGCCACAGCCCT
AGAAGAGGCACTTCATGTCACCAGGAACGGTAGCTTCGATCCCGAACGCGGTTGCGACTA
TCGCCCGGGTGGTACAGATCTGAGGCAGAGACTGGAAGAGCGGTGGAACGAACTGGTCTT
GGCTACTACGGGCAAGGCCAAAACCGAAGCCACCGATGGTTGGTTTCGAACCCTCTACGA
GAAACACACGGAAGCCGGAAGGTACTACCACACCGCGGTTCATCTCTGGGAAATGTTCGA
GCTGCTCGACATCGTTGCCAATGCTTGCGTGAACCCTTCTGCTCCCGGATGGTATGTTCC
GATGGCCTGGGCGGTGCTCTTTCACGATTCAATCTACGATCCCAGGTCGAAGCGAAACGA
AAAGGACAGCGCTGAATTGTTTCGAGCCTTTGTGCACGCTGACAAGGCAGTCGCCATGGA
CAGTACAACGTGCAACGCCGTGCTGACAATGATCCTGGCAACCGAGAAACACCAAGTGCT
AGCCAATGGCCGGGAGGAGGACGAATTCGTGGCGATGCAAAAGCACTTTTTGGACATTGA
CATGGCGGTACTGGGAAAGCATGGCACCGCCTATATGAAATACGCGGCTCTGGTACGCAA
GGAGTACAATTTCGTCCCTCGAGATGTCTATTGCAGCGCCCGAGCCGACATTCTCGAAAA
TTTTCTAAGTGAGACCAGCAACGAGAGTGGTACCACCACGGGGGCGAAGCACGTGTATCT
GACAAATGCATTTCGGGAGGCCTTCGAGACCCGGGCGGAAGACAATTTGAGGGAAGAAAT
CAATCTTCTTCGAAACAACACAATTCCAGAATAACACTGAACGGTCCTTGGGCTAGCGGA
GAGGAACACGGAGATGAAAACACATCAAGGCAGGTTGTTGTAGCGAAAAATACAGAGATA
CAATGGGTTCTAGCCTGGTCTATTGTTTTGTACATTGGATTTCAGTTTTGGGGATTCGAG
TGACAGCTACATGTACATATCCATTCGTCAATGCAGCTGCTCTGATTGGGCACAAGAGTG
CTGTGGCATTGCGGACTCTCTCTATTCCCCGAAGAAGAAATTCCCCGTCGCTTCGCCTTC
CGGTGACGAAAGTGAATTGCCATATCGACGATTGTGAGTTACAGGAACGAATACGTCCGG
TTGAGCTGGCTGTTGGTGAAAGTCTCCACCGCCAGCATTTTGCTGCTGCTGGTCGTTGTA
TTCGGTTCCCATGAAGTTGGACGACTCCCAAAACTGACCATTGATGTCATTCTGTTTCAG
TTGTTGCTGTTGCTGATCCTGGCTGACATGACCCTGACTCTGACGTTCGTTGAGAGCGTT
GTCGATAGGGCTGGCTTCGTATGGACTGGCAAAACGGCTCGCCTCGCTTTCTGAAAAGGG
ATCACCCGCCGGGCTTTCAAAATCTTTGGCATAAGATTTCGATTGTCCAGAGGATTCGAA
CTGCCGGTCATAGTGCTGCCTTTCATATTGTTGCCCGTGGTGTCGGGATTGCCTTTCCGA
AGTTTTCGGTAGGGCGTCGTCGGGGTGCATGGAGGAAGGGCTTGATTTGAATTTTTCGTG
GCCTCGGGACGATCGTTGCACCGGAGACGAGGAGGTTCTTTGTGGAGCCTTATCGGGAGA
CGGCATTCTGGAAAGGCGATCCAGAAAGTCTCTGAAATCGTCGCGACTCTTCCTTGGCAT
GTTGCGGGTATCAACCGAGCGATTTTCGCTGTGGTGATCTCTTGTTCGGCGGCGTTTCGA
TCGCCTCTCTGAACGTCTTCGGTTACTTGCATCGGTTGGGCTGTCGCCGCTCAGATCGAA
GGTCCTTCTCTCGCCTCGGCCTTCGTTCGAGTGCGACGGAGATTGATCGTACACACGCTG
TTGCTGATGCTGTTGAGAACTGCCAGTCTGTTGCGGATACGAAGGTTGAGGAGGGGGTCG
ATTATACTCTGTGCTGGGATTGCTTCCATTGACGGTAGGAGCCATGCCACCTTCGCCCGT
GTTGCCATAATATGCGGGGTATCCGGCCACACCGGGGTAGTAATTTGGTTGAATGGGAGG
AACCCCTGGATAAGCCCCAACCTGCCCCGGAGGCGCTTGCTGATAGGGGTTAATTCGGGC
ATCTTGGGTGTAGTGCATGGGATGAGCGGCGAACGCCACTGGAGCTCCAAATGCAGGAGG
AGGAGCAGCAGCAGCCTGTAGATGAGGAGGAGGAGGTGCGCCGCCATAGAACGACGGGGG
TGCATGTGGATATGGCAGAGCAAATCCCGTACCCACGGAAGCCGGAGCAACCGGTGCCGG
ATAAGGTGGTGGATATGCTGTTGTAGGTCCTCGATAAAATGGATGACCTGGAGCAGCAGT
GGCTGTGGCGAATGGGCCGGTCTGCTGCGTAGGGACGGCAGCAGTCGCTGGGTCCTGATC
TTTGCTGAGAGATTTGTTAATTTGTTGTTTGTTATTAGCAGCATTCCCTTCGACGTTGTC
TTCCTCATCTTCCGAATATCCGTCCTCGGAAGAGCAGCTTGACGAGGAATCTGATTCTTC
GTCTCCATTTCCAGTTTTGTGAGCGGGAATGGGGGAGATCCCGTGGCGTTGCTTTAACAA
CGCATCCGTCATGTTGTCAATAAGAGATTTTACTTGCAAGAGGGAGGCGCCGACCGAGGA
TCTTTTCTCTTCTTCCGACATGATTGGGAATTCCATCAGCTTAGCAGCCTTGTATTGCAA

TTGGTCCGGACCGTGGCTGGTACTAAAGTTGTTCAGGACGGAATAGGACAGCAGCGCGGC
GGCCTTGCCACCAAGCAACTTATTGGGTTTCTTTCCTTCCAATGCAGTCGTTAGAACGGC
CTTTGGGATTGGCTCCTTATGATTTACATTATTTCCCTTTGTGTCGCTGCACGCGCTGGG
TTCCTTCTCGTCGGAGGACGGAGAGATGCAGTTCTTACGATTGAGATGGGCTTTGGCTTG
CTCGATAGACTTGAAAAACACATCATATTCCTTTAGGAATTCGGCCCTGCCTCTCTCCTT
CGTCTGGCGATACTTTTGGAGGTGACTCTTGGTTCTTTCACGAGTTATGTATCTCGGCTG
CTTCCTCATATTCTCCATGATGACCGAAGGGGAACAATTCTTGAGGCCGATTTCGAATAT
CGCCGCAGCGAAGGCCTTGTGCTGTTCCAATGACCATTGTGGCGGCTTGTTTTTTGTCAC
AGCAAGCATGTTGTTCGATGTATCACAATTCTCGTCACCTCCGGCGTTATTTTCGGCCTT
GATTGCGGCAGCTTTGTCACCGTCGGTATCATCGGCATGGGTGCTGGGAATCTCCTCGTC
GTCATCGAAGTGTTCGAGTTGCAGTACACCATCATCTCCGCATTCGAGGCCAACAGAGTT
CAACATATGCACCGAGACTTTATTCTTGTTTCCCGAGCTATGGCTGTCCAGGCGTGAACT
GTAGTCTTTATTCTTGGTAGATCCATTTTGTTTTGCCAACCCCGTACCGCTGATCAATCG
AGGAGGAACGTAATGGTTGCTTATGGAAGCAGTTACGACTGTAGGCGGATGGCATGACAT
TGCCATCAGCGTTGAGGCTGACGCATTGCTGGTCACCTGAGATCTTGAACAGTCCTTTAT
AAACGCGTCGGTACCTTTGTCGAAGCGAGCTCTTTTTCTTCTCTTCGAGCGAACGTGAGC
TTTTGAAGCTATGTCGGCTATAGCATCGAAAGCATCACTACATGTTGTTTCATTACCGTC
CCTGTGCCCCATTCCCTTTTGTTTTATGGTGGCCTTGTCTTTAGATTCAGTGGCATTCGT
TTCGGCCTTGGCAACCACCGAAGCTGGTGCTTTCGAAACATTTTTCGCACCAGCCGTATT
AAGGCTCTTCATAGTTGTTGATGAGGCCTTTACGGGTACAGTAGGTTTTCCAGGGGTCTC
GTCTGTAGGTTTATTGCACCCAGCATCCCGTTTCTCGTCAAGAGCGACATCGTCATCTTC
TATCTTTATCACGGGCGGTTTTCTGTGTGTGACCGACTTCGATGCACTTCCTTCCATCGG
ATAATGATAGACCTCGGTTTGGAAACCGCTCTCCGCTCCACTGCGTGTTGAAAATGACGA
CGGTATAATCGAAACGGACCGTAACAGTACCATACCTCTTAGAGAGCAGGGATTTAGTTG
TTCCTAGGTGCGTTTATCCTCTTTTGCTATTCCCCCGAGTTCACTGATGACAGAGCGTAT
CGGATAGACCAAATTGCCGTTTTCGATCATTAGAGCACCCGGTAGGTAACGGGTATTAGT
TTGTCCTTCGCTTCTTAGCAAAAAATGCAGGGTTGGAATTTGCAAGAATCTGCGATGCTA
AGAATTTGATAAAATCCTTCTTCTCATTTTTTGGGTGACTTTTTGAACGTGCTCGATGCA
TGGACGCTTTTCCTCCAATGAAAACATACCTTCTCGATCGAGGACAAGCAAATTAATTAT
TATCGTTACGTACGTATACATTATTATGAAT
>comp2553_c0_seq1
GTTCACGACAACGAAAAAGAGTCTTCCCTTCGATTCCCTATTGAGAAGGCTCTTTCATAT
CACACAACACGTATATTCAACCATACATTCAGAGTAAGACTTGCAGATTTTTTCATCTAT
CTTGCTCTACTAATTTCAATCCGCGGAATCGTTGTTCGCTCTACTCATCAGCCAATCTAA
CAATCGACAATCGTCTCTTCGCGGTATAGAACGTGACTCAAGTCGAAACGATATCACATA
CTGATAGTACACATCGTACCCCTTAAAAATGTCCGACGACGATTACTCATACGAATACTC
TGAAGAGGAAGACTATGAGATCGGGGATGATGATGATGACATGGATTGGAATTCCTCCGC
AACTTGTACCATTGCATCTGAGAATCCGAACGCGGCTCCCACACTTGGCCTTGCAGGAGT
CTTGAACGGAATCCGAATCTCACCCGCGGATGATCTCCGTCCAGAGATGAAGCGTCGTCT
CAAGGAAGTGACAGAAATCTTGGATATTCCGGAAGCGGCTGCTTCCGTGCTTCTTCGTGA
ACACAAGTGGTCGAAAGAACAGCTGTTCGAAGTCTTTTATGCAGATTCAGAGAAGTTACT
AAAGAAGTGCGGGGTCTCCAACCGCTGCGACCCCAAAGATCCGATGAAGACAGATCCGGA
CGACGACAACTGCTGCCCAATTTGCTACGACGATATGGAAGATGGTCAGAAAATGTCGAT
GCCTTGCGGACACGAGTTTTGCATGAGTTGTTGGCATGATTTTTGTGCTAACGCTATTGA
GCAAGAGGGACCCTCTTGTGTTTATGTGACATGTCCCGAGGCCACCTGCACAGAAGTCGT
GACCGAGGTTGAATTTCAGAAGGCGGCACCGGATTACCTCCAGAAGTTCCAAACCTATCA
GCTTCGCAATTTTGTTGAGTCCAATAGGCTCACTCGCTGGTGCCCGGGCAAAGGATGCGA
TCGTATCGCTTGCGCTCCCACAGCTTCGGCGATGGAACAAGACGGCAACGTTGCCAATTG
CGATAGCTGTTACACTAGTTTCTGCATGGTTTGCGGCGAGGAACCACACGCACCTAGTGA
GTGCAAAGACTTGGCAAAATGGAATGAAAAATGCCGCAACGAATCGGAGACAGCGAATTG
GATTCTAGCCAACACCAAATCGTGCCCGAAATGTGCCAGCCGGATAGAGAAAAACCAGGG
CTGTAACCATATCACTTGCCAAAGATGCAAACACGAATTCTGCTGGATCTGTTCTGGAGA
CTGGGCGTCTCACGGAGCAAACACCGGAGGATACTACAAATGCAACAGATACGATCCAAA
GAGTACTGAGACGAACGATAATTCAGCTGCTGCAAAGGCGAAGCGAGAACTCGATAGATA
CCTCCATTACTACAAACGGTATCATGCACACTCGGAGGCCCAAAAATTTGCAAAGAAACA
GCTCAAGGAAACCGAATCGCGTATGGTGATGTTGCAGGAATCATCGGATAATTCCAAGTG
GACAGACGTTGAGTTTCTCAAGACGGCCAACGAGCAGCTCGTAGAGTGTCGAAAAGTACT
CAAGTACACTTACGTTTATGCATACTACATGAATCCGAATGACACGCTACAGAAGGAACG
CTTCGAGTATCACCAAGAGATGCTCGAGAAATTTACTGAAAATCTGAGTGAACAATCGGA
GAAGCCTCTGGACAAGATGGACCGAACCGATGTCGTTAATCAAACGCGGGTTGTTGACCG

TTTTATGAAGAATATTCTGAAATATGTAGATGAGGGTATGGAAGGGTGATTTTTGATTGG
CACGTATTAATTTACTCATTTATATTTTGGGAATCATTGGAAACAAACTTAAAAGGACGA
TGGCACCATTCGATACACATGGTCTCGATATATTCGTATTCTTTACTCTTTGATGGCTAT
ACGAGACTTTTTGTGATGTAAAATTAAAATAGACTTAATTTGAAGAAAAAAAA
>comp2553_c0_seq2
GTTCACGACAACGAAAAAGAGTCTTCCCTTCGATTCCCTATTGAGAAGGCTCTTTCATAT
CACACAACACGTATATTCAACCATACATTCAGAAACGTGACTCAAGTCGAAACGATATCA
CATACTGATAGTACACATCGTACCCCTTAAAAATGTCCGACGACGATTACTCATACGAAT
ACTCTGAAGAGGAAGACTATGAGATCGGGGATGATGATGATGACATGGATTGGAATTCCT
CCGCAACTTGTACCATTGCATCTGAGAATCCGAACGCGGCTCCCACACTTGGCCTTGCAG
GAGTCTTGAACGGAATCCGAATCTCACCCGCGGATGATCTCCGTCCAGAGATGAAGCGTC
GTCTCAAGGAAGTGACAGAAATCTTGGATATTCCGGAAGCGGCTGCTTCCGTGCTTCTTC
GTGAACACAAGTGGTCGAAAGAACAGCTGTTCGAAGTCTTTTATGCAGATTCAGAGAAGT
TACTAAAGAAGTGCGGGGTCTCCAACCGCTGCGACCCCAAAGATCCGATGAAGACAGATC
CGGACGACGACAACTGCTGCCCAATTTGCTACGACGATATGGAAGATGGTCAGAAAATGT
CGATGCCTTGCGGACACGAGTTTTGCATGAGTTGTTGGCATGATTTTTGTGCTAACGCTA
TTGAGCAAGAGGGACCCTCTTGTGTTTATGTGACATGTCCCGAGGCCACCTGCACAGAAG
TCGTGACCGAGGTTGAATTTCAGAAGGCGGCACCGGATTACCTCCAGAAGTTCCAAACCT
ATCAGCTTCGCAATTTTGTTGAGTCCAATAGGCTCACTCGCTGGTGCCCGGGCAAAGGAT
GCGATCGTATCGCTTGCGCTCCCACAGCTTCGGCGATGGAACAAGACGGCAACGTTGCCA
ATTGCGATAGCTGTTACACTAGTTTCTGCATGGTTTGCGGCGAGGAACCACACGCACCTA
GTGAGTGCAAAGACTTGGCAAAATGGAATGAAAAATGCCGCAACGAATCGGAGACAGCGA
ATTGGATTCTAGCCAACACCAAATCGTGCCCGAAATGTGCCAGCCGGATAGAGAAAAACC
AGGGCTGTAACCATATCACTTGCCAAAGATGCAAACACGAATTCTGCTGGATCTGTTCTG
GAGACTGGGCGTCTCACGGAGCAAACACCGGAGGATACTACAAATGCAACAGATACGATC
CAAAGAGTACTGAGACGAACGATAATTCAGCTGCTGCAAAGGCGAAGCGAGAACTCGATA
GATACCTCCATTACTACAAACGGTATCATGCACACTCGGAGGCCCAAAAATTTGCAAAGA
AACAGCTCAAGGAAACCGAATCGCGTATGGTGATGTTGCAGGAATCATCGGATAATTCCA
AGTGGACAGACGTTGAGTTTCTCAAGACGGCCAACGAGCAGCTCGTAGAGTGTCGAAAAG
TACTCAAGTACACTTACGTTTATGCATACTACATGAATCCGAATGACACGCTACAGAAGG
AACGCTTCGAGTATCACCAAGAGATGCTCGAGAAATTTACTGAAAATCTGAGTGAACAAT
CGGAGAAGCCTCTGGACAAGATGGACCGAACCGATGTCGTTAATCAAACGCGGGTTGTTG
ACCGTTTTATGAAGAATATTCTGAAATATGTAGATGAGGGTATGGAAGGGTGATTTTTGA
TTGGCACGTATTAATTTACTCATTTATATTTTGGGAATCATTGGAAACAAACTTAAAAGG
ACGATGGCACCATTCGATACACATGGTCTCGATATATTCGTATTCTTTACTCTTTGATGG
CTATACGAGACTTTTTGTGATGTAAAATTAAAATAGACTTAATTTGAAGAAAAAAAA
>comp2554_c0_seq1
CGACTCCATCTTATGCACTCTAACTCGGTGCTCCCATCGTATCAGGCATTATCGCCGTCA
ACCAGAAGGGTGACCCTTCTCAGTAGGAATATATGACCTTACACAATGTCGGAAGGTACC
GATTGCACTGAGAATCCCACAGGATTGGGCGCCGATAATTTTCTCTCGAGGATCACATTT
AGGAAAGCTTCACCAACAGATATTCTGAGATGCGTCGAGATGGTGAAAAAAGAATCTAGG
TCGAAAGGTTTAATGTTTGTTTCCAAGAATGAACTTGAATACCGTCAGCATTATGCTGCA
AACTACTACCGATGTGCGGTTTACGAAGACGAGGATGGCAATAATGACGAGGATACGATA
GTAGGCTTCATCACTGGTATTCGAGTTGGGGCGATAGAAGAGGCCGACAAAGTAGTGGGA
CATGCACTCACCCACGTTGTCCCCCACGATCCAAACGGCGAGGTATTGATTATTCGATCC
GTGATTGTCGGAGAGGAGTATCGGAAACAAGGAGTGGGCAAGGCGATGATACAAGATTAT
ATCGGTGCAGTGCGGGAACTCCCGAAACAATCGTCTTCAAAGCCCAAATCATCGAAGAAA
AAGCCAATTCAAAAAATTGTTGGACTTTGTTGGGATCATGTATTGCTGCCCTTTTATCTT
GCCTGCGGATTTTCACTCCAACAGGCTCATAAGGTTACTGGAGCATGCACAGAAACTGAG
AAGAGCGATGAATTACGACGGGCGTATTTCTTCGAATCATCGCTCGCTGCTACCTTTGAC
TACGGAATTTTTGGAGATGGCAATAATGACGATGGGTGTAACTATTACATCGTTGATAGT
TTCGCCTCCGTCCCAGGAACTGGTAATCCTGCTTCTGTTGTTTTACTGAGAGAAGAATTT
GACCCCATGGTGAAACACAAGTGGATGCAAAAAGTTGCAGCTGAATTCAACCTTGCAGAA
ACTGCCTTTTGTTGGCCGAAGGGTAGTAATACAGGCAACGATCATGCAGATGCTATAGCA
TTTTTTAAGAACGAAAGTCACTGGAATATTCGGTATTTCACTCCAACTACCGAAATGGGT
CTTTGCGGACATGCTACTTTAGCTTCGGCTGCTGTTCTGTACCAAACTTTGCCCTCAGAA
GTCCTCCCTTCGGGCACGGCAGTTGTATTCCATGCAAGCGAAGATATCTTGACGATGAAT
TTAGAAAACAATGGTGAAAAAGAAGGAATTTCTGCAGTTTCTTCGTCACAACGGGTCTCA
AAAGTGTCCATGAATTTCCCCCCAAAACCAACGAAGGATATTTCTACACGAGAAGAAAAG
GCTACGGTGCGAAATATGCTGAAAAGTGCATTTTCGATAGATTTAGAGCCATTGTATGTT

GGTCTCTCAGACATCGGGGACTTATTGGTCGAGTTGTCACCGAAGACATTTAGTGAGATT
GGCTATGACTCCTTGAACTTCAAGGCGTTTTTCGAATGGGATGGATATTATCGAGGTATC
GTTATTTGTTGTGCAGCCCCAGAATCTGCCGTCAAGGATAAGACCGACAAAAGCATAGAA
ATAGACTTTTTGTCTCGATTCTTCGCTCCCAAAGCTGGCATCGATGAAGACCCAGTCACT
GGAAGTGCGCATTGCAGTCTTGGACCGTACTTCGCAAAAAAACTTGGGAAAAAGAGAGTT
GTTGGGCGACAAATGTCTGTGAGAAGTGGTGTTGTGGAATGTCTAGTTGCTCCCGATAGA
GTGACGTTGACTGGCACGGCAATCACAACCATGCGAGGACAACTATTCATGTGAAGATGC
TAAAGATGACGCTAACAAATATGGACCATAAAACAAAGCTAAATTTTACAACCCCAAATC
ATAAACAAAGACAAGTTTAGATGTCGTGAGGAACCAGTAGATATGCTGACCTTATATACA
GA
>comp2555_c0_seq1
AAGAGCTCAACAATAGCATGGATGACCGCGCAGTAAAATAAAATATTACGTATGAACCGT
ATGAAGAAAAATAACGTTAGATGCTGGAAATGTGAATAGGGTTACGGAAGCGAATCAGAT
GAATACCGAAGTCGCAGCAAATGCATTGCACCGGGCATATGGTTTACAATCGAAGTCTGC
ACACCAAGACCCGAAAGACATCACTCAGAATATCACTTCGAAGACAACGACGATTAGGTC
ACTCCGACCATTGTGAAATCATGATTCGGTCGAGGGCAGTTAATGGCAACAATACCGTTG
GGAAGAGCGCAGGAACCTCCGCGAAACGAGGAACTAGACCTGGCATAAGAAAAGCTCCTC
GAAAAGAAAGTGCCAATCCTTTCGCAGCAATACTGTATGCGGTGCTCTTTATCATGATCT
GTGCGACTGTGGGGGGATTTTATGAAACGAGTTCTATGAGTGCTTCAGACAAAGTACCAC
CGCCGAGACAGTTGAAGACCAATCCCAGATTAATTCTTGACAAGATTCGCAAAGAGTTCA
ATGCACGTTACAATGTTAGTGGCAAAATAGATGGCCAAAAAATGCTAGAAAAGGGATTGC
ACTCTTTTGGTTCAATTGACCATACCGCTCGCCGCATCATGGAGGCCGCCAGGGCACAGC
GTCCCTTCGTCATGGCCTTTTCGGGTTACTCTATCACGGTCGGACGAGGAAATTTCTATA
AACAATCCTTTCCGTTCGTAGCAGAAGAAATCCTCAAAGGACCGATGCAAGAACTTTTCA
ACATTCCATTGGTGGTCCGCAATGCAGCCATTGGTGGAATTCCCTCATTTCCCTACGGAT
TTTGTATGGAACATTTCTTGGGGAGCGACCCGGACGTGATCTCGTGGGACTACAGCATGA
ACGAAAAGTCGAAAGACGCCAGTGTTTTCGAGGCTTTCTTGCGGCAGGCCACGAAACAAC
TGCCGAAGCGACCCATGGTTATCATGATGGATACGAATGCCAACCGTATGAAAACCCTTG
ACACATACACCCAAAATGGGTGGCTCAAGGACGCCATTGCGATCGGTAACAAGGACATTT
TGAAACAAGCAAACATTTTCGAAGGTGATGCGCAAGATTTGCCAACTGGATTTCAAGATT
GGAATGAATTTGGTGCGCCCGCGAATTGTCCAGGACGAGGTAGCTGGCATCCAAAAAAGC
AAGAGCATTCTATGATGGGATGGATGATCGCTATGCATTTTATTGAGGCGATGGATAGGG
CCTTGAAATTGTCGTCCGAAGAAGAACCAAGCCAGACCTCGCGCATGCTGTCGGTGGAGA
TGCCAAAACTAGGAGCACCCATTTCGAAATCATTGCCTGCCAATGACCCAGAAGTGACAG
AACTTTTGTTTGGGCACGGAGACAATCATATGATGAAGGACTTGTCTTGTCGAACTTCAT
TTTTGCCCGCAACTGATCACGACAAAACATTAAAATCCGTGGTGGTATCTGGATTGGCTG
AGGGAGATCTAGACATTATGATTGACAGAACTGACGAACACTACAAAGAGGGCTGGGTTC
TTGACGTGAGCAAGGTAGAACGTGATACGAAACGAAAGGTGGAAAAAATGCGGGGGGCTAG
GATATGTAGACATGAAAATAGCTATGTATGGGATCCCGGAAAGTGGGAAATTGCGGCTGT
GGCTCCCTTTTGAGGGAGCATTGCATGATCATGCTCATCACGGACACGGTGACAACCATG
TTCTGGATACAAATGCCAAACATTGGTTCGACGACTTTATTATTTGCGAAGCAAACGATA
AGCGTGATGACAAAGCTTGCAAACTGGATCGAGACTTGGAAATCATCGTCGGCGGCGTTC
CAGTGACCAGTGAAAGTATCCACATTGTCAAGGACGCCGCGGAATACCTCAAGCGACCAA
CGTGTGTCAATGTTGGAGTTCCCGAGACTGCAAAAAATAACAACATTGGGTGATCTTCGCA
CAACGAAAGGTAAAGCTTTATCGGGAGACGAAAAAAAGAAGTTCGGTCGGTCGTATGGGG
ACGATACGTTCGGAATGGTGGTGGACGTTACCGCGAAATCCAGGGTAACGCGTAAGAATG
GCGCATGCTGCCTGTCGCATATTGTATGGGAACAGCACTAGCGTTAGCTAGGTCGACCGA
AACAACAATTATTGATGTGAAACATGGAATGCATTACACTTTTCGTTCCATTTCGACTGG
CATGATTTCCTTCAAAATGTCGAACAGTTTGGAAACAGGTGAAAGACAAATCAAGACTTT
TTTGTTTAATAGTACTATTTGCACATTTGCAAAAGTTTACACAATCTTTAAATTCCAGTT
CTCATCAGGCTCTTAAAGTAGTCGCAGCAAACCAATGGTATTTGCAAC
>comp2555_c0_seq2
AAGAGCTCAACAATAGCATGGATGACCGCGCAGTAAAATAAAATATTACGTATGAACCGT
ATGAAGAAAAATAACGTTAGATGCTGGAAATGTGAATAGGGTTACGGAAGCGAATCAGAT
GAATACCGAAGTCGCAGCAAATGCATTGCACCGGGCATATGGTTTACAATCGAAGTCTGC
ACACCAAGACCCGAAAGACATCACTCAGAATATCACTTCGAAGACAACGACGATTAGGTC
ACTCCGACCATTGTGAAATCATGATTCGGTCGAGGGCAGTTAATGGCAACAATACCGTTG
GGAAGAGCGCAGGAACCTCCGCGAAACGAGGAACTAGACCTGGCATAAGAAAAGCTCCTC
GAAAAGAAAGTGCCAATCCTTTCGCAGCAATACTGTATGCGGTGCTCTTTATCATGATCT
GTGCGACTGTGGGGGGATTTTATGAAACGAGTTCTATGAGTGCTTCAGACAAAGTACCAC

CGCCGAGACAGTTGAAGACCAATCCCAGATTAATTCTTGACAAGATTCGCAAAGAGTTCA
ATGCACGTTACAATGTTAGTGGCAAAATAGATGGCCAAAAAATGCTAGAAAAGGGATTGC
ACTCTTTTGGTTCAATTGACCATACCGCTCGCCGCATCATGGAGGCCGCCAGGGCACAGC
GTCCCTTCGTCATGGCCTTTTCGGGTTACTCTATCACGGTCGGACGAGGAAATTTCTATA
AACAATCCTTTCCGTTCGTAGCAGAAGAAATCCTCAAAGGACCGATGCAAGAACTTTTCA
ACATTCCATTGGTGGTCCGCAATGCAGCCATTGGTGGAATTCCCTCATTTCCCTACGGAT
TTTGTATGGAACATTTCTTGGGGAGCGACCCGGACGTGATCTCGTGGGACTACAGCATGA
ACGAAAAGTCGAAAGACGCCAGTGTTTTCGAGGCTTTCTTGCGGCAGGCCACGAAACAAC
TGCCGAAGCGACCCATGGTTATCATGATGGATACGAATGCCAACCGTATGAAAACCCTTG
ACACATACACCCAAAATGGGTGGCTCAAGGACGCCATTGCGATCGGTAACAAGGACATTT
TGAAACAAGCAAACATTTTCGAAGGTGATGCGCAAGATTTGCCAACTGGATTTCAAGATT
GGAATGAATTTGGTGCGGTGAGTCGATTCTTTCTTCGATTGTGCCTATTTTTTATTGGAA
GTACTGAACACGATGAATGAGATCTTCTGGGAAATTTTGTGTTGCAATGGATGCCTTCAC
AAAGATGTTACATACATATTGTATTGGCAAGAACAAGTACTCGCGAAGAATTATTTTCGC
ACGTTTCTGCTCGTTCTCTTTTCTTTTACTGTATCCTAAAACTTTCCTTTTGTGACGAAA
CTCCACTTATTTACAGCCCGCGAATTGTCCAGGACGAGGTAGCTGGCATCCAAAAAAGCA
AGAGCATTCTATGATGGGATGGATGATCGCTATGCATTTTATTGAGGCGATGGATAGGGC
CTTGAAATTGTCGTCCGAAGAAGAACCAAGCCAGACCTCGCGCATGCTGTCGGTGGAGAT
GCCAAAACTAGGAGCACCCATTTCGAAATCATTGCCTGCCAATGACCCAGAAGTGACAGA
ACTTTTGTTTGGGCACGGAGACAATCATATGATGAAGGACTTGTCTTGTCGAACTTCATT
TTTGCCCGCAACTGATCACGACAAAACATTAAAATCCGTGGTGGTATCTGGATTGGCTGA
GGGAGATCTAGACATTATGATTGACAGAACTGACGAACACTACAAAGAGGGCTGGGTTCT
TGACGTGAGCAAGGTAGAACGTGATACGAAACGAAAGGTGGAAAAAATGCGGGGGGCTAGG
ATATGTAGACATGAAAATAGCTATGTATGGGATCCCGGAAAGTGGGAAATTGCGGCTGTG
GCTCCCTTTTGAGGGAGCATTGCATGATCATGCTCATCACGGACACGGTGACAACCATGT
TCTGGATACAAATGCCAAACATTGGTTCGACGACTTTATTATTTGCGAAGCAAACGATAA
GCGTGATGACAAAGCTTGCAAACTGGATCGAGACTTGGAAATCATCGTCGGCGGCGTTCC
AGTGACCAGTGAAAGTATCCACATTGTCAAGGACGCCGCGGAATACCTCAAGCGACCAAC
GTGTGTCAATGTTGGAGTTCCCGAGACTGCAAAAATAACAACATTGGGTGATCTTCGCAC
AACGAAAGGTAAAGCTTTATCGGGAGACGAAAAAAAGAAGTTCGGTCGGTCGTATGGGGA
CGATACGTTCGGAATGGTGGTGGACGTTACCGCGAAATCCAGGGTAACGCGTAAGAATGG
CGCATGCTGCCTGTCGCATATTGTATGGGAACAGCACTAGCGTTAGCTAGGTCGACCGAA
ACAACAATTATTGATGTGAAACATGGAATGCATTACACTTTTCGTTCCATTTCGACTGGC
ATGATTTCCTTCAAAATGTCGAACAGTTTGGAAACAGGTGAAAGACAAATCAAGACTTTT
TTGTTTAATAGTACTATTTGCACATTTGCAAAAGTTTACACAATCTTTAAATTCCAGTTC
TCATCAGGCTCTTAAAGTAGTCGCAGCAAACCAATGGTATTTGCAAC
>comp2556_c0_seq1
TACAATACTAGACTACAGTACGAGTACTACGTGCATCCTGCTAGTAAAACGACCGTATTC
ATCTCTTCAACCGTGACAGCGTCTCTGCATTGGAGACATCTTGCAGGCCTCCTGGCAGTA
TTGTTGTTGGCACAGCCGCTCCGCATTTTGTGCGATGCATTCTGCGTCGGTGTTGCTCCA
ACCCCGGCTCCCACCACTGCTCTGCACGCGAGGACCGGTTTGGGCCCGTCGCCGTGTGAT
TGGTTTGTTTTGTGAAACAATACAGTACACGGTGTTGGTTATCGAAAGCCCGCCGGCAGC
ATGAGCGACGGGGATCCGAACGGAGAAGGAGAACCTTCCTCCCCTCCCGTCCGGTGCTTC
CTTCGGATCTCGATCGGCGGGGAGGTCCTGGAGGACGACGTCGTGGTGGAGCTGGACACG
AGGCACCGCCCGAAGACGTGCCGGTCGTTCCTGGCGTTGTGCGGCGGCACCGGGGACGGA
AGCGCCCCAAGGGTCAAGACGACCAGGGGTTCCCCTTTGCCGACCTACCGCGGGTGCGAG
TTCCACCGGGTGGTCCCCGGCCTCTGCGTCCAGGCCGGCGACTGGGAGCGCTTCGACGGG
ACGGGGGGCTCCAGCCCGCTCTACGGCAGGAACTGGGCCGACGAGGGAAGCGCCATTGCA
ACCAACCAGCCCGGCTTTCTCCGCCACGACAAGGAGGGAATCCTGAGCATGGCCAACACC
GGAAAGCCGGGCACCAACGGGTCGCAGTTCTTTCTGACCCTCAAGCCGGCCCCCCACCTG
GACGGGGGGCACGCCGCCTTTGGGAGGGTGGTGGCGGGGACCGAGTCCCTGCGGAAAATC
GCGAGCGTCGAGCGCGGGGAGAGAGACAAGCCCGTCTCCCTCCAGCGCGTCGTCATCGAG
GACTGCGGGCGCCTCTTCGAGAGCGGAGGGGGCGAGCTTGGGGGGAAAAGAGACCGCAAG
AAGAAGAAAAAGAAGAAAGAGTCGAAGCAGAGGGACCGGAAGAGGCGCCGGGGGGGCTGC
AGGGGCGACAGCGACGAAAACAGCAGCGACGAGGATCGACGGCGAACGCACTCCCGAAAG
AGCCGAAAGAAAAAGCGACGCCGGCGTGGTTACGAAAGCGACGACTCGAGCGATTGCGAT
GGATCGGTTGTCGGCTCTTCCTCCAGCGATAGCGATCGCGGTGGTCGCCGGAAACGGTCA
GCGGATAAAAAATGCAAAAGAAAAGAGCACAAACAGCGCCATTCAAAACACGGCAAGAGA
AGGTCGTCGTCGAGACACACCTGACAAACAACGCGGTAGCCAATGTGCTAGCACGGAGCG
GTTCGCTCCCCCACCCGCCGGACGAGCGCTCCACGCACCGGCTGGAAAACGAAGGGCACA

CGCACTCTGGCATCCTACAAAGTGGGGCCCGCCATCGAACTATAGTGCATTACAATATGA
GCGAACAACACAATGTCGTAATAGTCTCCCCTAAGTAATTAGCGCGCCGGTCTCGTTGCT
GCCTTTTTGCGTCAAATGCTGTACAGGAGGCCCGGCGCGGACTTGTCCTCGAGCGAGGCA
AGAATGGGCTCGTTGCACAATTTCTCTCCCTTCTCCGAGGGATTCATGTGTTCTTCCAGC
TCTGCCGCTTTTGTTCCTAGCCGCTTGGTTTCGGACTCTCGCCTCCGAGACACCGCGACC
GCTAGCTCTTCGATGCGCTGTTCCCGGAGTTCCCCTTGATCGTCTCCTGCCGC
>comp2557_c0_seq1
CGAAACTATATTGTTGTTCAGGACGGCGTAATTGTTCAGGACGGCGTAAAAGGGAGAGAG
TAGAACGAGCGGCTGCGATGAAAAATACATATCATTGCGAACGAGAAACAAAAATCAAGC
GGAACAAAGAATGCCACAAAATCCGTTTCGGCTTCACAATGACAACGGCGATGAAGCCTA
CGAATGTCGCAGGCGACAGTTCTACCATTTGTTCGGAGTGTTTCTGATATTGGGTTTTCT
CTCTTCGGAAGCTTCGGCGTTTTGTAGCAGTAGCCCTATCGTTGGAATTCCACGTGGGAA
TCACGAAAACTTTCATCAGCGTAGCACTGTGATCAAAAACAGAAAATTTTCGACAGGAAC
AAGCACAACAATTCCCACAAGGACTAACAACCGAGTAAAGCTACGAAGCACAGAATCAGA
AGGAGAGGAGCAACGATCGCTGCTGGAACAAATCAGATACCGTCGAAAGACACGATCTGA
AATTCGGCGAGACCGACGACGAAAGAAAAGGAAACTTTCTGAAACTAATCCGTCGCTCTC
GACTACTGTCCCGCATTCCGGCCGACATTTCGTTCCTTCTCATCCGAGCTATCGTCGCCC
GTTGGTCCAGCGATTGTTTTCGAAACTGCGATTCAGAAAACGATTCATGGAAGGCTGGTA
TTATCGATTGACTCTGAAAGAATATGATATTGATGATTGCAATATCAAAGGAAAAAGCGA
AACCAACGCTGTAAGTTTTGCCATCATAATTTCCATCGAAGATCCAGGCATCAAATCGAG
CAATTTGCGATTGGCTTGTTTACAAGCGATCGGCCCAAACGATCAATATTTGGTACAATC
GACAAAAGACGATACAAAATTCTGGGCTTCCAAAAAGAGCCAATCGCTAGGGTGTACATT
TAACGAACACGACAACAACAATAACACTGCCCATCCAAAATCACGACTCGATCCGGATGT
GTGGAAGGAAACGGTAAAATCTGGCTTTCAGATAACTGACGACTCGTCGTTCCGAACGGC
ATCTACACCCAAACATTTGTACAAACCAAGATGGATGGGACGGATTCATGGTCACGATGG
ATCGCATGGTGGAGTCCTAGAAGGCCAGGGCGTCGGGGGCACGCTCGAGTTCGATTTTGA
GTTGGATCCAGACTCTGTTTGTGGATATGGCGGGTACACTACCACGATCGGTGATGCAAA
TGCAACTTTTGGTCATACAGCAAACACCACAAAGAGTAACCAAGGAATCCCTGATTTTTC
AACGACGAACGGAAGTGCTGGGAAAAGGCGGCGGCAGAGCCGGGATGCAAACCGACAAAA
AAGTACGGGAGGGTGGTTAAGTTCCTTTCCCGTTTTTGAACCTCACTGGCAGGTTACGAT
AGCCGACGCGTTAGCGACTGGGAAAATCAATTGGAATGGAACAGAGTACAGATTCAAAGA
CCAATCGTTCTATGCCGAAAAGAACTGGGGAGATGCCTTGCCTTCGAAATGGTATTGGAC
CCAATGCAATTCTTTCGACAACATTGAGGAAGAATTGTCAGTAACGGCCGGAGGAGGTGT
CAGAAAACTGCCATTTGGGAAAGAAGAAGAACTGGGGATGATCGCAATACACCACAAGGG
TCAATTCTACGAAGCTGTTCCGTGGAAAGGAAATGTGGAATGGAATGTTTCGACCTGGGG
AGAATGGAGTTTATACGGCGACCGCCTCGAGGAAACCATAGATCACGACACGGGTCGCAT
AACGCCAGCATTTGCGGTGGAAATTCAATACCGGTGCGATCCAGATAAGCTCCCTGGATT
GGTATTTCGGGCGCCCACACCAAAGCAAGGGATGGTCCCTTTTTGTCGAGATACCTTCGA
GGCCGACACCGTTTTGACCCTGTGGGAACTGAAGCGAAACGAGAACACCAACGAATTCGA
AAAGATAGAGCCCCCCATCATCGATCGCGCTAGGTCAAAAGAAGGTGGTGCCGAGATCGG
AGGGGGGCCATGGTGGGAAACTTGGAGTGGAAAATCGCAACTGAAACCTCTGATAAAGAC
GTTATTGAATATTCCAAATCGCTTTCTCCGCTACTGACAGGTACCTCGTGTCGCGGGAAA
AAACGCTGCGACAACCCGTGAGCGAGGGACTTGTGAAGGTCACAAAGTTCGTTTAACTTA
AGTGTGTTCAGTTTTAACAGACAACGGCCATCACATGCCGGTGTAGTCATTTTCTTCATC
CAAATATTTATTCGAAGGCCCTACTCTAGACGTTTCAATTCAGATACTCTTTTTTGTT
>comp2558_c0_seq1
CACACACATCTCATAACATCTTACCATAAGATTCGACCAACTACACACAACAATGCAACG
TCAACAATCATTCGTGGGCCTCGAACGCTCTCTCATGGTCAAGGCCAAGACCTGCGACCG
TCTTGTCACAGAGGTCATGCAGGCGAAGAAAAAGATGAAGGAAGTCGATAGTGCCAAGCA
GACTTTCTTGAAAATCCAAATTCCAAAGATGGAGAAGCAAATTGCGGAACTCAAGGTCGA
GCTCGAAGGTCTCTTGGACGAGGACGAAATTGATCCCTGTGGAGAGTACTACGAATTGGC
CAAGAAATTACTCGCCTAGGGTTTCCATTTGTCGAGGTGCGGTGTAAGGGAAAGAAGAAA
GGAGGAAAAGATATAATTGGGGAACGACCAGTAATTTTGGTCAGATGTATTTTGAGAACG
CTGTTGTCAAGGGATAACTCAGGAACCGATCAAATTTGCGGTTTTCTTCTTTAAATCAAG
CTTGCTTTCTGTCGTTCAATTACTTGCCAAACCGAATGTCACAGATGGTTAACCGAAGGA
AATGGTCGTCGATCTTTTAATTTATTACTTTGTATGTACTCGTACTGTTCTCGAGTGGAT
ATACTTCTAAATTAGTTCATTTATATTATTGTAAAGAAAAAAAA
>comp2558_c0_seq2
GTCAAGGCCAAGACCTGCGACCGGTAAGCAGTCTTGTGTTCATTTTGTACTCTAATCTGG
TCGTTCATGAAATCGAGTCGGGGGAGCCGTATTGTGACTTTCTCGTTGCGATGGATCCTG

GAAGTGATTGCTCACCAAGTGTGCAATCGATTCCACCGAAATCCATTCTCATTCTTTTGG
ACCATGCGTCATTTTTACTGTGATTATTGACGAATAATAATACCTCCTATTTTTCGGGAT
GCGACGGTTTTTAATCGCTCGACCGTACGCCGTACACACGCAATCGACGAACCATCGACG
CGATCCAAACTTTTCTCCATCAGTCTTGTCACAGAGGTCATGCAGGCGAAGAAAAAGATG
AAGGAAGTCGATAGTGCCAAGCAGACTTTCTTGAAAATCCAAATTCCAAAGATGGAGAAG
CAAATTGCGGAACTCAAGGTCGAGCTCGAAGGTCTCTTGGACGAGGACGAAATTGATCCC
TGTGGAGAGTACTACGAATTGGCCAAGAAATTACTCGCCTAGGGTTTCCATTTGTCGAGG
TGCGGTGTAAGGGAAAGAAGAAAGGAGGAAAAGATATAATTGGGGAACGACCAGTAATTT
TGGTCAGATGTATTTTGAGAACGCTGTTGTCAAGGGATAACTCAGGAACCGATCAAATTT
GCGGTTTTCTTCTTTAAATCAAGCTTGCTTTCTGTCGTTCAATTACTTGCCAAACCGAAT
GTCACAGATGGTTAACCGAAGGAAATGGTCGTCGATCTTTTAATTTATTACTTTGTATGT
ACTCGTACTGTTCTCGAGTGGATATACTTCTAAATTAGTTCATTTATATTATTGTAAAGA
AAAAAAA
>comp2559_c0_seq1
AGCTATGCCTCCTCTTTTATCTGATTGCCGCCGCTCCCATCCCTTTCGGTTGGTTTATTC
TTGATTATTCTGGATGATGGAATTCAGTTTGCTGTCGAATCGATCAAAGGCAAAGGTCAG
CTTCATGTTGCGGGGGCTACGCTTGAATTCCATTGGGTGGTTCAGCTTGTCCATCTTCTC
CACCTTCCAGTGACCAGCATAATAAAGCTTCTTTTCGCTGTTTTCTGGTTGTTTAGGGAC
ATGGAAGATGGGAATGCTCTCTTTCCACTCCCCGACCTTCTCGGCTGTCGGCGTCGTCTT
CGGCCCTCGTCTCACGAGGCAGATCTGTTTTTCGCAATTCCGCTCCACCAGGTTATGCCA
TCTTTCGCTTAGTACAAGAGTGTAGATCTTACCATCCTTCTTGCAGACATCAGGCCGCCC
AAACTTGGACGGCCCATGTACTTGACCATTCCTTTGATTTTGTTTAGCTGCTTCCGGGT
CCGGCCGATGGAACAGTTCGACCGCTGCTGGGGGGGTAGGTAGACGATTCTGGCGGCAGG
GGTCTTGGACTTCTTTTTCGATCGGGATCCACCATCGCTCGAGGGGAAAGGCAGAAAACC
ACGAGTCGTTGCGTCAACGTTCTTCTTTACAGAGGATCTTCTTTCGTCGCCTTGGCTAAC
GATGCCTCCGTTGCTAGTTGTGGACAATGCCCTGGATCGTTTACTTCCAGACGAACCTAC
TTTCTTGGTAGATTCCCCGCTATCCTCGTCATCAGTATAAGTTTCTTCCGTGAGGTCCAC
CATCACTGATTTTGCGGCCTCGTGATTTTCTTCTGCTGCATTTGATTCGGCCCGAAGTTT
TTCTAGCTCTTGAGTCAAGTCTTTTAGGTGTTGTCCGGAAGCAGCTATTTCCGCTACCGA
AGCAGCTATTTTCGCTTTCGAAGCAGCTATTTCCGCTTCCGAAGCAGCTATTTCCGATTC
CGAAGCAGCTATGCTCGCTTTCAAAGTAGCCATTTCCGATTCTTTCTCGTTGCCCTTTTC
TTGCCGGAAATCGCTTACTTCTCTGAGAAAAGGTTTTTCCCGGGGGTGACAGTGGTAATT
AAGACTTCTCATTCCGTCTGAAATTGTTTTTCCGAATCTACTTGTTAGCTATCACTCTGG
ACCAAACCAAAGAAATGTAGACTTATCAAATCAATGTGAAAAGAAAATTTGCAGCAAAAT
ATCTGAAAATTCTATAGCTTGATAGCCAGAATATCTTGTAGCAGTTTCAACTTACACTCA
ACGGATCCACGTTCATACCGCACCGTTTGTTGCCTCTAAACGTGTGAAACAAACATTCTA
AACATGGATCGAAATCACATGACAAATGTGAATGCACACGTGAATAAAAAATGCGATTAG
ACAAGTTCTACTCACATTTCTTCACGGAATACCAATTGTTTTTAACTGATGGATTTTTGG
CTTTAGTGGTAACTCGCTCACAAGACAAAAAATGGATTCCAACAGAAATATGCCGTGCAT
TGGACCATGATGTAGCGCGTGCCACAGATAAAAGCACTATTGACGCACTAGAAGATGCAG
TGATTTTCGAGAAGAACAAAATTAAACTACAAGCCCGGAAC
>comp2560_c0_seq1
CTGCTCGATTGTTGAATGTCGGTAGAAGATGGAAATATAGCATTCCGATCGACTGTTTAA
ACCTGACCACGGTAGATCCCTAGCCCAGAGTCGCAATGGATACCTCAGAGCAACGCGCCC
AATCTACGTCTTTTAAGTTTCCGTTTACGGACAACGAAGATGCAGACCTGAATCTATCTA
AGAGTTTCCATGAATTTCGAGCACCAAAAACACCGGTGGGAGCTCCAAGCTACAATGAGT
TTGCAGATGTCAATTTTATAGCCTCTCCATCAGATATGAACCTCGGCCTTAGCCCAATCG
CCGTGCTCCGAGACAGCGATGCAAGTGTAGATAGTTGCGTAAAATCATCGGGGGAAGAAT
GTGAACCACAGATACCAATTTCAGTGGAGAATACGAATGATGCCACAAAGAGTGACCTAA
GTACAACAGGATTGGAAATAGATAAATTGATACCGCCTGATGATCATTCCTCGCCATCCA
AAACTGATGACTTACATAGTGCGATGAAAAAAGTTGGTGAACGCTCAAGTAACTTTATTG
ATAAGATCAGAAACGCTGCTCACAAAAGAAAGGTTGCGGTCACCCGATCACGGGATTCGC
TAGTAGCTAAAGAGCAAGAACAGCTCCGATCAATTGCAGAATCGAAAACTCGATTGCTTT
CATTGAACGAGGAGCTGCAAATAGTGGAAAAGGAAAACGAAGAAACCACAAAAGAAAGCG
TCAGCAATACGGGAAAGTCGAAACCGAAACATTCGTACAAAAATAACAAAGGTGGTTTTG
GTGGGGTTGGCGTTCCAAAAGTGGAGAAGAGACCAACTACAACGCCACTATCACCAATGT
TGGGTTCTCGCAGAAAAGGGGGGGGTAGAGCACCCAGATTGGTGGAAAATTCCAGAGGGC
CATCGGGGAGGGGATTGAAGAATGGCCCAACTGCGAAAGCTATAGACAGGAGCAACTTTA
GCACCAAAGGTCTCAAGAAAGTTTCCATTAGAGATCATAAAGATGCTGCTTCAGGGACAA
CAAATGGAAGATTTGCAAAACGAGGGACGGATTCGTCGAAATTGGAAGGCCCAAACGAAG

AAATTTCGATACGAAATTTCACTGTCGTTCAAGCGAATGTGAGGCGATGGAACGCAGGGC
AGATAGGCATTCCAAAGGTATCAAAACGCGCCGTGACAGTTCCTGTATCGCCTTGTCTTG
GACCAAAACGTCAATCTCGGATAGCAGGAAGTCACGATGAAGAAAACAGCTCCAACAGCG
GTAGGATCATATTGAAAGTTGAGGTCGATGCATCGAAAAGGGCAAATGGATCAATATCAT
CACAAAACGAAGTAATGACACCCTCATCAACGAGAATGTCTCCACTTTTAGGACTCAACC
TTATTGATTCTGTTCAAAAAGTCAGGTATGCATGCAGTGAGTCAGATCGTGAATTAGCTC
TGAAGAGTTCTTTCAAACCGTTCGTGCCTCGTAGCACTACGAGAGCAAAAATGAGAAGTC
GTTACGACATCCTTCGCAAAGAAAAGCAAGAGATAAGACGCAGCGAAGAACGCGAACGAC
TGCAAGCTCAGATCAAAGTTATTCATCGAGAATTGAGGGTTTTGGGCAAAGATTTAACAT
AAGACACACTATATCATGTTCTAAAAATCTGATCCCGTTATA
>comp2561_c0_seq1
GCGTTGGTTGGGAATGGTAAGGCGGCACCATATTTTTACTTCTTGGATTTTGGTTCAGAC
AGATTTTTTTCCTAATACGTATCGCTGCCGATATTCTCTTCCTCTTAGTGTGTTCCATTG
TATTCTGGAGAGCAAGTTTGGTATGCCGAGGCCCTTACAACTTTTATTGCCGACCTAAAT
CGTTGCGAGCCTACTCTGTTTTTGTCGGTCCCTCGTCTCTGGACTAAATTCCAAGCCGGA
GTATTTAGTAAATTGCCGGAGAAAAAGGTCAATTTGTTGCTTAGTATCCCCTTGATCAAC
ATTCTCATCAAGAAGAAATTGCTGAAGACATTGGGGCTTTCAAAGGTTCGATTTGCAGGG
TCAGGAAGTGCACCTCTACCGCAGGATCTATTGAATTGGTACAGAAGTTTGGGACTTGAA
CTTTTGGAAGGCTATGGAATGACTGAAAATTTTAACTACTCGCACTTCAGCCAACCTGGT
AAATCCCGTGTTGGATACGTGGGAAATACATATGATGATGTTGTGTGCCGAATTGCTGAT
GATGGTGAGATCCAGGTCAAGACACCCGGTAAAATGATGGGATACTTCAAGAATCCCGAG
GCGACTGCCGAGACGATCACAGAAGATGGATATGTCCGCACTGGAGACAAGGGAGAATTG
GATGAGCAGGGGCGTCTCAAGATTACCGGACGTACCAAGGAAATCTTCAAGACCTCCAAG
GGCAAGTACGTGGCACCCGCACCTATTGAAAACCTACTGATTTCCAATCAATACGTCGAG
TTAGCGTGCGTCGGAGGCAAGTCGCAACCTCAGCCATTCGCTATTATTCAACTCTCCGAG
AGTCCCAAGGCGAAAGCCATCGGATCGGAAGGTGAACGTGAAGTGTTGGGTAAGGCGTTG
GAAGAACATTTCATTAATTCCGTCAATTCGAAGATCGATGGACACGAGAAGCTTCAGTTC
GTGGTGATCGTCAAGGATGACTGGTTGCCCGAAAACGGGTTTTTGACACCTACCCAAAAG
ATCAAGCGAGCCACGATCGAAGACACTTATGATCCCAAGGTTGAGGGATGGTATGCAGCC
AAGAAGAAGGTGATCTGGGACGGATTCTAAGCTTGGAATTTTGTTCCTTTATAAATTTTA
GATTTGGGCGTTTCATTCATTATGGAATCAGATCTTTGGGTTTCAAACGTTACAACGTCT
GTCCCATCAATTCTGTCCTTTTCGGTGTGTTTTCATGTACTACATTTTCCATGTGCATGC
TTCCGGATTCCATCGGTATCGGAAATATGTCAATCGTCTCTTTCCTTCCATTGAAGATAG
CAACTTCAGTTCTATATAGCTTCATGTCATTTTATCATGTTCATTGATTCAGTCTATATT
TATTTACATAGTACTCGTTGGAAAGCAGCAGAAAACTAGATCAATTCTTCGTGCTGAACA
AGGTTATTGTATTAGAATTCTGAGCCAAGAACTCAAAAAAAA
>comp2561_c0_seq2
GCAAGCATTGTCAAGATTAACCAATTGTCACAAATCCTTTCTAGCATCAATTTTTTTTAA
TAATCATTGAAGTACCCACCATGTCGGACGATACAAAGACAATCCTTGACTTCTTTTTCG
ATAATGTGAAAAAGCTTGGTGCCAAGACTTTCTTGACCCAGCCTATGGGCGGAGACGTCG
TTAAGACTTTCAGCTATCAGGAAGTTCTTGATGAGGCCAAGAAAGTGGCTGGTTATATTG
AATCCAAAGGGTATCCACCAAAGTCGCAGATCGCGATCTGCTCCAAGAACTGTGCTCATT
GGCTCATTGCCGACATCGGCATCATGCTTGCTGGTCATGTCTCTGTCCCAGTTTATCCAA
CACTTACCGCCGAAACTACGGGCTACATTTTGGAACATTCTGAATCCAAGATGGTTTTCA
TCGGAAAGCTCGACGAGAAGCCTTGGCAAGAAATGAAGGGAGGAATTCCCAGTTCAATCG
ATGCCGTGTCTTTTCCTCTTTGTCCAAAGGACACCTTGCATGCCAAGAAGTGGGACGATG
CGATTGCATCGAGTTCTCCCATTGCGAGTCCAGTCTCTAGAACTAAGGATGAGATGGCCA
CAATCATTTACACGTCTGGAAGTACCGGAAAGCCTAAGGGTGTGATGACGTCGTTCGGAG
CCATGACCGACACGACGAAGGGTATTTGCAAAGAACTGAGCGTCACCAGCGACGATCGTT
ATCTCTCTTATTTGCCAATCGCACACGGTATGGAGCGTTGGTTGGGAATGTGTGTTCCAT
TGTATTCTGGAGAGCAAGTTTGGTATGCCGAGGCCCTTACAACTTTTATTGCCGACCTAA
ATCGTTGCGAGCCTACTCTGTTTTTGTCGGTCCCTCGTCTCTGGACTAAATTCCAAGCCG
GAGTATTTAGTAAATTGCCGGAGAAAAAGGTCAATTTGTTGCTTAGTATCCCCTTGATCA
ACATTCTCATCAAGAAGAAATTGCTGAAGACATTGGGGCTTTCAAAGGTTCGATTTGCAG
GGTCAGGAAGTGCACCTCTACCGCAGGATCTATTGAATTGGTACAGAAGTTTGGGACTTG
AACTTTTGGAAGGCTATGGAATGACTGAAAATTTTAACTACTCGCACTTCAGCCAACCTG
GTAAATCCCGTGTTGGATACGTGGGAAATACATATGATGATGTTGTGTGCCGAATTGCTG
ATGATGGTGAGATCCAGGTCAAGACACCCGGTAAAATGATGGGATACTTCAAGAATCCCG
AGGCGACTGCCGAGACGATCACAGAAGATGGATATGTCCGCACTGGAGACAAGGGAGAAT
TGGATGAGCAGGGGCGTCTCAAGATTACCGGACGTACCAAGGAAATCTTCAAGACCTCCA

AGGGCAAGTACGTGGCACCCGCACCTATTGAAAACCTACTGATTTCCAATCAATACGTCG
AGTTAGCGTGCGTCGGAGGCAAGTCGCAACCTCAGCCATTCGCTATTATTCAACTCTCCG
AGAGTCCCAAGGCGAAAGCCATCGGATCGGAAGGTGAACGTGAAGTGTTGGGTAAGGCGT
TGGAAGAACATTTCATTAATTCCGTCAATTCGAAGATCGATGGACACGAGAAGCTTCAGT
TCGTGGTGATCGTCAAGGATGACTGGTTGCCCGAAAACGGGTTTTTGACACCTACCCAAA
AGATCAAGCGAGCCACGATCGAAGACACTTATGATCCCAAGGTTGAGGGATGGTATGCAG
CCAAGAAGAAGGTGATCTGGGACGGATTCTAAGCTTGGAATTTTGTTCCTTTATAAATTT
TAGATTTGGGCGTTTCATTCATTATGGAATCAGATCTTTGGGTTTCAAACGTTACAACGT
CTGTCCCATCAATTCTGTCCTTTTCGGTGTGTTTTCATGTACTACATTTTCCATGTGCAT
GCTTCCGGATTCCATCGGTATCGGAAATATGTCAATCGTCTCTTTCCTTCCATTGAAGAT
AGCAACTTCAGTTCTATATAGCTTCATGTCATTTTATCATGTTCATTGATTCAGTCTATA
TTTATTTACATAGTACTCGTTGGAAAGCAGCAGAAAACTAGATCAATTCTTCGTGCTGAA
CAAGGTTATTGTATTAGAATTCTGAGCCAAGAACTCAAAAAAAA
>comp2562_c0_seq1
GCTTGGATAGAACTTTTCTCGACAACATCGCGATATCGTTAATTAATCGAAGGGACTCTT
TGTCGATGCGATCTTACCGTGCATTTGTTACAGTAGCCAAAAGCTTTAGGAGTTCGAGGC
GTCTGTAATCGGACTATTCTTTCTTCGCCGAGTCCTCTGGAGACTAAAGTACGGTACGTA
CTCGTACTCGTATTTCGTTGGATTCCAGTAAGGTTTACGTAGTTCAGTACGTACAAGTAA
AAAATAACCAGAGAAACATGAAACATCGAAACATGAGCACCAAGCTCGAAACCGTCCACG
TTACTGGCAGAACCAAATGCATTCTTTAAGTTCAACAGCCAGCGGCAACCAACCAGCGAC
ATCAATCACATCATCAAAAAGCAAAAACGCATCAGAAAAGCCACTGCTCCCCTTCACGAA
CGATTTTCGAAGGTCTCTGACAAGGGAAAGAGCTACTACTAAGCTAATACAACTAATTCC
ACCTAAACAGGAGAACACGGAATCATGTCGAAACGATCCGGTGGCATGAACCTTGGTGGC
CTAATGGCCAAGAAATCGAAACTAGATAAAAAGACGGCCGCGAGTGCCGAGGCAAGCCGA
ATCGGTATAGGCGGAAAAGGCCCAAGCAATCCGTACCTAACAAAACCAGTCCCTCCCGAA
CAAATGATCCAAATCTTGCTTACGCAGTTTCGCAACGCTGCTCGAAAGCGCCACGGCAGC
AACGGCAGCAAGCCATATGCGAGCCCATTTTGCGAAATCGAAGCACGCATCGGTATTCTC
AAGGTCCATAACCGTAGGGTTACTTCGAGTGGCCCCAAACGGGTCAACGGGACCGTCGTT
CGGGCTTTTGACGGAGGGTCTACGCCGTCCGGAGTAGAGCCTCCATCCATGGTCAGCGGA
ATTTCTCGAAGCCACTTTGTCGGATGGACACTGGCAGGCTTGTCGGAAGCGTCGCCCTTG
TCCTCTGCACTTGGGGAAATGTCCGAGAACAGCAAAGAAATCAAGAAAAAGCTAGACGAG
CACGAATATGTCGAGACCGTATTTGCCGGCTATGAGGGGGATCGACGGGTTTCCTTTCCG
GGGTATGTCGATGTGGAACACCCACCCTCGGCTCCAGTTCCGACCAGATCGGGCAGCATG
GAATACAAAGAAAAAATTGACCAGCGCGATCTAATTATACCGGCTGCTAAGTATGATTTG
CGAATCGGATTGGCCACCGAAAAGATCGTTGATCCGAATGTTACGACTATCCCTCCGGGG
TGGTCGTTGCATCGGGTGAAACGACGACGATCCTACAAACGAAAAGACAAGAACATGGGA
TGGCAGATTGATGTCACCGAGGTCACATCGACGCCGAGGCACGATCTATCGAAGAAAACG
GTCGTGTATGAAATCGAAATGGAATTGCAGGCAGATGTCTTGTTGAACCTCATCAACATG
GAAGATAAGGACCAGATACGAACGGAGACACACAAATTATCACAACAGCTATGGTGGATC
CTCTCACATCTCAATCCATTGGAAGAAACTGTGGATGTTGAAGAATACCTGATCGACCAT
CCCGACAAGAAGGCTGTTCAATTGGCACTGGCAACCTGTGGGGCCCTCAAAAAGTTTATG
GACGGTCGTCCATCTCACGCCCACGGGCAACCCATTGAAGTCGGGAGTCTTCCCCTGGCA
AATCCAGGGACAATTCCACCTCCTTCGTTGGCAAATACCAAGTTTTGTGGCTGTATGCCC
ATTGGTTTTTCCCGACACAACATCGAGGACGTTCAGCGGGGGAGCAAATACTTTTTGAGC
GAAAAAACTGATGGTGTTCGTCATTTCATGATATTTACGGGCGAGACCGTAGTACTGGTC
GATCGCGCCATGAGGGGGAAACAATGCATACCAATCGCGAAACACCAACAAGAAAAGAGC
GCAGAACCGATGGCCTCTGTGATTCCGTTGATCAAAGCCGGCACCGTATTTGATGGCGAA
GTTGTGATGCACCGGGGAAATGGCAAAAAACACGGCCCGAGGCCGTTGTTCATTGTCTTC
GATGTCTTGACTATCGGACCGAGCGCGGTCGTTATGCATCTACCTTTTGAAAAGAGACTC
GAGCACCTAAAGAGAGCCACTTTCCGAACACCAACAGCGAACCGCGACATGTTTGCCGAT
TCGGCGATTGCCGATTTGAGCATTCCCTTGCCGCTGGTGCGGAAAAACTTTGTCCAGCGG
ACGGACATTGCGGAGCTCTTGAACAATGTGATAGAAGAAAAGGGATTTCGATCATATCGA
CGACTGCCTATCCACAATCACTTGACCGATGGGATCATCTTTCAGCCTAATCTTCCGTAC
AAGTGCGGGACAGATGTGAATTTGTTGAAGTGGAAATACCTAGATACTTGCACCATCGAT
GTCCAACTCTTGCTCGAGGGTGGGTACAGGCCCCGAGGTGGAGTCGAGGAGGACGACGAT
CCTCTGAACAGGAGTGGGAGGGCCCGACAACACTTCTGTAGACATGTCCCGGTACGTGAA
ATTGCCGGCCAGCGAACGATACCGTCTCGAAGCCGACAAAATGGAAGTGAAGTCGAAGAT
CCACATTGCCGAGGTTGGGTTCGATCCGCTGTCGGGAGAGTGGTATTATCTAACCATGCG
TCCCGACAAGATCGCTCCGAACCACATCAGCACTGTGCTTGGTACCCTGCTGGAATTGTC
GGAGAGCCTCACAACCGAGGAGTTGCAATATCGAATGAGCGTTCCTCCAGGACAGCGCGA

CACGTATCGTAAGGATCTAAAGGGAATGATGAAACAGCTTCTAAGCTACCAGAAGCAAAG
CATTTTCAAACAGCAACAAGCAGGCAGGCGATAGGCATTGGGATGCAGGAGAAAAACGCG
ACGAAATTCAGAAAACGTCTGCTCATTTTCTTGGCTTCTTCTATGACTCGAGTCTTTACA
AAAAACTGTTAATTCTACGAAGCGTCAATAGAAAACTTTGCACTTTGTTTCGAGGAACAT
CGAGGAAGAGTTTATGTCGCTCTTTGAGGTGTAGGTGCTTGGCACGATTTTCATACTAAC
TTACTATATCAACTTCATAGAGAACACACACAGCGCCC
>comp2563_c0_seq1
CAGCATCAGGAACAGCATCAGGAAACACAGGAGCAACAAAATCTAGAAGCTTCCATCGAG
ACGCCATTGAACATGGAACCCTATGTTTCATTCAACAGTGACAGCAATTGTAACGACACA
AGTACAAGCACCACTGCTGCGAATCACACAGAAAAGTTGTGCCGACGATTCGAACGATTG
GAGCCATATGTTGAGCAGCCGACACCTCTGAATAGCAATCCTCGATACCGTATTCGAGAA
ACGAGACAGGAGCACGAGTACGAGTATGCGCACGAGTGCCCACCGAAGCTCCTGTCGAAA
CAAGAAAAACCCATGCCAGTTCATGGTCTTGCTGACACCAACAGTGACCCCAGCGACCCA
CCAAACCAACGAAAAATTAGTTTTTCTGAGCGACTCCCAGAGGTTTCCTCGGCCTGTCCA
AGGGCCTTTCGGAACATGATTCCCCCCACGTCGTGCCTCGTCGAGGAACTACAAAAGGAT
CCGTCCTATGCACACGCCCTGGCGGCCGGCACCATCTGGCAGTCCCTGGTGGGACAGCAC
GTTCGCTTTCCCAAACTGTGGTACGACGGCGCCGAGCCGGCCCGGCCCTTTCTAGGATGC
TACGATCCCAATCGACGCAACAAGTGGTCCTACGTGGGACGGCACCGGATCGAGGGCGAT
CCCCTGCTCAATCGAAGGTACGTCCGTTCCAAGAAATCAAGCGGGAAGATCCTCTTGCAC
ATAGTGTGCCGGGACGTCCTGAATCTCACCGGCGGTGTTGATTTGGAGGACGAAGCAGCG
ACGGAAGATCTGGTTGTCGGGGTCTTTCATCCGAACGCCGAGGGGATCCATTCGAGACTC
GGCAACCCGTCGAATCACGGAAGCATCGGCATTGACTGCTGCCGAGACGTGTGGATTGCC
CACAAATCTAGGAGGATTGCCCACAACAAAAAACGGACGGCCACTCGCATCGAATCCTTG
CTGCGGAATCTCAACAAGAATCGGGTCGATGAGACCCCGCTGGGGGGAGATTTCATGCAC
AGCGAACCAGCACAAACAAAGCGAATTCGAAGGACAATCGACAATACAAACGTGAACGTC
ATCTTTGGGCGTGGGCCGCCCAAACACACCGTCTTTGTGCCGGAACACAAACTCTATCGG
ATGTTGGCACCGTTGGCCGAAACCGAAAACAAAAGAAACAACAAAAACATACCGGCATCG
GTCCTTCTTATGAGAACCTTTCTTCGGTAAAGAACGAAAGAGCAAAGACAGATACGGAAA
TCGAAGCAGCGAACGCAGAACACCCAGAATCGAAAAAAAGGAAGGGCGTTATTCATCTAT
TCCTACACGGAAAGTACATCGGATCCCGACTAGAGCAGCAACACTGGAAATCATCGTTTC
GTTCTTTGACGCGAGGCCGGGCGAAAGGAAACGGTGTGGTAATTCTTACAATGCCATCGG
ATGCGGTCGGACGTCGGACACTACCGGATTGCCAATCTAAAACCTGGACGAACGACGTTC
CTGTTGTCTAAGAAAAAGGCACGGATCGAACCGACGGATGGATGCGATTGCCCTGCTGTA
CCATGTCATGAACCGCGCCGCGGTCGAAGACAATGATACCCCACTCGGTTCGTGACCCGT
CGGAGCATGCCGAGCGAGAGCACGGAGGCAGTGGCGGTACTGCTTGGACGACCACACAGC
ACTGCATGGTTTGTACCAACTTGTTGGCCGGCGCAATTGTAGTACTATATGTGTTAGATT
TCAATATTATCCTTAGAGTGAAAAAAGTCGGCATGCTGGAAACCTTTCGTTTGTGTTCGT
GGATTCGTGTGTTTGATGCACGCGCAAATACTCCATGC
>comp2564_c0_seq1
TTTCGACATCCATTTCGCCATCCACTTCGCCGCCATCCATTTTGACATCCATTTCGCCAC
CCAGTTCGCCATCCATTTTGCCATCTCTGATCAAAGAAGCGACATCCCCTCCAGTCATAA
AATCGAGATCCACTTCGCCGTCCACTTCGCCGTCAAAAAAGTCCAAGGCGACGAAAAAGT
CCAAGGCCGGGAAGGCCCTCCGCGCCTCCCGCCCCATTACCCTCAACTAAGAACCAAGTC
GCTATGAAAGAAACAAAGGCTCTGAAAACGTCTTGGAGTTCAGATATAAGGATACCAATA
TCAATACATCTAATCTATCCGACTCCATTCATCATGGAGGGAGTTGCAACCAGTGTAGAA
GGTAGAAACAGCCATCAATTGGTAATGCAACACGTCGGAAATCTTGTCGTTTATGACATC
AACAGACATGCTCTTTGGGGTTGTTGTTAACCTGGGAATAATGGATGGATCCTTACTGCG
TGTCAATATGTGGTTTGCAATAGAGGAGCACACAACTAGCAGGAGATAACATCGGCTTTA
GTGGCCATGCTCGTTGAACTTAGTGTAGAGGAGAATTGCCTTCATTCTAGTATGCTTCCC
AGGCTAGACACTAAGGCAAGTTTGTGGATCAGTGTAAATGTAAAT
>comp2565_c0_seq1
CTGGCATCGAACTGCAAGGGCCGGGTGAGCCATCCCAAAACACAACACAAGAGCAAAGCT
TAACACCACGAGCAACGAGGCTCGCAGAAAACTAGACCGGACTTTAACCACGAGTCCACG
CTGTCATGCCGAGGTTGCGACCGCTTAAATCGAAGCGAGTGCCCTGGAGCAAGCGAAGAC
AATTTCAGAGAAGAGGCGCTATCCTCCGATGTTTCTTCTATGGGATGCTGTCCCTTTCGC
TGGTGGCCCAGGCACGAGTTTTGTGGAAATGGCTGCCAAAAGTGGTCGGCACCGCGAAAG
ACGACGCGGTGGGCGAGTTTCCGACCACAATGTCAAATGCATCGCGAGATGTCATGGCCG
AACAACAAGAACAAGAACAACAACGCGTGTTTCCAACCACCATGTCAAATGCATCGCGAG
ATGCCGTGGCAGAACAACAAAAACAACAACGAGAAGGAGAACCAAACGCGCAACCGCCGA
GCTACCGCAGCTACGAGCCACTGTCGCTGCTAGAATTCGTCACGGCCTTTGTCGAGCGAG

ATAGTTCGTCGTCGAACGATCCCGGCGAAAACGAGGAAGCCTGCGAGCTTGACACCTCCT
TCAAGGTCGTCGAGGATTGGAAAGAGCACAAGATGGACGCCACCTGTCGCAGCAACACTA
CTTCCCAACAGAGCCCGGTCGTCCACAAGTACGTTCTCGATCGGTGGGAGGAAGAGCCCG
TGATTTTCCGGTACGAAAACGCAACCGCTCGAATCCCCAGCGGGCCGCCGCGGCACTCCG
ATATTCCGTCGACCGCCCTGTGCTTTCCGGTGGACAATGCGGAAAGCTACCCTGCGGGCC
TGGCCACCACCGGCAGCAGTGCTAACGGCACCAACACCACGTATATCGACGTGGACGCGA
CGCTCATCCGGACGTTGGTCTTCGACCCCTTCAACGCATACGAGCGCTTTCATGCCTACG
CCAACGCTGCCATGATCATGCTCATGTTCGATGTCCGCAACCCCCAAATTGTGATGCTGG
GCAAGCCAGAAAGCAAATTTATCTACGACGAGGGCGACCGGGACTTCTGGCGCCTCTTCT
CTGACCTCGAGCCGATCTACATACCCTGGGGCAAGGACGTGGGAATTCCCGAGAATGCCA
ATGCCAATGTCACCGCCCACGACAAAAACACCATCTACCGATTCCGCGAGATCATCGAGG
CGCCCAACGCCGGCCTTTCCATGGTCAACACCAAAAGCAAGGCATCCGGGATCCGCGGTC
GGGCGACGGAGCACCATTGTATGAGTTCCCTCTTTCGCGGGGTCATGGCGTGGATCGAGA
CGAAGACATTCCTGGCCACCAATGCGAAGGCAACGAAAGCGATCGACGGCAATCGCGGAA
AACAGAACGGCATTGGCAACAAGAAGGTTGAGGTATTTTCTCGTCGCGCCGGAAACCATT
CTGGTGAATCAGACAGATCCACACCAACCGAAGGCTCGACCGAAACCGCATTTGCAACCG
CTGCCGCTGCTGTAGCGGCTACCGCAAGGACAACCGAACCCGAGGGAGCACCCCTTTCCA
AGCAACCACCGCGCCTCGTAAAAATCTTGTGGTCCTCGAGACGGCCCTATTGCTGCCGGG
ACGGAGGCATCTGGTTCCCAAAACGGGTGCTCATGCGCGAGACCGAGTTTCTGGAAGCCC
TCCTCGAGGCACTGTACGACAACGACAAAACGACAACACGCGCAAACGAATACGAGATCA
CGAGCGTCGAGTTTGGGTCCCTGTCGACGACGGAGTCCATTCGCATGGCATCGGAATCCA
CCGTTATGGTAGGCTTGCACGGGGCAGGCCTGATCTGGTCGGCCTTCATGTCCGGCGAGC
GCGGGGACGACGGCGACGACGACGACGACGGCAACTTTGACTATGACGACGAGGCCAAGG
TGGAGGAGATGATCCTCAAGGCCCAGCTCCGCCGCAACGGGGGGAAAAGCCGCCGTACCA
GCACTTCTTCCGTCGGATCGAACTCGAACTCGAACTCGATCCCCAAGCCCGGCCAGAGCC
GCGTGTCGAACGCCCTGAGCGCCGCCATCCGCCGGTCCAAGAGGGACAACGAGGACATGC
TCGCGGCCATCACGTCCAAGATCTACCCGGTCGCCGACATTCTCTGCGTCCAGGGGAACC
AGCGCAACTCGAGGGCCGTCGACCTGGACCCCTTCCTGAAGGAGGACAGCGTCGTGGAGG
GGATGGACCGGGACTTTGCCGCGGCCTTTCCCCCCTCGCAGATGCGGTGCCTGGCGCTCG
TGTCCCACAACGAGATGAAGTCCACCATGCGCAAGTTTGTGGTGGCCAACAAGAACCTCC
TGAAAAAGTTCCGCCTCACCGGGACCAACAGCACCATGACGATGCTCCGGGAGGTCTTTT
CCGACGAGCCCGCCGGCACCGTGATGTTCGGCCCGGCCTGCGCGTCCGGCCCCCTGGGGG
GGGACGCCGAGCTGGTCTCCCTGATGGTGGGCGGCCGGATCGGCGGGATCCTCTTCTTCC
GGGATCCCATGAGCGCCCACCCCCACCGCGCCGACATCGACTGCCTGGTCCGGCAGGCGC
TGGTCCACAACACCATGATGGCGGAGACCCCCACCAGCGCCCTGATGATGTGCCACTGCC
TCCGGGCCGCCCTCAAGGGGGAGGGCAAACCGGAGATGATCCCGTCGTTCTTTTTCTCGC
TCCAGTGCCCCTCGGTGGAGGCCTACAAGGCGGAGCAGAAGCGGGTGGTCGAGATGCAAA
AGAAAATGGCCCGGGAGTCGATGACGACGAGATCGTCGGTGACGTCGTCGGCCCTGGCCG
GTGCCAAGGACTACCGGTTCTCGTGCCTGTCCGGGTGAAACCGAGACGGCCGGATGCAGA
GGCAGAGGCAGAAGCGGCAGCACCAGAACTAGAAGCAGAAGCAGAAGCAGCAGGAACGGA
CGCCCGGTGGTGCAGCCGGGTTGCGGGACTCCATTTATTTCTCCAAACGATTTGCACCGT
AGAAGAGTATTAATATTAAACTGTAATACAGTGTAATACAGTAATATGCAACACGAATAA
GACGTGTTTTACTGTCTATCGATCAGAGTACCGAATCGAATTGATTTCAATCGAATGGAT
TTCAATCGAATCAATCGCGAAATCGTACGGTACTCGTACCTTACAGCGGGGTGTTTGCTT
GGTAACGGTATCCTAGGTAAACCCGAAAAGGTTCTTGAAAATCCTATCCAACCCCCAGAC
TAACTTAGACCCCACCTCTTCCTCTTCGAGGACCAATTACAGTC
>comp2566_c0_seq1
GAACAAGCTCCCATATCTTTGGATTGGGTCATTGCTTTGCCGTGCCTTGCTAATTTTGTT
TATGATGGTTGGTAGTGAAGCCATGACTGCTTCCGTATGTTTTGGGAAGTTCTGCCTGTT
TTTGTTGTTGTTCTCTCTCTGATACTTTTAAACCCTTTTTCTTTGCTTCAATGCTGGTTC
TTGGTTGTTGACAGCTGGGAACGGGAGCCTTTTCGACCGTTCGAATGGGATACCATCGAT
CTGACAGCGAGAAAACATATGCTATCAAGTGCGTCAACCGAAAGAAGCTAACGAACGAAG
ACGAGGTCGCTCTGCTGGACGAGGTTTCCATCCTGAAAGAAATGAAGCACGTGAACATCA
TTAGACTTTTCGATTTTTTCAAAGAACCAACATCATACTACCTCGTCATGGAAGAGATGG
ACGGAGGCGAGCTTTTCGATAGGATCGTCGCAAAAGCATATTATAACGAAAAGGAGGCCC
GGGATACTTGTAAGATTCTGGTTGACGCCGTTGGATACCTGCACGAGAATCAGGTAGCGC
ACCGGGACTTGAAACCCGAAAACCTGTTGCTGGTCAGCAAAAACGACGACTCGCAGGTCA
AAATCGCAGATTTTGGATTCGCAAAGAAGGTGCTAAAGCCAAAGAGCCTCACTACACAGT
GCGGGACACCGGGATACGTTGCGCCCGAGATTCTCGAGGGACACCCGTACGACGAACGAG
CCGATATGTGGTCCGTCGGCGTCATCCTCTACATTCTCTTGGGAGGCTACCCCCCTTTCA

TCGAGAACACTCAGCGAGAGCTCTTTCGAAAAATCAAACGAGGAGACTACGAATTCCACG
AGGAATATTGGGGAACGGTTTCGAAGGAAGCAAGAAACCTAATCAAATCCCTGCTGACCG
TTGATCCAGAACTCCGACTCAGTGCCCAGCAGTCGCTCGAAAATCCATGGATTTGCGGAG
ATGGCAAGACGCTGGCAAAGAGAGGCCTCGACAAAAACCTCGAGTCACTCAGGGCCTTTA
ACGCCAAGAGAAAGTTCAAGGCCGCGGTATCAACAGTTGTGGCTGTCAACAAACTCAATT
CGCTTGGAAATGCCTTTAAGAACCTAGACATGGCCGACGAGGAGAGTCCATAGATAATAA
AATTTATGTTTCAAACTAAGAAACGGGTGCAGAGTATCATTTGTCGTGGACCTACGTATC
GAGACGGTGTTGAGTTTTCCTTGTAGCTATCGGAAATACGATGTTTAGATCAGCGAGGCA
AGTACCAATAAACTTTGGGAAGACTTCCTCGGTTGACATTTTGGTCTCTTTCATTCATTT
GGGGCTCAACATGGTCCAGACAGTAACGAAACAATTTTATGACGTGGTAAAATTCTTTTT
GCGACAGTATTATTGGGAAATAAGTGGTCACCACTATTTTTTTGAATGAATAGGTTTTTC
GTCTTGAAAGATAACTCTGAGACATCGCTGCTGCAAGATCAAACTGTGGTGTGATGAAAT
CAAACAATATCTTTTTTCGCAAAGAAGCATTTCTTGCAGCTGACAAAAGTTCAACCCCGG
AGCAGTGCTTCAGTTGACTTTCTCCGAGATTCCATACGTTCACACCTATATTTATCATAG
AATTGTCGTACTTCATATGATGAAAATATTGCATCGTTCATTCTTGTTG
>comp2566_c0_seq2
GGAGGAGTTTAGCGAGCATTTTTCTCGTTGAAGGAGCTTCGCTTCGATTCGTTCCTAACG
CAATCAATTGGCAAGTGTCACTTGGCATCTACAACTCGGCAAATCGAAGAGTAATAATTC
GAAATCACAGTTCTCCCCCATACCTCGTGCCCAAGTGACAATAGAATCTGAGCCTCTTTA
TTTTTGTGTTTTTTTTTCTATCACAGACGAGTCTCTTTAACCCTTCTAGTAAAACACTTG
ATTTTACTATCGTTACCGCAGATCTTGTCGAAGCTTGGAAAAGATTTGGATTCCGACGAA
ATCTAGACTCAAAACTAGTTCCCTCCGACGAAGAGAATCCATAATCTACCCAGAAAACAA
CGAAGAAGAAATAAATTTTTAAAATAAAACAAAGTGGAATACAAACATGGCGTTCGAGGA
ACACGACGACACACCCGTCCCGAGCTCCAAGAGCGGGAAACACTTCGAGGAGTTATACCG
ACTGAAAGGAGTGCTGGGAACGGGAGCCTTTTCGACCGTTCGAATGGGATACCATCGATC
TGACAGCGAGAAAACATATGCTATCAAGTGCGTCAACCGAAAGAAGCTAACGAACGAAGA
CGAGGTCGCTCTGCTGGACGAGGTTTCCATCCTGAAAGAAATGAAGCACGTGAACATCAT
TAGACTTTTCGATTTTTTCAAAGAACCAACATCATACTACCTCGTCATGGAAGAGATGGA
CGGAGGCGAGCTTTTCGATAGGATCGTCGCAAAAGCATATTATAACGAAAAGGAGGCCCG
GGATACTTGTAAGATTCTGGTTGACGCCGTTGGATACCTGCACGAGAATCAGGTAGCGCA
CCGGGACTTGAAACCCGAAAACCTGTTGCTGGTCAGCAAAAACGACGACTCGCAGGTCAA
AATCGCAGATTTTGGATTCGCAAAGAAGGTGCTAAAGCCAAAGAGCCTCACTACACAGTG
CGGGACACCGGGATACGTTGCGCCCGAGATTCTCGAGGGACACCCGTACGACGAACGAGC
CGATATGTGGTCCGTCGGCGTCATCCTCTACATTCTCTTGGGAGGCTACCCCCCTTTCAT
CGAGAACACTCAGCGAGAGCTCTTTCGAAAAATCAAACGAGGAGACTACGAATTCCACGA
GGAATATTGGGGAACGGTTTCGAAGGAAGCAAGAAACCTAATCAAATCCCTGCTGACCGT
TGATCCAGAACTCCGACTCAGTGCCCAGCAGTCGCTCGAAAATCCATGGATTTGCGGAGA
TGGCAAGACGCTGGCAAAGAGAGGCCTCGACAAAAACCTCGAGTCACTCAGGGCCTTTAA
CGCCAAGAGAAAGTTCAAGGCCGCGGTATCAACAGTTGTGGCTGTCAACAAACTCAATTC
GCTTGGAAATGCCTTTAAGAACCTAGACATGGCCGACGAGGAGAGTCCATAGATAATAAA
ATTTATGTTTCAAACTAAGAAACGGGTGCAGAGTATCATTTGTCGTGGACCTACGTATCG
AGACGGTGTTGAGTTTTCCTTGTAGCTATCGGAAATACGATGTTTAGATCAGCGAGGCAA
GTACCAATAAACTTTGGGAAGACTTCCTCGGTTGACATTTTGGTCTCTTTCATTCATTTG
GGGCTCAACATGGTCCAGACAGTAACGAAACAATTTTATGACGTGGTAAAATTCTTTTTG
CGACAGTATTATTGGGAAATAAGTGGTCACCACTATTTTTTTGAATGAATAGGTTTTTCG
TCTTGAAAGATAACTCTGAGACATCGCTGCTGCAAGATCAAACTGTGGTGTGATGAAATC
AAACAATATCTTTTTTCGCAAAGAAGCATTTCTTGCAGCTGACAAAAGTTCAACCCCGGA
GCAGTGCTTCAGTTGACTTTCTCCGAGATTCCATACGTTCACACCTATATTTATCATAGA
ATTGTCGTACTTCATATGATGAAAATATTGCATCGTTCATTCTTGTTG
>comp2567_c0_seq1
AGAAAAACATAGTTTTTGCAAATAACTTTTCCTTAGTTTTCCACTAACGGTCACTAAAGC
AATATTCCAGGTCAGTAAGAGACAAAATAAGATGGAGGCTGCCTCACAAAATCAAGTCGG
TGCTAATAACACAAGAGAAGGAAGAGGGAATGGCAATAATCGCAATAACAGGCGACGCGG
GGGACGCGGTGGGCGCGGAGGACGCGGTGGACGCGGTGGTCGAGGCGGACGAGGTGGACG
CGGAGGGAGGAATAGGAACTCTGAGAACGGAGAAAACAGTCAAAATAGGAACTATCCCAA
AAAGCACAAGGAAGAGAACATGGATTCTTCCAATACCAATAACAAACGTCCCAAAAAAAT
TAAGAATACACCACCTGCACGCAATGAGAATGGTAGCACGCATGGCGGATCTGCTTCCGA
GTCGAAGAAATTGGCGCACATCACAAACAACAGATTCGCCGATCTCAAAATCTCTGCTGA
AAGTCGACGCGCAATGGCAGAAGTATTTCAGTACGAATTCATGACGGCTGTGCAGTCCGA
AACATTGCCTTTGATCTTGAAGGAGAATAAGGACTGTCTTGCTAAAGCGAAGACAGGAAC

AGGAAAGACTCTCGCGTTCATGATCCCAACCATCGAAAAGATCACTGCATCCCTGTCGAA
AAATGGAAAGCAACGTTCTGATATTTGTTGCTTGGTCATTTCTCCCACTCGCGAGCTTGC
CCAACAGATCGGATCCGAAACTGAGAAACTTTTGTCCTTTCACAAGAGCTATCTTAGAAA
AGTCGTGGTGTGCGTGGGTGGAACTAACAAGAACAAAGATATCAAGTCCCTTGTTGGGAC
TACGCCTATCGTTGTAGCAACTCCGGGTCGATTGTTAGATCATTTGCAAAATGGTGGTCT
TGCAGAGCGTATGAATCGCCTCGATACACTCATTTTCGACGAAGCTGATCAGTTATTAGA
CATGGGATTCAGACCAGATATTGAGCGCATCCTTCGCCTGTTGCAACCCAGCCAAGAAAC
ACGACAAACTCTGTTGTTTTCAGCCACTATACCTGATTCGGTCTCTGAAATTGCTGGTAT
TGCCATGCGTGGCGACTACAATTTTGTAGATACCGTTGGCGAGGACAGTGAGCAAACTCA
TTTGCACGTTCAGCAACAACTCATGGTGACTCCCCAAGCGCTATCGGTCCAAGCTCTGGC
CAACGTTTTGGAGCGTGAAACCAAAAGCAACAAAGATTACAAGATCATTGTGTTCTTTAC
AACCGCACGCCTTACAGGATTTATGGCAGAACTTTTCAACTCAGTACAAAAAGAAACCGG
GTATGATGTCCTCGAAATCCATTCTCGCAAATCTCAAAAGCAACGGGAACGAGCCTCTGA
AGAATTTCGGCGGAGAAAGAATGCTGTAATGTTCAGTTCTGACGTTACGGCACGTGGTAT
GGATTATCCAGATGTTTCTTTTGTTTTGCAAGTTGGTTTGACCGATCGATCTCAGTACAT
TCATCGACTTGGACGAACCGCTCGAGCTGGAAAGGACGGAAAAGGGGGACTTTTGCTAGC
GGACTACGAAAGAGATTACATGGTGAAGAAAGAACTTGCCGATATGCCGCTTGAAGTAAC
GCCTGTCCCCAACCTAGATCAGGCAACCGCTGCTATATCTAAAGCAATTCAAAATGTAAG
CAAGAAAAATGACTTGCGCAACTCTGCTGAACAAGCCTATCGAGCCTGGCTCGGTTATTA
CAATGGTCACCTGAAGAAAGTTCGGTGGGACAAGAAGACTCTTGTTCAACATGCTAATAT
GTGGGGCATTGAAGTCGTTTGAAAGAACAGCCTAGTCTCCAGAAAAGGACGATTGGCAAA
ATGGGACTTCGGGGAACTCCTGGTCTTGTCATCGAGAACTAAACGATACGATAACACACA
ATAAATGCATTATAGAATATTGTTCAACAAAC
>comp2568_c0_seq1
AAAAAGTACTGTTATTGTCATCATCCCCTTGTTTGAAACCAGCCCACGAACAACCAGACT
CCTGCGAATCTATATCACAGGCAAGATTTGCAACACTGGATCGAATCGTCGTCTTGTCAA
TCCACCCAAAAGAATGCTGGCTCTGCAGCGGATTGCTCGGACTCGCCCCTCGGCCACGTC
GCTGTTCCCATCCCGAGGTGTGTCGATAATCCCGTCCGCGCATTCGTCATCGCCGTTCTC
GTCGGTTGCTTTGCGATTCGACGAACGTCTGGGCCCGCAGGATCGAAGGATTCCGCACGA
TCGTGCCCGTCCTTCTCCCCGGTGCGTATCCTCGCTTTCGCCGTCCCCCGTGCCGACGTC
GCTGCCACCACTCCCGGGCAAGGTACTGATTGCCAACCGGGGGGAGATTGCATGCCGTGT
CATTCGAACCTGTCGTCTCCTAGACATCCCTACGGTGGCCCTGTACAGCCTTGCCGACGG
CCCCGAGTCCTTGCACGCGAAAAGCGCCGACGAGGCCTACCAGATTGGGTTTGGGAGCTC
TCCGAGCGAGAGCTACCTGCTCCAGGAAGAGGTCTTGGAGATTGCACTCCGATCGGGCGC
GGAGGCCATCCACCCGGGCTATGGCTTTTTGAGCGAAAATGCCGAATTTTGCCAGCGGGT
CCAAACCGAAACAAATGGCGGCGTGAAATTTGTTGGGCCCCCGCACTCGGCCATTACTGC
CATGGGATCCAAGTCAAAATCAAAAGCGATAATGGAAAAGGCGGGTGTCACGACGGTTCC
CGGTTTTTATGGAGATAGCGACGAGGAGCAAGAACCATTGCACCTACTGGAACGGGCAGT
CCAAGACGTGGGATTCCCTCTCTTGATAAAGGCTGTCATGGGAGGCGGCGGTAAGGGGAT
GCGATTGGTGTGGTCGGAATCGGAATTCCTCGAAAAACTCGAGGCGTGCAAAAGGGAATC
CCTGAATGCCTTTGGAGATTCGAGGGTTTTACTGGAGCGATACTTGCTGCAACCCCGGCA
TGTCGAGGTTCAAGTGGTTGCAGATGATTACGGAAATGTGGTGAGTTTGCACGAGCGGGA
TTGCAGTCTGCAGAGACGGCATCAAAAAATTATAGAGGAAGCCCCTGCCTCGGATCTCTC
TGAAAACTTACGCCAGCGACTGGGAGAAATGGGTTGCCGGGCTGCCAAGGCCGTTGGCTA
TGTGAATGCCGGCACGGTCGAGTTCCTTTTGGAATCCAACAAAACGAAAGAAGATCCAGA
ATTTTACTTTTGCGAAATGAACACGCGCCTTCAGGTAGAACACCCCATCACCGAGCTCAT
CACCGGAATCGATTTGGTGGAGTGGCAACTCCGGATTGCTGCTGGAGAATCCCTTCCGAT
CACGGATCCGGCCGCCATACCCTGCCGGGGCCACGCCTTCGAGGCGCGCATCTACGCCGA
AAATCCGGGCACCGGAAACTTCTTGCCAACCGCCGGAACGGTCTGGCACCACGAGCCACC
GGCACCTCCAAACGCGGTAGGCGTTGGGGAGGATAACTTGCGGGCCTCGAACGGGGTTCG
GGTCGATACCGGGATCCAGGCCGGCCAGGAGGTCGGCGTGGATTACGATCCGATGATATG
CAAGCTCGTTGTGCACGATGAGTCCCGCGAAAAGGCCCTCGAGAAACTGACCGATGCCCT
GAAGCGGTACCAGCTCTCGGGGGTTCCGACCAACATTGATTTTCTGATTCGGTGTGCAGA
GCACAGGACCTTTCGGAAAGCAGGAGCCACCAGCACCGGTTTTTTGGACGACCACATGGC
CGAGGTCCTTCCACCGGTGGAAAATGAACCCCCCGAGCTTGCCATCGCCATCGCTGTCTT
CGCCTCCCTGCTGCAATCGGAGGGACGGATCGGCGTTTCTGATTTTGAGGCAGAACGCCG
GATACAAACCTCGCCATGGAACTCGCTCTCGGGCTCCTGGAGAAACGGCGGACATTCTTG
TCAAAAACTTGAAACGGCGGACGGGACCTCTGTAGAAACCACGGCTTTTCGGGACGGGTC
GTTCGAAATCCGCATCACGAAGACCACGGAAGAGGAAAGCAATGGCAACGGCACTTTGTT
TCACATCGGCGGCACGCTGTCTTCCGACCAGTACATGCAAATCGTCGTCGACGGGTCGAA

GACGATGGCGCTAACGGTAGCTCTCCGTGAGATGGACGGGCAATACCAGGTTTGTATGTG
GCCACAATCCCTTTTGCTGCGAAACGAGGGCCACTATTTCTGGGAGGTTTCCGTCCCCAA
TCCACGGATTCCGACCATGGCGCAAAACGAGACTGGTTTCTCGTCGGGGCAGGGAGTGGT
CTCCGCTCCGATGCCAGGAAAGATATCAAAAATAAACGCTTCCGTTGGGGATATGGTCCG
GGAGGGAGATGTTCTTTTGGTTATGGAAGCCATGAAGATGGAGCACACGGTGCCATCGCC
ACAAAGCGGTATTGTTTCCAGCATTCCGTTCCAGGTGGGTGATGTGGTGGGAGACGGAAC
AGTGCTTGCTGTCGTCGGGTCCGAAGAAATGAAAGATGAAGCAGAAGCCGTCTAGCTGGC
GCACAATGGATGATACAAGTCTATTGTGCATTGAGGGACCGTGTGAACGAATCATAGCAA
CAACACTATCTAGATGATCAAAATTGGATTAGAGCTTCTAAGATGGTGATAATAGTAATG
CAATACGAAATGAAAATCAAGAGCTCAGTTTCCAGCTTTGTCTGACGCAATAATGCTAGA
ATGAGATGCATTCTCTCTCCTCTCATTTCAAAACATGTCTATTACTCTTCCCCTTCCATA
TCCTCATTAGCAATCGCATCGTCCGATTTGCTGATGAGCAATTTCCTCGGTGAAATGGCA
AACTCGAACGGTACATCGTGTGGCGTCTTT
>comp2568_c0_seq2
AAAAAGTACTGTTATTGTCATCATCCCCTTGTTTGAAACCAGCCCACGAACAACCAGACT
CCTGCGAATCTATATCACAGGCAAGATTTGCAACACTGGATCGAATCGTCGTCTTGTCAA
TCCACCCAAAAGAATGCTGGCTCTGCAGCGGATTGCTCGGACTCGCCCCTCGGCCACGTC
GCTGTTCCCATCCCGAGGTGTGTCGATAATCCCGTCCGCGCATTCGTCATCGCCGTTCTC
GTCGGTTGCTTTGCGATTCGACGAACGTCTGGGCCCGCAGGATCGAAGGATTCCGCACGA
TCGTGCCCGTCCTTCTCCCCGGTGCGTATCCTCGCTTTCGCCGTCCCCCGTGCCGACGTC
GCTGCCACCACTCCCGGGCAAGGTACTGATTGCCAACCGGGGGGAGATTGCATGCCGTGT
CATTCGAACCTGTCGTCTCCTAGACATCCCTACGGTGGCCCTGTACAGCCTTGCCGACGG
CCCCGAGTCCTTGCACGCGAAAAGCGCCGACGAGGCCTACCAGATTGGGTTTGGGAGCTC
TCCGAGCGAGAGCTACCTGCTCCAGGAAGAGGTCTTGGAGATTGCACTCCGATCGGGCGC
GGAGGCCATCCACCCGGGCTATGGCGTGAGTCTTCTTTCAGTTGTGTGCAGAATTGTCAA
ACTTCAAAGTGCGTCTTTCTCGTTTCTCCAATCGACAGGTCACACCAAACGCGGTCTTAC
TGAACTCGATTGGTTTTGTTCCCGTTTCACTCGTTGATCTCCGCCCCCGTCTAGTTTTTG
AGCGAAAATGCCGAATTTTGCCAGCGGGTCCAAACCGAAACAAATGGCGGCGTGAAATTT
GTTGGGCCCCCGCACTCGGCCATTACTGCCATGGGATCCAAGTCAAAATCAAAAGCGATA
ATGGAAAAGGCGGGTGTCACGACGGTTCCCGGTTTTTATGGAGATAGCGACGAGGAGCAA
GAACCATTGCACCTACTGGAACGGGCAGTCCAAGACGTGGGATTCCCTCTCTTGATAAAG
GCTGTCATGGGAGGCGGCGGTAAGGGGATGCGATTGGTGTGGTCGGAATCGGAATTCCTC
GAAAAACTCGAGGCGTGCAAAAGGGAATCCCTGAATGCCTTTGGAGATTCGAGGGTTTTA
CTGGAGCGATACTTGCTGCAACCCCGGCATGTCGAGGTTCAAGTGGTTGCAGATGATTAC
GGAAATGTGGTGAGTTTGCACGAGCGGGATTGCAGTCTGCAGAGACGGCATCAAAAAATT
ATAGAGGAAGCCCCTGCCTCGGATCTCTCTGAAAACTTACGCCAGCGACTGGGAGAAATG
GGTTGCCGGGCTGCCAAGGCCGTTGGCTATGTGAATGCCGGCACGGTCGAGTTCCTTTTG
GAATCCAACAAAACGAAAGAAGATCCAGAATTTTACTTTTGCGAAATGAACACGCGCCTT
CAGGTAGAACACCCCATCACCGAGCTCATCACCGGAATCGATTTGGTGGAGTGGCAACTC
CGGATTGCTGCTGGAGAATCCCTTCCGATCACGGATCCGGCCGCCATACCCTGCCGGGGC
CACGCCTTCGAGGCGCGCATCTACGCCGAAAATCCGGGCACCGGAAACTTCTTGCCAACC
GCCGGAACGGTCTGGCACCACGAGCCACCGGCACCTCCAAACGCGGTAGGCGTTGGGGAG
GATAACTTGCGGGCCTCGAACGGGGTTCGGGTCGATACCGGGATCCAGGCCGGCCAGGAG
GTCGGCGTGGATTACGATCCGATGATATGCAAGCTCGTTGTGCACGATGAGTCCCGCGAA
AAGGCCCTCGAGAAACTGACCGATGCCCTGAAGCGGTACCAGCTCTCGGGGGGTTCCGACC
AACATTGATTTTCTGATTCGGTGTGCAGAGCACAGGACCTTTCGGAAAGCAGGAGCCACC
AGCACCGGTTTTTTGGACGACCACATGGCCGAGGTCCTTCCACCGGTGGAAAATGAACCC
CCCGAGCTTGCCATCGCCATCGCTGTCTTCGCCTCCCTGCTGCAATCGGAGGGACGGATC
GGCGTTTCTGATTTTGAGGCAGAACGCCGGATACAAACCTCGCCATGGAACTCGCTCTCG
GGCTCCTGGAGAAACGGCGGACATTCTTGTCAAAAACTTGAAACGGCGGACGGGACCTCT
GTAGAAACCACGGCTTTTCGGGACGGGTCGTTCGAAATCCGCATCACGAAGACCACGGAA
GAGGAAAGCAATGGCAACGGCACTTTGTTTCACATCGGCGGCACGCTGTCTTCCGACCAG
TACATGCAAATCGTCGTCGACGGGTCGAAGACGATGGCGCTAACGGTAGCTCTCCGTGAG
ATGGACGGGCAATACCAGGTTTGTATGTGGCCACAATCCCTTTTGCTGCGAAACGAGGGC
CACTATTTCTGGGAGGTTTCCGTCCCCAATCCACGGATTCCGACCATGGCGCAAAACGAG
ACTGGTTTCTCGTCGGGGCAGGGAGTGGTCTCCGCTCCGATGCCAGGAAAGATATCAAAA
ATAAACGCTTCCGTTGGGGATATGGTCCGGGAGGGAGATGTTCTTTTGGTTATGGAAGCC
ATGAAGATGGAGCACACGGTGCCATCGCCACAAAGCGGTATTGTTTCCAGCATTCCGTTC
CAGGTGGGTGATGTGGTGGGAGACGGAACAGTGCTTGCTGTCGTCGGGTCCGAAGAAATG
AAAGATGAAGCAGAAGCCGTCTAGCTGGCGCACAATGGATGATACAAGTCTATTGTGCAT

TGAGGGACCGTGTGAACGAATCATAGCAACAACACTATCTAGATGATCAAAATTGGATTA
GAGCTTCTAAGATGGTGATAATAGTAATGCAATACGAAATGAAAATCAAGAGCTCAGTTT
CCAGCTTTGTCTGACGCAATAATGCTAGAATGAGATGCATTCTCTCTCCTCTCATTTCAA
AACATGTCTATTACTCTTCCCCTTCCATATCCTCATTAGCAATCGCATCGTCCGATTTGC
TGATGAGCAATTTCCTCGGTGAAATGGCAAACTCGAACGGTACATCGTGTGGCGTCTTT
>comp2569_c0_seq1
CGAACCGTAGACCATTTCAAAATCCAAATCGACGTTCGACTTTGACAGCTTGCACGTTTT
GTGAAACATTGTTGGTGAAAGATAGCACGGGTGAGACGGAGCTGCCAGCTTCACTCTACA
ATACATTCGAGAGAATACACGTTCACTAGTGCTCGGACTAGGACTGTCTTTCGTATCACG
CGTAACCACTAGAAGCTCCATAGGGCAATTGTCACAGTAGCAAAAGAAAACAACCCAAAC
CAACGAAGAAGGTTAGGAAATAACGAACAATTCAAATAAATACTCTGCTCTACGCACTCT
ATTTTGCACCGTACTTCGACAGATCAGAAAACACAGTAAAAATGAGCGTCAACGATGATC
TGCTCGTCGACGGCTCGATAGAGGGGGACCTGATGGCGGCAATGAACGCGGCTCTGGCAG
AAAGCGGCGATGACAACAACTCGAACTCCAACTCGAACCACGGGAGCGCCTTCTTTTCGG
AGACCGACACGGCTTCGATGCGTTCCATCGACGTCATGGGGGAAGCCATGGGTCTGCTGA
TGTCGGGGGAAAATGACTACGAAAACGACTACGAGAGTGAACCCACCAACGGCAACATGA
ATATGGACGGCAAGGAGATAAGAATCGACTCGGGGAACAACAACTACGGTGGAGTCGGGG
GCGATATTGACGACGAAGAGGAAATCAATTTCGACGATTTCATGGCAGGCAGCAAGGGAT
CCGGCGACAACAACGACGGCAAGAGCATGGACCAAGACATTTACGCAATCGACGGCGGGG
ACCAACCACAAGGGGGGGAGAGCAAGGACGACGATGCCGGTGCCCCTGGCGCGGAGAGTT
CCAACGCAACCACCGAAACGGCAGCGAGCAAGGAGCCACCACCGGCAGGACTAGCCGCCG
ACGTCGTGCCGCTGAAGCAGTTCGAGAACGCCCTGGCCCTCATCCAGGACCTCGAGAACC
GGATCCAGGTCCTGGAAACCGACCACCAGTGCCTCACCGAGGAGAACATGGTTCTGAGGG
AACAAACCCTGAGCCAGGCCACCGTCCTGGCGGACCTCGAGGCAAAGCTGGCCGCCTTCC
CGAAACTCCTAGAGGAAACCGCCCGGGAGGAGGCCCAGGTGGCGGCCGCCAAGGCCGAGA
TCGAGACCAAGTTCATCTTCTGGAAAAAGGACATGGACCGGCAGGAGCAGGAGCTGGAGG
AGGAAAAGCTCAAGCGAACGAGGAAGCACTACTCGGCGACGACGGACTCGCTCAAGCAGT
CGGACTTTCTGAAGGACGTCGTGGAGCGGAAGGAGCAGGAGAACGACGACGGCCACCACC
AAGAAAAACCAAACGGGCCCCTCGCATTCCTGCGCGGCTGGGGAAATCGAAACAACAAGA
AACACAGCAACAGCAAAGCCAACGGGGACAAGAACGATGCCGTGCCCAGCGGCGAGAGCG
ATGCATCCACTGGGGGTGGTGCCTCTGCCAATGGTGGCAGCAGCGGCAGCAAGGACCACG
GCAGCGAATCCACAATAGAGACATCTAAAGGGGACTTCAACATCAACATTACACTTTCCA
CCTCCAGGAGTGGGGACGACCTCCCCGATATGCCGGAACAAAGAAACGGAAAGATCCGTC
CCTCGCTACCGAGTAACGAGGAAGACGACGAAACCGAGGTGAACAGCAGCAACGCTATCA
AAGGAAACCACGGTCACGACGAAAAACTGCTGGACCTGCTGACGTAACGCAGCCGCAACA
ACGTCGCGGCAAGATACCTCCGTTTCTGATTGCTCCTTCCTTCGTCCAACCACTCACAAC
AGCAAAGCCAATCCGAAAGGTACCTGAAGATTACGATGCGGAATTGTTTTCCTTTCGTGG
ACTTTCTAAACTGCCATTCGCCGGGAAATTTGGAAGGAACGGCATGGTCTGATCTTGCCA
ATAGGATACTCCAATGACACAAGGGAAAAACAACCCACTCATCACACCGCACCAGACCGT
AACGATCGATAGTGCTATCTGCTCCATCTAAGGTCCGATTGAGCTAACCATTCACAAATA
TTATTCGGGGCGGAGCTCGTCTGTGGTTTGCATCGTAACACCGGTCCTTAAAACACAACA
CACCAAACCACTTTCAACGGAACAAAGGGATTGACTGAGGCAGTTTTCTTAAACTATAGA
CATAATAAAATAGCAGTTGCCTAGGATAAAGG
>comp2570_c0_seq1
CAACATCAAGAAACAAAGCGAGTTCGAGCATCGTGCTCGTTCCAGTTTCCCAACTCGTAA
CGGTAGTAAATGCCATTGTCTGGTCTGAAATTGCACCAATCAACGGTGGCATGTATCATT
CGCTGTAAGCGGTGATTGATTTTCCAGTTCACCAGACACAGACAACCTTCACAAAATGGC
ATGGTCAAGTCCAAAGAATAGCGGAAGGAGGACGAGGACAACTCGAACCCCTGCCGGGCC
GATCTACTACGACTCGAAACACTCAGTTGCCATTCTCCTTCAGTGCAACGGATCGGCATG
GCTCAGGGTAATACCCTGGTGTATCCTCAACGCGGCATTCACGGCAGCATTAACGGCACT
CGAAACGAGATACGACCTGCCACTAGCGCTGCCGGGCGAATCGTCGTCGCTCGGCCACGA
TTTTACCGGGTTGGTGCTTTCCTTTTTGCTGGTGCACCGAATCGGCTGGGCACTCCGCAG
GTACTCTCGATACCAGGAATCCCTCGGCACTGTCCTGCGGGACGTCCGGGGCCTGACCCA
GAAAGCCATCGTTTTCTCTCGGCGAAACAAGAACAACGACACCTCTGCCAAGGAATGGAG
GTCCGAGATCGCCTATCGGAGCCTGCTCATGGCCAGAATGACCACCGCCAATGCGGAATT
CCCTGTTGCGCAGATCCCCGCACACGAGGTGGCCGAGCTCGACGGCCCCGGAGCTCCAGTT
TTGTACTCCCGATCGGGACTTTCTGTACCACGCCGAGATTCCGTACAGCAAGGGGATCCA
CTCCTTGTTGCGGATGCCCTGGAAGATTGCGGAATTGATTCGAGAGACAATCTGTGGCCA
AGAAGAACGACTGGTCAAGCCAATGAGCACAGACCACGAAATGAATCTGCTGGCTACCGT
GGATTCTATTCTCGCTGGCTACCACGGAATCCAAAACCTTATGCTGACGCCCGTCCCCTT

CCCGCTCGTGCAAATGACCCTCACCCTCACCATGGTGTACGTTTTCACGCTCCCCCTGGT
GTTTTTAAAGGACGACGCCCAGCGGAGCCTGCTCGGCGACTGTTCACGCGTGTTTTTTGC
CACGTATTGTTTCCTCGGCCTGGCGTTTGTCGCCCAGGACCTGGACGATCCCTTCGGGTG
CGGCCCGAACGATTTCGACGCCGCGGGCTGCTTCGGGTTTGCCCTGGACGACGTCGTGAC
CATGATCCACGACGCGGACGGCCTGGAATGGGCCGATGCGCTGCGGTACAAGTGCAACCC
AAACCCCTCGAGGGCCGTCCACGACGAGATGGTGGGCGCGATGACGGGAGAGCGCGAGGG
CTTGCTGCCAAAGCACAAACACGGCGGCGGCGACGACAATCGCCCCTGGAAGCACGCCCC
GGTTCCGGACTGGATGGCGTAGGAGAAGAGCTGCCGCTGCTCCTCACAAAAGATGGTGCA
GGGTTTCTTTTTGTGCTTGTGGCGTTGCCGTCAAGCCCAACGTCCAAGTCCGAGACCAAG
CTTGTAGTTCCATGGAACTTTTGCGGTGCCTTTGTTCGTGCTGGTTGCAGGGATTAGTTC
CATACGGCACCGCCTTTAGAGGACGACAAGCCACAGATGTGAAGTAAAATTGTTTAATAT
TTCAATACTTGTTTTAAAA
>comp2570_c0_seq2
CAACATCAAGAAACAAAGCGAGTTCGAGCATCGTGCTCGTTCCAGTTTCCCAACTCGTAA
CGGTAGTAAATGCCATTGTCTGGTCTGAAATTGCACCAATCAACGGTGGCATGTATCATT
CGCTGTAAGCGGTGATTGATTTTCCAGTTCACCAGACACAGACAACCTTCACAAAATGGC
ATGGTCAAGTCCAAAGAATAGCGGAAGGAGGACGAGGACAACTCGAACCCCTGCCGGGCC
GATCTACTACGACTCGAAACACTCAGTTGCCATTCTCCTTCAGTGCAACGGATCGGCATG
GCTCAGGGTAATACCCTGGTGTATCCTCAACGCGGCATTCACGGCAGCATTAACGGCACT
CGAAACGAGATACGACCTGCCACTAGCGCTGCCGGGCGAATCGTCGTCGCTCGGCCACGA
TTTTACCGGGTTGGTGCTTTCCTTTTTGCTGGTGCACCGAATCGGCTGGGCACTCCGCAG
GTACTCTCGATACCAGGAATCCCTCGGCACTGTCCTGCGGGACGTCCGGGGCCTGACCCA
GAAAGCCATCGTTTTCTCTCGGCGAAACAAGAACAACGACACCTCTGCCAAGGAATGGAG
GTCCGAGATCGCCTATCGGAGCCTGCTCATGGCCAGAATGACCACCGCCAATGCGGAATT
CCCTGTTGCGCAGATCCCCGCACACGAGGTGGCCGAGCTCGACGGCCCGGAGCTCCAGTT
TTGTACTCCCGATCGGGACTTTCTGTACCACGCCGAGATTCCGTACAGCAAGGGGATCCA
CTCCTTGTTGCGGATGCCCTGGAAGATTGCGGAATTGATTCGAGAGACAATCTGTGGGTA
AGTCCGCCATTCGATTGCCCGCTTTTTCGGTTCAGATTGCCAATTCGGTGCAACAAGCAA
GTCTAACTCTAAATTTCGCACGGATTCTTCCACGCAATCCAATGCTTTCTTTCGCCTTGC
CCCCCTGGTTTGCTACAGCCAAGAAGAACGACTGGTCAAGCCAATGAGCACAGACCACGA
AATGAATCTGCTGGCTACCGTGGATTCTATTCTCGCTGGCTACCACGGAATCCAAAACCT
TATGCTGACGCCCGTCCCCTTCCCGCTCGTGCAAATGACCCTCACCCTCACCATGGTGTA
CGTTTTCACGCTCCCCCTGGTGTTTTTAAAGGACGACGCCCAGCGGAGCCTGCTCGGCGA
CTGTTCACGCGTGTTTTTTGCCACGTATTGTTTCCTCGGCCTGGCGTTTGTCGCCCAGGA
CCTGGACGATCCCTTCGGGTGCGGCCCGAACGATTTCGACGCCGCGGGCTGCTTCGGGTT
TGCCCTGGACGACGTCGTGACCATGATCCACGACGCGGACGGCCTGGAATGGGCCGATGC
GCTGCGGTACAAGTGCAACCCAAACCCCTCGAGGGCCGTCCACGACGAGATGGTGGGCGC
GATGACGGGAGAGCGCGAGGGCTTGCTGCCAAAGCACAAACACGGCGGCGGCGACGACAA
TCGCCCCTGGAAGCACGCCCCGGTTCCGGACTGGATGGCGTAGGAGAAGAGCTGCCGCTG
CTCCTCACAAAAGATGGTGCAGGGTTTCTTTTTGTGCTTGTGGCGTTGCCGTCAAGCCCA
ACGTCCAAGTCCGAGACCAAGCTTGTAGTTCCATGGAACTTTTGCGGTGCCTTTGTTCGT
GCTGGTTGCAGGGATTAGTTCCATACGGCACCGCCTTTAGAGGACGACAAGCCACAGATG
TGAAGTAAAATTGTTTAATATTTCAATACTTGTTTTAAAA
>comp2571_c0_seq1
GCGGTAGCATCAAGCATCAATAAGTTGCGGTGTGCCCGGCCAAACCAACAGCGTTGCCTG
ACACTTTTGCAGTTGCAAGGAGATAGAGATCAAGGAGAGCTCCTGGCTTTGAGCGCCTTG
ATACCGAGAGATTGAATAGAGCGATTACCGGAAGTCGAACTTTAACCGCCTAGATAGCTG
TTGTACTACTAGTCGCTACTCAAGTGCAAAAAGTGATTCTGAATATCTGCGCAATTCCTT
TGGGTTTCTTTTAGGATACTTTCCAGGAATTAGAGACTTGCAAGTTGCCGGCTGCTTGAT
GGTCTAAAGAGACAGATACTGCATCCCGGCAAGTCACAAATTTTAGTCACAAGAATTTTG
TTTCTTGTGCGTTGTTTCTCTACAGCAATAAAGAGAGAGGGAAGTGCCAATATTTCCATC
CAGTGGGCAAACAAAACAATTCATACTGCACATCGCCGTCCATTGAAAGCACCAAAATGG
CTTCTCCAACTCCATCTCCCAGCAAGTCTTGGATGACAGACTCACAAGGGAACCATTTGT
CGAAAACCGGAATGACCTCAACCCCACCTGGCTTTGTTGCTCAATCAGGTTTGTGGTCCG
AGAATGGTGGAATCGCTGCTACCGGTAGCCAAAATGCGGATGCTGTTTCGACGGGCGATA
AAACACGGCCGGAGCGCAGGCGGAATGAAAGGACCTCTAGTTTCAACAATATTGCAGCAG
CACTTGGAAGCGGGCTCGCAGAATCCATGGAAGATGCAACTCGTGGGGACCATATTTCCG
CATCTCATCACGGCGACAACGCCAATCATAACCAGAGCAATATTTCGAACGGCCCTGCAA
CTCTGACGGACAGCTTTTTCAACCCAATCGAGAATAACGGCATGGCCTATGAACGCCACA
AACGCCACTCTGCCTCGAGACTTGTTGGTGTACCTGGAGCGTCTACTTCGTCTGCGACCA

GCAGTCACAAGACCCCCGAGACAGGACCATTGTTTGTGGCATCTTTGAACTCGGGTGAAG
CACGTTCCAAGTCATTTAATAGCAGAAATGGTGGAAGTCACCTAGTCGGTGACCAAAGTA
ACATGAGCAGTTCTATCGACAAAGGCCACTGTCCGGGAGCGATAATCAATGATTCATCGA
TTGCTGCCTCTCCTTTCTCTCAAGATAAGAATGCCTCGGCCTTTCATACGTCGTACGATG
AAGTGCAGTCAAACTCGACTATGCAAACCAATCGAGGTCAACTCAACAACGTGGGTGATA
CGCAAGGAAACGGTCACATTAAATCGAGGTTACCTGTGACAAAAGATATCGGTTTGACGG
TCAATGAACCATCTGATTACTCCTTTGCAGACAGTGGAGGAGTCTCAAACATTCCAAATC
GCAATTTTTCTAATTTTGGTATGAATAGGGTTGGAAAACCTGACGTGAATTTTACATATG
GCATTCAGCAAGACATGCAAAACCTCTGGTCCGACGCAGGTGGTAGCAGAGGTGGAGGGG
GGAATCTGAGTCCCACTGGTAAACCTCAGTCGTCCAATCGAACTATCGAAAGTATGGACG
GTGGTGGTGTCGGGATCATCGACGGAAACCGCCATCGTGGATCCTCACCACAAGGCATAG
ATGCGGAAAACGATTTACGTCCCTTTACATGGGATACTAATCAACATGAAACTAGTAGAA
CGTTGGCGATTTTCAATGCAGCCGCAATTCCGTGCAATCAAATCGAGGAGCTGTGCGACA
ATTTTGGAGCAATAGAGAATTTCCGCTCAGAATTTTGGGAACGCGTTGGTGTCATCTTCA
TCAGCTATTTTGATATGCGTAGCGTTCAGTTTGCGGCTATGCAGTTGCCGAGAAGATTGC
AAGGGTTGCATCCAACGGGCGGAAACATTCATATCAAATATTGTATTCCAATTAACTCGT
CTTCTCAGAACGATGAAAGTTTGGTTGTGATCAACGATTTACCTGGTCAAATCGGTCAAA
GCAACCTTGGGAGCTATTTGTCGCAATTCGGAGCGATACGCTCTCTGCGATCAGGAAGTT
ACGGATCTTTTATTGCAGAATTCAATGATCTCCACGATGCGAGACAAGTGGTTTACGAAC
TCGAAACTCGTCAGCCCTGGGGACCCGACGTCAGCATTGAGGTAGGTATTAGAAGTCCTG
CTGATCGAAAGAAGGGACGAGAACTCCTCGCTCTTTTAGGTCGTTGGCGTACCAAAGGTG
GTGCAGGGCAAGGAGCGCCACCAAACAGAGGAAGGCAACTAGTCAATACACCTGATACGT
ATCGGTCACGTCCTGACTCTAGAGGTATGGGGGGCCATAGAGATGGTAGATACGAAAGAG
GTATGCATAATGAAGACCATCCTTCACAGCTCGGTTATGGTCCAGGGCCTCAAGATGCGA
GATATAATCACGGTGGTAATACTTATGTCGTTCATCACAACCAACACCAGCATCCGAGTT
ATTCATCTTATAGAGGCTCTCATCACCCCAATGATCATATAGGTCACGGCGGACATGGCC
ATCATGGAACTTACATGAACCGTCCAGTGAGTCACCAGCCGTCACCTCATCAACAACAGT
ACTACCAACAGCCTCATATAAATCACGGTAATTTTCCTGGGGGCAGCTCAGTAATTTCGG
CTGGAAGTTCTCGCCATACAGGTACATCAGGATACTACACAGATGACAGGTCAATAGGAA
GTCACACTAGTCATGGTTCAAACATGCGAAGCATCAACTCCCTTGTTGACAGTATGGCAG
GAAGTTCTAGGGACGGACAAAGCCAGAATCTGATACTAGATTTGGAATTAGTCGAGAATG
AATTGGATAAAAGGTCTTCACTGATGGTTCGAAATATCCCCAACAAATACACCCAGCAAA
TGTTATTGAATGAATTTACAGAAAACGGCCACGGTCCTGGGATCATAGATTTCTTCTATT
TACCAATCGATTTCAAGAACCGATGCAACAGAGGTTACGCTTTCATTAACTTTGTGGATT
ATCGTGACATCGTGAAATTTCACCGCCAATACTATGGACAGCACTGGCGAACATTTAACA
GCGATAAGATTTGCGATATAACTTATGCAAGAATCCAAACCAAGGCATCAATGCTGAAAC
GTTTTGAAAACTCAGCTCTGATGGAGAAAGACGAAGAATACAAACCATTGGTTTTTGTCA
GCGATGGTCCAGAGAAGGGTAAGAGGCTCCCTTTCCCTGTTTCTTCGAGGGGGTAGCGAT
GTATCTTGTAGGCCATAATTCAGGATTCGTAATGATTCATCTACTGGAGTTGGCTACTTC
GAAGCTGTTGCATACGCTAGCCTGGATTTTTACATCATTTTTGATATTTTACGGAGCGGC
TGGCAATGAATAGTCATCGTGATAGTCAATGTCCAAGAGAATAATAGAATGTATTTGATT
TGCAGAATTCTGTTCGTTTTTGATAAAGAATAGTTTTCACTCAATAGCATTTTTTGATTT
CGAGTCGTATGGTGGGATGATACTGATATCTCAACAACTAATGCATCCCCGAACACAATA
ATGCTACAAATTTGTAGCCTAAATCTCGTTGGTATGCCCACCAATTTTCTTCGTAAAGGA
AGTCCAACTCTTGATCCTTCTACTGTATTCTTGTAGTTCATAGAGTTGGCATTTGACAAA
TGGTTTAAACTGCAATCCCCAAACACCCCACAAGAATACTCCTCTATTTTATTATTTTTT
TAATGATTCAGTGCCCATCACTTTTGAGCTTCAATTGACATCCCAGT
>comp2572_c0_seq1
AAATGTTTCGAGTCCCTTAGTGATCGAACGCCAAAATCTAACACAGAAACAACATTCAAA
CATGGTTTTTGTACGACGAGGCTTTCGCACAATCGGATTACAATGATCTTCGGCCTTCAC
CAACGAGAAACCGAACCACTCTGATACAAGCATACATACATGCAACACTTTTCTATTACG
GTGGAACTTGACGAGCTCTATCTTCCACCTCTCCTCAAGCTCCAACTTCTCTGTTAGAAT
ACAGAATAGCCTAGAACAATATCCTAGAACATTGCGATGATGCTAACGTCCCGTCAAAAA
ATACGAAACTGGTTCATCGTAGTATCTTTAGTGACGAAAGAAGCAAAGATAGCAGCAGTC
GGCGCATCGTCACTGGTTGGCTCTGCTTCTCCGAATCGCCGACACTCACTACGCACAAAC
ATGTGGGCCGATGAAGATGGCAAAATCTCCGGCCAGCAGCAACCCGAGCGTTCTCTTCAG
GCATACACAAACATGACGGCTCAATACGAAGACGGACCTGCCGTGAATCAAGATCGCGAA
GTCGATTTCGAGTATTGCTATGGGGCCTTGCAACGCGCCGATTCTAACAACGATGGCCGT
CTCGAAACAGCCGATTATTTATCTTTTGTTCAGGACTATGGATCAAATGCTGAGTGCTTG
GGTAATATCATGTCGTTGCCTGTCATGCCAATTGAACTAACGGCGTGCTGGAATCAACTG

AGCTGTGAGTGCCGAAATCGCGGTGGTGCACCGGATTGTTGCGAGAAAGAAAACGCCCAT
TTGCCTCTGAATGGAGTCAACGCTGTAGATCCAGCACCCTCTGGACAGACAACACAGCAA
CCGCCAAAAGAAGAATATATCCAAACGGAACAAGATTTTTTGAGGCAGGCGTGTGTAAGG
ACGGACCAGTGCATCATTTCATTTTGCGGATACCCCCCTCCACCCTTGCCAATTCCGCCG
CCGGTCCCACCAACTCCTCCACCGACAATTCCTCCTGTACCAGTTGAGAAAAATCTTGAA
GTGGAGACCAATTATTTGTGGTTGCTATTCTTGCTACTTCTGCTTTGCTGCTGCCGCAGG
CGCTGGATATTATGTCCTGTTGCAAAAGATGACGACGAGGAAAACCAAGAGCGTGCTCCC
GATGATCCAGCTGCCATGGGTAGCCTTCCCGCGGATATCGAAAACAGGCCAGATGGTTTC
GAAGGGGACTACAACGACCCTGATGCCAATAAGATGAGCATGAAATCAACGGCTGCTACT
GAAGTTGGCGAAGGGGGAGGTATTCGGTACTACAGAGTGGTACAAGAACCGGAATACGAA
GAACCTCCTCCACCTCCTGAGCCGAAGATTCTCGACCAATACGATAAAGGAGTGCCAGAA
GGTGATGGAATGAAACTAAAACACATCGAAAAGGACGGGCCTCCTCCCCCCGAAGATGAT
CCATACGCATTGGAACATTACGAACCCGATGGTGGTGTAGTGGAACACAAACGAAAAGGC
AAATGGTCCTTTTTAGCTGGCGGGGGCTACAAGAAGGAAGAACGAGCCAAGAAAGAAAAA
TCAGATTGGAAACGCCCCGGGTACAATCGAGCTGCGAAGAAAGAAGAAGATGATCCGGAC
AATCGTCGTGATCGAAACCTAAATAAGTACGAAGGAGGTGCCATATTTGACCACCTGGAC
GAAACCGACGAGCCAGCTAAACGGGGTGCCGGAAACTCCCTTGAGTGGGTATTTTCGAAT
ACACTCAACACCCTCGAAAACAACGAAGATGATCTCCGATCAAATCCATCGAGTTCGTCC
CAGAGATCATAACTTTGAATCAAGATGAAACCGTTCTTTCAGGCAAAGCAAGTTGTTCGG
TTGATGGGATATGACTGGAGTTAACGTAAGGCGACGATTACGTGGGGAAATTGACCAAGC
ACGAATGAAGAAACCCACGGAAGCCACGAAGAATCAACTGGGAACGAAGAAAGCTCGACG
CTTCGTTCTGAATATTCTTCGTCATTCATGTATAGAAATGTATTTTTATCTGCGGTGGCG
CCCTAGTAAGGTGTTGTCCTAACTCCACGCAGGAGGCTCAGCTCAGACTCATCTGGCCTT
TCCTTCTCTTCAGAGTTTAAATTAGATATATTGCCCTATTTGATTATAATATATATCGTC
GTTCCCAGATCGAAGTACGTACTCTTCCGACTCGAGAAGAAGCACTATTTAGATGAAGAG
TGAGGTGAGCGATTGATAGGTATGCTAATAGAAAGAGTTTCAACCACATTGATAGTAGCA
CTTTAAAATCGGCAGGAAAGTAC
>comp2573_c0_seq1
AACGATGAGGAACTCAACAAGCTCCTCGGAAACGTTACTATTGCGTCCGGAGGTGTCTTG
CCCAACATCCACGCTGTCCTTCTTCCTAAGAAGACAAAGATGAACTCCAAGGCCTCGCAG
GACTTCTAAGCGATTATCAGTCAGTTGTACAGGAACAACACATTGCTACCGCCGCAACAA
ACCAAACCAAAACCAAACCGGAGATTTTAATCTCCAC
>comp2574_c0_seq1
ATGCACCGTAAATGAAAACATTTTATTGCATTTTCTCCCATAGAAGATAACATCATACTG
TATTTTTTGTGAACGAATTTGGGTCGAATAGCAAATAATCATGCCCAGCTTGAAAAAGAA
AAAGTCAAGCTACGAATCAACAGGATTCGTACGACGAATGCTAAAAAAGGGTTCTAGTTT
TAGATCACTTAACAATGGAAGCGGAGACTCCGTAATGAGCTCCTCGAGCGGTAATCAGCC
GTTTGGTAGACTCGCTGGAAATGAAGGAAGCAAGTGGAGGCCCCGAAAAAACCAATTTGA
ACGACTAGATGGCGATGACGATGAAAGTATAACAGCAGGTTTGGAGCTTAAGAAATCTTT
CTCTTCTTCGTGGGGTGTGGAACACATTTCCACATTTCCTTCCACAGAAAGCGAAAGTGG
AGAGTACAAGAGAGACATCATGTTCGACAAGAAGGCAACCGCACTCATTAATCGAACCCC
AAGCAAGGACAGGGATAACAAGGGCAGTAAAAAGAGAGAATTCAGTCCCATCAAATCACC
TGGAAAAGATACATCAGTGATACGAGGGCCAAGTTCTACAAAGAAGATTCAATCCAAGAT
ACAGGAAGAGGAGATTAAATGGGGCAACAACGATAATGCAGATGTGGTTGGCTTTGGTGA
CAATAAGACGACTGATGCTAACCAGCATTCATTTTTCCCTGTTGCTTCATCCGCTGCAAC
TAATTCCGCATCTAATTCCTTTGGTTTTCAAATCAACCCAATTGGCGGGCATTCTAAACA
AAACGTAACAAAGAAATGTCATACCATTCCTTCTCGTGAAGAACAGTCATCGGCAAATCC
GCAATCATCGTACAGCTCTGTCGCAAGTGATCCGACAGATTTCTTTTCCAAGGAACAAAC
GACAAAGCTAACTATGAGTAATCTAGCGAAGATGGATTCATCATTAGATAACACACATAG
TATCAGCGCTGGTGAAAAAGAACCTGCGATGGGCGGTATTGAGAGAATGGTCAAAGAACG
CAAATTTTATCAATTTACCATCGACGAGGACAAGGCCTCAACCGTGGTGGAATCAACATT
CCAACTTCCCGCCGACTACCCATACGGAGACAGAAACTACAGCATAGACGATGATGAAAC
CCAGTTCAGCTGCAGTGAAACTAGCCAAGAAACGAGAATCCAACAGCGTTCAAAGACGAA
TGAAATTGTCTCTAAAACTTCTGAAATTGTTACCGATGATAAATTGAACCCCATCCATAG
CGGCCAAAATGCCGATGACGTGAACGAATTCGATGCCTTCTTTCCAGGTACTGAAGGCGA
CGAAAAGGACGGGCAGAAGGAATTTGACGCTTTCTTCCCCCTTAGCACCAAGAAAGATAG
CTTCTTTGATGAGTGCTCTTCAAACCAACAAGAATCTCGACAAAAATTAGCAGCAACTGA
ATCTGACATCTCGTCTTCCTGGGGTGATGCGTATTTTGAACGTAGCTACAAATCGTCGAC
TGAGTTTGAGGGACGTTGCAGAGCACAACGAGGTCGTTGTGATGAGCGCCGTGTACAAAA
AGCGAGCACTAAATCTCTTGCGTCACAACAAGTCAGTATAACATCTCTTTCCTATAGTCC

TAGCCCTGTCCATAAGGCTAAAAGTACTGACATCCCTCAAAATGGCCGTCAACCGTTGAC
ACATAAAAATACTCAATCAGGCGCTGAGAACAATAATATAGTGCCGTCTGGAAAGAAGGT
GGTCCAAGGGAACGATTGGTTCAGATCGGAAAACTCACAGAGGAAAAGTTCCGAATTTCA
ATTCGACGATTTTGTTGCTCGTCCACCATCATTTAAGAAGAGAAATTCTTCTATTGCATC
CAGCAACATTCAGTTGCCACCATCTGACCCTTTCGAATCTCTTCCGAACGGAGCTAGTGC
AAAAGAAAACTTCGCTGTGCGATCCAATCGACCTGAAGATTTTGGTGGTGACTGGAGAGC
GTATCAGAAAAATCAATATCCTATTCATCGTAGAAAGTCTTTCGAAGAAGAGGACAAGGA
CGACGACGACAACGATAGCTTCGACGATGTTGGTCCTTGGGAAAAGCAGTCCAGTTTTGG
TAATGGAATTTCTAGAGCCCAACTAAGAAATTCTGGCAATAGATCTCGTCTTTCTAGACA
CAATTACTCCCCTGCCAATCGGCCAAGCAGAAATCAATACATTGACGATGGCTATGTAGA
CGATGACAATAGATCTACGGGTGGATTTTCGTTTGAAAAGCCTTCCAGATCGTCAGCTGG
TGCTGGATCCTATGCAAAACCCTTGGCATTACCGAGCAACGCTATTGTGGCATCGATGCT
GTTTCGAACGCACTATGACATCGATCAAAACGATGTTGAAGAAAAGATAAACAAATTTGA
GGAGGAAAACTCGAAGCAAAGAGAAATTCGTCATCTTCATGGTGATATTCCCGACGACGT
GCATGCAGATACAGAAAATATGACTACTGTTTCGTCCTTTTCAGATGCCACGAGTGCATA
CTTCCAGGAGTCTTGGAGGAGACCTTCCCGTGACTTGGTCAACCATTTGAGCAGTGCTCG
CGCTCTTGACATGGACTATCCCTCTGCGCGACGCTTTCCGACAAGGCAAGTAACACAAGC
AGAACAACAGTTACCGCCGGGATTGTATGAAGCCTAGTGAATCTATATCATGATAGATCA
GCATAACGATACACTATAAGTGCATGGTACAACTCATGCTGGTCGTGAGGTTGCATTCTG
ATCGGTGACAGTACAAGCTGTCACCACAAAAATTCCTGCTGATACAAAAAAGAGAGATAT
GAAAAAATGAGATTACGTGTCGTGTATTTTTTTGCTGAATATATATTATGTACTATTAAA
TATTTGAGTATCAAAGCATTTTGTGTAATCACAGTGGATCGTGCCATTCACGATACAGAG
AATTGAACTCAGAGTCTACTATTGCATGGGGCTAGGGTGCTTGACCATATATCTCATCCT
GCCATCACA
>comp2575_c0_seq1
GCTTGCTCAAATTCAAGAAATCCGATGCCAAACAGCCTCCCTCTCCTCCGACGCCGCTAG
AGCCCACCCCGACCAACGCAGAAGCCGGCAAGGTAAAGGGAAAGGGCTTGCTTCGATTCC
TGGATTCGAAAAAGGAGGAACCTCCATCAGAGCCGCAAGAACCTGCGCCCAAAACAGGCG
TGAATCACAGTGAAGCAAGCAAGAGCAAGGGGTTGCTCCGATTCCTCGAACCTCGAAAGG
AGGAATCTCCTTTGAAACCGGAAGAAATTCCTACCAAAAAGGGTTCGAATCACAGCGAAC
CGAGCAAGGGCAAGAGCTTGGGCCAATTCCTGGAATCTGGAAACAATGAAGCTCCATTGA
AACCGATAACGACGACGAACTCTACTCATAGCCGATCCACGCAGAGCCGATCAAATTACA
GTATTGGAAGCAAAAGTTCATCTGGAGGCACCAAAGAAAGTGTTGAGGAGCTTCCATCGA
AGTCGCAATATCCTCCTCCCAAGTTAGGCTCAAAGAATCCCAACTCTGGAGCCGGCGCAC
CAAATTCGCCGTGGAAGCCAAAGTCCAGGGACGATTCAGCCGAGAGGCCTCATGCTTCCT
TTCTTGCGAATGCTGGAAAGCCAAGGAAGAGAAGCACCGGCACAGTTCCTGTTCTGGAAA
CGATTCAGGCCGACAAACAGAAAACCAATTTCGTGAAATTCGCAGCTGAAACCAATTTTG
CGAAATTCGCAGCTGCAACCATAAGCGAGTCCGATGGAGGAACCAAAAAACAACCCAACA
GCAAAAAGGTGAGGAACTCGGAGAATAAAATCATAAAATCCATCACGTTGGATGACGGGG
CGGAAATGCATTGCGTTTGCGCCATTATGTGAATTCGGTTTGCAATTCGATTCGATTGCA
GTCAATTCCATTGCATTAGTGATGTCGTGCGCACAGGTGTGGCCTGGCAAATCGAACGAC
TCATTCGGGTACAATGAACAGCACTCAAACCGGTTCCCCGTATCGTGGGGGGTGCCGTAC
CGTACCGTACGTACCGTACGAGAATCTCTGTTGGAATATTTGTTCGTTTAGCATGTTTCG
ATCTATTTATCTGTAGAGCATGACTATCGTAAAGGCTAGCTCGGTCCTGATTAGAACCTT
CCCTGGACAAAAACAGAAGCAAAAACAACAGCGACTACCACTGCCACTATGATTCTACAC
TCAGTGGCACCACAATCATGACAAAACCTGCCATCATTACTTTTATAAATATATGTAAGA
TATAGGTGTCCTTGTACAACACGATCTTTCCGATATCGAAGCATTCGAAACCAAGGCTAC
CTACTTCTGTCTCTTGGATCTTGTTGGTATCCGTTTGGCTAGTATTGCTACGACGAAAAA
ACGTAGTACATCTTGACGGCAAGGCATAAATGCGGAACTCTTGGACCTTATTGCTCTCGA
ATTACAAAGATTCGCCCAAAAAAATCGAATCTGCTGGCGATTTGAGAAAAGTGTATCGGA
CAATTGCGTACGATGATGCTAATTATATATTCATTCAAGTTACGCATTGTCAAAATATTC
GTGCGTCTACTATCTTTATATTATATTGATAATAACCGTATATCTTGGCGCTTAACGTCC
>comp2576_c0_seq1
GGACATTGACTACACCCACCAGGGTACAATTTTCAGAAAAAAGAATCAATACCATGCTGT
TCAAACAAGCAACCACCTTACTTGGTTTCCTTGCTGCCGTTGGAACTACAACGACGGCCT
TTACTCCATCGTTCGGCGTCTCTCCTCGCACAGTATCTAGCCTCCGTTCCACCACCGAAG
ACATCGAAAAGAAGAATGTAGAACAAACCGAGGACACCTCCCGTGAAAAGGTCATGACCT
TCAGTTACGACATGTCTACAGAAACCAAGTACGAAAAGCCAACCTACAAGGGAACCGGTA
ACGGTTTGTCTGGAGACTCTGGGGAATACGACGTGATCGTGATTGGAAGTGGTATGGGAG
GATTGACCTGCGGTGCCCTTTCGGCGAAATACGGCTCGAAAGTGTTGGTTCTAGAATCTC

ACATCAAGGTTGGAGGATCTGCTCACACGTTCACCCGTGTCCACAATGGCGAAAAATACA
GCTTCGAAGTTGGTCCTTCCATTTTCGAGGGACTCGACAAGCCGTCGTTGAATCCTTTGC
GAATGGTGTTCGACATTCTCGAGGAATCCATGCCGGTCGAAACCTACAAGGGATTGGGCT
TCTGGACTCCAGAGGGATACTGGCGATTCCCAATTGGTGACAAAAAGGCTTTCGAGGAAC
TTCTGATGGAGCAATGCGGTGAAGATGGACCGAAGGCAATTGCTGAATGGAACGCCTTGA
GAGAACGTCTCAGAACCCTTGGAGGATCCACCACCGCGGTCGCACTTCTCAATCTTCGAC
AAGATGCTGGATTTCTCGCCACAACAGCGGGCGCCCTTCCGTTTGTTGCCACCCATCCAG
ATGTCTTTGCTGATTTACCTTTGACGTTCGATGACTTGTCCAAAACAGTTGACGAAATTG
TCACTGTCCCATTCGTCCGCAATTTCATCGATCTCATGTGCATCTTCTGCGGATTCCCTG
CAAAGGGTGCCATGACTGCTCACATTCTTTACATTCTCGAGAGATTTTTCGAAGAAGATG
CTGCCTTTTCTGTGCCTGTCGGTGGAACTTCCCAGTTGGGTGAAACCATCCAACGGGGAC
TGGAAAAATTTGGCGGAAAGCTTCAACTCAATGCCCACGTCGACGAAATTCTTATCGAAG
ATGGACGTGCTGTTGGAGTTCGTCTTAAGAACGGAAATGTCGTCAAGGCGCGAAAGGCTG
TCGTCTCGAATGCTACGCCATTTGATACCGTCAAATTGATGCCCGAAAAGGAGGACGAAC
CCAAAAAGCTTACCAAGTGGCGAAATGATCTTGGTACCCTGCCTCGTCATGGTGCCATCT
CCCACTTGTTTTTGGGGATTGACGCGAAGGACTTGGATCTTTCCTTTATCGATGACCCTG
CCCACTTGGTTGTACAAGATTGGGATCGATCGATGCAAGATTCTCAAAATCTCTGCTCGT
TCTTCATCCCATCCCTTCTCGACAAGTCGGTTTGCCCCGAAGGAAAACACGTCATCCATG
TTTACTCTTCCGGTGGAGAACCCTACGAACCATGGGAGAAGCTTAAATCTGGCTCGCCCG
AATACGAGGCGTACAAGGAAGAGCGTGTCGAGGTCTTGTTCAGGGCTGTTGAAAGATGCA
TTCCCGACATTCGTGACCGTCTTGAATTCTCGATCATCGGAAGTCCTCTGGCACACGAAG
CCTTCTTGCGAAGAGATCGCGGTACATACGGTATGGCGTGGGCCGCCGGTTCGTCCGCTC
CCCAAGCTGGACTGTTGGGCAAGGTCTTGCCCTTCCCCTTCCCTAACCTGAAGACACCCG
TCGATGGATTGCTGCGATGTGGTGACTCTTGCTTCCCTGGAATTGGCACCCCGTCTGCCG
CTGCTTCGGGTGCCATTGCCGCAAATACTATGACGCACGTCGACAATCACATCCAACTCC
TCAAAGAGGCCAGCCAGAAGGATCCGATGTACAAATTCTTGGACCCGGGATTCATGGCCG
AGGTTTACAAACCTTTGGTCCAGGGATTCACACCGAGTGCCGAGCTGAGAAACGAACGCT
TTGCTGCCGGGGCCGGAGTTGCCCCTGTGAACCACACAGCAAATAACAAAGCAAGAGATG
CTGCCGCTGCGGAAAAATAGCTGAAACACTAGTCTTATTTTAATCTATAGGTCGAACGCG
GTAATAGCAGCTCTG
>comp2576_c0_seq2
CAATTGCTGTGAGTAAAAAAGTTCCTGCGCACTTTTTTTTGAATCGTTGTGGAAATAGTG
TTGGTAACTTTTCGTGTTTGACAGTGAAAGAAATTGAATATTATTTGCTTACATTCCTCC
TTCGCTCAAATCGGAAAAATTGAATGTGAAACGTTCTAATGCCGATTTCAATGAAACAAA
CTCAACAACCACAGGAATGGAACGCCTTGAGAGAACGTCTCAGAACCCTTGGAGGATCCA
CCACCGCGGTCGCACTTCTCAATCTTCGACAAGATGCTGGATTTCTCGCCACAACAGCGG
GCGCCCTTCCGTTTGTTGCCACCCATCCAGATGTCTTTGCTGATTTACCTTTGACGTTCG
ATGACTTGTCCAAAACAGTTGACGAAATTGTCACTGTCCCATTCGTCCGCAATTTCATCG
ATCTCATGTGCATCTTCTGCGGATTCCCTGCAAAGGGTGCCATGACTGCTCACATTCTTT
ACATTCTCGAGAGATTTTTCGAAGAAGATGCTGCCTTTTCTGTGCCTGTCGGTGGAACTT
CCCAGTTGGGTGAAACCATCCAACGGGGACTGGAAAAATTTGGCGGAAAGCTTCAACTCA
ATGCCCACGTCGACGAAATTCTTATCGAAGATGGACGTGCTGTTGGAGTTCGTCTTAAGA
ACGGAAATGTCGTCAAGGCGCGAAAGGCTGTCGTCTCGAATGCTACGCCATTTGATACCG
TCAAATTGATGCCCGAAAAGGAGGACGAACCCAAAAAGCTTACCAAGTGGCGAAATGATC
TTGGTACCCTGCCTCGTCATGGTGCCATCTCCCACTTGTTTTTGGGGATTGACGCGAAGG
ACTTGGATCTTTCCTTTATCGATGACCCTGCCCACTTGGTTGTACAAGATTGGGATCGAT
CGATGCAAGATTCTCAAAATCTCTGCTCGTTCTTCATCCCATCCCTTCTCGACAAGTCGG
TTTGCCCCGAAGGAAAACACGTCATCCATGTTTACTCTTCCGGTGGAGAACCCTACGAAC
CATGGGAGAAGCTTAAATCTGGCTCGCCCGAATACGAGGCGTACAAGGAAGAGCGTGTCG
AGGTCTTGTTCAGGGCTGTTGAAAGATGCATTCCCGACATTCGTGACCGTCTTGAATTCT
CGATCATCGGAAGTCCTCTGGCACACGAAGCCTTCTTGCGAAGAGATCGCGGTACATACG
GTATGGCGTGGGCCGCCGGTTCGTCCGCTCCCCAAGCTGGACTGTTGGGCAAGGTCTTGC
CCTTCCCCTTCCCTAACCTGAAGACACCCGTCGATGGATTGCTGCGATGTGGTGACTCTT
GCTTCCCTGGAATTGGCACCCCGTCTGCCGCTGCTTCGGGTGCCATTGCCGCAAATACTA
TGACGCACGTCGACAATCACATCCAACTCCTCAAAGAGGCCAGCCAGAAGGATCCGATGT
ACAAATTCTTGGACCCGGGATTCATGGCCGAGGTTTACAAACCTTTGGTCCAGGGATTCA
CACCGAGTGCCGAGCTGAGAAACGAACGCTTTGCTGCCGGGGCCGGAGTTGCCCCTGTGA
ACCACACAGCAAATAACAAAGCAAGAGATGCTGCCGCTGCGGAAAAATAGCTGAAACACT
AGTCTTATTTTAATCTATAGGTCGAACGCGGTAATAGCAGCTCTG
>comp2577_c0_seq1

GTGGTACATTCTACCCCACCCGACGATGGCAACCCTGGTTTGATCCAATCAACTCCATTC
GATCCAACACCTAACACAGTTTCCCGCACTAGCCTGAGAAGCAGTTTCCCCTCACTAACT
GGAGAAGCAGCGCATCCAAGATGATGTCAACTCTAGATACACTCGACATTGACATCGGGA
GCAATCCAAATGTCTTGTTCGGACAGCAATTAAATACTAATGTGAATATAAATAGTGATG
AATATGCACGGTGCAACCGGTGTGGTTATGGCGGATGTGATGTTCGATTTCTTAGTTGCG
GCTGTACCCTACATGCGCGATGCATGGTACTCAACTCCGATGGTCCCTTGAAGAACTGTC
CCTCTTGTAGCCGCCCGGTTACCGGCATGTCACTCTTCCCAATGTCCTTTCGGGAGATCG
ACGAGGCTCGCAAGAACAGCCAAAGCCTAGCGAACGCCAACCGCGGGAAGAACAGAAAGC
GCAAGGGCGCGCCAGGACAAATAAGTGACATTGACACCGTGAATGGTGTTTATGTTTCTG
ATCGTCGCACAGGTCGATGGACAACAGAAGAAATGGCCTACTGTGATAAGCTGATCGCAA
AATTTGAATCTGGGGAACTTCCAATATCTTCGGGTCTCAAGTTGAACGAGTTCCTTGGAA
ACATGTTGAAGTCCAAGCAGTCTCGTCTAACAAAAAAGATGAAGAACGCGAAGTTGAGTT
CGAGAACATTCCAGCGTTGTGCACCCGGCATCATGGACAACAACGAGGTTCGAGAGTTTT
CCGAACTCGAGGACTTGTTCTTTCAATCCATTCAATGCCAACAGGAACGTGCAGAGATCA
AGTTCCACATGCAGAGAGAATGGCGCGAACAATTTAGCCTCTACTGCTCTTCCGTTTCCC
AACCCCTAGATGCCGATTCTTGGTTAAGCAGTGTGGAAGAAATGGACAGACGATCTTCGC
ACGCCAAGGATGCTGCCAGGATGGCGAAGCGAAAACTGATGATGGGTTTCGCACTTCGAA
CAGACAGCATGAACCCCTGCAACGGAGTGATTATTGAGAAGACACCATCTGATCAGCGCA
TTGCTCTCGGTACGCACGGTATCGAAAACGATATCGATGAACAGAAAGAGATCCTTCAGC
TCCTGACCGAAGAAGCAAATCAGATAGGTACCACTGACATCAACGGAAAAAGCAGCCTTT
TGCATTCCGCTCCCTTCTTGGCGAAAGCCGTTGTCTACCTAAAAAGACACGATGTGCCTT
TTGAGCATGTTGAGCTCTGGGTACCTTCCTTTGTCCCTCAGGCGAACGGACAAACCGCTG
ATGGCGCCGCTTCTAACTGCCGCCTGTGTTTTGCTGGTAGTGCCACAGTGGACGAGGTAG
TTGAGGAGGGTTCTAAAACAAAGAAACCCCTCTCTCAGCAAAGTCAATTTAGTTTGCAAG
CCTTCGGGGATTACAGCCAAAAGTTTTCCTTCGATGTTGGCTGTGGTCTGCCAGGACGCG
TTTATGAAAGTGGGCGTCCAACATGGGAGCAGAGTGTTCAAAATGCACCACACCATCATT
TTGAACGCTGCGGGGGAGCTTTGCAGTGGGGTATTAAGACCGTATTGGGAATTCCAATTG
CGAGTCCAAATGTTGGGCGAATCGTCGTGACACTGTATTCGTTGCACGATCGTCCCAAGG
ATCAAGATCTTGTCGGGCGATTATCGGAGGAATTTACCAGACTTCTACCTTCCCCAAAGT
GGAAGCTGGTGGTTGATATCGGTGAACCTCCACCTCAACCGCAGCAAGTCGGATCCACAG
GGACAGGGTCATCCGCTGACATGAACGGAGTGGACAAGGGCTCAGAAACCGTAAAGGATG
ACACTGGGCGTGATCCACGTATAGACGATGTTGTCTCTCTTCTTGGAGAGCTCATGCCGT
CAGATCCAAACTCTCCATTAGCACCTTATCTCTCGGGATTCATGTCCTTGAGATTGCTTT
TGCTTCGATCTTCTCGATCACCAGAGGAAGAAGATCGAGCACGTACTATGCTTGATTCGT
ACACATCCTACTCACAGGGTGCTAGATCAAAGGCGGATATAGGGATCATGTTGGCCCGCG
ACTACATGTTCCTATCACAGAGCAATGGCGCCACCAATAACACAATTCAACAACCACAAC
AACAACATCCAATCCAACTTCAACAGCAACAAGCCAATTTTTTTAATCTTGGGCAGGGTA
GCTCTGCAACTCTGTCCGGCAATTCTCTCGAGTTTGATGTTCACAATTCACCTATGCTTG
GCCCCATCTCTGCCAATGACACTTTATCTATCATCTCGAACTAATTTTTTTGATGACGAG
TCTTCACCAGTTTTCAACTCGAGCATTTTTATTTTGCTTTGTGATACATCATCCATTTTC
CATCCATTTATCGATTCAACATTTTAATTTCGTGTTGGCCAATTTTACAACAATCCATCT
CACAAACGGTTACGAAGTGTTACGAGAAATTTTATTTATAATTTTACATCTGAACTTTCA
ATTTTTCCTTTCAGATGATCAATTGATGTACTAATTTTAACGAACAAACAAATAAATCCA
ATTTTATTTTAAAAAAA
>comp2578_c0_seq1
ATTTCGAATTAGCCAGCGCTGAGAAACCCACTTGGTCGAGAAAGACATCTTTAGACTTCA
CATTGAAAGGCAGCTTGTCGTGCTAAGGTTGAATAGTAGCCTAATTGCTTGTTTCTGCCT
CGCTCTCCAACAAGAACAAAATGCTGTCGACACAGAAAACTTGCATTCTGTGGGGAATCT
TGCTCTCTTTGAACTCGCTTCCTGCTACGGTTGGTGGCTTCGATCTACCAAAGACAGCAG
GTCGATCGAATGGCTCTGTTCCTGGTCACAGGATAAGGACACCCAGTTCCACGTCGCTGG
AGTCCGGCGATACAAACATTTCGACCAAACAAACCGGGAAAATGTCGCCTCGCAGCAGAC
AGATTGATGAGGATTTGCTTAGCCAATTGAAAGACGTCGAGTCGGGAACGGCTGCTCGCA
GGTTGTTGGAACGTGCTTTATTTCTCGATGCAGACGGCAATAATGCCAAGCGACCCTTGT
TCGGATCCAGTCAAATCCCACCCGGCCTCTCCGACAGAAGCATATCCGACGGAGACCTCG
CAATCCAGACCAAAGTACGAAACATGAAATACGGAATTTTTGATCTGATCGACACGAACG
GGGATCGCGATGCAGATAGAGCTTCGGCCGCTGTGTTCGGCACATTTGTTGCTTCGAGTT
TGAGTGCAATCGTGGCGAACGAGAATCTGCCTGGGCCGGAGATTTTCCGATTCGCAGTTG
TATGGATACTATCTTTCGCACCACTTGCACTAGTTGGCTTTGGCATTGGTTCTACGGATA
AGTTGCAGGCATTTCTGGTTTCGGTCCAACGCCAAATATTTCCTGTTTACCGCAAACGAA
TGGTTCAACACGAGGCAGGTCACTTCTTGATAGCTCATCTTCTCGGATATCCAATCGCAG

GATATTCTACAAATGCAGTAAAAAATGCAGTCGAGTTTTATCCTTTGAGTGATAAGGACG
TAGGGAAAGACAAAGCTCGAATGTTGGGGTTCGACCGAAACATCTCCCGCGGTGATGAGA
GCCAAACAGAAAGGGCGAGACCACGTGTTAATGATGCCCCTTTCTTTAGTCCCGAAGGAA
GCGGAGCGATGGACCTTGAAACACAATCGGTCTTTAGAAACAAGAAAGATTACAGAAACA
ATCCGTTTCTCAAAATTGCAACGGAGGATCAACCCAGGAATTCTTGGCCGTACCGCGGAT
TCGACCATGGAACTGTGGACAAGTTGGCGGTTGTTAGTTGTGCGGGTGTTTGTGCTGAGA
TCATATCCTATGGATATGCAGAGGGTGGTCTTGCAGACATCAATCAACTCCAACAGATTC
TCGCAAATGCGGAACCAGAACTCACTGAAAGAGACAAAGAAAACAGGATACGGTTCGCCA
TGGGTTACACTATGACTCAACTACGGCTTCATTTGGGCTGTCTAGATGCCCTTGCAGATG
TCATGGATAGAGGTGGAAGCATTGCCGAATGCGTATCGGCGATCGAAAGCTGCACGAACG
TGAGTGGAAACAACGGGATAATTGGAGAGGACTACGAGGTCCGGCGGAGGAAAGAACTGA
AATCTTCGGCGTTGGGTATATTCGAAAAAATTCTTCTAAATCCCAAGAACGTCGATGCGG
AGGAAGACCGATTGGTACAGGGAGCTGGGGGTGGTTATCTAAAAGAAAAGCCCAGGTTCC
TTCAAATCAGCGGCGACGATCCTCTCTATATTGCAGGGGGCATTGCCTGCCTGTTTTTGG
CTTACGCGACGAGTGGAGGGCTTTCCTTGCATTAATAGCCATAGAGCTCGTGTTCTAGTA
ATATAATAGGGTACTAGGGCATAGCAATACTC
>comp2578_c0_seq2
ATTTCGAATTAGCCAGCGCTGAGAAACCCACTTGGTCGAGAAAGACATCTTTAGACTTCA
CATTGAAAGGCAGCTTGTCGTGCTAAGGTTGAATAGTAGCCTAATTGCTTGTTTCTGCCT
CGCTCTCCAACAAGAACAAAATGCTGTCGACACAGAAAACTTGCATTCTGTGGGGAATCT
TGCTCTCTTTGAACTCGCTTCCTGCTACGGTTGGTGGCTTCGATCTACCAAAGACAGCAG
GTCGATCGAATGGCTCTGTTCCTGGTCACAGGATAAGGACACCCAGTTCCACGTCGCTGG
AGTCCGGCGATACAAACATTTCGACCAAACAAACCGGGAAAATGTCGCCTCGCAGCAGAC
AGATTGATGAGGATTTGCTTAGCCAATTGAAAGACGTCGAGTCGGGAACGGCTGCTCGCA
GGTTGTTGGAACGTGCTTTATTTCTCGATGCAGACGGCAATAATGCCAAGCGACCCTTGT
TCGGATCCAGTCAAATCCCACCCGGCCTCTCCGACAGAAGCATATCCGACGGAGACCTCG
CAATCCAGACCAAAGTACGAAACATGAAATACGGAATTTTTGATCTGATCGACACGAACG
GGGATCGCGATGCAGATAGAGCTTCGGCCGCTGTGTTCGGCACATTTGTTGCTTCGAGTT
TGAGTGCAATCGTGGCGAACGAGAATCTGCCTGGGCCGGAGATTTTCCGATTCGCAGTTG
TATGGATACTATCTTTCGCACCACTGTAAGTTTTGATAACTCAATCGTTGCAAATACATC
GATGATACATTGTCGCTATGTCTGATCAAAATGCCCGTGAAACAACCATTCTCCGGCTGT
AATGTAGTGCACTAGTTGGCTTTGGCATTGGTTCTACGGATAAGTTGCAGGCATTTCTGG
TTTCGGTCCAACGCCAAATATTTCCTGTTTACCGCAAACGAATGGTTCAACACGAGGCAG
GTCACTTCTTGATAGCTCATCTTCTCGGATATCCAATCGCAGGATATTCTACAAATGCAG
TAAAAAATGCAGTCGAGTTTTATCCTTTGAGTGATAAGGACGTAGGGAAAGACAAAGCTC
GAATGTTGGGGTTCGACCGAAACATCTCCCGCGGTGATGAGAGCCAAACAGAAAGGGCGA
GACCACGTGTTAATGATGCCCCTTTCTTTAGTCCCGAAGGAAGCGGAGCGATGGACCTTG
AAACACAATCGGTCTTTAGAAACAAGAAAGATTACAGAAACAATCCGTTTCTCAAAATTG
CAACGGAGGATCAACCCAGGAATTCTTGGCCGTACCGCGGATTCGACCATGGAACTGTGG
ACAAGTTGGCGGTTGTTAGTTGTGCGGGTGTTTGTGCTGAGATCATATCCTATGGATATG
CAGAGGGTGGTCTTGCAGACATCAATCAACTCCAACAGATTCTCGCAAATGCGGAACCAG
AACTCACTGAAAGAGACAAAGAAAACAGGATACGGTTCGCCATGGGTTACACTATGACTC
AACTACGGCTTCATTTGGGCTGTCTAGATGCCCTTGCAGATGTCATGGATAGAGGTGGAA
GCATTGCCGAATGCGTATCGGCGATCGAAAGCTGCACGAACGTGAGTGGAAACAACGGGA
TAATTGGAGAGGACTACGAGGTCCGGCGGAGGAAAGAACTGAAATCTTCGGCGTTGGGTA
TATTCGAAAAAATTCTTCTAAATCCCAAGAACGTCGATGCGGAGGAAGACCGATTGGTAC
AGGGAGCTGGGGGTGGTTATCTAAAAGAAAAGCCCAGGTTCCTTCAAATCAGCGGCGACG
ATCCTCTCTATATTGCAGGGGGCATTGCCTGCCTGTTTTTGGCTTACGCGACGAGTGGAG
GGCTTTCCTTGCATTAATAGCCATAGAGCTCGTGTTCTAGTAATATAATAGGGTACTAGG
GCATAGCAATACTC
>comp2579_c0_seq1
CGCAGATTAGACTTCCATGCAACATGTACTTATCGCCGAGCTTCGTTGCGGTGTCTTAGA
GTCTGTTTGTACCCTAGAAGGTTTCGGACGGAGGGATGACACTTCGTTGATCAACGAACT
CTTGCGGACAACCGCGGCCGAGCAACGCGAAATGATTTTCTTGGTTTTTCTTTCCTTTCC
TTTCCTTCCTTTCCCTTTCCATTTGTCACAAAGCCATCTAGCCATCTTGCCGAGTACAAG
AAAATTGCTTTTCTTTCCTTTCGTCACGAAAGCCATCTTGCCGAATTTAATTTATAAAGA
TGTACCTGCAAAGAATTATCCATCCGGCATTGCCTGGGTTCTTCTTCCTCCTGATTGCTC
GCTGTGGAGTGGCAACAGCCCAAAAACTCGCAGTGTTTGTCGGGCCCCACGAGACGCACA
CCGGCACGACCACTTTTCTTTCCAAGCATGCAGCGAGCGATGGAACGGGGGACCCCGAGT
TCAATGGTTGGAGCTGGCCTACCGTTCATGAAAATGACATAGATTCGAGTCCCCAGCACG

CCTTTGATTTTCTCGTCAAAGAACAAGACGACGAGGGCATCCAGAATGTATTGATCGATG
CAATCCGTTCTGCACGGAAAACTTCCCAGAAAGGAACGTTCATCGGTTCGCTTGACTTTG
ACAAGGTTGGAACGAATCCGTATTCGGGCTACGATGCTGTCGAAGCGTTGAAGAGAGTTG
TTGACGAATTGGGTGTTTCTACAGATGATGTCATCGTGGTTGTAACTTATCGTACGCCTC
GCGTCGATCAATGGGCCGATGTCTGGAAAAATCATTTTGAAGCAGAATCTTACCAAGATT
TCATTTGTTCCGACGAACAAAGCAAGAAGCGCTGGGAATGGCTTGATACCTCAATGAATC
CCTTTAAGATAGCAAAAGCGTATCACGATCGAGATTGGAATGTTCTAGTAGTTGACAAAG
AGAGCACGGTTCGTTCTGGTAAGGACGTAGCAAACGTTGTCGCTTGCTGGGCAATGGAGG
ACGAGAACTGCCAAGACGGATGGATCAACGGCTTCGATCGCTCTGTACAAAATATCACGT
CAACAGGCCATCTAATTGATGAACTCGAACACACAGATCATAGAAACCTAGAGAGGTTGT
TCCTGTTGCGTGATTGCTACAACGTGGGTCAGCTCAGCGGTTCTTCAAGATTTGTTCTCC
TCGATGATGGAAACGAAGGATTTTCATCGCTGGCCAAAGAGTGTAAAAACTGGTCTGCGA
ATTCATACAAAATGCTTTCCAATACGAGCTTCTTGATGAATGCCATCCAATCTCAAAAAT
ACTGTGAACAGGACGACATCGACGTTCCAAGTTTATTGGCGGAAATATACGAGTCTGACG
TCCTCGAAACCCTTGCATCACTTGGATCTAGTACGGAGGAAGACAGATCTTCTTCGAATT
CGGTCTCGGAGGACGATACCGAAAAGGAACCTTGGGTGTCTCCTGCAGACGATAATCCTA
ATAAACGACTGGTGGTATTTGTTGGTCCGCACGAGACAGACGCTGTCGGCGTGACCAAAT
TTTTCGTCGATTATGCATCGACAAGCGGAGATTCCGAGCGTTCGTCGAGTTTTGATGGTT
GGATTTGGCCTTCTGTCAATAACGACCTCATTAAAAAACCTCCCCATCGTGTCTTCGACC
TACTTGTCCAATATCCCTCGGACGAAGATATTCAACGCGCTGTGATCGAGGCCATCGTAG
ATTCCTGGAAGATCGCAACGAATGGGGTCATCATTGGTTCACTCGATTTCGACAAGACTG
GAGAAAATCCATATTCAGACTACGATCCGATCAAAGCCTTGAAACGCGTGGTTGAAGCAC
TCGGAATTTCAAGTAGCCAAGTCATCGTATCCGTGACCTACAGTTCGCCGCGTATTGATC
AGTGGAGTGCCGTTTGGAATAACCATTTTGATACAAATGGGTACGAGAATTTCGTTTGCT
CCGACGGAGTCGAATCCTCAAAACGCTGGGAATGGCTCGATACGGTTATGAATCCTTTCA
AAATAGCGAAAACGTATCGCGACCAGGGATGGGACGTTGTTGTGATTGACCAAGAAGGCA
CGATCAATGACGGCCTTGAGGTAGCCCATGTCATCGCTTGCGAGGTCATGAAAAGCGTGA
AGTGCGATGGCGGTTGGGTCACCGGTTTGAAAAGTGCTGTGAGTAACCAACTCATAACCT
ACCCGATGGATTCACTGGCAGAGGAGGATCGCTGGGATTTGGAACAAATCTTTCGAGAAC
GTGATTGCTTTTTCAAGTATCAGTTGAAGCATGAGTCGAATTTCAGTATAATTCACCAAC
ACACCGCTTGGAGTTCTTGCTCGAATCAACACCAGTCGTTTTACAGAAAATTTGCCAACA
CCGACTTTGTGCTGGATCTGCTGAGATCACAAAAACAGTGTGGGGAGAATATTGTCGATG
TTTCGAAGATGCTGCGGAAGAAAATGACCCCCAATGAGCAGAAAGAATTACTTATCTTTG
TTGGCCCACACGAAACTTCAGCGGTTCCCGTCACAAAATTTTTTGTGGATCACGCATCTG
ATAAAGGGGGCACCAACCACTCCCCTAGTTTCGACGGTTGGGCGTGGCCAGTCATTGATT
CCGAAATCCTTGGCGACATCCAATCGCATAGAACTTTTGATCTCCTGCTGTCGGATGCCA
GCAAACGACCAGTACAGAATATTATTCTGAATGGAATCCGTGATAGTTGGAACAATGCTC
TCGATGGCGTTATCATTGGATCATTGAATCTCGACAGGGTTGGCGAAAATCCTTATTCTG
GTTACGATGCCCTTGGAGCAATACAGCGAGTCGTAGAAAAGCTTGGAATTTCCGATGATG
ATGTGACTATAGCATTGAATTATCGTTCCAATCGCCTTGATCACCTTAGCGCTGTATGGT
GGAATCATTTCGAAGCAGAATCTTTTGAGGAATTTGTGTGTTCCAATGCACAAGCCGAGA
AACGATGGGAATGGATAGACACAGTTTTGAACCCTTTGAAGCTAGCAAATGCTTATCATG
AAGAGGGTTGGAAGGTTGCAATGATCGACCAAGAAGGAACAGCGAATGCTGGAGGCGATG
TAGCCCATGCTCTTGCATGCAATCTGATGATTGGTGTAGACTGTGGCACAGGACGGGTAC
GAGGATTAGAAAACGAAACGATAGACGTCCCTTCAACCTACAAAATTGAAGACTTAGATG
AGCTTCAGCGCACAGAGTTTGAAACATTGTTACAGATGCGTGACTGCTACTACAGGTACA
CATTGGAACAAGACGGCAAGTTTTTGATAGTCAACAAGGATGCACTATGGGGTTCGTGTT
CACGAAAGAACTACGCTCTTTATGAAAAGTTAGCTAACACCGACGTTTTACTTTATGAAA
TGAGGTCACTACAAGGTTGCGGAGATATTGAGCCGTCGATTTTTTCTGGGAATCTTTTCG
ATCGCGATACCCTAGCCTCTTCCAAGAGCAGTCGTACAGTGGTGATTGGATCGGTTGTAT
TTTCAACTGCAGTTTTTGCCCTCGGTGCTGCCTTTGTGTGGCAACTGTTAAGAAAAGGTC
GGGAGGCAAAGAGAAATGGAACGGAAGGGATATTCAGGGACGGCACACACGATGCCAGCC
AGGAACCCGCTTTCCACGACAGGAAAGAGAACGATGTGGAAGACACCAAGTACCAAGATG
GATTTGATGATGAAAACGAGGGGAGCAAGCCCGAAGCAACTCATGAAAAGGTGCTAGAAG
AATACGATGTGGACGTTCATGTTTAATAGTACGGTTCGAATTAGACGATATGTGGTGATA
GGCCGAGCTTTCTTGTGAGTCATATCGTCGCGCCTATATCTGGTTTTGCAATCAAATAAT
ATGAAAATAAATTTGATAAACTCAAGTTTCAGCAAAAGAGAGTATGGCAGGTTTTTACTA
ATTTATGTCACTTCCAGCCACCGAGTCGGCATACTAGTACTGCTAGTAGTAGTTGACAAA
CAGTAATTTCTTGTATCTTGTACTAGTATTAGCCATACAGATGGATAGTATTTGAGATGT
AATTTTGCTGCCCTCATTAACTGATCTTCTAGTGGA

>comp2580_c0_seq1
CTACAAAAGACTACCGTAGACTTCGCGACACTACACTAAACCACAGCATAGCAAAGCATA
GCATAGCAGAGTATAGCACGGCACAGCACAGCACACCATACAAGTACCATACCATACCAT
ACATTACCATACCGTTGCTGTTGATTCAACATCGTCACCACACCAAGGCACGACAAATCA
CGCAATAATAGTACGAGTACTGTATTGATTGCGTCGTTGTTCGCTCTGGGTCTGTTCCTG
ATAGCGAGTCCTCTGTACCACACATCGCACTGCAACAGAATACCGGATCTAGCTTCTGGG
TCTGTTGCTTCTGCTGTTTCTGTTTTCTGCGCAAGAGAAGAAGAATTGCGTCGGAGAGTA
CCGGTTACTGTTACGTACTCGTACTTTACCATATTACAAAGTACCGAAGTATAAGCATAC
CTTGTAGTGTTGCATCACATCGTGCCGCACCAACACTCGTAGCTGCAGGAAGACGAATAG
CAGAATCGAGGCGGCAAGGAAAGTGAGCACTTCGCGCAACGGATCCTTGGATTGCGAGAA
CAGTACCTTGCTTTTGTTTCTGTTATGCAGTCCGTGGTAGCAGTATACAGTAACAAACTA
GAAGTACTACTACGATACAATACGATCGAACACGCCTCATTACGAGCAAGGAGCGGAAAA
AGCATTATCGCATGCTGATTGTCGTCTGCAAACCCATTTCAGAGCGAGCAAGCAGAGACC
GAAACACATCACGCATACGCACATACGCATACGCGCATACGCATACGCATATAGAAAGAG
AAATAGAGGGAGAAGATCAGTCCTACAAGCAGATTGATCGTTTTTCTTGCCAGTAGGATA
TCCTTGTTAACGATTTCATCTTTCAAGAGAAAAAGGAAAAAGGCAATCCAAGTCGTCGGA
GGAAAAAACTCGAGACAAACGAGTGAAGCGAAGTGAAGTGAAAAAGAAAAGAAAATCAAA
ACAAAACAAAACCGAAGCGAAGCGAACAGTTGCTTACCCGCTACGAATCAAACACCCCGA
CCAGCAATACATTCCCTTATACGCAAGGGAAAACAGACGCAAACATGGCGGAATTATCAG
CCACACCTGCTGCCATTCCTGCTGCTTCAACGATCCCTGTAGAAGCAGAAGCAGAAACAG
AAACACAAGTGCCAAACGGAAAGGAAACGAAAGATCCAACAAATGGCACCTGCGATGCCG
ATGCAGATACAGAAGCAAGTGCAAATGCAAATGAAAATGTCAATGGGACCACAATGAACA
CGAATGCTCGGCTCCGTCCAGAATTGAAAGAAAAGATCCAGAGCTGGATTCTTAGTTTCT
ATCACCTAGATCCTCGTTACAAGATTCTTACGTTTTTTAACTTGGTCGCTTCGGAAGGTG
CCGACAACCTTACGGACGATCAGATCGAAGAACATTCGTATGCGATGATGACTACGACGA
GCACGACCGGCGGTGCCGCAGGTAGTCTAGGGAACAGCGTAACTTCACAGCAACGACAAG
CATCACCCACCGAGGAGCCCACCACGCCTGTGTCCGCCAACCAATCGTCACCTGCTTCTG
GCAAAATTCCTCCCGCCAACAGCACTTCCAAAGACAAAAACAACGGCAACAACAGAGATG
TATACAAGGTTAACGCACATCTGATGAGCCGGCCCAGCGCACGGAACATTCGGCCGTCCA
CTCTCAGCTTGCTGTCGAGAATCTTCAACGCTACATCTATTCTGACCGTCTGGAGACCGT
GTTCGAATGATGCCATGCGGAAAATGATGGAGGGGGCCGGTGTCGGCAAAGGTCTGGACA
TTAAGGGGAAGTCCGCCAAGAAGGGAACACTGTCAGGCTTTGTGCCCTTTATGCAGATCT
TCGACAACGATCACAAGAACGAGATCCAACCCATCCCGGTCAAGGCATTGATGCGTGTCT
ACTACAAGGACGAGAAACTCCGGGATCACGTGCTGGGGGTTCTCCAAACCTACCTAGAAG
AGGGGCCGCCGCTCAAAAAGAACGGAGGCAATGTACCTCCTCTGTCTCCCGTGGGAAAAT
TGATGGACGAACCAATCATTACCGACGAAGTTGTTGCCTTTCGGCAAGCACGCACCGCGA
TGCGTTGTTCCACCATCGACGAAATCACCGAAGACGACGATCTACACAACGAACAAGAAC
AAGAAGAAGGAACAGGCAAAGAAAAGAAACCTTCTCTTCCAGACCACAAGAATCCCAATT
CGTCGAAGTACGTTCCCCCTAAGCCCCTCAAGAAACTCAAGAAATATACCAAGGTCGGAT
TCTATGGAATCGAGTGCACCCAGCGTCTCTTTTGGCTCGCAACCGTCCAGGATGCGGATA
TTTCGAGATCCGGAACCGGAACTGAAACCGGCCGACCTTCCCTTCCGGGGTTTCAGGACG
CCAACCTGAAAACTCTCAAAGTGGCCCACACCCAGCTACCGAAACCAAACCCGATGCCCG
TCGTGTTGCAGTACAAATCCTACGAAGAAGTAGTCGCAAGCAGTGATGACGAGGACGAGA
ATCAAAACACCAACCATCCACTGGATGATTGCCTAGATCCCCGATTTTTGGTCATGGCCT
ACGAAGAACAAGGGACGATAAAACCGGTCGTTTCTGATTTCGACGGCTTTTTGTTGGGAT
GGAGACGGGAAGCTCTGTGGTTCGGATGCTGTCTTCCTCGGTACCAAGAAAATCTGATGA
TGTGGTGCGTCGAACGCATCGAAGAGATATTGGACGATCAAAAGAAGAACCCCACCAATA
GCGATACATGGACGCAGCGCTGGTTAGATGTTCAGAAAAAGGCGGCACAGCAAGGAAAAG
TGTTCGATATTCCCGAATACGGCTTTGGGGATCCAAAGTCGACATCTATCATGGAACACG
CGGCTTTGAAACTCAAGTCCACAGGCGCGGTCCGCCACGGATCGGAATGTTTCAACTACC
ACTTCCCCCAAGAAATCGACGAGACGTTTCTCTTGATTTCCGATACCCTTAAACCTGTTC
CTTGGAAGTATGTCAACGTCAAGGAGCTACAAGATATTTTGTCCCAGAAGATCGCAGAAG
GCTTCGTGTTTCCGTTGAATCCGAAATGGATTCTTTGCGATCCTGGGTGGAAAAAGGTGT
ACGATGATCTCATGCGCAGCGACGCCCTGTATTCAGATTTGTCGAAAGACATATGGTTCC
CACCCTACAGTGGCATTCGAGAAAGAATTGAGAAAATACACAAGCTCCATCCAAACGGTT
TCCAGCGATGCACCAATTTTTCCAAAGCCAAAGGATTGGACAAGATGAAGTCACACTCGT
CGACGGGTCGCCGCCAATCGGCTTCGAACATTTTGCGGGACACGCTTGACGAAGGTCTCG
GTTCGCAGCTTACTGGAAATGCATATGCTGATCTGGCACAACTCGAGCTCGACGATTTCA
TTGGCCGGACGAGCGCGATGCCGGGCCGAGGCATGGAACTGGACAGCGCAATGGGCAAAA
ATATTCGCGACGCAATCAAGGATCTCTCCATCGAAGAACTCGACGACGACTACGCGGAAC

AGCCACGTAGCAGCAAAGGGGTCGGCGGCGTCCCCTTGTCACAACGGTCCAGACAGGAAA
AGCGACCGAGCGGTGTTTCGCACACCAGCAGCATTGCAAGTAGTGTGGGAAGCAGCGGGA
CGAGCCAAAGCAATAGTAAAACAGGCCCAAGCAGCTCCAACAACCAAAGCCCTGGGCTTT
CCCTCACCTCTAGCATAAGTAGCGGCAGCGGCACCACCCCAACTACCTTTCGGAAGCTAT
TTCGTAAGGTCCGTAGGCGTCCGAACTCCATGGAATTTACTAACGAATCTTCGAACAACG
ATCAGCACACAACGTCATCCGAACCGTACACCCGTTCAAAGACGACATCGTCTCCAGACC
TCCGACTGGGGCAACAAGCCAGAGCAGTTTCGAACCCGAAGGATTCGAGCAGCGCCAATC
CCAAGAAAAAGACAAGTTCGAAAGATACCAACAAGCCTCGCCGCCATTTCAGCAAGTGGT
TCAAATAACAAACCACATCAACAAGAAACATCAACATCTGTCACTCTGAGGACGAAGACC
CTAACACAAACTACGTCACAAAACACATCACAGCACGTCAAGTCACACCACACAATAAAA
CGAACGAGCCAATAGAAGAAAGAAAATTAGCCAACAAGCATTTGCAATCGTGGCGGGTTT
CATAGTGAGGAACGTTCAAACACAACACTAATGATTTCACGATTCTAATTCTAGCAAAAT
ACTCGTTATCACTTAGCTAATGAGACAAGCGTCAACTATTTTCCTTCCATCACCGCTATA
AATATGCCTAAATTTTTGATTGATCGACAAATCCCAAAGAACAATATATTTGGCCATGGT
GCTCCACACATCTGCTTGGATGCAAGGAGTTGGATAGATGTCAACCACAAAACCTACTAT
TACTGCTGCTACTGCTACTGCTACTACTACTACACAACTCTTACTAGTTT
>comp2581_c0_seq1
CAAGAATCGAATCTCCGTCGTCACCGAGAGGAACTTTATTCCGCTTGATAATAATTCTTT
GACACTCTTCAAACCAAAACAGCTCTGCATCACGATACCATCCAGAACGCCAGAAGGAGC
CGATCTAAATCATCCGCTAGCAACACCCCAGAGAAAAACAATCAAGCGAGAGAGGAGTTT
TTGGGACCCCACCGAATTCGTTGTCGCCGATTTACTACGAAGCTGTTCTCCAATCGTCTA
CTAAACTGATCTCGCTGGCATCATGTCGGACCCATCCTTTTTGTTCGACAGCGGAAGCGA
TAGCGGATCTCTGCTCTCGTCGTCCTCGACCTGCGAAATGTGCGGATGGCTGGCCGCCTT
TGCTGCCCTGTTGGCCTTTGGGTCCTTTGGGGTCCCAGTCAAGTCCAAGGTCGCGCGGTC
CATCGATATTGACCCACTAGTGATGCAGTCCTACAAGACAGCCATGTGCTTTCTCACTTC
CTGGCTCTTTCTCCTGATCCGGGGGGAAGAGGTTACCTTTACACCCTGGGGGATCGTCTC
GGGTTTGTTCTGGGTCCCCGGTGGGGTGGCCACTATCTACGCCATCAAAACGGCCGGGCT
GGCCATCGGAATCGGCGTGGGCTCCTCATTTATCGTTCTGGTCTCCTTTTCGTGGGGAAT
CTTCTTCTTTAATGAACACGTTCACAGCCGGATCCAGGCCTGCTCTGCCGTCGGCTGCAT
GCTCTTTGGACTGGCGGGGATGGCCTACTACTCGGCCCCGACAGTTGCCCACGCGTCGCA
CCCCCGCGCGCCCGAAGAGAATCCCCGGTCTGGCGACTTTGACGACGCCTTTGACGATGC
CTACTACCACGCCGTTTCGCCGGAGAATCTGAACTTTTTGCCGCCCAGCGAGGCCTTTGA
AACGGAGTACACCTCCATGGACGTAGAGGCGGATGCGGCCGAGGAACACGGGGCAAGCAG
CGACAACTGCAACAATTGCTTGGAGGACAACGACCACATCGACGAAAGCATGGAACGGCT
AGACGTTCCGATGATCCAGACAGTGGACTATTCCGAGGAAGAAACTCCGCACGTCGTCTT
TTGTTGGGGATTTCTGAGGTTGCAGGAACGAACGCTTGGAATATTAGCAGCCCTGGTCGT
AACCGGGATATGGGGAGGAAGCATAATGGTACCTATGCAATTTGCTCCGAATGTCGACAA
GGGCTTGCCCTTTCTGACCAGCTTTGCCATCGGAGCCACACTCGTGACCCTGTCGTTCTG
GTTGTTGCGGTACCTGTGTCTTTGCCACAAGCACAGCTACTCGTTTTCCACCGCGTACCG
GGCGCTGCCTTCGTTCCACTTCAAGAAGATGTGGCCCTACGGGTGCGGCTGTGGCCTGCT
CTGGAGCATCGGAAACTTTTTTTCGATCGTGGCCGTGGAGAACCTTGGGGAGGGCGTCGG
ATACAGCGCCTGCCAGGCGTCCATGCTGGTCAGCGGGCTGTGGGGGATCTTTTACTTTAA
GGAAATCGAGGGGACCGACGCCATTCTGAAATGGTACGCCTCGGCCTCCATCACGGCCCT
CGGAATAATCCTCCTTAGCTACGAGCACCACGAGCTGTAGCGATACGGTTCGGTTCGATT
CAATTCTATTTGATTTGATGCCAGTCACATCGCAACCGCAACAATCACCAAACTCTGCGA
GTCGCAAAAGTGGATGAATTATATGCATGTATTGCTGTAAACGTTCCGAGACAACACAAT
TCTGGTCGAGACCATTCCTACTTGCACAATTAGTATGGCGCATTTTACTGTATGTACAAA
CAGTTGCCGTACTATGGCTAGCTCGGGTAATTTAGTTAAGTGGCAAAA
>comp2582_c0_seq1
CTCTTTGACAACATCAAAGTAACTCCAACTATCTTTCACTAATCAAACCTCTACAGTACA
TCACCATGTCTACAAAACTTGGAATCAACGGATTTGGACGCATCGGACGTCTTGTCTGCC
GTGCCGCCATCGAAAACAAGGACGCCACCGTCGTTGCCGTCAACGATCCTTTCTTGTCGG
TCGACTACGCTGCCTACCAATTCAAGTATGACTCTGTCCATGGACGATTCCCTGGTAGTG
TTGAGGTTGACGGAGACTGCCTCATCGTCGACGGAACCCGAATCAAGTTCTTTGCCCAGC
GTGACCCAACCGAAATTCCTTGGGCCGAGGCAGGTGCCGAAATTGTCTGCGAGTCCACCG
GTATCTTCACCACTATCGAGAAGGCCTCCATGCACTTGAAGGGAGGCGCCAAGAAGGTTG
TCATCTCTGCTCCTTCGGCAGATGCCCCCATGTTCGTGATGGGAGTCAACAACTCCTCGT
ATGCCGGCCAGAATATCTTCTCCAACGCCTCGTGCACCACTAACTGTCTTGCGCCTCTTG
CAAAGGTATTGAACGACAAGTTTGGAATCGTCGAGGGCCTCATGACCACTATCCACGCCG
GAACCGCCAACCAGTTGGTGGTCGACGGACCCGCCAAGGGAGGAAAGGACTGGAGAGCGG

GACGTTCTGCCCTCAACAACTCCATCCCCGCATCCACCGGTGCCGCCAAGGCCGTCGGAA
AGGTCCTCCCTGAACTCAACGGAAAGCTGACCGGTATGGCCATCCGTATCCCTACCGCCG
ATGTTTCAATTGTCGATTTGACCTTCCGAACCGAAAAGGCCGCCACCAAGGAAGAAATTC
TTGGCGCCCTTAAGGAAGCCTCCGAAGGTGACATGAAGGGAATTCTTGGATACACCGAAG
AGAAACTCGTCTCGAGCGATTTCATCCACGATTCGGCCTCGTCCACCGTCGATGCCGGTG
CCTGCATCTTCCTCAACGACAACTTCCACAAGGTCATTTCGTGGTACGACAACGAATGGG
GATACTCGAACCGTTTGGTCGATCTGGCTGTCTACACCACAAATGCCTAAATTTGGAGCC
GAAGATCGCAAGACCATCGTTTTTCTGACGCCCCAGCATTCAGTATTGCAACGTTGAAGT
TGAGATGAGGATTAGGAAAATATAGAAAGTTTGTGCACTGTTTTATTTACAAACATAATC
TAAAATCTATTACTAATCGCGGTTTCTGCGGC
>comp2582_c0_seq2
CTCTTTGACAACATCAAAGTAACTCCAACTATCTTTCACTAATCAAACCTCTACAGTACA
TCACCATGTCTACAAAACTTGGAATCAACGGATTTGGACGCATCGGACGTCTTGTCTGCC
GTGCCGCCATCGAAAACAAGGACGCCACCGTCGTTGCCGTCAACGATCCTTTCTTGTCGG
TCGACTACGCTGCCTACCAATTCAAGTATGACTCTGTCCATGGACGATTCCCTGGTAGTG
TTGAGGTTGACGGAGACTGCCTCATCGTCGACGGAACCCGAATCAAGTTCTTTGCCCAGC
GTGACCCAACCGAAATTCCTTGGGCCGAGGCAGGTGCCGAAATTGTCTGCGAGTCCACCG
GTATCTTCACCACTATCGAGAAGGCCTCCATGCACTTGAAGGGAGGCGCCAAGAAGGTTG
TCATCTCTGCTCCTTCGGCAGATGCCCCCATGTTCGTGATGGGAGTCAACAACTCCTCGT
ATGCCGGCCAGAATATCTTCTCCAACGCCTCGTGCACCACTAACTGTCTTGCGCCTCTTG
CAAAGGTATTGAACGACAAGTTTGGAATCGTCGAGGGCCTCATGACCACTATCCACGCCG
GAACCGCCAACCAGTTGGTGGTCGACGGACCCGCCAAGGGAGGAAAGGACTGGAGAGCGG
GACGTTCTGCCCTCAACAACTCCATCCCCGCATCCACCGGTGCCGCCAAGGCCGTCGGAA
AGGTCCTCCCTGAACTCAACGGAAAGCTGACCGGTATGGCCATCCGTGTAAGTATAGAGG
CGCATGTCTTTATTTCGAGGTGAATACAAATATTGAACAGAATCACATCTCACCCAAATT
GCTGCACTTTCACAAACGTACTGCTCGATACTACCACAGATCCCTACCGCCGATGTTTCA
ATTGTCGATTTGACCTTCCGAACCGAAAAGGCCGCCACCAAGGAAGAAATTCTTGGCGCC
CTTAAGGAAGCCTCCGAAGGTGACATGAAGGGAATTCTTGGATACACCGAAGAGAAACTC
GTCTCGAGCGATTTCATCCACGATTCGGCCTCGTCCACCGTCGATGCCGGTGCCTGCATC
TTCCTCAACGACAACTTCCACAAGGTCATTTCGTGGTACGACAACGAATGGGGATACTCG
AACCGTTTGGTCGATCTGGCTGTCTACACCACAAATGCCTAAATTTGGAGCCGAAGATCG
CAAGACCATCGTTTTTCTGACGCCCCAGCATTCAGTATTGCAACGTTGAAGTTGAGATGA
GGATTAGGAAAATATAGAAAGTTTGTGCACTGTTTTATTTACAAACATAATCTAAAATCT
ATTACTAATCGCGGTTTCTGCGGC
>comp2583_c0_seq1
TTCTACTTCTCTGTTGTTCTCTCGATTGTTACCGATAAGGATACAATTTCGACATGCACA
AGGACTAGGGATCGCTCACGATAGTTTACTTGACCAGAAGAATTTACATCCTTGTGGTAG
ACATCGGATAATCACATACATACGGTAGACAGAAATGAAGCTTTTGATTTACTTTTCTCA
TCTTCAAATTCTTTTTCTTGCGAGATATTGTGGTGCGTTCTGTAGTAGGGCGAACTTATG
CTCTCGAAAAGTCCATTCTACATCACCCGTAATCAAAACAATAATTTGCTCTAGTGGTCC
TACACACTCATTTGTTGAAGGGGAAGAATACGACGATCATTTCGCCGAAATTGAGGCGAT
GGGAGGAGACCCATTTTTTATGGATGAAGAGGAATACAAGGATACAATTCCAGAGAAAGT
GGACGCTGCAAAACTATTTGAAAACTCTCAATTGAGTGAGCTGCTTGCTAAAGCCAGCAG
TCGCTCTGCAACAGACGGATTGGGACCTACTCCGAGTCAAAGGAGCACTGTTTCCAACAA
CAATGATTCAGATTGGGAGTGGGATGGAACGGTTGACGAAGATGCTCACTTAGGATTGGA
TTAATTACTCGATAGTTCATGGTTCAGAATTGGTGAAGACTATGACCCCTATCGTGAAAT
TGGCGAAGAACTATTTAAACGTAACTATATTGTTAACTTGGATCAGTACAATCAAAATGT
ATCATGACAAGAACCTGAG
>comp2584_c0_seq1
CAAACATCAGAATTTTCTTGAAGTTGATCGGATGACCACGTAATTTATGTGAAGTTACCG
ACCAATCATGTGGCCCGAAGGAAGTGACGAAGAAACGACGTGTAACTTCAGTACTTGGTT
GATATGGTAGGTCAGTCCACCGTACTATAGAAAGTACGTAACAGGATGATGATGGCACCA
AGAAGTTATTTTCACTTGTCCTAAGCATAGACACGGACTGCTGCACAGCATTCTGTGGAA
TAACTACGAAGTAGTTCTCTGTGCAACAGCAACATCATTCGTTCACTTGTGCAACCACAT
TTCGACGCAGCATTGGTGCAGATGCTGTCTTTGTCGGTGTCCTAAATCAAGACACGTTTT
ACCGAAGACGAAAACAGACATACAATGACAACCATAACGAATGCTTTTACATCTTTCCCC
GACATAAATAGCATTGAATTGAACCACATACCAAGCCACGAAGTGCAATCAACTATTTGT
CATCCTCACGTTCTATCATCAAGTACTGTTTCCACCATGTTACCGGGCGACAACGGAAAC
GGGAATGACCACGGAAAGGATTCTGATAGTGTTGGCAGCAGCAGCACTATTGGTTGCTTC
GATCTCTTACCTCAGGTCGCTTTCTCTCCGAACCGATTGGCATCGAAAGTTGCCTCAACT

TCCACCGTCCAAACCGGCATGGATTTAAAGACGATCAGTGTTAGCCAACCAACTTCGCCC
ATTTTCTCAACTCCGGTAGGGGGTAGCAAGGATTCCTTCACACCTCCTCCTCCGATGACT
AAGAATGTCCACTCCCTCGATCGCGAAATGCGCTCCAGGAAAAGAAGACGGAGAAACATT
AGCGGTTGCGAGGACGATTACTCACTCCTCGACTGCCCCCAACTTATCATGTCTCTCAAC
ATTGAACGAATCAGTCTCCCCCCCCTATCAACCCTCGAGGATTGCGTACACGTCGGCGAG
GCCTGTCTTCCCGATACATCTTTTCTTGCCCGAAAGAAATCAAAGAGGCTGCGGAGAGGC
CTTGTCATCTGATGACGACGAAATTTGGCTTCGTAGTACAAAATCAAGCTAATTTATTTT
TTGGCCACCAAGCAAAGAACCGCCATTAATTCAACCGCATGCTACGGGAAGGGGTCAGCG
TTGTGTCGTTTTGTTCAATATTGAAGGACAGGGTCTGGATCTAATTGAATTCTCCGAGGT
ATGGAACTCGAACGAACGCATCAATCCTTATCATTGATCACAATACGCAACAGAGAAAAC
AATTTGGCGTTTTCCGCATGATAATCAAGATTCATTCGTTTCACCGTTCCATCCATTTGA
ATCCCAACGATCGCAATCAATCAATCAAAATATATGTACCAAAAAATTGTAAATACGTTC
CAATCCATTGATGAATACTTGTCCGACTTTTCCAACCAAAAGGAAGAAAACTGTGTTGTT
GTAAATTTGTCCAACTGTAACTCCATAATCAACTGTCAGTAACTCAACTGTAATTAAAAT
CATTTACAACG
>comp2585_c0_seq1
AAGTTCACATCGGTTCCGTATGCTAAGGGCTTTGATTGTTGGGACACAACTAGTGCTCTA
ATCGCTATTTCATTCTACACTCTGTTCTTGCTCTCTTTTGATTGGACAGGTATGGTTGGT
GTCGTCGAAAACGAGGCGGTGGATCACGGTATCGATTCACCCTTGGCTGGTTTGGGTTAT
GGATTGCCGATTTCACGTAGTTACTGTCGATATTTTGGTGGAGACCTGAGCATTATGAGT
ATGGAAGGTTACGGAACAGATGCTTTCGTGTATCTTGCGAGATTGGGAAACACGAAAGAA
CCTGTTCCAATTTGAGGCAATTCATTTTATCCGGATAGCAATAGAGTGAGATTGGGCAGT
TGATAAAATTCTGTATTTTTGCAACTAGTTCACCTCAACGAACTTTCCAGGTAAATGCAG
GTGTCTCTACTAGTAGTAGTATTTTCAAATTTAGACCAGCTCGCAAACAAAACATATACA
GTACTTCCGATGCTGGTACTACTACTACTACTGTTGTTCTTGTTGTGCATTTTGATTTTG
TACAACCTCTGTAATTTTTTCAAAGTGGGAGGTACTAAGACTAGTACTAGTAGTATTCCC
CTATAGTAGCACAGCATTCCCACTAGTAATACTTGTATCATCAC
>comp2585_c0_seq2
CTCTGTTGCAAAGGTAGAATTCCTTTTCGAACCAACTGGGAAAAAGTTTTGAAGCCAAAG
TGAACTTTCTTTTGTCATCTCAAACTACGAACGAGATAGACGGCGACTTACATTGCTTCT
GAAGAATTTCCAACTACTTCGTGACCATTCCAAAGTTCCTTTATTCTACAGTCCAGTACA
ATTCAATAGAGGGATAAAAAAAGATTGAATTCAGCCGATACCAAGAATCAACTTCTGAAA
TACTGTACTAAGGTTCGACATCACCATTTATTTTTCTGCGTGGTTTGCACTGTATTGACG
AGGATGATTTTTCTCCGCAATGTACAGTCGTCCATCAGTAGTCGAGGATCGATGGCAAAC
GCTCGAAGATGGAGTTTTCTTTCTGGGAAACATCAGAGCGAAGCAATGGGGACCATAGGG
GTGGGCCGATCATTCTCACGCTCAACGAAGACATCAACATCGATTATGATGATAAGGACC
CCGACAAAAAACATCGTAAGAAATCCAAGCCATTCTCGGAGTAGAATTTTTTGTAGCGTC
CGCGCAAACAGTACCAACCATCAGCACGTGGCTAAACAGAATCTCCGCTCCGTGCGTTTC
AACTCATCGTCAATTCAAGTCAGTACTCCGAAAGTTGACCGAGATCTCGAATATGACGGA
GTTCCGAATGATGTAGCGAAACTCATAAACGATCACGCAAAGCAACCTCCGACTTCAGTG
AGCTTGCAAGCTTTAATGAGGACCGGTCGAGGAGAATACCTCGATCGACACTTTGAAAAC
GTAGCGATAAAAGAACACACTGCAACGGAATTGGTCTTGATGCAGGTTGCCTCTTTTTTG
CGACGGGAGCTTCCCATTCGATTAGCTCATCGCATTCAGGATTTGGAGAAGGTCCCGCTG
ATGAAAGATATGTCATCCGTAAAAGAAGTAAAACAATTGTACATACAATCATTTTTAGAA
TTGATCGATTTCGACAAGAAGATCGTTAGTGCCGATCAGGAGGAAGGTTTTTCGAGACTA
GTGGAGGGAATTTACGAGCGACACTCAAGAGTATTGGTACAGATGGCAAGAGGAGCCTAT
GAATTCCGCCACCACATCCATACAAACTCTACCTCAAAGAGTAAATCCAGTAGAAGGCCG
AAAGATGTGTTTGCTCAAATGGAAGATACGCACGCCTTTTTGGACCGGTTCTATTTGTGT
CGTATCGGCATTCGTGTTTTGATTGGTCAGTATCTCGCATTGCGACAGCCTCCGGTTGAC
CATTACGTCGGTATTATATGTTCAGTAACATCACCCTACGAAATTGTAAAACGTGCTATT
GACGATGCGGCATTCATGTGCACGAGAAAATATGGAGATGCCCCTGAAGTTATAATGAGC
GGAAGATTAGATTTGACCTTTCCTTATGTTCCGACCCATTTGCATTACATCATGCTCGAA
CTGCTGAAAAATAGCATGCGGGCAACCGTAGAGTGGCATGGGTCAGATGCTGATTTCCCG
CCAATCAAGGTCATAATTGCTGATGGCAGTGACAACGAGGATGTAGTCATCAAAGTTAGC
GATGAAGGTGGAGGAATTCCAAGAAGTAATGTTGAGAAGATTTGGTCATATTTGTTTACT
ACAGCCGATCCATCCATACAAGAAGGTATGGTTGGTGTCGTCGAAAACGAGGCGGTGGAT
CACGGTATCGATTCACCCTTGGCTGGTTTGGGTTATGGATTGCCGATTTCACGTAGTTAC
TGTCGATATTTTGGTGGAGACCTGAGCATTATGAGTATGGAAGGTTACGGAACAGATGCT
TTCGTGTATCTTGCGAGATTGGGAAACACGAAAGAACCTGTTCCAATTTGAGGCAATTCA
TTTTATCCGGATAGCAATAGAGTGAGATTGGGCAGTTGATAAAATTCTGTATTTTTGCAA

CTAGTTCACCTCAACGAACTTTCCAGGTAAATGCAGGTGTCTCTACTAGTAGTAGTATTT
TCAAATTTAGACCAGCTCGCAAACAAAACATATACAGTACTTCCGATGCTGGTACTACTA
CTACTACTGTTGTTCTTGTTGTGCATTTTGATTTTGTACAACCTCTGTAATTTTTTCAAA
GTGGGAGGTACTAAGACTAGTACTAGTAGTATTCCCCTATAGTAGCACAGCATTCCCACT
AGTAATACTTGTATCATCAC
>comp2586_c0_seq1
CGACCACCTTTTTTAAGAAACCATGCTGCGAACAGGACTACAAGCCGGCCTCCGAGCCGT
CTCTCGGAAGGCCAACAACAATGCCGGAGCCCTGCGATTCATGAGCAAAGAAATCAAGTT
CGGAGTCGATGGCCGCAACGGAATGCTCGAGGGAGTGAACACCCTGGCCGATGCCGTCCA
GGTAGGTGTCGGTGATGTATTGCACCGCGTTGTTTTGGCTTGTTTTGTTTCGAAACCCAT
CTGCCGAGTCACACTCGTGTTTCCCGGGAATCGGTAACGAGAAAATTCCGTTTTTGCGCG
ATTGCGTCATTGCAAAAGGGAATCCGAAATCCAATCTTGATTTGATTCGACGAGAACCCA
GCCGTCGATTTGGGTTCGTGTTTGTGTCTTTTTTGTTGGTTTTGGTTTCCACGAAATTTT
GGATTTTCACCAATCCCTTTTTGTTTCCTCGCCGTGAATCTAACACTAATCTCGTTCCGT
GTGTGCCTGCTGTGCAACGCAACGCAACGCAATGGACCCACCGAACCCAACAGGTCACCC
TCGGACCAAAG
>comp2586_c0_seq2
CGACCACCTTTTTTAAGAAACCATGCTGCGAACAGGACTACAAGCCGGCCTCCGAGCCGT
CTCTCGGAAGGCCAACAACAATGCCGGAGCCCTGCGATTCATGAGCAAAGAAATCAAGTT
CGGAGTCGATGGCCGCAACGGAATGCTCGAGGGAGTGAACACCCTGGCCGATGCCGTCCA
GGTCACCCTCGGACCAAAGGGACGCAACGCCATCATCGCCCAGCCGTACGGAGCCCCCAA
AATTACCAAGGACGGTGTCACGGTCGCCAAGTCCATCGACTTTGAGGATCCCTTCGAGGA
CATGGGGGCCCAGCTCATCAAGAGCGTGGCGTCCAAGACCAACGACGTCGCCGGAGACGG
AACCACCACCGCTACCGTTCTGGCCCGCGCCATCTACTCGGAGGGATGCAAGGCCGTCGC
CGCGGGCCTGAACCCACAGGACCTGCGCAGGGGAATCAACATGGCCGTCGACTCGGTCGT
ACAGACCCTCACGGACATTAGCCGGCCCATCACCAGCAAGGAAGAGATCGCCCAGGTCGG
AACCATCTCCGCCAACTCGGACGAAGAAGTCGGTGGCTTGATCGCCGATGCCATGGAGCG
GGTCGGAAAGGAAGGAGTCATCACCGTCCAGGACGGAAAGACGCTCGACGACGAACTGGA
GGTTATCGAGGGAATGAAATTCGAGCGGGGTTACATCAGCCCCTACTTCATCACGGATGC
CAAGACGCAGAAGTGCGTCCTCGAAGATCCCCACATCCTGCTCGTCGAAAAGAAGGTCAG
CAGCCTCCAGCAACTTGTCCCCATTCTGGAGTCGGTCATCAAGAGCCAGGGCTCTCTGCT
GATCATCGCCGAGGACGTCGAGAGCGAGGCCCTCGCCACCCTCGTCGTCAACAAGCTCCG
GGCGGGCATCAAGGTCTGTGCCGTCAAGGCCCCCGGATTCGGTGACAACCGAAAGGCCAC
GATGCAGGATCTCGCCCTCCTCACCGGCGGAACCGTCGTTTCCGAAGAAACCGGTATGAA
GCTCGAAAACACCACGCCCGAGATGCTCGGACGGTGCAAGAAGGTCGAGGTCGACAAGAA
CAACGCCATCATCCTCGGGGGCGTCGGTGATTCCGAGGGACTCAAGGAGCGGTGCGACTT
GATCAAGCATTCGATCGAGACGACGAAATCCGATTACGAGCGAGAGAAGCTCCAGGAACG
ACTGGCCAAGCTCTCGGGCGGCGTCGCCGTCATCCGCGTCGGGGGCGCCTCCGAGGTCGA
GGTCGGGGAAAAGAAGGACCGCGTCGTGGACGCCCTCAACGCCACCCGCGCCGCCGTCGA
GGAGGGCATCGTCCCCGGTGGGGGCAAGGCCCTGCTCTACTGCTCCACCAAGCTCGAGGC
CGTCAAGGAGGAGAAGGCCAAGAACATGGACCAAAAGATCGGTGTCGACATTGTCCTCAA
GGCCCTCCAGGCCCCCATGAAGCAGATTGCCACCAACGCCGGGGCCGAGGGGGCCGTCGT
CGTTGGCCACCTCACGGAGTCCGAGACCCCCATCGAGACGGGCTTTGACGCACAAAACGG
AGTCTACACCGACATGTACGCCGCCGGAATCATCGACCCCACCAAGGTCACCCGAACGGG
CATTGTCGATGCCGCCTCCGTAGCGGGTCTCCTCACCACGTCAGAGGCAATGATCGTGGA
CAAGCCGATGGAGGGCGGGGCCGCCGCTGCCCCACCGATGGGTGGCATGGGAGGCATGCC
CGGCATGATGTAAGAAAAAAACCCAAGGAACCACCGCAACGGGTGCGCAGCTGGAAACAA
CCATCGGAACGCACGCACGCACGCGGGCATGCATCGATCGGGAAGGGGCGCGGCGCAGGA
CAAGCCATCCCTCCGATCCCCTCTCGGAGTTCCGGAACGCGGCGGGGCGACACACAAACC
CTGCTTGCGGCGATCGCATCGCGGTGCGGAATGGTTATGCTTTCGGGAGCCGTAGCCTTT
TTTGGGGGTTTTAGTTGGACTAGGTATCATTATAATTATTAGAAGAACATACAGAAC
>comp2587_c0_seq1
TTTGATCAGAGAAGCATGTATTTTTTGTTTTCGCACCCATCTACCCCTTTTCTCTCGAAT
AGGTACTGTAATAGGTAACGGTACGTCCGAATGTATCTCCCGGTGATATTATCTTCGTCA
CATGACACCTGAATGTCAGCACAAGAAACAGAGGGAAATCAATAAGTACAGTTGAAGAAA
GAAAGTCTGCTCATCGGACACACAACAGAACGAAAATCTGGAAGAGGATTCAGTGGATAC
AAAATGCTGCGAAGAAATCCTTCTGAGGTGTCTTCGGCGCGAAAGACAGAACTCGATAGC
TTTGAACATGACAACGATAAAGGATCGAAAATGAAACGTCAAAAAGATTCGCAAACGAAA
GCAATTTTTTGCTGCCTGGCCATAATAGCGTTGCTCGGGCTATTAGCAAAGGTTCGAGTC
AATCACATGCAGAGATATGTACCAAGAAGACTTCGAAAACAATCAAACCACCACATACCC

GGAGAAAACAAAGTGGGAAATTCCAACACTAAAAGTGCGAATGATTTCCTTCCGCCAAAC
TCGCTCTACAAGCTGAAGGTCGAAGACATTCATGGCAAAATCGTGAACTTTTCTAGCTTC
CGAGGAATGGTTACGTTGATCGTCAATGTAGCCTGCTCGTGAGGCAAGACACACATTTCA
TACACCGAATTAGCCGAGTTGCAAAAGAAGTACCGTAGCAAGGGTTTTTCGGTTTTGGCT
TTCCCAATTAGTGATTTCCATCAAGAATTGGGGTCCAATGAAGAAATCCTTTCGTTCGTG
AAGGAGAATTTCCCGCAAGTTGATTTTCCTCTATTTAGCTTGAGTACACTTAACGAAAGC
CCGGTCTATAAGAATATTCGGAAACAGAAGCCAGATAAGCATGTGAAATGGAATTTTTAT
AAGTTTCTTGTGGACTCGAATGGTCAAGTTGTTGATTCGTACGATAAGAAAGTTCGCCCA
TTGCAAATAGCAGATCGTATTGAACAACTTATAGCTGAAGTCTCCCGAGATGGTGGACAG
AAGCTTGTGACGTCTTGAACCCTAGAAGAAACACGATATAAGTACAGTGAACATTCTGTG
GATGAAGAGCAAGGTTTAGCGATATTGGTCACATGATCAAATACATCATACTATCCTTGT
TCCGAGAACAGTTGTTTTCATTGTAAATATGTGTTTCACCAAGACGAGGTGTTGTGTTGT
TCGATCACATTAATTCGATGATATTGGGTGATTCCCGTAAATATAGAATCTATGTAATTA
CCTAAAATTTTCATTTGTGCACGCAG
>comp2588_c0_seq1
TGGAAAACACCATCCAAACGATTTCCACTATAATATTGGTTACAGAACAGGGACCATCCA
TCACATACTGATAACACACTCCACACCATCTCTTGTTCGATTCTATGCCCTGGTTATGCA
CCGCAGTAGCTTTTACTCTTCCACTGAATAACTTGAAAGGCCAAAAAGAATCTCGAGGTA
TCGAATTGAATGAAGTAAACGAGTTCGAGTATAAAAAGGAGCATTCTTCGTTGTTGGCGA
CATTAGATGTGCACACGAAATAGTTGGAGTTATTGGCAACGGCAGCATCAGGCGTAGTTA
GAGCTTCAGAAACAGCAACATCGAAGGATAAGATGAAAAAGAAAGAATTGGACTTTGAGC
GCATTGCGAGGCAAGCTCGCGAATTTCGAGAACAAGAAGAGAAGCGGATAGCCTCCGAAA
AAGAAGACGAGGCGAGGCGAATTGCTGCCGAGAAAGAGGCGGAACGAGAACGAATCGCTC
AAGCGCACGAGGAGACGTTATTGAATGCCGAGCATAGCAGCATCGGCTCTTCCCATTCCT
ACGAAAGTAGAGCACCAAAGCCGAGTCCTGGAACAGTAAAGCGTCGAACGGAATGGTTAC
GAAATCTAGCCAAAGATGCCGCTGAGCACCGTCACCTTGAGGAGGAATTCAATTCAATGT
TGGTAGCACGACTAAAGCGTCATCCCAACGAGGCAGCGAAGGATGCAGAAGCGGCGGTGA
AAGCTGCTTTGACCTCATCTTCACCTCGTGATGACTTTGACAGAGATATAGAAAAACTTC
GAGACCAAACAGCACATATTGCCAAAGAGGTTGCGAATGCCGAGCGTAGACTTCGAAAGG
GTTCGGAGGATTTGATCGAGGAATTTAATCAGATAGAGGCAGATTTGGAAATGGAGATTG
CAGAATATCAGACGCCTTCCTCTGATGCCAATTCAAGTGATCTGGATATAGAAGGCCACG
AGGTGAATGGTGTAAAACCCAGAGATTTAATGGAAGAATATAGTCACTCCGACGCACCAG
ATTTGACAACAGTCGAATCAATGACAATGGAGACTATTGAAGAAAATATAGATGCACCTG
AGGGAAATCTGGAACCACAAGGTGATTCGGATATTGAAAAACTCGAAACATCGCCGGGGC
AACAAATACATTCAGGAGGTCCTGAATACGGAGCAGAAGCTGATCCAGCGCATACCACAG
CAAAGACTGTTTCTGTCGTGTCGAAGTCGATCATAGCTGAGCCATCGACAATGGAGACTA
CAGAAGAAAACGTGGATGCACCTGAGGAAATTCTAGAACCACAACGTGATGAGGATGCCA
GAGAAATCCAACCATTGCCTAAAAAGAAGTCGTATTCGAACAATCTTGAATTTGGAGCCG
AAGCTGATCCAACAGATATCACAGATGGTACGTTCCCCATCGCATCAAAGTCGACCATTG
CTGAATCAACATCAATAGAGACTTTAGAAGAAAATTTTGATGTGCCAGAAGCCAATTCAG
AACTACAGGACGGTATGGGTGTTGGAATAATCCAAATGTTGCCCAAACAACAAATGCATT
CGGATGGTCTTGAATACGGAGCACAAGCTGATCCAAGAACGCAGGCGCTGCTAGCTGAAG
ACGATTTGGTACCCACGGGAAAAAAATATCATGCAGACGAGAATTTAAGGATTAAGGACA
GAAACAAGAAGAGTATAGTTCTGGATGGTTCGATTCCAGTTGATCGGAAAATATTTCGAA
GAAACAAAAGTTCTTCAAAAAATTACGAGGATGAGGACGGGGAATTTGGTTCTGAATTTG
TTCACAAGGAGGCTACTTTCGAACTTCCAATGGAACTTACAGAGAATGGGCTGAAGTTGT
CATCGAACACAGCAGAAGTGAATATGATCACTACTATCCCACCTTCGCGGCTTGATGGCC
GAATCAGTGGCATTCTGGAGGGTAGATCGACCTTGGGACCTCATTCAAATACAGCATCAG
GGTCGTTAGCTTTGGAGTACAAGTCTTCGAAACAAAGTCGTCTCACTCTTGGAATGATCC
GAGGATGCGAACCCCATCATCCCTTAATAACAATTGGAGGCCGGTTACTTCGTCAAGGCA
CCAGCGTCGGTGTAATCTTTTACCACAATGCACAATTTTTGCATCAAATGTTGCTTGAAC
ACTCCCTATGGGCCATCTCCTTTCGTCACTCCTTTCCTAATTCAAGATGGACTCTTTCGT
CACAGCTCTCACGACGGAAAGACCTTTCCTTGTCCTTAGTCAATGGAAACAAACTCTCTG
GTTTGATTGGCTGGAATCTACTCAAACCAAAGCAGTTTCACGCTCGGTTTGATGCCCGCC
CCAAGCTTACAGAGTATCGAAGAGCGCACCTTTACTGCCAGTGGAAAGCATCGCCTGGGC
CTGGTCTCTGGAATGTTGGATTATCTCTCGTTCAGTCACTCCATTCGCAGATCGCAACTG
TTGGTCTGGGATGCCGTTTGTTCTCCACAAAGGGACTCGAGTGGGTCATTTCGTGGTCTC
GGGGAAATTCCACTATCAGTATACCAATCCTCTTATCAAAAAGCATGGCACCAAGTACAA
CCATTGGCCAAACATTTTACTTTTCGCTCATTTCTTTCATGATGCAAGAATATATTGCAG
AAACCTGGGGATGGATCGGAAACGACAGCGAAGACGACGAAGATTGCAAGGTAGAAGATG

CATCGATAGCGCTTCGAGCTCAAAATCTGGCCAAGGCTCGGCAAGATGCGGTCATCCAAA
AGGAGCTCATGGCTAGACAAGCTCGTCGAAAGACTAAGGAAGAAAAGGAAAAAGATGGAT
TGATTATAATTGATGCCATCTACAAAGTCGAGAACGGAGAGGAATGGAGTGTTACCGTGC
CGTTACAGTTTTGGGTGACGAATTCCACTCTCACGCTACCGGCAAGACCAAAGAGCGAAC
TCTTGGGGTTTTATGATGTTGCAGCCCCACTCAAAAACAGCAGAGTGTCGTCCATCGCGG
GGAATCAAACCAGTACCACGTCGAGTAGTTCTGGGCGTTGCTTTCCGACTTTAAGTGAGC
TGTGGCACGATCTTCTTGACCGGACACCAGAGGATTCGCAGTCAAAGAAGTCTGAAATCA
GTTCTCCAACGTTAACTGTGAGTTATGAGTTCAGAGGCAAACCGCACGTCGTAACGATCA
AAGATCGAGACGAACTAAGGCTACCACAAGTCTAATTTGAGAATGTAGATAAAATCAGAG
AAGAATGAATAAATTCGTTCCAAAAAGCTAAATTAGAAATAAAGAACGCGCACGATAGCG
TTTGAAGACTTCAGACTCTAGACGTCAACAGCAATCTTCCATATGGCGTTGTGATGCTAG
GAAAGCCACAACATAATGTGCTCATTGGTGATTAAATTATCGCAGAGAAAAATTGAAACT
GAGTGCCCGTGTCTATTCCTACAGTTAGTATTCTCTCCAGAAAAATTGTGTGATCGTTTC
TGCTCTGCTTTTGGTTGATAGGAGAATCGCATATGCAGCAGGTGTGAATAGCAATTTATC
AAAATTGAGTGTGTGCTTTTATGAGGACTCAAATGGCTCACCTCAAACATCAGGT
>comp2589_c0_seq1
CACCAACACACAAACGCCGATACCAATTCAAAATAGAAACAATAACAATGAATCTTTTAA
CTTTTATCTCCGCAACCCTACTCATCATCACGTCGGAAAAACTAGCAAATGTTGCTGCTG
CCGGACCCCAGCTCCGCGGCAGAAGCAACACCGGGCAACTCCAGCGCAGGATCCTAAACG
ACGATTCCGGTTGGTGCCCGACGTACTTTCCCGGAACCAACACGGATTGCCCGCTGACCG
GAATGTTCCGGGGAACCTGCTACTATAGCTCCAGCGCCAAGGAGGGCGAGACCCCGAACA
CGGACTCGCAGGCGTGCTCGTGCAACGCCGAAATGGCCGGAAAGTTCTGGTGCGAGCCGA
GCCCCTTCCCCGGCGGAGCCCCCACGCCCCCGCCGTCCCCCCGCGCCCTCCCGGAAGGCA
GCTGGTGCCCCGAGCTGTTCCCCGGCAGCGACACGGAGTGTCCGCTGACCGGAATGGTCC
GGGGAACCTGTTTCTACACCTCGAGCGCCCCGGCCGGAGAACCCCCCAACAACGACCACC
AGGCCTGTGTCTGCAACGGAGACGGAACCAGCAGGTTTGTTTGCGAACCCAGGCCCTTCC
CCGGCAGCGGGCTTCGACCCCCGGCAACGGCAAAAGATCCGGCAGAACCCACCACCGGGA
ATCCCACCCAGTGGTGCCCCCCCGACGTCCCCAACACGGGAGACTCGTGCGCCTTGCCCG
CCGGGAAAACGACCGGATCGTGCACCTTTATCCGCAGAAACACCATCACTCTCTGCAGCT
GCTCGTCGGACACGCAAACATTTGTGTGCAATTGAGACCCGACCAGACCCGACTGCATAG
AATAGAGCAGTCCGCCGGAACCGAATCTTGGGGAAAAGATCACAGAGATACTAGCAATCC
GTCTGATCCCGCTTCGGATCTCATTGAAAGGCCTCGCGAATAGTCGAAACCTGAACTATA
TTGCACGAAGATAAAACATAGATACAATAATAATATAACATCGATATTACCACACA
>comp2590_c0_seq1
TTTCTGGTAGATCACCTTCGACCCATTGACCAAAGAACGCAACAACAGTATCATGAAGAT
TTTTGCCGCACTATCAATGCTACTCGCATCGGCCTCGGCCTTTGCTCCCAGTGCAACCCA
GAAGAGAGGCACCGCAATGAATGCCCTTGTCGACGATGCCTTCGGTGTGAGCATCGAAAC
TGGAAATAAGGTCCCACCTCTTGGTCGCAAAATCCTTGAAGACGCCGGACCTGATGGAAT
CAAGTGGTTCCAAAACGCCGAAATTAAGCATGGCCGAATTGCTATGGTAGCCACCATTGG
ATTCTGGGTACAAAAGCTTGGGGTTCACTTTCCGCTATATTTGGGACCCTCTGGATCGAA
TGCCTTCAGCCCAGACTCGACCGCCTCCAACTGGCTGCTGTCGTCCACCACTGGCGTTAC
ATTCCAGGACATCGCCCAGGCTGCTCCTCTTGATGCCATTAACATGGTTCCTGTTCAGGG
TTGGTTTCAGATCTTTTTGGCCGCTGGATGGTTTGAATGCATCGCTTACAACCGCCAGTG
GGCTGAGGGACGAGAAATTCCCGGTGATTACGGATACGATCCTCTGGGATTTACCAAGAA
GGAAGGAGGTATTGCCGGTAAAGACTTTGAAAGCCTTCGTATGAAAGAAATCAAGAATGG
ACGAGTTGCAATGATGACAATTGCTGCATGGGTTGCCAATGAAGCCATCCCTGGAGCTCT
TCCTGTGTGGCATCCGTAATTAGCTAGCCTCAGCTGAAGCGAAAGCAAAGATGTGTCTTG
CGGCGAACCACCAGGCGGCCAGAGAAGGATGTGCATGTCGAAACCCGTGCTGCCATATAG
TGCGCGCATGTGGTACCATGCATATTCTAAGATTTTCATAGATCGATGATGAATAGACAT
TATTGTAATTGACACTAGTACCAT
>comp2591_c0_seq1
CTGAAGTCCTACTGCCCACACGTTGTTTTTCGCGTTACATCCATTACGACAGAAATCTAT
TGACAAATCCGACGATCGTAGACATACTATCTCGACAGGCTCTCATTCACAGAATTTCTT
TTGAACTTCCGAACAGAAAAGAGGAAAATGGAGCTCACTCCTTCTATCACTGGTCCGGTC
GATGGATTCATTGACTTTATCTATTCATTTGGACCTCTGGCCGAAAAAACTGTGGTTTTG
GTTCCAGATTTTATCAAGGCTCAGGTGGGCGGCGTTGCAGACGCAGCTGGCATGGACATT
GAAACTGTTTCTTATGTGCTGGGTATGTTTTTGTGCTACCCTCTCGGTATGCTGATGGCT
CAGATTCCATTTGGTACTTCGAGACATATCTTTTCATTTCTCCTTGGAGCCTTTCTTCTT
CAATTCACTCTAGGAGTTCAATGGATCCATCACTTGATTAGTTCCTTGATCGCATACGGC
CTAATCTTGGTCTTGCCGCGAAAGACTCTGAAGACGGCTTTGCCTGTTTTTGCCATGACC

TACATGACATTGGGTCACCTTCACAGACAGTACATCAACTATCTCGGATGGGACTTGGAT
TTCACCGGTACGCAAATGGTTTTAACACAGAAATTGTATATGATCGGATATAACCTCTAC
GACGGCGAACTTTTGGCAAAGGGGAAGGAGGACCGAGCTGCTAAGAAGTGTGCTCCTTTT
GCTTTGAAGAACACGCCCAGCTTGATTGAGTTTCTCGGCTACACGTTTTGTTTCGCGAAC
TTGCTTGCAGGTCCTGCCACTGAATACGCTACCTACTTGCATGCAGTCGATGGTAGCATT
TTCAAGACACCTGATGGAAAAACAAAGCTCCCAAGCAATATTTTGCCCACACTGAAGCCC
TTCCTTACTTGCCTCCTGAATCTTGGAATTTTCTTGACACTTAGTGCCAAGTTCCCGTTG
CTCGACACCTCTGATCCGCAACACAACACCCCCTACATCTTGACCGAGGAATTTCTCAAG
AAACCTATTTTCTACCGTTATTTCCATACCTGGATGGGATTGTTCGCTCTTCGCCAGAAA
TACTATTTCGGATGGAAGAACGCTGAAGGCGCTCAAAATATTTGGTATGCAGGATTTGAT
GGTTACGACGAAAAGGGAAACGAAAAGGGATGGGAAACCTCCAACAACATAGATATCATC
ACTTTTGAGACTGCTCCAGGTGTCTCCATCATGTCGAGGGTTTGGAACAAGAAGACATCA
TTGTGGTTGTCGAGATATGTGTACATGCGAACAGGCGGAAATCTTATTGCTGTCTATTCC
ATGAGTGCATTCTGGCATGGATTTTATCCAGGTTACTACTTATTTTTCCTCTCGGTGCCC
ATTCCTTCGTTTTGCGAGCGATTGGCCAAGAAGAAATTGTCACCCTACTTTTCGCCGAAT
TATTTCTCGCCTTACGGTATCGTATGTGTACTGGTTACAAGCATCACCATGAACTACCTG
ATTGTGTCCTTCACTATGCTTGCGAGTGGTTGGTCGTTCGCTGCGTACAAGAGCTTTTAC
TATTTTGGACACATCGGGTGTGTAGCGTTTTATATTATTCTTACGATGCTTCCGTCTCCA
AAGAAGGAAAAGAAAAAAGAGGCTTAGAATATGGCAGCATTGTCAACCGAGCAGTACTCA
CAGGTAACAAGTATCAAGATACTGATTAACGAATCAATGACTTTTCTCTTTGGGTACCTC
GTTAGTGCATGTGCAGAGGTCGGATCATTGTGGTCATTTTATGCTCCAAATGAATATTGA
ACGTGCGACTGAGATTGACTATAATATGTATTGACGAACGTTATCTAAAGCCTTCGACGC
AATACAGCGACGATTTCACTATGATCTGGTTGTCTGAAGGGCTCTTCTTGACAACGTACA
ACAATCTGGAAACTATTTTGTTTAATCGACTTGTGTCACTATCGTTCCCGCTTACATGAG
TAAGAGCTGTCAAATCCGTACTCAGCCCGGAGCTGGTA
>comp2592_c0_seq1
ACTACATCCAGACCAAAATAGGAGGTTGACAACAAGCACCCGAGCATTGCAAAGACGAAG
TTTTCACAGCAACGACGACCAGGAAGCAAGGCAAAAAGCCATGAAGGAACCGTGGTCCAA
AAAGCACAAAAAGGATTTCAAGGGAAGGCAATTCAGCCTTTCGAATTCGTTTGCACAGCC
CCTTTCCCAGCCCGAGCTCGTCGCTCTCACCAAGGCCCGCAACGACCAGGCCCTCCTGGA
CGACTACGAACACCACGATTTGGTGTATACACCGAACGGTGGCAGCGACGACCTCAAGCA
AGCCATTAGCGAGTTGTACGGACCGAAAATTTCGAAAGAAAACATTCTCGTCTTCCCCGG
TGGACAGGTCGCTATCCAGGTGGCTGCGCAGGCGTTTGCCCGGGATTGCCATTCGATCGT
CTTTGTTCCGGGGTACCAATCGACCGTGGAATCCCCGGAATGGGCTCTCGGCAGCAAGGG
CGTCACACAGCTACAGCGCCGAGCGAGCAACCGATGGCAGATCGAAATCGAACAAGTGAA
AGCATCTATCCAAGAAACGACGAAATACATGGTGATCAACGAGCCCTACAATCCCGGAGG
AATCGTAATGAGCCACGAGAGCCAAAACGAGCTGGTCGAACTCTGCCGGGAGCATGGGAT
CACCATCCTCTCGGACGAGGTCTATCGGTTGCTGGAGCACGATCCGAAGGATCGAATCCC
GGCCATGGCTGATGCCTACGAACGAGGGTTGTCGTGTGTATCCATGTCCAAGCCGTGGGG
TGCCTGTGGCATTTCAGTTGGTTGGCTGGCCTGCCAAGACAGAGAAATGATTTCCAAGCT
CTGGAATTGCCAGTATTTTGGAACGGCCTGCATCGGCCGAGCCTGCGAGATCCAAGCCAT
AATGGTTGTGCGGGCCTCCGAGACTATACTAAAGGACCGGGTTTCCATCATCCGGGCCAA
CAAAGCCCTGCTCACGGAGGTCATCGAGGAGAAGTATCCCGACTTTTTCGAGTGGGAACC
ACCGAACGCCGGTGCAATTGCCTTCGTGAAGTTCAAGGGACCCCTAACATCCCTGGAACT
AGGGGATTTGTTGGGTGCTAGGGGGATATCGATCAAACCCGCCTACTGTTTCGTTTCCAG
CGATAATGGTGGCAAGGGGATACCGCCCGAGGTGGACTACTTTCGGTTGGGATTCGGAGA
GCGAAAGATGCCGGAGGCTCTGGACGCATTCTGTTCAGTCGTGGAGGAATTCAAAGATGC
CTGGAGGCAAGAAATGGAAGGAATGAAGAAATAAACCGATGTCTACTTTGGACCACCAGA
AAAATTCAAACGTCATCGACTTTCAACGCGATGCTGCAACATAATACTAGTACTAGTAAA
TGTTTTGATTGATTCAGGCATGTTCGCAGTTTTCTTGTCCGCAACTACACGTAGTGAGCT
TCCAAACCAATACTGGATGAGTTCATTACAGGAATAATGCACCCCCGCATCGTATGAGAG
TACACACACTCAACTGACATCGCATGCGATACGTTATTTCGTGGTTAGCAAAAGGACAAG
TACTATTCCTCGTTTTACTACTACTACTAGTGGGATCGTAGTTACTTGTACCATCACGCG
GTCAGAATAAAGGGGAATGATTTGGTGCATGCTGCTGGGG
>comp2593_c0_seq1
CTTCATTCGTGTTTGAGAAAAGACGACAACCGATTCCGCTGGTGTGCGAACAGTACGAGT
GCTCAACTCACCGCTCAATCTCGGCAAACTATTTCGAATTCAGTATCGACGGTAACCCAC
ACACAACGCAAGTTTCCCCCTCCCAGGTGAGTGCAGCCTTCCTGTCTTGTTTGAACGAAT
GGATCACCAAGGAGTTTCGTTTTCCTCCTGCAACACCACATTCCGAAGACGATGACGAAC
GATGCGGGCATCGCCTTCTATTCTGTACCTTCCGTATCGCACCGCACGCATGGTACTACT

TTAGTCCGTCTCCGAGCGCCGGGTTCTGTCATTTTCCCAGGGTATCCCCAACCCTAGAAG
AATTTTCAGATTCGACCGTTGGGTGCGCGCTATTGAAGGAGCAAGCTCTGTTCGCCGAAC
CGCTTACGAGAGAATAGTGCGTTCCGTGCACCCGAGCGATCAAAACTGCGCGCGTGCGCG
GAGAAGAATTTTGTTTTGCCACGATGGTGTGCGGCCGGCACTGCCGCATCGAGTTCGTTC
GGGAAACGAGAATGACCGATCGATGCGATGCGCCAAACACCCATGGTGTGTTGCTGCCGG
GAAACAACGACGTCGTCGTCGTCGTCCTCTATTCACATACTGCAACCGTAGAAACTTAGT
ACATTTCGTACGACTCCACGGAGGCATCGAATGAATCCAATTTCAATCCAACAACGCCTC
AAGAAGCTGATAATCACAACGGCAAAGCCGCATTACAACGCCATTCAATTCTAACCTCCG
AACGTTGCCTTCTTGCAAATCCATTGCTCAATCGATCACCCATCGTAGAGACAACGAAAA
CGAGAAACACATTTGAGAGAAACTTCTAACAATCACCAAGTAAAATCACATAATATACAC
AAATTTTTCACTCAATAAAACAACAAAAACAAGCACAATGGCGGCACGAGTTGATGAAGA
ATACGACTACCTTTTCAAGATTGTCTTGATCGGGGACTCTGGCGTGGGAAAATCTAACCT
CCTAAGCCGATTTACTCGCAACGAGTTCAACCTCGAGTCCAAGTCGACCATCGGGGTCGA
GTTCGCCACGAAGTCCATCCAGGCCGAGGGCAAAACCATCAAGGCACAGATCTGGGACAC
GGCGGGACAGGAACGCTACCGGGCCATCACCAGCGCCTACTACCGGGGAGCTGTTGGTGC
CCTGCTGGTCTACGATATCTCGAAGCACGGGACCTTTGAGAACGTTGAGCGGTGGCTCAA
GGAACTCAGGGACCACGCCGAGGCCAACATTGTCGTCATGCTCGTGGGCAACAAGAGTGA
TCTGCGCCACCTGAGGGCAGTTGAGACCGACGAGGCCATGTCGTTTTCGGAGCAACACAA
CCTTGCCTTTATCGAAACGTCGGCGCTTGACGCCTCCGGAGTCGACACGGCCTTTCAGCG
AATCCTGACGGAGATCTACCGGCTCATGTCCCGGCGCAACGTTGCCGCCGAAGATGCTGC
GGGTGGACCCTCTCTGTCGGCCGGCCAATCCATCAGCCTCTCTGGTGGAAACAACAACGC
CGGGCAGGGAGAAAAGAAGGGTTGCTGCTAGATTATATGGTATTATATAGTGTATGGAAA
GTTGAGTTGTCCGCGGGGTGGGAGTGAAACGGACGGAGCACATTGCATTGCGTGGCATTG
CACAGCATGGCATACCTTTTTGCATCCATGCTGCAATGGTACCAAATCTTCCCCCCTTGG
CATCGTGTCGTAGGAATGAACGAATTATCTATGCCCAGATGTAATCGTCGATGATTCGAT
TCCCTTCGATCCCTCTGTGTGTAAGCTGGTTCACCTCGCATTATTTCACACCGCACCATA
TCACATCCATACGAAATATTTTATTGTCTTTGTAACACAAAACACGTCTATCTTCGATAC
TAGTATCACA
>comp2593_c0_seq2
CTTCATTCGTGTTTGAGAAAAGACGACAACCGATTCCGCTGGTGTGCGAACAGTACGAGT
GCTCAACTCACCGCTCAATCTCGGCAAACTATTTCGAATTCAGTATCGACGGTAACCCAC
ACACAACGCAAGTTTCCCCCTCCCAGAGACAACGAAAACGAGAAACACATTTGAGAGAAA
CTTCTAACAATCACCAAGTAAAATCACATAATATACACAAATTTTTCACTCAATAAAACA
ACAAAAACAAGCACAATGGCGGCACGAGTTGATGAAGAATACGACTACCTTTTCAAGATT
GTCTTGATCGGGGACTCTGGCGTGGGAAAATCTAACCTCCTAAGCCGATTTACTCGCAAC
GAGTTCAACCTCGAGTCCAAGTCGACCATCGGGGTCGAGTTCGCCACGAAGTCCATCCAG
GCCGAGGGCAAAACCATCAAGGCACAGATCTGGGACACGGCGGGACAGGAACGCTACCGG
GCCATCACCAGCGCCTACTACCGGGGAGCTGTTGGTGCCCTGCTGGTCTACGATATCTCG
AAGCACGGGACCTTTGAGAACGTTGAGCGGTGGCTCAAGGAACTCAGGGACCACGCCGAG
GCCAACATTGTCGTCATGCTCGTGGGCAACAAGAGTGATCTGCGCCACCTGAGGGCAGTT
GAGACCGACGAGGCCATGTCGTTTTCGGAGCAACACAACCTTGCCTTTATCGAAACGTCG
GCGCTTGACGCCTCCGGAGTCGACACGGCCTTTCAGCGAATCCTGACGGAGATCTACCGG
CTCATGTCCCGGCGCAACGTTGCCGCCGAAGATGCTGCGGGTGGACCCTCTCTGTCGGCC
GGCCAATCCATCAGCCTCTCTGGTGGAAACAACAACGCCGGGCAGGGAGAAAAGAAGGGT
TGCTGCTAGATTATATGGTATTATATAGTGTATGGAAAGTTGAGTTGTCCGCGGGGTGGG
AGTGAAACGGACGGAGCACATTGCATTGCGTGGCATTGCACAGCATGGCATACCTTTTTG
CATCCATGCTGCAATGGTACCAAATCTTCCCCCCTTGGCATCGTGTCGTAGGAATGAACG
AATTATCTATGCCCAGATGTAATCGTCGATGATTCGATTCCCTTCGATCCCTCTGTGTGT
AAGCTGGTTCACCTCGCATTATTTCACACCGCACCATATCACATCCATACGAAATATTTT
ATTGTCTTTGTAACACAAAACACGTCTATCTTCGATACTAGTATCACA
>comp2594_c0_seq1
GAGGATTTCGGATTTTAAAGATCGCATGATGAACCTTCAAAATTTTGTATGGAAAATAAT
CTTGCAGAATTCCTCCCCATTTGGTCGTGCTGTCTGTTTTCTTCCAAAGCAGTCATTGTC
ATGACGAATCGATGGCGATCGTGCAGATCAAAATTTTGCTACCAGAGTTGCCCTGTTCAA
GCAATCAACCATGTTGACGAATCTCATGACCAGCATGATTGATACTTGCCACCCTTCCAC
CTACGGTTTCGCCTCAACATCTGAGCAAAGAAATCTAGCCCCGCCCAGGTATGTCAGATC
GGAAGAAATACATGAAGCAGCCTTTGCTCCAATCCCAACCAATAAGTGGTGGGGTAATCT
AATCCACCAAGATATGAACAACCCCGACGGACCGATTGAACCGATTTGGAGCAATCCGTA
CGCCATTCGTGTCGTTCCCGGAAAAGGTCTGGTTGTGAGCGGAATCCATCGCCATTACGG
ACCGTGCCTGGAGATTAACCCCAAGGTCAAAGCTCTGAAATATTATGCCCACGCATTCAT

TGATGACATTATTCTGTCGGCGGTGGATCTGCCTGCCGGTCTAGATGTATACGGCTGGGA
TGATTTTGGCGTTCGGATCCAACTCGGAAAAAGAATGAATTCGTCTCTGGTGAGAGGCAT
GGCTTTCGTTTCTGCGACTTATGGTCTCGGGACAAGGCCTCGAATCGAAACCATTCACGC
TATTTCATCGATCAATGGCGAAGCACCAAGCCCTGATGCATATTACGCAGTATCACCAGG
CTCGAAACTCGTCCTCGAATTCAACAACGGTCAGTTCTGGACCGTGTACGCCGACCAAGA
CATAAGGTTTTCGAAGGAAGGCTCTGCCTTAACCGCAACCGATGCAGTCGATACAGCCAC
CACGGTGCGAATTGCGAGGGTGGAGAATCCGGAAAATCGGTTGGATGTACTGGACGAATA
CGCGAAATGCGTAGTCCTGGGGGGGCGCGTCCAATTCCTCAGCCACAAGCGTTACAATCT
CGAGTGGGAGACGGTTGGGGAAGGATCATTTCTCCATTTTGCACTCGAACACCACCTCCA
TTCCTTTCTGGAACCCCCTACAAATGTTTCCATTTTGGCTGAATCCACTACCCATGGCCG
GATGCATGGCATTTGCTCTTCTAGATCGCGGGATAATGGGCGCATCAGAGTCTGGGAACT
TGCGGAACCGAAATCAATGCCAATCAGATTCGAGCCGCAGCATGGAATTGATCCAGCGTA
TGTGAAGGAGTTTCGAATCGACGAGCGCTTGAAAGAGGAGATTGAATCTAGCTGGGTGAT
TTCAAGGGAAGGGAGTTACTACTTCAACGGGAAGTTGATGCAAAAGTTTGCCTCCCTTTG
CCTTATGGCACACGACGACGCCATCAACAGGGACGACAAAATAAAACGAATCTGCCTCGA
AAAGTTAGCATCTCTATGGGATTCTTTTCTGTCAAACGGAGACGGTGAATGGCCTCATCC
TCTAGTCTTTGATGAAATCTATGGGGGAATCGTCTCTTCGGAGGGGTTTAAGAAGAACGA
CCAAAACGTTGATTTTGGAAATACAGTCTACAACGATCACCATTATCACTACGGTTATTG
GCTGGTATCTGCTTCAATCTTTTTGCACCTTCACCCGGGTTACCCAAGAGCGAAGGAACT
TCGAGGGAAAATAGACGTTCTGGTAAGGGACGTCGCGAATCCAAACGCAAGCGATCACTG
GTTTCCCAGGTTTCGCCATTTCGATTGGTTCCTCGGTCACTCCTACTCGCATGGAGTGAC
ACCCATGGCCGATGGGAAGGACCAGGAGAGCATCTCGGAGGAAATCAATTTTCATTACGG
ATTGATGCTCTGGGGCATGGCCACAGACCGACTGGATGTCGAGCAACTGGGCGAGTTGTT
GCTCAAGATCAATGCCCGCACGGCCCGCACCTACTTTTTCATGGCGGACGATAACGTGAT
ACACCCAAAGGAGTTCGTTCCCAATAAAGTAACCGGAATCTTTTTCGACAACAAGGCGGA
TTGCACCACCTGGTTTTCTCCCGAACGTCATTGCATCCACGGGATCCAGATGCTACCGTT
CTCGCCGGTCACCCCCCTATTTCGCCCTCAGAAATTCGTTAAAGAAGAATGGGAAAGCAT
CCTAAGCAATCTACCGGTCGTTTTGGATCCCAAAAGCAACCTCAATTCGTGGCAATCCAT
TCTGTTTGTGAACAGCGCCCGAGTGGTTTCGAATAGAGCGAGAGACGCAATGAAGCACCT
GGAGTACGTTCCAATGGACAATGGATTGTCACGTTCGTGGGCCCTGTACATGGCAGCGAC
ATTGTGCGATGCGTAGCATACGACTACAGAATTCTGCATCTAGTGAGAAAGTAATGAGAG
AAATCATGCTTTGCAGCTTCGTGTTACTTCATATAGGGATTGCAACGAATATCGCAAAGT
AGTAAATGATGACCAACCAACAGCCAAAGTAAGCAAAGAAATTTGCATTCCCGCTGCTCT
CCGGTTGAATTAGAAGTACATATTTACCTTATCAAATCTATGATCCAACAACCTCAAGCA
GCAATAGTAGCTCAACCTCTCCCCTTGCATTATGATTAGTAGCAATTGGATTGCTGCTCT
CCTTCTTGATGTCTGTGTTTATTTGGTGTTACTTTCTTCTCTCTTTCTTGATGCAGCAGC
AGTTGACACTATGAATGATGTTACATCCCAAGCAATGCCTTCCAGC
>comp2595_c0_seq1
GGCCAATCAACACTTTCTGCTACAGAATCTTGAGACCATGTTTACAAGAGTTGGAAAGGT
CGAAGTATCATTAGCAGTATTTTAGTTCCTGAGTAGAAGTTACCTTCTGTTGGGACAAGC
ATTGATGCATTCTCAACCCAAACAAACTTACCAATTGTAGGAAAGTCAAGGACACCAGAA
AAATGACGCCCAGTTCGAATGATCCCGCGGAGGCCAGGAAACGCCGAAAAGAAATTATTC
AATCGCAGCGCTCTGAGAACGAACTAATTGCAAATGGTTCAGTGGTTGCATCTTCTAGCC
ATCCTCCAGCCATTTCATCAGCGATGGATGGAATCCAAGTCAATAGTGCAGTCGACCATG
TCGAAGGCCAACCAACCAACATTTCTAATAATGATCCCAGTAGCAATGGGGCCGACAAGA
AACCTCCGAAGAAACGAAAACTTGGTTCTGGAGACCCCAATGGGAGGAAAAATAACAAGA
AAGATAGCGGCAAGTCAAAGAAAACCCAGATTCGGTATGATCCGGATGTCCCCATGAATA
AGGATCAGCTTGCTGCCTGGCGAAGGGAAGCCAGGCGCGTTCGTAACCGGGAAAGTGCCG
CAGCAAGCCGCCAGCGAATCCGCAATCGCATTTCTGAATTGGAAGAGGAAGTCGGAGAAT
GGAAATCGAAGTATTCCATTGCACTCCAGAGATTGGAAGCACTGGAACAACACATCGCGG
CGAACGGGAACGGAGAGAGTAGCATAGGTGTACAGGGAGAGCAGGAACCAAAGGAAGAAC
CACAAGAACAGAATCCACCGTCAGACCAGCCCACGGAACAGCATCCACAGGAACCTGGCC
AGCAACCCACAGAGCAGCCGCTAGCCAGCTCTGATAATGCTAATGTCTGAAGATTGCGAC
AACATGATTAAGCTCTGATTGGATGATTTCGTAGAAGTTTCATGACAATCATCTCAATTG
CTCATCCTGAGAGTTGGGAAAAAGAAATGTTTGTGAGATTCTCTTCATACTAGACTGGGA
AAGGGTGTGAGCATGTTACATCGACATATGGATGTGCTCAATGCAACTGAATAAG
>comp2596_c0_seq1
AACCAAATGATGAAGCTATACACAAACACTTTTGTCATCCTCTTGACTCTCGCCCTCCTG
GGCATTTCTTCTGTTTCGGGAAACGCCGGCAGAGAAAACAATGATAGAGTTCGTGCCCTC
AAGGCAAACAAAGCCAACAACTACGAGTACTATGGGGCGAAAAACACGAACAACGATTTC

GAAGTTATCGATCAGAGATCTGTAAATACAAATTCAAATCATAAAGTAGGCAAGAACTCA
GGCAAGAACTCAAACACAGGGAGAAATGAAGTGAACAGCGATTTCGATGTCATCAGAAGT
CATGACAAAAAACACAAAGAAGGCCGTAACAAGAAGAAAGATAAACCCAAGAATAAACCA
CTTCCTCGGAATATGCCACCCAGCCCCACCCCAGTACCATCTACCAAGTCACCTTCGTCT
CCCACACCATCTGGAAAGGGTAGAGGAAAAGGAAAAGGAGGAAAGGGCGGGAAAGGCCGC
AAAGCAGGAAAGAAGGGAAAAGGTAAAGGAAAGGGCGGAAAGGGCGGAAAGGGTGCCAAG
TACAGAAAACCCGATACCGCTGCTCCTATCACTGCTGCCCCCTCTGGAGCTCCTTCATTC
GCGCCGTCGGACGCGCCTAGTCCGGCCCCCTCAGCTTCACCAAGCGTTGCGCCCCCGCCG
GATACACTCATGCCAACCCCTCTAGGTTCTACAGCATCTTCCTTGGTATCTTCCGTGTTT
GTGAGTTCCGAAAGCAGTTCCGAATTCACTTCCGTATCCAGTACCGAAAGCAGTAGCGAC
GCCGCTGCCACCAACGGAGAGGGTGTGGGTGCCGCTGCTGGTGATGGTGTTGGCCACAAG
CGAAAAAACAAGCGAAAAAACACGCGAAAAAACAACCGCAATCCCAACCGTCACAAAAAG
GGAAGGTGGGTTCTGGTAAAAACCAAAGACGGTGAGTGGAGACGAAAAACGAAGAATCAT
CGCAAGCGAAATGAAAACAAAGGTGTCACTGTCAGCAAAAACGAAAACGAAGAGCTCGGA
GACGAAGAAGTTTCCATCTTCCAAGACGAAGGTGAAGACAATGAAGAAGAGGAAGTCGAT
GAAGTGGAAGTCGAAGAAGAAGAAGTAGAAGAAGAAGTGGAAGAGGAAATGGAAGAGGTG
GAAGATGAAGGCGAAGAAGCTCCTCTTCAACTGTAAGCGTAACATTTACGAAAACAATGT
CGATATTCTTGAGTCGGCAAGAATGGCTAGTTCCCCTACAGATCTATTATCAAACACGCA
TGATGAGCACAAAAAGTTCATCGGCACTTTCGTCGCCACCTTTGAAAACGAAAGATATAT
TTATTTATTATACATCACACAAAAAACAATGGTCATCGTTTTCATGTTCCTTCGGATGGA
TCACTATCTAAACAACAATGAATCGATCATCAGAGGAAAAGGGAAAAAAAAAAACTAATA
AAAAAAACTTGTAAATTGGATTATTTAATACGATTGGTACCGTAC
>comp2597_c0_seq1
CACTCGAAAAGCACAATCGCAAAAACCAACAACCATGACATCATTCTACTTCAATCGCAG
CAACAGGACTAGGCACCTTTTGGTGCTCGGACTATCTATCGTGGTATTTGCCGTCCTCGG
AAATTTCGTGGAAAGCATCAACCTTGTCGCAAGAGTAGATGAGGATTCTAAATTATTCAG
CAACATCAAAACTGGCGACAATATAGGCCGCATTCGTGGGAGCAGGGTCCGCAGAGCTTT
GTCCTCCGATGACGATGAAGGGAAAGGAGCAAAGGGCAGCAAAGGGTCAAAGAACGGAGA
CGACGACGACGACGACAGAGGTACCAAAGGTGGAAAAGGCTCTAAGAGCGGAAAAGGATC
CAAGAAATCCTACTATGGGGACGATGATGACGATGGAAAGGGATCCAAGAGCGGAAAAAC
ATCGAAAAGCGCTAAAGGATCCAAGGGCGGAAAAGCATCGAAGAGTGCAAAAGGATCAAA
AAGTTACTTTAACGGAGACGACGACGACGACGATGATGGAAAAGGATCCAAACGTAAGAA
GCGGTCAAAGACATCTAAGAGTAGGAGCAAGAAAAGCCACAGCAGCACCACAGAATCCAG
GATCGATGTTGAGAGCTCCTCCGTAGATAAACCAAAAAATTCCATGACCTCTCAAGAAAA
AGATCACAAAGAACACATCGCCGATATCGGAGAGCAAGGTCGGGGCATTTTTGCTGGCAT
GCGACCTGACCTATGGGGTGAGCTTAACGAGGAAGATCAGAGCGACAAAATCGTCGTTGA
CACCACTCAATCCCAACAACAAGATCTTTCTCCGAAAGAGGATAGCAACAGTGCTGCAAA
CGATAGAATTGTCGAAAGCACCCAATCCCAATCCCAATCTCAATATTCAAAGGAAGACAT
TGGTGGTACTGGCGAGGTAACCATCGAGGATACAACCCAATCTCAAAAACAAATTCTAGA
GGACGACATTGATGGTACTAGCGAAACAACAGTTGTCGAAACTACCCATTCCCAAAGTCC
AAATGAAGACATTGGTGTTGCTGATGCTGTAACCGATATGGATACAACCCATTCTAAAGA
CAATATTCTAGAGGATGATATTGGTGACAATAGTGCGACAGCCGTTGTCGACACCACCCA
ATCCCAAGGTCTAAAGGAAGAGATTGGTGGCACTGTCGAAGAAAACACCGAGGATACAAC
CCATTCTAAAGAACAAATTCTAGAGGACGATATTGATGGCAATAGTCAAACATCTATTGT
TGACATCATCCAATCCAAAGAAGAAAATATTTCTCCAGAAGAAAATATCCCTGAAGGAAC
TGGTGATACAATTGATGCCGACACAGCTCCATTGGAAGAAGAGAACAATATTCGTACCGT
TCAGCTTTTTCATCCAGAAGCTTTGGATGAATGTGTAACATACGACAAAGAGCTCGAAGA
AATGACCATATTCATGTCAGAATGTTCAACATTAAACAACGATACGAGACACGATGATTA
CTGGGAAATTCTTCAAGCAAGAGTATCAATAAATAATACGGAAGGAAATACCTTCCATTT
GCGTCACAAAGCGACTCAATTGTGCATACCGATGAACACGGAAAATCCTGATGCTTCCTT
CAATTGCCAGCGGTACTTTGGAATGAATGAGGCAATTGCCGATTCTTTCAACGGTTTGAT
TGACTGTGATAGTGGTGTTGCTGCCGTCTTTGGAATGGGTGGGGTCGCAAATTCATTATA
CCTTAAAAATGCCGGTTGTTCTGACATGGCGATATCAGATAACAAGTCAGATCTGGACAT
CGTATTTATCACCTATCAAAATCCCGTCGAAGAGAAGCGCGTGGTGGTTTTAGGTCAATC
AATTTTGTTAGAACTTGGAGGTGCTGTGGCCAGCTACGATTTTAGGACCGAGTGGGCCTT
TTTGGACGTGAAGGATATTGATAGAGAGAAGAAAGCGGTCGCACCGCAACCACATTCCGA
TCTTTTCGATCATATCTTTCACCTGAACGATGGGAAAAGCAAAAACGGAAGCTCCCCCGC
GAAGAGGACGTAAGATGGACGACAGCAATGCAACAAGTTGAATTGATCGGGAAGTGACAA
AGAATGTACAGGGGAAAATCTGTTACTTTTTGCCGTTACGGCACCTACTTATCTAACTGC
CATAGAATTGTAATCCTGGATCAGTCACTAAGAGGGATTCAACCAAGGCTTCCGTCACTT

CTGGTTGAAATAGGGATAATCTGGAAAGAAGTGTAAACATCGAAAGCAATCCGACAAATT
GTCAATGTTTAGGAAGGCACGAATCTAATTCGTTGATTGATTCATTCGATGAGGAAGCAG
AATAGAATTTTACGTGAAAGTAGCACATAGTAACACAGAATCTACTTCTAGTCATATTCA
TACGCC
>comp2598_c0_seq1
ATCGGTCTTGCCTGGCAACGCAGTGGATACTTACTAGACTGCGAGCCTAAGCTACTAAAG
GACTAAGCGGAACGCTAAAATTACACCAGGAGCAACACTCCTTCATATTTACCAAACTAT
TTGCAAGCAAGGGGATAATCCTAATTTCTCTCATCCGTGGAGCAATCTTCCCCGCTACTT
AATACTATGACTACGGCTGCAAGGACGACTGCGAAGTCGTCCTCGCCAGGGCCAGCGCCG
TCGTTGTGGACGGCCCTGGTTCGTTCTCCGTTGCTTCCACACGCAACCTTTTGGGGAGCA
GGACTCGCGGTTAGGGTTTCCTTTCCTGCCGCGTTGAAATGGTTGGCGTACGACAGCATA
TCGACACACCTGCTGACCGTTTCGTATCCCCTTTGCGCTACGGTATCCTTGATCCATCGC
GACCTCCAGCAGCGGCATAAGCAGCACCAAGCCAACAACGACCACGATCCAGCAAACGTC
AGAGCGAACTCCTTGCTCCAGGTTGAACGACAGTACTGGATCGAGTACTGGAGTGTAGGA
TTTGCAGGCGTGCAATTTCTGCAGCAAGCCCTCGAGTTGTTTCCCTCCTTGCATCGTGCG
GGACTTCAGGAGTACTCAAACTTGCCGGTGCTTTTGGCTCAACTGAAACTGATGTTCTTT
GTATGGATATTTTGCATGCAGAGCGTTTTGGCGGGGCACCAGAGATTCCTAGGCGGACAA
CAACAAAACGAGAAATGGAAGAAATTCGTCCCACTAACTTTTCTTACCAAAGCCATCGGG
CCCAAGCTGATGGGGTTGCAAATGGCTATCTCCGAACGAATATCCAAGGAAACATGGCAG
CGGTTGATTCACAGCAAAGTGCAGCGTGTCCTCGAAGTCTTGGTCATGCTGCAATTCTTG
AAGGAGACCTCGAGGGATTATCTGTTGCAACTTTTGGAAGAAGGAAGATCTTTGTTGCTT
CTCTCCGTTTTTGCCGTGTTGCCCTCATCGGTCACCCAGGTGGGGATTTTGTATGCCAAA
TACGTTTTCCCCGCTGCCCGTTCGCTGGTAGCACGGGGCGATGATCTCGAGGTACTGAGT
TTGAAATATTGGGTCTTGAACAACCTTTTTTCCCTGTCATTATCGTTCACTTGGTGGCTT
TGGTGGTGTGTGCCGTTTTCGACCCAAGTTTTGCTCGGGTTGTGGTGCTTCGCAACCTTT
CCATCGACGATTACTTATTATTATTCTTTGATTGAAATGGAGCTCATCACATTTGGCATC
CTTTCCGGCGAACCAAAGTTGTCCGTCAATAAGACAATGACTGTTCAAGCGCTGAGGCAT
TTTGTCAAGCGATTGCCGCGAGACAAGCAGGCGCAAAGCTTCCAGTTTGAGATCGACGAT
GATTCGATCAATCAAAAGAGCAGTGGTGATAATGGCGATGACAGTAGCATTGGCAGCCAG
GATACGACAGATTCCGAAAGAGAGAGAAGAAGACAAAGACGTCGCGAAAGGAGAGCCAGG
AGGAAACGGGAAGCAGAATCCAAATCAAAGAAGGCCACGCCGCCACCTCCTCTCCTGTCG
GAGGACTGCATCACGCAATATGCAAATGATGAACAAAAAGATGATGGAAAGTACAGCTCA
TTTGTCAACGAAAAAAGGCAGAGCGAGAGCGACGAAAGAAATCAGTACAGACATTGCAGT
ACCGTAACTGAAGAGAGAGACACAAGAAACGCTACTCAACTGCAACCCCAACAAGTGGAG
ATTAGGCATCCCACGTCGATGGTGTCGCTTGTGGATGTTGATTACGATCACTCCTTATCT
CGTCCAAACAAAGTAGAGGAAGAAATGTCATCTGATTCTCTGGCTTCCATGTCGGATGAT
ATCAATAATTTGATACCGGCACCGACTCTATCAATGAACAACGAAATGGAGGATAGCGCT
ATGGTCTCCATTCAAGCTGATGATTTTGATGATAAGGATGGCGATGATAGCGACAGAGCC
CTACTTCCGTCCTTGACAATAGGCTCTAATTATTCTGCTTCGCAGGTACACACTGATGCA
AAATCAATGGGCCTCAATATTGCAACGCCAACTGAAAATGTTACCGGGGAGGTAGAAGTT
CAGATGAGGACGACACGGGGGCAAGCGAGATCGACTACAACGAGCACGAGACCAACAAGC
TCTATTTTCCCAGCGACTTATCCAAGGTCATCGTCAAGAAATAGCCTACTACTGCTGCCA
TCGGCTTCTATAGAGAGGAGTTTTAGTCTAGTGAATTCGATGTCCATCCCGGAAGATAAG
CTTGCGCCAAAACCAGAAACAAAATCAAAAGCTCACTCCTCCGATAACAATGACGACGGT
GATTGTAATGACGGCTTGAATATTTCTGATGTTGACAGTACGTCATCTAGCCTGGTTGAC
GATACAGAGTCATTTGAAATATCAAATCCATTGGATGGTAATGAGAAGCAATCATCCAAA
GCAATCAAGCAGGATTATGTATGTAAAAAGAAGCCAACCAAGACTGTAGACGTAGATGAA
GGTGTTTCTACACCCCCGCGTCGATCGTCGCGTTTAGAAAAACTTCGCGAATGGCAAGAA
GAGAAGAAAAAGATGAGTCCTTCGAAAGCCTCCAAAAGATTGGAAGCAAATGGCGGAAGC
AAGCCATCGAAAAAAACGTCATCTTCGACCGCCGAGAAGAAAGGAAAAAAACCGAAAACG
CATTCGAGTTCTTCCCCGGAAAAGAAGAAAAAATCCAAAGATAAATGCCCTTCCAAATCT
GTTAGAGGATCGGAAGCAACCAATGGAGTCAATGGCAATTCAAGTAGGAGATCTACATCA
AAAGCGTCATCTTCGGAGAGAACAAGTGTCTTTGACAAACCGACGGCCGCTTCACTGACC
CGACGAGTTGAGAAGAAGTCCACGAAAACTCCTCGTGACAAAAGTCGCACGCGTGCCACC
ACAACATCCACTGCCAAAGCAGCATCCCCTGGAAAGACTACTAGAAGTAGCAAAAGTAAG
GGCGAGGCGCCAATTCCAGAATGTACGGAAGTAGGCCGAGATAAAAACAATTCATTTGAT
TCTCCTAATCAGAAAGCAGCTGGAGGAAAACCACGAAAGATTCCTAACAGCAGACGAAAG
AAAAAAGATATTTCTTCAGTCTTTGGTGGTGTATTGAGTCGAAAGTAGGGATCAATACGC
AATCAAGCCATGTTCATCAAATTTTAAAAGAATACAGCAACTATGCTGTACCATGGATTG
TTTTATCGAGTGGCAAACCACAAAATTGTCCCTTATGTACTATACTGTTCAAAATAAATA

TGCACTTCCACACAA
>comp2599_c0_seq1
CACAACTCGTAATTACATTCAACAACATATCAAAATGAAGTACGCTGCTGTTTTCGCTCT
CCTTTTGGTTTCGGCCACTGCCTTCAACGCCCCTCAGTTCGCCACCCGTGCTGTTGGAAA
GGCCCCTGCCACCAAGGCTGTCAAGAAGGCCTCTACCAAGGCTCCTCCTGAAAGTAAGGG
GTACCCTTCTTTCGCAGAGAAGGCACAAAACTTCAAGCTCGGAGGGGGAAAGATTTCTGG
AGGAGGTGCCGGACGTACCATCACACCTGTCTTCGTCCACCCTACAGTCAACACTGATCC
TATCCCTGACTTCGACTACAAGGAAGCCGCTAAGGCCCGTGTAACCCCCCAGAGCCGAAC
TGACTACGTTTTCGACGATGGTCTTACCGAGCTTGAGCGCAAGCAAAAGAGCACCATCCC
TGCTTTCCTGACTGGATCTGCCCGATCCAGAGTCGAGGCCGAGGCCCCTCGTGATGATAT
CGAAGAACAAGAGTACCCTTTCGGGCTTTCTGGAGACCGATTCCAACTGTTGTTCATCTC
CGTCTTCTCTCTCTTCGCACTTGTCGGAAGTCTTTCCGGATCTATTAGCTTCGATTAAAT
AATCACAAGCGTAATGAAATGAGTCGACCGATTGGAATTGCGGCGATTTTTGCAAACAAT
AAGAACACGTCACACACAAATCCTTCCCATTGTTTGGTTTGATTCGGTCTTGTTCGATTT
CTTGTGCACTCTTCATCTTGTAAGATGTGCCTCGTTATTGATGCGCTACGATATAAATCC
GTTTCTCCCTACTTTTTAAGTTGAATACTTTGATATAATTAATTAGTTTGGTAGAGGAAC
GATGGGTTATCCGAGCTCTTTGTAGCATTTGAGTAGATACGGGCCTGAATTGATGGCAAG
AGTCGAATCTGGAGATGATTTAAGGCCCATTGGTAATCGTAAGCATCTGTAGTTACCATG
GGTCGTGGTGAGTGTGCAAATCTTTTGGCTTTCCTCATCCAGTTCAAAGCAGTAAAACAT
CATAGACAGATCAAGAATCTTAGTGAAGTGTTCATACTTCTGGTGTCTGTTCATGATGTC
TTGGATTCTGGGGAGGTTAAACTGAGGACGCTTGATCCTTTTGTTTAGCTCTCTGAGATC
GACAAGCCATCTGACTCGTCCATCCTTTTTGGGAATGATAAAGGCTGGGAAGCCCCATTC
TGAATTGCCTGTAGGAGCGATCACTCCTTCATGTACTAGATGCTGGCACTCCTTTTTGAA
CACCTTCTGGTGTCTGTAGGGAACTGGATATGGTCTTATGTAAACTGGTTTGGCATCGTC
TAGGAGATCAATGTGGATTCTTTTGTGAGGGTATCTACCAAGTCTGCCATCAAACAACTC
CGTGTGTTTGGCTAGCATCTCCTGGAATGCTAGTCTTTCTGATTTGGTCATATGCTCCTG
ATGGTTTGCAACCTCTCCTGGAGTCACTAAGTTTGCCATCAAACAACTCCTGG
>comp2600_c0_seq1
TTCTTCTTCTTTGTTTGTGTCTTCAGATTCCCAAGGCGCAACCGAGCCAGCTCAATGGGC
CACAGCCCGCTTCGTTCAATGGCGGTCGGTCGAGTACCACCTTCTTCGGCAAGGAACTCC
CAAGTGGTTTGTATGTATGCGGAGACCACATGGCTACCGCAACGCTCAATGGCGCGGCGG
AATCGGGGGTAAAGGCCGGAGACGATGTCTCCAAGGCAATGTTTTCGACGGGTGCTCCGT
CGCCGTCAGCAAGCGAAAGCGATCCCGAACCCGCAGCCGTAGCGCAATAAATAGTAACGC
CCC
>comp2600_c0_seq2
GCACGACGACCGCCATCGCAACAAAAAAGAACGATCGAAGGAACGGAAGACGTGAACCAT
GGTCAAGATATCCTCAGCCGCATTGCTGCTGTGTTTGCAGATGCAACCGGGAACCGTTTC
GGGGTTTCTTCCGGCGAGCAAGAACCTGAACGCGAATGGCAACGGCGCCGTGACGTCCAC
GACGCTCGCCTTTGACTGGTTTGGGCTTCGCAAGAACGGCAACAGCGAGAGCGTCGCGAG
CATCTTCGACTCGTCGACCAATATGATTGTGAACACGCAACAAAAGGACTACGTGACTGA
TGGGGACTGGCTGAGCAAGAACCTGCGCAACAAAAACAACAAGAACAACGACGCCGAGTC
CGCCGACGTTGTCGTCATCGGTGGCGGAATCTCGGGCCTCGCCGCCGCCATTACCGCCGC
CGAGGCCGCCAAGAGTGCGAACGACCCAAAGAAGATCGTCCTGGTGGAAGCCAACAACAA
GTGCGGGGGCCGGGTCAGCTCGGTAGAAACAGACGACGGCTTTGTTCTCGACGAGGGATT
CGCCGTCTTTATCGAGGAATATCCGGTAGTCAAGAAGCTTCTCGACTACGACGCCCTCGA
GCTCAAGCGGTTCCTGCCGGGAGCCCTCGTGAAGCTCAAGGGTAACGACGAACTCGCAAG
GGTGGCGGATCCCCTGCGCAACCCGGAAGACATTTTCGCTTCCGTCCTTTCCCCCATTGG
ATCCCTTATCGACAAGATCAAGGTGCTGCCGCTGGTCCTGAGCGTCCGTCTGGGATCTGT
GGAATCGATTTTCGCAGAGGCCGAGACCGACACCAAGACCGCGCTGGTCGAGCGCTGGGG
CTTTTCCGATGCCTTTATCGATTCCTTCCTGAAACCCTTTCTCGAGGGGATCTACCTGGC
CCCGCTCGAGGAGCAATCCTCCCGAATGTTTTCGTTCGTCTTTAAGATGTTTTCCGATGG
ATCGGCGACCCTGCCTAAGGGAGGAATGGTGGCCGTGACGAACCAGCTCGTGGAAAAGGC
CGAATCGCTGGGCGTGACCATCCTCACCGGGACACCGGTCGCAAGCGTGGCTCCCAAGGA
CGACGATGCCGGATACGTGATCGAGTGCGTCGACGGAAGCAACCGGTCCTTCGACACACC
GTCCGTGGTGGTGGCGACCGACGGCAAGATTGCCAAGAAGATCATCGGCAACATCGAGGG
GTTCGAATCGCTCAGGGACGAACCCGACCAGGTCCAGCGCTCGGTGGGGTGTGCCTACTA
CGGGTTCGAGGGACCCGCGCCGGTCGAGGATCCCATTCTGGTGCTGAACGGGATGAAGGG
CGAAAACGACGATCCCGTCAACAACGTTTGCTTTCCGTCCGTGGTCAACGACGGCTACGC
GCCGGAGGGATACAACCTGTGCAGTGTAACGGTTCTTAGCGATGCCATGGACAAATACAT
GGGCAAGCCCGACGAACTCGACCAGGCCATCCGAAAGCAACTGGCGACGTGGTTTGTGGA

TCAAGAGAGCGACATTTTGGAAAACTGGGACCTGAAACAGATTTTTTACATTCCCAAGGC
GCAACCGAGCCAGCTCAATGGGCCACAGCCCGCTTCGTTCAATGGCGGTCGGTCGAGTAC
CACCTTCTTCGGCAAGGAACTCCCAAGTGGTTTGTATGTATGCGGAGACCACATGGCTAC
CGCAACGCTCAATGGCGCGGCGGAATCGGGGGTAAAGGCCGGAGACGATGTCTCCAAGGC
AATGTTTTCGACGGGTGCTCCGTCGCCGTCAGCAAGCGAAAGCGATCCCGAACCCGCAGC
CGTAGCGCAATAAATAGTAACGCCCC
>comp2601_c0_seq1
ATATTGAAAGAATTTTAGGAGAAAAAACTATGTCAAAAGTTGTTGGTATTGATTTAGGTA
CAACGAATTCCGTTGTAGCAGCTATTGAAGGTGGTCAACCAAGTGTTATTATTAATAGCG
AAGGGTTACGTACAACTCCTTCGATTGTAGCATACACAAAAAAACAAGAATTATTAGTAG
GTCAAATTGCAAAGCGACAAGCTGTAATTAATCCAGAGAATACGTTCTTTTCAGTAAAAC
GATTCATTGGTTCAAAAGAAACAGAAGTTTCAGCAGAAGCAAGACAATTACCATACACAA
TTACAAATGATTCGAAAGATAACATTAAGATTAAATGTCCTGCTTTAGGAAAAGAATTTA
GTCCAGAAGAAATTTCGGCTCAAGTTTTACGAAAATTAATTAATGACGCATCTGAATATT
TAAAACAAGACGTAAAACAAGCTGTTATTACTGTACCAGCTTATTTTAATGATTCTCAAC
GTCAAGCAACTATGGATGCGGGTAAAATCGCTGGTGTTGAAGTTTTACGAATCCTTAATG
AACCTACTGCAGCATCATTAGCTTATGGTTTAGACAAAAAACAAAATGAAACAATTTTAG
TTTTTGATTTAGGTGGTGGAACATTTGATGTTTCAATCTTAGAAGTTGGTGATGGAATTT
TTGAAGTTTTATCAACGGCTGGAGATACTAAGTTAGGTGGTGATGATTTTGATAAAGTAT
TAGTTAATTGGTTAATTCAAAACTTTAAAGATAAAGAAGGGATTGATTTATCAACGGATA
TTCAAGCATTACAACGTTTAACAGAAGCTGCTGAAAAAGCAAAAATGGAATTATCAACAG
TTCAAGAAACAAAAATTTCTTTACCTTTTATTACGGCAGATGCTTCAGGGCCAAAACATA
TTGATGAAGAGTTAACTCGAGATAAGTTTGAAGTTTTATGTGAAGATTTAATTGAACGTT
GTCGAATTCCTGTTGAAAAAGCAATCAATGATGCTCGATTAGACAAATCAGATATTGATG
AAATTGTATTAGTTGGTGGTTCAACACGTATTCCAGCAATTCAAAATTTAGTAGAATCAT
TAACAGGTAAGAAACCAAACCAATCTGTAAACCCAGATGAAGTAGTTGCAATTGGAGCTG
CAGTTCAAGCTGGTATTTTAGCAGGTGAAATTAAAGATATCTTATTATTAGATGTAACAC
CACTTTCATTAGGTGTTGAAACACTTGGTGGAGTAATGACAAAAATCATTGCAAGAAACA
CAACGATTCCTGTGAAAAAATCAGAAATGTTCTCAACAGCGGTAGATAACCAAACAAATG
TAGAAATCCATATTTTACAAGGTGAAAGAGAATTAGTTTCTGCAAATAAGAGTCTTGGAA
ACTTCCGTCTAGACGGTATTCCAGCTGGAAATCGAGGTGTTCCACAAATCGAAGTAACAT
TTGATATTGATGTAGATGGAATCTTATCAGTAACCGCAAAAGAAAAAGAAACAGGTGTCG
AACAGTTTGTAACAATTCAAGGTGCTTCAACATTAAGTGAAAGCGAAGTGGAAGCTATGT
TAGAAGAAGCAGAGAAAAATGCAGCTGCTGATAAAATCATGCGTGAAAACATTGATCTAA
AAAATCAAGCTGACTTACTATGTTCAGAAGTAGAAAAAGAACTTCCGAATGCAACTGTAT
CAGAGGATGAAAAACAAGCTATTACTACATTAGTTGGTGAAATCAAAGATAGTATTCAAA
ATGATAACATTGATACATTAAAAGTCTTAATTGAAGATCTAAAAGAAAAATTAAATGGTA
TGAAATCTCAAGAAGTAAATCCTGAAATGTAATTCCATAAAACTCCTTTCTCTTTTTTGG
GAAAGGAAAATCCTTATCTTTTCTCCAGAAAAGATAAATGTATAAAATTAATGGGTCTG
>comp2602_c0_seq1
GAAGAAGAAAAAGAAGAAGAAGAAAAAAAAGAAGAAGAAACAGAATGGGAAAGACGCAGA
ATCCAGAGCCGGAGGAAATAATACAAGCAATCAACACAACTCGGCTTTGGATCATTCGAA
TGCGGCAACAGTAGAAGAGAAGGAAGACCCGCTTGTCACGGCACTTTTGGGTATGGGATT
CGCCGAAAGCCAGATCATGGCAGCCGTCAAGGCCTGTGGTGGAACGCATCGTGCAACAGC
CGACGATCTCGTCACGTGGATTTTAGGACAAGGCGAAACCGAAGAAAGCGCTAACGAAGC
AACGAGAATCCCAGATGGAACGGACCAGCTGCAGCAAGAAAATGCCGCATACGATCGCAC
ACGGACAGAAGATTCGCTGCCAACTAACGAAAAAGAACAGCAGAAACAGCAACTCGAGGC
CGTCCGCAAACAGGAAGAGGCTGCGCGCAGACTAGCAGCAAAACGCGAAGAAGCTAGGCG
TCGAAACAGGGAGTGGAACAATCGAGAACAGGCACGGCAGCAAAAGGAAGCGAAGGCAAA
AGTCGCTCAGAAATTCTTCAATGTCCCCCGTCCTGCGGTGCCGCTTCCTGTTCCAAAACC
AATGGCAACACCCGACCTGCCCGTACCTAATGTTTCGAAATTGAAAGGACCTCCTCGACA
GCCTAAGCTAACAACCGGTACATCGGGATTGCCAATTGGTGGTAACAAATGGGGAGTTCC
ACCGATAAGTGCTAATCCAAAACCGTTTCCTGTCTCTGCTAACCAATCTAAAAGCATTGG
TTCAAAGCCAACATCTTCAGCGGCTCCAACACCACCGACGGCAACTGAACAGCATGCATT
GCCTGTTTCGAGCTTCCTCGCTTCTCTCGACGACGACAAAACGGTATCATCGTTCGGTAG
CAGTAGGACCATGTCCGTGTCGTCGAAGGAGTTTGTTCCCATTGCTTCCCAGCCATCTGT
ACCACCTCCAGGGTTCTTGTCAACATCGGCTGCGCCTATTTCAGCTGCTACTAGATCTCT
TGTACCCCCCTTGTCAGAAGGCCCCATGGGAGCTCCATTTTCGCCAGAAAGTCGGCAGGG
AGAAATACGAGCTACCGCAAAGGAATTCGTGCCATCGTTTACAATACCGGGTAGTGTAAA
TCCTAGCGCTTCCCTACAGAAAGACTTTGCTCCTTTGAACGATCTTGTTGGGGAGCCCCA

GCCTTCTGAATCACACGGTGTAGGATTTCCACCCGGTATATTACCGCAACATGGTGCGTC
GAAGGTGGGCCATACTAGCTCCCTTCTTGGACCGAGCCTTCAGAGTTCTTTTGACAATGT
ACGACCGATGAATTCTGGAGCCGCAGATACAATTCTGTCAGGTGCTTCGTTTGTGGATGG
ACCAAAGTCGGAGGAACCCCCAATGTCGATTCCTTTTGGATTTGGTCAGGCCGATTCGAA
CGAAAGCAACGACATTGGTGGCTCTAGGCTTCTGAATTCGATTTCCAGGAACAGGTCCAC
CGGATCGGCATCGATCTGGGGAGATGGCCAGCAGATCTCCTCGCTGGAAACCACTCTACA
GCCTTCCTTCTTTGGTGACGGAACGACAAACGAGTCGGACGACAATCAAAAGAGTATGTT
ATGGAACGTTTCAACGGGTGGCAATTCTAGCTTGCCGAACGGTGGCGGTCAGGGATCGAT
TTGGTAGACAGAAATGTGCTG
>comp2603_c0_seq1
CCTGAATATGGGAGGGAGATAGCTGCTGAAAGGTTCTACTCATGCTCGTGCTCGCGCTGT
ACAATCCTATCTTCAGACTGCGGAACGACTAAGTACCATACTCTAATAAGGGATACACCA
AGTTCGAAACCTCTCGTCTTCCACTCTCTCTGATGGACTACAATTTTCCATAAAGATTCC
TCGCAAATAGACTCTTTATTCAAGATCTAGTGTCTATTCTGTGCAATACATCGAAGGCTA
GAACACTGTTCGAGACAGAGAGAATCATATTCCTCAGTTCACAATGGCGAATCCATTTAT
CAATTCTTCTTTCACATTGCGTCCATCGATGAATAATAGGGACATCCCAGAGTTGACACT
TCGCCAGACCGAGGTCTCCAGAATCCCAATGGAGAGAACCGAAACGAAGGAAGGAAGGTC
ATTCAAACGCCAACGGTTATTTATGGATCGCTTCATGTCTACAGCGTTTCCCTTCGGTTA
TCGCTACAGTTGCAACGATGTTGATTATAGCTACGATGACCAGTACCTTTCAGACGAAGA
AAATGGCGTTCCATTAACAACACGGCGGAGAGTCTCGAGTAGACTTACCTCTTGTCGTAG
GATAAGTGATATAAGCATCGGTAGCAGCAGTAATGGTATGAACATCAGCGAAGCTGATTA
TTGGAAGACCCGCTGCCTTGATGTGCAAGATACGTTTGAGAATGCACAAGCTCGCTTGAG
AGAAGCGGAAGAGGACCAGAGACAGTTGCGACAGCGAGTTCGCGAACTTGAGGAGCAGCT
CTTGCTCCAATCGTCATCAGGAAACAGTAACAGCAATGGTGGGATTGGAAATGAAGATTA
TGAGGCTGGCAACACGGAAGAAACTTTCGAAACAAGGGATAGGGACGCTAGCAGTAATAT
AGAGGGGAAGAACAATAGAATCGCACCGAAACATTTGGGGAAAGATGATGGACAATGTGC
CGAGGATACAGGTCGGAAAAATAAGAAGGAACGACCACTCCCTGCCCTAGTAGTTGAGAT
CAAGGAGAATCACCAGGCAGTCTCGTCCTGTTTTTACTTGACAGACGGGGAAGGATTGAA
CGAGTCCTATGATCAAGAGATGGAAGACGTTTCAATGGATGTTTATTGCGGCGACCAGAT
GCTTGGTGATATCGATGACGATCGTAACGACATAAATTTTAACCAGCAGCGTGGGTGAAA
GTAAAAATCATAGCGAATACTGTCTGTATACTAAAAAACCTTCTAAAGCAAGATCGGTTT
CGAAATGAAA
>comp2604_c0_seq1
AATCTCCTCTACGCAGTAACACTTCAACTTGAGTGCCGAACTTTTCTTCGTCGAGAGCAT
CGTTTGCCTCCCGTTTTCTCCTAGACGACCCACTATTACTCCTCTCTGTCAGCAGTTCAA
GAAAGTCTCCATCATAGTGCAGTCGAACTCAGAGAGTACCTTTAAAACCAACCTAGATCT
ACAACATTTTGAACCTATTCCATTGTAACAGTACGTTGAATCGGATCGGATTAGCTGAGA
TATCTGCTCATCACACTCGATTATCTTTCTGAAAAGAAACAACATCGAAACCCAACGGAA
CGGAATTAGTAGCGAATAACAATTTAAAATGAGACTCTCGTTCGTGGCCTGGATGGCTTT
CCTGGCTTCGACAACTTTTGTTGATGCATTCACATCTCAGAGTGGCAATACCAATAGGCG
TATCACTCAGTCCCCCGGCAAATCGGGTGCGAATAAGGCACTTTATTCTCTGTCAACCAA
ATCGGAAGATGATGAAAAGGTTGCCGGAAAGATTCTTGAGGGTGGAAAAGTTATTGATTT
TGAGTCTACTCGGGGTCCTTCCCGCGCTGAAAACGCTCTAAAACTTGCAAAGGAGGAAAT
CGCAAATGGGACCTTGAAGAACACGCACCTATCCGATAAAATACTTGGGATCAATTCCGA
TGTCATTGCCGAAGTAGGGCACGACCTAGGATTATTCGCATCAGATGACGAGATTCAGAA
ATGTGCAGCCTATCTGAGGTCCAAAGCTGCATCAGGTTTCTTTGAAAAACGCCCAGGCTC
CGTTGCGGTTACCTATGACGAAATCCATAGCTCACCTTCAATAGGTAGCGAACAAAAGGC
AGGCGTAGCAAGTATTCTGGAGAAAGCATTCGTCGAATCGGGGGAAGTGACCAGTGCATT
TGCAAAAACATTTTACTTGGGAACGCAGCTCATGCCAGAAGCACAACGCGAGGCGATCTG
GGCTATTTATGTCTGGTGTCGCAGAACTGATGAGATCGTTGATGCCCCAAGGGACAATGA
CGATGAGATGCTCTCGGACCTCGGCATATGGGAGACACGCTTAGAAAACCTTTGGAAATA
CGGAGAGGTGGAAGATGTTTTTGACCTGTGTCTCTTGGATGTTTTGGTCAAGTATCCGTC
GCTGCCGATAACGCCGTTTGCTGACATGATACGTGGAATGCTCATGGACGTTCCGGAGCT
TGGAACAGATACATACGATGTATTCGACGACCTTCATTTGTATTGCTATAGAGTAGCTGG
AACTGTTGGATTGATGTGCTTGCCAGTCTTCGGATGCTCCGAAGGTTACGACGAAATAAT
CGCAAAAGAACCTGCACTGTCGCTTGGAGTTGCCTTTCAACTTACAAACATTCTTCGAGA
CGTTGGTGAGGACGCCACAACGCGTGGTAGAGTTTATCTCCCGATTGAAGATATGGAACG
ATTTGGTGTCACAAAAGAACAACTATTTGAGCAGAGAGTAGACGAAAACTATATCAACAT
GATGAAGTTTCAAATTGCGCGAGCCAGAATGTATTATGAACGAGCACGTCGTGGGGTGTT
TATGCTCTCCGAAGAGAGTAGATTGCCTGTGCAAAGCGCCTTGGATGCTTACGGAAAAAT

TCTTGACAAGATCGAGGACAACGGCTACGACACTTTAACGACCCGTGCGTACGTAGACAA
ATGGGAAAAGTTATCTATAATTCCATTTTCGTGGTACCGAACTCAAGATATTTCAAGAAC
AGTGCCGCTCCCAGGTGACAAGCAAATCCCATCTCTAGAGGAACCCACACCTTGAGCCGT
TCTTGACTAGGTTGGAAACTAGCTACAAAATCGTGATCGATCATACATTACGCCAGTGTA
GAAAATAAACCAGCGCATACTTCTTTGGAATAATCCATTCAATCAAGATGAATGGTGCTA
CAGAAGGCCCAGGAATCACAACTCAAATACACCAAAAATCGATTTCTATTTCAAACAAAA
AGACAATTCGAGAACGCGACAATACTGTCCAAGTCTTTTTGACTTGTCACGGCAAACGCC
ACATCGATACTACAAGTATAATAACCAAATGGATCTACAGTTCATGAACTACTTAAGTAG
AATCTCTATTTGTTTAATTTTCAATTCTTTTCTGGGCCATAATCGCAAATACGCATGCAG
CAGCAATATCTGATTATCTTGATCAACCCCAACTTAATGAAAACGTTCTAAGAAATCTTC
AGACTAAGA
>comp2604_c0_seq2
AATCTCCTCTACGCAGTAACACTTCAACTTGAGTGCCGAACTTTTCTTCGTCGAGAGCAT
CGTTTGCCTCCCGTTTTCTCCTAGACGACCCACTATTACTCCTCTCTGTCAGCAGTTCAA
GAAAGTCTCCATCATAGTGCAGTCGAACTCAGAGAGTACCTTTAAAACCAACCTAGATCT
ACAACATTTTGAACCTATTCCATTGTAACAGTACGTTGAATCGGATCGGATTAGCTGAGA
TATCTGCTCATCACACTCGATTATCTTTCTGAAAAGAAACAACATCGAAACCCAACGGAA
CGGAATTAGTAGCGAATAACAATTTAAAATGAGACTCTCGTTCGTGGCCTGGATGGCTTT
CCTGGCTTCGACAACTTTTGTTGATGCATTCACATCTCAGAGTGGCAATACCAATAGGCG
TATCACTCAGTCCCCCGGCAAATCGGGTGCGAATAAGGCACTTTATTCTCTGTCAACCAA
ATCGGAAGATGATGAAAAGGTTGCCGGAAAGATTCTTGAGGGTGGAAAAGTTATTGATTT
TGAGTCTACTCGGGGTCCTTCCCGCGCTGAAAACGCTCTAAAACTTGCAAAGGAGGAAAT
CGCAAATGGGACCTTGAAGAACACGCACCTATCCGATAAAATACTTGGGATCAATTCCGA
TGTCATTGCCGAAGTAGGGCACGACCTAGGATTATTCGCATCAGATGACGAGATTCAGAA
ATGTGCAGCCTATCTGAGGTCCAAAGCTGCATCAGGTTTCTTTGAAAAACGCCCAGGCTC
CGTTGCGGTTACCTATGACGAAATCCATAGCTCACCTTCAATAGGTAGCGAACAAAAGGC
AGGCGTAGCAAGTATTCTGGAGAAAGCATTCGTCGAATCGGGGGAAGTGACCAGTGCATT
TGCAAAAACATTTTACTTGGGAACGCAGCTCATGCCAGAAGCACAACGCGAGGCGATCTG
GGCTATTTATGTCTGGTGTCGCAGAACTGATGAGATCGTTGATGCCCCAAGGGACAATGA
CGATGAGATGCTCTCGGACCTCGGCATATGGGAGACACGCTTAGAAAACCTTTGGAAATA
CGGAGAGGTGGAAGATGTTTTTGACCTGTGTCTCTTGGATGTTTTGGTCAAGTATCCGTC
GCTGCCGATAACGCCGTTTGCTGACATGATACGTGGAATGCTCATGGACGTTCCGGAGCT
TGGAACAGATACATACGATGTATTCGACGACCTTCATTTGTATTGCTATAGAGTAGCTGG
AACTGTTGGATTGATGTGCTTGCCAGTCTTCGGATGCTCCGAAGGTTACGACGAAATAAT
CGCAAAGTGAGTATTGCCAACATAAATGACTGACACAAATATGCGAGAAAGACCTGCGTA
TTCCACTCAATCTTTTTTGTCATTCCTTCTATTTTACAATTCGAAAAAAATTGAAATTCA
GAGAACCTGCACG
>comp2605_c0_seq1
ATCGAAACCGATTGATCCGCAAACCCACCAAGACGCCGACCCTCCGGTCCCGCGACCTAG
CCCCAGCAATTCGAAGCTGTTGTGGACGCCATTGCCAGGCCTCTCTTTGCTAGCCAGCCA
GCCAGCCACCCAGCCAAACCAAACCGAATAGCACGGAACGAAAGCAGCACAAAGAAGAGA
GTGCCAGTGCAACACGATACGCCGGTGCGACGCTAGACTCATTCCGGCACGAGACCCGAG
ATTCGTGGCTCGGACCCCGCTGGACCCCCATTCCCAAGGATCCTTGATTGCTCGCAGGAA
CACACCAAGCACAGAACGGAACAATGCCGCCCTCTGCTGTAATAAACGACGACCAATCGC
AGTACTCTTTGTGCTGCGGCGCCTTTGACATCGGTGAGATCGAAATCGAAGTCCAGGCGG
TGGGCGGAGACTGCAACAGCGTCAAGGAGACACAGATCGTCGTGTCGCCCGGGAGCGCCA
CCACCAAGAACGAGAATGTCACGAACCGGTGCGATCCTTCGCAGTACGATTTTGTCCGGG
ACGGGAGGAACCCGCTGGACGGAAGCCAGGCAGGGAGCTTCGATTCAACAGACGAAATCA
TCGAGGGATCCAACGAAATGAAGCTCATGAGGCGTGTCGTGGAGAAGCAGCGGCTGGGAA
TGAAGGGTCCGCGACACCGGAGCTTCATCCAGCAACCCATTTCCATGGAACGGCAGGACA
GCATGTCGGACACCAACAGCGTAAACGCGAACGATGTGTTTGTTTCGATCGAAGACTCGA
AGGAGATGGAGTACCTCGATGTCTTGGACCAGCACCTGAACCAAACGAATCCCAAGGCCC
TCGACTACGAGCACCTGGAAGACGAAACGGAAGATTCGCACCCAAACAAACAGAGGTTTC
AAGCCGACCTGGACGACATAATCGAACATCTCTGCGCCGTGCCGTGTGCCGGATACCTGC
CGGTCGTGGACGGGACGTGCCTCGGCGACAGCACGGAGGCAGGCCATCCAAGGTCGGTGC
TGCGCAGGCCGAGGTACAGTTCTCCCGGCATGTATCCGCCCGCTAGCGCTCCTCTCAATC
GAGCTGCAGACGGAACAGGAACCACAAACTTTGATCAACCCATACTGATACGATCCTCGT
CCAACTACGATGCTACCGGTGCAAGCCGAGACACGCAACAACGGCACTCGGTGCGATTTC
AAAACGTGGACATTCGCCACCTGAAGATGACCCTGGGAAACCACCCCTCCGCGACGACGG
GACCACCTGTCATGCTGGACGGGGAACTAGCCGAACCCCGCCATTCGGCGCGCCAGATCA

TACTGCTCGAAAAATACGAGCAGACGAGGCCGCCGCGAAGGAAACGCCGCCAGCTCAAGC
TGACCCTGCAGCAACGGCACAACATTCTGGTGAAGGAGCGCGGCTTTACCTTTGACGAGG
TCAAGAGCGCGTGGCAGGAATCGCTCCGGATCCGGAAGCAACGGAAGGAAACACTGGAGA
GAGGCCTGGCGATGATGAAGTGGGACGAGGTCTGGGAGTCGACCTGCCGAAAGTTCAACC
GCTTGGTGGACGGTACCCTGTAACAACACACCTCAACACGCCGGGTATCCCATCGCGAAC
GATTGCCGTGCGGCCGACCGCTATAGTTCGGGACTCGTGGTGGGAGCTATGTACAGACCA
TGCAATGAAATTCATAGAAATAGTACGTACAAACAATGCTTTAGACGGCTAGTAAGCAGG
TTCATCCCAGAAGCACACAGGATGATCCGATGCGCAGCAGCGTTTAATGTAGCCAAAAGT
ACGGAAGCAGTATCCTTTTGCAACTCTGCTAGTTGCTATTCATAGTACAGTAATCTACTC
TAATAATACAAGGGATAGCACCGTTGTTTCGTTTCGCGTCCGGAAACCGCCGTGGGGTTC
GGCGGGGCCGCCGTCCTCCGGTGTTCGGTCTCTCGGCGGTGTTGGTGGGTTCGTTCCGCC
AGATCGGGTTGGGGTCTGATCGGAGTTCCGTTCTCTGCGATCAAGAGTAGTTGGAGCGTC
CGGTTTCTTGGCGTTGGCAGGTCCCGGGTCATGCTTTACAGGGGTGCGCTGTTCACACCA
TCACGACAGTTACAATCTACTGGAAGCTACAGTACTATTAAACATTAACTCTTATACAAT
TGGTAATTTTATGCATGACACAATACAACATTGTAGTCCG
>comp2606_c0_seq1
TGGTTATCAAACACACAAACGAAAACATCATTGTTACTCAGGCTAAGGCTCTCAGTAATT
TATTATCTCCTTTCTTGTGGAAAGTTCTTGTGGAAAGAATACAGTTGATGTGCTGGCCAA
AATCGGCTTGTCAACGCTCCCGGCATGTTGAAAGATAATTCGACCAAACCAGGCCTTAGG
ACTTGCATGGGAACCATTACACGCGATAGAAGTAGTGGCAGAAAACCAAAACCTCGATTC
GATGGTCCAATGTCTCCATCATACTCGAAACGCAGTACTGCATTGCTCAAGAGGGCGACC
GAAAATGGCCAAGGAAAAACGAGTGTCAAAAGTACGAAACGAAACGAACGAACGATGCTT
ACATTAAAAAATTACGAAACTCCCAATCCACGAAGGGCGAGCTGTGTGCCCGAAAAACCG
ATTCGAAGAGTTTCCTTTGCGGATCAAAAGACCGTAACAATATTCCAGCGGATACCGAGA
AAATTTGCGAGAGATGTCTGGTTCACCGCAGTCGAAATGGGGAGGTTTCGAATGAATGTT
TTGAATTCCGACGATCTCAGGTTTAAAGTGAAACTGAAGTCTGCTCGGTGCCACAATCAC
ATGAGACGCGTTCTACTGGAGCATCGGACCAGCAAAGGGGATATAGCGAACCAAGCATTA
ACAACATCAATTTATTACGAGAATAACGTCAGAAGGATTTCCAGTATTTCCATGAAATCA
AGCGAGAAACCGAGAGAAGCAGCTATCGAAACGGCAAACAAGCTTGAGCAGGAGATTGTT
GAGCAATCATTAATAAATCGAAGGGTGTCGAGCGCATGTTTTGGATTCAGCCATCGATGG
GTATTTGATTATTATCTAGGTCAGATGGTGGACAATATGTGCACAGTTATGTAGCCTATA
GCATAGCTGGGCGAGTCCAATAGGTTTCCCTCGGGGTGACATCACAACACACTAGAATAG
GACTTTTTGAAAGCACAACGAGCAATTCTATTTCATTGTGGATGAAAGCGGAAACACGAC
TATCAAGAAAAGGAGGAAGAAAAGAGCACGAAGATAAACATGGTTCGGTATTCAATGATG
CGTGTACGGTACAGAATAGCAATTACTTCTATTGAGGTTGACTCATTCGAAATTGGAGGC
TATCATGCAATGACGCCTATAACAGTTTCACCCGACCTTTTCTTTGATTGCTACTTGCAA
TTTAGATGAGACAGCATTTGCACCCAGTGTCGGTAGTTGTTGTAGTAATTGTTGGAGGGA
AAGCCGTTTTGAATCGTTTTGAGGCATGCGCTGCACCAGTTTGCACGTGCGTGCATGCCT
GAAGGTGAAAAACAAGTGAGGGACCCAATTTTGCGAGGTTTTTCAACGACTCATCCGAGT
ACACCACTATGCGCGCGTCAGAAATTGCAAAATTGCATCACTTTCTTCTAAAAAAAGTAC
GAAAAAGCAATAAAATTACTTTAATAAAAAATACTGTAAGCTAAAGCAGA
>comp2607_c0_seq1
AAACACTTCGTCGTTCCATCAGATCACTATCTTTCCGTGTCGGAGCACAAACCACACGGC
TTTCAGAATCACCAACACACACTGCACTTCGATACACTTAACATGACGGCGTCGGGTGGA
TCAGTGCGGCGGTTACTGGCCCAAAATTCGGCCGTCCTAAGGCGTGGGGCCGAACACGCA
AGGCAGCAAATATTTGGTCACGTCCCCATTCTCGAGGGAGCTGCTGCCGGGAACAAGACC
GCGAAGAAAACATTTACGGGGCCCTATCTCGAGAAATACTACCCGACTTCCATCAACCAC
CACGCAAGAAAAGTTCATGACGGATGGGAAACCGAACAAGAGGAATACAGGCGCGTGAAA
CTAACCCAGCGGCGACGCAAGGGAAAGGGCCCACCCAAGAAAGGGGCGGGCGCTCGAAGT
GGGAAAAAGCGGTAACCTTAATTGCGGTGAGATCGGAATTTGAGGCAAAATGTTCTGGTG
AACGCCATGCATTGGCTCGCAAAATATTTACATAGTAACAGAGGGGGAGAAAGAGACAAA
AGATTTCTACCCAGAAACGAAAGCGCAACGTGACGGAAATTATTTTTCAAGATATCTTGC
ATGACAAAAATTTTACTACGTATTACTGTTGTAGTGTGTAGAATTCTAGTAGAACAGGGT
GCACTTGTCTCCATAAC
>comp2608_c0_seq1
CGTGGTACACTTAGTGTCAATACGTCGCTAAAAAATCATACCACGATAGCGCGACCACAT
TCCATCTTGCAACTGCATTGTTCTATCAGAACAGTAGAACTTTGTCCTTCCACCATGGTC
CCGTCAGAACTCTAGGGAGTCCCATGTCTGGATGGAAGAATTCGAACAGATTTTTTGATG
CCTTTCCTATCAGCCAATTTCTTCCAAACCTTTCACCTTTCGAATGAGTTGTACGTCCAA
CACGAGTTCAAAAAGTGTCGAGTAGTGCATGCAACGCCCAAAAGAGTTTATTACGTTTTG

TTTAGATAACTGAAGTCGAGTCTATCGCACACAAAAGTAACAAAGCAATCTACCACTCAA
CCGAAACTCTAGCTGCGAAGTCGTTCCATAAATTTATTTGATCAACCACAAAACAACATT
CAAAATGATCGCAACAAGCATAAATTCAATGGATTCTACTACCAGCATGAGGATAAAAAG
TGATGATCAGATCCGCCACCGACTGATACGCAGCCTTGGTATCAACCATTCACCAAAATC
AAATATGAATGGCCGGATGATGCAGAAAAACATTCTTACCGAAAGAACGCAAGAAATACC
GCTGAAGAACCACTGCCACAACAAATCGGCACCGAAGGCCGATTCTAGAATCCAATTTAA
CTCTGTTGTTAAAGCGATGCCTATTCCTTCGCATCAGGATTACTCAGACCGAGTGAAGAG
GCTGATATGGTCGAACGGGACCGAAATTCGACAGAACGCGCAAAGAAATGCAAGGGAATT
TGCATTCGAGGGATGGAGCGCACAGTATGTCCTGGAAGAAGATCAGATGTATCTAGACGT
TGGCTCTATGGAATTCATTCATCCTGTTCATGTAGTGGGATTCTCGTAATCTGACGAAAA
TGTGTTGTACTTCGGTCGCAAGAAAGGGGCAAATTTATTGCATTCTGGAGGACTTTGTTT
GGACTTGCACATACTTCGACATAGTTACATTGCTGTACATAATTCTGAGCGGAGAACAGC
CCAGGCTGAGCGTAAACTATGAAAAATAATGTATCATTACAACCAATGCAATCAATACAC
TATGCTTTTGCTGGAACATACTGAAAGATCGATAACATCATATCATCGGCATAGCGACTA
CACATGTTTTGTTGAACCGTATTCTAGCATGCCAACCCTGCCTTCCTTCATCCACTAATG
ATCTGAATATTCCGGTAAGCTTAGTCCTATTACCTCATGTCTCTCACTACTTCGACAAAT
AATACGTACGGTAGGAGGCGAATGTGTTTCAAGCCAAACTGTTACTCGTGACTCAAGAAA
ATGACGTTTCAATCATATTTTTGGGCGACCAGTACAGGCGGAGATTATAATATCGTACCG
TACGTCTCTGTTGGAAGCAATCATCTGCAGCACCATCATTTAGAAATACCGATCCAAATT
CATTTGGAAAGTCTACACCTCTAGTCGCAACAAAATAGATACAAAACAAATAGTAATAAT
GGCCCAATCCTGTAGATATCTCTGGCTATTGCCTCTTTCGACCCTGTTTGGTGTTGTCAC
CAGTTTTGTGCCTCCATTTACCATGAGTTCGATGCCTGTGTACGACGCTGTCGACGCGAT
GGAGCATACTTGTACTGTGTCGACAGCATCAAAATCGCGACTTGTCGATGCTACGAATTT
TCTGGTGTCAGCGGAAGGTAGCGAAAGCGTCCACACGGGTCTTATAAATTGGGAAAATCC
AGGGGAAGCATTCGCAGGTGCAGTCACGCTCCTCTACATTGGGTTTTCGATTTTGGCTGG
CATCAAGTACGTCGTGATAGATGGTTGGAGGCCCAAGCTTTAGCACTTTGCTTTTCAATG
AAACTCCAAACGGAGAACAGAATTTCGCCACTTGTAATGTTTTGGGAGCAAGATTTGGGA
AATGAATCGTCCAGTATTTGCAAGTGCAAATTCCACTGCCACCACTGACCAGAACGCCTG
ACATTAGACCGTTGTTCAGAAAGAATAGATGAATTATTTCTAGGAGTGGTAGTGAAATCG
AACAATCGGCATCTTCTAAGTAGTTTTATTGGCAATGCACCAGTGCACTACTTATTCCGT
CCTAGACTATATTTCCTGATAGAACCAGATTTACTCTTTAAATAATCTGAATTATCGACA
ATCCATTAAAATCCAAAAATGCTACTGCTGAAGTATTTGAAGTCTATCATGATAGTAATA
GTGGTTCCGTTGAAGATAAATCCAAGGAGAGTCACTTTTGATAGCAACATAAGGGGGATG
CAAAATACCGAGGATCTTGAGATGGAGGATGCCGAGAATTGATGCTGAATATGGACACGA
CAGAGTATGGAGGTCAATGAATATGGAAGTGGTTTGGCTACAGGGGGG
>comp2608_c0_seq2
CGTGGTACACTTAGTGTCAATACGTCGCTAAAAAATCATACCACGATAGCGCGACCACAT
TCCATCTTGCAACTGCATTGTTCTATCAGAACAGTAGAACTTTGTCCTTCCACCATGGTC
CCGTCAGAACTCTAGGGAGTCCCATGTCTGGATGGAAGAATTCGAACAGATTTTTTGATG
CCTTTCCTATCAGCCAATTTCTTCCAAACCTTTCACCTTTCGAATGAGTTGTACGTCCAA
CACGAGTTCAAAAAGTGTCGAGTAGTGCATGCAACGCCCAAAAGAGTTTATTACGTTTTG
TTTAGATAACTGAAGTCGAGTCTATCGCACACAAAAGTAACAAAGCAATCTACCACTCAA
CCGAAACTCTAGCTGCGAAGTCGTTCCATAAATTTATTTGATCAACCACAAAACAACATT
CAAAATGATCGCAACAAGCATAAATTCAATGGATTCTACTACCAGCATGAGGATAAAAAG
TGATGATCAGATCCGCCACCGACTGATACGCAGCCTTGGTATCAACCATTCACCAAAATC
AAATATGAATGGCCGGATGATGCAGAAAAACATTCTTACCGAAAGAACGCAAGAAATACC
GCTGAAGAACCACTGCCACAACAAATCGGCACCGAAGGCCGATTCTAGAATCCAATTTAA
CTCTGTTGTTAAAGCGATGCCTATTCCTTCGCATCAGGATTACTCAGACCGAGTGAAGAG
GCTGATATGGTCGAACGGGACCGAAATTCGACAGAACGCGCAAAGAAATGCAAGGGAATT
TGCATTCGAGGGATGGAGCGCACAGTATGTCCTGGAAGAAGATCAGATGTATCTAGACGT
TGGCTCTATGGAATTCATTCATCCTGTTCATGTAGTGGGATTCTCGTAATCTGACGAAAA
TGTGTTGTACTTCGGTCGCAAGAAAGGGGCAAATTTATTGCATTCTGGAGGACTTTGTTT
GGACTTGCACATACTTCGACATAGTTACATTGCTGTACATAATTCTGAGCGGAGAACAGC
CCAGGCTGAGCGTAAACTATGAAAAATAATGTATCATTACAACCAATGCAATCAATACAC
TATGCTTTTGCTGGAACATACTGAAAGATCGATAACATCATATCATCGGCATAGCGACTA
CACATGTTTTGTTGAACCGTATTCTAGCATGCCAACCCTGCCTTCCTTCATCCACTAATG
ATCTGAATATTCCGGCGGAGATTATAATATCGTACCGTACGTCTCTGTTGGAAGCAATCA
TCTGCAGCACCATCATTTAGAAATACCGATCCAAATTCATTTGGAAAGTCTACACCTCTA
GTCGCAACAAAATAGATACAAAACAAATAGTAATAATGGCCCAATCCTGTAGATATCTCT
GGCTATTGCCTCTTTCGACCCTGTTTGGTGTTGTCACCAGTTTTGTGCCTCCATTTACCA

TGAGTTCGATGCCTGTGTACGACGCTGTCGACGCGATGGAGCATACTTGTACTGTGTCGA
CAGCATCAAAATCGCGACTTGTCGATGCTACGAATTTTCTGGTGTCAGCGGAAGGTAGCG
AAAGCGTCCACACGGGTCTTATAAATTGGGAAAATCCAGGGGAAGCATTCGCAGGTGCAG
TCACGCTCCTCTACATTGGGTTTTCGATTTTGGCTGGCATCAAGTACGTCGTGATAGATG
GTTGGAGGCCCAAGCTTTAGCACTTTGCTTTTCAATGAAACTCCAAACGGAGAACAGAAT
TTCGCCACTTGTAATGTTTTGGGAGCAAGATTTGGGAAATGAATCGTCCAGTATTTGCAA
GTGCAAATTCCACTGCCACCACTGACCAGAACGCCTGACATTAGACCGTTGTTCAGAAAG
AATAGATGAATTATTTCTAGGAGTGGTAGTGAAATCGAACAATCGGCATCTTCTAAGTAG
TTTTATTGGCAATGCACCAGTGCACTACTTATTCCGTCCTAGACTATATTTCCTGATAGA
ACCAGATTTACTCTTTAAATAATCTGAATTATCGACAATCCATTAAAATCCAAAAATGCT
ACTGCTGAAGTATTTGAAGTCTATCATGATAGTAATAGTGGTTCCGTTGAAGATAAATCC
AAGGAGAGTCACTTTTGATAGCAACATAAGGGGGATGCAAAATACCGAGGATCTTGAGAT
GGAGGATGCCGAGAATTGATGCTGAATATGGACACGACAGAGTATGGAGGTCAATGAATA
TGGAAGTGGTTTGGCTACAGGGGGG
>comp2609_c0_seq1
AGAGAACACACGAATTGCACAGTTCTGTCATGGACGTTTGGATCAGAGCCGTCCAAACCT
GTGTCTTATTTGCTTCCTTGGTTCTCGTCGGAAATTTGTGTCCTTTTTCATTTCGAGCGC
TTTCACGTCGTTCATTCTTACCAAAACCAGGTTTCTCATAACTAACTTTATTCGTGAAAT
GAATGTCGAATCTCCACCACAACGCATCATCCCTTGAATTGTCAATCACATCATAATTTA
GCTCTCCGCCACAAATTCCTCAATAGTCACCGCGAAAACATCCAAATCTCCATTGTAACA
CCGTCTCATTGGGTTTCCACACCACACAATTGTATTCCCAACATGATGATCTACCAACAA
ACAATGCTTGGTTCCACCATCAAGCCCACCCCATACACTCCCGGTCATCCGCGCGAGGGT
CCCAGCAACAAGAAGGGAGTAATGTTCAGTTCCTGCACCAAGGATACATCTTCCTCGCAC
AGGAGACGCCGACCTTCGAGTAGCTCTGTTCCTATCCCCCATGCAATGTTTCGATCCCCT
TCGGAACTCCAGCTCTCCATGGACGAGCAAATGGCGGACGAACGCGATTTCGTCTTTTAC
GCCCGCCTTATTAGAGGCATCCGAGAGCGCACGCTCCGTGTAAACACGAGTAAAAACGGC
GATGATAACCAACCCCAACTCTACCAGCAATACAACGAAGAGACGGAGCGTTGCCTGACG
CACATTATGCAAACGAGATATCAAGAACAAGATCGAGAACCAAAAGAGATTTATGACAGC
ACTTCTAATGAGAACTGTCTCGAGAACTTGGATGGTTGCTACGAGTCTTCCTGCTCTTCC
TCTTCTACTTCGTCCTTTGCGTACCAACGAGATTTTGAAGGTGACGGGGACGATTTTGAC
TCTGGGGAGGACGAGTTCATCTTTGATCTCGAGCTATGATGAATCTAACCGACGCTGCTC
GGACATCGTATGCTACGCGAGCATCTAGGGAACGCTAACCACACACGTCTTGCACTAATT
TTGAAACACAAGACCAATATACACAATTTTACGTCGAAAACAATCGAACAAGCTACACCA
CCGCACCGCTAAAACGGCTATCCATCCACAACATAAATGAAACTAGACCACA
>comp2610_c1_seq1
ACCGGTACCGGTGCGACGAACACTTCATGTTTACTTTCACCTGAAGGAGTTCAGACAGAG
CCTCGTATTATACTCTTTCCACCCTTGCCATAGTCAAAACTTCACCACCGGTTCACAAAA
ATGTGCTGATTCATCATCGATTGCGCAGCGTCCATGGCAAACGCGGTCACCCGAGCCTTG
GGATGCAACAGGACAAACCGCCCGTCCTTCGCTGCCTCGTGTTCGGCTGGGCAGTGTACC
GCAAATGCCAGGGATGTAAATGGGAATTGCAGTGTGTCGGCAAACCTTAAACAACGAAAA
CTCGGCTCCAATGAAGAGGGGAAAACAACAGCAGCAACACTTTCATCGTCTTCCGTGACT
AGTCAAAACATGCTGGAGGTGCCATAGGAGACATTGTGAACGGTCATATTTGATAAGGTA
GCGAATATCTGGTCTCGGAGAGTATAGCCAACCGTTTTCAACAGTAGCAAAACACAATGC
AAACCGACAACTGTTATAAAGTTCAATGCTTTGAATGACAACATTTCCTTGAGAGAACGA
GCTTATTGATGATGGTTCAGTCGGCAGATGGTAGCAGTACCATTATCTCAACCCATTGTA
GTTGTCTCAATATGAGTTCTCCCAAAGCAGATATGTTCGAGAAAGGCTGACTTCCTGATA
ATGTGTCTGATAATGTGTCTATGTTGCGTGCAGACGGTCTTAAAATAGAAAAAATAACTT
TGCTGGGTTATTGTAACATCTGTAATAGAGCTCCTCCTCTGGTCGTGTCTCCATTAACAC
TCCTCGGAGGGAGGCGCCGCCCGTCGGAGCATTCTTCCCTCCGGAAGTTATGGTTCGGGC
CAGCATAGCGCCTCTTCTGGTGGTTCATGAGGTTAAATATGATATTAAGATTTTTATTTT
TTAATAGAAAAAAA
>comp2611_c0_seq1
CAACAATCACCACGCTGTGGCGAGCGAGTCCGAGGAAAAGACATCGGTAGTACAGGGCAG
TATGGACCGCGCCGTAGAGGCGTGAAAAATCCCAATAGAAAATAGAATACAGTGATGAAG
AAATCGCCGTCGTCGTCCGCTCGAAGATGACCAGCGGCATAAGGCCATCCGTGGCCCGAA
CAATTCTCGGTGGACAAAACACTTTACGTACATGGCACACTCGCAGGACGATTTTTAGTG
AAAGTTTTCCACCAGCATCGGTTCCCTTGTGTCTTTGCCGCCGTCGAAAGAGGGGCCGTT
CCGACAGCTGGGTGTCGGCCTTTTCCTCTTCGCCTTTTGCGTCGTCGGGACAAGATCATG
GGTACGCCAAACTGAAAAGGGATGCCTCATCTTTGGTGGACCAAGCGTCCTTCAAGATTC
GAGGTTCGAAGCACCACTCAAAACTCTTGCGGCGCCACGTTGCCATCTTTTGCGAGGCTG

TCCGAGCGGGTCTTCTCCACAACAAAGGCGAACTCTATGGTAGCGATCGGTACTCGAACA
ACTCGTGTGAGGCAACGAGGAAGCTGACCGCCAGTCTTGGGGGGATCGGAGCCGATCGGG
GGATCATGCCGGATTTTTCGAATCCACGGGCTTTGCGGGGGTACACCCTCGGCCATTTCG
GAAGAGTCCGTCTTCTTTCGCAGTTGGTGGCGCAAACCGAACCCGATTGGCTGGCGACTA
GGGTCTCGCAGATCTTCTCAGACGAGGTGAGCAATCGAGCCGATGATCCTCATTGCTCTT
CTTCTCTGAAGGGGGGAAGTGAAATCGCCCTGGCGAGCCTCGGTGGGGGCTGCGGATACG
ATTTTGTGGCCATGTCGGCACTGAGCGAGTTTCTGAAAGGACCAAAGATTGCAGCCACCG
TATTCGAGTACGAACCAGCCTGGAAGGACGTTCTTGGGGATGTCGAAAGTGTTGTTCAAG
ACGTGGTTGCCTCCAAAGACAACCGCGCCGTCTCGCATGCACACCGGTGTGGGTTCGAAT
CTTGCGACATTACGCGCCCGCTGGATTCGTCTTCAAACGAAGCCCTTGCATCGGTAGTTG
GATCGACCCATATGTTTTCGTGCTCGTATGTGGTAGCAGAAAATGCTGTCGGGTTGCGAA
CCAACAAGTACGAGTTCTTTCGGCAGTTGTTCTCGGAAGCGTCGGACGGGAGTCTTTTTT
TCTTTACGGAGACCACGCACCGGCTGTGGCCCGACTTGATCGATGTGGCGATGGACCACA
ACGACCATCTCTCTACCATAAAAATCTCGATACCGCATATTCGATGCGGGAAGAGCGGGT
GGCAACTCGCACTTCTCAAGGACTCTGCCGGGAATGACCACGGCGGTAGCAAAACTTGTT
CACTCGAGGAACACCAGAGAGTGATCTACGAACGATTTCGAAGTGACAATCTCGCTCACT
TGTCGCGTCTAGATCGGGGATGGAAACGGGACAAGAAGAAGGCGAGGGGCGCAAAGCAAT
TGTCGCACAAAGATCAACGAAATCAATAATGATAGGTTATGAAATACGTGACGGTGCCTC
ATCATGGGCAATTCTGTGGTTGCGACTAAGTGTACTGAGGACGAATGCTGAGAGTTGGGT
TGCAAAGGCCCGTATCTCTCAATCTTACTGGAACGTAACTATGATGCATATAGAATCATG
ATTTCTGCCTTA
>comp2612_c0_seq1
GACGAAAGTAGTAACGCGAGTCGTAGTCTTTCTGGAAGCGCACAACATCCATCACACTCG
GTCTATTCCATCAATTGACAGAAAGTTGTTACATACGGTAGCTTTCCAGTTTATAACTTG
TGGGACACAACCCGTGTTGCTCCGATGATGCCGTTGCACCGTCGGCACGCCGCTGGAACT
ACCAAGGCCGAATCATCCTCGTCACCGCCGTCGTCTCCTTCGGACAAAGACGGTCGAGAG
GGGAGCATGAAAACAGGGGATGGCCATTTGGGCAGTGGTTTTTTGCCGAATCCCCCTCGG
TCCATAGTTTTCCTCGGTTTGGCTGTTCTGGAAGTCACAGTACAACTGGCTGTTATTAGG
AAGTCAGGGATTCGATCAGTCGGCCACCTTTTGACGGTCATCGTGTCAGCCTTTGTTGCG
ACTCTTCCTATGCTGCTCACATTGGCGGTGGGTATGGGAATCTCTGGAAATCCAACGAAA
GATTTGCCGCACCAGAGATATATTCCTCTTACGGTGGCAGCGACAGTATTTGCTAGTCAG
CTTCCTATCTTTATCAGTTACGCCATGGCGGCTACTGGAATTGTACTATTTGGACTGGCT
TCTAGACCAATAGAGGAAGAGAAGGCCAAGAACAAAAGTGATACTGGGACAGACACGAAG
GGCAATCAGTCGCAACCCAAAAAAAAAGAAATGTCTGGTCAGCTCAAGGCTGTATTCTCA
GTATTTTTAGTAATTGCTGTTCTCTTGACGGAAAACTTTTTTATCTGGGTCGTTTCGGCA
ACCTACAAGCCGAGTCAAAACCTGAGCACACTTCCTACTCCCCTCCAAGACAACGGACAG
ATTTCACTCCAGTATCTATTCCACGAGCTTTTGAATATCACGAAACGAGACGTTGTCAAG
GTCCGAAACATTATCAACGTCGAGGCAATTCTCGTGTCGGGACTTGCCCTTTCTTTGGTA
GCCATTGAGCTACAGGGACCCTCTATGCAACGAAATTTGTGGTCCATGGCAATGCGAGGT
GTTTTGACGCTTGCTGCGGCACGCTCCATCCGAACATTTAGCTTTCTCATCACCGTTCTT
CCAAGTCAAAATCCTCAATGTTTCTTCAGCCACTTTCCCATGCCCCCCAAGGATTGGTAT
TCCTGGCTCAAGGTCGGATTGATTCCTCAAATTGATGGGGGTTGCAACGATCTAATTATT
TCGGGTCATGCAACCGTGACTTCCACCATTGCATGTGTAATAACTAGTATTGTTGGAAAA
CCCTTGTTCACAATTGCAATATGGATGTCCGTTACATTGGATTATTTTGTCGAAATTTAC
GAGGGCTTTCACTACAGTGTCGACATGTTTCTAGGGGCGATGCTTGTAAACTTTCTTTGG
TCTTCCTTATCTTTTCTAGAAGATGTCAAGAAAGGGCATGCGGCTGTGGAAGAGAGAAGA
TTCCTCCCTGTACAAGATGCTACACCTTCGGATGTTCTTCGATACGCTATTCCTGCTGTA
GGGCTCTGGATGCAACTCGTGGTCATTCCGCATTCGTATGGACTCTACAGTTGCGTGTTG
TACACAATCGTGGTGGTATTTCAGATTTCAAAATTTGGATTTCAACAGTATTCACAGCAC
TGCCTCTTCTGTTTATTATTTATGACGCTAGGGCTATTCCTGTAGAATGTAAGAAAACAT
CATGTACATTCTTGTGAACCAGGAGCGGCACTATGAAGGCCAGGAATATTGTTCCGTACC
GGTCTCATTGAGGGCTGGTATAATTTTTTCCACAAAAATATAGGATATTCCTTGTTTTGC
GTTCCATAAGCTATCGAAACATATCTAGGACCCACAGCGTTCAAGCATTCTTTCAAGAGC
AATCATGGTATTAAAAAGTAGAGAACCTATCGCATCAAAACAGGTTACTGTATTATTGAA
GCGATCATCTAACAAAAATACCTAGACCTATCACATTTTGTTGTCAAACTTATTTCCCTC
GAGTTCCAGCG
>comp2613_c0_seq1
AAAAAACTGGGTACGGTATGCGGGCTCGAATCCAACGCACTAAACGGCTTCAAAACTGTT
TGCGCCTTGCGTGGCAACGGACCTCGAAACCAACCGATGAGTACCAGTACTTTAGGTCAT
CGTCATCGGTCATTGTCAACGAACTGCAAACATCATCAGTGCCCCCACTTTTTGTCTTGA

CTGAAGCATAGTTCTATAGAGGTCATTAAGAACAAATCAAGATCCCATAATATGCCTTCG
AAAGATAGAATGGTCGGTATGTCTCGCCATTGCTTCTGGAAGTTGTGGTTGCCCCGATGC
AGTCAACGGATTCAAAACGCGCGGTAATTCGTAGTAGTAATCATAGTCAGATTATCAGAT
TACTACTAGCAGGCAGGGTCAGTATCACGGGAATCTACACTTAATACTACGAGTACGTGT
ACGACTACGTACTGTTGGTATCCCGCGTCAAGGGGTTACGGGTGATGAGTTTTTGTTTCA
TTTGAGCATGATTTTTGATTTTTGTCTTCCGAGTTGCACGATTTGCACGATGCGAGTACC
TTTGAGGTGAATTCTTGCAAACTTTGTACGGAAGGATAACGTCAAAGAAGCCTCGAGTGA
GGGTCTTGGCTAATTTTACAAGAATCCCAGTGAATTCATTGGAAAAGTTTCTTCACAAAA
TGAATGCCGGCACCACGGATCCCATCTCTCTTCGCAACAGTTCCGCGATGGGAACAACCA
TGGGAACAACTTTGGTGTTCACGATGGGATCCAAGGTAGGAGAATGAGTTTTGTCAGTCC
TCCACGCACGGCACCGCACTCGGAAGCAATGTGACATTCTCCCATTGCGGTAAGGGTATC
ATCAATTGTCTCGTCTGCAATACAAGAAATACATGTCAGACAAAAGCGATCATCTATACA
ATATTATTGCACCCGTGCAAGACACTGAATACGAGGAATGAACACAAAAGCAGCACTTAC
TATAACGATCATCTACCCTGCATACAAACGAGTCAGCCATTGGCTACGTACATTACGAGG
AATACATACAGCATAAAATTATTGTTTCGAGACGATCATCTATCATTTATAACGCGAACA
GCACGGGGTAAGCAAAGTACAAGGCGTTCGACACTCTGCCTGATTCTGGGTGTGATCATT
GAACGGACAAACCGAACACGAAATTGCTCGGAAGACGGATCTGGCGGGGCGTAATCATCC
AATGATCTCCGAAGCCTGACCCTTCGGCATGGAACGGCACACCTCGAATAACTATTTCTA
ATTGCATATCGGACACCGAGACAAAACAAAAAACAAGCAACAATTTTTCTTTACACGAAA
CTCCAAAAGAGTCCTTCGGATCGATTCGAACGGTTGTTGGTAATGTCGGGGAGGAGCAAT
TCTTTTGTTCCCTTGTTCGTCTCTTAAAAACAGGCGTACTTCGGTTAACTTCACGAATCA
TCACGGAACCCCACAGCCGAAGGAAAGCACTGTACTTTACATTCATAACATTATATTAAA
TAGCAACGTATAAAACCAAATCCGTTTCAAATTGTTGTGGTGCGTTATAATCCATGCTGG
CGAAAACCATCTATCGTTGGTGGAGTCGTTTCGAATCAGAACGTACAATAAACAGAAATA
TAATAAGCATTAATACCAGGA
>comp2614_c0_seq1
TAACAACTTTGGTTTCCCACAAGTGTCATCTGACAACCTCCCAAACAGCAACCTAAAATT
TCTAGAAATAAAAACATCCTTTCACGCGAATTACATACATATCAGTTTGAATTGGCAAAA
TTAACAGCTCTTTCGGAGAACGACTTTGAAAATGGCACCTCATGAAAAATGTAACGAAGA
GGATGATATGAAGGACATCACGGAACCACGAGAATCAGATGTGCTATGCGGTAGAGGAGG
TGCTGCATTGAGGCATGTAGGGAATCAAACTTATCGGAGACTTGTGAATCTGAACAAGCC
TCTCTATATCACTTGTCTTAAAACAGAGAAACTGAAAATCAGCAGGTCAATTGTTGCTGC
CATCCGGGAGCAAAACGGTAGATTTTTGGAACGCGACGCCCAAAAAGGTGTTTGGACTGA
TATAGGGGACAAGAAGGCAATTGAAAAGACTTCACAAGCTCTTCGAGAGGGGCAACCTAA
ACTCAAGAAAAAGCTGGCTGCGATGGGACAATTGCCTGCAGAGCAAGCGAATGGTGGTGT
TGACGCCACTCGTAATAGTTCTACGCAACAACAGGCTGTGCAACATTATGGGAATGGTAT
TTACAATCCACGTGGCGTACATCCCAGTATCAGCAACGTTATGAATAACAATATTAACGA
TGGCATGGATATGAATTTTAATATGCGCGGTGGCATGAGGATGAACAGTGGTACGAACAT
GAATGCGAACATGAACATGAATATGAATAACTATGATCGGGCTGGATCAGGGAACTTTTC
GAGTTCGACTATGTTTGCTTCGATGCAACAGCAGGTACAACAGCAGCAATATGACCGATT
CAATCAGATGGGTGGGAACCACATGGGAAGGGACACTACTGGGAATATGAGACATGGTAT
GATGCAAAATCCTGCAAGACCACATCGTGACGAGAATCCTGCAAGACCACAACGTAACGA
TTTTGACGACGACAATGATTTGACAATGATCCGACATTTGAGTTTGGATTCAACTCCTCA
ATCCATACCTAGCTGGACACCGAGCGTGAGATCAACGGATTCATTTGCAATGTCTATTGG
AGAAGACATTCCGCAAATGGTTACCAATCGGAAAGGATCACGTACCCTTATCCGAAATCC
GTTTAGTGCACCTTACCCTTGTAACGAGAATAGAAATAGCAACGGAAGTGATTTTTCTGC
CTATGGCAATGGAATGGGAAGGGATGGAAGCCAAACGAGGTCCTCTTCTTTTGATATAGA
GGACGTTGGACCGTTTGATAGATCGAACGAACCACATCGACGACAGCAGCAGCAATTTGT
AAGGACTCACGACTATGGGCAAAATACTCTTAACGAAAGCAATCAAATGAAAACAAACGA
CAACCAACGGTCGCCCACTACTTCCATTAAAGATCGACGACGCAACTTCGCAAGATTGAA
GGATGTCCGAGCATCTTCATTGCGGAGCGGATCTTCCCGTAGCATTGATGACATGCCAGA
TATCCATATGGTTGATAGTCAATTTTCTTTGCTTTCGAACCTCTCGGCTCATGGTTCAAA
ACATCAAGGTCAAAATGCCATCGACATGGCTATTAGCACCCATCGTGGTGGGCGGAAAGA
TTCTATGGGGAGTGGATATATCGGTGTTGCAAGTCGACGGTCAGTAATGTCAGGTTTGTC
GAAAATGAGCGCGCACTCAGTGGACTTAAACGTATTTTCAGGGTTGTCGCGGAAGTTCAG
CGGGGGCACTAATCATACAGCATCAAGTCGATCTATGATAAGTGAATTCAGTGGAATTGA
CGAGGACGATTTTGCCGAAGATGATTTTGATTTCGATATACCAACGAAACCAAATCCCTG
AATATGATTCTGACAGGATTGTGATACTAGACACAAAATGAAACGGGACAGACTATCTCA
AAGATCTTGTTTTGGGACCTGGGCCAAGTAGAGTAATTGGTTCAATAGACCAGAGAAACG
ATACAGGATAGACTCCTCCTACTGTGTTGAGTCGGAGAATGAATGCAAAAGTTTCAAATT

GCGATCACCCATTGTTTAAAACCCAATTTTTCTCACGGAGGACTTCAGAGGTGGAAGTTA
AATTTAGTCATTGGTGATTGATACATGCATTTACTTTCCATGGACTAAATCCTCTAGTCG
GGACAACTGCAGGTAATATGCAAATAGTAGTCTTCGTTAGCATCTGTGAGTACAATCCCT
GTCAGTTCTGCTGCTATTTGCCTAATCACGCTTGATCTCAGAAAAAGGAAACAGACATCT
ACTGATACAAAACAGCACATTTTTGCTTATTCACACTTGATCTCGTATGAGAGAAACAGA
GCAAGCTATTGATACAAATCAAAAAGCTTGATTTACTGCTGTGTTTTCATTCATAATTTA
CTTTGCAACAACTAGTAATTTTGTTAATTTTCAC
>comp2614_c0_seq2
TAACAACTTTGGTTTCCCACAAGTGTCATCTGACAACCTCCCAAACAGCAACCTAAAATT
TCTAGAAATAAAAACATCCTTTCACGCGAATTACATACATATCAGTTTGAATTGGCAAAA
TTAACAGCTCTTTCGGAGAACGACTTTGAAAATGGCACCTCATGAAAAATGTAACGAAGA
GGATGATATGAAGGACATCACGGAACCACGAGAATCAGATGTGCTATGCGGTAGAGGAGG
TGCTGCATTGAGGCATGTAGGGAATCAAACTTATCGGAGACTTGTGAATCTGAACAAGCC
TCTCTATATCACTTGTCTTAAAACAGAGAAACTGAAAATCAGCAGGTCAATTGTTGCTGC
CATCCGGGAGCAAAACGGTAGATTTTTGGAACGCGACGCCCAAAAAGGTGTTTGGACTGA
TATAGGGGACAAGAAGGCAATTGAAAAGACTTCACAAGCTCTTCGAGAGGGGCAACCTAA
ACTCAAGAAAAAGCTGGCTGCGATGGGACAATTGCCTGCAGAGCAAGCGAATGGTGGTGT
TGACGCCACTCGTAATAGTTCTACGCAACAACAGGCTGTGCAACATTATGGGAATGGTAT
TTACAATCCACGTGGCGTACATCCCAGTATCAGCAACGTTATGAATAACAATATTAACGA
TGGCATGGATATGAATTTTAATATGCGCGGTGGCATGAGGATGAACAGTGGTACGAACAT
GAATGCGAACATGAACATGAATATGAATAACTATGATCGGGCTGGATCAGGGAACTTTTC
GAGTTCGACTATGTTTGCTTCGATGCAACAGCAGGTACAACAGCAGCAATATGACCGATT
CAATCAGATGGGTGGGAACCACATGGGAAGGGACACTACTGGGAATATGAGACATGGTAT
GATGCAAAATCCTGCAAGACCACATCGTGACGAGAATCCTGCAAGACCACAACGTAACGA
TTTTGACGACGACAATGATTTGACAATGATCCGACATTTGAGTTTGGATTCAACTCCTCA
ATCCATACCTAGCTGGACACCGAGCGTGAGATCAACGGATTCATTTGCAATGTCTATTGG
AGAAGACATTCCGCAAATGGTTACCAATCGGAAAGGATCACGTACCCTTATCCGAAATCC
GTTTAGTGCACCTTACCCTTGTAACGAGAATAGAAATAGCAACGGAAGTGATTTTTCTGC
CTATGGCAATGGAATGGGAAGGGATGGAAGCCAAACGAGGTCCTCTTCTTTTGATATAGA
GGACGTTGGACCGTTTGATAGATCGAACGAACCACATCGACGACAGCAGCAGCAATTTGT
AAGGACTCACGACTATGGGCAAAATACTCTTAACGAAAGCAATCAAATGAAAACAAACGA
CAACCAACGGTCGCCCACTACTTCCATTAAAGATCGACGACGCAACTTCGCAAGATTGAA
GGATGTCCGAGCATCTTCATTGCGGAGCGGATCTTCCCGTAGCATTGATGACATGCCAGA
TATCCATATGGTTGATAGTCAATTTTCTTTGCTTTCGAACCTCTCGGCTCATGGTTCAAA
ACATCAAGGTCAAAATGCCATCGACATGGCTATTAGCACCCATCGTGGTGGGCGGAAAGA
TTCTATGGGGAGTGGATATATCGGTGTTGCAAGTCGACGGTCAGTAATGTCAGGTTTGTC
GAAAATGAGCGCGCACTCAGTGGACTTAAACGTATTTTCAGGGTTGTCGCGGAAGTTCAG
CGGGGGCACTAATCATACAGCATCAAGTCGATCTATGATAAGTGAATTCAGTGGAATTGA
CGAGGACGATTTTGCCGAAGATGATTTTGATTTCGATATACCAACGAAACCAAATCCCTG
AATATGATTCTGACAGGATTGTGATACTAGACACAAAATGAAACGGGACAGACTATCTCA
AAGATCTTGTTTTGGGACCTGGGCCAAGTAGAGTAATTGGTTCAATAGACCAGAGAAACG
ATACAGGATAGACTCCTCCTACTGTGTTGAGTCGGAGAATGAATGCAAAAGTTTCAAATT
GCGATCACCCATTGTTTAAAACCCAATTTTTCTCACGGAGGACTTCAGAGGTGGAAGTTA
AATTTAGTCATTGGTGATTGATACATGCATTTACTTTCCATGGACTAAATCCTCTAGTCG
GGACAACTGCAGGTAATATGCAAATAGTAGTCTTCGTTAGCATCTCACATTTTTGCTTAT
TCACACTTGATCTCGTATGAGAGAAACAGAGCAAGCTATTGATACAAATCAAAAAGCTTG
ATTTACTGCTGTGTTTTCATTCATAATTTACTTTGCAACAACTAGTAATTTTGTTAATTT
TCAC
>comp2615_c0_seq1
CGTTATTCGTGCATTTCCTTCCTGTACCGAGAAATAGAAAGGAAGAAGAAGAGATATGTA
TCGACTAGAGGTCTCGCTGCTAGCGCTGGTTCTAACAGTAGCAGAAATCGTTTTCATCAC
TGACAAACATGCGAAGGTAGCTGCATTTCGCTGCGAGTCGAAAATCGCCACGCAGAACCA
ACAACTACAGCGAGAGCGAAGGAACGTTTGTTGTTCTCTCCTGCCTTCCTCTGTATTGTT
CCCTAATGTATCAACTGGAGCGTTTCGACAATCATCTATGTTTGCGCGCATCTATGGCGC
CGATGGAACTGTGGTCAACGACGACGAATTCGAAAACACAGATTACGGCTCTGACAACAA
AAACGATACTGAAATACTGGAATCATTGACGAATGCTGTGGATGACCAAGCGGTTTGGAC
CAGGATTGCCTGTGCATTTGCGCCACCACCCCACGACCGATTGAACCCAGAATTGGTGCG
AAGAGCCGAACTAGTACGAGTGGGCGGCAAATCCATCGATGTTGCTGTAGCGGTTCCGGC
ATCGGTTGGTCCCACCGCAAGCCCTTCCGCTGATCAACTCGCCCAGGTCTTGGTAACGGT
TGGATATCCTCAATCGTGGGTAGACAGCAAAGATCTGAACATGTTGACGCAACAGATTCG

CGTCTTGGACGGTCACGCAAAAGATAGGCTGTCCGAGCGAGATGTTGGTGAGGTGGGTGT
TGGTGACGACTCTCAATATTACGAACGGATTGTGATAGAAAAGCGATGGATGGAACGACT
GCAAGACGAACCAGACAAAAACGAAAGTCTACCCCAATGGTGGACAACGATTGCACCTCA
ATCGTCGCCCCTGGAGTTGGTGGACGAATGCAAGCTGTTGAAAAAATTGTTGAACGAAGA
CGAGTTCGAAGATGAATTGAGAGCTTTGTTTATGACACATTATGAGGACATAGGTGCCAG
CATCGACTTTTCAGCGGGTGCTGTTGCCCTTCGAGTAGCTGTTGCATCGATCGGATCTTC
GGGTTTATATCTAAAAGCACGCATAATAGATACCGCTCACGATGTTGGTAGTGAAAAGTT
ATCTGCTGTCTCCATTCCATACGAAGAAGATAACAGCGACAAAACACGAGTCGAAGTTAA
GACAGCTGGTGAGCTGAGAGAGGCCGTGTTATTATTGGTCGAATCAGTTGTTCCCATACC
GGTGCCCAAGACTGAAACAGTTAAAAGCGTCGGCGCGATTCCAACAACAAATGATGACAA
GGGAAAGTTCACACCCTTCAACGAACAAGTGGTTCAAGTCGTCACAAATTTGGAAGAAAA
TGCGAAGCCAAAGGCTACCACGACGGACAACGACTCCCCAGCAGAAATTGCCAACAAAGT
GGAGGAAGACGATGTGTTGCCAAAGGCTCAGTCGTCATCGCGCAATTCGTCTTTGGCAGA
CCGCCGTACGTATTATCAACGAAAATCTCCCGAAGACGAAGCCAAACTTTCGGCAAAATA
TGCAGCTATCGAGGACTTGGGAGAACGAGCCTATACAATCCTGTGTGATCTGGAGATGAT
CTAAAGAATAAGCTGTGAATTACACTTTTATACAGAGACAATTAGGTGTCCTCGCACCAG
CCGATAGTTGACTTTTTTATGTCATTCTACTACTTGATACGAGTACGAAAATCTAGGTGA
TTCTTCTGCATCTCTCAAATCGAAC
>comp2616_c0_seq1
GTTTCCTTCCAAGCTTCATTCGAACAATCCATTAATTTGAAACAGCTCCATCACAGTCTT
CACTCTATGATACCGACAAATTCCCCATAAATTCGAAAATATTGTTTCCGACAATTTCAG
TACAGTAAAACATGATAGAAATGCCTTCTGCGATACTGGGAACCATATTTTACAGAGGCA
TGACGATGGGATTGTTCTCGCTAGCGTTTTGCGCCTTTGCTTTAGCTCCGCCGGCGTTCG
CTCTCGCTAAAGACGAATTCTCTCCCTATGATATTAGCGAATACAACTTGGATAGATCTA
TATCGAGGATACTACAACAAAGTAACAACGATTTTGATCGAGTGAATGAATTGTTAGGGA
CCGTGACGATCAATATCGAAGACGATATGACGATATCTGAGAAGGTCGGCTTCATCAACT
TGAATTTAGTGGTAGACAGCTTGGTTTGTCGAGATATCAGCATTGGAAATATGAACGTGA
ACCACGTTACGGAAGATAATGTTATCGATGTGGACATATCGCTTGACGAAATCGATGTTG
CCTGCGACATTGAATATCAATACGAATACGGATTCTTAAAAGGAACCGGACTGGCAGAAA
TTGTTACAGATGGAAATATGGCCGATACTTCCATGAGATTTGTCACTAGCAACAAGGGAG
GTGATATTTCTGTGACGTCATCCGTAACTAAATGTGTCGTTGATGTTGAAATCATCGACG
TGAATTTCATAGACGCTGATTTCGCTTCAAATATCATCGGGCTCTTTGAACAGTATTTAC
GAGATTTGATCGAAGAAGAAATCGAAGGATATGCTTGCTCCCAGTTGTCTTCGACGGGGG
TTACAATGTTGAATGATGAGGTTTTCACTATGATAGACGAGACATTGTCTGATTTATCCG
CTACAGAAGATACTGGGTCATCTCTTCAGCCCAACGATTACGATACGACAACAGCACTCA
ATTTTAAAGACATCGCCGAAACACAGATTGGAGAATTATTTGAAACAGCACTTGGTGAGA
TTGATCAACTCTTTGGAAGCAACGAAGACAGCGGTAGTGAGCTTGGTATAAATGCTCTTT
TGAAATCTTATGTACTGAACGAAAATGGCGCATTAGTCGTGAACGTCGATGAGTTACTTG
GACAAGACGCCCTAAACTTTGAATTACACGACAAACTCACAAAAACAAGCATTGCTGTGA
AAGAAGTGAGAATATATGGCTTGGATAGCATGATAAAATTCAATCCCCTAGTGGTGGTAG
GAAATCATTCTCTAAGTAATGAGTTTGTGTGGGACTATTTGAACATGGAAATTGATATTG
CATTGGACATCCAACCTTCGTCGTTGGAAAAAGCACTATTAAAACTCGAAACCGATTCTC
CATCCAAAGGTATTAACGAAGAAATCACGATTAGATTCGGGGTCGAAGACGTTGCTGTAA
TAGCAAGGATATTCCTGTTGATTGACGTGAACGCATTGAGCGGATTCACAATCGGATCTG
TATTGGCGTTGACGAATACCGCAGATTATTCTTGGCTGCTACCTTGTTTGATGTCAGCCA
TTCAAGATCTCGTGTTTACAGAATTGACTGTTTCTTCCAAGCAGATCAGTTCACCAACAT
TGGAAGGATTCGTCGATGATGGCCTCGATCGTATCCTGTCCGACTTGGCAGAAATTGCTT
TCGAGATGTATCATGACGTCATTGTGGAGGACCTCCTTCCCAACATGTTTCATAACACTG
TCAAGACATTCGTCAATGACCAGATCTCTTTGATATTGGAAGATAGCAGTAGCAATTGTC
CAAAATACGAGAGCGTTTCTATCGACGGTAGCGAAGAGTATGTAGACTTTCGAGAGTTTT
TCGATTCCTCGTTCTCGACCGAAAGTAAAAGCCAATATGGAGAGTTTCCGTCTTTGTTGA
TGGATGCGGTGAACAAGGGACTGCTCGAAAATGATCTGACGACGGGGATGCCTAAGATTA
ATGAAGTATTGGTTGATCCATTGACCAAACGGCAATCCGGGACGGAGGGCACTTTATTGT
TCGGTGGAGATGGCGAAGATATATTTAATATTGTTCAAAGAATTAATCTCGGTGGATTTA
ATGCCAACGTTCGCTTGAGAGCGAGCGACATCAAAATTGAAAATCTCAATACATTGTCAG
CACCATTAGTCATGCTGCAGCCCGTTCCAAATGAACCCTACATGTTAAACAACAACCTAA
CCATCGGAGTCAAAGATCATCCGCTAAGAGTATCAGCGAATGTCGCATTTGCTGTAATAG
ACGAGGTTCAAGGAACTGAAATAGCGAACGAACTAGACATCAGCTTTGACATGTATTCAG
CCAATGCGATGGCAACTATAATGCTAAAAATCCTCAAATCGAATCTGTTGGAGTTTCCTT
TGATCGATGTTTTCGATCTAAATTGTTGGATTGCAACAATACCCGCACCAAGCTTGAATG

CACAAGGAGTTACCACTGGGGAAGACGAGGTTTCGGCATCTGTCGTTGATTTTCAGGCCT
CCGTGGCTAATCTCAACCTGAACGTGAGCTGTGTAGAGTGCTCGAGTCCTGGAATCGTCG
AGTTCACGGAAACGTTGATGTTATCTGGAGAAGCCCAAAATGATGTTACCTTTTTGGCCA
ATACATTGTTGCTATCCGCTGCCGATGTGATAAAGGGCAATTTATTGCAAGTCCAAATTG
ATCGATTGCTTAATGATGCCTCCAAACAATGTCGCCACAGTCCTACGTACGATCCCAATT
TTAGTCCCAGTAATATTTCTGCGAAGAAGAGGAAGTATAAGGATTTTGACACAATTCAAA
TCGAAGACTCCGCAACCTTTTTGATCCTTATCGGCGTGATCTTTTTGTCGTTGATTTTGG
TAGTAGCAGCTGTCGTTTTCTCGATTCGCTGCGTCACACGGCGTCGTCACAGACGGTGGC
TTTCGACGCTCCCTATAGAGCAAGTCGAAGTACTAAAGAATATCCAAAGAAACGAAGATT
CAATGGAATTTAACTTGAATTCAACTACGCGGTCGATGTTCCAGAGCGCAGAGGATATTC
CATGTGTCCTTCGCTGGGGTATGCCAATCATCATTTTGGGAAATATCGCTTTATTCCTCT
CGGGCCATTTAAGCCTCGGTGCAACCGTCAAGATAGTAGCTACAATCGCTGGGGAGACCA
TCGAAGTTGATGATTTCTTTGAATTTTCTGTGGCGAAATCGACAATTGATATCTGGAATT
CAGGTGGCAAAGCGCTCGCCATTCTTATTCTTATTTCTCCGGAATCTGGCCTTACACGA
AGCAAATGGCAACCCTCGTCTTGTGGTTTACCCCGACTTCGTGGGTGCCGATATCCCGTC
GTGGATCAATATTAGTGTGGCTTGATCGGCTGGCAAAGTGGTCCATGATCGATATATTTG
TTTTGATCGTCTGCTTAGCCGCCTTCCGTGTCACAGTGAATAGTCCAGCCAACTTTGCCA
TCTTGCCAAATGATTTCTATTCTCTAGACTTACTGGTGGTTCCGCTTTGGGGGCTGTACG
CAAACCTGATCGCTCAATTGATATCGCAGATCAGTTCACACTTTATTATTCATTATCACC
GTAAAATCGCGGAGAAAGCACGCATTTCATACCTGAAGACGAGTACTAATCTTTCGCTGG
AGAAGATTGAAGATGACTGCGACCAGAAAGTGTTATTGCGCACCCATAAATTCAGTCGAC
CACGCCGTGGAGAGGATGAAAAATTAATTGTTCGACCTTGGACGAGTAGCATGTTGATCT
TTTTGGTGCTATCGTTGATTGCATGTATCATTGCTGGATGCACCCTGCCTTCTTTTTCGA
TCGAAGTCTTTGGGTTACTCGGTATCGCTGTAGAGATGGGGCAGGAATTTAGGGAAGCGA
ACACAAATCACAGTGCATTTACAGTGATTCAGTTGATCTTCGAAGAGGCACAGTTTTTGG
ATACAGTGAAAGATGTTGTCGGCTTGGGTTCTTTTTCGGTGATTTTCGTTGCTACTATTC
TTCTAGTTCCTATCGCTCAATCAATTTTCTTACTTGGGCACTGGTTTGTTCCAATGACAA
TGAAGCGCAGGAGAAAAATGATTGTTCTGAACGAAATATTCCAAGCGTGGCAATACGCCG
AAGTATACATTATCTCTATTTTTGTTGCCAGTTGGCAACTTGCGCCTATATCATCCTTCA
TGGTCAATTCTTATTGTGGCTCATTGGATGGATTTTTTTCTGAGTTGGTGTTCTATGGAA
TTCTGAAAAACGAAGATGCACAGTGTTTCAGTGTAAAATCATCCATAGAAGATGGATTCT
TTGTTCTGGCGACAGGAGCTGTAATACTTGCATTGGTGAATACTTTTGTAGTTAAAGCGA
CCATGCAATACTTTCAGGATCAAGACGAATCTAACAAGCGGATTCAAATTGAAACGTATA
CCACTAGATTGGAAAGTGGTGAAAAGGGACAAGAAGCTGCAAATGAAGATTCGCAGGTGA
CAATCCGCCCTGTCCCCGTCTTATTTACGGACACATTTCGGTGGCTTTTGAGGCAAGATG
AAGAAGCTAACAAGGTGCTCAATTCCCGAGAGCCAAGCTGTGATAGCAACTCTAGCATGC
ATTGTTCAAGTGCTAAGGTTCAGTAGTTTTACAACAATAGTATTATAGATTTCTTACAAC
ATGAAAAAAA
>comp2617_c0_seq1
CTCTTCTCTTCCGTCGTAGCAACCTCGATCAAAAGTTACAAAAAATAGAAAAGAAATAAG
AAAAATTCAAAGCAGTGGAGCGAGGAAATTATCACAAACAATAACGAACAATGTCGGCTT
CTGGAGTTGCTCGGAGGAGTTTATCCACCGCAATGCGTACAGCAGGCGCCTCTCGGGTGT
GGTCGACAGCCCGCTCCGCTCCTGCGAAGTATCCCCTCGCATTCGGAGTTGTCTTGTCCG
GATTCAAGACAAGTTTTTCTGATTTGCTGGTTCAAAAAGTAGTGGAGCGCAAGGAGAAAA
TCGATTGGCGACGAAATGCTGCTTTTGCAGCGTTTGGTTTTGTCTATTTGGGAGGTATTC
AATACACGCTTTACGTTCCAATTTTTGGTAGACTTTTCCCAAACGCTGCCAAATTTGCCG
CCAAGCCCCTCAGACAAAAAGTGAAGGATATTCGAGGATTATGCAATTTAGGATTGCAAA
CTTTTTTGGATCAATGTGTTCACCACCCTCTCATGTACTTTCCTGCCTTTTACATTACGA
AAGAACTTGTGATGGGCGGAAAAGGCAATGACGGAAAACCAGACATTAGTCGCGTTTTGA
GTGACTACCGCACGAATATGAAAGAAGATTTGATAGCTCTGTGGAAAATTTGGGTTCCAG
CAACGCTTGTCAATTTTGCTTTCATGCCAATGCACCTTCGAATTCCTTTCGTGGCTGGTG
TGTCGTTGCTGTGGACCGCAGTCTTATCAGCGATGCGAGGCGGAGATGCGATTCATGCAG
ATGATATGGCTGGCGGTGTTGTTACGAACGCTTCGTATCAAATTTTTATGGAAGGCCTGG
ATGCTTTTAATACAACACCAGTTGAAATGGATCGGAATCTCGATCACGTGAGCATCTCGG
CAGCGGGCAAAGACAGACCAGGAATGGTTGCACAGCTTGCTAGTCATGTCGCCAGCCAAG
GAGGAAACGTTACACATTCAAAAATGGTGCGGTTGGGAGATGAATTTATAATTCAAATGC
ATGTAGCGCTGCCGAAAGAAGAATCAAGCTCTTTTTTCAAATCTCTGAACAACGGGAATC
TCGAAGAACTTAATATACAGCCTACGAGGCTGACTCGAAGAGACACGGATGAGAAGAAGA
AGGCTGTGATGGGAATGAAGATTCATTGTGTTGGAGATGATCGCCCAGGAATTTTGGCTG
ACGTCACCAATCGCATTGCCAGGAAAGGTATGTCACTCGAAGACGTTGCAACCTCGATTC

GATTGAGTACTACAGGGAAAAGAGAATTCGTGATCGATGTCTTAGCTAGCTCACCAAATG
TGGAAGACAAGGAAAATATCGATGAGTATGTTGCAGATATTAGTTCCATGGAAGAGGATT
TGAATCTCTCGAAGATGGATATCCGTGTACACACTGCTTAAATATCTGGTGATACGAGGT
TGAAGTCAGGTCTTGTTTTTTTTAGCCAGGATTGAATTCACCACCACATGTTTAAAATTT
GTGGGAAGTTTGGGACAAAATTATACAGTAATTGTAACATAAGTAACATACCATATTATT
G
>comp2618_c0_seq1
GGTTCTTTCTACATTCACCTTGGGAAACCGGAGGTCGATAATGTGTCATTGTGCTAACAC
ATTGAACTACCAGTAGCAGCGCAAATTAGAATCCGAACTGCAGGAAAGTATTTGATCCCT
AAAGCACGAAGAGAAGGCAAAGTCGAGAGAACAGTTTTTTTGTGATATAACAGCGCAAAC
TCCAAAGCATTGACAGCATCTTCTTTTCGAGCCAGCAACAAATACGCCAACAAATACAAG
ACAGCATGGTCTGGAAAGCCGGTTCCCTGAGCCGCCGGGGAAGCAGAGACAGCGATTGCT
CCTCCTACTCTTCCCTTCCGGAAAAATCCCCAGCCGAAACCGAGGTCCGAAGGATTGCCT
CCAAGATTGGCCTCCCGGAGGACTCGGACCTACTGGCAACCGTCTGCCACAAGATGCGAA
AGGAATACGTCGTGGAAGCATGGCAGCTCCCCTCCCTCGATTCGAGGCAGTGGGAGAAAA
TGAATGCCCCGATCGGGCTGGCGGTGGCGGTTCAGCACCTGTCTAGCCAGCGTCTCCGGG
AGCAGTTGCTCCAATGTCGAGAGGAGCAGCAGATCCAGGAGGACATTGACGCGGACATAG
AGCGGCAGATTTGTCTCACGCTGCCCTTTCGAGGACGCAACGAGCAGATGGGACGCTCCC
ATGTTTCCGAGCGGTGCAATCCAAACGGTGACATTTTGGCGTCCCTGAGCTCAACGACGT
CCGACTCAAGCGTCGTTTCGGAACGAGGGAAATCGAAGGAAACGATCAATATTTTCACAG
AGGCAGAAACGAAAACGGAGTCAGATCTTACCGGCCCCGTGCTTTTGCCCAATGCTGACG
AAACATCTAAAGAAATCCCTCGTCTTGCCCCGCCTATGAACCCAAAGCCTTCGTGCACCG
GCATTGATACACCACGGGAAGACATCGATGAAGATCCAAAGGAAGACCAGCAAAAGGAAA
TAAACCAAGGACTCTTTGATTCCGATCATGGTTCCGAAACTGCAGAATTCAGCGAGCCAT
TCCCAAAGTGGCTGAATCTGTCTACGGTCGCTTCGGGGCAACACGACGAGAAACCACAAA
AACTGCTGTCAGACGAGGATTACGGCGAAAAACCCGACGGCAGGTTCAACGCGATTGCAT
CTCCTACGGAAACCGAGGAAACCAAGAACGGCTCGACCCACGATATTTCTAGTGGCAGCG
AGGGTCTCGAAACTAAAGCGTCGCCAAGGAAGCAATTGTCGGTTGTAACACACCGAGAAC
CAAGCAAGGACGACGTTGAATCGGTCCAGGAAACCTTGGCGTTGAACGCGTCGTCAAAGC
CAGTTGGAGACGAGACGAACGCTGAAGAATCGCAAATCATTCCTGGCGACACAATCGAAA
CACCGAATGAAACGACGGTGCCAGCATTGCCGAGACCTGTCGAAACACAGCCAAATCCAG
AACCAGAACACGCCGAAACCGAACCGAGCGAGAACACCGAAACAGACAGCACAACGGTGG
ATAACACCCGTCTCGCAAGCGACGAGAACCAAGAACCGAAAATTGCATGTCCGCGCTCGA
GTGGTTCGCCGCAGGCCAAAGACAAGACCGGGGAAAGAACGCTGGAGAAAACCGACAGTG
ATTCGGAATCGGAGGACGCTACCAATATCTTTTCGGGGCTGGGGTCGTGCGTGTCCTTTT
CAGAGGAGGAGGAGGACGACGTGCTCGCCTTCGTCGTCGAGAGAGAAGCAGCGCAACAAA
AATCCCTGCTTGGCCCTATTGCGGAGGAATCCACCAACACCACTCCTGCTGCCGGTAGGT
ACGAAACAAACGAAAACGCCGCTTCTCCGGTTTCGGGAAGCGACGATGACGAAATCACGG
TGGCTGTTTCCAACGTCAGCCAGGCAGCGAAAAAGTCCGGATCGAGGAGGGCAAAAGAAA
CCTCCAAGAAATATCCCTACGACAACCGAGGCGATCAGAAGATTGATACCAGTGCACTCC
AGGTAATTCTTGTCAGGCTGCCCGATGTCGACCACAGATCCATTTTGAGCCAGTTGCTCA
TCATGGCCAACGCCAAGGACAAGGCTTCTCGCATAACACTGGCATTCCAGATACAGGATC
TTCTTCTGGACCTAATGGAAAAACACAAAATGGAAGAAGGGTCTGAAAAGGCGGTGCAGC
TGATTTTCCATCTCGCAAGACTCAAGAAGCAATACCGGGTGATCTTTGGCAAGTCTCTCT
TCAAAGCCATGTCGGCATTGTACAAAGAGTCGGAAAAACGAAAGGAAAAAAACAGCATTA
CTAGTGACAAGAAGGAAAAGAAAAAGGTGATCGACCCCGAGCACCTTCCCGAAACAGAAA
GCAGAGCACTGGAACAGAATTCACCGAAACCCACCATGGAGCAAGCCAAGATTGGTCACG
CGGCCGATGTCCTCTCCGCGATACAATCGCCTACGTAGCCCGAATCCATTTGTCCTCCAC
CATGTTTGAGTGGCGAGTGGCACAGAATCAAAGTTATAGTATTTGATCGACAGGCGCATC
AAATAAGGTTCACAACCGACAGAAGATTCGTTCGCCATGGGAAGTTTTGAGAAGAAACAA
TGGTACCGAGATCATGTCTCTAGTTAACATCAAAGTAGAACGCGGCATTTGTTCAAAATG
ACATTACCAGAAATATCCATTTGACCTCCCTAAAGGGAAACGATTGAACTCTTCCGGGCG
AAGTAACCCCATGGAGCTATGTGGCAATTCATATTCGTCATTTACTACAAGAGCAATTTT
TACAATGTTCTATGTATATCAATTTAGTCAGAAGAAGTCCATACTTCATCATCCGAAAAA
ATGAATTCGCCGTCTTCGTCGAAGTCGTCTTCGTCCATGAAGTCCCATTCTTCGTCCATA
AAACCTTCCAATTCGTCAGAATCCACATCCAAGAAACCTCCTTCATTGAATGGTCTCCAC
GCGGGTATCTCGTCTTCCTCTTCACTAGGTGGAATGTAATTGCCGTTGTCGTCGTACATA
AGGTCCTTCTCCAAAAGTTCCTGCGGAATATATTGATTGTTATAATACGAGTTGAACTCA
AAATCATATTTGTCGACCATCACCAAGTCGCCATCTTCAGC
>comp2619_c0_seq1

CAATCGCAACGGTAACTCGTGAAACCAAACGATCAAAATTCATTTCCTGTACGGCATTGC
ACGTGCAAAAAACTGTCGAATTCCGAATCTTACAGTAACAAAATGTATTCGTAAACGTCA
TTGTATCCTTCGACAAACAAAACAAACAGCTGCAACCAAGTACAACCCTGGAATAACACC
GCGAGCCAACGAGTATTACAAAAACTACAGCTTTACAGAGTAAGGTCAATCTAAAGGGAT
GAGCTCGGATGCAAATCGAAGTACGCCTCCACCGTCGCCAGCGCCAGCGCTCCGGCGAGG
TATCCACCACCCCATGAGTCCTATAGCTATTATGCGAAAAACATTGCGGCGACGATCGCG
TTCCGCCCCGCCAAAATTGAAAAAATCCTTGATTGAAGATCACTATAATGTCGAAAAGAA
CATTGTGGGCAAACGATTGGCGCTCCCTGGTGTTCCCAATTACGAAGATGACTGGGCTAG
AGATTCGCACGATTTCTTCAACTTGATTGTGTTGATTCCAATCATCGTGCTGAACGTGAT
GAACTGGAATTGGGACCGGTTGTTTACCCTAGGAAAGAAATACCACCTAGCAGATGCCTG
GACCGGTGAATGGTTTGATATGTTCTTTTGGGCCACCGTGACCTACTTCGTGACTGATTT
CTTTTGGGTTTTGATTGTTCCAAAATGCGTGAAGTCGCCGATGGTAATCATCCAGCATCA
CATTGTCACGATGCTGTATCTACTTCTTCCTTTTTACCGGCCAAAGTATCGTTGGTGCAT
GGGTGTCTGTATGAGTGTTGAAATCAACACGTGGTTCTTGATCGCAAGAAGAGTCTTCAA
CAAGCAGGGCTTTCCGCCATGGATTATTGATCTAAGTTTTGTTTCCATCAGGCTGAAAAT
TATCAGTATTTGTTTCTATGTGACATGGTTTTCGATTCGAGTATTCTTGTATCCCATGAT
GCTCGTATACCTTGTCAAAGAGCACCATAAAGAGTCCAAACGAGTGGGAACGTATATCAA
TGTTGGTTTGATTCCGCCAATTTTACAGTCCATCTTCTGTATTTTGAATTTGAAGTGGAC
ATTTGACTTGATCATGTCAAAGGTGCGATATTTCAGACGAAAGAAAGCAAATAAGGTCGT
GGGAAAGAGATTGGATTACGTCGACAAAGGTCTATGAGGAGAAAAACGACTTTCGAGAGA
TTCGCAATGACCGGAGGTTTTTGAAAGGGGATTGAAAATGTCTACACATCCAGATTTCTT
GTTTCAAATTGGCATTTCTGTAACGCAGAAAGTGACCTGGGTTTTAAGAATGATTTTTCA
TTACTGTCAAATTTCAAGTTGGCGTGCACACAGAAATGCAGAAAACAGGAGGAGGGACAT
ATGTCTAGGAAGTTAAGAACTCTCTGTCCCTCCAGCTGAAGGTTAAATATGTCACCTACC
GTATGTATGAATATCAACTAAAATTATTCTTGTCTATTATTGTTGAAACCTTGGCTGCTC
GAATCCATCACATACTTCTAAATTATAATACGCTG
>comp2620_c0_seq1
TATGACTGTAAAATACACAACATCTACATGGTGAGAAAATACAACTGAAACTCTGTAGCA
GTTGACCATGTCAGATGAAACAAATCAAAGCAACCCTGCAATGTCTGATCATAGAGCCAA
TAATCAAACCAATAATGACACCGATCGGAATCGAAGGCGGCAAGAGCAGCCGCAAGCACG
ACAGATTTTCGGCGGGCTACCCGCTCAGGGCTTTCAATTTGCTCGTCGCCATATGAATCC
AAACAATGTAGCTCGGATCCAGACAAGTGGATTGCCTATGAATCTGGACAGAGACATTCA
TGGTAATCCATTACAAAATGCCGCCTTGAATGGGAACAATGGCAACGGCAATCATGTTGC
AGATCGAGATATGAACGGAAATTTGAGTTTCCAGCGACTCCGATGGGGAGGAAGAGGGCA
AATGGCGAGACAACTGCAGCTACAGCAATTACAACGTCAACGACGGCAAAATCTTCAACC
GGGATCAATGCCACCAAATGGTGAGAATCAAGCAGGATCAACCACTTCCGTTCCCGTCCA
CCTTGGAATGGTGAGAGTTCAACTCGAACCTTTGACTCCTCAACCTGTTGCTGCCTCCGT
AGCAACGAATGCTACCGAGACTGCAAACCCTTCAGGATCAGGCACAAGAGTACAAGACCC
ACCTCAGTACGAACGATTTAAATGTGGAATATGCTACGAATACATGGACGACCCTGTAGG
ATGTGGCAAATGTTCCTCCAGATTTTGTCGAGCTTGTCTCCAGCGGGTCATTGATTCCGA
TCGAGAGAATCAACAAGTCACAAAATGTCCCGTTTGCCGATGTGAATGCGATGCGGCAGT
ACCCGACTATGATCTTTATGGGAAAATGAGTGAGGATAACGTCCCTCGGCTGCCATGCCG
TTATGTCTCTGTCGGCTGTCGGGAACGAAATCTCCCTCTGTCAGAAATCGCTAAGCACGA
ACAAGAATGCGAACATGCCCTCGTGAAATGTCGATACGCAGCCTACGGATGCAAATGGAT
CGGTAAACGTGGTCTTGTTCCAATGCATGAAGAGTATGGATGTAAGTTAGCACCCGCCGG
GCCTTTCATCCAGCACTTCCGGCAAACTATGGCGGATCAAAAAGTGAAGCTTGACCTATT
GGCCCAACAGACCTCGGGAGCGGTTCGAATGAGTCACGTGCTTCGACATGCGTATCTTCG
AGACAACCAACGGAAGAGCCTCTCAGATGTTTTGCGGCTGTTCCAGTATTGCCACAAAGT
GACAAGCTTAACTCCCCATTTTTTCTTGACCAAAGATCTTTGGGTGTCCTATTGGAGAAA
TCACGAAACTAGGGCAGCGGTGGTCAATTTTTGTGTCTGCTTCCCCTTCCTATCAGCCGC
TATCGGTGTGGTAGGTCATGGGACAAACTCCTTCTTCGATATATTTGAAGAAATCTCCCC
AGAGAAGACGTTGTGGGCTCTGACAAGGGCTTTTGAAGGAGCTAAAACGAATATTACGAA
CATGGCAGCACCTGAAACTATGGCAGTTACAATCAAGTCTCTTCAGAATTCGCACACTAT
AGAGCTTTGGGAAAACGCCTTCTTGGGATTGTGTGTTGGTAGCCTAGGAATGCTGGTGCT
AATGTTAAATTTTGTTGACACTAAATCAAGCATATCGTGGGACAAAATATCGATCCCTAT
CCTTGGTGGGAGATTTCCTTTGATTGGCGATGCAATGGCACTTTGTATTTTTACCCTTCT
TTTGGCGATGATGGAATATCATCAATCAAATATAAGGGCATGGATTATGTGGATGGCCAT
AGTTCCAGCATCGACAATTTTTCCAGCAACAATACTTTCGATATCACACAATACAGCTCG
CCTGGTGACTGGAACACCGACGCCAGCTGCTTTCGACATGATGAAAATGGCAAGATTGGT
CGAGCCATGTATGTTTGGATTGCGATTTAGTATGCTGGTAACATACTTTGGTATGATGCC

TGCATTAGACGCTTCTGTTCTTTTGGCTTTGGTGCCACAGTCATCAAAAATCTACTTGAA
GAATTCATTGCTCATTCACACGCCTCAATCAGTATTCTTTTTCTTTGCGGCAGCAAAAAG
CTCCTATTGGATAATCAAGATAAAGATACACTTGTTTGATAGTGGCTTGGATCTGTCGCC
TCTGAAAAGTGCGGCAATAACCTTCGAAAGCGCGCAAAACTTTTTTGCCTCTTCTCCAGA
TGCTATCGCTTTATCAGGCGTTTTTGACGACATTTCCACATCGCTCTTGGCAGTGGCAAC
CCTAATGGTTACGAGTTATATTATCAATAGCTTTTTTGACCTTGGTATTACGGTGGGAGA
GTACATCTCATTAACAAGTCAAAAAGAGGTTGAGAATCTATCAAGGGGTACGTTCAAAGA
ATACTCCTCTGTCGGCATTTTTGTCTTCGGTTTGTGGGCTATGATCAATACGCTCTTGGT
TTACATATAATGTAGCAGCGCTTCGATAACGAACATGGATATTACTTGATAGGTTCAAAA
TAAAGCTAAAGCTTCTTAAGTTTATAATGAGCTCAGAGGATTTACGTCCAAAAACTCAAT
AGGAACAATTTAGAAGCAGTAGTCCTCCTACTTTGTGAGCTCTACTACGAAACCATTAAT
GAAAGGAAGTGAATGAGATACATTAAGTGAAGCAATAGTACTAGCACCACCATGC
>comp2621_c0_seq1
ATCTCCTCATGCTAACCGTCATCACAATTCTGGATGGTAGTACCTGTATTTCGATTCTGC
TCATTACAATACAAACCTCTCGAGTGTCAAACTCATTCTACCTCGACGACCCCAGACATA
AGAGGAACTTCGACACCATGGGCCCCAAAGAAATGTACTTGATTGTGTACAATCTAAGCT
GTTGTGCCGGATGGTCGCTCATCCTGTTAGCTGCCATTCGATCCCTAGCCAACGGTGTAC
CGCAGGACGGGTTAAAGGAATCATTAGCTAATGTGTATCAAAATACCGACATGCCTTTTC
AGGTCGTATCGTCTGCAGAACCTGTGGGAGACCTTCTTTTCTATACACAGTCGGCCGCGC
TGCTTGAAATCTTTCATGCAGCAGTAGGATTGGTGAGGTCTCCGGTCGTGGTTACGGCCA
TGCAAGTCATGTCAAGAATCGTTGCCTTGGTAGCTGTCACGTATTCACCTGAAGCTCAAA
GTCAATGGGGTGCTGGTGTCATGATTATTTCCTGGTCGTTAGTTGAAATCTTTCGCTACC
TTTTCTACGCATTCGGCTTGGTTACGGGAGATTCAAACAAAAAGACCCCTTATCCCCTCT
TTTGGCTGCGATACTCGCTGTTTGCCGTTTTGTACCCAACGGGGATTACAGGCGAACTGA
CTTGCTTCTTTAGCGCTGCATATGACCCCGCATTTCAGTCTACTTTCTCTGCAGATATCG
CTATTTGGATTTACGCTCGCATTCTCCCATTGGTATACGTGTTCGGATCGCCTTTCATGA
TTATGAATATGGTAGGCAATCGTAAATCAGCCTTCAAAAAGCGATTTGCCAGACCTCCTC
CACCGCCTCGTGGTATTGTTTTCCCCGTCGAAGAAGGCAAGAAATCGAGATCCTCTACAC
CTGTCAACAAAGAAATTTTGGCATCTGCGGTTGGCGCCGTTAATCCGAGCAAGGCTGAAA
AAATCCGCTCTGCTAAGGGATACCGTTTCACATATGTTAAACATTTGACCGGACTCGTAG
AGGAACAGTGCAAATCTCCAGAAGCATGTCTGAAGATCGCTCAAGCTGGACTTGACAAGG
CCTACGATATTTTCGAGTTTGTTGCCCCCGACGGGTCTGCCGTAAGCGTCAGAGAAGCAA
TGAGCATGAAGAATACTGAGAAATTCTGCACTGGATTTATTAAGGGTGAGGGTGCAAAAG
ACACCACAATGATGGAAGTTCCTTACAAAGGAACAATTCTTAAAGGACAGTCGTTGAAAG
ATCAAGTAAAGAAATGGGTTGATTATGGTACCATTGAACCCAGTGCCGGGGAGGCAATTA
TTGGCTGTGTCGATAACCCGAATTGGTGTGACCTTTCGGATAAGTACTTTGTCTTGCTAG
GTGCTGGATCGGCGATGGGGCCTCTTTTAGTCTTACTGGCTTTGGGCGCCAATGTAATTG
CCGTGGATCTTGACAGGCCAAATATCTGGAAACGATTGATCGGACTTGCCAAGAAATCTC
CAGGTACCATAACATTCCCTCTAAAGCAAGAGCAGTCGTCTTTGGATAGTGATGACGCAT
TGTATGCAGCTGCAGGATGTAATCTCTTCACGCATACTCCTATGATTCGAGACTGGCTTA
TGGATCTCTATCCAGGAAAGAGTTTCACAGTTGGAAGTTATGCTTACCTCAATGGCGCTT
TGCACGTGCAAGTGTCACTTGCTATGGATGCAATTACCAGAGATCTTAGCGAGAAACGTC
CGGGAACCAGCCTTGCTTACCTTTGTACGCCGACTGACTTGCATCTTGTACCAAAGGAAG
CTTGGGATGCTAGTGTTTCGAACTTCAAGGAATACGGAAGTCGTCTTCTATGTATGTTCG
TAAGGTTGCTTAGTTCGGGTAGATTGTTAAAGAAGAACGCCAAAGCTCCAGTTGCTGGAG
AAGGAGGAGACTTTTACATGATCAACGGAGTTAGCGTCGCACAGGGGCCGAACTATATTC
TTGCCAAGCGCATGCAGCACTGGCGTGCCGTTGTTGCAAGAAGCAAAGGCTGTATTGTGT
CTTCAAACATTGCACCAGCTACTTCTACCGTATCTGTTACACAGAACAGAACATTTGCAT
GGGCCTATGAAGGCATGCCCTATTTTGAGCCCTATGAAATTTTCGCGCCCGAAACTTCGA
ACGCTGTCATGTCGGCAATTTTGTTCTCCGATCTCAACAGCCCGAAGAGCGTTGGAAATC
CTAAAACTTCGTTGAACAATCCCAACCAGTTGTTCTCCCAAGGATCTTTCCACGGAGGTA
ACTGGCGTTGCGCGTACGAGGTGGATAGCATTGGTGAAACGAGCGTTCTTATTTACTTCG
CCCGCATTGGTGCTCCGTACATCGCTGGATCCGCTGTAGTCTTAGCGGCTGTCGCATACA
AAGTTCTAGGATATTGATTTTTCCATGATAGCGAAGCTTAATGCACCCAAAACAACATTA
TACATTGCTTAATGTTGAAGTTAAATTAAACAATTATCTTAACGTGAAGTCATTGTAAAC
>comp2622_c0_seq1
GGTGCCTTCATTGGCAGAAACAGACGAAAAGTTTTCTGTCTGACAACAGAACAATCTCAG
AGTCGATAGGAACAATCCTTGTTACTGTGGAGCACTCACAAGCATACGGAAGATTTCAAT
CGAAAGTCTAATTCGGAGTGCCTTTGAGAAAATACAGCTGGCTCAAACCCAAGATGAACC
TTGTCGCCAGAGGCTTCGCAACATTGTTGACGCTTGTGATAGCGAATTCCATGACGAATG

TGGTTGATACATGCAAGGCTTTCGTTCCAATGTTATCAACTCAATCCGTTCCCAGTGACC
TAAGATGGTCATGTTTCAGAAAACCTGTCAGACTACATCTGCAAAAAGGATGGTTCGCCG
AAGACGGGGAAGACCGAGATGAAGATATGTTGGTAACGAGAGAGGATTTGAATCGTGATT
TACTCGGAATGGAGCCTAAAGTGAAACGAAAACGGAAGAATGGCAAAGGACAGGGATATA
GGCCGCTTGACAATCGGGATCATCTACCATTTTCAGTCAGAACAAAGACGCCAGATGATC
CATACAAAACTGAATTTCAGAGACGAAAGGAGCAACAAATGATTGACAAGAGCAATGGAG
ATAAAGTAAAGGGAACCGACCTCGACCGCCACATGCTTGCATCAACTAGGGGTAATTTTT
TCTCGAAAAGAAAGAATGAGAAGATAAATGGTAAATCTAAAAAGAAACTTTCAGATGATT
CGACTACGCCTTCGAAGCTGATCCAGCGTCAATCGAGAAAGAAAAGCAAGAGTCAAAACG
GAAAGTCAACGACGACAGTGGTGGGAGAATTTCAACTTGACAAGTCAACAACGAGTGGTG
ATATCATCGTGATAGGGGACAAAGAGTACCTAGTCGAGACTGCACGCTGCCAGTATAAGT
ATGCGGGAGGACAACGCTTCGTCATGGTCCGGAAAATTCTTGAGGTGAAAGAACTAGCAC
GGGCTAAAAAGGAAGATGACTTACTTCGGCAATATGAATCGAGCCCTTCCGCGTTCACGT
CACTTTCGAATGATTTCGAATGAATGAAGATTAATGATTTCTCTCAGTTTTTATCAACAT
TATTGGATGGAGACTAGTTACAATGGTAGTTGATCTTCTCAAACAGCATTTATTTGATAT
TTTTTCCCGCAC
>comp2623_c0_seq1
GGTTTCTTGTTTCGCAACTCGCTACCGTGTAATAGCAACAATTTCATTCAATCTTCAATT
GGTGATATTCATTAGAATTTGGATACGTTGTATACTTTACAGGTCCATCGACTAGGTAGC
TGAAGCAAAAGGTTCATCTGCATATCAAACAGAGCATTTTGGGTCTTACGGTATCGATTT
TCAGATTTTGGTGATAGAAAACAAATATTTTTGCAGAGATAGAGAACCTATCCATCACTC
GTCAAGGCCAAAATTTAATCTAGTCAGTAGATCACAAAAGTTACACTCACATCATACTTC
AGAAAAATGGATGGAGAGACAATGAGAAAGGAAATGATGAATGGGGGCAATGGTGCGCAA
GAATACGAAAAACACCCTACCGGCTACCAAAACCACTCACAGCATATCCAGCGCAGCCCT
TCGATGGACACAACCAATAGGCATCATCACAGACACAACCAACAACAATTCGCTAAGCCC
CTGCACCCGACTCAGCGCCATCCAGAACAACAGTACCACGGAAAAGCACCGAGCCACTAT
CCTCGGAGATACGACAACAGGGGAGGTTACCCAGCCTATCCACCACCGGCTCAAATGATG
AATCAGAATCACATGAATCACTATCATCCTCACTATTCACCACGATCTCGTTCACATACC
CCCAGGGTTCAAACGGTTGTTTCTCACTCGTTTAGCATGGAGGAAGAGCAACGGGAGGAT
GGTTCGAGTCGCTATTGCGGAGGGCACCACCATCGCAGTCCAAACCAGCGCGACACGAGT
AGCTATCCTGGGGACGAATACAGATCGCCATCGGGAGAATTCAGTGCCGATCGATCGTCA
CACCAACATCAACAGCGAGATGCTCAAGATCATTCGTTCCAACCGCCACCACCGTTACCG
CCACACCACTACGGCAGCAGACGCGACTACCAATACGTTAACGACCGTGAACTGCCACCG
GTGCTGGAGGAATCTAGAATTCAAATGTCGCCTGGTGGGATGATCTCTAGAACAGGCGAC
CAAGTTGTTTCGAAGAGGGAACCACCAAGTCCTCCCGTCGAAGGACAGGCTCCTGGAATC
GAACGCTCGCTATCGTCTGTATCCACCGGAGGACCTTTGAAGCGAAGTTTCTGGCATCAT
TCTCGCCCGGTAGATGCAATGAATGGAAATGACGTATCTATGCCAGCATCGTCATCACCC
TCTCTACCTTCCGAATTTCTACCTCCGAAACGTAGCAAAACAGGCGAGTCGAAATCCCCC
GGATCACCGAAACGAGAGAAGGAGTACACAATGACACCGCGTTCTCAAGCCTCTCCGAAC
AGCGATCGGGAACACCAACAGGAGCATGAGCACGAGCAGCAAATAATGGATATTCCTGGA
GGGAATGGTGGCCTGGGTTCTTTAGGTGGTGGAATTCCTACATCTACCTCCGATATAGGA
TTATCTCCTTCTGGTGACTGGTACACTAACCGCGCCCGTTCCATGTCATGGGAAGCCCGT
GAGGATTACTACCGCCGAGACCCTCGTTCTGCTCCTTCTTGGTCTTCGAGGAGTCCAAGT
GGGCGTGACGGTCCAGCTCCCGTTAACAGTGGAAGTGGATCACACTGGATGGAGGCTCCG
TATATGCCATCGCCGCGAGGGAGGCACACAAGGGATGGAAGTCATTACGATATGCATTTG
CAGCCTTGGGAAGCGTCTCCAAGAGACTGGGGACACCATGCCCATCACCTTCCCCACCAT
CCTCACCAGCCACCTCATCTTCAGTATTTAGCATGGCCTGGCAGGAGCAATGGCAACGGA
AGGGAAGAAAGCGAAGAAGGGAAGAAATTCAGAGGCCACGAGCATGGGGCATTCGATAGC
GAACAGAAACAGTCTCATTTGCACTTTCCAGTTCGTGGTGCACCACCTTTGCTTCCACAA
CCGCATGGAATGGGTCATATAATGGGACCACCCATGCAAACAGGCAGCGGTGGAGTGGAT
CCATCAATTATGATGGAGGGGCAATCAGGGGATATGATGAACAGCGATAGAAGGGGGGTA
GACAAGAGCGGCCCAATTAAGCTTTTGGCTCTCCCAGAGGATCGCATTTCCTTGAGTGAA
ACTCTCTGTATTGTACGCGAGAATGTTGAAGTATTTACGGCAACCATCGAAGACGTCGAG
GCACCAGCCCCAGGCCGCAAACATGCGGTCACCGTTGGACAAGTTGGTCTCCGCTGCATC
CATTGCCGCCACACAACTAGATCGAGTGAGCGCGTGAAGAGAGCTGTGTGCTACCCATCC
TCAATAAAGCGAATCTACCGCACCGTTATTGATATGAAGCTGGACCACTTCCTACACTGC
AAATTTGTACCACAGCCCTTGAAGGATACACTTCAAGCTCTGAAAGCTAACAACACGAGA
AGCACTGGAACTACAATGCAATATTTTATTCGTGCTGCAACATCTTTGGGAATGGTAGAT
GGTCCAATGGGAGTACGTATCGCAGATAGTAGTATACCTACTGTACCTATGTCACTTCAA
TCTAGTGGAAAAAACAAGTTACCGAAGCCATCTTCCTTACCGACAAAGGGTAAGGAGCAT

GTTCCTTCCAAGATAAATTCAAAGAATCCCGGGGAACAATCGGAATCTTCCTCTGGCAGA
GATATTCGTATCAATCGAACGAGTTCATCGATGTCTACAGAAGGCACCCCTCAGACCTCG
GTTTCAACGGACGATCTCCCATCGGAGCTATTTACGGGCAAAGTCGCTCTCTCTGTTCCA
GAGGACAAGATGTCGCTGAGCCCTCTCAGATGCTTTCTTCGTGAACAAGTTTGTGCCTTC
TCTGCTACCGAGGAAGATATTGCTGTGAGAACTCCGACGACGTTCGCTGTTTCCGTTGGT
CAGGTCGGAATCGGATGTATCCACTGTTTGGGCCAGCCCGCCAAACTGCGTTTAAACAGA
GCAGTTTGCTTCCCTTTTTCCATCACCCGAATCTACCAATCCGTGGCAGACATCCAGCGA
TTTCATCTGGGTGAATGCAAAATGGTCCCGAAGGACGTGAAGGAGAAGTTTTTGGAACTA
CAGAGCCAGAGCTCGAAGGGCTCCAAGGGTCTCGCCACGCGCCAGTATTGGGTGACATCC
GCTAAAAAGATCGGCCTTGTCGACACAACGAAGGGAATACGGTTTTGCAGGGACCCTGCG
ATTCCTGAGAACAAACCCGTTTCGCTCGACATTCTGGCACAGGTGGCTTCTAATGTGACC
ACAGTGAATAAGCCACTTGTTCTGCCGGAAGACAAGCCGCAGATCGCAGAATTCCTGTTT
GTCGTAATGGGGCAGCTGCAGCCCTGTCGTTTCACTGAGGCCGACCGGAACAAGAGGCGG
CTGAAGGACGTGGGTTGTATTGGTGTGGAATGCAAGCACTGCGCCGGTCAGGTAGACGGA
CGAAAGTTTTTCTGGTCGAGCGTGAACGCGGTCGAGTCAAACTTTGTCTCGGTGCACACG
CACATGATGGAATGCCGAATGGTTCCAGAGGAGACCAAGGAAGAGCTCGCCAGGCTAAAG
AAACTGCGGAAGGAACAAACCGCGGCTCTGAAAACTGGTTCCCAGAAGTCGTTCTTTGCC
AGGGTCTGGAAGAGACTCCACGAAGAACCAAAAAAGGATGAGCCAAAGGACGAACAAAAG
GACGGTGAACAACAAGATTCCACGACAAAAGAAGGATGTTCGGAAAGAGCAGCAGAAGCA
CCAATCGAAAAAACAGCAGCTGCAAGCGAAAAAGCCACCGCAACCACGGACTCTACTGCG
AAGGCAAGCTTCAGCGTGAGCTTGTCACAGGACACACTGGACTTGGCGATGACGACCCCG
ACCACGTCGCCCGCGTCCGTCGAAAAGCCGTTCACGGAAATCAGCGTGGGGAAACTTTCG
CCGGGTTCACCCGATTCTACCCAGAAAATCGAAGCCGTCAGCGATAAGATGGCGGTAGTG
AGGGTATAGTACAAACCAAATCAAACCAAACCAAACCAAGGGAAGGGAATGGAACGATTG
GGTTGGTTTTCATGTATTTCGTTCCAAAGTATTTATCATGCTGGGGCATGATTGAACGGT
ATTACAATGTTATGGTGTTTGGGTGCACCCACGGTGTCGTCTCTTTCTCTAATCTTATGG
CCTATCCGGTATACTATTCGATCTAGAACGGACCGAGGGGCTACATTCATAACAGCAATA
AAAATAAACTTCGTAGAATACATTCC
>comp2624_c0_seq1
CGATAAATAACGATACAGTAGTACCATAATCGAACCAAACACAAACCGAAAAGTCTAAAA
CGTCGTTCGAGACAAGCATTTCAGAAAACAAAAAACAACAAGTGGAGGCAAAATATATTG
ATCCTAATCTGTTTGGAAGTCCAACGGACAACCCAGTAGTAGCACACACAAGCACCATGC
AAGACAACACGACTCAAGAAATCGAGGATAAGCTCTCGGAGCGGCTTCTGGGACCTTCCG
GCGATGGCGATATGCACCACGCCGACACCAGTTCGTTCCGGATCGATGGGAGCCACAACG
AAGATTTTTCCGCCACCGCGATCATGGAAGGTGGAGAGATCATCGTCGGTTCAGGGGCCG
GGTTCGGCGGGCAGCAGCCCCTACAGCCAGAATACCGCGACGCCCCCTTTGCCCTGGCGT
TTCTGGCGCACCTGGGGTTCGTGTTCTTTGTTTCGATGGCCTGGGGAATTTCGGCCATGC
GCTCGTCGGAACCAAGCGGCGACGACGCAAGCAGCAGCGGCGACAGCGGCTCGGTGTTCT
TCTCGGGCCTGTTGTGGATTGCGTTCTTTACGTGCGCCGCCAGCATCGGAATCTCCGCTG
TTTCCCTCCAGGTCATGATGCACCACGCCGAGACACTCATCCAGTGCAGCCTGATCGGCT
CGTGCGTGTTTATGGGAATGGCGATCGTCTTCTTCTTCGTCGATGACGTCGTTTGGATGG
CCTTTGTCTGGATGCTCAGCCTGTTCCTTACGGCCTGGTACGCCCGCAACGTGTGGCACA
CGATTCCCTTTGCGGCCTCCAACCTCCGGACGGCCCTGTCGGCAATCCAAACCAACGGCG
GGGTCTGCGCCCTTGCGTACGGAATTGCGATCCTGGCATGCGTGTGGACCGTGGTGTGGG
TGCTGGCAGTGGTCGGGGTCAGCTTCAAGGAGGCTTCGTGCGTCGCCGGCGTCTGCAAGC
CCGACATCAACGCCTTTTCCGTCTTCTTTTTGATTCTCTCCTTCTACTGGACGATCCAGG
TCTTCCAGAACGTCCTCCACGTAACGGTCAGCGGCGTCGTGGGAACGTGGTGGTTTGCTC
CGCAGGATGCCCTGTCGGTGTTCTCTCCGGCAATTCTCGACTCGTTCCGGCGGGCAACGA
CCTACAGTTTCGGAAGCATCTGCATGGGAAGCCTTTTGGTAGCGGTCATCCAATCCCTGG
AGAGTCTGGCGAGAAATGCACGCCGAAACAACCGGGGTGGAATTCTGTTGTGCATCATCG
AATGCATTCTTCGAATGGTTTCGAGAATCGCCGAGTACTTCAACCGATGGGCCTATTGTT
ATATCGGCCTCTATGGCTTTGACTATATTTCTTCGGGAAAGAAGGCCATTGAATTGTTCA
AGGCACGAGGTTGGACGACCATCATCATGGACAATCTAATTTACCGGTCGCTGAGGCTGG
TGGCCATCGTGGTGGGTGCCGTTTCCGGCGTGATCGGTATGCTGCTGGCCAAGGCCACGG
GCTGGGTGGCGACGACGCTGGGAGAGGACTCGGACGGCCAGGTCTTTATCCTCTGCTTCC
TGGTCGGCTTCTCCATGTCCTTTATCCTGATGAAAGTGGTGCTGTCCGCGGTCGATACGG
TCATTGTGGCTTTTGCCGAGGCACCGAACGAATTCAACACGCACCACCCGGCTCTCTACA
ACAACATGGTTCAGAAATGGAGGCTGGTTTTTCCGGAGGAATGCGGATTTTGATCGAGCG
AGCAATTCTATTTTTTGTGAAGCCGCTTCGAGAACCTCTGGGAGTCGAACCAAACCGCCG
GGTCGTTTCTAATCCGGGAACGAGCGTTATCTCCACGCGCCTCCCACCTGTGAAGCACCG

CCTTGTGCATAAACCTCACGGGGCTATTTGCTTTTCTTTTTGTACACTAGTTTTACGTCC
GCATTGTAAGC
>comp2625_c0_seq1
AAACTTCAATACTTGGTTTAAGTTATCATTCAACATTAGTATTAGTTGAAAAGAGATCCC
TAACTATGGCAGCTGTAGACACTGGACTCTGGGGTCCCGAAACGAGGACTGCGAAGACAG
GAAAGGAGTCTAAAATCGAGGCTTGGTGGGAAGTGAGAAGAGCGGGACCAGCTGAGCCAG
CGCCTACTAGAGGCGCCACTCCGCTCGTTGAGAGATGCATGCGAGTTCACAGTTTCAGCG
AGGACTTCGCAAGACGTGTCCTCAAAGGTTACCGTCAGTTCATGGAACTGAAATCGGTTA
TGAATGATTGGCAAGAATTGAAGTTGGCTCCTTCTAATCCAATCAACCAAATGTGGGAGT
TGCATTTACTTGATAATCTGAATTACACCGAAGATTGTCTTCTTCTGTTTGGTCGTGTCA
TCGGTCACAATCCTGACGCCGTTCTCGACGGAGCAGCAATGCGGGAACGTATAATGACTA
CGCAAATTGCTTTCCAGGCGCGATACGGTGATGATCTGGATCAAGACGTTTGGGACTTTG
GTGACGATTGGAACAAAGATTCTATGGCTCAACCATCTTTGAATAAAAAGTTTGATTCTC
TCGAAGTGTCTTCTCGTGGACGTCTTCCAGTCGCTGGTCGAGGTGATGATATGAGCGCCA
GCCGTGGTCGCGCGTCTTCGAAGGGACCTGTCTCTCCGCGTAGAGTGAATGCAGCGAAAA
CGCCTACTTCAGCTTCTCCCGGAGGGAACGAGCCAATTACCATCTTTCTTCGGGACCAGA
GCACTGGTGAAGAAACGTACTTCTCTTTGCGATACAAGAGTACATTGCGTATTGTCTTCA
CAGTGTTTGCCGAACGACGTGGATTGAATCAAGATTCTCTTAAGTTCAACTACAACGGAC
AGCAACTAAACGGATATGAGACTCCTCACAGTCTCGCTCTTGAAGACAGAGCACGAATTG
ATGTATCAATCACACGATAGATATTTGCAGTGGTTGCAACAGCTGACCGAATAGCATTAG
CGACGACGGTAAGAGAGGAAGGTCTCCTACAAAGTGGATAGACCCAAAGGCTTCTGCATT
GAAGCAAATTGATATCTCCTCCATGTATTTATGATTATTAATTTGTACCAGATTATAACA
GAATTTTCGAAATGTGAGCAACCTCTTTGTTGGCCCTTGTAGTAGATCGTTTTGGTGCAA
TAAAGAAGTTATCCAATACAAAATTGCCGGACGAAGTTCAATCAGGTTTTCCGCGAAAGA
ATTGCGAGATTTCTTTTGCAGGAATCGAAAGACTTACTTACTGTGTGGTATTAGTAGAAC
GACGGGTTTTGTTATTGTAGACATACACTTCATTTACCGCACCAAAAAGTATTATTGATG
ACGGTAGGTTGACTCTTGTTCGGAGTGAAAGAAAGAACTTCTTCATATATTGGTCAACGC
AGCGAAAAATCAAATATAAAGATATTAGTTGGTCTTGTTTGTAGTGAAATAATAAAAACA
AATTCTTATAGAGCTTTAATTTTTCATACTGTAGTAC
>comp2626_c0_seq1
AGACTGTTGTAATAAATAACATCGAGCAACAAACACCATGTTCAAGGCAACAATCCTAGC
AACTCTTCTTGCGTCGGCTTCGGCCTTCGCCCCTTCTCAATTCAGTGGACGTGCGTCCAC
TGTTGTCAGATCAGAGGAGGCGGCTGAAGAAGTAGAAGCCCCTCCCGCCTACGTTGCCAG
CACTGCCCTTCCTTTCCTTGAATACCCTCCTAATCTTGCTGGATATGTTGGTGATGCCGG
ATTCGATCCATTCCGTTTCTCCGACTTCGTCCCTATGGACTTCCTTCGAGAAGCCGAACT
TAAGCACGGACGTATATGCCAGCTCGCCGTTCTTGGTTTTGCTGCCGTGGACATGGGATT
CCGAATCTACCCTCTTCCTGAGGCGTACAAGGGACTCACCTCGGTCACTGCCCACGACGC
CCTTGTCGAACAAGGTGCCATGAGCCAGCTGTTCTTGTGGATCGGTCTTGCCGAAGTCCT
CGGAACCGTTGCTCTTATGCAAACTTTGCAGGGATCCGGACGTGCAGCTGGTGATTTTGG
ACTTGACCCTGTTGGTTTCCTCGCCGGAAAGTCGGAATCCGAGATCAACGAGATGAAAGA
AAAGGAAATTACCCACTGCCGATTGGCTATGATGGCGTTCAGTGGCATGGTTACACAAGC
TGTTTTGACCCAAGGACCTTTCCCTTACGTCTAAGTCTGGAATCGTAGCGTGGGGCGCAA
CGAGACACGACTCGATGTACAAACCACACATGGACGACGGTGTTCTACCACCAATGAATA
GACATGCCCACTTCTATGCGTTGTATTTCTAAGATACCCGTTCCCTTCTTGAGTGTTCCG
TGCGCATAGTACTGTAGGCAGACTTTCGTCTGAAGTCTCATGTATCTATCAACCTGCTGT
CACAGCCGCAGCATTAATAACTTTTCGACCTAGCTACTTTAATCGGGATGTTAAATACGA
GAAGTTTCT
>comp2627_c0_seq1
CGCAACGTAACTACAGTAGGTTACCTTATCATCCCCCGTAACTCTCGAGGCGTTTTTGTT
CCGCTTCTTCGAGAACAAGACGCATGCTAGCTGAACTTGCATGCAGGTGGCATCAACGGC
TTCCAATCATCCATCATCCATCAACTAAGAGAACAGCATCTGGTGTTGATTGACCCTACC
GTAGGCCACTTGACACACCAGGCAAATCGTACCCCTACCCTTATCCAAGCCATAAAGTAC
CTACCGATAACAAATAAAGGAACGTTCGGAGTTTGAACACACACTCCGAAGTGCCGATCG
ATTTCGCAAACGAGGTGGTTACTCGTAACACAAGCAGGGTTCGATAGATAATCCGCAATC
GACAGGCAAAGGCTTGGATTCGTTCATCGGCAGTCATCGGAAGCAAGTAGATCGGGTATA
GCTCGGCTCGTCACTGCACAACATAACAGGCAAGCGCGAACGCCAAAAATGGAAAACACC
GGCAATGGCTGCAGACGTAGCCACGACAACCCCTCCTCCAGTCCCATGCACATCGGCATG
CAAGGAGTTGGTCTCAATCCGTTTTCGGCGGACGAGATCCAGGAGTTGATGGGGCTGATT
CCCGGCAGTGATCAACACTCCCACCCCGGGACAGCAGCACATGCCCATTCGCATTCCTCT
TTGTCTCATGCGCATGCGCCTTCGCCTCTGCCGCATGCCAACAGTCTCGGAAACGACATG

AGCGTTGGGGTGCACAATCCCCTCCAACTAGGCAACAACAATACGAACCAAGGCAGCAAC
ATGAGAGCAGCCGCATCCCATGCCGCCGAGATGGGCTTGGCTTCTCTTCCGCCCCACCCC
GGCCACGGGATGGTCCAGCCAACGTCGTCCCTATCGTCCATGCCGACCATCGGCAACGGC
AATCACGCAACCTCAAACAGGAGGCACAGCAGCGGAAGCAACACCAGTACGGCTATCGAC
GAGGGAAGCAAGAGCCAGGCGCGTTCCGAGCGGAAACGAAGCCGTGAAAAACAGCGCCGG
ACCGACGTGAACAAACAATTTGGTGAACTCACGGAAGTCCTCAAGCGCATCGAAGCGGAA
GAGCAGCAGATGCAGCAGGAGCGCGCGGCGCGAGAGGAAGCCGGTGTCGACGGGGCTGTC
AACAACGGCAACGGGAGCCAGTCGTCGTCCTTGTCGTCGTCGCGCATGATCCTCCCGCCC
TTTTCACCGACCAACCGGGTCGATCTGATCGCGCGTACCATAGCTCATCTCGAGCGCCTC
AGCCACCTGACCAAGAAACAGGTCCACGAGATCAAGGCGCTGGACGATCAACTCAAATCA
GCGCAAAAAGCGGGCGAAGATACGGCACAGAAACTCAAGGAGGCTGTTTTCCATCATCAG
CAGCAGGCCACCGTCCACATGGGCAACAACACCGGCGGCATGATGATGGCCCCCGGAATG
ATGGGGCAACCGATGAATTCGATGTTCAATCCCATGGCGATGGCGAACGGAACGAACGGA
ATGATGTCGATTCAGCCACAGAAACAGCAGATGATGATGATGGTTCCTATGATGATGCCG
CCGCAAGGAGGAGCCCCGGGGACACCCATGATGCCAACTGGCAACCAACCCTTTATGATG
ATGCCTCAGGTGTTCTTGCCATCGCAACAGCAGCAACA
>comp2628_c0_seq1
TCACAAATAGCGACTCCAACAAACGAAAAATCACCTTCATTTCAAACGATTAAATTTTAC
TAAATTTCAAATACAATGAAGGTAAACACCATCCTCGCTGCAGCAACTCTTCTCCCCCTG
GCTTCGGCCAGAATCCGTGGATTCGATGCCAGTGATACAATCGAGATCAACGACTTGGAA
GAGCGCCTCCTGGCGTTCTCCCTCCCTGGGGAGGCAATGAACGTCACCGACCCTGCCGAT
GACGATTTCGATGCTGACTTTGAAATCGAAACTCCTAAGGGAAAGAAACCCAAGCCCGGA
GCTGCCAAAGTAAAGATCGAGAAGGGAAAGAAACCCAAGAAAGCCGACAAGTCAAAGAAA
TCTAAACTCGCCAAGCCAGATAAAAAAGCCAAGTCAGCGAAGGAAGCCAAGTCAGCGAAG
GTAGCCAAGGAGGGAAAAGCCGAGAAGTCAGCGAAGTCAGCGAAAGAAGGAAAGTCAGCA
AAGGAATCGAAATAAGCGTCTTGTTTCAGCGAGGAGGAACCGCTCCAGCTAAATCAGACC
AGTCATTGGAAACAGGAAGAAGACGATCCGCAGCTTCAAACGGTTTGCGTCGTAAGTGGA
GTCCAGGCGCTGGAAATGGTTCGATTCGTGCTGAACCACCGGATGTATTGACCACTTCTA
ATGCGGAGGCGGGATAATATTTTATGTAAAAGTTGTTTGAATAGAAAAAAA
>comp2629_c0_seq1
CACGAGTTGCGTAAGAATATCCTCTTGATCATAACAAAACGAATACATTCGATAGTACAT
CGGACACTACGCACTGTATTGGTTCCCTGCTCAATCATTTGAACTACTGACTGCATTTGA
ATACAACGGCTACTTGTTCGCAGTGTTTCTCGGTCGATCTTGCGACTTCAATTTGAAATT
TTAGTATTCAAGTCAAATAATAAGAATTTGTAATTACAATCATGGCGAATGATGAAGATC
TGGGAACCTTTGGGGTTTCCCGTGGGATGAACGAGAAGGCCGCCAATAAGCGTCTAAACG
AACTTCAGCAGAAGAAACGTTCAACCTCTGATAAGGATATTATTTCCAAATACAATGCCG
ACCGACAACAAAAAGACCGTCTTAGACATTTAGAGAAGGATATCACTGTTCCGACGGGTC
GAATGAATGGATACCAAAAACGTTCGATACAAAAGCGAATGAATCGAAAAGACAAATCCG
TGCACTCCAAACTCAAAATTTACAAAGCTAAGCGTTATGAGGCAGCTGTGGCTGCTGCTG
ATGCACAAATAATTTTAAACACAGAACAGGCTGGTTTTTTGGAGGCCGAACACGAAATGG
AATTTACGTCGAGTCTCTCACAAGTAGAGCTCAAACGTAATCACCTCGATGAAAACACTG
CGAGGCATATCTATGACGTCACCTTACCTTCAGGACCATACGGTTGTAAATTCGATAGAT
CAGGTCGATATTCGATATTATATGGCCACAGAGGCCAGTTGGCTATGATGGATGCCCACC
ATCTCAGTCTCCATAAGGAATTTGACGTGCGAGAACGAGTGCGGGATGCCTGCTTTTTGC
ACAATTTCAGCTTATTTGCGGTAGCGCAAACCAACCATGTTTATATTTACGACGATACAG
GGGCAGAAGTACATAAATTGAGCGATCACCATGATCCCATGGCCATGGACTTTTTACCGT
ACCATTGGCTGCTCGCCAGTGTAGGTCGAACTGGACATCTTGTATATCAGGACACATCTA
CGGGAGGATTGGTGTCACAACAGCGTACGAAACTAGGACCTTGCAATGTCCTCCGTCAGA
ATCAATCAAATGCCGTTATGCATTTGGGACACTCAAACGGTACGGTTACACTATGGTCGC
CTGCTTCCAATAATTACTTGGCAAAAATGCACTGCCACAAAGGGGCACCAATTACATCCA
TGGCCATCGATATATCAGGAAACTATATGGCAACAGGGGGTGCGGACCGTCAGATCAAAA
TATGGGACTTACGAAAATTCCAATGCACCCACTCCTATTTCAGTATCGCTGGAATACCAA
CTTCCTTGGACATATCGCAGCGTCGTGTCTTGGGAGTTGGGCACGCTGGCCATGCAACAT
TTTGGTCTCCGGAGGCTTTGTTGAAAAAAGTAAAAGAGCCATATATGCGACATTCCGTTC
CGGCGTGTGGACCTGTGGAAACTCTTCGATTTCGTCCGTTCGAAGATGTTTGCACCATTG
GTCATACCAAGGGTATGGCCTCTATCGTCATCCCTGGATCGGGGGAACCGAATTTGGACA
CTACTGAGTACAACTTGAATCCATTCCAAGACAAGAAGCAACGGCGCGAGGCAGAAGTAC
GTGCTCTTCTGGACAAATTGGATCATAATATGATCACGCTGGATCCTCATGAAATCGGTG
GTATGGAAGAAAGTACACCAGAAGTTCGCAATGAACGATTAAAGGACGCTGAAGAACTGG
CGAATGCTCACAAAAAACCTAAGAGGCAAGCCACCAAAAAACGAGGTAGGAGCAAGATTC

AAACACAACTTAGACGCAAACAACGAAACGTAATCGACGAACAAGTTACCAAATTACGAG
AAGCACGGGAGAAAGAAAAAGAAATGGCAGCTCTTTCTAGTGGATCAGAAGGTACAAACA
GAAAGCCCTCTAGCGAATCGCACACTGCAAATAATGCAGTTCCATCTGCATTGAAGAGGT
TCTTCTAAGTAATAAACAAATGTTCTTTGTTGGCATAGAACGAAACCACAATTGACATAG
TATGGTAGGGCCATTGATTTTTGCACCCATATGAATTATTACTTAATCTCAATTCAGCAC
AATGTAA
>comp2630_c0_seq1
ACTGAGCAAAAAAAATCAGCAAGCACAAAAACACATACCGTACTTCGTAATTAATATCGT
TGCACAACGACGAATGGGATCTACTTGCAGCAAAATCAACACAGCGACAACCAATCAACA
ACCAAAAAATTATCACCAGCGAGAATAACAAAAACATGGCCCCAATAGCATCCACTCTGGC
ACTCGGCGCAGGGTGAGTAAATCCGAATCGTCCTGTCGAAGTCGATTAAAGTCAATTCGT
CCATGCTGTGCTCTCTTTTCTTTAACGACAACATCCGTCCACCTTGTTTGACGTTGTGCG
AAGCTCACCGCGACTTTGTTTTGCATCTTTTCCTTTGCATCGTCGCCAACTATCATTCTT
TCTTTCGAACACAGCTGTTACTGGGGAACGGAGAAATACATTGTCAAGGACTTTCAAAAG
AGACACCCGGGCTGCATCGCCAATGCCTCGGTTGGGTTTATGAATCCCAACCCAAAAGGG
ATGAAAAACCCCACGTACCGACAGGTTTGCTCCGGCTCCACGGGCCATGTGGAGGTCCTG
AACATCGAATTGTCCGAAAGCGCCTCGGCAACACCGGAGATCTTTGAGGAACTGATGAAA
TTCTTTTACATGTTTCACGATCCAACCACGCTGAACCAGCAGGGGAACGACCGTGGGACG
CAGTACGGGAGCGTCGTCTTTGTCTCCTCCGAGGACCAGAAGGAAATTGCAGAGCGAGTG
AAGCGAGAACTCCAGTCGCACGTCGATAACCGAACCATCAAAACGTACCAACGCAAGAAG
ATAGAAACGGCCATTGTCGATTATACGACCTTTTACGAGGCGCATGCCGAGCACCAAGAA
TATCTGGAAAAGAATCCGTCGGGCTATTGCAACCACAGGTATCGTTTCAAAGATTGGCCC
AAGGCGGAATTGTAATTTGAATTTCTGTGCAACCGTGCCAACAACCTCGTCGCAGAAAAC
AATAGAAGAACAAAGAAAGAAACAAACAATCGACAACATCCAACGCATTATGAGGCATAT
CGAGTAGGATATTTCATAGTCCGAAGAATGCAATGGTCGGCAATCGTTTCTAGAGAAGGT
CTCCCGGAAGGACTAAACTGTAGATTGTGGAGCGCCCACCTGTCTTGCCCTGGCATTCGA
GTTTAAAATAGAGAAATGTTTCAATGTTA
>comp2630_c0_seq2
ACTGAGCAAAAAAAATCAGCAAGCACAAAAACACATACCGTACTTCGTAATTAATATCGT
TGCACAACGACGAATGGGATCTACTTGCAGCAAAATCAACACAGCGACAACCAATCAACA
ACCAAAAAATTATCACCAGCGAGAATAACAAAAACATGGCCCCAATAGCATCCACTCTGGC
ACTCGGCGCAGGCTGTTACTGGGGAACGGAGAAATACATTGTCAAGGACTTTCAAAAGAG
ACACCCGGGCTGCATCGCCAATGCCTCGGTTGGGTTTATGAATCCCAACCCAAAAGGGAT
GAAAAACCCCACGTACCGACAGGTTTGCTCCGGCTCCACGGGCCATGTGGAGGTCCTGAA
CATCGAATTGTCCGAAAGCGCCTCGGCAACACCGGAGATCTTTGAGGAACTGATGAAATT
CTTTTACATGTTTCACGATCCAACCACGCTGAACCAGCAGGGGAACGACCGTGGGACGCA
GTACGGGAGCGTCGTCTTTGTCTCCTCCGAGGACCAGAAGGAAATTGCAGAGCGAGTGAA
GCGAGAACTCCAGTCGCACGTCGATAACCGAACCATCAAAACGTACCAACGCAAGAAGAT
AGAAACGGCCATTGTCGATTATACGACCTTTTACGAGGCGCATGCCGAGCACCAAGAATA
TCTGGAAAAGAATCCGTCGGGCTATTGCAACCACAGGTATCGTTTCAAAGATTGGCCCAA
GGCGGAATTGTAATTTGAATTTCTGTGCAACCGTGCCAACAACCTCGTCGCAGAAAACAA
TAGAAGAACAAAGAAAGAAACAAACAATCGACAACATCCAACGCATTATGAGGCATATCG
AGTAGGATATTTCATAGTCCGAAGAATGCAATGGTCGGCAATCGTTTCTAGAGAAGGTCT
CCCGGAAGGACTAAACTGTAGATTGTGGAGCGCCCACCTGTCTTGCCCTGGCATTCGAGT
TTAAAATAGAGAAATGTTTCAATGTTA
>comp2631_c0_seq1
CGCAACGCCAGATTTATGACTTGGATTTGAAATTTGAAACGAATACATAAATACAGTAAG
AGCGTACGTATTATGGAACGGTTACTGGTACTCGTACTTCCTTGTGGTTTCTTCCATCGC
CTTCTTCAATCAATATCCTACCGTACGTCTTTGAAGTCTTATCTGCTGTTGGCTGTCTGA
ATCTGTCTGCGGACGGCCTTTGTGTCCAAGTGAAGTTCGCACGAGTTCTTAACGGTAAAT
TCTGTTTTCGTCTCAACATACGCAACAAAATTGCCGATAGTGCAAGGCACCGGACGGGCA
TTGCAACCCTCCGTCAAAAATCGACCCTTCTGCTCCCCGCAACCCTTTATCGTGTTTGAT
AGTCTTATCCTTTTGATAACTGAAAGGAATCGCAAACGAGCAGGGGCGCATACCATCCAG
ATTGTCGACCGGCGATTTTTGTAGAGAAACCTCCAGGGTCGGATGCGGGGCCACCGAACC
ATTCAAATTGTAGAGAAGAATGCCGCTGCATAGCTCGTGCACTTGTGGGGTTGAGAGGAA
GGAACTATAGAACGACCACCGTTGCAAGAGGGAGGCATCCGTTCGTGCCATGGCGGATTG
CAAGGTCTATTCGGAGCGACGATCCCGTTGCTACAACGTTTACGGGAACAACCGAGGGGA
AGAATGTGTGCACGAAGCCCTCACCGAAAAAAGATGTCTTAGCATGCTAAAGTGTCCCCG
CCAAGCGCTGGAATATTACGGCGGCGCCGGCATCGGTGGTGGCGAACAAAACGATAGTGG
CTTCTTTGCGACACACAACGAGCTTCCACGACGCGAATTCGGTGCCGGCGAAGCAACGAA

GAATGGGTACCCAAAAGCTCTCTGCGCTTCCTGGGCCGAATCCTTTTTCTACGTCGACAA
GGCGTTGGAATACGGAGAAGAGGTAGCGAGCCACCATAGAGAGGCCCGTCGAATAGTATC
CAGCGATTCCAAGCTGCGGAAGGAATGCCGTCGAATGGCAATGGAACTGGCGGATTGTTT
GCGTGCTGCTACAGCAACGAAGTAGTGGGGTTTCGTGACGAGCGCACGGTTCTGTTCAGT
GGCCAATACAAACACCGGAGAATTGTCAGCGCATGATAAACCAAATTCACAGCGATGAGT
GTCGACTTCTTCTAGTTTTTGTTGAACATTCTTCTTCCTGTCGCTATCCTCTAAGCTACT
AGCTCTTCAGATTTGATGATCATAAAAGAAAATTATATGAAGTTGTTGTGGTGGTGGTGT
TTCTTATTCTTCTTGCAGTGGACTGCAAGAATATTAATTGTAGTACCTTTTTCTTGCTTG
CTTCTGGGCTCATCCTATCACATTTAAATTGTTGGTACCAATCACTACTAGTAGTACTAA
AAAAATCTTAATATTATATTTAATCTCGCGAACTAGAAGAGGCACTACATGGCCAGAACC
AGAGCCTCTCCGGAGTAGGGAGAAGAATGCTCCGAGTACTACAGTATTTTGTTTTAATGA
TATCGTCGTCAAGAAAAACCAATCCTAAGCGGGAAGTCGTCGCCGAGGATCTGGAGATAT
TTCGGCGGAGGCTTTCGGTCGAAGGTTAAGGGATTAGAGGAGATGACACTCAACATGTAG
TAGATGGAATGTACTCGGTGCTCTCAGAATATAATGATAATGTAGTCGCGAAGAAGCAAT
CATAAACCGTGATAGGCAAAACAACTCGCATGGAGACGGCGA
>comp2632_c0_seq1
CGTAGACCAACTTTCATCGAGAATATTCCGCTAATTCCAACGGATCAAACAAAGGCAGAG
CCCTCTTTCGATCGAAAGGAATTTACGACAGAACTTCGCAGTCGGGATTCGGCAGCGGAA
GATAGATCCCTGTGGTTCGTTGGTCATGGGAGTTATTCGACATGCAAAGAGAAATCGGAG
TAGCACTTTCCACTTGCTGTTGTCACGTATACTTCGTTACGTTGTCCCTTGGTTTTGCTG
TTCTGGTCGCAACGGTCAGAGTTATTTATCAAGCCAGCAACGTTCTAACGGTTGCCGTAT
GCTCTTGGTACTAAATAGGAATGACTGACGCAAGCAGAATTTTGTTGTCAGTCCATCTAT
CAACTCTGAATGACGGGTGAACGATGAACGATGAACGATGAACGATCATTGCAAATTTGA
CAGTTTATTGTTTATTGTCAACATCGGAAATCTATTCCTTTTCCCACAACCGTTCATCTC
GGGCTTGAATGACGCATCCGATGTCATTGGAGTTTCTGATGTCTTATTGGCCAATGATAT
ATCAGTATTCTATTTTTGATGACGCATGAAAAATAATAATTAAAAACCACAGCGATCACC
AACTTTTACTTATAATTTACAATATGTCCGAACTGAGCGAAGAAAGAACATTGGCGAAAT
CTTTCTCAACGGTACGCTGTACAATCTTTTATCGACAATTAAGGTTACAAAGTAACTTGA
CATTAAAATCAAGAGTCCAAAGCGCCATGAACCTTTCGTTTTATCAACGAAAAAAATGAA
TCTTCGATGGAAACGGATGGTTGCGATTCTCCAAAATGCTACAGTAGTACTTCTACTAAA
TATCGAATATGCTGAGAAAAGTAGGCGATGACTTCCGAAAAAAATAGCAGAAGATAGACC
ATGTGGCAATGACTTTCTTTCCTGCACGATAAAAGGTATTAATGCGGTATTACCTTCAAC
TGTAACTATCAAAAGAAAACGTTTGACCCTAAATTAAAACTACTTCTTACTTGCATTTGA
GAAGAAACGATGAAGGTGCGCATAATCGTATTCTTACGAGCACGTGTGAGATGGATAGGT
CTTTAGAAGTCGTCCCGTAGGATTGCTACCGTATTATTTTTAATTTTTTTGGTAAGCACG
AGTAATACTGTTAGT
>comp2633_c0_seq1
CAATCACCATAGGTCAAGTCCTGACAAAAACATTGTTTCCTTTCCCTTTTTCACAAGAAG
AACTATCGAAAACCAACTACTTCCTTAGTTCCTTATTTTGCAACCATGACAATTTCCACA
CGACTTTCGAATCTCGTTGTTTTTTTTCTCTGTTTGGTCTCGGCTTCGAGGGAGTTCGGA
TTCGCTTCGGCTTTCTCGCCCACCGGGCTGCCACAGTCGTCCCGGTGTCGCCTAAAGCGT
CGGTTGCGGGTACAACGACCCGGGTCGGAATGGGGATGTTCGATTTTATCCAGAACGCCT
TCAAAAACGAAGAATACGACGACCGACTCGCAACCGCCTCCCACATCCTGGTCGACACCG
AGCAAGAGGCACTGGTCATTATGAAAGAGATCGAAGAAGGACAAACAACCTTCTCCGAGG
CAGCGAAGACGTACTCCAACTGCCCCTCCTCCTCTAAGGGGGGATCCCTTGGCCAATTTG
CACCGGGAACTATGGTCCAGGCGTTTGACGACGTGGTTTTTGATGAGAACGTTGCTATCG
GTTCCATCCAGGGACCCGTTTCGACACAGTTCGGGTGCCACTTGATTGTTGTGGAGGATC
GAACGGTGAATAATGTGCGCTCGGAAGGAGATGGCTTCTTCTAACAGCTAGTGGTCATTT
GTACTGCTAGTAGATGCATCTATTCTACTAGATCAGAGGGTAGACGACTGGATGAATGTT
GAAAACAGAAACGTGGAAGTTTTGGCTTAGATTTAAGATGTATCTAGAGTTATGTCTATG
AATATATTTCATTCTACGCAAGACCGAGTTATAAAGGCTCTAAGCTATTCGTTTTCATCG
TCTGCGGCATCTAGTAGCAATAGTAAGCTAGTTCTTCTCCTTGAAACGGT
>comp2634_c0_seq1
ACACGATCGTAAAGCAAATCCAACACAACTTCCCATGGTACGACTCGTTACACCCTTATT
TTCTGTCTTCTGAAAAGTGAAAAGGTTTCAAACATGTCCAACGACGTCATGTCAAGTGTA
GATGGATGTCTTTTGTCATCCAATCTAAGCATTGAATCTCGTGGAAGTGACATTCCTGAA
TCCGTTCTATCGGTGTCGGAGGCCGACGCTGAAGCTGAAGAAGACGAACCACTTCCCAAT
AACATGACTCGATTGGATCCCGATGAAGCCTCGAATGAAGGATCAGACGCAGACGAAAGT
ATAAATGAAGAAAAGAAACCATATCCAAAAGCGCACTTTAAGAGTGACGCTTCTACGAGC
TCAAAATCAGAAAAGCTTGGGGAATCGGATCCTCCCGATGTTCGCTTAGTAAAAAGAGAC

CGTGTTTCGACAAGCTCAGCCACAAACGATGGTTGTTCTTCTCAAGAATGCACGTCCTCT
TCGTTGCCACAGGCGCATTCCGCTTCTGAAATTAATTCCAACCAAATCCCATCCATGCCG
GGAGCCTTTGCAATCGAAAATCCACTGTTTGAATCATTGGAACGAAGAAGATCGGCATCT
TTTGGTAGCTACAGCGATTCCATTGCAACGTATGGACACAATACTGATGACAGAATTGCT
ATCTCGGAAGCAACACCACCACTACAGGAACAGATGGATCGTGACACGGAAGAGGGCATG
GTTGTGCGAGCCGTGCGCGTAGAGGAGCACGTATTTGAGGCAAAACTTGTTGATCAGAAT
AGCACTGCGAACCCTCTAAAGAGATTGAAGAAGTGTTGGTGGTTGATAGCAATTGCTGTT
ACATTAATAGTGGTGATAACGCTTGCCATACTTCTTACCAAGGAATCAGAGGTAGACGAA
ACAAACGAGGTCATAAATGAGACGACGATACATTCGACGGAAAGTTCTCGAGTAAAAGAA
CTGCGTGCAATCCTTGCTCCGCTGAATGGAGACGGTGTCTTCGACAAAAATAGTACTGAA
TTCTCACCAGATCGTTTGACTGCACTGAATTGGCTCGTTACAGATGATTATTTTTCAAGG
TATTCCTTCGATGAATTCAAGATTCGCCAGCGCTATATTGTAACTTTGCTGTACTTTAGT
ACAAATGGCCAGGAATGGATAAACCAGTTATCCTTTCTTTCAGATCGGGACGTGTGTGAT
TGGTCCGCAGTAAAGACTGTCGATCGTGGAAACCAATTGGAATCTTTCGTTATCACTGGT
GTCATTTGCAACAAGTTGGGCCAAGTAGAAACGATCAAGATAGGTAAGTACCAGTCGTTG
GTTTCGTCGTTTTGGAGGGATCTACCCGGTTCTCGTTGATTTGAAATATATCCTTCTCAC
AACAGGTTGAAACAATGATATTGCCGTGCGAACCAACTTGCAGAGGGGAACAATTTATCG
GGCACGATCCCCCCCGAAATACGCCACTTCTCCGATACCATTATGGAAATTAGCCTCGGT
GAGGGTATGCTTTCGGGGACAATCCCTTCCTCCTTTGAGGAGTTCAAGGCTCTTGAAATA
TTTGATGTGAACAGCAACTGTTTGAGCGGTCCTATTCCCAATCTTTCCCGTATTCCATCT
ATCCGACGTTTATCGCTTGAAAACAATTTACGACTCTGGGGGTCGCTAAATGGATTTTGC
GATCACAGCGAGTACAATAACCCCCAAATTGATTTCATAAGGTCAACGTGCGGCGGCTGT
TCTGGAAAAGAGAAATCCGCACTAATAGAATGCGACTGCTGCACTTGTTGCAACGAGGAG
GATTTGACTTGTTGCGACTCACAAGGAGTATCTTCGACGACGTCCCAGTACGGGTACTCA
TACTCCTCTCCGTTTGAGTGTCCCGCCTAGACACCATTTCATTCAATCCTACCCACCCGT
CACTTTGTATTCTTTCGACATGGAAGTAGTCTTTCATTTGTTGGTGAGATAGTCTTCCGA
ACATCTTGAAAAAGAGACACCAGGATAAACCTGTCGTTGCTGGACAACTCTTAAATGAAA
AGTAGACAATCGATTTCACCATGGATGGCGCCTTTTCGATAGCCATCACTTTCATCGTCT
ACAATATGACCTTCCAAAGACGCAAATCAACCCTCCATATCTTATCGACTACTCCCCAGT
TGCTGCTGGAAACTATGTGATAACATTTGCCTGAGGTTGAGTTGATGCATCTTGAATCTG
GGACATAATGATTCTTAATAAATTAGCAGATGCGTGATGTCAATAGAAGTAGACTGAAGG
TGCGACAAGTAGATGTTTTGAAATACGAAGTATTATATTATTATTATGTTGATATTTTTC
GAAAGAGGAGAAAGGCGCAATACCATGACAAGAACAGGAACCTATAAGTTCTAAGTGGCT
TTTCGCGGCAAAGTTCCTCTTTGACGAAGAAAACCACGAGTGGCGAGGTCCAAGAAGTGT
AACCAGTGCTCTTCTCCTTCTTTTTTTACTAATGCTAGAATCTACTATTATTGTAGCATC
AGCATCACTTGACAGAAAAAAATATTTAAGAGAGAGATTGCTCCAACAAGGGTCACCTAC
TAGTACTACTAATAGTGCCAAATTGAGAGTAGAGTTTACAATAATTCTATCCAAATACTG
AGATCAACATGGGGATAGAAAGTAGACCAGAAAACAGGGTGC
>comp2634_c0_seq2
ACACGATCGTAAAGCAAATCCAACACAACTTCCCATGGTACGACTCGTTACACCCTTATT
TTCTGTCTTCTGAAAAGTGAAAAGGTTTCAAACATGTCCAACGACGTCATGTCAAGTGTA
GATGGATGTCTTTTGTCATCCAATCTAAGCATTGAATCTCGTGGAAGTGACATTCCTGAA
TCCGTTCTATCGGTGTCGGAGGCCGACGCTGAAGCTGAAGAAGACGAACCACTTCCCAAT
AACATGACTCGATTGGATCCCGATGAAGCCTCGAATGAAGGATCAGACGCAGACGAAAGT
ATAAATGAAGAAAAGAAACCATATCCAAAAGCGCACTTTAAGAGTGACGCTTCTACGAGC
TCAAAATCAGAAAAGCTTGGGGAATCGGATCCTCCCGATGTTCGCTTAGTAAAAAGAGAC
CGTGTTTCGACAAGCTCAGCCACAAACGATGGTTGTTCTTCTCAAGAATGCACGTCCTCT
TCGTTGCCACAGGCGCATTCCGCTTCTGAAATTAATTCCAACCAAATCCCATCCATGCCG
GGAGCCTTTGCAATCGAAAATCCACTGTTTGAATCATTGGAACGAAGAAGATCGGCATCT
TTTGGTAGCTACAGCGATTCCATTGCAACGTATGGACACAATACTGATGACAGAATTGCT
ATCTCGGAAGCAACACCACCACTACAGGAACAGATGGATCGTGACACGGAAGAGGGCATG
GTTGTGCGAGCCGTGCGCGTAGAGGAGCACGTATTTGAGGCAAAACTTGTTGATCAGAAT
AGCACTGCGAACCCTCTAAAGAGATTGAAGAAGTGTTGGTGGTTGATAGCAATTGCTGTT
ACATTAATAGTGGTGATAACGCTTGCCATACTTCTTACCAAGGAATCAGAGGTAGACGAA
ACAAACGAGGTCATAAATGAGACGACGATACATTCGACGGAAAGTTCTCGAGTAAAAGAA
CTGCGTGCAATCCTTGCTCCGCTGAATGGAGACGGTGTCTTCGACAAAAATAGTACTGAA
TTCTCACCAGATCGTTTGACTGCACTGAATTGGCTCGTTACAGATGATTATTTTTCAAGG
TATTCCTTCGATGAATTCAAGATTCGCCAGCGCTATATTGTAACTTTGCTGTACTTTAGT
ACAAATGGCCAGGAATGGATAAACCAGTTATCCTTTCTTTCAGATCGGGACGTGTGTGAT
TGGTCCGCAGTAAAGACTGTCGATCGTGGAAACCAATTGGAATCTTTCGTTATCACTGGT

GTCATTTGCAACAAGTTGGGCCAAGTAGAAACGATCAAGATAGAGGGGAACAATTTATCG
GGCACGATCCCCCCCGAAATACGCCACTTCTCCGATACCATTATGGAAATTAGCCTCGGT
GAGGGTATGCTTTCGGGGACAATCCCTTCCTCCTTTGAGGAGTTCAAGGCTCTTGAAATA
TTTGATGTGAACAGCAACTGTTTGAGCGGTCCTATTCCCAATCTTTCCCGTATTCCATCT
ATCCGACGTTTATCGCTTGAAAACAATTTACGACTCTGGGGGTCGCTAAATGGATTTTGC
GATCACAGCGAGTACAATAACCCCCAAATTGATTTCATAAGGTCAACGTGCGGCGGCTGT
TCTGGAAAAGAGAAATCCGCACTAATAGAATGCGACTGCTGCACTTGTTGCAACGAGGAG
GATTTGACTTGTTGCGACTCACAAGGAGTATCTTCGACGACGTCCCAGTACGGGTACTCA
TACTCCTCTCCGTTTGAGTGTCCCGCCTAGACACCATTTCATTCAATCCTACCCACCCGT
CACTTTGTATTCTTTCGACATGGAAGTAGTCTTTCATTTGTTGGTGAGATAGTCTTCCGA
ACATCTTGAAAAAGAGACACCAGGATAAACCTGTCGTTGCTGGACAACTCTTAAATGAAA
AGTAGACAATCGATTTCACCATGGATGGCGCCTTTTCGATAGCCATCACTTTCATCGTCT
ACAATATGACCTTCCAAAGACGCAAATCAACCCTCCATATCTTATCGACTACTCCCCAGT
TGCTGCTGGAAACTATGTGATAACATTTGCCTGAGGTTGAGTTGATGCATCTTGAATCTG
GGACATAATGATTCTTAATAAATTAGCAGATGCGTGATGTCAATAGAAGTAGACTGAAGG
TGCGACAAGTAGATGTTTTGAAATACGAAGTATTATATTATTATTATGTTGATATTTTTC
GAAAGAGGAGAAAGGCGCAATACCATGACAAGAACAGGAACCTATAAGTTCTAAGTGGCT
TTTCGCGGCAAAGTTCCTCTTTGACGAAGAAAACCACGAGTGGCGAGGTCCAAGAAGTGT
AACCAGTGCTCTTCTCCTTCTTTTTTTACTAATGCTAGAATCTACTATTATTGTAGCATC
AGCATCACTTGACAGAAAAAAATATTTAAGAGAGAGATTGCTCCAACAAGGGTCACCTAC
TAGTACTACTAATAGTGCCAAATTGAGAGTAGAGTTTACAATAATTCTATCCAAATACTG
AGATCAACATGGGGATAGAAAGTAGACCAGAAAACAGGGTGC
>comp2635_c0_seq1
CATAAAACAAACTTCCAACCAACCTCAAAGAACCAAGGACGACACAATCACCAAACCTAA
ACACAGCAAGCCACACAGACTTGACTATTTACAATGATGCCAACTATTATTAACTCACCA
GTAGAGACAATCGTTACGAACGATGCGCCTCAAATCAAGATGATTGAAATCGGAGGACCC
GAGACCGAAACGGAGCAAAAGAGACGACGACGACGCAGACGTCGACGACCCCGAAGCAAG
ATGTCTTCGGCGACTCCGGTAGAGGGCTGTGAGAAGTCATTGTCGTGCGACGACTCGACC
TGCGCCACAGCGTCACTCTCCAGCGATGACATGTCTACAGCCACTCCACAAAGCCCCCAA
CAGCAAACACAAAAGCAACGAAGAAGGAACAACCAGAAAAACAAATCAAAGAAACAACAA
AAGCAAAACAAGAACAACAATGCCGACCACGAGGTGTCCCCAGAAGAGCAATCGAGGTAC
CTGGCAATGGACTGCGAAATGGTCGGTGTCGGTTTCGGGGGCAAGCGATCGTCTGTGGCC
CGAGTGACACTCGTTGACTGGGACGGTCGCATCGTATACGACGAGTTCGTTCGCCAGGAG
GAAGAAGTCACGGACTACCGAACCTTTGTCAGCGGAATTACACCCCTTGACGTAGAGGAC
GCGACCCTCACGCTCGAGGAATGCCGCCTGGAAGTCCAAAGCATGATTGCCGACAAAATT
CTAGTGGGACACGCCCTCAAGAACGACATGAGGGCGCTCGGGCTCACACACCACTGGATG
GCAACCCGTGATACGGCCAAGTATGAGCCTTTTATGCAGACGAGGTTCGAGGACAACATA
TTGTGGCCCCGCAAGCTCAAGGAACTCGCGCTTGAGAAACTTGAGCGAAAAATCCAGAGA
GATGGGGAACCCCACAGCGCGTATGAAGACGCTGCGGCGGCCATGGACCTCTACCGGCTG
GTCCGAAGGAAATGGGAGAAAGTCATGGACTACAAAATCAAGAAAACAAGGGAGATCGAA
AACACACGAAACTAAATATAACTATCATCAGGAAAACGATCGAACGAGCAGACAAATATA
TACCCCGACACGAGAGGTTGACACCAAACATTCACACAATCAACAACCAGTGTCACATGC
ACATAATATACTATTAATCAAATGACGTACACTTGACTACTACT
>comp2636_c0_seq1
CAAGCCGCAAACCACCTCATACATGATGTGCTTGCAAACATGGGAATAATCGATCTTCTC
CAATCCTCTTCGTTTTATCCGATACAGTATGTACTAGTGCTTAACTTTCCCTTACTCGAA
CTCTGTCTACGGTCTTTTGATCATAAATTGAAAAACCCTTGAATTCATAACAAAACGAAA
CTAGTTACCATGAACAGACTTGCTGCCCGCACCCTCATCCTCTCGATGCTTTTTGCATCG
AACGATGCCTTCTCGCCAGTGCGATCAAGTCAGAGACAATCAATTGCGTTGTTCGATACT
ACCAGTACACCAACCGTAGAGGCACCAACAGAAGAGGCGAGTGTACCAGATGTCGATTCC
TCGACAGCTCCTGCGTTGGAGAAGAAGAAAAAGGTCAACAAGAACGCCGAAGGTATTTTT
GCCCCAGCTGTCCTTCTGACAAAGGACATTGTTGGAGAAACCGAACTCAACAAGCTGAGA
GCCAAGATTATCGGTTTGCATTCCGACGTGATTGGAAAATTCACAGAAACAGCTCAGTCC
GAATTCGGAAATCAAGTGCTGAAGGTCCTGTTCACTCTTGCGGACAAGAACGGAGATGGC
ACGATTGACGAAGAAGAACTCGCAGAGGCCTTGCAATCATTGGGATTTGATTTCCTGAAG
GAGAAGCAAATCGCCGGTATTTTTGCGCGTGCTGATGCTGACAAGAACGGGCAACTCGAT
TTCGAAGAATGGCAAAAAGCGGCTCCCAAAACACTCAAAACCTCTTTGATCAAACTTGCG
AAGAAGAATGGTCATGATTTGGGATTTTTGGCTTAGGGCAAGACATGAAACAACATGTAT
CCACCCAAGCAATAGATAGAATCGAACCCATAACAATAGTGTACAATAAATAATAGGGAA
A

>comp2637_c0_seq1
AACAACAACAACAACGAGAGCAGCGTGCTGTTGATCTACCCATGGAGTCTGTCACCCAAC
CAGCCTCACCCGATAGAGACTTTGATGAAATATCGGAGATTTCGCGTGCATCAAATCTTT
CAGGATTGTGGAGCAGAGGACGAAACAGTCTAAACACTATTACCTCTAATCTCAACACTA
TTGCCGAGAACCAAACAAACAAAGGCAAGGCGCTGCTGTCGCCTGAACTCATAGGGCGAG
CAACCCATGTTTCTGGAAAAGTGAGTGGATTGTTGAACAAATTCAGAGGTGTACCCGAAG
ATAGTGAAGACGTTGACGAAATAGACGAGCAACACGAACACTTCAACGTGTCTCAGGATT
CCATTCAGTTACAAAAGCAAAATATTGATTTGTCGACACCTCTAAAATTAGTTCATTCAC
CATCTGCAGATAGCCACGAGGCTCTTCGTGCTTATCGAATGAATCGGGCATCAATGTCGC
CCGAAGAAACCGACAAAATGTCAGATGCATCATCGTTTCACGCTTTACCGGACCAGATTC
GAATGGTTGTTCAGAACTCAGGAGAAAAGCCTCCGAAAAAATCTCTCCGTACTTCTACCA
CAGCACCGACCACAAGTCAAACCCAACCCCTACACAATGACCAGTACGAAAGCCCTATTC
GTTCTACAATAACTCAATCTACGTTGTCGCAGTCCGAGTTATCTAACGAACAATACAAAC
GGGCGCATGGAAGGTTCATCAATAATTCAGCCTTGTTAGGAGGAGAATTTCAGCACGAAA
CCATTATTGACACCGACGGGACACAGAGCGATGCAAGTGGAACAGCCGATGTAAATCCGC
AAACACTGGCGAGCTTAATGATGTCCCCCGATATGCTACAGAAGCGGCTGAAACAAGCGA
TCGCTTCTGTGGAAAAGAGAAAATGGGATCAAGTATTGTTTTTGATCAATGCGAATCCAT
GGCTTGCGGAGATGAAAGAATTGACAACAAATCAGTTTTTGTTGCACAAGCTTGCTTTCT
TTGGCAAGGGAACTCCGTCTGCCCCCATGAGTCTAAGCGAGCAGCTTGTGGAGAAGTTTC
CAGCAGCTGTTTACAAGTTCGATCAAGATGGGAACGTACCTCTTCATCTCGCAGCAGCTG
CCGGCAATGTTCAGATGATCCAAATGCTTGGAGAGAAGTTTGAGAGCGGGGCATCAATTC
GAAACGAAGATGGAATGCTGCCTCTTCATTATGCAATTGCGTCTTTTGCAGAATTTGATA
GTGAACAAGTCGATGGAGTACATTCCCAGCCGTTGAGTGTGCTGAAGACATTGTTGAAAC
TATTCCCCAAAGCCGTTGCAATTGCAGACAATGACGGTAATCTACCACTCCACGTGGCTT
CTGAGTGTCTGGAAGGTGGAATTGGAGTTGATGTTATCTTTTTGCTAATGGATGAGGCAG
AGCGTCAACTTCAAGATCCCTACGGGGCTCGTTTTCGCAACAAAGTAAAAATAGAAGATC
TTATGGACGATGATATGTCAGCTCGAACAATGTCAACGGCGAGGGATACGGATGTGTCGG
CAATGGACAGTGAAATGCCGTGCACCGTGGTTCTCAATAACTTCAACGAGACTCCCCTTC
TGGTAGCAATAAGATCACATAAGGGATGGGAAATGATAGAGGCACTTGTTAGTGGCCCTG
GAGGACGCAAAGCTGCTTTGTGTCAAGACACGGACAAGAACAACGCGCTTCATTTGCTTG
TTGGAGAATTTCAAGATGCCACAGCTGCAATGTCTATTCTTAAAGTCGTTCCAGAATCTG
CATTGCAACGAAATGCCGCGGGAATCCTTCCTATTGAAATTGCATGCATGCAGCTGATGC
CAGAGGAAGTTGTTCTCGCAATCGCCCTTGTTGATCTGCCCGTCAACATTGATGATAGGG
ATGGACTAAAGGTGATCAAAGATCGCGGTGAAAGCTGGATTTTTTTGACTTGCGATAGTG
ATGATCACATGGTCAATGTCGTTGAGGAGATTGTGTCGATCTGCTCCTTCCAACAACTTC
AGGAACTGTGTTTCATGACAGAGCAAGGTTCGGAAAATGCAATAATCGATAGGGCCACTC
CAAAATGTCGAGAAGCATTGAACCGTGCATTGCGATTTCTTGGACGATTTGAATTTGTGG
GTGACGGGCCCCTATTCTCGGATATGCAAACTGGATTCAAGGCTTTCGATGCTCTGGATT
TTGGTGACAGTGAAGCAGGGAAAAGAGTTCTTCTGGAATGCTACAAGAACGAGTTTGATT
TTGAAAACAGGTGCTACACCATGTTCGAGGTGGAATTGGAGAGCAATTTCGTTGAAGAAG
TCAATGTCTATGTGGAACATTTGGATGACGATAGCGCTTCGAAAACAACCTCGGAAGAGA
TACAACAACAACGATGCGTTGCCATCGAACGCCCCCGACTTACTTTGGACAAAGTTGTGG
AGGGCATATCGAAAAATGGTGCCTATGAGAACAACAAACATTTACGGCTCAAATACTCCG
CGAAACTGTGTAGCGTGTTACGGCTGATCGGAAAAGCCCTCCGCCACTTGCATTCGGCTG
GTGTGGTGCACGGAAATGTCAGCTTGGAGACCTGTGGAAAGTTCGATGATTCTATGTGGA
AGCTCTCGGAGCGATTAGACGTGCAGGCCATAGGAAAACCGTTCGACCCCACCCGATTTC
GAAAATCTTTCCCTCCCGAGTCTCTCGAGTCGTTGCCGATCAACGATGGAGAACAACGAG
ATGGCATGGTGTTTGATTCCGACGGTCCCACGGTGACTTTTGTCACCGACATGTTGGCAG
ATCCTTCGATAGATATCTGGGCCTTTGGAAAAATTTGCTACGAGTGCTTGGTGGGCAAGC
CCCTGATTGAATTCGACGCAAGTAACAGCGACAGTCCATCGGACGACGTGATAGCCTTGT
TGCAAACCTCCGAATGGAATGAATCAAACATGGAAACAGTCTTTGACGATCTCGTGGAGT
CCGGCATCGGCGAAAGCGGGGCCGATATGGTCACATCGTGCTTATTCCCGCAACCAGAGC
AACGACCGGCCGACATGGACGAGATTCTAGACAATCCATTCTGGCAGGAGATTCGTCGCC
ATCGATCACCGAGAAAAGGTCGCGGGGGTCGAAAAGAAGGTAGTATCGAAAGCACCGTCA
GCCTTTTCACAGACGTTGAGAAATATGAAGTTTGAAAAGTTCACTACTTACAAGTGAGAA
AGGCTTCGAATCACAAAAAAAGTCTTGATTGTACCGTACGATCATACAGTTTGGTGCGACT
CGAACAACAGATATCTGGTACCAGGTAATGTGTAGGACTCGGTTTAAAATACTTGTTGAT
GCTGTTACTCGTGTGCATAGTTCTAGTTCCTGGATTCATCTTCTAAAAGAAAACATACCA
TCACCTAATGCTTTTCGCCTGGAACTTACAATACACAATACACCATTTGTGTAGATAATT
ATAAGTAGAAGATAGGAAAGGTATGAATCCATGTACGGTACGTTGGATTTTGGATTTTCC

TCAAATGCATTTGAGCAAGATTTTTTCGGTTCCTATGCTCGACGTACGTAGACTACTACG
AACGTACTTCTAGTACTTTTTGATGTTCGATGCGCTTCTACGGAGGTACGACTGACGACT
TCGAGTACCC
>comp2638_c0_seq1
TGTCAAACCCAAGCAAACACACAACAACTAACCAACCAGACAACATCACGTCGTCGTCTC
TTCCGCATTGGGTTTTCTTTGATCATTTGCACTGGATCGATTATACTTCAATCTCGAAAC
GAAGTGGCGACATTCTCTTTTATTAAGCTGCCCCTAGCGCGACACATTGTAGCAAGAATA
TGCTAGCGAGCAGTGCCAATAGTCGTCTTCCAGCGGCTGCGGGGACGGCCCGTCAGGTTT
ATGCATGGGGGGGCTTGCTGAAACAGAAACGCACCTCGTCAAAGGCGATGGGGACAGTAA
TGTCGGCAAAAAAACAGCATCGCCGAGTGAATGCGGGAGGTGTTGTTCACAATGAGTTTC
GTAGCTGCAGCAACGACAACAAAGGATTCTATAACTTGAACAACGGGATGGCGATCCGGC
GGGAATCCTTTCGAATGATGACGACCAACTCTGGTGACGAAAGTAAGGAGTCCAAGACTC
GAATTGCGACCGTTCGGCTGTATCGCATGCTACGGCGCTCGTGTAAAGGCCTTGCTCCAA
CAGACCAAGGCACCATTTTGATTCAAAATGAAATACTCGCTTCCGATTGGGGAAAGCATA
GCTTTCGCGACAACGATATCATGGACAATGACAGCGGTGACGAAGTAGATACTTTAGTGA
CTGAAAACCAGACAAATGAATTGATTAGGCTGTTTTTGCGCTGGAATGGTATCGATCCCG
ATGCGGGATTTTCAAAAGTAAACGAATGGTACGACGAGCTAATGAGCGGGGCTCGGTTCC
GAGACGCTAAGAACACCGAGGTCGGAGCCTGCTGGACGACCCCTCACCAACTGCGGGAGA
CCGTTCGCTTCGCCTTCCTCTCGTCTTCGAAACTCTCCACCCAAACCCCGACAGAACTAC
AGGGATTGGCGGTTCGTGCCATCCAGATGATGCAGGAGCAAAACACGCTCTGGAAAAAAT
CGTCCGTATCCACGACCCTCGATGGATTAGTGCGGGTGACGGCCACCAGTCGGTGTCTCG
GAACGGTATCGCCGGTGTCCTTTGCCGCTCCGTCCTCCCCGCTCGACCCTAAATACCGAT
TTGCCTATCGCATCCGAGTGGAGAACATCTCCCCCGAAACGACGGTTCAATTGCTTGGAC
GATACTGGCACATAACCGAGGAAGGTGACGGTAGTGACGAGGAGTCCCTCGAACCCATTG
AGGTCGACGCTCCCTACACGGGTGCAGTGGGGCAGCTTCCCGTGCTCCGACCCGGGCAGG
CTTTCGAATACGTCAGTGGAACCGATCTGGCCACCCCGGTTGGAACAATGAAGGGACATT
TTTACATGGCGACGGTTCCGGAGAAAACGAAGTCGGCCAAATCGGGAGACGACGTGGTGG
CCGTATCGTACCAAGGGTCAAAGGGCTCATGTGGCACTTCCTCCTCTTCTTCAAAAGAAT
CAGGAACTGGTGGCAACGATACAAGGTTGTTTCATGCGGCCGTTAAACCCTTTCGGCTCG
AATCTTTTGAAAAGTAACAGATTATGGAAAATCGAGACAATCCACGGCGATGTGAGCATT
TTTTCAATCTTTTCTTTCGGTAACAGAAGTATCTATGTTTGTTACAAATATTTCTTTTGG
CAAGTGCGTATCATTGATGCTTCAAACGAAGGGGTCGGTCGGTGTCAACGATACGAAAGA
CTACATTTTGTTCGCCTACCACTGGTGCTGACAAATTAAACGGATGACATTCCGTGTTGA
TATACCTAAAGCATTAAGACGATAATGAAGAAAAAACTAGTCACGCTTTCTTGCTTCGTC
TTTTGATTTAAAAAATATTGGTATGATGAGAATAACGACTAG
>comp2640_c0_seq1
AGCAATTCTGAAACCTGCTTCGGATCATTGATTGAATGTGTGTGTTGCTATCACTGCCTC
TGCCCAGACTTCGACTACCACTCCTTATTTTCTTTGCATTCATCGCAGGCTGTTCCTGTA
CTCCGTCGCCGATCACAACGTCCACCGCACTGCAAACCGGCAGCACCACCAGCATGTCCA
TCCAGCACACCACCCGTCCCATAACCACAGGCACCAGCGTGGTCGGCATCGTATACGATG
GCGGCGTCCTGCTGGCCGCCGACACGCTGCTCTCCTACGGGAGCATGGCGAAGTCCTTCG
ACACCCCCCGCCTGCACAGCATCGAAGACCGCATCCTGCTGGGAGCCTCCGGAGAATACT
CGGACCTGCAGGAGATCGTGACCAAGGTAAAGGCCCTGGCTCTGGAGGAATCCACGACGC
TGTCGATGGAAAGCATCTACGTCGACCGCAAGCTCTCGGCGAGGTCCCTCTGGAATTACC
TGCGGGCCGTCATGTACCAGAAGCGGTCGCGAATGAATCCCTTCTGGAACGACGTCGTGG
TAGCGGGCTTCGACGAGCAAACCTCGAAACCGTTTTTGGGAACCGTAGACCGGCTGGGAA
CAACCCTCCAGGAAAACCTGGTGGCGACCGGGTTTGGGGCCTACCTGGCCATGCCGTTGC
TGCGGGAAAAGTGGAGGCCCGACCTGAGCGAGGGAGAGGCCCGTGCCCTGCTGGAGGACT
GCATGAAGGTCCTCTTCTACAGAGACTGCCGGGCCTCGGCCCGCATCCAGCTCGCCAAGT
GCACCAGCGAGGGCTGCATCGTTTCGGAACCCTACGAGCTGGACCACTCCAACGGATGGA
ACGCTCCCAGCATGGTCCGAGCGTCCGGAGATTTGGACGGAGACGGGGGCTGGTAAACCA
CTTTTTCGTGCGAGTGAAGCACTGCACGCAACGACGCCCGCGTAACGCGACCTGCAGGAC
GAATCAATGATACAATAAAGAGTAAAACAAAAATAGAAAAAACAATCGAATGTAATGGAA
TATAATACAATATTTCGAAGGATGCAAATGCGCATGGTATGCAAAGGATGGAGCAACTAA
CAAAATTATGACCCGTTTCACGTTTCACAGCATCCAAAGAATAGACAGATGGCGGTTGTC
TGTTTCTCTAGGCCCAAACCAGTGCACCATGATGTCGTGAATATAGAAAAAGCGCAAGGC
ATTATGAAGCCATCCATTTCCCTTTGTTTTTGCTTGGGTACTTTCTGGAACGCTAGCAAT
GAAAAAAAAAAGTATCAACATTTCTTGTAGTACTAACATGTGAGCATCTTTCTGCATGAA
CATGAGGACGAGCCTTATGAAGCCTAGTACTAGTAGATGGTCAAGAACAAGACACAATGT
TGTCACAGCAACTACCACAATAATGAATGGTTTGGGCTAGGAAGGAGCAACTACTAGTAC

TGCCTGCTCTAGAGTAAGAAATGACAAAAGACTGAAAGACAGGAGAAATCGGAAGCCTCC
AAGCAACGAAGAGTTGAAGTTAATACTGAGTACATGTACTACTTCTTATTGGACATCACG
GATGAGGATGAGGATGAGGATGACACTAGTGTTGGTGATGTTGACATTGATGTTGTTGTT
TCTCCAAGTAGACTGGTGCAACTGTAAATAGCAACCTAACTGTAACAACTGCTGATACAA
CCACAACGAGTGGTGATGGCCAAGTGATTGGACCAATGGAACAAGCAGTCTAGGAAGAAA
AGAAATAAGAATACTACTAACAAGTTAAACATGGGGGA
>comp2642_c0_seq1
CAACAACAACAACAACAACAAAAGTACTTTTGGCTCGTCGATTGACAACGCTGGGAGCAA
CATCGGCAACAGCAACAGCAGCAACCTTATTCGGCACCTGTCGCAGTTTTCGCGACTGCA
TTCGAAGGATTCGAACTCGATATCCGGGCTCGGCAACGGGATAACAGCTTTGTCTGCACG
AAAAAGGTCCTGTTGTAATAACTCACTTCAAGAACAACATCAACTACAACAACATCAACA
ACAACGGCTACAACATCAATTACAACAACATCAACTACTACAACAACTACAACTGCAACA
ACAACAACAACAACAACGAGCACCTACCATGGACACAAACATGAACAAGAAAATTTACAC
AAAAGACTACCCTCAACACAAACGCGACTCCAAGGGACAACGCGTGAAGAAAAAGAAAGA
GGTGAACAACTGCAGGGAGTCGTTCGAGAATTCCGTGAGCGAGCTCGACAATTACGCGGT
AGTTGTTGCCAACCCACAGCAGCGTCGACAAACGAAAGGCAACCGGAGGTTTCCTCGTCC
GCTCTCGAAACGGCGAAAGGTCGGTGGGGACCAAAGAGTCGCACACTGGCCCCTGCCCGG
AAATCTGGGCATGCCCGTCCGATCGGAAATGAACGACGAAACACCGCAACATAAGCAGCA
ACCACGCCCGGAAATATTGGTGCTGGACGACAATACCGACGACGATGCCGACGACCATCA
CGAGGAAAGAGATGATTTGGTGTTCTGATCGATTGTCCGTCTATATCGCGTGTTGTATTC
CGGCGGTGTACAGTACCAATCGTGGTTGTCCCGGAATTTAGATTTTGTGTTGCGGCATCG
CCTTCGAGGCAATCGATTGCCTAGATACAAAACCACGGTTGGTTTCTCTTTGTATCTCAT
CGATAATTTTTATAATACATATTACGAAAGAGCTACAGAATATTTGAGGCCATCGATCCA
TCTATGCTCCTCTAATATGATCACGTTAACTAGACAAATTGCGAACCCGAGGCTTAGAGT
TAGTCACGTCAATTTTTTATTGTTGCTGTTGTCCCTGATCGTTACCGCTAAAGTTTTTGC
CTTGTTTCTGTCTGCGGTGTTGCTGATGCTGTTCATCTTCGTGTTGGTGGTGGTTGTGGT
GTTGATGTTCTTGGCGACGGACACGTCTGCACCTTGATGATGCGGAAGGACGGGGGAGCG
AGGATGAAAAGGAGGACTGCTGCAGCGACGAAATGCCGTTGCGTCCTCTAGAGCTACACC
TTGAACCGCTCCCT
>comp2644_c0_seq1
GTCTTTTGGAGGGAGTTAAAAAACGTTTTTAAAACCCGTCGCATGAAATAAGTATTGCAT
CTGAATGTAGTTGCCAAAGGCCTATCATCGGTCACAAGCTTTCTCAGTTTGCACATATTA
TGAACGAAGGTGTGACTAGTACCTCACCAACAGTTATCATTGTTTTATCCACAATATTCA
AATCCAACTTCTGGACGCAAAAATTGCTTCGATTATCAGCAGTAACAGTGTTTGCATTTG
TTGCGACTGAAGTAAGTGAGAACGTCAGGAAGAAGCCATCGAGCACAATAATAGATTTGA
GGTCAACCAGTAGCAAGCTGGAAAACGAATCTAAGCAATGAAGATTTTCAGCTCTCCCGC
ATATGCCATTTTGGTCTTCTCGTGCCTCCTCTACGGCACCAGTGGTCAAGATCCAACTGA
AAGTCCTACCCCATCTCCAACACAGGCACCAACTGCA
>comp2646_c0_seq1
AATACGGTAGAGTACTCTTTCTGTCATATTGCAATACTCGAACGCTACCACACCGAGAAA
CGAACCGAAAATGTCGAGAGCATCACAATCTGCGGTTGCCGGCAGAAGCGACGGCAGAAA
CGATGGCAGAAATTACGGCAAAAACAATGGAAGAAATAACGGGAGCCACGACGGGGCTTC
GTTCCGAGGAGTCGGGGCGTTCTGCGGGGCTCTCTGGACACCGATCGCCTTCGGGGCTTC
GGTTCGTCGCCCTTTTTTGTCCCGCTCGTTGTCGGTTGGCGATTGTAGCAGTAGTGATTG
TAGTGATAGTGATTGTAGGGATAACACTAGCAACCACGATGGAGACGACGAAACAGAAGC
AGAAACCGCGTGCGCCTCTCCGTTCTCTCCGTCGGCGTCTTCTTTTTTCGCTTCCTCGGC
CGATGCTGCTGCTGCAGATGCCCATGCCCATGCCCGTGCTTGTGCTCGTGCGTGCCGAAC
GCAAAACCAAAGACAAAGACAAACCGCCGGCCTTCTGCAGTGGTACTGCGGAAACGCGGA
TGTCTCGGACCGCATCGGGAGAACCATGTCGCTTCTCCTGGTCACCAGGTTCCTCTTGTC
GGTGGTGCTCGCTGTCGTCGCGGTCGAAGGCGAAGGTGCAGACCACGGGGCATACGCAAA
CACATACGCAAACGCATAGGCATAGGCAT
>comp2647_c0_seq1
CTAGCACCTTCGAACCTAGCACCCACGAACCTAGCACCCAAGATTCTAGCACCCACGAAC
CTAGCACCTAAGGACCTAGCACCTTCCCTGGCTCCAATGATAACAACAACAGCATCCTCC
CCAGTTATGACGATGAACATCGCATCTCCGACGACGACAACGCTAGGCCCCATCCAACTA
ATCGAGGACGGAACAGAACAGAACAGGAAACAAGTGCTGCTGCCACGATTATCGCTTCGT
TTTCGTCCTTTGTCGATTGTCAGCGGACGCGAGGTTGCGCCGTCGTTCGCTGTGTCGGCA
GTGGCTTCTGCCTGATCAACGTCCGGTGTCCGCCTTAGTCTACGTCCCGCCATGCCCTGG
CCTTCCTCGTTGCCAGAGTCACAGGGCCGCCGTCTAATTGTCGTCTCCGATCGTTAGGAC
TTCCACCGACGCGGAGGCGGTTGGTGAAGGGGGCAGAAAAAGAGGAAAACGACGGCGACA

GCGTCGGCATCCATGGCATCAGCCGGGAACCAAGCCCGAGGAGGCCGACATGACCTGGCT
GGACTCAACAAGGGGCCGACGGGGTTGGAACGAGCCGTCCGGTCCTTGTTCAGCATCGAG
CTCTAGCAGAAGGAGCCCGGAACGAAATTCAACGAAACGCAATGCAACGCAACACAATGC
CGGGAGAAGCTTTTAAATAACGCACTGCGCTGCACTTGTCGGAGGATCGGCCGAGCAAAA
GACTGCAGGTATATATTCCAAAGCTATGAACGAATAGGCACCCTAATGCTAGCTTTCTTG
TTGTTGGTAGGAAAAATAAGGAAGCTAATTCCAATACTAGCACTAGTACGGGGACTTAGC
TTTCGTACCGTTTTTCCTACCGGTAGTAAAGGACAAAAATTAAAAATAGAGTACTTTGTA
AAACCTGGA
>comp2649_c0_seq1
ACGCTACAAAGTTATCTACCGCAACTACAATAGCGAGTGGCCACCAGCACTACGTATTGG
TATCACGACAAAAGGGACAAGAAACGCACCATGTCGTCCCCATTCATACTGCGATTGGCG
GGAAGCACGGCCGCTAGGAGAAGTTTGGCGGCTTCCTCTGTACACGCCGGTTCCTCTTTT
CAACTCCAGGCGTCAACGCTGGAATTTCCGTGCCGGCCCGAAACAAATACGGCTGCAGCC
ACGTCCAGTCCAATCGCAGAACAGCGTCGTTCGTTTTGGATGGACGTCGTGCGGATCAAA
AAGCAGTCGCCGCGTCGATCTTTCAAAGACAAGAACGACGGGAGCAGCAGCAGCAACCAG
AAGATGGCCCACGAAGACCCAGAAATGGTGGTCCGACGCCACACAGACGAGGTTCGGGCC
GATGGAGAGAACCTGCTGGACCTCATGTTCTACAACGACCGCCACGAAAAGGCGTGGATG
AAACGCAAGCGGATGCAAAACCTTAGGAGATACGAATTCGACAAGAAACACGTGACGGAT
CTGGCCAAGTACATTGCCTTTGTTCAGGATTATGGTGATGAAAACGATGACAAGGATGGC
GATGGATCCAAAAAATCAAAAAAGAAATAGAAATAATTATTACTGCTGAAACAACTGAAC
GGACGAATTAACTAAGAAACCAAGGTGTTCGGTGTTGAAAGCGGATGTGCTTTTCGGGGT
GCATTGGGAGCTTGAAGAACGGTTCTGCAACAACAACGAGACGGAGTGAGACTAATGATA
AACGTTACTGTATCCATGACGATGGGATCACAACGTGTAGAAATTATAAGATTGAATGGG
GATGAAACGATCAGATGGGTACAATGTTATTACGAAGGAAGGAATGGCAGTGCAAGAGTT
AGAAAAGGATGCATCACACAAAAACCTTTCCAAGTACGTTTTCTGGATGAGGTCTCATTT
GATGCCCTTGTGGTTTGGTTATTCGACTAGCTCGAAAAAGTTCGTGTGTGCACACGCATG
CGCACATTATTGTAAAGTAAAACTAGCAGTTGTCAGGAATTTTGGCGTGCCTTCCATACA
CTCCGTTCTATTAGATCGATTTCCGACCCAACTGCGCATGCATTCCTTCGGAAAACTTTC
GGAAAAAGTGTTTCTGAGATTCATCGACCGAAAATACAGCATTACAAAGACAACTGATAC
AACGATACGTAGAGATATTTGTCGAGGTTAAAATTCGTGTAGGCTGTGCTCCAATCGGGG
CCTCCCGACTGCTTTCACCTGGGACGACACTTCAGTCGCCGTCATAGTTGTGGCATCGAT
ATCAATTTGTAGTATACCATAACCACAATATACTCATTCAGTACTTGGGTTTGTTTCGGC
TGTATTTGTTTTCTTTTCTTCACCGTGATTGATAGTTACCTACGGAACCGGTTCCATCTT
TAATTGCCAACTCAGTAGCCCTTACTTGATGCATCAACGAGATAACACGACACATACGCT
AGATTAAATGAAT
>comp2650_c0_seq1
GAAAAACTGATAATCAAATGAATTGCTAACAAGAAAGAATAACAATACAAACAAAAATAA
TTGATCGCTTAACAGGGACTATGACGAAATCTATTCCGCTCTCCTCATTTGCAGTGCTAG
CATTGGCCAAATTGGCCGACGCGTATGACAGTGGTTGGCATCACCGTCAAACCACCACCA
ATTGCTCAACTATCATGGAAATCATTTGTGACGTTGACAACAATCTTTCGACAGACACAT
TCTGCGAAGCCGTTGAGATGGCTGGACTTGACTTGGATACCGAAACATGGACCGTCTTCG
CACCGGTCAATGACGCCTTTCATTTACTCCCCGATACCATTGTGGAAGACATGCTCGATG
GCGATGATCCAAGGGGCTTGATTGATCTCCTAGCTTACCACACTGCCGAGGAAAAGAAGA
TCATGAGCACCGATTTGAAATGCGACACACGAATTTTCATGAGCAACGAAGAATTTACTG
TCACAATCTGCGAAGGTGATAAAACCTTTCAGGTGGGTCTAGCAAATCCCACATCAGACT
ATCCACAAATTATCACCGCTGATATTGAGGCATGCAATGGAGTGATTCATACTCTAAGCG
AAATCATGCTATACAAGGAGTATGATGTTTCCAACAGTACTTTTGTTCAGGCGGCAGTAG
ACTTCACTTCCGGCCCTTCGAGTTCCCCATCCGACGGGCCCTCTATGGAACCATCTGTTA
GACCTTCGAGCTCCCCTTCTGATGGACCTTCTATGGAACCATCTGTT
>comp2651_c0_seq1
CCGTACCGCACGGTGGTACCGTAATTACTTCCTGTTCTTATTTTTTGGATTCAACAGATT
TCACGAAACGATATCTCCACACATTCTTCACTGAATTCGAAGGAAACAGAACACGGAGGA
GCAATGGCATCGCTCAAGATGTCGACGATATTATTTTCTTTTTGTATGCTTTGCATTAGC
AACCATGCACTCGTAGCGGCCCAGTTAGAGCTGTCTATCGATTCCAACGATAGAGATTAC
GATTGCGATGTGGGTCCCGATGCGTTGACCAATGCCCATGGGAAAGATATTCCACGGACT
TGCATCGATGTTCCGGTCGGTGAAGGATCGACTGCCACGACCGTGGAGCGATGCTACTAC
ACATACGTTCCGGAATCGTGCGTGGGTGATGAAAACGGGGAAAATCCACTGGATCTTCCT
GTTGTCTTTGATATCCACGGACTCACCAGCTGTGCGCTTTATTCGTCCCTGTACACGGGC
TGGAAGGAGAAGGCAGAGGAGGAGTGTTTCGTAGTGGTGTGGCCCAGTGGCAACGTTAAC
GACGATCTGTTGATCAACGGCTGCTGGAATCTTCCGGCGTTCTTGCGAGACGAAGGATAC

GGCACGGAAGGCGGAAACAACGTGACGACGGCCCCCTGTTGTTGCTTCAAGAGCGACGAT
GAAATGGGTTTACCATCGAAGGAACCCAACGATCCCTTGTTCCTGAGGATGGCTATTGAT
TCCGTTGCCGAATCCTTCGATATGGAGAACAGCAGCAACTACTACGGCAGCATTTCAATC
GATCGATCGCGTGTGTATATGGCTGGTCATTCCAATGGTTGCATGACGAGTCTTTCCATG
GCGGCCCTCCATTCCGACGTCGTTGCAGCGGTGTGCTGCCACGCGGGAACGCTTCTGACA
CCCTTCCCGGAGCAGTACACCCCGGTGCCCATTTGGATGGCCCACGGGTTGAAGGACGAC
GTCGTGAGAGCTGATGGCGTTGTCGCTTTCGATCTTGGGCCGTTGTTCGGCAAGCTGGGG
TCCTGGTCGACGAACGACGTCCTGGACTACATTGGGGGCCGCAACGACTGCGGGGAGGCG
GAGACGGCCGACATCGGCGAGAGCGGTGCGACCTACAGGCGGACCAATTGCACGAACAGT
GCAGACGTCGAGCTCGTCATGCTGTACGAATCGGGCCATTGGCCGTACGCCCCTCTCCCA
AACAGCATATTCTCGTCTTCCGTGGACGCTCCGGTCGACACTACGGCAATGGCATGGGAA
TTCTGTAGCTCCTACAGCAACCCCCAGCAGCAAGGGTATCCGTCCGGAGACGACTCGCTT
GAGAACGAGATCGACGACGAGATCGACGACGAGATCGATGATGATGCGGGGGAGCCAGTG
GAGAACGAGAAGGAGCACAAGAAACCAGAAACCGGTGCCGTTCCCGTGGACAACCAAGAC
GCCAACAACGCCAAGGCCGTGGAGGACAACGAAGAAGCGATGGATCCTCTATTGAGTTCT
TCTGCACCGCGATGGCGTTCGATGCACGAGGACGGAACAATTTTGTCGGCCACTGTAATG
ACATTGGTTCTTGTTCTTGGTTGTTTGGTTTAGTACTGGCACTGGCATCCCATTGTATTG
ATCTTTGACAACAATAATAACATTTTGATTTGGTGCAATTGTGTCATCAAAATTTTGTGC
ATTCATTATCCATTGGTTTGTGTTTAGCTACTGCTGTGAAAAAAACGAAAATAAGAATTA
CACATTATGATGGTGTAGTATGGTAGTTTACTGTAATAGTAGTGATTCATTCCCGTTTAG
TTCTCATTGTTACTACTGGGAAACAGTACTTCAGTAACTTCTAATAGT
>comp2652_c0_seq1
CAGCAACAACAACAACAACCACCACAACAGCAGGAACAACCACGGGGCGTTTGGGGGACT
GCTGGGTTCCTTCCGGCGAAACCGAGATGGAGCCAGCAGCCGCGACAGCACCAGCGAGAC
GCCGAAAAAGCAGGCCTTTGGGGGGATGTTCGATTCGTTCCGCGTAAGGCGTGGCGAGGT
TTTGGATGGCGATGGCGATGGCGATGGCAAAGACGACGACAAAAGCAACGACAAAGACAA
AGACCATGACAATGATAACGACGATGATAGTGACGATGATAGTGACAAAGACAATAACAA
AGACAATAACAAAGATGACAACAAAGACAACAGCGACGACAAAGACAATGATAATGGCGA
TGGCGATTCTGGCCTCGATGGCGATTCTTGCATCGACGGAAGCAGCCTCGGCACAACCAA
CGAATCGCAGAGCCTCCACGAGCACCAGCCTCCCACCGGTTTCCCCGAGGTGATATTTGT
CATGATCCAGTCGATGACCAACGACGGCCGGGACCTGAGCATCGACTACAAGCTGTCGGG
CCCCTTCTTCCGGGACGTCCCGACCGTGTCGTCTACCTACGACGAGATCTTTTTCCCGGC
CCCCCTCGCGCCCGACCCCCACACCGGGAGGGGGGACATTGTGGTCCCCAAGACGGCCTG
CAGCGCCTTTTGCACCACGAACCTCGACGTCCTGCTCCGGAGGCTCTTTGTGGAGCAGCT
GGTGGTCTGCGGCCAGCTCACGGACCAGTGCGTGGAGTCGGCCGTCCGGGACGCCGCCGA
CCTGGGTTACCTGGTCACGGTGGCGGAAGATGCGTGCGCGGCGCACAGCGAGAAGGAGCA
CTACAAGGGGCTCTTTGGGATGAACGGCTTTTGCCGGATTCTGACGACCCGGGACGTGGT
CCGGGAACTCGAGCGCGGGGACGGGTAGGAGCCGGCAGGTCCGCACGGAGGGAGGGAACG
AATCGAGCGAACAAACGAACGAATCCGCCGGGAAGGGAGTACGCAGCCCCACAGCGGAGG
CGGACAAGGTTTGGCTCCGATGCTCTCCGCCAGAGTTATGACCCGATGGGTAGCTAGGAA
GACTACCAGTGTTTCTGCCTTGTCGCAGAGAAAAGTGCAAAGCTACCCATGAACTGTCGA
AGCCGGGGCCGGGCCACGGTGCATGGATTCACAAGTACTATCGTCTCTGCACACCCCAGC
CGGATCTTTCAATCCTGCCAAAACTTCTGTGTGTGGAAGGGTTTCCATAGAGAGATCGAT
GCGTGCATTCGTTTCGACTGCTACAGTAACAAATCAAAGACAAAGTCGATTTCGGCTTGG
GTCTATCAGGCTGACGAGCACAGCCCAGATCTGCCCACTGGGAGCAAACCATCGTGACGA
CACAAGAAGTCCTACTAGTGGTAAACCACCACTGCTATAATGTTGTTGTAGTCATGTTTG
CTAGCTACTAATATTACTGTCTAATACTACCAAG
>comp2653_c0_seq1
CCATCCCTGCCGCTCCTGCCGCCATCACCAAGGAAGAAGTCCAGGGACTTTTCTACAAGT
GGAACAGTGCCTTGGCTACCTTGGACCCCGACCAAGTTGCCGCCCGATACTCTTCCAAGA
CTGCCCCTTGCTTGTTGCCAACTGTTTCGGATGTTCCCCGTACCGACTACGACTCCATCA
AGGCTTACTTCGTTGATTTCTGCAAGAAGAAGCCCCAGGGAGAGATCCTCGAATCGTACG
TCACTGTTGGACACAACTGGGCTATGGATGACGGTATCTATGAATTCACCATGGGAGCCA
CCGGCGATAAGGTCAAGGCCCGATACTCCTTCGTCTACACATTCGAGGATGGTGAATGGA
AGATTGCCCACCACCACT
>comp2653_c0_seq2
CCATCCCTGCCGCTCCTGCCGCCATCACCAAGGAAGAAGTCCAGGGACTTTTCTACAAGT
GGAACAGTGCCTTGGCTACCTTGGACCCCGACCAAGTTGCCGCCCGATACTCTTCCAAGA
CTGCCCCTTGCTTGTTGCCAACTGTTTCGGATGTTCCCCGTACCGACTACGACTCCATCA
AGGCTTACTTCGTTGATTTCTGCAAGAAGAAGCCCCAGGGAGAGATCCTCGAATCGTACG

TCACTGTTGGACACAACTGGGCTATGGATGACGGTATCTATGAATTCACCATGGGAGCCA
CCGGCGATAAGGTCAAGGCCCGATACTCCTTCGTTTACGTGTACGAAGACGGCCAGTGGA
AGATTGCCCACCACCACTCCAGTCAGATGCCTGAGG
>comp2654_c0_seq1
GTCTTCGACATCCCGTGCGTACAAGACATGGAAAGACTCGAAAGCCCACAAATCGGAGAA
GTCTGCCAAGGCAGGCAAATCTTCCAAGAAGGAGAAATACGAACACACTTACCAGCACGA
ATACCCTTACACAAAGTCCGCGAAATCCGGAAAAAAGTCCAAAAAGGCCAAGTCCGACAA
GAAACACAACGTTCCCTCCATGGCCCCCAGCGTGAGCATGCACCCGAGCGCGAGCCACAC
ACCGACGGCAACGACCGAGAGAGTCGAGGTCGTCGCTAATGCGCCCGACACACCCGATAT
CGATACCCAGAACTCATCGGCCACCCCTCCGCCGAGCCCGCCTCCCACCTCTCCGCCAAC
CATTTCGCCAACGATCTCGCCAACGACCCTACCCCCAACGTCCCAGCCCACCGGTGTTGC
TACCACAGCCGCCACCACGGCCGCCACCCAGCAGGAAACCTCGGAAGAGACCTGGATGGA
CACGTGGGAGACGACCTTTTGGACGACTCTCGACGGCGAAGAAGACGGCGATGGTGAGGG
CGACGGCGAGGGCGGAGAAACGGCAGACGCCAGCATGCTCTCGCCGTCTTCCGGCGATTT
CGGTGACTTCTATACAATCCTTCCCGATAGCTTTCATACTCTTTCTCCCACGGCATTTCC
AACGGCGTCTCCCGGTGCCGGTGCGGGCCACCCATCGCCCGGCGGGGGCCGGAGCGAGGA
AGCCCATTCCCCGACAAGCCCTCCGACGATCGATGCCGAGGGCTTTCAGACCCTCAATCC
CACGGTATTTCCAGCGGAGTCCGATGCAGATGCCGATGAATATCCCAATGCATATCCCGA
GTTCCATGCGTATGCCGATGCATATGCAGCCAACCTGTCGCCTGGCGAGGACACGAGCGA
AGAAACCCATTCTCCGACGAAACTCCCGACGCCTCACCCCACGGGCTTTCCAACCGGTGC
TCCCTCGGAATCTCCCACGAAAGCTCCAACCGATTCTCCCACGGAAGCCGACACGGAAAA
TCCGACCTATTTCCCTACGTTTTTCCCCACGTTTTATCCCACGTACTACCCCACGGATTT
CCCGACACTCTCGCCCGAGGGAGTCGCGGCAAAAGAAGAAAGACTTCGACTGATGAGACT
GAAACGAAGGCGGAAACGACGAAACCGGCTGCGTGAAGAGCAAAGGGCAGCAGAAAAGAC
GATCACTATCTAGAAAAAGACAAAGACGGCAGGGGATATTCGTTGCCCAAAAGACCAAGA
AACAGATAATCTTCTGGTTCGTATTGAG
>comp2655_c0_seq1
AGACCAGTTTCGTATTGTTACAGACAAGAATCAAGTCATGAAATTCCAAACTGCAAGCAA
CGTCCTCGCTATTACTGCCCTGGCATCGAAGGCTTCTGCGTTCGCACCTACATCGAACAA
TGTCAAGTCGACCGCGCTTCTAATGGGAACTCCGGGCATGGATTTGAGTGGAAACTCTTG
GAAACCTGATTCCGAGAAGATGGGTTCAACTGATACCGGTGATTACTTCCCTGAGGGATA
TAATCCCGAGGAAGAAATTGCCTTCTCCAGTGGAATGATGGGATCCCAATCTGGAAACGC
AGGAAGTCGCGATGGACCACAGCTTCCCGGACTCGAAAACTTGGGAGCAGATGCTGTTGT
TGCGGGTGGAATCTCCCTCGACCCCAACATTCCAGAGGGCATGGAATTTGTTCCGGCATC
GGTCCCCGACGGTAATTTCGAATTCCAGGTTGCCTCTACTGGAAGCGGTGGATCCCTTCA
GGTCATGATCAGTTCGCCCTGCATGACGTTTGAGGATTATTACGCTGCCTTTGCGCCTGG
ATCCCATCCTTCGCTATCGGTAGGGCCTTCTGCTGGTCGACTGGATCGACGCAGTGGTGA
CCCCACACCTTTGGAAATCACTTGCGAACCTAATGGGGTTGGAGGAGTCTTGACCGGGGA
TCTTGTTATCAACCTTCCAGATGATAACTCCAAACTCTCTTACAAAGTCACCGTCAACTC
GGTATAGATAAAGATTTGGTGTAAAACAGTGCAAGTTGGAGAATCACTTGGTGTTTCACT
CATTCCAATTGCAACGATTTGTCTATTCTCAAGTGTTGTGATGCGCTTTCCCATTACAGT
ATAGGCCTTTCTTCTTCGCTAAATATGCGTGCAAATTTTTTT
>comp2655_c0_seq2
ACCTGATTCCGAGAAGATGGGTGTGAGTATAAGAGTTATACGAAGAAATGAGTGCAATCA
TTAGTTCGGAGGTAATTGTCTGGGATTCGACGTTGAACCCAGAGACTGAAAAATTCATTC
ACATAATTTTCTGGAGAAATAGTGTTGGACAATTTGCTGTGGATCTGTGCCGAATGAAGC
CAATCATGGCTGTGATGATCCCAATTTGTTTGTGTTTCATATCTTTCTAGACCATGTGTA
TCTGACTCCAAGGCTTCGCTTTATTCCACCTACACTTACCGACAATGACTCTTGAAATTA
TCGATTGGTGAAATGAAATGGACTGAAATAACATAAAACACAATGTAGTCAACTGATACC
GGTGATTACTTCCCTGAGGGATATAATCCCGAGGAAGAAATTGCCTTCTCCAGTGGAATG
ATGGGATCCCAATCTGGAAACGCAGGAAGTCGCGATGGACCACAGCTTCCCGGACTCGAA
AACTTGGGAGCAGATGCTGTTGTTGCGGGTGGAATCTCCCTCGACCCCAACATTCCAGAG
GGCATGGAATTTGTTCCGGCATCGGTCCCCGACGGTAATTTCGAATTCCAGGTTGCCTCT
ACTGGAAGCGGTGGATCCCTTCAGGTCATGATCAGTTCGCCCTGCATGACGTTTGAGGAT
TATTACGCTGCCTTTGCGCCTGGATCCCATCCTTCGCTATCGGTAGGGCCTTCTGCTGGT
CGACTGGATCGACGCAGTGGTGACCCCACACCTTTGGAAATCACTTGCGAACCTAATGGG
GTTGGAGGAGTCTTGACCGGGGATCTTGTTATCAACCTTCCAGATGATAACTCCAAACTC
TCTTACAAAGTCACCGTCAACTCGGTATAGATAAAGATTTGGTGTAAAACAGTGCAAGTT
GGAGAATCACTTGGTGTTTCACTCATTCCAATTGCAACGATTTGTCTATTCTCAAGTGTT
GTGATGCGCTTTCCCATTACAGTATAGGCCTTTCTTCTTCGCTAAATATGCGTGCAAATT

TTTTT
>comp2656_c0_seq1
CCGCAACAAACTAATCTTTATCTTCGTGTTTTAGGTCCAGTGGTATCTCCCCCTGTCTTT
CACTCTCTTCTTGAATAAATTGAAATTTGCAAGCATGTCGGCGATGGCAAAGGCACAAAA
AGCCAAGGCTGAGGTTTTCATCAAGGAGGCCGAGGCGTTGTTATCGAAAAAGGGGTGGTT
CTCGTCTTCGAAGGAACGAAATGCAGAAGAGGCTGCTGAGACCTTCGAGAAGGCGGCAAA
CGCATACAAGGTTGGTGGTCTCAACCAGGAAGCAGGAGACGCATATGTCAAAGCTGGCGA
AATCTACAGGGATAAATTAAGTAACTTCAACAATGCATCGAAGTCATTCAACAACGCTGG
GACATGCTACAAAAGGAGCAATCCCGCTGATGCTGTAACTGCGTTTCAGCAAGCTGTCTC
GTTGTACACAGACAACGCCCGAATTATGCAGGCAGCCAAATTGATGAAGGAAATTGCAGA
ACTTTACGAAACCGAAGAAATCGACGAAGAGGACAAATCCCATGTTGTGTTAGCCATAGA
AGCGTATGAACAAGCGTCAGAGCTTTTCGCAATGGAAGATTCAAAATCTTCTACGTCTCA
ATGTCTCAGCAAGATCGCTGAATTGTGTAGCGCTGCTCTAGATCCTCCCGATTTTCGTCG
CGCTAGCAAGATGTATGACGATCTAGGACGACGGTGTCTTTCCAGTAATTTGTTGAAATT
CAATGCTAAGGGATATTTCTTACAGGCCGTTCTATGTCATTTGGCGAGTGGTGATGATGT
TGGTGCAGAACAAGCTGTCGCCAAGTACGAAAGTTTGGACTACACGTTTGCGGACTCTCG
CGAGGGGAAATTTGCTCAATCGTTGATCGAATCTGTTCAGGGCTTTGATGTAGAAGAGTT
TTCTAATTCTTGCTACGAGTACGATCGAATCTCGAAGCTGAACCCCTGGCAAACTTCGAT
ACTCGTGAAGGTCAAGCGCAACATTGATGACGGCGGAGACGGTGGCGATGACGACTCGGA
TGTTGATCTCACATAAAAAGTCAACATTAATCGACTAGAAGGGGTGCGCGGAGGTTTCTC
ACGTACAACAAGATAGGAAATCATTTTTCTTATTATTATTTCTCGGAGATAGATA
>comp2658_c0_seq1
CCAAACACCAAGTACCAAACACCAGACACCGGCTGCGTGGGGCGAGAGACGACGACGACG
ACGACGACGACCAAACCAGGGTTTAAGACCGACAGACACAACACCGAACGTGGTGCGGAG
AACGCTTGGACCTCGGATTCTCTACTGACTCGATGCGATGCGATGCGGATTCTGTACTCC
TTCGAATTGATGCAGATCCGGTTGCATTCGGAGCATCGGTCCGTCCCGAAACGAAACCGG
GGAGCCGAGTACTGCAGCACACCAAGGGTTCTTTGGCACCGGGGGTTGCAGTTGTGTCGT
GCGGCCTCCCCGCCGCAGGTTTGAAACGCGGAGACCGGCGCTCTTGGTCTTGCGATCCGA
AACGACCACCACCGCGATCGGCCTTTTGGACCACACGGCAGTTTGCTCTGCCACCGGAGG
AGTACCGTGCGTACGCACGTACTACTGCCGGGTCGGTTCCGCAACACAAACCACGCAAAC
CACTGCCCCCACACTGCTGCTTGTTACAGCATCGAGCAGGAGAACGACCATCCTGCACAG
TTTCCTAGTACGCACAATAACAACACCGTGCACACGGTACCACGCTAGACGCTAGACGCT
AGACGCTAGACCCTGGTGTATGCCTGTGTGTATGTATGCATATATGTAGGTATGTATTTA
CGGTAGGACTAAGTAAAACTCAGGAGTGCCTGAACAATAGTAGAAGTAGTAACGAACAAT
TGCAC
>comp2659_c0_seq1
CGAATTTCAATGGGCGAAACTCATCTTCGTCGTTGCTTCTATCATCTTCATCTTCATCTT
CATCTTTCTCAATTCAGCGTAACAGCCATTTGGAGTTAGCAAAATTTTTATCACTCTCTG
AGCCCTTTCTCTCATTCATTCTAGTGGGACTATGGTATCAGCCACCCGCCTTAAAAAAAA
TGGTTCGGCCTCGAGATCGAATGCCAATTTCTTTCAGTTGTCTCTTGGAATGTTTCTTGG
CGCATTAGGGATGAATCTATGGATTGGAAGTGACGTCGATCCCACGGCATTGTGGCTTGA
TCAAGATAGAAATTCAATTTCCGAAAAGAAAGTTGTAACATCGTTGAAGAGTATACCTGA
AGTGAAGGAAACGAAAGGCTGGCATTCGATCCACGTGTTTTACGGAGAGAAAGAAGGTTT
GTTCAACGACGCACCCAAGTCTATAACACAATCGCAGGAATCATTTGCACAAGTCGGACA
AGATTCGATCATTTTGGACTTGCTTGGATCAAACGGATACTTTATTGATCTTGCAGCGAG
TAAGTGAGGCTTTCCTTAGAAATATGAAAAATGGTCATTTACTGTGAGACTCATTGACGA
CTCGGTTTCGTGACGTAACAATTTCGTTAATTTCAGATGATGCTTTGGATTTGACTAATA
CGCTAGCTCTGGAACGTAAGGGATGGACAGGCTTGTGTGTGGAACCCAACCATGCCTATT
GGTATGGTTTATCTCATAGAAAATGCACGGTTGTGGGAGCATTGGTGAGCGGTACTAAGC
CCGAACCGGTGAAAGTTAAATTTCGAGGTGTGTACGGAGGAATCGTTGGTAAATTATCCG
CAAAGTTGGCCAACCGGAAGAAAGAACCTGATGCTCCAAAGGTAACAAGATACACGGCAC
CAATACTGGAAGTGCTGCAGAAATTCGATGTTCCGGAAATAATCGATTACTTGAGTTTGG
ATGTCGAAGGTTCCGAATACGACATAATGAAATATTTTCCATTTGACTCCTACACCATTC
GGATAATGACCGTTGAACGTCCGAACAAGGAGCTGAAAGCGCTCCTTGAAGAAAAAGGAT
ATGTTTTCTTGGCAGAGCTGGCACCATGGGGGGAATATTTGTGGGGTCACAAGTCGAGCG
GATTTACACCAGAGCACAACAAAATAAAATCGATAAAACCGAGAACATAGGAGAGACATT
TTCTCAATTAAATCCCATGTTTGATGTGAAGTAGCTCAATTGTGTAACCTCGACAATCCT
CATTATCCATAGGAATAAAATAATTGATCATTAATCGCAT
>comp2659_c0_seq2
CGAATTTCAATGGGCGAAACTCATCTTCGTCGTTGCTTCTATCATCTTCATCTTCATCTT

CATCTTTCTCAATTCAGCGTAACAGCCATTTGGAGTTAGCAAAATTTTTATCACTCTCTG
AGCCCTTTCTCTCATTCATTCTAGTGGGACTATGGTATCAGCCACCCGCCTTAAAAAAAA
TGGTTCGGCCTCGAGATCGAATGCCAATTTCTTTCAGTTGTCTCTTGGAATGTTTCTTGG
CGCATTAGGGATGAATCTATGGATTGGAAGTGACGTCGATCCCACGGCATTGTGGCTTGA
TCAAGATAGAAATTCAATTTCCGAAAAGAAAGTTGTAACATCGTTGAAGAGTATACCTGA
AGTGAAGGAAACGAAAGGCTGGCATTCGATCCACGTGTTTTACGGAGAGAAAGAAGGTTT
GTTCAACGACGCACCCAAGTCTATAACACAATCGCAGGAATCATTTGCACAAGTCGGACA
AGATTCGATCATTTTGGACTTGCTTGGATCAAACGGATACTTTATTGATCTTGCAGCGAA
TGATGCTTTGGATTTGACTAATACGCTAGCTCTGGAACGTAAGGGATGGACAGGCTTGTG
TGTGGAACCCAACCATGCCTATTGGTATGGTTTATCTCATAGAAAATGCACGGTTGTGGG
AGCATTGGTGAGCGGTACTAAGCCCGAACCGGTGAAAGTTAAATTTCGAGGTGTGTACGG
AGGAATCGTTGGTAAATTATCCGCAAAGTTGGCCAACCGGAAGAAAGAACCTGATGCTCC
AAAGGTAACAAGATACACGGCACCAATACTGGAAGTGCTGCAGAAATTCGATGTTCCGGA
AATAATCGATTACTTGAGTTTGGATGTCGAAGGTTCCGAATACGACATAATGAAATATTT
TCCATTTGACTCCTACACCATTCGGATAATGACCGTTGAACGTCCGAACAAGGAGCTGAA
AGCGCTCCTTGAAGAAAAAGGATATGTTTTCTTGGCAGAGCTGGCACCATGGGGGGAATA
TTTGTGGGGTCACAAGTCGAGCGGATTTACACCAGAGCACAACAAAATAAAATCGATAAA
ACCGAGAACATAGGAGAGACATTTTCTCAATTAAATCCCATGTTTGATGTGAAGTAGCTC
AATTGTGTAACCTCGACAATCCTCATTATCCATAGGAATAAAATAATTGATCATTAATCG
CAT
>comp2660_c0_seq1
CCAACAAACCCTACCAAAGCACCGACAAATCCAACAAGAGCACCAACAAACCCTACCAGA
GCGCCAACAAACCCAACAAAAGCACCAACAAATCCTACCAGAGCGCCAACAAACCCCACA
AGAGCACCAACTAATCCCACGGCAGCCCCACAGCCAGATGGAAAATGCCCAGGAGAGGTA
GAATTCGTGTTTTCACTAGAGACGGACGACTTTCCCGAAGAAACTAAGTGGGTGTTGAAG
AGAAACAACAGAGTTGTCAAAAAACGTCGTTACAAGACCTATGAGAACCCCCTCTCTTTC
TATGAAGAGTCCATTTGTGTCCCGTTGACTGGAAATTATAAATTCGTCATCGTAGATCGA
TACGGAGATGGAGTTTGTTGTGACAAGGGAGACGGCAGTTATGAGATTTCTTTCAATGGA
GAAGTGAAGAGACGTGGAGGAGATTTTAGAAGAAGACAGAGAACAAAGTGGTCTGCGTAG
ATATTTGGCATATAGTGAAACGATAAGCTCCGGTTGACTTACCAAGACAGTCTATTTACT
AGTGGTTTTCCACTGCTGCATAATATGCTTAAACAATGGTGTTTTTTGCATGGGTTTCAT
TCTCTCGAAGCGACAATGCCTTTGTCAATAGATCTAGTGAATAAAAATATTTAATTAAGT
TCGAAATTC
>comp2661_c0_seq1
CCCCTCCTGCTGATGGCCCTGGGTTGGATGAAGAGAATGATGAAGATAAAGATTTAGAAC
TACTGGAAGGAATTTTTGATGTTGATGAGCCTATTGTCCCTCCCGCTACTGACGGCCCAG
GGGTTGAGAATAAAAACGAGGAGAAGAACGTTGAAACCCCGAAACAAATTGTTGAGGTCG
ATGAACCTATCATCCCTCCCGCAACGGAACCGCCGAAAGAGATCAATGTGCCTTTGGATC
AGCCATTGACTCCTCCCACCTATGCCCCTAGGCTTGACGATGAGAAAGAAGACCAAGACG
TTCCAACACCACCAGTCATTCCTCCCATTGCCGAACAAAAGCTTGAAGAGGACGAGAATC
TGGATGAAATTGGAACCGAGCCTCCCATCTACACCCGACCACCTTACGAGGAAAAGAGGG
TGGAAGACGAGATCAACCAAGAGGAAGACAACCTTGTCTCGCTAGAATTGGTATCCGATG
AACCGCGAGTCGGTTGTGCTGGAGATGTTAAGATTCTGAGACAAAAGGGTAGTGGCCTTG
TATCATCCGAAGATCCAATGCAAAATGCTGTAAATATTGTTCGGCAAGATATTTCGACCG
TAACCGTAAGCCTCAACCAAGTCTGGAGTCGCGAAGACCAGGTCCTGGATTCCGTCTACT
ACCAGTTTTATGCGCCCGGGAAAAACTTCTGGGGCAATGCCAAGTGCTACGAGCAAGCGC
AGGTGCCCTTTTCTCGATCCGATCCGTTCGAGACGTTCGAGCTTGCCTGCAATGTTTTGA
ACCCCTACGCCGAATTACAGATCTGTCTTGTCGACAACGTAGAGAACAGTATTCTATTGC
CACCACGAGTGGTAGAGGATCCTCTGTCGCTTGTACCCAAGTGTTGCTCTCCGGACACAT
CCATACCCCGGGAACAACAAGCTGCCACTTGTTACATCATGGAAGTCAAATGCACCCCTC
CAACCGCCAACTCCTGTTAGATAAATTGAGGGGCCAGGGGAGAACGAAAAGAATGAGCAC
GAACACGCTTGGGTCTGTTCGCACCAATAGAATTACAATGTAGAATAAGTACAACATAGA
AGTATCATAGATCATGATTAAGCTGATTAGATTAGAA
>comp2665_c0_seq1
TTCAACGTTTGTACCAACAGCCATACCAACATACCCACCGACACCCAGCCCTGTTGCACC
AGCTCCGACACCACGTCCACCAACAGCGCTTCCTACACAATGTGGATGCGATAGACCTGT
CGAGACGGTGACGAAGAGATTGTGCAGCAAGAGGGTAGTGAAATTGGGCTGCCCTGCGGT
TTGCAACCGTCGCTGTACTTGCAAGGACAAGAAGGGGTTCAGAATGAAGAAGGTCAGGTA
CAAATGCAAGAACGTCGGCAAAAATGAACAGCAGCCCACCTGCGACAACAAGGTGAAGAA
GAATATTTTTGTGACTGATCTTTGCCCCAAAACGTGCGACTCTTGTTTCGTGATGTAAAC

AAAGATCAGTAACAGTCAATTTCATCTCATAGAAATAAAGTTAATCTTTGCCTACGGTGT
GCATTGAAAAAGAAATGTGAATACCCACAACACTCAACCGACAAAAACGAGTTCAATTTC
AGTTGCTTGAAATGGAATGAATAAACGGTGTTTTGAATATCAAAAGCCGATGGTTTCGCT
TGCGAGTAGCTTCTACAATTTGAATGAGCTAAATGGAAGGACCCTTCGATGTAGTGGCAT
CCTGTGGCTAAGTGAGCCGTTAATGTCACGATGAATGTGCCAACTTTTGCCAGTCAAACG
ACTGCTCTCGAGTTATCTGAATTCAGTAACTAGACCGGAAACTAATTCGTGCTAGTTATG
CTGGTGGCGATTTCATAATTTTCATTAGTCAATCTACGAGCCGAAGACATTATTGTATTC
AGATATTTGGATTACGGGATGATTGAGCCAAGAGATTCAATGGAATCTCTCCAGCTAGAT
AATTACGGAAATAACAAATCAAAATAACATGCAGTC
>comp2666_c0_seq2
AGGCTGTTCCGCCGTGACAACCTTTTCGTGAGACGAGACGAGACGATCACGCGACACGAC
GCCTACACCCATTCCACAGAACCTCCAACCCGTTTCCGGCTCTTGCCAACAACAGAGCAG
AGCAGAACAGAGCAGAACAGAACAGACACGCCCACACCGCACCACAACAAACCGGGTGCA
CCAATCCCCGCCCTTCGCATCCAATCCGCTCTATCGAACACGGGATCCGCCAGCTTCTTT
GGCCATCGAGAGTGTCCCTGCACCTAAAAGAACGATCGAAGAGTGCCGCACCGAACAGAA
TCCCCGCCGGAAAAGATCCCGGACCAGAACAATCCGACGACAGGGATACCAGTAACAACA
GAAAGAGCCACGGCCATTGCAGGCAACGCATTCGGCTTTGCCTTCCCACACCCATTCATA
CACCCATTCCTACACCCATTCCTACAGACATCCGCATTCCACCCACCATGATGCACGAAT
CGTACCCGGACCCGCGCGGGGAGGCCCCCGGGAACATGGCCGCCCGGACCCGGCGGTCGA
TGGTAAAGAGGGAGGGCCTCCTGGCCTCGGTCGTCCGGGCCGCCTCGGCCGTCAACAACG
CCCTCCTCGAGCACTGCGGGGTCTGCACCGTCTACTGCGACAAGCAGGTCCAGTACGAGG
ACTACGTCCAGCAGCAGTACGAGGTCGGCGTGGGCGCCATGGAGCACCTCTACGCCCAGC
TCCACACGGCGGCGGGGGAACCGGGAGCGGACGCGGGCCTTGACGCCGGCGTTGACGCGA
ACGCGGAAACGGGCCCCTCCCGGCAGCGGGCCCTTGCCCCTTCCCCGCAGGGTGGCGCGG
CCGGGAGCGAGCCCTCCCGGGGGCCCCACCGGGCGATGGCCCCTTCCGACCCCCGGACGG
CGGAAGCGGAGCGGTACTACCGCGGGGTCCTCTTCGACGAGGAGTCGGTGGTCCGCAGCG
TCGGGAGCCGCGGGGCGGTGGACGACGGGGAGTAAGCAGAGGAGAGGACGGGACAGGACA
GGACAGGACAGGACTCCACCGAAGGGTGCCGGGCTTTGCCGGAGCGATCGAGCAAGCAAG
CGACCGACCGGTGGCATCGCGGAGCAGAGAACAACCACCATCCACAAACCACCACAAAAC
GAAAGAAGGCACCGTGTTTCGGCCGGCGTGGGCACCCCGGGACGTGTGGGTGGTGTATTG
GACTGCATTCCATTGGATTGAATTGCATTGGATTGAATTG
>comp2668_c0_seq1
CCTCGGTGGGTTCCTCGGTGGGTGGCCCGGTCGGGTTTCTTGTCGGAGCCGTCGGCACGC
AGACCCCCTCCTTCACCAGCTGCGGCGTGTCCCAGGCAAAGAAAAAGTTGGACCTCCCGT
ACCAGCTACAGTGCTGTTTCGGGGCGACCTCTAGGAGCTCAAAGGGGCAGTAGGCCTCAT
AGGTAAAGGTCCAGCAGGCCGTGTCGACAAAGCCAAAGACGACGCTCCGCTTTCTCTGGA
GATCGGTCAGGCCGGCCGGGCCGTTGTCTCTTCCCGGGTTGTCCCAGCGGGTCCCGTCGG
TCGTCGCCGAGAAGACGGTGCGGGTTCCGGGCCTGCAGGGGGGAGGGGACACGGCTTCTG
CCCCGTCCCCCTCGCAGGCCACACCGAGCTCTGTGGACGGGTGCAGGACGTATCCGCTGG
CTTGGGAAGCGTCGATGAAGAGCCGCTCGTGGATTCCCCTTTTCCCCCCCGAGTTCTTGC
GGCGGTCCAGATCGTAGACGGACCTGCCAGCGGTAGAGATGGGTGCATGGATGAACAGGT
GTGGACAGACCATGCATTTTCATGTATGCATGTTGACGCATAGATGCATGAGGAAATGCG
TGGATACACACGTACGCAAGGTATAGAACGAAATGATCGTAGCAGGTGGAACGAAAATGG
ATAGGAGTGAGTGGCAGGGTCCTGAGCAATACGTGCTTTGTGGCACGCAAGCATCGACGC
ATCGAAGCATCGACGCATACACACACACAAGCATACAATCGGAACCACAATTCCCGACAG
CACCAAACTCCAACCTCAAACCTCAATGCTCGGCA
>comp2669_c0_seq1
TGACGATGACGATGACGATGACGACGATGCTCAAAAGGATGACGACCTCAATTTTGACGA
CGACGACGACGACGATGCTGTTGAACTTGACGACGATTCTGCGCTTGGGAAAAACAGTTT
GCCGGGATCGACCATGACCTATCAGGGTGTTCGTGGCGAATGGGAATTCTCCGGCATCCA
ATCCGATTGGGGTCCCGCCCACGCCGGTTCCATGGTGTACGATTTTACGAGAAATTCTGT
CTACCTGGTCGGGTCCTACTTTCCGGACAGCGAGAACGATTGGGTCCGGTCCGGATGTTT
CCTCGGCGAGATCCCGATGTCCGACATCAAGGATTGGAAGACCGTCGAAAGTCCCCTGCC
GCCGAGTGCAAAAAAACCGACTTCCGTCGAACCATTATGATGGCGTGGAGGTTTTCAGTCT
TCCAGACTCTCTGGACCCGCGCGAAGTTTTGAGTTGCCATTCCATTATGTATGACGACAG
AGATGACTCGAACCATTACCTCTTCATCGGTGCCGTCGATGAACCTGACGCCCAGATTCG
TAGCGGAACCATCAACGGATTCGTCAATGCTTTCCAGCGCAGCAGCACGAATCCCGATTG
GGAGCTCAAAATCAACCCGACGGCTTTGGACACGGCCAAAGCGGAGGATTTCCGTGACAC
TAGCCCGGTTCCTGTTCGGTACCCCATTGCCATGACAACTGGGTTTTACCAAAACCAAGA
ACACCTCATGGTTGTCTCGGTCAGCAGCAACGACCCGCTTTTGACCGAAGAGTACGTCGA

ATCGGGTGACGCCAACAATGCCCAAAACTTTAAGAACACTATGGTGCCACCCGGTCTGCG
TCCCTACAATTCATTCGCCAAGCGTGGAAGCAACTATTTCTATTCCCACCAGTTTTTCAC
CTACGATAAGAGGAAGAACGATATGGAATTGACCTTTTCGTTCGATGCGATACTAGACAG
CGACGAAACCTATCCGACCGGTGTGGTCAACCTAGTTCCCAACAATATGATAGCCGTAAT
GGTGGGACACTTGAAGGGAAGAGGTCCCAGAGTATTTAGATTTCCCAAACCCTTGATGAA
CGGGGTCGAACGCTTGGACGACTTTGACGGATTTGTCCAAAACTTTTTGTTCGCAAATGG
TGTTCAGCCGCAGTTTAGGGATAGCCTTCGTGTTTCCTCGGTGGAAATGAATCCACCCCT
CGATGATTTCATCCACGGCATTTGCTTAGGCCCCAAAAACGAGATAGGCAACCACGACAG
CTATTTTATTGTGGGATCGACCTACGGAACTATGCCCGCCGGAACGCTTCAGGATGAGAT
CACCACCAACATTTTGAGCGGCAAGTCCAAAAAGAGCATGGACGGGAACCGCATCGACAA
ACTGTCGGCCTTTGTGTCGAAGGTGGAATCGAATGAAATCGTTTGGACTACCCAGTTGTA
CGCCGTCCCCGAGTCCAAAACGACCCTCAACGGCGGCAAGACCGAGGCCTTTGGCTGCCA
CCTGATCGAATCGGACCCGACTACCATGTACGTGGGTGGAAACGTCTACGAGGGAGGCGT
CATGGATTCCAAGTTCGAATCCGCAGGCGGTGACGATGTGTGGGTCGCCCAGATTGATAC
CGCGAACGGAGAGCCCCGTTGGATCAAGCAGGTAGGCTCCGCGGGAACCGACCGAATCAG
CCACACCAACGGTGTCCAGGCCGCCCTGAACGGGGATTGCATCTTGTACGGAGAAACAAC
CGGAGAACTCTACCGCGAAAAGAGAAAGAACGAAGAAGACAAGTACACCTCGGACGTTTT
CGTGACCACCTTTGGCAAGAACGACGGCTTCACCGGGCCAACCATTGGCAGCCAGGACTC
GACCTCCAAGAAGTCGGGAACCATCATCGGGGTCGGAACGGCCCTGGTCGTCGTCAGTCT
TTTGATGTGCGCCGGCGTCTTTTACAAGTTCCGCCGCCCCACCCGCCGCGGAGCCTCCAA
GAACGCCAAGGCCGGCAGCGACGGTATCTTCAGCGGCAATCTTGCGCCGGAATACCACGA
CGACGAAGACGCCCAGGTGACGGACGCCGCGTCCAACGGGGCGTCCAACGGCTCGACCAG
CGGCTTTAGCGACAACGCCCCACCTCCGCACGTCTTCCAGGACGACCCGAAGATTGCACC
GGAGAAGTCCTTCGTGTAGGCGACAAAGCAAGGCATAGCACGGCACGGCACAGCATGGCA
CAGCACAAAACGACTTGTTTCGCCCGCAACGCACGATGGTGCTTGCGTCCATCTTCACAA
TAATTGCACACGCACACGCATTCGCAATCTTACGAACACCGTTTTGCTTCCGCACAACAA
CGGTGCAAAACAGTAATCGATCCCGTTGGGATCGAATTTCCAAGAGAGCGTGACCTCAAA
CCAACGAGAATTCATGGAACAAATTCATTTTAAATACAAAACGATAAACTAATCATGAAA
GAAAAAAATCAC
>comp2670_c0_seq1
ATTATGACAACGAAGATGATTATTACCGCACAAGAAAAATGGAAGCGCAGCGGAAACAAA
ACGCAATTGGAGGGATAGGAAAAAAATTAAAACCACACATCCGCGAAAAACCCACTCTCT
TGCTCCCGACACCTATCATCGTCATGGGATTTCCCAAAGCGGGGACAAGTTCGATTTTTA
GGTTTTTCCGCAAACAGGGGTTGCGTTCCCAACACTGGTACTGCTGTGAGGAGCAATCCA
CTAGGATTCAAGGAGGACCTCTCATGGCAGACTGCTTGCTTCACAATTTGAAACGAGGCA
ATAGAACCAGTATTTTCAGTGGTTGCGGTCACTTTGATGTCTACAGTGAGATCAATGGAC
CGCGTAGATTAAAGTACCATCCAAAATTTCATGACAGGAAGGGTTACCTCCTGGATGATG
GCACCTACGACTTCGACGGTCCGGGACAGCGGATTTTTTTTCCACAGCGATTTAGAATTC
GAGAAATCCACGATTCGTACCCTAATGCGACCTGGATTTTGAATTGGAGAGATTATGATC
CTTGGATTGATTCGGTTTTGAAATGGGGGACCGGCGACAATCTGCATTACCAATTCTTGA
ACGAATATTACATTCAGGGTGTCATACCCAGCGTTCCTCCCCCCGAG
>comp2670_c0_seq2
ATTATGACAACGAAGATGATTATTACCGCACAAGAAAAATGGAAGCGCAGCGGAAACAAA
ACGCAATTGGAGGGATAGGAAAAAAATTAAAACCACACATCCGCGAAAAACCCACTCTCT
TGCTCCCGACACCTATCATCGTCATGGGATTTCCCAAAGCGGGGACAAGTTCGATTTTTA
GGTTTTTCCGCAAACAGGGGTTGCGTTCCCAACACTGGTACTGCTGTGAGGGTAAGTGAG
TCGTTGGTTCTTAATTTTTCTGGACTTAGCAGCTACTTCCGTACAATTAGTCATTGCTAT
AGACACCTTTTTTACTATGATACCTTCACTACAATGCTTTGTCGATAGCAATGGCGATGG
TGACGGTGACATGGGATTCGATGCATACCTCATTACAATCGTTACTCGTGCTTCTACTTC
TCTTTTTTTGAACAAGAGCAATCCACTAGGATTCAAGGAGGACCTCTCATGGCAGACTGC
TTGCTTCACAATTTGAAACGAGGCAATAGAACCAGTATTTTCAGTGGTTGCGGTCACTTT
GATGTCTACAGTGAGATCAATGGACCGCGTAGATTAAAGTACCATCCAAAATTTCATGAC
AGGAAGGGTTACCTCCTGGATGATGGCACCTACGACTTCGACGGTCCGGGACAGCGGATT
TTTTTTCCACAGCGATTTAGAATTCGAGAAATCCACGATTCGTACCCTAATGCGACCTGG
ATTTTGAATTGGAGAGATTATGATCCTTGGATTGATTCGGTTTTGAAATGGGGGACCGGC
GACAATCTGCATTACCAATTCTTGAACGAATATTACATTCAGGGTGTCATACCCAGCGTT
CCTCCCCCCGAG
>comp2671_c0_seq1
GGCATACTCGCCAACGTCACCTGCTTACAGCCCAACAAGTCCAGCATACTCGCCCACTTC
GCCTGCGTACAGTCCCACAAGTCCGGCATACTCGCCCACTTCGCCCGCTTATAGCCCAAC

CAGTCCGGCCTATTCGCCAACTTCGCCTGCATACAGCCCCACCAGTCCAGCTTATTCGCC
GACTTCCCCTGCATACAGTCCAACCAGTCCAGCGTA
>comp2672_c0_seq1
TGAACAAGAACGTGAACAAGAACACGAACACCAACACCAACACCAACACGAACGCGAACA
CGAACGCGAACGCGAACACGAACATGAACATAAGCACGATTGAGGAGAGTAACTCGAATG
TGCACGTGATCGCCAAGTCAACCACAAACTCTCCAATCGTGGTAATTGATGAAAGCGAAG
AACATCAAGGGGAGCAGCAGCACCTACACCATAAGCGGGAGCAGGAACCACATGACCACC
TGGTGGCTAGCAAAAGAGTCAAGCGCAGAGGCAGGACCAAGTGCAAGAATGCAGCTAACA
AGGGGGCTCCAAAACGCCCTCTCTCGGCATATAATATATACTTTCGAGAAGAACGAAAGC
GGTGTCTTGCACGGGAGGAGGAGGAACTCAGACACGTTGATGACGATCAGATGGGATCCA
TCTCAGAAAAGCCACAGAAGAAAAGATTCAACTTTGAAGAACTCGGCAAGATCATCGGCA
GGGGTTGGAGGGCACTGCCAGCCTCAGAGAAACGAAAGCTCGAAGAACGGGCAGAGGCCG
ACCGGGAACGCTACCGGACGGAAATGATCGAATACCGAAAGGAGAAGCGCCGCAAGGACA
GACAAGAAGACGAGCATAGCGTAGGAAGCATATTTCACCGAAGCGTGCTGAACAACTATA
CTAACTTTCATCAATGCTCTTCTAATCCCCCGACTTTTCACAGAAACGCTGCTGCGATGA
AGCAAGGCCAAAATATGCAACCCTATTCGTCGCCAATCGCATCGGTTCGATCTGCATTTG
GTGCTCCTGCTCCTGCTCCCGTCCTGATCGCACAGATGCCTCTTCATCCTCATTATCATT
CACATCCAGGGCACCCACAACACCATCAACAATTTCAATCTTCAAGAGGAGAACGATTTC
CCTTTGTGCGTCATGCAACCAACTGCCAACAGTATCCTCCACAGCAGCAGCAGCAGCAGC
AGCGACGGCGACAGCGACGACGATTGCTGGTACACGAGGCCGAAGAAAGAACGCCGGTGG
CAAAAGATATGACACGGGTGTGATGTAATGGGATGCGATGCGATGCATTGCGTGCGTGGA
TACAGCAGACCAGTATTATACTAAAAGGAGATATATTACAAA
>comp2673_c0_seq1
TGTCGTTCATGCCAACACGTCTTTCCGATTGAATTCGGTTTTCTCAATTCGCACACACGG
TGATATATCAAAAAGTAGATAAAAAGATCTGCCATTCTACCTTTCCATCGAGAAAGGTTT
GCTTTTCGGAGCGAAAGAGGGAGAAGCAAAAATAGTCGTGCAACATGGGAAAGGCGATAG
TGCGAGTGACAAGTGGGAACAAGCCATTATCAAAACGCCGAGCTCGTCAACCCCCAACGA
CGACCTCGAGCCGTAGAAACAAGAGCATCGAAAACAGCAACTACGAATTTTTTCCCGTCC
TACCGAGACTAAGCTTCAACCACCGTGAGCAAGGCCGTGGCAATGAGACAGTCGAGGTGG
CAAGAGAGAACACAGAACCGAAATGCAGTTACAGATCCAAGGACTTGGGACTAATGGTTT
TGAATCCAAGGGAAGCTGCCGAGATCGATGATCCAGGAAATCCTATTGGGAACTCGGTCA
ACTTCTGTGTCGATAATATCTTTTCCATCAATACCGCGACCATCGACATATCCACCCTTC
TCCAATCGTGTTACCGGATTGATGTTGGGGATGTGATGGTGAATTTTTTCCTCTGTGACT
ACTCTGGCGGAGCATCACCGAAGACGGTAAAAAGCAAGGGACCACCAGCAATCAATACTA
TGAGAGCGGACGAAAATGACCGATTGGATGCTTTGGCACTGTCATGTAACAAGAAGGAGA
AGGCCGGATGGAAGAAAACGCTGAAAACAATCCAAGGCAAAAATCTGTTAAGATCTATGA
GTATGAAGCGCTGGCCAGCGCACGGAGATTGCGAAAATCGCCTGGAGGTTGCTATTTCTT
CTGATAGCGACGACACGATCTTTTCTAACCTAGAAGATCCCGAGAAGTGGGATATCAAGA
CATCATCGAAGTCGAAAACGATCAACACAAGGAAAGCTCACAGCAAATCATTGGCATCTC
CAGCCACCAAGGAAGAATGCGACAAGAAATACGATTTTTTTCACGAAGATACAGATAAAT
CGCTGGATACCTTTGATTCGTTTTGGAACTATTCTATGTTTACCTGGAACGAAGAAGTTT
GTGCCGAAAACGAAGTCCACAGAAACGATGGAGAGAATAAAAAGGTTACTTGCAAGGATG
GCACACAGAACCCCAACCCAATCGCAGCAAACCATGGCGCAAGAGAGGACAATGAATCTC
AAGAGCAGGAAAACGGAGTCAAGTTTGGGTTGGAAACGACTCGAGACGAATCCAACGTGA
GACATCGAAAATGGAAGAATATAACCTCCTGGAAGAAATTTGAACCATTTCGTGTAAGCT
CCAAGTTCAACAACGCTACGGGAAAAGATTCGACCCCTTCCTCGGGACAAAAACTTGGAA
CAAATCCAATTCAGAAAGGCTCGTATTCAGAACGAGAAGATATCTCAGGGCAAGCCTTAG
AGAATCCAGAAAATTCCGTGGACAAAATGGATGTTATTGAGCGGTCAAACCACCAGAATA
AAAACGAAACACAAGATCAAGGAAGCACTAAATTTGCTCTTGATGACGCCAGATCAAAAT
CGCTTGTATCTCAAAGAACAGTAAGAAATCTTCGAAAACGACGATTGCAGAAAGCTTTGG
GACTCACAAGAAGCAACCGATCAATCTCCTCCATGTCGAAGCCAAAACGTCCTTCCTGGA
AAAAGATGTTGATGGTCTCCATTCCTTCCACCCGTCGCATATCTTCAAGAGAGGTGGCAA
TTCCAGCGGATATAATCCACGTGATCGACAAGTCACAAGAAGTGCATGAAGTGAAAGAAG
AGGATATACCTACTCCCGGACCTATCATAAGTGAGGAAACTACAGACAAATCTGGCTTCA
A
>comp2674_c0_seq1
TTCCTGTTCAGCTCCATCCGTATCAGAGGGAAGAACACACTCTCCGCAAGAGAACAGTAC
AGTGACATCATTTTTCACTATCATTGGAGAACATTGCAGTAGTTTCTGTTGCGACCATGC
CTTCTTCTATGGGAGATGTGACTGCCATGGGGAATGCTGGCAGTGGCATGGGGGTTCCCG
GCTCTGGCTTTCCGCCACAACAGCACCATCACCATCCCCACCAAATCTCACAGTTTCTCC

CTCCCATGGATCGATGCGCAGGTTGCGGTGCAGTAGACACACCGCTATTGGCTTGCGACG
ACCTTGACAGGGCAAGCGGCAAGAAAAAGGATATAGTTATAGATCCTTCTCAACGATTGA
GGTTTTGTGCTAATTGTATGATCGGGAAAAAGGTTAGGGTCTTCTGGCCAGTCGATAACA
GCTGGTATATCGGAACGGTTCAGCAATTCAATGCAGTCACTGGAGAACATCTATTGAAAT
ATGAAGATGACGATACCGAATGGGTTCATATTGGAGAAAGTAACACGACGATGAATCCGT
CCGATTTTACTCCTCAGCAACCTCTATCAGCTCAAGTTGGTATTGTTGCACCGCAACCAA
ATAACTTCGTTCCAAGGTCTGAAGCTGGTTCTTCCCCGGCTGATAAAAAATCTGCGATGG
TACCGGCGTCCCGAATCAAAGATGGAAACACCACACCAACAATTGCGAATGCTGATCCAA
AGAACAGAGTTGGGTCGTCATCAGCATCCGGTGGGCCCACGACCACGAAAGGAACCGACA
CTCAATCAGCTGCTGGCCCTGGACTAGAAACAGAAAACCCGTCCAATGCACTTCATACAA
CGTATCGAACATTCGGTTCAGCGCAAATACAGGGAAATTCGTACCCACCCAATTCCTCTT
CGGGACAACGCCACCTTCCTCCTCATCTTGCTCACCACCAACCTCATCACATACCTCAAG
GGTACGGTCACGGGCCGGGAGGTCCTCCACAGCCAACGCCTTATGGCAGTTTCTCGTCGT
CGTCACTCGGGCATGGGCCACAACCTCTAGGGTACGGGTACCCTGGTCACCCTGGCATGG
GCATACCTCAACGTCACGGAGCGGTTGGAGCTTTACCACCGCACCATTACGGTGGAATGC
AAGGCCACCCGCCTCATCCTCATCGGATAGAAAATCCTGCTCAAATTTCTAATGCGCCAG
GCTCAGGGGGGAATGTTGGTGCGAATGGAAAGAAAAAGACAGGGCCTAAAGCTTGGACGA
AAGAGGAGGATGCACTGTTGCTCAGTATTGTTCAAAGCATGCGAGTGCCGATGAAATGGT
CGTTGGTGGCACAAAGCTTGCCAGAGCGAACGGGAAAACAGTGCAGAGAACGCTATGTAA
ACCACTTAAACCCGCGTCTCAAAGTCGCCGACTGGAGCCCGACTGAAGATGCCACCATAT
TTCACTTATACAACACTATCGGCAGTCACTGGGCAAAAATGTCTAAAATCATTCCCGGTC
GGACGGACAATGGAATCAAAAACAGGTTCCACAACCTGCGAAGGCAATTGGAACGAGAGG
ACGAGCACCGGCTCCGTCTGAATGCCTCAAGAGATTTTCCAGAAGAGATCCGACTTGAGC
TCATCAGAGAGTTTCCCGAGGAGTTACGAGGGAAAGCGGCTGAACTATGGGACATTAAAT
CTGGGTTGAGCGTTTTAGCCGCGCAAAGTGTATTGGGTGGGGATAGAACCAGAACAGCAG
GGCGTTTCGGACCTTTCCGTGAGGCAGTACCCGGAGAGGATATCTGCGTCCGGTGTGGTT
TTTGCCTTCCTAGTGTTCAAACGGGGAGCCAAATCTGTACAAAAACGGGTTGGTGTCAAA
GCTGCGCACGAATTCCACCGCACGTGAGCGGAAATATGCTGCGGGAATGCTTGAACCTTC
GCAGGTGTCAAAATGCGGAAAAAAGGAAGATCATCGAATCATGGGAGATACCCGATGCAA
TTAAAAACTCCAAGAAGAATAAGCAATAGTTTTAAGGCTATCAGGATCGGATTATATTGG
GGCAAGTGAGGGGAACGATTTGTCGAATGCATGCGCAACTTGCGAAGTAACCTTCCTAAC
AATAATATTTCAATTCGAATGGTTGCTACGCTGATGATTTCTTACGCTAGATGCGTCTTC
CTCGTTTACACAGCTTCCCATCAGCTTGTAACTTCTTTTGTTCTCAAAGCACAATGTATC
ATGTTTGATATCCTTGTCTTTGCAAACCAAGTGTCAACCTTGGGTTGAACCTATGATAGC
GAAAACTGACAACAAAACATGCTTAATTGACATTTTTTTTGTTGGATGTAAGCCCAAATC
CACATCATGTTCTGAGGAATGAATCAAGCTCTGTAGCTTGTAGTAGTATAATTGTTACTT
GAGTCATGGTAGATTTATTCTATATCCTCTTGCTGTTATTGATTCCACAATGATGTAAAG
GGGTTTGATGCTTCTTTCTCTGCTGACGGAGAATCGGTTATGCTCAAACTACCTTCATTT
ATTTACATGATGCTACTAGCAGTAAATGAACAGCAGTTTACCCTCAAACTTATCAAAGAA
ATTACTAGTAAGAGCACAAGAAC
>comp2675_c0_seq1
AAAAAATGCAGGCAGACCGAGCAGTAAAAGGAATAGAACTATGTCCAAAATCGCCCTCGT
GGACGATGACAGGAATATTCTGACATCCGTATCCATGACACTCGAAGCCGAAGGCTTTGA
GGTTGAAACCTATAATGATGGCCAACAGGCCTTTGACGCCTTTAGCAAGAAATTGCCCGA
TATGGCCGTGCTCGACATCAAGATGCCGCGCATGGATGGCATGGACCTGTTGCAACGCCT
GCGCCAGAAATCGCAGATGCCCGTGATCTTCCTCACCTCCAAGGATGATGAGATCGACGA
GGTTCTGGGCCTGCGCATGGGCGCCGATGACTATGTGAAAAAGCCGTTCTCGCAGCGCTT
GCTGGTGGAACGGATCCGCGCCCTTCTGCGCCGTCAGGATGCAGCCAATGGGCATGTCGT
GGTCGAAACCGAAGAAACCAAGGTGATCGAACGCGGCAATCTTTTGATGGATCCGCTGCG
CCATTCGGTCAGCTGGAAAGGGCAGGATGTGTCACTCACCGTGACCGAATTCCTGCTGCT
GCAGGCGCTGGCCAAGCGGCCCGGCTTCGTCAAAAGCCGCGATCAGCTGATGGATGTGGC
CTATGATGATCAGGTCTATGTCGACGACCGCACCATCGACAGCCATATCAAGCGGTTGCG
CAAGAAGCTGCGCATGGTCGATCCTGAGTTTTCGGCAATCGAGACGCTTTATGGTATCGG
ATATCGGTACAACGAAGAATAATGGCTCTGGTCGAGCGGATAGGGGTGCGGAGCGTGGAG
CCGCGAAAGGATAACGACCTTGTCCTTGGCGATGATTTTGTCGCACCGGATCGTCTGGAC
GAAGGAGAGCTGAATGCCCGCCGCGCCAAACGCGGCTGGTTCAGCTTTCGTGACTCCTCG
CTGACGCGCAAGATTATCACCTTCAACCTGATTGCGCTCAACGTGCTGGTTGTGGGTATC
CTCTATCTCAATTCGTCACGCGACAATCTCGCGATGCAGCGCGCCAACAGCCTGATGGTG
CAGACAGCCCTGATCGCGGATGTGGTCGAGGCGCGTCTGCCCGTCGGTGCGCCGGTCAGT
TTTGCAACCGGCGATGGGGTGGATATGGCCACCACGCTGGACAATCTGGATATTCAGACC

GGAACAGAGGTGTTTTTGTTTGATCGCACCGGCATGTTGGTGGGGCGCAAGCAGGGCGAT
ACGATGGGGCAGGACCGCACTGCGACCCTGCCATCAACGCCAATGACGGATTTTCTCAGC
CGGGTGTGGGATCGGATTTCAAGCCCGTTTTCTGGCGTGGATCAGGTCTCTGCCAGCCGT
GTGGTCGAGGATGTGGCCCGCGACATGGTCGATCTGGCAGTGGGCGGCGGCGTAGAAGTG
CGCACCGCCGAGACCGCGCAGGGCGTTGTTCTGACGGTGGCCGCCCCGGTGATGCAAGGC
AATACACCTGTCGCGGTGGTGGCGTTGGCCAATGCCAGCGGCGAGATTGACAAGCTGGCA
AGGGTAGAGCGCGAGCGCGTGCTTCAGATGTTCCTGATTGCCACTTTGGTATCGGTGGGT
CTCAGCCTCATTCTTGCCTCGACCATTTCGAACCCCTTGGCCGATCTGGCGGCGGCTGCG
GAAATCGGGCGCGACCGTAACAAGGGCAAAGGGGCCAAGGGACGTATCCGTATCCCCGAC
CTGACCGCGCGCCCCGATGAAATCGGGCGTCTTAGCGGTGCGTTGCGTGGCATGGTCTCG
GCGCTTTACCACCGGATCGACAGCAACGAGCAATTCGCCGCTGACGTGGCCCATGAGATC
AAGAACC
>comp2676_c0_seq1
AAATAATTCCAGTCGAAATTGTCAATAGCACGTAGTGTTGGACTTGACAACGAAAGAAGA
GAAAACACTTCCTGAGACAAATAACTTAACACCATGAGCGACGAGAAAGCTGTCGAAGAA
CTGGCAAAGAAAGTCGACCGATGGACGCTGCAGGACGATTCTACCATTGATTATGGGTAT
CTAGCCCAAACGTACGGCGCTGGCATCAAGGAAGCCAAGGAGGGCGACAAAACATTTTAT
CTGACGACCGCCATCAACTACACCAATGGGCCCGCCCACATGGGCCACGCTTACGAGGGA
GCATCCAGCGACGTTATCAGTCGATTCCAACGATTGAAGGGCGACAGGCCCTGTTACTTT
GTCACCGGTGCCGACGAACACGGACAGAAGGTCGCGGCCGCGGCAGAGAAAGAAGGAAAG
GACGCGCAACTCTACTGCGACAAGTATGTCGATGGGTTCAAGAATCTTAACCAGCGCCTT
TTGGTTTCCAACGACGACTACGTTCGAACGACGAGTGCTCGCCACAAGCGCGTGGCACAA
GAACTCTGGAAGCGTTGCGCCGCCAAGGGTGACATCTACCTCGATGCCTACTCTGGATGG
TACAACGTCAAAGAGGAAACCTTTGTGACGGACATGGAGGCCGAGAAGAACGATTTCAAG
GATCCTTCCTCGGGAATTCCACTCAAGCGAGTCGAAGAGGAATCGTATCTCTTCAAGATG
TCTGCATATGCGGACAGGCTCATCGAGCACATCAAAAACAACCCCGACTTCATTCGCCCC
GAACAACACAAAAACGCTGTTCTTAGCCGGTTGACATCGGATCCTCTTCGGGATCTATCC
ATCAGCCGCACCACTTTCTCGTGGGGTATCAGTGTTCCGGAGGGATTCGACGACAAGCAC
GTCATGTACGTGTGGATCGATGCCCTCTCGAACTACCTGACTGGTATCGACTACTTCGGA
ACCAATGGAGACGGAAAGGAGGGGATCGAAAAGTTTTGGCCCTGTGACGTCCACATCATC
GGAAAGGACATCTTGTGGTTCCACACGGTGATCTGGCCGTGCCTCCTCATGTCCGCCGAC
ATTCCCCTGCCCACGTCGGTCTATCTCCACGGCTTTGTCAACGATCGGGAGGGAAAGAAG
ATGTCCAAAACCGTTGGCAACGTGGTGGATCCCCACGATGTGCTAGACCAGTCTCACGTT
GACTCCTTCCGATGGTACCTCTGTAAGGAGGCTCCCTACGGAGGAGAGTTGTCCTACAAT
ATTGACAACATGCGGGAAATGCATAACGCTGATTTGTGCGATACGCTTGGAAACTTGTTG
AACCGCGCTACCAATCTCTGCAAAAAGTTTTGCGGCGGTGTTATTCCCGACGTTCCTGGT
CCCGCCAATCCCGTAGTCGACTTGGCCTCCATCATTGACAATTACACCGCAAAAATGGAC
CAGTTCGACTTGCAGGGAGGCGCTGCCATTGCGATTGCGGGATTCCGTGATGTAAACGGT
TACCTACAAACCGAGGCTCCCTGGCTCAAGAAGGGCGATGAGCACGCCGAATTTCGTCAA
ATCGTGGTCCGTGCCACACTGGAGGCCATTTATGCATTGGCCCATTTGTTGATGCCATTT
TTGCCGGTGGGATGCAAATTCATTTTCAACAAGCTTGGAAAGGAACCCGTTTCGCTCACT
GAGCTGAATCGTGATTGCCGAAGCCTTGATACCGGTGTAAAGATTGAGGTGGGAAGAGTG
CTCTACGAAAAGATGCTTTCTGACGCCGAAAGGGACAAGAAAGCCGGTGGAAAGAAAAAG
GAATCGTACGAAGAAGCCCAGAAACGCAAAAAGGAAATGAAGGCCGCAGCTATCGCTTCT
TCCGAAAAGGGAAGAGGAAAGGGCGACGCCAATCAGTCCGACTTCACAAAGATGGAAATT
CGCGTTGGAAAGATTATCGAGGTTTGGAATCACGAAAGTGCCGATAAACTGTTTTGTGAA
AAGATCGATTGCGGCGAGGAAACCGGACCGCGTGAGATTGCATCGGGACTGCGGGATTTT
TATTCCATTGAAGATATGAAGGACAAACTTGTTCTTGTCGTTTGTAACCTGAAAGCGTCC
AAGATTTTGGGCTTCAACAGCAATGGAATGGTCCTTGCTGCAAAGGAAGAGGATGGCAAA
ACCGAGCTTGTGGAGCCGCCAGAGGGCTCTCAAGTTGGTGAACGAGTTTACATCGAAGGA
TTGAGCGGTGATCCTTTTTCTTCCAGTCAAGTGAAAAAGAAGAAGATTTGGTCCAATGTT
TCGAAAGAGCTCAAAACAGGCGAAGGCGGAGTCGCAACGTGGCAAGGAAAAACCATTCAG
ACTGCAAGCGGTCCATGCAAAGTTGCGAGCTTGGTTGGGGCTCCTATTTCATAAAACCTA
AGTTTATCGGTCGGTTCCGCAACTAGCAGTATTATGACTTGGATTTTACAAGCAATCGAA
TCAATAAACTACAGTATCAGTATTTCA
>comp2677_c0_seq1
CGCACATCATACTTCACAAATCGAAATCGATCTCGATAACAACATCCAAAAGGACAACAC
CAACAACGCGGTTCAAGGAACTAATTTTTGATCTTACAACTAACAATTTCAAAATGAGAA
TCTACAACGGAGGTACTTTCACAAACAGCTACCGCAGCGATGCGATGGAAAACCGCAAGC
TGAAAATCGCCCCCTCCCAGAAGCTACCTTGTTCCTCGTTCCTTCCGGCAACCTCGAAGA

GATCCATCGTCCTTCCGGAGGATCAAGAAAACTCGGCACCCAACAAGAATTTCGAAGCCT
CGAGCAACGCTCGGGTTCCGAAAAAGCCCCTCTCGATGGATCAAGAAAACTCGTCGAATT
TCAGTAACACCAGCGTGACAGCAGCTCCCATGGTTCCAAGCAAAGCCCTTTCCGAGAAGC
CAGCGATGCAAAAGGGATTGTCGGAGAGGACCCTCGGAGAACCCATCCCGGAGGGTGGTG
TCTGCAACCTCGTCTACGAGCCAGACTCGTCCGGTCGCCTGGTTGAACACTTTACAAAGA
CCGAGCTCCATGGCATGGCCGTCGTGGGCCGCTGGACCGCGGGCACCGGCAAGAAGATCG
CCGGGTTCAAGTTCAAGCGCAATGCGGGCCGAAACGTCCTGATCGGCAACTGCGCCGCGG
GAGTGCTCGGCCGCAAAAACTATTGCAACGGTTTTTGTCAGTTTGTAAAGCAGGCATACC
TAATGAACGGCGAGGTCACCCTCTACGATATCCCGAAGGGATTCAAGGCAATGCCCGTCG
ACGTCTACCTCTATTACGACGACAACCGTCCCGGCCACCAAACGGTCAAGCTTGTACCTG
GAAAGTCGCTCCCGACCGAAAACCTTCTGGCTGTGTCGTGCCTTCCCAAGAACACACCTT
TCTACGAGACCGAGACAGTGGACTTGTTCAAGTGGCTGCATGACGCCGAGGTCCAGGGAT
ACTCCAGCAAATGTTAACTCCGCCGACTACCTGTATATTGCTATGACACCATATGCTGCA
GTACACAAACGCAGCATGCGCTGATGACTTCATGGACGATATATCTGCTGCACCGCCATT
CAATTGAAATTACGACGGACGAATGGCGACGACTGCGCAGTCGCAACTCTCATCGCAACA
TGCCACAAAGTGAAATAGAACAAACCAATACCCTATAGATCAAAGCTGTTTTTCAC
>comp2679_c0_seq1
GCTCTTCATTATCCTTTCATCTACCGCGACGGGTAAACAACGAAGACAAAATATGGTCCC
ATAACTCTTCATTATTCTTTTACCGAATTTGATTTTCTCTCCATCATTTCTAAGCTTAGT
TGCTTCTGGAGGAATTTGAGTCTCCCGGCAGTTGGAGATGTCAAAGAAATATCCATGGAT
GTGACGATTGATTGCACAATAGTGAAAGCAACATGCATGCCATTGATGTAGCTGCATTTC
CAAAGCGAATCACGTACTTGAACAGTTACCCGTAACTGTATTCCAATCGCTATAGTTCTT
TACATAGTTTCCATTACTAAACACACCCGCTTGATTGAAATTCATGCTTCCGATGCTGCT
GCTCTACGCTATCGGCTGGTAGACGAATAATCTAGAGTGTATGAAAAAAGGATTCATTTG
CGGCGCCTGGCTTCTTAAAAACTCGTACAATTCCTATTTTTTAACCATAATTCGTTAGAA
GTAGTTACTGTGGCAATGCATCCAGACTAGATTGCACCATCTTGGCTTGGCTTTCTAGTT
CTGCCAACTCAGCCTTGGCTTTGTCCACAACTGCAGCAGGAGCTTTGTCTACGAATCCTT
TCGAGCTAAGTCTACCAGCAAGTTTCTGGATCTCAATTTCAAGTTTGCCTTGTTGCTTTG
TCAGACGGGCACGTTCTTTTTCTGGATCGATTAGACCAGAGAGCGGCAAGAACACTTCCA
CACCGTCTTGAACAACGAGTTGTACCGACTCGTCATTATTAGCCGCCTTTGCGTCGTCGC
TGCCAGCTTCAACCACCTTGGCATCTTCGGGATCTAATCTCGCGAGAAGAACAAGCGATT
TCAATTCCGC
>comp2680_c0_seq1
TCGTCATTGCTTCACTAGCTGACACCAATCGCCTTCCCACAACAAAGACGATCTTGATCT
TATCAAAACAATCTGGAAGAGTCATACAGACGAAAGCAACATGTTTCGAGAACAACTTCT
ACGAGAACTGTACGAACAGCCCAAGGGCAAAACTCTGTTCTTCACAACGTCTGCCTCCAG
CAATCTGAAACTGACCGAAGGCAAGGCTTTTACGGGATTGTACAGGTGTACGCTCGAGAC
ACCAGAGGACTTGTTCGACGACTTTACGCACTACGGAGAAGACGAAGAGATTTGCAACGC
CGCCACCAAAGTCGGCGATGGCCACAACTTTGGTGGCGCCGATCAGTCGGAACTTTTGTT
GCGGAATCTGCAATTCGACCTGTCGTCGTTGCGGGAAAGTCCTCTGGATAGAAAGAAGAA
GAAGAACTATCTCTATGCCAACACCCACAAGGCTCACTACTTGAAGGGAGAGGATAGCGA
TTGGGAAATTGTCCGCGCCCAGGTTCCCGATGCCATCGATTTCGACAAGAAGATTGTGAA
TCACGATGGCTCTCTCAAGTTTGAGTCGGTCTTCAAAACACAATCGGTCATCGATTGGAA
CGAGTGTGACTTTGACTGCTGCACAAAGGCCGACTACGAAGATCTAGGCTTTCACAGCAA
CGCCAAGGAATTCGACTCCAACAACGAATATCATCACACATATGCGAACACGATCGTCCG
ATCATTCGTGATCGACGAACACACCGGAGACACGTTTGTTTCGTGGGAGGGCTTTTACAA
GGACTGCGGGGACACCTTCTCGTCGAGCAAGAAACTTATGTGGACGCTCGGCGTGTCAAG
GCTCAAGACAGAAGAGTCGGATTGTGTCTTTTTGGACACGGTGCAAGCCAACGATCACTT
TTCGCGTTCCAGCAACTTTCCACGATGCACCGAACCGGTCTCGATTGTTTACCAGAGCGC
ACGGGGCCGAGAGGCCGTCCTGCCCTACGGAGGACTGGCCATCATTCCGGCATCTCAAAC
CGGCTCTAGGAGAACCTTCTTGATGAGCGTCTTATTCAGTCCTGGTATTGATACCGGAGA
GCTGACCTCGCGTGTCTGGGCCTTTCCCGAAGGGGCCAATTCGATCAAGGACGGATTGAA
GCGCCAGGATCTGACCGGGACTGGAACGGTCATTGATGGCGCCCACTTTGATGCGAGTGT
CTGGGACGGGGGAAGCTTGCGATTGAATTACAACCCCCGGACACAAAAGCCGGACTACTT
CTGCAGGACCGTTTATGAAGCCGGCATCGAGTGCATCCCCATTGACATTTCTACAGACGA
GCAGGGACTGCCGGTGGTGAAGGCTGCCTCACAAACCAAGGATGTGTTTCTGGGCAAGGA
GCAGGTGAAGAATTTTTGCCGCCTAAATGTTGCCGACAAATCCTTTGCACGCCAGTTCAC
TCTCGTTACCGGCCTGGACGTTCAGTGGGACCCCGTCACCGGAGAGCCCCATCGGATTTT
CTTCGGATGCTGGGGTGGAGAAGACGGGACTGGAAATTTCGGTAGCGTCCAGAAAGATGG
CCTCCATCTCAAACAAGTGATGCAGAATGCGTATGCCGATGCGGTCCTCTTCATGCCGGA

CGAGTTGCAAATCACGGTCAGAAGCAGCAGCACCGGCAGGGTGGCCACGGCTGCGGCGAA
AGCCTCCGGCATTCCATTACAGAACCCGCTCTCGTCGCAACCCTTCGCCGCTGTATGCCT
CTTGTCCATCCTTTGTGCTGTTATGGCTACTATCTACAAGCGGAGTCAGAAGGCAAAGCA
GTATTCATCCCCAAGCGTTCTGCAGCAACAAACAGGATTCGGAGCGCCATACATCGAATT
GCAAAGCATGTACAGCAACATGGAATCGTCACCACCACTCCCACAATCGCCAACACCCAT
CGTCTGAGATTATGGCATTGCTATACAATCATCAAGGAATGGCAAGAACTGCCTTCGTAT
CTTATTTCCAATATGCGAATGTACATCCGCTGTTTATGTACGGAGCAAGTTTTTACAAAA
TATTGACTATTAATACTAAGTATTGAGAACATCAAACGTTCTGTGCATCCCCTCTGGTGC
AGCAACTGCTTTTAGATTAAGCCTTCTCCGGAACCTGTTTTCTTGTGAAACGTACCTCGA
TGCAACTCTTACCACGGCGCGAGGTTGCATATACATCGATCATCTGCCGCACCCCTACAG
CGGGGTAGACGCTGGTGTGGCGATTTGTGTCCATGCAGTGAGATAACGACAAAACTGCAC
CGACGTCCAACAATTCATGGAGGCTCAAAACGAAACCATAGAAATCGACAAACACGGTGG
CTCAAAATGCATTGCGTTTGCTATTGGACAATTTCTGTACTACCGGCTTGTCTTAGTATT
CGTGAACAAGGAACCTTGTAATGGAGATTGTGGATCTCCCTACACAGTAGGAGACCGACG
TGATCGCCTCGGCCGTGTGCATGTGTGCGTGCGTGTGGAGTCTGATTTGGTCGGCGGATG
CTCGCTTCTATTTCCAACCCGTGATTCAATTTTCAGAAGCGGTGGTCGGAGGCTCGGTAC
CGGAGCTGGTTCCTGCCGAAGTTTTGAGCTCCACAATGAACACCTTCAGGGGATGGAGCA
GTGTCCCGTCCCCGAGGTTGGAGTGGCGAGTTGTCTGGCGAAGGAACGAATACACTGGTC
CCATGATCGTTTCTTGTTTGCTGCTGACCGGACCCAGGCCCGGCACAAAGAATCTCCATT
CTGCATCGGAAGAAATGGTGTCCGGTACCAGTTCTTCTTGAATCACCAACCTAGCGTCGG
AAAATGTATTCGTTTTTCTCGATGCCAGTTTTATAAAGCCCACGTTCTTGAACGAATTTG
GCAGCGTTTCTCCATCC
>comp2681_c0_seq1
GAACAGAACCACCACACCAGGCGGACTCATTCTAACACAGCACCAACCAACGCGGTAATA
CAATTCCATTGCTATCAAGTTGCAGTCGAGCGCAACCAGAAAAATGAAAAGCAAAGCAAT
ATGCGTCGCTGTTTTGTATTCGCATAAAATCTTTTAGTGTCTCAACCAAAAAGGAACAAT
CACAACCAAACAAAACTAAAAGAAACAAAATGAAATCGTTTGCCACCGCCACCACCCTCC
TCGTCGCACTTTCTGGGTCGGCTTCTGCCTTCTCACCTGTCCGTGGTATTGCGAAGCCTT
CCTCGCTTTCCGCTGCCAGCGTCGATAGCCTCGTCGGTGCCCTCGAACCCGTGGGATTCT
GGGATCCCCTTGGATTCGCCGCGAAGGCCGACGACAATACCCTTCGTCGATACCGCGAGG
CCGAGCTTACCCATGGGCGTGTCGCCATGCTTGCTACTGTTGGGTTTCTCGCTGGGGAAG
CCGTTGAAGGGTCCTCCTTCCTATTTGATGCGCAGGTCTCCGGACCCGCCATTACTCACC
TCTCCCAGGTCCCAGTTGGCTTCTGGCTCGCCCTCACCATCGCCATCGGGTGCGCCGAGC
AAAAGCGTGCCACCATCGGGTGGGTCGAGCCCGAGAACGTTCCGGTCGACCAGCCCGGTT
TGCTCCGCGAGGGATACACCCCGGGGGACATTGGCTTTGACCCTCTTGGACTCAAGCCAA
CCGACCCTGAGGAACTTAAGATCATGCAGACGAAGGAGCTTCAGAACGGACGCCTAGCCA
TGCTTGCGGCTGCTGGCTTCATGGCTCAGGAACTCGCAGACGGTAAGGGGATCCTCGAGC
ACTTGTTTTCATCGTAAACAAAGCAATCAAAATGTGATTTGACAGAATAGAATAGAACCA
AAATCAATCCATGCAGAATGCAGCTCTCCATGCTTATCTCCATCCATCGATGAATCAATA
GAGTATGATACGTGCAAAACAAAATGCCAACACAGGAGAAACAGGA
>comp2683_c0_seq1
ACCGGCATGGACACCGGCATGGATACCGAAGAAGACGGAACCGCCAACAACCCTCTGGTG
GTGTCCTCCGAGGAACCCTGGCGAAGCGATCCCGGGGAAAAGGCCTCCAGCCACCGCTGC
AGAACGACAACAGTTACAGCCACAGCTACAGCCACAGCACCACCAACAAAAACCACACCG
CACCAAACCATCAGACACTCCGAACGGAACCAGCGACGGGAACAGCAAAAGCAATACCAA
CAGCAGCTGCAACCACCAACGATTTCCATCTTTTCTAATCCAATCGTTGTCAGGAGAAGC
GACGGCAGTGCATGGAAGGAAGGAGAAGGCGTCACCTGCTGTAGCAGTTGCAACACCAAC
ACCACTACCGGAAAAAACCTCCCCGTGTCGCTGGACGTGAGGACCTGCGAGGAGCTCTCC
TACGCGGTCATCTACAACCTGGCGCTCTCGCACCACCTCCGGTCCGAGGAGCTCGCCCTT
GGCGCCGAGGAACCTTCCTCCCAACCCCCTTCCGGCCGGGCCCGGGCCTACCTGCAAAAG
GCCTGGTCCCTCTACGAGTACTCGCACGAGATCTTTCGGAGGCAGAGTAGGACGATCGAC
CTCTTGTTCCCGGCCTTGCACTCGATGGCCCTGGCGAACAACGTCGCGCAGATCCACCAC
GTCCTCGGAAACGCGGACCAGATCGGCCTGTGCATGCACCACATCATCTCGATCGTCCTG
TACATGGTCGACCGAGGGGAGTTGGTCCTCGGGGAGGGGACCGCCGGGAGCAGCACCCAG
GGCCCTTCCCACGGCGACCCCCTGCCCGGGGGGACCGACCACCGCAGCGAGATCGAGCAC
TTTTTCGACACGGCGCTGCGGCTCACGTCCAAGGACCGGCCCGCGGCGTCGGCGTAGCAC
GCATGTGTTGCGTTGCGTTGCACGCACGGATGGACCGGGCGGCGCAAGCAGCGAAGAACC
AACCGCACGGGGCGAGGCACAAACGAAGGACAGCAAAAGCACAGCACAGCACTGCACAGC
AAAGCAAAGCAAACGAACAAGGAAAACCCGCAGCGGCACGCCAAGCCAAACACGACCTGT
AACCTAACCATTTCCCATGCAGGGGCACACACACACATTCACATTCCAACCCTGCAATAT

TTGTAAACTAAGTAACGAAACGAACCGATTTTAGTCACCTTTTGAAACACCAACCAACCT
ACCTACACAATTGTATACAAATAAGAGATTCAATCGATTGCAACACCGCAGAATCCGATG
CGTTCGTTCCGTTTTGCACGCATCCACGCGAATGCACCCATCACATCGCATCGGATCGGA
TCGGAAATGATGGAATCTGCTCTGCTTCGTTCCATTCCTATCGACACACGCTTCATTGGT
TTGTGTTTGTGAGTGTTTGTGATGCATTCGTACTGTGGGGGTGTGCAGTTCGATACCACC
ATCGATGGGCCAGTATCGGACGCACCGATTCGGTTCCCAAATAAAAAACAATCCTACTCC
>comp2684_c0_seq1
CTGCAGGTAAAGGCATTTTCTCGAAATTGGAAGCCCTCAGTAATGTTTATGATCATCGTC
AAACTGACTTTTTGTGCACTGAGTCTCATCTTCTGGATTTAGGCGAGAAACAAGAAACCA
TGGTGGAGCGGAGGTGCATTGAAAGGAATCGATCTCGTTCCAAATGTAGTATCCTATCCA
ATGAATTAAGTCATTGTCGACGTCGTCAACCTCGGAGATTGTTGGTGGCGTCGTCGCTAT
TTCTCTCGCTCCTTTCCTACGCGATATGTTTTCCTTTCGGCAGTAACACGATAATGAAAG
GGAAGCCTGATTGGGGGGCAGAAGCGGGGACGAAAAGGAAAACGAACAGATATAATGGGC
GTTCTTTTTATCACAGGGAGCATTCATTGTCCTCTCGATATACGTTCGTTTCGAACGTTG
CAGTGCTATCCCTGTCATCGGGTTCTCATTCGCGTGAGTCGGGTATTTCTCGGAACAACG
TGAGCACGGAAGAGAAAGAAAATAAAAATAGGAGCAACATAATAACCATCGAAACTGTGG
ATAGCTCAGGACAAGAACTTCCATTGCGAACAAGAGAATACCGTCGCCGAAAACACGAAT
GGGCGGAACGTTATACTAGCCAAGAAGGTTTGAGGGAAGCCTTCGGAAGAAACCGGAACC
GATGGTGGGGGGACCTCGATGCGCAAACAGCAAGGCGTCTCTACAAGAGATTATTGTTTC
CTGCTGCATTAAGTGAACTAGTCTTGGAATTGGGAGACGAAATAGTGAGACCAGAAGAAT
TGGCACCTCTTGCATACGAAGCCCGCAAAGCAGCAAAAATGTACGTCCGGGAAAGATGTA
GGGTGCCTTCTCGCGTTGGAGCGTATCTGATCGATGGCTTTCGACAATTGTACAAGTATG
GAAAATTCCAACCAAATGGTGCTTCGTATGAACAGATTTGGATTCGGTATTATGAAGAAT
ACGGTAGCAGCAATCACACCGAAGAGGAATTGATTGTATTGCTTACTCAGAATGATAATC
AGGACACAGATAGTGATGTTGATATCGATAATGATGACATAGTTCTTGTTGAGGATATAA
TTGATGACATTGACGATGCGGTGACCGAAGACGAAATAATAAAGCGGACTTGCCAAAAGA
TACTCGAAAAGTCTTGCACGACAAACGGAGCAATCGATCGGTTCTACTTGAGAAATTTCG
ATCAGAATTCGTTGAGGGAGGAATGCAGCAATCTTCAAGCTGAGAGCATCAGCAGCTGCG
ACGCGAATCAAAATGATTGCAATAAAAAAGTAAGAAAGAGTCGCGGGTCAAGATGGAGGA
ATAGAAAGAGGCGAAAAGATCGCTACAATGATCTATTTTTGGAAAAGATCACGAACACTC
TAGAAAGAGATGTTCGAAAACTTTTAGAGCCTCCACCCCCTTTGCTCCACTCTCCTACTG
AGCAAGAGGAAGAGGGGACTCTCTTTGGCCAGTTCATGAAAGAGCGTGCGAAATGCAATG
CAGAAATCGATAGAGTGGGCGAATGCGAAACGAATAAAAAAGAAGGCGACTGGTCATCTC
GAAGGATGACCCGACAAGAATATCACTCTCTGAAATTGTTTGCAAAGGCAATGAAACGGT
CGCAGCAACAATACAGTGAAAGCAATGCAAACGATGCAACGAAAAGAATTTCTATTGACG
ATATTGCTCTAACTGAGCGCAGCGAAAACATCGATATTGTAACTGTATCCAAAGCTGCTG
AGCCAGAAAGAACTCGAAACAAAGACCGATGATTTTGGTGTTCAAGATCGATAGTTTCAC
TGGCAATCTTCTTTCCAGGTGCGATTGCCATGCATAAATTATAATTGCACCTAAGCTATA
AAGATTAATCGACAGTTATCAAGTGGCACTAATGATGTTGTGATTAATATTATTGTAACA
TTCAAAAACTTGGGC
>comp2685_c0_seq1
CAAAGTGCATTGCTGGTCGAGAGTCTTGCACGTCACAGAGAGAGAGGACAACCGAACAAC
ACCGGCAAGGAGGTTTCGTCGCCAATAATCTCCCTCGAAGTTGAGTATTGTAGTTGACAC
AGCAATCTGCCTGTATTTGTATTCATAGACATATTCATATTCCCACCGCAACTCTCCTCG
GCATACCGTCGGACGATACCATCGGAGGAAACCATCCACAAAGTTTTCCGACGACGCACT
GCACCATAGCACAGAACAACACAGCACAGAAACAATGGCGACAACGATACAACATCGTTC
GAAGAGACGAGGCGAGAGGACCCCGCCCCTTGTCGGGGTTCGATCCGCGGCGGCTCTGGC
GGTGTCCTCGGCGCTCCTTCTTGCACTCTCGGCCACTCCATCGGTTGCCTTCCAGTCAAA
GGCGACGTCCGCTTTCGCCAACTTTCGAAGCACCGCCTCCTTTTCTCCATCGGTCCGGCT
CTTTGCGGAATCCTTCGAAAAGGACAGCAGCAAGGGCAACAGCGACAACATCACTCCCAA
ACAACAACCCTACGTGCGAATGACCGAAGACAACCAGTCGAGCGCCACCAGGGGTAAGTC
TGCCCTCCTCCCTTCCATCGGCGACGTCGTCCGGTACACGGATCTCGACGGCGGGGATCT
TTCCGGCCAGGTGCTGGTGGGAAAGATATCTTACGTCTTTGGCTCCAAGACAAGCGGGTA
CAGCGTCGAGCTCAACGAGCTGGAGGACGTCGGGGACGGGTACTTTGCCGAGTACAGCAG
CGCCCTGCGGAACCGCAAGGGCCGCAAGACGGAGCGGAGGCTGGACCAGGTCAGCCCGCT
CGCGGCCTCCTTTGTCGGCTCCGAGCAGGCCTACAAGGTCCCCCTGGACAGGGACACGGG
ACTCCCCAGCCCCCGCCAGGCGACCTACGACATCGACCGCTACGAGGGGCCCGCCTTTTA
CCAGACGGTCGACCAGGACGTCCTGGAGAGGGACGCCGAGGACTATTCCGCCCTCAAGTC
TAAGCTCTTCCGGAACGTGGCGCTGACCGGGCTGGTGGGAACCATCCTGGCCAACGCGAG
CTACGGGTCCGAGCTGGCGGTCATCTACTTTTTCGGGTCGGTGGCCTCGCTCCTCTACCT

CTTTCTGCTAAGCGTCAAGACCGATACGGTGGCCGTCAACCAAGAGGACAACACCCAGAA
CCTGGTAGGGACACCGATTGCCAACCTCCGCTTCGGTGCCCCCGCCCTGGTACTGATCGG
GGTCAGCCTCTGGAACAGCCAGAAGATGAGCAGCGGACTCGGCGGAGCCGACGATTTCCA
GATGCGGCTCTTCGACACGGTCAGCAGGGACCAATTCGCGGCCGCCGTCCTGGGATTCTT
GACCTACCGGCTGCCCCTCTTTGTCGGACAGATCCGGGACGCATTTCAAAAGCTCGAGGC
CGAAACCGACAACACCACGGATTCTTCCGGAATCCAGCTGCCGGGGAGTGCCGGCGTGGC
AGCCAAGCTCCTGGGCCGCGGAACCACCGCAGGAACCGATTCGTCATCCGTCGGTGGCGA
CGACAGCGACCTGGTCACCGTCCTGCTGGTCTCGGGGCCCCAGGCCACGGGCCGATCGGA
GCTCGTCGGGAGGCTCCTGGAGGATTCGTCGAACCCGGACCCGGACTCGTCGAACCCTAA
TCCGCCGCTGGTGCCCCCGGAGCGGGTCTGGAAGAGGGACGACGGGGCCACCTTCGAGCG
CCTGGCCCGCCGGGGGGAATTTCTTGACGCCACTACCATCGACAACGACATTGCGGCTAG
CGACTCGGCCTCCGGCCTGACGGTGGGGGGCATCTTCGACGCCGCCAGGGGCCTCGTCGA
CGGCAGGGAGGTCCCCAACAAGAACGTAGTGGTCGTCGACGCCAGCGTCGAGCTGGCCAA
GGCGCTCGCTACGGCCAAAAGCCTTCCAGGCAAGACCCGGCTGATCGGGATCTGGGTCGG
CCTCGAATCGGTGGCCGATTTCAGAAAACGGCTCGAGGAGGACATCGACACCGGGGTGCT
GGTGGTCCCCCCCGACGAGACGAAAGAATCCTTTCTGCGGGCCAAGATCAAGGAGATCGT
TGGAGAGATCGATTTCGGACTGGGGGCCGGGATTTTCGAGTTTACCATCCTGAACGATGC
GAGCGATCCCGAAAAAAGCCTGGCGGAGCTGAAGGAAGCGGCCTCGTACGCATTCAAATA
ATGAAACAATCGACGGGATGGGATGGGATGGCACAACAAAAAGGCCAATGGGCCAGCAGC
CACACCAACCGCTGTTCTCCCTCGAACAGCCGGCTCGCCAGGAAGCAGTGGAACCGACAC
GGATTGCACTTCTACAACGGTGCAGTGCTGACCGCGCGCAAGTTTTTCTGTACATAACGG
ACGGAATAGATTTATCAACTAGTTGCTTCTTGCCTCACATACGTATCTAAACACATCGTG
ATCAATGCTGCAAAGCAACGCCGTAGTAGGGGGTTCGCGCTTGCAAATTCGCCTGGTGGC
AATCTTTCGAAAGGAACAAAAGTAAATACATACTGCGGACGGAAAATTTCCCCTACCGTA
TGCCGGTGCGACAAGAAGGGATCTGTGTGAAATGTTGATAGGCTCCTTAGTGTATGATAA
TACGCGGTTTACAATAAACTTAACAGAACCTACCAAAACAAGCTACACGAGAGTCACGTA
AAAGAACTACGCAATATGAAGATCAACTACCATTCTGCAAGCGCCTATTTCCTTGCTGGT
GTCCTCTCCAAGGGCGTGGCAAACCCCGAGACCAACTTCCTTTCGGACCTCTTTGGGGAC
GAAGCCTTCCTCGAGTGCGTCCAGGACACGACGGCCCTAGAAAACAATAATCCAGATCTC
GCTGGGGCGGGGGAAGCATTCTTCGGTTCCTTCGAGGTGGGTCCACTCCAGATCACCGAA
GAATCAATTGGTGTGGACGTAATTTTCAACGAGACAAAACGTGACCCATACAAGGTGGCC
TGCATGGCTGCCGGGGGACTCTACGATGCAATGGATGGAAGCATCACTTGCGAACCCCAA
CCCGACCTTAGAGGACCGCTGCCCGAACGCAACAGCGGGCGAATATCCTTCCTGAATTAC
GGCCAATGCGTTGCCGACACGGAAGAATGCAAGGCCATCAACGTCTGGAATTTCCTTCGC
GATTTCTTTGAGGC
>comp2686_c0_seq1
GTTTTGTTTCAACCAAACAGGAAGATACAAACGCAATCTTGTTCAGAAGGTCATTCACAC
AGACTCATCCACGCACATAACATGGCTAGATTATCTGTAATCCGACGAAACCAGACAAAT
GGGTGGTCGCTGTTGCCGTTTTTGTGCTGGTCTCTGTCGCTTTGGAAAATGCTCCTGCTT
TCACCGGTTGTCGAGTCCTTTTCGGACGCTCTACTTCTGGGAAGACAACGAAGCGGCGGC
GGCAGCAGCGGTGCCTTTTCATCGTCCGCATTGTCTTCCAGCGAGGATTGCACTACCGGT
ACCATGAACGTCGTCGACGAGGGTGCCATCAATAGTCACACAAACTCCAGACGAGCCGCT
ATCGGAAGGCTAGTGGGATCGACAGCAGTGGCCGCCTGTGCCGGGATTGGTCTTTCACCC
TCACTCGCCGCTGCCGCTTCCAGTGACACGGATTCCGTCACCAAGCAGACCAATCTATCT
GAAGAAGAATTGCTTGCCATTGTTGTCGAAAAAGACCTTGTTGCCAACCAGTTCTTAGTC
AGCGGGAGGCTCACCCGCAGCATATACGATGAGGCCTGCGTCTTTACCGACGAGATCGAC
ACCTACACCCTCGACAAGTGGATCACCGGGACATCCCGCTTGTTTGATGCCAATCGGTCT
AGGGTGACCCTGGTGCCGGGATCTGCCACCATCAGCAAGAGCGATGGCTCGTTGTCCTTT
CGCTTCTACGAGTACCTCTGCTTCAACATCCCGTTCGTGAAACCGATCACCTACCTGAGC
GGAAAACTAGTCCTAGAGCGGTCTCCCTCCACCGGTCTCATCACCTCCTACAAGGAGATT
TGGGACCAGGATGTGAACACGGTCGTCTCTCAGAACTCGAAACTCTTCACGAGTGGCCTC
TCCAATGACTCACTCGACGCGGACCTCGAAACCTTTGGGGCAAAGATGGAATCCCTGTTC
GATTAGATAATGTTTGAATAGAAGCGAGTCGCGTGGAGTCGAATCGAATCAAATCAATGC
AGGGCCTTTGATCAACCCTAGCGCACCGGCATGCACATCAAACGGGGACAACAATGGTGG
ACGGAAGGAATCAAATGCTCATACGAAGCCAGTACTGAATGGAAGAAAAGACAAAACGAA
GCAATCAACAAACCAATCATTCTTCTATCAAACGCAATTCCTCAATTTGTGCTATTTCCT
AGTAACTAGTAACTAATGGTACATGAACCATCA
>comp2687_c0_seq1
GCATGCTTCCTTTTTTCAGAAACCATTACATCATCAACTCTCGCGGTAGTGTTGCATACA
ATGGATTGGTTTGCATCGCTAAACCCACTCCCAAAACAACAGCAAGCAGTAATTACCGAA

ACAACCAATAATCGAAAGAAAAGTCACCCATAGGAACACACGTTTCCCCTCACGAAAGAA
TTATGGCGAAAAGATCGCACAATTATTTTCTGGAACAGCATCCTCCTCCCCCTCCGCCTC
CGCCTCCGTTGCCTTCCTCGAATCCTTCCTCTCGTGATGCGGGCATTTCTAATGTTCGTG
ATCCACCTGGAACTTATCAGTATCTAGATCATCAACCTTATGCATACAATTACAGCTACG
TCAATGAGCGTGGGCACAGTGTCCGTGGCGATTCTAGTCTTCTGTCTACTATTGCTTCGC
CTTCGACTACTCAGAATATGCCGATCCCAATATTCAAAGGCCATATCAATAACAGCCATA
GTGCAGCGAGCAGTAATATTGCCAACATCCAAAACAAGAATTTACAGACATGCCTACCTG
TGCAGATATCACAGAGCTCCTCTGGAAAAGACTTTGCAGAGACAACGACAAAATCCGCTA
AAGCTCCGATGGTCTGGACATGCAAGGCCTGCAACGTGACGCTCGAATCCGAAAAGGCAT
TCAAGAGCCATCGAAAATCCCACGTCAGGTGCTCGGATTGCTCCTTCGAGGGTGCTCCCA
AAATTGTCAAGGCTCACTACCAGGCAACTCACGGCAAGTTTTCGGGATCGGGCTTCAAGA
CTATCACGGTGGGAGTCCCAGGCTGCCGTCCCCAGAAATTTAATATCTGCGTCGGCAATC
GGCCCGAGGACATAGAAAGATGGATTGCGGAGCGAAAAAAGAGGTTTCCCCGAAAATCTG
CTTCGGCTACGGAGCACAAGCAGGAAGGGACTAAGCAGAAAGTACACGGTGCCGAAGTAA
GGACAGCTGCTCAAACAGGCCTTTCCTCTTTGCTGGCGGGATACGGATCCTCATCCGACG
ATGACAGCGAGAACGAAAAGAAAGTGGAAGCCGATGGCAAAGAAGGTTCTGTATCTTCGA
GTCACGTTGTGGAGAATGCAGTTGGCAGCGACCATACTGAACTCAACAACAGAGAGCAGA
CCAACTCGGGTCCGATCCCGCCACCAGAGAAAACAAAGACGCGCCGGCCTTGTCGTTTCT
TTTTCAGTAATGGCTCTTGTCGTCATGGCAGTAATTGTCGATTTAGTCACGATGTAGAAG
AAGGACAGCCACTACAGAGCCATTCAAAACAAAAAAATCGAAGCCAGCCTTCCAACACCA
GCAATAGGAACAACAACCACAACAGTCGAAAGCGAAAGCGCGGGGGGCATACGTCATCGG
ACACACTCCTTCGCAAACTTTTCGAAAATGATATGGAACGAGAATCATCCATGACAATGC
AACTGCTGAAATTCATTGTCGACAACAATTTTTTCCTAGACAAGGATACCAGTGCCAGGC
CAAAATAACATCGCGAAACCAATGTCCTTCCAACTGTAGGGATGAATACTAACCCAAAAC
GATAATATTGTGACCATCGAGTGGCCAAATGATAGGTTCTTGAACAATCAGAAGACAGGG
AAGAAAATGAGGGTCGTTTTTTGCATTGCGCAACTCAAACTAATAATCTTCTCTTTTCAC
AGGCTGCGTATTTTTAGATGATTTTCTGAAAACAGATCGCGTAGAGCATCATTAGAACCC
CAAATTACTATTACTAGTTGCTAGCGAAATCTCTAGGTGTACGTTTGTTTCTTTGATTTC
GAACAAATTTTGTGATTCGCGGCTCTGTTCATTTGTTCTCCAATACATTAACCAATTTTC
GGAGTAACAGAATCATTGCGCAACCG
>comp2688_c0_seq1
CACAAATGCACTTGTATTGAAGAAAGTGGGAACCTTGGACGCCACGACACGCAAGCCGGA
AAACAAATATTGACAGAAGCGGTCAACTTCACAAGAGCACCAAACAAACACAACCATTGC
TTCCTACAGAGAATAACCACAGTTACTACTAGAATGCCACCGAAATCCTCTGTATCGACA
ACCTCGATTTGTGAAATGAACATGAAGGCTGCAGCTGATTACGACCCGAAGGCCGTTACC
TCCATGTTATCGGAAGAATTGATGAAACTGAACTCTGAGGATCGGAACCAAATAACGGAA
GAGATGCACGGAGTCCGAAACATGTCGCTGCAAGAGACACCCGATGTGCAAACCAGAGCA
CTAGGAATGCTGCAAAACCAATTAGACCGGATGCCACCGTCGAAAAAGGCCGCGTATGAC
AAGGCGCAAGGCCTCGAGAAAACCTACGTGAACGACGATAAATTTCGGCTTCGTTTTCTC
CGGGCGGAGCTGTTTGATGTCGACCGGGCCGCGGAACGAATGACGTCGCATCTGGATTTG
ATTCTGGAGTTGTTTGGCGATGAGGTCCTCGCGAGGCCTCTGAGGACCACTGACTTTAAG
GGAAAGTTAGTAAAAAGGATCCTGCGAGGTGGTCTAGTACAATTGCTCCCTTACCGAGAC
CGCAGTGGACGGCGGGTGATGGTGATTCTCTCTGACGTTATGTCCTATAGTCACATCGCG
AGGGTCAAGATATTTCTTTATGTCTTGACGGCTGCTGCGGAGTGCGAAGAGACGCAGAAA
AAAGGGGTTATATTCGTCATGTGGCCGGGGACGGATCGAACGATTCGGATTCCGGAAACA
GAAGAAAGGGCCATCTGCAGAAAGTCATTTGCGGCTTTTCCTCTACGCGTGGTCAGCGTT
CACTTTTGCTGGCCGGACACGCATTTCTTCCATATGGTGAAATACTTTTTCGTCCTTGTG
ATGGGACCTTCTCTTAGGGTTCGAGTAAACTTCCATAGTGGAGAACGTCAGGAACTAACA
TACAAGCTCATGGGATTTGGTATCCCAGTACAACTGCTGCCAACAACGGAAAGTGGTGCC
ATCAAAACAAAAAACCATACGCAATGGTTGAAAACACGATGTTTTTTCGAAAAGAACGCC
GGCAAGAATCCGACCACAACACCCGGCGAGCTCATTGGTGCCATTGAGTGTCCAGCCCTA
CACGATGTTCTCTACGAGAGAAGCAAGCCATGTACTTTTCACCCTGGAAATTCTTTCTTC
AAGGAACTAATCGAGGAAAATAAAGAAGAACATGCAGTACTGTCGCAAACCGGAAAGCGC
AATTTCTCGTGGTCCATCGTCGATAAAGTGGAGAAGAACAATGGTCGCTTCTTAACGTGG
GATCGACGAGGCTTTTGGATCAAGCTCGACGACCGATCTGAGATTCGATTGAAGGTGGCA
ACATCTCTGAGGGACTTCAACAAACACGCTCGTGCCCTGGCCAAGTGCCAAACAGTATCG
AGTGTCTGCAAGGTCAGTTTCGACGATGGCCAGTATCTCAAGAAAAGGCGGATCGTTGTC
ACCGACGACGAATCCTCGAATGTTTCTCTTTGTGAGAGGGCATCAAATTGCGAATCTTTT
TATGGAACCTGCCTCCCAGATTTACCAATATTCAGATCGAAGGGATACGACGAAACGAGG
AATAGGTTGACCTCGGGGTTTCCAATCGACGACATTTCTCTTCTTTCATATACCCCGAGG

AGTGCATGATTCAGGCTACCACAGTGCATATTTTACAGTCACTTCACATAAATATTATAC
TTGGCTCGGTATCTTTTATATCTGATTGCATGAACGACAGCACTTGTACCATATTTTTAA
ATTATGTAATCAAAATGTATTTATATTTTAA
>comp2688_c0_seq2
CACAAATGCACTTGTATTGAAGAAAGTGGGAACCTTGGACGCCACGACACGCAAGCCGGA
AAACAAATATTGACAGAAGCGGTCAACTTCACAAGAGCACCAAACAAACACAACCATTGC
TTCCTACAGAGAATAACCACAGTTACTACTAGAATGCCACCGAAATCCTCTGTATCGACA
ACCTCGATTTGTGAAATGAACATGAAGGCTGCAGCTGATTACGACCCGAAGGCCGTTACC
TCCATGTTATCGGAAGAATTGATGAAACTGAACTCTGAGGATCGGAACCAAATAACGGAA
GAGATGCACGGAGTCCGAAACATGTCGCTGCAAGAGACACCCGATGTGCAAACCAGAGCA
CTAGGAATGCTGCAAAACCAATTAGACCGGATGCCACCGTCGAAAAAGGCCGCGTATGAC
AAGGCGCAAGGCCTCGAGAAAACCTACGTGAACGACGATAAATTTCGGCTTCGTTTTCTC
CGGGCGGAGCTGTTTGATGTCGACCGGGCCGCGGAACGAATGACGTCGCATCTGGATTTG
ATTCTGGAGTTGTTTGGCGATGAGGTCCTCGCGAGGCCTCTGAGGACCACTGACTTTAAG
GGAAAGTTAGTAAAAAGGATCCTGCGAGGTGGTCTAGTACAATTGCTCCCTTACCGAGAC
CGCAGTGGACGGCGGGTGATGGTGATTCTCTCTGACGTTATGTCCTATAGTCACATCGCG
AGGGTACGTCGTGCGTAGCGGTGTATCATTAAAGCGGAGTTTGGGTTGCAACCCAGCGAT
TTCCACCAAATATCTAACATTGTATTATTACCATACCGTGCTGCTTTCCAAATAACTTCA
CAGGTCAAGATATTTCTTTATGTCTTGACGGCTGCTGCGGAGTGCGAAGAGACGCAGAAA
AAAGGGGTTATATTCGTCATGTGGCCGGGGACGGATCGAACGATTCGGATTCCGGAAACA
GAAGAAAGGGCCATCTGCAGAAAGTCATTTGCGGCTTTTCCTCTACGCGTGGTCAGCGTT
CACTTTTGCTGGCCGGACACGCATTTCTTCCATATGGTGAAATACTTTTTCGTCCTTGTG
ATGGGACCTTCTCTTAGGGTTCGAGTAAACTTCCATAGTGGAGAACGTCAGGAACTAACA
TACAAGCTCATGGGATTTGGTATCCCAGTACAACTGCTGCCAACAACGGAAAGTGGTGCC
ATCAAAACAAAAAACCATACGCAATGGTTGAAAACACGATGTTTTTTCGAAAAGAACGCC
GGCAAGAATCCGACCACAACACCCGGCGAGCTCATTGGTGCCATTGAGTGTCCAGCCCTA
CACGATGTTCTCTACGAGAGAAGCAAGCCATGTACTTTTCACCCTGGAAATTCTTTCTTC
AAGGAACTAATCGAGGAAAATAAAGAAGAACATGCAGTACTGTCGCAAACCGGAAAGCGC
AATTTCTCGTGGTCCATCGTCGATAAAGTGGAGAAGAACAATGGTCGCTTCTTAACGTGG
GATCGACGAGGCTTTTGGATCAAGCTCGACGACCGATCTGAGATTCGATTGAAGGTGGCA
ACATCTCTGAGGGACTTCAACAAACACGCTCGTGCCCTGGCCAAGTGCCAAACAGTATCG
AGTGTCTGCAAGGTCAGTTTCGACGATGGCCAGTATCTCAAGAAAAGGCGGATCGTTGTC
ACCGACGACGAATCCTCGAATGTTTCTCTTTGTGAGAGGGCATCAAATTGCGAATCTTTT
TATGGAACCTGCCTCCCAGATTTACCAATATTCAGATCGAAGGGATACGACGAAACGAGG
AATAGGTTGACCTCGGGGTTTCCAATCGACGACATTTCTCTTCTTTCATATACCCCGAGG
AGTGCATGATTCAGGCTACCACAGTGCATATTTTACAGTCACTTCACATAAATATTATAC
TTGGCTCGGTATCTTTTATATCTGATTGCATGAACGACAGCACTTGTACCATATTTTTAA
ATTATGTAATCAAAATGTATTTATATTTTAA
>comp2691_c0_seq1
AGCAGTTCTCCAGATATCCGAAACGCAAGGACGCAGCAATGCAAAAACTTGTCACGATTC
CTGCTGTCTTCTCCAACAACTCCCACCCCAGGCAAACAAGTTTTCTGGAATCCGTCTATG
GCCAACACCGCGTACCATAGATCAACCGGGACAATGAATGCTCTTTCTTCCGTACCATGC
CGCCGTAGCTGCGTTCTTTTCCTGTGCCTCTTGCTTGGATTGATTGCTACGTCCGAGGGA
TCGACGGATCCGATCGACGGAGCTAGGTTCCATGCCAAGAGAATGAGCCGCAAAGAGAGA
ATGGCTCTGAGGCAGAAGGAAGCGGAACGTTACGCAGAACTCCGTGCGTCGGGCCAGGAC
GAAGTCGGACACACACCGGAGGCAGCCGCAATGAGACAGTACCAGCTGCGAAAGAAGATG
AGGAGGGGGGTCTTTGGAAAGCGCAAGACGGACCTGGCGTCCATCCTCTTTCCGGGAGCG
ACCCCGGAACCATACATTGCTGGTGAAGAAGTCCTGATGTACACGGACAACGTAAACAGC
AGGAAGACCCAGGTTCCCTACGATTTTTACAAGCTGCCCGGCTGTGCCATGCCCAAAATG
ACCGTCTCTCGCAGGAGAAGAGAGCGCAAGAACCTCGGCGCCCGTTTGCAGGGCTTGGAG
CTCAAGCCCGCTCCATACACCATGAAGGTAAAGGAGGACTATGGCTGCACTCCCTTGTGC
CAGAAGATGATCACCCCCAGGAGGGTCGGATTTTTGAGACAGTTGATCGAACGCCAGTAC
AGAGTTCACATGACCTTTGATGGTCTTCCGGTCCTCATGAGAAGCGAAAATCACAACTAT
GCGATTCGAGGCTTTCCCGTTGGTTTCAAGGCTCCTCCAAGCTACACAGGATTGAAGGTG
GAACAGTTTTATCTCTACAATCACTTGAAATTTACCATCACCTACCAGCAGATGGACACC
GGCGAATTGCACATTACAGGATTCGATGTGGACCCTGTCAGTATCCGGCACGAAATTCAG
CAGGAGGACTCCTTCGTTGCCATGTCCCAGGAAGTTTCTACTTGCAATACCGACGATGCA
CCTCCCGTCAAGAACGATCTGGAAACGTACCTGGCTCTTGACAAGAAGGCCGGTGCCGAA
GGTTTTCTCCCCGTCATTTATTCTTACGAAGTAGAATGGAAAGAATCGACTCTTCCCTGG
ACCGACAGGTGGGATATTTACCTCATCGGAAGCCCGGACGACGATATTCACTCGTTTGCC

ATTATCAACTCCCTTATGATCGTCCTTTTCTTGACGGGAGCAATCGCCGCCATTATGATC
CGAACTCTGCGACGAGATATCGCTGGATACAACCAAATGCAACAATTCGAAGAATCTGGG
GAAGAAACCGGATGGAAATTGGTCCATGGCGACGTCTTCCGGCCCCCGGCTTCCTGCCCT
ATGCTTTTCAGCATTCTGGTGGGCACCGGTGCACAAATCGGATTCGCTTGCTTCCTCACC
ATGATGTTCTCCATCGTTGGATTGATCAGCCCAATGAAGAAGGGCCAGATGCTGACCGCC
ATCGTTTTGCTGTATGTGTCCTCTGGAGCCGTGGCCGGCTATATTTCGGCTCGTGTGTTC
AAGTATTGCGACGCCAAGGCCTGGAAGCAGAACACCTTTCTCACGGCCGCTGGATTGCCC
GGAGTTCTCATTGGCATTTTCACCGTCCTCAACGTCTTCCTTCACGTTGCCGGCGCGGCT
ACGGCCATCCATTTCTTTACGCTTTTGGCTATTTTGGGACTTTGGATTGCAATCGCGACG
CCGATGGTCTTCGTGGGCAGCTACTTTGGTTTCAGAGCCGAAAAGATGCCCGTTCCCGTA
AAGGTCAACTCCATCGCACGGTACATCCCCCAGCCTCCCTGGTACGCAAATCCCCCTATG
AGCTATATGCTAGGAGGGGTCCTGCCCTTTGGTTCCGTCTGCATCGAACTCTTTTTCATC
ATGAGTGCTCTCTGGCTCCACCAAGTGTACTACATGATGGGCTTTTTGCTTGCGGTGCTT
CTGATTCTCGGAGCCACCTGCGCACAGGTCTCCGTCGTGATGACCTATCTGCAGCTCTGT
GCCGAAGACTACCACTGGTGGTGGAAGTCGTTCTTCAACTGTGCCAGTGCTGGTTGCTAT
CTGTTTTTGTACAGTTTGTGGTTCTTGGTGAGCCGGCTGGATTTTGTCGGAATCCTTCCG
GTTGTGGTCTACGTCACGTACATGGGGATGATCTCTCTCTGCTTCGGTCTGTTCTGCGGT
TCCGTCGGTGTCCTCTGCAGCTTTTTGTTCATCAGGAGCATCTACGGAGCCCTCAAGGTC
GACTAGTCCGGTTGCTGTGTTTTCGATAGGAAGTATAGCACTGTACTACAAGTTTGTACT
GTACAGATGAGGTCTACATAAAATTCAATGGTATTACGGGAATGAAAGACCCGGCATTGC
GTACTGTATTCTCCAGTGCATTTCTAGTGGTGTATGTAATATTGTAGTAAACACCACAAC
AATTAAACGAATGCGGAC
>comp2692_c0_seq1
CTACGATTCAAACAAGGAAAACAATTTCGTTCCCAACATGGCCGACGAGGATAGCAAGGA
GCAACAGCATGTTTACGTGAAACTTGTAAGCGCTGAGGGCCATGAAACATTTGTTGACCG
AGCGATTGCGATGGGGGCCAGCGCCACCATCCGCACCATGCTGGAAGGACAATTCCGCGA
GGCGCAGGACAACGTTATCCGCTTTCCAAACATTACCACAGCAATCCTAGAACAGGTTGT
CCGATATATGCAATTTAAAGCCAAATACACCAACTCTTCTTCTCGGATTCCAGAATTCAC
CATCGAACCGGAATACGCTCTCGAACTTTTGATCGCATCCAAATATCTTGATTGTTGATT
TTGCAACCTCGTTAAGTGTCCAGCAACAGCGAAGAAAGAAGGATAGGAATTGGATTAGAT
GCATCTAAAGACACAAAGATTTATGAAATGATGAAAACTTGTATGTCAACATTGCTATTA
TAATATTTAACTGCTATGGTATTTCTAATTCTACTCGTTCACGACATGGGTGCTTTTCTT
TCAGAGGGAAGAAAACGATTTTCAAGAAAAGAGTGTTGATAACGAATCACGGCTGTCTTC
ATTCAAAATGTGCTTCACGCTTTAGTATTCAATCTTCACATATTGCGTGCTGGAATTTTT
TTTGATAGGGCTTTCATGCTGATTGCTATGCGTACGATCATTGACTGTTACGAAAAAATA
TTGTACCTGCTCTAAAAGTCTCTGTTGCTGTTTTCTATCCTCTATGTTCTAAATTCATTG
ACTCATTGAGCTTCCGAAACTAAGTTGTTAA
>comp2693_c0_seq1
AGTGAATACTTTGGACTCTTGGAAAAAGGATTGAATCTAAGCATTGTCAGAGAGAACTGA
AATTGTGACCTGTAGTGTGTAAAGAACTTTGAAGACAGCAGCAGCGTAATACTGGCACTA
GACAGACAGTGCCCGCAAGAACAGACCGTCACGTCCATTAAATTTTTCGAATCTACGAGC
AGACCGTCGCAACAAGTAGAAAGATGCCGTCGGCGAACGACTACAATGACGAGCGCCATA
AGAAAGAAGAGATGGCCCGTCGGGAAAAGAGAAAGAGCGCCAAATCTCATAAATCAGATG
CACAAAGAGAGAAAGAAAGAAAAAGAAAGGAGCACATTGAAAAGACTCTCCGAAAGCACG
GAGGTGAACGAAAAAAGCAGAAGACGGAACCGGAAGCAACATCCTCTGCGTTTCAGTCTC
TCTTCAAACAGAGAGCACAAGGTTTTGAGATTGCATTCAAGTTCCGAAATGCTCCTCCTC
GTCCTCCAGTCGGTCCCTGTTTTATCGGAACAAATCTTGACGAAAAACTTAGAGAGATTA
GTCAGTACAAGCCTTTGAATGCTGTCGAGGTCAATTATCGTTGGAAATTGCACAGTGAAC
CCGACTTGGGTGTGCCTCTGGCTCCATCTGCAATGGACCCAACTTCATACAAACAGAACC
AAACTGAGGAAGCCCAATTGCATCCTGACGATCTGAAGTTGCTAGAGTGGAATGGTTCTA
TGGCAGATACAGCTGCCGAACAGTTGAAGAAGAGACGTGACAATGCACGTGCGATTGCGA
GGTTGGCCCTAATGGGTAAATCCCCAGCCAAGTTTCTCATGGAAAAGACGAAAACCACAA
GATCTGGCCCAGCTTCGACAAAGAAAGCTTTCTCTCGTGTATTGGATGAGGGCATGCAGA
GTTGGATGAAGAAGACTACCTATATCACTAATGACTACGGCCGAAAGGTACACGACTTCA
AGTCGCTTGCCAAGACGAAACAAGAACTTGAGCGAGAATTGGAGGTTCGACAACAAACAA
TCATGCAACGTCGATCCGCTCCTGTTGTATCGAGGACTTTTGAAGAGGTTAAGGCAACCG
TTCAAAATCACCCCAACAAGAAAGGAGTTACACCAGTGGCCGAAATGTCATTTTTTGCCCA
ATATCGAGAAATGGGGCAATGCGTACACGTATGTTGTCGTTGACAAAAGTCCTGGTGATA
ATTCAACGTTCCTGAACAGTTCGATTGTCGCTAATGTCCAGAAGAAGGATGCTACTGCAC
GTATGACATGTGAATTGTTTGGACAATCTGAGAATGATTCCGGATCCTACCTGGGTATGC

AAAACTACGAATTGGATGTCGTTCCTCTGAAGGAAGGGGATGCACCGCATGCTAATTTCT
GTATATGGGTTGATCCAGAGAATAACACAGCATTATACACCCCTATTTCTTCGCGAGTTC
AACTTTCGAACGGAAGGCCAATCCGAGGAAAGAATTACAAATTGAACGTTACGAGAAGAG
CAATGAATGATGAGGATATGGCGGAGATTAACGAACGCTTGGCTGAAGTCGATGCCGATG
TTGAAGAAAAAATAAATGGTTCGAATAGTGCCAACAACAAGTCAGGAAACAACATTTCAA
TTAATGACGATGATGACGATAGCGATGAGGATATGTAAACCTAATGGACACTTCTTGCTG
CCTAGGGATATATTATTTGAAGTCATGCCACGGCGATAGAGAGTATCACGATCGAGCTCT
AAACGGGATAAGAATATAAAAGTAC
>comp2694_c0_seq1
GGCTTGCATTTATTACATATCCATAGGTCGGAGAAAACAAATAACAGCAGCACTGCTGGG
CAAGAAATCCGAAGGATAGTCGTCGTCGACCGATAAGAAGTGGAGCGCGAAGTTGGAATC
AAGATGTGATCTTCATTTTGATACTTAGCAAAGCTAAAATCTGTTTCTCCGAAAAACGTT
TCTGGTTTACGTCTCAAACGGCGATTCAATGGTTTTTTTTTTCCACGATAATTGATAGAA
ACCGCGTTCTATCAGCCACGACAACAAGGCGATACACTCATCATGATTGATAATACGAGG
GAACTATACTCTACATGCCCAACTTCACGATCTACATCGAGCTTGTCTCATTTCAGATGA
AATACTCTTTTCACAAGAAAAGTTAATTTAGTTGGTTCCACATCAGAACGAAAAACTTCG
AAAGAAACAATACCTAAATTACCACCATGAAATCATCATTTACTATTCTACTCGTCTCTT
TGATATTCCAAGCACTTACGAATACATGCGCATCATTGGAACAATCATTGTCGTCATTGT
CTCGATCAATGTCTAAGGGTAAGAGACTCAAAGGTGTTCCTCGCCAAGAGCACAGAAAAA
TCCTTCACAATGTAAAATGTATTCTATTTCGAAAAGAAACCCGATACTTGTCGACCAAGT
CTACAGATCCTCAAGCAACAATGGTTGCCTCCGAAGAAACCTGGACATGCGAACTAGAGA
ATGCCAGTGGTCACGACTTTGTCCCTGCCACTGTGGAGCTCAAAGGCATTAACAACCAAT
TTCTTGAAAGTCAAGGCGCCAAGTCTGGCTTTAGCGCCTTGGTTGCTACCGAAGGCATTG
TCGAAGGAGGCGACGGGACAATGGCGATGACCATTCCACCAGCGGCACTCGTGAAGGTCC
TCGAACAACCTACCACTCTTGGCAACAAGCTACGACGACAGAGTGGGCGAAAGCGGCGTA
ATCTGGCTGCTACCTCTGGTACTCTGAAAACACTTGTGGTTCGAGTGATCGCGAAGAACG
GGATTGGACCTATTGCGAACACCGTTCAACTGCGATCCGACGTGTTTGAAGATAAAGTCT
GCCTCAAGTCGCAATACGAAGCTTGTTCCCACGGCCGACTTAAAATCGAACCATTCACGG
GCTACACTGAAACGAATCGGTACATCAAAGATGGTGTTGTCAGCGTAGGAGTCGACGTGG
CACCTATCGAAGGAAATAAAGACAACTTCGAGGTTGCGGCAAAAAATAGGGCTACAGAAA
TTTATGGCGATCTCGGATCACAATTTGATCTTGTCATGTTCTGCATTCCGCCCGGAACGG
GTCCATGGCTTGCCTACGCCTACATCAATCGTTTCGATTCTTTTTTTAATGACGAATGGT
GTTCCAGTGTTAGCACACAGGTGCACGAAGTCGGACACAATCTTGGCCTCGCCCATTCAG
GAAAAGATACCGATGTTTACGGCGACCAATCTGGTATGATGGGGTTTAGCTATAAGGACG
ATGACTTCCCGCTCATGTGTTTCAATCCAGCCAAGAGCTACCAGCTCGGATGGTACAGAG
AACAGCAAGATTTTGTGAATCCCCTGACGGACATTTTGCCCATGAAGTACTCTCGAGAAT
ATATATTGAACGGTGTAGACGACTTTGATAGAGGCGAATCTGGCGTGAAGGAGGATCTGA
TTATTCTCCGACTGAAGCAGCAGAACTCTCGGGAAGATTACTACATTGGCTTCAACCGTA
AAAAAGGCAGAAATGCGGAAACAGTGAAGGACGCAAACTGCGTGACAATTGTAAAAAAGA
CGAGTGGTCCTAATGAATATGGCCAATCTTGGCAAGTATCTACGCTTTGTGAAATTCAAG
ATTCATACACAATCAATAACTACGACGAATCTTCATTCGATGTCACCGTGAAACTAATAT
CCATCATCGGTAAAGATGCCAAAGTAGATATCATCGCTTCAAATTCGGCTCTATGCGACA
CTGTTAGAGATTCAACGAGTCTCCGTTACAAAAATAAAAAGAGACGAAACTGCAATTGGG
TATCAAAGAGAAAGTCGCGTAGATGTTCTAAAACTTGGACGAACAGGCCGCTGTCTGAGT
GGTGTCCGCTAACATGTGGTTCTTGTTCGGGACAATTGAATATTTATAGTTCAGGCTTTG
GAATGAACGATTCTCTTCCCAGTACTTTGTGCGTGGATGCTCAAAATGTATCGTTTCAAA
ATAACTTTGCAAAGAATTGCGGTTGGGTCGGAAAAGAAGAAGAGAAGAAGGATGAACGCT
GCGCGAAGGAATGGCTTGGAATAGCACTTTCTGAGTTTTGCCCGAAAACCTGTGGTTCTT
GTTGAGCTTTCTAAAATATTCATTACGATCAGAAATAGTACAAAGTATTTTAGCATTTGA
CAAGTCCTTAAATATATTAATTAAAAACATCGTTTGGCATTGAAACGTATTAAACGCTGT
TGTCATACGGTCATTTCTCTTTCGCCACTAACAGGTATTCCACTCATTTACTTCCAGCAT
CTAATAGGAGTATCTAACATAACAAGAAACAAACTGAATAAGGAAGAGC
>comp2695_c0_seq1
CACCTCCTCTACACCGAATTTCCACCATGGCCGCCCGTCCTCTCGTTAGCGTTTTCTCCC
TCTCCGGAGACAAGTCCGGAGAGACCACTCTCCCCGCTGTCATGACGGCTCCTCTCCGTC
CCGACATTGTCCAGTTCGTCCACACCAATGTGAACAAGAACAAGCGTCAAGCGTACGCTG
TCTCCATCCGAGCCGGAAAGCAGGTTTCTGCTTCCTCGTGGGGTACCGGACGTGCCGTTG
CCCGTATTCCCCGTGTCGGAGGAGGAGGTACCTCCCGATCCGGACAAGGAGCTTTCGGAA
ACATGTGCCGTGGAGGACGCATGTTCGCTCCCACCAAGACCTGGCGCAAGTGGCACAAGA
AGGTCAACATTGGCCAGAAGCAATACGCCGTCGCCTCTGCCTTGGCTGCTTCTGCTGTCC

CTGCCCTAGTCATGGCCCGTGGACACGTTGTCGATGATGTCCCTGAAGTTCCTCTTGTCT
TGGACACCTCTGTCGAATCCACTAAGAAGACCTCGGCCGCTAAGGACATCCTTTCCGCCG
TCGGTGCCCTCGATGATGTCGAGAAGGCTGGAGACTCCAAGAAGATCCGTGCCGGTAAGG
GTAAGATGCGTAACAGACGTTACGTCATGCGACGTGGACCTCTTGTTGTCTACGCCGAGA
ACGATGGAATCGAACAAGCATTCCGCAACCTTCCCGGTGTCGAGCTTTGCTGCGTCGACC
GCTTGAACATCTTGCAACTCGCCCCCGGTGGACACATGGGACGATTCTGCGTCTGGTCCC
AGGCCGCCCTCGACAAGTTGGATACTATCTACGGAGAATCCGGAAAGCGCATTCCTGCTG
CCGCAATGACCAACGCCGACCTTGCCCGTATCATCAACTCCGACGAAATCCAATCCGTCC
TCAACCCCGCCAAGGACGGTTCTGCCACATTCGAACCCAAGGCCAACCCTCTGCGATCCA
TTGTCGCTCTTGAGAAACTTGATGCCCACGCTGCCAACAAGAGACGCATGGCCCAACAGG
CTGAGAAGGACCGTGAAGCTAACAAGGCTGCTCTCTTGAGCAAGAAGCGCGCTGCACGAA
CTGCCAAGAAGGCCTACAATGCTCAAGGAAAGGCTTTCATCGCCGAAGCTTCTAAGCAAG
GAGATGTTTGCGCTGACGGTTTCACCATTGAGTAAATTATCTTGAATAGATAGTGGAATT
GTATAACCCCCTA
>comp2696_c0_seq1
AAAAAATACCAAAATGAAGTTTGGCGTTGCGACAAAGTTGTTACTTGCTGCATTGGCAGC
CGGGACCATGCCGGCTATCACAGCACAAAACTGTACGATTGTTCTCCCCGAGATCACCGA
AGTCATGGAGCTTGCATTCCCAACCTGGAACGTCGACAAAAAGGGCAAAAGAGTAGAAGG
CGGGGGCAAGTGGAATCCGAATTATCTTACCAAGAGATGGAACGGTCTTGATCCCGCCCT
CGGTGGATACCCAACTCCTATCGATACCAGATACCCATTTGAATTTGCTGCCGCATTTAA
AGGACAACCCGGAGCTGGATCTCCACACCACTGCCCCGAAGGCACAGATCCACTCACCCC
GGTTCAGTCATGCCCTAAGATCATCACCGATAATGATGATGGCCCATATGGCATCGGACA
TGTCCCACCTCCCATTGCCCTCGCAGTTGTGCGCAACAATCTTCTTGATTGCGAGAACGA
TGCAGACATTGAGAATTGGTTTGATTTCGAATCCGAAACGGCTCCTTGCGACATCAAACC
ATCTGTTCTTGCGTCAATGATTCGAACCAAGTACCCTCGTGACCCTGAAACTGGTGCTGT
CGATTACCCTCCTCCAGTCATTGAAGGAGAATATGAATACTACGAACTCGAATTCCCCAG
CCCACAAGGACCTCCCCACTGGTGTACGGACGAATTCCTCGCAACTGGACAATGGGTCGA
TTACTGCCCTTACATTTTCGAAGGTCCCAACGCAGGACAGTACCGCCACCCCCACCTTGC
CTTGTCGGCAGTCATGCAATTCGTTGCGAACAAGATCAACCCCGAGGTCTGCGGCCCAGA
ATGGGACGACCGTGGTAATTATCCCGCAAACCCTCCTACCTCCATCGCATGGTCGGTAAT
GGAGTCCGAGGACGGCGATGCCCAGCCCGCTCTGCCCTATTTGTGGCCCGAAAATGACGA
CCCGCTGATCAAGGATGTTCCTGGATTGGTCTTGTTCGATCCTGCCGCGGAACCTCCGGC
ACCACCTACTCCCCCCGAACCGCCCTCTGAGGACGAGGTTACCCCGGTCCCAGAACCACC
TGCCACCGAAGCCCCAGAACCACCTGTCACCGAAGC
>comp2697_c0_seq1
CGTATGGCGGATGCCTAGGCTCTCAGAGGCGAAGAAGGACGTGATAAGCTGCGAAAAGTT
ACGGGGAGAAGCACATATTTTATGATCCGTAAATATCCGAATGGGGCAACCCGGTATGTT
GAAGACATATCACACCGTAAGGTGAGCAAACCCGGCGAACTGAAACATCTAAGTAACCGG
AGGAAGAGAAAACAAAAGTGATTCCGTTAGTAGTGGCGAGCGAACGCGGATTAGCCCAAA
CCAATTTTGTTACGGCAAAGTTGGGGTTGTAGGACCACGATATTCGAATCTAAGTGAACT
AGAACTGTTTGGAAAGACAGACCAAAGAGGGTGATAGTCCCGTATGGGTAAGCGAAGATA
AGATAGTGGTATCCTGAGTAGCGCGGGGCACGTGGAACCTTGTGTGAATCTACCAGGACC
ATCTGGTAAGGCTAAATACTCCTGAGAGACCGATAGTGAACTAGTACCGTGAGG
>comp2697_c0_seq2
GATTGATTTCCGTGCGAGACGGAAGAAGTTCTTTGACATATTGAGAGTGAATACATGAGA
TCGAGTGTAAACTAGAGATAGTTTATATCGTAATAGACATTTAAAAAGCGAATAAGAGCG
TACGGCGGATGCCTAGGCTTTCAGAGGCGAAGAAGGACGTGACAAGCTGCGATAAGCTTC
GGGGAGGTGCACATAACCGTTAATCCGAAGATTTCCGAATGGGGCAACCCGGCTAGTTGA
AGACTAGTCACCGCAAGGAGCAAACCCGGGGAACTGAAACATCTAAGTACCCGGAGGAAG
AGAAAACAATAGTGATTCCCTGAGTAGCGGCGAGCGAAAAGGGAATAGCCCAAACCAAAG
TTGTTTCGGCAATTTTGGGGTTGTAGGACCACGACATCTGTTGTTCAACGAACTTGAATG
AACTGGAAAGTTCAACCATAGAGGGTGAAAGTCCCGTAAAGGTAAGTTAGAATAAACGGT
AGTGGTATCCTGAGTAGCGCGGGACACGTGAAATCCTGTGTGAAACTGTCAGAACCATCT
GATAAGGCTAAATACTCCTGAAAGACCGATAGTGCACTAGTACCGTGAG
>comp2698_c0_seq1
CAACTTGGCTGCTGTTTCTGTTTCTTTCTCATCCGGCACAATCCATTGGATTTATTGAGA
CACCAGGGAAAAGAAATGCATTTTTTTCAAAACAGGAATCGCCCTCGCTCGTCCTTTGAA
TTAAAGGTAGGGAAATCCCATTTGCTCGAAGTTACCGTCCATCTCGTCACGCGCAGACGC
GATGTTGCCGTCCGGCGGAAAGTGAACCGCAACCCGGAATGGGACTGGTACAATACGAAC
AAAGATGACATCCACAGAGAATTCTTGGAATTGTTGGAGGCATCGGTGCTGTCGAGAATG

TTTGGAGACGAAATCGAAGGCTACCATCGGAAAAAGAACCCTTCTGTGATGGAAGGAAAC
AACGGGGAAATGGGCTTTGGGAAAGTAGGATCCAAGAATCGCGTCATTCGGAGCAAGGGC
AACGGCATGGCGAGCGGGACGAGGAAGCGAAAAGGGACGACAGTTCCAAGCAATTCCAAG
AAACATAAAGTCTTTCTCGAGACCAACAATGATGGGGAAAACAATAGCAAGCCCGAGAAG
GACATTTATTTTTCCTTTGGCGAATTGATCCAACTCGCATACAAGAAAGAGCCCATCAAA
GACGACAGAAGCTCGCGAACCATCATTTTCGAGTCCTTACCATCGAAATCCAATAGAAAG
ATCGCTGATGGACACAACGGCAACAGCGAAACAATAACGCGGGCGGCGGGAGGAGGAACA
TTTCTCGACCGGCAGAAACTCTCCCATCGACTGCTTGTTTGGATTGGCAAAAGCGAAGCA
GCAGCTTCTGCGGGTGGTGGACATTCACACGCCACAGCGGCCGAGGGAATGTACCGAAAC
GAAATGGTTCCCATTTCGAGCCTCTTTCGAAAACCACCTGAGTTTGTGTCTCTGTCGGAC
GACGATGACGGAGACTAGGACAAACGGAGTGGACAGCGGTCGAGGACCCAATCTGCTTCC
CATCCCCTTCTTGGTATCTCCCAGAAACACTTCTTGAAAAATTGTCTACGGGTATGTAAT
ATCTGTTTGGTATACCAATCAGTTACTACCCAATATAGCGTACGAGACAAGGCAAAACAA
TACAAAAATTAACTGGCCGTTCTTTTATGTCATTTCAGCATTTGGTCCGGTCCACTCGAA
TAAATAATATACGACCTCTTATTGATTACTCTGCCTGCCTACGCTCTTGAGCACCGAGGA
TAATTTAAAACCCTCTCGCTGCTTGCCTCGGCTCTGTGCCCGCTTGTTGACGTGGCCGTT
GATG
>comp2700_c0_seq1
GGCGGTTTGGTTCATTTCGATGGATCAACGTCTGTATATCAACGCTACTAGGGTTTTGGC
CCGATGATGTAGTGTCAGCTTCACTGTCTTGTATGGACTTCGGTTTCTTTGGAAACTGAT
GTGCATCAGACAGATGACTTTCAATGCGGTCGTTTCCTTAGCCGGGTCGACCATGCTGCT
GAGTGATTGTGTGTGGATTTATTCGCACTCAAGTAGCGCAGCGGTAGTGCATTGAAGATG
TGCAGAGGTTCGAACGTCAAGGATAAGCAGCGATGCTTTTCAACTTGAGAGTTTGATCCT
GGCTCAGAACGAACGCTGGCGGCAGGCCTAACACATGCAAGTCGAGCGAACCTTCGGGTT
AGCGGCGGACGGGTGAGTAACGCGTGGGAACGTACCCTCTTCTGCGGGATAGCCACTGGA
AACGGTGAGTAATACCGCATACGCCCTTTGGGGGAAAGATTTATCGGAGGAGGATCGGCC
CGCGTTGGATTAGGTAGTTGGTGGGGTAATGGCCTACCAAGCCTACGATCCATAGCTGGT
TTTAGAGGATGATCAGCCACACTGGGACTGAGACACGGCCCAGACTCCTACGGGAGGCAG
CAGTGAGGAATATTGGGCAATGGACGAAAGTCTGACCCAGCCATGCCGCGTGCAGGATGA
CTGCCCTATGGGTTGTAAACTGCTTTTATACAGGAATAAACCCCCGGACGTGTCCGGGGC
TGAAGGTACTGTAAGAATAAGGATCGGCTAACTCCGTGCCAGCA
>comp2700_c0_seq2
GGCGGTTTGGTTCATTTCGATGGATCAACGTCTGTATATCAACGCTACTAGGGTTTTGGC
CCGATGATGTAGTGTCAGCTTCACTGTCTTGTATGGACTTCGGTTTCTTTGGAAACTGAT
GTGCATCAGACAGATGACTTTCAATGCGGTCGTTTCCTTAGCCGGGTCGACCATGCTGCT
GAGTGATTGTGTGTGGATTTATTCGCACTCAAGTAGCGCAGCGGTAGTGCATTGAAGATG
TGCAGAGGTTCGAACGTCAAGGATAAGCAGCGATGCTTTTCAACTTGAGAGTTTGATCCT
GGCTCAGAACGAACGCTGGCGGCAGGCCTAACACATGCAAGTCGAACGGTAACATGTCCT
TCGGGATGATGACGAGTGGCGCACGGGTGCGTAACGCGTATGCAACCTACCTCTATCAGG
GAGATAGCCCGGAGAAATCCGGATTAATATCCCATAGTATTACTGATTGGCATCTATTGG
TAATTAAAGCTTCGGCGGATAGAGATGGGCATGCGTCCTATTAGTTAGTTGGTAAGGTAA
CGGCTTACCAAGACAGCGATAGGTAGGGGGCCTGAGAGGGTGATCCCCCACACTGGTACT
GAGACACGGACCAGACTCCTACGGGAGGCAGCAGTGAGGAATATTGGGCAATGGACGAAA
GTCTGACCCAGCCATGCCGCGTGCAGGATGACTGCCCTATGGGTTGTAAACTGCTTTTAT
ACAGGAATAAACCCCCGGACGTGTCCGGGGCTGAAGGTACTGTAAGAATAAGGATCGGCT
AACTCCGTGCCAGCA
>comp2701_c0_seq1
ATTTGGAGAGAGTTGTAATTATATATAAAAGCTTTGGAATCAATCATTAAACCAATCGAT
CCGTCCATACTTTTGTCTCAGATCAATGAACGACATGTTGCTCAAACGCTTGATGACGAC
GAATACTACTAGCAGGAAAAAGACGGTGGCAACGACGACATCGCCCTTATCGCTGTCGTC
TCGTCGCGGGACAACCGCAGCGATAACCAAGAAACACAAACGTTACGTTTTGTTTGTTTC
GTGGAAACAGCAATATGAAAAGGATCCGACCCGCACCGTTCTCGCTGCAGCGATGGCAGC
ACTGATCCTTGCCGCGGCGGCAACGGCGATTGTTGAAATTTTTTTGGTGAGTAGCACGAG
CCTTCGACCCAGATCCCCAACTGTTTCCGTTTCGAGGCTTGTTGCGAAATCTATGGCATC
GATCTCGATCTCCTTCGTTAACGGTCGAGGAGTTCGTCGTCCCCGGGGGCATCGCTACTA
TGACTATTGGAGAGACGAGGACCACGAAGATTTCGAACCGTATCCCTGGAAATTTATGAA
GCGCCCTTTCTCTAGCAGCGGGAGCAAGAACGCTGATGTCAATAATTTGGGGATCGCGCT
GGATGACGAGACCTATGTCCCAGAGCAAACTCTTGACAAAAATAGCAACGCCAACATCGA
ACGAAAGCTTACATCAAAGAGCAAGGAGGATGTCATCTTCAACGATCGATCTTCGTTCTA
CGCCAAGTTGCGCCAATCCTACGAATCCTTCTTTCCTCCACCCTATCTCTCAAGCAGCAG

TAGGAACGATGCAAACGCAAAACGGGCCCTCCAAGCCGTCGAAGACCTCCAAACATATAC
TAATTTGCAAACCTACCTTTCAGCATCGAACCCTGTCAATCCAGACAATTCGGATAGCAG
TGGCAACAGCAGTGGTCACAATCTGTACTACGACATTCACAATTGTACCGAAGAACCACT
TCCGGGGTACCCGAAGGAATGGAATCTCCTACATGACGTCCTGGGCCAATGGCCGGCGGA
CGATCCCGAGTTGCCCGCATCGGGGACTCTCTACCAGGGACTGTGTGTTTTTGATTTGCA
AGCGAATCCCGACGATCTTCGCAAGGCCATTGCGTACCGGAACGCCGAGGTCCCCTTTGT
GGTCCGGGGCGATCCGGCCGTAGCCCAAACCGTGGAACGGTGGAATGCTCCACACTACCT
CGAAGCGCTCCTGGGAGATCTCCCACACGGCACCGAGACCAGTGCCTCTGGAAGCTTTAC
CTATTGGACGGCAAACGGGGTCGACAGCCGCAAATATCCCATGGACGCTGCCTCGTACAA
AGCCCCGACTGACCTCACAGAAATGACCTTTGCCAAGTGGATGGCCCTTGCCAACGTGAC
CGATCCGCAATTGCTGGGGCCGGAGTCCCCCCATTATTATTTCCGTGTGGTGGGATGCGC
TCCGGCACGTCCCCCCACTCACCACAACAACGGACACGGGGCCGATTCCTTCGAGGATAA
CACGTGTTCGATACTGCCGGGAAAGGCCCGACACGTTCCGTATCTGGCCGATGAGTTGCC
ATTTTATACACAAACCAAAGCTAGTTTGTACGTCAAAGAGCCGCTGGCGGAAAAGATTGC
CCTGCAAGGTCCCGCCGGCGCGGGATCGAAGGCACTGAACTGCCGCTTTGGCATGAAGGG
TGTCATGACCACGAACCATTTCGATGGGGAACGCAATTTTGTGACCGTCCTGAGCGGAGA
ACGTCGCTACATTCTTGCCCACCCAAGGCAGTGCGGCAACATGGCCCTCTTCCCTTACGG
GCACCCGAGCGCCCGTCACTCTTCTGTCGACTGGGCTCGGCCCGACCTGTTGGCGCACCC
GCGGTTCGAGGCGGCGGAGGGGAACGAGGTCGTCCTACAGGCGGGGGACTCTTTGTTTCT
CCCGTCCCTGTGGTTTCATTACATTGTGAGCATGTCCACTAACATCCAGTGCAACACACG
CTCAGGAATGGACGGAAGGCACGTTTCCGACCTAGACGAGTCGTGTGGGTGGTGAATCCG
TTTTTGGTTCCTGGTCGTTCTTGTTGTTGATGATGAAGTGTAACAACATAGCGATGAAGT
TACCATGTCTGCTGTTTCCACGAGGAGTACTAATTCATTCTTGTTGTTCTTTTGGTGATG
GTTACTACTACTAAAATTTACTATGACCTAGTACTACTACTATCACTACTAGTACTACTG
ATCACCAGTAACGCCAGACATTATCCTCATCTTGTACAGATTGTATTGTACTACTGTACT
ACCTGGTACTGTATGGTGATAGGTTGCTGGTACTGCTTACTCCTTCAGGGTTCAGGGTAT
CAGGGGTGAGATAGAATTAGTCCATCTTGTTGTTCTGTCTCATATGGTAGTACTAGTACT
AGTATTTTACTTTGCAATGCTTACAATAAGACAAACAAATACTACAAGTACCGTAAATGG
AACCAGCATTTCTGGAAATCTACTGATTTGCTTAGAAATATTGTTCATTTCTGGAAATCT
ACTGATTCACTTTTGGGATAAGAAGTAGTAATGACAATAATCTTTTATTCCTACTACTAG
TCTTTTAATTCACTAAAATGAATAGTTCAAAATAGCATGAGGGGGACTCAGGGCTCTTGC
GCTTGCTCCTGTGTTAAGTCATTTAATCCT
>comp2702_c0_seq1
AGCAAACCGGTACGAACCGTGCTGCAGTTACATTTGGTACGATACGAGTAGAACGCTCGA
ACGATACCAAAAGTGCCAGTACTGGAATCTACAGTAACGTACCGTGCGTGCTTTTACTTC
ACCGTTTTAGCTTGTCTCCAGTCTCCATCCAGCCAGGGGACTCACAATAGGACAGTACAT
ACTCAACGGTACAAAAGTACGAGCACGAGAGCGTGAAAACAAATTGTCAGACACACAACC
AAACGAAAAAAAAACAATCGTTTTTGTTCAACCATGGCCAAATTTCCCTCGACCTCGTCC
GCGGAGGAGGACGAATCCGACCACAACGACGAACAGTACTCGAGAGAGGCGTCGGAAGAG
GAGAACATCGATACGAGCGTTTGGTCTCCTAGAAACAAAATCATGCTGCGATGCCTGACG
GCGGGAGTCCTTCTCTACGGGGCATTGGTGGCGTATACGGACCACCAGAATGCCACAGTG
GGAATCCACACCAACGCCAACATCGGTTTCAACAAAGTAGAAAATGAACCCAGAGAATTG
CACCCCCGTCGACGCTTGATGGCCGTTATGGGAGATGTGGTTCCTTCCTACATGGAAACC
TTGATGAGAGATTTGAAGGATCGAAAGAAACTCTTTGAGGACACTCCACCAGAAGAAATA
AAGTACTGGTTCGAATACACCGGACCATTGCAAAAATACTTCTATCGCTTTAGTAAATCT
CGAGGCAAGGCCGATTACTTCGAGGGCCGCGATGATTCTGGCGTCACATCCAGTCGGTTT
ACAGAAGAATTCTTAGGGGGGTCGGAACACAACCGCATGTTCAATGGTGGAGCTGCTGGA
GAAGCACACAAATCGAAGACTGGGTCCAATCAACACAGGATGCAATACCGGTTGCTTGAA
AACTGTTTGGCAATGGGAAGCCCATCACGGCCCGATACTAACACACCTGTCTGGAAGGAG
TGGTATGGTAACAAAGACGGTGTCGAGCACGATACATACGGGATGGACCGCTGTCGGGTC
TTGTTCACACTCAACACGGTTGGTGCCGGACGCGGGACTACAATGATATGGGAAACTTCG
CCACCCGCCTTGCCTCATGCGCAGGGCCCTGATCGATGGATGTCCGAGCAAGAAACATCC
GCTACACATATCCAACGCCAGCTTGAGGAAATCGATGCCATGTGTGCGACCAACCAGGTC
GCCGCCCGCGTTCAGGTGTTGGTGTGCGAACCCATTTCGGCTCGTTGGACCGAGTGGATG
GATTCCCTCTTGCCCCACTGTGGGGGCAAGGTCAGTGACCTATCTCAAGTGGACAAGAAA
ACTGCCAAGGTTGAAGATCGGGCCAGTCCCATTCAGGTGGTGGTGGTCGCCACTGCGGAA
TCCGCGGGAGTATGCCACGACCTCAAGTCGGGATTTATGGAACGCCGGACCCAGGACCTG
ATTTTAGCACCACCATTTTTGGGTGACGGAGCAAAGACAAACGGTGATGTATCCATCGAT
AATGCAGCTATAAAGACAACTCACCCCGATTTGGTGAGCAGCGACGACCATGACATGTAC
GTTGGGTTGAAGTGGACCTCACTTGTCACCATGAGGGCCCTTTCTTCCTTTTTACAGGCC

TCTGCAGACATTGCGGCGGGGGCGACCTCACCCGTTAATAAGGTTCAATCGTTGGAAGAC
ACGAGTGTCATCAATGGCCCTTCGAGCCCCTTTCTGATCCCTTCATTTGTCCGAGTATCG
TACGTGAGCGAGGAAAATGCAAAGGTGGCCAAGTGGCGCATGCATGGAGATTTCTTCCAC
CCAGCGGCTTGGGAAGTCTGCAAGGACTCGTTTGAATTGACCTGGATTGATCACTCTCTC
AGTGGGTCCGGCGGAATTAACGGCTTGTGCGACGATCCGTCTACAAAGACGGCCATGAAG
CGTCGTTTGAAGGAGGCTGCTGGCGATGACAACAAGGTTGACTGTGGCCAATGGTGGGTG
TACATGACGGAACTTCAGATGCCGACGTCTCCGGATGTATACGCCAATGAAGTGACTCCG
CCCATCGTCCAGGGTGGATCTAACTTTGTATGGATGGTGACCGAACGTCAGCGAAGGGAA
ATCGTCAGTAGACAAGTCTGTATGAAGAAGGCTTCTAGTTCCCCGAAAAAGACAAGCTA
GAGGCAGAGTGCCCGGCACCTCCCCAGGCTGTCATGCCCCTAGGAGACCTAGAATCCTTT
TTGATCAATTACTCTCCCAACACGGCCCTCGGCAAGAAGCACGTGAAAGAACGACGCCCG
GGATATTCGCGCCAGACCGAGCGAGAGGCCGAGAGGGAACTCGTAAGGGAACGCAAGCAG
CTCAAGGGACAGGAAGATATCGAAAAGGCCCTGTTTGATTACACTGTATATCCCGCCGCC
CTCTTTCGGAAGGATGCCACCCTTTCGGCGGCCCGGGCTCGCGATGCCGTTTTGGTGGAC
CAAAAGAAGAAGAAGGGAGACGACGACAACGAGGAGGTCATTGGTCCCCAATTCATCAAG
TCGGCCTACTACGACGTGACGGTCACCAAGCCCCGATCCCCTGTCTTGGGATGGTGCCAG
GCCATTGCCCGCAAGGGTCTGTGCACTCTTTATGCCGACTATTTCCGAACTTCTTCCGAT
GTGAGGTGTTTGGAGGCCTGCGGAATGAAGAAGAACTTTTCCAAAATTGAATGAACAGAA
ACGAAGTAATTTATCAAGCGATAGATCTAGAACCAAACTCCCTCGTTGAGTCGTGCCGAG
CCACCAAAATAAAAAACGCAACAGACTCCAACGAGAATGTATCCCAACGACTGACCAGTG
TTTCAGGAAGTGTTCTAATCACCGTTGTTATTTTAGTAGCTATGACATTTTTTGCGCTAA
ATACTACATCAATTGGACTATGCTAGCCTGCCTGAATGATTCAATTCTATAGAAGTCGTA
ATTTGTCAA
>comp2703_c0_seq1
GAATTTTTTAGGACCACGACATACCTACAATACCAGAAATAAATTCACATAGTATCACCA
TGAGAATGAATGATAATTCTACTCCCGAGAAGAGGTCACGGAAGTGCTTCTACTCAGGTG
CCTTGCTTCTTGTCGTAGTATTGGCTGCCAGTAGTGCTGTCGTCGGATACTTCATTTTCA
AAAAGAAAAACGATGATACTCTGAGCGTTGAAAGCAGTATGAGCGAGCCATGCCATCTTG
CTGTCAAAGACGCCCACCAACCCATCTTGGAGATCACGCTTTCTGCAGAACAATCGAAAA
CTATGTTAACTGATACTCAAGCCAAAGAGTTTGAAACAGCTCTCCTAGAGGCCTACAATG
ATGCTTCCGGTGGCTGCTCCGACGAATTTAAGCGCTGGATGTACGGCACTAGCATCGTTG
ACCAAAGCCTAAAGGAACACGTCGTCTTGGAGAATGAAGGGGACTCGTCCATCTCCCACA
CATTTGATAGCGAGTACAGCTTGGTACTTCAAGTGGAAACCATGATTTCCTGCGATGGTT
GCCCGGATGATTTAGCCTTCGCGTCTCAATACCCAGAAACTTTTGGAGACGTAGCGTCAT
CGAATAGAAACTTGGGTGTTGGGCTTCCACTAGGCCGTTTCTACAATGCACTTGAAGATA
TTGTTCATACTACACTGAAGAAGACAATCACCAGCGTGAAAGTGACCCCGAACGCAGGTT
CCAAGATTCAGCACATGAGCTATTACACAAAGACAGCCAAGGCAAATTCAGGTGGTAACT
ATTATACCTCCGGGAGGGCAAGCTCAGGCAACTATTATGGCTCCAAAGGTTCGAAAGGGA
TAGCGTCTGCAGTCGCCTTCGGAATGAAGGGAGGGAAAGGATCGAAGGGATTCAAAGGCA
ACAAAGGATCGAAAGGATCGAAAGGATTCAAAGGCAGCAAAGGATCGAAAGATGCATATG
TTCCTCCGACACCTGCACCACAAGAAGAGGCTTCGACGCCATCTCCAACTCGATTTGTAT
ACACTCCTCAGCCAACAGAGAAGCCAACTCATCGACCCACTCCGCTGCCAACTCCTCGAC
CCACTCCAGATCCAACACCACAGCCAACTTTGCCTCCAATCGAAGATACTGTAATCCCAC
CTAGTCCTCGTCCAACGCCGTTGCCTACCCCTGAGCCCACACCTGCGCCGACTCCAAGAC
CTACACCCGCACCTGTTGCATTACCTACAGAGCCACCAACTCGGTTTGTATACACTCCAC
AGCCCACTGCTTTGCCAACCAACCCGCCTACGCCAATCCCTACTCCGAGACCCACCCCAG
ATCCAACACCTCAGCCGACTGCTCAGCCGACTG
>comp2704_c0_seq1
GAACTTTCTCCTTTAGATAAAACCTTCATTGCAGAAGGTGCGAAAGGGCGGAAAGAACAG
AATGAATACCGCAGGACCCCATATGTCCATACCGTACTATACTCGTACTGTGGACAACGT
TGGTTATCAATCAAGCACAAATCACAAGGTGCATGTGATCGATGACGTTAGCTATGAAGG
ATCATGTTGTTCAATCGAGACCTTTTGCGTAGTGACTGAAGTCGGAACCTCATGATTCCG
TCCGAAAAGAGAATGAACCGCACTAATAAAGTACCAAACTGAACGCTAAAATCGAGCAAA
ACACACCACACAATTATTTCATTTGATTTCAAAAAAGTGTCGTGGTCATTAAAAGTGAAC
TAAAAAGGACCCTACAATCCAAATCAAGTTTGGAAGTTATTCTTCCTTCAGCGAACAATT
AGAACGATTCAGTTGCAATTCCCTTAGTGGAACGTGTCGACCATGGTATACAGAAGTCCG
TATGAAACGGAAAAACAACGACTCGAAAAAGACTTGGAGAAAGAAGTTGAAGAAAAGGGA
TTTCCTGATGTCATCATTACGTTGTACCTCAGTCAGTTCATATATCACATGGGTGACATT
CGAACCAGAACAAAGGAGTTGGGATATGGTAGTGGTGCAGAGGCAAGAACCGAACTGATC
AACGAGGTATTGAGGGATGCTGAAGCTTTCCAAAAACTAAGGGACGGAGGCAAATCCATG

GCGAAACTCATGAGGGGAAAGCTCCATCTGAATGATATCAACCATGATGGCCTACCTGAT
ATTCCCGATATTAACATGGATTTAGTTGAACATGTTTTGAATAATGAGAAATACAAAGAA
GTGAGCGAGTCCTGTGCTGATGAGCTGGATGTTATGGCAATTCATTCGTTCAAAAAAAAT
AAAGTTTATCTATACAAGTTTGACGCGATTTCTGACAAGACAGATCTAGTGCATGCTATT
GCTGGAAGAGAAAAAGAGAAGAAAGATGGCACTAAGTTTTCGGATCTGTTTCTTATTTTT
CGTGGCTCTGTCACATTGAAAGATTGGATTCAGAACCTTAAAGCTCCTCTCGTCAACATC
GATATTCAGAAAGAGAAGAATTCTGGAAAATGGTTCTTGGTCGAGCAGTGCACGTACAGT
AGAATTGAGATAGAGAAAGAGTTAGCTTCTTTGCTAGAAGATGCAATTGGCGATACCCCG
ATCAAGGTCCATCGAGGTTTCCTCAGTTATTTATTTGGCAAGACCAATTCGTCAACACCG
AAAAAGAAAGATAAGACAGATGAAAGTGGTCGAACCAAAAGGGCCAGCACCAATGGTACA
GTCTCTTTCAGAAACGTTAATGTTTCCAAGTACCAAAGCATTAAAAATGATCTCCTCCTA
TTGTTTCAGACTCAGAGAAGCTCCCTATATATTACAGGACATTCATTGGGAGGAGCCTTG
TCGACGCTCGCTGCTTTTTTCCTCGCTTGTGATGTAGAAGTCCATGATAACAACAGGTCA
TTTGAGGGATTCAAATGCATTACGTTCGCTTCTCCTTTGGTTGGTAATATCAGTTTCCAG
CGAGCTTTTGATGTTCTCCAAAACTATCGGCAAGACAAGAGCTCCCGGAAGTACGCTGCA
GGTGTTGTTCGACTTCGGCATCTACGCTTCACAAATGATCGTGATCTCGTTCCGCTATCA
CCTCCTTTGTCAAGATATAGGCACACGGCTTGCATCAACGTTCATTTGAATCTTTACAGC
CCTCCCTTGTCTCCCTCTTGGTGCAGGATTCGCAAAGAAGAACCCCATAGAGACATATTT
CATCGATGGAATTCGGATAAATCAGATAAACAGAACAAGCGAAAGCTTGGCCTACTTCCT
CCAACGAGCACTTTGTCAGGTTTATGTTCAACAACGTTTCCGTTTCTTTGGGTTGTATTC
AATACGGCACCCATCATATTGGTCATATTGTATCTGTTGGTACGGCCAGTCGCGAGTTCC
CTATTTTCTGTTTCTATCTTTAAAGAATTCTTTCATGCCATGCCGATTCAGATCCAGAAA
TCTTTCATAGAGTTTTCGGTGCGTGTTTCCGAAACCCTTTCGTCAAAGGCTATCATCTGG
ATGTCATGGGCTGGGTGGCTTACGATGGCAGGATCACCTAATGCTGATTTTAAACGCACT
GTAGCGGTGATGGTTGGGGCTCCTCTCCTGGGAGTCGGGACACTGTATCTCGCAAACGAA
CAATATTTTGACTACTTCACCGAAAATGTGGAGGCATTGTTTGTCGTTGGAATGCTCTTG
TGGCAAATTGTAATACTACCATTTCGAAGAGCCGATGAGGTTGTTTCTGCTTCCACCCAT
GGGTTGGCACAATATTACTTGCACATTAAAAAGAGGGGTGAGCCGTTGTCATCTTTGAAG
AAAATCGTTGAGAACAATTTAGCCATGAAGGGGGACAAAGCCTACGGCGAAGACTCCTCT
TCGAAAAAAGTTGTCTGAGTCCCAGTTTGATCGTGAAATATTTTCTATGTTGCAGTACTC
GTCTTAATTAAATAATTATAAATTATGGTTGATGATATGAAAAAA
>comp2704_c0_seq2
GAACTTTCTCCTTTAGATAAAACCTTCATTGCAGAAGGTGCGAAAGGGCGGAAAGAACAG
AATGAATACCGCAGGACCCCATATGTCCATACCGTACTATACTCGTACTGTGGACAACGT
TGGTTATCTAGTATGGTATCTTGCGCTTCAATCAAGCACAAATCACAAGGTGCATGTGAT
CGATGACGTTAGCTATGAAGGATCATGTTGTTCAATCGAGACCTTTTGCGTAGTGACTGA
AGTCGGAACCTCATGATTCCGTCCGAAAAGAGAATGAACCGCACTAATAAAGTACCAAAC
TGAACGCTAAAATCGAGCAAAACACACCACACAATTATTTCATTTGATTTCAAAAAAGTG
TCGTGGTCATTAAAAGTGAACTAAAAAGGACCCTACAATCCAAATCAAGTTTGGAAGTTA
TTCTTCCTTCAGCGAACAATTAGAACGATTCAGTTGCAATTCCCTTAGTGGAACGTGTCG
ACCATGGTATACAGAAGTCCGTATGAAACGGAAAAACAACGACTCGAAAAAGACTTGGAG
AAAGAAGTTGAAGAAAAGGGATTTCCTGATGTCATCATTACGTTGTACCTCAGTCAGTTC
ATATATCACATGGGTGACATTCGAACCAGAACAAAGGAGTTGGGATATGGTAGTGGTGCA
GAGGCAAGAACCGAACTGATCAACGAGGTATTGAGGGATGCTGAAGCTTTCCAAAAACTA
AGGGACGGAGGCAAATCCATGGCGAAACTCATGAGGGGAAAGCTCCATCTGAATGATATC
AACCATGATGGCCTACCTGATATTCCCGATATTAACATGGATTTAGTTGAACATGTTTTG
AATAATGAGAAATACAAAGAAGTGAGCGAGTCCTGTGCTGATGAGCTGGATGTTATGGCA
ATTCATTCGTTCAAAAAAAATAAAGTTTATCTATACAAGTTTGACGCGATTTCTGACAAG
ACAGATCTAGTGCATGCTATTGCTGGAAGAGAAAAAGAGAAGAAAGATGGCACTAAGTTT
TCGGATCTGTTTCTTATTTTTCGTGGCTCTGTCACATTGAAAGATTGGATTCAGAACCTT
AAAGCTCCTCTCGTCAACATCGATATTCAGAAAGAGAAGAATTCTGGAAAATGGTTCTTG
GTCGAGCAGTGCACGTACAGTAGAATTGAGATAGAGAAAGAGTTAGCTTCTTTGCTAGAA
GATGCAATTGGCGATACCCCGATCAAGGTCCATCGAGGTTTCCTCAGTTATTTATTTGGC
AAGACCAATTCGTCAACACCGAAAAAGAAAGATAAGACAGATGAAAGTGGTCGAACCAAA
AGGGCCAGCACCAATGGTACAGTCTCTTTCAGAAACGTTAATGTTTCCAAGTACCAAAGC
ATTAAAAATGATCTCCTCCTATTGTTTCAGACTCAGAGAAGCTCCCTATATATTACAGGA
CATTCATTGGGAGGAGCCTTGTCGACGCTCGCTGCTTTTTTCCTCGCTTGTGATGTAGAA
GTCCATGATAACAACAGGTCATTTGAGGGATTCAAATGCATTACGTTCGCTTCTCCTTTG
GTTGGTAATATCAGTTTCCAGCGAGCTTTTGATGTTCTCCAAAACTATCGGCAAGACAAG
AGCTCCCGGAAGTACGCTGCAGGTGTTGTTCGACTTCGGCATCTACGCTTCACAAATGAT

CGTGATCTCGTTCCGCTATCACCTCCTTTGTCAAGATATAGGCACACGGCTTGCATCAAC
GTTCATTTGAATCTTTACAGCCCTCCCTTGTCTCCCTCTTGGTGCAGGATTCGCAAAGAA
GAACCCCATAGAGACATATTTCATCGATGGAATTCGGATAAATCAGATAAACAGAACAAG
CGAAAGCTTGGCCTACTTCCTCCAACGAGCACTTTGTCAGGTTTATGTTCAACAACGTTT
CCGTTTCTTTGGGTTGTATTCAATACGGCACCCATCATATTGGTCATATTGTATCTGTTG
GTACGGCCAGTCGCGAGTTCCCTATTTTCTGTTTCTATCTTTAAAGAATTCTTTCATGCC
ATGCCGATTCAGATCCAGAAATCTTTCATAGAGTTTTCGGTGCGTGTTTCCGAAACCCTT
TCGTCAAAGGCTATCATCTGGATGTCATGGGCTGGGTGGCTTACGATGGCAGGATCACCT
AATGCTGATTTTAAACGCACTGTAGCGGTGATGGTTGGGGCTCCTCTCCTGGGAGTCGGG
ACACTGTATCTCGCAAACGAACAATATTTTGACTACTTCACCGAAAATGTGGAGGCATTG
TTTGTCGTTGGAATGCTCTTGTGGCAAATTGTAATACTACCATTTCGAAGAGCCGATGAG
GTTGTTTCTGCTTCCACCCATGGGTTGGCACAATATTACTTGCACATTAAAAAGAGGGGT
GAGCCGTTGTCATCTTTGAAGAAAATCGTTGAGAACAATTTAGCCATGAAGGGGGACAAA
GCCTACGGCGAAGACTCCTCTTCGAAAAAAGTTGTCTGAGTCCCAGTTTGATCGTGAAAT
ATTTTCTATGTTGCAGTACTCGTCTTAATTAAATAATTATAAATTATGGTTGATGATATG
AAAAAA
>comp2705_c0_seq1
ACAGAACGTTTTTACAAAAGATCGGACTACCGTGTTAACCGATCAATAGAATAATATACT
GGACTAATCTAACTCCAACATGCGCTTTTCCACTGCCACCATCGCTGCCCTAGCAATCAT
CGGGTATGCTCATGCACAAGAACCATCCTCGACACCGTCTGGGATGCCGTCTGGGATGCC
CTCTCTCTCTGGGATGCCCTCCATGGTTCCCTCAACGGTTCCTTCTGGGATGCCCTCAGG
AATGCCCTCTGGAATGCCCTCTGGGATGCCCTCACTCTCTGGGATGCCCTCCATGATTCC
CTCAACGGTTCCCTCTGGGATGCCCTCAGGAATGCCATCTGTCTCTGAAGTTCCCTCTTC
TGTTCCCTCTGGG
>comp2705_c0_seq2
ACAGAACGTTTTTACAAAAGATCGGACTACCGTGTTAACCGATCAATAGAATAATATACT
GGACTAATCTAACTCCAACATGCGCTTTTCCACTGCCACCATCGCTGCCCTAGCAATCAT
CGGGTATGCTCATGCACAAGAACCATCCTCGACACCGTCTGGGATGCCGTCTGGGATGCC
CTCTCTCTCTGGGATGCCCTCCATGGTTCCCTCAACGGTTCCTTCTGGGATGCCCTCAGG
AATGCCCTCTGGAATGCCCTCTCTCTC
>comp2706_c0_seq1
CATTCGAACAAATCCGTGATTGCAGCAGGCAGGGTAGGCAGAGGACGAGTAACCCGTACT
TGCACTGCTACTCCTACTCACTCGCATGGGGGTACTAACGGTATCCGTTTTGCCAGGGGC
CCAGATTTTATTCGTTCATCTTCAAGATCGTGATCAAGCCGAGTTCTTGACGCCAAAAAA
GCAGGCACCATCGACCGCAACAGTGACTGAGACACTACAAGCACAAGCTAGATACACAAG
AGCAGCATCGCACGGCAGGATAGAAGCAACAAGAACCACCGCCGGCATCCGCAAAAAAGC
ATCACCACCACTCGAGCAGACCGTTACACTACGCTAGACTGCTCTACACTGCACTGCATT
TCGGCACAACACTCCACTCCACGAGACCGAACAATACCACACAAGACAAGACAAGACAAG
ACAAGAGATGAAGATCGCCTCTCGCAACTCGGCACTGGCGGCAGCCCTCTGCCTCGCGGC
GTCGGCCCTGGGAGGGGCCCTGGCCTTTGCCCCCCACCACGCCGCGCGGAGGGCGCCAAC
GGTCCAAAGCAGCAGCAGCAGCACCTCCCTCTCCGTCAAGATCCCCCGGGTGGAACTCCC
CGAGGCGGTCGCCAGCCAGCTGGGCGAGTTCGACCTGAAAAACCCCAACGCCATGAACGA
CGACGACTACCGGAGCTACTCGGGCGCCGCCATCGCCGGGACCCTGGCCTTTTCCCTCCT
GCCGGGGGCCCTGGTCTCCGGCATCTACAGCGACCTCGGGCCCGTCGCCATTGCCACGCT
CATCGACTTTTTGTTCTCGGCGATCGTCGGCGGCGGCCTGGCGATCTACCTGTCCCTCCG
GAAGGACCAGATCGGCGAGACCGTCCGCTCCTACGGCAACCAGCTGCTCTCGGCGGCCAA
GGACGCCACCGGGATCGGGACCCTCCGCTACGACCTCCCCGTGGCCGCCACCGACGTCTT
GACCGGTGAACTGGGCCTCCTGGACCCCAACAAGATGGACGAGGACGACTACGACGGGTA
CTCCGGGGCGGCCGTCGCCGGGACCCTCCTCTTCTTTCTGCTGCCCGGGGCCCTCGCCAC
CGGCGCGGCGGGAATCCTGGGGGACTTTGCTTCCTCGGCGGCGGTCGATTTCGTGCTCTC
GGCCCTGGTCGGGGGGGGCGCCTGCATCTACCTCTCGCTCCGCAGGGACGAGGTCGGGGC
GACCGTTAGCCAACTGGGAGGGCAGTTCCTGGACGTCGTCGACGGGGTCCTCGGAGCCGA
GGAGGACACCAAGCTGCTGGAGGAGTAAGCAAGCACGCACAAGCGTTGTTCCGCAAGCAT
TGTTGTGCCTCGTGCTTTGCCGCGACGTGTCTGCCCGTTGCAACACACACACACACACAC
CGCGGCGAGTGCATCATCCCGCATCGATGGAACAGGGAGAAAACAAAAAAGCCACTGTTT
CGAAAGGCCGGGACGAATCCATAAAACCAGACGTATAGTACTACTAGTAGTTATAAATCA
TTAGAAAATTTGTTGCAGTGGCA
>comp2707_c0_seq1
ACGAATACTACCGCGTTCACGTTATCACATGTTTCAACCACACCACACATACGTCGGCAC
GCACCACATCAAGTCGCTGCACCATATAGTGCAACAAGCCATCCGAATACAAACACGCTA

GCAAAACAAATCGGCAACGATACAATCTACCGTACTTGGAACAAGCAACCTTCCGTTAAA
AGCTTCATCGAATCAAAGAGTACGCATAGTGGACGACATGAATCGTGCATTCCGATACGG
GGAATCGCCTTTTTCGTGCTCACCGACAAGCACCAGGGACACCGCCTACATAGTAGAAAA
GAGTCCGACCAGAGGCGGCAAAGCAACCATTTCTATGATGCCAAACACGACGAATGCGAC
AACCAGCACGACAAAGAAACTGCCCCGTTTCCACTGCTCGGAGAAAAACCACAAACACCA
ATCGACGGTGGATGCTCCCTCGGCTACGACCGGAAGCCAAAGGAGCATCGCGGTCCCGCA
GTCGCTTGCGAACCTAAGGAGTCAGCTGAATTATTCGTCTTCATCGTCGGTGACGTCGTC
CTCGTCCTCGTCGTCTTCACTGGAGGGAGGTAATGTCATCGCGTTGCACACGAATAACGA
TGCAAATGATTTTGCTGTGCAGGACCGAAGAAACAACAACAACAGCAGGAACAACAACAA
CAATACAATGCCAAGAAGGAGGCTTCACACTAACAGTTTTTCACCGTCCGTGACACAACG
GCAAACCCAAAAGCATCCAAAAGAACAGCCACAGCACTACCAAGAGCAACAATACCATGC
ACAACAAGGCATTTTTCGACGGCGTGCCGTACCGCTACACTACGAACAACGCCCTTTCAA
GCAGCAACGGAGTATGTCTCAGAGCCCCCACCGCCCCCGAGACACGTTGCGAATATCTCC
GGAACAACGGGGGCAGCAGCAACGCAACACCAAAGAACCATCCAATAGCAGCACAGCCAA
CATAGCAATGCTCATGTTGCCGAATTTCCAGGATCCTTCTTCGTCTACGGAGCAAGTCTC
CGATGCTCCCCCGCCCCCACCCCTGTGGACGGAAGGATTCGCGAAGGGCCTGGCAGATTC
CATTGCACGAACACCGCAGCCCCTTGGCGCCAACAGGGCGCGGCGAAAATTTTGGTCTCG
TCTGAAACCCCAGGATATCATCCGGGTTTCCTCGCTCAGCGAGGACAGCTGCGAGGATAC
CAGCAATACCGGGAGTGCCTATTTTGAAGACGAAGACAAGCACGAAGACGACAACGGGGT
GCTCGGATGCGGATCTTTCTCGAGTGTGACGAGGATCTGCGTCCGTGGAAAGGGGACCAA
GCAGTATGCCCTCAAGCACCTCAAGCAGGACCTGCTTCCCCCGAGGCTGTCCGAGGACTG
CCTGACGAACGGCTCCACCGCCGCCTTCGAGAGGGCGGCAAGAGAACTCGCACGGGAGGC
GAATCTCCTGTCCCGCTTCGGGAACGAACACCCACACATTATCAACATCGTGGGGTGGAC
GGACGGTGGTGCCGCCTCCTATGCGGCATATATGAGGCACGATGCGTTCTTTCTGGTCCT
CGAGTTGCTGCAGGAAGAAACCCTCGACGACCGCATCGAGCGCTGGAACCACGACGATGC
CCAGCTCCGGCAGAAACAAACGGTGGTGACAAACCCGGACAACCCGAGGCTTCATTTCAT
TCGGCGGAAGATCGAACAACTGACGATCTGCAAGCAGATCGCGGACGCTCTTTCCTATAT
CCACTCCAAAAACATTGTTTACCGAGATTTGAAACCCCAAAACATCGGATTTGCGATCCC
GACCAAGAAGAGCAGCAGTGTCGCGAACGACGGTGACAGCAACAACGTGGTGGTCAAGCT
CATGGATTTCGGACTCGCGAGGGAACTCCCCACGAGTGGAACGATTCTTCCCAAGCAGCA
ACAGCAACAGCAACCAGCTAGATCTCAACCACCGCGATCTCCAAACAAAAACAACAAAGA
CCCGACATTTTCAACCCCTTTCAACGGACTGTTCGACATGACCGGTACGGTCGGGACGCT
GCGATACATGGCACCCGAGGTGTGCATGCACCGTCCCTACGGACTGGAATGCGACATCTA
TTCGTGGAGCATTGTTGCCCATGAGATCCTATCGCAAACGAGACCCTACGAAGACATGAC
CCCCGATATGTACCAGACCTTGGTTTGCTTGCAAGGCGTGAGGCCCCAGAACGGTAGCAA
TGGCAGGGGCATGCCCTCCGAATACACGGTCCTGCTAACCCAGGCCTGGAGGACCGACCC
ATCGAAGCGGTTGCGTCTGGATCGGATCATGCGCCAGCTCGATTTGTTCCTGCAAAAAGA
AAAACTCATGCTGGAAGCCCAGGAGCTCCTATTGGGCATGCCCGAGAGCAACGAATCGCC
CCTGTCGCTTTCCCTCTCCTTTGCAGAGAGGAGCAGCACCAGCAGCCGGAGCCACCATCA
CCGCCATACGCGTGGAATCCATTGCGGATATCGCGTGCGGTCTCCCCACTACCAAGGTGG
AAAGGCGAAATACGGCAATAGCAGCAGCAGCAGCAACGGCAACAAA
>comp2708_c0_seq1
AGCCGAGTACGGAGTGTGTGGTCGGATGCAACTGAGTATATTCACAACGGTAAAAGCGGG
CTGGGACGGAGGATGCGAAGGATGTCTGTCGTCTCACAGTCGGCGATGAGTCCCATGTTT
TTTCTTGTTACATGGATGGAGAATTCAACGTCGAACGCTCACGGACGGTTGCATAGGCGT
AGTAGTATCACTTCGCGTACGTACTCGTACTCGTAGAGCATTGTACAGTTTTTCTAAACG
TGGCCTTTGCGGCACGAAATTTTCCACGTGGAGGTACCGGTAATGTGAGGGTTTTGGGCC
TAAATTTTTTCGAAACCTCCGCGCGACCACGACCCCCGTTGAAGCGTCTGACCGTCAGCC
GCAGGGGAAGGAGATCGTACGTGTTTCCCATCTTCAAGACAATAATCTGTTTGGCATCTC
AGCATCGGTCCAACGGGAGTTGTGCGCGAAGGCATTTTCAACTCTTAACAGAAAAAGGTA
CTGATGTGTCCTTGTCGTGTCAACTGATATCATGGATATCGATGCATGAATGAGTGAGTG
AACTCAATGTTGAGTGTGAATCAATCTCTGAGCAACAGAAGCAACAATCAACGCCAAACA
CCGACTAGACACGAAAACAACATATATCTGTTTTTACATCTAATCATCGATTGTTCAAAC
GAAGAATCAACACATTTGTAGAGTAGTGTCAACAAAATACTGCGGGTATTTTCACAGAAG
AAGGGAACATTGTTTCGACAAAACAAATACTGAGTCATGAAATTTACTATCGGATGCATG
CCGCTACTGCTAGCGCTCTCGGACGTCAATGTTGATGCCTTTGCGCCCTCAAAATTGGTC
AACACCGCTGTCAAGTCCAGTACAGCCCGGGCCTTTTCTCCCATGGACCCGACTTCTTTT
CAGGAACTCCCCCACCACGTCCAGTCCATCCAGGACTTTATGTCCACCCTTTCCGTAGCG
GACGTCGATGTTGGCGCCGGAATCGAACAGGTCGCCACCCAGGTTGCCACCACCGTCGAT
CCCGCCACTGCCGAGGTCGCCACCGAAGCCGCGAAGTCGAATGGCTGGTTCGGCTTCCTA

ACAGGACCCACAGAGGCCTTCCTCGAGATCATCCACAGCGTGCTCGTTGGTGTAGGACTC
AACAGCAACGCCTGGGGTATCTCTATCATTGCCCTGACCCTATCCATCAAATTGTTGACA
TTCCCCCTCACAAAAAAACAGCTGGAAAGCACCCAAAAAATGCAGGCTCTGCAACCATCT
TTGAAAGAGGTTCAGGCCAAATACCAGAGCAATCCAGAGGTCATGAACCAAAAGATTGCC
GAGCTCTACCAAACTAACAACGTGAATCCCCTCGACGGTTGCATTCCAAGTCTTGTCCAG
ATTCCCGTATTCATCGGTCTCTACCGTGCGGTCCTCGATCTTGCCACCGATAATGTGCTC
GACGAACCGTTCCTGTGGTTGTCTAGTCTCGAGGGTCCTACGTACGGAACCGATCCCACC
AAGGGGTCTGCCTGGTTGTTCGACAACTGGGTCAACGGAGCTCCCTCTCTTGGATGGGAC
GAGACGGTTTCGTTCTTGGTCCTTCCCGTTTTCTTGGTCGTTTCTCAGTTCGCATCGATG
GAGTTGATGACTCCCAAGGAGCAAAAAGATCAGCAGCCATCCTTCCTGAAGGTGTTGCCA
CTGATGATTGGTTATTTCTCCCTGAACGTTCCCTCCGCCCTCTGTGTGTACTGGGTCACA
AACAATATTGTCACCACCGCCTCTTCGCTCATCATCCGCAACAGCCTGAAAATGGAGCCC
GCCGCGGTCGTCGCTTCGGAAGCCACGGCAGCGGCCCCCGCCAACCCCAACGTGTTCACC
CCCCCTACCATTCGCGAGAAACCCGCCGGATTTGGAGAAGCGGCCCCCTCTGCCTCCGTA
GACGGTGTTACCCCCATCACCGGCTCGGGTGATGTCGTTGATGTCGAGGTCGTCTCGGAG
ATTTCTTCGAACGTTGAAGCCGCTGGAGAAGGCATGTCGGCTCCCAAGAGCAAGAAGCGC
GGAGGCAAGAAGAAGAAGAAGAGAAGAAAGAACTAAATGAAGCCTGAATAAAAAACTAAG
TAGCATCCGTGCCCGAGCAAACGAAAACCTGATTGTTTCTCTTTCCAAACGAATTCTTCT
GTCTAATAACTACAATGCTGTATCACCAAAGAGCGCTCGTTCAAACAATCAAAAATGGGA
TCACACAAGCAATGCTGTTGCGAGGGCTCGAAGATTGTTATGAATAGTATGCAGGCCATG
ATTATTTAATTTTTATAAAGCCTCTTGAGTTTTTCAAAAAAA
>comp2708_c0_seq2
AGCCGAGTACGGAGTGTGTGGTCGGATGCAACTGAGTATATTCACAACGGTAAAAGCGGG
CTGGGACGGAGGATGCGAAGGATGTCTGTCGTCTCACAGTCGGCGATGAGTCCCATGTTT
TTTCTTGTTACATGGATGGAGAATTCAACGTCGAACGCTCACGGACGGTTGCATAGGCGT
AGTAGTATCACTTCGCGTACGTACTCGTACTCGTAGAGCATTGTACAGTTTTTCTAAACG
TGGCCTTTGCGGCACGAAATTTTCCACGTGGAGGTACCGGTAATGTGAGGGTTTTGGGCC
TAAATTTTTTCGAAACCTCCGCGCGACCACGACCCCCGTTGAAGCGTCTGACCGTCAGCC
GCAGGGGAAGGAGATCGTACGTGTTTCCCATCTTCAAGACAATAATCTGTTTGGCATCTC
AGCATCGGTCCAACGGGAGTTGTGCGCGAAGGCATTTTCAACTCTTAACAGAAAAAGGTA
CTGATGTGTCCTTGTCGTGTCAACTGATATCATGGATATCGATGCATGAATGAGTGAGTG
AACTCAATGTTGAGTGTGAATCAATCTCTGAGCAACAGAAGCAACAATCAACGCCAAACA
CCGACTAGACACGAAAACAACATATATCTGTTTTTACATCTAATCATCGATTGTTCAAAC
GAAGAATCAACACATTTGTAGAGTAGTGTCAACAAAATACTGCGGGTATTTTCACAGAAG
AAGGGAACATTGTTTCGACAAAACAAATACTGAGTCATGAAATTTACTATCGGATGCATG
CCGCTACTGCTAGCGCTCTCGGACGTCAATGTTGATGCCTTTGCGCCCTCAAAATTGGTC
AACACCGCTGTCAAGTCCAGTACAGCCCGGGCCTTTTCTCCCATGGACCCGACTTCTTTT
CAGGAACTCCCCCACCACGTCCAGTCCATCCAGGACTTTATGTCCACCCTTTCCGTAGCG
GACGTCGATGTTGGCGCCGGAATCGAACAGGTCGCCACCCAGGTTGCCACCACCGTCGAT
CCCGCCACTGCCGAGGTCGCCACCGAAGCCGCGAAGTCGAATGGCTGGTTCGGCTTCCTA
ACAGGACCCACAGAGGCCTTCCTCGAGATCATCCACAGCGTGCTCGTTGGTGTAGGACTC
AACAGCAACGCCTGGGGTATCTCTATCATTGCCCTGACCCTATCCATCAAATTGTTGACA
TTCCCCCTCACAAAAAAACAGCTGGAAAGCACCCAAAAAATGCAGGTATGTGTGTCCCTA
TTTTGTTGTTGTTGTTGTGCGG
>comp2709_c0_seq1
TTTAGGGTGCCTAGATTCATTCAAGGAGACGCCATCTGTGAATTTCTGTTCCGATGCTAC
TGGGATACTACGGCAATCTGCAAGGGAACGACACGACGACACATGGTCTTAGTCTCAGAT
TACCTGATTCCACATTACCACTTGCTGATAGACCTTGCTTCGAGTTTCGTCTGCCACATT
GTTCCTCATTGCCGACACTACTTACGCATTGCAATACCCAAGGAGGATTTTAAAATACTG
TTAGCCTTGATACAAAACAAGGTAGCTCACCGAAAACAACACCGTAGTAGTTCCAATCGT
CGAGATCTCGGGACACATATCACTAAATTCGAAAAAAGCAAAGCGAAACTCAATATAACC
ATGCCATCGAACTCGAACACGCAACTTCTAGCCATTGCCGGTGCCGTTCTGGCGATCTCA
GCCCCGGCCTCCGGTTTTGTCCCGAGCCAGAGGCTTCCGGCGGCCACAAAGAGCTCGAGC
AATCTCTTCCAGTCTTCCCGGTCGGCCGTACCGGACTGTGCCCCCATTGGGGAGGTCATC
TCGGCCGTGGGCTCGGGTCTCGTCGACGAGGAAGAATTCACGCCCGCCAAGGAGAAAATT
ATTAAGGAACCAGGCCTTTGGGAATACAACTTTGGCCTCCAGGACCCCGGGCTCGCGATG
CCTCACGGAATCGTTAAAGACGTGACGGAACCCGAAACCTTTGCCATCACGGAGGAGCAG
ATCGCCACGCTGGAAAGGGACGGAGTGGTTCACATCAAGGGCGTCTTTGACGAGCAATGG
GTGGATTACCTGCGCAAGGCCACCGCCTACCAGGTCGACGAGCCGCATTTTTGGGCCTTT
GCTGGAACCGCGTCCAAACTCTACGACTACATTCAGCGAAACGTCTGGCAGACCAACAGT

GCCTTTGCGCGCTTTTACTACCACTCCGCGATGGGTCACGTCCTTGCGCAATGTGGCCGC
ACCGACGAGATCCGAGTCAGCACGGATCTGCTCATGGTCAACCCCAACAAGGGCTTCAAG
TGGCACCAGGACAACCAGAACGGGCCCATTTCTTGGGAGGAGGGCCTCCGCTTCTGGATC
ACCATGGACGAGACGCCGAAGGAATACGGAGCTCCCGTATACTTGCAGGGATCCCACAAA
AACGATGTGGTCGACGAACAGGCCGTCTTCGTAGACATTGAACAGGACGGACTGGAGGAT
TACAACACAAAAATGCTCGAGTTCCGAACGATGCCCGGTGACATGCTCATCTGGCACCCA
AGGACGATCCACAAGGTCGACGGACCCGCTGACGGCATCTGGACTTCCTACCGACGCGTG
CTGGGGGGAACCGTGGCCAAGGCCGGTAGCGTCTACCACGACAAGCGGGGCAGCGGGGGC
GTTCTGTCCGATCTCGGACGACACGGGCTTGAGGACGGTAAAGCGTTGAAGTCTTCCTTT
TTCCCCGTAGTATACCCCAAGTTTGACAGCGCCGAGGCCGCCGACCGCGACTCTGGGAAC
GTTGGACGAAACCCCATGGACATTGTTTCGAAGCTGGGCGGCCTGGCGACTAAAGCCTCG
GGAGAACGCTTCAATTCTTTCTTCCAGGTGTTGGGAAGCCAGAGCAATTCGAAGTAAAGA
TTTCAATCTCACATCAAGCTCAGCAAGTTGAGAATTGAAATTGGGTGTGTCTGAGTGTGT
AGAACGGGTAGACAAACAACTAAAACAAAACGAATCGGCGCATCCGTGTTCGTACACCAC
TCCACACCCAAAGGTAGGCAGCCAGGCCGCGCACAACACAGAACAGCACACACGCACAAA
AATTCCGAGAAATGGATTCCACTATTTCTGGGAACAGCAGGAAGCACTCCCTTTCTTGCT
TTGTATATTATTATCATTATTATTCTCGTATGTGCGGTGTCCCCCAAGGAGAAGGGCTCG
GAGTACAAATCTACTCTAGTGGTGTGCTTGTTTTTTATCCTATTTAACAACGGTAGAGTA
ACATAGATTGGGGTTAATGCGATGAAATTTCATCAAAGATTTTCTACAAGGCAGAATGAA
TACTTTTTTTAGGTAAAGCTATGATGCTCGAAAATACTTTGCTCTATGGAGAGACACAGA
ATGTTGAGTCAAAATTTCTGTGATCAATTTTGCTAATCAAACTTATCATCAGTGTTGTGT
TTGATTGAAATGCGGCGACGGTATTAAGAAAACGGTACGATGCAATATCATTAGAACACA
GTTTCTGTCTCCATGGCGGGTCACAGTTCGGTTCGATCAAAAACTCGACACAAACCAAGT
CCAACTACGCTGCGAAATCCCTAGATAGAAACAAGAAGCGAGGCTCTGCTTGACTGCGAG
TCGAATTGATTCCATATGGGCCTCGACGGGATGTGATGTAGCGAGGATATATTACTTTCT
TCTTGCCTGCGATCGGAGTAGGACATCTGTGGGTTGCAGCCGTGCACGGCATTGCAGCAA
ACTAGTTTCTTGCTCCATTCGTTTCGTTTCGCCTCGTCTCGTTATTGGGTATCCGTATTT
CT
>comp2710_c0_seq1
ACACCTCCTAGTAGCGACAATATATTTACATACAAATATCGGTTTTCTCGAACCAACAAA
GACTCTGCACACACACTAAAAGCCGCAAAGACAAGGAGGGAAAGAATACGATAAAAGAAA
ACTAGAGAGACGATGAAATTCTCGACGACGACGGTGACGTGCTGCCTGTTTGCTGTAGCA
GTGCGGCGTTCGAACTACAAGTTTGCATCTGCCAGTGCGGTAGTTGAAAGCGACAACACA
GGCAACGAAAACGAACGCGATCTGATCTCACTGAACGGAAGTTTCTTTGGCATCCCGGCG
CCAACGCCAGCGACACTAGCTCCGGTGTCAACAGAAGAGCAGGACAAAAAGGACGAAATC
TCGGGACGATACAAGAAAGTTCCAAAAATGCCTTCTCCTTACCCAGCGCCAGAGCCCAAT
CCTCCCAGAAACGACGAACCGACTCCAAACCCCACACCACGACCGACGCCCGTGCCAACT
CCGCGTCCAACACCAAGAAGAACTCCGGAACCAACACCGTACCCAACGAAGCCACCTGTA
GAGTATAAACCGTACTATCCTACCTATAAAAGACCTACCATTCAAACAAGGCAACCAACA
CCCGAACCAACCCCGGCTCCGACCCATAAACCTACGTCGCATCCAACGGCAAGGCCTACA
TCAAGGCCTACCGATCATCCGACGTACAATCCAACACCGGGTCCCACCCCGATGCCAACT
TCTCAACCGACGAGGCCCCCAAGTGCCTCTCCAACTTCGTTGCCTCCAACTCATAACCCT
ACCAGAAATCCTACACACAGTCCGACTCCGCGTCCTACCCTTTCTCCGACTGCTTCTCCC
TCGGCAAAGCCCACCGGAAAACCGTCGCCCCTCCCCTCCCCTGCACCTACTCCGAAGCCC
TCACCAGCGCCGACGATAAGCCAGGAACCCACGACATTGATACCTTCTATGGAACCGTCC
GAGAATCCAACTCAATCGGAAGAGCCATCGGACAATCCTACCGCATCGGAAGAACCGTCG
GACAATCCCACCGCATCGGAAGAACCT
>comp2711_c0_seq1
CAGCTCTTCAACAATGAGGTACAGTAGTTCGTTTTCAATTATAGTGCACAGGCTTTGTTT
TACGGAATTACACGTTTTTCAATAACATACCCAACGCGTTCGCAGCAACCACTGTTCCGT
ACAACAAACACAATCTGTATTGTTTTCCCAACGCGACTTCTTCCCAACGCGACTTCTTTC
AGATTCGAATCAGAACGTTTTCTCCAGCGTGCTCACAAAAGAAACTATCCTCTAAGTTGG
TTTGCTAATTTTTCTCAACGTGTTCGCAACAATCCCAAAAGTCTTTTCACAACTGCCTTT
CCAACGTTTCCACCTTGATACAATACGTCCCACCATGAATACAACAGAAGCAACTCCTCT
CAGCCGCACCAAAAAGCGGGTGAGATTCCTGGAGGACGTGGAAGTCATCGAAATCGGAGG
AGCATCCCAGAACAAGGACAAGGAAAACGACAGCGAAGCCCACCTCGAGGGCAACAATCG
CACGGCACTCGAAGAACAACGAGATAGGATTTCTACCGACAACAGCCAACCTGTATCGAA
CATGTCCAGAGCTCAACGAGGGCATTTCCTCGTCTTCCCGTCGTGTCCGATCACGACTTT
CCCACTACAAGGACAGAACGAGGTCTTGCTCACATCGCTGGCGCCAACCTCGGCACGCTT
GTTCGATCGATGGGCCTGCGGTGCCTCGCACCAGCAGCTCGATCCCCGTTCGTTTTCCGT

GCTTCCACCGACACTCCCCGTTCGCAGGGTCGATGATACCAAAGCGCAGTGCACGAGAGA
CATTATCCAGAGTGCCCTGGATTGTGTGACCGCCGATTGTCCAGTCCTTCTAGTAACGGA
CTGATCGAAAACGTTCGGAAAAAATACGACGAATGTACATAGCAGTTCGTTCTGTTCGACT
CCCTCGATAGATAGCAAAAGGCGGTATCTCCTATGGTTGACCAAAAGCACAGCTCGGTAT
CTGGAACCGGGCCCTACGAATTCACCAGGAGTAGCAACCCTACAACAAGATCATGTAAAA
AAACCTTCCTCCCAAACTCTGCGTGTTTCCACATCATTCTACATAAGCATGCAAGCACAC
AGACGACCATGCACTCTTACGAACACACCCACTTGAATACTTACATATACATTACTAGTA
CTATTATTATTATAACTTAGCGCAC
>comp2712_c0_seq1
AGACTTTTTGCTGTTCCAACCGATAATATCGTAAAATGAAGAACGTAGGACAACAGGTTA
CTTACTCTTCCGCTAAAAACTCGTTTTGAAAAGAACTCAGGCGCGCGATGGGAAGAATAC
AGCTTCAATTGTCGGATCATTCTGAAAATCACCTTTTGTCAATCGACATCTACTTATTGG
GTTCTTTCTTGAACCTCAACAAAAGAATTTGTTACTTCAAGGTTCAGTCGGTGATTCACA
TGTATGATTCTCCAATAGAGGAGTTCACTTGTTCGTTTGTTTTTCGCCTGTTTCTTATGC
CATCCAATTAAAAAATGCACGACCAAAGCTTATGTTGTTCGTGACATAACCAATTTCTGG
CAATGAAGCGACTAAAAGAATATAGCTTTTCACCAATTTTATCCAGCGCGTGCTGTCGCC
GTACTGATAACCAAATTGCTTGTTGGTGACATGTAGCGCCAGCGGCATATTGACGCTGCT
TTGCTTGGGGACCTTCTGTATTTTTTTCATTCCATCGTCCATCACGATCTCCGAGTCTTC
TCCTAGACACGGTACTGGTACTCGAACAGTGTACATAATTGCCATCTTCCGTAATCCTCA
CTTAGCCTTCACATCTGAAACATCTGTACAACTGCAAATTTACGCAAAGCACAAAAGGTC
CCATAGTCAACACACAAGAATACAGCCAAAGAACAACGCAATCCATAGTTTTTGCCGTTT
CTTCGTCCAACATGAGAAAACCCATCAGTCAGGAGGCGGAGGAAGATTGTGGTCAAGATC
AACAAGAGAGGTGCAAGAAAGGCTGGGAACCAATTCTTTGCAAGAAACTTGCGGCGGGCC
ATGCTTCCTTCAACCACATGAAACGCTTTTCAGAGAAGAGTAGGTTGGATCGATTCGATG
ATGAAGTGCACAGCGTCCCTTGGTCCGCGATACGTAAGAAAAACGTGTTGGGACAAGGTG
CGTATTCTATGGTATACAAAGTACAAGTGTCGATGAATGTAAATGATGCAAGCAAAGCCA
CAACTTTGGACAACCAGAAGTATTATGCACTTAAGCACATCAAGCCAGATATTACGGATG
AATCGAAAGATTTTGATGTTGCCGCTGTCGATCTAGCTATAGAGGGGGAAATGCTCTCCA
GGCTTCGCCACGATAACATCATCAAGCTGCACGGAGTCTACTCAGGTGACCCTAAGACGT
CCTACGTGGATTTCAAACACGGATATTTCCTTTTGATTGATGTATTGGGAGACACGCTGA
CACGCCGGTTAGCGAAGATGCGGAAGTTGGTCAAAAAGAGAAGGCGTGGAACTGCTCCAT
CTGTTCTAGATTTGATTCAAAATGTAGCAATCGGGATTGCCAAGGGTTTGGAATATCTTC
ACGAAAACAATGTTATCCTGAGAGACCTCAAGCCCGACAATGTTGGATTTGATGAGAAAA
ACGCTCCTATCATTTTTGATCTTGGGTTTGCTCGCGAATTACATACGGTTCACAAGTCTG
AGGTCGCAGGTAGCATTCGGTATATGGCCCCAGAAATCGGACTTCGGCAGGGGGCCAAAC
TTGCCTCAGACGTCTATTCGTTTGGGGTGTTATTGTATGAGCTATGCACATTGGAAAAAC
CTTACAAGCAGTATAGGGGACGTACAGAATTCATTCAAGATGTATTTGTCCACAATTATC
GTCCGGATTTGACGAGTATATCCTCGAAAACGATGAGAGACTTAATTTCAAGCTGCTGGG
ACGGAGACCACACCAAGAGACCAACCATGAAGAGGGTTGTAAATATACTCCGTGTTGAAA
CAGCATTACTCCAAAGAGAACGAATGTCTACTAAAAGTGCGAAGGTCTCGACGCAACGCA
GAAGTGATAATTCTGTATCATCTACGCACTCATCTCTATCCTTTAATGCGTTCGGTTCTG
TCAACTCTTTCCGCTCCTCGACAAAGAATTTGGTGAAGCCTAGCACTGGTTCCACAACGG
TTCCTTCGTGTCTGCTGACCAGTAGTTCGACTGATATAAAAAATTCTTTTGCTCGGAGGA
GCTCTATCATACGCCGGATGGGTAGTAAAAATTTTGATTGGAATAACAATCCTTCAGCCA
CTCCGGAAAACTCCACCGTCAATGACAAAAACATGGTCTTTGGTATTGCCCAACGAAGAC
GCAATTCCTTTACTTCTTCGCTGCTTGATCGAAGAAAGAGGAGTACCAATTCGATTGCGT
CGTCGACGTCTGCAAACGACGTAAAAGAGTCCAGGTCGGCAGAGCGAATCGCTGCGGATT
ACACCAGAGAGTTGCCGGCACCAAACACAAGACTCTCTAATCGTTATCCCACACGAAGAA
GCTACGGTAGAGGCTTAGCCCGTGGTATATCTTATGAATCTGAAACGTCTCTTGCATCTA
CCTCTGGTGATTCTTGCGCTACTTCTTACTCAACGGACTTGTCTACGTCCAACTTCACCT
TGGGCGAGTTCAGGGCGCGAGTGCTGGGAAGGTCTCGGCGGAACAGAACTTTTTCTTCGG
GGTCGTCAAGTTCTTCGAAAGACCTTTTTAGAATTGATGTATGTCATGGTTCAAATACAG
ATGATGAGAATATTTCTGGAGACAGTGACTTCGGTGATGATTGCATTGAAGATATGTCTC
TCCTTACGAAGAAGGAAGTCAATAATGAAGGAAAACAAGAGGAAAGGGCTTCACTACCTA
AACTTGTACATCCATAGCTTCTTCTATATAGAAGGCCTGTTGGGGCCAACATAACTACAT
TTTAATTTTCATCCAAACTGGATGCAATCATAGAGTAAATGATAACATGATAACAACAAT
TTCAAGCATTT
>comp2713_c0_seq1
GAAGTATGTTCAACCTTCGGCTTCATTGATGGAGAATGCACCAGATTGGGACGCTGTTCA
TAATGCTGCGACATCATTTAACCCGTCAGTTGTGAATGTAGATATAACAGAAACCAAGCC

AGAACCAAACGACGAGTCCATAGTGGAAGAACCCCAGAGGAAGAAGTATGTTCAACCTTC
AGTTTCATTGATGGAAAATGCACCAGATTGGGATGCGGTTAAGCCATCCCTTTCAACTTC
CAACCCTTCGGTCGTTAGTGTCGTCGTATCTTCGAGTCCAGATGCCAATTCGAAGACTAA
TGTTGGAAAGAATAAAGAGATAGATAATGTGACTGGACAACCTAAGAAACGACGGTACGT
CAAGCCTTCTGTTTCCCTGATGGACAGTGCGCCTGACTGGAACGCTGTCCAGCCTTCCCT
TTCAACATCCAATCCTTCTGTTGTCAATGTTGACGGAATTAATAGAATCAGCGGTGGAAA
GCGTTTGGTTCGGTCTTCCGTCGTCAAACCGGATTCGGAAACTGATCAGAAGACCCGTGC
TGCACCAGCCGACAAGCCGTTTGAGTACAAGCAAGTTCAGTCCATTCCGAAGGAAAATCC
CGCGGGTGCGAAGCCTGTCCGAGATGAAGAAGCAAAAACTAAACTCCCTGCTTCTATTGC
GCATGCTGCTGGAAGTACTTCTGAAGGTTGGACTCCTTCTGCTCACTTTCCAACTGATGA
CAAGACCAGTAGCCCTGGTGATCAGGACTCAAATACGAAAGAGAGCAGGACTAGGAGTGC
GCAATCGAGCACGATGGAAAATTATGATCCGGATGCCTTGCCTAGACCAACAGGCGTCAT
TCCTCTCACAGTTTCTAGTAGCACACGAGAGAAGACTCAGTTGTTTCGCGAGGAATATTC
ATTAGAAGCGGTAGAAGAAGATGCAGAAAATGACGGAGAAGAAGAGGAAAACCCATCATC
ATCCTCACGCAGCGAACCGTCGTTGTCGGAACCAGGGAAGAAGAAACTGTTCTTGTACGA
TGGCGGGAATCCTCAGCCGAAAGAACTCTGGGAATTTAGCATCGATGGGTCTGACACGAG
ACTTGATCAACGTTCAGAACAAGAAATGAACACAACTAAGAAAAAGCTAGTCACCTACGA
TGGAAATTCAGGAAGCGAAGCTATCAGAAGCGCTGTTGTGGATAACAAGGACAAAAATAT
CAGTGGAAACGGAAATGGTCGAGAATCAGAGAATGGTTCTAAGTTGAAGGAAAAAACTGT
TTCTCCCTTTACAAGGGAAGTGAGTGATAACGATCAAGCTGCTGGATCCTCTGCGACAAA
ACCAGTGAAAAGTTCAACAAACGATGGTATTTCTATTGATGCAGAGTCAGAAGATGTTGA
GACGTGACAAGCATACTAGTGTTATAGTTAGGGATTTTTTAGTCTCCATGGTCCAAGTG
TTGATCATTTATTCATTATGGGTTACAGAAAGAGTTTCAGTTCAAAAGTTACAAGGGAGA
GCAAAAGAAAACTTAATTTAATTTTGTCTTTTATTTCATATGTAAAAAAAA
>comp2714_c0_seq1
CGAAACAAAACGAACAGTACACCACCGCACGCAGGCTCTTCAAAGGTTCGGGAAGAATCA
CCTTTCTCATTTGATTTCAAAACGCAATAATCGGTTGCGTTCTATTTCTCATTACGGAAC
TTTACAGTTGGCACCTACCCGGTACCTACCGGTAGAATACATGATACACAGCCTACGTTT
CAACCCGAGACGGTTGTAAAACTCGACCTTTCACACCATACGCAAGCAGAAGTGACGCTT
CCTTGTTCTCAAAGTTATACGCGAAGCTGGTCCGAGAAAATCGATACAACCGAACTTCCC
ATCATTAATTCATTGATCGCCCTCGAAAGGACGGATTGACAGAACGCGAAAGAAAAACTC
TGTTATGGAGGCAGCGCCAAAGAACACACGGGTTTCCTTCGGGAGACCATCCGAGCCAGC
CACAAACTACACAGCAACAAACATCAACGACCACCGAACCAGCACGGATGCACCACACAA
ACAGCAGCAACGAAACGAAGCAATAGAGGACGCAGCCGAAGGCGCTACAGGGCAACAAGA
ACAAACGAATCAGACCAGGCAGTTGTCACAGGTAGAGCAAAATCAGAGGAGCATGGTACA
ACGGAGGAAAGCCTCCCAGTCGACACCGAATCTACAGAAGATGGAACTCAATGAACGCAA
TCGTGACCACCACCCGCCAAAACGCCACAGCGTTGCTACGTCTGCCAATCTCGGAACCAC
TGCCACCACGAGGAGCACCATGCGGCGGTGCACCTCGGTCAGCAACATGAAGCGGTCCAG
GAGCGAGCTGATTCTGCTTTCACCGGAGTTCCGGACACGCCTCATGAATGAGCAAAAGGA
GCGGGATCCGCTCTTTTACTACCGCGTTCAGAAAACGATCGGAAACGGGTCGATGGGATC
GGTGGCCCTGGTGAAGAAACGCAGCCAGACCGTCGGCGGATCGGCCAGGAAGGTCGTCCG
GGAGGCTGTTCAGACTCACAAGCGGAAGCAGAAATGCTTCCAACTACCGTTTGGGATTGG
GGGTTTGTTTCGGCTGTGCATCGAGGACGAGCTGCGGGTTCAGGAGGACAAGGCACCTAC
GCACAGTAAAACGAGAAAGCAGAATCGTCACCACCGCGTTGGGGGTACCAAGAATGCCCT
GCTATCGGTCGAGACCTGGCTGGGATCTTCGGAGGGATCCTCGGACGTCTCCTCCTGCGA
CTCGACGTCGACGGCGGGAAACCCCGCAAAACAGGGGCGACGGAGGTCCAGCATCTTGTT
CCCTGCCGACGATTCCACCACGGCGGCTTCCACCGCTTCGGTCCGGTCGGCCCTTGACGT
CGAGTATGCCATGAAGTCCATCCACCTCAGCCGCATAACGGACGATACCTTTGTGCAGGA
ACTCGAGAACGAGATTGCGATCCTGAAGAAACTCGATCACCCCCACATCGTGAGGCCACT
CGAAACCTTCCACTACCGAAACCAGATATTTATTGTCATGGAACATTGTAGCGGTGGGGA
TTTGTATTCTCGGGACCCCTACACGGAGGAGGAAGCAGCGAGGCACATCTCCTCCATCCT
AAGCGCCGTAGGTTATCTCCACGCGAACAACGTTGCCCACCGGGATTTAAAGTACGAAAA
TATACTCTTTGTGAACGATTCTCCCAAAGCCGAAATCAAGCTCATCGACTTCGGCCTGTC
GAAGGTCTATGGAGGGGGAGTGAACGGAAAGAGCCAGACGCTGACGGAGGGGGTCGGAAC
AATATACACCATGGCACCGGAGGTTCTTAAGGGGAGCTACACTAAACAGGCCGACGTTTG
GTCCGTTGGAGTCATTGCGTACATGCTGCTGTCCTCGCAGATGCCCTTTTATGGGAGGAA
ACGGCAGCACATTGTCGAGCAGATTCTGGCTGGAAAGTACGACTTCCGGGGCAGACGTTG
GAGAAGGGTTAGCACCCCTGCCAAGGACTTTGTTCGGGATCTTCTGGTTCTCGATCCCGA
TGAGCGGCTAGATGCCGAGCGCGCCTCGAGCTGCGTCTGGCTCAACAAACGCTTCTCGGC
CTCGGTCCGCGGTCCCCGCGAATGCGAGATTCTGCAGGCGCGCAACTCTATGATGCGCTA

TGTTGGCTACACTAAGCTCAAGAAGATGGCCCTTATGGTTGTTGCCCACCGATCGACGTG
CGAGGAAATCGGGATCCTGCGCAAGGTGTTCCAGAAGTACGCGTCGATTGACAAGGGTGG
GTGCATCTCGTACGAGGGATTCAATTCAGCGTGGAAGGAATCGGGCCTACCGCCTGAGGA
CACCCGGGCTATTTTTGATTCGGTGGATTTGGATGGATCAGGTCTGATTCAATACACAGA
ATTCCTTGCTGCCACAATTGAAGCCCAGGGTGCCATCAGCGAGGAACGGCTGGCGGAAGC
CTTTGATCGATTCGATTCTGATGATTCCGGCCACATTTCGGTGGACAATCTTGCCGAGCT
GCTGGGCAAGGATTTTTCACGACAGGAAATTATTGACATCATCGGCGACGCGGTCGATCC
AGATAATGGTGGCGACGGAACCAGCAACCAAATGATCTCCTATTCAGCCTTTCTATCCTT
ATGGGAGAAGAACCATGAAAAGGAGGTCCGCGCCAACAAGTTGCGCATGCTGGGCAGTCA
AGCCAATCTGTGTCAGCTGGGTGACGATGGCTTTGACGATGTTGAAGACACAGACGTTGA
TTCACCACACTACTCCATGGAGAATTGTGAGGAAGCAGCTAAGGCTCGGGCGACCTTTCT
AATGGACAAACACGGAACCCTCAGAAACAGCAATGCACAGGAGCAGAAAAAACTGATTGT
CGTGGAGCAACCGATCCTTGAAGATGGTGCCGATTTCGATGATTCATTCACCGTGGACGG
TGGCATTCAGATATAGCGCACCAATGAGAAGGAACATACCATGGAACAGAATACGAACAA
TCAGTCTGATGCACCCCTTTTGTCAGGTCACAGCAGCAGCAGCAACAACTACAACTACGC
AACTCATTTTTTCATAATGCACCAATAATGGATCAGAACGAGCAAGCTATAGTCAGCGCA
AAAATAAATGACTGCCTCGATCCATGGAGATAACATATGAATCATAATTTTTTTCGAAGG
ATGAACCCATTATGGAAACGAATGTACCTAGGAAGCATTGCTGTGTC
>comp2715_c0_seq1
CTACGAAGTACTTCTAATATGAAACCAAAAGAAGAGAAGCGTAAAAACGGTTCTGACCAT
CGCGGTGACAAAATCTTTGCTCGAAACATAGCTATTGCATAGCACTATTGTTCTCGCCCG
AAAATATATCCTAGTTTGCACAAACATCGAGGTGTTGATCGATCATAATGACTGATACCG
TGGCAAGAAGATCTATGAATCTCGCAAGGAGTGAGAATGGTGAGTTTGTGGAGAGCATTA
ATGCGTATCGATGTGACCCAGTTGCTCTTTGCGATCTGACCAATCGACTTTCCCCCGCTC
TATTTGTGTCAAAGATTCAAGAAGCCCCGTGTCCGAGCGTCACTGCTCAATTCTATTGCA
AAAGCACGTACCAGCTGATTTCGAATTTCCCACCGAATGCAGCAAGTTCGATAGTTTTCG
CGCCGGTCTCGTCCGGCGCGATAGCAAACAAAAAGAAGAAGCTATTGCATTTGCCGGAAT
GGATCCTGGCTCTGCGGTCAAGGCAATGACTGCTAGGGAGCAGGGACATCCCAGCGAAGC
GCAACTCATTCGTCGAATGTCTTCTAGAAGGAACTCAAAAGAGTCTTCGTGGTTTTGAAT
ACCCTAGGTTTGGAAAGCATCCTCTGTTGACTTGGAATACATTTTTCATTCCTAAATACT
GCAGATTGATGTAGACAAAGTGTCGTTTGCTTCTCTATGACCGAATGCTTGCCTGCTCTC
CACGCCTTCTTTGATCCCCCTTAACACGCACTAGCGCGTAATCAATAAGTCTTCTGCCCA
TTTGCAAGATTTATGAATACACATCCACAATTACATGCTACACTACGAATTGAAGAATAC
ATATTCTACGACGCTGCGTCACACCAATCCTATTGCAATGGCACCTCCATCGTGAATACT
GCGATAGAAAAAATGATGTTATCTTAATTATACAAAAAGATTGATTGACAGCAACAATGA
CTTGGGTCATATTTGTGCTGGAGTAAACGTTAAAAAAAGTTAAATCTATTCTTTTCGAG
>comp2715_c0_seq2
CTACGAAGTACTTCTAATATGAAACCAAAAGAAGAGAAGCGTAAAAACGGTTCTGACCAT
CGCGGTGACAAAATCTTTGCTCGAAACATAGCTATTGCATAGCACTATTGTTCTCGCCCG
AAAATATATCCTAGTTTGCACAAACATCGAGGTGTTGATCGATCATAATGACTGATACCG
TGGCAAGAAGATCTATGAATCTCGCAAGGAGTGAGAATGATTCAAGAAGCCCCGTGTCCG
AGCGTCACTGCTCAATTCTATTGCAAAAGCACGTACCAGCTGATTTCGAATTTCCCACCG
AATGCAGCAAGTTCGATAGTTTTCGCGCCGGTCTCGTCCGGCGCGATAGCAAACAAAAAG
AAGAAGCTATTGCATTTGCCGGAATGGATCCTGGCTCTGCGGTCAAGGCAATGACTGCTA
GGGAGCAGGGACATCCCAGCGAAGCGCAACTCATTCGTCGAATGTCTTCTAGAAGGAACT
CAAAAGAGTCTTCGTGGTTTTGAATACCCTAGGTTTGGAAAGCATCCTCTGTTGACTTGG
AATACATTTTTCATTCCTAAATACTGCAGATTGATGTAGACAAAGTGTCGTTTGCTTCTC
TATGACCGAATGCTTGCCTGCTCTCCACGCCTTCTTTGATCCCCCTTAACACGCACTAGC
GCGTAATCAATAAGTCTTCTGCCCATTTGCAAGATTTATGAATACACATCCACAATTACA
TGCTACACTACGAATTGAAGAATACATATTCTACGACGCTGCGTCACACCAATCCTATTG
CAATGGCACCTCCATCGTGAATACTGCGATAGAAAAAATGATGTTATCTTAATTATACAA
AAAGATTGATTGACAGCAACAATGACTTGGGTCATATTTGTGCTGGAGTAAACGTTAAAA
AAAGTTAAATCTATTCTTTTCGAG
>comp2716_c0_seq1
CGACAGAAAGAAAACCAAGTTTAATTCATTCTATAAACATACTATATCAAAATGAGTGAT
AAGGAACATTTGTCTTTGGTTGTCTGCGGACACGTCGATGCCGGTAAGTCGACTACTTGC
GGTCACTTGATTTTCAAGCAAGGAGGAATCCCCGAGCGTGAGATGGCTAAGCTTAACGAG
CTCGCCGCCGAGAAGGGAAAGTCCTCCTTCGGATTCGCGTACTACCTTGATACGTGCAAG
GAAGAGCGTGACCGTGGTGTTACCATCCAGTGCAACACCAAGGAGTTCTTCACTGAGAAG
TACCACTACACCATTGTCGATGCCCCTGGACACAAGGATTACATCAAGAACATGATCACC

GGATCTGCCACCGCTGATGTCGGTATGCTTTTGGTTCCTGCCGAGAAGGGAGGTTTCGAA
GCCGCCATCGCCAAGGCCGATGCCAAGCTTGGAGTTGATGAAGGTCAGACCCGTCAGCAC
GCCCGTCTTCTCTACCTTTTGGGTGTTGAGCAAATCCTTGTCGGAATCAACAAGATGGAC
TCCTGCAACTGGTCTGAGGACCGATTCAACGAAATCAAGGAGGAATTCGTTAAGATGCTT
CAAATGATTGGTTTCAAGCCAAAGAAGGTTCCTTTCATCCCTTACTCCGGATTCCACGGC
GACAACTTGGTTGACGCTGTTGACCCCGCCAAGGCTCCTTGGTACAAGGGATGGACTGCC
AACAAGACCCCCAAGGTCAAGATCACTGGAAAGTCCATTGTTGATGCCCTCAACAACTAC
ATCACTCCTCCCAAGCGTGAACCCGATGCCGCCCTTCGTCTTCCTGTTTCCAACATCTAC
AACATTAAGGGAGTTGGTCAGATCATCTGCGGTACCGTCGAACAGGGAACTCTTCGACCT
GGTGACCAAGTCGGATTCGTTCCTTCCAACCTCAAGGGAAAGAAGATTTTCTCCATCGAA
CAGCACAAGAAGGTTCTCGAATCTGCTGGACCTGGTAACTCCGTTGGTCTTTCCATCAAG
GGTATCGCCAAGGATGAGAAGGTTAACCCTGGTGACATCATCTACAACGAAAAGGACGGA
GAACTCAAGCCTATCAAGAGCTTCACCGCCCTTGTCGCCGTCCAAGAGCACCCTGGTGTC
CTCAAGCCTGGATACTGCCCTGTTGTCTTCTCTCGTACCGCCAAGGTTGCTTGCAAGATG
ACCAAGATCTTGTGGAAGATGGGAAAGAAGACAGGAGGTGCTAAGGTCGACAACCCACCA
GAACTCTCCCAGTTCGAGAACGCCGAGATTGAATTCGAACCTACCGCCCCTCTCTTCGTC
GAACCTTTCGCCCAGTGTGCGGCGCTTGGACGATTCGCAGTTATGGACTCTAACAGATTG
AAGATGCTTGGAAAGGTTTCGGCTGTTGTCCACAAGGACTGAACAGCATTTTTTCACTGT
GGGATGCTTAAGGAAAAGAAGTATGGATGGATCCAACATTTATACAGTCACATAATACGG
AACTAAGCTAAGCTAATTACAACTAAGTAAAAGGGTTTAACTTCATTCACAACAGACACA
TCTTTCGATCCCCTTTCGCAGTTTAAATTCTGAAACATTTGTCGTGCAATTGATCAGCTC
CGTAGATTAAGACCGCCTCTAACTAATAAGGCATGTTTATCTGAAGGATGATGTAGGTTG
TTCCACCATGACTTTTTTCTGATCGGCATTGGTCACAAGTACGAGTACCAGTAGTTCCAC
TACCGTATGTTTCCGTTAGAAAATACGTCTACCTGAGATTAGAACGATAATATTATTAGT
AAGTGGTAGAAGTACAGCGACCCGTATTGCACGCTACAGTACCATGATAAACCCCAAATA
CAATTATTTAGGGATACGGATAATTGCTCCA
>comp2717_c0_seq1
CCAACTAGGACCGTCTGACTGCTGGTTTCGGATCTTTGACACGGTCCTTTCCATCCAAGT
GAAGAAGGAAAAAGGCGACGCCGAAAACCGTTCCGAGTCGCTGTTCATACCTGTTCCCGC
AAACGAGCATTCATTGTACGGAGATTTGACACCAAGGCAAAACATGCTGCTATGAAATGA
AGCAGTTGGATCGTCATAATGTTTGCATCCGTTGATGCTGAATGCTTCTAGTATGGACTC
TTATTTTGGACACCAACGGGATGATTGCTGGTACAGCGACGAAGACGAAGGCAGCCTATC
ATCCCGATCCACCGATGAAGTTCTGAGCGAAACCTGGCTCGATTGTCTTGCGAATCGATA
CGGGTACTCAAAGATCGACGCCTCCTCGTCGGCCACACCCATGGCATCCTACCGCCGAGG
AGCATGCCGGTTGAATTTCTGGCTGACCACGGGGACCGTTGGATCGTATCTCGAGCACCC
TAGGCAGGGGAAGACCCAGTTGTTTCGCAGAGGTATCGACCGTGATGAGGCGGAAAACAT
TTTTGAAAATCCCAGGATGCATACCGGAAGAGGTTATCATACTAGGAATGGCAATGGTGG
CAGTCGAGGCCCTTGTCGGTACGGGCAGGATTGCCATAGGGCCGATTGCTGGTTCGACCA
CTAAAAGAGTGGAATCTTTGGTCGAGGCATGCAGGCAAATGTGTAGATAGCTCCGTGGCG
TCATCCAGTCCAAGCGCTTGCACCACGCAGGTACCGGTGTGCACACAACGGTAACACAGT
GGTCCCGTCATAATAATTTACAATTAGTTTCTCTGAAAGTACATGC
>comp2718_c0_seq1
AGGAAAGGGAGGAAAGGGATACTATGGACCCAAAAACTACGGATACTATGGACCCGGATA
CTACGGAAGCAAGGGAGGAAAGAGCGGACACGATGGAGGCAAACATTACGGAAAGACAAA
AGGCAAAGCTAGAAATCTAGGACAACAACCATACGGAGGCAAGGCTGGAAAATCTGGAGG
TTACTATCAATACGAAAAGTGTGAGATCGTTTGTGATGGCGATTGGTGGTAGATTTGATC
ATTAAACTTGTCGACGACAAGTACAAAAAAGATCGCCGAATGAGCGGCGAGCTCGGGTCG
CCAGTATTGCTGTATGCCGTCGCAATACGGACAGAATATGTGGCAAATCAACTGCTCCAG
GAAAAGTTTCATCTCAGGTTGTTGGCATGGTCGAGTGCAATTTTGTTTTGCGCTGTTTAT
CGATTTGTTTGTGCCAGCTGTTTGACCTTGGATGACTTTTTGAGCAAAGGGACGCAAGTT
CTTAAAGCACTGTAATATGTAACAATAGGTCAAAGTAACAAAAAAAACAATACAGCA
>comp2719_c0_seq1
GACCCGTTTCTTGCTTCCAGTCCTTTTTCTCCAGAGAAGTTACATTGGTCGTTCTAAGAA
GATTCGACACGAACTGGGTGTTCTTGAAAAAATTTCAGAAGAACACCTTCAATCATCACA
ATGTGTCATAAAGTGTATCATTATGGCATCGTGGTTTGTTTCTACATGTAGGTTATGCTA
GGTGCTGACGCTGATTGCCATAAACTTGGAGAAAAAACAGATTCGTTCCGAGACAATCAT
AAACTAAATTGCAACTCACCACGGCAATCAACATCATTCACGATACGATATGAATAATGT
CAGCGACGACGACAGGAGAAATTGGACTTGCAATTTAGTACGAGTAGTAGAAACAGACCC
TAACTTCAGTCCTGCCACTGCTGCTGTCGAAAGACTGCAACTGTTCCCCGAGAGAGAATG
TAAACGCATACGGCTTCTCTCCGAAAGGGACGACGATTATTACGAAGCCATCGACCAGAT

TTGCTACCATAGCCCAAGTTGTATCCTTCAAACAACTGGAGTGGAAGAACGAACAGGAGG
AGCCTTTGACATGACACCACTTTCTCAGATCTCAGATTCGATAGCAAAGGAACACGTTCA
ACCAATCGAGACAAATGATTTCATATTTCAACCGAGTGCTGATAGCAATGCAAAGAACTC
CTTTTCTGACAGGACAACAAACAAAGATCGGGGTATGATCGCGAAGGAACGAAGCACATT
AGTCCGGTGCATTGCATGGGATTCTAGGCTTGCTCTACTTGACTCCTCCTCATCCTCTGT
GTCGAATCGTGAACGATTCTATCCAGCGAGATGAATGCGCTAAAGATGTTTCGATGTAAA
ATGTATGTAAAAATATACAACTGCGGTCGATTGGGATCGTGACGTCGGAGCCTGCTACCT
GCTGTAACGCATCGCTGGTTTATTTCGACAATTTTCACTGTTCTATTCCATGTTGATATA
AAAGTCAACCGGTCGAGCATGCACATAATCACAAGAATATACTATTTTTGAGCTGTTGTA
AAAGAAGGGTACTGCTAAAAAATACGACTGATGTAAACTACAAAAATTCATAACTAATGC
AATCAATGCTTTTCA
>comp2720_c0_seq1
AATTTCGAAAAAATAGAGGACGACAAACGAGGCGGCGGAATCACCATTTTATCGCATCGA
CGAGAGCAAAGAGCGCAAGTGCCATGTCATCTGGAACTTTATCTCGCGATGGTTCTGCCG
AATCGAGCGCTTCAACTACCGCACCTAGGCGTCGACGAAAACGCCCGCTTACCAATACCA
ACACCGACAAACTCTGCCAAAATGGCTCGGTTCTGAGCAAATGGATATATCGATTCAACT
TGTGGACGGGTCTGTACATGCTGAATCCGTACGAGAGATTCGTGTTCCACTTGGTTGGTT
GGTTCTCGTTCACCGTTTCGTTGCTTTATGTCTACGTCTTCTGGAAGGGCTTCATCGAGG
GTTTTCGGCACAGTACATGAACCGAGCAGTACGCGGAGCGGTCGTAAGGACAGAGCGCTC
TCAAATCTCCTGCGAGATGGAATCCGGGGCGAACAATTGTGCACGATGGGAAAAGATCAA
AGGATGCTTGCAATGCTATACACGCGTTCGATTTAGGCTAGACGCGAATAGTACTTTCTT
CTCGAAGAATGGATCTATTGCAAGCCGGACCATCCGGGGTTTCTAATGCTAAATTGACTT
TGTACCACTCAAAGCGCAAAGCGAAAAAATGTTACAAACCTTTTTTTAAACTATTTTGAA
ACCATTCGATGTAGCATCCACTCTGCAGGAGAAAAAATGAATTGTTTGGGGATGCCTAACG
TGTGGAACAAAGTAACTTGAAGCGGTGTTTCGTTGTCACATTCCATGGAATTGCCCACCC
TCCTGCCTTTTGTCTCCGTTGCCGATAACAAGATTCTTTTGGCAAGTCCATGTGAAGCCG
AGTAAAATACAGCGCGTATATCCTAGGTCGTTCCCTGCCAACCCACGGATGTCCTGGTTT
GCGCTAATTGCGATGACTGTGATTCTGTTTTGTGTTGCTGTGGCATCAATGAAAACAACA
AATACATTATGCTTGGGAACAACGTAGATGTATGCCTGTGCGGTTTGTATCAATGGCAAT
TAAGTATAGTATTGAATACAATTCATTGATTGAGTCGGGATACCGCAGCATTCGTAACTT
TGGGCGTGGTTTTCAACTACTCTACTAGCGAGAAAGACATGATTCACCATCCCTATCTTC
GTAATGTACCTTCTAACATTAAATTATACTCCGAA
>comp2721_c0_seq1
TTTCGAAATCGGAAATCAAATTCAAGTACTTGTTGCTCTCGTTGATGTCGTTGAAAGTTC
GTCCAAATAGAGGGGTCGACGAGGTGCAAAACCGAAAATAACTTGAAACGAAAGTACAAC
AGTAAAACTTCACAATCGTGCTGCCTACCAATCTCAAACCAACGGATTTTCTGATTCATG
TCGACAACCAAACACTACGGCAATCGAAGCAATTGATTCGAAGCCGAAAGGATTTTGTTC
GCACTAAGAATCAAGAAATCGAAGGAATTGTCAGCGTTTTTACTCCGAGATATACTTCGA
CACTGTTTTAATATTGTATTTTGCAATGGCTCATCTCGAACAGTTGACTCCACAAGAGCG
AGAATGGGCACTCGCTTTGGATGAAGCGATTTACGGGGAAGTCAACCAAGGAGTTGGATG
GAGTCTTTCTCCGAGAGGGAATAGCAGCAATGATCCTAAAACCACGCCGACGACAACAAA
TTCGCGAGCTGCTACTGAACTGTACGGCAAGCCGTCGGACTTTGAGATTGTAGCACACGC
AATCCGTGCCAAGGGCGATGTCCAAAGAGGACTTAGGCGACTCAAGCGCATGAAGATGTT
CAAGGATGAATACAGCGTTCCCGACTTCGACTTGGATAGTAGTAGTAACGATCCGCTGGA
ACCTATCCTCAGAGTGATGAAGAAATTTCATTTGGCCTACCCCCATTTTATACAAAAAAT
TGGGATGGACAAATTTGGACGCGTTGCGGTCCAACTTCAGTTGGATGGACTTAGATGGTC
GCTTTCGCCACCTTTCAATCACACTGAAACTGAACGATTTCAAGCTTTCTGCTTTTTATT
GCACGCCACACAACCTACCCTTGAATCAATTCGTCGCGGAACAGTATGGATCGGGGATCT
CCGAGGTATTTCGCAACGGCCATCTTCTTCTATGTTTCGCGGCGCGCGAATGCTATTTAG
AGATTCCTATCCTATCAAAGTCGAAGATATACCGGTTGTAGATTGTCCACCTAAGTTCTC
GGTTGCATATGCAACCACATACCCCTTTTTATCGAGTCATTTTGTCGACAAATTTGTTCG
AGTTACACCGGAGGTACTCCGACAACATTTTCCACCTGAATTATTGAGCGATCGACTACG
AGGAATCAACAATACGAAGAACGATAACATCAACAAGCGACGATTTCGATCTAAAAAGGC
TAGCAAGAATAATGCAACCGATCAGAATACTAACGCTGTCGATATTGCTTCTATGGATGG
AGATGAATCTGAAGCCTCGTGGGAAAATCTTGACGATGATGACGATGACGTCGTAAGTTA
TGGTGGTAATAACGGTAATAATTCTGGTAATGGGAGTGTTGGCAATGGGGAAACGGACGA
GAGTGAGGTTTTGACGCGAATAGAGCGACTCCTTCGAATGCGATTCGAGACAGAAAAAAA
TTTTCGACTGGCTTGATCGTTGTCTTCAGACACCATAATTCTTTTGCAGTGAAAGCATTT
CAATGGATCCAAGGTCGTTGCAGATCTGTGGTAGCAATAATTTCCCATCACTTTTCACGA
ATCAGAATTGCTTTACAAAGGTATCTCTTTTTCCTTTCCTTTGGCATAGCAAACCATGAT

ATGATCTGCGTTGGTAGAGTGAGTCATACGGAACTACGGTTGCTGACTTCACTCAAAGCT
GACTCTCTTTATTTGAGCCTCATCTCGCATAGCAAGAACCTATTCAATTTCTCTCTCCTG
CAGCGTTGGCTTCTCTTGCACCATTTTGTTTTCCTAATCTTTTATGTAAACAATCATCAA
ATCAAAAACTTATACCGGTCTTCCATCCACCACAGCTAGTATGCACCAGTCACTTCCCTC
CTACTTGCACCAGTCACTTCCCTCCTACGACAACAACTACTAGTAGTAATCATCTTCATC
CTTGGACATGCTTGGAACCAGGGCTCTATTTGTCAGTAAACATCATGACCAGCAACTATA
TTTCAAGGCACCAATAGTAGACGTGTCCCTCCAACACTCACTTATTCCCCAATGATCCTT
AAGCAAAAGGATTCTTATCTCAAGCTAACAATACTTTGCTGTCAAATTGCCTCACCAGAG
TGTATGAGAAAAGCTTCATGCAAGTATATGCATACTCCAACAAACGTCCAATTACCCAAT
ATTTTCCATCTTTGCCTTACTGTAAATAACCCCTGGAAAGCCCCCAATTGAACATTTGTC
ATAGTCCTGCTTCACTAATGAAAATTTTCAAAGAGGAGAGTTCATCTGGCAAAGAATCTT
TTGCTTTTGGGTCATAAGTTTACATCAAAGTACTACTGGTATGCTTTTTTGATGATGGTG
ATAACTCTTGTTAGTGGGGTATCCATATTCTACACCTATTTTTCTTGTATCTTTTTTGGT
ATTTTTTCAACCACTCTGCTCCTTAATGACTTTTGGGATTGTTTATTTGTCAATTTTTTT
TCGTGCACAATGGAGAAGGATTGTAGTAATTTGATTTTTGTTGCAGACGTGATCGGATGA
CAAAGTTAAAGATGTGAAATAGTGTCGCTGTAAGTGACGTTTCAATCGGAAT
>comp2722_c0_seq1
CATTGTTCAAAACAAGAAGATTTCCAAGAACAACCAACAACAGAAGTAATACTACCCAGG
TATTATTACAAACACTTAATCCAAACTCTTTTATATTCATTCGTATACTCATTCATTTCT
AACCATGCCACTCGGAGGACGATTGCGACGAGAAGGCCGCAGAGAGGCCCGCAGAGAGCA
CCGGGAGGAACGCAAGGAGCACATACCCCCCGCTGTGAAAACCGCCGTTGTCATGGGAAC
CGGAGCCGGTCTCGGAATAGCCGTTGCCCGCCGCGCCAAAGCCAAACGCAGCGGCTCCCC
CGACTCGAGCTCCTCGTCCGACGAGGACGACAACGCCCCCAAGACATTTGCCATGAAGGA
AAAGCTCCTGGCCTTTGGAGACTCCTTCACCATCAACCGCGTGAGCCGCCGCCACCAGAG
GGGAAGACCCGCCTACTATGCCAACAACAAGGTGATGCGCCTGCGAGAAACCTTCCACCT
CCAGGCCAGCCGCTCGGGACCGACGCTCTATTGCATCCAGGACCGCAAGCTCCGGATCCG
TGATTCCATGGCCATCGAGGACGCCGACGGAAGGAAGGTCGCCGAGATCAAGAAGAAGGT
CATCGGCATCGTCCGGGAAAACTTTGTCGTGAAGATCAAGGACGAGACCAACTGGCAGAT
CCACGGATCGATCCTGGAGCACGACTTCACAATCAAGGAGAACGGACGAGAAATCGTTAA
GGTGCACAAGAACTGGATCGCACCAATCCGCGATTGCTACTTCATCGACATCCATGGCAC
GGACGACGTGCCCCTGGCCTTGACGGTCGTCATTGGACTCGAAGCCATGAGCGAAGATTG
AGGACCGAAGGGTTGATCGTCTGCCTTTGTGGGGGCTTCGTTAAACCTATTTCCTTAGAC
TCTTCGATGGTTGGAAACGAAAATAGAATCGACCATTCATGAGACGTTGTGTCGTTCAGT
TTGTTTCTGAAACTAGACGAATTCAAGCACCAAACCACACCATGTAAACTGAGTAAAAAG
CATTAGCGTAATTCTGAAACTTAGTAGTCTACGATATTACTAAAAAA
>comp2723_c0_seq1
GCCCGGACTAGATCGGAAAGGTTCGATCACACGTAGAATCAGCAGAAATCACTTTATTTG
CATCAAGCTACGAAATCCATAAGGGATGATTAGAAATACACTGGTGCAAACGAAATGTTA
CAACAAGAAGTAGATCGATAGAACATTCACGACAAGGAGCCGGTTCCAAAGCGGGAATTA
TAGCAGAAAAAATACAGATAAAAACATTCGGTATTGCGACTATGTCGGATCACTCCGAAG
TAGAAGAAACCCATCGCCGACCGTCAGTAATAGAAGTAGAGGGAGCGAGGGACGATGTCA
TCGATGATGTCATCGATGACGGCACGGAATCTCGTGCATCGATCACGACAGTCCCTTCGC
CATCTAGTCAAGACGAACACGGAGATTCTCGAACCACTGCCTCTTCCCAAACCCGTCTCG
CGAACGACGCTGCCGATTGCTCCATCGCCCCTCCCAGAGACCACTCGTATCTGGAAGAAT
CTCATCCTTTATTACCGGATGAAGATCGCTATTTAAGGATGGCCAGCGGGAGAGCTCTGC
ACAGAGATTCCACGGGTCTCAAACCTACTGCAAAACGTACACTTGAAGTGGCCGTCCTCG
AGTTGCACGGGGTGGTACTGTTTCCGGGATGCACCATTCCGGTCAAACTGCAAAATCGGT
CCATGATTAGATACCTCGGACGGCAGATACGATTGTGCAGGAATGATCCCGTTGCGCAGC
CAGTTGTGCAGCTGGGTATACTGCCCTACGAATACCAGTCCCCGGAACAGACATTGCCGC
AACAACACAGGAGGCGCAGACGAACCAATGAACCTCAAGAAGAACCACAAAGAGCGTCTC
AACGGCGAGGATCGTGGATGCGGCGAGGATTTTCCTTTGGGAATCGTGAGATTCGGGCAA
CCGAGTCCAGTCACGCCCTGCGGCGAAGCCTCACCCGTCTGCAAGACGAATTCCAGTTCG
ACGAGAACATGGATCTGGAGGACTCGGAACGAAGCTCGGGAGACGATAGCGATGGAGACG
TTGGTTTTGACGAGAACAACGATGAGCATTCGTTGTCACGTCCGCCGTTGAGTCCAGAAC
AGTCGCACCCCCTGGTCGGGCGAATCGGGACGATTGCAACGGTCCGGCACACACATGAAC
GAGCGTCATCCGTGGGTGGACATGGAGGGAGCGATCCCAGTCATGGGCGTTCCCAATCCT
CGTCCACTCTCTGGGGACGCTATGAAGAAGCCCCCGAGCTCGTCTTTACCGCGGTGGGGA
CGAGCCGCTTTCGCATCCTGTCGTGCCTCAACGACGACGAGAACCTTTTTGAGGTGGAGG
ACATGCGCGACGAACCGTTGTTATTGCCACCCGTTCCCTTTCGGCTTCTTCCCTTCCGGG
GACAATGCGTCCACTTTGGTAAGGTCAACGACGATGACGAAAGCGACGATGGCAACAACG

ACGACGAGCATTTGGGAAAAGGCTCGATCAAAACGGGCCGCATCAGAACAAGTGAATCGG
ATGTTGTTGAAACCAGATCGACACGGATGCAGGCGCCAATCGAACGCATGGCCTGGAATC
TCTCTAGGCTCACACCGGTTCCGTACTTTGTATATCTCCAATTGATGCCTTGGAGCCTTG
TTTGGAAAATAGCTGAAGCGCTCCAGACGAACAATGGACGGGGAAATCTCCCCTCCCTTG
GAGATGACTCCGTCGTGACGAACCGAGAGAATCTGGAACCGACGAAGTTTTCCTACTGGA
TGGCTAACAATGCACCCTTCTCCGAAGCAGAACGGAAGAAACTATTGAAAACAAGCTCCG
TGCTAGAGCGTCTTCTCTTCATCTGGAAGAAGGTCTGTAGATGGACGGCGGAAAATGCGG
AAACCCGCATCTGCTGCGAATTGTGCGAGACAAACCTCACCGGCGTGTCGAATATCTTCA
CGGTGGGTGGCGCCGAGGGGACCACCTCGACTTACGTCAACGGCCACGGCTTTATCCACC
AGATCACCACCCTCCGTAACGTGGACGACAGCAAGCTCTCCTTCCAGGGTCTACCAAGCA
CAGAAAACAGCTACTTCCCGGGTTACAGCTGGCTGATCACCTCGTGCTACAGGTGTGGAT
CTATTCTAGGATGGAAGTTCCAGAAGGTGGGAAATTCGGGGATCGATGCCAATGGTGGTT
ACGAGAATGGCAATGGAAATATCGTCGACAGAGACACCCCCCAACGCCCTCCACATTTTT
ATGGCTTCATGTCCTCGAACGTGAAAATCAAAAGCAGGAGTCGATAATCTTTCTTCTACA
TCTGGATATTGAATCAATCATCAGGCCTCATACTAGAGAACATCAAGACTAAACAGGGGG
AAAAAATTAACATGAGTTAGTCTCGAACTAGGTATTAGAATACATTTCACTAAGCACTAG
GTGAAGTTCGTTCCCAAGTAGTAAGCACTAATCGGGATTCGCAAGGACTTACAACAAGTT
TCAGTTTACTAAGAGCCCCAGGACCTCAACCACAGCGCATGTCAGCCAAGCCACTTCACT
TGAATACCTCCATGCTGGAGTTATACTCTTACTAGTACAAATAAGTACCAGTACGTACTT
CATTGTTGCAGGGTAGAGCAGAGAGTGGCGTTCTTTACAGTAAGTCGTGTTAGTCGTGGA
TAGAACTTGCGAAAAAATAGACCACAAAACTGGTCATAACTTTGTTTTCTGATGATGAAC
GGGAACACTTTTTCATTACAGCATACAACGATTGCTATAATCTTAGAGAGGCAAAGGAAT
GCATGCATTAACTTGCGTCCGCGATGGCAAACCCACTAGAACACACTTTCTCACCTGGAA
ACGATCATGAATCCAATGCAATGATGAGGATGGCTGATCCTTCACAGACTTTCTCTCTCC
TCAAGAGTTAAACTTCAAT
>comp2723_c0_seq2
GCCCGGACTAGATCGGAAAGGTTCGATCACACGTAGAATCAGCAGAAATCACTTTATTTG
CATCAAGCTACGAAATCCATAAGGGATGATTAGAAATACACTGGTGCAAACGAAATGTTA
CAACAAGAAGTAGATCGATAGAACATTCACGACAAGGAGCCGGTTCCAAAGCGGGAATTA
TAGCAGAAAAAATACAGATAAAAACATTCGGTATTGCGACTATGTCGGATCACTCCGAAG
TAGAAGAAACCCATCGCCGACCGTCAGTAATAGAAGTAGAGGGAGCGAGGGACGATGTCA
TCGATGATGTCATCGATGACGGCACGGAATCTCGTGCATCGATCACGACAGTCCCTTCGC
CATCTAGTCAAGACGAACACGGAGATTCTCGAACCACTGCCTCTTCCCAAACCCGTCTCG
CGAACGACGCTGCCGATTGCTCCATCGCCCCTCCCAGAGACCACTCGTATCTGGAAGAAT
CTCATCCTTTATTACCGGATGAAGATCGCTATTTAAGGATGGCCAGCGGGAGAGCTCTGC
ACAGAGATTCCACGGGTCTCAAACCTACTGCAAAACGTACACTTGAAGTGGCCGTCCTCG
AGTTGCACGGGGTGGTACTGTTTCCGGGATGCACCATTCCGGTCAAACTGCAAAATCGGT
CCATGATTAGATACCTCGGACGGCAGATACGATTGTGCAGGAATGATCCCGTTGCGCAGC
CAGTTGTGCAGCTGGGTATACTGCCCTACGAATACCAGTCCCCGGAACAGACATTGCCGC
AACAACACAGGAGGCGCAGACGAACCAATGAACCTCAAGAAGAACCACAAAGAGCGTCTC
AACGGCGAGGATCGTGGATGCGGCGAGGATTTTCCTTTGGGAATCGTGAGATTCGGGCAA
CCGAGTCCAGTCACGCCCTGCGGCGAAGCCTCACCCGTCTGCAAGACGAATTCCAGTTCG
ACGAGAACATGGATCTGGAGGACTCGGAACGAAGCTCGGGAGACGATAGCGATGGAGACG
TTGGTTTTGACGAGAACAACGATGAGCATTCGTTGTCACGTCCGCCGTTGAGTCCAGAAC
AGTCGCACCCCCTGGTCGGGCGAATCGGGACGATTGCAACGGTCCGGCACACACATGAAC
GAGCGTCATCCGTGGGTGGACATGGAGGGAGCGATCCCAGTCATGGGCGTTCCCAATCCT
CGTCCACTCTCTGGGGACGCTATGAAGAAGCCCCCGAGCTCGTCTTTACCGCGGTGGGGA
CGAGCCGCTTTCGCATCCTGTCGTGCCTCAACGACGACGAGAACCTTTTTGAGGTGGAGG
ACATGCGCGACGAACCGTTGTTATTGCCACCCGTTCCCTTTCGGCTTCTTCCCTTCCGGG
GACAATGCGTCCACTTTGGTAAGGTCAACGACGATGACGAAAGCGACGATGGCAACAACG
ACGACGAGCATTTGGGAAAAGGCTCGATCAAAACGGGCCGCATCAGAACAAGTGAATCGG
ATGTTGTTGAAACCAGATCGACACGGATGCAGGCGCCAATCGAACGCATGGCCTGGAATC
TCTCTAGGCTCACACCGGTTCCGTACTTTGTATATCTCCAATTGATGCCTTGGAGCCTTG
TTTGGAAAATAGCTGAAGCGCTCCAGACGAACAATGGACGGGGAAATCTCCCCTCCCTTG
GAGATGACTCCGTCGTGACGAACCGAGAGAATCTGGAACGTAAGTAGTTGGTTCCTCTCA
GCTTGTTGCCACGATCATTTTGGTTTGTGAGTCTTTCGGTTTGATCGATCTTGAACCCTA
CGAGTATCTAATGGATTTTGTCGTCTGCTTTCTGTTTTCGTTCGCTTGAAATTTCAAACC
AGCGACGAAGTTTTCCTACTGGATGGCTAACAATGCACCCTTCTCCGAAGCAGAACGGAA
GAAACTATTGAAAACAAGCTCCGTGCTAGAGCGTCTTCTCTTCATCTGGAAGAAGGTCTG
TAGATGGACGGCGGAAAATGCGGAAACCCGCATCTGCTGCGAATTGTGCGAGACAAACCT

CACCGGCGTGTCGAATATCTTCACGGTGGGTGGCGCCGAGGGGACCACCTCGACTTACGT
CAACGGCCACGGCTTTATCCACCAGATCACCACCCTCCGTAACGTGGACGACAGCAAGCT
CTCCTTCCAGGGTCTACCAAGCACAGAAAACAGCTACTTCCCGGGTTACAGCTGGCTGAT
CACCTCGTGCTACAGGTGTGGATCTATTCTAGGATGGAAGTTCCAGAAGGTGGGAAATTC
GGGGATCGATGCCAATGGTGGTTACGAGAATGGCAATGGAAATATCGTCGACAGAGACAC
CCCCCAACGCCCTCCACATTTTTATGGCTTCATGTCCTCGAACGTGAAAATCAAAAGCAG
GAGTCGATAATCTTTCTTCTACATCTGGATATTGAATCAATCATCAGGCCTCATACTAGA
GAACATCAAGACTAAACAGGGGGAAAAAATTAACATGAGTTAGTCTCGAACTAGGTATTA
GAATACATTTCACTAAGCACTAGGTGAAGTTCGTTCCCAAGTAGTAAGCACTAATCGGGA
TTCGCAAGGACTTACAACAAGTTTCAGTTTACTAAGAGCCCCAGGACCTCAACCACAGCG
CATGTCAGCCAAGCCACTTCACTTGAATACCTCCATGCTGGAGTTATACTCTTACTAGTA
CAAATAAGTACCAGTACGTACTTCATTGTTGCAGGGTAGAGCAGAGAGTGGCGTTCTTTA
CAGTAAGTCGTGTTAGTCGTGGATAGAACTTGCGAAAAAATAGACCACAAAACTGGTCAT
AACTTTGTTTTCTGATGATGAACGGGAACACTTTTTCATTACAGCATACAACGATTGCTA
TAATCTTAGAGAGGCAAAGGAATGCATGCATTAACTTGCGTCCGCGATGGCAAACCCACT
AGAACACACTTTCTCACCTGGAAACGATCATGAATCCAATGCAATGATGAGGATGGCTGA
TCCTTCACAGACTTTCTCTCTCCTCAAGAGTTAAACTTCAAT
>comp2724_c0_seq1
CTCAAAAGAGCCAAGCCATGTCCGTTCGGTTGGATACTATCCAATCTCTCGGGAGAAACC
TGAAGGATTGGTGCCGACATACCCTTCCCCTGATTTGTTTGGCCCGATACTCGTCCGATC
GGCCCATCGATTGGTTTTTCTTTTGGATCGGTTACAAGTGTTGTTGTAGGTGCTGGGGAT
TGGGGAAAGGTTTTCGAACAAACAGGAATTCAAATGCGAGTGCGAGTGCGAATAAGAATG
TGTTATCTGTTGATGGAGTGGAATTGGATAGTCCCACGGGAAAGCTCAAGAAGAAGGGAC
AAACGTTACAGGAAAGAGATACGAGCGATACCTTTATGCACAATTCTATCGAAAGCATAC
AGCTCACGGACCTGCCATCTGTCGTGATTTCGAACGTGGCTTGTTGCTTGCATGTGAAAG
ATATGACGGCCCTGGATGTTGCAATCCGGTCAGGTTGTTCGGCTGCTTTTATTGCTTCCG
ATGAGCAACAAGAAAGCCAAACAACAACAATAACACAATCAAGACGCGACGCTTCAGTAA
TTCTACACGACGTTTGGAAGCGACTCTGGTATCGCGATTACGGCGACGTCTTGCTCAGGT
GGAGAATTTCTCGGGAGGCACTTCAACGTTCTCTGGCGTCGTTGGGAACAAAGGTATCAC
GTTCTTGCGACAGGAACCAATGGCTGGAAGAGGAGCTCTCGCGTTTTCTCGACGACAACA
AGAGCACGAAAGACTTTTATTTTATATTTGGGGAATGCTACATGGACTATATTTTGGCCG
GGAACAACAAAGCCGACGGCTGCTACCTCGGCTTACACGGGCACGTCTTTGATTTTACTA
CCTTTGCCGACTACCACCCCGGCCTCGTCGAACCCATTTTGAAGGAATGCGGAGGCGATG
CTACGTTTTTCTTCGAGGACCTCCCCCACTCGGAAGGGGCCCGGAGGATCGCCGAGCGGC
TCTGCGTGGTGGTGCACCACGGATCCATGGACCGTTCCAGAGACGGCGAGCCCAGTGGCG
GCCACCGGTGCGGCCTGGAGCTGGTGAAGAGCGGGGCAGAGGACGCCTCGGAACTGGAGC
GGGTGCTTCGATCTTCGAACACCCGCCCGCCCCCCCTCGCGGACCAAAAAGGAAGCGGCC
GGAGTCGAAACCACCGATGGAAGGCGCACCACGTCCTCCCTCGCCGCCGCATGCCTATGC
GGCACGCGACGCTAGAAAGGATCCGGACCAAGTTCCACGAAGAACGGTCGTCGCAATTGG
AATTGCTGCGGCGGCAGCTACAAACGCAACAAAACCGGTTTTGGAAGCAAGTAGACAAAG
AATCGTTGAAAACCCGCATTTATTACGATCCGTTTCTTGAAGAATGGATTCGATGGGCCC
CATAGTGCAGTGAAATTGCGGGAATGCTGCAGCACTAGGTTGAATTGGACTGGAACAGAA
ATGTGGTCCGATAAAGAAAGAGCTTATACGCTTTCGAAACAATCCTAACCGCACAGAACA
AAATTGTTACATTGGAACAACATTTCTGTACAAAGTAACTAAAACATAAGAAGAATAGAG
TTCCATGCATCACAAAATTTTACATTACATTACGTTACATATAGAGTGATAGGAACGTTT
TGC
>comp2725_c0_seq1
CGTTAGGCATCTTCAATCCTGTTGCAACAAGACGAAAAGTTTTCCCACAAGTCACAATAC
TAAAGAAATTTCGTGCCAAACACCATTGGAATAGTTGTCGATAAAATTAATTTCGTCATC
TGTCACACAAAAGAAATCAAAAGATCAAAAGCGATAGTTTTCACCAATACGAGAAGATTT
TTCGTCGAAGCCTGAATTGAAGTGCGGTCATTCCAATAATAATTTGGATTTTTTCGTTCT
CAAACCCATTAGGCTTTCAATCAATCTACAGCAAGGATGGAGAATACATTCATACCCCAA
GCAATCGAAATTGTTGGGAAAGCAATCGAGGCTGATAATGCCGGGGAATACGAAAAGGCA
CTCGGCTTGTATCGAGACTCTCTGGGACGATTCACTATGGGACTGAAATACGAAAAGAAC
GAGGCCCGCAAAAAGCTCGTTTTGGAACGTGTGGAAGGTTACATGAAGCGCGCCGAAGAA
TTGAGAGATTATGTTACCAAACAGAACGAACTCGACAAAAATGGTGGCGGCGGATCGGCT
ACGGCAAAAAAGGGAGACAAGGAAGACGATATGGATGCAGAAAAGGCAAAAATGCGGGGT
GCTCTCGCTGGTGCCATTGTTGAAAAACCAGACATCACGTGGGATGATGTGATTGGACTT
GACAATGCGAAAGATAGTTTGAAAGAGACCGTAATTCTACCAACTCGTTTTCCTCAACTC
TTCACTGGAAAACGAAAACCCTTCAAAGGAATTCTCTTATTTGGTCCTCCTGGAACAGGT

AAATCGTATTTGGCGAAAGCCGTTGCGACCGAGGCAGACTCTACGTTCTTTTCCGTCAGC
AGTGCCGATCTCATTTCGAAATGGCAGGGAGAATCTGAAAAGCTTGTTCGGAATCTTTTT
GAAATGGCTCGGGAGTCGGAAGGAGGCCGCGCCATTATTTTCATTGATGAAGTTGATTCA
CTTTGTGGAAGTCGTTCCGAGGGAGAGTCGGACTCGATGCGTCGTGTCAAGACCGAATTC
TTGGTTCAAATGGATGGTGTAGGAAAAAGCGATTCTCGGGTCTTGGTACTGGGTGCGACC
AATATTCCATGGGAACTAGATGCCGCAATCCGACGTCGATTCGAGAAACGCGTTTACATT
CCACTTCCGGAGGCCGAAGCACGAACGGCGATGGTAAAACTGAATTTGGGTGACACACCA
AACGAACTGACAGAAGAGGAATTCGAAAGAGTCGGTGAAATAACTGACGGCTCGAGCGGA
GCCGATATTAAGATCCTGGTCAAAGAAGCACTCATGCAACCATTAAGAAAGTGCCAAAAA
GCCAAACAATTCTACGTTGATAAAGAAGGAAATTATCATCCCTGCGATAGATATCCGAAC
TGCTCTGCTTGCCCTCCCAAGCTGTCCACCGACAAGCCAGGAAAAGATTATACTTGCAAC
CGTTGCGGAGCGAAGCGCATGTCACTGTGGGACGTTCCAACCGAAAAGCTGAAAGCGCCG
ATGGTTCTCTACGATGATTTCAAGAAAGTCATGAAGAGTTCTGTCACTTCTGTATCTCCA
GAAGAGCTAGAACGATTCAAAGAATGGACCAAGCTATTTGGTCAAGACGGAGCATAGTGA
TATACACGTAATATTTCGCATGCTCTGCTTTCGATGCTACAGAAAACCCGT
>comp2726_c0_seq1
CAGGACGCAACGTAAGGTCCCTGATACTGTACTGTACGGTACAGTACGGTGCCTGCCATT
TCGACGCTGCACGACGATCCGGCTCTTTTATGTGTGTGTTTCCTCTGCGGTACGTAGTGA
GTACGAGTATGTTTCGTACGTTTCGGTACTTTACCGTACAAGAGGAAACAGATTCCGGAC
GGGCGGATGGCATCGGGGGAGAGAGTCTTGCGGGAAGGACGCACCCCGGAGAGCGTCTGG
TCGTGGATTCTTGCGGTTGCGGTGGCTGCTGCGGCAATTGTCGCACCACTTGTGTGTCTG
TGTGTGCTCGCGTTTGTTCGTATCTGTGTGGGTGGTCGTGGATGCCGTCGTTGCGACAAC
CGTGATTCCTCGTTCGTCTCTGGCGTCCCGGTGCATGTGCCTTTCTATTCCCGGTTTGGT
CGTGTTGTGTTGCGCCGTCCTGTCGTGTCGTGTCACACCGCATATCCCGGTTCTCGGGGC
GCGGGACTCCACCCGCTGCCCGGGTTCCGTTCGGAAGCAGAAGGAGGCACGCCCGGTCGC
TGCTTCGAGCGGGATGGTTCGAGAACCCCCACCGATTGCTTCGCGGTCGCATCGTGCCGT
TTTGGCACACCCGCTTGTTCCCCGGTTGCCCGAATTCCGACTCTGTTCCGTGGGGGCGGT
CCGGCAACGCAAGGGAGGTGTCTGCCCGTAGAAGCTCATCGAAGAAAAGACATCGATTCA
CCATCGGTACCGGAATGGGAATGAGAAGAAAAGAAGGGCTCTTCTAGGCTTTTTTTCGCA
GTAACTAGCAACTTTTTTAGTGGATGATAGCATACGCACAGTTTTCAGACGGCCTTCAAT
AATGCCAACTAGGGCCGTGTTGTTCTATTTCTACTATAGTATTGCGGGAGCGGTGCGTTC
CTTCTCCTCAAGACACTTAGAGCACTGCTACACGCAACCGCATTTTCCTAGCAACGGTGG
CAGCCTCGACCAGAGCAGAGCCACGATGCCTGCCACCAGCAAAAACACCATCCGAGTGCC
TTGGGTTTGTTCGTCTGTAC
>comp2727_c0_seq1
ACTCGATCGAAGTTGCACTCGGTAGACTTGAATTTCATCATGTGCAAACCTGAGATTTCG
AACGAGAAGGCTATCGAAGCCATGGGAGGTCAATTCTGTGTCAAAGATTTGGCGGAAGAA
GACAAATGGTTAGCAACTTTCGACTTTCCTGCCTTTTCGAAAGAAATTAAGGAGTTAGGG
AAGAAACTAGAGAAGGAACAAGGCCCTGACGATGTTGCCCACCTAAACAAAATGATAGGG
TGGTCCAACAGGTTCGCTGCGATCGGTCTTCTCACCATGGGTTTCGGGGTGAATCCTATT
ACGGTTATCTGTTTGTCGTTGTACACTTTCTCGCGGTGGACCATGATCGCACATCACGTA
TGCCACGGTGGGTATGAGAAGTGCCATCCCAACAAGAGCCGATGGTCTCGATTCAAGTTC
GGGATTGGTACTTTTTGGCGCAGATTTAACGACTGGTTTGATTGGATGATGCCGGAGGCG
TGGAATGTAGAGCACAACACCCGCCACCACTACAATCTTTCCGAGATTCACGATCCTGAT
CTAGTGGAGGAAAACGCCGCCATGTTGCGCAACCCAAACTTGCCCATTGTTGTCAAGTAT
GTCGGTGTGGTGTTCCTTGCATTGTTCTGGAAGTGGTTTATTACGCACCCAATACTTAT
AAGGAACTCAAACTAGCCAAGATGAGACGCGAAGGAAAAAAGATCCCCGATGACGTCGAT
CCTGCTGGAGCCGTGACAATTCGTTCCTGCTTAATGGGGACCAACTTCTTCTACTCCTTC
GCTGAGTTGTTGGCTGTCGTTCTATTGCCATACCTTGTGTTCCATTTCTTCATTACCCCT
CTTCCCTATTTATTTTTGGGCAAGCATTTTGGATACGAAGACACCCTGTACTGGAACGCT
GTCAAGAATCTCTTTCTGGCTGAACTCTTGACCAACGCCCATGGATTTTTGGCTGTTGTG
ACCAACCACGCTGGAGACGATATGTATCGCTTCCGCAAGGGATGCCGACCCTTTTCTGGA
TCTTTCTTCCTCCGTCAAGTCCTCGCGAGTGTCAACTTCAGCATGGGGACCGATGCTGTG
GACTTCTTTCACGGGTGGTTGAACTATCAGATTGAACATCACATGTGGCCCAACCTTTCA
ATGCTTAGTTATCAGAAATCGGCCCCACTCGTTGAGGAGATCTGCCGCAGGCACAACGTG
CCATACATCAAACAAAACGTCTTCTGGCGGCTGAAGAAGACCGTAGATATCATAGTGGGC
AATACGTCTATGAGATGGTTTCCTGAAGATTTCGAGGACAAATTCCTCGACACTGATTTG
AAGGTCGAGAAGGCAGCATACAAATCTGAATAGTCGGTTTGGAGCCAAGAGATATACAGT
ACTTGTTAAACTACACTACGAAGTATTATTATGATCAAAATTACAGGGGC
>comp2727_c0_seq2

TGCTCGAGGAGTGCTAGCAGGAAGACATCGTTGCGACATCGTTGAGAAGCGTGGGCGTGG
GAACTGCTAAAATGTTGTCTGAATGAAGTCTCTCACGCAATTTCTTGTGGTCCGGCTCAA
ACTTGATTCTGGTCGATTCCTCTCACTGGTCCAAAATCCACAGCCACCACTACAATCTTT
CCGAGATTCACGATCCTGATCTAGTGGAGGAAAACGCCGCCATGTTGCGCAACCCAAACT
TGCCCATTGTTGTCAAGTATGTCGGTGTGGTGTTCCTTGCATTGTTCTGGAAGTGGTTTT
ATTACGCACCCAATACTTATAAGGAACTCAAACTAGCCAAGATGAGACGCGAAGGAAAAA
AGATCCCCGATGACGTCGATCCTGCTGGAGCCGTGACAATTCGTTCCTGCTTAATGGGGA
CCAACTTCTTCTACTCCTTCGCTGAGTTGTTGGCTGTCGTTCTATTGCCATACCTTGTGT
TCCATTTCTTCATTACCCCTCTTCCCTATTTATTTTTGGGCAAGCATTTTGGATACGAAG
ACACCCTGTACTGGAACGCTGTCAAGAATCTCTTTCTGGCTGAACTCTTGACCAACGCCC
ATGGATTTTTGGCTGTTGTGACCAACCACGCTGGAGACGATATGTATCGCTTCCGCAAGG
GATGCCGACCCTTTTCTGGATCTTTCTTCCTCCGTCAAGTCCTCGCGAGTGTCAACTTCA
GCATGGGGACCGATGCTGTGGACTTCTTTCACGGGTGGTTGAACTATCAGATTGAACATC
ACATGTGGCCCAACCTTTCAATGCTTAGTTATCAGAAATCGGCCCCACTCGTTGAGGAGA
TCTGCCGCAGGCACAACGTGCCATACATCAAACAAAACGTCTTCTGGCGGCTGAAGAAGA
CCGTAGATATCATAGTGGGCAATACGTCTATGAGATGGTTTCCTGAAGATTTCGAGGACA
AATTCCTCGACACTGATTTGAAGGTCGAGAAGGCAGCATACAAATCTGAATAGTCGGTTT
GGAGCCAAGAGATATACAGTACTTGTTAAACTACACTACGAAGTATTATTATGATCAAAA
TTACAGGGGC
>comp2728_c0_seq1
TGGCAGCAAGGTAGATCCAGACCCGAGGTCAGGGTCTATATTTCCTAAAAGATGACCGGA
CAATTGAAGGTCCAATCCATTCTGACGCTATTTTGGCTAATGGGCTACCTTTCCTTCACC
CGTAGTTTCTGTATGGGCAAGAATTTGTTTCCGTCGAGGGCATCAACAATTGCGTTTTCA
AAAACAACGAGTCAATCGCAAACCACTGGAGTCAATTCTCGAGTCAATCATTCGGTTTCA
TTCTCCAGCAAGGTCTCTATTTCAGATGCTTACGATGGCGGGAATATCGAGCTAGCCAAC
ATCGAAGATGGCCCAACAAAAGTAGTCTCGTTACGGATCAAACCTGATCCGTACACAGAA
CTCGAGAAGAAGTCGCACATGCAATACTTTTCCTTTCGGTCCTCTATTCACACCAACAAT
GCGGAAGATGACAAGAGAGACGTCCCCGTCACATACGAACTACTAAACGCTGGAGAGGCG
TCGTATGCCAAAGCCTGGAATGGTTCCACTGTTTTTTACTCAAAGTCACTCAGTGATCCC
GACTCTTGGAAGCGTGCACAAAGCACTCGTTTTGTAGAAGGAAAACTCGTTTGGGACCAC
ATACATAGCGAGGAATCAGAGAGTGTTTATTTTGGCTATTTCCCACCATTTTCCTACGAC
CGACACTTGGAGCTTGTCGAAAAATGCAGCGAGTTAGCTACTGTGTTTTCACTGGGGCAG
ACCTTAGAAGGTAGGGAAATTGAGTGCATCCAGGTCGGTACCGGAAAAAGCATCGGGTGG
ATCATCCATCGACAGCATCCCGGAGAACATATGGCCGAGTTCTTTGCAGAGGGCTTTTTG
AAGCGCCTCCTCGGGCTCGACTCAAGCGGCGATACCGATGGTTTGGTCCGGCAGCTGCTC
TCAAAATACACATTCTACATTATTCCCTCTATGAATCCCGACGGTGCCGTTGCCGGAAAT
CTGCGGGTGAATGCGGCAGGTGCGAACCTCAACCGCGAATGGTGCAGCAGCGGAGACGAC
TATGAAGCTCCCACTCTAGAGCGATCGCCAGAAGTCTACCACGTCTTGGAAAAAATGAAG
GAAACTGGATGCGACGTCTTTTGTGACATTCACGGCGACGAAGAAATCCCCTACAACTTT
TTGGCACAGCCGGTGGTTCCAAACTGGGGTCCACGTCTAGAAGCTCTGCACGGTGCCTTC
AATGCGGCCTACCGTCGAGCCAATCCAGACATGCAACAAGAATTTGCCTACGAACCCACA
GAATACGCAGAGGGAGATATACTCAACATTGCCAACGACCAAATCGCCCATCGTTTTGAT
TGTCTATCTGTTACACTGGAAATGCCATTTAAAGATTGCAGGTCCAATTCCGATCCGGAA
CGGGGCTGGAGTCCAGCTCGCTCCCGGGCACTTGGAGCCTCTTTGTGCGAGCCACTCTTG
TATATTCAGCCCTATTTGCGCGCCGAGGCTCTAGACGAGAAATCCTTCGCCGACAGTTTT
CCAGAAATCGATCGGTACGTACTCCCCACAAGCGAATACAACAAATAAACGGACGACGGA
GTTCGAAACCGCTAATATGCTATAATACCGAAGCCAACAAGTGCTTGGCGTAGAATACGG
AATGCATCTAATTAGTTTAGTGCATCAGCTCCTACTCTAGTATGGTTGGTATACTAATCA
GATATGTGCAACTTTTATCATATCCCACAATTCGTATCTGTTTCTTCAACAAAAATGAAT
GTCGACAAGTGCATCATTCTGCTTCTCTTTTAGTTGTCACCGACTACTGTCATCAACTGC
TATCACGTCAGGAGTTTTTCGAACGCGAGCAATAACTCATGCCAAGACGCAATACCA
>comp2729_c0_seq1
CCAGTCTTGTTTTCTGTCTCCATCGCCATGATAACCTTTGTTGCAATTGTTGACAACATA
ACACACCAAAAGAACGATGGATGTCGAAAATGATGACATATTTGGCGGCGTTTGCTGGGC
GAAGCCACAATCGTCGTCGTGTGCTTCCGATGAGCATAAAGCCACCGCTTTCCGTGGTGA
CAGTGTCGGAAGTAAAAGTAACAAAAGTACAAACGTGTCAGAACTATTCGGAGCGCCAGA
TGGCATATCTTTGCGAAGAAAATCTGCTGTATCCACACCGGTCAACGGTGACGAATTTGT
CAAAGTGTGTGAAATCAAGACCGGGTGGCTCAACGACAGCAAGTCCAGATTGTCGTTGCT
TCCGACTCTAGATTCGCAATTTGCTGATCGACTAGAACGACTTCGAACTAGGGAAAGTGA
TGGGCCAAATGAAGAAGACGAAGGAAAGGAGAATATGAAGACTAACAACAAGGAGAACAA

CGATGAAAGTGACGTCAAAGACGGAAAAGAGTATCCAGAGCAGCAGCGGAATGAAGAGGA
AGATTGCTCATCTATTTTTAAAAAGGGTACTGGGCAGATATTGCAGCCGCAACATGAAGG
AGCAACCATTTGTGACCATGAATCGTATCTAAACGAAAAGGTTGATATCATTGAGACTAT
TCCAAAGATTCCGGCAATTTTGATAATGGATCACATCGCATCGTTCGTAAACGACCGTAT
GGTATGGAATTCGTTGGCTGCATTGAGTAAGGAAACCTATGCAATCACAGTGACGCCATC
CCATTCGAAACAGAACAGTATTTATCGTCCTTGGCCCCGTGTTCGGTGGAGATTAGCTGC
CACAACAGCGTCCATGGACGCAAAAACATATTCCCAGAATCGAAGAAATCATCTAACATC
GACATCACAACAAGTCTCACACCGTCGGTGGTCTCGAATGCCGGCCCGTGGGTCTGTCCA
AACATCCGCTAGACCTTGGACCGTTTGCTTCGGAACCAATTATTTAGGATGTGGCACCGA
TCGAGGGGGCGTGTTGGTACGAGCCGTCCACAACGGATCGACAATGTCTGCCCAGGGGCA
TTTCGGCCGGATCAACAGTGTTCAGCTCTTTGCTGAAAGCTGGTTTTTGTCTGGGGGAGA
CGACAAGGTTGTTCGTATATGGAACATTCGAAAGAACAAAATCTCTTGTGAAGCGGTCCT
CAAGGGCCACATTGGTAGTATCACATCTATTGCAGTAGTCTCATACCGAAACACTCATTG
TGTAGGTAATGGGGGTGGTGAAAATTCAATGTCAGGTTGCATGCTAGTAGCCACGGCTGC
TTTGGATGGCAATATTCATCTGCATGTGGTCAATTACAAGGACGACCGAGTGATATCCAC
ACAACACATGGTCCACTTTGAAGATAGCCAAGAACCTATCCACACTGCAAAACAACCAAT
TTATTCCATCGTCATGTTTGAAAAGGATGGGCATACGAGTCTTGTTTCAGGTGGTGAAGA
TGGATGTTTGAGAGTTTGGGATGTGGACGAAGCCATGGATAATGCTCTTGGCAATCATGA
AGGTATCCTTGGCCAATATCCCAATCGCGCTTCCGATAAAACAGACGAGCTGTCGATCAG
GGCAGCTCCCAAAAGAATAGTTCGCAACGCTTGTATTTTTCGAGATGATGGAGAAATCAA
GAGTATTGCAATTTCTCGCGATAAGCAACAGATCGCAGCTGCTTATGGCAGGACCATTTG
CCATTGTGCCCTTTCTGTAGAAACGCTTTCCAAGTTTAATGACAGCCATCATCATTACCA
GGCACACCACCAACGAAGACGACTTCGAACCAATCAGAACCATTTTGAGAGAGAAAATTC
ATTCGAAGCAGAACACCAGAACCATCGTCACGGCAATGATGGTTGGCGGCTTTTGAAAGG
TCATTATTCCGGTGACATTCGTTGCATTTCGTTTTCCTCCGATGGAAATTCCATCGCTTC
AGCGTGCAGCGATGGTTCCATTCGGCTCTGGGACCTCGAGAAAGGATGTTGGAATCGAAA
GTGGAAGGCACACAACGGCTTCATGGTCTGCTCGATTGCAGTTTCACCTGACGGGCAAAG
TTTGCTCTCTGCTGGGTCAGATGGAACTATTGCAATCGAGGGGCTCTTATGATTCAGTTC
CCCAAATTGGTCAATGTGTAGATAGAGCAATGACAAGAAGATAACCTAGCGGATAACGTA
CCAAAAGGCTTCCCAGGACATGTATTGATAGAACCTCATCAGGAGACTGGAGGTGATGAA
AAGACGAGAAACAGATATGTTCTATACGAATATTATAGGAGGTAAAGTACCCATCTTCTG
TTGGTTCATGAAGTGGATTATGGGAGCAATAAATCCAAAAACTAGAGAGTTCAAGAAAAA
GATATACGATATACAGAAGCAAGTTCAGGGAGTTCTGTAGCGCGAGCGACAAGGCTTTGA
CATTGGTTCTTGTGTTCAACGAATAGGACTCATGGACTAAACCCAATGACGCTCCCAAAA
AATCAGAGAAAAAGAAAAGGGTTTAGCGGCAGGCCTTCCAGGAGAGAGGAAGGGTGGAGT
ATCCAAGGAAGAACAATGTACAGAGATACTTTCGAGGAAATACAAAAACGAGGAAGGGAA
GAACAAGAAGAATCAAAAGACGTGGAAAATGAGCTGCAAGAATCCTTCGTCGACGAGGAA
GTTGTCCTAGTACTACTACTTCATGCTACTAACAGCAAGTTCCATAGAGTAACATTCTTG
TATTCTAATACTTTGAAATACAATGTCTCA
>comp2730_c0_seq1
TTCCCAGAACATAATTTCCAGTCATTTTGTCGTCTCATTCACTCGATCTGTTTATTGCGA
TCAATCAGGAAACGAAAAGAATGCAATCATAACATGAAAAATCATGACAATATTATTATT
CTCAATTGCCTTTCATCCTTCGGCTTCCACACAACACTTTTTTCATCATTGATCCTGCTT
ATGGCGCTCGCGTTTCCAGGCCATACTGTTGCGATGCTCATTCCCTCACATTTAAGTCGC
GCAATAAGAAAAAACGGAAGCAGTCACAAAATAACATCGATCCAGAAAGATGATACCGAA
TAATCATAATGATGTGAATGAAGCCTTGCGATCGGTCAATCCATGCACAGATCGGTTATT
GTTCAAAGACCAAAAGCACAAACATCATCACCTGAACGAACAACACCAACTGGAATCGCA
TGAACAAAGTAGCAGCGTCATCATGTGTAGCAAGGATCAAACTAGCAAAAACAATACCGA
CCAACAATACAGGGACGCCGTGAGATCCCTAGGTCCCTACGATGTCGTTTGTGGGAGAGG
GAGTGTCGCCTTCAACAATATCGGGAATCGTCGGTTTCGGATAATAATCGGGATGAACGT
CGACAAATACAGCGCAATCGATGGACGCTTCCAGAAAGGAATCTTTATCGGGAACTTGGT
CGATGCACTCGTCTGTGAAATGGGCGCGCGTTTCTTCAAGCAAAAAAAAACTGATGGGCG
ACTGGTGGAGCTGACGGAAGCAGAGATAAGAAAAAAGGTCGGCCACGCTTTGCGGGACAT
GTTTGCTTTCCAGGAGAGCCAACAGCAACAGCAACAACAAGACGTTAAAGAACAAAATAT
GATGGCAGGGGATACAGCGACGTTAGCAATACCTTCATTGCTATCACTGGCTCGATCGCC
AGCTCATCACAAGAAGAGCAACGATATGAACTGCAGCTGTTTGCCTCATGAAAGAACGCA
ACAATCTCTCCCGCAAAGTCTCCCACATTTTCCTCTTCCGACGTCCCTCTTGTTTCAAGA
GTCCATCGAATCTATCGATGTTCCAAGGAACTACAGGGCCGACACTAATAATGGTTTTCA
GGGAACAAATAATTTTGAAATCCAGAACCGTTCCACCGAGATCAGGCAACACGGTTTTTT
CCCTGTGTCGAACAGAGACAAACGCCAACGGCAGAAAGAACTGCAACGGCGGCTCCAGCA

GCAGCAGCAATCTGGTCATTTTTCGATGAGAGAGCTTGAAGAGAGGCTATCGGCTACTAC
CAACATTAGCAATAGTGAAAGGGAACGTCAAGGAACAATCTGAAACAAGAACAGTGGTTT
TCATGAGAGGCCAAGTTACTCTCTACATGCGATCGAAATAGAAATACATCGCAAGAAAAA
CGCTGAGAAAAATAACTACAAAATTTTGATTTTTTAAACAATATTATGGGATGAACGAAA
GAAAACATTGAGTACGAAAATTAGGAACCAAAAAGTGAAGTAGGTCCGACGTGCATTAGT
GTCGATGTCAAGGGAAATTTTCAGTTATATAACAAATAAATAGCAAGTAAGGGGTAACAA
ATTAGATTGGGATTCAGAAAAGGACAATGGCGCTTTCATTTTTCCCACTCCAATTCAAGA
TTCCAGCTTCATATCAAGCATTATGTAATTTAGGAAAAACTGAGGAACAAATCTCAAACG
AA
>comp2731_c0_seq1
GAAAGCAGCAGCACTGCAAAACATTCGCAACCAAACGAAACGAAACCAATCGCAGTCGCA
ACCACAGTCGCAATCGCAACCCAACCAAACCAGCGGTCTGAATTCTCTCTAAAGCATCGG
CGCAAATAAGCACGTTCGTAGCAACCGACCCGGCACAAACGCGTCCCGAAACCAAACGCA
AACCCAAAACCCAAACCCACCCACACAAACACTACAATCTACGACAAACCTACGACTCCT
CCAAGATGTTCGCCACCAAGTCCGCTGTCCGATCGGTATTCTCGAGGACCCGACCGACCG
CCCTCCGATCCTTCTCGAACACTTCCGCGACCCTGGCGGATCAATACGACGTCGTCGTTA
TAGGCGGAGGCCCCGGCGGATACGTCGCCGCCATCAAGTCGGCCCAGCTGGGACTGAAGA
CGGCCTGCGTCGAAATGAGGGGCCGCCTGGGAGGAACCTGCCTGAACGTGGGGTGCATCC
CCTCCAAGGCCCTCCTCACCTCCACGCACCACTACCACGACGCCAAGCACCACTTTGCGG
ACCACGGGGTCGTCATCGACGGAAACATCAGCATGGACGTCGACAAGATGCAGGACACCA
AGGCCAAGTCGGTCGAGGGACTGACGGGGGGCATCGAGTACCTCCTCAAGAAGTACGGCG
TGGAATACCACAAGGGGAAGGGAAGGATCACCGGACCCAACGGCGTCGAGGTCGCCCTCA
ACGAGGGGGGCAGCGAGAGCCTCGACACCAAGAACATCCTCATCGCCACCGGATCCGAGG
TCACGCCGCTGCCCCCGGTTCCCGTCGACAACGAGGGAGGAAAGATCGTCGACAGCACCG
GGGCGCTCGAGATCCGCAAGATCCCCGAGAAGATGGCGGTCGTCGGCGGGGGGGTCATCG
GACTGGAGATGGGATCGGTCTGGGCCCGCCTCGGAGCGGAGGTCACCGTCATTGAGTTCC
TGGACCGGATCCTCCCCAGCAACGACCACGAGATCGGAAAGAAGTTCCAGACCACCCTCA
AGAAGCAGGGATTCAAGTTCAACCTCAAGACCAAGGTCATCAAGAGCGTCGTGGAGGACG
ACGGCGTCGTGCTGACCACCGAGCCGGCCAAGGGCGGGGACGCGAAGGAAGAAAAGTACG
ACGTCGTCCTGGTCTCCACCGGCCGCCGGCCCTACACGGAGGGCCTCGGCCTGGAGGACG
TGGGCATCCAGACGGACCAGCTCGGACGCATCGAGGTCGACTCCCACTTCAAGACCGCCG
TCCCGTCCATCTACGCGATTGGCGACTGCATCAGCGGCCCCATGCTCGCCCACAAGGCCG
AGGAGGAGGGCATCGCCGCCGTCGAAACCATTGCCGGCTTTGCCGGCCACGTCTGCTACG
ACGCCATCCCGGGCGTCGTCTACACCTACCCCGAGGTCGCCAACGTCGGAAAGTCGGAGG
AGGAGCTCAAGGAGGCGGGGATCGCCTACAACGTGGGAAACTTTCCCTTTTCCGCCAACT
CGCGGGCCAGGGCCAACGGGAGCTCGGAGGGATTCGTCAAGGTCCTGGCGGACAAGGAGA
CGGACCAGATCCTCGGCGTCCACATCATCGGTCCCAACGCCGGCGAGATGATCGCCGAGG
CCGTCATCGCCATCGAGTACGGCGCCAGCTCGGAGGACATGGCCCGGACCTGCCACGCCC
ACCCCACGCTGAGCGAGGCCTTCAAGGAGGCCTGCATGGACACCTACGACAAGCCCATCC
ATTTCTAGAGCAGGAAACAAACACAAGATGTGATAGCAACCGAAACACCAAACCACACAA
CAGGCGAACCAAACAGAACCAAACGAGACGAGAGTCGGCAACGAAAGCGTGGCATTCCGT
TCGTTCGCCACCATGTAGTATCCGTGCCGCATTGCATTGCATTGCAATGCACAGCATAAT
AGTATACAACTTCTACTAGACAGTGAGTTAAGTTCC
>comp2732_c0_seq1
CAAACGCGTTGACACTAAAGTATGGAGAGAAACAAACAGCACGATTCCGAAAAGATGAAG
ATGGAAGAAAAACGAACCATAAATGCAGTGTTCATCGGTCATGCCAATGTCGGAAAAAGT
ACAATATTTGGTCATTTGATTCGAGAATGTGGAGGAACAGACAATGCATCGATTGAAAAG
TGCGAAAATGATGCCGAAGAAAAGGGTGAGGGATCTATGAAATATACTTGGCTCCTCGAC
CAAAGTGCGGCCGAAAGAGAACGCGGAACCATGATAAGCGGCAACCTCCGAAGACTCGAA
TCTACCAAGTATCATATAAATTTGATTGACACACCGGGTCACTGCGATTTCATTGAAAAC
ACGACAACGAGGATATCACAAGCAGACGTTGCACTTTTATGTATAGATTCGTCGACAAGT
GGCTATGGAAGAGGAATGGATGAAAATGGACAAACACGGCAGCACCCCTTGTTAGCCTAC
GCATTAGGAGTGAAACAGATGATTGTTATAGTGAATAAAATGGATGGTGATTCTGTCAAT
TTTTCGGAAGACCGCTGGAAAACAATCCGGACTGAAGTTTCTAACTACCTCAGGATAGTT
GGTTATAGGCCGATGAAAATTCCTTTCATTCCCATATCCGGCTGGCTCGGCGACAATCTT
ATCTCGAAGTCGATTCACATGCCATGGTACGATGGACCAACCTTATTGGAAGCTCTTGAG
GATGTCAATGTTCCAAACAGATTTTCGACCAAACCTCTGCGAGTCCCTATTCAAGATATA
CAAAATGCTGGACCATCTGGAACCGTTCTAATTGGGCGTGTCGAAACTGGTGAGCTGAGG
CCTGAGATGGAGGTTCAATTCGCCCCATCTGGAATAATTGGCACCGTTCAGTCGTTAGAA
GTAGAATGCCGGGAAGTTAGTGAGGCCATCGGAGGTGACATTGTTACTATCAACATGCAA

GACAAATCAAAACTTTCGAAACATCTTTGCCGTGGGCACATATTGTCGGACTCGAGTAAT
CATCCTGCCCAAGGTGTTTCTAGCTTCCAGGCACAGTTAATTATTATGAAGAATTATCAA
GGAGAAATTTCAATAGGATACACACCCATCATCGATGTCCATACATCTCACACGCCATGC
AAATTCGTCGACATCAAAACGAAGATGAATTCGCGAACCGGACAAGTTGTGGAAAAAAAT
CCCAGAAGTTTAAAAGCAGGAGATGCTTGTATTGCGGAATTACAACCACAGACACCTCTA
TGCGTCGAAGAATTTAAAAAGTTTCCCCCTCTGGGGAGGTTTCTTGTCCGTGGAGATTCT
GGACAAACTGTCGCATTCGGAGTTGTCAAGTCGGTAATTTATTGAAGACGACAAGATTAT
AAGAAGCCAGGTACATAAAAAATTTCCATTTGTTTCAACGGATAAAAGGGTACACGTATA
AGCTGTCCTCTAAATTTCAAAATGGACCGTCATGATCACAACGATAGAAATGACATTGAA
ATTTGCCATAGATGAAATCATGTGCTGAGTGCTAGCATCAATAGAATGACTGAAAATTCT
CTAGTGCCTGTGTAAAGAGATAATCACAGAGCAGAGATCGAGGACCGAAATGCATCGTAG
TTGATAAATATTGTTTATGCGTGTGTTAGTTTCTGATCGAAG
>comp2732_c0_seq2
CAAACGCGTTGACACTAAAGTATGGAGAGAAACAAACAGCACGATTCCGAAAAGATGAAG
ATGGAAGAAAAACGAACCATAAATGCAGTGTTCATCGGTCATGCCAATGTCGGTAGGTGT
GGGCTAAATGATTCATTGGCAGGATGATGCTTTGTATGATATCTTTTCCAATCCCAATGC
TCAATGCTGTAATTGTTGTCGTACAATCTACAAGGAAAAAGTACAATATTTGGTCATTTG
ATTCGAGAATGTGGAGGAACAGACAATGCATCGATTGAAAAGTGCGAAAATGATGCCGAA
GAAAAGGGTGAGGGATCTATGAAATATACTTGGCTCCTCGACCAAAGTGCGGCCGAAAGA
GAACGCGGAACCATGATAAGCGGCAACCTCCGAAGACTCGAATCTACCAAGTATCATATA
AATTTGATTGACACACCGGGTCACTGCGATTTCATTGAAAACACGACAACGAGGATATCA
CAAGCAGACGTTGCACTTTTATGTATAGATTCGTCGACAAGTGGCTATGGAAGAGGAATG
GATGAAAATGGACAAACACGGCAGCACCCCTTGTTAGCCTACGCATTAGGAGTGAAACAG
ATGATTGTTATAGTGAATAAAATGGATGGTGATTCTGTCAATTTTTCGGAAGACCGCTGG
AAAACAATCCGGACTGAAGTTTCTAACTACCTCAGGATAGTTGGTTATAGGCCGATGAAA
ATTCCTTTCATTCCCATATCCGGCTGGCTCGGCGACAATCTTATCTCGAAGTCGATTCAC
ATGCCATGGTACGATGGACCAACCTTATTGGAAGCTCTTGAGGATGTCAATGTTCCAAAC
AGATTTTCGACCAAACCTCTGCGAGTCCCTATTCAAGATATACAAAATGCTGGACCATCT
GGAACCGTTCTAATTGGGCGTGTCGAAACTGGTGAGCTGAGGCCTGAGATGGAGGTTCAA
TTCGCCCCATCTGGAATAATTGGCACCGTTCAGTCGTTAGAAGTAGAATGCCGGGAAGTT
AGTGAGGCCATCGGAGGTGACATTGTTACTATCAACATGCAAGACAAATCAAAACTTTCG
AAACATCTTTGCCGTGGGCACATATTGTCGGACTCGAGTAATCATCCTGCCCAAGGTGTT
TCTAGCTTCCAGGCACAGTTAATTATTATGAAGAATTATCAAGGAGAAATTTCAATAGGA
TACACACCCATCATCGATGTCCATACATCTCACACGCCATGCAAATTCGTCGACATCAAA
ACGAAGATGAATTCGCGAACCGGACAAGTTGTGGAAAAAAATCCCAGAAGTTTAAAAGCA
GGAGATGCTTGTATTGCGGAATTACAACCACAGACACCTCTATGCGTCGAAGAATTTAAA
AAGTTTCCCCCTCTGGGGAGGTTTCTTGTCCGTGGAGATTCTGGACAAACTGTCGCATTC
GGAGTTGTCAAGTCGGTAATTTATTGAAGACGACAAGATTATAAGAAGCCAGGTACATAA
AAAATTTCCATTTGTTTCAACGGATAAAAGGGTACACGTATAAGCTGTCCTCTAAATTTC
AAAATGGACCGTCATGATCACAACGATAGAAATGACATTGAAATTTGCCATAGATGAAAT
CATGTGCTGAGTGCTAGCATCAATAGAATGACTGAAAATTCTCTAGTGCCTGTGTAAAGA
GATAATCACAGAGCAGAGATCGAGGACCGAAATGCATCGTAGTTGATAAATATTGTTTAT
GCGTGTGTTAGTTTCTGATCGAAG
>comp2733_c0_seq1
CGAATCCCCTGTTTTCTTCTACTCGCTAACATGGGCTTGGAAATTATCATTGACGAACAA
TTCTGAAGGTGTCGTAACACCTGCCAATAATTTTTCAAAAGAAGGTTAGTCGGCGTCACA
TCGGCCTGCTGCTACTCAAATCGCAGAAACAAAGCATTTATCGTGTAGTCCTAGAGACTC
TCTTCCCACGAAGAAAACTATTTGAACCCAGATATATATGCATTGGAGCAAAGACAAATC
TATAGGAAGAAATTGTTTCGAACCATGAAATTTTCTATTTGCTTTTTCCTCGCTGCTTTT
GCCAAGCTTGCGACCGCGCAAAGGAGTGACATCCATGTATTGATGTATGAGACGGACTCT
TCGATGGAGGACAACCCAACATCGCCGCTGCATTTCTTCATGGAACAAAGCAACTTCGCA
CATCTTGCCACAACGGTATATGGTGGAGGCCTCGAATACCGTGGATTTGGAGACAAATAC
CAAGTTCTGAGGGAAATCCTCGACGTGATTGATCCCAGGAAATTAGTAGTCGTGGCAGAT
GCTCGTGACGTAGCACTCAATATTCCGCACGAGTACGAACTCGCCAACGAAGCGATCGAT
CGATTCGTTCGCTACTTCGAAAAACTCGCGAAAAACAATCCCCACGCCGTCGTCATGTCT
GCCGAGAGCCAGTGCTGCGTCTCGGCCATGACACATGCCCATCCCGGTGATTATTTTGAT
CCAATCACCAAGAAACGAAACAAGCGCGCTTGTTCTTCGGGAAGCCCGGGCTGTTATTGG
AGCCAGAATGAAAACATCCACGCCTGGGTGGATTTTCAACGCCAGCGGGCCTTCAACCAA
ACCGGAATCGAACGCTCTGGGGAGGATGTCGGAGAGGTTTACCTGAACGCCGGTCTCATG
GCGGGATATCCCGAAGATATTATGAACATCTTGGACCTACTCGATATCGAATCATATGAA

GACGATCAAGCAGTGTTGTCCGGTCTGATGTACCAGTTTCCTGATAAGATCATTCTTGAC
TACGACCAAGCTATGTTTGGAAACAACCAGTGGCCCAGAGGATTGAGTGAAGGTTGCATC
TTCGAAAAAGGAGAAAACAACGCCCTCATCCATCGCCAAACAAAAGCCCAGCCTTTAATC
GTTCACACTCCCGGAAAGTTCTACGGATGCCTTGACATGCTCATCGAAGAGTTGGGAGGA
GTAAGCCAACAAAGATACCTCTCTGACAGCCCGGAAGGAAATCCAAGAGTTCTTGAGAGA
GAAGGAATCTCCGCCGAATCAACAAGCTTTTCTGCATTCCCTCCCCGCAGTCTCCAGAGC
ACAGAAAACTGCGAATGTATTTGTGAAAGTCGAAGGCATCTTCTGAGTGGCCTCTGGGGC
GTAATGAAGACTTATTTCACCGATGAAATCCACCAAAGAACGGAAGAAGAGAAAAAGCCA
AAAATTGAGGACGAAAAGGAACTTTCGCGGCTCGTGATTGAAGATTCTTTCGTCGAATTT
ACAGAGTCCACAAACTACGGAAACTACGGTCAGTATGGTCAATATGGCAACTACGGTCAG
TATGGTCAGTATGGCCAGTATGGCCAATATGGCAACTATGGTCAGTACGGAAATTATGGT
GCTGCAGCAGACATTTGTGTAGACTTCTGCATTGACAAATGCTCCGAAGACGACGAGACA
AACTATGGTAGCTACGGAAACTACGGTTCATACGGAAACTATGGTAGTGTTCGCAGACGG
TTTGTGTAGAATGGAAACAAAGCATCAGACAAACCGAACTATTCAACTCAACTCCGATAA
GAAGCAAGGACGAGTGCTTCTATTTATGATATTGTGTGGCAGTCGAATATTTACAACGGA
TACTTCGTAAATCGGCTTCCGATGTGTCACCCTATGGAAAAATGGAAAATATGCAGTAAT
GAAAGTGTACTAGATATTCAAATTAAAACTATCGGAATG
>comp2734_c0_seq1
AGCTTCCTTTGTGGGTGAACACGGCCAAATAGTGCCTCATCGGTGGGATCTGCCCCGGAA
GCGTGGTACCTTCGTGGAGTCAAGCTGTCGCATCGGCTTCCGTCGCGACCGCTCTGGCGA
GGCAGGAATGCCAGCTCCCGGGAGCCCATACAACATAATGCCCTTCCAAGGTCCCCCAAC
AGCCGAAAAAATCGAGCCGGCTAACCCATAGCTTCGTTCAAGGCATCGCCACACGACGTC
CAAGCCGATCCCACCGTCCCGTTTTGCACCCCAAACAATATCGAAACGACCAAGAAACGA
ATGGCCCGTGTTCCAAATTCTTTCAAGATGCAATGGGCCCGAGGGGTGTTCGATTCCGCA
TTCGAGGGAACATGGCACATTGGAAATGAGAAAGGATGATCGGATCGGGTGGCATGCAGA
CACTTGAAGCAGAACGCCGGTTGGAACCTAGAGGATCGTCCCCGACCCTCTAGGTTCACG
TAAGACGAACCAAAACGATATGGATTGCCTGAATTCGCAAATCCCTGCCTCTCGACGAAA
AAGGTGTCAACTGCGACGATGACTGCAACGACTTTGTCTTGGTGGTCGACTGCGATGGAA
AGATTGGGAACCTTTCTCGTCGATGGATGCCAGTACGTTTGTCTGTAGCTTTGACTCGTG
GAAAAGAATCTCCAACGGCTCTTCGATGCAAACCTACTTTGAGGGGAGTGCATAATCTTG
AGTATTTTGGCAACGATGAAGACGATCGATGCGACCGCGAGGATCCATTTTTCAACGACC
TTCAAGATCATGGTGTTGTTTGCAAGATCGAGTTCGAGGGCGTCGTCGTTTCGGTTGTTC
AGCGTCGTCACGAGCACGTTATCTTGTTTTTGGATCGTCAAATCTTGTGCTTCTGTGCTG
TTCGGGGCTGGAATGCAGGGTCTAGAACTCTAAATTGTTAGGTGATCATATAATTTGAGA
GAAAAATATTCCGACGAATTTGGTTTATGAATTTAGTTCACTGTAGAAGAGGAATCGAGA
CTCCCAACACTATCCTACACGGAGTTAGAACGAATTGGCTCGTCGTCGAACAACTGCCAT
AAATGAAATACTCTCTTTGTGGTGTCTTGACATGCAAAAATACATTAATAAAATCCATTC
GACCCTTTACTGCGTGTGCTTGCTTGCTACGCTACACTGCTTGCTCTACTGCTTCTCCTT
CTTGAACATGAGAAGCATGCGGTACGAAACGGCGAAACCTCCAAAGACGTTGATGGTGGC
TACCGAGATGGCGAGGCATCCAAGGATGTTCGAGGGAGAACCACGCTCTCCGCTTACCAT
GAGAATACCTCCGAGGATAACGACCCCCGAAAGGCTGTTGCTAATACTCATCAACGGACT
GAACAGAGCAGGCTCCACGGCCCAGATCAGGTAGTATCCAAGGAATCCAGCGAGGATGAA
GTAGAGGAGCTGAGTCACGAACGAAACAGGAGCAAAGAATGCGAGAACTACGAAAAAAAG
CATCATGAGGAGGAATCCGAGGATTTCGCCGATGGAGGTGAGG
>comp2735_c0_seq1
CGAGAATCCAACTCAATCGGAAGAACCTTCGGAGTTCCCCACACAAAGCGAGGAGCCCTC
CGAAAGCCCAACGATTGTGTTTCGATTCCCTACTCTGTTCAGCCTCTACGAAATCGTATG
CAGCGAAGCAAACGCAAACATTTTTAGCGACTTTTGTGACTTGATCAACGCCGTACCCGG
ATTGGGACGACTGCTCGGCCAATCCTTGTTTGGGCGCAAGCTTCAATCGGGCGCAAAACG
ATACACGCTATTTGCACCCACCAACAATGCCTTTGAGTTTTACGACATTCCACTTCCCGA
TCTAACCAATTTAACGATTGACGACAAAAGGCTCGTGAAATTCCTCGCAACGTACCACAT
TGTTGAAGACTTGTATGATTATGATGAATTGGATTGCAATCAGAAATTGACTATGTACAA
TGGGCTTGAATCACAAACACGGTGCAGCATCAAAACCACGGGAATAAATATCGGAGTGCA
GCAAAAATTTCAGGTCGGTACCGACAGCCAAGCGTTTGGAGATGACAACGAGGACGTGGT
TCTGAGCAAAGCGGATGCAAGAATTACGAACAAGAACGTCGCAGCCAGCAACGGTATCCT
TCATGCTGTGAATCGATTCATCCTGCCCAATTTGACGATATACGAAACCGACGTTCCTAC
CAGCGAACCTTCCGACTCACCTAGTTCGTATCCAACCTCAGGGACTACGTTTTCCACAAC
TTCGTTGGAACCATCCGGAGTAATTAAGCCTCCGAGTCCGGGTCCCGTTCAGCCTCCTAC
TGAAGCTCCTACCAAGAGTCCCAGCAAGGCTCCCAGCAAGCCTCCCAGCAAGCCTCCAAG
TAAGCCTCCCAGTAAGCCTCCCACCGTGAATCCCAGCAAGGCTCCCAGTGCAGAACCTTC

CCCGGCCGCCAGTGATGATCCTACCGTGGGTGGGTCTGAGGTTCCCAGTGAGGCCCCAAG
TGAGGCTCTAAGTGTGGCTCCAAGTGTGGCTCCAAGTGAGGCTCCCAGTG
>comp2736_c0_seq1
CGGGACAGAGCATCGGATCGCACTTGCATTGGCATCATCTTGATCAATTTGACCATGATG
ACGGGACAATTTATTCGTTCAAAATTAGAATGACGATGAGCTTCGTCAGGCCTTGCATTG
TCTCCGGATTGATGTTTCATTTGATACATTCAACAAGTATCTCTTTGCGTTATTTGGGCC
CCGTGTTGACACTGGTGGTGGCTGCCAACGGTTTTCTCGACTCTGTTGTGACGACCCCCC
ACAGATTTATGTCAACGGCGAGGCACTGTGATGAGACAGCAATGGGTGTTCCTAACGATT
TAGTAGAAGCTGGGAAATCCTCCCTCGACGAACTCCACTCCAAACCCCGAAGTCTGCGTC
GTCAAATTCCATACGTCGAAACATTTGAAGAAAAAACTGTCCGTGACCTCTGGGAGAAAG
ACCCATTCGATGGACACAAGTACGTCGTGAATGCAGTCATCATTAGATCCAGGAACAATA
ATGAAAACAGACGAAGCCCAATCCTAAATTGTTGCGATCGAGAACCAATGCATCCGCTAT
CATTGTCAGGTTACGAAGACTCGTTCGATCGAATCTATCAGCCATTCTCCACTGAGAACG
AATCAGATTCTACTCGTGACAATATTAGTGGCGGCGGCAAGAATAGTTTAATTGAAGGAG
CAACAACTGCTTCCATCGCTGCCAGCCCTTTCGCCTGGACTCCTCCAAAGAACCAGAAAG
AAGAGGAAAAGATGCAAAATTTTTATTTGCAAGTCGCCTACGAAGGAAATGCATTTTGTG
GATGGCAGACCCAGCTGAAGAATTTGGTAAAACCTTCCGTGCAGCGGACCTTGGAAGAAT
GGTTGACGGTGCTCCAAAACAACGGGCATGCACTAGAAGCTGACATTATTGTAGACGCTG
TAAAAAATGCCGACAGGAAACGAGAGAGGCGACTGAAAAAACAAGGGAAACGGGAGACGA
AAAATGTGACCAAGAAAGACTCACGCAGCGAGAACTCGTTTTCTACCGCACCTCGGATCA
GATGGGCAGATCTTCCGGTTGCGGGCCGCACCGACGCAGGAGTGTCCGCGATTGGACAAG
TTTGTCGGTTCCGAACACATCGGAAGGACCTTACAGCCGAAATGATCAGCGACTATTTGA
CCAATAGAATAACAACAGCGCCTGAGATCTCCCAATCCCTGGGTATCACCAAAATAGTAA
AGGTTTCAAGATCCTTCCATCCAACCTTTTCGACCTCATGTCGAGCCTATGCCTATCTGA
TAGATGTCGATACGGAGCACAATAGTGACTGCGACGGTGTACTAGAGCTATTCAAAGGCG
GAAGAGCCAAACATCAAATCTCCTTGTTAGACGGCATGTTGCAAAAGCTAGAAGGGAAAG
CATTGGACTACATCGGTCTCTCCTACGGAAGAGTGAAGACGCTCAATACGATTTGTACAC
TGCACCATGCAAGGGCGAAATTGGTCCGGTACGAGACTTCTGCCGGAGCAAAAAGCCGTG
CGCGCACCGCCGTGTGCATCGAGCTGGTCGGAGATCGCTTTCTTCGTCGCATGGTGCGTC
TCTTGGTCGAAGCCTCCGTGCGGCTCGTTTCCACTGCGGAGAAGAACGCCGAGACCGAAC
CGCCGGTGATCGGTGGCATCGCAGATTGCGGAGCCACCACAGACGCCTTGTTGCAGCTCG
TCGAGAAGCGGGACCGAACCATAGTTGGTCGCCCGGCTCCCCCGGATGGCCTCGTCTTTG
TCGGAGCAAGATTGCCCTTGGCCTAACACAGCCATGTTTTGTTCTTGATCCGGCGCTGCT
TTGTAAGGATGACAATTCATCCTAATGAAATTCCTCTGTTGCGCTTCTGCAAACGCTCGT
GGACATAGAAAACGTAATGATTGTTTCACGTAGCCACATGAATATGCAATACAATAAGGT
AGAAAGCTGTTCGTTCTTGT
>comp2737_c0_seq1
AATCATCTCTGGTCTCTCCGAGAAAGTCGACACCGTGCTTGTTTGTTCTAGCTACGAACA
AGACCCACTCCCCACCGTGTACCACCGCATCCACCAAACCCGCGATTGCCAGCGACCCGG
ATACAGAAACCCGTTTCCGCACGGTTCGATACAACGCAACACGAACCAAAGACCCGAACG
CCCAAACCGAAGAAAGAACAACGACCATGGGATCCGCCCAGAGCGCCACCGGTGTGGGCA
TTTCCAGCGGCTCCGATCCCTCGCTGCCGCTCTCGGCGGTCGGAGAGGGATTCCGCGGCA
CAAGGGCCGGGGGCGCAAGCGAGGCCGACCGCGAGAACGACAAACCGAAAAAGCCAGCGG
GCACCGCACCGGCAGGGACCACCGGCGTCGGCGTCGACGTCGACCGCCGCTGCCGGAAGC
ACAAGCGGGCCTGGGGCAGGTGCGTCGCAGACCACTACGAGAAAAAGTTTCTGCCCGGGA
AGTCCCTGGAACCGGAGCAGGACTGCGACGACTTTTTCGAGAAATTCCGGGACTGCTACA
TGAGGGGGATGCTCGTGCAGCGCCGGCAAAAGGGCCTGCCGCCGCCGAAAAAGGATTCCG
CCCTCCACGAGTACATGGTGGACGAGGGGATGACGGACGACGGCGAGTGAGCGAGCGAGT
CGATGGGTGGGTGGGTGATGGGTGGGTGGGCCGAGGAAGTTCGTTTTGAAACATCGAAAG
AGAAACCGCGAAAGTTTCTCTACAACGACAGAAACAAAGCGTGGTCTTTGTTTGCTACAG
TGGTTTTTTTTTTCTGAAACCGAGGGGCAAACAAAATAGACGTGGTGTTTGTTTGCTGCC
ATGGTTTTTTTGCGAATCCAAGGGCCAAATAAATTGCAATTGAATGGGAAAATAGCCTTC
CTTCCACCCATCCACACCAAGGTCAATGCTCCTATTTCCCTTTGCTGTTGCAATCTATTT
TTGCAACCTTGTCCGGCACGTGCATTTGCTGCGACAGTGCACGGCGCAATCGGGGGCTGG
AAAATCCAGTGCCAACAGCGGAAGAACCGAATCCAACGAGAGACGGTTCGATCGCATCGT
TTTCCACCGCGTCGGATCCGCGAAACTTGTTGGTGGCGCACGCAGCACTCGATGCACAAC
CCATTTGGGAAAATCGTTCGATCGGTGCATTCTGTGCCATACATTGTAACAACACTTATT
CTATTCAATACCTTCTCTGGAAAGACAACCATACTCTACTGTAGGCTTTGGTGGATTGCT
AGGCAGAAACTAGCAACACCAGCATTCTCCACCGCCTTTGGTGTCAAGAGAGTGCACATC
ATGCAGTAGTCTGCTAATATTAGTACTCCGAAACGCGTTGCTAGTAGCCAACGAGAAGAA

ATCTTTTGTTTTTCTTTGGATAAGTATTGGTGTATTCAAGGCCACTGGTACATTTTGCCC
AGCGCACGGTTTGTTGTGTGCTAGTACTGGTGTAGTGGTGTGCCCATGAACAAGAGTAGG
AAGTGTTTGAGTACAGGAGATCCCCTATTTGACGTCTCTTCCTCCAGATTCCCTCTCCTC
CTTCTTGTTCTTCTCCTTCCCGGTGTCTTCTTGGACTACAGGCATCTTCTTCCAAAATAT
TGGACATGTGGGACAAAGTAGTGCTGGCAGTGAGGATATTGCCTAATTTTGTAGCTCTAC
TAGTACTAGTACTTATGTAAATCAAGTACTAGATTTGTTAGTATCTATTGTGTCATTATC
ACCATAATTGTTTGATGAAAG
>comp2738_c0_seq1
TCGATTCATAATTGGTCGGTAAGAAGATGCAACAACTGTACTTGCCAACAACTCTTAAAA
TAGATTCTATCATGACGGAAAGGTGCAGGAGTTCAGTGCCCAAGCGCGCTTTCCTTTGCT
CTCGAAGCGATCAAAAGAAAGAGACAAGCGCGGCGGTGTTGCCCTCCAAGGTCCCGATAA
TTGGTTTGGGATGCTCGAGCTTTTCCAGCTTCTTTTGGACCAACGAGGAGCTTGCGGCAG
AAACAAACGACGGCCAAACGAAAACTGCAATCACGGCCGAAGCGTTGACTCGAGATCATC
CAAAAGTACAAGAATGGATCGAAACGATTCGGTACGCAATCCTAGACTGTGGAATATCTT
TGCTGGATACCGCCCCCTGGTACGGGCACGGGACGTCAGAAATCGTCGTCGGGTGGGCCC
TCAAAGAATCGTTGTCGGGGTCTGACGCTGACAGCAGCAATTCCAAATTAATCGACCGGG
AAGATTTGTGCGTCAACACCAAGGTTGGTCGATACGAGGCCGATCCCACCCATCAGTTCG
ATTTTTCTCGAACCGCGACGCTGGAATCTGTCCAGCGAAGTCTCCAACGTATGAACTGCG
GATATATCGACGTGCTACAGCTGCACGATCCAGAATTTTCTCCCACTCTGGAGATCCTCC
TGAAGGAGACCATACCCGCCATGGTTGAATGTCGCTCCAATGGTTGGTGCAAGGCACTCG
GGCTGACGGGATATCCACTGGAAGTCCAGTACCAAATCCTGCAGCACTCGCTGGAAACAT
ACGGGGGTGAGGAATCCGTGCGGATCTGGGACCAGGCACTAACCTATGGGCACTTCAACC
TTCACGACGCCAGCCTTGTACGGCGCCGAATCTCTCCGTCGTGTGAGTCCTTCGCCGAGT
GCTGCCACCAGAGCGGGATGGGACTGCTGGCGGCGGCCCCTCTTTCGATGGGGCTCCTAA
CGAATAATATACCACCGGACTGGCACCCTGCAAATGGTTCCGAGTTGGCGAAAGCCTGCT
TGGAAGCTGCCCGCATATGCCGTGACTTGGATGTGGACATTGCCATTCTGGCGCTCTTGT
TTTCTATGTCGCATCCGAGCGTTCCGTGCACAATTCTTGGAATGAAAGATGTGGAACAAG
TGGAAAAAGCATCGGCGGTCGCACGCCGATTTGCAATGGTCGATTGGACGATACCGGACC
TCTCGCAGGAGCTGGTCTTGTCGTTCGTCCTGACCGTGCCCGAAAGCAAGGCGTACCGGA
TCCTGAGCGACCGTGAAGCCGGCCCTTTTGCAGAGGTGTGGAAACGAGAAACAGCCGAAG
ACTACGACGGGAACGGTATATTCGCTCCGAAGTTCCAATGGGACGGTGTGCGTGAAGCAC
ACGAGTTTTGGAGAACCACCGGGCAGTCCTTCGAGGACTGGCAGGACAAGTCCTTTCAAT
AGTATTACTACTAGTAGTAACTACTAGACTCTCGTAACGAGGTGACTGACATGCATATCT
CCTAGAGCAGACGAGGTCTGGTAAAATTTTATTAGAAAATGAAAGA
>comp2739_c0_seq1
TAGTAGTAAAAACAAAAGGATCTAAAAAATATCTTTCGAACACCCTGTGAATCTGGGAAG
AACAGAAACCTTGAGATGGAACAAACTATCAAGATCAATAGAGAAGCGGTTACAAGAAGT
AGGAGTTGTGAATGCAAAGAGGAATAAGCGACTAGAATAACAACTCCCGGCCTAATTAAA
CAACATTTGAGCTTATTCGAGTCATAGAGCACTGCATTCTAAAGCGAATACAGTCGATTG
CGGGCAATGAGTGCCGTTCTCAGATTTTGGAGCTGCGTTTGGTTCTGTTTCTGTCTGCTC
TGCGAATCAATCGAATTCAATGAAAAGAAATTGCACGATCTCTCTAAAAGTTCAGATATC
GATGGCAATGGGATCGGCAAAAATAAAGATCCGGTCGCCTTCCAAGAAATCGAAGTTAAT
TCACTGCTTACAGAGTATTTGGAGTCACCGCTAGGCGTCGACAAGGATGTTCCTCGTGTC
TCCTGGCAAATCCAGACCAAAACCAGCGATGACCAAAAGCACGGTTTCACGGCCGATGCT
GTTGCGACCGGGAATGTTCGTCGCCAAGTCTTTCAGGATTCATACCAATTGCATGTGTCG
ACTTCATCAGCGTCGTCGAGGTGGAAGGATTGCGGGTGGGTTGCATCGAACGAGACTAGT
TTGGTTCCATTGGAATGCAAGGGTCTGACCCCAATATCCACGAGTGACATGAATGTTTCG
TGGAAGGTCTCCGTCAGGCTCGACGATGGCTCGGTGATCGAATCGGCGGTTGCCTTCTTT
CGAACGGGTATCTTCGGTCCGTGGGATGCCCAGTGGATCAGCGGGGGTGGTTGGGACAAC
ACCGACGAGAAGGAACGAACTCTTTTCCGCAAGGAATTTTCGGCAGGCGCTTCCAACGCT
GGCGCTGTTCCCACCAAAAACCAGCCCGCCCGTCCTCATGCTACATTGTTTGTGGCGGGA
CTGGGATACAACCACGTCTATTTGAATGGAGTTCGAGTCGGGGATCATGAATTGGATCCT
GGATGGACGAACTACACAAAACGGGTATGGTATTCGACTTACGATGTGACGGACCTAGTT
CGATTCGCGGATGCCGAGGAAGGGGCTTCGAATGACGATTTCAACGACGACGATACGAAC
AATGTCGTAGCCGTCATGCTTGGCAATGGCTGGTGGAGTTGCGGGCCACCACCAGGAACG
GGACAAGATTACTGCACAAGTGATCCACCACAATTTATTTTGCAGTTGCACATTGACGGA
CAACCCGTTCTCGTATCCGACGAGACGTGGCTAGCTTCGGCCGATAGCCCGATTCTCTAC
AACTCCCTATACAACGGAGAAATCTACGACTCCAGGATCGCCAAACGCATTCGAGGTTGG
AATACTCTGCACTACGATGACTCTGATTGGGAGGTAGCAACATTAGCAACAAATGATGCA
ACTATGGCCACCCTGGCCTCCCAGCTCTTCGAGCCTATTCGTCACATATCATCCAGAAGA

CCAATCTCCATTGTGGTTGCTGGCAAAGGCGACAATCTTACCCAGGTAGTTGATTTTGGT
CAGAACCAGGCAGCAGTGGTGCGATTGAAACACTTCGTTTGCCCCGCTGGGACTACGATT
GCATTGCGGCATGCGGAACTCATCATGCATCCACCCTACGGGGAATACGACAATGCCACC
ATTTACGTTGAAAATCTGAGAACCGCGAAACCGAACGACTACTATATTTGTAATGGCGAT
CCGAAGGGAGAGTCCTACATTCCATCTTTTACACAACACGGTTTCCGGTACGTGGAGATC
ACTGGTCTAAACTATACACTGGATCCTACAGACGTCGAAGCAGTTGAGCTACACACGGAT
GTCAAACAAACATCCTTCATTCAGTTTGCCGATCCGTTGCTGAATCAGATCCAACACATG
GTGATGTGGGGACTCAAAAGCAATTTTATGAGCGTCCAAACAGATTGCAATCAGCGCGAC
GAGCGAAAAGGGTGGATGGGAGACGCTGCGTTGACTGCCGAAGCAGCCGTTTTGAGCTAC
GGAATGGGAGCTTTTTACACTCACTGGCTGATGCAAATGGTTGAGAATCAGAGTCCCGAT
GATGGTAGTATGCCCAATATAGTACCCCCAATGGGCAGAATAGAAGGTGCTCCCAACTGG
CAAACGGCTTATCCGATGATTCTATGGGTCATGATGACGTACTACGGAGATCGTCAGCTC
ATCTTCCAGCACCACAACTCTTTAGTGAGGTATTTCGACTTTTTGGAGGCAAGCTACAAT
AAAAATGGTTTGAAAAACTTTCGGACCGGATTCGGAGATTGGGTGCCACCATGGCCTCAT
CCGAAAACCGATCTACACTTCATGGGAGCATTTGCATTTTTGGGAGACCTCAAGCTAGGT
ATAGATATCTTTCAATACTCAGATCACCCAGATTCTGACACCCAGGTTGAACGCTTGACC
AAGCTTCGAGCCCAGTCGTCACCAGACTTTCATGCTGTCTTTTTCAATTTTGCAACGGGT
GCATATATGTCTGGGCTCCAGACCGAACAGGCTTTAGCGCTGTACCTCGGCGTGGTTCCC
GACGAATTGCAGGATTCTGTGTTGTCCTACCTAATTGATGACATCGAAATCACAAATCAC
GGACACACAACCAGCGGTATAATTGGTATCAAGTATGCCATGGAGGTTCTTTCGAAACTC
GATCGGGGCGATGTAGCACTTGATCTGGCATTGCAAACAACCTATCCGAGCTGGGGTTAT
ATGGTCAAAAGCCAGTTCGAACCTGCCACTACGGTATGGGAACTGTGGGAGTCCGATTTT
GCCGCACCGGACATGAACTCTAGAAATCATCATATGTTTGGGAGTATCACGGGTTGGTTC
TACAAATATGTGGCTGGCATCCAACCGTTGGATCCTGGGTTTTCTCGCGTCCAAATCAAA
CCAAATCTTCTTCGTCACGAGAATTTTACGGCAAGCGTATCGACTCCTCGTGGCAAAATT
TATCTGAGCTACCTTAAGAAAACTGCCAGCGACAAGAAAAACTCTGTTGTTTTCATTTAT
GAAATCATATTACCACCATGTACGAGCGGAACAGTCTACTTTCCTATGGCTACGGACGAC
AAGGAACTTGCTCTCTCTGGCGAGAACAACTATGACTTTTTGATCCGAGAAAGCGAAAAT
CCAATTTGGCTCGATCAGATGTTTCTCCCTGGTCACGACGGTGTGCTTCATGCTGAGATG
GTCGGAAAATACTTGAAGGTAGAAATTTCTAACGGAAGGTTTACTTTCAGCATCACGGTA
TCAGAAACGGCATCAATCTCAAAGAAATTGAATTGATGAAATGAATTTCCCTATGATCAG
TCTACTTTTTTTACTGGCTCAAAATGATATCTACAGGATGCTTTGTAATCGTTGATAATG
AATTCAAATTAATCCAGATAGGACCTCGACCGTAACTCATTTGGAAAGGATGAATTCCAT
TTTTCCATTAATTATTGTCAAGTTGAACTCCGAAAGCTGGCTTGGCTGCAATTGCTGGCT
TATGGAAACGATGTGTTTGTCATCATGCAACAGCATCATGCAACAGCGCTTTGATACCTG
GTAACTGGATGACTC
>comp2739_c0_seq2
TAGTAGTAAAAACAAAAGGATCTAAAAAATATCTTTCGAACACCCTGTGAATCTGGGAAG
AACAGAAACCTTGAGATGGAACAAACTATCAAGATCAATAGAGAAGCGGTTACAAGAAGT
AGGAGTTGTGAATGCAAAGAGGAATAAGCGACTAGAATAACAACTCCCGGCCTAATTAAA
CAACATTTGAGCTTATTCGAGTCATAGAGCACTGCATTCTAAAGCGAATACAGTCGATTG
CGGGCAATGAGTGCCGTTCTCAGATTTTGGAGCTGCGTTTGGTTCTGTTTCTGTCTGCTC
TGCGAATCAATCGAATTCAATGAAAAGAAATTGCACGATCTCTCTAAAAGTTCAGATATC
GATGGCAATGGGATCGGCAAAAATAAAGATCCGGTCGCCTTCCAAGAAATCGAAGTTAAT
TCACTGCTTACAGAGTATTTGGAGTCACCGCTAGGCGTCGACAAGGATGTTCCTCGTGTC
TCCTGGCAAATCCAGACCAAAACCAGCGATGACCAAAAGCACGGTTTCACGGCCGATGCT
GTTGCGACCGGGAATGTTCGTCGCCAAGTCTTTCAGGATTCATACCAATTGCATGTGTCG
ACTTCATCAGCGTCGTCGAGGTGGAAGGATTGCGGGTGGGTTGCATCGAACGAGACTAGT
TTGGTTCCATTGGAATGCAAGGGTCTGACCCCAATATCCACGAGTGACATGAATGTTTCG
TGGAAGGTCTCCGTCAGGCTCGACGATGGCTCGGTGATCGAATCGGCGGTTGCCTTCTTT
CGAACGGGTATCTTCGGTCCGTGGGATGCCCAGTGGATCAGCGGGGGTGGTTGGGACAAC
ACCGACGAGAAGGAACGAACTCTTTTCCGCAAGGAATTTTCGGCAGGCGCTTCCAACGCT
GGCGCTGTTCCCACCAAAAACCAGCCCGCCCGTCCTCATGCTACATTGTTTGTGGCGGGA
CTGGGATACAACCACGTCTATTTGAATGGAGTTCGAGTCGGGGATCATGAATTGGATCCT
GGATGGACGAACTACACAAAACGGGTATGGTATTCGACTTACGATGTGACGGACCTAGTT
CGATTCGCGGATGCCGAGGAAGGGGCTTCGAATGACGATTTCAACGACGACGATACGAAC
AATGTCGTAGCCGTCATGCTTGGCAATGGCTGGTGGAGTTGCGGGCCACCACCAGGAACG
GGACAAGATTACTGCACAAGTGATCCACCACAATTTATTTTGCAGTTGCACATTGACGGA
CAACCCGTTCTCGTATCCGACGAGACGTGGCTAGCTTCGGCCGATAGCCCGATTCTCTAC
AACTCCCTATACAACGGAGAAATCTACGACTCCAGGATCGCCAAACGCATTCGAGGTTGG

AATACTCTGCACTACGATGACTCTGATTGGGAGGTAGCAACATTAGCAACAAATGATGCA
ACTATGGCCACCCTGGCCTCCCAGCTCTTCGAGCCTATTCGTCACATATCATCCAGAAGA
CCAATCTCCATTGTGGTTGCTGGCAAAGGCGACAATCTTACCCAGGTAGTTGATTTTGGT
CAGAACCAGGCAGCAGTGGTGCGATTGAAACACTTCGTTTGCCCCGCTGGGACTACGATT
GCATTGCGGCATGCGGAACTCATCATGCATCCACCCTACGGGGAATACGACAATGCCACC
ATTTACGTTGAAAATCTGAGAACCGCGAAACCGAACGACTACTATATTTGTAATGGCGAT
CCGAAGGGAGAGTCCTACATTCCATCTTTTACACAACACGGTTTCCGGTACGTGGAGATC
ACTGGTCTAAACTATACACTGGATCCTACAGACGTCGAAGCAGTTGAGCTACACACGGAT
GTCAAACAAACATCCTTCATTCAGTTTGCCGATCCGTTGCTGAATCAGATCCAACACATG
GTGATGTGGGGACTCAAAAGCAATTTTATGAGCGTCCAAACAGATTGCAATCAGCGCGAC
GAGCGAAAAGGGTGGATGGGAGACGCTGCGTTGACTGCCGAAGCAGCCGTTTTGAGCTAC
GGAATGGGAGCTTTTTACACTCACTGGCTGATGCAAATGGTTGAGAATCAGAGTCCCGAT
GATGGTAGTATGCCCAATATAGTACCCCCAATGGGCAGAATAGAAGGTGCTCCCAACTGG
CAAACGGCTTATCCGATGATTCTATGGGTCATGATGACGTACTACGGAGATCGTCAGCTC
ATCTTCCAGCACCACAACTCTTTAGTGAGGTATTTCGACTTTTTGGAGGCAAGCTACAAT
AAAAATGGTTTGAAAAACTTTCGGACCGGATTCGGAGATTGGGGTACGTTAATATACATC
ATACAACCTCAAACCTCAAAAAATCAGATAGCATTGTGCTCACAGG
>comp2740_c0_seq1
TACGGATAATTCAGATTCAGAACCTTCGGTCCAAGCAATATAGTCCCTTTGCGAGAATTT
CCGAGGCTAGACGCTGCCCATGTGAACAAAGAGTTCAGGAAAGCATTGTTGTTGTTTCGC
ATTGCATCGCGTTGTGCTTGCGTCGGGTCGGGTCGGGTCGCGTTTCGTTTCGTTTTAATG
CATATTCGTTTCGTATTGGTTGGGAGCATTGAATACCGCACGGTAATCAACTGTGCTACG
TACTGTACTGAGCACCTAACATAGCTTGCTAAGACACGAGTACCCAGTTGGTTGTCGATA
GATTGATTGCTTCGCCTGTTTGTGTGTCGTGTTCTGTTGCGTTTCATTATTCGCGGGATT
CAAGAGTGAATAGGAGAAACGTTTTCGAGAACGTTTTCCGCAATTGCTGGTGTTAAGCAA
TTTTGTCTGATATACGCTCCAAAGCAATCGCATGCGCAACGACACACATGTGCAAGCCCA
CCGCCTGAATCGAATAGAATCTAAACGAACCGATTAACTGTGATGACCAAGCCCGACCCT
CTCGACGATGTCTTCGCAGATGCCGAGGACGACGTATACGGCCGGGTTCGAGCACCAAAC
TTCCACGATTGGGAGCACCACAACGGTCGTCGTCGCTTTCTTTCTCGCTTGCGGTACCTC
GGTAGAGATAAATCGAGGTCCGGAAACGGTAACGGAGGCGGTGGCGAATGCTTGGGCGTT
GGTGGATACGACGGCCTTTCCGAGTACGATGCCCAAGCCGAGGTGACCTGTTTCCGGCCC
ATCCTGCCGTCTCCGCTTCGAGAGCTGTCGTACAAACCCCGAACCGGTTGGAGGTACAAT
CCCTCCGCAAGAAATGCACCCTTTCTGGATCGATTAGGCACGGGGATTGCCGAACAAACG
GGACGCCGAAACAAAAAACAAACACGAACCAACCATCCGCTACAAAACAAACACAGGGAA
ACAACCACCATCAACAGCAGTGGCAATCAGAATGCAGTGTCGAATACAAAAGCGCAATTC
AACACTGATGTTCGTCTTCCAATGCCGTCTACGTCCATGATCGTACCCGTGGCGTGGACG
TCGGGCCTCCCTATAGAAAAAAATACCTGTAAAAAATTCACCGACCAGACATTCACCACG
GCCAACAACGACAGCGCCGACATATCAACGGCCCTGGAACGAAAACGAGGGAGGGACACA
GTTGACACTACTGGGAATTTGCCTCCCAGAAAAATGCAACGCTTTTCGGATAGTTCAACG
GTCGTGACAAACTCGACCGGAGGCGACTCTTTGAGCTCCTCTACTATCGCATCGAACAAG
ACCAAGCCGACCGGAGCGGCACCACTAGGGAGCATATATCCCAAGGCAGCTCTCTACCAA
TGGTATGGAAAGAAACCCCGGAAGATGCAGGTATCGTGCGATCAGTATCTTACGTGGGAA
TGCGATGGGACGGCTCTGCACCAACCAAACTTCACCACCGTTTTCATCTGCCCGATGACC
AAGGAGGCCTTCCTGGCCGGGCCCCTGCGCATTGCCATCGGAGACCCTAATTCCTCTCAG
GCGATAACCCCCGACAAGGACGGTCTCTATTGGTATCCTCGAAAAGTCTTGGCCGAGCAC
GCCGCCGCTGCCAGGGCGTGGGACAGCTTGTTGTGGAGAGATACCGGCGGCTGCGACGAA
ACCCTTGTGCGGTTTGGTGGAGCGGAGCCCTACTGGCCGTCGCAGCGGCCCGTGTGGCCC
TTCCATCGGATTCCGGCTGCGGTCCTGGAGCGCCTTCCACCGGATCAACGCCCGGACGCG
GCTGCGAACGGTGCAAAGAAGGAAGAAGACGAAGCGATGGCCTGTGTCGCTTCCGGGGCC
GATCCAGCGAGCGCGAAGCATGGCGCAACGAACTCAACTGTTCAATGCGCAAACAATGCT
GGGAGCAACCGAAACCTGATTCCCGGAGGGAGGGGACAGGGTCGAGGAGGACGACATTCC
CGGAGCAACAAAAGGGAAGAGGATTTTGCGATGAGAAAGCGAGAACGAGACCAATACACG
GCTCGTCGAAGGCAAAACAATCGCAGTCCTTATGGGAAACCAAACAACCATTATGTACGA
CCGCAGCAGCAACAGCAGTGGCCACCTCAACCAGCCGGCCACAATTCTCAGCCGTATTCT
TCGGGACGGTCTCACGCATCGATGCCTTTGGGTGATATGCAACGCCAAGATCGATACGAT
TACTTCCCGAGAGAAAGACATCAAAGCCGAAATCTTACGCAGCTGCAGCCATACCATAAT
CAATTTCAACACGTCGGTAGCATGCAGCAACACCAACAGCAGCCTCATCAGATTTACAAT
CAACAGCAGCCGCAACAATACAATCGGCAACCGTTGATACCGCCGCCGCCGCCACCGCTA
CCACCAGGACTGCCGCCACGAGTGATACCACCGCCGCCGCCTCCCCCTCCGAGCAATCGC
CTTCGGCCTTGACCAGAATAGATCGCTTAGCGACTACGACAACTAGATAGTGTCTGTATC

GATGGAAGGTATATTGGGGATCGTAAGGAATGGATGCCAGAAATGTAGCAGAAAGGTTTA
GTTTCCCGTGTTGATAACTTTATCCTGTCCCTATGCGCACTGATAGGAGTGTAAAGAAAG
CAATCGAATACAACGATAATAGAGCTAAGAAATACATAATTGAGCCCTTGTTGCGGA
>comp2741_c0_seq1
ACAAAATTCATACAGAACTATTCACCATGAACGCTCTTCGCCTTTCGTCCAGGCGCTTAG
CGCCCCAGGCATTCAAGTCCATTCAGAGTGTCGCAAGACCAAACAAACTATCGTCGGGTA
AGTGTGAAACTCTAGTGGTCCGAAGAGAAACGGAAGACTCTTTCCGTTCGGATACCGAGG
GATTGAATTGTCTTCGGTTTTTGCACGGCAGATGGCGTTTTTGGGGGTAGAGTTGAACTC
GGGACTGAACGCAGATATGCCGGTTGAAGATCTATTCTAGAATACAACACCAGTTTGCAA
TTGAAATGCATTAGCATGTTTGACGATAGATAATTACAACAGGGCATACCACTTCCTCGC
AGTGTAGATCTGCAGACGGAGATGCAGTCCATTGATCAAATCAGCAATCAATTCGTGAAT
GAGAGTGCGCGTTGGCGAGGAGTATCTTTTTCATCCTCTTCATTTCGACTTACCGACATT
TGGCCTCTTCCACTTCTTCCGTAATCACATTTTTCAATCATTTGCAATGGTGCAGCTTAC
TCGTTTTCGGGACTCCGAGCCTTTACCAGCACGTCTTCGTCATGGAAAGCGAGCAGTGGC
AGCGGGCCTTCTGTTCCTGTGCCCTCGGGTGTCGAAACGATGGACGATTACGGGAGTGTC
GACAATGGGTTTTATGATGATCGCATCGACAAGGTTGCAGAACCAGAGCGCCGTGCATTC
GCTTATCTCATGCTCACTACTGTGCGCTTTGCCTATGCTTCAGCTGCCCGGTTGCTAGTG
GTCAAATTGGTGGCCTCCCTCTCCATTTCCGCCTCGGCTGCCGCCCTGGCCACGGCCGAT
TTCGACCTTAGTGCGGTTGAATACGGCGAAACCCTGACCGTAAAGTGGCGTGGCAAACCC
ATTTTCATTCGAAAACGAACAGCGGAGGAAGTCGCAGAGGTTCGAGCCGTACCAATGGGT
AGTTTGCGTGATCCCGAAACCGACGAAGATCGAGTATTGACCGACGAATGGGTTGTCGTC
TTGGCGATTTGTCCGCACTTGGGATGCGTCCCCGTTTCCAACGCCGGAGAATTCAACGGC
TGGTTTTGCCCGTGCCATGGTTCGCACTATGATATTTCGGGACGAATCCGCAAGGGACCG
GCACCACTCAACCTGGAGGTTCCTCCTTACAAGTTCACCGACGGCGAAAATACCCAATTA
CTGATCGGGTAGACAGACGGAACAGATGGTGTGCATTTTCGAATGAAAGACCAAATGGAA
CAGGAGAAGCGAATCTGAACGGATCGTTGGTTGTTGATCCAACCTTAATGATTGGAATTA
AAGTGTAATGTCTACAGCGATGTTGTAGTGTACCAGTGAGATGGAAAAGAACAAAGATCT
AGTTTTTAATGGAAGTGGTGTAGAAACAACGAAATAGAATTCTAGATAATAATTTGGATT
TCATAAAAGGTACCCTATAT
>comp2741_c0_seq2
ACAAAATTCATACAGAACTATTCACCATGAACGCTCTTCGCCTTTCGTCCAGGCGCTTAG
CGCCCCAGGCATTCAAGTCCATTCAGAGTGTCGCAAGACCAAACAAACTATCGTCGGCTT
ACTCGTTTTCGGGACTCCGAGCCTTTACCAGCACGTCTTCGTCATGGAAAGCGAGCAGTG
GCAGCGGGCCTTCTGTTCCTGTGCCCTCGGGTGTCGAAACGATGGACGATTACGGGAGTG
TCGACAATGGGTTTTATGATGATCGCATCGACAAGGTTGCAGAACCAGAGCGCCGTGCAT
TCGCTTATCTCATGCTCACTACTGTGCGCTTTGCCTATGCTTCAGCTGCCCGGTTGCTAG
TGGTCAAATTGGTGGCCTCCCTCTCCATTTCCGCCTCGGCTGCCGCCCTGGCCACGGCCG
ATTTCGACCTTAGTGCGGTTGAATACGGCGAAACCCTGACCGTAAAGTGGCGTGGCAAAC
CCATTTTCATTCGAAAACGAACAGCGGAGGAAGTCGCAGAGGTTCGAGCCGTACCAATGG
GTAGTTTGCGTGATCCCGAAACCGACGAAGATCGAGTATTGACCGACGAATGGGTTGTCG
TCTTGGCGATTTGTCCGCACTTGGGATGCGTCCCCGTTTCCAACGCCGGAGAATTCAACG
GCTGGTTTTGCCCGTGCCATGGTTCGCACTATGATATTTCGGGACGAATCCGCAAGGGAC
CGGCACCACTCAACCTGGAGGTTCCTCCTTACAAGTTCACCGACGGCGAAAATACCCAAT
TACTGATCGGGTAGACAGACGGAACAGATGGTGTGCATTTTCGAATGAAAGACCAAATGG
AACAGGAGAAGCGAATCTGAACGGATCGTTGGTTGTTGATCCAACCTTAATGATTGGAAT
TAAAGTGTAATGTCTACAGCGATGTTGTAGTGTACCAGTGAGATGGAAAAGAACAAAGAT
CTAGTTTTTAATGGAAGTGGTGTAGAAACAACGAAATAGAATTCTAGATAATAATTTGGA
TTTCATAAAAGGTACCCTATAT
>comp2742_c0_seq1
CCAGAGCAACAGCGTATGATGGACATTGAGCATCGACGGGTGGTCCTTGATCCACCATGG
TTGGTTATTGTATCGACGTATAGGTTTTGAATCAATGGGGAGGGTTGGCCGTGCGGTATG
GTCTTGCAGCACTGAGCAATGTCGGAAGGAGGGCATCAACAACAAAAGGTGCTCTCTCCT
GCGAGGAGCGAATTCAATCACTTAGGGAGAAAAGAGATGCCTCGTCAAAAACTGGATAAG
TCTTCGTCGCACACATTTAGCTGATTCCGTTTCCGTTTCGGCAGGCTTGTGCGGCATGGT
TCGCCATATCATATCTGCGTGCTCGTTTGAGTTTGTACCATACGAGACCGTAGAAAGTAC
AGAACGATATTGGGGGGGATCGGGAAAATATTTGAGTTTGTCTTCGTTTTAAAACTAAAT
CCCCCAAATAATCCGTCCAATATAAAATTATTCATACTCGTAGATATTGATATGAACCGC
CTCCCAATGAAGCTGGCGAGCTTTTGTATGGTCTCTCCCTTGTTTTTCACGAGCAAATTC
GGCTCAACGACAGTAACAGAACCAGGAGAGAAGCAATTATCGTCAAGGAAGGCATCCTTT
TCAAACCCCGATGCCATTGAGACAACTGTAAACTAGAGCAGTGTTGACCGAGAACACCAA

CAATAAACAATATACTGAATAATCAAGATGACAGAAGTAGTAGCAGGATATGATGTATGC
CCATCGTACGCGACAGTGTTCGGATACCTGGGTGCCAGTGGATGTATGATCCTATCTAGC
TGGGGTTCTGCCTGGGGAACCTGGAGAGCTGGACTAGGAGTCTGTCAAATGGGTATCGAT
CACCCCAAGGGAATTATTAAGAACATCGTTCCTATTGTCATGGCCGGTGTTTTGGGTATC
TACGGTCTGATCGTATCTGTTATTATTACGCAATCTATCTCTAGTCCCGCAGGAGGCGGT
TACAACACATACAGTATCTTCAATGGATACGTCCATCTTGCCGCTGGAATGTGCTGTGGA
CTTTCCTGTCTAGCGGCCGGAGGTACTATTGGAATTATTGGAGATGCCGGAGTCAGAGGT
TTCGGACTAAAGGCGGAAGGCGGACGAAAGTGGTTCTGGTCCGAAGACCAAGGTGATGAC
GGTGACTTCGGAGGATCCAGCAACGCCGAGGCTTCAAACAAACTCTACGTCGGTATGCTT
ATTATGCTCATTTTCTCGGAAGCTCTTGCACTCTACGGATTGATTGTCGCACTTATCTTG
AGTCAAAACACCTATGTATGCGCGGCATAAGAAGAAACTATTGTACTGTAACTAATATAC
AGCACTGCAATGCAGTAACTAGCCATTTCTATATGCAGAAATCATTATCCTGCCGAGCTA
AAAATTGACAATGAAAGAATGATGACTTACTTTTTAAAATCAATTATATTTCTCTTATCA
TCGAATACGGTAGTCTCAATGTTGATACTGCAAGTCATGGTTCAGCCTGT
>comp2743_c0_seq1
GCAGCAACCCACAAAACGGTTAAACTGTTGAAACTTCGATATTTCAGCGTCCGCAGCTTT
CCTCTTTTTTCTAATCCAGAACAAATTTCAAAATGCCTCTTTCCCCCGAACGCAAGGCAG
AGTACTTCGACACCTTCAAGAACCTCTTGAGTACCTACTCCAAGTGCTTCGTCGTCGAAA
TCGACAATGTTGGTTCCAAGCAGCTCCAGGACACCCGTAAGACCCTCCGTGGAAAAGCTG
AGGTCATGATGGGAAAGAACACCATGATGCGAAAGGTCATCAAGGAATACTGTGAAGAAA
ACCCTGAATCCGAAGCCATCAAGCTCGTCGACGAGTGCCGCGGAAACGTTGGATTCGTTT
TCACCAACGGTGACCTCGGTGGTATCAAGGAGATCTTGGAGGCCAACACCCGTCCTGCCC
CTGCCAAGGTTGGAGCCGTCGCCCCCGTCGATGTTGTTGTTCCCAAGGGACCTACCGGAT
GCGATCCCGGTCAGACCGCCTTTTTCCAGACCTTGCAGATTGCCACCAAGATTACCCGTG
GTCAGATTGAGATGGTTGCCGATACCCACTTGATCCAAGCCGGAGAGAAGGTTACCGCCT
CGCAGGCCGCCCTCTTGCAGAAGCTCGACATCTGCCCCTTTTCGTACGGTATGTTGTTGA
AGTCTGTCTATGACAACGGTTCCCTCTTCGACGCCAAGGTCTTGGAAATCACCGACGATG
TCCTCACCGCCCAGTTCTGCGAGGCCCTCGGAATCCTTGCCTCCATCTCGTTGGCCGCCG
GATTCCCCACCCAGGCGTCCGTCCCCCACTCCCTCGTCAACGCCTTCAAGATGGTCTTGT
CCGTCACCATCGAGCTCGATACCTACAGTTTTGATGTTGCTGACCAATACGAGGAATACC
TCAAGGATCCTTCCAAGTTCGCCGGATCCGGTGGAGGCGGCGGCGGTGGTGAAGCTGCCG
CTGAGGTCGTCGAAGAAGAGGAGGAGGAAGAAGAGGAAGAAGCCCCTCAAGGAGGTGGTC
TCTTCGGAGATGACGACGGCGATGGTGACTACTAAGCCTTCGTTTGTAAGTCTCCCTCGA
TCCATTTTGTTCTGGAAACGATTGTGTGTTAGGTTGAGAATGCCGCTGAGTTAACACTTT
AAAGCACACTTTTTTTTTGGGGAAAAA
>comp2744_c0_seq1
CGTTCTACTTACATGGTTGTTCTAATTAGGAGTACCATGCGGCTTTGTATTGCGAGCTTT
CGATCTTGAGCAAAAACGCTTCAAATACGAGTACAGTACTTCTACTCGGTACGTTACTTG
TTCTGAATACTCTTTCCTCTAGTGTTGGATTCTAATCCCCTCTATTACAACCAAGTAACG
CCACCATGTCGAACTCAAAAGACATCAGTGTCGATGATGAAAACCAATGGATGATCGATG
ACTCGAACATCGATAACGACGGTTACAGTGGAGGTGACAGGGAAAGTAGCGATTACGATG
CGGTAGACAATCCTGATTACTTTAACGACGAGCAAAATTCTTCGAATAGAGACTTAGGGG
CATACGACGATTCTCAAAAATTCAAGGAGGATGGGGGCACTAGAATGGAGATCAGTGACG
AATGGTGGGAAGGCAGAGAAAAACTAATAGCAATCGTTGCAGTTTGTTGGTGCTGTCTGT
GTTTCATCATTATCGGGGTCGTTGTGGGTATCCTCGTAGGAGGAAAGAACAAAACGAGAG
GTCCCGGTGCGACCCCTAGACCTACTCTTCATCCGACTCTTGCTCCGCAACCTCTCCCTC
CCTCTCTAATATCGATTAAACCAACTGAAAGAGTAACCCAATTAACACTGACAGATTCCC
CAACGTTTGAACCAACATTGTTTCCGACAATTTCACCGACAGTTTCCCCAGCTCCTACTA
TATCAATTCCAGAAACCTTGGGTGTCATTGCGAATCAGGACACGTTTGTGATTTTGAATG
GGCACCATGACATAATGGGGGAGGAGCATGGAAAAATGGACACTTTCGTGGTTCAAAATG
GCCCCGTGAAAGACGAAACTGTACCGGATACCGTTGCATTGATTACTTTCCCTTTTGACG
CTGTCCCTGCGTTTAGTAGACTTACTAACTTACCGGTAAGCGCCGCACTACGACTTTATC
ATGTAGTGCCTGAAGAAAGCCGAGGTACGGCCACGTATACCATTGTTCGCATACCAGAAA
CAAGAATGGCGATTGAATATTTTCACGGATTCTCTTTCCGTCCACCTGAGGACGATGCCG
ATGGTGTTCTGGTTGGACCTTCTTTCACGGTGAAACCTGATGACGAAGTCGTCGACATTG
ATGTTACAGAGGTCTTATTTGAGTACGAGTTCGAACCCGAAGAAGAACCCAACCAGATGA
TGCTCATGATACAGAATCGGGGCCCCGAACAATTGCAAGGTGGTGATGTATTTTACTCAA
GGGAATCTAACACTCCACCTGAGCTTTTGATCGACTTTGTCGGAGGTGATCCGTCAGATA
TTACAATTGTTAATTTACCCGGAAGATGACAAGAATCGAATGATTTCGTTTTGGAAAAAT
CAACAATCTGCAGCTTCAAGGTGTTGGGACCCTAAGAATATATATCATGATCGTACTACG

AGCTTTCGTACTGCATCACGGGTATGCATCATACTTGTCAACGATGTAATGTTTCCATGC
TAGAAGGGCATGATATGGCAGGGAACTCCCCAGGTACAACATCATTTTTGAACTCCTAAA
TTGTAGTCTATGTATGAACTTACATCGAAATGAAAATGGCACAACGATAGATAAATTAGA
CAGGTATTGAAAAGAAGT
>comp2744_c0_seq2
CGTTCTACTTACATGGTTGTTCTAATTAGGAGTACCATGCGGCTTTGTATTGCGAGCTTT
CGATCTTGAGCAAAAACGCTTCAAATACGAGTACAGTACTTCTACTCGTAACGCCACCAT
GTCGAACTCAAAAGACATCAGTGTCGATGATGAAAACCAATGGATGATCGATGACTCGAA
CATCGATAACGACGGTTACAGTGGAGGTGACAGGGAAAGTAGCGATTACGATGCGGTAGA
CAATCCTGATTACTTTAACGACGAGCAAAATTCTTCGAATAGAGACTTAGGGGCATACGA
CGATTCTCAAAAATTCAAGGAGGATGGGGGCACTAGAATGGAGATCAGTGACGAATGGTG
GGAAGGCAGAGAAAAACTAATAGCAATCGTTGCAGTTTGTTGGTGCTGTCTGTGTTTCAT
CATTATCGGGGTCGTTGTGGGTATCCTCGTAGGAGGAAAGAACAAAACGAGAGGTCCCGG
TGCGACCCCTAGACCTACTCTTCATCCGACTCTTGCTCCGCAACCTCTCCCTCCCTCTCT
AATATCGATTAAACCAACTGAAAGAGTAACCCAATTAACACTGACAGATTCCCCAACGTT
TGAACCAACATTGTTTCCGACAATTTCACCGACAGTTTCCCCAGCTCCTACTATATCAAT
TCCAGAAACCTTGGGTGTCATTGCGAATCAGGACACGTTTGTGATTTTGAATGGGCACCA
TGACATAATGGGGGAGGAGCATGGAAAAATGGACACTTTCGTGGTTCAAAATGGCCCCGT
GAAAGACGAAACTGTACCGGATACCGTTGCATTGATTACTTTCCCTTTTGACGCTGTCCC
TGCGTTTAGTAGACTTACTAACTTACCGGTAAGCGCCGCACTACGACTTTATCATGTAGT
GCCTGAAGAAAGCCGAGGTACGGCCACGTATACCATTGTTCGCATACCAGAAACAAGAAT
GGCGATTGAATATTTTCACGGATTCTCTTTCCGTCCACCTGAGGACGATGCCGATGGTGT
TCTGGTTGGACCTTCTTTCACGGTGAAACCTGATGACGAAGTCGTCGACATTGATGTTAC
AGAGGTCTTATTTGAGTACGAGTTCGAACCCGAAGAAGAACCCAACCAGATGATGCTCAT
GATACAGAATCGGGGCCCCGAACAATTGCAAGGTGGTGATGTATTTTACTCAAGGGAATC
TAACACTCCACCTGAGCTTTTGATCGACTTTGTCGGAGGTGATCCGTCAGATATTACAAT
TGTTAATTTACCCGGAAGATGACAAGAATCGAATGATTTCGTTTTGGAAAAATCAACAAT
CTGCAGCTTCAAGGTGTTGGGACCCTAAGAATATATATCATGATCGTACTACGAGCTTTC
GTACTGCATCACGGGTATGCATCATACTTGTCAACGATGTAATGTTTCCATGCTAGAAGG
GCATGATATGGCAGGGAACTCCCCAGGTACAACATCATTTTTGAACTCCTAAATTGTAGT
CTATGTATGAACTTACATCGAAATGAAAATGGCACAACGATAGATAAATTAGACAGGTAT
TGAAAAGAAGT
>comp2745_c0_seq1
CAAGCTCGTTACATAACCTAAAATATTGTTGGTTGCCGATTGATTCCACCATGAGCGTTT
CAACCGATGGTTTGAGGCAGCGAAAGACCGCCACAGGGCAGGAGACTAAGGCCGTTGAAA
ATAAGAAGACTGCAACCCTAACCGAGGATGATGGAACCAACTTCGTAGAGGGAGGTATCG
AGCAGAACGCTTTCTTCTTCGGTCTGCTTGTTGCCGCTCCCTTTTTGTCATTGTTGCTGG
CGTATTTGACAAGCGAAGAAATGGCAACTGAGTTCCCGGGATATGCCACCCATCCTGTCA
CGGTAATGTTGGGCGTCTGTGTGAGTGATCCTGCTGGATGCGCTTCCAGCGTTGTAAAGA
GTGGATTGTCGGTTGTCCCAACTCTGCCAGCGACACAATTCGTGCTGTCTTTTATGGGCG
TCGCTTTGGTGTTGGAACGATTTTTGCCGGGAAAGATCGAATGTGGACCCGAAACATTGA
CGGGTCACATCCCCAAGTATGTCGACAATGCTGTCTTGCACTGCGTTATCTATACTGCTC
TATTTTATCTCGGTTCCAATATTGGTTATGGAGATCTCTATGACTTTGGAATTATCTACG
ATCTATTTCCCGGTTCGGTCGCCTTTTTGAATCTTTTCGGTTTGGCATTTTGTGTCTTTT
TGACTCTGAAAGGAATCTACTTTCCTTCCACTCAAGATTCGGGAAGTTCGGGGAGTCTAT
TCAAAGATTTTGTTTGGGGGACAGAACTCTACCCACGAGTTTTTGGATTGGATCTGAAGC
GATTCGTCAACTGCCGATTTTCTATGACGTTTTGGCAACTGGCCGGCCTGAGTTTCTGCT
ACAGATCCTACACTCTCCACGGACACACCATGGACTGGGGGCTTTTCTTTTCGGCCCTGT
CTCAATACCTATATCTGTTCAAATTCTTCCTGTGGGAAATGGGGTACATGCGCTCGATCG
ATATCATTGTAGACCGCGCTGGATTTGAAATTCAATGGGGGTGCCTGGTATGGGTACCGG
CTGTCTACACCCTTCATTCGCGCTTCTTGGTTCAATACCCGTCCGGTCTCTCCTTTGCGG
CGGCGGCGGGCCTGTTCATCTTGTCGATGACCGGAGTCGTCTTGAACTACATGGCAGATC
GTGAACGCGATGTCTTCCGTGCCACGAACGGAGAAGCCCTCGTCTGGGGAAAGAAACCTT
CGTTCATCAAGGCCACTTACACGACCATCGACCGCAAGACCGGAGAAAGTTCTCAGAAAA
CATCCTTGTTGCTGGCCAGCGGATTCTGGGGAGTGGCGCGCCACTTTCAATATTTTTTTG
AGCTTACTGCGGCATGGAGTTGGTGCCTCTTGGCCAATCCGCTCCAGAACGGAGTCTTAG
TTCTTAGCTACGCTGCGTTTTTGACCTACCTTCTTATCGACCGCGCCGAACGGGATTCCA
AAAAGTGCCATTTGAAGTACGGAAAGTACTATGAAGAATATTGTCTTTTGGTTCCCTACA
AAATCCTCCCTGGAGTCTATTGAGAAAGCTTGATTTTAAAAAATCATTCTATAATTGGAC
TCGCTGGAAGGTATTTGTTTTCTCTTCGCCTATAAGATGCATGTTTTTAGACGCAAAAAT

TACCCAGTACAAA
>comp2746_c0_seq1
CGGTCAACGGAGCAGACCAGGGATTCTGCTAGGGGGGAAACTGGGAATTTAATACAGAAA
CAACGCAAGGATCAACCGAAGCGCTACTACTCGTACCCACGAGACAAGTCAAGTACAAAT
ACCAACTCTCTACTTTACAACAGTCTAGGAAAAGGAAGCGACATCAGAGTTGACTAGGTC
TCCCCACGGCAGCACAGAAAGGCGTCTCTGCACGAACGGTTCATAAATCAACGATTGTCT
TCGGATCGTGGAGTCAAGAAGTCTCTGACCATGCTGAAGTTAACAATGAATCCGAGAAAG
CGCTCGAAGCGGATGAGAGCAGCGCAATTCGGAAAGCTCTATTATTCCGGGCAACCAAAC
GGTCCTTCACGGATGGAAATAGAACAAAAAGCAGTCGAGCATTGTGAATCGTTGTCGGAT
AGTCTCGCGGTCCTCCAAAAGCAAGAGATTGCCGCAACGTACAAGTGCAAAGACTACCTA
GCGATGACGGAGTTTCGATTGAATATATACGATCTCCTTCCAAACGGTCGAATGAATAGC
GACATTACGGCCCAAAATCCTCAGCAACAATCCCAGCCACAGTGCCCCGTCGATTCCCAG
ATCGACGAGTATTGCCGAGAGCAAATCGTGGAGTGGTCTTTTCGCGTGGTGGATTACTTT
CACATCGATCGAGAGGTCGTGGTCTTTTCTCTTTCGATGCTGGATCGCTTCCTGGCCTTG
TGCAAGTGTGACCGGGCCACCTTTAAGCTGGCGGCCACCACGACCCTGCACCTTGCTGTC
AAAATCTTGCACCCCTGCAAGCTTGCAGAGCTCGGCATCCTCAGCGATCTGAGCCGTGGA
GAATTTGACATGAAGGACGTGGCATCGATGGAGACGCACATCCTTGCCGAGCTTCAGTGG
CAGCTGCACCCTCCGTCGCCCATTGCCTTTTGCACCGTGCTTCTGGACCTCTTTTTCAGC
TCACAAAAAATGGGCATGGTCCACACGGATGTGAACGACCTGTACGATATCTCATCCTTT
TTTACGGAGCTGGCCCTCTGCGACTATTACTTTGTGGGCGTGACACCAAGTATAGTTGCG
ATTACTTCGATAGTCAACGCACTGGAGGGAATGTTTGGCCCCGAGAACAAGGTGGCGCCC
CGCATTCTGAAACTCGCCCGCGAAACGAACATATATCAAAACCAAGACCTGTCCCAGATA
TCCCACCGGTTATGGGAACTCTACGAGCGAAGCGAGGAGTGCGCCCTCCACAACAACGTG
GACGCCATGGAAGTCGAGAAGACACCGGAAACCGCGACCGTGCCTCCCGCGAGCGCCGCG
GAGGTCTTTGTCCGCAAGGGAGACTGCGGCGCCCAGGTCGTCTCGGACACGGGCTCGCCG
GTGTCGGTGTCGAAGCACGGAAAGACGCTGTCCTCGAACGAGCTCATGTGCGCGATGCGC
TCCCAGACGCTCCGCAACGGGAGCTGGTGAGCATCTGCGTTGTTTCGCAGCGCACCACCG
AGGAACCGCCCGGTGTGTCCCACGAAGGCCACGGTATCGCAACAAGACATGCTATTGTAT
TACTGTACTTATTCTCACCGACCTTTATCCCCATCCGGGCTCTGGTATAGACACCCGGAA
GCCCGGTTCATAGAAAACTACCATAAAAATAACAACTTACAATAAAGAC
>comp2747_c0_seq1
CGCTATCTTGCAGAACTGTACACATCTCTTAGCAAAGAAGAATCCTCATCATGGCAGAAG
CTACAACCACCACCAATGCCTTGTCGGCCCGAGAGCTCGAATGGGCTAAGAATTCACCCG
AGCTGCAAGCCAAGCACGCCGAAATCAATGGCCCAATCATTCGAACCCGCTTTCCCCCGG
AACCCAATGGATACCTGCACATGGGTCATGCGAAATCCATCAATATGAACTTTTCTTTGG
CTTTTGAAAAACTGGGCGTTGCACCTGAAAACCGACGCTGCATCTTTCGATACGACGATA
CGAACCCCGATGCCGAATCGAAGGAATACATCGATTCCTTTGTGCATGACCTGGAGTGGC
TTGGGTGGACACCCGAACGAACTACGTACAGCAGCGACAACTTTCAAAAGCTTTATGAAC
TCGCTATAAAGCTGATCAAAGATGGCTTGGCATATTGCTGTGACATGACCAAGGCCGAAA
TGGAAGCCCAACGAGAATTGGCCATGCAGCGTGTCCTCGCAAGAAACAAAGGAGAAGATC
CTGACGAGGTTGCTCCGATCCCTTCGGAAGATATTCTTCCCGGACGAAACCGCGATACAA
GCGTCGAACGGAACCTTGACATCTTTGAACGCATGAAAATGGGTTTGTACGACGAAGGAA
CCTGGACTCTTCGTCTCAAGATGGACTTTGAATCCTCCAATCCAAACATGTACGATTTGG
TCGCTTACCGAATCAGATATACGAGCCATCCACATGCGGGAAATGGCTGGTGCATCTACC
CCAACTACGATTATACGCACGGAATCTGTGACTCCCTCGAGGACATTGATTATTCCATTT
GCACTCTCGAGTTCGAAACCCGACGCGAACCCTATTTCTGGATTCTTTGGGCACTCGATA
TGTATCGTCCGAACGTCTACGAGATGTCTCGATTGAATCTCCAGTACACCGTTCTAAGTA
AACGTCGCCTTCTCAAACTCGTCAACAACGGCATTGTGCGTGGATGGAACGACCCTCGAA
TGCCCACTCTTTCGGGCTATCGCCGAAGGGGATTCACGGCAGAAATCATCAACAAGTTCT
GCACCGATCTCGGGGCTACTCGTGCCTCTAATGTCATCGAGATGGAGAAATTATATACGA
CTGCACGCGTGGCCCTCAGTGAAAAGACCCAGCGTGCCATGGCTGCTCTTGAACCAATCA
AAGTTGTAGTGACGAATTGGGAGGACGCAAAGGGAAATGGAGGCGAGGGTATGGTATTTG
AAGTACAAAACTCTCCAACGGACCCTTCTATGGGAAGCCACACGATTGAGCTGACTCAAA
CCTTGTACATCGATTCCTCCGATTTCCGTCTAGTGGATGAATCCAGCTATTATGGTTTGG
CACCCAACAAAGCAGTCGGTTTGAAGTATTATGGTGGAAACTTGATTTGCGACGAGGTCG
TTTTCAAGGAAGGGGCTAACGGCAAGGAAAAGTCTCAAATCGTAGAATTAAAGTGCCACT
TGGACGGGTCGGACGACCGTGTCAAGCCAAAGACCTGGATCAGCTGGGTCCCGGCCCAAA
CTGCAATTCCGTGCGAGATCCGTGTCTACAACCATTTGTTCACCGTTCCCGAGCCGTCGG
ATTTGTGGGAAGAAGAATCAATCCCAAATCAGAAATCGTGTACGAAAATGGTTTCATCG
ATCCGTCCGTTCTTCCACTAGTTGACAAGAAAGATATAAGTCTGGTCAAATCTAACCAAG

CCCTTCAGTTTGAGCGAATGGGGTACTTTGTGGTCGACGTCGATTCCACCTTCGATTCCT
CAACCAGAACGGGGAAGTTGGTCTTCAACCGCACCGTCACATTGAAGGAAGAAGTTAGCA
AAAAGAAGGCCACTCTCAGCAAGCGCGAAGAGGAAGCCAACGAGGCACGCAAGGCAAAGC
AAAAGGCAGATCTGGCGGCAAAGGAGGCGCGTTTGAAAATCGACCCCATTAACTTTTTCA
AGGAAGCCGAAGAATTCAAGGGCAAGTACTCAAAATTCGACGACAATGGCGTGCCTACGC
ACGACGCCGAGGGAAATGAGCTCACCAAATCCGCGATGAAGAAATTGAAAAAGGAGCAAG
ACAAGCACGTGAAGCAATTGAAAAAAGCAAGCATGAAAAAGTAAGAAGAATACAGGTTGC
GGAAATAATTCCTGTAAAAAAATCGAATCATATTAATAGCACAGTTGAATCAGATTGCAT
GAATAAGGGTTGCAGTCGTTGCAGTTTCCACAAATCAGAAGGCGCGTACCAATAGAAACC
AATAAGAAGTAGGTGAAGCATTTGTAAGAGAATTCAACCATTTCTCGATTGCGTTGCATA
TTATTGTTAAAATAGGAC
>comp2748_c0_seq1
CTTCAACCGAATTGTTTTCACCACCAAGAGAGAAAAGTAGTTTTGTCTTCGTGCGAGATA
AAGAGACATTACATCCTCCTCAGTAAAGGGATACCCGAATAATTTGAAAGTAATAGACGA
TGACCACAATTAACTCAACGACATCTTTGTCTTCACCTGCCCTCGTGATGGAAGAAGGAG
CGGACACAATCACTCAAGTTGTTGTGCACCCATTGGTCTTGTTGCATGTTTTGGACCACC
ACACCCGTCGACAGGAAGCGTCTGGAAGGGTAATTGGCACTTTGCTAGGTAGACGAGATG
GAAAGAAGATCGAGGTGACAAACTGCTTTGCTGTGCCTCATGCCGAAAGAGGCGACGAAG
TTGCTATTGGCAAGGATTTCAACAAACAAATGTTGGCATTGCACCTTCGAGCAAATAGGA
AAGAGACCGTTGTGGGATGGTATGCGTCGGCTGCCCTGGCGGACGGAGATAACGAAGCGC
CGGATTTGATTGCCAATACGTCATCATTGATTCATGAATTCTATGCGGAAGAATCGGACG
AGGGAGATCCGGTCCACCTTGTAGTTGATACGCGACTCATCGAAGATGCAATTACGGCCA
GAGCTTACCGAAGCACACCGGTAGTTGTTCAAGGAGAGCCGATGGCCAATATGTTCCATG
AGTTGCGTTTGACGCTGCAAAATTCAGAACCAGAGACACTTGCTCTCCATCGAATGGTGG
CTGACAGCAAGGAAAAATCGACGGAGGAGTCATCAGAAGAGCCCTTGCTTGTATCAATGG
AAACGCTGTACAATTTGTTGGAGACCGCCTCGAATTATGTTGATTCTGTCGTCGAAGGAA
AGACATCGCCCGATGCAGAAATTGGTCGTAAAGTTGCAGACACATTGGCTACCGTTCCGC
GCATCCGCCCTGAAGTGTTTGATAAGCTGTTCAATGACTCGTTGCAAGATATGCTTATGG
TCACCTATTTGTCGAACATCACACAAACGCAGCTTTCTATTGCCGAAAAACTCAATGCGA
GTTTGGGAGTTTAATAATTCAAATGTACTAGTAAAAATCCTTTTATTTTGCTTCCAG
>comp2749_c0_seq1
ACCGGCGCCGGGTTGGTAGAAATCTTTGGTGGGGATCGGGAACCGGTTAATCGCCACTAC
CACAACATTGCGAGCTTGGTGGGGCTCCCCTATCGATATACGAAGTTCTACACCGATTCG
CCCGGGCACAACGTAGACTGGAACCAGCAAGATGTTCTGAGGGTAGAGGAGATGATCCGC
GCCCTGATCGAAGAGCAACGGAGCAGCAGGAAGAATGGTTGAATGACCGCACCAGTATCT
TGCCTGCTCGTTTTCTTCAGAGTTCTGGAAACAACTCCTCGGTGTCGCCGATTTCCGATT
GAGAAGCAGACCTTCGGCCAAACTCATCGATGCGATACAATTAAGAGTAAACGCTGGTCG
ACCAGACTCCCTTGCACATTTATACTCAAGGAAACTCGTTTAGTATCTTCCAAGAAAAAG
TGGGCACAAAATAGCAAAAGAAAAAAAGGGCGCTCTCAGAAGCTCGAGTTACCCATTCGT
GAAACTAACGAAACGATTTCATACAAATCCAAAACTGTATTCATGAGAGAGTGTAATAAT
TCTATTTGCATTTAGCAGCAAGATATGAAAAACAGGATAGCTATCGAACATTTTTTTCAT
TACAGTAGTACCATACTCA
>comp2750_c0_seq1
CACCGAACGAACACCCCGCACAATGCATCGGTCGTCGCGATCGCTAGTGCTTCTTGGATC
GCTGTATATTCTAACAAAGATAGATCCAATTTTGTCTCTTCTTCATCCATCCGTAGTTGA
GAAACATCTCATCATGGTGTCTCTGTACGGCCAGTCGAGCATTTCGTTCCGCCGCTCAAC
CTCACGCCTAGCGAAAGCTGCCGATAACTCTGCAAACGGACGTCGTCCCGACGCGATCGT
GACGAACTGCAACTTCAACGCCAACAAGAATCCCTTGCACCGTCCATCAGATCCTCGATC
CGTGGTTGGAAATCCTTCTCAACTTGACGAAAAGGCGTCGGAGCTTGCGGAATGGCTCTC
TTCCAAACGGAATGTCTTTTGTCTGACGGGGGCGGGATTGTCGACCGATTCAGGGATCCC
AGATTATCGCGGTCACAAGGGATCCTATCACGTAGGCCACAAACCGATCATTCACCATCA
ATTTATGGAGCATCCTCACCAACGGAGACGGTATTGGGGGCGCTCCATGGTGGGATGGAA
AACATTTGACCAGGCAAAACCCAACGTCGGGCACTATTCGCTTTCGGCTCTGGAACGAAT
GGGTCGTTTGGGTGTGAACATGGAGGATAGAGAAGATTTTTACGACGAGCAGGATCGAGA
TGAATTTCTATTTACTTCGGGACAACGGCGGTTGACGGTGTTGACACAGAACGTGGATTC
TTTGCACGTCAGGGCCGGATCGAAGGACGTCCTTGCCATTCACGGAGAGAACAAAATTGT
TAGGTGCATGAACTGCGGGCATCGAATGAAACGGAACGACTATCAGGAGCAACTGGAAGA
GATGAATCTAGCTTGGTTGATGAAGCACCTAAAGCTATTGGAAGAAGGCAAGGAATCTTC
CCGCCAGCAAAGCCAACTGCGACCAGACGGAGATGCGCAACTCGTGAAGGCCAACTACGA
CGATGTTGTCCTCCCTTCGTGTCCTTGCTGTGAAGATGGTTTTTCAAGCCCGACGTAGT

CTTTTTTGGTGACACGGTTCCCAAACACAGGGTTCGAATCTGCGAGCAAGCGGTAGAAAA
CTGCGATGGGATCTTGGTAATCGGAAGCTCTCTGGCTGTGCATAGTGCCTTTCGTCACGT
GCGGGCCGCCTCCAGAAAGGGAACCTCGGTGGCAGTCCTTAATGTTGGAGAAACGCGGGC
CGAGGCCGAGAAATTAGAGGGGCTTGTCAAGATTGAATCCCCGATCAGTGACACACTGCA
ACGTTGCGTGGAAATGATGGGGGGTGCGTTGTAAGCACAAAGTTTCCACTCTGAAAATCA
TCACAGCATGTGCTTTTAGAGGCTAGAGGTTTCGCATTTTTATTATAATTAGTCTTTTTG
TAGTAACTACGTATAAGATGCACA
>comp2751_c0_seq1
CAGAGATTGCGCATTTCCAGAGAAAAAAATAATTTGCGCCTTCGTCGTGCTAATTGTTTG
ACGTATCGTACCACAGTGTGTCGATGTGAGACGAACGTGTTATTGCCAACCACAAATATC
TCGAAACACATCTCCACTTTCCTACTACTCCGAGACTAAATCCGAAGTACCTAGCTCACT
TTGAAGACATCCATTAGGACTCCTTTGTCATCAAAACACAAACTATCTTCGAAGAGTCTG
GAAGATTCACACACAACAAACTAATCGCGAGATACAATCGATCCATCGAATTGCCAATCC
AATCCAACAAATCCCTCCATTCTATTCTAGCTATATTTCACTTCGCCAGTGATTCATTCG
TTATTCCATACCCCAAAAACATCCAGAAGAAAGAACTTCACGATGAGTCGATGGGCCGAT
TCTACAGACGACGAAGATGATTACCTACACGATGGTGATCACGAAGATTACGTCGAGCAC
GCTGTTAGTGCGGATCAACTGAGAGAACCAATGCCACTTCCTGAGCACAACGTGCACCTT
CCCGACAAAAACATTGCGGAGGGCGCCGATGACGAAGACGCCGAAGCCGAGGCCGAGGAA
GAAAGCGAAGAAGAAGAGGAGGAAGAAGAGACCGAAGAGGAAAAGGCGGATCGACAGCGC
CGCCTCGAGGAAGCAAAAGCAGCTGCCAAAGCCAAAAAAGAGGCGGAGGAGGCCGCGGCA
AAAGAAAAGGCACAAAAGAACGACCTTGACAACCTCGATGACATTTTGAATGAAATGGGC
ATCGAAGCAGCCCCCGCGGCAGAGGAGCAAACGGTAGCCGCTGACGACAATGAGGAACCC
GAGGCCGGAGGAGGCACTAACAGCCGCAGGCGAAAGAAGAAGAAAAAGAAGGGGGGCCCT
GCGGCTGCGGAAGCCGCTCCTGCTGCTAGCGAGGAACAGCCCACCACGGTGGACGTTGCC
GCCGTGATGAAAGCCAAGGCCGGGAAAAAGAAGTCGACGACGAGTGCGGCTAAGGCTGCC
GCCCAGGCCGCGTCTTCCGAAAGCAAATCCGCGAAGGGAAAGAAAAAGAAGCTGAGTAAA
AAAGACAAGGCCCTGCAATACGGACGGTAGTGCCGTTGTGAGCTAAGCATGAGGTACTTT
AACTACTAGTGCCTGCCTTGCTGCAACCGATGCAATCCCCTACGGACACAACGCTCCAAC
GGTTTTGACATCTCTAGAGAAATGAATATGCTACTGACCATTGTACATATATTCATCGCC
CAAAATAAACCTAAGACCAATCCTTTCAAATCCAT
>comp2752_c0_seq1
ACAGAAGAGTACGAGTTCGCACTGCGAGAAACAATCTTTACAGTTCTTATTTCATCTCAA
GACTAGAGTAATCTCGTTTGATCGTTGATTCTTCGAATTATCCGGTCATCTGCACGTGTA
AACCTCGCCTCCGATGTTTGCGACAGTAATGGACCAGCAACAGACAGACTACCAACGGCA
ATGGCCAATGTCTTCACCAGGTGGAGTTACGTTCCAACAACAGTTTCCCGCTCACATCTG
CACGACTACTGTATCAGGTGCGCCATCTTCCACGAAACGTAGTCGATCCTGTAGCGAAGA
ATCATCGGCGGCGTGTTCTTCTACTAACTGTGGCAACATAGTTACAGATCCTATCGCACG
ATCATCACCATCGCCATCATCCGTATCATCATTAGTTCCGGTGTCCTCCACACCGAAGAA
ATCGAGACCCAATAATTACTTCGCACCCCGGTCGATCAAACCATCGGTTCTGACTGCGAT
GGGCACCCTTTCTGGGGCCAGCAATGGATCTAATGGAATACAACAAGACTCTTCTTCATC
CGTTCCTATGAGAAGAAGATTGTCAGGGGGACAGATTGATCAATATATATTTGGGGAACA
ACATGGCAGAAGTGGTCCGGACACAGACCCAAACCGACCAAGAAGCATGAGTTTTTGAAT
TTTTGCATCTCAGCTTGACTGGTAAGAGAAACTTTTGTTCACACTTCGAGTGCCGGGAAC
TCCAACTGCTCGCAAAGTACTGCATGCGGGAGCCTGCAATCTTTCCCAGAATACTTTGTA
CTGTTAGTAAATTAAAAATAATGTAATACCAAAAAGAAGAACAGAAGAGTAGACGATTGG
ACATTCATGCAAAAGTGTTCCGAATGCCTTCGAATTTTGCACTTGCATCATATATGGTGT
CATCGTCAGAAAAAAATGAAAACAACAGACGAGATTCATATCAATACAGATTATGAATGTT
GTCGACATCGCACTCGCATGATACGTGTTTGAAAGGTATTTGAAAATAATTTGAAAACAA
ACGAAACAGCTATATTGCTTTGACCTACTTTGCGTCCGGTATAATCGATCCACGGCTCTG
CCAG
>comp2753_c0_seq1
AACAGAACTGTCTGTTTCCGCAACGAACAGAATGACAAATCCATACGGTACGGTACGGGA
ACTTCTCGACTTGATGTAAAACAAGGAAGCATCTCTACATTCGCTACCACATCTGTCAAA
AGTAGGGAATCGTCCGAACACAAATATCTATCAGTTCCTTTGCAAGATGAAAGAACGGTG
CATGTCTTAGCTGCTTATTTTAAATTACTTACTTCCTCGCGAGGGTATTTCTGTCCTCGT
ATACTATTCCAACATGGCCTCTTCGAACGGAAGATCTCCGATCATAAAGCGGGAAACAGG
GCTCAAGGGATGGGTAGATACACTACAGACCCTATTCGTCTGGATGGCAGTTGTCTTGTC
GCTGTGCTGTTCAGAAACAACGACCACTACCTTTGCATTTGTGGGCTATTCGCCGAAGAA
ATCGACAACCATGACCGTCCAAAACCCTTCTCCAAAATACAACCTAAATACGAGGGCAAA
TAATTTGATTTCTCGAAGTAGCACGAGCGACGACGAAGTTGAACAGACGGCCACTGCCCC

AACTGCCCCAAAAAACCTTATGGACGACGACGAGGACACGATGGTCGAACAGCTGCAGCA
AAAGAACACTGCTGTTCTGAGTACCAAAGAGATCGATTCGAAAACTACGAGCCAGCGGGA
CCAGCAATTGGACCTAGTCGATGCCATCTTCAAGGGCGACAGTCCGAGTCGACGCAACGA
TGTAAAGAAGGTTGAAGACGACCGATCCGTCCTCGAAAAGATCGATAGCTTCCTGGACAA
GCCATTCTTTGATCCCGATGCCTACGACGACGACGACAATTCCTTCTTTGGAAAGTTTGC
CCGCCTCGTCAAGACGGACTACGAATTCTTCGAGGCGGTCTTTGTGGCGTGTTTCTTTTT
GCTGCTCATCACGATCACCAAAGACGTCTTGCGGGCGCAAATGGCGGCGAGCTCGGTACC
CAGCGGACGGCTCTTTTGAGAATCGAGCACAGATCGGTGGGTTTGCAAATGCGTTTGACG
TTGCTCGAATCCTTCTGAGATGCACAAAAATAGCAAAACACGGCACAGAAGCAAGATCTG
AAAACCATCAACATGACGGGTATAAATTTCGCAAACATCTTTTTTCGTGTTGTCACGAAC
TCACGCACCTGTAAGAAAATTTGAAAAGCGGACTTGGCATTTCAATCCTTACCGAAGCCT
ATCGTGACAAATTATACCTGAGATAATCATATCATACCCTAGGGCATAACCTTGGCGATA
TCAGAAGGGCTTTGGATTGGGCTGAATTATTGTCTTAGCCTTGTCTTAGCCATGTTTGTG
CACATTCACAAATCTCGTCTTCTAAGAATAATTAATATTAAAATGAATGACCCACTACGA
AGTGATGAGATG
>comp2754_c0_seq1
TCCCAACGAATCGCCGTCCATCGATGCCTGGGATGGGAATTCTGAGAGTTGCGTTTGCTC
CGGATCGGAGGGCTTCTGCTGTACCGTACACACATGTACAGAAGAGAATCCCCTTGGGCG
GTCGACGAACCACCGGCCGAACATCCCGAAGCGACAAACCCAGCTACAAATGCTGGTGCG
ATCGCAACGAATGATGCTGCTCCAACTGCTCCCGATGCCGCCGTGAAAACAGCGGCACCG
GCAGCAGTATGTATCAACAGCTGCACCAACGACGACGACGACGACCTATAGGGTCCGCCA
AGGTCTCAACGACAGAACGCCCCCATCCATCGACACCATCCGACCGGGGGGCTTTCCAGA
AAACCAATTCGTGCATCGCATCCAAACCGGCATCATCGAACGAATCGACAACGCCCACAG
GAATTCCTGCGACGATTCGATCCGGATCGAGCAGCAGCCAACCACGGGCAACTCGGTCCC
CACACTCGGTTCACTCCCTACACGACACCCTAATTGGACTATATCCCGGTGGCGATGGAC
CTGTCGGACCCAAGCGAGATCTTCCACCGGATACAGAAGAATCCCCTAAAGGTGGAAGAA
ATCGCTGCCAACGACCGGTCGTTTTTTCGTAGGATGCCTATCCTATGAAAAAGCCGACGA
GTTCTTTTGCGAACTCGTATACCGCTTGGCGGAGGCCAAATGCACACGAATCTGATTGAA
GAAAGAAAGATGCAATGCTTTTCCATCCGTGTTGTTTCCCCGGGCATCTGTCGCTTGAAG
ATATCTTGGTCCCGCGCCATGACTTTCGTTTGTTTACCTGGTCCAGTTCGTATTCTGGTT
ACCAGAGGGCAAAGAGAGCAAAAGATCGTCGACGTCAACGACGACAACGAGAATGCAGCA
TCCCTAACAGTAGTAGCAGCAATCTAGGCACTCCGATATTCGAGATCAGCTGCCAATAAT
AAAAAATCTAAATCGAACCAAGGTTGAAAAGCACTCCGAGAGAAACAGTAACGATTCCAC
AGTATCCTAACGGAAATCATTTGGTACGTAACTATTCGTAATCCTAAAAGTTATATGCAA
TGGTAAAAACCGAGAAACGACAAAAAGCAAGCCGGGGCAGCGGCGGCAACACTACGGGCA
AAACGCGAGCAAGATTGCAGAACATGAAGTTGGTTTGGACAACCCCAACGCCTCAACCGA
AAGGAAACATCGGCAGAAAAAGATTGGGACGGA
>comp2755_c0_seq1
CATCAACCAAGAACCACCGATTCGGCTTTTTGTCGACACAAACAAAATATCACTCATATT
TCCAAATCTTATCCATCTACATCCATTCCTTCCAATTTTGAATACTAAACACTTCGAAAC
ACTTCGCAACCATCAACTCTCTTTGAAAAACACTATGATATCCGTTTCCTCTCGAAAATC
ACTGGGGGTTATTGCCCGCCGCATTCGTGGTTCCTCCAGCAATCGCCTCTTTTCGACGAG
GCCAATCGCCGGTGGACGCGACGTCGTCATCGTTGGAGGTGCGCGACTGCCTTTTGCACA
AGCCTCGACCATCTACCAGGACGAAATGGCCGTCGACCTCCAACGTCTTGCCATAAAGGG
ATTGATCGACCAAACCGCTCTGTCGAAGGACGCCATCGATTACGTTGTGTGCGGAAACGT
CATACAGGAAGTAAAAACCTCCAACATTGCGAGAGAGGCGGCCATCAACGCCGGTCTGCC
CTACGATATACCCAGCCATACGGTAGCGCAAGCCTGTATTTCGGCCAATGCTGCTATTGC
GACGGGTGCATCGGCAATCCAAGCGGGGCACGCAGACGTGGTAATCGCGGGCGGCGTCGA
AACCTTTTCCGACGTTCCCATTCGTTTGACACGGCCGATTAGGCAGAAACTCATCACCCT
GCCCAAGGCCATGAAAAAGGGCGGTCCCATCGGAGCTCTGAAACACATGTTGAAGGGGCT
CAAGGCCAAGGATCTTGGCTTGGAGACCCCAGCGATTGCCAACTATACGACGGGGGAGGT
GATGGGAGTCAGCTCTGACAAGCTCTCAGCGAAATTTGGTGTCTCTCGCCAGGAACAGGA
CGAATTCACGGTGAGGTCCCACACCCTCGCCCAAAAAGCACACGACGATGGGTTCTACAA
GGAGGAAATCATTCCCTACCGCGGATCGACCGCGGAGAACGGCATCAAGGGAGACTCGTC
GATAGAATCTGTGAGCAAGCTCAAGCCGGCCTTTGTCAAACCCAACGGGACGCACACGGC
CGCCAACTCGAGTTACCTCACCGACGGCGCCGCCGCCAGCTTGATCATGAGCGAAGAAAA
GGCCAAAGAACTGGGGTTCAGGCCGCTGGCGTATCTGCGCGACTGGAGTTTTCGATCCTG
CGATCCGTGGGAAGAGCTCCTGCTGGGGCCCACGTATTGCTCGCACGATATCATGCAACG
CAACAAAATGAGCATGAGCGACATCGGTGTCTTTGAAATCCACGAGGCTTTTGCCGGACA
GATCTTGTCGAATCTGGTGGCCATGGACAGCGCCAAATTCGCGCAAGAAAAGCTGGGCGG

CAACAAGGTCGGGGCCGTAGACGTGGACAAAATGAACACGAAGGGTGGGTCCCTTTCGAT
CGGGCACCCCTTCGGTGCCACGGGGTCGCGGTTGGTGACCACGGCCTCGAGGCGGCTGCA
GCAAGAAGGAGAGCGGTTCGCCCTGATCGCGGCGTGCGCCGACGGTGGACTGGGCCATGC
GTGTCTGCTGGAGCGATACGATTGAAACCAAAACCAGCAATCGCAGTCCTTTCTTACTAT
TATTATTGTTTGAGCAGTGTAACGAATGTAGAGCACTCATTACGTCCGTTTTGAACTAAA
TAAATCCCGACTCAATCCAAAGCGCTCTGTAGAGAAAAAATAGAGCGCATGCTGTAGGCA
ATACAACATGCAAATGAATCCAAACTGCCAGAATTCCACAACAGCAACGAGGCAATGTCT
TTGAAATGGATTGTGATGCTTGCAGCTTTTAATAATATTATTTCATTACAAC
>comp2756_c0_seq1
AAACGGTCGAAGTCACTTTCTCTCTCTCTCTCAACCGATCTGAAGGTTTTCGCATTCCTT
TCGCTCCCAAAATTCCAATCCATCAGAAGTGTCATAATTCAAAATCATTGTCTCCAGCCG
AGCCCCTTCCATTTCCGCAACATATACCTTATAATACCCACCGCTAGCTACACGCAACAC
TCGTAGCCTTTGTGACCTCCGATTCCCCAGCACCGTAGCGCAACAGTTTCCAGAAGGAGA
AAACAGTCAGAATGATCCACCCTCCTCCTGTTGCAGACGCCGCCTGTCCTTGCAAGAAAC
GAAACGTCAAGGTTGGCAGCGGAAAAAGATGTCATGTCCAAGCAACAGCCGATTCCTCGG
CCCGAATTTTCTCCTGCGTCGCGTTCCTGCTCCTTCTTTCCGCTGCGTCTCTTCCAACCG
TCGATGCCCAGAAAACAACCGAAACGCTCGCTTCCCCAATTGCCACGCAGATCCTCCCGC
CGACCCCGATTCCAAGCTTTTTTCCGGGGTCCAACGAGAACGTGGAGGACACGCTGTGCA
CCCTGGACGATCTAGAACGAGCCAATGATAGCCAGCTGAACGTTATATTCCACGAGCTGG
AACAGACCAACTTCTTTCAGCACTTTGTGGTCGACCTTGAGGTCAAGTGTAACATGTTCA
AGGACGAAGAAGCCAAGAGCAAACCAGCGGCACCAAAAGCACCACCGAAACCAGCCTTTG
GCACGGGACAAACATCGCAGACGTCTTCGTTTTCTATGGATTCGTTCCCCTGGGGAAACG
ACGAGGACGATGGCGACGACTTTCTTTGCACAGGTGGTGCCGACGAACTGGACGAAGATG
CCGTCCCGCTCTGCTCCGTGGATACCGGTGACAGCGACGACATGCTTGCGGGTGGCGGCT
TTTCGGCCTGGGAGGAAAAGACAGACGAGATTGTGATGGATGAGGATCCTCCCTGCGATT
CCGATGAAGAGGAACAAGACAGCGGTATCTTTGACATACCCGACAGCTTTTGGATGGACA
TTTGCAGCAACATTCGCAAGGGAGAAGGAACCAAGGTAGTCAACCTCGAGCTCAATCCCG
AACGGAACACGTATTACAATGGGTCTCACGTGTGGGACGCGATTTACAAGGAAAACCGCA
TCACCACCGACGACACATGCTTGGAAGAGCGTGTCCTCTACCGCTTGCTGAGCGGCATGC
ACACCTCCACTACCCTCAGCATTGCTAAGAATTACTACCCTCCCTCCAAACGCAAAAATC
GAGAAAACTGGGAGGCTAACCCCCAGTACTTTATGGAACGATTTGCCGACCACCCCGAAT
ACATTCGCAACCTGCACTTTTCGTACATAGTTATGCTGCGTGCCATCAAAAAGGCTCGTG
ACTACCTCTACAACTATGATATTTCTTCGACAATGCCACTGGAAGACACGGCATCCACGA
ATATTTTGTTGAGACGTTTATTGGATACTACTATTTTGAGATCCTGTTCCGACGTGTTTT
CTGCCTTTGACGAGTCGGTAATGTTCCAAGAAGACGACAACAAAGAATTGCGAGAATCGT
TCAAGGAAGTCTTTCACAAGATATCCGGCATTTTGGATTGCGTCCAGTGTCAGCAGTGCA
AGCTCCACGGAAAACTGACGATGCTGGGGTACGGAACTGCCCTGAAAATCTTGTTTGTCA
AGTCGCCCAAAAACTTGGTGCTCGAACGAAACGAAGTGGTAGCGCTGATCAATACAGCGG
CAAGGCTGTCCAAGAATCTCGTCGAAGTTCGGGAACTCACAACCATGTACTGGGAAGAGG
AGCGGACTAAAATCGCCAACCTAGAGATCAAGACTGAAGTTTCAAAAGGCGCTTCGGTGG
TCCCCGACAAGCTGCCCTCACAGGTCTCCTCTACCCCATTCGATAGTTTAGACGTATTGG
ATGTTACCGTTGGTGTCATTTCTAATGCTGGGCGACTAGAATTGATTTCGATGGAACGAG
AGAAAGAACTGGTCAATATGGCACTGGCTAGACATCCAGAGTTGATGATTCTAGGCAAAC
ACTACAGCAAGGATCCCGAACGATTTGTTGCCATGGCAACAGATCTAGGGGAATTAGAAT
CTGGTGGAAGGGCAGCAACAAAGACTTCCTTGCCAGCGCTTCCGCCCGATGCTATTGTGG
TTGGCAGTGGACTTTCTGGTCTGGCAGCATCGCTAAACATTCTGGATCGAGGAGGTACGG
TCGTCATTCTAGAAAAAGAGCACCTAGTTGGTGGAAATTCCAACAAGGCAAGTTCCGGTA
TCAATGGATACTGTCCTCACAACGATACTTGCAGCGACTCCATGGACGCATTCAGGAATG
ATACGATCAAATCGGCCGGCACAGTAGCAGACTTGGATCTCATCGATGTCTTAGTGACAA
AGAGCTCACAGGCTGTGTCTTGGTTGAGAGACCGTGCAAACGTAGATCTTTCTCTCTTAG
CTCAACTTGGAGGTCATCGTTACAAGCGCACTCATAGACCGAGCAACGGCATGGCCGGAG
CTGAAATCATTTACAACATTCAAAAAACTGTCCGATCTTTCGAAAAGACAGGTCGAGTCA
AGATTCTTGTTGATACCCAAGTAAAGGAACTGCTCACCGAAGACGGTGGCGACCGAGTAG
TAGGAGTCAGGGCTGAATCCACGAAGGATGGAACCGTTCAAGAAATTCTTGCCGACAACG
TTATCCTTGCAACCGGTGGCTTTGCTTCGGACCGTTCTTCCGGATCGTATTTGGATCAGC
ACCGTCCGGAACTCATGTCCTTTCCAGCCACAGCCGGTGCGTTTTCAACCGGGGATGGTA
TTACGATGGCGACAACCCTGGGTGCCGCAACCCGTGATATGGACAAGGTTCAGATCCACC
CAACGGGCTGGGTCGATCCAAAGGATCCTGGAAACCCCTCCAAAATTTTAGCTGCTGAAT
TGATGAGAGGAGTTGGAGGAATGCTGATTGATCATTCCGGCCGGCGTTTTGCCAATGAGT
TAGGAACTCGAGCTTACGTAACCGATCAAATGTTGAAACACGACGAGGAGTTTGTCAAGA

CTGGAAAATGGAATCCCTCTACCGAGGTCCCCACATTTTTCCTTGTCTTGTCTTCATCCG
CCGCTGCTGATGGAAAAAAGCATGTCGATTTATACTCGCACAAAGGTTTACTGACAAAGG
TTGAGGGCGTGGACGCCCTTTCCAATCACATGGGTATGAGCACGGAAAAAATTACAAAAA
CTATTTCGCAGTATCAGTTGGCTGCCAAGAAAGGAATCGACGAATTCGGAAAACAATCCT
TTCGTGGTGTGCCCGCTGAGAATTTGAAAACAGAAACATTTTACGTTGGTACAGTTACAC
CAGTCCTGCACTACTGTATGGGTGGAATCAAAATCAATCCGGAGTGTAGCGTTATGAGGG
AGGATGGAAAAATCATAGAGGGTCTCCATGCTGCTGGGGAAGTAACAGGTGGAGTGCATG
GTGCCAATCGACTCGGGGGAAATTCTCTCTTAGAATGCACTGTTTTTGGAACCGTTGTGG
GTCAAAAAGTGCCTATCAAGGAAGAAGGGACGTACCGAATGGTCATTGGGGATGAAGAAA
TAAGTACAGAACAGTTGGATCCTGGTACAGTAGGCTCAGACGAGCTTCCGAACATTCAGA
TGGATGAATTGGCCAGGCACAATACAGAAGAGGATCTTTGGGTAGCGATTCACGGAACTG
TCTACGACTTTACAGAATTTGCTCATGAGCACCCAGCTGGCTTTAAATCAATATTTGACC
TTGCAGGCACAGATGGCACGGCTGCCTTTGATGCGGTGCATAATTTGGGAATGCTTGACG
ACTTTGAACAAGACCGACGAGGTATTCTCGTATGAAGAAAAGAGGGCTTGTATACCCAGA
AGAAAAAATGGTAGTTCCTTCATGAATACTTCGCAGACACATTTGGCACCTCCACCGTTC
ATTACGTGCACAACTAGGCAATGCTCGCGATTTTAGAAACATACACCAGGCATTCGTGAA
TACACATTAGAAGCTTGCCTGCTTTAGAAAAATAGTATAAGTGCGAAGTAGAGCATTATG
AATAATTTCTAGAAGTATCAAGACGACCTTGAGGTAAAGTATCAAAACAACGAAGGTTTG
AAAGGGTGCGTTCTTGATGAGAGTCCCTGCGGATAGACAAAATTGTTTTTTATAGTTATG
ATGGAGGCCTGAATCTCCTTGATAAGTGCGAAAGGTCCAGAAGACTTCGCATTCATAGTA
TCGAAGGCCAGAAAATAATAAAGTTGAAAACAATGGTACGATGAAGAATGTGTTGGTCGA
AAATTGATATACTTCTAGTAATGCATTCCACAAAAAATAAAGTTCTTGAAGTTTATGAGG
AATAATAATAAAAGGTTCATGTTTATTGAGTG
>comp2757_c0_seq1
CACTTCAATCCTCACAAACAACTTTTACCAAGATATCTTCCTGACAGAGATAGCACCGCC
TAATTCATCGCTTTTGTTTTCTTCTTCACAGAATTAAACCCTCATCATCGAACTTAAGGA
GTTGTATCTCCACCATTGAACCGACTTTCAGCCCTTTTTCATCGGTTGTCAATTGACTTC
CTTAGAACCTACCTGTCTCCACCGACCACACATCTGAATCTACGCAACAAAACGAACCAC
CGCTTTCAACATTTTGAACATAACACGTTGAATTCTAGGTTTTAAACGCACAGCAAACGT
ACAAAGTATAATTACACAAAAGATGAAGATAGCATCCACTGCCGTTGCCGCCATGGCCCT
CGCTGCCGATCCTACTGGAGCCTTTTCGTTCACGGCACCCGCCGTCGGCAAGACCGTTGC
CAATAGTAGTAGCAGCAGCAGCTCCCGATTGAGCATGGTTTTAGAAAAACCTAAGGCACC
AATCAAGAAGATTTCCAAGCTTGAGATTTTGAAATCGGAATCCGCGAATCTTTTGCATCC
GCTCAAGGAGGAATTAACAACCGAAAGCATCGGGATTTCCAAAGATGCGTACCAAATTAT
GAAATACCACGGCTCTTACCAACAATCCAACCGAGAAAAAAAGTCTAAGGTCAAGGATTA
CCAATTCATGCTCCGTCTCAAACAACCTGCTGGAGAACTTCCGCCCGATCTGTACCGTCT
AGTTGATGATCTTTCCGCAAACCACGGACAAGGGGATTTGAGAGCAACCACACGCCAATG
CTTCCAGATACACGGAATCATGAAGGGATCACTAAAGACTGTGATTTCTTCCATTATGAA
CATCGGATCTTCTACTGTTGGCGCTTGTGGTGATGTTAACCGTAACGTCATGACAACTCC
TGCACCATTTGTGTCGAAGGACTACGAATATGCACGCGAGTATTCAAAGGTCTTTGCACA
TTTGTTCCGACCCATGACACCTGCCTTTTCTGAATTGTGGCTAGATGGAGAAAAAGTTGC
AACTGCCGAACTCTGGGGAAAAGAGGTCGAAAAGCACAATATTGACGAGGCGATGACCTA
CGACAGCGGACGAGGAATCATCCTGGACCACCCAGTTGAGCCTCTGTACGGAGACCGATA
CCTTCCTCGAAAGTTCAAAATCGGTGTTACCGTTCCCGGCGACAACTCTCTCGACATTTA
CACGAACGACATTGGCTGCGTTGTTATCACCAACGATGAGGGTGAACTCGAGGGATTTAA
CGTTATGGTAGGCGGAGGCATGGGTCGAACTCACAACAAAGAAACCACGTTCGCACGTGC
CGCTGATCACATGGGATTTGTTCCCAAAGAAGACATTATGGAATGTTTGAAGGCCATTCT
CGCTGCCCAGCGTGATCATGGTAATCGCGATGTCCGAGCAAATGCCCGCATGAAGTACCT
TGTTCATACTCTTGGCATTGATAACTTTACAACCCTAGTTGAGTCCTACTTTGGCAAGAA
AATCCAACCGTGGAAGGAAATCCAAGAATGGAAGTACAACGATTGGATGGGATGGTGGGA
ACAGGGAGATGGAAAACTCTTCTACGGACTTCACGTCGACAATGGACGAGTCAAAGATGA
AGGCGACTTCAAACTCAAATCCTGCATGCGAGCCATTGTCGATAAATACAACTTGCCTAT
GATCATGAGTCCCACCCAGTCCATTATCTTCAAGGATATCAAACCCGAGGACAAGGCCGG
ATTCGAGGCCATTCTCGCGGAGCATGGCATCAAACCCCTCGAGGAAATCGATCCCCTCGC
CCGTTTGTCCATGGCCTGTCCTGCGCTACCCCTTTGTGGTCTTGCCCAGACCGAGGCAGA
GCGCATCATGCCCAGCTACATCGAACGAATCCGTGCCGTCTTGGGCAAGCTTGGCATTGA
CGACGAAGAAATTCTCATGCGTATGACTGGATGTCCCAACGGATGTGCCCGTCCCTACAT
GGCCGAAATGGCCTTTGTTGGAGACGGCCCGAAGTCCTACCAGATCTGGCTCGGAGGCTC
TCCAGTTTTGACCCGAACTGCCTGGCCCTTCAAGGCGAAGGTGAAGAACGACGATCTTGA
GGTGATCGTCGAACCAATTCTGGCCATGTTCAAGGAACAGCGACAGGAATTCGAAGCCTT

TGGGGACTTTTGCCACCGCGTGGGAGCCGATGCCATCGAAAAATTCTCGGAAACATACGA
ACCCATTGCAGCATAATCATAGACAATAAATACCTTTCCTACCGTATAACACGATATAAT
AATAAAATAACCATAATTTGGA
>comp2758_c0_seq1
CAGTCGTCCAACAACACACTGAAAGCAGCAGAGCCCGCTCGTACCCTACGATTGCAGAGA
CAGCACCAAGACACAACCTCGCTACGAGATAGGCTTCGTCATTCCCATTGACTGATTCAT
CTCTCTTTCGTAGATACTGTCGGTATTGAAATCCTCGATCGTCCCTCCAGTAGATACGGT
TAGTTCGGGTACCCGTTATTTCGAAATACTAGCGTAGACAGCGAGGCAAAGAGGCCAATA
GAGGTACACTGAAGTCAGAAGGTAGAGATCCTATATCGTACTTCAACCACTGGCTGCAAG
TGTGAAAGCGAACGAAGAAAAGAACAAGCAAACGAGACAGCAATCCATTCATCCATCTTG
CATAAAACCGCGAAAACCATGAATTTCGGAGATGAAACCGGCATGGAGGGCCATGGAATG
ATGATGGCGGAACAGCAGCAACAGCAATACGGGAATGACGAAATGATGATGGATGCCGAA
GGAGGAGGTGGTGGTGGCTACGAAGGCGAGGACGGGATGATAGACATCGACGAGGTTCCA
GTCACGCAGGAAGACTCGTGGGCCGTAATTTCTGCGTATTTCGAAGAAAAGGGTCTCGTC
CGGCAGCAGCTCGATTCCTTCGACGAGTTCATTCAGAACACGATGCAGGAACTCGTCGAT
TCCTCGGGAGACATTCGGGTCTCCCCGGAACTCCAGCACATGGTCGGATACAACGACGAC
GAGGATGCAGACGACGTCCAGTTCGGGGAGACCAAGCCCGTCTTTGAAATCAAATTCGGC
CAGGTCTATCTCTCGAAGCCGACCACGGTCGAAAAGGACGGAACCGTTACAAACATGTTC
CCCCACGAGGCGCGTCTACGAAACCTGACCTATTCGGCACCTCTCTACGTGGACGTCATG
ATGAACCAGTACCGCGTTCCGCGGGATATCAACATTACTGACCCGGCAGAGGACATGGGA
GAGGCCGAGTCCACGGAACATGCCAAAAAGGAATTTCTTGGGTACGTGCCGATCATGCTC
AGGTCTCTTTTTTGTGTGCTCAGTGACAAGGACGATACAGATCTAAGCGATCTAGGGGAG
TGCATCTATGATCAGGGTGGGTACTTTGTCATTAACGGAAGCGAAAAAGTCATTATTGCG
CAGGAACGCCTGTCCAACAACCACGTCTACGCATTCCGAAAGAAGCAGCCCTCAAAATTC
TCCTGGGTCATCGAGACGCGATCGCAAGTGGAGAACTCCACCCGACCCGTTTCGACGCTG
TATCTCCAGATGTACCAAAAGGGTGGTCGGGGAGCCATCGAGGGAAACCAGATTCGGTCC
ACCTTGCCCTACATCCGGACCGATGTTCCGGTGGTGATTATTTTCCGTGCCCTGGGATAC
GTCGCCGATCGGGATATTATCGAGCACGTTGTGTACGATCTCACCGACGGAGAAATGATG
GACTTGTTCCGGCCCTCTCTGGAGGAAGCCTTTGTGATTCAGCGACAGGAGGTTGCCCTG
GATTTCATCGGACGGCGTGGAAGCGCGAGAGATGTTACCAAGGACGATCGTATTCGCTAT
GCCTCTGCCATTTTGCAGAAGGAGGTTCTTCCGCATGTTGGCATCGAAGAACATTGCGAG
ACGAAAAAGGGTTTCTTCATCGGATATGCCGTCCACAAGCTCCTTTCCTGCAAACTGGGA
CGCGCCGAAGAGGACGATAGGGATCACTTTGGAAAGAAGCGCCTCGATCTAGCGGGACCT
TTGCTTGGGGGACTCTTCCGATTGCTTTTTCGAAAACTGACGCTGGACGTTCGTCGGCAT
TTGCAACGATGCCTGGACGAGGGGAAACATTTCAACATTGGTGCCGCTATCAAGAGCAAT
CACATTACGGACGGTTTGAAGTACAGTCTGGCAACGGGAAATTGGGGAGACAAGGGAGCT
AGCGCTCCAAAGGCAGGTGTGTCACAGGTCCTGAATCGACTGACCTACGCTTCGTCCTTG
TCGCATCTAAGGCGTTGCAACACGCCACTTGCCAGAACCGGGAAACAAGCAAAGCCTCGT
CAGCTGCACAACACTCACTGGGGGATGGTGTGTCCGGCCGAGACTCCGGAGGGTCAGGCC
GTTGGTTTGGTTAAGAACCTAGCTCTTATGGCCTATATTACAACCGGTACCGCCCAGGTC
CCCGTCATGGAATTTTTGGAAGAATTCAGTACAGAGAATTTAACAGATATTTTGCCGTCG
GTGGTCGCCGATCCAAACACCTGCAAGATTTTTGTCAATGGTAATTGGGTTGGAATTCAT
CGCGATCCACGTGCTTTGGTGGACACTTTCCGCTCTCTCCGACGAATGGTTGATATCGAT
GCGGAAGTGTCGATTGTTCGAGACATTGCCGCAAAGGAGGTTCGCATCTATACAGATGCC
GGTCGCATCTGCCGACCCCTGTACGTTGTCCAAGAACAGCAATTGGTTATTAAAAAGCAG
CATATTATGCAACTTCAGGGCTTGAGTGGCGAGAAGAAGCTGACCTGGACGGATTTGCTC
ATGGAGGGTATTGTCGAATATATCGATACAGAAGAAGAAGAAACCACCATGGTAGCAATG
GATCCGTTGGATCTAGATACTGACGATTCCTATTCTTCCACTTATACTCACTGTGAAATT
CACCCATCCATGATTTTGGGTGTTTGTGCCTCTATCATTCCCTTCCCGGATCACAACCAG
TCTCCTCGTAACACCTACCAGAGTGCTATGGGTAAGCAAGCCATGGGAATCTATGCATCC
AACTATCAGGTCCGCATGGATACCATGGCCCACGTTTTACACTACCCACAAAAGCCATTG
TGTACCACTCGGGCGATGGAGTTTCTGCACTTTCGCGAGCTGCCGAGTGGGGTCAATTGT
TGTGTCGCTATCATGATTTACACCGGATACAACCAAGAAGATTCCCTCATCATGAACCAA
TCGGCTATCGACCGAGGTCTCTTCAGAAGTTCTTACTACAGATGCTATATTGATCAGGAA
AAAGCAAGTTCTGTGGGTACCATGGGATCTTTGAATTCTGAAATCTTCGAGAAGCCATCT
CATGACAAGACCCGTGGTATGAAACACGGTGAATACGGGAAACTCGACGAAGACGGTTTA
GTAGCACCCGGTACTCGTGTTTCAGGAGACGACATCTTGATTGGCAAAACAGCTCCGTTG
GATTCTACTGCCAACATGCCCTCTCGATACACCAAACGCGATTGCAGTACATCTATGAAG
GCAAACGAAAATGGAATTGTTGATAACGTTCTAATTAGTACCACCAAAGAAGGATATCGC
TTCACCAAGGTTCGAATCCGAAATGTCCGTGTTCCACAGGTTGGGGATAAGTTTGCCTCC

CGTCACGGTCAAAAGGGTACCATTGGTATGACCTATCGTCAAGAAGATATGCCTTTCACC
GTGGAAGGTATGGTGCCTGACATTATTGTTAATCCTCACGCCATTCCATCGCGTATGACA
ATCGCCCAGCTTATCGAATGTTTGCTGGGCAAGGTCGTCGTTTTCCAGGGATGTGAGGGT
GATGCTACGCCCTTCACTGACGTCACAGTCGAAGATATCAGTACACGCCTCCATGCTATG
GGGTATCAAAAATTTGGAAACGAGCATTTATTCCAGGGCCACACTGGGCGACCTATGTGC
GCGCGAGTCTTTTTTGGTCCCACATTTTATCAGCGTTTGAAGCATTTAGTCGACGACAAG
GTTCATTCCAGAGCACGTGGTCCAGTTGCCATGCTAACGCGCCAGCCCCTTGAAGGTCGT
TCCCGTGATGGTGGCTTGCGTATGGGAGAGATGGAACGTGATTGTTTGATTACCCATGGT
TGTGCGAATTTTATTCGCGATCGATTCTTTTGTAATTCAGATCAATACCGAATCCATATC
TGCGAACGTTGCGGTTTGACGGCTCAAGTCAATTTGAAGAAGATGACCTATGAATGTCGA
ACGCCTTCGTGCCACGACAAGCCCACTGCTATTTGTCAGATCGAAATCCCTTATGCTTGC
AAGTTGCTCTTGCAAGAAATGATGTCGATGTGTATTAGCACAAGAATCTACACGGATACA
AGGGTGACACGAGACAATTCATACTAATTACTACAATTATTTATAAAATAAAACATATGA
TGATCAACACCAGTATCTTCAGGGACATTGATCATGCAGACTCGTCTTTCGCATTTTCTA
T
>comp2759_c0_seq1
GACTTTGAAACCACGAAATCCAAATAAAAAAAAGACCGCAAAACACTTGTCATAAAATAT
AATACAAATCAAAACAAAATGTTTGCCACTTCAGCCGCCGTCAAGGCCATCGCACGCCGC
GAGGCAACCCTCAAGGCCGCCGTAAATATTCGTGCCATGTCAAATCTCAGCGCATACGAT
GATTTCGGAAAGAACGTATTCACTGGAAAGGTTGCCGACGAATATTTGAAAAAGCACGGT
GCCTCGGGAGAAACCCTCAAGGACCCCAGTTGGGTTAACAACGACGCCGACAAGGTCGCC
AATGCAGTATTTGATTGGGCCCTCGACCGTGGTGCAAATGTCTTCTGCCACTGGTTTCAA
CCCATGGCCTCCAGTGGAGTTCGTCACGGATTGACCGGACAAGTCCAGAATATGATGTTG
AAATTCAACGGCGACGGCGAAGTCGAACAAGATTTCAAGGGAAAGACTCTTGTCAAGGGA
GAAACTGATGGTTCTTCTTTCCCCAATGGTGGACTCCGTGGAACTCACTGTGCTGGAGGA
TACCTTGCGGTCGATACCTCGTCCCCTATTTTGTGCCGTGGTGATACAATTTTCATTCCT
TCAGCCTTCGTTTCGTACTACGGAGCTGCTTTGGATGAAAAGACCCCTCTTCTCAGAGCG
AACTCTGCCCTCGACAAACACGGAACCCGCCTTTTCAGCCTCCTCGGTGGAGACGCAAGC
AGCGGAGTTCAGTCGAACATCGGTCTCGAACAAGAAATTTTCCTTATCCCTCGTGAAGAA
TACTACCGCCGGCCCGATCTTCAATTGACGGGACGCACCATCATGGGAAAGAACGCCCCG
CGTGGACAAGAGATGTGCGATCATTACATGGCCCCTCTTTCCAGCGCAACTGCTGCCTTG
GCCTGCATGCAAGAAATCCAAGACGAATGCTGGAAAATGGGAATTCCTCTTAAGACTCGT
CACCGAGAAGTCGCTCCCAACCAGTTTGATTTTCCCCTCTTTACGGAACCAACACCACCC
AGATCGATCAAAATGTTTTCGTCATGCAAATTATCGAAGAAACCGCCCCTAAGCACGGTC
TTGCCGCTCTTCTTCAGGAAAAGCCATTCAATGAGGTTAATGGATCCGGAAAGCACAACA
ACTGGTCCATCGCTACACGCTCCGGTGTCAACTTCCTCGACGTTGAAGACGTTTGGGAAG
CCACCGGAAACCCTGAGGCGTTCCCTATCATCATGGCTGCTATTGTTGCCGCTGTTGACG
AAAATGGTGACCTCATGCGTGCCGCCATTGCCAGCCCCGGAAATGATTTCCGATTGGGTG
CCTGCGAAGCACCTCCTGCTATTGTATCCACCTACCTCGGTGAAGATTTGACCTCTTACC
TCGAAAATTCATTGCCGGAGAAACCTCCGAGTACAAGCCTGGTAAAAAGATGCTCGACCT
CGGAACCCGTGAGGTCCTCCCATTCGAGGTTCCTGCCGAAGATCGCAACCGTACTTCTCC
ATTCCCTTACGGAGGATCTCGATTCGAATTCCGTGCCGTCGGATCATCCCAAAATGTCTC
TTTGGTAAACACCGTACTCAACACCATTGCCGCTGAGAAATTCGCCCTCTTCGCCGACCG
CATCGAAGCCGGTGAGGCACCCGCCGATGTTGCCCGTGAGGCCCTCAAGAAGCACTGGAA
GGTCATTTTCAATGGAGACAACTACGATGAAGCCAATCAACAAATGCTCACCGATTCCGG
TGTCTGGCGTATTGATTCTGGTGTTGAAGCCATTGCAACTCTTTCCAGTGACAAGAATGT
TGCTCTATTTGAAAAGATGGGTGTCTTCAACGGCGAGGAATTGTCTGCCAGGCAAGATGT
CCTCCACGATCACTACACCGGAACCGTTGAGATGGAAGCCTTGTGTATGGTTGACATGAT
CAACCAACACGTCATCCCATCCGTCAAGGCTGCTGGAGTTGGTCCTCTCGATGAATTGTC
CGCTGCCGTTACTACCATCACTGCTGCCGTCGCCGAGATCCACGCTGCTGAAAGCTCCAT
GGACAAGGCCCAACTCGCTCGCGTCCTTCGTCTCGAGACCATGATTGACATCCGCGCCAC
ATGCGATGCCGCCGAAGAAGTTGTCCCTGCCGAAAATTGGACTCTTGCCACTTACAAGGA
ATTGATGTTCCTCGACTCACACGTTGACCCCGAACCTGATTTTTTCGGAGAATAAATTCT
GGTTAAATGAAGTCTGTAGCATTTGCTGACAATGCAATTTGCTCTGTGATATCGCCATTC
GTGGGGCAACTTGAGAATATCTCATATTTTTTACTTCGTAATTTAGTGCAGCATTTTTAG
AAAATAAAATGAAAATATTTTTTTTCTATTGTTTTCTAGATTCAACAAACATTTTAAGCTT
CATTCTAACGCACAACTTCCTGGAACACCATCTAAAGTTGAAATAATTGTCTCACGCAGT
GGGATGAATTGGAATAATTTGCACCTGCAGTAGTTGGCAAATGTCTTGCGTCATGAGTTC
GAATCACAACGCTTCAGTA
>comp2760_c0_seq1

CACACCTCGAACGCATGGAAACACCTGGACACAATAGATTCAAGTACAGGTTGTACCGGA
TACAAGGTACAGCAGCACATCTTGAAAACTCAACTCCATAACTATCGTCATACAACCACG
AGTACTCGTACCCGACCAAAGGCTATTGCAGAGTGATTCCTTTCTGAACCCTGGCCTATT
GCGAGAACACGGTTCGATTAGAGGACAACAAAAGATAGTCGTGGCAACAGTATCTGGCGT
AGGCCAATCATTCCCAAAACACAGCAGTAGCATTTTCCCAAAGTTGTGTGCAATAGTCAG
TCCATCGCGATGTCGGCTGCTGCAAAACCAAGCGATATCATCGTGAAGTTTTTGCAGGAA
ATTGTTGAATCTTGGAAGAGCAAACGCTATCAAAAGGTGTCTCAACAATGCGTGGCCCTG
TTGAAACGAAAAAAGACGATCGAGAACCTGGGTATGACCGTCCCGCTCCAGCGGATTCTG
TTGCAGGCATACATGCAGGAAGATAAATACGAAAAGGTTCTGGAATGGGACAAGGCATCG
GCCTCCGCGACGAAAGGATGTGAGGATTTGATTCTGTACGCGAGGTACCGAACCGAAGAC
TATGCTAATGCGTCAAAACAAGCTGCGACAGCTGCTGCTGGAACAAACGATGGCCAGGAT
CACAGCTGCGCTGTTGCGTTCAAAAACCTGCAAGCTCAATCCGACTTCCATTTGAATCGA
ACGGAGGAGGGGCTTGCTGGCTATTTGGATTTGCTTTCTAACGAAATCAATGAGAACGAT
CCGGAATCCAAAATGGAGATTTTGACGAATGCCCTGGCGCTGATTGCTTCCTCTGCGTGC
ACTCCCGGAGTGGCCATCCACATGGACGAAAACAACGAAACCACGAAATTCTTGTTGGAG
GAGGCAGAAGCCGCGCTGTCGAATGACGACGACGCCGTGGACGAGGAAATGCTTGCAGAT
CTGGCGTCCAATCTGGGTTGCATTCGATTTCTCACGGATCCAACCACCACAGAAAACAAC
TGGCTGGAACAAGCATCGGCAGCAATCGAAGCGGGGGATGGTTCTTCGGAGCAAACCAAT
CTGCAATGGAGCAAACACTTTTGGTACCGTGACATCGAAGACATTACTTATGAAAATGTA
CCTTCGTCTAAAGCAAGCAACCAAGTGTCTGTGGTGCAATCCATTGCAAAGATCAACCAA
TCTTTGTTGGACGAGAATCTTTCGCGATTGCCGCCCCAACCGCATCCGAAATGGAACTTG
TTGCAGGTGCGGATGTATTGGTACGATCGAGCCATCTTGCAACTCAAAGCAGGAAAGTAT
GTGGAATGCCACGACTCTTGCCAGTCTCTTAAGAAAACTTTGTCGAACAAGGGTGCGTCC
GACAAGAAGAAAAAGAACAAGACCGGCTCGGGGGGAGCAGAGGCAACAGGCGGTGCACAG
AACGCATCTTCCCCCGCAGCCTTGTGGTGGGAAACACGCGCAGATGTCGTGCTGGCCTAC
GCGCAACAAAACCAGTCCAAACGCAAAGATGCCTCCGTGAGACTGAGCAATTGCATTCAT
AACTTGCAAGCAGCAGCATCCTCGTCGTCTTCGTCGTCCATGGTTCTAGATCATGCGATG
GCGCATGCATTATTGCATCAATTCGTGATGGAGCAGAAAGGTAACAAGAAAGATAGACAG
AGGCAAAAGCGTGAATTGTTGAGTTTGCTGAAGTCGTTACCAGATTCAATCCAATCCCGT
CCTGCAGTACAGCTCACGATGGATGACTTGGAAACTGTCGTCAACAATGATTCTGAATCC
AACAATAACAAAGCAAATTCTACGCCCAAAAGCCCTCTGGAAGAAGCCGATATTCTATTC
GGTCAAGGGCAATATGAGGAAGCTTGCAAACTATACGAAGGTGCTCTGTCGAATGGAAGC
GATGATTCTATCTTGGATTCGCAACTGCGATACGTCCAAGCTTTGGCTATGAGTGGTCAA
CACGAAGCTTCGCAAGAATTGTGGCAATCTCTGGAATCTTCGGTGCTCGGAGAAAGTACA
CCAGTGACCGCATCCCTTCCCTCCGGCGATGTGCTCGAAACTAAAACGTTACCGCGTGGG
ACTTCTAGTAACAGATCTTCGATCAGCAAGAAGCTAGCAGCTGCCAGTAATCCTTTGGAA
GAGGAAGAAACCGACAAGCCATCGCGAGAGAAGATTCTCCGGCGTCGCGCGCGCAAAAGA
GAGGTGTACCTCAAACAACTGGAGAGCAAGGGAAAATACAACCCCGACCGTCCTACCAAA
CCCGATCCTGAACGATGGATTCCCAAGAACGAACGCAGCCGCTCTGGGAGAAATCGGGGA
GGAAAAAGCAACAAGGGCCTCAATTCCGCACAAGGGGGAGGATCTCGGTTGGACGCCCAG
CGTTTGGATGCGGCAGCTCGCAGAGCAGGCAAAGTTCCGGTCTCAGCTGGGCCATCCTCT
GCCAATTTGAAGGTTTCTAGTGGAGGACGCAAGGGTGGACGAAGGCGTTGAACTGAGAGA
GGTGATAAATGAATCAATTAGTGCAAAACGATGGAACAAAGGAATGGAGCTAAATTGCAC
ATTTTATTTCTCTTCAAAAATGTAAACCATCTGCAACTGTCGTGATTTCCGGCATACGCA
GGGTCGACCTTATTCTTCACGATATTGCCTAGTGGCCTTTTTCAATCCCTTCTGCTGGAG
TAGTAACGTGTGTCCATTCTTTTCGAAACAGAAACGAATAGAGCTATGATACGACGGATG
CAAAAATCCACGCTGACACGTAGATAAAAATAATAGCGGTACAAAAAGGTTCAGGTCCAT
TCCGTCTCCCAAAATAGTATTGAAATATTTGAGTAACTGTACAGAAC
>comp2761_c0_seq1
AAGTGTCGAAAACAATACTGTACTTTGCACTATCTAAAATCAGTCCTCGTGCGACATCTT
ATAGGGTTAGCCTAAAACACTTCAGGCAATCACTAGAAGCTAGAAAAGAAACACAGAATA
TTCCTTTTCTGATCGTTGTCTTTAATTCTCCAAACTTCGCTCGAAATCTCAAACGGCACA
GCACACCAAATCAGACACTCTTCTCGAGAGGACACAAGTCGAATCAATCACACAAAGAAA
AAGCACAGCACTTAATTTTTTAATTTTCCAATATGATGAACTTTTTCGGGAAAAAGAAAA
AAGAGGTGAACACAACCGTTTCGTCGACATCGACGTCAAAACCATCGGATGCGCAAACGA
CGATCGTAAAACTACGAGAATCGATCGCCAACCAAGACAAGAGAGAGGATCATATTCAAA
AGAAGATCGATATGATGGTGAAAGAAGCAAAGGCTAAAATGGCCAAGGGTGACAAGAAAG
GTGCCGTTTATGCTATGAAACGAAAAAAGCTCTATGAGCAAGAACAGGCGAAGATTCAAA
ATGTACGCTTCACACTTGAAACGCAGGTTATTAATCTGGAATCGGCGCAACAGAATGCTC
AGACATACCAGGCCCTCAAGCAAGGAAATACAGCAATGAAGGGAATCCGAGAAAACGTTG

GCATCGACCAAGTCGACGATGTTATGGACGAAATCAAGGAAGAAATGGAAATGGCACAAG
AGATCAACGATGCCATTGCTCAGCCTGTCGACCCGCTAATGACCGACGAAGACGAACTTT
TGGAAGAACTCAATATGCTCGAGACGGCAGATTTGGAAGCCGAGCTACTCAAACCCCCAC
CGACGGCCGTGCAGGCAATGCCAGCCGCTCCGAACTCGAAACTTCCAGCGTTGGAGAAAA
AAGAGGAGGACGATCTGAGGGCACTGGAAGCGGAATTGGCTGGATTGTAGAATTGATTCA
AATAAGTGGTTCAAATTGTCGCAGCAATACCTTTCGCACGAAACGATCGATACTGTGATG
TTTCAATGTCGCGAAAAAATAATAACAAGGACATAATCTGCCAGAATGATAATTTTGGGT
GCCACCAACAACAATGTTACAACTTGTCTTTTCGAATATGCTTCGTATTTCTTAGAAATC
CGGTGAAGCACAATCATTTGTAGATTCTTTATTGAAAGTACAGTACTAACCTAGCTTTCG
AGAAATAGGCCCTGCTGCTCACTTTCCAATATTTATTTGCGCTATGAGTTGTAATAGCTT
CTTTTCTTCTGTTTTTTCACAGGTTCGGTAATTGTTTTTCTTCTGATATTTCACAGGTTC
GGTAATTGCGAGGGTGCAAATCAAACTAATAAAAATTAATTCCGCTGCATGCGTGTTTGC
AACAACAAGCCATTCTTCAATGAATAATTGCATCAGAAAACACTTCTAGGGAGGCCAAGT
ACCTCCCTCAGCGAGGATTCTTTCCTTCTTGGCCGCTCGTTCGGCTCGAATCTGCGCAAT
TTCTTCCTCTGTCCTCTTTGGGCCTGGCTTTCGTCCTCTCCGCTTCCCCCATTTTCCC
>comp2762_c0_seq1
CTAGTAGTTCGTTATTCAATACGCAATACACTTGGAGTAACAATACCTGCACAGTGTATG
TCCGAACAAGTGTTAAAACACCTTCAATAATATCTGATGACGGTTTGGGCCGGAATCGAA
TGAATGAAGCGTGATTAGCCAATAGATTGGTTATCTTTGTGCAACATAGTCGATTGTGAA
TAAATGAATACGGTACATTTCAAACCGGATCTGAACACGGCGAATTGGATTTTGGGTGAG
ACGAGCCAACAACGCAGGAGGAAGACACAAGTTGGTGTGTAAGACAGGATAACTTTCAGC
AGCTTGTACAACCAATCTGTAGTATCTTATTGTTGCATTTCTTTAGTGGCCGAGTGTGAA
CGAATCGATCAATCGATCACGCCATCAATGATCCAACGAAAAAAACTGACAAAAACGATG
TTAGACGCAACCCGTACCGAGAAAGGCAAAAAGATCGACACAACGCCACTCAACACCAGA
GAAATACTGAAACAGCTCGTCGCCCATTTTCGCCAAAATATTCACGTCAAAATTTTTGCA
GAGTCGTATTACTCTAGTTCCGATGAACGTTCACAAACAGACATACAAGTGTTGTCCGAA
GATGCAGACGACGTGGAACAACAACAGCTAGATGATTCTGCAGCCTCACCGATAGTCCAA
TTCTGGTTAGATCGATTGCTAACACTGGATAAAGCTCCTTGCAACGATGAAGGAATTGCA
GGTGTGATTTTAGAATGGATTCGTGGGTACGAAGAATCCGTACGTCCTATCGTGTTCCAT
TCGACAGGTTCCGATGCAAGCCCTGCGTTCCATCCCGATTGCACATATGTGCTGGAAAAC
TACGAATTGATTTTCTCGAAGATATTGTCCAGATTGGCAACTCTTCCTGATGATCCGAAA
GGGATTGGTCTTTCCCTCACCCTCCCGTTGACACCAGTGATTCCTTCATTTTCTCTACAA
CAGCAAACTCTTCAAAGGGCAATCAAGATTTTTTCCACCGATGTGACGCTTCTAACCGTT
AGCGCGAGGTATGATGTCGAGGGACGCAATGCCAATGACGACAAAACAAATGATCTTCGA
AGAAACGTCGCACTACCAACCCTTTTAAAAATCCTTGCCAACACTCTCGAAACCATGCGT
CTCCTGTTCGCGACCAAAACTGTCGCCAACAAGGCCGTCGGCAACCCAAAAACACGCGAC
GAAAAAAGCTCTTTGAGCGATTGCTACGAATGCCTCCGACGATGTTCGGTGACGTTGCTT
GCATTCGAAGTTCCAGAAATAGACGACGATGAGAGCGTGGTACGATCGGCTTCGATCATG
CTATCGCCGCATCAGCTGGCGCCATCTTTATCGTCATCGACGTCCTCGGACGATTGCCAA
TCCACAGACTTAGTGATTGTGAGACGGATGTTCCAGCATGTTGGATACAATTGTCCTTGT
TCTATTGATGTTGACGATGTTTTTGATGTATCGAAAAGCAAAAATTCATCGAGCGACTTT
ACAGACGTACGAAGATACATTGACGGTCCACTCGGAAATGACTCGTCTGCATACGACGAT
GTTGATGACCAATATTGGAATGAATTGCAATCTTCTTTGAAGAAATCTGTATCTTTAGCA
GGTTGGGTCGATCCGACTTTGAACAAGATGACATTCCCAGTTGTTCGAAGCACTTGGCAC
ACGATTGCAAACTTACTGAGGGAGGTGTCGAGGGAGGCAAAGATCCCGTTCCGTGAACAG
GACGAAGAGATTGCTTGTGCAGATATTAGCGTTCCAAAATGCCACCGGCGGCAAGTAGAC
TTTGTTCTGAGGACGCTAGGTGGTGCTGGTCCGATGCTTCGGGACACCCGAGCGCATTTC
TTTGGCCGAGATATGCACAGCATGGAAACACTCGTAAAGTTGCGAGCAGACGAAATTGCC
GGAGGCAATGACTCCTTCGCCCCATCGGTTTTGGTGCGGATGGGGCGCAGGGTTACCAAA
GCGCCAAAGAAAAGTGGTTTTGATGAAGCACCTTACGAACAAATTCCATCGAGGATTGCG
GCTGCTTTGAATGCTTTCTATTTCTTGCTTCTCCCTGGTATGAGCGGTGGTGTAGACAAA
CACTGCGAGGAACAGATACTGAATGACGTCTTCCCGATTTGCGCCGCGCTGGTCGATTCC
CATAGCTCTTTCTGTTGTGTTCTGGGAGCCGCAGGATTCATTCGAGTGATCGAAGTTCTT
CAGCCACGAAAGAGGGGCCCGGGTTCGACATGCACCAATGAAAAGGCATGGATAAACTTT
TCAGATAATGCGTTGTCAGTTCTCGATAAGGCTAGTAAGGCAAGTTCTGATTCCGGGGTC
TTGGTTGCAATTGGTCGAGCGCAGGCAATATTACTGGATGTGATGGTATTACAAGAAAAC
GAGAAAAAAGAGGAAGTTAGAAAGCAAGCAATAACGGGGGAAACGGGCAACCACTTCCGC
CTAAAACGAAGAGCTACAACCAGACAGTGGCTCGCGAATCTCGAGCGGTCGTTGCACCGT
TCCACAGGAACCAAAAGACACTCTTTGGATTTACTATTGGGCGGTGTGATTCCATCGTTA
TCGCAGCATGCCTTGGATGAAGAAAATGGGGCAGATGGTATGGAGATGGGTCGGCTGGGG

CTCGCTGCATTGTTGCCGTTGACCACAAATAGCATGGGCTTTACAGAACAACCCGGGGGT
GACGTCGTTGGCAGAAAAACGCAAATGGCTTCGATGGTGGCCTTGATCAACCTGATGTTC
GCAGCTCACCCAGTTATGCCTAGTCACGGAGGAAAGATCATGAGTCATTTGTTGGCAACT
GCGGCTGATTCTGCACCGGGAGCCGATAGATCTGATGACGAAAGCCAAAATTCCACAGGA
AATCTACCGACGAAAGATTCGATCAGAAACATGGCGATCCTAACAGCCGCCATTGCCCTT
GTTATTTGTGGACAAAGATTTGCGGGTGAAGTTTTGGAGTCTATCGAAAATCATCGAGAA
CAATACCAACATGAGTTAGTTGCGGCTGTATCCGAAGTCCGCAAGCTAGCCCACTGCTTG
ACGGCTAGCTCTTAGCTGGTCAGTCGATAATCGCAACGATGCATTAAGCAAAAGAACATT
TTGACATTTTTCCAATAGACTGAACATCAATCCGACGAATTCTATAATAGAGATAGACTT
GAGAAATAGAGATAGACTTGAGATTCGGT
>comp2763_c0_seq1
CGTGGAAGAACTACTGAAAAAAAGGAGACGAGGTCAAAAGATTATTCAACAAACGAAGGC
TGTTCATTCGGAATATTAGTAGTAGCAACATTCGTTACCATTGTGATCACATCGTGAACA
AATTGTGTCCACATATACTCGAAGAAGGAAAACATTAGCACTGTACTATGTCGTCGACAA
AAGAGCAACCCGAATCTCTCGTAGAAAATGCCACTCAAGTTATCCCGGCGAAGCCAGACA
TTAGTCTTGATCAGTACAAAACGCTTCACAAACAATCGATCGAAAACCCGACAGATTTTT
GGGGGAAAGCCGGTAGAGACCACATTGATTGGTTCCGGCCTTTCGACAAGGTTCTCGATG
GAGGATTTGAACACGGCGACGTTCGATGGTTTGAAGGAGGCAAACTAAATCTCAGTTATA
ACGCTTTGGATCGTCATGTAAACAGTGATTCCTCTAATGACCTCGCCTTGATTTGGGAGG
GTGACGAGCCCACCGATGTTCGAAAGTTGACATATGACGAAGTCCTCCGCAAAACTTGTC
AAATTGCGAACGCTATGGTTCGCTTGGGCGTTCAAAAGGGAGATATTGTCACCATTTACA
TGCCTATGATCCCTGAATTGAGTATGACGATGCTCGCATGTACTCGGATTGGCGCAGTCC
ACTCGGTCGTCTTCGCTGGCTTCTCGGCCGAGGCTCTTGGGCAACGAATTTCGGCGGCCA
AGTCAAAACATTTGATTACGGCTGATGTTGGTCGCCGGGGTGGCAAGACCATTGCTCTCA
AGGAAATTGTCAATAACGCCTTGACCAAATTGGATTGTGATTCCATCGTTGAGCACGTCA
TGGTCTGGGAACGCTTCTACGATGGTGCGGAATCATCTGCTCCCTATGAAATGAAGAAGA
AAGATATCAGAATGGATCTTTTGGTGTCGGCCCAACGTCCCTACTGCCCGCCGGCTCACA
TGGATGCTGAAGACAACTTGTTCATTCTGTACACATCGGGTAGCACTGGTAATCCCAAGG
GACTAGTGCATACAACCGGCGGGTACGCTTTGTATGCTAAAATGACGACCCAGATTACTT
TTGACCTGGCACCAGGCGATATCTTCGCATGTGTAGCTGATTGTGGGTGGATCACTGGAC
ACACTTATGTTGTGTACGGGCCACTCTTAAATGGTAGCACTTCCTTCATGTTCGAATCTA
CACCTGTGTACCCAGATGAAGGAAGGTATTGGGATATGGTCCAGCGTCATAAAATTACAC
AATTTTACACAGCCCCGACTGCCATTAGACTTTTGATGCGTTCCGGAGACGAACCTGTGA
AGAAATACGATTTGAGCAGCCTCAAAGTACTTGGATCTGTTGGTGAACCAATCAATCCCG
AAGCTTGGCGCTGGTATTACGAAGTCGTCGGAAAGTCAAACTGCACGGTCGTGGATACCT
ATTGGCAGACTGAGACAGGTGGACACATCATTACGAATTTACCAGGGATTACACCTATGA
AACCAGGATCTTGTACTTTGCCTTTTTATGGGATTGACACAGCAGTACTGTGCCCTACTA
CTGGCAAGGAACTAACTGAACCTGACGAAAACGGAAATTTCCATGGTGTCATGGCCCTGA
AACAACCTTGGCCAGGTATGGCGCGTACGTGTCTTGGTGACCATGGAAGGTTTCTCACGG
TTTACTACAAACCTTATCCCGGATACTACTTCACAGGAGATACCGTGTCTCGGGATTCGG
ATGGTTATCACTTTATTATTGGACGAATGGATGATGTAATGAATGTGAGTGGACATCGCA
TTGGAACAGCTGAGGTTGAATCCGCCTTAGTTGCTCATCCAGCTGTCGCACAAGCTGCTG
TGGTTGGTAAGCCCCATGACATTAAGGGCCAGTCTATTTGCGCCTTTGTCATGCTTACTG
CAGGATTCGAAGAATCTCAGGAACTTATCAAAGAACTACGTGCGTCCGTCCGAAGCGAGA
TCGGGGGGTTTGTAGCCCTGATATGATCATTGTGTCACCAAGTCTGCCCATGACGCGGT
CAGGAAAGATCATGAGACGTATTCTGCGCAAGATTGTGTGTGGTGAAACAGATAGTCTCG
GTGATACTAGTACTCTGGCTGACCCATCTATTGTCAATCTTTTGATCGAAAAGGTAGCTT
AATCATCGGAGAAGAAGGGATTGCGAGGTTTCGAAGACGATTCGAATCATTTTGTGTTGC
TTTAAAGCGAGAAAGGGACAACGATCACATCTACTTCGCCAGAGGGACGATGGTCTAATT
GCTCCTTCTTCATTGTGTTGACAGAAAAATATGCTTAGAAGAGAACTTAAAATTCTTCTC
TCGGTTCATACACAAAGGTTATTTACTTAATATACTAATAATTTGATTAAAGAAAAGCTG
TACTGTTGGTTGCAGAATAACCAC
>comp2763_c0_seq2
CTCGCATGGTAAGTCAATTGCTACAGCCACATGCCTTCAAATCTCATTAAAAAATGGATC
CATATTTATTTTTCCAATTCACTAATACATAGGTACTGCTATATTCTCTACTTCCTAAAA
ATTAAGTACTCGGATTGGCGCAGTCCACTCGGTCGTCTTCGCTGGCTTCTCGGCCGAGGC
TCTTGGGCAACGAATTTCGGCGGCCAAGTCAAAACATTTGATTACGGCTGATGTTGGTCG
CCGGGGTGGCAAGACCATTGCTCTCAAGGAAATTGTCAATAACGCCTTGACCAAATTGGA
TTGTGATTCCATCGTTGAGCACGTCATGGTCTGGGAACGCTTCTACGATGGTGCGGAATC
ATCTGCTCCCTATGAAATGAAGAAGAAAGATATCAGAATGGATCTTTTGGTGTCGGCCCA

ACGTCCCTACTGCCCGCCGGCTCACATGGATGCTGAAGACAACTTGTTCATTCTGTACAC
ATCGGGTAGCACTGGTAATCCCAAGGGACTAGTGCATACAACCGGCGGGTACGCTTTGTA
TGCTAAAATGACGACCCAGATTACTTTTGACCTGGCACCAGGCGATATCTTCGCATGTGT
AGCTGATTGTGGGTGGATCACTGGACACACTTATGTTGTGTACGGGCCACTCTTAAATGG
TAGCACTTCCTTCATGTTCGAATCTACACCTGTGTACCCAGATGAAGGAAGGTATTGGGA
TATGGTCCAGCGTCATAAAATTACACAATTTTACACAGCCCCGACTGCCATTAGACTTTT
GATGCGTTCCGGAGACGAACCTGTGAAGAAATACGATTTGAGCAGCCTCAAAGTACTTGG
ATCTGTTGGTGAACCAATCAATCCCGAAGCTTGGCGCTGGTATTACGAAGTCGTCGGAAA
GTCAAACTGCACGGTCGTGGATACCTATTGGCAGACTGAGACAGGTGGACACATCATTAC
GAATTTACCAGGGATTACACCTATGAAACCAGGATCTTGTACTTTGCCTTTTTATGGGAT
TGACACAGCAGTACTGTGCCCTACTACTGGCAAGGAACTAACTGAACCTGACGAAAACGG
AAATTTCCATGGTGTCATGGCCCTGAAACAACCTTGGCCAGGTATGGCGCGTACGTGTCT
TGGTGACCATGGAAGGTTTCTCACGGTTTACTACAAACCTTATCCCGGATACTACTTCAC
AGGAGATACCGTGTCTCGGGATTCGGATGGTTATCACTTTATTATTGGACGAATGGATGA
TGTAATGAATGTGAGTGGACATCGCATTGGAACAGCTGAGGTTGAATCCGCCTTAGTTGC
TCATCCAGCTGTCGCACAAGCTGCTGTGGTTGGTAAGCCCCATGACATTAAGGGCCAGTC
TATTTGCGCCTTTGTCATGCTTACTGCAGGATTCGAAGAATCTCAGGAACTTATCAAAGA
ACTACGTGCGTCCGTCCGAAGCGAGATCGGGGGGTTTTGTAGCCCTGATATGATCATTGT
GTCACCAAGTCTGCCCATGACGCGGTCAGGAAAGATCATGAGACGTATTCTGCGCAAGAT
TGTGTGTGGTGAAACAGATAGTCTCGGTGATACTAGTACTCTGGCTGACCCATCTATTGT
CAATCTTTTGATCGAAAAGGTAGCTTAATCATCGGAGAAGAAGGGATTGCGAGGTTTCGA
AGACGATTCGAATCATTTTGTGTTGCTTTAAAGCGAGAAAGGGACAACGATCACATCTAC
TTCGCCAGAGGGACGATGGTCTAATTGCTCCTTCTTCATTGTGTTGACAGAAAAATATGC
TTAGAAGAGAACTTAAAATTCTTCTCTCGGTTCATACACAAAGGTTATTTACTTAATATA
CTAATAATTTGATTAAAGAAAAGCTGTACTGTTGGTTGCAGAATAACCAC
>comp2764_c0_seq1
TGCTGTGCCCCATCAAAACGATTGTTGACCGTCGGAATCACAGTCTATCAAGTACTTTGA
TCCTCTACTATCGCGTTGAAGCAACATTCTTTGATTTATAAAGAGAAGAGATTTGATCAT
GGGAGGCCGTAACAACCCCGCCGCAGAACCGAAGACAACTCAAGACGTGGATGCCATGGC
AGCCGCCAGGATCCCCTTGGCCTATCGAGACAAGTGCGGACACCTTTTGATTGATTTGAA
CAAGTGCCGACGAGAAAACAAATTTAAGATGTGGGAATGTGGGCATGAGCGCCATACATA
TGAGGAATGCGAATATTATGCCTGGATGGATCGTTGCGCTCAGAAAAAGAATGGATCCGC
CTAAGGCGAACTATCGCAGCAAGTCCATGATAAAAATATCATCTGTGATTATAGCGTCTA
CGACGATTTGAATTATTTTTGATCGGGGAAACGCATGAAAGTGATCCAGGGATGATTGAG
CTGTTCATGAAGCTACAGCGACACGCAAATACTGAATGCATTCAATTTTACGGAAAAGAA
AGTTCCACGAACTTTCTTTTTATGAATGATCGTAGGATTGGAGTCACTCTCTAAGAAGTA
TTGATGTTGATTGACAGCATTAAAAAACAATAGAAATTTTTAAACATCTGCA
>comp2765_c0_seq1
CAAGCTTTCTCGTAATCATCTTATGATACAACCCCTTTTTATTTAACAAATCATCTATTG
CTACATCAGTATCCTTGACACAGGGTGTGAATGTGGCCACAGAGAGAAAGCTACGAATTA
CGATCAGAAAAAGAAAAATCAGTGAATTCTTCAGTGCTGCCATTGTTATCAACGCGACTA
AAACACAAATATCGCCAACTTCTAGACGAGTGTCCACAGCACAAAGAACAAAATAGTGGT
AGAAAGATGAAGTTCGAGAGTGGAGTACTGTCATTGGCCTTGGCATCATTGGTCGCTCCG
ACGGAGGCGTTCCAACCAAGCACAAGATCCTTCCATACAGCAAGAGCCCAAGATCAAATT
GTAAGGCAGTCGACCACCCCTGAAGTAGTGGAAAGCGCGAAGACGGCTGGCGATGATTCT
GAGTTGGAAGCCTTAACAGCAGATATTGTCTCGAAATTGCGCTTTCGCGAGGTCCAACGG
GAACTGGAACGCAGAGAGGTCGATACAACTGGCACATTTACAACCATGCGAACAAGGCTG
ATGGAACTTGCCGATGATGCCAATAACGGCGAACAGAACACCGAGGGAAGCAACGACGTG
CATGTTATCAGTAATGATGCTCTGAATACGGCATTCAAAGATACGGGAATATCATTCGAA
GATAATTCGGATCCAGATTTCGATTTCAACAATTTGCTAACAGAAATTTCATCTCTATCG
GAGAAGGTACATTGGAAAACCGCCGCTCGAAGATTCAAACAATTGATCAAGCGTTTCGGA
CAGAATGGACCAGAATCCAGAGAAATCCCTGAATCTGTATTTACGGAAGTGCTTACGGCT
TACATGCAGAGTAGACTTCACGGAGCTCGTGCTGCAGAACCAGCACGACGGGCGATGGAA
GAGATGATTTCGCATGGATACATGGTCCCCTCAGAGATTGCAAACTCATGCATTAAGGAC
TGTCTAGGGTTTGAAAAGGATGGTAGTCACGAAGGATTTGCTGGAATTGATACCGCGTTG
GCGATGTTGGCAGCGGTGGAATTATCGGAAGAAAACCCTACCCCAATCTTCGTGGACACA
TATGAGAAACTTATTACCACGTTGGCCAACGAAGGATCGATCGATCATGCTCTTCAAATG
CTTCGAACGACTGTGGTGGACAAGGCTGACACTCCATCACTGAAGGTCTTTGCCGATGTT
GCTTCAGCATGCGTGGAAGGCTCTGACGAACCAGAAAAACTGATGACAGGCTTGGCATAC
GTCAAGGCAACTGGATACGAACTGGACAACATTGCGTCTACTGTTGACGGCCGAAGTATT

CTCGCCGCCGGAGTCATTGCCGCCGAAAAAATGAACAATATTGGTTTGGGATTACGTTTT
TTGACGGCTGCCAGTCAAGCTGAAGGGTGTGAACCAGATAGAGGAGATACTCTAGTTGCA
AATCTCTCCCCGGCATCTCAGCGTGCGTGTACAATCATCCATCGTCGAGCTATTCTTAAA
GCCGTCGAAAATGGCAGCTGGAAACTCGCTGTGAAACTATTGGAACTGATGCTTGAAAGA
GGGTTGACGCCATCACCAAGCGTTTGGAGAAACGTCGTCATCTTGTGTACTAAAGAAGAA
AAGAGTAGAAAAGCCACGGCATTATTGTTGGACTGGATCGACTTGTACAATCAAGAACGT
GCAGAGAAACCCCCACTGAGAGTGTTCAATTCGGTAGTCAATGCATGTGAAGTGTGTGGA
GAGGAAGAGTTGACAGTGAGGGTATTGGATGCAATGAAAGAGACCCACGATACGGAGGGT
AACCTTATTACATTCAATATTGCTCTGAAGCGACTAGCGAAACAAGGCAATTCAATGGCT
TGTGAAGGTATCATCATCGGTATGCTTCAGGGTGGAATTGAACCTTCTGTTGTCACGTAT
ACAACAGCCATTGCTTCTTGCGTAGCGGATCCCAAGAAGTCAGATGTTGCCATTGAATGG
ATCAAGCGAATGAAATCAAGATTGGTTCAACCGAATGTTATCACGTATAATACCGCCTTT
GCATCTTGTTTGGATGGTACTATCGAGGGTACGAAGCGAGCAAGTGAGCTCGCAGTTGGT
ATGATCAATGACATCAAGTCTCAATTAGACGCAGGAATCTTATCAAAGGATGACTACACC
AACATCATTCCAAATTTCTACACCAGAACTCTCGCAAAGAGCCTTGTAGAACAAGCAGAT
GCACAAATGAAGAAAGGTGAGCTCGATGAAGACGACGCCGAGTCAACCATTCGTGGACCG
TTCCTCGAATTGGTCGATTTCTTGAATTCTGATCTGGCCCAACTAGCAGAGAAACAGAAG
CAATATGTGAAGGACCAATTAGATGATAAAGAAGCTGATATCGAAACAACAATAGAAAAC
GCGGAAGAGGAAGTGGACGAAGAGCTGGAATATTCTGTAGCAATCAGCACACACAGGGCA
GCTATGGTTTAGAGGGAGGAAAGAAAGGGCCAGCAAATAGATCAGTGACCCTGGATTAAA
ATTATAATTTTTCACTAGCATCTTCTCGC
>comp2766_c0_seq1
CGTCCCCGCCGATTCATTGAGAATTTTGAGTACCCTTACCTAGCACACCTAGCTCCAACA
ATCCGAATGCACCAGCACGAATTTCGGATCCTCTCGTTCTCACAACGCAAGAAAGCCGAA
AAACGGAAGGAGAACACGCCAGCAGAAAGAGTAATATCCGATACCATCGCTTTGTTCTCA
CATCCAACACAACAAACACAAACACATATTTTACCAACGAACTACCCGTACCGTGCCATA
TCCTTCTCGCACACAAAACAACCCAACGAACGAACGATCCAACGAACCGGCACGGCCATG
AACCAGTCCATCGACAAAATTGCGCACCTGCTGCTGGCGCGAAAGGCAATCCAATCCCTG
CCCGCAAACGCAAAGCTCACACTCGTCGAGCTCAACGCGATGCTGAACCACGCCACCGGA
ACGAGCACCACGCCGATCGCAATCGCCGCCACGGGCCGCCAAGCCGACACCAGCGAGCGG
ACGACGGTTGCCACCGGCCCGGCAGCGGCGGCAAGCCGGGGGGCCGAACCCCACCTCCGC
GCCCCGTACCCACCGCCGCGGCGCATCGCAATGCGCCTGCGGCATCTGTAGGACGGGCAC
GACCGCAAGCCAGCCGGTACGCAGCGTGCTCGGTCCCGCTTGTTGCGGTTCCGTGCCCGC
TGCGGCGCAGCGCAGCGCAACACCACACGGCAACGAGCGTGGGACGGGGAAAAAAAGGCG
CCGCCGACAAGACGAGGCGAGGCGGGGCAAGGCATTCGGAGGGATGGATGCCGGATGGAT
GCACACACCGATGCGTTCCAACCAGGGGTTGGCCGTGGGAACCGATCCGGTACGATTCTT
ACGGGGGGGGCAGAAGCCAAAAACTAGCAACACGTAGAGCAAAATAAACATTTAAACCAA
TAGATACATACAGTTGAC
>comp2767_c0_seq1
CTTCTTCAGGCGTATTCGTATCGATACAGTGCTTCATTGAATACTGATAACAACTATCTA
CTGGTAGCAATACATTTTACTTGAATAGACCAGAAAGAACGATGCAGATATATCTTTTCT
TAATTGCTTGCTACCTGGGGTTTGTTGCGTCGTTTCGAACTAGCAGCGCTCCTTTGCGCA
GTTCTTTCTCGCGCCTTTACGCCAACACAGGCCAGTCCTATCAGGCATCTCTCCCGTCGC
CTTTGATTGACGCTCAAGGGAACGTAATCGAATCGGCTGAAGCTATTGCCGGTCTGGTAG
AAGGTCAACGGGTAGCCCTCTACTTTGGTGCCAGCTGGTGCAAGGATTGTACAAACCTGG
AATTTATGCTTGCGCAATACCGAAGTGCACTCGCCGATTCTAACCAGGTACGCTGTCCGT
GATGTTTGGTTTTGAATCTTTTGTCTCCCATGCCCAACAAACAACTGAAATCTGATTCCG
TTGCGTTCAATCTCTTCAGC
>comp2767_c0_seq2
CTTCTTCAGGCGTATTCGTATCGATACAGTGCTTCATTGAATACTGATAACAACTATCTA
CTGGTAGCAATACATTTTACTTGAATAGACCAGAAAGAACGATGCAGATATATCTTTTCT
TAATTGCTTGCTACCTGGGGTTTGTTGCGTCGTTTCGAACTAGCAGCGCTCCTTTGCGCA
GTTCTTTCTCGCGCCTTTACGCCAACACAGGCCAGTCCTATCAGGCATCTCTCCCGTCGC
CTTTGATTGACGCTCAAGGGAACGTAATCGAATCGGCTGAAGCTATTGCCGGTCTGGTAG
AAGGTCAACGGGTAGCCCTCTACTTTGGTGCCAGCTGGTGCAAGGATTGTACAAACCTGG
AATTTATGCTTGCGCAATACCGAAGTGCACTCGCCGATTCTAACCAGCCCATTCAATTAA
TCTATGTTCCTTGTGACCAAAGCCAAGAGGACCAACTAAGTCAGATGCAGAAGCTCGGAT
TGGAAGTTGGGGTGCCCCTGGGCGAAACAGCTGATGCCTTGAAACAGCAGTATGGAGTTT
GGCCGGATGTGGACATTGAACGCTTCGGCGGTATTTCAAGAGAAATCGCTATCGAAGGCA
CAGAGACTATCGTGGCCGTGAAAGGCAGCGAGGGGGAGGAAGTTGCCACGGAAAGAGTAG

AAATCATGGGGAAAGTGGAAGACACCAGGGACGAATATGGTCGTCGATCGGGCATTCCTT
CCTTCGTCGTTCTGGATAATACCGGAGCAGAATTTTGTTTTCTTAATGCGGAGCGAGATA
GCATCAGCGTCTTGGCGGATTGGCCGCTCGATGACCCTCAAAATATTTGGTAGACCGGAA
GAATCAACTTGAAGAACGTTTATGTATCTTTTTACCGGGCGAGCGAAAGCATCTCTAAAC
CTCTACATCGGGTGACGCTACTAGTACATTGTCATAAACTCTGCATTTATATGTACAAGC
TACGACATGCATGAATTTCGATTAAAAGTAATTTCTTCTGCATCCCTCTCGTAAACCTGA
ACATCGAACCTGGTTATTCGCAG
>comp2768_c0_seq1
ACCGCAAGCAACACCGCAAGCAATACCGCAAGCACCACCGCAAGCACCACCGCAAACAGA
ACCGCAACTAACACCCAATCCAGCCAATCCTGGTCCCCATCTCCGGTGAAAGCAGCACCG
GAATCCAACCGCTCGAAGGCGTCATTTCCAACTCCCACTCTGGGGATCGAGGTCCAGAAT
GCCGTTCACGGGCGTTCCGAGGCAACAGCGAGTGGCACGGAGATAGTATTAACAAGAGAC
CGCGACTCTGTCGGTGACAAAAAGACTCCAACACACTATCGCCACCAGCCACCACAGTTA
GAAACAGATGACAACACATGTGCCACCAGTAGCGCATACACCGCTACCAGCAGTGATGAA
AGCTGCATCAGAACAACACACGGCGGCCGTAGTACCCACGAGATGACGATGATTCGCACC
CAATCCATCCGTCGTGATCAAAGCCACGTTCCAGGTCACTCCTCGCAGCGACCTGAACAG
GCTCCACGAACAATCTTCTATGCCTTGCCTTCTTCGTTAGTGCAACAACCACAACTTGTG
TCGCAACTCCCGAATCAACGACTCCAGCCTCCCAATCACCACCGCCCTCGCCGACATGTG
CGTAGTCACAGCTTTGGTTCGACGTCGCTCTCGTCATTTGGTGCCAGTAGTTATAACAGC
AGCAGCAGTAGCGAGGACTCAAATATAATCGCAAACACAACGTCGACTTCCTCTAGCAGA
GGCAACTCGATTTGTCAAAACAGTTCGAGTCATTTGGACTCTGACCAAGTACAGGAACAA
CCGCAGATGACCTTTTATCTCCGACCATCCGTAACAACCGTGAAGCATGTTCTCAAGGAA
GACCACCAATCGCAACAAATGCATGAAAACAGACGGCGTACGCCTATCCAAACATCCACT
TGGGACAGCACCCGTGACCAAACAGCACTCCCCGCCGTCACCGCCAACGCCACCGCCTAC
AGAGAGAATTATTCCGCTTCAATGAAAATTTTGCAACGGATACCGGGAAGAAGCACCGCA
AAGATCGCGGATGAGATGACGGACGAAACCTCAACCTCCGCAGCATCAACATCAATATCA
ACATCAACATCAACATCAAC
>comp2769_c0_seq1
TTTTTCTGTTTCCAAAGCTTCGAAAACGAGCCACAACACGCGCACACGATCGATCGAACG
TTTTTCCTAACCCAGAAACAGCAGCAACAACGACAACAGCATGTGCAACACCACCGTCCA
TCCCCCCGCCGTCGGAGCCGGTTCGACCCATTCCGCCTCCTCCACGAGTTCCCAGCAGTC
CCACTCGCACTCGTACTCCCACGAGTCGACGGGCAAGCTCGTCAGCATCACCGTCCGCAA
GGAAAACCCCAACGACAAGGCGGGTATCCAGCTGGAGCAGGACAAGTACGGCACCGTCCA
CGTGACGAACATCGCGAAGAACGGGCTCTTCGGGAACTCCCAGCTGGAAATCGGCGACAC
CATCCTGAGCGTGAACAGGAGGCGCCTGAGCAAGGGAGAGGGCCCGAAGGAAATCCTCAG
GGCGGTGCACAAGTACAAGACGATTTCGATCTCCATTCGAAAGGCACCTCCCAAGGGAGC
CCCGCTGGTGGGACGCAAAAAAGACAAGAAAAAGAACCAAGAGAAAGACAACAACCGGGC
GAAAATCGATACGTACTACAGGGGATGCTCCAAGTACAAGGAGGACGGGAGTATCAACGT
GCAATACGATGAGGACAGCGCCGACGACGAGAAAAAGATGCCAACCATCACCATTTCGGC
CCAGAAGGGCTGTCCCTCCAACTGCACCAACGAAGAAAGCAAACCTGGCAACCGACGGAC
TTCGAGCAGCTCGCGGAGCACGACCCCCAAAAAACGACGCAGCCTCCCGAGCGAGGTCGG
CCTGGTGGGCCTCGAACTGGCCGCGGAGGGCAAAAACAGGCTCGTGGTCCGGGAGATCCA
ACCAAAGTCGATCTTCAGGGGCACCAAGCTCCGTGTGGGAGACCGCATCCTGAGCATCAA
CGACATGTCGTTCCGCCAGTACATCGACGCCGAGTACGCCCAGTCTGTCATGGACAACGC
CCCGTTCATGGTCACGCTGGTCATCGAAAAGCCAGAGCTCCGCCGCGGGAGCCGGAAGAA
AGAAGAACTAGACCACAGCGAGGGCCAGAGCGATGAGGACGAGTTCGGCAGGGACACGCC
CCTTTCCAAATACGAGCTCAAGATTGAAACGGACTTCCTGATCGAAACCTACCGCCCGGC
CACGATTACTGCCCCCAAGAGCCGCAGGAGCCAGGACTCGGGGCTGACCTTCAAGCTGGT
CCGGACGACCCGGCCCTTCGGGAAGGACAAGAAGGGGAGCAGTGCCATGCGAAAAAAGAA
GGCCACCTGGGTATACGTCGACAAGATCGACCCCGATTCCCTCTTCGCCAAGACGTCCCT
GGAAGTCGGTGACAAGATCATTAGCATCAACAACATCGACCTGCGCCACACACCCGATCC
GAACCTGGCCTACCTGACGTGCAGTAGGTCCACCGAAAGCATCGCCCTCGTCGTTTTGAA
GGACGAAAAAGTTTTCAAGGAAAAGAAATTGTGCTTCGATAAATCCATCACGAACCTCGA
GTGGAAGGTTTAGAACAGAATCCGACTGTTGCTATTGCCAAGCATGTGGCAGAGCACCAA
TTTTTGAACGACACAAGAACCATACTACTACAGATACATACAAAGATATAAAAAATATAA
AAAATAGTGCCATCTTCTTTCAAGAGTCGAAAGATCTACTCATAAACTATTATAAGTAAA
AGTTTCGCTACAGCA
>comp2770_c0_seq1
TGTAACTAACGATTCATAGCTATTGTGAATGATCACTCGACCATTCTCTATCTGTATCTC
GACGAAGCCAAAAACATGACTGTTGATAACAGCCATGCTGCGACTGCCACCGCGGATGAT

CCCCTGGCCAGAATGACGAGCAAAAACAATCGGCAGCAAAGCGCCTCAAACGACGAAATC
GACGCAATGGTTCCATCCAAGACGAACAACGATGCCGTTGGCAATAAAGGCGATTTCCAT
AACAAGGAGAACAACGAGAATCAAAGCGCTTTCGTTCTCAATGGTCCGGAAACATCTGCT
TCCGCCGCGAAAATAAATTCCACAATTTCGCAAACGGACCGAACACACACCGCAAGCATC
GTTGATGCCCCGCCGATCCTGGCCCGCCCCCGCGCCACCCTCGCCAGGGAGATCTCGCGG
CAGGTACTGCGGCACAAGCTCCAGAAACTCCGCCACCTGGTCCTGGAGGGGGTGGTCTCG
GAAGAATATCTGGAGCAGCACATCATGCACCCCAAGCTCCTTGACATGTTCGATCCGCAA
ACCGTTACGTACAACGGAGGCATTGCCAAGATCAAGGAATGGAAGATTTCGTGCTACCTG
GAGGTCATGGAGGGAGGCGTCCCCTGCACCAATCCACACACGGAGCTCCTAGAGGTCTTT
CGGCCGCTCCTGAATACCTGCGACGACCTCTTTCTGGCCTGGTACAAGCAACAGCACGCC
TGCAACAACCACCGGACGAACCGCGGGCACTCCGCGAGGCGGACCTGCGAGCGTCTCATG
ACCTTTGTCACGCGTTACACACCGGCCCCGGGGGAGCAGGCCCTCCTAAAGCACGTCGAC
GGCGCCGGAAAGGTAGACGGCTCCTGCGTCCTGGCCCTCCCCGTCGACCGCTGGACGGGG
CCCGACAATTCCTTTTACGGGCACGGGGGCGGCCTGACCTTCTGGGACGGAAGAGAGCCG
GTTCCGGAGGACGACGGGGGAGACAGACCCGATCGCAGCGATGGTCATCCGAAGCGGACC
AGGCCGAGAACCCGCCCGCGCGAGATCCACTACGATACCCGGCCCGGGGACATTGCCTTT
ATCGATCGGGCCGTTTGGCACCAGGCGGATCCGATCACCAAGGGGACCCGGTGGGCCCTG
GTAATTTTCTACAAGGTGACGGAGCAAGAAGACGACGAGAACGACTGTGAACAGGAACGA
AAGTGATGCCGGGCGATTTTCTGCTGTAGCGCTGGGGTGCCGGGGGAGCGACGACTCAAG
CCATGCATTGAGTGAAACAAACACCTAGGAATCCGTATGCATCCGTTCCCTTCCGCGAGC
AATATCACGGGGGAGTCTAGCATGATTTTTGGAAGCAACGACAAGCTCTTCGGCTTGGTC
GTTTTGTCCTTGAATGGTTGACTCCTCGATGCAATGCAACAGTACACCACGCACCACCTT
GATTGGACTTTGTTCGTCGCGTCGTTTCGGATTTGTGCCACACCCACGTGCATCATATCA
TAATAACAACAGCGGGACAACCGTACTTGTTGGAGGAGTGCAAGGTGCCGGGTTGGTTGT
CCTGTTTCGGTTGGGTTGCATGCATGGCCGGTATTCTCTGTTGTGTTCAAGGATGAGATG
CGTTCTGGTACCGGGCCGACTTTTTGTAACCTGTGCCAAACCTCCGGATCGGCTTTGATG
CAGATGTAGAATTCGATACTAGGGTGCTGGAACAGACCTCTGTCATACGACTATGCTACA
GCGATGTAACTACTTTAGCAAATTCAAGCTACAAGCACACAGCG
>comp2771_c0_seq1
AACTTGACGAAACAAAGTATCATAGTTTGGCATCAAATATCGTTCGTTTGACGTGACACT
CGAACCCGCATTCACTCTTAATCGATTCATGAAGCACAGTCCCATATAATGGGCAGCGGT
GCAAAGCGACTTCTCCAGCGTTGGCTCTTGGCTCGGCCCCTACCGTATTTGAAACAGGGC
TCGACGATTTCAATTCGATCAAAATTGAAAACTGATCTCCGTATTCGATCGCATTGGAGA
GATGACGGGGAGCTTTCCTTTCTCCAGCATTGTCACCTTCCAAGTGGCAGTGCTACAATA
GACAACGACAACCAGTCCGCTCGTATATTTCTATCTAGAAATGAAGACAAGATGACTTCA
CTCGGTCGCGAAGATCCCCATTTATCGATAGATATACTGGAGAAAATTGAAGTGGAAGAT
TCAGTATCAAACGGACATATCGGCAGCGAACCCATAGTGACATCACTATCATTGCCACCC
CCGACAACAATAGAAACTTCAGAGGAAGAGGTAGGAAATTTGCTAACGATAGATGTCCCA
GATAAGATAAATATTGACTGTAACCTCACAGAAGGGGGCTCTATTTTTGTGGAAAATAAA
ATTGAGGGGGACGTTCGACTCAAAACAGCACAAGGTAATATTTCGGTGAAAAAGCTCAGA
GGACACCACATCGAGTTAGATACAACGTCTATCCAATCCCAGGGTCAGAAAGGAGAAGAG
GGTAACAGCACGATAACCCCATCGACTATTTTCGTTTCAGACGTTTTGGAAAGTCAATCT
CTCCGACTTGCAGTGGATAGATCGTCGGTAGATAGGATCAGAGCCAAACGCATTCATGCA
AACACGATGGAGATCCAAGCAGGAGGAGTAGGTGAGAAAGATGAGGGTGATGTTCCAAAT
TTGACGACGTTCAGAGAATCGCTACTTGATGAGGACGACTCCGGAGCTATATGTGACATT
GGCTCCCTCTATCTCGTAGGAGATGCAACTGTTAATGTTCAACAGTCTGCTGCCAGTGAT
AACCGGAATCATGGACAGCGTCAAGCAGTTCGTATCAAATCTCATCACGGCCATGTCCAC
GTCCAAGTTTCAGCCCCTAAACCCAACATCGACAACGAATTAACTGGGGAAGCGAGACCC
ATGGTTGATTTAGGTGGCGTGAATGGGAGTTGTGAGGTGTTTGTCTCAAGATCCGATAGT
GATAGCAAGTCAGCCGATGATGAATGGACTTCGTGCCACGTTCACTTTGACAGTATTGCT
CCCGATAGCGTGAGCCTCATTGACGCCAAAGGCGGCAATGTGCACGTAACAGTAGATCGA
AAGGTGGAGTCGGATGTCCGACTCTTTTCTTCTCCTTCCGACGCACATGTAGACACAGAA
ATTTTATTGCTAGAAGACAACGAAGATGGTAGCCTCGCGGACGAAGTGACGCACATGATC
CAGGAAATTGAAGATAATGCGACGTCTGTAAATTCCAAGGCACTTCCAAAAGCTGACAAA
GGTGACTTTGATATTGAGACGAATCGGATAAAAATAGAGACACAAGCCTTCACATCACGA
GACCGAGACGAAAGCGGACAGCTTCAGCATTGTCAACTTGTGGATGGATGGGTCGAGAAC
ACGACATCAGAGCCAGACAGCCGTTTCGACCGCAAGATCAGAGGCAACAGTGGAGGAGGC
AAGATCAATCTCGGCGGAGCACAAAATCAGGCACTGCACGGCTTTGTGTCAGATAGAAGC
GGTGCGGCCGAGGCGGCCGGAGAAGACTTTGCGCGCCCGTTGCTGGCGGTTTCTTCTACG
GGAAAAATCGTTCTTGAAACCTTAAGTTGGCTCGGGAACATAGCCCGTCGGTATGGGCTC

GATGAAAATCGAAGCGAGGACGACTTGGGTAGACAAGCGACGAGACGAAAAGGATTAGGA
AGAGTAGTTCCTAAAGAAGATTAGAATCTTTTTGTTTCTTTCTCAAGAAATACGTTATGA
GTGGGGATAATTGTTATTGCCCGTACTGAACAATGATGGATTGAATAGTGTCACAACTTT
TTCATAATTACAGACATACCGGTAGAGGAACAGGATTAAGGATTCAGTAGGTTTGTTGAT
TGGCATACAGTTATAAGATACGAACCATACGATATCAGGTACTTCCTCCGTCCCTGAATG
ATTGTACCCTAACACCTTTACTACTG
>comp2772_c0_seq1
ACACCGACACCGACACCGACACCACCAACACGAACTCCAACTCCAACTCCAACTCAACTC
CAACTCCAACTCCAACGCCAACTCCAACTCAACTCCAACACCAAAAATGGCATCCACCGA
GCCAGAACTCCGGCAGGCACCCGACTCTGCCGCTCCCGGGGAAGAACCCCCGGTGGTGGT
GGAAGCCGACGCCGACCTCCTGGAACGGGGGATCTGCCGGGTGGAGCTCCCGCCGGGGCT
GGACCCCGAGCGCTGGGCGGCCGAGCTGTCGAGGCCGACCCCCCTGAACTTTGGCTTTGA
GGGGGACGGCGAGGCCTTTTTGTACCGGAACATCATGGACGAGCCGGGGTTTCCCTTCGA
TTTGGTCTTGCTTGGGAGTGGCGGAGACGGAGACGGAGACAGCCTCTCGGAAATCGGGCG
GGCGGTCCTCGAGCACTTTCCCGTTCTCCCGAAGGGCAGCGGGGACGGAGGAATCCGCGA
TCCGCGGAAGGTCCTGTCGGAGGAGCTCCGGCTCGACGACGCCTTTTGCGTCCACTACCA
CGAGGACCAGCACGACACCCGCGGAAAAAAGCACAGGGACCCCAGCGACATTACCGTCAA
CCTCTGCCTCCACAAATCGGAAAACGCGGAGGGGAGCCTCGTCATGTTCCACGGTGCGAG
AACGCTAAGGAACGCGGGCAACAGCGACAGCAACAGCGACAGCGGAAGGGCCCTTGCCGG
TGGGGACCGCCACCGCCGCTTCCTGGTGCCCCAGAAGCAGGGGCACGCGACCCTCCACTT
TGGGGACCACCCGCACGAAACGACGGCCCTCACCCGCGGGAGCCGGACCAACGTCATCCT
CACCTACGTCTACACGGATCCGAGCCGGAGCGACGCGACCACCCGGATGTGCTACTGAGC
AATGCTTTTCGGCGGAACGGACCGTGCCAGAAAGCCAGCATGCTCCCTTGTCTCTCGTCG
GGAGAAGAACGACAAAGCTGTGCATCGACTCGAGAAGCTTTTCGGGTGCGAGAAACCCCT
GCTTTCCTTTCCAATGGACCTGGGCGGCATCCCTGTCGGCTGCGGGAGAAGAACGACGAC
GGGACAAGCATTCCTCGCCGCCCTTGCCGACCGATCCGCGCGACGCCAAGGGACATCCCG
CGGACCGAACGAGATGCGAAGATGCGGGCTCGCGGTCCGATCCGAGGCAAAACGATGGGT
CCGCAAGGGACCGGGGTCGGTGGCACACGGATCGGGCCGATCCATCCGGGGGGGACGACC
CATTTGCTGCCAGCGACGACGGACGGGAACTCCCATCCTCGCGCAGCAGCCCAGGGCATT
GTTTTTCGTCTCGGCAGAACGAAAGGCTGGAATTCACGAAAGAAATAATTCTTGCATGGG
TGTTTGATACATCAGATACTGTAATAATATTATCAATAATAATAATAATATACTATTGGT
GTTCGTTTTGTGGGGGAAAGGAATGATTTGTCCGGATGGGTGTTTGTGTTACAGTTTGCA
CTCAGCGAACTCGGCAGAGAGAAACACCGTTGTGCCAAATTATTCTGGTGGTAAGAAGTC
TCGAGTTGATAGCAG
>comp2773_c0_seq1
TTGCGAATCGCATCGGATCGTGCCGCCTGACATTGCCGTGCGGTCTTCCCGAGATAGGTC
AGCAGGCCCGCAAAGACTAACCAGCCATCCAACGAATCACCATGACGGCTTCCAAGCGAA
AGAACGAGAACGTGGACTACGTCACCCACGAGCTGACCAAAATGGTGATTGTAAACGACA
GCAACCACCAAGAAAACGAAAACGAAAACGAACAAGGACCCCCCAACCAGGGCGTCCACC
CGACGGCGAGGACGCCGATGGAGCGAATGAAACTCCTCCGCGAAAGGTGGGGAGCGGCCG
GGGGGCCTTTCGCCGTTGGCGCTTCGGAGGGCGGAAGGGACGACGAGATCGCCCTGGCCA
TCGGCTCTTTCCTGGAGGGCGGCGCGGCCGAGGAGGAGAGAGAGTCCGAAATGCTCGGGC
GGGCCGAAGAAGTCCGGGCCGGGCTGGCCCACCAGATCCAGCGGGCCAGGGAGCTCTACC
GGGAGGAATCCAGCGACCTCTCGGACCTGTCCAGAGAACTCGGCGGCCTCAAGGAGGAAA
AAAAGGCGCTGCTGCGGGAGATCGACGAACTCGAAAGCCGCCAGCGCGCCTCGCAGGAAA
ACATCGCCGTTTACCAGGCGGAAGCGGCGCAGGAGCTGGACGAGATCATGGACGTGGAAG
AACAACGAAAGCAGCACGTCCCCCGGTTGAAGATGACCATCTCCCTCTTTGCCTCGACGA
CGGGCATCAAGTGGGACTTTTGCGACCCCGATCTGCTGTCGGGCCAGGTGGCTGTGCCTT
CCCAGAATTCCTTCAAGCTCTTTAGCATTGATCCGAGAGATTATTCGCCGGTGGAAACGG
CAAACATTTTGTGGGGCTTGATGGATGGGGTGGAGCCCCAGTAACCTTGTTTTCATGCAT
GTATGTTTTGGCTGCATCTGAGACGGGGTAATCATATTAGAAAGATGCCGCATCGTGCAA
ACGTTGTTGTTTTCAACACTCAATAGTGGTTTTGTTTGTAAAAAAATATTCTGGTGTCTC
TGTTGCGATGATTCATCAGTACTGATGCCCACCAACTTGATGTTTTTTTATTTTTGGCTT
CCGTGGCGCTAGGCAGTTGCGATGGTTGCCCCTGCAACTGCTACGGTATTTTGCG
>comp2774_c0_seq1
AGGAAAAGAGCAATCCATCAGCAATCACAAACCCATACATTCGTCGTCGCTGAAATATCG
GCAGCTGCTTCCAACGCACATTGTTGTCAAAAGACACATAGCAATAGCTAAGAAGGCCAT
CGAGAACGATTGTCGTCGCCGCAGACCAAAGACACGGTTTCCTCGAAGAAACCTACCAAC
CACACCACACCACACCACAAAGGAAGCATGCTGTCGCTGGCCTCCCTCGGTCGCTCCCTG
GCGCAAACCAGCAAGACACCGGCGGTGCAACGCTGCCTCTCCCGCCGCGCCGCGTCCGGG

CTCTGCGGGCTGCCGGACGAAAGGGATTCGCACTGGCGTTTGCGCTCGGGCTCGGAGGCC
GCGAGCTCGCAGGAAGACGGGACGGCCCAGGGATCCCCGGGGTTGGCGAACCCGGGGGAT
TCCCTCGCGGACCACCCCTTTTACGACCAGACGGCGGCCGCCGAGCAGATGATCTACACC
ATGGAGAACTCGTACTACTTTGCCTCGTACAAGGCCGGGAGCGGCAAGGAGGGACTGGGC
AGCTGCTAGCCGCGCGAACGGCGGGAAAGGGAAGGCGAGGCAAACCAAACCATACCATAC
CATACCATACCAAATCAAACTCGAAACCCCAATCACCAAACACAAACCAAGAATGTGGCA
GTTTGGAAAGTTTAAGTACTGTACATACTCTATCCGACTGCAAAACACGACCGGTCGCTT
TCCATCCATCCATCGCAAGCACGCATGCATCGGCCTCGAATAGGACAGGACAGGACAGGC
TCGTGTCGCGGTTCTGCCTCGGAATCCGTACCGGGAAACCCCTGGTGCTTCCTATGGAAT
TTGGGACTGGTGTGCAGTGCAATCCTTTTGTGCCCACCGGTAGAGCGGCTCGCATTCTTG
TGACACCCCGGTGTGGGGTTTGAGGAACGGCCGAGGACACCAACGATTGAAAACAATTCT
TGGAGAGATGCTCCGACCAACTAGACCTGCGCGGCAGCAATACTACTGCTTCCTAGGTGT
CATATAGAAGATTATTATTAGTATTTATGAAGTGGTTCTCGATTCGAAACTAAAAAGTGT
ACCAGTGCCTTATCAGATACAACTACTTACTAGATGTCATTTAGAAG
>comp2775_c0_seq1
ATTCGCTGAAGACACAGAGACTACTATACCACGAGGAATAAAAGTATCTCGAGTGCTCAG
AACACAATGAAAAATGGTTCATGTTATCGCAGTGTACATCGATGTAAAATACCTTTTTTT
TGGCTAGTAGCATTCTGTTGTCTTCTGCTTTTGAAGAGGACTTCGTCTACACTCGATGGG
TTCAACTTATTCGTAACTGGGTTTGTCGTCCCGTCGATGACTCTCAACCGAAAAACGAAT
TCGGCTACCGCTTCGTTTCTGAGTTCACAAACAATTGATATAGTAAAGAAGCGAACAGTT
ACTCCCAATGACTATAACAGAAAATCGAGACTCGCAAAAACCATCATGTTCTCCTCCTCT
TTGGACGGGGATGGGGATGACCTTAACAATAATGAAAACTCCCCAGACAACGCGTTCCAT
CTCATCACGGAGTACCCCAAACGATCGATTCTTTTCTCAGTCCTAATGACTACTTGTGGA
GCCACACTGGGGCCGTTTTTGGATTCCTATCATAGTGCCTTTGGTGTCTTGGCTTATGAC
GAGCCAATCCGATTCATTTTGTGGGGAAGCGAAACTTATCCCGCGTTGACCACCGCTTGG
TGGGTTCCTGAACTGTTTGGACTTGCGGGGTTTCTTATCGGATGGCTATATATTTTGTTT
GATGCCACGCTACTGAATGCATCCAATGACGAGCGATTACGACCTACTCCATCGAAAACT
CTTGTAGGGATATCATTCTTCACACTGCAGTACTGGTTAAGCGGAATACTGTTGCAATCG
AATACATTGGACCGAACTGGGATTCTAAACCTTATGTCACTTCTTGCCGCTTCTGGATTT
CTCGTTCTAGATGGAACATTTTCGGGTCTTACTGTTAGTCTCATAACGTGTATAGGAGGA
CCGCTTATCGAAGCTGGTTTGATCACCGCTACGTCGTCGGGACTAATGAGTGGTGGCTAC
CACTATACAGATTTGGGAGAAACAGGATTCTTTCCATTATGGATTGTCCCTATTTATTTC
CTGGGAGGCCCTGCCAATGGTAACTTAGCTAGGGGCTTTTGGAACGCGTTGTCGGGTAAA
AGAGGAGGGGGGGAAATGGTGGCTGTCAATGAACCATGCGAAGTTTGTCATAGTACTCGG
CGCACCCCATGTCCAAACTGTGACGGTGTCGGAACCTATGTTGCAATGGGGAATCGAATT
GTACAGTGTACGAGCTGTAATAGTCGTGGCTATGTAATATGCCGGGCTTGCTTCGACCAA
TTCGATGACGATCCACACGACATCGACGGGATCCGGGACATAATGAATCGAATGCCAGAC
TACTACGAGTTTCGTAACAATT
>comp2776_c0_seq1
TTTGTATCGTTCACAACGAAACAAAGTGTCAATAATTATGCCCAGAGCTAAGCACGCAAT
TTCGTACTTCCGAAACAAAGAATGAGCGTTAGAGCTGCAAGAAACACCTGGCGTCATCCG
TTGCAAGAATCTCTAGAATTCGACGTACGAAAACGAAACTGCAACACCCAATCTGTTTGG
TGCCCTACCGGTCAGTATCAAAATGATTCGTCTCATCGTGATGGGTATTTGGAGTGATGA
AAAGATCTGAATTTTCTTGTGACGACCGTCACCACTATTCTGTAATCGAGCCCAAAGAAG
AAACGTTACCTAGAACCAACTACCGTGGTCTGATATAATGCCTATCATTGCCGTAGAAGA
GGATGATCAAGACGAAGTGATTCGACTTTTGTCCGGTGCTGAAATTCCAGATATCAAATC
CAATGCTACTATTTCGATCGAAACTCCACCCTGTACGGATGAAGAGCAAAAACACAATGA
AGCTATTGATATCAAGGGCCAAGATCCCGACTGGGATGACAACCAAAGTTTCGGTAGCAC
TTTTGAGCTTTGCAGTATTTCGTCATGCACAAATCTTTCAGAGTACAACAACAACGAATC
CTTGAATAAATCCGTTGGCAGATCCACGCATTCTATTGGTAGATCTGGTCATTCCATCGG
TAGATCTGGCCATTCCATCGGTAGATCTGGCCGATCCATAGGGAATAGTACTTCCTCTCA
CCATCGATACACCATTGTCCGAAGAGGTCCTTCTACTAGTCAGATCCAAATGAATAGATC
TATCGGTAGCAGCAATTCTTCGGGTCGGAAGATCGGTGGTCCTAGGAAGAGCTCACTTGG
ATCAAAAATAATCGATGATGTCATTGTCATTCCCAACACGGAAGAACGAATCGACACCAA
ATACTTCGTGGATCATTCCCGCGAAATTGGACGCGGAACTAAAACGATTGTTCGGAAGTG
CATCGAGCGTTCTACTGGCAACCGATACGCTGTCAAGTCTGTGAGACGAACTGACAAAAC
CGAATACGAACACATGCGGAACGAAGTCAATCTTCTGACGGTGTTGGATCACCCTAGCAT
CATCAAGATCTATGACACCTACGAGGACGACAAGTTCCTGCACATCGTGGTCGACATTTG
TAAAGGTGGAGAACTCTACGATTACGTGGTGAAACCGACAAGCACCAACAAGAAAAACGG
GCAGCAGGAAGCCGTCCCGAACTGCCCTTCCGAGGAGGTGGCTGCTGTCATTGTACGCCA

GGTAGTCGATGCGGTTGCCTATCTCCACGATCATGACATTGTACATCGGGATCTCAAGCT
CGAAAACCTTCTCTTCAAGTTGAAACCCAATGCCAAAAACAAGCAGAGTCTAACGGAGAT
TCGTGTTATTGACTTTGGTCTCTCCCGCAAGTACAACACACACCACCGATTCTGCTCGCT
CAAGAAGCTTACTTCCTTTGTCGGGACCAAACTCTACGTGGCACCAGAAGTTCTGAACCA
TTCTTACACGCACGCGGTAGATCTCTGGTCCGTCGGCGTCCTCGCCTACGCCCTGCTGAG
CGCAAGGGCTCCGTTTACGGGTCGAAACGACCAGGAACTCTTCGACAAGATCCAGCACTG
CGGAGAGCAACTGAAATTCCCGAGTCCCGATTTCGACGCGGTTTCGGAGGAAGCCAAGCA
TTTCATCCGTAGCCTGCTGGTGAAGGACGAATCCAATCGACCCACCGCGTGCGAGCTGCT
CGACCACCCGTGGATGGTAAAAGCCGACCAGTGGAAGGAAGAAATCGACGCGGCCGCCGC
TGCGGACGGAAATGCGTCTTTGTTCAAGAAAATCCTTGGCCGCTTTGGAAAGGGAAAGAA
AAAGTCAAGAGCGAAAGGAAAGAACGAGCGAGACCACGGCCCCAAGAGCAACGATTTGGA
CGGCAGAGTTCAATAAATTTTTGTCTCACAGACTAAGAAATAGGCAACGAAGGTCTTCTC
ATCGAGCCAGCAACCACCTTCATTTTTGTTTAAAAACACCGAGCCGATTAATTTTGGAAG
TTGACTCTGGATTTCATGCAATACACACAAACGCATTTCCGTGCAAGAGACATTCGGAGA
ATAAACTACTTCAACAAGAGTTGACGGTTGGTCAATCATTTGTTGCAATTGGGTACTACA
CGTCAACCGGACCCTAATCAGCAGAATGTATCTTGTTAACAAAGCGATGACCAGAATTTT
CGATTCTTCGTCCTCGTCCTTTTTGTTCGACCTGTATTTAAATGCAGCAATACGTTTCTA
GAGAATCCAATGGGGAGCATCGATGCTGCGATATTGCAGGGTACTAAAGTTATTTTTGAG
AACAGCTTATTGGATATACGAGCCAATAAAGTAAATTGCCCTACGACACCAAGTTTTGTA
TTACAACGTAATATTTTAATCATA
>comp2777_c0_seq1
GTTCCTTCTGCTGTTGTTATTGAATTTACAGAAGTTGCCAAGGAGAACCAGAAAACGAAC
GTCGATACAGTAACTCCATTTGGTGGACGGCAGTATTCGAGCATGAGGTTTTCTGCCGCC
ATCGGTGCCTTATTGGTACTGCAAAAGTCTGCTTCGTCTGGGGCGGCATCAGTCAATCCA
GCTGGAGATACATTTTCACATCAATCGCATCACAATAGTAAACATTATGCGAATCAACTT
CCTAAGGTTGAAGGAACAGGCGCAAGGTCGTCTAGGGTTCTTAGAAATCTCCCCAGCAAC
CACAAGGAGACAGAAAGCAGGCAGAAACAGCCTGAAATATCGAGCTCTCCTCTAGCGATA
AGCAGCGCTTTAGTAGATCTCGGTGAAGACAAGAACTACGAGGAAAATTTTGGCAGCATA
TTTCTTGGTGTGTTAGGAACAATTCCAAACCCTTCTGCTGATGACACGATAATTCGCAGC
CTTCAAGAAGACGCGTCTCAATTTGATCATGTGATGAGTGGCATTTGCAATGAACCTAAT
ACCAAAGGTGTCTGCTCCAAATGCCAATATTCTTCCATTAACAACGCAGAAGATGGATTG
TTATGTAGTGCAGATGTGGATTGCAATAGCTACGTTTTTCCAAACGAAGACAGCGAATAT
TTTTTGGTCGCTTATTTTCAGTATTTGTATCATGTTTGTAGAAGCGAAAACCTTGTCTGC
GAATCCCCGTGTGAAGTTGATGTCACTACGAAACGGCTTAGCTGGAAGGATTGTTTATAC
ATATATGATCCCTCAGATTTTTACACATATGATCCCTCAATATATGGCGGTTACACGATA
CAAAATCCCGAAGAGTTACTCGACCAAATCTCATTCGAGGATTTAGAAAACGAAATCCAC
GATGGTGAAGCAGTAGATGAAGTCTCAGTCGCCTATCTAGTCAGTAATTACAACCCGCCC
AGCTTTGAAAGCTTATGCGATATCTTCTCATCAATTGAAGACGATGCTGGTAATTCTATC
TGCCCAACTTGTATGTCTTCCACGATCGTGGGCGAGAAAGGTCTTCTTTTGTACAATATG
GAGGTTGACTGTGACGAGTCTGCGTCAATCAACGAAGATCTAGGAAATTATCTCACCCAA
ATCTTCGAGGGATATAGTGCTTTTTGCGATCCCTTTTCAAAATACTCAGATTATTTCGTG
TGCGAAACGGGATGTGAGGTTAACGTTGATGCCAAATCAATGATAGCGGAGGGATGCTAC
TTCAAAGAAAGTGCATTCAACTATATATATGCCACGCATGTTGTTGCTGCTACGACTGAT
ACGTTTTGCAGCTATTACGGTGCTGGTGGTGATGGTCACTGCAGAAATTGTGGTATAACG
TATGTGGACGAGGGTGAACAGACGAATGCGGTATACGATGTTACCATAAATAGTTGTGGA
AGTTTCGAAGAAGCAATGAAGGGTGAATATAGTATGAGACATGCGTTACTCTATTCTTTT
TATTGGATTTGTTATGCTGGCACTTACTGCACAGGAGCTTGTGAAGTTGATGTTGATAGT
ATGACGATTTCAATGAGACATTGTTCTCCTAACAACTACTCAGTATCTTTCACAAATGAT
GAAACCGATTCACTTGGTGGTATACCCAGCACCGTTGGATCGGACATCGTACCTGGAGAG
TCTGAGATGACAACAGATGAGTCTGAGGATATGGGGGACGAGCCCGAGAATAAGACCGAT
GGGACTTCGCTCGTGACTATTTCTTTAATAGTCAGAGTGCCAGTCGATCTTGAAGCTATT
GAAGAGAAGGTTATTGCCGTAGCTCAATCGATACTGGATGACGAGTCGATGAGTAGACGA
GGGCGCCAACGAAGACTCTACAAAGTCACCTATCATCGATACTTGAAACCCGACCAAAGG
CCTAATCTCACCGCAGTTGCAACGATCGTACCAGCCTCCAGCTGTCCCGAATACGACAGC
TTCGATTTATCAGCCGGTTCTCAATGTATTGTTGTTTCTCTACTAGTCGAGGGCGTTGGA
AAGAAGAAAATTAGAAAATATACAAAGAGTATTGAAGACTCTGTCGAAAATGGTGTGTTT
GAGAATGAGTTACAAGAGGTTGGTTTAGATACGACAGTCAGAATGCAAACAAACAACGAA
GCTGGGAACCTGTCGGATAATACTTTGATCGATCCATCTACAGCAACACCGTCTACAGGG
GGCCCATCTTTACTAGCACCATCAGCAGCTCCAACAATGGTTCGAACAAAGGAACTCACA
GTGACTCCAACAATTCATACAGTAATTGATACAGCAATGGATACAGCAATGGATACAACA

ATGGATACAACGATTGCCCCAACAATGGCACCAACAATGGCTCCAACGAAGGCGATAACG
AAAACACCTACAAAAGATCCAACACCAGTCCCAACAATAGTTCCAACGGCGACTCCAACA
AAGGCTCGATCTACGAAGACCCCAACTACGACGGCTCCCTCTGCGAACATGATATCTGTG
AAGGATACATCTACAACGTCTCCATCTACCAATACCCCTGATATTTCTGATTCTCTGACA
TCAAATCTCGATTTAGTCGCTACCGATCCATGCCCCGGTTTCGCGGATGACTGCTCCAGG
TGTGTAGCAAATCCAGCTTGCTCGTGGTGCCTCGAAACCAGTGTTTGCTTCAATGCCAAT
GTGGAAATCGAAGCCGCCCCGGTACAAAACGGAGACGGCTCATCCATTATTGCAAGGAAA
TTCGAAAACCAATGCTTCGGGACCATGACACAGTTGGTGTCGGCTTGTCCTATTAGTTCG
CAGAATCCAAGTTTGGGTGAAACCGAAGGCGGTAACTACAAAGCAACGAATTTGGATGCT
GACGTGGATGCGGAAGGGAATGTGGGCCCAGATACGAACGCTGATGATACATTGAAAGGC
ACACACGAGACAACCACCAGTAGCTCCACTGCTATGAATTTGGATGCCGCCAACGATGAA
AATGTAGACACGGATGCAAACGCCAGGGCTTCATTCGAAGGCATAAACGAGACAACCGAC
ATAGCCCAGTCCAGCAGCAGCTCTATAAATACTGCTGGGATGTTGCTGGCATCGGGGGTC
TGTTGCATCGCGACTGTGCTGGCAATGAGACACATGTAGATTGACATGGACACGAATACC
ACCGAACGAGTCGGCACAATGATATTCTGAATTGGTGTTTGTCTTCACACTCGATACATT
CAACACAGCTAATTCCAAGGGAAACTTCCTTGAAGCTAGACTTGCTGCAACTCAATTTTG
AATTCTTTATATACGCATTTTAAAAATTACTATATGAAGTTCTCACTGATCTATCCGAAT
CATTTCTCTCCCTATCGTCTCTTGTACGTATCAATGAGAGATTCTTGTTTTGGACAAACG
ACCACCGCACAAGTCGATAAAGACAGGTTTTCTAGATCGCGCCCTCAGAGTGCCTTCGAA
TGCTTTCCTAGACCTCTGC
>comp2778_c0_seq1
CTTACGGTGTGACGAGAAGGTAAACGTGAAAACAAAAAACAGCAGAACCCTTTGTTTTTG
AATCAAAGGAATACGAGTTAAAAATGGCAAGCAACGTGGTCGATAATGTCTTCCTCAGTA
AGAGGACTCTGAGAAACAAGGCGGTGTCAATGTCCCGATTTCTTTGTTTTGTCGTCGTGT
TATGTATGCTCTTTTTCTCAAGCACGGCCAATACCGTATCGGCATCCGCCTTATTCGAAA
ATTATAACAGCAAAGGCACTGTATTTGAGGGCCAGTGTCCACCTTGCAATTGCGTACCCG
CAAAGACGGAGCAAATATGGAGCTGGCTTGTGTACTTTGCCACTACCCTCGAGGTGTTAG
TGCGTGGATTCTCTCTGGGATTTGGTGCCCGCATGCTCAAGTACATTTTCTTTCCCGAGG
AGAGCGAGAAGTTAGAGGGTACGACGGATAACTTGTTTGAACAATTTAAGCGCCGAGCCA
TTGTTTCGATGATCTCTGGCCTGTCCGTGGCGGCCTTCATCCACGGCTCGTCGTTGTTCA
TACCTACCCAACTGCGAGACAATTGGTTCGATTATGTCGATCAATGAAGCATGCCAAAAT
ACTGTACACGAAATGCTTTCTATTTGAAGTGCGTCTTACCGTATCGTTGAACTCTCCCAT
AAGACTTGAATTCATCTTTCGTATATGAGCAAGTAAGTTAGGAAAGAAGTTTCGTTGTTG
ATGATCTCACACTAATTCAGTTGTATCAACTAGTCGACTGGCAACTTCGTGTGCACTCGA
CATGTGAAGTTAGAAGGAAATAGCGGTAAACGTACATAATTAAGTTTAACATTTGGAAAC
AAGTCGTGGTTATTTTAGGCAAAGGTCAGAAAACACAACTTGAACAGTTCAATTTCAAGG
TTATGTCTTCTTTGTTCCAAAGAGGTACATAACTCATTTAGTATTATTACACCACCGTTT
CTGATTGAAAGTAGATGAAAAGTCGAGGTTTTATAGTATAAAGAGCTTCGTTTACCTTCG
AGAAGGAACATGTCTTTCCAGACAAGCATGTTTGTGAAGCTAATAGATGGTCCGTGGCTT
GTTGTTGTGAGATAACTTTATTCTCTTCCTGGTAGCAACTCGGATACCAGAATCCAAAAA
CCCAACGACTGTAAAGG
>comp2779_c0_seq1
GCCTTAGGGAAATCAAGTTGGGTTGAAAACCACATCTCACTCATTTCGTCTGCAATCTTG
ATCAGTTGAAACCAAAGCTTTCGGTTGCATGTGAACCAACTTTCAGTCTTTGTGAACGCA
GAAAGAAACCAACAGAGAAACCATGGGAAACTCCCATCCTCAAAACAAAACTGGTCGGAA
GGTCGTCGAACAAAAATTAGAGAACGCAAAAAAAACCGGAGTTCTTTCGCTTTGTGAACA
CAAATTAGAAACATGTCCCAATCAGGTATTTGAAATTTCAAAGCTTACGACGCTCGATCT
GTCTAAGAACAAGCTCGTGAAACTCAATCCAAAACTATCGACTCTGAAAGTTCTCAAATC
ACTGAATGTAGACAACAATAAGCTTATGCCTGGAAGTATGACTCCTATATCATCGTTGAC
AAAACTAAAGACACTTTCTGCAAGCAACAATCTACTAGGTCGTCCTGTCACCAACAAAAA
CAGCAAAGCTCCTGCAGCTCCCAGTACTGCGAAAAATACTGACATATTGCCAACTCCATT
GCCCAAAGGCCTTAGAACAATACTGCTTTCGAACAACTCGCTGACCACGATACCCCGGAG
CATTGTCAACAACAGCAACCCTTTGAAATTATTAGAAAAACTCGATCTTTCCTTCAACAA
TCTCGCTACAGTTCCGGAAAGCATCGCCAATTTAGTAGCTCTTAACGATTTGAATCTCGA
CAGCAATGTGATTGTCAGCTTGCCCCCGGCCATCGGAAAACTAAAAAGACTGAAGGCCCT
ATCACTGCGAAACAACCACATCTCGTCTGCAACTACTCCCCAACCCTTGCCGGAAGAACT
CTGGACAAACACGTTGTTGATCGATATGAACTTACACGGCAATCCAATGACGAGCACGCA
ACTCAACTCGATGGATGGATATGACGCTTTTCTGTCGCGGCGTCGGGAGGTCAAGAACAA
GGACATACTGGGCGGTGCCCTTACCGATCTAGATGGATGTGGTTTGAAATAAAAATAATC
GCGCATTTTTTGTAAATTACGACCACAAATGCCGTATGGATAGACGTACAACCAATGTAA

AATAAAGTGAGAGATTAAGC
>comp2780_c0_seq1
GTACAACTAGAATTGCAGCACGCAACAGAACAGAACAGAAAGTAACGTGCTACGAAACAA
CCAACCAGCCAACAAAACAAAACACAACCAACACAACACCGAAGCATGCCTCGGTGGAAT
TCACTTTTTCTGCTGGCCGTGGCAATCGCCGCCACCGCCGTCGATGCCTTTTACGTTCCG
GGCGTCCGCCCCCTGGAACTGAAGGAGGGAGAACCCGTTGAGATGAAGGTAAACGCCCTG
ACATCGGTCCACACCCAAATTCCCAAGGACTACTACCGCCTGCCCTTTTGCGTGCCCTCC
GGGGGACCCAAGATGGCCTCGGAAAATCTGGGAGAATTCCTGACCGGAAACAAGATCCAG
TCCTCGCCCTACAGCATCAACATGCTCACCGACGCGTACTGCACCAAGCTCTGCCAGAAA
ACGCTCAGCCCCGTCCAGGCCATGACCCTGCGCAAGCACATCAAGTACGGGTACCACAAC
AACTGGATCATTGACAACCTTCCCTCCGCCGCCGTGGGCCTCAATTCGCAGACGAAAAAG
AAACAGCTCCACTACGCCGGTGGATTCCCCATTGGATTCCTGGACGCCAACACCGGACAC
CCGTACATCTTCAACCACGTCAACATCATGATCAAATACCACCAGTACGACGAGACCAAA
TCCGAGTACCGCGTGGTGGGCTTTGCAGTGGAACCCATGAGCATTAAGCACAGCTTTGCG
GGCGGATACGAGTGGGACGGCGACGACCCCGAGGGATGGAGCAAGACCCTGTCGAGCTGC
ACCGACGCCTCCCAGCACCTCCACACCAGCCGCGAATCCATCGCAGCCTCCCAGATCATT
GCCCCCAATGAGTCGATCATCTTTACGTACGATGTCATCTGGGAGCAGTCCGATATTGCC
TGGTCGTCCCGATGGGACATTTACCTCTCGGAAGACGACCTGGTACCGGCCGAGGTCCAT
TGGTTTTCGATCACCAACTCGATCCTGGTCGTCCTCTTTCTGAGCCTACTGGTGGTGAGC
ATGCTGGTCCGAAACCTCCGCCGGGACATTGCCGCCTACAACGCGCTGGCCGCGCTCGCC
GACGAAGAACGGGACGAGGACATGGACGAGAGCGGCTGGAAACTGGTCCACGCCGACGTC
TTCCGACCCCCGGACAACCACCCCATGATGTTTTGCGTCTGCTGCGGAGGGGGCGTCCAG
CTGGCCCTCACGATCTTCCTTTCCATCTGCTTCTCGGCGATCGGATTCCTGAACCCGGCC
CGGCGTGGTTCCCTCATGAACGGAATCCTCTCGTTCTACGTGCTCAGCGGAATCCTGGCG
GGATACGTCAGCAGCCGGCTCTACAAGGCCTTTAAGGGACGCCTCTGGCAGCTGTGCACC
GTCGTCACCGCCCTGCTCTTCCCCGGGGTTGCTTTTTGCATCTTTTGCTTTTTCAACATC
ATCCTCTTCTTCCTGCACTCGTCGGCGTCGGCTCCCCTGCTGGACATTATCATTGTCGCA
GCCATGTGGTGCGGGGTCTCCGTCCCCCTGGTCTTTGTGGGTGCCTACTTTGGCTACAAG
CAAGAGTCCATCTCCTTCCCAACCGTCACGTCCACGATCGCCCGTGCCATTCCGGAGCCG
CAGCTCATTATGCACCCCATGGTGGGAGCCCTCGTGGCCGGTGTCGTCCCCTTTGCCGCG
GCCTACGTCGAGCTCTTCTTCATCATGACCTCCCTGTGGATGGACCAATACTACTACGTC
TTTGGATTCACCCTCATTGTCTTTTTCATTCTTATGGTTACCTGCGCCGAGGTCACCCTT
CTCTTGGTGTACAACCAGCTTTGCATCGAGAACCATCGCTGGTGGTGGTTCTCCTTCTTT
GCTCCCGGATCGACCGCCCTGTACACCTTTATCTACAGCATATTCTGGTTCAAGGCCCTG
GAGGCTTCCAAGATGATCATGACGTACCTGCTCTACTTTGGATACATGGGACTCATCTGT
TCGGCCATGTTTTTGGTCACCGGAACCGTCGGGGCCCTGTCGTCCCTGTGGTTCGTGAAG
ACTATTTTTGGGACCATCAAGGTGGACTAAGAGACAAATCGGTGGTCCGGGTTGTCGATC
GAAGACAGTCTTTGTTTCTCGGCGTGGGGCGGCAGAATTTTCACCGAATCAACCGAAACA
CGTGGCCGCTGCGAGTTTGATCCGTCACAAAAACACTCCTCGAAGCAAACGCACCGAACA
CCAATACGTTTGAATTAAGTATTTTTAAAACTCATTTTATTGCTTTCAAGATGCATCTTT
CGTGGTGCGTTTTTGGCGTCGTGTCAACCAGATAGCTCGGACGGTGCCTCAAGAGTTCTT
CTGAATCTTGCTTTTGTGCGATTTTGTACTTTTCAGTCTTTTTCTGGGTTTTCAGTGGAG
ATTGTTCTTGTCGGAGAAGTTCGTAGTCTTGTACCGTAGCAACTCATACAGAACCGTGAT
CCGTGATGCTACGAGTATTCAAGCAAAAGTGAAGTAATGTACTGGCTAACGTACGGTACC
AGGTACGTACGACAGCGTTGCGTTTTTCATTTAAAAAATTATAAACTATAAAAAAGTTTC
TCGAAGCGCCCCTCATCCTTTCAATGAACATCGGTCACCCAGTCAGACCTTGAGAATTCG
TTGTTCGAGTGTACGAAGATGCCAGAGGAAACGTGCGTACGTTCCTCCGAGGCTCGGTGT
GTACTCCCGGTACGAGTACCAGTACGTACTCGTACGAGAAAAATGTGAAGAATCGAGACT
TTCGAGAAAACTGAGGGGAAAATGTTGCTACTTGTCTTTCCAGAGAAGTGTGTCTCATCT
CCCGTTGTTTGGCGACGATCCATGATAGCACGCGAAAGACATACTATGACACTGATATTG
AAATTTCAGGCAGTCGTTTCTACACTATTAATTCTATCTCTTCTTCCCGAAACAACAAGA
GCAATGGCATCTTCGGCAGCGGCTTCGACGATCTCGTCGTCGGTTCGAGTCGCCATCATC
GGCGGCGGGGTCTCGGGGTCCGCTTCGGCGAGACGGTTGGCCCAACTGGCACCATCGGCA
CGGATCACACTGTACGAGATGGGTCGTGGTCTAGGAGGTAGAGCCTCTACACGGAAGACC
CGCTCGGTGCCCCATATCTACATCAATCACGGTGCACCGTACGCCGATGTGCGGTCGAAG
CTGGGTCGTTCTCTAATATCTTCCCTTGGTCCGTCGGGCACAGCGCCCTTTTCCGGGGTG
CGAGGATTCTTAGATTCAAAAACGGGGACTTTCGCACCCGGAGATGGAGAGAAAGAGGGA
GTGGACTATGTAACAGGCGCTAAAGGCGAAATGTCTCAGATAGCGTCTGCCATGATTTCT
GGCATTCCATCTATCGACACAAAATTCAAGACGATGATCCGTGGTCTATCTCGGTCACCC
AGTGGAGAATGGCAACTTCTTGATAAGTCAGAAGAGGTCATCGGTTCCGCCGACTGGTTG

ATCGTTGCGGGCAGTGGTGTTGCACACCTTCGGTGGTCGAAGACCTTTGGTGGTGAACCT
CCGTTGATTGCTGCAGAGAAAGCCCGACCGGATCCAAAGCTTCGTGAGGCATTAGATGCC
ATTGCTTCTCACGAAGCTTCCCCCGTCCTTGCTGTTTTCTTTTCGTGCTCGGGACCCACT
GCCCGTAAATGGTTGTCCCTAGATTTTGATGTTGCCGACGTCAACGGGAGTTCAATTCTG
TCTCGAATCATGATACAAGGAAAACAGAAAGATAAAAATAGCTCTCAATCTGACGGCGGT
GGTGACGAATGGTGCTCGGTTGTTTTGCATTCCACAGAAGAGTTTGCATTGCAAAATTCT
GGTGTATACGGCTCGTCTTCCAGTGCCACCAGGATAGGAGATGCTGCCTCGGATGCTTCT
CGAGAGAATTCCCTTATCTCCGATATGATGGTAGCGATGAACGAAATACCGGGCATGCCT
GAAATTAACACCAAACTACAGGATGAATCATATTCGTCACGTTATGATTATGGACCAGTA
ATCCATAGATGGGGGAACGCCTTCCCGAAGGGTGATGCACTTGCGGAAGACCTAGCGTTC
CTGCCAAGTTCGCGCATTGCATTTTGTGGCGATTACGTTGATACTTCGAATCCAGCTCGA
TTTGGAAGCTTCGAGAGTGCCTTGCTGTCTGGTACTTGGGCTGGAGAACAAGTTTCACAA
TTCTGCCAAAAGGACTAATGCGTAGTAGCTTTGAAAAACAGGAATTCTTTTAATTACATG
CTATTTATTAGTTTCACC
>comp2780_c0_seq2
GTACAACTAGAATTGCAGCACGCAACAGAACAGAACAGAAAGTAACGTGCTACGAAACAA
CCAACCAGCCAACAAAACAAAACACAACCAACACAACACCGAAGCATGCCTCGGTGGAAT
TCACTTTTTCTGCTGGCCGTGGCAATCGCCGCCACCGCCGTCGATGCCTTTTACGTTCCG
GGCGTCCGCCCCCTGGAACTGAAGGAGGGAGAACCCGTTGAGATGAAGGTAAACGCCCTG
ACATCGGTCCACACCCAAATTCCCAAGGACTACTACCGCCTGCCCTTTTGCGTGCCCTCC
GGGGGACCCAAGATGGCCTCGGAAAATCTGGGAGAATTCCTGACCGGAAACAAGATCCAG
TCCTCGCCCTACAGCATCAACATGCTCACCGACGCGTACTGCACCAAGCTCTGCCAGAAA
ACGCTCAGCCCCGTCCAGGCCATGACCCTGCGCAAGCACATCAAGTACGGGTACCACAAC
AACTGGATCATTGACAACCTTCCCTCCGCCGCCGTGGGCCTCAATTCGCAGACGAAAAAG
AAACAGCTCCACTACGCCGGTGGATTCCCCATTGGATTCCTGGACGCCAACACCGGACAC
CCGTACATCTTCAACCACGTCAACATCATGATCAAATACCACCAGTACGACGAGACCAAA
TCCGAGTACCGCGTGGTGGGCTTTGCAGTGGAACCCATGAGCATTAAGCACAGCTTTGCG
GGCGGATACGAGTGGGACGGCGACGACCCCGAGGGATGGAGCAAGACCCTGTCGAGCTGC
ACCGACGCCTCCCAGCACCTCCACACCAGCCGCGAATCCATCGCAGCCTCCCAGATCATT
GCCCCCAATGAGTCGATCATCTTTACGTACGATGTCATCTGGGAGCAGTCCGATATTGCC
TGGTCGTCCCGATGGGACATTTACCTCTCGGAAGACGACCTGGTACCGGCCGAGGTCCAT
TGGTTTTCGATCACCAACTCGATCCTGGTCGTCCTCTTTCTGAGCCTACTGGTGGTGAGC
ATGCTGGTCCGAAACCTCCGCCGGGACATTGCCGCCTACAACGCGCTGGCCGCGCTCGCC
GACGAAGAACGGGACGAGGACATGGACGAGAGCGGCTGGAAACTGGTCCACGCCGACGTC
TTCCGACCCCCGGACAACCACCCCATGATGTTTTGCGTCTGCTGCGGAGGGGGCGTCCAG
CTGGCCCTCACGATCTTCCTTTCCATCTGCTTCTCGGCGATCGGATTCCTGAACCCGGCC
CGGCGTGGTTCCCTCATGAACGGAATCCTCTCGTTCTACGTGCTCAGCGGAATCCTGGCG
GGATACGTCAGCAGCCGGCTCTACAAGGCCTTTAAGGGACGCCTCTGGCAGCTGTGCACC
GTCGTCACCGCCCTGCTCTTCCCCGGGGTTGCTTTTTGCATCTTTTGCTTTTTCAACATC
ATCCTCTTCTTCCTGCACTCGTCGGCGTCGGCTCCCCTGCTGGACATTATCATTGTCGCA
GCCATGTGGTGCGGGGTCTCCGTCCCCCTGGTCTTTGTGGGTGCCTACTTTGGCTACAAG
CAAGAGTCCATCTCCTTCCCAACCGTCACGTCCACGATCGCCCGTGCCATTCCGGAGCCG
CAGCTCATTATGCACCCCATGGTGGGAGCCCTCGTGGCCGGTGTCGTCCCCTTTGCCGCG
GCCTACGTCGAGCTCTTCTTCATCATGACCTCCCTGTGGATGGACCAATACTACTACGTC
TTTGGATTCACCCTCATTGTCTTTTTCATTCTTATGGTTACCTGCGCCGAGGTCACCCTT
CTCTTGGTGTACAACCAGCTTTGCATCGAGAACCATCGCTGGTGGTGGTTCTCCTTCTTT
GCTCCCGGATCGACCGCCCTGTACACCTTTATCTACAGCATATTCTGGTTCAAGGCCCTG
GAGGCTTCCAAGATGATCATGACGTACCTGCTCTACTTTGGATACATGGGACTCATCTGT
TCGGCCATGTTTTTGGTCACCGGAACCGTCGGGGCCCTGTCGTCCCTGTGGTTCGTGAAG
ACTATTTTTGGGACCATCAAGGTGGACTAAGAGACAAATCGGTGGTCCGGGTTGTCGATC
GAAGACAGTCTTTGTTTCTCGGCGTGGGGCGGCAGAATTTTCACCGAATCAACCGAAACA
CGTGGCCGCTGCGAGTTTGATCCGTCACAAAAACACTCCTCGAAGCAAACGCACCGAACA
CCAATACGTTTGAATTAAGTATTTTTAAAACTCATTTTATTGCTTTCAAGATGCATCTTT
CGTGGTGCGTTTTTGGCGTCGTGTCAACCAGATAGCTCGGACGGTGCCTCAAGAGTTCTT
CTGAATCTTGCTTTTGTGCGATTTTGTACTTTTCAGTCTTTTTCTGGGTTTTCAGTGGAG
ATTGTTCTTGTCGGAGAAGTTCGTAGTCTTGTACCGTAGCAACTCATACAGAACCGTGAT
CCGTGATGCTACGAGTATTCAAGCAAAAGTGAAGTAATGTACTGGCTAACGTACGGTACC
AGTCAGACCTTGAGAATTCGTTGTTCGAGTGTACGAAGATGCCAGAGGAAACGTGCGTAC
GTTCCTCCGAGGCTCGGTGTGTACTCCCGGTACGAGTACCAGTACGTACTCGTACGAGAA
AAATGTGAAGAATCGAGACTTTCGAGAAAACTGAGGGGAAAATGTTGCTACTTGTCTTTC

CAGAGAAGTGTGTCTCATCTCCCGTTGTTTGGCGACGATCCATGATAGCACGCGAAAGAC
ATACTATGACACTGATATTGAAATTTCAGGCAGTCGTTTCTACACTATTAATTCTATCTC
TTCTTCCCGAAACAACAAGAGCAATGGCATCTTCGGCAGCGGCTTCGACGATCTCGTCGT
CGGTTCGAGTCGCCATCATCGGCGGCGGGGTCTCGGGGTCCGCTTCGGCGAGACGGTTGG
CCCAACTGGCACCATCGGCACGGATCACACTGTACGAGATGGGTCGTGGTCTAGGAGGTA
GAGCCTCTACACGGAAGACCCGCTCGGTGCCCCATATCTACATCAATCACGGTGCACCGT
ACGCCGATGTGCGGTCGAAGCTGGGTCGTTCTCTAATATCTTCCCTTGGTCCGTCGGGCA
CAGCGCCCTTTTCCGGGGTGCGAGGATTCTTAGATTCAAAAACGGGGACTTTCGCACCCG
GAGATGGAGAGAAAGAGGGAGTGGACTATGTAACAGGCGCTAAAGGCGAAATGTCTCAGA
TAGCGTCTGCCATGATTTCTGGCATTCCATCTATCGACACAAAATTCAAGACGATGATCC
GTGGTCTATCTCGGTCACCCAGTGGAGAATGGCAACTTCTTGATAAGTCAGAAGAGGTCA
TCGGTTCCGCCGACTGGTTGATCGTTGCGGGCAGTGGTGTTGCACACCTTCGGTGGTCGA
AGACCTTTGGTGGTGAACCTCCGTTGATTGCTGCAGAGAAAGCCCGACCGGATCCAAAGC
TTCGTGAGGCATTAGATGCCATTGCTTCTCACGAAGCTTCCCCCGTCCTTGCTGTTTTCT
TTTCGTGCTCGGGACCCACTGCCCGTAAATGGTTGTCCCTAGATTTTGATGTTGCCGACG
TCAACGGGAGTTCAATTCTGTCTCGAATCATGATACAAGGAAAACAGAAAGATAAAAATA
GCTCTCAATCTGACGGCGGTGGTGACGAATGGTGCTCGGTTGTTTTGCATTCCACAGAAG
AGTTTGCATTGCAAAATTCTGGTGTATACGGCTCGTCTTCCAGTGCCACCAGGATAGGAG
ATGCTGCCTCGGATGCTTCTCGAGAGAATTCCCTTATCTCCGATATGATGGTAGCGATGA
ACGAAATACCGGGCATGCCTGAAATTAACACCAAACTACAGGATGAATCATATTCGTCAC
GTTATGATTATGGACCAGTAATCCATAGATGGGGGAACGCCTTCCCGAAGGGTGATGCAC
TTGCGGAAGACCTAGCGTTCCTGCCAAGTTCGCGCATTGCATTTTGTGGCGATTACGTTG
ATACTTCGAATCCAGCTCGATTTGGAAGCTTCGAGAGTGCCTTGCTGTCTGGTACTTGGG
CTGGAGAACAAGTTTCACAATTCTGCCAAAAGGACTAATGCGTAGTAGCTTTGAAAAACA
GGAATTCTTTTAATTACATGCTATTTATTAGTTTCACC
>comp2781_c0_seq1
GTCGTGCTCGTCGACACTACCATTTTCTTCCCATCATCGTGCAGAAAACGATCGATCTGC
ATCGTACAAGATTCTGAGTCAAATCGCCTCCGTTTTCTGGGATCTATCTATGGGGGAAAA
TGATGTTTCATCGGAATTGTATTCTCGCAATGATACTTTTATCACGTCGTCGCCATGTAT
TATCATTTCTTGGACAGAAGAATTGCCTGCTGAACAAGCAGAGAACGCCCACATTGAAAA
ATACGATTCTGAGACATTTTAGCTCATCGATAGCGGTTCGTCCTGGAACAGAACCGTCCG
GCACCAAAGTTTTCATGGGAAGCGATTCAGGCGACGAGACAAATAACACCGACGATAGAA
CCCTAAAAGAAGATTTACTGCATCCCGATGACTTTGGACCGATCCCACATCCGAAACACT
TGTCGCCTTCCTCCGCGATGACTTTTAAGGAATGCCCCCAATCGTTTCTGTTTCAATACT
TGTACAAAATAAAACAACCTACAAATAAGATTCTGGCCAAGGGATCGATGTGTCACAGCG
CCCTCGAGCACGTTTTCGATTTGGATCCCAAAGACAGGACACTGGAAAACCTCCAGAACT
TGATGCGGGTTGAGTGGGCCGAAAATCGATTGGAGGAGGAGTACAGGTTCTTGTTCGAAG
AAGAAGAGGAGGACGCCGAAACGGGGAGCACGAATGTTCGTCGGGACATAAATGCGGAAC
GGGAATGGGGGCAATCCGCGTTGCAGCTACTGGAGAACTACTACCAGAGCGAGGACCCGC
GAACGATTCTGCGCCCTAATCCGCACAAGAGGGAAGTTTGGGTCAGTGCCGACCTTACGG
TAGACTCGAGCCTGGGTGTGACGACACCAGCGGGCAGAGCAGCGACAAAAACACAACCGA
ATCCCGAAACCTTCAAGGTCCGGGGAATCATCGATCGACTCGACATAGTTCGAGAGGCTC
AGAGGAAGGTCGCCCTGAAAGTCATTGATTACAAAACCGGCAAGGCACCCAATCTGAAGT
ATTCTCCTGCCATGAACCAGAAAATATTCGAAGAAAAGTTTTATCAGCTCAAAATCTATG
CCCTGTTGATGCGAGAAAAAACACACGCTGCCGCAAACGACAAGGATAGCATGGACCTGC
GTTATCTCAAGCTCCACTTTCTCACTTCCGAGGAGGGACCGGCAAAGCCATGGGAAATGG
ACCTTGGAGAAAACCAGGAGATACGTGACGACGAGTTGCAAAAAGTGCACAAAGACTTGT
CCCAGATCTGGATGGACATCGGTGCGTTGGTAGACAAGCAAGATCCAAAGGCATTTCAGC
ATTGCGATCGACCTTTTTGCTTCTGCCACGTGTGCAGAGACCGCTTCGTTCCTGGTACGG
TATGGGAGCGCCCCGAGTAAAAGTTAGAAAAAGCCGCTAGCGACTCGTCTTTTGCGATCA
AGAATCATTTTCTTTATCTGGTTTGATGCTCCAGCTATAGCATGGGACACTACGAAAGGG
AATGCCAATTATTATTCATGCTCAATAGTAACTCTGCTTATCACATGAATATGATACAGA
AACATATTTGGAAGTGGCAAGTTTCCTGCTGCCCGGAAATGTTAACCCGATCATGAGCCG
ATAGACAGTACTTCGGTAACTAACACCAGTGATTGGGAAAAGAATGAAGTTGAAACGATT
TTGGCTCCAATTGTTCTATCATGGTATAGTGGAGCCGCTGGTAGAGTAAACTGATAAAGT
GCTCTCGACTGCATATTGGGATGAAAAAACTATTGACAGATTATTACAGGATAGGCTTCG
GCCCTTGGTTGGACATTGTGTCAAGCCAGGAAAATTGATGTGCAATAATTAATGTCAAAT
CGGATGCTAATGTTGGCTATGGTGGACTGGACTACCACTTTTCATTTGTGCACCGGAGCC
ACTGACCTTTAAGAGTTCTCAGACTTACCAAAGAAACAAAAAAAACTATTTTTTTTGTAG
AAACCATGTAGGCCTCAGTATCCCATTTTTGCAAATAAATGAGTATCTTGACATAAAACA

AATTATTCATTCTTAACTTTAAAATTCAG
>comp2782_c0_seq1
CGAACCTTGAGGCACCCGTTGAGATTATTGTGAAGTATACTACTTACAACCTATACTTAC
ATAACAATAATATTGTTGATGGTGCGATACGCATAATCTAACATGACAACGACGATGATG
GTGACGCCGATTGCAACGGTGTTTGTTCTAGTGCTGGCGCAGCACGGTTGGACCGAATCA
TTTCGAGGTCATCACGGAGTTTCGTTCCCTTTCAGAATGGGATGTTATCACCTCGATGTA
CTACCAAGGAAACCAACAGCCCTCTTTTCAAAACAACATCACAATCACTTTCAGCAGCAA
TTTCCAATTCCGGCCATGCCGTCGGATAATTTCAGAAAGATGGCACAGTCTCAACTGGAA
CTTCTTGCTAGTTCTCTTTCTCGCGCCGATGATCCTTCGATTAGCAAAGTCCAGTCAATG
ATTCTCTATCTTCCACAAGAAAATTCTTTCACTGGACAACTAGAATTCACTCCTGCTATC
TTATACCCGGATCCAAGCACCGAACGAGTCTTCATCGCCTCGGATGCTGCATCAGGGCAA
GCTCCGACGTTACCGAAATCTCTGTCGAAGTTGCCGGGCTTTTCTTCTGCCCCAGATTTA
CTACCTGGATACCCCATGCTTGCAGGTGGTAACGATCCTGGGGTAGGTACTGTTGAAGAA
GTATTATGTGATATTCGATTGAAGACGTCGGCCCTTTCGATTCCACTTTTATCTGGTTCA
GAAACCGTTGGCGTACTTTTGGTATCGCCAACGGTAACACTCGATAACAAGAGCAGTGTC
GAAAACGGCACATCTTGGACAAAACGAGAGAGAGAGAGCAAGTTTCCCTGGCTGCACAGAGT
TTGTCCATGGCTCTGAGAATGGACAACGAGCGTAATCTCCTTCGGGAACAAAATAGTGTT
TTTCGGGAGAATCTTTCCGATAGTTTGCATCAAATGAAGAATCCTATACAGGCCCTGAGA
ACTTATGGCAAAATTCTGCAACGACAGGTTGCTGATACAACAAGCGACGGTAGTGGTACA
CCTGAACTCTTGGAGTTAGCCGAGAGACTCATGGTTCAGAGTGATCGGGTGGTCGATATG
TTGGGTCCAATGGATTCATTGGTTGAAACGTTGGATCAGCCAAACTCTGTTGTGATGCTT
CCCCCTGCAATAAGTAACAATACTGAGAATGTGCAGTCGATGGTACTTTGGAATGACCAT
CCTTCGAACAATAGCGCCATGGTCCCAAGGTCCAATCCAGAAGCAACTATCGACGAGAAT
GACCCTCAACCTCTAGCTCCAGAGAAGATTACCATCGAAAACCGAAAAGAAACCGAAATA
GATGGCTTGCAGGCAACCGTAATTGGGGATTTCGAAACCGAAATGGTGTTTATTACTGAT
GTTCTTGAAAGCGTCTTCGACACTTTCGAAGCGATTGCATCCGAAAATGATATTAAGTTT
GAGGTCTCAATTGAAGACATAGACGATCTACCCGGTGTATTCGTAGCTCCCAAGAGTTTT
CAGGAAGCTACAAGCAATGTCTTGGATAATGCTTTCAAGTATGTTGTTCTACCGAAGGAT
GACGCACTGTTTACAAGAAATCCCAATCCTGAAGTTCGAGTTCGAATATATCCCAATGAT
CACGTCGAAAGGCCCGGCGTAACAGTTCTTGTGGAAGACAATGGGCCAGGAATAGCAGAA
GACGATCTCGACCTCATTTTTGAACGGGGATTTCGCAGTGACAAAACGTCATCGGACGTT
GGGGGAAGAGGAATAGGGTTGGGATTGGCAAGAGCTCTTATGAAACGAATGGGAGGATAT
CTTGGTCCAGCAAGCGATGACGACATGTCGCATTTTACAGATATTCTCGATGGTGCAAAG
ATGAAATTAGAGGTCAGCCGGAAGCCGTATGCATCGCATCGATAACATGTGAGTCCCCGC
AGTGATAGACTTAGATATTAGAATGATAAAACATGAACACTATTACCAAAACGAA
>comp2783_c0_seq1
GGAACTTTTTTCTTTGGGACTGAGCAGACAGTGAGAGACCCTGTTGTACGGTAGGACGAT
GAATCAGGAACCAAACTGTCGAATCATCGATGAATAAATGCATGCCTGTTTTCTCCGTGT
CACTCAGAAAAAGGATATTAATTGGAACAAAAATCAATGAAAGAGAAAGAGTAGAGGACA
ACAAGCACCGCTACGACAAATAAAAACAATACCGGCATTACAACAAATGATGAACCAGTG
TGGCCCAGTCGGATACTCTATTGGTGGATACAACAACTGTAGACAGTTGTCGAACCAGCA
GCAAAACCATCAACGACCAAAAACTCGTAACGATTACTTTGGAAAGTTTCGGTTTCTCAA
ATTCGACTTGAAATGGCCGGAACTAAAAATGCCCGACATGCTGAAGGACTTTTCGGTCGA
TCACATTTGCTATTCGGGTGAAGTCATCGACGAGGCAGCTTCAACTACCAAGAGTCACCT
CAGAAAAACAGCAGAAAGTGCAAGAGAGCTTCCCAAACAGGGAAAACAATTCGCCGACTC
CCAGGCCGAAATCCTTCATAGCCATTCCAAGGTCTTAAAAACGCAGTTGTCGGAAAATTG
GAACTCCTCGATTGCAAGCATTGCGTCGATGCCCTCGGCCATCTCAGAGTCTTGGGACTA
TCACGCAAATATCTATCACGAACAACAAAAGCAACTCAAACAACAGCAATAATTTGTCGG
TTGTTATTCAAGTGCACTTGAAACGGGTGCAAAGGTTGGTCCTTCGTGTGTTTGACTGTT
TGTGACTATATAAATTTGCTGTATCTCTCTGTATGATGAACAGTGATAGAAACTGATTTC
AGATTTCGAATCGAAGACTTGGCATGACACGTCACGACAGAATGATATGCAACACAATCT
GTTTTTGTGATTCGTGTCTTTCGATTTCTGAGGCATCGTCACTCGAATTGTATTCGAAAC
AAATAAAATGAGTATGAACGGAATCTAAAATGAAAGGCTTACTCGTGAAAGCTTTTGTCG
GATCTCGCAAGTATCTATGGTGAGATATTATTGTGTGTGAAGCCATTGTCCGATAGTAGT
ACATACCTACGAAAAGACGAAAGATCCGTCCGTCGTATAATACCGGGGAAGAATGCATCT
CCGCTCCTTATTACTATACTTTCAATTCATGAAGGTGTCAAATTGAAAAAAGTGCACGGA
TGTGTTTCATAAAAGTCAATTCGATAAAATAAGCAAAAGGCAGAACA
>comp2784_c0_seq1
CGATCGCTCATCACCCCACAAACCGCTACTCCGAAAAGATCTGAACGCATCCAACAGTAA
GGTATAACGAGTAACCGAACATTTTGCATCAGATAACGACATAATACAACTCGACTCAGT

GTAACACATCCACAAAAAACCATGAAGTTCCATCTTTCCCTTCCCTTGCTCTCAATTGCC
TTCATCGAATCTACTTCGCCTGCCGCTGGTGCTCAAGTAGACCTCATGCCCGAAGGCGAG
GATATTTATTACTTCAAACCTTCGGAAACTGCCGCTGCTGCTACTCCCCCTGAGTGCAAC
AAGGAATACGAAAGCATACTGGATATCTGCGAAGCCGGTGCCTATGATCTCACTGAATTT
TGCCGACTCCTCGCATTCGTGAACTACTTGGATCTCTCCGATAGCAACAAGTCCTTCACT
ATATTTGCCCCTACCGATGAGGCCGTAAGTCGCCTATTCGACGACGTCTTGGATTCCAAC
GGAATCGGTTCGACCCTGATGAGGGAAATCATTCAATCTAATTACATCGATGGCCAGGTC
GAAACTAGAGATCTTGAATGCAACTCCAAGCCAAACGTTGTATCCAGCGCTGGGAAAGAT
CCGCCGATCAAATGCAAGGTCGACATTACGGGAAATACTGTTCCCTATATCAAGGGGAGG
GGAAATGCGCGTGGTTACATCCCACGATTTACCGACCCTCAAAACCCCATCTTGACTTGC
AATTCGAATATCTACAAGATTGATGAAGTGGTGTTGTTGACTAATTACGTTTAGAAAATG
AGTATCAAGATGCCCTTGGGGTTTGTTCCTGTTTTCAATCGAAACGAATTGGTAGAGGGG
CACAGTAATGTGCATTGTAACCACTCATCTTGAGAGCAGATTTGCTCATCGAATCATAGT
TTCATCTTTGGATTACTCAATTCCAATTGTGCACGTGTATTAAGTCTACAAATTGAATAG
ATTGGATATTACCAATTGGTAATGTTCAAATTTTTAATTATGATTGTTACTCACCATCAG
CG
>comp2785_c0_seq1
GCGAAGGATTATTTTTTTCAACGTTGCATTTTATCATTACTGTATAATTATTTTCTCACC
TTGCATGGAAGATCCGTTTCACAAGAAGTAGTACTGATACATCGGACATTATGATAGGAT
TTTACAATTTGCTTTCGATCTTATTCCTCATTGCTCTGACCGTAGCAACTGATTCAGAGG
AAGGCTCCCTCATCGCTATTGAATCTTCCAGAAAAGGTCATCCTGCCCGTGGTCTCAAAA
ACTCCAAGAATCAACCTGGTGACAAATGCATGGAAATTGAGGCCGAGATCGAAAGAGTTA
TAGAATTATACGACGCCTTGATTGCGGAAGAAGAATTGGGCACAAACGATGTTGGAGTTC
AGGCCAGCCCCATGTGCTTTTTTTTCAACTTTTGCTGTTGCTTATAACTGTGGAGCCATTG
GAAAATGCGCCATTAGTTCATGCCTTTCAAATCTTTGCGGCACTATTAATATGTGCTTCG
TAGGCCCACCATCTGCTGTTTGTGCTGCAGCTATCACAAAAATGATGGCAAAATGCAAAA
GGGGATGATCCTCGCATTGCATGTCGCGAATAAACACGTCCATATAGACTGTACGTAATA
TTACTTTGATGACACTTTGTAGTATGGTTCACTTCTGCTCTTTTGTGCAAGCAC
>comp2786_c0_seq1
ACCTCCTCAAGCTGAGAAGGGACCATTAGTAACCACTGAACGATCAAGACACCAAACCTA
TCAATCTGCCAAAATGAAGTTTACTTCCACCATTATCCTTTCATCTTTGCTCTCTGGAGT
TTCTGCATTCGTTCCTGTTGCAAGACCTACCGTTGCTACATCATCTTTGGCTGCGACCGG
TGTCGACGACAAAACCGAAGTCCGCGAATATTTCAACACGGAAGGATTCAACAGATGGAA
TAAAATCTACTCCGACAGTGATGAAGTCAACTCGGTTCAATTGGACATTCGCACTGGTCA
CGACCAAACTATCCAAAAGATTTTGAACTGGGTCCAAGACGATGGTGATATCAGTGGAAA
ATCAGTGTGTGATTGCGGTTGCGGTGTCGGAAGTTTGGCAATTCCATTGGCGCAAATGGG
AGCCAAGATCTCAGCCAGTGATATCTCTGATTCAATGGCCAATGAGGCCGCAAGACGTGC
TAAAGAAATGGGACTCGAAGCAAAATTTTACACGTCCGATTTGGAATCTGTGTCAGGAAA
GTATGACACCGTCACTTGCGTTGACGTTGCCATCCATTACCCCACGGATAAAATGGCTGA
AATGGTCGGTCACTTGTGTTCCCTTGCAGAAGATCGTGTTATTATCTCTTTTGCACCGAA
GACATGGTATTATTCCTTCTTGAAGAAGGTTGGTGAGCTCTTCCCCGGACCCAGTAAAAC
GACACGCGCTTACTTGCATGAGGAAGAAGTTGTTCGCAATGCTTTGAAGAAAGCCGGCTT
CGAAGTTGCACGGGAAGAAATGACAGGGACCAACTTCTATTTTTCCAGATTGTTGGAAGC
AAAGAGAGTATAAAGCATAGCTGATAACATTCCTTAATATTTTTATACTGTCTTCCACTT
CTCATCGGAAAAAGATTTTGTCTCCTTGGATTGCTGCCGCTGATTTCTTAATGACAACAT
AACTAACTTCTTTCTTGAGAAGTGCTTCGAAAATTCGCATCATCTGTATACTGCAACTCC
CTGGGAAAGGTAGTTCTTAAAGGTGCCTTCGCAGGCTATATTTTGGCGAAAGATGATTCG
AA
>comp2787_c0_seq1
ACGACAGGGAGCAGGGAACATCCATCGAAACCATCAGTCCAACCCTACCGGTACGGTGCC
AACATCAATCCATCATCAATACAGTAGTGAATAGAACAATCCAGTTCGTACACAAATCAT
ACAAGTTGGTCCATCATGGCTTCATTGCAGCTCTCCAGACACGCTAGAAACCTCGGCGTC
ATTAACAGCCCCGCCGTATTGTACAAACGGATATTGAAGGAACTTCCCCGCGTTCTGATG
ATCTACGATATCGACATGCCTATCGAAAAGGCGAAGGCGACTATTCGTGACCGATTCTAC
GACAACAAGGACGTTAAGGACCACCGTGTCCAGGAAATGCTGATCGAGACGGGGTATTTC
GCTTTGGAAACGTCCATGCTTCAGCACAAACAAAAGAACCATCTTATGCACTTCCTAGAG
GGTTACACAACCCCCTTGGAAGGGGATCGGAAGCGATTGGACCAGGATTCGACGATCCTG
GACCAGTTTGCAAGAAACTAAAATGATATTTGGAACTTGGATTATATAATCCATAGTAGT
TTGGTTAAATGGATGGAGAAAGAATGCTGCTAGACAGACAATCTATTCCAGGATTACACC
AACAGCATATCATTCGGCTATTCATATAGAGTTCGAGTCGATCACAACGGAGTTGTGAAC

TCAATGCTCTCGATGATCACTCGACGGTCACTCGTCGAATAACTTAAAGATGCATGAAAG
AAAGATGAACAATTTTTTTCGAGTGATAGCCATGTAGGGCATGAATACATGTATTGTGGC
GCA
>comp2788_c0_seq1
TTTTAATCTCAACATTGCGAATTGGAGTCAGTGTCATAGAACAGACCTTTCGAACCATGC
AAACCTTCTATTCTCTTCTGCTATTCTCAAAGCCAAGACCAGTGGTTGTATTATTTCTTG
CGGCATCGGTTGTAGTGGATTCCTTCGCTCCCGCAACGATACGTTCTGGTGTTCTGCAAC
ACCACCGAACCAAAAATGCGAGCGCGAAGACAGAGAGGGGCACACTTCTCTTCGCTGAAT
CTCCGCAGTATCAGAAGCAGGAAGGAATATTGAAGCGATCAGAATGTGTGGGCAAAGGGA
GCTACCTACTATACATCGATTGCGTTGATGACGACACAAATGGATACAAACCAGGGCATG
TTCTAGCGCTTGAAATCCAACCTCCCGCTGTGGATGGTTGCGACGATGTCGAGGTGTCTG
AAACAATGACCATGAACGAAAAAACAAGAAAAGACATGGAAGCGAACGAAGGTTGGATGC
GGGGGCCGTATACGGTAAGCCGTGGGTACGGCACCAGCTCCGAGATAAATGGCAAGGCAA
AAAATGACGGCTTTCAGGTCTTGGTCAAGGAAGTAGGTTACAAGTCCCACGTCTTTTCGA
CCTGCCCTGAAGGAACTCCCGTTCGCTATGGTGGCAAGTTCAAGGTGCCAATTGCGGAGG
GAATCTTGGAAGCCGCGGAAACAAGCGACAGTGAAGATCGTGGGGCAACAAAGCGGGTGA
TTATGGTCTCGACTGGGGTTGGGGTGGGGCCTTGTCTAGGCGCTTCCGAGATATTGGTGA
ACATGCTGACCTCTTCCGACGCAAAGGAATGCGCAATTGATTCCATTGATCTAGTGACGA
GTTTCCGAACCCGAGAGGAAATCGCCATGGCTTCGGATTTGGACAAATTGCAGCAACAGC
AACAAGGTAGAAACCCTGAATTTCACTGGGAATCTATCATCACTGGAGAAACTGGACGGC
TTTCAGCGAGTGGCCCAGAATTCTTGCTAGATCAATACGTAAGTCCTGCCACAAGTACTG
GTTCCGCCATTCGAAACACTCATTACCATATCATTGGAAACGGGCAACTTGTCAATGAAT
GGACTGCCGGTCTGGAACAAGCAGGGGTTCCCTCTTCGAGGGTCACCACCGAAGCCTACT
TTAATCACAAGGGCGAAGCAGACCCCGTTGCCGTAAATGCGATCTGCCAGGCGATCCTCA
GATTAGATCGGGATGCAGAGTCGAAAGAGGTGGAAACTGCAGTAGAACAGGCAGCAAGAG
ATGCATAGGCGTAGGACCGACAGAAAAACAATCTAGCTCCCTGCAAAGCCTGAAGTATTA
ATCATTCATAAATACACAAAATGTAAAAATACTTCTCTATCTGACACACACGTTACTTGC
AAAATGCATAGTGTACACTTCTATGTTCTGTCTTTTGAAATACCACCTAATCGTTAACAT
TTTTTGTGATTGTGGAATGATATCACATCACAATAAATGGTCAGAGAGGTTTGTCGATTT
CGATATTTTCAAAAGAACAGAGGCAATCTCCACGGCTAATGGTTCATTCGCTC
>comp2789_c0_seq1
CGACAAAAGCAAAAGCACAAGAAGCAACAACAATACATCGACGTCCGCTCTCTCGTCTTT
ATCACTCCAAAAGAACATTCGTTGTTATTCCAACCCAAATCACGTCCTCACGCCAACATT
TTATTCCATCATGCCTCCAAAACGAGTAAAGAAGGTAATGACATTGCCCATCAATGTTAT
TTTTGGGCACTTGCAGAAAAAAACAAGAGTTCGAATTTGGTTGTACGAAGATACGAAGAC
GCAAATAGAGGGGCAAATCATTGGATTCGACGAATACATGAACATGACCCTGGACGACGC
GGTAGAAATCGGAAAAACACGAATCGAGGTCGGAAGAATCTTGTTGAAAGGGGACGCCAT
TACGGCGATACAGGAAGCAAATCCAGTAAAGAAGGAGTGATGGGACAAAGAGCAAACACA
CCGAGAAAGTTGAAATTTCCGAAACACCTGGAATTATTTTGGTTGTTGTTTTTTCGGACC
GTTCGATTGCGCTGGATTCTGTGCCGTGGGCTCAATTCCCGCTTTGTTGAAGATTTGGCA
GGACAGCTCTCGGTTGAGAAAATCTGTTTCTTGCCAAAACACAAAGAAACATTCAACAAT
CAGTTCTTCCGATAAAACTTATGAATGTGCTGCATCAGTGGGCAGACGCTAGCACGAGAC
GATGCATATAATACTAACACTGAAATGAAAATGAAAATGAACACTACAAGTAATGCGTTA
CCGATTCCATTTATTTTTGACTTTCGGTTGCACAAAAGGGCACGCAAATGCATAGCTTAT
GTGCAGAAGCCATGATGAGTTTGAGTCGATTCCGATCGCATCCAAGCGGTAGATGAATGC
AGTCGGGAAATCGTGTTCTTTGTATGGGTAGCTAGGACACCG
>comp2790_c0_seq1
GTCGTACTCTGGGAGACCGTTGGTTCACCCACATGAGTACAGTCGAAACGATTGAATAAC
GGGACCCAGCGCACAGATTGTCCTCTTCGGTGCACAAAAACATCGACGAGGTGTCGTTGT
CTCCAAGCCAGTGCAGAAACCACAGACCAGATCTCTGGTTAACGCGGTTGTTCCCGAGGA
GCGAGCAGAGACAACCAACCAGCCAGGACATTTTCTTCGGGATAGTTCCTCCCCCAGGAC
AGCTGCACAGCGTTTGTGTTGTGCCGTGCGAAACAGAAACAGAAGCCGACGCCAAACTCG
GCCTTGTCGTGGTTCGCCATGCCGATCAAGTTTTCAAAAGAGACGAGCTCGAGGTTGTTC
CGATACGTCAAGTCGGTCGACATTCGATTCAACCCCTTTGACAACAGGACCACCTCCGCG
AGGGAATTCTGGAGGCAGATGCAGGCCACTCGGTACCGCAAAATGAATCCCAGGCTCGAG
ATCAACACGCACGTGCTGGGATCGGCGAGCCCCCCGGAGATCGTTCTGAAGCTCATCGAC
GACACGGAGAAGCGATACGACAGCCGCAACTATACGGCGAGCGAGATTTTCTTCGACGTC
CACCTGTCTCTCGACAACCTAGACAACGAGTTCGAGCTGAGCGGGAAATCTCTGGACGAC
TAATGTCGATCGATCGATCGGTCGATCGATCCGGCCCAAGCGGCAATTATTGTAGCGTAG
CATAAGAACGAGCGAACACTTAAAAAACACC

>comp2790_c0_seq2
AACAAACCATTGCGCCGGGTCTATGGTTTTGGTTCGATTCAAGGCAAAGCGCTGTTGTAG
CGGCGCCGGATCGCTTCGCCTCGGCCGCCAGTTGTGGTTATTGTTGCTGCTGTTGATTTG
GTGGTGGCAGAGTTTCCGAACCACCCGCCTTGTGTGCCCGCTGCCTGCTCACCCACTCGA
CTCACGACGCGCTTCGCCTTGCGTTCCGTTTTCTCCTCGACTTGCTCCCGTCCCTGCCGC
AGCCTTTGACAACAGGACCACCTCCGCGAGGGAATTCTGGAGGCAGATGCAGGCCACTCG
GTACCGCAAAATGAATCCCAGGCTCGAGATCAACACGCACGTGCTGGGATCGGCGAGCCC
CCCGGAGATCGTTCTGAAGCTCATCGACGACACGGAGAAGCGATACGACAGCCGCAACTA
TACGGCGAGCGAGATTTTCTTCGACGTCCACCTGTCTCTCGACAACCTAGACAACGAGTT
CGAGCTGAGCGGGAAATCTCTGGACGACTAATGTCGATCGATCGATCGGTCGATCGATCC
GGCCCAAGCGGCAATTATTGTAGCGTAGCATAAGAACGAGCGAACACTTAAAAAACACC
>comp2791_c0_seq1
CTCGAAAGGTAAAATTCTTGAGCGTGTTCTTCCGTTGTCATCACTGAGAAAGAATGCCCC
GGTGCAATAAAAATTCGTCACCGACGTCTTTTGAGCTAGCACTGTCATTGTCCGTCATTA
CTTCATCTCAGAAAGAAAGTAAAAGTTGAGACACAGACTGCGCATTCGTGTACTAGAAAA
CAATATGAAATTGACGAGTATGATGGGTCAAACCGCAGCCATTGCTTTGATGCTGGTGAA
GACATGCCTTTCATTTCGTCATTGTACTTTCCCCGCGCTGCCGTCTCTGGGATTGAGAGT
TGAGGATCGAGGAAGTCTCAACCCGTTGTTTTATTCGAATCAAAATGGGGATGATCCTCA
GGAAGACTGGCGAGATATCCGTGCTAAACTGGTAATGCAGTACGACAAAGGAAATGATCA
CACTACTACGAATAGAAGCAATACTACATGCATGCCACCAATACCAGGCATGAATCGCTG
GGCGTATGATTCGGGGGATGTCATTGAAGTTGGTTCCTTAATTGTGAGTCACCCATATCA
GGATTGGGGAGGACTCAAGAAACAACATTTTCACAAGTGCATAATTCTAGTAATTGAGCA
TTCCCAGGATGGCACGAAAGGAATCATTCTGAATAGAAAGTTACAGGATGAACAACACAA
GAACTGGATTTGTCATTTTGGAGGTGACACCGAAGGAATCGGTTCGAGCGACGGATCTCT
CGGGTGCTTGTTTCGACTCAACGACTTGTCGCGTGTAAAAATTGGTATGGCCTTGTCGAT
TTTGAACGATATTGCAATTATGCCCTTAGACATCGCACAATGCCTGGTGGACGAAGAAGA
AGCCAAAAAAGAAGATTTTAGACTCTATCACGGATATTCGGCTTGGGGACCCGGTCAGCT
TGAAAAACAAATTAAGCGAGGCGATTGGTTTATGGTGGCAACCGACACCGAGTCTCTATG
GGACATTATCGTACAAGAATCTACTTTTTCGACAGGAACCGACAAGTGGGTACAAATGAT
GAAGCGCATAGGAAAAGAATCATTGATGATATCCTCACCAAACCAAAAATTTGAAGATGA
TATGTTAAAGCAATATATGAGATTGAGGCTACTCAAGAAGTCGAGTAATGAACTTCAGAG
ATCAAAATCGAGCCGACAGCAAATGCATGCAAGGAAGAAAAGCGCTTGTAACCTTTCTCC
TGGAACTTTGGTTAGATCATCCACTACAATTTTATTCGATGAACAGGTCTTTCACCAGTC
ACTCGTTCTCATCTTACAAGACAATCCAGAGATGACTATTGGGGTTGTACTCAATCGACC
ACATTCAACTAGCGTCAGTATTGGTGGCACGAGCCTACCACTGCGGTACGGGGGAAGATT
TGAATTGGACGACGAAGGCATTCCAGAGCTCTGGTTTCACTGTAATAATGAAATTCTTCA
AGAGGCAAGGATTGGAGAACCTGTCTCACAAGAGGGGAAAGCACGCAGTATCTTTTGGAA
ATGCACCAGAAAAGATGCAGAAACTGCTATCAAAACAGGATTGGGACGCGCTGAAGACTT
TTTGGTGGTCTCGGGACTGTCGGTGTGGGAGAAGGAAGAGTTGACAAAACATGCGGGAGA
GACATCTTGTATAAAGCTCGATGATTGCTTTTCCAAAGTTGACGAAAACTCTATCGACAC
AATTTGGAAATTGCTGATGGTTCAAGAGCCTTTGAACAAAAGAAATGCAGCTGAAAATCT
TGAGGCAGCGAATACCGCGTGGATGCTTTCGGGCGATGCGGGTTGGATGCTTTCCGGTGG
TTCAAAACAGTTCAATTCCCGGGTCAGTTCCGATTTCGATAAAAAAGAGCAACAAGTTGT
TCAAAGTTTAGCTTATAGTGCCTTGGACAGATGGATTCGTACATATTTATTAAAACATTG
ACCTTATCGAATACTGCAACGGATGCAGTATGAATCGATTATGTGTTAGTGTTCAAAGTT
GAGTTTGACTCATGCAGCCG
>comp2792_c0_seq1
GCCAACCAGACGACACGAAGGAGGAAGAAATAGTCGTGACGACCAAAACAGAATATGAAA
CATGAAAGGGCAATATGAAAACTCTCATTGGTAGTGATAGGAATATTCGTTTGGGACAAA
GATGGCAGGGGGGGGATGGCCCCTCTACATGCATGTCGTCGAAAATTCCCATCCATTTCT
TGAAAACACTTTTTGCCGTATTTGCAATGACGCTTAGTAAAGCATCGGGCATTACTGTGA
ACGGCGTTCTTTTCTCGGCCCCCTCTGGGGCCGTCTCTAACATCGGTGTCGCAACACAAA
CATCACTGCCATCAACGACATGTTCTCGATCTGTTGTTGTATTTATTCACGGCAGGCTAG
AGGCGGATCCAGCAAGCGATGACCTAACGATCCCTCGACCAGCCGAATTGTATTCGGATT
TCTGCAAAGGTTTAGCAGCATCGATGATGAGTGACGTTTTCCTTGTAGATTACCACGAAC
ATTACAGAGACTGGAGACGAGAGCCACGTGATCTTGAACTTGGGCCAGTTTCATCGGCAG
TAGGTGAAACAATTCGAAAGCACATCAAGAGCTACAGCGATCCGATGAATGCTACTGCTA
CTTCAATCTCTGTAAAGATAACACTAGTTTCCTTTTCGATGGGGGCAGCAATAGTGCTCA
AGCTTCTTAAGAACAGTCCTATGTTCGAATCAGCTTCAGTTTCCGTTCCTTCTTCTATTT
TGAAGAACGGAATAAAAATCGAAAGAGCGATTCTGGTTGAACCCGTTTGGCGATGCTGGT

TGCCATTTGCAGTCTCGAGAGCCGCAGAAGCCCGGTACTCTGCAAATGCCAAAAAAGTTG
AAATCAGCAAGGTGCCTTCTCTGGTGCTGGTAGGAACCAACGATTTGGAGGTTCGACATG
ACATTAATGGAGGAATGGGTAGCAATGGTACTCGCAACCTTGGTCAAAGTGTGGCTCGAT
GTCTGCAACCATTCCTGCCAAATCTATCCACAATTGATATTGCTGGAGGTAATCACTTTG
GTCTGTGTTCTGGACCCCCTAAGTTTATGGTTTTTGCCAGCGAAGGGTTATCATCTGGCA
AAAGTTCGGAACGCCTTCGAAAAGAAATGATTGATCATATATCAGAATTTTCTGCACAAT
CGCCAGAAAGTGACGATGACAGTGACAGTGACAGTACGGCAATATAGAACACCATAAAAT
TAGTTTTTCTTTCTGTGGTTCTGTGGTATTTGATTTCTGCTGCTTGGTTGATTGCTGCTG
AGATTACTGTCGCTTCTTCTAGGGATTAACTTGGTCACTGCTTGTTTCAATATTACAATA
CC
>comp2793_c0_seq1
CAAAACTTGTGTTGTACGTACTGTACGACACTTTGTTCTACCAAAAGCTAACTTCGTCTA
GACTCCATATCCATCACTTTCTGTTTTCTTCTCAACACCTGTAGGAAAAACGAGTTGATA
AGTTTTGAAAGCGATAGTAACCATGAAATTCTCTCACGCAACAACTCTCTTGGCCATCGC
TAGCGTCAGCACCGCCTCGGCCTTTTCGACGAGATCGGCATCCGTCACTCGATCAACGAA
GGCGAGTGTCAAGACGCCCCAATATGCCGTTTCAGTCGATTTGCCCGATTCCACAAACAC
AGCTGTCCCAGAAGTAAAGAAAGTCCCTGAAGCGACGGAGGAACCTTTTGATATGACCGG
TGTTGCATTGAGTGGGCTCGAGGGAAAGGCATTACAATGGAAGGAATCGGATTTCCCAAA
AGCTAAGGATCTACGAGCTGTTATTCCAAAGGATTGCTTCGAACCAGAGACCAAGACTTC
CATGAAATACCTGAGTGTCTCAGTCATTGGCACTACCCTTTGTTCTTTGTTTGGTGCCAG
AATGCTCAATGTGTTCAATCCCGGGAGCGTGGCAAATCTTCCTTTTTGGGCTGCTTATTC
GACTGTGACCGGAACCGTCGCTATGGGGTTGTGGGTTCTCGCACACGAATGTGGCCATGG
AGCTTTTTCCAAGAACAAAAAAATTCAAGACACTGTTGGATACATCTTGCATTCACTTTT
TCTGGTTCCTTACTACAGCTGGCAACGATCGCACGCTGTTCACCATCAATACACCAACCA
CATGGAATTGGGAGAGACGCATGTGCCCGAAACCGCCTCTGGCCACTCGCTCGGCCTGCG
GAATAGCTTGGTTGCAAAAGTAGGAAAGAGTGCAGGGATTAAAATTTGGGGATCCTTACA
GGCCTTTTTGCATCTGATCATCGGATGGCCTGCCTACCTCCTGATCGGTGCCACCGGTGG
ACCCGACCGCGGAATGACGAACCACTACTGGCCCAATCCATTGACCACACCGAAGCAGCC
TAGACGAGAACTTTTCCCAGGGAACTGGAAAGCAAAGGTTATGAAATCAGACATTGGAGT
AGTGGCCGTGGGTGCTGCAGTTGGAGCTTTGGGATGGTTCAAGGGGCTACCTTTGATGAT
GGCCATGTACGGTGGACCTCTTATTGTTGTCAATGCATGGTTGGTCTTGTACACATGGCT
CCAACACACGGATGTTGATGTTCCACACTTTTCCTCTGACAAGCATTCTTTCGTCAAGGG
TGCGCTTCACACAATTGATCGTCCCTACGATAAACTTGATCCATTTGGATTCATCGACTT
CTTGCACCACAAAATTGGTACAACTCACGTTGCACACCACTTCGACTCGTCAATCCCTCA
CTACAAAGCCCAAGTTGCTACCGATGCCATCAAAAAGGCTTATCCTAAGATGCATTTGTA
TGATCCAACTCCAATCCCTAAAGCCTTGTGGAGAATTGCAAAGGGTTGTACTGCTGTTGA
AAAGCGAGGCGACAAATGGATTTGGAAAAACAGCGGCCTTGAGCCACTGCTGTAAAAGAA
ATAAGCGTAGATTCTTTCCCAATCAAATCACCTTTTTGATATGAAATAGAAAAGCTAGAT
CAATGTAATAACGCACCAATACAGAAATGAGTTGCATTTTAACGATAGAATCATCTACCA
G
>comp2794_c0_seq1
CTTTTAATCTTTTCATCTCCAGTATCACCGCCGCCACCCAAATCATCATGAAGCTATCCT
TTTCATTGCTATCCCTCGCACTCGTTGCACTCTCCGCCTCCTCCGTGTCTGCTGCCGGGC
TCTCGACCCGAAAGCTGCAAGAACAGTTCGAGGGCACCGACAACAGCCAGTACAACGCGC
CAGACGCTCCATCTTACTGCAAGAAAGACCAGCAGCCAAACAGCGGCGACAAGTGCCCTT
CCGTGGGCCTCCAGTGCGCGTTCGACAACAACATTCAGTGCATCTGCGGGGAAGGAGGAT
GGTCCTGCGGTAACCCAAGCCCCACCCCCACCCTGCCCACGCCCATGCCCACCCCGTCGC
CCATCTCCAAGCCGACCTGCAACGGGTCGGACCCCTGCGTGACCATTCTCAAGGAAAACG
AGGAGGTGGACACCGAAAACATCGCCGTCACGGACGAAGAGAGCTCTGCCGCCGGCGCCG
CGTCCGCCGCGGCGGTGATGGTCGCCGCCGGGGCCGCCACCGCCCTCGTGCTCTAAACCC
CGCGGCCTTCTGTTCGGGATCCACGTGTCGTGCGCGTGGTTCCTTTGTTTGTTTATGGAA
TGGATTTATTCGTTCGTGCATTTATTCCTTCCACCATGCCGCCGAGCAGCAATCGAATTT
ACGGATTAGGGTTTCCAATGGATGCGTTGTTCGGCGAGATAGCATCGTTCATGCTGCAAC
TTTTACAAGAAGACGGATCATTGGACACAATCGACGGTTTCGATGGATTGTATTTCTTCT
CCGTTATCGGTATTCTATTGTACGTACGATGCCTGTTCACACCGTTTTTTGAAAACAAAA
GTAGGAAAGTACAATAAATGATTAACAAGTTGTATTCTACTAGAGTCAACAAAATACTGC
ATGGACTGCGTAGTCGACATCATACTGGGTTTTAGGCTATTGCTCATGCCTCATGGGGAT
AAATGCAAATCATCCCATGTGCTAATCCCTACATCTCTTCCATACCATTTTCTGTGGCGT
TAACGTAAATAGGCCAATAGGACCC
>comp2795_c0_seq1

TTCGGATTGAATAAGATATCCTGCGAATACCAACTTGAAACCTCTGTCATCAATCATCAA
CATCGTCCCCCGCATGGATTGATTTCTCCATATCAATTTCATCGACAAAACAGACAGCAA
GCCACCTAAAAACTTCCTATCTACTGAAGATTCATTAGTGATCATGCAGATGCAAGATTC
CCAGCTTAGGAATTGTTTGCGACAAGCATTTTTTGCTCCTCGGCTGCTGCTTCTCGTGCT
ACTAATATGTGGTATTGTTTCCAGCACGAACGCAAAACCAGGCCCGAATTCAATGTTGTT
GCGGGTAAGATTGGCTGATGGTTCAATGGAACGAGTCCAAGTCGAGGAAGGAAAGGAAGA
TACGTTGACACTAGAAGATGTTCTGAAACCATTTGGTGTCTCCGATGATGCATCCATCAC
CATTGGTACGAGTAAGGAAACTGCGATTAGGGATTCTTTCTTAGCGAATCTTGGCGTTCG
AAACGGAAGCATGATTACGGTCATGGCTCCCAAAGAGAAAGAATCTGGGAATGGTACTAC
CAAACCAACCAGTAGATTTGCTAGTCGCAAAGCTAGTGCGTCCAAGGTTGGAGGATGGAA
CCCCTACCCAGATCTTGCCAAGGACTACGACTCGGTGGTTATTGAAGCTCAAAAGAGACG
TGCCTCTCGACAGAGCATGTCATATGATGCTATTTCCAAACTCCAATCGTCTCTTCACGT
CGTCGATCCACAAAAAGAAGGGCCACTGCTACGGGTTTATATGTGTAGGGTTTCGGCGGA
AAGATTTTATTCCAACGGAGTTGTCACAGCATCCAAATCGAAAAAGAAACAGCAAGGCCC
AACATATAACTGCCGGGCCGGATTGTTGCTGGGGACAATCCAAAAGGAACGTGTCGACCA
ACGGCCCAAAAAGGCGAGGACCTCGCTTTCGAGTCAAACGGCTGATTCAGAATACTGCAA
GGTTGCCAAGATCCAGGCTATATGGGAGCCACCAGGACAGAAACCTTCATCTGATAAGCT
TTATGATGCCAAACTAGCTGCGTCTCTATTGGATAGCAGCAAAAAGCGGATGAAAACCAT
TGCCAACCATTTAGGTTTGGTGCCGGTGGGTTGGATTTTTTCGTATCAAGACGATCGATT
AAAAGAGGAGAACTCCTTTTCAACAGAAGGCCCTCAAGCTCTGTTTGGTTTGGATGTAAC
TATCGGTGCAAAGCTAAAGATTCAAAATATGGAGGACCGCCGGGCTATCAAAGAAGATGA
TGTGGATCTCCAGGGGGACCGATTCGTCACGCTGGCGATGGACGCCACTTCCGGCGCTAC
CGAGGCCTTCCAACTGAGCGACGTCTGTGTCCAAATGGTTCACGAGGACATGTTTCAAAA
GCTGACAGAAACTGACGGTACGTCGGAAATAACGAATCCACCTATCGTCCCCGTGCGCCA
TGCAGTTTTGGTCGATGGCCGGGAAACCAAGCTTCTCGATTCTGTCCTTTGTTTGGTCAA
TACTGCTCTCTTGAGTCACGTCGGGAGCTACAGTGGACACGTCACAGGCAGCTCCGTTTC
CGTAAAAAAGATTAATGGCTCTCTTACCAACAAGACTAAAAAATTGTTGCTCAAGGCCCT
CGAGTCGTCGGATGCGAACGACTTTTTGCAAGAAATTTGCGACTTCAATATCTTGATGGC
TCTGGACCAGTTGCTATCTCAATCGCAAGTCAAGTCCTCGAATAACGATGACCATGAAGC
TGATATGGAAATCCTGTGCGGCCTCGTTCGAAAATGGGCCCGAGGGCAAAAGAAAGGGAC
TGCGATCGATCCGAAATTCAAACGAAAACTACAGACGTATTTGAGTACATAGTTCAAGTT
ATCTTCTGGGAAAAGGAATTGATTTTTTAGGCAAATTAAATGATAAGCATTACAAAATTC
TTGGACTACAGAAACCAAAGTCTATAAAGTCTCACATCTTCCATCCCCTGGCCGAAAGCA
TTACTACCATCAAAACAATGACGTGTAGCTTGAGTATTGGAAGATTCATGATTATCACTA
CACACTTAAAGCGAACAATTTTTCGAAC
>comp2796_c0_seq1
CAGAGTTCGAATAAACAAAGTAGGAAGAATCAAGATGAATACCTTTTCCATTGCATGCAT
CCTTGCTGTTTTGGGAGTCGCCCATGGTTTCTCCATGACCATGAACGCTGCTGAGCGTAC
CTACATCATGATCAAACCTGATGGAGTTCAAAGAGGAATCGTCGGTAATATCATCAGCCG
ATTTGAAACCAAGGGTTACAAGTTGTCTGCCATGAAGACCAAGCAAGCAACCAAGGAGCT
TCTCGACGAACACTACAAAGATCTCGTCGATAAGCCTTTCTTCCCTGGTCTCAGAGATTA
CATGATGAGCGGCCCTGTTGTCTGCATGGTTTGGGAAGGAAAGGAAGCCGTCGTCACTGG
ACGAAAGATGTTGGGAGAAACTAACCCATTGGCCTCGAGCCCAGGAACCATCCGAGGAGA
TTTCTGTATCGAGGTGGGACGAAACATCTGCCACGGAAGTGACTGCGTTGAAAACGCCGA
GAAGGAAATTGCTCTTTGGTTCGACGATGATGAAGTGATTGACTGGACAAGCCACTCCAA
GGACTGGATTTACGAGTAAATACTCTGTCCTCCACTTTAGAATTTAAAAGGATAATACTA
CTTTCATTCTATTGGTGCG
>comp2797_c0_seq1
CTGTACCTGAGAGGGATCAAAGTGAAGGTGATATTTCACATGTTCTTTGAGAAATATCAC
GTCTTGATTATCAACAGAAGAAGTCTGCCTCGCTGTAGGAAGAAAAATAAGAGCATTCAA
TAATACGTAAACGATGTCCCGTCCCGAATTGACCGGCCACGCCTCAAACTTCTACAATGA
GAAAGAAGCGAGAAAGTACAACTCGAGCTCGCGTATGATCAACGTTCAGCGAGAGATCAC
ACTTCGTGCCATTGAATTGTTGCTACTGCCGCCAGATGAAACATCTCTTGTTCTAGATAT
TGGATGTGGAAGTGGCCTGAGTGGTGAGGTGTTGGAAGAGCAGGGGCACGTATGGGTTGG
CTGTGACGTTTCGAGAGATATGTTGCAAGTAGCGCACGAAAGAATGGAAGATTCTTATAA
AGTGAAAGACGACGGTGACAGCGGGATGAGCGATAACAGCGACGACGAAGACGATGACGG
ACTTCCATCCACAGGGGATCTCATGCACCACGACATGGGTACCGGACTTCCCTTCCGGCC
GGCAACCTTCGATGCGTGTGTGTCCATTTCAGCCCTCCAGTGGTTGTGCTATAGCAACAG
TGCATCTCAGATTCCTAAGAGGCGCCTGGTAAGATTCTTTTCAAGCCTGTACTCTATTCT
TAAAAAATCGGCACGTGCCGTGCTTCAATTCTATCCCGAAACCGCCGAACAGGCTGTGCT

GATCAGTGAATGCGCCACTGCCGTCGGCTTCGGTGGCGGAATCGTCGTCGACTATCCCAA
TTCCGCCAAGGCTAAGAAGCACTACCTAGTATTGAGCTGCGATCGTACTTGTCGAAAACA
GCTTCCAACTGGCATGACTGGTGACGAACGGGTTGAAAACAGCAATAGGCATGTTCAGGT
TTCGTCTTCGAGTTCTGGAAGAACGCAGAGAGAACGCGGGAAAGAAAAACCCATCAGAAA
AAAGAAGGGTGTCAAGACAAAGGAATGGATTTTGCACAAGAAAGAAACTCAAAGGAAAAA
GGGAAAGAACGTGCGTCCCGATACCAAATACACCGCTCGACGTCGACGCGGCAAGTTTTA
ATTGCGATAAAGCTGCTGATGTATTTGTTAGCATACTCCAGTTTTATATAATCGAGTCTT
CAGCTGCACGTAATGTTGTTGCTGGTACAGATAGTAGTATTGACTTTACAAAGCATCCTT
CCGTCAAACCACACACCGCTTCTGATGACGCAATGTGTAGTTTGCTACTAGTGGTCTCTG
CTGCAGCACTTTTCTTATAGCAGTGTTTTTGTTCCTGAAAAAACTCCATCTTTATAATAG
TACTAATAGTAGTACTACTACTTCTTTGAGATAAAACACTTGCAACACTAAAA
>comp2798_c0_seq1
CGATCGATCCTCCTGGACGAGCTGCGGCTGGTAGAAATGGAAACGGGAAACTCGACCCAC
TGGAACGACACCGAGGTCGTCGGGGATCCCTTCGGCCTCCCCGACGACGAGGAATCGCAG
TCCCAGCCGCGCGAGGCCCCCGACTACGAGTCGAGCGCCTCGAACCGCCCGGCTTCCGGG
GAAAGCACCGATGTGTCCATCCCGAGCGACGACCCCCGCAAGAGCATCGTTCACAAGCGA
CAAAACCTCACCATGGCAACGGATTCGGCAAAGTCGCTCCAGTCGAGCATAGACTTTGCC
GAGCTGCAGCTCCAGGAGGTCATCGGAGGGGGTGGCTTTGGTCAGGTCTGGAAGGCTACC
TGGAGGGGGACCCCGGTGGCCGTGAAGGTGCTGACGGGATCGGCACAGAACACCCACATT
GCAAAAGCCATACTGGAAGAATTCAAGGCCGAGATCAACCTCTTGAAGGGCATGCGCCAT
CCGAACATCTGCCTCTACATGGGTGCCTGCATGACTCCTCCCAACCGCGCCATCATCACC
GAGCTTGCTGCCAACGGTTCCGTCTGGGATTCCCTGCGGCTCCCCCTCATGCCGCCCTAC
ACCGCTTCCGACGGCACCCCCAACGGCTCGTGGCCGTTGAGCCTGTACCTTCCGAGCCAC
CACGGCGTCCCGCCGACGAGCAGCGGGAGCGTCCTCCGGGCCACGACGGAAATCTCCGCG
CCGATCCCACCGAGGGGGACCTGGCCGTGGGAACTCGTCAAGCGGGTCAGCTGCGGCGCG
GCCCGCGGCATGGCCTACCTCCACAGCGGCCACCCGCCCGTTCTGCACCGCGACCTGAAG
AGCGCCAACCTCCTGCTCGACGAGAGCTACACCACCAAGGTCTGCGACTTTGGCCTCAGC
CGGCTCAAGGCCCAGGCCCGCAGCATGACCGCGAACTGCGGGACGGTCCAGTGGATGGCC
CCCGAGGTCCTCGCCAACCGGAGCTACGACGAGCGGGCCGACGTCTACAGCTTTGGGATC
ATCGTCTGGGAGCTCTTGTCGAGGGAATGCCCCTACGAGGGAATGACGGCGATCCAGTGC
GCCCTTGCCGTCCTGAACCGGGACAAGCGGCCGGAGATCCCAAAGTGGTGCCCGCCCGGC
CTGCACGCGCTCATCAAGTCGTGCACCAAGAAAGACCCGTCCGAGCGGCCATCATTTGCG
GAAATCATCGCTGCCCTGGACGCCATGGGGTGAATGGGACGAGACGAAACGAATCGAAAC
GAAACGAAGTTGTTTGGTTGAAGATGGTTTCGGGGAGCCTTTCAATAGACTAAGAATAAT
TGCGTCCACTAATGTATTTGAAAAATGCCAGCAAACTAGATTGTTCTTGCCTTCGTGCCG
TTCTATTGTTCTACTAGGTAACCCATA
>comp2799_c0_seq1
GTACCGTACGGTGACGCTTCTTGTTGCAAATCCGGTGCACACTTCCAGTGAGTCCTTTCA
GTCTACTTTCCCTTGATTTCCTTTCTCTCAGAATTTCGATGACGACACTGTTGTTGCCGC
TGTATTTGACCCAGCGGTGATCTCTGTCAAGACCGACAAACTTCAGAGCAACAACGAAAA
AAGAGCCATTGTCTCACACAGAACGATGATTGGCGCTGCCCTTCTTGCTGTATCATTCGC
GTTAACACTTCCATTTGTTCGCGCATCCAATGATTGGATGTGGATAGAAACGCATCACTA
CACTGGTGAGCGGAGAGGACTACCCAGCAACGATTCTTACCTGTCAAGGAGGAGGCTTGA
AAAAGAAACAAGCATCGGCAGGGACAAAACAATTTCCGATACTTCCTATGACTTTCCCTT
TCAGAGTGCCGACATTGGTAGCAGTAGCAGCAGCAGAGACATTGGTTCCAAACAACACCA
ACGTCAAGCTTTTCCTTCTGAGACAGAATCCGGTAGGAGGAATTTTTTCTTTCCGATCGA
CTTTCGACGGCAGAAGGGTCGGCTAAACGTTGGGTTTTCGTTTGGTGACGACGATGGAGC
CCGAAAGCGCGGACAACGAAATCGGCCCCCTCGTCGAAAGTCCCTTACCAATGGCATCAA
GGATCGCAGCTTTCCACCGATGTCGATTGTATCTGGTAATTCGACCACGATACCTCCAGC
GATGGCATCCGGCGGGGACCCAAGTGAGGGAACTACCGCTACGACAGCGTTGGTGGAGAA
AACAAACCACGCTTTGGAAAAAAGACGAGCCACCTCTGCTACTCTGCAGCACGGAGATGC
TTTCAGGGCGAGATCGAACTACAACGACAGCAACGTCGAGCTTTCTCTCTCGCTCGATCC
GTTTTCTCTTGCGTCTCTGGTTCGAAACTTGGTGGCGAGCAGTGTGGGCGCGGGCTCGGT
TTATGTCGGAACGCTCAAGCTATTGGGGCCCATGATACTTGCCAAGCAATGCCTGGCGTC
GGTGGGATATGCCTTCTACGATTATTACAGCGGGCGATACCTGCCCCAAAAACAGCAGAA
ACGGGTGCGCTGCGTGCAGGAATACGAACTTATTGCCGCGGGAAGGGCAGCATTGCGAAG
CCTTTTGCAATTCCTTTGCATGGGCCTCGTTGGTCAGGTTGTAAGGCTCGTGCTCGACCC
CCACCACCGGAGTTGTACTCTAGGTCCGCCATGGCCTTGCCATTTATGGTATGGAGTGGT
GTGGCTGGCCTCGGTTTGCGCGGCTGGATGCGCGTGCGAAGAGTGGGGGTTCGATTTTCT
CGCGAAGAAGACCGGCAATAGATTCGCTCCCTCGTCTCTATCGGTCCTTTCAACTGCGAC

CGATGCCGGTCCGAGGGCTGACTTCTTCTTTTCATCCAAACATCACGTCCGCGAAGACTG
CTTGCGGGGGCGACGGAAGCGAAACGTGGTCGGGCCACAACGGCGGTTCGTAAAAGGACC
GAGCCGAGATCCAGAAAACTGGCTGAAGAGCATCATGTGCATTCTCCCGGACAGAGAGGA
TCCAATTCGTCCCCGGCAAAGTGGTACCAACAGCGGTATTTGCAGAAAAGTTTCTGGTGA
TGTCGATGTTCTCTTGTTCCCGTCTGTGTGGAAACCCTTGAAGTTTGTGATGTTTCTAGC
TTTTACCAAGGCTATCTACGAAAGTTTTTCGGATTCGCCGCTCAGAGCGTCTGAATCTGG
AAAACTCGATCTCGTACTGCGAACTTTTGTCCTTCAACAAACGTTGCACAGCGAATGGCA
CCGGCTGTTTGTGCAAGAACGGATGCTCTCATTGGGGGCTTGTGTGAGCGTGATCGGGCT
GGTGGCTCTGATATGGTCACTCTACGCGGTATCAATCGTCGATCGCACCGCCGCTCTCTT
GCTGACCCCATTCGCTGCAGCTCGGTTGATCACAAGCTACGTAAACTTTTTGGTCTCTTT
CGATCGATCGACTTTTATCGACAATGGCGTTGCTTTCTCCTGACGGTATTCGCACGGTCT
GACTTGGCATTTTGTGGGGTTACACAGCATTGTGTGAGGGCCAGTAGTTGCGGGAACTTT
CCTTTTTTGTCTCGGTACACATGATGCTGCTACTGTTGTCATATTTAGTAAAAGAGAGAC
TAATATTTACAGTGCTAGAATAACGTCTTCTACAAC
>comp2800_c0_seq1
GTTTTCGTTCTTACAACGCTGTCAAGGATTAACTTTCTAATCACAACCTTCCCAAACATA
AAAAGTACGACATGATGGTAGGGTGGACTTCGTCATTCCAGCTTCTGCTTTCGGCGATGG
TTGTAGCTACCCGAGCATCAGCGTTCACTGCTTCCTCGTCGCCGTTCGCCATTAGATCAT
CCGCCGCGTCTAGGACGAACAGTATGGCATCTGCGGGGGACACGCCAGAAACGCTTCCGG
AATTTGCCAACGCCGAAGATTATCTGAAATACTTAGGCACGGTTAGTCAATTGCCTCTTG
GATTTGCAACTGGAACAGCCGACGGGACGTTCATTTCCGTTGAAGCACCAGGATTGGGAA
ATTTGAAAATTCGAGCCACCGTCATTCAATTAACCGAGGGGCCTACGGAGAATTGGGCTG
CGTGTTTTACGTCCAACAAGTTCCCTGGAGCTCCCATTAAGGTGGGCCGAAAGCGTCTCG
AGGCTGGTGGTCCAATTGAAGCCCTCGTTATTAATAACAAGGTTTCCAATGTTTGCTCTG
GAGGTGACGGAGAAGCCGATGCTGAGATGGTATGCGAGGCAGTTGCAAAAGCGTTGAATC
TCGAATCAAAAGATTTGGTGTTACCTTCCAGTACTGGAGTCATTGGATGGCGTCTTCCCG
CAAAAGAATTGGCGACAGAAGTTGTTCCGGAAGCCATCAAGAACATGCAAACGGATTCTG
TGTTGAATGCTGCCGAGGCCATAATGACTACAGACAGATTTCCAAAGGTTCGAAGCATTA
CCCTTTCTAACGGAGCCAGGGTTGTTGGGATCGCCAAAGGTGCGGGAATGATCGAACCGA
ATATGGCAACCATGCTCTCTTATATATTAACTGACGCTACTGTGCCAAAGGAAACGCTTC
AGTCTATGTTGGGATCCGTTGTGAACGATACGTTCAATTGCATGTCCGTCGATGGTGATG
AATCGACTTCGGACACTGTCGTGTGCTTGGCATCAAATAAAATTTCAGAAGACATCGATC
TAAAGGAGTTTGAATCCGCCTTGAAAACTGTCAGTGAAGGACTTGCCGCCGACATTGTCC
GAAACGGAGAGGGGACCTCCCACGTGATGCGTGTTGTAATCAGCAACTTCCCCGGGACGA
AAAAAGATGCACGATTCCTTGGACGACACATTGTCAACTCTCCTCTTTTCAAATGTGCCG
TTGCTGGAAACGATCCCAACACAGGTCGATTGGCGGGTGCAATTGGATCGTACATGGGAA
AATACATGCCGGACGCAGACACTACCAAAATGTCGCTGACACTTGGAGGACGATCAATTT
TCACGAAAGGAAAATTTGTCTTGGAGGGCGACAAAGTGGAGAAAGAGCTGAGCAATCACA
TGAAGAATGCAGAATATGGTGATGACACCCATTTTCCTCCGCACCAGCGATTTGTAGAAA
TCGGGGTTGATTTTGGCATCGAGAATACCGACTTCGAGGATGTCAGCGTGCTGGGAAGTG
ATCTAACGAAAGAATATGTTGTGATCAATGCTGACTACAGAAGTTGATGGTAAACCACAT
GTATGCCGGACGTGTTCAAGACTTGAATACAAAAATTCAATGCTCGAACTACAAACATTT
TAAAATAGAAATATACACCTCGTTTTTCCGCAAAAGGGAACGTTCAAACGTTCTTACGAA
AATACTTCCAAGTCTATTTGGCTTCAGCAGCCTTTTTAAGCTTCTTCGCCTTCATTTTCT
GGATGGCCTCTTGCTTGATGACTTGTTTGAAAAGCCAAATCATCTCGCTTTTCTTTTCAG
ACAACTTTTCCACCTTGTCCTTCAGGTCTGCTTGTTTCTTCTCTAGAACCTGAACGTTGT
TGCCACCAGTAGAAGGTACGTTTCCTTCCCCTGC
>comp2800_c0_seq2
GTTTTCGTTCTTACAACGCTGTCAAGGATTAACTTTCTAATCACAACCTTCCCAAACATA
AAAAGTACGACATGATGGTAGGGTGGACTTCGTCATTCCAGCTTCTGCTTTCGGCGATGG
TTGTAGCTACCCGAGCATCAGCGTTCACTGCTTCCTCGTCGCCGTTCGCCATTAGATCAT
CCGCCGCGTCTAGGACGAACAGTATGGCATCTGCGGGGGACACGCCAGAAACGCTTCCGG
AATTTGCCAACGCCGAAGATTATCTGAAATACTTAGGCACGGTTAGTCAATTGCCTCTTG
GATTTGCAACTGGAACAGCCGACGGGACGTTCATTTCCGTTGAAGCACCAGGATTGGGAA
ATTTGAAAATTCGAGCCACCGTCATTCAATTAACCGAGGGGCCTACGGAGAATTGGGCTG
CGTGTTTTACGTCCAACAAGGTAGGTCTGAAACATCAGTACGCTTGATATGACACTTCAA
GTAAAATAAATGGCGAAATACAGTGTGCGCGACAAGTTGAAGATATATCCTTCTGATTGA
TTTCCAAATTTTTATTATCGTCTTTTGTTTGTCGACACTGACTTGCAGTTCCCTG
>comp2801_c0_seq1
CAAGCCGACTCTGTACGTCCGATCATACACCTCTGGAGCATCCTAGGGTACGTTGTTAAA

CATCGACAGGAGCAATAGAAACAACAATAGCAGTGGCAGCAGCTGCAGCAGTATATAGCA
GGATCGAAAACAGAAAGAATCGGTACAGAAGAAAGGAAAAAGGGAAAACACAACACCAAC
AAAAACAGCAAAAATACAAGAAGATAGAAACCATGAACGTTGTCAAGTCACGCAGCAGCA
ACCTGCCCCGGGCGTCGACATCACCGGACAAGAAGTTTTCAAAGGCTGTGACGAACATCA
ATCTATACCAAGAAATCCCGACCACGGAAGTTAGTCTAGACGATTTCGAGGAATTTGCGC
TCGATCGGCTGAAGGTTCTGAAAAAAATCGAGCAGCTCAAGATCCGTCACGTGCACCCAG
ACAACTACAAAAAACACCTCGATCCGCTGCTCTCGAAACTCAACGACCCCACCAAGGACA
CCATCTCCCACTTTGTGCTGAGGCTGGCCTACTGCCGATCGGAGGACCTGAGGCGGTGGA
TCCTGGCGCAGGAGGTAGCCCTGCTCAAGCACCGTCTCCACCACAGCAGGGTCGGAAACG
CCATCCCCAACGCCCTCCGATCGGTGCTGCCCAACGCCCATGTGATGACCAGGAGCGAGT
TGGAGCCCCTGCTGCCCAAGATCCTGGCGGCGACGCCGAGCCTGAACAAAAACCCCCTGT
CGGTGGCCAGCGGTGGCGAAGCCAGCGCGATCGGCCAGGCCATCTACCGCGTGCCCTTTG
CGCAGGCGCTGGACCTGGTCCAGCACCGCCAGGTCTTTGTCAAGGCGGGCAAGGCCTACA
TCCCGGAATCGAAGCTGCTCAATCTGGTGACGGCCCGGTTCCGGACAAATCTCTCGAGGC
AGCTGGCCCTCCTCAGCGCCGTCCCGTCCACACCCTCCCTCCAGAGGACCACGGGCTTTC
TGAAGAACGTGGCCACGGTGCACACCCAGCAAGAGGATTATTCTCGTACCAACAAGGACG
GACCCAACATGAACGCCCACAATGTGCCGAGCCACCTGAAGCACATGCCGCTGTGCATGG
CGCAGCTGCAGATGGCACTCGGAACCGAAAAGCACCTGAAGCACTGGGGGCGCCTGCAGT
ACGGCCTGTTTCTGAAGGGGGGCGGGATTGTCTCTAGACGATGCCCTCACGTATTTCGAGC
GCATGTTTGTCGGAAAGGACTTCAACAAGGAATATTCCTACAACTTCCGGCACATGTACG
GGAAGGAGGGAAAGCGCCAGTCCTACCCCCCCTACACGTGCTCGAAGATCATCAACAGCA
ACGCACCGAACGCAAACGAGCACCACGGCTGTCCCTACAAGCACTCGAGCGTGCAGGACC
TCTCGAGGATGCTCTCGAGGCTGGGGGTGGCCCCGAGCCAGCAGAAATCGATCGTCGCCC
AGAAACAGTCCCACAACTACCAGCTGGCCTGCGTCGAGCACTTCAAGGCCGTCCACCCGG
GCGTGACCCCGTCGGAGAGCAACTTCGGGAACCACCCCAACGCCTGGTTTGCCTCCAGCG
TCAAGGCAGCCGCGGTGAGTGGCGGCGGCACCACCTCGAGCGAACCGGGCTCGCCGTCGT
CTCCGGGCGACGCGACGATGAAGGACGCCGATCCCGTATCGGTGTCGCCGTGATACAGTG
CCGTTTAAGAATCAGATGAATGGGAGAAAACCATGGAGTGGGCACATGCCAAACGAATTA
ATTAGGTTCTACACTTCAAGCAAAGGAAGCAGCGGGATAATGAAATCGGAAATCGTTTCA
ATCCATTGACTACAATGGTGTGGGTTAGGTTGTAATGAAATAATATTATTAAAAGCTGCA
AGCATCAC
>comp2802_c0_seq1
AAAATAAAAGTACAACTACAAAGGGAATACAACGAAACTGAGAACCGACAGCGGGATTGC
GTTGCTCCTTCGTGTAATGTTTAATGTATGAGTCCTAATATTTTGTAACGATGAGTATGG
CGAATGCGAAGGAGGCCTCTTCGACCGGGACTTCGGACGATCCAATTGCTTTACTCGACG
ACGACAACGACGACGACGATGATGGCACGGTAGCTTCGATTGGGGCGGTGAGAACCAACA
CGAACGAAAGGAAAAGATCGGCGTCTCCCGGGAGAGAGTGCAGCTCTTTGGAGTCGGTTC
CACGCAAACGATCCGTCCGAGGAAACCGCGCCGTTTCTCCCCAAGAGAGGGCGATTCTTC
CACATGACGATGGCTGCATGCTGAACCCGCGCCACCCCATCAAAATATTTGCGACCGATC
GAGACAAGACCACGCGGCGAAACCTTAGGCGTCGCTTGAAGAAAGGCGAGGTCGACAGTG
ACAAAGATGGCTACCACTATACTAACACTGAAGATGCATATCGTACCCATTGGTCGGTAT
TTCACTGCTGGACCTTTCGCGAAATGTTGGGATTCGACCGCTTTTCGGGAATTCGGTATC
AAACCCAGGCAGATCTCATTGGCAGTGACACATTTCCCGGTGCTAAGACCAACAAAATTA
GCCATGGGAATCCTGTGGGTATCGATTGGATCTTTATCACGACCTACATACTTGATGTGG
ACTATCTGCTTCACGAACTCCCGGAGTTGCTCGATATCCCGCTCGTCGTCATTGTCTACC
AATACAAGGATTTCAGTTCGAGAGAAGAGGCATGGATCAAGGAGGTTTCCTCAAAGAAAG
AACACAACCTAGCTCTGGTTGAGCGGAATCCACGGGACCCACCCCGAACCGAATCCAATC
CCCTGAAGGTGCGAATGCCCTATGGCTGTCACCATACCAAAATGACGCTGGTTGGCTATT
CCTCGGGGCGCCTGCGGGTTCACATCCACACCTCGAATTTACGGCAGGGAGATGTTCACG
ACAAGTGCCAAGGGGCATTCCTCCAAGACTTTCTGCCGAAAACCGAGGACCAGCTGTGCA
ATTTTGTCACCTCTGACTTTGAGGAAAGCCTCGTGACGTATCTGGAATCTTACCACTTTG
TTCTCCCATGGGTATGGAAGAAAGCTGAAATCGGACGCGGTGAGGGAAGCGAGGCAACCA
CTCTTGTCTCAAACATACAAACCTATGATTTTTCTAGCTCCGTGGGGGTGCTGGTTCCAT
CTGTTCCGGGTTATCACAATGCGTCTCTAGATGCACGACCTTCACATCGAGGAAACAGGG
AAATCTATGGCTATCTCAAGGTCCACAAAGCGATACGGGAACACTGTAACTGCGACAGTA
GCAGTGCTGGTCCAATTGTGTGCCAGTTTTCTTCCATGGGGGCTCTATGGGAGCCGTATC
TCGACAAGGTAGCCGCAGCCTGGAACGCAGGTGCAGCCAACTGTGGTTCGAAGACCGTGG
CGACGTCGCGGCCTCCAAAAAAGGGGGAATTGGCGGCTTCCTGGCTGCGATTTGTCTGGC
CCACAACTTTAGAAGTCAAAGAGAGCGTGGAGGGACCGATGGGGGGTAATTCGGTTCCAG
GGACGGCAAAGAATCTCCACAAGACGTTTGTCCGCTCCCTTTTGCACAAGTGGAGGTTTC

TTTCCAGTAATAATGGTGGTAGCACCACCGGTATGTACGGGGAAAACCTCGATAGCACCG
ATTCCAACCAACGACGTTGGAAAGAGGAGGATCCACTCGACAAGGGATGCCACGTCCCGC
ACATCAAATCGTACTACCAAATTCATGAGCTTGGTGACAGGTACGGGCGAGATAGTATGC
TCTGGTTTGTCCTGTCCTCGCACAACATGTCCGTGGCGGCCTGGGGACAGATTCAAAACC
GTCAAAAGGACCTCGGGAGGAAAGTACTGACCGTTCAGCACTGGGAGCTGGGCATTTTTT
TGGCTCCTTCCACGCTGGGAGTTCGTTCCATGGGACCGACATTGGAGGGATCCAGGCACC
GGCAAGCATACAACTGCAAAACTGCCAACGATGATCCCATCAACGAAGCAACCCGGGCAG
TAATTCCCCTGCCATTCAAATTCCGACCAATCAAATACCAAGATTCTGATAAACCATGGG
TTGTGGGGTAGAAAACCGCATGCACAATGAAATTGGGCTGAATTTTTTCTTGATTTGGTC
TCTGTTATAGTCAAATCCACGGAACAAAGTGGTAGTAAGTGCTGCTACTATTTATAACAA
TAAGGCAGTACACCACAATAGTGAGCAAATATGACTGTTTGTGAAGAATGATTGTGAATA
CTGCTACTACTACTATACCGCTAGTACTACTAGGTATTCCTACTACCATAGTATCACACC
ACCATGATCATTGTCCTCATCTAATACTTGCGCATCCAATTCTAGGATATGATATAAACT
TGTGTTTCAGTTTTTTCTCAATTTGATATTCAGCAGGGATACAACTTTGCTAATAATCAT
CCAACCATCTCATTGTATTACTTCTTGTTTAAAACTCCTTTGTTCAGAGATTTGTCCACC
ACACTACACCAAGAACAAATAGAAACTACTACTACTTCTAATCTAAATCTTATGCAAGAA
GAAATGTGATTCTCACAAAGAAAAAGCAGTTTCCATGTATCATGTCCATGATGAAGTGGT
GCAACATGAAGTACATTCATTCCTACTTGTGATCCACGTAAAAATAATCCAATTATCAT
>comp2803_c0_seq1
TGCACCGTCTGCACCGTCTGCACCACCCACACCAAATCACCGGACCACTCCAACCAAAGA
ATACTTTCACGGATTGTTTCCTCTCACAACAGATTGCGAACACAACCATGATGTCGACAA
GTGCAAGCAATCGGTTCGCGAAGTCTTTGCTGCTGAACGCGTTGCATCGATCGTCCCCAC
GAAAGCCCCTTCGGCATTCACTCGCGTCTTTCTCTGCCCTCGAATATGCTCCAGGAGCTG
AAAGAAGCGATGTTGCACGCAACAGAGGACTCGGACTCGGCTATTCCTTTTATTCAACAA
CCCGTCACCTAGCCGGGAAGAAGAGAATCGAAAAAGAATTCGAGTACGAACCCCTCTTCC
AGTCGAATGTTTACGATACGAGCTTGGAAGCAAACGCGGAGACAGAATTCGTGCAAATCT
TGCCACCCGAGGGACACGTCGAAACCGTCGTCGTGGACGGAGAAGAGTACCTGCGCCTCG
TCGATCCAAAACGCACGCTGTCCGAGTTGTCCGAAAGAGCCTTTGGAGACATTGCACACC
TCTTGCGTCCGAAGCACCTGGGCCAGTTGCGATCCATCCTGGAAGATCCCGAAGCCTCCA
AAAACGACAAGTTCGTGGCTCTGGAACTCTTGCGAAACGCCGACATTGCCTCCGGAAGGA
TCCTGCCGGGATGCCAGGACACCGGTACCGCTATCGTTAGCGCGAAACGTGGCCACCGAG
TCTTGACCGACGGTGACGACGAAGAATACCTCAGTTTGGGGGCCTACCAAGCCTACAAGA
ACCTGAATCTGAGGTACTCGCAAGTTGCGCCCCTGGATATGTTCAAGGAGAAAAACACCG
GAACAAACCTGCCGGCACAGATCGATGTTGCGGCTGCCAAGGGAGACGAATACAAATTCT
TGTTCATGGCAAAAGGAGGTGGTTCGGCCAACAAGACGTTTTTGTACCAGCAGACGAAAG
CTCTTCTAAACCCAGGGAGTCTCGAGGAATTCTTGGACAACACGATCAAAAGCATTGGAA
CCTCCGCTTGTCCCCCCTATCACCTGGCAATTGTCGTCGGAGGATTGTCGGCCGAACTCA
ATCTGAAGGCAGTCAAGATGGCATCCACCAAGTTTTACGACGATCTTCCCACGACCGGAT
CGGAGGGCGGAAGGGCCTATCGCGACGTTGAGTGGGAAAAACACATCCTGAAAATGACGC
GGAACATGGGAATCGGGGCACAATTTGGTGGCAAGTACTTTTGTCACGATGTTCGCGTGA
TACGCTTGCCACGCCACGGTGCCAGTTGCCCCGTCGGAATCGGTGTGAGCTGTTCGGCCG
ATCGACAAATTCTTGCCAAAATCAACAAGGATGGGGTATTCGTCGAGCGTCTGGAAACAG
ATGTCGCCAAGTACATCCCGGACGTCACGGAATCCGATCTGGCGAACGACGACGACAACG
ACACGGAGGCTGTGTCGATCGATCTCGATTCCATGTCCATGACTGAATTGCGGGAAACCC
TAGCCAAGTATCCAACGAAAACCAGGTTGAGCCTGACGGGAACCATCGTGGTGGCACGAG
ACATTGCCCACGCCAAAATGTTAACAAAAATTGAGTCGGGCGAGGGGTTGCCCGACTATG
CCAAGAACAATATTATTTATTACGCTGGGCCCGCCAAGACTCCCGAGGGATACGCTTCCG
GATCCTTCGGTCCGACTACCGCCGGAAGAATGGACGCCTACGTCAAAAAATTCATGGAAA
ATGGTGGAAGTTTTGTGACCCTGGCAAAAGGAAATCGGAGCAAGGCTGTCACCAATGCAT
GCAAAAAGTACGGTGGGTTTTACCTGGGATCGATCGGCGGTCCCGCCGCTATCTTGGCCA
AAAATTGCATTAAGAATGTGGAGGTTCTCGACAACGAAGAGGACGGGATGGAAGCCGTTT
GGAAGATAGATGTCGTCGATTTTCCCGCCTTTATTGTTGTCGATGACAAGGGTGACGATT
TTTTCAAAGAGTGGTTGGGTTGAAAAATGGAATAAGGAATCCATTGACTTGCTGAAGTAA
GATTTCAACAGGCTAGAATCTATTTTCAATGATAAATATAACTTTACAACTTGAGCAACA
TTTGGG
>comp2804_c0_seq1
CGACGACCCGCATACCATTGCTCCTGTATTCGTTTGGTGCACTGATAAAACACACTACTG
CAGCATTCTTCCATTCGATCGATCTCGCTTTTCTTTTCCAAAGGAAGTAAAATGTCGTTC
AGGCAGACCTCGTACCGGAGAAGCGCGGTTTCGCTATGGAAGGTGATTTTCAGGCTCGAG
GGAAACATCATGTTTGCTGTCCTTCCTCTGTGTGTGATCAACTGCCTGTTACTGGCCCTA

GTATCGTACAGCCGAGAGAAATTTCGCTTGGGATTTTCACCGAACGGCCACGGGCTCCTG
ACACTGCTGGTCTCCTTTTTGGTTGTATCGAAGGTCAACCTGGCCTACGATCGGTACCGG
GCCGTTCGGGAATACGCCGGCCGTGGATCCACGCTGCTCAGAGAACTGATACAAATGGCA
ATGGCCATATCCATCGCACACAGCAATAACAATATCGAAAACAGCGAGAGGGAAAAAGAA
CTCCGACAGTGGAGATTGGAATGCGTAGAAAAGGTAAAGGAGTTGTTGAATTCTTCGGTG
TGCGTTCTGCAGGACCGATCGCTGGCCAAGCACTTTGCCTACAACAAGCCCGTGACGAAA
AGCGGACCCTCGATTCTCACATCGAGCAGCAGCTACGATTCTAGAACCGTCGACCTCAAT
GGTGGGTCACGAGACGAGGATGCCGAGACAATGCTTCTGGATCCCCTGCTGCAGATCCAA
TCACTGCGACTGCATCTCTATTGCACCCCGGCCATGGGGTTCGAGCTTCTAGAACGGGTC
CACCTGACGAACAAGCTCCAGGAATACGCAACATGCTACACCTCGCTGCTCACGCTGGCA
TCGACTCAGCTTCCTTTTCCGCTGGTGCAAATGGGGAGGGCCTTTCTGTTGATATGGACA
TTCTCGATGCCCCTTGCTTCTAGAAGGGCCCTTTTCGGACATGTGGTCGGCCCAGACCTT
TTTGTTCTTCCTCACCTATGGCTTTATCGGTCTGGAGCTGGTATCCATCAAGCTGTCGGA
TCCCTTCGGAAGTGGGCGGGACGACGTGCCGATTTCGGGGATCCGCGACGCGGCGATCCA
GGGCATCGATAACGACCTGAGGGAAATAGCGGGGATGCAGGCCACTATCAGCGAGCGGCG
CTTGAAGTTTTCCCGGCAAACAATAAGGCAGCGTCGGCACCAATCACACGCACAGGAGAC
AGAGGCTGGGGATTGTCAGGACCACCACCACGACGACCAAAACTATGGCTACCACGCAAT
GGCCGGAGCCGACAATGATATCGCCTGAAAAACGGCACTCGACAAACCGAAGAATCTGGT
AACATTGACAATAGACCACAGCACGCAAGCATAAAAGCATTGATTGTTTGGGAGCTTGCA
AAAGAGAATTGTTTGTTTTCATTCCGCTGTCCTCGCAGTGCGCAAAAAATATTACTTCAT
TCTAGATTATCGATCTTCGAGCTCGAAGCCTTCCTCCTGCTGCTGGTGAATGCCTCTGTC
AAAATACTGACGTCCAAAAATGATTGAAACGTCCCCACTAAAGGAAGTATCCAGTAGTTG
AGCCTACAAGTATGTGAATGGATATCTTTAGATTTAGATGAAATAAAAGGCTAGATTTGT
CACTGAAAATAATATCCATCTGTAGTGCGATAACTCACCGGAGGACTTCTGAGGAACTAA
TTCGTGGGTAGTAAATGTGAGGTAGTACACACGC
>comp2805_c0_seq1
GATCCTCAGCAACACCCGTCACCAGTAATCGCCGGTTGCTCGTTCTGTTAGCAATGGTTG
TCGTACCCGTACAAGCATTTATTGGAGGCAGTCGTGTTGCTCTTCGCTCGGCATTGCCAT
TCACGTCCACCACAGCATCCTTTTCGGCAACAGTGGGATGCCACCACCCTCGGTTGCATT
CGAACGATTCCAATTCCAACAGTTTCTCGGATTGCAAAAGCTTTGGTGCGGCAATGGCGC
GATGGCATTCTACCAGTGGTCGGAGAACCACCTCGCTGTTCGAATCGGTGGCAAATGGTT
ACTATCGTAATACCAATAGCGATGAAATCCCACTGGAAAAGCTCTTGCAAAAGGCTTCTG
GCATCGTTCGGGACTACAGCAGAAAAAAACCGCAATAGCGATTCTGATTCACCAGCAGTAT
TTTCGCTCGAGGCCCTCGGAACCCAAGCAGTATACGAAAACACGCTGTTCTTCCCTTCGT
TTTACAATGCCGGTGTTGGCGCCAACAAAAAATCATCGCCATCATCGTCCACCACTGGGA
TGACGGAGCGTGGGTTTTCCAACTGGATCCTTCCCGGGAAAATCATGGTGGGGCAATACC
CTGGAAGGGTTCCGGAGAAGAACACCCCGACCGAAACAGAGGTACGGGCGCACCTTGGAG
AAGTCCTGGAGGCCTCGAGACGTAGCGATGCCACAACCAATGGCGGGGTTTGTTTCGTGA
GCCTACAATCGGAACTCCCGCCGCAGGACGACTACGATAGCTGGAAGGCCAGCGGCGGCG
AGATCTACCTCGAGGGTGACGCCCGGAGCGAGTGGCCGAACCCTTTTTCGCACTACGCGC
CTACGGTGGAGTGGGTTCGTTCCCGAATGGAAACGGACGGCGACGGGGACTCTGCAGTGG
AGGTCCAATACCTGCACCACCCCATTCGGGATTTGTCGGTTCCTTCCAATTCCAAGCATT
TACGGCTCTTACTCTGGAAGATACTAGAGGTTCTAGATAATAGGGAAAGCGATGGCAGCA
ACAGCGATAGCATGGTATACATTCATTGCTGGGGCGGCCGGGGCCGGGCGGGCCTGACCG
CCTGCTGCCTCTTGGCGCTCCTGCACTTTGCCTCCGAGGGGGGCACGGACGGCGGCGGCA
ACCACGACGACAACCTCAGTGCGATCTTCGACATTGTCCAAACCGGGTACGACTCCCGGC
TGGGATCCGAGCACATGCCACTCGCTCTCTCCAGGTCCCCGCAGACCGATTCCCAGCGAG
CCTTTGTGGCCGGCTTTTACGGGGAGGTCCGAGCCGCCGCGGCCGAAGCGGGGAAGAACG
GCAACGCAAAATGAACCGAACCGAAGTTGAACCGAACCCAAATGGATGGAACAAACGAAT
AGAATAGATCGATGGCTACAAAAAATACGAACCGCCGCATTACCTGTGCTGGCAATCGCA
AGTACAACCCATAAGAAACGCACACGTGAGTAAACATAAG
>comp2806_c0_seq1
GGCAGATCGTCGCGTTCGCCGTCTCGGAAAGAACCAGCGCGGCAAAGGAACGCCCACCCA
CCCGCGGCAAAGGCAACCCGGCTCACCCCTTCCTTGTTGTGTTGTGTTGTGTTGTGCTGT
GTTGTGTTGTTCGTTCCCGCCCCGCCCCGAACCGGATCGCACGGACCCCCCGCGCGCCCA
GATCACGTACTTTCGGTACTGCCGCGACGCCATGCTGGAGGAGGGGGCCAGGTCCCACCT
CGACGCCTCGGAGCACTACCGGGAGGCCCGGGAATTCGCCCGGTGGTCGGCCGAGGACCG
GGCCAGCAGGCGCGCCGACCTGCCCCCGCTGACCCCCGAGCAGCGGGAGCAGATGAGGAA
CTACCTGCGCATCATGCAGGAATACCACGCCTTCAACCCCGACTCGGACCTCGATCCGGC
CACCGGGAAGCCACGGTGACGCAAGCGGTCCAAAGGAAAGCATCGGAACACGGACTCAGA

GGGATTTCCGGTGCGGAACACGGTGCGTTGCCAGAAACGAACCGAACCGAGCGACGGCGT
TGTGTTTTGCTCGCAAAAAGGAGGGAAGAACGGGTACAGGTGGATGGTTGGGTCTCGCAC
AAGCGAGAGAAGATCGTAATAGACACGATTCGAGGAGGATCGATTTCCTCTCCGCTCTTA
CGGTACCCATACTTATCGAGCAAGAGTATACGTATATCCTGTAGAACGTAATTATTGGTG
CGAGAAGCATAGTCCACCGTCGAGACGTGAGGTGTACATGCAGTGCTCATTCTTCCTACT
ACTACAACTAATAGATGAATGTATTTTTGAGCAGAA
>comp2806_c0_seq2
ACCGGTTCGAAGTGGAGCACAGCAGAATCGAACCACACAACCGTCCGACGAGGAAACGAC
ACTCTCCGCAAGCGCCCCATCCATCCAAAGCAACAGAAAACAACGCGACACTTCTTTGCT
TCGGATCGAAAGAACCGAGGGACACCGATGGAACCGAGCGAGATTCGGACGGGAACATAG
AAAAAGGGAGAACCGATCTAGTTCGCAACCGCAATCGAAAACCGAACGAACCCGGTGGAT
TCGTCCTCGGGCTCTCGACGAGAACCGAACAGGTGTTCCGGTAGCAACAAAAGCGCAGAA
GCAGCGAATTTCTGCGACGAAACAAACAAACAAATAAACCGAACCAAACCGAAACGAACA
TGTTTCAGAACTTTGGAAGGCTCTCTGCCAACCAGAGGGTGTGGGGTGCCATCGGAGGCG
TCATTGTTGTGGGAACCGCATTCAAGATCACGTACTTTCGGTACTGCCGCGACGCCATGC
TGGAGGAGGGGGCCAGGTCCCACCTCGACGCCTCGGAGCACTACCGGGAGGCCCGGGAAT
TCGCCCGGTGGTCGGCCGAGGACCGGGCCAGCAGGCGCGCCGACCTGCCCCCGCTGACCC
CCGAGCAGCGGGAGCAGATGAGGAACTACCTGCGCATCATGCAGGAATACCACGCCTTCA
ACCCCGACTCGGACCTCGATCCGGCCACCGGGAAGCCACGGTGACGCAAGCGGTCCAAAG
GAAAGCATCGGAACACGGACTCAGAGGGATTTCCGGTGCGGAACACGGTGCGTTGCCAGA
AACGAACCGAACCGAGCGACGGCGTTGTGTTTTGCTCGCAAAAAGGAGGGAAGAACGGGT
ACAGGTGGATGGTTGGGTCTCGCACAAGCGAGAGAAGATCGTAATAGACACGATTCGAGG
AGGATCGATTTCCTCTCCGCTCTTACGGTACCCATACTTATCGAGCAAGAGTATACGTAT
ATCCTGTAGAACGTAATTATTGGTGCGAGAAGCATAGTCCACCGTCGAGACGTGAGGTGT
ACATGCAGTGCTCATTCTTCCTACTACTACAACTAATAGATGAATGTATTTTTGAGCAGA
A
>comp2807_c0_seq1
GACAAAGGCATAAAGCGCAAACGATTCTGCCATCAAGAGAGCTTCGTCGCACAAAACCAC
GTTAACAAGATGGCAACAACAATGGGCGAAGCCTGCGACAGTCTATTGATTGGATCCTTC
TATTTCATCGGTATCAATGCATGGCCTACCGTACTCGATGATCTCACTAACAGCACCACA
CAACGACGCGGTGAGGTTTTGATGTTTGGATTTCAGGATATTCCGGGAGGACTCGATTTG
TACTTGACAGACCGAGCCTGGACAGACGAAGGATTTGTTCCCAATTTCGTTGGGAAAGAG
GGCATCCTCAAGTTTACGACTCCGCCACAAGGTGTGCCGTCTGGTGTTGCTTTCGGAATT
GGGAACAATACCCAGATTTATAAATACGCAGAGGAATGGATGGATGAGGACGTAGATGGA
ACACAGGCGGAGGTTGGTGGCAACGAAACACACTACTTCGATTTGGGATTGGACGGCGAC
CAGGTGTTTTTGTACTGTGTCGGCGGTGACGGGAAAGATCGTCCCATTGCAGGGATTTCT
TACAACGGGGACTTTGCCTACCAAGACACCTATGGTACTAACAAGAGTTCCGCGCCCGGA
TCTTTTCAAGAGCATGACACCGAAAGCGGTGCGAACCGCACGACGTTGTTGGTTATGCCC
ACCGTCGCAACCGAAGACGAAACAAGCAAAATTTTCTTGGACTGGCAATACGTTGGCCCC
ATGAGCAGCCTCAACTTTTTCGAATTGCAAGACGCCATTCGCGACACCGAAAACAATTGG
CTCGGAAGAAACCCAGACGGGACGACATCGGGTTCGCCGTCAAACACATGCGGAACAATG
GTGGTGCCAAACGTTCTCGCTTTCTTACTTTTTTGCTTTCTTGCCCTAAAGTAGAAGCAC
AAGCAGTAATTTCGATGACGAACTCGTACTAGTTGTTTTCATTTGATTTTGGTCGGCGCA
TTTCAGATACGATCCCTTTGGGAGAAGGTGTGTTCAAAGCCGACAGATGCACCCAGCAGT
TCCGGCTTACCAACTTTGCAAGAAAATTGGCAGGGAAAAAAATGCGTCGGTTGCTTTTTT
GCATATGATTATGTCCTACCGCATTCGCTTTGGTATTCAATGTATCTGAAAAGTGTATTT
CTCAGATACTTAGATCTTATTCGATCGCATCAGTTGTGCACAATGGAAGCTATCAATGTC
TAAACATAGAGAGAATTCACCTACAAGGAATTCAAGCCACTATCATGTAAAGTACAAAGT
ATTTTGTACTTCTTAATTGTCAAAACAAGAACCAACACAATCCCATTGTCCGTCAAAAAC
ATTTTTTTCTTAGGGACTGGTTGCTTGAATCATATCTTGTAGTTTAGCAGCATACAAAGA
ACTCATCACAACTTTGTTGTGAATCTTATTCACTGAGCTGCTTCCCACTACTAGTAGTAC
CTGTAAAGTATAATATCTATGAGCAGTGTCAATTAGTAGTATGATGAAAACATGGTACTA
CTAGTATGATATGTGGTCTTTAGACAGCAGCCCCACATCCTTCTTGATTTTTTTGTGTCT
TCTTCAATTGAATGCAATGGCATAAAAGCTAAGAAATCAGGAAAGGTGTTTGAAACCCAT
TCCTATCAGCAATAGTACTACTTCTAGTACTAGTACTAGTACTAGCAAACCTTTTTCTTA
TGAAAGAAGAATACATTGTTGCTTGTTGTTCCTAGTACTACTACTAATTGATTCAAGTAC
ACCTCTCCTTGCCC
>comp2808_c0_seq1
CAAGCTCGCAACGTATTTCCAACGGCAGCAAGAATTGGGCTGGGAAAAAAAGACTTGTCA
AAACACAAGGAACGTTCCAGAAGCCATAATTATTGAACATAGCTACCAAACGACGCAGCA

CAATAGAGAACCGGAAGCACGAGAGGAGTACATTAATAGAGCACCACAACAATATCTTGT
TTGATCTTGTTGAACACGTAAACCTTAGGTCCCAAAACAGCGAGAGACAGAGAGAGAACC
AGTGTGTGCGTATAGGAAAGAGAGGGAGAGAGAGACAGAGAGAATGCATGTTGCCCCCCC
CCCCGCTCTGGTGATTTAGATACAATTCTCTGTATTCATATTAATTTTGCGTGGGACCAG
TGTTCCAACATCAGCGCCTAGCTCTAGAACATGTCTCCTGCGATGACCGACGCAGATGAA
ATTCTCTCTTATGCAACAGGGACCAGTAGTTTGAGCAGCAGTGGCAAATTTGCAACGCAC
GATGTCTATGACGAACATTTAGAGGATATTCACGCCAATCTGAGCAAGCGGGAGTCTATT
CGTACCTCGCTAATTTCAAATAGGAACAATAGAGTGTCCAACGGAAAACAAGATCCTTAT
GACGAACTTGAACAGGACATCAGGGAAACGGTTCGTTCAAGCACAAAACAAAGCAGGAGC
AATTTGCCGTCCCACAATGGCAGAAGGAACAGCAACAATTCAAATAAAGAGCAAAGGGAC
AAAAAGACGAAGAGTTCCGATCAAAAATCGAGTCATTCGGGCAGAACACGATCCAAGATG
CGGTCTTCTGGGAATGAATCGTGTGCGAGCACCGTGGCATCTTCAATGTCGATGCTTTCC
AAGGATGATAGCGAGAATAAAGATACAGGCCATCGTCGAAATCGTAAAATGGAACGCACG
AGTTCTGGTCGACGCATAAGGAGTAATAGCGACATCAATCGATTGCTACAATACGACGAG
ACCGGCGATTACTCAACCGAAAAGAGCAGAAGAGGATCGTCCACTCGTAGGGGAGGAAAA
AATATTCGGAGCTCTAGTCGCAGCGGACACCGGTCCCTGGCTGGAAGTGTCAGAAACAGG
ACCCAATCATGTGAAAGAAGAGTGGGACGGTCGACTTCCGAGTCGGTTCCAAAAGTGGTC
TTGGACGACAGTAACCGCGATGATTTGGATAAACCCAGGCGACGGTCGTCGTCGCAGCAG
CACTTTCAATCCAATCTCAAGAACAGAAGACGTTCAGACGAGCGCAGGAACGATGATCGA
TATGGCAAAAGCAGCTCGAGCCTTCGTTCAATTCGATCGATTCAGAGGCGACAATCGGAC
CAGGAGAATAAAGAAAAAAGAAGAGAAAGAGGCAAAAGTTTGGATGCGTTGGATAAGTTG
CAAAGAGATTCCTTTGAAAAAGCTTGGGCAGATGATCGTCCTATCCTGGGTCAACGGAGC
AGGAGCGAGCGAGAAATATCAAACAAGTCCTTTTCTCGAATTGAAGGCGTCAAAGGTGAT
TGTTCGACAAGAAGTGGTACTTTCCCAGCGGATAAAGGTAAAAAGACTAAGCTCGAAAAA
ATTCACGAGCTTCAGGGGAAATGCGACCTCTACAAAGCAGAACTAAGAAAATTGACGGAT
GACAGGCAAAGATATCGTCGAAAGTTAGATAATAGTCGTGAAGAAGTAATCTCTCTAAAG
AAGATCGTGGACGAGTACGAGGGAGATATCACAGCGCTGCAAACAAAACTTGCTGATGTA
CAAGATACATTACAAATGACGAGGAGTGCACAGCGAAGCGAGCTTACGGAACTCTCTGAT
GTTGCGAAGGAGTTGGCAAGGGTCAACATTGATTACGCAAAATCAGTTGAAGAAGCTCGA
AGCACAAAAACTGAACTAGAAGGCGTGAAACAAAAACTGGCGGATCGAGAGAAAAAAATT
GCTGAACTTGAGGAGGAATTGCGCAAATCCGTAGAAAACGTGAAGCAGTTCGAGGCCGAT
GTGTTGTACGCGGATGAACACATAGACAAGCTCGAATCGGAGGTTAAACGCCTGGAGATA
GACCTGACTACATACAAGGACGCTGCCGAACGAGATTCAGTTTTGAATTCCGATGGGAAT
CAAAACGGAGATCATCTACGAAGGGCGAAAGAAGAAGCTGAAAAACGGAAATTTGAAGAA
CAGGAAAATCGCATAAAAGAAAGGAATAGGATTTTAGAAGAACAAAAGCAAGAATTCGAA
GCAGAGAGAAAGCGATGCTATGAAGAACAAAGGAAGAAAGAACGCGATTTTGAAGAACAA
CGAGCGCGAGAAGATGATAGGTTGAAGATTGCAGAAGCAGATCAGCAAAGAAAGGAAGAA
AAAATGAACAAAATCTTGAAAACCTTGGAAGATGAAAATGCTGCATTAAAAGGAAGATTG
AAGGGCGAACAGTTGGATTCTACCATCAAATTACAGAAGAAGGATAATGCTATTGGCGAA
CTGCAAAAAGAAGTAGCACGACTCACTAAGGAACAAAAACATCATGATTCTGCCCCTAAT
AATTCACCCGCTCTCCTCCAAGAAATTGAAAGGCTGAAAGCTGCCGCCGCGGTTAAAGAG
GTAGACTTTGAAGATGCGCATATTAAAAATATGGAACTCACAAATGAAGTGAGCGACCTT
CAGATTGCATCAAAGGAAATGGTAGCTCTTTTGAGTAGTCTTGAGAACGAAGTGGTTGAA
CAAAAATTAGAAGTAGATAATCAGAAAAAGAAAACTATGGAATGGCAGAAGAAATCCGGT
GAATGGTCGGATAAAGCTTTCAAATGGAAAGAAATGTCCGAGTATTGGGAAAAGAAAGCA
AAAGAATCGAACAACCATGATTCATGCAGCAGTTTTGACGAAAATGTAGTCCAAACAGAT
CCACAAGCTCTATTCCTTGCAGCTGCCGTGGAAAAGAAGGTCATGAATACATCGACAGCA
AACGGCAATGGTTCTTGGCATTTGCGACGACTATTTGGACAGGCTCCTGAGAATGGCGAT
GAAGCACATGACATTGTCGAGAAGCTTGAAAGTGAGAATTCCATGAAACAAACAGAGATC
AATAAATTGAAAAGCGAACTGGTGAAATTACAAACCAACTTTAAGGGTGAAGCATATAGC
AGATCGCAAGAATACGAAAAGCTAAGAAAGGAAAAGAATGACCTGGAGGTAACGAACGCC
AACCTCCTGAAGGAGCTTGAGTTAGCTCGCAAACTTAACCAGACTATATCCGACTACGTG
GACTAAATCTGTGCATTTTGTTTCAGCCAGAAAGTCCAACGAATGAAAGGAACAGCCTTC
ATGCTGAATTCGTTTAAATTCAACGCCTTGTTGTTGGCAAGCGCATGCCAAAAGAATATG
GCTTCTTTTTGTGTCCAAGGTTAATTTAGGAATGTTTTTGTATTCCCGAAGGAAAATTGT
TTTCATGATCAACCTAAAATAATAAGTCTTTTTATAATATGAAAATACGGCAGCGGATGC
TGTAGAACCAAAACCAAAATAC
>comp2809_c0_seq1
AAAACGAAACACAAAACAATACAACACAACGCACCATGATCACGTCGTTCACGATCGCTG
TCACAGCGGTTGCTCTCACCGAATGGAAGAAGCTCGAAGGGAACGGCAAGAACACCACCC

ACCACCAGAAGGACAAGGTCAAGGACAAGGACACCGGCAAGCGCTACGGCAGCATGCAGC
GAAAAGCAAGCAAGGCCAAGAAGAAGGCGTCGCGCCCGGTGGCGGTGGCGTCGTCGTACT
TTGTTGCCGGAGACATTCTCTTCGATCCGACCAAGACCAGGGCGAGCACAGCCAATAACA
ATGGCAATGGCAATGGCAATGG
>comp2809_c0_seq2
ATCGCATCGCATCGCATCGCATCCAATTCCGTCACATCAGGATGAACCAAGAACTCTAGG
TCGTGGGGAGTTTTAGGAAGCAAACAGACAAGCAGACAGGAGGAACAGTCGGTGCGGTTG
CTCTCACCGAATGGAAGAAGCTCGAAGGGAACGGCAAGAACACCACCCACCACCAGAAGG
ACAAGGTCAAGGACAAGGACACCGGCAAGCGCTACGGCAGCATGCAGCGAAAAGCAAGCA
AGGCCAAGAAGAAGGCGTCGCGCCCGGTGGCGGTGGCGTCGTCGTACTTTGTTGCCGGAG
ACATTCTCTTCGATCCGACCAAGACCAGGGCGAGCACAGCCAATAACAATGGCAATGGCA
ATGGCAATGG
>comp2810_c0_seq1
CTGGCTTGAATCAGGGTTCAACGTCACGGTTCACATCGGATCGCATGCATCGTGTCATGC
GATGGCAACCAATCGAAGGCAACACAATATTTCATCACAAAAAGTTAGTTTTCTTGTTGG
GTTCTTGTGTTTGAGCTCGGGTGCTTGATTCATCCTCGCTCGACCAATTCATCAACAAAC
GAAATCTGGGAGCCCTTTCACGGACCAAAACCATGAACAGAGTGGTCCTCCTTGCGTCGA
GAGCTCCGCGGGGTACGACCATCCCGCTGCAAACCAAATCCCCCGCCCTGCTCCGAACAA
ACACTTTCCGGTCGGCAGGAGCGATCGACCCGGCAGCAACGATTCCGGCGGCGAGGGGTC
GTTCGATCGCGACAGCTAGCAATCCCGCAGAAGAAAGCGGTACTGGCGAGAGACGCAGCA
TAAGCATGAAAACCCATCCCCCGTTTGGATCGGCACCAGGGCCGAGGGCAGTCGGGTTCC
CACCGGCGATAGCCTTCCGTCGACAAGACGCCCTCCACGACCAGCAACGCCGCTTCTTTT
CTTCCCCTCCTCCGGGGGCCGGCCAGAGCCTCGGAAGCATCTTCGGGACCGATCCATCGG
GAGGTTCGTACCTGGAGGACTTCACCATCGATCTGACCGAAATCGCCAGGGGTGGGAAGA
AACAGGGAGGCGCCGATTCCCCCGGCGGGAACGGAAGCGGTGGCAACACGAAAATGGATC
CCATCATCGGCCGGCACGAAGAAATCGGGCGCTGCCTACAGATACTGGCCCGGCGCACAA
AGAGCAACCCGATTCTCATCGGGCAGGCGGGGGTGGGCAAGACGGCCGTCGCGGAGGGCC
TCGCCCAGCGCATCGTGGGGGGCCAGGTGCCGGAATCCATGAAGCACAAGCGGGTCCTGA
GCCTGGACGTGACCGGCCTGGTGTCGGGTGCCTACATGAGGGGACAGTTCGAGGAACGCC
TCAGGGGCATCCTCAAAGAAGTGACCGAGTCGGACGGAAGCGTCATCTTGTTCGTAGACG
AAATCCACCTGATCGGAACGGCCGGAAAGGGAGAAGGCTCGGTGGACATGGGAAACATGC
TCAAGCCGGCCCTGGCGCGGGGTGATTTGCAGCTAATCGGTGCCACGACCCTCGACGAGT
ACCGCATCCTGGAAAAGGATCCGGCGCTGGCCAGGCGGTTTCAGGCCGTCTACGTCGACG
AACCTTCCGTGGAAGACACCTTGTCGATTCTGCGGGGCCTCAAGCCCCACTACGAGCTGC
ACCACGCCGGCATCCGAATCAAGGACGAGGCCCTCGTCGCGGCGGCCGAGATGAGCGACC
GGTACATCACCGATCGGTTCCAGCCCGACAAATCCATCGATCTCGTGGACGAAGCCTGCA
GCCAGCTCCGACTGGAACAGGAATCCAAGCCAGAGGTCTTGTGGAAGGTCGAGCGGGACC
TCATGACGAAGCAGATCGAAATGAGTGCATTGCAGGGAGAAGACGACAAGAATTCCATCA
AGCGCAGAGAGACGGTAGCCTCGGAGGTAGAGGAATTGAAGCAGCGAGCCCAAAAGCTCA
CCGAAATGTGGCAGGAGGAGCGAAATTCCCTCACGCGCGTCAAGGATCTCAAGCAGGAAC
TGGCAGAAGCCCAGCAGGAAATGGAAATTGCGCGATCCAAGGGGGACTTTGCCAGAGCCG
GGGAACTCCTGCATTCAAAAATTCCGCATTTGAAGCACGAATTGGAAGAAACGGAGAAGG
AGGAAGACGATGCGTCAGCCACGCAAGGCAGCCGCCTCTTGAATGATTCTGTCGATGCAA
GTGCCATTGCCGCAATAGTTGCTCGATCTACTGGGATTCCGGTATCCAAAATTACGGGAA
CAGAGTCCCGGAAGCTGCTTGACATGGAGGACAAATTGCGACAGCGTGTGATCGGCCAGG
ATCATGCACTGCAAGCGGTGTCCAATTGCGTGCGATTGTCGCGCACGGGGTTGCAGGCAC
GGGACCGGACCCTCGGAAACTTTTTGTTTGCCGGACCGAGTGGGGTTGGAAAGACGGAAC
TGTGCAAAGCACTGGCAGAGTTCCTCTTTGATGACCAAAACGCAATGACTCGCATCGACA
TGTCGGAGTTTGGTGAAAAACATACCGTCTCTCGATTGATCGGATCTCCACCCGGATACG
TTGGATACGAAAAAGGCGGAGTTCTTACCGAAGCAGTCCGCAGACGTCCATACCAGATTC
TTCTGCTCGACGAGTTCGAGAAGTCGCACCCGGACGTGTGGAATCTACTGCTGCAACTGT
TCGACGACGGTAGACTGACCGACTCTCATGGCCGTGAGGTTGACTTTTCGAATGTGATCG
TTGTCATGACCTCAAACATGGGAGCCCACGTCATATCAGAATTGCCGAGCCATCTGAAGG
GAAACGAACCAGAGGTCAAGGAATCCATAATGAACGTCGTCCGCAACACTTTGAGCCCAG
AGTTGCTGAATCGCATCGATGAAACAGTGATCTTCAACCGTCTTCAACGCGAACAAATGG
GTCAGATCGCCGGGATTCACCTCGAGGACATCGCGACGCGTTTGGAGGAAAACCATGGCA
TGGCATTGAAAGTTAGCAAATCTGCTCAAGCTTGCATCGAAGAGCACGGTTTTGACATTC
GATACGGTGCGCGCCCGCTAAAACGAGCAATCACAAAAGATATATTGAATCCATTGAGTA
AAGCCATTCTTGGAGGCGAGATTCGTGATGCGGATCTTGTTCGTGTTGTGACCCGTGGAG
AGGCCATCAATCTCCAGAACGAAGAACGCCATCTTGGATGGGTGACAGGTGCTGACCCCA

GATCCACGGATAGAAACGACGTTGTTCTTCTGAAGAATCACGAGGCTTACACGGACGACG
ATATTTCTGGTTCTGGAAAAGTGTCCGGGGAAGATGATGACTACTATAGCGACGAGTGGA
GAGCCTGATCAATAAGCACAAATAAATACACAGTAATGTTAAATAATGTATGATGCCCAT
TTAACTGTAAATGGCAAAACTTGAATTCATTTTCAAGTGATCTCAGTCGTTTAACATTTT
GATTGGACAAGCATTCTCCAAATTAAGATTGCAGGATCATGTCTGGATATGTTTTCACAA
CGCTTCCTTCCATTAGAAGGTGTGGGTACCTTTCTCAAAGAAGAGTATTTCGGATTGCCT
TGTATCTACTACCAGTAGGTCCAATGAATTCCAAATATTCATCAAAATCAATAGTTCCAC
TTTGATCGATGTCTGCAGCTTGCTCAACTTCTTCGCTTTGAATTTTGGAAAGAGTTATTC
CATACGAATAAAGTGCTATACGCAATTCCTTTCGCGACAGTTCTCCATCACGGTTCGTGT
CCATGAGATAGAAGGACTGCTTTGCATCCTTCAACCAATCTTCACCACGTTTCTGGATCC
ATTTGTCTTCCACTCCATTCGAGGAGCAAATTGGCATTAGTGCCTCTTCTTCTGCGTAAG
TCATTTGGCCAGCTACGTCAACAAGATGGGTCTTGTAGAACGTGTAGAAATCATAAAGTG
CGTGCGTTATGATTGGCACGGCTAAATTCCCTGTTAAAAGATACAAAGTACAAAACGTTC
CTCCGTTAATGATTTGCAAAATCAACAGTGAGAAATTATCGATGAATGAATCTTTGCCCT
TGAAAAAACCAAGAGGGTTCGTATGAAGTACTGCAAACAGCAAAGTCGAAAGTAATGCTC
CAGTAAAAATGTCGCCATTGCCGTAGGCATTTGCAAATCCTGGAAGAGCTATAGATTTGA
GCAACAGATATATATTAATAGTGAGTGATAGTGCCAAATCAGAAAAATATGTGTTTTCTT
TCACCAGGAAAACCCACCTTGCCCACGAAATACGGTTTCCTCAACAATTCCGGTCGATGT
CGCGAGCAAGAGGGAGACCAAAAACGCCATTATTGGTTTTTTCCTCGGTCCAAATATTCG
CAAAACAGCCATATTGGTAGACAAATTTAAGCCGGAAACAAATGGCGATTCGGATGTTTC
GATAGCTCGCGATAGCTGAAGCAAAGGAATCAAACCGGCAACGCCAAAGATGATTGCTCC
CGGGCCAAAATGTGCTTGGTCGGAAAGCAATCTGAATCCAGTCCCTCCGGTCCAAATTTG
GTAGCCAATCAGAGCCTGATTGGCTACGTTCTGTAGAACTAGCTGGTAGTTGATTGATGT
CGACCCCTCATCGAATGACGGCGGTTGTTCGTCTCCTTCCGTGACCATGAACAATGGATA
CCGAGCACCAACACGTGCTGGTGAAATGTAGGGATCTATATTCGACTCTGCTCTGCCTTT
GAAAGCTAAGGCATCACGTTGCTTAATAATCTCTTGACACCGGTGATTCCCTCTATGGAA
TGGAGTCCGGTGAATGATGTTAGGATCAAATTGAAATCCATAGACGCGAGCCCACGGCTG
CTGTTGATAAAGATGGTGAAAACCCAGAAGTATCAAGGCGACGGCGCTGGTGGAAAACTT
GTTCTTCGTCATCATTGGTTTTTAATGCTTCGAAGACTTACTTCATCTAGAAGTAGCAG
ACGAGTTCTACCTGTTCCTCGGAGAGCTTCGGTTTTCCTACTTCCTTTTCTTTTGAAATT
AGATGCAAGTATCGTGGTCACATGCACAGCACACGTACCGTACCGTGCTCTTTTCCCGGG
AACTCGTACCCAACGTGGTCAGGTGTATCTTGACGGTGTCCTTGCGTGCGAGTACGTATC
TACTCTATCGTATGGTACCGGATTTGGTGCGGTATCTTCCAACGAGGAACATGCTTTTTG
GAGAAATCTTTCAACACGGAATT
>comp2811_c0_seq1
CACACACCAAAGTTCTTGGTCGCCGGCGGAAGATCTGAACAACCTAGCTCCTGTGAGAAA
AACGCCACATCGAATCTTGATTCATATTTCCTGCATTCCTAGAAAATTTCCAAAAGATAA
TCAAAATGGCATCTATTTGGGCACGATACAACAAATTGCTGGACACCCAGCCACTTCTGA
CCAAGTGTCTGACCTCCCTCACCGGGTTCACCGTCGGAGATATCTTGGCGCAGAAATTCA
TCGAGCCCAGCGACAAACCGTACGACATTATGCGTACCATCCGCCTGGGATCCTTCGGGT
TTCTCTTCCACGGAAGCACCGGCCACTACTTTTACGGAATGCTCGACTCGAAGATGCCCG
GAACCAAGCCCGTCACCGTTGCTTCGAAGGTTTTGATCGACCAGACCATCTGGAACCCCA
TTTTTGGATGCTGTTTCTTTGGATTCCTGAACTTTGCCGAGGGCAAGACGTGGGACGACT
ACACCAAGAAACTCAACTCCGACCTCAAGACCGCCGTCATGGGATCCTGGGCAGTTTGGG
TGCCGGCCCACACGATCAACTTTGCCTTTATTCCTCCCTCCCAGCGATTGTTGTACATTA
ACACTATCCAGATCGGATACAATATCTTCCTTTCCTTCCTTGGAAACAAGAAGGCGGACG
AATAAACAATAATTCATAGACGTGTATGTATTATTTTTTTGGAACGGCAGATGCGAGAAA
TAAGCATGATATGTGCTATTGTCTACCTTTTTTTAGATTGGTTGTGCCCTTGGGGTGGTG
TGTTGCGTTCTGCTGCAAGCGTTGCGTGGGGCAGCGCGGAGGCAACCCGCTCAATCCGAA
CGCGGCACTGCTCTAGTACAATATATGAGAATAATGCTGGTGCCAAGTAATCTCAAGTAC
GACGATTAGATAATATCAGTAAGGTAGTCTTCGACGAATAGAGTGAAATTTCTGAAAAAC
CACGTCAAAATGAAGGAGCAATAACTTTGTTACATTGTATGGTTGTGGATGGGTGGAGAA
TGGAAAAGGTATTCGGTAGCTCGTGGTGTCTCACTCTATCCTAGGAAGCTGGCTGTCGGC
ATGAAGAATTTAGAACATGGGAAGCACCCAAGGATAATGCCCTCGTTGTTGTAGTGTCGA
TGAGAGCCCTGCTTACTTGAGTACGAACTGTA
>comp2812_c0_seq1
CACAAGTGAAAGTACGAATACCTCGTGAAACTTCGTTTGGGTTCTGCTGTAGCTGAAACC
ATACCAAATACAACGGCTCTTGGTTGCGCAGGAGCAGACCATGCTGGGCACGAAACGACA
CCTAATCCTGGAGGTAGTGGTGGTGGTGGTTGCCTTGGTTTGGAATCATTCCCTCGAGGC
GACTCGTTTTGGTCAGGGCGTTGACGCATTCCTCGCCACTGCCGTCTTCCCAAAGTTCCC

CCGTGGTTGCACCGCTCCTCGGCAACCTCAATTTCCGTCGATGGGTCCGTTGTTCTCTCT
GGGGGACTCCAGAGCTCCGCCGGACACAAGAGAGACGAATCGCGCCGGGAACGGCTCCGA
CGCAGTGGAGAAAAAGAACCGGAGAAAGCGGTCCCTGTACTCATTTTCGGAGGCCCGGAA
GATTGCGCGGGGCCACGGGTTTAGCACCAGGGAAGAATTTATCGAGTACGACTGCGCCGG
TGCCTACCAGGTGCCGAAGAACGCGGACGAGGTCTGGTCCGAGGACTGGACGAGCTGGGA
GGATTTTCTTGGCCTCCGCCTCGCCGAGTTCGAGCAAGCCCGGGATGTGGCTAGATCCCG
GGTGGGACCGAATTCGACTACGAGCTGGAAGGTTTCTACGGAGGAAGAATACCTGGAGCT
CTTCCGATCGAAGCAGATCGACGACGACGACATTGCCAGCAGGCTGCCCTATCGGCCCGA
TCTCTTTTACAAGAACAAAGGATGGACGTCGTGGGAAGATTTCCTGTCCCCGGGTAGCGA
CGAATAACAGTACGATACAAAGGAAATGTGATCCCGAAGTTTCCCGTAGCTAGTATCTCT
ATTTTTTTCTTCTATAGCATTACTTGCAAATCAAAAA
>comp2813_c0_seq1
CCTGATTATTGTCAAATTGCGTCGCACAAAAGTTACGAAGCAATAGACCATTTCACCAGT
AGTCTACCCCATTAATAACCTAAACAATGGGAAAGAAGCGAAACAATCGACGGCGGAAGC
AAAATTATCAATCTGGCTCGAGCAATACCCCGCGCCTGGGAAGCAAAGGATGTTCACTAT
GTTACGATAGCAGTATTCCGGAGAACCTGAATGCCCACGTCAACGACAGTCAAACATGTG
CCGACATCCATCTTCAGTTGGCAATGTTGCGATATGACAACGCCATGTGCTCGGTGGGGC
AAGAAAAGTATCAGGAACTATGCTGCACAAAAGTAGACACAGGAAATGGAGGCTCCACGT
TGGGTTTCATGCTTGGTGCGATTGTAGCGGGTTTCTTTCTGAAGAATATGTTCACGCTCA
GGAAACGCCGAGTTGAGGAAATGAAGAGCATGGATGGTGGCATGGAGCTTCCAAGAACAG
TTAGTTTGGGTGCTCGCAGTCGCAGCAATAGCAGTCGAGAGAATCAAGATATACCATCCA
CCTGGTACGTCAGGATGGACGATTCCAAATCAAAATCGAGTAAAAGCAGTAGTCGATCGC
GTGGTAAGCCCATGGCCTACATTACCAACAGTAAACGTTTGGAAAAAAATAGAGGCACGT
CGCGATCCAAAGCACTACTTTCTACCAGACGACCTCGGAGCCGGAGTCGACCAAGAG
>comp2814_c0_seq1
TGTAATTGCCATGAAAAGCAAATAGCGCGAGAAGAATCTGGCTAGACAAAAAAAGCTTAT
TCTGCCCCATTCTCTCCGTTCTGTTGTGTAATAACTTACCTTCAACTGGTGCATCGAACG
AGTCGTCGGTTGTGTTGGAGACGCCAAAAGCACTCTGGAGAAAGGCTGACTGGTCAGATC
ACTCCCTCTCAGAGCATGGTCAATGTAATTGTACGGTACCTGAAGTAAATAGAGATAGGT
ACTGCGAATAGATAATGCAAAAGAAGATCAGAGACACATGTCACTAGTGTATGCAAAGCG
CATTTCAAAACTCATCATCTTTACCATCACAATCGCCGAAACCATGACCACAAGCGTCTG
CTACCCGGACCAAGACTGCTGCATAGATACAGACGAATCATGCGGTATGGAAGGCACTCA
TTTCTCTACCATCTCATCGGTCTTCGTCCCAAGATTAAAGCAACTCCTGCGGAAAAGTGT
GAACTCATTGTCCCCGTCCACACCCTTCGAAGAGAACGACGAGACCGCGATTGCAACACT
AATTTTTGAATCTATGTACACGCCTGGCCGTGTGTACCATAGCATGCAGCATGTGTTTAA
TATTTCAGACAACTGTGACATAGAAACAGACCCCATGTTGCTTCTTTCGACTCTCTTTCA
CGACGTGATTTATTATACGGTGGACAAATCCTTTCTGAAATCACAGCTCGAACTCCTCGA
TAATGTCCTCGCTTTCGAAGACGCAGGGGACGAACCGAAGCGCCTGAAGGAGCCTTTGCT
ACTATCACCTGATGCCCAAGAAGATCCTCTCTTGGATATGATATTTCGACTCTTTGACTT
TAAACCAGGGACGCCGCTGCCATCCTTTGGAAGCAATGAATTTTTGTCCGCCGTGATCGG
CGTCCGTGTCTTATCAAAGTGGTTGAGTGTGCCGCAGGTGACACAACTCACAGCAGCCAT
CGAAGCAACGGTTCCCTTCCGGCCTCCCTCGCCCGATGGAAAGACAGCGATGGATCGTCT
CTACGATCGCTTGATCACAGTGGCTCCCAACCAATCTGAAGACTGGTTCGAAGAAACGAT
ACATTTGTCGGCTCTTATGGCCAACTGTGACCTCTGTTCGTTTGATAGTTCTAATTTCGA
TTTCTTTTTGGATTCCAACTGGAGTCTTGTCCCAGAATTTAGACCGGCCCTTCTAAAAGA
AAACTGCCCTCTCCAAGAATACTACGACGAATTTCTCGCGATGGAAGGGAGAACCAAGTT
TTTGATATCCGCCATTCCCAACATATTTCAAGTATTTCGGAATGTACCTTCCGAAAGCGA
ACTTGCTGATAAGAGGGCGAGTACTCGCGCCAACTTGGTCCTTGGAGACGACTACGCACA
AGTGCGACGGCTACAACTGATGGTATTGATGGAATTCGTCACGATGGTCGGAGAAATTCC
CGAGTCAATCCCTGGACGTCAATTTCTTTCTCTAGAGCTCAAGAAATCATCGTGCAGTGG
CGAGGAAAGCCCGGTCAGAAAGCGATTAGACCCAGGCCGACGCCAGGGTTTCCCCTGGGA
TCCCGCAAGGTGTTCGCTAGGGGCTTTTCTGTATGACCATCTGGGAAAAGAGGGTATTGA
TACGGCAGTAGAAGTTGGGAAAACACAAAAACCTGGGAGTGGCCATCTTCTTGACCATCT
CCCAAACGATGTCGTTGCCGTCGTAGCAGCTTCTTTGGAAGAAATTTTGCCCGACCATTC
TGAAGCGCTGAAGAACATTTCTGATAAACTGGGGTTTAGTCAAACATAGGCTTAAAAAAA
TTAAG
>comp2815_c0_seq1
ACTGCAACCAACATACCACCCTCCAACAGTCCCTTCCTCTACAAAGCAACGCAAAGAAAG
AGCAATTATGGGAAGCAACGGTTCCAGCAACAGAAGGAAGAAAAAGAGAGGGATCAACAT
CGAACGAGTGTTGTTTCCGCTGGTCATCCTCATGGCGATCGGTCTGCTCTTCGTGAACAT

CTCGGTCATGGTCGAACACAGCAACAAAGGGAAACGAAGAGGCAGCGACGAGGCAGATTC
TAGCATTCGGAAATCAAGGGGCATGAGCGAGAGCAAAGAGAAACAGAAACGCGGGAACGG
ATCAGATTCCCATTCGAAAGATGTACAGAAAGATCGATTGCCTCCACCCGAAACGAAACC
CCACGACGAACCGAAACCAAGCAAGCAGGAAACTCCGCAGACCAAAGCTGCGCCCGGGGA
GAACAGCGGCGGTGACATTGACGAACACATGCTTCGGATCCTCCGGCACGTAGGAATCCA
CAACTCCAGTCAGCTGACGGACCAGGAACTCTCGGCGCTGCCCACCTGGAGCCAGGTCGT
GGAAAAGTTTGGAAACAAGGGCCCCGTGATGCTCGGGCGCGAGACGTGTTCCGAGTATCG
CTCCAGCGTGGACCGGAAGCACCGAAAACTGGGCGTCGCCGGACCCTTCAGTTCGGGAAC
ACATTATCTCCACACCATTCTCAGCAACAATTGTAAGAGCCTAGAAGATAAAGGCAGGGT
CATGTTCCAGGTCCCTTGGGGCAAGCACCAATCCGCCAACTTTCGGCTGGTGCACAACGT
CGCCGTGAGCAGCACGCTCAAAAACGACCACAAAAAGAACGAGACCACTGGCAAGCTGGA
CCACCCACCCCTGGACCCGAAACACCTGAAGGAAAACAAGAGCGTGCTACCGATCGTTGT
TGTGCGGGATCCGTACAGTTGGTGGCAGTCCATGTGCAGGGTCCGGTACTCTGCCCATTG
GTTCCACGTTGTACCCGATCACTGCCCGAACTTTGTTCCGAACGAGGTCGAAAGAGAATG
GTTCAACAAGACCAAGAAGGAAATCAGAAAACACTACAACAACGACCCGTGGAAGGTCGA
CAACGTGGTGGACAAGGCCAATTTTACGCTCGACAAAACCGTTGTTCCGCTCTGGGTCCG
ATATCACTCGGAAAACCGGCAGCACAAGTCCCTGGCGCACATGTGGGCCGATTGGTACAA
CGATTACTACGAAGCGAACTTCCCCCGGCTCATGATCCGACTGGAAGACCTCGTGTTTTA
TCCCCACGAGACCCTGAAAATGGTTTGTGAATGCGCCAACGACGACGACGACGGCGACGA
ACAGAAGGCTTCCTTTGAGTACGTCGGCGACGAGAATCTTGAGCTGTCCCTCGAATCGGC
CATCAAGGGCAAGGGGGACAAGGTCGACAATATTCATGGAAAGGACCGTACCGGATTGTT
GGGCGCGATGAAAAAACACGCCGGAAAATATTCCGACGAGCACCGGTTGACGGGGATGAC
GCCTTCCGACATGGAGTTTGCAGAGGCCGTGCTAAAAGAATCGGATGTCTTTAAGGACCT
GGGCTACCAGCGTCCAGTGCGCCCCAGTGCCTAGGCAGACGGAGATAAACGGATGGATTG
GATGCGATCGGATCGGATCTTCCGGCCACTTGTGGTGATTGATTCGATGCATCCCAAATA
ACAGTTCCATCGGAATCAAAAAAACGATCGAAGCATTTTCATTTTCGACAAGACTAAATC
AAATTGCTCAATTAAATGTTGAAGGATTGGAGTACAGAACAATAGACTGGAGATTTGTAA
AGTTGAGAGACGCGAATTTCAATCCGATGCCATGATTTTTTCGACGGAAGGAGCATTCCT
CCAGAAGGACAATTGGATATAAGCTTCTGCGCGAATGGGTAGAAAACAATGACTCATAGC
ATTTTTCACCCATTCAGTTCGAATCTAGTGAAGTGCAGTACGTACTTCATTTTAATACGA
TCCAGTGCAGTTTATCCTGCTGGAATAGCAGATCGGGACAGCCAAAATATAGAAGAGCCT
GTCGTAGTTTTAAACCTCGTAGCAAATAGATCTACTATCGATTTTAGCAGTGTTTTAC
>comp2816_c0_seq1
ATTCAACAATCCAACCTCGTCTAGTGCCAGAGTGCTGCAGTTGCGCCATCTCACGAGCAT
CTTCTTTGTGAAGGATCTTGTACCGTACCATACGCTGCATACTTACCAGGGCACTCGATA
AAAATCGGATATTGCATCGTCGTCGTCGTAAACATGAACGCTCTCAGAACATCCATTCGC
CACTTGCGGGGGTTTCGTCCAACCCCCATAGGACGACAGCATTGCACGTACTACACGCAA
ATCCGGCGTCCTGTTGTTCCAAAACGAGCTAGGCAATTCTCGTCGGTTCCGCAGCACGCG
GCAGAAGAATGCGATGCGGCTGGCGGCATTTCCCAGGAAGAAATCGAGTGGCAGCAGCGC
TGCCAGCTGGCGGCTTGTTACCGGGTATCCTTCATGCAGGGCTGGCACGAGAACATTTTC
AACCACATCACGCTCAAGGTTCACAACTCGGACGATATCCTGGATGGCCCGCACTTTTTC
ATCAACGAATTTGGTACGGGCTTCGACGAAATGAGGGCCTCGTCGATCCTCAAGGTCAAC
CTGGACGGGGACGTGGTGGACGGCTACGGGACTAGCGGGAGGGTCTTCAAGCCGGGCTTT
GTGATCCACTCGGCGGTCCACGGGGGCCGGCCGGACACCCACGCCGTGTGGCACGTACAC
CCCCTGGATACGACGGCCGTGTGTCAAACGAGGTTCGGCCTGCTGCCCATCAGCCAGGAA
TCGGCTCTGGCGGCCAAGCGCGGCCTGTCCTACCACCCCTTCGAGGGCACGGCGAATTCC
ATCGACGAGCGGGAGCGGCTGGTAGAAAGCCTGGGACCGACCAACCAGATCCTGATGCTG
GAGAACCACGGCCCGCTGGTGGCGTCCCCCACGCTGCACCAGGCCTTTGCGGGGATGTTC
TTTCTGACGCGGGCGTGCACCTTTCAGATCCGGGCGCTGGGCAGCGTCGGCGGGGACCTG
GGCAAGATCCACGTGCCATCGCGGGAAGAGGAGGACGAGATACTGGCGCGGTCGAAAAAG
TTCGACGACGCCCCCCAGGAGGGTGTCCAGCACGACTCCGACAAACTCATGTTCGAGTAT
GCCGTCCGTGCCGCTGAGCAAGCCTTTGGCGCGGACGACATCTACCGATAGGGCAACAAA
CAGCAACCTCATTTTCCATTCGCATCCCCACAAACAAGAGAAAGGAAACACAGGAGCGGA
TAGAATAAGGTATCGTATCTGTTCGAATCACATCGCAAATAAACGCTACTGCAATAATGG
CATCAATGGGATTGATTAGGGAAGCGAATTGCATAGGATGGTCTGGCATCAGAATGTCAA
AGTCGATTGATCCAATGCGCACAAGTTTTTTGAACCAGGCGGACTAGAGAATTCAACTTA
AACCGATGCTGCTGCTAGCCCCAAACGATACGGGCACTGTTATAATGACTAATAATGATA
GTATGTATTCGCTTCTAATTCATCTATTTACAACCCAGATAAAGCAGCGTATTATCTTTC
TGTGCTCCAAGTGCGAAGACAGGCAGAAGAATTCGTTTTCTGCTTTTCGGTCGTACCATT
ATTATAACACTAGTCTACC

>comp2817_c0_seq1
TTTGTAATGTTTGGGTCCCAAGCTGGCTAGCATGTTGACCGCTGCGGGTACTACTAGACC
ATACTTGTACCGTACAGTCGATCGACATCTTCTGCAATCCGCGGGAAATGGATACCCGTT
TAGCTCTTTCATCGGAAGCGCACAGTTTTACTCGCCAACGGCAATACCCGTAGTAGTATC
CTAGTTGTCCGCATTTGCGACACAACGCACATTCCTGGAACAAAACAACTGTTGCAACAT
TCCATACCCCAAGGAGACAGCAAGTCAAAGGATAAATACACTAGTATCGAAAAATGACGG
CTGCCACTAACCGAGATAACTCTGTGAAGGTGAAGAAGGCTTCATCAACGACCAAGGTTG
CGGGCCCGAAAACAGCGAGTTCGGGGATCATGCACCAAGTCATTATGCCGAAAACCCTCC
CCACTGCTCCACCGACGTCTCAACCATCGAAGCCCGTCAGTTTGCCAGCCAACAGAACTG
TTGCCACCGTAACAACCACCCACCGCTCCAATTATGCTAAGAAGACAAACGGCCGGGTCT
CGAAGAGAAACGGGGTCTGGTACCTGGGAGGCAACCACGTTGTGAAACTCCTCACCAGTG
TCGATGGTGATCTAGATCATTCTATTCTCTCGAAGCTAGCAGCTGTCCCGACAGTGGTCG
GGGAATTGGCCCTCTCGGTGGAGTCATTAGTGTCCGATGATATCACCGTGGGTGGCTTTG
AGAACGATCTCCTAGCGAAGATCGCTTCCAACACCCTTACAGCCAAGAGGGAGATCATGG
CAGCAAAGGCAGCAGCCGCACCCAAAGCTACACCGATCTGCACCACGCCAACTCCGCTTG
GAAGCACTTCGATGGGAGCAGCAGCCGCTGCATCCACTCACAAAAAAGCCAAGGGAGCCC
CGTCCTCGGGATCGGAACAGGTTCCGAATCCACTTCTGGCAAACTACCAACCGGTTCCGA
ATCCCCTAGCTATTCGAATCACACCCCCAGCTTCGCCACACACTTCGGCCGCTACCGAAC
AATCCTCCGCAGGCAGTCCCAAGACTACAAAGACACTCCAGACCAAGGGACTGCCCTTGA
TTCATGCGCGGTGATGCGGCTGTACAGCCCACCAACCAACGCAGCTGGTGATGCTATCCA
TCCAGTTAAGAAACAAAACGATAGAGTGCTTACATTTTCATAGCACTAGTGCAAACGGAT
CCATTAATACGATCGATCCATTATACTTGGAGACAAGAAACTACACAGCTGATTTTTTCA
TCGGGTCGGAGAAGGGAAGCTGATGACCGGGAATACAACCGAGCTACAACCTGTTGTAAT
ACTATATACGCAGCATGCACGCTGTAATGTCGCAGCCCTAGACTCGATACGAGCGCATGC
GGATGTGAACGAACAGAGGACCTACATTGGTGGAGGAAAAAGCAAACATACCGATGGAGC
AATGTCTCCTACGATTTTGAATACATACTTTTATATACTTTACGAGTTGAAAATTAGGCA
CAATAATAGCATGGCA
>comp2818_c0_seq1
ATAGAATTTCCCGAAAGGGAGCACTGTACGCGTAGGATGTGCTCGTTGTCGCATGCTCGG
TGGTACGGCGTGCTAAGGAGCGCTCTGTGTGGTGTCACGCAACGAACGAAAAGAAAGAGT
TAGAGTAACCACACGGAACAATTGCATCGACGCATCGATTCGTGGATCATTTGCGGCTCC
TTCCGCCAAAATTTAGTGATACCGTACATACCCCTTACGTTCGTGCCTTTCGATTTCGAT
TCGATTGCCGTCGTGAGGTTGTTGCGAGTAACGAACAAAAAGAAAAAGCAGCAAAAAACC
TCACACACTTGCATGCTTCCATTCCGATAGTCTTCCTAGCCATCGTTGCATATCTTACAC
TCGCGCACACACAACCCACCAAGCAAATCTACTAGGCCACAATGGCGGAACCCACGATCT
GTCTGCGGAACTGGCACAGCGGGAGGCCTCTCTCCCCGGGTCACAAAAAGAAACCAAGGG
GTTCCTTCCCGTCCCCCTACGATCGAGACGACCACGAAACAAGGATCCGGCACTATCCCC
TCTCCAAGCAGCTGGGGGAGGACCTCCGGCGGGCGTGGAGGAACCACCGGGCAGCCACGG
CGATGCACCAGAGCTGGACGAGGAAGGACCACCGGCACCTCCCGGACGATCGGACCGCGC
TGATCGGACCGGAAGCACCCCGGTGGCGCGCAAGGTAACCGCCCAGGCCGGCACCGGATC
AAGGGCGAAAGCGAAACGAAAGCGACAGATACTCGAGGCCTACCCACGAATCCAAGCAGA
CGGAGGTACTCGTAGAAGCAACAGGTGTTTGCTTGTCCCTCGGTTTTGATGCATACCAAA
TTCGATTCTCTTCTTTTCCATTCGATTCGATTCGATTTGATTCGA
>comp2819_c0_seq1
TTCTCCTGGACCATCGCCAGCACCCTCTCCGTTTCCAACCCTCGCCCCAAATTCGGGACA
GCCAACACCTGCTCCCTCTCCTGGTCCATCGCCAGCACCTTCTGCAGAACCGTCTTCCCC
TCCATCTCCCGCTCCTACGGGAGCATGTAGTATCAGTTCATGCCTCAACTGTACCGCCAT
CGTTGAAGGAATTGAGCTAGATTGCGCGGATATCCCGGAAGAAAAGCAACCTGTTTGCTT
TTGTCCAGAGTGTGTCCGAGAACTTCAGTTCACGTACACAGGAAGATCATGCCCAGAAAA
CCAGCTTTCATGTACAGACAACAGTCCTAGCCCATTTGTTGCCGGTTACAGAATCACGGA
CGCCAACGACCCCACTTTGGTTCTTGCAACTGGACAAGCACAACAGCAGGATACGATCGT
TGTCACATCGCCTACTGGATGCATTCCCGATACACTTTCAGTCACTATTTCGGTTCCCAC
TGGTGCTGTGACGCAAACTTTCTTGATCGATGCCCGATGTGACGGTGGAAGAGGATTGAC
TTTGCTTGGAAATTACGGTGCGTTTGAATCCGTGGGTTACACTTGTGATGCCACTGACGT
CCACAACTGTGTACAGACCGTGACCTACGGAAACAAGGTTTGCAACGATGGTCCCGAACC
ACAGAAGATCTATGAGTTTGATCTAACAACCAAAGAAACCAATACGGCATTTGATGAGAG
TTGCGATCTCCTCCAGGATGGCTCTATTCCAATGCTTGGTCCTGGTGAATGCTACTATGA
AAACAAACCTTTCGAAGTGAACAGATGTGTTCAATCAAACTACTGTGTTGACGTTGTAGC
AAATGCAACTGACGTCCTCACAGGAATTCCTCCCAGCTGTCCGGCTTCGGATGAAATCAA
ATTCGGCTGGCCAGGAATGCCCCTTCCTCCACCAACTCCTTTCCCAACTGTTATGCCTAG

CCCCCCCCCTTCGCCTGCCCCCACCTCGGCTTGTGTTATTGATGTTAAAATTGATGGATG
TCCTGCGCCTAATATCTCTCTCGATAACAATTGCGAAGGTCGTCCACAAACTATCACCTT
CAAATACACTGGTGGAGACTGCGAGCAGTCAAGAAACTTTCAGTCTCGTCAAAAGTTTAT
GTGTACACCTGCAGAGGGACCATCCGCTCCAACCACTAAAGGAACTGTAAACTACATTGA
GGCTGTTCCAAGAGGAGGAGGTGATCTCTATTACGCTGGTAACGTTGCAGTTGGAGACAA
CTACATTTTGAACGAAAACAAGCAGTTCGACAAACTTTCCGCTGACATGACTATCACGAT
TTTTGAGTCTCAGGGAGGACCTGTTCTCCAGACTGTTGACGTGCACTTGTCTTGTTCGCA
AGCTTTGTTCCTCTTCGATAAGTTTGGAGCAAATCAGGTTGTCCGATGGGTGGAAACTGA
TGGACGTGTGGTTGAAGCACAGGCTTTTGTTACCACTGACGATTTCGTTTTGAATTTGGA
AACCTCTGAGGTTACTGGTAGTGGGGGACCCATTACTCTTACCGAAATGTCTGTTCTGAC
GAACGCTCAAGACCTACCAATTGACTACACGCCTCAGGTGGCCGGAAAACAACTGATCCC
TGGACAGCCAATTAATCTCCCTGGTTTCCAACTTGACATTGATCTTACTCAAAGAACTGA
GTACACTTTCTTCACAACTATCATTGGACAAACAAGTGACGGAACACCTTGCAACGGATC
AGACTTCCACTCTTGTGTTGTTGGATTTAATTTATCTCCATCTTTCCCCACGGATGTTCC
AACCCCAAGACCAACTATCACCCCTTTCCCTACGGGCTCTCCGAATTCTACAGCCTGCGA
GATTGCATCCAACATCGAGTGTACTGTAACTCGACCAGCCAATCCAAAATTGCCTCTTTC
GTGTAGTGATCTTTCTGGCAGTGTTTCGGCAACCTGTTCTTCTGAACTCCTAGCTGCTTT
CCTCGAGTACGATGGATCATTGGGACCACGCGTGTTCGTTAACCCTACTTGCGGCAAGAA
CGAGTTCTTTGGTCAGTTCGTCAACGCAAATGAGGTTGTAGAACTCAACACGCGAGCTGC
TGATTTCTGTGACGGAGCTGTCACCATTGGTATCTATGATGCTGATCCACTTCAAGGAGG
AAGCCAACTTGGATCTGCTGATGTCGAGCTCTCTTGCCCAGGGCCTTGGACTATCGGAAA
CACGATTGCTCCTGGATTGACGTTGGCTTACTACGTCAGCACTGTCAACAATGGTCTCAC
CTTTACTTACAACTTCCTTGACGCCGAGGTACAGATTGATTACGTCGGTATCAACGCCGG
TGCAGGTCCCCTCACTGTTTCCGGTGGAGACTACTCTGGTACTTCTCCGTTCACATCAGG
CGCGATTACTGGTGGAAGCACAATTACCGGACGTGGACGTCAAGTTCTTGCAACCGAGAC
GCAGACCATTTCGCTCCCTGGCACTGGCGGAACAGCCTTGGACTTCAGTATGTCTGTTGA
CGGTGCGACTGCAAACCAGTTTGCTCTCCCTTGTACCACCGCCTCCAACTACCGACTCAA
TCTGTAGGAGTGTTAACTATTTGGCTAATCGATGATGATTGATAGCATCTACACGAGTAT
GAAACGAAAGACTCGGCGTGTTGCAAAATAAGATCATTGTGGATCTAATTGATGTATTTG
TTTTGAAAAAAAAAACATGAGTTCAGATTTCCTGCGGGGTTTCCTGCGAAATTATTTTGGC
AAGACGTACAAAAGGAAATTTGATCTTGTAATTAAATGTGAAAGAAAAAACTATTATTTT
AAAAAAATCTTCTGAAACGAAA
>comp2820_c0_seq1
CATTTTCATGATGATTTTAGATCTTCTGTTCTCGGTTCTCATTATTGACACATTCATACT
ATGAATAAATCGCTCTCCCCTCACCAACGACATTAATTTAAAACGAGAAACAACAAATAT
TTCCAGCATGACGTCAGGCGTACCCGTAGGCGATAGTCCATTTGGTCCTACCGAAATGAC
ACCCGAAGAACAGAGACGACACATGTCTGGACTCTGCTGCTTCAACTGGCTGGGGCTAAC
GTCTCTCGCAGCCTTCATCTGTGGAGTTTATTCCTTCGCATACTGCAACTTCTTGTCTCG
ATACGTCACATTGAGCGATAATTATTCCCCCGGTGACTTTGCAGCCGCCTGTAGCGACTT
GGGATATGAAGATGGAGACTCCTTCGATGCGCAAACTTGTCAATCCATCATGGAGGACCA
CGCCATTGGTTTCTCCTATTGGCAAGCCACCATCCCAGTTGATCAAAGAGTCTGCTTCTC
GTACACTCAGCTAACTCCATGGGGATATGCATCGCCAGACTTTGACTCGAGCTTCTTCAC
GAGTCGTACCTTTGCCATTATTGGATATGTCTTTGGGGGCATGGCTTGGTTCACCCTTGC
TTGCTCGAGTTGCTGCATGATCGACCAGCAACGCCTCAAGGGAATTGGATGCTATTTCAT
CATTGCGAGCTTTTCACAGGGTCTCTCACTTGTGATGTTTCGTTCAAACATCTGCGAGAA
AGGATTCTTTGAACCATACTTCGTTCCACCAAGTCAGCGAGACAACGAAACGTACCTTGA
AAGCTTCAATAGCGTTGTTGCGGATGTCGAGTGCGAGTTGAGCTTTGGATCGAAGATGGC
AATCAGCGCATCTGTCCTATATTTTATCTGTGCCTTGATGGTACCATTTTCAGTGGTTCC
GCTGTACGAACAACGATACTACCGACGCGATTTTGATGGCGATCAGCAACAGCGAGGTCA
AGACGAAGCTTCTCCGTCTCCACTTCCGGAAGGTGAAGGTCCGGTCGTTCGAGGACACGT
GACAGGAAAGGTTTGATTCTGTGAAACGATAAGTGAAAGCAAATCCGGATTGTGCCGAAA
ATAATGCACATCAATACCCTTTTAAGTGATTCAATGTTTTATGTCCATCAAAGTAACAAT
GGCTTACCTTGTAGATTCTGTAGAATTGCAGCAAAAGCACTGACCAGCGCTGACGGCATT
CATTATCAAATTACATTCTCTCTTCCACCATCTTCTTAGTTTTTTATTTAAGTAGCACTT
AAACCTTGCGAGTTTCATG
>comp2821_c0_seq1
TGAAAACGGGGTCGAATAATTTATGTCAAGTAGGAGGAAGAGAAGAGTCATTTTTGATGA
CCAGATGGTCTTTCGATCTTACTGCCGCCACTTTGAACTCCACATTTAAAGTGAAGGTAG
CTTACCACGATTTGCCATGGTCTTTTCTCCGCGAGAAAACAACGAAAATTTGATGCATTT
ACGAATACTTATGGAGACGAGCAACTTGTGTGCTTGCTAGTCTCCGCGACGACGCCATCA

ACATCTAAATCCAAGTCTACAAGACAACGTGCAGTACGAAGGTCCGGAAGAGGAAGCGAT
CGCTCAAGAAAAACTGCATTAAGCCGTACAAAAAATCAGGAGGGATTGTAACAGCACGAG
TATTGAAAAGTCGCATAATCTGGCAATCATCAAACTTTTTTGCTTCAATATCACACATTT
AGCCAAAACTACTTACCACTGGACTTTGGCATGACTTCTTTAAATAATGATGGCATTATG
CGTCGTGGAAGTTATAATCAGGGCCTTTCGAATTCGAAGATTGAGAGCGCCAGAAGGTTT
CTCTACGACGAAGACGAGTCGTTCGGCTCGGTGAACTTCAACAAGTCTGTAAGAAGGAAT
ACCAATCTTAGCGGATTTCCCACAGTGACCTCGGTCCCTTACGGCAACGAAAACACGAGA
GGGCGCGACTACAGCTCGAAAAGATGTCTGTGTGCGATAGTTTTGATTGGGATTCTTGCC
GTGTGCGTGTGGACGAGCTTCGTGGTAGCAAGTGGCAGGGAAAGCCCCGAACACGTCGAC
GAAAAACGAATCCAGGAATTTCAAAGCAAGATTGTTGAGTTGGGACTGAGCTTGGAAGGG
GACATCGAAACTACGGGGACTCCTCAGTATCATGCCTTGCAGTGGCTGGCGAATGACGAC
GGAGCCAAGCTAGACGTTGCAGATGCCCATGCGATGCAACGGTACGCACTCGCCGTTTTG
TTTTACAGCACGGCCGGCACAGAGGACCATATCGACCCAAACGCCAATGGCCAGTGGGTT
AATCAAACCAACTGGATGACCGGTGCCGGATTCTGCAGTTGGCACGGGGTAAAATGTATT
GCAGACTCTACCGAACACCGCAACAACGAAGATGGAGACGTCCTCGCCCTAGATTTATCT
CAGAATGGATTAGACGGTGTCTTGCCGTCGGAACTGAGCGCTTTCTCGCTCTTGCAGCGA
CTAGATTTCTCCCACAACGGTCTGACGGGAAATTTACCGAAGAACTTCGAAACCTTGACA
GGGCTCAGGAGTTTGATTCTTAGGAATAACAAGTTGAGCGGGACCATTCCTAAGGGTTTT
GGAATCAAATTGACGAACATGGTATGGTTGAACTTGGGTGAAAATCAATTGCATGGTCCC
ATTCCTAGTGAAATGGAGCATATGGTTGATCTGCGATCGTTGGGATTGGATAGAAATCAA
TTGAGTGGCAAGATCCCCGATCTGGAAGATTTGAGCAAACTCAAAAAGTTGTATTTGAAA
GAGAACAACTTAGAAGGGCCCTTTCCAGGAAGTTTGTCCAAGTTGACATCGCTCGTGGAA
ATAAATTTGAGTGGAAACCATTTGACTGGACTTCTTCCCTCAGAAATTGAAAAATTAACT
AAACTCGGTGAGTTGGTGCACGATCAAGCTTTTTCTGAATGAGCATTGATTTTTTTCTTT
CCACATGCGTAGCGATTTCTTCCATTGGATTTCTCGTTACTCATTAAAATGTTAATCGTG
TTCCTTTACAACAGAATTACTTATACTGAACAAAATGAACTTGCGAGGCACCATCCCGGG
AGAAATTTTCAAGAAGGTGACCCGACTGACGGAACTTAATCTTAGCAACAACCACTTCGG
TGGACAAATCCCAACCTCCATCGGTCACCTGAAAGATATTCAGGGATTTTTCTTGGGCGA
AAATAAGTTGACGGGAACTATACCGCGACAAGTGGGACTTATGGTCGATTTAAAACGATT
CGAAATAGATGGCAACAACATTGGCGGAACGATTCCTAACCTTATTTCTGCTCTAAATAG
TCTGGAAGGATTCAAGCTGAGTCGAAACTCGATAACGGGTACGATCCCTCCGGACATTGG
GAATTTACATCGATTGGTTGAATTGGAATTCGAATCCAACAAACTGGAAGGAAAAGTCCC
ACCAGAAATAGGCAATCTGGTAAATCTCAAGCAAGCTCGATTGTACGATAACAATCTTAC
AGGAGATGTGCCAAAAGAAGTGTGTGCATTAACTAAAGACGAGGATTTGGCATATTTGGG
GGTCGATTGTGCTAGCGACAAGGTAAATTGCGATTGTTGCACAAAATGCTTTTAGATTTT
TGTGGAAAAGGTCTATGAGAATAAATAGCGTGTAAAATTAGAACTGCCGTAGAGACTAAA
AAAAA
>comp2821_c0_seq2
TGAAAACGGGGTCGAATAATTTATGTCAAGTAGGAGGAAGAGAAGAGTCATTTTTGATGA
CCAGATGGTCTTTCGATCTTACTGCCGCCACTTTGAACTCCACATTTAAAGTGAAGGTAG
CTTACCACGATTTGCCATGGTCTTTTCTCCGCGAGAAAACAACGAAAATTTGATGCATTT
ACGAATACTTATGGAGACGAGCAACTTGTGTGCTTGCTAGTCTCCGCGACGACGCCATCA
ACATCTAAATCCAAGTCTACAAGACAACGTGCAGTACGAAGGTCCGGAAGAGGAAGCGAT
CGCTCAAGAAAAACTGCATTAAGCCGTACAAAAAATCAGGAGGGATTGTAACAGCACGAG
TATTGAAAAGTCGCATAATCTGGCAATCATCAAACTTTTTTGCTTCAATATCACACATTT
AGCCAAAACTACTTACCACTGGACTTTGGCATGACTTCTTTAAATAATGATGGCATTATG
CGTCGTGGAAGTTATAATCAGGGCCTTTCGAATTCGAAGATTGAGAGCGCCAGAAGGTTT
CTCTACGACGAAGACGAGTCGTTCGGCTCGGTGAACTTCAACAAGTCTGTAAGAAGGAAT
ACCAATCTTAGCGGATTTCCCACAGTGACCTCGGTCCCTTACGGCAACGAAAACACGAGA
GGGCGCGACTACAGCTCGAAAAGATGTCTGTGTGCGATAGTTTTGATTGGGATTCTTGCC
GTGTGCGTGTGGACGAGCTTCGTGGTAGCAAGTGGCAGGGAAAGCCCCGAACACGTCGAC
GAAAAACGAATCCAGGAATTTCAAAGCAAGATTGTTGAGTTGGGACTGAGCTTGGAAGGG
GACATCGAAACTACGGGGACTCCTCAGTATCATGCCTTGCAGTGGCTGGCGAATGACGAC
GGAGCCAAGCTAGACGTTGCAGATGCCCATGCGATGCAACGGTACGCACTCGCCGTTTTG
TTTTACAGCACGGCCGGCACAGAGGACCATATCGACCCAAACGCCAATGGCCAGTGGGTT
AATCAAACCAACTGGATGACCGGTGCCGGATTCTGCAGTTGGCACGGGGTAAAATGTATT
GCAGACTCTACCGAACACCGCAACAACGAAGATGGAGACGTCCTCGCCCTAGATTTATCT
CAGAATGGATTAGACGGTGTCTTGCCGTCGGAACTGAGCGCTTTCTCGCTCTTGCAGCGA
CTAGATTTCTCCCACAACGGTCTGACGGGAAATTTACCGAAGAACTTCGAAACCTTGACA
GGGCTCAGGAGTTTGATTCTTAGGAATAACAAGTTGAGCGGGACCATTCCTAAGGGTTTT

GGAATCAAATTGACGAACATGGTATGGTTGAACTTGGGTGAAAATCAATTGCATGGTCCC
ATTCCTAGTGAAATGGAGCATATGGTTGATCTGCGATCGTTGGGATTGGATAGAAATCAA
TTGAGTGGCAAGATCCCCGATCTGGAAGATTTGAGCAAACTCAAAAAGTTGTATTTGAAA
GAGAACAACTTAGAAGGGCCCTTTCCAGGAAGTTTGTCCAAGTTGACATCGCTCGTGGAA
ATAAATTTGAGTGGAAACCATTTGACTGGACTTCTTCCCTCAGAAATTGAAAAATTAACT
AAACTCGAATTACTTATACTGAACAAAATGAACTTGCGAGGCACCATCCCGGGAGAAATT
TTCAAGAAGGTGACCCGACTGACGGAACTTAATCTTAGCAACAACCACTTCGGTGGACAA
ATCCCAACCTCCATCGGTCACCTGAAAGATATTCAGGGATTTTTCTTGGGCGAAAATAAG
TTGACGGGAACTATACCGCGACAAGTGGGACTTATGGTCGATTTAAAACGATTCGAAATA
GATGGCAACAACATTGGCGGAACGATTCCTAACCTTATTTCTGCTCTAAATAGTCTGGAA
GGATTCAAGCTGAGTCGAAACTCGATAACGGGTACGATCCCTCCGGACATTGGGAATTTA
CATCGATTGGTTGAATTGGAATTCGAATCCAACAAACTGGAAGGAAAAGTCCCACCAGAA
ATAGGCAATCTGGTAAATCTCAAGCAAGCTCGATTGTACGATAACAATCTTACAGGAGAT
GTGCCAAAAGAAGTGTGTGCATTAACTAAAGACGAGGATTTGGCATATTTGGGGGTCGAT
TGTGCTAGCGACAAGGTAAATTGCGATTGTTGCACAAAATGCTTTTAGATTTTTGTGGAA
AAGGTCTATGAGAATAAATAGCGTGTAAAATTAGAACTGCCGTAGAGACTAAAAAAAA
>comp2822_c0_seq1
TGAAAGCATCAGAGGCAACTCACCGAATACCTACAAAGACACCAAAGGACATGGAAGACG
ATCGATTACTTCCTTCAGAATAGAGAAATGAATCGTTGATCGAACTGTTCTGCTGACCAG
TCAAGAAAAATACGAACAAATACAAAAGCATGACTTGGTTTCGAGCGAACCGAGCTCTTT
GGGCTTGTCCCAATACTTTGCCACCAGATACTGCTAGAACAATATCAACCACAACAAGAC
GAAAGAAAAATGTTGAACACCTCATCGAAGAATATCTAGGCCAACGAACAGAAGATGATG
GTTGCAGTGGAGAAGAAAGTTCGGACGTTTTGGAAAAAGAAGAATGGATGCACAACGACG
ATTATCCTCTCGAAGAGAAGAACGACACACCAGCAGCAACACACACCACCGACGAAGACA
ACTATGGCTACTTTGAGATCTCGTTGACGACAAATGCCATTCAAGATATGGTAGACGAGG
GAACGATATGGGTTGCCGAACAAGCAACTAAGAATTGCGAGTGCGAAAACGGGGAGGGAC
TCCTCTCCTCTGCCGCCCCGCTTGCAGCCGGTCCCCTGGTGTTGTGGCTGCGCGATCTTC
GGCCACAGACCGCCCCCCAACCAGATCCCAGAGATGACGACGCTCAGATGGATCACAAAC
GTATAGGGGAGATAGAGTCGGGGATGTTTTCTGCGTCCATTTCGGATGGTACCAATTGGT
GTTCCGGAGCTATCTGGCAAACTAAGGAAGGCCAACTAGAAGACTTGTCGGAGTGTGTCC
TTTCCATCTCTGGATTTGTCCTTGGAGTACATTGTGCGGCTTCCCTCGAGTGTCAGCCTT
TGTCTCCAGATACTGCAAGAGGCGATAGAAAATGTGATTGCGATGACAACTGCACCGTTG
CGAGCGGCGGCAAGAGGAAACCAAAACCAGTATCTTGTTGTTCATTAAATTCCTTGGATG
GAAACAAACGGCAAACTTCTCCCTATATTTACATCACGCGCTATGAGGTTCTTTACGAGA
AGCGATCAAAGAATCAGAGCCATGACGATGATGATAGCATCAATGATATCCAATTCACGG
AGCAGATTCGTTCACAAACGTGCGAACTCCAAAAAGAAGGAATACTTGCTGGTTTATTAG
CATCGAGAAAACTCGGTACGGACGTCTTGATCGGCGAAGACAAGGGTAAACTAATATGCC
ACAGCTACCATAACCGTCACGACCATCTTTGTCAAGAAGCAGCAATGCTCAAGAAAATAA
ACGAAAATGCCCTTGAAGTAAGAAATTTTATGGAACAGCATTTCGCCATCACGACACTCA
AGAGCAGGAAAGAAGATGCGTCGTTGATGGCGTCTTCCTCTTTCAGCCTCATGCTGGGAT
CGCAGAAATGCTTCCAAAACTATTATTCAGCTCTGAACGAATTTGACCAGGGGCAGCGCG
AGAATCTATTCACCTCCCTGAACGACACGACGGAGGAATCTTTTGTTGACGGCTCTGTGG
ATCCAGAAGAGACGGCAAGCGACACCCGCAGAGAGCAGAAACGGATCCAGAACAAAGAAA
CCATGGAATATGCATTGCAATGCACGGAGGAGCGGCTCTCCCCGGAGTTGCTGTTGCGCT
GGCATGCTTGGCTCCTGGGGGATGGGCTAGAGCAAGAAGCAGGAGCGTTTCGATCCGAGA
AAATCTCAGTACGCGTCGGAGCCTTTTGTGCATCCATGGAAGACCATTGGTTGCCGAGAG
TGAGGCAGGACCCAACCAATGCCGTTCGGATTGCGACATTTGCCGCCGCTGTGAAGCTCG
GTTTCTTGGACATGCGTCCATTTGAGGCCGGGAATCAGCTACTCGCAAGGATCCTTTTCT
GCTGGAGCCTTCGGCGGGCTGGTTTGCCCTTTTCTGTGATCACCTATTCCAATGAAAAGC
AGAAATTGGATTACTACTCTTTGATTGAATCGACGAGACGGAATTTATCTCTCGTGCCTC
TAGGACTTGTTGATGCTGACATAATGACACGGATATTACTCTCTACTGGTGGCCTTGCTC
CTCTTGTTGGTTTCCTTGTAAACTGTATTGCTTCCTCTGCAATTGATCTTTGCAGATTGG
TTGAAAAAAAAAAACAGCATGGCGTCGGAAGAAACTAATGCTCGTTTGGTTCGATTGGCCC
GAGAAAAGGCCGCTAAGGGGACTTGTATTATCTGCTTTGACGAGAACCCAAATATCGTAA
CCATGTGTTGCGGAAAGCCGGTTCATTTCAATTGCATGGCAGAATGGCTCACCTCTAATT
CTTCGTGCCCTCAGTGCCGATCGGATATTCCGTCCCTTTCTACAAGAAGGTTTTCTCAAC
CAGCAAGAGAGAATCCAGTGGTGGAATATGGTCAGGACCGTAGTTATCTCAGAAATTTTC
TGAGCGAAGAATTTTTGTATGCTCACTCTTCATCGAGCTCATCTTCATCATCGTCTAGCT
CCTCTTCGTCGTCCTCATCCTCTTCTTCCACTGAATCGGCCTTCTTCTACAGCGGTCTAG
ACTATGGTGTAAACGATACGAGCAGTGCCAACAGCCCTGTTGAAAACGAGAGCACGAACA

GTATCAGAAATCTATTGCCCTACAGAATCGATTCACCAGTTCCAAGCCAGATCCAAACGG
GCTTAAACTATGTCACAAGTGATGAAGATGAAAGTCAGCACAATTATGTCACTGACGATA
CCGACGATAGCGCAGATATTTCACAATCAGGGAATAACGACGATGGCGATGGACAGTACT
CTTTTGATTCAAATCATTCAGCGAATTACTCGACACATTTTGAGTTGACTCGGGGCAACA
GTGCAGACAACGATAGTGGGTGGGAGTCCGAGAGCACTGCCTTGAGCTTTGCCAGAAGTG
GGCGTCTCGAATTGTGCGACGACAGCGCGGCAAATAGTGAGCCCGAATTGTCCGATGATG
GCACAGAAAATAGTGAACATTATTCCTGCAGCGACGACGAACAGTCATCTCTGGGAGACC
AGGAACACTTGCATCGTTTTCGAATGTCCTTTCTGGATCCAGAACTATTGGGCAACGCCC
AGGCCCTCGAAAGCCGAAGAAGCAGAAGAACGTACTTGCACCGCGAATCCAGCGATTCAA
TGGTGGGTCAGGAACGGTTGCGGTTCTTGCGTGACGGCGAGGGAAACGGTTCTTTGGAGT
TTGCACTACCCGCCGACCCTCGATACCTGCGTGTCGGTGACGGCGATTCGTCACATGATG
CAAACCAATTGGTGTCGAGCATCATCGAGGGGAGCCTGACAAACGCCTTTGCAGACATGC
GATGCGATGTTACTCCCGCACAACCGAACGACCCCGTTCCCGATAACGACTCTGTCACGA
TATGAACGCAATCGAAGCGCCAACCAGTGAGAAATCTTGTTTGGTCCTGGCGGTATGGTT
TTCGATCCCCACGATTCCCAGTAATTTCATTTGCGCAAAAAGCCAATGGTCATTCTGCAC
AGGTGGGATAATAGTCTACGATCTTTGAGAAAGGTAGGTTGAGAAGTTGCAAAATCTACA
CTATCGTTATCATGCTATGAAATAGACATAAACATTAAAAATAAAAACTACATTTCCGGA
>comp2823_c0_seq1
TGCATCTCTGCGACTGCAAGGAGATTTTGATATAGGCACGATTGCGCTGGGTGGCACCAG
AAAGGTATTGAAATACTGCACCCATCGCTTTTTTGTTGCCTGTGCGCATGAGAGCCTCAG
ACACAACCGCTAGTTCGTTCGGCCGCCAAGGAACGCTACAGCCACAACCGAAAGAGAACT
CCTGAGTCTAGCGAGCGAGCAAACGAACGAGTGGCAGAAAAAATACAACCCATTGTCGTA
TTTGAAACCATGGGAAATCGGTGCAGAAGAAGAACAAGGAACGGACATCCACCGTGAGCG
GGACAAACGCCGACACGAAGCGGGACGAGCCGCCGGACGGTTGCTGTCGTCCTCCAAGGA
TGGTACGTTAGTTTCGTACAACGAGTCCCAATGTTCCGTAAAATAATAGAGTATATATAG
TATTTTTTCGTCATATCAAAAA
>comp2824_c0_seq1
GTACAAATACGAGTACGAACGTAGCAGCCTTTCCATTCGATTTCTTGGATCCCTCGATTC
GGTTCCGGTCTTTGTTCCTTCCACCCCGATTGCAAACAAGAATCCACAGCAGCATCGCCC
ACGGAAACGCGTACAACACAGAAAATAATCAAACACATCCGCCTGCAACCCCCATTCCAC
AGAAATCCAAACAAAACACACACAGAGAGAGAAAGACAAAGACACACACACACACACAGA
CACACGATGATGCTCCGAATCGGTATTTCACTCCTCGCCTTCCTCGCGGCATGGGCCCCG
CTGTCGTCGGCCCTTTCTTGCCCCCCGCAGAAAACGTCGGCTGCCAACCACCAATCGTCG
TCTCTGGCTTCCACACGCCGCTCCTTTGTCGGCGGGGCCGCAGCGGCCGCCGCGGGCCTC
GTAACTTCGGGCCCGTCGTTCGCTTCCGAGCCGCCCTCTTCCACCGGGCTTCCTTCCAAG
GGGGCCAGGGCTCCCTCCTTTGAGCTACCCAATTCACGCGGCACCGGTCTCGTTACGCTC
GACGGCCTCGTCAAGAACTCCAAGTGGACCGTTCTCTATTTCTACCCGGGAGCCTTCACG
TCCGGCTGTACCCTCGAAGCCCGTGGATTCCAGAAGGACCTGGAGGAGTACCGCAAGCTC
AACGCGCAGATCGTAGGCGTCAGCGTCGACCCGGTCGAGAAGAACGCGCAGTTCTGTACC
GAGGAGAAGCTCGACTTTTTCATGCTCTCCGACAAGGGCGGCATCGTCTCGAAAAAGTAC
GGAAGTGCCCTGTCCATCCCCGGGTTCGGGACCTTTTCGAACCGGCAGACCTACATCATC
GACCCCAGCGGTGAGCTGCGGTGGATCTTTACGGACGTCGAGTCCAAGATCGCCCGGCAC
TCGTCCGAGGTCCTGGAGAAGCTGGCCGAGCTGGAGCGGGCCTAGTCGCAACAACAAATC
ACCAACACGGAACAAACAACGGAAAAGAGAAACAAAAACGAACGCTGGTGAAGTTGAAGA
AGACGGGGATGCATTTTGTACCATTAGAAACGGAAGGTAGGAGTAATCTGCTATATCTCA
AAGAAGGAAGACGAACAGATAGATTTCAAACATGTTGCAGGAGGAAACGAAACGACGCGT
AGAAAGAAGAGAAGAGAAATGCGTTGAACGATTGTTTAGGGAATGAGACGGAACGAAAG
CAAATGGAAACATGGTGTTCTTTTGTTTCCTTTCTGGTGGAAAGAAAGTGCGTCAGTTTC
GAGGCAATCCAATGTGATGCTAAGGTTCTAAGCCCCACTCTATAACTGTTTTCGATATTA
TATTGAAGCTGAAGAACCGGCTCGTAGCACCCTATAACTGCAACCAAAAGAAAGACCGAA
CAAATACTTCTAATTGATAACTACAAAATTCTTATTGATTGATATAACTCAACAGATG
>comp2825_c0_seq1
CGTCTGGGCTCTCCCTCGACGGCATCAAACATGATACAGTCATAATATGAACGAAGTGCC
ACAGAGACGCAACAGAAGAACAAGAAAACAGAAGAAAATCATCGGTAGAAGTAACGTTTA
CCTCCTATTGGAAAATTAGGTTTCTGGAAAAAACACGTGAAAGGCAATAGTAAACATACC
GAAAGAATAAATGCTGTTTGGCATGAGGTTGGTGAAAACGATTTCGAGAAAATCTTTGTT
GGCCTTCGGCTACCCAAAATTGGAGACCTATCACCCGGAACTATCTGTCTATGATGTGGG
TGCTGTCAAGGTTCGAATGTCACCCGAACTTCCCGCGTGCCAAATATTTTACCCTGTGGA
GAAAGACTCTGAGAAGGATCATGTCGACGACGTCGTTCCATATTACCGGCCAGAAGCCGT
GGAAGCTTTGGTGGACTACCTCAATGGTTTTGGAGATGGAATAATGCAAATGTTGAATGA

AAAATCACATCCTCTTCAAAATACATACGCCATTGCTCCGTTAATGGTTGATAGCAACAA
TGGCGATTCTATGAAATTTCCCCTCGTCTTGTTCAGCCATGGTCTTAGTGGAAACATGGA
AATGTATTCTCAAATCTGCGCTCAGCTTGCTTCAACTGGATGCGTTGTAGCAGCGCTAGA
GCACGAAGATGGGAGCGCTTCCTACTCCACAAAACTTTTGAAGGACGGAACAGTTGAAGC
AATTCCGTACAAACAGCCGCTCCCAAATGCCGAAGTCCCCTACTCACGTAAAAAAGTCCT
AGATTTCCGAACCCCAATGCTCGAACAGCGGGTCAACGAGTTGCGGAGGATATATAACTA
TTTTCGGGATGAGAAAACTTCTGTGGACGAAACAATAACGACAAACTGCTATGACTCGAA
TAGTAGATTAGTTCGTAAAATTCTTGCCATCGCCGACCCGACCAAATTGCACCTGGTAGG
GCATTCCTTTGGAGGAGCGACGCAATTACTAGCAGCACAAAAATGGATTATGGAAGGTCA
CGATGAAAAAGTGCAAACCGAGGCAACAACAGAGGCGCATCCCATGTCTATGAGGCTTTC
CGACAGCGAGAATCCCGCCACCACTTCAACGATGACAATTGATACTTCTACGTCCTCGTC
GTCACCCCCTATTCCCCAGTCTGTAATGGTATTTGATGCCTGGAATTTCTCTCTCTCGGA
CGAAGTACTCAACCGGGGAATCTCGATTCCTTCCAGTCCTACGGGCTCAACAACAGCAGC
TCCCACTATCGTTTCTGTTCTTTCGGAACAATGGGAACGGACGAATCCTGAAAAAGAGCA
AACATTGCAATTCTTGCGCAATTGTCCGCCAAGTACGACGTATTCCTACCTTGCAAGGGA
TTCTGTTCACCAGTCTATTTCTGATACGGAATGCTACCTACCCAGTATTGTGGCCCGGAA
ACTAGAGAACCTTGGCCCTTCGGAAGAGCGGCACCAAACAGTTCGTGCGGTGGTAGAGGA
GTTCGTCAGGCGTACGAAGTCAAGTGGGAATGACTGCAGTAGCAAGAAGAGAGGTAGTGA
CGACAACACATTAATAGAATTGCCGCTAAGAAATATTCATATTGGATAGATGTAATCAAC
ACCTAAAGAGCAACCTCAGATGTCGCTGATATTGAATGAATTGAAAACAAAAAAGGCTAC
GGCATCGCCAATATACCAACAATGACAAACGCACTTCATGTCGAATAAAATGTTTACTTT
TTGAGACGAGTTCTTGGGCTCACTTGCTACGTATGAGAAAAGCTCCCATGGAAAAGCTCC
CATTAATTAGAAAGAATTTTGCAGCTTGCTTGAAACGTCGGTATCCTCAACTAAACTAAC
GCAACAATTCTTATATCAATCTTGATCCATTCTGTCATACTGAAAAGCATTGTGTTTGTT
GTACGGTAC
>comp2826_c0_seq1
ACCGGTACGATCCTTCAGATCGAGTGTAGCAGCGAGTAGTTTCAAAATCACAACATAGAC
AGGACTGCTTCTAGGCAACACCCAAGACAGAACGAAAAATGTTTGGGAACAAGAAGAAAC
GGCAGTCCGCACCCGGAGATGTCACCGGCACCCGCTTGAATGCCAAGCGGCGACGCCTAG
AAAATTCACCATCGAGAACTACCGCACCTGGTGACAGCGAAAGTCCCTCTGTGCGTTTGA
CTGATAAAGAAGTATCACATGCAATGCTATTGAGAGCTGCAGAGGTCGGACTCGATTCCG
CGATAAACGAGGCAAAGATGGAGAAATCGAGAAGCAAAATGGTAATCACTCCTCAAAGTG
TTATTCAGACCAAAAAAATCTCATCGGTGTCAGATGACGACTCAAAAAAAGCAGGCAGGA
CAGGTACAACCGTGACCGAGAGAAAACCAGATCAAGCCATCCAACAACTGGATCCAAAAT
TAGCTGCCACGAACATCTGCGACTTTCGGAATGATGTCGATCTGTCGCCAGACGATGATG
ACCAGAGTTGCTCGTCCTCATCGTCTTCTTCCTCGTCGTCGTCTTCCTCATCGGAAGAGT
CATCTGGTCTTTTGAGTTATCGTTCGAATAGTTCGAGGGAAGACGGCGAACAGAAACTTC
CACCACTCAATGTTGGTGAGGAATACGAAGGGATTCAACGGCTGGAGAAAAAGTTGAGTC
TCCGAAAGGAACGATTGAAAAAATTGTGCGCTGGCCGACCTGCTGCCTATGCAAGATATC
TTGAGGCAAACGAGGAGGCGGCCGGTGATATTCGAATTGCTGAGTACAAAGAAAGCTACC
CGGACGAAGACAAACCACTGTCTTGTGGATTGCCGCGCTCGAAGTTAATTCGAACCTGCT
CGTCGCGGTTGGGCCTCCTCCAATTTGAGGCTATGCATACATTGCCCTGCTTTTATACGA
TTCTGGTCGTAATGGCAGTCCATTCCACATTTTGTGCCTGCACTGAAACAGTTTCAAACA
TTTTGTATGGCTCTATTTTTGAAAACATTGTTAGCAAAAACGTCTACAATGCTTTTGTGA
TCGGCTTTGGACTCGCTCTCTTGCGTATCAATGGTCATTGCTTTTGGTATACGGGAACAT
CGAATGACTATACTTTGATTCAAATGGAAATGGGTAATCGGCTTAAACTAAAAAAGTTTG
ATGCTCGTGTACTAAAAAAAATCAAGGAATCTGTTGCTTGCTCGGCGTGCAACATGTTTG
GGTATTACTTGATTTGTGTAGGGATAGGCCACTTTCACTTCAGACACTTTGATAATCTGA
TGGATCAGTTCGATCTTTGGCTTGACAGCATATGGATTAGGGTTTCTGAAATTGTCAACG
ATGAAATGGCGAAGCAAGTACCTGAATCTGCAACATCAAACCTGGAACCATTCGGTTGCA
CCGCGGTCCCAGAAATAGAATCGGCAATAAGTCCAACATGCCAGGTAGCGGTCGACGTGC
TTGCGTCCCCCAAGATCGAAGCTGTTACATCTCCCATCACGAGACAGTTGTACCACTTTT
ATTGCCTAGATTTCACAAACGAATACAAAAGCATGCAGCTTTCGTTCACGGATTGTTTTT
GCTGGTTTCAATTGCCTTGGCTCGTCGGGTTGGTCAGAATTTTTTGACCTTGTGCGACTA
ACCGCATATTTGGCTACCTACACCAGAAATCCATTTGCTACTGAAAATATTACCACATTA
TGAATCCTCATGAAAATATAGTCCAGACGGTGACAAAAATGATAATGGTAGTACTTTTCA
TTATGCTATGACTACTCACAAGTTGGATAGTTTTACTAGTGTGAAATGAAATTAGATTTC
AATTTTTGGCGAAAGAATTAGATTTCCAGACCCTCCTCCTGGTACTGAAAATTGTGATTG
GTTTTGATTATTTCTATGTTTTCGGGAATGACCCAATCACCCGATGTCTGACCCCCGGCG
ACAACTGTGTTGAATTCATGTATTACGTCATCAAAAATAGTTAAATACAGAAAGCCTAAA

AGGGTTCTCCTCCACTGTTGACTAGATAAAACTATAAAGAATGAGGACATTG
>comp2827_c0_seq1
AACGAATTTTACCTGCACAACCCGCAGAAGACCATCTTTGCCCAGGCCAAGCGAAGAAAG
ATCAACGGACCAGAACATCAAAAGTATTTTTTCAATGATGACAACAATTTGCAAGAGACT
CTCAGTCCTCTCCTTACTCCTCGCGATCACATGGTCGACTACGTCGGGATTCTCGACTGT
AACGTCAAGTAGCCATCGTGTCGCGACGACGAGTATATCGGTGGCTACAGAGGCTTCGAA
CGATTTTGGATCCGCTATGCCCGAGGACGTTGATCCCCATGATATTATCGGTGTCAAACC
CGAACTTCTTGCGATTGGCATCAATGCCACCGAATTTTTGGAATGGATCGGAACAAAAGA
GGATTTGATGGAAAAATTGCAGGCCGATTTCAAGTCCTACAATTCAGAGCGAGTCGAAGA
AGAAGCCGACAAATTTATGATGGATGCTGAAAATGTTAATATGTACATCAAGTATCTGAA
GGATAGGAAGGCAAACCCAGCAAAATATGCAAACGAAGCCTTGGAAGCTGAGTTGTCCTT
ATCCAACCCGAAGACAGCAGCTACATATGCGGCATGGCTGATTGGTGGTGTATCCTTCGG
TGTTCTCAGGGAAAAATATATCGATCCAAAGTTGGCATCGGGAGAGTGGAAGGGTATCAC
ACTCGACTTGCCATTCCTGCACAAAGCTGACGCAGCTGCTGAGGCCGCAAGCACTCTTAC
ATCGAAGGCAAATATTATTGTAGAAGGAATCGCAAACATCAACGATTCTGTTGATTTATT
GGGTTAATAATCGTCGCTGCACTACTCATGAACCTCGAAGTGAATATTTGCGACTTCGAT
TTGAGTTTACACCGCTCAGATTGTCGTGGGCGATTTGTTGAATGCATTGATTAGCAATTC
TTGAGACGATTGGCATTGATGCTGTCCCTACAATACTTGGCAGAATCCTGGTTCGGTTCA
CGTCCCATTCTCTCGGGAATAATAACAGCGAGACAGAAGCTATGACGCAGAACAGTATTT
TTCTTGAATGGGCTCCTGTAATCATCAGGAACGAAACCCATTATCTTATCTCTCAGAGGT
ATTTACTAGTGCCGTCGTAGAACGGAATTAGAGCTATTATATCTACATCAAACACTAGAG
GTTTGCAAACCTAAACCCCTTTTCGTACGGGAGGTTCCGCCTCTCATGACCTGAATCTAC
TAAGTACGTAACTACAGCTGTAGGTTCTACTTCGTTTGTTTCAGAAGGACTCGACAGACA
CTCGTTCAACAATATTTCTACTGGGAATCGGAATTCTGCCTGTTCTTCGCTGGCACCTGT
GCAAACAATCACAAAAATGTTAGTAGGGTC
>comp2828_c0_seq1
CCACCATATTGAAGAATCACATCCATCTTATATTGTAAGGACCATGTCCTTTGCATCCGG
AGCTAGGAAAACCCTTCATCGTCGATCAACTCTTCTCCAAGCCAACGTTAGCGTCTTGCG
CCAGAAGTTGGGTTTGATAACCGCCCTGCGGAAAACATTGGGACCGAACCAAGCCAATAA
AGGATACGCACAGTCATGCCCGATTGTGGGGGCCTCCATTGGACAGCACTTTCGTCACAG
TATGGACCATATGGAACGTGTGGCCGACATCATAGAGGTTCAGAAGAAGGAGAACAACGG
AGACGGTGGTTTCAGCCGGGAGATTCATTACGATGTGCGCGTGAGAGGTGGTTCTGATGA
ATATGATATGGATGAGGCGGAAGCACGAATACGGCGAGTAATACAGCCTTTTCGAGACGG
CATTGATGCAATCGTATCTTTCTGTCCTACAGATGATGGTGGCGACGATACGGAAGTTTG
CAACGACAGTAATAGCGTTCTGGCTTGCTTTCTGCTTTCGGGAGATCCAAAAGAAGACGA
ATTCATGTTGTCGAGCACTGTCGAACGTGAACTTGGATTTGTGGCGCATCATGCGATCCA
TCACTTGGCGATGGTCAAGATAATAACGATGCACACACTAAAGCTTTTACCACCAGATTC
TATTCCAGAGGGGTTTGGGATGGCCCCGAGTACCATAGTGTACGACCATAGCCAAAGGTA
ACACCTATCGATGGTCAAGCAAGCATTAGAGATAAATCTCAAACATGACGGTAGTAGTTT
GATGAGACGAAGATATGCCTCTCTGGTTGATGCATTGGAAAATCGCCTGGAGATCAATGT
GCAGATTGTTGAGGGGAAAGATCTCCACTCGAACAGTTACCAGTGCTTATTAGGCTGAAG
AAGTCGAGTAGTTGACTGCACTCTTAATGCCAAGTGCTGCTTCCAATTCATTCGAGCCAA
TTAGCCATAGTTTGGAATTAAATCTTCGTTCTTACCCTTCTTGACGCCATTGAAAAAGGA
AGGAAGGAAATACACACACTTTGAAGAAAACTGGATTGAATTTCAGCCAAGCGAAAAGTG
AATGCAATAGAAAGGCAATTGGAGGAAAGTTGGGGTTGAATACAAAGCAAAATTAAACAC
TCAAATGTTAAATATTGAAACAGTTTAGATGTTTTATAAAAATTGGTTGGCTAACACAGG
CACTCACTACGGTGCAAAAACTAAGTGAGTGCATAGGTGGAAAAATTAAAAAGATTTCAG
GAGTG
>comp2829_c0_seq1
CAAAAATACCGAAACATGAAACTGTCGCTTTCTGTATGTCTTGCGGCTACTGCCGCCGCC
TTTTGCCAGGGCTTCACTCCTACGGCATCCTCCACACGACAAAGCACAGCTATTCGGAAT
ACGCCTGCCATGGGCCCATGGACCATGATGCCTGACGAACCGACACCCGAGGTCGACCCC
CGTCGCCGCAACCCCGATGAGCAGCCCCCTTCCAACCCCGTTCGAATGCCTGTTATTGAC
ACCGATAATGGACAAATGGATGTTCTCTCACGACTCCTCAAGGATCGTATTCTCCTTTTG
GGACAGGGTGTCGATGACGAGGTTGCCAACGTTTTGGTTGCCCAGTTGCTGTACCTTGCG
AACGAGGATCCCGAGAAGGACATCACCCTCTACATCAACTCGCCCGGAGGATCTGTTTCT
GCTGGTATGGCCATTTACGATACCATGCAGTATGTCCCCTGTGACGTTTCCACCGTCTGC
TTCGGAATGGCCGCCTCAATGGGAGCTTTCTTGCTCGGAGCCGGTGCCAAAGGCAAGCGA
CGAGCTCTTCCGAACAGCCGTATTATGATTCACCAACCTCTAGGAGGCGCCCAGGGTCAG
GCCGCCGACATTGAGATCCAGGCCAAGGAAATTTTGTTCATTCGCGAAGTCTTGAATACC

TACATTGCCGAGTATTGCGACCAACCCAAGGACAAGATCGAGGAAGATTGCGACCGAGAT
TTCTTCATGACCCCCGAGGAGGCCGTCGAATATGGTATCATTGATGAGGTCGTCAAAACC
AAGACCTCGCACATTTCCAAGCCTGCAATGCCCGCCCTATAATATAATTTTGTTGTATTG
ATTAGTACTTTTGCATGCTACTA
>comp2830_c0_seq1
ACCAAGCGTAGGCACGCATCACCTAACCATTTCGTTTGTTGCTTCATCTCTTGCAATCGA
TGCCATGAGGTTGCTAGCGACTATTTTGATGCTCGCGACGGGGCCTTCTTTCGTCTCTCG
TTCTTTCAGCCTTGTACAGGCGTTCCACACTACACAGCCACCGGTGCTCGGGGCCGCTTC
GTCGAAGCGATGCGGATACCGACGGCTTTCAAAAACACCTCCACTCATGCGCACTGAGCG
ATGCGAGAATAAAGCCTGGAAGCCGGCCTCCTTGTCCTCGCCATTGTTCACTGCCTCGTC
CCTCGACGGTGACGACAGCCCGAGAAACAAACAGAGCCGTGTTCGCCGTTTCTTCGATAG
AGCCGTTTCGTTTCTTGGGGTTGGAAGAGCGCTTTCGCGATCTATTCGTCGCTGGATGGT
ATCGATGGTTCTATTTATGGCAATTACTTTCTCGGCTCTGGATCCGGCCTGGGCAGCGGG
CAGCGGACGAGCGGGCGGATCGTTTGGGAGGTCGGATCGGTTTCGCCATCCGCCTGTTTC
GAAGGTCCACCGCCCTTCCGGTGACAATCGCAGGATCCAAAGCCGGCAGCACCGTCCGGC
TCCGATTCGTGTCGTGTATCCACCCGGCCGCTCGAACACGAACCGCATTCGCAACCACTC
CTATCGCACCTACCATCGACCGACCGGCGAAAAAGTGACCGGCGAGGATGTAGCTGTCGT
AACGAATACCGATGGAACGACAAACTTTGTGCGAAAGGTAAATCCACATCCTTTCCACGA
TTCGCGGGCTTCGGACATCACTGTAGCGGTATTATCCACGGCATTGGTGACCAGGGGGGT
TCTGAAACGAAGATCGGACAGAGAAAAATTCGGAGACGACGACCTGAGCCATCCTCTTGG
TCCGGGCCTGTCAGTGTGGAGTTTGACCGCGTGTTTGAATGTGCCCGACATCGGTGCTCC
CACGTCCGTTGTGCAAAAGATACAGCGATTGGCCGAAACGACGTCGACGGTAACCCGAGA
GGGATTGCAATCGCTGATTGCCGAAACTGCTTTGGAACTCGCACGTCAGGTAGGCAAGGG
CACCGTCGCGTCCGTCGAATCACAGTACCATCACTACCGATCGAGCGACCAATCCGTCGT
TCGAGCTGAACGGCACTACAACCGTATCTCGGCAAGAGAACGCAGCAAGTTTGAGCGGGA
ATCCTGGAGCAACTACAACGGGAAGATAAGACTAGACGATGTTTCGGAGCGAGACGCTTT
GTCTCCCTCTTCTGCTGGCGATAGTATCGCATCGCTCGCTCTGGTTCAGATTCATCTCGT
CATTGAGGGAAACACTATGGAGCCCTTTGGTACTCGTCAGGTTGAGACACGAAAATCTTT
GCGAGAAGCATTGGTACAACTCTCCGGGGATGTGACGGCGGTAGAGGACTGTGTCGTGGC
AGGGGAAGTCCTTTGGGCTCCCCAGTTGCAAGAGCAGCAGGAGGTGATGACAGAAGAATA
TGTTTACGCGAACTATCCTAGGTTATGGCCGTTGGACTACACTAAGTGACCGCAATTTTC
CCGAGATGGAATGAATGGTTGGCTAACGATAAATTATGAAAAATGGTGAATTTGTTTTCG
ATCACTACAGCCAATGTGAAAGGCAAAGAAACAGTTGGGGTTGCTAGTGCTGGCGGATGT
GCTTTGAAGCAGTAGCTCGTACTTGTAGAGGAAGTGTATGAAGTCTCCTGTTTCTTTCTC
GCAAAACTTCTCTACAGCACCCGGTGTCCTATGGATAACTAATATTCGTGTTTCTGTTTC
AGACAATGTTTCAAGAGAGGCCTTGTCATAGTCTTAACCCTCTC
>comp2831_c0_seq1
TGTCACTCACAGCAAGATAAATACACCGTTTGCCGAAACGCCATCGTCAAAAGCACAACT
GTTTGACTTTGCCCAAACATTTAGAGGTGCAGACACATACACCTGTAGCGATAAGAACGA
AGGAGTCTACCGAAAAGGTTTTGTGTCAAAGCAGCTATTACTCTTGTGAGCTGACAGAAA
AAGCAGTTTTGTCGATATTAAAAAGCATCGAGCGACATTCTAGGAGTAATCTACAAGTAA
CAACACCTCAACCATGTCAGCCGCCTTTTTGGTCGAAAGTCGAGGCACCCCCAAAAGGAT
GAGCGCGGAATTCAGCAGGGAGGTGGAAGAGGATGGCCTCAAGTTCAGCGTCGAAAATGC
TGCTGAAGACATCGTCAGCACCCTCTTTTGTACGTATGAGCCGCCTACATCAAAAAAAAT
GCAGTACAAGGCCGCAAAGGCCTTTCGCAAGCACCCCGAACGGTACAAAAATCGGCCGCA
TGTCCCAAAAAGTATTCTCAAGTTCAAGGTACCCACCCCCAAGATCTTGCGAAAAAAGCA
CCAGAAACTCGAACCGGAACCGGCGCAGGACGAGGGCCGCGACGATATCGACGGAGCCGT
GCTTTCTACTACAACAGATGGTGGTAAGAAATCCGTGAGGTGGAGGGACCAGCAGAAACC
CTACGAAGATCGAGAAGATCGCACCGATATTGAGAACACGGTTGCGGGTCTTGCCTCCAA
GTACTGTGGTGGGGCGTTCAACCATGGCGATGACATTGCCGATATTCTCTCGAGTCCGCA
GGGACGGGCGGCCCAGACCTTCTTTGGGGGTCCGGAAACTCCCGGAAGCGTGGCCAACAT
GACCTCGAGTTTGTTTGGGGGAACACCCGCTGCAGCGAACACTAATGCTAATAGTAGCAG
TACTGGTTCCGTCCACACGGACACCAAGATATTCTACGACGACTCGGGGAATCCCATCCG
GGAAGACGATGTTCCAGAAGACGAAGAACAAGGCTCCTACTTTCCCTCAACGCCGAAGGA
CGCAGCCGCCTCCGTTGCGACAGAGAATCCAGCATCTCCACAAACCGCCACTCGACCTGG
CGGTAACGAAGGGCAACCGAATCTCTTTTGCAGCAACATTCCCTTCATGGATACCGTCAT
CACAGGTATCGGGATTGCCGGAGTCATGGCCGCTTCCGCTGCCGTCAAGGCTGGGGTTCC
CGAAAACTACTGTAACCGTGTCTACACAACGAAATCCCAGAGATCCGCCAGCCAAGAACA
AATCTCTGAAGAACTCAGGCAGGCCCAGGAAACCCTCGGTGTGTATGAACCGGAGAGCTA
CGATAAAGAGCCTGACCTTTCGTCGGGCAGCGGGCTCAAACGCCAATCGACACCCCGAAA

CGTGCGTCGCAAGGGAACTCCACGCCACATGGACCCCGAGGATGAGCTCCGAGAAGATGT
CGAGGACAATGAGACAGTTGAGGACCAATCACAAGGTGGGAACAATAACAGCAATCCATA
TTCAAGTCGGCGCTCGTATGTCAACAGTGATATTACAATGGGCAATACCACCGTCAAGCA
TTTTGGCAATGGAAACGCCAGTCTTTTCGACGATATGGAAGAGGATGGTGCCAAATCTCC
TTCTATTTCCGGAAGATCTGGGCGGTCGCAGTTTGCGTCGATGGTGGAAGAGCTGAAGCA
TGTTCAAAAAGAGAGGCGTCTTGGAAGTTCCGTCAAGAGCCATGATCTCAAGAAGCAAAC
AACCAAAGAAATTGACCATCTCGGAGTTTGTGTATCGTCCATAAAGAACGATTACCAAAA
GTCCTGCAAGAGCCCCAAGCACTCAGTGGTGACTTCGAGCTATCTAAATTCTATCAATAA
ATCTCCTGTTGGTAGTCAGTTCAATGGTGCTGGCCTCCCTGCTACTCCAAACCAAAACCG
TCAACTCTCGGATCCCGATCTCTTCGAAAAACGCACTGGTTCTGTGGCCAGCTTGGCCAA
GCAGTTCGAGAGCCCACGAAGTAATCGGCTCCCTATCACTGGTCGCAAGAGTGTCGACCC
GAGTGAAAGCCGATTCTTTGATGGAAAGGTAGACTTGTTACCTCCCACACCTAGAAGAAA
GTCTCCCGAAACTCCAACTGGATATTCTATGGTCGGAAATAAACTCAAGGTGCACGACTG
GGACACTCCTTCGCCGCACTTTAACAACAAGGGAACTCCTTCTCCACGTTTTTCTAGCAA
TCGGTATACGGTGAAAGATATGATGAGTCCCAAATCTGGAACCACGTATAACACAGAAGC
CGAGGTTCGGGAGGCACCCTCCTCGATTTTAGACAATGTTGCAACCCAAGACAGGCCCAA
TTTTGGGGGCCAACGACCAGCATCGCTCGTGACGGGTCCTGAAACTGTTGACGACGATAC
TGTAGAAGAGAAGAAGGTGTCCAAAAAGAAAAGCAAGAAAAACAAGAAAGGCAAATCGAA
AGAGCAACCGGGTAGGAGTAAATCCTTCACCGAAAAACTCAAGAAGTCTGTAGAAAAGGC
ACAGATCCGGGTCAAAAATGCCACGAGGAAGGTGGAGGCGTCGAGGATACCGGACAAAGA
ACAAGACACCCTTTAATTTGATGGTGCTGTGATCGAAGTCGAACCTGACGCATTAACTAT
AATTTGGTGCGGTACAGAGAATTCACTCTGTAACGCCGCCTTGCTATCCAGAATGTGAAG
AAGAAATTGTATGATTCGATGACATCCATTGAATGAAGGGTTATAATTTATAATCCTCCG
ACTTTTTGTTGCTACTGGCTAGCATCACATCCTCGTGCTTGCCGATTTGCATTGTATTTC
TAAGTATATTTATTTCTAAAGAGGAGACGCGTTTGTTGTAACAATAATATTAATTCAATT
CTTTTGATCTGCAAAGAACAAGTTTTGTGTTATTTTCATTTGTATGATAGATTTCATCAA
ATCGGTGCAATGACGTACGCTACCAATTCAAAACAAAGCAGCCATTGTTCGGCTCCATTC
TTTCTTCGAAGTAGACACAGGGGAAAGCACTCATACTAGCACAAACCTATTTGCGCTGCA
TTACTACTTTTCGGTTAACACATAGTCTTCCTATCAGC
>comp2832_c0_seq1
AAAGGTTTGGCGTTCGGTAATAGACGTGTGAGTACCTAACAAACTTTGGGAGTCGACTCA
GGGGCAAGGGGTCAGACAAGCGAGGAACAGAGGGAGAACGATAGCTGAATAAGATATGGG
ATCTCCTCGGAGAAGTCGATCCTCGAATTTACCCCTCAAGTATTTGGCGGAAAACGAGGA
AGAATCCAGCACAATGAGTGCTGGTAAAGGTGCCACGAGTCCAAAAGTGAAGGGTCTGAT
AAAGAGGTATAGTGGCGCTCCCATCCCCCAGGAAGCAATAGCATCTCCTTCAGTTTATTC
CTCTTCGACAACAGCTGGATCGCCAGCTCCCAAAACAGCCCTTCACGACCAACTGCATGT
GTCCGTACAGGATGAAGACTTTGACACTCGTATCACGACTCCTACCCGTCTTTTCAGAAA
GCCTTCTCTACTCTCCCGATTATCTTCTACGATCAGTGATAATGGAACGACCCCCTCCAG
GCAGAAACAACGGAAAGAGTTGAAGAAGTTGAAGAAACAGCAGCGACGTAGAGACGAACG
GAGGAGGAAGAGTGATTCGCAATACTACGCTACAACCGGCAGTGTTTCCAACGAGAAGCC
CACGAAGGCATTGAATGGGAATCAGGTGGTTTGTTCTCCGACCGCTTCGGTCCAAACTTC
AAACAGTACTAGCCGAAAGTCGAGATCCAGCATCTTGTTTCTGAAACGGGTTCTTTCGTC
TCCCCCGATACTCCGGCAGCGATTGCGTAGCTCGAGTCATCTCTACGGCAACAAGTTCAA
GGAACGCGATACATCCGATGGTAGTTGTTGCCAAGACAACAGCAACAATGATAATATAAG
CAGCAGTTCGAGAAGCAGCGTGGAACTGAATCCTAAAACGAACAATGATACTGAAATGCC
AGAACGTATATCGATCGTTGCAAAGTCGGTGTCTCCTACCGCTTCGAAGAATAGACGGAG
GCGGACGGAGCTTGAAGAAGAAAGCGTAGGAAAGCCTGCCGATGCGATGATCCGAAGTGT
GAGCTGTAACGCAACATCCATATTCCACCATTCCTTTTCAATGGAATCGATCTGTGGCGT
TTCCATGGGGGAGCTTCACAACAGCGACATGAGTAACCTTGTCTTCGAGGAGTCCAATAG
GAAATTAATGAGTGCCACAAATTATGGCAGTGAGAGTAGACAAGGAAACCTCATGAATGA
TACGCTTGGTCAACAGAGCAACCTCGATTACTCATACTGTGATTCCATGGGACCCATTTC
AGAGGATCCCACGGTGCAAGAATCCATCGAATGCATTTTTGCCTCGCAATTAGAGGATGG
CCTGAATCTATGGGACGACGAACACGATGGCAGTGACGAGAACTGTACCACTGAAATAAT
ACTTCAAAGACATAACCCTTCGTTCGGAACCCTCAACCGGCCGCAGAACCCGAGCTCCTC
CTCTTCGCCACGCGGAAACTTGAATGAGTTGATTTCCCCAGCCGGTTTCCAGCAATCGAG
AATGAATCGGAAGGACCGAAAGAACTCTTCTAGAAGCCTCTATGCCAATATGTCCCAAAA
CAAGAGACGATACGAGCAGGCCAGCTTAGTCTATGTTGGAACATTTGATCCCTCCTGTCC
GTCTCCTACCCATACTATCAGTCAGGATAATGTTCGTAATATTTCTACCAATGACAACAG
TTTGGACAGCGCCACTATTTATGATACTATCAATCCACTTCCATGCAGGTGCGCTACAAA
CTTTTTGCCCGCAATCGAGCCTAAAGATTGGCCACAGGCACCCATTGCCCTTCGACCAAC

CCCAGGAAGCGGTACGAAAGTAAAAGCAATCCGACTTTGCAATTCCGACGAGCCCCTGTG
GGTCCCAGGCTCCCATTTGACGTGGTCTCAAAGTTTAGCTAAGCGATGGGGCAGAAAGTG
CGAAGACCAATCTCCTCACTTTGCCTCCTGCGAAGAATGTGTGATGCTGCCTATCAACAA
TGGAAACGAAGCACCGGGAGAATCACTTGTCACTGACTTTGAGAGCGAATTGTTCGAGGG
GACGTTGTTATTGCGTTTGCGCCATTCTGATGGAACAACTCCCGAGCCTTATGACGACTC
CAAGGGTTACTTTGCGGGTGTCAACCGTCGTTATCAAGTATGCATCCGGGGGCGCTTCAA
AAAGACCTTGTCCTTCACGGAGTTGAACAACGGGTTTCGACTCCGTCGTAAATTTGGAAA
GCTTCCATCGAAGTGGGTTTTGAAAAGTGCACTCAAGGTTGTTAGTTTTTTTGCCCCCCA
ACTTGATATCAAATTGGAGGGTGTGGACCAGCCATACTCGCTTACACCGCTAGGGAGCAC
ACCCCAATGCATCACGATCGACGATGCCGACGTTCTTTACAACGAAAAAAACAAGGAAAT
GAGCGGCTGTTCCGGTCTAAGCGACGGACTCAACAGTATCAACAAAAATAATCCGTTAGA
TGGTACCAATGAGGAACCCATTGAAGCTCATCGGTCTTTGATCGGGGCTGCTCTGAAAGC
GGACACATCTCTCCAGCGTGCCAAGATGCGCAAAAAAGCTTTCGATAAATTGTTTGTACA
AAAATCTCCGTGTCCGAAGACGGATCCCTCAAAGATCTATACATTTGAATTTTTGCAGCA
CATGCTTAACTTTCAAGATTTTACCGTTGAACTGGGGAGTGTCATGGGATCCCTCGAGTT
AGGAGACATACTGGACGGCCAGCCCTTGCAAGTAATGGCGGAGCACGGACCTTCCAAGAT
ACGCTTGTGGTCCTTTGACGTTTGGAATGAATGCTTGTGGAACAGAGCGAAAGAACACGA
CAAAGCCGTAGCTATTGCTGCGAGTAAATGACATTTGTAGAGGGCCAGAGCACAGTCCGA
TATTTTTTGCTAGGTATTGTTAGCTTACCGAGATGCATGAAACACAAATTGGAAATCAAA
TGCCAAAGCCGGAACGAAGGTATTATTGGACAGCAATTACAACATACTTGTAGTAGAATA
CTATTTCATTAAACATACCAGAAATAGTGAAG
>comp2833_c0_seq1
AACAACAGCAACAACAACAACAAACCCAAACCCAAACCCAAGCGCAAGGCGAACAGGGCG
GGCAAGCGCCAGGACAAGGTCGACAAACAGAGTCCGAAAAAGACGAGCGGGAACAAGCCC
GGCAAGAGAACCCCGAACCACAAGCAAGGCGTGGTCGGCAAGAGAAAGAGGAAGAACAAG
AACAAGAACAAGAACAACCAAAGCAACAACGACAACACAAACGGGCTCGATCGGGGCGGG
GGCGAATCCTCCTCCTTTCTGCTGGGGTCGGGGTTTTCCGCGGTCGACATCGTGTACGTT
CAGTGCCTCGACTGGAGCAAGGACATTGGTTGGAACGACAAGTGGAAGGACAACGACGCC
CTGGCCAGCTACGTTGCCGCCTGCAAGTCTCGGGCAGCCTATGCGCGAACACTCGCGGTC
GCAAAGAACGAAGGGAAGCACGACCGTAGCGGCAGCAAATTGTAGCGTGCCAGGAACCGA
GCAATCAGCACCACCAACACTGCCGGATCGAGGAAAACGGGACGAGATGCGTTTTTGGCT
TCGTTTTTGTTTTCGTTTTCTCTCCATCGCAATAGATGCATTTCGAGAATGGATGCCGTT
GTGTCACCGATGGACACATCGCCCAAAAAAAGGAAGAGCAAAGTCAGCGAAGCGAAGCAT
TCCACTTTTCTTGTGTGCCGACCGTATCGGTCCATGCCATAGTCAGGAAAAGTCAAAGCG
AGAAAAAAATCGATTCGAATCGATTGGCGGGTTTGTGTTTGGATCACTAAGCGGGGAGTGG
GCATGTGCGTAGTTGCGTACTCCCGCACACAATCCTTCTATGCAACCGCTAGAAACGCGT
CCAACCGGGACAGCCAGTTTTGTGGCAAACAATCCACCGATAGTCGACCTCTTGCAGCGA
ACCAAGGCACGGCTAGGTATGTCTACGTGTGGCTATTCCCTATAAGGCAAGATGCGATGC
GATGCTAGAGGGATAAAATAGCAATATATTCAGTATAGAAGCGATGGAGTCTCGATGCGA
TGATAAATGAATAAAAGAATTAATAGTAACAGCAGCAAATTATCC
>comp2834_c0_seq1
CTTCCAAGCCCCAACGCAAGGTAGGATCAGTGTGGTCATAGAAACGGTGCCGTTCAAGCA
CTGTACAGTATTTAATTTGCAAGACGAGCATAGACTTTTTCCGATAGCTCACGAAAAAGG
AGATAACGCATTCATTTGAAACTTGGGAGAGAAGATAGGGCGCGTCGATTCGATTGAGCT
CCACTTCCGACCAACCTCGCGAAACTATTGAACACAAAGATTGATTCAAATACTGACTTT
CCAAACGTTTTCATCGGCAGGAAACGATCGCAACTTACGTCAATCATTCCGCCATAAGAA
CTCGCCACCTGTCAGACATAAAAGCAGCACGATACTGCAACAAACGCCATCCAACTACGA
TCGATATCTTCCAATTTTTAAATACAATCAGTAAGGGTAATCATGACCGGTATCCAGAGC
AGCAGCAGCAGTGTGCTGATCTTGTTTGTCTTGGGATTTGCTAGCCTGGATTGTACAATT
GTGTCTTCCTTTGTGCTTCCTTCCACCAATACACGAAGCACTATAATACGGCAAACTGCG
AGTGTTGGTACACGCAGTGTCAGCACCTCCCGTGTGCGCAGCAACAATCTCCTTCTGCGC
ATGTCCGAAGATGACGACGACGACGACATGAGCTGGCTCCAGGGGCCGCCGGAGGGAATA
CCCGAGATGGAAACCATCGAATGTGACGTCGCAATCATTGGAGGTGGCCCTGCGGGATGT
ACCTGCGCACTCTATACCAGTCGTGCCGATCTCAGCACGGTCATTCTCGATAAGAACGAG
GCCGTCGGTGCACTGGCGATCACATCCCATATCGCTAACTATCCGGGAGTCGATCGTTCC
ATGTCTGGAATCGAACTTCTCCGCCAGATGAAAAAGCAAGCCGTTGAGTACGGGACACGG
ATCTGCGATCGGGCTCAGGTTTTTATGATCGAGATCCAGGACGACGGAGGCGACGACGAA
AATGGTTCGAAGAGCAATCCCTACAATAAGATTGTTTATACCCCCGATTACACTGTCAAA
GCCCGATCCCTGGTGCTTGCCACAGGTGCCATGGGACGATCCATGCCGTACCTTCCGGGA
GAGGAAAAATACCTGGGACAGGGAGTAAGCTACTGTGCCACTTGTGACGGTGCCTTCTAC

CGCGGCAGCGAGGTAGCCGTATTTGGATCCACGGGAGAAGCAATGGAAGAGGCCATGTTT
CTCACAAAATTTGCTTCGACGGTCCACTGGATTACACCCACCGACGTAGTAGGGTATCTG
GACCGACTCGACGCGGGCGGTTTTCAGAACGTTGAGATGAATTCCATGTTCGACGAGGCC
ATTCTTAGGGAGCTTCTTAGTAAACCCAACGTCAAACACCACAACAAAGCTCGCCTCCGG
GAGGTCGAGGGAGATGTCTCAGGCGTGACAGGAGTCAACGTCCAATTGATCGGATCGGGA
GAGGAATTTACCATCCCCGTCGAGGGCGCCTTTATCTATGGTGCAGGTGGTGGCTCCAAG
CCCATCACGGATTTTTTGAGTGGTTCCGGGGTCGCTCTGGACGAAGCCGGGGGGTTGTCA
CGGAGGATACGATGGAATCGACTTCGCACAAGGGGATTTTCGCCATTGGGGACATACGGA
ACACACCATACAAGCAGGTAGTGGTGGCAGCCTCTGATGGATGCGTCGCCGCCATGAGCA
TCGAAAAATTCCTCAAAGGACGAAAGATCATCAAGGTCGACTGGGTTCACAAATAAATAA
AAATGATGTAAAGCAAAATAAGAGAAATTCAATCCAGTCACAAAGCAATTGATACTAAGA
CAATCCTATTTGCGAAACATTCGTGGAACAATGGTATCATCGATGAAGTGTTTCATTATT
GAAAAAAATAGTGAACGAATATGTTCCTATTGGGTTTTCACTTGAATGCAATGGAAAGTT
GTGCAAAAGACCAGTCGTAGACAGAATAATTTGAAGATGTATCTACTTCTAGTACGTACA
TTTTTGTTTCAACATCAAAAATAAGAAATAATTACTGTATCCGATTTTGAGAATATTTTC
TTCCAATTCGTGGAAGCCTGTGTCAAATCCCAATCCGATAATGGGAACTATTCTCTGAAA
TCACGCACAGATCCGTCAATTCCACTACAATTGAGTGGTGGGAGGGGAATACGTGGAAAG
TTGGCAGACTAAATCTTAACTTCTCCTAGTCGCCGATGAGTGTACGAAACCCACTATTCG
AAGGTGGATCATGCGGGAAAGCTTTTCAAAAACTACCTCGTAGTAAGAATTATTCTTATG
ATTACTGTAACGCATGGTATTAATGAATGAATCGTATCGAAATAAGTTTCGGCA
>comp2835_c0_seq1
GGAGTAACAAGAATTGAGCACAACAGTATCACTGTTGCGAAATGAGGAAGTAGCATGACG
AACACTGATAATGATACCGTTACAGAGAAACGAGAAAATGGTGTGATGAAACGGATTGCG
AGCATTAGCGGTGCGAATGGCGAGAAACAAGGTGATGCATTTAAACTTGCACCGTTCAAT
CCATCCAGTGACCAGATTCAGGAAAAAGCCACATCCATGTTCGATCTGGGAGAGCATGAT
GTACTTTTTGATATCGGATGTGGGGATGGCCGCCTGCTATGTTTCGCCGCAACGAAGATA
CCGGGTTTGAAATGCGTCGGGATCGAGATCTTAGAAGAATTCGTTTCTCGGGCACGGGAG
CGTGTGAAGGAATGTAACTTGGAATCTCGAATCGACATTAGATTAGAAGATGCTACAAAA
TTGGTAGAAACAAGTACTGCCGAGATTGGAAAAGGCATAAAAGCGAACGATGAAGGGGAA
AAGTGTTTGTCCGACTTGACTTTGTTAGATGACGCTACAGCGCTGTATCTATTTATCTTG
CCCAAGGGCATTGTCAAAATAATGCCTATTTTGCAATCTTTGAAAGAGAAGAGAAACGAC
GAGGGCAAGCGACTAAAGGTATTGAGCTACATGTTCAAGATCCACGATTGGGAAGCAACC
AAAACTGATGTGTCAGGGAAAGGAGGCTGCCCGGTCTATTACTATGACTTCCAACCGTAA
AACACGCAGCTTCCAACTTTTCAAGGTCATTGCTGCCGATAGTTACTGATATCAAACTTA
AAAGCATTGCTAAAATCATACTATTGAATGACTTAGCGCTATCGCTTTCTTGTCTGTTGC
TAAGCGAAAGTTATGCGGATGATCAAAATAAGATCTGGGGTACAGCCGAGGAATGAAATA
TTCAATGATGGTCAATATAAATGTCGACGTTGCCTTCAAAGAATTCGCTTTCCCAATACT
TCAAACAACTTTTAAAACCCCAGCATGCACTAGTTGAAGAATCTTCTCTCTAGTTTGCGG
GTTGCTCATGGCCCGGTTAGCAGCAACGGGATTCTCGTGGAAGTCACGCATTATTTGCTG
AATGATTGGATCTTGAAGCAATGCTTCAATCCCAGGGTCCTTGAACAACACGGAATTCCA
TTTCACTGTGTGATCTTTCTTTGTCAGTATCAACACAATCCTATTCTCTTCGATTTCATG
TCTGGCATGGGTGATCTCGCTCGTCAGTTTTTTGAATGACAGCATGAAATCTTCAGATTG
GTAATTTTTGACAATCAGTGACAACGAACGTTCCCTCGACTTAAGCTCAATGTCCTTCTC
GGAGCATTTAGCTCGGACGTCTTGAACGTTTAGAAATATTCTGACTAGTTCCCCCTCGTC
GGAAAAAGTATATGAGCC
>comp2836_c0_seq1
CCCAGAGTAGATCTGTCGGAATATTATACCCAAGCTGTGTCCTTGTCCTTGTAGAGCCAA
CCACAAGAGTACAACCACAAGATTTAGGGATCCTTCACGAGGAGAAGAATCGGGAGGACT
TCAAACCCGGAGTGCTTTGTCAAAGATACAACAGACTTCACAATGATCGCTCAACTGTTC
CGTTCTCCCGTTTCTAGCCTCTCGTCAAGGGCACGGTTGTGGAGCCCTAGCCTTGCGCTG
CTGCTCGGTGTTTGGATGGCATCGATGCAGTTTTCACCGGGATGGATCGGTGCGGGGATC
GGCGGCCCGTGTTCGATGGACGGGGCTGGGCTCGGGGTTCTGGCCTTCCGGCCAAGCCCG
CAGCAGACCACGTGGGGATTGCGTGTCCGCCGGCCATCCGGCCCGTGCCGGGCCACCGCG
GGCCGTTTCCCGGGCGGATTCCCCGTGCTCGCACCGGCACCGGGTGACCGCTTGCGTCCC
GGGTGGCAAAGCCCGGGCAGCGGCCGGGTCCGACATCCAGGGACGGCGGACGCGTATGGG
CACTGCCGCCGCCGGGGCCTGTTCGATCCGCTGTTTGGGGGCCGTTCGAGCGAGGAGGAG
GACGGTGGCAGCGGTGCCGACACACACGGTTCGGACGATGCAAGGAACAGACGAAAACGA
TCGTTTTCGATGAAACGCATCGGCGGGAGACAAAGACGAGGACGGCGGTTTCCGGAAGAA
GCGTCCAAGCCATTGCCGCCAACGACAACCGACTCAGGGGACAAGCGGCGCTTCCCGGGC
GGGTTTCTGATATCCGCACTCCTGGCCCTCACCCTGCTAAAGAACCTGTTGCTCGGCTCC

GGCGGTGGCGACGCTGGATACTACTATTATTCGTATTCGAGCAGCAGCAGCAGTGTCTAC
GAAACAACCATCGATCCGGACGGCAGTCGAAGGACGACCGAAACCTCCAGGAGGGTCACG
TCGGACCTGAAATCGAACATTCCCGGGCTGACGAAAGATTCGATCCCCGGGGCCAACCAA
AACCTTGGCATCGTGATCTACGACGATGGATTTTAGAGCCACGGACGATGTGTTGCGTTT
CGCCGGCCTGGCATGGACCGAGGCGAGAGAAGCCGTTGCTTCCATCGATTGCCGGTTTCG
AAATCCATCCAGCTCTCAATAATGAATGCACAGAGTCGGCATGATGGATTATCGCTTTGG
AACAATGCATTGTACAAGAACGCCCAAGAAACCGCAAGGGGACAAAGAAGCGAGACGAGT
CCCGTACGATGACCAACGAAGGTGCCCGGCGGAAGCCTTTACGGACCTGCCAACCAAGGC
AATGGAACGAAAGCGTGCGGTACGTACGCGTCGCACCAAAAACACGAGATGGGCATCTCG
TCGGCGTTCCCATGGTATCACAAACAATCGCGAATCCAGCCAGCTCCGCCCTGCCCTGCC
CTGTCCTGCCTCGCATTCGGATCGTTCCCCGATCCAAGCGACCAACGCAAAAATAAAGCA
ACA
>comp2837_c0_seq1
CGATTGTCGTAACGACTTTCAGCGTTGTCGAGGAAGGAATTGCAACACCCAGGTTGGTTC
TTGGAAGCCGGGAGGGCATGGTGGTGCTACATTCAAGAAAAAGAACCTTATGATGTACTT
CTAGTTGTTTTTTCCTCGGATATGGCTTCGTTCGAGGGGCAAGAGAGAACCAAACAAGCA
AGAATGTTCAACAGAGCAATGGCATTCTTTGAAGTCCTTAGTGCGATCATTTTCTGCAGG
TCCGTCTCCCATTTGGAAGAATAGTCTAGCCCCGAAGCTCGTTGCGATCGATACTCTCCA
AGACAGTATGTGATAATCCAAATTACAGTCTCATGATGATCAGAGGACTGCACAATCGCA
ACAGTTTCGATCCACTCTTCGGAGATTGTCGAACGCCTTTTTTCTATTTCGTCCCCAACG
GTAGCAACAGCACGTCTTACATCCCTGCCACTCTGTGTGCTCCGTTGAAGGATTAAAAGA
AACGTAATTACGTGCTCGACAACGTATTTGATAACCAAACAGCAAGAGAAACTGATGATA
TTATGATAGACTGCGTACGACTTGATAGACGGTGTATGGCCACCCGGTCTTTTCGATTTC
TTTGTCGGTTTAACGCATTTGCCCGTTTGTGGTTGTGGAAGCAGTCCATCCTAGAGATTG
AATAAAGTATCTGTTGTAAAAGAAGGGAGAC
>comp2837_c0_seq2
CGATTGTCGTAACGACTTTCAGCGTTGTCGAGGAAGGAATTGCAACACCCAGGTTGGTTC
TTGGAAGCCGGGAGGGCATGGTGGTGCTACATTCAAGAAAAAGAACCTTATGATGTACTT
CTAGTTGTTTTTTCCTCGGATATGGCTTCGTTCGAGGGGCAAGAGAGAACCAAACAAGCA
AGAATGGTGAGATGCAATTTGCTCTATTTATCTCGGTGCTTCTTTTGACGATATTCTTAC
AGTTGAATACAAAGTTCAACAGAGCAATGGCATTCTTTGAAGTCCTTAGTGCGATCATTT
TCTGCAGGTCCGTCTCCCATTTGGAAGAATAGTCTAGCCCCGAAGCTCGTTGCGATCGAT
ACTCTCCAAGACAGTATGTGATAATCCAAATTACAGTCTCATGATGATCAGAGGACTGCA
CAATCGCAACAGTTTCGATCCACTCTTCGGAGATTGTCGAACGCCTTTTTTCTATTTCGT
CCCCAACGGTAGCAACAGCACGTCTTACATCCCTGCCACTCTGTGTGCTCCGTTGAAGGA
TTAAAAGAAACGTAATTACGTGCTCGACAACGTATTTGATAACCAAACAGCAAGAGAAAC
TGATGATATTATGATAGACTGCGTACGACTTGATAGACGGTGTATGGCCACCCGGTCTTT
TCGATTTCTTTGTCGGTTTAACGCATTTGCCCGTTTGTGGTTGTGGAAGCAGTCCATCCT
AGAGATTGAATAAAGTATCTGTTGTAAAAGAAGGGAGAC
>comp2838_c0_seq1
CGATTGCGAAATTGACTTTCTCCCGAATAGCAACACAAATAACTTGTTTCTAGATACGAA
ATCATAAACGGAAACAGGCAAATTACTACCGGAGCATTCCTAACAACAAAAGCAAGACCA
ATAATTAAGAAAACTTCTTACCCCTCGAACTTGAAGCATGGCAGAATCGATGGAAATGGT
AGACCAGTGCCAATCGGTCACGCCTCCGGAGGTTCTGAATACGAAGTACAAACCGATCTC
AGACCCTAGCCTTCTTCCAAAAAAACGATCCCAGATTCCCGGTGCGTGCTCCAACCTCGT
CAATTCCATTGTCGGTGCTGGAATAATTGGGATTCCCTTTGCCCTCCAGCAGAGCGGCCT
GGTCAGCGGCATTATACTGCTGTTGCTGGTGGGTTATTTCACCGACAAGTCCCTGCGCAT
GCTAGTGAATCTGGCGACCTCGAGTTCTAGTCTCAGAGGCAAGGGTGTGTGGACATACGA
CGATTTGATGCGCCTCCCTTTTGGGCCTCCGGGAAGAACTTTTGTCTTGGTGAGCATGTT
TGTCACGGCCTATGGGGCAATGGTTGCCTACTTGCTGATCATCAAAGACACTCTACCAGT
CGTCTTGGGCCTGGTAGACGACGGTGACTCTAGCGTTGGTGGATTTCTTGAACGGGAGCT
CATAATGATCGTCACGAGCTTGGTAGTGGTGGTTCCCTTGTCCATGCAACGAGATTTTTC
CTCCCTAGCCTTCACCTCGAGCATCAGCGTGACGGCAGATATGATTTTGGTAGTCTTTGT
TGCAGCTTTTGCGCCTATCCAGGAGACAGTATCGGCAGCGGGAGGTTTTGGTGAAGTCAT
TAAGAACTCTATCATCGACAAGGGTTTTTTCATTGGATTTGGCGTTTTGACGGTTGCTAT
GTGCTGCCAGCATTCGGCCTTCATTGCAGCTTCGTCTCTCGACAATCCGACGCCTAAGCG
TTGGTCCGTCGTCACTGCCGTATCGTTGACGATCTCGGTAGTCTGTTGCCTTGTTCTAGG
TGTTTCGGGTTATCTTGGATTCTTGGATGACACCCAAGGAGATATCCTCAACAATTTTGG
GGTAGATACGGTCGTCGCCAATACGGCAAGAGCCCTGCTGGCTCTGACCATGTTTTTCAC
CTATCCCATGGAGGCCTTTGTGGCTCGGCATGTTCTGGTACAGCTCTTGTGGAATGGCGA

TATGGATGGCTACATTGTCGACACGGTGGCGAACGAGCAAACCGGGGAAGAAACAACCTC
CCAGCGCAAGAGCAAATGCTGCTATGTGTTGAACAGAAGGCACCAAGTTACATTCCTCAT
TTATTTATTGACGTTGATGCCGGCATTGATTGTAGACGATCTCGGTCCAGTCCTTAGTAT
AACGGGGGCGATTGGTGGCTGCTGTCTCGCCTACATTGGCCCGGGATTGGTGTACCTGGG
TATTCACGGAGATGACTTTATAAACCGTTTGGCCAACAGAATTCTGGCCTTGAATACGAA
CAAGGCGTCGAGTTCAAATGCTTCTGAAGTCGATTTGCCGCTAGAGGGCGACGCCACAGC
GCAAATGTCGCCCGGGGGCAGAGCGGTCGATCCCCGCCAAGTATTTTCAACAGAATACCT
CTCTGGCTACCGAAAGCCCTGGTTTTGGTATGTGCTGGGCATGCCCCTATTCGTAAAAAT
CGCGTTGAAGGGATCGAATGGTTTGAGCGAGCGATTGGCTGCTTTGGATGGGGAACACCA
CAGACCAAACTCTCCCACAACACTCAGTGCAGATAACTTGGGTACTAACGATTCCCCTCC
GGCAACCATCAATGACAACAATGAGTGCATTGTGGAACCCAGCCTTGTGGACTATGGATT
TGCCATGTTTTTCATAGTCTTTGGAGCAATTGCCCTGGTTGCGGGGGTTGCCTCGAATGT
TTACGTCCAAATCAATGGAATCTTTAATATTCCTGCCTAACTAACTCTTCGTGGTTTGTC
ACCATACTAGTTTTCTATCCTTATCCATCAGAAACTGCAATTGTTAGAGAAAGTCTGAAT
TTTTCGTAAGATTCAGCTCTCGTATAGGGGCACGAAAAACCAATGTCTTGTGTGATATAA
TTCACCAGGTGATCGGAAACATCTATCGAAGATTCTGAAGCAAATGTTAGATGTAAGGCA
AATCGCATTGTTTTAATACTAAATTAGGATTCGACGA
>comp2839_c0_seq1
CGCAATGTCTGACAAAACACTACATCAAGAATATTTTTTGGAGCTCGACGTCTTCGGAGC
ATAGCTACATCCAATTATAATGGTAGCGACCAAACGGAATTTGAGGACTTCGAACTATCG
AGATCCCGACGGCAGCAACCTCTCGGAGAATAGTGGTGGCAACTGCCCTGAGAATCGTTT
TGACAAGCTGATTCATGACGTGAGCGATCACGGTATCTACGACAGCAAGGGCGAAAAATC
GATGGCTCAAAGTGCAGGCCTCTTTCTCAAGATAGGCGTCGGGAGTGCCGTTTTTATTAT
GGTGCTACACTTTCAGACATCCTGGATCCAAATATACCACGACCATCAGTTTGTCATTGG
AGGGGATGAGACCCCAAAATTAAACGGAGGTGGCGGATTTGATAGTCGTCAAAAAATCAT
CGGCCTCGAAAATCACGATCGCAAAGATCAGGGATACGAACTCTTTCCATTTCTAGTGGA
TGCCGAAAAGGTTGCATCTTCGCTGTCGTCATCGACCGCTGAATTGAACTCTGTGTTGTC
GACGCCAACTATGCCTGACTTCCCTTTAGTCCCACGTCTCGAAAGATGTTTGTTGAAGTG
GAAACCTCCACATCCACCACGGTCGCAAGAAGACAAGAGATTAATGTTTTGGATGCCATC
TTATCCATCTAGCGGAGGCTCATGTCCCACGAAGAAAGGGGACATAGTACGGCAAGTCAT
AGATGGGCTCTTTCTCGGCGAACGGATCGATGCTACAGCCTACGGAAACCCGACCAAAGA
TTTCCACGCTTCTTCGAATAATCTGAAACGTTGCATCGGTGTGAGTGAAACGATTGCATG
CACGAGCTCTCATCCGCTGGTCTCGACAACGCCTCACTTACGACACCACGTTTTTCGAGG
CGCTGTCATTGTACCGATCAGGAATCCTGCTACTGTCATCCCGCAACACTATACCTCAAA
GGACATGGCTTACAGACAAGCCAGCGGACAAATCTCTGTCGAATCATGGAGGCAGATGCG
GGATTCGTATTTCGAGGGAAGTCTTGCAGATTGGATGAGAATCATTCGATTCTGGAGAGG
TACCAAAAGTCGTCTTGGTGAACCAGCATCTTATTACCAAACCGAATTGTACGTTCCCTA
CGAAGATCTCATTACAACCGATGTTGTAAAAGCATCTGCGATAATAGAAAAAGTATCCAA
TCTTTTAGGTGGCGCTGGGTCAACCGAGATAGTGAATGCTTCGAATGCGACGAGCTCTGA
TTTGACGAAGCATGGTTTCTTTGAAACATCCAAGTCTAAGGCTGACTATGAATGCCTTTG
GTATCGCGTGGCACATAAAGAGTGGGAACGATTGCAAAATGTTATTGGACACTACATACC
AGCATACACAGTACGGCAGAAAAATATGATGATTCAAAATCTAACAATATACGCAGACGA
AATCGAAGCCGAAATTTTAAACGACAAGAGTGATTTGGCCCTGGCATCCTTGCTACGTCG
ATATGCCTTCCAAATAAAACATTATGTGGTTGTCGAAGATGTTATTTATACCCCTCAGTA
GAACAATGAAGCGGTTGGCAACTGATTCAAACCCGTCGTGCTTTTTCGGTCGTCACCACA
TGACGTGTATACGGGAGTGCATAGAAAATGTTGGAGAACTGTACCTGGTCTTGTCTTCAG
GAGAAATGTGATCAGTATGCCACCGCGTTGCCCGCCACTGTTTTTTCAATTTGTTGTTCC
AATCAAAATCTATAGCGCCAGTTTTAAATAGTGTTGTTACTCCTGCGATGGTGCCGCTTA
ATCAGGAAAAAAATATATTCTTGCTGGGTCAATTGAGGCTACTTCTTGAACTACTAGCAT
TATTTTTATTCTGAAGCAGAAGACTTCTTGTATGTTCATACATCGCATGACATATTCTTT
CGTATTATTTCTTATTAAAATACTAGTGATACTAACATCACAATTATGATAATCGTCATG
GTACGGAATAAAGAGAAATAGCAGCAAAC
>comp2840_c0_seq1
CAAATACATACACCACCACCGACATGGATGCCCTGCTAGCGGGATACGGGTCGTCGGACT
CCGAGGACGGTCCCTCCCCGGCTTCCGGGAACAGGGACGGCGAGACCGGAGAGCCGGGAC
CACAAAAAGAGCAACCAAAGCCAAAGCCAAAGCCGGCAGGGCTCGCGCTGCTGCTGGGGG
GGGGCGCCGACGACGGTTCGTGCTCGTCGTCGTCGGAGGGCGCTGCGCACCGGGGGCAAG
CGGGCCGCGGCGGCTCTCCCGCGGAACCGGCTTCCAAGAAGCCCCGCCGCGGCGGGAACG
AGGGGAACCACGACACCAACGGCGGCGGCAGCAGCAGCCGTTTGTTTCTGGCACGGCACC
GCCTTCCGTTTCCGCGGCTGGCGTCGCTGCGGAGCGGTTCCACGAGTCCGTCGTCGTCGT

CGTCCATGGTGCATCGGAGCAGGGACTCTTTGTCGCGGGAAGCTCCCCGCCACCGCGAAA
AAGGTTCGCCCGAGACAATAACGGCATCCATCGGGAGCAAACAACACCACCAGCGGTTTC
GGTTTAAGCAACTGGCGGCGCTGGTCGCACCTTGTGGCGGCAGCCACAAAAGCTGGGCCG
AGGCCATCAAGAGCGAGCAGGGATTCCACAACCCGGGCGTCTTTCAATCGGTGATGGAGC
GCTCTGGAATCACCGAGCCCCTGGGGAGCCGGGCCGATCCCGCGTGAGGGAGCGATCGAT
CGATCGAGAAAAAGGCCGAGAAAAAGGCCGAGCGACCGGCGAAAGAACAAGCAAACGGTG
CGGCTCGGTACAGGCGCCGTGCGTGCCCGCCGTCCCCCGGACACTAAGACAGACGAGGCA
AAGGATTCGACAAGCGCGCGGGATGGGGATCCTTTGCGGCGAGCACAAGGCCACCACGGC
ACACGACAAGCCATTCGTTTGGCACGGGGAGTTTCTCCGATCGCCTCCCAAGCGCTCCAA
TCCGTCCATACACTACAGTAGTTCAGTTTTTTGCGTTTCAAAA
>comp2841_c0_seq1
AAAACCGACCTTCTGGTGCCAGTATCCAATCCATCGTCGAAATTATCTGAAGGTGTAGTA
ACAATAGTCTGCAAAGATAAAAAACCAATATGACGTATCGCGCTCGAGGTAGATCACTGA
TAGAAGTCGTTCTGGCAGTATGTTTTGTGGTATTCCTAGTGGGTTTCATAACCGTTCTAT
GCATTGGTAATTACTTCCGAGCGAAACGAGCGCAGGAACGACAGAGGCAGCGGCGCCTTG
CGAAGCTTGCGGCGTCAAACAATGCAGCGGCGGCTCAGGGAATGTATGCAACGGATGCCA
ATTATTCAGCATCGTACGAAGCCCCGGCACACGTGGCGGGAGGTGGTGGTGTTCAGGCAT
AAGGCAATGCAAACTTAACGGCCTATCTTTCAAACAAACAATAGGAGCGAGCAGGACTTG
AACCATTTCGTCGTTGCCGTCAGTATTCTGTTTCTCTGAATAGACAGAGAAACCAATTGC
AATCCGGGGATCGCGATTCTACATAGTAATTCGGCAACTTTTCGTAAGACGGAAAGAATG
TTCTCCCTACTCCATACACAATAATGTATATTAGTTCTGAACTTTACCCATCA
>comp2842_c0_seq1
CAAGTTAATTCGCGAACAAGATACCAAGATGGCAACGCAAACATGGGACGGAAACGCCAT
GTCCCAGGAGGACATGATGATGAAGGACACTGTACTCGTGCTGGATGACGATGACAATGT
CATTGGGTCGGCCTCGAAGAGAAAATCCCACGAGTTCACGCCCGAACAACCCCGAGCCAT
TTTGCACCGAGCTTTTTCAGTCTTTATTTTCGACGAATCGACCGGGGAGCTTCTGCTGCA
ACAACGGGCTTCGTCCAAGATCACGTTCCCAAATGTGTGGACCAACACATGTTGTTCTCA
TCCCCTTCATGGCATGGAACCACCCGAAGTTGACGGACCGAAAGATGTAGCGGACGGAAC
TGTTATGGGAGTCAAGAACGCCGCCATTCGAAAGCTCGACCACGAACTTGGAATTCCTGG
ATCTGAACTAGACATTAACAAATTCAAGTTCTTGACGCGTCTTCATTATTGGGCCGCAGA
CACTATCACACATGGTCCGGATAGCCCATGGGGAGAACACGAGATTGACTACGTCCTTTT
CTACACCGTCTCTTCGAAGTCGGATCTTACTATTAAAGGTCACCCCGACGAGGTAGACGA
CGTGAAATGGGTCACTCGTGATGTTCTCATTGAAATGATGGAGGATAAATCGTTGCTCTT
CTCGCCTTGGTTCCGTCTTATTTGTAAAAAGTGGCTTTTGGATCACTGGTGGAAGGATTT
GAAACGGACCATGACAACTGATGATCACGTTGATTTGAAATCCATTCATTGTTTTGATCC
TCCCAAGGAACATCTAGGTGGTGGTGGCAAGGCTGGCCCACTTTTTGGAGCTGAGGTTGA
AGGTGATGAAAGGTAAATAAGCATTACCAGTTTCACTGCTTTGCACGCGCTTCCAATTGA
CAAAATTCTCTGATTAGGACGGAGAGAGAGAAAATATGCAAAAGTGATTACGCTAAACAA
TACAACCATGTTTAAACTACTGCAGTCAATGTACC
>comp2842_c0_seq2
CAAGTTAATTCGCGAACAAGATACCAAGATGGCAACGCAAACATGGGACGGAAACGCCAT
GTCCCAGGAGGACATGATGATGAAGGACACTGTACTCGTGCTGGATGACGATGACAATGT
CATTGGGTCGGCCTCGAAGAGAAAATCCCACGAGTTCACGCCCGAACAACCCCGAGCCAT
TTTGCACCGAGCTTTTTCAGTCTTTATTTTCGACGAATCGACCGGGGAGCTTCTGCTGCA
ACAACGGGCTTCGTCCAAGATCACGTTCCCAAATGTGTGGACCAACACATGTTGTTCTCA
TCCCCTTCATGGCATGGAACCACCCGAAGTTGACGGACCGAAAGATGTAGCGGACGGAAC
TGTTATGGGAGTCAAGAACGCCGCCATTCGAAAGCTCGACCACGAACTTGGAATTCCTGG
ATCTGAACTAGACATTAACAAATTCAAGTTCTTGACGCGTCTTCATTATTGGGCCGCAGA
CACTATCACACATGGTCCGGATAGCCCATGGGGAGAACACGAGATTGACTACGTCCTTTT
CTACACCGTCTCTTCGAAGTCGGATCTTACTATTAAAGGTCACCCCGACGAGGTAGACGA
CGTGAAATGGGTCACTCGTGATGTTCTCATTGAAATGATGGAGGATAAATCGTTGCTCTT
CTCGCCTTGGTTCCGTCTTATTTGTAAAAAGTGGCTTTTGGATCACTGGTGGAAGGATTT
GAAACGGACCATGACAACTGATGATCACGTTGATTTGAAATCCATTCATTGTTTTGATCC
TCCCAAGGAACATCTAGGTGGTGGTGGCAAGGCTGGCCCACTTTTTGGAGCTGAGGTTGA
AGGTGATGAAAGTAAAAAGCAAGGTGCTTACGGAAAGATTAAGACTCACAAGGAACCTCT
CGTCAAACAGCTATCGCATCTCGATGAAGTATTTGCTGCGGTTACTTTTTTGTATGTGAA
GCCATTGAAATCCAACCTCGACACACCCTACATCCGCGAAACTTTTGATGCTGATGATCT
GGCATTCTGCGATGAGATTTTGGTCAAGGTATCTCGTAGTTTTGCGGCAGTCATTCGCCA
ATTGCCAGCGACCATGTTGGTAGACATTCTCATCTTTTACCTCGTCCTTAGAGCCCTTGA
TACGGTAGAGGACGACATGACATCTTTCCCTAGTAATGATGTTAAGATCAAGCATCTTTT

GAACTTCCACAAAACGGCCCTCGCTGATCCGACTTGGACAATGGACGGAGTCGGTGAAGC
TGACGAGAAACGGTTGCTCCAACAATTCGATAAATGTCATCGTGTGTTCGCAAAACTCAG
TGACAAGTCACGACGTGTAATTGTAGACATCACACAACGCATGGCTACTGGAATGGCTGA
ATTCGTCGGCAAGGATCTTGGACAGGGTACGACCGACATCGATCAGTACAATCGGTACTG
CCATTTTGTGGCCGGACTAGTCGGTGAAGGACTCAGTCGCTTGTTTGCAGCCTCTGGACT
AGAGAAGGCATCTTTTGCCAGTGAAGTTTTCCTGAGTGACCAGATGGGATTATTTTTGCA
AAAAACCAACATTATTCGAGATTATTTGGAAGATTATGTTGATGGCCGTGCCTTTTGGCC
TCAGTCGATTTGGAAAAAACATTCCAAAACAGGAGAATTGGGGTATTTTACTGAACAAAA
CGACCCTGAAGTCCGAATGCGGTCGTTGGAGTGCCTCAACGAATTGGTAACGGACGCTCT
AGAACTTGCACCTGATTGCCTACTCTACTTGTCCAAACTGGAATGTGCTGAAATCTTTCG
ATTTTGTGCAATTCCTCAAGTAATGGCCATTGCTACCTTGAACTATTGCTACAATAATGA
TGACGTCTTTACTGGTGTTGTGAAGATTCGAAAAGGAACCTCCTGCAAACTTATTCTTCG
CACTAACTCACTGGACGAAGTTCATGATACATTTTATCAGTTTGCCCGCGGCGTATTAAG
AAAAGCCGATGCGAACCGATCGCAGGGTGTCCTCGATCCATCCTATGCTCGAACCATCAA
AATTTGCAAGACCATCATGGATATTACTGCCGACGGACACAACAGGGAACGCAAGGCCCG
TCAGCTGCCGTGGATGGCCTCGCTCTTGGTGCCCGCAGCCGGAGTTGCCGCATCGATGTT
GCTAAAGATTGACGTTACTAAAGACATGCCCAAATTCCAACGAAGTCTAGCACTTCTTGG
ACTCAGCGCATTTACCTTGACTCCTCTCTTCTTCCAAAAACCATCTGATTTAACGGATTC
GAAATTTTTGGAAAATAAGGGCAAATAAAGCAACTAGGAGCTATTCCATTCAATGTTAAT
TTAATGGTATTTTCC
>comp2843_c0_seq1
CCAGAGCACCCCAACACTTTCGGGGTCCGCAAAGTCCCAGAGACAACGGTTCAAGCCGCC
GATAGTTCGACGAGGAGCACAGGAGAGCAGTTTGTGCTGCTGTATCTCGCCGTTCCACGC
TAGTACAACACCCCTGTACTGCCGTTGCATATTTGCAAGCACTGCCCCCGTTCTCGTCGG
TTCTGTTCCAACACACCACACGACACGACACGACACGACACATCGCGACACAAGAAAACA
AAGCACCAGCATGCCGCGGAAGGATTCGTCCTGGATCGAGGCCAGCGGCCTCTACGGCAC
CTGGACGTTTTCTACGGAGACCATGACGGACGTTGGATACTACAAGGGGACCGCCTACGT
TGTGATGCACCTGATGCCGGATCCGATGCAGGTGAAGAGCTGCGCTCTTGGAAACGGCGG
GAGCACCGAGGGCAAGAGCTGCCGCGAGGCCGACAACAAGGCCTGCAGCCACGGGGGGGC
CTCCGGGGACGAGCGGGAGGAGTGCGTCGATACCGAAACCAGTTCGCTGGAGTGGAGCGA
TGTGACCGCCTTTTTCCAGTTCGGCTTTCGGCTCCGAGAAGGAATCGTCAAAAAATCCTA
CCACACCATAAATACGTCCGATTGGTTGGGACCCTACAACTTGATTCTTGCTCCGATCGA
AAAGAACCACTTCCAGCTCACCAGCGCTTCGGAGAATGTGCAGCACGTGCATACCACGAA
GGAGCTGTTCGTTTCTAGGTACGTCAACACTCCCTGGACGTCCACCCAGCTGCAGCGGCC
CTTTACGACCGCGTCGATCTTCAACTTTGACGAAACCGTTCTGGAAACCGTTGCAGGGAC
CATCGCCATCAACGCGACGATCCACGAGCAAATCAAATGGAGGGGAGGAACCAACGACGA
TCCGTACGATAGATACTCGAAGACATGCGCCCATGGAGACCTGAACCCCCTGCTGCCGCG
GATCCAGATCGAGAAAACCGTCACCCAAAAATCGGCTGAGGCACCATCCAAGACGAGCTA
TTCTATGGCGGTTGATTATTCCATGCTCAGCGTCCCCGTCCACGGAATAGACGATCTATA
CAAAAAGTGGCCGTCCTTCAAGCGCCACCTGGGAGACAAGTTCCCACTGAACGGGCCGTG
TACGTAACACAATGCAATTCATGTTAGTGATCGACAATTGCTTCGTGGGGAGACCTGTAC
GCATTCTCACAACGCTCGTCTTTTCCCCACCATTTCTATCCCTACACTATGATTCTCTTC
GTCCACTGCCTTCTAACTTTCTAGATTGTTACGGAAAGGACCCCAATCGCGCCTTTGCCA
TCCGCCTTTCGTACGACAGCACGGAATCGAACACCACCTCGATATGGAAGGGCACCAAGG
GTTGCCGAGCCTCCTTTCCCTGCTGGGAAGGCGACGCCTCGGGAACCGCCGCGCCGTCGG
CGCGGATCCCAACCGATAACAACTCGCCGGGCGCGTACGTGCTGGATCCGATGGGCCTCG
AGTTTGCGCTGCTCTACGGGATGGGCTTTCTGCTGGTGGTAAGCCTCTTGTTCAACTGCC
AGCTGGCCAATCGGCTGCGGTCGCGGCAGGTGCTGCCGGAGCCCTCGCGGCAGCCACGGC
CGGCGTGGAGCGGCCCGGGAGATCCCGCCGCGAGTGGCAGCGGGAGCGGCAGACGAAGGA
ACAGCACCGGCAACAACCCAAGCCGCAACGAATTGGAGGAACCGCTCTTGGACAACACCG
ATGCTTCGGCCGGCGACGAGGAGCTGTTGGCGACTTCCTTCGACAACCCCCCATCGGCAT
CGCAAGTAGAGCATGGAGTCGAAGGATCTACGGAAGAGGGTGTCTAGCAGAGCCTACCGC
AGGTGCCAGAGCACGTGGTTTTGGATTGGTCTCTGTTCGAAAGCTATGTGTGTGAAACAA
CATGGGATGACGGAGCAACGCTATTCCTAATGCACATGTGCTTGTGCATCGAATATACAT
TCCTACTTCATTTGAAAATCGGCCGCCGATAATTTGTAATAGTACATGTTTTGAAATGCA
ACACACCAAGTACTACCCAAAGTGAAAAGAAATTCACCCAAAAATCAAGAAACAAATACT
ACAAAACACCTCCTTTCTTGAGGAGGCCTTCAAATCATACTTCTGGTAACAATAGCTCTA
ATGCCATAAATTCAAACCTGTGACTAGTAGAAACTATTTTTAAAGTAAAAGTCAGGTTAA
ACAATAA
>comp2843_c0_seq2

CCAGAGCACCCCAACACTTTCGGGGTCCGCAAAGTCCCAGAGACAACGGTTCAAGCCGCC
GATAGTTCGACGAGGAGCACAGGAGAGCAGTTTGTGCTGCTGTATCTCGCCGTTCCACGC
TAGTACAACACCCCTGTACTGCCGTTGCATATTTGCAAGCACTGCCCCCGTTCTCGTCGG
TTCTGTTCCAACACACCACACGACACGACACGACACGACACATCGCGACACAAGAAAACA
AAGCACCAGCATGCCGCGGAAGGATTCGTCCTGGATCGAGGCCAGCGGCCTCTACGGCAC
CTGGACGTTTTCTACGGAGACCATGACGGACGTTGGATACTACAAGGGGACCGCCTACGT
TGTGATGCACCTGATGCCGGATCCGATGCAGGTGAAGAGCTGCGCTCTTGGAAACGGCGG
GAGCACCGAGGGCAAGAGCTGCCGCGAGGCCGACAACAAGGCCTGCAGCCACGGGGGGGC
CTCCGGGGACGAGCGGGAGGAGTGCGTCGATACCGAAACCAGTTCGCTGGAGTGGAGCGA
TGTGACCGCCTTTTTCCAGTTCGGCTTTCGGCTCCGAGAAGGAATCGTCAAAAAATCCTA
CCACACCATAAATACGTCCGATTGGTTGGGACCCTACAACTTGATTCTTGCTCCGATCGA
AAAGAACCACTTCCAGCTCACCAGCGCTTCGGAGAATGTGCAGCACGTGCATACCACGAA
GGAGCTGTTCGTTTCTAGGTACGTCAACACTCCCTGGACGTCCACCCAGCTGCAGCGGCC
CTTTACGACCGCGTCGATCTTCAACTTTGACGAAACCGTTCTGGAAACCGTTGCAGGGAC
CATCGCCATCAACGCGACGATCCACGAGCAAATCAAATGGAGGGGAGGAACCAACGACGA
TCCGTACGATAGATACTCGAAGACATGCGCCCATGGAGACCTGAACCCCCTGCTGCCGCG
GATCCAGATCGAGAAAACCGTCACCCAAAAATCGGCTGAGGCACCATCCAAGACGAGCTA
TTCTATGGCGGTTGATTATTCCATGCTCAGCGTCCCCGTCCACGGAATAGACGATCTATA
CAAAAAGTGGCCGTCCTTCAAGCGCCACCTGGGAGACAAGTTCCCACTGAACGGGCCGTA
TTGTTACGGAAAGGACCCCAATCGCGCCTTTGCCATCCGCCTTTCGTACGACAGCACGGA
ATCGAACACCACCTCGATATGGAAGGGCACCAAGGGTTGCCGAGCCTCCTTTCCCTGCTG
GGAAGGCGACGCCTCGGGAACCGCCGCGCCGTCGGCGCGGATCCCAACCGATAACAACTC
GCCGGGCGCGTACGTGCTGGATCCGATGGGCCTCGAGTTTGCGCTGCTCTACGGGATGGG
CTTTCTGCTGGTGGTAAGCCTCTTGTTCAACTGCCAGCTGGCCAATCGGCTGCGGTCGCG
GCAGGTGCTGCCGGAGCCCTCGCGGCAGCCACGGCCGGCGTGGAGCGGCCCGGGAGATCC
CGCCGCGAGTGGCAGCGGGAGCGGCAGACGAAGGAACAGCACCGGCAACAACCCAAGCCG
CAACGAATTGGAGGAACCGCTCTTGGACAACACCGATGCTTCGGCCGGCGACGAGGAGCT
GTTGGCGACTTCCTTCGACAACCCCCCATCGGCATCGCAAGTAGAGCATGGAGTCGAAGG
ATCTACGGAAGAGGGTGTCTAGCAGAGCCTACCGCAGGTGCCAGAGCACGTGGTTTTGGA
TTGGTCTCTGTTCGAAAGCTATGTGTGTGAAACAACATGGGATGACGGAGCAACGCTATT
CCTAATGCACATGTGCTTGTGCATCGAATATACATTCCTACTTCATTTGAAAATCGGCCG
CCGATAATTTGTAATAGTACATGTTTTGAAATGCAACACACCAAGTACTACCCAAAGTGA
AAAGAAATTCACCCAAAAATCAAGAAACAAATACTACAAAACACCTCCTTTCTTGAGGAG
GCCTTCAAATCATACTTCTGGTAACAATAGCTCTAATGCCATAAATTCAAACCTGTGACT
AGTAGAAACTATTTTTAAAGTAAAAGTCAGGTTAAACAATAA
>comp2844_c0_seq1
TCAACAGCGTGAGATTGTGAACACTGTAGTTAATTCTTGCCTTTAAAACAGAAGCTACTG
GTACAATTTCTTAATTTGATAGAAGTCCGAAGATTGCACAACAATGGCGTCCTCAGGAGA
ATATGTCGAATTGCATGAACTGGCAAAGGGATGGAAAGCTGCGATCGGCCGTGCCATGGA
TCCAGCTACGAAACATGAAGCACAAATTCGCAACGACCGAGGAAACCTGCCTCTCCACTC
TGCTACTTCTTTCCGTGCTCCGATCGACGTCGCAGAGGCACTTCTGCAAGCCTATCCAGA
GGCCGCGTCAATCACAAACAATTACGGAAATCTCCCCCTTCATTTCACAGCATGGAAGAA
GGGACCTCTCGAAGTAGAACGTCTCTTGCTGAAAATCTACCCTGAAGGTGCAGCGCAAAA
GAACAACCATGGCAACCTTCCTCTGCACTATGCCGCTCACTACAATGCTCCTCTCGAAGT
TGTAGAGGCGCTGTATAATGCATACCCGCAGGGTGCAACCCAGAAGAACAATGACAACAA
CACTCCCCTCGATCTGGCGATTGCTGACGGAGCCAGTCCAAACGTGGTGAGCTTGTTGCA
GGGAAAGAAAGTTCCACCCACCGAGGATGAGGCTTTGGAGAAGGCCAAGAGACGTTACGA
GGCAGCAGAACGCGAATTGGCACGACATATGAGTGAATCCACAGGTACAACCGAAGAAAT
GGAAGAAGTATTGGCCTTGCTGTTGGATATTTATGAGGCACATCCGCATGCGCTTTACTC
GGCCGGCATCAATCCCGAAAACGCTGGAACAATGGACTCCATGATGGAAGAGGTAAGTTT
TGTTTTGTCACGAAAGTGCTTGCCACTCAACGATCACGTCAAATCAAGTATCATGGAACT
AAATTTGATTCTGATGCTCCATCCAAATGATTTAGGTTCGAAAAGCAGCTGCCGC
>comp2844_c0_seq2
TCAACAGCGTGAGATTGTGAACACTGTAGTTAATTCTTGCCTTTAAAACAGAAGCTACTG
GTACAATTTCTTAATTTGATAGAAGTCCGAAGATTGCACAACAATGGCGTCCTCAGGAGA
ATATGTCGAATTGCATGAACTGGCAAAGGGATGGAAAGCTGCGATCGGCCGTGCCATGGA
TCCAGCTACGAAACATGAAGCACAAATTCGCAACGACCGAGGAAACCTGCCTCTCCACTC
TGCTACTTCTTTCCGTGCTCCGATCGACGTCGCAGAGGCACTTCTGCAAGCCTATCCAGA
GGCCGCGTCAATCACAAACAATTACGGAAATCTCCCCCTTCATTTCACAGCATGGAAGAA
GGGACCTCTCGAAGTAGAACGTCTCTTGCTGAAAATCTACCCTGAAGGTGCAGCGCAAAA

GAACAACCATGGCAACCTTCCTCTGCACTATGCCGCTCACTACAATGCTCCTCTCGAAGT
TGTAGAGGCGCTGTATAATGCATACCCGCAGGGTGCAACCCAGAAGAACAATGACAACAA
CACTCCCCTCGATCTGGCGATTGCTGACGGAGCCAGTCCAAACGTGGTGAGCTTGTTGCA
GGGAAAGAAAGTTCCACCCACCGAGGATGAGGCTTTGGAGAAGGCCAAGAGACGTTACGA
GGCAGCAGAACGCGAATTGGCACGACATATGAGTGAATCCACAGGTACAACCGAAGAAAT
GGAAGAAGTATTGGCCTTGCTGTTGGATATTTATGAGGCACATCCGCATGCGCTTTACTC
GGCCGGCATCAATCCCGAAAACGCTGGAACAATGGACTCCATGATGGAAGAGGTTCGAAA
AGCAGCTGCCGCAGACAATTCGCATGCAAATGGCATAGGTGGCATTGAAGACTATGCTGG
TCGCAATGAGAATGAAGAGGAAGCGCAACTTATCGAAGACGCTTTGTGTCCCCCTGATGA
CACAGTTGAAATAGCATTGAGCAAGCTCGTTGGTCTCGAGGCAGTCAAAAACCAAATCCG
TGGAATGCGTAGAACGATGGAACTGGTCAAGACGGAGCAAAATGGAAAGCTCGCTCACCC
AAGTCACATCGTTCTAGAAGGTAATCCGGGAGTTGGGAAGACTTCTGTTGCAACCATCCT
TGCAGATGTTTTGTATGAAATTGGTGTAACCAAGACGCTAAATTTCATGAGTGTTGGTCG
AGATGAACTAATTGACCGCAAGAGTGAATCAAGAACCATTGCAAAGACACGGAAAGTGTT
AGAGAGAGCCAAAGGTGGTGTATTGGTTTTAAATGAGGGCTACACTTTGTTGCCAAGTAC
GGCCCGTCCTCGTTCACGGGATCATGGAGGTGCTGCTCTAAGGGAAATCGCCCGTACCTT
GCCGTCTGGAAATCCTCTTATCATTTTGACAGGAAGTTCCCTTGAATTACAGCGTATTTT
GTCCAGTGAAATCGGTTTCAAGAGCCACTTTCTAACTCGAATCGAATTCTCCGACCCCAC
CCCAAGTCAGGTTGCCCGTATTTTCATTGGCAAAATGAGCGAAAAGGGTCTGGTGCCAGG
AGATGGTGTCACAGTGGATTATCTTGCTGAGTTAATCTCTACAAATACTGATGAAGATTG
GAGGTTGGAACGCAACGGCCGTATTGCTGACTTGCTCCTGAATGGAGTAAGGGCAGAATT
GCGAAAGAGAATGCACTTCAGTGATGACTCAAGCAAAGTATCGTTCAGTCCTATCAAGAT
GATGAGTCCAACAGCCGCGTCAAGGATGCCCGCTGCTACTCCTGATGAGATTTTTGTGAC
CGTTGAAGATGTGCAAAATGCTATCGTTAATGGAATGTAATAAAAAGACATAGTGCCCAT
CAAATTCTTGAAGCATTCTTTTCGGAAATAAAAGCACCTTCGTTCAATCTTTGTGACAAT
TATCTTTATAAATTTAAATGTGAGTTTTTAAC
>comp2845_c0_seq1
GGATGTGTAGAATCAGCGACTGAATGATGCTGCCCCGTTTAGACGAAACATCTAGTAGGA
TTTGCTTAGACAAAACAAAGTCATCATGAAGGACCACGCCGTAGTCGTCACCCCAAATAG
AGAGACTCTACGGCACCAAGTAAACGTTGCCGCGTTTGCCGCCTCTCAGGCATCGAAAAC
ACCTCTGTCCAGTCGCCCTTTGACTCCGGGTAGATCGAGGCGAGGGGGAGGAGGTGTCTT
TCGGTCGAAGAGCCCCGGCAAGAGGCATGCAGCAGGCTACCGTGATCTCGCGCAAGAAGT
CTATGATCGAATGGGAGTCAACTACGTTCGAGGTCAAAATTCCATCGATTTGTATGACAG
TACATCTTCCATCTCGATCTCCTCGACGAAAAGGCACTTCCAATCGCCCGATCGAGGAAG
CCCTGTTTCGTCTATCACATCGAGGACCTCGGGCCTATCTGGTGTGTCCAGGGCTTCGGT
GACAAGTGAACATCCCAAAGAGCGTGCAATCGGGGGACCGGGCGTCGCATCGCAGCGCAA
TGCTTACTCGTCTGTCAGAAACCGCTATTCCCCTTCTGCTGATACCCGTTCCGATCAGGA
TGAGAGGAATTGGAACAGGAATCCAGGGAGCGAGGATGAAGAACGGGAATCTTTTCGTAG
TGCAAGATCTGTCAAATCGATGGTGAGTGTTTTTAGTGGGGGCAAGAGTACGGGGAGTTA
TGGTAGAAGTGTGGCGAGCAATAAGAGTAGCTACTCCGCTTTTAAAGCACCACCAAATTT
TGTCAATGCTGCTTTTAACCAAGGACAGAAACGAATCTCAGATAATGATAACGACGTTAA
CACCAGTCTTTCGTTCGGATCTCTGGACGAAGGTCGGTGGGTCAACAAGCAGCAAAATCA
ATACCCCCAAGAACAACCGCAACGGCAGGTATACCGGAGTCGGTCGTTCTCTTCCAGTCG
GAGATCGTCCTTCCAAAATTCAAAAGCTTTTTCCTCGTCGAGTATGAGCTCGAGCCATCC
CAATAACACGTCGTGCGACAAAATGATTGAGAAACTTGTCAAAGAAAAGGTCGAGGAAAA
GCTTACCGAATTGGATGCAAGCTTCGAGGACAGGCTTCGTCGAATCGATACAGATATCAA
TTCACGGCTGAACGACATCGAAACAAAATTGAACATGATCTTGAAGGTAGAATCATCACC
GGCGCTAAGAAAATGGGAAAAGTCTTCCAACAGGACCCCGAAAAACAAGCGCAGTTTCTC
TTACGAAAGGTTGCGTCTGTAATCGTTTTGCGTTGGGAGCCAGACAATTTTTGTGTGATT
GGCAATGGCGGGATAGTGTTGGTTTCACAGCCGTGCGTGAAAAACTTTTGAACCTCACAC
ACCAACGTGAGTGATTCTATAATTCATATAGAGGAGAAGAGAGAGTGAGAGAGAGCCTCG
GTCCGACTTGCTCGACGGGAAGTCCACTAAAGAGTTGAACCGAAAAAAATTGTTGGATAT
GCTTCTTGCAAGTTTAGAGAAATTGTGAGTTATGCCCAGAAAAAACATCGGGAAATGGAT
CGAAAATCAGAGGGGACAAAGCACCAGCAAAGGGATACAACGATTATCGTCATCATTGGA
CTTTCCATTATCTTGTATGGAAAGCTGCCAAATGCATACTGTTCACAACGTAAGCAGCAC
TAGGAA
>comp2846_c0_seq1
CCTTCATTCGAGCAAGTTAGTTATATTTGTTTCATCCAACAACCACCCAAAAAAACAAAC
TCAACTACCAAAATGGTTTCATTCCGCCGCAAAAAGAACAAGGAAGAGGGGAAGGAAAAG
TCTGGCAAGGCCCCCACCCCCAAGGAAGAAAAGCCTTCCAAGAATGCTCCCGCCAAGCCA

CCCACCCTCGATGACATTCCCGACCTCATCGACACCATGCTCAACGCCAAGGGCGAGAAG
AGCGCCGCCGCTACGAAGCGCGTCTACGAGTTGTGCGACGTCGGCCACAAGCAAAACCGT
GTCCCTATGGTCTGCAGCGGAAAGTACGACGTCCTTACTCCCCTGGCACAGTGCCTGACC
CAAGAGAGCGGAGACGGACGCCACCTTGCCTGCCTCGCCCTCAACAACCTCTCCATCCCA
ACGGAGAACAAGCGCGTCATGGCACTCGGACCCGCTTCCAAGGACGTCATTGGGGGATTG
TGCAAGGTCATTGCCGAAGACAAGCAAGAATCCTACCTCTGCTGCATCTGCTTGATGAAC
CTTTCGTTCCTTGAGGCCAGCATCACAACCATCCTCCAACACTCTCCCAGTGCCGATGGC
AAGGAAATTCCACCCCTCGAAAACCCAAACTCCCTTCTCCGTGTCTTGGAAAAGCTCCTC
ACCAACTCCCCTGCCGCCCCCAAGGCCAGCAGCGGCAAGTCCGAGGGAGTTCGATGGGCC
TGCGGTCTCATCAAGAATCTCGCAAAGAGCGAAGAAAACGCTGCTCTGATCGGACTGACC
GAGATTCCCAAGTGCGTCGTGGAGAACATCCGCGCCGCCACCGCCCCTCCATCCCGCTGG
ACGTCCAACTCCCTCGAGGATTTCTCTCTCTTTGTCATTCTCAACCTCGCACAGTGGCCT
CAATCGCGCGAGTCTCTCATCAGTGCCGGTGCCGTTGATGTCATCAAGTCCATCATGGCC
GAGGGAGATCTTCAGGGACTCAAGGCCACCATGGCCTGTGCCTTCCTCGATGCCGCCTGG
ACCGACTTCCCAGAGAGCGGAAACCCTGCCGCCAAGGCCGTTTCCGAGCTCATGACCAAC
ATTGTCGAAAAGAAGGGCAAGGACGGACAATACGCCTACGGTGTCTTCAAGCTCTACACG
GCCACCAAGGCCTACGCCGATCTCTCGAAGGCCGCCGGAAAGGCCGACTCGGAAGCCGGA
GGCGAAGCCAACACCAAGGTTCTCGCCGTTCCTTCGGCCGTTGCACTCAACTTCCAGATC
GTCTCCGACCTCGTTCTCTCGGCGATGGACGATACCGAAGGTGGCTCGAAATACGTTCCC
GATGCCATGTCTGCCGAACACGCCGTTACTGCCATCGAAGCCATGCTTCCCGCTATTCTT
CAAGCCGGAGATCCTCCGCGCAAGTCTGTCCAAAGCGACAAGGCATGCGGCGAAGTCAGC
CAGATGCTTTTGACGTACGCTGATATCTCCGGAACTTCTCCCGAAGCAAAGGCAAGTGCA
CGGGCTGCCGCCGACCAAATCAGCAAGGCTTCTGGCTCGGCTCGACCGATCCTGGAAATT
TCGCACGATTTGTGGGTTCAATATCGCAAGAGAGAAGGCCAACCACTCGACCAATTCATG
TCCGAGAAGGGCGTCGAAGTCGACGAATCCGGCCCACTCGACTTCTTGCCTTGCGCAAGT
GGGGACTCGGGCTGCGTAATTGCTTAGAAAGGGAGCACAAAGATGTACTATTTGGTTCGT
TTATCGACGCTCTCCGGCAAACCGAATTTCGCACGCAACACAAAGCCAGCCAATGACTTG
CATATTATATTAATCTTGAAAAGTAAAATTTGTATTCTGAAATTAAAAAA
>comp2847_c0_seq1
TTAAATGTTTTTTGAAACCGCAGCTCGATCATTCCATCCCTGCGCAGGAAGGAAAGTGTT
AGAGTACTTCTGTACGGCAATCGGACTACTATACCCGTGCAATCGTCTGTCGAGGTTAAT
AAGTATTCAAACGTGTACTTTCCGAGATCTTCTTTCTGCTTGGAAGCATTCGGAGGAATT
CCAAATTTAGAATGTCATGAAAGGCAGATGGAAGGAAGAGAGGATGGAGTCCTCTCCACT
TTACAGCACGACTCCTGCGTCAGATTCCTTTTTTCAGGTTTGGATCCGCGCCCAGAAAGC
TTTGGCAGCTTTTTTTGTCCGGGAATACTCGGTTCGTGCCTTGCCGTGTGCGTCACGAAC
GGATCTCTTCCTGGTTTCCGTCTCGCAGTTGATAAATTCTCGAGAATGTGCAGAGTACCG
TGAGGGATGGTTTGGCAACGAGATTCTGTGGCACACCAGCACGAGTCATAATTTCCAATT
GTTTCCGCAGAAGGACCCTACAACAGAAAATATCACATCCCCTCCTCTTACTCTAAAAAT
GGTTAAGGAATTTCATCTCAAACAGAAGTTCAAGCTCGTCTCCAACGACTACAGGATCAC
CCAGAACGGTGGCGCGGTCTACCAGGTCAAGGGCAATGCGTTCAAGTGGGGAAACCAGGC
TTCGTTCCAAACCATTGGCGGGGAAGAATTGTGTTGCTTGAAGCAAACAAATGCAACCAC
GGTCACCCCGTGGAACAACTACGAATGGTTCAAGGACGGCGAACTCTGGGCGACCGCCAA
GCAAGAAGACTGGGGCATGGCCTCCAAGAAAGAGATCGACATCGACATCCCCGGTGACAA
CGACTACAAAATCAAGGGCGATCGCATGAGCTGGAACTTTACCATCTTGAAGGGCGACGT
CAAGGTTGGCGAGATCAGCAAGAAGTGGGGCATCCTCGACAGCTACGGAGTCCGCGTCGA
AGACGACGCCAACGAGGTAGACATCTTGATGTGTGCCATCCTCGTGGATGCGACCTACCA
CCACAAGGACGGCAACAACAATTAATGGAAGAACGATTGTATTGTTCTCTTCGTTCCACA
TGATCGTTCTGGCGGCACCAGCCGCCCGTGTGCAATCGAGAGCCACGATTCGATTGCACC
GACCGAGAGCCGCCGAGTCGAAGCTCTCTACGGCGCGGCTATTCGCTTTCTTGTTTGAAC
CTATTTTGTTACAATTTTGAGACATTGTGAACTTTACATATTACTGTACTAGAGCAATTT
ATCGT
>comp2848_c0_seq1
GGTCAGAATAATTCTGTGTGTCGAGAGACTTGATAGCAGGAGACTATCTAGTTTAATACT
TCACGCTTCACTTTGACAAGCTAGAAGTATTCCATTAAATAATGGTAGCAGACGGACTTG
TTAGCGGCATTTCGCAGTGGATCGACAACCTCATTGGTGTTCACGGGAGTTCGGGATACG
TCTTTATTTTCTTGCTTGCATACATAGGATACAGATTCATGAATAATTCGAGCGAACCCT
TCAAAAAATCCTCCCAGGTCGAGGAGGATGAAGAAGAGGAGCCCGATCCTCCCCGAAATT
TTACCTCGCTGCAGCTTGTGCACTTCAACGGAGAACCCGACGAACGGACTGGGGAAGACA
AGCCGGTCTATCTTTCGATCAACGGCATCGTCTTCGATGTTTCGCAGGGCCGAAACTTTT
ACGGCCCGGACGGCCCCTACGCAGCGTTTGCCGGAAAGGAGTGTGGGGTTGCCCTGGCGA

AGATGAGTTTCGACTCTGAGCACCTCAACAATCTTGCTGGGTGCAAGGATCTCAACTACG
GAGAAAAGGACGAGCTTGAGAACTGGATCAACAAATTTACCTATTACAGAAAGTACCCCA
TCAAGGGCCGGTTGGTCCCCGATGACATTCTGGATCCACTGAAGAGTCGAACCCTAACAA
AAGAGGATTTGGCCAAGCACATGGGAGATGCAGAAGAAATCCCCGAGGGGTACGCAGCGG
TTCCGATCTATATTGGTGCGGGGAACAAGGTATTCGATGCGTCTTTTGGCGGGGTTGAGT
TTTACGGACCAGCGGGTGGCTACAATAAATTCGCAGGACGAGACATTTCCAGAGCACTTG
CAAAAATGAGTTTCAGCCCGGAGGATTTAGAGAACACCAACACGGACGACCTGGAGGAGA
AGCAGAAAAAAGTCCTTGACGACTGGATCAAAACCTTCGAAGTCAAAAAGGGATATCCCA
TTGTAGGAAACCTAGGAAAGAGCAGTTGAAAATTGATACTAAATGGTACTGATATGCTAT
TAGAGAAACGATCGACGTCGTTTTCATTTCTGGTTGTTGAATTTCGTAGTAGCCGCAGCC
TAAATAGAAAGATATGTTTAAATCGAAGGTGATTTTAGCTAAAATTTGGGAATGTGTTTT
TATGTGAGTATTTTCATGTCTGTGGGT
>comp2849_c0_seq1
AGCAAATGTAAAAAAGCAAGGTTTAAAAATTACATATGAGATAGTACGCTTTTGTAGTAA
GAAATAAGCAGCGAGCACCCCGTTTTGCAATAATCAAGTAAATTTCGCCATGGTTCTCGA
TTTTGGACTTACCAAGAGCAGCAGCGTTGCCCACAGCATCTCTTCTACTAAACTCAGGAG
TCTCAAGAGCAAAAAAAAGAGAGAAAAGAAAAGACAGGCATCTTCAAGCGATTACTGTGG
TTCGTCATCATCCTCATCCGACGAAGACGAAGGATATTCCTCTTCCAGTAGCTGCGACGA
GGAAGATATCCTTATTTACGAAAAGAACGTGCAAAGAAGTAGAAGAGGCAAGGTTAAAAA
AAACAGCAGCGAAAGCGATAGAAGCAGCAAAAGACATCATTTGGATCCTTCGCTTGTTGA
GAGAATGAGTAGCTTCAACATTGATGAGCAAGGGTCGGCCTCGATGGCACTTGCATCGAT
GTTAAACCTTGAATATTGCGAAGACGAGATCGCAAACGACCATAACTTAAATGTGAAACT
AGCTGCTGATTTGGAAGCCGAATTGGTCCAAAAACAGCAACACGCAACTGTTAACGACGC
CGAAGGATACGAGAATTATGATGACGAAGAAGAAGACCTAGAATCGAGCGACTTTTTGGA
TGCCCTCGACGAAGTCCACACCCGCTTTTTGCTGAACGTCCCTCCCGAAGAATTATCTTC
GTCCGAGCGAATTTTTTTTCAATTGGAACAGGCATGGTGGTATTACGAAGATCTAATTTG
CGACAAGCTGGAAGAAGACGAAGGTCAATGCCTCTTGCCCCGACATTCCAACCTCAAGGA
TTTTGCTGAGCGCCTCTTTGACTTTAGTCCGCTCCTTGGAGGTCTTGACTTTGACAAACT
TTGGAATGAATTCACCATTTATAAGCGCAAAATTTCAACTTACGGCTGCATTTTGCTCAA
CGAGGACTGCACCCACGTTGTAATGTGCAAGGTATACGAGAGCAACGTATGGACCTTTCC
TGCCGGAAAGATAAACGAAAACGAGATTGGAATTGAAGCGGCCGCCAGAGAAACCTACGA
GGAGACGGGCTTTGATCCACACTGTAACTATGGTCTGACAGGCCAATGGAACTCTCGAGA
GGAAGATCGGGAAAAGCATATCACCTGGATGCATCCACTACAAGATCCGGACAACATGCT
GACATATGTAGAGCAGAAGAGTGGGAAACGGCGGACGTTTTATGTGTGTTGCGGGGTTCC
CGAGGATTATCCCTTCCAACCAGTCTGCAGGAAGGAAGTCGACAAAATAGAGTGGGTGGA
TCTTCGGGACATCAGAGAGTATGAAACATTTGCTGTATTGCCTTTTGTTGGAAAGCTGAA
GAAATGGATAAAGCGCAAAAATAAGAGCGGCAAAAACAACAAGAAAGTTAAGAAGATCAA
AAAGAAAAAGGAACGAAAAGCAAACAAATCGAGAACCAAATCTCGTCCCAAATCTAGGGA
GAAGTCCTCCACCAGAGATAAGCAGTCTTCCTCTAGACAACGGTCGAGTCGGAAAAAAGA
CAGGGAACAGCTCGTGGATGCCGGGCTGATAGCGTCGATGGGCGATTCGGCGCGTTGGAC
CGAGAGGGAAATGTTCGAGGCGAATTCCAAGCTCCAGGGTGGCCGAGTTGTGGAATATGA
TGGGAATCCCCATTTGTTCGTCGAAAAGGGCTTTGGTATCGACGACAGTGCCACAGGCCA
AAAACGAGTAGATCCGCATTCCTTCCGAGTCGTGGGGGGGTCATTTATGAACTCCAAGCA
CGGCGATAAACTCGCCGAGGTGAACGATGCCGGTGCGAACTATCAGCCACTTGTTCGCAA
CAGCAACGAAGACAGTGGACTCCAGCCCTTTTTCAGCGAGCACGGTGCCACGCCTTGGGG
AGAAGTTGTGGACGAAGCGAAATCGGAGGACGATGAGGGCAGTGACAGCACTAGATTTTC
TGGTAGCGACGGCCCAGCAGGAATTGAAAATAGCAACCGCCGAGCGAGTCAGGATCTACT
AACCATGTTACAGAAGGGAGCACCTGACAAGTCGAAGAAATCAAGAAAGTCGAAAAAATC
GAAGCATCAAAACCAGCATCAGCAACCGATATCTGGGGACTACGATTACGATAATAGCAA
TCATGACGACGAACTAATGATATTTGCCACCGACAAGGAGATCACCGCCAAGCGTCAGCG
CGATCACGAGCTAAAGAAGGGAAAAGGAAATGAATATTCGTCGTCGCTGGTATCGCCATT
GAGGTCTTCTTCTATCAACACTGCTACTCGCGACGATGCGCGACGGCAACGTCTCCTTGC
TCAGTACGAGGAAGACATGAAGTTTGTTCACAAGTGGGTTGCCAACAGCCTCCCAAATCC
AATCAATTTTAAGATTCGCAACGTTGATGGTATAATTGACCAGCACTGCGGGGGGCCAGG
GACGAAAACAAAACGAGGGAGCATAAAAAATTGAGGAATCCAACAGCTGCCTCGTTGAAT
CCATTGTGCACGCAATTTTATATAGAAAAGAACCGTCGAATAACGATAGAACTTAACAAA
ACGATCAATAATTCAACAAATTCGATCAATCATCAACAGTAACGGTAGTGAATGCAGTAG
ATATGGTGC
>comp2850_c0_seq1
CTTTGTGTTGCTTCTTCATAAACCGAACCAAATAATCGAACAACGAGCACAAGCTACTGG

TTTCTGTGGAAATCATTTTTTGTTTTTCGCTTCCGATCGCAAATCCACAAACACTCAAGC
CATGATATCCGAGTCGTTGCTGAACGATATCACCGACGTTGTTGATCTCTCTAAGAGCAA
AAGGAACACAATTGCAGAGTATTCCTCAGCGCCGTGTTCCAGAAATGGGATGGTAGACCT
CCTGCTCCCACGGAAGGAAAAGAGGCACACCAAACATGCTGGGGCAGATACAGACTCGAG
TCGTCATTTGCACAACTTCCACCTTTCCGAGACACAACGTTGCTCTTCGGCAACCCAAAA
AACGCAGGCAACAACCGATACTGATACAACCGACGACGACGATGATGACTATGGATATGA
CGAGGAAGAAAGATGGCTCCGCGAGAACAGAAATTACCACCAGCTGCAACGCGATTCAGA
GCCAATTTTTAGGTCAACAGTTTTAAATAATGATGTGTCGACTTCGTCCATTCAGCCGTT
GAGGGAACGGCGGCGCTCCTCGATGGGCAGAGAAACACCAACTGTTCCCCCTCTAGGATA
CGAAAACCAGGCAAATAATAGCCGCAGTTGCTGTATGCGGCTGCCCAAGAAGTTTCGGTT
GTACGACCCCGATCTCTTTTAACGACAAAGATGGATCGCCAGTCACATTTTTGCTTGACG
AATATTGAACTCGCTAACGATGGACTACTGTGCAGAAACAAACAAACAAACAAACAACTT
CAAGATGTATCGGTATTTGACATGCCCTTGAAGCGATGGTCTCGAAATGCAACGGCACAG
ACTTTTACGGAATACATGTTATCGCGACTGGACAGACATTAGCTGCTTGTAGGAAGCAAA
CTAAAAGCGCATAGCGATAGTATTGTAAATTTTTCAACTAGTTCATCCTTTATTTACTCA
AAAAAAAAAAAAA
>comp2850_c0_seq2
CTTTGTGTTGCTTCTTCATAAACCGAACCAAATAATCGAACAACGAGCACAAGCTACTGG
TTTCTGTGGTAAGCCTTTTCGACTCAAGTCGTAACATAAAGTTTTCTTCTGACACAACAA
AACGGAACAACAGGCGGGCTTGAACTTGTTGACATTGCATTGCATCATTCAGTTTGGATT
TTGCGAAACAATCCATCACACGATAACACATTGTTCCTGACCTCCTTGACAGGAAATCAT
TTTTTGTTTTTCGCTTCCGATCGCAAATCCACAAACACTCAAGCCATGATATCCGAGTCG
TTGCTGAACGATATCACCGACGTTGTTGATCTCTCTAAGAGCAAAAGGAACACAATTGCA
GAGTATTCCTCAGCGCCGTGTTCCAGAAATGGGATGGTAGACCTCCTGCTCCCACGGAAG
GAAAAGAGGCACACCAAACATGCTGGGGCAGATACAGACTCGAGTCGTCATTTGCACAAC
TTCCACCTTTCCGAGACACAACGTTGCTCTTCGGCAACCCAAAAAACGCAGGCAACAACC
GATACTGATACAACCGACGACGACGATGATGACTATGGATATGACGAGGAAGAAAGATGG
CTCCGCGAGAACAGAAATTACCACCAGCTGCAACGCGATTCAGAGCCAATTTTTAGGTCA
ACAGTTTTAAATAATGATGTGTCGACTTCGTCCATTCAGCCGTTGAGGGAACGGCGGCGC
TCCTCGATGGGCAGAGAAACACCAACTGTTCCCCCTCTAGGATACGAAAACCAGGCAAAT
AATAGCCGCAGTTGCTGTATGCGGCTGCCCAAGAAGTTTCGGTTGTACGACCCCGATCTC
TTTTAACGACAAAGATGGATCGCCAGTCACATTTTTGCTTGACGAATATTGAACTCGCTA
ACGATGGACTACTGTGCAGAAACAAACAAACAAACAAACAACTTCAAGATGTATCGGTAT
TTGACATGCCCTTGAAGCGATGGTCTCGAAATGCAACGGCACAGACTTTTACGGAATACA
TGTTATCGCGACTGGACAGACATTAGCTGCTTGTAGGAAGCAAACTAAAAGCGCATAGCG
ATAGTATTGTAAATTTTTCAACTAGTTCATCCTTTATTTACTCAAAAAAAAAAAAAA
>comp2851_c0_seq1
CCACGACGAGTGTCCGTTCCTCGGTCTACATACCGGTACACACCGTCTTCTTGCGAGACA
CACCAGGCATCCAAACCAACACACTCCACAGACCAAACGGCAAACAAAACCCACCCCGAA
CTCTGTTTGGAATCCAGACCCGAATCCGGCGGACAGCACCAAGAGCCAGCAACTGCAACT
GCAACCGCAACCACAACGCAAAACACGCAACGCAAAACACGCAACGCAACACAACGCCAT
GAATCGATCGAGCCCGGGTGCCCTGCTTCTGCTGCTGGCGGTCGCGAGCTTCCACACCAG
CGAGGCCTTTACGGCACCGGTGGCGAGGACCCTTTCCCCGGCCCTCCCAGTGTCGTCCCT
CCGAACGGCTACGGCCGTGTTTGCGGCCGCCGGCGACGGGGAAGCCCCCGGCGAGACAAG
GGCCGCCCCCAAGCGCAAGCGCAAGCGAAAGAAAAAGGCGCAGGTGGTGGCCGAGACGGC
TCCGGAGGAAACCGAAGAACCCGGGGCTCCCCCCCTTGTCCCCGACCTCCGGCCCCGCGA
GGACGCCCCGGTGGTCCTCGAGGTCCGGAACATCGTGGCCCCCGAGGGGGACCCCGAGCC
GAGCGCCGTGGCCAAGGCCGGCGCGGTGCTCTCGTCGGTCTCCTCGATGCTGGGGTCGTC
CAAGGGCGAGCCGGGGGCGGGCAGCGAGCGGGCCGTCTCGCCGCCCCCCCCGGGCCTCGG
CGGCCCCGGCCGGTCCCTCGACGACTCGCTCGACCAGCTCCTGGAGGACGCCCGGTCCAT
GTCGGAGGAGGGGGGGGCTTCCCCCGGGGGAAGCGGCGGCCTCCTGTCGGACGAGGAGGG
GACCGGCGTCAAGCAGGCGATCGGGAACGCCCTGTCGACGGTCGTGACGGCGGATTTCTT
TCTGGTCTGCGGCTTTCTGGCCTGGTTCCTCCTCGGGATCTTTTGCAGCTACGTCCTCAA
GGACGACACGGTGCAGATCGCCTTCAACAACAACTTCGAGCGGCTCGTCCAGCCGGCCCT
CGGGGTGCTCATGATCGCGGCGATCGGCGGGAACTTTTTCAAGGAAGAAGAGGAGGAGTA
CGACCTGTAGGAGATCTTGGAAACGCGGATGCGGAAGGAACGGGAAGGCTGTTGGCGATG
CCGCCGGCTGCACCAGACGCACCACAAGCGCGGTCCGAGGTCTCTGCCCAAGGAATCGCG
TCCTTGTACAGCTACCGGGAACACGCTACCGATTTTTCCAAAACTCTCAAGCATCTTTGG
ATGGCTTGTTGCGATGCACGTACTGTAGTAGAGCCACAAGACTTTTCCAACAGTAGGGTC
GAGAGCAAATCCAAATGGCCTA

>comp2852_c0_seq1
GACTAGGTTACAGAACGGCATGAAGCACGTTGTCACAACAATTGATGGGTTATCTATCTA
CCTTCGCTAGAACTGCAGTGCCGTTTTCGCTACCGGCTGGAGAAAAGTACTCGTACAACC
ATGACTGGACAGCAGGAGAGGGAGCTGTACTGGTACGATGACGCAACCTACGACATTGGC
TTTTATAACTATTACCAACGAACGGTGGATCCAAGCCCATGGGCGTTCATAGTTTGCCTC
TTGTTCTGTATCATAATGGCGCTGCTGCTTCCCCGCTATCTTGCGTACTATCGATCAATT
CATACGAGTAACTTAAAGAAAGCATATTCAAGCAATAATATAATACAGCTCTGGTCCCAG
GCATTTAGGGCAAACGACGCGGTTCCAAACGGCAACAACGACGAGACGATCAGGACGCAG
AACAAAAGAAATATCTACCTTCCATCGCTAAACGAAGAAAACGAGTCTGGTGAGAATTCG
GAGAAGGACGAGGAAGCCAACGGGTCATTCCGACGGAAGGTGATGAGGGAAAGGGCGTTG
CAATCACTCTGTCACTTCGTTCGCGCACCAAAAAGCGAAATCGACAATTGCATCAATAGT
CAATCCAAGACTGAAGAGACGCGGGTTCCCACAACAGACTATACTAGCATGGAAGATCGT
GCATGTGACGACGATGCTAAGAACGACATCGATTCTTCGGGGTATGGAGAATTTGTAACC
ACGGAAGCTGAAATACCACCAAGCCAGGATCTGACGGAACCAAACGATGCGAACGATCCA
CTTCCTCCCGAATATCTGTATGATGACCTCACCAGGGATGCGGAATCATGTGTATCATCT
GTTTTGTCTGAGAACACGAATCCTTTTCACATAATCAAGAACGATTTCAGAGATTTCACA
AGAAACAGACATCCCGACGAAAGTGTATCTGTAGCTAGCGGCTGGGAAGAAGACTTTGAT
CCGTCGACGCAAAACACGATATACTTTCATCCCGCAACAGGAGCTAGAACCACAGAAAAA
CCCTTGGAACCACATTCTGAGAACTTTCGCCCGTGGTACCTGTTCTATACCTCGCCTTCC
TTTCGAAGAAAAATGCGAAAATGCAGCAAATGGGACGACGAGATGCAGAAAATCATAGGC
CTCGCCCTCCCGTACACTGCTCACACGATAGCCGTTGATATATTTGGATTGCTGGAGGTT
GGTGTCATTGGAAGATTACTAGGTACGACCGAGCTATCTGCCTACTTTGCCACAGAATTT
GCTATCAGCTTTGCAACCATGTTTTTTCATGGCATTCTGGCGAGTTTAAAAGTTCTAGTG
AGCCAGGCCCACGGAGCAGACAACATAAGGTTGGCAGGAACCTATGTCCAAATCGGCGTG
TGGACGCACCACCTATTTTCTTTGCCGCTGGTTCTCTATGGTTGGAATCATTTCGATGAT
GTCGTACTCCGTTTGGGTCTTGACGAAGGAACAGCGGAGTCTGCCGACCAATACGCTAGA
TTTGCCTTGATCTACGAAGCGATAGGAATTTACGACGGTGCCTTGCACTGTGTTTTGGAC
GTCACTGGACATGAAAAATACTCGGCAATATCGAACGGATTCCGTGCATCTTTAAGCTTC
CTTGCAGTCCTCATTGTCTCTGTTATGTTGAATAAGGGAGCTGAATTGTGGATGGTAGGA
GCAATCCATTTGGTCGCGAAGGTGTTCTTTTTCTTCTTCAATATGTCGGTAATCCTATAC
CAAAAATGGCTGGATAATTACTGGAGTGAAATGGTGGGTAACAATCCGTTCAGTCACATG
GCCCCGATTAAAACCTTGTGGAAGGCTAGTCTCCGTCTTTCGTCCGGTCGAGTGATTGAA
AATTGCGAATGGAACATTCTTTTTGTATTCTCTGCTATACAAGGTCCTGCAGAAGTTGCA
ATCTGGGGTTTGGTTGGGGAGCTTTGGGACTTTGCTGACGATATTGTCATAGCTGTTTCC
GATGCATCCAAAGTCCGATGCGCACATCTCTTGGGGTCGGGTCTACCGGAACAGGCGAAA
TATTGTTCGGAAAAAATCGCTATTTATAGGTGTTGTTACTTCGATAATCATGTCGGTATCA
CTTGGAATTCTGAGATCGAATATACCGAGATGGTTGACTAATGACTTTACATTGCAACGC
TTATTAGGGGATATGATTCCGATGGTATGTCTTGCTGTGGCGACCCTATCGTTTGGAAGT
CTATGCTCCTCTATATTGTGCGCTCAGGGTCGTGCACATCTGGCAACTGCTGTCACAGGC
TTTGGCAGCCTTGCAATAGCTTTGCCACTTGCATTGATGTCAAATTTCTGTTTCAATTTG
AATCTGCAAGGCCTCATAGCTTCGCTCATCATTGGCTATGCTACTTCTGGCTTCTGCAAC
TCAATTCTGATGTTGACATCAAACTGGACGCACATCTCGAGAAAGGTTAGAAAAAGGACG
AGTCGAATCGAAGTGAAGCTCGAAAAGGATAAGATGCTGTATAATGCCACAACAGACACA
GCAGTTGTGGATGGAAAGGTTGAAGCTTCCGGGGATTTAACTTGACGTGCAGTTTTGGTC
GATACGAACGAGGTACAAATGCAGGAAATACCGACACGACTCTTCCAGAAAGTCCACCAT
GGTCGATTTTTCTTTCTTGTTATGACATGGTCGGAAAGTTCATATTATAAGGAAAGATGC
GGATCGTGAGCAGACAAGACTTCATCTGTTACCAAGATTGGAAAGTATTGTTTTCGACAC
TACAAACTTAGCCAGAACAATCTTGCAAAAGCTCCATCCATTCGCATTGCAGAGACACTA
TTTGATGAGGAAACTCTCACTGATAAATTCACAATCAGCGATTTATTGCGCGTCGGTTGA
AAACATAAGGTACAGCTATTTGTACAAGACCAAGAGTCATGGAAAGGGTGGCTAACCTTT
CAAGTTTTCTGCCATGTGCATCACTTTTAAGTGAAGCTCTATTGTGATCTCCACAATCAT
TCTTCTCCTTTTGTTAACCAGGAACCTATTGATAAAATACATTGGTCCCA
>comp2853_c0_seq1
AATCAATCCAAATCATAACAAAGTCGGAAGTGATAACAGAGCTACCTTACAATCCATTCA
GAAACGCCCCAAACGAAAGAAAACAGAAACAAACAATAACAAACGCATTACAATATAGCA
ACCAGTCGCAACACGATATCAATAAATTTCGCTGTTGCTGTCTGTCATAGCATTGCGGTT
GTTTTTTTCGAAAATGCTGATGTTAAAAGTTGCTTCAGTGAGAGCATCACGCATGCCTAT
TATGTCGATATTCCATATTATATTTGAACTTCTATTTCTATTTGCTCTTCTAAGTGATTG
CTGGGCAAAGAATCACGAAAGCAACTGCGATGCAATCTCGGATGGCAGCAATCCCAATAA
TAAATCTTGTGTCATTGATCGCTCGGGTTTTGGTCCCATTAAAGTCATCGTTCATGGCAC

CAAGAACGATCCTTTTTGGCAACAAGTGAAAGCAGGAATGGAACAAACCGCAAGGGACAT
GAAAGTTGATTTAGATTTACTTCTGTATGACGACGAAGATCCCACGCTCGATGCCTCGAC
GTTAGCAAATGCCATGGTGAACGACATTCGAAAGTACGACTACCTGTACAATGGTGCCGA
ACCAGACAATGATCGCCGCCAACGACGCCGTCGGAATAGCTTTGCAGATTTTACCAATAG
GGGAGATGGGAAAAACCAAAATGAAAACGAAAACGTGGAGCCACGTTGCGGTTTTGGCGG
TTGTAAATCAAGAATAGAAGGCGGCCAAATAAGTACAAATGATGCTCTCTCTCCCCGTCC
GGCAGCAATGATTGTGAGCCTTCCAGGGAACTCAAAAGTAGAAGATGCTCTCCGAAGCAT
ACAGAAGGATAGCAACAACGACATGCCAATATTTGGAATCAACGCCCTCGCAAGCAACAG
AGAAAACAGCAGTATTGAAAAAGAGGGATCATCCTCTTTCTTTCTGGGGACAGTATCGAT
GAACGAGACCCAAGCCGGAGTCATGGCTGGAAATCATATCCGAGAGCTTTTGCTTTCAAG
AGAGGATAAAATCGACGGCGCCGTTACCGGATTGTACGTCAATCATCGATCTGACGTCCA
TGCCCTGAAGGAAAGATTCGACGCACTTTCCTCAACCTCTAGTGACATTGTTCAAACGTG
GGAAGTGGCATATTTGGAAAAAAATACCACCGAAGAAGATTTCATGGCTTTTTTTGAGGG
ATGCAGACACACCGTCATACAACTTGCGGGAGGGAGCGGTATTGTCGAGTCAATCCTTGA
GGCGTTGGTGGCAAATGGTTGCAATATTGAAGACAATCATATTGTTGGTACTTTTGACAC
CTCATCTCCCTTTATTTACGATGCCATCACCGAGGGCACAATTGACTTCGCCATCGCTCA
GCAACCCTATTTGCAGGGAAGTCATGCTGTGCTCATGGCAGCTTTGTATGCCACAACGGG
ACAGAAATTGGTTGGCGGAGCAGAAGCTGCGAACATTGAAACTGGTCCAATTTTGGTAAC
CGAAGAGAGCGACGACTTTCCTGGAAAGCGACACCAAATTTGTGAATCCGAGGGATATCC
CGTCTGTTGGCCTGGGACCGGTAGCAGTGAAAAATCAGAGTGTCCATGTACAGATCGATC
CAATATTTCCATTGCGGTCGTCACTCACGACGAATCATCAGATTTTTGGAACACAGTGTT
CTCTGGTATTTCCCAAGCTGCAACCGACTTCGGCATCTCGGTTGAAAGAAATCGCTACAA
GCGAGTTGGTCCCAACCATGGACGGCAGCGACAGCATGGGAAGCAGACAAACTTGCGTCT
GAAGCACACCTTCGACATCAATCAAGCCTGCCAATCCAAAAAAATTGATGGCCTCATCGT
GAGCCTACCGGATGCAAGCATGATGAGTAGTTTGGTAGGATGCAAGTCTCGGGGAATTCC
ATATCTGGCGCTTAATGCGTTGGCGGACACTAACCAGAACCAAGACATCGTTGAGAAATT
ATCTCCCACAAGGCCTTCAGTTCCCTACGTAGGCCAGAAAGACTTCGAAAGTGGCTTCGA
AGCAGGAAAAAGGTTGATCGAGGCCGGTGTAGTACAGGGTTGGTGTCTGATGCATGACAA
TTTTGACACGTTAAATGATCGATGCGATGGAATGAAGGAAGCGTTCCTTGAAAACGAAAA
AACGGAGTTCATGGGAATCATATATGTACCAAACAATAACGATGCGTTGACATACAAAAC
TTTGGTGGAAACAGCATTTAATCAGGACGGCAGAGGAGACGACTGGAGTGGGTATGGTAT
CCTATCTACGGGACGAGAACAAATCCCTTCCCTTCTTTCACTATTGGAGGACCACCCAAA
GATCCTTGCCGGGACATACGATGTTGAGTCTACTCTCTATAAAGAAAACAACGATAGTGC
TCTAAGAGATCAGATTATCTTCGGAATCGATCAAAATGCTTACATGGAAGGTTATCTCTC
AGTTGCCACGATGGTGTGGAAGACAAGTACTTCGGAGACGCACACAATTCCCATCTTAGA
GACTGGTCCGAATTTTGTTTCACAAACCGATTCATCTTCTTCCCAAGTGATCGAGAAAGA
AGAGCATTGGCACCAACAATGCCGGGACAATGGATTCCGATTTTGCGAATCCAGAAAGAG
TGAACCTCCGCCCATCGAGGAACGACCTCTCACATTCACTGGCAAATGTAGTCAACTCGA
TCGAAAGTGCGGCGTCTGTGAGGGCGACTGCGATGACGATACACACTGCGGGGAAGGTCT
TGTTTGTTTTGTGCGAGACTCTCTAACAAGTGGTGTCTCCGACCCAGTACCGGGATGCTC
TGGAAATCCCATTGAATGGAGTGCGAAGGATTTTTGCGTGGACGCTCGGCTCTACGAATG
CAAAGATGTCGATGTGTTCGAGGTACGAGTGGGGAATTTTTCCGTCCAACGGACGTGCTC
CTTTGTGGAATTGGACAAGCGACGGTGCTCGGAATACGGGAGGCTTTGCCGAGCCACATG
CGGGTATTGCCGTAAAGAAGCACGCGAAGACGACACAACCGCATACTGAAGCGATTAACA
GACCAACAAATTGAATTGTATCATCAATAAACTGTAATATAGATTAAAAAGCTTCCAAGA
GTTCCATAACCCAGTCATGTGGAGAACTTAACAAGGTGGTCAACAATGATCTACGAAGTG
ACAATTTTTGCGTCATTCTGGGCTACTGCACTTGTTCTGACTAAGAATTCATCGTTATGA
AATTCTATCATTTACCAACTCATTCTGGTATTCAATTACGAGCTTCGAAATTGTTAAATT
TGAGCCATGAATAATTAATATAGAAAAGAAGAACCAATAGCTAAGATGGAGGATCTTTCT
ACTATCACAAAAATAAAACAAACACACTGACTACTCCATTGGACCTTGCAGCAAGAGCAG
GAAGAAGTCTTCTTGTGACAGTTCTTCTTTGTTGGCATGTGTACTACTACCACTAACTAC
TAATAAAGTACTATTGGTAGTAGTAGCTAGTAGAACACTACCAAGAGTACTCTCAACAAT
TGGCTATGGTCCACTGCACCAACACTGTTGACATGCGCAAAGAACAAAATAAGAATCTCC
TAAGATGATGAGAACACCACAACTGGAATTGAATCTTCTAGGGGTAAAGAAGCAAAATAT
T
>comp2854_c0_seq1
TCACACATTCTTCCTAAAACCCAACGAAGCGACAGCAAGCAAACGAAGATAGTAATATTC
GACGAGTTGTTTGCTTTTCACTCCGCCTCGCTCCATTTCTAAGCAAACCAACACACGACG
ATTATGGCAATTTACAACACGAGTTCGAACAACAACCATGCCAGCAATGCTTCTGTCCTT
AGCATAGTTATCGGAACCACGTTTGTGGTCGGAACGCTTTCGTCCGTTGGATGGTACTAT

TACTTCACCCGTGCGACAATAAGCAAAGACAGCGGGGATGACAATGGCAGCAACAAAAGC
AGTTTGTCCCCCGCAAAGCCATCGTCGTCACTCATCAAACCACCTTTTCCGCAGACGCTC
CGGGACATGCTGTCCGTGTGCCGACTGGCCTACCTTTCCACCGTCGACAACGCAGCGGGA
AGCTCCCACTTGAGCCTGATGCGTTTCACATACCTCCACGACGAAAGTGACGGCGAGGTC
GTCATCATGAGCACGAATCGTAAGACGAAAAAATTCGATATGCTCTGCCGCCAGAACGGC
GTGGCCCTGCTAGTCCATGACTTTGACGGCATCACGAAGCAGCAGCTGGCGGCTGCCGCA
GCAAACGAGATAAACGACGACGACAAGGCCGACCCGGAGCACCCCAAGATCCTCCGGTCC
ATTACACTCAACGGAAACTGCCGGATCCTGGACGGTTCTTCCGACGAGGAACGCTACCGA
CGGGCACACCTGGCGCACAATCCCGACTATCCCCAGTTCATCGTCGGGGACGACATTGCC
GTCCTGTGCATCGACGTGACCTCGGCGCGGATCTGTGACCTGAACGACCGGGTTGTTCAC
TGGAACGTAGAGGACGCGGCACTCGCGGCGGCGACTACAGCAATGCCACCGTCGCCACTG
GCGGAAACGGCAAGGGTTTAGAAGAGGACAGGGAGGATCGATAGGACAGTCACCCGACAT
CCCTATGCGTACGAATGATACAGCTAGATTTATTCTATGCAATACTAGAAGCGTGGTAAG
ATATTCATGAGAAAGAAAAGCCGTCGTGTTTCCATCACGAATGTGGGTGTGGTCGTAAGA
GATAGTGGATCTTCGTGCGGTGTCTTGTCGTGTGGACGCTTGGGTTTTGTGATGAGAACA
TTGCTTTTGTGGTCTTTCCGTTCTGGCGAAACTAGCTGCTCAGTTAACTATCAAGCTTGT
CTTGGAAAGAATACGCTTGGAAAAGATTGCAAAAAGGAGGTTGAACGATCACATGGAAAC
AACGAGATCTATACGGTGGATGGGCAAGCAATGCACTGACTTGCTTGCTCGACAATCGTG
ATTTTACTTCGCTTGTTTCCTATTCCAGTCGTATAAATGTTGGTTTGGTCTGGTACCATG
GTGACTTGAAGAGTCTGCACTGCTCTACTGACTTCAATTGGAAGTACACGAATCAAGATT
TCTCCCGTGCAATGTGATGGAACTCGAATGATAATACAAACCCAACGACAACCTAGACTC
AGAGTAACCAATATGATATTGCGTGAAAATTTCTCATATCACTGCTAGCTTGATATCAAA
TTTGTGTATTTTCAACAGTAGCAATGTACGTACCTCTCCACCAGCATCTTTGGTCGATGT
AACTGACCGAGGCGTAATTTTGGTTCACTTGTTCCGAGATGAAAGCTCCATTCCCATATC
CTTGTTGTGATCTTTCGTCCCTCGTTCATCAGGATCTCTTTGCCACTTAGAATCATAGAC
AATCTCAC
>comp2855_c0_seq1
CAAGCAATAACAATGTACAATAGCTGTGAAGAAAGCAGAACCTTTAAGAAATGGACACCG
ACGAAGACATGGATGGCATCAAAGCCATCCGGACGTATATCGACCGGATGGTAGAAGGAG
ATGGCAAGTGCAGAGGAATGAAGGTGTTGTTATTAGACGCTTCTACGACACAAATCGTCT
CCGCTGTATATTCGCAAACTGAAATATTGAGTAGCGATGTATACCTGGTATCCCGGTTAG
ACGACACCAAACAGCAAAAACAACAGCAATCTTCTGGGAAGTCCTCGCACCTCAAGGCTG
TATGTTTCGTCCGACCGACGGAAGCGAGTGTTGGAATGCTAGTGCGGGAGCTGAAGGAAC
CTCGATTTTCTGAGTATCACTTTTTTTTTAGTGGAATCCTTACTCAGGGCCTTTTGAGAT
TACTAGCCGAATCGGATATGACTGAACGAGTGAAGCAAGTTCAAGAGTTTTATGGTGATT
TTGTTCCCATCAATGATGACTTGTTAACATTGCAATGCCGAAATACCCTGTCGATGTCAA
TGGCTGCGGGGACATCATGGGCACCCCAATACGCCCACTTATACGAACGCAACCTTCGTG
GGCTTCAGGGCATGTTGCTAGCTCTCAAACGACAACCACTAGCAGGTATTCGATATGCAG
GGCAATCGGCATGTGCCGAAGAACTTGCCAAGGATATTTTTGATGCGATATCGACAGACG
AGATTTTCCACTTCCGTAAGAGGAGACGAAATAGTGCAGGAGCGCTCTTGTTAGTATTGG
ATAGAAGAGACGATCCTGTAACGCCCCTATTGAGCCAATGGACGTATCAAGCCATGGTAC
ACGAGCTGCTGGGTCTGAATAATCACAGAGTTGTACTCAAGGGTGCTCCCAATATTGCGA
AAGATTTGGAGGAGGTGGTGCTCAGTTCGTGCCAAGACGATTTCTTTCGTGAAAACCGAC
ACAAGAATTTCGGAGAATTGGGAGAGAATATTCAGAAACTGTTGCAAGATTATCAAAAGC
AAACTGCGCAGCATAATACATCTATCCTCAATACCATCGAAGACATGCAAGCATTCATGG
AGAAATTCCCGGAACTGCGATCACGATCGCACAACGTTTCAAAGCACGTTGCAATCATGG
GAGAGTTAGCGCGACTTGTAGATGTATGTTCTTTGTTGGATGTATCCCAATTTGAGCAGG
AACTTGCATGTGCTGATGATCATACGGCGCATTGGAGAGAACTCATGGAGAAATTAGATT
CACCAAGTGTAAAAATACCCGACAAATTACGCCTTGGTCTTTTATATGCGCTCCGTTACG
AAACGTCTGGGAATATAAACATGGTACAGCATGCCATGAAGAAGGGCGGTGTCCCTCCTG
ATATGGTCAATCTATTACCAGTCATTCTTCGGTACGGCGGCAGCAAATCTCGAGGTCCTG
GACTGTACGGAGATCCAAACAACATTATGAGCAATTTGACGAAATCATTTATGACTAGTG
TTCAAGGAGTTCAAAATGTCTATGCGCAGCACGTCCCAGTTCTAATGGATACGATACAAT
CCATAACAAAAGGAAAATTAAGAAAAGAGACACACCCTTTCGTTCCAGGGTCGATCAAGA
CAGTTCCAGGTGTATCTCCAGAAACTTTGGTACCCGATGAAATCATTGTGTTCATGGTGG
GCGGTATCACCTATGAGGAGGGCAACAAAATCGCCGAGTTTAATCAAGTTCACCGGGGGA
AGTTTCAAGTTTTATTGGGAGGAAGCACGGTACACAACAGCACGAGTTTCTTGGAGGAGT
TGCGAATGACAAAGTGACAGTATTACTTCGTATTCTTTAGGAAAACTTTGCCCTTGCGTA
TGTTCTTTAATCTCTCCCGGTCGATATGTTGTTCTTTCCATAACCTTTTGAATTTACAAT
TTATTGTGAGAGAAATGGAGCTGATGATGAAAGTAATACTATATGCAGTTGTTCTGATAA

CCTTTTCAACTTGATTTTTCAATTGACAGAATACTAGGCATCGTGTTGAGCAAACAAAGA
CAATTTGCCAGATTTAGAGTAAATCTGTTTTGGCAATATCTTTGTGGGATCAGCATTTTG
TTGTCAGCTTTACTAAGGTTATCTATTCTGGTAGCAATGTGTTGCTTGAGTTGCTGTAGT
TTGAGCTGGTTGCTTTAAATCTACAAGAACTTTGAACCCCCCTAAAATCTACTTGAGGAT
TCTTTTATGATTTGTCATTTTTTAATTATCTCTAACTTCTCTCATCCCAATCCAATTCTT
GGGATATGGGGATGTACCTGATTTGTCACCAAATTATTAAAATATATTTCAAGAAAACAT
CATTAT
>comp2856_c0_seq1
ATCTACGACGTGCATACAGTGTTGTACGGTTCGCAAATCACCATAACTAGAAGTACTTCA
TAAACAAAACTTGCCAACATGTCGAACGAGAAGAAGCCACTAGTAGGTGTACTATCCATC
CAGGGAGCCTTCGAAGAGCACCAAGCGTGCTTGGAGGCATCGGGATGCCGGACGAAACAG
ATCCGGACTGTTGCAGATATGACAGAAGGAGGTGAAGCGCTGGACGGAATGGTCCTTCCG
GGAGGAGAATCGACTGCTATGGGATTGATTGGAACAGCTACCAAAGAAAGCGGCGGTAAA
AGTGTCTGGGATGCTCTAAGGGACTTTATCGAAGTTGATAAGAAGCCTACCTGGGGGACC
TGTGCGGGAATGATCTTGTTGGCCGAACGATGCGTCGGAGCGAGCGCCGTCATTCAAGAC
GGCCAGGCACTGATTGGGGGTATGGACATTTTGGTGTGCCGGAACTACTTTGGATCACAA
GTGAGCTCCTTCGAAATGGCAACTCCTCCTCCGCCGACAAGTGGCGGTGAGGATGGAGGC
ATGGCCTCTCCTTATCCCGGGGTATTTATTCGAGCTCCCGCCATTCTGTCGGCAGGGCAG
GAAGTCACCGTCTTGGGCAAGGTAGTTGCCACACCCTGCCGCCAAGCTGCAGTGGTCTTG
GAAGAGCTGGAACGAAAGATCGAAAAGGGAGAAACCGTCGTCCAGATGGGAGTCGTCGAC
GCCTTGGAACGAGATGGATCGCAACTTCGGCACCGGGAGGTTGTGAAAGCATCACCCTGT
GAAACAGGAGAAGAGAAGAAAGAACTCGAGCAAGATGCATCTATCGAGCTTCCGGGTGCT
GCCGATGGGAGCAACGCCCGCGAGGTTATTTGTGCCGTGCGAAAGAGCAATCTATTGTGT
ACTGCCTTTCACCCCGAATTGACAAACGATCATCGTTGGCATCAATACTTTGCACAGATG
GTGAAGGAATCCGTTGCGTCAGCTTGAGTCGATTGCACCGCTCTAGTACTCGAACGATGA
AGATTCAGTTGACAGTTGGACAAAATAGAAGAATAAAACTTCAGTTCGTGTCGCCATCGC
GCATCGGTGAAAGATAGATCTCATCTCACACTTGACTCTTATTTACACGAAATTCCAATC
ATTAAATATTATTTTACAATAAAAAATCAAGTCCTCGACTATTAATCTACGTGCGGAATT
CAAGTTTCCTCCACTGCAGATTGAAAAATTCAATAAATCAATACCACTGCCACCAACTGA
AATTTCAATCACAAATTAACGAAATCTAATTTAACAATTTACAAGAACAGATCG
>comp2857_c0_seq1
CAACGAACCCGAATGGTAGTCAAACTTCGTTCTCAACCCTCTACTCGTAGTTGCTTCTCT
TCGATCCGATCGGTTTTTCAAATGGTAAGCACAACAAAACTCAAACTATCTATCCGCATA
GTGAATCAAACATTGAAATCGAATCGAAACGAAACGAATTAAATCACATTGAATCGAATC
CATCGATCTGTCGCTCGCTGTGCCCCATATTGCATGGCAAGCACGAAAACCACCATTTTA
CGTGAGAAGCATCGTTGCCGTGACTAGAGGAATAGAATGAACCACTTTGTTGCGCGGTAA
CCAACCCATTTGGCAGTACGAGCTTCGAATTCCGCACGGATCGGTGCCATTCAACGCAAC
AGAATCCTCCCAATCCATATCTCGAACAGTCCTAAATATCTCGAACAGTCCTAAATGTTG
TCTCAGACGCCGAACGCTTCTTCCTCTTATCTGCCCACACAATACCGCCGGAAACTCCCC
CGCGCATCCAATGCCACCGCCGACAACAACGCAACGACGATAAACACCCTGCCACTGGAC
CAAAAGGCACCCTCCAAGAGCAACCAGGCTCCCTCCCCCACCACCTACCGGATTTTGGTG
GCGACCTTTGTGCTGTACTCGGTCTTTCTCGGCTGGTACGCGTCCACCCGCCCCAAGGGC
AAGTGGAAGTCGCCCTTTTCGTGGCACCCATTTTTGATGACGGTTGGCATGACGGGATCC
ATGGGGATCGCCGCGGTCACCAAGAAACTCGGGGGCTACACCAACACCAAGCTGCACGGA
ATCCTGGCGAGCCTGGGCTTTTTCGTCTCGCTCGGTGGCGTGTACGCCATTTACCACAAC
AAGAACCTTTATCAGCGGCCCCACTTTACGTCGGTCCACGGGAAGGTCGGGATCGCCGTG
GTGGCGATGATGGCGCTCCCGATGCTGGCCGGCGCCGTATTTCTGCACCCCGACTTTGGG
ATCGACAAGACCAACAAGCTGTACCGCAAGATCCACAAACTCGCCTCGAGGGTCTTGATG
GCGGTGGCGTGGGGGAACAGCCTGTACGGCCTCTATGGAATGCGGTCGGAGCACCCCATG
GAGCTGATTCTGTACGGCGTTCCCCTCGTGGTCCTGATGCCCATGACGCTGCTCTAAAAG
GACTCCAAACCGTATCGAACCGTATACTTTGCAACACTTTTCACAAAACCAAACCAGATC
GTATACGCAACAACGACAAAAAGCAACACATCGGATTTGGTGGCTCGATTGAGCTTTCCC
ATAGATCATTATAATAACAACATGATAACTTGCTTTTGCCAC
>comp2857_c0_seq2
CAACGAACCCGAATGGTAGTCAAACTTCGTTCTCAACCCTCTACTCGTAGTTGCTTCTCT
TCGATCCGATCGGTTTTTCAAATGGCACCCTCCAAGAGCAACCAGGCTCCCTCCCCCACC
ACCTACCGGATTTTGGTGGCGACCTTTGTGCTGTACTCGGTCTTTCTCGGCTGGTACGCG
TCCACCCGCCCCAAGGGCAAGTGGAAGTCGCCCTTTTCGTGGCACCCATTTTTGATGACG
GTTGGCATGACGGGATCCATGGGGATCGCCGCGGTCACCAAGAAACTCGGGGGCTACACC
AACACCAAGCTGCACGGAATCCTGGCGAGCCTGGGCTTTTTCGTCTCGCTCGGTGGCGTG

TACGCCATTTACCACAACAAGAACCTTTATCAGCGGCCCCACTTTACGTCGGTCCACGGG
AAGGTCGGGATCGCCGTGGTGGCGATGATGGCGCTCCCGATGCTGGCCGGCGCCGTATTT
CTGCACCCCGACTTTGGGATCGACAAGACCAACAAGCTGTACCGCAAGATCCACAAACTC
GCCTCGAGGGTCTTGATGGCGGTGGCGTGGGGGAACAGCCTGTACGGCCTCTATGGAATG
CGGTCGGAGCACCCCATGGAGCTGATTCTGTACGGCGTTCCCCTCGTGGTCCTGATGCCC
ATGACGCTGCTCTAAAAGGACTCCAAACCGTATCGAACCGTATACTTTGCAACACTTTTC
ACAAAACCAAACCAGATCGTATACGCAACAACGACAAAAAGCAACACATCGGATTTGGTG
GCTCGATTGAGCTTTCCCATAGATCATTATAATAACAACATGATAACTTGCTTTTGCCAC
>comp2858_c0_seq1
CAACCGAACAAGGTGCGCTCCGTTACTCCTTTGTAATATCTGGTCACATACAATGCCTCC
ATTCAGAACGCTCTGTATACGTACTAACAGTAATTACAATTCTCTCCTTCTGTTGATCAT
GGTCAACGAACGACACTGTTGCTTGCAATAGATCCTCCAAATCAAGGAAATGGATTCGTT
TTCGGAATACCTCGGAAAAGATTTTGAATGGGATGAGCTCTCTCAGGAACTGAGAACACA
GTTGGGCCTTTTCAGTTCTACTGCCACTGAATACTAGCATTGTAAGTTGCAGGCTACGCA
GTCATGATCTCTTACTACAAAATTCTATGCAATTTCAAGTTTCATTAATTATTAGAAGAA
TTTTTCGTATCCAAGAAAAATGGGAGACGAGTCGAAATGGCTATGTTGAGCGTGACTTGT
TCCCATGGTGTTACAACCAGAAGAGCACACATCTCAGTGCATCCGCATTTTCCAACTTGT
TCGATCGTTCGATGCAACAAAGATGTTTCTCTCCATTGGTAAGGCCTAAAGATATTCACT
TTTTGGTAATGAAGTCAATACTAGTGTTCACATTACAATCACGCTGTTTTGCCTTAGCTT
ACGATCTGCCTAGATAAAGAAGCTGAATTGCGAGCTTGCTTGCATCAGCTATCAATTAAT
TTCAGAAGGGTTCCAATTGACAGTTTTGTCTGTAAGATATTGCATCTCTACCAGATCATT
GATAAAAATATACACAACATACTTTGATTCATTTCACCCTGAAAATGACCATTGTAAGAA
TAATAGAAACTCATCACCCAACTA
>comp2858_c0_seq2
CTTACTACCAGCACCTCCACAACTTGCAGTCAAAAATGAACCAAGAACCGACTGCAATAC
CACGGATTAGGCAAATAGGCATGAAATTATGAGAGGAAGTACAATGAGAGGCACCAACAA
CGAAACTTTGAATCAGAAAAGACCATGGACTTCGCAGCTGTTCAAAGCTGAACAAAATAT
ATTATTATCCTCTCGGAAACTTGAACGTGCCTTCAGGGAGATAAATGACGAGTCCTCCGG
CGATACCACTAGGAATGACCTCAGTGTCCCAAACCCCATGAAATTGATTCATCGTTTGAC
AGCTCTCGAAATATCAATCGGGCAACTAAGGCAAGACTGCGAATCAATGTCGAGACATAG
AAATCAAGTTGTCCAGTCTGTCGTTTCTGATCAAAACAAGAATATCCTCCAAATCAAGGA
AATGGATTCGTTTTCGGAATACCTCGGAAAAGATTTTGAATGGGATGAGCTCTCTCAGGA
ACTGAGAACACAGTTGGGCCTTTTCAGTTCTACTGCCACTGAATACTAGCATTGTAAGTT
GCAGGCTACGCAGTCATGATCTCTTACTACAAAATTCTATGCAATTTCAAGTTTCATTAA
TTATTAGAAGAATTTTTCGTATCCAAGAAAAATGGGAGACGAGTCGAAATGGCTATGTTG
AGCGTGACTTGTTCCCATGGTGTTACAACCAGAAGAGCACACATCTCAGTGCATCCGCAT
TTTCCAACTTGTTCGATCGTTCGATGCAACAAAGATGTTTCTCTCCATTGGTAAGGCCTA
AAGATATTCACTTTTTGGTAATGAAGTCAATACTAGTGTTCACATTACAATCACGCTGTT
TTGCCTTAGCTTACGATCTGCCTAGATAAAGAAGCTGAATTGCGAGCTTGCTTGCATCAG
CTATCAATTAATTTCAGAAGGGTTCCAATTGACAGTTTTGTCTGTAAGATATTGCATCTC
TACCAGATCATTGATAAAAATATACACAACATACTTTGATTCATTTCACCCTGAAAATGA
CCATTGTAAGAATAATAGAAACTCATCACCCAACTA
>comp2859_c0_seq1
TGAAAATCGCTGGTTGACTCTTGCAACAGTACTCACAAAAGAGTTGCCCGAGTTCGCAAG
GTGAATGCCACGGAAGCAGACAAAGCCGATTATCGAAACACATAGTAACGCAGATAACAA
TTAACTCAAGTGGTTGCGAAGGGACGAGGACCGTTCAGGAGTTCCAGTAAAACGCAACAG
AAAAATCCCTGAGTACTAGTGATAAAGACAGAGCAGTAAGAGACAGAGAGACACAACCAC
AGAGAGATTGATTCCGAGCCAGAAGAAAAGAACACATATTCCAACAGCAAAACACGAAAG
CAAACACAGTGACCACTGCAGACAACAACCAACGCTACCGACCTAGAGCCTTCTTCCTGC
CAACACAACCTATCGAACCAAAGGGAGTCGAATTTGGTATAGTTCGCACGCACCTATTCT
TTTCCCTTCTCGATCCGAAGTATCACCGTCGCCGAAGGCAACGAAACCAAACCGAACCGG
ATTGGATCGAAGCAAAACACGGCTCGTTCGAAGTCTCATGTCCAACAGTCCCCCTGCTAA
GCATGCTTCCACTCCGATGATGATGGCAACCACGGCACATACTACAACATTACCAAACCA
GCAGGGGCTGACTTTGAACGGCTCACCGCAATTTCTGCGGCCCAACGTCGTGTTGGGCGT
CACCCAGCTTCCACAGCATCAACAACAGCCACCACCACAGCAGCAGCACATGCGGGTCGT
GAGTCCTGGGGTGAGCCCCATAGCACAACTGCCCCTGCAGCACTACCAACACAACACTAA
CCCCTCACCTCTTCCGCAGTACCTGCCCGTTTCCACTGGCGTTCCAAACCACGGCCACAA
TCCCGCAATGGCGTGGCAGCAGCAGCAACCCCACCCGTCGATGCTCCCGTTGCAGCAGCA
ACAAGCGCCGCATACAGGCTTTACCCAGCAGCAGCAGCAGCACGTTTCGATGCAGGTTCC
TTTGCGATTAATGCCGCGCCCAAATCTCGACGATATCGAGAACAGCAACAGAGACGATGT

CTCCTCGGTGCCCTGGGGATCGATCCGGCCCGATCCCAAGTACAATCCGGCCAAGGATCC
CCTCAACCCGGTCAACATCGACAAGCCCTTTGTTCCTCCACTGGAAATCACCATTACGAT
AGTACCATCAAGGAGGAGGAAGTCCATAAACCGCCATTCGAGTCCCCCGCCACAAGCGTG
TGTGGCTTCGGACCGTTACCCGTCGATAACGCCCACAAATCAGGAGTCCGCCTCCACGGA
ATGGCTGCAGCTACAGGTTCCGCCTCTGTCCACGGGTCTTCCCGGAGGCGAGGTTCACCG
GATCCCCGTGCTCGGACTGGCCAATTCCGGTTCCATGGACGTCTCGAACACGAACAACCC
GGACTCGAGGAAGAACAAGTCCTTTGAGACCATCGACGACGAGAATGATGAGACTGCCAA
CGCGAACGACGACCTCCTGGAGCAGTTCTCGGACCTCGCCACGTACACGGCCGTAGACGC
GAGGGGGAACCAATACTATCTCACGAACGAAACAGACGACGACGAAGAATACCTCGAGCT
GAGGGGCGATTTGTTGGAACTCCAAAAACTCCAGGAGCTCCAGCATCCGTCCTATTACGA
CGGAATCGCGGTCCAAACGAGGTCTGGAGACGCGGAAGCAGAGGTACCCAAGAACCACGG
CAACAGCATCGCCCACAACGCCGGCAACACACCGATGGAGGTCATCGCCGAGGGTGCTTC
TCAGCAGCGCAGGGGCATGGTGCGAAGGGAATTCAACCGCCGGGTCTGGCGCTTTAAGAA
AAAGAAGGCGAGCCCGAGCGACTCGGAGGAACTTGCTCCCGGAGGCGAGCTCGCCCCCGA
CGTCGCGGATCACGCCGTGGCTGCCGCACACCTCGAGATGCCAAGCATCGTTGCGGACGC
GGAGGCCGACGGCAATCCGGGCAAGCAAACACTCGTGAGCAAGAAATCCATGCACTTTTA
CTCGCCGTCCTTTGTCACCCACAACTACTACAACAAGTCCAAGATGATGACAATGGTGAG
CGGCCAGGAAAACGGCTGCTAAGGCGGGTGTCCGTCCTATCGTTTTGTTTCGCTGCAGTT
GATGTCATGGACATCGACTGCACACACGAACCAAACCACCCACCGACCGCGACGGCGGGA
CCGCAGGATCCTACACGCAACGCAAACCTGATGTGTTACGCTGCGGTACGACTATTATAT
GTTACATTTTCTTATCATCGTGAAACACCCACACACACGGTACATATTTTTATAACAGGC
AGCACTATCGTGCTTGCATAGAGTCTAGACAAGCATAACAGCGCAACAACAAAATTAACA
AAAACCATAAACAATAGCCATTACAACAACA
>comp2860_c0_seq1
CTTCATTCAAGAATACGAAGTCAGGTCTTGCATAACAATGAGTCTGTCGTTTGTTTCTTC
GGCCGTTCAAATAGGGACCTCCGATGGTGGTTTCGAAGAAAAACCAATTGAAAACAAAGA
AACCGAGGCCGTGAATCGTCGCAATGCTCACAAGCCACTATTCGAGCAACTCCGATCTAA
CCAAGAACAAGATCAGGAGAAGAAAGAGGAGCAGGAAAGGCTTTTGATGCGGAGCACTTG
CGCATTGGACGAAGATGATGTCGCTCACTTGAATGCTATCGAGAGGCAGCGATCTGACCG
AGAACGAAAAATTCAAGAACGAACTGAAAACGAGTTAAAGGAATTTCGTGCCGCTAGGGC
TCTGAAGAAACAAGCCGCGCTCAGCGGAGAGGACAAAGATGACGAGAATGATGAAAGCAA
CGCCGAAGACCAAGCTGCTAAAATATTCATAATGAACGGTAATGCATCGGAAGCGTTAGC
AGAAAAGAGAGAAACGAACCCCGGTCAGGCCGGTTGTAGTTTACCCGTCATCAAAGTAAG
GAAACGGAAACGGCGCACAAATCCACTCGATACGCAATCAAAGCAAACGAATATTGCGAA
GGGGGAAGCTTCCAAACTAGAAAAAGGCTACACATCAGACCTGAACGAGGTCAATCCTTC
GTCTGAAAAGAGAGTTGAGATGTCATCTGCGGATGTGAAACGTGACGAAAGTGCTGGTGG
ATTAGGTAGTCTGCTCTCTGGCTACGGGTCATCTTCAGATGAGGATTAAAACTTGTATTC
TACTAAAAAACTCACGATTCTTTATGCTGGAA
>comp2861_c0_seq1
CAGATTGAAACGCTAATCCAACTGTGGAGAATCGTATGGTACTGTGAGACAGTAAATCAC
AGGTACAACTGAAGATAGATTCACAGTTGATTGACACTGGTGCTGTTTTCATCTTGTGTA
AGAGCGCCTTCACCATAGCGTCTTAATCATAAAGAACATCTCGATCGAATCACCATAGCG
GAAATTTTAAAAGAAAAAGTCAGCCAAAAATGAAAATAAGAAGTCCGATGGCACTGGATG
CCGGGAATTCCTCACCAAAGAGAACAATTTGGTTTATTCTTCCTGCAGCATGTGCCATGG
TGTATTGTATAATGATTAACCTGAACGAAGATTTGCAATACGAATTGTTATCAAATAGAG
AGCCTGCACAACAAGTATTGCGTGCCGCTACTACCGCCACCACCATCATAAAAAAGGAAG
AAGAGCATTCGACATATGAATTGGCTCGAAGAGAGTCACTTGGATTTTTCACGGACATTC
CTGAAGAAACGTGGAAGCGACACAAGAAACGATTCCAACTGACTCAACCCAATTACGATG
ATTCAAATCAAAGAGCTTTTGAACGACAATCGAGATATTCGAATCACTTCTGGGCAGGTC
ACTTTGAACCAGAATTTACCTGTCCCCATGAATTTCGCTTGGGAAAGCTTGGGGACGGAG
GGAAGTGGATTTGTGATCCGCATCGAATCACCCAAACAAACAAAGATGGGAAATGTCTTA
TTTACAGCATTGGTAGCAATGGCAACTATCAATTTGAGAAGGAAGCCTTCAACCACGTCT
CAAAAACTTGCGAAATCCACACCTTTGACATAAATAAATTTGGCCAACGAAAAGACTTTG
CGGCGTTAGCGCAAGAGTTGGGGGTGAACTTTCATCACTGGGGTTTGGGAGAAAAGACAA
AGGTATACCAAAACATGAAAACTTTTAAGGAAATCATCCTAGAGTTGAACCACCAAGATA
GGGTAATCGACGTAGCGAAGATTGACTGTGAGAGATGTGAATACGCTCAGTTTGAGCAGT
GGTTTAAAGATTGGAAGGAATTAAACATGGTGGTCCGACAGATCATGATTGAAATTCACA
ATTCAGATTTGCCGGGCGTTGTGAATATTTTCAAAGCATTTCAATCTGCTGGTTACGTAA
TGTTTCACAAGGAGGCGAATTACATCAACGAAGGAAAGGCAATTGAGGCTGCATTTATTC
TCCTGAGTCCAGATTTTCAAAAGATCTAGATCACGCATGGTACATGGTGCACGGTGCATG

CGGAACCAAAGCTAACAGACGACGTTGGCTTGCCCTGAACATAACCAAATCATCCGTTCG
CAACGACAAGTTGTTATCTTTATAACACAGACTGTCAGATCAAGTTCCCAGTACACAAAC
TCATTGTGCTTGTGCCAAGAATAATTTGATAGATATATGAATCTCCTATTAGGAAAATGA
CAACTTGAAATGAAAAATATAGAAATTGAATAAGAACTAAGCTCATGAGCAGCTTGATGA
CAATTAAGAGGCTTTGTTGGGAAATATTTGCTTTGTTCAATGCTCAAAGAACTCAGAGGG
AGAATTTCTAATGACATAAATACTAAGATTCAAGATAGAAAAATTGCATCAATTGTGCAA
TACATTTGAGATAAAGTGCAATACATCATGCTCATTGGTAAGAAGTGTTATTGTCC
>comp2862_c0_seq1
AGCCTCCTCTTTAAAACACCTCACATCGCTCTGAAGGACATCGCTAACCATTACTTCCGA
TAAAAGTAATAGCAGTGAAGGACGTCTGCTTCTATCATCTGTTGTAGATGAGAAAAGGTA
CGATCCCATGATAGCCATGAATATAACTGTCAAGAAACGTAACGCTAGGGATATCGGGGT
TGGTTGTTGTACTTCGCAGGAGGACATGCCGCTCAAGCAACGAAGGCTCAACCCGAAATC
CCCGAGTTCATCGACCTCTTCAATATCATCGGTGAAAGCCTTTCGATTCATGATGATTCC
TCGAATGATAACTTCTTCCCAAGAGTCATCTATACCCGTCCTTAGTCCCTGTACATACAT
GTCCATAGCCCTCAAGAACAAGAAGATAAAAACTCGTGTCGATTCCTTCAGCTTGGAACA
AGCACTATATTTTGAACCCTACCAAGAAGCAGAGATCCATGTAGACCTTCTTAAGGCTCT
TAGAGGAGCCGATTTGGAGAAACTACGGACTCTGAAAGACGAGAAAGAGAAAAACTACGA
TCTTGATGCTAGGAATCAGTTTGGGGAGAACCTGGTTCACTTAGCTTGCCGTATGGCGCT
CAGCTTAGATGTGCTAAAGTTTCTAGTCGAAGAAACGAAGATACCAGTGAACGTGAGAGA
TCGTTTTGGAAGAACTCCTCTACATAATGCATGCATGTCAGCACTTCCGAACTTTGACAA
CATTGATTTTCTCATAAAGACGGCGCCCAAACTTGCTGTCTTCGAGGACGATAAAAATAA
AATTCCTTTTGAGCTGATACCTCAACGAGTCTTCGAGCGCTGGATACGGTACTTCTCAGA
GATGAATGTACTCAAGAAACTTACTACAGAGCTTGCAAAGCACGAAGGCCTTTCGAACAA
GTGATCTACGTGACTCGGTCTGGTTTGAATGAATGATCAATCCAAGTTTATTTTGTCTTC
ATCAACCTATCAAGGTTTTACGAAATATACGAGATGAGAAAAGTGCCAATGGTATGGACA
ATATTACCACTGTGCCCCCTCCAAAAGACCGAGGTATTCAGCGATGCAATGTCACACGGC
CCGCTATCATTGATTGCGTCGAATCGCGATCCCAAACATTTCACTCACCATGATTGAAGT
GTGCAAGCGATGTAAGACTAGCATTGCACCATCTGAACTGCAAGACACGAAAGCTAACGG
CTCGATCACTTATATTGTCTGATCAACACCTTTAGCCGATAGGGTTCACTTCAAAGAAAC
GGTGAACAAACCTCGCGTCTGCCGCATTAATAGCCAGCAAATCTGGTAGAATGCCCTCGA
AGGCGGCTTACCGATGCCTCTCATAATCTACAATATAATAAAGATTTTTGAAGTACAAA
>comp2863_c0_seq1
CGTCTCTTCAATCCATCCCTCCGACCATCCACCATGAAGACCTTTTCCCCCCAACTGCTC
GTAACCTTTGCCGCTCTCTTATCGGTAGCCTCCGCGGCCTCCGAGGCCGGATCCGAAGCG
GCCGTGGCGCTTACTCCCCCGGAACCCAGGGATCTCACCCAGTCTCGACGAAAACTCGAC
GACCAGGAGCCCTGGGACACCGGCACGAAGGTCTACAACGAATTCCCCAACCAGGGCTGG
TGGTCCGGCACCATCACCGCCTACGACCCCGTCGCCGAGATGTACACGGTGACCTGGGAG
GACGGCTCCACGGACTACTACGACGACGGCGACAAGATCGACGAAATGGTCGCCTACGCC
CAGAACGACCCCCAGAACAACCCCGCCGGCGGATCCACGGACCAGTCGGGTTCCTACCCC
ACCGGAACTGCGGTCTCCATGTTCGAGGACGGTGAGTGGTACGACGGCGTCATCATCAAA
TACGGAAGCGGCACCTACACCGTGGAGTGGGACGAAGACGGCGAGATCGAAGAAATCGCC
GCCGGATCCATCATGGACCAGATGGTCCAGGACGCCCTCGGCGACGACGACGAGGCCCCC
GAGGGTTTCGACGGCGGTCCTTCCGACAGCGGTTCTTCCTCGGGAGACATTGCCGTCGGA
ACCCCCGTCTCGGTCTTTGAAGACGGAGAGTGGTGGGACGGAACCGTCTCGGACTACTCC
GACAACACCTACACCATCGAGTGGAAGGACGGAAGCACCAACGAATACGATGACTTTGGG
GACGACCTGGAGGAACTCAAGCAGGCCGTCGAGGACTACATGGCAGACGACGACGAAGTC
CCGGAATCCGTCGGCAGCGGACCCAAGTTCCCCCTCGGCACCCCCGTCTCGGACTACGAG
GACGGCGAGTGGGTCGAGGGAGAAGTCGTCGACTTTGACGACGGAAGCTACGTTGTCGAG
TGGGAGGACGAGGACGAGCTCGAGTACTACGAATCTAGCAACGCCGAAGACATGCAGGCC
CTGGGCAACATGGCTTCCTACTCCAACAAGGACGACGACGCCCCCCCCGACTCGTACTTT
GAGGAAGAAGACCTCTGGAAGGTCGGAACCCCCGTCGCCATCACGGAGGACGACAACCTC
TGGTACGGAGAGATCAGCGGCTTCAACAACGGCGAGTACTCCATCGACTGGGAGAACGGA
GAGCGGGAGTGGCTCGACAACTTCGACCTCGTCAACGAGATGGTCTCCAACGCCGCCCTG
GCGCCCAAGAAGGGCATGTCCGCGGTCGGAAAAACCTTTTTGTCGCTCTTTCTGATCGGC
GTCTGCGCCGCCGGCGCGGTCTTTGGCTACCGGTTCTACGAAGAGAACCAGAAGCAACGC
CAAACGCGGGAGGGAGACCTCTCCCTGGAGGACGGCGGCGCCCGGTCCTTCCGGGACAAC
GCCGACGAGCTCCCCAAGATCATCTAAGCAACCACGCGCCCACCAAACCAAATTAAACCA
AACCGCATGGCTTCTCTGCCACCATACAAGCTTCCCACTCGTACCACTATACATTCCAAC
ATTTAGACACCACTGCACAATAATAATAATACTATAACATTAGTAACATACAGCGA
>comp2864_c0_seq1

GCTGAATGTCGATACTAGTGTTGCGTTCCATTTCTTCGTAAAGGTGGTTTCATTGCCTTG
GAATTTGCCCAGATCATGGAGGGCCTTGGAACGGAAGTAACTCTCATGTACCGAGGCGAT
ATGTTCCTTCGGGGATTCGACAACGACATCCGAAACCACCTTTACGAAGAAATGGAACGC
AACACTAGTATCGACATTCAGCTCAACACGGATCCTACCGAAATCATCAAGAACGAGGAT
GGATCGTTCACAGTGGTCACTGGAGATGGAAAGAAAGTTGACTGCGACCAGGTCTTGTAC
GCCACCGGTCGCAAGGGAAAGATCGAAAACCTCAATTTGGAAAGTGCCGGCGTCAAAACC
GAGGGATCCTTCATCCCAGTCGATGAGTACTCTCGAACCAACGTCGAGGGAGTTTATGCT
GTCGGCGATGTGACAGACCGCATTGCTCTGACACCCGTCGCTCTCATGGAAGGTCACTCG
CTCGCCGATACCGTCTTCGGGGGAATGGACCGCCCCTGCGATCACGAATTTGTCGCTTCG
ACCGTCTTCACGACACCAGAAATCGGAACCGTCGGATACTCCGAGGAAGAAGCCGCTGCC
AAGTACGGCGACATCACCGTTTACAAGACAAGATTCCGACCTATGGCCCATTCGTTCCCG
AAGAGCGAAATGTTCACAATGATGAAGATCATTGTCGATACCGCGACCGACAAGGTGGTT
GGTTGCCACATTGCTACAGATGGTGCCGGAGAAATGATGCAGGGTGTTGCCATTGCCGTG
AAGATGGGAGCCACCAAGGCCGACTTTGATAAGACAATCGGTATCCATCCCACGAGTGCC
GAAGAACTTGTTACGATGCGAACTCCTTCGTACCACTACAAGGACGGGAAAAAGGTAGAA
GAGTAAACGAAACAATAAGCAATTGTGAAATATCGAAAATGTACAAACCGAATGGTGCAT
AAATCAATCTGTGCACACGCTTTTTCTTTTCCTTTTGTAGATTGATTAGCACCAATTTTG
CACATAGCACAGCGTCACAGAACTCACTCACAAATGCAAATAAATATTTCGATTCCTGTG
GATGGTTGCCAAATGTCGAGTGCCTTCACTTATTACTGGCAAAGTATGAAAGATCAGAAA
ATGCTGCTGCTGTTGAAGTGGTAACATACAGAACAAAATCAAGATACTGTGATAGTACTG
CAGAGTATTACAGATAGAAAAATGATGTGTCAAGTGTTATGTTGGATGATTCATATGATA
TAATGCACATGGTGAATAAATGCATATACTATTACTACATTCATGGTGGGAATCCACCTT
AGTTGACATAGATCATGGTAAACTCAGAATGTTTGGTGCTAAGAATATTGGA
>comp2864_c0_seq2
CGCAAAACACGCATCGTCGTCTGAGATAGACAGATAGATCCAATCAATCCCTACGGCATC
CGGTCTCTCAGTTAGCAAGGAATCAATCCAGCGAAACTACGATTTCCCACTAATACCAGC
AACCATGAAGATCCCTGCCGCCTCCCTCTCGGCTCTATTTGCCACACTGACTGCCTCGAC
CGCGGCATTTGCTCCCACGTCGACGGCCTCCCGAAGTTCCACCGTTGCCTTTCGTCGGGC
TTATTCTCTAGCGATGGCCGATAAGCCGTACGAACCCGATCCCGACTTCGATTACGACAT
GGTAGTGATTGGAGGTGGGTCCGGAGGTGTTCGCGCCTCTCGAATTGCTTCGGGATACGG
CGCCAAGGTTTTGTTGCTGGAGGGCCAGAACCAACACGGACCCCCTAACTACAGTGCCAT
CGGAGGAACCTGCGTGAATGTTGGCTGCGTTCCGAAAAAGCTCATGGTATTCGCATCTCG
CTACCCCGGTGCCATTGGTGAAATGACTGGATACGGATGGGACGGAGCCACGCCCGGAAT
CTTTTCGTGGGACAAATTCATGGAAGCTAAGAACGAAGAAATCACCCGTCTCAACAACGT
TTATTCCAAGTTTGTTCTCGGCAACGCTGGCGTCGAAACCATCGAGGGACTCGGAAAATT
GAACGGAAAGAATTCCGTCGAGATCTCACTCACGGGTGGTGGCACCAAGACGGTTTCCGC
CAAGAACATCCTGATTGCCGTCGGTGGATGGCCCTTCAAGCCCAATATTCCTGGAATCGA
GCACACCATCACCTCTAACGAAATTTTTTACCTAGAGGAACAACCCAAGCGCATGGTCGT
TGTTGGAGGTGGTTTCATTGCCTTGGAATTTGCCCAGATCATGGAGGGCCTTGGAACGGA
AGTAACTCTCATGTACCGAGGCGATATGTTCCTTCGGGGATTCGACAACGACATCCGAAA
CCACCTTTACGAAGAAATGGAACGCAACACTAGTATCGACATTCAGCTCAACACGGATCC
TACCGAAATCATCAAGAACGAGGATGGATCGTTCACAGTGGTCACTGGAGATGGAAAGAA
AGTTGACTGCGACCAGGTCTTGTACGCCACCGGTCGCAAGGGAAAGATCGAAAACCTCAA
TTTGGAAAGTGCCGGCGTCAAAACCGAGGGATCCTTCATCCCAGTCGATGAGTACTCTCG
AACCAACGTCGAGGGAGTTTATGCTGTCGGCGATGTGACAGACCGCATTGCTCTGACACC
CGTCGCTCTCATGGAAGGTCACTCGCTCGCCGATACCGTCTTCGGGGGAATGGACCGCCC
CTGCGATCACGAATTTGTCGCTTCGACCGTCTTCACGACACCAGAAATCGGAACCGTCGG
ATACTCCGAGGAAGAAGCCGCTGCCAAGTACGGCGACATCACCGTTTACAAGACAAGATT
CCGACCTATGGCCCATTCGTTCCCGAAGAGCGAAATGTTCACAATGATGAAGATCATTGT
CGATACCGCGACCGACAAGGTGGTTGGTTGCCACATTGCTACAGATGGTGCCGGAGAAAT
GATGCAGGGTGTTGCCATTGCCGTGAAGATGGGAGCCACCAAGGCCGACTTTGATAAGAC
AATCGGTATCCATCCCACGAGTGCCGAAGAACTTGTTACGATGCGAACTCCTTCGTACCA
CTACAAGGACGGGAAAAAGGTAGAAGAGTAAACGAAACAATAAGCAATTGTGAAATATCG
AAAATGTACAAACCGAATGGTGCATAAATCAATCTGTGCACACGCTTTTTCTTTTCCTTT
TGTAGATTGATTAGCACCAATTTTGCACATAGCACAGCGTCACAGAACTCACTCACAAAT
GCAAATAAATATTTCGATTCCTGTGGATGGTTGCCAAATGTCGAGTGCCTTCACTTATTA
CTGGCAAAGTATGAAAGATCAGAAAATGCTGCTGCTGTTGAAGTGGTAACATACAGAACA
AAATCAAGATACTGTGATAGTACTGCAGAGTATTACAGATAGAAAAATGATGTGTCAAGT
GTTATGTTGGATGATTCATATGATATAATGCACATGGTGAATAAATGCATATACTATTAC
TACATTCATGGTGGGAATCCACCTTAGTTGACATAGATCATGGTAAACTCAGAATGTTTG

GTGCTAAGAATATTGGA
>comp2865_c0_seq1
ACTACGATTACTGTTTCGTCCTAACGCAGAACAATTGACTGCTCAACACCACAAAATTGA
ATAGCACGTTCAACAAACACAAGCTGAAGAACCAATTTTCACATACATACTCACACGTTC
GCATCATGTCTGTCAAGGTCTACTACTTCAACGCCACGGGTCGTGCCAACCAGATCCGCC
TGGCCCTGGCCGCCGGTGGAATTGCCTTCACGGACGACTTCGCCAGCTTCCCCCCCTCGG
ACGAGAACAAGGCCAAGTGGAAAGCCCTGACCAACGGCAACTCGACCTTTTCGGTTCCCA
TTATGACGATCGATGAGGGAACCGACTCCCAGAAGGTCTACATTCAGTCCTCGGCGGTCC
TCCGCAAGGCGGCCCGCATGGGGGGCCTGGAACTGACCCTCGACGACGCCGACGACGCGG
CCTACCTCCAAGACAAGCTCATCGCCGACGCCGACGACCTCCGGACCGCCTCCTACCGCG
CCTTTAAAATGTTTGGCGCGTCGGATGGCGCCGTCCAGAAGTTTGTCAAGGAGGTATTGC
CGAAGCACGTGGACAATCTCGAGCGGCAGTTTGTGGCCGCCGGTGGCGATTATTTTGGGG
GGTCTTCTACCCTCAGCATCGCCGACGTTGCGCTCTACGACGCGATTGTCTTTTTTGGCA
CCCGGCTCATCGACGGCGCCGAGGGCGTGGACAACCCGTGCGGGCCCGCTCTGACCAAGT
GGATCGAGCGCGTCGAGTCCAACGAGAAGATTGCCGCCTACCTCGCCGGCGAGCAGTTCA
AGGCCGTGGCGATGGTTTTTGACAAATCGGCCCTTGGCTTTTAAATCAATCCAATAACCA
CTAGCAAGCAACCATGGGCATTGGTGTTTGCTCTTTGTGACCGGATGCTGTATGGGATTT
TTCGGATTATATCAGGAGTATGCAAAACAAAGAACGGTAGTAACAGTCACTATTTCATTT
GCACCCCCGGCAAAACTAGGAAGGAAGGAACGAATGAACCCGTGATCTTTGTCAGCGGTA
TTCATAGAGGATTCCACACCATAAAATTTTATAATGAATAGCCGGCCATAAGATTTGGAC
GGCCTCGATGGGCGAGTTTTATCGTTTTGTTTCTAAAAGAAGAATTTTGCAAAGCACGTA
GAGTTAGTTGTCTTCTAGTATCTTCTAGTACACCTTGAACATCAGCGATAACAAAGGATA
ACAATAGCAAATGATTGCTTATTATCACCTACCCGTACACAATCAGTTTTATTCAATATC
TAGACCCCAATTTCCAG
>comp2866_c0_seq1
CCTTGCTCGACCGTTGAACAACTCCCAGATTCCGTCTGAACAGAAATACAAACTATCTAA
AAAATGGAATTCCATGCAAGAAGCGTGCAAGAGGCACCACAATAGAGCATTGGCCTTACG
TTGCTGGAACGCAGCAGAGCTGTTGAGAGGGAAGAAACAGCAAACAGTCGAATACAATCG
ATCGTGGAGGAAAGACTCATTAAGATACGTTAGGTTGAATACGTTACGTGACGAGGGCTC
CGCTGACGTGACGGGTGGCAACGTGATTCAAGAAAGGGCAGGAAGAGAACTGACGAAACC
AAACGGGGTAATCGATCATGGCAAATACTTCTACACGGCAAAGGTCGTTGACGTTCGATC
CCTATGTGTATGCACCAGACGATGAATTGCTAGATAGGACAATGACAACGCAACAGACAG
AAAACCAGGAACAAGACCGTGGCTCTACTCGGAATAGACTATTGAATGTGCAAGCTTTCG
AAACAATTTCAACATCAGCTCCTATTGCTTCTCTTTCGACTGACGACGACGAAGATCGCA
TTCCTCCTCCCATCGAAGTTGTAGGTGCGAAAACGTGTACCAGTAGAGCTGATAATCATC
CGGATTTTGCATCATGGAAACGGGACGTATTCGGAACGCTACCATCATCCTCGTCTTCAT
TCTCACCAAATAGTAGAAGAGAACTTATTGATTGGAAGACGGCTCAAATCAGCAAACGAC
TAGAAAAACAGCTCCGATTACAGGAGCAGCTATTGCGTACAGTAAAGAGCGACATAAGCA
ACGACGACAGCAACGAGAGCAGTTATATCAACGTTTTGAACAAAGAGTTGAAAGATAACA
ATACATTTGAAACAAGAAACTCTTCCTATATGCTTGGCGACGAATGTAAAGAAAGCAACA
ACGAGAACCAGAACACACAACCTATGTCAGAACAAACGACGATCACACATTCCATCTGTA
AGATAAAAAAATTTCATCCAGAATCTACCGTCACCAACGTTTACGTTCGACAAGAAAAAG
AAGGAAAGGCGAGGAGGTGTCATCAACAAGATCAACAGTACTTAACAATGGCAGTACCTC
CAAACTCACCAAAAATGAAGAATCATACAATCTCTTCTCTCTCGCTTAAAAGAACTGGTA
TCCCCGAAAAGAAGAACAATTCGACACAGCTTGCTGCAATCATGATGGAGCTGGCTGAGC
TCAAGGATTGGGTAGCGAAAGACATTGATAATGCGATTGCGGACAATGTTGCTGATTCTC
CGACTTCGCTCCCATCGATTGCCACCCGTCTCCGTCAATCGACGCCTCCGTCTTCACCAA
CATCGCCCGCGCCTCCCGCAAGTCGTGACAAACCCGAGCTCCAGATCCCATCTCTTGATG
TACCGAACCTTGTGTCGCCGCCATTGTCGCCGGAAGCAGACCACAGTACTAAGAATGAGA
ACAATGAAATGATTAAGACCGAGAGCCCTCTTAAAGTCGAGACGAACATGGACGCTATGG
AAGCAACCATGAAATCGAAAGAGAAAAAGAGCGTACGGTTCGCATCTCCAGAGATTACCG
ATACGATTTACCGACCCTTTACACCTCAAGAAGAAATCAATACTCTATTCTTTCAGGAGG
ATGAACTGTTGGATTGGGAGAACGACGAAGAAACCACCGTTAGAGATCGCTTCGAAGTCA
TTGTGACAGAAATTGAGACCTCCCATTGTTCTGAAGTCGAGTCAAAGCAACACAATGACA
ATAGGCTTGTTACCGGTAGCTTTTTGAATATTGGTACCCCAATTATTTCGTTTCACAATT
CGTACTCGAGTTCCTTCCAAGAAAGCAGCGACGATGATAGCTCGCATTATGAGAACAACA
TGTACGAACTATAATCATCCATTCAATTTTCCAAACTATTGGCAGCTAGACGTGCAGGTG
CTGATAGGGAGGAGTGTACCATATTCTTGTTATTGAGCCGTGAATTCAACTGCGCATCGA
CCATGAAAATGTTTGGCAAAAACTAATTTGATGAAAAAGAATTTTATGTTATATTTATCT
ACGACGATAGAATAAAGCCGTTGCTAATAAACATTAAGAGCAAGTCATTGGTATAAGAAA

CCAGAAC
>comp2867_c0_seq1
CAACAACAACAACAACAACAAAAGAACAATCGCTACAGTAGTTGTTAGAATATTTCGATT
AGTCGAATACTATATCAATATCAAGTAGTCATCGAATACTTTTGCGTGCTCTCCGGCTCC
TCAAACGCTACAATTTTTATAGTATCGCATAGCTAGGCTGTTGTTCTTATCACTGTTCCC
TGTACAGCACAGGAAGAAACCAGATCTATCAACACACTCCCACAGAATTCTTGACAACGA
ATTCTGCTCTGTCTGTTTGGTTGAATTCCTAAAAACCGAACCCAGCCCCAGCAGGGACCT
AAGAAAGACCAAGCTCTCATACTCCACCACAAGTAGTACCACTCCTCCCCGACACGACCA
TGGAATCATCGATGGCATCCTCGATGGCATCCTCTCCACCAAAACCACCGCCGGAAGGAT
CCGTCGCTTCCGAAACCGCGGCGGGCAAGAGCAATCCCCGAACCTCTACGACCGGACCGC
CATTCCTGAACCGTGCTCCACGAAAGGTGTCCACCGAAAACAACGCAGCCGCTGGCGCGG
CGTCTGGGACACTGGAGTCGACAGCGACAACATCGCCGATGGAAACGACCGGTGGATGTG
GCAGCCCGGGGGGCTGCGTGCTTTCGGATTCCCCGAGATCCGGGAGGCCCGCATCCGGGT
TCCGTGCGAAGCCATTTCCTAGCACCTGGAAGCGCACCGTCACATCGGCTTGTTCCCCCT
TGGCCAAGTACAAACCACCCACCAAGAACCCGCTGGCCTGGTCGTCTCCTTTCGGCAGGA
AGATGGAGGCCCGTTTTGGACGCATCGGCGATCCTCTGTCACCGGCCTACCGCCACTCCC
TTTATGTCAAGGAACGACTCAAGCGCCTGAGACAACGTAAGAAGGAGCTTCTTTCTACGC
TGTCATCTTCTCACTCCTGTTCCTCTCGGCCTTGCACCGATTCAAATCCAAAGGAGCGGA
CTGAGGAAATTGCGAAGCCCGTGGTCATTGATTTATGCAACGAAGACGACGATTGCAAGG
AATCTCCGAAGGAGTCACTGCGGGATGCTCCCAGTAGCAGTGTAAAATCAGCACAAACAG
GGGGAACGCCGCAGAAAAATGCAGTCCTGACAGGCACTGCCACGCTGAAACCAAATACAG
AGACAAACCCTACTGAGGATGGTATTGGAAATCCCAATAGTCGATCTTGGAGGAAGTTTC
CTCTCGAACGAGTCACGGTTGAGGGTCCAGATATTGGGTCAAAGACGTACTGTCGACGGG
AGAACCAGCCTATTATCAAATACAATAATGATTTGGGATTTCGAATTGTGCTATCCGAAT
CAAAAGAGAAGGTCCGAATAATTGTTTCCCACGTTTTGTCGTTTTCTCATCATTGCCGTC
GAGATTTGATCTCGAGGATCGAGATTCCTGGAGTGGGTAGTTGCGTGCTTGTTCATTGTT
ATGCGAACATCAACCCGGCAGATATCAAGAATAAAATGAGAGGGAAAAGGTGTATGGTGA
CACTGGAATTTCAGGACCACGGTGACTTTCAGAATATGGTAGATATTGCCCTTGAGTACT
GCTCTCCGGGAGTGGTCTCGGAAGCGGAATGGTTTCATAAGGGGCTAATTCTGGCCAATT
TAGGATACAAACCCTCGTCCCCAACATCATTGCGATCAAGATTAGCACTGAAAAAGGATG
AAGCCCATGGTGGGCCTCTTTTAAGGCGTCTCGCTACAAGAGCATCGACCTTGACTGCAA
CATGGGAGAGAGTCCAGAAACAATCCAAGCGTCCATATGAAACCTCCAAGGACAGAACCA
TAGCCAGTAGGATCAACACAACTTTTACATTCGATAAAACACCCAGTGATTTTGGCCAAC
CAGACCCCAAGAAGCAACGTATTGCCACTGAAGATGTCACTACAGGAACTGACATCGATA
AGCACTCCCAAGCCACTGACAAAATGTCCGTGGATTCCGAGTCAAAAAATCCTTCAGATC
CTCATCCCGAAGAGATTGCTCGCTATACCAGTGCTGATAACCTAGTTGGTGACTCAGTTT
CTGGACTGCTTTCGCCCCAACTTTCTCCTTCGCCTTCGCCTTCGCCTTCGGACACGCGTG
CCTCGAAGAAGTTCGAGTCGCATCCCCTCCCAGACGCAACTTCAGCCAAGGGGGGCTACG
CATCTGAGGACAACAAATCTATAGGTGCGAAGGAGACGGCTGGTTCGCAATCTCATACCA
ATGGCAAAGCAACCTCCAACAAGGAATGCTCATTTGAATGGGACGGGACCTTTCAACTCC
TTCAACAAGATTCCTCGAAGCCGGAAGATGATTCCAAACCTACAGGTAGCGATGTTGATG
CTGACGTCAAAGATGTTGCGAATGAGAAAGAGGCCGATGATGTTCGACAGGATCAAGGCA
GTAGTCTTTGTGACGAGTCGTCCAATAACGGCGAATATCAGTCTTCGAACAATTCGGATC
TTGATCGGGCGCCTAGGAGCAGTGTCGATCACAGTGGAATCCAAACGACCCACAAGGAAG
ATGCACAGACTCAAACTACGGTAGAGAAAGAAGCCATTGAATATCATAATGTTGCAGAAA
ATGAGGCAACGTCCGCTCAACGATACAATGACAATAATGAAGAAAGGCTGCCTATTGTTC
GACAAGTTCTGACTCTCTTCAAGGGAATTGCATCCAGTATTGCGGAAAATGTCGGTGACG
TTCGCAGCGCACAGCAAGAAGACGAAGCTGAAGACAAAGACGAGGAGGATGACAAACGCC
CTGTAGGAGGTGACTCCATTCCCCAGGAGTGCGTTCTTATGCCACCGGAATCCAATTCAG
AATCCGACGCAGATTCTGAGGATCTGACGGTTAACTCCAACCAGGATAGTGAACAACAGA
ATCACAACCCTCTCATTTCCATTTCTGTGGGAAACCTTCTCGTGTTAGCCTTGCCTTTGG
TTGGACTTGCGGGCATTTTCTGGAGATTACGACTCTTTGATGTTTCAAATTCAATTCTGG
AAGGATGTTTCAGAACTTTTGTTCAGTTGCATATATTGGGATGGTTGCTATCGCCAATTT
TCAAATGGGGTACCAGGAATCCGCTACTCATCGCATGCTATGCTTTGTTCATGATTTTAC
TGGCATCCCATGAAGCGTCATCCCGAACAAAGTACACATACGACGATCAATTCTTTGTGA
TTGCACTTTCTCTTGTCGTCAATGTCGGTTGGGTGGCTCTCGGGGCCTTTGGACTTGTCT
TGAAACCAAAACCATTATGGGATCCACGTTATGTCCTTCCTATTGTTGGGATGCTGCTTG
GAAATTCCATTAATGGCATTTCTATCGCGCTCGATACGGTCACTACGTCCTTGGTAGAGA
GGCAGGCCGAGATCGATCTTTACCTGAGTTTTGGCGCATCGCAGTTCGAAGCAGTTTCCG
GCATTGTCGCCCATGCGATTCAGAAGGGATCCACGCCCGCTCTTAATATGATGTGCGTGG

TCGGCATCGTCAGTATTCCCGGAATGATGACCGGCCAAATTCTTGGTGGAGCGAGCCCGA
TGGTTGCTGCGCGTTACCAAGCCATGATTATATTTTTGATTGCGCTCTCTACCCTATCGA
CGATTCTTGTTAGCTCGTACCAGACGATTACCTCGATGTTCTGCTCGCATCAGATTCTGA
GGCCCGAACGGCTGGTCAGGAATCACAAACGGAGCCTAGCTCGCTTGATCATGTGGCTTT
GGGGGAGTATTTTCGGAGCCACTGATCAAATCCCAAGTGGTGCAAATATGCTTTCGCATG
GAACCGAATATGTGGGACAGCCTTCGCTAATGAATCTACCC
>comp2868_c0_seq1
CTTTCTTCCACATTACGAATACAAATTGTGGAAGAGGAGATGCATACTGCAGGAAAACGA
CCTTTTAAATGAGAGGAATAGACAACGATGACCAGCAATAGCGATCCATTTGATTGTGCA
TTTAACAGCGATCTTTCCGATGTGCAAAGCACCTTTGATGAAGTAGTATTGGGAGGGGAA
GTCTCGGCCATCGAAGAAGGCCGGGTCCTAGGAAAGGAAGAAGGATTATTGACCGGATTC
CGAGAGGGCGGTGTTCTCGGTCACAAGACTGGGATCGACTACGGAATGGAGATCGGTTTT
GCGTTGGGGCTTTTGCAGGCCGTCCGAGAATCGGTGTTGTTGGAGGGCAGCGATTTGACG
TCTTCGTCGTCGTCTCTCGATCGGATACGGAAGAGCGTCGACGAACTCGAAAAGGCCATT
AACGATTTCCCTTCCAGCGAGGAGGATATTCGAAGACGTCTATTTCAATCGTCAGACAAA
GAAAAAAACGTCCATTTCAGAGATAGAGATTGCGACGAAGAAGATGGGAAAAACCACAAC
CAGCAAACACAAGCAGAAGAAGATGTTCGAGCACAATTACAACGAATCCGGGCTCGGAGT
AAGGTATTGACCGCAAGACTAGGAATTCCCCGTCACTCTCTGAAAAATTCGATGTCAGAA
GCCGCGAAGAAGATGGCACTATCGACACGGCAGGAAGAAACCGGACATGCTTTTGACGGT
AGCGGAGAAAATACCCAGGAGTGGTAAAAAAGACGACAATGATGGCATACTAAACACAAG
CACACACACATACACACGCAAAACACAACGCGCAGACAAACCTCGAACTTTCCCCCCCCT
AAAGAAATAATTCCGTGCCAAAATGAATAGAGGGATCGTGAAAGAAACGAACGAAGCTTC
TGGACTCAAAACAGATGGATATGTCCTTGAAAGCGAATGTTACTGAGACCTCTCTTGTGA
ACATTATTGAAACACGTCATGGGTTTCTCCACAAAAAAGGCTATCGATGGTGTTCCTGAA
ACTGTAAGCGATGGAAAATATACTTGTGCTTCGTGGGTGTTCCTCAGCACGGCCCTACTA
CAAGGCATACGTCCCATGAACCAGAAGGTATACCCGAATTTAGAAACAATCATAGAAACA
GGAAATCCACGGATATACCCGAATTTAGAAACAATC
>comp2869_c0_seq1
TCCGAGGTAGAAGAACCTCCCGAGATTGACGAAGAACAACCAGAGTCAGCGGATGATGCC
TCCGAGCAATCCCCCGAGGTAGAACAACCCCCCGAGATTGACGTAAAACAACCCCGCGAT
GAAGAGGAGTCCAAATCTATCGAACAAAATCGCTCGGATATCAAATCACTCATGAAGTCG
AAAAAGGAAAGCGACACCTACTCCCTGGCCGAAGACGTGTACGCCTTCATTTTTGTCGCT
CCGGTATCGAGCTATCCGTTCATTTTCTCTTCGTACGTGGTCCTCACCAAGATCATCTGC
TACGCAATTCTTGTGGAGGACATCAGTTGGGAAAGCACCGTTAGATACAGCACAAATTCT
ACCGCGGTCAAGTTCTTCTTGGTTCCAGTCGCAGTGGCGATGCAAGAAGATTTGATTCAG
TGTTTCTCTAGCTTTGCAAACATCGTGTACGATCCACAGATCCTCGAAACCCACAAGTCC
GCAACGAAAGTCAAATACATGCTGTCGTCTTTGCTGAGGCTCATAGACGGTATCCTTTCG
CTTGTCGTGAACTTTTATGTAATGCTGACCACTACCGAAGTTTTGAGCGTCTTTCTCAAC
TTTGCGGCCCTCCAATTCCTCCAATCGATTGACGATATTTTCTACGAATTGGTGACCCTC
GGATTTTTTGGCGACGAGATGGAACACTGGGCGACCCAGTGCGAGGAAGTTGAGCTACGC
CGACGCGTTGGCCCAAATGACAAACAGATATTCAAGCGTTTGAAGATGAGTCACTTAGAT
TCGTTACTGTTTGGATTCACGCTGCTCGTTTGTCTTATCACTTATGCGATCACTATGGCA
GGTGTGAAATCATACTTCAAATATTATTCACCACCAGAGGAATCTTTTTAGATTGTTCTT
ACGATGCTGTGCGAATCAACAAAAAAGAATGCAGATCTGTAGCATTAATAGACCTTGTAA
GTGATGTTGGGTACGATACGTAGTATGCAACTACTTCAAGTGTGATTTCATTAGCGAAAT
AGTTTTCTATCCTATCAGTAACACTGGCAGTACTACCAATAAGGTCCTGTCTTCACTATA
AGATCATACTACTAGGTTCTTCCTGGTGACAAGCAGAGCAGGTACTATTGGAATGGTAGT
GCTTGAGACAATACATCTTCTATTTGGTCCTCAATGAGACCCAGGCCTTGCAAGAAGAAG
AACAGCAAAATAGACCCACTCCAAAAGATAGAAGAAGAGGTGAGAAAGGAGTATTGTTGT
TTGTTCCATATATGGTATGAATGAAAACTGATACTGGAAAACAATATACTAGTAGTAGTG
CACGGTGAGGGG
>comp2870_c0_seq1
ATTTTCAACTTCCTCTGCAAACATGCCGTTTCCGCTCACAAGCAGTATCCAGAATACGGT
GTAAGAAGTCAATCGAACGTCGCCAGTGACGTTGTCGAAGAGAGGATCTGTCGGAGAGAC
TCAATAGTTAACTGTCCCCTAAACACCGTTGCTCTAGCATTGCTGGAAGAATAAGATTTG
AAAGTAACACCATGACCGTCGCTCCTGCAATTTTTCCAACGCATTCTGGTGATCGAGACG
CAACGAATCCCACTATTGTACGACCGACCGAGGCTCAATTGAGGTATGCACAATTTCATC
ACAAACGGCGAGCCCAAGCCGTGAAGCACCGGATCGATCGGCTCCACCGAACGCCTCTCC
AAGTGACTGTGGTCCTTGCACAAGAGCTGGAGGAAGACAACGATAACTATAGCAGCAGGA
GTGACTTTAGTGGGACAGACACGAGTGACGTCGACTTGCGTGCCGTCCGACTTTTGCGTC

CAAAAATCACGTCTGGACGCCGAGCACTGCTAAAGAAATCAAAGCGAAAATATCGTCGAC
GAAAGCGGGATATTGCAAAACACGGATACAGAGGTTGCTTTTTAGACGACAACGAGGATG
ATCAGCTGGAGCGAGGAGAAGCATCGGAGGGTGATGAATTCATTGACAAAGATTGGAACG
ACATTGACAAGGAAGAAAACGATGCTATGCAAGAGATATTCATGACACGGCCGAAACATC
AACGGCGCAGGGCCCGGCGGCATCGAACACCAAGCGGTGGCAATGCCCAGAGTTACAAAC
GATTCCAAGCTGCGTGCCATGCCATGATGAGGAACATCCAAACCTCACAAACGAAGGCGG
CCCTTCCAGCAATAGAGGTTCCGTCCAAGCGCTGGACACGGTCGGAAGAACACGTTGCTG
CCGCGGCCGCCCAAACGACCGACTTTTATCGGTATCGAACCGGATTGAACGCTAGACGAA
TCAAGTCGGGCCGATCCGCTGCACTCTTGCTGAACTATGCATACTACCAACAAAACCAAA
AGGACCATGATATGGATCGGGGTGCTACCTGGTTGACCCATTTGCCGGCAAAACAATCCA
AGAGGGGCAGCAGCAGTTATTATTTTCACAATCTCGATGGACTTCCACCTCTGATATCCT
CGGCAAACGATGAAGGAGAGGATGAACAATATTACGATGGCGATGGCAACGAATCCTCTA
TAACCTTATCCATGAGGCAACCGGGACCAGCAAAGTTTCATCGCCTTATGGCCTACGTGC
AGCAACGACTCATCGAAAAAGAAAAAAAAGAAAAGGAACATGACGAAAACAACCACCAGC
AAAAAATTTTAATGTTTGAAGGAATTCAAAATAAGAGCAGCAGTCCGGAAAAGAGTCGAA
CTAGAGAGATTATCGAATCCCTGGAACCGAAAAGCGAAAACACTAACAGGAGCACTCGGA
TTCGACAAATGGCTGAGTCTATAGAAGGAAAGTCGTCGAGGGGAACACCGACTATCACAC
TGAGAAGTGGTACACCGTTTTCTGGATTTTCCTCCCAAGGATCGCAACGAAATAGACGGG
GCAAATCGCGGTATGTTCCGAGAGTGAGGCTTCGCGACTTTGTTGTCGAAGGAACTGACG
AAGACGACAACGGCGAAAAACCAACACCTTTGCCCCCCAGGAAATTGTCGGAACGATTCC
AACAAGAAGACGACGATGGCAGCAGTGATAAAAATGAATTGTTCTCACCTCCAAAAATAA
GACTCCGAGACTCTTTCATTGGAAACGAAGAAAAGGACACGGAAACCAATAACCGGACTC
GAGAAATTGCTCTTGCTTTTGAGGCTGATCAAGATAACGGTGTAGGGGGAAGCAAAGTCA
ATCAACTTCGAGAATCGTTGGAACCCAAAACTACGCCGAAAATAAAGCTAAAGAAAGATT
TTTTCGGGAACGACACTGATGAAAAAAAGATAACCGAGCGAGAACCGTCTCATATTTCCC
GATCAAACGTTGATCGGCTCTCCAGACAATTTCAAGGACAGCAAGAGCGACTTGAAGGCT
TGGATAGTCCCTCGACTGAGGTGCTCAATGAATTTTCGTCGCACATAGATCCACGCATCT
TTGAACAATTCCAGGATGAAGGGTCTAGTAAGCGTCAGTCGGCAGAAACAAATGTGACTG
AATTCTTCGCTCGATTGCGCGGGGAACAGCAGCAGCAGCAGCA
>comp2871_c0_seq1
GCTCCGATCATTGTTCAGAGGCTCCCGCAAAACTGCCGCACACCGAATTATACCCGTAGC
ACCATTTACTAGCTACAACCTCCTGCTGTTGAACAATCCACAAGAGCAACCAGCTGCAGA
AACCACCACAGCAATTGCAACAAGCATAGCTTAGTTGCCACAGTTGTGTCCAGTTCGTTC
CAGCAGAGCCGCAGTAGATTTCAAGTCATCCGACGTCATTTATTGGACATATCGATCCCG
ATTAGAATTGTTTCGTTACCGATTCCAGAGAAACGGAAAAGACCTGTTAAGTTTCTTTCC
TTCTCGCCATCGCTAAAACCCCCGACAAACACAAGAAACAGACGCATATCACGGCTGTTT
GCGCAACAGAAAAACTTCGGTTTCTCGAAACTCCAAGTTCGCCAACCACGCACTGACAGT
AGCTGACAAAAGCACGCGAAGACTTCCAAAGCCACGCCCTTTTCATTCCTAGCTTCTGCA
TCTTTTCTCATAAACTTCGTTTCATGCGAGAGAAAAAAAGAAAGCAGAAAAATACAGCGA
TCCATTGACTCTTGCAATACCTATTTCATTTTGTTAACGATACGAAGACAGCAAGGCTAA
GCATATGGTAGGATACCGAAATAAGACAGCAAAATCCAGCATCGCAATGGATATTGACAA
TCAGAATCAGCGATCCAAATACGGAAGTGCCTCTCACCAGCAGCACTATCAGCACTCCGC
ACCAGGATCCTCCTCTTCCTCGAAACCGGCTTATGACAGCAGAGGAAGTCGATCAAACCG
GGGAACACGAGATGACATGGTTGAAAACGAAGGACCTCGCGGCGGCCGCTCGTCACAGGG
TTATTCACGGTCTTCCCGACAGGGAGGTAGCAGCAGATCCTCGCGATACAATGTTCAACA
GCACCAAGAACAATCATCGGGTGGAGGATCGAGTTATCCGCATTCCTCTTCCTCTCAATA
CAACCATCACCAATCTTCTAGGCAAGGACCAACGAATGCTCCTCCGTCTTCAATGATCAC
CCCGTCAGCGAACCAGCACTTCACGCCTCCAGGAAACAATCCCTACGGAGGACAGCAGCA
CCGCAACAGCCATCACCATGTGGCTCCGCACACGCCCGATCAGTCCGTCCAACCGACATT
TCCGCCGCCGGCGGTCACCTCGGAGTCACCGCCCCCGGCGGAACAGACCCAGCGCCGTCC
CGCGTACCTTCTTCATCGCGACCTGGTGATTGGAAAAGGATCCTACGGCCAGGTTTGCAT
CGCTTCACGCGGAGAAGCCGAGGGCCACATGTCGGGAAGCGGACGGTCTGGAAAACGAAA
AAAGTTTGCCTGCAAGTGTGTCATGCTGCGGAACGATCCGAAGTACATTTCCAAGTTACA
AGAGGAAGTCAATGTTCTTCGCGAACTTCGTGGCCACGACAACGTGATTCGATTGTACGA
TGTCTTTTGTGTCGACAATGAGCTCTTCATAATAACGGAGCTCGGAAGAGGAGGTGACTT
GTTCCATTTGTTAACAACCCATCCCAAGCACGGAGTCACTGAAGCCTATGCCGCCAAGAC
TGTATCGGAAATGCTTTCGGCGGTAGCTTTCTTGCATTCGAGAAGCATATGCCATCGAGA
TCTCAAGCTTGAAAACTGGGTTTTGGAATCCGGAAAAGACGTCTGGAGCCCGTTGAAACT
CATCGATTTTGGTCTGTCAACACACTACAGACAGGGCGAACGACTCACGCGAGTGGTTGG
TTCATCGTACTATGTTGCTCCAGAAGTATTGAAGAAGTCTTACACGGAAGCCTGCGATTT

GTGGAGTTTGGGAGTCATTGTTTATATGCTTCTCAGTGGAGCTCCTCCATTTTATGGCAA
GAACGACGAAGCGATCAAGGCATCGATTGTCCAAGGGGAATACACCTTCCCGCATGAGCT
CTTCCGGGACGTTTCGGACGAGGCCATGGCATTTGTGTCGACTTTGCTTTCGTACAATAT
CGAGTACAGATATACAGCTGCCCAAGCTTTGACTCACCCTTGGTTGTCCGCCAATTGCGA
CTCAGAGGCATTGTCAAGGGCAAAGGACGGCGCAAGAGGGGTGGGTATTATTATTGATGG
TTCCAAACCAGAACCTATGCAACTGTAGGGTAGTACGGTATTATTAGTAAGTTGCATGTA
TCGTGCAACATATGATGACAGTGTTTATATAAAAACAATGCAACACAGCAATGCATCGCG
ACGATAACAGCTATTATACTATACTAGAATATAACAACTGCAATT
>comp2872_c0_seq1
CAAAGATCGTCAGCAGAGAGAGAAACAAAACCGACGACGAATGCGGATCTGCTCTGCATC
GTTACACCCGAGACTAAAAAGAGAAGGCAATCTGCCTTGTGACTCAAAAGAGAAAGCTGC
TAGGGACGAACGAGCAGGCTCGTGAATCGATCCCTACCTTCTAAAATGGTTTGGCGATCG
AGTGTTTTTGCTCGCCGCATCCTTCGCAGCAGAGGGATCGCTGGCATCAGAACAGGTGCG
TCGGCTTCGTCGAATAGGTTCCAACACAACGAGCCGTCTCTGAATCCTTCGACGGTGTCC
CTGGCGGTCTTGGCAACAGCGACGGCGACCTTTGGATTCTGCGGGGCCGCCACGATTCAA
AACAGCGGGGATGACCACGCCAGCCACGGTTTCGGCGACGGCTTGGTTCGGTTCCAGCAA
TCGATGGCGCCAACGGCCCTTTGCGAGAGCAGCTACAAGAGCGAAAACCGCGACGGGGAA
CCTCCCCACAAAAAGGAAGCAAAGCTGAAGCGCAAGAAAACGGCCCTGAAAGTGGCTACC
AACCTTCGAAAAATGAACACGAGAGAGGAAATCTCAAAGCTGAGGCTGATCAAGGACGAA
ATGCTTCGGCGGTGGGAACGAGACGAGGATGGCTGGAGGGATCTTCCCGCACGGGCATGG
CCGGAATACCAACCAAACTCGGAGCAACTGGGAGCGATTGTTGCCGAAATCCAAAAACTG
GGTTGCAACGACAAGCTGGCGACCAGGAACTACGCGAGCGGCACCAACCAAGCCGACGAC
GAACAAGCGGAGGGGGCAGCCCACTGCTACAAGCTGCTCTTTGACATGGCTACGGGCTTG
GTTTTTTACAACGTCGATCCGGAAGCGGGATTTGCGCTGTACGAAAAACTCGCCAAGCAG
GGACACACGCCCTCGATGGTGGCCTGTGGGATCATTCTGATCGAGGGACTGGGGGTGCCG
CCCCAAGAACTGCAAGGCTTTTCGTGGCTGGAAAAGGCCATGGCGTCCTCGGAAGACGGA
GACGGCTCGGCGGCCCAAGCCATGTACGAACTTGGAACCGTGTACTACACCGGAATCGAT
GGGGTCGTGGAGGAGGATCCGGAGAAGGCCTTTGCGTTGTTCGAGCGCGCCGCCAGGCTA
GACCACACGGCCGCCCTTTACATGGTTGCGGATTGCCTGGTCGAGGGAGAAGGGACGGAG
CGGAGCGTGGCAAAGGCGGTTCCGTTGTTTTACAAAGCCGCCGAGCGGGGGCACCGGTAT
TCTCGGCAACGCATCCGAGAACTCTTGGCCCGAGTCGATTACCCGCTATAGCAACATTCC
ATTGAGTAACAGTTCCGATGATAATACATATCAGAGCTTCTTCGTCTAAACTTTTTCTCA
GCTCGGAATAGAAACAAACTATTGTACTGAATATAATATTTTCATAAATTACTTAAAAA
>comp2873_c0_seq1
GACGGCACAAACTAAATGTATTGTTGATCGTATTGCAATGGCGAAGAAGTGATTTATCAA
AGAACAAGTACGACAAACATACTATACGACGATGACGTATATGCCGATGATGGCCTTTTT
GAAAAGCTCTCTGTTTTTCCTCCTCTTTCTTTACGCCGAGTCGTTTTCCCACAATTTGGC
ATTTTTGAGCAACTACAATCCTTCATTAAGTCGAAGTCGAAGATGGTCAACCAATGAGGT
TGCCTCGCAAGATACAGTAACCGATCCGAGTAAAGATGAAATCACCACCAAAGTTGTTAT
GGATGAGAATAACAGCGAAGACCTTGAAGCCTCTGTCATAAAAAATGGCTACCCGATGAA
CAACAATTTCAACGGTTACAGCATGACTTCGGGATACAGTCCGTTCTTGGACCAAGAACC
ATCGCCAAAATGGCTAAGGAAACTCATAGGAAAAGATAGCGAAAGCATCAGCAGTAGACA
GGATATGGAGGAAGATGGATATGCTGAGATGCAGCGACGGAAGCAATCTCTACTGAAACG
GATTGCGAAGTTTCCTTTGCGCACTGCCAAGAGACTCGTTTCGACGAATTCAATTGAACC
GGGAACGCTGATTTTGGTGCGCCACGGAGAAAGCTCTTGGAATAAGAATAAAACTTTCAC
AGGTTGGTCAGACCCAGATCTGACGGATCAGGGAAAACGAGAGATGGAACACGCAGCGAG
ATTGCTCATGGAGGGAGGCTATCACTATGATATCAATGTGGTTTTTACGTCAAGATTGAA
ACGCGCGATCCGCTCCTCTTGGATCTTACTGCAGGAATTCAACCGAGTCTACTTGCCCGT
TTTTAAAAGCTGGCGATTAAACGAACGATTTTATGGAGCATTGACGGGCTTGAACAAAAC
TCAGACAGCTGAAAGGCTTGGAGAGGATTTAGTCCAAGGGTGGCGTAGTTCTCTTCGCAT
TCGACCACCCGCACTGACCGAAAATGACCCGTTCTACCCCGGGCGTGACAGAAAATATGC
TGATTTGACAAAAGATCAATTACCTCTTACTGAGTCGCTTCTCGACTGTATGGAGAGGAC
GAAACCACTTTGGAACGATAAAATTCTGTTTGAACTTAAACAAGGAAGGAATGTCCTTGT
TGTTGCACATGCGAATACGATTCGAGGTTTAGTAAAAACTATCGATGATATCGGTGAAGA
AGAAATTCAGAACGTAGCGATTCCTACGGGGATTCCGATTGTATACAAGTTTGAAATGCA
GACAAATGGTCAGCTGATATCCATTCCTCCAAAAAGTGAGGAGAATTCTGTGAGTCAATT
GCATATGAAAGGAAAGTTTCTAGAAAAACCTGGTCTACTCAAGGAAGCCTTGAAGTTAGA
AGGGAATTGGAGAGAACAAGTTCCCGGCTATGATGCAACAATGTCGCGCCATAGTAGGCC
CATGACTGGACTTGAGAGATCTTTGTATAAATTGAATGCAGTAAGGGAACTCGGGGAATG
GGCCACGGACTTTATCGATTTGGATCCAGAAGAAGACGACGGCAACGACGGCAATGGTAT

ATTTTTTACAGCAGATAAAAATGAATCCAGCACAGTTGCTGGATTGAAAGAAAATTCTAG
TAATGAGGACATTCCTGCCAATGACCAAGATGCAATTCCTACATTTTTCGCTCAGCCGTG
TATAACCTCCGCCCCAACATCTGCTGTTTCGAAGCGCCAAGACTCCGTAATTGTAATTAT
TCGTCATGGTAAAACACAACATAACAAGCTTGGCCTGTTCACAGGGTGGGAAGATGCGCC
TTTAGCAGATGAGGGAATCGAAGAGGCAAAACTTGCAGGTCAAAATCTGAAGAAGCACGG
GTTTGAATTCGATGTTGTGTACACCTCTTGGTTGAGTAGGGCTCTCGAAACCGCTTGGCT
TGTCTTGGACGAACTGGATAGTCTTTGGCTTCCAATTGTGAAGACTTGGCGCTTGAATGA
ACGGTACGTTTGGAAAAGGGTATTGTGTTATGTCAATTGTACCCTAGCAACAATCATGGT
CCTTTTCCCTCTAACCTACAATTTTGTATACCATTTTCTGCTTCAATTTCACCTTCGGTC
ATGTTCACGTTTCTTGTCCCTTTCAAACAAGGATGTATGGTGAACTCACCGGACTTTCGA
AGCAAATGGTGAAGCAACGTCACGGAGAGAAGCAATTCAAGGCGTGGAGAAGAGGCTTTG
AGGTTCAGCCTCCAGCTGTGTCCTCGTTTTCCCAGAACTATCCCGGCAACGACCAGCGAT
ACCGTTATATTCGAGATGTTCGTTATTCGGTGAAAGAAAGTGCCATACGTTCGATTGAGA
AAGGCAAACTGAGACTGGCGAGGAAACTCCCAAAAACTGAAAGTCTGAAAGACTGCATGG
ATCGAACGATTCCTTATTTTCTCGAGAGAATTGCCAAAGACGCAGTAGACCAGAATAAAA
GGGTTCTAATTTCAAGTAGCGAAAATGCAATCCGTGGCTTATTAATGCATTTATGCGATA
TCCCTGAGAGTGAAATTACAGGTTTAGAGATACCAAACGGTCTCCCTTTGATTTATGATG
TCAAATCGAAGTGTGTAAAGCTTTTGGATGACGGTACTGGACGTGATCCCTTAGAGGTTT
ACAATTTTGGTCCATCTGCAAAATATTTGTTCAGACCTTGCCAAAACGAAGATGGGAGCC
TCGACGAGGAATGCACTATCAACTTTGATAGCCTAATTACTACGACCGCAGACCAGGATA
CTTTAAATTACATTAAAAGTAAGACGGGTCGATAAAAGATAGATTTTGAATCACAGATAT
CAGTGCCAAC
>comp2873_c0_seq2
GACGGCACAAACTAAATGTATTGTTGATCGTATTGCAATGGCGAAGAAGTGATTTATCAA
AGAACAAGTACGACAAACATACTATACGACGATGACGTATATGCCGATGATGGCCTTTTT
GAAAAGCTCTCTGTTTTTCCTCCTCTTTCTTTACGCCGAGTCGTTTTCCCACAATTTGGC
ATTTTTGAGCAACTACAATCCTTCATTAAGTCGAAGTCGAAGATGGTCAACCAATGAGGT
TGCCTCGCAAGATACAGTAACCGATCCGAGTAAAGATGAAATCACCACCAAAGTTGTTAT
GGATGAGAATAACAGCGAAGACCTTGAAGCCTCTGTCATAAAAAATGGCTACCCGATGAA
CAACAATTTCAACGGTTACAGCATGACTTCGGGATACAGTCCGTTCTTGGACCAAGAACC
ATCGCCAAAATGGCTAAGGAAACTCATAGGAAAAGATAGCGAAAGCATCAGCAGTAGACA
GGATATGGAGGAAGATGGATATGCTGAGATGCAGCGACGGAAGCAATCTCTACTGAAACG
GATTGCGAAGTTTCCTTTGCGCACTGCCAAGAGACTCGTTTCGACGAATTCAATTGAACC
GGGAACGCTGATTTTGGTGCGCCACGGAGAAAGCTCTTGGAATAAGAATAAAACTTTCAC
AGGTTGGTCAGACCCAGATCTGACGGATCAGGGAAAACGAGAGATGGAACACGCAGCGAG
ATTGCTCATGGAGGGAGGCTATCACTATGATATCAATGTGGTTTTTACGTCAAGATTGAA
ACGCGCGATCCGCTCCTCTTGGATCTTACTGCAGGAATTCAACCGAGTCTACTTGCCCGT
TTTTAAAAGCTGGCGATTAAACGAACGATTTTATGGAGCATTGACGGGCTTGAACAAAAC
TCAGACAGCTGAAAGGCTTGGAGAGGATTTAGTCCAAGGGTGGCGTAGTTCTCTTCGCAT
TCGACCACCCGCACTGACCGAAAATGACCCGTTCTACCCCGGGCGTGACAGAAAATATGC
TGATTTGACAAAAGATCAATTACCTCTTACTGAGTCGCTTCTCGACTGTATGGAGAGGAC
GAAACCACTTTGGAACGATAAAATTCTGTTTGAACTTAAACAAGGAAGGAATGTCCTTGT
TGTTGCACATGCGAATACGATTCGAGGTTTAGTAAAAACTATCGATGATATCGGTGAAGA
AGAAATTCAGAACGTAGCGATTCCTACGGGGATTCCGATTGTATACAAGTTTGAAATGCA
GACAAATGGTCAGCTGATATCCATTCCTCCAAAAAGTGAGGAGAATTCTGTGAGTCAATT
GCATATGAAAGGAAAGTTTCTAGAAAAACCTGGTCTACTCAAGGAAGCCTTGAAGTTAGA
AGGGAATTGGAGAGAACAAGTTCCCGGCTATGATGCAACAATGTCGCGCCATAGTAGGCC
CATGACTGGACTTGAGAGATCTTTGTATAAATTGAATGCAGTAAGGGAACTCGGGGAATG
GGCCACGGACTTTATCGATTTGGATCCAGAAGAAGACGACGGCAACGACGGCAATGGTAT
ATTTTTTACAGCAGATAAAAATGAATCCAGCACAGTTGCTGGATTGAAAGAAAATTCTAG
TAATGAGGACATTCCTGCCAATGACCAAGATGCAATTCCTACATTTTTCGCTCAGCCGTG
TATAACCTCCGCCCCAACATCTGCTGTTTCGAAGCGCCAAGACTCCGTAATTGTAATTAT
TCGTCATGGTAAAACACAACATAACAAGCTTGGCCTGTTCACAGGGTGGGAAGATGCGCC
TTTAGCAGATGAGGGAATCGAAGAGGCAAAACTTGCAGGTCAAAATCTGAAGAAGCACGG
GTTTGAATTCGATGTTGTGTACACCTCTTGGTTGAGTAGGGCTCTCGAAACCGCTTGGCT
TGTCTTGGACGAACTGGATAGTCTTTGGCTTCCAATTGTGAAGACTTGGCGCTTGAATGA
ACGGATGTATGGTGAACTCACCGGACTTTCGAAGCAAATGGTGAAGCAACGTCACGGAGA
GAAGCAATTCAAGGCGTGGAGAAGAGGCTTTGAGGTTCAGCCTCCAGCTGTGTCCTCGTT
TTCCCAGAACTATCCCGGCAACGACCAGCGATACCGTTATATTCGAGATGTTCGTTATTC
GGTGAAAGAAAGTGCCATACGTTCGATTGAGAAAGGCAAACTGAGACTGGCGAGGAAACT

CCCAAAAACTGAAAGTCTGAAAGACTGCATGGATCGAACGATTCCTTATTTTCTCGAGAG
AATTGCCAAAGACGCAGTAGACCAGAATAAAAGGGTTCTAATTTCAAGTAGCGAAAATGC
AATCCGTGGCTTATTAATGCATTTATGCGATATCCCTGAGAGTGAAATTACAGGTTTAGA
GATACCAAACGGTCTCCCTTTGATTTATGATGTCAAATCGAAGTGTGTAAAGCTTTTGGA
TGACGGTACTGGACGTGATCCCTTAGAGGTTTACAATTTTGGTCCATCTGCAAAATATTT
GTTCAGACCTTGCCAAAACGAAGATGGGAGCCTCGACGAGGAATGCACTATCAACTTTGA
TAGCCTAATTACTACGACCGCAGACCAGGATACTTTAAATTACATTAAAAGTAAGACGGG
TCGATAAAAGATAGATTTTGAATCACAGATATCAGTGCCAAC
>comp2874_c0_seq1
TTTGACTTGGATATTCCAAAGGTGTGTTTCAATAAATCAATTCTTTACTGTAACCCTTCT
TTCGAAAAGCGAAAATATTTTCCCACGTCTTTCAAAAATAGGAATTCTGACTTTCTGCCC
TTTCTTCAAAACAATATCACGATATCACACCAATTACCACTAAGACGTTGGAAGCCGTTA
CCGAAAAGACTAAGCTCTTGTTCATCTGTAGCCCCGGAAATCCGACCTGCAAAGCGATTC
CCTTGTCGGATGTCGAAAAGATTGCCTCCGAAACGGAAAAATATCCCGGTTTGATAGTCG
TCGACGAAGCTTATATTGATTTTGCTGGTGAAGGGGCTTCGGCCGTTTCGCTGTTGAAAA
AGTACAACAATATTGTCGTCTTGCAGACCCTGTCGAAAGCCTTTGGGTTGGCCGGTATCC
GGTGTGGATTTTGCATCGGCCCTCCCGATGTCATTCAGTTGATGAACAATGTCAAGGCTC
CGTACAATGTCAACTCCTTGACCAGTGAGGTTGCCATCAATGCAATGAACTCAATGGACA
CACTGCGCAAAAATGTGCAGAGCACCCTGGACGAACGAGCGAAGGTTCGCGCGGCGCTCG
AAGCACTCACGAGCATTGTTGTCAGTGTGTATCCATCCGATTCTAATTTTTTGCTTTTCC
GTGTGAAGGACGACCTGAAGGCGAACGATGTCTACAAACGCATGGCCGACACCGGGGTCG
TGACGCGATTCCGGGGCAACGAAATGCATTGCAACCAGTGCATCCGAGTGACGATCGGCA
CCCCGGAAGAGAACCAGAAGTTCTTGGCCTCACTCCAGGCGACATGGAAAGAACTTTTGG
AGGCAAAATAAACAGAACGTGGTTGGCGGTCCCAAGATAAATTCAATACCTGCTGCTTAA
CAAAATGGGAGCAAATATAATGATAGTAGCAGTTATTCAATTTCTTCACTCACAAATACA
TCAAAAGAATTCAATCAAAATAGAGACCCAAACTTCATCCAAACCGATGGGAATCAATTG
TCGGCTTTTTAAGGGACAAATATTTATTGCCTACAACCACTCCTATGCGAGGATTCTGAA
TGCAACAGATTCACTCTCAGCTCAATTAAAAAATTATGAAGATGATAGGGAGGAAGTAGA
TGTACATCAAGATGACGCATGTTTGGTTAGTAAGGATGATGCCTTCACAATCATAGGACC
CTGTCACAAATAAAATAATACCTTGTTTTGTCAACTTGGATATCTAAAGTAAAAATAAAG
TTATGAAC
>comp2874_c0_seq2
GCATGGTTGGTGATTGGCCATTGAGGTTTCTTCGCGCCAAACATCATCGTCATCGACGTG
GTCGAACGAAATTTGACATCCTTTCTGTGACGATTATTTTTTTGAAGTGCATGTTTGACA
AACGGATATGAAAACGCAATTGCAAATGACAACAACCAATTACTGGAACACAATACTTGA
CACCCAAACCATACCGTCCAGTAGTGCATCGACAGATACGGCACAGTTACAACAACGTAA
ACTCTCATTTACTGCATGAAATTCTACCGACGAAGTTTCCACATCTCGCTATTATTTTTT
CTCATTGAAAAGCATTCTGCCTCACCGACACCATTCTCCTCGACACACCAGCACCATCGA
CGATTTCCATTTGTTTCATTCGTGACAGTCGTTGGCAACCACAAAGAAAAACACGGCCTC
AACAATTTCGAGCGAAATCAATCTGTACTGCAACAGTCGCTATCAAAACTCCCGAACGAG
CCCATCGACACCGAACAAACCAAATCCATAAGCAACATGACAACTTCCACAGGAATCGCC
GCGAAATTGGCACGTCCCAATATTTTAGAGCTCCAGCCCTATCGTTGCGCTCGCGACGAT
TATTCCAACGGCGTTTTATTGGACGCAAACGAAAACGCGTTTGGCCCTACATCGCTTCCG
ACTAAATCCTTGGACCCCTATGATAATTCCAACACACTCGAACGATACCCAGATCCCTAC
CAGAGCAAACTAAAGGAATTGTTCTGTCGGGAACGCGGAAACGACCTCCAGCCCTCGAAC
GTCTTCGTTGGTGTTGGTTCAGATGAGGCCATTGATTTGCTGATGCGAATTTTCTGCACC
CCGGGTACCGGCGGAGACAACATATTGATCACCCCGCCAACCTACGGCATGTACAAGGTT
TGTGCCAAAGTCAACGACGTGCAGATTGTCTCGGCTCCGCTGACACCCGATTTTGACTTG
GATATTCCAAAGACGTTGGAAGCCGTTACCGAAAAGACTAAGCTCTTGTTCATCTGTAGC
CCCGGAAATCCGACCTGCAAAGCGATTCCCTTGTCGGATGTCGAAAAGATTGCCTCCGAA
ACGGAAAAATATCCCGGTTTGATAGTCGTCGACGAAGCTTATATTGATTTTGCTGGTGAA
GGGGCTTCGGCCGTTTCGCTGTTGAAAAAGTACAACAATATTGTCGTCTTGCAGACCCTG
TCGAAAGCCTTTGGGTTGGCCGGTATCCGGTGTGGATTTTGCATCGGCCCTCCCGATGTC
ATTCAGTTGATGAACAATGTCAAGGCTCCGTACAATGTCAACTCCTTGACCAGTGAGGTT
GCCATCAATGCAATGAACTCAATGGACACACTGCGCAAAAATGTGCAGAGCACCCTGGAC
GAACGAGCGAAGGTTCGCGCGGCGCTCGAAGCACTCACGAGCATTGTTGTCAGTGTGTAT
CCATCCGATTCTAATTTTTTGCTTTTCCGTGTGAAGGACGACCTGAAGGCGAACGATGTC
TACAAACGCATGGCCGACACCGGGGTCGTGACGCGATTCCGGGGCAACGAAATGCATTGC
AACCAGTGCATCCGAGTGACGATCGGCACCCCGGAAGAGAACCAGAAGTTCTTGGCCTCA
CTCCAGGCGACATGGAAAGAACTTTTGGAGGCAAAATAAACAGAACGTGGTTGGCGGTCC

CAAGATAAATTCAATACCTGCTGCTTAACAAAATGGGAGCAAATATAATGATAGTAGCAG
TTATTCAATTTCTTCACTCACAAATACATCAAAAGAATTCAATCAAAATAGAGACCCAAA
CTTCATCCAAACCGATGGGAATCAATTGTCGGCTTTTTAAGGGACAAATATTTATTGCCT
ACAACCACTCCTATGCGAGGATTCTGAATGCAACAGATTCACTCTCAGCTCAATTAAAAA
ATTATGAAGATGATAGGGAGGAAGTAGATGTACATCAAGATGACGCATGTTTGGTTAGTA
AGGATGATGCCTTCACAATCATAGGACCCTGTCACAAATAAAATAATACCTTGTTTTGTC
AACTTGGATATCTAAAGTAAAAATAAAGTTATGAAC
>comp2875_c0_seq1
CGGGCGGAGAAGGAAGGTTCTTTCAAAAACGTTCAAACTACCGTCGCTGTTGATACGCAA
CATCGGTTTAACCACGCAAATCACAATACTACGTGCATGCAGTGTATGACGATATACCAT
TTTCCACCATGGCGTCCCGAATGGCTATCAAGACCATTGCAGCAGCACTGGATGCGACAG
CTTTGCGAATCCCCGATCGAGTCGCCCTTGTTTCTGCGTCGCCCGAAGCACTGATGCAAA
CAGTTACGACAACCGATAGCAGAGGTATGAATGAAAGCCTTAATCTCGATCGCTATACTT
ACGGCGACTTTCGAGACAAAACAAATCGCTTGGCTGGGTTTCTTAGGGCGTACGGCTACG
AAAAAAACGATGTAATGATGAGCGATTTGCCGAACGTGGCCGAAAATCTCATGCTGCAGA
TTGCCTGTAACAGGATTGGTGTCAGCTATGCGACTGCAAAAGGATTGGAGGGGATGGCAA
AACTGACCAAAGTGAAAGGCGCAGTGTCCGCCACCGGGACTGGGTTCCTAGCCGAAACAA
GCTTGTCCCTGCCATATTTAGGTGGGGATTTCTTGTTGGATCTGATTCACGGCACGACGA
ATAGTAGTGATGAAATCAGCGGTTTGTTCGGGTTGGACGAATTTGATCTCGAAGACAATG
AACAAGATTTTGCTTGCGGTGACGAAGACGAAAACACCAAAAAATTGCCGCCGCATGCCT
ATTACAACAGCACGAAACCGTTCACGAACGAAGAGGCTTTAGAACTGGGAGCAGATGCTG
CCTGGGAAGTGGCAATGGTCGAGGAAGACTCCGTCTGCATCGCTATTACGCTTTGCCACG
CCTTTGGGATGGGATCGGCGGTGTGTTCAGCTTTGAAGGAAGGCGCCACGATTGTTTTGC
CAGCGGTGGGGGGCATTCGTGGGTGTGGCGTTCCATCCCAGCGAGCCGAGGCCACCCTTC
AGGTGCTGGAATCCGAACAATGCACTCTCCTCTTTGCCGATACGCATACATTGAAAGCAT
TCCCCGACGAAGAATCCATCGGCTACAATCTGGCGCTGAAAGGTGGGGTTTGTAAGGTAG
GGAGCGGATCGCAATTCTTGGAGGAGACGGTGAAGTGTTTTGGAGCAAGTCTCCGAACTT
TGGGCTCGAAAGGAAATAATTGGAATTTAGAATCCTAGTGCAGTCATCAGATCGCACCAA
TGCCAATTGCTTGCTATCGTGCATCAGCATTCACAGGGCTAATCTTCACTTCAAGAATAA
ATCTTGGCTATTCATAGAAAACCTAGTCAATATAAATTCAAGCAAAGCCTGGTGGTGTGG
TTTGGCGTATACTGTAGTTTGATTAGCAATGCAATGATGGC
>comp2876_c0_seq1
TTCGAAAATCCCTACCGTATTGCTCCTCCGTTGCTGATCTGATCTGATCTCGCATCGTTA
TCATTATCACCAATTACTCATTCATTCATTAATTCAAAACATTGCAGATCTTCGGAGAGG
AAAAGAAAAGATGACATCGTATGTGCAGTACAATCCAACCATGGCAACGGCGTCGATGAC
GATGGCCTCCCAGTCTGTTGACCACTACTCAAGTGACATGTCGTCATCAATCGATCCTTT
TGTAGAATCACGGGACGTCTTGATCGAAAAGATGAAGTCTAACTTGATTCGATACGCTCG
TTCCAATGATCGAAGAATGGTGCTGCGATGTCTCAAGGATGCCAGATTCTTATCTTACGT
TCCTATGGATGCAGCAGACGAGCAATTGCGTATTCCTATTTCTGAATTGGAGAAAGAGAT
ATCCAAGGAAGAATTCCTTTTGAATGGCATTAGTCTAAAACCTTATGATCAAGCTCAGGC
GAAAAATGTCTGTCGTAATGAAGGAACAAGCAGTGGCAGCTTAAAGATGATCGGGACTTT
GGCACAGATTTTATGCGAAGGAACTACAATGGACGGAAACAATTTGTATGAACGTGTCAT
TAATCGCGTAGCAAAAACTTCTGCAGAACCTGAGGTATACTATCACATCAATGCTATGAT
GGGAAATACTACTTTGAGTGTTAAGCAGCTGTCCCCGGAACAGATTTCCAAAGAGGAGGA
AGCAAATGTCACCGATTTGAAATTGTACGAAACTGGAGGACAAATACAGATGGTTCTGGA
AACGACGCATAGTTATGGTTTGTTTCGTGAAAAGGATGCAATTATTAATCGTCCTTGGAT
CATTGTGCACTGCAAAGTCCACGAGCGCGCTAACTTATCGAACAACGAAAGCTTTCGAAG
TATGAATATTAAGACGCCAAGTTTGTACTAAAGTAATGATAGCGAAAATTGAATCGTTTG
GATCATTTTTAAACAAGGATTCAAAAAGTTTCGGTCGGTGTGATGATATTCTCCAAAATT
CCATTGACATGATGGAACAAGAAAAAGAATGAATTATTCGATGAACTGGTAAGACGAATA
TGTGTAATATAAACTGTGACGGAGACAGGTATAGCCAAATCAAAAATGTTTAGTGTTAGA
TAGATGAAAAGGCAAAAAGCCATCTAGCATATATGTTATGACCCCCA
>comp2877_c0_seq1
CTGTATTTCAGAGAAGACTAGAGCCGAATGCGAATGCCCATCAACCACCTCCGATTTCAT
TGCATGGCATCAAATTCACCATGTCTACCAACGAAGATTCTCCGAGCGCGAAACGGCGCA
AAGCGCTGAAGGATGCCGATTCCGCCAACCAGCTGCGAGTCCTAGAATCATGTCCCCTGT
CCAAATACGCCGATATCGCCGAGCGACTCTTGGGACACTTCCAGAATGCGGTGGACGGGA
GGAGGCTGGACGAGGCCTACGTCTTTGGTTTGCGGTTTGCCAATCTTTGCATTTCTGGTT
TGCCACAGCATCCGGAATGGAGACGCGAAATCGGCAGCAAAACAAGGAAACGCTTGACCT
CCCAGGTCGGTGACGTTTTGAGCATGATGGACGTTATCAAGCAACGAATGGATGCGGAAG

AGCTCATGAAGATCAAGGCAGAGATGATGGCGAGAAAGGCAGAAGAATTACGGAAGAAGG
AAGAGGAGGACCGCCGGAATCACGAGTTTGAAGAAGCACAGCGCCGAAAGCAGCAAAAAC
GCGACGCTCTGGAAAAAGAACGGGCGGAATTTCTTGCCGAGCAACGAGCTCAGAGACAGA
AACAAATCGAAGAACGAAAAGAAATAGAGAAGAGAAATGCCGCAATCAACAAAAAGAAGG
AGATCGAGCAGAGCGCAATGGCAAAACTCAAGGCAATGCAGGCCCGGGTGCCATTGGCGG
AGACGAAAGAGGAACAATCAGTCGCAGTGAGCGAAACTAATGTGGAAAAGCCTTCAAATA
CCAAAGCCAAACCAAATAGACTGAAAAACTGGTTTGGTGGTGGAAAGGACAAATCAAGCA
AAAAGAAAAATGAAGAGAAATCCAAATTGTCGGCCCCATCCGGCGCCAAAGATAGCGATG
AGATGGTTTCAACGAAACCAACAGTGACCGACAGAAATGTGGCCACGGCCGAGGACCGAT
CTGCTGTCACCACGCAGGAATATCCTCCTACCGAAGCTATCCAACTGCAACAACCTGAAC
AAGAAACATCTTCGAGTTCGACAGCACACGTGACCGGTAGGAAGAAGGGAGCGAAACCAG
TTGAGTCTGTAACAGGCGGTCCAACAATATCTAGAAGATCAAACAAGAGCCAATCAGTGG
ATTCTTCCACCATATTGAATCCCTTGCGTTCAATGAGCAAAATAAAACCGAGGAAAAATA
CGGATTCCAAAAATACGAAGAACAAAAATGACAATGAACAGGAAGAGTCATCGGCTAACT
CCAATGAAGTGTGTAGCAAAAAGCATGTACATCATTCAAGTGAAGAAAAACCGATCGGAT
CGACTTTGCCCTCCTCGTCGTCACCAGAATGTAATGAAGGAGTAACGACCACAAGTGATG
TTAGTAGCACAGCTGTTACTATGATCCCTTCACACCTAACACCTCTGAGTCGAAAGGAAC
AGGCAACCATTCACAAACTCCAACGAGCCATCACCGTTCAGGAAGACAGATTGGAGGAAA
TTGAAGGGAAACAAATTCCACAGCTTCTCGAAAGAGCCAAGGTATTGCTTAAGGAAAAGA
ACAAACAGGAAGCATTGAAATGCCTGGTTCACAAAAAGAGACTGGAGCGCCAAGTGGATG
CAATCAAAGCCGCAGTCTTCAACATGGAAACCCAAATGTTTATGCTAGAATCGGCATTTG
AAGATCGTCACGTAAAGAAGGCGCTCGACGAAGCAGCTAGTGCCATTGCTGGATACCAAC
AAATCATTGGCGATCCAAAGGCAGCCATGGTAGATCTGACCAACATGAGCGAGTCGCTGC
CAGAGCTCGACGTTGGAGAATCTACCGACGAGGAGCTGATGGAGGAGCTGACGGAATGGC
TTACGCCCGAGGAGAAAAAGAGAGCAAAGGCCAATGAGAATACTTATGCTGACAAAGAGG
ACCTTTTGCTGTTGTCCATGCCAGAGTTTCTACCGGCAGCACCAGCTGATGCGCCGGAGC
CTCTGTCGGCTGGTCGAGCCCCCAATGCCGTGGTAGAAACTAACAACTGATCCTAAGGAC
TAGAATAGTAGATTTCCTCTATCTTGAGAGCGATTGTAACAACGGCGCAGCAGAGAAGGA
TATACGATATGCATTGTAATTTTGTTTATCTCGTTGGACGCTGTCTTCTGATCAGCGACG
ACACGCATATCCGTATGACCAATGGGATGACGAACTTTGGACTCTGCACTTGAAACGATA
TTTAGCAACAGAAACGGATGTTTTGAATTGACAATTTTGCCTTTCAAGGCATGGGCAATC
CTGGAAGTTGCCCTACTCTACCATGCTTTTGATATCCCTTATACTACTACTCTTACTAAC
AAGTTCAGAAAATCCCAAAAGCAAGTCATCAGCTCCTCCTACTATTTTGCAAGTTTTATT
ATTCTTGTTCTTCGTCACTTCGTCTTCACTAGAATGTATCTTCCCCATCTTGGGTCAATT
ACCTTGGTTGGTTTGTCCAAAACGGTGCCTATCAGTTTCGCTCCCATTCCGGTGTCCTAC
CGTACCATGCTACTGATCTTATTAATTCGAAACATCTACCGTAGTACGAATACTCCACTA
TTAGTACTAGTAGTACTAGTTCAATTTGCTTGGGACACGTATGGTAAGGATTTGTCATCG
>comp2878_c0_seq1
GGTGTAATCGAACCATCGAAACAAAGGTGCTGTCCAGGTGCAACCCAATTCCAGGTCATA
GTAAAGTGTTCGTTGTGTGAGAGTGACGATAAAAGTATCAGATCATACAACCAACAGTCG
AAAGAAAAGAAGGAAATCCCGTTTCCAACAACAAGATGCCATTTGGATCGCAATCATCGT
TATCCTCGTCTAGCGATGAATCCGTCTACGGTTCGATCAATGCCAACAACGATGTTGAGA
TGGCGCCTAGTGATCCGATCTTCACCGGTGAGGCACGCAGCGGGAAGAACTCCAACGTTT
GTGGTGAAAAAGTCCTCTCTCCCAGAACCAGGCTCGTCATTCTCGGTGCTGTTCTCGGTG
TTGTTTTGTTAATGCTTGGGAGGAGAACAGCGATGCCGGCTTCCCACCAGTTGAAACACG
GCAAAGGTGCCCGCATCAAACTCTACAACAAGGACGACTCGGGTATTGGGTCTCTTTCGA
ACGGTGCTGGGAACGACCCATTTCAATTGTCGCCCGTCGAAAACTTGGGAATGCTGAGCA
TCGATCGAGCCGACGATGCTCTGCCTTCACCAATTTGGGGGAATCATTTGAAGAAAGGAC
TACCACTCCCTACAAACAGCTGGTACCTGAATCTTGTGTCTCATCGAGCGGCCTTCCGAC
CAGACGATTCGACACGCGTCTACACGGTCCCTTACATCATTGACACAGCGTCGCCGTACC
CGGGCATGGAGGGACTGCGCGTTCACTGGCCGGTGGTTCAAGCTTCCGATGGAAACGTGC
AAATGGTGAACGATTTCAAGAACGCTCTTTGCATCGGATCCGACGGCTTTAGTAGTGAGG
ACGGTGACGATGGTATCGACGGAACATACAGGGTTAAGGACGGTGCTTTGTCGCCGCTTG
GGGTTGGATTGGAATGGGGAGCTGGAAGTGATACCAATAGCAGCAATATCATGTCGACAA
ACATTGTTCGAGGTATGCCGTACGCGACCATGGTGTATAGCGACTCGTCCAACATGGTTC
CGACGATTTACAGCTACAACGGATTGGCAAGTGACGTTCAAATCGACCGCACCTCTACGT
TTGATCCAGTGACGAGCAAGTTCCCGAAGCTTGTGTGTGGAATCGAGGGCACTTCCAAAA
AAGGAATCTCAGCCACGGTCCACAGCCACCTCCATTTGCACATGATCAACTCCGATTTCA
CTTGGATCGTCTTTTTTAACAAACCGGTCAAGGTATCGTGCGTGACAAACAACGAGCAAG
ACCCAAAAGTCCGTGACTTCAAAATGACCTTTCTCGACAACGAGGACGACACAGAGGCAG

GAGACACCGATCTGGTGGTCCGTATCGCACTATTGAACCAGTGCACAACCGGTCACGCGT
CCATCAAAGAACATTGCAAGGAAAGAAACGCCTTGGCGGACAAGGACGGCTACGAACTTT
TGTTGAAGAACAACGCTCATCTGGTCCCTCAATCTCCCACCATAGATTTCGAATACGCCA
GCAAGTCGAAGAACGACGACGACGTTGCAAAAATTAATATCGACTGGGATGTCACTAACA
CCAAAAACGCTTCGAACGATGACGCCGATCTCCTAATGTTCGCTCTGCCTCACCACCAGG
ATTCTTTGTCGTCGGCAGATAGAGAAGTCAAAAATCAATGTGTCCATTCGTTCCACGGAA
AAACATGCTTGGTGAGTAGTTCAAATTGGACTCTGGAAGAAGATCTTGGATCTCCGCTGT
CCTTTGATGCACCTCGACCACCCTCGGCGGAATTCATTCCGACCATTGCTAAATATCTGA
AGGAAGACATTAAATTCGAAATTCCGAAGAATACAATGCGGGGTGCTTCCGATACATACT
TCAGCGGAAAAATTCTAGGGCGATTGGCTCGGATCGTTGCGATTGCCTCAGAACTCAGAT
CCCTGGCTTCGGCATCTTCCGTAGAAGACATCCAGGGCCGATATGACATGAAGTTGGATT
CGAGCATTGATATGCTGCAAGAATCCATGAAGGCGGCAGCGAGCGCTAATCTGCCATCCT
CTAAGGAATTTAAGGATGCACTAGATCAGCTCAAGTCGGCCGTCCAGGCCTGGTTAAAAT
CGGATGCAGATGCACCCTACATTTATGACAAGAGTTGGGGTGGACTCGTGAACTGCGGGT
GTTCTTACGTCGGGAAGATGGACAGGGGTTACTGCAACAATACTTTCCCGGAGTGCCCGG
CTTTGTTGAGTGTAAACGAAGATTTTGGAAACGGTTACTATAATGATCACCACTATCACT
ATGGCTACCACGTTTATGCTGCTGCGGTCGCTGCAAAATTCGATCCAGCGTGGGGACGAA
AACATTTCGAGGAGGTTATGCTCTATATCCGAGATTTTGCGAATCCAAAAGGAGATGATG
AATTCTTTCCCCAGTACCGTCAAAAAGATTGGTTTCTGGGATCATCTTGGGCGAGTGGAA
TCATTTCCGCCGAAAATTCACCCCATGGACGGAACGAAGAATCTTCGAGCGAAGCCATTT
CAGCGTACGAAGGTGTGACCCTATATGGGTCCGCGATGGTCGATGCATTCGAAGAAGTCA
AAACCGACGGCGGCGATGAGGAAATCACCACCAAATTGGAGACTGCGAAACTGATTCGAG
ACAGTGGGCAACTCTTAACGGCGTCTGAAATCAGTGCAACGAATCGTTACTGGCACGTAT
GGTCAAGTGATACACACAACAATACGTATCCTGCTGAATACACGAAGCCGGTTGTTGGGA
TGCTGTACGATACTATGGCAATGTTCCAAACCTGGTTCGCTCCGTGGGCCGTTGTATCAT
ATGGTATCCAATTGATTCCATTGACACCCGTTGCAGAAACGCGTGACGACCCGGACTGGG
CCGCCGAGCTCTATCCGTTGTACGACGAAGCGTGTGACGATGCAGGAAAAGATTTTTGTG
AAGCCAACGGGTGGTCGATTTTACAAGCCGGTTTATTGGCAACGGCGGGAAACCAAACCG
AAGCCCTGAATCAGGCGAAAGCGGTACCAAGAGAGGTGTTTGCCACCGAAGGCGGTGTAG
GAAACTCAATGAGTAATACGATATGGTACATTGCCACAAGAAAGAAGATTGTTTGATTTG
ACGTACGACGCGAATGGTTATCACATCAAAGTAATGAAAGATATCAAAGATGGGCTATAA
TGATGCTATCTTGATCGAATCCCATTCACGAAAGTCGAATTGAGTTTTCAGTCTACTTTG
TTATCATCGCTGATGGTAGAATCGGATGCTTTATGTGTGAAGGTACCGGTACATCTTGTC
ACGCGAGCGAAACCATGGAAACGAACGGAATACGAGCTTGCTCTTTTTTGCTTTTGAACC
CTGCACCTGGCTACATAATATCAATAAAATCTTTCATTACACGT
>comp2878_c0_seq2
GGTGTAATCGAACCATCGAAACAAAGGTGCTGTCCAGGTGCAACCCAATTCCAGGTCATA
GTAAAGTGTTCGTTGTGTGAGAGTGACGATAAAAGTATCAGATCATACAACCAACAGTCG
AAAGAAAAGAAGGAAATCCCGTTTCCAACAACAAGATGCCATTTGGATCGCAATCATCGT
TATCCTCGTCTAGCGATGAATCCGTCTACGGTTCGATCAATGCCAACAACGATGTTGAGA
TGGCGCCTAGTGATCCGATCTTCACCGGTGAGGCACGCAGCGGGAAGAACTCCAACGTTT
GTGGTGAAAAAGTCCTCTCTCCCAGAACCAGGCTCGTCATTCTCGGTGCTGTTCTCGGTG
TTGTTTTGTTAATGCTTGGGAGGAGAACAGCGATGCCGGCTTCCCACCAGTTGAAACACG
GCAAAGGTGCCCGCATCAAACTCTACAACAAGGACGACTCGGGTATTGGGTCTCTTTCGA
ACGGTGCTGGGAACGACCCATTTCAATTGTCGCCCGTCGAAAACTTGGGAATGCTGAGCA
TCGATCGAGCCGACGATGCTCTGCCTTCACCAATTTGGGGGAATCATTTGAAGAAAGGAC
TACCACTCCCTACAAACAGCTGGTACCTGAATCTTGTGTCTCATCGAGCGGCCTTCCGAC
CAGACGATTCGACACGCGTCTACACGGTCCCTTACATCATTGACACAGCGTCGCCGTACC
CGGGCATGGAGGGACTGCGCGTTCACTGGCCGGTGGTTCAAGCTTCCGATGGAAACGTGC
AAATGGTGAACGATTTCAAGAACGCTCTTTGCATCGGATCCGACGGCTTTAGTAGTGAGG
ACGGTGACGATGGTATCGACGGAACATACAGGGTTAAGGACGGTGCTTTGTCGCCGCTTG
GGGTTGGATTGGAATGGGGAGCTGGAAGTGATACCAATAGCAGCAATATCATGTCGACAA
ACATTGTTCGAGGTATGCCGTACGCGACCATGGTGTATAGCGACTCGTCCAACATGGTTC
CGACGATTTACAGCTACAACGGATTGGCAAGTGACGTTCAAATCGACCGCACCTCTACGT
TTGATCCAGTGACGAGCAAGTTCCCGAAGCTTGTGTGTGGAATCGAGGGCACTTCCAAAA
AAGGAATCTCAGCCACGGTCCACAGCCACCTCCATTTGCACATGATCAACTCCGATTTCA
CTTGGATCGTCTTTTTTAACAAACCGGTCAAGGTATCGTGCGTGACAAACAACGAGCAAG
ACCCAAAAGTCCGTGACTTCAAAATGACCTTTCTCGACAACGAGGACGACACAGAGGCAG
GAGACACCGATCTGGTGGTCCGTATCGCACTATTGAACCAGTGCACAACCGGTCACGCGT
CCATCAAAGAACATTGCAAGGAAAGAAACGCCTTGGCGGACAAGGACGGCTACGAACTTT

TGTTGAAGAACAACGCTCATCTGGTCCCTCAATCTCCCACCATAGATTTCGAATACGCCA
GCAAGTCGAAGAACGACGACGACGTTGCAAAAATTAATATCGACTGGGATGTCACTAACA
CCAAAAACGCTTCGAACGATGACGCCGATCTCCTAATGTTCGCTCTGCCTCACCACCAGG
ATTCTTTGTCGTCGGCAGATAGAGAAGTCAAAAATCAATGTGTCCATTCGTTCCACGGAA
AAACATGCTTGGTGAGTAGTTCAAATTGGACTCTGGAAGAAGATCTTGGATCTCCGCTGT
CCTTTGATGCACCTCGACCACCCTCGGCGGAATTCATTCCGACCATTGCTAAATATCTGA
AGGAAGACATTAAATTCGAAATTCCGAAGAATACAATGCGGGGTGCTTCCGATACATACT
TCAGCGGAAAAATTCTAGGGCGATTGGCTCGGATCGTTGCGATTGCCTCAGAACTCAGAT
CCCTGGCTTCGGCATCTTCCGTAGAAGACATCCAGGGCCGATATGACATGAAGTTGGATT
CGAGCATTGATATGCTGCAAGAATCCATGAAGGCGGCAGCGAGCGCTAATCTGCCATCCT
CTAAGGAATTTAAGGATGCACTAGATCAGCTCAAGTCGGCCGTCCAGGCCTGGTTAAAAT
CGGATGCAGATGCACCCTACATTTATGACAAGAGTTGGGGTGGACTCGTGAACTGCGGGT
GTTCTTACGTCGGGAAGATGGACAGGGGTTACTGCAACAATACTTTCCCGGAGTGCCCGG
CTTTGTTGAGTGTAAACGAAGATTTTGGAAACGGTACGTCTACATAGCTCTTCAGATCCA
TGTTTGTTGCAATTACAATTCGATTATTGGTTGGGACAGACTGGGATCCACACTTCCGGC
TGATTTGGTGCCCTCTCGATTTCTTTTATTCTCCAACAATTTAGGTTACTATAATGATCA
CCACTATCACTATGGCTACCACGTTTATGCTGCTGCGGTCGCTGCAAAATTCGATCCAGC
GTGGGGACGAAAACATTTCGAGGAGGTTATGCTCTATATCCGAGATTTTGCGAATCCAAA
AGGAGATGATGAATTCTTTCCCCAGTACCGTCAAAAAGATTGGTTTCTGGGATCATCTTG
GGCGAGTGGAATCATTTCCGCCGAAAATTCACCCCATGGACGGAACGAAGAATCTTCGAG
CGAAGCCATTTCAGCGTACGAAGGTGTGACCCTATATGGGTCCGCGATGGTCGATGCATT
CGAAGAAGTCAAAACCGACGGCGGCGATGAGGAAATCACCACCAAATTGGAGACTGCGAA
ACTGATTCGAGACAGTGGGCAACTCTTAACGGCGTCTGAAATCAGTGCAACGAATCGTTA
CTGGCACGTATGGTCAAGTGATACACACAACAATACGTATCCTGCTGAATACACGAAGCC
GGTTGTTGGGATGCTGTACGATACTATGGCAATGTTCCAAACCTGGTTCGCTCCGTGGGC
CGTTGTATCATATGGTATCCAATTGATTCCATTGACACCCGTTGCAGAAACGCGTGACGA
CCCGGACTGGGCCGCCGAGCTCTATCCGTTGTACGACGAAGCGTGTGACGATGCAGGAAA
AGATTTTTGTGAAGCCAACGGGTGGTCGATTTTACAAGCCGGTTTATTGGCAACGGCGGG
AAACCAAACCGAAGCCCTGAATCAGGCGAAAGCGGTACCAAGAGAGGTGTTTGCCACCGA
AGGCGGTGTAGGAAACTCAATGAGTAATACGATATGGTACATTGCCACAAGAAAGAAGAT
TGTTTGATTTGACGTACGACGCGAATGGTTATCACATCAAAGTAATGAAAGATATCAAAG
ATGGGCTATAATGATGCTATCTTGATCGAATCCCATTCACGAAAGTCGAATTGAGTTTTC
AGTCTACTTTGTTATCATCGCTGATGGTAGAATCGGATGCTTTATGTGTGAAGGTACCGG
TACATCTTGTCACGCGAGCGAAACCATGGAAACGAACGGAATACGAGCTTGCTCTTTTTT
GCTTTTGAACCCTGCACCTGGCTACATAATATCAATAAAATCTTTCATTACACGT
>comp2879_c0_seq1
CATTCAGGGGCTATCGATGAACTACGTACAGTAGGATTGGAGAACAGTTCAAAAGTTAAC
CAGAACAGTGGATCGCACACTTTTCCTACCGGTATTTTCACGAAGCGTCTGTGATGTAAA
ATTTCTTCGGAAAATAGTTTCAAAAACAGTGAGGGATGGGCCATGATTCGCGGTGTGGGT
ATTCAACCTGTCGAGAAAAATATCCTTGCTTCATTGGCGTTCTTGGGAGCCATTGCTCTA
TCCCGCCAGTACTTTTACGATTTTAATTTTTTTCCCCGCGACAATGGATGTCAAGCAAGC
ACTATGAATGGCGGTTCACGAGAAGAAGAGACCGATGATGGAACTCCAGAGGGCGGCGCC
GCCGACGTGAACATCGATGGAAATGCAGTTGCAAACGATTTGGTTGAAGACAACAAAAAA
AATATTTGTTTTCCATGGGAGCCCCATTATTCTCCTCCAAAATGCAGGGACACCGAAAAT
CTCAATCCAAGGAAATTCCCGCTAAAGAAATGGCATGAAGTTTCTGCACTACACAAGATT
GATTATGATAGTGCTCACGAGGATGACCACGGCACAATTTCTACAGAAACGTGCAAAACT
GAGCTTGATTTTCTGTCATCCATGACTTTTGCGAATGGAGGACTGCGATCTCCCTCTTGT
CCTTGCTGTGTTTGATGCTCAGCGAACAGCGTATTTGAGTTTCACGAACTTCTTCGCTAA
TATTGGGCAGTGGTGAACGAATGTATTTGTCGACACTCTGCGGCTTCAACTGGAGAGGAG
AAAAATTGAGAGAAATATAGCCATTTGCACACCTTCTGCATAAGCGCTTCTGTCTGTGAA
TACATATTCCTGTCAGAAACGATAATTTACTTCGCAGAAATAAGGTTACATTTTGTGGGA
ATACTTTAATGATACGCAAGTCATAAAGATGATCAACAAATGACAACAGAAATTGAAAGG
GTTCTAGAGTGAATCGCCCAGGTCGTTGAAATGTTACAACGAGGTTATGTTCAGTTCTTA
CCCTCATCAATGATCGGAACTTTCCATCAAGCACACCAGGATATCAGTTACAATTGAATT
AAAATTCATTATCTTATGTGGTTATGTCGTTTCAAGTGTGTCGAAGTATTTTTAATGTCC
GCCAGTAAGGTACTAGTAGTAGGATCCATATGCTCTGTTCA
>comp2880_c0_seq1
CTCAACGTATCTTCTCTCATAAACTAAGAGTTGAATACAAACAAAAGACAACAGACAACA
ACACTACAAAACAGACGGAAATTGCCAAACAACACATTGCAACCCTTCCCTAGAGCAACA
TGGACAACAAAAACACTAACGATGCCGACACCGATCACAAAATAATGATGGAATCCACTT

CCACCGCCAATATTGAAAGCGCAAGTAGTTCCGCGGAAGCACATCCGAGCGAGCCAGTGG
CTACGAGCAACGGCATTGTCACGAAGAGCAACGACGATACCCACAATACAATACGGAGCG
GCGACGAAAACGACGACGACGAAGTCCTACAGGAGGAAGACGACGAGGAACAAAACGACA
CTGTTCCCACTTCACGCGCATCCTGCTACAAGAAAACCGCCGTTGGCCTTGTTCTTCTCG
GACTCATCATCTTCGTCATTGCCGATTCGCTAACGAACCAGCACATCAAGACGGGTCTGC
TCGCCTTTTTGGAATGGATCGAGGACCACCTGGTGGTGGGCGTCCTGAGTTTTGTGCTGG
TGTACTTTCTGGCCACGGTGTTCTTTATCCCGGGCAGCATTCTGACGCTCGGTGCCGGCT
TTGTCTTCTCGTCCGCGATGGACTCGTTGTGGATTGGGGTCTTGCTGGGAACCGCTGCCG
TCTTTGTGGGAGCCTCTCTGGGTGCAATCGTGGCCTTTTTGCTCGGACGGTATCTCTTTC
GGGAAGCGGTGGCGGTCAAGCTGACGCAAAAATACAAGATCTTCGAGGCCATCGACCGGG
CCCTCGAACAGAACGGACTAAAAATCATGGTGTTGCTGAGGCTGTCTCCGATTTTCCCCT
TCAATGCCCTCAACTACAGCATGGGCATCACGGCCATCCGATTCTGGGACTACGTTCTGG
CCTGCCTGGGGATGCTCCCCGGAACGATCGTCTATGTGTTTCTGGGGGCCTCGGCCGGTA
GCCTGGCGGAACTCGGGGGCGACGAAGACGAAACCGAGGAAGACGACGACAACAAGACGG
TCACCATAGTGGTGATCGTTGTCGGGGTGGTCTTTGGGTTTTTGGCCGTCGGTCTGACTT
CGTATTATGCCAAGCAAGAACTCAACAAGGTGCTGGAGGAAGAGGCGGAACAGAACGACG
AAAGCAACAACGGCGTCGACGAGAACGCGGAGGAAGAGATCAGTGTCTGATGCGATGCTA
CCGATGCCACTCTCGAACCTTCGTACATACCTATACTATACTAATATACTGTAATCCTAA
TCCTGCG
>comp2881_c0_seq1
CGAAATTTTTTAGTTTGAACTCCCTCTTCTCAGTTGTGTCGACCTTCTACGGTACGTACC
GGAACTTGTATTTACTTCGTCCTCTTCCCATGCAGATTTGACGAAGCATCATCTGCTACT
ACCACATCTACCAATATCAATAGGCATTACTAGTTGGTCGAAGAGAAGTTAAATCAACTT
TTCTATAAACGACATACCTTTTGGGAAAGAGGAGATCAGATTGCCGCAATCGACATTTTT
CTTGAGTCTTGACTTTGCGCTTCCACCACCAACTACCGGTTTGAACTCGAGAAGTTGAAG
GAATCAAAGCATCCACTTCACGCGGCGTACGATACCATCGAAATAAACAATCATGCCGGA
CTCGGCACGGCCAACAAGAAAAAATGGACTCTCCCCGCCGTACACCCCTGCCCAAATATC
CACTTGGGTTCTCCTGCCCTTTCTGGTTCTGGGATTCCTCTTCTTTGTGTCACCCATCCT
GCCACTAGCGGCGTCGATTCCTTGTACAATCCTCTTTGTCCTCTCTGCAATCGCCTCTAT
ATATCATGGATACATGGCAATGTATATTGATCCATCCGATCCACGGCTCTTAAATGCCAA
TCATAGCAACAATCAGAACCAAAGCGGAAGAGGAAACGGAGCTTGTGAAAACTGTTATGT
CGATCCCGAAGAACCGACCAAACAATGCTGGATATGCGATGTTCAAGTTGGAGAAAAATC
AATGCACTGCAAATTTTGCAACAAATGTGTAGATCATTTTGATCACCATTGCATGTGGCT
GAATACATGTGTCGGCAAAGCCAACTATTCCTACTTTTTCAGAACCATGGTTTGCATCCT
CATAATGTTGCTTATTCAAGCTATCATCCAAGTTTTTTTGATTGTTGCTATTTACGTAGG
AATTGGGGAAGTTAAACGAAGAGCGTTAGAATGGTTTTCTGTGAATACAACAACACCGGT
GGTAGTGGGCATGGGAATTTTCTTGCTTTTTGATTTAGTGTCCATATCATTGATAGGACA
GTTATTGATATTTCATTTGAAACTTCAAAAAGAAGGCATAACGACGTACCAGTTCATAGT
GCGAGACAACAAAAATCGACGCGAACGAACCAAAAAAGAATCCGACCTTAAACTTCGACG
GAAAAGTGCAATCGAAAGGGCAAAGGAGGAGGGGAATAAATGCCTTTTGTTTCGACTCGA
GAATGGAGGTTTTTTGCGTGAAAAGTGCGGCTTGTCCTGTTTCGACCCTCTGTCGCTAGA
CGAAGAGACTACGGAAAATAACAATAGCACAGACGCGAATGCTCAAAGTAATGGTGCGCC
ATATTAATACCTCATTATTCAGGGGATGAATGAAATGAAAGAATTAATTGCAACATGGAA
TAAATCATTGAACCAATCTATTGGATTTTGACGAAAAAAAAAAAAACTGCCCTTTGCATGC
TCTATAACGCTCACTGTGTGAATAATATATTCTTCCTAAAAACTCATACTTTCA
>comp2882_c0_seq1
AGAAAAAATGATGCGAAGGAGAGCGCGTGAGGTCGGCCTTTCTCCGATGTAAGATTTACC
CAAGTTTTGATTTTTGCTTTGCTCGCTCTGCATTGTTTTCGCACGATATCATATTTCCAA
CGACGACGCGGTAAGAATTACTGTTGCAAAAATATTGCATCTTCTGAGAAAGTGTCCTTC
ATTCAGCGACATACAATCCAGCCAAACCATGAGCTTTTCTACAACCACCTCCATCACACA
AAAAGATGTATCCCTTTCCTCCGAATGGGCTCCATCGGAATGGGCCGAGACAGACACGCA
AATGATCCCTGGAAACGTCGACGGAGAAATGCTTCCTGCCAGTGCTTTCCAAGCTGCCGG
CAAGGTTCTCAAGCAGTGGCTATCTGATGGGCAACAAGAGCCTGATCAATGCTATCAAAA
CCACCTAAACAACTGCAATTCTGCTATTGCAGTGATTCCATGCGATTCATCGGCATCGTC
TACCGAACACGATGATGCGACCGCTGACCTGGCCAGTCTCATGGAAGTCGACGAAGAAAT
CGGCGAGAACCTTTTAGGCGATGAAGTTTTTGGTGGTCCATGCGAAATAACTGAAGATAG
CCAACTCCATTTCGGTTACTCGTTATCCGCAGTATCTCACGAAGACAAAACTCTCATGAA
CTCGATCTCGGCTATGAACATGGAAGATGACATGAGCGAAGAGGATCGTCGCTTCTTGGC
GCCTTTGCTTGTGGAGAATACCTCGACATCGATGGATTACGATGCCGGTTATATTGTCAC
AGACGATGAAGTTCTACATCAGCCATGCGCATCATCTCCATCGGCGATCGTGGACCAGCA

GCGATACCAAGAAATCATGAAGAAACTTGAGGCGTCTATGAACCGATCTCAAGAAACGCG
CAAAAGCCTGACCATGAAAACGCCAAAAACAGAAAAATACAATCGCCGCCGAAGCATAAC
TGGTGTCCTCTCTTCCATCGAGTCCTCTTCGAGACAACTCCAAAATTTCTTGCAAACGGT
GAAGCAAAGGAAGCAAAGAACTCCAGTTTGAATTGAAACTCTTGTAGAATGCGCCTACGC
AAAGCTTCGCAGTTAGATCCGACTGAACTAATGATCCATCGAACAACATTTTGATTATAA
ATGACTGGCTTTACCTGCCCCTCACATGCCAATTGTTATCACGAGATCATCAAGTCAAAA
TCAACAAGTCATTAACCGAACGGCCGATCCTGTCGTAATAAATAATTCGATTTCTACATT
TTATCGCCTTTTTCAAAAGTGAACATCGTGCTTCGCATTCCTTCAAAGCATTCAAAATAT
TCCTTTCAATACGAAATGCAGCTATTCATATATCCAATCAAGAAAGCATCCAGAGCATCA
CAAGCGCTGCTTCAAAGACATTCCATAGAAGGAGATTCGATATTATATTTTTCATCGTAA
TGAAATATCACGCACCGTGAAGAGTATTTCAGACAGAAAAAGGTCTTTTGTTTTCAATGC
ATGGTCATTAATGCAAGGTCTACAAAGGTGTCTCCGAGTGATATCCAATGTTGGACAACG
GAGGTGCAATTGAAACAATTAATGATTCGATGCTTACAGCTGTTTACACTGTACGACTGA
ATGTCTCAATTGGGTGATTCCTATTTCAAAGGCATTTTAAACCGATCTAGGCTCTCTAAG
TCGTTGTAATCAGCGATGACTTTGATTTTTTTGCGAGCGTCGGTCATGGATCACAGCCTT
TGAAATCGTGCTATCGTGCGTTAACGCAAAAGTAATGACTGGAATTGTGAAGTATGATGA
AATAAGATCTACCGAAAACAATTGGAAGCGCTTTAAACCCAGAAGAAACGGAATCATTTC
ATTCCAAAATCAACAATCAATCCGAAAAACTGTTAGTTGTATGCAACAATAGAATGGATA
CTCTATTCTCTATCGCAAATTGATACTCGTGCTGTGGTTCGAAAATATTCAATTTCACAT
GAAGTATTCGATAGATACAATTTGGAAAGCCGTATGTTACTTTATTAGCTATTTATGGCT
GTCAATACGGAATCGGGAAAGAGAATCCTGAATTATGTACTGATGTCGAGTATGGTATCG
CCAATGCGATGGGTGATTTGTTGTGAATAGTTATGCGCTGAAACCGAAACCATTCATATT
AATCGTGAGCCAACCAGTCACTTAACCTTTCTCAATGACTCGATTGTTTCTGGATGATCA
CTGCAACAGGAAAGCATGAGCATAGTAGCTCATCATTGCTTTCGGGCTACATAAGTAGAT
TGATGGCATGTAGATTACTGCGTAAAGCTAATACGATATGAAGAGTATTAATCGCTTAGT
TTCTCTATGTCGAAAA
>comp2883_c0_seq1
TGTGCAGCAGCATTCACTCCGGGCACTACGTCTTGTTACACAAACAACGAACAACTAGAG
TTCCTTCTGAAATAGGCGTAAACCATCCCTGCAAAAAGCAGAAAGACATAACCTTGGAAG
TATCCTGAACGGTGAATGGAGCTGTAACAGGTACAACTATCAAACGACAGCTATTGCATT
TTGATTCAATCTGAATTTGAATCGAACGAGCATTACGATGTCTACCCCTTGGAATCCTAA
CAACCACGGGTTTACGTCTGGTCCGCCAGGAAAGAATTCACCTCCAGGTTCAGCTGCCGT
TGCTCTAAGGAATGGCAACAGCAGCACAACATTTGATGCGAGAAACAACATAGATAGTAC
CGCATCGTCCGAAGCAACACGAAATCCGAAAGCGGGACCCACAAAGGAAGAAAAGGGCCA
AGGGCAGGAAAAATCACAACGGGCTACTCCGATGATTCCCACTCAGCAACAAATGCGCGA
TCAGCAACGTCCAATTTCTTCCACTTCAACTACTTCGGCAACATCTTTCCACGCTGGTTC
ATCTAATGGAATTCCTCATCCGCCCCACCACCATCCGCAGCCATCCTCTGGGATGGGTGG
CAATAATAACTATCACATGGCACCTCAATATCGTCATGCACCTAGCCACCACCATCCTTC
CCATGGTCAACCCAATCATTTTTATAACGGAGATCAGATGATGGGTGCGGTCCGAGGCCA
AGGACGCCCGCACGATCCAGATCATGCCATGCAAAACAATAATTTCCACCATCACAACTT
AAATCGCCCCACCGGGAGATATCCTATCCAGCATACGGTCCCAGAAGGATTTGGACACCA
TCCGCATCCGCATGGACATGGACATGGACATTTCGGTCGTCCTGGTCCAGCGAGAAACTA
TCATCAACACCAACAGCATCAACCTCAGTTCCATTCATTTCCACAAGTACCTCACACAAA
GGGGGGTACAATAACGCAAGGAATGGGGCAGCAGGTAGGCAGCCGTGGGCCTTCACCTAC
CCACCCGCATCCGCAATATTTATCGCAGGGTCGGAAATCTGTGTCACCAATTCCCCCTGG
CGTAGCAGTAGCGATCGCGCAGTCTCAGCCTTTACGACGTCTTTCACAGAATTCAAGCGC
CAACAACTCTACTGATTCCAGTTCTGGCCCTGTCGTGCCCCACGAAGATACTGGCAATCC
AAGTAGTAACCGGGTACCCACTGGGTCGTACCAATCGAAACCAGTGACGTCACAGCAGAA
CCAACAACAGCAGCAAACTAGTGTCGGACATTCTGCTCCACTAAGGCCACCCTCGCAACA
AGAAATGGCTGATATATCGTCACAGGACCAGTACCGCCATCGTGGTGGCCCTCCTGATAT
GCACCAAGGATATCAAAACCAACCGTATCAGAACCAGTTTCCACAGACTGATCATATTCA
TTACGGTGGTCAACCGCCGATGCACAACGGAGGTCACCCGAACAGCATGCAATCAGATAT
GCAAAATTACAATTATCATCATACCTCACATGGCCCATCAACATCAGGACCTCCTCTTGC
ACACAATCACTACCGTGGTTATCCAGATAATGGGATGCCTACTGGTTATCCCCATGGACC
TGGGGGACACCGTGAAATGTATCCGCATCCTTCCAATCAACATTATCATCAGCAACACCA
ACAGCCACATTCATACGGAAAACCTCTCCCTCCTCCACCTGCTCCGCAACGTATACCAGG
GCATGGCTTTCAGCTACCGAAATACAATTCAAACCTGACAACGAGTGATCCAGCAACATC
TGACAACAAGAACGAGTCCAGAAAGTCCACCAACCGTTCTCCATCGCCACCACCATCTCC
TCCACCTGCACCCTCTGCACAAGCATTACTACGAGCAAGGCTACGCCAACAAGAGGGTGC
GAAGAAAACAACAGATGAAATGTCAACATCATCTCCAACTACTGGAGACATGAGCATCAA

TAGTAGTACAAACGAAAACCCTAGTCGTAGAAATAGTAGTGGAAGTCACTCTTCGATGGT
TGGCAACAAACCCACGGCCCTTGGAGATCGGGGAAATGTTGCTGTAACTTCCCGTGGTTC
TACATTGGGAAAGAAGCAGACGAATCAGCCCGTGTCAAAGCCAGCAAAACAAGAAGACGT
TACTGCGGATGCTGCGTCAATATTACTGCAACTCGGCTCCATGAACCGATCCACCGGTGG
TACTGAAAACCAAAATCCTTGCAGTCATCCTTCACCCCCAATCCCAAGTTCTGTTCAACA
CTCATCTTCCATGGACGATGTCTCAGACGCCGGAACCATGCTCACGATGCAAACTGATGT
TCCAGATCTCACCAGCGGTCCATCTTCGGATTCCATTGATACAGAGATGAGCGGTGTCAG
CGCAAGTAGACCTATGGATACGCCGACGAAAAGTAATCTCACAACAAAAGTGACCACGAA
GCAAGAACCCGATACGGATTTCCCATGCCCTGTTCCTGACCGCTATCCTACAAGACTGGC
TCTTCCGTATGATAATGCGAAACTGAACAACCTCCATTGTTTTTTGCGATCAGAATTGCT
TGAGATTTTTGTCGTCAGCAAATCAAACAAGAAGTCTCCATGTCATTCTCCGAGCTCCTC
AGTGGGTCGTGTCGGACTCAGATGTGTGCATTGCGCAATGAATCGAATGTCTTGTGCAGA
TGCACCCATGTCTGTCTTTTATCCGAAATCTATAGCCGAGATTTATCGACTTGTGACATC
ATGGCAACGTTGTCATTTGAGGAAGTGCAAGAAGATTCCTCCTGGAGCCAGAGCTGAGTG
GCAAAAACTACGCGATACTGATAAATCCAGGGGAAAGACCCATTATTGGGTAACTTCGGC
AAAGCAGCTAGGACTAATGGATTGTCAATCTCGGGCAGGAGGCATTAGATTCAAGCCAGA
CTTTGACCCCAGCACTCTGCCACCAAATACCCCACTGACTCCATCGGCGAAGGAAGCCAA
AGAAGCCGCGGAGAGGGCGGCGGCAATTGCTGCCAAGGCTCAGAAAATAGTCTCTTTGGC
TACCGCCGTGACAACTACCAGCATAGCGGCAGCTTCAGCAAAATTAGAGGAGAGTACAGT
CATGACAGATGCACCATCCACTGAAGTTTTGGCCTCAGCAGCAGTTCATACCTAAGGAAA
GGAACTAAAAAAAAATTTCAACACATGATGTAATTGGGGTGGCGTCTGAAGAATGAAGGG
GTAATTCATATACGACAATTAGAATAGTAGGAGGTTCCAGTGCGATCTCTTTTTTAAAAA
ACTACGTTTCAAAATAAATTAAAGTTCATTTTTACAATAAAAAAA
>comp2884_c0_seq1
AAACTACAGACAAATCTGGCTTCAGTGAAGAAACCACCGACAAGTCAAAAGATACGCTTG
ATTATTCAGAAAGCGATGATGATTCAAAAAGCGACAACGAAGATTCCACCAGTCCAACTT
CGAATAGAAGTAATGTAATCACAATGGCAGACCTTTCGTCTACATCTGTGATGGGGCTTC
AGCATTGGGCTTGGAACATGGGAGGCAATAAAAACTGTGATTCAGATGAGAAGGTCAATC
TTGCAGTTTCTACTTTGAAAGAAGTTGATGATAAAGCTTCTTCTTCTATTGGCACGAAAG
ACAACCACGATGCTTGGATTATGAATGAAGACCAACTGGATTCGTTCGCCGAAGATGATG
ATAAGGAGGACGAAAATACGAACGAGGGCAAGGAGCAAAGAGATGTAGAAGGGGATACCC
ATGATCTCATCAACATTGAAACGAATTCACCGAAGTCTAATCTAGAAAGCGAAACAGACG
TAACTAAAGATAATACTTCACCTCCACAGGCGCAAGACAATCGAATTGCATCTCCCAAGC
GCGAGCACTTTCTCGCAGCACAAAAGGCTCTCTCAAAATCTTTCCGCAAATCTCTGTCAT
CGATCAAGAAGAAAAAGCCACACTCAAGTGCTGACATTGCGAGCAATGTTCTTGTGAACG
CCACTGTGACGATGGACGAAACTCCCGATCCGCCCATCGAAACCGTGTTGATGAATTCTG
CCGTTGAGGATCGAAACCCATCCTCTACCAACACTCGGATTCCAAAACGATTCGGCGCAA
ATCGCGCCAACAAACGAAACAGCCTTGCCAATAGTGGCGTTGCATTCTCGAAGGCCAGAA
AGTTCTGGAGAGAAAAAACACAAGAGAAAACACGAAAACCGACAGGAATTGGCAGGAACG
ACACAGTTCGAACACGAGATGTGGAAACAGTACACAAAAGCTCAGACGACTCGCACAGTT
CCAAAGATGTCCTGACGATTGAAAACAATACGATTGCCGAGACTATAATTTCATCGCATT
ATGATCCCGTAATATCGCCAAGTAGGGAAAATTCCTTCCTAGATGACATCCGCGACATCG
CAATGCATGGACTCCTCGGTGCTGAGTACGACGACTACGACGCGGATTCACTTGGAACGA
CTAATACTTCCTTTTCTTGGGTCAGAGATGACGATGAAACTGTTGAATCGCTGAGAGATG
ATGGTTCACTCCACAATCAATCGCATCAAGTTATCCGAGAGTGAGACATGCATGAAGTTT
GAACTGAAATCGCTGAAATTTCTTTGAGACATAAATTCATGGTTTAGACACACATGGATT
ATCAATGAAGTGTTGAGTTCTGGTACCATTTCACTAACGTGCGTGAGATATGTGCGACAC
AGCGGCACTGTCGAGCCACAACAACGAAAGTACCTGCAATATTTTAAAGACGCATTCAAG
GGAATGATGAATTTTGCAGACATGACATTGAGTACACATTTGCTATGGCAATGCGATCAC
GTTTGTTCGAGCGGTATAAATGAGTTTAATCTGTTCCATACTACGCGTGGCTATGAATTT
AGATGCATTGCTCCGGTCCTTGACACTTATCTCCGCATTCTAGACAATATGATGGTACCC
GGAGAAAATTCTCGGAGATAGTTTCATATTTGTTCAAGGGCCGGAGCAATGCACCTGAAT
TTTGAATAACCCTTTTTTACTATAATAAGTAATATATGGATATTATTTTAGTACTACCGT
ACTACCTTTAAATTAACGATGCGCTTGTTCCAAGATTATGACATAAATGGGTACTTTCAT
TGCAGTACTTGTCACGGG
>comp2885_c0_seq1
CCGTAAAATGTGGCCCAGAGTCATTAAAATACCTAGCCGTTGTTCATAAAGAATTCCTCC
CGGGGTTGGTATGTACCGCACGTCTTGACACGAAATGTGTCAGAGTTCGTTGTCATTGGC
GATCGCATGCATATCGTAGCTATTACGGATATTGTACTCGTACTCATACTCGTACCGTAG
GGCAGGACAATTTGAATTAAGTTAGATGTCGTCCCCGCGACTATCATGCTACCGTACGGT

ATCGCACGAGTATGCGCTACAACTTGAACTGTTTTGATCTCGTCCTCTCCGGGAACACCA
TTTGAAACTTTTGGATCTCCTCCCCGTGTGGACACAAATTGTTGATTTCCCAACCACAAA
AGCTCCAAACCAAGCCAAGCCAAGCCAAGCAGGACAACTCAAACACCACCAATCGAACGA
TGTTTTTCCAACACAAGACCCTCGCTGCCTTCCTCCTCCTCTCCTCCTCTCCCTTTGCAG
ATGCTCAGTGCTACGAGTCCAGCGTTTGCCTAGACGTCATGGGAAAACCCGGTGAGTGCA
CCTCATCGGAGGGCCGAACCTTCCCGATCTGCTGCCCCGACGATGCCACCCAAACCTGGA
ACCCCACCGATGTGTGCACGATGGGCGGCGACCCCACTCCCACCGATGCCGGGGAGGCGG
CTCCCACCGGGGAAGCGGAAGCGCCCGAGAAGCTTTGCTACGAAATAGGAACCGCTATAT
GCTGGGAGCTCGGAGTCAAAATGAGCTCGTGCGTGACCGGCGACGGGAGAGAGTTCAATG
CCTGCTGCCCCAAGGACGTCACCGAATCCTCCACATGGAATTCAACCTGCATCTACGGAG
ACGGAGGCCACGAAAAATCCGCTGCACACGCCCGTTCTCTCGAGACCGTGGTCGCCCCTTG
GTCTTGCCGTCGCCACCACCGCCGTGGCGTTTGCCGGTTTCTGAGCAAGCGAGGTTTGTC
GGTTGCTTTGATCGACACCGGCATTGGCATCCAGCGACCGCCGGGCAATCGGATAATCCC
GTGATTATGAAGATCCTTACTTTTGGCCACCAATGATTTCTGCTAGTTTTCTTCCTTGTT
GCTACTTCTTCCAGTGGTTGTAGGGCCTGCATTTCCTGCTGCATTCATAACATTACTAGT
AGTGACACGTTGGTCGTAAATGGTAGTATACTCCAGTTTAAGAAAATTTGCAGTGTCCTT
CTCTACACTTAATACTAGTAGTACAAAATATCCAGC
>comp2886_c0_seq1
CAAAAGCACCTCCAGCAGTCAGTATTTTGGTGAACAAAAACAGCAAACAAACTCTCTCGC
GAAGTAAGATAACGTCATGCACAGACAAGCATTCTGACACATTCGCTCGCCATAATTCGT
TGTGTCTACAAAGGGTCCCCTGAGGTATCTCTTTTGATTGGACATCATCAGTCGCTTCTG
GTTACCAAACTTTATCAGTGACGGGGATCAAACATCGACAGATGAATGTTCTTTCGCTAA
AGCCATATATCCAATAACTTGTTTCCCACTCGAAATCAACTAATCCAGTGAGTATTCTCT
ATTTCTGAATGTAAGCTAGCGCTTCGAGATGGGAATTCTAGATGAAAGTCTGGTATCTAC
GGCTTCGACTCTGATCGATAGCTGTTCCATATCACTGGACCCGTCTCTTGACAACAGCCG
CTCGGATCACCCGAGCGCTGAAAAGGAGAACAATGGTTTGAACGTGAATCCAATTCATGG
GCGAAACACGAAGAGTGGCACGAGCTCGAAGCGCTTTGTTCGAGCCATAAGCCACGCTTC
ACAGATCGTCGAGAAGCGAAATGCTAGATTATTATCTCCCTCTGCGACCAAAACCCACCG
GTTTTTGCAGTCTGAGAACCATTCCGTTTCAGAAAGCACAACGACTACAAACAACTCCAA
GCTAGATATACTTCGCGCAAGGACTAAGCTATTGAGGAAGCAAGGCAGCCAAAAGATTTC
CGAGAGCCGAGCTTCTCTTCGCAAGAACCGAACGGTGCAGGTTGGTCTGTCTCCGCATCT
CCCTTGTCCGATGAATCAACCCGAAGCTTTGTTGGGAAATACCCCAAGACCGGCAAAAAC
GAATTCTGCGTCTATCATGAGGGGCAAGATACTAGGTGCCTCCAAAAATATGGATCTTCC
TGTTTTATTGCGTCGCTCAAACAGTGTTATCGACCAACTAACACTAGAGATGGAAACAGC
CGGTGATATTATTAACCAGACTTATGTCAGCGAGAATTCCGACTCAAGGATGATCCTGGA
CACCAGCAGCATCTACGATACGCATGTCCTTGTTCCTCCCTCTCCCATTATGCAGACCTC
TCACGACCAAGATATCGAAGACGACACCAGCAGTATTCACAACATTTCTGTTGACGACTA
CTGGGATCAATCAACCATATCTGCTTCGGCTCGGGATGCTTCATCAATCTCTGAACAGCT
CAGCCCCACCCGATCGAATCTTGAAGAGCCTCCTCCATCTCAATCCGTCAATTGCTCGGG
ACTTCCGCCTCCCGAAGTTGTGGTGAAAATAACTACTCCCACGTCTGCTAAGCTCTCCCG
GTCTCGCGAACTCGCCGAATCGCGGCACCGAAGGATGAGTCCACCCTCAATTTCACCGAT
CAAGGGAGACTGTGCTTCCATGTACAACACAGATCAAAAGTGTGAAAAGCTGTCCTTCGA
AAACGATTGCCTGCGACGGCAGCTAGAGTTGCTCGAGAGTCGGCACCGAGACTTTCAACA
GAAACTCGATAAACAGATGCTTAGCCCCGAAAAAGTACCCGGTGCGTTGCGAACACCGGG
AAGCCCGTCTACTGCCACTTCCACCTCCAGTGGGGCGTCAAGCCCCAACAGCTACTACAA
GTATCACAAAAGAAAGAGGAGAAGAAGCGATCGCCGTCGCCTTGCTCTGGTATTGTTTTT
CGCCCCATTTCTCATATCGACGAGTCTCTTTTTCTATCGAGCCGTCCGCAATGTCTCCGA
TGCTCGGAGGAGAAATCGTGACAATGCTTTGAGGAACAGTGGGCTTGGATCGATCCCTTT
TTTTGCGGATACTAATTCTAGGGGACCGCAAGCGACTTTGTCGAAACCAGCGCTGAGTGG
TCTCGCTTCATTGATGCGTGAGGGAGTCCTACGCCAAACATCCTTGGGTCCAATTTTGTT
CCGTGACAAGTCGAATGGGAGAATCCAAAGGGACAACATAAATTTTTCTGGCGATTGGAT
TGGTGAAAGACACGAGACTTTCAGTGCAGAAGGACAGAATGAGAGTTTTGACTATGCAGA
TTCTCTTCCTGTAATGGCTCTTCCAAAATACCGAAGAATAGAAGGCTCTGTAGGTGGAGA
TAATCCCGATTCGACCCAGTCTGGAAGAAAGGGCAGAAGTGTGAAACGATGGTTTCAAAA
AAGGTTGTCGAAGATGAAAATAGCAACCCATAATGACCCTGCCGGGGTTTGGCTAAAACG
AGCTCAGCAAATCAAAACCAGAGAACTGCATGCGGAGCTCTGAGGCTGCAACAAGATGGA
TGGTCGTGGATCCTCTTCTAATTATCTTGACGCATGATTGCTTTCGTGCGCAATCCTGCA
AGTTACTCAGTCCTGACTGATTGTATATATACTTCAATTGATTTCTTTTTTTCAGCTTAA
CAGTGGAGGAGCTATCTCTGTTCAACTCATCAACACTGAGATGGAACTACTGCAACATCA
ACGTGGGATGCTACTGTCATGTTTATATCGGGCAAAAAATACTGCGTCCCGAGACTGTTT

TCAACTCAGATGACTCTCATCCGACGACGAAATGCCGATCTGCACCATTTCATCTAAAAC
ATCGCAATAACTTCTTTCCCCTGCTCAAATTGTGGGATGACGCATTTCAAATCGACTGTA
CTTGAACTGCTAAGTCGACGTCAAAGGTTGCGCCGTGGCAATGACGCCTTGCTGGCCTTT
AATTGTGTGGCATGAACTATCACAGTACTAGATGAACTTTTGATCGCCCACATGCATCGA
TCTTGGAGCCGAAGTGATCATGTTTCTGCATTGTCCAAGGTGTTGAAGAATATTGCTCTA
TTTCTCTCTTCTGCTTAAATTTAGGCCTCCATTTTGTTGCTTAGTCTCACGCCTTGAAGG
TTTGATCAACGATCCCCTTCCCAGGACAAGAATGGCATGTTTTTCTCCACTCCATCTGCC
GTTAATTGCATAAAAGATACTTTATAAGCACACCTTACTGATCTCGCTGGTTTGGATTCT
CGTACACTCCAAGTGCTAGGTCTCAAAATAATAATTTGCATTAAGCATCATACGAAAGAA
TACGCATACCATAAGTCTTCAGATGATAATGAATTCAAAAAGATACCAAATTCGTTGTTT
TCCGTTGACACCTGAGACACCCTTTTTGACGCATGTATTCCATCTGTCACGATGCCATCA
AGAGTTCGGCCACAAACGGTATCCTAACGAAATATTTTGTCACAACGAGCGATTTTCGGT
AGGAAAAAACCCGCCGTTT
>comp2887_c0_seq1
AGTCGTTGTTGGAAGACTGCATCGAAGAATCCATATCGCACGGAAATTTTATCTTCGATG
AGCCGAGGGAGAATGATGAAGCAATCCTTCGCGTCAACTGCAGCAGTCCTGGCCCTGTCG
TCGTCCTTTTCGTCGTCCCTGATCGGAAGTGTCCATTCCCTATCACTGAGCATGAGTGCA
GCCTCGTCGACGAGCAAGGCCGGCGCCAAAAGCATCGTGGTGGTCGGTGGGGGCATCCAG
GGCACCTCCGTGGCCTACCAGCTGGCCCAGAAGGCGACGGCCGAAGAGCCACTCGAGATC
ACCATCCTGGAGGCCAAGGAACCCGCCTCGGCCGCCTCCGGAAAGGGGGGTGGCTTCATG
GCCCGATCGTGGGGGGACGGTTCCCCGACGCAGGGCCTCCACCACCTGGCCTTTGACATG
TACGAGGACCTCGCGAAGGAACTCGGTTGTTCCTCCTACCGGAAGCTCCCCGTCCTAAGC
GTTGCACCGGAGGGCGGGGGTGGCATCCGGGGGAGCAAGGGAAACCCCCTGATCCCCTCT
TGGCTGGACGGAGACTCGATCGGCCGGATCTCCCCGATGGGATACGGCGACGACACGGCC
CAGATCACCCCCAAGGAATTCGTGGAGAAAATGCTGGAGGCGGCATCGGAAAATGTTTCG
GTGGTACTGGGAACCTGCACGGGGGTCGATGTCTGCGAGAGCAGCGACGAGGACGACGAA
CAGTCGGTCACCGGTGTGAGGTACCAGCCCCGCGGAAGCGAGGACGGCACCGAAAAAGTC
CTCCCTGCCGATGCGGTGGTGGTGTCGGCAGGGCCCTGGTCGTGCCAGGCCGAGGACTGG
TTCGAGGGATCGGGCGTCCAGCTCCCGATGGAGGGGGTCAAGAGCACCAGCATCGTCTGG
AAGCAGCCCGAGGGCACCAGCGAGGTGGACGCTACAGCCCTGTTTTGCGGGGAAGACTAC
CGATTCAACACACACCTGGAAGTCTATCCCCGGCCCGACGGAACCATCTACATCTGCGGG
ATCGGTGGATCGGACTACATCACCACGCAGGAACTCAAGCAGGGCGCCTACCGGGAGGTC
ATCGAAGCGAACGATTCCCGCGTCAAGGCCGCCTCCGATAGCTTCCGGGAGCTTTCGAGT
CTGTACCGGGAAAAGGGTGAGCTCGACCGCACCCAGGCCTGTATGAGACCCTGTCCACCG
GACGCCCTCCCCTACATGGGGGCGATCCCGTCCTATACCAACGCCTTTGTCAACGCCGGG
CACAACTGCTGGGGGATTGCCTGGGCCCCGGCCTCCGGCAGGGCCATGGCGGAGCTCGTC
CTGGACGGGGCCTCTGAGAGCGTGGACCTCCGGCCCTTCGATCCGGCGCGCTTCACACCC
TCGGCCGGCAAACGCGGTGGCCGCGGCCGAAAGAAAAAGGGAGCGAGCGTTGGCGAGCAG
TGGTAGCCAGTGGGGAGAACGACGGTGACGGCGGTTTGCGCTGGTTCGCTCGTGCGGAAA
ATAGGTCCCGCAAAGACCTCGCATTCCCTACATGTGTGGTTCGTAGTGTGTTGCCTGGTT
TTCACTGACCCCATTGTTTTGCCAGCTACCACAGACAACTACACTGCGCCTTTTTAAAAA
AATTAAAGGCTTGTGTACCTCACTTTTCAAATGAACTCACGAGAATAACGTACTAGTACT
ATCATCAACAATTGTTGTTGTCCTGTACTTTGGCGTGCCAGAAAGAAATGTGGTAATTTA
AAGCAATTATTTTACTGCAAGTATAGATATAACAC
>comp2888_c0_seq1
CCTGGCAAGCGTCGGGGACTGCCGGAGGCTGATGAGGGCGGCGGACGACGCCTTTGGCAG
GCTGGACGTCCTGGTCAACGCCGCCGGCACCACCGACCGGGGCTCCCTGTGGGACACCAC
CCCCGAGGACTACGACCGGATCATGGCCGTCAACGCCAGGGCGCCCTTCTTTCTGATGCA
GGACGCCGCGCGGATCATGGAGCGGGAGAGCACCGAGGGCTCCATCGTCAACGTGTCCTC
GACGGCCTCCTACGGCTCGATGCCCATGCTGGCGGCCTACGGGATGTCCAAGGCGGCCCT
CAACGCTTCCACCAAGAACGCCGCCTACTCCCTCGCCTGGTCCCGCATCCGGGTCAATGC
CCTGGCCATTGGGTGGATGGACACGCCCGGGGAGGAGGCCGCCCAGAAGGGGATGTGCAT
GCCCAATGGCCACAAGGGCGGGGGCCAAGGATGGAGGGAGACGGCGGGAAGGGGCCTGCC
CTTTGGCCGCCTCCTCCGGCCGGAGGAGGTGGCCCGCTGCATCGCCTTTTGCGCCTCGGA
CGAGTCGGGAATGATGACGGGCTGCGTCATCGACTTTGACCAGTCCGTCTGGGGGGCCGG
GAATCCCCCGGTTCCCCCCCGCAAGGAGGAGTGGGACCGGGCCAGCGGCATGACCTTTTC
CTTCGAGGGGAGGGACCGCTTCGTCCCGGTGCCGGCCGCGGAGCAAAAGGCAGTAAAGAA
GGCGGCAGAAAAGGCAGCAGTGGCAGTGGCAGTGGCAGCAGAAAAATCACCACCAGCACC
ACCGACACCGGCACCAGCTCCAGCTCCAGCTCCGGCCAGCAAGCCCGCCGGAAGAAGGGC
GGGGAGCAAGTCGCCGGGCCGTTTGCGATGGCCCCCGCCGCCTCCCGTAGCAAAAGACGT

CGAGCCCGAGTCCTACCGGCGGGCCTCCGAGGTTTTTGCTGCCGCGGCCACTCCCGCAAC
CCAGGACAACAGCAACAGCAACAGCAAGAGCGAGAATTACGGCTACGACAACAACGGCGT
CGACACCTCTTCCCAAGCATCGATCAAAGAGCCAACAGCGGCTTCCGAAACGGCGAGCGA
CGCATCCACCGGGGCCTCACCCACGCCCACGCCCACGCCACCACCGCCCGAGGAGGACGC
CGAGGACGCCGCGAAGGACGACGCCAGGGACGCCGCGAAGGAAGCCTACTTTTCGATGCG
CACGCAGCGGATGGGGGACGTCTACCGGCCCGACGAGTACCTCCCCGGCACCGAGCCCAG
TGGCTATTTCGTCCCCACCTCCCCGGCCTCGAGGCGCAAGAAGAAGGCCGGCAGGAAGTG
GGGGGGAAACCCGGACTGCCACGGGAAGTCGAGGGTGGACGAGTGGCTGGGCTCCGGGGT
GGCCCCCAAGCTCACCTACAGGGTCAAGCCGGCCTCGCGGTAGGCCGGGGTGGTCAAGTG
CACCGGCACGAGTGCGACACAAACCAGCGCAGAAGATTCCACCACACTTCTGCCATGTAC
AGTACAAGTAAACAGTAAACTACGACCGTAGTAGTATAGGCCAGACAATGCCAAAAGAG
>comp2889_c0_seq1
GTCGCTTCGTGCCGTATATGTTCGATCACCACTGAACCAAAGGTTGCTGGTTTGGACTGG
TCTACCATACACGAGATTGGAAGACATACAACAATGCGAAGATGGTCGTTCATATCACCT
CAGGCGAACGGGGAGGGCGGTGAAGCAGCTGCCGATCGGGACGAGGCAAATGACGGGAAA
AAATTGCACCATTATTTGCAAGACCCAGAAGAAAACGGAGTTGATTGCTACCAGAGTTGT
GATTATTGGACACACGAGGACCCGTTGCCATCTGTCCCATCATCGTCGTCGTCTATCTCA
CTGCCGAATGGTCCCGGAAGCGGCCTGTCGCGAAGGGGGCAGAAAGCCGCGACGATTTCG
GCTTGCTTTCTCAAGGACTACGAGCGAGGCCACCCGCCAACGCTGTCGAGCGACTTTTCC
GGCATCACCCGACGCCAGTTGCTGCTCTACCGCGTCAAGCACTCGTTCCACTGGAACATC
TTTGGTCTGTGGCTCGCCACCGCTTTGTTGTTCGTCCCTTCCTTCCGTGCGAGGGCAGCG
ACGCTGTTCTTGCACGCGTACGCAATTCTGGCCTTTTCGGCGGACTTGTACATGGTGGAC
CAGTTTCGCTGCGAGGACACCGAGCGGGAATCGAATCGGTGGCCCTTGGGTGCGGCGGAT
CGACCGGGGAGACTTTTGTTTGGTTGCATGGAAGGGTTCCTGTTCCTGATGGGTCTCCAA
ACGATCGCGAACGCAATTGTTCCAAACAACACCGTGGTGCACGCATTTACCCTTGCGGTT
TCGGTCTTGAAGCCCCTCGTCTTTTTCTACCAAAGCAGGCGGGCGAGAGACGCCCTTGAA
GCACTCATCCGAATTTCAAAGAAGCTGCTGCGTGTCATTTTGATCGAAATGTTCTTGATA
TTGACTTTCGCGGCAGTGGCCTGTCGTCTCTATTACAACTACGACAGCTTCCAAACACTG
CCTCAAGCTTGGTTATCACTATTTGCCCTATCTACAACCGTGGTCAACCCAAGCATATGG
ATGCCTGCGTACAATGAATCTCCCTGGAACGCAGTCTTTTTTGTCATCTTCATCGTCGTT
AGTATTTTTTACCTGCATTCTTTGGTATTGTCGGTTGTCTTTCAGGTGTTTATTCAATCT
GCAACCGAAGTCCACCGCCAATCCGCCACGGACAAGGAGAACTCGCTGAAACTAGCATTT
CTTGCGCTGACCTCGGCCAGAGGCAATGCTTCCAAGACCTTGGGCGACAAAAACTTTTCA
CCCGATAAAAATGCATTGGTAGATGTGCGGTTGATTTGCGAGGCCTTTCGATTGCTGAGG
CCTCACTACGGCCAACAGAAATTGAACGTTCTGACGGACATTATACTCCCTCCGAATTCG
GAAGGTGGCAACGAAGCCACGCAACACAACGGGCGAAATGGCGAGGAAAAAGACCGCAGC
AACAAACACAACATAGACTTTACGGAGTTCCGAAGTAGAATACTGCAGGCCCTGAACTCA
TCGGTTCGCACCACGAGGACCCACAGCACCGAGGGGATGGCCGTGGAACTCTTGTCCATT
ACGGTGTCGATCGTGAATTTTCTCTACGTGATGATCTTCGCGTCGCGCATGCGTCCCCGT
GAGGAGAGCACCGACAGTGAGTTTATAATAGGAAGTGTCATAACCCTACTCACACTTTGC
GAGGTCTCTTTGCGCTATAATCCGTGGCAATTCTCGAACAGAATCAATCCAATCACCCGG
CTCAACGCCGTATTGGATGGAATGGGATGCCTCGGCGGAATTGTGTCATTTTGTGGCATA
GTCGTCTTTTTATGTGGCAACGAAATTGGGTTGGAATATCTTTTGGTCGGTCGAGCGATT
GGTATGATTCAAAGCATGCGATTCTCTATTTGGTTCCGGGAAGTGCTCCAGAGGTCGCTG
TACGTACTGCCCTTTCTTGCCGGCCCGATTCTTCTTATTTTGACGGCAATGCATGTCTTT
GTATGCATCGGAATGGCGTTATGGGAAGGGGCTGTAGATACGGAAGAGCAAGCATCCAAC
GAAAACGTAGAATATCTCTACTATCTCAACAACTTCAATTCGTACGCCGAGGGATGCATA
ACTGTATTCAACGTGCTTGTCGTGAATGATTGGCACCAGATTGCTAATGTATTTCTCTAC
GGAAACAGGTGCTCGAGCCCGTTTGTTGTATATCTATACTTTGTGATGCTTGTCATCGTT
GCGGTCTGCATTTTGTTGAATGTTATTGTTGCTTTCTTCGTCGAAACTTTTGTCACTCAG
CTGAACCTGGTGGATGTGGAAAGTGACTCGGTGAATAGCGACGTAAAAGTTCGTATCGAC
GAGTCTACCCCCGCAATGCAACCGACTGATATGTCTACACACATTGCTCCCATTGCTAAA
TCGTGGATTTTTGATTCCAGCGACCTGAAGAAGACCAGCGTGAGATCGCTCAGCTGTCCG
AATACCGGTGTGAAGACCGACTACGATGTTATTCGAAGAGAAGGATTTGACGACATTATG
CAATCGGTCGGAAACGACGAGGACAAACAATTCGAAGACTTTGCAAGAGACCTTCTCGGT
GCACTGGAACTGGTTCGCGCTTTGACACCGGATAGAAACATCGGACATCTGCTTTGTTGC
CACCAAAAGGGAATTCAATTTCAATCGAGCGGTTTTTCTCTTCTCACAAAAGACTATATT
CCTGGGAACAAGGGGATTGAATCCATCGTTGCAGAAACCAAAGACGCGCTGTTAAACAAT
GCGAACTCATCCGGTCGAGTGGGAAGATACATTCCAAATCCAAATAATGATTCGAAAGTA
CTGCAAATTGATGGTGCACTGTTGCCACAGAACAATGAAGTACTGATGCTGACAGCAATT

GAAAAGAAACTAGAGAACTGACAGGGAAGGAATTCTGAACCATGGGGGAGTGGAAATGGG
CTACGAAAAAATCAAAATGCTGTACCATTCAGTAGTTTGAAAATATTTCGTTTGCA
>comp2889_c0_seq2
GTCGCTTCGTGCCGTATATGTTCGATCACCACTGAACCAAAGGTTGCTGGTTTGGACTGG
TCTACCATACACGAGATTGGAAGACATACAACAATGCGAAGATGGTCGTTCATATCACCT
CAGGCGAACGGGGAGGGCGGTGAAGCAGCTGCCGATCGGGACGAGGCAAATGACGGGAAA
AAATTGCACCATTATTTGCAAGACCCAGAAGAAAACGGAGTTGATTGCTACCAGAGTTGT
GATTATTGGACACACGAGGACCCGTTGCCATCTGTCCCATCATCGTCGTCGTCTATCTCA
CTGCCGAATGGTCCCGGAAGCGGCCTGTCGCGAAGGGGGCAGAAAGCCGCGACGATTTCG
GCTTGCTTTCTCAAGGACTACGAGCGAGGCCACCCGCCAACGCTGTCGAGCGACTTTTCC
GGCATCACCCGACGCCAGTTGCTGCTCTACCGCGTCAAGCACTCGTTCCACTGGAACATC
TTTGGTCTGTGGCTCGCCACCGCTTTGTTGTTCGTCCCTTCCTTCCGTGCGAGGGCAGCG
ACGCTGTTCTTGCACGCGTACGCAATTCTGGCCTTTTCGGCGGACTTGTACATGGTGGAC
CAGTTTCGCTGCGAGGACACCGAGCGGGAATCGAATCGGTGGCCCTTGGGTGCGGCGGAT
CGACCGGGGAGACTTTTGTTTGGTTGCATGGAAGGGTTCCTGTTCCTGATGGGTCTCCAA
ACGATCGCGAACGCAATTGTTCCAAACAACACCGTGGTGCACGCATTTACCCTTGCGGTT
TCGGTCTTGAAGCCCCTCGTCTTTTTCTACCAAAGCAGGCGGGCGAGAGACGCCCTTGAA
GCACTCATCCGAATTTCAAAGAAGCTGCTGCGTGTCATTTTGATCGAAATGTTCTTGATA
TTGACTTTCGCGGCAGTGGCCTGTCGTCTCTATTACAACTACGACAGCTTCCAAACACTG
CCTCAAGCTTGGTTATCACTATTTGCCCTATCTACAACCGTGGTCAACCCAAGCATATGG
ATGCCTGCGTACAATGAATCTCCCTGGAACGCAGTCTTTTTTGTCATCTTCATCGTCGTT
AGTATTTTTTACCTGCATTCTTTGGTATTGTCGGTTGTCTTTCAGGTGTTTATTCAATCT
GCAACCGAAGTCCACCGCCAATCCGCCACGGACAAGGAGAACTCGCTGAAACTAGCATTT
CTTGCGCTGACCTCGGCCAGAGGCAATGCTTCCAAGACCTTGGGCGACAAAAACTTTTCA
CCCGATAAAAATGCATTGGTAGATGTGCGGTTGATTTGCGAGGCCTTTCGATTGCTGAGG
CCTCACTACGGCCAACAGAAATTGAACGTTCTGACGGACATTATACTCCCTCCGAATTCG
GAAGGTGGCAACGAAGCCACGCAACACAACGGGCGAAATGGCGAGGAAAAAGACCGCAGC
AACAAACACAACATAGACTTTACGGAGTTCCGAAGTAGAATACTGCAGGCCCTGAACTCA
TCGGTTCGCACCACGAGGACCCACAGCACCGAGGGGATGGCCGTGGAACTCTTGTCCATT
ACGGTGTCGATCGTGAATTTTCTCTACGTGATGATCTTCGCGTCGCGCATGCGTCCCCGT
GAGGAGAGCACCGACAGTGAGTTTATAATAGGAAGTGTCATAACCCTACTCACACTTTGC
GAGGTCTCTTTGCGCTATAATCCGTGGCAATTCTCGAACAGAATCAATCCAATCACCCGG
CTCAACGCCGTATTGGATGGAATGGGATGCCTCGGCGGAATTGTGTCATTTTGTGGCATA
GTCGTCTTTTTATGTGGCAACGAAATTGGGTTGGAATATCTTTTGGTCGGTCGAGCGATT
GGTATGATTCAAAGCATGCGATTCTCTATTTGGTTCCGGGAAGTGCTCCAGAGGTCGCTG
TACGTACTGCCCTTTCTTGCCGGCCCGATTCTTCTTATTTTGACGGCAATGCATGTCTTT
GTATGCATCGGAATGGCGTTATGGGAAGGGGCTGTAGATACGGAAGAGCAAGCATCCAAC
GAAAACGTAGAATATCTCTACTATCTCAACAACTTCAATTCGTACGCCGAGGGATGCATA
ACTGTATTCAACGTGCTTGTCGTGAATGATTGGCACCAGATTGCTAATGTATTTCTCTAC
GGAAACAGGTGCTCGAGCCCGTTTGTTGTATATCTATACTTTGTGATGCTTGTCATCGTT
GCGGTCTGCATTTTGTTGAATGTTATTGTTGCTTTCTTCGTCGAAACTTTTGTCACTCAG
CTGAACCTGGTGGATGTGGAAAGTGACTCGGTGAATAGCGACGTAAAAGTTCGTATCGAC
GAGTCTACCCCCGCAATGCAACCGACTGATATGTCTACACACATTGCTCCCATTGCTAAA
TCGTGGATTTTTGATTCCAGCGACCTGAAGAAGACCAGCGTGAGATCGCTCAGCTGTCCG
AATACCGGTGTGAAGACCGACTACGATGTTATTCGAAGAGAAGGATTTGACGACATTATG
CAATCGGTCGGAAACGACGAGGACAAACAATTCGAAGACTTTGCAAGAGACCTTCTCGGT
GCACTGGAACTGGTTCGCGCTTTGACACCGGATAGGTGCGTTCGCTTTGTCTCGTCGGTG
ACGCCTGGCATAGTATTACGGTAATATTGACTTATGTTATATTCTACTCAAACGTTGAAT
TGTTTTGAATTTTTGTGCTCGGATGAAGCAGAAACATCGGACATCTGCTTTGTTGCCACC
AAAAGGGAATTCAATTTCAATCGAGCGGTTTTTCTCTTCTCACAAAAGACTATATTCCTG
GGAACAAGGGGATTGAATCCATCGTTGCAGAAACCAAAGACGCGCTGTTAAACAATGCGA
ACTCATCCGGTCGAGTGGGAAGATACATTCCAAATCCAAATAATGATTCGAAAGTACTGC
AAATTGATGGTGCACTGTTGCCACAGAACAATGAAGTACTGATGCTGACAGCAATTGAAA
AGAAACTAGAGAACTGACAGGGAAGGAATTCTGAACCATGGGGGAGTGGAAATGGGCTAC
GAAAAAATCAAAATGCTGTACCATTCAGTAGTTTGAAAATATTTCGTTTGCA
>comp2890_c0_seq1
TCACACACCACGCCAGAAACACCTCCGGAAAACAAATCCAAAACTAAATCCTTGGTGGTA
AGCTCGGCGAGTGTGGCAACCATTTTAAGCAGGAATCAAGACAACAAGAACAACGACAAC
GACAACAACAACAACAACCGGAACCTGATCCTCGATGCCTTTTACCAACAGCAACAGCAG
ATGTATGCCGTGAACAATGGGAACCAAAACAGCAGCGGCAACAACTATTCGAACCCCGAT

TCGAATTTTGTTTACACCAACACCCAGAGCCACGGCGCCAACAACAGTCATCTCAACGGG
AACGCGGCGGCCGCTGTCTCGGAGACCTTCCACAGCTACTCGCTGGATACCCAGGGACAG
CTTAGCAACTTTATGAGCTTCTTTGGGCGAAACAACCACGGCAACAGCAACCACCACGCG
AATATCCGCGCTATCCAAAGCTTTCCACTGCCCCATCCATATCATCTTCACCAACAGCAG
CAGCAGCAGC
>comp2891_c0_seq1
CTCTTACCGAGTTGTCAAACTAATTGAAATTGAAAGGGAACATCGGGGCAAGGAGAGACA
AAGCTTTGGACAATGATTGTTGGCTTTCTCGCATTGTCGTATCTCTGTTCTTTCTACACG
GCAGCATTCTCGATCGACCGCGGCAATAGCGCGCCAAGCACCGGCGTAAGGGGTCCAAAC
GGTAGAAACAGTTGTGCCTTTCACCGCCAAAGCAGCAGTCACGTAACGAGTTCAGTTTCA
TTGAATACCCAGCTAATCAGTTCAGAACTAGAGACACAAACATCGAACATGACAGACAAA
GTGCTACCTTGGAGAGACACAAATAGATCTCCTCTATCGGGAACCGAATTGCGAAACCTC
GTGGTCAGAGCCGCTCTTCTGGAAGAAATCGAAGACAACGAAGACGACGATTCTTCTTCG
TTTCTAGACATTCCAGGGGTTGCGTGGCTAGAGCACATCAACCTGATTGTTGGTAAAGTC
GCGGAAGATCGCTCTGTTGCAGAGTCTTTTTACTTGGATGTCATGGGAATGACGGCAGAC
AAGAGCAAATCCTTTCACGTGAATCTTGGACGGCAGCAGTTCCATTTAGCGACGCCCAAG
CGAGACGATGAGGTGGCACACCGTATCCATGGAAGCATCGGATTGGCGGTTCCCGATCTG
GAGGCACTACTTGATCGACTTCGAGATGCAAGAAAAGATGCTGAGGGAAACAACGACTAC
GGCTTTCGTGAGACACTTTTTGACTTTTACGAAGAACACGACGAGAACGACGACGAGGAC
GACAAAATTGTCCACGTCCGATGCCCCTGGGGAAATGTATTTCGTTGCTATTCAGTAAAA
TCCCGTGGTACACCACCTACGCCCTTTGCGAAACCAGAAGAGTCCCCACAAAAAATGACC
AACCTGCATGCACCACAACTCGGCATCCACGGGGCCGACAAAATGGGCGTCCTGGGAGTT
GGACTGCCAGGAATACGTTTTGTGGAATTGATTCTTCCCAAAGGTGTATCGTCGCATCAA
TTGTATGGCTTTTACAAACGTATGCTTAGGTGTCCAGCACAGGTTCTACAAACGTCGTGC
GATGGAAAAAAAAGAGATTGCTGCGTCGTTTCCGTCGGAGCTGGCGTTCATCTGGTGTTT
GTAGAAGGCAGCATTGGTTCCGAGAACGAAGAAGAGGCAATTGCACAACGCCTAATGAAG
GGGGTTCACATTTGCATTTATGCCCACGATTTTTACGGGTTGTATAAGCGCCTTTCCGAC
AAGTCCCTAATCTGGTCGAATCCCAGGTTTTTCCATCTTGATACTTGCGATACGTGGGAA
GAAGCGAAGAATTCACGAACCCTTCGATTTCGACACATTGTCGACGTCGAAAGGGAGGAA
ACAAGCAATAATAATGGCGAGAACAACGGCAACAGCAATATTCTAATTGAACTCGAACAT
GAGACTCGACCGCTACGGCATGGGCAATTTATGAAGGTTCCACACTACGTTCCAAAGTAA
CCAAAGCCAAGGATGAATAACCAATACTATGACTATAAATACAACTTCTCAAGAGCTAGG
AA
>comp2892_c0_seq1
CCGAGTTGATATTGTATATGATTGTTCCAAGCAGGCAATAGACTGCAGTTTGAATGATTT
AATTGAACCGATTCCAATCGGAGAAATCAACGTTGCCTATATTATCGACGTGATCTGTGC
AGTGCATACATGAGCATCTGGAAGCTTTGACTGCATCATCCAATTTATCTATTTCTGGTT
CTCCCTGACACAGAAAATGACCGATGGCTTTCGTGGAAATTTCGTCAGCTCGAAGAGAGG
ATTGCTGGCGTCTGTGTGGTCCATCGTGACAATACTGACAGCTATTTCTTTTCTTACGGC
ATTTGTATACGCATTGAGCACATCGGACGACGAGAATGACGAAAATTACTACAATAATAA
TGAAGGGGAAGGAAGCCAAGACCAGCGGGAAGGCCACGATGCAGAAGTGGCTGTGACATC
TCGGGCGTTGGCGTTTTCGGCACTCTGGACAGGAGTTTTAGCAGCCCTGTTATCCATTTT
TGGAACGGTTATTCTGGGCTGGCAAAGTCCAACGGGGCAATATTACACATGTTGCAGTAG
TTCGGTTCATCGTACTACGCCACTAGGGATTGGTTCTTTTATTGGCGCACTCCTTATGTT
TGCAAATTTGACTCTATTGTGTTCTGTTCTATTTGGGGAATTTCAGGTTCGAGACAACCG
AGAAGGAGAGGATAGAGCAGGAGGCGCTGGAAATCAAATTGATGGGCAGCGAGAGACCAG
ATCAATGGCCTTTTCGATTATGTGCGCGGTCCTAACAGTTTTATATGGTGGCTTTGCTGC
CTTGACGTTAGCGTACGCACCGGATGTCATAAACGAGTATGCCGTCGATGAAAGAGACGA
GGTTTTGATGACATCGACTCGGAATAAAAAGGGCCATTTCAATGCGGCCTACGATGGATA
CATAGGGGAAAGATTTGGTGTAGGACGAACAACTTTAGCGTAGTTCAATTGTCAAGTTCA
TGAAAATTTCGATGATAGGAACCTGTACTCTTTTTTGCTGCTATCGAAATTATTGATGTT
ATGTATTACTAGATATGTTTTTACCAAAAGGTAGCAACCCACTTCTTATGAAATATTGGA
TTTGAGATTCATAGTTCAGTACAAGATGCCGGGAGATGAAATACAGTAATACCGTATTAT
TGCTCTGACTATCCAATGCATACGTCCTCTCTAATATAACCATCAAATAAACTACTCTGA
AGT
>comp2893_c0_seq1
TTTTGGCTAGAAGCAGTTGTCATCGACATTAGAATACTGTGATGTCATTAAAGAAAACAT
CCTCATTTGAAGTAGCCAAGAACACTTACGATACTGAGAAAATGGATTCTACAGAAAAGA
GTTTTTCTAAGAACGGTGAAATCGAAGTTTCCATCAATTTGTCGGTTGATGACAAGACCC
GCCACTGGGTATACTTCAGAAGCAATCCAAGAACCGATGAGGAACGGTTTAATTCGATAC

ACAAGGATGACTTTGGTAGCGCAAGTGTCCAGTGCAAACAAACAATTCTAAATGGACGCA
CAAATCGGGATTCATTGACACTAGATGATGCAAGTTTCGAACTAGTGGACGACTTTGATG
TTTCGAAACTCTTCTCAAACCAAGACTTTTACGAAATGCAATCCGGAGAAAAAAATTTCA
ATGGCTATCACAAGGCAGTTGAAGATTTCGTCAAGACCAAGTTGGGATGCGACAAGGTAA
TTTGCTTTCATAGCCAAGTCCGCAATGCACAGAGAACTTCTTCCGACGGTGTTGAAGGAT
ACGCCGGTGGTAGCCCCCATACCGATTCGAGTCCGGTTTCAGCCGATCAGTTAGCCCTAC
AGATGTTGCAAGCCAACAACGACACCGGAACATACCAACGATACTGTTATTTGAATTTAT
GGAAGAACATCTCCGATACACCCATTCAAAATGATCACCTGGCAATGATGGACGAACGTA
CCGCGGTGAAACCCGATGATTATATCACCAAAGATTTATTTGGTGATGGCTATTCTGTGG
TACAGTATGGATTGAATGCCCGCCACGCGCATTTGCATAAATGGTATCATTTCCCACACA
TGACTTCGAAGGAGGCGATACTGTTCAAGCAAATGGATAGCGATCCAACAAAAACCGGAC
GAATTTGTTTCCATATGAGTGTACACGACATATCTGCCGACAAGGATAATGCCCAGCAGC
AATCACCCCGCGAATCTATCGAGACGCGCATGATGTGCTTTTGGAAGGACGTGGCTGCCT
CCAATGGAAGTAGCAACGGCAATAATGCAGGCAGTAGCATGCCGACCCCTACCAACATTC
ATGAAGATATGATAAAAGATCCGGAAGTCTTTGCTACCGAGTTGGCTGCCGGAGGAGCTA
CTTTGGAAGGAGCTACTGTTTGGAGGCTATTGCTTGCTTTGTTCAAGAAAATTCCCCTCC
TAGGTTCTTTGCTGTTTTCCAGTGATATTTCATCTTCTCGTCCCAGCAAATATTCTGGAA
ATCCTGAAGATTATTTGCCTCCATTTGTGGAAGCAATGAAATACTATGATTCGTGGCCGG
CATTCGGAAAGACGTATGTTGAAAACCAATTCAAGAACTCCGCCGATTACGATGCTGGTG
TGAAACAATTGACGCGACTTCTTGTCAATGATGCTTACGGTAATCTGAAGACCAGAAGTC
TCAGGCCCTTGGAAAAGGATGCCGTCGTACTCTGTCTGCTTTCCTATGAACCATATCTAG
CAGAAGTGAAAAAATGCTTTGGTGATTTAATGTAACCGACAAAGAATACAGCCGGTCGCA
TTGGAGGATTCTGTCGTCTAGGGTTATCCGTGGAGAAAAGATATTCCCCAGTGCCATGCA
AAATATGGACAGGTCATCCATAATATACAAACCCAACGGATCAACAGTGGTTGGTGTATT
CAGAAAGATGCTAAGTCCACAAAAAAACGAGTAAACGATGTCCTTCTTTGTGTCCTTTCT
GTGGCCAGCGCTATCCTTGTTCTTTTCATTATTTGTTGCTGGCAGTGAGATTATATAATT
CCCAATACTACATCAGATACATAAATGCAACACTTTTGCAGCAATTAAAAGAGGATCTAG
GACAGAGATTCTTACTTTTATTATTTTAATACTTCATCATCAATCTAATATGTAGATAAT
GATTTCATCAATTTGTGAAGATATTAGTAGGGTGTGGCTGAATTCTTAGAAACTCAAATT
TGTACGCGCAACAAGCAAGGAAATAGCTATTTCTGAGCAGTTGGTGGTATTGTGTTGTTT
CTTTCTTGTCTGCTG
>comp2894_c0_seq1
CATTTATTGATGGGTGAACCAACCCGCAAGGTACAGATAACGCATTCCATCAAACCAAAC
CAACGCAACTCTGAAACACCATCGTGACCTTTTTTGTTTGTTCGGCATCGGGTTCCACCA
ACTTTTGCGCCCGGGCATTTGTCGTCGTCGTTGTCGTTGTTCCTCCGTATCAAACGACGA
ACGCAAGAGCAAGAAGTACTTCCGCGCATATTCTGGGTCTGGGGTTGAATACAGCTCATC
CTCTCGACGCAGTGCACCAATGGTCGGTTTGACAATGCGTCGCTTGCCTTCCGAGAGGCC
ACTGAGCCAATACCGCCGTTGCGGTTTTCTCTGGACGATCCTTCTGGTCCTCCTCTTGCC
ACTGGGGACCCTCGGGCAATCCTGCATCGACGATTTCGAAGCGATTTACAAGCGGGAAGC
CGTCGTGACGGACACCTCCGTTCCGCGCCTGTACATTCTTTGCCCGCGCCACGTGTACGA
GATTGGGAAGCTCGATTTCGATGGCAACCTGGTCCAGCCTCCGGGGGGAAAGGCCGTCAT
GCCGCCGCTTCCCCTCCGGCCCAACATGACCATTCGGTGCGGAGACCAGGGTCTGCGGGA
GAATGTTTGCTGGGTCAAGGGAGGAGACCTGCACCTGGACGGGACGAAAATTTTGGGGAT
CACGGACGACTCGCTGGAAAACGTCCGCATCGAGGGACTCGTCTTCATCGGGGCCGGGGA
ATATTCGTTCTGGGGCAACAAGCCAGGATCGGTCACCTTTCAGGACTGCGAGTTTCGAGA
ATTTACGAATTCCTCCGCGCCCATCATGCTGGACTTTTACGATGCCTCGGCCCCTTCGGC
GGAACTCGTGACGACGTTTTTGGATTGCGAATTCCGGAACAATCGCTATTTTGGAAGGGG
CTCGCAGAGTGCTCTGATATACGGCAACAGTGGGCAAAATCGAATCAACGTCACGAGCAG
TCTGTTTGAAAACAACGACATGATATGGAACAACACCAGGCCCGACACGCACAGCTACAT
AGTCGAATCTCTGGGTCCCGTGGGCGTATCAACAACGTGTTTCTCGGACAACCTGGTGGG
CTCTTCCAATGTCGTGGTGTTTGGGAATTCCTTCCAAAACGAATTCAATTACGTTCGGAA
CAGCAGCGGAGGCCTTTGCCCGTTTTCTTCGGTCTTTGAGAACATGCAACAATTCGAAGA
CTTTGATCCCACGTGCGTCGCCGCGAGCCAGAGTGTTTGTAGCCGGTACGTGACGCTCAG
TCCGACGCCGGCTCCCAGCCCGAGTCCAACGGTATCCCCGGCGCCGACCGCGTCCTCGTC
GGGGAGCCCCACCCGTACCCCCCAGCCCACCATTACGCCGGTGCCCACCGGGGAACCCTC
CGCGAGGCCCTCGGGACGGCCGAGCCCCTACGGAGCCACGAACGAGCCATCGATGGCTCC
GACGGAGCCTCTGCTCGAGTTTTTCTGGCCCACCATTATGGAGGAAGACCCGTTTGCGTC
GTCGGCCACTCGCGTCGGTGCCGGTGCCACCAATTTGGCGATGCTGTGGGCGTGGAGCAC
GGCTCTGGTGTGCTGGTCGTTGTAGAAACCTCCCCAGGGGATTGCGGTGCGGTGCGACAC
GGCTCGGCTCTCACGCGTCTTTTTTTCGAACCTATTTTCCACCATCCAACCGATTGGTTC

GGCGATCGATTGCTGCGCTGTGAAATGTTCCTGCTGCCTATTGCTTGTTTCTGGTGCTCT
GCCCTACCGCAGTGGCATGGTGATTTACCGTCCCCCGCCGCCGTCTGCCGCTGCCGCTAG
GTATACGGCCGTCGCGGCAGTGGTTCTGGCATGGACGGGCACGGTTCTGGCCGGTTGGTC
GTTTTGGTAAACCGTGGCGATGCTTTGCTGTTGTGGCACGGCCCGAACAGCGGGTGAGGG
CACGGTGCTTTGTGCGGCACGGCGGGTCCGGAACGGTTGGACGGCGGGCAACCCCGGAAC
AAGGCTTGTTCCCACCGGCGGACGAAAAGGAAAGAATTACTATACTATTATTACTATATT
ATTTTTATTATCATCATTACTAGCAATTGAAAAA
>comp2895_c0_seq1
TGAATTTCCTATCGGAGATCTCAATACACCACTTTCTTTTCCTCCATAGCCCCTGTTGAT
AACCATCTGATACACACAAGGACCAAAGAACAATCGCATCGAGAAGCTGTAACAGTCTAG
ATTAGAAGCACCAGGTCCCGTTCGCGAACGAAACAAGAGACAGACATACCGTACATTCCC
TGCCAGCAGCAACAACAAAACAAGAAGAAATAGCACCAAACCCAGCAATCAAAGCAGGAC
AGAAATTGCCTGTTTTGTTTTTTACCGCTGCTGCTTGCGGAAGTCTTCAACAAATTCAAT
TCCGAATCCAAATTGACTCATTAGGTTTGATCGATCGATCGATCAATCCATCAATCCATC
AACCCATTCATCGAACCTCCTACAAAGAATAATAGAATCCTATTGGGAAACATACCGCAC
CCGATTAGAAGGATTATTTCTGGAGTAGAAAGGAACCAAAGCAACAACTGCAAAACATCG
GATAATCCTAAGCCGCGATATACGTGACGATCGATCCTTGGCATTGTGATTGGCAGTATT
AGTTTTGTAGCTTGTGCCCATTTGAATTACCACAGTGGCCAAAAACTTCCAGACTAGAAG
CACACAACACAAATATACACCTGTTCAATACAATGTCGTCTTCTACCAGCGCCAGCGACA
CATCCGACGTTACGGAGACCATCACCAAATCCAACATCGACGACATTGTGGAATCCCTTT
ACAATAACCAAGATGAGCTCATCGGATATTCCTGCAAGCAGATAATGGAGAAAAACACGG
TCGAGGAGATGGATCTGGTCTTTTACATGGACGTATTGAGCCCAAAGGACGACGAGAGCG
GCAACAGCGACACAAACTACCAGCTAGCCGCAATCGATGCGACGCAGTCGGTCGTGTTGG
AGGAAGTTGCCCTGGAATACCAGATCGATCCCGACATTTCAAGGGGGGTCCGGTGCTTCG
ATCTACCCGTCGACGGATCGACATGGCTGGTGCAGATGTCGATCGAGAAGAAGGACTTTG
TTGAGGTTACAATTTTTGGTGGTTGCCGGAAATTGGAGTGGGATGATGCAACGCAGGACT
GTAATTTTTATGAAGCCCGCCTCAAGGGATCTTACATCGGTGCCGTTCCTGTAGCCGACG
ACGGAACCGAATTTTCCGATGTCGTGGGCTTCTTGAAGTCCCTGATCAACGGTCCCACTA
TAGTGGACAACCTAAACAACAATGGCGACGGCGGAAACGGCTTGGACAACATTTTTGGCC
AAAATGCGGCGAGCGACGCCTATAAGATGGCCTTTCTGGGGGTCCCCCGGGCAGAAACGA
ACCCCAACTTCCAGGCCGAGGAGGGTCGAAACGACCTTAAGCCACCCGTCAACATCAAAA
CAGGCATTGATGCGAGTCAGAGCACAGCTCAACCCAACCGCAACACCATTACTGTGGTAG
GGGGGATGCTAGTGGCCTGCTTCTCGATTGCCTTTTTGATGGTGGCATACATTCTCGTAA
AGCGCCGACAGTCCTACCGAAATGAGAGGAACGAGGATGAGGAAGAAAGGAGGAGCGCGG
AAATATCGATGGCGATGGCCTCGGGAGCTTCCGCCGGGAACTTTCACCTCGACACGGTGG
ACAGAATCGACCGCAACGACGAATACAATCACGCTGACGACGACGGTTATGGGTACGACC
GCAATGATTATGACGATGACAACGATGGCGATGACGATGATGATATTCGCGAGGACCAAG
CCGGCGGTGGACGAGAGATGGATCCAGAGTCCTTTCGGGACGACGATGCCGGTCCAGAAA
TGCAGCAAAACCACCTTCAGCACGAATCTTTAAGGTCCGCACAACGACCGCAACAAGAAT
ACGGTGACCATCCCATGTCGGCCGAAGCCATCCAGATGGACTTGGGGAATACCCTCAAGG
GCCAGATGATTGGGTTGCACGGAAACACCTCGAAGAAGGTCCTCCCCATGTGTGGCCCTT
CAACGCGGGGAGGAAACGGAGGCAACCAACACAAGGTGCAACGTCTCGGGAACCGAAACG
CTGGGTCCTTCTTCCACCAGCTCAGCGACGACGGAGACCACGATTCGTTCGACGACTCGG
ATTCGTGGGCCCAAACTGATGGGACCATCGGGTCGCTGGAACTCCAGCTAGAACCCATCA
CCGCGGAGGTATAGTTCGCAAACATACATTCTATACTTTGTTCATACCACATCGGTACTA
CCGTTGTGCTCAATGCGGCAATAAAGGCAACATACTTTGACAGGAATGCTGCTCAATGTG
CCTCGTCGGAATTCCATTGTGGCGCACATCCTATTTTGTCTATCAATATGATGCGATGTT
AAGCTCTTTCCGTTTGAATAGTGTGTGCACGGGTCGTAGGTTGGTAGGTCACGTTCTCAG
ATTGAATACCACATACCCGTGGTTTTTCTTTACACTTCACAGAAAACATGCGAAGAGGAA
CTACTATAATAAGTTTATCTTCCTATTTGCGTTGTTAAAGCCAGAAGAAAAAGAATCATA
TTAAAAACTAATGCTAACAATCAACACAGACAAGAAGAATATACTACTAGTAACACCTGC
TGATGGAGGAGTTGATTCGTCAATACAAACAGCAGGAGGAGGCAAGATCAGCAGGCCTAT
ACCTAATCAAAATGATATTTTCACTTCTTTTCTTTAATC
>comp2896_c0_seq1
GAACAACCGGAATCTCCTCTCGCTCCCATTTTGGCAAACCCCGTGCTTCCAGCTCGTCGA
AGCGTCCTTCGCATCGCACCAACACCGCTCCGAAACTCCATGACGGCCATGCTGCTGTCG
CCTCCGTGCTCGCACGAACTCAAAACAACAAGCGATGCGTGCGGAACCGAGAACGACGGC
TCAAGGAGACACGCCTTGGGCATCGAAATCGAACGGACCAACAACGAGTTCGCGGAGGAA
AGGTGCCGAGAGATCCTTTCCCACTACGAAGAGGAATCCCACCGTCCCGAGCACGAAGAC
GCGCGGTGGCTCGTCGGACAGCTGGATCGAGAGGAGCAGGAAATCGCCGCCCGGTGCTCC

TATGCCTATTGGTATCTCTCGCGGCGCTCGCTCGAGGAACCTTCGGCGCAAGCCGTCAAA
GATCCCGCCCGGTTTGCGAGCGCCATGCAGGAGGCACGCCGCCACTTGGACTGCGCCGAC
CACCGATCGGAGGTCCTAAAGGTCTTTCGGGAGACCCTGGACTTTCACCGGTCCCACAAA
ACAGCGCTCTACCGAACCTGCATGGTTGACGCTCCCGGCGCGGACAAGGGCCTGTCGGAC
TCCGAAAAGGAACTGAGCAAGGGACGCAGGGAACGCATCGGCGACGAAATGAAGGACCTC
CAAACCAACGTCGTTCGGGGCCACGACCGGGAGGACCGCGCGATTTTCTTTGCCTTTCCC
CGCCGAAAAGCCGGGACCTCGGAGGACGCCTTTGTGGATTCGCTGGTGTACACCGTGGAA
CGCGCCGCGGCCTCTTCCGAGTTCCAATCGATCGGCCGCCAGGACCAGATCGTCGTCGTT
CTCGATACCAAGGGGAGCTCCTGCCCGCCCTTCAAGACCCTGCAGGCCGCCGTTGGAATA
CTCCAAAACTTTTATCCGGGACGCCTGAAAACATGCGTGATTCTCAATCCCCACTACGTG
GTCACGGGAATCTTCCGGATGATCAAACCCTTTATGCACCCCCTCACCTTCTCCAAGTTT
GTGCTGGTCAAAGCCAAGCGACAGAGCGGCATTGCAGAGGAAGACTCGGTGCTGTCCCGG
TTGATCGACGAGTCGCAGGCCATGCCCGAAATGATGCCGGGGCGTGGGAAGCTCGCCTCG
AAGGTAGACGTGGACCGATTCTTGTACAAAGTCCCTTTTTATCGGCTCTACGATTGCACC
GACCCCGAGCACGACCCGAAGCTGCTAGACAGGGAAACACACTCGACAACCTCCACCACG
CTATCGCTGTCGTCGTCGTCGTCGTCCCTGTCCCTGTCGTCCGATGCCGCCGACGAAGAC
GAACACGAATGCGACGAGGCGACCGAACCGTTGGCAAACCTTGAAAGGGGAACCACCAAG
GCCAAACCCACCCGAGTGGCGCTGCGTTCCCTGGCCGTGGGGCAGCTCTCCGTGCGAGCC
AGCCCCGGATCTCCCATGGTGGTCGATCTCAGAATCGTTGGCTGAACGACACGAATGGCG
GCACGACCGGACGAAACACAAAAAAAGACGACACCGTGCACCAGAAATGCTTTCATCGAT
TTCACTCGGGGATAGATACAACTTGTTCGAAATAGATAGGAACGATTATTTTTTGCGAAC
AGAACCCGCAATACGAGTAGTTTTGTACTAGTGCATGCCTGACCGATTATCGGGGGGAAA
TTCGATGCAATCAACGAAACCCCAGCCTTGCTCAAACCCATACCCAAACCACCCAGCCTA
GCTCTGCATCGTACCAAAACAACCATTACAACCCTGTATTTCTTGAGGGAATGCATCCCG
AGTATTTAGGTTTGTATCCCAATTTGAAATGGAAGATGCTCCCAAGTAGCAAGCAACAAG
CACCAAAGAAACAAAGTCTGTACAACTATTTGTAGTAACTACTATGAGTGTAAGGGCAAG
CAAATGCACAAATAGTCTTACAGAAGCAAACACAAAAGGAATCCCAAGAAGAAATAGAAT
GTTCCCGTGTCGTTAGTAGCACACCACAGG
>comp2897_c0_seq1
TTGAAGCTTCTGCCATCACTAGTGTTCTATTGAGGTTCTCATGTTGGAAATGCTGAAGAT
CAACCTAGTCTTTAGGTGTCAAGTAGGACTACTTCGCTAAGAGTGATCATATTCGCTGCA
TGACGACGGTCTCACAAAACCGAAATACAAGAACATAAGAAAGAAAAAAGTAACAAAACG
ACCAACAAACGAAATATAAAATAGTAGTGTCCGGAAGAGCAGAATAACCACTAGATTTTC
ATTTCATAAGACTGTACCGCAATGACGACCTTCCAAGTAGCATCTCGTCGCTTCTTTTTG
CCAGCGCTTCGTCGGAGCTCCGTCACTCTCAACACAGGTCGACGTACAATGATGGCATTT
GAAGACCACGCTCGTCTAAGGGGAAAATTCGAAGAAGACCGATACCTGAAACAACAGGAA
GAGCAATGGCGCCAAAACTTACAGGCCCTTCGAGCTCAAGAAGAAAGCAAGCGCGAGAAA
CGTCATCAGGAAGAGGTCGTCGAACCAGTCAAAAAACACCTTGAAGAAATCTTGGCAGCG
TCTGGAGACAAGATTTCTGACGAAGGGCTGGAAAATCTTGCCAAGTTCCGTCTAAGTCTC
TGAGTGTCACTAATATATCTGGTTCCGGAACCGGGAGGATGTTACAGCGCTGTAGATTGG
TAATTCAAGCAGCTCGGAACTTACTATTCCACCATTCTTAATCTTAGTAAACCCCGAAGA
ATGATGCAGTCTCTTGTTTTCTTTCCACCATACTGCGTCACTTAAAAATGGTTGTGTCCT
TTTGAGTTTTCGTGTCTCGTATCTTCTACTGGTAATTCAGCTTGCAAAGAAATCGTATCC
CCGCACAATTCACTTGGGGTTCATTCCAATCGAATGACTCCGAACAACCCAGGTATATTT
ATTTGATCACAATTGATGGTTGTTGCATTGTCCGTCAAGCAAGACAAAAACATCTAGTCG
AAAATATTAGTTGTTTGTTTTAGATAATGTAAAGATTTCATTGTTATATAAAAA
>comp2898_c0_seq1
GTTCAAACTTCTATGGCAAAAAATAAAGGCACTCGAATTTTAATTACTTTAGAATGTACA
GAATGTCGTTCTAATTTAAATAAGCGTTCACCTGGTGTATCACGATATACAACGCAAAAA
AACCGTCGAAATAACCCAGAAAGATTAGAGTTAAAAAAATATTGTCCAAATTGTAATAAA
CCTACTACACATAAAGAAATTAAATAAGTTATGGCAAGACAAAAACAAAAAATTTCACCG
ATTAGTCTAAATCAAAAAATTGATTATAAAGATATCGATTTATTAAGATTATTTGTAACT
GACCAAGGAAAAATCCTTCCACGTCGTGCGACAGGAGTAACGGTTCAACAACAACGAAAA
TTATCAAAAGCAATTAAAAGAGCTCGTATATTATCGTTATTTCCATTTGTTGCATCAGAT
>comp2899_c0_seq1
CAGAAGAGCGCCAAAGAAAAGAACAGCAGCGGAAAGAAGTAGAAGAGCGGCAAAGAAAAA
AACATCAGCGGAAAGTAGCAGAAGAGTTACAAAGAAAAGAAAAAGAGCGAAAGGAAGCAG
AAGAGCGGCAAAGAAAAGAAAAAGAGCGGAAGAAAACTGAAGAGTTGCAAAGAAAAGAAA
ACGAGAGAAAGAAAACTGAAGGGTTACAAAGAGAAGAAAACGAGAAAAAGATATCAGCAG
AGTTGTCAAGAAAAGAGAACGAGCAAAAGAAAACTGAAGAATCCCAAAGAGAAGAAAATG

AGAAAAAGGCAGCAGCAAAGTTGCAAAGAAAAAAGAACGAGCGAGATAAAACCGAGGAGT
TGCAAGGAACAGAGAAGATGAGTGTAATGAATGAAATTACCAAGGATGTCTCAGAGAAAT
CTCCTGCTACTACTTCTCCTAAGTTGCAGTCAATTAATCGGAAGATCGTTGCGATGGACA
AAACTGTATCTGATGCTCAAGCGATGCTGGCAAAGGGTATGATTCGAGTTTCTTCGGTCG
AAAACAATGTACCTAGTCCAATAATGAACTCTGACGTACAAAATTCAACGAAAACTAATG
GGAAAGCTTCTTCCGACATTACTATTTCCGCCGTGCGCAAAGGATGTCTCTCGGCGAAAA
CAGTGGTTCCTAGTAATAAGGTACAAAATGCAGTAGTCGAGGGTTCATACGCAGATGCTA
CAAGAACTAATGCTTCAACTAATCCCGTTTCTTCGGAACCTGCGGAAAAGATATCAGATA
TAGATCGAGACGATCCCAAATTGACCAAAGGCACAGTTGTCATGAGTGACTCAAGTTCAA
ACGACCCAAGGTTGCCTGAAAACAGGGCATCCGGATCATCTGAGTCAGAAGAATACATTC
CAAAAGGAATGGGCAGCAAAATAATGGGTAGGACAACCTCGACTCTTCCGAATCCAAATT
CTATTCTTGCAGGGGTGAGTAAAGGTTTACAAAGTTTGGTGACTGGGAGGCTAGCTCAAG
GGTCCAGCTCAGATGAGAAAACTCTCTCAAGCGCCTCGGAGAAAATTTCTTGAAGGGGAA
AAGCTGGCAGCGATAACCCAATCCCGATGGATTTGGATTAAAATGAAGAGGCTGATATCT
CTGGGAGAAAGCTTTTAGGTGCGTTAGTTTGTTATTTCAAGGCAGTGGTAGATGTTGCAC
TTGAAAAGAAGGAAACAAAAGCACTTTAATCAAATTGATCGAACATGGGTGACAGGTGAT
ACTGAAGCAAAAGCTGATACTGAGTGATACAGTATTGAAAAATGTAACGATTACGTCACA
GGGAAAGCATAAGCAAACAAATTGCAAGATCATAATTGTATCGATGTTAATTTTCAAAGC
CCTATTTCATTGCAATTTGCAAATCAAGTACTATTGATGAACAATTGTGAGAGCCTGTCT
TGATTGAGCTTGAGGGGAAATAGAACCTACTTGTACTGATCTCTTCCTAGACAAAAATGT
TTAGAATCGCTTCTGACAATAATTAGACAGCCTATTTTGAAAATGACAAACATTGATCAT
CTATTGAGAAATAGTCAATAATGGCATTTAAATTCTTTTCCTGGTAATTGTAATAAAATA
CTGTATGGTGAGTACTTGGCATCTGCTGAATAGGCACTGATCTCTCAC
>comp2900_c0_seq1
CTAATTTTGGCAAACGGTTCACTAGCCGACTGCGCACAGCATAGATAATACACTCTCAGT
CTCATTCAGTTTGTTAGTGTTCTAAGTAGCGAGCGAACTAGGACCTCATTTTCTAATCCT
GCACTCGTGACGTTAAAAATCAAGATTGCGTTCTGACTACTTGCGTTTGCGTAAAACGTC
TTGGCTTTCTCGCGTCAGTGTTCAGAGCAACCCCGGGATCCGCTCAAAGGGTACTTTTCG
CCCTCTTTCCCTAACCGATCGCTCATCTGTATCCAATAGTAGGGAACCCTTTCAAGATGC
AGCCGAATGTCAATGGAACCACTGGAACGAAAAGTAGAGAGGCCAACATGGAATCTCAGC
GGGGCAGAAATTTCACGATACCACAGCCTATAGCGGAAGAAGACCAACAGCAACAATCCG
AAAAAACGAGTGTTAGAAAGAATCTCGAGAAGGCATCTTTCATGGATTCCGTGAAGAAGT
TTTTTGAACCTTGCGTCGGTGCTGTAGATGCTGCTTCCTTCTACATTGGCGAATGCCGAC
CTTCGGACTACGAAGCGTGCGATCCCAACCAACAGGACAAGGTTGCCGAAGACGTGATAA
TGAAGCTGAGAGAACGCAACGGCGGCCTCAAACGAAGGAACGAGACCCTCGAAATCCCAA
CCAATGGCATGTTGTTCGAGGACGACGATGTTTCCGCCATATCTTCCCACACCCTCGAAG
AGATGGAACGGCTGAGGCTGGCCCAAAACAGCGGAATCACGAACTTTCAGATCGCTCCGG
GCAAGCACCAACCCGGAAATATTCTGCACCCGAGGCCGACGGACGATCCCACAAGCCCAC
AAGACGCCGCAGACCGTTCTGACGCGGTGAAGTTTCGAAATTGGAGCACCCAAAAGACAC
CGAGCACCGCGAAACCACAAGAAAGGGGCACCAACGAAGGGAATATCACGCTCTGTGTGT
CCGTTTCGGACTCTTCGAGCAACGAAGATGGCGAAAAGGCGTCACCGAAGGCTTCCTCGC
CGAAGGAATTCTCACCGAAGACATTCACATCGCCACCCTCGTGGTCAAAGAAGAGCGGGA
AAAATAGGGCCTTCAAGACACACCCCGTTGGATAGAAGAAGGACGGAACAAACCTATTCC
GTGTCGTCTATCCGTTTCAATTTTTGAACCGCCGATTTGTCCGAAACGAACGAATCGGTG
CCATCCGCGTTCTATGATACCAGAATTCGTTCCATTCGGAAATTCCGCAAGATCTGATCG
ATAGACTTTTACAAAATTCATACAGTCCAACCCCTCACGAACTACACCATTACAGTATAC
ATATACGTACATTAAAATTGCATGTAAGATTGAAATCATGACGAGCAACATGCACACAAG
ACCGACTATGGGTCCAGCTATTCAAGCAAAACGCAACTGCATCCGAGAAAAGATGCAATT
TTCCTACATATCTCAGTTGAAACGTTTCGAAAAACAAAACATAAGGTACTAGAAAT
>comp2901_c0_seq1
ATCGATTTGGTTGCACGTTGACAGTACAACTCACAGTCCGTACTTGTGCCACTCCATACC
GAATACTCGCACCGTCTCAGCTCTCGACCCGACAGTACGCACAATAGAATGATTCATCGT
CGTTGCTGCCATCATCGTCACGATCCATTATGAGCAAACCAAACGACGAGACCTACAGCA
ACAACCACGGTGCCCCCGCGGAGCTTCCCGCTCTGGGCCAGCACCAGCAGCCTCCCTCGT
CTTCCTCGGGAATGCGGCGCGGAGCTGCGCCCGGCAGCGGGGGTGTCCTGGTCAGCAAAG
CCGGCGGGGGCAGCAACAACAGCAACCAGAGCATAATGCCCACGGCCTCCAATGTTTCGA
TCGGAACCTCCACCTCGCAGCAGCACCAGCACCTATCGGTCGAGCGGAGTCCCATGCCGC
CCATGATCCTGCGGAGCCTGGGCGATCGCTCTAACGAAAAACGAAAGAACGCCGCCCTCG
AGATCGAAGCCCTGGTGAAGACACTCCAGGAAGCCAACAATTCGCTCATGATCCAGAACG
TCATCGAGATTCTCTCCAAGGATTTCTGTACCAGCATGAATTCGAACTATCGGAAGGGCG

GACTCGTCGGGCTTGCGGCCACGGGGATTGGCTTGATGGGACACTCCCGGACCTTTTTGC
CGGTCTTGCTGCCACCGGTCTTGCATTGTTTCGACGATCCCGAGTCGAGGGTGAGGTACT
ACGCCTGCGAGAGCTTGTACAACATTGCCAAGGTTTCTCGGCAGGGGATCCTACAGTACT
TCAATCAAATTTTCGAGGGGCTGACCAAGGTCATAGCCGATGTCGACGTGGACGTTAAGA
ACGGAGCGCAGCTACTGGACCGTCTCATCAAGGACATCGTCACGGAGGCCGACGAGGCCT
TTCAGGTTGACCAATTCTTGCCGCTCCTGCAAAAGTACATTCAGAGGACCAACCCATACA
TACGACAACTCATCGTTGGATGGGTGACCCTGCTGGATTCGGTCCCCGACATTTCCATGC
TCGATTACCTGCCCGATTTTCTAGACGGTCTGTTCAACATGCTCAGCGATTCGAACCGGG
AGATCCGGCAGGCCGCCGATTCGGCCCTCTCGGAGTTTTTAAAGGAACTTACGAGCAGCA
CCGTTATGGAATTCGGACCCATCATTTCCATCCTTGTTCTTCAGTGCCAATCTAAGGAAC
GCCTGAACCGTCTCACTGCTACTACCTGGCTTTCGGAACTCATCCACCACCCTTACTCCG
GCGGGGATGCTCTGCTGCCATACAACTCGGAAATACTCGGGGCAATCTTGTGTTGTATTT
CAGACGGAGAAGTGGAAATTCGCATCGTTGCGGAACGCACAAACGATGATTTGCTGAGTC
TTGTCAAGGGAACGTCTAGCCCCTTCGAACTTCGGTCGCTGCTCGAGACGCTGACGAGCG
AACTGCTCAACAAGGAGGACGTCCCTACCAAGATGGCGGCCCTGGGGTGGATCAACATGC
TCATGGAGAAACGAAAGGGGGACATGAACGCCTTCATCGAAGAGCTGCTGCCCGTTCTCA
TCCGGACCCTTTCCGATCCGAGCGATGCGGTGGTTCTCCTCAACCTGCAGGTCTTGTCGC
GGATTTCATTGGCGCAAAGGGACGAGAAAGGCGATGCCGAGGAATCGGCCGAGGAACAAT
TCGAGCTGGTCCTCAATGCTATTCTGAGTCTGTTTGCGAAGGACCGCCAGCTTCTCGAAA
CAAGGGGTTCCCTGATAATTCGAAAACTTTGTGTTCTTTTGAATGCCAAGAGTGTTTACA
TACGGTTGGCCAATGCATTGATGCAGTACGAAAAGGTCAACGAGAAGATTGCCGACGCTG
GAACACTGGAATTTGTCAGCACCATGGTGCAAACACTCAACCTGATTCTTCTTACCGCTT
CCGAGCTGCACGATTTGAGGTCGATTCTATCCAAGAGCTTCACAATACCGCCCGAGAACG
ATAGCAAGAAGAATAATCCCGAGCACGATCAGAGGAAACGGCCCCAAAAACAAAAACAAA
AACAATGGTCGAGGCAAAAAAAGAATTTTATAGAAGATAGTCTCAAAGTTTTTGTGATCC
TTTTCCGGTGCTGGTCGCACAATCCCGTTGCAACGTTTTCCCTGTGCCTGCTTGCACAAG
CGTACGATCTATCGTTTGCTCTTGTGAAGAGGTTTTCCAAAATGGAGGATGTCTCTGTCG
GGTTTTTGATGCAAATCGACAAGCTAGTATATCTGTTGGAAAGCCCGATCTTTGTTCATT
TAAGATTGAAACTTCTCGACGTTGAATCTGAAAGCCATGCCCAGTTGCTGAAGAGTATTT
ACGGGATTCTTCTTTGTCTGCCACAGGGGGATGCATTTCGACTTTTGAACAATCGGCTCA
CAACCGTATGCAACCTACGGGATAATCTTGGTCTGACTCCAATGCCTGATGCACCGAAAC
AAGATGACTTGTCCTTGCCATCTACAAGCACCGTTTTCGTTGGTTGCTTCCCCTTAAGTT
TGGATGCGCTATTAATACGGTATGACGAAATTTTAGAGATGCACAGAGTAGCCCAAGAAC
AATACGACAATTTTGTTTACGATGAGCAGCAACGACTACAGATGCAGCTAGCACCTGGCT
CGCAGCAGATAGGTTTGTCGGGTAGCAGCCAAGGTGGCCGAAGTCATCAAGCGGTACTTG
CCCATGGCCCATTGAGTCCGCAATATCAACAACACGAAAGGCGTTTGTGGGATTAAATAT
ATTCTGATTTTCGATAGATACATAATTTGAGCAATAAATTAAATTCATGTTCTTTAC
>comp2903_c0_seq1
GGTTCATCACTATCAAAAAGAAGACATTTCGCTAAGCGTCACAAAAAATTGAAAGACTCG
AGCTGTCTGTGGAACACCACAACTGGCCATCACCAAACTCAAATACAAGCAACTCTACAA
TCTCCTCTAACGAGATTGTTTACAGAGTTTGAACGTATTATAAACCGTCATACGCATTTA
GTCATCAGACCATGTTTGTCACGAAGAAATTCGCCCTTTTCATTGTGTCCGCCGCCTTTT
TGGCATCCTCCGTTAGTGCTGGAGGCGTGCGCGGTGTCCCAACCGAGGACTCAGCACCCG
CCAATCATGGGTGGGGTAGAGAACTGAAGAAGGATCAAAACAAACCAAACAAAAATCAGA
ACAATCCTGCGGTCGTTACGAGGCCTGCACTACCACCACCTCCAACCTCGCCTCCTGTTC
CTAACCCCATCGATGTTCTTGTAGGCAAATGCAGCAACCCAACCTTGACCAGTTGCGTAC
TCAGGAGCGGCGCCAACGAGAGAGTATGCTTCACTTGTCTTTATTCCATTTCCCGATTGT
CATCGGCCGGCGATTCCGCTGTCGTTAACTGTGGCAGAAGCCAATGCGGTGGTTGCGTCA
ACGAAGCGAGAGACACATTCGGATGCGCTACAGGAGGTAGTGCGCCAGCCCCAGTTACAA
CTCCAGGTGTGCCAGCTCCTGTTTCTGTTTCAAGCCCCGTGCCAGCTCCCGTGCCAGCTC
CAGTTCTTGAGGAGATTGAAACCTTCGATTTTACCACCCAGGGATGTCCCGCTCTAAAGC
CAGTAGGCGGAGCTGTTTGCTCTGTACCTGCGCCGTTCCAGTTCCAACAATGCGACTACC
CAACGGGATCAGGTGTAGGAGTACGGTGCATGTGTGCCTATGGGCAGTTCATTTGCAACC
CTGCACCTATTTCACCTACTCCAGCACCGGTGCCTGCCCCTACCGCTCCTACCGCTCCTA
CCCCTCCGGTTGCTGCACCTCCTGGAGTTTGTTCTCCAACTTTGCCTAACAGTGGCGACA
CCTGTTCCACTGGAGGTGCCCCTTTCGCCACATGCTGCTACACCGGAATCAGCAACAAGC
CAGCCAATTATGCCCATATTTGCAACTGCCTCAGTGGCGAAAACAGATACATCTGCAACG
GTGGCGTTTCTAGCCAGTGCACTAGTGTCATCAATGTAGTTGCTTCTTCTCCGGTTCCGG
CTCCGGTTCCGGCCCCTATCGCCACCATGCCCACTCCGTCTCTGGTTAACGCTCCCGCAC
CGACCGGCTCAAGCTCCTCCATTCCCGAATTCTGCCTAGCCCTGCCTGGTGTTCCCAAGG

ATGGCGAATCCTGTGCAGGTGTCTTGCCCGCAGGAATGGCAAGCGGAGGCTGCGGAGGAG
AAATCATCGAAACAGATAACATGAACGTAACTACGGTGACAAATGCTGCATGCACCTGCA
CTGCCACCGATCCTGCACCCGCGTGGTCGTGTGTGAAGATTAGTGCCGTCGCTCCTCCAA
GCTCAGGATGCCCTCCCCAAGCTTCCCCACCCAATACTGGCGACTCTTGCGCCACAGTCT
TGCCAGCAGACTCCTTCTTGACCTCGGTGGAATGCAAGTACTCCAAGACAGTCACGGACA
GCAGCCAACTCTCCACAACCACCGAAAAATTCTGCACCTGCGAGAAAGCTACTCAAATCT
TCACTTGCACCGATGCTACATCGCAACTCATTCCTCCTGTCTCTATCGAAGCCCCAGCCG
ACCCTGCTCCAGCCGATCCAGCCCCTGCGGATCCAGCACCCGCTGACCCCGCCCCAGCCG
ATCCAGCAGCGCCTGCTGCCTCCTCTCCGACCATCGTCCAGGACGGTTGCCCCTCTACAA
TTACATCGGGTACTCCCTGCAAAGCCTTCATCCCTGCTGGTGCGAACGAACAGTCTTGTA
TGACTTCTTTGACCACGCAGTGTGATTGCCCAGGTCAAGAATCTGCCGATCCCGTGTGGA
CGTGCCGCACTGTCCCTTAATCTTGTCGAGGGAAGGACGGGGGCCATCAAACATTCAACA
ATGATAAGTGTCGAGATGACTGATATCCCTCGAATATTCTCGAAACGATGACAGAAGAAG
ATGAGTGATTGTGTCAAAAAAAATACAAAATCAAAAAGGAAAAAATATTTGTGTGAAAAG
AAAGTAGTAGAGATACCGAAAATACTGGCAGAGAGCAACACGGTGTGGTCACAAACACAG
TTGAAAAATTTCTCTGCTGTGAAAAACAAGTTTCTTGCAATTGTGTGTGTTAATTGCTTC
AAATCTATTTTTATCGTATTTATTGAGAGCAGTTTTTCGAATTTTCGAATGCCGTCTTCA
CGACGATACAAATAGTCGTAGAAAACGTCTTTTTTTCTTTGAATGCACCGCCGTCTCCAT
GCGAGTTGTTTTGCCTATCACGGTTTATGATTGCTTCTTCGCGACTACATTATCATTATA
TTCTGAGAGCACCGAGTACATTCCATCTACTACATGTTGAGTGTCATCTCCTCTAATCCC
TTAACCTTCGACCGAAAGCCTCCGCCGAAATATCTCCAGATCCTCGGCGACGACTTCCCG
CTTAGGATTGGTTTTTCTTGACGACGATATCATTAAAACAAAATACTGTAGTACTCGGAG
CATTCTTCTCCCTACTCCGGAGAGGCTCTGGTTCTGGCCATGTAGTGCCTCT
>comp2904_c0_seq1
CCTCTCTTAAACCATGATACAGTAACTGTTGCAATAACTGTTAACTTCTATCAAGTCCTT
CTTGGAAGCCGAACACAATGAAACAGGAGACCGATTTATGATTCGAAGTGAAACCTTAAT
ATCTTACAACACTACAATCGATAACACAGTAACATCACCACAGAAGAAGGGTAGGCGAGG
CGCTGCGCTGTCATCCGAACACGATCATGATAGACCAGACAGATGTAGTGAAAAAGTTTG
CTTCGCTTATAGGCACAAAACAGTCATCAACAGAATCGCAAGTTTCTTCCTCGTTGCTGA
GAGCTGAGCCCAAGGCCTCGAATTCTAGAGAAATGTTAATGTGCATTTCCAAATCTGTTA
CAATCGACGAAGTAGTTTGTACGCTGCCATCTCTTGACATGAGGAAATTGGATGAAGAAC
CAGATTCAAATCCGTTGCCCGAAGTGGGAGAACATTTCCCGATGAGGATGGAAGCTTCAG
TTCTTAACAATGCTGCTGATGCCTCTGCTAAAGCGGCTCAAGTAGAACAGAAAGCAGCAC
AGACGCTTGGATCATTGACAGCTTGGACTAGGTCCTCGGTGATTTACGCTTCGAAGCAGA
TATCCGAAAATGCCGCCGAGTCATTTTCTTCGCTGGTGGACTCTCGCGTCCGAGCCTGGA
CTCTCCTTTTGTTGAGGCATTCGCTTACAACTGGGGACGACGCCAGTCGCTCTCGGCTAC
TTAGTATGCTTGCATCCACCATTAAAGTAAATTCTGCAGAAACATCCTTCAAACCCCAAC
CTCTGCCGCCGAACTTAGCGAACATGGCTAAGAATGAAAATGATCCCGATGTCATACTCC
CGTTGATTTTTGAAATCGACTTAAAGATAAGTGTGCGAAACAACGATTGTACCGTAACAC
TAAGAGCACCCGGTACCATATCAGCTGACTTCGATCGCCTCGAGAACAAAAAAATGGGGA
TGGGATTAAAACGTGTTGATGTACAATTGAATACTGGAACACTGCTGGAGTCCATGGTGA
ATGAGGCACGGTTGGTTGTGTTCAATGCAGTAGAGGCTGCAGCGAAGTCAAACTCTGCGA
CGAAAGGGAATGAGCACAATAGAGTTAATCCTTCTTCACAGTTGTCGTCGGGATTTTCTA
GCTTTAAATTATCCGCCCCTTCGACGAAAGGTTCTAGTGGGGGCCTTCCGCCTCCCTCTC
CACAAGGGGGAAAGCATCAACCCTCGCATCGTGCTTTGAAGGCACAATCTTCATCGTTGA
GGCTTGGCAGTGTTTTGTCGTCTTCCACCTCCACTTTTCAGAAAACAAGATCTGTTCAGT
TCAACAGCTCAGACTTGAAGCCGAAATCAAATATGATACCGACGCCCAAGCGACAAAAGT
TGGCGACAGCTGCTCGCTTGACAAGCATCAAATCTTTTGGTCGACCCCATGGTGGCGATT
TTGGGGCGGAAGGTGGCCCGAAAAATGCAACATTTGGAGACTTTGGCGGCGGTAGCCAAA
GGTTCTGGGGGCGAGACGGAAAGCTTGCAAGCAAGCCGAAACCGATGCTACCCTCCGAGA
CAACCGATATGATGACTTCCCAAACACGAAGAAATGCAAACGCTTCGTTTGATATTCAAA
AGCCTCCTATTCACTTAAGTAGCGGGTCCGATGTTAGGATGGGTCTAGGAGGAAAGAAGA
AGCCACCAGCTGAGAATCAAGTTCCACCATCCATTCCACGGACTGCAACCGCGCTTGAGG
CCATGTACATAAAGAAGTTTGGCGGGAAATTCTGAATTTCATAATAGAATAGGAAAATAG
TGAGTTGTTGATTTGTGCATATTTTTGTCAACAGCATTAAA
>comp2905_c0_seq1
GTCAAAATTCTCAAGACCAGAAGGAATCGTCATCGTCGTCGACCAAACAGCATACAAAAA
CATAAGTGACAGTTCCAAATGGGGACAACTTCTACGAAACTAGCAACGTCTGCACCGAAA
AAAGGGATGAAATCCTTCTATTCACTCTCCGCCGTCACTGGGGACGGTTCTATTCAGAAA
ATGGAAGAATTCCGTGGGAAGGTCGTGTACGCCACGAATGTTGCATCCAAGTGAGGTGCG

ACCCGCCGCGAGTACGCTCAGTTTGAGAGCTTGAATGAGCGGTGGGGTAAGAAGCTTATT
ATTTTTGGATTTCCAAGTCGAGAATATGGCTGGCAGGAGTTCGACAATGACGAAGAAATC
AATGAATTCGCAAAGTCAAAGAACTTCCCTGGAATCATGATGAAGTTGGGCAAGATCAAG
GGTGACACAGCTCCTGAAGTTTGGCGTTTTTTCAAAGATGAAACAGGCGCCGGTGATCCC
ATGTGGAATTTCAAAGGAAAGTTTCTTGTCAGCAAGAACGGAGAGGTATCTGTGCCGACG
GATTTAGAAGCTGACATCGAACGCCTCATCAACGAAGACAGTAAGGAATAATTCTGTTGG
CAAAGAGATGGCTCTGTGAAAAAGAACGCACCCTCAGTATGAATCACTGCTAATGACAAA
GAGCAAAAGAACAGCCTCGGATGCAAAACGATGCAACTTTTCAGTCCTTTCGAAAGACGG
TATAACACTGATGCATAGTATCTTTTCTACACCCAAACTTTAAGATTCTCTGTCGAGAGA
TTCACAACGACCATAGTAAAATCAGAC
>comp2906_c0_seq1
TCCTGCTTCCAACGTCGTACCTCTGCATATCAATTTGGCGAGGTCGATCTAAATTTATTC
TCTGAAAGGTTGGGCGAAACAAAACTTCAAAGTATGCACACCATTTCCAGATTGCGATTC
TTCCTTCATCATGCTGCCGTGTTCAGCTTGATTTTCTTATGCCTTCATACCGTGGTATGC
CTCCAATCGATATCGTCAAAAAGCAGACGAAAAGTTTTGGAAACTATGTCAAAAAGTGTT
TTGACGATATCCTCTGCCAGCACCATCTTAGTAGGTGATCCAATTGTGGGGTATTCACTC
ACACCGAAAGAGGCAGAAATTGCGTACGATTCATATGCGGCAAACTACGACGAACTCGAC
GGAGGGAAGGCGGCAGATATTTTTGGTCTCGACGAAGCCCGTTCCGCCTTGTTTCGTCAG
GCGCGTGGCTCCGTCCTCGAGATTGGTGTCGGTACCGGCCTTAATTTAGAAAAGTACGAC
CCGTCCAAGTTGGAATCGCTGACCCTAGTTGATATCAGCCGAGGCATGCTTGGAGAAGCA
CAGAAGAGATTTGAAGCTATTCCAGCATTGCAACGTGTTCCCGTTCGCTTCATCCAAGCA
GATGCAACATCGGGATTGACTGTACGATTTGGAGATGGCTCATTCGATACCGTTGTCGAT
AGTTTTTCTTTTTGCGTCATGGGATCAAAGGGTGCTCAGTCGTGTTTGGAACAAGTAAAA
AATGTAGTCAAGTCCAAGGAGAACAATGGCCAAATCTTGCTTTTGGAAAACACGCGTTCG
TCCAACCCGTTGCTTGGACTTTATCAAGATGCGACTGCAGAAGCAGCAGCATCTGCGGGT
GGGAAAGGATGTCTTTACAATCAGGATGTCTCAAAAATGATTCAAGCAACTAATGGAATA
GAAATAATGAAGGAAACCTCGTTTGCTGGTGGCGTCTTCCGTTCTTATTTTTGCCAAGTC
GTTACTTGATTCGTTGGAAACTTCGATGCGCACAAACAAACGTGAGGTACGTACTCGTTT
TCGAATTAACAATTTTAACACAATAATCGAAACTTGATTTTTAAAAAGCATTTTGATTCG
AAACTTATGCATTGACATTCGGACCTTACAGTATATCAA
>comp2906_c0_seq2
TCCTGCTTCCAACGTCGTACCTCTGCATATCAATTTGGCGAGGTCGATCTAAATTTATTC
TCTGAAAGGTTGGGCGAAACAAAACTTCAAAGTATGCACACCATTTCCAGATTGCGATTC
TTCCTTCATCATGCTGCCGTGTTCAGCTTGATTTTCTTATGCCTTCATACCGTGGTATGC
CTCCAATCGATATCGTCAAAAAGCAGACGAAAAGTTTTGGAAACTATGTCAAAAAGTGTT
TTGACGATATCCTCTGCCAGCACCATCTTAGTAGGTGATCCAATTGTGGGGTATTCACTC
ACACCGAAAGAGGCAGAAATTGCGTACGATTCATATGCGGCAAACTACGACGAACTCGAC
GGAGGGAAGGCGGCAGATATTTTTGGTCTCGACGAAGCCCGTTCCGCCTTGTTTCGTCAG
GCGCGTGGCTCCGTCCTCGAGATTGGTGTCGGTACCGGCCTTAATTTAGAAAAGTACGAC
CCGTCCAAGTTGGAATCGCTGACCCTAGTTGATATCAGCCGAGGCATGCTTGGAGAAGCA
CAGAAGAGATTTGAAGCTATTCCAGCATTGCAACGTGTTCCCGTTCGCTTCATCCAAGCA
GATGCAACATCGGGATTGACTGTACGATTTGGAGATGGCTCATTCGATACCGTTGTCGAT
AGTTTTTCTTTTTGCGTCATGGGATCAAAGGGTGCTCAGTCGTGTTTGGAACAAGTAAAA
AATGTAGTCAAGTCCAAGGAGAACAATGGCCAAATCTTGCTTTTGGAAAACACGCGTTCG
TCCAACCCGTTGCTTGGACTTTATCAAGATGCGACTGCAGAAGCAGCAGCATCTGCGGGT
GGGAAAGGATGTCTTTACAATCAGGATGTCTCAAAAATGATTCAAGCAACTAATGGAATA
GAAATAATGAAGGAAACCTCGTTTGCTGGTGGCGTCTTCCGTTCTTATTTTTGCCAAGTC
GTTACTTGATTCGTTGGAAACTTCGATGCGCACAAACAAACGTGAGTATATCAAAGCTAC
GATGTTAACCCTTGCAAAATGTTAATGCCAGGTACTATGCTATTTTTTTCCTCGTTTGTG
CATTGAC
>comp2907_c0_seq1
CGACAATAACGTCATCTTTTCTCATATTCTATCAAACTTGTTTCGTAAAAGTATTTTACG
ATGGCGTTCTCAAGTTTCTTCAAGGCCGCCGCCTTCTTGGCAGTGATTGTTCCCAATGAA
GCCTTCGCACCAGCCTCTCTTGGTGTTAAGACTTCTAGCTTGTCCATGTCCAGCCAAGCC
CAGGATGTCGACAAGAAGGAGGCTTCTTCTGCCGCCCAGCGTGAAGTCGATCTCGACCTT
TACCGTAACATTGGTATCATGGCTCACATTGACGCTGGAAAGACCACCACTACCGAGCGT
ATCCTTTACTACACCGGAAAATCTTACAAGATCGGCGAGGTTCACGAAGGTGGCGCCACA
ATGGATTGGATGGAACAAGAACAAGAAAGAGGAATCACCATCACATCGGCTGCGACCACA
TGCCAATGGAAGGACTACCGTATTAACATCATTGATACACCTGGTCACGTTGATTTCACT
CTCGAGGTCGAACGTTCTCTTCGAGTTCTTGATGGTGCCGTCGCTGTCTTCGACGGTGTT

GCAGGAGTCGAACCCCAGACCGAAACCGTCTGGCGACAAGCCGACAAATACAAGGTCCCT
CGTATGTGCTTCTTGAACAAGATGGACCGAACTGGAGCCAACTTCTACTTCTGTGTCGAC
ACCATTGTCGATTTGCTCGGAGCGACACCTGCTGTTCTCCAGCTTCCTATTGGTTCCGAA
GGAGATTTCTTGGGAGTTATCGACCTTGTCACTAAGGAGGCCATTGTCTGGCGGGGAGAA
GATCTTGGAGCCAGTTTCGATGTCATTCCTCTTGCCGAATGCGATGATATTCCTTTGGTT
GACGATGAACTCAAGGCCAAGGCCGAAGAATACCATGAAAAACTTGTCGAACTTGCCGTT
GAACAAGACGAAGATGTTCTCATGGAATACCTTGAAGGAGAGATGCCGTCTGTCGACACA
CTGAAGAGACTTCTCCGTAAGGGAACCTTGAACCTTGACCTGGTTCCTGTCTTGACAGGA
ACAGCGTTCAAGAACAAGGGAGTCCAGCCCTTGTTGGATGCTGTGGTCGATTACATGCCT
TCCCCTCTTGATGTGCTTGCCATTGCCGGTGTCACCGAAGACGGCGAAGAAACCGAACGT
AAATCCTCTGATGCTGAGCCTATGTCTGCCCTCGCCTTCAAGGTTGCAAATGATCCTTTC
GTTGGTACTTTGACTTTCACCCGTGTTTACTCTGGTGTAATGGAAGCCGGAACCAGCGTG
TACAACTCTGTCAAGGGAAAGAACGAGAGAATTGGACGTATGTTGCAGATGCACGCCAAT
GACCGTACTGAGATCAAGTCTGCTGTCGCCGGAGATATCATTGCTTTGGTTGGACTTAAG
GACACAACAACTGGAGAGACTCTTTGTGACAAGGACAATGCCGTCATTCTTGAAAAGATG
GAGTTCCCTGAGCCTGTCATCAAGGTTGCTGTCGAACCCAAGACCAAGGGAGATCAGCAG
AAAATGTCCGAGGCTCTCATCCGTCTCGCAGCCGAAGATCCATCGTTCCGATTCGCTCGT
GATGAAGAGACCGGCCAAACTATTATCGAAGGAATGGGAGAACTCCACCTTGAGATTATT
GTTGACCGTATGAAGCGTGAGTTCAACGTCGAATGTACCGTCGGGGCTCCTCAAGTGTCG
TACCGTGAGGCAATCACAACCACCGCAGAAGTTGACTACACCCACAAGAAGCAATCTGGA
GGTTCTGGTCAATACGCGCGAATCAAGCTTGTCTTCGAGCCTAAGGAGTTCAGCGAGGAA
GAAGGTGCCATGGACTTCGAGTTTGTTTCTGATATTAAGGGAGGAGCTGTTCCAAAGGAA
TACGTCCCTGGTGTCCAAAAGGGAATTGAATCTGTTCTCGGAACCGGTGTCGTTGCCGGT
TTCCCTGTATTGGGAATGAAGGCTACGCTTATTGATGGTGCTTACCACGATGTCGATTCT
TCCGTCATGGCGTTCGAAATTGCCGGACGTGCCGCCACCCGTGAAGGTTTGAGAAAGGCC
AAGGCTCGTTTGATGGAACCTCTGATGCAAGTCGATGTGTCCACACCAGAGGAGTACATG
GGAGATGTACTTGGAGACATCAACTCTCGCCGTGGTATGGTCGGTGAACTCGGAGAACGT
GGTAATGTTAAGACCATCAGTGCCCAGGTTCCTCTTGCCAACATGTTCCAATATGTTTCT
ACTCTTCGATCGATGAGTAAGGGACGTGCCAATTACTCCATGAAACTTGCCAACTATGAC
TTCGTCCCTCCTAATATCGAGGAGGATCTGAAGAAGGGATTCACTGGCGTTGAGGAGGAA
TAAACAAATAATATTGTAGTTTAAAATAAAATCTTCTTGCACTTCACA
>comp2908_c0_seq1
CTAGTGTTGAGAATCAACGAAAATCAATCACTGATTGTGGATACCATACCATTGAAGTCT
TGTACATTGCCAAGCTAAGGTAGCTGACACAAAACGAGACACCATCAAGGTATTATCAAG
CAAGTTCACCTGTTTCGCCTTATCCAATCGGAAAATGATGACGACAAGTCGATCCCACTT
TTTTGCGCCGACGAAATTTTTTGTACTCTCCGTGCTGGTGTTGGTTGCGGCATTTTCACT
CGCCGATGCATCGACACTAGCCAAGACCTATGTATCGAAGCAACCCGGGACACAGCCCCG
TGGCACCGAGTACGTCGTATCCAGCTTCTGCAGCGACGAAACCAACGACGAACCAGAAGA
ACCCGTGGCGTTCGTCGCAAAGAAACGCCATACCTCTCCGCTAAAGGCTGCGTATCAATG
GTACCTAAAATCTTGTACCGAGACACCGTTTCGGGCGAAAGGCCTAACGTCGGCAACGAT
TGCTGCACTCGGTGACGTGATTGCACAGCTGATAGAAAAAAAGGCCACGGCGAAGGCCTT
GAATGCGGCCCGCGTCGCGACCTTCTTTTTCGTCAACATGCTTTTTACGGGTCCGTTCAT
CCATCTGTGGTACACCTTTCTGAATACCGTCGGAAAGAAGATGGACGAACGCTTCTACAA
CGTTTCCGCCCTCAAGAAGACCGTCACACAGGTCTTTCTGGACCAAACCCTGGGCACCGC
CGTCTTTTTCCCGCTCTATATCTATGTGTATGGTGCTTTCGAGTCGGTGATACAGATGCA
CCGCCTTCCAAACTTGAGCCACGCCACCGAAAAATGCCGAAACCACCTCTGGGGGATCAT
AATGACCCAGTTTCGGGTCTTTCCATTATCAAACATGATCAACTTTGGGTTGGTTCCGTT
CGAGCTCCGGGTCCTGTGGACGAGCACGGTTTCCTTGTTCTGGAACATCTATCTCTGCAG
TGTCGTCGGTACGCGGTGATTGGCTGGTTTGCTAACATCCGTAGATGAACGATGTCTATC
ACACAGGCCAATTGGAATAAAATGTATAATTAATCAACAGTTCAACCACAGC
>comp2909_c0_seq1
CGGTTCTAAAAAAATCTCGCATTCGATAGCAATTGATAGTTGAGTACTAGAATGAAAAAG
ATTGAATAGAAAGGTATCGTAAGGAGCAAGGAAACAAACGATAATAGAAACCAGCATATA
ATGCCCCAGCAACACTACATTTTTGGATATGGTTCGCTGATTTGCGCCAAATCTAGGGCA
GCGACAGCTTCCGCACTCGGCGTTGTCGGCGAAGTTGAGGCCGACGAAGCCGGAGATCGC
AACAAACACAATACGGGCGGAATTCCCGTTCGGCTGAACAACTGGATGCGCTTATGGAAC
CTACAGGGTCCGAACACCTACCTCGGGGTGCAGAAGAGCAACACCAATACCAATTCTTCT
TCTCCGAGAGTTTCCTGCGTCGGCGTTTTAGTCCCCCTGCCGTCTTCCAAACAGAACGAG
ACCGGCGGCAGCAAAAACGATGAGGATGAAGTCCTCGAGGCTCTAGACCGTAGAGAAAGG
GGCTACTACCGACATAGTGTGGATTTGAGCTCAATAGAGCGAGTGGACGATCTGCTCCTG

ACGAACATCGCCGAGAGCGAACGCTGTGATCAAGCCATCGAAGACCGGTACTATAAACAC
ACGTTTTTACGGCGAAAGAACGACACTTCCACCAACGGCAACGACAACACAATTCGATCA
CCGACGCAAAACGATGATGTTGTTTGCAAAGTCTGGATCTACGTCCCACAGGATTGCTAT
TCGGGCATGGCCTGCCCCCAAAAACCGATCCTGCAATCTTACGTCGATATTTGCCTGCGA
GGCTGCCTGTCGATTTCCGAATCGTTTGCGAGGGAATTCATAGCATCGACCTACGGTTGG
TATCCCGGCCACGGCCATCTGTTATTGACGGGTCATTCCGTGGGCGAGAAATGCTTCGAG
TCCAAGCGTTCCGTGGACGAAAGCAACGCTGTCGTTTCGTCTTGCATAAAACACTGCTGG
ATCAATGATCGGGAACGACCGATCTACGTGAGGGCCGACAGGAATTTTTCGATTGGCAAT
TCAGAACTCTTGGACGCTCTATTTGATCCCCAGCTTCTGGAAAGACGTCGGTGATGATCG
AAAGGGATACAGGAATCCGTGGACACCAAGGGATCGGGCTACATCGTAGCAGAAGAACGA
AACACGTTAAACGTAAATGCTTCCTTCGCACAACACGGAACCCAGAGTTACTAAATAGCC
AAAGATCTGCGGTGCTGGAGCCACACAGTAAAATCAAAAGGATATGGTTTCATCCTAAAC
ACATTTAAGAGAACCAAGATAACGGAAGGCGCAGCAACAATTTCTGCCTTGCTTGGATTT
GTGAGAAGAAAGAAATCCAAACGCGACACATTTCCTAATCAAGCGACAATTCACAACAAC
ACGATTGCATCTACTCCTCCGACCTCTCAAACGTAACAGAATGCTTTTCTCAGACATACC
CATTGGGGTATGCATCTTGATTATTCTGTCCATGGCAGACTACGAAACGGCTAGAGAAAG
CACATGACATCATTTCAAAATGGTGTTCGACGTAAAGAACATAATTCGAACCAATCCGCT
ACCCTACCACACTCCTTAGTCATTTTTCTCTCATCTTGGAACTTTTCCCGTGAACTGCAT
CAATGACGTTTCGGAAAGTCTTGCTATCGTTACAAACATTGTATCTTTGATGCCCTGCTC
CATGCATGCTCCTTGCACTTGTAATTTCACACTAGCACCTCATTACATGCACTTTTGCGA
CATCTCCTCAATGGCGGAGACAGCTCTACGTTGTATCCTTGATTGGGAGAGAAATGGGAG
GTTCTTTCTCCAACAGTTTCAAAGGACAAGAGCAGTAATCTTTGCGAAAGAGTCGCCTTC
AGTGATGTTCTCTAT
>comp2910_c0_seq1
GAGACAATCTAACTTTCACCATCACATCACAGTAGCGAAAGAAAGATCATCGAAAATAGA
CAATGGGACGAAGAAGAAAGAGGCTACAGAGCGAGGACTACGACGATTCCCTGGAACGAT
CGTTTTCCGCCAAGCGATCGAAACCAGAAAACGATGATTCGGGGAAAGGTGGTGCATCGA
AACAAGATGTAGGAGCAAGCCCAGCAAAATCCGGCGAAACGCAGAATCACAACGATAACA
ACAGTAAAAAGAACGACAAGGACAACATCGAGCGTCTCAGAGAGAAAAAGCGCCTAAAAA
AGCAACGACAAAAGGAAAAGAAACTCGCAGCCCAAAAGGAACAAGAAAAACTAAAGATTC
TCCAGGAAAAACAGAACAAAGAACGCGAAAAACGAAAACAAAAGCAGAAAAAACTGAAAG
AGCTCGAGAAGAAAGTGTCGTCGGATACGAACACTTTCATCAAAACCTCCATGGGCGTTC
GATACATGGATGTTGTGGTCGGGACGGGGCCCCTCCTCGTGGATCGGAAGAAGATAGTTT
GTCAATACGTGCTAAGGGCTAAAAATAAATACGGCAAGGTCCTAGATTCCGGCGAAAGAT
TTTCCTTCAAGTTTGGCAAAGGAGAGGTCATTCCAGGATGGGAAATTGGATTGAAAGGAA
TGAAGCAGGGCGGCAAAAGGCATATAATCGTCCCGCCAAATGCAGGATACGGATTTAAAG
ATATCGGCGGAGGAAAAGGAGCTATGTTGTACTTCGAAGTGACGCTGGAGAAATGTTAGG
GGAGGAATAAGTCAATATCAAG
>comp2911_c0_seq1
TTGCTTTTGACTTGGTGTCATGTGACCGGAAAGGCCAGAGCGTGGACTGTAATTATAAAT
ATCAAAGCCCCGAGTCAGTAGATAGTCCTTGATGCCGTTTGGGTCGATGCCTGATTCGAC
CTTCTCACCAATACCGCCCGCGTATTCAAAGAATTCTTCTCCGCCATAGATGCTCTTACG
ACCTTCTGGAGTCATGAGCTTTTTGTCGCTGAACGTAAAGTAAAGATAGTGTGCGGGGTC
CAGCAGTTTTTGGCCCAAAGTACAGAGGCTGGCGAACATTCTGCAATGATGAAAACAACA
ACAAAATGACAATGAGGAACAAAGTGCACAGTTCAATTCTGAACTCCAATCTATGCTTCT
CTTGCACTTACTTGTCAACGCTATCATCCGATAGATACACTTGTACACCTTCCCAGAGAA
AGAGCGTAGGTTTTTTTGGATCGAATTTGTCGCAACCATTCATGACGTCTGCAACGCTCT
CTTTGTTGAAATCCGTCGAGAGGTGAACAAGCTCCTTACAGAGAGGCTTGAGATCCGCAC
ACTTTTCCTGCTTTTCACTGGACATGAAGGGCTGATCAACTTCTAGAACAAGAACATCCT
TCAGAGACTTCAATCGATATGCTCGGGTATCATAGCCCGCTCCCAAAATTACCAATTGAC
GCACGGGCTTGTTCTTTCTGGAGGTCAAATCCAAAATGATTTCGTCAATGGTCTTGGTCC
TTGTAATGAGATGACCCAAGGCACCAGGGTGCTGGACCTCTAGTTTGAGGGTCATGAGGT
ATCGCAAGACAGCGCTTTTGACAACTATATGGAGAACACCCCTTTGGACAAACCGAATGG
CATAGGGATCATCGATGTGCAGCCGAGAATCCGAGGACGCGTTACAAAGTGCACGAAGTG
CAACAACACCTCCGGCTGTATTCTTGTGAGAAGTCTTCTTGCTAAATTTCTTCATCGCAT
GGGGAAAAAGAAAGTCAAGGAATAGTAGAATGGGTCCCAAGAACAACCCAACCATTATCT
CGACAGTGACTTGCACAAACATTCTTGTCGGGAAACCATCCGAAATGGACGAAGATCTCC
ACCGGCGCATTTCCTGAAACGATCCATAAAACAGTGGTGCAACAAATGCCAAGCACAGGA
GTGCCTGTTCAGTTTTCCCGCCATCGCTGAGAACGGCAACCACTGAGGAAAAAGCGTACG
CAGAAACAATTATTTTCCAGGAACCGACATTGGTACAATGAAAAAGATGTTTCATTGTCG

GAATTCCGGACAAATTATCTTCTCGGGTCAAGAAATGTCGCAAGAAGGAATAGCAACTCC
GAACAAAGTTCAATGAATAGATGGTCAAATGAACTACTACTGCCAACCATAGCCAATCCG
ACATCAGAGTAATTGGATAGCACCAGGCATAAAGCCGCCCCAATGCGAGGCTCATCGCCA
CGATGTGATGCGCCCAGACGACGTTTGGTAAATCAAAATGAAATGCGAGTATTTCAGCGG
CCGTCAGAGTACATTGGATCAAGAAACCCATTTCCTCTCTGTAGCACACGCGAGAGAATT
CATCGGGAGGCAAGATGACTCGAAGATCTTCTCCTTCGAAATTGGCATTGGACATGAGCA
TGGTGTTCGCTGCCCGGATGTAATTTGGAATGTATACCGCCACCAAGTTGGGCAGTAGCA
GACATGACGTGAAAAGTCCGAAAGGGATTTTTAGCAAGTATACGGCAGGTCGTCTCGCCT
TTTTCCCCTTTTCATCGTCAAGTCTCGTAGGAGGAGGTAAGATGGAGCAATATTCCTCGA
GAAACTTGATGCCGAAGCCAAAGATAATGACGATGAACATCATTATGCCAATAGGATTGG
GCAAATGCCCTTCGTTTTCATTGCAGTAGTGCATTCTTCCAAGTTGCTGAGAAATGAGGT
GTACAACAAAATTAATTGAGCCTCACTGTGAGCCTAAATCACGAGTAGGTGTAAGATTAT
GGTCCGAACAATTGCTTCTTCGTGCAGGAATTATGTGCAACGCATTGCTCTTGGAAGA
>comp2912_c0_seq1
TAAAACAAAGCATACGTAAAAAAAATTACACCACCAAAGAAAACCATCACGCAATTTCAC
CGAAATCCTCAGCACGACAAACCATACGAAACAATGGAGTGGCCTTCCCTAGAGTTCCAA
TCGGATCTCCTCAACTGCATCGAGAACGACGAAATCCTCTCGCACTCGTCGCAAGTGTTG
TCCGATCGATTCCTTTCCGATCTGGCGTCGTCCATCGTCACCAAGGAGATCTACGCCCGG
GCTGCAAGCGCTGCCCTGGCATCGGACACCAGCAGAATTGTGGCCCGCAAGAGCAACACC
GGGGAAAACGAACTCTCCGAGAAAATGGTCGTAGCTTTTCTGGACAAACTCCGCACATCC
GCTTCCGACAGCGACGAGTGGCATGCATACCAGTACCGCGAGACCGCCCGCGACGTTACG
GAGCTCATGGCATCCCTCGATCCACTGGCCACGCTGAAATTCTGCAAAGGTGGACTGGAC
GCCGTCCACGATTTGTTAAAGTACCGGTTGCCAAGCAGCAGCAGCAGCGATTCCCCCACT
GACGGATCAGCGGCACTCTTGAATGCGAAAGACGCTTACGTATTGTCTCAGTCGTTCCCG
AAGCTCGATGCCGATAGCGTGACCGGCACACGGGCCCCCGATCTCGAGTTTCGGTTTGGA
CTTTTGCACAATAACAGCAAGGTCAATAGCAACAGCAACGTCGACTGCGATGTCAACTCC
CTTTACGGCATGGAGGCCTGCGATATCGTCAACGCGCTTCACCAAGACGGAACAATGGAG
ACCTCGGCGGCATCACTAGCCAAGAACACGCTCGCAAATCCCAATCTGGTAGCACAATTA
TCCGCATCGAAAACGACGAACTCTACAGGCGAAAACCAAACCGAAACGATCTTTGTCGTT
TTGGGGTGCGACCATCCAATGAGCCCGGCGAAGCCGCTTTTGCGGATTCCCGGTGTGAAA
GTTCTAGGAATCCCCTCTTCTGAAGAGGGTTTGGAAGATATCCTAGATTACGTTCGATAC
AACAGTCCCGACGATACCACATTCGTCCATCCGATTTGGAACCAAAAGGACAAGAAAGGA
AACTACCACAGAAAAAGAAACCGGACCGGCAACAACTTGATTCTTTCTAGGGGTCCACAA
GTAGCCCAGTGGCTTATAGACCACGCAATACTCGCAAAGAAGGACGGGGATGAAATCACG
ACTGATAGTAAAGCAAGTGCCAATTGCAGAAAAAAACTTAAACTCGTTTTGGTTCCCATG
GCCATGCCATCACTGCCCCCGGCAACCACTTCCCGCAACAGTGATTCGCCCTTTGCTTTC
GTCCCTGAAGCCAGCGTCCGGTGGGCTGCGTCTTCCGACTTAATCGTTCAGCGGGTTGTG
ATGGCGGCCGAAGCCGCAAGCACCACGTCTAGGCAAATACAGTGCTCGCTGTGGTCCTAC
CAGAGCTCAAGCACCTGCATGGTCGTCCCTCCGACGTCTACCACACTGAGCACCGAGTTG
TTGCACAAGCGCCCATCTCACGAACAATGGCTCCACACACTAAGCATGGGCACTGTTTTG
ACTCCGACAATAGAAGAGGACAATAACCGCAACAAGCTTCACAAGCATCACACCGAACAG
CAAGATGACCATTCGAGATCGACTGCGGTTCCTTCCGGAAGCTCCGAAAGTGATATCGAA
GCCAACACCGTGAGGAATCGCTTCGTGGGTGCTGTTAGCAATACAAACCACAACTACTCG
ATCGTCAACGGAATCGTGACCTCGGAAGGTCCTCACCATATGCTGTCGGAGCACATTAGA
TTGTGGAGGGCCATGATAACACACTACAGCGCCGATTTAGAAAATAATTCCTCCCAAAAT
TATATTGGAGATTATGACTCCGACTCTGACGACGATGATGATGGTATACAGCGGAAGAAC
AGTGTCCACGTCTTTGCCCCTCACGTTCCAATTGTTTCTCGTGGCTTATTGACGGAAGCT
ATCGAAAAAGCATCACCATCGACACCAAAAATATCGGCAGCGAGGCCGAACAAGATCACC
GCACCTCTGCAGATTCTAAACTCGGGGACAATGGCCTCTCTGATGGCAGCCATCGCGCTA
GCGGGCTTTACAGATCCCTTGATCAATCAACCGATGCCAATGGGTCTAATCGACGAGAAA
GATGCAAATACTGCCGAAGCAACAATAATTACTACCCCCTTTTGCCTTTTTTGGAACGGT
AGCGTTCATTCAGGCGTTTGGAACTGCCCTTACACACTCGACAGCATCTTTGGAACGGTG
GGGTATTTCCTCGAAAAAGTGTACGGGTATTGCGACTCGGCCCTGGCAGCTGCCTCTAGC
GCGATAGCTATTGCTAATGCGAATGCCGCGGTGTCGGGTCTCTGGAATAAAAACAGCAAC
GATACCCAAAACATTGAACAAAGAATCACGACGAATACTAAATCAACAGAAGCAGCAAAG
AGTGGTCGAGACAACAAATTCAACTATGCAAACAACGACGACCACTGTAACACATCGATC
GTGGACATGGTTCCATCTATCATGAGAGACGAAGAACGACAAGATCAACAATTGATGCCA
AATCTGGTAAAGGATCGATTGGAAATTCTAGCATAACACAGTAGAAGTACTAACGAACTG
TGTTGGATAGCATAAGTTTCTATAACACTAAATGAATCTTTTGTTCGTGCAAGCGGAAGC
GATAGTCGATATGACTGTTTCAATTGGATTCCAATAGAAAGGAAAAGGAAGGAATGCATG

ATGCGTGTCACGCTGTTCCATTTAGGGTTTGTACGCTTTCAGAAATCAATTCAAATAAGA
AAACGAGCATATAGAAACCAAGTAGTAGTGCTGGCATACATTTTCTTGCCTTCCAACATT
TTGAAAAGCGCTATTTCTACTTCAAATTCTTGGTTGACGGATGGAGTACCTAGGCGATAA
AACAAGACTGAAGTTCGATGTCAACATAAGTTCTATTACTACAAGTTGGTTCCCAATACA
ACGGTGGAAAAGACGAAATCAGTGTTGTTTCTGTCCCCATTAAATTTTCGATACAGAGGG
AAGAAAAGAGAAACCGGAGTTCACGAAGTTCCGGTTGATCAATTCGTGTGCTTGAGGTAG
AGAGCCACGAAATGATTTCATTGCAGACCTTTTCGCAACAAAATGTCATACATTCATGCT
TGGCTGTCGAAGGATCATGCATCCATGGTAGTCGTAAATTAATTTATCCTAAAATACCAC
>comp2913_c0_seq1
GCAAAGAAGAAAGGGTATACGATACAACACCACACATTCAAGGAATCCCGTCACCGCAAA
ATTTCAATAGAACCAAACAATGTCGACCACAATCGTATTTTTGCTCCAGGCCGCACTTCT
CGTCGGTGCATGCGTGGCGTTTCAGCAACAGGTCCTCCCGGGAAAGGTTTCGTCGAGGCA
GACAATCCAGAGCGTGCCACTGATGATGGCGGAGAGAAACGAGGACACTGAGAACGCTTT
TGGCAGAAGGAACTTTCTAGCGACCTCTACTGCGGCCGTGGCAACGGGTTTGCTCTCGTT
TCAACAATCCGCAATGGCAATCCCGATGGCCACCGTCGACGAATTCGATACCATCCTGAG
GGACTCCCCGCTGTCGGTCAACGTCGTCGAGTTTTCCGGTCCGCGGAGCGAGAACGTCGT
CGTGAAGCTCGTGGACGGAACCGTCTTTGGGGTGAAGGACATCGTCGAGTCCCCGACCGA
CCCGCGTTCTCCCCTCAAGCTGGCGGCCGACTGTCGAGAGGCCAACGTGAAGACGAAGTT
TGTTGATTTCGAGGCTATGCTCTCGGGATCTCCGAAAAAGAAAAAGCTGTACACAAACGA
GCGGGTCCAGAAAGCTTACGAGCTGCAGGCGGCACAAAAAGAGCGAATCCGCCAGGACGA
GCTGGAGCGGCTGAGACAGGTCGCTCTGATGGAAGAACAAGAAAAGGCCGCCGCCGCCGC
TGCTGCCGCACCACCGGCTGTTTCCGAATAAACTTCTTGCGCTGCTAATGCAGCTACCCA
AAACATAAAAATCAAAGATGAAGGAAACGCTGTTGTTGTGTCTTTGGGTTTTCATTTTAT
GCCTTAGTTCTTTTTCCTTTACGGGCAGCAACATTTCCATCGCCTCTCAGAAAAATTTCT
AAAGATGTTTTGGTTTAGACAGTACATGCGGCTGCGCGCTGATATCTATTAGATTGTCTC
TTATTTGCGTAGTGGAACTTTCAGCAACAGCTTGCAGTAGCGCCAATAAAAC
>comp2914_c0_seq1
ATTTCTAGCCACGACACATTGCGGTGGTTTGCTGTCACAGGCAAGAGCCTGTCCCGGTGC
AGTCTCCCACCTTTTGGTTCCATCCCATCCGAAGAATTCCTTCGCTGTACGCCGTTCCCG
AAGGATAGCGTACACCTGCGGCATCCGATCCAAAAAACCACCCGGCTGGAAGAGACGTCA
CGGTCCTCGACTCTGACACACGAAACAGAACGTTCCGACAGCTCTTGCCGAGATTCCTGT
GCCCTGCGTTCCATCGCGGGGCAAAGCGTTTCGTGGACCATTCAACACAGAGGACGAACG
CGACAGAGCCGAACCGCAAACCTCAGCGAGTACTGTAGATATGGCACGACCAAAAATTTG
AATCGAAAGATATTTAGTTCTTACGTTATGCTACTCGGCGCAGATCGTTTCGCGTCTAAC
TGCTTGGAAACCAACGTGCGAAAAAAAACGATGACGCAATGACAGGGGGCGCGTCGTCTC
CGTCTGCGTTTCGGCGACCGACATATTTGAACAATCTTCGGTCTGCCGGTGTCGTAAACT
GCTAATCGTAGTTCTTTCAACAATGAGTTAAACATAATTTTCCACAGTAGTACTAGTATG
ATGAGTTTCACCTCAATACTAGTATCAACAGTAGTAGTAACAGTAGTCACAATACTACTA
CTAGCCAACGCTACTTGTATTTTGGTAGTACTAGTGGGAGTGGTGTTGGTACTACTAATA
GGATGCCTAAAGAGAAACCATTGTATCGGTATCGGTTTGCATGATAACCAAGGAGTGATA
GAAGAAC
>comp2915_c0_seq1
CGCATTTTTCATCAGAGCCCCAGATAGTGATTATTTGTTGGCATTCAAAGATCGACTCAA
TGGAACTTAACTACGAAATGAACAGCCGCCAACACTACCAAGACAACATGACAAAACTAG
TGTCAAGTCGCCGAGGGAGTAATTTGCTTTTGCATAATCTAGAAGAGAACGATGTTGTCT
TCGGAACCAAAGGGAACGGACGAAATGAAATCTTTCTCAAGGTCCTGGAAAGCCATTCCG
AACAATACATGCAGCTTTCGAAGTTCCAGAAGATGGGACTTATCCAGCAAATCATTCGAG
AGTGGAACGGTAACTTTTACATTCTCAACTCAAAGACAAATGACTTGCATCTTGCCAAGA
GAAAGGATCACGACCTTTCTACCACAGATCCGTCTTCGAAGAAACTGTACACGAGTGTTC
GAAGGATGATGAACTACGTGAATTCAAAGGTCCAGCGCAAACCCCAGCACCTGACCCGAC
CCACAACGAATCTTGCTGGTACTGCCATCATTGACCGAACGACACAGATCATGAAAGTGA
TCCCAACGATCGCTATCGGAGCTCTTAATCCTCAGGTCCAGAAGGGACAAAGAATGAACA
ATGAGCAGCATTGCTCTGCTCCCACCACCGATGCTGCGGCTGCGGCAGCTCCCGACAGGC
CCCCGACGGCGATGACAGCCATGGATTACGTTCGCAAAGAACATATCACTCTTCCGCTAC
CCGGTGATATTTCATCGTATCCAACATTCGTCCCGAAGACCATCAACATTTCGCCACTCC
AGAGATCCGTGATTATGGTCACTCCGGAACCGTCTCCTCGATCGGCGTTTTTGCGACCCC
CACCGATCTCGCCGCTCCCTCTCTCGAAGCCCGATATCCCATACAATAACACCGCGTGCA
CCGCACCTCTTTCGCCAGAAGCATCCTTCTCATCCGAAACAATTCCGGCGCAAACAAACA
ATGGCGGTTCTCTCCTCTCCAAGAAACAAGTCACCACACCCGTTGTTCCTACCACAAGAA
TGCAAGCAATCGAAGAACAAGGTCCCAACGCCAAAACGACTGACGTTCCCGGTAATCTAG

AGCATTCTGCGATTCTCGCTCTAATATCCCTGGGTGCCTCGCCCTCAAGCTAAACGAAGT
TACCAAAGCGTTTTTGGATGACTGCGCATTTTCACTTCTGGTTCGCTATCTGCACCATCA
CTCCATGCTATTCACAGCCTCCAATAAATGACTACATTGATTGATTGGCTAAAAAACTCT
GCACAAACATTATATATACTTGAAACTCGAGCTGATCCAAAACGAGCAAAAGAGGTGCTA
ATACTCTCGACACTAGGATCTAGGAATCAAAATAGTATTTGCAAAATGCTATTCAATACT
TAAAGTAATAATTGCATCATACTAGCA
>comp2916_c0_seq1
AGAAGAAGAATTGCAAGAAACAAAAAAGAAAAACAAGAAAATGGAGAGATGGCTTACTAA
AATTGTTGAAGAATCTACAAAACGAGAAAATCTCTTAAAGCAAAAGCTTCGTGACTTGGA
AGCGAATGTCGGAGACACAACAGACCAAGAGAGGCTCCTTCAAGAACAAGTCAATGGATT
GGAAACGACGAAAAAAGAACTCGAAGAAAAATGCTCTCGTGCTCTTGAGCTGGAAAGCTT
CATGGAATCTGCATTGGAAAAAGAAATCAAGCAACAAGAGCGTTTGACGAAACGATTTGT
TCAATTGAAAGCAAAAGCGGAGAAAACTACACAGATCCATGAGAAAGAGGTGAAGCATCT
CAACTCACGTGTATCAGACCTGGAGACACAGCTAGCATCATCGGTGAAAGATGACGAACA
GCGCAAGCAGCTTGATAATCGTATAACGGAACGAATTGCCGAGCTGGAAGCAAAGGCTGA
ACAGATGCGCCGCGATGGCAACAGGAAGAAACATTTCGAGTCACGCATAGCTGACCTAGA
GGCAGAGCTAGCGTTGTCGTTGCAAAACAACGATTCCAAAGAGGAGGGAAGAGAGGGGCA
GTACAATCCCAGTAGCTTCTATCAAGACCTTATTAAGGAGAGAAACAATCTGGCTGATAG
GGTCTCTGAGCTCGAAGAAGAGCTAGAAGCGGAAAGAACAGTTCGAGAGGAGTTTAAACA
AACTGTCGACGAGTTGAATATTGAAATAGAAACACATGCGCAAAAATATAAAGCAATCGA
AGCAGAAAATCTCGAGTTGGAAAAGGAGTTGGAACTAGGAAAGTCTGAGCACATTCGTCT
GAAAAAAAATGTTGACGAGCTAAGGGATGAATTTGAAACGATAACAGAAGACTACAATTC
AGTCACTGCTATTAACCTGGATCTTGGGCAAAAACTGGTATCAGCCAATACAGAGCGGAA
CCGCCTAGAGGAGCGTCTTTCTGAGCTTCAGGATGTACTTGACTCGAATACAAATGAGCG
TGGTATCGAAGAAATTCCAAAAAGCATTCCAACTGGGATGCAGAACGAGCTTGAAACACT
AGTCGATGAGAATAAAAAGAAGCAAGAGCAACTCAATTCACGCATTTTCACTCTTGAAAA
TGACCTGGAATCAAGCTATCAAGAACGGAGTCGTCTTGAGGATCGCATAGTCGAAATGCG
GAAGGAAATTGATTTGGAGAAAAGCGCGCAAGTTCAAATAAGTGGTGATGTTGCTGCGCT
CAAAATCGAGCTTGAACGGGTGAGAAACATCACTCAAACCCGAATTGCGGAACTTGAGAC
GGAATTAGCATCGGGAAAGGCAGAACATGATCGTCTCCTTCAGGTTGTTCGCGAGCTAGA
AGATGAACGCAAAGTGAGAGGAGAAAAACACGACAGTGCAGCGAAAAAGGTCGTAGAAAA
GCACAGTAAGGCAAATAGCAAGCTCGAGGCCCGTAATCTGAAACTCGAGGAAGAGCTAGA
CTCGTATAAAAGAAAACAAGAACACCTTGATGCCCAAATTTTGGAATTGCAGAAAGAACT
CGAAATGGCTACTGAAAGAAGTAGGAAGGCACAGGAAATACGGGATGAAAATGACCTCAA
GTTCAAATACGAACTGGAAGCAGAAAAGACCGAACAGAAACGCCTAAATAAGCTTGTTCA
TGAACTAGAGGATAAAGTTGAAAAGGCGGCAGGGAAATATGAATTCGAGCTGGAAGCAAA
AAGAGCCGAACAGGAGCGTCTGAATGACATCATTGAAACAATGCAGAACGAGGTTGAAAT
GAATACAGAAGAGAACGATAAAATTATCGAGAGCCTGAATGGTCGCATTGGCGACTTCAA
AAATGAACTTGAAGCTCTCAATGCAAAGATTGCGGAACTCGAGCAAGAGCTAACAATCCT
TGAGGAAGAGCCGAAGGACGCCACAAATAACGGCGCCCAGATAATTGACCAAAACCTTAC
TGATCGTATCCTTGAGCTCGAGACCAATCTAGAGTCAACTTTGGACAGAGAAAAGAAAGA
ACAAGAACAGCTTATTGTCCGCACTTTGGAACTTGAGAAAGAATTAGAATCCACGATCGA
GAAAACGTTGAAGAATGAACAGCATTTGTACTTTCAAATATTGGAAATGGAGAAGGAGTT
AGCCTCCAGTCGAGATGAGGCAAGGAATCAAGAAGAGCGTTTATCAGCCCAGATACGCGA
GCTAGAAATGGATATAGATACGACGAAAGAAGAAGGACACGAAAAGGAGTGGGTTTCAGA
GCTAGGAAAGGAGTCAGAGAAAATGGAATCTCTCATAAATATGATTGATAGAGAGCGAAA
GGAAAACGGAGTTCTCAATGAGAAGTACTCGGGATTAAAGCACGATTTTGAGTTATACAA
AAAGAAAAACAAAAAGCTATCTTCGCAATACGTCAACATCACACTCGAGGGGAACCGACA
TCTTAAACTTCTCCTACGTCGTGTCGCCGAGCTCGAGCAGGAGATTCTGCATATTACAAA
AAGCAATGCTACCGAGAAAAAACTCACCACGAGGCACGACGAAGAGCTGGACAGAGACCT
TCGAGAAAGAGCCCAGAAAAATGAAGCCGAGCGAAAAGATCTGAACGAGCTTGAACCCGA
AAAGAATGAAAAGAATCGGGTCCGCCCTAGCGGCGGTGAAGTAGACGTTCGACGAACTCA
AAAAGAGGATGGACACCATTCCGACAAAACTGAAGCATCCACGTCTTTCGCTACTACAAT
CGAACACCAAGTTGCGAATGACAAACTTAGTGCTCATTTAAGAAACTCTTCACCCAGTAA
TAGGGAAACAGACAGTCCAGAATCTTCAATAGCAGAAGAAGAATTGAAAGCCCCAAGGAA
TCAAAAGTTTACGAAATCTATGTCAGAATCGAAAGCTGACTTAATGGAAGAATATCTTCA
TCCACTTCTAACCGAAACCGAACAGAAACTCGGCGATGAGATACCAAGCAACATTATCGG
AAGGATACACGATGCCTTTCTAGACAATTTCACGAATGGAAAATATGATTTAAGATGGGA
AGAGTTTGAAGAAATTCTTGAGGAAGCTTGCCAAGGACACGACGAGGAGACGTGGTTCAA
TATTCAAGAGGCTTTTTTCACTGTATGGGAGACAGCAATGAAAAACAATCCAGATCGTGA

TCGTGAAGTGATCACAAAATGGGAATCATTTACGGATTAGGACACAAATCACTGGGGGTG
TTTTCATTTTAGAGCTCTGGGGTGTCTGGGGAAATAAATCTAGATAGAATCTGCCG
>comp2917_c0_seq1
AGTCACGACAATAAAAACAAGAATTTTTCAAAAGCAGAACACATCATCATCAGGGCAACA
CCAGCATCAGCAAGACATTCCAAAGCAGCAGAATCATTGCTCTTCAGTCCTTAGTTGCAA
ACGACTTCCGAACGATCCCCCTAATATAAATAAAGAACAAAGAAAATAGAAGAAGCCATC
GAGAAAAATAAGACTTCACCAATGCCACTTTCACCAATGAGACCAACCACTGCGAACAAG
AGCAAAAGGTGCAGAAATACATCCAGCAACATGCCTGCAATGACGACGAGACGGACAGCG
ATCGGCATTTGTTGGGTGTCTTTGGTGCTCGCACCATGGGGTTGCGTCGAAGGATGGGTT
GCTCCAAAATCAACCGCACAGAAAACACCAAAATCCACCGCACCAACTTCCAGCAACACC
CTGTTCTACAAGACGTGTGGATCGGACGAAACCTTCTCACGCGTCGCCAAACCCGTGCTT
CCGGGCATTCACGAAACGGTGCACGAAGAACGGCAGCCGCGCAAAGACGAAACAGAGGAT
CCGGTGTCCAGAGCGGCAAGCATCCCCTGGATCGGAGACGAACACGATTGCACCGGCGAT
GTCTTTTTGCCCCACTGGAACTGGCAGCTGGACTTTTTTAGGACGCACCTGACCAACCTG
CGGTTCAACCCACCGGGCGATGCTAGCGACGAAGACCTCTATTGCATGGACGACGGGACC
AAGCGCGTCTACACGATTTCGCTGTCCTCGGACGAATATCGGGATATTCGAATGACGTAC
ATGGATTTCCCTTCCTGCAAGACAGTCCGTTGCCTGGCGTATCCCTCCGACGAGAGACTT
CCCATCCTGGGCATGGGAATCATGAAGTTTGGCAAGCACCAGCACATGGCCGTCCTGGAC
TACCAGCCGCTGAGCACTAAAGAGGAGGACCACGACGTCAACGATGCGTACACCTCGGAG
CTCCTGCGGATGCGGGCGGAGAACCCCACCATGAGTCAGCCCCATACCTACCGACACTTT
GTGACGGAAGAGCGCAAGTACTTTACAGACTTTCCGCTGATTGGCAAGTTCAACGACAAG
GAGGATCCCGCGTCACCGGAGCAGGGAACCTCTGCCTGGATGCGGGAAATGCACCGCGGC
CAGAGGGAGTTTGTCGAGACGCACGTCAAGCTCACCCAGCGCCACACGGAGGCGGACCCG
GGCACCACCGACGACGATGCCAGCGTCAGGGAGGTCCACTCGGAGTTCGACACGCACGTT
TCGAAGAAGGAACCGGCGGGGCCGATGCTGACCGCCACCTACGGCGACGAAATCGCGCAC
AGGCTGGTCCACCGGGTGATCTTTCCGCTTTCAAGGAACTGAGCGAAAGAGCGCCGAGCC
TCGCCACCTCGCCGGGATAGGGGGAAATGTACAAACCAAAAAAAGGAAGAAATCGCGGTG
TAACACTACACTGTCCTACCCTGTTGTCGACTCCAATGTACCATTATTGCACAAAACCAA
AACAACCACTCCTCAACGATGTATGTTACATTAGATAAAAAGTAATTGCAAAC
>comp2918_c0_seq1
TCACAAACCAACAGCCCCAATTTCAAATCTAATCCACGAAAATTCACAAGTATGCCGACA
CAAGATTCCGCGAACCTTCGCCTTACCCCACAGCAGCGGGAGGCAGTGGAAAGACGGTCT
CGTATGACCCAAAGAAGGCAAGAACTCACAAGTCGGATACGAAAGCAAAAGAAAAGCAAA
TACATACAGAAGAGAAGAGGTTTACAGTGTGGCGGTGTTGATGTTGTCAATTCCGCCAGA
CCATTGAATTTATGCGTTGCAGAGTTCAGAAACCTGCTGATAGCATACTGCCAGACTCCC
TCGGCGCCGGAACGATTGCTTCCGAGTGTGGACAATTTCGTTGCGAAAACAAAAATTACC
TCCACCGCCGTTCGAAGCGCCGAAGACCCTCTCGTCCTATTGGAGGCAAACGATGAAGAT
CTCGCGCTCAGATTTCTGGACTGCCTGCGGCAACAAGCAATTGACATTTTTCCCAATCAA
AGCAATCCAATTTCGTTTCAAAGCATCCTGAAGATTCTTGTGCATCTGACATCAATTTCT
TCCGGTGATGCCATGGAATCATCGATGACACCTTCTGTGGAATATTACGGGCGCCACCCT
TCGACGTGGTCGGAATTGATAGTTTCGTCAACGTCTTCTCCAACGATAACTGCTGCCCAA
TCAATTGAGAAAGCACCTTCTTGGTTGGATATCTTCGTCCAAGGACTCGCATCTTCACGG
GAACCCGAGCTGGTAAGCCTCGTTCTAGGAAACTTGGTGGGAGATGATGGCGCAGCTCAG
AACGTCTTCCAGGCTATATCGAAAGATTTTAAGTCGTCAATGGTAGCCGGCCTCGTTCAA
TCAGTGTCACCCGCTACTCCAACAGCAGCTTGGACGCTAACGAACATGATTCGGAATGAT
CCTCTTTCATACGCTAGGACCTACTGTTCGGAAAACCTTTTGTCAGCTTCGCTATTGGCG
TCATGGCTCAGAGAACCGTGCCTCGTTTCTCAGACGGCGTGGATGATTGCATCGTTGACG
GCAAGGGAAGAAGACGCGGTGCAATATCTTTCTGCCCAACATTCCACTCCGGAGCACTCT
CCCTTTCTTTCAGCCATCGTCGAGTCTGTTCAAAACCCATTGCAGACCGATCAGATCTTG
CCGCTTGTACAAGCGCTGGGAAATATTTCATGCCACTTGACGTTAGTAGCACCATTGCTG
ACCAAGACTGCACCTGCTCTGGTCCCACTTTTACAGCACATACTGACGACGACTCCGTCT
CGGAATCCAATCCTGGCGAAAGCCGCATGGGTCGCCGGATGCCTTCTCGTCGATGTCGGA
ACAGAAGCCCATCCCTCTACGACCGTTGCCGCACCAGCTTTGATTCCTGCCTTGATAAAT
CGCCTTGGGGACGTTAACGAAGAAATTGGGGGATCAATGGGAACCATGACGCTAGAAGAA
GAGCGAGAATTTGCGTCTTCCTTGTGGAACGCTTTGGATGTGCCACCAGGTCCTGGTGGT
CCGCAGGAACTACCCCAGAATTCCCTCTGGAGCAATCACCCGACACAGTCATCCCCTGTT
CAGTTGCCTTTTGCTCTAAATCTTCCAAGATCGACATTGCGATCACTCGTCCGCCTAATC
AATTCAAATGATAGCGATGCTGTTCTCGCGGGAGTGCATGTTATAAGTTTGTTGTTCCAA
CGCGAAAATGATCACCAAAATTTACAACGAATCTTGGAAGAAGAAGAATTGCCTTGTGCT
TTGGAACAAGTCTGCGATTCACAAATGGAAGAAGCCGCTGATGTGGCAGCCGATTGCTTG

GACGATTATTTCTACAATGAGAACTATGACAACGATGAGTTGGTAGAAACCCCGTCTCCT
TGGGCTGGTGGACCGCCAGTGCCTACAACATTTCATTTTGGTATTGGTGCGGAAGCTCAC
GCAACGAATAGAGGAGGGCGGGGCCGAGGCCGTGGTTCAACAATTCCGGCATGGATGACG
AAGTGATACGAGCACAACGTCGTCGTGGCAATCGCCACACTTCCACCAAGTCTAGAATAC
GATTGTTGTCCCCCAGATTAGAGTGAACCAAACGTAAAAACGAGCTTAAAATAGATAAAC
TAACTAATGACAC
>comp2919_c0_seq1
CGGAACATCATCGTGCGTACAGATATCTGTATTGTGCGTTTTCCTAAACTTCCACGGCAT
GAAGGAAATTCGGTGTCTGCAAACATTTAACGATCAGGTCTAGCTGACCGGAATCAATCT
TCCTTTTGGAGTTTAAAGCTCGATCACCCAGAGCGATGAAATCAGTGTACATGGGACTCA
TCTCAACACTCTACAACACATTATCTACCCGATAATAGTTTCCATTAGCATTCGCTGATA
GAATAGATGGTAACAACACAGTACCTGCAACGATATTATTCTCACAGAACAACACGATTA
CTGCACTGTTACACTTGCGAGGAGCTTCGCACTTGGCTTTAGTCATACATCATACTATTA
TTATCAGTTCGAGAACGTGACACACCGAACTGAATCGGCATTTCGCTATAGTGAAGTGAG
AACAGAAGCAATAGTCATTAATAAACAGATTATTCGATACCAAATCTATTCAAAAGGGAA
AAACACAGCTTCATATTGCAAAATTGTCACATGCAGGCTACAATTATGTTACCTTTGAAT
ATAACAGATACAGAGAAACACAGCACCGCCTTTCCACAACCTCCGATGACTATATTATCA
ATCGAAGAAGGTCAGCAGCAGCAACAGCAGCCACACTATAATCGCATTCATCAAAGTCAA
CATAGTTACAACAGCAAAGAGAGCTTTCGTTCATCATCTCATACGGAGTCCACGGTGTGC
GAATCCATTGATGTGCTGACTTCGGAAGACTTCATCATGCCCAGCAACAGCCAAGATGAG
AGCGCGATCTACTATTTTGGATACGGTCCCATTGTGAACCCTTTAGTCCGGTGCCGACGG
GGATGCAAGATCTCACCCCATAACGTCCGCCCTGCGATTTTGTACGACCACCGGCTGAGG
TTTGTAGAGGGCGGCACCGCAAATGTCGTTCCCATGATGGGTTGGGATGTCAAGGGAGTC
CTGCTCCGGTTTGACAGCGTTAATGAGTGGGAGAACTTTCGAAATTTTGATGCAGGGTAT
GATATTAAGGAGGTCAGCGTCTCGATCATCGACGAGACGAACCTGGACCCCAAGAACAAG
AACTCAAGGACCTTTCCGACTGAGGTGAATAGAGGAAGCGATGAACATCTAATAGAGGAA
GAAGACCACAAGGATGAAAGCTACAACGGGGAGCGCAGAAATGGTTCCGATCTGATACGG
AACCGCCATTTGTGCAAGTCTTGTGCTTTCTCGACTGCTGACTCAGGACTTGTTGGTGAC
GAAGACGATGAGGGATCATGCTCCTCGGAAGAAGAATACTCCTGTCCCTTCACCATCGGG
GCGAAGTCAAGGTCCCAAAAAGAAGATCCCGCGACTGTTCGATGTCTGACCTTCGCGATG
AGCGATCAAGGATCTCAACATCAAAACGCGAGGATTTCTCTCTCTGGAAACAATGGCGAC
TCCTGCAGCGTTGATTCGGTGTTTTGCAAGCCCCAAGAACGCTACCTCAGGCTCATGATG
GACGGATTGCGTGCACTCAAAGTGGATGAAACCTACATTAGAGATGAGGTATTAAGGGTG
AACTATATCCCAAGTGAGCGCGACCGAGTTGCGGATCAAAGTTATTCATACAAGAAGTTT
CCTGTGGCGACAAAATCCAACAAGATGCCAAAGATCTCTTTTGCTAAATATAAGAGCAAA
CTATGCAGCAATAGCAAACGCGGAGAAAACGCAACTGCTACTTATTTCATCTGCAACCGC
AAAGTTTTAAAAGTCAATCTGCTATCGGACAAACTCAGCAGCGATAATGATCTCTCAATG
TATACTAACAATGCCTGCGTAAAATGGTTGCGGGCTCTCGGTCACGGGAAGGGCGATATC
ACTCTTCTGGTCCACCAAACTTTTGTTGATCCAGATTGCCTACACATCCCTTTGGTGGAT
AATGATGACGACTTAACTCCTATGCACTACGAATGGGCCGAGCACACGATTCTCCTCTAC
CTAGAACGGGGTGGTGTCACGGCGACGACGGTCTACGAATTAACCGACAGCGGTTGCAAT
TCCAACGCCAAGACTCGCAGGCGCTCTCTATCCACCAGCCTCACAAGAGTTGGCAAGTCC
GTGCCCTCTCTAACAGCCAGCATGGGGCGACGATTCCAAAAGAACCGTTTCAGCGATGTC
GATAGCGGCCACTCTGAATCTTCCAACGCGTTACCGAAAATTACTTCGCGATTCTCTGCC
CCGACCATACTCACGACGAAATCGAGCACGGCAGAAGCACCAACGGAAGACTTTTCGACT
ACTTCGATCAAGGCCAAATCGAAACTCTGTGGTATTACGACCCGACTAAAGAACTTACGA
TTGTCCAAGAGTGAGAACGCCAACGAAGTCTGATCGCATTGATTGAGGAGTCATAAAGCA
ACCAAATCTCAAATCTGGTATATTTCAACGTTGTGATGCTACTTGAAGTGGCAAGCTGGA
CTTGCTTCTAATCTAATCTATCATGGACCTGCTAGATAAGTTCGTCGTCGAGCTTTTGAT
TTTTACCAACTCGAATCTGGATCACAAACCACTCATTTGCGGCTACGTGAATCCAAGAAA
AGAAACTCACCTCACCTCACCTCACCTCACTCGCATAGTGATAAAGTGATACACTATTTT
CTATTCGTGGAATCGTTGCTATTGCATCCTTGCTATTGCGTACTCGTACTGAGAGATTGC
AAGACAAAATGATATGACAATATACAAAGACCGAAACTAAACAATCACATTGAAAAAGCT
>comp2921_c0_seq1
CTCCTCATCAGTCCGGAAGAATCAAGACAAGCCGCACCCAGCGACTGCTTCAACCTCTCT
ACAAGTATCACCCACAGTTGATCGACTCCGTTCATCGCCAGCGTTTTCTCGATCGTTGCT
GCCAGCAGATACCAAATCACGAACCATAGATAGACTAGAACAACACACCCCAGTTACCAA
CAGATCGTGCCTCAAAAAATGTCGTCGCAAGCCCCGAGTGTTGCCCAGGAAGTCTCGGCG
AGTCTTTCGGACGGTCCCAGCACCGAGACACGGAATGCAGAAATATGGTCGACGCGGCTA
CAGCGCGAGCTTCTAGCGTTGACCACGGACAATGCCTCGGAGGAGGCCAAGACGGAAATG

ACCGCCGTCTTGCCGCCCTTTTGCTCAATAAAGGAGCACGCTTTGGACATCGAAGCCGGA
AACTGCACCGTCACGTGTCTCCTGGACCTTCCCAAGGCGGCGAGCACCGACGGGGACAAG
GCGGAAGGATCCGAAACAACAACTGAGGAAACTGACAAGGCAGACGCTCCCATCGTTGTC
ACCTTTGACGTGTCGCTGCAAAAGAAAGCCGACGGATCCGTGGACACCTCGGCCGTTTCC
TATCCCTTTCTGAAGCCGCTCGCCTGCCTCGCCAGCGGTTGCGGAAACTTCCCGGAGGGA
TCGACGATCCGTGACGGAGACGCGATCGACATTGAAATGGACTGGACGCCCAGCCTGCAC
CTGTCGGACGCAATCCTGAATGTTTCTCTCAAGATCAAAGAGTGCTTGTCCCAGGGCGAA
GCCTTTCACGCGGCAGAGGTCTCTTCGAAGGCCTCCAAGGCCGAGCAGGCCGTCAACGAA
GCCGTCAACAAGGCCAAGACGCTCGGGGCCAACCTCGGTGCGAGCCTACGAAACCTGGCG
CACAACGCCACCACCGACAAGGACGGCGAGAAGAAGAAGATCCGGCTCCCGATGGGACGA
AAGAAAAAACAGCGCCCCAAGGCTACGGCCAAAGAGATCCGAATCGGAGACGAGATCAAC
ATGCTCGAGGCCCCCTGGGTGGACTGTCAGGGCGTGTACTCCTGCAAGGCCATTCGCCGG
CCCAAGTTTGCGGAGGAAGCCATTGCCCTGGCGGACTCCAAAGACAAGATAACGAAGGAA
GAGGAAACCGAACAGGTTGCAAGTGCGGGCTTTGCCGGCGCTGGACTCATGCTTCAAAAA
TTCACCAACTCTACCCGTGGACTCCTGGAAGAGTCGTTCTTAATGATCACCGAATCCCAC
ATTATCGAAATGAGATCGAACAAATTGAACCTGAGCACCGGTACCGTATCCTTTGCCATT
CCCATCGACATGATGGCCAAGCTCAAATTCCGGCGACAGGAATCCATTTCCTTGTTCTTC
AAACCCGCTCCTGAGGATCCTCTCATCTACATGTGTCCAGACAGCGCGGATTGTGTCCAC
CAAATCCAGACCGTTTTGAAACGACACGGTGTCAAGGGAAAGCACACCAATGCGGCTACC
CACCGCGCCATCAACGAGGCGCTTCAGTTGGTTCAGGAGATCCAAACCAAGGAGCTTGCA
CTGAAGCACGATCCCAACATCGAGCGGGTGAACCAAATCATGGATCTATACCGACAGGCC
GCGGAACGCTTCGAGGTGGCCGGTGATGTTCGACACGAAGAAGTCGTGACCCACATGCGC
AAGTTCTTGGCGTTGCCCCTCACCGTCAGTGTCTTGGATGGAAGCTACGTCACACCCACG
CGCAATGCCGACAACGAACCCATTCCGTCCCCGGTGACCAAGGGAGGCGCGTACGACTCC
AACATGGACTCGCTCCTTTCCAAAGCCGAGACCGACCTGTCTAACATGAAGGGTAACGAC
GACAAGGAACTGGACGATCTCTTTGCCGACGGGACGGACGATGCTCTCGGAAGCACCGCG
GATACCATTGCCGAGTTCGAAAACATGCTCAAGGAGGCCGACAAGGAATTGGCCGAGCTG
ATGGCATCATAATACCACTGCAGGCATACCACCCACCACCAAATGGATGCCATATGCAAT
CCCCGGTACGGTGGTCGAGAAGGATCGTTGGATCAAGCACCCGTGGACCCTTGTTTCCGA
GACAACGAAAGTGAAAACACTCTAAAATCCAAATCTGAGATGCAACTCTACCCTAGTGCC
AAACACAAAAACAATAACATCTGTGTTAGAAGTTATTGGTTTGTTTGTTCGAAAGCTCTT
TCTGTTGTAGCTTGCTATTCTAATAATAGTGGCACTAGCTCATACATTAAACATGCATTT
AAAACATATTTTTTCTTAA
>comp2921_c0_seq2
CTCCTCATCAGTCCGGAAGAATCAAGACAAGCCGCACCCAGCGACTGCTTCAACCTCTCT
ACAAGTATCACCCACAGTTGATCGACTCCGTTCATCGCCAGCGTTTTCTCGATCGTTGCT
GCCAGCAGATACCAAATCACGAACCATAGATAGACTAGAACAACACACCCCAGTTACCAA
CAGATCGTGCCTCAAAAAATGTCGTCGCAAGCCCCGAGTGTTGCCCAGGAAGTCTCGGCG
AGTCTTTCGGACGGTCCCAGCACCGAGACACGGAATGCAGAAATATGGTCGACGCGGCTA
CAGCGCGAGCTTCTAGCGTTGACCACGGACAATGCCTCGGAGGAGGCCAAGACGGAAATG
ACCGCCGTCTTGCCGCCCTTTTGCTCAATAAAGGAGCACGCTTTGGACATCGAAGCCGGA
AACTGCACCGTCACGTGTCTCCTGGACCTTCCCAAGGCGGCGAGCACCGACGGGGACAAG
GCGGAAGGATCCGAAACAACAACTGAGGAAACTGACAAGGCAGACGCTCCCATCGTTGTC
ACCTTTGACGTGTCGCTGCAAAAGAAAGCCGACGGATCCGTGGACACCTCGGCCGTTTCC
TATCCCTTTCTGAAGCCGCTCGCCTGCCTCGCCAGCGGTTGCGGAAACTTCCCGGAGGGA
TCGACGATCCGTGACGGAGACGCGATCGACATTGAAATGGACTGGACGCCCAGCCTGCAC
CTGTCGGACGCAATCCTGAATGTTTCTCTCAAGATCAAAGAGTGCTTGTCCCAGGGCGAA
GCCTTTCACGCGGCAGAGGTCTCTTCGAAGGCCTCCAAGGCCGAGCAGGCCGTCAACGAA
GCCGTCAACAAGGCCAAGACGCTCGGGGCCAACCTCGGTGCGAGCCTACGAAACCTGGCG
CACAACGCCACCACCGACAAGGACGGCGAGAAGAAGAAGATCCGGCTCCCGATGGGACGA
AAGAAAAAACAGCGCCCCAAGGCTACGGCCAAAGAGATCCGAATCGGAGACGAGATCAAC
ATGCTCGAGGCCCCCTGGGTGGACTGTCAGGGCGTGTACTCCTGCAAGGCCATTCGCCGG
CCCAAGTTTGCGGAGGAAGCCATTGCCCTGGCGGACTCCAAAGACAAGATAACGAAGGAA
GAGGAAACCGAACAGGTGAGAAGACGAACCGCGTCCTTGCTGGACCAAGACAGCGATCAG
GGCCCAGTCCCGGATGCCTTTGGAGATTACATGCGGCAACAGGCCGGTGGCGTCACCCAG
GTTGCAAGTGCGGGCTTTGCCGGCGCTGGACTCATGCTTCAAAAATTCACCAACTCTACC
CGTGGACTCCTGGAAGAGTCGTTCTTAATGATCACCGAATCCCACATTATCGAAATGAGA
TCGAACAAATTGAACCTGAGCACCGGTACCGTATCCTTTGCCATTCCCATCGACATGATG
GCCAAGCTCAAATTCCGGCGACAGGAATCCATTTCCTTGTTCTTCAAACCCGCTCCTGAG
GATCCTCTCATCTACATGTGTCCAGACAGCGCGGATTGTGTCCACCAAATCCAGACCGTT

TTGAAACGACACGGTGTCAAGGGAAAGCACACCAATGCGGCTACCCACCGCGCCATCAAC
GAGGCGCTTCAGTTGGTTCAGGAGATCCAAACCAAGGAGCTTGCACTGAAGCACGATCCC
AACATCGAGCGGGTGAACCAAATCATGGATCTATACCGACAGGCCGCGGAACGCTTCGAG
GTGGCCGGTGATGTTCGACACGAAGAAGTCGTGACCCACATGCGCAAGTTCTTGGCGTTG
CCCCTCACCGTCAGTGTCTTGGATGGAAGCTACGTCACACCCACGCGCAATGCCGACAAC
GAACCCATTCCGTCCCCGGTGACCAAGGGAGGCGCGTACGACTCCAACATGGACTCGCTC
CTTTCCAAAGCCGAGACCGACCTGTCTAACATGAAGGGTAACGACGACAAGGAACTGGAC
GATCTCTTTGCCGACGGGACGGACGATGCTCTCGGAAGCACCGCGGATACCATTGCCGAG
TTCGAAAACATGCTCAAGGAGGCCGACAAGGAATTGGCCGAGCTGATGGCATCATAATAC
CACTGCAGGCATACCACCCACCACCAAATGGATGCCATATGCAATCCCCGGTACGGTGGT
CGAGAAGGATCGTTGGATCAAGCACCCGTGGACCCTTGTTTCCGAGACAACGAAAGTGAA
AACACTCTAAAATCCAAATCTGAGATGCAACTCTACCCTAGTGCCAAACACAAAAACAAT
AACATCTGTGTTAGAAGTTATTGGTTTGTTTGTTCGAAAGCTCTTTCTGTTGTAGCTTGC
TATTCTAATAATAGTGGCACTAGCTCATACATTAAACATGCATTTAAAACATATTTTTTC
TTAA
>comp2922_c0_seq1
ACCGAACCCTTTCGTTTGTTGCTCGCACCAAACCAAACCAAGCCAAACCAAGCCAAGCCA
AACCAAGCAGAACCCAAAAATTAAAACACAATGTCGGAATCTCCCTCCTGGCTCGATGCA
CCCGCCCCCGCCCCCGCGGCCGCTGCCCCCGCGGTAGAGCCGATGAGCATCGAGGGCACG
GGTGCTGCACCGACCGCTGCGGCCGACGAGAAGGACCTGCCGTCGATCATTCTGATGATG
CGCCTCCTCAACCTGGCGGCCGCCGCGGCACTCATCGTCGTTTCGAGCCTCCAAATCGTC
GGCATACCGGCACTCTCGAGCATAGTCCTGGCGGTCTACACCATCTGCGGTGGTTTGTTG
ATCTGCTGTCTGGAAACCCAGCTGAAATTCCTCCGCGTCATGATTGCCGTGAACTTTGGC
TTTTTGTTCAACTCCTTTTGGCGATTCCTCTTTTACCTCATCCTGGGATCGGTGTGCTGG
GCCTTTGGCGGGATCATGGGAAGAACGATGGCCTGCGTCATGGGAGGCGTCGCCCTCTTC
AACACATACGTCCTGTGTCGCTATCCGTCCTACCGCAAGATCCGGGAGCAGATTGCCGAG
GAGGAAGACAAACGAATCGAGGCCCGGGTTTCCAAGGAGGTGAAAACGCAGGCCTTGAAG
CAGATGGCCAAGTCCGCGACCGGGAAGTAAACAAAGAGAACCGAAGCAAACGCAAGAAGA
ATCGGGTGCGTCGTGCCGCACCCGGTCTTTTTTGCACGGTCGTGGCTCGGAGGACGAGTT
TTTCTACGTATGTAATGAAATAATGAAATAAACCGAAGAATATTAGCAGAAAAGGTTCCG
TAG
>comp2923_c0_seq1
ACTGAACCCAAGAACGGTTCCACAAGTACAAGTAGTCGTACTAAATCAAAGAGGACAGCA
GCAAGTCATTTGAAAGCATTGGTGGTATCGAAGACGAAAACTTCAACTGAGTAAAACAAT
TATCTTGATTCCTCGATCTTACGAAAGAAGAGAAAAACTAAGAAGTAGTTAAAGAAAGCA
CTAAACCCCACGATTCAAGCTGCAATCTGAAATCATCAGCATCGTTGCATCCAGCGGCAA
CGGCAATCACAACATCAACGTTTACTCATAGTAGAATAAATTCCGATTGCCTACACGATA
GCCGTTAGGCGAAGGAACGTTCAAGGATGTCTCGACGCAGAGGAGAGTTTGAGCATCGCA
CCTCGATCTTTGAAGGAGCCTTTCGTGCTTGGTCTCACTTGGGAGGTCTGGAACCACAAG
AGCTGGATAGCAAGGCCTCTACTGATCTTGATCCTCTTTCGCATCCAATCGAAAATGACG
TACGTTTGCAAAACAAGAAAGACGGCAAAGCGGGATCGAATGCGGATCCGAACGGCAGTA
AACGCAGGCAGTTCCCTTACGAGCCTCTTCCCCGCTCAGGTCTAGCACATCCCTATGTTC
AAGCAATTTTGAGCCCTTGGCTCGGTTCCGATGCCGATAGTGATGCCATTCAATTGGGAT
TGACAACCCTGCGGACCTGGTGGCAGCATCGTCGCAAGGGAGAAAGTGGATCGGCGATTG
CGGCCTTGGGAACAGAAAAAATGAAAGGGGTAGTGGAAGGATATACCCGTCATTTTTTTA
CTCTGGCATATTGTCTAGTAATCAATGACAAGGAACAACCACCGAGGACGCTTCACGGAA
AAATGAAAGAGCTTGAAAAGGTTCGGGATAAGAAAAAGAAAAAGCGTTCGCGAGACACTG
AGAAGGGAGACAACGCGAACGCTATGGGTATGAATTCAATGGGTAGGAGAGGTGTTGACT
TGAGCGCAGAAATAATGAACAACCCGAATCTATCTCCACTTGAACGTCTTCATATGGCGC
AGCAAAATCAATTAAATGGGGCAGGTGGAATCAAATCCAGCCTTACGGAACCAGGACAAA
TGATTAATGTTAAGAATAAATGCGTCCCTGGCGTAGTGGATTTGCCTGGCATTGTTGACA
AGGTCAGTCGCGCAGCCACGCAGCTAGCACATTCTGGTGGAGGAAGGTTATTATCAAATC
CATCTGCGGTGGCATCTATGGGTGGGATGAATGGTGATAATGGGGAGCATTCGGACACCG
TTCCAATTATATTCGTAACAGAAAATAGAGAAATTATGATTGCTATGGCGGTGGAAGGCA
TCACATGTGCTCATTGTGTGAAGATTGTCGAGACGGTACTGAGGGGATGCAATGGAAACA
AATCGCCTATAGACGGACTGATTGACGCAGCTGCTGATAGAACTCTGAATGCAATTTTGA
TTCGAATCGACCGAGTAAGGAACGCAAGACGTATTTCTCTGGAAGCGACTCGCAATCTTG
CAATGGTTGGATACCAGGTTCAAGTCAAGACCATGAGAAAGATCGTGGAAAAGAATGGAA
ATTCAACTAAAGTGCCACAGGATGAAATGTACATGGCTTTCGAAAGAATGGGTGCAATCG
ATCCGAGGGATATATTTGATTGGAGTGCTCCCTGTTCCTGTCCCGACAATGGCGTCGCTC

GCGATGATTGCCTTAGGTAAGAAAATGGGGTGAATCACTTCTCTTTCATGTTCGTTGAGC
ATTTCATTTTGGCTAGTTCGACCGACAAAATTCTCAAATTTGGTCGATAAATTCTTTTAC
CTTGATTGTATCAGACACGGGCAAATGAACAACTCTCTGCTTGATGCATTCGAAACAAGA
GCGGCAGGTGTCCGTCGCTTTCTTTCAGGTGATATGCCGCAACCGATACAGAAAAATCAC
ATCCCACAGCGAATGATGATGAATAATCACTTTCCGCAACAAAGGCAACAGCAGGGAATG
ATGGATGATATACAACCATTAAATGATACAACTGGTATGGGATTGGACATGTTTGGAGGA
TCAAGTGGAATGAACAACATGGTTGCTAACATGAATGCTCCCAATAGTGGCGGTGACATT
AGAGGATCGAACATGGGTTCGGGAAATAATATGAACAATAACCAAATGATGTCCTTTGGG
AATCACCGCATGTCCCTAATGGGACGTATGTCACAGAATCCGAATAATAGAATGTCTTTA
ATGGGACGTATGTCAGAGATATCGTATGGCCGCGCGATGTCTGGTCTTTCGGCAATTTCA
ATTGATTGGGAGAACATGGAGGATTTCGATGTCAATGTTGATCATAGCGAGCACATAAAC
AACAACATGGGAGCTGGGGCGGCTGGTGCAATGGGAAGAGCTAGAGGGCCACGTGTTGCA
CCTCGGTGGAGCATGGCGCAACATATGAACATGAACAACAATGCTGGCGGTCAAAATATT
ACAGATAGCGATGCACATGTTCAATTTAAAATCTAGTTGATGAATTTGTTGGAGCAATCG
TGAAAATATTCAGAAATTAAAATACAGTTGTGGGCAAAACTGTATCTTCGGTGTGAAAAA
AGAGCAAAGTCTATTGCTTAGAACCGTACATGGTGTGCATAACTTTTCCACAGTTTTTGT
ATAATGAGACCTTTAAAACTTTCAGAACGGGATGCTTTCTCCGATCTATAATGGGTGTCA
CGTACCTAGTGACCTTTCTTGCTGACCGCAAGTCTCTAACATAATTACTCTATAGGATTG
TGTAAAATTAGTACCAGA
>comp2923_c0_seq2
ACTGAACCCAAGAACGGTTCCACAAGTACAAGTAGTCGTACTAAATCAAAGAGGACAGCA
GCAAGTCATTTGAAAGCATTGGTGGTATCGAAGACGAAAACTTCAACTGAGTAAAACAAT
TATCTTGATTCCTCGATCTTACGAAAGAAGAGAAAAACTAAGAAGTAGTTAAAGAAAGCA
CTAAACCCCACGATTCAAGCTGCAATCTGAAATCATCAGCATCGTTGCATCCAGCGGCAA
CGGCAATCACAACATCAACGTTTACTCATAGTAGAATAAATTCCGATTGCCTACACGATA
GCCGTTAGGCGAAGGAACGTTCAAGGATGTCTCGACGCAGAGGAGAGTTTGAGCATCGCA
CCTCGATCTTTGAAGGAGCCTTTCGTGCTTGGTCTCACTTGGGAGGTCTGGAACCACAAG
AGCTGGATAGCAAGGCCTCTACTGATCTTGATCCTCTTTCGCATCCAATCGAAAATGACG
TACGTTTGCAAAACAAGAAAGACGGCAAAGCGGGATCGAATGCGGATCCGAACGGCAGTA
AACGCAGGCAGTTCCCTTACGAGCCTCTTCCCCGCTCAGGTCTAGCACATCCCTATGTTC
AAGCAATTTTGAGCCCTTGGCTCGGTTCCGATGCCGATAGTGATGCCATTCAATTGGGAT
TGACAACCCTGCGGACCTGGTGGCAGCATCGTCGCAAGGGAGAAAGTGGATCGGCGATTG
CGGCCTTGGGAACAGAAAAAATGAAAGGGGTAGTGGAAGGATATACCCGTCATTTTTTTA
CTCTGGCATATTGTCTAGTAATCAATGACAAGGAACAACCACCGAGGACGCTTCACGGAA
AAATGAAAGAGCTTGAAAAGGTTCGGGATAAGAAAAAGAAAAAGCGTTCGCGAGACACTG
AGAAGGGAGACAACGCGAACGCTATGGGTATGAATTCAATGGGTAGGAGAGGTGTTGACT
TGAGCGCAGAAATAATGAACAACCCGAATCTATCTCCACTTGAACGTCTTCATATGGCGC
AGCAAAATCAATTAAATGGGGCAGGTGGAATCAAATCCAGCCTTACGGAACCAGGACAAA
TGATTAATGTTAAGAATAAATGCGTCCCTGGCGTAGTGGATTTGCCTGGCATTGTTGACA
AGGTCAGTCGCGCAGCCACGCAGCTAGCACATTCTGGTGGAGGAAGGTTATTATCAAATC
CATCTGCGGTGGCATCTATGGGTGGGATGAATGGTGATAATGGGGAGCATTCGGACACCG
TTCCAATTATATTCGTAACAGAAAATAGAGAAATTATGATTGCTATGGCGGTGGAAGGCA
TCACATGTGCTCATTGTGTGAAGATTGTCGAGACGGTACTGAGGGGATGCAATGGAAACA
AATCGCCTATAGACGGACTGATTGACGCAGCTGCTGATAGAACTCTGAATGCAATTTTGA
TTCGAATCGACCGAGTAAGGAACGCAAGACGTATTTCTCTGGAAGCGACTCGCAATCTTG
CAATGGTTGGATACCAGGTTCAAGTCAAGACCATGAGAAAGATCGTGGAAAAGAATGGAA
ATTCAACTAAAGTGCCACAGGATGAAATGTACATGGCTTTCGAAAGAATGGGTGCAATCG
ATCCGAGGGATATATTTGATTGGAGTGCTCCCTGTTCCTGTCCCGACAATGGCGTCGCTC
GCGATGATTGCCTTAGACACGGGCAAATGAACAACTCTCTGCTTGATGCATTCGAAACAA
GAGCGGCAGGTGTCCGTCGCTTTCTTTCAGGTGATATGCCGCAACCGATACAGAAAAATC
ACATCCCACAGCGAATGATGATGAATAATCACTTTCCGCAACAAAGGCAACAGCAGGGAA
TGATGGATGATATACAACCATTAAATGATACAACTGGTATGGGATTGGACATGTTTGGAG
GATCAAGTGGAATGAACAACATGGTTGCTAACATGAATGCTCCCAATAGTGGCGGTGACA
TTAGAGGATCGAACATGGGTTCGGGAAATAATATGAACAATAACCAAATGATGTCCTTTG
GGAATCACCGCATGTCCCTAATGGGACGTATGTCACAGAATCCGAATAATAGAATGTCTT
TAATGGGACGTATGTCAGAGATATCGTATGGCCGCGCGATGTCTGGTCTTTCGGCAATTT
CAATTGATTGGGAGAACATGGAGGATTTCGATGTCAATGTTGATCATAGCGAGCACATAA
ACAACAACATGGGAGCTGGGGCGGCTGGTGCAATGGGAAGAGCTAGAGGGCCACGTGTTG
CACCTCGGTGGAGCATGGCGCAACATATGAACATGAACAACAATGCTGGCGGTCAAAATA
TTACAGATAGCGATGCACATGTTCAATTTAAAATCTAGTTGATGAATTTGTTGGAGCAAT

CGTGAAAATATTCAGAAATTAAAATACAGTTGTGGGCAAAACTGTATCTTCGGTGTGAAA
AAAGAGCAAAGTCTATTGCTTAGAACCGTACATGGTGTGCATAACTTTTCCACAGTTTTT
GTATAATGAGACCTTTAAAACTTTCAGAACGGGATGCTTTCTCCGATCTATAATGGGTGT
CACGTACCTAGTGACCTTTCTTGCTGACCGCAAGTCTCTAACATAATTACTCTATAGGAT
TGTGTAAAATTAGTACCAGA
>comp2924_c0_seq1
TCGATACTATTGTTCTTGATGACAATCCTCGTTTGGGAACAACTGCCCGTGTTGAAAGTA
AACCCGCTTGATTCGATGGTCGTCAACGCCCTGGCGACCACGAACAACAAGCAATTACGA
ATGCAAACCTCGTCTACCTCGCCGCCGGTCTCATCGCCAAGAATAGCTCCCCCGCCACGG
CTGCCTCTGAAACCGCCCTTTCCCAACGGTCTTTGTGGTGGTACGATGGTGGACATTGTT
CCAGAAGAAACCTTTGGCGTCGATGTCAATCTTGCCAATTTTAATTTTATGGGTGACATG
GTCCTGTTGCCACGAACGATTCGGATATGGCTCCCACCAGGATACGATGAAAACGATAAG
GATGGAGGACATCCCGTATTGTATGCTCACGATGGACAAAATGCTATGGAAGACGAAGAG
AGTTGGACGGGGGCCTCGTGGAGATTAATTGGAGCCATCACTCGACTAGCTGATCACAAG
CTGATCTGTACATCATCGAGCCCCGCTACGAATAGCACATCCAGCAGGAACAACGGAAAC
GAAAGCCGACAACCAACATCATCTTCCTGTACCAGTGACGGAAAGCTTCCCATCGTTGTG
CTGTTACCCAGTGCCCAGGGTGATTGGCTGCCGGGAATTCGACGACGACATCTTGAATAC
GGAGACATGAATTTTCCCTTCGCGCAAGCCCACGCAGATTTTGTTGCCAATACGGTCAAG
CCCTTGGTAGACAATCGGTTCAGGACCAACCCTTCCGCGGAGCGAACCTTTTGCATAGGA
TCGAGCCTGGGGGGTCAGGCATCCCTGCATTTACTGCTGAGACATCCCGACAAGTTTGGT
GGCGCAGCCTGTTTGTCTCCAGCATTTGGGCCCGCGATACTGAATTTCGTCGATTCTTCC
AAGGGAGGCAAAATACTACGAGAGGGGAAGAAGAAGGTTTACTTGGATATCGGGGGTGAC
ATGGACGATTCTAAAAGCAGCGACTTTGTTCGCGTCTCGCCTATTGACGTATTAGACCAT
TTAACACCGGTCCATGGATGGAACCCTGGCTATTTCTGGTTGGACACCCAACTTCAAGGA
CAGGTAACGGCTATGCGGAAGGCCCTTGATCGATCCGGAGCTGATTACGAATATCGACAA
TATCCCGGAGGTCGGCATAACGAACGAGCCTGGGCCTTGCGGATTGATAAGCCTCTTTTG
CATTTGTTTTCTGACAAACGTAGAAAGAAAGAATAACAAAAAAAACACTACGAATAGAAT
GGCT
>comp2925_c0_seq1
GATCAAACGAAGCAACTAAGCAAGCTGCAACGCTATCTCTTGCCGTCATTCAATACGAAG
TGTATAAGATAATACGGACACAAGCAATTGCCTTAGAGAAGAAGCAGAAATGGCGCCTGG
AGCGGAAGGGAAAGGCGTTGTTTTCTGTTGTTGTGTGCATGCCTACCTACTATGAATGAA
GAGTCTTCTTCCTCCTCCTCCAAACGGCGTGGGCCGACGACCTATCCTCCTCCAGACGCA
TCGGCGGCTACCGATGATGCTACGGAGGCGTCGCTAACGCGGAGAAGCAAGAACGATCGG
ATCCCCTTGTCCCTGGGAAAAGCCTCGCTGAGGATCCGGCTAGAGGCGTACTACAGCTTG
ATATCTCCAGCAATACTGTCCGATCGGACTGCGTGGCTACGCAAGTACGATCAAATCTAC
GAAAAGGTGAGCACTGGCACACAAAAACAGGAAAGCAAGCGCGCTGTCCTGTTCTGAACC
ACATTCATTCCGTTGTTTATTGTCATACCAACCTGGAAAATCACCACTGGCTTTTAATGC
TCTTTTGGATCGTTTCTTACGACACACTCTATGCGACCAAATCGAACCGAATCAGTACGG
AGGAACCCATTCGGGAGAGCGAAAACTAGCAACCAAGCTGGCGAAAAAGTATGGAACGGC
CGTGCGGCTCCTCTTGGTGTCCGATTCGTCCTCCCTCGTTCCGAGGTTTCCTACGGATAG
CAACCGCAGCAATGATCTCGCTAACACGAGGAAGAGCAATCTTCCACGAGACGAATCGTG
GTACCAGCTACGGAAGGAGGAAGCTGGAAGTGGAATTGTCGACTTTTTGTCCTCAAGGTT
CGACCCGGTCGAGGCACTTACGGGTGCTTCTGGAAGGGGCAGTGTTGCCAGAGCCAATCC
GTGGATGGAAGAGCTCTTGCCGAAGACCATTCTAGACAACATCGGAAAGTGCGCTGCTCT
GCTGCCCACCGATGATCCGTTGCGAAAGGAAAATCATCTCCAGCTGCAGCTTCGGAAATC
TGCTGCGTCGCGGAATAGTTCTTCGCAGCAGAACCAGCTTGCCCTGGGCACGGCGGCGGC
AGCAGAATCCGTACAGATACGGCCCACCACCTTGCGCAAGCAGACCAACCACGATCTACA
TCCTTTCGAGGCGATTGCCTCGCACCTCGACACCGGGCCGCATTCTCTTCTTTTTCGGTT
GCGCAGGGACCGAAAACGCGCCAGGGTCGTCGTCCGGTACGTCAACATGGTCCGGGGAAC
GATTACGGGGAGCCTTGTGGCCTTCGACAAGCACATGAATCTGATACTGAGGGACGTTGA
GGAAATTTATTCCCCCCGCATCAACGACAAAGAATGCCGTCTTTCAAACCTGGAACTCGA
ACTTGAGCGAAGGCGGAGGCTATCGCTTGATGCGGAGGATCTGGGCACGAGTCTCGAAGC
TGCAGCAGCAACTGCCACGAGCAACCAGAGTGGGGGATCCTCGATTGATTATAGAGTTGG
AAAGGGATGTTCTGCAATACAGGAGCAACATCAACACCAATCGGGGAACTGGAAGGTGCG
AAGACGACAAATGAAACAACTGCTGGTGCGCGGGGATATGGTGGTTTCGATCTACGAGGC
CGCCCAAGAACGAACGAATGTGCAGAAATCGAGATATCGGAAACGAATCGCAACATCAGC
GTTGGTGCGAGCTGGAGCATTTGGAACGAGTAAACGTCTTACAGAAAAAGAGGCAAAGTA
GTCAACGTAAAACTTTTTCGACAGCAACGACTGAAACAGGGCCAAGGAGAGTGGGATGGG
ATGGATTAAGAGGCAGCGACAATTGGTACAATATTCATGCACCTAATTCGATTCAAGAAC

GTGCCCTGTTTAGGAATGATGGTGGCATTTTCCCTTGCTCGTTCCGCGCATTATCATCTA
CTCCTTCTAAAACATAATGATAGAATGGAACCACA
>comp2925_c0_seq2
GATCAAACGAAGCAACTAAGCAAGCTGCAACGCTATCTCTTGCCGTCATTCAATACGAAG
TGTATAAGATAATACGGACACAAGCAATTGCCTTAGAGAAGAAGCAGAAATGGCGCCTGG
AGCGGAAGGGAAAGGCGTTGTTTTCTGTTGTTGTGTGCATGCCTACCTACTATGAATGAA
GAGTCTTCTTCCTCCTCCTCCAAACGGCGTGGGCCGACGACCTATCCTCCTCCAGACGCA
TCGGCGGCTACCGATGATGCTACGGAGGCGTCGCTAACGCGGAGAAGCAAGAACGATCGG
ATCCCCTTGTCCCTGGGAAAAGCCTCGCTGAGGATCCGGCTAGAGGCGTACTACAGCTTG
ATATCTCCAGCAATACTGTCCGATCGGACTGCGTGGCTACGCAAGTACGATCAAATCTAC
GAAAAGTACGGAGGAACCCATTCGGGAGAGCGAAAACTAGCAACCAAGCTGGCGAAAAAG
TATGGAACGGCCGTGCGGCTCCTCTTGGTGTCCGATTCGTCCTCCCTCGTTCCGAGGTTT
CCTACGGATAGCAACCGCAGCAATGATCTCGCTAACACGAGGAAGAGCAATCTTCCACGA
GACGAATCGTGGTACCAGCTACGGAAGGAGGAAGCTGGAAGTGGAATTGTCGACTTTTTG
TCCTCAAGGTTCGACCCGGTCGAGGCACTTACGGGTGCTTCTGGAAGGGGCAGTGTTGCC
AGAGCCAATCCGTGGATGGAAGAGCTCTTGCCGAAGACCATTCTAGACAACATCGGAAAG
TGCGCTGCTCTGCTGCCCACCGATGATCCGTTGCGAAAGGAAAATCATCTCCAGCTGCAG
CTTCGGAAATCTGCTGCGTCGCGGAATAGTTCTTCGCAGCAGAACCAGCTTGCCCTGGGC
ACGGCGGCGGCAGCAGAATCCGTACAGATACGGCCCACCACCTTGCGCAAGCAGACCAAC
CACGATCTACATCCTTTCGAGGCGATTGCCTCGCACCTCGACACCGGGCCGCATTCTCTT
CTTTTTCGGTTGCGCAGGGACCGAAAACGCGCCAGGGTCGTCGTCCGGTACGTCAACATG
GTCCGGGGAACGATTACGGGGAGCCTTGTGGCCTTCGACAAGCACATGAATCTGATACTG
AGGGACGTTGAGGAAATTTATTCCCCCCGCATCAACGACAAAGAATGCCGTCTTTCAAAC
CTGGAACTCGAACTTGAGCGAAGGCGGAGGCTATCGCTTGATGCGGAGGATCTGGGCACG
AGTCTCGAAGCTGCAGCAGCAACTGCCACGAGCAACCAGAGTGGGGGATCCTCGATTGAT
TATAGAGTTGGAAAGGGATGTTCTGCAATACAGGAGCAACATCAACACCAATCGGGGAAC
TGGAAGGTGCGAAGACGACAAATGAAACAACTGCTGGTGCGCGGGGATATGGTGGTTTCG
ATCTACGAGGCCGCCCAAGAACGAACGAATGTGCAGAAATCGAGATATCGGAAACGAATC
GCAACATCAGCGTTGGTGCGAGCTGGAGCATTTGGAACGAGTAAACGTCTTACAGAAAAA
GAGGCAAAGTAGTCAACGTAAAACTTTTTCGACAGCAACGACTGAAACAGGGCCAAGGAG
AGTGGGATGGGATGGATTAAGAGGCAGCGACAATTGGTACAATATTCATGCACCTAATTC
GATTCAAGAACGTGCCCTGTTTAGGAATGATGGTGGCATTTTCCCTTGCTCGTTCCGCGC
ATTATCATCTACTCCTTCTAAAACATAATGATAGAATGGAACCACA
>comp2926_c0_seq1
CAAAGAAGCACACAGACATCAGTAGTTGATAGAGAACAAAAATGGTTCGCAGCAGTCTCT
TCTCGGCAATTGTTTCCCTTCTTTTGGTGGGGCCTTCCCATGGATTTTCTCCGTCAACGC
CATCGCCATCGACGACGACAACACGTCTGAACGGCTTCGGGGATGCATTCAAGAATGCAT
TTGCCAATGACGACGATCTTGGAGCTCGAAAAAATGAGGGTCTCTCCGGTGGTCCCAACT
ACAACGAATCGGTCACAATGAATGGAAAAGCAGTCCCAGGCTGCGTCGTGGGTCAAAAAC
TAACGGTTGTAGGGGGAAAGGCGAGAGTCAAGATTCCTGTGAATTGCCAGGCCGGAGATT
GCGGAACCTGTGTGGTGAAGATGAACGGGCGAAAAGTGAAGGCTTGCCAAACACAAGTAC
CTAAAGGAAAGTGCAATATCCAGACCTTGTAATTTATTCTAAATCGAAAAAATAAACTTT
GTCGTGAAAACAATTAAGAAGAAAAAATTGCTTGTAGATTTACCTCTCTTTGTGGATTTC
GATCTACCATTGTTCTGCCTACTGTGATCCAATA
>comp2927_c0_seq1
AACAACAACAACACCAGACCTCTCCCACCAACGGCACGCAATACCAACGAAGGCCTCCTA
CCTTTCCCGAATTGACGCGAAGCAAGAAGTAGAGAACCTCGAAAAGAAATCAGTTGCGCT
TGGGCGGCTGAGAGGCATGAATGCAATGAAGATTGTAAAGCCATTGATTCAAGAGAAAGA
AATTGTCCGTAAGCAGTTCATCCGCGCGTGCATCGCGAGCAAGCAATATATCAGAGACGC
ATGAATATTTTTGATCTCGTAATTCGAAAAAGACGCACAGACCCACATATTTTCAGACCA
AGACGAGATATCTATTGGTACGTCTTGCGAGAGAAAAAGAATTGCGAAAAACAAACATCG
CTAACAATTACAACTATTATGACTATCAATACTTATATCTACTGAAAAATTTGGGTAGTG
GAGATGGCATGAAATTCTTGTTCGGTTGGCTGTTCAGAGAATTTTCGAAACAATTATAAA
TTGATATTGAGAGA
>comp2927_c0_seq2
AACAACAACAACACCAGACCTCTCCCACCAACGGCACGCAATACCAACGAAGGCCTCCTA
CCTTTCCCGAATTGACGCGAAGCAAGAAGTAGAGAACCTCGAAAAGAAATCAGTTGCGCT
TGGGCGGCTGAGAGGCATGAATGCAATGAAGATTGTAAAGCCATTGATTCAAGAGAAAGA
AATTGTCCGTAAGCAGTTCATCCGCGCGTGCATCGCGAGCAAGCAATATATCAGAGACGC
ATGAATATTTTTGATCTCGTAATTCGAAAAAGACGCACAGACCCACATATTTTCAGACCA

AGACGAGATATCTATTGTGGAGATGGCATGAAATTCTTGTTCGGTTGGCTGTTCAGAGAA
TTTTCGAAACAATTATAAATTGATATTGAGAGA
>comp2928_c0_seq1
GAAGCATCAAGCATCGCCAATCAAATACAGTAGTACGAAGTACTTTAGTACAGTACTTTA
CTTCGTAATTGACTACTGTGAAATTTGAAGCGTACTCGTACCTTCAATAGATATGTAACA
TATCACTATGAGATTCTGGTTGCTTGAAGAAGTCATTTTATGACATCGAAGGCATTGAGA
ATTATTATGCCACTTGAATGATTGTTTAGAAAAGAGGAGTCATTTTCACGAGCACAACAC
GGAAAAGCGAATTGCTGATATCCTCCCCACAAGACCAATACGACTTTTTCTACCGCACTG
TGCGAACCTCGGCATGAACGGTAACAACGACAAAGCGAACGGCACGATAGTTCCGCACCA
GAAACTAGTACTCTTACGGCAAATCGAGATTACAATATTTAGTCTGTGGGAAGACAAAAT
ACAAAATACGGTACAAGGGAACGTGCTGAAGTTAATCCCAGAGAGGAATAAGAAAGAACA
AACTGCAACTCCCAATAATTTCCATAGATCTTTATCGAAACCAAGTTCAAAGCCTCCGAG
TAGACGGTCATATGGAAACCAAAAAACAACATTGTTAAAAATAAGAACATTCAGAATGAA
CTTTCAGCGACCAAGAAGGAGTAGCCGGCGATATGCGGGCGACAGAGCCATCGGAACCGA
GGCGGTTGCCAACCTTTCTAGTATGAAACAAAGAAGATCGGCAGATGGGACATATCTACG
CCGGCGTTATGCAACTATTGCCGTCGGTCTCGTGGTGGTGATCGGTGTGATCATTTTTGC
CGTGACTAGTACGAAGGCGAAGGACGAACTTCCAGTCAGCGTGGCGGAGATCACGGAAAA
AGTCATTTCAGAGATTACACACAGTCACACGGATTCTTACGAATTGTTGACTAGCCAGAG
TTATAGCGATGCGAACTCTACAATTGGCGTCAACTCTACACTAGCAGTTTATGAACACAA
AAAGTCAGGCATAGATATTGTAACAATGATTCCCATTGATCCGTATCAAGATGCGACTTT
TGGTATCAATTTCAGAACGCCTTCGGAAAGAGATGATGGGGCACAATATGTTGTGAAAAA
TGCTGTGTTGGCGGGCTCAGAGAATTACCCCGTCAAAGATCCCCTTAATCAAATGAAGCG
TGGGTCTCTTCAAACCTATTCGGATTCTTGGATCGAACGAGATCGCACTTCGTTTGTGAT
GGCATCAAGAAACTTGGCCGACTTTCGAAACAATTTAAAGGTGACTGTCGATGCTGTATT
CAATCCACTTTTCATCAAGGATGAACACAAATGGATATACCGTCAGGAAGGATGGAGGTT
AGAATCTCCAGACAATGTCAATTTGCTCATCAGTGGAAACGCATACGTGGAGGCCAAGGC
AGCACAAATGAACCCGGAAGAGGTTATGATAAAACACATATATAGCAACCTCTTTCCGGG
TCATGTATACGGCAAGAATCCGAAGGGAGAAGCTTCAGAAGTCGTAACAATGACGTACCA
AGAGGTCATTGCATATTACGAAACCTACTACCATCCATCTAATGCACAAGCGTTCTGCTA
CGGTAGTCAGGAATTTATTGACGTCTGCTTGAACGAATTGGATGTTCAATTGAGTGAATA
CGAAAATAATACGTCGGTTGGAAATCATTCCCAGATACCATGGCAAGAGTTGACAGAAGT
CGAGGAGCAAATTAAAAACATTACCTATCCCGATTATCAAGAACAAGTCGATTATCGTTC
GGTCATAGCATGGATCCTCAATGACGAGCCTATGGACCTCCGTACCCAAATCGCTTGGCA
GTTGATCTACGAGTTGCTGGCAGGCTCGAATACTGCACTTGTACCAAAAATTATTTTCGA
TTCCAACCTGGGGACCGATATTGTGACACACTTCCAGCATTCGCTTCAACAATGGGTCTT
GGCACTAGGCGTGAGTGGTATTGAATCATATGAAGATGTAGCAACTGCTGATCGTAAAAT
CATGCAAGAACTCGCGAAGGTGGTCAGTAAAGGCTTCGACTCTGACGCCATGGAGGCTGC
GATGCACAAAATGGAATTTAAATTTCGTGAACAGAGCCCTGATGATGTTCCCCTAGGAGT
AAAATATTTTAGAGATATTTTGAGGAACTGGAATTATGGTCAAGCACCATTGGAGCCGCT
GAATTCAGCAACAGAGTTCCTCAACCTGAAAGCGGAGATCCAAAATGAAGGCCAAGAATT
TCTCTTGACACTCATGACCCAACGAATGATGGATAACAAACATACTGTATCACTAAGCCT
CCATCCAGACACCAACTATGCCATTCAATACGAAAGACTGGAACAAAATTGGTTGGATAA
CTTGGACACCTATCTTAACAAGGAAGAGGGATTGAAAGTTATGTCGGAAACTCAAAAATT
CAAGGAGATGCAAGAGAACGGCGATTCGGAAGAAGAATTAGCGAAGATTCCTCGTCTTGA
AGTCTCTGAGCTCAACCAGACCATGTACATTCCTCCGTCCGATCTCATTAAGAATCTGTA
TGAAACGGATGTTACTGCGATCAAGCACAAACTACCTTTCACTAACGGTATTGCTTACGT
GGACTTCTCCATTGACATGTCAAATATGGATTTTGATGATGTCGTTCTCATCCCTCTGTT
TTGCCAATTGCTTCTAGAAGGAGGGACGGAAGTCCACGACGATGTTGAGATGCAACGGCA
AATTGATAAATACACGGGGGGTGTAACCATTACCCCCATAATCGAAGAAATCGTTCAGAC
GGACGATCACGATTATTATTTGGTTCCAGATGGGAAACATTTTGTTACGAAATTGATCAT
CAGTGGATCTTGTATTGCGTCCGATACATGCCTACCGATGTTAATCCTATTTCGCCAATT
TGTCTGGGATTCCAATGTTCGCAACAAGGAGAAAGCTATTGAAATTTTGGAGGCAAGAAT
TAACGAAATGGAACAAGATGTTCAAATCAACGGACACAAATATACGACCAGTCGTGTTGA
GTCTCGGTACAGCCTTTCTGGGTTCGTACGAGAACAATGGTATGGTATTACACAATTAAT
GCAAATGAGAAGAGCGCTTGCACAGATCAAAGACAACTTTACTGAACTTGGTCTGCGTCT
TGTCAAGATGCAGGATGAACTTAAGCGAGGGCATCACAACGGAATGCACATGTCAATCAC
TGGAGACCACGAAGCACTGACTGATATCAAAGGTCCATTGAGTAACTTCATGAAAAACGG
TCTCCCGCTTGCCACACAAAAAACACCTTTTCCGAATTTTGCCGAAGAAGAGCACCCGTG
GGTCCCCAAGGGCATGCATAGAGTCAAAGAAGAGTTTTCATCCGAAACTTCCAACCAAGC
CTTCACTGTGCCTACTCGAGTTAACCATGTTGCTAAGGGCGGAATTCTGTACGATGTTGG

TGAACGCATTCCAGGAGCCGACATGGTTGTTACTCAGTATCTTGGTGGTTATTACCTATT
CAACGAGCTTCGCTTCGGTCAAGGAGCTCAAGAGGCGTGGTCTTTCTTAGACCTTGATTC
AGGCGTATGTATATACCAAAGTGATCGCGACCCTGGTATTTTTAGTACCCTTGAGACCTA
CGAACATGGGGCAACTTGGCTTTGGGAACAGGTTCACAACGGAGAACTTCCTTTGGAAGC
AAAGAGTGCCATAGTTGGTGCCATTGGCCGTATGGATGCCAAAATAATGACCGAACCCAA
AAAGTTGGGGAGACAAGCGCTAATTAGTCATTTGAAAGAAGACAGACATGAGTTTAGACA
ACACTGGCGTAATCAGATCCTTAATGCCAATGCTGATGATTTTATGGCAATGGTCGAGCG
TCTTGGATCTTGGGGACATCCATCGGTGTGCGTCGTGACAAGTCCTGAGGTTTACGATAC
AATTGATCAAGAGGACTTCGCAATTTCGAAGTGCGATTACTCTGGCTACCAGTGCTAAAA
GAAAGACTTCTACCAAAAGAATGTCGTATCGTCGTTCCAACAACTACAGGGGTTTGAACC
AGTGATTCATTGTATAAGTAGTTGTATTTTTTACTAGGAAATGCCTAAATTGTCTTTGAG
TGATGGCGAAACGACTTTCATGCTTTCTTCCATTTGTTCATATAACTTCTTGACATAGTG
GG
>comp2929_c0_seq1
CTCCCATCACCGAATTTCCAGCATTGCACTCTGCATCAAATACGAGTTTTACTACTTTTA
GTTCTACCAACAAAACTAAGTAGTTCAAGTTGAGATATATTCTGATAATTCGGAAGAAAG
AAGAAACATGAAGCTCTTTTCTAGAATTGCAATCCTTGGGCTAATCGCCCAGATGAGGGA
CACTGAGGCATTTGGTCTCGTGTCCAAACCCAATGCGCCAATTTTCACCGGATCGTCTAC
GAGCCTTAATGCCGGGTCCGAGGTGTTCATGCCCGCTCTTTCTTCCACCATGAAGGAGGG
AAAGGTTGTTTCCTGGTTGAAATCCGAGGGAGACGAAATCGAAGCTGGTGAAGCAATCAT
GGTAGTTGAATCCGACAAAGCCGACATGGACGTTGAAGCCTTCGAAGATGGATATTTGGC
AAAAATCATTGTTGACGAGGGAGAGATGGCCCCTGTCGGAGCTGCCGTTGCCTTGATCGC
TGAGACCGAAGGCGAAATCGATGAAGTAGCGTCCGCAGGAGGTTCTGCACCAGTCGCTGC
TGCAGAGGAGGCTGCACCAGCCGCCGAAGCTGCGTCTGCAGGCGCCCCGGATTGCGAATT
CAGTCAGATCGATATGCCTGCTCTCTCTTCCACGATGAAGGAGGGTAAGGTTGTTTCGTG
GTTGAAGGCCGAAGGTGACTCCATTGAATCGGGTGAGGCAATCATGGTTGTTGAGAGTGA
CAAGGCAGATATGGATGTCGAGGCATTCGACGATGGATTCTTGGCCGCCATTATCACTGA
TGAGGGTGAAACTAGCTCCGTTGGCGCACCTGTAGCATTGATTGCCGCCGAGGAGGCTGA
TATCCCTGCCCTCAAGGCGTATGCCGCATCTTTGTCTGGTGCTCCTGCTGCAGCTGCTGC
ACCAGCTGTTGCGGCACCTGCCGCCCCCGCAGCCCCCAAGGCTTCGGCAGCTCCTGTTGC
CCGTGCTCCTGGAGCCCGTGTAGTTGCCTCTCCTTTGGCCAAAAAGAAGGCAGAAGAAAT
CGGCCTCGATCTTAGTACCGTTGCCGGAACTGGTCCGGATGGAAGAATTACCGCAGCTGA
TGTCGAAAACGCTGCCAAGGGCGGTGCTCCTGCTGCTGCCAAGCCAGCCGCAGCCCCTGC
TAAGCCTGTGTGGACTCCCGCTGCTGGTGTTATCGCTGCGACCCCCATGGCACGAGTTGC
CGCTAAAAAGGCGAAGATCGACCTTGCAACTATTACAGGAACTGGGTCGCTTGGACGTGT
CACAGTTGATGACGTGAAAATGGCGACCGGAGAAAAGAAGGCAGAACGCAAGAAGGCTCC
TTCTGGAGAAGCTGCTGTTGAATTGCCAGATGGTTTTGTTCCTTTCTCTGGTATGCAGAA
GGCTGTCAGTGGCAACATGGTTGCTACTTTAACTGTCCCATCCTTCCAAGTTAGCACAGA
CATTGAAATGACGGCATTCGAAGCCATGTACCAGAAGGTCAAGCCAGATGGTATTTCTGT
TTCAGCGATGTTGGCCAAGGCTTGTGCTTTGGCAATCGAGAAACATCCTATTATCAATAG
TGCCTACTCTGATCAAGACGGTGGTGGCATTTTGTACAAGGGAGATATCAACATTGCCAT
GGCAGTTGCTATTGACGGAGGACTCATTACTCCTACACTCATGTATGCTAACGAAAGACC
TATCAAGGAAATCGCCGAAAACTGGAGGGAATTGGTAGGAAAGGCCAAGACTGGAACTCT
TTCACCTGCTGAGTACACCACTGGTACATTCACAATCTCTAACATGGGAATGTTTGGGGT
CACATCGTTTGGTTCTTTGCTTCCAGTTGGACAAGGAGGTATTTTGGCTGTTGGAGCGAC
ACAAGATAAGGTCGTGCCAGACTCCCAAGCAATCATGGGAATGAAGAAGGCCCGACTAAT
GACTGTCACTTTGACAGCCGACCACAGACAAATCTATGGATCCGATGCTGCTCTCTTCCT
CAAGACTCTGAAGAGTATTATGGAAAACGACGTTGCCAAGCTTGCGAAATAAGTCTTGAC
TAAACTAGAAACGTCTACAATTGCGGAACCAAGTGCACTAAAACAATCTAGTCACAAAAA
ATATCGCAGATACTAAGTGTGGTTGCGT
>comp2930_c0_seq1
AAACTGATATCATTTGCGGAACGCCATACAGTACATACCGGTGCATGTCTTGGACTGAGG
CCAGAAGGGAAACCAATCTCGTTGTATTCAGATTGTGCCATTCGAAAGCATGGAAGATAC
GGTGGCAAAACAATCCTCGGCAGCAACGTCTAGCTCCACGTCTTCCGAGACGTGGAACGT
GGCTGTGAAGAATAGGGGGCCATTTGACGATCGCCGAGGATCCCTTAGAGAGGGGGGCTG
GTGGATTGATTTGGATGAAGCAAAGAAGCTCGGTATGGATGAAGAATGGTGGACCGAAAC
CGAGGAACCTACAAAGAGTCCGGTGGCTTTACCCAAAGCAAGCCCAACGATCTATCATAG
CTGGGGCAATGTGAAATATCGGCCGAAAGGACCGATCTGGCTTTCCGAAAGCGGAACCAT
GTACCTTGGACCATGGGCCGAGGTGCGACAAGGACCGGGAGGAAGAACTGTGCGTGTGGC
AAAAGGAATAGGTATATCGTGCCAAAAAAATGGCCAATGCCACATTGGAGAGTACCGGGA

TGGCTTATTCCATGGATGGGGTCGCTCTTTTTGGCTGAAAAATTCTTTGGTTTGGAAACG
AAACATCAACGAGGAAGAGAAGCAAATCATGGAGACGCGAGTTGTTGTGACCGGTGACGG
TCAGAAAACCGAGGAATTAGTGGGATTGCCCTACGAATACCACGGCACCTTTCGCTCAGG
TTCGAAACATGGCATTGCAGGAAAAGTGGTTTTGAAAGATTTGACAACGCGGGTTGGATC
ATGGGTAGACGACATCGCTGTTCTTCGCAAATGGTATCGCATTCGTCAAGACCTGGACCC
TGTCTTAGAAGAGTATCAAGCAGACTGGTGGAAACATCATGTGAAATTGGACTGTGTTCC
AATCGATTCTCCGCAATCGCGGTATATAGCAAGTAAGACAAGGAAAAATGCAAAACATGA
ACTACCTTCTCCCTCGGTGCTTTCGCGGCGAAAACGTTCATGTCGAGCCCCAAACCGCAA
AAAACATAAGAGCTCGAAAACGAGAAAATGTGGTGAGGAATCCCATATAGGCACGGAGAC
AGACCATCGAGGCGAGGATGAAACCGACAACCATCCCAGAAGCGAATACAATCAGGATGC
AGAATTCAAGCAAGAAAATTCTCTTGGCGCCGGCACGGAGGTCATTGACGTGGATGCCTT
GGACCCGGATGGCTTAGTTCCCGATGAACCTCCTTCCTCCTTAATGCCCGAGCGAATAAT
CTCCGCGGTCGGCAATAGCAACAGGCATCTCTCGTCGCAGGTCTTTCCCGATGCCTACCT
ATCCGAGGCCGAGGAAAGCAACTTTCCGCTCCACACCGCCGTCAAGTTTGGTGACGAAGA
CTTTGTCGACGAAGAGCTGGAAAAAATCGCAACGGGGACGGGCCCCGAGGGGAGTCCCAT
CGGATCGGATAGGAGCTACGATTCTGCCCGGCTCAATGCAGTCGACTCGAAGGGACGCAC
GGCTCTCGATCTAGCCGCGCTGACGGGCCAGGTGGGACTGATCAAAAAACTGCGCAGGGC
CAAGGGGATCAGCTTCTCGTTCAAGCCGGGTCCCCGCATGATGATCCTTGCTAAGAAACG
CTCGAAAGACGTGGCGAAGTACTTAGAGCAGGTTCACGAGACAGTGGAATAACTCCACTT
CGCGCAATGACCTACGAGTATACATGAATTGCATAAGTTACATGAATTTTGAACAAGTTA
CATGAATTAAAATCCTTTGTTGGC
>comp2931_c0_seq1
CCAAACTTAACTGAACTCTCCGATGAGAATCGTTCCGACAAACTGCAACAGACTCCCCGC
AAGTACTTTCTGACGGAATCCAAAACCCTCTGTACGCACAAGGGCCAAAGCGTTAAAAGC
GAACGATGGTGCAGAAAGCCTTTCTAAAGATCCGGGTTGGCGAAAGGCAGGTCCTGAGCT
TCGATTTGGTCCTGTACGACGATGACGTTCCAAACACGGTCCGAAACTTTGTCCACTACC
TCGAAGAGCGAGGGTCCACCGGATACCTGAACTCGCGCTTTCATCGCATTATCAAGGGAT
TCATGGCGCAGGGAGGGGATTTCGTCAACGGAGACGGCACCGGCACGGCAAGCATTTACG
GTGGGACGTTTGCGGACGAGAATTTTCTCCGCCGCCACGACCGACCGGGGATACTCTCGT
AAGTGCTGCACCAGCGGGGGAAATCGGCCGCTGAACGAGATCCACTTTCCATCCAGAGCA
TCGACAGTCATTTGAAAACCACTAACGCTGGTCGTGTTTGTTGACGAAATTTCTCTCTGT
TTCATTTGCGCGAAGTATGGCCAATAGCGGAAAGGACACCAACGGCTCGCAATTCTTCAT
CACCTTCCGTCCGACACCGCACCTCGACAACAAGCACGTTGTTTTTGGCCATGTGGATCT
CTCGTCAACGTCGAGCAAGGATTCGTTGCGAATACTGGAAAACGTACGAACCGGCCGGAG
CGATCGACCAGTGACACCCGTCCAAATCGTCGACTGCGGCATCATCTCCGATGATGAGGG
CAACGACGAGAGCGAAAAAGGTGCCAAAAAGGGATTGGTGTCGGGAGAGAATACAACAAA
ACACCCAACAGCGAAAGATGACATCGAAGTGGAGTTGCCGGTGGAGGAAGAGGAGGAGGT
ACCTCCAAAGACCAAAGCGGAAGCTCTGAAGCGACGTATGCGAAAACTCAAAATGAAGAT
GAACCAAGCCCGGCAACTGAACAAACAAGAAGTCTTGCGAGAGGGAGAACGCCTCGGAAG
TGTCGAGGGAGCTGCCAAGGCACGCAAGCGACAACAAATTATGGACAAAAAGGCCAAGGA
GCTCGAGTGGAAGAAAAGAAACAGCAAGGCCCTGGAAGTTGCCGCGGCCGCTGGCTTGGA
CGGGAAGCACCTGGTGGAACAGGCCGATGCGAGCCTCCGGAAAGCCTACAAGAAGGAACA
AAAGGCCGTTTCGCAGCAGTTTGCCGTGAACGACTACTACAATCCGGAGGGACAGCACAG
AAACTACGAGCGCAATCTCAAGAGCCTGTCGCGGGCGGCCGGGCCGCTGCCGGAGGACGC
GGTGACGAACGGCCACGCAACCTTCGACCCGACCACGGACGAGTCGGACGAAAAGCGGGA
GCGAGAGGGAGCCCGCAACCTGGCCAACGAGCTGAAGCGGCGAATCGAGAAGCAAAAGGA
GAAACGAAACCGCCTCGAATTCGAGGGAGAGGACGTCACCTGGATCAACCAGCGAAACAA
GAGGTTCAACCAAAAGATTTCCCGCAACTTTGACAAGCACACGGCGGAGATCCGCCAGAA
CCTAGAACGGGGAACGGCCTTGTAGGCGCAATGCATCCAGGGACGGGGCGTGTTTTGTGT
TCATTAGTATTGCTAATATTACATCACTATCATCTTTATAATCCTTATTTTTTCACTCC
>comp2931_c0_seq2
CCAAACTTAACTGAACTCTCCGATGAGAATCGTTCCGACAAACTGCAACAGACTCCCCGC
AAGTACTTTCTGACGGAATCCAAAACCCTCTGTACGCACAAGGGCCAAAGCGTTAAAAGC
GAACGATGGTGCAGAAAGCCTTTCTAAAGATCCGGGTTGGCGAAAGGCAGGTCCTGAGCT
TCGATTTGGTCCTGTACGACGATGACGTTCCAAACACGGTCCGAAACTTTGTCCACTACC
TCGAAGAGCGAGGGTCCACCGGATACCTGAACTCGCGCTTTCATCGCATTATCAAGGGAT
TCATGGCGCAGGGAGGGGATTTCGTCAACGGAGACGGCACCGGCACGGCAAGCATTTACG
GTGGGACGTTTGCGGACGAGAATTTTCTCCGCCGCCACGACCGACCGGGGATACTCTCTA
TGGCCAATAGCGGAAAGGACACCAACGGCTCGCAATTCTTCATCACCTTCCGTCCGACAC
CGCACCTCGACAACAAGCACGTTGTTTTTGGCCATGTGGATCTCTCGTCAACGTCGAGCA

AGGATTCGTTGCGAATACTGGAAAACGTACGAACCGGCCGGAGCGATCGACCAGTGACAC
CCGTCCAAATCGTCGACTGCGGCATCATCTCCGATGATGAGGGCAACGACGAGAGCGAAA
AAGGTGCCAAAAAGGGATTGGTGTCGGGAGAGAATACAACAAAACACCCAACAGCGAAAG
ATGACATCGAAGTGGAGTTGCCGGTGGAGGAAGAGGAGGAGGTACCTCCAAAGACCAAAG
CGGAAGCTCTGAAGCGACGTATGCGAAAACTCAAAATGAAGATGAACCAAGCCCGGCAAC
TGAACAAACAAGAAGTCTTGCGAGAGGGAGAACGCCTCGGAAGTGTCGAGGGAGCTGCCA
AGGCACGCAAGCGACAACAAATTATGGACAAAAAGGCCAAGGAGCTCGAGTGGAAGAAAA
GAAACAGCAAGGCCCTGGAAGTTGCCGCGGCCGCTGGCTTGGACGGGAAGCACCTGGTGG
AACAGGCCGATGCGAGCCTCCGGAAAGCCTACAAGAAGGAACAAAAGGCCGTTTCGCAGC
AGTTTGCCGTGAACGACTACTACAATCCGGAGGGACAGCACAGAAACTACGAGCGCAATC
TCAAGAGCCTGTCGCGGGCGGCCGGGCCGCTGCCGGAGGACGCGGTGACGAACGGCCACG
CAACCTTCGACCCGACCACGGACGAGTCGGACGAAAAGCGGGAGCGAGAGGGAGCCCGCA
ACCTGGCCAACGAGCTGAAGCGGCGAATCGAGAAGCAAAAGGAGAAACGAAACCGCCTCG
AATTCGAGGGAGAGGACGTCACCTGGATCAACCAGCGAAACAAGAGGTTCAACCAAAAGA
TTTCCCGCAACTTTGACAAGCACACGGCGGAGATCCGCCAGAACCTAGAACGGGGAACGG
CCTTGTAGGCGCAATGCATCCAGGGACGGGGCGTGTTTTGTGTTCATTAGTATTGCTAAT
ATTACATCACTATCATCTTTATAATCCTTATTTTTTCACTCC
>comp2932_c0_seq1
GACTTCTAATACAACTACTTTTGCCTCTTCACCAGTCAACCCTGTTGCCATAATAAGATT
AAAAGGTTATAATTAAAATGAAGTTCAGCATCGCATTCACAGCTCTCACCGCAGCCGTTG
TCTCGGCAAACCCTTTTGCTCCTGCCAAGAGCCCCAACTCATCGAAGACAAAGTACATGT
CGAAGTTGATGCGTAATGCTCGCCCTACACCAAACTCCCAACTTGGACGTAAACTTGATG
AAGAAGAGGCAGAAGCAGAAGAATACGAAGTTGACATCTCGGGATACTCGCTGAAATTCG
AACAATGTCAATTCGTCAAGGCGTATGATGACGAACTCGCTGGAGAAGACTCCGATAGCG
TTCTCGCCACTCGAAGGTTCGTCATTTTCCGCCTGTGTCCTTCTGACGCCTGCGACTCGT
GCAATTACAATTTCGGTGAGTACATCGTCGACCTCGAGGAATACTTGGAGGCGACTGTAA
ACTACCAGCAAGAAATGCAAGAAGAGATGTGCAATGCTTGCGAGGAAAACTGTCAATGGG
AAGAAGACGAGGAAGAGGAGGAAGAAGATAACGGACGTCGTCGCCGTAAGCTTGAAGATT
ACGGCTCCAATTGCAATACCTGTTACGAGGAATGTATGAAGATTGAGAACATGGAAGACA
ACGGATACATTGACGCCACCCAATTTCTTGATTGTCAGATGGTCTACGATCCCGAGGATG
ACGGCCTTTCTGCCCTTTACGCCGGACCTATGTGTGCTAGCAATGGATCTAAGATCAAGA
TTGGAATCTTCACTGACGAAGAATGTAATGTTCCTGATTCCGACAAGGACGTAGACGATT
ACCTCATGGACGGAGACGGTGTCAGCATGAAACTGTCCCATGCTCTTTTGAAGACTGTGT
ACTCCAGTGAGTGTGTCTCCTGCTTGGCACCACAAGAAGAGGAAGAAGACGACGAAAATG
GAGATGAAAACGATAACGGAGATGAAGAGGAAGTCGAAGTCCTCGAAATGTGCGAACAAC
TCTACCAAGCTTCCGCCAAGTGTGAGAAGACCAATGGATTTGATAACGGATACGCTAGCT
ATGACGGATACGAGAACCAGCTTGCGCAAGAAAGTGTTGTATGTGATTTTGTCGAATCGT
TGAAGGCAGGAAGTTATGACGAATCTGGAGAGATTGTCGTAAACGGAAAGAGTTCTTCTG
TTGGTGGAGGATCCCAGACCACAGGAGGACAAAAGTTTGCCCTGACTTTCTTCATCCTTG
GAACTGCTGGACTTGCTGGATACGCTGCCATGCTTCATTCCAAACTTACAAAAGGTACTA
TGGGCCTCGCTGAAACTGGAGGATCCTTGGCATAAACAACAATCTCTTGATTTCTGATGA
TTGTGTTGCATGGCAGGCCTTCAGACTGACAACAGTAACAAGCAACAACCTAATTTGTAT
CATTAAGTCCTCCCATTTTATGGCTTGTGTAATTGTGAAAACACACTAAAACATAAAAGA
ATCACTAAACACTTAAAACCAATTTCACTAACGAGAAAGAAGTGAACGAATGTCATGAAG
TACTGTACGGTTGAAACACTTCATTGGATATGCTTGGGAAAATAGAATAACTTTTGTGGT
TTCAGTTGAGTTTTGACATTTGAAGGCTTATCTGTCAACTGAAGGCCTGCGTATTTTATG
TTCCACCATCAATCTGATTTTGCTTTTAGTCTCCATTTCATGTTACTTCTAGCTATCCAT
GCACACGGGTATTCAATCTCATTCTTGTTGAACTGCAAATCATTGTCTCTTGGAATCAAA
GATACTTCATCTCAGATGAGGATGTAAATCATAGTGTTGA
>comp2933_c0_seq1
ACGACGACGACGACGATAGCAACGAGGAGGCGTCCGGGGGGGGTGACGACGAGAGCGAGA
GCGCAAAGCCAGCGATGCCGCCGAAGCGCAACACGGGTTCGTGGTCGGTCGAGGAGCAGG
AGGCTGCCGCCAGGGGCCTCGTGATCCACGGGACGACAAACACAAAGGCGATCGCGCAGA
TGGTCGGAACCCGCAACACCGATCAGGTCCGCCGCTACATCGGTCGCCACTACCACAAGC
TCCTTGCGATGGGAACGAATTCCGAGGAATCGTCGTCCGACAGCTCCTGAGCCGCGATGA
GAACGAGACGAGACGAGGGAACCGAGCGAGGGAACCAATCGCGAAAGCAATCGCACGGTT
TCTTGCTTGTGCAAGCTAAGTATGTGTAAGGATTTTGGAGTGCGACTGTGCACGATGGGA
AATTTCGAAACCGATGAAAAAAAAATCTCGGATGTCCGGATGGAAGCAAAGAAACGAACG
AACTAGCTATGCCTAAACAGATGCAAAAATCGGTAGAGCCTAGATTGTAATTTGATTCCA
ATTTGAATGGATTAGGGTAAAATAGGAGGCAAATAGATGAAATCTAGTAAGGTTGGATGA

GAAGGGGTGGGATTGGATTGGAGTGGGATGAACACGAAACCAACTAACTAAAAGCGACAT
AATGAGAGGCAGCCTCAGATTTACCATCGGTTCGAAGAGAAAGAAATGTTTGTCGAAAAC
TGCCTTGCCTACGAGTGTTTTCAGTGTGCCTGTGTATGTATGTCTTGTAATGGAAAAGAG
AATGCCTTTTATGTCGTTCAGTTTGTTGGTTCTGTTGAAGTACTAGCAGTATCTTTCTTT
TCTTCTCTCGTGTAATACATTGAACGAAG
>comp2934_c0_seq1
GTCGGGTTCTAGGATCCATTGACAAACAGCAACAAAAAACCCTCCGCTTCCACCGGTAGT
TCGAACAACAGAAAAGACGGAATGGTGTTCCTTGAGATCTTTGTACCAAATCAGATATTA
CCCTTCGTCGTAGTGACACATTAATCTTCCACTCGGGTACATTTCTCAACTCCTGTCACT
GTTCAAGAGAACAATGTCACCGGACATCCACATACGACTATCGCTGCCAACGAGGCCGGT
GCCTGTACTAATCAACGAAAACCTGGATGACGAAATTCTAGTACCGAGCGAAAGAGCTTG
CCCTAATAGGATGAGCTCCCCGCCTCACTACTTCTCGCCCAATGATCATTTTCGAAAATC
TATTATGAAGTGGAAAACACTGGATGAAAGTTTAAAAAGTACTGATGAAACAGAGGAGAC
TCGCGAGGGAAGGGGGATGAAGAAAAATTCACTACAATTTACTATCATTAACATCAGAGA
ATATTCAAGGACGGTGGGTGATAATCCGTCATGTAGCTCCGGGCCTCCTCTGAGGTAAGA
ATGTGTAGCCAAAGTTTGCCCGAAGCTGTCTGTAGCTGGTTGGTCGCGTTGAGTTAGATA
TATTAAGTTGATCGTTTCAAAGCTCAACTTTAATATTTCAATTTTGCAACGCTGTTTCTT
GCATTATCGCGTTTCTCCTTTGCTCTCTAGCATATCATGGGAATATATGAATATTGGAGA
GATTGATGTAGATGAGTATGAAAATATGAGACCCCCGAGACGCGTCCAGAGAGAAATGAT
CTTGCCCCGAAACATGCGTGAAGATATTCTGCGGTACGAATGTAATGCGAGTCGACAGGA
AATAACCGAATCGATTCGTCAGAATGTGAGAATCAAGAACCAAAGAAGGACAACCATCAA
TAACCTAGGCAAAGCAGAAAAACTTGAGGAAGTAATGGAGAGTGCGAGTAGAAAGTTAAG
GCGCTTCGTCACACGAAAGAAGTCTGTGCATAAACAAGTTGAAGATCTTGAAAAAGAAAT
TGATGTGTGTAATCGAAGAGCACGAAAACTGAATTATCTCGAGAAAACTATGTCTCAAGG
TCGAGAAGAGTCATTCCCTGGAGAGAGCACGCTGGATTGTGCTGATAGTGTCAGTGTAAA
TTCGTAAATGTTCCTGATAAGGTCCATTGACGTGATTTCGCTCCCTCGGAATGGATAGGC
TTTTTTGTTTTCAAGCCAATTTTCAACCAGACCAGGATCCAAGTACTACGTCAACATGCA
GAACGAAAGATTATTTCTTTCTCTCTTTTTCTATTTTGCAAATTTGCATTTTTCTGTAAC
TTCGCAGCCATCTGATGTGGCCTTACGTGTGTAAAATACAGCAATCCGGACTTTGCTGAC
CGAATTATTTTGAAGCTCGAAGTTTAAAATTCACTATTATTTATATTTTGATACCATTTT
ATCCTTATCGGTTCAAATACGGAATATAATATTCGATTTTGCATATTATTGGGAGGGGTC
TCTGCTTTTTTTCTCGAACAATATGCTTACTATTCTCATCTTTTCTGCCTTCGTACTAAA
TCTTCATTCCAGATGCACGATGTTACCAATAGTAACAGTGATCGCCAATGTGACCACAAT
ACACAAAGCAACCAACAGATGAACTAATTGTAGGCTTTCATACTACTGTTAATGAACGAA
TATTTGGAGGCTCAAACTGATACATTTGTCAACCACCAACACAAATGATATCCTTGTTGA
CACTGTCATGGCATCACTTTTGGAAGGGGAAGAGAATCCTCATTCCTGGGCCCCCCTTGT
ATCATTTCTATCCTCTGGTTGTACTCATCAAAGATTCACTTCTATCACCCCTTACATGTT
GCTTCCTGGTGGTGGGATTCTCTTTTTTGAGGATTGCTTTTCAGTCATTTCCCCTAGTAT
GCTTTCCCCCTGACACTTATGATGGTGGACATCAGCAACCTAGATCCCAACCCGAAGAAC
CCTAAAAGAAGAAGAAAATGTCAGCTCCGAAATTTGATCAGCTGACCTAGTATTACTCTT
GCTGTTTCCACTCACTATGACCTATAGTAGTAACTGCTTGAGTTTCAAGATGCAAAACAT
ATGTAATAGTCTGGCCATTTCCCTGATGTACTAATCTATTCTGATATTCACATCATATCT
CAACACCACCAAATTTTTATCCAAGTTTTCTTCTCATGCTGGTACAGAAAAAGCTCAGGG
AACCATAGAAAATCAAAAATCATTTTTTGTCTAATTGAACCACCATGTAAAGTGTTGACA
ATAACAACTGACATTGGTCCATAAAACTTGAAACCCAAGTTTCAAGGATACTATGCAGCA
GGCTGTCTGTCTAAACTCCTCAGTCCAAAAAGCTACAATCACTTACTAAATTTCTTCTCC
CTCTCCCAGCTCCTAAACATAAACCTGATATGTTCATCACCAACACCAATCCCCCTGCTT
GTGATGCATTCTGCTGGACAAAGCATCTCAAATCCCCAACACTGACAACAGCCAATCCCC
AGGCAATTCTGACACAGACTTAAATTTCTTGCACTTTTTCATTCCCTCTGCTGGTGCTTG
GTGCTTTTGTTGTGGTCCTTTCTGGTGCTTTGTCACTGCATTCTTTGTTTTGAAATACTG
TTCTAGATGAACTTTGCTATTGTATGAGTAGCTCCTTGTTGTGCTTGTCAACAATCTCAC
CAAAAATCCAGCTCCTCCTAGTTCAGAATGTTTGGTTCTATC
>comp2934_c0_seq2
GTCGGGTTCTAGGATCCATTGACAAACAGCAACAAAAAACCCTCCGCTTCCACCGGTAGT
TCGAACAACAGAAAAGACGGAATGGTGTTCCTTGAGATCTTTGTACCAAATCAGATATTA
CCCTTCGTCGTAGTGACACATTAATCTTCCACTCGGGTACATTTCTCAACTCCTGTCACT
GTTCAAGAGAACAATGTCACCGGACATCCACATACGACTATCGCTGCCAACGAGGCCGGT
GCCTGTACTAATCAACGAAAACCTGGATGACGAAATTCTAGTACCGAGCGAAAGAGCTTG
CCCTAATAGGATGAGCTCCCCGCCTCACTACTTCTCGCCCAATGATCATTTTCGAAAATC
TATTATGAAGTGGAAAACACTGGATGAAAGTTTAAAAAGTACTGATGAAACAGAGGAGAC

TCGCGAGGGAAGGGGGATGAAGAAAAATTCACTACAATTTACTATCATTAACATCAGAGA
ATATTCAAGGACGGTGGGTGATAATCCGTCATGTAGCTCCGGGCCTCCTCTGAGCATATC
ATGGGAATATATGAATATTGGAGAGATTGATGTAGATGAGTATGAAAATATGAGACCCCC
GAGACGCGTCCAGAGAGAAATGATCTTGCCCCGAAACATGCGTGAAGATATTCTGCGGTA
CGAATGTAATGCGAGTCGACAGGAAATAACCGAATCGATTCGTCAGAATGTGAGAATCAA
GAACCAAAGAAGGACAACCATCAATAACCTAGGCAAAGCAGAAAAACTTGAGGAAGTAAT
GGAGAGTGCGAGTAGAAAGTTAAGGCGCTTCGTCACACGAAAGAAGTCTGTGCATAAACA
AGTTGAAGATCTTGAAAAAGAAATTGATGTGTGTAATCGAAGAGCACGAAAACTGAATTA
TCTCGAGAAAACTATGTCTCAAGGTCGAGAAGAGTCATTCCCTGGAGAGAGCACGCTGGA
TTGTGCTGATAGTGTCAGTGTAAATTCGTAAATGTTCCTGATAAGGTCCATTGACGTGAT
TTCGCTCCCTCGGAATGGATAGGCTTTTTTGTTTTCAAGCCAATTTTCAACCAGACCAGG
ATCCAAGTACTACGTCAACATGCAGAACGAAAGATTATTTCTTTCTCTCTTTTTCTATTT
TGCAAATTTGCATTTTTCTGTAACTTCGCAGCCATCTGATGTGGCCTTACGTGTGTAAAA
TACAGCAATCCGGACTTTGCTGACCGAATTATTTTGAAGCTCGAAGTTTAAAATTCACTA
TTATTTATATTTTGATACCATTTTATCCTTATCGGTTCAAATACGGAATATAATATTCGA
TTTTGCATATTATTGGGAGGGGTCTCTGCTTTTTTTCTCGAACAATATGCTTACTATTCT
CATCTTTTCTGCCTTCGTACTAAATCTTCATTCCAGATGCACGATGTTACCAATAGTAAC
AGTGATCGCCAATGTGACCACAATACACAAAGCAACCAACAGATGAACTAATTGTAGGCT
TTCATACTACTGTTAATGAACGAATATTTGGAGGCTCAAACTGATACATTTGTCAACCAC
CAACACAAATGATATCCTTGTTGACACTGTCATGGCATCACTTTTGGAAGGGGAAGAGAA
TCCTCATTCCTGGGCCCCCCTTGTATCATTTCTATCCTCTGGTTGTACTCATCAAAGATT
CACTTCTATCACCCCTTACATGTTGCTTCCTGGTGGTGGGATTCTCTTTTTTGAGGATTG
CTTTTCAGTCATTTCCCCTAGTATGCTTTCCCCCTGACACTTATGATGGTGGACATCAGC
AACCTAGATCCCAACCCGAAGAACCCTAAAAGAAGAAGAAAATGTCAGCTCCGAAATTTG
ATCAGCTGACCTAGTATTACTCTTGCTGTTTCCACTCACTATGACCTATAGTAGTAACTG
CTTGAGTTTCAAGATGCAAAACATATGTAATAGTCTGGCCATTTCCCTGATGTACTAATC
TATTCTGATATTCACATCATATCTCAACACCACCAAATTTTTATCCAAGTTTTCTTCTCA
TGCTGGTACAGAAAAAGCTCAGGGAACCATAGAAAATCAAAAATCATTTTTTGTCTAATT
GAACCACCATGTAAAGTGTTGACAATAACAACTGACATTGGTCCATAAAACTTGAAACCC
AAGTTTCAAGGATACTATGCAGCAGGCTGTCTGTCTAAACTCCTCAGTCCAAAAAGCTAC
AATCACTTACTAAATTTCTTCTCCCTCTCCCAGCTCCTAAACATAAACCTGATATGTTCA
TCACCAACACCAATCCCCCTGCTTGTGATGCATTCTGCTGGACAAAGCATCTCAAATCCC
CAACACTGACAACAGCCAATCCCCAGGCAATTCTGACACAGACTTAAATTTCTTGCACTT
TTTCATTCCCTCTGCTGGTGCTTGGTGCTTTTGTTGTGGTCCTTTCTGGTGCTTTGTCAC
TGCATTCTTTGTTTTGAAATACTGTTCTAGATGAACTTTGCTATTGTATGAGTAGCTCCT
TGTTGTGCTTGTCAACAATCTCACCAAAAATCCAGCTCCTCCTAGTTCAGAATGTTTGGT
TCTATC
>comp2935_c0_seq1
GCTAAATTTTGGGGAAAAAAGGATTCTTCCTCTTGCATCAGAGACGCTTTCTCCGGAGTC
TTGACGATCTTGAGGTTCTAAACGAAGAGAGAGAAAGGAAAGGCTGGGATAGCTTTCTGA
AACCTGCGTTCCATTCCCACACCTTGCTACTATTGGGCAATAACGCAACACAGCAGCGCC
AACACACTTTGCTAGTGAAAGAGCACCATACTATATTATCACAACAAAATGACACGGCAA
CTGCTACTTCCAATCCTTGTTGGGGCGATCGCCTCGATGAACCCTGCTACCGGTATGGTC
TTGCGCGAACCTCCTAAGGTTTCCAGCCCCGCTGGAAGCGCCGTCGAATACGGCAAGTGG
TCGCCCTCCTCGTGGCGCACCAAGGAATCGAAACAAATTCCCGAATACCCCGACCAAGAC
GCACTCAAAGCCGTCGAGGACCAACTCTCGAAATGTGCTCCGCTCGTCTTCGCCGGAGAG
GTTCGAAACCTGTCGGATCAGTTGGCACTTGCCTCACAAGGAAAGTCGTTCGTTCTCTTT
GGCGGTGACTGCGCCGAATCCTTCGACGAGTTCTCGGTTGACCACGTCCGGGATACCTTC
CGAATCATCCTCCAGATGGCCCTGATTTTGACTCACGGTGGATCCAAACCCGTCGTCAAG
ATCGGACGCATGGCTGGACAGTTCGCGAAGCCCCGATCCAGCCCCACCGAAACCATCGAC
GGTGTCACCTTGCCATCTTACCAGGGAGACAACATCAACCGGGAGCACCCCTTCACGGCA
GAAGCCAGACAAGCGGACCCCAAGCGAATGGTCGAAGCCTACGATCAGTGTTCCCAAACC
CTGAACATCCTGCGTGCCTTTTCGAGCGGGGGATACGCCAACTTGTCCCGCCTCCACGCC
TGGACTCTTGATTTCGTCGAGGCAACACCGGTCGGATCCAAGTACCGAAAACTCGCCTCC
GTGGTCGAAGAATCGCTGCGATTCATGAACGCCATCGGAGTCGATCTGGAATCGCCTGCC
ATGCAACAGGTCGATTTTTTCACTGCCCACGAATGCTTGTTGCTTCCCTACGAAGAAGCC
ATGACCCGCATCGATTCGACCTCTGGAAAATACTACGATACATCGGCGCACTTGCTCTGG
GTCGGCGAGCGAACAAGACAACCCGGATTTGCTCACTTTGAGTTCGTGCGTGGATTGGAG
AATCCCTTGGGAGTCAAAATCAGCGACAAGGCCAGTCCCGAAGACGTCCTTGAGATCTTG
AACACCTTCAACCCAGACAATATTCCGGGACGGGTGACGCTCATCACGAGAATGACCGCC

GAGGGAATCCGAACGAAGCTTCCCGCTATCATTAAGGCCGTCCAAGCCGAGGACAAGCAC
GTCGTTTGGGTAAGTGACCCGGTCCATGGAAATGGATTCCAAGCAGAAAACGGATTCAAG
ACGCGCAGCTTTGATGCCATCCGCGAGGAAGTTGTCGCCTTTTTCGATGTCCATCGCGAA
TGTGGAAGCCACCCCGGTGGCATCCACCTCGAAATGACCGGAAAGGACGTCACGGAATGC
CTGGGTGGGGACACGAACGGAGTTACCATGGACGGGGAACGAGGACTGGCCTCGAAGTAC
GAAACCCACTGTGATCCACGATTAAACGCCATGCAAAGCCTGGAACTGGCCTTTTGCGTT
GCCGAGAAAATGAGAGAAGCCGAAGCCGCCAAGGAAGAAGCCTCTAATTAAGTAAGAAGT
CGGGTCTGAAACCGTGCTTCTGAATCGGTTTTGGACGATTACATTCTGCGCAAGGTTGCA
TGCATAAATAAGGGTGACTACGCCAAGAAAGAAATCACGAATGGATAATTGGGATCTTTC
TTGTCTTCCCATGGAATAAGCATAAAATATTTTACATTTTTTCCAGAAAAAATATTTTAG
AAATGGATTTTATGTGTTGGCGCCAAACCTTCTCGACGAAGTAGACTCATAGCATGAGGA
TTGCCC
>comp2936_c0_seq1
GTATCGTATGAAAGCAGTTGGTTTGTGATAGCATCGTTTACGGGCACGGAAGATTCAGCC
ACAAATTTATCGCGACGATCACTCAGTAACAATTCGGTCGAGAGTAATTGCGACACCAGC
GGACGACGTTTGATTGACGTTTAAAGCAAAGCAAAGCACACACGTAACGCCAACTGCGCC
AGATCGGTTGACATCCACCGCGTGCGACGAAATCTCTTTGAATCGCATCGGATTCCATCG
CACAGCATCGCCCACGGAAACAAGTAGACCTATAGCCGGGACCGTCTCGACACGAAGCGC
AACACAACACAACGCAACACAGCTCGGCACAACAATGGAAACGTCCGCAAAAGTAACGCT
GTTCGAAGATATATTCGAGGTCACGGCCCTGAACCCCGACGGATACAAGTTCGACCGGGT
GAACCGGATCCAGGCCGTCGGGACCACCTTCGAGTGCGACCTCCTGCTGGACATCAACTG
CGAGATCTACTCGCTCCGGGAGCACGACAAGGTGACCCTGATGCTGGCGTCGACGCTCCA
CCTGGACGGGAGCCCCGCCGACCACTTTTCGTACACGCCGAGCTCGGAACCGACCCTCGC
GGACAACTACGAGTACGTCATGCACGGGCGGGTCTTCGACATCAGCTACAAGAAGGACGG
AACGGCGGTGCTGGCCCTGAGCTTTGGGGGATTGCTCATGAGACTGACCGGGGACCAGAG
GCACCTGGCGAACGTGCTTCCGGACATGAGGTTGTACTTGCTGCTCAAAAAGGACTAAAC
AATATAATTCTACTACGTAACAATACTGTAATCATGGGTGACGCCGT
>comp2937_c0_seq1
GTTGCTACGAACTGTAGTGAAGGAAACTTTGGAACTTTCATAGTGTGATTGTTCGTGCTA
CGATAAAATATAATAACTAAACCATAACAATGAGCTCTGTCACGTGTTCGTCAGAATTTT
GCAACTCAGGATGGGGGGAAGTCCTGACGCATATGTCGCCTTACGGATTCGCGAACTTCG
GTGTCGCGTTCGGATTAGGACTTTCTATTATCGGCGCCGCATGGGGAATCTGGCTCACAG
GATCGAGTCTTGTTGGAGCTTCTGTAAAAGCTCCTCGCATTATATCAAAGAATCTGGTAT
CTGTGATCTTCTGTGAAGCGACTGCTATTTACGGTTTAATTATGGCTATCATCTTAGGAA
ACAAGATCAAGGAACCCGAAACTGTTTCATACTATCTCGAAGAAGGCTGGGATTACAATG
GCTACTACTACGCTGGATATGGAATGTTCTCGGCTGGCCTTAGTGTAGGATTAACAAATG
TTGCCAGTGGAGTTAGCGTTGGCCTAGCCGGTTCATCTTGCGCGATTGCCGATGCTCAAG
ATGCAAGTCTCTTCGTGAAAATTTTGATCGTAGAAATTTTCGCGAGCGCTCTTGGAATCT
TCGGAATCATTGTTGGTATTATTCAATCGAATGCTTGCACATTCCCTACGTATGCTTTAG
GTCAAGAGTGAGCGCGAAGAGCGTTCTCTCGTCAGGAATGAAACGAATCATTTCTTTAGG
CTTGTAATTTTAAAATAATTTTATGAAAACATGCTTTGAAA
>comp2938_c0_seq1
AGAAAATTTGCGTTGCCGACCAACACTTTTTACACCTCTTGTCAACGTCTTTCTTCCATC
ATAACTCAATACAGCGTTCTTTGAAATAGCTAGTTAGTTCACATCCTACCCACTTTGTGA
ACGAGATCTTTCAACTAAAGATATACTCGGGAAATGAAGTTCATTTTAACCACGGCTTTC
CTTGCCTCTTTGGCGCTCGGACAGAACAACCATGCCTCCAACAAAGATGCGGAGGATTTT
GCTTACTGGAGAGATTTAGTCGACGCTGTTGACAGTATGCCACCGACATCAGCCCCGACG
AAGTCCCCCGTCACCCCCACAGATGCACCAGTTACACCTACGGATACCCCGGTTGCTGCT
GTTACTGATGCACCTACGGATGCACCTGTCACCCCAACGGATGCACCTGTTGCTCCCACG
GATGCACCGGTTGCTCCCACGGATACGCCAGTTGCTGTTGTTACGGATGCACCGGTTGCA
CCCACCGAAGCACCTGTCACTTCAACGGTAGCTCCAACGGTAGCTCCAACGATAGCTCCA
TCCGTATCTTGCAAAACTATTGCTGAGGTTATCACCACCACACCTGAATTTGCAACGCTT
ACAACATTGGTGGAAGGAGCTGGAATTTTGGATAATTTTGAACAAGGCACTTTAACTTTA
TTTGCTCCGACGAACGATGCAATCGCAGCGTTACCCTCAGAATTAGTTGACACCATTCTT
GAAGACAAAGAACTTCTCGAATTAGTCCTTTTGGGACATGTTGTTCGAAGGCAACAAATT
ATGGTTGAAAATCTTGAGTGTGAAGGTGGGCCGATTTCTTCGTTGACAATGGCAAATGGT
GCTGAGACTACAATCACGTGCGGCATGTCACTTGATCCTGAAGGTAACGAAGTCATCGAT
ACATTCATCGAGGATAATGGAGTTCCTGGGAAAATTGTTGGACCAGATGGTATGGCATGT
AATGGTGTAATCCAAGCAATTGATGCGGTAATTATTCCGGACATTCAAACAACTGAAGCC
CCGGTTGCGCCAATTCCAAAACCTACTCCTGCTCCAGTTACTCTTCCAACTATTGCACCA

ATTGCCCCAACCATTGCACCAGTTGCCCCAACTGACGCTCCAGTATCTGGTACGCAAGCC
AAACTTTCTACGTTCGCCCTTAGTAATGGAGCTGAATTCGAAGACACCACAAGTTACCAG
TACAAGGCCCTCAAACAAACGGAATCTCAAATTGGTGTTGATTCTTTCACAGATGCGAAG
CTTGTACAGTATTACGCACTATACTGTATCTTTTTTGCGACAAATGGGGTGCCTAATGTG
ATTACAGATGCTGATCCTCGTTTTGAAGGTATTCCTTTCCCGAATTGGTTGATTACAACC
GGATGGGAAGCAACAGACATAGACCCCTGTGATTGGTATGGTATTATCTGCGACGCCGAA
TCGAGAGTTAGTATCATCGATTTGTTTGAGAACTTGTTAACTGGATCTTTTCCACCGGAG
GTAGTACTTTTATCTCTAGATGGTCCTTTCTCGACTGGAGCAGGAAATTTGTTCCGCATC
GATCTTTTCAGAAATGAATTCCTTTACAACAATGCCGATAGTTCATGGATGACAGATCTG
GGTTCCAATATGACTACCATCATCGCCGAGGAGACCGCCTTCAGTGGTGATATTCCCCGT
CTTCCAGAGAATCTTGTCAACTTTGACATTTCATTCTCATACTTTACAGGAGGCCTCATC
GATACCAATTTCGAAAGCCTCCAAGAGCTGGCATTTATTGATCTTGATGGAAATGCATTC
AACAGCACTATTCCAGCTGTTTTCGGTAGACTCCCCAGTCTTGAATTCTTATACCTCTCT
GATTCCTTTGTCTCTGGTGATATTTCATATATGCAAGGAATGCCTTCCATTCGTGAACAT
TGGATCGACACCAATCCAGGGCTAAAAGGACCAATTCCAGCTTATATTGGAGATCTAACC
ACTTTGGAATCCTGGTCTATGGCCTTCAACTCTTTGACCGGAACCATCCCTTCAGAACTT
GGAAATCTTGTAAATATGAAGCAAATGTGGTTATACGCCAATGAGCTAACAGGAACAATT
CCATCCGAGCTTGGAAATCTTGACACTATGCGAATCCTTGAGCTTGAGGGCAACAGTTTT
GTTGGAAGCATGCCCGCAGAAGTGTGTGCGAATACAGTCTTCCCTACGCAAATTATTGAA
AAGCTGGGTGCTGACTGCGAGGACGAAAATTTCGAATGTTCTTGTTGCACATGCTGTAGT
GTATTGGAATGCAGCGCTGAATAGTCGCAATCAAGTCACAACTAGACGCAAACCAAAAGA
ATTCAATTGCCTGAGTTATTAAAATGGGAGTTGGAGATCATTGTCATGAGATGCAGTGAT
TACTTTCATAATTAGACATGTCATACCGTAAACAAGAAAGGAAATCAGATGCAACGCTTT
ACAACCAAGTAATAGAGAACAGGAACTTGCGGTGAATAAGGACCTTACTTCGATTATCGA
AAGCAGGAACCTACAGTGTACACGTAACTTTAATAATCCGCAAGCTTTGGTTGTTG
>comp2938_c0_seq2
AGAAAATTTGCGTTGCCGACCAACACTTTTTACACCTCTTGTCAACGTCTTTCTTCCATC
ATAACTCAATACAGCGTTCTTTGAAATAGCTAGTTAGTTCACATCCTACCCACTTTGTGA
ACGAGATCTTTCAACTAAAGATATACTCGGGAAATGAAGTTCATTTTAACCACGGCTTTC
CTTGCCTCTTTGGCGCTCGGACAGAACAACCATGCCTCCAACAAAGATGCGGAGGATTTT
GCTTACTGGAGAGATTTAGTCGACGCTGTTGACAGTATGCCACCGACATCAGCCCCGACG
AAGTCCCCCGTCACCCCCACAGATGCACCAGTTACACCTACGGATACCCCGGTTGCTGCT
GTTACTGATGCACCTACGGATGCACCTGTCACCCCAACGGATGCACCTGTTGCTCCCACG
GATGCACCGGTTGCTCCCACGGATACGCCAGTTGCTGTTGTTACGGATGCACCGGTTGCA
CCCACCGAAGCACCTGTCACTTCAACGGTAGCTCCAACGGTAGCTCCAACGATAGCTCCA
TCCGTATCTTGCAAAACTATTGCTGAGGTTATCACCACCACACCTGAATTTGCAACGCTT
ACAACATTGGTGGAAGGAGCTGGAATTTTGGATAATTTTGAACAAGGCACTTTAACTTTA
TTTGCTCCGACGAACGATGCAATCGCAGCGTTACCCTCAGAATTAGTTGACACCATTCTT
GAAGACAAAGAACTTCTCGAATTAGTCCTTTTGGGACATGTTGTTCGAAGGCAACAAATT
ATGGTTGAAAATCTTGAGTGTGAAGGTGGGCCGATTTCTTCGTTGACAATGGCAAATGGT
GCTGAGACTACAATCACGTGCGGCATGTCACTTGATCCTGAAGGTAACGAAGTCATCGAT
ACATTCATCGAGGATAATGGAGTTCCTGGGAAAATTGTTGGACCAGATGGTATGGCATGT
AATGGTGTAATCCAAGCAATTGATGCGGTAATTATTCCGGACATTCAAACAACTGAAGCC
CCGGTTGCGCCAATTCCAAAACCTACTCCTGCTCCAGTTACTCTTCCAACTATTGCACCA
ATTGCCCCAACCATTGCACCAGTTGCCCCAACTGACGCTCCAGTATCTGGTACGCAAGCC
AAACTTTCTACGTTCGCCCTTAGTAATGGAGCTGAATTCGAAGACACCACAAGTTACCAG
TACAAGGCCCTCAAACAAACGGAATCTCAAATTGGTGTTGATTCTTTCACAGATGCGAAG
CTTGTACAGTATTACGCACTATACTGTATCTTTTTTGCGACAAATGGGGTGCCTAATGTG
ATTACAGATGCTGATCCTCGTTTTGAAGGTATTCCTTTCCCGAATTGGTTGATTACAACC
GGATGGGAAGCAACAGACATAGACCCCTGTGATTGGTATGGTATTATCTGCGACGCCGAA
TCGAGAGTTAGTATCATCGATTTGTTTGAGAACTTGTTAACTGGATCTTTTCCACCGGAG
GTAGTACTTTTATCTCTAGATGGTCCTTTCTCGACTGGAGCAGGAAATTTGTTCCGCATC
GATCTTTTCAGAAATGAATTCCTTTACAACAATGCCGATAGTTCATGGATGACAGATCTG
GGTTCCAATATGAGTAAGTGTGGTCACGAACAATTCAGATACTCAAGAAACCTCAATTGT
TTATGTACAGTGAATCTAACACAATCGAGTCATTTACAATCAAATTCTCGATTAAATTTT
TCAGCTACCATCATCGCCGAG
>comp2939_c0_seq1
TGCTTCAACACTTGGTTATGGACAATAGACAATACGGTATCATTTCTGTTCCTATCAAGG
TGCAGATTTGCAGCGGAACACCACTGAACAGAGGAGAGAGCCTTTCATTCATTCTCAAAC
AGAACGCGAGCAACCCAATCCAAACACAAGCGAATACCAAACCGTACGGCATGCTGGGTT

CGGCACCGACGCCGCGCCGGTCGAGGCCGAGGAGAACGAGCCTTCGACTCGTCGCCCTTT
TGTGCTGCCTTTTTGCAAGCATGAACACAACCACCGTGCGCGGTGGTATTGGTAGCTGGA
GAAAAAAAACAAGCCAAGAAGAACCAGACGAGGGAGAATCAGGTGTGGTCAATGCCTCGT
CGTCTCCCGAAGCCAACGGCAGCAGCAACCACAACCACATGTCGAACAGCGAGCATCAAT
ACCACAAGACCTACCTGGATCTTTTCGGCGAGACGGCCAAGGACTTTTTGACGCGGGTTG
CGCCAGAGCGCATCTCGCGGATCGTTCCCCCAGAGGAACCGGGCTGCCGGTGGGACTGGA
GGTACCTCCGGTGCGAGCCCTACTGCGCCTGCACCCTTTCGCCGAAATTCCCCTGGGACC
TGCACCTCGGGAGGGCCTGCCGCAAGAGGGGGGGCGCAGAGGGGACCGACGGCGGCACCG
AAGCCGGATTCGAGGAAGGCGCGGACTACCGGGGGCTCTGCGAAGGCGGCGATTCGCGGC
CCGGGTCCACGCTCTTGTCGGTGCTCCGGTCGCCGGCTCCCTTCCTCGCGAAGACCTCCG
GGGCGGCCTGGCGGATCCTGCGCACCAGGGCCGATCCGGTGGTGGCAAGGGCGGCGGGCG
AGCTCGAGCGCGTGGGTGCCGGGGTCTCGGCCGTCGTCTGCGGGGACCTCAGCGATCGGT
GCAAGGACGGCGGCGACGGAGCCGAGGGCCCGCCGCCGGTCCTAGCGTGGCAGGAACGCC
TGGTGTGCCGGGAGATTGCCAGGGACTGCGGGCTCGTCCCTCCGAGCCGGGGGCCCGACC
CGGCCGAAGCGTGAGACCGCAGCGGGAAAAAGAGCCGTGCAACGCAACACAACGCAACAC
AGGATGATGCGACACGATGCGATAACATACCATACGCTCCAAAAGAACCACGAGTCCGCT
CCGTTTCACATACGAGTGCTGTGTGAACAGTCGGGGATGGGTTTGGGTTTGCTTCAGATT
CTAGGCATCCACCGGATGGGGTGGCGCGACGAATCGCCTCGGAAAGCACAAATGAACGAA
TGCCGATCATGAATCTACAATGCTAAGGCAACCGAAAGAGGGCTTGGAGAGATCCACCCA
ACAGCCATTCATGAGCTATTCGTGCGTCCGTACACCCAAAGTGCTAGTACGTGTATCATG
CCAATCGATGCAATTCACGGATAAAACGCATCGGGAGATTCGCATAAACAAAAAAAAGCA
TCGACAACAACATGTACACCACAACAGAAAATGTTGTGGATGGATAATTTGCTGC
>comp2940_c0_seq1
TCCAGAAGTTCGAGAGCTCTAAACCATAAGGCTCTTTGGTTACTCTTTGGTTACTGAATC
TTACCGGTACATCCACTCGTTGCACAAAAAATACTTATCCTACAAAGCGAGACAACCTAA
AAATGGGAGCAGGACGAGAGGATGAAGAGATCACCGATCTTCTCACGAAGTTGTATACTA
TACAGGAGGATATCGGCGCGAAGCCGAAGACCTCTGAAGATGAAAAGCGGGCGAAAGCCA
ACAACACGGCCAAAATGGGATCTTCGAAGAAAGCGGAGAAGAAAGGATCTAAGTTTTTGG
AGTTAAAATCGACCATTGTTGATCGTTTGAAGACTATCCACCAACTTTTGAAAGATACTA
AGAAACTCGAGGGAGCGGGATACGGAGGCGACAACGCAAAAGATATCATCAAGATGCAAG
CAGAAACTCGCGAAAATATTCGACAGGCAACAGACGAATGGAGAGAATTAGAAGCTATTT
ATAAGAAAGAAGCACGCAAGAAGAAGAGCAAATTCACCCCTGAAGAACTAGAGGTTCAAT
CGGAACTGACCAAACGCCTCTACGCTGAGATTGAAAAGGTTAAGGACGCACAAATGAAAG
GATACGCTCGAAGCCGTACCCCCGATGCTGCTGCTGCCCTAAACACAAAGGCGCTGGCGT
ATAATCCGTCCGGTTCTTATGGTGGCAAGAAGCCGTCTTGGGGCGGCACCGGTGGAGGGG
TGGCACTTACCGGTGATCAGCAACAGCAGATTATGCAAATAGAGGAACGCGATGCCGATT
TTGATTTACAGCTTGATGAAATCGGTGAGGGGATTCAAGACTTGGCGGAAATCGCTCAGC
AACAAGGCGAAGAAGTTCGTCTCCAAAATCAAATGCTGGACAAGGTGGATAAAAAGCTCG
ATAAGAATCTTGAGAGAATGACCACTGTGAACGCTAGAATGAAGGAAACTCTTGAGCAAG
TCGGGCGCTCCGGAGACAAAATCATGGTAGACATCATGTGCATTGTCTTGGCAATAGGAT
TTGCAGCAGTCATTTGGAACTTTTCCAAGGTAAGTTTTCACGCAGTAGTAGTGTCTTGGA
ACCTATTCCTTCGACATGATTTGACGTTCACCACAATTTTAACACCGTTCTGGTCTTGCT
GCCCGCTTTCACAGAAATAACTAGGTCTGATTGAAATCGAGAGACCAAAATCTGGAACAT
GCGAAAAGAATCGTACACAATGCAGCCTATGCTCTATTTTAATAGCGTTCATTGTGAACG
TCAGCGCCATGAACGACTTTTTTATCGATGCATCAGAAGTCACTTTTTATGCCAATAAAA
TAAATACACAAAACATTGTAG
>comp2940_c0_seq2
TCCAGAAGTTCGAGAGCTCTAAACCATAAGGCTCTTTGGTTACTCTTTGGTTACTGAATC
TTACCGGTACATCCACTCGTTGCACAAAAAATACTTATCCTACAAAGCGAGACAACCTAA
AAATGGGAGCAGGACGAGAGGATGAAGAGATCACCGATCTTCTCACGAAGTTGTATACTA
TACAGGAGGATATCGGCGCGAAGCCGAAGACCTCTGAAGATGAAAAGCGGGCGAAAGCCA
ACAACACGGCCAAAATGGGATCTTCGAAGAAAGCGGAGAAGAAAGGATCTAAGTTTTTGG
AGTTAAAATCGACCATTGTTGATCGTTTGAAGACTATCCACCAACTTTTGAAAGATACTA
AGAAACTCGAGGGAGCGGGATACGGAGGCGACAACGCAAAAGATATCATCAAGATGCAAG
CAGAAACTCGCGAAAATATTCGACAGGCAACAGACGAATGGAGAGAATTAGAAGCTATTT
ATAAGAAAGAAGCACGCAAGAAGAAGAGCAAATTCACCCCTGAAGAACTAGAGGTTCAAT
CGGAACTGACCAAACGCCTCTACGCTGAGATTGAAAAGGTTAAGGACGCACAAATGAAAG
GATACGCTCGAAGCCGTACCCCCGATGCTGCTGCTGCCCTAAACACAAAGGCGCTGGCGT
ATAATCCGTCCGGTTCTTATGGTGGCAAGAAGCCGTCTTGGGGCGGCACCGGTGGAGGGG
TGGCACTTACCGGTGATCAGCAACAGCAGATTATGCAAATAGAGGAACGCGATGCCGATT

TTGATTTACAGCTTGATGAAATCGGTGAGGGGATTCAAGACTTGGCGGAAATCGCTCAGC
AACAAGGCGAAGAAGTTCGTCTCCAAAATCAAATGCTGGACAAGGTGGATAAAAAGCTCG
ATAAGAATCTTGAGAGAATGACCACTGTGAACGCTAGAATGAAGGAAACTCTTGAGCAAG
TCGGGCGCTCCGGAGACAAAATCATGGTAGACATCATGTGCATTGTCTTGGCAATAGGAT
TTGCAGCAGTCATTTGGAACTTTTCCAAGAAATAACTAGGTCTGATTGAAATCGAGAGAC
CAAAATCTGGAACATGCGAAAAGAATCGTACACAATGCAGCCTATGCTCTATTTTAATAG
CGTTCATTGTGAACGTCAGCGCCATGAACGACTTTTTTATCGATGCATCAGAAGTCACTT
TTTATGCCAATAAAATAAATACACAAAACATTGTAG
>comp2941_c0_seq1
CAGAAAAATTCCATTGCACAAAATCGTACCTTTTCACCACATGGAAAGGTTGTCCATTGC
ATCGACCACTGACCAACTCCCGAGCCCTAATATTGATAACTTATAATTACATGTTGCTAT
ACCTGACGATAGTATTCCTGTTCTTCTATAACACCAACGCGCACGGAATCATTATGGACT
TCACCCTGAACACAATTTTGGGCGATAGCGATTGTGCAACGCTGTTGGCGAGGAGCCTTT
TCTTTGTGTCGAGGGGCAATCAAAAGAGACTCGCTCTGGGGGGAACATGTGGTGATCGAA
CAGCAAATGGGATGGAAAGAAAAACAGATACGGATGCATGTGTGGTGAGCTTTCACGAGC
GAACCTCCAAGAAGAGGGGCAACAAGTTGTTGGGATCGGGGTTATTTTCCGTTGTCAAGC
ATCGCGACTCGCTGGTTGTATTGGAGAACCAAAATCGGCAACAGCACCTCAAGCACATCG
ACGATGACGACGAAACCGCCTCTACAGTTGACATGGACGAGGATGAATTTCCGGTCGCCG
CTTCCTCCTCCTCGAACCATCGCGAGAAAAAAGAGAGGAGGTCGGTGCAGTTCGAAGAGG
AGTTGGTGACGGCTGTGTACACGCGTCCCCGAACGACAAAGGAAGACAAATACTACCTTC
ACTACGACGAATACGACTATATGGATTTCAAGCTGGAATACAGAGATCAGTTGCTCCAGC
AAAGAGATCGGGAGAGAAATTCCAAAGGAAGAATTAGCAACCAACAACAACAACTGCCTT
GTTACAGGAGGAGTCCCCGCAAGGTTTCCTTCAAGCGAGAAGTCGTTGAGAGTGTCCACC
CCGTGATGGATCGAAGCCAACGAAAGAAAATCCAGAAGGATCTCTTCTACACGGAAGAGG
AAATGAGAACGTTCTTGGACGAGTTTGTCGTTTCCCTCCAAAAGCAACAACAGCAGCAAC
AACAAGTAGTGGTAACCTCGTGATTGTCCATGCAACGCAACACAGCGATATGAACGAGGT
GTCATTTGTTGTCATTTACAGGCATCACTGAATGTCTCTTCGCAAGTGAAAACCATTATT
AGTGGTTGCGATCGAACCCTGCCTGTCTGCTACAAGATGTACAGTACGATCAGCGTATCT
TCCCGGCGGATTAAAGATTTCGAGAGCGAGGTGAACCACTCGGTGGCAGCCTTGGATACG
CAGCACGCGTTATATCTGCTCCACAACGAACGAACAAGTATGCACTGTATTGTTTTGTAC
ATGCCTATATATTATTGTAAGAGATGACGATGATGATGACCATCCTGTGGGCACGTGGAG
CCTGGAAAAAATCTATGATCATCAGCATTGGGCGAGCTACTAAGAAACGAATATTCACGGC
TCAGCTGGTGGTATTTCCGCACACCAAACAATTCAACTCAAAAGTAGGTGATGACACGAG
ACGAAGCAACACATTCGTAGATGGCTCCCATAAGAATCAAACCACAAGCCAAATGCCTGT
TTCTGTCACCCCCTTTTCACTTGATCGATGCCGAACGAAACACAACAACAACGCTACTGC
CAACGCCATCACACGATGGACGATGATGGTTCGTTCGATCGATCGATCAATCAATCACAA
GAACGCAAACAACTTGCGATTCAAAATGTTTATTAATTAATGCTAAATAGTCTGTTTTAT
ATTACGTACACCTC
>comp2942_c0_seq1
AATCAGCCTATTGGTGATTGGGATACGTCCAGTGTTACTGACATGAGTGGGATGTTTTCA
AATACAACAAGTTTTGACCAACCTATTGGAACATGGAATACTTCATCTGTCGAAGGAATG
GGTCGCATGTTTTATTCTTCGCATTTCAACCAACCCCTAGATGAATGGGATGTGTCAGCT
GTGGCTCACATGTTTCTCATGTTTGACGGAGCTACTAATTTTAATCAATGCCTAGGCTCA
TGGGCTGAAAAGACTCCAGACTATGTTTCTGTTTTTGGCATGTTTGACAACAGTGCATGT
CCTAACACTTCTGATCCAGATAAAACGAAGGGACCCTGGTGTCAAACGAGCAACGACTCT
TGCGGTCACCTCGTTTCTTCTGACTGTGCGAACGAGAAAGGCTTCACAATCAAACGCAAA
TATATTGAATGCAACGAATTGTCAGAACTTAGCGATAGAGAAAGGAATGCATTATGCAGG
AAAAAGGTACCAAAGCAGGCCTGCCCTGGGGTGTGCGACCGTCGGTGCATTTGCAAAGAT
AAGAAAAGGTTTTCAATCAAGAAGAACAGAAAGCATATTTTCAAGGGATCATACAAGTGC
AGAAATGTTGGAAAACAGAATCAACCATCCTGCGATACCAAAGCAAAGAAGCGCCTACTC
GTAAAGGATCTCTGCCCAAAGAAGTGCGATTCTTGCTTGACTTGAGGATCAATACGTGCT
CATGAAAACTGCCACCGTGCATCGGCATTTCCGGCAGGGCGAAAGCAAAGGTAAACTTCA
AACTCGTATTTCCCGACAGATACAATATATTTGCAAACACAAAAATCTTTCAAAATTTGA
CAGAGGAGTAGGATTTCATAACGAAACGAATAGCATCAGTGCACTACCCGGAAGATAACA
CACACACACACGTTCCGTCAAATCTTACACGAGGGAGTCTATTCATAACTCACAACCGCA
GTGCTACGGTTTGTTGTATTCTACGTGCTTGTTATCATT
>comp2943_c0_seq1
ACCATCTCAAATGTTGTCCTTGCGTCGGCTGGAGGGTGAGAGTACCTTAGTTATCAATTA
TCGACAGAAGTGCATCCAGATCGAAGAAAGGACCTTTGTTCATTAGCGGCAAGCCCTCGC
TGGAAGCCAACCAACCAATCAATCCTTAATGACGCAACAGAAAGTGTAAAACACACAAAA

CGATACGAGCAAAACAACATGCCATCTGGGAAGCCAAGATTGAACACTCCGTGCTATCGG
TGGGATTTTTCGGCCAAGGAGTGCCTGTGCCTCCATTCCAATCTGATGAAGACCACTTCC
AACCACAAGAAGGATGATGAAAATGCCTTCCTGTCGGACAAGTCGCTCTCGGTAGTCCTG
CTGCACGGATCCTGGAACCACGATTTGTCCCCGGCCCTGAGCGACGAGGACGTCTCCGAG
CACGCCGGAAAGGAAGAAATCGAGGGACAGAACCGCCGCGAGACGACGAAGCAAATCGTC
ATGCAGCAGATCGCCTTTGCTTTTTCCGCGGCACGCGACTGGGCCGTGAAACTGTCCAGG
GAACAGGCTGGAACCGATGCCAGCATCGCGAGGGACATTTTGGAGTACGCCGATTGCCGC
ACCATCGGAGGATCCATGCTGGTGGATTCCAGCGACGATGAGACGATCGACATCGCCGTC
GATCAATTCCTGGCGCCGGAGGACCTCCCGGCCCTGGTGGTGGTTGCCAACGGTGACGTC
GGGAGCCCCGAAGCACTGCTCCATCCGGTACGGGGGCTGGATTCCAGAACCCTCGGTGCC
TTTCTCTCCACGCCCAGTTCGGAACCAACCTACAACGAAGCTTCCTTCACCGTTCTGAAC
GCACTCAGAGACACCCTACGGAATATCTTCGAGGGAAGGCCCGCGGACGCCGGTGGCGGC
GAAGCCGGTAAAACCAAGACCGGTCCGAGCCTGGAGGGAGAGACCGAAGCGCTGAGGATT
TTTGTCTCCGGCGATCGGTCCCAGGTGGGGAAATCTTCCGTCTGCATGGGCCTGATTGGG
ACCCTGGTTCACGATCTTGGCTATCCTCCCTCCTCGCTGGCCTACATCAAGCCTGCAACG
CAGTGCGAGGCTACCCAGCTGGTGGCGCTCTACTGCGAGGAACTGGGTGTCTCCCACCGT
GCCATCGGGCCGATCGTCTACTACAAGGGCTTCACCCGGGCCTTTCTGGCGGGGGACACC
GCACCGAGCGAGGTGCTCCTGGAACAGGCCGGGGAGGCCGTCGATGCCATTGCCGAGGGG
AAGAGGATCGTGATTGTCGACGGGGTGGGATACCCCGCCGTGGGGTCCATCTGCGGGACG
GACAACGCGTCCGTGGCCGCGGCCTGCGGGCCGATACAAAGCCAAACAAGCCAGGGCCAG
GGCAGGCGGCCACCCGTTCCGGTCCTGCTGGTGGGAAAGAAGGGTGTCGGCGATGCCGTC
GACTCCTTCAATCTGAACGCGACCTATTTCGAATCAAGGGGGGTTCCGGTGCTGGGATCG
GTCTTCAATCGGTTGCCCCTCGAGGGGTACTATTCCCTCGACAACTGCAAACAGGCAGTG
GGATCGTACTTTGAACAAAAAACCGACAAGATCGCCTTTGGGTTCATCCCCGAGGTCGCG
AGCATATCGTCGTCGAGGGAGGAGGGGGCCGCCCTCGAACTGGAATCCGCCAAGGAATTC
GTAAAGACCTTTGCCGATCACGTCGACGTCAACGCAATCGTAGAAGCGGCAGCAAAGGCA
ACGGCGGTACCGGTTCGCAAAATAATGGCGCCTCCTCTTTCCCACAAAAACCTCGACCAA
AGAACGCACCAACCAATCACGCGGGTGGTATCGGAGGAAAGCCCGACGAAACGGCCCAAC
ACCGAAGCCAGGGATCGGCAGAAGATCCGACTGACGAGGGCGCAGGTCGAGGAGGCCGCC
AAGATAGCCGGAGCGGCCGGTGGATGAGGCGCAAAGTAGCAGCAAAGCCGTGGCTTAGAG
CGGAATCGAATCCGCCCTTCGAATCCGCC
>comp2944_c0_seq1
CACTGACAAAGGACAATATTATCAGTTCGGTCGAGTAATCGTATCGATATAGGTGGCAAT
TGTGCCATAGACTTATATCCACACCCGTTCTCTATCATACAGTGGAGAATCACAGACCGT
GAAGATCATGAGCGAAACAGAAAGTATTGATGTCGCTTTCAAAATGGTGGCGATATTGGG
CTCCATGCTCGCCGTGATCGTAAGCCTGGTGGCAATGCAGACGAAAATCAAGGGGGGCGA
TTCCCGATGGCTACCGAACCCAACGTCGGAGCCATCAAAACGAGCTTATGAAATTTACGC
CCTCTGTTATACTCCAATCTGGATCTTCGCTTTCGGCTGTATCGTGGCCTTCGGGCTATA
CGAAGACTTTGACAAATGGAGCTACATGAAGGTGTGCGTGGGACTATCTCTACCATTTTT
GCTGCAGCCAGTAATTTTTGCGTCCGCTGGTACTCCTGATTCCCCAGACGAGAAGCGTCC
CTTGATAGAGCGCTATTCGTTCAAAGCAAACGTGTGGATTGCGGTGTACAGTTTCATCGG
CAACTACTGGTATACTCATTACTTCTACTCTGTCCTCCGCGCGAAGTACACTATGCCGGC
ACATCGACTCAACAACGTTCCATTGGCTTTATATTTTGCTACGCATTTCTACTTTTCGAC
CTACCACGTCTTTTCGAATCTCTTGTTGAGAAAAGTTGTGACCACCTACGTACCTGGAAT
GGTCCGAAGCATCCTGTATGTTACTGTGGTTTTCACATTTGCGTATTTCACGGCTTTCAT
GGAGACGCTTACAATAAGTGCCTACCCAGATTATTCATTTGAGGATAGAAACATGGCCTA
CACGGTCGGTTCCGCGTTCTATGGAATATATTTCATTGTTAGTTTTCCGGCATTTTTTGT
CTTTGACAAAGATATCGACAATGCAGAAGCGAAAAGAAAAACTTCTGTTTGGGACACCAT
TGTATCCAGTTGTGGGTATGGAATGATCATTATGATTTTGCTCGACATCGTAAGACTGCA
CTTGGGCATCCCACTGGTTATTGGATCATCCGATGACATTTGTTCAATCAACAAATAAAT
AACTAAATCAATGTAAACTAGTTCTTCCAACTGCGCAA
>comp2945_c0_seq1
TGATTCGTTCCAGTACTCTGCATTATACGAAGAGCACTACAAGATATCAAACGAAAGCAG
AGAAATCGATTGTAAAAATTAGGACAAGATAGTGAATAGGACAGGACAATGGATATGAGC
GAGGCCTGTTACGCAGCGGTACCGCATGAATCGTCACCGCTCATCCGTAAGAAAAAGACG
AAGTTGCTACCTGTCCTACTTTGGGTAGGACTAGCATACGCAGTATTTCAAGAAACTTCA
TCGCACGTTCGGAAGTCAGTGAGCGTAGCAGCAGAAGTAGTTAGAAGTTCAACAAAAAAT
CGTGGAAATCATTACAACAATGATCCTGTTCATTTCGATGATACAGATACTTTAGATATT
GATGACCTTCATGAACCTGTGCATCGGAGTTTTGGAAGCGACGTCGATATTGACGTAGAC
ATAGACTCTGAAGGAATTGATTGGGACAATGACGAAGACAAAGATGAAAATATCGAGAGA

AAAAATTCTGCACGCACGGGGCCGAACATCATACTTGTAGCAACAGTAGATGGAACAATA
GCGGGAATTTCAAAAGAATCGGGGACTGTTTTGTGGAGGCAATCGGAGGACATATCATCT
GTGCCCTCTTCTTCTTCCTCCTCCTCTTCGTATTTGATGATGGAGTCAACCAAAAATGAT
TCATGCAGTCCAATTTTAAAGCCCCTTGTGAGTACCACAACAACCAAAAAGTCAGCTTCC
GATGCCTCGTATAAAGTTGTTCCGTCCGTGGACGGCACCATCTACACAACAACAAACGAT
ATGACCATCGCCACTCCTGTGAAGGATCTCGTCGCCAAATCCCCCTTTTTGGATCAACAG
GGTCGATTCTATGTCGGTTCCCAGTATGCATCGACAGCTGCTTTAGACGGCACTACAGGA
GAAGTGCTTCGCGTTGTTTCGTCAACTGCTACGACTATTCCAGACGACGAAGACGACAAT
GAATCGTCTCTACCTACTTTGGATGATCCGAACATAGTTTGGATCGGTAGAGTCGATCAC
GAGGTATCGATTCAAGATGCAAAGACGGGATTTACAGATGCTCAATTCTCTGTTGGTCAA
GTCATGAGTGTAGAAGATATGCGTGGAATGATGAGGGAGAGTGTTTCGAATCCTGAACAA
ACGATACGCCCGAATGGCTCGTCCTTGCTATCAAAAGCTGTCGCAACCACGGTAAATGAA
GCCATGGATCTTCCAAACTCCTTGGTTGCAACTCCAAATGGTAATGCAGCTCTGTGGAGC
TTTGAGAACAATAAATTTGCATGGGTTGCAAGCGAATCCTTGAATTCACCTATTGTATAC
GCAATGGATGCTGAAACGGGAGTGACTTTGCGTGTAGATATCATTCCTGACGTTCCTGAT
CCAAAAGCTAACATCCGTGATCTAACGCGAGAGATGGAACGCCAGTCAAGGATGGCATCA
GTAACAAACACTCCGGTAGTCGGTGCAATGTCCAATGGTCAACTTTACGCACTTCCATTA
CGAAGTTCAGATCAGTCGACTAGTAGTGCCACTATAGATCAGCTTCCTACCCAACCGGGT
GGGAAATTGATTAAATCACCTCTCCCAATTGGTGATGGAGAAAACGATTTTCCAGATTCA
AGCCTTGTCATTCAAAAAGAAGAAGAGGGATTTTTCCATCCAAAATATGGCTACATCTCT
CCAAAGGACTTGGGTGGTTTCGTTCATCCCGTATATGGTTATGTTTCACCACGGGACCTC
TTCGGAGCCCCAAAACGTTCAAGTAAGACCTCCTCCCTCTTTCGAGTCTTAGGATCATGG
TTGCCGCCAACAATCGCCCTTTTGTTTGTTATTTCGTTTGAACTTGGTCGTCGGAAGCGT
TTGAAGGATGAAAAGAAAGGTGCACGGAATTCTCTCCACCCCATTGAGCAAAAGGACCAG
ATCAATGGAGTTATCTCCTTTAACGAAGACTCTGGTGGTAAATACGGAGCCTTACAGAAG
TCACATCATCAAGAGGTGATCTCAGTCAGCGAAGACGTACTTGGATATGGAGGCCATGGT
ACAGTCGTTTATAAAGGTGTTCTAGATGGGAGAGATGTCGCAGTAAAGCGAATGCTCAAG
GCGTACCATGCTAGTGCCGATCGTGAAATTTCTCTACTTATTGAAAGTGACGGTGATCCT
AACGTAGTCCGGTATTTTCTCAAGGAGGTACGGGGTGACTTCGTTTACCTTGCACTGGAG
CTCTGTGATTTATCATTGCATGAGCTGATTGGTGTTCTTCGGGAAAAGCAACAAGAAAAA
AGTTCTTCGATACTGCCGAATGCTGACAAAGAAGCATGCGTTCTCTCAAACGAGAGCGTG
GGAGCTACTACTAGAATCTTATTGCAGATTGCTTCAGGAGTGAAGCACCTGCATTCTCTC
CGAATCGTACATAGAGATTTGAAACCAGCAAATATTTTGCTCGCAGTCTCAAAGAAAGGA
AAGAAAAAAGTAAAAGAAGAAGATCCGATCTTTGCAACCTTTACCCGCAACTATTATGAT
GCAAAAATCTCAGATATGGGGCTAGGAAAGCAGCTGATCGGACAAAGTAGTATTGGTGCT
AGCTTTATCGGGGACTCTTCTTTTCGCGGAAACAAATCGGGCGTACACTCAATCGGAGTA
GGACCTGGAACTGTGGGGTGGCAAGCACCTGAAGTAATGGCGATGCGTTTGACATCTGAC
ACATCTGCTCGATCCACAGACAGTGCTAGCAATATAGGGGAATCAAAGGAAGGCCAAGGA
AACGACATTCTGCCAAGTATTCGGACAAGCAGATCTGTGGATATTTTCTCTCTCGGGTGC
ATTTTCTACTCTCTATTGATCCCGGGATCCCACCCTTTCGGTGAATGGTTCGAGAGGGAA
GCCAACATAATGCACAATCGACCTAATTTAAAGCCACTGACAAGCATTTCATCAGAAGCG
TATCGTTTGATTCGCTCGATGCTGGATCGAAATCCAAGACTACGCCCTACAGCGAAAGAA
GTATGCGAGCATCCATTCTTCTGGAATGCAGAGAAAAAGCTGACGTTTTTGTGTGATTTC
TCAGATCGATTAGAAACCGATACCATTGCACCGGGGTCATCGAATGTATTCGGGTCTTTC
GCTATTGAACGTGGGGCACTTGAAGTTGTGGGAACCTCTTGGGAAGAAAAGCTAGATAGT
TCACTCGTTGACAATGTACAAAAGTTTCGGACATATGACTATTCCTGCGTTCGAGACCTG
TTGCGACTGATTCGAAATAAGCATCATCATTTCGAGGAGCTGCCAGAAGCATTCCGTTTG
AATGAGGCTCCAAATCAGAATGCACTATGTAAATACTTCACAAAGATATTTCCGGCTTTA
ATCATGCATTGTTACACATTTTGTGGACAAAATTTAACGGATGACGACCCACTTGTATTG
AAGTTCGTCATACCTCCGGCGAAGTTTCAAACCAGAGAATTTTCTACTGCACCAATATCT
GTCTCCGTCAAAGAAGAAACAATTCCGACCAATCATAATGAGGCTGTCATCTCTTCCGAA
GAGTTGAAAAATGATTCGACCGATACGCAATGCTTGGAGATTGCCCAAGTGGTAACATGT
GATTCGAACGACGAAGTCGATGAAGATATTATTGTATGGGAAGGCAGTATTGCTGCTAAA
ACATTTAATAATCGCGGTTGGAGCAGATCGCAGGATGAATGGTCGAGACACATCGAACCT
TTTTATAAAAAACGTGATCCAGCCTCAAAGCGATGCATGGAGGACCCCAAATTTCGAACC
AGACTTTGCAATCACTGGGATATGAGTCTCGGAGCATTTTGTCCAATGAAAAAGAAAAAT
AAATGTGTATTTGCTCATGGACCAGCTGAGTTGCGGGTGAAAGAAGGCAAAAAGAATCGA
TGGGGAAAACTTGTCGACAAAAATGGAAACAATTCGAATCCTTGGCACTGCGGAGGAGAG
GACACTTATGGTGCGGCAAATTCAATAGAACTAGTCAGAAAAGAAGAGGGGAAATGGAAT
GCTTCGAAAGATAGAAAGAAGAACCAAGGGACAACTCTAAGAAAAAAGAAACCGGGATCA

AACAGTAACCAAGTCAAACAAAAGACAAATCCAAAGGCACCAGCGTGAAGAATACGATGA
AGGCGTCGGTTGGCAAGAATACAATCGTGACACAAAGTTTGAAACAGGCCATAGCGTTTT
GGTATCAAATAAATACTAGGTATGCACTTTTATGGTAGTACCAAAACCTAAGATCGGAAA
AACAGCAAAATTGCTGTTGATCTTAGTGTCATTTAAATTGTTAATGCTGGTGAAATAAGA
TTTAGTATGACGTATTAAATCTTCTCATGAGTTGGTAGACTGATTAATGGATCAATTGAT
TCCCATAGCAGCCCTTTCATCTGGTAGTATCTTTGATCTCAAATCAAATACATCCCCACA
GGTAGTCAATTGTGGGGAAGAAACTTGCTATTATTTGAGTCAAAAGAACAACCAATTGTT
TGTTTTCTCTTCAATGTTAAACACTGTTTTCTTCTCATTGTTTCCACCCAACAATGAATC
TAGATGTAGCACAAACAACTTTAGCATGCATGGCTTTCAGGTCATAGTACTCTTTTACAG
GCACTGTCCCTGTGCACGACGATGAGATTGCACCTCTCAATGGAATTGGTCTTGATTCCA
TCTCAACTCCTCCAGTTCAGGAACACATGAGTGAGAAGTAGTACCTACCCAAAAC
>comp2946_c0_seq1
CAAAAGAAATACGAAAAGAAATAAGAAAAACAACTAAAGACAAACAAAGAATAAAAAAAT
ATGATGAAAGCCGATATTGAAACCAAAAGAATTCAAAATTCGAACGAGAACGATGCTACA
ATGGACAGCAATACTGACACGTCCAAGAAGAGAAGACGCGGGTGCTTTGGTCTCATCCGT
CACTATCAAGTTACGGGTATTCTAATTTTCGCGGTCATAGGAATCGGAGCAGGAATTGGA
ATTTCCTCATGGCAGCCAGAGAATGAAGATGCGAAAGATATTGCCGTCAAGTGGATAGGT
TTAGTCGGCGATTTATTCCTTCGAGCGCTACAGTGTTTTGTCCTACCATTGGTTTTCGTC
AACGTCATAGTCGCCGTTGTTGAGATGGTTAGTGTTGGAAAGGCAAGTTCGGTCGGATAC
ACCGTCATAGGATTGTATTTCGCGACTACACTGGCTGCAGCTTTATTCGGAATTGTATCT
ACTCTCGCATTTATGGGACTCTACAATACAGAAGAAGACATTCACGAAGAGGAGATTTCA
CCGTTGTACATTCAACTGGGGTGCAACGACGCTGACTCGGTTTTAGCAGAAGCCAAGAAT
GGCACCATTACCTGCATTTCAAAAGCATTGGCGGACGGTATCGACTCTACCTTTATTCTT
CACGATCTCAACAACACGCTAGTTGAAACCAATAGCGCTGTAACCCAGGTTTCGATTTCG
GACACGGTCTACGAGGGAATTTTCGAGAAAATCGTTCCGAACAACATCATCGGCGCCTTT
GCAGACAGCAATTTTACAGCTATCATCTTCTTTGCTGTCCTCTTCGGTGCCGCACTAAGT
CCTGTCATTCTCAAAGAGAAGAACGGAGGTGAAAGTAGCCTGATGACTATGCTGAAAGAA
ACAGACTTGGTGTTCCAACGCATTATCCGCTGGATTATTGATCTGACACCATTTGCCGTC
TGCTCCTTGATTGCCTCTGCTGTCGGAGAACAAGAAGATCTCTTCTCTATGTTTTCAAGC
ATTGGTCTCTTGATGGCATCGACAATTGTGGGATGGGGTTTGCAGGTAGTCGTCATCTAC
ATTGGATTATTTGCTCTGCTGACCAAGTCGAATCCATTTGGCTATTTGAAGCAGCTTATC
CCGGCACAAATGATGGCGTTTGCTACCTCTTCCTCAGCTGCAACCATCCCAGCATCTCTC
GCAGCAGTCAAATCCACGGGGAGGGCACCCGACACGATTTCCCGATTTGTAATTCCTTTT
GGTGCAACGGTCAACATGGATGGAGGTGCTGTGTACTTTGTATGTGCCTGCATTTGGTTG
GCGGTGTTAAATGGCGAGGATGTGACCGTATCGTCTTTTTTCATGCTCATCATCATTGCA
ACCATTGGAAGCATGGGGACAGCTCCGGTTCCATCGGCAGGTCTGGTATTGATGATTACA
GCCTATAACACGGTATTTGGAACCGCGGATGTTCCTCATGGTTTTGGGTATATTGTTGCC
ATTGATTGGTTCATGGACCGATGCCAAGCAGCATGCAACATCACAGGCGATTGCATTGTG
ACCGCAATAGTTGCTCATAGATGTCCAGTTGACGAGACCCCAGTGATCATGGTGCAGACG
AATGACGACGACAGAGATTCATTGACCACCGAAGAGGAAGACGAGCAGTGCAGTGAAGTT
TAAACGGAAACTGAAGTTTTGCGGACATTTTTGGCAACATTATCGGACAACTCTAGGAGG
ATTTCAACATCAGCAACAAGAAGGATCTTGCTCCTCCTAAACAATTTATACTAATGATAG
AAATGCATATACTACTAGAACTCAATTTTGTGATA
>comp2947_c0_seq1
GGCAAGACAAGGCAAGGCAAGCCAATCCAAGCCAAGCCAGGGCAAGGCAACACGCAGGGG
GTGTGTTCCCGGAACGGGACCGGGGTTGCGGCACCAGCGGCCGCGTGGTGGCACTTGCTC
TTGCTTGGTGGCTTTTCGATTCGTGTCACAAACAAGCAATCAATCAATCAATCAAGCAAG
CAAGCAATCAAGCAAGCAATCCATGAAACCAACCATGGAGAACCAAGAACCAAGAACCGA
CGTGGCGTCGCCGGTTGCTTCCTCGGAGGCAGCAGCAGGAGCAACGCAGCAACAACATCA
ATATCAACAACACCATCATCAACACCAACACCATCAACAACAACACGAACACCAAGCCGT
TCGGGGGGACGTCCCCAGTGCCTCCCGCAAGCGGTTCCTCGCCCAGAAGCTGAACAACAG
CGCCGCCCTGTGCATCGAGATCGGCCGGTACGACCGGGCCATTGGGTCGCTGCACAGGGC
GCTCTTGCTGTGCGAGAGCGCGGGCGGAGGCTTGCGAGCCAGCGAAGGTTCCGGGAAAGG
TTCCGGGAAAGCTTCCAGCGAAGCAAGGGTGCGGCGGCTGCCTCCGCCCACCGAGGCCTG
TGCGGGCGACGCAGACCCGGAGGAGGGGAGTGTCCGGGGCCCTCTGGGCTTTTCCCTTTC
GAGGACAGAAACAGAAACAGAAACAGAGACAGAGACAGAGACACCTACAGCCACAATCCC
AAGCACTGCAGGCACAAGCACAAGAACAAGAACAAGCACTACAGCCACAATCCCAAGCAC
AATCCCAAGCACTGCAAGCACTACAACGTCCACCTCCACGAGAACCACGCTGGACGACTG
CATCGCCTTTTCGGAGGCCACCAGTCTCGTCCATGCGGTCCGCTCCAAGAAAAGGCAGCT
GGAAGGCATGGGTGGAATCGGCAGCAAGAGAAGACGCCTCGGAGCGACCGAGAACGACAG

CTACAGCCATTGCAGCAGCCACATCCATTGCAACAGCAACACCCAGGTTGCCCCCCTTCA
CTTTGTATACTCCACACCGATACGAATACGAGTGACTCCAACCAACAAAGAAGCGGAAAC
GGGATCCATCCTCGGCCTCGTCGTCCTTTTTAACCTGGCGCTGGCCCACCACCTCCGGGC
CACGGAGGCCAAGCAAGGTGTCCATCAAGGCGTCCATCAAGGC
>comp2948_c0_seq1
CAATTCCCAACACAAACAGGAGATTCCGGACGCTAATCTCTGTTTGGATAAAAGTCTAGT
TTGAAACATATCTAGCAAAAGTTAGAATGGCCGACGAACCAAGTGAACCCCCAGCCGACA
CCACTCCCGACGCGAGCGATGATGCTCCTGGTGGATTGACCGATGCCGACATCTCGTCCA
ACTGGGACGAGGTTATCGAATCCTTCGATGGCATGGAGCTTCCCGAGGCACTCCTTCGTG
GTATTTACTCCTACGGTTTCGAAAAACCTTCTGCCATCCAGCAGCGTGCCATCAGACCCG
CCATTTCGGGCAGAGACTTGATTGCTCAAGCTCAATCCGGTACCGGAAAAACCGCCACCT
TCGCTATCGGTTGTCTTGCCGTTATCGACCAGAGTGTTCAACAATGTCAAGCCCTGATCC
TGGCCCCTACTCGTGAGTTGGCTTGCCAGATTCAAAAGGTTGTCTTGGCTCTCGGTGACT
ACATGGACATTAAGGTACACGCTTGTGTTGGAGGTACAGCTGTCCGTGACGACATTCGCA
CCCTCCAAAACGGTGTCCATGTTGTTGTTGGAACACCCGGTCGTGTGAACGACATGATCA
CCCGACGTGCTCTTCGATTGGACTCCATGCGCCAATTCTTTTTGGATGAGGCCGATGAGA
TGCTTTCTCGTGGGTTCAAGGAGCAAATCTACGACATCTTCAAGTTCCTTCCCGAACAAG
TCCAGGTCTGCCTTTTCTCTGCCACCATGCCCTTGGACGTATTGGAGGTCACCAACCGTT
TCATGCGCGAACCCGTTCGCATTCTTGTCAAGAAGGACGAGTTGACCTTAGAAGGTATTA
AGCAATTTTACATTGCCGTTGACCGAGAAGAATGGAAGCTCGAGACCTTGTGCGATTTGT
ACGAGACTTTGACCATTACCCAGGCCATCATCTACTGCAACACCCGACGCAAGGTCGACT
GGTTGCAAGAAGAAATGCAAAAGCGTGACTTCACCGTCTCGTGCATGCACGGAGACATGG
ATCAGCGAGAGCGTGACATCATCATGCGTGAATTCCGTACCGGTTCCTCCCGTGTTTTGA
TTACCACTGATTTGCTCGCCCGTGGTATCGATGTTCAACAGGTTTCCCTGGTCATCAACT
TTGACCTTCCAACAAACCGAGAGAACTACATTCACCGTATCGGTCGTTCCGGACGTTTCG
GTCGTAAGGGTGTGGCCATCAACTTCCTCACGGAAGGAGATGTCCGTTATCTTCGTGATA
TCGAACAGTTTTACACCACTGAAATTACCGAGATGCCCATGGACGTCGCTGATCTTATCT
AAGCCGGTGCCGTCGGAAGAACAAAGCTTCTTAGTACGACGATACTAGGGTGAAGGCGCG
ATGCTCCTTTTTCGTCAATTTTATATAACGAGAAAAAACTGTTACGTGATAAAAAATAAT
TATTTGGGAC
>comp2949_c0_seq1
AGCCGAGTGAAGTACGAGTACACGTACAGGCAATAATTGTACTTCCAAGCAAGAGGATCT
CAGAAAGAGGTAAGGTACCGGTACGTTCCACCTCTGGGTGTGATGGACCGAAAATGCATG
CAAAGCAAGGCTAAAACATAAAATTCGCGATTCCTTCGCCGGTGCAACAATAGTCATGTT
CCATTGACAGTCACCTTGATTTTGCTGTCAGTGGCGACATCGCCACACGGTGCTGTACAC
GCGCAAGATTTTGGCGTGGTACTCGCACCGTACTTACAGCACGGCACTTGCTCTGGTACG
GTACTCTTCGCCTCTGCACTCACGAGTTCCAATACCGTGCCGGAACGGCGAGACGACAGG
ACAACAACACCTCTGAAGGCATGCTGTCTCCGTCGAACCGTGACCGCCGGTGTGGTGCGG
TCCTGCCGTGCCATACCTTGCCGGGGTGGATGTTTTCATAATATTATCTCGTGGAATCGA
TCCAGCGGATTCTGTTCCATCTTCTTTTCGCATTGATTTCGCAGCGCTGTTGTCTCGACG
GCCGCGGCACACGCCTTTCAGATAAAGCTAGAGAACAAGAAAATAAACAGAGAGGAAACA
AAAGCTCGTGATAGCATGACGCCGTCAACAGATACAACGAACAACGATAGTGTGCTCCTA
GCACTGCCATCTGCAAGAACGGATTCGGAACGTGGCAACACCAACACGGTTCGCGAAGAA
TTCGAACACCACCAACTCGACAACCACACCTGCATATGCATATTCGACAACGACCTCGTT
GTGGAGACTATCTTCTCCTTCCTGGACGCTCGGTCGCTGCTCTCTTTGTGCTCCACTGCC
AAGGCAACACACCATCGCTCCCGCCTGCGATACGATCACGTTCTTCGATCGGCGCGGTAC
GGTGCGGATTCGCCCGGTTCTGCCTCGCCTCGCCCACCAACCGTTTCGAAAACACGGGCC
GACCACCACGCCTTTTTGGTTGTGGAGAAGCTGGTGTCGGTCTTTGGCCTCGGCGGCAAC
GAAAACGAAAACGGCAGAAGCAGAAGCACCAGGAACACCAAGAAAGCACTGCCACAACCG
TGGGTCCAACCACCCACACCCACACCGATGCGGCTGCTGCAATTGGCGAACGGCAAGAGG
TGCGAACGGTGCAGCCGCAGGCTGTCCCCCTGCTACGCAATGGCGGGAAGCACGGGGCAG
GCCCGCATCATCCTCCCGAGCCTGTCACTCGGGGTATTTTGCTGCACGGGGTGCATATTT
CAACCGACACAAACAAGGGCTTCGACAAAAACGACGACGACGACGACAAGCCAAGCAGAA
ACAGCAAAAGCTACCACCACCACCACGACGACGATCCCATCGATTAGTGCTAGTGCTAGT
ACCAGCAGCACTACAACCAAACCATCCGGGCTCGTGTTCAACGATTGCAGAAGGACAACG
CCGAAGCCGAAGTCAACAAGAACACCGACGATGACAGCATCGGAAGAGAAAACACCATTG
GCACCAAAACGAGGATTAAAAACAAAAGCATTGCCATCACCAACAGCCATGCCAATGGTG
ATTTGCAGAAGTGGCGGTCGCCGTCCCTTTCTGTTGGAGAAAAAGAACACATTCGTTCTG
CAAGAAGAGCTTCGTTCCTTGGCCTCTCATAGCGACAAATTCATGGCGACGGAATGGTGG
CTTCTTTTGGGTCAGAGAAATGTTGTGATAGTAATTTCAAAGAATAACACATACCGTACA

TGACAGTTCACTTGCTTACACGTTAAAGTACGTACGTACTCGTTCTCGTACTTTTTGAAC
TAAGATTTCTTCCATGGTTATTTTTTTTAATTCAGCGCAGCATCGCGCAGCCCACCAAAT
TATCCCTTGCATAGATACCT
>comp2950_c0_seq1
CGCTTCATCGCCTATCGTAATACTCTCCAGAAATTGATCACATTCATCAATTGCATCCAA
TCCGAAGTCAGTCCTCTCCATTGCTGTTATAATCTTGAAACAAGAAGTCAATACGGCAAC
CATGTCACGAGGTTTCCTTTTCACCATCCTTTACTTTGCCAGTGTCATCCCTTCCGTGTT
GGGTTCGGCTAAGTTGGTTCCAAAGACATCGACGGCTTCTTGTACCGGGCACCTGAGCGA
CCTGATATTAACATCGATAACATGCGATCATTCAAATAGTGGGTGCACGTACGGTTCTGA
AGTTTTTGTAACTGGACAAGTTACTTCGAATGCCGACCTTCCACGTCCGTTAAAAATCTC
CGTATCTAGAACGTTTCCCTCGATTTACGGCATCGGAACTCAAGTTTCCAGTAGCGAAGT
GTATGATCTCTGTAGTTCGGATGCGATCAGTCTTGTCGATGATAATAGCGGCAGCAGTTG
CCCAGGTGCAGGTGTGTACAACTTCAACTTCCTTTTTGACAATTTCGGTACCTCCAAATC
GTGGTACGCTGGATGGCATGGATTCAGCATGGGTATGGCGGTTCATTTGAAACACGAAGG
TGGCGGTAGTGACTTCGCAACGTGCACACTCAACGTCAGTGTTGAGGGAAGCGACGAAAC
CAGTTACGCCACCAACGCAACTCTCGTGAGTTTCGCAACACTAGGCTTAGCAGGATTATG
CGTGGGTATGTTTTCGAAGAAACGAAAGGAACGCTTGTTCAAGTGCGGTGAGAACATCGA
AGATGAGAGAACAAAAGAACTTGCCACAAACTTTGAGCTCGTCCGTGACTCTACAGGTGC
CATGGTTTAAGCCCAGTAGTTCCGGAAGCTCTTTACCACAAGATCTTCTTTTATGTTCCA
AATGCTACATGTCCAAAACATCAGGAGTTTGAAAAAAATCATTTTACACTAA
>comp2951_c0_seq1
CCGCAATACGGGACGGCAAAATCGACGGGACAAGCCCGACTCCATCTACCTTCGCACACG
GCATACTCCAACCCGCAACAAATAATAATAATAACGATCATGGCACGGTGTACGGCGGCA
TTTGCCCTTCTCTTTCTCTGCTTGGGAACCACGTCATGGGCCTTTACCACGACGAATAGC
CCGCTTGCGGCCGTATCGATTGCCCGGACGCGGCGATCGCAGACCCCCCCGGTCGTCGTC
CTGGGGGCGGGCGGCTTCGAGTGGGAGGACCCCACCGACGAGCAGTACGACCAGGGCGTC
GAAAACCCCTTCAAGAACCCGGAGCTCCTCAAGGCGATGAAAAAGGGCGACGACGAAAGC
GGGGACGACGAAAACGCCAAAACCATCGATCCCGCCCGCCTGCTGGGCCCGAGGCTCCAG
GGATCCAACCTGTACCTGGTCGGAATGATGGGTTCCGGAAAGTCCAGCGTCGGAGACAAG
CTTGCCAGGCGAATGGGGTCGTACAAGTTCCTGGACACGGACGAGATCATCGAAAAGGCC
ACCAAGCTCACCATCAACGAGATCTTCGAGTCGGAGGGCGAGGAGGGCTTCCGGGACGTC
GAGTCCCAGGTCCTCGACACGGTCCACGCCTACGTCCGGTGCGTCGTGAGCACCGGCGGC
GGCCTGGTGTGCAAGCCCAAGAACTGGGGCAAGCTCCAGACGGGCATCGTCGTCTGGCTC
GACGTGGCCCCCGAGGTCATCCTGAAGCGGGTCGAGGGGAACGAGGACCGTCCCCTGCTC
AAGACAAAGGACCCCCTGCAGACCCTGAAGGACCTCCTCGAGGAGCGGACCCCCAAGTAC
AACCAGGCGGACCTCCGCGTCGAGATCACGGAGGACATGGACGAGGACGCGGTCGCCTCC
CGGATCGTCCTCGACCTGCACAACTTCATCGACGACAATCCGCCGGCGTGGAAGCTCGCG
AAGCAAAGGCGCAGGAGGAGGGACTGGACTGGGTCCAATGAGAAAGGACAAGAACCGAAG
GATAGAACCAAACCAAATTCCCACTGTAAAACAACTACACAAAATGCTGTCATATCTTT
>comp2952_c0_seq1
TTCTTCTTTGGATTATCTTCAGCGAAACCTATTGAACTCCGTTTCTCAAGTCTAAACTCT
TTCAAGGGCAATTTCTCCGTCAGTCACGGAAGCTGCTTGGTTGGTGGCATGGAAGGGGAA
AAAAGGAGAACGAGAAAATTTATGACAGCTCTCGATACGTACGAACGTACGTACTTACTA
CATACCATACGACTCGTCCTGCTATGCTACAGAATGAGTTTGCCTTGAATTCAATTCAAG
TCTTTCAGTACGAGTACCGTACGTAGCGCATGGTATTTTTTACGTAAATTACGTTAATTA
CCATCCGGATCAGGATTGAAACTTGCGATGACAACAACAATTCTCAAACGTGGGGATGAA
GATGATATTCGTGATTCAGATAGTTCGTGACGGATGACCCCCTACCTTTTTTTTGATTTT
CAGACAACAAAACATAATTTTTTTGGAACATTGCATTATTGCCGTCACAGCAAAATATAT
GTCGTGCCGTATCCGAAGATAGACAACTGAAAGAAACAGTAAACGTGGGAATCGTTATTG
CGTGCATACAATATGTACCATCCTTTGCCACTTCGTTTCCTTTGTTCTGTCTTCATTGTG
CTGGTAGCAGTTAACCAACAATGCTGCGATGCTCAGCAGCAAAATCCGTTGACGATGAAC
GGGGGTTCGTTGTTGGCGATGGCCGGAGACGGCTGTGTTGCACTGGCTGTAGACAAACGG
TTTGGTTCGGGACCGCAAATGGTGAACATCGCTCCTCGCCACGTATGGGCCGCGCATCCA
AATCTGATGCTAGCGTTTTCGGGCCTGGAAGGAGACGTTCAGAGTCTCTCGGAAGAACTG
CAGCTCGAGGTGGCCGCGAAACGAAACCGGGCCCTGGGCTTTAGCGTTGGTGGATTGAAA
ATCGGGAACGAGAAGGGCAGCAATGATGAAATGGATGAGCGGCGGTTCCGAATATCTCCT
CCCGCACTGGCATGTCTGACAAGTCACGTATTGTACCAGAAACGAGGTTACTATGTGGAG
CCCTTAGTGGTAGGTTTGATGGAGGGAACATGGGCCCCATTCCTGTGCGCCATGGATATG
ATCGGAGCACAATCGTTCTCGCAGTCTTTCATATGTTCCGGTGCTGCATCAACGTCGTTG
TATGGAACGGCGGAGGCGCTCTGGAAACCAAACATGAAACCCAAGGATCTACTAGAATGC

TGTGGGAAAGTATTTCAGAGTGGTACGTTCACGAATTGCTCATTCATAAAGCACGTCTTC
CTTTACTATTGCTGTGTTATAGATTTCCACTAAAATATTCTTGATGACACCAATTAACTT
CCTTTTGTGTTCTCCCTGTCCGGTTGGAATTAGCGCTCGAACGAGACTGTCTGAGTGGAT
ACGGAATTTTGATCTATCTGATAACGAAGGATGGGATAGCAGAATACGATTTCGCAAGTA
GAAATGACTGATGATTATCATTTGATATCTGGGTCTTAAAATTAGCCATGAGCTCAGCAA
AAGTCATCTGTTCAAAAAAGTAGAATGGGAATCTTTAGAACAAACATTCAATTATCTCTG
TATCTTTCTGATTGATTCCTCTATTTGATTGAGAATTGGAAAATGGGATGGTCGATTTGA
TCTAGGAGGAAAGCATTCATTTTTTTGAATACTCTGCGGAAGTAATTCAGGTCTTCAATC
AAGGTGAAGAGGCATTCCGTCAACTTGTTAATTTCATGATGCGAATGCCGAACAATTTTG
CTGTAATCCAGTATCAAGCGCTTGAGTTTCTGACGATTACTACGCTCTTTGTTTAATGAC
ATGTGGCGCAATCCTTGGCATTGAAAATACTTTCAATTTTGAAAAACACCTCTGGAAGTT
TCTTTCGTCTTTGGAAATACTATCACTTTTTCCACTTCACCTGCAAAAGTATTGCAGAAA
TTTCATGTTTTTTGCTGTATGAAGCAAAAGTTTCTTCTAGCGCTTAACGCATGCATAAGC
AAATAACTGCTTCTTAGCTTACTTTGGCTAATGATTCCATATTTTGTATTGTCAGCAGAC
AACACTAAATATCAGATGCATTGTCATTTTGCACAAGGGGTTTGCTGCATTTCCAGCGAA
GTATCTGTATTCTTAAATACCATCTTTTCAGCGAGGTGAAGTGATGGGTAATGACGGTGA
ATATCATAAAATATTGTACATCTATTGTCTGCATTTGGGTTAACAGGAATGAAAATGGAA
AATGCTTCAATTCTTGATTCTTTTCTGCTCGTACCGTACGGTACCATACGTGATGTGGTT
TCTTCCTGTCATTTTTGGAACGTCAGAAAATCATGGAAAAAGGCTATGGTCTAGAGCTAC
TAAGTACGGGCCCCA
>comp2952_c0_seq2
TTCTTCTTTGGATTATCTTCAGCGAAACCTATTGAACTCCGTTTCTCAAGTCTAAACTCT
TTCAAGGGCAATTTCTCCGTCAGTCACGGAAGCTGCTTGGTTGGTGGCATGGAAGGGGAA
AAAAGGAGAACGAGAAAATTTATGACAGCTCTCGATACGTACGAACGTACGTACTTACTA
CATACCATACGACTCGTCCTGCTATGCTACAGAATGAGTTTGCCTTGAATTCAATTCAAG
TCTTTCAGTACGAGTACCGTACGTAGCGCATGGTATTTTTTACGTAAATTACGTTAATTA
CCATCCGGATCAGGATTGAAACTTGCGATGACAACAACAATTCTCAAACGTGGGGATGAA
GATGATATTCGTGATTCAGATAGTTCGTGACGGATGACCCCCTACCTTTTTTTTGATTTT
CAGACAACAAAACATAATTTTTTTGGAACATTGCATTATTGCCGTCACAGCAAAATATAT
GTCGTGCCGTATCCGAAGATAGACAACTGAAAGAAACAGTAAACGTGGGAATCGTTATTG
CGTGCATACAATATGTACCATCCTTTGCCACTTCGTTTCCTTTGTTCTGTCTTCATTGTG
CTGGTAGCAGTTAACCAACAATGCTGCGATGCTCAGCAGCAAAATCCGTTGACGATGAAC
GGGGGTTCGTTGTTGGCGATGGCCGGAGACGGCTGTGTTGCACTGGCTGTAGACAAACGG
TTTGGTTCGGGACCGCAAATGGTGAACATCGCTCCTCGCCACGTATGGGCCGCGCATCCA
AATCTGATGCTAGCGTTTTCGGGCCTGGAAGGAGACGTTCAGAGTCTCTCGGAAGAACTG
CAGCTCGAGGTGGCCGCGAAACGAAACCGGGCCCTGGGCTTTAGCGTTGGTGGATTGAAA
ATCGGGAACGAGAAGGGCAGCAATGATGAAATGGATGAGCGGCGGTTCCGAATATCTCCT
CCCGCACTGGCATGTCTGACAAGTCACGTATTGTACCAGAAACGAGGTTACTATGTGGAG
CCCTTAGTGGTAGGTTTGATGGAGGGAACATGGGCCCCATTCCTGTGCGCCATGGATATG
ATCGGAGCACAATCGTTCTCGCAGTCTTTCATATGTTCCGGTGCTGCATCAACGTCGTTG
TATGGAACGGCGGAGGCGCTCTGGAAACCAAACATGAAACCCAAGGATCTACTAGAATGC
TGTGGGAAAGTATTTCAGAGTGCGCTCGAACGAGACTGTCTGAGTGGATACGGAATTTTG
ATCTATCTGATAACGAAGGATGGGATAGCAGAATACGATTTCGCAAGTAGAAATGACTGA
TGATTATCATTTGATATCTGGGTCTTAAAATTAGCCATGAGCTCAGCAAAAGTCATCTGT
TCAAAAAAGTAGAATGGGAATCTTTAGAACAAACATTCAATTATCTCTGTATCTTTCTGA
TTGATTCCTCTATTTGATTGAGAATTGGAAAATGGGATGGTCGATTTGATCTAGGAGGAA
AGCATTCATTTTTTTGAATACTCTGCGGAAGTAATTCAGGTCTTCAATCAAGGTGAAGAG
GCATTCCGTCAACTTGTTAATTTCATGATGCGAATGCCGAACAATTTTGCTGTAATCCAG
TATCAAGCGCTTGAGTTTCTGACGATTACTACGCTCTTTGTTTAATGACATGTGGCGCAA
TCCTTGGCATTGAAAATACTTTCAATTTTGAAAAACACCTCTGGAAGTTTCTTTCGTCTT
TGGAAATACTATCACTTTTTCCACTTCACCTGCAAAAGTATTGCAGAAATTTCATGTTTT
TTGCTGTATGAAGCAAAAGTTTCTTCTAGCGCTTAACGCATGCATAAGCAAATAACTGCT
TCTTAGCTTACTTTGGCTAATGATTCCATATTTTGTATTGTCAGCAGACAACACTAAATA
TCAGATGCATTGTCATTTTGCACAAGGGGTTTGCTGCATTTCCAGCGAAGTATCTGTATT
CTTAAATACCATCTTTTCAGCGAGGTGAAGTGATGGGTAATGACGGTGAATATCATAAAA
TATTGTACATCTATTGTCTGCATTTGGGTTAACAGGAATGAAAATGGAAAATGCTTCAAT
TCTTGATTCTTTTCTGCTCGTACCGTACGGTACCATACGTGATGTGGTTTCTTCCTGTCA
TTTTTGGAACGTCAGAAAATCATGGAAAAAGGCTATGGTCTAGAGCTACTAAGTACGGGC
CCCA
>comp2953_c0_seq1

TGAAACTGAAGTAAATTGGTTGTGACGGCAACGACACAATTGCAACGCATCAACAATAAC
ATGCCGCTTCGTCTAGATATAAAGAAAAAGCTATCGGCATCCAGTGAGCGCGTAAAAAGC
GTTGATCTGCACCCTACAGAGCCATGGGTGTTGGCAGCTCTCTACTCGGGAAATGTCATG
ATCTGGGACTACGAATCGGGAAGTCTTGTCAAATCCTTCGAGGTATCGGAACTTCCAGTA
CGATGCGCTAAGTTTGTAGCCCGCAAGCAATGGTTCGTAGCGGCTTCAGACGACATGCGA
TTTCGAATTTTCAACTACAATACGATGGAAAAAATTAAGGAGTTCGAGGCTCACGCTGAT
TACATCCGTTGCCTAGAAGTCCATCCATCTCTTCCATATGTATTATCTAGTTCCGATGAT
ATGTGTATCAAACTATGGGATTGGGATCGTGGGTTTGACTGCACGCAAATATTTGAGGGA
CACGCGCACTATGTTATGCAGGTAAAGTTCAATCCTAAGGATACTAATACATTTGCCAGT
GCCTCTTTGGATCGCTCTATCAAGGTGTGGGGGCTCGGATCTCACGCACCGCACTACACT
CTTGAGGGTCACGAAAGAGGAGTCAACTGTATCGACTACTATCCTTCTGGTGATAAACCT
TACATTTTGAGTGGAGCCGATGACCAAACGGTGAAAATCTGGGACTACCAGACCAAATCT
ATCGTTCACAGTTTGGATGGACACACGAATAATGTTTGCGCTGTCATGTTCCACCCCAAA
TTACCCATCATCGCGTCCGCTAGTGAAGACGGAACTGTTCGAATTTGGCAATCCACTACC
TATCGCGCGGAGACGACTCTAAATTACGGAATGGAACGTGCCTGGGCACTTGCTGCATCC
CCTCAAACCAATAAACTTGCTATTGGATTTGACGAGGGTTGTGTTTGCATCGAATTGGGT
TCGGATGATCCTGTTGCTTCTATGGATGCAACTGGAAAGGTTGTATGGGCAACGAACAAT
GATATTCAAACCACCAGCGTGAAAGGATTGGTTGGATCCGGAGAGGACGCATTGCCTGAT
GGAGAGCGTCTCCCTGTAGTGCCTCGTGATCTTGGATCTTGCGAGCTGTATCCTCAGATG
CTAAAACACAACTGCAACGGACGATTTGTAGCTGTTTGTGGAGACGGAGAGTTTATTATC
TACACAGCTCAAGCCTTGAGAAATAAAGCATTTGGTCAAGCGCTCGATTTCGTCTGGTCT
GGTAACGGAACTGGAGACTATTGTATTCGTGAATCTTCCAATTCTATTCAGACCTACAAG
AATTTCAAGGAAAGCCAGACCATCGTACCTGCGACGGCCAGTGCCGATGGTCTTTTTGGA
GGAACTGCAATAGGTGTCAAGGGATCGGATGGTGTCCTCTTCTATGAATGGGACTCTGGT
GAATTTATTCAAAAGATTGAAGCTATTCCGAAAGACGTTTACTGGAGTGATAGCGGCAAC
ATGGTCTTGCTTGCCACAGAAGACTCTGCTTACGTTTTAAGCTACAATTCTCAACAAGTA
TCGCAGGCGATTGCAATGGGGCAAGTGAACCCAGAAGCAGGTATCGAAGGATCATTCGAG
CTGCTTTATGAGATCAATGACACTATCACCTCTGGTAAATGGGTTGGAGATTGTTTCATT
TTTATCAATGGCGTTGGTCGATTGAATTACTGTGTTGGAGGACAAATTGAGACGCTGGTG
CACCTCGAGACTTCTGCAGGAGGTCAGAGTCAGCATACCATACTTGGATACTTGGCGAAA
GAAGACAGAGTCTATTTGGTAGACAAATCGTTGAACATTGTTGCGTACAAGGTCATGTTA
TCCGTCCTCCAATACCAGACTGCTGTAATGCGAGGTGATTTTGATGCTGCGAATCAACTC
TTACCAAATGTACCAGAGTCTGAATATACCAAAGTCGCTCGTTTCCTCGAATCCCAAGGA
TTTAAGGAAGAAGCCTTTGCAGTGACAACTGACCCGGATCATAAATTTGAGCTTGCACTT
GAACTTGGACAAACTGAGACCGCACATGCACTACTACTTGAAACACCGGACGAGGATAAG
GATTCAACAGAGACTGAATCGAAATGGAAACGACTTTCTGATGCAGCACTTAAGGAAAAC
AATCTGGAGCTTGTCGAAGCTGCCAGTGTAGCTAGTCATGATTATTCAGACCTTCTGTTG
CTTTATTCAGCCATCGGAAATTTGGCTGGTATGGAGACACTTGCGGACGCTGCATCAAAG
GACGGTAAAACAAATGTCGCCTTTGTTGCCTACTTGTTGACAGGTAACGTTGAGGCGTGT
GCGGAACTATTGGTAGCGACGAAGAGACTACCAGAGGCAGCCATGTTTGTCCGTACGTAT
CTACCGTCCAAAATTGATGAGATTGTAGCTCTATGGAAGCAAGACTTATCTACTATTAGT
GAAACGGCAGCAGAAGCTCTCGCTACACCTTCCTCTAACCCCGAGCAATTCCCTGATTTG
AGCATTGGCCTTCAAGTGGAACAAATGTTCTTGCAACAACGTGAAGCAACCAAATTAACT
GGAATGCCCGGTGTGAATTATCTTTCTGCTAAGGACGATCTTGAGTTGAATCTCATCGAG
CTTATCAAAGCCCGTCAAGGGGCAGGGGAAACGAAAGAGCCTGAACCTGCAGATGTAGTT
CCCGCTCCACCGCAAGACCAGGCCGAAGAAGTTGGTGAGGCCGAGGTGGAACAAGCTATT
GTCGAAGACGCAGCTGCTGTGGAAGCTGCAAGAGTTTCTGCAGAACTAGCACAAGCGGCT
GAAGCAGAAAGACTGGCAGCCGAGGCTGCAGCTGCTGAAGCTGCAGCTGCTGAAGCCGCC
GCCGCCGAAGCTGCTGCGGCCGAAGCCGCCGCTGCGGAAGCTGCTGCAAAAGCAGAGGAA
GATGACATGGCCGACGACTTCGGAGATGACTGGTAATTTACCTATTTAAATCTAGACATG
AACACCACGA
>comp2954_c0_seq1
CACATTTTCTTTTGTGTGGTACCATTACTTCTAGTATCCCTTGGTAACATTCAAGAATAA
AGCAAGCAATATAAAGATGGGTTGGTTCGACTGGATGTACAGCGCTCTTGCGAGCTTAGG
TCTCTATCACAAGAATGCCAAGATTTTGTTTCTCGGCCTGGACAATGCGGGAAAAACGAC
GCTGCTCCACATGCTGAAAGAGAACCGGGTGCAGGTCCACGTGCCCACGCTGCATCCCAA
CACGGACGAGCTGATCATTGGCAACATTAAATTCAAGACCTTTGACCTCGGGGGCCACGA
GACGGCCCGCCGCCTGTGGCAGGACTACTTCACCACGGTCGACGGGGTCGTCTACATCGT
CGACGCCATCGACCGGGGACGCTTTCCCGAGGCCAAGCAGGAGCTGGACGCCCTGCTGAC
CACCGAGGAGCTCCAGGACGTTCCCTTTTTGGTCCTCGGAAACAAGATCGACATGCCCAG

CGCCGCCAGCGAGGAGGAACTGAAATACGAGCTCGGGCTGATGGACACGTACGGGAAAGA
CAAGGGACCCGACGGTTCCGGAGCCCGCCCCATCGAGCTCTACATGTGCTCCGTGGTGCG
ACGAATGGGATACGCCGATGGATTCAAGTGGTTGTCTCAGTTTTTGTCGTAAGCGATCGG
ACTTGACTCGACTCGACTTGACTTGGTTTCCTTTGCTTTCATTCAGCCCGTCGAAACGAA
ACCCTCGAACGTGCGGTACGAATGCATTTTTGGTGTGGTGGTTTGGAGGGATTGGACAGC
AATCGCACGGTGTCTGGAGGCAGGCTGCAACTATGTATAGACGACAAAAGTGAAAATAAA
CAAAACGTGGGCTGTGTTGGAGAAAAGTTTTCGTGCTTTTTGTGTGGTGTTCTTTCGCGT
ATCTTTCGTTCCGGTATTTTTTCATGCTATTCCCGTTGCTACCGGCTGTCCTTGAAATTA
GAGAAGGAGCACACCAGGGCAACAACAAGCAAGAAACGTGGTGTGTAGTTGTTCCATTCC
TTCTTTCATTCACTCGTACAGTGCTGCATACATTCATACACATATACACACCCCTCTTGC
ATCTCTACAGAGAACGTCTCGGACACAACAAATTACTACTATCATTAATGCATCTTCCCG
TTCACGACACCGAAAGTTTCGGAGAAACGATAAACATAGACCTCAC
>comp2954_c0_seq2
CACATTTTCTTTTGTGTGGTACCATTACTTCTAGTATCCCTTGGTAACATTCAAGAATAA
AGCAAGCAATATAAAGATGGGTTGGTTCGACTGGATGTACAGCGCTCTTGCGAGCTTAGG
TATGTCTTCAGGGTGCAAACGATCGATTGGAAGAGAAACGTAATTGCTGGGGCAGTCTTT
TTTTGGTTGGCGAGTACCAAACGTGCAACGACGAAGGACACGTGGTTCGTCAATGGTTCA
GCCGCAATCTTGTTCCTGCCGCACTGAATCAGAAAATTCGATAGCTCAGTGTTTGATGGA
AGGCCAAGGAGCTTGCGGCAATGTCTGACTCGTGCAGGGATGAATTCTGTACGCTTTTGC
AACGTCGACGGGAGAACTGGTCATCTCCGATCGGAGTTTTCCCTGTAGAAATAGGAAAGG
TCGCGCGATGCATGCCATCCCAAGCCATCTACTGGATGCCATACCACACCACTCCGAAAC
GATGCCCCAAGTGGTTCCTAACCAATTTGTTTCTTGTGTCCGTTCCCGTTTCATCTCCCC
GCAGGTCTCTATCACAAGAATGCCAAGATTTTGTTTCTCGGCCTGGACAATGCGGGAAAA
ACGACGCTGCTCCACATGCTGAAAGAGAACCGGGTGCAGGTCCACGTGCCCACGCTGCAT
CCCAACACGGACGAGCTGATCATTGGCAACATTAAATTCAAGACCTTTGACCTCGGGGGC
CACGAGACGGCCCGCCGCCTGTGGCAGGACTACTTCACCACGGTCGACGGGGTCGTCTAC
ATCGTCGACGCCATCGACCGGGGACGCTTTCCCGAGGCCAAGCAGGAGCTGGACGCCCTG
CTGACCACCGAGGAGCTCCAGGACGTTCCCTTTTTGGTCCTCGGAAACAAGATCGACATG
CCCAGCGCCGCCAGCGAGGAGGAACTGAAATACGAGCTCGGGCTGATGGACACGTACGGG
AAAGACAAGGGACCCGACGGTTCCGGAGCCCGCCCCATCGAGCTCTACATGTGCTCCGTG
GTGCGACGAATGGGATACGCCGATGGATTCAAGTGGTTGTCTCAGTTTTTGTCGTAAGCG
ATCGGACTTGACTCGACTCGACTTGACTTGGTTTCCTTTGCTTTCATTCAGCCCGTCGAA
ACGAAACCCTCGAACGTGCGGTACGAATGCATTTTTGGTGTGGTGGTTTGGAGGGATTGG
ACAGCAATCGCACGGTGTCTGGAGGCAGGCTGCAACTATGTATAGACGACAAAAGTGAAA
ATAAACAAAACGTGGGCTGTGTTGGAGAAAAGTTTTCGTGCTTTTTGTGTGGTGTTCTTT
CGCGTATCTTTCGTTCCGGTATTTTTTCATGCTATTCCCGTTGCTACCGGCTGTCCTTGA
AATTAGAGAAGGAGCACACCAGGGCAACAACAAGCAAGAAACGTGGTGTGTAGTTGTTCC
ATTCCTTCTTTCATTCACTCGTACAGTGCTGCATACATTCATACACATATACACACCCCT
CTTGCATCTCTACAGAGAACGTCTCGGACACAACAAATTACTACTATCATTAATGCATCT
TCCCGTTCACGACACCGAAAGTTTCGGAGAAACGATAAACATAGACCTCAC
>comp2955_c0_seq1
CAACAGTAGAAGCTAACGATAGGGGTGCAAGGGGTCCAGTTTTGTCGATGCGAAAATCGA
AACCTTCTTGTTTCCGACCCATAGTGCAATATTAACTGCAAAGGAGGTCATTAAGGAAAC
GAATATCCTGGGGTACGAATTCTCACTTGGACAAATTGCCTCTCCATATATTTGTAACGA
TATTTCCATTATCGGAATCGTTTTAGCAGCAGACCCGTACCGCGTCATCGCTGCAATCTC
TGTTTATCAAATCTTCGGGTAACCTGTTTAATCTTCGTCACTTTGCTAGAACACTCGTTG
CGTCGTGAGAACATAAAAGCCACGAAAAATGTCGTCTAAGAAGTCATCGAGCAAGTCTTC
AAGCAAGTCTTCTAGCAAGTCTTCTTCGCCAAAGGTGTATTTGACACCCCAGATTGAAAA
GCAGGCGAAACGGCGGGAAAGTCTCTGTCCATCTCTCTTACTCGAAGACCACGTACGCTT
ATCGCAAAGCAAAAAAGGTAACGGCCTTGCCGCCATAACTAAATCGTATCAAACTAACAC
GAAACTCTTGAATTCGGAGGTTTCAGATTTGGAAAATGACGCACAGGAGCTTCAGTTCAT
TGTTGACAGTGTCCAGAAGTATCTCTCCAAGATTGTGGATGAGAAAAAGCAAAAGTCGGA
CCTAATATCGATGAAGTTCAAGCCGAAAGGTTGTACATGGGTATTGGAAATGAATCCAGA
TGTTGGAGCAACCGATTACATGATTACAGTTGTGTGCTACGCCAAAGGAGAAGGGAATGG
CATCACCACGTTTGTCGTCGATACCAAACTCGTAGGAAAACAAAACGATTCTACTACATC
GACGACTTTGCCCGGGTTTCGAGAGGCCAAGGGCGTGCTCGAATTTACTGCGTTGATGGT
CAGAAAGGGAGTAGTGAGATGCGAGTTTCCAGTCTTTCCAGAAGGGCTGAAAGATAAGTC
TTTCCGAGAGATTTACCAATTGAATGCAAGGGTAAGGCAAAAATCTTTTTTTTATTGATT
GTTTACATGCAAACATGAACGGTGGAATAATCTCGGAGTTTTCACATGTGTTCGTGGTTA
TGGATAGCATTTTTTGATTGTGCGCCGTTCTTATGAAAATTATTTCTCCATTTGATCTGT

ATCGCAACCTTCTCGTTGCCGTTGCATCGTCGTGTGACGGTACTTCTTCCGACTGTATCG
CAGCTCAAATCTGGTTCCTTCGCGACGGTTTGCCGAGGAACACATCGGGCAACTGGGAAA
AAGGTGGCGATAAAATGTGTGCTTCGAAAGGATCTCCCTCCGAATGATGACGCTGCTATA
TACGAAGAAGTGTCAATTATGTCGTCGCTATCGCATCCCCATGTTGTTCCGCTCATTGAT
TTTTTCGAAGAAAAAGATTGTTACTTTCTTGTTATGGAGCTCATGAGCGGTGGTGACCTC
TTTGATCGTATCGGGAAGAAGAAATCATATTCCGAAGAAGATGCTAGAGACCTTTCTCTT
AAAATGCTCAAGGCCATGGCATATTGCCACGCGCACAAAGTCGCCCACTGTGATATGAAG
CCAAAGAATCTCCTTTTGATGAGCGAAGACAATCATTCTTACATCAAGGTGGCAGATTTT
GGGTTTGCTGCTCATTGTCATACCGAGAAATGTTTGACGAAACAATGCGGCACACCATTT
TTTGTTTCTCCAGAAATTCTCATGAGACAGTCATATGATCAGCAAACAGATATGTGGTCT
GTAGGGTGTATCATGTTCTTATTACTCAGCGGGAATCTCCCCTTTAATGGTAGAAGTCAA
AAGGAATTGTTTCGAAAAATCGTATCGGGCAAATACGAGTTTAAGGAAGAGGGTTGGGAC
GGCGTTTCTGAGGATGCGAAAGACTTAGTGAAGAAATTGCTAGTTTTAGACCCCGATGAA
AGAATTACCGCTTCGCAAGCCTTGAGGCATAGATGGATGAAGGCCTCGAGAGACCGATTG
GCTCGCATTACTCTCCAGGGAACTTCACAGAGGTTGAAGACTTTCAATGCTCGAATGAAA
TTGCGCTCAGCTATGATTGCGGTCGAGTGGGTGGCACAGTTGAAAAAGACTTCATGGGGT
TCAAAGAAAGTTGTTCGGGATTGATCAAGTGACAATCTTATTCAACTACTTTTGATATCT
GTAGTCAAATAGAAATATAAAAATGTGAACAATATCTAAATATTGAAGTGGAAGTAATAA
CATTGTCATTTATTTACAACGCGAGCAGCCAGCCCCCATGATGATTGAATAAGAAAGGAT
TCACTTCAAACCTTACCTTCGTTACATCTAATACCGACAGATTAACAAATCGAGAACCTT
CCGATTTGTCAAACGGGATGGGTTTATTCGCATATGAGCGTATCTGACGAGACTCACCGC
GCCGCAAACCATGCTTTTAAAATTGGGTCCCTGCAATAGAAGAGGCTCACCATTTTTGGT
TTTCAATGCTTAATTCAACTTTTCCCTTAATCGTATCATTGATAATAAG
>comp2955_c0_seq2
CAACAGTAGAAGCTAACGATAGGGGTGCAAGGGGTCCAGTTTTGTCGATGCGAAAATCGA
AACCTTCTTGTTTCCGACCCATAGTGCAATATTAACTGCAAAGGAGGTCATTAAGGAAAC
GAATATCCTGGGGTACGAATTCTCACTTGGACAAATTGCCTCTCCATATATTTGTAACGA
TATTTCCATTATCGGAATCGTTTTAGCAGCAGACCCGTACCGCGTCATCGCTGCAATCTC
TGTTTATCAAATCTTCGGGTAACCTGTTTAATCTTCGTCACTTTGCTAGAACACTCGTTG
CGTCGTGAGAACATAAAAGCCACGAAAAATGTCGTCTAAGAAGTCATCGAGCAAGTCTTC
AAGCAAGTCTTCTAGCAAGTCTTCTTCGCCAAAGGTGTATTTGACACCCCAGATTGAAAA
GCAGGCGAAACGGCGGGAAAGTCTCTGTCCATCTCTCTTACTCGAAGACCACGTACGCTT
ATCGCAAAGCAAAAAAGGTAACGGCCTTGCCGCCATAACTAAATCGTATCAAACTAACAC
GAAACTCTTGAATTCGGAGGTTTCAGATTTGGAAAATGACGCACAGGAGCTTCAGTTCAT
TGTTGACAGTGTCCAGAAGTATCTCTCCAAGATTGTGGATGAGAAAAAGCAAAAGTCGGA
CCTAATATCGATGAAGTTCAAGCCGAAAGGTTGTACATGGGTATTGGAAATGAATCCAGA
TGTTGGAGCAACCGATTACATGATTACAGTTGTGTGCTACGCCAAAGGAGAAGGGAATGG
CATCACCACGTTTGTCGTCGATACCAAACTCGTAGGAAAACAAAACGATTCTACTACATC
GACGACTTTGCCCGGGTTTCGAGAGGCCAAGGGCGTGCTCGAATTTACTGCGTTGATGGT
CAGAAAGGGAGTAGTGAGATGCGAGTTTCCAGTCTTTCCAGAAGGGCTGAAAGATAAGTC
TTTCCGAGAGATTTACCAATTGAATGCAAGGCTCAAATCTGGTTCCTTCGCGACGGTTTG
CCGAGGAACACATCGGGCAACTGGGAAAAAGGTGGCGATAAAATGTGTGCTTCGAAAGGA
TCTCCCTCCGAATGATGACGCTGCTATATACGAAGAAGTGTCAATTATGTCGTCGCTATC
GCATCCCCATGTTGTTCCGCTCATTGATTTTTTCGAAGAAAAAGATTGTTACTTTCTTGT
TATGGAGCTCATGAGCGGTGGTGACCTCTTTGATCGTATCGGGAAGAAGAAATCATATTC
CGAAGAAGATGCTAGAGACCTTTCTCTTAAAATGCTCAAGGCCATGGCATATTGCCACGC
GCACAAAGTCGCCCACTGTGATATGAAGCCAAAGAATCTCCTTTTGATGAGCGAAGACAA
TCATTCTTACATCAAGGTGGCAGATTTTGGGTTTGCTGCTCATTGTCATACCGAGAAATG
TTTGACGAAACAATGCGGCACACCATTTTTTGTTTCTCCAGAAATTCTCATGAGACAGTC
ATATGATCAGCAAACAGATATGTGGTCTGTAGGGTGTATCATGTTCTTATTACTCAGCGG
GAATCTCCCCTTTAATGGTAGAAGTCAAAAGGAATTGTTTCGAAAAATCGTATCGGGCAA
ATACGAGTTTAAGGAAGAGGGTTGGGACGGCGTTTCTGAGGATGCGAAAGACTTAGTGAA
GAAATTGCTAGTTTTAGACCCCGATGAAAGAATTACCGCTTCGCAAGCCTTGAGGCATAG
ATGGATGAAGGCCTCGAGAGACCGATTGGCTCGCATTACTCTCCAGGGAACTTCACAGAG
GTTGAAGACTTTCAATGCTCGAATGAAATTGCGCTCAGCTATGATTGCGGTCGAGTGGGT
GGCACAGTTGAAAAAGACTTCATGGGGTTCAAAGAAAGTTGTTCGGGATTGATCAAGTGA
CAATCTTATTCAACTACTTTTGATATCTGTAGTCAAATAGAAATATAAAAATGTGAACAA
TATCTAAATATTGAAGTGGAAGTAATAACATTGTCATTTATTTACAACGCGAGCAGCCAG
CCCCCATGATGATTGAATAAGAAAGGATTCACTTCAAACCTTACCTTCGTTACATCTAAT
ACCGACAGATTAACAAATCGAGAACCTTCCGATTTGTCAAACGGGATGGGTTTATTCGCA

TATGAGCGTATCTGACGAGACTCACCGCGCCGCAAACCATGCTTTTAAAATTGGGTCCCT
GCAATAGAAGAGGCTCACCATTTTTGGTTTTCAATGCTTAATTCAACTTTTCCCTTAATC
GTATCATTGATAATAAG
>comp2956_c0_seq1
GGAAAAAGTTGTCGGCGAGATCGCTCCGAATTCACTGTCACATAGAGGAGGATGCGTTGG
ATGAGGTTTCTCTCCGTAATAGATCTTGCGAACGTTTTTGCGATTCCTGTTTTCTAGTTG
AGTCTGTTTTGTGGCGTATTCAGCACCTTTCTTGACGTGATCAGCTTATGATGGTCTCCT
CTTCTGGTTGGCATCCAATCTAAATTGGATAGATGTGAAGATCTTAACAATCTCTCATAA
TCATGGATGAATCGGGTTAATATACGATCGACCATTGTAAACAATTGAAACATTTTTCGG
AAGACAATCATGACGGTCACGATGACATCACGTTTCTCTGTATCTAAGATTGTGTCTTTG
GCATATAGAACAACTACGGTCTTAATTCTGCTGACAATAAGTTGCCATGAAACATTTTTC
GTCACAGCCGATGAACCAGAGTCGCGGGTCGTTGGCGGTCAGAATGCCGAAGTGAATGAA
TATCCATTTTTTACAAGTTGGGGTGCAAGTTGTGGAGCAACTTTAATTCACGACGACATT
TTGCTTACAGCTGCACACTGCAACCCTGTCGAAACAAATCAAGTAGTTGTACATGCATAC
CACAAAGGAAGGCCGTCTGTCAATTCACAATTGAGATGGGTAGAGAGAAGAATTATTCAT
CCACAATACAATGTAGACACATGGAATTATGATGTAATGCTTCTGAAACTAACGCAACCA
GTAAATTCGGTTCGAAAAGTGAAGTTGAACAGAGATACAACAAAACCCGCAGTACTCGAT
ACTGTCACTCCGCTTGGTTTGGGCAGAATAGCCGAGGTAGATGGTGCATTCCCAGAAGTC
TTGCAAGAAGTTAACGTAAGAGTCATTCAATCGAGCAAGTGTAACCAGGCACCTATGTAT
CCGGGTTGGATTCAGGAAAGCATGATTTGTGCCGGAGTGAGCGGTGGAGGACAAGATGCC
TGTTTCGGTGACAGTGGGGGGCCTCTGCTATATAGAGACAATGCAACTGACGAGATAATA
CAAGTTGGCGTTGTTTCGTTTGGGACCGGATGCGCTAGACCGGATAAACCTGGCGTATAC
CATCGCATTAGTTCTTCATACGACTGGATACAAGAAAAAATATGTGAATTTTCCTCCAAT
AAACCGTCAACTTGTCCCGGTGTTATTTCTGACCCAATTCCTAGTGCCAATCCAAGGGAT
ACAGCAATTGATGGAAACAGTACCCAACTAGAAGAATCAGATCAACCGGAGCTACTCCAA
CAACCAGAAGATGGTGAACAAGAACAAGTTGTTAATCTCTTCACACATCCCGAGAATCCA
CCGCCTCTTCCACTTGGATTGTGTGAGGGTGACTGTGATCAAGATTCCGACTGCGCAGAA
GGTCTCTTTTGTTTCTTTAAGACGAATGGGATAGTACAAAATGTACCGGGATGTGAAGGA
AATGACAACACAAGCACAGATTTCTGTGTCGACATTAAATATAGACCTGAGGGTACAACT
AGTAATCCTGTCCGGCAATCAATTTTTTTTTCAAATCAAAAAAATTTGAGAGGCAGTGTA
CAAAGTGGAAAACTTGATAAGCTCCAACCGACCAGCAGAATGAGCGGTCTACTAAAGGGC
GCCTTTAAGGAAAGCATACCGTAGTGAATTATTCGTTTTGCTGGAAAGAGAACGAGTATG
AAGGGATCAATGGGCAGGATCGAAGTGAAGTCCAAATGCATTCGATTGTTGGAAAAAACT
CCACTTTTCAAAAAAAAAGCACTATAGGAAGAGCGACCAAATCGAATATTAGCAAAAACA
TAGAGACAACGCACATGTCTCAAACCAGCCGGTTGCGATAGAATTGAAAAGTTCAGCTGT
ATAAATTGACTGTAAAATAGACCAAAACCAACGGCAAGC
>comp2957_c0_seq1
AGCAAAACGACGACAACCAATACGTTCTTGCGGAAGCTAGTACCGTTTTTGCGCGGTACC
ATTCGGAACCGTATCCGACCCCTGCCCATACCTACCGAGTTTCTTGGATTTGGTCTTGCC
AATCTCTTGCGGGGGTTGGATAATTGCTCCGGCTTGTGATCCACTCGGAGCCGTTCCAAA
AGCTGCACCCGATGCAATTGTGTCGCATGGCCACTGAACTAGCAAACAAACAAATCCAGT
AAGCTACAAGACGCCGTTCGGAGTGATTGTTCTGATTGTTCACCTCCCTTGACCAGACCG
GTCGTTCGTTCACAAACGAGAACAACGAACGAACGATGGCAATAAATAGGAACAATTCTT
TGCCAACGGGGTCCGAACAATCCGGATCCACGATGTCGGTCCAGGACATAGTGATACGAC
GGCAGCAACAACTGCTCCTCGGGCGTGAAAACATCAAGCTGACCGAGGACCAGCAGCGGC
TTTTTCTCCAGCACGGAGCAAGTGACGGCATCGTCCAAAGTAGCAGCAGCGGCGACAACG
CACACGCCGTTGCCCCCGGTTACTGTGGCGATTCGTCGCCCATCGGTCTGTCTCCGAACA
TCTCGCCCACCACCGTCATAACCAGAAAGGCACTGGCTACTCGGCCACCACCAGGCACGC
CGTGGCCACTCGCCGCCACACAACAACAACAACATCAAGAGCATCGGCATCGGGACCAAC
AATGGCATCAGCATCAACATCAACAAGAACAACAAAAGCGACATCAACTGCTACTACAAC
AGCAATATAACCACCATTATGAGCTACATTATCAGCAGCTGCAGCAGCAGCATCAACAAC
AACTACAACAACAAAAAGAGCAACGACAGAGACAAACGGAGCATGAGGGTGATTTTATCT
GCTTGCACCCTAGGGACGATACACGGTCCCGTGTCCGTTTTTCAACGGCTCTTTCCGTGT
ACGATCATTTCGAAGACGACGACAACGACAGCAACGACGGTTCCGATCGCGCAACCAGGG
AAAACCACCAGGGCGGCAGCCCGCTGGGGATCATCAACGAACAGACCGCAAACGCAACCG
CAAGCACAGCTGCAGCGAGGACGCTCCCCCACGACTTGTTCCTGCGCCGCCGGGCCCGTC
GCTGCTGGTACTCCCGCGAGGAACTCGCGGCGATCAAGGCCGAGCGCAAGGCGATCGTGA
GAGCGATCCGGGGGAACGCCCGTGGCAGCGCGGATGCCCCGGGGCACGAGCTCCGCGGGC
TGGAAGCCTCTCTCTCGGTAGGTATCTGTCGTACCATCCTGTTATGCTGTGCCACAATGC
CTTTGTTTTCAAGTCCACGAATGCCAACGGGTATGCATAAATATACAAAAGACCAGTGTT

CGTAGCTCATGTTCCCATTTTCTTTGTCGTGTTTCCCCATCGCTCAGCCCGCAATCAAGG
AGGCGACCTACCGAAAACGCAGGCAAGTCCTGGAGGTGGTGCTCGGCGAGCAACACCGGC
AGCGAACACAAACGCATCCTACCAATTCCAATACTACGAATACTACTATCAGGAGCAGCA
CCGGTCAACCCCTCGCCCCCGACGACGAAAAGCTGCGGCTCGCCAGCAGGGAGGCCACGG
AGTGGTTCCGCAACCGGGCGCTCGAATCCGCAAGGCAGGACGCGTCCGATGCCCGCGAGT
TCTACGAGAGCGGCGCCAACAACCACGACGCAACCAGCATGCTGTACGAGTCGATGTCCT
CCCTCCACGGATCCTTCGCCCTCATGGACATCGACGACGGCGCCGCCGGGGTGATTTTTG
GCAACGAAAACGAAAACGACAACTCGACGCTCCGGTATTGGGCGGACTCCTCCTGGACCA
GGAATCTCATGGAAGAATAGCACGAAAAATTATCGATTCCCGAAAAAACTTGACCTGCTA
TTATCTATATAGTTTTGCTTTAACAGTTTTGGAGTGCCAAAATCTATCATCCTGTTTGGG
GCTT
>comp2957_c0_seq2
AGCAAAACGACGACAACCAATACGTTCTTGCGGAAGCTAGTACCGTTTTTGCGCGGTACC
ATTCGGAACCGTATCCGACCCCTGCCCATACCTACCGAGTTTCTTGGATTTGGTCTTGCC
AATCTCTTGCGGGGGTTGGATAATTGCTCCGGCTTGTGATCCACTCGGAGCCGTTCCAAA
AGCTGCACCCGATGCAATTGTGTCGCATGGCCACTGAACTAGCAAACAAACAAATCCAGT
AAGCTACAAGACGCCGTTCGGAGTGATTGTTCTGATTGTTCACCTCCCTTGACCAGACCG
GTCGTTCGTTCACAAACGAGAACAACGAACGAACGATGGCAATAAATAGGAACAATTCTT
TGCCAACGGGGTCCGAACAATCCGGATCCACGATGTCGGTCCAGGACATAGTGATACGAC
GGCAGCAACAACTGCTCCTCGGGCGTGAAAACATCAAGCTGACCGAGGACCAGCAGCGGC
TTTTTCTCCAGCACGGAGCAAGTGACGGCATCGTCCAAAGTAGCAGCAGCGGCGACAACG
CACACGCCGTTGCCCCCGGTTACTGTGGCGATTCGTCGCCCATCGGTCTGTCTCCGAACA
TCTCGCCCACCACCGTCATAACCAGAAAGGCACTGGCTACTCGGCCACCACCAGGCACGC
CGTGGCCACTCGCCGCCACACAACAACAACAACATCAAGAGCATCGGCATCGGGACCAAC
AATGGCATCAGCATCAACATCAACAAGAACAACAAAAGCGACATCAACTGCTACTACAAC
AGCAATATAACCACCATTATGAGCTACATTATCAGCAGCTGCAGCAGCAGCATCAACAAC
AACTACAACAACAAAAAGAGCAACGACAGAGACAAACGGAGCATGAGGGTGATTTTATCT
GCTTGCACCCTAGGGACGATACACGGTCCCGTGTCCGTTTTTCAACGGCTCTTTCCGTGT
ACGATCATTTCGAAGACGACGACAACGACAGCAACGACGGTTCCGATCGCGCAACCAGGG
AAAACCACCAGGGCGGCAGCCCGCTGGGGATCATCAACGAACAGACCGCAAACGCAACCG
CAAGCACAGCTGCAGCGAGGACGCTCCCCCACGACTTGTTCCTGCGCCGCCGGGCCCGTC
GCTGCTGGTACTCCCGCGAGGAACTCGCGGCGATCAAGGCCGAGCGCAAGGCGATCGTGA
GAGCGATCCGGGGGAACGCCCGTGGCAGCGCGGATGCCCCGGGGCACGAGCTCCGCGGGC
TGGAAGCCTCTCTCTCGCCCGCAATCAAGGAGGCGACCTACCGAAAACGCAGGCAAGTCC
TGGAGGTGGTGCTCGGCGAGCAACACCGGCAGCGAACACAAACGCATCCTACCAATTCCA
ATACTACGAATACTACTATCAGGAGCAGCACCGGTCAACCCCTCGCCCCCGACGACGAAA
AGCTGCGGCTCGCCAGCAGGGAGGCCACGGAGTGGTTCCGCAACCGGGCGCTCGAATCCG
CAAGGCAGGACGCGTCCGATGCCCGCGAGTTCTACGAGAGCGGCGCCAACAACCACGACG
CAACCAGCATGCTGTACGAGTCGATGTCCTCCCTCCACGGATCCTTCGCCCTCATGGACA
TCGACGACGGCGCCGCCGGGGTGATTTTTGGCAACGAAAACGAAAACGACAACTCGACGC
TCCGGTATTGGGCGGACTCCTCCTGGACCAGGAATCTCATGGAAGAATAGCACGAAAAAT
TATCGATTCCCGAAAAAACTTGACCTGCTATTATCTATATAGTTTTGCTTTAACAGTTTT
GGAGTGCCAAAATCTATCATCCTGTTTGGGGCTT
>comp2958_c0_seq1
ACGAGAGTGCATCTGTACCAACGCGCGGTACTATCAGGTAACTGGTATTGCGTTCTCCGT
TTTCTTCATTGTGAAAAAGTATTCGTACGATAGCTTCCATTGTGTCGTCTAGTTTTCTTT
CTTTCTCCACTCCCTGGCCGGAACACATTCAATAATCTACAAAATTATATCACAATGCAT
CGGGTATTAGCATCAGCACTGGTTCTCGCCGCAACGGCGTCGGCCTTCGCGCCATCGGCT
CAGCAGCAGCAGCGACAAGCCCAGCACGCGGTCTCGACCATGCCCCTCCAGGCCACAAAA
CTCGCTGCTCTGCCTACCATTCCCAATCCCTTCAAATCGCTTCCGTGGAACGTCGAAAAA
GCCCAAAAGTCCCAGGAGCGCAAGATCAAGCTCGAGCGCTCGAAAATGCACCGCGAACTC
GGCATCGCCCAGGACGCAACCTACGAAGAGATTGTCGAGGTCACCCAGCGAATGATCGAG
GATGCTCCCGACCGAAAGCGACAGATCCAGATCGAGGTCATGAAGGACAAGATTTTGCAG
GCCCGTTTGAACGAGCGCCTCGCCGGTTTGGTGTCGGACATTGTCAGCGACGACGCCAAG
GCCCAGGATGACGAGTCAAACCTCGAAGAGCAAGCGATCGCAGAAATGAAAGACAAGAAG
GCACCAAAGGAATGGAACGCCCCCGCCTGGACACAGGGACTCATTGTCAAGCCCGACCAG
GCCCACATCAAATCGCAGGTTCAGATCTGGGGTGTCATGACGTTGATGGGAGTCGCACTT
CCCCCCTTTATCCAGTACGCCAATCGTTTTTCGTGGATGGTCTGCGTTGCCCAGCTTTCG
TTCCGGGGCATGGCCAAGCAGGAAGGCGGAGGTGGCGGCTTCGGTGTGATGCGAATGGGG
AACGCACCCGGTGACAAGAAGCACCTTAAGGTTGCCTGGCTGTTGGGCATCACCATCACG

ATCGTCGGTGCGGTCGCCACCTACGGATTGATGCCAGCCTGGGCCAAGGGACACCGAATG
ACGCCCGCCATTGCCTTTGGAATGCGAAACTTTATCTACGGCGTAGCGTGCTCCTACCTC
CAGCCTTACAAGGGATAAATAACGATAATGCTACACTCCACTGTACTGTATATAGAAATT
CACTAATCA
>comp2959_c0_seq1
CAGTGATGATGGCAGTGATGATGGCAGTGACAATGGCAGCGACAATGGCAGCAACGATGG
CGGCAATGATGGCAGCAACAACGGCAGTGACGATGGCAGTGACAGTGACAGCAACAGTGA
CAGCAACAATAGTAGTGGCAATAGCAGCGACGATGGCAGCGCCGATGGCAGCGACGATGG
CACCAATGATGGCACCAATGATGGCAGCGCCGATGGCAGTGACGATGGCAGCGATAGCGG
CAGTGACAATAGCAGTAAGAGTGGTGGCGCTGACGCCAGCAATAGCGATAGCGATAGCGA
CGACGACGACGCGTCCACAGAGCCATAGAGAAACCAGCGACACCATCCGAATCAACGGTC
TCCCCCAAAACACATTTATCAGACTAAATGGATGAACTTAAAGATCATACTGTTCCCATA
CTCTGTTATGGGTTGTATTCAAAACTTTTTGTTTGTGGTTCAAGAGTTCTTCTCTGAGAC
AAATTTGGTTTCAGATTAGGGCCGATCACAATTTCATAGTATAGAATGATTTAGAATGTC
TTGCAAATTAAAAAA
>comp2960_c0_seq1
CGCCTTTTTGACCATCACTATCGTCTTCAAGTCCCTGCACAGCCAGTGACTCACCAGGGG
ATCCATCGACTCGCATCTCCATTGCCTCCACTGATAGAGGATCCGCACCGTCTCGCGTCT
CCGTTGCCGTCACTGATAGGGGACTAGCGCCTTCGAGTTGCTGCTGCCTCCATATATTGA
CCCTCTTGCCCTTGGCGCCTCCGTTGCCTCTACTGTTAGGGGATCCATCCGCCCTTGGCG
CATCCGTTCTTGCCTCGATCTTATTCATTTACAGTACGAATACACTCCCTGTAGGAGTTC
ACTTGTACGGTGTGGGTTCGGTGCTGAAGAATATATTTTCGTATTGCAGACTGCAAAGTC
ACAAAGAACTCAGTCTCATCACAGAAGTTCTTCAATCTGTCTCCAAAGAGTTTAGCAAGA
GCAGAAAAGAGCTGAAAATTTAGAAAATGTTTTCAAAACTTGCCACTGGTAGGTTGATCG
GGACGCCAATGCGATCACTTCGCACAAAGTTATGTGTGCCGTCGGGTCAATTTACGAGTC
GGTTGTTTTCGTCTTCTTCGGAAGAAGTATCGCCTCTCGTCGTTAGCATCGAACTGGTCT
CAGATACAATGTGACCCAATGGTTATATCGGGAAGAAACACCTGAATAGCGCAATCAACG
AAGTGCTACAAAATCCTATTCCCGATGCGCCAAAGCCGCTGGCATTCTCTATTTTACGGA
TTCCATTCTTTTTGGAACCCGGCTACGACGAAGACAAAGTATTCGTTGAGACCAATCGGG
AACGGTTGGTAAAAAAATGGGGCGGGAAAGCTGGTTGGGACAACCAAAAGCGTAGCCACG
ATCTGAAGGGAAGAGGATTAGAGGCGGGGATCCCGTATTTCAATCTGGATAGAAAGGCGT
CGAGTACGATGGCATCTCATCGGCTGATACAACACATTGGAAAGAAGTATGGATTGGCAA
CGAGTGAGGCTGTCTACGACAAACTCAATGTGTACTATTTTGTTGATGGCCACTCATTGA
ACGACAGGCCTAGGTTAGCCCAAGTTGTTCACAAGGTACTGAGAGAATTGATACTGGATA
ATGCTCCCATTGAGGACGAGCTTCTGGAGTTTTTGAACGGAGACGAAGGAAAGAAAGAGA
TTTCATACGCCACCCATGCATTGCAGCAGCTTGGAATTCACGGAATCCCAAAATTCATTA
TTGAAGGAACAACAATGGTCGATGGCGCGGCACGGCCAGATACGTTCGTACATATTTTCC
GAGAGATCGAAAAGCGCGGAACTGTTCAGAGCGGTCCAATTTTCAATGATGTTTTAGGGG
TGAGTGATGAAACTATCCAAGTACCAAGCCATTCTTTGTCAAGTTAATGCAGAGGAAAGG
CTGAGATTGGCTTATTTGCCTGTATACTGGTACTGCAAAGGTTTCCAATATTCTTTGAAG
GAGAGACGAATGACAGCAATTATACAGTGAACACTGGGAGACTTGCCAAGATTTTTTCGA
AGCTGTGTTTCTCTAAATGGGTTCTCAGGGTTGCTTGATATGCTATTTGCCTTAAATGAT
TATAATGATCCCTTTCAGTCTTATCGTACGCGCGAGGCGATTCCC
>comp2961_c0_seq1
CACTCTCAACAATGAGAACGGAGGAGCCAACGGTACTACCCTTTGTGTCATCTATATATT
TTTGAAACGTATTCAAGCATGGGAGCATCATTCAGCACAGCTGGAAACTCTTCGGTTGTA
TCACGCTGCGACGAAAGGAGGGCGGTAGATAATGGTGCAAATAACCCGCCTCCGATGAGA
CGGGTGATGACGGTCGAGGAAAGAATGTGGCAAAAGTGCAAGGACGAACCTTTGGTCCCG
ATAGGATGTGCCGCCACTGCGTACTTCTTGGCATCTGGCATCAAATCGTTTCAAAACCAA
GATCCTCGCAGGGGGCAAAAGATGATGCGGCTTCGCGTAGGAGCTCAATTTGCAACCCTA
GCAATGTTCGTGTTTTATGTCGGACTGGACAACATCAATTTCGAGATTGCCCCACAATAT
CAAAGAGCGAAAAAAGAGGAGGAGGAACGACGACTGAGAGAGCAGCAGCAGTAAGAATGA
ACTAGAATTCGAACATTTTGTTCGAAACTATGAAGCTTTTGAGGTTGGGCTGATACGACA
AAAATTCGTGGTTTTTAATATGATCGCAAAGCAATGTGACTCGTAGAA
>comp2962_c0_seq1
CTCAATCTTTCTCGTTTCTCCAGATCTTATTCTACAGAACGTACTCGCTGTATCCTCTCA
TATTGACATTTTGATCTCCATCTTTGCAAAGGATTGACAACTGAAGACCATGCCTGAAAC
AAACCCATACGTCGACGATGGTGAGGGATTTTCCACAACCATGCTTCACCATGGGCACAA
ACTGGACACAGACAACAGAGCGCGTGCGCCACCAATATTCCAGTCAACATCCTTTGAATT
CAAGTCATCGGAGCATGGGGCCAGTCTCTTCAGTCTGTCAGAATTGGGGCCGATCTACAC

ACGTCTGATGAATCCCACGACGCATGTACTGGAATATAAAATGGCCAAGCTCGAGGGGGC
TCCTTGCCCGGCGCATGGAGATTGCGACAACGCTACCACCCTCCCAAGTGCTCTTGCTGT
AGCCTCGGGCATGTCGGCGCAAATGCATGCCTTAATGACCTTCATGCAGGCGGGAGACAA
TTTCGTCACTGCTTCTGAGCTATATGGAGGCTCTTTCACACAGTTTGCTTACTCCTTCAA
GCACATTGGAATCGAAGCCAGGTTTGCCAACGTCTCCGACCTTGATCAAGTAAAGAGCCT
CATCGACGACAAAACTAAGGCCATTTACATCGAGACACTCGCCAATCCGAGCGGAACGAT
TCCGGATTTCGATGCAATCAAGGAGATTGCCACAGAGGCAAAAATCCCTATCGTGTGTGA
TAACACTTTTGGCATGGGAGGTTACGCATGTCGTCCGCTCAAACTTGGTGCTGACATCGT
GGTAGAATCGGCCACTAAATGGATCGGTGGGCACGGAACTTCCATCGGAGGAATCATCGT
TGATGGTTCCTCTTTCGACTGGTGTGTGAAAAACGCCGACGGCTCACTCAAGTTTCCACT
CATCGGTGGTCCTCAGGAATCGTATCACGGAGGAGTCTTCGCAGAGCACCCAATCTTTGG
GGTCAAGGCGACCAACACGGTTTTCATTCTTCTAGCACGTGTCAAGACGCTTCGCGATAT
GGGAGGATGCATCTCTCCATTCAATGCATGGCAGCTCATCCAGGGGATCGAGACACTCTC
TCTTCGAGCGAAGGCCCATTGCGAGAATGCCAACGAACTTGCGTTGTGGCTGTCAACCCA
TCCGAAGGTGAAGGAGGGTTCTGTCCTCCATCCCTCTCTTCCTTCCCATCCATCCCATGA
GAAGGCTAAAAAGTACTTCCGCCCCGGATGCTTTGGGTCAGTTTTCTGCTTTGAGTTGAA
GGGAGAGGATCCCGATGACGAGAAGGCGAAAGGATCAAGCTTCGTCACCAATTTGAAGAT
GTGCGCCCATCTTGCCAACGTCGGCGACTCTCGAACGCTCGTGATTCAACCATCCGCTAC
GACGCACCAGCAACTCAACGCTGACCAGCAAGCTGCTGCTGGGATCAAGCCCGCCTCGAT
CCGCGTCTCGGTTGGCTATGAGGATCTGGAGGATATCAAGGCCGACTTTGAACAGGCTTT
TAACGCATAGGCTTTTCAAGCTGGAAAAAGTCACTTATATACGTTAGTGTTGCCAGGCGG
CGAAGGTATTCAGTCGATTGTGCCTGTTTCTGTCCCACTTCTAGGAACTACAAAGTTTGT
TTCATTTAACTCTATGTCTATTAATTTGAAAAATAAAGGAAGAAACTACATGAAATAGCA
ACTTCTTGCAAACAATGGCCAATCACTCTCAGTAATATCTTCTCATCTGTACTTCGATTT
TACTTGAAGCAGATTTCATAGGAAGAAATCGCTTAGAACGCCAG
>comp2963_c0_seq1
CCGTTCCGTTCTTCGATCGCCTCGATCGCCCATGGTGTTGCAAACCACTTGCCAAACGCA
AATTCCAGCCGGGCCAGCGGCATTCTCCTGACAGACACATGGAAAACCGCGTCCGATGGC
CAAGCCACGAACGCGGACCAGCAGGAGCTCCCGGCCCTCGCTTCCCCGGACGATCTGAAA
GACATCCGGAACCACGCCGTAAAGAGAGCCCTGATGGCCCTCAAAACAGCCCTGAAGCTC
TTTGCCGAGAACGGCACGAAGCCGGACGACCTGGCGCTGCAATCGGCACGTCTCTCCTTT
GCGTACATGTCCCTGGAGGACAACGACTTCAAGACGGCCCTCAAGTACTCGATGTTGGTC
CTGACCCACAGCAAACCGGACGAGCCGGGCACGAAGGGAGACGGGGAGGAGGACCCCTCG
GAATCAGCCCGCCGGATCCTGATCAAGCGGCAGGCCGCTACCGCCCGCATGTACGCATCG
GAGGCCAACGCCTCCCTCGGAGACCCCATGGCCTCCATGAAGGTCCTGGTGGGCGACGGG
AAGGACGACGCCTTTGACCGCCTGTCCTCCGACCTGGGCGGGGTCACCCTCGGCATGGCC
TCGGCCAACCACAACGCCAAGGCCCGCCTCGCCAAGGCTCAGACTATGGTCCGATGCTCG
GCCTCGGCCGCCACGGCGGCGCTGGGGAACCTGACCGCCTCGAAGCAGCTGGCCATGTCG
GCCCAGGCCATGGAGAACAGCTACTCGTCTTCGAGGGAGGGGACCTCCCAGGCCCGAAAG
GCCCTCATCTATTGCATGCTCCGCGAGGGAAACCACGGGGCGGCGCTCAACCTGCTGCGG
TCGGCCCACTGAGAGCAGCGAACCCATCCGTCTATCCATCCATCCATCCCTCCGTCCATT
CATCCAAGGGGCGTGTGCGGCGCACACCCGGGGAACTCGTGAATCTCGCTACCGAATGGG
ATCCACGCACGGCTTGGTTGCATTTGGGTAATGGGAAAAGAAAGAAGAAAGCAAAAGCCG
>comp2964_c0_seq1
CAGAAACCGAGAATACCATAATTTAGTGTAATATTTCGAGCAGCAACTTAAGGAAACAAT
CTACACTACCATTGATCAGTCAATCATAGACGACACTTACAATATCGCATCCTAAAAATA
ATGCTAGCACAACACTCTCATTCTTCTCCGACAACTTCCATGATGAGCTCACTGTTGCTG
ACAGAGTTCTTCCTTACCTGCGAAGTATCAACAGCTACAGCATCTGAGTCTACGTCGACA
ATGCCGATTCATGCGCAGTATGGTCTGCGTCATCAGCCTTGGACATCAGCCGTGGACTCA
TCATCTACATCTTTTTCGTGGTCAGATTTTCCCCTCATTGTTCACGACGAAGAATACAAC
AGCGACGTCAATGGGCTTTTTCATGCTTCGAAAACGGTAACACCTATGGATTGTCCCGGG
TTTCCATTACATATGACATCGACAACATCGTCCACATCGTCCACATCGTCCACATCAATA
CCGACCAAGAATATGAAGAAAAAAAGATCGGTTCGATTTGCACCATTCGTAGAAATTCGG
ACGCATTCTCAAATATTGGGAGATCATCCGTGCTGCGAGGGCGGACTGGCACTGGAATTG
GGATGGGAGTACAACAGCAGCTCCGCTCTCCTACCTGAGCAAATAGTAAGAAATGTTGAG
AAGAAGGATTGTCAACAGAGTCAATTCAAAATCATAGGCAGGAGTAGCAGCGGTGAAAGT
ATGACGGGAACACGCATACCCCACCGACGCTCGTATTCGGAGAGGAAACGTCTCTTGATC
GAGGTCGGAGGCTACACGTCGGACGAATTGAATATGAAAGACCCTAGGAAAGAATATTGT
CTCGTTGGCAGAAACTGCTCCGATCAAAAGAAGAACAGTACCAACGGCATGACGGCTGTA
TTGCAACGCCAAACACATTTGTCGCGTGTTGGATCGATCACTGAGTCGCTCTGCTCCGTC

GTTGCTGCAGGGGCGTGATCACATGAAGCATTGCAAATTTCATAAATACAAACGAATCAT
CATAGCATTATAGCAGTAGTATTACTATGAACAGTTTATATCAGCACTTATGGATAATGT
ACTAACAAATATGGCCTCTGCGTATCTCTCTCAAGGAGGCCCAACCCATGGATGATCAGC
ACTCCGGCGAACAGGATACAAATGTCATACAATTGGCTTACTTCGACGATGAAGCACAAT
TTAGGCTGACTATTTACCAACAATACAATGATGGGGAGCACTACTACTAGCAGCTACTAC
CAGTTGTAGCAGATTACTAGTAGTACTAGTAGCTAGTAAGGAGAGGC
>comp2965_c0_seq1
CTATCACCGCAGTTTCGTGCTTATTCTTGATCCTTTGTACCAGTACCGATACGTAAATCT
CGTACGTATCGTACAGAGCTCTTTCACAAAGATTCCCGTTTGATCTCAAGTTCAGAAATA
CTAGGTGCCAAGCTCCGTGCCGCACCGAACTCATCTCAGAGCAACTCTGTACTACACCAT
CCACCACTTCCTAATAATCAGACGCTCAAATCGAATATTTTCCACAATGAAATTTCTATT
TCACCTTTCGACCCTCGTGTTGCTGAAATCGTGCGTCTCGTCGGCGTTTGTCTGCAACCC
GCAACGCATCGCGTCCGCAACCACGCGGTTAAGCGCAGCCAATGGTGATGTCGATTTTGA
CACACCGGAGATGGGTGACGTCGGTTTTGTCCTCCTTGCTGGGGGCACGGGGAGCCGAAT
GAAAGCGAGCATGCGTAAGTTGTTGACTCGTTTGTGAGTTCCCGGTGTTGCTTTGGGTCA
AACGAGACGACCGGGATAGAAAATTGTTCAAAATTAAAAAAAGATTCTGTTGCGAATTAG
CAACTGCGCCAGAGTTACATAGTAGTCTCAGGTACTGTTGTAAAATGATTTTCTTATTCT
TATCGTTTGAACGGGGCAGCAAAACAATACCTTCCACTGCGAGGAGTACCTGTGTTGCAC
CATTCGCTCGATTTATTCCTGGAAAAGCTAGTAGACCATTGTAAAAGCAGTGGAGACTCA
TCGCCTCCAGAGAATGTTGTACTCGTCATGGACCCGAAGTATCAACCCGAATACCAGGCT
ATTGTTGATAGATACCCCGGTCGTCTTGAATTTGCAAACCCCGGAGAGGAACGACAAGGA
TCGGTCGAAAACGGTCTCAACAAACTTGTGGAAATGTCAGAAGGAAAGTGCAAATACGTT
GCTGTTCACGACAGTGCACGACCCTTGGTGACTATCGGAGAAATATGTGATGTCGTCAGA
GATGCCAAAGAACATGGTGCAGCCGTATTGGGAGTCCCATGCAAGGCCACCATTAAAGAA
AGTGAAGATGGTTCTTTTGTCCAACGAACAATCGAGCGATCTAGACTTTGGGAGGTGCAC
ACTCCACAGGTAATCCTAATCGAAAATTTGCTGAAAGGATTCAAAAAGGTCGCGGAAGAA
AATTTGGAAGTTACGGACGATGTTTCTGTCATTGAGCAATTGGGAGAAGCGGTGAAGCTT
ACACGAGGCGAATACACAAATTTGAAGATTACAACTCCGGAGGACATGGATGTCGCTGAG
GCCATCTTAGAAGATCGGAAAAGTAAGCCTGTCAATATGTTGGCTTAAAAAATCAAATGT
GGTTTCATGAAGAATTTCTCGAATCCGCTCATTGTCTTGATAGAGAAGCAGAAAAAAGTG
TTATATGCTATGCATAAACTTCGTATTATTTATATCATAACGAATAGTAACTCTCCTATT
ATGACAACA
>comp2965_c0_seq2
CTATCACCGCAGTTTCGTGCTTATTCTTGATCCTTTGTACCAGTACCGATACGTAAATCT
CGTACGTATCGTACAGAGCTCTTTCACAAAGATTCCCGTTTGATCTCAAGTTCAGAAATA
CTAGGTGCCAAGCTCCGTGCCGCACCGAACTCATCTCAGAGCAACTCTGTACTACACCAT
CCACCACTTCCTAATAATCAGACGCTCAAATCGAATATTTTCCACAATGAAATTTCTATT
TCACCTTTCGACCCTCGTGTTGCTGAAATCGTGCGTCTCGTCGGCGTTTGTCTGCAACCC
GCAACGCATCGCGTCCGCAACCACGCGGTTAAGCGCAGCCAATGGTGATGTCGATTTTGA
CACACCGGAGATGGGTGACGTCGGTTTTGTCCTCCTTGCTGGGGGCACGGGGAGCCGAAT
GAAAGCGAGCATGCCAAAACAATACCTTCCACTGCGAGGAGTACCTGTGTTGCACCATTC
GCTCGATTTATTCCTGGAAAAGCTAGTAGACCATTGTAAAAGCAGTGGAGACTCATCGCC
TCCAGAGAATGTTGTACTCGTCATGGACCCGAAGTATCAACCCGAATACCAGGCTATTGT
TGATAGATACCCCGGTCGTCTTGAATTTGCAAACCCCGGAGAGGAACGACAAGGATCGGT
CGAAAACGGTCTCAACAAACTTGTGGAAATGTCAGAAGGAAAGTGCAAATACGTTGCTGT
TCACGACAGTGCACGACCCTTGGTGACTATCGGAGAAATATGTGATGTCGTCAGAGATGC
CAAAGAACATGGTGCAGCCGTATTGGGAGTCCCATGCAAGGCCACCATTAAAGAAAGTGA
AGATGGTTCTTTTGTCCAACGAACAATCGAGCGATCTAGACTTTGGGAGGTGCACACTCC
ACAGGTAATCCTAATCGAAAATTTGCTGAAAGGATTCAAAAAGGTCGCGGAAGAAAATTT
GGAAGTTACGGACGATGTTTCTGTCATTGAGCAATTGGGAGAAGCGGTGAAGCTTACACG
AGGCGAATACACAAATTTGAAGATTACAACTCCGGAGGACATGGATGTCGCTGAGGCCAT
CTTAGAAGATCGGAAAAGTAAGCCTGTCAATATGTTGGCTTAAAAAATCAAATGTGGTTT
CATGAAGAATTTCTCGAATCCGCTCATTGTCTTGATAGAGAAGCAGAAAAAAGTGTTATA
TGCTATGCATAAACTTCGTATTATTTATATCATAACGAATAGTAACTCTCCTATTATGAC
AACA
>comp2966_c0_seq1
GCTCAAGGCGACAAGAATAATCACTGTCGAACCGAGTCTACCCAGTTGGGCAATCCACTA
TGATATGAACGAATCATGTCCAGAATGGTTTCCTGCCGGTGTGAAACCGCTGTGCGCGTG
GTGCGGCATTTGGCAGATCGTGGTGACTCATAAAATTTTCGATGGTTTGATGTTGTACGG
TTCACAGTATTGTCTTTCTACATAGATTGTGGTTGTAAATATTCGATGCAGCCAACACAC

TGGACGTCCCAGATTTCATGTATCGTGAAACCATGGGGCAGCATCCACACGGTAGCGATG
GCATCAGAATACGAAACGGCCCCGCCACAAAATCAAAGATAAACGTTTGCTCAAATGTTT
TAGTGCCCATGCAAGTTTTTCCGTTCCTCAATCGTACGAGGAAAAAAATGGCAAGTCGCC
GAGAACTCATAAATTGTTTTGAATTAAGTACCTCCAAGAAAGCCAGAAGAAATAGACGCC
CCAGAAACGCCATTCGAATCCAATCAAGTCATGCCAAACACAGGTGGTTGTAACGCGAAA
GTTCGCTCTACTCAAAAGAAGATCGAGAACGTCTTGGTAGAGTTTAGAAGTGTGGGAATT
TCGCAAATCCCAAAGGATCGCCTCTGTGAACTAACTGGATACAAGTGCACATCTACCAAG
ACATGGGCCACAGCCTTTAAGGTCGCAAAGGAAGAAGGAAGTATTCAAGTTTTCAGGAGC
AATAGGGAAGTGATGGTACAGATTTCGGATTGTCCAAAATCTCTCATGCTGAACCCTAAC
AATATTGAAGCCTTACTTCGTAGATATTCAAAGGAACTGAAGATTCAACAAACTACATTC
GATGTATTCGACGTACTCCTCGGTCTTGGTATCGGTCGTTATATCGATAAATCAAGACTT
GCCGACGCTGTTAACAGGAATATCGCGACAAAGAGTTTCGTGAACTCGTGCAAATTGTTC
CAATCCCTTGGTATGCTCGAAACACGAAGGGTTGGTCAACGAGGTAATTCTACTTCGTAT
CGTTTGGTTCAGTCACTCCTCTCTCGTGACGGTGCAACTGCCGACGCCAGTGTTGCAATC
GGAACCAAGCCTCAAGGAAATATTTTCACAAATTCTACTACCTTTAAAGAAGTTTCATCG
CCACCGATGAAATGCGAAATACCATCTGTAACTGTCAATGGCACAAGATTACGAGGTGAC
AAAAACCCACTTCTTTGCCATCGGGATCGGGATCAGGGCACTGAAGATCTTGTAGAACCC
AATTCAAACTCTCCTTCGCAAAGAACTGACGGGATTGCGGACGACGATGAGACCAGTAAT
TATGAACGAATCGTCACATTTTGAACATATTTTCTGTCTTTGCTTCAGACCTCGGAAGCA
CCAAAACATGACTTCAAAGAGGAGACAAAAAGTAGTACATTCGAATTGGCACATACCTTC
AAAGTAAATCCTAGATATTTCACTCTTGCCACTCTTGTGTGATTCTTGGTTTCTACTAAG
TAATCGATAGACACTATTCTGAGATGTGGGAGAAATCACATAGTTACATCAAATGCTATC
TTATTGTTATTGATATTACAATTATACTGCTCTTTGCTATTATTGTATCATTCGACTCGT
GGATTCTGTGTGCACACAAGGCG
>comp2966_c0_seq2
GCTCAAGGCGACAAGAATAATCACTGTCGAACCGAGTCTACCCAGTTGGGCAATCCACTA
TGATATGAACGAATCATGTCCAGAATGGTTTCCTGCCGGTGTGAAACCGCTGTGCGCGTG
GTGCGGCATTTGGCAGATCGTGGTGACTCATAAAATTTTCGATGGTTTGATGTTGTACGG
TTCACAGTATTGTCTTTCTACATAGATTGTGGTTGTAAATATTCGATGCAGCCAACACAC
TGGACGTCCCAGATTTCATGTATCGTGAAACCATGGGGCAGCATCCACACGGTAGCGATG
GCATCAGAATACGAAACGGCCCCGCCACAAAATCAAAGATAAACGTTTGCTCAAATGTTT
TAGTGCCCATGCAAGTTTTTCCGTTCCTCAATCGTACGAGGAAAAAAATGGCAAGTCGCC
GAGAACTCATAAATTGTTTTGAATTAAGTACCTCCAAGAAAGCCAGAAGAAATAGACGCC
CCAGAAACGCCATTCGAATCCAATCAAGTCATGCCAAACACAGGTGGTTGTAACGCGAAA
GGTAAGTAGGATCACATACAACTAAGATATGGGGGTGGCAATACACGGCAATGAAAGGAC
CATGCATTCAGCCCAAAAATCTCATTCGCCGACGTTGCATTGGTGTAAAACATAATTTCC
TCTCTTTGCTTTGTTGCAAAAATCAAATGTATAATCTTCAACTCTCGACTCCAACGAAAC
ATGACATGAAAATGCAATGCGGCGACACCTAAAACAGTTCGCTCTACTCAAAAGAAGATC
GAGAACGTCTTGGTAGAGTTTAGAAGTGTGGGAATTTCGCAAATCCCAAAGGATCGCCTC
TGTGAACTAACTGGATACAAGTGCACATCTACCAAGACATGGGCCACAGCCTTTAAGGTC
GCAAAGGAAGAAGGAAGTATTCAAGTTTTCAGGAGCAATAGGGAAGTGATGGTACAGATT
TCGGATTGTCCAAAATCTCTCATGCTGAACCCTAACAATATTGAAGCCTTACTTCGTAGA
TATTCAAAGGAACTGAAGATTCAACAAACTACATTCGATGTATTCGACGTACTCCTCGGT
CTTGGTATCGGTCGTTATATCGATAAATCAAGACTTGCCGACGCTGTTAACAGGAATATC
GCGACAAAGAGTTTCGTGAACTCGTGCAAATTGTTCCAATCCCTTGGTATGCTCGAAACA
CGAAGGGTTGGTCAACGAGGTAATTCTACTTCGTATCGTTTGGTTCAGTCACTCCTCTCT
CGTGACGGTGCAACTGCCGACGCCAGTGTTGCAATCGGAACCAAGCCTCAAGGAAATATT
TTCACAAATTCTACTACCTTTAAAGAAGTTTCATCGCCACCGATGAAATGCGAAATACCA
TCTGTAACTGTCAATGGCACAAGATTACGAGGTGACAAAAACCCACTTCTTTGCCATCGG
GATCGGGATCAGGGCACTGAAGATCTTGTAGAACCCAATTCAAACTCTCCTTCGCAAAGA
ACTGACGGGATTGCGGACGACGATGAGACCAGTAATTATGAACGAATCGTCACATTTTGA
ACATATTTTCTGTCTTTGCTTCAGACCTCGGAAGCACCAAAACATGACTTCAAAGAGGAG
ACAAAAAGTAGTACATTCGAATTGGCACATACCTTCAAAGTAAATCCTAGATATTTCACT
CTTGCCACTCTTGTGTGATTCTTGGTTTCTACTAAGTAATCGATAGACACTATTCTGAGA
TGTGGGAGAAATCACATAGTTACATCAAATGCTATCTTATTGTTATTGATATTACAATTA
TACTGCTCTTTGCTATTATTGTATCATTCGACTCGTGGATTCTGTGTGCACACAAGGCG
>comp2967_c0_seq1
CGATCTCAACGATGCAAACGCAAATCAGCAAAGAGACCGATAACCTCATAGACACCCAGG
ATGGCTGGTACGGACGAAACGATCCGAGGCCCTTGACACGCGGTAAACGAATATTTATTC
GCACCATCCTGATCGTTCTTTCCCCGATCGTCTTGGCTCTGTTCCTCCTGTACGTTGCAT

TCACTGCTGCCTTGTTGTGTGTTGTGACTGTAAACGACTATCAAATCAAATCGAGCAAGG
TGCTGTCCTATGAAAACTGGTGGAGGGCTCCCGGTAAGATGCTCCGGGTAAAGAAACAG
GGGATAGCTCCAGCGACATTGTCATTTCCGCGGACATGGTGAACGACGACGGAGAGCTCG
ACACCGGCAAGAACACCAGCCTCCGAAAATCGATCGAGTGCACCCTCGGGGAGCACCTTC
GGAAGGGGCCCCACGGTCCAGGGATCGCGGCCGACGAAGACCGAGACCGGCCCCACTACC
TCCTGCACGGCTGGCCGGAACCCTTCGACCCCGCCGGCGACGAGCTCTGGTGCACCCACG
CCCGTACGGCGACGTGGGCGGCGATCTACAGCTGGTCGGTCCGGCTGCGGCCCGGGGGGT
TCCTGAAGAACTGGAACCAGGCCATCGATCTGGAGACGGCCCCCGAGGTCTTTGGTTACG
TGTATCTGTTCGAGATCGGGAATCCGGACTTTTACGACAAGGACCGGATCGCACGCGTCG
GTGCCCTCGCGGCGAATGCCCCCGACCACACCCCCGAGACCGAGAACAAGGTCTTTGCGC
GGTACAACTACGGCCACGTCAAGTACATGGGCGACTTTCCCGAGGGAGACCCGGATGCCT
ATGTCTTCAAGGGCGAAAAGGTGATGTCGGAGGCACGGCTTCGGCTGTTGCACACGTACC
GCGTGATCAACCCGAACTACGCCTAGAGGAATGGCTTGGGATTCTGTGTGCTTCTCGGTA
TTTTTGGTTGATTGTGTGAAACAAACACGGTTTGGTTTCTCTTGGACTGCGAAATGGAGC
AGAGAAATGAGACTATGGGTAATAAGCGCAGAATCGATTTCGAGGTATATTTTAATGGGT
ATTGTTAAGCTTGAGCATTAGAGCAAACGACTGTAAAACCCAAAGGAGTGTTTTTCTTTC
TACTTCGAAGTACTTGTAAGTTTTGCCTTAATTCGGCAAGGCAAATCTCCGCAATGGATC
TAATGCGGTCGTCCTCTCCGGCGGCGTCAAACAAGTCGTAGATGGATGCGTGGAAATGGA
CCCAAACGGATATGCCTGGCGTGTGCCCCCGAACGACAACCAAACCAGGGTGCCTCCCGC
C
>comp2968_c0_seq1
AGAAGAACAAGCGTATCTCACATCTGTGAATGCCTCTAATCGTGCGACCGTAGATACTCT
GCAACACATTAAGATATCCAACCTGCATTACGTTTATTGAACGTCTTGGCGATGGATAAG
GACTTAGAAGTACAAATTTTGGCAACAAAAAGATTAGACGACCCTGCATCTCCGCGTTCT
TATGGTGCCATGTCTGATACAAACGATTTTGGCCATGGTGTGCCCAATGCCACGGGGGAT
CGAAAACAAGCCACGAGAATGACTACTGGTGCCATCAACAGTGAAGGACCCCAATTTCTT
TCAGACGAAGTTCTCCGGCCTCCCACAAATAAGAAATGCAGGCATTATGTCCAAATAGAC
GACGCAATCGAGCAGATAGGAATAGGCGCGTTCCAATACCAAGTTCTGTTGGCGGCGGGA
CTTTGTTTCATGTCCGATGCCATGGAGGTTTTGCTGCTGAGTTTTCTGTCTACCGTTTTG
AAATACGAGTGGGCTTTGTCAGAGCGGGAGGTAGATTCGATCGTGGCGGTCGTTTTTGCT
GGTTCCTTGCTGGGAACTTTGGTGTTGGGTCTGGCAGCCGACAAATGGGGTCGACGACCT
ATCTTTGCCGTTACATCGTTCATTATTGCTTTGTCGGGTATTGCTACAGCCTTTTGTCGG
AACTACTTGGAAGTCGTCACGGCACGATTCTTCGTTGGATTTGGTGTAGGGGGTTTAACC
GTGCCCTTTGACACCTTGTCTGAATGTCTGCCGACTGCGTCCAGAGGGAAAAATCTTTTA
TATATTGAATTTTTCTGGACGATCGGTACTCTCAGCGTTCCAATGCTGGCCTACTTGACG
CTGGATACGTCTACCAAGAATGACGACGAAACCAACTGGCAATTGTTCGTGATTCTATGC
AGCATTCCCTGTTTCGCCAGCACCATTCTAGGTATGGTGTTGGTTCCAGAGTCACCAAGA
TGGTTGCTGGAGCAGTGCTCGGATCGTCCTGAAGACGAATCCGGATCCGAGAAGGCGTTG
AAAATATTGAAAGACGCGGCACGGAAGAACGGGATTAGCCAAAACGTCATAGATGAAGGG
TTGTTTCCACCAACATCAAGATTGGTCATATCCTGGGACGATGTTGCCTCGATTGAAAAG
GTCCTGTCTCCTATCCAGGCGGGCAACGGTGATAACAAAAGTTATCCAACAAGTGAGACT
CCTGTGCAAAGCAGTGCTGGTGCTGGAGACATCTCAACGCTATTTAGCACGCCCAGCCAA
ACACGTCTCACTCTGTTGTTGTGGGCGACATGGTTTGGAAATGGATTTTTATATTACGGG
GTTATAATTGCAGTTTCGATTGCTTTCACGAACGAGAGAATAGAGGAACAAGATGACTTT
TATGTAGTGAACGACGATACCGGTGGAAACGGCAATGATGATACGAATTCGTCGATGTCA
ACTTCCTACAGTTTTGATTTCGCTGCCATATTCATAACAGCTTCATCTGAAATCTTTGGT
TTGGTGGCAGTGCTGTCTACTGTGGATCGATTTGGACGGATTCCATCACAAACAATGTCG
TATAGAATTGGTGGCATCGCTACTTTTGTGATGGGACTGTATGGTGCCATTGCCTTTGCT
GCTAACGACAACCAATACCATCGATCTTTGCTCATTTCACTAGCATTCGTCGCTCGAATG
GCAATGATGGGCGGGTCGTGTACGACGTGGGTTTCGACCAGTGAAATACTGACAACGGAT
ATTCGTACCACAGGGCACGGAACAGCAAATGCCATGGGGCGATTGGGTGGATTTACTTGT
CCCTATTTCATAACTGAAGGCAACTCTTTGGCGTTGATCGGAGTATTCGTGTTATTTATA
TCGGTTGTCACCGCCGAATGTGCAAAGCGATTGCCAGAAACGGCCGGCAAAGCCATGGGC
GACATCAAAATTGAGTCAACCATTGAGAGCCCGGTGTCAAAAGACAATGTTGATTGCCAA
CAAAGAGCCGCGACGACATACTATCGCGATTTATCGTGAAAAGCTATCGCAGATCGGCCT
CGGGAAGATTCGAGAGTAGCGAAAAGGATCAGAAAAGAGATAATAATAGCATTTGTCACT
GGTCTCTATGATCTTAAATTGCGCTAACGATATGTTTTTCAGCTGAAAAAGTTCATTACA
ATGATGTCTTCAACCTCGTGGAGTTCTTAGAATCGCCCAGCTTTCAAATCGATGCTTTCC
AACGTTTATCCAACATTGATGGACATTAGGAGACTTCACGATTTCAGAGATTTTGACTTC
AAGTTTGGTCCATCAATCCTTGATCACAGGGCTAATAGGTAACGTATTTATCGAGTGCGG

```
ACTCTAAGCCCTTCCGAGTATTCATCAGAAGTGCCTCAAGCAAATTGTTGAACTTGCTGT
TTGAATTGGATGAGGATAAATTATAAGAATGGGGACATCCGTTTTGCCTTTTTCAAAACT
TGTTTTCAAATGCTATGTGAATGGTGGTGCATAGTTCTAGTTTTGAAAGGATGCCGAATA
ATGTCATAAATATGATTCATCACTCCCCTCCTACTACT
>comp2968_c0_seq2
AGAAGAACAAGCGTATCTCACATCTGTGAATGCCTCTAATCGTGCGACCGTAGATACTCT
GCAACACATTAAGATATCCAACCTGCATTACGTTTATTGAACGTCTTGGCGATGGATAAG
GACTTAGAAGTACAAATTTTGGCAACAAAAAGATTAGACGACCCTGCATCTCCGCGTTCT
TATGGTGCCATGTCTGATACAAACGATTTTGGCCATGGTGTGCCCAATGCCACGGGGGAT
CGAAAACAAGCCACGAGAATGACTACTGGTGCCATCAACAGTGAAGGACCCCAATTTCTT
TCAGACGAAGTTCTCCGGCCTCCCACAAATAAGAAATGCAGGCATTATGTCCAAATAGAC
GACGCAATCGAGCAGATAGGAATAGGCGCGTTCCAATACCAAGTTCTGTTGGCGGCGGGA
CTTTGTTTCATGTCCGATGCCATGGAGGTTTTGCTGCTGAGTTTTCTGTCTACCGTTTTG
AAATACGAGTGGGCTTTGTCAGAGCGGGAGGTAGATTCGATCGTGGCGGTCGTTTTTGCT
GGTTCCTTGCTGGGAACTTTGGTGTTGGGTCTGGCAGCCGACAAATGGGGTCGACGACCT
ATCTTTGCCGTTACATCGTTCATTATTGCTTTGTCGGGTATTGCTACAGCCTTTTGTCGG
AACTACTTGGAAGTCGTCACGGCACGATTCTTCGTTGGATTTGGTGTAGGGGGTTTAACC
GTGCCCTTTGACACCTTGTCTGAATGTCTGCCGACTGCGTCCAGAGGGAAAAATCTTTTA
TATATTGGTAAGAATAGAAGAGAAGTCTCGATTCAAATCTCCTCTCTACTCCGTTCGCCA
TTTTGATTCATTTGCTGCCATCTCCTTACATTTTTTT
>comp2969_c0_seq1
AGCTTTTTCAACTTTCCAAAACCTACATGTAAACGATCAAAGCCATGGTCCGAACCCGAA
GCGATGACAATACTGTATCTTACAACAGCGATGGCGCCGGCGGTGCTGCGTGTAGGCCTC
CTCGAGGAAAAACATATCTAACGATCGGTCAAGATTTCTTTTCCGTCGAGGAATATGTTT
TGAGTCAGTACAATGCCAGTCTGCATCGCCCCGATGGCCATAATGTATACAGCGAAGTCG
GAAACTCTAGAAGACAAGCCCCAATACTCCCCTTGCCATACTTCTATCCGGCGGCAACCA
TGGTGTATACTGACATTCATGAACTGAAGGGTTTAGATCAACCGGCTGATTACGGAAGCG
GGATTGAATATGTGAAGGGCCTGGCGGATGCATTTCCTGAATCTGGATTGCAAATCGGGC
TGTGGCTCAATGGCTCTGCGGGATGTCGAGACATCGTTTCGGGTGTACTAGACGACCAAA
CCTATCGGCTGTTTGACTTTATTCGGTCTGAACTTCCGGAATCGTTGCCACGGGTGTTTT
TGCGTGTAGGATACGAGTTTGACAATCCTTGGTTTGGCTATAGTGACGATCCTGCAGCAT
ACCAAGAGGCGTTTCGGAAAATAGTCCATGCCTGTGAGAGACAAATGACGACGAAACAGT
GTCACGAAAAAGTTGATTTCGTATGGCATTCCTGGGCAGCTCCACGGGAAGATACTTTGT
CCCTCGATCAGTTTTACCCCGGAGACTATTTTGTCGACTGGGTAGGTGTTTCGATCTTTC
AACAAGTGTACCCATGGGCAAACGACGAAAAACAGAACGAGAAAGGAAATTTTGCTGGAG
GAGATATGAAAGAGGTGAAAGAAGTCCTAGAATTTGCGACGTCGAAAGGTATACCAATAA
TGATTGCTGAATCTACTCCTTTTGGAGGAATGGATGTGGCAGAATCAGATGTTGCAAGGC
AATATATCAAGGACGACGCAAATAAGGACGATGTTTGGAATATGTGGTTTCAAAAGACGA
TCGACCTGATTGAAGAGTTCGATATTTCAATGTGGTCGTACATCAATTGTGACTGGGGGT
CGCAACCGATGTGGCATGGTGTGGGGTTTGGAGATACGCGTATCTCTAGTTCGAAAGTTG
TTATGAAGCGATGGTGGGAACAGGTTTTGAGCAACGACTCTAGATTCTTGGTTCGCATTG
AAGACTGCAATGAAAATTCACGTCCAAGAGGTCATATCAGAGAAGAAGATGAAATGGCTG
TAGCGCGTAGCGCAACAACTGCGATTGATGGATTGTTCCATAGTGATGGAAGTGTGAATT
ATATGACGTCGAACCCAAGGTGCCTGCAACTACTTTGCATCGGTGGTGTTCTTTTGGTCA
CTGTTGCTTTCCTCTTTTTACTTCAGATAAGACTCTGGAAAACCATAAATCGAAGACACT
GCAAAGAAAGAGCGTCAAGACCCAACTGTCATTGCACCCACTATGGTGCGGTAGTCTACG
ATGAATAGAACGCTTCGAAAATGACATCGTGTGCAATAAAATTTGTTACCTTGGCTGATC
TTATTTGATAGACACTTGTCGAAAAAATAGGCCTTTTGCAGATTAAAATTACTATTCAAT
ATGAAGACGTATTCATTCCAACTGCAATCGTCAAACGTAATCATTGTAACTATAGTTGTT
AACAAATTATTTAATGCTCCTCGCC
>comp2970_c0_seq1
ATTGGACACTGCTTCCAGTGAGTATCGAGCCAGCGAAACACTTCTGCTGTGTGTTGCATT
TTTGAACAAGATTTGGCTTGAATTGCACCGGGTACTCATTGCAATCACTTATTCTCCTTT
GTTCACACACCCCGCATCCAAAATAATGCGAAACAAAACACCACCAACCACGACTCGCAG
TTCCTTTCCCCGGGGTCGGGTGGATCGATCGGATGTCGAAGAATGATCCCGATGCGTCAA
TTACCTTAGCAGACGGAAGGACTTACCAAAACGCATATGCGGCTATTCTAGACAACGACC
CGCTGGGTAACCCTTGCCGTAATTTTAATGCTTGGGTCAACTCAATTTTCTTTTCCAGCA
ATGTCGCTGCGTGGTTCGTGCATTTACTAAACGACAATGTTGAGTACGCTCATTACCTCC
TGTGTTACCTACGAAACTTCATCGCTGGCATGTTCGTATACTACGGAACTGCAGGTTCCT
TTCACTACTTTTGTTACGTTCATCCCATGTCCAAAAAAACGTTCGAAGAACAAGGGAGAA
```

CACGACCCAGCTGGGAGACGATCAAGGGCCAAATAAAATTGTCCCAAGCTTCCCTTACCA
TCTACACAATGCTACCCGTGTTTGACGAATGGATGATCGAATCTGGCTTGACGAAAGTTT
ACTTTACGGTGGATGAAATCGGTGGGTGGCTGCCTCACGTTGGGATCATGGCCTTTTACT
TTGCGTGCGTCGAGATCGGAATTTATTGGATGCATCGCACCCTTCATACGAACAAGTTCC
TTTATAAGCACATCCACTTGAAACATCATGATTACAACAAACCCGAAACGCTGACCCCGT
GGGCGTCGATTGCCTTCCACCCGCTGGATGGTATTTTGCAAGCAAGTCCATACGTTTATC
TCTTGCCCTTCATCCCATGCCATTACATGACCCACATCTGCATGGTCTTTTTCACCGCCA
TTTGGGCAACTTATATTCATGATGCTATGGACTGGAACGTGGACCCTATTATGGGAAGCA
AGTACCACACGGTTCATCACACCCACTACATTTACAACTATGGGCAGATCTTTACTTTCT
GCGATCGGTTCTGGGGGACACTGAAGCTGCCGGATGGACCCACAGGAATGCAGAAGCCAC
GCACGGCCAAGAAAGTGGATTAATGCTGGTGATTCGGACAGGGAAAAGACTCAAGAGTAG
ACTGGGATGTGACATGCCCTGTAGTATTGAATGTGATTTCTGTCGATAGAAGTGATTTTG
AAATGTTATCCTGTCGCCGAGTCTATGTCTCTGTATGAAGTGTCTTTTGAAAGTCACATT
AATGTCATCGATATAACTAGATCCAACAGTTTCAACGAGCGCTGAATTAGAATCACAACC
GAGTCAGTTATATAGAAATCACCAATAAATTGAGTTCCATCCAACTTTGCAGGTGCAAAC
CGGACAATTATCTGTCAAATGGTCGTTTTCAATTTTACTGCTGAGAACTTTTGCGTCAAT
AGCTACTTGCAGATAACATGCCTCTTTGGACTCTGCACCAAGAAGTTATGCTTTTTCTTC
TTGTACTAGTACTAGTAGTATAATGATAGTAACCACCAGCCAGCACACACA
>comp2970_c0_seq2
ACTCGATAGAAAGGAGTTTTCGTTTTTGCCTTCGGCATTCCAGTACGGTCGTAGGTAGGG
AAGAGTAGTAGAGCTCACCCCTAAGCCTTGCAAGCTTCGTCCATCGAGCCTGCTCTTGCT
GCGTTCAAACCGAAGTCTTTCTCATCCGCCACCATCGTCCCTGCCTTTCCCCTCCTTGCC
CAGGTGTCGAATAACTGGAAGACAACAGCTTCGGCATTGTTTCCATCGAAAAAGATAGAA
TTAACATGGTCTTTTCTGTGTCACGTCAAACAGCAGAATTGTCGCTGTTGGTGTCCTTTG
CGGCTTTCACGGTGTGTTGCGCCGGATTGTTCGAGCGGAACAATGAGACGCTAATGGCTG
ACGAAACATCCGGTGGATTGGACACTGCTTCCATTCCTTTCCCCGGGGTCGGGTGGATCG
ATCGGATGTCGAAGAATGATCCCGATGCGTCAATTACCTTAGCAGACGGAAGGACTTACC
AAAACGCATATGCGGCTATTCTAGACAACGACCCGCTGGGTAACCCTTGCCGTAATTTTA
ATGCTTGGGTCAACTCAATTTTCTTTTCCAGCAATGTCGCTGCGTGGTTCGTGCATTTAC
TAAACGACAATGTTGAGTACGCTCATTACCTCCTGTGTTACCTACGAAACTTCATCGCTG
GCATGTTCGTATACTACGGAACTGCAGGTTCCTTTCACTACTTTTGTTACGTTCATCCCA
TGTCCAAAAAAACGTTCGAAGAACAAGGGAGAACACGACCCAGCTGGGAGACGATCAAGG
GCCAAATAAAATTGTCCCAAGCTTCCCTTACCATCTACACAATGCTACCCGTGTTTGACG
AATGGATGATCGAATCTGGCTTGACGAAAGTTTACTTTACGGTGGATGAAATCGGTGGGT
GGCTGCCTCACGTTGGGATCATGGCCTTTTACTTTGCGTGCGTCGAGATCGGAATTTATT
GGATGCATCGCACCCTTCATACGAACAAGTTCCTTTATAAGCACATCCACTTGAAACATC
ATGATTACAACAAACCCGAAACGCTGACCCCGTGGGCGTCGATTGCCTTCCACCCGCTGG
ATGGTATTTTGCAAGCAAGTCCATACGTTTATCTCTTGCCCTTCATCCCATGCCATTACA
TGACCCACATCTGCATGGTCTTTTTCACCGCCATTTGGGCAACTTATATTCATGATGCTA
TGGACTGGAACGTGGACCCTATTATGGGAAGCAAGTACCACACGGTTCATCACACCCACT
ACATTTACAACTATGGGCAGATCTTTACTTTCTGCGATCGGTTCTGGGGGACACTGAAGC
TGCCGGATGGACCCACAGGAATGCAGAAGCCACGCACGGCCAAGAAAGTGGATTAATGCT
GGTGATTCGGACAGGGAAAAGACTCAAGAGTAGACTGGGATGTGACATGCCCTGTAGTAT
TGAATGTGATTTCTGTCGATAGAAGTGATTTTGAAATGTTATCCTGTCGCCGAGTCTATG
TCTCTGTATGAAGTGTCTTTTGAAAGTCACATTAATGTCATCGATATAACTAGATCCAAC
AGTTTCAACGAGCGCTGAATTAGAATCACAACCGAGTCAGTTATATAGAAATCACCAATA
AATTGAGTTCCATCCAACTTTGCAGGTGCAAACCGGACAATTATCTGTCAAATGGTCGTT
TTCAATTTTACTGCTGAGAACTTTTGCGTCAATAGCTACTTGCAGATAACATGCCTCTTT
GGACTCTGCACCAAGAAGTTATGCTTTTTCTTCTTGTACTAGTACTAGTAGTATAATGAT
AGTAACCACCAGCCAGCACACACA
>comp2971_c0_seq1
AATAACCAAGAAAAAATTACAATTTACAACTTCACAACTCAAAACCTAAACAATAAACAT
GACAAAGACACTTCCAATCATTATTGTCGGGGCAGGCCCCTGCGGTTTGGTCACTGCATT
GACCCTAAAGAAATACAACGTTCCCTTCGTGATGATCGAGAAGGCATCCCGGTCAAAGAT
TTGTTCCAATGCCGGAAGCGGATTCGAACTGGCACCAACGGCCGTAGATATCCTACAAAA
CCGACTTGGGATCGATGTGACTAAGATCATGTCCTACTACCAGGGTATGGCGATCGTGAA
CAACAATGGGAAACTAATCCGGCACTCGAGGATCCCGGACGGTTACACAGGAGGATCCGC
CAACCGGGCAGATATGCAAATTCATCTCTTGGATCAACTCTTCCCGTCTCCCAAGGACGA
AGAAGGGGTTCTCTTCTGTGGATCCGGCCTCGAAACATACCGAGAATTGCACAATGATGA
CCCAGATGGCGGAAGGGTGGTGGCAACATTGTCATCGGGTGAAGAGATTTCTGGTTGTGT

CCTCCTGGCCTGCGACGGGATACGTTCGCGGGTCCGGGCCGTCATGCACGGAGGTTACGA
CAGTACCAAAGATTGGAAGACAAACACCGAGACAGCAAACACCAAAGATCCCCTCCATTT
TTGCAACACGATAGTTTACTGGGGAAAGACAAAGGTTCCGAGGGGTTCTGCCCTGGAGCT
CGAGTTCACCAAGACACAGATGGCTAAGAAAGCCAAAGACAACGAAAAAACAAAATACTG
TACGTCGTTTGCCTTTACAATAACAACGCCCAGCGCACCAGCCAGTTTCTTCATTATTCC
TTCTCAGAACGGGACAGAACTGAACTGGGCTGTCAGCGTACAATCTGAAACTGATAAGAG
GAGCAAGAACAATGATGGGACGGACCTCACACGTCGGGGTGGTGGCCCACTAACGGAAGC
GGAAAAGAAGCGATTGTTCGACTTTACAAGCGATAAGAACAGCGAAAGTGTTGTTCGGGG
CATCACAAACTTCCCGCTCATACAAGAGCTCTTCAAGATGACTCCCGCCAGTGACATCAC
CGAGGCCGGTCTTTTTGATCGAGAAAATCTTGATCTCCCCTACGCAAGTGAATCGAAACG
AGTGGCATTGCTGGGTGGTAAGTGAGCTCGAGAAAGCATTGATTTGAAAGAGATTGATTG
AATGCTAACGAAATTCATTTTTCTGGATTCGTGATGAAATTGTCCTAAACTATAACAGAT
TCTGCCCATCCTCAAACGCCTTTCTTAGGCCAAGGGGTTAACATGGCCATCGTCGACGCC
TATGTATATGCCACCAATCTTGCAGTTGCCCTAAAGACCAAGAAGAAAAGCCTCGAACAG
TCGATATCCGATTGTACGACGGGCAATCGCCACGGAGAGGCCAAGAAACTTGTTGAAGAT
GCCCGGATTGGGTGCACAATGATGACGTCCCAGAACATCTTTCTATACTGGTTCATGTTT
CTCTTCATCCGGTTGGCCTCCACCAGTTCCTTTATGAATCAGATCATCTCGCAAGACAAG
AGCAACAGCGACTTTTTGAAGCTCCTTGATGAGGAACACTGCAGTCCCAAAGAACAAGAA
AGCATGTCTCAAATGAGTGCGTAATCAAATACACCGTAGTATTGTATTGAATAATTGGAA
ATAATAAGAGTGGAATTATTTATGATACTCTTTGACTTCGTTCTATTCATTTTTTTGGAA
TTTGGTTACATATGGTGTGCTGTCTAGGACAACCAACTTGGCAACAGATCACATCACATC
ACTGTACTTCACGTCCTGCCAGATTTTGACCAA
>comp2971_c0_seq2
AATAACCAAGAAAAAATTACAATTTACAACTTCACAACTCAAAACCTAAACAATAAACAT
GACAAAGACACTTCCAATCATTATTGTCGGGGCAGGCCCCTGCGGTTTGGTCACTGCATT
GACCCTAAAGAAATACAACGTTCCCTTCGTGATGATCGAGAAGGCATCCCGGTCAAAGAT
TTGTTCCAATGCCGGAAGCGGATTCGAACTGGCACCAACGGCCGTAGATATCCTACAAAA
CCGACTTGGGATCGATGTGACTAAGATCATGTCCTACTACCAGGGTATGGCGATCGTGAA
CAACAATGGGAAACTAATCCGGCACTCGAGGATCCCGGACGGTTACACAGGAGGATCCGC
CAACCGGGCAGATATGCAAATTCATCTCTTGGATCAACTCTTCCCGTCTCCCAAGGACGA
AGAAGGGGTTCTCTTCTGTGGATCCGGCCTCGAAACATACCGAGAATTGCACAATGATGA
CCCAGATGGCGGAAGGGTGGTGGCAACATTGTCATCGGGTGAAGAGATTTCTGGTTGTGT
CCTCCTGGCCTGCGACGGGATACGTTCGCGGGTCCGGGCCGTCATGCACGGAGGTTACGA
CAGTACCAAAGATTGGAAGACAAACACCGAGACAGCAAACACCAAAGATCCCCTCCATTT
TTGCAACACGATAGTTTACTGGGGAAAGACAAAGGTTCCGAGGGGTTCTGCCCTGGAGCT
CGAGTTCACCAAGACACAGATGGCTAAGAAAGCCAAAGACAACGAAAAAACAAAATACTG
TACGTCGTTTGCCTTTACAATAACAACGCCCAGCGCACCAGCCAGTTTCTTCATTATTCC
TTCTCAGAACGGGACAGAACTGAACTGGGCTGTCAGCGTACAATCTGAAACTGATAAGAG
GAGCAAGAACAATGATGGGACGGACCTCACACGTCGGGGTGGTGGCCCACTAACGGAAGC
GGAAAAGAAGCGATTGTTCGACTTTACAAGCGATAAGAACAGCGAAAGTGTTGTTCGGGG
CATCACAAACTTCCCGCTCATACAAGAGCTCTTCAAGATGACTCCCGCCAGTGACATCAC
CGAGGCCGGTCTTTTTGATCGAGAAAATCTTGATCTCCCCTACGCAAGTGAATCGAAACG
AGTGGCATTGCTGGGTGATTCTGCCCATCCTCAAACGCCTTTCTTAGGCCAAGGGGTTAA
CATGGCCATCGTCGACGCCTATGTATATGCCACCAATCTTGCAGTTGCCCTAAAGACCAA
GAAGAAAAGCCTCGAACAGTCGATATCCGATTGTACGACGGGCAATCGCCACGGAGAGGC
CAAGAAACTTGTTGAAGATGCCCGGATTGGGTGCACAATGATGACGTCCCAGAACATCTT
TCTATACTGGTTCATGTTTCTCTTCATCCGGTTGGCCTCCACCAGTTCCTTTATGAATCA
GATCATCTCGCAAGACAAGAGCAACAGCGACTTTTTGAAGCTCCTTGATGAGGAACACTG
CAGTCCCAAAGAACAAGAAAGCATGTCTCAAATGAGTGCGTAATCAAATACACCGTAGTA
TTGTATTGAATAATTGGAAATAATAAGAGTGGAATTATTTATGATACTCTTTGACTTCGT
TCTATTCATTTTTTTGGAATTTGGTTACATATGGTGTGCTGTCTAGGACAACCAACTTGG
CAACAGATCACATCACATCACTGTACTTCACGTCCTGCCAGATTTTGACCAA
>comp2972_c0_seq1
ACGAAGACAATACGAACTACGTCGCGAACCAAGAAATACTTCGCCAATCCGAGGTCGGCC
TAGCTGCAAAGAGCGGTGAAGATCTCGCGGCCCCGATCACGTTCACTCCATACAAAAGCG
AGGATCATACCAATTATGTTGCCAACCAAGGGAAACTGAAGCGATCCGAGGTAGGCGATG
CTGTGAAAAGTGGCGAGGATCTTGCCGCACCCATCACATTCACTCCCTACAAGAATGAGG
ATCATTCCAATTATGTTGCCAAC
>comp2973_c0_seq1
AACCCATTCGTTGGGCAATCAACAACTTCCAACCATCGAACCATCGAACCATCGAAGCAT

CCATCGATTTTTTTTCGACCATGGTGTTCGTGCCGATGAATGCTTCCGATGCCAAAGCGA
TCCGGCACCTCTTGACGGCCACCCAGCGGTCGGTGGCGCCTCCGCGGAGCGAAACTCCAC
GAGCGAATGCCAACAATGCTCCGTATCGCTACCGCTTCGACGAGTTTGATGACTACGACG
ACGACAGCGACGACGAGCGCGATTTCCGAGACCTCTCCCACTATTGCTCCATCGATCCCA
AGACGGGGAGGGTCACCGAGCTGTGGCTGGGAATCATCGGGAGCCAGAACGCCAACCATC
CGCAACACACCACCGCCCAAGGTGCCGCAGCTGCAGGCAACTACTACTGGACGCTTTCGG
AGGCCGATTGGCAGTGGCTGGCGCGGTTGGACCAGCTGAGGCACCTCAAGGTTACCCGCT
GTGGTGCCCTTCCCTACCGACAGCTAAAGGAGCTGCCCCACCTGGAACACATTTCCTGCT
CCGAGTGCGGAACCAATCTCGACCCTCTCGGTGCGGGCGACACCGATGAGGACGGTGAGG
ACGGCGACGGCGGCGACGACGACGAGGGAGAAACGATCGCCGACGACGACACACCGCCGT
GTCGTTCTCGGTGGAACCCGCCACAAACAGACAGCCACCGAAACACCGGAATCGATCTGC
CAAACATAAAGAGCCTGTCGCTGAGATGTGCCTTCCAGCCCACCGCTCAGGGGATTCGTT
CCCTCGGGTCTGTCTTCGACTGGATCAAGCACCACCTTCCGGGACTGGAACGCCTCACCA
TCTGGAGACTCGACCAGCCGCCGAATCGGTGGAGCCCCACTCCGCTGTCGGCCTACCAGC
GCACCACCAACAAGAGGCTCATGGTGGAAGCCCTTCTGGACGGCTTGTCGCCGCTTTCTC
CCGGTGCCACCAAGGATGCCGACGCGTGCACCAAAAGCAATGCCTGGAGCAACAGGGTCA
CGACCCTCGAGCTCAATCGATGCGGGCTGACCTCGTCGGACTTTCGGCGATTGGTCCTGG
ACATTTTGCCGGGCTCCTTTCCCTTCCTCCAGCGCCTGTCGCTGGCGAGCAACGACATCG
ACGAGCTGCCGCCGCTCAAGCATCCGAATGCCTGCCCGAACTACCACCAAGAGCCCGAAC
GCAATTGTTTCCACAAATACGAATACGAATGCGAAACCGAATGCGACGACCCGACGTTTT
CCAGGACCAAGCAAAAACTTTGCCGGCTGCGGCGGTCACTCCAACAAATACACCTGTCGA
ACAACCCGATCATGGAATTTCTGGGGAAGCACGGGGACGAAGATCCACTGCCGTTGTCGC
GAGAACAAATGGAAGGGACGTCGTGGTCGTTCCCGGAAGAACGTACACCGGGGCGGGAAT
CGGGGACGCAACCGCGGAAGCACACACTGGAACAGCGCCGGAAGCGGTTGTTGCACCGCT
TGCTGGAATCCGTCGACGAGAACCACACCACCGCCACCGAACCGTCGCTTCCGGCCGTCT
TGGAGCGACGGGTTCGCCGACTGGTGGAGCTCGAACACCTCCGGGACTTGCTGGAGCTGT
TCCCGCGGTTGTCCCACCTGGGGTATGCCTCGTGCGTCCACAAGCTCTTGCGATCCGTCG
TGGGAGACGTCGTGGTGGACCACAACAACGACGGCAACGGCAACGGCAACGACGAAACCG
AAAACCGGTGGTGCGTGTTGCTGCCCTTTGGCCTGTATGCGATGGACCTGCAACGCCAGA
TCGAATTCTACCTGCGTTTTAACCGCGGAGGCCGACGGCTGGTGGAGGGCATCGGGTGCG
AACCAAGGGCACAGCGCAACGAAGAACGACGAGACGACGGCGACGACGACGACAAGAACC
ACGGGAACGCCGGGGGCCGTCTCTTGGAATTTTTGGCTCTTTGGCCAAACATTCTGGAAT
GGTCGTACCGGACGTCCTCCGGTGGGTGTTTTCTGATGCCTCTTCCGCCCCTCTCGTCGT
CGTCGCATGCGGGTTCCGCTGCCCAGGCAGCACAACACGCTGTTTCCACGCCAACGCACC
ACTCCGCCGCATTCTCCAACAAGAACGCAACGGCGTTGTACCGATTGCTGCGGGAAGGTC
CCGTATTGTTTGGCAGCAGCGAACGTCATTGACGGAGCAGTGAATGGCACCTTATACAAA
CACCACCAAAC
>comp2974_c0_seq1
CGGTTAGTTAGTTAGCATCACATACCCACATTTCCGTTCCGACTCCTTCTCAACACTCCA
GAAGAAGTTTGTTTGTCCAACAAACGCCGTCACATGCCGTGCCCAGAGCTGCTGTTGCGA
GACGAAAAGACGACAACTAGTATGCCATTGAGGAGCAACAACGCGAGGAAGATCTAACAG
CACAGCAACAACACAGCAGCAAGAGCGCAGTCCAACACCGCAATCCAACAGAACCGGGAA
CAACACAGGAGGTTTCAGAGGGCGGAACGAGGCACACCGATACAGTACGGCCCAGAAACA
CGCACCGTTCCTTGTCGATCGATTCGGTGAAACCAACAGCAACCGGCAACAACAACAACA
ACAACA
>comp2975_c0_seq1
GATGCGATGCGATGCAAAGCAGTGCAATGCAATGCAAAGCAAAGCAAAGGAATGCAATAC
AATTCAGTTCAATTCAATGCATCACTGTACAATGCAATGCAGCGCATGCCATGTGGAATT
TTGCGAGTGAGCCCTCGTTGCCATACAAATCATTCATCGACCCTTTGGCCAAGATTCCAT
TCATTCATTCGGTCAAACGACGTGGCACATGTTGCGTGCTACATGCGCAACTATTCGAAA
CCCTTTCAAATGGAACGAACAAAACAGAACAGAACAAAACAAAACACAAAACTGCACGAC
AAGACTTGGTAGAACAAAACAAAATACAAAACGTTGCCGCGTTTATAAGCCAGCACATGC
TGCCTACACACATACAGAGATCATTACTTTCCGATTCCTTGCATGTATTGAGCAAACGGT
AACATTTACACAGAACAAGGACGAGGCGGGTCGTGGACGATTCGGAGAAACATGTATCAC
ATGGTTTTATTTTTGCGTAGTACATTGTAATTGTATTGCAGCCAATTGGGTGTAAAGAGT
AGATGGGCGGAGTGGGACGCATTGCGATTGATTTGCGTCGACTCGACTCGACCCGGCTTG
GCTGGCATCGCATGGCAACGGCATCGGGACGGAATCTTGCGGTGGGTGGCGATTCGGCCG
AACCGGCCGGTGCCTGCCACTGCCTATTGCGTCTCCTCCGTGTCACCAACGGCGGTTCCC
GAAAGCCCGCGAAGGCGCGGCGCAAGCGTGGGAATTTTACGGCCGGGAACACCGGCCCG
GTCGAAGAGACAGACAAGCAAAAACACGGGCACGAAACCCCACCATTGTCGGATGGAAAA

TCAATGCCGAGGCTGCTAGCAATGTGCGACGGATGGAACGATTCGATATCGAATCGAATC
GTCTCGTCCCGTTCCATGCTGGTCCACATTCACGACAGAGTAGTACTTTTAATAACAATA
GCCTCCATTCATTGTGGAAAGAACTTTCTGTATATGCTGTATCCAT
>comp2976_c0_seq1
ACTCAACATGGGTCTCAAGTAAGTGTGAACGTGCAATAGATGTGGGATATAATGCTTGAA
ACACGAAAAAGGAAAGAGAGGCAGAGAGAATTTATTTTGCTCTTTCGATTGACAGTAGAA
GCTACGAACACGAATATCTCACCTTAGTTTATGGTTATCGTTCCAATATTTTCGTATCGT
CATCTCCATTTATTCGCATGTGCGCTGAACCAAACACCGCATAAATCATTCTATATCGCA
ATATGATACGTAACAGCGGTATGGATTTGATCATCGATGGCTGGTTCCACGAACGTGGAG
TGCTCTGGCCCGGACAAGCCATGTCCCTCAAAGTAAAAAAAGTGCTTGATCATTACCGTA
CCGACTTTCAGGATCTGTTGGTCTTCGAGTCGGAGAACCACGGAAATGTTTTGGTATTGG
ACGGTGTGATCCAAGTGACGGAACGCGACGAACATGCCTACCAGGAAATGATTGCACATA
TTCCTCTCTTTGCACATCCGAATCCCAAGAAAGTCCTGGTGATTGGTGGTGGAGATGGCG
GGGTGTTGCGAGAAGTTGCTAGGCATCCCGGTGTGGAAGAAATTGTCGAATGCGAAATCG
ATGAGGGAGTCATTATTGCGTCGAAAAAGCATCTGCCGAGCTTGGCAGTGGGTTATGACG
ACCCTCGTGTCACCGTAAAAATCATGGACGGAGCAAAATTCATGGATGAGAATCAGGATG
CCTTTGACGTGATCATTACCGATTCCTCTGATCCTGTAGGGCCTGCATCTGTACTGTTTG
AAACACCCTTCTACAATGCCATGTACAAGAGTCTTCGAGAAGGCGGCATTGTCTGCACGC
AGGGAGAATGCATGTGGCTTCATTTAGATCTCATCAAACCCCTGGTGGATTCAATCAGCC
AAACCTTTTCTTCTGTCGAATACGCTTACACAACAATCCCCACATATCCATCCGGACAGA
TTGGCTTCATTTTGGCTACCAAGGGAAGGAAAACCTGCAAAGTCCCAGAACGAGAAGCTG
ACATGACTCCAGAATTACAGAAAAAATTGAAGTACTACACTCCTGCGCTCCACAGAGCAT
CCTTCGTCTTGCCTGCTTTTACCCAACGTGCAATATTTGGAGAAGACACCGTATAAAGCA
TCAAGAAACGAACCGTACACTACGGAAGCCTAGCTTTGTAATCTCGACTTTTTATGATAT
TCCTCTAGACTGATCATTTCTTCAATCGAAGGTGTCTGCTGTGACTCAACGACACGAGAA
TTTTCCTGCTTCATTATATGTTCAGGAGCGACATTGTTTTGCCAAAGCTGCGGTACTGGT
ACTTTCACTTTTTTCAGCATCACTAGTTATACTTTCTTCTTCGGCCTTGTTCTGCTACTC
CTGTTTTGCTAGTACTACTATTACTTTGTGTTCTTGATAAAATGTTCGTCGTTGTGCTAA
TGATGGTTTTGTACAATATCGCAGTTACTAATAATATTGTATGTAATTTCTTGGACAACA
TCTGTGCTACCACAAGCAGCTGACTTCTTCCGAGTTTAATATTCCAG
>comp2976_c0_seq2
TTTCTTTCAGACAAATAAAAATTGCGAACACTCAACATGGGTCTCAACGGTATGGATTTG
ATCATCGATGGCTGGTTCCACGAACGTGGAGTGCTCTGGCCCGGACAAGCCATGTCCCTC
AAAGTAAAAAAAGTGCTTGATCATTACCGTACCGACTTTCAGGATCTGTTGGTCTTCGAG
TCGGAGAACCACGGAAATGTTTTGGTATTGGACGGTGTGATCCAAGTGACGGAACGCGAC
GAACATGCCTACCAGGAAATGATTGCACATATTCCTCTCTTTGCACATCCGAATCCCAAG
AAAGTCCTGGTGATTGGTGGTGGAGATGGCGGGGTGTTGCGAGAAGTTGCTAGGCATCCC
GGTGTGGAAGAAATTGTCGAATGCGAAATCGATGAGGGAGTCATTATTGCGTCGAAAAAG
CATCTGCCGAGCTTGGCAGTGGGTTATGACGACCCTCGTGTCACCGTAAAAATCATGGAC
GGAGCAAAATTCATGGATGAGAATCAGGATGCCTTTGACGTGATCATTACCGATTCCTCT
GATCCTGTAGGGCCTGCATCTGTACTGTTTGAAACACCCTTCTACAATGCCATGTACAAG
AGTCTTCGAGAAGGCGGCATTGTCTGCACGCAGGGAGAATGCATGTGGCTTCATTTAGAT
CTCATCAAACCCCTGGTGGATTCAATCAGCCAAACCTTTTCTTCTGTCGAATACGCTTAC
ACAACAATCCCCACATATCCATCCGGACAGATTGGCTTCATTTTGGCTACCAAGGGAAGG
AAAACCTGCAAAGTCCCAGAACGAGAAGCTGACATGACTCCAGAATTACAGAAAAAATTG
AAGTACTACACTCCTGCGCTCCACAGAGCATCCTTCGTCTTGCCTGCTTTTACCCAACGT
GCAATATTTGGAGAAGACACCGTATAAAGCATCAAGAAACGAACCGTACACTACGGAAGC
CTAGCTTTGTAATCTCGACTTTTTATGATATTCCTCTAGACTGATCATTTCTTCAATCGA
AGGTGTCTGCTGTGACTCAACGACACGAGAATTTTCCTGCTTCATTATATGTTCAGGAGC
GACATTGTTTTGCCAAAGCTGCGGTACTGGTACTTTCACTTTTTTCAGCATCACTAGTTA
TACTTTCTTCTTCGGCCTTGTTCTGCTACTCCTGTTTTGCTAGTACTACTATTACTTTGT
GTTCTTGATAAAATGTTCGTCGTTGTGCTAATGATGGTTTTGTACAATATCGCAGTTACT
AATAATATTGTATGTAATTTCTTGGACAACATCTGTGCTACCACAAGCAGCTGACTTCTT
CCGAGTTTAATATTCCAG
>comp2977_c0_seq1
GGACCGCGAAATGGAACGCCTGGAGGTCGCCCACAACCGGCGCGTGGCGATTCTGGACCG
GGGGGTCTTTGCGCTGGAGATGGCCTGCCACGTCCTCACCGTGGCCCATTTCTGCCACAT
CTGGAGCCTCAACGGCGTCCAGTTTACCCTCATCGACGGCGTCCTCGCCCTCCACCTGCA
CTCGGCCATCTCGTCGGCCTGCACCAAGCTGGCCCGCCGTCGGAACGTCCACAACATCGC
CCGGGACCTCCAGGGGAGGTTTCCGAACGCCACCGACGAGGAGCTGGACAAGGCATCGGC

GGCCGGCGACGTCTGCTGCATCTGCCTCGGATCGATGACGACCGGGGGGAGCGTGAAGAA
GCTCCGCTGCGGCCACCTCTACCACACGCACTGCCTCAGGGAGGTCATCGAGCGCGCACA
GAACCTGCGGTCGGCCCGGTGCCCCCTCTGCCGGGAGCCCCTGCTGGACCGGCCGCAGCC
TCCGCAGGGGGATCCCCCGCCGGCCGACGCCGAGACGGACAACGCCAGAGCCACGGCCAC
GGCCACGGCCAATCCCGAACCCCCCCGGGCCGGGGGTGCCGACCCGGCAAGGAGGCCCGC
CGCCGAGGCGGGAGGAGGGGGCGGGGACCCGGCCCGGGGGGAAGGCGAGCGGGCCTTGTT
TCGGTTCTCGACCGAGGGCATCCTGCCGGTGTGGGTCCCCGTCCCCGCCTTTTCCTTCGA
GGTGGTCCGGCGGCCCCCGAACGGGACCCAGGGAGCGGGGCCGCTTCGGAACGAACCCGC
GGCAGCGGAGCCGCGCGCGCAAGCCCCCGGAACGGGGGCCGGCGATCTTGCGAACGCACT
CGGGCGGCAGCAGCAACACGAACACGAACCGTCGTTTCTCCAGCGCGTCCTGGCGCTGGC
GGGGCTCGTGCCAATGTCGCCGGAGGAGGAAGCCCGGGCGCTCGAGCAACTGGTGGACAT
GTTCCCACAATACGATCGCCAAGACTTGCTCGGGGAGCTGAGGGACAGAGGGTCCATCGA
AGCCGTAACCGAATCGATCCTGATGGGGATCTTTCGGGGGGTTCCGCGGAGCTAGAAAGT
CGTCCTGGCAACCGTGCTCCGATTGCACCATCGCGGCCGCGTGCGAAAACAAAACAAAAC
AAAA
>comp2978_c0_seq1
CCATCCTCGAGAGGCGGCGGGCCGAGGAGGAACGGCTGCGGAAGGCCCGGGAAGCCGAGG
ACGACCGGCTCCGAGCGGTCCGAGAAGCCGCGGCCGAGCGGGACCGGGCGGAACAAGAAC
GGGCGGCGGCGGTTCTCGAGCGGGAAACCGAGAAGGAGCAGGCCGCCACGAGGGACCGGG
AGGCCCGGGCGGCAGAGACCGAGCGGCGACGCAGAGAACGGGAAGCCGCGGCAAAGAAAG
CGACCGAATACATCGACAAGGCAAAGAAGCTGGTCGGGCAGCTGGTCCAGGTGAGGGCAT
CGGTAGAACCCTTCGAGAAGAACAAGACCGTTGGAAAGCGGCGGCTCGGAATGAAAAAGA
TGGCCAACGGAAAGGTCAACACGCTGAACGACAATCCTTCCAAGATCCGGGAGGTTGCAA
CCCAAATCTCCACAGCGATTGCCGCGTACGAACAAGAAGATGGACAGATCAAAACACAAT
TGCAGCAAGGTGCTCCCGGGCTGACATCCGACATGGCGGTTGGAAGGCGATATTTTGTGG
ACCTGCTCGCGTCGACGGCCATGAAACGCGTCCAAGCCGAGTCCTTTGGAGGAACCAAGG
GAGATGGGTTTCCCTTGGCGGCAATGCTTGCGATGGTATCGGTGGAAAGCAAACAGATCA
TATCCGTCCTGGCCGCACACATATACACAGTGTGTCCTGTGGCCATCCCCACGCTTCCGG
CACCCAGGGATAATGCCAGCGAAGACGACCTGATGATCGGCCTCGGGATGCAGAGAAACC
AAAAGACCGGTGAATTCGAGACGTTCCCACGGTTTTTGGCACGGACGGAAAACATTGTAT
CGTTCGTGGCCGCTCTCCAATCGTCTCTGCCGAGCTCCCACCAGCTGATGGGCGGACACG
CCGGAGCTATGTTGTGGCTGAAGCGATTCATCGATCTTCTGCCACTGCCACCAACATCGC
CGCTTCCGCTCACGACGGCACCCGTGCTGGGGGCCTTTTTGACGGTTGCGGGGCACATGA
TGGCCAACGCCCACGCCGACGACTTCAGAACGATGCTCGCCACCATCCAGAACGACGTCA
TGAATCGCCTGGACGAAGGAGAGCTCGGAAAGCCGTCCGCGATTCGATTGGGAAAGGTTG
TCGAGGGAGGATTCGATTCCTTCCGAAAGAATCTGCCTCCCAAGGCCGTTCCGGAGCTGT
ACTACGGAGCGTCGGAACATTCCCACAAGAAGGAAGGCGTCGTCTCGTTCGGAGGAACAA
TAGGTGGGGAGGAGGACGTTCAACCCAAAAAGCCACAGGGGATGCAGCAGCAGCCGCCGC
CACAAAACCCGTTTGGCGGCGGAGTTTCGTTTGGCGGACCAAGTAGCACAGGCGGCGGGA
ACCCTAATACGTTTAGCGGCGGCGGTGG
>comp2980_c0_seq1
ACCGTACTTGTACTCTACCCTCCGTCATCTGAGAACATTTCCCGAGTTCACAATACGACT
CCCGATAGTATCATTTAACTCTTTGCCACCATGAAATTCGTTGTCCTCCTCCTGTCGACC
CTTTCCTTGGTTAACGCTTTCGGATCTTTTGGATCCAAGAAAGCTTCCCCCGCGGCTACC
TCTCCCTTGCCGACCTTCGACAAGAACACCGGAAAATGGTCGCCCGCGCCCAACGCCGAA
GAACCCTACGGCATTGGCAGAACGTTGTTGGGAAATGGACCCGTTCCGGCCTTCACTCGC
TTGACCCAATCGGAAGATTACATGCAGGCCGTCTACAAGTTCCAAGCTAGCGAAAAATGC
ACGGTTCGATACGCCCAAGGCAACATGGACGCCTACTTCGAAAACCCAAACGACTGGGCC
TACCAACGAAAAGTTGAGGAAAGCGGTGGCTACAAAAAGAAATATGGCGAGCCAATTCCC
CAAAAGCAGGTCATCCTGACGGTAGTCTGGGGTGTTGGTATCACCGGCTTGCTCGGCAAG
GGACTCTTAACCTTTGTGAGTTACAAGTTAGGAAACTAAAAACAATGTTGTGAAAGATGA
AGTGATGCGGTTTGGTGTGGTGTGGTTTGGTGTG
>comp2980_c0_seq2
ACCGTACTTGTACTCTACCCTCCGTCATCTGAGAACATTTCCCGAGTTCACAATACGACT
CCCGATAGTATCATTTAACTCTTTGCCACCATGAAATTCGTTGTCCTCCTCCTGTCGACC
CTTTCCTTGGTTAACGCTTTCGGATCTTTTGGATCCAAGAAAGCTTCCCCCGCGGCTACC
TCTCCCTTGCCGACCTTCGACAAGAACACCGGAAAATGGTCGCCCGCGCCCAACGCCGAA
GAACCCTACGGCATTGGCAGAACGTTGTTGGGAAATGGACCCGTTCCGGCCTTCACTCGC
TTGACCCAATCGGAAGATTACATGCAGGCCGTCTACAAGTTCCAAGCTAGCGAAAAATGC
ACGGTTCGATACGCCCAAGGCAACATGGACGCCTACTTCGAAAACCCAAACGACTGGGCC

TACCAACGAAAAGTTGAGGAAAGCGGTGGCTACAAAAAGAAATATGGCGAGCCAATTCCC
CAAAAGCAGGTCATCCTGACGGTAGTCTGGGGTGTTGGTATCACCGGCTTGCTCGGCAAG
GGACTCTTAACCTTTACTTCTGGAGACTTCTGTCTTTCGGAAGCCAATGCTGCAACCAAT
TATTGCAAAGTGTTCTTCTCCTAGACTAGCTCAGAATTCGCGCTGTCACTTCTGGTTGGA
AGGGCGATTGCCAGCAATTCGAGAGTCAAGATTTGGGGCATGATAAGCTCCCTTTGATTG
TGTTCAACATCGTTGGATTCTGTTTAAACAAACAATACGCAAAAACAATTGGAGCAGCGT
ACTCTACTCCAGTTGCTAGTTCATAAAATAGAAGTCCACGGAAGAAATATAAAATGTAAT
TAAAATATTTAGCTGCTGGG
>comp2981_c0_seq1
AAGTTGTAATCAAAGCAATACGAGCTATGCCGACAACCCGACATCCTGCAAAGAAAATGC
CCGTTGTCGGTTCTATGGCACTCGGTGCCGCGCTAATCTTTACCATTGCAGTGGGCATTG
CAGTTTTGCCAATACCAAACTATGCTTTTACTCCCGCTGTTTATCAGTATAAGAGTGACC
GAACACTAATGCAAATAAATTTTAAAAATGACAAACTGGTTACGGGAAAGAACTTTGAAT
TATCGGCGAGAGACACGATTTTCTCGCTCGTCGAAGAAGACGACGGCAACGACGACAATA
TAGAGAACCCTGACATTGATATTGCGGATCTTCTTGCAGACAAAGAATTTCTGGAGAACA
ACGTAAAAACTGATGATGGAAAGGATTCAAAGAAGAAGAAAATCGGTGATCCATTACGGG
AAGACAACGGTGTTCGACCTTCCATTCACCCTACCGCGATAAACGCAATTGTCGATGCCC
TGAAGGCCCGTGCTGAACAGAGCAAACAGGAAATGGACGACGATGACGACGACACGATGC
ACTTTCGTGCCACCGAAACCGTCGAACCACTCATGGTGATGGTGACGGCTGGGCAATTCG
CTTCCGACGCAATACAGAAACGACAAGAATCCAGCGAGGCTGACGGAATGCTGATGACCC
AGGACGAAGAACAGACGCTCGCCGGAAGGATCATGGGAGTGATCATGCGGCTCGATGCGC
TTGAGGAAGAACTGGCCGAACGCGTCTCGGGCGTGGAATGGATCGGAAAGTACGACGAAT
GGGGAAACTTTGGGGTTTTATCCGACGAGCACCAAAACAACCTCGCTCGTGATAGCGATA
GCGGCGACGACGGATTGGCAGACGTTCACGCCAAAATTCTCGATGATCCGCTCTTTTGCA
TGAATCGAGCCGAATGCCTGCTCGCGATATTTCTTCAAGAAGTAGAAATTCCACAACTGG
AGAAATTGAAAGAGACCGTCCCCGACGGAAGCAAGATCGACTTTCTTGATCAAGATAGAC
TGGAAGTCGTAGTTGGATCTGACAAGTCGTAATTTACTTTGATCAGCTTTCGCTCAACAA
CTATCAGTACTTTCGCAATACGCATTCCTTGTACATCGCAACATGAAAGGGTGTTGCCTT
TTGTGAATCCAAAGCTGTTAAATTGTGGCAATTACTAGAAGCGGAATAGAATTGCAGCAG
TTAAATGGAATTTGCTGTATTGGGGCTACTACTATTAGTGCTGAAGTGTTTGCCTTCATA
GAGATCAAATTAATTTCAAGAATAGTTCTGCTGTTGCTACTATTACATTCAATGTGAAAT
GAAGTGAGAAGGTCTTTTAAAGCGAGCAGACACCAATAATGATCAAAGAAGTTTGACTTC
TTCAACCTGTCCCAGCAGCAGCAGTTGATTGAGATTGTTATTGGACCCAGATGTCATGAA
AAGATAGGAAAATTTCTGGCAACAAGGAGGACCCGTCTCATGGTTTCAATAGTAGTGAGG
AAAGCAACAATATTTCTAGTAGTAGTACTCCTACTACTAGTACAAGAAGAAGAAGGAGTG
AAGAAGAGGATGCATCCACCACCAATCAAGAAGGGACTGATGGATCTTCAATTTGGAGCA
AGCAAGAATAAGAATAAGAATTGATTGCTGGTGAGGAGGACAGATTCTTCATTTTTTGGT
CATGGCAAAGAAGAACAGGAGGAACAAGAAGAAGCAGC
>comp2983_c0_seq1
AAAAGAATTATTATACACCGAAACGGACGCTGTTTTCTCCCTAGCCATTGCTATTAGTAG
TAGTATTCTAGACGATCGTTGGATGGTTGAGCACTATTCGTTGTGTCTTGCAGAGACTTA
TGGCGATTGCCAATTGCACCTACGAAAACCGCAAGAGTAGCAGTACAGACCAAGCATACA
CCGATACCTCTGCAGCAGATTCGTCCGTTTGAACTACTAGCAGTATGCAGAACAGACAAG
GAAGGCATTGCATGACCACCAGCCCACCTTCTGAGGGAGGTCCTCATTGACCTTTGTCTA
CCATCACCACCAGTACTACTAGTAGTACTAGCCACCCACAATTCACAATCTAGTTCTACT
ACTACTACTACTAGTAGTCTCTCAATAGTATGTACCAGCAGCAGAT
>comp2984_c0_seq1
ATATGATGGCAGATTACGAACAAATAGCGACGTTTCCCTCCTTACCACCGCATGCTATAA
CAGTGGAACAACAGAGGGTCCTTGAGCCTCTCAGCTACCCCAAAAGTATTGATACGCGGA
CATCGCATTCTCGGCAAGTCGTAGCGCTTCTTCAAGACCTTCATCAATCCATAACACCTC
CCGAACGATGGCAAAGCCTGCAAAGGGCGCTGAATCTTGTTTCCCATGATGTGACAGAGC
TTCATGACTACGAAATAAGCCTGGGGCTTGATAAGGCACTTTGTCTGAAACTCGGATATG
TTGTCGTAACAACCAAAAGCAATTGCGCCTCAAAGGAGGTGACCTTGATTTGTAGTTGTT
TGCTACAAATTTTCAAGTGCTCGTCGAAAAATCGATTACGTTCTTTTCACCACATCGGTG
CCACTGAATTGCTCCCAATACTCATACAGTTTTGTATTGGCATCATTAAATGCAAAATAG
ATATGGAAGATCAGTCCCCTGAACATGATGACGGACTCGTTTCGATCGTTCGAGTATTAA
GAGTGTTTTCGAAATTGATTCCAGCGAAGTCTTTTTTAATAAACTATCTTCAAGGAGCAT
ATCTCGGCCATTTACTGAGAGATATGCTAATATGGATAGACGATCCTGCGTCTTCAATTA
TATACTCATCAACGGAAGTTTTATGGGAAGCTATTGGTGTCTTAAAGGATTTGACGTTCC
GATCACAGACAAACGACAAAGAAACATTATTGCACTTGGAAGGTGGGGTTCTCTTAAAAA

TTTTATCAATCTGCTGTGGAGGAATAAAGGCATCGCATCCACGAATTCAAGAGTGGTGTA
CTGCTGTAATTTGGAATCTAGCACTAGATCCTCAAATCTGCCGGAAATTAGTGTTTGGAA
ATGATACCCATGAAAGTGGTGGAAATGTTATTTTGGACGGGCTCCTTGAAGTATTGACCC
ATTATTCTGCAGATAGCAAGATATCGGGATATTCCCTTAAAATCAAACGAAACGCGATGA
GTGCAATTGGGAATAGTTTTGGATTCCCAGAACTCTTCGACTCTTTTCAAAAAAAAAATGC
ACAGATAAATCCTCTGAAATACTGCAAAGGTTGATGAGTTTGGTTCAAAACGACTCCGAC
TCGGTAGTTCGTAGACGAGCTATGCGGACCATAAGGTGTCTAGCAAGCTCTGGAGTAAAT
GATACTATTCTTCTAAGAGATCTCTCCTCTTTATTGGTCGACATAATATCGCAAAAAGGA
TCCGAGAACGATGAAAATGATGAAGACATGCATGTACAAGTTTGTCAAACAATTATCATA
CTGAAAGAAACTATTGACGCTAAGATTTGGCCTCGATTACAAAAGTCTCTTGTATGTCGA
ATAGAAACTACAACATCAATCAAAATAATTTCTGCAGCAATTGTGTGCTTGTCCAACTGC
TTCAGTGATGAACATTCAATTCAAAACTGGGATGGTTTCTCTGATACTTTTTGGACAAGT
CTTGAAAGAACAACAACAGCTAACTTAGAGGCCCATGAATCAGTATCAAAACTGCTCCTT
GGAATTGCAAATAACGAAGGAAACATAAGAGATGCTGGGGATCGAGGTATAAGAAACCCA
AGTATTTTGACAAAAACAGCGGTCGTGAACACTCTGACCACAATTCTTTTGGAATCAGAG
TGCAACAGGGAAGAACCAAGGAATCAGGCTTTACAAACTGTTCAAATATTGGCCGGGAAC
GAAATCAATAAACGACCACTAGCTGAAAATGATAGGCTGTTGTCTGGTCTGGTAACTCTA
TGCCTTACACAACCAGATCCCGGTATCAAAGATTCAGCGAAGGGAGTAATATTGCAACTA
GTGCCTGAATTGTAATATATTTTAATGGCCAGAGGTAGTCATGCCTTTGAACTATAACAA
CGGGTTGTGACCATCATTAGTGCCTTTGTATCACAGGTTTTCATTAGA
>comp2985_c0_seq1
TGCAATAATTGGTTCGTTCTCGTAGTTGGTTGTTGATATCGTAAATCACAACCCGAACGC
GAAGAAGAGATACAAAGTAGTCGTAAGATTGTAGAGTTCCTCTGTAAACTCCGTTATTAA
AAACGTACACAGCGGCAATCCTTCCCCTTTTCGGAACAATGAATGTAACGATGACAACGA
AAAATTGGCTCCTTTTAGCTTGCGCACTTCGAATACAAGGCGGGAATGGGTTTACTCAGA
CGAAGTTGGTCCACGTCAACAGCGGAGTCGACAACTTTGTGTTAAGGGCCTCACTCACGA
ATGATTATGGAAAAAGTTGCTCTCGCCGCTCGTGGTTGGCCGATGCTTGTAGGACGATCG
GAGTTGCAACTGCATCTGTAGCAATAGGAACATCAGTGAAACCAGAAAAAGCGCAAGCCA
CGGAAGAGCAATTTAATCCAGACGACCCCGAATTCAAGGAGATTTACAATAATCCTGCCA
CTCCTCCTGCTCCAGAAGAAAGAAGTGGATTAGTTGTTCTGAGAGTCGCAGAGGTATCAC
AATTCCAAGAAAAAATTCTCCGTGCGATTGTCAGTGGCGAGCTAAAGGATGTTATGGTTA
GCCCGATGCAATTTGCCTTCGGAACTCAGATTTTACTAAGAAACTCAAATTTAGATGGGA
ATATGAGGCTTATGATTCAAGAAGAGGTACCGAAACAAAAACGTGATTCCGCTAGAAAGA
ATGCTGCCAATGTCATGAACACGCTTCAAGACATTGCAAAATACTCTGCGTCAATCCAAA
GGGATTTCGAACCGTTGGAAATGGTGGAACTCGCTGATATGTACAAGGTTGTTCGAATAA
ATTTAAATGAGCTGTATGAATATCTTCCAGAGAAAGAGCGAGAAAAGTATTACGGATACT
TCGTAGCAGTAACAGAGTACGAGAAAAAAGTTGCCAATGGAACGTACAATCCTGATATTG
ACGGTGTACTCAAATTCGATAATTAAATGCAATATTTTACCTATGAC
>comp2986_c0_seq1
ACAGCAGCAACAACAACGACAGCAACAGCAACAACACCCTTCGTGTTCTGTTCAGCGGCG
CGGGTCCCTCACTCCCCCGAGGAAATCCAGAGAGCTCTCGTGGAGGGACCACGAACGGAA
AGCGGCAACGCACTCGCCCGGCTGTTCCAGCACCTCGGCGGAAACCCTGTCGCAGCCCTC
GCTGAGGTCCCGGACGCCACCGAGGAGGCTGAGCGCGAGGTACTAGCCGCCAGCCGAAAC
ATAACAAAACGAATCAGATCGAATCGAATCGAATCG
>comp2987_c0_seq1
AATCTATGCTCTGTAATTTCAGACTACAACAATCTTTATGGTCAATTTCTAGCAGAGATT
GGAGAAACGCTCTCACTTCCGACGTCTGTTGAGATGTATCGACCAACATCTCGACCTTTA
GAGACACCGTTAGAGACCTCTCAACCAACATCTCGACCGTCAGAGACACCGTTGGAGGCG
TCTCGACCAACATCTCGACGGGCAGAGACTCTGACAGAGACACTGTCAGAGACACCCATG
TTGATGAAGTTTCGACCGACGTCTCCACCAACGTCTCCACCAATGTCTTGACCAACGTCT
TGACCAACGTCTTGACCGATGTTTATCAAAACGTTGACACCGCAACCGCAACCTTGAGGT
GATGATTTGATGACATTTCGACCGACTTTGAAACCGACGTTGGAACCTCAACTGCGACTG
CAACTCCAACTCCAACTCCAACTGCAACCTCCACCACAACCTCAACTTCAACCTCAACTT
CAACCTCAACAGCAATCTTTGGACGACGTTTGGACGGTGGTTGGACCGATGTCTGTTGAA
AACTTTAAACCAACGGATGAATCGACGGTTGAACTGATCGATCTAGGGACAGATGAACCA
AATCAACAATTGATTGAAGAACCAACGGATCCAGGGACGGAAGAACCGACGAATCAAGGG
ACGCGTTGAGCGACGAATGAACGTCGGGCGTTTCTGCTGTACAAAATCGTTCTGAAACGT
CCCAGACTGCTGAACAGAACGGCGCTCAAGATTTTATTTGTTTCGATACTTCCCACAAAG
ACCTATCTTTGTGAACAGGTGCCTTCACAGATCCGTCGGCCTTACCGTGCAAAGATCCAT
CACAACATATTTTATCTACGTTGGTGCGGAAAGGTGTCCTTATGGTGTATTGTTGCAAGC

CCACCTGCCTTCACGTGCATGTGTCCATTACAACGTATTGGATTCACCTTAGTGCGGGTC
CTGTTTATAATGCGGAAAGGTTTTTACGCGGTCTAGTGTGTCAAGCCCACCTGCCATAGC
GGGCTGGAAGCCACTACAATATATGCTATTTACCTCATTGCAGGTCCCATTTAAATTCAT
AGTTACGCATGTGCAAAATGTTGGAGTACGAGTACTACCTCATTGCAGGTCCTGTAATTC
TTAGTTTGTTGTGCATATGAAAAATTTTATGTACGA
>comp2988_c0_seq1
AGAATTTTCTTCGAAAAACTGTTTATTTACAACAATCTGCTCAAGTTCCAGTACGTACGG
CACCTTACAATTCTGACGACAGTAGCTTCGTGTCGCAAGATAGAATATCCATATCCTAAC
ACCTCATTTTCCAAGGATCAAGGCAAAAAAATGATGAACACGATGAAAACTTCCTTCCTC
GCTGTAGCCATGTTGATGTGCGTCTCGTACATCCATGCATCGGTAGTAATCGAAGGCGAC
AGCGTACGAAGTGTTGGCAAACCTTACTACAGATTCGAAGCGTCTTCTCACGAGGATCGC
AACTTGAAGGAGCGCAACCTGAAGAAAAGCAAAGGAGACAAGAGCAAGAAAGGAAAGGGA
AGCAAATCCAACAAGGGTGCCAAGTCCGACAAGAAATCCAAAAAGAAATCCAAAAAGGGC
GTCACGGAAGAGCCATCCGCCGCTCCATCTGCGGCCCCATCCGCGGCTCCTTCCGCATCT
CCAACAGCGGCCCCAACAGGTGCTCCAACTGGTTCCACAACCGTCTCTTCCACCGCGACT
TCTTCTCAAGAAGGAAGCACTTAATTTTGAATGGAAGCCACAACTGTCATGGCTGCGAGT
GCACCGCGATACTCTGATCGGAGCGAGATCAAATTAACATCTCTATTGCATCCGTCGTGT
TTTGCAGTAATCCTCCGGCATCAATCAAGTCTTTGGATCTTTGGATCATAGTGTGTAAAA
TCGTTTTTAGTCTTAATGTTAACATATTGTACAGCGGAAGACCAGCTGCAGCATTTTTCT
AAGAAGATACCAACAATGAATCCGATGAGACACGCTGCTGTGATCACATTTCGTGTCTTT
CCATCAAGCCCACCATTCCCTGACTCCACTCAATGGGGCCGCCTAATATTTCCTTACCGG
ACGAAAAAGAAGACGACGGTAAGGACGAGGCACCTCAGTACCAACTGGAAGAATGCATTT
GGGCTCTTGCTTTACAGTATTGATATCATTTCGAAAGGCCTCTTCTTCACTTGAGAGCCG
AAGTTTCGTACCTGCAGTAGTATCGTACCGTATGTAGCTTACAATTTCCGCAGTAGAGAG
AAACTTCGAAGTACATATAGAACCATTATGATTCTGTATCGGTACGTTAGTTATTTTGTA
CGTACCCGTACTTGTACGTGCAAGAATGAAATGTTGCTCATAACCTGTGAAATGTTAATA
GACTCCTAGTTCTGCATGATTACGTG
>comp2989_c0_seq1
ATTAATATTCGTCGTTCTAAGCGAAAAAGTTTCGGGTATTATCATGCTTCCACCACAGTA
CAGCGCGACCCCAACCACGATGGACTGTAATCAAGGAATTTATCTGGAACGACTACTTCA
GCAACAACTATGCCAGCGAAAGAATGGGAATAACATCAATTCGACTGTAAACAAAAATAT
CAGCGATGGGAACAGTTCATTTGGTAATTGCTCAGCTCCTTCAGGATTCATAGCAACGAA
TACTACATCGATCGGAGTATCCAATACCGGCATCGGTCTCGGAATGCACCAGCAACAGCA
CTTTCAAAATCGCATGATTGCGTTCCTAAAGGAACGGCAGCAACAACGACAGCTTGTAGC
AAACGGGATTATCTGCGCCCCTTGCGATATGACGTCCAATCCTAGTATCCCAGGCCTATC
CAATATCGCTGGGTTGGGTCACCAATTAGTAGGGAGAGATTGTGGTGGCATTGGACAAAC
TATTCCTACGAGTGCATTAATAGATGGGATCAGTAGTGGTGGTCGTGGAGCTAGAAATGT
CAGGGGTTTGGGGAACAGGGGTTTCTTATATCCTAGCACTGAACAAGATTTTCTCTCGAG
CTCCAATCCATATACTGCAGTGGGAGAAATCACTGGGGAACATAACACTAAACTTAGAAG
CAACGGATCTGTTGGTGCTGGATCCCTGAATTCTTCCATGGGGGGGTTATCTCAGTTTTC
TATTCAACATCAAGCACTGACCCAAGCTGCATTGAACTCTTACCAGAAAGCGAACGAAAA
GAGGTTGACTGATGTGATCTTGGCCAAGCAGGCTCAAGTTGCATTTCTCGAAGCGGCCCA
GGTCCACGTTCCACGGACTATCCGCCTTCCTTGCGGTGCAAGAGGAATGAAAGCTGATCA
CAACTCATCTACCGCATTCTTTGACATACCCGAAAACGCCCGACACGGGCAACATCTGCT
TTGTTCGCACTCCGTCTGCAGAGCAGCTGGCGTGAAGTTTAGGTATTGCTTCTATTGTAA
AAAGCCCGTTACGAAACAAAACTTTAGATCCCGTCACTTGCACGCCAACCTCGATCCCAA
TAACAAAGAAGCTGAAAGAAAACCAATCGATAAATTGAAGGAAGAAAATAAGAGCAAAAC
GGAAATCTGTCAAAGTAAGGAAAGCAATGCGAACATGCCGAGTACATACAAAGCTACGAC
GAAACCACAAATTGCGGTTTCAGAAAGTAATTCCGTCAAACTTTCAAAGAGCTTGGGTGA
CAAAAATTCGATCGATTCTTCTCCTGCGCTTCCAAGTATCAAACGTAAAAAAGAAGAGAA
CAGCAGAAGCACAGAAGCTGAGCGAGCTCAAAAGATACCTAAATTATTGCTATCAGAGTT
GGGGCCATGATACGTCAAACCCAACCAATAGGTCGAGTTTTGGGACCACGCAAATTAGTT
TCTTTTGAAGGTATTCAATCTTGGACCCGAAAGGTTTTTTCTTTTTGTCGTCCCGAAATG
GATGTTTCTGTTTGCATTATTAGAATTTTGGAAGAAAGTTAGACACGACGGAATGTCAGA
AAGTGGCCCCTTTGGGCTCTAGAGGTCAGTGATATGTAGAAGGCAGAAAGATGGGAAGTT
GAGGCAGCTCACCACAAATGCTTGCCAATTTCGCTGGCCGATGGTTCGACTTCTGTAGTG
GATAATGCAATGAGACTATGTCCTTATGAATGGCTAGAACCTCTCAACCAATGAAAAAGA
AATGGGTGGGATGATTGTAAAGGTTCATTTTACTCGATCAACTACTAAACCGTATGAAGG
CGTATGCCAACTTCATTTTGTTTGTGCAAGTGAACCTTTGCAACAATTTTGAGCCCGAGG
TAAGGGTTTTGC

>comp2991_c1_seq1
AACCTCACCATGGTGTCCTCCGAGGGCATCGCAGAGGCCTTTTCCCGGACGGGCATGGCC
CCGCTCGGGGATCCCTCCGGGCCCTGCTCGGACCTGCGGAGGAACCTGGTCCTCTCGGGA
ATTTCCGCGGAGGCCCTCAACGCCATGGTCGGCCACGAGGTGGCGATCGGAAGGGAGGGA
AGGGTCTTTGTCCACCGCCGGTGCGTCCCCTGCCGGTACCGGGAGGCCGCCTGCAAGCGC
AGCGGCCTCACGAACAACCTGTGGGGCGTCTCGGGGATCAGCTGCGAGGTCCTCCCCCCC
CGTTCCAAAACCGCCCCCGGGGGCACGACCATCCAGGTCGGGGACGAAGTCGCGGTGGTC
CCGGGAACCCACGCGCCCGACCGCATCGATGTCGGCCGGACCAAGCCCGGGTCCTTTGTC
CGGCCCGCCGACCGGTCCGCGGAGGACGTCAAAAAGTCGATCACCCCGCCCTACATTGCC
GCCCTGGCGAGCCTGTGGGACCCCGAGGGCTTTGCCCGGGTCGAGGCCGCCCACAACTCG
GTCGGCCTCCGCTTCTGGACGCCCGAGGCCTACCAGGTGGGACTGCTGGCGGCGGCCTTG
[truncated: 589,796 more chars]
