# Supplementary material for: Transcriptome sequencing of three Pseudo-nitzschia species reveals comparable gene sets and the presence of Nitric Oxide Synthase genes in diatoms
Source: Sci Rep. 2015 Jul 20;5:12329. doi: 10.1038/srep12329 (PMC4648414; doi:10.1038/srep12329)
Supplement: Pseudo-nitzschia multistriata peptides [file srep12329-s15.pdf]

***Pseudo-nitzschia multistriata* peptides.**

>comp162_c0_seq1; 44 1 433 LEN=527; translated
XMWPWELWIFALALIVSAALLILLHALKAWNERILFVKLTEMGLCILFFIAPMITSNFFH
MHHWYAGWLMGMHFNYDVWWSRLAMAWCWGMYVNGVAVYGRDPVMTCEYSYFLSLDNRCP
YISCYLEGLADENNTEVVEMVPVDW
>comp235_c0_seq1; 438 1 1332 LEN=1332; minus strand; translated
XGPSGEAYGPSFPFNLMFKTSIGPEGTSVGYLRPETAQGLFVNFRRLLDLNAQKMPFAAA
QIGLGFRNGMLYFEGSGNRLKERKIVFDSQNEFADLFCSHSNSTEIAPRSGLLRVREFCM
GEIEHFVNPDDKSHPNFSSVAEKELVLFGRDDQLGTGKTKTLKIGEAVKGGLVNNETLGY
FMARTQLYMEKIGMDPERLRFRQHLATEMAHYAADCWDLEIKSSYGWVECVGHADRACYD
LDVHAKATNTSMVATVKYDTPKEIEVAKLKFDRKKIGMVFKKDARTVSGALDKLAESWKD
FEPIAKALEADGKVTVDGFEVTKDMVSWEQSTKKVHELKFTPSVIEPSFGMGRILYSLLE
HSFYIRENDEQRCVMKFKPQVAPQKCAVLPISSSPEFNAIVDGIAASLMEYDLATRVDKS
TAALGRRYARSDEVGVPFAVTVDF
>comp236_c0_seq1; 20 1 209 LEN=209; minus strand; translated
VPTVSNFFPIERYYEASDKVFESFEMVFNNRQLDNAYVYGRRYCTFYVKGISKHDYYRSQ
NSEARRNQAX
>comp292_c0_seq1; 18 1 248 LEN=248; minus strand; translated
XGDINSTEVFLLRDAVHKLHKDLDMPLVGVYFSFDVEESFREARKDHLYEVVERYKAENK
TNNPYVPERFPEQTMVNILKMWH
>comp406_c0_seq1; 52 1 156 LEN=298; minus strand; translated
AGAGACCLLLPPPLVIHERPRQEQTVTYTQGPDGKVQETHVAVVKGTPVEP
>comp436_c0_seq1; 55 1 237 LEN=237; minus strand; translated
GGVSVVDGHDESDSSSPLAKVSRWRYLLSSQQRVLLVLVLFVASAAYWAGNCFVNRQREI
QRTWEERLRLEELQMAKVL
>comp645_c0_seq1; 89 1 752 LEN=752; minus strand; translated
XALTTCTRVVTDQITIDSSFCSVMPNTCQQDPTVEYKTDIEEPMSSRALLRPRVLTRDPP
SDPPGTPSNISPKKQIPGHHRREQRVAFEKNSVKELMYRVILFFPYVAIMIMCKVLVSNI
TEHETSVVSLHGATQERAEATTMTAPTGSRPWMVVHVGPPKTATTTIKKGLLEVAEKLAL
DDNVYFVGGIQSSRVDVNVGVDTEFKVYRNADTFPSNHESAMQSNFEEFSQALEGHLRNG
HNVVISNDMYTX
>comp689_c0_seq1; 25 1 210 LEN=210; minus strand; translated
RKRKVKAGRQKRKHTGYTLFMQETYPKAKIENPDLPPKALISIMAKLWRNLAAGGKREWK
ERAETVNSDD
>comp778_c0_seq1; 94 1 265 LEN=265; minus strand; translated
XGGAPGGDQNYYNLDYRDSNRRMDRGGGLMNYRPNYNQRGGNEYDNQRGDEYYYDDPYDS
SGSFYNHEGKRVRRFDPVYDRPDGYYNPX
>comp882_c0_seq1; 125 1 247 LEN=247; minus strand; translated
REGTGASGKRFSSRYIGSMVGDVHRTLLYGGVFGYPGDTKNPNGKLRLLYEGAPMSFIME
QAGGISSTGKERVMEITPEYVHX
>comp889_c0_seq1; 90 1 174 LEN=266; minus strand; translated
RECVLRGATSREELVHATVSKMRSVTGAEEDVCIAILKRHRYDVTEAVEAYLDGQTR
>comp936_c0_seq1; 82 1 289 LEN=289; minus strand; translated
XEKIYSLSKLEELYLFSNSFEDQLSNGSFGKLLNLKVIDVKNNLLSGPLPDALFSTLTNL
EVIDFHQNALNGHINDVVAKGHSLRYMDVSSNLLSGG
>comp1141_c0_seq1; 125 626 771 LEN=771; minus strand; translated
MSHTDEEWEQIRIPIPLSVEEDYEIVIPPSIVPSSMDAHDSSPRTARSG
>comp1194_c0_seq1; 8 1 218 LEN=218; minus strand; translated
GGGWDSIFENDQSSKVLNIDTAAKLFDTSTKITDAEWPTLKQIASPCPTLKVIGAVTQGM
VKREELKRMRELG
>comp1198_c0_seq1; 33 1 268 LEN=268; minus strand; translated
XALLFYHVAPNVYRTQDLFCNGPNRYVRMLRLGTSRTRCEGNRRTAQLGTCNGRGNIPYF
TQRDILASNGIIHVISNVMIASPDGTLQGX
>comp1252_c0_seq1; 69 1 337 LEN=337; minus strand; translated
XGSQNEATAQKSSGRFSDEKKMELEDNDDDVVDVKRKVSEDGSLKHANVSRCDTSLMCGE
VVGEDGSIRCVXTINKRNQARQPRFGWIAMNEANAAQNTKGHVARENPVESAA
>comp1480_c0_seq1; 35 1 406 LEN=406; minus strand; translated
DGQLWRVDEKGNKIKRVRRKSTANATNTYENCAETTTGHSVEEKVEGRLGRKIRGGRRRN
VPSDWSLTEDSGASSVLSYKSNDSRVRTRGRPFSREALDRLDNSTHGSDNGSLSHDEIPS

KPRRSQSNEKNSDAKX
>comp1578_c0_seq1; 49 1 213 LEN=213; minus strand; translated
XEVANGLIDYCICVEMVMFAIAHSFTFTYKEYLPSTVENAIGDYEQVQQHNQPIDDAEDN
SRNDNNDGDQGX
>comp1614_c0_seq1; 128 1 327 LEN=327; minus strand; translated
XTGETEGDKSGAVGGDDQNEYELDPEKRREARKMRRIMANRRSARESRERRKKLLTDLQD
SVENLTTDNAVLTKENFSLRRELITLVEQSGGVASLSMIPNIQASLESAR
>comp1664_c0_seq1; 350 91 1335 LEN=1503; translated
MKSKLISRFSPIGVIIIAGLFCIEVDLCYSFALVGGQFELKNHNQLLMAADKNPVTVSNE
NNGSGSENSKGGKVFDEASYEKERLAKDAQAMDAMKVVAEKELSKVDTGSVGELRSPWKW
VLRKRIWDFMEENDIARFPRPVHHRIPNFVGADQAARRLSELPEFRSASTIKVNPDTPQR
PVRHFVLEQKKVLLTPQPRLRTGFFSTIEMKELPSLVEIKECTNSKGVVKYGTPVTLNEE
YTVDLVVVGSTAVCPRTGARVGKGEGFAELEWGILSAQGNLEAKTCLVVTTVHDCQVIED
ENDMPEGWALTKHDVPVDIIVTPTRVIHVFDRAPKPSGILWDLLSPQKLAAIRVLRQLKQ
ETEDKLGVTLPSGPDELLPPVASRNNKNRKKKNNRRRKPRQQQRKGSETPPAKK
>comp1667_c0_seq1; 476 1 1481 LEN=1481; minus strand; translated
XFEKHTDFMNAMESNDKMSATKSIGSNNTIYQKEPNLESKDEGRHGSKKEVLTISNETKI
EGRIAILNIQSEKERTTEEMEQFRTETEIDTIELLSPIQSEKEMISENYEQTGTKAEIYS
SFKKEEENISGITEQTVIEEISEKTEIRAEIPRSPLQSEKKMIDEMLGKFLKTMESMDDD
KFTDDDAQVLNSSRTGYIDSSSVLDDSEPRNHYSSSPDNTSLKSSPRFGYDESGFSSPAR
SLLEQSNFYKPSKSEIASQSNYWSNLSEADSNGEASLLKTKSISIMPSFRPLDESRSRIA
SPDLNLSFVRSSENDFLAATWIDSSGSHGALSRILSKDEPGIRTASKEDCSMQDNSLCIM
DNAKGTNYVTDTPRPLNPIAAVLSRGFSKDVVAQVENNGQDMSREGNKKSIKVIDATDSS
KARNPFAAVSYQAFSGNTVTQVDDNGQDLLSKVIDVLAEAMNKSEKMIDITESSRPYNLN
AVVLQGTSEDEIASX
>comp1667_c0_seq2; 469 1 1543 LEN=1543; minus strand; translated
ELSSDNTNLDTNSIKVDVHMEHIVPPAFMNAMESNDKMSATKSIGSNNTIYQKEPNLESK
DEGRHGSKKEVLTISNETKIEGRIAILNIQSEKERTTEEMEQFRTETEIDTIELLSPIQS
EKEMISENYEQTGTKAEIYSSFKKEEENISGITEQTVIEEISEKTEIRAEIPRSPLQSEK
KMIDEMLGKFLKTMESMDDDKFTDDDAQVLNSSRTGYIDSSSVLDDSEPRNHYSSSPDNT
SLKSSPRFGYDESGFSSPARSLLEQSNFYKPSKSEIASQSNYWSNLSEADSNGEASLLKT
KSISIMPSFRPLDESRSRIASPDLNLSFVRSSENDFLAATWIDSSGSHGALSRILSKDEP
GIRTASKEDCSMQDNSLCIMDNAKGTNYVTDTPRPLNPIAAVLSRGFSKDVVAQVENNGQ
DMSREGNKKSIKVIDATDSSKARNPFAAVSYQAFSGNTVTQVDDNGQDLLSKVIDVLAEA
MNKSEKMIDITESSRPYNLNAVVLQGTSEDEIASX
>comp1682_c0_seq1; 324 1 1492 LEN=1748; translated
XGQQPIDRLVQYKACASAWATTRSNGARNDDSNAAPPTQSKQKKKRPSMPPKTTILPYDP
LFGEQGILSSSDEEGDDDAKEELNLDKPVVNSIWGSSIGTNTNRYLLLDIPKKKKKKGKD
KKNKGKPIQNVPKKRKGEKSPNDCAKLFVQSVSSPQEGYAIAVRYLVWGGVGGAPAERGV
LIPGSEVAFPATPQGVSNCWQRYSPLIHVITRTWPGFAACILTHLVDFVLSIEDSSAVCE
NESLDAGSIRKLYFLYAWIRLILSQRFMAALDRNFSVKTTTTKNSNPLDLPLAHLDHLES
LGYPLNSLLDRCRRCNSEKHDNSNGENGDASGRSFVNNSDSMGTSRDIVRDLESILGAKV
SPNFGYSDNTFVPSNTNNTVEPNPLSRSTENDDSINIQVEHAKTSGEMSLDEMEAMLLSE
DNNNNGQEGAKAEHPPTASHLEDAMKATYMTSGTSAGACMEMAEQQRQVDLNMRRPAWIR
CQRWDECAIGTLPGYPT
>comp1697_c0_seq1; 146 17 505 LEN=505; translated
MKVIEGASRVAFLAFFLSHIPITLFVDAQGLFGPHYPKLLTDLIAWYCGLFGDVLMKNAP
SGEYAWFSSLIGCEILFQLPFFIVAARTIMTAGKCNGSSTTNPSDGGSTPQESYPEWFRI
ACIVYGSHVSTTLVPILSCFVWSEEMTTGQKCATTASKYRIES
>comp1697_c0_seq2; 219 17 592 LEN=718; translated
MKVIEGASRVAFLAFFLSHIPITLFVDAQGLFGPHYPKLLTDLIAWYCGLFGDVLMKNAP
SGEYAWFSSLIGCEILFQLPFFIVAARTIMTAGKCNGSSTTNPSDGGSTPQESYPEWFRI
ACIVYGSHVSTTLVPILSCFVWSEEMTTGQKCATTAIYSPYLIFPLWLLWLAVKETFVDA
MDEKRSSGKRD
>comp1702_c0_seq1; 234 287 934 LEN=1251; minus strand; translated
MSDASATAPTGTKQVVRAMALIAVLAGCTWFSPAPNPAGGGHGLVLLHLLAFSSWFGCSV
WVSFVAGIVMFKTLPRHTFGRLQSRLFPAYFVFSAVSILVAIATSGALASPGGEEAAAPY
SLVVVLVCILANLFCFEPATTRVMYQRHAVEKRLGTGHEIGKIRPDDPKKANDPELRALS
KTFGMLHGVSTLLNLLALGFGCAWLSRCAEQIVVA
>comp1708_c0_seq1; 43 640 983 LEN=983; minus strand; translated

MAYAMLNAFGKHQRSLAAAAAVLRGYNSVRPIGPEERRYLPLLIACRLSCSATLGAYSYA
QNPGNEYLLLHATPAWNALELIWGTDPERRSSVLETVQEVFDLACSSCETDKKTR
>comp1718_c0_seq1; 219 609 1324 LEN=1324; minus strand; translated
MMSKATGTLYSILVSLVLVLCCSKCHAFLPQQTKAPNTINTQIGWFGQQKTSAPGHPLSM
GLVDDFVASTDEKTRKSRNEKYLAKLQERVTRINGLEETIEELGDDELQAKTESFKERLK
NGEDINGPLLVLSFAFFDLYLTGXVLELRHYDVQILGGLVLHDGRLAEMATGEGKTLVST
LPVYLNALTGETSFVVTVNDYLAKRDMEIMGQVHRFLGLTVGLIQSEMKEEERRVAYEN
>comp1733_c0_seq1; 215 1 514 LEN=600; minus strand; translated
XGSEADNVCDADAEKEPETEIEVGTTNGDDDHGQEAEMQAEPKEKSEKPAYIPGMFVINN
TETAAEEEEKKSETSPDDVGGEAKSPAEEGGEAKTDSGVETPNVRKVTYMNKHSKFAAMD
DNSCTTDEVKSNKKKVKGMPRKANKTHSVSQLHYNILFCNTTCQYTYLDHK
>comp1812_c0_seq1; 525 1 827 LEN=881; minus strand; translated
XGGGDVFSSPLVPAAAAPTNQVTQGALKNAIPPPPKSAPQATTPATLAEELMKNPAPVAT
KTTTSNDDILALFGSPKPKQNQPQSGFGMQAMNQMGPGGGFNANSMTSMNGMMPQQHVNM
NAFGNRQNMMTQQQHQQMMMMQNNNNVNNMMPNSNQAMANQMMQQQMMMQRQMMMMKQQQ
QMQNQFQMNNAGAMNNTNMFNSPNGINNSMGRMANNNNHMNSAIKGMQNMNMGNNIASSQ
SSDDGGFGTPMGGSSQQQTMKDDAFSSLGGMNAFR
>comp1818_c0_seq1; 718 1 1765 LEN=1814; translated
XEYCGAFSANGGNSLGTLIAHQQVGKEQTKQAVGFRRRSSLALGAERDCRVAISTNSSDS
VAGEPYNENDTSHNDLEAVKSLTRTAMYRCGTEEGLDALKSLRELCDNRLPFDFDSVHRR
QTYGGRFSSPDPRNAAIDQGEETISLLPGFLPTQAVGDFLESVRCMEEQGWMSTNPDSVD
GLPSLHLNLVSQGKPLFDETPPSCDDSRESPSEYCDEDSFESQIFRLYDIVRPYVYEELL
PKVDQLLNNANKSTPRKRKLRVSDVFLRRYGQDICGDVTRNGISIHYDVYSRVTAVVALD
EVAAEGDNGLFTLTVDENTGETSNHKALRRFFPLRTGDCVVHTWDVLHGVDVQPGLDRTS
LIVWFDEVAEGEGQNEAESVSPWLSLESQNAPPGVTTLQNGNDVRQFVLASALSSLEGDE
DTELEEILLYLRSASRGNTFALTRMGSICDEGALSSPELQKEAVQLLDRLRPFKNLPEIL
QGLLEGNDAQNQLACRFWFEASLSGNPLAQKALANEIMFRASQSERPDQRLLAAVLFALA
SQQEDEDGPSDSLSRVIEHDLAARNVQSQEEFLASPVVQTAKAAFGAA
>comp1834_c0_seq1; 519 608 2302 LEN=2504; minus strand; translated
MEEDRIIIDSHIRFGNRWAEISKMLNGRTDNAIKNHWNSSMKKKIEKYLERKRVDPTAPV
VDESGRFLLRREDIEGCLQALQQSGSNSKSIKNQAKSYAGRPLGSTRLNHMHPNRMVPLA
TPMSAAQNPAAYNSMMKRQYDAMMVGAYPGMGYTPKSVKRVKSSSEYNKNSKLAEMSPVN
KKILEEFLSELKGGYIKGVYHSALERRRIVEKAVKSGSIGELNALGLSQSEYSRLQKAVP
MNQSRRHLDGPKWATPQQQLYPHHHRYSNGYMVPPHPMHWGHPSPLYPMAQLFPGYPPVR
PPPTTPLPTNNLPTPSSTKDSTQLQQASSKIETLSNSLSLKHSPILRTKDNTVQKDTKSK
DNFELGESSDLGYTLDEYALLGSPCAKGSKSNCFSPFLIPTPNKSSTPGMCTSWGGDDAK
LLHDTFSKGYVSSSKSSSANTPRVFFKDDLTEQSILLDSGTKVKHTNEETPFQSDFTMTP
GRPKSRIGVVTGSGRERIRGAMVDERDNLLSTAILATPKSPKSGNVQEIDHSLHHIDAYS
VIKSPLHFGSPIVKAAARSPMRLL
>comp1834_c0_seq2; 567 246 2183 LEN=2385; minus strand; translated
MERDGEKCKERYDFLRQSQGGRGPVPWTRQEDEQILALVAQHGAKKWSSIANSIAGRSGK
QCRERWHNHLNPNINKSKTWTMEEDRIIIDSHIRFGNRWAEISKMLNGRTDNAIKNHWNS
SMKKKIEKYLERKRVDPTAPVVDESGRFLLRREDIEGCLQALQQSGSNSKSIKNQAKSYA
GRPLGSTRLNHMHPNRMVPLATPMSAAQNPAAYNSMMKRQYDAMMVGAYPGMGYTPKSVK
RVKSSSEYNKNSKLAEMSPVNKKILEEFLSELKGGYIKGVYHSALERRRIVEKAVKSGSI
GELNALGLSQSEYSRLQKAVPMNQSRRHLDGPKWATPQQQLYPHHHRYSNGYMVPPHPMH
WGHPSPLYPMAQLFPGYPPVRPPPTTPLPTNNLPTPSSTKDSTQLQQASSKIETLSNSLS
LKHSPILRTKDNTVQKDTKSKDNFELGESSDLGYTLDEYALLGSPCAKGSKSNCFSPFLI
PTPNKSSTPGMCTSWGGDDAKLLHDTFSKGYVSSSKSSSANTPRVFFKDDLTEQSILLDS
GTKVKHTNEETPFQSDFTMTPGRPKSRIGVVTGSGRERIRGAMVDERDNLLSTAILATPK
SPKSGNVQEIDHSLHHIDAYSVIKSPLHFGSPIVKAAARSPMRLL
>comp1835_c0_seq1; 518 1 1561 LEN=1578; translated
RGAEYAVNFVPKIKLEIAVSAAMADQVVETITKTARTGKIGDGKIFVLDIQQAVRVRTGE
TNDDAFERAGERINTMNSMLKLGLAATLIALPQMGLTQEAAAEAVPSFDEIGPYIMTTLL
FCMAGFLVFFMAAGFAMLEGGLVRSKNVTMQMTKNIVLYSFATIMYWLVGFNLMYPGDGW
LVAGWVGPFFAMTELDPVGVAAADAALDYASIGSDFFFQLVFVAATASIVSGALAERIKL
WPFLVFVIVLTGFMYPISGSWQWGGGWLSEAGFSDFAGSTVVHSVGGWAALAGAIILGPR
LGKYGKDGKVTPMPGSNLALATLGTFILWLGWFGFNGGSQLAMGTVGDVSDVSRIFANTN
MAAAAGSIAALILTQVLYKKPDLTMVLNGALAGLVSITAEPLAPSLFGALWIGAVGGVIV
VLAVPMLDKFKIDDVVGAIPVHLLAGIWGTLAVVFYGEASLGTQLLGIFAYGAFTFTVSI

VIWLIIKGTMGLRVDQETEINGLDMAEMGMEAYPEFSKS
>comp1840_c0_seq1; 308 1 1408 LEN=1408; translated
GKSMSPVAISQFNHSRRARSPSSGRNESITSSATTAAATEPERMVWRGRAQDTVVSSGTN
PNTLTRKPKTINRDNRDSNNNSRVQKDNANTNDGDSISRIKNNNNTICGNRRRQCCPGST
AWIVAWIVLVLCRQLMTETGLLRTALASYGAPCHYFDRGGWTEQGDECGYGRGNGRNRPS
RSAHGSSVDASSNQTLGSDNDKNSSSINITVPSLPSALDLLNPPFKKNYTIRVLGGDSIG
DIYNSNCNGSDTAVTTTSRKNKNSNNRNIIVTAYYKIPSKHTPERYRDWMKNFLGRFRDP
VVAFTSPDMVDSLAALRCPLSNSSSSNSTSDKDLNRSLSQSPACFPTVLVALPLEELPLA
ELYPKSFWVDQLDRDPEGKIHAPLRGDPRVGGRFESGELYWIWLSKAWFVHEAVGLLMQE
QHQGANEEKKSVEEEVAATTRLTSTATAAEGEKEHCPERDKRGDVFAWIX
>comp1841_c0_seq1; 292 1 897 LEN=1482; translated
NDNDDDNDETNAAPSSPRVKFRGIDASTTGGGSSGGDSFVGGNPLDRFLAVVASDSFSIA
SGAIGLLAVVVHRWNLVLAASDGSTAASIAAAEALTYQTRTDLLGVFAAGSVLLNGVTKL
DVTTALAESVVLEGKALPSPESVGGASGERPGPEVAWGLESVLGATPARSAVLLGSDKDG
GWVVLHRAGVVPPVPAPVPGKTPILDRVGSPGNTRETYLPTLQALPGRTELNYLPPNAQM
AVMLPIAAGGEAQSENESESESGSGNSVLVLGGNAAKSFTPRDIAWSRIVAERIGESL
>comp1843_c0_seq1; 300 1 835 LEN=1110; translated
XRKVEVAAEEAAEDETEALEELEALPDGVFVEAPEEGDMAAVHGSSWGSLDGRHKRNPGP
PGSKNGGRASKDQDGDVEMEPATKKTKLGTEQDGAQGAKPGGPKGGSGEGEENDDALSDE
FVYVPSFYPRPPSTKVVVDDRRTVVDETDDEEQRLLQQTRTQLRQEQQVSYASSLAARAF
PGAALRDTIDIDSSRGVRTSLVQLGSGHSHYWGSGWDEAAPKGQRPASASAIAVPMGRKP
AASGNTVPLGEPILPMSRASGSRVSRVLEGSMDAAAMQ
>comp1844_c0_seq1; 505 104 1830 LEN=2015; translated
MKTPRSLLAALFATVHLQLPPPCHSFSAKLTTNPKAAPGTNNVNFEDWITDAPEEVREWK
DLTVRGDIPDYVRGTWIRNGGGIWSGGDNGDGGESFSHIFDGFAKVSAYRFGNGRLRHQA
RFLEGKWYKKFQSDGSLPPSIGTGPILDASGTPKVGAARTVGAILNVATAFDNAXGKHLG
FRSALEDSRSQKGGGLTDAPPRVNLDYDTMDTLSSSTINPLAKGIRGFELLITAHPLYSL
ESDDTYNIAVEIGLGLPNARVNLIKETPLGDRSVVGSFATDDGIPYFHSFGLSRNYAVVV
VQPLRIDPGNLSGLLEKGFMRSMKSVEKTRVVVLDLKTGECVLDQSVPEPLYFYHAVSTA
EVDHGRSLSLRLCAYKSSDQLTGENQFMRLERCRRGKEWRNKLDKGGRFCDVVCDLEKGT
FSLRWDDRIRQGFELPVTRYSRAHGGSVEERRAAADAPIDGHPRYVYSFGAFALGAEEYD
AWGLFKFDTERREVAASYRCDSVYVSEPVFVPDPSGSREDDGVLLTQAYFGAERETRLLV
LDARTMEVVAEAGTGMRTPMDFHGGWIPEGGGGRP
>comp1847_c0_seq1; 323 1 1191 LEN=1251; translated
XLSHGDIGPTGSAGNFCAVGTFGPGIEIWNLDVMNALEPSCVLGGEDTSMADDIMRLNMM
GASEKKHSNTLSPQGRSLKPGSHTDAVMALSWNKVHRQVIASGSADCTVKLWDVTQAQSD
KANAATLKHHEDKVQSVLWHPKEGTLLATGSYDRTVGLVDARSTENVKTVKIPGDCESLA
WDPFNPQYLSAASEDGTVTCWDVRKFSTKTPLWSIVASEFGGVSDLAYNTHVPGMMATSS
VDKTVTIWDTATQSPTAKMNKDMKVGKLFTLKFYPSLPWLMGCAGGSKEMAIWDMSQDDT
IQKCFGDRIDHSSLVQTDERDRKPQNEISFDSLMNSTSVEKTNGKSKGKSSISNKKKKKK
KAHEPADDYCLKKTDEMIVNEFFRDDNFQYMWNNQM
>comp1848_c0_seq1; 483 317 1621 LEN=1621; translated
MAAANATLVFHSNAEEGECNNDSKRLVTENNILVNTGANHTIRMTTTTRRIRTRARKQHI
LLSPSLFLVLVTIALFQTVDPLLVRAFCPSPGIESISYSRWKQSIGRPRFLSTSFLKLST
VTPPKTVLPSRAELESMKVVDLKELIKDSGLNERGLFSRLKRKQDLVDFLVQAGSREQQT
TTAAAKEKSQIISDQPKEQQTQPKTRRKPLGMPSKKSGARQDAATTTTTANKQQDTGTAS
HNAVADEESLPLPKSPLQVIYDRVHEQYPPLKYLSEGNNTLLGEQDIRQKYHPMLQHAAK
AITSKRDKNNKDQNDDQDGKSDDGESETDDDTYTKPALSGDMDLIFVGTASCTPSITRGV
SCTALRLHSLAKQQTQSANVRVDARKPSKGNTKQSGKNFDVAQNSQSNLGTWLFDCGEST
QVRKGKELRRSEKVS
>comp1848_c0_seq2; 478 317 1600 LEN=1600; translated
MAAANATLVFHSNAEEGECNNDSKRLVTENNILVNTGANHTIRMTTTTRRIRTRARKQHI
LLSPSLFLVLVTIALFQTVDPLLVRAFCPSPGIESISYSRWKQSIGRPRFLSTSFLKLST
VTPPKTVLPSRAELESMKVVDLKELIKDSGLNERGLFSRLKRKQDLVDFLVQAGSREQQT
TTAAAKEKSQIISDQPKEQQTQPKTRRKPLGMPSKKSGARQDAATTTTTANKQQDTGTAS
HNAVADEESLPLPKSPLQVIYDRVHEQYPPLKYLSEGNNTLLGEQDIRQKYHPMLQHAAK
AITSKRDKNNKDQNDDQDGKSDDGESETDDDTYTKPALSGDMDLIFVGTASCTPSITRGV
SCTALRLHSLAKQQTQSANVRVDARKPSKGNTKQSGKNFDVAQNSQSNLGTWLFDCGEST
QLQIQKTS
>comp1851_c0_seq1; 155 1 608 LEN=608; minus strand; translated

XRLEYLEGGISSGFVKVEPTKEKPLFFRYGPRENRKEGELVQVPMDVSSMDSRSGFILLA
DKATVWVWHGKEVGTTKRDLCGRGLCVCVCVYVCVCARSVDWESAVDAFLDRRMLVCLFL
LSILFWCPGKPELTRLSSKHEMPFAQMKIMDKIACTQRGEELCTLGTVTVLDQGDCDDDE
EFWNYLYHGGSDKAVGSRSIGTCX
>comp1852_c0_seq1; 250 105 2116 LEN=2116; minus strand; translated
MHHHRVATAALLAWNFAAVTSFAVRGGSRNVRWEFTGSDHRRSNCRIWEQERLSATHRGE
SHTECSQGPLRVGIAGAGAVAFGTASILSKNGHESILWSPSGKGTADLVSRDNGSGDEGP
STVSSTGALRHEFDPEIASSAEELVKKSDVLIIALPANGHKQIFDALAPHLSSSSSDSSP
AAAGAPPKKKHIIVSSHASFGALYLSQFLHQLGNHHHTITSWGTTVCTARRTSGTSVDIK
TIRKSVDSSCLPEGDSASSLELCERLFPDTEFRPREGLLAITLSNLNPQNHLAIAMGNIS
RMDKGEEWYQFQNITPKIGGFLEALDKERLEIASALGLDVKTVYDHFSLSFHVPIPASGS
LSEMCQNIYEKGNDVHGPTAADSRYVTEDVPYGLAPVVALGKLVGRPAVLHESGMRICDA
MYGRNFSAENDLLGAIGLETIGLDELGEAARTGKLPGQRLLAGSRPLSPAHTHDVQXVGL
PAAGLGLARKIDSHPAPTIEGSLRLAPNPAAPDANENAEQTPIHRTAHRDKIRFGCGSFL
APGDESLQGHPLGDPAQVQRLLLGGQRLFLPRELHTAGARKELGSEPGRGRRHPAVDGGV
HHARRQVGPVGARVVQVFSEQPVPERNVFLPEDKECVSHGRDPRPLAGRELPSEDPRVVL
VALRQAFRKGX
>comp1855_c0_seq1; 682 220 1986 LEN=2064; translated
MRYDLAFFLCCCRSFLGDGENDDGEASGFYGLLGVESDATPDQIKRAYKRRSLQMHPDKL
AQRGKPVTDEDRARFTRMKDAYECLSDPHKRETYDAIGEKGMKWLDEPFSVDPQELAGNF
ARSSVLDRSKIFAIFVGLAVALLALPVAVCLHLDGDLGDDASWSVTLVPLWIWNAMALFY
HSRVLTMPTVSAPDGVPPEEWIDPFPMKDRIRSLFRFLLLVSAEILLALKLDEVLECAWG
LVFLPVYLWEAANVHEQWPLSRLRTLTVEDLEAALGKPLGECTADETEAIRRAYVVVPSA
ESPEFAEAEERKARAARELAKSAARAIVLSALAVRLDTRADWNWWLVFLPVWILSLGACL
TDYLSFAEASASAAEKDPGLFGGDRDDPEAAATTRASFGASGSAAAAETTSAVQSGESGG
PGSGGTATNYGSVGADGAATPSAAAAGSDLSEEEKDRLREHVMNQGSECCTRCCWRGCFL
LLMGLVAAKLQGAAFSAFWIISPFLFAAGIILLCLGFAIFGVSETTDGEERGAEFYSHPA
ASDITTNAQPSTNIVLPVAPGTPDTRGLLATAGGSSASENEMEIRGLD
>comp1856_c0_seq1; 237 49 624 LEN=767; translated
MTTDKDHTKSNEIYHFPTHASYLVPVVIKETPHHGEGQRGVFAKEPIAKGTKLWVWTHRV
KSIHRDDLEDYIQKTFGNDLEETRVFLRQGFVLPPKHSSETMSPPQKQGETTPRNASNDD
NFFHSNPTDAGRLTNHSSDPNTGPDGALRDIDPGEEMTMDYSFHGNPEWYQAICARFGVM
TEAQIAALHHT
>comp1858_c0_seq1; 410 103 1974 LEN=2391; translated
MGPLPTTSFLSKSKREEEKDQDEVDEFKNKILRNQSCRQWLVSESIKIAFLLSFGVVLLQ
WKIMKQVDVTEDLSFQPFVSGHRALKTLNAPLGEKNDNPKYRNCPFRDSPIVESIYVYPH
PGSPEWKGDILSNYTRIQTNSSSEDFIVYPWIDNDLRCKAAGVGPYDGTSQLVQYNTELL
VRDIITHPDSCLRTNDPEKATLFYVPYLPSAEYKNGTLFGKDMSQSKYGKAIQNIITKQE
YESWEKEFGLTSEYWKRRNGSDHILVFSEPMHGLWHPKSRRGNFHFLHSQFQTHSPIVIS
NELSKTFIDMYPLCARKNILVPYPNTNGRWFNGALDNEAVAMMTEHSGIRKLSESPGAIQ
SEIDLAKQSELKETSNGSNSTPPRLLGYYYSGGNHGTCVDLRKSMKDEFRCSPSGKMVKK
YKKALNNYAHGYRQSTFCPCPGGDSPSAKRNFDSVFAGCIPVILSEDFVWPFTSEFDRSS
NSGSESGIVALNPYDFSIRLNAKDHWHSKFDSPKNCNPKINTGEGHNETDHRSLQSVLEA
VPSKELARLRQGLARAAEVYSYYRKRPDLPDEPLREGLLPDGGAAHMLVRALAERASGAL
WNACRQEMKGKDISKDSVKKFKC
>comp1864_c0_seq1; 288 32 1608 LEN=1608; minus strand; translated
MKFLQITVATISSFSFLVEEVHGHGYMFEPISRNYYSNQFGLTWGTEPNIPMKEYCFHCL
NTKGVGSVCGTSEQGVNYDSWLDSLQQPISWNSNGNVYQEGGIITVETFLTAHHTGHMEL
RACPMGRASTQECFDQHVLEFVEDVNNFDVKMPKGKYAFNSADRAKLDKNVPSTDVIGCY
RFPFLDENYPERGYYYGSQAFNNNRFAMNFRLPQGLTGEEVLLQWLYDVLFELIYDLFXM
IYDPPNISDNMXNFYDLLVTANSCSPEGYAEYYSLNSHLPQEFWNRQLSTCTPEQFPPEF
YTGDSPERFVNCAEISILPNDNNIHIDEPAPTPTPTPEISTQSPVQVISVPDVSPPTPIA
TPITNESESRVIAYVGNWQACPTDEQIAQYTHVVIAFAVSYQWSPGKNICSETCEIAMPP
VCENVNRPDLIAKWKGMGKKVILSFGGAGMGGSWAGDNNDCWDYCFGRETKVIDRLTTIV
NEMNLDGIDIDYEYFYSDNQNGSGFSKGAEARNFLTEVTVGLRNSX
>comp1865_c0_seq1; 428 81 1556 LEN=1570; translated
MDFSLPHVHEDSLATLSKIRHIDLPAKDERGGSTGNGEPSLDELLIEEANNAAASLFLGK
WNNDNKENNSGEDAMDCGDNSSKSNNSSLTVNERRMLMNSASYPERSTPQTEKITEDSLV
VIFESFDNLKFCYPKKGAIFSNRNGHFHHEDFIGKPFGCKIRSRNNQGWGFVYLLKPTPE
LWSRSLNHRTQIIHFLDASMIVFYLNIRPNMVVCESGTGSGALSHCIMRSIAPRGMLHTF

EFNQKRAEDAKREFASNGVGHLSKVHHKDVCGKHGAGGFDQPQSSVDAVVLDLPEPWLAV
PHAAYCIKRNARLVSYSPCIEQAQKTIETLKKNGFHSIKTMEFRLKENYVDEVEYSMPPT
EKRQRVEDNRVYPREDKEIASGIAADAAASNQSEKPVVETKEESGICKDSPTEESTEQLK
TGDATKIGETQPAPKGAEKNPEPSSSEATAPAAAKRKAPKTKLVARPFVIMRGHTAFLTF
ATSGNGLRPKP
>comp1867_c0_seq1; 831 91 2352 LEN=2468; minus strand; translated
MNSSLSSWNHEYSIVAHDDHGIRLRLDPRQFTCPLNIEEAILTSIEGRQWRLLDRPIFVF
FDTESEQPQQHRQGPALEEREHKRLXASTPIKASMSTSTSTIAPWPTKMVESHGDYRDEA
WLEELAHNKDTTYKNQSSLPQLPVPAIEDTLSRFLPTALPLAESEEEKRALENAVQKFPA
QAQKLQERLLERAASVQKNSSWLQHWWNTLGYLQVRDSVCINVSYYFQFADDRTLAGKGS
KGNSLQVQRAASILYATGQYRNIVASGRLAPETLGRKNIPLCMTAYKYMFHACRIPLPQQ
DSYRIYDPHHYKHAVVARKGRFYKIPLIDESTNEVLSVESLEGHLSQILELADEEDSKTE
APGLGYLTGDHRDAWATARAALLELPGMAEAMEVLESGCVLVNLDDASPSSRAQCGKQLL
CGNGDASNRWFDKSVQLMVCPNGKAGLLGEHSMMDGMPVVGLANHIVSTTYQQARDESTT
GNSGLETFGGVADIFGSIEMTPKVLEMIETSKGNFREWTTGKHDLEVQSFWGYGSGFMKK
SGFSPDAYVQMAMQVATARLFGKQVGTYEASQTRVFRHGRTETTRTVSPASAAFVQSLLG
RNGGGQPLSATDKIALLNKACKSHARYIGNAAKGLGVDRHFLGLSMLVRDGEAAPDLYSD
PVFVRSKKWRVSTSNLTHPKLDNWGYGEVVPDGVGLSYSVHKDRVIFGITALKEHGGWPE
RLSQYLEDALVEMRLLVEEAAASDAAPPTRSKM
>comp1868_c0_seq1; 468 87 2348 LEN=2954; translated
MQCGANGIWDETGCFVYEITAPHRICVRNSPCSSPKPRQIQDFYYFECGELVSIDLIRHN
REDYTDPIGSNHSVGLHPGAHASITRRRRRRSDDGPFLRLSDGSGWILARSDGQDLAKRV
YVEDANHDSDEKRSRTPFWNFRADNLPLGIELRRHPNEELRNPNDTNRFSSPGSGGIRYL
PMQRIVCDRKITTASGVSFFRVQGTCGWVFDRRSSNGSNHTGQEPYREMMIRDDLVDTGL
FAFRVTSTEPIAVRRSCHVGDGPNNLGPTVVQHGDTLLVDVVRRSPLQDANGNGPFLRLV
DGSGWLFQHRRGVPGSLLEEVPIENGVFRLRILQVPDHTATNHGQPFLDRYQHRGLSSKV
TNNYNSNNNLSPRLKQGEIVSCDRKVVVPSPTSSAGICWCRQVGTDQWIMVKQQTNEDDD
EDMHDGGHIVAEIVPEPEHPASFLRERTAGDVRKRETYASSNGAKRLQQQPWSPRLVRSY
ACMVPGLEEIGFDRQHQILSFKSSNGVIFNIYCENRVIGILSVRENTIQGCVQRYHRNCS
DLELREILQDTKTCRGEGVHRCDKRARSFMPPSPIRTRFLADEEDCNNSEELSLRENQNI
ITPDTSSSSSAIGTGFEMHQDYNKDDDTIDTELECRMDLVDCQRKIQNLLYRQGNILRAI
KVYEEERMRDAEAMRAKSERIRIEHRENQRQVEQLQHQRRVEEDDRQARWRRTCRQLVSE
NVFVGAFVGGVVGLVLSPYCGPLLVQKLGLQHL
>comp1872_c0_seq1; 421 439 1410 LEN=1616; translated
MITRTRTTSTLFAAAIKATALCWAVLCSSSSHDSHSMAFVQSFSPTPLANGAEPRGTTIA
SSNSGLSSKTALSMAGGVRRRGLEQRREGATPTEGGMTLYLKAAEDGTSVGDCPFAHYIR
MILEEKNLPYTVQPCASNDDKPGWLLEYYEGKMPCLRHKKEAYVESNVIAAYLDYFFPSG
ESDADHKAALKDAEDAMDGFFPAVAGYLKDKESDAEAELSCEPLAALREKLGALEEHLGN
AGGEDCNLDGTSTSFGILDCRLVPQLYHLKVGIDTFKEHGKPDLESEFPTVHAYLSRSMT
RESFVNTMYPPETIEWGWTNARQ
>comp1875_c0_seq1; 566 203 1372 LEN=1612; translated
MKTTDRMAVFFLSLLSLNSAAGFTSSAIRTSTNRVSSPLAPFPPSANKQERQTPLIRCNE
KPGWLDDAMDGIPSGDFDEESYRNRIDLRGGIAGFSVDPELGFVCILVAGGEDSNQQHNK
HWMPVVVSPVDTDRPKSAEALTCVQLAGGLDLGTAILPPDSLARLVAEHDAEEQSGEGTA
RDKGAPKRLSLTKITAAPNPDASENAKAGIEEEAQELVATSPEREKAISDALPKVETAVK
TLPGLQETTTESVREAMQRFADEKGAVDRDAFSSILDSLRSLNTPAISLAPPLFRLDVSV
IDGNGISQVTVDTTNSMIALGLAMRYKVTVDMEEKYRHPRGGKGVDELLERFPVFRPIQE
LNEDSRIVDGFIPSMFEKARSIDNDMKGE
>comp1876_c0_seq1; 698 44 2179 LEN=2474; translated
MSQVEETPASLKDTKDAASTVQKSPKADSILTNLDATIAGFGDGHIFPDTDTSPASSCCS
SSSDKTDHRANALGDGIVERFSPPGLEEPFKQLHSILFPALHRGHAKDGIDPGRAGPATL
PLSSPSSKFQNPISSSSAAILMGVKGSGKTLLLERVLAACREQLQYNPGGGRRRLLYRMV
EINGIVCRGEDVASVVYEIIRQLSDIAYRSIQTTKAEESPLKATRKIRRTSGDDSDTTGK
RGFSSDSILRHNDGKTEEGNETGRLGGNEKDDSGDESIFVHDDDDANTRKRKARRQRRKQ
RKIDKHMLRLRRATFNSNLSLLESILEFAAADDIPIILNLDELDSFTDEGERQMLLYHLL
DRVATPGSNLVLLGTTSSFSALTLLEKRIRSRAEGTSKIIYLRTPPTYHGLLELLDYKLK
GCVARKDILDRLAHPKDIEAKAREIVSKKGLGARNGHANHTEESTDEIVKLSVAMEREFR
VGRDTRWFSRVIATSLSLYRHDCIMAMSFNDSSVSSGSPLSDGAPPNFCAKYIRDAMAMM
GASVLETMAGSGQQPDLCIVDGVAASPRLQALLDLSTPQVALMLSARRILKREEQRELVV
LAPLTLERMLKEYESFRRGSKSMGNSKLLKRAASHLMEQGLLVPSMDHSGGGPLQYDVSK

MYRTLDNYTLLRLPLHIPYDINRELGKALEQNLLECPTALKEWARFISKAA
>comp1881_c0_seq1; 109 1 733 LEN=1320; translated
XSLHRPPEQIAPHRITGTCRTQETERPKRPWLSETGPAAVPPASRPGRNGSVRADSAPPQ
TAPPSTAREPPARRLSRTAGSSPASDTXISASVTERVPWGFVCGYPVKTGSGDLYGGGHY
LEAASSNETPQGRAVLEAALRFGTGGTLPFCPCKRGYKPVSEGPPPRRMVACSFHEAARR
KRLARDKGKLRGGEADPSELFWSPKLSSEESRAELPPPPGSSREKAREKSSEKGDGQHDT
KTAT
>comp1884_c0_seq1; 92 308 653 LEN=653; minus strand; translated
MAAYTTPRGPHIIFLLSPFALRAMSEAQYVSTGQIGGEQGLSHYGLGLGKYTHFTSPIRR
YADVVVHHQLLQTLTLKADDFPLRAPPGFGRKALKSLPESKTISIIQGEGIEDAKX
>comp1885_c0_seq1; 349 109 1066 LEN=1066; translated
MASSRWKRFAFFEKNAISLPSEVLEDLLPAGEYEYSNSGGRGRRSEEGPSNSKDKVRMVV
TTAALPLDSKPGHGATAGMPNQGSGGNANASGGGEYTVALNEMWSGLAACNPMENIPGGY
AGGTNQTITLPSQAQLFEDDSNVVSSSSGAAVDGLVLAFVTSRDTNRVHCFDISVRCNHR
SSGGAPGIEPSADGDTQASGKKASDPEDLDGWRGYLAPIQMGTRATSDSRAAAGRSSDGR
ASADTDAARPQEEPREGIVGIATCRGTSGHRPMHMACITATNLVVCVDPHLYLSCRRPLS
TPENNEAPSFTLGSNWNEAX
>comp1886_c0_seq1; 382 150 1481 LEN=1481; translated
MRPPISKTKVLVLLAVFVFVVVSTPSASGFCFVHPHRKSPSAQPRQLVSSLQSRPVDVNH
KLEKEGDDVDSLNQREKPSNNTTKQQREAAKNKTPVHLLAGFLGSGKTTTLKHVLENKED
VRVGVVVNDVASVNIDAKLIAQKDASSADAFSSEATATKPPIKASRNDVVELQNGCACCS
LAGELFDSIESLLRPRIGSGDSEATSPYEAIVVELSGVADPLAIRNHWREAVATDSHPGV
TNQAELGRILTIVDATTFGTDYMTYDVLRDRDGWLGGESSTTINTSERQRLQPNNNCSGN
KQVAYLLVEQVEAADLVVVNKQDLAGETKTDVARRMVEGLNEEAEILTTNHGKISATKLL
GLPWPSDLEESDSREQEEESADGSHSHSHTHQHSSRKDADSPSLDAYSHDHDAACDDADC
TDPSHSHSHTHSHTHSHSHSHSHS
>comp1888_c0_seq1; 225 1 544 LEN=1140; translated
XHQGTSPHSLHHHDPAESAAAFAASIALAGSVVGVNGIGIGAHGDLASHQVTDSAAVEAA
VAAALAATDAKSNAYHHHHHAGDLVGPNHILHGTGDPMEAGASAANGSMPVGAGLPPAAV
GSPRASAPAAAPARGDVGLGLALDAAAKLAAAAAAGGDVVLAATTAVSTGGPVGSTAKQG
A
>comp1890_c0_seq1; 127 1 305 LEN=305; translated
KGSKGGYYSIAKPSYGPSYPTYPSYPSYGEVSYSYYGGKGSKGGKGSKGGYYTPSYKPSY
PSYPSYGGNCYEVCYGYGGKGGKGSKGYYGGYKYGYGSVGYG
>comp1891_c0_seq1; 438 297 1286 LEN=1741; translated
MTPSASASPNGHSTSSLLLAASSFPEKEAKKLLEVEEKFSFSDRSKLEERLKMEGFALCQ
EVIMVDWYYDRLVANSECLASVLPLVRNDHWLRYRQITSRNCIECVDGEAEWQLKRGTSI
GGGDGGATVYEEIEGTEALKIACSLANRELSKRDEANPTKNETALLLLFEGESFPVPPVD
SCDLQPFAKIVTHRAKWEQRQGQQPTSGTKTTNASLFSKLVVDLDTTPDGFAVGEVEALV
EAYGKTEGTSTAVYQEEAVAMARTDIRELLERILKGDDNNDDRSTPTRSPSMGKLEQFLF
LRQPLIHKVCVESGVIPQPPFSAEEEPSS
>comp1892_c0_seq1; 281 133 1359 LEN=1433; translated
MARRFSRPLVPHILLALVSCAFLGSTRVKLTIDALVVHSSATSFIGRGDDGNLVQLRGIR
FDCEGDFENEQQATVVSNIDKITPIMSTSSTRRNLFANTCFLAAVATTLELPASAAEPVR
AKDTDSVLAIAKRKLRPKPPKILRRKLSQDFAVLLMRSSYNALDEMDCVAMDQFQRDFFL
IRVAEYETYTKNLGAGMVTQGDLTDPFYFDFISFAQYKTINREVTQDPPYVFEEQQVPPE
GSSEPSMNPNGTARFVPVIVQRDPTLTNKMLIPTFDKKVGSKILDKLEENFANTESKIPN
IGYKPDSVALLSGIRQIVNLFLIGGYAFKGDVKTIAPESNGVLATYVLSLSAPATLWSGK
VLQVEGDPLDNNFLLKTLAEYIRRSGYINIKSSIKYEGTGEEITITVQ
>comp1897_c0_seq1; 235 1 1708 LEN=1742; translated
XLALRQQQQQHVLFQAQEEQSIAVTLSPAQWFESKLCKRKNGSNSYWRGSLFNERDHRRH
QIIANKKLSNLNGRFFQRHCLEFVKSLSMSSLDRIARKRRSIFHIALAREYGLCGREQFE
RGVSRFFYVKQLLQDPDIVSPHRKSISSLSVDLTGRFLLAGSADATITIYDVSKWGRSES
ISKDDANSQRYSYSPVAKSIKVPAVPDVLQLPAGHSASITYTQWYPTDPGVFLSGSSDGT
ILVWDTHKMKPVLRVHPFRDNSTGWVSAHLRTGGDHSMIAAGSWYESEIKLVDIRSGACS
HQLVGHGKGISALKWSSSNPNVVASGSRDSTVRLWDIRKSGSRACVTVLDREQVSSSANS
FGLSSSESTSVGYASDYSHLRREPTWARKQRRQQIPGIAPNNYDHVQHNGAKSHRAGHVS
GLSFFSSGHYLCSVSGLDGELLLWDLRSGCLLPSKFVAPGKSSAGSPNQRRTAIIAEGSG
YTDSCNKSSTIWISRRGVLHGFSTEGGTPKQTLKGHLTNITSLEAMEPGGKLLSGSADGM
ILAWGQPQGALEVGDATMKEIVQQDIDDW

>comp1898_c0_seq1; 466 1 1067 LEN=1256; translated
XAAAATTTAAPTSPHRRRHPTGSLSKRPRSGVVLLLIAILLGSTTHTMLPASARAAASAA
GRTALRRSSSSSAGGDKVTTIVFDVDDTLYDVASGFTAHRNGEVVWRFMVDRLGFPDTAT
ARAVRDEYFAVYHSTAKALTVARAEGRFPEGAPAFDPKDLSSYWVDHLDYGKLFGTRAHP
TEEAAKKAKAAFGAALAECPCELVAFSNGPRSYVLKVLDRLSLLELFGTGRSSRDGALVW
GVDDVLPHCKPEAAAFSAVFGELNAKRRDAGHHDEVVPEECVMVEDSMKNLRAAKALGMK
TVLITGTGEEGRILPGDAPSSEDPAVDAAFATVEEMVDGLPGLWRDPPVFEPPKG
>comp1899_c0_seq1; 401 111 1598 LEN=1718; translated
MTRCSTKRKRSAAFCTNGNSNHGKEGRAIVGNISFPPIATTKIASDSPLDEIKFSDWQSA
RVCSIREDNEPKHCQSSLLSFFAKAKRMKAAQSSPQKRLQKNDNDNSKSNSRGFWCSKEN
RASLVSSLPTAPSCSSSFRRQRNPSIGKTATEKRSQKSSKPKAKSLTQVYIDCGQSSFGQ
ILCDKCGMLYMPGIAEDELQHKQFCQAYTHGIPCVRGNVKGGKYVPDTGSVNALAAREKG
RKRNGAATVVLWRPGTKCHNRARSKQRQQHPEGFAVTTTKDAEISKSNHNKNLPSQWPLL
AQMISKDLGTNEETTLNHIANQMVFLYIESDNSKYLSTSQIHRASAPGRSRILGVATVQV
LSRRIPAYEMISLHERSLTSVEAKLGIGLLWTHPVARQRGIATRLVDAARGHAIFGMKVT
KTEVAYSSPTQAGYDFAIRYSCCRNEKNGIVSSRSDENDDCKGCDGEHVAIHPKEQSEAS
LCRAVKGPLVYEIDL
>comp1900_c0_seq1; 762 81 1637 LEN=1788; translated
MKFSVAATALALSSATTVSCFSIAPNGRSSLASLRVATSSEEAVDSSVNGDDDIVRKSDE
EVFSKHKRPTSFATKCASLYHPNPGAERGLAPPVYFGSTFLLDDAAHGARLHAKREEPYT
DDDGFVYSRWGAPTNEAAAVQIAALEGVDDPNKGALGKCLLFNSGMSAITSALMAVLRAG
DHAIFPYTVYGGTHEFAENFCKEWGIEVTYIDASGVDGPENYKKAFRENTKVVYTETPAN
PTMRLTDLEGVGRVVTEHYGAAEDTPGSDPNARKRPWVMVDGTFATPYHQRVLDIPGVDV
SIHSATKYLGGHSDILAGSVTSRSEPFLHGCAKVQKLITVPLNPMDSFLLARGLRTLDVR
MQRHGENAIKIARMLEEHPDVESVFYPGLDSHPDRSMSDGIYSSGRDEDDEDGFNQTYGG
MVTFIMAGEGDIALQRARNVCEHLRVINLAVSLGGVESLVEHPASMTHGMIPREQRLAGG
LADGLIRLSVGLEKAKDLIDDLKSALDRCDEEGCDLDI
>comp1905_c0_seq1; 238 235 805 LEN=1759; translated
MEFAAARELASTWAETGVIDPREERLLALLAGIPEDLRQTMMEVSIKCMPSKMDLADGVA
LVSALAAIGIINLRDNLRIDFEKHSKHTAPVWFYQMDDEGRVKRLRIGGSMIYFDGRNDE
DNFDLPSEIAHLDQLEELSVVNCRSLPIDIQHLTNLEILVFLRCDFNQQPFWQHGRVDEY
GLLELNSST
>comp1906_c0_seq1; 174 1 1067 LEN=1067; translated
XESSRRRTEHDDAALHKVASRKMYLRITLFIICMCRVVDAFTTRSSLCHYAVPSKCSATP
KESSSAFPKVRSIPPSSLFLSSENNENALYVPKGITLKIAFDSQGGVADLSSEKSERFTC
GESLDMVHRLRRVSDAVLVGRSTVEIDDCTLTVRRVPLLPLDKEPSNGTAPKAKKKQPFR
VVLDPNLDLELDQFKIFQDGLETIVIHALSDSDLVDAGSKSTSADEMGNSYFVKSTKKEF
PAVTFLGLQPIIDQDKLRLCTRQVRDILAEEFGIHHIMVEGGPNTALQFLRDKTVDRAIL
VYAPISFKDPLLSNISTSVLEQAGLEPIEGYKLGVDSLKCYSRPELSWPSTPIKSWX
>comp1908_c0_seq2; 200 155 595 LEN=985; translated
MIGDRTTAFQSIVLVSRPGRRNRGKFRTNQCHNHNFIHNCRDASLFASPSGKEDIDFNDE
LDDEKFNEVVSEQPIEFEWRVLQQMLGINIFTVILAALIVFFLSMNIILGPGWLGSTIGI
PGTGNIQEVSPTLPGSLDLNRPEYRL
>comp1913_c0_seq1; 196 1 506 LEN=550; translated
XPSSGPSSGPSSGPSSGHLVSEDDEDEENDSDCEDDSAFLFRGREGKNCNWVKKKARKFR
RFCRKRSNGISVADFCPMACGMCNEDDNDSDCKDDSAFLFRGREGKNCNWVKKKVRAFCN
KKQMASKSKSRVQLLAMHASFSTTMPMLKKMIQLTPNFFIYNLDLSHI
>comp1914_c0_seq1; 448 404 1810 LEN=2138; translated
MANQLNRHRGNNYRVHQFQTEQHKPRNRGSLLFLFFILITLGLYSTLSIVYIHRLTLYSR
PENAISEKTTHEDSSVASLKRKENDVSKSKGEGEKIESSSQKFSFKSRDRDHDKVLRSLQ
ELTRSPPPGSIVCPSPHMTPIYDRILFSTSPRSENTTAVSLGGADRKIPRIVHFSFNNRC
VPNDLATSIRLWSDALPDHSIFFHDDEAVKRLMEGDDRSNQSPLWHSSEHFPNLRDRMPC
VTSTGAMLIDVWRMLVVWTYGGIYTDIDNWPGPKFNSQTIREEDSFFSLSDGKNRCSQWL
FGMAPQHPIAIFTLQDLTRRLVLIKNVARPRVVRITGPMTLKTAFQKFKQLLKENATIFG
ESSYFTQIQKVDLATSDQYARGSLGGTFDEIVDHYYDEIHNTTYINITKRERAELISGIE
HWTKQVNRTSKVSANKTEGESLNTVDKNLPLPPYAGVSCSDYLDALNK
>comp1914_c0_seq2; 453 404 1828 LEN=2064; translated
MANQLNRHRGNNYRVHQFQTEQHKPRNRGSLLFLFFILITLGLYSTLSIVYIHRLTLYSR
PENAISEKTTHEDSSVASLKRKENDVSKSKGEGEKIESSSQKFSFKSRDRDHDKVLRSLQ
ELTRSPPPGSIVCPSPHMTPIYDRILFSTSPRSENTTAVSLGGADRKIPRIVHFSFNNRC

VPNDLATSIRLWSDALPDHSIFFHDDEAVKRLMEGDDRSNQSPLWHSSEHFPNLRDRMPC
VTSTGAMLIDVWRMLVVWTYGGIYTDIDNWPGPKFNSQTIREEDSFFSLSDGKNRCSQWL
FGMAPQHPIAIFTLQDLTRRLVLIKNVARPRVVRITGPMTLKTAFQKFKQLLKENATIFG
ESSYFTQIQKVDLATSDQYARGSLGGTFDEIVDHYYDEIHNTTYINITKRERAELISGIE
HWTKQVNRTSKVSANKTEGESLNTVDKNLPLPPYAGVSCSDYLDALNNFLSFHV
>comp1915_c0_seq1; 432 486 1489 LEN=1931; translated
MKAMYPKIMKKKKLRLLQGKTLREAPRNASDRQHQQPYRSLLDDEETTISNTWKSSGSNT
SEDVDANVEGGTEDIQEDCEEENEPKVSEQMPTERETKEIESVNNIVFTSDSIGQIEVSS
AQVAEEEEHETKVEECTDNRKSASWTPFLKKKIQATVKKSYTIVAGQYCEKNHTRAIAMT
KHRSKMWTEAMSGIRVGIKVDESSMTTLTAVEGDSDSSTASNGNTASYNSFFLLEESAAA
SQIENWLADHKEGNVVSSHVSKNTTGFEVQRLRAGHIIITPDEATDPMNIIKPQPYVSDT
SETILTNDGTNLLKTVECGATTIPIQSFAKLIIV
>comp1917_c0_seq1; 188 1135 1678 LEN=1678; minus strand; translated
MQASVGVLIGLLQKVGPVDKILMRIQKCTVKPTDFLVLTTTLSAAISIGNVLRNDLLASM
NHTTAPPQQGPNSFSCDHQYSFFSELCIECNVEVMIDLRERITNTIDSELTLEEKGASVV
IRHGFHEQLDGWKEQYECLEETLAEAAKDLYHKYGQQLDGLTVIFIPQVGYLVGLNETLV
LX
>comp1918_c0_seq1; 479 91 1362 LEN=1362; translated
MKPFGISCTRRSQLLVILSTLFVTLAGAFHTESLLRHPKLAASRQEKIIQSTTSVRPHVR
SNGTCDICKSFDTLALRAGGDDGALEKKPNKVSQIIQYCNKNYFLVGMFVAVSLAKALPA
LGKNGGILRPEVLIGNYGVALIFLLSGFSLQTSDLAKAISNVKLNGLVNLLLFGFWPFCI
GVPLRFLFTDIFPTLIPPALADGILITTTLPTTVNMCIMLTSTAGGNAASAICNAVISNL
AGIFLTPLLLMKFFGASIHLPFGQMVLKLCKKVLLPVTIGQLLRTNGQAKAFYKTHSKKF
KRLQETVLLGIVWNAFCNAFSDGLGIQLKHSLVLLALLPTLHIVTLGAFFKFFSLPFLGF
SKGETIAGMFCSSQKTLAFGLPLINTIFQGNPNLASFCAPLMFIHPLQMAIGSFLLPTIQ
KYTG
>comp1919_c0_seq1; 145 1 589 LEN=970; translated
XQQQQARYSNGSSTAGSSSAENHRASESTDCVCLGHTRNNSSCQKSSSNNNNHDHNHNHY
NENYSDEAIEELFHTEKDADGDSPRLVIWPELDIMCRHDKGKAFFSDSRNTEFESIADCG
HLFHSDGRIILEIAXGEAERMPARFFAVLADERARNGWNGSCSSLAQTNDSPKPSLRNDK
SMEQNQQSAGYVRVLN
>comp1920_c0_seq1; 120 144 923 LEN=923; minus strand; translated
MSVEGFAFFPAGHRSEPPPPRFLPERAPGAVVLALVPPLPRFGSRGRWQGPGPATPKKLF
MVLRSVIAHAIVRGQTGAXVLVGKAPADTLPGPSQGIELGPQRPRGLVVRGKPDPDDPPA
PDPGPDDLQGPLWLGPHPDRLLEGRQHVEDGAVHQRHLLAAGGEALAVAKAVRFPKGGPP
GGFHGLDGAGQVALLRFGGRCHVAVTEVPAGQDEPVRLVEVPRALVGEARPRVRPDDLHQ
SQGIQGGGLVFRLEDGPVKLX
>comp1923_c0_seq1; 286 40 798 LEN=946; translated
MRRGTKVRVKGLVKAAKHNGKIGIVTKATAPGEGQRVGVKLQDGSGVLAVKIENLEEIKS
SPRTTATVSKPKSASASTPASTLPKERTLKRDNALLREVDGSPDPNVLALYYHFADRAFD
CFNASEYNVQMLRYYEKGLSVRLICPRMIGGNEYFLVGLQHAQHDKNSLCEVAFNCGRSF
TGISMLVKKRCFVCHKPLPGNSQCPSCLCACFCTDSSCINRNSTIRSDHERLCKKIDLTK
VVVEKESLQLLQ
>comp1924_c0_seq1; 615 492 2292 LEN=2292; translated
MVNKMPGSNLNSSRSTGGAAAILSRKRLPASGAGEESNGVRTTNNESRTTTIGRGDGNSI
RDANTGIENNNSSSARSSTSASTNSNNGSTRKMRRGSFRRTLMLQRNKRSNNGSSSSHSK
REGNAVDGGATPATAVTNQLPVSKRNSTYHDQSSAPVPLKGSVGASVAFALEERETGACS
PTLTSSNVTTPVRGSQQRHERLPRGHIETIRKHYGSNREGVLDIYEDVLKISPDASDREI
RIAYFRRGREILGDNASSIKINKKNQKAIKQLDPAIKSRFQAVSMAYEILSTPAWKEAYR
KQGGILNTPGARASPLVPKVVVEQDGVGLSHPRITVGKKKQQEQEPFDPFVSYPLASRAS
FWENTNANTGNPSAMFPTTFSSDPVLPAPSTASRSPKRLVGALRKSKFLGNSGDVDNSNS
SRSRRQRPSSASVRWKDHVEELIFVNHPDEHGSSDEESSEEADYYSSDDENLRGDDSTGN
DAGNSRASSRPALPVAVDLYSEPADPGYNAQENNSNNSLSYSSRGSGSTRRRKKNKARIV
IDSEELESHLKLMDSEAEKHFVQDFWDNFEESMDGILTLVDSIGGGGGAAADISKASNSN
X
>comp1926_c0_seq1; 298 1 1328 LEN=1450; translated
XHNECCLNTLLTNTDENKKSLIMPIDTRILSAFGLVQMLLLGNSNGWTHPGTNTANISSC
YCGAIRRRWGQSNKMYSSSAENLQSICSYTHQKQRQSLRASTIAAAATVQNQPLAPGLPK
ELEKLNLFEPKPLEYNNDGDEVHGQTVERVSMDPPIFIFRKALTPKQCRGIRQTAFSMVE
MEQGKTNTCKDDNDEIHEEVSTASDQERKKEQHRKHPRRFSNVGWLSNEMPMVEQVARNG

HGVFLNGMPFHSTRGIEPLQVVHYYDVGGEYINHHDSNNRLLTLLYYLNGVGETWFPLAD
NHDVVVRDSREADLACRDKSPDRDGVLVTCRRKSSIIERGGDCGDLQLDSKGCSGPVVAH
VEEGDAVAFYNYNLDGSMNWKAFHSGLPITETDLESPDGENSASKDEDGVHKNDGGKWIA
NHFFHFVPSHVPETPPPTPVEK
>comp1927_c0_seq1; 383 106 2154 LEN=2332; translated
MEEDGIVVREISTIARDDISGEIDHEGSVTKSSFQQKPNSNPTTEAENTRKKPSIRHAEK
RRTSTCRFADRVAQLSVDHYKRTVPADRRPAQTCIATIVAHCRKHNGGRDAGEEHEDGVD
VNGTGTGEADVLWVLAMGIGTKFLPESRLQRERENEGQEGYGSRVRDMHAEVLARRAFRR
QLTLEIQEDLRLPAEEHERQKERPSNRVLVRSEDNSSTVCYRIRPGVTVHMYSSSAPCGN
ATLKRFCKMSKEKFRDDLGPDEWPSDAHDPPSGHSIKLGEFSLLVKQDSSSSSNPQTNEA
TSNSTTNSEEKNKNTSSKMDANMDYGSKPKRTRLAGDRPPALRTNISKASATSQGRKTVS
RRAKPWPATLSDDWTPPGTTIVGFRHKGSIHTCSDKICRWNYLGMQGSLLASLMEEPIYM
STLTVGRKLSGPVCRRAVCCRLDTRCRQPNLAIPLNNSIPLTGRTNSNKIQYRVNHPAIM
GTSVYLDTGVVDTSNVEERGQDVRFHSSFVWAWWAGSSQCGVNSNKIPNTDCYGILECIE
SSSGLLADLSAEVTAENDIDKGIRSTTSVPQVSTKSLTSAFLQVFQEASGNGSYTKNTAG
YASSAASYENSNDTKLTPEGVTPESWENDTNTLNTLRSVKKRFSPIHEEVKDRLFAEHRV
LSQWRRRCYQDICTEAINTMSS
>comp1934_c0_seq1; 288 1 1324 LEN=1397; translated
XTTTAMRSSTGTGGAGDEPPVTHVVVAGAGVIGVSTAYYLAKNFGVATTLVDPTGTIAPA
ASGKAGGFLALDWNDNSPALGPLARRSFALHEELADDLGADTISYRRLECVSVPVGENGG
RRPAGRKLEGIEWAEGANAMGDASVLGGTRTIAQVHPKKLCDAMWERVLELAPESKLVRG
RVVAPEDKDGDAGADAAVAARLEDGSLLEGDALLYSCGPWTATNMYGTKYHSLVVPTTRV
LHQCVFFSGLGDPEVYVRGDSTAYCTGFPDPPVRVAEHPGEEEVRPEAIDRIRESVRAAS
SFADGASTSYLVLGDDGDDGRQAPLEQACYLPGTADGIPMMGRVPGKERTYVCAGHTCWG
ILLGPASGEAMAHEIAAAXGERKLPALRGPGDVFPRAVRKAARAGPGFRSGAVTAERARR
RTTKRERGGFAGSPVRTSFTS
>comp1942_c0_seq2; 273 533 1342 LEN=1342; translated
MRQPQQIWGGSQGKKRRMKCTDTSLPYYSSLSLWHPLDFTQSTVPIGITLERHCHNNPHE
PIIGGNAFHNLNFEVNVHGIHLASPGERLHMHQLRCAKRAAETFHNVFLSNSKKLLDDGM
DLIAGYYGASLQRQSNRDFPQTNFSESIHTAKKEGNHFSGMLLLQILYLLLKDGGALLRP
YYDRQEIDGRIYDLELILGMEEFLKWSGTPCKIFHLQQNINGRNLNNAFKFPKGRTGANN
LDKMVVHFINNINNHLGRNEGNSKNLIKNH
>comp1945_c0_seq1; 216 106 618 LEN=996; translated
MTTPIHGKPTEGMCCLCTMEDITEENGNYVEYQTYPSLTWHPCGYERSTVEHLLKTQFGA
YLERVKTTNCQAELRRLLRDGPPVWLTDPHALKLPGAGEDKETTTITEATTTTPATDTHV
CSLWYAGDNTVVSAKLNGAVEGGDRENLWNELKEFLVEEGKEPGDDDDNN
>comp1946_c0_seq1; 116 139 642 LEN=714; translated
MGKKSKRPNRRQPPPATAGAVSPAAATAPAPATATASPPVYFYPSDGPSDVELLQTTSRS
LQAKLDLLTRLGGENDRAGFVREFVPLDLPGPDAAGYLEDLTTAPEAEGQWRNLVAEIAA
IRCGRGVEKIGGDQVSRAVFHFEHPLLEKCDREVAFCCVGGEWRAEG
>comp1950_c0_seq1; 68 1 319 LEN=765; translated
XMYWSHAGKHVGIEPAGPFWASVPRPSMEEFFARTQNPSECARILREDFCTEEFGDRRQE
VVFIGVGLRRSEIEEALDGCLLTDGEMDDYRGHLEEKNRLLAKPAA
>comp1951_c0_seq1; 502 1 1189 LEN=1282; translated
XTMQATVTRFVFATAVIAAGCNAFVPITTTLSSARQGTTKDGDDVLLRASEEDSSWSSTS
TDVSGNAVIERTILPDGVILNEKLVLRDITPEERGKGGVEVGQGGKIKALEVLARIPRDL
IISTLDVPDRAIEAASGARNLTWATDLTAASLSALHPIEEELAASASVQARKEWVQSWQA
GGWATDGADLGPPDANFGSKDVTGSLLSTGTDNDHNIYAKFRMPCHPVLLRASLGLQVLT
KCTEEKAREALTARGFTYRSMRDALEPLVLESTERAKGSKREKRCWDVADTLSRVLSRAT
TLQLEETNDSEELSLAYAIVPLHEQLEHSVKENSKLVSNNNGDEILLVATRDIGEGEAIT
RDYNSSPKLLNDKSEGALHLLLQFGLPPKAWPNSPN
>comp1952_c0_seq1; 406 1 1829 LEN=2187; translated
XDATIPSLAVKKRPHNADVEISTSPSGSSLEYSMSSSFSSGEDLGDLEDVPMLSEFDTLQ
SNDRSNRNSRDLVQRRRASFAPRYRPAIAFIALAFSSYIYIAITSQSVKDPVKTAVSESV
VDRHKDNKIHGNATTKEEESSSLRKFHSHSTSPVLVSTPRRSPNPSALPAPEPTLKPSPN
PSVPSVPEPTQKPSPNPSEPPVPEPTQKPSPNPSLGLPTEPPSSHPISKPNVASPLNNRG
GKNVEAQRRKKNLVLHVGPQKTGSTTLQDAWAPYGILGQPLKNDNYLYAFVHRNSGYFNC
DQNKWGGLENCKSSPRLKRLLQTAKNNGRNLLLSDENLGDRFSPALRDVIDDNDWDVTVI
VVYRRIHQWLVSWYNQINKTTNKDSNGNILFDKNGNAYRKEHTVWPDKGGSHIPAFSTWY
KEFTQNWDPSEFVEKHRTIEFINAYNSSFSNVVVHNMHEDGDMVKNFMCNSVPDAHHCCN

RLKYDSNRSPRDKNMSVNLNIDILAVKAREIGAMKKSISRKRAVSEIERYTKVFNKRIPL
KCDNDVISEIKNWLLESEKAVFHETWSDQQTSDLEDTFDAYLPSGKLCDVDFENIFNDEQ
WVGFFKSLG
>comp1956_c0_seq1; 227 304 1083 LEN=1261; translated
MLVTIAILDSLLDSDPESVLFMLYIIYSILGSFLNSRAVPEASAPHLLDQVRAAETGIAG
LPIEGVPVPPSPCGRTLGETAATCGWAVAWGAYKNNHYHINYHHHYHHHGDRPFSSPRTT
LRRYLGTTVRRAAFLAWSSAYGRWVDLREEAAAFHRSAVEKAAGEGGLFGHWGAAKGRFC
ESLCQGIEPLAATQRYALSLAQTAATAATLAASLGAAGIEHCLRVGAGAGAGENQNEREN
QNQDERGSTPRSETQTADL
>comp1957_c0_seq1; 299 1 985 LEN=985; translated
XDSDGGGKPSAVPATAAAAPARGLSLPPAASPAATTATTTATATATTAAKKGSTRADRTK
RVIGRKVLKLSAVLPEHILNQLQLAGGGGSSGRKNRKSGDGTNSDSDSDSSEDNPDTDSS
DDEDTGAQSRKTPGSRRKKGANPGANPGSTNAPPRDFSIDKGLMGLLKELSKSGSSSQPK
NKKILGGGAEESSGSATTGDENPTPETEPKPTQKPKPKSEPLGAAFLATTVETTTRKRRQ
PVAVRNIHDAEDSAVKPDASVHASASASASPRTPSVSVPRPSVAPPPPQPRARAASSMGR
SAAPQVRGSSSTSYNSGSHPASHYRIQQX
>comp1960_c0_seq1; 288 118 1070 LEN=1070; translated
MSRRNQDQGGSKPSIAFHMIRHAESNNNKVYGDAKRLYRYGSDDYDSEGFRRYVDAHRRA
DPHLSPLGVEQADALASEYLNPALFDCQASSPVRILCSPMLRTLLTIRPALQRLVVSSSQ
TAGSGGSGFRQKVHMVVVAHYHEIEGCHDKGVAQPGMSPNEIRDCLFRDLLCSSGGKSSS
ESNDTMPISQSDIDEIVTLEFVGFDHPHEGWYGGTGHEARVDAEARAAKYCLWLTDYLDS
LMYGNGDGDPDETGDSNNNGDLFDVGFLLPGEELEDQHDRFARPLRRRRTVLAIGHGDFM
SVVLKRLVGGYVGCGRGG
>comp1964_c0_seq1; 427 138 1799 LEN=1992; translated
METSCEWNGRVKNVVRRSGCDGVLNKLKGYEDHLSSVAMSIERHENSRRNASQNKQNESE
GSGNWTERFISKTNTNPTTTSTIISQLNDYHRHGALAVIDARRDTNWNGRNKVTPPSGAL
KSPWEKQTCESLSDTNLQRKHPGDPPPLQLSGSNPAFTGTISRSSMSVATTQNSLLSLPG
KKGVPHIYHDYSNVPDTIGVVRKKTGGVTQPFPEKLHLMLDNDDDPSIVSWLPHGRAFLV
RKPAEFTAQIMPKYFRQTKLTSFQRQLNLYGFRRLTQGADAGAYYHELFLRGRPQLCLRM
QRQKIKGTGHKQPADVQTEPNFYTMPLPKRYEQTKKKEKEQTDEVSKVAKVRTVSHSGNS
MLKDSSFGNSSFAHLSPGSRGVHGAAHLLNGMKSTICSMKGRSLVDLKSLLGKSAISSTV
SVSEMNVQQSNRTSSPTSSLSTTQSSHHTSSPILTANYELYVPTQRSMSLLGRVQQFSKP
KERKRSSDMAEAPGEYLWPATRKSTIGSSGHTIATFGSSSITYSSSPPPTYLHLDFEDSS
KQGGIDQPEKHQV
>comp1969_c0_seq1; 850 64 1461 LEN=1803; translated
MAMKKQKAGKGKKTKHEEIVEEDEDESDDQSEADESDSNDDDDSDSESGSDGDEDVAASL
FKGANEEEESENDDDEDDTDDEDQPASALPSEPYTFDLRNMLAINTDQLEVGSLHKSSKK
KKSDAGGNEEIPLANAAFEVNEEYLLSKATDGCTQLIEALWKLPTEASDAGPLVTLPTYD
EVRLPRAMPPPPPKQETKWEKFAKAKGIPLNKEKRSRKVWDEGTGTWMFRHGYQKANAKE
GEWPIMEVGANDDPFDDPWEKQREEKRARLDRNMERRLKNEERAGNLTKGATNRTMKARE
QAYKAGKEAGRKDSRSTPVGLPIDLKNSNDPRKAADNKLRGKSSTLAALSAVQRSTASLG
KFDKMREGEPERKKVLSKTKKRKYENPTDKKVLQSEGDSSMKILNAVMNGGGAAKAKAIR
KGQLAKGETAYDYEFNDGLGASSFRKKKGRAGVGKMKKMTKKRAK
>comp1972_c0_seq1; 211 181 1014 LEN=1014; minus strand; translated
MSPITPSPLSGWLGSTLRCSKCKHVRPIQNAPFLDIPLVPTSVPNYLSKHMDPQQNLFLP
ISHTSLLARWKIASRILHVSNGSKMLNAGPVRFDXEIANLEEEALMLQGAVGTTERRRNK
RMGGSNKRTSELEPFEQTKCLRDDLIKVEKRLFRLKTMDPDDDSVDPSLAPFVSEVDNMI
LFDSSDHDENISIQRFEAKKCLLLTRSPSILCCHVQRRYYDPFTNRMEKCVQFVEFPLIL
DLSPYCAYGQKSITPWAAGSCMKDVRTNERRPPWNKRNX
>comp1974_c0_seq1; 294 96 1197 LEN=1436; translated
MGRLSRDKRDVFYRRAKEKGYRARSAFKLLQLDSEFNLFDGVTRAVDLCAAPGSWSQVLS
DKLVVSKRNSAPDETKTEETSNATIVAVDLQPMAPIDGVLCIQGDITSYETAQAILKPFG
CGSEGGTKDGGETRAELVICDGAPDVTGLHDLDEYLQAQLLISAVKITTHVLVDGGTFVA
KIFRGRHASQLYSQLRLLFDRVSIAKPTSSRNSSLESFVVCQQFNKRGSADLRNLSLDRD
TVLDVVEGSPSLPKCIPPFLTCGDLSGWGEAPGTIMDADKSYPMEEEDYVQPIHPPIAPP
HMQAIEEKRYNARNRDDEFIIQFFVRDLTWDYERCSAASSNGSGATREMLHGTATTTHEI
WYKRSE
>comp1975_c0_seq1; 418 1 1169 LEN=1360; translated
XLLCTVQYLARKGKSSSVPTDCKETTNQVPYTENCRARHWQEANRRTQLYGVNDTDIEYA
ETLSEHNNNSSNNDKADDGNDYDEDDDNFAMAKAKTLVRMVSWIVLALGIATAPRGVRSL

SPGSTDPLPLGSTGTASEPTAVSPEPIPTRRRLLRAAAMAAAAGLSGTRSADAACLTGDT
RPVCIGIYKVPQDAAFSNYISTPEQLKEFAPDLTYVPPTPAPSSYKQALEILEGQRVVAE
GIRRNVAAGRLEDAGVGVLGLIPQVTASGRFVVDELSARVYRKQQAAKKLAAAPPPPADG
EEPEAVREYDENGYKLPTTADELAVDMASNQMDYVAGYFGECDICIGQGIRGELGVSAVA
QLTILASLKDATIALDDFVATAASLGARM
>comp1976_c0_seq1; 475 1 971 LEN=1137; translated
XNEQDRTSIHEAMEQQSISVSKAGIVTSLQARCSVIAAANPIGGRYDSSNTLSDNVELTA
PILQRFDILCVLQDTVDPVLDERLAKFVTSSHMSAVATRDLNNGEATLPPSTSVAQQQEG
IIDQELLRKYIQYARTNVKPTLRGNAFDQEKIASLYVALRRESKNSGGVPIAVRHVESIM
RMSEAHAKMHLRDYVRDDDVDAAIKMMLESFIVAQKFSIRRSLRRSFAKYITSGEDRAHL
LLHILGDMMRKEQMYQVIRLRQKGQSEDLLGRLEVPVDELEARARERRIYNVTDFCKGPS
FQEAGYFLDEKRGLISRSAVIST
>comp1978_c0_seq1; 306 234 854 LEN=873; translated
MEEVGTARGQFFLWFFGSSGGGGIALSSFPRMYEQVAYIQSLKGVGPSLGGDTLGISPLC
GYPQDIAVKDVEKVANSKLTIDQMVEKFPQEGNYLASKGYITFSAFEQANEGCNPLAIRA
VFDTFSQSTNISDPRVAQEKLDLYKQDPFAINGALFKAKVTGFSAVFTLLVLLGLAESIA
FGHAKDGWFHDWTWSDGILNIPDFWI
>comp1981_c0_seq1; 139 1 937 LEN=1497; minus strand; translated
XEKLMFSEDGGHLPWSNSTANAKSTPTEPAMHLISRFVDDFLFISPDKAAFQGFLDRTHE
GITELGARVNSKKTLVNVRSFVQRNADDGRTQSISLSVSSKLNRSGSVLFPWCGFLFDTE
TGEVFCDYERFQGGKLRDGITIDYDGNEGRKMEFRVRTFVYPRCLPILYDSSINTFETSV
GNFYQMMLFAACKTAEYLRGHITHNVDHVVRCIRGLPQYAVWSIRCRFEKSPGTTQDFGA
VFAINGHLASVLSWKAFCDVFSYLQGFAVITRELREEASSSSRTMTVRKRQQVQRIVSRA
FEDFQIRHMIEK
>comp1985_c0_seq2; 383 1 1031 LEN=1066; translated
XLVKIDRAILRIPTLAIHLETAEERKAFKINKENHLSPILAQAAQKALSGDTDDSESGDN
GKVSSKKDDEDGWTEYQEPLLLQLLSSELGIPIDDIVDFELSLFDIQKASLGGIHSEFVH
SARLDNLASCFIAVQALMDCAQQDGFLENDEDVSMVVLYDHEEIGSTSAVGAASPILGEA
VKRISSVLNEEEQAVAPHDLYEATVRHSFVLSSDQAHALHPNYASKHEKNHQPQMNAGMV
IKRNSNQRYATTAATGILMREIARRSNLPPVQEFIVRQDCGCGSTIGPLISSSTGIRAID
MGCPQLSMHSIRETMGVCDLSNGLELFKAFFMKFREVDKSLEK
>comp1993_c0_seq2; 112 612 819 LEN=819; minus strand; translated
MLLSQMPIPVRVELFLPNRTNKKKKKPPNGAVRANKDDRGLVDRIRLFEEHGITSFNIVN
KDKTDKDSVX
>comp1995_c0_seq1; 608 178 1205 LEN=1205; translated
MCGPTNRLPPVQDGPAVDARSSSGKPPLPNPATPTTGHQAPPGSSKRSSAVPSGILHVSP
AVAAAALAAFVSVAPSYLYVNRGFTVWFHWASLACVLTRSHFLAQALVLCGTSICLGWYA
CLVHEYAVHGRVFDALHKNMPGALVDRMLLPGGGGGESGGSPNGVLDFESTGSLLAMAVS
HVLDFLXPSVPDLLFLEKAPPAPKPIQCWRQQQKQQQQEEEEVDPEEEEESDDGDDDDEL
NGSYDDDDYNEEFEEKDEDGSHEEDEERYKSEEGGDENVEQTIQNQESPKTEPLEEDEET
EIEEDEDDDDDYLEDEDEPDNPWGIKPIDDKEELRLQQQQQQQ
>comp2005_c0_seq1; 422 1 691 LEN=850; translated
XMHDVLDAQYVYDTTKDESYLRRVVMPLEVLLTKYKRIVVKDSAVNAICYGAKFMIPGLL
RFADGIELNDEVVLMTTKGEAIAVAIAQMTTSVMATVDHGVVAKIKRVIMERDVYPRRWG
LGPMAQRKKSMIKEGKLDKHGKPNDRTPSDFLTSYKDYSKSKPPQLSGEGSTPAAAGVPS
SPSGDVSMDGGAGKRAKESDGNSSDEQPAKKKEKKEKKEKKEKKKKKKE
>comp2006_c0_seq1; 229 90 882 LEN=882; minus strand; translated
MNSEFVPIYQSVSGCLSLSNSLLLLSVGGAIFFSPRKALSVLLDYKNSYYNHSYRLESHV
IDLSDLELIRGSLNHEDSIGSACDMMAAVILAQGLGCIMMLYPLISRGSSSALAIWNLRT
SITIQLITGLLWIVSALYDDRGNEAVLVNKKKYVGFYSQAHGDPIVGAVQRRTTFGLFLI
GFVMFITACLGMMISFWPVATAENTRLHSERSEMSRERTRHQESVDGNEDLTAPLLPTAQ
NFENTSQTSLQYEEDNSDQLQASNX
>comp2007_c0_seq1; 175 310 697 LEN=697; minus strand; translated
MRRAVPVHRNTATAAIRIPRVVTASETAFGPFGATAESVPLAAHRHFGRLVQSVDGPNHF
VDGHVDAFREARPAIALPGRQEVVLEEEPLPEQPLADGQPVLFLLLVKARPDPDVLRVVV
VVVVVVAVV
>comp2008_c0_seq1; 429 160 1212 LEN=1263; translated
MIRHTLFLLLLGIPAICYAFVSPVPQSHRSIASGLVGLNVAAPLEQDTREDEQQLREREE
KRQKRKLRKEREKQERNSPVGKLKFKLLKQLKAVAKDYEMLQDGDHIMVCVSGGKDSAVL
LVLLMHLQEQLKPIGVDFKLTAVHVDQKQPGYDGEPLVKWFDELGVDYEILEEDTYSVVV

DKTAAGKSFCTMCSRLRRGILYSKALELGCNKLVLGHHADDSLETLVLNMLHAGELKAMP
ARYTSRRGKLAVMRPLITCMEDDIEEYAMLSKFPILPCNLCKNQNGLHRPQVKLLLSTLD
SFTPDAKKNWLTSLSNVQPSHLLDQNLRKAVGMDPITGEEEEETEEQNVP
>comp2009_c0_seq1; 1614 335 4291 LEN=4361; translated
MAQYSKPSVSVRRRTRQNRIMPTVRGFFLFTIALISRFGYCSSKKDDLQPYHQLVPRVHA
GVEQDFHELMSEMHQAEDSGSSVERMMQSKEDLAFCYDALYLADMNNDNMVNRDEFVTFA
QIMGPTGFLPNANVFQDLPLILQSNFIILSCLCLQMPTFDGGNVVDPQCCAVNPHIDVDG
SQPGEIPTSQQSQYLYQVCFLTETSIERLLLSETPTTEPTKKIIPTSPPSMAPTNTPTNE
PSDEPTNTPTVEPTAEPTEMPTDTPSAHPTAEPTGTPTAEPTSKPTDEPSPELSDQPTDT
PTKEPTEKPSGEPSGEPTKTLTDEPTISPTVSPTLFPTTSEPSMRPTTSPTEKVEPTVSP
TVQDINVTPVPTQSPTVTSVPTPSSVVEVAIAKYGIAIADGKTEEIPQSSYESDLIESMN
IVAEDVADDLNSSLNEGDRLLQEQRESGTALRRSLQQNHRKLESSLAVKLPTVIDDIAGS
ECPSFVPPNDECKAVTASVELDIAAVGINSSTDIKNLFEERLNSAIENGALQAALVEVNP
NSVVSIINGAAPGPPTPTASPVTLTPTTVATVEPVPISPGGIAGLVLAGIGGLIIGFLII
TYLRRRGATDGGVNAVGAYAVPAEESADVEEALLKNTEAQEIDLGSREDSSFDKDEKYET
LHSQMPTIVDSGPPMVDTGTVATAAFIGTGGALAAAAALESNDNQASLDTSPGKKKRASG
NLYGSDEASSAGESGWSSNQDGSSANTSMDASLDSIPQSAISGVSPVVLGDIAAVGRDSE
TVDQASPSPASPERLQVSTEEEQVNYVSQSLSPTNTISPENSRSAQSQTNSQENQSYTGT
TGTEGSIQSTYSELDEAIQKGDWAAVGVTAALLASQAYGEESTQGSSKNSSVRFIRKDAP
LDPERAAELDLLVESGDWEGVVAAAAKFDAQEALRGDPQSPQNSAGSAVSVSSAAGSNNS
SALSGSVDGSSNGTIPSNMSGSQTIDTTTSPSTYTATGLTATSDTASTRSKARKLNEIRD
EVEALVNAVVPEEAENIDEMMTQFRGREEELVETLRSMQERLVAQKARKESQKQAKRQAK
EYVQDKKKQESAGDTDVATDAADDMWIKEIEGSSGAADALAGTSVARGEALETPFEVDED
AEAKAMKTQLKEAISNEDWENVAEAAAGLSSHAFKKPGSSQDSRSEASNRSVDINALVDE
GDWDGVVAAASKYREAASTPGSSSNAETDIPDDSTIERRRKRREERLKEEEEALAQAQIW
DAIADQTKVDNKAEGSAAGLAADWAIDQSLAALKKAEEEGDEDSQESDEDQRNDNESL
>comp2010_c0_seq1; 200 1 1091 LEN=1603; translated
RVRVRIHPNPIPHCCTTAEQSNSNAASDREHREKRHKRKNLEASIMTGLAAAAPSVVLGG
AITGSGSSFAAAGASLALAVFRGHRDRGGGDDPFLHGPPHRRGRQGWRSENTEPRARDTA
GSNGNNHRNPLVGVYHAWLHLPQVFRFFVAGNLGNLGFFYLEKVFFQCLSHILIASTEVY
SFLLDGIEKYQDGLSFFSAYLVQIVTTHLLYAFLVYGMDTIDTYEKYCLTLWGQFKVYGF
GLVGATGLNSFLIGRGFEKFAAFWITTASFAVINYVLVSRVVSGVVEASEAAPPGDAGAS
PGPWGKRRKQKQRQRKSLGFAERVGRGGGRHPQPPVAASAPLATASGXAGMPGGGVQLLL
FEL
>comp2011_c0_seq1; 468 336 1946 LEN=2097; translated
MTETTKTTLLAGQQTKLTKNGEQVLRVLESRSKGDLPMFVCEEIQNSIVALMIANGFLDK
WKTAFMSVSTRTNSAIQDEDVLSLLSSILEDEPECRDSEIAATMTRLLISRETVIQEGIN
DIAESFLANLENTVHQLLCDEFNPERADRGFYLDWFDEAKITAAIRLVPSVLSTKWNGAY
PIHWQTWKLQETSDEDATSYSLKSLSLVPMLAKLGAELGCRFRDEIEENNNDSYNNDSYL
AMRGGLLSKDLDGLNVLQNLCLYPWVCFSHDEEHQKLCDETTSRTLQTLKEQQLLTKEDI
RDHDLVWNLFCLSTTRGETDRRNHSYFPEQRFRFFSDWDPTTLATPIGGSVPLFGACTKS
YDATKPRLPIHWSMGFSNESMRAFSIVLKAGMKHYPEKIGFVFANEDGPYSCGYRNSDNG
TKMNRRRAKRRKRHNVPYFEACETYGAHRVKTEVLDRLTTTTAATDTNRSNDKQLSPEGF
LLSAMTDETMDRSGLHLLLQHFSGALSQILTKNERISKRAALETISNTKVGQVQSK
>comp2012_c0_seq1; 580 242 2623 LEN=2873; translated
MDEEVVCPLCCEELDISDRQFFPCKCGYQVCMWCWHRIRESESGLCPACRTPYGDDPHEF
SAVDVEDVLKANKEKEAAAKKERDRQRHQSNEGYISEVEGLAGGRQVGQMEIPKDRTQLA
NMRVIRRNLVYAVGLPPNISTEEQLRKPEYFGQYGKIAKIVINRSQTTSGGDARRASASA
YVTLVHKEDTLACILALDGFYMDGRNVRSSYGTSKYCSAFIKNVRCNNPECTYLHNMGLI
EDTFTKQEIQAGYVTSGRDVLARQQQIVQQALSAASGAAGTTPSYDEAVKNPTTSLVPTP
PTITHVRSAAAAAASVQTIGSGFPSISAAGAGSSSSASSNHMNAASRVASSSVISASKNI
GPAGALSSASKSAVASLPMSQTARKNMPGSINGTNSGSMSLAPAGATAASVVAGVHSVSS
HSEPPAPHTTLTPLTPLKRSTTASKGAPKTNSASDVQKYSNSRTSTKKTSGLKSASSQPA
ASNPEVGISSIRNNVPGQLEGIGGDVISAPVSVPPPINRELASTNGIPRGPQLKFGGDGL
GGLGGEVFDGPLLSSSNTRSAIGSGKGKWDSLNDAGFPGNTKAQSWGNGSQNALPGANRD
SIGGGVIGGGPIGNFHGSGSSALASMLGINLPTGSGSLRETSSQLWSSEPGRQSSMSSFN
NDSLPLQGVIGGATKASNNPIGGVPIGGMNSQIPANPNVSNSSDIALLQSLLPGVHITSG
GNYQGSGFGSIGSSNNNPGRGPSHHKNGGSASLASGDTHQGFGLQQSLGGQPIGTIGQNQ
GHGKQRQAPGSIW
>comp2012_c0_seq2; 624 242 2695 LEN=2945; translated

MDEEVVCPLCCEELDISDRQFFPCKCGYQVCMWCWHRIRESESGLCPACRTPYGDDPHEF
SAVDVEDVLKANKEKEAAAKKERDRQRHQSNEGYISEVEGLAGGRQVGQMEIPKDRTQLA
NMRVIRRNLVYAVGLPPNISTEEQLRKPEYFGQYGKIAKIVINRSQTTSGGDARRASASA
YVTLVHKEDTLACILALDGFYMDGRNVRSSYGTSKYCSAFIKNVRCNNPECTYLHNMGLI
EDTFTKQEIQAGYVTSGRDVLARQQQIVQQALSAASGAAGTTPRRRTGGGGPSGTGKASS
NPIFPPPSYDEAVKNPTTSLVPTPPTITHVRSAAAAAASVQTIGSGFPSISAAGAGSSSS
ASSNHMNAASRVASSSVISASKNIGPAGALSSASKSAVASLPMSQTARKNMPGSINGTNS
GSMSLAPAGATAASVVAGVHSVSSHSEPPAPHTTLTPLTPLKRSTTASKGAPKTNSASDV
QKYSNSRTSTKKTSGLKSASSQPAASNPEVGISSIRNNVPGQLEGIGGDVISAPVSVPPP
INRELASTNGIPRGPQLKFGGDGLGGLGGEVFDGPLLSSSNTRSAIGSGKGKWDSLNDAG
FPGNTKAQSWGNGSQNALPGANRDSIGGGVIGGGPIGNFHGSGSSALASMLGINLPTGSG
SLRETSSQLWSSEPGRQSSMSSFNNDSLPLQGVIGGATKASNNPIGGVPIGGMNSQIPAN
PNVSNSSDIALLQSLLPGVHITSGGNYQGSGFGSIGSSNNNPGRGPSHHKNGGSASLASG
DTHQGFGLQQSLGGQPIGTIGQNQGHGKQRQAPGSIW
>comp2013_c0_seq1; 345 81 965 LEN=1415; translated
MSTSTKTKTNTFPWVLHEMLETAHEKGFDGIVSWLKDGKSFKVHDPASFVEKIMRKFFQQ
SKYKSFQRQLNLWGFQRIAYGPNKGGYYHPEGFFIREKRCKIHMMQRQRIKGCVLKDKSN
KNAKIDDDHVEKGNITEIDNTVIEASGAASIISQGSLVDINNDALNVGRDSAEPFLPPLK
SLQSSISDEESSSLLQLELKDEFSIDPEVGDCIGFAGRSFFFTEEEAVENTPCTVSSSMT
LARRLHPQQRVALPPVSVLLPNTYLCTANMMSQISRRRVSIGIFGAACKPTTGC
>comp2013_c0_seq2; 277 493 1068 LEN=1518; translated
MMQRQRIKGCVLKDKSNKNAKIDDDHVEKGNITEIDNTVIEASGAASIISQGSLVDINND
ALNVGRDSAEPFLPPLKSLQSSISDEESSSLLQLELKDEFSIDPEVGDCIGFAGRSFFFT
EEEAVENTPCTVSSSMTLARRLHPQQRVALPPVSVLLPNTYLCTANMMSQISRRRVSIGI
FGAACKPTTGC
>comp2014_c0_seq1; 996 212 2227 LEN=2407; translated
MGLFGKKKSKASKEQASSYSPPDVVSSSTEVEAVVPGEDGVMVSGNGSVTSLGSDDSKKE
YLIDGEEEIESNTTDQIMEFVNKTKATAISYKDKGMEQITVISATVLAWWFSLSKVTQVA
LLAGAAIGAVTIVVAAAAGGRSPAFPSHINVAFAGNSYFYANDLPRFVESIGNGHVTQDS
VVHNSASILELVMTGNGMWNKWSTQNGMINGAKFDTYRGTTEYLYDMGACSVPQLLTGSD
QMITPGNQLGAFVNDGQNPCFQQDAYREYKDSVTLKHGWDFVVITDQPKAMAFDESRHDA
LMAFNYTYGPILKKKHISPIIVQPHAYSSSGVNSASTNDLATFTALIMEGAQIYRKYLNK
RIGLFAKAHVAPVGNAYLAIYEESPNDLWPKLFLDDGVHPSAYGTFVYGCVIYATMTGYM
PKYKRVVVDDMENSALFASARRVQSANSAAGFPTKDEAATLYKIVKKVAISGYKPKALRG
FKIQEDAADFLDSTSSNVYDGSYASGNSYASNNVYNEYDSYNQYNGYQNNAVNNNDYNVN
NQYDNYQYDNYQENNGYNNNANGNYQENNGYDNNENYNGDYNNGNNYQVNNNYEDYQENA
NGNYNNANQQNMDADADANENNNNGQYEAAQQYYNARSYQNHNSNNVNGQYGGGNQNKQF
YYNDLKDNDGN
>comp2015_c0_seq1; 365 285 1217 LEN=1362; translated
MAISTELKSDVKITGKAPYKKRLEGINASPALYDLWKSAYSTHVTTKKDSSVEDSFRASA
TPSTKTEAIDVLRNAGDPSKIKRKKNKPAVVPIRVLQSIRLLLPLAGEESSSLDSALSSS
ASLQLAFTPPPPAPELTEERKKYLARMEKLRLKAEETKYTRITTNIKDQRQDDDKTAKSM
TYAASVGINMIVAPISFGAFIYFFSGGVFDYLFPREEGDDEFDKNPTGVDIKRVILAVIS
GVGMMIIEMVLFVIRSHEFETHTTKKKKKRGVQPFGSYSANSAMTYTDGNLDRKINKTID
REDPAVKKNK
>comp2017_c0_seq1; 556 259 1539 LEN=1630; translated
MSGKGAYRSMGTGNDGGRVTKKVGLRLRLMGLFRKKGGVSSDEKKLIHHESASIDDSTAC
ESPSKESDDSCSEQFDVYFASSFLPHVDTNGKKKAPIGTKKKSVHWKAFEDAKEMAIPPS
PPKLVRTRKQPLPNGYATNKAHKTESTPTSPRNSSQNEVQVPCVPLENDYNNLTTDVQAP
PEEIVAEKANESPSNALNTTAELLSFDDDLIDTVDKSQADFNKMLDRMSSESKSIRMMNP
FSDETVLMSLPEAGTAESESMISKPMGGIVKYFSKLKAAVGETSNPSHDGEGDTCTYGTT
ATSVYPELLKTKMDSIAKAFCSVELESTAKNSELPTNHLCTFIECTDPTNDIPMGTLPEH
LHAPLNLNSPLNRSWFSLNEDSDSISKLSMPIGTNDMDLSVTSEMSPTPLMEKEPIAGFS
HGETHL
>comp2018_c0_seq1; 382 151 1407 LEN=1527; translated
MTVTCNSFACLFLLCLVATAKVSSSSQRATDADSHDTMDVPTGALHNGKKIPLVGLGCAS
GVRREHVSRALSIGYRFLDTAQSYNWGYHEDEVGTAAKKFMDDNIDEEIFIQTKVHPEDL
GYEATKRAVYQSLERLQVRTIDSVLIHKPHCWTGACTKEPEGTWQESWKALEELYDDGII
AHSIGICDVSDDLLDELLSQRIKPHIIQNWMDPFHQDKRMRDRIQSHGILYQAYSSLGTQ
WHHHKGHSKNPVMNHPVLQEIAEAHGDVGADVGQVVVNWATTRHGISVLPASTNPGRQEG

NLRNSFRFSLTDEELDRIDALDGRVPKAKSSNEVSITFTSDGDTASGTVDVYWIGNDGIE
EVPVGSVESGRSLFLNSYHGHVFRFKDKENNVYSDHTIERQAGIQQVHVITPIDDEEL
>comp2019_c0_seq1; 301 118 1569 LEN=1614; translated
MATAPRTTQQYPIAGPTAAGGTNEVATAIPALKVMRLQKPELDMTKAGTLKPSGCLLGSS
LCLPDSFGIIHVGETFTAYLGALNVSKRLPVTNLTITAQLQTPSQRWHMASKTLDECNAA
GGCELLPGQGIDSIVSHALEEAGQHILRVEVGYGGNGGPMKSLRKFYRFQVSNPVVMSEL
TYRTGDSSCFVSISLQNNGHESKGGLTVSSAEFEAAPGLVVEKAIACAPKTDDTNKIGAP
TNHLGEGLDMFDNSGRLEPGDSKRYIFKVGVSKADSASGASVKGIAAGDQLGKAIFTWRK
ACGEMGRMASSPVVCPMLHPNLDPNDPGTTMQGTGTDFVVHVQGSSLSIDVATHAAGRTT
NSPRDGTTLDLLLPLTVEPVNPCKQLRVGVPVDFAFLVVNHSPEFVTPQLQFRLEHMKGI
AVCGPSFKNLEEIPGNGGSTTVLVRFIALSTGLAQLTGCCVLNLATGHVIAQPPLMNIMV
IEE
>comp2020_c0_seq1; 612 90 1589 LEN=1936; translated
MIATKQKRGLASPLKKATACILFLAIAAQPSFATRFGVLESLPRTRKQAFLSKKIADAPS
WKTIPRGGDIQEDVSSPTATDDDCAFAVVASAIKGLASFMKGPKADTMLLLSTTALNTPL
SKMIGLSPILGYLALGVLFGPNGLSLVKDIHTTELLADIGIVFFLFEMGIHLDFKTLMSM
KRDVFGLGGCQFLVTGAVVAAICKFCFGLSSAAQIVLGGGLALSSSAFVLQLLKDKDQMG
TKFGRSSFGVLLLQDLAVVPLLVVTPILAGGGGGLAEAFGAAGVKAAIALGTIAVVGKFF
LKPFFDSVAKTKNQEAFVGAILTTVLGMSFLTEGLGLSNTLGAFLAGMLLSETDYRHQIE
QEIAPFRGILVGLFFFTVGFEIDLELIGSNPGRIVSIVLGIASIKCVIASVLASMFGLDK
GTALRLGLILSQGGEFAFVAFRLARSCGILSPELTKLMLTCVSLTMGLTPFLDDVGAKMA
IALDDAPSTGKGKGKKNR
>comp2021_c0_seq1; 70 1 1411 LEN=1674; translated
XWAKELYEINVHEREVINDELHGVSVSFHPNPEQNWTEKQYDYYMRMFQAEIDTNIPTED
KEAYKLGLDLGSSYIVSLEFRFRFIRAEQYDIQNAAIRYCNCLSFLFEVFGEKSLLRPLL
INDLTKNEMKFLKEGYIQILPSRDRIGRRILAQLGSYGGSRYTEFEKFRVSIYLCFSVLS
EDVTSQHRGVVSLGSFTIEAESFMWKELRVISKLIKRFFAAVPLRWSATHLCIPDDPVYH
LVKSCTLFFLGKNGRRILRIHVGTPMECDYKLRCFGIPTEDIPRTCTHKIKLKNHNRLIV
VRKAIDQFREQSMRPNNVVNMSFPGVECPEINCVLFGKCAYSYPGNIEFRGFLREIHTKH
RKVDLENQGMIYDITQRVIEELPSQNFRFMMYDRNTLFYNEVTNYEEIWKLVELSVRDYR
KRSRARAMTNEARLSISKIIDKSGSEDETGMNVTHRSSCACLKGEYRSTAW
>comp2022_c0_seq1; 415 1 1653 LEN=2079; translated
GEKMMMTILCGKVPLAQLPTLSVVVLSLLIIQNFTTRCEANLKLPTFVASHMVLQREPLS
SRIWGWADPGSLVRVSLDSKIYTDTNAQPDGSWIIELPPQPAGSDHKILIADGETSITLQ
DVAFGDVYLCSGQSNMELSLPAVFNASAEIEDSSNYPNLRLATVDKVTSDTPQKDAPSKS
PDYAWARSGPDAMNLNDTFKYYSATCYFFGRELYKALDSEIPIGLVTSCWGGQTVETFSS
PDALADKSCGGTRPVHGTDGGGGRNGTTVVTSGPGPRPTQLWNAMIHPLVNMRFAGSVWY
QGESNAFDPESYACRFPAMIRDWRLKFGLSDLSFFFVQLAGFHGTKDDWPGLRAAQMAAT
QLPRVGYAAAIDLGDPSSPSSAIHPRRKLEVGRRLSLSARTIQYGERGGLVSTGPILAGV
QQLPTFSTTHTIRLSFSPGTADGLHVHGSPECSLCCEKLPFRVLDANGTWSRVDGAEVRD
SEIDLSSNVGPVFGVRYAWELYPECLVYNGEGGPDDHAGIAGAPWEWCSEPSGDPAWTGT
ACKVPASAKG
>comp2023_c0_seq1; 482 34 1101 LEN=1384; translated
MLLGVKLFIIGFICCSLSGVSAWVVPTSNRLCSTRRTQSRATATVPYQLQQKHGSPSTRR
GNTRLYNKLLEFLSPYDSKIPEELRDEIYKAEANTPAAKDRGTRVAIYATMALFGIILAS
FNGFLTDMRDRETAMNAATVVASAADPGTPNSLYVLEANGFGWVLSNPIFKFLFTNKIGG
IITLLFGGGSGLMAEAELDSQRINAEKIYEELERRREQKQTKKTGGGGMSKSKKKRRSGK
EKKRLAAISEVVMDDTTEATSVLSKESTPAEETSSTNDVETTGMGGESDDKKGVFGKMKD
LYEKADSMAASQALILNKNLEEAGVVEKITDETGLKVIGREEGKKIEEKKASDDN
>comp2024_c0_seq1; 302 349 942 LEN=1060; translated
MSFQHLTQIFLLSCAILCATSFVSVDAFSPKAPTSSSVEPSSRREWLEQASFGGVAAIVG
AVATTTASFPQAARADSGEYVAELQSSLAKLQPIPKMLEDQKWDEVRTILKTPPVNKLWN
LGDSQNTVLKLVKETGNVELFEIKDDLAYHLQMCDQLTYDNNFIYYQPGNGKLKIKEPID
AINKALVSLKSIIDESS
>comp2025_c0_seq1; 216 311 1369 LEN=1745; translated
MASPRCSSFAFETGFPGSPHSITSNHGSNGPSEITARSLARSCGLLSATLWIGYTLGASK
APPTAPVLSSDLNVLASLRVLADRFEEWIQWIVVPLYLLGTVTYLSVRTYNDTDWTLVLS
STKTSEKKFRYSLLPSLRMASTETIPEEPEEEGLKEETTTPLSVTPLPSQKYDKPAPIDM
NGTFKVVENHNFAEFLNAQGLPWFLCKAASKARPTHTFSLPTYSDLTITIKGIIESETHY
TIDGPCTETTIRGRVFRDSVNYLYEESDEGENDGVCVGLRTTKVSVGEGYAVHVQRRIVP

AGATYAPPKGQHAYDLGTPADVDRLLMTNTLVYDEPAKGGSGVVASQLFHRQ
>comp2026_c0_seq1; 550 315 1882 LEN=2105; translated
MVVYNSXEFEHKQQEHFFSTKSKIPSVPSENASPSSTTKTEEDQPPSIATTGNIASHLAP
IPTERDLAIVGGVFALTSAKRDKLGELRNALGEGDLTLDDKATDEELMDLYLADGLKLIT
KCTDAALDLSGTKDEMKLLSDFSAWLDSQHDPSEDDELSRTAMEIKLELISAKLDNLLGE
VPADTCLRHVRDFLDPETIPEELGEAGEDENLLCSLGGAESDALDVGSPFFRNAVARYRL
LLTKATVDKVSASWDVLTTVSDGDIDRAASEGIALVPELETVSISGVLGFLQKTATGSCS
ERITAAWNLMDRDRDGSLDEAEMNHVVHLCLGIETDALQVLFREAVDAFPVRAPLSAIGA
GNEKDGGDGDVDNAGAVGRQGWKARRREKKTRKTLAKMFERACQRHFDRELEIDHRLRCV
YAWANKADQGNQLKSVLVDEQAGWSGRKRYVELSPKISEAEFREVQEIHFRHLDKLGTEL
VTSFREDLWVLQGKGRERKDLVKNSFLFLGAVSAIDTAILLL
>comp2027_c0_seq1; 149 147 619 LEN=619; translated
MAIRYEAPPYAPQPGGGGSLSITVKPERWRLFAYGFFWSLMIFAKLVCATLVEPIIEAGA
PPDTPAERLGCGPFNREGGPFDLEYGMGFTLDESHLTQIFGYANSCVVWDYTPAREMMAM
YFPFFEYSLVIYLFLDYTSTMLSYRRGELPEWYWKLMX
>comp2028_c0_seq1; 82 231 989 LEN=1269; translated
MATRCAFENSPEVGVFSALTNSYALAGIGGSENFYSTLEAELIEHIPVIHATIGGGRYVG
RTTAGNVRGLLVPNTTTDQELQHLRNSLPDKVVVQRVEERLSALGNVICTNDHVALIHPD
TDRETEEIISDVLGVEVFRQTVGGQALVGTYMNISNQGGMVHPRTTVQDMEELSSLLQIP
LVAGTVNRGSDVLGGGLVVNDWSAFCGLDTTGTEISVVESIFKLQRNRGQDQSMQPMDIV
QDMRSSLIDQLS
>comp2029_c0_seq1; 463 74 1171 LEN=3199; translated
MMINIKGSMVSAAFVLLLLLDRAKESSALSIRDKSRGGSGRRDFIEGIATATATALILPE
QAHATRAVGGAEIECREAGNCLEKFELDGALGWNWGAKDRCDATDPNCGSNGKLRDSPLV
GDSVPDRMGYEITHVVQMSLAIGRSDEDCTVRMGLYGNDVAVSVEEFLNIVSPSGLRTTS
ELMFENGMGVAAIPVSLTRGGILGQIVPGQRLDFGIPLQSAAYARSKGMANAGDNFVPQP
RPRELKDVPVLRKHDVPGLVSIPAKGIGYGGTGFESEDECFESAFQIIASANPAMDKENR
RVIGQIVDDESMQSLARLASLPTKKGFRGVIPGQNSGPPLIKVVLTDIQVAKVQSQPSQG
SPVES
>comp2030_c0_seq1; 154 248 562 LEN=562; translated
MFSHGLPMQVTINQRQSECLYETLNEGEAVTASVFILAGSQLMATYSLEGPVTDLSTDSG
IALYESALAFERTGGSEGSELRISGEVDFEHLNDEINDDDDDDDD
>comp2031_c0_seq1; 504 283 1416 LEN=1783; translated
MGSSGSKHTTTNTTNTMPISPSQFSFFQTLTARCDDSNPPPPPPPPGSDSAPPPPPPSSA
NDANDDSIAKAAAMASQMMNPGPLEQAPADMKRLVQLDTHDGFRCDINKQISPYLAVVHS
FWLGTNMLPDGQKRTYTWMAQVADESSFCFSRLDPSQSSVDGRIIKTLLGGIATAKLQLG
LSAEGQADQCLLEVDVNAMSWNGNLKYGSMGGGAVYGCSFHQGITPNLSMGGDGMFVAAN
GNMMSNYLLKYSFGASGESTSANEPPSSAVCLQFAPSGPSPMPLTLNYKRTVTPKRVTLG
AELSCDPLSMQSHCVLGGEFKFSRSKLHVAVDGNLKMQSLLETPLGKEPGQPRLSLSAEL
DHLRDEMKFGYGLAIDG
>comp2032_c0_seq1; 270 119 957 LEN=1003; translated
MKLVIATRLHLGRASSPPTEESLHSVLANLRGVCDDNGLRQDYETEVLIAVDATPRLEGY
DYVEAVRSALHSNSANEANGSNHIHILPVTPWGKFVPALNSLVIHAKTQQEADLIMFVSA
EVNLSCKTVRTLCQQVSDNKNVIVAGAVLNGHQYHEIESSSDERGRVVPLTGRTTPWNTL
CVWDLHKLSMTGFSIISDLGKSAGVEECAAIALLQNLFPEAEARLVNVDGTKWEEEFQDE
ERRKWHEEKMKSKVERPRRQLELLGLSGTXSPLLTAHVE
>comp2033_c0_seq1; 528 216 1640 LEN=2082; translated
MRFPRNESMSATPMLLILVLLAAVIEVDAGAVMGKIRSGNSNPNSRSLNIENQSGRKVDL
YWVNMFEKPEKFVPQFVEDGVSVGCSYGADKSISSYVGHTFEIRELHSKKTEACIFDECR
KVRYTVSNRQEQKIVINKDFTLTVIDERQKAYQKADEMYSRCKEKADAVVDPLGSIELLT
NCMQEEMTQKIDVNKEERSFQSKIQRKMVADLVPFECGDVNKTDSREIMNTTWSYYDSND
ESNKDYTVRTLHELPTSKIVVVDDFIAEGTCNILKSLRQEISDGKIGIPTMIATNESKES
KRMLEEFYKIYDLIKTIFPDWDELEFLSDTLFDFFKDSDGFQTPSQLCVGPEEVAGAHDE
INAGKHPKCKIPGGEPVRASSKRFVVEEKGSKKGPKRQVAQFFLFCDEPEQKLGGVHFPY
AGVYYVAPKAGKVVGAIHRHANDPSDELDGYVDEYHFCPNHDLYTHTINHNIPAE
>comp2034_c0_seq1; 985 214 2400 LEN=2611; translated
MASSTVATNPMLEQSGLPKFETIEPKHLTPAVEELLEKLENDFVEMEDTLSKKDGAVEFD
EVLPVVERMQYPIGYVWGLASHLNGVKNGDELREAYESNQGKVIESMTKFSQSKALYDAL
SAIEKQWAEDGDQCDSFLSKQKRRAVENSLLEMKLGGVGLEGADKERFNEIKLQLGKLSN
KFSNNVLDDTKTFSYTVEDPAIMEGVPESAKALWANSHAQFIKSEAKEGEDVPEVDLDAA

TKSGPWRITLDIPSYIPAMSHLADRGIREKVYKAYISKASEKSDGKNNVPLIYEILTLKQ
EMAKLLGFSNYAELSLAKKMASSVDEVTELTNLMADKAVPAAKKELEEVTALAREKGGDE
YSTENLEKLEPWDVTFWSERLKESKFDMTQEELRPYFALPAVLDGMFGLLNRLFDVDVKA
ADGEAEVWNEDVRFFKIFDSKTDKHIASFFLDPYSRPENKRGGAWMADCIGKSEATNNDI
PVAYLTCNGSPPVGDKPSLMTFQEVETLFHETGHGLQHMLTDAQVGDVAGINGIEWDAVE
LPSQFMENWCYDRPTVFGFAKHWETGEPLPEEKFEKLCAQKIFNSGMQTCRQLYFGQLDL
ALHSDFDASAGEEGKGIFELQREIASKYTPHSMPLEEDRFLCSFGHIFAGGYAAGYYSYK
WAEVMSADAFGAFEDVGLDNEEAVRTVGKKFRETVLSLGGGVPPADVFKEFRGRGPSPEA
FMRHNGLE
>comp2035_c0_seq1; 585 1 2659 LEN=2708; translated
XDDDDDDDSIEDEIWDEHGKKIAYQQKQLGRRRRRFAPAYNIWEKPALRPIPKKVAKRAG
QQAVRSMMKHGSVMIPQILSDATMEDLREFIVDKNERVLGTPDQYPMSASHHRISYGIES
TEHPSVVRALKEIHDHGVFAELIQNLVGDENPALSEITAITAWGGAEMQSWHPDVKPDGN
GVMFGRTYSHSYSLFLPLQDTVGEMGPTDLCPGTHMCADEDLWDMCEENKIGLHEIRKAK
PRHLYDPSVEGTKEYLEDLSKEGTWRAGDGALLNQQGWHRGTAHTDEDMDDRVVFIVSFL
KRPSAADPRQLARGTYFHQKWLNWGSTWQDMADAAESLKRPWNILRCLHLYKASDRSWGY
DLFTATTLRIANEQMGGEAGDLEGLIDNVMIPLRYPEWLQGTIDYDSEEAWQMYLRETIQ
KTHGFLYRVNAIGLTGFMVAMFIIAILAHLYNRFSGQKNRERSVIIPVLKASARRLVWTH
GSIFAFGLYVWHVLIRSSQWAIDIDSGTTLMRPFPPDPIYRDVDPGVQGGNTTLPRRSDV
LIGTRYHTNAIGAYRTWLDYHPGNSVFNDFVDTYGGNGNFYHSLLQLNKESHAVSQLPLS
LADQLASVALNTIQSRHDGRFLMQDYRSGDWKVLSEAESKKYIHKRLFVGSKDTLLGALN
DETELMLNRYRFGVERKTFSMAWNSQLFVADLSESLFSPANKKYLKENLNQEMVRDSSTS
SLAFRSILPHSSFRLRKRKKVHENANISHNNSRIRRYMVGDSLPSLFPGMEIYYSIDNSR
DGSSDEIFFATLIGISSKKTYDGRARYHIAFDGSEVPEIETTELGRVKRQSIRTRPPITE
GSRVMVGDVAGTILLVLADGSIDVVFDEDGEIDTDIPYTDYELFVE
>comp2036_c0_seq1; 578 383 2491 LEN=2674; translated
MVQVVQVVLKWSKEVIEFEIEVPTTPNSFTGRSFKDRVHSLTKVPVDRQKIVVAKTKSKP
TPSGTGKTKNPIKWWKGILKDDFDFASAIAASINTDNSTRIELRLTLMGTAEVLESNSNR
EKTKFIEDMSLSEKKEAEQREWMNSMESVAAMIPALQVPPLDRQPPGNTNGDGNGNSSGD
PMQSSPPTIVSEFTESRAYDRLVHGYSQLRIDALLREQTRGDLPGADAEEPGSLSTPRLH
GRPVMTMGLELQRAYVNDLAVLKDGTLLSALDDGHVQLWKHCRRICDIVHKPGQFGGSQF
FSGVDSVLALDDSNSPSSPFAFCTAGMGTVRVWDSDGDALLGLVPSPLPNTSPTGLVRIP
TNSPDALCLAARFRIAWPASRRPRLVPQDEAGRRRLIEIERSEAEMNDALTGMSKTVQVF
FSDGGSKTQGVKNFFLTAPAPVTALASWKQGDDSILAVGDTQGGITLQKIATSEPGSNGS
QANISNNKNNSTLRIQEVRRIQFASSENASETCSSIVYLQYMAETKQLLVSTKEIQLTAL
TNPSSNIPIPILTLIPSAGPQAVHSIDMEAVLSDATPRPLSSNPLLFSLAGHKDEVHCIM
SLPNGDLLTAGGKHDATTQIWSRSQLQSATTRTETPASVPILAKAATVNLCKDAGYIFAA
EVLEDFKSKGDGSGDEPNNKKEQKSSPFAIAVARYNVVKIIV
>comp2037_c0_seq1; 523 221 1903 LEN=2044; translated
MRQNKSKKKTSLPSRISAEHCSKKNDKTSSARRRSSPERRSAKESRRQTGRGSSRRSKAM
SPSGGGPRLPLSPRSSTAAKSPTSIKSQTSSFSRRSKIEPLLSDDELRIAVTTSSKIEKS
TENYTPPVAGNRSAVETENPKSLASGASSPRKLKPPSEITARVAQTKQYKPQPVAETASI
EDSAENDRYELAESKLAAASQGMFEVKNITINETSPTGQHRGSSALEAGLFTDGSGTFGT
DTYASDTFADEMSFSFESHQRPKKKSYRKYRFSRSWVTTDASSFDDVSVIVKQRVAWGCI
FLSAVQFAILTTQVLMCGIAKLNINPTIGPYPDAFSEWGGKNAYLLVEGHQYFRLVTPTF
LHVGYLHFLMNAFFQLETCAYLEREWGFNVWIFIYLASGLGSCLAASAIDPNVIGVCSSG
ALMGLFGARIAQAIVWYSFEPRHEYIGQGALLFERLGGIVCSAAVVFLLTFLTYIDWSGH
LGGLATGFLVGLVVFPFALEGRSTKAVLRFTGVLGLLIGGVVLGVLLFHYAELDEELANA
CNYFRSLYTEGYNCECQAFD
>comp2038_c0_seq1; 649 72 1937 LEN=2032; translated
MTSQTNFGTNENSGAVLAPLMSRGDPAMGAIKYVKQPGPAPAPALQNNGGQSTANATVVQ
MPALAPQPIAPPAPVNAAGVSQPPPPMVINMQQQQQMHNMQQQLHTMQAQLQQKMQPKLI
PMNTRPKVPAAAAAAATTVNHVPSHAAPNGTSSASVVGTDPGTLRTGGGGGQLLQFPWKL
HEMLHLAEKNGKTDIISWLPGGNGFKVHNKELFCEEIMPGYFASQKYKTFQRSLNLWGFE
SVAKGPDRGACYHQYFVKGHPXVVPQHVSSRFVLSFLFSFAFVRCCLHSEGKLCFSSTDL
FXELISMFPSYLDSIPGLXVKIKGQSNPRTPAAAQAAAAAAAKRAALSSFANQQHQQQAV
TAPVPHQQQQQHQQPINMMAINQFMPTMPGVVTPIVGIPNFGMQMNGATAASATTNPYAF
AAMNQFVTMNALQQMNPTLAAAAMQNPAMNAMLTAQVAAAAAIQAQATQLQQQQALGADM
AAGQQVAPSIAPPTTPFSPVGPQYPPPMSSAPTSTAAVHPEPAPTATVPTSTSTTSKSNS
NGLLTLSNANGNDHSSEAKNKTQSNADANFAEYLARKSAMDRHIEQLSDPSAPVPTVAAS

NNNNKKTGSTNSAGSDGAPVAV
>comp2038_c0_seq2; 764 72 1790 LEN=1885; translated
MTSQTNFGTNENSGAVLAPLMSRGDPAMGAIKYVKQPGPAPAPALQNNGGQSTANATVVQ
MPALAPQPIAPPAPVNAAGVSQPPPPMVINMQQQQQMHNMQQQLHTMQAQLQQKMQPKLI
PMNTRPKVPAAAAAAATTVNHVPSHAAPNGTSSASVVGTDPGTLRTGGGGGQLLQFPWKL
HEMLHLAEKNGKTDIISWLPGGNGFKVHNKELFCEEIMPGYFASQKYKTFQRSLNLWGFE
SVAKGPDRGACYHQYFVKGHPELCHSMTRVKIKGQSNPRTPAAAQAAAAAAAKRAALSSF
ANQQHQQQAVTAPVPHQQQQQHQQPINMMAINQFMPTMPGVVTPIVGIPNFGMQMNGATA
ASATTNPYAFAAMNQFVTMNALQQMNPTLAAAAMQNPAMNAMLTAQVAAAAAIQAQATQL
QQQQALGADMAAGQQVAPSIAPPTTPFSPVGPQYPPPMSSAPTSTAAVHPEPAPTATVPT
STSTTSKSNSNGLLTLSNANGNDHSSEAKNKTQSNADANFAEYLARKSAMDRHIEQLSDP
SAPVPTVAASNNNNKKTGSTNSAGSDGAPVAV
>comp2040_c0_seq1; 292 127 1008 LEN=1230; translated
MSAVFLPQSRIKEVHIEGLAMMKIVKHCQESLPNMVQGALLGLAVDGDILEVTHAFPNPA
GKTDDDIDEEYQVEMMRMLREVNVDNNCVGWYQSMYLGVYSTASVLENQFSYQTEVSPNA
VVILYDPLQTANGNFCLKAFRLSDECVEMKKSGSNDFLKPSNIFEELPVRCTNPSLATAM
LQDWTPKSGIALDRLDMSTQPYLEKHLESLCGWVDELAGEQQKFQYYTRHLARGNNKSKA
SWAAPEAPKRMESLLVTNQIENYCEQMDKYVGGGLGKLFLAGGLHKGERSTSA
>comp2041_c0_seq1; 587 42 932 LEN=1039; translated
MKFTAFAALALASSTEAFSPVTRSNGASATALEAVRQPIMAGNWKMNPATEDDAISLSTG
LTKLLGDETCPLDDENPLCTEVVLFPPFPFISKVKDAVEEVGITVGAQSIFFEDKGAFTG
SVSASMVDSIGCEYVLCGHSERRTIFSDDDDAINRKVLKVLTSGMKPILCIGETKNEYDL
NIKNEVCAMQLSRDLAGVTKEQMADVVIAYEPVWAIGTGLVCGSQEANDTHAFVRTILAR
MYDDEVAQATRIQYGGSVTPDSVDELMAMPEIDGCLVGGASLDAEKFGRIVNFESK
>comp2042_c0_seq1; 270 1 456 LEN=551; translated
QQQQQQQHHHHHQHQQPPATKKQKKPLSRKRQMEQMLRAGKLDEVRGDHEVRGVAHVYQQ
QGDYAAAVAASSSASSTVRVVPTSAYDAATGTTATSTSVTSRQKNSNQLNSLLANAASLE
SQRAQNPRYAGGSLGAGQGSHRATAKRRYGW
>comp2043_c0_seq1; 143 385 978 LEN=1322; translated
MQCTSSSVAQHRIAPCPTRPLGAPTGVVGPEAAHRCGEKRDGRAAPSFQIPVRWNGMVWF
GMGWFPDNTTQRSTPGGGSTEQSPREAGEASDRTHPAGGEGEQTSTLFAGRTPVLPRTKG
GRGGSSAAQRNGREGNGGGINDSKETQRPTRCRHRTKAEAQKAMCFRHKGRCGHQKRCSI
RGREAIRYGTIEQGKTE
>comp2044_c0_seq1; 476 1 1689 LEN=1721; translated
PVSFEHNLHSFPYGQRAMIDHIVQNADILQNVLNFLANDGDSVARFALSSKELYDKILRS
ETIINPTLWKELLRQRWKRTMRRPMVASSNADDNQNVTVAELSYRELYVAKRQADAEAIR
LLEEMTSDLLVVLRLNEPKSVLTKKNPHIGKASNHNCWYHLLRDRADFYDVMKATAMRYA
RPTAASSAHDRLMGFCAARCFQSMHFADCLWEWRRLAEKEEEERLDRQQQRELSPQPSLS
QMRACQLSASNFLEQFALLVCEIQKSPLQHLEEPNGCACTEYNANDEITGIGFRNTMRYS
HQATKSMDAIAKVCRERIKEKSEANASTSTKLAVINDVLMNDYGFAGNSGDYYNFRNVLL
DEVVESKTGMPLTLCVLYSCICRRLGIPVHLIGLPGHVVLGFHTDDESIDFDEIHSFIDV
FHGGRILSRDDCRRIVENYRMPWHDEFLNPIPTTMIIQRIFNNLRNCHERAMMNPNPPMF
CSDLMFQQHILGMIHRSPPELGTILLERLTEDLPNVLSPELLHAYHLLSPEELGNGPVVN
ATHARMLLELSSYAGLSYNNYA
>comp2045_c0_seq1; 658 29 1429 LEN=1662; translated
MKFSIAALTTFATATSAFTHTSSFTGKTSLNAASNGSNMDMRYKVAVVGGGPSGACAAEI
FAQEKGIDTVLFERKMDNAKPCGGAIPLCMVGEFDIPETVVDRKVRRMNLVSPTGAEVSI
GDTLQPDEYIGMCRREIMDTYLRDRAIEYGAEAINGLVTRIDVPADHKTSESQYTLHYQK
FEPGKSSGTAAEMKVDMVIGADGANSRVAKAMDAGEYNFAIAFQERIKIPDDKMVFYEEM
AEMYVGDDVSPDFYGWVFPKYDHVGVGTGTVVNRPAIKQYQKAIRDRAGDKIAGGKIIKV
EAHPIPEHYRPRRVQGRMVLVGDAAGYVTKCSGEGIYFAAKSGRMAAEEIVACMDGGKKL
PTQEEIENTYLKKYDKAYGPTYAVLDILQKVFYSNNGAREAFVELCDSKYVQQVTFDSYL
YKKVQGNNPLDDLKLLGETIGCLIKGYSNAKPDAVFSNPVESQKRL
>comp2046_c0_seq1; 700 1 1805 LEN=2032; translated
XVQLLFRMQSLRTQRRGRPFSRIAAVAVAAIVSIVATALAPVFGFAPQAFVSSNKPTRFF
EKYVDEPICYRTERYSRLISFSSKSGSLDVKLLEEIRSMRVKELKAELTERRISTADVFE
KEELVQRLYEAKLKSPDPTETASASTKRTSSDTSSSSGTSTGVLCADLFFTSVDSGRSMS
GMDGNSQSVTIKVEDVSDPYPTIAIDVYDSSGSSSGSFPLKLLVDTACSGLVLRPSVVQR
HSSCLKSYSTPVTMTGGAGAAANTGLTQIQSYSLRDSAGTQPKRLGPLPAAVQDIGALPL
ELDGILGLSLLSQFACTELDLENARLNLYETEYRPPLANENKEIVVAEAELSPTKLGIWT

VDVSFAVGGRTGRPIKMLVDTGAMATFLTWKGLDDMGLGLSRSSPSVRELTQPMGAMGSD
NVAMSLSHRIELDADAPISFGRKSGRLQQEQLGLSIASKRKDGKEPSLPIDIGNIGILEN
QLASDNVVGILGMDVFSKASRIRMTFRGPVPRITFYQKKETTLSKDEMKDATAAADVEPE
KHTAKTEDAAPVDPVEPAAAPPTKGLEARGEEPETKNAVPTMVEAPTRKKKKKKKKRRYD
D
>comp2047_c0_seq1; 282 85 1179 LEN=3159; translated
MSRCILASNKSLRLARHLAAPLQLLPDAVGATAGVDSPSTVNSEGNDLYNKSYFHFRRRF
SSVVRDQATSTAPMKTENIQNQGLARTDKSIVRRLFEKYSMSKQKDRILMAESLFQAATT
QASDPRWYGPGRIGRDFRAYQALLTMHIWFLHRRLVTDEADPHTAALIQEELFDILWIDT
SNRMRAHGVNEMLINKNLKKVQQYSFMHMFHYDHCYTGDLLENAADRLEALKMTVKTHVL
LLPSLTDKDAADGSGGDAAVNEEALQKHVEHDDHAERIAWYIETQFQNILHDLPESFFQK
ARIAWVDLPSFEHMVDGNTGKELTMQPINQDDLLPTNWTKSIANDGTYYFWNIETREVQW
ERPE
>comp2047_c0_seq2; 282 85 1179 LEN=3032; translated
MSRCILASNKSLRLARHLAAPLQLLPDAVGATAGVDSPSTVNSEGNDLYNKSYFHFRRRF
SSVVRDQATSTAPMKTENIQNQGLARTDKSIVRRLFEKYSMSKQKDRILMAESLFQAATT
QASDPRWYGPGRIGRDFRAYQALLTMHIWFLHRRLVTDEADPHTAALIQEELFDILWIDT
SNRMRAHGVNEMLINKNLKKVQQYSFMHMFHYDHCYTGDLLENAADRLEALKMTVKTHVL
LLPSLTDKDAADGSGGDAAVNEEALQKHVEHDDHAERIAWYIETQFQNILHDLPESFFQK
ARIAWVDLPSFEHMVDGNTGKELTMQPINQDDLLPTNWTKSIANDGTYYFWNIETREVQW
ERPE
>comp2048_c0_seq1; 517 1 1746 LEN=1983; translated
SPTNSEKKDSWDDTDGFQKQASFSPTNSEKNNQNKNHTSFSGDNAWNVSVSSPVGQSPSK
LNSSNEQFSRNRDTDPFRNSSAIGGSSYVSAGSSSTGLPAENRSLTELLEAAKSKRKDSR
RSYGNSATRLTQHNQSTSSRMSSASVNSAPAITASYLRQHHNLGGNNVAGDDPLSPRDGT
SVSDIIQSLEDTDRMKRAEQITSSHRSMGDAGVVATARAAKERLRERRRRERESAGIYTT
AGRSANSLRHSSSRDHGSDSDESENGNEASESWLFDQVTGAMGPVGIAADLESLSGRSNR
SKNSHGGKSHKSSGTSNRRRSSRKTSSTRRHRSDRSVDSHGSRTSRYSHRSTKSFLSQMS
EQSRSVANDLLRLEMQLAMVGNNNVNGGGKEATMREIDSLPVVQSARTGMSSSASLGGTS
RASGRSRRSVTSSHHNSSSSSSVLRRTKTTIVAPPGKLGIILANKADSKGTVVSGVRTSS
VLVHKISPGDRIVAIDGEDVSRMTVSEITTIMSRKAEYERRLTVMTIPRIGSPSNGNTNS
GYADQQKMRSPNRVRSSPRVASISDSKFDSAGSGSNYHSVR
>comp2048_c0_seq2; 1045 490 3705 LEN=3942; translated
MGILKKIFRGNKKKTREFQDGGSRPISDPSFPSQTSYSQTQSQATTRGGEWRGTMVGDST
IPNGMKIVGSASTSTASSYPNNNLMLNTNNNNINNTSSHPQQQLMGGYRSPRSTGPVDTD
DEGIIVPENETEAFYDNARNQTQEQLRLAYEMSKANKSNNNSANISSDSSNSSFALTSNQ
LQQWDHQQRHSSTHQFSSSPTTNNNNPKPVYTMSSDLPSMNPQQPSGLDDTMRSVSSASS
FNLSTDAEDSEYENLKRTYYPPGQLTSNLGIGLSALTALDTSAIDAAGSIASYTTDEDRS
KIFPNLPTDDDMTLGTDASSKRSEPPPSLLDFPQSRLYASTNVGSSPYGAQVTVPMHAAL
AINTNQRNSENNTNTNSRNKPGGTPRDFTSPRSPKASKLEHKLEKNHSSSPKKNMSRNRN
NDSSGDDGFGNISTNRSHESNGSGNHRQNKSPTAAAISHSSVEDSGFADFGNFADFADFP
RGEGVDDSGWTTVKEEKKDSWDDTDGFQKQASFSPTNSEKNNQNKNHTSFSGDNAWNVSV
SSPVGQSPSKLNSSNEQFSRNRDTDPFRNSSAIGGSSYVSAGSSSTGLPAENRSLTELLE
AAKSKRKDSRRSYGNSATRLTQHNQSTSSRMSSASVNSAPAITASYLRQHHNLGGNNVAG
DDPLSPRDGTSVSDIIQSLEDTDRMKRAEQITSSHRSMGDAGVVATARAAKERLRERRRR
ERESAGIYTTAGRSANSLRHSSSRDHGSDSDESENGNEASESWLFDQVTGAMGPVGIAAD
LESLSGRSNRSKNSHGGKSHKSSGTSNRRRSSRKTSSTRRHRSDRSVDSHGSRTSRYSHR
STKSFLSQMSEQSRSVANDLLRLEMQLAMVGNNNVNGGGKEATMREIDSLPVVQSARTGM
SSSASLGGTSRASGRSRRSVTSSHHNSSSSSSVLRRTKTTIVAPPGKLGIILANKADSKG
TVVSGVRTSSVLVHKISPGDRIVAIDGEDVSRMTVSEITTIMSRKAEYERRLTVMTIPRI
GSPSNGNTNSGYADQQKMRSPNRVRSSPRVASISDSKFDSAGSGSNYHSVR
>comp2049_c0_seq1; 190 1 365 LEN=588; translated
XRIYTPIDSTKMGKKKGANEKKGAKIFKTKCSQCHTVEEGGAHKQGPNLYGFFGKQSGGQ
DGYSYSAANKKSGVIWEDQSLFDYLENPKKYMKGTKMVFAGIKKPADRKDLIAYLKKTCT
A
>comp2050_c0_seq1; 260 163 962 LEN=1253; translated
MIGSLIGQEVTNEXLQNNNDKINPLLLWIDGNNVRGIGKFEWNAVELQHRVVRFCHKYRV
PNAIVVWDHGSHQLSCSKQYHFNDSGKEIASSEADVPYSKDDKSFCVNLITLFSGIRQRA
DDVLVKESNHLVSLSFRTNVESDYNTKLNWSSNAFVTNDTELNYKLRRQAVPNSPSIRLS
RRRRRDAGKIYNSDSSSNNEVKDTSNPLFCDSTGFVDLLCEISREYDMAMNDVDQEASML

IEKAKDSLRECSKGQRRGYNPRREKTW
>comp2050_c0_seq2; 321 163 1262 LEN=1347; translated
MIGSLIGQEVTNEXLQNNNDKINPLLLWIDGNNVRGIGKFEWNAVELQHRVVRFCHKYRV
PNAIVVWDHGSHQLSCSKQYHFNDSGKEIASSEADVPYSKDDKSFCVNLITLFSGIRQRA
DDVLVKESNHLVSLSFRTNVESDYNTKLNWSSNAFVTNDTELNYKLRRQAVPNSPSIRLS
RRRRRDAGKIYNSDSSSNNEVKDTSNPLFCDSTGFVDLLCEISREYDMAMNDVDQEASML
IEKAKDSLRECSKGQRRGYNPRREKTWERCVQAETLRRFIIDQEERVTKEKVNETSPSDT
NDAVFVANYLNELQTKRGYISPLHEENREDSYETFLPFLAPSRLDKRQRRLLDRYNALVK
NGEITS
>comp2051_c0_seq1; 916 126 2603 LEN=2762; translated
MSMHVIKRDGRMESVHFDKITSRIDKLAFGLDTKHVEPPLVAQKVIQGVYPGVTTCELDE
LAAQTAASFATQHPDYSILAARISVSNLQKSTCEVFSELTEALYNYIHPTTGKAGPLVSK
EYYETVMKHKDVLDNAINHDNDFEYDYFGFKTLEKSYLLKLNGKIAERPQCMLMRVAVGI
HGDDIERVLETYKGLSERYFTHATPTLFNAGTNNPQMSSCFLLTMKEDSIDGIYDTLKSC
AVISKYAGGIGLATHNIRASKSYIRGTNGTSNGIVPMLRVFNNTARYVDQGGGKRKGSIA
IYLEPWHADIFAFLDLRKNHGNEADRARDLFYSLWIPDLFMERVKANGTWTLMCPDECPG
LPDCHGQEFKELYEKYEKEGKGRQTIQAQQLWFAILDSQVETGTPYMVYKDHCNNKSNQQ
NLGTIRCSNLCTEIVQYTSPEETAVCNLASISLSKLVRPLTNNGPLIFDFEKLRQVAMIL
TRNLNRVIDRNFYPIPEAKYSNMKHRPIGLGVQGLADAFAMLKLPFDSPEAAQLNRDIFE
TIYFGACTASCDLAKTEGHYESYPGSPVSQGKLQFDLWGVTPSDRWNWAKLKAEIAEHGM
RNSLLVAPMPTASTAQILGNNESTEPFTSNIYNRRVLAGEFTIVNKHLLRDLTSLGIWNE
SLRNRLIADRGSVQKIEEIPKKLRDVYKTVWEVPQRIILDLAADRSPFICQSQSLNVHIA
DPSSKKLTSMHFYAWKKGLKTGMYYLRSRPKADAIQFTVDQEKLAADTKKKANISSDKEN
KKKVSNIPPMESLKKKKPMVEAKPLVDSATLGQAEEEEVCLNCGA
>comp2052_c0_seq1; 275 110 1912 LEN=2461; translated
MAIKEELLFSLSITTGTLSLVGSCLLIATILRSRKKLSLPYRRIIFGTSVFDVFQSLGLT
SSIFLSPKDVEPPVPYAIGNTSTCTIQGFLTTIGAIGVPIYLCSLSIYYFCTIPLNMKAR
TFKKIEPFLHAAPLVYSLSVGFYALANQYYNNAGSVCYVAPSPLTCEILPGVECNRGEGA
SKFRVRFLSFPSMIIFVIVCTTMAVITYSVKKQERRQRRHLFPLHGGGEVSDASSPVWRR
LKRIGSVFLDVLFCVSLSRKNENENENDTGIDLDGTASRGENECPSEPKAESARTEMSPG
PSQRISSSSRRRSSTRIRETTTQALLYVGCYVLSYGFVWAQSIYSLATNLPPPKAFTLLS
SIFFPLQGFLNVFIYCRPHIVSLRRNFPDDYSWFQAFVKVLKSGGDDPLTVQERRMIRRI
SSSITVSHPSTRRPSMSAGMGDYEADSQMTVTMTSRTEIFERRSSITSRTTADLEEVPDN
IQDSEKAEMGFSKITIPQGDAEQRSXNHDCNRIESSGENRQQSESSEPEVDDSDHYRRPS
ARDEPGNKTSVTPENNLSDVEIIPESLEDFIHEAEEALDDALHEVLELAREAENEANGEV
>comp2053_c0_seq1; 508 235 1926 LEN=2116; translated
MTASDLFFPLVATSAASAVAGLVLLQTDMVSRKILDPDQLNTKRRSESRYSWKKSVDRAK
LLMDYSLAGGQRILTDDPEIVMKHTEYISKEDELDSLIKKIVDRKDSESRFYALKIDKDP
LDASEEFYLYIIKEQEQKNVKTLYQIPLVKFGLKLAQAFEDQLTTTALCFVADASSGKAT
SMLESLVTESKSGVAVVSEPLWMIQIARLAEASIVAKSKIQKLIFALCRLDAWSVRHKVG
DAKTVMFTLPGQAVVGTLLPLVQSAFPEDRHVFAYDGCVESVKRGIYAERMYKHNKLIPD
LKSIIQGMGKDPLRITTPLPSNSPLSHDASLGGRGGAMERAFSQVPVKQARIVEAWLSSI
DAYFKLKNEETTNGYVPYCFKLGFLTSPVGDFEYGTDSYWSLNSFLQYVTGCQGRKVQEH
VIDAAREWMKDYNSEQKAEDGSFETSVILSEHERKMIENCCFQHKQILIGNKTLQDTVLP
KEHWTMKQASRNGCSCCGPDPYDQMEEEESDVMEEAKSNNAMTGNFGAGKPNMTIGMNAN
SSRQTGYVDATQVFAFDPTRFTT
>comp2056_c0_seq1; 170 917 1585 LEN=1695; translated
MNRMLPVDIRAWNLERASPEEVAETGSNFTLPGVYNWNAMRSSTSKLYSYRICVGDAMDP
SERFHRWQLPWRGLDAARLQRILKKFEGQHDFVCFAGALEQQQKKTGIALGTIRTIHEIR
LVKEVDIDRNRFDINLSDMGGDPSEHYYRIDIYLDGALYKMVRNIVGTAIDVCRESVSEE
VLSDLLDRPKELKYSRKNNPCVPAPPNGLTLERVFYSDHEHF
>comp2057_c0_seq1; 176 520 660 LEN=1313; translated
MEKSTAYFLHQNKKTETKDPSNMDMFINNQFELYVKAMPWDQHSSL
>comp2058_c0_seq1; 395 197 1195 LEN=1277; translated
MAGNTGISPFLTLLILGLVEIVKPELLNMGKTIEIILASWWSIAVLGVLTIVEMVGKCIP
AVDEIIDSVEVFIVPVISILASMATMGLLPDAGTPQSDAGLGQDINVISILDTEYSGDGY
RNLQDQVGKANLAGIEDIPSNELGEGFLTFMKCSLVAFGMLLALLIHFFKMLIRLSSMVC
TAGCCQPCITVVEFLIVISGVILAILIPTFAVMACIAFLVAAGYVVVVKCCKTKDGKNHT
TTSGVEVEVVGNNPEINDIECQPSACRTSNATIPTHVVLYPAETKVEFVRPVSPTTPTIT
PKEMIRNSSIGELVVVPLSPIQAKKDFQATTY

>comp2059_c0_seq1; 877 64 1842 LEN=2011; translated
MATPFSVPFVVSNPDSWGPPPPGADEGNPNGPPSSTKFPDLPYSPFGRSDRLGRAADFTN
ISRGDRSRFNRHGRYDKNDRNDRRSKNDNDSEDEGEDFQLVDTSKATTQKRFVPPAAKRR
QHTARLRQVNARRQNQGGPSFSLSKQEGFGGRGRGAGGRGGRGGRGGRGRGRGGRGYNNN
RIDRQPSVAVQPDWVKVEELDLAKVTKNLSGTSSSDSKKSKKALEESIPTPQDVLWCGFL
DQYNDAYDKVTARQPAPLKRMATKEFYPVTTTDDPVLEKLAVDGIGGAGEGDKRSIFVTD
NILAHLMTCSRSVYPWDLVIQKTAGGLLFLDKRDNSQLDYLTVWETAYNAPQPAKEGEDA
SANINTPERLGLEATTINQNFSQQILKKSGRKNMDLPNPFFDEDDADGMEPASVAYRYRK
FALDENTTLVCRTELHGLVKKTQYMTAFALNEYIPPPQNGMPSAAQTKTWRDTIDSSRGS
VLANELKNNSFKLAKWTAQSLLAGADQMKIGFCSRGNPKSAYEHVILATQFYRPKDFAQQ
ITLNESQMWAMLRMFVAMFAKQEEGKYVLMRDPNKAVLTMYKVPQETFEEDE
>comp2060_c0_seq1; 430 148 1390 LEN=1721; translated
MKAESVETFEMILVHCSYLIAIAALSHLADYAKGFPSFLQTRGSSNHGRTLFVSGCAPLA
LIKDDNGHLDDEEGTANWTMEEDWALVDNLPLFTVSTTTETKTFWTQLWSANSAFFSGKE
PEDLYQRTLELQQERQATVAIMEKSENRRGRRSLLVFGDSPPVLENWKIDVEQQDNRAVG
QIGNGNSEGRRTIWFHYHSIGRLEGDPFADGSSRVVSLFPGGYIEAVGGRIYELGQPMMF
DKWHTKDGNKLRPNTQEISRDSVREDGEKPSIPRWLLPGGTAAMSALVSATILSACIGYG
AGLSMIRDQPSPYSAWPVPNMLTVETVLAGPSNSNGVKMLPPSSSSTEAAVTKSSAIKTS
NLNQAPSTEELRARAQYRVLREERLLEKISQRLEIDKQTLKQLDDQQSSGLHP
>comp2061_c0_seq1; 195 221 661 LEN=915; translated
MTVLSKSSGEEYKKQNYTERMQKTGRPVSPHVTIYALPVPAFASITTRVTGIMLSFGAFG
VGAIDLMGGPGASLSLMESIGSSGFLVASPAKFAVAFPLVYHYLGAVRHFVWDYFPDKYL
NNEDVQKSSIALIGSAGLISLGFVFV
>comp2061_c0_seq2; 150 221 316 LEN=579; translated
MTVLSKSSGEEYKKQVRLSVDLGRLSFYRAG
>comp2062_c0_seq1; 172 202 1432 LEN=2008; translated
MRLPKASQSTSEVSDRSSLRFHLNGNIYPALVITCLVLRFAVLNFESGLSEYSRAASNVV
KFDDRNTTHGPPVSSRHIYRSANDTAYTWQGNIWIPPLGVPHYTGAAVRCLFRSENTIWV
GDSTIRQEYHTLRSFLEAANPGNIEASEFVDGAFNPNLGHKIPFLFTARHGVSGSGDRCL
RVRPKGPSEDPNATLAIATYNQDPSKHSEDCISTGRNDLYRLNCYRDGIQWIDQDLRKPH
YKHGLRSWFSLAIISLGVWDTDPGNRDKCASLDRLEELLGKIEKEVSGPDLFVIWQAHAP
ASYEGPTNGAEALSLGNRARAWFLEHQPEHMDFIDLGSILVGGNRSFWPNRITGDHTAHY
GFEARALGGQLVADALHGGNNGMGSIALRTPNADGSTRMPAGGAKRNNP
>comp2063_c0_seq1; 344 63 905 LEN=1401; translated
MLSSNFRRTVLSSRNLWARSFATGPASGGSVSDEMLQKLGSLSTQALVDGLWVMGWPPAQ
IDGARPLTSGMKCVGRAVTLNFVPARPDVAQDKPPGGESPEYEAFELCGPNEVLVMSSVG
PAESVGGDIKFLRLKQREIGGLVTDGSVRDTDEIINYGFPCFSDSTTARQGPAAMQPWEC
NGVISVSGVTVRPGDAIVGDQDGVVVVPAKVADTVYSIAHGREVIEEIIKEELIKNPGPP
GKFYPFMSGKIKVDSPLGELLASKGITPENSNQFEGTADW
>comp2063_c0_seq2; 134 63 589 LEN=589; translated
MLSSNFRRTVLSSRNLWARSFATGPASGGSVSDEMLQKLGSLSTQALVDGLWVMGWPPAQ
IDGARPLTSGMKCVGRAVTLNFVPARPDVAQDKPPGGESPEYEAFELCGPNEVLVMSSVG
PAESVGGDIKFLRLKQREIGGLVTDGSVRDTDEIINYGFPVKFFVDTPDLLMIVGY
>comp2064_c0_seq1; 144 384 1101 LEN=1101; translated
MCLDRRSHSQGHFLSTGFRGKRVRWGGPHVAYTGFEGTEELVYRVRSSIRSEDPLVPLVS
ASAFRGACTVYYEATIQGGGSVGIAAADDGLHAFVRHRHRYGFLSRSRPVGSLPVSYGFH
GGDGRIHFHEGSAGATGSLAFDEPFGAASDATTLVGCGYDAARGLLFFTRNGRLLGVCPR
GIRCLGYCAAVSLTHPGGSATFNFGSAPFAFDLELFCAQRARFLQNCENNKNENNDENNX
>comp2065_c0_seq1; 615 156 1815 LEN=2023; translated
MLLTPAIFTTGFFLSIFVSLGLLTDVVRADLKYYLYIEDEGALHDTINKDGAAGEVVVLE
SVGSLPFEKSSYSAVGGSCPTVTKAGGMNSRYLCIGSHEKSNENVVFFPIAPAPGHDSTT
ASILPLRFGSLEESHGTSSGLLLFRNALAVDWLVPTTTLNEGTVSLNAISYGRDNSRDGF
NMEWLRSAASGYKDGQIVAEWNLLTEAGGDPILDGGSIQFIKGKPLPAIEQKQLSDGGNK
TLAAANISNSSQPETSAVTCQSITQNLCDDKNNDKFSTICSLLMQVGLDDVLSSPSSNSY
TLYAPTNEAFSRAFGKNASPALLSGHDLTKLLLSHLFVGDNIKSSQTQSISGQNPLRYED
MVCGAKQIMASQQVTKIGCDANAEVSYVTGAGNQNMYKPSFLAVDQETCNGIIHTLDYVI
LPASTEIGDEQIQHHHDHHVVDIDTTGGPGEGKSSTRKNNLDESALISFFNDNIGQVDDA
EDNMKTTSSTAATIEANNEGGPTFGNNDVHPVRRKDVNPVNTPQTNEQPRKIGDGELGRD
PVNKIWAKDSEG
>comp2066_c0_seq1; 356 1 1953 LEN=2225; translated

RGHWCLHRSDPRGIGSIPPPGDHPSLIGSSNPFHSMCVDPSVLSNNAPQHDGNNDNDNIN
HNHQHHEDSQAPCARTIRNQRQAAGSGGRRRTNGDRGVLLPKRGGTGHRKRPCRVGMPTL
REGGLETLRNGGLAPSNSSGHGVFDLLFRETADGPVQQVLMDGIRDAWKNSGRGIVLGAE
SFLGVGVGPEESGGRAPKTVYRLMENLSLRSGDVTLVLVYGTPRIREWASVWLGRSSPGT
YEDFLCGDAWADQRLEHSETTMNPFRVAEAYLSRGWNVFVLDEAAARASGLDPAHVLACE
ALEGVSCEGGWIAGLEGETSGIPPSYEIDGLDRSEESDLEKLFLARDCCYRAGLASTKGS
GTLRTSIGASAWKDCRSLSCGAGAGENLTDPSFFLRALRSQKGCGGEALDLSGLLTGGKR
PPDAAAPLLPVAPVVVVATATALSTLAGLLVWWVRHRWRSGKPGPLRGASDGLFSGETLG
RPGGPAPPASAFSGRLCNACRFVRADPACVFCGEGTVASPPGGMGREIERRTRECLGRAK
GRTDRGGRCPVDASHFDTNLRKTRHQSRSHKRKQMLQQLQVHRQRDEEAEEGAARESRVV
RKPKELLVTKTETGEKIYEVAERESSALWSGPCEGNPQAIVFENDERTLL
>comp2067_c0_seq1; 291 1 961 LEN=1203; translated
XRPTPTTGDGNRDLGTDTSPHRQLNTTIEEGVVPTQPRIAARSTHPPKMPRMTRIRSLVA
FLLLGACALLSNGVCHAFVSHRTAARPVPNNRFHRGRSPAGSTDTRLCAIPPEILVDVEG
SRVQFFGWFFGASGGMGIAVGAFPRMYEQFRALRALRGCEPSLGGERVGLSPLCGYPEDL
CVRDLEKVVNNKLSIEQIVEKFPIENNFLSRNGYITFLAYERANKGNNPLVVRAVFDSFA
QSTDACDPNVAQDKIDSYKEDVYQINGTLLQSKLVGYSAIGTLLFLLGLALSVMVGQLKN
GWFPSWTLADGIFNIPDFWI
>comp2068_c0_seq1; 590 103 891 LEN=1651; translated
MSTKRKFVADGVFYAELNELLIAELAEDGYAGVEVRTTPHRTEIIIRATRTQNVLGENGR
RIRELTSVVQKRFGFADGAVELYAERVQNRGLCAQAQAESLKFKLLGGLAVRRACYGVVR
FVMEASAKGCEVIVTGKLRGQRAKGMKFGDGYMIKTGHAGQVYTDTAVRHVMMRQGVIGI
RVSIFLPHDPKGQLGPKIPLDDVVTIMEPKEEVDPAALLAAQEAQQQAAALAAAQAQAAA
ELAAQQQAQVEVAAVGDAGMAM
>comp2068_c1_seq1; 806 145 1353 LEN=1666; translated
MILVSFKSFPRWLAATAAISAFALPTTEAFHPVSLQSTTGVSHSLGVTPAPSVMVAGGAR
QHAHNRPLTVMYRIRCENKYYQLEELEDADNCTTELFLKEDGTMDIMETDGPIFKKAVGH
WEIKENQFAMTITKTFVTGTDNKSDPTGMGEFSFDVERVFEGEMTVVGGTEVAINGKILA
EDVVTSKMEKEVGFFNMIDGTDQRLERRPDARPKNIGVIPSPIDSNDVKGHDGIPKEWLE
PQAPQVPQMDAFGGGSFGQDAQGYGGGGAGYGQQQQQQASDWGSGSAGYEQQQQQSPGIS
EYGNPGTFGQESQGYGGGGGYGQGDNGTNDPGPSFADYYGQQQQQEQQQEGYYGDGSQQQ
QTEYEQTNGDGNTGGSWDDYYNSQGGAGVSPMGGGGGPNPWP
>comp2069_c0_seq1; 385 1 1374 LEN=1586; translated
FSIMVASAVFITDLQGKAIISRNYRGDVVMNKVIERFATYLVDVEEEAKKPVFHVDVNGD
VQSGENVGETGTGGEHYVYVSHQNVYLCAVTTKNSNVFLIFTFLYRLAQVFKDYFGSLEE
ESIRDNFVVIYELLDETMDHGMPQSLDSNILRSFITQDANRMAEDSKSKPPVALTNAVSW
RAEGIKHKKNEIFLDVVEKLNLLVAANGTVLHSEINAAVKMRSFLSGMPELKLGLNDKLM
FEATGRANQAKGKSVELEDIKFHQCVRLARFENDRTISFIPPDGEFDLMTYRLNTHVKPL
IWVEAVVEPHKGSRIEYMIKTRSQFKSRSVANNVEIVIPVPPDVDSPSFKSSIGNVTYLP
DKDAIVWNIKQFHGGREYLMRAHFGLPSISAADVDPDGQKKGENSWKKPISVNFEIPYFT
VSGIQVRYLKIIEKSGYQALPWVRYITANGDYQLRMS
>comp2070_c0_seq1; 403 87 980 LEN=1129; translated
MMFGAKLPSNRENGSLSSLLFLQTSALLKKAPIFVLLICLPLATSFVSPRSAFSINNHLC
HYRKVGRKLYAADSAISADDPIPPARARLLPSVLRFRGAVDEGYGRGGKKLGVPTANLPS
SLFQNALEDVGTGVYFGWVALERNYDNDIEFHKAVVNVGFSPTFEGEENKEKIIEAHLIM
EDGDEKDTGDNTNNAEKNETTSDKAFIEDFYGVPMRLQLVGFLREEKKFDSFPELIAQIH
ADVKDAKLSLECEPYKACQADQFLCIKTSPAWIGSGGGDKTASWENAPLEEFLDTLK
>comp2071_c0_seq1; 198 48 446 LEN=640; translated
MGVLTVKVVKASHLADKDLMGKTDPYVKLELEQDNLIRDKDYGYQKTSVKQGDVNPVWNE
SFNFNIPTLDNMVLTLRVYDSDIGSKDDKAGWCKIKLEHEGISSSPKRIEKTIDRNLLRA
NGTITVDISYEE
>comp2072_c0_seq1; 386 1 1006 LEN=1244; translated
XQTKPNQTTPSKAKQSKPNAKPNQSDRTATTAAMMLLQRAILGASLLLAPALAFSARKGS
ELVRSAASSPTSSSSSPLSRSALSVGAVWTEDEDYYGDDSFLLGRAGACADSESCSLEEA
RACLDGVLRSQADCVGDALGSSPLCGNADKVAEVVAGLRQKIAREERRLAPLRAAFHLAN
LSVGAWVMWTVLHGVAAVPNVPVDGLSMVGTLPEGQAPETWGVATILPQEWYWSVRDGYF
PSLLAEWAKHGGLSVDLSAFDEKVVPFSPQEWVWAAQNGSLGTMLGEHLRYGGYLVDSGY
DTEGLSPLTPQEWAWSVRGGYFGDALRHFYRNGGI
>comp2073_c0_seq1; 301 148 576 LEN=673; translated
MIKPDGVQRGLIGEIIKRFEQKGYKLVAMKLTSPTKEHMENHYADLKEKKFFPGLIEYMT

SGPVCAMVWEGKNVVLEGRKMLGATMPSESAMGTIRGDFCIEVGRNICHGSDAVESAKHE
IAMWFPEGVISYAATEAQWIYE
>comp2074_c0_seq1; 285 1 1787 LEN=1872; translated
XTMLDIAKHAATDEVRLNQLDPDFAALSFYKLFGHPTGLGALLVRRPSVEVLLEASTVGG
GHYQGGGSVDLVLPFRGYTVPKPGLASLASGTCHFRGIADLSHGFDALERLGGMPAVHRH
AVSLAGELARRLSEMRHANGRMVVRIYGAWAGRGGEHRQNNGIGPTVALNVVRSDGSPVG
YSEVSKLASLHRPPIQLRTGCFCNPGACQRALGVGEETALEHYRTTGHVCGDHIDLDGGT
PTGAVRVSFGKDSLWEDMDVFVRFLETTFRNHNPETSPVVTGGNAPGHCEPKSGPEESKV
SVSELYLFPIKSCAAQRAPKWPLVLPSGKLRYDREFALVDRSGTVLRLQKHPKLGLVSPV
VDLEAGTMTVSAPGCEDLVVGLSETLYHGGENAVRVCGDTCGGRVWGDASVSDWFASFLG
IECWLARYDSSLATDRVIKDRPGFSNEQPILLVSESAVATLNGVLSEQDQPPCTAKRFRA
NIVVKGHTTRGGHPASDRCHGHAEDEWKTVRLSRNHRLEFSVEGDCPRCAMVDYDPETGQ
KGKTLRALASYRRRNNGRIVFGIFLRASEGILPDTSSLGAGNGDRGEEGSDAGTIW
>comp2075_c0_seq1; 222 1 948 LEN=948; translated
XKTANKHVYFHQRISHRLIIHSGTTIPEALPQENRNKLFRDSNHGQRNIRTEETPLSSAR
EAKVEHFRDDENKPDHLRGIFGLGCIDLGLQNNYLNQLSQGGQNFQQEPTEYYQPAAPEP
GSYAADSYEAEVVPTEENKSNEFNPAYYQPPPRIRLGYGYDHQDNRKGGMAPLKPGVDTT
PNGASATMGRGKMEMLSQLLNAKASAEVASMGREKMQMLSQLLNASPSGESATMGRDKMQ
MLNQLLSSKASAEVASMGREKMQMLSQLLNASPSGESATMGREKMDMLNQLLNSKPSDEY
ASMGTEKMEMLNQLLN
>comp2076_c0_seq1; 299 283 951 LEN=1032; translated
MECEREKIESSETSSENDAFDDDKKYKSSTKRKKENRKSERKSEDRARKKERLGNSRGKG
NNEDDYSDRSSSRESASEDNYRRRDRKRKRHAKKQRGSSRKASRSYFSDDSSDDDESSIS
NSSETDSYRNEARRKQKKDGRQRRRKRKEKRNYRYDYSSSSSSDDESSNHKKRKHKKGKK
KKKKKKSSKSEERRSVISGKKIKLHIEKTDEDLLEKKPERIIW
>comp2077_c0_seq1; 396 122 1351 LEN=1449; translated
MSNRLGRATGTVVNASQLRLILMGAIGALFLNNLMISTSHTNRIIHENLDGSDMIPPEFY
SSSQRQTKEETEEKFPLEVGRKLLTDDDDETIRRLRNSRTLMGIFSSDNMFDASHRKWHR
NLFNNVWKDDRVCTLNQFRNSDDIDFQKKCELIYTFVTGANEDPNAPTERLEEFDTVDTP
IELPGGYKSPMKEDINWDDVTHLNIRENMNDGKSPTWFYFGSKMAKHFEKTETPIDYIVK
FDSDSMLKLHDFLEFAHNRLPPAPYNKNIFAGALRDKRPWYFKDSKLGHNQADMPRYESH
WGTEYDGVHLYLAGQCYLMSADLAAFVAEEAPFSKIRVAKGGYLEGHEDHDVAAMVYHNP
SPISLITIGKSQRFWEHPVKGAPRYNKIVARETARVNKKPFEGKILKLY
>comp2078_c0_seq1; 446 239 1396 LEN=1396; translated
MNRSSQYLPNIGVPCHRGASSSSSSKQFSKDQKKTKTHYIRPEDLRPYDILCGRDKATFN
NIGNRRFRVSIGINIPRYEAAKSKAQKADVIKSVCNVLQKDVGVRFLKKTKSPSTKVSGD
DECEDYYYIELDETEARKKVGHALRDMSVARQQLRQKREVAKRQQGSQKNIENIHNKRYK
LTTDKQNDGVINFPMTENFQAESYTAQNNIEDTLLEPLPIIDNLYNDPLLPSQIQLESRE
GPEEPRRLSLQLPQQRRPEESRRLSLQLPQQLRLEEPRRLSLQISQQLQPMQTSDDAFSQ
IEQQRIQFQQRQQQFREQIQMQLQQQRNRQRNQKQHRQFDQQHHQPHAISMQDIPQLEQQ
QYDRHRMLLLLQQEQQEQRHQQQQQQ
>comp2080_c0_seq1; 314 240 1187 LEN=1299; translated
MIKNSVTVSDHNHDGDNDDEYFMRKSLEVAKRALDIGEVPVGCVIVLDRHHPAVVKKKDK
IICKNETNESCSIGEERRGVVISHGANQVNATRDATRHAEVVAIDRLLTDGVSSDKMRLP
PDLARKGQSSHAGDRVPYSVQKARNDQWEDRWINEPSNSGHWTNSFGWRNNFDCNGASLG
QDEDKEHISIEREIRSEDIFRHCDLYVTCEPCIMCAAALATVGIRKVIFGCKNDRFGGCG
SILHLHQVEKEDTATLQKIGRGGCDNNPTTMTNKSVGYEIKSGVLEEEAITLLRSFYDRE
NFHAPDDKRRRKDQA
>comp2080_c0_seq2; 228 240 695 LEN=973; translated
MIKNSVTVSDHNHDGDNDDEYFMRKSLEVAKRALDIGEVPVGCVIVLDRHHPAVVKKKDK
IICKNETNESCSIGEERRGVVISHGANQVNATRDATRHAEVVAIDRLLTDGVSSDKMRLP
PDLARKGQSSHAGDRVPYSVQKARNDQWEDRW
>comp2081_c0_seq1; 449 95 838 LEN=937; translated
MKFTLSILCLVVAYVFSAEATSLRGKSIPRKETLMQAFLSAAQSGDRRLDQYNYNQYATD
DNQNQGDDQVDDSYGDDYVFYDDYALQNCEEGDEDCETVAKYTAYDDTYLANCEEADEDC
NNAAAYMTAKKAKEEEELNSNEWSLSNLTDKYNSMSRSSKTWTIVLAVWFTALLVTTSYF
CCCRQSLSQREKSRGRRLRESFLGGNRRGDSDGAKTSDERKRGRSKFKFFGRKGNKSKRG
NSRGPVY
>comp2082_c0_seq1; 478 336 2807 LEN=3227; translated
MRITNGTLRCSSSRRGNFILLFFAFLVQSSSDALFCNAEVPVGSYGTASESSDRNSGIDG

GLWKVPTLNSESGPQANEIYLNYRCPVGDLSPVMLEEDTGTYVLFRQSPHSNETEENLVL
IDGGEKLNRSRGNIYSGDNIDSSKEKFGADDNSVLDHIRAGTRRYLRGAKMSRETENRIL
VSTPIFVASGFVEEAPTSENSMRFLSTYRKNSLVADRRDLRDAKNSNDETSADLESYSTS
ELLMLRPCGCFDEFGTFGGVSEGSDSDINQIGDPLLTFCPASSDWCAYTTSYDTDASSRS
PKLECFSMAKDERVALNAWPLLWIMYAVVLCSFCCSFQGRILLDFVRGTILYYVCGCGDR
YRNRQMRNINRYNQFVVQEILDEERVDRENVVRHRTRNQNGNWMNFDPPRIPPTITTSTH
HEGLSQDNDNTEGSNPITEGRGDHPDVSIGNNRLVTWWRGVVWPWQTSWNRYIKYNLLLR
VEWMAIRDEIAFIESRQERGLPHAIYEMKTRRWSSTEEDTSRSSCCRTGNEYDHSIDAPH
CTICFTELEDGDRVGDLGCIHVFHVDCLKMWIQKRNSCPLCNIPIANRRRLSSEELLVKN
NNNDNRGAQNESPQEGHRNNHGRTIEEGSLEAQGSSLSPTIRNTDHGSPSNHQRRLSHLP
VTPSTIADDVDAYSTFSEEIESVVQLDDDDDESTFGRRTLEIGEESGNGEAEQTTVAVES
SLLAATTRLSASSAVTEENEDKNSIMSIEDEYCNIDDFLLSKDNQGRSVASDHSEAASFA
SSARAENSVDDEVDLPSPPRHVRRLSSEAPSSSVMLPNHQRQY
>comp2083_c0_seq1; 191 228 599 LEN=1280; translated
MIQAVFRRYMVLQSMEREGVTTSYIRNRKRQRKASTKMSYFQTSTVDETAPDLGFGCCAM
GFDLCGNDYDAADLAAYRTFQRRQYEERKKAQKEREEFLSRSYMEQKGIDTSIMLRGQSL
IDA
>comp2083_c0_seq2; 369 253 1086 LEN=1767; translated
MGLFRKKNRGADADFDIDFDDYDNSSIPREVNTKGARHSRSRSLGKIFGGSSSKKTDKKG
KRKSGKGNNNSGSMEKINYSSSTSTSSNRDARDDRPQTFRASTSKAKSETKSRRHNEDYG
QVNSLCSIPPTAADAAFHGPPRFDWIDIEFNAATMIQAVFRRYMVLQSMEREGVTTSYIR
NRKRQRKASTKMSYFQTSTVDETAPDLGFGCCAMGFDLCGNDYDAADLAAYRTFQRRQYE
ERKKAQKEREEFLSRSYMEQKGIDTSIMLRGQSLIDA
>comp2084_c0_seq1; 369 308 1306 LEN=1566; translated
MTRPTGPLGIDPLAFTFSLFGLLLLFVLYLILPRGLRKQYFGAYPKRHAWSARSRMRRNR
GYGDHSLGGGSITGASSVATGTTFGTHGHPGSHRQLQGMGLGASHSVAGSTIGGTTAYSN
MTGRRGFDEEQVVFGGGAAGAAGTNQMLDIQQNEEIVISAAMQQLRDPGVLIVAHGSRGK
PKTVRLQLTESAVTWRTETRKKGVAKDAKPKMGKLHQVPLSHIMYVDVGKQTTALRRVEN
ASVNDALCFSLLTKEGSLDLEANSPRERDALVSCFSLVLDEVHAQNWRDIYRGPSSDMPS
SFDEIDQQQQQQQPQGTQGQRQHDPTAGRGHP
>comp2085_c0_seq1; 558 230 2389 LEN=2504; translated
MGFKRIGRGNNLSGIFHLSVATVVVAILRSNSNGTPSVFCSAWVSTSSSSTNGGTCSSST
FSALNNGATARSPSGRISDKIGDRRYWMILNAVVDDSRQGGNDTLSTSTTTKSQGGRKSS
TISTSKIKKTNRIYINVDRWNSMFERLKEYKKTYGNTNVPKKYNDGGTPALGNWVSKQRF
EALVYQNTKGEEGQITPERISKLESIGFVWELGRQDEWEEKWLSMFSELEEFQKKYWSTK
VPILNNKELNFKSGSGLEDPMSSLGRWVKTQRIQRNKLLRNEAAPAMTKERLEMLESIDF
AWSGKQASDYDKWLNMYFKLMFYYQRHGTTVVSDSQGSSRLLKWTEEQRRFYRDLMTTNS
TMSASKTGAAATILNTLTEHRINLLNDINFDWSHCHEDRTWDDMFNELVEYYVNFNSTLI
STSINERLGKWTILQRDKYNAGSKDLDRQKITKLNTINFDWNATEDVNWNAMMYRLEAYK
RKYDTTIVPNHDGGDRPLANWVNNQRVKLGKYVINEDGEFYDDHDLVERIALDLGCSPET
EFEPKGFIQKLSPSTKATRIRRLNEVGFVWDAREASWQDMYQRLLVYKEQNNGSTMVPFV
PQDHRSNNKSSGSTEQQSESLGVWVKVQRHKKQLGEMSEKRLVLLNEIEFVWDPQEAQWA
EMFDRLCKYQKENNGCTRVPRAYSPDPELGAWLNTNRRAYKRKTLPQERIDRLDSIGFVW
>comp2086_c0_seq1; 517 128 1516 LEN=1773; translated
MTTGSMLRGRMLGAELAHSDALRYLTTYNNSTVADSESDTSNVMNTAKDYASRIKTATSD
LTSTHPRDWTHDQYLGVVTGFVAIWTLLLLLCTCRKCCDRRRKIQRKSVDDSYMNMSFAD
NKQALLNQHRSGPYKYDMVRPRVVRGTATDAGGIASVESPSGYDYDAMDPNRLPMGARLH
GANKLRSDGLSGRGVRVAIIDSGVDHTHPGLHDKVRTKRWFRHGTSLLEDDHGTHVAGTI
HYVAPEAELFDYRVFGKDGDFTGDVAIAKSIRSAVDIDKCHVINLSLRCSYPVLPAITKA
VQYAKRKGVHMVCAAGNDGDGNGNTDELYTFPAQFDETISVAAVRKEAGLPPANFSESNA
KVSFSGIGKDVISLKPGGGHQIMSGTSMAAPHVTGLIACLLSDEPTKYTDYQLRKLLREK
HTVDIAEKGFDKQTGAGFVTYLVAEQGEDEESNSLFDLLALV
>comp2087_c0_seq1; 222 64 393 LEN=606; translated
MASMQFNTFDAFEDAEDKGGGASEEKVHVRVQQRNGRKCITTVAGLADDLDIKRICKAFK
KNFNCNGAVQKDEDAGEVIQLSGDQRTNVKQFLVDQEICHALSIVLHGF
>comp2088_c0_seq1; 1147 1 3439 LEN=3503; translated
XQQQQQQDGHRPGEKTRRKSTTRVESEAMSPSSRRSSSRRSSSRTNIGVPETLEVVRRKP
SRHGSNSSNHCGDDNSIVSAITMDVHLFHPYSSRDDNFAFNTNYSEEEEEEEQRIPARPT
HQRRPRPEKSGRSKQKGTGNNGSKSIDNNMNNIGIGQFHTALRRRGSHSDESSHRSDSSQ
HSRHRKSHSMRGDSERNLYPWDYGEGQEMGSGAMHQQLNPSTNITPVLECDALMRSVSSN

EDTYFFDRKSVSNTKTKKIGSEVGIDFQPDENANHSRRSRQTGMQQSIDFATGSTANNRN
TENSAHAVRSEYPPKPHDKTAIEDDNDGFPSFIMDDYDYNDYNDDYDHNQNILRQKQQQH
FQQLLKNDTNPIPHPPITSVSSSSSSSGRIHNGTNARQNSLFTPITKQDITDIQGILHCL
NLEECNPDVREDALCKLFDMIQNMSRAGDRHTREFILEHNVIEAATKAIWADMQIVEVQE
AAMQLLLLISATTTLIGGRDGDHEHGKSSGSGGSNDSNLLSKNESVCDSILFAMQNHATE
PGIQLKGCLIFASLAGSLPDESAAGTNSDGSLSGAMTMVLKAMSNHGNSRSIRKAGLQAL
HHQCLLSIHVENNKRSLASGKLENGMAAVDVLIYAMEELQQDLVAMDWACQLCWCLTARK
DLLQELEESSLHEAILSVCQHYMTSPAGISVVEASIGTIGNLAYLDKKRKELLNIGAVEL
VLDGLRYHSNHFGISYEAASAIENFALPPDIRGSSSMVSKADAVQMLVHGLKMFKSEHEY
VVQVFRALACIATQSQSSKETICTEEILVLVLESIRKHKTPEIIATCSTLVATLATGDSN
TQSELMINNGIVDILIQCMELTNGEKAQDAACLALRNLSCQIHDSEPLLKNAKTLSLIIS
AMESHRSSLSIQSSACCIIWNLLSKTEEKQLVFNSKIVPSITSAMQSHFESGDLLELACG
ALWVFVDTFNDQKLFVGKEVIDMVACAMVMHPTKTPTLENACGLLSHLSSEESLAEAISK
AQGVNIVAEAMCNNGSSISLLEAGCLTLKNTTIACPSFARDGSVAISTVINALKENVNHT
SFVVEACDLLWVLASEDDNARSKILALDGISILMHCLEENSNIPQVETSALGAFNQLAKS
NTVNVA
>comp2089_c0_seq1; 243 101 508 LEN=824; translated
MKSHLFSAPSLLLATALFLAKAASVSGMGFFNSLMAHIQENAVEEIDRMTDSSGKGIYFS
SGGASTGGVNRYAQPERFTAFPGSSSKNDEERIHQRKQLLAKERRTKGYLPPMGVTQSYS
SHDEELIERLVELQN
>comp2089_c0_seq2; 243 73 480 LEN=796; translated
MKSHLFSAPSLLLATALFLAKAASVSGMGFFNSLMAHIQENAVEEIDRMTDSSGKGIYFS
SGGASTGGVNRYAQPERFTAFPGSSSKNDEERIHQRKQLLAKERRTKGYLPPMGVTQSYS
SHDEELIERLVELQN
>comp2090_c0_seq1; 278 24 677 LEN=826; translated
MIAINNLMLTKQTVLKNKGIMMNVNSCKKSRNEINPNDVLCGRGGLTNSNIGNKRFRSVV
AKYQQEYLHARKNDKKLIARRIITEIKENDGRFLQRSSDPSIWSEISMKKALEKTSQALR
EGLDVRHKTVRPEKLVHKIDIDSNRWRTPKKTEARVVEGVVIESPSKNEIVSGEDVPNFV
QESETHEFDPIFTFSHAMISEQKEHIQESESTNIEAI
>comp2091_c0_seq1; 607 288 1679 LEN=1724; translated
MGQDEKTQEEMDKQIEIFKVKKLIKNLEAARGNGTSMISLIIPPGDQISRVNKMLSDEYG
TASNIKSRVNRLSVLSAITSTQQRLKLYNKCPPNGLVMYCGTVITEDGKERKVNIDFEPF
RSINTSLYLCDNKFHTEDLNELLIDDEAFGFIIMDGNGCLYGTVQGSSREILHKFSVDLP
KKHGRGGQSALRFARLRLEKRHNYVRKVAELATQLFVPDGQKPNVQGIVLAGSADFKSEL
MRSDLFDQRLHKIVVKMVDVSYGGENGFNQAIELSADSLANVKLMKEKKLLQKYFDQIAQ
DTGQYCFMVDDTLRGLEMGAVEDLIIWDNLEIQRYKVRNIQTQEEKVLHLTKEQEGDDKF
FRDAETGAELETIDKEPLVEWFAENYKDYGCNLEFITDRSAEGTQFVKGFGGIGGTLRWK
VDLAELNSLEEAANMDHGVGGDDSDDDSDDGSEYGFDDGDFGF
>comp2092_c0_seq1; 427 88 1152 LEN=1374; translated
MKLTKAQNGNKTTRTYAYSTIFLLCASMLYFREGMSLGEYNLYDDAAKMYNSAVVGVGSD
SSSAAVAEKSHMLSLEPSKSSLHAEWKLWEEMNPSEQDEAIQKVGAYMTKYGKLIYPNAK
RRQTIKQGTCELVEFASGHALCGPKPPNGCSFFSFGINDDPSFDQKLAELWGCRGFAGDP
TVPHPSKLHPLVTFHNVGASMLQDNEERLANKGGAEEWWSTSMPKLRYWLGLERVEIIKM
DCEGCEFALARDILREDPTFLYNVDQISIETHVSKAWMTTREHFYYFALHFALLEEAGFK
MEWSSIFGCSKRHEVPGCIPELEQFGFPCGYDPWPGRPNVVLGRSCHDFLWKRY
>comp2093_c0_seq1; 424 159 1022 LEN=1132; translated
MAVQATNVVLKENERIPGKGRYIYSYDDDVANKCGVVVMIGVGTAMPASAYRILSKKTSA
GASIVTVFFDHNSGSLDKTDDKKYADFYNAFTENLSEYVPVCEGKSPKIIAGGHSAGGRA
GLQAMMHRGIEPDGFIGLDPYQASKDRIRGIGAKAAPATEIASDLPVLSWGFERRTCAVD
PKIAGKSQYNLSGENNRVFFRANNGSARGVGHCDFTDSGCGPLPFCPNPQNDDVKDVVAE
SIKVFAAGVVAGSISKSNFGFDTTNPSVPIDVFVNSEDVFAPSEAEL
>comp2094_c0_seq1; 386 1 924 LEN=1159; translated
IINDQQLEATLVEPIGHRTDIPCPRKHLGKFEMIAICQTRSLLASSLLLSVAVCLSQAFF
LPSATHGSHRQTHNERGLSNFNLPLRESDFLALSAKKKRRRRRKDNTATPSNEQTEDVED
LSSFDETLGPGELPEFDLGEEEEEKEKPKKKVNPDEITANMMGSGSSGSKTVDELISDRA
LESKLEFDDNGDASIPDFVDLAQASSTTPTYSPDDPSMTAGVGKKKQRRAERVANAIAAR
EAEEEEKSFLEKFPQFLNEKGEVSAIKILEQGAWIGIFLLIGWELYINSPFFERAAPMAP
IVYEILM
>comp2095_c0_seq1; 350 169 1491 LEN=1940; translated
MSSRVCFTRGLSSLQIGIQGRRNLLFATEACSSRRYTNADDTFVSAACIGRDAQRVSATE

VELPLADGMVVRGQRWIHLDRLQNKNQAATTAPTVAVGGENPVTTRILALHGWLDNCRSF
YCLAPRLVEKLGTRAELVAIDLPGHGRSSHRPIDGPTAVLSEGAYYIAEILDALEWGQDA
NANSSSKCNGAKEDQTKVTLLGHSMGGGMAVTYAGVFPEQIGNIVSLDMFGPEPGEPEDA
AAHIRSHVVQRRGLGPLGRPHVLYPSLERVIERRMKSARMAPGGHQYLSIEAATELVTRS
TVPVYASDDDEAAEESFPKGYRFRHDTRMMWPSLQYLTAEQIASILGRVDCRVCLLAAED
GYPFRQDRVDRALACLEPEVYKVLPGSHHFHADPDTAEAVADAICEFLRGGKNRESTNTT
RGARKNASPSQEATATYGMQ
>comp2096_c0_seq1; 300 275 1144 LEN=1247; translated
MPIHTARHRTPISVVAFALLLSIGSTRSNRQVLAAPAPTCQSFGESWYFPNSCVADCTAQ
SDGSSTMDTDRSRNVNGDLKCYCTGRESPLCADDPMCSDLEILPGSAKESCEAVCGSSGG
SSDVVVVDDVEYAGDASAANKNQTHFRVGCSCDGGATQVCGTDYVLFSDLTYLKWCTRKA
DNNSLQIKSAEACDAYCTKTNPLFDGGIWNDSAKSCACKEDSMEVSAVACDDSSANTNDG
SGLGNPCYENVGVSTADCSSSSNANGEQPSTTQTLVSSAAIMGIWLLPW
>comp2097_c0_seq1; 118 116 364 LEN=752; translated
MPFVHITWLPKTCRNAKVRKEVAAAVMKAMTSHPEADVAPENLVVRFSEAVDGFPLPKGH
SENPELSAPQTPAGEAFLKDRE
>comp2098_c0_seq1; 337 188 898 LEN=1024; translated
MKPRISLSNTAIRAEVDGMPEADIAAGVPDTAAPQAADAVSEAVGAATSPAAPATVDTAA
NIITPNQFAPPVAPKIIDPDAPMPLPVAPLTDLDTIALVAGQENYGLAIVLLGEAIWSFS
KAPSVDHGLKTLVPAVLAAAALFVASGPMITSGDAGSISTGLFIATGVSVFMGACYAARL
SAPFSPSPKEIPALGLVVAFAGFFSFSQNLVVDGFVTLPSLPHVDLPSLPSISLPF
>comp2099_c0_seq1; 501 1 1210 LEN=1351; translated
XVFVPPRYNRALISIVRFQTKSGMIGHKIMQNDDGNRGVVDDKIPTDIAADSGAVTVPST
QTATSEDRIVESLYRHVIESMCQDVASNMHELIKTGAGDVVPSSWKLFMGTKIPTRRELY
PELYNGSDFNAASEGNKNEEDTREGGDEAGENMDPKTLNRIRMTDEEIQGLLDKYAVDLP
TSSSYSNVSPTVSRKRRTPRDMAEEEEERRLLSKIKKGKESNYKDDSEDIEDDVTNGDDD
DDDDEDFKMDDAEGDDDDDDDLGGAQKKKRTLSSSSATPSTDSIAEPAITTDLPSSSSNS
ISNINSHHPKTDIWGKTPSKEPKNRLCLCQLCGRLVSTSRFASHLDKCMGLSTSRGGPSS
ISSSTASXEKYVIVVRNAHEKSKKGKLTYEEVMQKAQITSKEI
>comp2100_c0_seq1; 315 474 1490 LEN=2059; translated
MTAPEAPTTTAIGDDTKPFPPASSLKDEETTSLRSNASSCTSLNSSCSHDTDTSCARALP
PSLDPSTKKRKRVSFSLQQTAYFSPEQMLSKQERKSQCWYSEVELSLSREEARAAIAALH
HQLQLDAAAAAAASAGTHGKRQPSPAQPTELSNEYGSWVLRCPHDETKIVCLRGIEKYAD
AAAKYAGQKRLVDSVLQQQSLNSEDIHVALVSRTLSQPFTEVARYYAVKSAEELDLCRKH
EQEKERNQTEERKQQEEVATVLLLIMGQQENREGTLHEPPHHPPGTPSPSPSSSKLQEDS
PIVTPRGSASGKRSSSFSNSPLCEQRNVKPCIRAVSDR
>comp2101_c0_seq1; 273 118 960 LEN=1016; translated
MITNSQRHSDERTRTRLLNRLGYFKTPGGGTAGLSSSELSSVISQSTISCTNFSFPLANH
HPDNSQNLTLLGDAAPVMVNLNDNLNEGDCRHDDDVPYFKSSSPTSCMIDISSTIRSSST
SHQSYNKSQRRNYRPPEPPPTDITAITTATYNSTSVQFNEKAAVLLIPSRYQYSDRIKKC
MWSSKQEMTEMTKRNATEFAAEGWDWKNVVMERDMHFDKVTGTLVHPCHLNDKRLYMLYD
EIDRDAYDKDDYQAYDCEQEYCFQPLERKSHVMARGNMTR
>comp2102_c0_seq1; 944 220 3063 LEN=3249; translated
MSSIMVRSSTTDITETGHDRYTIAETMDDSFDDDDDEYESQYNSSRHERTIISESTYSGT
YGTTSYMSEMSEVLDEDASFIDDDDDYLSLGSEAMVGDGWVDTACGCLDRPVSTYQNMCS
RKQTSLLRHSRGKPRPNFNQRSAVIPTSKLTMSAKKRRNFRLKDRSVCEHGERDIEDVED
SDIKTDARRSDEIKMMRIQPRESYQLEDDFDVRKVRDTEKTRNKNSSKSMKVKREEAAVS
HNDEDEPNVKQEDRPEKASEKKMVPATESKTEDDPVIKNIEAPPPMERGIEAEGIEISAQ
MSGISTRDRSVKRDPPTESEAVATREDQPADYYPENYSNLEAPASQVEGEERSKDEDASE
EDVTLPSPPSFGDKKTSGLETNRNGNVSPIPVEEISRNPSDMVVLSSTSQNEDFARTPSN
ENQKEVGDGASTSNNIEIIDVLNLSIDDEDSWTPVARHDVEKGTATGKNSNEFSRRSMNS
NRALHPMNSPSMEERWIEEEAKLMKKVKALGLTNSRPQNHTVMESAEDVWNEERMKLQKP
SDDIFDDPKAREAFLDRRQGRLRDAVMISRNKMLSKNGTSITDSLDGVGKQVFPRSQSTG
RNPPPSPEMRTKIMQSRSSSRDRITRNRSMDPGSASLLPSRSKDLVAVRSIEEGQRMRSS
NRASNKQRTFSNSHDPIVVHSYVGDTLSRGPYDELQGRNQHSRTTDRRNRPSTPSRSRST
SIRSEPIVAADLTNVDYPMNSHRFSDERDDQESLTTANPNSIESSQTNVFSASEKLLKLE
QKIERQLRQVRGGVQEDLERSREIRRIESKLSQKLKSFRTDGADLQTLSSRDIKRLEKQL
AQKLSGDNEKRASRLKKIKRRGVSSARSHAHSSGMLTDDQQQLSLASSAGRDRAGSAEVV
TTPSGKSLSRRSSRSQESRSEKSKYQTLQGLRSRYVRRGYSRMPETD
>comp2103_c0_seq1; 1093 61 2055 LEN=2436; translated

MKFVAAAVACIAGSTAMTLATAELRGMRNLEQDHKKMLRRVPPGFDINQFTQPPEVADDS
SGTVVEVTSPPSAAPVLPDCTPVDGAPSRTEEYVVVQYDSSANDKVINFDKKEDQIVLDV
LYEFAYKNATGPCTQKGAVNEVFDAEFLAFNEDDSTFLLRIGRITNSIGEMFSGTSESCG
PIVYNSTDPGSVDLSVRRLEEQCECPLPTAAQLVAGYSAALANEYKDEGLGENFIVGTQQ
LCAEEGCKADKKGITTYSSAGVCLGDMEALTNVPSSSPTDFPTDFPTKFPTDKPSRKPTA
KPSKSPTEGPTKEPTFEPTPSPSAEGDTPSPTAEDTSSPTADDTPSPTITNTTSSRFLVE
EELVVSQNQDAYAYLTEAQAKQDVCSQIDWKLVNNEANRRMCKDYLELPYAKTDAKKCAE
QSSETKVPKKFFSVARTSHESLVHTGVSASNPDFERYHFSDSEALEFIEENCGADVARAY
QCLAPPAFRADLFRFCALYSNGGVYLDSDILPLVRLEDLYDPCAVATVGHDWPQGRPQKQ
MKILASQKNAPIFGCMLQKIVGNVANEFYSDNALAITGPIALQECYELHSEGVAITYHDT
RQAAFPFTGMRDVHGHSVLALETPPTVERENYKTDVDSHEVYRPNCPLHTNIAKKVAATV
VDNM
>comp2104_c0_seq1; 430 115 1134 LEN=1341; translated
MKFSAISLHTCLAATALFASNCNAYTPGVPKVTPVTQSNALDNNADIAMSQYEAALKAAF
EQASSTAAPDAPEAESSTASATFAEQVEEQEQAIDQYEAALKAALESTSAAAGAPSSISF
GEADQNGPNWESISENISSQIEKAESKIGAPLSAAAKGEIVGTVLAGSAVLGTAAINSPL
LMGAALGYASTHVLSGKRGEDLARVSKDAFLSAVTFTQTQLDQEEGDISKASARIMQHLQ
HEAQNKAMEVGKVAQTQAHQVTRDITNAPTHLVEEGKKIIASEDFKKMPNRTFKAVQAFL
GSDEVKRAKESVVKSIKDGLESDELKAVKDRATRSLKEI
>comp2104_c0_seq2; 437 115 1155 LEN=1362; translated
MKFSAISLHTCLAATALFASNCNAYTPGVPKVTPVTQSNALDNNADIAMSQYEAALKAAF
EQASSTAAPDAPEAESSTASATFAEQVEEQEQAIDQYEAALKAALESTSAAAGAPSSISF
GEAEPVANQDDQNGPNWESISENISSQIEKAESKIGAPLSAAAKGEIVGTVLAGSAVLGT
AAINSPLLMGAALGYASTHVLSGKRGEDLARVSKDAFLSAVTFTQTQLDQEEGDISKASA
RIMQHLQHEAQNKAMEVGKVAQTQAHQVTRDITNAPTHLVEEGKKIIASEDFKKMPNRTF
KAVQAFLGSDEVKRAKESVVKSIKDGLESDELKAVKDRATRSLKEI
>comp2105_c0_seq1; 432 129 1704 LEN=2050; translated
MARKLFAPLVSISPPVLLCRSGISLGTSNNIAPSSKSTTRLLLNIPKLSHLSLTTTARTT
TQSRRVLPRTNHFYTAKKWFGNIHSISGEPIIKSTDYKSSRVEQAFDDGSLETTRAPSLK
TNSIPMIRKAGSQRLRNKDCFAASASQQLGEMSPNIKTKDENRYSVVIDNGFMKISMIPT
LTLSDGKTKRMDGDFCNNCDSPNNFEKVMQSPSNRDENEFKKQKIEIPETVLQYQRQLRK
TQAAKRASTPLLKSQLQVVYNDKHIVVVNKPSGVLTVPGINSNPSMLTLLHKKYEHEMDP
QMKPEHMIVHRLDMDTSGIVMYAKTKKALLSLQALFRERDGVMKYYEAVVCGHLNPDVER
GSIDLPLQRDHRFPPFMRVATPKSEHEAQQVVKDLKNAGWKKIVKKKPKPSKTLFEVIER
EYVYCDGYAEVDENLCGAKEKQREVKRYPVTRLKLTPITGRTHQLRVHCASIGHPILGDP
AYGIYGEASANGGFEEDLMDELIRDRASINLQLSLNEYAKAKAK
>comp2107_c0_seq1; 227 1 1006 LEN=1075; translated
XKGIDTRTYVRSTRSSPPPIGFRAVPYRIVTVYKEISLRSIGHISYIDSFQPFPMRRGWK
VPALYLATTCTSSHFALGFVSHQLSTAAKASAWKPASSLIYGSPSSGSTFTRRNMSSEVV
PAKDYGPTDQSWRSLLEVSMARSRKIRGSNYVQISTATKDGEPRCRTVVFRGFLNNKNLP
EDHPMSASGNVDGKPCVMKMCTDLRSNKVAEVAHQPISEMVWWFPKTSEQYRVRGSLLLV
GDDSGDRDLQIARKELWGNLSDPARESFLDDKLPVPVGGRDDEGKVVPVPENFLLMLLDP
IHVDFLRLTGAQYRQIDERQSGPDGSTWSAKSVKP
>comp2108_c0_seq1; 362 87 1670 LEN=1778; translated
MNGETFNVDPDFSVIFGCRSSLSLVGLTTAVVGYWLMERKWDNEGTGALEKPDQRVENEA
GNGIYIHMYDPNRVVAQGFHPSGERDLVRVRTDDSEDKRYHPGQAASNSTEPIQSLEAAK
YYSSPDILYAKLAHALPVPRTMLVGFGLWSLSFFLDPSLGGFRFYANFWNSCCFIFSGLL
GAILGFPMRTATLERDAEQKKKAMAAFVACSVLLWIVAIVDPEVDGPWYFNVFGVLFILL
SYPAFMASRKMGYTWFTEGKPRSTASITIQNMGPLLLIFGVFLLWVGTNAIYMVDLNQSY
IPFWTTAVRGWVVFIAGMFLIVPAHMALDRAFDQGSQPVVPGFRDTYVYKMDGTTFAALA
RENITRFDLAWMARLLETPLLGSIGWILMGLCSFMPFGIMDPTIQKFFTMFLCFAVPPVQ
FVLLTPAFWRSDRADFLKWMRVYCALMVALVVAIGHSSGIAFLFSFIGMGLIYGGKRKDF
HDERKRGNLWLTESPPSANPSPQVYGLGQPLHVLGWILFCTAMSVPM
>comp2108_c0_seq2; 221 87 770 LEN=911; translated
MNGETFNVDPDFSVIFGCRSSLSLVGLTTAVVGYWLMERKWDNEGTGALEKPDQRVENEA
GNGIYIHMYDPNRVVAQGFHPSGERDLVRVRTDDSEDKRYHPGQAASNSTEPIQSLEAAK
YYSSPDILYAKLAHALPVPRTMLVGFGLWSLSFFLDPSLGGFRFYANFWNSCCFIFSGLL
GAILGFPMRTATLERDAEQKKKAMAAFVACSVLLWIVAIVDPEVDGPW
>comp2109_c0_seq1; 277 128 847 LEN=1448; translated
MISEIANSLFLTYLASEYILSSPSAASSSSKSLSTSRTLKSANANSYENHCKKASLEKDN

CVVLGFLLSMVGIANLLDHHYYLSVVWESATELFPNIPSYYIQIPAKLLTAATSYVLALY
VDIHRMRKSDNSRRSKHRIKKIPFRTVFLPRVGWAFLKILPAYPFLAVLISFGFLFVVDL
WDFVFHLPLDWLNNPIYYGTLYGPFAYTYVYVKREIVHRNNDECTGTDGGESTTESSFV
>comp2110_c0_seq1; 647 83 1963 LEN=2062; translated
MPSKSRENARSTDGSSDVDRERTGDQSSTITSLLIVILSLVFVVIFGGSLIGGILSGDPS
NVFFITIVAASAFAISVALGVSVRYVLTQRSKRQRAEHGDDYFAKDNHVRETFSGSSDEE
NHLCANNRPSDYDPRDGHYPPIESEIKEIEAKSVFGEMSALSPTTYDGGSLSTYRHHIIR
DNHHRGFDFSSITSAKHGDNASGRQDPPEGTGLATIQAAWNTRAVSREPSAPKFSTEGET
IVDNDYSKDANDDCDEKSSRSRKSARSRGDLEEKSVRTERSKYSQRTKSSSKSRSRSKSP
SNLRNGKSPANSEANSFFSSGRKEKEATVHPVPPMSTRPGATSSVKPSAVPSTPAASEAG
SFASSFMGLFAKGKKTASEAGSTSMSSSDKSKTEPKEKDPDGKKKDRKPPMVRPPMMPSN
KKGGDGLQSVGLTGPSVSTPSNNSTFTIPPPRVVPRTPAGGDGFSEFQSVVESTYSNGVA
SSTFDPQARERALQKLRRNQAAIGAAPIGAAPLGATPVDAGDMPIYYTDDDDKASSVGGN
SAAQLNSYDVFAPPGALGIVVDTTDKGCIVYSLKKSSPMLGLMHKGHLIIGLDNFDVRNM
TASSLTKLMAKKSDQQERKFTLVPNN
>comp2111_c0_seq1; 380 280 2107 LEN=2851; translated
MTEESGISSSVKKRCLGFPAIFGVSAVFLFLWRGTTVALCDAFFFSLPVATREFGAHPCN
INFDSEHGRLRIQRVKEPTKKHDQNTCSGRIYSCHSSITSHHNEGGDDEEEDKNEDNINT
SRDYSFLMVRSSSRRKMLRSTPGTIAAAVFGTTSLAGVPSITFGEESDSSENISTTYTSQ
NADSSIRDRNKINDIATGRKNESSLHRVLESSSVYRVKTAPTVRLTGRRTPVVPALDAKP
PSVHRMASQNLLRDLQSKRALVLGEHHPDPRDHLLQAALVRDLHKARRHSGSGSSEMAVG
LEAVQQQFQPFLDAYVEGILDNDELKVATEWESRWYWSFDAYLPVLRTCRDLGIPLLALD
LSSEDRTTVERGGLESLSLDRLNNYVPDPDAFCSFGSTAAYRAYLDYTLRPPYELLQRMA
KQGPASSNSSGIPFSTFVENAERRGDGLGLHTVAPPPPRRIAGGIVGTNHASFGCGVPGR
IARTLASNSGEKRGRAAVASVLINPEPLNTGTELRICNKDGTLATNDYDDEESPTRIGFL
PNSGNQVCIENSTDLQNYALQFQFASFSLSPSQQQGILQEATLAQQTAEGENVLGLADYL
VFSPKEKT
>comp2112_c0_seq1; 285 274 1155 LEN=1554; translated
MAASIAGENCSFKVQSTKNIKEIKAILFDMDGTLLDTESLADKAILMALFGCSDNDDDSD
QPRLPESLRSKPPTSEWRLPWELKKKILGLRGKEWAPICLDFARQHCERSLPTAEDLINL
WEQNLNGMCSEIEACEGAKALVKSIISSCNSSNSKIPLAIATSSRMSGVDKKRTRHEKDL
FECIDEIVTGDDPAVKNGKPAPDIYLEAARRLGVDPTECLVFEDALSGVKSGKAAGCQVV
AVPDPRFSNKERTRLFLNEGGADVVLTSLWEFDGREFGMDFDMNEIAQSRGQQ
>comp2113_c0_seq1; 393 319 1239 LEN=1736; translated
MKFMENEKLNQISSELSEAVLKDSHRMIQGRIEAYTMKRAGNDKKFAHALGQKYIAEIED
LQEELAATVERKRRRRSNSESFLFTTTKKSKLSLPSTPESTKAPSKRERAKSFDCTLDST
SKTTLGDFSEISTRKLMTDLILTLNASFPDYDFSTVKPNQFQKVKMADVRKSIHENLSEF
ASQRSSPNFLEELWDALNDVIDLQEANVYSFDYEFADDDESGNNSLWNFHYLFVNKSVRR
IVFFTCSERISDDMTGAQGSADEVDDTSERVEYYARENVYTPVDDVDWDPGDNIAGGMSV
EVTIGG
>comp2113_c0_seq2; 393 535 1455 LEN=1952; translated
MKFMENEKLNQISSELSEAVLKDSHRMIQGRIEAYTMKRAGNDKKFAHALGQKYIAEIED
LQEELAATVERKRRRRSNSESFLFTTTKKSKLSLPSTPESTKAPSKRERAKSFDCTLDST
SKTTLGDFSEISTRKLMTDLILTLNASFPDYDFSTVKPNQFQKVKMADVRKSIHENLSEF
ASQRSSPNFLEELWDALNDVIDLQEANVYSFDYEFADDDESGNNSLWNFHYLFVNKSVRR
IVFFTCSERISDDMTGAQGSADEVDDTSERVEYYARENVYTPVDDVDWDPGDNIAGGMSV
EVTIGG
>comp2114_c0_seq1; 1039 17 1900 LEN=2004; translated
MKLRIASFLSVAAATHAFVVDTKTSQRHNGHVLYSTTAATLGSAPPTEAAETFGTVVGDT
RGAALRLENVAISRGATPLLNNINWSIQPNERWGIVGINGAGKSTLLGAITGTVRMDEGK
ALVHSNVRVGYLRQSAVSGSTKTVAEEARSEMTHIEEAKIELEAATKVVEDGDYSEEALD
KLATAQEHFETIGGYQQEQMVDSVLKGLGFEPEDSDRLCSDFSGGWQMRIALARLLLSKP
SLLLLDEPSNHLDSSAKDWLGKYIASYDGSVVLVSHDVSLLDVSVNSIAEIAGNTVVEYR
SCSYNKYLEEKEFRAASAQAEYERNLEEAARLQAFVDKFGASATKAKSAQSRVKMLEKMK
KEGKLDPPPVAVVARERDPELVLPPPPKPNGEYLMKMKDATIGYDPSEPPLLKNINLKIP
RGMKLLLRGPNGAGKSTLLKALRGNVPEMIQSGERVENDRLFLGTFTQDLAQELDPDSRA
VDLVTDYARSGSDGNIRVTDEAARGVLGRLGLGGEKPLRKVSALSGGEKARVALGMFALK
ASNLLMLDEPSNHLDVGCISGLAKALSEWGGKDGAIVVISHDRKFCEKVGFTHVGTVMDG
KLVLEQRSLRDSDWDQYDINAASRSPA
>comp2115_c0_seq1; 141 184 429 LEN=778; translated

MAPGGGVMLIPFVRVVISILLVLTICGFVADVARIHMVVLGCLSGGLLFSLSMFEREFNK
VKNRGPPSTTTTKLANSNKTD
>comp2116_c0_seq1; 476 124 1374 LEN=1866; translated
MWLTHAGLGVAIAVAAFAVWTRGSSSPLSLPWQGGFVTVIDPCPSFETERVDLPSPADEN
DPLLVDAEHVLLGLEAPEDGFRLDGTGNGDEWIWSMGNGDLLRVNLRTMVALPFAHTGEI
HEECGAMHMESRCGRPLGLLKLPPEDYDRYQRYYDSPVATKQDKGNGSEPAEAETPLCLV
ADAYKGLMLVFSRGTIVPLLEAVAGQPLFFANALARAGDGTIFLSDSSSRFRRSDVLLET
LESRPTGRVVSWNPDTEVSRLVASDLPFPNGLVLRDNDKSLLVALTTRHQIIRIDLSNHG
ESGWVAPEPVLFAALPGIPDNLHVFHSNEWNRSVLWVGTSTKSSPVVRFLNRWPQIRKLV
ATLPRGMLLKCFKKYGLLLALDPDTGELLRAYQDPTGTTGYIAGIHFDDSYAYLGSW
>comp2117_c0_seq1; 371 115 855 LEN=1518; translated
MYLTRSEYDRGVNTFSPEGRLFQVEYAIEAIKLGSTAVGIQTKDGSVLAVEKRLTSPLLD
PDSVEKIAEIDSHIGAAMSGLVADAKTLVDHARVEAQNHTFTYDEPIGVEPLTQAVCDLA
LSFGEGSDGDSSKRMSRPFGVALLLAGYDDLEGSQLYFSDPSGTFVRYKAKAIGAGSEGA
QSNLEESYSDSMTLKEAEELALGTLKQVMEEKISTDNIELARVTPEGFQIVKHEEVAAIL
ERLTTG
>comp2118_c0_seq1; 281 90 1010 LEN=1550; translated
MSFEKTSPGTTAASCSPPKPLPEYVSDELEYLQPSQVDEAARLLADCFLDDSNPSPQYSW
IFEELLLTEEEEERAPRAAGNHKEDPRIGSKRRSRQRDALRWIFRKNLEIELEHDAAQNR
TTACRCSFRRKPNQPPEMVCFFILKDVQSPTTNNSGSTDKKDGESGDGTKISTWTMLRNG
ILWFPILFGWRVFYRLLVVKNYFDTATLELLSSNHVVRSCRALERVVVHPSLQGKGVGTF
FLRKALFGDGTDDGSAGNGNSGGGSVILHTQEERNVAFYSRLGFVVVKKDRPFSGGTNWA
MVMTSS
>comp2119_c0_seq1; 267 1 904 LEN=1022; translated
XMGSGASSALTLQKNHRRRIRFQTKYENIFDMLKSTIQSKRSSTEKLSMFIGSTTMPRRR
NPIILWYMAFLLVLSTANCVESFSVCFGLGALLTQPKGLITTSTKGDIPALLEASDFFVD
AFWVGKVGGGANELSEKQRRTLSASQFYEFRSRYAGAGRGQSELVTCRLPSGDLTGCAGI
EVSPIPDKGLKGLNTKSGPLMSNLAVGRDFRRKGIAEKLVTEVERIVRYEWGYDDCYLYV
EERNKPAVRLYQKLGYKKLWVDKEASTLLPSSNGKLTNSATNIVCMKKRLDLGIIGRLLP
F
>comp2120_c0_seq1; 366 110 1159 LEN=1233; translated
MGRFFKKFRQRIESRIDAAVAEATGGGGFGIANQTAAQLSFEEQKPDSMRQKDVRMISGC
EDSQTSADVSNVAHFSLPDPAGRAGGACTSTLLNILYKDETVPEDTLSFTQVLELMRSDL
RRGGYSQIPQLTSLNKIDVETDFQLVPSTATGVHRAVMVGINYVGHSPGELRGCHNDVLN
MKKYIMEVHGFEEENIVILMDDGIHTSPTKENMIAAYEQIVADSEEGDAIFLHYSGHGTK
LVDQSGDEDDGYDEALVPLDFRESGMILDDDLFDILIARLSDGVHMVSLMDCCHSGTILD
LPYIFKPMQDGSMPQEMQLDDSVNLDGILQQFGGQALGMLTNFLNSKLE
>comp2120_c0_seq2; 361 110 1334 LEN=1408; translated
MGRFFKKFRQRIESRIDAAVAEATGGGGFGIANQTAAQLSFEEQKPDSMRQKDVRMISGC
EDSQTSADVSNVAHFSLPDPAGRAGGACTSTLLNILYKDETVPEDTLSFTQVLELMRSDL
RRGGYSQIPQLTSLNKIDVETDFQLVPSTATGVHRAVMVGINYVGHSPGELRGCHNDVLN
MKKYIMEVHGFEEENIVILMDDGIHTSPTKENMIAAYEQIVADSEEGDAIFLHYSGKSRN
VMQELYFIQELVIATVQGKNAILASCFLILSLGCIDMLTNFCAFLSCTGWQPAGHGTKLV
DQSGDEDDGYDEALVPLDFRESGMILDDDLFDILIARLSDGVHMVSLMDCCHSGTILDLP
YIFKPMQDGSMPQEMQLDDSVNLDGILQQFGGQALGMLTNFLNSKLE
>comp2121_c0_seq1; 348 148 1259 LEN=1393; translated
MANVTDPLIKSIQGSDPQNLMEYITRQKIYDSRFWKEQCFGLTVADVMERAVETLTCVGG
TYSGMGKPTKFLSLVLKLLQLQPDYELIRTFLEQTHFKYLRALGAFYLRLTGRPQDIYEL
LDPLYADYSKLKYRGVNEWKLVHLDEFVDELLTNSFCCGIAMPRLPARETLQEAGYLEEG
PRRTALHDALTRAGGIKEYLRIKVDEEASARKSGINDAVNDSAFGGAISLXGKRYGKLKP
IERGEDDENGNTNENLESTRDNEVVGGDNHETESERGTGQNNSEGRLGKRKAADTDADSV
EAKKKKKKEKKKSSKYGSLFKSSAVDSSKNSNSMLPKDEEGTQSSKSVDQNSDEYWNELR
SSLGMNPLKK
>comp2122_c0_seq1; 265 27 803 LEN=1601; translated
MVPPLARTSIQTLQILCQKHGWGYLFGADEATGDWFLDIFYGLGEWKRFMVPIPKQALHG
KTLVSEAALVGLREIIEREESKRILSFKDVFPNPIVIYDSHSKSTWERFWNDSPTEVGVD
CEGNSISPPVLVQISTENYTILETPLDGHLSENLQRLLGMKSIVKIFCDNYSHRDKICLG
LNVYGDYNERDQVSKKTHIVDIEILSNRVIGPSKTPRGLSKLVCLCFPELNVRISKSGKS
MKQRFRNIGKFVEIEQGK
>comp2122_c0_seq2; 290 27 938 LEN=1460; translated

MVPPLARTSIQTLQILCQKHGWGYLFGADEATGDWFLDIFYGLGEWKRFMVPIPKQALHG
KTLVSEAALVGLREIIEREESKRILSFKDVFPNPIVIYDSHSKSTWERFWNDSPTEVGVD
CEGNSISPPVLVQISTENYTILETPLDGHLSENLQRLLGMKSIVKIFCDNYSHRDKICLG
LNVYGDYNERDQVSKKTHIVDIEILSNRVIGPSKTPRGLSKLVCLCFPELNVRISKSGKS
MKQRFRNIGKFVEIEQGKAEPLKGICDLNDTQRQYAALDSWCTLKIYQRMVSIMKKEIAG
QVN
>comp2123_c0_seq1; 357 188 1231 LEN=1490; translated
MSVLSRSRPIRWGILSAGKISSDYAKAVAVTEGSKVAAVAARSLANAESFAEKHGISKAY
GTYEELLRDDSIDVVYIGSIANQHYPLAMQSLLAKKPTVVEKPLTLSYQDTNSLVSLAKQ
QNTFLMEGLWTRCFPAMRKVQSLIQQGTIGQISTIQADFGWSTKDCGPEDRIWAPESGGM
ILDIGMYMVQLGQVGFSGSPPSRVQAMGNFRNGVDHTVLTNIQYGSSGFLQFIVTGDANT
EERVVVQGTKGRIILEPPAHVPTVVKLQADAGRGVIEEEVFEFPLPDDSYTTWNYPGSIG
FTHQIQSVTDSLINGEKECQYFTHQDSLEVASVVDQILQQVHNQQKE
>comp2124_c0_seq1; 271 266 535 LEN=535; translated
MGQSASTGGGAEGPPDLSSTIVIAETDEEDDERSGSDRDAFFSDAKAGGGVEGIGGKNAD
ETDSGSTWSSSSDSDSSSTHSGSSSSSSSS
>comp2125_c0_seq1; 338 349 1535 LEN=2128; translated
MMFRLMFRLDKAVLTTLLLSWTMSYFLCGKDAAVLVEASTSFSMESMMENGSTTISQECL
PWWQRQIDVAEVFLVCDHQSGDGEDGDDQYYYTNRASCRTGDLGKVALGFTVKNQLPYDS
TLFVSIKTHYYGRSATIRNRTDVCSFSNLGYINADGAFYSSGSNNDDNGDADDGGEGGDG
SGNPLCPIQAGMHYMLMESFTLQDSSASRTSNWQEFKPDLSVSFYLSSDESSPPIGCVLT
GTRAKHALDQRRSRDGVIALIVSVFSFVIVFGLCLHNQKQRKKAVELLENNRVAXDDSAV
QLSKEQQRWILYGADDGFVEPRAQATAGGRPVSRGALRQSPREPATGSAPRSSAAVAITT
ESLLATARIVFPETHSCPDGVGTARAQALTSKALE
>comp2126_c0_seq1; 560 170 1762 LEN=2649; translated
MRTAAQAGGTIRRRTRIATNDGGSDNNDGFGLPNPSRVRRQRTRNRRSNGALGFAGAIVV
AIAIAGGWTLPGVPFSAAEALAVSGTGLPPAAALRSSGRSERGGHPREQAKEQQQPSPPL
LGSCRQGFPGSRIHQNRLARTRLHLFFNQRDENGNLVQKPAKRAKPSSDLLDELRSPGIS
FPKPIRLTGSSSPKEPSAGTDSPSLVLRYMTTEDLRALVPMCINEFGSISQQPPPEDTHA
KKGLRGLVPRWIQDPKRIPELWEGKSFELLIYLTLRLKLMQGGGNSGKNSGISSGNSSGS
NGNPVANSGNPGSIPNDMVMLVLCEENEPKPQEQSKSLWRKPKPAAAIAAATTATTVEAT
QSQPLSPLSPQQRVVGMIELSLQPPDADRNPPALPLPRWFKAALARQTTIDGRLQGWITN
LLVDDDRRGRGYSKLLVAACEGIAKHSWGCSSVYLHADADISSGRIPQSLYEGMGYEAVV
GSTQKNPNGARSDGDGGEYAWAGVAGKELERFTAIRMVDGVALLCYSKKL
>comp2127_c0_seq1; 222 699 1115 LEN=1410; translated
MSKFTFCLLTVLVAACSAFAPTSQLSSATRQASMTSSPFALNAVEDYDSETYWEDEYPPS
KVLGPIMSKMPSGLLAALSVVFLTSLVLSISGSAALQQEPGAFENGSWVKWYYVLGSFGG
PLAWGTHVAAWIQRKNGM
>comp2128_c0_seq1; 696 123 2252 LEN=2876; translated
MPMLTLQQARSDAVANEAEQRTTSPEHKRLLSERGEHDTTIGESKKRRKLSESLDTELVV
TGSSDDKTSKKIQNQKETIDGVTMLKPSDKKKVDPTELASAFALASLASLSPGGSSVGSA
SGISTPPRESREANVIRKTDSKLDDEVRKAASFESETRSPKASEHPLSPEQLSLREQEDH
SASSTVVNSPAGNANRRVSFAPNTKSAEESKVPSTPTTSASSSGELATSTSHRSLAMPQE
SPGGKNGRSGRSSNSNTTINTSPNSMMQMSPRPHRMYGGRGLPPFVRTPPQVNQMNHRLD
YGTPPPPPHAPYHHRLPPPGTNGSMPPGSFRHGPPVLHHRQQFGYPGAPPGHRFSPHQQY
FPPRYLMQPPLLHRTNSYGVGMVPSAPTPPNQQGAPATTKCNQWVCDYCNVASFSSYEEA
CAHEEICKKAGPKAAAQLLSVSRSYSSASMDEDSVVSSNMSMMVGGHHLPANNPHLHPAF
GNAMEAARRHHHASLALKMGHQQHRMQIDGNNSMISGSNNGSSSSNNNRPGKATPVSAGK
GNSREEQQPMTEEERRRQEMHLHSLGGYIVYPNDVEMIPPYVHFLMRQVEPCLFTEADRF
VARSKGPVGYPGFQCRHCHGHAGLGKYFPVSSKSLSTNSTSQNIHAHMLKCRKCPDNVKE
QLVQLKIEKSRAARLEPGWRKVFFDKVWKRLHREAGDNDEQATCKTQSN
>comp2129_c0_seq1; 564 1 1383 LEN=1653; translated
QNTTQHNTIQQPRHKMSRKALSVDTGDDHQQVEGTMSGVDTNANTPVADNKSGSSLFRRS
PQSKTQALRPPSPPKTSLAAENERLRNELRVLRRQIQEMEEFQDWKQQDSNEQHRHTQQY
QQHHLLQSSFSSHDYHGRASKNTSNKVIVELPEMFQSSLPPPTSESEICNHRRDGTVKEK
HSISTADDQETVEPILDIEAHHQSAAGLHHRNTTMITSNHTKKKKEVLTEETASSSASSC
GNSLGETDVEAIALIEGIDSHGNKTNTHHNKTFWSSLLDRAGWLIGLLIFQSLSSFILAR
NESLLTHHSVIVQFLTMLVGAGGNAGNQASVGVVRGIAVGTIRRSNARRVLLREFAMGTC
LSIILGLAGFVRAKVFAVPWLETMAITTSLFLIVFISVVVGATLPLCMEAVAIDPAHSST
TIQVVMDITGVIITVKVSSLFLDSGGDWLGAVLSMDGETR

>comp2130_c0_seq1; 1130 40 1479 LEN=1578; translated
MKIVSSLAFAAFLATTEAFGPAQPLKGVVSNGAANGMTMRYGIKDQALRGRVCAIVDANP
TKEIVEEELLSDSMSEAIKGANWKLRKALIRKVTNQAARYDVAVDPAFGKADTQAEREAK
EAVAGAANKAAKVAAIKAAADEFAANQAARKERRAAHKARAAEALAARKSGAPAPAAEEA
AAPAPAAGGEITPKTIKALRDLTGAGMMDCKKALTESGGDQEAAAEFLRKKGLAQADKKA
TRVAAEGKIAVSTGSDGKAVMVEVNCETDFVGKDSTFLAYCDRVAGAASASGTVVVDDLM
NEEVDGESLEATRQALVSKIGENIQVRRLESRGDGSTTVGGYVHMGTIGVLVEIEGGSEQ
LCTDIAMHVAAMNPPFATADDVPQEVLDNEKRILTEQAIESGKPEAIVEKMVEGRIRKFL
EEKCLVSQNYVKDSDLTVQQLLDKNDAKMIGFSRLVVGEGIEKKVDDFAAEVASMAAGN
>comp2131_c0_seq1; 249 536 1735 LEN=2057; translated
MASEDDTDNMASAGLELNQLHPKAVMWIPRIVGVITLVCGASMISMAWKRSDRLFHRLVL
AMAIHLAILGIALIIGAAAIPESLSNSDEPSESILGYHGTEATCTAQGFVFYVCFMASMM
YYCSFSAYSYLGVPRNFVSRGFEVAGKYIHAFVNFFALVTAVAIVLLDGFNNTGLGFCGP
ARSDISTYKLFLMVPLMLEIVFSSVVMVLLYGKVKRNQAMIFINARSVARQATIYLAVLY
WVVVPVLLSQCISWATNGGHGRSFMTFSVVNFSLFGLWSMLSYWYFSVEQRHHKKRRRGD
DDTADPTNTFFATERTQMDSMRGRSSNCIFSSKEFNMSRAKGESEAPNSQTRTQRFSFNI
FDGTNASGRFASFVFSGDDEDEEADKIESDKWKDIQDHV
>comp2132_c0_seq1; 540 1 869 LEN=869; translated
XKNNEEEGTSKEDEKKNNNGNEDNEDQDISKEKDQKNNDGNKDNEDQGISNEDENVNGIV
DEENNGGNKENEDEDISNEDEIFHDIIDMENDDGNKDKDTISDEEEMKDNKEEEMINNNG
NKDNKNKDITSDEEDKKNKNKNKDENKNKDKNKNKNKESEDKTDPPTTAPSASPSHAWSK
TPSIEPSSFVYGEREAPITSPDIISSTDSPTFFLVNQLEPTTYPTMIYKNTIPSFVAVNI
GVTPVPSSFPSDKPSIHFSNSPSDGPTREPSLNPSDAPSVSPSGGPSQAP
>comp2133_c0_seq1; 388 590 2107 LEN=2248; translated
MIVIDSTHTESSPLLIKKDGNDQISIGTDKTYNSSEDFSAVGSDGFRPDPSSVVILLHRG
NDNESERKDHNDGDHTKLKSNNCEEPLQKSLIEGLAEEFHDITEAALNEVYYVPDDENYM
IEMGLTRNLSILHDDVVEASVLLQGSLLLTSEDNYDTEAGDDNCAERFVPGAVAAAVIVP
LGAYLLLFSAIVAMSTIGPLLELQGDTTATMKVLWRMIGTSLLLAPRALIDIYRQAGVLR
LTPPQWITFFLSTFWFVVMAIGFCLSLDYTSIGNAVILSNSQALMLLSGRMCTGAPVTML
EFTGAIVAFGGAVLCSRDAADTMDDDKDESIGFGTTITGDLLALASAIGGVGYVTLAKTS
RAQMSLFVFMFLTMALGAVIVVLFQVAVLKEIVTWDRDVVHGVWGFLVWDRFDRLPLELV
VVLICNLVGTMGYVRAMPHFDAIVICSVQLLEPTIAEFLSYFAGVGVLPGLLGWIGNAAV
AGGTLVVVSESQKFHDASKKTSDVH
>comp2134_c0_seq1; 387 269 1548 LEN=1806; translated
MATPTEVLSQGVSEEKNEDKNSDGESNTKPGSVHLMPSNNAIPFPWKLHRILGDAETQNF
SDIISWVPTNNGFKVHKPKSFDTDIMPTYFNNTKYKSFQRQLNMWGFDRVGSGPFKGAYL
HQYFVRGNPDLCERMHRTKIKGIHSKKLRKNRFQASHGSDVRSSHSSMMTMDSGMPCFPS
PLDPHESFKAAAQKVADLERKKEEIQRKLEMVSNRANAAVASNALANMSGMIPRVSINSE
MQIGQASTNNYRRNDDFFPLPGAGEGDSLMFGGQTFVFADNNQNQQQQQHHQSPRHRVAR
RYSLEPKGPESDEYVLTELEQGMHGNNIGMKTSEEDFLMPTPLPPNMDVIKVTTKFPNST
PAPLVIGLDKPKRRFSFLSTPVENPLDXALRDTASPKSCDEQQHDEQVDYEQFRQPEWHD
DDELEL
>comp2135_c0_seq1; 325 1 650 LEN=891; translated
XFTQDTINKFKTTTSKMVISPSIVSYGAADGIGTMMTEGVRTLASQPAPWLLQSHLETIM
LGSVILDAMAGGIIFIFSNTIMKTFAEFDDEIGINVMVLLNKIIINPLFAFVFFGGLISA
YPAIVMMTRPDEFSKPARYYALASTLVFFFGEFLVTVTQNVPRNDALMAADPTSDEGVEY
WRKDFLGDWVAWNTARCIASVVASILGGLSLMAMRS
>comp2136_c0_seq1; 192 1 566 LEN=1658; translated
XEMPEQIYDTQKSKISQANYVTIGMVSASLGFACKENMQYVFQSQYVGDELLTLFLRSLL
ALHPLCAAIQSLGVIKRDILGDPSSSLGRILLPAVILHGAFDFAEMLLAYFLYINDLEEQ
GTFAPSSAQDTGSQPSSGIGIVSPVGVGGIFMIVGLMYYFFRARLQQNQLALAHMSESTS
ALELSSFV
>comp2137_c0_seq1; 156 60 303 LEN=303; translated
MGQSASKGLSKAAEKAARQAPAKLKRPPIPSRVLRTQEASAQNPESFLRGQGIGAQDLRD
RGQEMYLQHAQQQRQQQQQQQX
>comp2138_c0_seq1; 390 1 1118 LEN=1240; translated
XRTAAAANSLLRRMSVRARLSGSVDGALFMSKYERLNQVIDSPRHKHRRARGSRHERGQP
YTGNDNTQGHNHDLFLDEETVEDEVGGNADSSSSYREHIPSPTNNVVMDPTLFRANRSGQ
RVQVRSSMFFEQDGRMLMRLPRDQVRLVMDYNLEPGILSVEQWRSKEEADRTIERMAVSA
DAATPRTKGGPNHGSQTERRRRDREKRRLRREKERERRLRRRQKRKEKKQKKKLLREKAA

AATAKNDTDGSATEDRDASGHDPGDSSSSSSSSWSSSDSDASSSCDTYLDYDLEEGRPGL
AELPELKYVITVPDDLYRRMVGEMSAKIFPPAWGFFKCCHQESERADIKLALVILAVVMA
LLFVGSLEWRTT
>comp2139_c0_seq1; 421 179 1099 LEN=1240; translated
MLGENFTGATKVSSLATSLFNESLSDYIDYHKPDPIARRQLESWKMSWSEDTNDSRKSND
NANDKKKHLDWKPTNAIEGKLEICGEYVAATIKMECFWTDWNPAVNDDKIEDGRLCFSCK
LISGRLIDKKDGEERTERKVRKKMLTRLRGDSYISKMLAMTEGGKSDTKDIQTHHQLCLA
QASIYVNATKFEMEERVDVSESVAETLRRSLWPSIHSSLDVVEVILALPSLPCRTPGSKT
RATTRLANRAKLRMLEDAMLDECEKEGDDQIIEDLNISNEQASSKEDIIEHESADSSSRK
KKKSKR
>comp2140_c0_seq1; 443 347 1354 LEN=1399; translated
MIAAKGFGAASAEKKTEPPRNAADSNTQNDNNSGEIYSKPSMYDMAFGYRNFEEEVDFLM
DQHRQLNPDNALPKRVLELAAGPARHCIEALGASYIESATAIDNSADMIKYAREIAAEEL
KSDDYTFEEINGADDVTKDSAYEHRVNSLNYVQADMTNFSFPGDDEAQLFDSAWILLGSL
QHLTTNEQVISCFKCINRVLRPNGTFILELPHPRETFSMVECTRNGWEIPLEDESGGTCG
ELKIVWGDDDDDFDPITQVRQFTVSMELTGENPNDAPIQSVREVVPMRHFTSQEIDMLAR
IAGFEVMSMHGALQFDVDVNDDDQAFRLVCVLQKK
>comp2141_c0_seq1; 296 77 628 LEN=1906; translated
MTVAGIMRQYQVVGRKAPTDDVPNPEAYRMIIFAPNAVIAKSKFWYFMHQFRKMKKTTGE
ILDCVEIVEKNNRIVKNYGIWLRYNSRSGTHNMYREFRDVSLTGAVSQLYDEMAGRHRAR
PRSIQIIRTAVVGAKDLKRVNGMQFAQANVKFPLPHRVASGKRGQKALSSSTFTTVRPTT
FLG
>comp2141_c0_seq2; 258 77 628 LEN=2054; translated
MTVAGIMRQYQVVGRKAPTDDVPNPEAYRMIIFAPNAVIAKSKFWYFMHQFRKMKKTTGE
ILDCVEIVEKNNRIVKNYGIWLRYNSRSGTHNMYREFRDVSLTGAVSQLYDEMAGRHRAR
PRSIQIIRTAVVGAKDLKRVNGMQFAQANVKFPLPHRVASGKRGQKALSSSTFTTVRPTT
FLG
>comp2142_c0_seq1; 512 519 1763 LEN=1959; translated
MPKRKAGPKFYAVASGRIPGIYTTWAECQTQVKGFSNAKFKSFGTVEEAESFVKKENPNA
GEKTHRKPQGSVGALVSEKATSVKKQKHNLGKPSSSFAGYVARNMERSEVRADPTLKRFV
EAEIDKILFSEQHTDAAGTDWSIVPLVPLPATDPPATEFEKGSRTVGSSCNSDPDSDGDN
LARTPPPHKLSFHVMFDGGSRGNPHGHAGAGTYVVTRSYSRSGPQSEEPDNAAAKGARKT
PKTTRPKESLAEERANIRTYLGMGELTNNQAEYQGLLTGLEYVSEALKRVDRLASVELLV
QGDSDLILKQVRGEYACKSPKLRPYHSRAVALIEDLSRDCRRRLGCAPEIALEHVYRNHN
EVADALANDAMDARRSWTTTTTKKTGATAPGEKTTNDQTVDGRGPGNDDGAIEV
>comp2143_c0_seq1; 568 45 1523 LEN=2864; translated
MRNSNDNNKHNGNSHSTSTLEDRHFLSDPRPVRDLKLPTWEVDIHREGVDMPIMTLRVDS
TVLPPRKNKKKKVLWKLPDNIDTAPKFGEQDKRRVLELFREKKKELKKLKNKQRAYRRAS
SMESMNSSGAGSIDLSTHAGGGGKDDLNSNAEGLPPEANGKDKQHENQKQCQPTGTNEKN
NGNNSNPDVAIVASDGLNPSSSKTIEERGGRRGALSPPGFGNPPPRTSPLKLAPPGLPSK
NNEHLAENNCKAANGHSSSLSPATSNSTSSQPPLPSQNGHHPQQHLHHGAQHSLTPPPPP
PPGLQNIHDLNQDGIRAPKAIPPQQPPMAIGPCFVLPPNAPPGMSIGKFVTETYYLSLRN
GLVRELDAYYYQNGGERSGADVAKVCKAVTVGGAHAFCTNQQDRLMQLNTFSGTEMRIKG
VQEQLTVGNGVLVLITGVSIRFSAPNGSSPQQLPFCHTLILAPVSTRTPTADSSTIGYQI
LNDNLVILTADE
>comp2144_c0_seq1; 310 176 604 LEN=1063; translated
MAYLLPHLHSGWEVDQAIVTAEDRVVVIRFGHDNDATCMVQDEILMSIADKVKNFAVIYL
VDITEVPDFNNMYELFDDCTTMFFYRNKHIMVDLGTGNNNKINWALADKQEMIDIIEMVY
RGARKGRGLVVSPKDYSTKYQY
>comp2144_c0_seq2; 165 360 518 LEN=977; translated
MVDLGTGNNNKINWALADKQEMIDIIEMVYRGARKGRGLVVSPKDYSTKYQY
>comp2145_c0_seq1; 834 537 3572 LEN=3846; translated
MGSDSDNDPNLLGDDAEKDASNGRKREKTRSLSPCPSFLPFDVEDKKCAVYVGDIPFNGR
NRCRICFGNGDRSIDRGSIQDCDNDTVDSNIASDDDILVSSDRSNPKNKDGACACETELK
AYIRGRIFAAYKTPIRYIGIRSCRFFALPARSTSSSTSSSTSTEKNDAEDDEDTTKPCDT
NSNLHRRSDRRLRIAACIEFENPRLAEYSLGLKYTTFSFRSHPTVRAALKFRPWAIESRL
PQQLTPRMPPKHQNSKEQQLDPPKNVLPRPISSPPHRRNLVCDIDCNSSDEQSPSLSISF
SPSPPTSSCLVLITNIPSDVTCDELVAFLVERMNRAFRGKPSPTILRLAIRKPKAMDADR
SDHATDRNDQSRPIRNHACVEFQDARAAFRATTLKHRRWHAKPQECETENNVGNGFGAGG
CSDTKGSDKTRDSARAPCLGIEIWDPTKHDVNDFFIGNESSIPEAINTDDGTEDDISESS

NLVAEKDEKSDGVRTASELTENEIEPKLKTLFEELKEDGFLVCKHASINQKLDHLFAIPG
SGKKASMCQGYAFRGRICRAYRRSLGIDNNNNKRGSASSNHSCPYVHIDSLEDLEDPLDV
LAILYFVHNNDDINFVPGHGWTPQEYLQQQQFKLSALPPSQHQQPTQSKSMEEVVEKLQN
DLSEVRTQLAITQQELLKSENANEYLLEKQSKAALIEPSNTSSKQEVQEKDTIRSLQDEI
ARLKIHLDEKDQNLKASERDAAEIRRECFALIEQSRNSTKQVANNHISDDNLKSDIMKLK
EELQQTRSDIQKLDQTRYFNPNSHVGHVWAQGKMDNFIQTSVPALMELAGGTQNEQQQQD
AMYHSLNNSLSSLTRNGTNESKTTESVKSVALQAEIAAVQSLYGREAVVTSSGSSTTVTR
YIILPVLNSQAGDITVTLIITLPKGYPSQGVVEIDANALPHSFPRIKECISSLLLVCQWE
AEACTGSPAVIRIMKKVEQWVQNDWEILQRKLTSMGHGMNEETRKTFFHSM
>comp2146_c0_seq1; 668 232 1731 LEN=1957; translated
MGKFVRRSYKTAGKRSSGGFGCRFNPHHTAFVLLGLAMILLRHNQSKEIFSRFSDLDVGT
LANPLPNTATATATAPHQGSIRATTGAPPPQAAASNATPTTECTREELLTIRKHLNPEFC
PTAREFAYFQFCSLTQATKCVDASNWLEDYYRDLRKEGSLPDAFLGLSVGCNKGFDALNT
LRMGTHDTAFTKKAWKQSMELDGAKLSESVCNQDIVDEFQVSLEDNSNPADAAADAFATG
EMHCFEPMPPTVKKLQHAAKQLGYDQKGFKVVHAGVSKTPGTAWFATVGSESGVENVGMD
NCEAMGPIRKAKYCTQVPVVSLKDYVKQHIDGDNDNDNANGKKRTIHHLSIDVEGFDADV
LSGTGSDVLKRVEYLEFEYNWMGSWKDQHLYDVVTALDQISMTCYWAGTRRLWRITGCWM
RYYDIHTWSNVACVNRNLVPKLAAKMEDVFRATLGDSAITAHTFRNPVWRRANHWMRGLK
NDPMLATEPHEAMVSTEYL
>comp2147_c0_seq1; 382 249 2115 LEN=2115; translated
MLSGAARKMAGLSATNPALVRIRTNTSGMCNRTFNSTCNSMLRLRSILDLEGRGKGPCVR
RFSTAPAIVGDDDSLGHRQGHSHSHSNSNSPIQVKAKQPSYKDAIRGGPKEILHPTEQTT
KHNHQPPTTKAGDDCKQEQKQKHPGQNSRKARTNTTPDRLVDFGDISRAMVAIRSGIRRT
KCTQSFFLSELCRANIFLKPEVHQFTGSFKERGARNAILSLLKEQESPRVIAASAGNHAL
ALAYHGKQLGVPVTVVMPTVAPLLKVDRCRQFGATVLVEGLHIGESKTVAEELAAREGLT
YVNGYDDPPIVAGAGTIGMEIVEDVPDADVVVVPVGGAGLIAGVSCAVKTLKPCTRVVGV
EPERCASYTEALRKGHPFDVSASGRSRHKNQNQTTLADGLAVPEVGSHAFALARQHVDEC
VLVDERAISLAVLRLIENEKIVVEGGGATGLAALLPGGPLSGRPEIVGKNVVVPLCGGNI
DTAVLGRVLERGLASDARLCSFTAEVSDRPGGIEGLTKVLRGQGASIKDLYHERAXAAQQ
RRPGEGPLRGGAPREGPRPAPPAVPRGGRAFDRVEPADALLASACGRCQGCVGTLWEYYV
VSSARILVELETGRDTMRRTIVA
>comp2148_c0_seq1; 567 406 1521 LEN=1735; translated
MSTEDDFTVPSSEEEEAGLADMKKVFETEMTDDQTGKKKSYWSFAMETMGMASGKDGDED
LWLETVEQGVPILKKYIREKDAKLAEEQEQAKKKLPFTSYMDLVPLDEFHTTQTNFLKSF
LKWSIKDKEDIVEGSEGANLVVNASKARRRLDSYFEWMKDNMAEDMAENPLTFESVKEVH
KAWGLEATHSKDDQLIWWFDLGTMHTKSIKSLAPQDQMRYIVWFSHLMMFDHRGQDNGLL
LVEALDNLGFWKSMTLLPMELGAKMDRFTIGVLPIKMKGIYMFGCARWVHLMVGLMKPFM
SKKMRSRMIIVTEKMAPEPQKYCDDNFGQGSIPDGFMSLNGGSKKDALIHRLNKKGKQKE
KKAATKAKEEE
>comp2149_c0_seq1; 714 224 2545 LEN=2647; translated
MPNNNGDDNDSSEELTAKKMDSVLNRVSFFEKSQEIMQSKIARTPPRKKETKKKEDPSPT
PEPEKVPEKSRESSTMSRVQQWESRASGNNETKKGSFSGTETSSSANRDISLGFSGTSAS
SSSEDTDEQEVLMHSMTNDDDIPLEELFSNAASLNVRLETANSANSPPQKLFSKDGEEIV
ENTKKQDENKESPIAEPTAVRTVLYDSDDDEMDVESGQGLARYMPAAGFMAIPYSNGKRK
KDADGMSVVSDDISIAANVGILNTKFEMLRYTVPKPDYKEYPPDNSTERKYKVIIMLCCV
LLIGVISVIIGLLASRRPEILPSPVVTTAPMTAPSSPITQKPSLAPVDMVLVEEGPSLPP
VSVIPVAKEPSLAPVGMIPVAKEPPVEIGLPPIKYKFKNTATIVGQADGDRLGSVISMSS
DGTFLAALSNTPNAPVQTFQRDENSGDWVPTMATPVDNATISTNTSLAFGSDISSATIPE
DGTPVLAISSQTQVQVYQLVGRSWVNRGQPILWDVDTVAYSAMTLSSDASTLAMGCVDDN
GVIMSVQIYRYDLSSKEWTPIGGNPLLYDAQNRHVGGNQRRTLLSISLALSRNGSVLSVK
EWAVADPQVVVQTFGFKDLSSGRDDHSGSWEAMGGQLMHQFGPASVALSEDGYTLAVVAT
HPGEAVVYRWIEGYWKKISGDANHGYLPSGGSSVSLARNGSRILIGNLQKDEARIYDYSN
NETWLRTTTLWGSGSSGFGTSVAMDESGSTLGIGAPSLSSRNGNNLGQVRIYS
>comp2150_c0_seq1; 197 1902 2760 LEN=2858; translated
MKIYRRANTELSTETTFQQVESEEDDHQLFFVTSYSDFEKLAHGPRGSEAKHSVHVYRFS
PSDGSLVLLNIQGDANVIKNPAFSRHHPRLNVVYTCTEDCHENGQIIAYKLKQDGSLEQF
GEAVDAGGTSTCYLTIDKAHRNLLAVNYWNSTLVTIPMDPETGALVGGVKNVYDPNMGKS
MVACAKKDGGVNHSCNDASTISARQADPHSHALVLDPFVGRVAYVPDLGKDLIREFYYDA
ADGKIAIELNVMPSGLCTGKPDGPRYFDFHPEYNIGYVVNELAPP
>comp2150_c0_seq2; 371 1902 3458 LEN=3758; translated

MKIYRRANTELSTETTFQQVESEEDDHQLFFVTSYSDFEKLAHGPRGSEAKHSVHVYRFS
PSDGSLVLLNIQGDANVIKNPAFSRHHPRLNVVYTCTEDCHENGQIIAYKLKQDGSLEQF
GEAVDAGGTSTCYLTIDKAHRNLLAVNYWNSTLVTIPMDPETGALVGGVKNVYDPNMGKS
MVACAKKDGGVNHSCNDASTISARQADPHSHALVLDPFVGRVAYVPDLGKDLIREFYYDA
ADGKIAIELNVMPSGLCTGKPDGPRYFDFHPEYNIGYVVNELSSTVAVFEINRQLLNEIY
AASKNGEDMNRFRGQSTLRLVQSIKTIPQAFPTTMNTCGRMCVHKSGRFVIVSNRGHQSI
TVFRVKRKGSKRGELQTVGCFHTRGETPRHFQFDNSGQYLLVANQDTDSIAVFNFNLSNG
ELKYSGNEYRVPSPNFVCCCPTYAEDDSDLYHENMGSSISAVTSIQADENSSPACSDSED
STLPTWTDKSSDDDVKAELAKAREEIEALKKLLMERTQ
>comp2151_c0_seq1; 606 71 1438 LEN=1486; translated
MRRPTSKPPVTRLRSPSRSSTYKTSSKQNNGAAALEDERFKQFLKNADEIESIEHFMYWK
DEFFTTFRPYLDPLGTATAREADEELYFELEKLANACVHTNELVKEGKISPEKSSVKGQK
SINEMIASTCTCEQHIDRYFPKTQEEELTCGYSKFELAAVLVRDGFRVYGMMVATRNYVQ
GLVIEGGALQKILKSNQMSILQYYFRCLDTFAQTMADLGMFKLMVRCEELYSVRPRRKKN
KTKKFNKHGAKDALSDTSCEGEAINGGRMFQKSKLNYRAIMDKGWSNGLCLGQEILGPLK
FSKKNDKKKKKGGKKKKRLIKENNKDANCKSGEDNEVASNDETNFSPEAGSSSSIEDDSV
DETAETVDELMSMASSSEDEEEDEEDVIYYFDPVRKIVGKVSRKKCLAEITIVSVDSETG
TERAEGVVEEWDSRDDEIGKQDLILNLKEILRSKA
>comp2152_c0_seq1; 390 251 1015 LEN=1206; translated
MSKHADTHVDVDNDQEKDSDDTDIGNSPNARRSLSQRYNDRCLSCKMLLGIVPFVVLAIV
SPVDEELSVWLALALSLSLNLRLLYRGCYDPKYPKWYWMEMALCLSLLGIGIFNGITGVA
VSLLQIIPSTAMFLAIPISMMFEDPFLYRELAPTVTPDVSSSPGFMQLCQYLSAYWMFVM
FLQSVSMWVSLLFLTSLTSGEPSEPIPYLILSAVLPIGFIILGFLTTKPLADYLKSKGEE
KDGDEENGDVTCDV
>comp2152_c0_seq2; 311 376 840 LEN=1031; translated
MALCLSLLGIGIFNGITGVAVSLLQIIPSTAMFLAIPISMMFEDPFLYRELAPTVTPDVS
SSPGFMQLCQYLSAYWMFVMFLQSVSMWVSLLFLTSLTSGEPSEPIPYLILSAVLPIGFI
ILGFLTTKPLADYLKSKGEEKDGDEENGDVTCDV
>comp2153_c0_seq1; 579 257 2047 LEN=2235; translated
MATPVSTEAPLNGLVRSGSDQERDLQAKMQHMIRLQYAEQTISNTNANANQDLAGGDGTK
GHQTGAPLENDREDDGSRGGRGLTMTKRGKTKTHANLENLGNDTDTNSVDNNAEVGKGTI
VFSGSLRGSMLPPGIVDDRLDQNATATKGLEREHQRYQMPSHSTTLESQQQRLLSSAPPQ
QIYSGYGSVHEQNHEFHYGNPDYNNIDDEDYYFDEHGGFRRDGDHRTTYCGQLFGCLVSP
IANLVGQENLRRSFCYGAIDGLLTGSGIASALWGLGLLSVRTKTELRVAFVVVTLAACTA
DALCMAMGHVWTTYIVTSNHAWERSHERRLLEHDKADSKGRLVDMLLERGMLKIDAMSLA
DTLEGYPDLFVSALVGDSLLSSGIQDALLDERTDDGRNYPREHYSPGTYSDPATDFDGGG
AFFGSFGSWKFPMHYDSDERGEFRNIEDRHARVVYSESQKEATFMMVGFAFFALIPSLLW
LFLPRWFNNPANNGDFGQTHAYSSLSSQEAYSGVGDGETVNVPSLIILILSSIIWCLGVW
KSSFVDSNWVVFGLETVAVLILCISSAYWIAVLLVHFLGLEYLILSGETSLGSPDL
>comp2154_c0_seq1; 436 28 1251 LEN=1468; translated
MKVLSTLYLASVASAFTTQSSHRSPLAVASRKVKQSTVARSLLPNKQQQSDASPFAIDVE
SSTVLDAKGTSKTAEDGSAGVRQLLGVRGADKSDDIWKIRLQLTKPVTWIPLLWGVMCGA
AASGNYHWIWNPFDPNDRNVLLGLEDTAKAITAMVLAGPFLTGYTQTINDWYDREIDAIN
EPDRPIPSGAIAEGDVIAQIWILLLGGLGLSYGLDVWCGHEYPINLYLAIFGSFISYIYS
APPLKLKQNGWAGNFALGASYISLPWWCGQAVFGELDRPVFWILPVLYSIAGLGIAIVND
FKSIEGDRAMGLNSLPVAFGVDGAAKICAGSVTITQFGVAAYLYSINEPIYAAVLLALVL
PQAYFQATLLLPDPIENDVQYQASAQPFLVFGILATCLCLGHHEFPV
>comp2155_c0_seq1; 139 155 499 LEN=834; translated
MKSTILFCLLSVILGMFFAPQSASAFAPVSVAPVALTPTTSTQLFFFGKPKDDGSPGDYV
CKDCGYVFTKGPKAWAALDDSKYKCPPCGAPKFRFKKVPKGSESGKVEVKKSWF
>comp2156_c0_seq1; 304 311 1108 LEN=1608; translated
MSRSPLAAPIAPRPARANWRTATKRQILGNFQQGSPSMSSLSSAKIDGSNTDHIQMLIGG
LQEKHDQMIAFLKSDLDDCKLEQEEVLSTGLIKLPKAVRNMSVRDFNRQHGCDLLALLKS
KDGVILSKNSVNGINNIPKHPNNTTACMIDANKKRCYETPAPRMRRPGAPMGTILRTARR
GEELYSQNGSPVAATEPGTVIATVCKKRRGNESANVEINIGEGQYISLNDPSGISELDAQ
MKQTAASQLKVLQDQMASLMAQLQH
>comp2157_c0_seq1; 139 281 841 LEN=1119; minus strand; translated
MCDPRPTVLHSKAIATDTRVPCRFLAGNGFAWIRRSFFHRNSASLSSQHEQGDAKQSDRK
KVRLRHRSVPSDASAKHPSPPRSGKAGRPTPPRLPPGSAPGAASVLSQGRGVVHAHHPAL
AVGHGDRRPVLLQAQSGLAGFPGQPDRLRGQQPQLPQPRGSLLVSRAAPGIDRLLFFPPV

LVAGGV
>comp2158_c0_seq1; 580 84 2252 LEN=2401; translated
MLRQRAQFLISRASTSVSGRNGSQNSARSLFSRRSTGSSERFKLSTRATSTLSSSAHSED
AGSSLWNTASAAALIAAAAATTTFVSSDSTTDCCGIVGVIGTDKNADAREFLLDGLTILK
NRGYDSAGIATVPSDGSALSITKYASDGDNADSIELVAKHSQNHGHFTGIAHTRWATHGG
KTDENAHPHLDSSGKIALCHNGILNNANDLRRELQSEGHTFTSQTDTEVIVKLVGKYYAE
GRSENITLKDATQKALDRCDGTWGLAIVCTDTPDEMVVACNGSPLVIGLGADRTFIASET
SAFNRYTKNFISMKDGEIGVLHADGRNLDLSRKQIAPDQQVKLSPDPYPHWTLKELNEQP
EAIAQALNFGGRMSTDRVTLAGLDRNEEKMSKVKHMTLSACGTSLNAAKYGERIMKHLGS
FDSVASIDAAETESKDFPCMDRPETTGLIVVSQSGETKDVHRVVKNAMEKGIVSMSVVNA
VGSLIARTTKLGVYCNAGRENAVASTKAFTTQVTVLALIACWFRECRDRLSGRPANMEAR
LVKDSLMRLPITIGMTMKTCQEQCKKVAERLMDKEHCFVLGKGYAEPVAYEGALKIKEMC
YLHAEGYSGGALKHGPFALIEGEKGKFGATPIIMFILDDQHAHHMRSAAEEVKARGAELI
IITDKPSLARDLDDNPIVIPQNGPMTALTGVIPLQLIAYELALLRGINPDTPRNLAKAVT
VD
>comp2158_c0_seq2; 569 84 2201 LEN=2506; translated
MLRQRAQFLISRASTSVSGRNGSQNSARSLFSRRSTGSSERFKLSTRATSTLSSSAHSED
AGSSLWNTASAAALIAAAAATTTFVSSDSTTDCCGIVGVIGTDKNADAREFLLDGLTILK
NRGYDSAGIATVPSDGSALSITKYASDGDNADSIELVAKHSQNHGHFTGIAHTRWATHGG
KTDENAHPHLDSSGKIALCHNGILNNANDLRRELQSEGHTFTSQTDTEVIVKLVGKYYAE
GRSENITLKDATQKALDRCDGTWGLAIVCTDTPDEMVVACNGSPLVIGLGADRTFIASET
SAFNRYTKNFISMKDGEIGVLHADGRNLDLSRKQIAPDQQVKLSPDPYPHWTLKELNEQP
EAIAQALNFGGRMSTDRVTLAGLDRNEEKMSKVKHMTLSACGTSLNAAKYGERIMKHLGS
FDSVASIDAAETESKDFPCMDRPETTGLIVVSQSGETKDVHRVVKNAMEKGIVSMSVVNA
VGSLIARTTKLGVYCNAGRENAVASTKAFTTQVTVLALIACWFRECRDRLSGRPANMEAR
LVKDSLMRLPITIGMTMKTCQEQCKKVAERLMDKEHCFVLGKGYAEPVAYEGALKIKEMC
YLHAEGYSGGALKHGPFALIEGEKGKFGATPIIMFILDDQHAHHMRSAAEEVKARGAELI
IITDKPSLARDLDDNPIVIPQNGPMTALTGVIPLQLIAYELALLR
>comp2159_c0_seq1; 357 150 1214 LEN=1430; translated
MATFDTKHCWVRDLKTGTFLANSRESVRVMGVVTHISDFSMEDKALLHPLMKCPNWSSPI
ERPITSVNNRGEALSQTQNSGTDGGNEAVFLSVTIDDGTDSIVVCAPRQMIESPSSEIVI
DIGTTYDWVLKLRQNSSTKQWFADTLIKIENPIDEHYRWMELSHHDQMSCSQSSPSSSSL
RVQYSNLCHRAGFPTRRKNAPEVYRLIRLNAQLQREQQQEQTGKPIAKRFRNSIPQKRS
CLRFNSDRLPLRLQNRPKGLQRSFVNTNRIIPARRSLNSKLQPTKLHTGTYCLQTQSSAK
TKEQPPQVQSLNGLLLKDLAAVLQKTQRDIQEMTEDLQMEGKIYQNERGEYLPL
>comp2160_c0_seq1; 261 73 1167 LEN=1373; translated
MSSPATQSAIIPPGSRSDENRLASCLVLLNEVDEWISGPMFRLGLPKWFEFWYSIPACFF
GTTFTSVAGPVWIALLALVWGQHDEKETCGAELEDNIFLLKIITFSVTVGYVVMWGLWQI
GGHEYLAGIAFWNGNVYVLGVPFNIAVLAYILNGFPYSSYQGSETNCSAEYKKNIFSMAI
YPLFLWPILITLLNWMKSTSKRSRPALKDIERKNAEGCGRGTSENWTQKKKFPAMTQLLA
KYNGDASFPSGDVLCATMVALSIFNISSDVSHAYRPSHLASTIVILAGTGRMYVLAHHFF
DVMVGAILPFLAHRLATKLGCGMFDMEWWHPLLVLGIFFVINSITRRKSDEKKSTKNPLA
EKEA
>comp2161_c0_seq1; 281 357 1648 LEN=2577; translated
MRASSSVSTPPSSVSRLPCASFCDFEASFKDMFRCQGVEFAIGDGNDILAGRDSDTISAT
SSASTVSQRSILVIGSDDAIEVDVDVDVDADCESARDYLERVSNNRSKNTDTSSASQQEE
QRDETSSSGSSSPRPSPRGKSKGNKKHHKLYHIIRSRSSSNSNSEVPSSPRRRRRPSFGS
YLKRNDSDSTDSTRSLSEDESERQPREQEKQPHDLEPKDSDDTRDSCEPDSGREERQAQA
QPKPTASPLPRNGALDXPSGLDSPAQGTVCGPDDCRQRQDPHLCPVHGLCGPGRAPPGPP
ENPPAAEAEGEHPGGGGNDRSLLEAGGSPEPAPVLVEFESAFALLGVVVLLLFRFRIDRS
LQRPRKEAALAEGPAKGGAADRAIGQQRAIAPGLKRVLRLSVRNETAAIRYDTIQHHIAR
HRTTKEHTQH
>comp2162_c0_seq1; 411 250 1062 LEN=1229; translated
MCQSFRSLRLLNRGMILKSDLLGRQIFDEAMSGQQIKSRLLASTGACSMRSYAMTPTRLF
GSKKGVPGNPLGTVSIYNDQEALKEIDEDALQRTVHRISKIIGYETYDVTLLLVDDDEMR
ETNLETRGINSPTDILSFPFHSREDNKAGLLKEPEFDIPDYYTLGDMVVCVPYVIRRCKE
DILFRKEQAEGATNDEKKSEYGDDDSDLIDDDDRGVSGAMANIDDPEKRIRMLLVHGMLH
LVGYDHIEDDDYVEMVEREEELLKELGDLL
>comp2163_c0_seq1; 1301 523 4603 LEN=4793; translated
MITEVPTSSFPETEGSSTRKPRAAQQQQHSINITSIGHLNSYRSPSPFGSALKTPLKSGS

IMRTRSLDDHHQEQQKQINCNPPLSQDIEMTGNSFCEGGSASSCCNDDKDEGTTVATALH
SSFDISLLRGGGASCSSGDLTLFENGLDEEEGDSQVIIHTRNSCFDPGDLTPSAKSDSIH
HHYQKLRQPKLSSAKEVSYIDLKPPATVRKPKISAKSRKDRRRSSDTATTVGTHLSTGSF
AFPMSKTPARTPGRPLRHGKNTNARTPGKPAGLHRSPAPSSTAKRRRDPLAHPARLSPMR
RYGTLSPSSCAATPAANTPTKSQPPPPFSLSHPAALAGKGGADKIRSLSTQTPIHQNLSS
DPHGAFLKKLPYHTGTAAGATPLETTFEVDGDSVDTNSPARFRFTSFPASLPRVHHANHY
PRSTPPVCPGSVRKRMSFDTEEENANTEDAKNTATEHIDDEIENDPVGLNYSIHDAVNQS
KDDDGTHNTSLSSLSVDGGHHQHLPHLPHQHPPHFASVPMKNLLLFPGEQDDENRQNELG
TNMFGYSDDEEDDVSSKFVGEDIRTSASPDQEGTVRRTRLNFNVFLSPSSDKKNLENSQE
NGFGGDRQIQLMSNADLSRPYDPQNPIHSFHSSLSSHTQINASAHTNEENLMVGTSSSSM
SPPASVVKNVNSNANSLPSTPREVHLHFPPESECSPILGVPKHDFVEPPSCSRALMLNRQ
QLETISSVRKNGTSSPIHSASKHLSHQKEDSTSSISSSSESTTTTQKRRLRPMPDMSAFE
NVKASSTRPADRSIDDNNTATDSRGAGSLTRRERACPPTPQRTPAWANDGGGRPIFPTRK
QNSLIMTKVLLSCPSQVLEGRSSLETSVLDEDSKHSGSRNSFSNSCDSINYPNGEDNQNL
PEKSTNEDGTTNMDVEKCEQPLGLSTLSTGNAHEERKVTHDQIAAPPKLMRRLPSNPDIN
AVISFSDDFEILGSLGSGTFADVYKVRQKSDDHLYAVKRNRRQFRGKRDRDVALAEVQSM
QRLQSVFATSNASGERNHSENSNNSSSDSKRNCYSLYLLFFYRAWQEDGYFYSQTELCCR
DTCRELLDSLRFLWNSAKKKYPRLVKTLPPPSGVEAGSSMDAFGRLVPNLTVWKICHDIS
AGLSHIHSHGIVHQDIKPSNIFFVTNARFGAMCKIGDFGLAGSIGSSGEGQEGDTRYMPP
ELLASATRHSSSDIFSLGLTLYEIAMDDHVEMPSEGPRWHQLRSSDEPKLPACRGDNLQR
LLQCMTNPDERKRPTADTILENTNVKIAGHEVDTFLQNYIRDVESFDRQEEERLAMERSD
DQTPRNDHRSAAVRSPSLSMLLPVAPCMFSPPSSKVIHS
>comp2164_c0_seq1; 946 110 2167 LEN=2715; translated
MSAPNKDDMETTVSRSIDGYSDENDGNSSSTNGINRDRYYDGVYYGDSDDDPAALLLALD
EAERNESSFDILADHGEFSYSEHDQRMYTPALDIDALLPPDNNMLPISEEEGEVGGGTAS
SSLYEIPSGYRNNRSLAETQGEDELDDVVDDEDMSSQTSSLSGSEPSRSIQEKVVEINTF
LDDAMQQQPSNKANRGFAKTGSGANLWNRFSAKGSSFDENASAVSDASFLKKGTVSLVQE
LVAADASYLNSKRKSGGSPTEDPEALATALLPSAPVGEEVDAGADTIQQKRYFVRRAIWC
AILIIVLISAAGTTAGLLIAKNRRNNNETTTEVVVSPGETTEKKNETSFDSTNNSSALDE
VNNSSIGFSNSSSNSSDANDQNGPSSSSLLNPSMRPSTAPWITSTNFPSTMPTNTISLSP
VQTASVEPSVRDDNDTNEPSKEDNAEITGDDVIDKSDRPWDDDDFQPIEIQGDDDFYKNG
EEEDNDGIKGGGFEDDKPVDQPSGDDDFQPIEMLGEDDFYTGIEPQDPLHDNTMGVPSAV
VHDKKQKQTKESKINSTKKKKSKSTKTRTKKPKSTRTKEQKNEIPHSEDSVDGSLLYGSA
EAVNIKQRSFFSKGRNIDPQPSRYRTYDRRYPQPQLHNQYYGKRPQKFWYHKYDSNPYLS
VKTFRKQPHKWRQQQKQWRRIPRGY
>comp2165_c0_seq1; 361 213 1061 LEN=1564; translated
MMENLVPFVAGASYGLTTVVVGQPLDTVKVRMQGLPSAANSSGVAVAGDLFAKEGVRGLY
RGGLPLFVGGAFMRSAQFGVSGAAKVWLEENTNGGKPAPRLFGILDWQVMIAGAAGGVSR
GLVEIPTDFFKTRRQVEHGWSIREVMDGTGVTLARNTVLYTAFMFYLDMAKLLCRQGYVP
SLLMADDRENLSPFVKGAVCANLAWLTCWPADVVKTQRQSGNYASRSNTELLMENLRNGR
LFRGIVPGLVRSTIANGSSMVVYEYVLTTLTKKFDLERRDLA
>comp2166_c0_seq1; 260 445 1080 LEN=1487; translated
MIRMNSILFVLASTILVFGCESHAFIPLLRSASTLRPAPTSEISYGRKTVVVSYSFYPVV
EAVEHIGYAAKVLIEHDVGKPLGSIQRIICVVDPDVEARFLVDITHLLLDFTVIFNFDGA
LLNFAQVVGRIAFIAIDFLPGHEFHYEEMAVQLVLLGVSIKRMAFAETKDSTPSALEKFD
QLDHVGQETQCATDTDFSTVDESMLVEACEE
>comp2167_c0_seq1; 133 399 653 LEN=928; minus strand; translated
MPRSISESSPHWSDSAVVSPARRCVYTMLRRTSTSSPSRSTLLKAEFTGAKSVTLPPSFP
DKKFDRSTSLTSSQNLLKLFIEQM
>comp2168_c0_seq1; 274 556 918 LEN=918; minus strand; translated
MVGLKLGGEEGLLDGASDGASDGAADGSEDGATDGDADGASDGAADGSEDGAADGDADGA
ADGAADGAEEGAKDTVGAPDGDSEGDAEGAAEGDAEGVAVGDAEGAADGAAEGEAEGIAD
G
>comp2169_c0_seq1; 373 1 1895 LEN=1972; translated
XKLSADRLDDFLPTVVLSNLRELTLTNFEGLTDTHVHVLLLSTTTMLLGAGAANCKPGRR
NLPLGSPNQNQRQPHSTGRQGNQWTRLVLDGCHGLTSHALTSIAKLCSQDHLRELSLRDC
PGIDTIAPLSELLVTKLGAKKATTATTMATTAATTATNTNTNTHSHRDELLRPSPVPLSA
LVPAEALGRKLRDFSDGECSTRAARVPAPMARLFETPPAASVETTHRRGRGSPPLSSSTL
ASLFDLPGTSPTRRKTAESLVLLPPPPPVLALGESGAITSAGSSPLRLRPPPRLPSDPLS
HRRSSPGLETQPLLLSCADGVTPKPSPPRKHQLVPEKETAAAAAPSMAHASRRYHRHKQS

LSKEAALPFPPGLLPSKNRDEWERVCRLHSFAPTFECRDHRSDNRANGNPGPSTALNDVR
PPSFFGEGTTTVGSLEALDLRGTAVRPREIVGVLWSLTHHRPRKKPLMSVGARTCFPNQS
DSNTASAWCRVWFEELHLNGGLDYYSCRRRNEIRVNLRTGAGEDATHATIDAAGACFLPA
TRWWDGAMAESDSCPGGTGWTGEDRRDLEASLAMDEMELRIGGEKAKKKKKHAIATNYEC
NATQYNNANRAKRKEPSTCGKSIVLRFVHYT
>comp2170_c0_seq1; 220 274 531 LEN=610; translated
MQNEAGENVDLYVPRKCNWTNRLVEAKDHSSVQVNVANVDPVTGLATGDATPYCLVGYIR
KISEGDMALTALVEKNDAAMFASEE
>comp2170_c0_seq2; 220 42 299 LEN=378; translated
MQNEAGENVDLYVPRKCNWTNRLVEAKDHSSVQVNVANVDPVTGLATGDATPYCLVGYIR
KISEGDMALTALVEKNDAAMFASEE
>comp2171_c0_seq1; 740 181 2136 LEN=2196; translated
MSDALPPCRLLFRSKEGIEIFSVRSERDAEPLPPATDRKFLLKGASSFQTMLPDGSAVLV
HRPDKGIVKIDLDGNDTQNSDQPFFPNTSKVQIMDVSPKGSYLLTWERGSANESENNLKV
WNTSTGECVTGFRRKMINRNCWPYLQWTHDEKFAFLLTTNEVRIYPGTFPQKAETRFVDK
IRIPQITQMSLPSSTATDSVGKILFAAFIGKDKNKPARAALYEYPPANESASPYPAIASK
SLFNAEEMTVHWSPRGDVALIALQSSVDNTGESYYGSSSLFLMSQQTSNDAIAVPLPQEG
PVLDVGWMPNAEKPPCFAVAAGRMPSMASLHNGRDGKATFLFGNRHQNTVSWAPHGRFVN
LAGFGNLAGGMTFWDKNKGKQIPPGTPVTASETTQFGWSPDSRLFVVATTSPRMNVSNGV
NLYRYNGEKINNNVLPWNNENYLPDRLLQATFVPAAPEVVYPDRPQSPSLKDLGTGPDAA
AASAAAAPKPAGRYIPPSQRNRAGRGGGNSLADRMRAEKEGKMLGAQKVTKTTPGMKRGV
VGATGKVVVGMAPPEQKEGKSKSALRREKQQQKKKEEAARKALEEKLAAEQKAQEEANKP
VDPVKRAKKINKVLRQIDELKQKDPSELNEDQAKKVASEPSLREELAKLSV
>comp2172_c0_seq1; 191 599 1132 LEN=1875; translated
MGAVLAKLLQVFWTKKLDIVVIGLENSGKTTLLSVLAHGEPVETVPTIGLNVKVFKKGQV
NMKCWDIGGQEQYRSEWSRYTKGCDVVLYVVDAAAPQKLGAAKKELHKLLDDGSIGSTPM
LVCANKIDLSPHVGEAELIDKLQLNYVMETPWMVLPISALKCTNIEQVVEWLTSQGK
>comp2173_c0_seq1; 621 52 1245 LEN=1645; translated
MDILQREMKRKKEALLKAKKEADASSETSSAGDGRKKRKYLKVSDLRRIEDEEQEEKEKM
RWDRNTNSNRKAVSISSASKETDDDDAISQARMKFKNAKKGKSSTTSSPSSSPAATVRDK
IDSSPASITQKLREMGLIVRYFGEGNTARLERLKNALEYQSKTLEGLSELEEFRLGKGHG
IRNPFLENEKQFKNNQHQMPGMGINDKGSLENSRLERKKQGKGKHATKSDVDGENEEDQS
DPPKFIYKYLKGLLKEWEVELAERAESVARSVAGRNESKTLKQCKDYIRPLFKLLKSRKL
EEGLQLHLLKIVKFAKQGEFVKAHDSYIDVAIGRAAWPIGVTMVGIHARSGRAKIESANV
AHVMNSELQRKYLTSVKRLLTFDQNRRTDVDPSKKVR
>comp2174_c0_seq1; 681 287 1615 LEN=1706; translated
MAKTKTKAATKGGSKREKPPALDQYKMPLIALCLSMLGYHMFRGMMAKEIKRVDMTATAN
ELELREVFFGEDVNTGSTNYAVLCYPENAVFPVSSVFQDAANDGSAPCQFRLMDCDAAMP
FSEEGKSVVKRFGLNKKTRPVVFVSGAVGQPKQVPAKYLKTGAMLTKALKNLLEPRAEKI
ETTQDLRSKCLDKDICGLMLKGGKVSPTYAKEAMQKLVVEYPKVTFAAVDSSVLYVKGIE
AEFLPELTPGLPRFIVFQKTSGGVKSDSSRLKSTAIPLDVDVAYGPMSNLVASVVSGKAE
MTKIPASPTIKTRTKKLEKSENEKRQRRKNKTNGSGDSSSDSGTPHSGANDGSREGRRAE
RERRRAEHRKNNPNYREKTPEEIKEMERKRRIRMEEEAKKWNMDAEDPSEDSSSGEYMVE
DEGFDEDEENLDEEDEDVMDLD
>comp2175_c0_seq1; 658 577 2031 LEN=2504; translated
MGLFGRKSNIGRDEKQQQLKPPSMYEQGGPNDNADDVDLSESMNDDYDDFHEYNHDDDDA
SVASHMEEMTTDPHDDYFDFMDPEAMANAASASASGPQQQLQEVSRSSFPQRHTNGDKYY
KNASPSFHSSHDALKRSNSSSSKNCAADHENDTNAIPERTNSATALVHNSNERSNGHSRK
REDPLVPVTSSLSPAATARSVSSXEHGHRQSLPVPQLAFDVQDALEVPVVPDPTLALALA
LEEILPVRRRWVEEQDETRREAEKWRGPEKPTFGNGKASSTTALEKQEQVAPSPAAKARA
SAKSSPSGDLVEQQQHEILAMQIQQLSLLQQQREQEQQHPSPAHPIDESETTRDLVKTFI
GEIWNRGEISSISKVCSPRLRFNSESGLEKVGHEGFSQMVLSVRSSLEDYHCEIHSMVVE
GRKCFCRLKFTGKHVGTLLGCPATHKLVSWSGASEFTVCPKRHQILKVWELGDIKTLEKQ
LKID
>comp2176_c0_seq1; 391 1 1229 LEN=1431; translated
XKAQQQHSQSLSPRHHPTILLFKTSLIVPLLAAVFFAMSLNPTPADAAAKRDAFAATHTT
TTGEAKEGDDSYGASHTSFYTTEEKKDSYSTLSKTLETKCSDKLVRQVILDMMGVCADIT
DALRVNLVTVEGSANTFGDSQLSVDVISDKLMWDACKKSSVIREGASEEDPIVHNVDIEG
ESGGEFTVCWDPLDGSSIIDNNWAVGTMIGIWPKKTGLIGATGRDQVTSLIALYGPRTTV
LVALDDGVYEFTYGADVPDEATTDGGDPTWICTRSRIRLKDDSKIFSPANLRSAQDLPGY

ARLVNHFMTNRYTLRYTGGLVPDVYQQFTKNMGVFCNPTSEKSPAKLRLAFEAAPFGLIV
EKAGGLTSDGVTGGSILDVKITAVDQRTALCCGSKNEVLRFNEFLELSK
>comp2176_c0_seq2; 391 1 1202 LEN=1633; translated
XKAQQQHSQSLSPRHHPTILLFKTSLIVPLLAAVFFAMSLNPTPADAAAKRDAFAATHTT
TTGEAKEGDDSYGASHTSFYTTEEKKDSYSTLSKTLETKCSDKLVRQVILDMMGVCADIT
DALRVNLVTVEGSANTFGDSQLSVDVISDKLMWDACKKSSVIREGASEEDPIVHNVDIEG
ESGGEFTVCWDPLDGSSIIDNNWAVGTMIGIWPKKTGLIGATGRDQVTSLIALYGPRTTV
LVALDDGVYEFTYGADVPDEATTDGGDPTWICTRSRIRLKDDSKIFSPANLRSAQDLPGY
ARLVNHFMTNRYTLRYTGGLVPDVYQQFTKNMGVFCNPTSEKSPAKLRLAFEAAPFGLIV
EKAGGLTSDGVTGGSILDVKITAVDQRTALCCGSKNEVLR
>comp2177_c0_seq1; 195 1 771 LEN=951; translated
AQHNTSPDVLRYHNSLLPHDTQENDCKARHIAPHRTAPVQRATMVRTGLLRQGTLRCRNS
FAAKAAAHSLPGTGTTGPSASSFGGARRSFLCQHRPGRPIAIKASASASATIHPNPLCAS
YRRSFAEGFASESDYHTAADETLEDLLDALEAAIEDHCDDMVAAGGGSENENDDEPEATL
ASGVLTVGLPPHGTWVINKQTPNRQIWWSSPLSGPRRYEHDGEDWVCTRAEGEGPKTLAE
ALEGEFEELYGISLGL
>comp2178_c0_seq1; 76 350 769 LEN=1230; translated
MTSRILSLARMIQSRTAAATRSVPAALSEPLKVLFGTTPCEDAAEDNEANHADRREHEEP
AAEQAPGLFAALVRGPAAAGPAAAANTPWNAREERKASIRDYYHRAIAEGREAAANNDRV
LRNPDLSVPDGEQYLYIAQ
>comp2179_c0_seq1; 877 187 2451 LEN=2757; translated
MPVQKILSTVGCILLLACSGSNAAAPAPKRTKKGVPKGAVSPRGALAHKDQLRKKTVEKI
EAAKRLNRREKKEVADAEMQTYKTLRREGADEVTNSAEAQMLRNYRLRNKKRKGLLRKKQ
TSLAQILFPGVSPEEYNFQEQVWIYSDLVESKKTQVPYEYYDLPGCPYQVESGIKRRRRE
RKNLGARLQGHDTKPAPFKLFAKQDKECTPMCLVTLEGKKLRWMRNLIQRQYRVQLQLDG
LPVLMRSSELNYAVRGYPIGFVSAAGGTVAKSKRNNADVYLYNHLKFAITYRTGEDFDGI
RITGFDVHPVSIAHDIPENKAVTETASSLSTCKGMGVENDPSSYLSLSFDKGNSVDVIYS
YDVTWIENDSLLWADRWDVYLVGAPDDDIHYYSIINSLMIVLFMTGAVATIVIRTLRKDI
AGYNELQTLEEAQEETGWKLVHGDVFRPPQFSPMLLSAMVGTGAQIGCAFGFAMLLAIFN
VTNSMRKGQILTSMILLYVLCGSIAGFVSSRIYKFCEATNWKMNSIATATGLPAVFVGMF
TLLNIFLSFAGAATAVSFLTILVLFLLWVTISAPMVFVGAFIGLKAPTIEVPTKTKQIAR
VIPEAPIHMNSGVMMLMGGVLPFGSVCIELAFIMSALWLHQIYYVMGFLLAVLGILAATC
AQVSIVLCYLQLCAEDHRWWWKSFNNCAMAGIYLFLYSLWFLSSRLDLVGMLPVIVYLTY
MSMISICFGLFCGAVGFMSSFWFTRTIYGQVKVD
>comp2180_c0_seq1; 471 294 2054 LEN=2339; translated
MEMKDIERSSQLEGSRNNNAQKLLDQECPIVWRWVPFHANHGDSGISSLSQPRVIYATSK
KVGKPEPSPLLTGGELREARELLIRSKVNSLIEATSATEPSPLSLRIPHVPRNIPPHVNL
VRHTQHQVLLESQGASGNASNGMNSSSKRAETAGGELEVANLLLNMGGGSSPKSGETAQK
KKQTTKKTALPSRPKGQTGKSAADKKPSLLEDTYLDNWVAGSISLHTPEDDDVLSPLHCF
MRRYCVEAFSATPEDVATPRYGKSHGFKVEVGQVGIRCLHCKDLPPSKRAERAVCYPSSL
RNIYHSIETWQRRHSLVCKCINPWVRKSILELMESSKTRAGGRRQYWEDSARRLGMVDTS
QGVRFCRKPGDLGPIPSPGSGRLPGEPDVPQHEPEKIVRKEDKDLVTDYLFLLMDQMQTC
QFTEEDRTGGRSKIKDNEVGFPGMECRHCQGRAGFGRYFPSSVAALSLANSDRNVYNHLQ
KCRKCPESVKTELIRLQKTQAQSKNRRGLRKLFFNRIWKRMHKVDKDDSENSRPSESGLV
GRQQLPQGRPGEFVQYSFSQLPPAIMNHSYQHTMHPQVAVHNFRRY
>comp2180_c0_seq2; 368 294 1364 LEN=2457; translated
MEMKDIERSSQLEGSRNNNAQKLLDQECPIVWRWVPFHANHGDSGISSLSQPRVIYATSK
KVGKPEPSPLLTGGELREARELLIRSKVNSLIEATSATEPSPLSLRIPHVPRNIPPHVNL
VRHTQHQVLLESQGASGNASNGMNSSSKRAETAGGELEVANLLLNMGGGSSPKSGETAQK
KKQTTKKTALPSRPKGQTGKSAADKKPSLLEDTYLDNWVAGSISLHTPEDDDVLSPLHCF
MRRYCVEAFSATPEDVATPRYGKSHGFKVEVGQVGIRCLHCKDLPPSKRAERAVCYPSSL
RNIYHSIETWQRRHSLVCKCINPWVRKSILELMESSKTRAGGRRQYWEDSARYVTV
>comp2181_c0_seq1; 301 58 672 LEN=1392; translated
MSDLSLQSKETACVFIEYQNEFTTEGGKLYPATKEVMEKTNMLENSTELLKFCREKGILV
VHVPISFRPGHNEIAKDPYGILAGIKDGSAFESGDSGSEIYEPMKPIEGELIVQGKSGLC
SFASTNLEFLLRQHNVKNVVLGGFLTNCCVESTMRTAYEKGFKVYTLNDCTAATSLAAHE
ASFEHNFGMFSVPTSSEDIKKAIQ
>comp2182_c0_seq1; 284 158 730 LEN=969; translated
MPTNAIHRNYRKTFTTPRRPYEKETLDAELKLVGEYGLRCKREIWRVQYALAKLRKSART
LLTLDPKDPKRIFEGQALLRRMSRYGLLADDELELDAVLQLTTQKLLERRLQTKVYKQGL

AKSIHHARVLIRQRHIRVGSQLVTVPSFNVRTSSEKHIDFSVNSPYGQGPPGRVAKKRAA
SRAAAGDDEE
>comp2183_c0_seq1; 414 98 1277 LEN=1371; translated
MSTDPEDRMNTQEDREARDGLLQLNTYLGGHADGLVSDDSSEEDFHQEEAVVPPPPPAAQ
PPVPPAPPTLPAASHPLFKTAQATLAKRKFVPLKEKQDLLLRIREKLSHDLIKAVFPGGD
ALSLEMQGVYHNITATGNTHHKRIGMESGLYSHNACQILYRNGDQEGEERPTTKKGEQKE
AAAVAELETVSRAVKKAMKAEETSNRLKGSKHKKGKVYWQQGAASRLQDQCSYDPAKLDR
FGCPICDHHSIMPVDDLDKIQKHNQGKRKEHDIAVAEHDAANRDKKGHPKLKGMLDVRMA
CYCCKFNCKLDPRGGTCPECSKPGVDYLEYENGVPVACNCPLCNCQCSIYFYLTDRFNIN
KNEERLRSGPQPEEEQYSGNPIFARSSTCRRP
>comp2184_c0_seq1; 316 448 1719 LEN=1956; translated
MASQYNNGNNNGNMSTMDATRSYVTARTAASQSVANHTTSGDTCSLAPPPGAMAQPQPAP
YPSFVKVCGCVEIYKPGREVLEYDYTYDDVSGKKPPQSYPRIRERGEFYIDSYNGEPWSC
SFGTAEKNGTWFNSSDQAGSIMSCLVWVLMLYSSVTVTLLTITEGIPVTCGMVYNVLCFM
ALACHAKTSLTDPGSVPLAAIPSERQRLMKDSHSMCGQCQTFKPPMSHHCRICNRCISRM
DHHCPWMNNCVGGNNLKHFLLFLIYTWTCAAYALALFGWNYFFCADEDCTFHPVVVHLVR
VMTMLCVGAFLFTSSMLMNVCYGLMTGIGTIDRLKKKATNTMNQSDEEPIPLKDVFGIQG
WITWPFPVDPIFEDFDQVMGYCLPQRLDRERQFMEYKRPIPEIQQVTSHEMELPKCINDL
LPV
>comp2185_c0_seq1; 564 97 1557 LEN=2106; translated
MKPSGSKGRNVSLFTTWLLIVGNQCTGLIGGEAFVITSHVKISAYCSGLGRRLENTFLCG
STGDNDDFSFRRTDGDARYWNLPNDFTMFLTQRAIQSFIFLTSQLRDPETCYWVEQFTEP
NLVRLDTKQSFSLGDTLPNLSDISSGKSDWRPNADGDDPKDDEDEEERRTLLLLKYHGLA
AMDTVRFPTWESYFEEMLEQPTDKWIIASDQSHIPDYTWEIDPSSLCSRILSVREQLSRE
FASDLQAVSNMGGQTLDWYWDRLRQSREDERREEGQQTEGKNSGENEDNPFVDPTSLRGS
RQNLLFLEINVDRPELDHKPSPLRRGNFDLLVLLATEEAIQRVLNRWQSKKSVGDLDGSE
FVSYEFLEDFYKERQHLFQGPLPSYGKADDILEELLSSALSFKGDGEVINPTKIAEVVLR
ERERVALDWKDIALDSPQDHMKIQKLRLSRMMMGNDNNGNGQQGESSRGESSSNVGYHDP
GPGAFE
>comp2186_c0_seq1; 576 191 2422 LEN=2890; translated
MEQINKKNLRRRSVDYGTKTNRSKLADDSKRLRLFNQSNPNKTQLTGDSRFVVAAKIATL
NNSAVDLYIKRDFENASGFFEEATLLRLISDSLFPPKVLFDLSRPPNNKMTWDKGTDAAK
TYTMGDSAVPLTSNYMYQRTEFDEGISLYCDTKVIHDGDHHLSAKATLLFNAGQARRELG
DLVGAVAFYGDALSVLLPNLETDEATHFSPHRIVVPVLHNLGQIAYRMGAASKAIWLYEI
ALVHCRVLNGERSLSVASTLNCLGVMHYHSPANDDSDQAIRYFLRALDVLHEVMGSASEY
EATILNNLGRAYVVHYNEFDLAFESYDQSLAIRKHYLGPDHVDYAATAFNAGQSLHHKGAL
YFDRAMELYGDFLRVALVRFSKKHRDVAVVLSGMAQIHQERKEYPKALKLYEEALEVAKE
ALGNFHLEVAVICNRIGNFHFERGHCDKALEAYKEGLHIELRLFERTHPNIIVTMSNIGG
ILRQKGSLECAIRIYKDVVKLQTMRYGKNSAEVATTLSVIGMIYDQNGNSDKALVTLQRA
LAIRRSATGVNDVDICTTLTLIGTILYRKSMVSTAIEFFSESLSIRRIKLGKNHRSIAYT
LYNIGLCHQSLGNLNQAVDCYKETLRIEKMVLGEDHKDISMTLLKLGEACKAKGDLDEAL
DVLNYALAIERSTKGQDNPAAVVRIINEIGTIHLEKGDFVPMMEAFCEASTLFQQAGLTA
ESIAMPGALYHIGIIFPSGAPAA
>comp2187_c0_seq1; 71 252 1079 LEN=2093; translated
MSYSSAARTNLSSQDVRISNYKLGEGAFRVCHEGTYVGGNRNGQYAACKRFKPQFRSMET
EYFEKEFMIADTAIHYAEQWNDICDPEDKILITKGHIIYSNSGIPYMAEPVIRDFKKFTS
NSGWIDESDIYTEAMEAFSHMTYHNSGGNHLVCDLQGRYRNNYRKSRFELTDVAICSVAR
EFGPADLGEKGIENFFANHQCNNFCFPHWRRPNLARRWFPSQPGTSMFPAGDVRLQRPSP
GTHFQLEGISVRPGAYQLTTTEDGLIRGLFPVPQT
>comp2188_c0_seq1; 338 52 732 LEN=927; translated
MVSLSSALYSHYTLSALAIALLVVLPFPLQAGGASARRKLTKKATQEPIPTPDPTPAPGP
KTLKLAFDALRLVSEDVASIGFESSSLSTGNPQPTFVGATTVSFLNLFPFDTVTAENVES
LHADPSSGGAIGTIEFPCIITNNGFSKGLCQGVVDFLLDGEPAGAMVSYGGHLINQEHYQ
GMIIGGTGALGNAIGGTYQSVKTNIPTADGTSVYVIENFVATFVLA
>comp2189_c0_seq1; 274 1 1195 LEN=1742; translated
XSSKKKPKQTTDIEMDQDTKKNTSKGEKKNMKSKKSNQNKKNKTKKDKKNDRKRDDVADA
IPNVLDVTPIVAVAPIAAVAIPLPDDIHPDQHYTPPVSAQSNTTDHQFPNEGSINPVIYP
VVSEIAAVAVPLPDDIHPNQHYIPPVSNQPSAMFPNQEGRNQPNIGSPISGNGNVKLKDQ
GYSSGLIYSIGLNNVICPLRIWVVDNSGSMKKRDGHRISENKRTKQPFLLECTRWAEMQD
TVEYHARMAAQLESPTVFRLLNKASTGQQQFSIGERGHQFIQEDLNIALETIWRTVPNGK

TPLVEHIYQIRSVIMPIRNSLCQDGQKVVVVIATDGLPTDSSGTICLERFKVALRSLLQL
PILLVIRLCTDMKSVVKFYHKLDADLEYKLEVLDDFFG
>comp2190_c0_seq1; 298 348 1235 LEN=1506; translated
MIVPFPPIVVEAALTASLLLNPMAADPIPPPMVQTQTKNYEFGNGSVRIDNRMKFPGMTL
EEPRFLGAGGGGSVWGMKRILDPGLDRSGDGNGNPGGVPKNVVVKISWLRSTDSVRNECD
VLSVMEQRGVTGVERCLGSVEYPYDSRRVAIAMEPLMEDDGDATTSSLDDLTPELALRSA
LQLGKTMAQMIAAGVVTTDLQPLVSRSTGEVLLIDMTEARVLALAESGDIAEGDKLLIDA
FCTEVLGLIPDSLLDDASESFVAELHRLEAKPGVRIDRRVKDRLRDLPITGVEKL
>comp2191_c0_seq1; 246 88 1131 LEN=1855; translated
MNTARNSFSAATGFGRVEASIFGESALIDRETGRVYWECGDSNTLYESITPKLDQYGVEH
AQATFLTIVITLAFLSVLSGWLQRVIAYYNSSVSTRSLSLLILLAEWTILKLPKIENKWL
VLASLIFYFYESYNCSTRHFLANAMSSSSELDTYIDSLRNEQPIVTWKVKTFHYELRRIF
AITTIIRSLFRSVKKTGAVQELSTSTSALPSSRPLDMGLPIKSARNTPPMFLITRKVVTN
EASANYMYTSCNDNTIAGVWTRAQTDSDDGSIVPFTKISLSKVLVLADKRSREHYFQQQS
DFVTKYGREDEFAEFSTHIDVTGYRPRLLAHNDCTQWKTRMFRTSIFW
>comp2192_c0_seq1; 1111 126 2687 LEN=3055; translated
MRFSGRQIFSAALFCTVSDAFVPRRNNFRVVAPQTDDHAAPLRVPFHPDFQVGGREPIVA
LHAKKKKGNAKAAALDALDALDDDLNAPMSKKELKELEKKKKKEEAAKAAAAVREEKAAP
PAKPMSKKEKMLAKALELEMMDEAAAAAKGEEDDKPKLSKKELKALKKKEEKMAAKAKKK
AEKMAAKNAGSEGEEGTNGAVNGDAAPAEPAVAVPEKKVKLTLEDKIRKERPPPRIRVME
GAQPGFVSLRMEDIGITFRNQEVLKDVTWGVQSGERIGLVGKNGAGKTTQLRIMAGELEP
TTGDVVKSSGDLRTAMLRQEFVDELVKERTLKEEFMSVFAEENQIVQDLKDAENRLETMS
DADPEEMQEILDRMQDLQQKAEDKDVYVLESKVKKTMDLMGFNDDEADDLVASFSGGWKM
RIGLGKVLLKDPNVLLLDEPTNHLDLESVEWLEAFLREQSIPMIIVSHDREFLDQVCTKI
VDAEGGICTEYDGNYSRFLQLKKARMDSWQSAYDAQEKKIKSERQWINKFQAKQPQVVKQ
RKDKLEKFMKSDEYVQKPPFTGKPFRFRFPPAPRLSPEVAQVDEMSHSYENEISNNRLFD
KVDLSIEKGDRIAVVGPNGSGKSTFLRLLIGREEADEGSAQIVGQNVEMAYFEQNQADAL
DLDKTVIQVIQGASNGQSYNELRALLGQFLFKGDDVEKKVEFLSGGEKARLSLCCMMLRS
ANLLILDEPTNHLDIPAKEMLEEALQHFDGSIMVVSHDRYFISKVATTIVAIEDKKLTKY
QGDYKFYMEKSKAIREKIEARYVKGLDRIGSAPEINLEELIKPKKKNFGGAKTANQVTRK
NKGVKNAKRQQQR
>comp2193_c0_seq1; 404 37 882 LEN=984; translated
MGSTKAARPSVSKPAHQKSYRNVDESGEEVKGFSEYMFEALHHFMMFSSYIPLFLGQFIE
DFQIAAAASLGVVVFNALLSFMMYKLGKTQSWPKPLDICFFVLFGIMVIVVFAYPASADF
WRFWQGVYFNFGSFLFLSVMWCFGMPFTETYVEDEYGKIGVTHPVCQWGVKINCGVWVAS
FFILGCLSLPAGIAQQLGKPSTDLLAISTYGTIVVMVSALSFSFMLFPWYMEKYEDPIFD
KFMDQILEWNAKHPDEDWAIEFEKAMAEEKAAAQAQLSQNV
>comp2194_c0_seq1; 190 63 428 LEN=1296; translated
MPSDSNYSAVARYTRQPLSKAARPVALHVVDESWASDTLSDDEVEITRHEAAVAPLLEEG
ELDADAIVVSSTGNSASAANNAAERRRREEGWNDLGLDQIDANGVGVSTSVPSRMSTQQE
G
>comp2195_c0_seq1; 572 1 1526 LEN=1605; translated
QQQQKQQQQQGNPLAVDIETETKLTKAQQELIEEQVEAEAEAEYGPDPSGDGDHHHHKSI
RYLSKSPGQQHFEYDEAYLARDYDANSEIVDVDPSQPLWDANGRRLSPPPYDPTGIACID
AGKIRRFRTAPEEGNKDSLYPTTFGVIGDIGQFEHSQQVLNHIRDHLQGIRAVVLVGDIA
YPDYDGRKWDTFFDFLDDQSNFDEVPLMVAAGNHDIDKQYSGNEIFLGYEHRFRMPRVHP
PELGVFDGDCEDGAMNMDAPPYPLPYEWGNSYYAFTYGPARHVVVNAYADMSPGSTQYEW
LEKELGSIDREATPWVFLTMHPPIYNTFSLHHKDPQIFAAREHLEPLFVEHHVNLVLTGH
IHAYQRTAYVAFGNLTDTGPMHVTVGAGGRKADAPFSSREPEEWLEVRDASMYGYGRLTV
RNETHADWRWIPIAPSDLHDYNQVRGEDDLHLPSLDHDREVIVNQYYXAPRRDGLREGET
HTQAGERRRGRHTNALLLPPSQLQLHVR
>comp2196_c0_seq1; 260 51 710 LEN=969; translated
MPDAAPTAMTTPLPYSIFTNGSACGPGGVEAVGVIQSLVVVDDKSQGAKAQAPAETQPPG
PAYSYFCETTTHTHTDPDNGGTIETTVYTKVGVTCSPLDQGDLALDAVTCEEAGCGSCET
AYPVAATLAVPVFAPLPAPDACYGITADATGVTVLNRFDAGADREGIETYWEVYKANSCL
GDLVEAASASASSAGEGRSGSVGSLVVAAALAASPFLLN
>comp2197_c0_seq1; 288 321 1202 LEN=1283; translated
MKFSTLVLVTFGSNALAFSPRSNDIKATKTQATSVADNEPIRQPIFDPLGLYPTNSHERR
FGMISALEQPLEKDETVIDPLSLYKDESQVTRDVEMSPSLPFLKRPEHLDGSLPGDRGFD
PFNFASDPSAMAWYRDSEIKHARLAMLAAVGWPLAELAHKSIATRFDLDPVLGIGGRVPT

VLNGGLGLTNPLFWVSAISAAAALEFVATKNGDSREPGDFGFDPLSLAGTNGQQSVFVRE
AEIFNGRLAMLAITGFVAQEFYTNTAVVNQTPIFFKPFSDVVAHFLGAGGGSI
>comp2198_c0_seq1; 705 83 2107 LEN=2232; translated
MRRVSSRQRLVSWDAFDATAVSDSGSGNDSSVGGFEEDDRSCASGYETAYSRSYHSSMAL
NMLCEGNLYISNSIDNTNSMEEITGIDATTELRRRKRDRKSSRSSPTERANSSKMVETPK
TFVFTTLSTSKSPKLVPSHSWETGKPPPPSSVGFDDMISDLHLKIFSFLDLSSLRSAMYS
SRHYRNLIISNDARNSLWMDHCEKIWHISEFMEESGQNQYHFQPHPKFTDNFHLPIAAHT
STVTNSTGFETDPVDTTNISLLLSLTPKDFPTCVDKDALKSRMQLSGTIRQTIPVDRQDD
EDQQVQYYKEQSTGRSLIRYTGYVGQGDRCIRSNRPIPRPSQRARLNETFESAKDINEIN
SSRSLMNSIGPSFLHIDDSHHPLLYSLLQKCSSKAMKGDGMKSSKSSSSSNSSVLSPPSP
SLAWTPFVVPFVDKSSSDDNSTFLNVTPRFISYYEVSILKQDGGENQADFPAERMVPPPL
HRRSNMDCVAVGLGTKDFNNRTRMPGWDRQSFGYHGDDGGIFHASGGMLKQYGPKFGPGD
TVGCGVDYISKGIFFTYNGKFLGYAWNNVSDDILNNDLYPVVGLDTNFAVHLNWGTSGPF
EFDLSKFIKKHEVAVMATYSLDGCSCLCPRDTTVTGATAAETDSSTKKVARSFSGRSTSR
RHRKGFIGKRNQSR
>comp2199_c0_seq1; 559 64 897 LEN=1164; translated
MRIISAALLSLASVNAFTPAVLRTHTGFQSTALSMSTVSQDELKKQVGYKAVDDYVKSGM
VVGLGTGSTAYFAVERVGNLLKSGELKDIVAIPTSERTKEQALSLDIPLVTLDTHSKLDV
AIDGADEVDPDLNLVKGGGGALLREKMVEVMADKFIVIVDESKLCDGLGPGFPVPVEITP
FCHEHTMRQVAALDSCAGCKPVLRMGSSSSNQPDGDEIAVTDNGNYIVDLHFEEPIKDAR
KMAADIKNVCGVVDHGLFCDMSTAVIIAGADGISVKE
>comp2200_c0_seq1; 319 1 908 LEN=1235; translated
XLFHSIAPIHITPLESIMSSPSSSSSTCHGRLAYGVRRLRPGAEPSFQKDFEAWLATHNE
SRLAAALAFPDPSDAHLLHLLVWDDNTTEEGADDVLLGPESDAFEEEDADGVLHVYGSGS
ATGGASNSTSTNRKLPPVHMQTPLAGYMRRRLRTTAASKDTGSLLLIGVFKRAIHSGKRD
DLAQAFQRVCDIWYKTVPGILAAMVFPDPTDTGFVHDIRIFDDKASYDAHVDKSNPELTR
AMEEWFSHYDTSIPHKGIMVAEDTSDPAMHTSSIKKRPVKVDFNVFHYGQGGCMGRCLHV
TF
>comp2201_c0_seq1; 1199 1 3307 LEN=3391; translated
XGSFFSQNTTLKLFNLEGNFVHDEGVQTIADGLQANPGGTKLEELYLGWNGIGDEGCLHL
ARMIETNSTVTKMGLGENDITSIGARALLNALAQNQTLQRITGLYHNMIDRKFVIECINR
ILYSHLETLPAELEQVRRAEFERLKLASMSATDTLLEPLGSHLPIPDEISEGELDWADRL
YNDETDLVESGVTIGEVPREETPSALGQVDTEEGDIDEAAEPKTIAMMSQFGKMPEPKSL
LDRLVVFQAAPLVYLHRETAFRQAIPLHDFDQETAIIQNSLNGCSRLDATIDMDVQPATA
ESFASFLASGRGRVLHFTGYGHPNHLLALEDEAGYLDDTFSLDTLPDLIKSANPPLDLVV
VNSFHSGRIGKAFLDAGVPHVVCCHHPETFRDKAANSFLKNFYRALATNKSLKQAFYHSQ
EAVRVEEISKDVDRYVLLPRKPEDDPYHDVPIFYTQPVPANTLDQDTDNLLLEDGRKLLP
KLPRHFTGREVDMCKVLDALRNQKLVRIGGIRGIGKSSLAAAVCRYVQKRSGFFEFDNVF
WLPAARGVVPDEDTLFADLVEYTSLMVKADHNISEDEDALECRERIEIELEGQSTLLAID
SRKFDSEVSAECLETFLSELLNTDLEVKVILINSNEEDEEATLRLAPIDFKSTSLLFGMV
SRFITANGCPAAQSPDEYADLIVPPSIAIGEPPKSAISVRQERLLSVMGDGIPLQVIRSA
KSMQANIFIRLISMANTPEVHVDSLDSLEVSISKWTSRMEHAIENKNFFRALDLQHVLKE
LEGQRSKFPSVDDLVAREKELHRKHTLCFKTRRYEEGNRIKREILVLKKEILRQKRRLAT
NRHITDAALTQSQTNKIADIQAQMKSIMKLANSSFSSLTDLQESPDKNEAVFRLGSAYHS
CQVRIYPGNVEDFDPGDDLGASICWTNECCDINLDEQGASLLSYGGQNLADDAETLPAIA
ETPWGTTKCGTGNAVIVGPGNYDDLRVHCVILAVGPISPTRDEVLDESDEDSLHYITVMM
RSCLRSSFILAKHSQVQSIAVPTLTTKVGSPTYESTLLTNLKLLVDEAKFSDLNTLHIVA
RSDEESTKLIDMALTMGLTMS
>comp2202_c0_seq1; 350 139 888 LEN=1193; translated
MDDSRISLVSTEEVLRSEAYMLFYRVVDHPYSKKLANQVKALNDSYDVSDSLKAKEEKEA
AVASRKRKEKETKDASIEIEKNSADASSKNSSRLSDRSIGSGPAASVKRNHRKRKAPEFF
CGEEWARSKTVIAEKNIARFRDVEGKVSRHITFTPEFEKLLSEHALKTNAKIDQIPSSGS
SLLDNCGDSSTEDINITLLRCFLAVSKNDGIVDFAPTPTPTMTTRSSAASRGKLQSSAIH
VVDPTDDLL
>comp2202_c0_seq2; 843 496 2787 LEN=3092; translated
MPQLMEDKNDGIGNDNDLMILPPRPPQRAVARAVSNLGNTCYMNAVLQALAHAPELCMAM
DVHGHRMRCPIYKENAEKMAALSAASSAPSSRSSSPDNTLNQNFDSNGELKSVVYTYSSTG
GAGTRKSTRRSARTNSPTSNENSEDKSVDDPNVTKFCALCETEHHMDRVHRNTHGSNMTS
NSKAVSPTNFVHGFIDHVAPASFRVGQQEDSHEFLRLLIEAMQKSCKQARRRKVTTIDQG
NPQDTPTESLPSEAESESANSLVKKQEESPDTEYPFQLFSGKVESKVTCGYCHTTSSTLD

PIEDLGLEVTPLAGTSLNAVGRRNAPSPVPTSSLADVSHALQRFACAEDLDAGYKCEKCG
KVGKATKQMRLASIPPILTLHLKRFRYGSSDPSMANAGGGAGLLGNGGSGYLPTRRSQRT
SEVSQLIASSGGVTSSGSGVDWFSGGKSGSAKIEGPSEFKAVMDLKPYLTEELQKKHGNM
LCRLFAVVVHVGKNSHSGHYLAYVRNISKNEWWKMDDSRISLVSTEEVLRSEAYMLFYRV
VDHPYSKKLANQVKALNDSYDVSDSLKAKEEKEAAVASRKRKEKETKDASIEIEKNSADA
SSKNSSRLSDRSIGSGPAASVKRNHRKRKAPEFFCGEEWARSKTVIAEKNIARFRDVEGK
VSRHITFTPEFEKLLSEHALKTNAKIDQIPSSGSSLLDNCGDSSTEDINITLLRCFLAVS
KNDGIVDFAPTPTPTMTTRSSAASRGKLQSSAIHVVDPTDDLL
>comp2203_c0_seq1; 400 139 1056 LEN=1420; translated
MIAPIDVAEQIEGRPKIDVDLLTEASVSGEVFSRRKSKVQFHAVEVREYARSIGDNPTAM
DGPPLGLGWTYQNLKRSSFSEKSQRTESNIVVTTGSETDTTVDDHDESITIPIDEYETAT
NERRRRRIMKLLKLRENATFQLKKQRSRATQRQASTGNYYYKSKEKDDKLDPNELTDEQI
NQLSATWLKIQPVPGKTRRKILLEATDMSQNQIEDTVRELAKLRNQRRSTQAMAETGLDD
FQAAVEFCVRRLRRIRLGMSKKREQELLWEKAEEYWKSIETPHFNRDSAPRSALKVCEVD
DKTED
>comp2204_c0_seq1; 369 72 1657 LEN=1667; translated
MADKGQRRLVLSEEDYTSKLSLIVARDYFPEISRLERENSLLDCRLAGDAIGAVAVRRAT
RNSLEEEEAAAARREGDDRDVLESESNENTLARRNPPGANIQIRKRMRPLEEETVTGFVM
RATNEDDDEFDSNLKQNIRENRQRLEAIYQTKSNPTSKTNENERPGGAISNLCLEMASDD
FAPESNRTAWKHKPLMRNGLFFNPTPMRNSSETDSDAIGNVREQALLKNSTALMVLSDGS
DDMPPPSKQHTSGLLSNRNAGKDKLTQNLLKSQLVEYIPKHKLEKKIEPSATRFPSNPSL
IPIPGSALHGSLDEETDSDSHTDYMSSTDASTDLDAPLRSLSEERRRFKRKKESYRQSYP
YVAMSSPRAKQQLPISTHGAIDGPPLVLSHQEEQLEKPESTSSFRMPSKNEREFAASNAE
KLMARRARLASPSSLRRSSLKSGKHSSMCQALGPGSLTPAALSLMKAKSIKLRNRDAFGS
ALRRSYTPQVXPPLVLVQEFSEKEKRKSTRGSCLQCDAPKIEIRKTLG
>comp2205_c0_seq1; 565 95 1318 LEN=1505; translated
MKFTVAATLLATSVTATSGSDQSVANDAASASHRGLAVSLEDRFEVTSVLKERRASKNKG
KVIRNLQRKLQQRQLGNEVEEDSQVHEEDLDLGIFSRGLQANATEPEEELSVMEQLIELC
GDTEETDESFSCTCADLNVEAYTASVTCAYAENCLSPTEDICGGNTTFCFLETYNLEVTA
PGTGSSAICYELTSPLSFNYCYGLTYTDESTTPSGCVLEVEGNQCNSCEFYFYDSQPNVT
CNNFDCSNADETIGSGIICGDDTIVSAKIEDHLTYAPLPCEGGCNICPSNGEMSLVDNNV
TMITGDVYQCWQLNLAAQAGYLQDMPGDLCNSLLEVVNEPCGCVVPPATEVPEVTPTSDE
DTNPEAAPTDAPMSSTEPNEASDGATVKSRFSFAVTALAATFSWMII
>comp2206_c0_seq1; 251 200 664 LEN=1211; translated
MMKHSTAMNSGKKTIVACLWMAVLSIAWSSNNAGVFVQGMGEKTSSLPLTANTPISCDEM
IILHRWEGSCCSLNVTNGGGCILNVFDGYCNVKGQEWTLKYNSTYDEKPCPPSEFSNTQL
GMKGEPTLPPSGSSSRFTTKAMVVLGASIFAALL
>comp2207_c0_seq1; 374 105 1253 LEN=1582; translated
MNNETLATIARENTSLALSTLAIAAAGAAVVFASTAISRKPDNSQNDKPIPLPVAPIGSK
FAILPKSGVTLRYTDTQEEQVTEKDSGESSSEQVTFVLLHGFAGCVETWELLTPYLVKSN
STTNESEVAVRVVAIDMVGSGFSDKPIGDDFDYAYRNQGKVVSEFISVLSLSNVVIVGHS
SGTVVGAATASQQQSNESGKTAVVGAVFMACALFRPKSDFFSKSWLKPLFRWMTKKMMAN
RKQSLERMHLPAHAERVLTDDFVEKFAAPTRLPNFHDALVETVMAKEAPYEELVDELINT
PSAPFPMLFVWGKQDTYTPLPAEQKDSIQQKLDTMNKTSEQERQPIVETTELDECHHYPQ
HEQPEALANEILTFVQKNVKLH
>comp2208_c0_seq1; 1180 105 1916 LEN=2256; translated
MTQSMRAIALLLVFGIVATIVQGYRAPSSLLSQSAATNANANAVVGGASPKLSPLMRSVM
NIRGGDEEEEESDGEEEEESDDDEETGLSSSGSGIDYVAVLEKALDVTKTKIVPTVIDYS
KKGFLAAKKTSIVVYGALHRAIQAGLEGEESEESDDEDDSDSDDEGEEATLVEKILTMTK
KTVKTVQRMVKAALTVPEGDETSEDEDEETIDLEEGEDDAIDTTEEGTEEGAATGPEETP
AAKDGNDDEAAVNDNDFGSYLSEAYEVDDLRNSGKSPGTVILDGSLQDALETARKQARML
LVFIPSEKPKSEQGGLSLFGGGRKGTEESEQNNLVAIESLLSKEVAKASNRKARKMQAED
FGSFAIWGTKAGSSEAAVAIKRLKVKEISAKGEKRAILCVVYPASSGGVIAPKVLAQHHC
NPPLKAESMASWMNALRKRHGKQYEVMQTEFTELQLYKERKEGYIDSVQSDNQRKAKEAQ
EEAERKAKEVKEAARQAEIDARRQELEQSLPEDIKSGSNVKKIALRFPDGRTGQRGFASD
QPLTVLFNWVDAMFEIERETVVLTTLNGKQTFSWEGDENTTTLEDAGLNKMTAFRVSQAA
KAD
>comp2208_c0_seq2; 230 1 765 LEN=1105; translated
XASSGGVVSIDSVRARWCRENDAKVSLFVFHSFFARLQPHVRFFRMYRLLTEFCGSFLFQ
IAPKVLAQHHCNPPLKAESMASWMNALRKRHGKQYEVMQTEFTELQLYKERKEGYIDSVQ

SDNQRKAKEAQEEAERKAKEVKEAARQAEIDARRQELEQSLPEDIKSGSNVKKIALRFPD
GRTGQRGFASDQPLTVLFNWVDAMFEIERETVVLTTLNGKQTFSWEGDENTTTLEDAGLN
KMTAFRVSQAAKAD
>comp2209_c0_seq1; 149 220 600 LEN=982; translated
MPDYREASNFCARYSIVGMIFMVLISMMLTYQPFYIGGIEDLDQARSNAYGGAGTFLFVF
ILSVVYLVFDALRGGGNNRNRRESRISRGNEYDAVSLSGLDGTEQMMENMANLELPPSVQ
EARFT
>comp2210_c0_seq1; 161 143 901 LEN=1228; translated
MRQSTLYCCSVLLSAVGTGPAMVDAFAPALQEQSNPIRLQATTKDSDSIDIDRRGVFRTA
ATAAVAAIAGTSISLPANANQAEGVNLQQYEDFTKAPEGFSYRDVNVGTGVSPSIGDRVV
YEWSGYTIGYFGRPFEAKGGPQGGAFDKNQDYSRTVLGKGEVVKGLELAMQSMKVGGIRQ
IIVPYGELGYPSTKEDASHDKIGPKPTTFSGERALNFVLDNPRVDRTLLFNVKMIRVDKA
DGKGGFTRGEKA
>comp2212_c0_seq1; 489 240 2921 LEN=3195; translated
MKSTREDSGRDKNDRGGRGKNGRKQSGNQGRGGGGGSKGGSRRSKNKGQNNGNRSSNRSN
RSSFSSSSSPLVYPQYATLEECLTRYNRGDRNLIRGTIRCLPVRDGPAFCTCDRGSQSKD
VVLDGPLERNRALDGDVVFVEIISKPPEEKEKGTAPSDRTPREEPGSETKEQESEDEWWQ
DDIDQVNLWDPVVPIAHKKLIPKSDSSSPSVAFDKEQKHGRVVFVVHPKEWSSEIKSKQN
SHSRDRSAPTKRIVGTLKRLKGGTALLTSSNNRSMQQFRLSNADAEKYKDAPEGSIFAGK
YTYGTWQETHKWPACSNLEQFGATGNIEDETAALLIEFGVDHGEFPSHVLEESQTVVASG
QYSNGTESGWRPTEDMYEGRRDYRNQRIFTIDPTTAKDLDDALHITDLGNGQIEIGVHIA
DVSFFVTPDCAIDVEAQRRCTTVYLVDRVIPMLPRPLCEVACSLNENVERLAFSCVWRMN
RDGSLVKGQDVWYGRTVIKSCARLDYSTAQNIIENKVAYPGENEIDEELWPVSRRPTGGH
TIGQVAADVRLMNEIAQARRRLRFRNGAVALDAVKLTFQLDDDRETPLLCEPYKIRDSNK
LVEEYMLLANFLVAQRLITNTGPRACLRNHEPPDYEGLEKAAALAMEAIGFRIDIESSEA
VQKSLNRLSQECQDPILLKCITELLKNPMKPATYIAAGTLSQPEWAHFALHIPYYTHFTS
PIRRYADVIVHRLLQASIDGEDAVEDFPMEVKEISAICGTCNEKKEGSRKAQERSDAVFL
ALYLRKFPKKRQLAVVTSIGMKAFTVFLPHLGISAMVYLDEHKHWIEAEAYEDPKFGRRI
KLKRTQTHKGERWKELLVKNFLRLRVTCGCSDKPPLNVNLKLEGPWEGDDETR
>comp2214_c0_seq1; 726 1 1651 LEN=1877; translated
XQQQRYNPSFEDDVFNEERYYSIENGYAISCYELAMALYQDLILTENEEIRKANDKAMEL
AESQLGLNEDATEDEIMDALLSQTTSGGMGGAGATSSFVSIPLDIGDDELQDLFGAGTGR
FSEAKEASLLEIEGFLDTAILTFLQHADYPPSGDGARSGGFFHGQTPPKKKKIVVVNGKR
KKSQEELRAEERLFHWRASLATAYQNSARIATVRNHHIRARELTNKALELYTKVVVPYYE
ETERGSGKGSKARENARQFVSSSMASTMTKEYAKTSIGELKKSLAATALYLGNYSESMGQ
YNKAMTYFQRNHLTPSPDGMYNTLQGDDVGMHQYREIVATYQKELEDYRGNVRAMYVYKD
DSYEAGVLLALASMTMAMGDLPVTIRSYEEAIEIYDRIQSNPDRKTRLGLASAHSGLSSA
LFFSRRFAHSGHHYGKALGHFRSVYGEGTPPQEEAELAIEDMKDELIQAYGEEYYESLYA
QYTAMGGGKGGGGPSGSGSGADGPGVSGNFDVVGYHNATFAAEESAAATAGGDDSDDDDD
HAHRAHSGEL
>comp2215_c0_seq1; 387 117 710 LEN=922; translated
MKYSVGLVLASLIPAASLTREGIGRRDAISAAASGVVGGFLSQVQPSFAAGTPPTKEELG
RIVLGYKQITALLENFEESTTVCRENGGECKRDAEPIRRVLGLRSTTDPLFQIEKVFNKV
KYMDDDIDLEVFFEATEDWNSAMTLSNSMAFISQFGEYNPGGGVEEVLKYLNESKKAVLV
AQSALKRIIDTLHLEGV
>comp2216_c0_seq1; 177 732 998 LEN=1278; translated
MALVPDFNPLAPIQSLQLVLLTLTGTQAGFCSSISPDSNCSSVTGRSLFQQPIRRTRGMR
RRVSDTEDADAGRWCMPLTQIADTEDVH
>comp2217_c0_seq1; 539 62 1498 LEN=1571; translated
MGKHALVLTVVLALAQTECCLGFQLARETHLPLSLASGLSKNDEPSNDNGCVSGLCNSEL
SRRDACRSVVGTALTMSSALLGGADPVFATKEDENGFAPAVRPTAYRVDSTIPPTLLPLS
SARKEKATLKALGGGSGTDKEEIKIDTLNLNNFMNKLVFGSIDFVSQTVSQSKDESKVGP
GYASFVCMGMPSKTTSNDIDLATSLMGSILQSRREELATGLGLSFCPMSSQPALDAFTKS
GDESTLQSALKGAGVGSETIDLYMPLIRYAKSKSVDFLALSPEFQDIEAARTKGLASVDF
ERRKSYVLDPEGFISMTQDPRYKLYTDRSLLKDFEPTNSEDNIKGFFAERIFVHEAGASA
VAKYTVNKPESMVVMISPISDVRYLQGINGRIPRVFGSLVEKNNANGVNSQTVTNKVSDN
AVTTILLNPTALDTLSAGEKLRLEIGTGPDTIDYQSKIADYIWFSSSPKVSLIPRLMN
>comp2218_c0_seq1; 428 103 807 LEN=1589; translated
MKFKLFDKLKKALKPKEYAAAVKLQTFFRGILARARVSRMVNKLITDILIRRGELPPDFY
EQEEKRKEEEKKKEEEKKKSATKGRGAMLEIQKGSVKDLTLKFDKNPPKPRETASSEACP

VEEEPPSEPKNSIYKSFRCEHNATKHVISSEDILNEQARRLNEEIEHLLLDIKRIGEKGK
KSVKFGELFDDDDVANYYEALVGTLKAAKKKGLISYEGQILLKGVHDDHEICIL
>comp2219_c0_seq1; 124 1226 1624 LEN=1729; translated
MILKQVPYTVTKNVSFDVFTKFFYSCLRNSGVATVSHAMKFTIPLVSAAIASVLSSLTSQ
PGDTILSLSCAHGGDYRTRDISRNILRSDRGIAGFFVGTRTRLLHVGMIVTIQLLIYDYV
KRICGIGATGLA
>comp2219_c0_seq2; 314 199 1518 LEN=1623; translated
MVRHLGDTYGVLKVATLIVCCFQESSSFNLHNNRLTHCHSTVTNINQNPSTELSNPIRQQ
RLLATSSGVGNNFEEALQHSQNHASSLNAAGGATWDDVLIPSLELPVNDHTKITAMDKLG
IAIFGGCSIGVFLSIVSKSAGSWRFFLAGGLCAAISHTIPTPVDVIKTRKQVDTRFVDKS
FVEAGRVILKEDGFSALWAGLGPTTFGYLLEGAVKFGVYEVLKPAIKSWLSVIASMSSFS
FLNSHFFSFVLSGIASGLAASFILCPMEVIRIRLVAEPNYTSGNWIRGGYKILKKEGVQG
FSKGLNPMILKQVPYTVTKNVSFDVFTKFFYSCLRNSGVATVSHAMKFTIPLVSAAIASV
LSSLTSQPGDTILSLSCAHGGDYRTRDISRNILRSDRGIAGFFVGTRTRLLHVGMIVTIQ
LLIYDYVKRICGIGATGLA
>comp2220_c0_seq1; 792 341 2995 LEN=3106; translated
MTPHHIFRHGRFHSTLRTLSLAGGSLLAGCQIASSPLLFENEDTCHCSLKVNNNADDTIC
TVRNRLREIHSVFSRFSRLLEANRSSLEPASKPASRIASENNTILATSVRQRKTCLEPGK
AIGSVGNTALYDFVIVGYGNAGQSALRTLQEQCPGARVALVDPLRSPNKGTKGKGVDTDY
FRDTVVEFDPSSKTLRLLAADPSRRAIRYKHGVLIATGARGAPPPLELFQEESLPRVLEL
RPTELFRNTKRPAMAPEKVRNAVAEAAAKGAKIAVLGSGWEALDLLLVAERACKSTKRRP
TMCFASSGIAWHTLPPYLSAELRKKLGKREVDVQDRSFVRYVADYQQSNRQQIELHTAKT
YDLMDTRRTALDLLVVAPDSFGDKGTAALPTMDVPESMKESSDGRPWYKTWSQMARRRNA
EPSALVCFEDDGRISVNAELHVASRIYAAGGCAKYPNSSTGHSCIAGEGSIDGSEAGRLA
ALNMSRDYRLATRASSSYGFSSSGDDIEEAHSFATNSLPIWRSDITSFPACNGDRISSLS
SIGVQALCVGKCDSERQSTRAFWWTNSSARRKMNRFIKDESESSQDDDGDDGKDEDVDEG
SIRRRKSLVISRHKTGRVNSRGLVKPVYGVGVVFYLDHYGRIQGIMTWGLPFADRPGGKI
NPELLEHMKHMIASNAGVSALDAEDNHQLVNLALGKASQKLVALSVKDQVSHTTRTWHGL
DGPIQGFATPLYRYTEVSNSRNKTVNVLKRKDGSGLGVLGEGLYVRDDFVLEEVADNGPG
DHLSEEENEDASPANIPTTMYPITVAPASHHDDAYGKKEISLETMKELNRYLAVQLRWEA
NENRARPGKEDPLWLRPGDERKNTSGKQNVIDAYRRIMFPHQSS
>comp2221_c0_seq1; 379 56 1435 LEN=1483; translated
MKYSFVGFLLAFPRYRGKLNSTTSLVAGFSPSPVTRRNYRNAIGYLTGCRSYVEKTARSR
RSSLSAQSGRDSRDNYGSNPDSIENKAGTNNNTKNCNWLVVGDGDLSYSASIAEYLANKG
IRLFATVLEEEEVHDSVYKRSSQNKAAILSYHNLHDSLVRSDTTGGDDTAATNLQSQHIV
LFGVDATHLIDYFPRTKFSTIVFNFPHWRGKTNAKRNRILLDSFMGSAAEVLAEEGKIII
SLCEGQGGFPASTAEEWKRSWLVPQYAAGHGLLLHKLKQYDPMYQQTSHRGKDRPWIKDG
TDQRYIFGFPNEGCIDEDLQISCRHELRVVLPKDFVEGSAVGRQEIIEGDAVFQLTKGFV
PDGIRMEVVTREILYPYFFDNEEVTLAIFLLNYSGEKAPLTRESADTIRSKVEASVQQDW
NLKVAKSGRLVSRPYPTHLLPALIRESSERNLTLTNSSP
>comp2222_c0_seq1; 504 129 917 LEN=1047; translated
MAELHPSVREKTDALDAAGNTTAAIGKGFAIGSAALVSLALFGAFVTRIRDSSGQTLFTD
GVNMLEPVTFSFLIIGGMIPFAFAALTMKSVGMAAMEMVMEVQRQFDEKPHLLDENPSER
PDYDACIAISTQASLKEMVAPGAMVIFTPLVTGILFGVTAVSGLLVGALVASIQLAISMS
NSGGAWDNAKKYIEKQSADSELKGKGSDIHKAAVVGDTVGDPFKDTSGPALNIVMKLMAV
LSLVFADTFYAINNGTGLLQLS
>comp2222_c0_seq2; 1075 35 2278 LEN=2408; translated
MSLLTGASGLIFGSAVVALLWGYLQFKIIAAIPVKSSSGSGEEEGLADQTDHVTATQRLN
EIYEAIYEGAESFLKAEYSVCFWFVVSFSVLIFILVSWGTGWDMSRGSFTAISFLLGAFT
SMASGYLGMKVAVFSNVRTTVSAQKAGWTECFNTAFRAGAVMGFALCGMGIFMLYGTALL
FKSHYPDAKEWILLTESLTGYGLGGSVIAMFGRVGGGIYTKAADVGADLVGKVVHGIPED
DPRNPATIADNVGDNVGDVAGMGSDLFGSFAESTCAALVIGSSIGLSGGWDAMVFPIIVS
AVGIFVCLVCSFIATHIWTVKQESDVERALKVQLISTTIVMVPAVYYAAMNYLPASFELH
ATVGDAVLTLTPLDATICVAMGAVGGLTIGLITEYYTSHSYAPVRELANSCKTGAATNMI
YGIALGFKSAIIPVVVLALVVYFSFDICDMYGVALAAIGFLSNLATGLTIDVYGPVCDNA
GGIAEMAELHPSVREKTDALDAAGNTTAAIGKGFAIGSAALVSLALFGAFVTRIRDSSGQ
TLFTDGVNMLEPVTFSFLIIGGMIPFAFAALTMKSVGMAAMEMVMEVQRQFDEKPHLLDE
NPSERPDYDACIAISTQASLKEMVAPGAMVIFTPLVTGILFGVTAVSGLLVGALVASIQL
AISMSNSGGAWDNAKKYIEKQSADSELKGKGSDIHKAAVVGDTVGDPFKDTSGPALNIVM
KLMAVLSLVFADTFYAINNGTGLLQLS

>comp2223_c0_seq1; 307 1 1788 LEN=1788; translated
RAMKKQLLYLILSLALVIFYGSCDNGNADPVIVDGPPVIESLTVNAGTIENTDFMATAVV
NDGIAGSTISTLATANWTITNGGTTVASGTESLSGDLATINIAVAGGFGVGDYNLDLVVS
DSNGNSSTENVSFTVELPLPDFDISGDWKLEPVAGALKVGPAQGDGSWWSSGAGDVAGRA
CLFDDVYTFTGNATGTFGVNVGDMTWLEPWQGMDPEGCGTPVAPFVSSTDFTYTYQNNQL
TLEGVGAALGLVKAHNGVELGNPADAPDQVTYEIASQTQEGSVRRMTLQIFSGGETWWEF
LLISGEPDAVPIDVVGDWTLEPVAGALGVGPGQGNVSWWASGDGDVTGRACLFDDVYTFD
ANGNFSMTLGSETWLEPWQGTDPEACGTPLAPHDGMGSYTYTLSGTDLQIVGTGGHVGLA
KVTNGSELGNPADAPGDITYIVSDFSVDGTTKRMTLQIFAGGETLVAVPADLAIIPLVHS
FYGXAPMRDGGGLIKPQLLKSMKKINSIFAAILVVCFYACGSDDGGETPGGGNTETITIP
TTGFTSPTSYPNMTLAWEDDFDGAVLNSAHWTHETGTGSNGWGNAELQYYRSENTTL
>comp2224_c0_seq1; 494 1 1279 LEN=1554; translated
XAFVLVLFPPMKDQNRFTKKTLTKPKLKMSAAAARRKKQLLARKKQQEEAAAAGHDALDP
VSAQLQKLLEDPEKLAEEATAYEALQLAQSQIRKKVHAGSYAEAIELACSASLIILEQGR
VSVASQLTALLANVLRETHTEETPALLEKLKGLHAAQEKAMDGKTGAEAVRLNRLQRDWL
RKCVQWSSELGKTRYGNLELQKLLAQQSWKLSQQLVAEGADENVDEEDNEVMELKADAVT
HMALAEEPMGIVEMLKTMPKPTAAETKSGHTCPPADRDALLTRAVLCLCAVENLRDASAL
CRAFLEQVEDRDADALTASYVSKDDGKAPSHIIFCCMLIRICEKDPRTGPLFSWLLRSFK
RELDGFYKVQIIQSYTSKIGKIYFNIQPPPSMMNMLENMMGSMGGGGGMGGLNPAMMQAM
MQNMQM
>comp2225_c0_seq1; 653 262 3198 LEN=3496; translated
MTCSMCSSAIHKALTGMTGINNVTVSLATNIASVEYVESETLTAEIIAEEIEDIGYDVTD
IIRKVPAYRRTRVLKSVEYAVGGMTCSMCSSAVTKALTGMPGVQTVDVSLSTNTARISYY
DDDQVENDTNEPLDYPGDFQEMIEDIGYDVNGVVDDCGEESLCENNDDNDVDELVPSEAE
NRLERILRQQNSELKARRNAFLWSFVGTFPILIITMVMPRVLHKDNFVCQFMRRHVDVFG
CKLLIEAIILWMLATPVQFLCGHSFYKTAFHGIRRGVLGMDVLVSIGTTSSYVYAFLAMW
KNDSGYRFFETSAVLISFVLLGKWMNAMAVRKTSQALTQLMKLQAKTAIKITIPPSTNSS
TWNPLTDPYSEEIVPIQKIKPGNIVKILRGANIPADGTVIHGELTVDQSMITGESIPVLK
SPDDAVIGGTICSETGTVEGDNNALTAAAFVRVTGVGADSALSKIVQMVQDAQSRQVPIQ
KLADGIASIFVPVVVVFSILTFLTWWVCCSTGRVPTGWYDKENPIAFSLMFGIAALVISC
PCALGLATPTAVMVGTGVGASHGILMKGGEALEVASKVNAVIFDKTGTLTRGKPAVTDFT
RVVSDEYLRSLMSNDVAQISADYFLLWLLGSLERNSEHVLATAIVKYAELNIESLLRSLP
FAQPSDFVPVTGRGASGVINGKTVAIGNRAFAELHTGSMKISSEAEECMQKLEGDGKTAI
LAAVNGVVCAVLGIADEIKTESPDAIRYLHDRGMDVWMVTGDSWRTAKAIANELQIPRAR
VIAEALPDSKINQVRKLQKEGKIVCMVGDGVNDAPALAEANVGISMGTGSDIAAEASDMV
LVGGNISGTCTALHLSRAIFRRIQWNFVFSMGYNIICIPLAAGIFYPATHVRLPPTMAAI
AMALSSVSVVCSSLALRLYKPPDIVARSSSSLCCKPLGQLRLLGRRTTEQEYEMIAQQPL
AWSDENSVGSDLFGDEMV
>comp2226_c0_seq1; 303 1 808 LEN=1088; translated
XKNFVKQSSERDSNYLCYLIKKMQKDGGDPDFRSCGLDRSCPRSVTTPQGQGVRLDQYQS
SITGNQNKRQRRQSRQSYSGPTTVAIVAQTCTTFCPASTRTSLAVIPDHDFEWYDLEAIR
KVDNKENTIIVGHHDYNAGLLFTTERQVLGKGAEMNMFNTSITIESDDSEGKSKDGKNSR
GKKGKNNKGNEGKNRQKRFFWEKNDNDIIEETPAEKEEREFRVQMQELEQEMSIDLSSQS
SGSKHNRNKNDSDNAFFATWRVGDPVIRY
>comp2227_c0_seq1; 366 249 881 LEN=1204; translated
MKMEDFGFLKGTGIGFEDLWEGEEVISESALEARLNKDGLRFRMNRTPEEADEVGRLEGL
PGVTVNLPIIGETFLGPPKVESIWEAIGFTATSNNEARQLEKKKAIEKCKIAKEGVSENQ
TMFGPGGGGADTRKDWLDKYGYPRLVGTGGIFYADQLSSDKEPSGGFNMGKSGSIWPVPD
VVKEGQYGGSMGWGMKKKGPAVDGLAKEKL
>comp2228_c0_seq1; 941 133 2442 LEN=2489; translated
MASEESKEIPTKKMGDLKIDAGGKSKSGGGGKKKEAKPQREWTPEMQADYDRILSVGEEC
QNPDELRGLILAKGRGSEHPTGFNLYDGFEPSGRMHIAQGVFKAMNVNKCTFPGTNSTFI
FWVADWFALMNDKMGGDLDKIKTVGKYLLEVWKAAGMDLSNVKFKWCSEEFTTHADKYWP
NMLDIARRFNVTRIKKCCQIMGRMEGSLTSAQVLYPLMQCTDIFFLKADICQLGVDQRKV
NMLARDYCVAAKIKNKPIILSHHMLFGLKAGQEKMSKSDPDSAVFMEDTAEDVERKIMSA
YCPTTEDEKTETDTGGEEDAGKASMQLKEIKIKNPCLDYIENIIFSPPGATFTAGGTNYT
DFETVRDKFLAGDISEEELKRGLIDELNKLLEPVRHHFTTDENAKDLLSKVRQYKKEAPP
KVTNVRRLNLVELSKVPAGSHLVFAPMPTATPTLQEAIDVLTLLRRAEEGQPCVLMLPDW
SARVTNACDGDTKSITAYFTILATALKALCAETMSKVQIILQSEAILSDPSNYWISVINA
GRHFTLKDVMGEMSDSDPVGWVIARLMLVGDVAGVEPKSVALLEGGNNIHADGASIGSKM

ITEFFQEKLVSLVQPSVTTGAGPNMLLQRRELEAHKTENDEYYLLDDPKVNGKSKMKKAF
CEPKNIEFCPPINVAAAFAFGLEPDSNGEMIISRTPENGGDAVFKNRSELEAAFADESLH
PGDLKAFASSTMVATLTKLSTAIKADGDSAKAGKTLKAMAKKLAKQKKK
>comp2229_c0_seq1; 207 1 815 LEN=1179; translated
XSSSSYSGSGAHSFQTKSALKVWTTTATEEEEADRQPDPSFDRRSLLLAGASSVATGFVL
NENKDALRRAPAIMTSIEAPSEAGGVVAPATTSVSSVEEALTIIESLGDKRFLHAVVASD
YKFLYEQTPRQNRSGAPKSVLSDIDAEAIFSKKKNVSSSPRSIVLAASGNLAPGKAPSLW
PLETARCSVAGSCDSIHYAWPEQGGAIPRRNNDEFAARSAMIVDGIDCGKMSLEDALEGD
MQVLVQAPTYLLVPSYMESNLRRGLQGAFLI
>comp2230_c0_seq1; 278 185 1003 LEN=1263; translated
MPYLSRACGAKIFYETKGPIHGIPVLLLAPGGMRSSIGKWEMSPYNPWMRLSSKNEKHGK
FLLIGMDQRFAGRSTGTVNKDDGWHTFLGDQLALLDHLRVPKCHILGSCIGPSYALQLMK
MAPERFGRCVMLQPIGLAHHTTEPGEAWEGLNSDASWDWVNSWADEMIGTKKSSDRSILN
DLYYNMFVAQPHREFVFSITRQDAARIQQPLLIFMGKDIYHPSETSRQISRIVKNTELVE
VWRDAGPEKINAAASKISSFLLTDTFSSTPPR
>comp2231_c0_seq1; 326 139 1323 LEN=1347; translated
MRTARNGPEQRTSGSCCLAVVPRLVLVAVCLPLSNGLVPSTSPATGRPPQRSSAAALREK
SGSGGFLEGLFGSTAPAKSPPRVRTPEGFVTPEPKPLQISESADPARFARNTLAFVIRLG
VGAFVLGWKIDTVFYKQPEDDGNDNDKDNGEPSGDGRKTRYSLDLGPFSIRDSSSVVLPK
PEQPLVLYEYEASPFCKRVRETINLLDLTVEYRPCPGARQGKFSDELFEMTGRRTVPYLY
DPNTGEGLFESSDQIEYLLANYGPADASTYDRKALWPVTLEPFSVATSTLCAILLGMPGK
ARRPDARPDNEEMLPLELWGNEASPFVRNVRETLGGLCLPHAMVSCPRGSSNRDRMVERT
GRFQVPYLVDPNTGIEMFESEEIVKYLEEVYTVS
>comp2231_c0_seq2; 293 139 1393 LEN=1417; translated
MRTARNGPEQRTSGSCCLAVVPRLVLVAVCLPLSNGLVPSTSPATGRPPQRSSAAALREK
SGSGGFLEGLFGSTAPAKSPPRVRTPEGFVTPEPKPLQISESADPARFARNTLAFVIRLG
VGAFVLGWKIDTVFYKQPEDDGNDNDKDNGEPSGDGRKTRYSLDLGPFSIRDSSSVVLPK
PEQPLVLYEYEASPFCKRVRETINLLDLTVEYRPCPGARQGKFSDELFEMTGRRTVGGLR
VPSFVPDGLPVFIVLPLAVPYLYDPNTGEGLFESSDQIEYLLANYGPADASTYDRKALWP
VTLEPFSVATSTLCAILLGMPGKARRPDARPDNEEMLPLELWGNEASPFVRNVRETLGGL
CLPHAMVSCPRGSSNRDRMVERTGRFQVPYLVDPNTGIEMFESEEIVKYLEEVYTVS
>comp2232_c0_seq1; 117 1 321 LEN=495; translated
VCSSDLEKSQSKIHDTSKVNSSNSTSNNISSLSSSLSVIEDAAQTHLTGRKRYRTVVPRR
VYLDSIAQQCDEEQDSFYVVQSDPETSRTGKRLLESFEEATTLNTF
>comp2232_c0_seq2; 528 179 1996 LEN=2170; translated
MSKDAPPCFSDGRKFPLISIPKPADRGVTNSNINDRAPPKSTSKRIRIRRRNPFSDGRRN
NFPDLDNFFKSTSESPSPGRLRVGHDSFHSHSRHDAYDDEDECYHLSDVGETSVLNMSIG
ARAALNEHARHVAKYGIDGEVAEKQESDESENQKNNSYEISSNAGTNVFLDHQWERLFDL
SQVNYSSEGSDSGGIPPTPNFGTINSKFDVVNASTDRSGYEESSIEVLTDVSYDYFNSSR
INLLVTPERNKNFRTAMIVTRDGNGVEDDLFGNHQNFLFDGAEADGLPYHDEIETSRVGS
CRFTHSSQEGAFVQDPWVSSFNIGDVSRISAHSVSNANCTPNTSFQNYHSHGKKGNKSND
NFNGPLSAHFGADSPSMILNDSSFSSIGGMGPSQLPFDNNGYTSPEIPFSPSNDQKSGIR
TKEKPSPDRSANSSSSIQSTVSSFIAEARTMAKQVVSDVEKSIGAMESLPVDFLRALEIG
RADTLSPISRALSTPPSSRRDGSKKEKSQSKIHDTSKVNSSNSTSNNISSLSSSLSVIED
AAQTHLTGRKRYRTVVPRRVYLDSIAQQCDEEQDSFYVVQSDPETSRTGKRLLESFEEAT
TLNTF
>comp2233_c0_seq1; 901 183 1925 LEN=2028; translated
MNRGGMQPGIIILREGTDTSQGLPQLVSNINACQAVADAVRTTLGPRGMDKLVAAGSKAT
ISNDGATIMKLLEISHPAARTLVDISMSQDASVGDGTTSVVLLAAEILTKLKALVEEGIS
PTRMIQTIRKTGRFAVEQVGALAVETDREEMLLKCAGTALNSKLISSHRDLFAPMVVSAV
TKLHEAGNVDEIMDWVSIKQIPGGDVRQSFLVEGVAFKKTFSYAGFEQMTKKFENPKILL
LNVELELKSEKENAEVRISSPKDYQSIVEAEWQVIYDKLDACIDCGANIVLSKLPIGDLA
TQYFADRGLFCAGRVADGDMKRVAKATNGSIQTTTNGLTESVLGTCGCFEETQVGDERFN
LFTECPSSLTCTIVLRGGTEQFIAESERSVHDALMVVKRTITSGSVVAGGGAVEMCVANS
LRRRALEIEGKEQMIVAAYAKALEVIPRQLCENAGLDATDVLAALRRKHFVEEGDENACW
YGVNIEEGGITNTFDLGVWEPSDNKMNSFQAATEAACVILSIDETVISPRSQDPAAQATG
QMGGGAGAMMGNAMDIANSGGPRTGQMGPGVSYMKGRGGG
>comp2234_c0_seq1; 716 74 1887 LEN=1944; translated
MKFVAACLLASAACPGVSAFAFHSTPSSNVAASTEVSMKRASLKPSFQLNAETVDSDTVA
GLPDTIETEYELLGLPARPGRPLKVAIAGGGVGGLTAAYYMLKKGFDVTVYEKTSEFARF

GGPIQFASNALSVLKEIDDRLFDRVMEKFTFTGIRTCGIKDGLRSDGSFRMTDDSLDYLV
NPDAPADWFVKFPLKECADLFGLPYTGVIDRPDLQEILLDECRQIKPDFIQNGNPVVGYE
NLPKGSGVSINLDDGSTAEADVLVGCDGIWSAVRNQMYNEGGVKSLSKNKKKRQGCDYSG
YTVFAGETVLKTPDYYETGYKVYIGPQRYFVTSDVGDGRIQWYAFFALPPGTKKAPSGWG
GTEREDQADPEENLVEYIKSLHEGWSDEIMTVLDATPPDSVSNXDLYDRAPEFFRSWADG
NVVLMGDAIHAMMPNLGQGGCQAIEDAYVLTENLANTKSTDKIEASLQDFYKKRILRVSA
VQFLSRLASDLIVNAFDTPWSPHDNLGKSWKSYLTFFWKPILQYAIFPAQFAFLYSYYPS
GRLDDVAEKLSDEWKSRHFEASEEAFKKAEEGFVRESGPSFFQKAEVEESKPLVAGLKSK
FSIF
>comp2235_c0_seq1; 517 382 2172 LEN=2816; translated
MTKQILLKTPRGNTAVIPVDHFVSKAKAGATATSGAKPTLAFSSPGERRTASVLSSSNYC
DYPHGHPATTEAEEEAAAIAAALDTSRIFEDEHEYDVDRESKEELLFHLIGASKRRDVGR
KRNLRSLYNLEVEGEDDGMEIETEYAHQHPHQNECQTHNNGWSSSDDEKEQTVVATRLHE
MDINRDDYYYYSGAGNRRLFKSPRRFYSHSGVRSSSAALVPTSPTPAVRRRQREDPGLAR
QADRPSQQGSSPPLMVAFYHQGRKVSLDRIWDWITGSSAAKSESGTTDDVGVAGRSNSET
ASSSSSTPLFPLYLTLSTSARLLGGIDRQNRVGSKFGGGGVSSSQNSERERKERLKQLAL
ESIDLAKDPYLMRNHLGTYECKLCLTLHTNEANYLAHTQGKKHQAGLAKRAHMEKLRKER
EEQLADRNNLKRPPPGGRFVCQPATTRKVPRIGRPAYQVFKSKDPDTSQRCLTFELTYKD
IETDLQPRHRFMSAFEQRVDLPRDKRYQYVLFAAEPYETIAFKIPNEPLDRDPGRLVTHW
DEDDKKFTVTLYFADGKDGGPSNENVTGGSHKEAASMEIDGSAAGTSVFAPALGSH
>comp2236_c0_seq1; 164 332 1441 LEN=1994; translated
MTPPQCSTNDDGGGFRLRIPLLDDDSYHDDSGNRVLSQKTIDKFRSDGFAVFPNVLSPDA
VDALNDRLEDVLRGVYDRGQKPDKTPRLLKSHKPQSLPRDESTYPSNVETENPEIAAAAE
IEPKGSGKKKKHRKKKGSPSVGPIGFSGNLENVKVLQIINIHKADSLFRRLETSASLAKI
VAELAGWEEGARLAQDQVWAKPPGAPPLVFHRDSPYFMFDPPDVVTVWLALDDMDGALGP
LEYVKGSHLWGDGRVGSAKQFFQADTRSLLRSAAEREGIPEADLEFVSMEGLERGGLSVH
HGRIWHGSAGNSSTSLPRRGLGLHFVPSTVRFTADTAKSKLWKSYVEDLEGDPSEVELPE
EDFPLSTAL
>comp2237_c0_seq1; 679 96 1412 LEN=1541; translated
MTEQTSGASQPPALSDLSSETLLSLGDSHFVDENYEEAADAYAAALSLFRETEVSSQVRA
LSHRSSAFFKLGRYEDALEDSQKALELLSNNKPSGLRMGEGEICHRRAGLAALELKQYQE
AKEALQAAAKLASLNQKKTNKELYEDLLQRCDAGLSASKKKTATSAAPLPDFPKNEDVPE
ATSMPSRVSSPSKAKTTTRPALAGVKGKTPKYQYYQSDKVMTISVLEAGVKEQDLTVRFQ
PKHLVVILRKDNTDFTVIAGNLYSEIDVEKSKAVFKDEKVLVKLRKVEHYEWPELLGKGD
DNNSSKATKKKASEEDSSSDTATNPKMIPSVPQDSKKNRPYASHRDWDAIEKAIEEDEKN
EKPEGDEAMNKLFKQIYGNADEDTRRAMVKSFQTSGGTVLSTNWNEVKDKDYEKERTAPK
GVEWKTWDGEKVPMKEDD
>comp2239_c0_seq1; 107 1 318 LEN=667; translated
NQSNSAFARACSLGTMADQKNAVWLHYLRSFLRSDQTDASAEKKRKSEAGLEEATKTNPT
SSDFYCEPNGPGRIPVAVHHPFVAMDQSSSSSPQPSNKRVCRSRS
>comp2240_c0_seq1; 688 60 1616 LEN=1803; translated
MDIEKQFSDLLQSVESLKADSVNEIRKLRVENEKLKEQLKVFSGGRKQKRMTTANAGFAE
WAQADFVRMAKNTDALHGMDNVEVADGEDGMVLTDSVAFDPEKSSYASGYDYKYLSAKEV
LITLHDVGDNNVNIDAGPGMWANYILSKERNSTLKCVDKAKLLKEGKYYHVKSIREGLDI
DTLVAYCKERNIKAICPTSVSDEIYLATHNKVLRGQGIYPFCCEDPQSYKTLDHKWLSYL
FCEENGIPQAKTLPLRVDTVDACRELVAETCAAGKPVFVKECFDTCAGDGVIKVDKIEEY
DDAVERITKGRGPQPADTSGIDQHIIVQAGHPGRIACCHNVFYKGNLVSLYITKENMKIM
DKLGELRWDMTIGTWSTKTDDLSTSLKLELDDPCDRLIRDQAITIMKKVGKALNYTGMCE
VEFICEHSPDAILNVLEFNPRFSGACHSFVGAGMVQDYMHVIGLMVNTNDDTDKVIAEKA
GTFHARSDTHLPKSNFRDYNTLKFYMPQLATIAKLGKI
>comp2241_c0_seq1; 635 31 1083 LEN=1120; translated
MNLFVTLLVALSVLATVPSTSANASFSRGVGAPLFGISRGGGLFGGKDDSKNNEASVAAS
GEKVMYPAMTQQEVEDWLEHIPVFAVTDSNGAGVVLKPDEDSSVFYFFLNPLAANATLTQ
LKSVNEDMDLKVSAFSLGKIWFKLLQSDADREVLLKKPGDDDDMTQATKGVEYRLVPDTK
DLLGARMLLTMNPEDGEALKNGQEMTPEMAQKAVEKAMNESPKFKETFNEIPVFTIAQMR
MQKQPAEGESSAEPVTLLPMYFSLQNMVGTWQQFMAQSAPELQGTEPAINLMALHDLVEM
MQKESEIDFRSVVLVPSIPEGPGGAAPQGPPVASAADPMASMGGATLGDI
>comp2242_c0_seq1; 389 90 1085 LEN=1271; translated
MSETNPEEGDPSAVSSKVEPTQAGDEQKVKKDDGTNKDKGKKKNPVQMTYVSATQIERMV
TVSLYMHNLSWKHLSCRQFYWFTMPQAVLTAVASVLAFVATSDALSDDEASKVILNLIVG

STASLVVFLQTMSGLKNYGTRAAMHQGAAVDLKDLLCDLHILKMKMSQVENIDQEYLDMM
RGIQPKDPEADAEADKEALKYSETFGTIQSRLTQTLNACKSNIPMEISAAFNGLRTTAHL
AMTKANFESFEKIYGHENFLNTYYSKADDILQGEILGSRFFPMKLPYSKVMVKKTMEILY
TQFKDYDRHDFGFDSSTDDATNDVEKQGESK
>comp2243_c0_seq1; 418 113 1052 LEN=1052; translated
MSAAKTMAALAAAVIASSMPAMAFNSVAAPRSAARRAAATATATTTRLYDSYDDQYAKFQ
NRGPPPSQQPAEAGAVEEEAPSAPVPSAYGSYLDNLGSAPVAEETEEVFYEATNSYEAPP
VFEAEPEPEPEPLPEPVFAAAPAVPQPVVAAAPPAAAKPPPAVSSRPPPIEYQSSPTLSA
ESSTLSVTLPVGLRPPKLGKSKDISYGEGSRKFRRTVYSHDDWVRHRDPDRFFRYIASVP
TSGIYRNLAREVWAVVAVTMLVLVYNGLITGYQDVSGALHEPVLSSPFLPPLTLPATPFT
LASPSLGLLLVFRX
>comp2244_c0_seq1; 121 646 765 LEN=2763; minus strand; translated
MTESVAPDTHESSHVREQGNHCFNMENYLVASRSKNERQ
>comp2244_c0_seq2; 197 1 1643 LEN=2681; translated
XFMSGPQLPAKSTIRKRSVAALESASNRKATSNERGISNIPRKGSPMAMTARPVRSSNKE
DNATANEAKSSSDAMASKSPKRQHYGFQPSANWMIRSPLLWAVAVVLPLFYWVYSKQGHG
TSLADATKTSDIPTGDRDTTTTTILEEYRTLIELSIPPGALTPGEHRKGRYGKVHATFCA
IDWDLQSANPSTVPMFKDLKGRSSSCKATKTVVTDFYKLARAARDYDAEQSDRNETDEAP
YPSGVVFHETRCGSTLVANLLAGSSPTMGTSRVYSESPPPIAALQACARNGGNACDPDLH
SSLIRDVFYVMGRRRPASPKAEQHRVFYKIQSIGSMSIDKFTAAFPTVPWIFVYRDTVEI
LQSHLQQGRTNPTNSQGQQKSTVIPKVCTRNYNLGLNRQPATTLEAMKDAGVAIDKPPSA
SLDPIEYCAAHLAGLSLAALHEHDRWQSNHKQAASGGGRFVEYSQLPDRLWNEILPDHFG
LGRPLPEQAISNMETIAGVYSKGRGKKANQEWASDSEAKQETASKQVTEAAKRFASGAYE
RMKALSS
>comp2245_c0_seq1; 192 127 870 LEN=1372; translated
MTVSSAYQKSPMSWTERPIPGLTITTAFAVWLGITTVLVPALIQNSYYRTESGKSPLLPP
ITILGWSIMFFNNLNVLIAFCEIALGNHIAFIQNHYQKLLKEYRDAKTGKPKKAEYLAGF
AYLNMPLSLRDLYDGGQKWSQMWSTYSLWDPSYSNPESFGFFIDVGNGWSTIVPCWTWNF
AILFPHYFARSNTDGTAVVLLGFIGACSYWQVLYGTIIYFLSFLWNKRYQYKPLVEVLGF
VGFANGIW
>comp2246_c0_seq1; 1070 225 2509 LEN=2509; translated
MTNESESVVKFIPFSSTVESPFWVRYCHLKLETIRLNEDPIDIRASYGAAAAATSAATGG
TNRIQCQESGLLLPGADDAGGHDAARESPIPNDRVACAGVLLGYNTKESFQKVDKNKLLK
DVFCDRFFGASSSSLAAPSLVLLTFPDLKQHKVLYWFGFPTIMARAPILASTGGSPGQSW
WAGLAEGLYAYRVREDALPPYFAVRAGGFTAGDDPGPLDIAAPLDRDHREALDLSEGQEA
VFGFVDPTAIGVASSSSSGSGTKTNDILMMGWPMRNLVAYLVLHLGLGGKKVRILSLRPG
GCPGLRGRIDATDSNEFGSMLSAVDGERLAENSVLMTVTLPHSRDYDYGKSDGKEDTKEP
TYKVVGWELNARQKPGPRWVNLKPLLDQNHLAIQAADLNLKLMKWRMIPELDVDLLQKNC
KVLLLGAGTLGCSVARTLLGWGIRHFTFVDYGTVSYSNPVRQVLFNLEDCHFDHGQGKPK
AEAAAAALKEIAADVVSEGIRLSIPMPGHASDNDESLAETVDRLDALVKNADVVFLLTDT
RESRWLPTVMAAHHDKILINAALGLDSWLVMRHGHGGADLGGDDNSSQSNRLGCYFCNDV
VAPENSTKNRTLDQQCTVTRPGLAPIASSMAVELLVSLLHHPSKQRAHAPAPGGNNSGNF
APTERSGSDSSPLGLIPHQIRGSIVTYTMMTPSVPAFRCCTGCSAPVLAAYDVDRFGLVS
RACASMDGSYLENLSGLTAFRAEAAEKLADLDELDWDDDDDX
>comp2247_c0_seq1; 366 56 1234 LEN=1313; translated
MVFGITIVTRVPLALIIYMGVVSLLLLRSILTNYKTAFDAIYRDQTPGIQALDYRTSVRA
NAFSQYLYRDLNATAYTWQGNIWIPPIGVPHYSGVATRCLFQSENTVMVGDSTIRQQYHT
LRSFLESENPDNIPVAQFGEGRDFYPNLGSKNAFETTMERKVLGSKGNCFRVRSTGTDGN
STSYSHHKEKHGPDSTSNSTPGRNYGYHSEECLETGKYDLFRLNCFKEAIDWLQRNSTIP
DIRHRYSFVVLSLGVWELDEYQKASCASLDRLEELLGLVARELSGPDLFVVWQAHAPDSS
HIGDVGADTLALGDRASAWFVENRPRYMDFIDVGNMLVRANRAFADQRIGGDHHAHWGFE
ARALQGQLVAQALHRKQQREGMDCVVPYTHSA
>comp2248_c0_seq1; 414 139 1284 LEN=1784; translated
MKSARGHTGSSSSPILIRGAMLLHRVAEFAIFAVLAIATTIFSNVVAGRGGVGPVAAES
STAGPFADAFAPTYIAPAISRHARHYVSIHTNTRPESPGGSSLAALAAASDVPRLGPDDL
ETLKSQGYVVVEDFLPSDSEWKNSLRSDVLQLREKNKFKIARIGQDSTNTLNEEIRVAET
CFLGRNKAELRDVPSADRERLYDVLDALRQDLEDHGAKGNKLDEGLSEFLYAYYPTGGFY
RRHRDAIKGSASFLREYSLLLYLNDDSYDAETDGGRLRIHMDSGGDFLPAGEAPLFQDVD
ACGGTLVLFESDRFPHEVLDTVRERFAVVGWYNRPMSIGDLASINSESGGVGDSASDPIR
LAGLAVAAGLVTVGLINLLGS

>comp2249_c0_seq1; 244 309 1412 LEN=1438; translated
MTAGLNSIRSSCRCGSLVIDVFGNPSAKGGGDNGNCTSRNRAINCHCRGCRRYHTGAYAS
FLETDWSNIFVRQGRDKIGKFSSDCNEFGPVERWFCKNCSSKLLLVPQHLFSSEETAHGD
GGDKVDDDDDKEEKKYAERKIAPDTKCLLNLGPVIDDTIHPDTANCWKEQLDRSENNLHF
DKTKSVWARALPENEDRDYIYDDDHCQTLPTPLKWFGGCACGACRYEFILTHPTEMQHCY
CRLCRELSGSPFMTWIPVEKRNFRWIVCTKGVETETLTSSSALQLVRTTPFGSRHVCNMC
QSVMTIVYDEQPDTIWPCAGSLDDSALPKSTEEMGETLSRVCHICCRYKPTWLRIRRDGM
ERIEEAC
>comp2250_c0_seq2; 495 146 1549 LEN=1734; translated
MEGGEIYRDNQPETDPSTVTNPYTKTKHQLEPSHRPKGKCDPDIEEGLLYSYSRNGSRNS
VFFGNGNGVNPVPSRNLGPIPAAPVSVSVEKSVEKPEPNDTDATSIHGCNNFDNGIQACT
ASTIINGSNSKSNIKSNSNNDTISFREILPRDRTRIQELFEEWFPVDYKDEFYDDLCQNQ
SMGSQRLYTLVATTTTTAAKAPLSPAPEPQRERLIACLLGCKLTASKLNRTSRRLLIPGY
ESRKTDRPAGRGAGGDTDPASDDENENDYHCDGEEDRRFLDRTEVFYIMTLGVVGEYRKR
GLASYLVERALEDQIGAAAATEHSDGKQPDGTALSNGTKGTDPTEATRPPCETAYLHVIV
DNEAAIKFYEKLGFVRLREIAGYYTIRDEKHASFLYAKFFDRASLEKRRARRARAAWKRW
VLAVPGRLLEALAVGPWLWSIWSSVLVRHGPPHEGRQGGGGGGMKHD
>comp2251_c0_seq1; 164 202 687 LEN=1079; translated
MAFVGHATSRQQSTTSLDALGRREAFFGITSALVAGTTLVYNPEAASAKYSDYARREKDW
HDRGEAGQIKVSTAKDLRRQLAEIVPQNSEGSKIFCPNGASAAVSPLMENKCSDTLMALP
SVYGRTQDSMGNSIPGFQKGYDWSASQSSSMSAAVGGFPKY
>comp2251_c0_seq2; 164 340 825 LEN=1217; translated
MAFVGHATSRQQSTTSLDALGRREAFFGITSALVAGTTLVYNPEAASAKYSDYARREKDW
HDRGEAGQIKVSTAKDLRRQLAEIVPQNSEGSKIFCPNGASAAVSPLMENKCSDTLMALP
SVYGRTQDSMGNSIPGFQKGYDWSASQSSSMSAAVGGFPKY
>comp2252_c0_seq1; 176 50 553 LEN=1020; translated
MKLRSHSIPSSSSTLSMQEVLSPTKIGESFLRTSRNLFDVAAETSERTLFVVPAGSIPET
ESRKEQIIQVVGMTVFFVVLPFVITILFCCVAYKHFNVTVERDDIRNATPRVPQRRSTTR
RELPTVELSLGDNDGDGEESCSETCTVMSQDMDLKSDIDAKQPDSVR
>comp2254_c0_seq1; 90 1 661 LEN=684; translated
XGFSKPLSPSEFAALRAYLREEDGAEIGEDEIPVEPANEASVLGGYADTEELNXEVEVLE
RDEVHGRHPGRRSLLGPPPVRPQPDPPGQPPGGQADPEAPPPPQQPGAPPEVHHPVGPDH
EPGPVPLGREGPAEDRQAHQAGPVPGPHPQQRAVRGGGPRERPRGRPGRGQGVGGRAEAE
GPGGREGAAAHKHERAAPVGSGTLERGHRRGAMAQRDELD
>comp2255_c0_seq1; 138 143 725 LEN=992; minus strand; translated
MLCWSLAIVKWPALSIVQEYLARRIRGVGKEFDRQHPAVPNAVGQQHAVVAPEEVDPAFL
RAFLGVHVDVHRRGLAHARHHACLRWEARSKVDVRPVHLPEAAVPQDGLPRPRVRPVHDA
GERPQGFGRDPGQQELRVDGGNAVALVRPYAEDRLPLPFAGGRVQVPRPVLEPEALLVAV
FDHPSDGFQRGPP
>comp2256_c0_seq1; 434 170 1714 LEN=1970; translated
MARKWAAKLDMYRKVPLDLAEGTRRGNIVSLVAIFVMSYLFYKETRDYFTTKLVPSLYLD
RRHSEQEQINVTFKITMMDLRCDFVEVDVVSVLGINQNATKFVKKVPLDANGVLNTMAER
NLDQKDIQDIALHDAAVTKSIEDLHVNGEDAIRLDSKTLGYALHENDLVFVDFFASWCSH
CRVLAPTWETLAKVMLDANEESEEEGGDEYGEQELRTAEALDVPVVIAKVDCVEHDKLCR
EQNIQAYPTLRLFVNGKPFDSGDYEGHRTVLDMVQYLKLAENKLGKEKKLSADGLHSALE
RHLEMSVEERHWAEAFQRLQKRDQDVSWNPMEHPGCQISGSLLLNRVPGNFYIEAHSPFH
NLAPHMTNCSHEIHYLSFEPTGVDLKEGDPLPPNFYKSNSPLNGNVYVTQKLHESYHHYI
KLISTNGNKFQVLQSSQLASYPHDDTPEAKFILDLSPIAVSYEKISRKWYDYITSLMAII
GGTFTVVGFLDSGIRVATRKRIKLSHNNKPRTIY
>comp2256_c0_seq2; 264 170 913 LEN=2056; translated
MARKWAAKLDMYRKVPLDLAEGTRRGNIVSLVAIFVMSYLFYKETRDYFTTKLVPSLYLD
RRHSEQEQINVTFKITMMDLRCDFVEVDVVSVLGINQNATKFVKKVPLDANGVLNTMAER
NLDQKDIQDIALHDAAVTKSIEDLHVNGEDAIRLDSKTLGYALHENDLVFVDFFASWCSH
CRVLAPTWETLAKVMLDANEESEEEGGDEYGEQELRTAEALDVPVVIAKVRDISTLSHNE
RQVVPFS
>comp2257_c0_seq1; 215 84 836 LEN=1652; translated
MPLLGVGFSMGSSGDGDSPAFGKSLDEIISDRRKEQAKASSGKSVAAKKNGKIEAKNKKT
AMADRSIATGRAKRAAAIKARRGLAGNGAGNGASNTAGNGAAKNNASSGKKKKAKASAME
IEKEVYRQSRKTSESKKRAEQKKTGGRLPPNSSLRAKNNTNKKSVEISPEAPAVVTIKQP
RKTQLMAAIKGMEQAGCPVPDGFQVAMQFIPSPVTKATKGMGKKSQAGSNKNNNSTNKRN

TSGRGRGGKK
>comp2258_c0_seq1; 610 68 2334 LEN=2686; translated
MSANENNNMMMGKIDDEVVELRFSNINVDTEELISGIISDEFEDIVEEYADATAERNTNR
GTKTANRLSAISFSKGTDGRPSILSVRPSLMSLGMSDRLLEKVMEDLSDPSLDPCDLPPQ
EQNSSDNASDTSWAMNVAAPEGTNGDLYNDDRHDSFGASPSVSAPTLADSPSSNPFTGVA
MESHSEQFHAGGNYAKGRISMTADPLVFGAGGIPIPIDAHNGENHYESTQPFADPAISQN
IGDQTYNDSISPPPLKRPFAMRFSVSRRTLNMLDDDFFHDFSPHSSGHENDEHPKPVNVV
SSEMQQTYLNETEEVFKAQELALTREMEKIAVHDQDKVNFEVHGIPHMGTLNVEPVNTDW
YLHQLEMELQSTIQDNSAFQEAQRMNPSYVNSKTFRLMFLRTFWQESTFDVKQAAAKIAL
YFSTKKLIFGGGEILGRDVRLSDLSVDDRAAMDCGALQTMPDRDAAGRVVILYAPGQRVF
NTVENWLRAMWYMLSVTAKDEDNQKSGMVMVVYLRGFTNRRDTFEQAKKITMVRDSIPLK
IVGLHYCYNDXVDESFGDGPKGAFLDPKPTKSYPRTFGKTRDICFRLETYGIHVDKCILL
ENGHLGMAWYNQWIRLRGVAEVEMVASSGTATTPRTIDTTIVPKKFDVLFGRGRNTREHC
GNLRCAHLVEMHQLEYENCSKPQKTALALKIVDMVKECGGKFLKKDRKYGWQEVPDTAAR
EKVSHFFRHLRSTKSSADDDKQPGVTESQKRSAPC
>comp2259_c0_seq1; 575 19 1014 LEN=1061; translated
MSGINETLTLRGTLKGHGDWVTSLATTPEDPNLLLSSSRDKSVVVWQLFNGSTPTTVNVE
DHEYGFARRALRGHSHFVSDVVISSDGAFALSASWDSELRLWDIATGKTTRRFVGHEKDV
LSVAFSVDNRQIVSGSRDGTIRLWNTLGECKFSISGNGEGQQGQGHTEWVSCVRFSPSQS
VPLIVSSGWDRLVKVWNLNNCQLRNDLFGHTGYLNTVCVSPDGSLAASGGKDGTAMLWDL
NEGKRLYSLDAGTIINGLVFSPNRYWLCAATEDSIKIWDLESKVVVDTLRPTEIEGAKIP
ACTCLAWSADGSTLFAGYSDNVIRVYTVTPS
>comp2260_c0_seq1; 269 206 988 LEN=1325; translated
MPSSSNGLSNESKEKTHYEVLGIEDPDRCELSDIKKAYRKLALKYHPDRVPVGEKESATV
KFREVSEAYEVLSDKKSRAEYDDSLRYGANGNRNTGQGRRPNYHGGYNTNHRHRYRDPFA
QFDDLFRNDPFFSESFRGMDDLFSKTFQQRRNGGHPNTKSKGWGEWMLDKLGVQMTTSST
STRTNRDGTFSSSSSTYSNSRSSYTSKSTRTIIENGRRITIQSMEKDGNRIEEKYEGNKL
VQRLINGRPQDIGRIAGGDL
>comp2261_c0_seq1; 754 303 2312 LEN=2429; translated
MLRRVTSTATASRTASSLLRRSNPSMAIGGNTRAKHPSSRAFAAATSAEKRALNAQQLKG
SSGKSSSTAAKESATGAAPTAESGGGGIGPVLALLVATGGAGAAYYNGLLDDFLGADSSP
LPQKLEDVKKPKKKKKAAEATDSPTPITNIEKKNANAAAAADESLSTKELLEVNEIIADV
IGEVNEEVAAVTEAIESIAKENEAALASVVATPVDITAKIEERPPVPAEAVPKEVKKKTP
EPEPVPEPQPEPEPAPVLMAYSTPASTLDEAKIMSEIAELKKALHEKSDKALTEAHSELA
KISSMDVMSLDLDQMTSSQLKVRLVQMAKDLEERTKWEAVRLQEFLSMKEKEVEDRYVLL
IKKLRIEAEHLQEEKLSEQRKALVAQAEEALKEQQARSEAFLENSMQIEAKAHAEDKAAF
EQKTDEAMSAKYEELYGKELAKIKDDFATKMNQRVQQMERLTKKLSDLETSLKDSQDFQV
GSLEAHRITAAAIALMEKLESGEPAGAAVNALEAVASENAVVSAAVKSLPNSVTRSGIMT
VQELQAGFEENIYPQCRRAANIPEGQDGLEGQLLGSIFATLRPAPGPEDVAPESEKNSSE
YVLARIRRYVRLGDLEKAIGETKKLSGQVAYTAKDWNSHARDRVAVEKALKVIRMECALA
NDNLSKAAA
>comp2262_c0_seq1; 309 86 1207 LEN=1580; translated
MYSANNFEDEPGLEGIDKFEDIPVPSEKVRFDGLDEAAAKAERRRKRRRRRNQRTGMIAA
CIILVCILIGVIISATVVEETEHMLYQDAYVPETEMPSTSPTTAKPTMHTTPSPTVARKP
TFSVKTPSPTIRHTPSPNLDLQTDSPTETPGPTSIMTDVYTFEPVGDTYMYLDGNHKVQA
YGHEDSLWVQRGNKESTLPGHEVTIPTIVGLLEFDTTQETPTNMALPKRSRWPAEAENNV
VVTLRLHHVAKSDSDELAVEDILPVNIEVYRLPNNHDWVVESLTGDDFQNSPKAAREGIL
VAQHVIEATDTLVDINVESAIFLPEDINARMYKDDQVLLLLKVYWEEAPNEGDSFETRES
DGRSPQLIFSNMM
>comp2262_c0_seq2; 267 197 853 LEN=1226; translated
MTDVYTFEPVGDTYMYLDGNHKVQAYGHEDSLWVQRGNKESTLPGHEVTIPTIVGLLEFD
TTQETPTNMALPKRSRWPAEAENNVVVTLRLHHVAKSDSDELAVEDILPVNIEVYRLPNN
HDWVVESLTGDDFQNSPKAAREGILVAQHVIEATDTLVDINVESAIFLPEDINARMYKDD
QVLLLLKVYWEEAPNEGDSFETRESDGRSPQLIFSNMM
>comp2263_c0_seq1; 256 528 1064 LEN=1846; translated
MRLIQSLACCLAFLSSGPPHTLLWWTVSLAVSASFASSTPHLWTSEHRRHASSSKMVPGG
QGASTRAIDALRTRKRCPLRDINDNDNDCECECEPVWDRDDGNYDGDGISIGINTNDEDN
FDASSSCWYPINRFALEKDHERRSVRAAEYAHGTADAKIGGESTDQIPSSTTGEGIFV
>comp2264_c0_seq1; 446 94 1743 LEN=1855; translated
MEFGTLHETKDVIDHNGKSIEVNHVDVRDKSRWDVGLDCINSLLRPGSATLEAMTDYQRL

GLKNLKAMIMLSGDMEDDTKNHIPQALLESSESASSDFLLAEYAGYDRRSQAKTLTNSIH
KVLSAKKCAIKMKKIASSNVNARESAESLFLESVDFESKERTNLPPHWFDLDNTSKLKLR
DLLSWENLSRWDFNVFEVDDTLKGKNTLIFVAWAIIGSPHSQYAMDMACSKIEGDTNTEV
EGLAERKGYDFVSALKIREKTLLNFLREIENTYRDVIYHNKVHAADVTQNLNALFQMGGE
FFSDGKLETFSMLIAAVVHDVGHPGVNNAFLIHSKSDLAIVYNDVSVLENMHLSTMFRII
TGNSRDPKIDVFENFLEEETEVARKLIIKAVLSTDMTKHFKKLNTIRGVILSQEDGEEVT
ESLPMDICFTSEILSFMLHAADISNPSRDTATAIKWADKVLEESFHQGDLEQSMGLPITP
LSDRNSTHREKSQIDFIKFIILPTFELLGQLIPRVTSEVIPKIFENLKYWQEQDLLRENE
VRSRRRSKT
>comp2266_c0_seq1; 599 369 2153 LEN=2823; translated
MTDTPQLAEIAIISPELGLKTDDVSDRRARQAAAEILASDHNNTNGDETAGLSSQPQQSS
VLEQDQGLLMDVLKELDRERQKRAELEARVRVLEQEVQFHRKKSTTKSNKSSKSSEASVR
DYTIVKTERDGYKEILEAITQDRPAFSTYAPDDNIQKRRFGRRNKQQPPSLPIHIVRLLE
VMPWDSRAQQYVFGEEHVYEWQFYSAGKKWQKELRYFPTFFKTLPIVVPSPGKIVSDDAA
TKKHRRFGSANIAPPKQCVLTDLEGKTILNIDKGYPLPEDGGGWAWVGAWKVEKNNDTDE
DGWTYSNETSIMSDPSTYYGEFRVPEQGTPNIAKRRRKWSRSRVLTDYPYASQMTREYLK
LITEKSIMDSNIVKLSGQLVDTKMNLTTLEAEKEQTDERIRTLERDLEDKNKILGLVEVG
AGLQLTKATSAAADTSDASIKSTDSANKVDEIRSAVTQWVSNTVQKADGDNNTVVSNGSG
EEAALITKLVQQQQQQLRLHDPKQQLFDSIRDKGTGLFEILKQKGEQLDKIKQGGGAGGV
GLPWQKKEEDADTDSKETEDPESSTSSAASAPTKSNVYTPSARMRFLSSGSSKD
>comp2267_c0_seq1; 603 198 1751 LEN=2035; translated
MVIRASSVVRLSRHWRATSARISSGASSRRLASAAAGSSCTGDSSTVNNLLLAAGAASIL
AAGVALGSANATATNTTDCEQDWGNLPAFGSSSDSIPGSEYSEKANGSNNNTILLSKIKR
KRTQQEIDDLEKERQKFEELLGQNILSTSAALPGGSDSKSTESVTTKKMYFYRTSQIETE
KKSKFILLAGPSSDQLGSDIAHLLGKELNNMDVGAFADGETKVELEDSVRGKHVYLVCST
SSNDAVMELTFMLSALRRASAKSITAVIPYFGYSRQDQQFGREPVAASDVAIMYEEMGVD
HVMCLDLHNDSLRGFFSPKIPVENLVPVPVAAAYFNEELVGDGEKITVVASHEGQVSRAT
IFRNVLQRLSGEDIELAFITKARQRRGEKKYTPQVVGNVKGRKCVIVDDLVNTGTTLESN
VEKLAEMGAKSIHAWATHGVFGPKELCQAREKIGSLKDLEYLLISNSVKYDGALPDKIRQ
LNVAPLLAEAIARSFHHGSVSGILNLNETIQERYDSR
>comp2268_c0_seq1; 458 1 2130 LEN=2344; translated
XAEGSRSLLLMRLHTVSDRFNEPVAYXMEFSRKRIALRTVTAVLFLGFLTVKQWRYIVLF
HTSSHPNDWQAQGRTEIDEAYLIQGKNQSLEYKTTIRSENEELRHHRPGVVVVNEIIHRA
FFGLGHRLHRSAASYHLARSLSSPGASRSPAGELDPVNTDHYNQQPTITHLRFHWESCLA
DGDQGNSSLHDEAGEKKEYNVFRFLFGEDLWELKDQPENSLMASTDSEGDSDKNVGVQNI
RNMIVLRNDIPGYIAGQLYKDLKLPVKMKPKEAPSRNNKADALSSATVALTDKLYEYDTI
LDKVMHSDVHFYRRLVDNYRFERELQDFQVQHKWGQRPLVVGLHLRAGNGEDAHFVKSGR
GASIEYNESTMITRLMMLINEVAAREILKAEHNEQDDALSPLLFVATDTAHLIPLVEKVA
KLNAHNDADSMHGLEGLRSLNATATNTRTKTLPLQIITWPQSYLPKNAGVGFDALKGKGD
RCLEGWKSAMSDILLLSKVDVLVAARRSTFTQSLPISLGFDRSRKYTEIARASDISSGIS
NDPESDRRRFSFCEVSESDTARITCFGSMQKWLFRGKDDESMTSKTYQEEDGKGTQASVD
PEIWTLSIPATTSSASDQPEKREQVEHKVTVLLPDVVPPNEMKEARDFLRREGSHGRKEA
VNEGKSREAIFRYGRSKIYKKYRAAHKTNDKTTSGWNLVLSHVGNKRIEK
>comp2269_c0_seq1; 411 116 1912 LEN=2085; translated
MAYHQTRRHCTISICGDRYRIYALFLVVATVTLSCSGFTSTAFIVNNAPKLTMLGHTSSF
TKTIQPKRFSRPSEKSNLVLPTTTSVASASSSFEPLFRGVKRDFENRCPEFKSDIVDGLN
AQSLATVFFLFFACLAPAVGFGSVLGGLTGNQMGVIEMVASTSLSGILYATFSAQPVQLI
GPQGPVVAFVAALFGLASSLGLDFLSLYAATGSVAAVCLAIFAFTSASNLVQKLSRWTDE
TFSVLVSVLFLAQAVGDVGATFALPASVVPSVLTKAMATALMTLVTCTSTFGTAVALKGL
PKTRYGTAAIRKQLSNFAPAIGVVVGSVVARAARIRWGVSLLALQLPAKFVTSTGRTWSL
LSTLASNSCSKLWLAAVPAGVAASILLYLDQNITARLVNHPRFKQTKGNRTSITDGMHGD
MLVLAGLTALSSFLGLPWMCGAPTRSAAHVRALATSDEDGTISGTLENRVSGFSIHALIG
LCAVAAAPRNLLATVPKAALSGIFLYLGFTSLQGLEFWDRIRGLFQDDVVLDKFDSVKRS
AITVFTMAQILCLWTMMQVTQSKYGVVSPLLIALLPFARWGLLRMGAVTKNDMKILDD
>comp2270_c0_seq1; 317 153 1166 LEN=1279; translated
METAKRLQGYQKRGYQWPVLEFVPNTPGWKKLMEDRLEQISNIEDPAERFKGYTATMYSG
IVIQNYTEHGFARTRISESLLKELQQGIRDGFEDRVNEGYTPSITGNQAWHIQRDDLTQK
VEDELHDKLEEWGRVDLDLTYVYGLRLFRNTTTIKMHLDKKGSHSMGYVLHVDSSDDYDS
DGDPWPFLIEDFHGRTHEITMVPGDLILFEAAKLVHGRPHQLNATWYCNVVGHYYPRDET
WASMNHVAEAEYAVPPKWANEPSGSSKADDAMVREIQFRGGLREPGCMDGWCRSTSLERI

QWKGPSGPSTHWIDANGESHEFMGRLKQKEEQKAVEL
>comp2271_c0_seq1; 485 1 1323 LEN=1400; translated
TGSNRNGKKVFSQQQLRANALLSSSGYLKDKTGISLITENMASITASSALVNVVDGAELC
LVRLLDESAAPGSIRPTFVQDCEACIAHSDASQLLKTIVSDQGAIAALVSLDEEAISAIS
LLAACLDRVEDSSAVILNNLADSIIAISTATGASSKAISLLATLYNMRSDPTEKVGLLVK
MIRLAVESGDVLESENSVLGKWMDPAQVETMLNEWNVEPTGRRELYQVAAQSSQKSPAAT
QQFTLLVVETYSKSDVDAAGLEAAKQAAIGAIRDPVTLFAQQRKILSLPAIQALEKSDAP
LFALLKVLQEGKLEDYNSYIKSNGGDSVLAQWQLSAEDCSRNMRILSLCSLAADHEEIPY
SVVAETLQADAGDVEKWVIAAVSSGLLSAKMDQLQQKVMVERSVVRKFDMDQWKALQSRL
HLWKQNVGGILEAYKQSLNK
>comp2272_c0_seq1; 808 121 1263 LEN=1508; translated
MAPSTEDTTPEATVAETTPAAPAEETAAKRPSEEEAENSAKKAKVDDSAAPAVDADAERL
AKTHAYGKLILFGEHFVVYKVPALVGAVAAYTDCSIKLEDAPADSKDESPTGIVEVIDNR
PAVPNYKTKKAEEGKKAIDLVLNHFKIFGKGKKATLTFGGNLTCASGIGASAAQVVALAR
AVKETLPEFAKLTDDEVNAAGYEGEKGYHGTPSGIDNTAATFGGLLKFQRTDADPIFEKK
TIKEPIRIVYASTGITSSTTEVVGDVKAKKEADPAWFDDLLKKYCEVATKGEKALEDGDT
ALLGSLLNENHKLLQDLTVSCKELDTLVDAARAAGAVGAKMSGTGRGGLMLALTPTEEIQ
NAVAEALEKIAPQVWKTSFQ
>comp2273_c0_seq1; 518 44 748 LEN=1007; translated
MPLLTATVVKEETSSKLGIAFERLAEDKAMTIKLIRDDSLFAGSDLCEGLIVASAVGKDM
EGMSPKDCADALREAPGGEEISLVCKGSKATIKKEKKKLLKKEPKIGISFKSTTSAPGKI
FISNIKEDSKFAGTDLTVGHQVLTINGQKCPGRVTEAVEMLTKKGVSEITLVTIDPEDEI
VVPKVKEEPVEEVVEEKKEDTEAAAAEEPKEAEEDLTPEGNKTILDTMFGVCMC
>comp2275_c0_seq1; 606 1 2033 LEN=2102; translated
RYRMTLKGTRQQIPQHRATASATNRDIEHQQAEQCTQSAEDXNKNNNHQSVVQQFERRLA
IMLFLLSRSRSTTIPVFLSFLLVGSCIFGVGSASETEHAPYLRRDSGSKEGSPISVVGDD
DPDIKEMYITQQLDHFDRTNEATYQQRYFVDYSRHGSKQSGSQETLPIISLLCVGGEGPG
FDESVLIDSVHCSGDMLELAKRIAQTKSHRVHLYALEHRYYGKSYPAFVDSKTGNKTSPL
TVQNLRFLSSHQALEDLAHFVHTMNKETSTDGDPSANTWVTFGGSYPGLMSMYARYKYPH
LVFASVSSSAPLQLRVDFPGYKAKQGWGLKYDKVGGSEECHRIVQAGHEQAVAVLHQPGN
KTNATEAGPVVLAKKFGVCDPGKALSMRRNQELLLGDGLVVIPSQGNDPKCTGGELCNIA
GLCRFMIQETEKMRNATAHNSTGKSNIELEVLAKVSQQQRSERTRGNEKTYKHNKRQQMM
QRMRLPRRLAGGGDNDDCVDVDFEGMVKYYSDLEVRPFGDRSWLWQTCTEFGFYQTCGDD
CPYAAHFHQVDMDLEICARLFNISSDRVYENVQSSLDAYGGKSFLESGATSRILTVNGNV
DPWSALGLEENDSTSFLLPVKMVDGASHHFWTHAVKDTDALEIIKIREYIYSCVMSWLGI
TVPHNPLLSLHASNVAR
>comp2276_c0_seq1; 481 399 1724 LEN=2592; translated
MASNRSRNRTQGRSHTIKVHDHDVLSGRGVNIAQHPGNERFRSLINASRDEKYCSDFTTE
EKKALAQEVIDHIESLDPPGRFLKREGVSQSSRGLEGPWEELTKKEASKKATQALRDCNR
PDRTGYAAQVKAPPDVKVTAEERRRSGISLKDHAAAKVEERSKQPKRNKNSSSSDNRKNP
PSSVTAASRKSTKRSIRGSSPANNYKKHRLEETPLSFLQEGPFGAQPELSDSVPFNEATS
STAFAETSVGATPGPPHETSVPVTETPVQYRPTFNQREPQDYGFHHQSHYQNQQQQHLHP
YSRSLPVPIMPNSSASYLPVDTGHHQFQSQESNEGDSVPYRAPYSPLVRNSPPRHGNGHS
EDVLRLPMDFRQTLFDGEEDDFESMAANAAAGLPHGHNEGGFDMMHHDAHHLDETNENIS
NGNVANASNQLILPWPSPEQK
>comp2277_c0_seq1; 141 119 454 LEN=682; translated
MLSPRSTIAAALLVLLLAVDPSHGLAGVVNRFRRSAPWGRAVARQRRTSTSTTSGVGLPV
RAAVAKQGLLTEPEPELSPHPPGSTPFDADAYRQQMTDLVYQRSMERIFRD
>comp2278_c0_seq1; 273 129 1121 LEN=1121; translated
MSPRSTLKLASILLLALSSSCAGVRSGAPYRAERSQRRGLNKRTRGIGGVSGRAEYGSKS
KSGGKSGSKSGSKAGKAGASASASTGSSSSSPNTGGDAVPASISPPTAAPGASEPEARAP
TPVYRPVPAPTERRPSFWYPGSDREPTEGRIDPSGGKLPNQNEVVAVVDEEDGGSPFGPG
SGEGKQDGSPSDAFQDLFDLVTGDGGGSGTSIGTETGDDQAQGGGPSGGSGNDTDGSPGD
NSDGRDPSTGGNDSRGSGLDGDQAGDSSQGTQTGISTGVSTGGSKEDSKESSTGSSTGDS
TGSSTGGSTEVSTGSSTGGATGGSTEGSTEG
>comp2279_c0_seq1; 216 62 539 LEN=539; translated
MNLSFVAILSVIALQSTQAMISPAVSCCEDNGGKYEIVDGSDGQSGVCRKDGVNTEAFYF
MDQNCPPPPTVSPSNAPTMKYSVYAVNNCDATVTANFVLRSSRRLGGNRNSQVEVQQGQC
TYVGPTNLDVVTYTSQGSYVSSGNDSCLNIGDSNNSDCNX
>comp2281_c0_seq1; 224 423 1225 LEN=1609; translated

MCSNGCVCPPTASWRGELSRLPPLRRATNRPGDEXALVGVMGVVAVTIDYNNRTSTGVLS
LGALSCDHCECEAVRRRSNGIPTSGDVFSVGKITHEPATKISFPALCNGMSLGGVGVRIK
YVFVKVYAVGAYFDPIAMAAVKKGTQADIEGALVDPTYPRTIRIVMNRGLSVDKFIAALN
EAIEPRLNGKNLETLDEFKELFPKIDLIEGDEIELTIRGDTLLLKTGLGVGTIRSQPFTE
AMCDVYFGKESVSPTLKEDVLKGIPAL
>comp2281_c0_seq2; 197 423 1368 LEN=1752; translated
MCSNGCVCPPTASWRGELSRLPPLRRATNRPGDEXALVGVMGVVAVTIDYNNRTSTGVLS
LGALSCDHCECEAVRRRSNGIPTSGDVFSVGKITHEPATKISFPALCNGMSLGGVGVRIK
YVFVKVYAVGAYFDPIAMAAVKKGTQADIEGALVDPTYPRTIRIVMNRGLSGEFLFFSSV
DLHVLFSDPFKLTEACPSXPPAPLVDVFMTIVFVEYCAVVDKFIAALNEAIEPRLNGKNL
ETLDEFKELFPKIDLIEGDEIELTIRGDTLLLKTGLGVGTIRSQPFTEAMCDVYFGKESV
SPTLKEDVLKGIPAL
>comp2283_c0_seq1; 158 232 450 LEN=1457; translated
MASYSSALALLLVLSNASFFANALDIEFDRIQCDETLSAYSSFSEIEVTCNDGKDSRCSF
GNDVNIKGMCKC
>comp2283_c0_seq2; 235 232 990 LEN=1295; translated
MASYSSALALLLVLSNASFFANALDIEFDRIQCDETLSAYSSFSEIEVTCNDGKDSRCSF
GNDVNIKGMLHYHNLYPYLTNNTGYASANLRLLSVEYNLFDLFPVDFCGDWAVPYNSTYG
HDCPDWDSYYAFDIDYKLPWDDDDITTWFATGWQGVSNLAIYSNQTVDSELITYCTMHWH
TYVTPSEDEDFRTLPSAAVTGIALASFLVALFCCCCYVNCCRRRQKHVTDRDYYDDEVSR
KSRYERYEEHAC
>comp2284_c0_seq1; 594 86 1564 LEN=1672; translated
MNEICGFSSSRRRRQRKNLLWNSIAVLISVGSMGSTYAEDFATQYPTTESTRFDDDDNSY
DDILFFDLPSMAITLDVDTEDNVLIPFEDKLTESIEHHLEHFYQKKLLTSEIGNPKFIHV
DLESNLLWTEVSMAPNMRQTDDVTFVKKYQVQGIFNSKIKLQVEPIVTEEGEKEKLSLLN
QVLMDLFFLEAFEDDYQWDLLHGFLLEPLLHDIINVEINIMDSGFVHPHDANGNLVFEQG
GDDFYSKSIFSLQTKDVHWIMVGVILATSFLVGLIALWICLCIATKGGKRHYCFRWLRGK
TTTYYDDKSLEGSSVTGSTSSHDSVSAESEATSLSYNLDAWANAITSITLRNPARRKKRG
AKVVKRPYFRPSDEHSSDLSCIAEGDNESCTSTVKSAGTSRSERSRRKRRNSKSSWKSST
AYDLPSLDSSSVSSALSSVIYEEEPEKETADSDENANGITEDDDGTKQSAPLLSERGLIR
LMPSDDEDDFKM
>comp2285_c0_seq1; 873 903 3017 LEN=3178; translated
MKTHEFDIRVALVGYVSVGKSTVLNALLGDRFSEVSLKRTTAGVNLFRISQPSQVEGKKD
LEGSATQEWSTLDDCKVMKGANATHEEISEDNKKLRSSDVVEEKTFDIQVDYQICEMRPD
TNLILIDIPGINEAEASKKYKDYVESNWDTFDCVVVVMDAIKGVNTEEQVELLKFVQQNN
QNTKDIPTIILGNKIDDPDDEDAIALMHETHTKTMEIFGHVDSKYMPHERKTKDNENNYF
GSNETDTAFIPISAKNAFTYRKAGRIDTDHLKDAKHRDLLNKIGNDELGRKWSKMDMDKK
VEAVTEILRDPAELDERLADTNFNTFLAVLSSFVGSDERQQDILRKQTELELARISHGVL
GEKAIAASILEVFKRMKPIGWVDTGILHTAFWGAFENFKCGALIDAFETDVSPKAFGRAF
TELEKYHDVASKLGWTKESERAIFEMKKMLRSQLVLLLQKVKNWSFHSFCMEAGVRLHSD
WGEHGCGLCSGYGCNTVYRYPQSSSRYAQSSSTERSYGVCRRSKFASWPDTLTIPSATTW
ANLSPQDWITILASLSLVWNQSWFIEDFGHEKLELEAALLRYRSFFYSSPVGTLSSDKFT
GNQDDDTALNEFILSHSYKDAIINIYGTKKSKKSESQSKNRFDLLLSDVIDTNGTDNAAI
TKVRMPMSLADPSHWGNLAWKYIKFCNKQEDMIDSREKKKPRLL
>comp2286_c0_seq1; 192 185 757 LEN=984; translated
MAMTNERWSAPGGTNENDALGAFVSNSTTQTDRSNKSDSLRQMLCQDIGTRTLVLTKGPS
SKARSLIRSMPIPSNNSLGSMSTISSTESLWLYGESQKLGSVTNISKNSLLRYGNMDSRN
SLLSLNNRWRATPPNAVNSNPQKIFSSSFNAPISTGNQQSRWKATQGGDQPIGMVTRKTS
ATYLFPAKSA
>comp2287_c0_seq1; 287 208 733 LEN=1234; translated
MSLLGFASHHALVNLQQSQKTLSIFEKNGGFSPTWKRRKRSDKTKVALLGGTSIATSNGD
PTFGGLATGILFIILCTAVGTFVFANQVYTPEIIEGAQMIRKSNRENEIRKLLEAVRSHQ
EDGKELIELRLPLETALGSTLEEYVAAVEGSEQKSDTIFTMADKDLVKILKQNV
>comp2288_c0_seq1; 210 1 836 LEN=1174; translated
XFTKPAPGSRERMRFDVYYSTGTVGTRLPLVLPPGEEPPRDKIGRVKKMTHLIRRDVSSP
EILEAIFSDPYAHGGARPREGTSNRARNDLRLAQQNDRFHRAFHDALHGIIDTTGDHPRS
HFLNWGTSRFLEQLARSVFSFRRIKSGSNQINVPPPHGGDEGEPPREGTEYIDMENGILE
VVQKMIQKSPRLSERWELKEKKQAASKSSGGSSSKTERHAGDHLACGGGRHGESSDEHDG
NEHEHEHDDGEGERMDLADEHEHDDNDDEEFGHRMLGL
>comp2289_c0_seq1; 663 1 1690 LEN=1708; translated

XDNPEVTQDYSEQPSSVLEXNLFLEDKIGGHYFNQIEKRTRRQQQQRKRHRSIMSTLRQV
RISQFALILLSVWIPRIVVDGFSSPTTQHRICGRKAPLFSSVEDKPQTSSVNEDEEDGPA
GIGGAEFFGGNKQKEELYDPVAEREAGKGIELKATSYNRFSLGVGAFDSVEVASMAQSLQ
AQINEVLYSDNKKLNRLLKRNQKDSESDQQKLDFPDVNFGRSLSWDTPMTVSSTNPFEEI
RAAKNFYQNIDLAIVGGRRVSEGVSELSWELSVVWPTFWSPRVVLSGSSTVTTASDKVTI
TKQVDRIFGADNNAALLSLLGGQITPRFWDWYHIGMTPSAEQVPRKVISQKGGMAVYQIP
PQLVTAPSLIETGTRDNRNAQMVPNHSFTCVIRTMGPKDDRDIYVPTTPIEVKIGRNDRS
QNDNAEDGKESDDRLLLSWSIPLSTQFQALNKNLLLPAEDDEDAEEGAFPECNYVWQPLR
QVATIKYGGNVQDEEISGIRKKLYEQVMKEGLKPKLDEAGKPKFFFWQNDVKACYIEDGL
GMVVYDWRPEFANSNEVGIELMV
>comp2290_c0_seq1; 254 494 1270 LEN=1693; translated
MFIFPPRSLVSSLYSKTKREWEKLAASVKGMVTIAYWDTEGGSRPPRLLGEYKGTPTIKL
FKPKRRQRKKGSSAEKDVVEYQHGERNAKDMRKFLEYQLPSFIERVKFGPDDLAKARSKA
DKYGLPVAVLFTSKASTSTTVKWLSVEFRRRMLLVEVPPTEKNKGIREALLEEFSAGTSA
DDSNSLPALYVIPPQEGPLSSETDKGTLQIYEGESFNRRKLQSFFDKTVLKKPVLEPLVK
PEEDPQPKKKESVGGDEF
>comp2290_c0_seq2; 209 1 1075 LEN=1498; translated
KCSHIQFLHTKVTTPLSKYDKNEAGNLRFLTVALWWQQRCPNRSNGRRTGMDGSFRVLSF
DNHYDEPIRVGLNSQSVVFLQCRDARFLELERNRGGQSAPCVGQHLPKGGGYCQLLQPEW
EKLAASVKGMVTIAYWDTEGGSRPPRLLGEYKGTPTIKLFKPKRRQRKKGSSAEKDVVEY
QHGERNAKDMRKFLEYQLPSFIERVKFGPDDLAKARSKADKYGLPVAVLFTSKASTSTTV
KWLSVEFRRRMLLVEVPPTEKNKGIREALLEEFSAGTSADDSNSLPALYVIPPQEGPLSS
ETDKGTLQIYEGESFNRRKLQSFFDKTVLKKPVLEPLVKPEEDPQPKKKESVGGDEF
>comp2291_c0_seq1; 656 56 1765 LEN=2040; translated
MATINGLYELARRPIKNGNGWFDSLPSLPDFSDEYELVRDTAGTLISNTYDEIEQRLPDD
LKNNYFVFEDQVGESLAVIGLGDVRPRTVIKWAAYLLVLSIVVLKYVSKMKRRRRHKMLY
GNNAFPPFTKASMAKTVSALVTNSQSPWFFKKCSEDINSSVFRLRLPFVKAPMVVAVGDL
VTAKEILQDPLTMKSESHFSSVSSIAGGPNIITSEGPQWKKSRKAVSPAFMKKHLDRMHH
VCREETEDWINNKLKRYIDKDKDFDIGKEMMRLTLSILCKAAFDYDTEVDERAAIASELE
IVTKEFGYDDMNRPLRVRFGILRSSTRRARLARTRIQDFAKKILHSHRKKKQTENSTAVD
GNETILSCIVKSRKYEDDSRRISDIVMFLFSGVENTAYSLAWTLFELARNPRVASELNSA
LNGNNDKRAQEMLKDVLREGMRLRPPLPSIGLRTVGRDFYMEDRSIVIPKGSQIIVPSLV
TTRFDVEDAESFRPSRWRDHPDKSFLLFSTGRRNCVGQSLALAEITWVLSRLCAKFEFHV
VDDGAIEYCGATMKCLGTRLKAKVVPKHL
>comp2292_c0_seq1; 492 254 1603 LEN=1663; translated
MATSSDNQTDLSRNHLFKIRTITAFVSLKDSDFHHDAVALKSKLETTSRALEMARQQLEG
KSSGSGYEIQTLRVATNPFPEYLLGSDSSSKTACDEAVVRERLKVLDDCFEKHGIAFVSL
GPACSQETVEVCTRIVDFSGRFSCSVQLASNDVALARVSAETILRNSKLGNPEDGGLANF
RFCVANVKPYIPFFPAAKARTEDDETFTIRFAIGLENGALLKDLLEKDAGRTIANILPPK
TLSPASGSSFAAGFAESVRPIQSICDNLVREWDGSSAALEYIGLDASFNPSLDEEGSVAS
AVEALDEVSRGFGGVGTIAATAAMTTAIQTKLPSEEEGFGIKITGYNGLMLPLCEDKRLA
QLATEGKLRMTDLLNVSSVCGVGIDTVPIPGDASVEELTSLLLDVAGLAERWNKPLSCRV
FPVPNKTVGEMTTFDSPFLVNSKILPVSS
>comp2293_c0_seq1; 392 1 1717 LEN=1752; translated
XQNFEINTAPNXMPPQRGTSNDRGVDSLSEPLVSESIDDTLGAAAESPEIASIANSKDIV
VRKCCKRGGENENNVVIRVNHNVFLNLLLSVLYGISNSLWSGTAYLAFLKKMGHGGNGPV
GNIEAVSGLASLVCALPIGYFADKMGKSIVIRSGGMLLFTTSILQIGILQWVGTDFDKNE
DDEDSNSNRNNTALWLMGAIMVLWGLGDGVVNGPCQALYADSTPEGQRSTYFTYQFACYL
SASAVGPLVSIVLFQVLGDEWDLYHLRLVIYTGLGMEVFNSWLMMLFDDSKALDEDQDTV
ESTDENDGDDASFDWSNEGIEQRLPSERDTTPTTLQKRQEWIPYLVFFSGLVSSLGSGMT
VKFFPLFFKDEIGMSPSQVQVTYVLVPLVMIVMGSLCTKIANAGVGRVQTTALYEVGGIS
LLLAMVVFKNYLDVHPFLLVPIYVMRTSLMNATYPLQQSILMDFVPKDKRARWKSLDSVA
SFGWCGSAAFGGWLADKYDYTHTFLLTAVFQSVSLGIWCCLLPLVPRVEGSELEEDGEEG
PLPDQAEAADDHSPGFSTTEENGLTELSEPLL
>comp2295_c0_seq1; 443 129 1382 LEN=1622; translated
MKHRNFPPSSSLMLVFFAPLIANLANSNYHHAVEGFSSPLLIKNASKPRIATPIFAAKGF
GGGGGFGGGGTKKKTKKQSGKKKGNRLADSLADRPSKPVAKKGQTYVKSEQDAMLEELAA
KSSRTPIGKAVNEAKRALHAAGIDTDVDPFWDLMPSLISSKFPSLQDSQLERVAGMVKHA
LHPDRQSLLDQSIVADEWRPHEEIHAYMPDLGETKPFWDSSELSICQKLSENYDTICSEY
QALLQDQKDRFQSVTSMNYESGWKTLVLFYNGHRIPDFPYHLCPKTTELMESLPLAGRIA

GFNRQQPQSGIPLHSDGNNMWLTLQMGIKVPEGEKAWIRVGPETKRWKEGECMLYDTTYE
HETFNESETEERVVLHIDFFNTLKMTPVEIEVMRYIYSMREEFMKAEGVSKVGNQIL
>comp2296_c0_seq1; 576 332 2497 LEN=2692; translated
MLRSGGKKFKAALTSGPCRRYLSSSPPVAAAAAASPGDHASSSSSSSIRYAMPLSMLSLA
VGFAAGRLSFRSPEEEERLKQLPKGNPLACCGDNHTTDDGSQAHSHSHSHSHTHSSNFWK
TTSCDAPQLSEEHQQLVKTLRRLVGKDCFLDGREINTETTAYLKGARMGKGTALAIVRPT
KLKQIQDIVQASVDAGCAVLVQGSNTGLTGASVPRPQSDGRPTVVISMKNFDTIFPIDGG
EKVVCLAGVGLASLKHFVADNFPDRESHSILGSTFLNPTTAAGVAFGSGGTQSRKGPAFT
DRALYLKVVPDKYGKHIVKVVNTLGIEDLDSEEGEFVANKALDGVIPTLDSYIDVVKNEQ
DNTMKKSNNTYGKDQCHDKKYKEKLCSLDNKMSRFNADTSGRDCNRSEGKVLILATVHDT
FKAPTKAKSLWISFDSLELATKFKTEVCLDSPEDLPVSCEYMDRDSFDVIDEAGRFMGHL
IKFFGSASPVVSAGWAIKLKIEALDIRGASTFPDTVQYYLNNFLPAVLPKEIMKMGYARD
HHIATTVGDFNGSLGRFEERFEKFCKENEGKIDVYECKTNSEKNGVTAFRFIAAAAFKTY
CVGTGLQGYSVDYALPKKDSSSPKLPESNQPVKRMRYSHFLCNVVHEDLAYEQGVDTHAA
KMELKHVVDETYHGRLPAEHGHGTEYHAPKDTQKRWMKMDPLNVMNPGVGGLSTKYRYEK
E
>comp2297_c0_seq1; 371 256 1416 LEN=1648; translated
MKLTTIATNAFLLVAAGSFQGSQAFAPTSHTQHKTSSQLSMAQSGTLPCRPIGVGHAAPK
TVVTNKDLEAVVETDDEWIRTRTGIAQRRLLVSGESLRELGVKASKDALEMAGVSAEDID
IVICATSSPEDMFGDATAIAAEIGCSKATMAFDLTAACSGFLFGTVTAGMYLSSPNTKLK
NALVIGADALSRVVDWEDRNQCILFGDGAGAMVVTNQNDSDDESDEITPGLLGYAAHSNG
EGYEDLNCVYKGTPVEVSTPDPITLENSEYNNLAMTGQNVYKFATREVPRVVTEALEEAN
MTADDIDWLLLHQANIRIMEVVAKRLKIPKEKIITNLADYGNTSAGSIPIALSEAVQSGQ
VKKGDVIACAGFGAGLSWGSAILKWG
>comp2298_c0_seq1; 449 1 996 LEN=1152; translated
QEPSRTRQINEDKQNQEDVASKRENLWKHRLPSNAPATQNPTARHTEPKYNTTQHNTSTN
EEGQRDMTETDEPMGESEGMSNPTTRRKKPVKPALKSSSSRSSGNNGTTDQDYDHHTTTD
DDSETDSRKTSSTGKFAKKRTKKANRTLKWDEAMIQEHDQLRGTRMKIEEANTPFAYYDS
GSETDGSFGSARGMSVASNDSGGSHRERGKGGPPNRAGGICWDALTNKLEAHAAVAASSS
SAASARFAPGSGGESEGDGDGHLRRRDEQRAELKRLEFQEHRKRHYNEMEVLRRFRREHP
DGEPTANGHGEDEGEDEDDYDEQNDGDDEND
>comp2299_c0_seq1; 431 190 975 LEN=1032; translated
MARPAEKARAMLNKWVKMREEGNATPSVRGARPKLASECEHLGDAEFYRTQINKEITGMI
AKIQNPALGEHTIRDLNDEINRKFREKHHWNKRIFQLSNGTIDYNKRERSARIEEGDVQF
LAVTNNGRKHGPTYRYFGAAKDLPGVKELLEQQQEKMRAMNRKKRGPHEIYKHVGVGYFG
WRDEEDGVLLELEEDAFRNHKRNKQQKTDANGTGGPAVQVTEVDLDRLLEKDYLADVPSQ
EEMKQIILAEKKKALLARFSV
>comp2300_c0_seq1; 245 95 952 LEN=1498; translated
MIHPAKRINQINLQIANEEYDEAIVSLVGILRTIKLVLSGDARISIESERVEIDRDNDQR
DDTTPSSGTSLEYDFFSTPKSPSILETADVGNECCHSSYYQPNPQRTRSDLALASHNKSD
ESTTYCSAQKHHPQIFRDPMMVRGNFFRASLDNKVCQQLSYVALYNLALSHHLKSMQLSR
KKSEKQTEYLQKALSLYEHFHEALMAQTASNSIPITHGMALLGNLGHIHRELGNQRSSET
CLQCLLSRTMYLVVTGQVDKMGDLMDTTFDIVFPCLSIGSPAPAA
>comp2301_c0_seq1; 411 116 944 LEN=1063; translated
MATYVVANDTNSRRRQSSQVILLSPEEILGLVDAVDEDEDNETEAKVAIIELAATSTTMS
VRVTSQQRHRRSGPSPTDLHESPQQPRRVSWNGEAMISSDDQGIISDNIGLYDDGIVDSK
DDKYGDDVSDDDSMTTNAPNPPSRETKNDLKTRSHYWNSLNDQPRDILQATRKNTNTNTY
HSTDYPMNARALPPAGIIDNPVFRPIDADREAGNNTKKLRIAAAKTANATTTTTQIAALF
LILPAQVHDTAIATPTITNSELHHRGLKFFSKLQD
>comp2302_c0_seq1; 320 1 1175 LEN=2061; minus strand; translated
XCACQFLTLIYNDGFSTRCLEFDKNCVDAIRLKYYLSKRIIEVDDLKLGAPVAMPILTEE
QRDYGRTFQWLGDPQRGLDPIELEREFHTKSKILLDEEKIFLALRSVSHIFLCTNLRVMA
ILVSGLSNMRFLYTSLPYRSIRYYSVYLGPASVGMKSSVCIYTRNRWNLASIGFSFLAGT
ADVLQLQEILSSFIVGCHTDPKVVFRPKTCIGYEKNPFGLNRFLPMAIEINPEQLNVQFH
HVYPLLLEEETVLKALEQGTKFFLYTNLRLIIVTGRMDFVSVPYKSIHGFFFETAARFDP
KASISLYTTSARCTQTDKPRVCNLLISSVNISPKQTDIYKMAHFILDHTIIHPPTNVGAA
PPRVIMATEAVQAQDSDEGIIVASEAVLIRK
>comp2303_c0_seq1; 298 404 1423 LEN=1684; translated
MIVENSWLHADDPVRSVTSKKTILSSRNQTSANEGRRESAPGRTLRFRKTTSILWIESAR
DMSDEEIDACYYQNRDYESFRSREKRMSRGFSAWSFMNGTKSEDLLGVETRMQRFHRRQR

SKNAVFAVIFEQELRQENGRDAHSSTGDDDIILAQVYQQYTMESTRLAKERAVLNASQVE
RLTLRTNGSLMNDVETEEMNKETKSDRTANVGSPWQIPVSNRNKTDRNRYSEKVRYVSCS
RPILPQQYSGMSTPDMKLDDQKESQDFRYEMQTEMQGRGSNYGSRRQEAPVQQQINRAEE
KFHYRRPTGVLRLKAQDECQSMPNYATAKNNYAGTLAQKW
>comp2304_c0_seq1; 623 194 2017 LEN=2063; translated
MRSVKYLVPCYLTASIVSIVAGFQQQQPNQQFRLQRRVVDPSTLSRQKMPAKTTLSKKSN
DAYSRTFGRTTPGSTATQLNALSSSPLGALAVLAGIVVVHESGHYLAARAFNITVEEFSI
GFGPKLAGFEALGNEFNLRALPLGGFVRFPENYDSEAVFENEELKRKAKRTLRREEKRNP
EDGNNWNWKDEALDAASLGYWGEKKFEEARIEKRIERENKVASTKPFWAGLFGASNVAAK
TNGGENNNSKEDGELVDIDKIYEDLDELEEFEVEYFEDPQLLQNRPWQERAVVLSGGVIF
NLILALSIYFAAIGPLPVGSSEGLPRAIFDNGVVVSQVPRSDGPSSGLLQRGDVITEING
RPVQVLGSKKTSVAAGQQQVTDVISAIRATPEGESVSISVLRGATKPVATTEASSLDAVA
TKQTTTETKTSKTTTTVRIKPSTADGSNVQSIGVMLSPRIERLERLKSDNPIVAFQLAWG
YLSDIFTQTLLGTLSVLTSSLSPSGPPPGQKISGPIGLISQGSNVVATKDWTAVLLFAAG
LSVNLGVINALPLPALDGGQLVFVVAEAITGKKVDQKLQEKVTSVAVLFLLWLSFSAAIG
DVGGLFQ
>comp2305_c0_seq1; 336 162 1115 LEN=1667; translated
MTPPLQNDKTFDVPKRLRGMFSPKRVQVLKKMGAGAIISSLPVLVWWKWAIDQRNKRAED
VRTRVRVPNVQTIDDLMIERCRPGDILLFDRRWEHCAAGPLAAFVCALGRTVLCFADNSK
AIGEGKFDHCGIVVPGYVKHESDRYDPTNLLLLEATAAEGIVARPLLTRLEMSESRSVVL
LPLALPGERRNDQFYESSSSVIATEQIVNKKLEEFRDNWVKLSKTMNYSTAHSTLGLIGA
IAYLLGYHTANPGPTSPSAWFVTKALVDAKVAQNVSERSAKEAKVEDFLRDYRFQDQDVI
RLRPGWRFLAPVSFRET
>comp2306_c0_seq1; 301 269 784 LEN=792; minus strand; translated
MMGMNMSRSGSSPEDEEFVVVACDLLDACVWRDRSSLSFRSVSSDVDGVVPDPSVSVGLV
PHPSSSSSLEENRVVENPDCMGEMFPEPEHLIAATPIVLHMVIDSAGDFNTATNASDVHA
DVNDSRSAISIAVEPWQRMMLLVLSFPISPLVIVFLLCSILRARIVPDSIL
>comp2307_c0_seq1; 332 150 1136 LEN=1369; translated
MEADNDKDDPSVETTTRSQDEQQLDHELKDVEGNKNIMPRSKDIISGNYGHRKLAPENHG
SIFWDCFVRSLSPLHAGGQLTKYEKSLLADMMMACVKKMGMRFVKRIRDESQSTGKEKLQ
SQEGHRSVWCELRTFNARSKTLNDLRRYPDYKWKTLHLERGDSSNIVASAKATASIVAET
ILSSKAKRINEAISVHEKKDFRKSVLDLDEKKEYYQYRFAMNAVEKHIPENGIFDAFDEK
SNLRSNNCTNYVRAESDNETDIFVAPGIATDMVLGQSLIMTQDHRCQQRWLREEHAKARR
EKLQHDNDLYCALFKNVFAVPNQELKPYW
>comp2308_c0_seq1; 398 279 1519 LEN=2141; translated
MKIVKKNISAKDGSGTVMLPPETPEDLWHSYNLLKKEDLVRCTTVRKVIKESSTGSTTSS
KKRLMLTILLGKIDFDPDALQVRLSGQVRNQNELVRLGAHHTLMLELHQNFTIEKXLLGP
DLPGDPRGSLQPDRQAKLAAIVMHSGLAHLCLVTGSLTITKARIKTNISKKRTGSSQNSK
AITKFFEAIYQAILRHVDFTKVKALLIGSPGYTKDDFFAYLKETCVKRDDKPLRDAVHQD
KFVLVRASNGHKYALEEVFSDPVIAKKIEDTKVVKEVAILGKFMRMMDTDPDKAYYGYSH
VKSAADQCAIDSLLITDELFRAPTPQVRCMYVDLVENVRSNGGTVYIFSALHISGQQLGQ
VSGVAAILRYPLPDLEELELEYEAAMMGNNNDESEDEDEGIDEVQEDLDDMHF
>comp2309_c0_seq1; 443 482 1327 LEN=1525; translated
MPPLSESGHGGDDVDGFQNGIERKSGATDLGFTSSLMESISAFESKIEAWAEHEKSKTDL
RAKSYNNTLMQQQAAIDAQVTELASVQRERGANNLISGVKEGKGDNSESNKENIASRRIS
LEEESTKVQVEILRLKTERDNREKRVKDIELEESKQSIRASDAAVLKRRAEEAKNTTIDD
LTRGVVNYKKLGLDFEQTGDGELEFKFDQLDPHDPSRLFSFTLFINDEDNYDISGCNPNI
IAENLTEILEETNASSDRDNMSTLARRMRRAFKDICEPERY
>comp2310_c0_seq1; 493 167 1627 LEN=1726; translated
MSDAISVLRGWSNSGKLSEVAFSDEGKLRLGETASLTASETVEVAVGGGKTCRYTVGSIF
LQLVDVNQSLLAYRKACKKHKVKDAVKAIDKAELIAFFKGGAGPKPAVPEHAREGAAPVS
SSKPAPPPPRPEGDRGRDRHSNREDKKRHRSSSERERERDRHRSSKKRELSSSRSSSRQP
ERKEKAHKPIDPEMLFSNLSVVVGKREIKAKRDAEQAKLHAALSTEGFGTVAKELIAEFR
SKHSMAHEIPVGDSASILKAGAGKDLSRILKLYSETMEKKSAPGRKPTSGSSRAKGFSDG
QKRSWRPYLVDKKPIIVLPKGMTAPITLVNAHKFFAESQFVSRTVMQKELRTKRQAPKTH
FERQVNARFGGGTVQYELMDNPRSKLHRPEDWERVVAVLALGQSWQMKDWPGPYSNPVQL
FHRVFGFYVGMEGDNVPKELQGWSVTQTRVSRDKRGLDSVTYASFWNALDEWMAIHKPEL
LPQPNE
>comp2311_c0_seq1; 421 1 1069 LEN=1605; translated
XSNWRFERVPSSKKTQDFVVDSDVQVKKTKSSKIFDSSPSESQEGGIFYFQRICSRNESH

KTKTAHDSYTRVSESSSDCAVTTNGDQQLPNFFEVNVDGDDLFADLEAVADSKDDSEISF
DDLMDIDVEDGASTEHIQNYNGHPYSDSIYDVDGFLNATVDNLRNASLEDDEDINMMSCD
DTASNNMNTYLSPVTSKASLSSTLPFGSLDSPTCVAQDMALSPIFSSSLSMPSASTRPAF
QQMRKIMTKQLMDINNGSMPRSLHKDSLLPCAEGMDQMYLDTLQKLSISMARSNRTRKSL
SIPLQTMQDGNDSHGLQRQQDYNNREYTRVNQVLKSVQTSSRHVDTCLQSVRMMSR
>comp2312_c0_seq1; 428 160 1911 LEN=2183; translated
MENNFGMICRNDGIGTQRAMLAFNRGRVYDCGRVPPIPRRSSDTTSAGAITLIRFLMTSH
TNHQRHRFSESGWRRRIEKGQILMDGIVVNDAEMELTKANVKQRVEYVRGPWREPIVAVV
VHDDTIDTGPEPSCQLKILYIDDHLMVVHKPSRLPTMPSQTYFEFSVLNALRQLHCMEKE
LSKDKEDEDNCEVESYSNTNANTDSISKNHCVVATSTVRRLIRSHCSALPQPVHRLGVGT
SGILLIATSLLGRQRLTDAMRLKSHNVNNNKLRKTYRALVYAYRRDERLSIATDDGSPEV
DGSSKERSTREGPKMLIPDFMMIDCPIGPVPFPIGGDSIHAACPISGDSNDKIDPLYSHV
HLHNQVSDVQENQPQERSTTASNNNKKIKHALSLVKVIRRNIPRHERNVDKENGGETDSD
TYSGHNFDTTATAVVEIEIPTGRPHQIRIHMAYAGYPLVDDPLYLPGGIPDVRPRPFKTR
KREEEDLDQSDETEDGGIDCDEDNGTHSTTMRVALPRDCGYHLHAYQITLEHPCSSRHSS
VATISTANADVSDAAPSAYAEECAVSSSSAMMTFTATPPRILR
>comp2313_c0_seq1; 382 191 1252 LEN=1341; translated
MEREARTKCKHHRQHKPQRKLADALRCAGFDIFHGPFSPKKYNDLLECEGLEGLSRLPEE
RRCTGSSGSNSSGSNSDYEAYLIGNTKHLWPVFLDWLQLRRQSAASTATATATTTASSIE
AHPLDTYERTAIERVVRESLGPDEEKGKGKDANCNTNCDTNACRCYELFFSSDLGTGLVS
MARVASCSGFSYLDPHTHLSIHPRYGTWHSYRAVLLLASEGLPPGLFETGTKTETPILTP
IQNPLSPIDARKSRKAFDRALAITTGTTTDELMDEAMEELCEMLGAGLYRDVRDERQEEN
DNDNNNKTKSKNETVAAAWIALRDSISIGREDYRFGTNQLLYHYTKDPRHLLP
>comp2314_c0_seq1; 405 157 1317 LEN=1454; translated
MPSSPDHDDSCITVKTTQGRNYSVERYYMLSEIEQHSIHPVYLNNHHSSSEVHDSNDISH
GVNENENSSCSRGSKKGHDISRKNNIKFPPFVILYLLWNAHIDEKTTATTASFQKGPFRE
AIQKLKCLLEMKRTSTVLNQSEDPPDTPRVYVVVDMLVQPSSVAKRSNALRSDEFQKHVS
NAEELARCILLSESVGNNKQQDGDCLGDTTPYMLSAIDLAGVTVGLTDHPRAAPGLESCL
DAILIGSRDRRKYSDKTGSHRDGVDLKKSCIGIVCNSIQDLIGFDEERETDAVQGVMQSR
TDGVRMDCHKETDGEEERKEEALESPLSSFAHESHRYWRVHNAGLPPEPTLEEKKFDAEY
GPIENNRITWILGTVIALAAYCWKAS
>comp2315_c0_seq1; 159 1 215 LEN=622; translated
XNNNNNNTKKGQGGNSNSNNNSNGGNGGAGSSGAGNNNNNKKKPRTKPKSDNRVNRQRDR
KMNKQGAFGVQ
>comp2316_c0_seq1; 477 160 1818 LEN=1818; translated
MMISRLAPATLQLLSAFWALAPVISTQSRDLQNGMGLGWNENPGSCHGTLFYFQSEVFVD
FGGFDTDVNDLTRIRWEELERSFRMSYNALSEDLCDNSYRRVVDVSINEQPDGDILIKRG
GNQYSLSYTVEAVCRGCDPSSVTLFSPPNPDYFRKLISSDGATIDSMDENPLNAVLQKRR
RNSEKSSKSEKGSKGSKSKAEKSKKSHYYRSGTGSVDEGTINGSENGKGTVAGKGVEAGK
GNDAGKGDGGEGDAVDGREDEYDCGCKSQYPKYRAPTEEEFATAYGFSIREIEEKPRAQR
HAGVIVDNVLKVTEIQDVSCSIVTETFESTVAIEFFGNYEAVTDQERSILEQNFVWAYNG
LQDHRCDSPEFRKATSADIFFMIDPIAKGETTFVYLLNVKGTCRGEGCSKSDDLLLFSAS
ETGGFFRNLQFVDGAECTCPLWGTAFGPPSTSEMEVRYFQVIQDLRFLDGPFTRLKSIDA
SGFVKEIGEGASTPFPTFRPTLPPAEVPAIPGVPTLKPVTLSPTREGETRSPTREPTDFP
TRKPVTLSPTREG
>comp2317_c0_seq1; 120 1 409 LEN=865; translated
XKISRSNKSVKTFDICVRRRESVSSILPKAIEXPKGWQIFLGASVAIFWGFSRLGAGNKK
RAEQQLFERQERYTLVSLLQNEADREYLMREKKLQKQEATIMSEVPGWEVGKSQYYNASR
WTPDHIMDSMKSNMKK
>comp2317_c0_seq2; 221 95 511 LEN=967; translated
MSVRPDPNFIPKQESGPLGGWAHTKVFHLTKGNRGPKGWQIFLGASVAIFWGFSRLGAGN
KKRAEQQLFERQERYTLVSLLQNEADREYLMREKKLQKQEATIMSEVPGWEVGKSQYYNA
SRWTPDHIMDSMKSNMKK
>comp2318_c0_seq1; 233 29 484 LEN=927; translated
MKIAAVLALVVSSVAAFAPNGQVRQTTELAAERREFMSSAAAALGVAGLTAFAPEAKADR
DYAGVGLLGGGNVVDVNNANVRVYLKMPGLYPGVAGKIASNGPYKTVGDLYGIPGLSGAE
KDLIKKYESRFTVTPPSADYVIDRINNGLYR
>comp2319_c0_seq1; 338 229 1638 LEN=1840; translated
MEGITHRQSSSSAIDVTTTDTIEESVFSPLEIEESSAISDIDGYDDDGVSPLLGNNDGNG
TSRTSSNTNGKELADHSHGAGPPGSAGTPQVIINIIISFVGAGLLGVPNAFSKSGWLLGS

ITLVLVSTLNVYAMLCLPRVQRVVQAKYPNEIVQSYGDVGRLILGEKGEKVIFLCLGISQ
AGFATAYIIFIAANLNSIYGWSRGLICATCVPGLLGLVQFRELASLSPFSLLANTANFCA
LSAVLFQDYEHYTPHNDAIHKADFGGVIYVMAVTIYSMEGVGLILSLKASCKQPQQFSYL
LIATLAAITTFMAIFGSAGYWAFGDATLAPITLNLANHWSATFVKCSLCLGLYLTYPIMM
FPIWAIIEDANTTQLSRISVRSAIVLVSALVAYSVPNFGKFLSLVGSSICTLLGFVFPSY
FHLHTFGKELPVWQRGLDTFLLVGGFSFGCMGTYQSVVAMMEGELEGHH
>comp2320_c0_seq1; 434 488 1492 LEN=1564; translated
MNRPSPNREKRNDRNRIKSVRRALSFDLGAPSGNSSHQFVERILHDCFSPRRYHRVAGDG
GSESNGKNNTNKNIYATVAGRESERSNRNDIFRPRTNRHHPGFRRGAHERVSHSFPSRFT
LLSLQEEREQALRDYKVTIEYKHLKSHAPGGVYLIPSMNSLRDFHGIIFVRRGPFTNGIF
KFRLNLPAKYNDTNMWPRITFSSHVFNPYVNEATGELDVQSAYPTWDPSRHYLVTVLTYL
KKIFYAKNFADARANTAAKELAESDPQAYKKKVEECVHESQKNVYVNEEGSTAKFTEEAV
AHRVLRDLLKHHIKNENQVSKQAVMTQIEKARKV
>comp2320_c0_seq2; 226 89 775 LEN=847; translated
MSTPSKTPTRIPISPIASEEEREQALRDYKVTIEYKHLKSHAPGGVYLIPSMNSLRDFHG
IIFVRRGPFTNGIFKFRLNLPAKYNDTNMWPRITFSSHVFNPYVNEATGELDVQSAYPTW
DPSRHYLVTVLTYLKKIFYAKNFADARANTAAKELAESDPQAYKKKVEECVHESQKNVYV
NEEGSTAKFTEEAVAHRVLRDLLKHHIKNENQVSKQAVMTQIEKARKV
>comp2321_c0_seq1; 418 1 1632 LEN=1915; translated
LESNPVKPNPIYLTNTPFHLNMPQLQAQQPRILAASSSSWMTKGNASWLATAVGGTAFVA
ASTASAWMVYTHRTKRLGGSIVSGLLSPLFRRSRSLRSSSSSSKPQIATAPKSYYLDYNG
TTPIYPEVFDAMEPYLKEHFGNPSSSHLFGTEPRIAIENARRQILTQLLGVKDTAESNPG
KNGLDLSAVWFTGCGTESDNLAVRLALMSAPSRSSASGGRQKHIVTCNVEHPAIENYLRW
LEEDQSDEIDGAVRSSGSIRVTRVPVNNEGRVSAEDVLKAITPDTVLVTLMLANNESGAL
QPVKEVAEECRRRGVLCHTDAAQAAGKVSIHLSDMGNPDMVTIVGHKIGAPKGIAALYVR
PGCLNEHGRAFRANQSGVLLIGGGQEFGRRGGTENTPYIVGLGRAAELAQEHLERNAAHT
EELRGRLLDNLERRLGAARVRANGPGDPCQRLPNTLSVGLDGIRSSDLLSSVGHVVAASA
GAACHSSGGSISSVLVAMEVPESFARGTLRLSVGPGTTLGDVDEASEIIARAALSQWGEE
GRA
>comp2322_c0_seq1; 380 1 1159 LEN=1159; translated
XEAARLQRAAEEAEAARIKLAEEAEATRLKKAAEEAEAARLRQEAQEAEEAQQRQAAEEA
RIAREREAERIRELEEQEAKDKARQEAIAREQRAVEILRQREEEEAAQLKAEELRLASGG
KATVAGVNILRDYVAKFGEAFNPAQQLEQNPNLEQLRTSLNQLGISPGVAGGLAAATLIV
GGGATAIKVLADQAESNIDDAREEDGAMGKGDESNSGGKDLLDSRIADLENKKEEKTTQQ
APTVRRTLRIVDDDINGNSPNGQSETSRPNADPPASIKFGDSPSQPQPKVSPPPVKKSSN
PMNTQGPLSSSSSSQKFDTQSDKNGNKVSPVKANVSDSSLNSSSPTSTSSSGFGVPPQKT
VGPSTLSPPPDTFGSPPSKTSSPFGVP
>comp2323_c0_seq1; 202 323 889 LEN=1214; translated
MKTAVRTTSSSSDDPNHRSNNRSEATTSNNNTHTTPTIWRSTYRYVWLSGIIGVVGLQSI
TLFRLTSGFTLDETGTLSNATNNAWSDLGIPEAVFLTGAALDRNRSNAESKAFAEALDKK
RWDFQRRQKLRKRLAFPGSNRTARFKERSRKFPIISSRSDATNESLSLHPVAAVYNNEST
KTSKAKET
>comp2325_c0_seq1; 322 87 1145 LEN=1409; translated
MSAAPSPLLGLRMSNFPRTPAAVSDRCGNTDRVKAMLNQSESTYRCNDYIRRRSRKDKSG
CHATVGEGASDSGCPELSVEQEQLDTVCREKMCEWSYRVCDHFQTGREIVAVSFSLLDRF
VDKCSCERSAFKLAAMTTLYMANKIYGGPQAISIGALAELSRGEFEPSHISEMEVIILKT
LEWRLHPPTVQCFIDAFFHYLSLPSHGAWSVAIYERATFFAELAVYDYAFVPKRKSLIAL
GCLMNAMEGMDNSLSAEQQRGFVDAVNATFDLEFTTDSIESVRNRLWYVYSMSAQYKEDD
AVAGAEPGDVVKKSVSQRTYGVSSTAATDLSSSPSTSPVSVAAASGPHHIAS
>comp2326_c0_seq1; 729 178 1833 LEN=2083; translated
MKSLSLVLAVFFLAILAVFVMNFVVLFWFFPVPESASRPSSAFAAAKGGGWFRTPGESTE
QQKHRKKQQRPPLRPSNKRHPLHHQTEQATEPPIIKPPDPTESATSDPIPAKNDNSTRKP
SVGECRSFGCPIYPPELTNPLQNATIRDALESSRLGGSPGSKSLLPDREKAASIGFAAAS
FATLSQKGESHTVNQDRAVYVPSFLSDLLVSSARKPSGAAAELPAPESFLVGLFDGHGEL
GHDVAEEVVEKLPVLLAEKLTKGLGGGSSSSNGEFDDFKRAVATDSYRDNSNNSTNTVNN
EAIVVHAKEYDKTDFIIRKALNETFLEVNKKGTSPTFYLGGCTASVALRWGSKLYVANAG
DSTTILVSAAEKKAPKKKETPSLDVKVEYQTRRDKANQPGERERIEKLGGKVHVNAQGFD
PRVIVYSHVAKDTIGLAMSRSLGDWEWKVAGVTAEPTIDVIDLSELSEPPAQERSLFLLA
ASDGFWDMRQQHFLATLSASSFVVPSRDTGNESEEEKLLRPLFHLYDIIRRITPKEQTGY
RDDITATIVRL

>comp2327_c0_seq1; 406 73 1251 LEN=2403; translated
MTFHSCSRRPTKIPTKMVPCARELLFLVCATLVILLGCLGSTDALAGPSQPRNNGPVTIK
DERIVLPNGVRAQVVSALPEGWPRQQNQDEILSKKGVDEEKPKPPILFLHGSFHGAWCWQ
EHYIPFFVERGHPCVAFSWKGTGGTPAGEGVTKVRVVEDHCADLEALLDCLPSILGDTGS
VGDESLRPIVVSHSMGGVVVMKFLEKMARSKDLQPSELFRGIVSMCSVPPSGNSKTTMRY
LRRSLVDTYKITVGFVLKKVITDDAICRECFFGGTGKGPEPGVSDEDLARYQGYFERDTR
AILDVTDLNQIVPSRSADSGGRAPFVADLPPCLVVGARDDFIVDAVANAETATYYGTSAP
VYVDSPHDVMLGGQWRNGADALHDWIQETFLA
>comp2328_c0_seq1; 215 177 395 LEN=395; translated
MASDGPPQPMPMNPGVPTASLSNPMAQQQQAPPQAMAMAMPPPSMMVQPTPMAMNVSMTQ
GMVQVQQQQQQQQ
>comp2329_c0_seq1; 434 279 1718 LEN=2244; translated
MVQQVTPLRSPGVEKDGQEFGLVPRYVLRKKKAVNKEGRGPTGDSNHVKNNQNGHHDMMK
DCDEEDTPQKTPQEVDKLLHETNSLLARRWRLSSLIDSERDKKVLLATEAYLPSKAADRI
EKNCKTKMELFGSELMDKKITLFEDLSSEKSEVGVEGGYVQLLPTRDLAGRAVLFFQYDQ
DLYSEQPINFRKILFYMMASALEDVDTARKGISMVVWNGAPTKWFDPFWRVFEKISAPFK
ISKLHYCQVSQYESSYQVLEKRERGETDVRIHKGSLQECMHGLVTYGVPSHFIPLNPLDG
SINLKEHLKWIELRRRIESFPESQQACIVLVPGQDDVLLGKRKMSMNGNVLYHQMIMKNI
KAYHEATSEDARDDILMAVWDSIQCRGGRFLLQVEDGQLWERLELQSALYKISQAFDFLC
MQLYAPKPTSSKDSSSKNSKDDEDIFFGWNDLLDGKKCYECFDLIHCKGDGFGKPAACW
>comp2331_c0_seq1; 214 178 457 LEN=457; translated
MNNSNNNKMNDNETNDNDTVSTSTSNDNDNEKGHGFARHHQWLPSGPAVLPLAQAAGVFL
AAGPRTGGAAPAGPGGSGVRLSDRGDAALGAPEX
>comp2332_c0_seq1; 315 311 2140 LEN=2322; translated
MVSFLRSTASKFTVLAVVFLGSGAAPIHAMEPELEDTEIGNDAPLDTICKDKPNWRYNDK
ERKTCEWIGKNPDKRARLCEKKRVKTNCPETCGVDCNDLINRNGPTDPMPPLEQTPPRDP
CKNDPSWRWNDKERKSCDWISRKPQNKERLCVKKRVREACPETCGVVCEDVSRNVPTDPI
PPPSSPITKCPKFLGAKVDSAVPAFDRIPSAESYFEEPTMDCSEYLDGLTCYTDYRYNGC
SDDTMQCTPSKASVCSNNVWQTSVAVNPALCGSDFGEMPYLSVFGEYCDPKECPVSQPKN
GSDCSSYEGEFSCMYNYKTFGCDGFDDSHGCFPTTSLKCTDDNKWQMSIFSIAKCPDGSP
QGQVACDPEECPLKEPMHGTDCSSYEGKDECHYDYKTIGCDADSLTCAPTKYYTCMEEPD
GKSDRWQLQYTMRPACYYEGADKSCDPSGCPLSAPIPGTDCSSYNGGKQDCMYNFKFDGC
SFDDLRCSPTSYFSCNSDFNTWEVAETKPLDCEVIIQDFEGISEDLPVGETCKLCPTVEP
ADLCPAKQPRAGTSCTSDKYDYEVGLVCSYDFKYEGCSTNKLKCKPHEFFECTEQDGWVV
KMTEPEPCN
>comp2333_c0_seq1; 178 1 732 LEN=1604; translated
RSTNDVHRHLFHFHLPEDQIFAYIMFSALARKSTSKSAAALTSTVRRTVGVRTFASEDSH
QPPVSVYGTAARYANATYVAASKAGALSQVEGELNGLAQAASDSPAFAGFLDNPLISRDE
KSAQIEALLKEKNVSEITTNLCTTMAGNARLNELPKVADTFATLMKAKRGEVEATIISAD
PLTKKETADVAAAIKATSQGASEVVISSKVDPSIIGGIQVQIGDQFLDLSIKSRIEEISR
TSI
>comp2335_c0_seq1; 38 1 809 LEN=932; minus strand; translated
XMANSLGVDLEQKIMEGQMTFDTLGDVVLACTGCVNPETCEKWLQQNKTATATPDFCRNT
DVFSLLMAGKHAERARSGPDPKRPCAPSEAGREGRCDAKEKAPAGKGHEEVPDGIGEPQA
LPPVKHHPKRIKQPSRQQQRKAMCRKNRRKRDKQRHADPSQRHIGQPSRWHLPALGPDLG
RGPNDGQHPDQRKHHIAKGSAQKHAERREGARDFHIDRSMIQTAQNGPRGGMGIKVIGRR
GPQHHQKRTGIDDGRGQRHPVHAQRPHGE
>comp2336_c0_seq1; 262 125 776 LEN=1000; translated
MNVEQRTMRWTNEPEKWKRKEDGIKVTCPMETDFWRGTMHGYNKDDGPFYWMIAENNFEA
RLSLKVKLKELYDQAGMMIRIDSENWIKLGICCYRDQPHVSCCFTRGVSDWSTHRLSQTK
VDHFHVWVKRKEEVIECFYSLDNENWTRIRQGYFSDRPRLKVGMMCAAPESAGFKVTFSD
LLIKNLDDDGDGDSDSNNKVWTTRGLEDEGLRGTED
>comp2337_c1_seq1; 752 1 2558 LEN=2870; translated
XATSERERQSTVPGNYRAEAGYGAGRMLPGDPHERTENRKRAHKRQEARHGRRPKATPAT
TVRNEAEAERVLEEFWSVLGSAASGEDPTEASEGTGKPKRGSSKGTNLEATSKSHKPHPS
AAATKTTPPTANEKTTEMVDTTTITDPKPTTSTEVSSALGGLKTIHRSNDPGETNHSLSL
PSLPIVGNEVVSFVAEEMPQICRFQLTMTLRQPSSSPPHERSSGGLDADSIVVSTTPLGR
AASRTIVIIRRREANKPGDVRASQPATDHTAALFVGEFPRKIEASDVTWRVYPSNTPGNE
SSAAVPIFSAVKIVFRLPYPYDPSVAPGSLGGSAIGNASSSTLDEINSVLCGSCHTPLI
LRRRHDCGAHDNDVEASSNNGRSNSHNKTAIHSSSSDTEPSQSDVGTIRRVFPLPVGHWD

EIAEYLICYDGQPVVDFSAGSECAEPSMALQDASLLCFHRRDVEHAVCALAVDGYGEPTE
RRKNDANRGEIDGFGDLGTFGTKPESSLFVLQEPQDSGGPNRNDPQGAPGDAKEEEHRPN
SEDEVLPSSAVVRGNRSWQDAADGDSVSLCCGRCCAPIGFASLGSPETWRFWKHRLCLPM
PKLPQENEQTRESEYRIGENPVEANHIPFQNASLLQPTKPFASCSSFLARELVRYAESKA
IFTFIVRCEETEDDDDDAEGGESTKSNSEIKCLLLRLLSWETAIATSFEINDSSLSSRRR
LRFQRVAKIVFEETIDPTASEAAAGASAGHNGNGSSATTQWFWGGVDLCCPPPSAAATKT
SAPKASSGERGNEGDDPVSTVRLQLPRLEYDRVLDDLVSGRSLFQKEVADATIMLKMGGM
SEGLGLTAVALE
>comp2338_c0_seq1; 161 497 1300 LEN=1927; translated
MRHAAKLARRVLDLACSVAKPGVTTDEVDTVVHEALLEAGAYPSPLNYVGFPKSLCSSIN
EVICHGIPDTRPLEFGDIVSFDVSCFINGVHGDNCATIIVGDEQENDEIGTDWRGVPYLG
SEYFENRPEADEQMIRSARRLVHAARESLYEAINRIGPGSCLTDIGAAVQDVADSYGYST
VAKYRGHGIGTDFHRPPFVKHYRNYDKLELLPGMVFTIEPMIVEGASDCFEWSDHWTVAT
SDGGMSAQFEHTVLIKEDGVEILTVPE
>comp2339_c0_seq1; 592 1 1401 LEN=1672; translated
TTTTNNNNEKNRPSDMVAREQESLLQEVWESCDSIAAKLGDGTVFFLAASLLIGAAAFAF
HPQTTIPSRPLFAPAIASVSTNSLLLPLHATTTDASDNGRDDDEEEEFFDASFSFSTERL
VSKSGLPVCRYALGGAARSTQPKSIVNEYRERLENAIVGANATPAAPFLFYYNPHRYPDF
MRGIRELANKDSGNNPIRREDLFLASGGTDRSQKGMEQRLRDALAYSGGDYLDLFVLEYV
CPEEAGDDSGDLVKALELARSWVRDGSVRYVGASTHSHSVGAWLGSLDRGEERDNDNDND
GASSPLPLLDAMMLRYNMGHKAAAEALSLPVCAERGIPVLAFTTTRWNRLQDGHPDWGQR
APTTPECLSFALSASSDERGASAGPDAPTTTEDGAGRPIEIVLHSARDAEELGEGMSCLD
SSFVPPTIDAGNRAAVAIWRDYGDLEWNDMDGFDEYPEEQQGTSIL
>comp2340_c0_seq1; 412 1 1437 LEN=1461; translated
SVSFLRTGHAENAIDFPIRRHLTIMSSSESILKLDGRNDASKSDCGDDSVLNYLETPLNA
CSLESFCNTIHSELCGEQQFIPLCLGCYQLNEITGNREGLLELYASTTESFGTGMHPPLT
ILGDGDGAPGVLDGKWSSRLPSSSTSIDFHNYYATAHANGEIWVHNITNKAVEIANDGVE
KNTNPFRATLAGKSRSPSENEGLCLALAWEDPLYRNGDSNCSTAVDTRIISSYSDGRTAI
HRVREHPPTSAEDATQTVELSLEHHWDSHTIFKCPAEVWCANFIDSNTVVTGGDEGSWKV
WDLRQPLIKPVYHGKNDFEAGVTVLSPHPRHKNLLAIGSYDETIALFDLRRMTTDSTSAN
KCPEELYHSEPLGGGIWRCKWHPRDDSRILVAAMHGGCVSGKFGGLDSANFEGQEAVIPT
SISFSIQKQFNLHQSMAYGIDWLVYPEGTTEDTSTEAAASCSFYDKAMYLWKTGPENI
>comp2341_c0_seq2; 132 1 294 LEN=779; translated
PVATRTHQSNFTSFVTMNRQSTSNDYAHDTTCRLSAEDKGMMAQVSMTSCNRIAGHSEET
AMKTNSNSPKLSTTPQDTSFMTEEKMKSIYMPKRIDF
>comp2343_c0_seq1; 251 90 734 LEN=1316; translated
MTFLDTPVCKCCGGNNKTTIGDVFKLIIPVAWSTVAFMACLAYLISPLSRGDTPRWGSAA
LVALSGAAGTGLSIAIYSFHRTTSSFAIDENYVESSNDGDGEREQDEDQQGTTKLEPPMK
KIQRQKMLQIHAVLALGLSILSIILAIFFGVFASNLNKCAHETCGADVSWSCITLVVSVL
WMGITYLGFKHLRQQLLPEKDATNNENDQGLEIP
>comp2343_c0_seq2; 251 90 734 LEN=1437; translated
MTFLDTPVCKCCGGNNKTTIGDVFKLIIPVAWSTVAFMACLAYLISPLSRGDTPRWGSAA
LVALSGAAGTGLSIAIYSFHRTTSSFAIDENYVESSNDGDGEREQDEDQQGTTKLEPPMK
KIQRQKMLQIHAVLALGLSILSIILAIFFGVFASNLNKCAHETCGADVSWSCITLVVSVL
WMGITYLGFKHLRQQLLPEKDATNNENDQGLEIP
>comp2344_c0_seq1; 228 117 758 LEN=1785; translated
MNRLRPLVARGLPSAAATATATTTRMAAWRRQRLARSHRSFQLRLGSSSHKHKDKHKPSS
SSAPRPGGTWIYLGWTVLGLVGVDQALQHKQASADAKARKALSVLQKDAEASAKADWERS
VDQRANTAPVGASRIARVDPCLDGTKMLTRHRGVQRGDAVDVLETGVGPAGRYHLCRWTK
GGANENAGDGSAVVVVGWYPIDYLEPLERGKHA
>comp2345_c0_seq1; 592 42 1277 LEN=1489; translated
MSFVSRWMQRISQRSRDGLALSRAMYLAVPRAAPANTTIPERIKAVLLEESRQPSNNSVA
ISLLKIDKEMGLVKYWGLAEYMRQKQDSFVLPSILSSNSNSSLPENPDSKVSVTDSPGTG
AAISTHSASSGAKVAFMITSPMKHDLINNLGYETNIIKGMTPQQASLVLHHRVLPESYDD
CISKLEEQFQEEQQKLQEQKTCLQEKEQFEMEKQPREVDVEDNLTTAEIAAIPSDNASNS
LYVASHDRDEGYDRDNRVNIDQKQLILSSSQNEELSSLSSDSRLPNDLATESPTLPSTED
RFIPESSVPKENSTDTIVRKEEEDMNLWYEVVEIKGEKASSAASDAGVYSNEVRHGLYRN
RDEAMLSLETRQMIKNRRELDTNDNGAKNSTETSSSFVLRPISDEDLRVQS
>comp2347_c0_seq1; 366 31 684 LEN=818; translated
MGRRPARCYRYQKNKPYIKSRYCRGVPESKIKIFDVGAKKAPVDLFPFVAHLVCDEKQQI

SSEALEACRVAINKHLVKTIGKEGYHIRMRAHPFHVLRANKMLSCAGADRLSSGMRHSYG
KPIGVAARVDIGQILLSVRSKDASEAHVIEAIRRGKFKFAGRQKILKSAKWGFTKYPREE
YVEKRRSGELSCDGNIVKVHNRRGLLVNSALYQAGQE
>comp2348_c0_seq1; 556 247 1881 LEN=2075; translated
MFSLIIWLSFAGATRCTAFVPSSTLSSLHGAHDLLLLNVATDDSADTITGENVIREVFPG
VHLPKDNVPLALNNDGVEAKTKKFVFKAAPLLFALNGKPKADGLLPLQNAYLEETLDDYT
IPDDPNYDFFPNNCNAKTLRYLLKVDPFFASALTKTRWGFELRSFDRRDPDCKNKPSLYR
EMTRTLTGVGRNVNFFFDKRMKLTGYKVYDDSKGSKIFEFKSQQDEDYWASAAIYNLFFY
ASSIHATIHVLHYLITSAFQYVSKDFEPMHQWAKFYANNIQQKYSQVGTLLILPAPEILD
TDQSKITALITGKLGFGSTAAITPILSRLLNTWGQNPSASSWIDSMVGISSKDMDRAGIL
TEFRKHVNLISPFAKEASDAFRNINPEKAAAVEERLKQYLKECGSFTSEINSLEDWIELM
SITADVHGSTLGYTRLATSSDVMRWRDIRDPNWDSLGLNMITVLLGTVCGMEEGRHTMTS
TVDAPYDPALQAVLDKYDEKATRLKEEYQENIQKDPDFSDYGWILSGFCTDGFDGKQLTS
ATYI
>comp2349_c0_seq1; 255 1 474 LEN=787; translated
KVSVARIPRITDTTLIHSTFKHPKMKFTILASLGFLATASAFAQQSCNRPSFTALNMASP
AEFVDSEIESNDVVVFSKSYCPFCTKTKELFDSLNVEYAVHELDQMGDEGPELQMALFKK
TEQKSVPNVFVKQQHIGGNDDTQAAAAAGKLQELLGL
>comp2350_c0_seq1; 1320 117 5179 LEN=5357; translated
MSPRRSLSKKSTVTVVHPKSGRSMDLSGEELDEFCRGHGLCRRCARVITHKRVFKLFGTN
HPKWEPLTVLKPTTAADEWNNNGDNKGGNQGGNHGNVEQYKKNDEIDNDEDDSYLVYKGY
CLGEGCYTMKEARRLLGETGDKNSRYNKENSGWRSRMTKSTRFKKKNPKRPGKRRTRTNP
ETGSVVTNAASLSSLRSSSSSSIVTTSSASTRHSIISGMSGLSGFSGMSGSVKSTRRSGR
SRRSTQRSSSRSSSDDDDDEHNRKEFSSEKNAVSHIVVERLEKLVSYDYFTVLDLSNIDM
SNSSKSTKNESNANLDALVKAIGIAKTLKAVVLEKCGLGDEGLEKLAGGLEQQIVTTSLH
KSKSADAGDVLLPSILRLRGNRIGNRGVQALEFLFRSSSTLKEVDLSDNHIGSKGACSLF
ESLAQNPNGIPLSVLNLSQNEIWELSSPMENNNASTFLYTNSTLKELNLDGNLLHDGGAE
FIAQSIRSNRRCALKRLHLGWNAIGDDGVRALGKALESNTSLEVLGLAENDITNTGARAL
LAALAVNTSVKEISGLYHNPIERKFIIVAIKRLLHRKSSVRGDKNNTDPARSLANSEHSA
AAAASTRTGATTAADNEDYEDSEEMIVPLKPAASNKAVVVVDDGFSRLQRSYEETARSSQ
GGDHHTHASTASHSGGSLALEAIENWDWGTFGIEEIERKISGDLDDNIEVQVDGYTDPSS
AKDSALSNSNTKRGLSADTKKLVDRMFVFQSAPLAYFDRKKMQHVGVPLLDFDLEARSLR
DVLPEDKNIDVVFQNATQNNFKLYFSKSLSPVMHFSCHGNDSDCLILENGFGYMQALPLD
SLKKMVAASKIKLVVVSSCNSRHLGEVFLNAGVPHVVALQRETTYSDEGPIDFARAFYGA
LGQRKTLREAFDIGINECKKFSKPPRLLDAYKLLPEHCSNHDVEIFFQQSPPPITESVLA
KDLSEDTSILPEVPKQFVGREVDLYEILESLRVDDVVRVGGAPGSGKKSILAVLSRYILE
RSRSLEIDSVYWFPTPPGVTPDPDSLYGDLCNVMKWIVEAEDDVWDEEDYAESRERIVIE
MEGKNSILVIDGRIFANEIAGEMLERFLTHILNEVNVKILLITATDESRVKSKRSKSEEA
TIHLGPLDYTSTVRVFGSACPLLSLNEKDCSCTVDTVEEFESCLLPNSKAAGPTVFVDPK
QDVVRSPHQSELYNLMGSGNPRDIARKAASCQQEDLYRLLRLAQRPVVSVASSTELERQV
AKWMARKEQAVQNHYYLRASDIDKTLQELEDLRKTYPSLTDMKTKERDLKEKFRALLKAK
RYDEANSVKRQLLTLKRSMVKEQSSSSSISSQSTAAQESILEIQERMKTMIALAETLGQS
MSSVGSPTSHLVQESPPAEAEFCLSDTCTLKISPGCLWTDFPCSKTGGTSGKDNSTVSAM
IVFTNEACDLTSNSKAISESGDTDRKLKLSEKIHDLETLTETQWGTVKCATGESVALDDI
GAGGNRNLCIVLAVPPLVTNVNEDAKDFDSDQHYVATQLRSAIRSSFRTVRRTNNKVTAI
GISTSPSLSLNGKGNRYRTNDELDSDENTSSFVTLKAIVDEICRGGGGKNNECLRTVHLF
PSTGIVESKMLIRTAFEMGLKADSKAIRVVTAREFEFLFLSCGMNNENDSNKSLKIITAT
IIFLPE
>comp2351_c0_seq1; 363 454 1296 LEN=1407; translated
MTLAPKLMPYTGNNLVPITSKVPAQQPRVVHTKAKPKTNKATTSIAMQTPSDSSAATVKP
KVTKRKPLDVLQTILSSADAVNGSSSENSSAAVQQQMTTPKSTSLTTPKALTFQKPSEVD
IAAYDLETVKAIRSNNLDLLRQLWQSGKSMDACNQFGESVLHMACRRGYRKVVEFLLVEV
KVRTDRCDDFGRNPFHDALWTSTPNFDVVDLLIDHADPALLLTEDVRGNTPFAYARNDHR
DQWNEFLEKRKGKLINRMRGSVDSNSDGGVDAAKKVEVVG
>comp2352_c0_seq1; 356 20 808 LEN=1486; translated
MSLSNLKCIVTGASSGIGKATSISLSAAGAKVVGVGRNEESLKELKNNGQIHDYVVADLV
EPGACERIVQEAVSMLDGELTTLINNAGVLKGGAAGAPGTDLSNYKYNMTLNTQIPFELM
HYSVPYLEKHPNVSSIINVSSVNGKQAFAGCLSYCMSKAALDMMARCASVDLAPKGIRVN
NVNPGVVTTNIHTAGGRMDETQYAAFIKRSVETTHPFAQSLGRIGEATEVAELIVFLAGE
KAKFLTGECIAIDGARQNLGSR

>comp2353_c0_seq1; 410 47 1216 LEN=1373; translated
MKDKILAAVYILGWALATGDCFSPNPATPISARRRQAEASVVVRSSNEDGDSDEPLVIPT
SDVVKKVAVTGATGKTGKLVVEELLSRNVQVVGLVRNETKAEELYGGSPSDMLDIQVCNL
SDPSSIEEALEGCDATIWCATGFSDAPGGPPTTTVMSSEQSIDVVGVPVVAKTMLRNSGS
EEREQAFPNVVMLSSAGTTRPGWDEEKKDRLRGCADIPIVRLNPFGILGLKAESEAKLRD
TGADYCIVRPCGLNDNWPEGARPVVSQGDVAVGRINRRDVSRILCDVLSLPEALHKTFEV
LGLGGYPPPSSIGPALERLRTDSEPPSEAEVEATYLALQQLLPGERQDAAALAMGQTYEQ
LDRGETGRLGERGTENVDAAGIKPTTAAA
>comp2354_c0_seq1; 318 64 882 LEN=961; translated
MPIRNTSTGSSSSGKKRPPVFPKRLYDMLQSAERDGYAHLISWMPDGNSFKIHVDGSPDP
EDEKAIVRLLKQVFNQTRFKSFVRQLQLYGFERTYKGPRRGECRHPLFIRGRRDLLDKKS
IDNFQQKSNDKTSRRSKHVRQLLQKSPSRAEVISFMRKNDSRIETYSMQSLENTASSPCQ
YLNTSVIPTKLHNLVLSDSDCSTKGSDSCNENYDDEDLSVIVSNPLLDDPPLNNYQQTVD
NLYGGDLISIHDSESVFPDWTGIELEILRSAL
>comp2355_c0_seq1; 579 1 3006 LEN=3407; translated
XATGNNEVLQNPLLLPLTPDSVYEFYVHELFPAVVSSLPILFYRIWNDALSWAWLAGHKL
YVFVQPTWMTNGGTIEANNETTANILMHLHLAEKDLGGMIQNIWEQAVQSSKQHVNATFF
FELLREHSLHSNSTTGPLELLARLPATANIIIQQEAAAVEKSFWTWFSREWPLMDWKIGV
FLWQTFGGVLLVVALLSIMPGRLHTWSGKLLRWPILGLTYLLINVELVVYVCIRLGIRLI
EAIVATPKHRALRAKMANESSTYEEWYGYASELDASQKRTLWQSTTTDETSYQYNWPLIQ
QLIADMRVARCDKKDPILALAVLQQCTRKNVGGIMNEDLFTKTYTGEPKFIVMEFIGEVT
KTLYWVTEQTLTLEEREELRNKTAGRSSTSRGGPKTYEQNLQQKSKAEHHKLWRNLVAFA
TLDFSTRTMSQNNEQEKEPLPSESEANDPGSPKSTGSGPQQPPVNRAQLLKFLKRAKHAY
GKTALCLSGGSMMGLYHLGHLKGLMETDCLPEIVSGCSAGSAIGAVLCTRTNDELHRDLD
PTIIGPRLKCFDRTWSECLRSVWKTGNLFDGDDXARDDQVVGRYMRWLCXHPFACKQICF
LDAVHGILTLWVLYTFLYSSMIARFGSCFCFCFFLGLARFQTLSMAPSPPSTQPKQQVTL
GDTTFEEAYQKTGRVLCIALAATAKKTPPILLTYISSPNVTIASAVVASAAVPGFVSPRR
LQIKDPVDGTIRSAGPETYFDGSIKHDIPTAGLSEMLNCHFFVACQCNPHIVPFFFNAKG
GVGRPTRWSGGEKETSWRGGFLLAALEMYIKNDMRAKFVFLRDLDAAVSFTGTMMTQSFE
GTTTIVPQVELRDFFTLFRDPSVHRLYHYLKVGSVAAYQHAAMIRCHYRIADALEECIER
LEAGGEKGQQQHHDHAARREAFGQYESPLKRRASRRACFETGSMRFEPGRCDLHTESQSH
SHRSQRSVPEIVSQRTDSDDSVSTASETGTLPPGGGMVHLAR
>comp2355_c0_seq2; 656 1 2776 LEN=3177; translated
XATGNNEVLQNPLLLPLTPDSVYEFYVHELFPAVVSSLPILFYRIWNDALSWAWLAGHKL
YVFVQPTWMTNGGTIEANNETTANILMHLHLAEKDLGGMIQNIWEQAVQSSKQHVNATFF
FELLREHSLHSNSTTGPLELLARLPATANIIIQQEAAAVEKSFWTWFSREWPLMDWKIGV
FLWQTFGGVLLVVALLSIMPGRLHTWSGKLLRWPILGLTYLLINVELVVYVCIRLGIRLI
EAIVATPKHRALRAKMANESSTYEEWYGYASELDASQKRTLWQSTTTDETSYQYNWPLIQ
QLIADMRVARCDKKDPILALAVLQQCTRKNVGGIMNEDLFTKTYTGEPKFIVMEFIGEVT
KTLYWVTEQTLTLEEREELRNKTAGRSSTSRGGPKTYEQNLQQKSKAEHHKLWRNLVAFA
TLDFSTRTMSQNNEQEKEPLPSESEANDPGSPKSTGSGPQQPPVNRAQLLKFLKRAKHAY
GKTALCLSGGSMMGLYHLGHLKGLMETDCLPEIVSGCSAGSAIGAVLCTRTNDELHRDLD
PTIIGPRLKCFDRTWSECLRSVWKTGNLFDGDDWLEMIKWFTLGDTTFEEAYQKTGRVLC
IALAATAKKTPPILLTYISSPNVTIASAVVASAAVPGFVSPRRLQIKDPVDGTIRSAGPE
TYFDGSIKHDIPTAGLSEMLNCHFFVACQCNPHIVPFFFNAKGGVGRPTRWSGGEKETSW
RGGFLLAALEMYIKNDMRAKFVFLRDLDAAVSFTGTMMTQSFEGTTTIVPQVELRDFFTL
FRDPSVHRLYHYLKVGSVAAYQHAAMIRCHYRIADALEECIERLEAGGEKGQQQHHDHAA
RREAFGQYESPLKRRASRRACFETGSMRFEPGRCDLHTESQSHSHRSQRSVPEIVSQRTD
SDDSVSTASETGTLPPGGGMVHLAR
>comp2356_c0_seq1; 925 173 1484 LEN=1484; translated
MKQDPMDAYRRERARDRSNSFDRHNNNNNRRSRNQNGPGGGGVSHYGPSPYGNNNSSNNQ
NKNHQYPPNSKYAPSSGSGGPPPPPPHNGMGGLMGNNGNLPPPPPPPPPRQAAHGGGPPP
PSQYGAPPPQQQMQQQQQQLPPPHTQHYGSQQPQQQQQPANNMYGGQQQPSYAAQSQQQP
HHQMMTPGSASVGVPPPATGVPSNNSYYGAQNPQQQQQYGGNVAASYSNPPATNAMAAQK
QPYMQHQQQAARVPPATNGYAPQHQQSQQQQQTGWQQQTQNQSVAGAAGLVDILGIADKA
ASAVQALQNQNRLKQPQQQQPYQQQQQQHQQPYQQGTQQQPAQQHAPPVQHHQQQSYQAQ
QHQQYQQQPQIQQPQQYQQAQVQQPAANNQYGAYGPPAQTSTPYSTGAPPYQQQAQQQQQ
QYPPQQGHYGNHMQQQQX
>comp2357_c0_seq1; 296 500 1873 LEN=2013; translated
MVEQLQLLPPDSVSLSPTEPQVRVVSPRSPKNGVEGTRWADHRFRPVHLNPEDWIALGST

LSMAELVDPNMSYDFSGNIGAKFSGRWHLEEDEDGRVVKLFINPFGWGGIYNDVFASIDD
VVGHIHRNDPFHLSEDISRLTKLTFLGLWNCRSLPSRGLSSLPCLQKLHLDNYMCPELLS
NFPSSMKLKHLTELVVDFNLTEPPAQFLEWITNKDMVPMLTEIEFVQTKEEHLPLLLDAL
LGAEIAFRGQLTSISFLESQFNGDKLETLLFDFLPRFPNVATLNVEHLKIESFRPILERI
EQGEAKSPVSGSLRDLSIDVSHILERIKTVAEEKEAVLRFLKLFGRLSRLGNGHPRWDSD
IRYALMKNAAGRRLLEYGTIGAPAGLPLALWANAIERASRREWWEERNAPATKVFYLVQN
AREIAMEASERYTKEGKAREREQAEGEAHAKRSRLFY
>comp2358_c0_seq1; 493 89 1333 LEN=1438; translated
MKFSLVALLAAAPRASAFAPQGAPSHLLSPSLSASAAPVEDYVDAKLTEHSHCTEPGDRD
ILVRAARGEVTERTPVWLCRQAGRYMEAFRAYSTRYPFRERSETPEMAVELSLQCHRKYG
MDGIIMFSDILTPLPTLGIDFDVVPGIGPVISTEVASESDVEALGSVEGVEAFDEKLPFI
REILGDLSREAEEKNTALIGFVGSPFTLAAYTIEGKSSKHCLATKKLMMEAARGETATMD
LFLDKIATMIGHYACHQIECGAQVIQFFESWAHQLSPKHFNDFAKPAAQKAIAIVKEKHP
DVPVIYFANGGSSYLELQRDMGADMIAVDWSVDMAEARRILGPDVAVSGNIDPTVLFGSK
EQIEQAVRDCIDKAGGPGNRHLLNLGHGVMQGTPEEAVGWLVDEAKRYTGKQAE
>comp2359_c0_seq1; 464 318 1433 LEN=1548; translated
MPLRASVGSVIERDRRHGMREHFLLALSAHDEAFQDWSSDSRIDVRNLLTQRAIQSFMFL
CEECRDPHSGKWIEDFLKTQNLLNYHGTGASFMADVPWDGPLLELMDQPEDVMIVSAKRR
GRGHGGWSKNNPYLQDRYVEFKITIDPASLTDRILAVREQLAAEWVNDLEILGRANQQIL
DSYFQLAKDERNQDPAMSPAVAFERTAVDALNSHTSFGASGSPFRMGSFDLLYNLCTQAS
IHRLLRSLGDKGDDANLELLRDFYVDRLEDFFDGDLPFGKADDFIEELLLLSPSIIHRDN
GNVALTDPFGISEQIIEFRNDIFSEWIEAMKLVPTAHQDGVRKRLLNKRMEAWNEYTRKS
KGSGNTGAGFQ
>comp2360_c0_seq1; 179 187 630 LEN=718; translated
MFRNNPETIRRVLLNAKTIAVVGASPKPERPSNFVMKFLLDHGYEVVPVNPGLAGKQIHG
QTVYASLSEIPVSGNKGIDIVDVFRNSAAVPPIVDEAISVGAGCVWMQSGIVNEEAAATA
TEAGLDVVMDACPKIELPRLLPTGSEL
>comp2361_c0_seq1; 401 156 1121 LEN=1121; translated
MSNGSGGVQCHYDVLGCSQDADTSAIKKCHRKLVLKYHPDKNAGDEDAAEKFILVQRAYE
VLTDPQERKWYDDHRDAILAGWSASEAANSNTADIMLFNVAPYMHPGCYSSYTDSTGGFY
QVYRGVFEHIVACERKQSETIVELPTTFGNSETSWSELSFFYKSWECFSSALNFAWEDTY
NAREDAPSRRVRRLMEEENNKARRTAKKAYKNDILQLVAFVKKRDPRYKAHTEEQERLKK
EREERQKQEKIDRKKEQQKAKEEWREASLREMEKAEEEDRLRGRIRLADLEDDYDYGGGK
KKKRGKKKNRKSMIKEEEKEEE
>comp2362_c0_seq1; 478 1 1769 LEN=1848; translated
XKKSEASRTYETSITQQMTTMLPTTPFSIDNKYKSTKNTKNKNNTRRPHDGPSNGTSGSV
GRPPMRRAAKTGRWQHKQRHYTQVQQAFRQKVVSANQNSICNTTNRRVGRRTLRQLLLPV
DASRTHGSANEDDSEAYIVPIFQRRYCWTTTQWETLWMDVWKRKSVKHSLGRLTCTTVPE
VEPMTTGTQIRYAHQQQTNSNGSNVGFNDNIGASPHDDDVPVRPRSVIIDGQQRFTTVTL
LLAAIRDELLSTGAITEDCGCNDSPGPSTFELKPEEAALVQSIHQMLFLDVPAMREWVET
ESTNHRALEDGTVLDFCRLVPTFCDRSAYLAAILPPSAPQVQRFLAKSYNPTWHRPLLAK
KYFETKIRDQLLENVSNQQNETTKAAFLGQLTRSLLDGIDMLYFPVDVSGGYKDGTEDTQ
VIYERLAIRDATWCKPKRHSEYHSMDGTDMIRNLFLGSMDTSQSKVDFYQNYWLPLERKC
YPNLGEDEDGDESKSISTFFRAFLDHQASILDGVHQKRLTEAVASPNPGLIGGRIYRDFE
LWMGLDLELWQQKQGATKPEPATTEDHTRDVGRRLLEFARDSITEDSLG
>comp2363_c0_seq1; 477 171 1548 LEN=1629; translated
MIYQNVFLATLALASTASNFGLVSAVRGERKYLRSNDQPLEQEQDERSLLFKRGRMGKNK
KDKDKKNNKKPGSSVNLNPQFQIIANPPTAAPAKTTTDLEDWKKACQGTPINNIDLCTLP
MITADATPENCMKCLFAFGSLSTSTAQGSASSCRNNEACGTRCSTDLLKPFFECGKRVDK
AYVDAGRPAVWDASTLNTSQLAEDAPGAQEPQEPQEPQGQGPASEVVIDTVNCPATLPES
GTNCTFVGDANIKSCPYYEYNPVPICTCVRGEDTWVCTNQGSGTLDVEKEDLTTIVTVTN
PLAPPLAPVADVGIGVDLSVNNLAINPNFCGAEAPMSGDACNPSGFEEFTAATTTTPSGP
SSARARVPPTPSRAPGGPSPPAPRCVPQGLRMRMRMRIPIPWRATTPAANNNREALRSAS
SRSFLVLGTEPMTHHDHGAGVLLDIEPNRIDPSTEEMI
>comp2364_c0_seq1; 458 1 1924 LEN=2866; translated
XDNSHPSQHNQKVXSNSFDRTEINLKIHQNTSAMEGANRDGSFFEDVISSDLALRIIQWL
NVADSGRLACTSQRYYYLVHQYRRWRGPEIAITTDLLTLAGIEQPTKTERVLSYSSDEIS
SAMAKDWKKKLQSKPNLILGFSTPSNLVDAIKKDTCFPVSPRTCEAISLNVVAHQRIQVY
QPELGQSGVQSRQPKLDYKSRASLMAMSFPGATIMPYLIRGQPQVEDLEFMEHRLRFHNK
SNDENFWKAMILYAVGSGVQASDQITTRIQALMPSAVIVGGACREGFVSMPTYTKEELSS

MSIKNIKYVLSHCCSNAIHEGVVDRDAAKNFLEKSELVDCIYAALEKERAVTSDRYSSVY
TISGAGILGVFLGGNVPIKSVVSRGVHSVLNNNGPPQPFSDLVVHETALSRPGDDDYLFG
GHDQPPIHLIRKIRDKATGVLYSPIELMVKYFPAAESQIDFLGLKRKGWDGFQLSSLNEM
TQALNAFAILTDGSEPELETLEEAEMDFFCLNGEACMEDMDRTMLKLKEQTKGEEILGAL
MYTCNGRGPEIGYLIPEEMSDAKRFARSFPAVPCLGFYAAGEYGPIALAGNENVFQKGRA
SLQGFTAVFALFIVPVRDLPSTYDWDDSIQNVEQFVRHRLH
>comp2366_c0_seq1; 149 1 495 LEN=700; translated
DILKAKPNRFQSPTKYFAMTSFKILFSFLLLAASARAAASDTIIRDLQKELKPQKFIRRC
MKTGVSEDECEELVEECIKDTETFRMCKAKFKECKTSTDKKGCRKSLVEEFPECEREKKD
KDCKQKIKQCKEQGIPKKDCLANLPDCKPSTVKNACKALFPKVK
>comp2367_c0_seq1; 161 1 387 LEN=387; translated
XKAEKSDKASKAEKKEGKSDKKEKKKKGDDESAAPTSSASPTSSASPTSPTVQPSGISPT
KAPVVTTKPPTTSPTTSPTTRPTTSPTVTSSSGGTSSTTSDGSGGGTAGNSDENTSETSD
SGNAAIAEMP
>comp2368_c0_seq1; 447 475 1695 LEN=2217; translated
MYQVLDVLPGWMATYRHCTEPLGVGCVGAFAYNVEQDGNNEDGTSDDSNTIVENRCPVEA
RQDGGSSCDERLLETTLEESGIRDGNAWRDLCFACTRVGEVAGIHDENGASNGPSNSNDE
HSLLVQTIKVRENDISAGSTPSQNSANRSGDNPRSDAIGDNSSDSSTTKNKKEKIKREID
SNDKEQTIVVETIKIRTADFLEDREPFSTTPDHGDAKNTTIHGKTGTKQNLWPIDPPGHV
GDKKEKQNGFHKLLLFHHKLNINLQNHMRQHKERQRITRGRIRRKDKESWTRNERDQAEE
QMLLLTPRARTEPSCRLSSNQTHPCRLESTAADVDLHMATEPPLKRSHSSPQPTLQRSFR
GSKSHKLSPFRVARSLSFSVMTSPFGKSKSHKTGKTPRFEYLKDTE
>comp2369_c0_seq1; 408 432 1249 LEN=1249; minus strand; translated
MEEGTVVAALSIAPSSMSPPDSSPVGAFVFFELSDFDAFVSVGALDFFTPTDFDALEETA
SVGTAVGDWVTVPPRAPSSTAPSSTAPPSGTWSVDESAVLDLLDFDNFSTAGTFDFLSGR
DFSALDLLNFSPFDLSDLGTFDLSDLSDLDAYDLPALESLVFSAIDLLDLPSLDLSDLPA
LLSRDPITPNEVFSGLLPPRNPRSLLRSCHSLSAFTHEARTTRIKMDNDVRRQLRQMLLG
VEVFIFVLIVSDNVQMEFMKSVELFDVCFVLDG
>comp2370_c0_seq1; 330 191 1033 LEN=1216; translated
MAPKTDTPVPTLLALLVACTVVALAAVRTADAFAATAPALRTAVSSRSALPARPRSKWDD
LVDEDDEKGSGYEGGTFESGIPVPPDMTYVERNVKRCHENFLNLRKVGGKDLCHDVYAKS
PLKKEEVWYVGKVAKITDVSLEDCIARQWNIIETHAANLRPIELYPQRGNLEIWTAPGDT
ELDVAYNRPELVMARMEKYEVLAKDLRNNLIGFQGEVYQEGEEGFRTWRKEDGSPARPEI
NPGGETRPPTEEEMEQLKAEAQRQNISFQDVYKESQKRMD
>comp2371_c0_seq1; 284 258 1079 LEN=1381; translated
MTLAIQVSTKTETPGDSYDESKLFNSSCPSKSGQEENNNEVCKYTKAESSISRLRNTTAS
VADALLKLGGSQDDVEPTLSNPLKRKRSLFVLGERKFTTYVKDAASTVSETDTESTDHFS
KRFRNDGCEQRRAPSDFKLNLPLSLSIPRTNGTGNEVDDALTFSTSEFQLPLQDFNERKC
PGELGPISSTLQDFRPLRARPHMPTFIVPEHPPPPQQNYYSPQLLPSDSPMMTSVVAADE
GCFVLHSYTIVPRQNLHPTPLPLMTNAPYPCYR
>comp2372_c0_seq1; 433 74 1123 LEN=1348; translated
MTKILFPLALIGSLVASLPQLAMAQSDLPEESTGSFSIDQFHESYDGELQSIAQKFLSEY
VPPESANELSPCQEEGAKTIADYCGEITEDSHPIYVWCKAAFETGLMNKYANPLRKYTIF
APSKAGFVNYYTELARTQNVPHETLMPILEYMELEGNIYGPNQLWCKKKYITTTDRIAGH
VKPPKIICLNDINGDPVTFLRGSDKESQKVFTPQFENPGDPIKVCNANIYPMNNMVLYKI
KGSNEGKGRKRRNRRNKNNGVTTAPVSTPIVGVAEVAAAIPAPLAPAAPVPAPVAVVAPI
PSPIAIVAPVPAPVAVEAPVPAPVAVVAPVPSPIAVEEAPVAATDPIQV
>comp2373_c0_seq1; 407 1 1423 LEN=1557; translated
XRSSKVKYNVQQTKQETILLLTTSEGIFSIPFVSHHYRKKLSQPTGINDMKGLYHPITAI
GVCIAAYCCVAKVHEILTFLDFFEKPSVHHDEACVKVMSIGASEDQTQFNSNSIVMTEWR
ATGYHPGEEKPPGALYVSSGFKQGTTAKSAQEFVENNLKIQELPIFNYPENIDFHPHGMY
IRKEDSTLYVINHAYENGGERIDVFGVGTADDGDDDTENDIPNRLDYQYSITSDWMNNDM
HGILNSLIVVEKNKFYVTQYLPIPERHDGWLSFERNIDIERFKTLVFGKRGTYVWYCEYD
DESGSLDCRKVADQFIGANGITHNEDYSKIFVGDYFTINVFDRDASSNELQNRTTIDVPN
FVDNLKFDKVSGNVYGGTINNLWYTVFVHPFPAESEQSTSGIMELAFDPQTSSWQAREII
SSSKLNAISNGLKMGEFYVMGAGGLGYPGLLVCPVIKEAGQSNTGGGKAYDSEL
>comp2374_c0_seq1; 225 262 704 LEN=798; minus strand; translated
MAMGANSSFRILSKAGQEGGAAATTTLWAALDEQVGADAFLHVFIAAGETLHRVTAQALG
STGAKSFGFDDSAVAAKAGNLAALSFLLVGCHAAAPRLGVHRKAACMADTKAIARFFVAK
LHVLAITGRVGTTKATIHASSTVSHDD

>comp2375_c0_seq1; 333 361 1277 LEN=1828; translated
MSXAETAAKHGGEVLLLNRPSERSVSSLEKLRAAVPDGKFVPIDCDLQDFSSVRAAAQTI
KDKGYKSIYCLSNNAGIMAVPDEITKADGFDKQMQTNHLSHFLLTKELFPLIVAASKEHG
DARIVQHSSGARHMTPNNGLEEKYFLKQEKDGMLGGDTENGFMKGGKWYRYHQTKLANSV
FTQCLHDKIQASENADCKNILSLCAHPGGSRTNLADHLPIDGIFMKALLNVVAYTFMQSA
EDGSMGLLKGMMDSRESVRGGTLYGPERFAGWAEPNPPKPYEVDPKAKEMLWSTSEKATG
VTFEI
>comp2375_c0_seq2; 328 1 1064 LEN=1615; translated
XNDNATTSIPNKTSNQNRIMGSKFTKFDAALPSVDGKVFAITGTTSGTGYVAAETAAKHG
GEVLLLNRPSERSVSSLEKLRAAVPDGKFVPIDCDLQDFSSVRAAAQTIKDKGYKSIYCL
SNNAGIMAVPDEITKADGFDKQMQTNHLSHFLLTKELFPLIVAASKEHGDARIVQHSSGA
RHMTPNNGLEEKYFLKQEKDGMLGGDTENGFMKGGKWYRYHQTKLANSVFTQCLHDKIQA
SENADCKNILSLCAHPGGSRTNLADHLPIDGIFMKALLNVVAYTFMQSAEDGSMGLLKGM
MDSRESVRGGTLYGPERFAGWAEPNPPKPYEVDPKAKEMLWSTSEKATGVTFEI
>comp2376_c0_seq1; 320 142 1247 LEN=1282; translated
MDESEPPTPKKTTRVFVLLGQSNMVGMGTVQGADTEGTLQHAVQQKKLYRYLVDAATGEW
KEVVDPSVRNIFTMGSGNDPRDGVRQNELLTVKHSRMIGPEIGIGCVLGRWRRSEAAART
PTPNGSSSSSNRSSHPNDEIETVLLKSCIGNRSLGWDLLPPGSPSFEHTEPSSGKKWTYA
GYGQSPSRWEAGTDPVPDPSWHAGEQYDGDVRRARRVLEGLSGDGENDGAVSVAGFFFWQ
GDKDRYDLAYAGRYKENLARLVRALRKDFGAPGAPFVLATLGQTARDQAGTGADGLIFEA
QTGLQELPEFSGNAFCVYSKPFCHGGASNGHYNHNAETYMDVGLAMGRAMVXPPADDATQ
SPGPTGIL
>comp2378_c0_seq2; 351 1 552 LEN=1267; translated
NNDDGNNAYGGDYGYAENNQENSYEGSYGNNDDGNYQDDGGYNNGDDAATYDGDYGNSNS
AYSNDDNTYYNSNSAYQANVYNGRDQYWDGQSVQMYTDDEEPVIEEEGEGGIKIFGKYAG
LSIASTFSLVFLLLMAAISTYVFLLLARGFNLIDFYHKYLCKRRPANEPKDDSFVKLEDP
VLS
>comp2380_c0_seq1; 209 114 746 LEN=746; translated
MNNFNRLWTILWLLGAASIPTTCTARISHEKQAERADFVRAMDRALGQKKKDDLFRSRLA
QLAVPASSNPRYVRSQDFNAEEPRRLEEYQDYAIDLSEYALKYIGCSNIRTWSDDLAAEN
DGGSDSVLKMNKFVVLRLCPRDSCSNYNQYGCLEQFGDYLIPMETYMQTMAETFFSQYQE
YCQTCYECMNTNYNNDDGGNYNNDDGNNYNN
>comp2381_c0_seq1; 191 452 1286 LEN=1286; translated
MTTRIFLFALAAVATTLPSSVATPIVDFDSSRIVNGVDAEPCQFPYFAQFESGCGGSLIA
PDIVLTSATCKPVNDEGVVVGAYRRGRDNEFGAKKRFCKEFVVHPNSMQRLKYDFALCKL
NKPVFIDESFVRLELSKNVSAPVGGTRTISIGMGWTNNKVDVLRNVTIPTRSNGDCKRDY
SWFDRRTQLCMEYYPTITLPPCNGVSGAPVVERVYQGDGFFVDVQHGLTSDGTYCTHGFN
ARISGEIEWIRSTSCLTLHSIGSMCDTITSPSTSPSISX
>comp2382_c0_seq1; 438 1 2455 LEN=2637; translated
XREECSSRLNSEYTRTQYTRSSTSDDSKSMKAWRASPNSNRLPMKSSCHSSRDSLVFEFL
PEATRELFHRNSIDAMDEDIMSSDGVHLAHVYLSQATSETEDAKNKSEPDQCSEGESKQR
QTQTPDAAKPIDGKIASNHLDENLLQVYRNKLTILTEANTEKERRLEELEKEHNDHNVQH
KEGIYWLRLQLDTTRRQKKNAEKRLAEMKKEFRKLGAGDASTSDEDDLTIGSDWDEKDIL
IAELRERLRKSEAGFGVMERQMATIESSSALVIKSLKEEIANIMEDRTRVELDLLNKLSD
LDTERQRRQLEFALELQDRDETIDALRRGQQASLCTADATSFSGTYSDASWCVHGVSSKS
RSESHADESFSNHQHHRPGGAELHRILEVASHDSEGACAGYGGGNDSGRSGELVWKERHR
IDSSICKMKTVLESTNASISNMEGIVKSFDPNDDAGGENKERETMMSILQSASLISEQVK
LSISLIELKLRNKSESLKSGVTVNEGHQECSSVDEKTIDTIEEIEDNALIELGKVEETLT
NQLKELEERAKQELMNQNPDIKNGSLPSLGDHCHKDTRSNTDDTLVISRGVLHRLEEELV
QFAECMQSKNDKIDELTAEIRKQKKRERDLKKELKAASRGTSSERSSGSKSTREHYRSRS
KSGDTKSTRSKRMPEDRTKDQKVEPKNLTRTRTFSHQPIELPAELQSKKEVLQITSIKLA
PDLLVPPGPIRDDETKGHAGSFSPGGHGCALSVQADFPEAQQEPVGIFLGHQPEARDRRR
NALGHPRGTIEAPLARRDRVMVCGGEGGRSRLCQSPRD
>comp2383_c0_seq1; 127 1 1135 LEN=1371; translated
SNQRKPRVALCASEVFTRPQFLSSGRLISNTAKTKQGRDEDEDQRRFPILHHGFFAALAF
VALTTKILPGGVGSVCGFLLSGDRVKNDSSSNLSSRHYVPVYDNQQCRMHAVHSSSEFNG
NDKQKQEYPQSPQPLQSREAFFGTLRLASLGAIVLSPIENAFAFDGGVGGLGKTKPETGV
ELFDETSAPIQNEKGIVSSEIKGKDGSPILVAFQTPWPLLPTSGALEARDIRSTESAFVQ
VTGLSAARSSDQKWKSKKEFEKVLMESVFAAQGKFGAYGQPYDIQAKKVARADGDESDAK
PTLFSVGFTTLTPAMRESERRALVAATQVGADTLVILVVTTTSAQYASNEKTFKAIASSF

EAIAAPESGLKNKRSSK
>comp2384_c0_seq1; 163 201 317 LEN=1885; translated
MRNEVPDDGATTSTIASDVFRKETKRMTQATTASMSKP
>comp2385_c0_seq1; 445 538 1277 LEN=1277; translated
MAKPVRTSTQRRRPVVETSADGAPSIPNKKSNKVRSKRKQKKALKPMYVVSYSDGGDDEA
NEAKRELYAPFRREFPDTAVRSSPKQKPKHKKDATASDHNRSHKNKGSKHSSLLCKHRNG
VVSSHARMSFAGEVLEGDRHGDNYFASKQGKTSSSAHPRQLGLPLMPTESSSSETISILS
DTVFCVGQNSADANAATAAAAATASPEEIQAFSAILDHMMKEGGAEDAKKKKNNNNNKKK
KDKKKKX
>comp2386_c0_seq1; 241 443 976 LEN=1389; translated
MANALCCVYTHVVSGSGITDVNSAFFEIYDGSTIEIAGCGTSKTSIQLMQDVMDSICDMS
MGSEDCYSQRVCIENAKRTSVPSTVPTPVPSAVPTPPRNNESECEDDEAFLFRGKAGRNC
TWIKKKRETHCSKTSRGKRVSESCPLACDQCSNNDNNSGCKNDSTFLFKGKAKKDCNW
>comp2387_c0_seq1; 475 231 1520 LEN=3255; minus strand; translated
MTVTSSQTRSFYGQRKILSFLQICLTVLLLVLFIAPRNHVQAFSTTSQAFATKPDQSVTF
SRHFAVWASIGESFVDETSNDDTSSSATASSLDWSFLDSVCLIHCPNGDVDGNRIQSTRN
ILNTINLLDSTTIKEFETDDEDRVRGCYTSHVSVFRDVLGANSRGSSVSPGGFDFFNVFQ
KVNARSIDSKNDGKDLNVLIFEDNLSLGNRILTQDTIDAVSDFIENKNSKWDVIHLAYIP
YVPDLKVSKTNEERIVKLSTSNSQSALGTTAYIINKRAMERIIEEDDQNGYTLSIPDKMA
VLFGDSRYALNPTAFVRAPATKSLVNPQLDDLRSILFRPTVVSIVQQILVVTGLSTTVLL
PLVILLLVLTSISSATSSVRAAIEFANYGTVDGPLLFPIMNSIISLFSLGLIIQGVLLAP
KPEEENDDE
>comp2388_c0_seq1; 414 1 1041 LEN=1203; translated
ARNVQGPKVSLLAMALLCVLLLFRTFSDPFPETQEQPMLRVPVANRLSHDYAITVSKTDP
SIAVSVYKQGDIVSNNIVKHGYWDGGLTEVVSAILDARRQHHPSTGNTQPQTIVDFGANV
GWFSLLAASKGHRSIAVEPMSSNRGALLQSVALNSFDDLIQVVAAALGDGSSDSPSSLCI
QPRVAGHNIGNGQTTTKVDDCGEVVQVRTLGSIIGNRKVDFVKADCEGCEANAIMGMKDI
IAGKSPPCSFAIEWRVETMKELGGNPTEVTKLLFQSGYLFFRQQCDLVTGEGLKLTEIPS
SLVLEKLPTGSMDLVLLHNSKRCFERDGKAFRYVMDRVASFQPVLL
>comp2389_c0_seq1; 331 189 1558 LEN=1643; translated
MSAEKVGKRAGIGFTYLVILVIPLLFSLRDHAIISDWTRTLELNSNVLPSGVSPWNRHGR
PQTQATALLEFDKFSRNSSCVYETEVVDEFCKNTTCDEHEFCAEGEDPGPCDEHEWDTED
EDGEEGCKMLWFAAIYDGGDEVACNEKGTGYKNFYSVALNXATVNAGESLQPVLLLGRLE
LEDDSETTTTTTSTTTTATKTASLSKVGRWAEQKGAWVFNVPRLSFQEIVNRHYPEMPPS
HRQGPWLRLEIPRLIKEHGLMDKAPNMCKRRFLYTDTDVMFPNKLKKANLKRLSDTLSND
DAAIMYGRESSKEAEAVNTGIMLIDVEKFGKEIPGMLAYFNETGQHVAFDQGLVNDYFNA
TDLGRSLLPIHYNWKSYWKLHPSTFEEVKIIHTHGPKPGKGLAEIGSCDISGLPNIPYYF
GFFRTGICCDRGKTAAWFMDSYEKLLAPSEDICGSA
>comp2391_c0_seq1; 1614 1 2334 LEN=2529; translated
EEEEKEEDRSDSNETPLLETVATATKPSNDDAARSFLEEFDVASKTDEISALLEENVALK
TTFSMVATDQSEVTYSDFWTRYYFRIADPDRVAETYGVYYRKELEAFEQRREEAEAAARD
AAANDNLARGISNVTNFFGGVVNRLTQEDDEGGADEAGVDESMDVDDESAPQTSGLGGFF
NSVTGGGVGRPPFVLNTAVSDDEYDDYQAPEEEDDDDSEVELGWDDDDEDLESDDDDEQN
TSATMQFKDGDDLSETVDFKDAEKEGLLDQLEQARAERDALQKTVAMQTEELKKARTADI
AAISPPAAVLEGGDKDEEVQKLSIQLFEKDSELAALRAKLEDDKNEAKDQQQQQETLEKL
LQSIAEKDAELEALRRSTDERIEMLQNTVDQKTTEQERLRTEWDSEKEALLLEKEANRQD
TSVVTAEKADDAEMLALRSKIAEQEQKIEELVASKDAAEKANSVEIVALRSKVAEQEQKI
EELVESTTAQEADGVEMASLRSKVAEQEQKIEDLVMSKATAQEADGAEMAALRSKVDEQE
KKIEELAASKAAATEELQHQTEQSAQEQEALRTTLQLKIEGLEQRLLVATNQEESDVAAM
EALISEKEATNVKLQTALAECQSKLAKVTERNSQDQAALRADLEAQIDTLIDQSSKRDDE
YKQTKKQHTLEVSELGEQLASSRAVLDLLKVSNIAEATNLLASLEANRSELERSKAELSA
ALTAPASPGSSSTGVQVRLPSGDDGEPVSMPSASPNEPGNNADADDDDDDGWGDDW
>comp2392_c0_seq1; 305 1 1183 LEN=1390; translated
XEELRRKRESEDNLQKLLKDAQSRFSTLHDQNEDISARNRELEGQIRKQAGSDHGQQSDA
DTTRTTQLTETISLQDEEIRRLRSELDRARPLSSPSSHAGSSRAASSLERELEAARGDLA
QKEHANTVLNKSLKEALGLLKPLQMHLEAAEMEKNETSKELRNLRKRFRQLQMGEMVDDQ
SRSTVGGRAIDGGVELAKIKEELEETVRQLELENSELHDALDDLTEGGNKQNNEAKTRHR
LVELNSLYEVTQNQLEDARVENHALLKALRQKEAEEIERRAEVVRLGEQLHKTESELTNA
KKIAQSALVKVEELTMSNXRAALDPPGLARECTPDRPLEPARGVLLLAAGPGFPGNQFAT
HERQAEKARHTGTGKNANFFGWNSLVVVCHANQT

>comp2393_c0_seq1; 590 58 2134 LEN=2322; translated
MEKATATKINTISVAEDGENKEIEVFSLNLRGATGRIELADGSGIFRSEGTELFDSXEDY
SKCNVPDSTFITTVHLSSLGASIVNFLSNADENDEVVDIVAGYKDAKTFHDTGNPPFFGG
IVGRVANRIGGGKFSLGKKSYSIFPNNPPNALHGGKVGTSRKIWAPEIIREGSALQFSLQ
SPDGDQGFPGDITIKATYILRPSFSPSGVLLQLDMHAELNNPGEGTVKETPINLANHSYF
NLGDQENGILDHYLQVESDSYTPVDKNFIPTREVRSLDDDGVMDFRQGKRLRDALEKYAI
EKMDLSAQQSQEHLAARNYSSLPIPYGFDHNYVVRKQRGTSLPKIGFLTYGLRRLTIYSD
APGVQIYTANNLGDSDQSSSVDVCKAEYGPWSAVCLETQHFPDSISSAKTASSSTRDFWA
GKCPILTNAEPTYNQTMVMHLEMDHSNAEASYFGSDTNGKKYSSIEEMWRNQDSSTWYTR
AKGWYEDNCPSTIDGVLGGIGHISCKDLEGSREFLKQLKLPSRADGKASVACECGAGIGR
VTKGLLLDFADRCDLVESSSRLLFSAPDHLGDSSSNKCRFFCAELQDWKPGANKYSIVWI
QWALCYLTDFDIVQFLRRCSLSLVEGGWIVLKENTCEDQAFVVDMDDASITRSLEYWLDL
IAKSGLQVKRLTWQNNFPDEIFPVPMIALQK
>comp2393_c0_seq2; 685 58 2016 LEN=2204; translated
MEKATATKINTISVAEDGENKEIEVFSLNLRGATVHLSSLGASIVNFLSNADENDEVVDI
VAGYKDAKTFHDTGNPPFFGGIVGRVANRIGGGKFSLGKKSYSIFPNNPPNALHGGKVGT
SRKIWAPEIIREGSALQFSLQSPDGDQGFPGDITIKATYILRPSFSPSGVLLQLDMHAEL
NNPGEGTVKETPINLANHSYFNLGDQENGILDHYLQVESDSYTPVDKNFIPTREVRSLDD
DGVMDFRQGKRLRDALEKYAIEKMDLSAQQSQEHLAARNYSSLPIPYGFDHNYVVRKQRG
TSLPKIGFLTYGLRRLTIYSDAPGVQIYTANNLGDSDQSSSVDVCKAEYGPWSAVCLETQ
HFPDSISSAKTASSSTRDFWAGKCPILTNAEPTYNQTMVMHLEMDHSNAEASYFGSDTNG
KKYSSIEEMWRNQDSSTWYTRAKGWYEDNCPSTIDGVLGGIGHISCKDLEGSREFLKQLK
LPSRADGKASVACECGAGIGRVTKGLLLDFADRCDLVESSSRLLFSAPDHLGDSSSNKCR
FFCAELQDWKPGANKYSIVWIQWALCYLTDFDIVQFLRRCSLSLVEGGWIVLKENTCEDQ
AFVVDMDDASITRSLEYWLDLIAKSGLQVKRLTWQNNFPDEIFPVPMIALQK
>comp2394_c0_seq1; 211 677 1300 LEN=1522; translated
MSGVDVGKAADEKHDATNVLLNAIEGLAFEETASVNTHGALAKSDSVTVGNTGEIRYENV
WQKMDGKARIGAKKLWDDTFGDAMPCKVKNERLGALCVVAFSGDEVVAVSTVVIEMNSSL
YCKVGWLRCLVKEEFRRRGIFTEIMSRCKIALEEWAIENPLAGLLAMGVMLELKFDGKNL
NNKDPVWPKTGLTLVGYTQRGVQIRIAW
>comp2395_c0_seq1; 479 402 2234 LEN=2464; translated
MNQGRLTINMFSNSVELSPESLERKTSKKIASNCLPPKSPASMNGKKLLSVVTNASSSSS
RRRGPSRRRSSSRRRSRSKSRNCRGNSSSDASCSSENSSSSDTDSLKLLPLSPRITDPSP
IHLTVDTDSDDFSNAEQFSLLSFEKSMKSNSTFSYLDKNSSELADRRNSGCQDNHTQNRR
RQHRRSHRREHSWNSSTPINLNTVDTGTSSKSCLTVPKILRRQRVIVADVASVGNASDTL
VSIVETSNGNGRGRITGRELHETAKVFMNEGEYQEALQMFEAILRAQLARFGCEEHSSVG
AALHNVGVVRLRIGEPLKAEEAFLRALTIRRNVLKSDHLDLAATLAKLGSARTSLGKFDE
GLFDLREALRISRKVLGRNHRNVAQILCHIACLYFEAGELFSAQATFQDALEIYRGVFQT
ESDRDACMAQLTETLCNIGSIQNKRKNFSGAIESFREALYLQRGILGHDHPKVIATLDNL
AYSFSKSKSYNQALICYREMLSAEISRYGSFTLECCDTLAKQVLILGKLKKVDDALQVVN
KYLESIQAKSEQEVSAANDEVAEEVNQMLLDLKTAKKKSSKPKTRRRERSLDCFDRILFE
KGKDKRASEL
>comp2396_c0_seq1; 209 148 1170 LEN=1250; translated
MEGPEANHGGRPKTPQEFLGCVPIGHPDSMMSVESVRKAVEEFRTKPTDVIVATFPKTGT
TLVTWICHLLRTGACPKKSLGFETLYEVVPWPTLSWDIGYDPNVQGSEFNPRVFKSHLRM
ASIYPSCKYVVTIRDPAKTALSFYNFFIAKKVPFLVSPEDGKLLMDVSTFLLDTPFVKGN
HPKRASIWDYYSEYHALMECPSVLMIIYEDFVQEMSSQTRILAKFMDIPEMNKDDADNEN
LIARTVSMSSKEYMAQHMSKFDEPYERAKSLRRAADISQLAPGAKIAVKKHVQKFDERAN
SFLEDQWKATMGTLGYDDYDSFCSTVRTRNKRLFGNRNIQ
>comp2397_c0_seq1; 431 1 820 LEN=1035; translated
XCRHCPDDDAMGGAASATSVFEVKTPVGFLCRHCPDDDAMGTDSLLMTAISQDCAGLCQK
EAAAELEVDFCNKIRSAQNSKYLSSAKSCAIRFDIEEKSSNNKRVSSATNKVATVDSTII
LKGVGTGDATKEEQAILAKAFVSAYNDVHWGANHYLTDAEIPFTAATGSPEGFLCRHCPD
DDSMGGAQQTNTLVLDIVSPVEAFLCRHCPDDDAMGSQFDIQSLNSNPFDKKAIEVAFCK
KIQNSVSDKLAATKSCSIAVESVIAAASNTKKW
>comp2398_c0_seq1; 656 73 2015 LEN=2326; translated
MEAHGNYKPGYDSEYDTAKNETTSDDHHHGSGSVEKEGKNDDYKISSYTKMNCPPSKQMR
QPLSSSSSQRSTRRLPSYNNKIVQESANNCINSRFDHDTFSTAKSMALKFANPCGLDMNQ
YNNNYCPPPRTPSTVGSSFSDDESVETSSSLAQHSSHPKRNNLKSRCSDDNRSGHPFLRR
TVIQRRSSRSSEAYSQFSTEKDEGGYPPLTRGIAPNFDKNDGEDVVIVVGDQEYPLPRTN

EKFVVKYLLSRQERVDDHFSYVDLSTHSPNEFEEVMEFFENNRNTETWDEIHWKNIHIIL
PWFVEFQSLPLMTAVDSFLCHNGLGSGGQGFGCNDNGNTKRNKHISLSNLLILTQIAFRC
GLESTKSHARRLLKQCLLEPRKEYGGSDVLDDSNHTNRPPMEAATEDIELEWTLHDLQIL
AQTLQAYSDLREYLWDTSVIVYLPHDLDISNSTELVSNALFPYLLREGMMQMMIVEGIRI
DSSADLSYSQANESSFNSSVSIGFSYSESTSCSDTTIPTNPAAQRKNLTQEEMRVHLNEI
VMYLDKFRAEKVARIEMHKEQLENSLLANESSFDDIGELSVDGVTNSRSSQDYGSRSDKY
DSSSSGKKIFNVLSGESEGGLNVCMLKNILKAHTTIWDDFVQKELRR
>comp2399_c0_seq1; 125 164 697 LEN=1097; translated
MAGYVTKPAAAVNKALVEFLPELDSDYATYPMKHDRWYEPDETGPKGEPCFIAKGGTSLA
TGSPPIKKDYVYCTTGGNGEGYYSLMCRTSYINLYYKLKSCAPQGTCTACPCFASKATRD
ALDRYDDCKRVVYMRQKCFPKPDDKLASDIVMGIAKDTANMVYHGTQNEQLIVNAVF
>comp2400_c0_seq1; 478 153 1673 LEN=2038; translated
MQTKGDDLDKINIRMKDDGTKSGICTCGGMSSSDMMTTVAMDEKLKTANSSGSGIGENNE
ELGAIHFEGAKKSPVWLESFFVNLMTRRNIRTSKDYKERHHQQLSQHGFLERHQHLLKLL
EVAYNANSDDIDHHKNENHGKCDAMVELCVECIDRVEAALEADTQRMYAEIEVYQKAVHV
AKNKTKSLQKTTEGVVGAGIAKATMGEDPTEDQAHGKNNIAIEEAYRHEISMLEQEAQTR
CDELLHYKNIHKEQTTITRELDLFEEAIQLEQNSLELQSQAFDNRRQILTKTLSEVQNEV
DRLTCVSLPRVLFDLQVDQVRGLHYPLINQLRLAFRPKGDVPAQEIQVAWSQATQLLLVL
GNLLDYPSSDWKLVPLADCAKLIYRKEIFNLEPGDCRSLMAWNALLDQIVKHALSITVGG
GNKVNLNSVSTDIYDQDSTSTSTNAKPPYISSPTMIGNTELARLDPIDHVSWSQVIHRMA
SNLLWLSNRASALAATQVGSIVHCVV
>comp2400_c0_seq2; 482 153 1483 LEN=1592; translated
MQTKGDDLDKINIRMKDDGTKSGICTCGGMSSSDMMTTVAMDEKLKTANSSGSGIGENNE
ELGAIHFEGAKKSPVWLESFFVNLMTRRNIRTSKDYKERHHQQLSQHGFLERHQHLLKLL
EVAYNANSDDIDHHKNENHGKCDAMVELCVECIDRVEAALEADTQRMYAEIEVYQKAVHV
AKNKTKSLQKTTEGVVGAGIAKATMGEDPTEDQAHGKNNIAIEEAYRHEISMLEQEAQTR
CDELLHYKNIHKEQTTITRELDLFEEAIQLEQNSLELQSQAFDNRRQILTKTLSEVQNEV
DRLTCVSLPRVLFDLQVDQVRGLHYPLINQLRLAFRPKGDVPAQEIQVAWSQATQLLLVL
GNLLDYPSSDWKLVPLADCAKLIYRKEIFNLEPGDCRSLMAWNALLDQIVKHALSITVGG
GNXVYPLPWNLINYEMSIFLREG
>comp2401_c0_seq1; 440 1 1643 LEN=1972; translated
XQKVSKVSYSNKLTATRMDLFQEEDWPKEADKMYERIEVLGKGSFGLVWMCERKTKADDE
FDDKYVAVKNIEIKEEKGKVYAQREISILSELKHPNVIRLIREFPIYNNISRLVALQLAR
GPNLQRVVSKRGALGLPLCRLISRELVAAVSYLHGRGVLHRDIKPTNLILENTEIRPVDY
YDYSRDSSIWSDGRDAEEMVERNKWKVMLVDFGFARALTANEIDFYRSEADDKTPDLAKK
SRRHMRNSITLESKGPPTQTKPPSAAVNVDDSDEELALITMAANSMRTNMMDNAAHQPSK
RRTSNVSNLEEIKEATNATNGTPTPTEDRVAPIKSKNRRESITQQKVRSMSALGTKAYAA
PEIKHDLRNKTIEDINKTNAALTECVADYGMIVDAYSVGWTLRVVLTGVPPNSTISKYMR
KYNGQKASAEFTDEVVCCCSFLGSPTDIEEENKTQTKPDFRVRDTDEIPRAATIFISQLT
KKDPEERMSIRQAQLHPWIKGDEQKGEPLYKVPQGDYPSHHGDPVVPLKCAANLTNIVEI
HNINKDQ
>comp2402_c0_seq1; 343 37 681 LEN=946; translated
MSEVQVNAVLSTQSADTVDSTQSRDANGNPIVSKSKARSLRRKRAKQRKKAAMQNGGQGV
EEKKTEPQDKENSGAQQPKGQAGAKKKKRHRKKKNKAQAGDGAKPDAIKNNNGNKVRSSS
AATSKNVEPKVTASTKLSESAKPKVTASTSEITKGTEETSPAKIDDLPVREETIAPVATS
VEIKSDTTQATIYDDDNENNIGEKEDCACACTLM
>comp2403_c0_seq1; 537 73 1578 LEN=1821; translated
MAYFAVAARRIATASSRRAVQSTANRSFSSNGADVLAGSGVYGGLQDQDRIFTNLYGEQD
WRLKDALKRGDYHMTKEIMTMGPDWIVQEIKDSGLRGRGGAGFPSGLKWSFMPKETDGRP
SFLVVNADESEPGTCKDREIMRKDPHKLVEGCLLAGYAMRARAAYIYIRGEYFNEAVVLD
EAIHEAYSAGLIGKNACGSGYDFDIFLHRGAGAYICGEETALIESLEGRQGKPRLKPPFP
AGVGVFGCPSTVTNVETIAVSPTILRRGASWFAGFGNENNRGTKLFGISGHVKNPTVVEE
SMSIPLQELIEKHCGGMRNGWDSLQACIPGGSSTPAMNKELCDEALMEFDHLKSLGTSLG
TAAITMFDDSVDMVAAIRRLAHFYKHESCGQCTPCREGTSWLEDVLIRMEKGDADKREIP
MLEEIARQIEGQTICALGDAAAWPIQGLLRHFKEDIEKRIDDPASFDHDAAFQTSWSGDP
FANDEWVDEFGDGLTYKKATA
>comp2404_c0_seq1; 354 1 714 LEN=839; translated
HRVSTVTAPGTIGTTTSLFHLTSDFLVKTTMADGEEPITSDDVANGTAGDSNASPNETEA
SALEDNIATKGRNSYYFAHKHKAKGPKWDGKAEPKSLSKEDMKALSLEDPTKLLEQSGKA
SSFAYHKSNITSYAFLNEEKQVKLYITLEGIGEQCKDEDIQLDWDESSLSLLIKNYSGTD

STGTEDRTLSFGRLTAKISNAKFKLKPDKIILTLKKEKAGMEWHTINDKGSPDHELV
>comp2405_c0_seq1; 821 113 1990 LEN=2244; translated
MLFRSIALFAALASPAFGFAPVSTPRPSVMALRVATDPIKSDDLVSKEANVKSVRRTKPT
KNPMSPEFEQIRSVPYNEAFPNSTKEHKTVTHEETGHVLNVPFRRVHLEDPDQKYLDLYD
TSGPQGHDPKIGIPKLRKDWVAAREGKFERYTQMHFARQGMITEEMLFVAEREGVEPEFV
RSEIARGRAIICSNKKHLELEPQIIGRMFKVKINANIGNSAITSSIEEEVEKLQWSTLWG
GDTLMDLSTGKHIHETREWIMRNSPIPVGTVPIYQALEKVDGIAEDLNWEVFRETLIEQA
EQGVDYFTIHAGVLLPYVPMTVKRMTGIVSRGGSIHAKWNIFHHKQNFAYEHWDDILDIC
AEYDIALSIGDGLRPGSIYDANDEAQFAELKTQGDLTRRAWEKDVQVMNEGPGHVPLHKI
PENMRNQLDWCNEAPFYTLGPLTTDIAPGYDHITSAIGAANIGALGTAMLCYVTPKEHLG
LPNRDDVKAGIIAYKIAAHAADLAKGLPGAQDRDDALSKARFEFRWNDQFNISLDPVTAR
AFHDETLDSDASKSSHFCSMCGPKFCSMKITDDVRQYAAENGYGVEEEAIAKGMEEMSDM
YKEMGNKLYLEDMDNVVNPLEDLTP
>comp2406_c0_seq1; 140 1 296 LEN=350; translated
XDNDNDNDVGFRHTVSFSVSFSSSLLAGAVDVLHPGGAFGSSPVSGAGVLCSARHNLGVA
ESKNKTMADRQCHAQSRNGNNEPEPEPATASTIQESKE
>comp2407_c0_seq1; 422 54 965 LEN=1137; translated
MVFGRKKKETTPAEGENDLALAAGAPDDASSTGDSVPPPPPEAFAGGKQNTESIEADQYV
VTMPQASGLADEAKEGKDDPDFYKKLGVILLCISTFFVLLGVSIKYGVSRNQRAENSIGD
PLLVESGPAEEGTSEPPRTVDQQSVPWEDTPSDPTGTNTTGVGVDAETAPGAGEKTVVPS
QVGTTGGTLPAPNPFAGCTADGIVLEATCVDGASLVSMDFCLANGGGATDEFWAWVETPD
AYARFVETDWGWLRDEASREVEGLPGGTYVIGLFSDGRQLLPEYPLLVSQEFTVSCPELE
VRN
>comp2407_c0_seq2; 470 54 1004 LEN=1176; translated
MVFGRKKKETTPAEGENDLALAAGAPDDASSTGDSVPPPPPEAFAGGKQNTESIAVADDD
DGDGESIEADQYVVTMPQASGLADEAKEGKDDPDFYKKLGVILLCISTFFVLLGVSIKYG
VSRNQRAENSIGDPLLVESGPAEEGTSEPPRTVDQQSVPWEDTPSDPTGTNTTGVGVDAE
TAPGAGEKTVVPSQVGTTGGTLPAPNPFAGCTADGIVLEATCVDGASLVSMDFCLANGGG
ATDEFWAWVETPDAYARFVETDWGWLRDEASREVEGLPGGTYVIGLFSDGRQLLPEYPLL
VSQEFTVSCPELEVRN
>comp2408_c0_seq1; 174 209 664 LEN=1018; translated
MATKRLRQLLSIQISRSIVCRRAAEFQPAEFANQTFSLSHRAFSDDRSGSSSEKPPPRPQ
SLGNEWRKQQLDKLENRFQGKLLETRTIESEEDLQPMWKEMEGRVTRRKARTLADTGGKS
GRRNIKKTDEDVWLQEGMYGTDDNKDASKKQ
>comp2409_c0_seq1; 427 106 1098 LEN=1418; translated
MAPSRKGRVSAPLLTAGLFLAFVAGAFSFVPQRRWAQGTSTRSSLCMSAESNASSNTKGK
LLVLGGTGFLGQTICRRALLEGYQVTSVSRRGLPPATKDNKSEQQQAGPIGSNSIDYRAG
DARKENTISSILREGGYVGVVHCIGLLLDDASGLGTYNRFVSGSGSVPDENSSYDSITRV
TAFNAIDSTIQYLNDQEGGPPGGRLPFVFTSAAEAGWPDVAGGSQIEKFLAPEWLQRYLV
AKRAVESKLLGTDAQKLLRPIIVRPSLIYSLDKPASYVPVGAFFVGNRLGLPFVDRPVTV
QSLANAVVKSMSKDSTVEGVLRYPEIDALN
>comp2410_c0_seq1; 461 202 1487 LEN=1559; translated
MSTTRSRTRARASISMALAVTLFLSLETSVVAMIRSVESASPLISKAPTTKNIDVDVDVD
VDSDGINDIEVNNSNCKQRGKMLKERFDEHDLLRAYQEIKAEYQEKAFGGSCDATDGSQS
IGKGQEEPPQWRVLATVNPNKGKNTNTEHDDELIVVSMLEHPSDPLCPYVKMETVIPVPV
EKCWNFLSLDRWDETMPRMDPFYEGLDVYGEYAVASGSGGEGDKSDHDAARCQLVRMILA
RKRTKRILAFGKREFVFVSVEDTPLQDGTWVSGTVSVQVDESSLKSPLKRNKSYTRAFQD
SIAFYKPLPSRAATEGKAGGGDADHRSGRTGLTIVCRIDLNDSNGRGGCIPMXALRENDR
GDGGEIRLEHAQVPAGRVCLMLQQSVEAIAVHLTRAAERQSNPPTVCIEEYYTWKMCVRH
NRTNAKKL
>comp2411_c0_seq1; 305 681 1214 LEN=1566; translated
MASKLGLEKPERVAVAVEGCYQNTGIATSVAASMFTGTQLATAIGVPLYYGLVEMIVLAI
FCLGCWKSGWTKAPPDENFFTMIGKSYEVEQARLESPNAIEVVHNNNPKDDGDIEDLVFN
QTIEGYQVDERSLQEKTNAGLMEQRQMNGSPLRARSSAESSRRIDDDTLKAEQGEFT
>comp2411_c0_seq2; 322 130 1317 LEN=1669; translated
MSRATEIGAHFLLFFLVFGMSATVDIGHMRKQLRNWKALMIGLCLQFVILPFVGFCIVKI
LNMDPAIGVSLLVVTSSPGGSYSNWWCSLFNAELALSVTMTGISTLLSVVMLPLNLVIYT
TGSYSDEVVKSLDWFALFLSLIVVLGGITSGVLMSAWYNSTRFNLMANKLGNLAGVSLVI
FSAIVSSTGQDTSIWNQSAVFYIGCAIPAIVGVILATYMASKLGLEKPERVAVAVEGCYQ
NTGIATSVAASMFTGTQLATAIGVPLYYGLVEMIVLAIFCLGCWKSGWTKAPPDENFFTM

IGKSYEVEQARLESPNAIEVVHNNNPKDDGDIEDLVFNQTIEGYQVDERSLQEKTNAGLM
EQRQMNGSPLRARSSAESSRRIDDDTLKAEQGEFT
>comp2412_c0_seq1; 438 162 1511 LEN=1745; translated
MLRKCLAGPCLLLIAVAACAISTGSSSFPATEALSSSGKPKRPIVSLTGSGDVSKFLYGK
LQRATSLYGSGLVGPATAVVHGDKDTRELLPKPVVASSSRDQRKDLDDMLWKQFGMATCE
GIVEREDLLTKMSYLKPRLADLQDTVLFLDATTSGMGKNSAGSGETGLGGLLSGFLGGGS
KPKPNSTPKNKPRSNKKKKSMQSDCWVDVDLVRSAMSRGSRVFVVCESSDTTRCAKALNK
CISAMDGDNSIVTLVSPEDGTILGGNEGEDTAWKSLRPQDLEGELLQAVSVRRDPVAERR
GGGPSGDNGSRARLAAASADYIATDSAKGLPLSRQDLAEVCVQCALRLPTTPPAPTEEPG
TDVDADTGSAPTGTPTLRVVRVIPRGTERAARDGIDWTERPNKNYFAMMGGKKAKAREGT
VASADWARTLEPLVDGGSIRVFPKRPSEM
>comp2413_c0_seq1; 241 114 1136 LEN=1309; translated
MDQDNDDNEKESKINRQSRGFLQNALILVATLTVVGLIMGYIHTHDPPQLSKNIQHLVEK
WEDNLVAHESAAQGQLRTPPIATKETPPEKTSKQTEFDLTFQVEELDQKVRARKSTEGVL
METDPEGLRLTKKLQAATLKLLKHRYGYGTKHERFRVRVDLVYPEVIVGEGESKKAFFVI
EMAPPSLIPCSVFYFMEMVRTYKSGSFHRNADHVLQINAQTEATAGHKSMPFQEYSPEHP
HKKYTTGYAGRPSGPGWYVSIKDNTKNHGPGSQQKHNPYEADANFGRVVEGIKEGVIDKM
HSVPQKGWLDKDDCIIIPKMTILVQAPDDELEWIEWTPDL
>comp2414_c0_seq1; 322 214 996 LEN=1093; translated
MKIPFQKLPEEREPITESVHVSKIRIVHKRPSTTSRRNSDTGPKRASHRSYARTKDTEVT
TTPKRASHLLCERTKDVEAASVETKSTLHRSQGNTKTIQPPSPTCVKEFARQRIVVEEEK
QGEIFEEEKHDEICEEEEANVSDGWASSHCTRYACVESFPDFFSDTEFLSSVQGNSRMKG
FKDFARVVNMKVEGFQEQYHVNHHALEKIFTLRHFCHNLPCTDLNACSPSAVDDATDAKD
ISMTVEFRSKNTPTIERIEI
>comp2414_c0_seq2; 343 94 945 LEN=1042; translated
MRFSFRQATDRAKEFCNEMQLRRMKIPFQKLPEEREPITESVHVSKIRIVHKRPSTTSRR
NSDTGPKRASHRSYARTKDTEVTTTPKRASHLLCERTKDVEAASVETKSTLHRSQGNTKT
IQPPSPTCVKEFARQRIVVEEEKQGEIFEEEKHDEICEEEEANVSDGWASSHCTRYACVE
SFPDFFSDTEFLSSVQGNSRMKGFKDFARVVNMKVEGFQEQYHVNHHALEKIFTLRHFCH
NLPCTDLNACSPSAVDDATDAKDISMTVEFRSKNTPTIERIEI
>comp2416_c0_seq1; 607 254 1639 LEN=2000; translated
MVQTIYSNAKVSRKCPHRRDPRNTNAAMEPNNKIGSRRISKRRRSAMARRAFSLSEYANG
IGTAVANLSVAFFLVILLLAAPSFVRATTLPFGRITKNAPQQASQSPLSSLKQPAFHMRA
GSTAAAAGFVTPTLELMPLSYYLRIALAGGLAGATGSAVLYPIDSAKTLRQSNPTAYSSV
RDALWTMLKSKNALRRVYRGILPAALGAIPSSALYFGAYESSKVTLKRLVFQKNHKNVDS
DDYRARLFVHGLAAASGNVISSFVFVPKEVIKQQLQIRASEATTGTVQAIGSIVRQNGIS
GLYAGYGATLLRNIPSATIRFVLYEELKRKWATAPSGESGSSPQTQKLRAAGTFLAGAVA
GATSSFAMTPIDVVKTRMATGSCPLGVRSCMLHVVGEVGWKGLYAGAGSRVLWSGAFSAI
GFGTFELVKGYLGVSSTSSKLSPSTSTKPTPFTTAKTSSSL
>comp2417_c0_seq1; 688 1 1917 LEN=2240; translated
ATRRSEFPSNDTEREIQQRYGKVMGSAVNPVLREGNSDRRVAPPVKAYAQKNPHRMGIWS
KASRTHVAHMTQGDFYGSEQSTIIGDKPTSVRIEFIPKNGSASRILKEDIPLEASEVIDA
SCMSAEALTAFLADEIEDAKDSDILFSIHLKATMMKVSDPIMFGHCIKVFFKDVFDKHGD
ILKEIGADPKQGLASILEAVENKVDDKKKKKEIMLDFKGSYGTEDRPWLAMVNSDKGITN
LHAPNDIIIDASMPVVIRDSGMMWNEDGELEDCKCVIPDRCYATLYQEVINYVKTHGQFD
VATMGNVSNVGLMARKAEEYGSHDKTFELEAGHVAVTDNNTGEVYFQHIVGDGDIWRMCQ
TKDEPVRDWVRLAIERGRATNDRVIFWLDKKRAHDRSLIDKVNTYLKDHDLSGLDISIMK
PVDAIRVTMERATLGKNTISVTGNVLRDYLTDLFPILELGTSAKMLSIVPLLAGGGLYET
GAGGSAPKHVQQFVEEGHLRWDSLGEYLALAVAFEQLGSTTDNGKAVLLGECLNKAVGRI
LNNRKSPSRKVNQIDNRATNFYVALYWADYLAQEDPTYQPMFDALSNARATIVEEFKECQ
GDPVELGGYYLVDPKKVSKAMNPSPTLNKILASIGETL
>comp2417_c0_seq2; 901 374 2821 LEN=3144; translated
MMHRAARAARSLSLANTRRPHTFAASKTAALSRMATKSFSTTRFAASDAASASDLDIPSA
PTSNHSHTALPGAQASIIYTETDEAPALATYSLFPVVNKIGALADIDIVPCDISLSGRVL
ALFPEKLEPNQRVPDNLAYLGELAKVPEANIIKLPNISAPLNQLEDCISELRSKGYDVPI
YPREPSNDTEREIQQRYGKVMGSAVNPVLREGNSDRRVAPPVKAYAQKNPHRMGIWSKAS
RTHVAHMTQGDFYGSEQSTIIGDKPTSVRIEFIPKNGSASRILKEDIPLEASEVIDASCM
SAEALTAFLADEIEDAKDSDILFSIHLKATMMKVSDPIMFGHCIKVFFKDVFDKHGDILK
EIGADPKQGLASILEAVENKVDDKKKKKEIMLDFKGSYGTEDRPWLAMVNSDKGITNLHA
PNDIIIDASMPVVIRDSGMMWNEDGELEDCKCVIPDRCYATLYQEVINYVKTHGQFDVAT

MGNVSNVGLMARKAEEYGSHDKTFELEAGHVAVTDNNTGEVYFQHIVGDGDIWRMCQTKD
EPVRDWVRLAIERGRATNDRVIFWLDKKRAHDRSLIDKVNTYLKDHDLSGLDISIMKPVD
AIRVTMERATLGKNTISVTGNVLRDYLTDLFPILELGTSAKMLSIVPLLAGGGLYETGAG
GSAPKHVQQFVEEGHLRWDSLGEYLALAVAFEQLGSTTDNGKAVLLGECLNKAVGRILNN
RKSPSRKVNQIDNRATNFYVALYWADYLAQEDPTYQPMFDALSNARATIVEEFKECQGDP
VELGGYYLVDPKKVSKAMNPSPTLNKILASIGETL
>comp2418_c0_seq1; 350 203 883 LEN=980; translated
MTILYALVSRQQTVLAENTNASTTGNFPTVTRVLLEKIPPQDGRMTYVYDEYVFHYVVEN
GICYLCMSDEPSKHRIPFAFLEDVKQRFLQQFGLEQAQQAIAFSMNEGFKAVLGERMDFY
GTEEADRSIDNIGTVKSQIDEVRDTMVQNIERVLERGEKIELLVDKTDRLNQQAFRFESN
SRSLRRTLYWRQMRTRAFLGAAVLAVLCLATVSACGFGLQRCRSSS
>comp2420_c0_seq1; 316 255 1073 LEN=1378; translated
MASNNGQTPGTTFPVMPLSIPKDIAQNEAALSSSLPSSSSSTTTISKPGVPGTPKSIYEM
IMSNPPPTSANYTPQAAMHRPVEYYSETNRVDRGRGYNKDYALDKLTRKSLPYFMPWKTS
GGPTEIPPNYAWLMDGCAGKAAIGVFGGGVMGLLMGVFLGALSDSQPPIQVAGGKDVPQA
PFKEQARYTMRATAEKSMYWCRNFAFITGVFGGSECLVEKFRGKHDMWNPVVSGCITGAA
LQAHAGPQAAAVGCGGFAAFSIVIDKIMGNYD
>comp2421_c0_seq1; 535 62 1186 LEN=1309; translated
MDSEAVSSHEDNPPSRVKNHNKYRKDKPWDNDQIDHWKNQPWNEEDGDQLVGGRLLEESC
FATLFPKYRENYLRQVWPVVTKALDGYRIACELNLVEGSMTVRTTSKTSDPYIILKARDL
LKLLSRSIPVAQALKILNDEWVCDIVKIGGLVRNKERFVKRRQRLLGQEGSTLKALELVT
GCFILVQGNTVSIMGQTYHGLKQARNVVVDCMNNIHPIYHIKRLMIMKELAKDEKLKNED
WERFLPKFAKKNVKRKKPTKKKTPKAYTPFPPAQQPRKVDLQLETGEYFVSEEQRKSQKL
AAKKEQAKLISLEKRKRRMEEDEIAPARKTDNKRKKMASTENANVDVQAFKSKIKSRSKE
VNPKDSKISDFVKD
>comp2422_c0_seq1; 1482 215 3532 LEN=3709; translated
MEDSDDARDMAIKEARLEQMLSEKLMQGYVLMESTCPKCSTPLVKNHQMVPRSLQCPSSD
QNDTRRVDKCVLLPQESFDQPFKPVEGVPVCVQCNSHVITQETEVSILEQCDSMKDKGSI
YVALEEITDDKPEIIHLDGAFEVSVSPSKERSNNTFRDGDNVTEQKHESPTRQMVDIETT
FDDGTTNYEITLSPREGEEGKPIVLDGDETKPIDLVEEDPLGSYAERRELATKVLGAKML
QGYTLQETTCEKCSMPVMSYKGVVKCVVCPALAKRAKKKLRQKQKHQEAALGGQQHEHEE
PSDVSEDLQHRDEQLDRIKTVRKEIEEKTHREEEADAKRAEEQQKAINHEKARILDNMED
DSKDHAVSQSGPTEDPQIPMSDEQAAELARLGAIAKEEQRKRKLAKEQRQKEEEAVELEA
QKKALQEIETKALAEELQRIEGMDKNTLQNDKALAMKKDLMVEEYRKRMLGKAALDNEVA
RLEEERLNEEMEARRLADEQRAESESRMIAALEADAAMKALAAEDAIRKAKEALAEVQST
KKQIISQTIEIAEKEVIAEAEQTIMANREDYTEPVILPTDSEIESERWETLRMEGRAIMT
RRILQGWEILPEACKGSECFMSPLIGRLGRKECCVCGGNGSGTDGAYSKQEVAEQQQSEA
ELAPPPSVKLETSDDECKGKDWYDMMDFEKKRAIYSKEIGKRMLLGWVLVDATCPKCGMP
MMMDDLGNTDICVVHGEMNKHFDASTIATKDMAALETAGEVSVEEVPEETGEQKKHVESG
SDGTSPELVVKRTDSDLCSTIQKQAKKQQTKRKPVTTSDPPAFKPQARVSVETARQISQA
SALVESARQIAGQPSQKVNVKDVIPIDTNDFADSSAFEKVTGAQGREETKPIDISIEMVA
NMFLKSPHGYDFQDFGHSMNVDEIKELVDIFLVTNVDNSVSDDFKLSVAQRILSKMNLDT
AKSRDDSLPLRIEVDEQQFNFNDIESDYTATKSTGKFGKRAKPRPEDQARGLPPVSPTKR
TTPRGGLNYVGSPTYRRSRSDDMSVASRASTVASDALESIYDRIDACKEKLLDPNNTLDE
QIATASLLERLAQAAVAVKEMEYVE
>comp2423_c0_seq1; 651 198 2411 LEN=2450; translated
MTIAGRFVPFVRSTKAFKSLSSSVTSRFNSNTGGRNRLFGTVARKSGTSVFHQLVSFSRR
ALSSSATVIHPLDPLLPDEITRASDAVRKHLGLTPDNAVQNLRFVSISLLEPPKADYIQG
VKTPRKAECITLNPQTGIASNFTVTLENNEAKIVESTEYPKGTQPLLTPEDCDLAEAIVQ
SSPEVARALRDRYGIIDSSRVACDPWSVHLASPADQDLVNWRDDGVPGRLVQTFLYHRQY
GDGLEDNHYAHPIDIVPVVDLNARKVVTIHGMERSPAPEIPTSSVQYHRDLVSINSYLET
EWRKDKLAPLDVLQPEGPSFTVTGNQVEWSKWKFRVGFNSREGLVLHEVEYDGRPIMHRG
SLVEMAVPYADPNEHFARKCAFDVGDYGLGFCANSLSLGCDCLGHIHYFDAILNDSQGNP
VELKNAVCMHEEDTGVLWKHVEYRNGHNESRRSRELIISSIATVVNYEYLFYWHLRQDGC
IDFKIKLSGELSTNLLSDGESVPSHGVMVAPNVNAQIHQHMFCARLDMSVDSHKNSISEV
DMYSEPLSDTNPYGNCFNVKETILEDEESAKRVYDANKARSWKIFNAEGKTNGMTGKPVA
YKLLPFSMGPSQPILLTDPSCAVTKKGEFATKHLWVTPHSDKERYPAGECTVLGDGSDGL
PDWTKANRNLQGEDLVLWHAFGVAHVPRTEDFPVMPCETTGFTLKPDGFFDGNPGIDLEP
TVNKASELAEGCCPNTK
>comp2424_c0_seq1; 363 164 814 LEN=1033; translated

MDRIFGKKKQAAAAPSLSDTSTNLGGRVDEMDKKIAGLEQELRVYKDKIKKARTPAAKNA
VQKRAMEVLKRKKMYEGQRDMVAGQQFNIDQASFGIESAKANVQTIAAMKGANQELKRTM
KKDLNIDDVDDLADDMAEMMDEFNEINEALGRNFATPEDIDEADLDAELEMLEDEMFEEE
AEAAEATPAYLQTNEMPDTPTTELPQAELGLPAVPN
>comp2425_c0_seq1; 207 112 725 LEN=774; translated
MIDAIHAGLDYLIANKDYLLLFCFSVDAVNTLISFPLIVWKGPKWLLSSTEPGAKEKLED
GDAGTEVVDLSDTDRKSFRVLWEIFTICYEGYFGFTASTLACLYTAPETRPLFAYPLFAL
YVYKAKAYMTTLSTDSAQARAKLGSILFFYWPCYGGYCLLHSIEWARGRAXRAGSGARAP
RRFGNTTTYNSDGAKQGMHKRARG
>comp2426_c0_seq1; 320 1 1619 LEN=2132; translated
XPNEVANDQATCCHPCLYDIASWPFLRNKSPIAHQRNEMSLSATIGTVSSSASPRKVAII
GGGAAGLVTARVLKRNGIESTIFEKDSNDTGVGGVWSYKQNSDTRPMYRGLRTNLPRELM
AYREFPWGGDGKTRSFVTHREVKDYLSRYSKVMGVEDTIRYDSIVTQLEILEIDEKKMEN
DEGNEQYSWPQIKVRWESVLDGINAGDRNSQSEVFDAVCVCNGHYSAPANPIISGMENFE
GNIIHSITYDDPSVFKDQTVLCVGGRASGSDLAREISHFAKHVYLSDTTCPALVDGKPII
EENVSWVPRTTAVLPNSRISFGPTCGETPKVDTIILCSGYDYEFPFINDNSNLDLSVVPG
ERRVKPLYEQLWHAQYPSLAFIGLQHSVVPFPFFELQAEAIASQLVGDSSSRSWTLPPMK
ERMECAEKDATSGGPKAPGRVQDTHYLGSYQWEECLKYARFAGVCDEDLKKYIMTNKAIY
DHSGEARKNLFPGGPDTYRYISYVRDDNNETFTAVSLHHEMDLEENLHPAHSDSSVKAS
>comp2427_c0_seq1; 372 98 966 LEN=1014; translated
MDDDADSFSAGDGCWQLFTNYGHTTSDADLSDNSGGGLYPHSRSPSQDDSSVFSGDTTST
TKSSHDVISTSSKNSLSLGTSLSDDALYYYNNAPFDNKIQELLLDLDLSDSSDLEQDELE
HLLHDSSVLMSPTTSTSSSKILPAGTHMSLDGDEKAHSNNDHEEECNNNVTQHQSPCVRN
FVQFFEQVQDTTSPPKRRGDQLCIGVLLILLEVSFMIISNNEELFGGFHLRMSRLEQSNT
VNKACISGVMVTWNGKKSLGDDAEEWSPPPLSSIHHLEEVQPQVPPML
>comp2428_c0_seq1; 549 1 1224 LEN=1240; translated
PKVDEATKEPNPDDDDNAVKLDKVGKNSTEDDDQYTKNDTDNNKTDDDETKPQILVETYG
SSSRNKNETLPFAARNTTNGAEVSNVTDVAIENSTMTSLYSLNGTDSILSLSAGTNNSSI
GNTSTLDSGILPTNTTKPSTDSSDKDIPTNSSAVANTTAPSTFAGENVAILAEDFNATAS
NASFLANDTVTTEGTTETIDANANTTLKRMNAPVIVIPDSDGNDGHSLSNNTNTSKTVSG
SFESSNNVTIGGGSSGSFSNSTVPLEIPTNDIRTVATEGKMATEKKKKGKSRARESDSTS
DTSAMTSSTEGGGASEAKETSESTESESKGETTGQTNLSGKTTSSSKKKKKSKSGTMKND
GSESRSANPVTSAVGTGGGDKKKKSKSRKGSKTQSIKAEELGGSIKK
>comp2429_c0_seq1; 312 275 757 LEN=1168; translated
MRIVVQKVKSASVTVNGSIVSSIGPGAMALVGIHENDTDEDLEYCAKKLLGCKLWENDAG
ALWRHGVKQRNLELLCVSQFTLYGKLTKKNQPDYKASMKAIPAKAMYEDFLGKLKSSYEP
EKIQDGVFGAMMDVELVNDGPVTIVIDSDAVSSSKTPPSD
>comp2430_c0_seq1; 514 1 1298 LEN=1711; translated
XHQKRNSTDQHRIESMNKNRSLPLFLSTATAYAFGFLLWMTATTIKECGGTVLALALATP
PTTIPVAVLGATGRLGKETVGALLERGIPVRALVRPSSVEKLKQQQNQNELPGDGIGILD
HPQVEIIEGELLDTPASGTYSDDTVLPSEELVECLTGCSVCIACYGATRKTKPSELLSWV
LSTKSNDDEPDPSEDTDPIHPKQINYRSMLALGKACERINSSNNNNKDTTRIEHIVRITG
KGEDPTGIFSVLLNGLGSFCKLWNYQGETVLRSKAFGGPNSGTNENEGSVGYTIVRPGVM
VEDLKEDDLKPGQRRYLELKGNGGNDLPVTPVTRPQIAGLLAELAVGEAAGAGRRRRVTL
AAMNVVAEDEAGASGSDLRDRIRGLASDSRSFPDSLVHEHKSATKRFFRRLAAAVAAVVV
AAVSLLGKVVLG
>comp2431_c0_seq1; 395 166 1188 LEN=1235; translated
MNESSPDPSPDTTDDAVLLERAEEFYGEERLLKAAEVLKQVKDKDNCLTDRHKRILRWAD
LIESGIDNLLQDPERDGSPWIKQREQHGHRDFLVFYQLTDNNKLIARIDCAFESSLLVPL
LSVFNESELFSSWMPCWERPVRLGISRSEKLKDEGRGKQIIRVTTSLAWPFADREVVFRV
MAADAIEEDRSCIAIQAVSQTSDDDPVIPKATREAVRVDFSNFMLIQACPPDHPCVARSK
HHSNDKDHEPLILMNLTLEVDPHLGVVPQSVQNFVTRTVIGRLWLALLHVAEDVRDGKRP
RHKEAIDAKPELYDWIKERVGVMIENLGKEKEKLGCEPSM
>comp2432_c0_seq1; 256 33 948 LEN=1292; translated
MDRRRQQKIQSVLLDSCAAGAVAMLYHKQPEFFNPWNLSPAHWVLGGLAIRLAINFYDHL
VVYAVDSLFGHKYLPTRTTGTPVRYVELDAKSVVFLSINAVHEWIFVQRLCHYLWHSQEV
SLDPADLGLLNTVGALWVMFVVLDLCYAPCHHLLHTPALYPLVHKHHHRQHYPTRGYLDA
GNEHPIEHVIGVLCTWTAVLAAVRLCGAHSLVVFLFFNVHAALAMLNHSPYDVEFDIIPF
GILTYSVANHEMHHRKFTVNYAQYSMWYDHLVKTFAGYEGPAVAKAVTKQLYTAGTEAGQ
RVRV

>comp2433_c0_seq1; 692 77 1189 LEN=1455; translated
MAVAMACSTEAFIQKGGVASNVMMLGKKSGGKIATAPKGAVKKSRGVKLSASKAADSEGK
SGLGETLQVGTYFALWYLFNIAYNIYNKQALNVLDYPWTIATIQMATGIFYFVPMWLTGL
RKAPKLSFSELKTLFPIALCHTGVHVGAVIALGAGAVSFAHIVKASEPVVTCLMNYLFLG
QVLPLPVYMTLLPIIGGVAIASMKELSFTVLALASAMLSNVSSSARGVLSKKTMSGKSIG
ENLDAQNLYAVLTAMSTLVLIPMMLAIEGTGFFSAFKNVVSAGDFTNKSLAALMALGGAS
YYAYNEVAFLALGKVNPVTHAVGNTIKRVVIIVASVVAFKTPMSTGSIIGSSIAIAGTLL
YSLAMNASKK
>comp2434_c0_seq1; 505 76 1289 LEN=1289; translated
MSPKDETTIPVVIVESHQHVLEHVHDVLRKKKLFDQPWSMVHFDAHPDLACTKSAPAIAC
FAPRQYYGMGNRNNKEPGDNATGNDLDGEADGEEERNLYELLDLTPSGIAEWIMPLVLAA
NLNRIEWIKPFFSKQIAEGFYRFEVGAEYQRNNNVDGNVDDGGTGGDDSSHRDEINSFLD
LPRDAKLKVNFDHPYYLDDLSVVPSEDLVLKQTLELKVSELQRRLPIEQQNDTSVFCGSD
KSGTRSSTNGSATKDDPRLWILDICLDYFACQNPFLSDLETKNESATRAFLDVMEGSIFN
HCTDEDKDGNSVVEISNNQPATNASFVYQTELVCFYKLLKELLLDLCGQECTERKYQEIS
HSKSGNSLVFDQISKYFGGKERAKVLIGTLVDEINANDDGRDDGR
>comp2435_c0_seq1; 133 79 1016 LEN=1664; translated
MLLSSNSVYVDGAILTFGMQFTGFVVAAVLQTETFYDVLGGINFLALAVLAFVSGQGTID
AGRNNVFVVLFAVSRGWLLLFLAWRAHHRKGDSRFDGVRNVPSTFFVYWMVQAIWVYCIS
LPLLAVAEHAHDHQPDAAADTGGVDGYSLLLVAMAMSIVAEIHSDVAKAXMGGEGPARRI
LSGGPLETLEAPELCRRDPDVGVCGGVRNLCLWLVLEGGHRCIGLAALYGADPAEYLGDG
RLERRGEKPAALLRRRNDRRELRGVPKDDTPTVSDTVRPLRESPAGVPAPDLLRMESLRV
QETVERGKRAMR
>comp2436_c0_seq1; 695 187 1869 LEN=1996; translated
MSSNRSLSENNGGAIPITRDDDPEAQGRVIDTMEEGRMSASLELEPRRESLLREDSVDRS
SSNRSSANSSRDDEGRARAVTMIGSVANLCSATLGAGILALPFAFYQAGIICGVLLLLIS
ALATSSSIGLLVEACDRYNLSTYEKIVEKGLGRNARKLVEISILVFCCGTAVGYVIAVGD
IMERVVPSLTTSQKQLSMSLVWCVAMLPLSCLKTMRSLECASSVGILSIGTLLAAAVVHL
VRGDGSGGRGNDYGYSDDETLLESFSSEPASIASLLGPAGGSWVSVLQACPIFFYAFSCQ
VNVAQIFDELPAVRGGEKLPTMNRVTSTGVLVCGLLYASVSLVTLVDFGASVEPNVLSNY
DLGRGAQPLLHAAFLGMALAVVMAFPLNIFPARVSLIQMWEETTKENNNASLSSSLPPEF
EAVRQPLLSEEHRANGGGLDYNSDGESSGAAGSGEDPLHDETGSHDDVDEPGGFRPVRHA
FVTLLLASLALGLALVVPNISVVFGLLGGSTSSLLGFVVPGLLGLKLDHTRVGAWVLVVC
GSMIGILTTGVTIYSMVHAS
>comp2437_c0_seq1; 396 213 1414 LEN=1673; translated
MGLMVSLMAKAVATGAAAGFASHKLVEGLSPGLPPPARAALALLSSLAGTAGYLRAWIQD
YWVDRSYRGDKDLTGTTAVVTGGTVGGLGFAAAELLYELGATVVVTVRTAEKGRAAVEGL
QGKACDTETEGRAFYVLCDFLSEDSVRACAAELRAKCGGGGIDFLVLNAGISGGGTGGSP
QAKPEAERDADAARVWMTNHVGPFVFANELMPLLVQAARKDPARHSPRAVWVSSGAHKRA
SIDWEDPFHPSFAASGMRVYAQSKLANILHAREYQKRVRECLNEGSKKEGGPDVRCFALT
PGAVWTGILGATNRPLLYPLLWATMRTPRTGAQVIKMACLDXGPGGGEYLSNCYAKETEG
KNGCSNDEAQWKKLWELTEGQVAAKAYADPSFFGAREKSE
>comp2438_c0_seq1; 112 174 398 LEN=398; translated
MKFSSSQTFIATATTMFSIATRRTAGFVHKNAVAKTATRAYSGSAANLAKDGQAEVILVG
CGAPNRGMGWYHAVQ
>comp2439_c0_seq1; 536 1 1340 LEN=1340; translated
XGNGNGNGSVGLPNDAQTHQKARRRSSFSSVMSALGSETKRLSCMKKRREKQAKHHQEGK
DEDRGICNGNVDNDNDKKEKKKTPTKDKDKDKDKRKKKREKKTEENKPRISENEGVSCSS
SCSSSSTAELPQDARVPNQQPTFVVSDPHGLVFHDKDRDKEKDRDDHETKNKSKKKKKEK
KKKKKTPKKKDGKRTQQGTNEKTGPPEEASACESHKHSCSHSCSHSCSHSQRRLPSDPSS
LGLDGAVSPADLVRRLRSIRSIHSTMSGSLALHSHHEGSLSVSRTDSRTGHSYNYGYNHS
GTESRSSCSSSCDSSCDSSTQSTTSHHSLENRENHNLFPVATKEGISQGTSEGTSSLSSF
RSHQQPLQMSQSSLFSSPPPLRFRRATRRSSLGSKSSATRMTVCSTIEETFSHGQEERLP
RPGSRRSLTPPRRRRFSHPQQQQQQQQ
>comp2440_c0_seq1; 215 1041 2404 LEN=2666; translated
MSSHSAGSSSSGGGGDPRQAQSTSSNNGNGNGNPRDPSGFLNTDEYGRPSESSAPTVVKI
VGTARDDLNGLLGFCTAFNRDRERYMVRMASVDPGNSPNATVMALKPANLTKASGLETYR
AMFQQLKTDPRVKRKLSEYYQQADTLCRGLPLPAGTKPEHAAGALLCVVVGLVYLLGLTR
VIMLSACLVMVGCILADDVLRHRKPWRVVLQNLPSRSNAVLSRQFPFLRGRLSDPVAAGL
VGLLVLWTLQSVLSRSPAPAPRSGTTGGVGSRSGRDLSASYASVNRDVLEEFYNLGFKDS

AEGRERGHSFAEEVEQLLGSSSSSSNIGDDGDIPYXVFGNTAPAADSTVRPGVPSKNLFP
ETGEHAHDGVFVLSVPHGHADRSRSLDGDFFRCPALFEHSALDATVAKGDASFFVLQPGQ
QPSVLEGSMEKMEFLQMVLLRCMESICDSRDDPK
>comp2441_c0_seq1; 590 100 1311 LEN=1494; translated
MSDDEYSAASEEVEEEEVTDLSNSDVCTKYQEASKIVNLTLEGLVAQTVVGAKVLDLCKF
GTTVMETAASKLYTKKVNGVAIDRGVAFPVCVSVNDIVCNCSPLNDPEPESIAMTPLKAG
DVVKFDLGCHLDGYIAVAAHTVVVKETADSPAPPEDEELAKCAVATYNAMLVAAATIAAG
KTNADVTKAVDKVAKAYGVTPISSVRMHQMKRYVLDGVKEVALKEPLPDDPEEEKVPDCT
FEQAEVYAVDVAMSTGDGKARPGTMRTTVFKRNVEQQYLLKVKAARQTLAEVDKKYPTMP
FTMRDLTDVRAAKLGINECVAHGLLTPYPSLHDYSGKVCHFKCTVLLLPSGTARVTGLKL
PEYFKTDVQADEETQKILAEIAEAEAKKAAKKAAKKRNKKKKK
>comp2442_c0_seq1; 288 199 1047 LEN=1194; translated
MLPKISFLLATAAFLRFAADRLGTADAYNVVRVCSNKHCSKRARGDVLQTIHNLLGTTGG
ETETRVVVEASGCLSHCDKGPNLEVKTKDGTTILQGMIDAQMCAYQLGEISSASPSFPPV
PKLLVAASKVIEQSERFASEGNHREQIRYLGSVIDKVGASPSLSPLTAANAHAHALRARA
HLKLACSEGEGLPPRSESFAASVRDARRVVQDFSEVSTPSSLSIAYRTWADALLEQQKET
EDEPSKQRDLGQVVAVLTQWHRVQPVYRTKLQGEIRGLLANT
>comp2443_c0_seq1; 528 387 2201 LEN=3158; translated
MSVKQKAQPQRVTLLTGSGHRVDADPQEQNHVTAVAKQKQQQQPPSRKIRRFASSTSTS
SLVRVVFFVVSVLMPMVYVKYHSELLPTFAVMIVEPKQQPNVSVSPLLNTSTRVSTPGAY
AAATGSQQKVNWSIGVGKNDSQTEIHSHTTTTAESIETVSKVALLPDNRGGNAKRNNKND
DGKQKRKKRRSTNLFKLESKSVSELLENDQGRAIVVLSMGKNKTHEEIAGQQPQPQQKGH
PAIGNQTQLSPLERFVFSARNIAKFRGWIVVVTDAPSLDDLEESINAALDENDANTLLVR
PRPGDLLEDRFHDEQMAYFRLKTFALDYIDMDPRLDGVRIVYYMDEAVLFGSMIHETMQE
LEFMYGIGRPTTEATKSDKTIGTAALQSSGDGTEALEDAVTKVQASSARGKMWMFRANHN
SSDHDHAMESGHIVLDRNISRPCLDRWRREFDEKAAGERIVMDQRLLSFLLEEEEEYKTT
TVHKTSKPLECSIQLMEQAPYADYPSVDSIRNQTYALIEHKKVRHPPMVHTGNVMRMEAR
SPEFIRPYLQDLLRLRGNEKDEKDEGLIGTDTFARPDTQEGIEETISSPTARVVAWLRTT
QQNT
>comp2445_c0_seq1; 276 224 895 LEN=967; translated
MSVLAMTRNGLKGRILVDGFALSRQSRARFSRSAADKVSSTSSSLSSSSDTINGGSSKVE
SDASVSTRLFSEEINVLYDSKCNVCKLEMDWLVSRDIRLNGPDNRKLRLTDLEDNFDETD
PANGGIDYDTGMAAIYAIKHDGTIYKGVPVFEISYDQVGLGWIWKINKVPAVNRFLNWGY
EIFARNRTKITRGTPLEELLRLNAEKKKAKAMEDCESCNNKII
>comp2446_c0_seq1; 426 78 899 LEN=961; translated
MKAAIFLWQLIVSAGAFSSSPVTGVGRTHSTPLRSTETDLSSPTGTSYTLDGEEIRGPIT
PLRNFILVKVKDTLTATSGGILLPDQSKERPTEGLVVAAGKGKIHPFTGIRITNPIKEGM
SVLYGKYDGKAIIYNDDSCQMIRDDDCLLYYSGVTMKLDAVFPVRDYVLIALDENPESLS
TSSGVVIASQVVADDVPCEGIVVKVGEGRLNSSGELSPSPVEEGERVKFKDYAGNDVTIE
GKQYSVVKMVDILCSLSKEVQSIRETGGDEEAP
>comp2447_c0_seq1; 628 320 1729 LEN=1939; translated
MAKDKDKDAAAVKIQAIARGRRDRKKVDGLLQGLIESLMASKEGKKKSGKKSKSKPKTKK
KEKKEKKKSAWCCKGDVSAVEEEIIEVVEIVTVVDSKPETTPKESTAKTSVPAKGAAAPK
ARTPKVSEFDRYNQQQKQEQAARAMARPPGLKPTKSWREPPSGGKAARPEWMEKAKSFRD
ASEKSAASSTAAASKGPVDPRKPPPPKPQGKHKSALVNRYLEAAARQNEGEKKNENEASK
IIETRNLEEEKAKKEEEDAKREEEEKAKAAEIEADRAKNAGKKKWKGNTTAYQNHPPNVP
KPWEKKSKQPPGSEVPSGSSDADVAAKPREPVPEGGDAKDKVASLNDNLMNKTSETTTGK
MTMSTLSPPSGYDESQGLPLWWMQLVPHNTNHKGNTGSAKGKNKVIYKIDKNHPPAKRAK
LQAISEGDEPEAPKTTSNRSAPASSSRKKKSGIFAKWFSRPRRRSMGEF
>comp2448_c0_seq1; 244 148 1305 LEN=1379; translated
MKKDGIAEPPGRSGYAHVSQRPGHAKHCREARGAVWKDLLAGGIAGTVGIGIGHPFDTIK
VRFQQGHTPPEAKRAGIPTARTPQPSPYRGLYRGLGAPLATAALVNASVFCVYGATARQW
DELFGTTTTWEESSTASAIGREAFCGGVTGFATSLLICPIELVKVQLQTLRPPRQGSFQP
SPPGTLSVARQILGSEHGLRGLYRGLLATALRQTPSLAIYFPVYRSLKDRLGGTGGTPTR
EPSWYSSALAGGLAGCVSWAVVYPIDVLKSRIQSLPMEAPARERSLRRVARQTLRNEGLV
RLMCSGGLAVTLLRAFPVNGAIFFVYELASERLAPLGNPLGSNGKSVIGAETSLAAAGIG
GASSRLRGRITSQVECSEFNATADV
>comp2449_c0_seq1; 286 227 646 LEN=983; translated
MKAMRSNFKYTTVLCCFKAKKQTKVSSLEFKILQLKKKFGVDYLTLVSENAPVEDLKDCL
KTALEELSNLQEEIDDTLDEIDEKEHKVNEKISRTKSADELFGPNKNKTVRSKPKQKKSR

RVDNSEKESDWRDDDGSFK
>comp2450_c0_seq1; 709 233 1876 LEN=2085; translated
MRALSVFRYALSWSLLRFLVAASASASASASAPALSRRFLRSLQQEQDAAPPVVRTDLLR
LVPFQIQIAIRDETTGEEAELVSEILYEGGARTLLTDTITDWLVSSFASKSTGAAGELLG
IGNATSFDSIALEMVGDSASTGTIDGQELSLVQVSFEGVSLWERTGTGTAPMEAENVELI
QRATFLEDKRLRDDLQAAIASLLYDLGLETTESITILDVRAYITPPGSILDGSGGGSDAQ
PTDSAGTQAQAGDSGADQNNNLEIIIIVAIVVACLAFALLIFAVVWAWRADRNDRGSSKS
SSASGGRKRTKKKNAYDSVRDDDAEAAAAAPAAGGKGGRRGGKKSTQQTAKQQQREREQP
PPQTRGSRGIFSKKNRESPEPASSPSRGGMVIGDTTGGRGVSVDISDNETDDDDTAGNAT
NDSYPILQPNDSVVSSDISSSLTAYYKSGMGYGGGSGSKNSGRSGARDFNDAASMSSMDS
YGYSLDGYAPSLGPAQGGYPVGPMQAAANAPIPIGTSQDEELANHDDFEAIRQLQEDESI
VDYQAEA
>comp2451_c0_seq1; 226 77 1432 LEN=1674; translated
MIAYRKHLLARNRGHTGEKITLQPPTPGDAKGKHTRTAGLSSCVRRLAMKFRFYSNKTMF
SIKTIALVVLALVCVGIWDRFFYRTGCVRLGGDQPWLPSLSSPLLPPPHPSTFAVVINTY
RRPERLRKTVRHYAETCGRRYGVGRVFVVWADQHIEPPTDDFFWGTGGPPLRALQGYNNG
DDTATGSDANRVPVEVLKKHKNSLNVRFEPIPQLQTRAGTTSVFMVDDDVRVACPSLVEA
FSVWRENPDTMVGFYPRLALPPIRKHDTNPYDAPAGHGDLLYHLWPKVYLAQKFNIILTK
ASFLHSRYLELYTSDDSFPREIKDYVDEHMNCEDIAMSVLVANYTNQYRTTSKDHTDGST
QNPSRPVLYVEGSVSDVGMFGGISSGPGHFATRTNCLNEITSIVRARGWGSPLERDFDLV
ENSCGQHSPGFWWQSAPSNSLEWLGLVNVLS
>comp2452_c0_seq1; 392 175 1065 LEN=1481; translated
MKDVKELADNVEDLKNDGMMNMDLAFDGMWSFSGYCGMMVQLAHFFVESKQGREENKGFG
ENDEDDEDEFLFVGSMDISAEETIIRCQENLEERNLHMADGVLRRLVEGCCPPKIRITRL
DEDTLKKLIDYKVQGNHFFGRHEFREAIDFYDLALSSIPETQRRLYIAPRNQIQEIVNVL
SNKAECLLRKVKYEEAAETATDALIFMADHEKSRIRRAKAGLEIGKYDRYEATSRGDGSM
TGVAFLVQSKYDLEEVLNAPDATLVGRQTAEEILHQVDKLLKGAKKKVLSKDPNPEW
>comp2453_c0_seq1; 413 1 1026 LEN=1079; translated
AIALDSNIDDNDNDGEANDDGEGTSSSPEGGRNVTTMLQKILSLGGSEEEGENGQAEAEP
APTPTPTPPVEPEEAVTDPEYPDAAVVSGASSRIPSVQHNGRLVCNLPDHPIHAGATAVV
AVLTGRILTVSNAGDSRAVLCRRRAGANPEANAGSDCYAYPLSYDHKPSQMHERNRILRS
GGFINHFGRINGNLNLSRSIGDLKYKQVPGIPPAHQMITAEPDIMQIALDPSVDEFFVLG
CDGIWDCLTNQQAVDFVAKRIDTMSPTEIGREMLDEIISDDPRVSQGIGGDNMTVMIVDL
QSKKRSRKSAAAPEAPQQGEPPSAGEPAGAEADQAPEAATE
>comp2454_c0_seq1; 463 125 1195 LEN=1495; translated
MNHTDGQPHRRNKGYRGEKTNTNKNNDEEIGINRKQHDRGTHCHTHQFQHFRMKRNILYS
SATALLFIVLTIYQFQDYFDERLQDKYWRAMLLLPLRYLRVKFMDQRPTRPKTFTLKATR
SFVINLDADKKRWRFFQESNQQQLQSITRFPATSLQTIHDDEREHYFLEKYQFLRNLIKN
EGKGSAACAISHLKLLLEFVSNTANDDDYIFVFEDDALLTSHLLQYGFVVGPDNADFILL
SEYSSAHIYVPFDKTDVNTNVNDLSKKSLEENLLQNYAEKINTATRVVSGYTAIGYIVTR
RGAKVVLDGMKKEEELPVDLYFFANPQIKAYLPLLGWPLVQHHQPSQSVRRDKNKE
>comp2455_c0_seq1; 298 128 1067 LEN=1067; translated
MEHQEGDDVSSTAEIILADLCKKETEAAELKEALKNLLDSHVRTKRRHAKELSEVHKQYS
KRLVQLEIDLETHDIHFTNYAKDQMGLETKAILEDKKECAWKKPKSDILAQQNLIAKLIA
ENADLEHRYKRDCTGHNDRILELENENRWLRRSLRRRVDSSSSTGDAESAGGQLALETDS
NCSLVVEKYEKQNTELLRSVDELKEKIRSLELKHVLAGGPTNTKEENGKELSCNDKDEKL
KDCKIALLKSKAIAYQARNIWPPDTECSAPPESLSSSSTAIKDLKEQIQELENILIRKDK
QLRSQSKRYRKTRX
>comp2456_c0_seq1; 330 196 1176 LEN=1235; translated
MTPLNPRRTLAILAILLQGAESYFNPNVYALPRSLSGELFAKSAICYNNAYGSFRIRQSS
PITSTCTKHTGSDFMKFPQPTTSTSSSLAVMNPVRRRSETGNGGRGTSPAFLNTFLNNVG
TLTESLVSATMKSLRKHWWSFPMIMALIPPYCVLFRGSYASMPHWWGVVNMDYVAAAKNA
NLIIAHFLGSNIAYAASGAYVLHKFHFFGRSKSGGLEFRLSRFSMLGIWILMAGMVSTIF
HSVQALGSYALAESMCYLDHAVAGSATFYFADTCGAPSKTTSIIGALALMSLVMSSPAYA
WIHSCWHYLSALVATRWAIDGSARMH
>comp2458_c0_seq1; 200 1 640 LEN=919; translated
XNNNNNNSRFAPERIRRVSSAGVTDRFVPGRENTDHKHDARHTVVPPRTGLALAVAPTAS
PLPSVRDVLLGRGRPLQKHPGNLRLRMLISDRLDEYESSTKPEKTWLAEDIVKEVRAGGG
RFLQKRKPTENAKAKEAGPGICVGVGVGISGERKNAAADDDTVNDGTVECGWEEVDDHTA
RLKVAYTFRTLRKTKTKAKTREPRQQQSTRAKP

>comp2459_c0_seq1; 390 113 1255 LEN=1963; translated
MVAILTRGAFLKAAALASSLTISPSSPAVDFDLASGTQTGGVVGTAQPGPFPSVSSTVVG
TIPSPLEDRQYAAAAAFVRREITRIVREENPSMAGSILRLSFHDATVRSVASDPSIGGAD
GSIRYELEWPENRGLAKPLAVVTSVYEEQLVRFSVPGSSPIEEENAPLPSCPLSFADTLA
LSGAAAVEAAEGPQIPVRLGRRDKEVADNRFLDRPIPGFTTMDQGPGDRASITSSLPSAG
LDSLGLRNYFRRLGLTESELVALSGAHDLGRHVTLLGMRKDCLRDLTRACLEDAPVLWPF
VTKDPDTLSNRYFATLLRWNKREIERGEASFIPTDVALVVDDGLKRHVVAFAKDEKLFFR
RFRTAYQKLVDSTASSQSRF
>comp2461_c0_seq1; 212 224 823 LEN=1482; translated
MSETTSTEPKPTAAMAKRGTKSEEKVKPDTDQNLCTATNNTNDQPSLNKKSVRFGSVFVR
LYGITIGCNPATSLGASVCLTWDYDELLPEPLDTREGKKPKRKKNTSPSTFYLSYYRREG
ILKRAGFSKEDFDRHEKLIQRDRLKRKISTYQCYPILFAKSFGLSRARRKSRTFVKKYRK
EKRNKKKQTINKPKEQARQ
>comp2462_c0_seq1; 397 273 1074 LEN=1333; translated
MSTEETNGGSGASDLPRIDGSETIGSTRWLKLETLSYTDATGSQRKWDYATRTTKASKSS
TDAVVIIPLLKRPYKKEGETGNEKDEEDKDDVLDTLLVEQFRPPVGRATLEFPAGLIDAG
ETPEQAALRELREETGYVGEACRIPPVVSRQVCMSPGLCDETVQCVVVVVDLDNPYNDPS
GALFEATPDEGEHITVHRVSLKDGFQTLLDKGSSPMAIMGLYLFAMGLEVGKTMGKESTT
ASVNGAHESLDMALVDHGIVVVRIEM
>comp2463_c0_seq1; 301 1 1066 LEN=1085; translated
XSKLSIDLIVPLISQEKDEYYYSMKKKSKSKTNRKESSEVDTMDSPVAEESTPIEDKLER
GNPNVEDGNAETSIFSTSDGKYRNKQRCLVLCLRGATARYRHLLEDLRTLLPHHKKESKL
DSGKGGAATAVNDIAQIRGCNTVLFMECRKRQDAYLWLGRTGDNHGPSAKFHLTNVHTMD
ELRLTGNCMKGSRPILTFDESFEKHGHLKLLKSLFTDVFGTPRGHPKSKPFVDRVMAFYY
ADKKIWVRNYQIVEEQPTNALEAHQAKKLSGQEESTSLVEIGPRFVLNPIRIFRGSFGGQ
TLFQNPDFVSPNEIRAVEKKRKGRSYELRKESQKMRKSRQEDYVLPEDPLNSVFR
>comp2464_c0_seq1; 475 67 1827 LEN=2194; translated
MRYDAATPSTVFGADDEEKENPDTVDLLLTKELLGLSFKERNAFQEEIHGVVCLAREETP
SLLKKSLERLQQEIDVMPDCRKEAYLKSQTLANVIKNNSPSCTTYVNTDGFRLRFLRFEL
FDATKAAVRMTKFLDVILDIFGEYALQRPIRLSDFSKMELRNLRKGRYQFLPYRDRAATC
GRRVLCVFPDQEWEAIPPRVRNKILIYLVWVAGEDVNTQKEGFVVVCWIDHTFKVSKSIK
VHTKDHQTLTVRISALHICSSDTPLHRFRRAIIAMRIGHNRSRLKQHVGESMELRYALQS
YGIPVDQIPITYTGKVKVTYFRQWLRLRDFVEGNRCDKINHSIELSRTNQVDDNNQMIFC
CDWNSCCEDKSNGNSCICYTAIESPYLNDIIFRKGANTSSHPGNAVMSRLIADKTRQELY
AQHEQQLVGENNKRTKMKLFVSEIIQEIREKNSTERRMCRFLVWDERGWWTEILNEYDIQ
TRVEYIARGIRNTVTKNYRSSTPLMQSPINMGYKCAYIVKEDERMMLGAFATQRQLKDDA
MFRYQDFCSGLDKRKQKRFKAMTNSSGDGDGIMMASETRFCGSYCF
>comp2466_c0_seq1; 177 114 914 LEN=1153; translated
MATATLLSIVLPGDDVTDKIVPTSLKKSIPKLGTGLRIERDGKEDGHERVFATCAGRLVH
RSNTYYIKQNVQRYRPLQEDRVIGIVEDRVASDGAGGDIYRVNIGGPHPAMLNNLRFEGA
TKRNKPNFTAGKLVYARIDTMPPSMDPTLSCQLGPHDAGVPRKDWMTNEGTYGELKGGTC
RKIPLGLARELLYPKNLVLNELSKSIAFEVCIGVNGFLWVHSPRPEYTILILNAIMNSQV
LTEAQVRGMVKSLVDTVNRQIEDEED
>comp2467_c0_seq1; 272 1 1175 LEN=2330; minus strand; translated
XYDLENRRNNGDEFDLFHKEGVDFLAPSVNFMEGPYPSTDSYQKIPARTGILIHQISHSA
PSMSFSVNLMNELSSAFLGGNGASGTMFDILPVKDINIRKIYLNLAVDETVNVELWTRTG
THISFENKPWLWQRSVVVSVDGNGRGKKSCMDIENLPLNANTRYGFYLVLTKGRFRYTNG
IAAGSVSVSNEDLTVYEGVGLTRPFGKVYQNRIWNGGIFYDVLSEDGERSGGRRLETTFD
GGSGQDGVMFDVLPKHDLDMDGFDLHLIDSDTVCVHVYTKEGPFTGSENKRDDWILLAKM
NVKGKGINVATKVLLDHSDVITLKKDKLQAFYIFIENGRGLRYTEGHGTGNPVRSDDNLT
IFEGVGVASQFGSIFQSRVWNGALHYRIKKN
>comp2468_c0_seq1; 171 1 720 LEN=1572; translated
DTFKESRYNTVLPYELHNEEVAHNGVRVSSTVTDFKRCDLFAVKIMGIPIPGDKFPGDQR
PLIRGGDIDVRHAASRILSSVSHTTTIKLERSNDKDENDYHHYCCYYTAQEYWLLALIVA
SMVCIIVHLRNGALYGDSHVQIMSKKYARIRYVLLGMSTIAAPILPFEHGVLVIAMLGNG
FHLFEVIRSHHDTTTVYQKLETTKRHEKRMPLEIDTSKYVDDCSGDLKIELAVKWDSTA
>comp2469_c0_seq1; 224 77 1258 LEN=1436; translated
MSQHESTIDERKSDYKVTTEQPKNDQVLSVEDWNDAYLAHQYRLSLPSSPTQSNFRVLAL
LFYEEKYDGNGSPMATSHLPPWVKQMVGDRNFIVGTNDEPGYIGGAICAERAAMVQLRFV
PAFRITKLVIATDCETVPISPGMLCREFLAGHGESVPWDLKVISTGSQCTRCKRKDEDII

RPLKGLQSAKEDGDKSYCCIEGHEGHSLPTLRTTIAELYPFPSPYTRLTASQSVALGTKF
GESSHRNRHLDYLDSTAKRLLELAISEAQQSVLNEAGRENHPIHFGAAVAFEDQSISTSN
QRSALEYGCSLDAVSQLVPYIEDSDCPPVLIVQVDQYGVAHSPFAPARAYLSEHGYEDCR
IIVHSTDINDVTCLDKWDLIKVRVANLAPTPPAW
>comp2470_c0_seq1; 230 71 613 LEN=613; translated
MSDTEVKTGEPTEWKAYSEYAIEVDPDQDDKAKTIKLCSLARPHMRAFHFSWWGFFVAFF
IWFAIAPLLSEIKVDLGLTKQEIWTSSIVGVGGTILMRFILGPACDKWGARIPMAVVLCA
ASIPTACTGLVKDATGLAILRLFIGSAGGTFVMCQYWSTSMFCKEVVGTSNALCGGWGNL
G
>comp2471_c0_seq1; 352 1 1614 LEN=2008; translated
TTPKPSFAQSHETKRDHCQSIARESVRETMVDSGNQDSPVMAKTERKRRKITVVQKAVFA
AATVTTMVTAGRLCSSSSSAWSSLFQRYSRNNSMDSAHGQTHTYLQTNERRRRLGEIEAN
MENDDFVAESQKKHHPMFSVSDTFTILGPTVFPKPTYVYGDDVPSDKDLVTYLQPTSGSH
RPDRDSVFVFAAEYKFDTYVLFLMSLYDTGFRGDVVFGISKMDWENKEVRKFLEHWQNAA
GKDEMTVVVYVVPYHCYNLEGEVVPSHRGGMRVCKCNDMFGLRAKGGDPDAPTIPIADKR
HGRTAQTIRYELYWIWSEHYDPDSWILLIDARDTIFQQTPFGRVPRRENHGDDGGVLIFF
GENADATSLGQSKYNRKWLTLAYGESVADAFYDKPTICSGSTMGEQKAIETYLRAEVAES
DESKTVIFGADQGFHNYLYYSNKMANAEAISQILVQDQGLGLVNNMGALRDKPLSEWGNG
RMLQEEASEAHPQNTVFNWDGTVSPIVHQYDRHKVLSKWWCKTKYKEYKNRWNEMKK
>comp2472_c0_seq1; 456 350 1549 LEN=1687; translated
MVLYGQVVAGPPGSGKTTYCTGMQEYLRLLGRNAWVINLDPANEVPSATKKSTVEKNGAD
VEGDEKDQDEFENDLPYECLLDVCKSVVNLSSVMKQLGLGPNGGLVYCMEYLEAHVDEVV
DMIEEQIREQGDNNAYLIFDLPGQIELYTHGTAVPRLLAKLVKVFDLRLVAIQLIDAHYC
TEPANFLSAALLGTTVMLKLELPTVNVLSKIDLLANYGPLPFSLEFFTDCQDLERLVPYL
QQQGDFDLNENEDDFTISTQQYFDEDNYADDPEYQRARKIKSSSPFFRKHTRLHKVMAEI
VEEFGLISFLPLDISSAESVGRVVAKIDKCNGYIFTQQVRGGSGSSSSSANGSNTTTAAE
DLFQCALESEPSNYEAIADIQERLSIGRAKGQQSGRRDT
>comp2474_c0_seq1; 485 320 1525 LEN=1600; translated
MNTSNITKNQSVGDMSDVTTVASSLGGSFPPSPASSVASESVRSWMDTELVEPHVGYKKV
LVTGGAGFIGSHVSEYLLARGDDVVIIDEMNHYYDVNIKESNLKLLKESYPEEGRVTIYR
GDICDRDLLERIFETENIKWVCHMAARAGVRPSIQDPFVYIHSNINATTHLMELSHKHGI
QNFVFASSSSVYGGSKSTFFSEDEVVDHPVSPYAATKKACELLGYTYHHLYNLKVTGLRF
FTVYGPRGRPDMAPFKFTDRIYRGVEMQQFGDGSSSRDYTYISDIVDGVVRAIDRPYDYQ
IFNLGKGDGTSLKKFIGLVQKYVGKKAIIKVLPDQPGDVPYTCADVAKAKHLLGYKSKVS
FEDGIKRTVDWYKRSYATQNNEQKNECQQTIPVVQMAQLAV
>comp2475_c0_seq1; 323 135 1111 LEN=1155; translated
MPPKKRPAGSSIIVPXNEEEKGSFRENKGGIKYAFRRKEEISEGWCHAALPGFTGPTEAQ
ARGIEQNNTRCIPKAEEKPLLVQVINELSKASKLKMEKSGVDNDLYYTLVSDEVAQKYIG
LDVSAKMVLQCIEKAGNNGIWTKEIRMQTGLQLQGLNKTFKQLEQRCLIKPVKSVNAKAK
KLYMLYDLTPSKEITGGVWYSGLEFDHEFISELRTFVMHCVRKLNGGKGVTLSEIKKKTE
QAKVSRVELGVDDIKQLVQTLVFDNYIEEGYRNDSDEVCYVYARPVSTTCDFKWWDALSP
DFHFRAIKFEDGVTLLPHEPHHHTA
>comp2476_c0_seq1; 409 142 756 LEN=1022; translated
MTTQSDGLALSGKIITSENGKSTVNSDNENISEKDASAASGKGSAVKKRNDNICNQNAVQ
DDDEDDQMMDIEVPSGMVFLEDSAGDDGRINLIQQKHHTKNLPQKPAVNLQVDDRAILDC
WNLTVASHEAAMVVTATNPTNTSASFDSSPSTTTTSLTSPFVTLLENEYRWNAKDKASTA
PDATDVFGSWEPKPLALPIWAIVP
>comp2477_c0_seq1; 516 71 1384 LEN=1443; translated
MLKLSTTVLRRKQREILRRLQSTSAKSASECMELEDKYGAHNYHPLPVVLSKGAGTKVWD
TDGKEYFDFLSAYSAVNQGHCHPKIIGALVDQAQNLTLTSRAFHNDALGEYCEFVTDYFG
MDRVLPMNTGVEGGETAIKLARKWGYQVKGIAPDKARCLFANNNFWGRTLAAVSSSNDPT
AYNEFGPYMPGFSLIPYNDVESLEKEFEKDAENIAAFMVEPIQGEAGVMVPDDGYMRKVK
ELCEKYNVLLIADEVQTGLCRTGYRLAVDHDGILPDILVLGKALSGGVLPVSAVLAKEEV
MLTLKPGEHGSTYGGNPLACKVAMTALEVLRDEKLAENSQVRGEQLRSGLEGLIGNSSVE
HVRGRGLLNAVIINQPKGGYGDTGKAWDLCVKMQNHGLLAKPTHGNIIRLAPPLTITEEE
IDQCLDIFERSVRELDA
>comp2478_c0_seq1; 864 100 1791 LEN=1886; translated
MSETKTPVKEEPKGAAFTEEDIPEDATWGEVLQSCCVHSPKEWGLIFIGVCVVVFFLYFF
LLGLDLLGSGAKVMGGCAAGSLFGDDTNPVAGLMIGILATVFLQSSSTTTSIVVSLVGAG
SVSVNQGIYMIMGANIGTSVTNTIVAMGQMGDGDQLERAFAGATVHDMFNFLTVAIFFPV

ELITGYLNWLTGLCVRNFTARDGEKWVGPIKKFVGPLTKKIIIANKNVIKDVAKGGNCEE
YYPIECEDMSAPTKKTCSRVGIIACDKGTGKCPAFFDANATLADDQVSGIAAFVLGLIIL
FVCLFGMVTMLQKMLLGISSRIIYKATNINGYLAMIIGVGVTILVQSSSITTSTFTPLVG
LGVIRLEQMYPITLGANIGTTITALLAALLSTTEALQVALAHLFFNISGIIVWYPVPFMR
NIPLNAARHLGKATRMWRGVPIVYILVAFFLIPLALLGISLLFTQGSTGLTTLGSLVTIF
LLGGIIYFIYWFQFTGGKNSIAECMAKRQTRRIAIEDLPSDMEALKAKIQSLEEHTGLLT
EEPEDEKEAPEEAPNSVSAEEQV
>comp2479_c0_seq1; 109 330 551 LEN=551; minus strand; translated
MTARGWFKMTGKSNSGCHPLNGGNTENIIGSCRLRFRFWLSSTTDSTTACFTCIYFPISS
SFHMTGDGMSYLFM
>comp2481_c0_seq1; 435 156 1370 LEN=1431; translated
MPAATNSGGGGGEQWAWLGLLKWTLAHTDGTRPSSESPTMMSDEDKAFLEKVMAEGIIDE
SERMKFVLQEFSKAMEYYKSQSQSDGQEAPTTSTTTEEKEQEPPLQPPAPPNEDDLEELL
QELRDIVEQVDYARGFVNLKGCRYLLGAIAAPEVPTTIRDACLGILSTLAQNNPPVQKEL
VELGSIKVLSDLFLSETQTDADADTATESGTRAVPLSTKTRTMQALSAIVRCYDLTEGVF
EGLPQAPAMMVQGLSTDPNVSSTSLRTKTLFFLRAFLTSDLSTPARAQAFRYAIAAVADT
DSLYLKASDESGNDWASIQLRESAVSLLGQLLERRLAVGLLLQRKQHLASRGVQRIRELR
ACTGEDAEATRTELQQWEAFLLLLARTEPEAPEEGASEAPKLLL
>comp2482_c0_seq1; 245 1 1537 LEN=1537; translated
XNSHNNTVPDQEKVREQQKDNKNDSSTSPGPLDYLVAPPPVILLCFHFPYLLTLSASTGG
FMVSWSRLGSLRTAATNKIVQKYESEIGPVVLNVLDFGAIGNGIDDDTDAVRRALQIASN
LAGGIVATILLPENHTFSTGPLNLTSHVTLQVDGTLRALNWTETTWPQIPPLVNYGRAED
GGFHLQYQAFLYAVNAHDIRIIGKGMIDGQGQPWWDAAQNHSSLLKAGRPNLIQLVRCRN
VEVTGVMLKDSPFWCLHPVLCKNVHIHHIKIRSHMYAFNSDGIDPDSSKNVLIEDNDISC
GDDHIAIKAGLCGKTAALDCGKTKEFNDGTYETVNVTIRRNMFRIGMGISLGSECSGGIR
NVRVEDNVIGFCHPGHCEVGCCGWSPALHLKTTGTRGGHMKNILFANNTIHNTTSVILVE
TDYQDDGKDRNDRPTLIQNLSFQGNSGVGTAKGIRFACSKYMPCRNVTFLNNKFDPETSV
ECENVEVIDQNQNNICSIKTLDDFLTVNNNKL
>comp2486_c0_seq1; 460 129 1526 LEN=1779; translated
MLSKATGDKASRAVIFLSRLHPTRRAAAFATFGYGYDRESRLSFSSPPSSIRRQDENDSR
NLPASPAQNDVPIDFAVSSKIEGEESQIATVTVRPGERLRAESGSMIYMTQGVEMATTLE
GSSSAFKRMMTGQNIFLTDFTYRDTNNENGEGKGTVALGTDFPSKIIRFSLEEHGGSLIA
QRGAFLASNTTVDIQMEFTKTMTAGFFGGQGFILQRLVGAGDVLIKAGGTLIERELEEGE
VLRATSGSIVAFEPTVSYDIQMMQGVKNVVFGGEGLFVTTLTGPGRCWLQGMPPDRMIAE
IASRIPSGGGFGPVIPVGGMGGTGGGAEGAEGGAGAPIDVPGTTDVDSGEEIVAAANEGL
PIDQEATTTMDDASTTFSNAVSSEIDDAFQETTFQDDGTDFSGPSFSDDTSFSTGNTFRD
ENFEQQEGEFFGGDDAEIFDSGATEEAAEAGSSILTTLWDIFNGD
>comp2486_c0_seq2; 460 129 1526 LEN=1938; translated
MLSKATGDKASRAVIFLSRLHPTRRAAAFATFGYGYDRESRLSFSSPPSSIRRQDENDSR
NLPASPAQNDVPIDFAVSSKIEGEESQIATVTVRPGERLRAESGSMIYMTQGVEMATTLE
GSSSAFKRMMTGQNIFLTDFTYRDTNNENGEGKGTVALGTDFPSKIIRFSLEEHGGSLIA
QRGAFLASNTTVDIQMEFTKTMTAGFFGGQGFILQRLVGAGDVLIKAGGTLIERELEEGE
VLRATSGSIVAFEPTVSYDIQMMQGVKNVVFGGEGLFVTTLTGPGRCWLQGMPPDRMIAE
IASRIPSGGGFGPVIPVGGMGGTGGGAEGAEGGAGAPIDVPGTTDVDSGEEIVAAANEGL
PIDQEATTTMDDASTTFSNAVSSEIDDAFQETTFQDDGTDFSGPSFSDDTSFSTGNTFRD
ENFEQQEGEFFGGDDAEIFDSGATEEAAEAGSSILTTLWDIFNGD
>comp2487_c0_seq1; 346 1 948 LEN=989; translated
AVGAAVGATAGSVRSHSLRWMVAGTSEKTTENNAALRDQTTEQKRGHGSNSTGRHRSMPA
SPDRHNRWNEMYRRLFRYKDRVGHANVPQHYRTSEKDHHQKLGKWLDKQRQLKAQGTLDA
GREELLEDLGVAWDVQSWKWDKMFRLLARYQAEHGHANVPQNYTVEVRTDKADGGDGDDD
RHGEASPSSANLGGWLKDLRQLKLEGRLDPTRQEKLEGLGVVWDMHSWRWNHMFRLLVRY
RERRGPSSVNTMKQSHKENGENLGRWCSRQRESFKKGDMDPERIAALEEIGFAWTVAASN
KARKKKKNSAKRTKK
>comp2488_c0_seq1; 227 139 1002 LEN=1345; translated
MRSTSRIFYITVAVICLVTTTVDGFAALRPGVTSTTKITSQLKSESSTTANNLAIVRQTQ
LFAGSNAGVVPGIGEEGCNLPSVSGINTKDDLTQAFVVKVVFLSLALGTFLFSSGLEQCS
GFLDSNFPSQYEFFRNTWPISFGLIFSLAGVTHFTLKEEYENIYPTRGAWGVWYLPGSRA
FHVAWTGAAELAGGLGLLAAGVSSFAGNPNLAPLTSAGLESDSAAALFLLVLAVTPANIF
MFTHGAKLPMDSEPLPVSFHGVRWIMQVVLLGFLYQMGQETFQALLS
>comp2489_c0_seq1; 505 114 1469 LEN=1748; translated

MIQQHRQLSQRLVSMPAALMVAASYFLLLTPPISAFLVGGTSTVLSRGSLSVATSSLQMT
ATNGARIDQHEETKDDDNYDVVKVDLTDGRDYPIYIGTSFTDEQISETLTSHIHGKQALI
ITNDKIAPMGFLEKYEKLLAPHKEVHTLVLPDGESNKNLEILQLILDKALEEKLDRKCTL
IALGGGVIGDMVGFAAAIYQRGVDFVQIPTTVMAMTDSSVGGKTGVNHPLGKNMVGAFHQ
PQCVFIDTDSLSTLPDRELQSGIAEIIKYGLIRDRDLFEWLEVNMEKLLNRDPEATRHAV
KRSCENKAEVVKADEKEAGVRATLNLGHTFGHAIESGSGYGTWLHGEAVAIGTAMATTMS
AQMGWIDNELVKRTYKLLEKAKLPTDLPLDSPMNREIFLKYMAADKKVANGQLRLILLKG
ALGNCVFTGDFDQEAMIDTIDEFVAECSGAQ
>comp2490_c0_seq1; 160 179 505 LEN=766; translated
MSSTSNNYTPPPRDTAAMKDTGREGRLSFRNFAENQLKKEFKAEAMKKCDLQIGALAECI
KDEGLFAPFNCRPIQKDVDECMMVYNSAERFKLYVKEHEDELQSKPFV
>comp2491_c0_seq1; 424 1 1025 LEN=1082; translated
XGNGNGNGNGESESESGPPGECKLAYRKFLLSSSDSLSQGWKDLYIDDYSLFAPVTSRED
DQMADYFNRPDVRSALHVTDTMEQSWPNPDVGFSYTKEYNACNWKPAEFIRYPNTTMVDV
YREIIPMLDRVWIYNGDTDPCVSYEGTREAVKQILVDELDGGSYRPWFYQQEAASLDVLA
EKSVLFGPDLVAQTLEQNQFAGEVTSYQNGLSFVTFHGSGHMVPQFRPQSSLHFLKRFLY
AADNGSALSPLLPTNATLLAMSDDDFEQAMDDWTESARDELSSGTSRAGHGANRAAGTPE
GRKERTNPDRGNELAAIGGADSLESLPASTNDSSTINVCVG
>comp2492_c0_seq1; 991 171 2657 LEN=2935; translated
MNDDETETIALAIKTLEKGVSRILKRRGETFNDNSRHRIQMLIEHARHVLLELFKKRRSL
KAFLRWFHKEWVRIEIDLAKHPVQILQHSMQHFLRCQDAFILLTSGECRHEYDMKLAKKT
KFRSRLSTLQERDDGYTALYKGTGVYSGYGFDEFNNEIVGIRALLVKELKKYHKAPWDYL
ERQGGTELLENLLDQHEREQKELQHALQSLKRLMIENMTKRLKPKNLKFATQDKLERLSL
STKGQTIDLERRGDAFQIDGPLLPNENKNPDDWHSFSLEIKEEMDQSSIPPSKIQKRRRV
IIDSDDGSDNDDEREDAPKKKGKRQNNKTSQASGLVVRVDSSRTNAEKEDSLAAIKSQVG
ISTQDLEQAREILEEENASTKKVFEDEKVLRLEKILRRVLARDEIDENEVWDARECLRYA
CMEAGNKYLWDSSVGCVEKAIENFEKAKEIVELQQKSQQKVTSSEASLFVQLNLFYLHGQ
ALVNIGISLVDTSHHRLSIPRVKVMRAIEEFEGAKKQMAKLRSLAGHTSQSTSRASVKEA
NSYILKSKQLESLACRWMGRGLWLISEEMKAVTSFEEASQFLNDDILRKWQRDVYFEIDV
FDITAEAIYATCDLADRCYSMMEALDHKSPSSRKKGHDLLEIITRALNRHIGIIESIELC
SSPVRTKHFRDEYDISSIEDVLLYRKNVMKWWKEQCENEMNDVKPNQNCNSRLPESRSDS
SSLKTKKILTSDGSRRKRYEKRRKASSHMHGNRSNNSQDFGQENDAVYALSGHYQPRPPV
KFRRWGDEYLIAQQQLAKSECDDPHEETNSSFLTYPSIAPPKPVEFQV
>comp2493_c0_seq1; 228 61 816 LEN=1209; translated
MSTILNESTQTRMLEWALALFSLTVLGCVMYKKSRFGAESEDIDESIHPNPTANTTTANR
KNVPKWEEQKRRAVIFKLFQTEHIQERVTCSRIRRSRSVSMGSATTKDSESLDGEEDRED
VCSLDGEEDHGIVMVGGADGVHSDGGTEPDDTDIETGGDPGGTGGKPVANLCAICLEEYH
EGDTIVWSSNKNCRHAFHRDCLTSYLVKINAKDTYESYPCPCCRQNFFYEDRGDGGKQNA
SASTGSEEEEV
>comp2493_c0_seq2; 181 483 938 LEN=1331; translated
MGSATTKDSESLDGEEDREDVCSLDGEEDHGIVMVGGADGVHSDGGTEPDDTDIETGGDP
GGTGGKPVANLCAICLEEYHEGDTIVWSSNKNCRHAFHRDCLTSYLVKINAKDTYESYPC
PCCRQNFFYEDRGDGGKQNASASTGSEEEEV
>comp2494_c0_seq1; 825 117 2087 LEN=2570; translated
MAVKNIDSSSGIGDYRPHEGTGRLMMFFMGFVAIMGLTFVSQLRDYHRTDHGGGGRSNAV
HLSQPSFPAADSKIAAGAEQMVEHLQRQAEQIAAETAMLLVPKGLSGAAEAKPEAKPEVK
PEAEPEAKPEVKPEAKPEDPPKPAEQKKPRRLNVALFYADDWTMKVLGKLDKNVKTPNID
RMADNGMIFTNNCVTTSVCWSSRSTLATGTYSAVHKHTFPFQEADFETEHWTETLYPLMK
RRERGNYYTGLFGKWHKLEKENELNEAFDESIRYYGSHWEERNGKMRHVTELNAADSLWF
LDKWDSRRRQVPATPEEGDDLTKPFFLTTSFFATHARDGEFPSYQPLNATREIEYPDSVI
IPKPKTATKKHWNDLPHFLRHDGNEGRTRWKKRFDPESFQANIKDIYAMATDVDAAIGTI
VDKITQLGVLGETVLIFTTDNGNMHGEHGLAEKWYPFEESLRVPLVIQDPRMPKERRGTI
SEAWTLNVDLTPTILGVANLPPSDFMQGRDISDLYLYKDEDKEKALSNTEVADKVAWRKD
WFYEFNLGMKDDASDHPWTNFIDASFALVTDEWKYVVWPQHDDYEQLFHRSVDPFDEWDL
LNKVLRGKNEEVEVEGNGDTVNGPFKDTVQTTRKMYEEMKQRYAELKKKAQAGERI
>comp2495_c0_seq1; 391 295 1854 LEN=2052; translated
MAFSSAGEAIVNAGEYILSPLKESSPSSRRNRVNFFKEEDDTEKEPPQVDIRPRVDGTGI
ELVANNDHCAARKENTNNKLGTSYLRGSMLMFDPIAAEAENGELEAMNLRVLRGESLPID
RTRVRIGVLNREGAAVDQIIGSTPSSEGTRDPIWGSRTCSWTLNAKESTKLLFTLVAADS
EKGSKVETKTNGAIKFCAEISVADLLSKRNPNFWINLKNEDDPCSSNNNGRIQIRLTRPT

QAKLHEQETCLRISPGPGLPRVQSYAIRKMALYSCSPVMLNVYDVSNNPRIQTVNNTTKA
LGYGGVFHAAIEIQGKEYSFGGTVDKKSKVSGVFQCAPKQCPMHSYRESVFLGDSEVNGQ
QIQSILAELRPKWLARSYNLFRKNCAFFSREFAIELGVGDIPEWVFSLATTAETIEPYLQ
KLNAYLKSRTNAASPDKRITKSTTTTSTKQKSSKRVSIDTDEDIKAHEVAKNTQEALLDH
AMAARIQRRVRASNVRRASMYRAQTQLKIEPVGFASASA
>comp2496_c0_seq1; 257 150 1211 LEN=1596; translated
MPNQFFITSPEIATVGQSGQAYLQRTVVIVLAVACIGINIFNLRADCRLVLFTWTVDLNA
YELERTANVTNTVSDVLGVVGIQLDNSNRNFDILDYYVEGDDGLYFGLNGFAFVNSKSSE
YWNKKYKCVPFDSLPETKITMTEEDVKFFDSIKTVAILSSYFGFAGVLCAFVEFLFCVFY
PSVIAGSVFLALAAFMNCVYLLLYYLGSVECFTAGMCKPDLNFFSYLFTTGGFFFASCLL
VFGFRTYPWFLRKNRDPANCKVVEADIVDEEGYDRSEHQEEQGGGVPIEAYRELEEELAE
TKRRMLRAERELQDYLAQKAGKTSPHTGRGHTESNASNDKRIAFRHYGSQPAE
>comp2497_c0_seq1; 497 574 2205 LEN=2441; translated
MLGEESAKPTDGTPSSKKGIFMWFKEIYSTCLLIFCSIIVLTVIFEKNTKISEATAWGAF
FLFWIALYWLSMVEGGQASLVGLPPVDMELYKESHPTTHKIMAIINKGETLDRYLMGRQF
MVLALVFVENLCVHPIDASLSILGMPIAINKIFLDTGLAVFFMTAMIGKISAQVNASRCM
LDYVNNFFAYFTMQTARLIEVSGLLHCCYPVQIFFAWAAGQPLESKEPPRSLVQNIFFWA
RVLVSTAILCFAFAVTLYAILNGQTTMWEGVPSWLTILLFFLFMSVVGMLEGMQIAFFAV
ARMTEAERARSTWAKRTCDVLFEGDGRNLPGFMIGRQMCVTLCFFIIARVTTVQLQAGDE
NIFGVSDATQAFFATGLLGALITTIVASIAWQLVASAFPMAFLSTPITYVLLRFCLALEW
TGICQGSWVVARIHRKIVKFKRDEVYIGTAEERAARAKANPSKFEEDKGEKYDVVPGHMY
PGVPTLPPDFKGVHRSLEEIEDLERDLLSRQQEIEGRLAELKAQKAILIGNGVESTRSVD
VEC
>comp2498_c0_seq1; 346 1 1744 LEN=1988; translated
XRFPSSKISDLGYPESISFGVIFDATTMKYYFRPQQLVGLSTILAFLASTCRASFPANET
TYLNFHIPTSLRHNKTWPHAASMFGRMGHGPEGSLVLPVKMYVDSRYLCNDNFDFTEVNK
QLKIPSDGGAPFILLVESGICTFVKKVRNAQHLGAAAVLIADMEHEHLPDNLELSGDILH
RAHPHHDTSNHTEHRNAFRMADDGSGRDISIPSMMIAKNEYDELKKVIDSKPNSTGIVVA
EMSWHVPKFVNKVILDLWHSPADMHSKQFLASNFSVIARTFDLNEMHGKTHDNDSYNENM
NLLQFREWPVLLDGKALGCVGNTDAPDEPCYLLCTNGGRYCHASHHLTDGKYIVKEALRR
MCIDKHYKSPKFHWDYIDHFSKFCWDSDYFANEKCIEDAYKHSKIDKKVIESCFDDSGDP
ESDHSNALLQHALDMQLKWGVHKSPSVTINHEISPLVEFQGLTPATILSGLCDAFAYGDK
PHVCYACQHCGDPVKCAQRSPMKCLSTDGVEKEDTSAHKEVNGGGKKHKKKRSFFWRHFF
IGVFVLGGLAGGYVYYQKMEENGSGGLGSYTLQDAFLSDSM
>comp2499_c0_seq1; 157 353 877 LEN=1001; minus strand; translated
MTNSPCCPSKISLMNVKIASQNTGVISTPKAGGILPFASLNKGSDGQATTAQGNSFRLVS
GYHDATTRHNIAKDIKFKNGPNTVAVGFTQASVSANNIPLPDEIATPTLSATEEAIVGST
IDRVLEDDLAAADATGDVGTTKAETPLMAVAAIIAQQITKEKRYILFYCLEYRR
>comp2500_c0_seq1; 81 1 549 LEN=1017; translated
PTPRDKHTGIIEPTHGLKRKPLRSPVEDTTMVRAANEKSAGFYFVLFLCFLLGAVCIYHD
LGVIAFVLAVFVGIFGFGLREKFSNETTASAYSVFNEDGRAIVGGFTSSQFERQLRGSVA
SQMASGAGGNPDASAASGPIARARTEGPARRTNVALSDAERTARRRAAAEAAERRFQAAK
EE
>comp2501_c0_seq1; 713 523 2553 LEN=2806; translated
MSTPGYRSIKARKCSRRVLFESSNGSVQPSVDDSDSDDWEDDASSSSSDSEMSRRHRWSR
QVTSRSLSAKNLKSSSWGIWGVYDSSRQMNYDGSLDGSNSMSNSNKRSDDSNKLDDDDID
IEIEGSDSDDESFLEQKQQDEKVKLKMVAILSTVVCLLLLMTAVGLGIALSKKKRRPPAI
PLNGLSDNNSSVFDPFAPSSTPSSSLAPSVAMNTSSNTTTIGTMNVTLDDFDPIFLNETD
DNVGANETSSPTQSTTIFTTDSPTLSPTTASPTKSPSNSPTQSPTRDPEMIIPDAVDLTP
AGDTYVVILPDGEVYEKESATPTLLVRSRNSAGNNGASLITQQVNEDEILAGDGLPLDAL
SYALLHFDMADYKWYEDGNAELLSTKANAYLCLEHVPNTRENDAWGNPNDQKIFSACRVE
NPILEEDWLANPAFQAELTGYKMPEDCFGGEVKKTFVSPDTGNVCIDVSMFVDNYPPFAE
VDPSDDFTLPDPDDEDLVETLGVRHRLLRPNIFKNMESTNETEAFSESATPRVGGNYKNM
LFMVAHIEPGVDASAEFYSRQDTDNAPKLYVTLETLTEAPTVAPTGSEYPSQHPTADATN
DVTLGSSGGVGTIGAVDGNATMVPTLVRPPPPARALRXDEIGAVAPTSRATTETGNGKAG
DETAAALAAAKDAIAV
>comp2502_c0_seq1; 402 227 1219 LEN=1240; translated
MIMVKPRRRLERVFIHVPALLSLLSFSLPLVCGFALTTMTMTTKGMNNLSSVEDFLLRSR
LEGIKKGIKKDYANSPGVKLLSGEVDEKSTMPTFIFPGAGGLDELVLELQATIPNSEVID
WKEYRGTLLTAGYDSEAVGEAIAELVLEQIGGDGEEINNGGYENEELESPSISSDSCSGV

RFVGISVGGFAANAAATIVHRQITERSQPKNVEKNKENDILNVHLVLLDPFCGRGVFGQN
YGRNNFGKYATSAIQILNTDDPVPTTNDPLSNCYCLDVTEAPEKQDFVVLPGDSMHSWPV
AYFARYYYKKCQDPSLTPMLFPRGVVEEVS
>comp2503_c0_seq1; 655 96 1169 LEN=1346; translated
MSGRCLKLLRQGRGGRQIRSAAISSFSTFSSSPVATATTSSGNDGGFRRLCSLPLPYGGN
LRGHPQYTHQPPLPLCQKRWRSGKMGHQVESLKEMAHRPEREAAQERRKKKKDRKAAKKA
GGKKQQASETDGPGGGADTAGGDEDEDEDDIFFGDEDEDEEAGDGDGEPTLPDPVAVRSK
MDAVVDRFSESLRSVRGAEPTADMFDDVMVQAYGAPTPLNAVAQVVIVSPTLAQVTCFDP
SVARDVVKAVQLTLELNPQLEEGGAGNIKVPLPRISKEVREKLSRSVKKRAESCKKRLRG
VRRKAMDAVKKGKDGKLAGVSKDDAFASGKEIEAASEAATERLKAVVAEKMDSIMAV
>comp2504_c0_seq1; 302 28 1110 LEN=1128; translated
MTATLERRESVSVWERFSAWITSTENRLYIGWFGCLMFPTLLTATSCYIIAFIAAPPVDI
DGIREPVAGSLLYGNNIITGSVIPSSNAIGMHFYPIWEAASIDEWLYNGGPYQLVVMHFL
LGVASYMGREWELSYRLGMRPWIFVAFSAPVAAASAVFLVYPIGQGSFSDGMPLGISGTF
NFMLVFQAEHNILMHPFHMAGVAGVFGGSLFSAMHGSLVTSSLIRETTENESTNNGYKFG
QEEETYNIVAAHGYFGRLIFQYASFNNSRALHFFLALWPVLGIWLTAMGISTMAFNLNGF
NFNQSVVDSQGRVINTWADIINRADLGMEVMHERNAHNFPLDLASGEVLPVALTAPAVNG
>comp2505_c0_seq1; 148 1 370 LEN=420; translated
XPYHRWNRVQPSIGLPSPTNPEDGQKMEHAASPGTGAAGNEPSACEKECSKQQDALTACM
NAIRDAREVVASGAGAGETNDDDGDVSRVDTSCLAPAVASWTECCALANNGAVESNEGHI
GIG
>comp2507_c0_seq1; 619 372 2621 LEN=2893; translated
MDDLTSIPWWQKNGNDEDLMRGDYDDAGDDDILSTEQPVYAREGDFHRERRKQNQNEYEY
DYEYKNQRHSKAGAIHTTAETNPRRTTGSRNERFVSEDPSANRRSDANASSTKNASTRRK
QSVSRTKSGNALRGGNASPEQAPEPERPPPAKRTSSSSSARRHSGDKSDKRSSNGDKIRK
QVRWICSSCTFVNARNCRVSCTKSTILTCEVCSVPYVLPAAAARSSGDVHDARSITEQQA
PQHAEDRTRRNSSSSLPQANTKNDTARRQKDSQSRHSKGDNSDSTSSTGASTSKSNGNNR
TYSISELVRCLGATGEDGSSSVSNLPPSLERRVQDFRFAQQKRREKHGDQKAWGIYGLYA
HLSDIRADLEWAEDAAWRRRRSKPYLSWKDFDAARDKGAHSRPYFTYGVMVLCTVMMLVT
LGVNGWKFEPLTVNPLIGPSSETLIACGARDTNLIVNENQWFRLFTPMVLHAGLVHYFVN
MLAMYFIGGAVEKAHGAAFTSVLFVIPAVGGNILSAICLPGFISVGASGGIFGLIGGCMA
DICLNWNLLFLKTTTDEDSRLRHLCVLLWLAVDIIVNCILGFTPFVDNFTHLGGFLYGFC
IGYASIERLATGFFGIRIGDSHWSRLKSVGLRYFGLVLSVVTIFATTIVLAQSDGLTSPC
HRCRYISCVPFPFGAERDKWWHCDDCDIVTADLFVATDGSGLYQQVDIHCPNGETVEVPI
WDEGIYDTEVLRRKLPSYCRGFCAEVFAH
>comp2508_c0_seq1; 461 89 1177 LEN=1372; translated
MLSRKVLSKPFLAKRLLDVASPSTAGAKSIRLVSTSQTKKGFPPPILFDYETIRNNLTVA
DAIPSVKEAFCALAAGKVDVPMPMHIGIEESLAAGPGDCHIKGGYVYGEKTFTVKLACVS
FYKNVTERNLPAGSGVFVVVDAQTGAPLAFFQENRIMTDLRTGAAGAVAIEYTTPNDDSN
VIGFIGTGAIARNMARAAAAVRPNFTGVAYGLDFPGAEAFAKEMSSELGLNFSVASSSEE
LCHRSNVIFTQTTGSEHVLELEHLRKGTTIIASGSDQPTKQELPNDVLASADKYIADLIN
QTSRVGELRGAIEANVMTTEEVYAELGEIVNGSKPGREDHEELIVVDLTGTGAQDAAIGQ
VAW
>comp2509_c0_seq1; 563 83 988 LEN=1192; translated
MVSSVLSILALLSAVQLSPVEVLAAEDVGLSWTIYHSWDANQQFVRRGSLTWGPAPVDES
KDDDGKKEFQILNDDASANLTAGDIRSMLEYGWYHIKIQSKDDENSFVFQTVPACNLRRA
NFKDQFDVTLPRSSLDDRQDPMTSFAYTPLVSPLAPKNCDDYDDESDGTTRSFSSRVSVQ
LDTPAMTIKNILPQSKPPPGIAFVKQPPGGSGEQRGGTDDGESYQDPEAPTTPPGPFGFF
SKYWYIILPMMILQVLQVPEEPPKEQQGQQQQQQGGGGGTIAPQAGASPQKVRRGKTSKK
N
>comp2510_c0_seq1; 513 14 1810 LEN=1957; translated
MSNWVRKKNDQHTSDIEPESTETFESDDEEEYDEDEDIEDISFWREHGLAILVAFAASII
VHFHNQNLISLSGFLNRLPFGTNSERHSHLDGFSRMANVSFCRQRIQPPASERLNSMDFY
IPRDHFESLLTFYVADLDEDDYYANTDIAVSSSRKEDLGARLKDNKEFECLSEQHHINPT
DKKLKGTTYFYKDPTLEEIYPELMKENTTPNLSMVKKSSNERKRQLEQPPLRFTGFAAKF
VNLDTDPVLLYWDGKGGHESSRKLVGEIPPMESIGTATMPGHSFHVTPIYDPSTVLNHWV
VTLDTALVFYEPKSATEMKHTLKAKDPKSYAMYQRQMVNKAFSRDYTIISGRTWLGHFPR
PFPLHYMHEASFIGQEHQVGGMTLKVASVRPRVFTIENFLTSEECEEVVRLSLEQGLTES
TLHSSPQATQNRDVSTRSSSNAWLSRETSSLTKMIFEKTAELTKIDRHLFQKFHESNAKR
HSIAESLQVVRYKKGEEYTPHHDFVSPSINSRHQPSRFATLLIYLNDVDEGGETRFPRAV

NNYNAEGLEIVPKTGKAVLFYNTLEDGNLDDLSQHGGNKVLAGNKWLANLWIWDPVIG
>comp2511_c0_seq1; 146 683 1111 LEN=1111; translated
MDRHIVTEFLRKVRTISDGHNAIGAPHHSWSSMNNPRGIPRNKSNCSNDCHSHTQNVNAT
QREQSSQDSHILHGRPQERMHHQPFFMEERLQSDLAVNLSSLSSNGSNGSKNTTSSKTSF
SNHIHMQPLTLHRGRQQQQQQQQ
>comp2512_c0_seq1; 471 65 799 LEN=1253; translated
MDRDSLVYKAKLAEQAERFDEMVQDMKEVAKQPQELTVEERNLLSVAYKNVIGSRRASWR
VVTSIEQKGDSDKMEIIKDYKAKIESELVEICNDILGIIEESLIPNSTSEEAKVFYYKMK
GDYHRYLSEFQTGDTRKESAGAALDAYQAASGIAQSDLPPTHPIRLGLALNFSVFYYEIL
NAPDKACSIAKQAFDDAIAELDTLNEESYKDSTLIMQLLRDNLTLWTSDQADSEVVDKGD
AEGE
>comp2513_c0_seq1; 374 180 1253 LEN=1772; translated
MEGAEGGEAVSRKVIVVGMNRPRLLKIISMVDDEVAGGNAPTGVEYVPCLAAMSSYEAEN
GDAVRYMSDFVFRDGSPMSSFFDDEAFRESLDTVVMVGYEWQENDKQLVEKYFESNLLSV
TIVCVSPNPGFSSLGEEMSHFKNLSDDEKERHTTNHTMGPKKMKRFILDTTTPLQTTTEK
KDSAEKDNAVESLDGEENEIEIDSPPDKPKEEEEVPPKKKIPVAIDHNATSFACRMCRTV
LFGESHLADDHKKALHSLKRTPNRASGTVASCQSFFCDESVLEWLAPDGADVEGKLACPH
CNFKLGHWNWSGAQCSCGTWVVPAIQIPMGKVDVILPPSERAKVSPIMIVAPKASYA
>comp2514_c0_seq1; 522 120 2084 LEN=2123; translated
MATNGLSRRRGTRRRKRNWGPLLLGGAVLFLFGIIGGSILCFVMLDVGHTGDSTVAVDAT
ADVTGDAKADAKADATADATADAGRSSFSRQKRHVRKRNILEYDPDDSKKIPPKSLDDFM
KEAKKKFMDHLVQEGTSGSFPYYPRDEVAKDHDFTTWTAPGGKRFEEYKHGDSPYTYAPN
ESDALARSRRYHVKKAMEHAWRGYEKYAFGMDELAPVSERGKIRWGGLGTTLVDSLDTLW
LMNMTDEFYRARDWCKTSLSHNKDVQVSVFETTIRSLGGLLSAYDWSQDEVFLDQAKDLG
ARLFKAFDTESGLPASQVNLRTGNAQKARWMSTGAVILSEFTTLQIENRALARFSGIRDY
KLKSEEVFQRMNRVENVKDGLYPWSIHGTKDGATIYNQKITFGAMSDSFYEYMLKIWIQG
GQTEPIYREMYDKSIQGMHDRLLSVSRPSELVFIADENNGRMDHKMDHLVCFMGGLLALG
AYTDPQGLDSKRAQRDLKTAKALTYTCYQSYARMPTGISPEFIQFHMDADDFGPGRGAPH
YLLRPETVESFFVLYHLTGDPVYREWGWEVFQAIERYCKTKVGYGELSDVQNLKIKPRDT
MESFFLAETLKYLYLLFDPETPIDLLHKHVFNTEAHPMRIFPVMDSDGIMDLIK
>comp2515_c0_seq1; 360 93 953 LEN=1275; translated
MRIVCVAFVLLHPAAIGHALLLPPPATRKLAVIGSIEPATRPSRSHTRNRFGPSSTCDTA
LSSKAPPEEYDDGIGDLQNQNNRNDGDDNNWQERSDDAATNYAEGESLSAEFFQNLRDRQ
EGGAVDANSSLSSLSRSPVSREQFDSGLFEPSEYDDNYTDARANAPPTPGTKKFTGRRSE
GSLDYFGASSSGNNQRSGGSNNSNNNNAVRQQMMRREYDLVSGATGRTALGLQAGLALTM
LVFFLYVGFSGGIVSGEGAVDADFGGDDLIQFEQIIPLPRDSDKSVW
>comp2517_c0_seq1; 694 155 1681 LEN=2291; translated
MSLTFDICFIHAIADLQNPGNLALCTTIDDIDAVGGDLHCLGWNGSRTFEDIDEKASRNP
GLDFLPADLDDKDEPLPNWRYENGLLRAPFQGSEDGVGGSRDLCLGPSDVALFEAPCKPY
ELKLQECPSESNVRNSIVLDEGYDNAVPADSAASSEEMAASRHMRFLVTGQGRVQSLAPV
LERRTGRDVIETKPFCLTMLNGNDDGLGKKPYLVPCQPENKDGKDTNHDGRHGLASPVQV
FGVYGTYSDSFVMVLQTVWGYNEVVKLAYQVGIPTGSMSSFTSSATIVIEQQVGSSNSHV
DNSTPMLVKEIPLVEDHHVANSISGILSFSSCQYEMVGPMTATLHSIRDDVNASNFEAST
ASTAASRAFFVVDDPQKESLNGFFEMFVIIFCSIAGCFTLSMVADWCLKDKIRAFGTLWG
ENSDGKTVCLDDDLDDNFDLEKDEGTEITSEPEKENERTADEDNNTEHASADDSSRNIDG
SDDYSMCSNENHGKADNEETPPLFPNDV
>comp2517_c0_seq2; 697 376 1881 LEN=2491; translated
MPVPTSLEEMNPGNLALCTTIDDIDAVGGDLHCLGWNGSRTFEDIDEKASRNPGLDFLPA
DLDDKDEPLPNWRYENGLLRAPFQGSEDGVGGSRDLCLGPSDVALFEAPCKPYELKLQEC
PSESNVRNSIVLDEGYDNAVPADSAASSEEMAASRHMRFLVTGQGRVQSLAPVLERRTGR
DVIETKPFCLTMLNGNDDGLGKKPYLVPCQPENKDGKDTNHDGRHGLASPVQVFGVYGTY
SDSFVMVLQTVWGYNEVVKLAYQVGIPTGSMSSFTSSATIVIEQQVGSSNSHVDNSTPML
VKEIPLVEDHHVANSISGILSFSSCQYEMVGPMTATLHSIRDDVNASNFEASTASTAASR
AFFVVDDPQKESLNGFFEMFVIIFCSIAGCFTLSMVADWCLKDKIRAFGTLWGENSDGKT
VCLDDDLDDNFDLEKDEGTEITSEPEKENERTADEDNNTEHASADDSSRNIDGSDDYSMC
SNENHGKADNEETPPLFPNDV
>comp2518_c0_seq1; 626 113 1369 LEN=1414; translated
MAESKPKLVLAYSGGLDTSVILKWLCEKGFDVIAFCANVGQFEEDFSAVEAKALAVGASK
CVITDLRKEFVTDYVFDCIKSNAIYEGRYLLGTSIARPCISKEMIRIANEEGATYIAHGA
TGKGNDQVRFEMCAQALSPTIKCIAPWRDAEFIESFKGRKDLLAYCEKHSIPVDAKPKSN

YSIDENLFHTSYESGCLEDAACGPEESMFKMTVSPQAAPETGEIVVIEFDKGIPVKITNK
SDGTVKTDPLDIFLYCNMIAGKHGIGRIDIVENRFVGIKSRGVYETPGGTLLRAAHMDLE
GICMDREVKRITEGMSNEFARLCYNGFWFAPEMELIRHSLDYSQRDVVGEVEIELYKGNC
TIKGRTSPNALYNADLASMDIEGGGDAFDYNPADAQGFIRINAVRLKTYAALRAKDSA
>comp2519_c0_seq1; 375 287 905 LEN=905; translated
MAGRKLIDTARSRAFRTLWMLEEIGIPYQHVAALPQSEQARRYNPLGKIPVLVEDDGFSL
YESGAILSYLGDRYRSNSGGGLVPRAGTRERAIYEQTMSVLLTELDAQGLWIHRKHEVMG
KFFTYVPDAVEHARKYFHKTNRVLIQQLKDNNNNSNKNKNKNNKGNVKNDMNERIGQRKK
KPGRKPKKVGKKVGKKNRNEDEEDEEX
>comp2520_c0_seq1; 297 1 843 LEN=1000; translated
QRLSQITIWKSSGFGASHRYKQMLFNSSPVGLFLLATAWSQPCYGFHVRQEQPRLPCSAI
SLFQESSCGFMEEKTDSFDASVSSRRDLFRNSAAVAFALLLNEGCSNQPAFAFDGSGASA
SAGYSPATKAEKKKKFQERVAADVKDFKALGKAIDKGETDGPEWVNFFILFQRREPDEVG
RTYAALADLRGLPGKKKGEYEGGDGLLLANTFTKPGKPPDNTPAVKSFLKLSKTFDAIEA
AGKAKDAAKAKKEWQKTSELFSQYLSDVELPGDLNDSLYK
>comp2521_c0_seq1; 666 287 2485 LEN=2956; translated
MKVLCFPSFFFVFHAAIAPVAFCVNSEDRSALSLGKEEVPVSSPWIDLSQLNSNPPQDNG
QEGSVQGRKAPKKSKSKSSKKGSKSPTDSPMSSPTSPPVSSPIDLACVDVFTGNRTYIPS
CAGAVNEIIVQCNSEGNCNYNQYVIEFDSDGNVILDNDTCLLLAEFSYNKVMVKSSQCEL
QLEPIQLSHVGTCNFPNKGLNYGAPNMLKANIRPDGTMDLFYSKDDGETFFDDVRVATMK
NEEGIRKVDPAIFRSAAQCETLNPAPYQIDELSSGYGPCRNDADCKQVNTDTWCSLDTGD
NFPTAYYCYDCLNNNHCIGGQTCETNRNSRSCVNPDINQVRGIAGITTGYDVFDADPLFT
AYASGLADQAEDAGAVNRRRFFELEWSSDPKYKVDLPSNKTYYEPIGFEVTAELVMTSQT
SVKAFASLDQFSEEQNKALGLGASGTIKGVQLGGSVGIKSKKRIAEDAHKNLVTVTAKSA
KQIYRFQIASNKKLKLTLSAEADLKKASSSKDWNDFFRMYGTHLTLGGKVGAWQRQIYSF
DENQRSQLTTTERTFSQSVNIGIPGIFGFDQDSESSESKEIGKKIQQLQVKQKTYSKGNV
DNVYDEAAIIQRRLEPICDHVGEVEGVTKDECQEALIEYCVERIKDTGQITTTKCELSGD
EFNFTCLYDNQCPRQETCNRGICTPRCKEDSDCGKFEECIDVDNVPSKECHPMKGESQLH
KHQCSYENNDTLW
>comp2522_c0_seq1; 1411 223 3357 LEN=3474; translated
MSQPYKASAEDVLKEYGVSLDQGLSEAQVLELRKKYGTNELEHEEKTPLWQLVLEQFDDK
LVQILLGAALLSFVLAFFEEVDHWVEAFVEPAVILLILIINAIVGVWQESNAENALEALK
KMQSLHTPCLRDGVWQDELPTEELVPGDIVKISVGDRAPADVRLCKLLTATLRSDEGALT
GESETVLKTTDSISSGEKISDQKNMVFAGTTISGGKAIGVVTSTGMTTEIGKIQSAVTEA
SEDAEKTPLNQKLDEFGDLLTQVITGICVLVWIMNFNQFFDPVFGGVFKGCIYYLKIAVA
LGVAAIPEGLPAVITLCLALGTKKMVKRNAIVRKLPSVETLGCCTVICSDKTGTLTTNQM
VVEALVVPDAQEGDLQEFSVTGTGYAPTDGVVQNYTSTTGNLEQMANVCSLCNEAVIRYS
EGKYVRVGEPTEAALKCLVEKMGLPGQSAPSNEVEVADYFGKSFQGQFDKEATLEFSRDR
KSMSVLCKNKATGDRVLFVKGAGELLLERCTSIMRPDGSVQPMSAKDRQTLADRVTDMSK
RPLRVLGMAIKTNMPPKEQLPSQLLNLDEYAQIESGLTFVGLTGIKDPPRPEVRSAIQRC
QDAGIRVIMITGDNKTTAEAIAREVGIFEHDEKVTGKSFTGAEFFHGSISDDQRAHLIMH
GGGSRVFSRTEPRDKQLLVKLLRSCGEVPAMTGDGVNDAPALKQASIGIAMGIAGTEVAK
EASDMILADDNFATIVSAVEEGRSIYSNMKAFIRYMISSNIGEVVSIFLTSTIGCPEGLI
PVQLLWVNLVTDGPPATALGFNPPDPDVMLKPPRGKDDSLITNWVFFRYMVIGLYVGFAT
VGIFVFWYTEYEGFVNWLGPSIMELLGSSYDGHSMVSYDQLTRWGHCRTGEEAVGTIFEG
FSVNDFDNTGNGLIEDFSTDPCSYFTKGKMKASTLSLSVLVTIEMFNALNAVSEDGSLLT
IPPWVNPYLLLAMASSFILHFMILYVPWMATVFSISPHDAVDWILVFLFSFPVIIIDEIL
KYVGRQMNETERKERERLRKEKEA
>comp2523_c0_seq1; 1645 218 3895 LEN=3954; translated
MALGSEPEGPPRSPEATIRVAGGDEAIKSTVKSLRKLMGGICDPRKNNADGSGEEEEDNQ
YDLYLHEQYQHYLHDQDRHQDRHQDQHQHQDQLQDQLQDQHRPTHDPTWSPPVRPSSPSA
TDGATTVVASSTSKNDDSNNPPPLVDGNGATATPSEEKDSLDSMDSAKKVLFPNEENDEP
LVDETTPIFEESCDNSIISNNSNTNLEIDPIPEQRHPRDHLHVWTRTLLSIAALVFLALG
TTILCMQTTDTEGLNINEVLDRIRKYRRTTHLQERSTIHPASVTNDVDSTIREASLPPKT
RRTTEEINDHAKEPNSWLDTMYDDAEEILRLTYDRTQLYLREAVGEILETLGHQKNKVLE
KIYEHHKTIQSEQFNEPELSLNGITKYYSELVDIRNVTVALELFLKYFSGNSTWEQMGIN
VISKEYFTQYIPKTLELVRGEANLEHVHKIWTSDLTKRIRNMRVFDEDDIYNKLNEIGDT
IARFYHESGLHQYVERLEQKLADAVGETNMTIVDAMRAEILDRLAEYYQNNPLSDYLQLR
PLLSSDIAGVDEATASGASSDECATTGADSTPERETDELDQAETGLDTDERGAEEEEVQI
VDRDPSGSDEESELLVDTVQDNPIETNDSSSIEEEEGGEGEESSPTTESNPSISDVDVVD

AELDDTLEPEDDSDATTTEDETIGKHADEAADLIESDQEPKLPADPTIADSSSGTDNSLL
TEMEGSIPKMESKPSLSDVGVIDAEFHATLEPDMNIEGSETNDKNSDETVNLNIADQEPD
VPAETATTDSVDTEQSLRTEIEEYNTTTESNPMDFDEGVIGVELDETIEQEKDPKISIPA
EEYDIINKKKPLVYDEVDLQNPNDSDQDMLVPKKALDGDSDGLSSGEEHSIKSDVKESLG
KTTTKEQAGEEHDDQPEGTDILDEASIASENPELTTDESTPDDSLADESSQGDERIPSEL
HNSIPMGTFPSENKEKGGEAKDADSDIQTVSSEDHGTGDEIGEDLEASSSQSIPPSDVSD
ADIKEIAVESPIQKPAELGAHDSKDEEDGTDGENLLVVSAETLDSQHSIIEEATPDSDKE
RNSVQGHDETDVHDQKDSGNPEILNEGNLISSDNERDSTNQSPQTPTSAQPARDVPDNIP
DMIKGIEYHMTKMEQSVAPDLDKKCSSVECMIEEIEAIVDRTRTESYAARKYDAADNFEE
RNPVVSKPLSVRRRWNSLQGWMQRR
>comp2524_c0_seq1; 376 531 1577 LEN=2336; translated
METSDGDDGCKIGSCRLSEIETKPITKAPSTVFPAVVACGYRRVGIAKSGNYFPAFLVAF
PNHRDSSNPETVQRKSAICFAWRSKRDFMLLARSSDSLVLPKAALKKVVDSNSVPWRRFD
MNLVGNNNTPYFLSDPKLPNHFSVRMKKSLWLLDHFLHRAETSATETAKAAWETFCRPSD
TEDLITPREFTPGADSTTNQRDGNIAKPKGEDTQAHSTETKINDPNTSFSSSHNENVTTL
ADSSRLGQYFASEENSRMVVECALEKILPLFHDGKNSDILFLEPSCGHGDIVASLVEALE
EHEISPRSVFIQGYDIDPCVIKTCQQSTTFHNSRSANSCKDSNDYRVLW
>comp2525_c0_seq1; 150 324 695 LEN=722; translated
MRAIVLNATKMGVYDVAKGQVVKSTGWTRKDPRTAFCSSFLAGFFMTCTVAPFDRVRTKL
MSQPTNEKLYTGLSDCFVKTVSQEGPLSLWRGFVPIWARFAPMATLQLLTIESLYAFFGF
DSI
>comp2525_c0_seq2; 199 209 937 LEN=964; translated
MGVIQTISDTAKNEGIVAFWKGLPWAYCREGSYTAIRLGAYAPVRDAIGAGAPDSPVYMK
FLAGAMTGAIGSIAGNPFDVLKTLSQTNKGKSVPLSTLVGQMYKDQGVAGFYRGVGVNVM
RAIVLNATKMGVYDVAKGQVVKSTGWTRKDPRTAFCSSFLAGFFMTCTVAPFDRVRTKLM
SQPTNEKLYTGLSDCFVKTVSQEGPLSLWRGFVPIWARFAPMATLQLLTIESLYAFFGFD
SI
>comp2526_c0_seq1; 601 125 1636 LEN=1711; translated
MTTKRAASVEKPPLSPVASVSRPISWWTICCSLLLLLPAVLEANKIDDDEQWGANLEDHP
DPNDEHYEGMIYVGKHDANGKRIGFDQDNGIRYIFGTSFDETHKDIEEKDLLHHPLRPVQ
DIPDHEREAVLRERRHGSLKQDGESGRYEMPKAFGHVDEHPLDGGVVPPKVVHVDPFFLD
ETLVTNKEFGKFVRATYYETEAEKYGWSFVLESMVDKAALMQHRRDQQQDEDLELENDPE
AEDWMAVDGAYWRRPEGPKSSYKYREDHPVVHVSHRDAAEYCKWVGKRLPGEWEYEAAVR
GKNTGPSNRTMFAWGDDDSDWELASKHANLWGPGAFPNENGALDGYRATSPVKHYPPNDL
GFYDTTGNVWEWMRGGKHKARIVRGASFVDSLDGSVNHAATLGARSTVHATTTTTNVGFR
CAKAPKRRTEYHFVTHDEATHGHLAVEDQFGKRDMIPQKGWEDQYHHVDEEENENDEDPD
NQYNNQLKKRRVIKRRERYSNEL
>comp2527_c0_seq1; 251 19 525 LEN=704; translated
MAFFLSCFQQTIVFGILNLISSVWGVVLAIGLLTGGALIGSDGVEEWEDEAGSASLYILG
GILMFVSVCGVGFYSFQIHAAKKFNVCGLKLVIAWDIFTICFRIAELIIQQQHSGDVGVM
INNLFWTVAIYGLSLYPIVGLVFEIQKGIMSEETYPREAYSCCCEPKV
>comp2527_c0_seq2; 271 247 843 LEN=1022; translated
MSSEEVKEVPVATPEAEVPMKDQEKIGAKCCGCCCDYRRATIVFGILNLISSVWGVVLAI
GLLTGGALIGSDGVEEWEDEAGSASLYILGGILMFVSVCGVGFYSFQIHAAKKFNVCGLK
LVIAWDIFTICFRIAELIIQQQHSGDVGVMINNLFWTVAIYGLSLYPIVGLVFEIQKGIM
SEETYPREAYSCCCEPKV
>comp2528_c0_seq1; 831 1 2000 LEN=2156; translated
XDNNYTDSYKDCVFTMAIMFNHSFPRLLVWVVAVLATLHGTAGEDATKAEAEPKVVASIE
VQLDPNRPMDGVDKEFGDDGEKFECKQWASEGECEKNPGFMLQNCAASCKAANTVIVSVY
DGEDVGVAAFRFGEEHSSAFGSPSETQESVLKVAQELQGVLDKGSYEPPKELTHCGKRQC
SAGKLWKRAEEMRKADMHDDAGADLIRALLKTGIEVDFVERCKRSLQWAFGSIQRQRERE
KRDAIEEAKLEARQAEERVAMEEALQRKKEYEADFVKFGVKVQEMVADGSKVSSASVGAD
GTVETETNNGLILEIMKSFVAEGPEGGNWSNVLQLIKKVRPSEKTTEILLIEARCHEMMG
DHKNALSAAGRLIQKAASYTPWVDDSPRMIAVTLGANAAMQLGLSENAISFYQTVLKFDP
EQKKTRKQYRGLKKVVKLMAKADEQIEKGYNKAASTFVDDCLSAMRGLDVDSPLFRSKIQ
LKQCTILSGMGKHEEALANCDSAVEQRAGNESVSDESKKEAYLVRAEALLLDMDYDEAVG
DFRMAFDLVPETQETMQEKHELNQRLKQTQRQQELWNGGVKDYQFNENTGYPDGRPPQRD
HAKILQLPIDLDQQSKEVKCAWLKKQFKTLVRKYHPDKYKGNKKRAARKFKEVKEAKEII
SKAWQC
>comp2529_c0_seq1; 592 104 2008 LEN=2218; translated

MRRNLKCSSIFQLVVIVAPVIALQLILVTQRLDGVVGVGVDDHQHLYTDSFPRNDEYNLM
ALEPETPFGTPYGGFSRATASRLAALPLKLPSISKVEKGENDQDDEDDGEFDPMYTTVRD
NAGRAFACRIFLEDELKASSLGDSMFDTPILKEKPNSSSKESSPSEERNEETSGSEQADP
SASQSLSEIGFDPVMVGHTIHQRLKKLTGLCAQLHPPGWWSYEWCHQENIKQFHVKVLQS
NGKAQKFELEGITNLGSLSERRIQVVNEDDDDDTNDNEDETDLAQGRKEIGRVIDTFVGG
ETCPETGEPRVTEATFRCCSERFLRKTKGGVLKNGNQVDTNIISIFEAKENRNAVCRYNI
TLCTPLLCDDFDDGTGEKDSKAGDTKTESPLEKAYKKATNGKGKNQINNELDPSTAENMS
VAEILEETFGQAGNFCIRQYTGGWWAYEFCPGSYVRQFHEDKSFKQDQFKGIVTAQKEET
AGPGVTEYFLGRFISENHKGVTKENEWEQVVNATSSSQGGSKPKPSADDKSPTVPFANAG
GNGAYYFQEYTGGDICDDVDVTDSAMKAGAFGEGGIERAITVRYSCNEDLSVAVKEDSTC
HYIIDITVPTLCHHPLFRAPVSKRQIVKCLPISS
>comp2530_c0_seq1; 881 1 2075 LEN=2142; translated
XSLSMQINPRVLISTNLGDTLELKTAQSTTTFMVNRIDTMMIMMAAALFAVGRQATHAFH
AAHSQTGLVGRTLSRTGSFAAETSPGQSFYNNKSITRMFSAATELSDEQKSVLESKIKDT
GDRIRALKDEGAEKETIAPIVQELLALKAELDPTFNKKPKKQQKKQKQQKKQKQKQKQTD
DDSSDFITARAVDYSKWYNDVIRVTGLAETSPVRGCMVIKPWGMALWDKVRTELDDRIKE
HGAENAYFPLLIPKSFLSKEAEHVDGFAKECAVVTHHRLKSSDEEDGGLVVDPEAELEDP
LIVRPTSETMIWYMFRKWINSHRDLPLKVNQWANVMRWEMRTRPFLRTSEFLWQEGHTAH
ATAEGARDDAKTMMEEYASLCENLLAMPVVRGVKSPSERFAGAEDTFTIEALMQNGWALQ
SGTSHFLGQSFGKAFDVTFQDADGEQKDVWGTSWGVSTRLLGALIMTHSDDEGLVLPPRV
APIQAVIVPIAPKKNDEEGKAAMNKALDDLVAGLKAKGIRVKVDDRDFMRNGAKYFEWER
KGVPLRIELGPRDIKNNSCVFKYRAGDNSDEKINVDLTDAASKASEGLEAMQQSLLDQAQ
SRLDNGITEDVSYEEMKTALESDEASSYPGNGLYLVPWKCDAANEDAIKTECKATIRCYP
LASNNDGQHEGKKCFYSGEDATHMALFGRAF
>comp2531_c0_seq1; 162 1 812 LEN=1175; translated
XLLQTKQFERSKMLFFSKFDCVFAAAVVVLGSTPFANAESCVDKHEKIADCMEWAWHGEC
DKNPGFMHLQCRKSCGLCKPSKSETDNEDTCVNWHAKCDDWATKGNSQVDNLCKGNWNKW
KNGKKLTGAYIVEMCPKACKVCDIHLDDRDIDLGIGLPQSFPGMEDDKELFNLLLGKVAE
TRAYVESIEDEEIREVCKMSHPNCARYALSSDCQTHFDHPIMKYGCAAACQTCEKLVEDN
GIFEARYMWGDALREFNEKKLAKETIKATV
>comp2532_c0_seq1; 420 45 944 LEN=1055; translated
MSVKNQNMPLATSAAAAPGLLTQVGMAGTSAVITVSFIHPIDVVKTRIQVSAEYSALGMG
GTVKKVMSDEGVIALWKGVNAAWLREASYTSLRLGLYEPTKVLFGANGSDAPFYKKFAAG
SAAGAIGSLAGNPFDVLKTKMMTAGGKEVPSITKTAGDLMANQGIGGFYRGIDSNIARAM
VLNGTKMACYDQTKEIVASTTGMSKKALPTQFLSAVAAGFFMTCTVSPFDMVRTRLMNQP
ADAKIYSNAFDCAMKIATQEGPLTFWRGFMPIWSRFAPTTTLQLVIFEQLRGIMGMKAL
>comp2534_c0_seq1; 488 92 1171 LEN=1263; translated
MTADSIEKSLKEEFPDATEAECFRFVQAAKAVHKKANAKLDKDKLIKDTASEKLEEYLEW
RSLYGLDYDRPAKDADDSSIWEWAARKALGAAETKKREAEALRAAVEAKRAADREARLKA
KRGDVDDKPPKVDYDAMREQVMREEELARTGGGDDGQTDDEENNDSKCIGEGKAESKTTL
PQVFFRRKDPKTGKLLCDKKGTELIHVLAARIDRFATDNETWALAVALYIDASVDRNSKH
SFAILVDARSGEGWPNPVILMIVSLIGQIVTEIEKRQPERCKSLIICPLPRALMMVWGTV
RGFYSAEMNELIIAFSGPSGIGSPLPKKKLDPHVEDATIEFFEQCRRDLFVCEVEDEKV
>comp2535_c0_seq1; 1216 358 3021 LEN=3118; translated
MKGKLKKFFSGRNLSSEISGSFDGDAAAHDSLLGSNKEEDDWDAWSIDKKNDDASDPGRG
RLDRRAGTRRSHSERPSRRKRSKSEVSKRLKRLEGDFMTALNSSKKKFSSKSVGGVFKGK
SSDVRSRIDSLALERSTSDSAFDTSNIDRSKSESNFGPKSTKKLRGGRRKSMLDEPTTNN
VSTRTRTRSLSRGRRSLKADDREGSSSQRSFSKARRSGSKKPSSKRSISKPRKSKSTQDF
PELIQRDSSEGQNSVSTMTKESKQKKKKKKDKNMDKDKDERKQAGDDFSVPSKVEMNGDC
ESQGSITMHDSIKLEEPTRSKKGKDKEKKEKKEKKKKKRSKSKSSFPDLPPDDFGDGLEG
LAEEFRRSKQAYSKTGSEGMPSGELPHGEDSNYTKVNSMFESAWTGMESGNANGDSRNPF
EDEKKHNTEDDILRQAVDFEVKQADTTNEKNEEILRLQQELSAALQKQLKMSEEHIEERN
EFMQVSRDLDRLKVELAESQAERGEVMEELKQRDEVIENDRNRIADLEQAIDDQLGKEEE
LVKNLHHSEEEIEHLLEEIQKFEKKLDNGETDGGGASFVELRNTKKELTDREEEVVALTA
KIGLLENELKESLTVPQLQIEELDQENKALQGRLKGERLEYTSKLSAKDDIISNLRSELN
TFTSTPDAQDLQSARQKLTEAREDATQVREDLARTRKYVEEIQGDREDLVEKYNLLKDKS
VFMEKTVKDLSEKSDNLAKKLLVWTEKTYDWKKKAEAAERKLQEQKASKDKGGSETKSAN
APGDPQTMFLQAAMEKGKPEKKSGSWGIFKNGSGDQDSSPEEGELRALRDKNLEFEAKIA
QLNSDLVKMQTAHKNELYSTKKKIAQLEGENDALVLQNATLSQLSQR
>comp2536_c0_seq1; 483 134 1701 LEN=1774; translated

MASVTYSSTRGGQSNLDFRTVVMTGLAHDRGLFVPDTFPTVEAEELESWRGLDYPSVAVN
VISKFVKEDQVPADKLEDIVRRSCAAFRHEEVTPVINVAGHKVLELFHGPTFAFKDVALQ
MLGNFFEYFLETGSNDGRLAVLGATSGDTGSAAIYGLRGKKGVDCVILFPKGRVSAIQER
QMTTVQDDNIHCVAIEGTFDDCQDIVKASFNSPEFRDRVKLGAVNSINWCRVLAQITYYF
WSYLRVTDEDEHKDAKVGYSVPTGNFGDILAGFYAKQIGLPVGKLIVATNENDILHRFFT
KGEYHREAINKTISPSMDICVSSNFERYLFFLAGNDSAKLAEWMTTFEETKKLTIEGELL
AKAQSDFSSARADTSMTLDIIREYNENHNYVMCPHTAVGVSAIHQTNDVVETMVCLATAH
EGKFPAAVNQAIDPLPTPPEQLACLLDLPTRLSECPNDVQHVHDFMEKKIAXENGESLKK
PNAFRVKDSQTEYEMEDKLCNFSSIEASNNVKAFLSRYNMNY
>comp2536_c0_seq2; 404 134 1818 LEN=1891; translated
MASVTYSSTRGGQSNLDFRTVVMTGLAHDRGLFVPDTFPTVEAEELESWRGLDYPSVAVN
VISKFVKEDQVPADKLEDIVRRSCAAFRHEEVTPVINVAGHKVLEVANLLQNIXKNKSEW
SASPEKADTRPLLMRLCDTFSLQELFHGPTFAFKDVALQMLGNFFEYFLETGSNDGRLAV
LGATSGDTGSAAIYGLRGKKGVDCVILFPKGRVSAIQERQMTTVQDDNIHCVAIEGTFDD
CQDIVKASFNSPEFRDRVKLGAVNSINWCRVLAQITYYFWSYLRVTDEDEHKDAKVGYSV
PTGNFGDILAGFYAKQIGLPVGKLIVATNENDILHRFFTKGEYHREAINKTISPSMDICV
SSNFERYLFFLAGNDSAKLAEWMTTFEETKKLTIEGELLAKAQSDFSSARADTSMTLDII
REYNENHNYVMCPHTAVGVSAIHQTNDVVETMVCLATAHEGKFPAAVNQAIDPLPTPPEQ
LACLLDLPTRLSECPNDVQHVHDFMEKKIAXENGESLKKPNAFRVKDSQTEYEMEDKLCN
FSSIEASNNVKAFLSRYNMNY
>comp2537_c0_seq1; 540 1 844 LEN=844; translated
XEKEAKEKAEREAKEKAKKEAKEKAEREAKEKAEKEANEKAEIEATAEAGEEANENTEGN
AAASATTISSPTKSTTEPATTSTVDESKAVAESGNNDGFLKQVQEDGFVNAFLKLDVTTQ
ATAAAGVLASGAAAALSIVKRDDDNGDDNGSGFGGNSSFGNQGRQNNQGGPSSFGNQGGN
PSFGNQRNQAGPQSFGNRNNQGGTPPFGSAPADASQNNNKDGKSLFSSFSFGNPPSAKNS
NSNNAGGLGGPPKKNGGFGGAFGSSQTSSKKQGGFGGGGGFG
>comp2538_c0_seq1; 432 38 1528 LEN=1528; translated
MVSATLKKYLKFLLFQCLILSVLGDDFDNAISAKIKSLAIRGASIAYYDSNKMPSPVTRG
YGQISSDSSSASVTPDTAFMIASVSKPFTASAVAVLVDKGIISLDDDICDVIPAGYSDTM
CRNQNHPNVKITWRMMITHRSSMKGNIPDAKDRWGDPISPSYGPSGGYTSDAPAVGNPTC
PLGEIGDFYHALLTDDPNATTDVGAGVILQGGKELDWFDLAISNGSMWSRSKPGQRRDYS
NAAYGFVSALIEFATGQSFPDFCREHLFEPLGMSHTEWFLDDLPDGSQVAVPVEKKRNGG
YMDVGHYCFIDYASGQLRTSANDIARWSNAMLDYGSPMLWSTAVGKEIVKCQEKNVNGEP
IGNNNCDFGYGWILLNNSMKQSTQEKWLKQGFSEYDWTDGVWHDGSEAGSQTNIVILPKA
GIFVAVLTNTDLNSETAAQQLTQAVVEAPLPSLPPTPTPLSNPTLAPQPTRAPASPTRAP
TNPTKAPTNPTRAPTNP
>comp2539_c0_seq1; 744 370 2724 LEN=3009; translated
MIQHVLFYPFRVLFSPYFSDDQLTSFLTGAFTVGLLWLLDGLYHSVPDHFTKDRTRSDGS
IDDDDDDDDLTFIDPWNDPLAYVSTQQRRMMRTSISTMVDIQKNIGELPKRLKREHWPWE
TIRRKVMGEESDRDEGKTEEERDSQTTENQGEEKRMKNLMSGESEEEASYQENKKEEKPL
CIGSIFGLDVGGTLAKLVYFEKKALDVDPLYTRSLREKHYAKALSAQTVLMARMDDNTDG
RAKRSASMSPMRKIPAPLGNRFRSSTAEEYEFKEEDSFEKRMLIPPSNEIPGVMRRSQTM
TGHIAHPVKIKESQNYPTKIESSLFVEEDGEETNIMPEHATKERNSTNDLQSLYAIRQES
LPDDVRQFRHVVDDSDDENDSVFKQTTSLESSSSGLPPSLNLKKRSLSMLDMTTAHDQEK
VEALNRFYDFARRLDTNEDSISDIQLSFYCRELGGEFHFISFETRKMKNAMDLIRFNKLH
MNIRNMGATGGGAHKFADKWERELAIGMIKQDELDSLVAGMQFVLSTVVGECYTFRPKQN
RTAETTGDGKNETADGGKNPKLEWWTRKVQRDTISYSSAYPYMLVTIGTGVSVLRVDGPR
KHERVSGSTIGGGTFWGLMRLLTDVENFEDAMKLAEKGDPTKVDMMVGDIYGEDSDALEK
LGLTADIVASSFGKLVSKEDPAKGLTQEDLARALLSLVTNNIGQVAYLNAKLHKTPRIYF
VGSFLRHNMISQRRLSYAIDYWSKGEMEALFLEHEGYFGALGAFLMTQNIEKKSGGFFQW
ENNP
>comp2539_c0_seq2; 669 370 2848 LEN=3133; translated
MIQHVLFYPFRVLFSPYFSDDQLTSFLTGAFTVGLLWLLDGLYHSVPDHFTKDRTRSDGS
IDDDDDDDDLTFIDPWNDPLAYVSTQQRRMMRTSISTMVDIQKNIGELPKRLKREHWPWE
TIRRKVMGEESDRDEGKTEEERDSQTTENQGEEKRMKNLMSGESEEEASYQENKKEEKPL
CIGSIFGLDVGGTLAKLVYFEKKALDVDPLYTRSLREKHYAKALSAQTVLMARMDDNTDG
RAKRSASMSPMRKIPAPLGNRFRSSTAEEYEFKEEDSFEKRMLIPPSNEIPGVMRRSQTM
TGHIAHPVKIKESQNYPTKIESSLFVEEDGEETNIMPEHATKERNSTNDLQSLYAIRQES
LPDDVRQFRHVVDDSDDENDSVFKQTTSLESSSSGLPPSLNLKKRSLSMLDMTTAHDQEK
VEALNRFYDFARRLDTNEDSISDIQLSFYCRELGGEFHFISFETRKMKNAMDLIRFNKLH

MNIRNMXVSSTEVLVFFVLVRVSRFSCAMLEISDRNRLMLITSNVSDAAATGGGAHKFAD
KWERELAIGMIKQDELDSLVAGMQFVLSTVVGECYTFRPKQNRTAETTGDGKNETADGGK
NPKLEWWTRKVQRDTISYSSAYPYMLVTIGTGVSVLRVDGPRKHERVSGSTIGGGTFWGL
MRLLTDVENFEDAMKLAEKGDPTKVDMMVGDIYGEDSDALEKLGLTADIVASSFGKLVSK
EDPAKGLTQEDLARALLSLVTNNIGQVAYLNAKLHKTPRIYFVGSFLRHNMISQRRLSYA
IDYWSKGEMEALFLEHEGYFGALGAFLMTQNIEKKSGGFFQWENNP
>comp2540_c0_seq1; 430 73 1101 LEN=1261; translated
MKYCQAAVLMLAAASGPVPGSSQLRMRTKTQPKRDEELQNRINDEEEVPFWTSALLNNLD
SMVAPTPTQAPPAGASIHPSLGDTFLETGFPTPTPTIIPGSISETDSPSSTPKSINIVDI
DNSSFFFSDILNVASYSTFCRAGEYTNVQIVSAREDVVYCITNFEGGEVEFGYEQDYPDR
PIMKLDITPPLVCGDDEVEVEDSISIDTPYKNYCGFFGKANFQPMAITVTDIDGENIGFI
DANITETASYTTYCVDEEYSNVEIVDINGDIYCVTVAPGNPTIEFVDNSGFMALIDVEPT
MSCNGIDGSISVPCGFEGVALTVWPNFISSDSNITDSNSTVF
>comp2541_c0_seq1; 207 1 538 LEN=787; translated
XTTQHDKLRYDTARPKHCCFPTEHEPRPENATHRNPTPAMDKFSPAHVGANELPDGFTQV
NPNGGGGPGGSEADFKKAAIQEQKQMILEQAMDLQALARLGRIRLVKPEKAAEIENTIVS
MAMQGKLPGKINEAKLVEILERKNRQDARSSEGASHGIQIQRKRYALDSDDEDDDDDI
>comp2542_c0_seq1; 774 67 3386 LEN=3686; translated
MIHSVFCLGASEPKSISLHRGELGKNKANYISAELNGRQWSDXNFYDEESSPTAGAITTA
SLPIPGRSFTLLKASTSASSLNVSSSSASSRKAEAHSVHTNQSGGNKFEGSMIAPSSHCG
TPPRKKWERSFNSFLKGKNPNLSPTMSSSPGSSINSMLPAEDMSTGTNGSGASQSTTGGG
MRGSKKKSSFSIIKGSKRLVSRNLSGKSGGLPSMFSKGGASSGRIQRGHQSMDALDVQLR
KGQQIAKNDSTTLARRSLPSTSLLAVVAPSATTHTRMHFPSSVAEYTRTGPLHSSGLQKE
VDCHGDNRSANTETTVSTTPPTFTSALDEQLRGSTSYNNMNATSTACHSPSFQINPVVSE
GEEQPNSASSAPQLTQLHRTRSNSRTHIYSSSSNSNSRTNTPVRQYNHNYHDELDFRNVP
EMPTLKGLRIQNKHPSSEFHDVGLNLDDSDNSLVPEPNNGDTNLMSQNNSSHYSFSNTTP
TIITDGRITEPPENLDGALETIGGMVTVHTSSSSHLLHKLGQTERTVFPASPETKKAFTK
IHNSSTDSESPFLGGNKASNVDLAGMYNSQERPVSSSNLLSKSLPTTTFAKDNIIHSNSS
RLLKPIQSADSWARGRRYLIAPAALAACPSSTIQGLSGLCTESVKEAATSTCPSKASGAI
ELGDAVMTYIGEKYHLSFGKFSSCKLVLRQNYLFEYDLSAPLTNLPRGIAHLQHATAHIH
KDFEDGLELHFYASPCAKVDHRVLLIRVKNKDERDHWITCLNKAARLQIEDLWEIDEDHP
LGSGRYASIFAARRKNSKTANNSDEEDRCDKERSPIDDGKYNCALKVINKNEFWRMVIKG
RERPDTIVRELAVQSTLTAKFGTKQNFLQIQGFFETSDKVVIELELLDGRDLFEYISSKG
VLEEGEAGCIIRDILVALDAMNRAGIAHRDIKPANILMTTSNESKYGVSVKICDFGMSTL
VGADGFVRGRCGSPGYVAPEIFKAGPGEGYRNKVDVFSAGVTFYLMLCGYEPFYGETEKA
LIEANKTAELDFPESEWGKISQDVKDLVELMLDKNPTDRLTAKEALEYPWIQGLEKNEKH
NTNRCPGGTNDDTNNPRLQGGICALM
>comp2543_c0_seq1; 279 376 1024 LEN=1024; translated
MSSSEKADGGWSISATGKRAASFISGLFFGAPGGKDGDENPQPSNKRRKTTTTTSSAEMD
LVLETVREHTARAEAREKAAAARAETIEALLEAVLERLEQRKRPAARETFSTPRKPARPL
PLNPNPPSASGQKLWTQQERSIVVKGLAAYGMGSAKRVAELLKHRNYSQTRGFMRRNADS
IRREIEATTGYATTEAEKQEILDSIAAENEEDGEDDX
>comp2544_c0_seq1; 86 1 639 LEN=639; minus strand; translated
EVDANKDKDLDKEPGAPMLFEKDAPAIAVVVHARLSNTRLDAETAAXQHPEERSAVDADA
GADARARARAFARAGCPTEQWLLLQRHLCSSCFQKEEWMSGFASVPCTDLWIYGTIGKAI
VWGDLESELLLGIPREWGFCSATDRWPVVFVLGVFGFSFLTSSVVGTVQVQVPSTGLYGN
RIVLLAPNTHTYATFQSTYVHSVLLLVPGSCLWX
>comp2545_c0_seq1; 233 1 801 LEN=915; translated
AQTSTGNLDQYSTVLTPDARPKRHTQTEPTQTKRKRNPTSRMADEGTKIESVGLAPEVFV
LVAAHAAVHPASPVHGVLVGSREGGRISVHGAYPVCHETPTGVLVETALSLVQSSLEEDT
SNETTTEIVGWFTAPELLHEKAPGPVALRIVASLAAATSGGGTDPVLLVVDNEALLALAG
GGGAVLASEAVRAFGKDLGRQWKEPVGSLAVESDAGAAEAAASAIREATGAAAGPFVRDL
VDHWKAGAASEWTTAASLAAFTKKHT
>comp2546_c0_seq1; 472 134 1738 LEN=2213; translated
MLPRFQLFTSSKLFGLSSSLLASSVRSFSSTPTTASELLRSSLKDPSLTEWPEDPSSPTD
EPRFPVFDPSSPTTLLAEISTRDPKAAIEKCSDALPAWRDETTAPHRASLLTKWSSLMTE
NADDLATIMTLESGKPLAESHGEINYAKGFLDYYAAEAIRPTGAGGGFLVPSPFPGPSGG
PKGQVMAVNQAVGVAAMVAPWNFPLAMITRKVGPALAAGCTTVAKPSELTPLSAIALKHL
ADRAGIPEGVFEIVTASTEGTPAVGTEFCTNPIVKKISFTGSTRVGKILMAQSSDTVKRV
SMELGGNACFVVFADANVDDAVNGAMTAKFRNAGQTCVSADRFLIHSSIHDEFVSKFSER

VKTLKVGPGMHPGTEMGPLINASGVQTITEKVKAAVKEGATIYEEVALPEKGSGPQFYPP
TVLTNVSADSEIWKTETFGPVAAIRSFETEEEALAIANDCNVGLASYMYTNDLSRAFNFA
SKMEVGMVGVNEGMISSTATPFGGVKESGIGTEGSPLGIKEYLETKYIFMKTAN
>comp2547_c0_seq1; 197 185 631 LEN=798; translated
MSCSAIRKSPDSMHAAAAPVRVAAPPPPHSHYFEESGPPIGAGSEKLHTKRQVKGAAVAG
GVTGLVLLGPAAGLLAAGGAALATTKGRGKLGRAARATGDSVSDLGRSAKKFDQKHGVRE
KTSRGIAKGCDWISKKLDKGKEATRKPY
>comp2548_c0_seq2; 171 383 916 LEN=1369; translated
MTFPLLLSDDGHSIQSLITTTTKQKRSSVLTRVWGKITESMKFDRKEIAKMGIDFGLTYN
MISNINGSITLSTAWYIASMKSGLSPLAPGNWRSLLAAYASLYVVAALARPFRIAFALGV
THKMEHFVQHVQKRIGCTRTNAIGLIFGFGVVMWITCCAAGVTLASTLAGVPVWKYD
>comp2549_c0_seq1; 189 199 766 LEN=881; translated
MSYRRGLLRRVGNLRISYNSWGNPRFFGNVGFDDFYNDYYYGNSWNVNFNWNIGAICVYN
DPFFYGNEFRNSYRRVREDANYIYYRANDGTNVSRDRMIRRRKASSNNVANGQTVKKVEE
EIHRREQINEDLFKTSLTTLEEELSLVQITIEREDRFKMNLTTGENPSSIQIITGKKERL
ETLLPKEK
>comp2550_c0_seq1; 403 216 1373 LEN=2362; translated
MVDTSAASECLELIYLSIFAYIESVLISFGIQHDTAIIEKNPREELECRNKTDCIDRCLG
TKRPVDLWELRELALSEGGLLRAGYRQKAWPKLVGLTEEDYIPELELSSTYSESPSSVRS
RNESALWSPSTTATTSTSCLIIEEEKKSEIEANVDLIRRDAGRSVIFRYHLGTSDANPIV
PPCASERLAQVLENTIRQGNSSLHYYQGLHDIAGVVLHNMDYQVDITSQILQRISHSHLR
DAMRENFSNINWFLNVLLPPLVERVDPSVHYSLQMANVELSTVCLPWMITWFTHDIHDPE
TAGRLVDAFLSGHPLMPMYFAMAIITHPLLKQELLNADPEDTSSIFMAIKSMPSRLKTKV
EEDEQLSEQQHVSLQEVLEDCITIM
>comp2550_c0_seq2; 450 216 1730 LEN=2250; translated
MVDTSAASECLELIYLSIFAYIESVLISFGIQHDTAIIEKNPREELECRNKTDCIDRCLG
TKRPVDLWELRELALSEGGLLRAGYRQKAWPKLVGLTEEDYIPELELSSTYSESPSSVRS
RNESALWSPSTTATTSTSCLIIEEEKKSEIEANVDLIRRDAGRSVIFRYHLGTSDANPIV
PPCASERLAQVLENTIRQGNSSLHYYQGLHDIAGVVLHNMDYQVDITSQILQRISHSHLR
DAMRENFSNINWFLNVLLPPLVERVDPSVHYSLQMANVELSTVCLPWMITWFTHDIHDPE
TAGRLVDAFLSGHPLMPMYFAMAIITHPLLKQELLNADPEDTSSIFMAIKSMPSRLKTKV
EEDEQLSEQQHVSLQEVLEDCITIMNRMPPRSLLDLIDSNIYSRSELLQRMSSISLFKAP
ASWSMASGGLPRSRNELNDIEGTTYCVRAKLASGVPMLMNTVSESTVIRRKSWGSRKYLR
KKKTQRMKKLTRIILGNTTNTKMS
>comp2551_c0_seq1; 433 62 1003 LEN=1286; translated
MIALKPFFLVALLAGTSSAFAVIQPASSIAKATTTSTILKLSGGAVGAGTAPELKPPPSL
YQGAVAAGVGKASKTWGDIFKLGIVSGCHIAFGAYLAITVGGACSGIAAENPGLQKIIFG
SFGLPFGLIMTLVTGGELFTGNTALVTAAYKEGKISGKDLIKNWVSSYTGNLVGSLLLAY
LAYKSGTLAASPASAAIATAKCSLPWDVAFVRGILCNWLVCMAVYMASGCASMAGKMTAV
YFPISAFVALGLDHSVANMFIIPLGMLRGADITIKAFLMKNLIPVTLGNIVGGALCVMAP
FGSVFGSWGKKSD
>comp2551_c0_seq2; 390 62 1242 LEN=1525; translated
MIALKPFFLVALLAGTSSAFAVIQPASSIAKATTTSTILKLSGGAVGAGTAPELKPPPSL
YQGAVAAGVGKASKTWGDIFKLGIVSGCHIAFGAYLAITVGGACSGIAAENPGLQKVRIG
VENMEQIIFSSGLLNRDQSFHRSLNEFDYRIQSVFQDLLKRHSSVSIYNQELFRVLTVVN
NLSSRLTFAFTFHKKIIFGSFGLPFGLIMTLVTGGELFTGNTALVTAAYKEGKISGKDLI
KNWVSSYTGNLVGSLLLAYLAYKSGTLAASPASAAIATAKCSLPWDVAFVRGILCNWLVC
MAVYMASGCASMAGKMTAVYFPISAFVALGLDHSVANMFIIPLGMLRGADITIKAFLMKN
LIPVTLGNIVGGALCVMAPFGSVFGSWGKKSD
>comp2552_c0_seq1; 927 455 3430 LEN=4711; minus strand; translated
MEGSASKSVTHRKPPVIKIEDDDVALDEKRDAGCNKPTDETPGKPTVPVKASSTTMKSLN
TAGAKNVSKAPASVVAKAETNATESKDKATIKQKGMGHRDGNETTCSDAFDAIADIASKA
HVRSKRRKRARFDKGTDAFIKDCSRSQVTSNASASTLMAMSCHPPTVVTASISNHYVPPR
LISGTGLAKQNGSTKNKDYSSRLDSHSSGNKNKVSVHMLNSVGLECGDDGVLQLEHFDDD
EEIPSTHADDTDGDKAAAIKAENNAGGDENCDTSNNMLAVTKNKPPQWSLEQHKAFAAAI
FEIGLKNCSPSVIMENMRKQPRYITRERTKSHLQKYRQTKERGRAEFLKEYDVFFKSIEQ
AKAHLNRKNCISPSSDEKEPSACSDTKGNNVNHKEPIPKAVLTTALEGKKPNKLLGGKAA
ALLSYSVLNNFSTSHGPDQLQYKAAKLMEFPIMSEEEKRSSVGASLLQVKSLIDNMTDAL
LKQRHGISPIPAHKTGNGDEESDSSSSCSSEDGYSEDEEDNVEGNAANNKQQINKSLSKD
QDPATAAVPTQQTGPFATATAAPGHPFYRGPTTAYPPPYPAPVAPASVGTGFALPYPHAP

PSFYGGAPPPPHLQAAAAPPPAFGAPVAFAAHPMHYTQDARINPYQQAPPGQVGAYPGVP
PIQPNYYPGVAGYPAYYGNTGEGGMAPTVNGSNPSTEYNRPPPQPSYPQQTGSSQQHQQQ
RVYDQSPSHSNEGRGERRTFDLSGDSPTDASNRRRSERRSKRRRTRDHHSENRSVDTRNM
PRKSRDDFRDFLDRLSRMPSPDKAPQRTSSSPVQRSSRGHEKFKSSPSSMHPDDALPKTS
ERQSRHHGQQYERQHYDRQFESSGQSKSYAKDFESPAGDPFSESEASRFASPYEASPIDN
ALNERQSQGHVSQDQQQQQLKQNDINGQFWESSNFMGTEYNDQQQQNAGGGDFHQQPAQP
DVFVPVTHNRRYGNSLSSPEGEATGNFFFGE
>comp2553_c0_seq1; 493 269 1789 LEN=1973; translated
MSDDDYSYEYSEEEDYEIGDDDDDMDWNSSATCTIASENPNAAPTLGLAGVLNGIRISPA
DDLRPEMKRRLKEVTEILDIPEAAASVLLREHKWSKEQLFEVFYADSEKLLKKCGVSNRC
DPKDPMKTDPDDDNCCPICYDDMEDGQKMSMPCGHEFCMSCWHDFCANAIEQEGPSCVYV
TCPEATCTEVVTEVEFQKAAPDYLQKFQTYQLRNFVESNRLTRWCPGKGCDRIACAPTAS
AMEQDGNVANCDSCYTSFCMVCGEEPHAPSECKDLAKWNEKCRNESETANWILANTKSCP
KCASRIEKNQGCNHITCQRCKHEFCWICSGDWASHGANTGGYYKCNRYDPKSTETNDNSA
AAKAKRELDRYLHYYKRYHAHSEAQKFAKKQLKETESRMVMLQESSDNSKWTDVEFLKTA
NEQLVECRKVLKYTYVYAYYMNPNDTLQKERFEYHQEMLEKFTENLSEQSEKPLDKMDRT
DVVNQTRVVDRFMKNILKYVDEGMEG
>comp2553_c0_seq2; 493 153 1673 LEN=1857; translated
MSDDDYSYEYSEEEDYEIGDDDDDMDWNSSATCTIASENPNAAPTLGLAGVLNGIRISPA
DDLRPEMKRRLKEVTEILDIPEAAASVLLREHKWSKEQLFEVFYADSEKLLKKCGVSNRC
DPKDPMKTDPDDDNCCPICYDDMEDGQKMSMPCGHEFCMSCWHDFCANAIEQEGPSCVYV
TCPEATCTEVVTEVEFQKAAPDYLQKFQTYQLRNFVESNRLTRWCPGKGCDRIACAPTAS
AMEQDGNVANCDSCYTSFCMVCGEEPHAPSECKDLAKWNEKCRNESETANWILANTKSCP
KCASRIEKNQGCNHITCQRCKHEFCWICSGDWASHGANTGGYYKCNRYDPKSTETNDNSA
AAKAKRELDRYLHYYKRYHAHSEAQKFAKKQLKETESRMVMLQESSDNSKWTDVEFLKTA
NEQLVECRKVLKYTYVYAYYMNPNDTLQKERFEYHQEMLEKFTENLSEQSEKPLDKMDRT
DVVNQTRVVDRFMKNILKYVDEGMEG
>comp2554_c0_seq1; 648 106 1794 LEN=1922; translated
MSEGTDCTENPTGLGADNFLSRITFRKASPTDILRCVEMVKKESRSKGLMFVSKNELEYR
QHYAANYYRCAVYEDEDGNNDEDTIVGFITGIRVGAIEEADKVVGHALTHVVPHDPNGEV
LIIRSVIVGEEYRKQGVGKAMIQDYIGAVRELPKQSSSKPKSSKKKPIQKIVGLCWDHVL
LPFYLACGFSLQQAHKVTGACTETEKSDELRRAYFFESSLAATFDYGIFGDGNNDDGCNY
YIVDSFASVPGTGNPASVVLLREEFDPMVKHKWMQKVAAEFNLAETAFCWPKGSNTGNDH
ADAIAFFKNESHWNIRYFTPTTEMGLCGHATLASAAVLYQTLPSEVLPSGTAVVFHASED
ILTMNLENNGEKEGISAVSSSQRVSKVSMNFPPKPTKDISTREEKATVRNMLKSAFSIDL
EPLYVGLSDIGDLLVELSPKTFSEIGYDSLNFKAFFEWDGYYRGIVICCAAPESAVKDKT
DKSIEIDFLSRFFAPKAGIDEDPVTGSAHCSLGPYFAKKLGKKRVVGRQMSVRSGVVECL
VAPDRVTLTGTAITTMRGQLFM
>comp2555_c0_seq1; 553 261 2141 LEN=2388; translated
MIRSRAVNGNNTVGKSAGTSAKRGTRPGIRKAPRKESANPFAAILYAVLFIMICATVGGF
YETSSMSASDKVPPPRQLKTNPRLILDKIRKEFNARYNVSGKIDGQKMLEKGLHSFGSID
HTARRIMEAARAQRPFVMAFSGYSITVGRGNFYKQSFPFVAEEILKGPMQELFNIPLVVR
NAAIGGIPSFPYGFCMEHFLGSDPDVISWDYSMNEKSKDASVFEAFLRQATKQLPKRPMV
IMMDTNANRMKTLDTYTQNGWLKDAIAIGNKDILKQANIFEGDAQDLPTGFQDWNEFGAP
ANCPGRGSWHPKKQEHSMMGWMIAMHFIEAMDRALKLSSEEEPSQTSRMLSVEMPKLGAP
ISKSLPANDPEVTELLFGHGDNHMMKDLSCRTSFLPATDHDKTLKSVVVSGLAEGDLDIM
IDRTDEHYKEGWVLDVSKVERDTKRKVEKCGGLGYVDMKIAMYGIPESGKLRLWLPFEGA
LHDHAHHGHGDNHVLDTNAKHWFDDFIICEANDKRDDKACKLDRDLEIIVGGVPVTSESI
HIVKDAAEYLKRPTCVNVGVPETAKITTLGDLRTTKGKALSGDEKKKFGRSYGDDTFGMV
VDVTAKSRVTRKNGACCLSHIVWEQH
>comp2555_c0_seq2; 491 261 2380 LEN=2627; translated
MIRSRAVNGNNTVGKSAGTSAKRGTRPGIRKAPRKESANPFAAILYAVLFIMICATVGGF
YETSSMSASDKVPPPRQLKTNPRLILDKIRKEFNARYNVSGKIDGQKMLEKGLHSFGSID
HTARRIMEAARAQRPFVMAFSGYSITVGRGNFYKQSFPFVAEEILKGPMQELFNIPLVVR
NAAIGGIPSFPYGFCMEHFLGSDPDVISWDYSMNEKSKDASVFEAFLRQATKQLPKRPMV
IMMDTNANRMKTLDTYTQNGWLKDAIAIGNKDILKQANIFEGDAQDLPTGFQDWNEFGAV
SRFFLRLCLFFIGSTEHDXMRSSGKFCVAMDAFTKMLQHIVLARTSTREELFSHVSARSL
FFYCILKLSFCDETPLIYSPRIVQDEVAGIQXKQEHSMMGWMIAMHFIEAMDRALKLSSE
EEPSQTSRMLSVEMPKLGAPISKSLPANDPEVTELLFGHGDNHMMKDLSCRTSFLPATDH
DKTLKSVVVSGLAEGDLDIMIDRTDEHYKEGWVLDVSKVERDTKRKVEKCGGLGYVDMKI

AMYGIPESGKLRLWLPFEGALHDHAHHGHGDNHVLDTNAKHWFDDFIICEANDKRDDKAC
KLDRDLEIIVGGVPVTSESIHIVKDAAEYLKRPTCVNVGVPETAKITTLGDLRTTKGKAL
SGDEKKKFGRSYGDDTFGMVVDVTAKSRVTRKNGACCLSHIVWEQH
>comp2556_c0_seq1; 211 301 1208 LEN=1733; translated
MSDGDPNGEGEPSSXSRPVLPSDLDRRGGPGGRRRGGAGHEAPPEDVPVVPGVVRRHRGR
KRPKGQDDQGFPFADLPRVRVPPGGPGLCVQAGDWERFDGTGGSSPLYGRNWADEGSAIA
TNQPGFLRHDKEGILSMANTGKPGTNGSQFFLTLKPAPHLDGGHAAFGRVVAGTESLRKI
ASVERGERDKPVSLQRVVIEDCGRLFESGGGELGGKRDRKKKKKKKESKQRDRKRRRGGC
RGDSDENSSDEDRRRTHSRKSRKKKRRRRGYESDDSSDCDGSVVGSSSSDSDRGGXPETV
SG
>comp2557_c0_seq1; 449 131 2156 LEN=2278; translated
MPQNPFRLHNDNGDEAYECRRRQFYHLFGVFLILGFLSSEASAFCSSSPIVGIPRGNHEN
FHQRSTVIKNRKFSTGTSTTIPTRTNNRVKLRSTESEGEEQRSLLEQIRYRRKTRSEIRR
DRRRKKRKLSETNPSLSTTVPHSGRHFVPSHPSYRRPLVQRLFSKLRFRKRFMEGWYYRL
TLKEYDIDDCNIKGKSETNAVSFAIIISIEDPGIKSSNLRLACLQAIGPNDQYLVQSTKD
DTKFWASKKSQSLGCTFNEHDNNNNTAHPKSRLDPDVWKETVKSGFQITDDSSFRTASTP
KHLYKPRWMGRIHGHDGSHGGVLEGQGVGGTLEFDFELDPDSVCGYGGYTTTIGDANATF
GHTANTTKSNQGIPDFSTTNGSAGKRRRQSRDANRQKSTGGWLSSFPVFEPHWQVTIADA
LATGKINWNGTEYRFKDQSFYAEKNWGDALPSKWYWTQCNSFDNIEEELSVTAGGGVRKL
PFGKEEELGMIAIHHKGQFYEAVPWKGNVEWNVSTWGEWSLYGDRLEETIDHDTGRITPA
FAVEIQYRCDPDKLPGLVFRAPTPKQGMVPFCRDTFEADTVLTLWELKRNENTNEFEKIE
PPIIDRARSKEGGAEIGGGPWWETWSGKSQLKPLIKTLLNIPNAFSATDRYLVSREKTLR
QPVSEGLVKVTKFV
>comp2558_c0_seq1; 206 53 319 LEN=644; translated
MQRQQSFVGLERSLMVKAKTCDRLVTEVMQAKKKMKEVDSAKQTFLKIQIPKMEKQIAEL
KVELEGLLDEDEIDPCGEYYELAKKLLA
>comp2558_c0_seq2; 203 340 522 LEN=847; translated
MQAKKKMKEVDSAKQTFLKIQIPKMEKQIAELKVELEGLLDEDEIDPCGEYYELAKKLLA
>comp2559_c0_seq1; 348 609 1547 LEN=1601; minus strand; translated
MATLKASIAASESEIAASEAEIAASKAKIAASVAEIAASGQHLKDLTQELEKLRAESNAA
EENHEAAKSVMVDLTEETYTDDEDSGESTKKVGSSGSKRSRALSTTSNGGIVSQGDERRS
SVKKNVDATTRGFLPFPSSDGGSRSKKKSKTPAARIVYLPPQQRSNCSIGRTRKQLNKIK
GMVKYMGPSKFGRPDVCKKDGKIYTLVLSERWHNLVERNCEKQICLVRRGPKTTPTAEKV
GEWKESIPIFHVPKQPENSEKKLYYAGHWKVEKMDKLNHPMEFKRSPRNMKLTFAFDRFD
SKLNSIIQNNQE
>comp2560_c0_seq1; 483 96 1622 LEN=1662; translated
MDTSEQRAQSTSFKFPFTDNEDADLNLSKSFHEFRAPKTPVGAPSYNEFADVNFIASPSD
MNLGLSPIAVLRDSDASVDSCVKSSGEECEPQIPISVENTNDATKSDLSTTGLEIDKLIP
PDDHSSPSKTDDLHSAMKKVGERSSNFIDKIRNAAHKRKVAVTRSRDSLVAKEQEQLRSI
AESKTRLLSLNEELQIVEKENEETTKESVSNTGKSKPKHSYKNNKGGFGGVGVPKVEKRP
TTTPLSPMLGSRRKGGGRAPRLVENSRGPSGRGLKNGPTAKAIDRSNFSTKGLKKVSIRD
HKDAASGTTNGRFAKRGTDSSKLEGPNEEISIRNFTVVQANVRRWNAGQIGIPKVSKRAV
TVPVSPCLGPKRQSRIAGSHDEENSSNSGRIILKVEVDASKRANGSISSQNEVMTPSSTR
MSPLLGLNLIDSVQKVRYACSESDRELALKSSFKPFVPRSTTRAKMRSRYDILRKEKQEI
RRSEERERLQAQIKVIHRELRVLGKDLT
>comp2561_c0_seq1; 302 439 1110 LEN=1482; translated
MTENFNYSHFSQPGKSRVGYVGNTYDDVVCRIADDGEIQVKTPGKMMGYFKNPEATAETI
TEDGYVRTGDKGELDEQGRLKITGRTKEIFKTSKGKYVAPAPIENLLISNQYVELACVGG
KSQPQPFAIIQLSESPKAKAIGSEGEREVLGKALEEHFINSVNSKIDGHEKLQFVVIVKD
DWLPENGFLTPTQKIKRATIEDTYDPKVEGWYAAKKKVIWDGF
>comp2561_c0_seq2; 639 81 1772 LEN=2144; translated
MSDDTKTILDFFFDNVKKLGAKTFLTQPMGGDVVKTFSYQEVLDEAKKVAGYIESKGYPP
KSQIAICSKNCAHWLIADIGIMLAGHVSVPVYPTLTAETTGYILEHSESKMVFIGKLDEK
PWQEMKGGIPSSIDAVSFPLCPKDTLHAKKWDDAIASSSPIASPVSRTKDEMATIIYTSG
STGKPKGVMTSFGAMTDTTKGICKELSVTSDDRYLSYLPIAHGMERWLGMCVPLYSGEQV
WYAEALTTFIADLNRCEPTLFLSVPRLWTKFQAGVFSKLPEKKVNLLLSIPLINILIKKK
LLKTLGLSKVRFAGSGSAPLPQDLLNWYRSLGLELLEGYGMTENFNYSHFSQPGKSRVGY
VGNTYDDVVCRIADDGEIQVKTPGKMMGYFKNPEATAETITEDGYVRTGDKGELDEQGRL
KITGRTKEIFKTSKGKYVAPAPIENLLISNQYVELACVGGKSQPQPFAIIQLSESPKAKA
IGSEGEREVLGKALEEHFINSVNSKIDGHEKLQFVVIVKDDWLPENGFLTPTQKIKRATI

EDTYDPKVEGWYAAKKKVIWDGF
>comp2562_c0_seq1; 457 505 2854 LEN=3098; translated
MSKRSGGMNLGGLMAKKSKLDKKTAASAEASRIGIGGKGPSNPYLTKPVPPEQMIQILLT
QFRNAARKRHGSNGSKPYASPFCEIEARIGILKVHNRRVTSSGPKRVNGTVVRAFDGGST
PSGVEPPSMVSGISRSHFVGWTLAGLSEASPLSSALGEMSENSKEIKKKLDEHEYVETVF
AGYEGDRRVSFPGYVDVEHPPSAPVPTRSGSMEYKEKIDQRDLIIPAAKYDLRIGLATEK
IVDPNVTTIPPGWSLHRVKRRRSYKRKDKNMGWQIDVTEVTSTPRHDLSKKTVVYEIEME
LQADVLLNLINMEDKDQIRTETHKLSQQLWWILSHLNPLEETVDVEEYLIDHPDKKAVQL
ALATCGALKKFMDGRPSHAHGQPIEVGSLPLANPGTIPPPSLANTKFCGCMPIGFSRHNI
EDVQRGSKYFLSEKTDGVRHFMIFTGETVVLVDRAMRGKQCIPIAKHQQEKSAEPMASVI
PLIKAGTVFDGEVVMHRGNGKKHGPRPLFIVFDVLTIGPSAVVMHLPFEKRLEHLKRATF
RTPTANRDMFADSAIADLSIPLPLVRKNFVQRTDIAELLNNVIEEKGFRSYRRLPIHNHL
TDGIIFQPNLPYKCGTDVNLLKWKYLDTCTIDVQLLLEGGYRPRGGVEEDDDPLKGVGGP
DNTSVDMSRYVKLPASERYRLEADKMEVKSKIHIAEVGFDPLSGEWYYLTMRPDKIAPNH
ISTVLGTLLELSESLTTEELQYRMSVPPGQRDTYRKDLKGMMKQLLSYQKQSIFKQQQAG
RR
>comp2563_c0_seq1; 256 1 1230 LEN=1838; translated
QHQEQHQETQEQQNLEASIETPLNMEPYVSFNSDSNCNDTSTSTTAANHTEKLCRRFERL
EPYVEQPTPLNSNPRYRIRETRQEHEYEYAHECPPKLLSKQEKPMPVHGLADTNSDPSDP
PNQRKISFSERLPEVSSACPRAFRNMIPPTSCLVEELQKDPSYAHALAAGTIWQSLVGQH
VRFPKLWYDGAEPARPFLGCYDPNRRNKWSYVGRHRIEGDPLLNRRYVRSKKSSGKILLH
IVCRDVLNLTGGVDLEDEAATEDLVVGVFHPNAEGIHSRLGNPSNHGSIGIDCCRDVWIA
HKSRRIAHNKKRTATRIESLLRNLNKNRVDETPLGGDFMHSEPAQTKRIRRTIDNTNVNV
IFGRGPPKHTVFVPEHKLYRMLAPLAETENKRNNKNIPASVLLMRTFLR
>comp2564_c0_seq1; 162 429 645 LEN=645; minus strand; translated
MGREARRAFPALDFFVALDFFDGEVDGEVDLDFMTGGDVASLIRDGKMDGELGGEMDVKM
DGGEVDGEMDVEX
>comp2565_c0_seq1; 1056 330 3013 LEN=3284; translated
MSNASRDVMAEQQEQEQQRVFPTTMSNASRDAVAEQQKQQREGEPNAQPPSYRSYEPLSL
LEFVTAFVERDSSSSNDPGENEEACELDTSFKVVEDWKEHKMDATCRSNTTSQQSPVVHK
YVLDRWEEEPVIFRYENATARIPSGPPRHSDIPSTALCFPVDNAESYPAGLATTGSSANG
TNTTYIDVDATLIRTLVFDPFNAYERFHAYANAAMIMLMFDVRNPQIVMLGKPESKFIYD
EGDRDFWRLFSDLEPIYIPWGKDVGIPENANANVTAHDKNTIYRFREIIEAPNAGLSMVN
TKSKASGIRGRATEHHCMSSLFRGVMAWIETKTFLATNAKATKAIDGNRGKQNGIGNKKV
EVFSRRAGNHSGESDRSTPTEGSTETAFATAAAAVAATARTTEPEGAPLSKQPPRLVKIL
WSSRRPYCCRDGGIWFPKRVLMRETEFLEALLEALYDNDKTTTRANEYEITSVEFGSLST
TESIRMASESTVMVGLHGAGLIWSAFMSGERGDDGDDDDDGNFDYDDEAKVEEMILKAQL
RRNGGKSRRTSTSSVGSNSNSNSNSIPKPGQSRVSNALSAAIRRSKRDNEDMLAAITSKIYP
VADILCVQGNQRNSRAVDLDPFLKEDSVVEGMDRDFAAAFPPSQMRCLALVSHNEMKSTM
RKFVVANKNLLKKFRLTGTNSTMTMLREVFSDEPAGTVMFGPACASGPLGGDAELVSLMV
GGRIGGILFFRDPMSAHPHRADIDCLVRQALVHNTMMAETPTSALMMCHCLRAALKGEGK
PEMIPSFFFSLQCPSVEAYKAEQKRVVEMQKKMARESMTTRSSVTSSALAGAKDYXVLVP
VRVKPRRPDAEAEAEAAAPELEAEAEAAGTDARWCSRVAGLHLFLQTICTVEEY
>comp2566_c0_seq1; 322 225 1133 LEN=1669; translated
MGYHRSDSEKTYAIKCVNRKKLTNEDEVALLDEVSILKEMKHVNIIRLFDFFKEPTSYYL
VMEEMDGGELFDRIVAKAYYNEKEARDTCKILVDAVGYLHENQVAHRDLKPENLLLVSKN
DDSQVKIADFGFAKKVLKPKSLTTQCGTPGYVAPEILEGHPYDERADMWSVGVILYILLG
GYPPFIENTQRELFRKIKRGDYEFHEEYWGTVSKEARNLIKSLLTVDPELRLSAQQSLEN
PWICGDGKTLAKRGLDKNLESLRAFNAKRKFKAAVSTVVAVNKLNSLGNAFKNLDMADEE
SP
>comp2566_c0_seq2; 350 407 1432 LEN=1968; translated
MAFEEHDDTPVPSSKSGKHFEELYRLKGVLGTGAFSTVRMGYHRSDSEKTYAIKCVNRKK
LTNEDEVALLDEVSILKEMKHVNIIRLFDFFKEPTSYYLVMEEMDGGELFDRIVAKAYYN
EKEARDTCKILVDAVGYLHENQVAHRDLKPENLLLVSKNDDSQVKIADFGFAKKVLKPKS
LTTQCGTPGYVAPEILEGHPYDERADMWSVGVILYILLGGYPPFIENTQRELFRKIKRGD
YEFHEEYWGTVSKEARNLIKSLLTVDPELRLSAQQSLENPWICGDGKTLAKRGLDKNLES
LRAFNAKRKFKAAVSTVVAVNKLNSLGNAFKNLDMADEESP
>comp2567_c0_seq1; 591 92 1822 LEN=1952; translated
MEAASQNQVGANNTREGRGNGNNRNNRRRGGRGGRGGRGGRGGRGGRGGRGGRNRNSENG
ENSQNRNYPKKHKEENMDSSNTNNKRPKKIKNTPPARNENGSTHGGSASESKKLAHITNN

RFADLKISAESRRAMAEVFQYEFMTAVQSETLPLILKENKDCLAKAKTGTGKTLAFMIPT
IEKITASLSKNGKQRSDICCLVISPTRELAQQIGSETEKLLSFHKSYLRKVVVCVGGTNK
NKDIKSLVGTTPIVVATPGRLLDHLQNGGLAERMNRLDTLIFDEADQLLDMGFRPDIERI
LRLLQPSQETRQTLLFSATIPDSVSEIAGIAMRGDYNFVDTVGEDSEQTHLHVQQQLMVT
PQALSVQALANVLERETKSNKDYKIIVFFTTARLTGFMAELFNSVQKETGYDVLEIHSRK
SQKQRERASEEFRRRKNAVMFSSDVTARGMDYPDVSFVLQVGLTDRSQYIHRLGRTARAG
KDGKGGLLLADYERDYMVKKELADMPLEVTPVPNLDQATAAISKAIQNVSKKNDLRNSAE
QAYRAWLGYYNGHLKKVRWDKKTLVQHANMWGIEVV
>comp2568_c0_seq1; 841 134 2575 LEN=2910; translated
MLALQRIARTRPSATSLFPSRGVSIIPSAHSSSPFSSVALRFDERLGPQDRRIPHDRARP
SPRCVSSLSPSPVPTSLPPLPGKVLIANRGEIACRVIRTCRLLDIPTVALYSLADGPESL
HAKSADEAYQIGFGSSPSESYLLQEEVLEIALRSGAEAIHPGYGFLSENAEFCQRVQTET
NGGVKFVGPPHSAITAMGSKSKSKAIMEKAGVTTVPGFYGDSDEEQEPLHLLERAVQDVG
FPLLIKAVMGGGGKGMRLVWSESEFLEKLEACKRESLNAFGDSRVLLERYLLQPRHVEVQ
VVADDYGNVVSLHERDCSLQRRHQKIIEEAPASDLSENLRQRLGEMGCRAAKAVGYVNAG
TVEFLLESNKTKEDPEFYFCEMNTRLQVEHPITELITGIDLVEWQLRIAAGESLPITDPA
AIPCRGHAFEARIYAENPGTGNFLPTAGTVWHHEPPAPPNAVGVGEDNLRASNGVRVDTG
IQAGQEVGVDYDPMICKLVVHDESREKALEKLTDALKRYQLSGVPTNIDFLIRCAEHRTF
RKAGATSTGFLDDHMAEVLPPVENEPPELAIAIAVFASLLQSEGRIGVSDFEAERRIQTS
PWNSLSGSWRNGGHSCQKLETADGTSVETTAFRDGSFEIRITKTTEEESNGNGTLFHIGG
TLSSDQYMQIVVDGSKTMALTVALREMDGQYQVCMWPQSLLLRNEGHYFWEVSVPNPRIP
TMAQNETGFSSGQGVVSAPMPGKISKINASVGDMVREGDVLLVMEAMKMEHTVPSPQSGI
VSSIPFQVGDVVGDGTVLAVVGSEEMKDEAEAV
>comp2568_c0_seq2; 780 134 2724 LEN=3059; translated
MLALQRIARTRPSATSLFPSRGVSIIPSAHSSSPFSSVALRFDERLGPQDRRIPHDRARP
SPRCVSSLSPSPVPTSLPPLPGKVLIANRGEIACRVIRTCRLLDIPTVALYSLADGPESL
HAKSADEAYQIGFGSSPSESYLLQEEVLEIALRSGAEAIHPGYGGVFFQLCAELSNFKVR
LSRFSNRQVTPNAVLLNSIGFVPVSLVDLRPRLVFDENAEFCQRVQTETNGGVKFVGPPH
SAITAMGSKSKSKAIMEKAGVTTVPGFYGDSDEEQEPLHLLERAVQDVGFPLLIKAVMGG
GGKGMRLVWSESEFLEKLEACKRESLNAFGDSRVLLERYLLQPRHVEVQVVADDYGNVVS
LHERDCSLQRRHQKIIEEAPASDLSENLRQRLGEMGCRAAKAVGYVNAGTVEFLLESNKT
KEDPEFYFCEMNTRLQVEHPITELITGIDLVEWQLRIAAGESLPITDPAAIPCRGHAFEA
RIYAENPGTGNFLPTAGTVWHHEPPAPPNAVGVGEDNLRASNGVRVDTGIQAGQEVGVDY
DPMICKLVVHDESREKALEKLTDALKRYQLSGVPTNIDFLIRCAEHRTFRKAGATSTGFL
DDHMAEVLPPVENEPPELAIAIAVFASLLQSEGRIGVSDFEAERRIQTSPWNSLSGSWRN
GGHSCQKLETADGTSVETTAFRDGSFEIRITKTTEEESNGNGTLFHIGGTLSSDQYMQIV
VDGSKTMALTVALREMDGQYQVCMWPQSLLLRNEGHYFWEVSVPNPRIPTMAQNETGFSS
GQGVVSAPMPGKISKINASVGDMVREGDVLLVMEAMKMEHTVPSPQSGIVSSIPFQVGDV
VGDGTVLAVVGSEEMKDEAEAV
>comp2569_c0_seq1; 777 342 1912 LEN=2192; translated
MSVNDDLLVDGSIEGDLMAAMNAALAESGDDNNSNSNSNSNHGSAFFSETDTASMRSIDVMG
EAMGLLMSGENDYENDYESEPTNGNMNMDGKEIRIDSGNNNYGGVGGDIDDEEEINFDDF
MAGSKGSGDNNDGKSMDQDIYAIDGGDQPQGGESKDDDAGAPGAESSNATTETAASKEPP
PAGLAADVVPLKQFENALALIQDLENRIQVLETDHQCLTEENMVLREQTLSQATVLADLE
AKLAAFPKLLEETAREEAQVAAAKAEIETKFIFWKKDMDRQEQELEEEKLKRTRKHYSAT
TDSLKQSDFLKDVVERKEQENDDGHHQEKPNGPLAFLRGWGNRNNKKHSNSKANGDKNDA
VPSGESDASTGGGASANGGSSGSKDHGSESTIETSKGDFNINITLSTSRSGDDLPDMPEQ
RNGKIRPSLPSNEEDDETEVNSSNAIKGNHGHDEXTAGPADVTQPQQRRGKIPPFLIAPS
FVQPLTTAKPIRKVPEDYDAELFSFRGLSKLPFAGKFGRNGMV
>comp2570_c0_seq1; 366 176 1402 LEN=1639; translated
MAWSSPKNSGRRTRTTRTPAGPIYYDSKHSVAILLQCNGSAWLRVIPWCILNAAFTAALT
ALETRYDLPLALPGESSSLGHDFTGLVLSFLLVHRIGWALRRYSRYQESLGTVLRDVRGL
TQKAIVFSRRNKNNDTSAKEWRSEIAYRSLLMARMTTANAEFPVAQIPAHEVAELDGPEL
QFCTPDRDFLYHAEIPYSKGIHSLLRMPWKIAELIRETICGQEERLVKPMSTDHEMNLLA
TVDSILAGYHGIQNLMLTPVPFPLVQMTLTLTMVYVFTLPLVFLKDDAQRSLLGDCSRVF
FATYCFLGLAFVAQDLDDPFGCGPNDFDAAGCFGFALDDVVTMIHDADGLEWADALRYKC
NPNPSRAVHDEMVGAMTGEREGLLPKHKHGGGDDNRPWKHAPVPDWMA
>comp2570_c0_seq2; 330 176 1543 LEN=1780; translated
MAWSSPKNSGRRTRTTRTPAGPIYYDSKHSVAILLQCNGSAWLRVIPWCILNAAFTAALT
ALETRYDLPLALPGESSSLGHDFTGLVLSFLLVHRIGWALRRYSRYQESLGTVLRDVRGL

TQKAIVFSRRNKNNDTSAKEWRSEIAYRSLLMARMTTANAEFPVAQIPAHEVAELDGPEL
QFCTPDRDFLYHAEIPYSKGIHSLLRMPWKIAELIRETIVGKSAIRLPAFSVQIANSVQQ
ASLTLNFARILPRNPMLSFALPPWFATAKKNDXVKPMSTDHEMNLLATVDSILAGYHGIQ
NLMLTPVPFPLVQMTLTLTMVYVFTLPLVFLKDDAQRSLLGDCSRVFFATYCFLGLAFVA
QDLDDPFGCGPNDFDAAGCFGFALDDVVTMIHDADGLEWADALRYKCNPNPSRAVHDEMV
GAMTGEREGLLPKHKHGGGDDNRPWKHAPVPDWMA
>comp2571_c0_seq1; 575 477 3236 LEN=3767; translated
MASPTPSPSKSWMTDSQGNHLSKTGMTSTPPGFVAQSGLWSENGGIAATGSQNADAVSTG
DKTRPERRRNERTSSFNNIAAALGSGLAESMEDATRGDHISASHHGDNANHNQSNISNGP
ATLTDSFFNPIENNGMAYERHKRHSASRLVGVPGASTSSATSSHKTPETGPLFVASLNSG
EARSKSFNSRNGGSHLVGDQSNMSSSIDKGHCPGAIINDSSIAASPFSQDKNASAFHTSY
DEVQSNSTMQTNRGQLNNVGDTQGNGHIKSRLPVTKDIGLTVNEPSDYSFADSGGVSNIP
NRNFSNFGMNRVGKPDVNFTYGIQQDMQNLWSDAGGSRGGGGNLSPTGKPQSSNRTIESM
DGGGVGIIDGNRHRGSSPQGIDAENDLRPFTWDTNQHETSRTLAIFNAAAIPCNQIEELC
DNFGAIENFRSEFWERVGVIFISYFDMRSVQFAAMQLPRRLQGLHPTGGNIHIKYCIPIN
SSSQNDESLVVINDLPGQIGQSNLGSYLSQFGAIRSLRSGSYGSFIAEFNDLHDARQVVY
ELETRQPWGPDVSIEVGIRSPADRKKGRELLALLGRWRTKGGAGQGAPPNRGRQLVNTPD
TYRSRPDSRGMGGHRDGRYERGMHNEDHPSQLGYGPGPQDARYNHGGNTYVVHHNQHQHP
SYSSYRGSHHPNDHIGHGGHGHHGTYMNRPVSHQPSPHQQQYYQQPHINHGNFPGGSSVI
SAGSSRHTGTSGYYTDDRSIGSHTSHGSNMRSINSLVDSMAGSSRDGQSQNLILDLELVE
NELDKRSSLMVRNIPNKYTQQMLLNEFTENGHGPGIIDDFFYLPIDFKNRCNRGYAFINFV
DYRDIVKFHRQYYGQHWRTFNSDKICDITYARIQTKASMLKRFENSALMEKDEEYKPLVF
VSDGPEKGKRLPFPVSSRG
>comp2572_c0_seq1; 566 421 1728 LEN=2303; translated
MWADEDGKISGQQQPERSLQAYTNMTAQYEDGPAVNQDREVDFEYCYGALQRADSNNDGR
LETADYLSFVQDYGSNAECLGNIMSLPVMPIELTACWNQLSCECRNRGGAPDCCEKENAH
LPLNGVNAVDPAPSGQTTQQPPKEEYIQTEQDFLRQACVRTDQCIISFCGYPPPPLPIPP
PVPPTPPPTIPPVPVEKNLEVETNYLWLLFLLLLLCCCRRRWILCPVAKDDDEENQERAP
DDPAAMGSLPADIENRPDGFEGDYNDPDANKMSMKSTAATEVGEGGGIRYYRVVQEPEYE
EPPPPPEPKILDQYDKGVPEGDGMKLKHIEKDGPPPPEDDPYALEHYEPDGGVVEHKRKG
KWSFLAGGGYKKEERAKKEKSDWKRPGYNRAAKKEEDDPDNRRDRNLNKYEGGAIFDHLD
ETDEPAKRGAGNSLEW
>comp2573_c0_seq1; 90 1 129 LEN=217; translated
NDEELNKLLGNVTIASGGVLPNIHAVLLPKKTKMNSKASQDF
>comp2574_c0_seq1; 963 101 2680 LEN=3009; translated
MPSLKKKKSSYESTGFVRRMLKKGSSFRSLNNGSGDSVMSSSSGNQPFGRLAGNEGSKWR
PRKNQFERLDGDDDESITAGLELKKSFSSSWGVEHISTFPSTESESGEYKRDIMFDKKAT
ALINRTPSKDRDNKGSKKREFSPIKSPGKDTSVIRGPSSTKKIQSKIQEEEIKWGNNDNA
DVVGFGDNKTTDANQHSFFPVASSAATNSASNSFGFQINPIGGHSKQNVTKKCHTIPSRE
EQSSANPQSSYSSVASDPTDFFSKEQTTKLTMSNLAKMDSSLDNTHSISAGEKEPAMGGI
ERMVKERKFYQFTIDEDKASTVVESTFQLPADYPYGDRNYSIDDDETQFSCSETSQETRI
QQRSKTNEIVSKTSEIVTDDKLNPIHSGQNADDVNEFDAFFPGTEGDEKDGQKEFDAFFP
LSTKKDSFFDECSSNQQESRQKLAATESDISSSWGDAYFERSYKSSTEFEGRCRAQRGRC
DERRVQKASTKSLASQQVSITSLSYSPSPVHKAKSTDIPQNGRQPLTHKNTQSGAENNNI
VPSGKKVVQGNDWFRSENSQRKSSEFQFDDFVARPPSFKKRNSSIASSNIQLPPSDPFES
LPNGASAKENFAVRSNRPEDFGGDWRAYQKNQYPIHRRKSFEEEDKDDDDNDSFDDVGPW
EKQSSFGNGISRAQLRNSGNRSRLSRHNYSPANRPSRNQYIDDGYVDDDNRSTGGFSFEK
PSRSSAGAGSYAKPLALPSNAIVASMLFRTHYDIDQNDVEEKINKFEEENSKQREIRHLH
GDIPDDVHADTENMTTVSSFSDATSAYFQESWRRPSRDLVNHLSSARALDMDYPSARRFP
TRQVTQAEQQLPPGLYEA
>comp2575_c0_seq1; 256 1 872 LEN=1620; translated
XLLKFKKSDAKQPPSPPTPLEPTPTNAEAGKVKGKGLLRFLDSKKEEPPSEPQEPAPKTG
VNHSEASKSKGLLRFLEPRKEESPLKPEEIPTKKGSNHSEPSKGKSLGQFLESGNNEAPL
KPITTTNSTHSRSTQSRSNYSIGSKSSSGGTKESVEELPSKSQYPPPKLGSKNPNSGAGA
PNSPWKPKSRDDSAERPHASFLANAGKPRKRSTGTVPVLETIQADKQKTNFVKFAAETNF
AKFAAATISESDGGTKKQPNSKKVRNSENKIIKSITLDDGAEMHCVCAIM
>comp2576_c0_seq1; 892 54 2120 LEN=2175; translated
MLFKQATTLLGFLAAVGTTTTAFTPSFGVSPRTVSSLRSTTEDIEKKNVEQTEDTSREKV
MTFSYDMSTETKYEKPTYKGTGNGLSGDSGEYDVIVIGSGMGGLTCGALSAKYGSKVLVL
ESHIKVGGSAHTFTRVHNGEKYSFEVGPSIFEGLDKPSLNPLRMVFDILEESMPVETYKG

LGFWTPEGYWRFPIGDKKAFEELLMEQCGEDGPKAIAEWNALRERLRTLGGSTTAVALLN
LRQDAGFLATTAGALPFVATHPDVFADLPLTFDDLSKTVDEIVTVPFVRNFIDLMCIFCG
FPAKGAMTAHILYILERFFEEDAAFSVPVGGTSQLGETIQRGLEKFGGKLQLNAHVDEIL
IEDGRAVGVRLKNGNVVKARKAVVSNATPFDTVKLMPEKEDEPKKLTKWRNDLGTLPRHG
AISHLFLGIDAKDLDLSFIDDPAHLVVQDWDRSMQDSQNLCSFFIPSLLDKSVCPEGKHV
IHVYSSGGEPYEPWEKLKSGSPEYEAYKEERVEVLFRAVERCIPDIRDRLEFSIIGSPLA
HEAFLRRDRGTYGMAWAAGSSAPQAGLLGKVLPFPFPNLKTPVDGLLRCGDSCFPGIGTP
SAAASGAIAANTMTHVDNHIQLLKEASQKDPMYKFLDPGFMAEVYKPLVQGFTPSAELRN
ERFAAGAGVAPVNHTANNKARDAAAAEK
>comp2576_c0_seq2; 637 171 1610 LEN=1665; translated
MKQTQQPQEWNALRERLRTLGGSTTAVALLNLRQDAGFLATTAGALPFVATHPDVFADLP
LTFDDLSKTVDEIVTVPFVRNFIDLMCIFCGFPAKGAMTAHILYILERFFEEDAAFSVPV
GGTSQLGETIQRGLEKFGGKLQLNAHVDEILIEDGRAVGVRLKNGNVVKARKAVVSNATP
FDTVKLMPEKEDEPKKLTKWRNDLGTLPRHGAISHLFLGIDAKDLDLSFIDDPAHLVVQD
WDRSMQDSQNLCSFFIPSLLDKSVCPEGKHVIHVYSSGGEPYEPWEKLKSGSPEYEAYKE
ERVEVLFRAVERCIPDIRDRLEFSIIGSPLAHEAFLRRDRGTYGMAWAAGSSAPQAGLLG
KVLPFPFPNLKTPVDGLLRCGDSCFPGIGTPSAAASGAIAANTMTHVDNHIQLLKEASQK
DPMYKFLDPGFMAEVYKPLVQGFTPSAELRNERFAAGAGVAPVNHTANNKARDAAAAEK
>comp2577_c0_seq1; 643 387 2324 LEN=2597; translated
MSLFPMSFREIDEARKNSQSLANANRGKNRKRKGAPGQISDIDTVNGVYVSDRRTGRWTT
EEMAYCDKLIAKFESGELPISSGLKLNEFLGNMLKSKQSRLTKKMKNAKLSSRTFQRCAP
GIMDNNEVREFSELEDLFFQSIQCQQERAEIKFHMQREWREQFSLYCSSVSQPLDADSWL
SSVEEMDRRSSHAKDAARMAKRKLMMGFALRTDSMNPCNGVIIEKTPSDQRIALGTHGIE
NDIDEQKEILQLLTEEANQIGTTDINGKSSLLHSAPFLAKAVVYLKRHDVPFEHVELWVP
SFVPQANGQTADGAASNCRLCFAGSATVDEVVEEGSKTKKPLSQQSQFSLQAFGDYSQKF
SFDVGCGLPGRVYESGRPTWEQSVQNAPHHHFERCGGALQWGIKTVLGIPIASPNVGRIV
VTLYSLHDRPKDQDLVGRLSEEFTRLLPSPKWKLVVDIGEPPPQPQQVGSTGTGSSADMN
GVDKGSETVKDDTGRDPRIDDVVSLLGELMPSDPNSPLAPYLSGFMSLRLLLLRSSRSPE
EEDRARTMLDSYTSYSQGARSKADIGIMLARDYMFLSQSNGATNNTIQQPQQQHPIQLQQ
QQANFFNLGQGSSATLSGNSLEFDVHNSPMLGPISANDTLSIISN
>comp2578_c0_seq1; 476 141 1775 LEN=1832; translated
MLSTQKTCILWGILLSLNSLPATVGGFDLPKTAGRSNGSVPGHRIRTPSSTSLESGDTNI
STKQTGKMSPRSRQIDEDLLSQLKDVESGTAARRLLERALFLDADGNNAKRPLFGSSQIP
PGLSDRSISDGDLAIQTKVRNMKYGIFDLIDTNGDRDADRASAAVFGTFVASSLSAIVAN
ENLPGPEIFRFAVVWILSFAPLALVGFGIGSTDKLQAFLVSVQRQIFPVYRKRMVQHEAG
HFLIAHLLGYPIAGYSTNAVKNAVEFYPLSDKDVGKDKARMLGFDRNISRGDESQTERAR
PRVNDAPFFSPEGSGAMDLETQSVFRNKKDYRNNPFLKIATEDQPRNSWPYRGFDHGTVD
KLAVVSCAGVCAEIISYGYAEGGLADINQLQQILANAEPELTERDKENRIRFAMGYTMTQ
LRLHLGCLDALADVMDRGGSIAECVSAIESCTNVSGNNGIIGEDYEVRRRKELKSSALGI
FEKILLNPKNVDAEEDRLVQGAGGGYLKEKPRFLQISGDDPLYIAGGIACLFLAYATSGG
LSLH
>comp2578_c0_seq2; 369 141 1877 LEN=1934; translated
MLSTQKTCILWGILLSLNSLPATVGGFDLPKTAGRSNGSVPGHRIRTPSSTSLESGDTNI
STKQTGKMSPRSRQIDEDLLSQLKDVESGTAARRLLERALFLDADGNNAKRPLFGSSQIP
PGLSDRSISDGDLAIQTKVRNMKYGIFDLIDTNGDRDADRASAAVFGTFVASSLSAIVAN
XESAWAGDFPIRSCMDTIFRTTVSFDNSIVAXYIDDTLSLCLIKMPVKQPFSGCNVXALV
GFGIGSTDKLQAFLVSVQRQIFPVYRKRMVQHEAGHFLIAHLLGYPIAGYSTNAVKNAVE
FYPLSDKDVGKDKARMLGFDRNISRGDESQTERARPRVNDAPFFSPEGSGAMDLETQSVF
RNKKDYRNNPFLKIATEDQPRNSWPYRGFDHGTVDKLAVVSCAGVCAEIISYGYAEGGLA
DINQLQQILANAEPELTERDKENRIRFAMGYTMTQLRLHLGCLDALADVMDRGGSIAECV
SAIESCTNVSGNNGIIGEDYEVRRRKELKSSALGIFEKILLNPKNVDAEEDRLVQGAGGG
YLKEKPRFLQISGDDPLYIAGGIACLFLAYATSGGLSLH
>comp2579_c0_seq1; 1086 300 3806 LEN=4116; translated
MYLQRIIHPALPGFFFLLIARCGVATAQKLAVFVGPHETHTGTTTFLSKHAASDGTGDPE
FNGWSWPTVHENDIDSSPQHAFDFLVKEQDDEGIQNVLIDAIRSARKTSQKGTFIGSLDF
DKVGTNPYSGYDAVEALKRVVDELGVSTDDVIVVVTYRTPRVDQWADVWKNHFEAESYQD
FICSDEQSKKRWEWLDTSMNPFKIAKAYHDRDWNVLVVDKESTVRSGKDVANVVACWAME
DENCQDGWINGFDRSVQNITSTGHLIDELEHTDHRNLERLFLLRDCYNVGQLSGSSRFVL
LDDGNEGFSSLAKECKNWSANSYKMLSNTSFLMNAIQSQKYCEQDDIDVPSLLAEIYESD
VLETLASLGSSTEEDRSSSNSVSEDDTEKEPWVSPADDNPNKRLVVFVGPHETDAVGVTK

FFVDYASTSGDSERSSSFDGWIWPSVNNDLIKKPPHRVFDLLVQYPSDEDIQRAVIEAIV
DSWKIATNGVIIGSLDFDKTGENPYSDYDPIKALKRVVEALGISSSQVIVSVTYSSPRID
QWSAVWNNHFDTNGYENFVCSDGVESSKRWEWLDTVMNPFKIAKTYRDQGWDVVVIDQEG
TINDGLEVAHVIACEVMKSVKCDGGWVTGLKSAVSNQLITYPMDSLAEEDRWDLEQIFRE
RDCFFKYQLKHESNFSIIHQHTAWSSCSNQHQSFYRKFANTDFVLDLLRSQKQCGENIVD
VSKMLRKKMTPNEQKELLIFVGPHETSAVPVTKFFVDHASDKGGTNHSPSFDGWAWPVID
SEILGDIQSHRTFDLLLSDASKRPVQNIILNGIRDSWNNALDGVIIGSLNLDRVGENPYS
GYDALGAIQRVVEKLGISDDDVTIALNYRSNRLDHLSAVWWNHFEAESFEEFVCSNAQAE
KRWEWIDTVLNPLKLANAYHEEGWKVAMIDQEGTANAGGDVAHALACNLMIGVDCGTGRV
RGLENETIDVPSTYKIEDLDELQRTEFETLLQMRDCYYRYTLEQDGKFLIVNKDALWGSC
SRKNYALYEKLANTDVLLYEMRSLQGCGDIEPSIFSGNLFDRDTLASSKSSRTVVIGSVV
FSTAVFALGAAFVWQLLRKGREAKRNGTEGIFRDGTHDASQEPAFHDRKENDVEDTKYQD
GFDDENEGSKPEATHEKVLEEYDVDVHV
>comp2580_c0_seq1; 901 1065 4019 LEN=4490; translated
MAELSATPAAIPAASTIPVEAEAETETQVPNGKETKDPTNGTCDADADTEASANANENVN
GTTMNTNARLRPELKEKIQSWILSFYHLDPRYKILTFFNLVASEGADNLTDDQIEEHSYA
MMTTTSTTGGAAGSLGNSVTSQQRQASPTEEPTTPVSANQSSPASGKIPPANSTSKDKNN
GNNRDVYKVNAHLMSRPSARNIRPSTLSLLSRIFNATSILTVWRPCSNDAMRKMMEGAGV
GKGLDIKGKSAKKGTLSGFVPFMQIFDNDHKNEIQPIPVKALMRVYYKDEKLRDHVLGVL
QTYLEEGPPLKKNGGNVPPLSPVGKLMDEPIITDEVVAFRQARTAMRCSTIDEITEDDDL
HNEQEQEEGTGKEKKPSLPDHKNPNSSKYVPPKPLKKLKKYTKVGFYGIECTQRLFWLAT
VQDADISRSGTGTETGRPSLPGFQDANLKTLKVAHTQLPKPNPMPVVLQYKSYEEVVASS
DDEDENQNTNHPLDDCLDPRFLVMAYEEQGTIKPVVSDFDGFLLGWRREALWFGCCLPRY
QENLMMWCVERIEEILDDQKKNPTNSDTWTQRWLDVQKKAAQQGKVFDIPEYGFGDPKST
SIMEHAALKLKSTGAVRHGSECFNYHFPQEIDETFLLISDTLKPVPWKYVNVKELQDILS
QKIAEGFVFPLNPKWILCDPGWKKVYDDLMRSDALYSDLSKDIWFPPYSGIRERIEKIHK
LHPNGFQRCTNFSKAKGLDKMKSHSSTGRRQSASNILRDTLDEGLGSQLTGNAYADLAQL
ELDDFIGRTSAMPGRGMELDSAMGKNIRDAIKDLSIEELDDDYAEQPRSSKGVGGVPLSQ
RSRQEKRPSGVSHTSSIASSVGSSGTSQSNSKTGPSSSNNQSPGLSLTSSISSGSGTTPT
TFRKLFRKVRRRPNSMEFTNESSNNDQHTTSSEPYTRSKTTSSPDLRLGQQARAVSNPKD
SSSANPKKKTSSKDTNKPRRHFSKW
>comp2581_c0_seq1; 286 263 1600 LEN=1848; translated
MSDPSFLFDSGSDSGSLLSSSSTCEMCGWLAAFAALLAFGSFGVPVKSKVARSIDIDPLV
MQSYKTAMCFLTSWLFLLIRGEEVTFTPWGIVSGLFWVPGGVATIYAIKTAGLAIGIGVG
SSFIVLVSFSWGIFFFNEHVHSRIQACSAVGCMLFGLAGMAYYSAPTVAHASHPRAPEEN
PRSGDFDDAFDDAYYHAVSPENLNFLPPSEAFETEYTSMDVEADAAEEHGASSDNCNNCL
EDNDHIDESMERLDVPMIQTVDYSEEETPHVVFCWGFLRLQERTLGILAALVVTGIWGGS
IMVPMQFAPNVDKGLPFLTSFAIGATLVTLSFWLLRYLCLCHKHSYSFSTAYRALPSFHF
KKMWPYGCGCGLLWSIGNFFSIVAVENLGEGVGYSACQASMLVSGLWGIFYFKEIEGTDA
ILKWYASASITALGIILLSYEHHEL
>comp2582_c0_seq1; 653 66 1070 LEN=1232; translated
MSTKLGINGFGRIGRLVCRAAIENKDATVVAVNDPFLSVDYAAYQFKYDSVHGRFPGSVE
VDGDCLIVDGTRIKFFAQRDPTEIPWAEAGAEIVCESTGIFTTIEKASMHLKGGAKKVVI
SAPSADAPMFVMGVNNSSYAGQNIFSNASCTTNCLAPLAKVLNDKFGIVEGLMTTIHAGT
ANQLVVDGPAKGGKDWRAGRSALNNSIPASTGAAKAVGKVLPELNGKLTGMAIRIPTADV
SIVDLTFRTEKAATKEEILGALKEASEGDMKGILGYTEEKLVSSDFIHDSASSTVDAGAC
IFLNDNFHKVISWYDNEWGYSNRLVDLAVYTTNA
>comp2582_c0_seq2; 471 66 1182 LEN=1344; translated
MSTKLGINGFGRIGRLVCRAAIENKDATVVAVNDPFLSVDYAAYQFKYDSVHGRFPGSVE
VDGDCLIVDGTRIKFFAQRDPTEIPWAEAGAEIVCESTGIFTTIEKASMHLKGGAKKVVI
SAPSADAPMFVMGVNNSSYAGQNIFSNASCTTNCLAPLAKVLNDKFGIVEGLMTTIHAGT
ANQLVVDGPAKGGKDWRAGRSALNNSIPASTGAAKAVGKVLPELNGKLTGMAIRVKYRGA
CLYFEVNTNIEQNHISPXIAALSQTYCSILPQIPTADVSIVDLTFRTEKAATKEEILGAL
KEASEGDMKGILGYTEEKLVSSDFIHDSASSTVDAGACIFLNDNFHKVISWYDNEWGYSN
RLVDLAVYTTNA
>comp2583_c0_seq1; 261 359 604 LEN=739; translated
MGGDPFFMDEEEYKDTIPEKVDAAKLFENSQLSELLAKASSRSATDGLGPTPSQRSTVSN
NNDSDWEWDGTVDEDAHLGLD
>comp2585_c0_seq1; 124 1 315 LEN=644; translated
KFTSVPYAKGFDCWDTTSALIAISFYTLFLLSFDWTGMVGVVENEAVDHGIDSPLAGLGY

GLPISRSYCRYFGGDLSIMSMEGYGTDAFVYLARLGNTKEPVPI
>comp2585_c0_seq2; 383 369 1851 LEN=2180; translated
MEFSFWETSERSNGDHRGGPIILTLNEDINIDYDDKDPDKKHRKKSKPFSDRIFCSVRAN
STNHQHVAKQNLRSVRFNSSSIQVSTPKVDRDLEYDGVPNDVAKLINDHAKQPPTSVSLQ
ALMRTGRGEYLDRHFENVAIKEHTATELVLMQVASFLRRELPIRLAHRIQDLEKVPLMKD
MSSVKEVKQLYIQSFLELIDFDKKIVSADQEEGFSRLVEGIYERHSRVLVQMARGAYEFR
HHIHTNSTSKSKSSRRPKDVFAQMEDTHAFLDRFYLCRIGIRVLIGQYLALRQPPVDHYV
GIICSVTSPYEIVKRAIDDAAFMCTRKYGDAPEVIMSGRLDLTFPYVPTHLHYIMLELLK
NSMRATVEWHGSDADFPPIKVIIADGSDNEDVVIKVSDEGGGIPRSNVEKIWSYLFTTAD
PSIQEGMVGVVENEAVDHGIDSPLAGLGYGLPISRSYCRYFGGDLSIMSMEGYGTDAFVY
LARLGNTKEPVPI
>comp2586_c0_seq1; 129 23 277 LEN=551; translated
MLRTGLQAGLRAVSRKANNNAGALRFMSKEIKFGVDGRNGMLEGVNTLADAVQVGVGDVL
HRVVLACFVSKPICRVTLVFPGNR
>comp2586_c0_seq2; 1096 23 1753 LEN=2037; translated
MLRTGLQAGLRAVSRKANNNAGALRFMSKEIKFGVDGRNGMLEGVNTLADAVQVTLGPKG
RNAIIAQPYGAPKITKDGVTVAKSIDFEDPFEDMGAQLIKSVASKTNDVAGDGTTTATVL
ARAIYSEGCKAVAAGLNPQDLRRGINMAVDSVVQTLTDISRPITSKEEIAQVGTISANSD
EEVGGLIADAMERVGKEGVITVQDGKTLDDELEVIEGMKFERGYISPYFITDAKTQKCVL
EDPHILLVEKKVSSLQQLVPILESVIKSQGSLLIIAEDVESEALATLVVNKLRAGIKVCA
VKAPGFGDNRKATMQDLALLTGGTVVSEETGMKLENTTPEMLGRCKKVEVDKNNAIILGG
VGDSEGLKERCDLIKHSIETTKSDYEREKLQERLAKLSGGVAVIRVGGASEVEVGEKKDR
VVDALNATRAAVEEGIVPGGGKALLYCSTKLEAVKEEKAKNMDQKIGVDIVLKALQAPMK
QIATNAGAEGAVVVGHLTESETPIETGFDAQNGVYTDMYAAGIIDPTKVTRTGIVDAASV
AGLLTTSEAMIVDKPMEGGAAAAPPMGGMGGMPGMM
>comp2588_c0_seq1; 1088 333 3215 LEN=3595; translated
MKKKELDFERIARQAREFREQEEKRIASEKEDEARRIAAEKEAERERIAQAHEETLLNAE
HSSIGSSHSYESRAPKPSPGTVKRRTEWLRNLAKDAAEHRHLEEEFNSMLVARLKRHPNE
AAKDAEAAVKAALTSSSPRDDFDRDIEKLRDQTAHIAKEVANAERRLRKGSEDLIEEFNQ
IEADLEMEIAEYQTPSSDANSSDLDIEGHEVNGVKPRDLMEEYSHSDAPDLTTVESMTME
TIEENIDAPEGNLEPQGDSDIEKLETSPGQQIHSGGPEYGAEADPAHTTAKTVSVVSKSI
IAEPSTMETTEENVDAPEEILEPQRDEDAREIQPLPKKKSYSNNLEFGAEADPTDITDGT
FPIASKSTIAESTSIETLEENFDVPEANSELQDGMGVGIIQMLPKQQMHSDGLEYGAQAD
PRTQALLAEDDLVPTGKKYHADENLRIKDRNKKSIVLDGSIPVDRKIFRRNKSSSKNYED
EDGEFGSEFVHKEATFELPMELTENGLKLSSNTAEVNMITTIPPSRLDGRISGILEGRST
LGPHSNTASGSLALEYKSSKQSRLTLGMIRGCEPHHPLITIGGRLLRQGTSVGVIFYHNA
QFLHQMLLEHSLWAISFRHSFPNSRWTLSSQLSRRKDLSLSLVNGNKLSGLIGWNLLKPK
QFHARFDARPKLTEYRRAHLYCQWKASPGPGLWNVGLSLVQSLHSQIATVGLGCRLFSTK
GLEWVISWSRGNSTISIPILLSKSMAPSTTIGQTFYFSLISFMMQEYIAETWGWIGNDSE
DDEDCKVEDASIALRAQNLAKARQDAVIQKELMARQARRKTKEEKEKDGLIIIDAIYKVE
NGEEWSVTVPLQFWVTNSTLTLPARPKSELLGFYDVAAPLKNSRVSSIAGNQTSTTSSSS
GRCFPTLSELWHDLLDRTPEDSQSKKSEISSPTLTVSYEFRGKPHVVTIKDRDELRLPQV
>comp2589_c0_seq1; 125 48 840 LEN=1016; translated
MNLLTFISATLLIITSEKLANVAAAGPQLRGRSNTGQLQRRILNDDSGWCPTYFPGTNTD
CPLTGMFRGTCYYSSSAKEGETPNTDSQACSCNAEMAGKFWCEPSPFPGGAPTPPPSPRA
LPEGSWCPELFPGSDTECPLTGMVRGTCFYTSSAPAGEPPNNDHQACVCNGDGTSRFVCE
PRPFPGSGLRPPATAKDPAEPTTGNPTQWCPPDVPNTGDSCALPAGKTTDRAPLSAETPS
LSAAARRTRKHLCAIETRPDPTA
>comp2590_c0_seq1; 406 53 739 LEN=924; translated
MKIFAALSMLLASASAFAPSATQKRGTAMNALVDDAFGVSIETGNKVPPLGRKILEDAGP
DGIKWFQNAEIKHGRIAMVATIGFWVQKLGVHFPLYLGPSGSNAFSPDSTASNWLLSSTT
GVTFQDIAQAAPLDAINMVPVQGWFQIFLAAGWFECIAYNRQWAEGREIPGDYGYDPLGF
TKKEGGIAGKDFESLRMKEIKNGRVAMMTIAAWVANEAIPGALPVWHP
>comp2591_c0_seq1; 546 148 1647 LEN=2018; translated
MELTPSITGPVDGFIDFIYSFGPLAEKTVVLVPDFIKAQVGGVADAAGMDIETVSYVLGM
FLCYPLGMLMAQIPFGTSRHIFSFLLGAFLLQFTLGVQWIHHLISSLIAYGLILVLPRKT
LKTALPVFAMTYMTLGHLHRQYINYLGWDLDFTGTQMVLTQKLYMIGYNLYDGELLAKGK
EDRAAKKCAPFALKNTPSLIEFLGYTFCFANLLAGPATEYATYLHAVDGSIFKTPDGKTK
LPSNILPTLKPFLTCLLNLGIFLTLSAKFPLLDTSDPQHNTPYILTEEFLKKPIFYRYFH
TWMGLFALRQKYYFGWKNAEGAQNIWYAGFDGYDEKGNEKGWETSNNIDIITFETAPGVS

IMSRVWNKKTSLWLSRYVYMRTGGNLIAVYSMSAFWHGFYPGYYLFFLSVPIPSFCERLA
KKKLSPYFSPNYFSPYGIVCVLVTSITMNYLIVSFTMLASGWSFAAYKSFYYFGHIGCVA
FYIILTMLPSPKKEKKKEA
>comp2592_c0_seq1; 441 101 1294 LEN=1660; translated
MKEPWSKKHKKDFKGRQFSLSNSFAQPLSQPELVALTKARNDQALLDDYEHHDLVYTPNG
GSDDLKQAISELYGPKISKENILVFPGGQVAIQVAAQAFARDCHSIVFVPGYQSTVESPE
WALGSKGVTQLQRRASNRWQIEIEQVKASIQETTKYMVINEPYNPGGIVMSHESQNELVE
LCREHGITILSDEVYRLLEHDPKDRIPAMADAYERGLSCVSMSKPWGACGISVGWLACQD
REMISKLWNCQYFGTACIGRACEIQAIMVVRASETILKDRVSIIRANKALLTEVIEEKYP
DFFEWEPPNAGAIAFVKFKGPLTSLELGDLLGARGISIKPAYCFVSSDNGGKGIPPEVDY
FRLGFGERKMPEALDAFCSVVEEFKDAWRQEMEGMKK
>comp2593_c0_seq1; 282 938 1591 LEN=1930; translated
MAARVDEEYDYLFKIVLIGDSGVGKSNLLSRFTRNEFNLESKSTIGVEFATKSIQAEGKT
IKAQIWDTAGQERYRAITSAYYRGAVGALLVYDISKHGTFENVERWLKELRDHAEANIVV
MLVGNKSDLRHLRAVETDEAMSFSEQHNLAFIETSALDASGVDTAFQRILTEIYRLMSRR
NVAAEDAAGGPSLSAGQSISLSGGNNNAGQGEKKGCC
>comp2593_c0_seq2; 237 256 909 LEN=1248; translated
MAARVDEEYDYLFKIVLIGDSGVGKSNLLSRFTRNEFNLESKSTIGVEFATKSIQAEGKT
IKAQIWDTAGQERYRAITSAYYRGAVGALLVYDISKHGTFENVERWLKELRDHAEANIVV
MLVGNKSDLRHLRAVETDEAMSFSEQHNLAFIETSALDASGVDTAFQRILTEIYRLMSRR
NVAAEDAAGGPSLSAGQSISLSGGNNNAGQGEKKGCC
>comp2594_c0_seq1; 424 215 2356 LEN=2746; translated
MIDTCHPSTYGFASTSEQRNLAPPRYVRSEEIHEAAFAPIPTNKWWGNLIHQDMNNPDGP
IEPIWSNPYAIRVVPGKGLVVSGIHRHYGPCLEINPKVKALKYYAHAFIDDIILSAVDLP
AGLDVYGWDDFGVRIQLGKRMNSSLVRGMAFVSATYGLGTRPRIETIHAISSINGEAPSP
DAYYAVSPGSKLVLEFNNGQFWTVYADQDIRFSKEGSALTATDAVDTATTVRIARVENPE
NRLDVLDEYAKCVVLGGRVQFLSHKRYNLEWETVGEGSFLHFALEHHLHSFLEPPTNVSI
LAESTTHGRMHGICSSRSRDNGRIRVWELAEPKSMPIRFEPQHGIDPAYVKEFRIDERLK
EEIESSWVISREGSYYFNGKLMQKFASLCLMAHDDAINRDDKIKRICLEKLASLWDSFLS
NGDGEWPHPLVFDEIYGGIVSSEGFKKNDQNVDFGNTVYNDHHYHYGYWLVSASIFLHLH
PGYPRAKELRGKIDVLVRDVANPNASDHWFPRFRHFDWFLGHSYSHGVTPMADGKDQESI
SEEINFHYGLMLWGMATDRLDVEQLGELLLKINARTARTYFFMADDNVIHPKEFVPNKVT
GIFFDNKADCTTWFSPERHCIHGIQMLPFSPVTPLFRPQKFVKEEWESILSNLPVVLDPK
SNLNSWQSILFVNSARVVSNRARDAMKHLEYVPMDNGLSRSWALYMAATLCDA
>comp2595_c0_seq1; 278 183 890 LEN=1075; translated
MTPSSNDPAEARKRRKEIIQSQRSENELIANGSVVASSSHPPAISSAMDGIQVNSAVDHV
EGQPTNISNNDPSSNGADKKPPKKRKLGSGDPNGRKNNKKDSGKSKKTQIRYDPDVPMNK
DQLAAWRREARRVRNRESAAASRQRIRNRISELEEEVGEWKSKYSIALQRLEALEQHIAA
NGNGESSIGVQGEQEPKEEPQEQNPPSDQPTEQHPQEPGQQPTEQPLASSDNANV
>comp2596_c0_seq1; 577 1 1176 LEN=1485; translated
NQMMKLYTNTFVILLTLALLGISSVSGNAGRENNDRVRALKANKANNYEYYGAKNTNNDF
EVIDQRSVNTNSNHKVGKNSGKNSNTGRNEVNSDFDVIRSHDKKHKEGRNKKKDKPKNKP
LPRNMPPSPTPVPSTKSPSSPTPSGKGRGKGKGGKGGKGRKAGKKGKGKGKGGKGGKGAK
YRKPDTAAPITAAPSGAPSFAPSDAPSPAPSASPSVAPPPDTLMPTPLGSTASSLVSSVF
VSSESSSEFTSVSSTESSSDAAATNGEGVGAAAGDGVGHKRKNKRKNTRKNNRNPNRHKK
GRWVLVKTKDGEWRRKTKNHRKRNENKGVTVSKNENEELGDEEVSIFQDEGEDNEEEEVD
EVEVEEEEVEEEVEEEMEEVEDEGEEAPLQL
>comp2597_c0_seq1; 1015 35 2053 LEN=2406; translated
MTSFYFNRSNRTRHLLVLGLSIVVFAVLGNFVESINLVARVDEDSKLFSNIKTGDNIGRI
RGSRVRRALSSDDDEGKGAKGSKGSKNGDDDDDDRGTKGGKGSKSGKGSKKSYYGDDDDD
GKGSKSGKTSKSAKGSKGGKASKSAKGSKSYFNGDDDDDDDGKGSKRKKRSKTSKSRSKK
SHSSTTESRIDVESSSVDKPKNSMTSQEKDHKEHIADIGEQGRGIFAGMRPDLWGELNEE
DQSDKIVVDTTQSQQQDLSPKEDSNSAANDRIVESTQSQSQSQYSKEDIGGTGEVTIEDT
TQSQKQILEDDIDGTSETTVVETTHSQSPNEDIGVADAVTDMDTTHSKDNILEDDIGDNS
ATAVVDTTQSQGLKEEIGGTVEENTEDTTHSKEQILEDDIDGNSQTSIVDIIQSKEENIS
PEENIPEGTGDTIDADTAPLEEENNIRTVQLFHPEALDECVTYDKELEEMTIFMSECSTL
NNDTRHDDYWEILQARVSINNTEGNTFHLRHKATQLCIPMNTENPDASFNCQRYFGMNEA
IADSFNGLIDCDSGVAAVFGMGGVANSLYLKNAGCSDMAISDNKSDLDIVFITYQNPVEE
KRVVVLGQSILLELGGAVASYDFRTEWAFLDVKDIDREKKAVAPQPHSDLFDHIFHLNDG
KSKNGSSPAKRT

>comp2598_c0_seq1; 1033 187 3168 LEN=3315; translated
MTTAARTTAKSSSPGPAPSLWTALVRSPLLPHATFWGAGLAVRVSFPAALKWLAYDSIST
HLLTVSYPLCATVSLIHRDLQQRHKQHQANNDHDPANVRANSLLQVERQYWIEYWSVGFA
GVQFLQQALELFPSLHRAGLQEYSNLPVLLAQLKLMFFVWIFCMQSVLAGHQRFLGGQQQ
NEKWKKFVPLTFLTKAIGPKLMGLQMAISERISKETWQRLIHSKVQRVLEVLVMLQFLKE
TSRDYLLQLLEEGRSLLLLSVFAVLPSSVTQVGILYAKYVFPAARSLVARGDDLEVLSLK
YWVLNNLFSLSLSFTWWLWWCVPFSTQVLLGLWCFATFPSTITYYYSLIEMELITFGILS
GEPKLSVNKTMTVQALRHFVKRLPRDKQAQSFQFEIDDDSINQKSSGDNGDDSSIGSQDT
TDSERERRRQRRRERRARRKREAESKSKKATPPPPLLSEDCITQYANDEQKDDGKYSSFV
NEKRQSESDERNQYRHCSTVTEERDTRNATQLQPQQVEIRHPTSMVSLVDVDYDHSLSRP
NKVEEEMSSDSLASMSDDINNLIPAPTLSMNNEMEDSAMVSIQADDFDDKDGDDSDRALL
PSLTIGSNYSASQVHTDAKSMGLNIATPTENVTGEVEVQMRTTRGQARSTTTSTRPTSSI
FPATYPRSSSRNSLLLLPSASIERSFSLVNSMSIPEDKLAPKPETKSKAHSSDNNDDGDC
NDGLNISDVDSTSSSLVDDTESFEISNPLDGNEKQSSKAIKQDYVCKKKPTKTVDVDEGV
STPPRRSSRLEKLREWQEEKKKMSPSKASKRLEANGGSKPSKKTSSSTAEKKGKKPKTHS
SSSPEKKKKSKDKCPSKSVRGSEATNGVNGNSSRRSTSKASSSERTSVFDKPTAASLTRR
VEKKSTKTPRDKSRTRATTTSTAKAASPGKTTRSSKSKGEAPIPECTEVGRDKNNSFDSP
NQKAAGGKPRKIPNSRRKKKDISSVFGGVLSRK
>comp2599_c0_seq1; 245 1 598 LEN=1493; translated
XTTRNYIQQHIKMKYAAVFALLLVSATAFNAPQFATRAVGKAPATKAVKKASTKAPPESK
GYPSFAEKAQNFKLGGGKISGGGAGRTITPVFVHPTVNTDPIPDFDYKEAAKARVTPQSR
TDYVFDDGLTELERKQKSTIPAFLTGSARSRVEAEAPRDDIEEQEYPFGLSGDRFQLLFI
SVFSLFALVGSLSGSISFD
>comp2600_c0_seq1; 123 1 290 LEN=303; translated
XLLLCLCLQIPKAQPSQLNGPQPASFNGGRSSTTFFGKELPSGLYVCGDHMATATLNGAA
ESGVKAGDDVSKAMFSTGAPSPSASESDPEPAAVAQ
>comp2600_c0_seq2; 1038 59 1813 LEN=1826; translated
MVKISSAALLLCLQMQPGTVSGFLPASKNLNANGNGAVTSTTLAFDWFGLRKNGNSESVA
SIFDSSTNMIVNTQQKDYVTDGDWLSKNLRNKNNKNNDAESADVVVIGGGISGLAAITA
AEAAKSANDPKKIVLVEANNKCGGRVSSVETDDGFVLDEGFAVFIEEYPVVKKLLDYDAL
ELKRFLPGALVKLKGNDELARVADPLRNPEDIFASVLSPIGSLIDKIKVLPLVLSVRLGS
VESIFAEAETDTKTALVERWGFSDAFIDSFLKPFLEGIYLAPLEEQSSRMFSFVFKMFSD
GSATLPKGGMVAVTNQLVEKAESLGVTILTGTPVASVAPKDDDAGYVIECVDGSNRSFDT
PSVVVATDGKIAKKIIGNIEGFESLRDEPDQVQRSVGCAYYGFEGPAPVEDPILVLNGMK
GENDDPVNNVCFPSVVNDGYAPEGYNLCSVTVLSDAMDKYMGKPDELDQAIRKQLATWFV
DQESDILENWDLKQIFYIPKAQPSQLNGPQPASFNGGRSSTTFFGKELPSGLYVCGDHMA
TATLNGAAESGVKAGDDVSKAMFSTGAPSPSASESDPEPAAVAQ
>comp2601_c0_seq1; 594 30 1832 LEN=1919; translated
MSKVVGIDLGTTNSVVAAIEGGQPSVIINSEGLRTTPSIVAYTKKQELLVGQIAKRQAVI
NPENTFFSVKRFIGSKETEVSAEARQLPYTITNDSKDNIKIKCPALGKEFSPEEISAQVL
RKLINDASEYLKQDVKQAVITVPAYFNDSQRQATMDAGKIAGVEVLRILNEPTAASLAYG
LDKKQNETILVFDLGGGTFDVSILEVGDGIFEVLSTAGDTKLGGDDFDKVLVNWLIQNFK
DKEGIDLSTDIQALQRLTEAAEKAKMELSTVQETKISLPFITADASGPKHIDEELTRDKF
EVLCEDLIERCRIPVEKAINDARLDKSDIDEIVLVGGSTRIPAIQNLVESLTGKKPNQSV
NPDEVVAIGAAVQAGILAGEIKDILLLDVTPLSLGVETLGGVMTKIIARNTTIPVKKSEM
FSTAVDNQTNVEIHILQGERELVSANKSLGNFRLDGIPAGNRGVPQIEVTFDIDVDGILS
VTAKEKETGVEQFVTIQGASTLSESEVEAMLEEAEKNAAADKIMRENIDLKNQADLLCSE
VEKELPNATVSEDEKQAITTLVGEIKDSIQNDNIDTLKVLIEDLKEKLNGMKSQEVNPEM
>comp2602_c0_seq1; 640 1 1687 LEN=1701; translated
XKKKKKKKKKKKKKQNGKDAESRAGGNNTSNQHNSALDHSNAATVEEKEDPLVTALLGMG
FAESQIMAAVKACGGTHRATADDLVTWILGQGETEESANEATRIPDGTDQLQQENAAYDR
TRTEDSLPTNEKEQQKQQLEAVRKQEEAARRLAAKREEARRRNREWNNREQARQQKEAKA
KVAQKFFNVPRPAVPLPVPKPMATPDLPVPNVSKLKGPPRQPKLTTGTSGLPIGGNKWGV
PPISANPKPFPVSANQSKSIGSKPTSSAAPTPPTATEQHALPVSSFLASLDDDKTVSSFG
SSRTMSVSSKEFVPIASQPSVPPPGFLSTSAAPISAATRSLVPPLSEGPMGAPFSPESRQ
GEIRATAKEFVPSFTIPGSVNPSASLQKDFAPLNDLVGEPQPSESHGVGFPPGILPQHGA
SKVGHTSSLLGPSLQSSFDNVRPMNSGAADTILSGASFVDGPKSEEPPMSIPFGFGQADS
NESNDIGGSRLLNSISRNRSTGSASIWGDGQQISSLETTLQPSFFGDGTTNESDDNQKSM
LWNVSTGGNSSLPNGGGQGSIW
>comp2603_c0_seq1; 393 284 1198 LEN=1270; translated

MANPFINSSFTLRPSMNNRDIPELTLRQTEVSRIPMERTETKEGRSFKRQRLFMDRFMST
AFPFGYRYSCNDVDYSYDDQYLSDEENGVPLTTRRRVSSRLTSCRRISDISIGSSSNGMN
ISEADYWKTRCLDVQDTFENAQARLREAEEDQRQLRQRVRELEEQLLLQSSSGNSNSNGG
IGNEDYEAGNTEETFETRDRDASSNIEGKNNRIAPKHLGKDDGQCAEDTGRKNKKERPLP
ALVVEIKENHQAVSSCFYLTDGEGLNESYDQEMEDVSMDVYCGDQMLGDIDDDRNDINFN
QQRG
>comp2604_c0_seq1; 473 329 1795 LEN=2229; translated
MRLSFVAWMAFLASTTFVDAFTSQSGNTNRRITQSPGKSGANKALYSLSTKSEDDEKVAG
KILEGGKVIDFESTRGPSRAENALKLAKEEIANGTLKNTHLSDKILGINSDVIAEVGHDL
GLFASDDEIQKCAAYLRSKAASGFFEKRPGSVAVTYDEIHSSPSIGSEQKAGVASILEKA
FVESGEVTSAFAKTFYLGTQLMPEAQREAIWAIYVWCRRTDEIVDAPRDNDDEMLSDLGI
WETRLENLWKYGEVEDVFDLCLLDVLVKYPSLPITPFADMIRGMLMDVPELGTDTYDVFD
DLHLYCYRVAGTVGLMCLPVFGCSEGYDEIIAKEPALSLGVAFQLTNILRDVGEDATTRG
RVYLPIEDMERFGVTKEQLFEQRVDENYINMMKFQIARARMYYERARRGVFMLSEESRLP
VQSALDAYGKILDKIEDNGYDTLTTRAYVDKWEKLSIIPFSWYRTQDISRTVPLPGDKQI
PSLEEPTP
>comp2604_c0_seq2; 352 329 1330 LEN=1453; translated
MRLSFVAWMAFLASTTFVDAFTSQSGNTNRRITQSPGKSGANKALYSLSTKSEDDEKVAG
KILEGGKVIDFESTRGPSRAENALKLAKEEIANGTLKNTHLSDKILGINSDVIAEVGHDL
GLFASDDEIQKCAAYLRSKAASGFFEKRPGSVAVTYDEIHSSPSIGSEQKAGVASILEKA
FVESGEVTSAFAKTFYLGTQLMPEAQREAIWAIYVWCRRTDEIVDAPRDNDDEMLSDLGI
WETRLENLWKYGEVEDVFDLCLLDVLVKYPSLPITPFADMIRGMLMDVPELGTDTYDVFD
DLHLYCYRVAGTVGLMCLPVFGCSEGYDEIIAK
>comp2605_c0_seq1; 499 324 1583 LEN=2200; translated
MPPSAVINDDQSQYSLCCGAFDIGEIEIEVQAVGGDCNSVKETQIVVSPGSATTKNENVT
NRCDPSQYDFVRDGRNPLDGSQAGSFDSTDEIIEGSNEMKLMRRVVEKQRLGMKGPRHRS
FIQQPISMERQDSMSDTNSVNANDVFVSIEDSKEMEYLDVLDQHLNQTNPKALDYEHLED
ETEDSHPNKQRFQADLDDIIEHLCAVPCAGYLPVVDGTCLGDSTEAGHPRSVLRRPRYSS
PGMYPPASAPLNRAADGTGTTNFDQPILIRSSSNYDATGASRDTQQRHSVRFQNVDIRHL
KMTLGNHPSATTGPPVMLDGELAEPRHSARQIILLEKYEQTRPPRRKRRQLKLTLQQRHN
ILVKERGFTFDEVKSAWQESLRIRKQRKETLERGLAMMKWDEVWESTCRKFNRLVDGTL
>comp2607_c0_seq1; 53 103 435 LEN=677; translated
MTASGGSVRRLLAQNSAVLRRGAEHARQQIFGHVPILEGAAAGNKTAKKTFTGPYLEKYY
PTSINHHARKVHDGWETEQEEYRRVKLTQRRRKGKGPPKKGAGARSGKKR
>comp2609_c0_seq1; 267 343 942 LEN=1132; translated
MMIYQQTMLGSTIKPTPYTPGHPREGPSNKKGVMFSSCTKDTSSSHRRRRPSSSSVPIPH
AMFRSPSELQLSMDEQMADERDFVFYARLIRGIRERTLRVNTSKNGDDNQPQLYQQYNEE
TERCLTHIMQTRYQEQDREPKEIYDSTSNENCLENLDGCYESSCSSSSTSSFAYQRDFEG
DGDDFDSGEDEFIFDLEL
>comp2611_c0_seq1; 358 147 1529 LEN=1692; translated
MTSGIRPSVARTILGGQNTLRTWHTRRTIFSESFPPASVPLCLCRRRKRGRSDSWVSAFS
SSPFASSGQDHGYAKLKRDASSLVDQASFKIRGSKHHSKLLRRHVAIFCEAVRAGLLHNK
GELYGSDRYSNNSCEATRKLTASLGGIGADRGIMPDFSNPRALRGYTLGHFGRVRLLSQL
VAQTEPDWLATRVSQIFSDEVSNRADDPHCSSSLKGGSEIALASLGGGCGYDFVAMSALS
EFLKGPKIAATVFEYEPAWKDVLGDVESVVQDVVASKDNRAVSHAHRCGFESCDITRPLD
SSSNEALASVVGSTHMFSCSYVVAENAVGLRTNKYEFFRQLFSEASDGSLFFFTETTHRL
WPDLIDVAMDHNDHLSTIKISIPHIRCGKSGWQLALLKDSAGNDHGGSKTCSLEEHQRVI
YERFRSDNLAHLSRLDRGWKRDKKKARGAKQLSHKDQRNQ
>comp2612_c0_seq1; 450 148 1509 LEN=1991; translated
MPLHRRHAAGTTKAESSSSPPSSPSDKDGREGSMKTGDGHLGSGFLPNPPRSIVFLGLAV
LEVTVQLAVIRKSGIRSVGHLLTVIVSAFVATLPMLLTLAVGMGISGNPTKDLPHQRYIP
LTVAATVFASQLPIFISYAMAATGIVLFGLASRPIEEEKAKNKSDTGTDTKGNQSQPKKK
EMSGQLKAVFSVFLVIAVLLTENFFIWVVSATYKPSQNLSTLPTPLQDNGQISLQYLFHE
LLNITKRDVVKVRNIINVEAILVSGLALSLVAIELQGPSMQRNLWSMAMRGVLTLAAARS
IRTFSFLITVLPSQNPQCFFSHFPMPPKDWYSWLKVGLIPQIDGGCNDLIISGHATVTST
IACVITSIVGKPLFTIAIWMSVTLDYFVEIYEGFHYSVDMFLGAMLVNFLWSSLSFLEDV
KKGHAAVEERRFLPVQDATPSDVLRYAIPAVGLW
>comp2614_c0_seq1; 578 152 1861 LEN=2434; translated
MAPHEKCNEEDDMKDITEPRESDVLCGRGGAALRHVGNQTYRRLVNLNKPLYITCLKTEK
LKISRSIVAAIREQNGRFLERDAQKGVWTDIGDKKAIEKTSQALREGQPKLKKKLAAMGQ

LPAEQANGGVDATRNSSTQQQAVQHYGNGIYNPRGVHPSISNVMNNNINDGMDMNFNMRG
GMRMNSGTNMNANMNMNMNNYDRAGSGNFSSSTMFASMQQQVQQQQYDRFNQMGGNHMGR
DTTGNMRHGMMQNPARPHRDENPARPQRNDFDDDNDLTMIRHLSLDSTPQSIPSWTPSVR
STDSFAMSIGEDIPQMVTNRKGSRTLIRNPFSAPYPCNENRNSNGSDFSAYGNGMGRDGS
QTRSSSFDIEDVGPFDRSNEPHRRQQQQFVRTHDYGQNTLNESNQMKTNDNQRSPTTSIK
DRRRNFARLKDVRASSLRSGSSRSIDDMPDIHMVDSQFSLLSNLSAHGSKHQGQNAIDMA
ISTHRGGRKDSMGSGYIGVASRRSVMSGLSKMSAHSVDLNVFSGLSRKFSGGTNHTASSR
SMISEFSGIDEDDFAEDDFDFDIPTKPNP
>comp2614_c0_seq2; 578 152 1861 LEN=2344; translated
MAPHEKCNEEDDMKDITEPRESDVLCGRGGAALRHVGNQTYRRLVNLNKPLYITCLKTEK
LKISRSIVAAIREQNGRFLERDAQKGVWTDIGDKKAIEKTSQALREGQPKLKKKLAAMGQ
LPAEQANGGVDATRNSSTQQQAVQHYGNGIYNPRGVHPSISNVMNNNINDGMDMNFNMRG
GMRMNSGTNMNANMNMNMNNYDRAGSGNFSSSTMFASMQQQVQQQQYDRFNQMGGNHMGR
DTTGNMRHGMMQNPARPHRDENPARPQRNDFDDDNDLTMIRHLSLDSTPQSIPSWTPSVR
STDSFAMSIGEDIPQMVTNRKGSRTLIRNPFSAPYPCNENRNSNGSDFSAYGNGMGRDGS
QTRSSSFDIEDVGPFDRSNEPHRRQQQQFVRTHDYGQNTLNESNQMKTNDNQRSPTTSIK
DRRRNFARLKDVRASSLRSGSSRSIDDMPDIHMVDSQFSLLSNLSAHGSKHQGQNAIDMA
ISTHRGGRKDSMGSGYIGVASRRSVMSGLSKMSAHSVDLNVFSGLSRKFSGGTNHTASSR
SMISEFSGIDEDDFAEDDFDFDIPTKPNP
>comp2615_c0_seq1; 589 278 1564 LEN=1705; translated
MFARIYGADGTVVNDDEFENTDYGSDNKNDTEILESLTNAVDDQAVWTRIACAFAPPPHD
RLNPELVRRAELVRVGGKSIDVAVAVPASVGPTASPSADQLAQVLVTVGYPQSWVDSKDL
NMLTQQIRVLDGHAKDRLSERDVGEVGVGDDSQYYERIVIEKRWMERLQDEPDKNESLPQ
WWTTIAPQSSPLELVDECKLLKKLLNEDEFEDELRALFMTHYEDIGASIDFSAGAVALRV
AVASIGSSGLYLKARIIDTAHDVGSEKLSAVSIPYEEDNSDKTRVEVKTAGELREAVLLL
VESVVPIPVPKTETVKSVGAIPTTNDDKGKFTPFNEQVVQVVTNLEENAKPKATTTDNDS
PAEIANKVEEDDVLPKAQSSSRNSSLADRRTYYQRKSPEDEAKLSAKYAAIEDLGERAYT
ILCDLEMI
>comp2616_c0_seq1; 1359 132 4646 LEN=4690; translated
MIEMPSAILGTIFYRGMTMGLFSLAFCAFALAPPAFALAKDEFSPYDISEYNLDRSISRI
LQQSNNDFDRVNELLGTVTINIEDDMTISEKVGFINLNLVVDSLVCRDISIGNMNVNHVT
EDNVIDVDISLDEIDVACDIEYQYEYGFLKGTGLAEIVTDGNMADTSMRFVTSNKGGDIS
VTSSVTKCVVDVEIIDVNFIDADFASNIIGLFEQYLRDLIEEEIEGYACSQLSSTGVTML
NDEVFTMIDETLSDLSATEDTGSSLQPNDYDTTTALNFKDIAETQIGELFETALGEIDQL
FGSNEDSGSELGINALLKSYVLNENGALVVNVDELLGQDALNFELHDKLTKTSIAVKEVR
IYGLDSMIKFNPLVVVGNHSLSNEFVWDYLNMEIDIALDIQPSSLEKALLKLETDSPSKG
INEEITIRFGVEDVAVIARIFLLIDVNALSGFTIGSVLALTNTADYSWLLPCLMSAIQDL
VFTELTVSSKQISSPTLEGFVDDGLDRILSDLAEIAFEMYHDVIVEDLLPNMFHNTVKTF
VNDQISLILEDSSSNCPKYESVSIDGSEEYVDFREFFDSSFSTESKSQYGEFPSLLMDAV
NKGLLENDLTTGMPKINEVLVDPLTKRQSGTEGTLLFGGDGEDIFNIVQRINLGGFNANV
RLRASDIKIENLNTLSAPLVMLQPVPNEPYMLNNNLTIGVKDHPLRVSANVAFAVIDEVQ
GTEIANELDISFDMYSANAMATIMLKILKSNLLEFPLIDVFDLNCWIATIPAPSLNAQGV
TTGEDEVSASVVDFQASVANLNLNVSCVECSSPGIVEFTETLMLSGEAQNDVTFLANTLL
LSAADVIKGNLLQVQIDRLLNDASKQCRHSPTYDPNFSPSNISAKKRKYKDFDTIQIEDS
ATFLILIGVIFLSLILVVAAVVFSIRCVTRRRHRRWLSTLPIEQVEVLKNIQRNEDSMEF
NLNSTTRSMFQSAEDIPCVLRWGMPIIILGNIALFLSGHLSLGATVKIVATIAGETIEVD
DFFEFSVAKSTIDIWNSGGKALAILILIFSGIWPYTKQMATLVLWFTPTSWVPISRRGSI
LVWLDRLAKWSMIDIFVLIVCLAAFRVTVNSPANFAILPNDFYSLDLLVVPLWGLYANLI
AQLISQISSHFIIHYHRKIAEKARISYLKTSTNLSLEKIEDDCDQKVLLRTHKFSRPRRG
EDEKLIVRPWTSSMLIFLVLSLIACIIAGCTLPSFSIEVFGLLGIAVEMGQEFREANTNH
SAFTVIQLIFEEAQFLDTVKDVVGLGSFSVIFVATILLVPIAQSIFLLGHWFVPMTMKRR
RKMIVLNEIFQAWQYAEVYIISIFVASWQLAPISSFMVNSYCGSLDGFFSELVFYGILKN
EDAQCFSVKSSIEDGFFVLATGAVILALVNTFVVKATMQYFQDQDESNKRIQIETYTTRL
ESGEKGQEAANEDSQVTIRPVPVLFTDTFRWLLRQDEEANKVLNSREPSCDSNSSMHCSS
AKVQ
>comp2617_c0_seq1; 537 111 1421 LEN=1561; translated
MSASGVARRSLSTAMRTAGASRVWSTARSAPAKYPLAFGVVLSGFKTSFSDLLVQKVVER
KEKIDWRRNAAFAAFGFVYLGGIQYTLYVPIFGRLFPNAAKFAAKPLRQKVKDIRGLCNL
GLQTFLDQCVHHPLMYFPAFYITKELVMGGKGNDGKPDISRVLSDYRTNMKEDLIALWKI
WVPATLVNFAFMPMHLRIPFVAGVSLLWTAVLSAMRGGDAIHADDMAGGVVTNASYQIFM

EGLDAFNTTPVEMDRNLDHVSISAAGKDRPGMVAQLASHVASQGGNVTHSKMVRLGDEFI
IQMHVALPKEESSSFFKSLNNGNLEELNIQPTRLTRRDTDEKKKAVMGMKIHCVGDDRPG
ILADVTNRIARKGMSLEDVATSIRLSTTGKREFVIDVLASSPNVEDKENIDEYVADISSM
EEDLNLSKMDIRVHTA
>comp2618_c0_seq1; 703 246 2438 LEN=3101; translated
MVWKAGSLSRRGSRDSDCSSYSSLPEKSPAETEVRRIASKIGLPEDSDLLATVCHKMRKE
YVVEAWQLPSLDSRQWEKMNAPIGLAVAVQHLSSQRLREQLLQCREEQQIQEDIDADIER
QICLTLPFRGRNEQMGRSHVSERCNPNGDILASLSSTTSDSSVVSERGKSKETINIFTEA
ETKTESDLTGPVLLPNADETSKEIPRLAPPMNPKPSCTGIDTPREDIDEDPKEDQQKEIN
QGLFDSDHGSETAEFSEPFPKWLNLSTVASGQHDEKPQKLLSDEDYGEKPDGRFNAIASP
TETEETKNGSTHDISSGSEGLETKASPRKQLSVVTHREPSKDDVESVQETLALNASSKPV
GDETNAEESQIIPGDTIETPNETTVPALPRPVETQPNPEPEHAETEPSENTETDSTTVDN
TRLASDENQEPKIACPRSSGSPQAKDKTGERTLEKTDSDSESEDATNIFSGLGSCVSFSE
EEEDDVLAFVVEREAAQQKSLLGPIAEESTNTTPAAGRYETNENAASPVSGSDDDEITVA
VSNVSQAAKKSGSRRAKETSKKYPYDNRGDQKIDTSALQVILVRLPDVDHRSILSQLLIM
ANAKDKASRITLAFQIQDLLLDLMEKHKMEEGSEKAVQLIFHLARLKKQYRVIFGKSLFK
AMSALYKESEKRKEKNSITSDKKEKKKVIDPEHLPETESRALEQNSPKPTMEQAKIGHAA
DVLSAIQSPT
>comp2619_c0_seq1; 274 314 1177 LEN=1535; translated
MSPIAIMRKTLRRRSRSAPPKLKKSLIEDHYNVEKNIVGKRLALPGVPNYEDDWARDSHD
FFNLIVLIPIIVLNVMNWNWDRLFTLGKKYHLADAWTGEWFDMFFWATVTYFVTDFFWVL
IVPKCVKSPMVIIQHHIVTMLYLLLPFYRPKYRWCMGVCMSVEINTWFLIARRVFNKQGF
PPWIIDLSFVSIRLKIISICFYVTWFSIRVFLYPMMLVYLVKEHHKESKRVGTYINVGLI
PPILQSIFCILNLKWTFDLIMSKVRYFRRKKANKVVGKRLDYVDKGL
>comp2620_c0_seq1; 558 68 2470 LEN=2695; translated
MSDETNQSNPAMSDHRANNQTNNDTDRNRRRQEQPQARQIFGGLPAQGFQFARRHMNPNN
VARIQTSGLPMNLDRDIHGNPLQNAALNGNNGNGNHVADRDMNGNLSFQRLRWGGRGQMA
RQLQLQQLQRQRRQNLQPGSMPPNGENQAGSTTSVPVHLGMVRVQLEPLTPQPVAASVAT
NATETANPSGSGTRVQDPPQYERFKCGICYEYMDDPVGCGKCSSRFCRACLQRVIDSDRE
NQQVTKCPVCRCECDAAVPDYDLYGKMSEDNVPRLPCRYVSVGCRERNLPLSEIAKHEQE
CEHALVKCRYAAYGCKWIGKRGLVPMHEEYGCKLAPAGPFIQHFRQTMADQKVKLDLLAQ
QTSGAVRMSHVLRHAYLRDNQRKSLSDVLRLFQYCHKVTSLTPHFFLTKDLWVSYWRNHE
TRAAVVNFCVCFPFLSAAIGVVGHGTNSFFDIFEEISPEKTLWALTRAFEGAKTNITNMA
APETMAVTIKSLQNSHTIELWENAFLGLCVGSLGMLVLMLNFVDTKSSISWDKISIPILG
GRFPLIGDAMALCIFTLLLAMMEYHQSNIRAWIMWMAIVPASTIFPATILSISHNTARLV
TGTPTPAAFDMMKMARLVEPCMFGLRFSMLVTYFGMMPALDASVLLALVPQSSKIYLKNS
LLIHTPQSVFFFFAAAKSSYWIIKIKIHLFDSGLDLSPLKSAAITFESAQNFFASSPDAI
ALSGVFDDISTSLLAVATLMVTSYIINSFFDLGITVGEYISLTSQKEVENLSRGTFKEYS
SVGIFVFGLWAMINTLLVYI
>comp2621_c0_seq1; 578 138 2417 LEN=2520; translated
MGPKEMYLIVYNLSCCAGWSLILLAAIRSLANGVPQDGLKESLANVYQNTDMPFQVVSSA
EPVGDLLFYTQSAALLEIFHAAVGLVRSPVVVTAMQVMSRIVALVAVTYSPEAQSQWGAG
VMIISWSLVEIFRYLFYAFGLVTGDSNKKTPYPLFWLRYSLFAVLYPTGITGELTCFFSA
AYDPAFQSTFSADIAIWIYARILPLVYVFGSPFMIMNMVGNRKSAFKKRFARPPPPPRGI
VFPVEEGKKSRSSTPVNKEILASAVGAVNPSKAEKIRSAKGYRFTYVKHLTGLVEEQCKS
PEACLKIAQAGLDKAYDIFEFVAPDGSAVSVREAMSMKNTEKFCTGFIKGEGAKDTTMME
VPYKGTILKGQSLKDQVKKWVDYGTIEPSAGEAIIGCVDNPNWCDLSDKYFVLLGAGSAM
GPLLVLLALGANVIAVDLDRPNIWKRLIGLAKKSPGTITFPLKQEQSSLDSDDALYAAAG
CNLFTHTPMIRDWLMDLYPGKSFTVGSYAYLNGALHVQVSLAMDAITRDLSEKRPGTSLA
YLCTPTDLHLVPKEAWDASVSNFKEYGSRLLCMFVRLLSSGRLLKKNAKAPVAGEGGDFY
MINGVSVAQGPNYILAKRMQHWRAVVARSKGCIVSSNIAPATSTVSVTQNRTFAWAYEGM
PYFEPYEIFAPETSNAVMSAILFSDLNSPKSVGNPKTSLNNPNQLFSQGSFHGGNWRCAY
EVDSIGETSVLIYFARIGAPYIAGSAVVLAAVAYKVLGY
>comp2622_c0_seq1; 284 231 1043 LEN=1152; translated
MTNVVDTCKAFVPMLSTQSVPSDLRWSCFRKPVRLHLQKGWFAEDGEDRDEDMLVTREDL
NRDLLGMEPKVKRKRKNGKGQGYRPLDNRDHLPFSVRTKTPDDPYKTEFQRRKEQQMIDK
SNGDKVKGTDLDRHMLASTRGNFFSKRKNEKINGKSKKKLSDDSTTPSKLIQRQSRKKSK
SQNGKSTTTVVGEFQLDKSTTSGDIIVIGDKEYLVETARCQYKYAGGQRFVMVRKILEVK
ELARAKKEDDLLRQYESSPSAFTSLSNDFE
>comp2623_c0_seq1; 819 307 3969 LEN=4226; translated

MDGETMRKEMMNGGNGAQEYEKHPTGYQNHSQHIQRSPSMDTTNRHHHRHNQQQFAKPLH
PTQRHPEQQYHGKAPSHYPRRYDNRGGYPAYPPPAQMMNQNHMNHYHPHYSPRSRSHTPR
VQTVVSHSFSMEEEQREDGSSRYCGGHHHRSPNQRDTSSYPGDEYRSPSGEFSADRSSHQ
HQQRDAQDHSFQPPPPLPPHHYGSRRDYQYVNDRELPPVLEESRIQMSPGGMISRTGDQV
VSKREPPSPPVEGQAPGIERSLSSVSTGGPLKRSFWHHSRPVDAMNGNDVSMPASSSPSL
PSEFLPPKRSKTGESKSPGSPKREKEYTMTPRSQASPNSDREHQQEHEHEQQIMDIPGGN
GGLGSLGGGIPTSTSDIGLSPSGDWYTNRARSMSWEAREDYYRRDPRSAPSWSSRSPSGR
DGPAPVNSGSGSHWMEAPYMPSPRGRHTRDGSHYDMHLQPWEASPRDWGHHAHHLPHHPH
QPPHLQYLAWPGRSNGNGREESEEGKKFRGHEHGAFDSEQKQSHLHFPVRGAPPLLPQPH
GMGHIMGPPMQTGSGGVDPSIMMEGQSGDMMNSDRRGVDKSGPIKLLALPEDRISLSETL
CIVRENVEVFTATIEDVEAPAPGRKHAVTVGQVGLRCIHCRHTTRSSERVKRAVCYPSSI
KRIYRTVIDMKLDHFLHCKFVPQPLKDTLQALKANNTRSTGTTMQYFIRAATSLGMVDGP
MGVRIADSSIPTVPMSLQSSGKNKLPKPSSLPTKGKEHVPSKINSKNPGEQSESSSGRDI
RINRTSSSMSTEGTPQTSVSTDDLPSELFTGKVALSVPEDKMSLSPLRCFLREQVCAFSA
TEEDIAVRTPTTFAVSVGQVGIGCIHCLGQPAKLRLNRAVCFPFSITRIYQSVADIQRFH
LGECKMVPKDVKEKFLELQSQSSKGSKGLATRQYWVTSAKKIGLVDTTKGIRFCRDPAIP
ENKPVSLDILAQVASNVTTVNKPLVLPEDKPQIAEFLFVVMGQLQPCRFTEADRNKRRLK
DVGCIGVECKHCAGQVDGRKFFWSSVNAVESNFVSVHTHMMECRMVPEETKEELARLKKL
RKEQTAALKTGSQKSFFARVWKRLHEEPKKDEPKDEQKDGEQQDSTTKEGCSERAAEAPI
EKTAAASEKATATTDSTAKASFSVSLSQDTLDLAMTTPTTSPASVEKPFTEISVGKLSPG
SPDSTQKIEAVSDKMAVVRV
>comp2624_c0_seq1; 671 177 1733 LEN=1931; translated
MQDNTTQEIEDKLSERLLGPSGDGDMHHADTSSFRIDGSHNEDFSATAIMEGGEIIVGSG
AGFGGQQPLQPEYRDAPFALAFLAHLGFVFFVSMAWGISAMRSSEPSGDDASSSGDSGSV
FFSGLLWIAFFTCAASIGISAVSLQVMMHHAETLIQCSLIGSCVFMGMAIVFFFVDDVVW
MAFVWMLSLFLTAWYARNVWHTIPFAASNLRTALSAIQTNGGVCALAYGIAILACVWTVV
WVLAVVGVSFKEASCVAGVCKPDINAFSVFFLILSFYWTIQVFQNVLHVTVSGVVGTWWF
APQDALSVFSPAILDSFRRATTYSFGSICMGSLLVAVIQSLESLARNARRNNRGGILLCI
IECILRMVSRIAEYFNRWAYCYIGLYGFDYISSGKKAIELFKARGWTTIIMDNLIYRSLR
LVAIVVGAVSGVIGMLLAKATGWVATTLGEDSDGQVFILCFLVGFSMSFILMKVVLSAVD
TVIVAFAEAPNEFNTHHPALYNNMVQKWRLVFPEECGF
>comp2625_c0_seq1; 409 66 980 LEN=1537; translated
MAAVDTGLWGPETRTAKTGKESKIEAWWEVRRAGPAEPAPTRGATPLVERCMRVHSFSED
FARRVLKGYRQFMELKSVMNDWQELKLAPSNPINQMWELHLLDNLNYTEDCLLLFGRVIG
HNPDAVLDGAAMRERIMTTQIAFQARYGDDLDQDVWDFGDDWNKDSMAQPSLNKKFDSLE
VSSRGRLPVAGRGDDMSASRGRASSKGPVSPRRVNAAKTPTSASPGGNEPITIFLRDQST
GEETYFSLRYKSTLRIVFTVFAERRGLNQDSLKFNYNGQQLNGYETPHSLALEDRARIDV
SITR
>comp2626_c0_seq1; 352 38 694 LEN=969; translated
MFKATILATLLASASAFAPSQFSGRASTVVRSEEAAEEVEAPPAYVASTALPFLEYPPNL
AGYVGDAGFDPFRFSDFVPMDFLREAELKHGRICQLAVLGFAAVDMGFRIYPLPEAYKGL
TSVTAHDALVEQGAMSQLFLWIGLAEVLGTVALMQTLQGSGRAAGDFGLDPVGFLAGKSE
SEINEMKEKEITHCRLAMMAFSGMVTQAVLTQGPFPYV
>comp2627_c0_seq1; 569 469 1658 LEN=1658; translated
MENTGNGCRRSHDNPSSSPMHIGMQGVGLNPFSADEIQELMGLIPGSDQHSHPGTAAHAH
SHSSLSHAHAPSPLPHANSLGNDMSVGVHNPLQLGNNNTNQGSNMRAAASHAAEMGLASL
PPHPGHGMVQPTSSLSSMPTIGNGNHATSNRRHSSGSNTSTAIDEGSKSQARSERKRSRE
KQRRTDVNKQFGELTEVLKRIEAEEQQMQQERAAREEAGVDGAVNNGNGSQSSSLSSSRM
ILPPFSPTNRVDLIARTIAHLERLSHLTKKQVHEIKALDDQLKSAQKAGEDTAQKLKEAV
FHHQQQATVHMGNNTGGMMMAPGMMGQPMNSMFNPMAMANGTNGMMSIQPQKQQMMMMVP
MMMPPQGGAPGTPMMPTGNQPFMMMPQVFLPSQQQQX
>comp2628_c0_seq1; 363 76 495 LEN=711; translated
MKVNTILAAATLLPLASARIRGFDASDTIEINDLEERLLAFSLPGEAMNVTDPADDDFDA
DFEIETPKGKKPKPGAAKVKIEKGKKPKKADKSKKSKLAKPDKKAKSAKEAKSAKVAKEG
KAEKSAKSAKEGKSAKESK
>comp2629_c0_seq1; 434 222 1988 LEN=2107; translated
MANDEDLGTFGVSRGMNEKAANKRLNELQQKKRSTSDKDIISKYNADRQQKDRLRHLEKD
ITVPTGRMNGYQKRSIQKRMNRKDKSVHSKLKIYKAKRYEAAVAAADAQIILNTEQAGFL
EAEHEMEFTSSLSQVELKRNHLDENTARHIYDVTLPSGPYGCKFDRSGRYSILYGHRGQL
AMMDAHHLSLHKEFDVRERVRDACFLHNFSLFAVAQTNHVYIYDDTGAEVHKLSDHHDPM

AMDFLPYHWLLASVGRTGHLVYQDTSTGGLVSQQRTKLGPCNVLRQNQSNAVMHLGHSNG
TVTLWSPASNNYLAKMHCHKGAPITSMAIDISGNYMATGGADRQIKIWDLRKFQCTHSYF
SIAGIPTSLDISQRRVLGVGHAGHATFWSPEALLKKVKEPYMRHSVPACGPVETLRFRPF
EDVCTIGHTKGMASIVIPGSGEPNLDTTEYNLNPFQDKKQRREAEVRALLDKLDHNMITL
DPHEIGGMEESTPEVRNERLKDAEELANAHKKPKRQATKKRGRSKIQTQLRRKQRNVIDE
QVTKLREAREKEKEMAALSSGSEGTNRKPSSESHTANNAVPSALKRFF
>comp2630_c0_seq1; 158 481 915 LEN=1169; translated
MKNPTYRQVCSGSTGHVEVLNIELSESASATPEIFEELMKFFYMFHDPTTLNQQGNDRGT
QYGSVVFVSSEDQKEIAERVKRELQSHVDNRTIKTYQRKKIETAIVDYTTFYEAHAEHQE
YLEKNPSGYCNHRYRFKDWPKAEL
>comp2630_c0_seq2; 190 155 733 LEN=987; translated
MAPIASTLALGAGCYWGTEKYIVKDFQKRHPGCIANASVGFMNPNPKGMKNPTYRQVCSG
STGHVEVLNIELSESASATPEIFEELMKFFYMFHDPTTLNQQGNDRGTQYGSVVFVSSED
QKEIAERVKRELQSHVDNRTIKTYQRKKIETAIVDYTTFYEAHAEHQEYLEKNPSGYCNH
RYRFKDWPKAEL
>comp2631_c0_seq1; 191 590 1045 LEN=1722; translated
MADCKVYSERRSRCYNVYGNNRGEECVHEALTEKRCLSMLKCPRQALEYYGGAGIGGGEQ
NDSGFFATHNELPRREFGAGEATKNGYPKALCASWAESFFYVDKALEYGEEVASHHREAR
RIVSSDSKLRKECRRMAMELADCLRAATATK
>comp2634_c0_seq1; 521 94 1359 LEN=2682; translated
MSNDVMSSVDGCLLSSNLSIESRGSDIPESVLSVSEADAEAEEDEPLPNNMTRLDPDEAS
NEGSDADESINEEKKPYPKAHFKSDASTSSKSEKLGESDPPDVRLVKRDRVSTSSATNDG
CSSQECTSSSLPQAHSASEINSNQIPSMPGAFAIENPLFESLERRRSASFGSYSDSIATY
GHNTDDRIAISEATPPLQEQMDRDTEEGMVVRAVRVEEHVFEAKLVDQNSTANPLKRLKK
CWWLIAIAVTLIVVITLAILLTKESEVDETNEVINETTIHSTESSRVKELRAILAPLNGD
GVFDKNSTEFSPDRLTALNWLVTDDYFSRYSFDEFKIRQRYIVTLLYFSTNGQEWINQLS
FLSDRDVCDWSAVKTVDRGNQLESFVITGVICNKLGQVETIKIGKYQSLVSSFWRDLPGS
R
>comp2634_c0_seq2; 578 94 1539 LEN=2562; translated
MSNDVMSSVDGCLLSSNLSIESRGSDIPESVLSVSEADAEAEEDEPLPNNMTRLDPDEAS
NEGSDADESINEEKKPYPKAHFKSDASTSSKSEKLGESDPPDVRLVKRDRVSTSSATNDG
CSSQECTSSSLPQAHSASEINSNQIPSMPGAFAIENPLFESLERRRSASFGSYSDSIATY
GHNTDDRIAISEATPPLQEQMDRDTEEGMVVRAVRVEEHVFEAKLVDQNSTANPLKRLKK
CWWLIAIAVTLIVVITLAILLTKESEVDETNEVINETTIHSTESSRVKELRAILAPLNGD
GVFDKNSTEFSPDRLTALNWLVTDDYFSRYSFDEFKIRQRYIVTLLYFSTNGQEWINQLS
FLSDRDVCDWSAVKTVDRGNQLESFVITGVICNKLGQVETIKIEGNNLSGTIPPEIRHFS
DTIMEISLGEGMLSGTIPSSFEEFKALEIFDVNSNCLSGPIPNLSRIPSIRRLSLENNLR
LW
>comp2635_c0_seq1; 263 97 1035 LEN=1184; translated
MPTIINSPVETIVTNDAPQIKMIEIGGPETETEQKRRRRRRRRPRSKMSSATPVEGCEK
SLSCDDSTCATASLSSDDMSTATPQSPQQQTQKQRRRNNQKNKSKKQQKQNKNNNADHEV
SPEEQSRYLAMDCEMVGVGFGGKRSSVARVTLVDWDGRIVYDEFVRQEEEVTDYRTFVSG
ITPLDVEDATLTLEECRLEVQSMIADKILVGHALKNDMRALGLTHHWMATRDTAKYEPFM
QTRFEDNILWPRKLKELALEKLERKIQRDGEPHSAYEDAAAAMDLYRLVRRKWEKVMDYK
IKKTREIENTRN
>comp2636_c0_seq1; 422 190 816 LEN=901; translated
MNRLAARTLILSMLFASNDAFSPVRSSQRQSIALFDTTSTPTVEAPTEEASVPDVDSSTA
PALEKKKKVNKNAEGIFAPAVLLTKDIVGETELNKLRAKIIGLHSDVIGKFTETAQSEFG
NQVLKVLFTLADKNGDGTIDEEELAEALQSLGFDFLKEKQIAGIFARADADKNGQLDFEE
WQKAAPKTLKTSLIKLAKKNGHDLGFLA
>comp2637_c0_seq1; 1249 1 3215 LEN=3670; translated
XQQQQREQRAVDLPMESVTQPASPDRDFDEISEISRASNLSGLWSRGRNSLNTITSNLNT
IAENQTNKGKALLSPELIGRATHVSGKVSGLLNKFRGVPEDSEDVDEIDEQHEHFNVSQD
SIQLQKQNIDLSTPLKLVHSPSADSHEALRAYRMNRASMSPEETDKMSDASSFHALPDQI
RMVVQNSGEKPPKKSLRTSTTAPTTSQTQPLHNDQYESPIRSTITQSTLSQSELSNEQYK
RAHGRFINNSALLGGEFQHETIIDTDGTQSDASGTADVNPQTLASLMMSPDMLQKRLKQA
IASVEKRKWDQVLFLINANPWLAEMKELTTNQFLLHKLAFFGKGTPSAPMSLSEQLVEKF
PAAVYKFDQDGNVPLHLAAAAGNVQMIQMLGEKFESGASIRNEDGMLPLHYAIASFAEFD
SEQVDGVHSQPLSVLKTLLKLFPKAVAIADNDGNLPLHVASECLEGGIGVDVIFLLMDEA
ERQLQDPYGARFRNKVKIEDLMDDDMSARTMSTARDTDVSAMDSEMPCTVVLNNFNETPL

LVAIRSHKGWEMIEALVSGPGGRKAALCQDTDKNNALHLLVGEFQDATAAMSILKVVPES
ALQRNAAGILPIEIACMQLMPEEVVLAIALVDLPVNIDDRDGLKVIKDRGESWIFLTCDS
DDHMVNVVEEIVSICSFQQLQELCFMTEQGSENAIIDRATPKCREALNRALRFLGRFEFV
GDGPLFSDMQTGFKAFDALDFGDSEAGKRVLLECYKNEFDFENRCYTMFEVELESNFVEE
VNVYVEHLDDDSASKTTSEEIQQQRCVAIERPRLTLDKVVEGISKNGAYENNKHLRLKYS
AKLCSVLRLIGKALRHLHSAGVVHGNVSLETCGKFDDSMWKLSERLDVQAIGKPFDPTRF
RKSFPPESLESLPINDGEQRDGMVFDSDGPTVTFVTDMLADPSIDIWAFGKICYECLVGK
PLIEFDASNSDSPSDDVIALLQTSEWNESNMETVFDDLVESGIGESGADMVTSCLFPQPE
QRPADMDEILDNPFWQEIRRHRSPRKGRGGRKEGSIESTVSLFTDVEKYEV
>comp2638_c0_seq1; 319 180 1517 LEN=1842; translated
MLASSANSRLPAAAGTARQVYAWGGLLKQKRTSSKAMGTVMSAKKQHRRVNAGGVVHNEF
RSCSNDNKGFYNLNNGMAIRRESFRMMTTNSGDESKESKTRIATVRLYRMLRRSCKGLAP
TDQGTILIQNEILASDWGKHSFRDNDIMDNDSGDEVDTLVTENQTNELIRLFLRWNGIDP
DAGFSKVNEWYDELMSGARFRDAKNTEVGACWTTPHQLRETVRFAFLSSSKLSTQTPTEL
QGLAVRAIQMMQEQNTLWKKSSVSTTLDGLVRVTATSRCLGTVSPVSFAAPSSPLDPKYR
FAYRIRVENISPETTVQLLGRYWHITEEGDGSDEESLEPIEVDAPYTGAVGQLPVLRPGQ
AFEYVSGTDLATPVGTMKGHFYMATVPEKTKSAKSGDDVVAVSYQGSKGSCGTSSSSSKE
SGTGGNDTRLFHAAVKPFRLESFEK
>comp2640_c0_seq1; 72 926 1704 LEN=1778; minus strand; translated
MLGXVPSVGVVQLVGFRNDAALAGALGELDAGRGPAVSVEEDLHAVLQQGTGLSLAQVGP
PLFPQQRHGQVGPKPGRHQVFLEGCSQPVYGSQKRFRGLLVEARYHDVVPEGIHSRPLLV
HDGPQVIPEGPRRELAVDVDAFHRQRRGFLQSQGLYLGHDLLQVRVFSGGSQQDAVFDAV
QAGGVEGLRHAPVGEQRVGGQQDAAIVYDADHAGACGYGTGGVLDGHAGGAAGLQCGGRC
DRRRSTGTACDECKENKEW
>comp2642_c0_seq1; 222 329 748 LEN=1274; translated
MDTNMNKKIYTKDYPQHKRDSKGQRVKKKKEVNNCRESFENSVSELDNYAVVVANPQQRR
QTKGNRRFPRPLSKRRKVGGDQRVAHWPLPGNLGMPVRSEMNDETPQHKQQPRPEILVLD
DNTDDDADDHHEERDDLVF
>comp2646_c0_seq1; 171 71 679 LEN=689; translated
MSRASQSAVAGRSDGRNDGRNYGKNNGRNNGSHDGASFRGVGAFCGALWTPIAFGASVRR
PFLSRSLSVGDCSSSDCSDSDCRDNTSNHDGDDETEAETACASPFSPSASSFFASSADAA
AADAHAHARACARACRTQNQRQRQTAGLLQWYCGNADVSDRIGRTMSLLLVTRFLLSVVL
AVVAVEGEGADHGAYANTYANA
>comp2647_c0_seq1; 97 557 909 LEN=909; minus strand; translated
MAGRRLRRTPDVDQAEATADTANDGATSRPLTIDKGRKRSDNRGSSTCFLFCSVPSSISW
MGPSVVVVGDAMFIVITGEDAVVVIIGAREGARSLGARFVGARILGARFVGARFEGAX
>comp2649_c0_seq1; 258 91 630 LEN=1513; translated
MSSPFILRLAGSTAARRSLAASSVHAGSSFQLQASTLEFPCRPETNTAAATSSPIAEQRR
SFWMDVVRIKKQSPRRSFKDKNDGSSSSNQKMAHEDPEMVVRRHTDEVRADGENLLDLMF
YNDRHEKAWMKRKRMQNLRRYEFDKKHVTDLAKYIAFVQDYGDENDDKDGDGSKKSKKK
>comp2650_c0_seq1; 345 81 767 LEN=767; translated
MTKSIPLSSFAVLALAKLADAYDSGWHHRQTTTNCSTIMEIICDVDNNLSTDTFCEAVEM
AGLDLDTETWTVFAPVNDAFHLLPDTIVEDMLDGDDPRGLIDLLAYHTAEEKKIMSTDLK
CDTRIFMSNEEFTVTICEGDKTFQVGLANPTSDYPQIITADIEACNGVIHTLSEIMLYKE
YDVSNSTFVQAAVDFTSGPSSSPSDGPSMEPSVRPSSSPSDGPSMEPSV
>comp2651_c0_seq1; 642 139 1593 LEN=1848; translated
MSTILFSFCMLCISNHALVAAQLELSIDSNDRDYDCDVGPDALTNAHGKDIPRTCIDVPV
GEGSTATTVERCYYTYVPESCVGDENGENPLDLPVVFDIHGLTSCALYSSLYTGWKEKAE
EECFVVVWPSGNVNDDLLINGCWNLPAFLRDEGYGTEGGNNVTTAPCCCFKSDDEMGLPS
KEPNDPLFLRMAIDSVAESFDMENSSNYYGSISIDRSRVYMAGHSNGCMTSLSMAALHSD
VVAAVCCHAGTLLTPFPEQYTPVPIWMAHGLKDDVVRADGVVAFDLGPLFGKLGSWSTND
VLDYIGGRNDCGEAETADIGESGATYRRTNCTNSADVELVMLYESGHWPYAPLPNSIFSS
SVDAPVDTTAMAWEFCSSYSNPQQQGYPSGDDSLENEIDDEIDDEIDDDAGEPVENEKEH
KKPETGAVPVDNQDANNAKAVEDNEEAMDPLLSSSAPRWRSMHEDGTILSATVMTLVLVL
GCLV
>comp2652_c0_seq1; 452 1 928 LEN=1474; translated
XSNNNNNHHNSRNNHGAFGGLLGSFRRNRDGASSRDSTSETPKKQAFGGMFDSFRVRRGE
VLDGDGDGDGKDDDKSNDKDKDHDNDNDDDSDDDSDKDNNKDNNKDDNKDNSDDKDNDNG
DGDSGLDGDSCIDGSSLGTTNESQSLHEHQPPTGFPEVIFVMIQSMTNDGRDLSIDYKLS
GPFFRDVPTVSSTYDEIFFPAPLAPDPHTGRGDIVVPKTACSAFCTTNLDVLLRRLFVEQ

LVVCGQLTDQCVESAVRDAADLGYLVTVAEDACAAHSEKEHYKGLFGMNGFCRILTTRDV
VRELERGDG
>comp2653_c0_seq1; 186 1 378 LEN=378; translated
XIPAAPAAITKEEVQGLFYKWNSALATLDPDQVAARYSSKTAPCLLPTVSDVPRTDYDSI
KAYFVDFCKKKPQGEILESYVTVGHNWAMDDGIYEFTMGATGDKVKARYSFVYTFEDGEW
KIAHHHX
>comp2653_c0_seq2; 224 1 396 LEN=396; translated
XIPAAPAAITKEEVQGLFYKWNSALATLDPDQVAARYSSKTAPCLLPTVSDVPRTDYDSI
KAYFVDFCKKKPQGEILESYVTVGHNWAMDDGIYEFTMGATGDKVKARYSFVYVYEDGQW
KIAHHHSSQMPEX
>comp2654_c0_seq1; 388 1 1213 LEN=1288; translated
XSSTSRAYKTWKDSKAHKSEKSAKAGKSSKKEKYEHTYQHEYPYTKSAKSGKKSKKAKSD
KKHNVPSMAPSVSMHPSASHTPTATTERVEVVANAPDTPDIDTQNSSATPPPSPPPTSPP
TISPTISPTTLPPTSQPTGVATTAATTAATQQETSEETWMDTWETTFWTTLDGEEDGDGE
GDGEGGETADASMLSPSSGDFGDFYTILPDSFHTLSPTAFPTASPGAGAGHPSPGGGRSE
EAHSPTSPPTIDAEGFQTLNPTVFPAESDADADEYPNAYPEFHAYADAYAANLSPGEDTS
EETHSPTKLPTPHPTGFPTGAPSESPTKAPTDSPTEADTENPTYFPTFFPTFYPTYYPTD
FPTLSPEGVAAKEERLRLMRLKRRRKRRNRLREEQRAAEKTITI
>comp2655_c0_seq1; 263 1 727 LEN=882; translated
XDQFRIVTDKNQVMKFQTASNVLAITALASKASAFAPTSNNVKSTALLMGTPGMDLSGNS
WKPDSEKMGSTDTGDYFPEGYNPEEEIAFSSGMMGSQSGNAGSRDGPQLPGLENLGADAV
VAGGISLDPNIPEGMEFVPASVPDGNFEFQVASTGSGGSLQVMISSPCMTFEDYYAAFAP
GSHPSLSVGPSAGRLDRRSGDPTPLEITCEPNGVGGVLTGDLVINLPDDNSKLSYKVTVN
SV
>comp2655_c0_seq2; 233 343 870 LEN=1025; translated
MXSTDTGDYFPEGYNPEEEIAFSSGMMGSQSGNAGSRDGPQLPGLENLGADAVVAGGISL
DPNIPEGMEFVPASVPDGNFEFQVASTGSGGSLQVMISSPCMTFEDYYAAFAPGSHPSLS
VGPSAGRLDRRSGDPTPLEITCEPNGVGGVLTGDLVINLPDDNSKLSYKVTVNSV
>comp2656_c0_seq1; 474 95 1036 LEN=1135; translated
MSAMAKAQKAKAEVFIKEAEALLSKKGWFSSSKERNAEEAAETFEKAANAYKVGGLNQEA
GDAYVKAGEIYRDKLSNFNNASKSFNNAGTCYKRSNPADAVTAFQQAVSLYTDNARIMQA
AKLMKEIAELYETEEIDEEDKSHVVLAIEAYEQASELFAMEDSKSSTSQCLSKIAELCSA
ALDPPDFRRASKMYDDLGRRCLSSNLLKFNAKGYFLQAVLCHLASGDDVGAEQAVAKYES
LDYTFADSREGKFAQSLIESVQGFDVEEFSNSCYEYDRISKLNPWQTSILVKVKRNIDDG
GDGGDDDSDVDLT
>comp2659_c0_seq1; 236 152 547 LEN=1300; translated
MVSATRLKKNGSASRSNANFFQLSLGMFLGALGMNLWIGSDVDPTALWLDQDRNSISEKK
VVTSLKSIPEVKETKGWHSIHVFYGEKEGLFNDAPKSITQSQESFAQVGQDSIILDLLGS
NGYFIDLAASK
>comp2659_c0_seq2; 317 152 1012 LEN=1203; translated
MVSATRLKKNGSASRSNANFFQLSLGMFLGALGMNLWIGSDVDPTALWLDQDRNSISEKK
VVTSLKSIPEVKETKGWHSIHVFYGEKEGLFNDAPKSITQSQESFAQVGQDSIILDLLGS
NGYFIDLAANDALDLTNTLALERKGWTGLCVEPNHAYWYGLSHRKCTVVGALVSGTKPEP
VKVKFRGVYGGIVGKLSAKLANRKKEPDAPKVTRYTAPILEVLQKFDVPEIIDYLSLDVE
GSEYDIMKYFPFDSYTIRIMTVERPNKELKALLEEKGYVFLAELAPW
>comp2660_c0_seq1; 193 1 480 LEN=669; translated
PTNPTKAPTNPTRAPTNPTRAPTNPTKAPTNPTRAPTNPTRAPTNPTAAPQPQDGKCPGEV
EFVFSLETDDFPEETKWVLKRNNRVVKKRRYKTYENPLSFYEESICVPLTGNYKFVIVDR
YGDGVCCDKGDGSYEISFNGEVKRRGGDFRRRQRTKWSA
>comp2661_c0_seq1; 417 1 980 LEN=1117; translated
XPPADGPGLDEENDEDKDLELLEGIFDVDEPIVPPATDGPGVENKNEEKNVETPKQIVEV
DEPIIPPATEPPKEINVPLDQPLTPPTYAPRLDDEKEDQDVPTPPVIPPIAEQKLEEDEN
LDEIGTEPPIYTRPPYEEKRVEDEINQEEDNLVSLELVSDEPRVGCAGDVKILRQKGSGL
VSSEDPMQNAVNIVRQDISTVTVSLNQVWSREDQVLDSVYYQFYAPGKNFWGNAKCYEQA
QVPFSRSDPFETFELACNVLNPYAELQICLVDNVENSILLPPRVVEDPLSLVPKCCSPDT
SIPREQQAATCYIMEVKCTPPTANSC
>comp2666_c0_seq2; 201 468 995 LEN=1240; translated
MMHESYPDPRGEAPGNMAARTRRSMVKREGLLASVVRAASAVNNALLEHCGVCTVYCDKQ
VQYEDYVQQQYEVGVGAMEHLYAQLHTAAGEPGADAGLDAGVDANAETGPSRQRALAPSP
QGGAAGSEPSRGPHRAMAPSDPRTAEAERYYRGVLFDEESVVRSVGSRGAVDDGE

>comp2668_c0_seq1; 104 263 815 LEN=815; minus strand; translated
MVCPHLFIHAPISTAGRSVYDLDRRKNSGGKRGIHERLFIDASQASGYVLHPSTELGVAC
EGDGAEAVSPPPCRPGTRTVFSATTDGTRWDNPGRDNGPAGLTDLQRKRSVVFGFVDTAC
WTFTYEAYCPFELLEVAPKQHCSWYGRSNFFFAWDTPQLVKEGVCVPTAPTRNPTGPPTE
EPTEX
>comp2669_c0_seq1; 763 1 2179 LEN=2472; translated
XDDDDDDDDDAQKDDDLNFDDDDDDDAVELDDDSALGKNSLPGSTMTYQGVRGEWEFSGI
QSDWGPAHAGSMVYDFTRNSVYLVGSYFPDSENDWVRSGCFLGEIPMSDIKDWKTVESPL
PPSAKNRLPSNHYDGVEVFSLPDSLDPREVLSCHSIMYDDRDDSNHYLFIGAVDEPDAQI
RSGTINGFVNAFQRSSTNPDWELKINPTALDTAKAEDFRDTSPVPVRYPIAMTTGFYQNQ
EHLMVVSVSSNDPLLTEEYVESGDANNAQNFKNTMVPPGLRPYNSFAKRGSNYFYSHQFF
TYDKRKNDMELTFSFDAILDSDETYPTGVVNLVPNNMIAVMVGHLKGRGPRVFRFPKPLM
NGVERLDDFDGFVQNFLFANGVQPQFRDSLRVSSVEMNPPLDDFIHGICLGPKNEIGNHD
SYFIVGSTYGTMPAGTLQDEITTNILSGKSKKSMDGNRIDKLSAFVSKVESNEIVWTTQL
YAVPESKTTLNGGKTEAFGCHLIESDPTTMYVGGNVYEGGVMDSKFESAGGDDVWVAQID
TANGEPRWIKQVGSAGTDRISHTNGVQAALNGDCILYGETTGELYREKRKNEEDKYTSDV
FVTTFGKNDGFTGPTIGSQDSTSKKSGTIIGVGTALVVVSLLMCAGVFYKFRRPTRRGAS
KNAKAGSDGIFSGNLAPEYHDDEDAQVTDAASNGASNGSTSGFSDNAPPPHVFQDDPKIA
PEKSFV
>comp2670_c0_seq1; 16 1 647 LEN=647; translated
XYDNEDDYYRTRKMEAQRKQNAIGGIGKKLKPHIREKPTLLLPTPIIVMGFPKAGTSSIF
RFFRKQGLRSQHWYCCEEQSTRIQGGPLMADCLLHNLKRGNRTSIFSGCGHFDVYSEING
PRRLKYHPKFHDRKGYLLDDGTYDFDGPGQRIFFPQRFRIREIHDSYPNATWILNWRDYD
PWIDSVLKWGTGDNLHYQFLNEYYIQGVIPSVPPPE
>comp2670_c0_seq2; 147 317 852 LEN=852; translated
MIPSLQCFVDSNGDGDGDMGFDAYLITIVTRASTSLFLNKSNPLGFKEXPLMADCLLHNL
KRGNRTSIFSGCGHFDVYSEINGPRRLKYHPKFHDRKGYLLDDGTYDFDGPGQRIFFPQR
FRIREIHDSYPNATWILNWRDYDPWIDSVLKWGTGDNLHYQFLNEYYIQGVIPSVPPPE
>comp2671_c0_seq1; 2 1 216 LEN=216; minus strand; translated
XTLDWLDCMQGKSANKLDWWGCMQAKLANRPDWLGYKRAKWASMPDLWDCTQAKWASMLD
LLGCKQVTLASMP
>comp2672_c0_seq1; 299 55 441 LEN=1122; minus strand; translated
MHRIASHYITPVSYLLPPAFFLRPRVPAIVVAVAVAAAAAAAVEDTVGSWLHDAQREIVL
LLKIEIVDGVVGALDVNDNEDEEASVRSGREQEQEHQMQIEPMRLATNRVAYFGLASSQQ
RFCEKSGD
>comp2673_c0_seq1; 531 165 1861 LEN=1861; translated
MGKAIVRVTSGNKPLSKRRARQPPTTTSSRRNKSIENSNYEFFPVLPRLSFNHREQGRGN
ETVEVARENTEPKCSYRSKDLGLMVLNPREAAEIDDPGNPIGNSVNFCVDNIFSINTATI
DISTLLQSCYRIDVGDVMVNFFLCDYSGGASPKTVKSKGPPAINTMRADENDRLDALALS
CNKKEKAGWKKTLKTIQGKNLLRSMSMKRWPAHGDCENRLEVAISSDSDDTIFSNLEDPE
KWDIKTSSKSKTINTRKAHSKSLASPATKEECDKKYDFFHEDTDKSLDTFDSFWNYSMFT
WNEEVCAENEVHRNDGENKKVTCKDGTQNPNPIAANHGAREDNESQEQENGVKFGLETTR
DESNVRHRKWKNITSWKKFEPFRVSSKFNNATGKDSTPSSGQKLGTNPIQKGSYSEREDI
SGQALENPENSVDKMDVIERSNHQNKNETQDQGSTKFALDDARSKSLVSQRTVRNLRKRR
LQKALGLTRSNRSISSMSKPKRPSWKKMLMVSIPSTRRISSREVAIPADIIHVIDKSQEV
HEVKEEDIPTPGPIISEETTDKSGFX
>comp2674_c0_seq1; 378 117 1887 LEN=2603; translated
MPSSMGDVTAMGNAGSGMGVPGSGFPPQQHHHHPHQISQFLPPMDRCAGCGAVDTPLLAC
DDLDRASGKKKDIVIDPSQRLRFCANCMIGKKVRVFWPVDNSWYIGTVQQFNAVTGEHLL
KYEDDDTEWVHIGESNTTMNPSDFTPQQPLSAQVGIVAPQPNNFVPRSEAGSSPADKKSA
MVPASRIKDGNTTPTIANADPKNRVGSSSASGGPTTTKGTDTQSAAGPGLETENPSNALH
TTYRTFGSAQIQGNSYPPNSSSGQRHLPPHLAHHQPHHIPQGYGHGPGGPPQPTPYGSFS
SSSLGHGPQPLGYGYPGHPGMGIPQRHGAVGALPPHHYGGMQGHPPHPHRIENPAQISNA
PGSGGNVGANGKKKTGPKAWTKEEDALLLSIVQSMRVPMKWSLVAQSLPERTGKQCRERY
VNHLNPRLKVADWSPTEDATIFHLYNTIGSHWAKMSKIIPGRTDNGIKNRFHNLRRQLER
EDEHRLRLNASRDFPEEIRLELIREFPEELRGKAAELWDIKSGLSVLAAKVYWVGIEPEQ
QGVSDLSVRQYPERISASGVVFAFLVFKRGAKSVQKRVGVKAAHEFHRT
>comp2675_c0_seq1; 674 41 1807 LEN=1807; translated
MSKIALVDDDRNILTSVSMTLEAEGFEVETYNDGQQAFDAFSKKLPDMAVLDIKMPRMDG
MDLLQRLRQKSQMPVIFLTSKDDEIDEVLGLRMGADDYVKKPFSQRLLVERIRALLRRQD

AANGHVVVETEETKVIERGNLLMDPLRHSVSWKGQDVSLTVTEFLLLQALAKRPGFVKSR
DQLMDVAYDDQVYVDDRTIDSHIKRLRKKLRMVDPEFSAIETLYGIGYRYNEEXMALVER
IGVRSVEPRKDNDLVLGDDFVAPDRLDEGELNARRAKRGWFSFRDSSLTRKIITFNLIAL
NVLVVGILYLNSSRDNLAMQRANSLMVQTALIADVVEARLPVGAPVSFATGDGVDMATTL
DNLDIQTGTEVFLFDRTGMLVGRKQGDTMGQDRTATLPSTPMTDFLSRVWDRISSPFSGV
DQVSASRVVEDVARDMVDLAVGGGVEVRTAETAQGVVLTVAAPVMQGNTPVAVVALANAS
GEIDKLARVERERVLQMFLIATLVSVGLSLILASTISNPLADLAAAAEIGRDRNKGKGAK
GRIRIPDLTARPDEIGRLSGALRGMVSALYHRIDSNEQFAADVAHEIKNX
>comp2676_c0_seq1; 909 94 2394 LEN=2487; translated
MSDEKAVEELAKKVDRWTLQDDSTIDYGYLAQTYGAGIKEAKEGDKTFYLTTAINYTNGP
AHMGHAYEGASSDVISRFQRLKGDRPCYFVTGADEHGQKVAAAAEKEGKDAQLYCDKYVD
GFKNLNQRLLVSNDDYVRTTSARHKRVAQELWKRCAAKGDIYLDAYSGWYNVKEETFVTD
MEAEKNDFKDPSSGIPLKRVEEESYLFKMSAYADRLIEHIKNNPDFIRPEQHKNAVLSRL
TSDPLRDLSISRTTFSWGISVPEGFDDKHVMYVWIDALSNYLTGIDYFGTNGDGKEGIEK
FWPCDVHIIGKDILWFHTVIWPCLLMSADIPLPTSVYLHGFVNDREGKKMSKTVGNVVDP
HDVLDQSHVDSFRWYLCKEAPYGGELSYNIDNMREMHNADLCDTLGNLLNRATNLCKKFC
GGVIPDVPGPANPVVDLASIIDNYTAKMDQFDLQGGAAIAIAGFRDVNGYLQTEAPWLKK
GDEHAEFRQIVVRATLEAIYALAHLLMPFLPVGCKFIFNKLGKEPVSLTELNRDCRSLDT
GVKIEVGRVLYEKMLSDAERDKKAGGKKKESYEEAQKRKKEMKAAAIASSEKGRGKGDAN
QSDFTKMEIRVGKIIEVWNHESADKLFCEKIDCGEETGPREIASGLRDFYSIEDMKDKLV
LVVCNLKASKILGFNSNGMVLAAKEEDGKTELVEPPEGSQVGERVYIEGLSGDPFSSSQV
KKKKIWSNVSKELKTGEGGVATWQGKTIQTASGPCKVASLVGAPIS
>comp2677_c0_seq1; 288 114 977 LEN=1196; translated
MRIYNGGTFTNSYRSDAMENRKLKIAPSQKLPCSSFLPATSKRSIVLPEDQENSAPNKNF
EASSNARVPKKPLSMDQENSSNFSNTSVTAAPMVPSKALSEKPAMQKGLSERTLGEPIPE
GGVCNLVYEPDSSGRLVEHFTKTELHGMAVVGRWTAGTGKKIAGFKFKRNAGRNVLIGNC
AAGVLGRKNYCNGFCQFVKQAYLMNGEVTLYDIPKGFKAMPVDVYLYYDDNRPGHQTVKL
VPGKSLPTENLLAVSCLPKNTPFYETETVDLFKWLHDAEVQGYSSKC
>comp2679_c0_seq1; 252 1 366 LEN=850; minus strand; translated
AELKSLVLLARLDPEDAKVVEAGSDDAKAANNDESVQLVVQDGVEVFLPLSGLIDPEKER
ARLTKQQGKLEIEIQKLAGRLSSKGFVDKAPAAVVDKAKAELAELESQAKMVQSSLDALP
Q
>comp2680_c0_seq1; 727 101 1927 LEN=2897; translated
MFREQLLRELYEQPKGKTLFFTTSASSNLKLTEGKAFTGLYRCTLETPEDLFDDFTHYGE
DEEICNAATKVGDGHNFGGADQSELLLRNLQFDLSSLRESPLDRKKKKNYLYANTHKAHY
LKGEDSDWEIVRAQVPDAIDFDKKIVNHDGSLKFESVFKTQSVIDWNECDFDCCTKADYE
DLGFHSNAKEFDSNNEYHHTYANTIVRSFVIDEHTGDTFVSWEGFYKDCGDTFSSSKKLM
WTLGVSRLKTEESDCVFLDTVQANDHFSRSSNFPRCTEPVSIVYQSARGREAVLPYGGLA
IIPASQTGSRRTFLMSVLFSPGIDTGELTSRVWAFPEGANSIKDGLKRQDLTGTGTVIDG
AHFDASVWDGGSLRLNYNPRTQKPDYFCRTVYEAGIECIPIDISTDEQGLPVVKAASQTK
DVFLGKEQVKNFCRLNVADKSFARQFTLVTGLDVQWDPVTGEPHRIFFGCWGGEDGTGNF
GSVQKDGLHLKQVMQNAYADAVLFMPDELQITVRSSSTGRVATAAAKASGIPLQNPLSSQ
PFAAVCLLSILCAVMATIYKRSQKAKQYSSPSVLQQQTGFGAPYIELQSMYSNMESSPPL
PQSPTPIV
>comp2681_c0_seq1; 328 210 857 LEN=1006; translated
MKSFATATTLLVALSGSASAFSPVRGIAKPSSLSAASVDSLVGALEPVGFWDPLGFAAKA
DDNTLRRYREAELTHGRVAMLATVGFLAGEAVEGSSFLFDAQVSGPAITHLSQVPVGFWL
ALTIAIGCAEQKRATIGWVEPENVPVDQPGLLREGYTPGDIGFDPLGLKPTDPEELKIMQ
TKELQNGRLAMLAAAGFMAQELADGKGILEHLFSS
>comp2683_c0_seq1; 259 1 1082 LEN=1500; translated
TGMDTGMDTEEDGTANNPLVVSSEEPWRSDPGEKASSHRCRTTTVTATATATAPPTKTTP
HQTIRHSERNQRREQQKQYQQQLQPPTISIFSNPIVVRRSDGSAWKEGEGVTCCSSCNTN
TTTGKNLPVSLDVRTCEELSYAVIYNLALSHHLRSEELALGAEEPSSQPPSGRARAYLQK
AWSLYEYSHEIFRRQSRTIDLLFPALHSMALANNVAQIHHVLGNADQIGLCMHHIISIVL
YMVDRGELVLGEGTAGSSTQGPSHGDPLPGGTDHRSEIEHFFDTAAAAHVQGPARGVGVA
RMCCVALHARMDRAAQAAKNQPHGARHKRRTAKAQHSTAQQSKANEQGKPAAARQAKHDL
>comp2684_c0_seq1; 448 294 1772 LEN=1935; translated
MKGKPDWGAEAGTKRKTNRYNGRSFYHREHSLSSRYTFVSNVAVLSLSSGSHSRESGISR
NNVSTEEKENKNRSNIITIETVDSSGQELPLRTREYRRRKHEWAERYTSQEGLREAFGRN
RNRWWGDLDAQTARRLYKRLLFPAALSELVLELGDEIVRPEELAPLAYEARKAAKMYVRE

RCRVPSRVGAYLIDGFRQLYKYGKFQPNGASYEQIWIRYYEEYGSSNHTEEELIVLLTQN
DNQDTDSDVDIDNDDIVLVEDIIDDIDDAVTEDEIIKRTCQKILEKSCTTNGAIDRFYLR
NFDQNSLREECSNLQAESISSCDANQNDCNKKVRKSRGSRWRNRKRRKDRYNDLFLEKIT
NTLERDVRKLLEPPPPLLHSPTEQEEEGTLFGQFMKERAKCNAEIDRVGECETNKKEGDW
SSRRMTRQEYHSLKLFAKAMKRSQQQYSESNANDATKRISIDDIALTERSENIDIVTVSK
AAEPERTRNKDR
>comp2685_c0_seq1; 736 275 2221 LEN=3194; translated
MATTIQHRSKRRGERTPPLVGVRSAAALAVSSALLLALSATPSVAFQSKATSAFANFRST
ASFSPSVRLFAESFEKDSSKGNSDNITPKQQPYVRMTEDNQSSATRGKSALLPSIGDVVR
YTDLDGGDLSGQVLVGKISYVFGSKTSGYSVELNELEDVGDGYFAEYSSALRNRKGRKTE
RRLDQVSPLAASFVGSEQAYKVPLDRDTGLPSPRQATYDIDRYEGPAFYQTVDQDVLERD
AEDYSALKSKLFRNVALTGLVGTILANASYGSELAVIYFFGSVASLLYLFLLSVKTDTVA
VNQEDNTQNLVGTPIANLRFGAPALVLIGVSLWNSQKMSSGLGGADDFQMRLFDTVSRDQ
FAAAVLGFLTYRLPLFVGQIRDAFQKLEAETDNTTDSSGIQLPGSAGVAAKLLGRGTTAG
TDSSSVGGDDSDLVTVLLVSGPQATGRSELVGRLLEDSSNPDPDSSNPNPPLVPPERVWK
RDDGATFERLARRGEFLDATTIDNDIAASDSASGLTVGGIFDAARGLVDGREVPNKNVVV
VDASVELAKALATAKSLPGKTRLIGIWVGLESVADFRKRLEEDIDTGVLVVPPDETKESF
LRAKIKEIVGEIDFGLGAGIFEFTILNDASDPEKSLAELKEAASYAFK
>comp2686_c0_seq1; 264 82 966 LEN=1233; translated
MARLSVIRRNQTNGWSLLPFLCWSLSLWKMLLLSPVVESFSDALLLGRQRSGGGSSGAFS
SSALSSSEDCTTGTMNVVDEGAINSHTNSRRAAIGRLVGSTAVAACAGIGLSPSLAAAAS
SDTDSVTKQTNLSEEELLAIVVEKDLVANQFLVSGRLTRSIYDEACVFTDEIDTYTLDKW
ITGTSRLFDANRSRVTLVPGSATISKSDGSLSFRFYEYLCFNIPFVKPITYLSGKLVLER
SPSTGLITSYKEIWDQDVNTVVSQNSKLFTSGLSNDSLDADLETFGAKMESLFD
>comp2687_c0_seq1; 368 183 1508 LEN=1886; translated
MAKRSHNYFLEQHPPPPPPPPPLPSSNPSSRDAGISNVRDPPGTYQYLDHQPYAYNYSYV
NERGHSVRGDSSLLSTIASPSTTQNMPIPIFKGHINNSHSAASSNIANIQNKNLQTCLPV
QISQSSSGKDFAETTTKSAKAPMVWTCKACNVTLESEKAFKSHRKSHVRCSDCSFEGAPK
IVKAHYQATHGKFSGSGFKTITVGVPGCRPQKFNICVGNRPEDIERWIAERKKRFPRKSA
SATEHKQEGTKQKVHGAEVRTAAQTGLSSLLAGYGSSSDDDSENEKKVEADGKEGSVSSS
HVVENAVGSDHTELNNREQTNSGPIPPPEKTKTRRPCRFFFSNGSCRHGSNCRFSHDVEE
GQPLQSHSKQKNRSQPSNTSNRNNNHNSRKRKRGGHTSSDTLLRKLFENDMERESSMTMQ
LLKFIVDNNFFLDKDTSARPK
>comp2688_c0_seq1; 293 202 1749 LEN=1891; translated
MKAAADYDPKAVTSMLSEELMKLNSEDRNQITEEMHGVRNMSLQETPDVQTRALGMLQNQ
LDRMPPSKKAAYDKAQGLEKTYVNDDKFRLRFLRAELFDVDRAAERMTSHLDLILELFGD
EVLARPLRTTDFKGKLVKRILRGGLVQLLPYRDRSGRRVMVILSDVMSYSHIARVKIFLY
VLTAAAECEETQKKGVIFVMWPGTDRTIRIPETEERAICRKSFAAFPLRVVSVHFCWPDT
HFFHMVKYFFVLVMGPSLRVRVNFHSGERQELTYKLMGFGIPVQLLPTTESGAIKTKNHT
QWLKTRCFFEKNAGKNPTTTPGELIGAIECPALHDVLYERSKPCTFHPGNSFFKELIEEN
KEEHAVLSQTGKRNFSWSIVDKVEKNNGRFLTWDRRGFWIKLDDRSEIRLKVATSLRDFN
KHARALAKCQTVSSVCKVSFDDGQYLKKRRIVVTDDESSNVSLCERASNCESFYGTCLPD
LPIFRSKGYDETRNRLTSGFPIDDISLLSYTPRSA
>comp2688_c0_seq2; 225 202 1869 LEN=2011; translated
MKAAADYDPKAVTSMLSEELMKLNSEDRNQITEEMHGVRNMSLQETPDVQTRALGMLQNQ
LDRMPPSKKAAYDKAQGLEKTYVNDDKFRLRFLRAELFDVDRAAERMTSHLDLILELFGD
EVLARPLRTTDFKGKLVKRILRGGLVQLLPYRDRSGRRVMVILSDVIPIVTSRGYVVRSG
VSXKAEFGLQPSDFHQISNIVLLPYRAAFQITSQVKIFLYVLTAAAECEETQKKGVIFVM
WPGTDRTIRIPETEERAICRKSFAAFPLRVVSVHFCWPDTHFFHMVKYFFVLVMGPSLRV
RVNFHSGERQELTYKLMGFGIPVQLLPTTESGAIKTKNHTQWLKTRCFFEKNAGKNPTTT
PGELIGAIECPALHDVLYERSKPCTFHPGNSFFKELIEENKEEHAVLSQTGKRNFSWSIV
DKVEKNNGRFLTWDRRGFWIKLDDRSEIRLKVATSLRDFNKHARALAKCQTVSSVCKVSF
DDGQYLKKRRIVVTDDESSNVSLCERASNCESFYGTCLPDLPIFRSKGYDETRNRLTSGF
PIDDISLLSYTPRSA
>comp2691_c0_seq1; 634 1 2346 LEN=2538; translated
SSSPDIRNARTQQCKNLSRFLLSSPTTPTPGKQVFWNPSMANTAYHRSTGTMNALSSVPC
RRSCVLFLCLLLGLIATSEGSTDPIDGARFHAKRMSRKERMALRQKEAERYAELRASGQD
EVGHTPEAAAMRQYQLRKKMRRGVFGKRKTDLASILFPGATPEPYIAGEEVLMYTDNVNS
RKTQVPYDFYKLPGCAMPKMTVSRRRRERKNLGARLQGLELKPAPYTMKVKEDYGCTPLC
QKMITPRRVGFLRQLIERQYRVHMTFDGLPVLMRSENHNYAIRGFPVGFKAPPSYTGLKV

EQFYLYNHLKFTITYQQMDTGELHITGFDVDPVSIRHEIQQEDSFVAMSQEVSTCNTDDA
PPVKNDLETYLALDKKAGAEGFLPVIYSYEVEWKESTLPWTDRWDIYLIGSPDDDIHSFA
IINSLMIVLFLTGAIAAIMIRTLRRDIAGYNQMQQFEESGEETGWKLVHGDVFRPPASCP
MLFSILVGTGAQIGFACFLTMMFSIVGLISPMKKGQMLTAIVLLYVSSGAVAGYISARVF
KYCDAKAWKQNTFLTAAGLPGVLIGIFTVLNVFLHVAGAATAIHFFTLLAILGLWIAIAT
PMVFVGSYFGFRAEKMPVPVKVNSIARYIPQPPWYANPPMSYMLGGVLPFGSVCIELFFI
MSALWLHQVYYMMGFLLAVLLILGATCAQVSVVMTYLQLCAEDYHWWWKSFFNCASAGCY
LFLYSLWFLVSRLDFVGILPVVVYVTYMGMISLCFGLFCGSVGVLCSFLFIRSIYGALKV
D
>comp2692_c0_seq1; 169 38 358 LEN=811; translated
MADEDSKEQQHVYVKLVSAEGHETFVDRAIAMGASATIRTMLEGQFREAQDNVIRFPNIT
TAILEQVVRYMQFKAKYTNSSSRIPEFTIEPEYALELLIASKYLDC
>comp2693_c0_seq1; 735 204 1658 LEN=1765; translated
MPSANDYNDERHKKEEMARREKRKSAKSHKSDAQREKERKRKEHIEKTLRKHGGERKKQK
TEPEATSSAFQSLFKQRAQGFEIAFKFRNAPPRPPVGPCFIGTNLDEKLREISQYKPLNA
VEVNYRWKLHSEPDLGVPLAPSAMDPTSYKQNQTEEAQLHPDDLKLLEWNGSMADTAAEQ
LKKRRDNARAIARLALMGKSPAKFLMEKTKTTRSGPASTKKAFSRVLDEGMQSWMKKTTY
ITNDYGRKVHDFKSLAKTKQELERELEVRQQTIMQRRSAPVVSRTFEEVKATVQNHPNKK
GVTPVAEMSFLPNIEKWGNAYTYVVVDKSPGDNSTFLNSSIVANVQKKDATARMTCELFG
QSENDSGSYLGMQNYELDVVPLKEGDAPHANFCIWVDPENNTALYTPISSRVQLSNGRPI
RGKNYKLNVTRRAMNDEDMAEINERLAEVDADVEEKINGSNSANNKSGNNISINDDDDDS
DEDM
>comp2694_c0_seq1; 550 447 2294 LEN=2569; translated
MKSSFTILLVSLIFQALTNTCASLEQSLSSLSRSMSKGKRLKGVPRQEHRKILHNVKCIL
FRKETRYLSTKSTDPQATMVASEETWTCELENASGHDFVPATVELKGINNQFLESQGAKS
GFSALVATEGIVEGGDGTMAMTIPPAALVKVLEQPTTLGNKLRRQSGRKRRNLAATSGTL
KTLVVRVIAKNGIGPIANTVQLRSDVFEDKVCLKSQYEACSHGRLKIEPFTGYTETNRYI
KDGVVSVGVDVAPIEGNKDNFEVAAKNRATEIYGDLGSQFDLVMFCIPPGTGPWLAYAYI
NRFDSFFNDEWCSSVSTQVHEVGHNLGLAHSGKDTDVYGDQSGMMGFSYKDDDFPLMCFN
PAKSYQLGWYREQQDFVNPLTDILPMKYSREYILNGVDDFDRGESGVKEDLIILRLKQQN
SREDYYIGFNRKKGRNAETVKDANCVTIVKKTSGPNEYGQSWQVSTLCEIQDSYTINNYD
ESSFDVTVKLISIIGKDAKVDIIASNSALCDTVRDSTSLRYKNKKRRNCNWVSKRKSRRC
SKTWTNRPLSEWCPLTCGSCSGQLNIYSSGFGMNDSLPSTLCVDAQNVSFQNNFAKNCGW
VGKEEEKKDERCAKEW
>comp2695_c0_seq1; 737 27 1175 LEN=1213; translated
MAARPLVSVFSLSGDKSGETTLPAVMTAPLRPDIVQFVHTNVNKNKRQAYAVSIRAGKQV
SASSWGTGRAVARIPRVGGGGTSRSGQGAFGNMCRGGRMFAPTKTWRKWHKKVNIGQKQY
AVASALAASAVPALVMARGHVVDDVPEVPLVLDTSVESTKKTSAAKDILSAVGALDDVEK
AGDSKKIRAGKGKMRNRRYVMRRGPLVVYAENDGIEQAFRNLPGVELCCVDRLNILQLAP
GGHMGRFCVWSQAALDKLDTIYGESGKRIPAAAMTNADLARIINSDEIQSVLNPAKDGSA
TFEPKANPLRSIVALEKLDAHAANKRRMAQQAEKDREANKAALLSKKRAARTAKKAYNAQ
GKAFIAEASKQGDVCADGFTIE
>comp2696_c0_seq1; 388 14 1116 LEN=1116; translated
MKFGVATKLLLAALAAGTMPAITAQNCTIVLPEITEVMELAFPTWNVDKKGKRVEGGGKW
NPNYLTKRWNGLDPALGGYPTPIDTRYPFEFAAAFKGQPGAGSPHHCPEGTDPLTPVQSC
PKIITDNDDGPYGIGHVPPPIALAVVRNNLLDCENDADIENWFDFESETAPCDIKPSVLA
SMIRTKYPRDPETGAVDYPPPVIEGEYEYYELEFPSPQGPPHWCTDEFLATGQWVDYCPY
IFEGPNAGQYRHPHLALSAVMQFVANKINPEVCGPEWDDRGNYPANPPTSIAWSVMESED
GDAQPALPYLWPENDDPLIKDVPGLVLFDPAAEPPAPPTPPEPPSEDEVTPVPEPPATEA
PEPPVTEA
>comp2698_c0_seq1; 310 1 918 LEN=1264; translated
QLGCCFCFFLIRHNPLDLLRHQGKEMHFFQNRNRPRSSFELKVGKSHLLEVTVHLVTRRR
DVAVRRKVNRNPEWDWYNTNKDDIHREFLELLEASVLSRMFGDEIEGYHRKKNPSVMEGN
NGEMGFGKVGSKNRVIRSKGNGMASGTRKRKGTTVPSNSKKHKVFLETNNDGENNSKPEK
DIYFSFGELIQLAYKKEPIKDDRSSRTIIFESLPSKSNRKIADGHNGNSETITRAAGGGT
FLDRQKLSHRLLVWIGKSEAAASAGGGHSHATAAEGMYRNEMVPISSLFRKPPEFVSLSD
DDDGD
>comp2701_c0_seq1; 304 293 2032 LEN=2670; translated
MAALILAAAATAIVEIFLVSSTSLRPRSPTVSVSRLVAKSMASISISFVNGRGVRRPRGH
RYYDYWRDEDHEDFEPYPWKFMKRPFSSSGSKNADVNNLGIALDDETYVPEQTLDKNSNA

NIERKLTSKSKEDVIFNDRSSFYAKLRQSYESFFPPPYLSSSSRNDANAKRALQAVEDLQ
TYTNLQTYLSASNPVNPDNSDSSGNSSGHNLYYDIHNCTEEPLPGYPKEWNLLHDVLGQW
PADDPELPASGTLYQGLCVFDLQANPDDLRKAIAYRNAEVPFVVRGDPAVAQTVERWNAP
HYLEALLGDLPHGTETSASGSFTYWTANGVDSRKYPMDAASYKAPTDLTEMTFAKWMALA
NVTDPQLLGPESPHYYFRVVGCAPARPPTHHNNGHGADSFEDNTCSILPGKARHVPYLAD
ELPFYTQTKASLYVKEPLAEKIALQGPAGAGSKALNCRFGMKGVMTTNHFDGERNFVTVL
SGERRYILAHPRQCGNMALFPYGHPSARHSSVDWARPDLLAHPRFEAAEGNEVVLQAGDS
LFLPSLWFHYIVSMSTNIQCNTRSGMDGRHVSDLDESCGW
>comp2702_c0_seq1; 888 274 2694 LEN=2949; translated
MAKFPSTSSAEEDESDHNDEQYSREASEEENIDTSVWSPRNKIMLRCLTAGVLLYGALVA
YTDHQNATVGIHTNANIGFNKVENEPRELHPRRRLMAVMGDVVPSYMETLMRDLKDRKKL
FEDTPPEEIKYWFEYTGPLQKYFYRFSKSRGKADYFEGRDDSGVTSSRFTEEFLGGSEHN
RMFNGGAAGEAHKSKTGSNQHRMQYRLLENCLAMGSPSRPDTNTPVWKEWYGNKDGVEHD
TYGMDRCRVLFTLNTVGAGRGTTMIWETSPPALPHAQGPDRWMSEQETSATHIQRQLEEI
DAMCATNQVAARVQVLVCEPISARWTEWMDSLLPHCGGKVSDLSQVDKKTAKVEDRASPI
QVVVVATAESAGVCHDLKSGFMERRTQDLILAPPFLGDGAKTNGDVSIDNAAIKTTHPDL
VSSDDHDMYVGLKWTSLVTMRALSSFLQASADIAAGATSPVNKVQSLEDTSVINGPSSPF
LIPSFVRVSYVSEENAKVAKWRMHGDFFHPAAWEVCKDSFELTWIDHSLSGSGGINGLCD
DPSTKTAMKRRLKEAAGDDNKVDCGQWWVYMTELQMPTSPDVYANEVTPPIVQGGSNFVW
MVTERQRREIVSRQVCMKKASSSPEKDKLEAECPAPPQAVMPLGDLESFLINYSPNTALG
KKHVKERRPGYSRQTEREAERELVRERKQLKGQEDIEKALFDYTVYPAALFRKDATLSAA
RARDAVLVDQKKKKGDDDNEEVIGPQFIKSAYYDVTVTKPRSPVLGWCQAIARKGLCTLY
ADYFRTSSDVRCLEACGMKKNFSKIE
>comp2703_c0_seq1; 490 60 1353 LEN=1353; translated
MRMNDNSTPEKRSRKCFYSGALLLVVVLAASSAVVGYFIFKKKNDDTLSVESSMSEPCHL
AVKDAHQPILEITLSAEQSKTMLTDTQAKEFETALLEAYNDASGGCSDEFKRWMYGTSIV
DQSLKEHVVLENEGDSSISHTFDSEYSLVLQVETMISCDGCPDDLAFASQYPETFGDVAS
SNRNLGVGLPLGRFYNALEDIVHTTLKKTITSVKVTPNAGSKIQHMSYYTKTAKANSGGN
YYTSGRASSGNYYGSKGSKGIASAVAFGMKGGKGSKGFKGNKGSKGSKGFKGSKGSKDAY
VPPTPAPQEEASTPSPTRFVYTPQPTEKPTHRPTPLPTPRPTPDPTPQPTLPPIEDTVIP
PSPRPTPLPTPEPTPAPTPRPTPAPVALPTEPPTRFVYTPQPTALPTNPPTPIPTPRPTP
DPTPQPTAQPTX
>comp2704_c0_seq1; 663 463 2478 LEN=2565; translated
MVYRSPYETEKQRLEKDLEKEVEEKGFPDVIITLYLSQFIYHMGDIRTRTKELGYGSGAE
ARTELINEVLRDAEAFQKLRDGGKSMAKLMRGKLHLNDINHDGLPDIPDINMDLVEHVLN
NEKYKEVSESCADELDVMAIHSFKKNKVYLYKFDAISDKTDLVHAIAGREKEKKDGTKFS
DLFLIFRGSVTLKDWIQNLKAPLVNIDIQKEKNSGKWFLVEQCTYSRIEIEKELASLLED
AIGDTPIKVHRGFLSYLFGKTNSSTPKKKDKTDESGRTKRASTNGTVSFRNVNVSKYQSI
KNDLLLLFQTQRSSLYITGHSLGGALSTLAAFFLACDVEVHDNNRSFEGFKCITFASPLV
GNISFQRAFDVLQNYRQDKSSRKYAAGVVRLRHLRFTNDRDLVPLSPPLSRYRHTACINV
HLNLYSPPLSPSWCRIRKEEPHRDIFHRWNSDKSDKQNKRKLGLLPPTSTLSGLCSTTFP
FLWVVFNTAPIILVILYLLVRPVASSLFSVSIFKEFFHAMPIQIQKSFIEFSVRVSETLS
SKAIIWMSWAGWLTMAGSPNADFKRTVAVMVGAPLLGVGTLYLANEQYFDYFTENVEALF
VVGMLLWQIVILPFRRADEVVSASTHGLAQYYLHIKKRGEPLSSLKKIVENNLAMKGDKA
YGEDSSSKKVV
>comp2704_c0_seq2; 663 484 2499 LEN=2586; translated
MVYRSPYETEKQRLEKDLEKEVEEKGFPDVIITLYLSQFIYHMGDIRTRTKELGYGSGAE
ARTELINEVLRDAEAFQKLRDGGKSMAKLMRGKLHLNDINHDGLPDIPDINMDLVEHVLN
NEKYKEVSESCADELDVMAIHSFKKNKVYLYKFDAISDKTDLVHAIAGREKEKKDGTKFS
DLFLIFRGSVTLKDWIQNLKAPLVNIDIQKEKNSGKWFLVEQCTYSRIEIEKELASLLED
AIGDTPIKVHRGFLSYLFGKTNSSTPKKKDKTDESGRTKRASTNGTVSFRNVNVSKYQSI
KNDLLLLFQTQRSSLYITGHSLGGALSTLAAFFLACDVEVHDNNRSFEGFKCITFASPLV
GNISFQRAFDVLQNYRQDKSSRKYAAGVVRLRHLRFTNDRDLVPLSPPLSRYRHTACINV
HLNLYSPPLSPSWCRIRKEEPHRDIFHRWNSDKSDKQNKRKLGLLPPTSTLSGLCSTTFP
FLWVVFNTAPIILVILYLLVRPVASSLFSVSIFKEFFHAMPIQIQKSFIEFSVRVSETLS
SKAIIWMSWAGWLTMAGSPNADFKRTVAVMVGAPLLGVGTLYLANEQYFDYFTENVEALF
VVGMLLWQIVILPFRRADEVVSASTHGLAQYYLHIKKRGEPLSSLKKIVENNLAMKGDKA
YGEDSSSKKVV
>comp2705_c0_seq1; 147 1 244 LEN=373; minus strand; translated
XPEGTEEGTSETDGIPEGIPEGTVEGIMEGIPESEGIPEGIPEGIPEGIPEGTVEGTMEG

IPEREGIPDGIPDGVEDGSCA
>comp2705_c0_seq2; 89 1 138 LEN=267; minus strand; translated
EREGIPEGIPEGIPEGTVEGTMEGIPEREGIPDGIPDGVEDGSCA
>comp2706_c0_seq1; 483 428 1288 LEN=1523; translated
MKIASRNSALAAALCLAASALGGALAFAPHHAARRAPTVQSSSSSTSLSVKIPRVELPEA
VASQLGEFDLKNPNAMNDDDYRSYSGAAIAGTLAFSLLPGALVSGIYSDLGPVAIATLID
FLFSAIVGGGLAIYLSLRKDQIGETVRSYGNQLLSAAKDATGIGTLRYDLPVAATDVLTG
ELGLLDPNKMDEDDYDGYSGAAVAGTLLFFLLPGALATGAAGILGDFASSAAVDFVLSAL
VGGGACIYLSLRRDEVGATVSQLGGQFLDVVDGVLGAEEDTKLLEE
>comp2707_c0_seq1; 734 338 2566 LEN=2566; translated
MPNTTNATTSTTKKLPRFHCSEKNHKHQSTVDAPSATTGSQRSIAVPQSLANLRSQLNYS
SSSSVTSSSSSSSSLEGGNVIALHTNNDANDFAVQDRRNNNNSRNNNNNTMPRRRLHTNS
FSPSVTQRQTQKHPKEQPQHYQEQQYHAQQGIFRRRAVPLHYEQRPFKQQRSMSQSPHRP
RDTLRISPEQRGQQQRNTKEPSNSSTANIAMLMLPNFQDPSSSTEQVSDAPPPPPLWTEG
FAKGLADSIARTPQPLGANRARRKFWSRLKPQDIIRVSSLSEDSCEDTSNTGSAYFEDED
KHEDDNGVLGCGSFSSVTRICVRGKGTKQYALKHLKQDLLPPRLSEDCLTNGSTAAFERA
ARELAREANLLSRFGNEHPHIINIVGWTDGGAASYAAYMRHDAFFLVLELLQEETLDDRI
ERWNHDDAQLRQKQTVVTNPDNPRLHFIRRKIEQLTICKQIADALSYIHSKNIVYRDLKP
QNIGFAIPTKKSSSVANDGDSNNVVVKLMDFGLARELPTSGTILPKQQQQQQPARSQPPR
SPNKNNKDPTFSTPFNGLFDMTGTVGTLRYMAPEVCMHRPYGLECDIYSWSIVAHEILSQ
TRPYEDMTPDMYQTLVCLQGVRPQNGSNGRGMPSEYTVLLTQAWRTDPSKRLRLDRIMRQ
LDLFLQKEKLMLEAQELLLGMPESNESPLSLSLSFAERSSTSSRSHHHRHTRGIHCGYRV
RSPHYQGGKAKYGNSSSSSNGNK
>comp2708_c0_seq1; 479 757 2076 LEN=2322; translated
MKFTIGCMPLLLALSDVNVDAFAPSKLVNTAVKSSTARAFSPMDPTSFQELPHHVQSIQD
FMSTLSVADVDVGAGIEQVATQVATTVDPATAEVATEAAKSNGWFGFLTGPTEAFLEIIH
SVLVGVGLNSNAWGISIIALTLSIKLLTFPLTKKQLESTQKMQALQPSLKEVQAKYQSNP
EVMNQKIAELYQTNNVNPLDGCIPSLVQIPVFIGLYRAVLDLATDNVLDEPFLWLSSLEG
PTYGTDPTKGSAWLFDNWVNGAPSLGWDETVSFLVLPVFLVVSQFASMELMTPKEQKDQQ
PSFLKVLPLMIGYFSLNVPSALCVYWVTNNIVTTASSLIIRNSLKMEPAAVVASEATAAA
PANPNVFTPPTIREKPAGFGEAAPSASVDGVTPITGSGDVVDVEVVSEISSNVEAAGEGM
SAPKSKKRGGKKKKKRRKN
>comp2709_c0_seq1; 182 361 1617 LEN=2522; translated
MPSNSNTQLLAIAGAVLAISAPASGFVPSQRLPAATKSSSNLFQSSRSAVPDCAPIGEVI
SAVGSGLVDEEEFTPAKEKIIKEPGLWEYNFGLQDPGLAMPHGIVKDVTEPETFAITEEQ
IATLERDGVVHIKGVFDEQWVDYLRKATAYQVDEPHFWAFAGTASKLYDYIQRNVWQTNS
AFARFYYHSAMGHVLAQCGRTDEIRVSTDLLMVNPNKGFKWHQDNQNGPISWEEGLRFWI
TMDETPKEYGAPVYLQGSHKNDVVDEQAVFVDIEQDGLEDYNTKMLEFRTMPGDMLIWHP
RTIHKVDGPADGIWTSYRRVLGGTVAKAGSVYHDKRGSGGVLSDLGRHGLEDGKALKSSF
FPVVYPKFDSAEAADRDSGNVGRNPMDIVSKLGGLATKASGERFNSFFQVLGSQSNSK
>comp2710_c0_seq1; 245 133 1047 LEN=1047; translated
MKFSTTTVTCCLFAVAVRRSNYKFASASAVVESDNTGNENERDLISLNGSFFGIPAPTPA
TLAPVSTEEQDKKDEISGRYKKVPKMPSPYPAPEPNPPRNDEPTPNPTPRPTPVPTPRPT
PRRTPEPTPYPTKPPVEYKPYYPTYKRPTIQTRQPTPEPTPAPTHKPTSHPTARPTSRPT
DHPTYNPTPGPTPMPTSQPTRPPSASPTSLPPTHNPTRNPTHSPTPRPTLSPTASPSAKP
TGKPSPLPSPAPTPKPSPAPTISQEPTTLIPSMEPSENPTQSEEPSDNPTASEEPSDNPT
ASEEP
>comp2711_c0_seq1; 179 335 844 LEN=1165; translated
MNTTEATPLSRTKKRVRFLEDVEVIEIGGASQNKDKENDSEAHLEGNNRTALEEQRDRIS
TDNSQPVSNMSRAQRGHFLVFPSCPITTFPLQGQNEVLLTSLAPTSARLFDRWACGASHQ
QLDPRSFSVLPPTLPVRRVDDTKAQCTRDIIQSALDCVTADCPVLLVTD
>comp2712_c0_seq1; 473 732 2717 LEN=2831; translated
MRKPISQEAEEDCGQDQQERCKKGWEPILCKKLAAGHASFNHMKRFSEKSRLDRFDDEVH
SVPWSAIRKKNVLGQGAYSMVYKVQVSMNVNDASKATTLDNQKYYALKHIKPDITDESKD
FDVAAVDLAIEGEMLSRLRHDNIIKLHGVYSGDPKTSYVDFKHGYFLLIDVLGDTLTRRL
AKMRKLVKKRRRGTAPSVLDLIQNVAIGIAKGLEYLHENNVILRDLKPDNVGFDEKNAPI
IFDLGFARELHTVHKSEVAGSIRYMAPEIGLRQGAKLASDVYSFGVLLYELCTLEKPYKQ
YRGRTEFIQDVFVHNYRPDLTSISSKTMRDLISSCWDGDHTKRPTMKRVVNILRVETALL
QRERMSTKSAKVSTQRRSDNSVSSTHSSLSFNAFGSVNSFRSSTKNLVKPSTGSTTVPSC
LLTSSSTDIKNSFARRSSIIRRMGSKNFDWNNNPSATPENSTVNDKNMVFGIAQRRRNSF

TSSLLDRRKRSTNSIASSTSANDVKESRSAERIAADYTRELPAPNTRLSNRYPTRRSYGR
GLARGISYESETSLASTSGDSCATSYSTDLSTSNFTLGEFRARVLGRSRRNRTFSSGSSS
SSKDLFRIDVCHGSNTDDENISGDSDFGDDCIEDMSLLTKKEVNNEGKQEERASLPKLVH
P
>comp2713_c0_seq1; 587 1 1387 LEN=1551; translated
XKYVQPSASLMENAPDWDAVHNAATSFNPSVVNVDITETKPEPNDESIVEEPQRKKYVQP
SVSLMENAPDWDAVKPSLSTSNPSVVSVVVSSSPDANSKTNVGKNKEIDNVTGQPKKRRY
VKPSVSLMDSAPDWNAVQPSLSTSNPSVVNVDGINRISGGKRLVRSSVVKPDSETDQKTR
AAPADKPFEYKQVQSIPKENPAGAKPVRDEEAKTKLPASIAHAAGSTSEGWTPSAHFPTD
DKTSSPGDQDSNTKESRTRSAQSSTMENYDPDALPRPTGVIPLTVSSSTREKTQLFREEY
SLEAVEEDAENDGEEEENPSSSSRSEPSLSEPGKKKLFLYDGGNPQPKELWEFSIDGSDT
RLDQRSEQEMNTTKKKLVTYDGNSGSEAIRSAVVDNKDKNISGNGNGRESENGSKLKEKT
VSPFTREVSDNDQAAGSSATKPVKSSTNDGISIDAESEDVET
>comp2714_c0_seq1; 742 365 2896 LEN=3167; translated
MEAAPKNTRVSFGRPSEPATNYTATNINDHRTSTDAPHKQQQRNEAIEDAAEGATGQQEQ
TNQTRQLSQVEQNQRSMVQRRKASQSTPNLQKMELNERNRDHHPPKRHSVATSANLGTTA
TTRSTMRRCTSVSNMKRSRSELILLSPEFRTRLMNEQKERDPLFYYRVQKTIGNGSMGSV
ALVKKRSQTVGGSARKVVREAVQTHKRKQKCFQLPFGIGGLFRLCIEDELRVQEDKAPTH
SKTRKQNRHHRVGGTKNALLSVETWLGSSEGSSDVSSCDSTSTAGNPAKQGRRRSSILFP
ADDSTTAASTASVRSALDVEYAMKSIHLSRITDDTFVQELENEIAILKKLDHPHIVRPLE
TFHYRNQIFIVMEHCSGGDLYSRDPYTEEEAARHISSILSAVGYLHANNVAHRDLKYENI
LFVNDSPKAEIKLIDFGLSKVYGGGVNGKSQTLTEGVGTIYTMAPEVLKGSYTKQADVWS
VGVIAYMLLSSQMPFYGRKRQHIVEQILAGKYDFRGRRWRRVSTPAKDFVRDLLVLDPDE
RLDAERASSCVWLNKRFSASVRGPRECEILQARNSMMRYVGYTKLKKMALMVVAHRSTCE
EIGILRKVFQKYASIDKGGCISYEGFNSAWKESGLPPEDTRAIFDSVDLDGSGLIQYTEF
LAATIEAQGAISEERLAEAFDRFDSDDSGHISVDNLAELLGKDFSRQEIIDIIGDAVDPD
NGGDGTSNQMISYSAFLSLWEKNHEKEVRANKLRMLGSQANLCQLGDDGFDDVEDTDVDS
PHYSMENCEEAAKARATFLMDKHGTLRNSNAQEQKKLIVVEQPILEDGADFDDSFTVDGG
IQI
>comp2715_c0_seq1; 199 168 598 LEN=1019; translated
MTDTVARRSMNLARSENGEFVESINAYRCDPVALCDLTNRLSPALFVSKIQEAPCPSVTA
XILLQKHVPADFEFPTECSKFDSFRAGLVRRDSKQKEEAIAFAGMDPGSAVKAMTAREQG
HPSEAQLIRRMSSRRNSKESSWF
>comp2715_c0_seq2; 199 168 503 LEN=924; translated
MTDTVARRSMNLARSENDSRSPVSERHCSILLQKHVPADFEFPTECSKFDSFRAGLVRRD
SKQKEEAIAFAGMDPGSAVKAMTAREQGHPSEAQLIRRMSSRRNSKESSWF
>comp2716_c0_seq1; 756 52 1422 LEN=1891; translated
MSDKEHLSLVVCGHVDAGKSTTCGHLIFKQGGIPEREMAKLNELAAEKGKSSFGFAYYLD
TCKEERDRGVTIQCNTKEFFTEKYHYTIVDAPGHKDYIKNMITGSATADVGMLLVPAEKG
GFEAAIAKADAKLGVDEGQTRQHARLLYLLGVEQILVGINKMDSCNWSEDRFNEIKEEFV
KMLQMIGFKPKKVPFIPYSGFHGDNLVDAVDPAKAPWYKGWTANKTPKVKITGKSIVDAL
NNYITPPKREPDAALRLPVSNIYNIKGVGQIICGTVEQGTLRPGDQVGFVPSNLKGKKIF
SIEQHKKVLESAGPGNSVGLSIKGIAKDEKVNPGDIIYNEKDGELKPIKSFTALVAVQEH
PGVLKPGYCPVVFSRTAKVACKMTKILWKMGKKTGGAKVDNPPELSQFENAEIEFEPTAP
LFVEPFAQCAALGRFAVMDSNRLKMLGKVSAVVHKD
>comp2717_c0_seq1; 143 233 664 LEN=826; translated
MDSYFGHQRDDCWYSDEDEGSLSSRSTDEVLSETWLDCLANRYGYSKIDASSSATPMASY
RRGACRLNFWLTTGTVGSYLEHPRQGKTQLFRRGIDRDEAENIFENPRMHTGRGYHTRNG
NGGSRGPCRYGQDCHRADCWFDH
>comp2718_c0_seq1; 112 1 226 LEN=537; translated
XGKGGKGYYGPKNYGYYGPGYYGSKGGKSGHDGGKHYGKTKGKARNLGQQPYGGKAGKSG
GYYQYEKCEIVCDGDW
>comp2721_c0_seq1; 356 326 1456 LEN=2572; translated
MAHLEQLTPQEREWALALDEAIYGEVNQGVGWSLSPRGNSSNDPKTTPTTTNSRAATELY
GKPSDFEIVAHAIRAKGDVQRGLRRLKRMKMFKDEYSVPDFDLDSSSNDPLEPILRVMKK
FHLAYPHFIQKIGMDKFGRVAVQLQLDGLRWSLSPPFNHTETERFQAFCFLLHATQPTLE
SIRRGTVWIGDLRGISQRPSSSMFRGARMLFRDSYPIKVEDIPVVDCPPKFSVAYATTYP
FLSSHFVDKFVRVTPEVLRQHFPPELLSDRLRGINNTKNDNINKRRFRSKKASKNNATDQ
NTNAVDIASMDGDESEASWENLDDDDDDVVSYGGNNGNNSGNGSVGNGETDESEVLTRIE
RLLRMRFETEKNFRLA

>comp2722_c0_seq1; 324 125 841 LEN=1067; translated
MPLGGRLRREGRREARREHREERKEHIPPAVKTAVVMGTGAGLGIAVARRAKAKRSGSPD
SSSSSDEDDNAPKTFAMKEKLLAFGDSFTINRVSRRHQRGRPAYYANNKVMRLRETFHLQ
ASRSGPTLYCIQDRKLRIRDSMAIEDADGRKVAEIKKKVIGIVRENFVVKIKDETNWQIH
GSILEHDFTIKENGREIVKVHKNWIAPIRDCYFIDIHGTDDVPLALTVVIGLEAMSED
>comp2723_c0_seq1; 447 222 2267 LEN=2899; translated
MSDHSEVEETHRRPSVIEVEGARDDVIDDVIDDGTESRASITTVPSPSSQDEHGDSRTTA
SSQTRLANDAADCSIAPPRDHSYLEESHPLLPDEDRYLRMASGRALHRDSTGLKPTAKRT
LEVAVLELHGVVLFPGCTIPVKLQNRSMIRYLGRQIRLCRNDPVAQPVVQLGILPYEYQS
PEQTLPQQHRRRRRTNEPQEEPQRASQRRGSWMRRGFSFGNREIRATESSHALRRSLTRL
QDEFQFDENMDLEDSERSSGDDSDGDVGFDENNDEHSLSRPPLSPEQSHPLVGRIGTIAT
VRHTHERASSVGGHGGSDPSHGRSQSSSTLWGRYEEAPELVFTAVGTSRFRILSCLNDDE
NLFEVEDMRDEPLLLPPVPFRLLPFRGQCVHFGKVNDDDESDDGNNDDEHLGKGSIKTGR
IRTSESDVVETRSTRMQAPIERMAWNLSRLTPVPYFVYLQLMPWSLVWKIAEALQTNNGR
GNLPSLGDDSVVTNRENLEPTKFSYWMANNAPFSEAERKKLLKTSSVLERLLFIWKKVCR
WTAENAETRICCELCETNLTGVSNIFTVGGAEGTTSTYVNGHGFIHQITTLRNVDDSKLS
FQGLPSTENSYFPGYSWLITSCYRCGSILGWKFQKVGNSGIDANGGYENGNGNIVDRDTP
QRPPHFYGFMSSNVKIKSRSR
>comp2723_c0_seq2; 393 222 1727 LEN=3042; translated
MSDHSEVEETHRRPSVIEVEGARDDVIDDVIDDGTESRASITTVPSPSSQDEHGDSRTTA
SSQTRLANDAADCSIAPPRDHSYLEESHPLLPDEDRYLRMASGRALHRDSTGLKPTAKRT
LEVAVLELHGVVLFPGCTIPVKLQNRSMIRYLGRQIRLCRNDPVAQPVVQLGILPYEYQS
PEQTLPQQHRRRRRTNEPQEEPQRASQRRGSWMRRGFSFGNREIRATESSHALRRSLTRL
QDEFQFDENMDLEDSERSSGDDSDGDVGFDENNDEHSLSRPPLSPEQSHPLVGRIGTIAT
VRHTHERASSVGGHGGSDPSHGRSQSSSTLWGRYEEAPELVFTAVGTSRFRILSCLNDDE
NLFEVEDMRDEPLLLPPVPFRLLPFRGQCVHFGKVNDDDESDDGNNDDEHLGKGSIKTGR
IRTSESDVVETRSTRMQAPIERMAWNLSRLTPVPYFVYLQLMPWSLVWKIAEALQTNNGR
GNLPSLGDDSVVTNRENLERK
>comp2724_c0_seq1; 154 1 1360 LEN=1623; translated
XQKSQAMSVRLDTIQSLGRNLKDWCRHTLPLICLARYSSDRPIDWFFFWIGYKCCCRCWG
LGKGFRTNRNSNASASANKNVLSVDGVELDSPTGKLKKKGQTLQERDTSDTFMHNSIESI
QLTDLPSVVISNVACCLHVKDMTALDVAIRSGCSAAFIASDEQQESQTTTITQSRRDASV
ILHDVWKRLWYRDYGDVLLRWRISREALQRSLASLGTKVSRSCDRNQWLEEELSRFLDDN
KSTKDFYFIFGECYMDYILAGNNKADGCYLGLHGHVFDFTTFADYHPGLVEPILKECGGD
ATFFFEDLPHSEGARRIAERLCVVVHHGSMDRSRDGEPSGGHRCGLELVKSGAEDASELE
RVLRSSNTRPPPLADQKGSXPESKPPMEGAPRPPSPPHAYAARDARKDPDQVPRRTVVAI
GIAAAAATNATKPVLEASRQRIVENPHLLRSVS
>comp2725_c0_seq1; 590 277 1617 LEN=1671; translated
MENTFIPQAIEIVGKAIEADNAGEYEKALGLYRDSLGRFTMGLKYEKNEARKKLVLERVE
GYMKRAEELRDYVTKQNELDKNGGGGSATAKKGDKEDDMDAEKAKMRGALAGAIVEKPDI
TWDDVIGLDNAKDSLKETVILPTRFPQLFTGKRKPFKGILLFGPPGTGKSYLAKAVATEA
DSTFFSVSSADLISKWQGESEKLVRNLFEMARESEGGRAIIFIDEVDSLCGSRSEGESDS
MRRVKTEFLVQMDGVGKSDSRVLVLGATNIPWELDAAIRRRFEKRVYIPLPEAEARTAMV
KLNLGDTPNELTEEEFERVGEITDGSSGADIKILVKEALMQPLRKCQKAKQFYVDKEGNY
HPCDRYPNCSACPPKLSTDKPGKDYTCNRCGAKRMSLWDVPTEKLKAPMVLYDDFKKVMK
SSVTSVSPEELERFKEWTKLFGQDGA
>comp2727_c0_seq1; 444 1 1353 LEN=1430; translated
TRSKLHSVDLNFIMCKPEISNEKAIEAMGGQFCVKDLAEEDKWLATFDFPAFSKEIKELG
KKLEKEQGPDDVAHLNKMIGWSNRFAAIGLLTMGFGVNPITVICLSLYTFSRWTMIAHHV
CHGGYEKCHPNKSRWSRFKFGIGTFWRRFNDWFDWMMPEAWNVEHNTRHHYNLSEIHDPD
LVEENAAMLRNPNLPIVVKYVGVVFLALFWKWFYYAPNTYKELKLAKMRREGKKIPDDVD
PAGAVTIRSCLMGTNFFYSFAELLAVVLLPYLVFHFFITPLPYLFLGKHFGYEDTLYWNA
VKNLFLAELLTNAHGFLAVVTNHAGDDMYRFRKGCRPFSGSFFLRQVLASVNFSMGTDAV
DFFHGWLNYQIEHHMWPNLSMLSYQKSAPLVEEICRRHNVPYIKQNVFWRLKKTVDIIVG
NTSMRWFPEDFEDKFLDTDLKVEKAAYKSE
>comp2727_c0_seq2; 366 222 1013 LEN=1090; translated
MLRNPNLPIVVKYVGVVFLALFWKWFYYAPNTYKELKLAKMRREGKKIPDDVDPAGAVTI
RSCLMGTNFFYSFAELLAVVLLPYLVFHFFITPLPYLFLGKHFGYEDTLYWNAVKNLFLA
ELLTNAHGFLAVVTNHAGDDMYRFRKGCRPFSGSFFLRQVLASVNFSMGTDAVDFFHGWL
NYQIEHHMWPNLSMLSYQKSAPLVEEICRRHNVPYIKQNVFWRLKKTVDIIVGNTSMRWF

PEDFEDKFLDTDLKVEKAAYKSE
>comp2728_c0_seq1; 409 52 1548 LEN=1857; translated
MTGQLKVQSILTLFWLMGYLSFTRSFCMGKNLFPSRASTIAFSKTTSQSQTTGVNSRVNH
SVSFSSKVSISDAYDGGNIELANIEDGPTKVVSLRIKPDPYTELEKKSHMQYFSFRSSIH
TNNAEDDKRDVPVTYELLNAGEASYAKAWNGSTVFYSKSLSDPDSWKRAQSTRFVEGKLV
WDHIHSEESESVYFGYFPPFSYDRHLELVEKCSELATVFSLGQTLEGREIECIQVGTGKS
IGWIIHRQHPGEHMAEFFAEGFLKRLLGLDSSGDTDGLVRQLLSKYTFYIIPSMNPDGAV
AGNLRVNAAGANLNREWCSSGDDYEAPTLERSPEVYHVLEKMKETGCDVFCDIHGDEEIP
YNFLAQPVVPNWGPRLEALHGAFNAAYRRANPDMQQEFAYEPTEYAEGDILNIANDQIAH
RFDCLSVTLEMPFKDCRSNSDPERGWSPARSRALGASLCEPLLYIQPYLRAEALDEKSFA
DSFPEIDRYVLPTSEYNK
>comp2729_c0_seq1; 655 77 2152 LEN=2790; translated
MDVENDDIFGGVCWAKPQSSSCASDEHKATAFRGDSVGSKSNKSTNVSELFGAPDGISLR
RKSAVSTPVNGDEFVKVCEIKTGWLNDSKSRLSLLPTLDSQFADRLERLRTRESDGPNEE
DEGKENMKTNNKENNDESDVKDGKEYPEQQRNEEEDCSSIFKKGTGQILQPQHEGATICD
HESYLNEKVDIIETIPKIPAILIMDHIASFVNDRMVWNSLAALSKETYAITVTPSHSKQN
SIYRPWPRVRWRLAATTASMDAKTYSQNRRNHLTSTSQQVSHRRWSRMPARGSVQTSARP
WTVCFGTNYLGCGTDRGGVLVRAVHNGSTMSAQGHFGRINSVQLFAESWFLSGGDDKVVR
IWNIRKNKISCEAVLKGHIGSITSIAVVSYRNTHCVGNGGGENSMSGCMLVATAALDGNI
HLHVVNYKDDRVISTQHMVHFEDSQEPIHTAKQPIYSIVMFEKDGHTSLVSGGEDGCLRV
WDVDEAMDNALGNHEGILGQYPNRASDKTDELSIRAAPKRIVRNACIFRDDGEIKSIAIS
RDKQQIAAAYGRTICHCALSVETLSKFNDSHHHYQAHHQRRRLRTNQNHFERENSFEAEH
QNHRHGNDGWRLLKGHYSGDIRCISFSSDGNSIASACSDGSIRLWDLEKGCWNRKWKAHN
GFMVCSIAVSPDGQSLLSAGSDGTIAIEGLL
>comp2730_c0_seq1; 282 443 1303 LEN=1682; translated
MCSKDQTSKNNTDQQYRDAVRSLGPYDVVCGRGSVAFNNIGNRRFRIIIGMNVDKYSAID
GRFQKGIFIGNLVDALVCEMGARFFKQKKTDGRLVELTEAEIRKKVGHALRDMFAFQESQ
QQQQQQDVKEQNMMAGDTATLAIPSLLSLARSPAHHKKSNDMNCSCLPHERTQQSLPQSL
PHFPLPTSLLFQESIESIDVPRNYRADTNNGFQGTNNFEIQNRSTEIRQHGFFPVSNRDK
RQRQKELQRRLQQQQQSGHFSMRELEERLSATTNISNSERERQGTI
>comp2731_c0_seq1; 790 1 1748 LEN=1956; translated
XKAAALQNIRNQTKRNQSQSQPQSQSQPNQTSGLNSLKASAQISTFVATDPAQTRPETKR
KPKTQTHPHKHYNLRQTYDSSKMFATKSAVRSVFSRTRPTALRSFSNTSATLADQYDVVV
IGGGPGGYVAAIKSAQLGLKTACVEMRGRLGGTCLNVGCIPSKALLTSTHHYHDAKHHFA
DHGVVIDGNISMDVDKMQDTKAKSVEGLTGGIEYLLKKYGVEYHKGKGRITGPNGVEVAL
NEGGSESLDTKNILIATGSEVTPLPPVPVDNEGGKIVDSTGALEIRKIPEKMAVVGGGVI
GLEMGSVWARLGAEVTVIEFLDRILPSNDHEIGKKFQTTLKKQGFKFNLKTKVIKSVVED
DGVVLTTEPAKGGDAKEEKYDVVLVSTGRRPYTEGLGLEDVGIQTDQLGRIEVDSHFKTA
VPSIYAIGDCISGPMLAHKAEEEGIAAVETIAGFAGHVCYDAIPGVVYTYPEVANVGKSE
EELKEAGIAYNVGNFPFSANSRARANGSSEGFVKVLADKETDQILGVHIIGPNAGEMIAE
AVIAIEYGASSEDMARTCHAHPTLSEAFKEACMDTYDKPIHF
>comp2732_c0_seq1; 290 22 1365 LEN=1662; translated
MERNKQHDSEKMKMEEKRTINAVFIGHANVGKSTIFGHLIRECGGTDNASIEKCENDAEE
KGEGSMKYTWLLDQSAAERERGTMISGNLRRLESTKYHINLIDTPGHCDFIENTTTRISQ
ADVALLCIDSSTSGYGRGMDENGQTRQHPLLAYALGVKQMIVIVNKMDGDSVNFSEDRWK
TIRTEVSNYLRIVGYRPMKIPFIPISGWLGDNLISKSIHMPWYDGPTLLEALEDVNVPNR
FSTKPLRVPIQDIQNAGPSGTVLIGRVETGELRPEMEVQFAPSGIIGTVQSLEVECREVS
EAIGGDIVTINMQDKSKLSKHLCRGHILSDSSNHPAQGVSSFQAQLIIMKNYQGEISIGY
TPIIDVHTSHTPCKFVDIKTKMNSRTGQVVEKNPRSLKAGDACIAELQPQTPLCVEEFKK
FPPLGRFLVRGDSGQTVAFGVVKSVIY
>comp2732_c0_seq2; 188 373 1467 LEN=1764; translated
MISGNLRRLESTKYHINLIDTPGHCDFIENTTTRISQADVALLCIDSSTSGYGRGMDENG
QTRQHPLLAYALGVKQMIVIVNKMDGDSVNFSEDRWKTIRTEVSNYLRIVGYRPMKIPFI
PISGWLGDNLISKSIHMPWYDGPTLLEALEDVNVPNRFSTKPLRVPIQDIQNAGPSGTVL
IGRVETGELRPEMEVQFAPSGIIGTVQSLEVECREVSEAIGGDIVTINMQDKSKLSKHLC
RGHILSDSSNHPAQGVSSFQAQLIIMKNYQGEISIGYTPIIDVHTSHTPCKFVDIKTKMN
SRTGQVVEKNPRSLKAGDACIAELQPQTPLCVEEFKKFPPLGRFLVRGDSGQTVAFGVVK
SVIY
>comp2733_c0_seq1; 507 265 1749 LEN=1959; translated
MKFSICFFLAAFAKLATAQRSDIHVLMYETDSSMEDNPTSPLHFFMEQSNFAHLATTVYG

GGLEYRGFGDKYQVLREILDVIDPRKLVVVADARDVALNIPHEYELANEAIDRFVRYFEK
LAKNNPHAVVMSAESQCCVSAMTHAHPGDYFDPITKKRNKRACSSGSPGCYWSQNENIHA
WVDFQRQRAFNQTGIERSGEDVGEVYLNAGLMAGYPEDIMNILDLLDIESYEDDQAVLSG
LMYQFPDKIILDYDQAMFGNNQWPRGLSEGCIFEKGENNALIHRQTKAQPLIVHTPGKFY
GCLDMLIEELGGVSQQRYLSDSPEGNPRVLEREGISAESTSFSAFPPRSLQSTENCECIC
ESRRHLLSGLWGVMKTYFTDEIHQRTEEEKKPKIEDEKELSRLVIEDSFVEFTESTNYGN
YGQYGQYGNYGQYGQYGQYGQYGNYGQYGNYGAAADICVDFCIDKCSEDDETNYGSYGNY
GSYGNYGSVRRRFV
>comp2734_c0_seq1; 204 1 358 LEN=1543; minus strand; translated
XLTSIGEILGFLLMMLFFVVLAFFAPVSFVTQLLYFILAGFLGYYLIWAVEPALFSPLMS
ISNSLSGVVILGGILMVSGERGSPSNILGCLAISVATINVFGGFAVSYRMLLMFKKEKQ
>comp2735_c0_seq1; 268 1 1010 LEN=1010; translated
XENPTQSEEPSEFPTQSEEPSESPTIVFRFPTLFSLYEIVCSEANANIFSDFCDLINAVP
GLGRLLGQSLFGRKLQSGAKRYTLFAPTNNAFEFYDIPLPDLTNLTIDDKRLVKFLATYH
IVEDLYDYDELDCNQKLTMYNGLESQTRCSIKTTGINIGVQQKFQVGTDSQAFGDDNEDV
VLSKADARITNKNVAASNGILHAVNRFILPNLTIYETDVPTSEPSDSPSSYPTSGTTFST
TSLEPSGVIKPPSPGPVQPPTEAPTKSPSKAPSKPPSKPPSKPPSKPPTVNPSKAPSAEP
SPAASDDPTVGGSEVPSEAPSEALSVAPSVAPSEAPSX
>comp2736_c0_seq1; 344 282 1766 LEN=1940; translated
MGVPNDLVEAGKSSLDELHSKPRSLRRQIPYVETFEEKTVRDLWEKDPFDGHKYVVNAVI
IRSRNNNENRRSPILNCCDREPMHPLSLSGYEDSFDRIYQPFSTENESDSTRDNISGGGK
NSLIEGATTASIAASPFAWTPPKNQKEEEKMQNFYLQVAYEGNAFCGWQTQLKNLVKPSV
QRTLEEWLTVLQNNGHALEADIIVDAVKNADRKRERRLKKQGKRETKNVTKKDSRSENSF
STAPRIRWADLPVAGRTDAGVSAIGQVCRFRTHRKDLTAEMISDYLTNRITTAPEISQSL
GITKIVKVSRSFHPTFSTSCRAYAYLIDVDTEHNSDCDGVLELFKGGRAKHQISLLDGML
QKLEGKALDYIGLSYGRVKTLNTICTLHHARAKLVRYETSAGAKSRARTAVCIELVGDRF
LRRMVRLLVEASVRLVSTAEKNAETEPPVIGGIADCGATTDALLQLVEKRDRTIVGRPAP
PDGLVFVGARLPLA
>comp2737_c0_seq1; 96 207 650 LEN=1761; translated
MGSAQSATGVGISSGSDPSLPLSAVGEGFRGTRAGGASEADRENDKPKKPAGTAPAGTTG
VGVDVDRRCRKHKRAWGRCVADHYEKKFLPGKSLEPEQDCDDFFEKFRDCYMRGMLVQRR
QKGLPPPKKDSALHEYMVDEGMTDDGE
>comp2738_c0_seq1; 312 72 1442 LEN=1546; translated
MTERCRSSVPKRAFLCSRSDQKKETSAAVLPSKVPIIGLGCSSFSSFFWTNEELAAETND
GQTKTAITAEALTRDHPKVQEWIETIRYAILDCGISLLDTAPWYGHGTSEIVVGWALKES
LSGSDADSSNSKLIDREDLCVNTKVGRYEADPTHQFDFSRTATLESVQRSLQRMNCGYID
VLQLHDPEFSPTLEILLKETIPAMVECRSNGWCKALGLTGYPLEVQYQILQHSLETYGGE
ESVRIWDQALTYGHFNLHDASLVRRRISPSCESFAECCHQSGMGLLAAAPLSMGLLTNNI
PPDWHPANGSELAKACLEAARICRDLDVDIAILALLFSMSHPSVPCTILGMKDVEQVEKA
SAVARRFAMVDWTIPDLSQELVLSFVLTVPESKAYRILSDREAGPFAEVWKRETAEDYDG
NGIFAPKFQWDGVREAHEFWRTTGQSFEDWQDKSFQ
>comp2739_c0_seq1; 868 247 3336 LEN=3615; translated
MSAVLRFWSCVWFCFCLLCESIEFNEKKLHDLSKSSDIDGNGIGKNKDPVAFQEIEVNSL
LTEYLESPLGVDKDVPRVSWQIQTKTSDDQKHGFTADAVATGNVRRQVFQDSYQLHVSTS
SASSRWKDCGWVASNETSLVPLECKGLTPISTSDMNVSWKVSVRLDDGSVIESAVAFFRT
GIFGPWDAQWISGGGWDNTDEKERTLFRKEFSAGASNAGAVPTKNQPARPHATLFVAGLG
YNHVYLNGVRVGDHELDPGWTNYTKRVWYSTYDVTDLVRFADAEEGASNDDFNDDDTNNV
VAVMLGNGWWSCGPPPGTGQDYCTSDPPQFILQLHIDGQPVLVSDETWLASADSPILYNS
LYNGEIYDSRIAKRIRGWNTLHYDDSDWEVATLATNDATMATLASQLFEPIRHISSRRPI
SIVVAGKGDNLTQVVDFGQNQAAVVRLKHFVCPAGTTIALRHAELIMHPPYGEYDNATIY
VENLRTAKPNDYYICNGDPKGESYIPSFTQHGFRYVEITGLNYTLDPTDVEAVELHTDVK
QTSFIQFADPLLNQIQHMVMWGLKSNFMSVQTDCNQRDERKGWMGDAALTAEAAVLSYGM
GAFYTHWLMQMVENQSPDDGSMPNIVPPMGRIEGAPNWQTAYPMILWVMMTYYGDRQLIF
QHHNSLVRYFDFLEASYNKNGLKNFRTGFGDWVPPWPHPKTDLHFMGAFAFLGDLKLGID
IFQYSDHPDSDTQVERLTKLRAQSSPDFHAVFFNFATGAYMSGLQTEQALALYLGVVPDE
LQDSVLSYLIDDIEITNHGHTTSGIIGIKYAMEVLSKLDRGDVALDLALQTTYPSWGYMV
KSQFEPATTVWELWESDFAAPDMNSRNHHMFGSITGWFYKYVAGIQPLDPGFSRVQIKPN
LLRHENFTASVSTPRGKIYLSYLKKTASDKKNSVVFIYEIILPPCTSGTVYFPMATDDKE
LALSGENNYDFLIRESENPIWLDQMFLPGHDGVLHAEMVGKYLKVEISNGRFTFSITVSE
TASISKKLN

>comp2739_c0_seq2; 583 247 2386 LEN=2386; translated
MSAVLRFWSCVWFCFCLLCESIEFNEKKLHDLSKSSDIDGNGIGKNKDPVAFQEIEVNSL
LTEYLESPLGVDKDVPRVSWQIQTKTSDDQKHGFTADAVATGNVRRQVFQDSYQLHVSTS
SASSRWKDCGWVASNETSLVPLECKGLTPISTSDMNVSWKVSVRLDDGSVIESAVAFFRT
GIFGPWDAQWISGGGWDNTDEKERTLFRKEFSAGASNAGAVPTKNQPARPHATLFVAGLG
YNHVYLNGVRVGDHELDPGWTNYTKRVWYSTYDVTDLVRFADAEEGASNDDFNDDDTNNV
VAVMLGNGWWSCGPPPGTGQDYCTSDPPQFILQLHIDGQPVLVSDETWLASADSPILYNS
LYNGEIYDSRIAKRIRGWNTLHYDDSDWEVATLATNDATMATLASQLFEPIRHISSRRPI
SIVVAGKGDNLTQVVDFGQNQAAVVRLKHFVCPAGTTIALRHAELIMHPPYGEYDNATIY
VENLRTAKPNDYYICNGDPKGESYIPSFTQHGFRYVEITGLNYTLDPTDVEAVELHTDVK
QTSFIQFADPLLNQIQHMVMWGLKSNFMSVQTDCNQRDERKGWMGDAALTAEAAVLSYGM
GAFYTHWLMQMVENQSPDDGSMPNIVPPMGRIEGAPNWQTAYPMILWVMMTYYGDRQLIF
QHHNSLVRYFDFLEASYNKNGLKNFRTGFGDWGTLIYIIQPQTSKNQIALCSQX
>comp2740_c0_seq1; 554 523 2472 LEN=2697; translated
MTKPDPLDDVFADAEDDVYGRVRAPNFHDWEHHNGRRRFLSRLRYLGRDKSRSGNGNGGG
GECLGVGGYDGLSEYDAQAEVTCFRPILPSPLRELSYKPRTGWRYNPSARNAPFLDRLGT
GIAEQTGRRNKKQTRTNHPLQNKHRETTTINSSGNQNAVSNTKAQFNTDVRLPMPSTSMI
VPVAWTSGLPIEKNTCKKFTDQTFTTANNDSADISTALERKRGRDTVDTTGNLPPRKMQR
FSDSSTVVTNSTGGDSLSSSTIASNKTKPTGAAPLGSIYPKAALYQWYGKKPRKMQVSCD
QYLTWECDGTALHQPNFTTVFICPMTKEAFLAGPLRIAIGDPNSSQAITPDKDGLYWYPR
KVLAEHAAAARAWDSLLWRDTGGCDETLVRFGGAEPYWPSQRPVWPFHRIPAAVLERLPP
DQRPDAAANGAKKEEDEAMACVASGADPASAKHGATNSTVQCANNAGSNRNLIPGGRGQG
RGGRHSRSNKREEDFAMRKRERDQYTARRRQNNRSPYGKPNNHYVRPQQQQQWPPQPAGH
NSQPYSSGRSHASMPLGDMQRQDRYDYFPRERHQSRNLTQLQPYHNQFQHVGSMQQHQQQ
PHQIYNQQQPQQYNRQPLIPPPPPPLPPGLPPRVIPPPPPPPPSNRLRP
>comp2741_c0_seq1; 184 640 1212 LEN=1460; translated
MDDYGSVDNGFYDDRIDKVAEPERRAFAYLMLTTVRFAYASAARLLVVKLVASLSISASA
AALATADFDLSAVEYGETLTVKWRGKPIFIRKRTAEEVAEVRAVPMGSLRDPETDEDRVL
TDEWVVVLAICPHLGCVPVSNAGEFNGWFCPCHGSHYDISGRIRKGPAPLNLEVPPYKFT
DGENTQLLIG
>comp2741_c0_seq2; 211 27 794 LEN=1042; translated
MNALRLSSRRLAPQAFKSIQSVARPNKLSSAYSFSGLRAFTSTSSSWKASSGSGPSVPVP
SGVETMDDYGSVDNGFYDDRIDKVAEPERRAFAYLMLTTVRFAYASAARLLVVKLVASLS
ISASAAALATADFDLSAVEYGETLTVKWRGKPIFIRKRTAEEVAEVRAVPMGSLRDPETD
EDRVLTDEWVVVLAICPHLGCVPVSNAGEFNGWFCPCHGSHYDISGRIRKGPAPLNLEVP
PYKFTDGENTQLLIG
>comp2742_c0_seq1; 193 688 1290 LEN=1490; translated
MTEVVAGYDVCPSYATVFGYLGASGCMILSSWGSAWGTWRAGLGVCQMGIDHPKGIIKNI
VPIVMAGVLGIYGLIVSVIITQSISSPAGGGYNTYSIFNGYVHLAAGMCCGLSCLAAGGT
IGIIGDAGVRGFGLKAEGGRKWFWSEDQGDDGDFGGSSNAEASNKLYVGMLIMLIFSEAL
ALYGLIVALILSQNTYVCAA
>comp2743_c0_seq1; 691 93 1055 LEN=1167; translated
MPLSPERKAEYFDTFKNLLSTYSKCFVVEIDNVGSKQLQDTRKTLRGKAEVMMGKNTMMR
KVIKEYCEENPESEAIKLVDECRGNVGFVFTNGDLGGIKEILEANTRPAPAKVGAVAPVD
VVVPKGPTGCDPGQTAFFQTLQIATKITRGQIEMVADTHLIQAGEKVTASQAALLQKLDI
CPFSYGMLLKSVYDNGSLFDAKVLEITDDVLTAQFCEALGILASISLAAGFPTQASVPHS
LVNAFKMVLSVTIELDTYSFDVADQYEEYLKDPSKFAGSGGGGGGGEAAAEVVEEEEEEE
EEEAPQGGGLFGDDDGDGDY
>comp2744_c0_seq1; 331 186 1349 LEN=1638; translated
MSNSKDISVDDENQWMIDDSNIDNDGYSGGDRESSDYDAVDNPDYFNDEQNSSNRDLGAY
DDSQKFKEDGGTRMEISDEWWEGREKLIAIVAVCWCCLCFIIIGVVVGILVGGKNKTRGP
GATPRPTLHPTLAPQPLPPSLISIKPTERVTQLTLTDSPTFEPTLFPTISPTVSPAPTIS
IPETLGVIANQDTFVILNGHHDIMGEEHGKMDTFVVQNGPVKDETVPDTVALITFPFDAV
PAFSRLTNLPVSAALRLYHVVPEESRGTATYTIVRIPETRMAIEYFHGFSFRPPEDDADG
VLVGPSFTVKPDDEVVDIDVTEVLFEYEFEPEEEPNQMMLMIQNRGPEQLQGGDVFYSRE
SNTPPELLIDFVGGDPSDITIVNLPGR
>comp2744_c0_seq2; 331 119 1282 LEN=1571; translated
MSNSKDISVDDENQWMIDDSNIDNDGYSGGDRESSDYDAVDNPDYFNDEQNSSNRDLGAY
DDSQKFKEDGGTRMEISDEWWEGREKLIAIVAVCWCCLCFIIIGVVVGILVGGKNKTRGP
GATPRPTLHPTLAPQPLPPSLISIKPTERVTQLTLTDSPTFEPTLFPTISPTVSPAPTIS

IPETLGVIANQDTFVILNGHHDIMGEEHGKMDTFVVQNGPVKDETVPDTVALITFPFDAV
PAFSRLTNLPVSAALRLYHVVPEESRGTATYTIVRIPETRMAIEYFHGFSFRPPEDDADG
VLVGPSFTVKPDDEVVDIDVTEVLFEYEFEPEEEPNQMMLMIQNRGPEQLQGGDVFYSRE
SNTPPELLIDFVGGDPSDITIVNLPGR
>comp2745_c0_seq1; 433 51 1523 LEN=1633; translated
MSVSTDGLRQRKTATGQETKAVENKKTATLTEDDGTNFVEGGIEQNAFFFGLLVAAPFLS
LLLAYLTSEEMATEFPGYATHPVTVMLGVCVSDPAGCASSVVKSGLSVVPTLPATQFVLS
FMGVALVLERFLPGKIECGPETLTGHIPKYVDNAVLHCVIYTALFYLGSNIGYGDLYDFG
IIYDLFPGSVAFLNLFGLAFCVFLTLKGIYFPSTQDSGSSGSLFKDFVWGTELYPRVFGL
DLKRFVNCRFSMTFWQLAGLSFCYRSYTLHGHTMDWGLFFSALSQYLYLFKFFLWEMGYM
RSIDIIVDRAGFEIQWGCLVWVPAVYTLHSRFLVQYPSGLSFAAAAGLFILSMTGVVLNY
MADRERDVFRATNGEALVWGKKPSFIKATYTTIDRKTGESSQKTSLLLASGFWGVARHFQ
YFFELTAAWSWCLLANPLQNGVLVLSYAAFLTYLLIDRAERDSKKCHLKYGKYYEEYCLL
VPYKILPGVY
>comp2746_c0_seq1; 272 286 1470 LEN=1669; translated
MNPRKRSKRMRAAQFGKLYYSGQPNGPSRMEIEQKAVEHCESLSDSLAVLQKQEIAATYK
CKDYLAMTEFRLNIYDLLPNGRMNSDITAQNPQQQSQPQCPVDSQIDEYCREQIVEWSFR
VVDYFHIDREVVVFSLSMLDRFLALCKCDRATFKLAATTTLHLAVKILHPCKLAELGILS
DLSRGEFDMKDVASMETHILAELQWQLHPPSPIAFCTVLLDLFFSSQKMGMVHTDVNDLY
DISSFFTELALCDYYFVGVTPSIVAITSIVNALEGMFGPENKVAPRILKLARETNIYQNQ
DLSQISHRLWELYERSEECALHNNVDAMEVEKTPETATVPPASAAEVFVRKGDCGAQVVS
DTGSPVSVSKHGKTLSSNELMCAMRSQTLRNGSW
>comp2747_c0_seq1; 744 51 2144 LEN=2358; translated
MAEATTTTNALSARELEWAKNSPELQAKHAEINGPIIRTRFPPEPNGYLHMGHAKSINMN
FSLAFEKLGVAPENRRCIFRYDDTNPDAESKEYIDSFVHDLEWLGWTPERTTYSSDNFQK
LYELAIKLIKDGLAYCCDMTKAEMEAQRELAMQRVLARNKGEDPDEVAPIPSEDILPGRN
RDTSVERNLDIFERMKMGLYDEGTWTLRLKMDFESSNPNMYDLVAYRIRYTSHPHAGNGW
CIYPNYDYTHGICDSLEDIDYSICTLEFETRREPYFWILWALDMYRPNVYEMSRLNLQYT
VLSKRRLLKLVNNGIVRGWNDPRMPTLSGYRRRGFTAEIINKFCTDLGATRASNVIEMEK
LYTTARVALSEKTQRAMAALEPIKVVVTNWEDAKGNGGEGMVFEVQNSPTDPSMGSHTIE
LTQTLYIDSSDFRLVDESSYYGLAPNKAVGLKYYGGNLICDEVVFKEGANGKEKSQIVEL
KCHLDGSDDRVKPKTWISWVPAQTAIPCEIRVYNHLFTVPEPSDLWEEEINPKSEIVYEN
GFIDPSVLPLVDKKDISLVKSNQALQFERMGYFVVDVDSTFDSSTRTGKLVFNRTVTLKE
EVSKKKATLSKREEEANEARKAKQKADLAAKEARLKIDPINFFKEAEEFKGKYSKFDDNG
VPTHDAEGNELTKSAMKKLKKEQDKHVKQLKKASMKK
>comp2748_c0_seq1; 482 120 1034 LEN=1077; translated
MTTINSTTSLSSPALVMEEGADTITQVVVHPLVLLHVLDHHTRRQEASGRVIGTLLGRRD
GKKIEVTNCFAVPHAERGDEVAIGKDFNKQMLALHLRANRKETVVGWYASAALADGDNEA
PDLIANTSSLIHEFYAEESDEGDPVHLVVDTRLIEDAITARAYRSTPVVVQGEPMANMFH
ELRLTLQNSEPETLALHRMVADSKEKSTEESSEEPLLVSMETLYNLLETASNYVDSVVEG
KTSPDAEIGRKVADTLATVPRIRPEVFDKLFNDSLQDMLMVTYLSNITQTQLSIAEKLNA
SLGV
>comp2750_c0_seq1; 352 1 1294 LEN=1404; translated
XTERTPRTMHRSSRSLVLLGSLYILTKIDPILSLLHPSVVEKHLIMVSLYGQSSISFRRS
TSRLAKAADNSANGRRPDAIVTNCNFNANKNPLHRPSDPRSVVGNPSQLDEKASELAEWL
SSKRNVFCLTGAGLSTDSGIPDYRGHKGSYHVGHKPIIHHQFMEHPHQRRRYWGRSMVGW
KTFDQAKPNVGHYSLSALERMGRLGVNMEDREDFYDEQDRDEFLFTSGQRRLTVLTQNVD
SLHVRAGSKDVLAIHGENKIVRCMNCGHRMKRNDYQEQLEEMNLAWLMKHLKLLEEGKES
SRQQSQLRPDGDAQLVKANYDDVVLPSCPCCEDGFFKPDVVFFGDTVPKHRVRICEQAVE
NCDGILVIGSSLAVHSAFRHVRAASRKGTSVAVLNVGETRAEAEKLEGLVKIESPISDTL
QRCVEMMGGAL
>comp2751_c0_seq1; 538 403 1110 LEN=1295; translated
MSRWADSTDDEDDYLHDGDHEDYVEHAVSADQLREPMPLPEHNVHLPDKNIAEGADDEDA
EAEAEEESEEEEEEEETEEEKADRQRRLEEAKAAAKAKKEAEEAAAKEKAQKNDLDNLDD
ILNEMGIEAAPAAEEQTVAADDNEEPEAGGGTNSRRRKKKKKKGGPAAAEAAPAASEEQP
TTVDVAAVMKAKAGKKKSTTSAAKAAAQAASSESKSAKGKKKKLSKKDKALQYGR
>comp2753_c0_seq1; 317 431 979 LEN=1392; translated
MTVQNPSPKYNLNTRANNLISRSSTSDDEVEQTATAPTAPKNLMDDDEDTMVEQLQQKNT
AVLSTKEIDSKTTSQRDQQLDLVDAIFKGDSPSRRNDVKKVEDDRSVLEKIDSFLDKPFF
DPDAYDDDDNSFFGKFARLVKTDYEFFEAVFVACFFLLLITITKDVLRAQMAASSVPSGR

LF
>comp2754_c0_seq1; 209 94 291 LEN=1233; translated
MYRRESPWAVDEPPAEHPEATNPATNAGAIATNDAAPTAPDAAVKTAAPAAVCINSCTND
DDDDL
>comp2755_c0_seq1; 425 155 1525 LEN=1792; translated
MISVSSRKSLGVIARRIRGSSSNRLFSTRPIAGGRDVVIVGGARLPFAQASTIYQDEMAV
DLQRLAIKGLIDQTALSKDAIDYVVCGNVIQEVKTSNIAREAAINAGLPYDIPSHTVAQA
CISANAAIATGASAIQAGHADVVIAGGVETFSDVPIRLTRPIRQKLITLPKAMKKGGPIG
ALKHMLKGLKAKDLGLETPAIANYTTGEVMGVSSDKLSAKFGVSRQEQDEFTVRSHTLAQ
KAHDDGFYKEEIIPYRGSTAENGIKGDSSIESVSKLKPAFVKPNGTHTAANSSYLTDGAA
ASLIMSEEKAKELGFRPLAYLRDWSFRSCDPWEELLLGPTYCSHDIMQRNKMSMSDIGVF
EIHEAFAGQILSNLVAMDSAKFAQEKLGGNKVGAVDVDKMNTKGGSLSIGHPFGATGSRL
VTTASRRLQQEGERFALIAACADGGLGHACLLERYD
>comp2756_c0_seq1; 1347 252 3875 LEN=4412; translated
MIHPPPVADAACPCKKRNVKVGSGKRCHVQATADSSARIFSCVAFLLLLSAASLPTVDAQ
KTTETLASPIATQILPPTPIPSFFPGSNENVEDTLCTLDDLERANDSQLNVIFHELEQTN
FFQHFVVDLEVKCNMFKDEEAKSKPAAPKAPPKPAFGTGQTSQTSSFSMDSFPWGNDEDD
GDDFLCTGGADELDEDAVPLCSVDTGDSDDMLAGGGFSAWEEKTDEIVMDEDPPCDSDEE
EQDSGIFDIPDSFWMDICSNIRKGEGTKVVNLELNPERNTYYNGSHVWDAIYKENRITTD
DTCLEERVLYRLLSGMHTSTTLSIAKNYYPPSKRKNRENWEANPQYFMERFADHPEYIRN
LHFSYIVMLRAIKKARDYLYNYDISSTMPLEDTASTNILLRRLLDTTILRSCSDVFSAFD
ESVMFQEDDNKELRESFKEVFHKISGILDCVQCQQCKLHGKLTMLGYGTALKILFVKSPK
NLVLERNEVVALINTAARLSKNLVEVRELTTMYWEEERTKIANLEIKTEVSKGASVVPDK
LPSQVSSTPFDSLDVLDVTVGVISNAGRLELISMEREKELVNMALARHPELMILGKHYSK
DPERFVAMATDLGELESGGRAATKTSLPALPPDAIVVGSGLSGLAASLNILDRGGTVVIL
EKEHLVGGNSNKASSGINGYCPHNDTCSDSMDAFRNDTIKSAGTVADLDLIDVLVTKSSQ
AVSWLRDRANVDLSLLAQLGGHRYKRTHRPSNGMAGAEIIYNIQKTVRSFEKTGRVKILV
DTQVKELLTEDGGDRVVGVRAESTKDGTVQEILADNVILATGGFASDRSSGSYLDQHRPE
LMSFPATAGAFSTGDGITMATTLGAATRDMDKVQIHPTGWVDPKDPGNPSKILAAELMRG
VGGMLIDHSGRRFANELGTRAYVTDQMLKHDEEFVKTGKWNPSTEVPTFFLVLSSSAAAD
GKKHVDLYSHKGLLTKVEGVDALSNHMGMSTEKITKTISQYQLAAKKGIDEFGKQSFRGV
PAENLKTETFYVGTVTPVLHYCMGGIKINPECSVMREDGKIIEGLHAAGEVTGGVHGANR
LGGNSLLECTVFGTVVGQKVPIKEEGTYRMVIGDEEISTEQLDPGTVGSDELPNIQMDEL
ARHNTEEDLWVAIHGTVYDFTEFAHEHPAGFKSIFDLAGTDGTAAFDAVHNLGMLDDFEQ
DRRGILV
>comp2757_c0_seq1; 737 353 2236 LEN=2302; translated
MALAADPTGAFSFTAPAVGKTVANSSSSSSSRLSMVLEKPKAPIKKISKLEILKSESANL
LHPLKEELTTESIGISKDAYQIMKYHGSYQQSNREKKSKVKDYQFMLRLKQPAGELPPDL
YRLVDDLSANHGQGDLRATTRQCFQIHGIMKGSLKTVISSIMNIGSSTVGACGDVNRNVM
TTPAPFVSKDYEYAREYSKVFAHLFRPMTPAFSELWLDGEKVATAELWGKEVEKHNIDEA
MTYDSGRGIILDHPVEPLYGDRYLPRKFKIGVTVPGDNSLDIYTNDIGCVVITNDEGELE
GFNVMVGGGMGRTHNKETTFARAADHMGFVPKEDIMECLKAILAAQRDHGNRDVRANARM
KYLVHTLGIDNFTTLVESYFGKKIQPWKEIQEWKYNDWMGWWEQGDGKLFYGLHVDNGRV
KDEGDFKLKSCMRAIVDKYNLPMIMSPTQSIIFKDIKPEDKAGFEAILAEHGIKPLEEID
PLARLSMACPALPLCGLAQTEAERIMPSYIERIRAVLGKLGIDDEEILMRMTGCPNGCAR
PYMAEMAFVGDGPKSYQIWLGGSPVLTRTAWPFKAKVKNDDLEVIVEPILAMFKEQRQEF
EAFGDFCHRVGADAIEKFSETYEPIAA
>comp2758_c0_seq1; 945 379 4107 LEN=4201; translated
MNFGDETGMEGHGMMMAEQQQQQYGNDEMMMDAEGGGGGGYEGEDGMIDIDEVPVTQEDS
WAVISAYFEEKGLVRQQLDSFDEFIQNTMQELVDSSGDIRVSPELQHMVGYNDDEDADDV
QFGETKPVFEIKFGQVYLSKPTTVEKDGTVTNMFPHEARLRNLTYSAPLYVDVMMNQYRV
PRDINITDPAEDMGEAESTEHAKKEFLGYVPIMLRSLFCVLSDKDDTDLSDLGECIYDQG
GYFVINGSEKVIIAQERLSNNHVYAFRKKQPSKFSWVIETRSQVENSTRPVSTLYLQMYQ
KGGRGAIEGNQIRSTLPYIRTDVPVVIIFRALGYVADRDIIEHVVYDLTDGEMMDLFRPS
LEEAFVIQRQEVALDFIGRRGSARDVTKDDRIRYASAILQKEVLPHVGIEEHCETKKGFF
IGYAVHKLLSCKLGRAEEDDRDHFGKKRLDLAGPLLGGLFRLLFRKLTLDVRRHLQRCLD
EGKHFNIGAAIKSNHITDGLKYSLATGNWGDKGASAPKAGVSQVLNRLTYASSLSHLRRC
NTPLARTGKQAKPRQLHNTHWGMVCPAETPEGQAVGLVKNLALMAYITTGTAQVPVMEFL
EEFSTENLTDILPSVVADPNTCKIFVNGNWVGIHRDPRALVDTFRSLRRMVDIDAEVSIV
RDIAAKEVRIYTDAGRICRPLYVVQEQQLVIKKQHIMQLQGLSGEKKLTWTDLLMEGIVE

YIDTEEEETTMVAMDPLDLDTDDSYSSTYTHCEIHPSMILGVCASIIPFPDHNQSPRNTY
QSAMGKQAMGIYASNYQVRMDTMAHVLHYPQKPLCTTRAMEFLHFRELPSGVNCCVAIMI
YTGYNQEDSLIMNQSAIDRGLFRSSYYRCYIDQEKASSVGTMGSLNSEIFEKPSHDKTRG
MKHGEYGKLDEDGLVAPGTRVSGDDILIGKTAPLDSTANMPSRYTKRDCSTSMKANENGI
VDNVLISTTKEGYRFTKVRIRNVRVPQVGDKFASRHGQKGTIGMTYRQEDMPFTVEGMVP
DIIVNPHAIPSRMTIAQLIECLLGKVVVFQGCEGDATPFTDVTVEDISTRLHAMGYQKFG
NEHLFQGHTGRPMCARVFFGPTFYQRLKHLVDDKVHSRARGPVAMLTRQPLEGRSRDGGL
RMGEMERDCLITHGCANFIRDRFFCNSDQYRIHICERCGLTAQVNLKKMTYECRTPSCHD
KPTAICQIEIPYACKLLLQEMMSMCISTRIYTDTRVTRDNSY
>comp2759_c0_seq1; 870 79 2215 LEN=2539; translated
MFATSAAVKAIARREATLKAAVNIRAMSNLSAYDDFGKNVFTGKVADEYLKKHGASGETL
KDPSWVNNDADKVANAVFDWALDRGANVFCHWFQPMASSGVRHGLTGQVQNMMLKFNGDG
EVEQDFKGKTLVKGETDGSSFPNGGLRGTHCAGGYLAVDTSSPILCRGDTIFIPSAFVSY
YGAALDEKTPLLRANSALDKHGTRLFSLLGGDASSGVQSNIGLEQEIFLIPREEYYRRPD
LQLTGRTIMGKNAPRGQEMCDHYMAPLSSATAALACMQEIQDECWKMGIPLKTRHREVAP
NQFDFPPLYGTNTTQIDQNVFVMQIIEETAPKHGLAALLQEKPFNEVNGSGKHNNWSIAT
RSGVNFLDVEDVWEATGNPEAFPIIMAAIVAAVDENGDLMRAAIASPGNDFRLGACEAPP
AIVSTYLGEDLTSYLENSLPEKPPEYKPGKKMLDLGTREVLPFEVPAEDRNRTSPFPYGG
SRFEFRAVGSSQNVSLVNTVLNTIAAEKFALFADRIEAGEAPADVAREALKKHWKVIFNG
DNYDEANQQMLTDSGVWRIDSGVEAIATLSSDKNVALFEKMGVFNGEELSARQDVLHDHY
TGTVEMEALCMVDMINQHVIPSVKAAGVGPLDELSAAVTTITAAVAEIHAAESSMDKAQL
ARVLRLETMIDIRATCDAAEEVVPAENWTLATYKELMFLDSHVDPEPDFFGE
>comp2760_c0_seq1; 957 310 2511 LEN=2867; translated
MSAAAKPSDIIVKFLQEIVESWKSKRYQKVSQQCVALLKRKKTIENLGMTVPLQRILLQA
YMQEDKYEKVLEWDKASASATKGCEDLILYARYRTEDYANASKQAATAAAGTNDGQDHSC
AVAFKNLQAQSDFHLNRTEEGLAGYLDLLSNEINENDPESKMEILTNALALIASSACTPG
VAIHMDENNETTKFLLEEAEAALSNDDDAVDEEMLADLASNLGCIRFLTDPTTTENNWLE
QASAAIEAGDGSSEQTNLQWSKHFWYRDIEDITYENVPSSKASNQVSVVQSIAKINQSLL
DENLSRLPPQPHPKWNLLQVRMYWYDRAILQLKAGKYVECHDSCQSLKKTLSNKGASDKK
KKNKTGSGGAEATGGAQNASSPAALWWETRADVVLAYAQQNQSKRKDASVRLSNCIHNLQ
AAASSSSSSSMVLDHAMAHALLHQFVMEQKGNKKDRQRQKRELLSLLKSLPDSIQSRPAV
QLTMDDLETVVNNDSESNNNKANSTPKSPLEEADILFGQGQYEEACKLYEGALSNGSDDS
ILDSQLRYVQALAMSGQHEASQELWQSLESSVLGESTPVTASLPSGDVLETKTLPRGTSS
NRSSISKKLAAASNPLEEEETDKPSREKILRRRARKREVYLKQLESKGKYNPDRPTKPDP
ERWIPKNERSRSGRNRGGKSNKGLNSAQGGGSRLDAQRLDAAARRAGKVPVSAGPSSANL
KVSSGGRKGGRRR
>comp2761_c0_seq1; 463 273 950 LEN=1558; translated
MMNFFGKKKKEVNTTVSSTSTSKPSDAQTTIVKLRESIANQDKREDHIQKKIDMMVKEAK
AKMAKGDKKGAVYAMKRKKLYEQEQAKIQNVRFTLETQVINLESAQQNAQTYQALKQGNT
AMKGIRENVGIDQVDDVMDEIKEEMEMAQEINDAIAQPVDPLMTDEDELLEELNMLETAD
LEAELLKPPPTAVQAMPAAPNSKLPALEKKEEDDLRALEAELAGL
>comp2762_c0_seq1; 845 418 3075 LEN=3209; translated
MLDATRTEKGKKIDTTPLNTREILKQLVAHFRQNIHVKIFAESYYSSSDERSQTDIQVLS
EDADDVEQQQLDDSAASPIVQFWLDRLLTLDKAPCNDEGIAGVILEWIRGYEESVRPIVF
HSTGSDASPAFHPDCTYVLENYELIFSKILSRLATLPDDPKGIGLSLTLPLTPVIPSFSL
QQQTLQRAIKIFSTDVTLLTVSARYDVEGRNANDDKTNDLRRNVALPTLLKILANTLETM
RLLFATKTVANKAVGNPKTRDEKSSLSDCYECLRRCSVTLLAFEVPEIDDDESVVRSASI
MLSPHQLAPSLSSSTSSDDCQSTDLVIVRRMFQHVGYNCPCSIDVDDVFDVSKSKNSSSD
FTDVRRYIDGPLGNDSSAYDDVDDQYWNELQSSLKKSVSLAGWVDPTLNKMTFPVVRSTW
HTIANLLREVSREAKIPFREQDEEIACADISVPKCHRRQVDFVLRTLGGAGPMLRDTRAH
FFGRDMHSMETLVKLRADEIAGGNDSFAPSVLVRMGRRVTKAPKKSGFDEAPYEQIPSRI
AAALNAFYFLLLPGMSGGVDKHCEEQILNDVFPICAALVDSHSSFCCVLGAAGFIRVIEV
LQPRKRGPGSTCTNEKAWINFSDNALSVLDKASKASSDSGVLVAIGRAQAILLDVMVLQE
NEKKEEVRKQAITGETGNHFRLKRRATTRQWLANLERSLHRSTGTKRHSLDLLLGGVIPS
LSQHALDEENGADGMEMGRLGLAALLPLTTNSMGFTEQPGGDVVGRKTQMASMVALINLM
FAAHPVMPSHGGKIMSHLLATAADSAPGADRSDDESQNSTGNLPTKDSIRNMAILTAAIA
LVICGQRFAGEVLESIENHREQYQHELVAAVSEVRKLAHCLTASS
>comp2763_c0_seq1; 551 168 2162 LEN=2424; translated
MSSTKEQPESLVENATQVIPAKPDISLDQYKTLHKQSIENPTDFWGKAGRDHIDWFRPFD
KVLDGGFEHGDVRWFEGGKLNLSYNALDRHVNSDSSNDLALIWEGDEPTDVRKLTYDEVL

RKTCQIANAMVRLGVQKGDIVTIYMPMIPELSMTMLACTRIGAVHSVVFAGFSAEALGQR
ISAAKSKHLITADVGRRGGKTIALKEIVNNALTKLDCDSIVEHVMVWERFYDGAESSAPY
EMKKKDIRMDLLVSAQRPYCPPAHMDAEDNLFILYTSGSTGNPKGLVHTTGGYALYAKMT
TQITFDLAPGDIFACVADCGWITGHTYVVYGPLLNGSTSFMFESTPVYPDEGRYWDMVQR
HKITQFYTAPTAIRLLMRSGDEPVKKYDLSSLKVLGSVGEPINPEAWRWYYEVVGKSNCT
VVDTYWQTETGGHIITNLPGITPMKPGSCTLPFYGIDTAVLCPTTGKELTEPDENGNFHG
VMALKQPWPGMARTCLGDHGRFLTVYYKPYPGYYFTGDTVSRDSDGYHFIIGRMDDVMNV
SGHRIGTAEVESALVAHPAVAQAAVVGKPHDIKGQSICAFVMLTAGFEESQELIKELRAS
VRSEIGGFCSPDMIIVSPSLPMTRSGKIMRRILRKIVCGETDSLGDTSTLADPSIVNLLI
EKVA
>comp2763_c0_seq2; 329 326 1648 LEN=1910; translated
MVWERFYDGAESSAPYEMKKKDIRMDLLVSAQRPYCPPAHMDAEDNLFILYTSGSTGNPK
GLVHTTGGYALYAKMTTQITFDLAPGDIFACVADCGWITGHTYVVYGPLLNGSTSFMFES
TPVYPDEGRYWDMVQRHKITQFYTAPTAIRLLMRSGDEPVKKYDLSSLKVLGSVGEPINP
EAWRWYYEVVGKSNCTVVDTYWQTETGGHIITNLPGITPMKPGSCTLPFYGIDTAVLCPT
TGKELTEPDENGNFHGVMALKQPWPGMARTCLGDHGRFLTVYYKPYPGYYFTGDTVSRDS
DGYHFIIGRMDDVMNVSGHRIGTAEVESALVAHPAVAQAAVVGKPHDIKGQSICAFVMLT
AGFEESQELIKELRASVRSEIGGFCSPDMIIVSPSLPMTRSGKIMRRILRKIVCGETDSL
GDTSTLADPSIVNLLIEKVA
>comp2764_c0_seq1; 112 119 364 LEN=652; translated
MGGRNNPAAEPKTTQDVDAMAAARIPLAYRDKCGHLLIDLNKCRRENKFKMWECGHERHT
YEECEYYAWMDRCAQKKNGSA
>comp2765_c0_seq1; 1003 247 2472 LEN=2549; translated
MKFESGVLSLALASLVAPTEAFQPSTRSFHTARAQDQIVRQSTTPEVVESAKTAGDDSEL
EALTADIVSKLRFREVQRELERREVDTTGTFTTMRTRLMELADDANNGEQNTEGSNDVHV
ISNDALNTAFKDTGISFEDNSDPDFDFNNLLTEISSLSEKVHWKTAARRFKQLIKRFGQN
GPESREIPESVFTEVLTAYMQSRLHGARAAEPARRAMEEMISHGYMVPSEIANSCIKDCL
GFEKDGSHEGFAGIDTALAMLAAVELSEENPTPIFVDTYEKLITTLANEGSIDHALQMLR
TTVVDKADTPSLKVFADVASACVEGSDEPEKLMTGLAYVKATGYELDNIASTVDGRSILA
AGVIAAEKMNNIGLGLRFLTAASQAEGCEPDRGDTLVANLSPASQRACTIIHRRAILKAV
ENGSWKLAVKLLELMLERGLTPSPSVWRNVVILCTKEEKSRKATALLLDWIDLYNQERAE
KPPLRVFNSVVNACEVCGEEELTVRVLDAMKETHDTEGNLITFNIALKRLAKQGNSMACE
GIIIGMLQGGIEPSVVTYTTAIASCVADPKKSDVAIEWIKRMKSRLVQPNVITYNTAFAS
CLDGTIEGTKRASELAVGMINDIKSQLDAGILSKDDYTNIIPNFYTRTLAKSLVEQADAQ
MKKGELDEDDAESTIRGPFLELVDFLNSDLAQLAEKQKQYVKDQLDDKEADIETTIENAE
EEVDEELEYSVAISTHRAAMV
>comp2766_c0_seq1; 243 28 708 LEN=918; minus strand; translated
MFILLYVLLVFGFCPPRKNRTGSVPTANPWLERIGVCIHPASIPPNALPRLASSCRRRLF
FPVPRSLPCGVALRCAAAGTEPQQAGPSTLRTGWLAVVPVLQMPQAHCDAPRRWVRGAEV
GFGPPACRRCRAGGNRRPLAGVGLAARGGDCDRRGARSGGVVQHRVELDECELCVCGQGL
DCLSRQQQVRNFVDGLVHGRAGSLDRSFVGLFCVREGYGTVRVVRW
>comp2767_c0_seq2; 169 102 833 LEN=1043; translated
MQIYLFLIACYLGFVASFRTSSAPLRSSFSRLYANTGQSYQASLPSPLIDAQGNVIESAE
AIAGLVEGQRVALYFGASWCKDCTNLEFMLAQYRSALADSNQPIQLIYVPCDQSQEDQLS
QMQKLGLEVGVPLGETADALKQQYGVWPDVDIERFGGISREIAIEGTETIVAVKGSEGEE
VATERVEIMGKVEDTRDEYGRRSGIPSFVVLDNTGAEFCFLNAERDSISVLADWPLDDPQ
NIW
>comp2768_c0_seq1; 271 1 1100 LEN=1100; translated
TASNTASNTASTTASTTANRTATNTQSSQSWSPSPVKAAPESNRSKASFPTPTLGIEVQN
AVHGRSEATASGTEIVLTRDRDSVGDKKTPTHYRHQPPQLETDDNTCATSSAYTATSSDE
SCIRTTHGGRSTHEMTMIRTQSIRRDQSHVPGHSSQRPEQAPRTIFYALPSSLVQQPQLV
SQLPNQRLQPPNHHRPRRHVRSHSFGSTSLSSFGASSYNSSSSSEDSNIIANTTSTSSSR
GNSICQNSSSHLDSDQVQEQPQMTFYLRPSVTTVKHVLKEDHQSQQMHENRRRTPIQTST
WDSTRDQTALPAVTANATAYRENYSASMKILQRIPGRSTAKIADEMTDETSTSAASTSIS
TSTSTST
>comp2769_c0_seq1; 525 101 1513 LEN=1695; translated
MCNTTVHPPAVGAGSTHSASSTSSQQSHSHSYSHESTGKLVSITVRKENPNDKAGIQLEQ
DKYGTVHVTNIAKNGLFGNSQLEIGDTILSVNRRRLSKGEGPKEILRAVHKYKTISISIR
KAPPKGAPLVGRKKDKKKNQEKDNNRAKIDTYYRGCSKYKEDGSINVQYDEDSADDEKKM
PTITISAQKGCPSNCTNEESKPGNRRTSSSSRSTTPKKRRSLPSEVGLVGLELAAEGKNR

LVVREIQPKSIFRGTKLRVGDRILSINDMSFRQYIDAEYAQSVMDNAPFMVTLVIEKPEL
RRGSRKKEELDHSEGQSDEDEFGRDTPLSKYELKIETDFLIETYRPATITAPKSRRSQDS
GLTFKLVRTTRPFGKDKKGSSAMRKKKATWVYVDKIDPDSLFAKTSLEVGDKIISINNID
LRHTPDPNLAYLTCSRSTESIALVVLKDEKVFKEKKLCFDKSITNLEWKV
>comp2770_c0_seq1; 398 76 1206 LEN=1784; translated
MTVDNSHAATATADDPLARMTSKNNRQQSASNDEIDAMVPSKTNNDAVGNKGDFHNKENN
ENQSAFVLNGPETSASAAKINSTISQTDRTHTASIVDAPPILARPRATLAREISRQVLRH
KLQKLRHLVLEGVVSEEYLEQHIMHPKLLDMFDPQTVTYNGGIAKIKEWKISCYLEVMEG
GVPCTNPHTELLEVFRPLLNTCDDLFLAWYKQQHACNNHRTNRGHSARRTCERLMTFVTR
YTPAPGEQALLKHVDGAGKVDGSCVLALPVDRWTGPDNSFYGHGGGLTFWDGREPVPEDD
GGDRPDRSDGHPKRTRPRTRPREIHYDTRPGDIAFIDRAVWHQADPITKGTRWALVIFYK
VTEQEDDENDCEQERK
>comp2771_c0_seq1; 560 109 1944 LEN=2186; translated
MGSGAKRLLQRWLLARPLPYLKQGSTISIRSKLKTDLRIRSHWRDDGELSFLQHCHLPSG
SATIDNDNQSARIFLSRNEDKMTSLGREDPHLSIDILEKIEVEDSVSNGHIGSEPIVTSL
SLPPPTTIETSEEEVGNLLTIDVPDKINIDCNLTEGGSIFVENKIEGDVRLKTAQGNISV
KKLRGHHIELDTTSIQSQGQKGEEGNSTITPSTIFVSDVLESQSLRLAVDRSSVDRIRAK
RIHANTMEIQAGGVGEKDEGDVPNLTTFRESLLDEDDSGAICDIGSLYLVGDATVNVQQS
AASDNRNHGQRQAVRIKSHHGHVHVQVSAPKPNIDNELTGEARPMVDLGGVNGSCEVFVS
RSDSDSKSADDEWTSCHVHFDSIAPDSVSLIDAKGGNVHVTVDRKVESDVRLFSSPSDAH
VDTEILLLEDNEDGSLADEVTHMIQEIEDNATSVNSKALPKADKGDFDIETNRIKIETQA
FTSRDRDESGQLQHCQLVDGWVENTTSEPDSRFDRKIRGNSGGGKINLGGAQNQALHGFV
SDRSGAAEAAGEDFARPLLAVSSTGKIVLETLSWLGNIARRYGLDENRSEDDLGRQATRR
KGLGRVVPKED
>comp2772_c0_seq1; 286 1 1379 LEN=1575; translated
XTDTDTDTTNXELQLQLQLNSNSNSNANSNSTPTPKMASTEPELRQAPDSAAPGEEPPVV
VEADADLLERGICRVELPPGLDPERWAAELSRPTPLNFGFEGDGEAFLYRNIMDEPGFPF
DLVLLGSGGDGDGDSLSEIGRAVLEHFPVLPKGSGDGGIRDPRKVLSEELRLDDAFCVHY
HEDQHDTRGKKHRDPSDITVNLCLHKSENAEGSLVMFHGARTLRNAGNSDSNSDSGRALA
GGDRHRRFLVPQKQGHATLHFGDHPHETTALTRGSRTNVILTYVYTDPTGATRPPGCATE
QCFSAERTVPESQHAPLSLVGRRTTKLCIDSRSFSGARNPCFPFQWTWAASLSAAGEERR
RDKHSSPPLPTDPRDAKGHPADRTRCEDAGSRSDPRQNDGSARDRGRWHTDRADPSGGDD
PFAASDDGRELPSSRSSPGHCFSSRQNERLEFTKEIILAW
>comp2773_c0_seq1; 486 102 884 LEN=1135; translated
MTASKRKNENVDYVTHELTKMVIVNDSNHQENENENEQGPPNQGVHPTARTPMERMKLLR
ERWGAAGGPFAVGASEGGRDDEIALAIGSFLEGGAAEEERESEMLGRAEEVRAGLAHQIQ
RARELYREESSDLSDLSRELGGLKEEKKALLREIDELESRQRASQENIAVYQAEAAQELD
EIMDVEEQRKQHVPRLKMTISLFASTTGIKWDFCDPDLLSGQVAVPSQNSFKLFSIDPRD
YSPVETANILWGLMDGVEPQ
>comp2774_c0_seq1; 167 208 549 LEN=1127; translated
MLSLASLGRSLAQTSKTPAVQRCLSRRAASGLCGLPDERDSHWRLRSGSEAASSQEDGTA
QGSPGLANPGDSLADHPFYDQTAAAEQMIYTMENSYYFASYKAGSGKEGLGSC
>comp2775_c0_seq1; 115 346 1342 LEN=1342; translated
MFSSSLDGDGDDLNNNENSPDNAFHLITEYPKRSILFSVLMTTCGATLGPFLDSYHSAFG
VLAYDEPIRFILWGSETYPALTTAWWVPELFGLAGFLIGWLYILFDATLLNASNDERLRP
TPSKTLVGISFFTLQYWLSGILLQSNTLDRTGILNLMSLLAASGFLVLDGTFSGLTVSLI
TCIGGPLIEAGLITATSSGLMSGGYHYTDLGETGFFPLWIVPIYFLGGPANGNLARGFWN
ALSGKRGGGEMVAVNEPCEVCHSTRRTPCPNCDGVGTYVAMGNRIVQCTSCNSRGYVICR
ACFDQFDDDPHDIDGIRDIMNRMPDYYEFRNNX
>comp2776_c0_seq1; 581 338 1936 LEN=2424; translated
MPIIAVEEDDQDEVIRLLSGAEIPDIKSNATISIETPPCTDEEQKHNEAIDIKGQDPDWD
DNQSFGSTFELCSISSCTNLSEYNNNESLNKSVGRSTHSIGRSGHSIGRSGHSIGRSGRS
IGNSTSSHHRYTIVRRGPSTSQIQMNRSIGSSNSSGRKIGGPRKSSLGSKIIDDVIVIPN
TEERIDTKYFVDHSREIGRGTKTIVRKCIERSTGNRYAVKSVRRTDKTEYEHMRNEVNLL
TVLDHPSIIKIYDTYEDDKFLHIVVDICKGGELYDYVVKPTSTNKKNGQQEAVPNCPSEE
VAAVIVRQVVDAVAYLHDHDIVHRDLKLENLLFKLKPNAKNKQSLTEIRVIDFGLSRKYN
THHRFCSLKKLTSFVGTKLYVAPEVLNHSYTHAVDLWSVGVLAYALLSARAPFTGRNDQE
LFDKIQHCGEQLKFPSPDFDAVSEEAKHFIRSLLVKDESNRPTACELLDHPWMVKADQWK
EEIDAAAAADGNASLFKKILGRFGKGKKKSRAKGKNERDHGPKSNDLDGRVQ
>comp2777_c0_seq1; 886 1 3159 LEN=3499; translated

VPSAVVIEFTEVAKENQKTNVDTVTPFGGRQYSSMRFSAAIGALLVLQKSASSGAASVNP
AGDTFSHQSHHNSKHYANQLPKVEGTGARSSRVLRNLPSNHKETESRQKQPEISSSPLAI
SSALVDLGEDKNYEENFGSIFLGVLGTIPNPSADDTIIRSLQEDASQFDHVMSGICNEPN
TKGVCSKCQYSSINNAEDGLLCSADVDCNSYVFPNEDSEYFLVAYFQYLYHVCRSENLVC
ESPCEVDVTTKRLSWKDCLYIYDPSDFYTYDPSIYGGYTIQNPEELLDQISFEDLENEIH
DGEAVDEVSVAYLVSNYNPPSFESLCDIFSSIEDDAGNSICPTCMSSTIVGEKGLLLYNM
EVDCDESASINEDLGNYLTQIFEGYSAFCDPFSKYSDYFVCETGCEVNVDAKSMIAEGCY
FKESAFNYIYATHVVAATTDTFCSYYGAGGDGHCRNCGITYVDEGEQTNAVYDVTINSCG
SFEEAMKGEYSMRHALLYSFYWICYAGTYCTGACEVDVDSMTISMRHCSPNNYSVSFTND
ETDSLGGIPSTVGSDIVPGESEMTTDESEDMGDEPENKTDGTSLVTISLIVRVPVDLEAI
EEKVIAVAQSILDDESMSRRGRQRRLYKVTYHRYLKPDQRPNLTAVATIVPASSCPEYDS
FDLSAGSQCIVVSLLVEGVGKKKIRKYTKSIEDSVENGVFENELQEVGLDTTVRMQTNNE
AGNLSDNTLIDPSTATPSTGGPSLLAPSAAPTMVRTKELTVTPTIHTVIDTAMDTAMDTT
MDTTIAPTMAPTMAPTKAITKTPTKDPTPVPTIVPTATPTKARSTKTPTTTAPSANMISV
KDTSTTSPSTNTPDISDSLTSNLDLVATDPCPGFADDCSRCVANPACSWCLETSVCFNAN
VEIEAAPVQNGDGSSIIARKFENQCFGTMTQLVSACPISSQNPSLGETEGGNYKATNLDA
DVDAEGNVGPDTNADDTLKGTHETTTSSSTAMNLDAANDENVDTDANARASFEGINETTD
IAQSSSSSINTAGMLLASGVCCIATVLAMRHM
>comp2779_c0_seq1; 362 143 1012 LEN=1100; translated
MGNSHPQNKTGRKVVEQKLENAKKTGVLSLCEHKLETCPNQVFEISKLTTLDLSKNKLVK
LNPKLSTLKVLKSLNVDNNKLMPGSMTPISSLTKLKTLSASNNLLGRPVTNKNSKAPAAP
STAKNTDILPTPLPKGLRTILLSNNSLTTIPRSIVNNSNPLKLLEKLDLSFNNLATVPES
IANLVALNDLNLDSNVIVSLPPAIGKLKRLKALSLRNNHISSATTPQPLPEELWTNTLLI
DMNLHGNPMTSTQLNSMDGYDAFLSRRREVKNKDILGGALTDLDGCGLK
>comp2780_c0_seq1; 592 106 2070 LEN=4278; translated
MPRWNSLFLLAVAIAATAVDAFYVPGVRPLELKEGEPVEMKVNALTSVHTQIPKDYYRLP
FCVPSGGPKMASENLGEFLTGNKIQSSPYSINMLTDAYCTKLCQKTLSPVQAMTLRKHIK
YGYHNNWIIDNLPSAAVGLNSQTKKKQLHYAGGFPIGFLDANTGHPYIFNHVNIMIKYHQ
YDETKSEYRVVGFAVEPMSIKHSFAGGYEWDGDDPEGWSKTLSSCTDASQHLHTSRESIA
ASQIIAPNESIIFTYDVIWEQSDIAWSSRWDIYLSEDDLVPAEVHWFSITNSILVVLFLS
LLVVSMLVRNLRRDIAAYNALAALADEERDEDMDESGWKLVHADVFRPPDNHPMMFCVCC
GGGVQLALTIFLSICFSAIGFLNPARRGSLMNGILSFYVLSGILAGYVSSRLYKAFKGRL
WQLCTVVTALLFPGVAFCIFCFFNIILFFLHSSASAPLLDIIIVAAMWCGVSVPLVFVGA
YFGYKQESISFPTVTSTIARAIPEPQLIMHPMVGALVAGVVPFAAAYVELFFIMTSLWMD
QYYYVFGFTLIVFFILMVTCAEVTLLLVYNQLCIENHRWWWFSFFAPGSTALYTFIYSIF
WFKALEASKMIMTYLLYFGYMGLICSAMFLVTGTVGALSSLWFVKTIFGTIKVD
>comp2780_c0_seq2; 737 106 4118 LEN=4178; translated
MPRWNSLFLLAVAIAATAVDAFYVPGVRPLELKEGEPVEMKVNALTSVHTQIPKDYYRLP
FCVPSGGPKMASENLGEFLTGNKIQSSPYSINMLTDAYCTKLCQKTLSPVQAMTLRKHIK
YGYHNNWIIDNLPSAAVGLNSQTKKKQLHYAGGFPIGFLDANTGHPYIFNHVNIMIKYHQ
YDETKSEYRVVGFAVEPMSIKHSFAGGYEWDGDDPEGWSKTLSSCTDASQHLHTSRESIA
ASQIIAPNESIIFTYDVIWEQSDIAWSSRWDIYLSEDDLVPAEVHWFSITNSILVVLFLS
LLVVSMLVRNLRRDIAAYNALAALADEERDEDMDESGWKLVHADVFRPPDNHPMMFCVCC
GGGVQLALTIFLSICFSAIGFLNPARRGSLMNGILSFYVLSGILAGYVSSRLYKAFKGRL
WQLCTVVTALLFPGVAFCIFCFFNIILFFLHSSASAPLLDIIIVAAMWCGVSVPLVFVGA
YFGYKQESISFPTVTSTIARAIPEPQLIMHPMVGALVAGVVPFAAAYVELFFIMTSLWMD
QYYYVFGFTLIVFFILMVTCAEVTLLLVYNQLCIENHRWWWFSFFAPGSTALYTFIYSIF
WFKALEASKMIMTYLLYFGYMGLICSAMFLVTGTVGALSSLWFVKTIFGTIKVDKRQIGG
PGCRSKTVFVSRRGAAEFSPNQPKHVAAASLIRHKNTPRSKRTNTNTFELSIFKTHFIAF
KIIFRGAFLASCQPDSSDGASRVLLNLAFVRFCTFQSFSGFSVEIVLVGEVRILYRSNSY
RTVIRDATSIQAKVKNVLANVRYQSDLENSLXRVYEDARGNVRTFLRGSVCTPGTSTSTY
SYEKNVKNRDFRENEGKMLLLVFPEKCVSSPVVWRRSMIARERHTMTLILKFQAVVSTLL
ILSLLPETTRAMASSAAASTISSSVRVAIIGGGVSGSASARRLAQLAPSARITLYEMGRG
LGGRASTRKTRSVPHIYINHGAPYADVRSKLGRSLISSLGPSGTAPFSGVRGFLDSKTGT
FAPGDGEKEGVDYVTGAKGEMSQIASAMISGIPSIDTKFKTMIRGLSRSPSGEWQLLDKS
EEVIGSADWLIVAGSGVAHLRWSKTFGGEPPLIAAEKARPDPKLREALDAIASHEASPVL
AVFFSCSGPTARKWLSLDFDVADVNGSSILSRIMIQGKQKDKNSSQSDGGGDEWCSVVLH
STEEFALQNSGVYGSSSSATRIGDAASDASRENSLISDMMVAMNEIPGMPEINTKLQDES
YSSRYDYGPVIHRWGNAFPKGDALAEDLAFLPSSRIAFCGDYVDTSNPARFGSFESALLS
GTWAGEQVSQFCQKD

>comp2781_c0_seq1; 296 1 1400 LEN=2069; translated
XSCSSTLPFSSHHRAENDRSASYKILSQIASVFWDLSMGENDVSSELYSRNDTFITSSPC
IIISWTEELPAEQAENAHIEKYDSETFCSSIAVRPGTEPSGTKVFMGSDSGDETNNTDDR
TLKEDLLHPDDFGPIPHPKHLSPSSAMTFKECPQSFLFQYLYKIKQPTNKILAKGSMCHS
ALEHVFDLDPKDRTLENLQNLMRVEWAENRLEEEYRFLFEEEEEDAETGSTNVRRDINAE
REWGQSALQLLENYYQSEDPRTILRPNPHKREVWVSADLTVDSSLGVTTPAGRAATKTQP
NPETFKVRGIIDRLDIVREAQRKVALKVIDYKTGKAPNLKYSPAMNQKIFEEKFYQLKIY
ALLMREKTHAAANDKDSMDLRYLKLHFLTSEEGPAKPWEMDLGENQEIRDDELQKVHKDL
SQIWMDIGALVDKQDPKAFQHCDRPFCFCHVCRDRFVPGTVWERPE
>comp2782_c0_seq1; 626 103 1905 LEN=1975; translated
MTTTMMVTPIATVFVLVLAQHGWTESFRGHHGVSFPFRMGCYHLDVLPRKPTALFSKQHH
NHFQQQFPIPAMPSDNFRKMAQSQLELLASSLSRADDPSISKVQSMILYLPQENSFTGQL
EFTPAILYPDPSTERVFIASDAASGQAPTLPKSLSKLPGFSSAPDLLPGYPMLAGGNDPG
VGTVEEVLCDIRLKTSALSIPLLSGSETVGVLLVSPTVTLDNKSSVENGTSWTKREREQV
SLAAQSLSMALRMDNERNLLREQNSVFRENLSDSLHQMKNPIQALRTYGKILQRQVADTT
SDGSGTPELLELAERLMVQSDRVVDMLGPMDSLVETLDQPNSVVMLPPAISNNTENVQSM
VLWNDHPSNNSAMVPRSNPEATIDENDPQPLAPEKITIENRKETEIDGLQATVIGDFETE
MVFITDVLESVFDTFEAIASENDIKFEVSIEDIDDLPGVFVAPKSFQEATSNVLDNAFKY
VVLPKDDALFTRNPNPEVRVRIYPNDHVERPGVTVLVEDNGPGIAEDDLDLIFERGFRSD
KTSSDVGGRGIGLGLARALMKRMGGYLGPASDDDMSHFTDILDGAKMKLEVSRKPYASHR
>comp2783_c0_seq1; 205 389 712 LEN=1247; translated
MPDMLKDFSVDHICYSGEVIDEAASTTKSHLRKTAESARELPKQGKQFADSQAEILHSHS
KVLKTQLSENWNSSIASIASMPSAISESWDYHANIYHEQQKQLKQQQ
>comp2784_c0_seq1; 270 142 774 LEN=1022; translated
MKFHLSLPLLSIAFIESTSPAAGAQVDLMPEGEDIYYFKPSETAAAATPPECNKEYESIL
DICEAGAYDLTEFCRLLAFVNYLDLSDSNKSFTIFAPTDEAVSRLFDDVLDSNGIGSTLM
REIIQSNYIDGQVETRDLECNSKPNVVSSAGKDPPIKCKVDITGNTVPYIKGRGNARGYI
PRFTDPQNPILTCNSNIYKIDEVVLLTNYV
>comp2786_c0_seq1; 355 1 853 LEN=1082; translated
XPPQAEKGPLVTTERSRHQTYQSAKMKFTSTIILSSLLSGVSAFVPVARPTVATSSLAAT
GVDDKTEVREYFNTEGFNRWNKIYSDSDEVNSVQLDIRTGHDQTIQKILNWVQDDGDISG
KSVCDCGCGVGSLAIPLAQMGAKISASDISDSMANEAARRAKEMGLEAKFYTSDLESVSG
KYDTVTCVDVAIHYPTDKMAEMVGHLCSLAEDRVIISFAPKTWYYSFLKKVGELFPGPSK
TTRAYLHEEEVVRNALKKAGFEVAREEMTGTNFYFSRLLEAKRV
>comp2787_c0_seq1; 161 136 501 LEN=783; translated
MASLQLSRHARNLGVINSPAVLYKRILKELPRVLMIYDIDMPIEKAKATIRDRFYDNKDV
KDHRVQEMLIETGYFALETSMLQHKQKNHLMHFLEGYTTPLEGDRKRLDQDSTILDQFAR
N
>comp2788_c0_seq1; 309 57 1268 LEN=1553; translated
MQTFYSLLLFSKPRPVVVLFLAASVVVDSFAPATIRSGVLQHHRTKNASAKTERGTLLFA
ESPQYQKQEGILKRSECVGKGSYLLYIDCVDDDTNGYKPGHVLALEIQPPAVDGCDDVEV
SETMTMNEKTRKDMEANEGWMRGPYTVSRGYGTSSEINGKAKNDGFQVLVKEVGYKSHVF
STCPEGTPVRYGGKFKVPIAEGILEAAETSDSEDRGATKRVIMVSTGVGVGPCLGASEIL
VNMLTSSDAKECAIDSIDLVTSFRTREEIAMASDLDKLQQQQQGRNPEFHWESIITGETG
RLSASGPEFLLDQYVSPATSTGSAIRNTHYHIIGNGQLVNEWTAGLEQAGVPSSRVTTEA
YFNHKGEADPVAVNAICQAILRLDRDAESKEVETAVEQAARDA
>comp2789_c0_seq1; 173 1 400 LEN=882; translated
XDKSKSTRSNNNTSTSALSSLSLQKNIRCYSNPNHVLTPTFYSIMPPKRVKKVMTLPINV
IFGHLQKKTRVRIWLYEDTKTQIEGQIIGFDEYMNMTLDDAVEIGKTRIEVGRILLKGDA
ITAIQEANPVKKE
>comp2790_c0_seq1; 181 319 663 LEN=751; translated
MPIKFSKETSSRLFRYVKSVDIRFNPFDNRTTSAREFWRQMQATRYRKMNPRLEINTHVL
GSASPPEIVLKLIDDTEKRYDSRNYTASEIFFDVHLSLDNLDNEFELSGKSLDD
>comp2790_c0_seq2; 153 311 511 LEN=599; translated
MNPRLEINTHVLGSASPPEIVLKLIDDTEKRYDSRNYTASEIFFDVHLSLDNLDNEFELS
GKSLDD
>comp2791_c0_seq1; 541 185 1786 LEN=2000; translated
MKLTSMMGQTAAIALMLVKTCLSFRHCTFPALPSLGLRVEDRGSLNPLFYSNQNGDDPQE
DWRDIRAKLVMQYDKGNDHTTTNRSNTTCMPPIPGMNRWAYDSGDVIEVGSLIVSHPYQD
WGGLKKQHFHKCIILVIEHSQDGTKGIILNRKLQDEQHKNWICHFGGDTEGIGSSDGSLG

CLFRLNDLSRVKIGMALSILNDIAIMPLDIAQCLVDEEEAKKEDFRLYHGYSAWGPGQLE
KQIKRGDWFMVATDTESLWDIIVQESTFSTGTDKWVQMMKRIGKESLMISSPNQKFEDDM
LKQYMRLRLLKKSSNELQRSKSSRQQMHARKKSACNLSPGTLVRSSTTILFDEQVFHQSL
VLILQDNPEMTIGVVLNRPHSTSVSIGGTSLPLRYGGRFELDDEGIPELWFHCNNEILQE
ARIGEPVSQEGKARSIFWKCTRKDAETAIKTGLGRAEDFLVVSGLSVWEKEELTKHAGET
SCIKLDDCFSKVDENSIDTIWKLLMVQEPLNKRNAAENLEAANTAWMLSGDAGW
>comp2792_c0_seq1; 370 75 1187 LEN=1322; translated
MKTLIGSDRNIRLGQRWQGGDGPSTCMSSKIPIHFLKTLFAVFAMTLSKASGITVNGVLF
SAPSGAVSNIGVATQTSLPSTTCSRSVVVFIHGRLEADPASDDLTIPRPAELYSDFCKGL
AASMMSDVFLVDYHEHYRDWRREPRDLELGPVSSAVGETIRKHIKSYSDPMNATATSISV
KITLVSFSMGAAIVLKLLKNSPMFESASVSVPSSILKNGIKIERAILVEPVWRCWLPFAV
SRAAEARYSANAKKVEISKVPSLVLVGTNDLEVRHDINGGMGSNGTRNLGQSVARCLQPF
LPNLSTIDIAGGNHFGLCSGPPKFMVFASEGLSSGKSSERLRKEMIDHISEFSAQSPESD
DDSDSDSTAI
>comp2793_c0_seq1; 562 143 1555 LEN=1681; translated
MKFSHATTLLAIASVSTASAFSTRSASVTRSTKASVKTPQYAVSVDLPDSTNTAVPEVKK
VPEATEEPFDMTGVALSGLEGKALQWKESDFPKAKDLRAVIPKDCFEPETKTSMKYLSVS
VIGTTLCSLFGARMLNVFNPGSVANLPFWAAYSTVTGTVAMGLWVLAHECGHGAFSKNKK
IQDTVGYILHSLFLVPYYSWQRSHAVHHQYTNHMELGETHVPETASGHSLGLRNSLVAKV
GKSAGIKIWGSLQAFLHLIIGWPAYLLIGATGGPDRGMTNHYWPNPLTTPKQPRRELFPG
NWKAKVMKSDIGVVAVGAAVGALGWFKGLPLMMAMYGGPLIVVNAWLVLYTWLQHTDVDV
PHFSSDKHSFVKGALHTIDRPYDKLDPFGFIDFLHHKIGTTHVAHHFDSSIPHYKAQVAT
DAIKKAYPKMHLYDPTPIPKALWRIAKGCTAVEKRGDKWIWKNSGLEPLL
>comp2794_c0_seq1; 246 48 536 LEN=1045; translated
MKLSFSLLSLALVALSASSVSAAGLSTRKLQEQFEGTDNSQYNAPDAPSYCKKDQQPNSG
DKCPSVGLQCAFDNNIQCICGEGGWSCGNPSPTPTLPTPMPTPSPISKPTCNGSDPCVTI
LKENEEVDTENIAVTDEESSAAGAASAAAVMVAAGAATALVL
>comp2795_c0_seq1; 567 164 1852 LEN=2068; translated
MQMQDSQLRNCLRQAFFAPRLLLLVLLICGIVSSTNAKPGPNSMLLRVRLADGSMERVQV
EEGKEDTLTLEDVLKPFGVSDDASITIGTSKETAIRDSFLANLGVRNGSMITVMAPKEKE
SGNGTTKPTSRFASRKASASKVGGWNPYPDLAKDYDSVVIEAQKRRASRQSMSYDAISKL
QSSLHVVDPQKEGPLLRVYMCRVSAERFYSNGVVTASKSKKKQQGPTYNCRAGLLLGTIQ
KERVDQRPKKARTSLSSQTADSEYCKVAKIQAIWEPPGQKPSSDKLYDAKLAASLLDSSK
KRMKTIANHLGLVPVGWIFSYQDDRLKEENSFSTEGPQALFGLDVTIGAKLKIQNMEDRR
AIKEDDVDLQGDRFVTLAMDATSGATEAFQLSDVCVQMVHEDMFQKLTETDGTSEITNPP
IVPVRHAVLVDGRETKLLDSVLCLVNTALLSHVGSYSGHVTGSSVSVKKINGSLTNKTKK
LLLKALESSDANDFLQEICDFNILMALDQLLSQSQVKSSNNDDHEADMEILCGLVRKWAR
GQKKGTAIDPKFKRKLQTYLST
>comp2796_c0_seq1; 326 101 559 LEN=619; translated
MNAAERTYIMIKPDGVQRGIVGNIISRFETKGYKLSAMKTKQATKELLDEHYKDLVDKPF
FPGLRDYMMSGPVVCMVWEGKEAVVTGRKMLGETNPLASSPGTIRGDFCIEVGRNICHGS
DCVENAEKEIALWFDDDEVIDWTSHSKDWIYE
>comp2797_c0_seq1; 355 134 1081 LEN=1373; translated
MSRPELTGHASNFYNEKEARKYNSSSRMINVQREITLRAIELLLLPPDETSLVLDIGCGS
GLSGEVLEEQGHVWVGCDVSRDMLQVAHERMEDSYKVKDDGDSGMSDNSDDEDDDGLPST
GDLMHHDMGTGLPFRPATFDACVSISALQWLCYSNSASQIPKRRLVRFFSSLYSILKKSA
RAVLQFYPETAEQAVLISECATAVGFGGGIVVDYPNSAKAKKHYLVLSCDRTCRKQLPTG
MTGDERVENSNRHVQVSSSSSGRTQRERGKEKPIRKKKGVKTKEWILHKKETQRKKGKNV
RPDTKYTARRRRGKF
>comp2798_c0_seq1; 353 1 1233 LEN=1407; translated
RSILLDELRLVEMETGNSTHWNDTEVVGDPFGLPDDEESQSQPREAPDYESSASNRPASG
ESTDVSIPSDDPRKSIVHKRQNLTMATDSAKSLQSSIDFAELQLQEVIGGGGFGQVWKAT
WRGTPVAVKVLTGSAQNTHIAKAILEEFKAEINLLKGMRHPNICLYMGACMTPPNRAIIT
ELAANGSVWDSLRLPLMPPYTASDGTPNGSWPLSLYLPSHHGVPPTSSGSVLRATTEISA
PIPPRGTWPWELVKRVSCGAARGMAYLHSGHPPVLHRDLKSANLLLDESYTTKVCDFGLS
RLKAQARSMTANCGTVQWMAPEVLANRSYDERADVYSFGIIVWELLSRECPYEGMTAIQC
ALAVLNRDKRPEIPKWCPPGLHALIKSCTKKDPSERPSFAEIIAALDAMG
>comp2799_c0_seq1; 354 206 1651 LEN=2196; translated
MIGAALLAVSFALTLPFVRASNDWMWIETHHYTGERRGLPSNDSYLSRRRLEKETSIGRD
KTISDTSYDFPFQSADIGSSSSSRDIGSKQHQRQAFPSETESGRRNFFFPIDFRRQKGRL

NVGFSFGDDDGARKRGQRNRPPRRKSLTNGIKDRSFPPMSIVSGNSTTIPPAMASGGDPS
EGTTATTALVEKTNHALEKRRATSATLQHGDAFRARSNYNDSNVELSLSLDPFSLASLVR
NLVASSVGAGSVYVGTLKLLGPMILAKQCLASVGYAFYDYYSGRYLPQKQQKRVRCVQEY
ELIAAGRAALRSLLQFLCMGLVGQVVRLVLDPHHRSCTLGPPWPCHLWYGVVWLASVCAA
GCACEEWGFDFLAKKTGNRFAPSSLSVLSTATDAGPRADFFFSSKHHVREDCLRGRRKRN
VVGPQRRFVKGPSRDPENWLKSIMCILPDREDPIRPRQSGTNSGICRKVSGDVDVLLFPS
VW
>comp2800_c0_seq1; 558 204 1547 LEN=1894; translated
MASAGDTPETLPEFANAEDYLKYLGTVSQLPLGFATGTADGTFISVEAPGLGNLKIRATV
IQLTEGPTENWAACFTSNKFPGAPIKVGRKRLEAGGPIEALVINNKVSNVCSGGDGEADA
EMVCEAVAKALNLESKDLVLPSSTGVIGWRLPAKELATEVVPEAIKNMQTDSVLNAAEAI
MTTDRFPKVRSITLSNGARVVGIAKGAGMIEPNMATMLSYILTDATVPKETLQSMLGSVV
NDTFNCMSVDGDESTSDTVVCLASNKISEDIDLKEFESALKTVSEGLAADIVRNGEGTSH
VMRVVISNFPGTKKDARFLGRHIVNSPLFKCAVAGNDPNTGRLAGAIGSYMGKYMPDADT
TKMSLTLGGRSIFTKGKFVLEGDKVEKELSNHMKNAEYGDDTHFPPHQRFVEIGVDFGIE
NTDFEDVSVLGSDLTKEYVVINADYRS
>comp2800_c0_seq2; 159 204 467 LEN=595; translated
MASAGDTPETLPEFANAEDYLKYLGTVSQLPLGFATGTADGTFISVEAPGLGNLKIRATV
IQLTEGPTENWAACFTSNKVGLKHQYA
>comp2801_c0_seq1; 480 213 1673 LEN=1868; translated
MNVVKSRSSNLPRASTSPDKKFSKAVTNINLYQEIPTTEVSLDDFEEFALDRLKVLKKIE
QLKIRHVHPDNYKKHLDPLLSKLNDPTKDTISHFVLRLAYCRSEDLRRWILAQEVALLKH
RLHHSRVGNAIPNALRSVLPNAHVMTRSELEPLLPKILAATPSLNKNPLSVASGGEASAI
GQAIYRVPFAQALDLVQHRQVFVKAGKAYIPESKLLNLVTARFRTNLSRQLALLSAVPST
PSLQRTTGFLKNVATVHTQQEDYSRTNKDGPNMNAHNVPSHLKHMPLCMAQLQMALGTEK
HLKHWGRLQYGLFLKGAGLSLDDALTYFERMFVGKDFNKEYSYNFRHMYGKEGKRQSYPP
YTCSKIINSNAPNANEHHGCPYKHSSVQDLSRMLSRLGVAPSQQKSIVAQKQSHNYQLAC
VEHFKAVHPGVTPSESNFGNHPNAWFASSVKAAAVSGGGTTSSEPGSPSSPGDATMKDAD
PVSVSP
>comp2802_c0_seq1; 353 111 2171 LEN=2759; translated
MSMANAKEASSTGTSDDPIALLDDDNDDDDDGTVASIGAVRTNTNERKRSASPGRECSSL
ESVPRKRSVRGNRAVSPQERAILPHDDGCMLNPRHPIKIFATDRDKTTRRNLRRRLKKGE
VDSDKDGYHYTNTEDAYRTHWSVFHCWTFREMLGFDRFSGIRYQTQADLIGSDTFPGAKT
NKISHGNPVGIDWIFITTYILDVDYLLHELPELLDIPLVVIVYQYKDFSSREEAWIKEVS
SKKEHNLALVERNPRDPPRTESNPLKVRMPYGCHHTKMTLVGYSSGRLRVHIHTSNLRQG
DVHDKCQGAFLQDFLPKTEDQLCNFVTSDFEESLVTYLESYHFVLPWVWKKAEIGRGEGS
EATTLVSNIQTYDFSSSVGVLVPSVPGYHNASLDARPSHRGNREIYGYLKVHKAIREHCN
CDSSSAGPIVCQFSSMGALWEPYLDKVAAAWNAGAANCGSKTVATSRPPKKGELAASWLR
FVWPTTLEVKESVEGPMGGNSVPGTAKNLHKTFVRSLLHKWRFLSSNNGGSTTGMYGENL
DSTDSNQRRWKEEDPLDKGCHVPHIKSYYQIHELGDRYGRDSMLWFVLSSHNMSVAAWGQ
IQNRQKDLGRKVLTVQHWELGIFLAPSTLGVRSMGPTLEGSRHRQAYNCKTANDDPINEA
TRAVIPLPFKFRPIKYQDSDKPWVVG
>comp2803_c0_seq1; 724 1 2003 LEN=2106; translated
XAPSAPSAPPTPNHRTTPTKEYFHGLFPLTTDCEHNHDVDKCKQSVREVFAAERVASIVP
TKAPSAFTRVFLSLEYAPGAERSDVARNRGLGLGYSFYSTTRHLAGKKRIEKEFEYEPLF
QSNVYDTSLEANAETEFVQILPPEGHVETVVVDGEEYLRLVDPKRTLSELSERAFGDIAH
LLRPKHLGQLRSILEDPEASKNDKFVALELLRNADIASGRILPGCQDTGTAIVSAKRGHR
VLTDGDDEEYLSLGAYQAYKNLNLRYSQVAPLDMFKEKNTGTNLPAQIDVAAAKGDEYKF
LFMAKGGGSANKTFLYQQTKALLNPGSLEEFLDNTIKSIGTSACPPYHLAIVVGGLSAEL
NLKAVKMASTKFYDDLPTTGSEGGRAYRDVEWEKHILKMTRNMGIGAQFGGKYFCHDVRV
IRLPRHGASCPVGIGVSCSADRQILAKINKDGVFVERLETDVAKYIPDVTESDLANDDDN
DTEAVSIDLDSMSMTELRETLAKYPTKTRLSLTGTIVVARDIAHAKMLTKIESGEGLPDY
AKNNIIYYAGPAKTPEGYASGSFGPTTAGRMDAYVKKFMENGGSFVTLAKGNRSKAVTNA
CKKYGGFYLGSIGGPAAILAKNCIKNVEVLDNEEDGMEAVWKIDVVDFPAFIVVDDKGDD
FFKEWLG
>comp2804_c0_seq1; 165 190 1288 LEN=1714; translated
MFAVLPLCVINCLLLALVSYSREKFRLGFSPNGHGLLTLLVSFLVVSKVNLAYDRYRAVR
EYAGRGSTLLRELIQMAMAISIAHSNNNIENSEREKELRQWRLECVEKVKELLNSSVCVL
QDRSLAKHFAYNKPVTKSGPSILTSSSSYDSRTVDLNGGSRDEDAXDNASGSPAADPITA
TASLLHPGHGVRASRTGPPDEQAPGIRNMLHLAAHAGIDSASFSAGANGEGLSVDMDILD

AXLLLEGPFSDMWSAQTFLFFLTYGFIGLELVSIKLSDPFGSGRDDVPISGIRDAAIQGI
DNDLREIAGMQATISERRLKFSRQTIRQRRHQSHAQETEAGDCQDHHHDDQNYGYHAMAG
ADNDIA
>comp2805_c0_seq1; 393 234 1334 LEN=1480; translated
MARWHSTSGRRTTSLFESVANGYYRNTNSDEIPLEKLLQKASGIVRDYSRKNRNSDSDSP
AVFSLEALGTQAVYENTLFFPSFYNAGVGANKKSSPSSSTTGMTERGFSNWILPGKIMVG
QYPGRVPEKNTPTETEVRAHLGEVLEASRRSDATTNGGVCFVSLQSELPPQDDYDSWKAS
GGEIYLEGDARSEWPNPFSHYAPTVEWVRSRMETDGDGDSAVEVQYLHHPIRDLSVPSNS
KHLRLLLWKILEVLDNRESDGSNSDSMVYIHCWGGRGRAGLTACCLLALLHFASEGGTDG
GGNHDDNLSAIFDIVQTGYDSRLGSEHMPLALSRSPQTDSQRAFVAGFYGEVRAAAAEAG
KNGNAK
>comp2806_c0_seq1; 85 212 439 LEN=816; translated
MLEEGARSHLDASEHYREAREFARWSAEDRASRRADLPPLTPEQREQMRNYLRIMQEYHA
FNPDSDLDPATGKPR
>comp2806_c0_seq2; 143 360 704 LEN=1081; translated
MFQNFGRLSANQRVWGAIGGVIVVGTAFKITYFRYCRDAMLEEGARSHLDASEHYREARE
FARWSAEDRASRRADLPPLTPEQREQMRNYLRIMQEYHAFNPDSDLDPATGKPR
>comp2807_c0_seq1; 321 70 780 LEN=1754; translated
MATTMGEACDSLLIGSFYFIGINAWPTVLDDLTNSTTQRRGEVLMFGFQDIPGGLDLYLT
DRAWTDEGFVPNFVGKEGILKFTTPPQGVPSGVAFGIGNNTQIYKYAEEWMDEDVDGTQA
EVGGNETHYFDLGLDGDQVFLYCVGGDGKDRPIAGISYNGDFAYQDTYGTNKSSAPGSFQ
EHDTESGANRTTLLVMPTVATEDETSKIFLDWQYVGPMSSLNFFELQDAIRDTENNW
>comp2808_c0_seq1; 879 391 3246 LEN=3502; translated
MSPAMTDADEILSYATGTSSLSSSGKFATHDVYDEHLEDIHANLSKRESIRTSLISNRNN
RVSNGKQDPYDELEQDIRETVRSSTKQSRSNLPSHNGRRNSNNSNKEQRDKKTKSSDQKS
SHSGRTRSKMRSSGNESCASTVASSMSMLSKDDSENKDTGHRRNRKMERTSSGRRIRSNS
DINRLLQYDETGDYSTEKSRRGSSTRRGGKNIRSSSRSGHRSLAGSVRNRTQSCERRVGR
STSESVPKVVLDDSNRDDLDKPRRRSSSQQHFQSNLKNRRRSDERRNDDRYGKSSSSLRS
IRSIQRRQSDQENKEKRRERGKSLDALDKLQRDSFEKAWADDRPILGQRSRSEREISNKS
FSRIEGVKGDCSTRSGTFPADKGKKTKLEKIHELQGKCDLYKAELRKLTDDRQRYRRKLD
NSREEVISLKKIVDEYEGDITALQTKLADVQDTLQMTRSAQRSELTELSDVAKELARVNI
DYAKSVEEARSTKTELEGVKQKLADREKKIAELEEELRKSVENVKQFEADVLYADEHIDK
LESEVKRLEIDLTTYKDAAERDSVLNSDGNQNGDHLRRAKEEAEKRKFEEQENRIKERNR
ILEEQKQEFEAERKRCYEEQRKKERDFEEQRAREDDRLKIAEADQQRKEEKMNKILKTLE
DENAALKGRLKGEQLDSTIKLQKKDNAIGELQKEVARLTKEQKHHDSAPNNSPALLQEIE
RLKAAAAVKEVDFEDAHIKNMELTNEVSDLQIASKEMVALLSSLENEVVEQKLEVDNQKK
KTMEWQKKSGEWSDKAFKWKEMSEYWEKKAKESNNHDSCSSFDENVVQTDPQALFLAAAV
EKKVMNTSTANGNGSWHLRRLFGQAPENGDEAHDIVEKLESENSMKQTEINKLKSELVKL
QTNFKGEAYSRSQEYEKLRKEKNDLEVTNANLLKELELARKLNQTISDYVD
>comp2809_c0_seq1; 260 36 322 LEN=322; translated
MITSFTIAVTAVALTEWKKLEGNGKNTTHHQKDKVKDKDTGKRYGSMQRKASKAKKKASR
PVAVASSYFVAGDILFDPTKTRASTANNNGNGNGNG
>comp2809_c0_seq2; 147 1 370 LEN=370; translated
XSHRIASHPIPSHQDEPRTLGRGEFWKQTDKQTGGTVGAVALTEWKKLEGNGKNTTHHQK
DKVKDKDTGKRYGSMQRKASKAKKKASRPVAVASSYFVAGDILFDPTKTRASTANNNGNG
NGNG
>comp2810_c0_seq1; 595 426 2948 LEN=4823; translated
MKTHPPFGSAPGPRAVGFPPAIAFRRQDALHDQQRRFFSSPPPGAGQSLGSIFGTDPSGG
SYLEDFTIDLTEIARGGKKQGGADSPGGNGSGGNTKMDPIIGRHEEIGRCLQILARRTKS
NPILIGQAGVGKTAVAEGLAQRIVGGQVPESMKHKRVLSLDVTGLVSGAYMRGQFEERLR
GILKEVTESDGSVILFVDEIHLIGTAGKGEGSVDMGNMLKPALARGDLQLIGATTLDEYR
ILEKDPALARRFQAVYVDEPSVEDTLSILRGLKPHYELHHAGIRIKDEALVAAAEMSDRY
ITDRFQPDKSIDLVDEACSQLRLEQESKPEVLWKVERDLMTKQIEMSALQGEDDKNSIKR
RETVASEVEELKQRAQKLTEMWQEERNSLTRVKDLKQELAEAQQEMEIARSKGDFARAGE
LLHSKIPHLKHELEETEKEEDDASATQGSRLLNDSVDASAIAAIVARSTGIPVSKITGTE
SRKLLDMEDKLRQRVIGQDHALQAVSNCVRLSRTGLQARDRTLGNFLFAGPSGVGKTELC
KALAEFLFDDQNAMTRIDMSEFGEKHTVSRLIGSPPGYVGYEKGGVLTEAVRRRPYQILL
LDEFEKSHPDVWNLLLQLFDDGRLTDSHGREVDFSNVIVVMTSNMGAHVISELPSHLKGN
EPEVKESIMNVVRNTLSPELLNRIDETVIFNRLQREQMGQIAGIHLEDIATRLEENHGMA
LKVSKSAQACIEEHGFDIRYGARPLKRAITKDILNPLSKAILGGEIRDADLVRVVTRGEA

INLQNEERHLGWVTGADPRSTDRNDVVLLKNHEAYTDDDISGSGKVSGEDDDYYSDEWRA
>comp2811_c0_seq1; 230 126 665 LEN=1172; translated
MASIWARYNKLLDTQPLLTKCLTSLTGFTVGDILAQKFIEPSDKPYDIMRTIRLGSFGFL
FHGSTGHYFYGMLDSKMPGTKPVTVASKVLIDQTIWNPIFGCCFFGFLNFAEGKTWDDYT
KKLNSDLKTAVMGSWAVWVPAHTINFAFIPPSQRLLYINTIQIGYNIFLSFLGNKKADE
>comp2812_c0_seq1; 226 101 787 LEN=877; translated
MLGTKRHLILEVVVVVVALVWNHSLEATRFGQGVDAFLATAVFPKFPRGCTAPRQPQFPS
MGPLFSLGDSRAPPDTRETNRAGNGSDAVEKKNRRKRSLYSFSEARKIARGHGFSTREEF
IEYDCAGAYQVPKNADEVWSEDWTSWEDFLGLRLAEFEQARDVARSRVGPNSTTSWKVST
EEEYLELFRSKQIDDDDIASRLPYRPDLFYKNKGWTSWEDFLSPGSDE
>comp2813_c0_seq1; 180 87 717 LEN=717; translated
MGKKRNNRRRKQNYQSGSSNTPRLGSKGCSLCYDSSIPENLNAHVNDSQTCADIHLQLAM
LRYDNAMCSVGQEKYQELCCTKVDTGNGGSTLGFMLGAIVAGFFLKNMFTLRKRRVEEMK
SMDGGMELPRTVSLGARSRSNSSRENQDIPSTWYVRMDDSKSKSSKSSSRSRGKPMAYIT
NSKRLEKKEARRDPKHYFLPDDLGAGVDQE
>comp2814_c0_seq1; 413 344 1789 LEN=1805; translated
MTTSVCYPDQDCCIDTDESCGMEGTHFSTISSVFVPRLKQLLRKSVNSLSPSTPFEENDE
TAIATLIFESMYTPGRVYHSMQHVFNISDNCDIETDPMLLLSTLFHDVIYYTVDKSFLKS
QLELLDNVLAFEDAGDEPKRLKEPLLLSPDAQEDPLLDMIFRLFDFKPGTPLPSFGSNEF
LSAVIGVRVLSKWLSVPQVTQLTAAIEATVPFRPPSPDGKTAMDRLYDRLITVAPNQSED
WFEETIHLSALMANCDLCSFDSSNFDFFLDSNWSLVPEFRPALLKENCPLQEYYDEFLAM
EGRTKFLISAIPNIFQVFRNVPSESELADKRASTRANLVLGDDYAQVRRLQLMVLMEFVT
MVGEIPESIPGRQFLSLELKKSSCSGEESPVRKRLDPGRRQGFPWDPARCSLGAFLYDHL
GKEGIDTAVEVGKTQKPGSGHLLDHLPNDVVAVVAASLEEILPDHSEALKNISDKLGFSQ
T
>comp2815_c0_seq1; 336 1 1594 LEN=2098; translated
XLQPTYHPPTVPSSTKQRKERAIMGSNGSSNRRKKKRGINIERVLFPLVILMAIGLLFVN
ISVMVEHSNKGKRRGSDEADSSIRKSRGMSESKEKQKRGNGSDSHSKDVQKDRLPPPETK
PHDEPKPSKQETPQTKAAPGENSGGDIDEHMLRILRHVGIHNSSQLTDQELSALPTWSQV
VEKFGNKGPVMLGRETCSEYRSSVDRKHRKLGVAGPFSSGTHYLHTILSNNCKSLEDKGR
VMFQVPWGKHQSANFRLVHNVAVSSTLKNDHKKNETTGKLDHPPLDPKHLKENKSVLPIV
VVRDPYSWWQSMCRVRYSAHWFHVVPDHCPNFVPNEVEREWFNKTKKEIRKHYNNDPWKV
DNVVDKANFTLDKTVVPLWVRYHSENRQHKSLAHMWADWYNDYYEANFPRLMIRLEDLVF
YPHETLKMVCECANDDDDGDEQKASFEYVGDENLELSLESAIKGKGDKVDNIHGKDRTGL
LGAMKKHAGKYSDEHRLTGMTPSDMEFAEAVLKESDVFKDLGYQRPVRPSA
>comp2816_c0_seq1; 335 428 1477 LEN=1579; minus strand; translated
MRLLFVALSVDVVRAKGLLSGTDGILEHEFVGVVLDTLLGGVVELFRPRQYLVLLFPRWH
VDLAQVPADAAQRPDLKGARPRQKEHPRKGLVQRGGRHQRAVVLQHQDLVGRSQAFYQPL
PLVDGIRRALEGVVGQAALGRQSRFLADGQQAEPRLTHGRRIQGVYVPHGVGVRPAPVDR
RVDHKARLEDPPASPVAVHHVPVQVDLEDRRGPHFVEARTKFVDEKVRAIQDIVRVVNLE
RDVVENVLVPALHEGYPVTSRQLAALLPLDFFLGNAASRIAFFCRVLRNRRELPSSFWNN
RTPDLRVVRAMLSSYGGWTKPPQVANGCSESVHVYDDDDAISDFYRVPW
>comp2817_c0_seq1; 281 294 1094 LEN=1516; translated
MTAATNRDNSVKVKKASSTTKVAGPKTASSGIMHQVIMPKTLPTAPPTSQPSKPVSLPAN
RTVATVTTTHRSNYAKKTNGRVSKRNGVWYLGGNHVVKLLTSVDGDLDHSILSKLAAVPT
VVGELALSVESLVSDDITVGGFENDLLAKIASNTLTAKREIMAAKAAAAPKATPICTTPT
PLGSTSMGAAAAASTHKKAKGAPSSGSEQVPNPLLANYQPVPNPLAIRITPPASPHTSAA
TEQSSAGSPKTTKTLQTKGLPLIHAR
>comp2818_c0_seq1; 125 402 829 LEN=885; translated
MAEXHDLSAELAQREASLPGSQKETKGFLPVPLRSRRPRNKDPALSPLQAAGGGPPAGVE
EPPGSHGDAPELDEEGPPAPPGRSDRADRTGSTPVARKVTAQAGTGSRAKAKRKRQILEA
YPRIQADGGTRRSNRCLLVPRF
>comp2819_c0_seq1; 886 1 2587 LEN=2842; translated
XSPGPSPAPSPFPTLAPNSGQPTPAPSPGPSPAPSAEPSSPPSPAPTGACSISSCLNCTA
IVEGIELDCADIPEEKQPVCFCPECVRELQFTYTGRSCPENQLSCTDNSPSPFVAGYRIT
DANDPTLVLATGQAQQQDTIVVTSPTGCIPDTLSVTISVPTGAVTQTFLIDARCDGGRGL
TLLGNYGAFESVGYTCDATDVHNCVQTVTYGNKVCNDGPEPQKIYEFDLTTKETNTAFDE
SCDLLQDGSIPMLGPGECYYENKPFEVNRCVQSNYCVDVVANATDVLTGIPPSCPASDEI
KFGWPGMPLPPPTPFPTVMPSPPPSPAPTSACVIDVKIDGCPAPNISLDNNCEGRPQTIT
FKYTGGDCEQSRNFQSRQKFMCTPAEGPSAPTTKGTVNYIEAVPRGGGDLYYAGNVAVGD

NYILNENKQFDKLSADMTITIFESQGGPVLQTVDVHLSCSQALFLFDKFGANQVVRWVET
DGRVVEAQAFVTTDDFVLNLETSEVTGSGGPITLTEMSVLTNAQDLPIDYTPQVAGKQLI
PGQPINLPGFQLDIDLTQRTEYTFFTTIIGQTSDGTPCNGSDFHSCVVGFNLSPSFPTDV
PTPRPTITPFPTGSPNSTACEIASNIECTVTRPANPKLPLSCSDLSGSVSATCSSELLAA
FLEYDGSLGPRVFVNPTCGKNEFFGQFVNANEVVELNTRAADFCDGAVTIGIYDADPLQG
GSQLGSADVELSCPGPWTIGNTIAPGLTLAYYVSTVNNGLTFTYNFLDAEVQIDYVGINA
GAGPLTVSGGDYSGTSPFTSGAITGGSTITGRGRQVLATETQTISLPGTGGTALDFSMSV
DGATANQFALPCTTASNYRLNL
>comp2820_c0_seq1; 463 128 1036 LEN=1279; translated
MTSGVPVGDSPFGPTEMTPEEQRRHMSGLCCFNWLGLTSLAAFICGVYSFAYCNFLSRYV
TLSDNYSPGDFAAACSDLGYEDGDSFDAQTCQSIMEDHAIGFSYWQATIPVDQRVCFSYT
QLTPWGYASPDFDSSFFTSRTFAIIGYVFGGMAWFTLACSSCCMIDQQRLKGIGCYFIIA
SFSQGLSLVMFRSNICEKGFFEPYFVPPSQRDNETYLESFNSVVADVECELSFGSKMAIS
ASVLYFICALMVPFSVVPLYEQRYYRRDFDGDQQQRGQDEASPSPLPEGEGPVVRGHVTG
KV
>comp2821_c0_seq1; 327 451 1662 LEN=2405; translated
MTSLNNDGIMRRGSYNQGLSNSKIESARRFLYDEDESFGSVNFNKSVRRNTNLSGFPTVT
SVPYGNENTRGRDYSSKRCLCAIVLIGILAVCVWTSFVVASGRESPEHVDEKRIQEFQSK
IVELGLSLEGDIETTGTPQYHALQWLANDDGAKLDVADAHAMQRYALAVLFYSTAGTEDH
IDPNANGQWVNQTNWMTGAGFCSWHGVKCIADSTEHRNNEDGDVLALDLSQNGLDGVLPS
ELSAFSLLQRLDFSHNGLTGNLPKNFETLTGLRSLILRNNKLSGTIPKGFGIKLTNMVWL
NLGENQLHGPIPSEMEHMVDLRSLGLDRNQLSGKIPDLEDLSKLKKLYLKENNLEGPFPG
SLSKLTSLVEINLSGNHLTGLLPSEIEKLTKLGELVHDQAFSE
>comp2821_c0_seq2; 365 451 2208 LEN=2278; translated
MTSLNNDGIMRRGSYNQGLSNSKIESARRFLYDEDESFGSVNFNKSVRRNTNLSGFPTVT
SVPYGNENTRGRDYSSKRCLCAIVLIGILAVCVWTSFVVASGRESPEHVDEKRIQEFQSK
IVELGLSLEGDIETTGTPQYHALQWLANDDGAKLDVADAHAMQRYALAVLFYSTAGTEDH
IDPNANGQWVNQTNWMTGAGFCSWHGVKCIADSTEHRNNEDGDVLALDLSQNGLDGVLPS
ELSAFSLLQRLDFSHNGLTGNLPKNFETLTGLRSLILRNNKLSGTIPKGFGIKLTNMVWL
NLGENQLHGPIPSEMEHMVDLRSLGLDRNQLSGKIPDLEDLSKLKKLYLKENNLEGPFPG
SLSKLTSLVEINLSGNHLTGLLPSEIEKLTKLELLILNKMNLRGTIPGEIFKKVTRLTEL
NLSNNHFGGQIPTSIGHLKDIQGFFLGENKLTGTIPRQVGLMVDLKRFEIDGNNIGGTIP
NLISALNSLEGFKLSRNSITGTIPPDIGNLHRLVELEFESNKLEGKVPPEIGNLVNLKQA
RLYDNNLTGDVPKEVCALTKDEDLAYLGVDCASDKVNCDCCTKCF
>comp2822_c0_seq1; 1075 150 3365 LEN=3600; translated
MTWFRANRALWACPNTLPPDTARTISTTTRRKKNVEHLIEEYLGQRTEDDGCSGEESSDV
LEKEEWMHNDDYPLEEKNDTPAATHTTDEDNYGYFEISLTTNAIQDMVDEGTIWVAEQAT
KNCECENGEGLLSSAAPLAAGPLVLWLRDLRPQTAPQPDPRDDDAQMDHKRIGEIESGMF
SASISDGTNWCSGAIWQTKEGQLEDLSECVLSISGFVLGVHCAASLECQPLSPDTARGDR
KCDCDDNCTVASGGKRKPKPVSCCSLNSLDGNKRQTSPYIYITRYEVLYEKRSKNQSHDD
DDSINDIQFTEQIRSQTCELQKEGILAGLLASRKLGTDVLIGEDKGKLICHSYHNRHDHL
CQEAAMLKKINENALEVRNFMEQHFAITTLKSRKEDASLMASSSFSLMLGSQKCFQNYYS
ALNEFDQGQRENLFTSLNDTTEESFVDGSVDPEETASDTRREQKRIQNKETMEYALQCTE
ERLSPELLLRWHAWLLGDGLEQEAGAFRSEKISVRVGAFCASMEDHWLPRVRQDPTNAVR
IATFAAAVKLGFLDMRPFEAGNQLLARILFCWSLRRAGLPFSVITYSNEKQKLDYYSLIE
STRRNLSLVPLGLVDADIMTRILLSTGGLAPLVGFLVNCIASSAIDLCRLVEKKNSMASE
ETNARLVRLAREKAAKGTCIICFDENPNIVTMCCGKPVHFNCMAEWLTSNSSCPQCRSDI
PSLSTRRFSQPARENPVVEYGQDRSYLRNFLSEEFLYAHSSSSSSSSSSSSSSSSSSSSS
TESAFFYSGLDYGVNDTSSANSPVENESTNSIRNLLPYRIDSPVPSQIQTGLNYVTSDED
ESQHNYVTDDTDDSADISQSGNNDDGDGQYSFDSNHSANYSTHFELTRGNSADNDSGWES
ESTALSFARSGRLELCDDSAANSEPELSDDGTENSEHYSCSDDEQSSLGDQEHLHRFRMS
FLDPELLGNAQALESRRSRRTYLHRESSDSMVGQERLRFLRDGEGNGSLEFALPADPRYL
RVGDGDSSHDANQLVSSIIEGSLTNAFADMRCDVTPAQPNDPVPDNDSVTI
>comp2824_c0_seq1; 213 247 945 LEN=1438; translated
MMLRIGISLLAFLAAWAPLSSALSCPPQKTSAANHQSSSLASTRRSFVGGAAAAAAGLVT
SGPSFASEPPSSTGLPSKGARAPSFELPNSRGTGLVTLDGLVKNSKWTVLYFYPGAFTSG
CTLEARGFQKDLEEYRKLNAQIVGVSVDPVEKNAQFCTEEKLDFFMLSDKGGIVSKKYGS
ALSIPGFGTFSNRQTYIIDPSGELRWIFTDVESKIARHSSEVLEKLAELERA
>comp2825_c0_seq1; 371 203 1609 LEN=1929; translated
MRLVKTISRKSLLAFGYPKLETYHPELSVYDVGAVKVRMSPELPACQIFYPVEKDSEKDH

VDDVVPYYRPEAVEALVDYLNGFGDGIMQMLNEKSHPLQNTYAIAPLMVDSNNGDSMKFP
LVLFSHGLSGNMEMYSQICAQLASTGCVVAALEHEDGSASYSTKLLKDGTVEAIPYKQPL
PNAEVPYSRKKVLDFRTPMLEQRVNELRRIYNYFRDEKTSVDETITTNCYDSNSRLVRKI
LAIADPTKLHLVGHSFGGATQLLAAQKWIMEGHDEKVQTEATTEAHPMSMRLSDSENPAT
TSTMTIDTSTSSSSPPIPQSVMVFDAWNFSLSDEVLNRGISIPSSPTGSTTAAPTIVSVL
SEQWERTNPEKEQTLQFLRNCPPSTTYSYLARDSVHQSISDTECYLPSIVARKLENLGPS
EERHQTVRAVVEEFVRRTKSSGNDCSSKKRGSDDNTLIELPLRNIHIG
>comp2826_c0_seq1; 428 99 1355 LEN=2092; translated
MFGNKKKRQSAPGDVTGTRLNAKRRRLENSPSRTTAPGDSESPSVRLTDKEVSHAMLLRA
AEVGLDSAINEAKMEKSRSKMVITPQSVIQTKKISSVSDDDSKKAGRTGTTVTERKPDQA
IQQLDPKLAATNICDFRNDVDLSPDDDDQSCSSSSSSSSSSSSSSEESSGLLSYRSNSSR
EDGEQKLPPLNVGEEYEGIQRLEKKLSLRKERLKKLCAGRPAAYARYLEANEEAAGDIRI
AEYKESYPDEDKPLSCGLPRSKLIRTCSSRLGLLQFEAMHTLPCFYTILVVMAVHSTFCA
CTETVSNILYGSIFENIVSKNVYNAFVIGFGLALLRINGHCFWYTGTSNDYTLIQMEMGN
RLKLKKFDARVLKKIKESVACSACNMFGYYLICVGIGHFHFRHFDNLMDQFDLWLDSIW
>comp2827_c0_seq1; 324 1 787 LEN=1350; translated
NEFYLHNPQKTIFAQAKRRKINGPEHQKYFFNDDNNLQETLSPLLTPRDHMVDSSGFSTV
TSSSHRVATTSISVATEASNDFGSAMPEDVDPHDIIGVKPELLAIGINATEFLEWIGTKE
DLMEKLQADFKSYNSERVEEEADKFMMDAENVNMYIKYLKDRKANPAKYANEALEAELSL
SNPKTAATYAAWLIGGVSFGVLREKYIDPKLASGEWKGITLDLPFLHKADAAAEAASTLT
SKANIIVEGIANINDSVDLLG
>comp2828_c0_seq1; 303 44 721 LEN=1265; translated
MSFASGARKTLHRRSTLLQANVSVLRQKLGLITALRKTLGPNQANKGYAQSCPIVGASIG
QHFRHSMDHMERVADIIEVQKKENNGDGGFSREIHYDVRVRGGSDEYDMDEAEARIRRVI
QPFRDGIDAIVSFCPTDDGGDDTEVCNDSNSVLACFLLSGDPKEDEFMLSSTVERELGFV
AHHAIHHLAMVKIITMHTLKLLPPDSIPEGFGMAPSTIVYDHSQR
>comp2829_c0_seq1; 398 1 822 LEN=863; translated
QKYRNMKLSLSVCLAATAAAFCQGFTPTASSTRQSTAIRNTPAMGPWTMMPDEPTPEVDP
RRRNPDEQPPSNPVRMPVIDTDNGQMDVLSRLLKDRILLLGQGVDDEVANVLVAQLLYLA
NEDPEKDITLYINSPGGSVSAGMAIYDTMQYVPCDVSTVCFGMAASMGAFLLGAGAKGKR
RALPNSRIMIHQPLGGAQGQAADIEIQAKEILFIREVLNTYIAEYCDQPKDKIEEDCDRD
FFMTPEEAVEYGIIDEVVKTKTSHISKPAMPAL
>comp2830_c0_seq1; 333 437 1549 LEN=1844; translated
MAITFSALDPAWAAGSGRAGGSFGRSDRFRHPPVSKVHRPSGDNRRIQSRQHRPAPIRVV
YPPGRSNTNRIRNHSYRTYHRPTGEKVTGEDVAVVTNTDGTTNFVRKVNPHPFHDSRASD
ITVAVLSTALVTRGVLKRRSDREKFGDDDLSHPLGPGLSVWSLTACLNVPDIGAPTSVVQ
KIQRLAETTSTVTREGLQSLIAETALELARQVGKGTVASVESQYHHYRSSDQSVVRAERH
YNRISARERSKFERESWSNYNGKIRLDDVSERDALSPSSAGDSIASLALVQIHLVIEGNT
MEPFGTRQVETRKSLREALVQLSGDVTAVEDCVVAGEVLWAPQLQEQQEVMTEEYVYANY
PRLWPLDYTK
>comp2831_c0_seq1; 702 254 2596 LEN=3098; translated
MSAAFLVESRGTPKRMSAEFSREVEEDGLKFSVENAAEDIVSTLFCTYEPPTSKKMQYKA
AKAFRKHPERYKNRPHVPKSILKFKVPTPKILRKKHQKLEPEPAQDEGRDDIDGAVLSTT
TDGGKKSVRWRDQQKPYEDREDRTDIENTVAGLASKYCGGAFNHGDDIADILSSPQGRAA
QTFFGGPETPGSVANMTSSLFGGTPAAANTNANSSSTGSVHTDTKIFYDDSGNPIREDDV
PEDEEQGSYFPSTPKDAAASVATENPASPQTATRPGGNEGQPNLFCSNIPFMDTVITGIG
IAGVMAASAAVKAGVPENYCNRVYTTKSQRSASQEQISEELRQAQETLGVYEPESYDKEP
DLSSGSGLKRQSTPRNVRRKGTPRHMDPEDELREDVEDNETVEDQSQGGNNNSNPYSSRR
SYVNSDITMGNTTVKHFGNGNASLFDDMEEDGAKSPSISGRSGRSQFASMVEELKHVQKE
RRLGSSVKSHDLKKQTTKEIDHLGVCVSSIKNDYQKSCKSPKHSVVTSSYLNSINKSPVG
SQFNGAGLPATPNQNRQLSDPDLFEKRTGSVASLAKQFESPRSNRLPITGRKSVDPSESR
FFDGKVDLLPPTPRRKSPETPTGYSMVGNKLKVHDWDTPSPHFNNKGTPSPRFSSNRYTV
KDMMSPKSGTTYNTEAEVREAPSSILDNVATQDRPNFGGQRPASLVTGPETVDDDTVEEK
KVSKKKSKKNKKGKSKEQPGRSKSFTEKLKKSVEKAQIRVKNATRKVEASRIPDKEQDTL
>comp2832_c0_seq1; 697 194 2851 LEN=3032; translated
MSAGKGATSPKVKGLIKRYSGAPIPQEAIASPSVYSSSTTAGSPAPKTALHDQLHVSVQD
EDFDTRITTPTRLFRKPSLLSRLSSTISDNGTTPSRQKQRKELKKLKKQQRRRDERRRKS
DSQYYATTGSVSNEKPTKALNGNQVVCSPTASVQTSNSTSRKSRSSILFLKRVLSSPPIL
RQRLRSSSHLYGNKFKERDTSDGSCCQDNSNNDNISSSSRSSVELNPKTNNDTEMPERIS
IVAKSVSPTASKNRRRRTELEEESVGKPADAMIRSVSCNATSIFHHSFSMESICGVSMGE

LHNSDMSNLVFEESNRKLMSATNYGSESRQGNLMNDTLGQQSNLDYSYCDSMGPISEDPT
VQESIECIFASQLEDGLNLWDDEHDGSDENCTTEIILQRHNPSFGTLNRPQNPSSSSSPR
GNLNELISPAGFQQSRMNRKDRKNSSRSLYANMSQNKRRYEQASLVYVGTFDPSCPSPTH
TISQDNVRNISTNDNSLDSATIYDTINPLPCRCATNFLPAIEPKDWPQAPIALRPTPGSG
TKVKAIRLCNSDEPLWVPGSHLTWSQSLAKRWGRKCEDQSPHFASCEECVMLPINNGNEA
PGESLVTDFESELFEGTLLLRLRHSDGTTPEPYDDSKGYFAGVNRRYQVCIRGRFKKTLS
FTELNNGFRLRRKFGKLPSKWVLKSALKVVSFFAPQLDIKLEGVDQPYSLTPLGSTPQCI
TIDDADVLYNEKNKEMSGCSGLSDGLNSINKNNPLDGTNEEPIEAHRSLIGAALKADTSL
QRAKMRKKAFDKLFVQKSPCPKTDPSKIYTFEFLQHMLNFQDFTVELGSVMGSLELGDIL
DGQPLQVMAEHGPSKIRLWSFDVWNECLWNRAKEHDKAVAIAASK
>comp2833_c0_seq1; 207 1 465 LEN=1065; translated
NNSNNNNKPKPKPKRKANRAGKRQDKVDKQSPKKTSGNKPGKRTPNHKQGVVGKRKRKNK
NKNKNNQSNNDNTNGLDRGGGESSSFLLGSGFSAVDIVYVQCLDWSKDIGWNDKWKDNDA
LASYVAACKSRAAYARTLAVAKNEGKHDRSGSKL
>comp2834_c0_seq1; 332 403 1736 LEN=2334; translated
MTGIQSSSSSVLILFVLGFASLDCTIVSSFVLPSTNTRSTIIRQTASVGTRSVSTSRVRS
NNLLLRMSEDDDDDDMSWLQGPPEGIPEMETIECDVAIIGGGPAGCTCALYTSRADLSTV
ILDKNEAVGALAITSHIANYPGVDRSMSGIELLRQMKKQAVEYGTRICDRAQVFMIEIQD
DGGDDENGSKSNPYNKIVYTPDYTVKARSLVLATGAMGRSMPYLPGEEKYLGQGVSYCAT
CDGAFYRGSEVAVFGSTGEAMEEAMFLTKFASTVHWITPTDVVGYLDRLDAGGFQNVEMN
SMFDEAILRELLSKPNVKHHNKARLREVEGDVSGVTGVNVQLIGSGEEFTIPVEGAFIYG
AGGGSKPITDFLSGSGVALDEAXGVVTEDTMESTSHKGIFAIGDIRNTPYKQVVVAASDG
CVAAMSIEKFLKGRKIIKVDWVHK
>comp2835_c0_seq1; 287 55 720 LEN=1398; translated
MTNTDNDTVTEKRENGVMKRIASISGANGEKQGDAFKLAPFNPSSDQIQEKATSMFDLGE
HDVLFDIGCGDGRLLCFAATKIPGLKCVGIEILEEFVSRARERVKECNLESRIDIRLEDA
TKLVETSTAEIGKGIKANDEGEKCLSDLTLLDDATALYLFILPKGIVKIMPILQSLKEKR
NDEGKRLKVLSYMFKIHDWEATKTDVSGKGGCPVYYYDFQP
>comp2836_c0_seq1; 214 163 1056 LEN=1503; translated
MIAQLFRSPVSSLSSRARLWSPSLALLLGVWMASMQFSPGWIGAGIGGPCSMDGAGLGVL
AFRPSPQQTTWGLRVRRPSGPCRATAGRFPGGFPVLAPAPGDRLRPGWQSPGSGRVRHPG
TADAYGHCRRRGLFDPLFGGRSSEEEDGGSGADTHGSDDARNRRKRSFSMKRIGGRQRRG
RRFPEEASKPLPPTTTDSGDKRRFPGGFLISALLALTLLKNLLLGSGGGDAGYYYYSYSS
SSSSVYETTIDPDGSRRTTETSRRVTSDLKSNIPGLTKDSIPGANQNLGIVIYDDGF
>comp2838_c0_seq1; 519 158 1960 LEN=2197; translated
MAESMEMVDQCQSVTPPEVLNTKYKPISDPSLLPKKRSQIPGACSNLVNSIVGAGIIGIP
FALQQSGLVSGIILLLLVGYFTDKSLRMLVNLATSSSSLRGKGVWTYDDLMRLPFGPPGR
TFVLVSMFVTAYGAMVAYLLIIKDTLPVVLGLVDDGDSSVGGFLERELIMIVTSLVVVVP
LSMQRDFSSLAFTSSISVTADMILVVFVAAFAPIQETVSAAGGFGEVIKNSIIDKGFFIG
FGVLTVAMCCQHSAFIAASSLDNPTPKRWSVVTAVSLTISVVCCLVLGVSGYLGFLDDTQ
GDILNNFGVDTVVANTARALLALTMFFTYPMEAFVARHVLVQLLWNGDMDGYIVDTVANE
QTGEETTSQRKSKCCYVLNRRHQVTFLIYLLTLMPALIVDDLGPVLSITGAIGGCCLAYI
GPGLVYLGIHGDDFINRLANRILALNTNKASSSNASEVDLPLEGDATAQMSPGGRAVDPR
QVFSTEYLSGYRKPWFWYVLGMPLFVKIALKGSNGLSERLAALDGEHHRPNSPTTLSADN
LGTNDSPPATINDNNECIVEPSLVDYGFAMFFIVFGAIALVAGVASNVYVQINGIFNIPA
>comp2839_c0_seq1; 454 80 1561 LEN=2009; translated
MVATKRNLRTSNYRDPDGSNLSENSGGNCPENRFDKLIHDVSDHGIYDSKGEKSMAQSAG
LFLKIGVGSAVFIMVLHFQTSWIQIYHDHQFVIGGDETPKLNGGGGFDSRQKIIGLENHD
RKDQGYELFPFLVDAEKVASSLSSSTAELNSVLSTPTMPDFPLVPRLERCLLKWKPPHPP
RSQEDKRLMFWMPSYPSSGGSCPTKKGDIVRQVIDGLFLGERIDATAYGNPTKDFHASSN
NLKRCIGVSETIACTSSHPLVSTTPHLRHHVFRGAVIVPIRNPATVIPQHYTSKDMAYRQ
ASGQISVESWRQMRDSYFEGSLADWMRIIRFWRGTKSRLGEPASYYQTELYVPYEDLITT
DVVKASAIIEKVSNLLGGAGSTEIVNASNATSSDLTKHGFFETSKSKADYECLWYRVAHK
EWERLQNVIGHYIPAYTVRQKNMMIQNLTIYADEIEAEILNDKSDLALASLLRRYAFQIK
HYVVVEDVIYTPQ
>comp2840_c0_seq1; 193 1 707 LEN=1003; translated
XNTYTTTDMDALLAGYGSSDSEDGPSPASGNRDGETGEPGPQKEQPKPKPKPAGLALLLG
GGADDGSCSSSSEGAAHRGQAGRGGSPAEPASKKPRRGGNEGNHDTNGGGSSSRLFLARH
RLPFPRLASLRSGSTSPSSSSSMVHRSRDSLSREAPRHREKGSPETITASIGSKQHHQRF
RFKQLAALVAPCGGSHKSWAEAIKSEQGFHNPGVFQSVMERSGITEPLGSRADPA

>comp2841_c0_seq1; 164 90 362 LEN=593; translated
MTYRARGRSLIEVVLAVCFVVFLVGFITVLCIGNYFRAKRAQERQRQRRLAKLAASNNAA
AAQGMYATDANYSASYEAPAHVAGGGGVQA
>comp2842_c0_seq1; 412 29 856 LEN=995; translated
MATQTWDGNAMSQEDMMMKDTVLVLDDDDNVIGSASKRKSHEFTPEQPRAILHRAFSVFI
FDESTGELLLQQRASSKITFPNVWTNTCCSHPLHGMEPPEVDGPKDVADGTVMGVKNAAI
RKLDHELGIPGSELDINKFKFLTRLHYWAADTITHGPDSPWGEHEIDYVLFYTVSSKSDL
TIKGHPDEVDDVKWVTRDVLIEMMEDKSLLFSPWFRLICKKWLLDHWWKDLKRTMTTDDH
VDLKSIHCFDPPKEHLGGGGKAGPLFGAEVEGDER
>comp2842_c0_seq2; 831 29 2308 LEN=2355; translated
MATQTWDGNAMSQEDMMMKDTVLVLDDDDNVIGSASKRKSHEFTPEQPRAILHRAFSVFI
FDESTGELLLQQRASSKITFPNVWTNTCCSHPLHGMEPPEVDGPKDVADGTVMGVKNAAI
RKLDHELGIPGSELDINKFKFLTRLHYWAADTITHGPDSPWGEHEIDYVLFYTVSSKSDL
TIKGHPDEVDDVKWVTRDVLIEMMEDKSLLFSPWFRLICKKWLLDHWWKDLKRTMTTDDH
VDLKSIHCFDPPKEHLGGGGKAGPLFGAEVEGDESKKQGAYGKIKTHKEPLVKQLSHLDE
VFAAVTFLYVKPLKSNLDTPYIRETFDADDLAFCDEILVKVSRSFAAVIRQLPATMLVDI
LIFYLVLRALDTVEDDMTSFPSNDVKIKHLLNFHKTALADPTWTMDGVGEADEKRLLQQF
DKCHRVFAKLSDKSRRVIVDITQRMATGMAEFVGKDLGQGTTDIDQYNRYCHFVAGLVGE
GLSRLFAASGLEKASFASEVFLSDQMGLFLQKTNIIRDYLEDYVDGRAFWPQSIWKKHSK
TGELGYFTEQNDPEVRMRSLECLNELVTDALELAPDCLLYLSKLECAEIFRFCAIPQVMA
IATLNYCYNNDDVFTGVVKIRKGTSCKLILRTNSLDEVHDTFYQFARGVLRKADANRSQG
VLDPSYARTIKICKTIMDITADGHNRERKARQLPWMASLLVPAAGVAASMLLKIDVTKDM
PKFQRSLALLGLSAFTLTPLFFQKPSDLTDSKFLENKGK
>comp2843_c0_seq1; 486 251 1847 LEN=2227; translated
MPRKDSSWIEASGLYGTWTFSTETMTDVGYYKGTAYVVMHLMPDPMQVKSCALGNGGSTE
GKSCREADNKACSHGGASGDEREECVDTETSSLEWSDVTAFFQFGFRLREGIVKKSYHTI
NTSDWLGPYNLILAPIEKNHFQLTSASENVQHVHTTKELFVSRYVNTPWTSTQLQRPFTT
ASIFNFDETVLETVAGTIAINATIHEQIKWRGGTNDDPYDRYSKTCAHGDLNPLLPRIQI
EKTVTQKSAEAPSKTSYSMAVDYSMLSVPVHGIDDLYKKWPSFKRHLGDKFPLNGRVRNT
MQFMLVIDNCFVGRPVRILTTLVFSPPFLSLHYDSLRPLPSNFLDCYGKDPNRAFAIRLS
YDSTESNTTSIWKGTKGCRASFPCWEGDASGTAAPSARIPTDNNSPGAYVLDPMGLEFAL
LYGMGFLLVVSLLFNCQLANRLRSRQVLPEPSRQPRPAWSGPGDPAASGSGSGRRRNSTG
NNPSRNELEEPLLDNTDASAGDEELLATSFDNPPSASQVEHGVEGSTEEGV
>comp2843_c0_seq2; 521 251 1702 LEN=2082; translated
MPRKDSSWIEASGLYGTWTFSTETMTDVGYYKGTAYVVMHLMPDPMQVKSCALGNGGSTE
GKSCREADNKACSHGGASGDEREECVDTETSSLEWSDVTAFFQFGFRLREGIVKKSYHTI
NTSDWLGPYNLILAPIEKNHFQLTSASENVQHVHTTKELFVSRYVNTPWTSTQLQRPFTT
ASIFNFDETVLETVAGTIAINATIHEQIKWRGGTNDDPYDRYSKTCAHGDLNPLLPRIQI
EKTVTQKSAEAPSKTSYSMAVDYSMLSVPVHGIDDLYKKWPSFKRHLGDKFPLNGPYCYG
KDPNRAFAIRLSYDSTESNTTSIWKGTKGCRASFPCWEGDASGTAAPSARIPTDNNSPGA
YVLDPMGLEFALLYGMGFLLVVSLLFNCQLANRLRSRQVLPEPSRQPRPAWSGPGDPAAS
GSGSGRRRNSTGNNPSRNELEEPLLDNTDASAGDEELLATSFDNPPSASQVEHGVEGSTE
EGV
>comp2844_c0_seq1; 421 104 955 LEN=955; translated
MASSGEYVELHELAKGWKAAIGRAMDPATKHEAQIRNDRGNLPLHSATSFRAPIDVAEAL
LQAYPEAASITNNYGNLPLHFTAWKKGPLEVERLLLKIYPEGAAQKNNHGNLPLHYAAHY
NAPLEVVEALYNAYPQGATQKNNDNNTPLDLAIADGASPNVVSLLQGKKVPPTEDEALEK
AKRRYEAAERELARHMSESTGTTEEMEEVLALLLDIYEAHPHALYSAGINPENAGTMDSM
MEEVSFVLSRKCLPLNDHVKSSIMELNLILMLHPNDLGSKSSCR
>comp2844_c0_seq2; 789 104 1840 LEN=1952; translated
MASSGEYVELHELAKGWKAAIGRAMDPATKHEAQIRNDRGNLPLHSATSFRAPIDVAEAL
LQAYPEAASITNNYGNLPLHFTAWKKGPLEVERLLLKIYPEGAAQKNNHGNLPLHYAAHY
NAPLEVVEALYNAYPQGATQKNNDNNTPLDLAIADGASPNVVSLLQGKKVPPTEDEALEK
AKRRYEAAERELARHMSESTGTTEEMEEVLALLLDIYEAHPHALYSAGINPENAGTMDSM
MEEVRKAAAADNSHANGIGGIEDYAGRNENEEEAQLIEDALCPPDDTVEIALSKLVGLEA
VKNQIRGMRRTMELVKTEQNGKLAHPSHIVLEGNPGVGKTSVATILADVLYEIGVTKTLN
FMSVGRDELIDRKSESRTIAKTRKVLERAKGGVLVLNEGYTLLPSTARPRSRDHGGAALR
EIARTLPSGNPLIILTGSSLELQRILSSEIGFKSHFLTRIEFSDPTPSQVARIFIGKMSE
KGLVPGDGVTVDYLAELISTNTDEDWRLERNGRIADLLLNGVRAELRKRMHFSDDSSKVS
FSPIKMMSPTAASRMPAATPDEIFVTVEDVQNAIVNGM

>comp2845_c0_seq1; 275 86 1216 LEN=1686; translated
MKDHAVVVTPNRETLRHQVNVAAFAASQASKTPLSSRPLTPGRSRRGGGGVFRSKSPGKR
HAAGYRDLAQEVYDRMGVNYVRGQNSIDLYDSTSSISISSTKRHFQSPDRGSPVSSITSR
TSGLSGVSRASVTSEHPKERAIGGPGVASQRNAYSSVRNRYSPSADTRSDQDERNWNRNP
GSEDEERESFRSARSVKSMVSVFSGGKSTGSYGRSVASNKSSYSAFKAPPNFVNAAFNQG
QKRISDNDNDVNTSLSFGSLDEGRWVNKQQNQYPQEQPQRQVYRSRSFSSSRRSSFQNSK
AFSSSSMSSSHPNNTSCDKMIEKLVKEKVEEKLTELDASFEDRLRRIDTDINSRLNDIET
KLNMILKVESSPALRKW
>comp2846_c0_seq1; 1067 73 1707 LEN=1850; translated
MVSFRRKKNKEEGKEKSGKAPTPKEEKPSKNAPAKPPTLDDIPDLIDTMLNAKGEKSAAA
TKRVYELCDVGHKQNRVPMVCSGKYDVLTPLAQCLTQESGDGRHLACLALNNLSIPTENK
RVMALGPASKDVIGGLCKVIAEDKQESYLCCICLMNLSFLEASITTILQHSPSADGKEIP
PLENPNSLLRVLEKLLTNSPAAPKASSGKSEGVRWACGLIKNLAKSEENAALIGLTEIPK
CVVENIRAATAPPSRWTSNSLEDFSLFVILNLAQWPQSRESLISAGAVDVIKSIMAEGDL
QGLKATMACAFLDAAWTDFPESGNPAAKAVSELMTNIVEKKGKDGQYAYGVFKLYTATKA
YADLSKAAGKADSEAGGEANTKVLAVPSAVALNFQIVSDLVLSAMDDTEGGSKYVPDAMS
AEHAVTAIEAMLPAILQAGDPPRKSVQSDKACGEVSQMLLTYADISGTSPEAKASARAAA
DQISKASGSARPILEISHDLWVQYRKREGQPLDQFMSEKGVEVDESGPLDFLPCASGDSG
CVIA
>comp2847_c0_seq1; 351 197 1045 LEN=1265; translated
MKGRWKEERMESSPLYSTTPASDSFFQVWIRAQKALAAFFVREYSVRALPCASRTDLFLV
SVSQLINSRECAEYREGWFGNEILWHTSTSHNFQLFPQKDPTTENITSPPLTLKMVKEFH
LKQKFKLVSNDYRITQNGGAVYQVKGNAFKWGNQASFQTIGGEELCCLKQTNATTVTPWN
NYEWFKDGELWATAKQEDWGMASKKEIDIDIPGDNDYKIKGDRMSWNFTILKGDVKVGEI
SKKWGILDSYGVRVEDDANEVDILMCAILVDATYHHKDGNNN
>comp2848_c0_seq1; 305 102 989 LEN=1167; translated
MVADGLVSGISQWIDNLIGVHGSSGYVFIFLLAYIGYRFMNNSSEPFKKSSQVEEDEEEE
PDPPRNFTSLQLVHFNGEPDERTGEDKPVYLSINGIVFDVSQGRNFYGPDGPYAAFAGKE
CGVALAKMSFDSEHLNNLAGCKDLNYGEKDELENWINKFTYYRKYPIKGRLVPDDILDPL
KSRTLTKEDLAKHMGDAEEIPEGYAAVPIYIGAGNKVFDASFGGVEFYGPAGGYNKFAGR
DISRALAKMSFSPEDLENTNTDDLEEKQKKVLDDWIKTFEVKKGYPIVGNLGKSS
>comp2849_c0_seq1; 940 110 2494 LEN=2649; translated
MVLDFGLTKSSSVAHSISSTKLRSLKSKKKREKKRQASSSDYCGSSSSSSDEDEGYSSSS
SCDEEDILIYEKNVQRSRRGKVKKNSSESDRSSKRHHLDPSLVERMSSFNIDEQGSASMA
LASMLNLEYCEDEIANDHNLNVKLAADLEAELVQKQQHATVNDAEGYENYDDEEEDLESS
DFLDALDEVHTRFLLNVPPEELSSSERIFFQLEQAWWYYEDLICDKLEEDEGQCLLPRHS
NLKDFAERLFDFSPLLGGLDFDKLWNEFTIYKRKISTYGCILLNEDCTHVVMCKVYESNV
WTFPAGKINENEIGIEAAARETYEETGFDPHCNYGLTGQWNSREEDREKHITWMHPLQDP
DNMLTYVEQKSGKRRTFYVCCGVPEDYPFQPVCRKEVDKIEWVDLRDIREYETFAVLPFV
GKLKKWIKRKNKSGKNNKKVKKIKKKKERKANKSRTKSRPKSREKSSTRDKQSSSRQRSS
RKKDREQLVDAGLIASMGDSARWTEREMFEANSKLQGGRVVEYDGNPHLFVEKGFGIDDS
ATGQKRVDPHSFRVVGGSFMNSKHGDKLAEVNDAGANYQPLVRNSNEDSGLQPFFSEHGA
TPWGEVVDEAKSEDDEGSDSTRFSGSDGPAGIENSNRRASQDLLTMLQKGAPDKSKKSRK
SKKSKHQNQHQQPISGDYDYDNSNHDDELMIFATDKEITAKRQRDHELKKGKGNEYSSSL
VSPLRSSSINTATRDDARRQRLLAQYEEDMKFVHKWVANSLPNPINFKIRNVDGIIDQHC
GGPGTKTKRGSIKN
>comp2850_c0_seq1; 223 1 682 LEN=973; translated
XFVLLLHKPNQIIEQRAQATGFCGNHFLFFASDRKSTNTQAMISESLLNDITDVVDLSKS
KRNTIAEYSSAPCSRNGMVDLLLPRKEKRHTKHAGADTDSSRHLHNFHLSETQRCSSATQ
KTQATTDTDTTDDDDDDYGYDEEERWLRENRNYHQLQRDSEPIFRSTVLNNDVSTSSIQP
LRERRRSSMGRETPTVPPLGYENQANNSRSCCMRLPKKFRLYDPDLF
>comp2850_c0_seq2; 244 286 846 LEN=1137; translated
MISESLLNDITDVVDLSKSKRNTIAEYSSAPCSRNGMVDLLLPRKEKRHTKHAGADTDSS
RHLHNFHLSETQRCSSATQKTQATTDTDTTDDDDDDYGYDEEERWLRENRNYHQLQRDSE
PIFRSTVLNNDVSTSSIQPLRERRRSSMGRETPTVPPLGYENQANNSRSCCMRLPKKFRL
YDPDLF
>comp2851_c0_seq1; 372 239 1342 LEN=1342; translated
MNRSSPGALLLLLAVASFHTSEAFTAPVARTLSPALPVSSLRTATAVFAAAGDGEAPGET
RAAPKRKRKRKKKAQVVAETAPEETEEPGAPPLVPDLRPREDAPVVLEVRNIVAPEGDPE
PSAVAKAGAVLSSVSSMLGSSKGEPGAGSERAVSPPPPGLGGPGRSLDDSLDQLLEDARS

MSEEGGASPGGSGGLLSDEEGTGVKQAIGNALSTVVTADFFLVCGFLAWFLLGIFCSYVL
KDDTVQIAFNNNFERLVQPALGVLMIAAIGGNFFKEEEEEYDXVGDLGNADAEGTGRLLA
MPPAAPDAPQARSEVSAQGIASLYSYREHATDFSKTLKHLWMACCDARTVVEPQDFSNSR
VESKSKWPX
>comp2852_c0_seq1; 579 121 2565 LEN=3050; translated
MTGQQERELYWYDDATYDIGFYNYYQRTVDPSPWAFIVCLLFCIIMALLLPRYLAYYRSI
HTSNLKKAYSSNNIIQLWSQAFRANDAVPNGNNDETIRTQNKRNIYLPSLNEENESGENS
EKDEEANGSFRRKVMRERALQSLCHFVRAPKSEIDNCINSQSKTEETRVPTTDYTSMEDR
ACDDDAKNDIDSSGYGEFVTTEAEIPPSQDLTEPNDANDPLPPEYLYDDLTRDAESCVSS
VLSENTNPFHIIKNDFRDFTRNRHPDESVSVASGWEEDFDPSTQNTIYFHPATGARTTEK
PLEPHSENFRPWYLFYTSPSFRRKMRKCSKWDDEMQKIIGLALPYTAHTIAVDIFGLLEV
GVIGRLLGTTELSAYFATEFAISFATMFFHGILASLKVLVSQAHGADNIRLAGTYVQIGV
WTHHLFSLPLVLYGWNHFDDVVLRLGLDEGTAESADQYARFALIYEAIGIYDGALHCVLD
VTGHEKYSAISNGFRASLSFLAVLIVSVMLNKGAELWMVGAIHLVAKVFFFFFNMSVILY
QKWLDNYWSEMVGNNPFSHMAPIKTLWKASLRLSSGRVIENCEWNILFVFSAIQGPAEVA
IWGLVGELWDFADDIVIAVSDASKVRCAHLLGSGLPEQAKYCSEKSLFIGVVTSIIMSVS
LGILRSNIPRWLTNDFTLQRLLGDMIPMVCLAVATLSFGSLCSSILCAQGRAHLATAVTG
FGSLAIALPLALMSNFCFNLNLQGLIASLIIGYATSGFCNSILMLTSNWTHISRKVRKRT
SRIEVKLEKDKMLYNATTDTAVVDGKVEASGDLT
>comp2853_c0_seq1; 947 242 3074 LEN=3661; translated
MSIFHIIFELLFLFALLSDCWAKNHESNCDAISDGSNPNNKSCVIDRSGFGPIKVIVHGT
KNDPFWQQVKAGMEQTARDMKVDLDLLLYDDEDPTLDASTLANAMVNDIRKYDYLYNGAE
PDNDRRQRRRRNSFADFTNRGDGKNQNENENVEPRCGFGGCKSRIEGGQISTNDALSPRP
AAMIVSLPGNSKVEDALRSIQKDSNNDMPIFGINALASNRENSSIEKEGSSSFFLGTVSM
NETQAGVMAGNHIRELLLSREDKIDGAVTGLYVNHRSDVHALKERFDALSSTSSDIVQTW
EVAYLEKNTTEEDFMAFFEGCRHTVIQLAGGSGIVESILEALVANGCNIEDNHIVGTFDT
SSPFIYDAITEGTIDFAIAQQPYLQGSHAVLMAALYATTGQKLVGGAEAANIETGPILVT
EESDDFPGKRHQICESEGYPVCWPGTGSSEKSECPCTDRSNISIAVVTHDESSDFWNTVF
SGISQAATDFGISVERNRYKRVGPNHGRQRQHGKQTNLRLKHTFDINQACQSKKIDGLIV
SLPDASMMSSLVGCKSRGIPYLALNALADTNQNQDIVEKLSPTRPSVPYVGQKDFESGFE
AGKRLIEAGVVQGWCLMHDNFDTLNDRCDGMKEAFLENEKTEFMGIIYVPNNNDALTYKT
LVETAFNQDGRGDDWSGYGILSTGREQIPSLLSLLEDHPKILAGTYDVESTLYKENNDSA
LRDQIIFGIDQNAYMEGYLSVATMVWKTSTSETHTIPILETGPNFVSQTDSSSSQVIEKE
EHWHQQCRDNGFRFCESRKSEPPPIEERPLTFTGKCSQLDRKCGVCEGDCDDDTHCGEGL
VCFVRDSLTSGVSDPVPGCSGNPIEWSAKDFCVDARLYECKDVDVFEDEWGIFPSNGRAP
LWNWTSDGARNTGGFAEPHAGIAVKKHAKTTQPHTEAINRPTN
>comp2854_c0_seq1; 240 124 921 LEN=1748; translated
MAIYNTSSNNNHASNASVLSIVIGTTFVVGTLSSVGWYYYFTRATISKDSGDDNGSNKSS
LSPAKPSSSLIKPPFPQTLRDMLSVCRLAYLSTVDNAAGSSHLSLMRFTYLHDESDGEVV
IMSTNRKTKKFDMLCRQNGVALLVHDFDGITKQQLAAAAANEINDDDKADPEHPKILRSI
TLNGNCRILDGSSDEERYRRAHLAHNPDYPQFIVGDDIAVLCIDVTSARICDLNDRVVHW
NVEDAALAAATTAMPPSPLAETARV
>comp2855_c0_seq1; 599 51 1817 LEN=2346; translated
MDTDEDMDGIKAIRTYIDRMVEGDGKCRGMKVLLLDASTTQIVSAVYSQTEILSSDVYLV
SRLDDTKQQKQQQSSGKSSHLKAVCFVRPTEASVGMLVRELKEPRFSEYHFFFSGILTQG
LLRLLAESDMTERVKQVQEFYGDFVPINDDLLTLQCRNTLSMSMAAGTSWAPQYAHLYER
NLRGLQGMLLALKRQPLAGIRYAGQSACAEELAKDIFDAISTDEIFHFRKRRRNSAGALL
LVLDRRDDPVTPLLSQWTYQAMVHELLGLNNHRVVLKGAPNIAKDLEEVVLSSCQDDFFR
ENRHKNFGELGENIQKLLQDYQKQTAQHNTSILNTIEDMQAFMEKFPELRSRSHNVSKHV
AIMGELARLVDVCSLLDVSQFEQELACADDHTAHWRELMEKLDSPSVKIPDKLRLGLLYA
LRYETSGNINMVQHAMKKGGVPPDMVNLLPVILRYGGSKSRGPGLYGDPNNIMSNLTKSF
MTSVQGVQNVYAQHVPVLMDTIQSITKGKLRKETHPFVPGSIKTVPGVSPETLVPDEIIV
FMVGGITYEEGNKIAEFNQVHRGKFQVLLGGSTVHNSTSFLEELRMTK
>comp2856_c0_seq1; 452 79 987 LEN=1314; translated
MSNEKKPLVGVLSIQGAFEEHQACLEASGCRTKQIRTVADMTEGGEALDGMVLPGGESTA
MGLIGTATKESGGKSVWDALRDFIEVDKKPTWGTCAGMILLAERCVGASAVIQDGQALIG
GMDILVCRNYFGSQVSSFEMATPPPPTSGGEDGGMASPYPGVFIRAPAILSAGQEVTVLG
KVVATPCRQAAVVLEELERKIEKGETVVQMGVVDALERDGSQLRHREVVKASPCETGEEK
KELEQDASIELPGAADGSNAREVICAVRKSNLLCTAFHPELTNDHRWHQYFAQMVKESVA
SA

>comp2857_c0_seq1; 205 415 1137 LEN=1302; translated
MLSQTPNASSSYLPTQYRRKLPRASNATADNNATTINTLPLDQKAPSKSNQAPSPTTYRI
LVATFVLYSVFLGWYASTRPKGKWKSPFSWHPFLMTVGMTGSMGIAAVTKKLGGYTNTKL
HGILASLGFFVSLGGVYAIYHNKNLYQRPHFTSVHGKVGIAVVAMMALPMLAGAVFLHPD
FGIDKTNKLYRKIHKLASRVLMAVAWGNSLYGLYGMRSEHPMELILYGVPLVVLMPMTLL
>comp2857_c0_seq2; 304 1 675 LEN=840; translated
QRTRMVVKLRSQPSTRSCFSSIRSVFQMAPSKSNQAPSPTTYRILVATFVLYSVFLGWYA
STRPKGKWKSPFSWHPFLMTVGMTGSMGIAAVTKKLGGYTNTKLHGILASLGFFVSLGGV
YAIYHNKNLYQRPHFTSVHGKVGIAVVAMMALPMLAGAVFLHPDFGIDKTNKLYRKIHKL
ASRVLMAVAWGNSLYGLYGMRSEHPMELILYGVPLVVLMPMTLL
>comp2859_c0_seq1; 690 518 2062 LEN=2371; translated
MSNSPPAKHASTPMMMATTAHTTTLPNQQGLTLNGSPQFLRPNVVLGVTQLPQHQQQPPP
QQQHMRVVSPGVSPIAQLPLQHYQHNTNPSPLPQYLPVSTGVPNHGHNPAMAWQQQQPHP
SMLPLQQQQAPHTGFTQQQQQHVSMQVPLRLMPRPNLDDIENSNRDDVSSVPWGSIRPDP
KYNPAKDPLNPVNIDKPFVPPLEITITIVPSRRRKSINRHSSPPPQACVASDRYPSITPT
NQESASTEWLQLQVPPLSTGLPGGEVHRIPVLGLANSGSMDVSNTNNPDSRKNKSFETID
DENDETANANDDLLEQFSDLATYTAVDARGNQYYLTNETDDDEEYLELRGDLLELQKLQE
LQHPSYYDGIAVQTRSGDAEAEVPKNHGNSIAHNAGNTPMEVIAEGASQQRRGMVRREFN
RRVWRFKKKKASPSDSEELAPGGELAPDVADHAVAAAHLEMPSIVADAEADGNPGKQTLV
SKKSMHFYSPSFVTHNYYNKSKMMTMVSGQENGC
>comp2860_c0_seq1; 345 1 769 LEN=812; translated
XFIQEYEVRSCITMSLSFVSSAVQIGTSDGGFEEKPIENKETEAVNRRNAHKPLFEQLRS
NQEQDQEKKEEQERLLMRSTCALDEDDVAHLNAIERQRSDRERKIQERTENELKEFRAAR
ALKKQAALSGEDKDDENDESNAEDQAAKIFIMNGNASEALAEKRETNPGQAGCSLPVIKV
RKRKRRTNPLDTQSKQTNIAKGEASKLEKGYTSDLNEVNPSSEKRVEMSSADVKRDESAG
GLGSLLSGYGSSSDED
>comp2861_c0_seq1; 420 210 1229 LEN=1676; translated
MKIRSPMALDAGNSSPKRTIWFILPAACAMVYCIMINLNEDLQYELLSNREPAQQVLRAA
TTATTIIKKEEEHSTYELARRESLGFFTDIPEETWKRHKKRFQLTQPNYDDSNQRAFERQ
SRYSNHFWAGHFEPEFTCPHEFRLGKLGDGGKWICDPHRITQTNKDGKCLIYSIGSNGNY
QFEKEAFNHVSKTCEIHTFDINKFGQRKDFAALAQELGVNFHHWGLGEKTKVYQNMKTFK
EIILELNHQDRVIDVAKIDCERCEYAQFEQWFKDWKELNMVVRQIMIEIHNSDLPGVVNI
FKAFQSAGYVMFHKEANYINEGKAIEAAFILLSPDFQKI
>comp2862_c0_seq1; 255 137 904 LEN=1379; translated
MNITVKKRNARDIGVGCCTSQEDMPLKQRRLNPKSPSSSTSSISSVKAFRFMMIPRMITS
SQESSIPVLSPCTYMSIALKNKKIKTRVDSFSLEQALYFEPYQEAEIHVDLLKALRGADL
EKLRTLKDEKEKNYDLDARNQFGENLVHLACRMALSLDVLKFLVEETKIPVNVRDRFGRT
PLHNACMSALPNFDNIDFLIKTAPKLAVFEDDKNKIPFELIPQRVFERWIRYFSEMNVLK
KLTTELAKHEGLSNK
>comp2863_c0_seq1; 1036 34 1467 LEN=1616; translated
MKTFSPQLLVTFAALLSVASAASEAGSEAAVALTPPEPRDLTQSRRKLDDQEPWDTGTKV
YNEFPNQGWWSGTITAYDPVAEMYTVTWEDGSTDYYDDGDKIDEMVAYAQNDPQNNPAGG
STDQSGSYPTGTAVSMFEDGEWYDGVIIKYGSGTYTVEWDEDGEIEEIAAGSIMDQMVQD
ALGDDDEAPEGFDGGPSDSGSSSGDIAVGTPVSVFEDGEWWDGTVSDYSDNTYTIEWKDG
STNEYDDFGDDLEELKQAVEDYMADDDEVPESVGSGPKFPLGTPVSDYEDGEWVEGEVVD
FDDGSYVVEWEDEDELEYYESSNAEDMQALGNMASYSNKDDDAPPDSYFEEEDLWKVGTP
VAITEDDNLWYGEISGFNNGEYSIDWENGEREWLDNFDLVNEMVSNAALAPKKGMSAVGK
TFLSLFLIGVCAAGAVFGYRFYEENQKQRQTREGDLSLEDGGARSFRDNADELPKII
>comp2864_c0_seq1; 426 76 906 LEN=1372; translated
MEGLGTEVTLMYRGDMFLRGFDNDIRNHLYEEMERNTSIDIQLNTDPTEIIKNEDGSFTV
VTGDGKKVDCDQVLYATGRKGKIENLNLESAGVKTEGSFIPVDEYSRTNVEGVYAVGDVT
DRIALTPVALMEGHSLADTVFGGMDRPCDHEFVASTVFTTPEIGTVGYSEEEAAAKYGDI
TVYKTRFRPMAHSFPKSEMFTMMKIIVDTATDKVVGCHIATDGAGEMMQGVAIAVKMGAT
KADFDKTIGIHPTSAEELVTMRTPSYHYKDGKKVEE
>comp2864_c0_seq2; 647 125 1711 LEN=2177; translated
MKIPAASLSALFATLTASTAAFAPTSTASRSSTVAFRRAYSLAMADKPYEPDPDFDYDMV
VIGGGSGGVRASRIASGYGAKVLLLEGQNQHGPPNYSAIGGTCVNVGCVPKKLMVFASRY
PGAIGEMTGYGWDGATPGIFSWDKFMEAKNEEITRLNNVYSKFVLGNAGVETIEGLGKLN
GKNSVEISLTGGGTKTVSAKNILIAVGGWPFKPNIPGIEHTITSNEIFYLEEQPKRMVVV
GGGFIALEFAQIMEGLGTEVTLMYRGDMFLRGFDNDIRNHLYEEMERNTSIDIQLNTDPT

EIIKNEDGSFTVVTGDGKKVDCDQVLYATGRKGKIENLNLESAGVKTEGSFIPVDEYSRT
NVEGVYAVGDVTDRIALTPVALMEGHSLADTVFGGMDRPCDHEFVASTVFTTPEIGTVGY
SEEEAAAKYGDITVYKTRFRPMAHSFPKSEMFTMMKIIVDTATDKVVGCHIATDGAGEMM
QGVAIAVKMGATKADFDKTIGIHPTSAEELVTMRTPSYHYKDGKKVEE
>comp2865_c0_seq1; 354 126 824 LEN=1277; translated
MSVKVYYFNATGRANQIRLALAAGGIAFTDDFASFPPSDENKAKWKALTNGNSTFSVPIM
TIDEGTDSQKVYIQSSAVLRKAARMGGLELTLDDADDAAYLQDKLIADADDLRTASYRAF
KMFGASDGAVQKFVKEVLPKHVDNLERQFVAAGGDYFGGSSTLSIADVALYDAIVFFGTR
LIDGAEGVDNPCGPALTKWIERVESNEKIAAYLAGEQFKAVAMVFDKSALGF
>comp2866_c0_seq1; 737 1 1874 LEN=2107; translated
XLARPLNNSQIPSEQKYKLSKKWNSMQEACKRHHNRALALRCWNAAELLRGKKQQTVEYN
RSWRKDSLRYVRLNTLRDEGSADVTGGNVIQERAGRELTKPNGVIDHGKYFYTAKVVDVD
PYVYAPDDELLDRTMTTQQTENQEQDRGSTRNRLLNVQAFETISTSAPIASLSTDDDEDR
IPPPIEVVGAKTCTSRADNHPDFASWKRDVFGTLPSSSSSFSPNSRRELIDWKTAQISKR
LEKQLRLQEQLLRTVKSDISNDDSNESSYINVLNKELKDNNTFETRNSSYMLGDECKESN
NENQNTQPMSEQTTITHSICKIKKFHPESTVTNVYVRQEKEGKARRCHQQDQQYLTMAVP
PNSPKMKNHTISSLSLKRTGIPEKKNNSTQLAAIMMELAELKDWVAKDIDNAIADNVADS
PTSLPSIATRLRQSTPPSSPTSPAPPASRDKPELQIPSLDVPNLVSPPLSPEADHSTKNE
NNEMIKTESPLKVETNMDAMEATMKSKEKKSVRFASPEITDTIYRPFTPQEEINTLFFQE
DELLDWENDEETTVRDRFEVIVTEIETSHCSEVESKQHNDNRLVTGSFLNIGTPIISFHN
SYSSSFQESSDDDSSHYENNMYEL
>comp2867_c0_seq1; 750 360 3821 LEN=3821; translated
MESSMASSMASSPPKPPPEGSVASETAAGKSNPRTSTTGPPFLNRAPRKVSTENNAAAGA
ASGTLESTATTSPMETTGGCGSPGGCVLSDSPRSGRPASGFRAKPFPSTWKRTVTSACSP
LAKYKPPTKNPLAWSSPFGRKMEARFGRIGDPLSPAYRHSLYVKERLKRLRQRKKELLST
LSSSHSCSSRPCTDSNPKERTEEIAKPVVIDLCNEDDDCKESPKESLRDAPSSSVKSAQT
GGTPQKNAVLTGTATLKPNTETNPTEDGIGNPNSRSWRKFPLERVTVEGPDIGSKTYCRR
ENQPIIKYNNDLGFRIVLSESKEKVRIIVSHVLSFSHHCRRDLISRIEIPGVGSCVLVHC
YANINPADIKNKMRGKRCMVTLEFQDHGDFQNMVDIALEYCSPGVVSEAEWFHKGLILAN
LGYKPSSPTSLRSRLALKKDEAHGGPLLRRLATRASTLTATWERVQKQSKRPYETSKDRT
IASRINTTFTFDKTPSDFGQPDPKKQRIATEDVTTGTDIDKHSQATDKMSVDSESKNPSD
PHPEEIARYTSADNLVGDSVSGLLSPQLSPSPSPSPSDTRASKKFESHPLPDATSAKGGY
ASEDNKSIGAKETAGSQSHTNGKATSNKECSFEWDGTFQLLQQDSSKPEDDSKPTGSDVD
ADVKDVANEKEADDVRQDQGSSLCDESSNNGEYQSSNNSDLDRAPRSSVDHSGIQTTHKE
DAQTQTTVEKEAIEYHNVAENEATSAQRYNDNNEERLPIVRQVLTLFKGIASSIAENVGD
VRSAQQEDEAEDKDEEDDKRPVGGDSIPQECVLMPPESNSESDADSEDLTVNSNQDSEQQ
NHNPLISISVGNLLVLALPLVGLAGIFWRLRLFDVSNSILEGCFRTFVQLHILGWLLSPI
FKWGTRNPLLIACYALFMILLASHEASSRTKYTYDDQFFVIALSLVVNVGWVALGAFGLV
LKPKPLWDPRYVLPIVGMLLGNSINGISIALDTVTTSLVERQAEIDLYLSFGASQFEAVS
GIVAHAIQKGSTPALNMMCVVGIVSIPGMMTGQILGGASPMVAARYQAMIIFLIALSTLS
TILVSSYQTITSMFCSHQILRPERLVRNHKRSLARLIMWLWGSIFGATDQIPSGANMLSH
GTEYVGQPSLMNLP
>comp2868_c0_seq1; 347 88 747 LEN=1176; translated
MTSNSDPFDCAFNSDLSDVQSTFDEVVLGGEVSAIEEGRVLGKEEGLLTGFREGGVLGHK
TGIDYGMEIGFALGLLQAVRESVLLEGSDLTSSSSSLDRIRKSVDELEKAINDFPSSEED
IRRRLFQSSDKEKNVHFRDRDCDEEDGKNHNQQTQAEEDVRAQLQRIRARSKVLTARLGI
PRHSLKNSMSEAAKKMALSTRQEETGHAFDGSGENTQEW
>comp2869_c0_seq1; 339 1 891 LEN=1332; translated
SEVEEPPEIDEEQPESADDASEQSPEVEQPPEIDVKQPRDEEESKSIEQNRSDIKSLMKS
KKESDTYSLAEDVYAFIFVAPVSSYPFIFSSYVVLTKIICYAILVEDISWESTVRYSTNS
TAVKFFLVPVAVAMQEDLIQCFSSFANIVYDPQILETHKSATKVKYMLSSLLRLIDGILS
LVVNFYVMLTTTEVLSVFLNFAALQFLQSIDDIFYELVTLGFFGDEMEHWATQCEEVELR
RRVGPNDKQIFKRLKMSHLDSLLFGFTLLVCLITYAITMAGVKSYFKYYSPPEESF
>comp2870_c0_seq1; 625 192 2203 LEN=2203; translated
MTVAPAIFPTHSGDRDATNPTIVRPTEAQLRYAQFHHKRRAQAVKHRIDRLHRTPLQVTV
VLAQELEEDNDNYSSRSDFSGTDTSDVDLRAVRLLRPKITSGRRALLKKSKRKYRRRKRD
IAKHGYRGCFLDDNEDDQLERGEASEGDEFIDKDWNDIDKEENDAMQEIFMTRPKHQRRR
ARRHRTPSGGNAQSYKRFQAACHAMMRNIQTSQTKAALPAIEVPSKRWTRSEEHVAAAAA
QTTDFYRYRTGLNARRIKSGRSAALLLNYAYYQQNQKDHDMDRGATWLTHLPAKQSKRGS
SSYYFHNLDGLPPLISSANDEGEDEQYYDGDGNESSITLSMRQPGPAKFHRLMAYVQQRL

IEKEKKEKEHDENNHQQKILMFEGIQNKSSSPEKSRTREIIESLEPKSENTNRSTRIRQM
AESIEGKSSRGTPTITLRSGTPFSGFSSQGSQRNRRGKSRYVPRVRLRDFVVEGTDEDDN
GEKPTPLPPRKLSERFQQEDDDGSSDKNELFSPPKIRLRDSFIGNEEKDTETNNRTREIA
LAFEADQDNGVGGSKVNQLRESLEPKTTPKIKLKKDFFGNDTDEKKITEREPSHISRSNV
DRLSRQFQGQQERLEGLDSPSTEVLNEFSSHIDPRIFEQFQDEGSSKRQSAETNVTEFFA
RLRGEQQQQQX
>comp2871_c0_seq1; 460 647 2128 LEN=2265; translated
MDIDNQNQRSKYGSASHQQHYQHSAPGSSSSSKPAYDSRGSRSNRGTRDDMVENEGPRGG
RSSQGYSRSSRQGGSSRSSRYNVQQHQEQSSGGGSSYPHSSSSQYNHHQSSRQGPTNAPP
SSMITPSANQHFTPPGNNPYGGQQHRNSHHHVAPHTPDQSVQPTFPPPAVTSESPPPAEQ
TQRRPAYLLHRDLVIGKGSYGQVCIASRGEAEGHMSGSGRSGKRKKFACKCVMLRNDPKY
ISKLQEEVNVLRELRGHDNVIRLYDVFCVDNELFIITELGRGGDLFHLLTTHPKHGVTEA
YAAKTVSEMLSAVAFLHSRSICHRDLKLENWVLESGKDVWSPLKLIDFGLSTHYRQGERL
TRVVGSSYYVAPEVLKKSYTEACDLWSLGVIVYMLLSGAPPFYGKNDEAIKASIVQGEYT
FPHELFRDVSDEAMAFVSTLLSYNIEYRYTAAQALTHPWLSANCDSEALSRAKDGARGVG
IIIDGSKPEPMQL
>comp2872_c0_seq1; 532 166 1311 LEN=1439; translated
MVWRSSVFARRILRSRGIAGIRTGASASSNRFQHNEPSLNPSTVSLAVLATATATFGFCG
AATIQNSGDDHASHGFGDGLVRFQQSMAPTALCESSYKSENRDGEPPHKKEAKLKRKKTA
LKVATNLRKMNTREEISKLRLIKDEMLRRWERDEDGWRDLPARAWPEYQPNSEQLGAIVA
EIQKLGCNDKLATRNYASGTNQADDEQAEGAAHCYKLLFDMATGLVFYNVDPEAGFALYE
KLAKQGHTPSMVACGIILIEGLGVPPQELQGFSWLEKAMASSEDGDGSAAQAMYELGTVY
YTGIDGVVEEDPEKAFALFERAARLDHTAALYMVADCLVEGEGTERSVAKAVPLFYKAAE
RGHRYSRQRIRELLARVDYPL
>comp2873_c0_seq1; 608 92 2795 LEN=2830; translated
MTYMPMMAFLKSSLFFLLFLYAESFSHNLAFLSNYNPSLSRSRRWSTNEVASQDTVTDPS
KDEITTKVVMDENNSEDLEASVIKNGYPMNNNFNGYSMTSGYSPFLDQEPSPKWLRKLIG
KDSESISSRQDMEEDGYAEMQRRKQSLLKRIAKFPLRTAKRLVSTNSIEPGTLILVRHGE
SSWNKNKTFTGWSDPDLTDQGKREMEHAARLLMEGGYHYDINVVFTSRLKRAIRSSWILL
QEFNRVYLPVFKSWRLNERFYGALTGLNKTQTAERLGEDLVQGWRSSLRIRPPALTENDP
FYPGRDRKYADLTKDQLPLTESLLDCMERTKPLWNDKILFELKQGRNVLVVAHANTIRGL
VKTIDDIGEEEIQNVAIPTGIPIVYKFEMQTNGQLISIPPKSEENSVSQLHMKGKFLEKP
GLLKEALKLEGNWREQVPGYDATMSRHSRPMTGLERSLYKLNAVRELGEWATDFIDLDPE
EDDGNDGNGIFFTADKNESSTVAGLKENSSNEDIPANDQDAIPTFFAQPCITSAPTSAVS
KRQDSVIVIIRHGKTQHNKLGLFTGWEDAPLADEGIEEAKLAGQNLKKHGFEFDVVYTSW
LSRALETAWLVLDELDSLWLPIVKTWRLNERYVWKRVLCYVNCTLATIMVLFPQPTILYT
IFCFNFTFGHVHVSCPFQTRMYGELTGLSKQMVKQRHGEKQFKAWRRGFEVQPPAVSSFS
QNYPGNDQRYRYIRDVRYSVKESAIRSIEKGKLRLARKLPKTESLKDCMDRTIPYFLERI
AKDAVDQNKRVLISSSENAIRGLLMHLCDIPESEITGLEIPNGLPLIYDVKSKCVKLLDD
GTGRDPLEVYNFGPSAKYLFRPCQNEDGSLDEECTINFDSLITTTADQDTLNYIKSKTGR
>comp2873_c0_seq2; 659 92 2647 LEN=2682; translated
MTYMPMMAFLKSSLFFLLFLYAESFSHNLAFLSNYNPSLSRSRRWSTNEVASQDTVTDPS
KDEITTKVVMDENNSEDLEASVIKNGYPMNNNFNGYSMTSGYSPFLDQEPSPKWLRKLIG
KDSESISSRQDMEEDGYAEMQRRKQSLLKRIAKFPLRTAKRLVSTNSIEPGTLILVRHGE
SSWNKNKTFTGWSDPDLTDQGKREMEHAARLLMEGGYHYDINVVFTSRLKRAIRSSWILL
QEFNRVYLPVFKSWRLNERFYGALTGLNKTQTAERLGEDLVQGWRSSLRIRPPALTENDP
FYPGRDRKYADLTKDQLPLTESLLDCMERTKPLWNDKILFELKQGRNVLVVAHANTIRGL
VKTIDDIGEEEIQNVAIPTGIPIVYKFEMQTNGQLISIPPKSEENSVSQLHMKGKFLEKP
GLLKEALKLEGNWREQVPGYDATMSRHSRPMTGLERSLYKLNAVRELGEWATDFIDLDPE
EDDGNDGNGIFFTADKNESSTVAGLKENSSNEDIPANDQDAIPTFFAQPCITSAPTSAVS
KRQDSVIVIIRHGKTQHNKLGLFTGWEDAPLADEGIEEAKLAGQNLKKHGFEFDVVYTSW
LSRALETAWLVLDELDSLWLPIVKTWRLNERMYGELTGLSKQMVKQRHGEKQFKAWRRGF
EVQPPAVSSFSQNYPGNDQRYRYIRDVRYSVKESAIRSIEKGKLRLARKLPKTESLKDCM
DRTIPYFLERIAKDAVDQNKRVLISSSENAIRGLLMHLCDIPESEITGLEIPNGLPLIYD
VKSKCVKLLDDGTGRDPLEVYNFGPSAKYLFRPCQNEDGSLDEECTINFDSLITTTADQD
TLNYIKSKTGR
>comp2874_c0_seq1; 253 1 851 LEN=1268; translated
FDLDIPKVCFNKSILYCNPSFEKRKYFPTSFKNRNSDFLPFLQNNIXDITPITTKTLEAV
TEKTKLLFICSPGNPTCKAIPLSDVEKIASETEKYPGLIVVDEAYIDFAGEGASAVSLLK
KYNNIVVLQTLSKAFGLAGIRCGFCIGPPDVIQLMNNVKAPYNVNSLTSEVAINAMNSMD

TLRKNVQSTLDERAKVRAALEALTSIVVSVYPSDSNFLLFRVKDDLKANDVYKRMADTGV
VTRFRGNEMHCNQCIRVTIGTPEENQKFLASLQATWKELLEAK
>comp2874_c0_seq2; 435 517 1659 LEN=2076; translated
MTTSTGIAAKLARPNILELQPYRCARDDYSNGVLLDANENAFGPTSLPTKSLDPYDNSNT
LERYPDPYQSKLKELFCRERGNDLQPSNVFVGVGSDEAIDLLMRIFCTPGTGGDNILITP
PTYGMYKVCAKVNDVQIVSAPLTPDFDLDIPKTLEAVTEKTKLLFICSPGNPTCKAIPLS
DVEKIASETEKYPGLIVVDEAYIDFAGEGASAVSLLKKYNNIVVLQTLSKAFGLAGIRCG
FCIGPPDVIQLMNNVKAPYNVNSLTSEVAINAMNSMDTLRKNVQSTLDERAKVRAALEAL
TSIVVSVYPSDSNFLLFRVKDDLKANDVYKRMADTGVVTRFRGNEMHCNQCIRVTIGTPE
ENQKFLASLQATWKELLEAK
>comp2875_c0_seq1; 400 129 1163 LEN=1361; translated
MASRMAIKTIAAALDATALRIPDRVALVSASPEALMQTVTTTDSRGMNESLNLDRYTYGD
FRDKTNRLAGFLRAYGYEKNDVMMSDLPNVAENLMLQIACNRIGVSYATAKGLEGMAKLT
KVKGAVSATGTGFLAETSLSLPYLGGDFLLDLIHGTTNSSDEISGLFGLDEFDLEDNEQD
FACGDEDENTKKLPPHAYYNSTKPFTNEEALELGADAAWEVAMVEEDSVCIAITLCHAFG
MGSAVCSALKEGATIVLPAVGGIRGCGVPSQRAEATLQVLESEQCTLLFADTHTLKAFPD
EESIGYNLALKGGVCKVGSGSQFLEETVKCFGASLRTLGSKGNNW
>comp2876_c0_seq1; 325 161 931 LEN=1187; translated
MATASMTMASQSVDHYSSDMSSSIDPFVESRDVLIEKMKSNLIRYARSNDRRMVLRCLKD
ARFLSYVPMDAADEQLRIPISELEKEISKEEFLLNGISLKPYDQAQAKNVCRNEGTSSGS
LKMIGTLAQILCEGTTMDGNNLYERVINRVAKTSAEPEVYYHINAMMGNTTLSVKQLSPE
QISKEEEANVTDLKLYETGGQIQMVLETTHSYGLFREKDAIINRPWIIVHCKVHERANLS
NNESFRSMNIKTPSLY
>comp2877_c0_seq1; 919 81 2030 LEN=2640; translated
MSTNEDSPSAKRRKALKDADSANQLRVLESCPLSKYADIAERLLGHFQNAVDGRRLDEAY
VFGLRFANLCISGLPQHPEWRREIGSKTRKRLTSQVGDVLSMMDVIKQRMDAEELMKIKA
EMMARKAEELRKKEEEDRRNHEFEEAQRRKQQKRDALEKERAEFLAEQRAQRQKQIEERK
EIEKRNAAINKKKEIEQSAMAKLKAMQARVPLAETKEEQSVAVSETNVEKPSNTKAKPNR
LKNWFGGGKDKSSKKKNEEKSKLSAPSGAKDSDEMVSTKPTVTDRNVATAEDRSAVTTQE
YPPTEAIQLQQPEQETSSSSTAHVTGRKKGAKPVESVTGGPTISRRSNKSQSVDSSTILN
PLRSMSKIKPRKNTDSKNTKNKNDNEQEESSANSNEVCSKKHVHHSSEEKPIGSTLPSSS
SPECNEGVTTTSDVSSTAVTMIPSHLTPLSRKEQATIHKLQRAITVQEDRLEEIEGKQIP
QLLERAKVLLKEKNKQEALKCLVHKKRLERQVDAIKAAVFNMETQMFMLESAFEDRHVKK
ALDEAASAIAGYQQIIGDPKAAMVDLTNMSESLPELDVGESTDEELMEELTEWLTPEEKK
RAKANENTYADKEDLLLLSMPEFLPAAPADAPEPLSAGRAPNAVVETNN
>comp2878_c0_seq1; 901 156 3116 LEN=3404; translated
MPFGSQSSLSSSSDESVYGSINANNDVEMAPSDPIFTGEARSGKNSNVCGEKVLSPRTRL
VILGAVLGVVLLMLGRRTAMPASHQLKHGKGARIKLYNKDDSGIGSLSNGAGNDPFQLSP
VENLGMLSIDRADDALPSPIWGNHLKKGLPLPTNSWYLNLVSHRAAFRPDDSTRVYTVPY
IIDTASPYPGMEGLRVHWPVVQASDGNVQMVNDFKNALCIGSDGFSSEDGDDGIDGTYRV
KDGALSPLGVGLEWGAGSDTNSSNIMSTNIVRGMPYATMVYSDSSNMVPTIYSYNGLASD
VQIDRTSTFDPVTSKFPKLVCGIEGTSKKGISATVHSHLHLHMINSDFTWIVFFNKPVKV
SCVTNNEQDPKVRDFKMTFLDNEDDTEAGDTDLVVRIALLNQCTTGHASIKEHCKERNAL
ADKDGYELLLKNNAHLVPQSPTIDFEYASKSKNDDDVAKINIDWDVTNTKNASNDDADLL
MFALPHHQDSLSSADREVKNQCVHSFHGKTCLVSSSNWTLEEDLGSPLSFDAPRPPSAEF
IPTIAKYLKEDIKFEIPKNTMRGASDTYFSGKILGRLARIVAIASELRSLASASSVEDIQ
GRYDMKLDSSIDMLQESMKAAASANLPSSKEFKDALDQLKSAVQAWLKSDADAPYIYDKS
WGGLVNCGCSYVGKMDRGYCNNTFPECPALLSVNEDFGNGYYNDHHYHYGYHVYAAAVAA
KFDPAWGRKHFEEVMLYIRDFANPKGDDEFFPQYRQKDWFLGSSWASGIISAENSPHGRN
EESSSEAISAYEGVTLYGSAMVDAFEEVKTDGGDEEITTKLETAKLIRDSGQLLTASEIS
ATNRYWHVWSSDTHNNTYPAEYTKPVVGMLYDTMAMFQTWFAPWAVVSYGIQLIPLTPVA
ETRDDPDWAAELYPLYDEACDDAGKDFCEANGWSILQAGLLATAGNQTEALNQAKAVPRE
VFATEGGVGNSMSNTIWYIATRKKIV
>comp2878_c0_seq2; 873 156 3247 LEN=3535; translated
MPFGSQSSLSSSSDESVYGSINANNDVEMAPSDPIFTGEARSGKNSNVCGEKVLSPRTRL
VILGAVLGVVLLMLGRRTAMPASHQLKHGKGARIKLYNKDDSGIGSLSNGAGNDPFQLSP
VENLGMLSIDRADDALPSPIWGNHLKKGLPLPTNSWYLNLVSHRAAFRPDDSTRVYTVPY
IIDTASPYPGMEGLRVHWPVVQASDGNVQMVNDFKNALCIGSDGFSSEDGDDGIDGTYRV
KDGALSPLGVGLEWGAGSDTNSSNIMSTNIVRGMPYATMVYSDSSNMVPTIYSYNGLASD
VQIDRTSTFDPVTSKFPKLVCGIEGTSKKGISATVHSHLHLHMINSDFTWIVFFNKPVKV

SCVTNNEQDPKVRDFKMTFLDNEDDTEAGDTDLVVRIALLNQCTTGHASIKEHCKERNAL
ADKDGYELLLKNNAHLVPQSPTIDFEYASKSKNDDDVAKINIDWDVTNTKNASNDDADLL
MFALPHHQDSLSSADREVKNQCVHSFHGKTCLVSSSNWTLEEDLGSPLSFDAPRPPSAEF
IPTIAKYLKEDIKFEIPKNTMRGASDTYFSGKILGRLARIVAIASELRSLASASSVEDIQ
GRYDMKLDSSIDMLQESMKAAASANLPSSKEFKDALDQLKSAVQAWLKSDADAPYIYDKS
WGGXRELRVFLRREDGQGLLQQYFPGVPGFVECKRRFWKRYVYIALQIHVCCNYNSIIGW
DRLGSTLPADLVPSRFLLFSNNLGYYNDHHYHYGYHVYAAAVAAKFDPAWGRKHFEEVML
YIRDFANPKGDDEFFPQYRQKDWFLGSSWASGIISAENSPHGRNEESSSEAISAYEGVTL
YGSAMVDAFEEVKTDGGDEEITTKLETAKLIRDSGQLLTASEISATNRYWHVWSSDTHNN
TYPAEYTKPVVGMLYDTMAMFQTWFAPWAVVSYGIQLIPLTPVAETRDDPDWAAELYPLY
DEACDDAGKDFCEANGWSILQAGLLATAGNQTEALNQAKAVPREVFATEGGVGNSMSNTI
WYIATRKKIV
>comp2879_c0_seq1; 278 163 736 LEN=1181; translated
MIRGVGIQPVEKNILASLAFLGAIALSRQYFYDFNFFPRDNGCQASTMNGGSREEETDDG
TPEGGAADVNIDGNAVANDLVEDNKKNICFPWEPHYSPPKCRDTENLNPRKFPLKKWHEV
SALHKIDYDSAHEDDHGTISTETCKTELDFLSSMTFAMEDCDLPLVLAVFDAQRTAYLSF
TNFFANIGQW
>comp2880_c0_seq1; 599 120 1190 LEN=1267; translated
MDNKNTNDADTDHKIMMESTSTANIESASSSAEAHPSEPVATSNGIVTKSNDDTHNTIRS
GDENDDDEVLQEEDDEEQNDTVPTSRASCYKKTAVGLVLLGLIIFVIADSLTNQHIKTGL
LAFLEWIEDHLVVGVLSFVLVYFLATVFFIPGSILTLGAGFVFSSAMDSLWIGVLLGTAA
VFVGASLGAIVAFLLGRYLFREAVAVKLTQKYKIFEAIDRALEQNGLKIMVLLRLSPIFP
FNALNYSMGITAIRFWDYVLACLGMLPGTIVYVFLGASAGSLAELGGDEDETEEDDDNKT
VTIVVIVVGVVFGFLAVGLTSYYAKQELNKVLEEEAEQNDESNNGVDENAEEEISV
>comp2882_c0_seq1; 443 209 1051 LEN=2356; translated
MSFSTTTSITQKDVSLSSEWAPSEWAETDTQMIPGNVDGEMLPASAFQAAGKVLKQWLSD
GQQEPDQCYQNHLNNCNSAIAVIPCDSSASSTEHDDATADLASLMEVDEEIGENLLGDEV
FGGPCEITEDSQLHFGYSLSAVSHEDKTLMNSISAMNMEDDMSEEDRRFLAPLLVENTST
SMDYDAGYIVTDDEVLHQPCASSPSAIVDQQRYQEIMKKLEASMNRSQETRKSLTMKTPK
TEKYNRRRSITGVLSSIESSSRQLQNFLQTVKQRKQRTPV
>comp2883_c0_seq1; 820 218 3175 LEN=3345; translated
MSTPWNPNNHGFTSGPPGKNSPPGSAAVALRNGNSSTTFDARNNIDSTASSEATRNPKAG
PTKEEKGQGQEKSQRATPMIPTQQQMRDQQRPISSTSTTSATSFHAGSSNGIPHPPHHHP
QPSSGMGGNNNYHMAPQYRHAPSHHHPSHGQPNHFYNGDQMMGAVRGQGRPHDPDHAMQN
NNFHHHNLNRPTGRYPIQHTVPEGFGHHPHPHGHGHGHFGRPGPARNYHQHQQHQPQFHS
FPQVPHTKGGTITQGMGQQVGSRGPSPTHPHPQYLSQGRKSVSPIPPGVAVAIAQSQPLR
RLSQNSSANNSTDSSSGPVVPHEDTGNPSSNRVPTGSYQSKPVTSQQNQQQQQTSVGHSA
PLRPPSQQEMADISSQDQYRHRGGPPDMHQGYQNQPYQNQFPQTDHIHYGGQPPMHNGGH
PNSMQSDMQNYNYHHTSHGPSTSGPPLAHNHYRGYPDNGMPTGYPHGPGGHREMYPHPSN
QHYHQQHQQPHSYGKPLPPPPAPQRIPGHGFQLPKYNSNLTTSDPATSDNKNESRKSTNR
SPSPPPSPPPAPSAQALLRARLRQQEGAKKTTDEMSTSSPTTGDMSINSSTNENPSRRNS
SGSHSSMVGNKPTALGDRGNVAVTSRGSTLGKKQTNQPVSKPAKQEDVTADAASILLQLG
SMNRSTGGTENQNPCSHPSPPIPSSVQHSSSMDDVSDAGTMLTMQTDVPDLTSGPSSDSI
DTEMSGVSASRPMDTPTKSNLTTKVTTKQEPDTDFPCPVPDRYPTRLALPYDNAKLNNLH
CFLRSELLEIFVVSKSNKKSPCHSPSSSVGRVGLRCVHCAMNRMSCADAPMSVFYPKSIA
EIYRLVTSWQRCHLRKCKKIPPGARAEWQKLRDTDKSRGKTHYWVTSAKQLGLMDCQSRA
GGIRFKPDFDPSTLPPNTPLTPSAKEAKEAAERAAAIAAKAQKIVSLATAVTTTSIAAAS
AKLEESTVMTDAPSTEVLASAAVHT
>comp2884_c0_seq1; 591 1 1244 LEN=1818; translated
XTTDKSGFSEETTDKSKDTLDYSESDDDSKSDNEDSTSPTSNRSNVITMADLSSTSVMGL
QHWAWNMGGNKNCDSDEKVNLAVSTLKEVDDKASSSIGTKDNHDAWIMNEDQLDSFAEDD
DKEDENTNEGKEQRDVEGDTHDLINIETNSPKSNLESETDVTKDNTSPPQAQDNRIASPK
REHFLAAQKALSKSFRKSLSSIKKKKPHSSADIASNVLVNATVTMDETPDPPIETVLMNS
AVEDRNPSSTNTRIPKRFGANRANKRNSLANSGVAFSKARKFWREKTQEKTRKPTGIGRN
DTVRTRDVETVHKSSDDSHSSKDVLTIENNTIAETIISSHYDPVISPSRENSFLDDIRDI
AMHGLLGAEYDDYDADSLGTTNTSFSWVRDDDETVESLRDDGSLHNQSHQVIRE
>comp2885_c0_seq1; 245 420 944 LEN=1236; translated
MFFQHKTLAAFLLLSSSPFADAQCYESSVCLDVMGKPGECTSSEGRTFPICCPDDATQTW
NPTDVCTMGGDPTPTDAGEAAPTGEAEAPEKLCYEIGTAICWELGVKMSSCVTGDGREFN
ACCPKDVTESSTWNSTCIYGDGGHEKSAAHARSLETVVALGLAVATTAVAFAGF

>comp2886_c0_seq1; 464 329 2263 LEN=3258; translated
MGILDESLVSTASTLIDSCSISLDPSLDNSRSDHPSAEKENNGLNVNPIHGRNTKSGTSS
KRFVRAISHASQIVEKRNARLLSPSATKTHRFLQSENHSVSESTTTTNNSKLDILRARTK
LLRKQGSQKISESRASLRKNRTVQVGLSPHLPCPMNQPEALLGNTPRPAKTNSASIMRGK
ILGASKNMDLPVLLRRSNSVIDQLTLEMETAGDIINQTYVSENSDSRMILDTSSIYDTHV
LVPPSPIMQTSHDQDIEDDTSSIHNISVDDYWDQSTISASARDASSISEQLSPTRSNLEE
PPPSQSVNCSGLPPPEVVVKITTPTSAKLSRSRELAESRHRRMSPPSISPIKGDCASMYN
TDQKCEKLSFENDCLRRQLELLESRHRDFQQKLDKQMLSPEKVPGALRTPGSPSTATSTS
SGASSPNSYYKYHKRKRRRSDRRRLALVLFFAPFLISTSLFFYRAVRNVSDARRRNRDNA
LRNSGLGSIPFFADTNSRGPQATLSKPALSGLASLMREGVLRQTSLGPILFRDKSNGRIQ
RDNINFSGDWIGERHETFSAEGQNESFDYADSLPVMALPKYRRIEGSVGGDNPDSTQSGR
KGRSVKRWFQKRLSKMKIATHNDPAGVWLKRAQQIKTRELHAEL
>comp2887_c0_seq1; 487 172 1446 LEN=1775; translated
MSAASSTSKAGAKSIVVVGGGIQGTSVAYQLAQKATAEEPLEITILEAKEPASAASGKGG
GFMARSWGDGSPTQGLHHLAFDMYEDLAKELGCSSYRKLPVLSVAPEGGGGIRGSKGNPL
IPSWLDGDSIGRISPMGYGDDTAQITPKEFVEKMLEAASENVSVVLGTCTGVDVCESSDE
DDEQSVTGVRYQPRGSEDGTEKVLPADAVVVSAGPWSCQAEDWFEGSGVQLPMEGVKSTS
IVWKQPEGTSEVDATALFCGEDYRFNTHLEVYPRPDGTIYICGIGGSDYITTQELKQGAY
REVIEANDSRVKAASDSFRELSSLYREKGELDRTQACMRPCPPDALPYMGAIPSYTNAFV
NAGHNCWGIAWAPASGRAMAELVLDGASESVDLRPFDPARFTPSAGKRGGRGRKKKGASV
GEQW
>comp2888_c0_seq1; 493 1 1518 LEN=1559; translated
XLASVGDCRRLMRAADDAFGRLDVLVNAAGTTDRGSLWDTTPEDYDRIMAVNARAPFFLM
QDAARIMERESTEGSIVNVSSTASYGSMPMLAAYGMSKAALNASTKNAAYSLAWSRIRVN
ALAIGWMDTPGEEAAQKGMCMPNGHKGGGQGWRETAGRGLPFGRLLRPEEVARCIAFCAS
DESGMMTGCVIDFDQSVWGAGNPPVPPRKEEWDRASGMTFSFEGRDRFVPVPAAEQKAVK
KAAEKAAVAVAVAAEKSPPAPPTPAPAPAPAPASKPAGRRAGSKSPGRLRWPPPPPVAKD
VEPESYRRASEVFAAAATPATQDNSNSNSKSENYGYDNNGVDTSSQASIKEPTAASETAS
DASTGASPTPTPTPPPPEEDAEDAAKDDARDAAKEAYFSMRTQRMGDVYRPDEYLPGTEP
SGYFVPTSXGLEAQEEGRQEVGGKPGLPREVEGGRVAGLRGGPQAHLQGQAGLAVGRGGQ
VHRHECDTNQRRRFHHTSAMYSTSKQ
>comp2889_c0_seq1; 702 1 2901 LEN=2996; translated
VASCRICSITTEPKVAGLDWSTIHEIGRHTTMRRWSFISPQANGEGGEAAADRDEANDGK
KLHHYLQDPEENGVDCYQSCDYWTHEDPLPSVPSSSSSISLPNGPGSGLSRRGQKAATIS
ACFLKDYERGHPPTLSSDFSGITRRQLLLYRVKHSFHWNIFGLWLATALLFVPSFRARAA
TLFLHAYAILAFSADLYMVDQFRCEDTERESNRWPLGAADRPGRLLFGCMEGFLFLMGLQ
TIANAIVPNNTVVHAFTLAVSVLKPLVFFYQSRRARDALEALIRISKKLLRVILIEMFLI
LTFAAVACRLYYNYDSFQTLPQAWLSLFALSTTVVNPSIWMPAYNESPWNAVFFVIFIVV
SIFYLHSLVLSVVFQVFIQSATEVHRQSATDKENSLKLAFLALTSARGNASKTLGDKNFS
PDKNALVDVRLICEAFRLLRPHYGQQKLNVLTDIILPPNSEGGNEATQHNGRNGEEKDRS
NKHNIDFTEFRSRILQALNSSVRTTRTHSTEGMAVELLSITVSIVNFLYVMIFASRMRPR
EESTDSEFIIGSVITLLTLCEVSLRYNPWQFSNRINPITRLNAVLDGMGCLGGIVSFCGI
VVFLCGNEIGLEYLLVGRAIGMIQSMRFSIWFREVLQRSLYVLPFLAGPILLILTAMHVF
VCIGMALWEGAVDTEEQASNENVEYLYYLNNFNSYAEGCITVFNVLVVNDWHQIANVFLY
GNRCSSPFVVYLYFVMLVIVAVCILLNVIVAFFVETFVTQLNLVDVESDSVNSDVKVRID
ESTPAMQPTDMSTHIAPIAKSWIFDSSDLKKTSVRSLSCPNTGVKTDYDVIRREGFDDIM
QSVGNDEDKQFEDFARDLLGALELVRALTPDRNIGHLLCCHQKGIQFQSSGFSLLTKDYI
PGNKGIESIVAETKDALLNNANSSGRVGRYIPNPNNDSKVLQIDGALLPQNNEVLMLTAI
EKKLEN
>comp2889_c0_seq2; 576 1 2664 LEN=3112; translated
VASCRICSITTEPKVAGLDWSTIHEIGRHTTMRRWSFISPQANGEGGEAAADRDEANDGK
KLHHYLQDPEENGVDCYQSCDYWTHEDPLPSVPSSSSSISLPNGPGSGLSRRGQKAATIS
ACFLKDYERGHPPTLSSDFSGITRRQLLLYRVKHSFHWNIFGLWLATALLFVPSFRARAA
TLFLHAYAILAFSADLYMVDQFRCEDTERESNRWPLGAADRPGRLLFGCMEGFLFLMGLQ
TIANAIVPNNTVVHAFTLAVSVLKPLVFFYQSRRARDALEALIRISKKLLRVILIEMFLI
LTFAAVACRLYYNYDSFQTLPQAWLSLFALSTTVVNPSIWMPAYNESPWNAVFFVIFIVV
SIFYLHSLVLSVVFQVFIQSATEVHRQSATDKENSLKLAFLALTSARGNASKTLGDKNFS
PDKNALVDVRLICEAFRLLRPHYGQQKLNVLTDIILPPNSEGGNEATQHNGRNGEEKDRS
NKHNIDFTEFRSRILQALNSSVRTTRTHSTEGMAVELLSITVSIVNFLYVMIFASRMRPR
EESTDSEFIIGSVITLLTLCEVSLRYNPWQFSNRINPITRLNAVLDGMGCLGGIVSFCGI

VVFLCGNEIGLEYLLVGRAIGMIQSMRFSIWFREVLQRSLYVLPFLAGPILLILTAMHVF
VCIGMALWEGAVDTEEQASNENVEYLYYLNNFNSYAEGCITVFNVLVVNDWHQIANVFLY
GNRCSSPFVVYLYFVMLVIVAVCILLNVIVAFFVETFVTQLNLVDVESDSVNSDVKVRID
ESTPAMQPTDMSTHIAPIAKSWIFDSSDLKKTSVRSLSCPNTGVKTDYDVIRREGFDDIM
QSVGNDEDKQFEDFARDLLGALELVRALTPDRCVRFVSSVTPGIVLR
>comp2890_c0_seq1; 229 1 490 LEN=490; translated
SHTTPETPPENKSKTKSLVVSSASVATILSRNQDNKNNDNDNNNNNRNLILDAFYQQQQQ
MYAVNNGNQNSSGNNYSNPDSNFVYTNTQSHGANNSHLNGNAAAAVSETFHSYSLDTQGQ
LSNFMSFFGRNNHGNSNHHANIRAIQSFPLPHPYHLHQQQQQQX
>comp2891_c0_seq1; 524 289 1500 LEN=1562; translated
MTDKVLPWRDTNRSPLSGTELRNLVVRAALLEEIEDNEDDDSSSFLDIPGVAWLEHINLI
VGKVAEDRSVAESFYLDVMGMTADKSKSFHVNLGRQQFHLATPKRDDEVAHRIHGSIGLA
VPDLEALLDRLRDARKDAEGNNDYGFRETLFDFYEEHDENDDEDDKIVHVRCPWGNVFRC
YSVKSRGTPPTPFAKPEESPQKMTNLHAPQLGIHGADKMGVLGVGLPGIRFVELILPKGV
SSHQLYGFYKRMLRCPAQVLQTSCDGKKRDCCVVSVGAGVHLVFVEGSIGSENEEEAIAQ
RLMKGVHICIYAHDFYGLYKRLSDKSLIWSNPRFFHLDTCDTWEEAKNSRTLRFRHIVDV
EREETSNNNGENNGNSNILIELEHETRPLRHGQFMKVPHYVPK
>comp2892_c0_seq1; 205 197 943 LEN=1203; translated
MTDGFRGNFVSSKRGLLASVWSIVTILTAISFLTAFVYALSTSDDENDENYYNNNEGEGS
QDQREGHDAEVAVTSRALAFSALWTGVLAALLSIFGTVILGWQSPTGQYYTCCSSSVHRT
TPLGIGSFIGALLMFANLTLLCSVLFGEFQVRDNREGEDRAGGAGNQIDGQRETRSMAFS
IMCAVLTVLYGGFAALTLAYAPDVINEYAVDERDEVLMTSTRNKKGHFNAAYDGYIGERF
GVGRTTLA
>comp2893_c0_seq1; 485 1 1714 LEN=2055; translated
FWLEAVVIDIXNTVMSLKKTSSFEVAKNTYDTEKMDSTEKSFSKNGEIEVSINLSVDDKT
RHWVYFRSNPRTDEERFNSIHKDDFGSASVQCKQTILNGRTNRDSLTLDDASFELVDDFD
VSKLFSNQDFYEMQSGEKNFNGYHKAVEDFVKTKLGCDKVICFHSQVRNAQRTSSDGVEG
YAGGSPHTDSSPVSADQLALQMLQANNDTGTYQRYCYLNLWKNISDTPIQNDHLAMMDER
TAVKPDDYITKDLFGDGYSVVQYGLNARHAHLHKWYHFPHMTSKEAILFKQMDSDPTKTG
RICFHMSVHDISADKDNAQQQSPRESIETRMMCFWKDVAASNGSSNGNNAGSSMPTPTNI
HEDMIKDPEVFATELAAGGATLEGATVWRLLLALFKKIPLLGSLLFSSDISSSRPSKYSG
NPEDYLPPFVEAMKYYDSWPAFGKTYVENQFKNSADYDAGVKQLTRLLVNDAYGNLKTRS
LRPLEKDAVVLCLLSYEPYLAEVKKCFGDXNVTDKEYSRSHWRILSSRVIRGEKIFPSAM
QNMDRSSIIYKPNGSTVVGVFRKMLSPQKNE
>comp2894_c0_seq1; 458 275 1585 LEN=2074; translated
MRRLPSERPLSQYRRCGFLWTILLVLLLPLGTLGQSCIDDFEAIYKREAVVTDTSVPRLY
ILCPRHVYEIGKLDFDGNLVQPPGGKAVMPPLPLRPNMTIRCGDQGLRENVCWVKGGDLH
LDGTKILGITDDSLENVRIEGLVFIGAGEYSFWGNKPGSVTFQDCEFREFTNSSAPIMLD
FYDASAPSAELVTTFLDCEFRNNRYFGRGSQSALIYGNSGQNRINVTSSLFENNDMIWNN
TRPDTHSYIVESLGPVGVSTTCFSDNLVGSSNVVVFGNSFQNEFNYVRNSSGGLCPFSSV
FENMQQFEDFDPTCVAASQSVCSRYVTLSPTPAPSPSPTVSPAPTASSSGSPTRTPQPTI
TPVPTGEPSARPSGRPSPYGATNEPSMAPTEPLLEFFWPTIMEEDPFASSATRVGAGATN
LAMLWAWSTALVCWSL
>comp2895_c0_seq1; 585 633 2174 LEN=2679; translated
MSSSTSASDTSDVTETITKSNIDDIVESLYNNQDELIGYSCKQIMEKNTVEEMDLVFYMD
VLSPKDDESGNSDTNYQLAAIDATQSVVLEEVALEYQIDPDISRGVRCFDLPVDGSTWLV
QMSIEKKDFVEVTIFGGCRKLEWDDATQDCNFYEARLKGSYIGAVPVADDGTEFSDVVGF
LKSLINGPTIVDNLNNNGDGGNGLDNIFGQNAASDAYKMAFLGVPRAETNPNFQAEEGRN
DLKPPVNIKTGIDASQSTAQPNRNTITVVGGMLVACFSIAFLMVAYILVKRRQSYRNERN
EDEEERRSAEISMAMASGASAGNFHLDTVDRIDRNDEYNHADDDGYGYDRNDYDDDNDGD
DDDDIREDQAGGGREMDPESFRDDDAGPEMQQNHLQHESLRSAQRPQQEYGDHPMSAEAI
QMDLGNTLKGQMIGLHGNTSKKVLPMCGPSTRGGNGGNQHKVQRLGNRNAGSFFHQLSDD
GDHDSFDDSDSWAQTDGTIGSLELQLEPITAEV
>comp2896_c0_seq1; 570 1 1485 LEN=2010; translated
EQPESPLAPILANPVLPARRSVLRIAPTPLRNSMTAMLLSPPCSHELKTTSDACGTENDG
SRRHALGIEIERTNNEFAEERCREILSHYEEESHRPEHEDARWLVGQLDREEQEIAARCS
YAYWYLSRRSLEEPSAQAVKDPARFASAMQEARRHLDCADHRSEVLKVFRETLDFHRSHK
TALYRTCMVDAPGADKGLSDSEKELSKGRRERIGDEMKDLQTNVVRGHDREDRAIFFAFP
RRKAGTSEDAFVDSLVYTVERAAASSEFQSIGRQDQIVVVLDTKGSSCPPFKTLQAAVGI
LQNFYPGRLKTCVILNPHYVVTGIFRMIKPFMHPLTFSKFVLVKAKRQSGIAEEDSVLSR

LIDESQAMPEMMPGRGKLASKVDVDRFLYKVPFYRLYDCTDPEHDPKLLDRETHSTTSTT
LSLSSSSSSLSLSSDAADEDEHECDEATEPLANLERGTTKAKPTRVALRSLAVGQLSVRA
SPGSPMVVDLRIVG
>comp2897_c0_seq1; 209 262 603 LEN=1014; translated
MTTFQVASRRFFLPALRRSSVTLNTGRRTMMAFEDHARLRGKFEEDRYLKQQEEQWRQNL
QALRAQEESKREKRHQEEVVEPVKKHLEEILAASGDKISDEGLENLAKFRLSL
>comp2899_c0_seq1; 343 1 1073 LEN=1668; translated
XEERQRKEQQRKEVEERQRKKHQRKVAEELQRKEKERKEAEERQRKEKERKKTEELQRKE
NERKKTEGLQREENEKKISAELSRKENEQKKTEESQREENEKKAAAKLQRKKNERDKTEE
LQGTEKMSVMNEITKDVSEKSPATTSPKLQSINRKIVAMDKTVSDAQAMLAKGMIRVSSV
ENNVPSPIMNSDVQNSTKTNGKASSDITISAVRKGCLSAKTVVPSNKVQNAVVEGSYADA
TRTNASTNPVSSEPAEKISDIDRDDPKLTKGTVVMSDSSSNDPRLPENRASGSSESEEYI
PKGMGSKIMGRTTSTLPNPNSILAGVSKGLQSLVTGRLAQGSSSDEKTLSSASEKIS
>comp2900_c0_seq1; 278 348 1115 LEN=1496; translated
MESQRGRNFTIPQPIAEEDQQQQSEKTSVRKNLEKASFMDSVKKFFEPCVGAVDAASFYI
GECRPSDYEACDPNQQDKVAEDVIMKLRERNGGLKRRNETLEIPTNGMLFEDDDVSAISS
HTLEEMERLRLAQNSGITNFQIAPGKHQPGNILHPRPTDDPTSPQDAADRSDAVKFRNWS
TQKTPSTAKPQERGTNEGNITLCVSVSDSSSNEDGEKASPKASSPKEFSPKTFTSPPSWS
KKSGKNRAFKTHPVG
>comp2901_c0_seq1; 766 150 2756 LEN=2817; translated
MSKPNDETYSNNHGAPAELPALGQHQQPPSSSSGMRRGAAPGSGGVLVSKAGGGSNNSNQ
SIMPTASNVSIGTSTSQQHQHLSVERSPMPPMILRSLGDRSNEKRKNAALEIEALVKTLQ
EANNSLMIQNVIEILSKDFCTSMNSNYRKGGLVGLAATGIGLMGHSRTFLPVLLPPVLHC
FDDPESRVRYYACESLYNIAKVSRQGILQYFNQIFEGLTKVIADVDVDVKNGAQLLDRLI
KDIVTEADEAFQVDQFLPLLQKYIQRTNPYIRQLIVGWVTLLDSVPDISMLDYLPDFLDG
LFNMLSDSNREIRQAADSALSEFLKELTSSTVMEFGPIISILVLQCQSKERLNRLTATTW
LSELIHHPYSGGDALLPYNSEILGAILCCISDGEVEIRIVAERTNDDLLSLVKGTSSPFE
LRSLLETLTSELLNKEDVPTKMAALGWINMLMEKRKGDMNAFIEELLPVLIRTLSDPSDA
VVLLNLQVLSRISLAQRDEKGDAEESAEEQFELVLNAILSLFAKDRQLLETRGSLIIRKL
CVLLNAKSVYIRLANALMQYEKVNEKIADAGTLEFVSTMVQTLNLILLTASELHDLRSIL
SKSFTIPPENDSKKNNPEHDQRKRPQKQKQKQWSRQKKNFIEDSLKVFVILFRCWSHNPV
ATFSLCLLAQAYDLSFALVKRFSKMEDVSVGFLMQIDKLVYLLESPIFVHLRLKLLDVES
ESHAQLLKSIYGILLCLPQGDAFRLLNNRLTTVCNLRDNLGLTPMPDAPKQDDLSLPSTS
TVFVGCFPLSLDALLIRYDEILEMHRVAQEQYDNFVYDEQQRLQMQLAPGSQQIGLSGSS
QGGRSHQAVLAHGPLSPQYQQHERRLWD
>comp2903_c0_seq1; 434 192 2000 LEN=2692; translated
MFVTKKFALFIVSAAFLASSVSAGGVRGVPTEDSAPANHGWGRELKKDQNKPNKNQNNPA
VVTRPALPPPPTSPPVPNPIDVLVGKCSNPTLTSCVLRSGANERVCFTCLYSISRLSSAG
DSAVVNCGRSQCGGCVNEARDTFGCATGGSAPAPVTTPGVPAPVSVSSPVPAPVPAPVLE
EIETFDFTTQGCPALKPVGGAVCSVPAPFQFQQCDYPTGSGVGVRCMCAYGQFICNPAPI
SPTPAPVPAPTAPTAPTPPVAAPPGVCSPTLPNSGDTCSTGGAPFATCCYTGISNKPANY
AHICNCLSGENRYICNGGVSSQCTSVINVVASSPVPAPVPAPIATMPTPSLVNAPAPTGS
SSSIPEFCLALPGVPKDGESCAGVLPAGMASGGCGGEIIETDNMNVTTVTNAACTCTATD
PAPAWSCVKISAVAPPSSGCPPQASPPNTGDSCATVLPADSFLTSVECKYSKTVTDSSQL
STTTEKFCTCEKATQIFTCTDATSQLIPPVSIEAPADPAPADPAPADPAPADPAPADPAA
PAASSPTIVQDGCPSTITSGTPCKAFIPAGANEQSCMTSLTTQCDCPGQESADPVWTCRT
VP
>comp2904_c0_seq1; 527 207 1775 LEN=1841; translated
MIDQTDVVKKFASLIGTKQSSTESQVSSSLLRAEPKASNSREMLMCISKSVTIDEVVCTL
PSLDMRKLDEEPDSNPLPEVGEHFPMRMEASVLNNAADASAKAAQVEQKAAQTLGSLTAW
TRSSVIYASKQISENAAESFSSLVDSRVRAWTLLLLRHSLTTGDDASRSRLLSMLASTIK
VNSAETSFKPQPLPPNLANMAKNENDPDVILPLIFEIDLKISVRNNDCTVTLRAPGTISA
DFDRLENKKMGMGLKRVDVQLNTGTLLESMVNEARLVVFNAVEAAAKSNSATKGNEHNRV
NPSSQLSSGFSSFKLSAPSTKGSSGGLPPPSPQGGKHQPSHRALKAQSSSLRLGSVLSSS
TSTFQKTRSVQFNSSDLKPKSNMIPTPKRQKLATAARLTSIKSFGRPHGGDFGAEGGPKN
ATFGDFGGGSQRFWGRDGKLASKPKPMLPSETTDMMTSQTRRNANASFDIQKPPIHLSSG
SDVRMGLGGKKKPPAENQVPPSIPRTATALEAMYIKKFGGKF
>comp2905_c0_seq1; 226 79 591 LEN=807; translated
MGTTSTKLATSAPKKGMKSFYSLSAVTGDGSIQKMEEFRGKVVYATNVASNEVRPAXEYA
QFESLNERWGKKLIIFGFPSREYGWQEFDNDEEINEFAKSKNFPGIMMKLGKIKGDTAPE

VWRFFKDETGAGDPMWNFKGKFLVSKNGEVSVPTDLEADIERLINEDSKE
>comp2906_c0_seq1; 423 226 969 LEN=1119; translated
MSKSVLTISSASTILVGDPIVGYSLTPKEAEIAYDSYAANYDELDGGKAADIFGLDEARS
ALFRQARGSVLEIGVGTGLNLEKYDPSKLESLTLVDISRGMLGEAQKRFEAIPALQRVPV
RFIQADATSGLTVRFGDGSFDTVVDSFSFCVMGSKGAQSCLEQVKNVVKSKENNGQILLL
ENTRSSNPLLGLYQDATAEAAASAGGKGCLYNQDVSKMIQATNGIEIMKETSFAGGVFRS
YFCQVVT
>comp2906_c0_seq2; 423 226 969 LEN=1087; translated
MSKSVLTISSASTILVGDPIVGYSLTPKEAEIAYDSYAANYDELDGGKAADIFGLDEARS
ALFRQARGSVLEIGVGTGLNLEKYDPSKLESLTLVDISRGMLGEAQKRFEAIPALQRVPV
RFIQADATSGLTVRFGDGSFDTVVDSFSFCVMGSKGAQSCLEQVKNVVKSKENNGQILLL
ENTRSSNPLLGLYQDATAEAAASAGGKGCLYNQDVSKMIQATNGIEIMKETSFAGGVFRS
YFCQVVT
>comp2907_c0_seq1; 1257 61 2343 LEN=2388; translated
MAFSSFFKAAAFLAVIVPNEAFAPASLGVKTSSLSMSSQAQDVDKKEASSAAQREVDLDL
YRNIGIMAHIDAGKTTTTERILYYTGKSYKIGEVHEGGATMDWMEQEQERGITITSAATT
CQWKDYRINIIDTPGHVDFTLEVERSLRVLDGAVAVFDGVAGVEPQTETVWRQADKYKVP
RMCFLNKMDRTGANFYFCVDTIVDLLGATPAVLQLPIGSEGDFLGVIDLVTKEAIVWRGE
DLGASFDVIPLAECDDIPLVDDELKAKAEEYHEKLVELAVEQDEDVLMEYLEGEMPSVDT
LKRLLRKGTLNLDLVPVLTGTAFKNKGVQPLLDAVVDYMPSPLDVLAIAGVTEDGEETER
KSSDAEPMSALAFKVANDPFVGTLTFTRVYSGVMEAGTSVYNSVKGKNERIGRMLQMHAN
DRTEIKSAVAGDIIALVGLKDTTTGETLCDKDNAVILEKMEFPEPVIKVAVEPKTKGDQQ
KMSEALIRLAAEDPSFRFARDEETGQTIIEGMGELHLEIIVDRMKREFNVECTVGAPQVS
YREAITTTAEVDYTHKKQSGGSGQYARIKLVFEPKEFSEEEGAMDFEFVSDIKGGAVPKE
YVPGVQKGIESVLGTGVVAGFPVLGMKATLIDGAYHDVDSSVMAFEIAGRAATREGLRKA
KARLMEPLMQVDVSTPEEYMGDVLGDINSRRGMVGELGERGNVKTISAQVPLANMFQYVS
TLRSMSKGRANYSMKLANYDFVPPNIEEDLKKGFTGVEEE
>comp2908_c0_seq1; 224 158 919 LEN=1072; translated
MTTSRSHFFAPTKFFVLSVLVLVAAFSLADASTLAKTYVSKQPGTQPRGTEYVVSSFCSD
ETNDEPEEPVAFVAKKRHTSPLKAAYQWYLKSCTETPFRAKGLTSATIAALGDVIAQLIE
KKATAKALNAARVATFFFVNMLFTGPFIHLWYTFLNTVGKKMDERFYNVSALKKTVTQVF
LDQTLGTAVFFPLYIYVYGAFESVIQMHRLPNLSHATEKCRNHLWGIIMTQFRVFPLSNM
INFGLVPFELRVLW
>comp2909_c0_seq1; 190 121 1074 LEN=1875; translated
MPQQHYIFGYGSLICAKSRAATASALGVVGEVEADEAGDRNKHNTGGIPVRLNNWMRLWN
LQGPNTYLGVQKSNTNTNSSSPRVSCVGVLVPLPSSKQNETGGSKNDEDEVLEALDRRER
GYYRHSVDLSSIERVDDLLLTNIAESERCDQAIEDRYYKHTFLRRKNDTSTNGNDNTIRS
PTQNDDVVCKVWIYVPQDCYSGMACPQKPILQSYVDICLRGCLSISESFAREFIASTYGW
YPGHGHLLLTGHSVGEKCFESKRSVDESNAVVSSCIKHCWINDRERPIYVRADRNFSIGN
SELLDALFDPQLLERRR
>comp2910_c0_seq1; 295 63 779 LEN=802; translated
MGRRRKRLQSEDYDDSLERSFSAKRSKPENDDSGKGGASKQDVGASPAKSGETQNHNDNN
SKKNDKDNIERLREKKRLKKQRQKEKKLAAQKEQEKLKILQEKQNKEREKRKQKQKKLKE
LEKKVSSDTNTFIKTSMGVRYMDVVVGTGPLLVDRKKIVCQYVLRAKNKYGKVLDSGERF
SFKFGKGEVIPGWEIGLKGMKQGGKRHIIVPPNAGYGFKDIGGGKGAMLYFEVTLEKC
>comp2911_c0_seq1; 492 196 1671 LEN=2038; minus strand; translated
MMFIVIIFGFGIKFLEEYCSILPPPTRLDDEKGKKARRPAVYLLKIPFGLFTSCLLLPNL
VAVYIPNYIRAANTMLMSNANFEGEDLRVILPPDEFSRVCYREEMGFLIQCTLTAAEILA
FHFDLPNVVWAHHIVAMSLALGRLYAWCYPITLMSDWLWLAVVVHLTIYSLNFVRSCYSF
LRHFLTREDNLSGIPTMKHLFHCTNVGSWKIIVSAYAFSSVVAVLSDGGKTEQALLCLAF
VAPLFYGSFQEMRRWRSSSISDGFPTRMFVQVTVEIMVGLFLGPILLFLDFLFPHAMKKF
SKKTSHKNTAGGVVALRALCNASSDSRLHIDDPYAIRFVQRGVLHIVVKSAVLRYLMTLK
LEVQHPGALGHLITRTKTIDEIILDLTSRKNKPVRQLVILGAGYDTRAYRLKSLKDVLVL
EVDQPFMSSEKQEKCADLKPLCKELVHLSTDFNKESVADVMNGCDKFDPKKPTLFLWEGV
QVYLSDDSVDK
>comp2912_c0_seq1; 645 94 2616 LEN=3240; translated
MEWPSLEFQSDLLNCIENDEILSHSSQVLSDRFLSDLASSIVTKEIYARAASAALASDTS
RIVARKSNTGENELSEKMVVAFLDKLRTSASDSDEWHAYQYRETARDVTELMASLDPLAT
LKFCKGGLDAVHDLLKYRLPSSSSSDSPTDGSAALLNAKDAYVLSQSFPKLDADSVTGTR
APDLEFRFGLLHNNSKVNSNSNVDCDVNSLYGMEACDIVNALHQDGTMETSAASLAKNTL

ANPNLVAQLSASKTTNSTGENQTETIFVVLGCDHPMSPAKPLLRIPGVKVLGIPSSEEGL
EDILDYVRYNSPDDTTFVHPIWNQKDKKGNYHRKRNRTGNNLILSRGPQVAQWLIDHAIL
AKKDGDEITTDSKASANCRKKLKLVLVPMAMPSLPPATTSRNSDSPFAFVPEASVRWAAS
SDLIVQRVVMAAEAASTTSRQIQCSLWSYQSSSTCMVVPPTSTTLSTELLHKRPSHEQWL
HTLSMGTVLTPTIEEDNNRNKLHKHHTEQQDDHSRSTAVPSGSSESDIEANTVRNRFVGA
VSNTNHNYSIVNGIVTSEGPHHMLSEHIRLWRAMITHYSADLENNSSQNYIGDYDSDSDD
DDDGIQRKNSVHVFAPHVPIVSRGLLTEAIEKASPSTPKISAARPNKITAPLQILNSGTM
ASLMAAIALAGFTDPLINQPMPMGLIDEKDANTAEATIITTPFCLFWNGSVHSGVWNCPY
TLDSIFGTVGYFLEKVYGYCDSALAAASSAIAIANANAAVSGLWNKNSNDTQNIEQRITT
NTKSTEAAKSGRDNKFNYANNDDHCNTSIVDMVPSIMRDEERQDQQLMPNLVKDRLEILA
>comp2913_c0_seq1; 309 80 751 LEN=1012; translated
MSTTIVFLLQAALLVGACVAFQQQVLPGKVSSRQTIQSVPLMMAERNEDTENAFGRRNFL
ATSTAAVATGLLSFQQSAMAIPMATVDEFDTILRDSPLSVNVVEFSGPRSENVVVKLVDG
TVFGVKDIVESPTDPRSPLKLAADCREANVKTKFVDFEAMLSGSPKKKKLYTNERVQKAY
ELQAAQKERIRQDELERLRQVALMEEQEKAAAAAAAAPPAVSE
>comp2915_c0_seq1; 383 108 1133 LEN=1407; translated
MTKLVSSRRGSNLLLHNLEENDVVFGTKGNGRNEIFLKVLESHSEQYMQLSKFQKMGLIQ
QIIREWNGNFYILNSKTNDLHLAKRKDHDLSTTDPSSKKLYTSVRRMMNYVNSKVQRKPQ
HLTRPTTNLAGTAIIDRTTQIMKVIPTIAIGALNPQVQKGQRMNNEQHCSAPTTDAAAAA
APDRPPTAMTAMDYVRKEHITLPLPGDISSYPTFVPKTINISPLQRSVIMVTPEPSPRSA
FLRPPPISPLPLSKPDIPYNNTACTAPLSPEASFSSETIPAQTNNGGSLLSKKQVTTPVV
PTTRMQAIEEQGPNAKTTDVPGNLEHSAILALISLGASPSS
>comp2916_c0_seq1; 1276 1 3220 LEN=3296; translated
XEEELQETKKKNKKMERWLTKIVEESTKRENLLKQKLRDLEANVGDTTDQERLLQEQVNG
LETTKKELEEKCSRALELESFMESALEKEIKQQERLTKRFVQLKAKAEKTTQIHEKEVKH
LNSRVSDLETQLASSVKDDEQRKQLDNRITERIAELEAKAEQMRRDGNRKKHFESRIADL
EAELALSLQNNDSKEEGREGQYNPSSFYQDLIKERNNLADRVSELEEELEAERTVREEFK
QTVDELNIEIETHAQKYKAIEAENLELEKELELGKSEHIRLKKNVDELRDEFETITEDYN
SVTAINLDLGQKLVSANTERNRLEERLSELQDVLDSNTNERGIEEIPKSIPTGMQNELET
LVDENKKKQEQLNSRIFTLENDLESSYQERSRLEDRIVEMRKEIDLEKSAQVQISGDVAA
LKIELERVRNITQTRIAELETELASGKAEHDRLLQVVRELEDERKVRGEKHDSAAKKVVE
KHSKANSKLEARNLKLEEELDSYKRKQEHLDAQILELQKELEMATERSRKAQEIRDENDL
KFKYELEAEKTEQKRLNKLVHELEDKVEKAAGKYEFELEAKRAEQERLNDIIETMQNEVE
MNTEENDKIIESLNGRIGDFKNELEALNAKIAELEQELTILEEEPKDATNNGAQIIDQNL
TDRILELETNLESTLDREKKEQEQLIVRTLELEKELESTIEKTLKNEQHLYFQILEMEKE
LASSRDEARNQEERLSAQIRELEMDIDTTKEEGHEKEWVSELGKESEKMESLINMIDRER
KENGVLNEKYSGLKHDFELYKKKNKKLSSQYVNITLEGNRHLKLLLRRVAELEQEILHIT
KSNATEKKLTTRHDEELDRDLRERAQKNEAERKDLNELEPEKNEKNRVRPSGGEVDVRRT
QKEDGHHSDKTEASTSFATTIEHQVANDKLSAHLRNSSPSNRETDSPESSIAEEELKAPR
NQKFTKSMSESKADLMEEYLHPLLTETEQKLGDEIPSNIIGRIHDAFLDNFTNGKYDLRW
EEFEEILEEACQGHDEETWFNIQEAFFTVWETAMKNNPDRDREVITKWESFTD
>comp2917_c0_seq1; 322 271 1400 LEN=1553; translated
MPAMTTRRTAIGICWVSLVLAPWGCVEGWVAPKSTAQKTPKSTAPTSSNTLFYKTCGSDE
TFSRVAKPVLPGIHETVHEERQPRKDETEDPVSRAASIPWIGDEHDCTGDVFLPHWNWQL
DFFRTHLTNLRFNPPGDASDEDLYCMDDGTKRVYTISLSSDEYRDIRMTYMDFPSCKTVR
CLAYPSDERLPILGMGIMKFGKHQHMAVLDYQPLSTKEEDHDVNDAYTSELLRMRAENPT
MSQPHTYRHFVTEERKYFTDFPLIGKFNDKEDPASPEQGTSAWMREMHRGQREFVETHVK
LTQRHTEADPGTTDDDASVREVHSEFDTHVSKKEPAGPMLTATYGDEIAAQAGPPGDLSA
FKELSERAPSLATSPG
>comp2918_c0_seq1; 506 1 1986 LEN=2113; translated
SQTNSPNFKSNPRKFTSMPTQDSANLRLTPQQREAVERRSRMTQRRQELTSRIRKQKKSK
YIQKRRGLQCGGVDVVNSARPLNLCVAEFRNLLIAYCQTPSAPERLLPSVDNFVAKTKIT
STAVRSAEDPLVLLEANDEDLALRFLDCLRQQAIDIFPNQSNPISFQSILKILVHLTSIS
SGDAMESSMTPSVEYYGRHPSTWSELIVSSTSSPTITAAQSIEKAPSWLDIFVQGLASSR
EPELVSLVLGNLVGDDGAAQNVFQAISKDFKSSMVAGLVQSVSPATPTAAWTLTNMIRND
PLSYARTYCSENLLSASLLASWLREPCLVSQTAWMIASLTAREEDAVQYLSAQHSTPEHS
PFLSAIVESVQNPLQTDQILPLVQALGNISCHLTLVAPLLTKTAPALVPLLQHILTTTPS
RNPILAKAAWVAGCLLVDVGTEAHPSTTVAAPALIPALINRLGDVNEEIGGSMGTMTLEE
EREFASSLWNALDVPPGPGGPQELPQNSLWSNHPTQSSPVQLPFALNLPRSTLRSLVRLI
NSNDSDAVLAGVHVISLLFQRENDHQNLQRILEEEELPCALEQVCDSQMEEAADVAADCL

DDYFYNENYDNDELVETPSPWAGGPPVPTTFHFGIGAEAHATNRGGRGRGRGSTIPAWMT
K
>comp2919_c0_seq1; 412 526 2373 LEN=2760; translated
MLPLNITDTEKHSTAFPQPPMTILSIEEGQQQQQQPHYNRIHQSQHSYNSKESFRSSSHT
ESTVCESIDVLTSEDFIMPSNSQDESAIYYFGYGPIVNPLVRCRRGCKISPHNVRPAILY
DHRLRFVEGGTANVVPMMGWDVKGVLLRFDSVNEWENFRNFDAGYDIKEVSVSIIDETNL
DPKNKNSRTFPTEVNRGSDEHLIEEEDHKDESYNGERRNGSDLIRNRHLCKSCAFSTADS
GLVGDEDDEGSCSSEEEYSCPFTIGAKSRSQKEDPATVRCLTFAMSDQGSQHQNARISLS
GNNGDSCSVDSVFCKPQERYLRLMMDGLRALKVDETYIRDEVLRVNYIPSERDRVADQSY
SYKKFPVATKSNKMPKISFAKYKSKLCSNSKRGENATATYFICNRKVLKVNLLSDKLSSD
NDLSMYTNNACVKWLRALGHGKGDITLLVHQTFVDPDCLHIPLVDNDDDLTPMHYEWAEH
TILLYLERGGVTATTVYELTDSGCNSNAKTRRRSLSTSLTRVGKSVPSLTASMGRRFQKN
RFSDVDSGHSESSNALPKITSRFSAPTILTTKSSTAEAPTEDFSTTSIKAKSKLCGITTR
LKNLRLSKSENANEV
>comp2921_c0_seq1; 1118 199 1992 LEN=2299; translated
MSSQAPSVAQEVSASLSDGPSTETRNAEIWSTRLQRELLALTTDNASEEAKTEMTAVLPP
FCSIKEHALDIEAGNCTVTCLLDLPKAASTDGDKAEGSETTTEETDKADAPIVVTFDVSL
QKKADGSVDTSAVSYPFLKPLACLASGCGNFPEGSTIRDGDAIDIEMDWTPSLHLSDAIL
NVSLKIKECLSQGEAFHAAEVSSKASKAEQAVNEAVNKAKTLGANLGASLRNLAHNATTD
KDGEKKKIRLPMGRKKKQRPKATAKEIRIGDEINMLEAPWVDCQGVYSCKAIRRPKFAEE
AIALADSKDKITKEEETEQVASAGFAGAGLMLQKFTNSTRGLLEESFLMITESHIIEMRS
NKLNLSTGTVSFAIPIDMMAKLKFRRQESISLFFKPAPEDPLIYMCPDSADCVHQIQTVL
KRHGVKGKHTNAATHRAINEALQLVQEIQTKELALKHDPNIERVNQIMDLYRQAAERFEV
AGDVRHEEVVTHMRKFLALPLTVSVLDGSYVTPTRNADNEPIPSPVTKGGAYDSNMDSLL
SKAETDLSNMKGNDDKELDDLFADGTDDALGSTADTIAEFENMLKEADKELAELMAS
>comp2921_c0_seq2; 1148 199 2097 LEN=2404; translated
MSSQAPSVAQEVSASLSDGPSTETRNAEIWSTRLQRELLALTTDNASEEAKTEMTAVLPP
FCSIKEHALDIEAGNCTVTCLLDLPKAASTDGDKAEGSETTTEETDKADAPIVVTFDVSL
QKKADGSVDTSAVSYPFLKPLACLASGCGNFPEGSTIRDGDAIDIEMDWTPSLHLSDAIL
NVSLKIKECLSQGEAFHAAEVSSKASKAEQAVNEAVNKAKTLGANLGASLRNLAHNATTD
KDGEKKKIRLPMGRKKKQRPKATAKEIRIGDEINMLEAPWVDCQGVYSCKAIRRPKFAEE
AIALADSKDKITKEEETEQVRRRTASLLDQDSDQGPVPDAFGDYMRQQAGGVTQVASAGF
AGAGLMLQKFTNSTRGLLEESFLMITESHIIEMRSNKLNLSTGTVSFAIPIDMMAKLKFR
RQESISLFFKPAPEDPLIYMCPDSADCVHQIQTVLKRHGVKGKHTNAATHRAINEALQLV
QEIQTKELALKHDPNIERVNQIMDLYRQAAERFEVAGDVRHEEVVTHMRKFLALPLTVSV
LDGSYVTPTRNADNEPIPSPVTKGGAYDSNMDSLLSKAETDLSNMKGNDDKELDDLFADG
TDDALGSTADTIAEFENMLKEADKELAELMAS
>comp2922_c0_seq1; 389 91 690 LEN=843; translated
MSESPSWLDAPAPAPAAAAPAVEPMSIEGTGAAPTAAADEKDLPSIILMMRLLNLAAAAA
LIVVSSLQIVGIPALSSIVLAVYTICGGLLICCLETQLKFLRVMIAVNFGFLFNSFWRFL
FYLILGSVCWAFGGIMGRTMACVMGGVALFNTYVLCRYPSYRKIREQIAEEEDKRIEARV
SKEVKTQALKQMAKSATGK
>comp2923_c0_seq1; 598 327 2496 LEN=2778; translated
MSRRRGEFEHRTSIFEGAFRAWSHLGGLEPQELDSKASTDLDPLSHPIENDVRLQNKKDG
KAGSNADPNGSKRRQFPYEPLPRSGLAHPYVQAILSPWLGSDADSDAIQLGLTTLRTWWQ
HRRKGESGSAIAALGTEKMKGVVEGYTRHFFTLAYCLVINDKEQPPRTLHGKMKELEKVR
DKKKKKRSRDTEKGDNANAMGMNSMGRRGVDLSAEIMNNPNLSPLERLHMAQQNQLNGAG
GIKSSLTEPGQMINVKNKCVPGVVDLPGIVDKVSRAATQLAHSGGGRLLSNPSAVASMGG
MNGDNGEHSDTVPIIFVTENREIMIAMAVEGITCAHCVKIVETVLRGCNGNKSPIDGLID
AAADRTLNAILIRIDRVRNARRISLEATRNLAMVGYQVQVKTMRKIVEKNGNSTKVPQDE
MYMAFERMGAIDPRDIFDWSAPCSCPDNGVARDDCLXVRKWGESLLFHXSLSISFWLVRP
TKFSNLVDKFFYLDCIRHGQMNNSLLDAFETRAAGVRRFLSGDMPQPIQKNHIPQRMMMN
NHFPQQRQQQGMMDDIQPLNDTTGMGLDMFGGSSGMNNMVANMNAPNSGGDIRGSNMGSG
NNMNNNQMMSFGNHRMSLMGRMSQNPNNRMSLMGRMSEISYGRAMSGLSAISIDWENMED
FDVNVDHSEHINNNMGAGAAGAMGRARGPRVAPRWSMAQHMNMNNNAGGQNITDSDAHVQ
FKI
>comp2923_c0_seq2; 675 327 2378 LEN=2660; translated
MSRRRGEFEHRTSIFEGAFRAWSHLGGLEPQELDSKASTDLDPLSHPIENDVRLQNKKDG
KAGSNADPNGSKRRQFPYEPLPRSGLAHPYVQAILSPWLGSDADSDAIQLGLTTLRTWWQ
HRRKGESGSAIAALGTEKMKGVVEGYTRHFFTLAYCLVINDKEQPPRTLHGKMKELEKVR

DKKKKKRSRDTEKGDNANAMGMNSMGRRGVDLSAEIMNNPNLSPLERLHMAQQNQLNGAG
GIKSSLTEPGQMINVKNKCVPGVVDLPGIVDKVSRAATQLAHSGGGRLLSNPSAVASMGG
MNGDNGEHSDTVPIIFVTENREIMIAMAVEGITCAHCVKIVETVLRGCNGNKSPIDGLID
AAADRTLNAILIRIDRVRNARRISLEATRNLAMVGYQVQVKTMRKIVEKNGNSTKVPQDE
MYMAFERMGAIDPRDIFDWSAPCSCPDNGVARDDCLRHGQMNNSLLDAFETRAAGVRRFL
SGDMPQPIQKNHIPQRMMMNNHFPQQRQQQGMMDDIQPLNDTTGMGLDMFGGSSGMNNMV
ANMNAPNSGGDIRGSNMGSGNNMNNNQMMSFGNHRMSLMGRMSQNPNNRMSLMGRMSEIS
YGRAMSGLSAISIDWENMEDFDVNVDHSEHINNNMGAGAAGAMGRARGPRVAPRWSMAQH
MNMNNNAGGQNITDSDAHVQFKI
>comp2924_c0_seq1; 275 1 1236 LEN=1264; translated
SILLFLMTILVWEQLPVLKVNPLDSMVVNALATTNNKQLRMQTSSTSPPVSSPRIAPPPR
LPLKPPFPNGLCGGTMVDIVPEETFGVDVNLANFNFMGDMVLLPRTIRIWLPPGYDENDK
DGGHPVLYAHDGQNAMEDEESWTGASWRLIGAITRLADHKLICTSSSPATNSTSSRNNGN
ESRQPTSSSCTSDGKLPIVVLLPSAQGDWLPGIRRRHLEYGDMNFPFAQAHADFVANTVK
PLVDNRFRTNPSAERTFCIGSSLGGQASLHLLLRHPDKFGGAACLSPAFGPAILNFVDSS
KGGKILREGKKKVYLDIGGDMDDSKSSDFVRVSPIDVLDHLTPVHGWNPGYFWLDTQLQG
QVTAMRKALDRSGADYEYRQYPGGRHNERAWALRIDKPLLHLFSDKRRKKE
>comp2925_c0_seq1; 442 172 1741 LEN=1955; translated
MNEESSSSSSKRRGPTTYPPPDASAATDDATEASLTRRSKNDRIPLSLGKASLRIRLEAY
YSLISPAILSDRTAWLRKYDQIYEKVSTGTQKQESKRAVLSEPHSFRCLLSYQPGKSPLA
FNALLDRFLRHTLCDQIEPNQYGGTHSGERKLATKLAKKYGTAVRLLLVSDSSSLVPRFP
TDSNRSNDLANTRKSNLPRDESWYQLRKEEAGSGIVDFLSSRFDPVEALTGASGRGSVAR
ANPWMEELLPKTILDNIGKCAALLPTDDPLRKENHLQLQLRKSAASRNSSSQQNQLALGT
AAAAESVQIRPTTLRKQTNHDLHPFEAIASHLDTGPHSLLFRLRRDRKRARVVVRYVNMV
RGTITGSLVAFDKHMNLILRDVEEIYSPRINDKECRLSNLELELERRRRLSLDAEDLGTS
LEAAAATATSNQSGGSSIDYRVGKGCSAIQEQHQHQSGNWKVRRRQMKQLLVRGDMVVSI
YEAAQERTNVQKSRYRKRIATSALVRAGAFGTSKRLTEKEAK
>comp2925_c0_seq2; 481 172 1572 LEN=1786; translated
MNEESSSSSSKRRGPTTYPPPDASAATDDATEASLTRRSKNDRIPLSLGKASLRIRLEAY
YSLISPAILSDRTAWLRKYDQIYEKYGGTHSGERKLATKLAKKYGTAVRLLLVSDSSSLV
PRFPTDSNRSNDLANTRKSNLPRDESWYQLRKEEAGSGIVDFLSSRFDPVEALTGASGRG
SVARANPWMEELLPKTILDNIGKCAALLPTDDPLRKENHLQLQLRKSAASRNSSSQQNQL
ALGTAAAAESVQIRPTTLRKQTNHDLHPFEAIASHLDTGPHSLLFRLRRDRKRARVVVRY
VNMVRGTITGSLVAFDKHMNLILRDVEEIYSPRINDKECRLSNLELELERRRRLSLDAED
LGTSLEAAAATATSNQSGGSSIDYRVGKGCSAIQEQHQHQSGNWKVRRRQMKQLLVRGDM
VVSIYEAAQERTNVQKSRYRKRIATSALVRAGAFGTSKRLTEKEAK
>comp2926_c0_seq1; 220 42 452 LEN=574; translated
MVRSSLFSAIVSLLLVGPSHGFSPSTPSPSTTTTRLNGFGDAFKNAFANDDDLGARKNEG
LSGGPNYNESVTMNGKAVPGCVVGQKLTVVGGKARVKIPVNCQAGDCGTCVVKMNGRKVK
ACQTQVPKGKCNIQTL
>comp2928_c0_seq1; 1010 596 3958 LEN=4142; translated
MNFQRPRRSSRRYAGDRAIGTEAVANLSSMKQRRSADGTYLRRRYATIAVGLVVVIGVII
FAVTSTKAKDELPVSVAEITEKVISEITHSHTDSYELLTSQSYSDANSTIGVNSTLAVYE
HKKSGIDIVTMIPIDPYQDATFGINFRTPSERDDGAQYVVKNAVLAGSENYPVKDPLNQM
KRGSLQTYSDSWIERDRTSFVMASRNLADFRNNLKVTVDAVFNPLFIKDEHKWIYRQEGW
RLESPDNVNLLISGNAYVEAKAAQMNPEEVMIKHIYSNLFPGHVYGKNPKGEASEVVTMT
YQEVIAYYETYYHPSNAQAFCYGSQEFIDVCLNELDVQLSEYENNTSVGNHSQIPWQELT
EVEEQIKNITYPDYQEQVDYRSVIAWILNDEPMDLRTQIAWQLIYELLAGSNTALVPKII
FDSNLGTDIVTHFQHSLQQWVLALGVSGIESYEDVATADRKIMQELAKVVSKGFDSDAME
AAMHKMEFKFREQSPDDVPLGVKYFRDILRNWNYGQAPLEPLNSATEFLNLKAEIQNEGQ
EFLLTLMTQRMMDNKHTVSLSLHPDTNYAIQYERLEQNWLDNLDTYLNKEEGLKVMSETQ
KFKEMQENGDSEEELAKIPRLEVSELNQTMYIPPSDLIKNLYETDVTAIKHKLPFTNGIA
YVDFSIDMSNMDFDDVVLIPLFCQLLLEGGTEVHDDVEMQRQIDKYTGGVTITPIIEEIV
QTDDHDYYLVPDGKHFVTKLIISGSCIASDTCLPMLILFRQFVWDSNVRNKEKAIEILEA
RINEMEQDVQINGHKYTTSRVESRYSLSGFVREQWYGITQLMQMRRALAQIKDNFTELGL
RLVKMQDELKRGHHNGMHMSITGDHEALTDIKGPLSNFMKNGLPLATQKTPFPNFAEEEH
PWVPKGMHRVKEEFSSETSNQAFTVPTRVNHVAKGGILYDVGERIPGADMVVTQYLGGYY
LFNELRFGQGAQEAWSFLDLDSGVCIYQSDRDPGIFSTLETYEHGATWLWEQVHNGELPL
EAKSAIVGAIGRMDAKIMTEPKKLGRQALISHLKEDRHEFRQHWRNQILNANADDFMAMV
ERLGSWGHPSVCVVTSPEVYDTIDQEDFAISKCDYSGYQC

>comp2929_c0_seq1; 1127 128 2032 LEN=2128; translated
MKLFSRIAILGLIAQMRDTEAFGLVSKPNAPIFTGSSTSLNAGSEVFMPALSSTMKEGKV
VSWLKSEGDEIEAGEAIMVVESDKADMDVEAFEDGYLAKIIVDEGEMAPVGAAVALIAET
EGEIDEVASAGGSAPVAAAEEAAPAAEAASAGAPDCEFSQIDMPALSSTMKEGKVVSWLK
AEGDSIESGEAIMVVESDKADMDVEAFDDGFLAAIITDEGETSSVGAPVALIAAEEADIP
ALKAYAASLSGAPAAAAAPAVAAPAAPAAPKASAAPVARAPGARVVASPLAKKKAEEIGL
DLSTVAGTGPDGRITAADVENAAKGGAPAAAKPAAAPAKPVWTPAAGVIAATPMARVAAK
KAKIDLATITGTGSLGRVTVDDVKMATGEKKAERKKAPSGEAAVELPDGFVPFSGMQKAV
SGNMVATLTVPSFQVSTDIEMTAFEAMYQKVKPDGISVSAMLAKACALAIEKHPIINSAY
SDQDGGGILYKGDINIAMAVAIDGGLITPTLMYANERPIKEIAENWRELVGKAKTGTLSP
AEYTTGTFTISNMGMFGVTSFGSLLPVGQGGILAVGATQDKVVPDSQAIMGMKKARLMTV
TLTADHRQIYGSDAALFLKTLKSIMENDVAKLAK
>comp2930_c0_seq1; 353 110 1672 LEN=1764; translated
MEDTVAKQSSAATSSSTSSETWNVAVKNRGPFDDRRGSLREGGWWIDLDEAKKLGMDEEW
WTETEEPTKSPVALPKASPTIYHSWGNVKYRPKGPIWLSESGTMYLGPWAEVRQGPGGRT
VRVAKGIGISCQKNGQCHIGEYRDGLFHGWGRSFWLKNSLVWKRNINEEEKQIMETRVVV
TGDGQKTEELVGLPYEYHGTFRSGSKHGIAGKVVLKDLTTRVGSWVDDIAVLRKWYRIRQ
DLDPVLEEYQADWWKHHVKLDCVPIDSPQSRYIASKTRKNAKHELPSPSVLSRRKRSCRA
PNRKKHKSSKTRKCGEESHIGTETDHRGEDETDNHPRSEYNQDAEFKQENSLGAGTEVID
VDALDPDGLVPDEPPSSLMPERIISAVGNSNRHLSSQVFPDAYLSEAEESNFPLHTAVKF
GDEDFVDEELEKIATGTGPEGSPIGSDRSYDSARLNAVDSKGRTALDLAALTGQVGLIKK
LRRAKGISFSFKPGPRMMILAKKRSKDVAKYLEQVHETVE
>comp2931_c0_seq1; 577 557 1585 LEN=1679; translated
MANSGKDTNGSQFFITFRPTPHLDNKHVVFGHVDLSSTSSKDSLRILENVRTGRSDRPVT
PVQIVDCGIISDDEGNDESEKGAKKGLVSGENTTKHPTAKDDIEVELPVEEEEEVPPKTK
AEALKRRMRKLKMKMNQARQLNKQEVLREGERLGSVEGAAKARKRQQIMDKKAKELEWKK
RNSKALEVAAAAGLDGKHLVEQADASLRKAYKKEQKAVSQQFAVNDYYNPEGQHRNYERN
LKSLSRAAGPLPEDAVTNGHATFDPTTDESDEKREREGARNLANELKRRIEKQKEKRNRL
EFEGEDVTWINQRNKRFNQKISRNFDKHTAEIRQNLERGTAL
>comp2931_c0_seq2; 723 1 1448 LEN=1542; translated
PNLTELSDENRSDKLQQTPRKYFLTESKTLCTHKGQSVKSXTMVQKAFLKIRVGERQVLS
FDLVLYDDDVPNTVRNFVHYLEERGSTGYLNSRFHRIIKGFMAQGGDFVNGDGTGTASIY
GGTFADENFLRRHDRPGILSMANSGKDTNGSQFFITFRPTPHLDNKHVVFGHVDLSSTSS
KDSLRILENVRTGRSDRPVTPVQIVDCGIISDDEGNDESEKGAKKGLVSGENTTKHPTAK
DDIEVELPVEEEEEVPPKTKAEALKRRMRKLKMKMNQARQLNKQEVLREGERLGSVEGAA
KARKRQQIMDKKAKELEWKKRNSKALEVAAAAGLDGKHLVEQADASLRKAYKKEQKAVSQ
QFAVNDYYNPEGQHRNYERNLKSLSRAAGPLPEDAVTNGHATFDPTTDESDEKREREGAR
NLANELKRRIEKQKEKRNRLEFEGEDVTWINQRNKRFNQKISRNFDKHTAEIRQNLERGT
AL
>comp2932_c0_seq1; 758 78 1355 LEN=1840; translated
MKFSIAFTALTAAVVSANPFAPAKSPNSSKTKYMSKLMRNARPTPNSQLGRKLDEEEAEA
EEYEVDISGYSLKFEQCQFVKAYDDELAGEDSDSVLATRRFVIFRLCPSDACDSCNYNFG
EYIVDLEEYLEATVNYQQEMQEEMCNACEENCQWEEDEEEEEEDNGRRRRKLEDYGSNCN
TCYEECMKIENMEDNGYIDATQFLDCQMVYDPEDDGLSALYAGPMCASNGSKIKIGIFTD
EECNVPDSDKDVDDYLMDGDGVSMKLSHALLKTVYSSECVSCLAPQEEEEDDENGDENDN
GDEEEVEVLEMCEQLYQASAKCEKTNGFDNGYASYDGYENQLAQESVVCDFVESLKAGSY
DESGEIVVNGKSSSVGGGSQTTGGQKFALTFFILGTAGLAGYAAMLHSKLTKGTMGLAET
GGSLA
>comp2933_c0_seq1; 73 1 379 LEN=869; translated
XDDDDDSNEEASGGGDDESESAKPAMPPKRNTGSWSVEEQEAAAXGPRDPRDDKHKGDRA
DGRNPQHRSGPPLHRSPLPQAPCDGNEFRGIVVRQLLSRDENETRRGNRAREPIAKAIAR
FLACAS
>comp2934_c0_seq2; 125 194 991 LEN=2586; translated
MSPDIHIRLSLPTRPVPVLINENLDDEILVPSERACPNRMSSPPHYFSPNDHFRKSIMKW
KTLDESLKSTDETEETREGRGMKKNSLQFTIINIREYSRTVGDNPSCSSGPPLSISWEYM
NIGEIDVDEYENMRPPRRVQREMILPRNMREDILRYECNASRQEITESIRQNVRIKNQRR
TTINNLGKAEKLEEVMESASRKLRRFVTRKKSVHKQVEDLEKEIDVCNRRARKLNYLEKT
MSQGREESFPGESTLDCADSVSVNS
>comp2935_c0_seq1; 565 229 1731 LEN=1986; translated
MTRQLLLPILVGAIASMNPATGMVLREPPKVSSPAGSAVEYGKWSPSSWRTKESKQIPEY

PDQDALKAVEDQLSKCAPLVFAGEVRNLSDQLALASQGKSFVLFGGDCAESFDEFSVDHV
RDTFRIILQMALILTHGGSKPVVKIGRMAGQFAKPRSSPTETIDGVTLPSYQGDNINREH
PFTAEARQADPKRMVEAYDQCSQTLNILRAFSSGGYANLSRLHAWTLDFVEATPVGSKYR
KLASVVEESLRFMNAIGVDLESPAMQQVDFFTAHECLLLPYEEAMTRIDSTSGKYYDTSA
HLLWVGERTRQPGFAHFEFVRGLENPLGVKISDKASPEDVLEILNTFNPDNIPGRVTLIT
RMTAEGIRTKLPAIIKAVQAEDKHVVWVSDPVHGNGFQAENGFKTRSFDAIREEVVAFFD
VHRECGSHPGGIHLEMTGKDVTECLGGDTNGVTMDGERGLASKYETHCDPRLNAMQSLEL
AFCVAEKMREAEAAKEEASN
>comp2936_c0_seq1; 186 335 778 LEN=827; translated
METSAKVTLFEDIFEVTALNPDGYKFDRVNRIQAVGTTFECDLLLDINCEIYSLREHDKV
TLMLASTLHLDGSPADHFSYTPSSEPTLADNYEYVMHGRVFDISYKKDGTAVLALSFGGL
LMRLTGDQRHLANVLPDMRLYLLLKKD
>comp2937_c0_seq1; 174 90 671 LEN=761; translated
MSSVTCSSEFCNSGWGEVLTHMSPYGFANFGVAFGLGLSIIGAAWGIWLTGSSLVGASVK
APRIISKNLVSVIFCEATAIYGLIMAIILGNKIKEPETVSYYLEEGWDYNGYYYAGYGMF
SAGLSVGLTNVASGVSVGLAGSSCAIADAQDASLFVKILIVEIFASALGIFGIIVGIIQS
NACTFPTYALGQE
>comp2938_c0_seq1; 836 154 2362 LEN=2576; translated
MKFILTTAFLASLALGQNNHASNKDAEDFAYWRDLVDAVDSMPPTSAPTKSPVTPTDAPV
TPTDTPVAAVTDAPTDAPVTPTDAPVAPTDAPVAPTDTPVAVVTDAPVAPTEAPVTSTVA
PTVAPTIAPSVSCKTIAEVITTTPEFATLTTLVEGAGILDNFEQGTLTLFAPTNDAIAAL
PSELVDTILEDKELLELVLLGHVVRRQQIMVENLECEGGPISSLTMANGAETTITCGMSL
DPEGNEVIDTFIEDNGVPGKIVGPDGMACNGVIQAIDAVIIPDIQTTEAPVAPIPKPTPA
PVTLPTIAPIAPTIAPVAPTDAPVSGTQAKLSTFALSNGAEFEDTTSYQYKALKQTESQI
GVDSFTDAKLVQYYALYCIFFATNGVPNVITDADPRFEGIPFPNWLITTGWEATDIDPCD
WYGIICDAESRVSIIDLFENLLTGSFPPEVVLLSLDGPFSTGAGNLFRIDLFRNEFLYNN
ADSSWMTDLGSNMTTIIAEETAFSGDIPRLPENLVNFDISFSYFTGGLIDTNFESLQELA
FIDLDGNAFNSTIPAVFGRLPSLEFLYLSDSFVSGDISYMQGMPSIREHWIDTNPGLKGP
IPAYIGDLTTLESWSMAFNSLTGTIPSELGNLVNMKQMWLYANELTGTIPSELGNLDTMR
ILELEGNSFVGSMPAEVCANTVFPTQIIEKLGADCEDENLNVLVAHAVVYWNAALNSRNQ
VTTRRKPKEFNCLSY
>comp2938_c0_seq2; 523 154 1761 LEN=1761; translated
MKFILTTAFLASLALGQNNHASNKDAEDFAYWRDLVDAVDSMPPTSAPTKSPVTPTDAPV
TPTDTPVAAVTDAPTDAPVTPTDAPVAPTDAPVAPTDTPVAVVTDAPVAPTEAPVTSTVA
PTVAPTIAPSVSCKTIAEVITTTPEFATLTTLVEGAGILDNFEQGTLTLFAPTNDAIAAL
PSELVDTILEDKELLELVLLGHVVRRQQIMVENLECEGGPISSLTMANGAETTITCGMSL
DPEGNEVIDTFIEDNGVPGKIVGPDGMACNGVIQAIDAVIIPDIQTTEAPVAPIPKPTPA
PVTLPTIAPIAPTIAPVAPTDAPVSGTQAKLSTFALSNGAEFEDTTSYQYKALKQTESQI
GVDSFTDAKLVQYYALYCIFFATNGVPNVITDADPRFEGIPFPNWLITTGWEATDIDPCD
WYGIICDAESRVSIIDLFENLLTGSFPPEVVLLSLDGPFSTGAGNLFRIDLFRNEFLYNN
ADSSWMTDLGSNMSKCGHEQFRYSRNLNCLCTVNLTQSSHLQSNSRLNFSATIIAE
>comp2939_c0_seq1; 160 261 1034 LEN=1495; translated
MNTTTVRGGIGSWRKKTSQEEPDEGESGVVNASSSPEANGSSNHNHMSNSEHQYHKTYLD
LFGETAKDFLTRVAPERISRIVPPEEPGCRWDWRYLRCEPYCACTLSPKFPWDLHLGRAC
RKRGGAEGTDGGTEAGFEEGADYRGLCEGGDSRPGSTLLSVLRSPAPFLAKTSGAAWRIL
RTRADPVVARAAGELERVGAGVSAVVCGDLSDRCKDGGDGAEGPPPVLAWQERLVCREIA
RDCGLVPPSRGPDPAEA
>comp2940_c0_seq1; 484 123 1037 LEN=1341; translated
MGAGREDEEITDLLTKLYTIQEDIGAKPKTSEDEKRAKANNTAKMGSSKKAEKKGSKFLE
LKSTIVDRLKTIHQLLKDTKKLEGAGYGGDNAKDIIKMQAETRENIRQATDEWRELEAIY
KKEARKKKSKFTPEELEVQSELTKRLYAEIEKVKDAQMKGYARSRTPDAAAALNTKALAY
NPSGSYGGKKPSWGGTGGGVALTGDQQQQIMQIEERDADFDLQLDEIGEGIQDLAEIAQQ
QGEEVRLQNQMLDKVDKKLDKNLERMTTVNARMKETLEQVGRSGDKIMVDIMCIVLAIGF
AAVIW
>comp2940_c0_seq2; 511 123 1055 LEN=1236; translated
MGAGREDEEITDLLTKLYTIQEDIGAKPKTSEDEKRAKANNTAKMGSSKKAEKKGSKFLE
LKSTIVDRLKTIHQLLKDTKKLEGAGYGGDNAKDIIKMQAETRENIRQATDEWRELEAIY
KKEARKKKSKFTPEELEVQSELTKRLYAEIEKVKDAQMKGYARSRTPDAAAALNTKALAY
NPSGSYGGKKPSWGGTGGGVALTGDQQQQIMQIEERDADFDLQLDEIGEGIQDLAEIAQQ
QGEEVRLQNQMLDKVDKKLDKNLERMTTVNARMKETLEQVGRSGDKIMVDIMCIVLAIGF

AAVIWNFSKK
>comp2941_c0_seq1; 480 174 983 LEN=1694; translated
MDFTLNTILGDSDCATLLARSLFFVSRGNQKRLALGGTCGDRTANGMERKTDTDACVVSF
HERTSKKRGNKLLGSGLFSVVKHRDSLVVLENQNRQQHLKHIDDDDETASTVDMDEDEFP
VAASSSSNHREKKERRSVQFEEELVTAVYTRPRTTKEDKYYLHYDEYDYMDFKLEYRDQL
LQQRDRERNSKGRISNQQQQLPCYRRSPRKVSFKREVVESVHPVMDRSQRKKIQKDLFYT
EEEMRTFLDEFVVSLQKQQQQQQQVVVTS
>comp2942_c0_seq1; 232 1 705 LEN=999; translated
NQPIGDWDTSSVTDMSGMFSNTTSFDQPIGTWNTSSVEGMGRMFYSSHFNQPLDEWDVSA
VAHMFLMFDGATNFNQCLGSWAEKTPDYVSVFGMFDNSACPNTSDPDKTKGPWCQTSNDS
CGHLVSSDCANEKGFTIKRKYIECNELSELSDRERNALCRKKVPKQACPGVCDRRCICKD
KKRFSIKKNRKHIFKGSYKCRNVGKQNQPSCDTKAKKRLLVKDLCPKKCDSCLT
>comp2943_c0_seq1; 639 199 2009 LEN=2009; translated
MPSGKPRLNTPCYRWDFSAKECLCLHSNLMKTTSNHKKDDENAFLSDKSLSVVLLHGSWN
HDLSPALSDEDVSEHAGKEEIEGQNRRETTKQIVMQQIAFAFSAARDWAVKLSREQAGTD
ASIARDILEYADCRTIGGSMLVDSSDDETIDIAVDQFLAPEDLPALVVVANGDVGSPEAL
LHPVRGLDSRTLGAFLSTPSSEPTYNEASFTVLNALRDTLRNIFEGRPADAGGGEAGKTK
TGPSLEGETEALRIFVSGDRSQVGKSSVCMGLIGTLVHDLGYPPSSLAYIKPATQCEATQ
LVALYCEELGVSHRAIGPIVYYKGFTRAFLAGDTAPSEVLLEQAGEAVDAIAEGKRIVIV
DGVGYPAVGSICGTDNASVAAACGPIQSQTSQGQGRRPPVPVLLVGKKGVGDAVDSFNLN
ATYFESRGVPVLGSVFNRLPLEGYYSLDNCKQAVGSYFEQKTDKIAFGFIPEVASISSSR
EEGAALELESAKEFVKTFADHVDVNAIVEAAAKATAVPVRKIMAPPLSHKNLXPKNAPTN
HAGGIGGKPDETAQHRSQGSAEDPTDEGAGRGGRQDSRSGRWMRRKVAAKPWLRAESNPP
FESA
>comp2944_c0_seq1; 293 128 1078 LEN=1118; translated
MSETESIDVAFKMVAILGSMLAVIVSLVAMQTKIKGGDSRWLPNPTSEPSKRAYEIYALC
YTPIWIFAFGCIVAFGLYEDFDKWSYMKVCVGLSLPFLLQPVIFASAGTPDSPDEKRPLI
ERYSFKANVWIAVYSFIGNYWYTHYFYSVLRAKYTMPAHRLNNVPLALYFATHFYFSTYH
VFSNLLLRKVVTTYVPGMVRSILYVTVVFTFAYFTAFMETLTISAYPDYSFEDRNMAYTV
GSAFYGIYFIVSFPAFFVFDKDIDNAEAKRKTSVWDTIVSSCGYGMIIMILLDIVRLHLG
IPLVIGSSDDICSINK
>comp2945_c0_seq1; 1339 109 4128 LEN=4735; translated
MDMSEACYAAVPHESSPLIRKKKTKLLPVLLWVGLAYAVFQETSSHVRKSVSVAAEVVRS
STKNRGNHYNNDPVHFDDTDTLDIDDLHEPVHRSFGSDVDIDVDIDSEGIDWDNDEDKDE
NIERKNSARTGPNIILVATVDGTIAGISKESGTVLWRQSEDISSVPSSSSSSSSYLMMES
TKNDSCSPILKPLVSTTTTKKSASDASYKVVPSVDGTIYTTTNDMTIATPVKDLVAKSPF
LDQQGRFYVGSQYASTAALDGTTGEVLRVVSSTATTIPDDEDDNESSLPTLDDPNIVWIG
RVDHEVSIQDAKTGFTDAQFSVGQVMSVEDMRGMMRESVSNPEQTIRPNGSSLLSKAVAT
TVNEAMDLPNSLVATPNGNAALWSFENNKFAWVASESLNSPIVYAMDAETGVTLRVDIIP
DVPDPKANIRDLTREMERQSRMASVTNTPVVGAMSNGQLYALPLRSSDQSTSSATIDQLP
TQPGGKLIKSPLPIGDGENDFPDSSLVIQKEEEGFFHPKYGYISPKDLGGFVHPVYGYVS
PRDLFGAPKRSSKTSSLFRVLGSWLPPTIALLFVISFELGRRKRLKDEKKGARNSLHPIE
QKDQINGVISFNEDSGGKYGALQKSHHQEVISVSEDVLGYGGHGTVVYKGVLDGRDVAVK
RMLKAYHASADREISLLIESDGDPNVVRYFLKEVRGDFVYLALELCDLSLHELIGVLREK
QQEKSSSILPNADKEACVLSNESVGATTRILLQIASGVKHLHSLRIVHRDLKPANILLAV
SKKGKKKVKEEDPIFATFTRNYYDAKISDMGLGKQLIGQSSIGASFIGDSSFRGNKSGVH
SIGVGPGTVGWQAPEVMAMRLTSDTSARSTDSASNIGESKEGQGNDILPSIRTSRSVDIF
SLGCIFYSLLIPGSHPFGEWFEREANIMHNRPNLKPLTSISSEAYRLIRSMLDRNPRLRP
TAKEVCEHPFFWNAEKKLTFLCDFSDRLETDTIAPGSSNVFGSFAIERGALEVVGTSWEE
KLDSSLVDNVQKFRTYDYSCVRDLLRLIRNKHHHFEELPEAFRLNEAPNQNALCKYFTKI
FPALIMHCYTFCGQNLTDDDPLVLKFVIPPAKFQTREFSTAPISVSVKEETIPTNHNEAV
ISSEELKNDSTDTQCLEIAQVVTCDSNDEVDEDIIVWEGSIAAKTFNNRGWSRSQDEWSR
HIEPFYKKRDPASKRCMEDPKFRTRLCNHWDMSLGAFCPMKKKNKCVFAHGPAELRVKEG
KKNRWGKLVDKNGNNSNPWHCGGEDTYGAANSIELVRKEEGKWNASKDRKKNQGTTLRKK
KPGSNSNQVKQKTNPKAPA
>comp2946_c0_seq1; 738 61 1683 LEN=1835; translated
MMKADIETKRIQNSNENDATMDSNTDTSKKRRRGCFGLIRHYQVTGILIFAVIGIGAGIG
ISSWQPENEDAKDIAVKWIGLVGDLFLRALQCFVLPLVFVNVIVAVVEMVSVGKASSVGY
TVIGLYFATTLAAALFGIVSTLAFMGLYNTEEDIHEEEISPLYIQLGCNDADSVLAEAKN
GTITCISKALADGIDSTFILHDLNNTLVETNSAVTQVSISDTVYEGIFEKIVPNNIIGAF

ADSNFTAIIFFAVLFGAALSPVILKEKNGGESSLMTMLKETDLVFQRIIRWIIDLTPFAV
CSLIASAVGEQEDLFSMFSSIGLLMASTIVGWGLQVVVIYIGLFALLTKSNPFGYLKQLI
PAQMMAFATSSSAATIPASLAAVKSTGRAPDTISRFVIPFGATVNMDGGAVYFVCACIWL
AVLNGEDVTVSSFFMLIIIATIGSMGTAPVPSAGLVLMITAYNTVFGTADVPHGFGYIVA
IDWFMDRCQAACNITGDCIVTAIVAHRCPVDETPVIMVQTNDDDRDSLTTEEEDEQCSEV
>comp2947_c0_seq1; 263 1 1012 LEN=1183; minus strand; translated
ALMDALMDTLLGLRGPEVVGQRQVKKDDEAEDGSRFRFFVGWSHSYSYRCGVYKVKGGNL
GVAVAMDVAAAMAVAVVLGRSEASSLAADSTHAFQLPFLGADRMDELVASEKAMQSSSVV
LVEVDVVVLAVLGIVLGIVAVVLVLVLVLVPAVLGIVAVGVSVSVSVSVSVSVLEREKPR
GPRTLPSSGSASPAQASVGGGSRRTLASLEXFPGTFPGTFAGSQASARALAQQERPVQRP
NGPVVPADLDAQGGAVVQLLGEEPLAGGTGDVPPNGLVFVLLLMVLVLMMVLLILMLLLV
APAAASEEATGDATSVLGSWFSMVGFMDCLLDCLLA
>comp2948_c0_seq1; 558 87 1322 LEN=1450; translated
MADEPSEPPADTTPDASDDAPGGLTDADISSNWDEVIESFDGMELPEALLRGIYSYGFEK
PSAIQQRAIRPAISGRDLIAQAQSGTGKTATFAIGCLAVIDQSVQQCQALILAPTRELAC
QIQKVVLALGDYMDIKVHACVGGTAVRDDIRTLQNGVHVVVGTPGRVNDMITRRALRLDS
MRQFFLDEADEMLSRGFKEQIYDIFKFLPEQVQVCLFSATMPLDVLEVTNRFMREPVRIL
VKKDELTLEGIKQFYIAVDREEWKLETLCDLYETLTITQAIIYCNTRRKVDWLQEEMQKR
DFTVSCMHGDMDQRERDIIMREFRTGSSRVLITTDLLARGIDVQQVSLVINFDLPTNREN
YIHRIGRSGRFGRKGVAINFLTEGDVRYLRDIEQFYTTEITEMPMDVADLI
>comp2949_c0_seq1; 343 616 1367 LEN=1880; translated
MTPSTDTTNNDSVLLALPSARTDSERGNTNTVREEFEHHQLDNHTCICIFDNDLVVETIF
SFLDARSLLSLCSTAKATHHRSRLRYDHVLRSARYGADSPGSASPRPPTVSKTRADHHAF
LVVEKLVSVFGLGGNENENGXKQKHQEHQESTATTVGPTTHTHTDAAAAIGERQEVRTVQ
PQAVPLLRNGGKHGAGPHHPPEPVTRGILLHGVHISTDTNKGFDKNDDDDDKPSRNSKSY
HHHHDDDPID
>comp2950_c0_seq1; 249 122 850 LEN=952; translated
MSRGFLFTILYFASVIPSVLGSAKLVPKTSTASCTGHLSDLILTSITCDHSNSGCTYGSE
VFVTGQVTSNADLPRPLKISVSRTFPSIYGIGTQVSSSEVYDLCSSDAISLVDDNSGSSC
PGAGVYNFNFLFDNFGTSKSWYAGWHGFSMGMAVHLKHEGGGSDFATCTLNVSVEGSDET
SYATNATLVSFATLGLAGLCVGMFSKKRKERLFKCGENIEDERTKELATNFELVRDSTGA
MV
>comp2951_c0_seq1; 375 100 1001 LEN=1079; translated
MARCTAAFALLFLCLGTTSWAFTTTNSPLAAVSIARTRRSQTPPVVVLGAGGFEWEDPTD
EQYDQGVENPFKNPELLKAMKKGDDESGDDENAKTIDPARLLGPRLQGSNLYLVGMMGSG
KSSVGDKLARRMGSYKFLDTDEIIEKATKLTINEIFESEGEEGFRDVESQVLDTVHAYVR
CVVSTGGGLVCKPKNWGKLQTGIVVWLDVAPEVILKRVEGNEDRPLLKTKDPLQTLKDLL
EERTPKYNQADLRVEITEDMDEDAVASRIVLDLHNFIDDNXAGVEAREAKAQEEGLDWVQ
>comp2952_c0_seq1; 249 553 1293 LEN=2355; translated
MYHPLPLRFLCSVFIVLVAVNQQCCDAQQQNPLTMNGGSLLAMAGDGCVALAVDKRFGSG
PQMVNIAPRHVWAAHPNLMLAFSGLEGDVQSLSEELQLEVAAKRNRALGFSVGGLKIGNE
KGSNDEMDERRFRISPPALACLTSHVLYQKRGYYVEPLVVGLMEGTWAPFLCAMDMIGAQ
SFSQSFICSGAASTSLYGTAEALWKPNMKPKDLLECCGKVFQSGTFTNCSFIKHVFLYYC
CVIDFH
>comp2952_c0_seq2; 256 553 1320 LEN=2224; translated
MYHPLPLRFLCSVFIVLVAVNQQCCDAQQQNPLTMNGGSLLAMAGDGCVALAVDKRFGSG
PQMVNIAPRHVWAAHPNLMLAFSGLEGDVQSLSEELQLEVAAKRNRALGFSVGGLKIGNE
KGSNDEMDERRFRISPPALACLTSHVLYQKRGYYVEPLVVGLMEGTWAPFLCAMDMIGAQ
SFSQSFICSGAASTSLYGTAEALWKPNMKPKDLLECCGKVFQSALERDCLSGYGILIYLI
TKDGIAEYDFASRND
>comp2953_c0_seq1; 1158 61 2976 LEN=3010; translated
MPLRLDIKKKLSASSERVKSVDLHPTEPWVLAALYSGNVMIWDYESGSLVKSFEVSELPV
RCAKFVARKQWFVAASDDMRFRIFNYNTMEKIKEFEAHADYIRCLEVHPSLPYVLSSSDD
MCIKLWDWDRGFDCTQIFEGHAHYVMQVKFNPKDTNTFASASLDRSIKVWGLGSHAPHYT
LEGHERGVNCIDYYPSGDKPYILSGADDQTVKIWDYQTKSIVHSLDGHTNNVCAVMFHPK
LPIIASASEDGTVRIWQSTTYRAETTLNYGMERAWALAASPQTNKLAIGFDEGCVCIELG
SDDPVASMDATGKVVWATNNDIQTTSVKGLVGSGEDALPDGERLPVVPRDLGSCELYPQM
LKHNCNGRFVAVCGDGEFIIYTAQALRNKAFGQALDFVWSGNGTGDYCIRESSNSIQTYK
NFKESQTIVPATASADGLFGGTAIGVKGSDGVLFYEWDSGEFIQKIEAIPKDVYWSDSGN
MVLLATEDSAYVLSYNSQQVSQAIAMGQVNPEAGIEGSFELLYEINDTITSGKWVGDCFI

FINGVGRLNYCVGGQIETLVHLETSAGGQSQHTILGYLAKEDRVYLVDKSLNIVAYKVML
SVLQYQTAVMRGDFDAANQLLPNVPESEYTKVARFLESQGFKEEAFAVTTDPDHKFELAL
ELGQTETAHALLLETPDEDKDSTETESKWKRLSDAALKENNLELVEAASVASHDYSDLLL
LYSAIGNLAGMETLADAASKDGKTNVAFVAYLLTGNVEACAELLVATKRLPEAAMFVRTY
LPSKIDEIVALWKQDLSTISETAAEALATPSSNPEQFPDLSIGLQVEQMFLQQREATKLT
GMPGVNYLSAKDDLELNLIELIKARQGAGETKEPEPADVVPAPPQDQAEEVGEAEVEQAI
VEDAAAVEAARVSAELAQAAEAERLAAEAAAAEAAAAEAAAAEAAAAEAAAAEAAAKAEE
DDMADDFGDDW
>comp2954_c0_seq1; 182 77 652 LEN=1186; translated
MGWFDWMYSALASLGLYHKNAKILFLGLDNAGKTTLLHMLKENRVQVHVPTLHPNTDELI
IGNIKFKTFDLGGHETARRLWQDYFTTVDGVVYIVDAIDRGRFPEAKQELDALLTTEELQ
DVPFLVLGNKIDMPSAASEEELKYELGLMDTYGKDKGPDGSGARPIELYMCSVVRRMGYA
DGFKWLSQFLS
>comp2954_c0_seq2; 155 616 1077 LEN=1611; translated
MLKENRVQVHVPTLHPNTDELIIGNIKFKTFDLGGHETARRLWQDYFTTVDGVVYIVDAI
DRGRFPEAKQELDALLTTEELQDVPFLVLGNKIDMPSAASEEELKYELGLMDTYGKDKGP
DGSGARPIELYMCSVVRRMGYADGFKWLSQFLS
>comp2955_c0_seq1; 465 329 2124 LEN=2509; translated
MSSKKSSSKSSSKSSSKSSSPKVYLTPQIEKQAKRRESLCPSLLLEDHVRLSQSKKGNGL
AAITKSYQTNTKLLNSEVSDLENDAQELQFIVDSVQKYLSKIVDEKKQKSDLISMKFKPK
GCTWVLEMNPDVGATDYMITVVCYAKGEGNGITTFVVDTKLVGKQNDSTTSTTLPGFREA
KGVLEFTALMVRKGVVRCEFPVFPEGLKDKSFREIYQLNAREGKNLFFIDCLHANMNGGI
ISEFSHVFVVMDSIXLIVAVLMKIISPFDLYRNLLVAVASSCDGTSSDCIAAQIWFLRDG
LPRNTSGNWEKGGDKMVLRKDLPPNDDAAIYEEVSIMSSLSHPHVVPLIDFFEEKDCYFL
VMELMSGGDLFDRIGKKKSYSEEDARDLSLKMLKAMAYCHAHKVAHCDMKPKNLLLMSED
NHSYIKVADFGFAAHCHTEKCLTKQCGTPFFVSPEILMRQSYDQQTDMWSVGCIMFLLLS
GNLPFNGRSQKELFRKIVSGKYEFKEEGWDGVSEDAKDLVKKLLVLDPDERITASQALRH
RWMKASRDRLARITLQGTSQRLKTFNARMKLRSAMIAVEWVAQLKKTSWGSKKVVRD
>comp2955_c0_seq2; 551 329 1912 LEN=2297; translated
MSSKKSSSKSSSKSSSKSSSPKVYLTPQIEKQAKRRESLCPSLLLEDHVRLSQSKKGNGL
AAITKSYQTNTKLLNSEVSDLENDAQELQFIVDSVQKYLSKIVDEKKQKSDLISMKFKPK
GCTWVLEMNPDVGATDYMITVVCYAKGEGNGITTFVVDTKLVGKQNDSTTSTTLPGFREA
KGVLEFTALMVRKGVVRCEFPVFPEGLKDKSFREIYQLNARLKSGSFATVCRGTHRATGK
KVAIKCVLRKDLPPNDDAAIYEEVSIMSSLSHPHVVPLIDFFEEKDCYFLVMELMSGGDL
FDRIGKKKSYSEEDARDLSLKMLKAMAYCHAHKVAHCDMKPKNLLLMSEDNHSYIKVADF
GFAAHCHTEKCLTKQCGTPFFVSPEILMRQSYDQQTDMWSVGCIMFLLLSGNLPFNGRSQ
KELFRKIVSGKYEFKEEGWDGVSEDAKDLVKKLLVLDPDERITASQALRHRWMKASRDRL
ARITLQGTSQRLKTFNARMKLRSAMIAVEWVAQLKKTSWGSKKVVRD
>comp2956_c0_seq1; 259 310 1647 LEN=1899; translated
MTVTMTSRFSVSKIVSLAYRTTTVLILLTISCHETFFVTADEPESRVVGGQNAEVNEYPF
FTSWGASCGATLIHDDILLTAAHCNPVETNQVVVHAYHKGRPSVNSQLRWVERRIIHPQY
NVDTWNYDVMLLKLTQPVNSVRKVKLNRDTTKPAVLDTVTPLGLGRIAEVDGAFPEVLQE
VNVRVIQSSKCNQAPMYPGWIQESMICAGVSGGGQDACFGDSGGPLLYRDNATDEIIQVG
VVSFGTGCARPDKPGVYHRISSSYDWIQEKICEFSSNKPSTCPGVISDPIPSANPRDTAI
DGNSTQLEESDQPELLQQPEDGEQEQVVNLFTHPENPPPLPLGLCEGDCDQDSDCAEGLF
CFFKTNGIVQNVPGCEGNDNTSTDFCVDIKYRPEGTTSNPVRQSIFFSNQKNLRGSVQSG
KLDKLQPTSRMSGLLKGAFKESIP
>comp2957_c0_seq1; 485 393 1880 LEN=1984; translated
MSVQDIVIRRQQQLLLGRENIKLTEDQQRLFLQHGASDGIVQSSSSGDNAHAVAPGYCGD
SSPIGLSPNISPTTVITRKALATRPPPGTPWPLAATQQQQHQEHRHRDQQWHQHQHQQEQ
QKRHQLLLQQQYNHHYELHYQQLQQQHQQQLQQQKEQRQRQTEHEGDFICLHPRDDTRSR
VRFSTALSVYDHFEDDDNDSNDGSDRATRENHQGGSPLGIINEQTANATASTAAARTLPH
DLFLRRRARRCWYSREELAAIKAERKAIVRAIRGNARGSADAPGHELRGLEASLSVGICR
TILLCCATMPLFSSPRMPTGMHKYTKDQCXVAHVPIFFVVFPHRSARNQGGDLPKTQASP
GGGARRATPAANTNASYQFQYYEYYYQEQHRQPLAPDDEKLRLASREATEWFRNRALESA
RQDASDAREFYESGANNHDATSMLYESMSSLHGSFALMDIDDGAAGVIFGNENENDNSTL
RYWADSSWTRNLMEE
>comp2957_c0_seq2; 548 393 1730 LEN=1834; translated
MSVQDIVIRRQQQLLLGRENIKLTEDQQRLFLQHGASDGIVQSSSSGDNAHAVAPGYCGD
SSPIGLSPNISPTTVITRKALATRPPPGTPWPLAATQQQQHQEHRHRDQQWHQHQHQQEQ

QKRHQLLLQQQYNHHYELHYQQLQQQHQQQLQQQKEQRQRQTEHEGDFICLHPRDDTRSR
VRFSTALSVYDHFEDDDNDSNDGSDRATRENHQGGSPLGIINEQTANATASTAAARTLPH
DLFLRRRARRCWYSREELAAIKAERKAIVRAIRGNARGSADAPGHELRGLEASLSPAIKE
ATYRKRRQVLEVVLGEQHRQRTQTHPTNSNTTNTTIRSSTGQPLAPDDEKLRLASREATE
WFRNRALESARQDASDAREFYESGANNHDATSMLYESMSSLHGSFALMDIDDGAAGVIFG
NENENDNSTLRYWADSSWTRNLMEE
>comp2958_c0_seq1; 573 175 1098 LEN=1149; translated
MHRVLASALVLAATASAFAPSAQQQQRQAQHAVSTMPLQATKLAALPTIPNPFKSLPWNV
EKAQKSQERKIKLERSKMHRELGIAQDATYEEIVEVTQRMIEDAPDRKRQIQIEVMKDKI
LQARLNERLAGLVSDIVSDDAKAQDDESNLEEQAIAEMKDKKAPKEWNAPAWTQGLIVKP
DQAHIKSQVQIWGVMTLMGVALPPFIQYANRFSWMVCVAQLSFRGMAKQEGGGGGFGVMR
MGNAPGDKKHLKVAWLLGITITIVGAVATYGLMPAWAKGHRMTPAIAFGMRNFIYGVACS
YLQPYKG
>comp2959_c0_seq1; 364 1 328 LEN=555; translated
XSDDGSDDGSDNGSDNGSNDGGNDGSNNGSDDGSDSDSNSDSNNSSGNSSDDGSADGSDD
GTNDGTNDGSADGSDDGSDSGSDNSSKSGGADASNSDSDSDDDDASTEP
>comp2960_c0_seq1; 349 609 1367 LEN=1605; translated
MXPNGYIGKKHLNSAINEVLQNPIPDAPKPLAFSILRIPFFLEPGYDEDKVFVETNRERL
VKKWGGKAGWDNQKRSHDLKGRGLEAGIPYFNLDRKASSTMASHRLIQHIGKKYGLATSE
AVYDKLNVYYFVDGHSLNDRPRLAQVVHKVLRELILDNAPIEDELLEFLNGDEGKKEISY
ATHALQQLGIHGIPKFIIEGTTMVDGAARPDTFVHIFREIEKRGTVQSGPIFNDVLGVSD
ETIQVPSHSLSS
>comp2961_c0_seq1; 222 79 474 LEN=588; translated
MGASFSTAGNSSVVSRCDERRAVDNGANNPPPMRRVMTVEERMWQKCKDEPLVPIGCAAT
AYFLASGIKSFQNQDPRRGQKMMRLRVGAQFATLAMFVFYVGLDNINFEIAPQYQRAKKE
EEERRLREQQQ
>comp2962_c0_seq1; 538 110 1510 LEN=1784; translated
MPETNPYVDDGEGFSTTMLHHGHKLDTDNRARAPPIFQSTSFEFKSSEHGASLFSLSELG
PIYTRLMNPTTHVLEYKMAKLEGAPCPAHGDCDNATTLPSALAVASGMSAQMHALMTFMQ
AGDNFVTASELYGGSFTQFAYSFKHIGIEARFANVSDLDQVKSLIDDKTKAIYIETLANP
SGTIPDFDAIKEIATEAKIPIVCDNTFGMGGYACRPLKLGADIVVESATKWIGGHGTSIG
GIIVDGSSFDWCVKNADGSLKFPLIGGPQESYHGGVFAEHPIFGVKATNTVFILLARVKT
LRDMGGCISPFNAWQLIQGIETLSLRAKAHCENANELALWLSTHPKVKEGSVLHPSLPSH
PSHEKAKKYFRPGCFGSVFCFELKGEDPDDEKAKGSSFVTNLKMCAHLANVGDSRTLVIQ
PSATTHQQLNADQQAAAGIKPASIRVSVGYEDLEDIKADFEQAFNA
>comp2963_c0_seq1; 284 1 1020 LEN=1020; translated
PFRSSIASIAHGVANHLPNANSSRASGILLTDTWKTASDGQATNADQQELPALASPDDLK
DIRNHAVKRALMALKTALKLFAENGTKPDDLALQSARLSFAYMSLEDNDFKTALKYSMLV
LTHSKPDEPGTKGDGEEDPSESARRILIKRQAATARMYASEANASLGDPMASMKVLVGDG
KDDAFDRLSSDLGGVTLGMASANHNAKARLAKAQTMVRCSASAATAALGNLTASKQLAMS
AQAMENSYSSSREGTSQARKALIYCMLREGNHGAALNXAAVGPLRAANPSVYPSIHPSVH
SSKGRVRRTPGELVNLATEWDPRTAWLHLGNGKRKKKAKAX
>comp2964_c0_seq1; 336 121 978 LEN=1307; translated
MLAQHSHSSPTTSMMSSLLLTEFFLTCEVSTATASESTSTMPIHAQYGLRHQPWTSAVDS
SSTSFSWSDFPLIVHDEEYNSDVNGLFHASKTVTPMDCPGFPLHMTSTTSSTSSTSSTSI
PTKNMKKKRSVRFAPFVEIRTHSQILGDHPCCEGGLALELGWEYNSSSALLPEQIVRNVE
KKDCQQSQFKIIGRSSSGESMTGTRIPHRRSYSERKRLLIEVGGYTSDELNMKDPRKEYC
LVGRNCSDQKKNSTNGMTAVLQRQTHLSRVGSITESLCSVVAAGA
>comp2965_c0_seq1; 283 748 1308 LEN=1449; translated
MDPKYQPEYQAIVDRYPGRLEFANPGEERQGSVENGLNKLVEMSEGKCKYVAVHDSARPL
VTIGEICDVVRDAKEHGAAVLGVPCKATIKESEDGSFVQRTIERSRLWEVHTPQVILIEN
LLKGFKKVAEENLEVTDDVSVIEQLGEAVKLTRGEYTNLKITTPEDMDVAEAILEDRKSK
PVNMLA
>comp2965_c0_seq2; 364 227 1123 LEN=1264; translated
MKFLFHLSTLVLLKSCVSSAFVCNPQRIASATTRLSAANGDVDFDTPEMGDVGFVLLAGG
TGSRMKASMPKQYLPLRGVPVLHHSLDLFLEKLVDHCKSSGDSSPPENVVLVMDPKYQPE
YQAIVDRYPGRLEFANPGEERQGSVENGLNKLVEMSEGKCKYVAVHDSARPLVTIGEICD
VVRDAKEHGAAVLGVPCKATIKESEDGSFVQRTIERSRLWEVHTPQVILIENLLKGFKKV
AEENLEVTDDVSVIEQLGEAVKLTRGEYTNLKITTPEDMDVAEAILEDRKSKPVNMLA
>comp2966_c0_seq1; 250 273 1344 LEN=1643; translated

MGQHPHGSDGIRIRNGPATKSKINVCSNVLVPMQVFPFLNRTRKKMASRRELINCFELVP
PRKPEEIDAPETPFESNQVMPNTGGCNAKVRSTQKKIENVLVEFRSVGISQIPKDRLCEL
TGYKCTSTKTWATAFKVAKEEGSIQVFRSNREVMVQISDCPKSLMLNPNNIEALLRRYSK
ELKIQQTTFDVFDVLLGLGIGRYIDKSRLADAVNRNIATKSFVNSCKLFQSLGMLETRRV
GQRGNSTSYRLVQSLLSRDGATADASVAIGTKPQGNIFTNSTTFKEVSSPPMKCEIPSVT
VNGTRLRGDKNPLLCHRDRDQGTEDLVEPNSNSPSQRTDGIADDDETSNYERIVTF
>comp2967_c0_seq1; 204 1 926 LEN=1321; translated
XISTMQTQISKETDNLIDTQDGWYGRNDPRPLTRGKRIFIRTILIVLSPIVLALFLLYVA
FTAALLCVVTVNDYQIKSSKVLSYENWWRAPGKMLRVKETGDSSSDIVISADMVNDDGEL
DTGKNTSLRKSIECTLGEHLRKGPHGPGIAADEDRDRPHYLLHGWPEPFDPAGDELWCTH
ARTATWAAIYSWSVRLRPGGFLKNWNQAIDLETAPEVFGYVYLFEIGNPDFYDKDRIARV
GALAANAPDHTPETENKVFARYNYGHVKYMGDFPEGDPDAYVFKGEKVMSEARLRLLHTY
RVINPNYA
>comp2968_c0_seq1; 650 112 2019 LEN=2558; translated
MDKDLEVQILATKRLDDPASPRSYGAMSDTNDFGHGVPNATGDRKQATRMTTGAINSEGP
QFLSDEVLRPPTNKKCRHYVQIDDAIEQIGIGAFQYQVLLAAGLCFMSDAMEVLLLSFLS
TVLKYEWALSEREVDSIVAVVFAGSLLGTLVLGLAADKWGRRPIFAVTSFIIALSGIATA
FCRNYLEVVTARFFVGFGVGGLTVPFDTLSECLPTASRGKNLLYIEFFWTIGTLSVPMLA
YLTLDTSTKNDDETNWQLFVILCSIPCFASTILGMVLVPESPRWLLEQCSDRPEDESGSE
KALKILKDAARKNGISQNVIDEGLFPPTSRLVISWDDVASIEKVLSPIQAGNGDNKSYPT
SETPVQSSAGAGDISTLFSTPSQTRLTLLLWATWFGNGFLYYGVIIAVSIAFTNERIEEQ
DDFYVVNDDTGGNGNDDTNSSMSTSYSFDFAAIFITASSEIFGLVAVLSTVDRFGRIPSQ
TMSYRIGGIATFVMGLYGAIAFAANDNQYHRSLLISLAFVARMAMMGGSCTTWVSTSEIL
TTDIRTTGHGTANAMGRLGGFTCPYFITEGNSLALIGVFVLFISVVTAECAKRLPETAGK
AMGDIKIESTIESPVSKDNVDCQQRAATTYYRDLS
>comp2968_c0_seq2; 226 112 709 LEN=877; translated
MDKDLEVQILATKRLDDPASPRSYGAMSDTNDFGHGVPNATGDRKQATRMTTGAINSEGP
QFLSDEVLRPPTNKKCRHYVQIDDAIEQIGIGAFQYQVLLAAGLCFMSDAMEVLLLSFLS
TVLKYEWALSEREVDSIVAVVFAGSLLGTLVLGLAADKWGRRPIFAVTSFIIALSGIATA
FCRNYLEVVTDDSSLDLV
>comp2969_c0_seq1; 485 45 1418 LEN=1705; translated
MVRTRSDDNTVSYNSDGAGGAACRPPRGKTYLTIGQDFFSVEEYVLSQYNASLHRPDGHN
VYSEVGNSRRQAPILPLPYFYPAATMVYTDIHELKGLDQPADYGSGIEYVKGLADAFPES
GLQIGLWLNGSAGCRDIVSGVLDDQTYRLFDFIRSELPESLPRVFLRVGYEFDNPWFGYS
DDPAAYQEAFRKIVHACERQMTTKQCHEKVDFVWHSWAAPREDTLSLDQFYPGDYFVDWV
GVSIFQQVYPWANDEKQNEKGNFAGGDMKEVKEVLEFATSKGIPIMIAESTPFGGMDVAE
SDVARQYIKDDANKDDVWNMWFQKTIDLIEEFDISMWSYINCDWGSQPMWHGVGFGDTRI
SSSKVVMKRWWEQVLSNDSRFLVRIEDCNENSRPRGHIREEDEMAVARSATTAIDGLFHS
DGSVNYMTSNPRCLQLLCIGGVLLVTVAFLFLLQIRLW
>comp2970_c0_seq1; 134 1 1163 LEN=1611; translated
XLDTASSEYRASETLLLCVAFLNKIWLELHRVLIAITYSPLFTHPASKIMRNKTPPTTTA
VPFPGVGWIDRMSKNDPDASITLADGRTYQNAYAAILDNDPLGNPCRNFNAWVNSIFFSS
NVAAWFVHLLNDNVEYAHYLLCYLRNFIAGMFVYYGTAGSFHYFCYVHPMSKKTFEEQGR
TRPSWETIKGQIKLSQASLTIYTMLPVFDEWMIESGLTKVYFTVDEIGGWLPHVGIMAFY
FACVEIGIYWMHRTLHTNKFLYKHIHLKHHDYNKPETLTPWASIAFHPLDGILQASPYVY
LLPFIPCHYMTHICMVFFTAIWATYIHDAMDWNVDPIMGSKYHTVHHTHYIYNYGQIFTF
CDRFWGTLKLPDGPTGMQKPRTAKKVD
>comp2970_c0_seq2; 236 246 1376 LEN=1824; translated
MVFSVSRQTAELSLLVSFAAFTVCCAGLFERNNETLMADETSGGLDTASIPFPGVGWIDR
MSKNDPDASITLADGRTYQNAYAAILDNDPLGNPCRNFNAWVNSIFFSSNVAAWFVHLLN
DNVEYAHYLLCYLRNFIAGMFVYYGTAGSFHYFCYVHPMSKKTFEEQGRTRPSWETIKGQ
IKLSQASLTIYTMLPVFDEWMIESGLTKVYFTVDEIGGWLPHVGIMAFYFACVEIGIYWM
HRTLHTNKFLYKHIHLKHHDYNKPETLTPWASIAFHPLDGILQASPYVYLLPFIPCHYMT
HICMVFFTAIWATYIHDAMDWNVDPIMGSKYHTVHHTHYIYNYGQIFTFCDRFWGTLKLP
DGPTGMQKPRTAKKVD
>comp2971_c0_seq1; 341 59 1165 LEN=1833; translated
MTKTLPIIIVGAGPCGLVTALTLKKYNVPFVMIEKASRSKICSNAGSGFELAPTAVDILQ
NRLGIDVTKIMSYYQGMAIVNNNGKLIRHSRIPDGYTGGSANRADMQIHLLDQLFPSPKD
EEGVLFCGSGLETYRELHNDDPDGGRVVATLSSGEEISGCVLLACDGIRSRVRAVMHGGY
DSTKDWKTNTETANTKDPLHFCNTIVYWGKTKVPRGSALELEFTKTQMAKKAKDNEKTKY

CTSFAFTITTPSAPASFFIIPSQNGTELNWAVSVQSETDKRSKNNDGTDLTRRGGGPLTE
AEKKRLFDFTSDKNSESVVRGITNFPLIQELFKMTPASDITEAGLFDRENLDLPYASESK
RVALLGGK
>comp2971_c0_seq2; 451 59 1543 LEN=1732; translated
MTKTLPIIIVGAGPCGLVTALTLKKYNVPFVMIEKASRSKICSNAGSGFELAPTAVDILQ
NRLGIDVTKIMSYYQGMAIVNNNGKLIRHSRIPDGYTGGSANRADMQIHLLDQLFPSPKD
EEGVLFCGSGLETYRELHNDDPDGGRVVATLSSGEEISGCVLLACDGIRSRVRAVMHGGY
DSTKDWKTNTETANTKDPLHFCNTIVYWGKTKVPRGSALELEFTKTQMAKKAKDNEKTKY
CTSFAFTITTPSAPASFFIIPSQNGTELNWAVSVQSETDKRSKNNDGTDLTRRGGGPLTE
AEKKRLFDFTSDKNSESVVRGITNFPLIQELFKMTPASDITEAGLFDRENLDLPYASESK
RVALLGDSAHPQTPFLGQGVNMAIVDAYVYATNLAVALKTKKKSLEQSISDCTTGNRHGE
AKKLVEDARIGCTMMTSQNIFLYWFMFLFIRLASTSSFMNQIISQDKSNSDFLKLLDEEH
CSPKEQESMSQMSA
>comp2972_c0_seq1; 89 1 263 LEN=263; translated
XEDNTNYVANQEILRQSEVGLAAKSGEDLAAPITFTPYKSEDHTNYVANQGKLKRSEVGD
AVKSGEDLAAPITFTPYKNEDHSNYVAN
>comp2973_c0_seq1; 909 81 2192 LEN=2231; translated
MVFVPMNASDAKAIRHLLTATQRSVAPPRSETPRANANNAPYRYRFDEFDDYDDDSDDER
DFRDLSHYCSIDPKTGRVTELWLGIIGSQNANHPQHTTAQGAAAAGNYYWTLSEADWQWL
ARLDQLRHLKVTRCGALPYRQLKELPHLEHISCSECGTNLDPLGAGDTDEDGEDGDGGDD
DEGETIADDDTPPCRSRWNPPQTDSHRNTGIDLPNIKSLSLRCAFQPTAQGIRSLGSVFD
WIKHHLPGLERLTIWRLDQPPNRWSPTPLSAYQRTTNKRLMVEALLDGLSPLSPGATKDA
DACTKSNAWSNRVTTLELNRCGLTSSDFRRLVLDILPGSFPFLQRLSLASNDIDELPPLK
HPNACPNYHQEPERNCFHKYEYECETECDDPTFSRTKQKLCRLRRSLQQIHLSNNPIMEF
LGKHGDEDPLPLSREQMEGTSWSFPEERTPGRESGTQPRKHTLEQRRKRLLHRLLESVDE
NHTTATEPSLPAVLERRVRRLVELEHLRDLLELFPRLSHLGYASCVHKLLRSVVGDVVVD
HNNDGNGNGNDETENRWCVLLPFGLYAMDLQRQIEFYLRFNRGGRRLVEGIGCEPRAQRN
EERRDDGDDDDKNHGNAGGRLLEFLALWPNILEWSYRTSSGGCFLMPLPPLSSSSHAGSA
AQAAQHAVSTPTHHSAAFSNKNATALYRLLREGPVLFGSSERH
>comp2976_c0_seq1; 416 243 1136 LEN=1547; translated
MIRNSGMDLIIDGWFHERGVLWPGQAMSLKVKKVLDHYRTDFQDLLVFESENHGNVLVLD
GVIQVTERDEHAYQEMIAHIPLFAHPNPKKVLVIGGGDGGVLREVARHPGVEEIVECEID
EGVIIASKKHLPSLAVGYDDPRVTVKIMDGAKFMDENQDAFDVIITDSSDPVGPASVLFE
TPFYNAMYKSLREGGIVCTQGECMWLHLDLIKPLVDSISQTFSSVEYAYTTIPTYPSGQI
GFILATKGRKTCKVPEREADMTPELQKKLKYYTPALHRASFVLPAFTQRAIFGEDTV
>comp2976_c0_seq2; 421 37 927 LEN=1338; translated
MGLNGMDLIIDGWFHERGVLWPGQAMSLKVKKVLDHYRTDFQDLLVFESENHGNVLVLDG
VIQVTERDEHAYQEMIAHIPLFAHPNPKKVLVIGGGDGGVLREVARHPGVEEIVECEIDE
GVIIASKKHLPSLAVGYDDPRVTVKIMDGAKFMDENQDAFDVIITDSSDPVGPASVLFET
PFYNAMYKSLREGGIVCTQGECMWLHLDLIKPLVDSISQTFSSVEYAYTTIPTYPSGQIG
FILATKGRKTCKVPEREADMTPELQKKLKYYTPALHRASFVLPAFTQRAIFGEDTV
>comp2977_c0_seq1; 127 1 1075 LEN=1144; translated
XDREMERLEVAHNRRVAILDRGVFALEMACHVLTVAHFCHIWSLNGVQFTLIDGVLALHL
HSAISSACTKLARRRNVHNIARDLQGRFPNATDEELDKASAAGDVCCICLGSMTTGGSVK
KLRCGHLYHTHCLREVIERAQNLRSARCPLCREPLLDRPQPPQGDPPPADAETDNARATA
TATANPEPPRAGGADPARRPAAEAGGGGGDPARGEGERALFRFSTEGILPVWVPVPAFSF
EVVRRPPNGTQGAGPLRNEPAAAEPRAQAPGTGAGDLANALGRQQQHEHEPSFLQRVLAL
AGLVPMSPEEEARALEQLVDMFPQYDRQDLLGELRDRGSIEAVTESILMGIFRGVPRS
>comp2978_c0_seq1; 641 1 1408 LEN=1408; translated
XILERRRAEEERLRKAREAEDDRLRAVREAAAERDRAEQERAAAVLERETEKEQAATRDR
EARAAETERRRREREAAAKKATEYIDKAKKLVGQLVQVRASVEPFEKNKTVGKRRLGMKK
MANGKVNTLNDNPSKIREVATQISTAIAAYEQEDGQIKTQLQQGAPGLTSDMAVGRRYFV
DLLASTAMKRVQAESFGGTKGDGFPLAAMLAMVSVESKQIISVLAAHIYTVCPVAIPTLP
APRDNASEDDLMIGLGMQRNQKTGEFETFPRFLARTENIVSFVAALQSSLPSSHQLMGGH
AGAMLWLKRFIDLLPLPPTSPLPLTTAPVLGAFLTVAGHMMANAHADDFRTMLATIQNDV
MNRLDEGELGKPSAIRLGKVVEGGFDSFRKNLPPKAVPELYYGASEHSHKKEGVVSFGGT
IGGEEDVQPKKPQGMQQQPPPQNPFGGGVSFGGPSSTGGGNPNTFSGGGG
>comp2980_c0_seq1; 175 91 579 LEN=634; translated
MKFVVLLLSTLSLVNAFGSFGSKKASPAATSPLPTFDKNTGKWSPAPNAEEPYGIGRTLL
GNGPVPAFTRLTQSEDYMQAVYKFQASEKCTVRYAQGNMDAYFENPNDWAYQRKVEESGG

YKKKYGEPIPQKQVILTVVWGVGITGLLGKGLLTFVSYKLGN
>comp2980_c0_seq2; 203 91 624 LEN=860; translated
MKFVVLLLSTLSLVNAFGSFGSKKASPAATSPLPTFDKNTGKWSPAPNAEEPYGIGRTLL
GNGPVPAFTRLTQSEDYMQAVYKFQASEKCTVRYAQGNMDAYFENPNDWAYQRKVEESGG
YKKKYGEPIPQKQVILTVVWGVGITGLLGKGLLTFTSGDFCLSEANAATNYCKVFFS
>comp2981_c0_seq1; 545 57 1052 LEN=1718; translated
MPVVGSMALGAALIFTIAVGIAVLPIPNYAFTPAVYQYKSDRTLMQINFKNDKLVTGKNF
ELSARDTIFSLVEEDDGNDDNIENPDIDIADLLADKEFLENNVKTDDGKDSKKKKIGDPL
REDNGVRPSIHPTAINAIVDALKARAEQSKQEMDDDDDDTMHFRATETVEPLMVMVTAGQ
FASDAIQKRQESSEADGMLMTQDEEQTLAGRIMGVIMRLDALEEELAERVSGVEWIGKYD
EWGNFGVLSDEHQNNLARDSDSGDDGLADVHAKILDDPLFCMNRAECLLAIFLQEVEIPQ
LEKLKETVPDGSKIDFLDQDRLEVVVGSDKS
>comp2984_c0_seq1; 232 1 1875 LEN=1968; translated
XMMADYEQIATFPSLPPHAITVEQQRVLEPLSYPKSIDTRTSHSRQVVALLQDLHQSITP
PERWQSLQRALNLVSHDVTELHDYEISLGLDKALCLKLGYVVVTTKSNCASKEVTLICSC
LLQIFKCSSKNRLRSFHHIGATELLPILIQFCIGIIKCKIDMEDQSPEHDDGLVSIVRVL
RVFSKLIPAKSFLINYLQGAYLGHLLRDMLIWIDDPASSIIYSSTEVLWEAIGVLKDLTF
RSQTNDKETLLHLEGGVLLKILSICCGGIKASHPRIQEWCTAVIWNLALDPQICRKLVFG
NDTHESGGNVILDGLLEVLTHYSADSKISGYSLKIKRNAMSAIGNSFGFPELFDSFQKKM
QDKSSEILQRLMSLVQNDSDSVVRRRAMRTIRCLASSGVNDTILLRDLSSLLVDIISQKG
SENDENDEDMHVQVCQTIIILKETIDAKIWPRLQKSLVCRIETTTSIKIISAAIVCLSNC
FSDEHSIQNWDGFSDTFWTSLERTTTANLEAHESVSKLLLGIANNEGNIRDAGDRGIRNP
SILTKTAVVNTLTTILLESECNREEPRNQALQTVQILAGNEINKRPLAENDRLLSGLVTL
CLTQPDPGIKDSAKGVILQLVPEL
>comp2985_c0_seq1; 333 171 986 LEN=1007; translated
MTTKNWLLLACALRIQGGNGFTQTKLVHVNSGVDNFVLRASLTNDYGKSCSRRSWLADAC
RTIGVATASVAIGTSVKPEKAQATEEQFNPDDPEFKEIYNNPATPPAPEERSGLVVLRVA
EVSQFQEKILRAIVSGELKDVMVSPMQFAFGTQILLRNSNLDGNMRLMIQEEVPKQKRDS
ARKNAANVMNTLQDIAKYSASIQRDFEPLEMVELADMYKVVRINLNELYEYLPEKEREKY
YGYFVAVTEYEKKVANGTYNPDIDGVLKFDN
>comp2986_c0_seq1; 22 1 276 LEN=276; translated
XSSNNNDSNSNNTLRVLFSGAGPSSPEEIQRALVEGPRTESGNALARLFQHLGGNPVAAL
AEVPDATEEAEREVLAASRNITKRIRSNRIES
>comp2987_c0_seq1; 268 1 669 LEN=1176; translated
NLCSVISDYNNLYGQFLAEIGETLSLPTSVEMYRPTSRPLETPLETSQPTSRPSETPLEA
SRPTSRRAETLTETLSETPXVDEVSTDVSTNVSTNVLTNVLTNVLTDVYQNVDTATATLS
DDLMTFRPTLKPTLEPQLRLQLQLQLQLQPPPQPQLQPQLQPQQQSLDDVWTVVGPMSVE
NFKPTDESTVELIDLGTDEPNQQLIEEPTDPGTEEPTNQGTR
>comp2988_c0_seq1; 300 163 564 LEN=1226; translated
MKTSFLAVAMLMCVSYIHASVVIEGDSVRSVGKPYYRFEASSHEDRNLKERNLKKSKGDK
SKKGKGSKSNKGAKSDKKSKKKSKKGVTEEPSAAPSAAPSAAPSASPTAAPTGAPTGSTT
VSSTATSSQEGST
>comp2989_c0_seq1; 323 80 1390 LEN=1872; translated
MDCNQGIYLERLLQQQLCQRKNGNNINSTVNKNISDGNSSFGNCSAPSGFIATNTTSIGV
SNTGIGLGMHQQQHFQNRMIAFLKERQQQRQLVANGIICAPCDMTSNPSIPGLSNIAGLG
HQLVGRDCGGIGQTIPTSALIDGISSGGRGARNVRGLGNRGFLYPSTEQDFLSSSNPYTA
VGEITGEHNTKLRSNGSVGAGSLNSSMGGLSQFSIQHQALTQAALNSYQKANEKRLTDVI
LAKQAQVAFLEAAQVHVPRTIRLPCGARGMKADHNSSTAFFDIPENARHGQHLLCSHSVC
RAAGVKFRYCFYCKKPVTKQNFRSRHLHANLDPNNKEAERKPIDKLKEENKSKTEICQSK
ESNANMPSTYKATTKPQIAVSESNSVKLSKSLGDKNSIDSSPALPSIKRKKEENSRSTEA
ERAQKIPKLLLSELGP
>comp2991_c1_seq1; 148 10 705 LEN=1774; translated
MVSSEGIAEAFSRTGMAPLGDPSGPCSDLRRNLVLSGISAEALNAMVGHEVAIGREGRVF
VHRRCVPCRYREAACKRSGLTNNLWGVSGISCEVLPPRSKTAPGGTTIQVGDEVAVVPGT
HAPDRIDVGRTKPGSFVRPADRSAEDVKKSITPPYIAALASLWDPEGFARVEAAHNSVGL
RFWTPEAYQVGLLAAALRVPLMLLLALVAGSALAGLGVLLLRSGGREAGEL
>comp2992_c0_seq1; 133 1 473 LEN=473; translated
XKRKDYKGRGEGKGYKAKVEGKGKGYGVKGDRKDSNGTKGNNKGSKAKTDLTEEGSDHSG
KGNANKSTKRSKDNKGNDKDRHLYQYDSGMNSRKTTITEEGEKGNIDEMIDQIIQAYFRA
SCEPTQSPTQATLPPSETINPQPDFPTKSSIPSVAPTV

>comp2993_c0_seq1; 376 1 1999 LEN=2066; translated
XLINKIKVLVGLKAYSTALHQELDLGTSLNVAIDCSSNFATKVLPHRYFENAAIMLSDTY
LLLNNAVATTSRILSLKTVRLLLVFATHLLLLTDAASRHGQRPHRNHHNLRYNNYRSTPQ
EKLDDSISNPILEALRAKGTVTSWGAAGPGVIATIMCRGGSIATKMPRSSKQGGKNISVS
TDMDGDSPSLLILIRVMFFSYYASLGSLLPYLPVYYHSLGHGGQVIGMLGAIKPFTTFLV
APLWGIISDQTGSPFTVLYFTFLVSLVGQLAVSWRHDPFWIMSMVFITAFFNAPVKSLID
SMVMSNIKNGKLYGRLRLWGQLGFGVGSSVVGLILSKSQQAQQQREVIGDGTITTAAILG
GSGTMMEKIDQIWQKITGYKLLFFTHALLSIPTLICIRAFHRMTTKDTAAAVEKPPKGAS
KKKGRASKSSENKPNVLEGIRVLFHNSDALLFFFLVLVVGVSSGVIENFAYVRLREVGGT
GKEMGLSRLVSSMAGAPMFWFSGPLTELLGADRVLALSLGNYVLRYFNYAFMQSPLQGLP
AEALRGATFAAFWSTCTIYASRIAPPGMQATMLMLLNAMYGGLGQSLGAIIGGKLQSKLG
TVRTFIYAGAFDACFVLLLIGYQVLEKVVASRTPKPSSCPKERVLGRLTGRDFEDDRDAR
KYIVT
>comp2994_c0_seq1; 477 173 900 LEN=900; translated
MASIAGVLICTVGFTDAFSAKQQSPLLSSTRLGLIPPPGSGYAGPEEEIDVMPDTYEPMM
EYPGTMRPGRTPENMPFHDLPIGDDDPDPVPWPHFQQIEWHHRWAPPHPHPTPMEEFIEQ
QGRWATPEMEAEMRTGSRRASRTRREMEEEQKRDTLIMDDDEDDDDFVDDFPQELGEGMF
GQLGSTDDAKITADAVSPDRMNDDKKKSKDDDDDDADDDAGLDDFLLDLGLDSLGDDDDD
DDX
>comp2996_c0_seq1; 393 1 1575 LEN=1686; translated
AFLYTGTTNMVASAQESDRGTTDKPRTAEMMMGAMRMSTYVGIGLGSLLFAFARPLLRAI
IGNDAIGPAVFASAMRYVRIRALGMPAAAILGSTQAACLGMQDVRSPLYVLAAAAVVNLF
GDALFVGHSHPLIGGTAGAAWATVLSQYAAVALFVRWLCGRAPEEREADVDSGPEVMNLS
SAILEITNPGERDSNSNARGGHRTDRRQRFRAALESFKLAARRKKTPVPGRSEARRSPLR
RLLPGRTAEKTSSGSGNESGLSFSTRGFLRNRFSAADLLRPPGAETRREFAPYVVPVTTT
QVGRVSGYIAMAHVVASSLGTVGMAAQQVVVSIFYCLCPIADSLSLTAQSFVPAVSERPD
GPEKASAMGTVLANFLKAGLVFGAAMVAAVGAIPLLAGWFTSDPSVVALVRSVVPLLLVF
FGVHGLVCGSEGVLLGRKDLGFLGKMYAAFFVAVPYFMMGVKRRALAGAAGLSSVWTVFV
GYQLARCGIWVARACALQARTRRSAAATAVRPFASEPESNKLAP
>comp3000_c0_seq1; 267 345 764 LEN=1057; translated
MKFSAIVALTTLATASAFAPTSSPKTSVVSLNAEAEQSRKAFFSVAGAALFGAAVAPGVA
GAMDQDNVSTPTEVWEVGKPKADVVNERFARYQNARNQNNSNFPPIKRLTLERKSPVTRL
DLNAPNFEGYKKSYPGLFK
>comp3000_c0_seq2; 223 1 443 LEN=736; translated
XAFEIIDKMKFSAIVALTTLATASAFAPTSSPKTSVVSLNAEAEQSRKAFFSVAGAALFG
AAVAPGVAGAMDQDNVSTPTEVWEVGKPKADVVNERFARYQNARNQNNSNFPPIKRLTLE
RKSPVTRLDLNAPNFEGYKKSYPGLFK
>comp3002_c0_seq1; 385 1 1500 LEN=1518; translated
KTTAEDSAVSRLIRLVEEAQSNRSPTEMMVDSFARSYTPFVVGAAAIMCTIPWYFGTETG
RMWTLRGLIIVVIACPCALTISTPVTYAAGLAATAKRGVIVKGGAKLEAMGSVDRVVFDK
TGTLTRGQFSVTHLEVVGRSRTRRQMLELLSLVQDRASHPIAASLVRAAKSEGVRTPKQT
AVSDHKILKGEGITARVDGKLQVYVGNQRLFERLGMLRGLSSEDKDAIEEWDDTGGTVGF
IGIENDGIIGMFCAKDSVREEAGDALDELKEALIETYMCTGDSDTTARAVSKEVGIPGHR
VQSRLLPEDKLRFVEGLRRPEPSKFGLCRKKKYVLFCGDGVNDAPALAAVDIGVSMGEGA
AMALEMSDVTLMDSNLTKLPYVIKMGQRVLKTVKENIVISFAAKFAVVSLTLAGKMTLLY
AIASDVGIMLLVTLNGMKLLPNDRFDLVPPKQSRRRGGTIASRLAKLKGLGKNAGFELVS
MNNTDDEPPEDLRVAGIV
>comp3003_c0_seq1; 460 1 1416 LEN=1508; translated
YALDGDHVVKALAGVFYDVIGWNPSDDGDGDENGSESAHGNSNGADGNENGGDEENSSGA
ENDDEPRFSPREAWKGPLTRRGRRWERFCRASLGRGNLPAESARCLEAVLTILRNLSFVS
ANLRLLAYSNDILSILVGCLYEPNQGDAGGDDGSNGIVLSALGILINLSPHLDVTGRKLF
CDKLFLRSTAQGAPPEDGAKLPDPSTFGQVVNGSWGFGSLLLAKKLDTKEDYVQDIDKEM
LLQVTKEYLVRVWAIFPALGSVLVDTRTPRAVVTMALELLQEFINQARVGVIGSVEDQDP
DEIPNTRAILVYIPDDLLARLSDFLYIPRLGSDAIEYVDPRVTIVTRVNPIKLTVGYDGS
VDTDVRDRALEVLVPLLELDSPGIAGRFGTKPGGSGLPNNKVFDAIFPILSTQSGRNEAP
LLATQLLRELSKARENRLGCLYLQERIVALASKEPRVAHLAFNHLYASEED
>comp3006_c0_seq1; 395 116 964 LEN=1159; translated
MMASRLPTMFRKNGLPAAAAAFAAASAASASVTLLDEEDVVHAPHYPWRHQGYFSSFDHA
ALRRGYQVYREVCASCHSLHYIRFRELVGVTHTKEEVKQMAMDVDVLDGPNDQGEMFERP
GKPYDVIPSPYPNKEAGRAANGGAYPPDLSLMVKARHNGADYVFALLTGYCDPPAGKEMM

PGLHYNPYFPGGAISMPPPLNDGGVEYEDGTEATISQQARDIAQFLSWTAEPESEERKKN
ACKYMVVLAFMALSVGYYKRWKWAPLKTRQIAYVKGVGGAPH
>comp3006_c0_seq2; 314 1 1330 LEN=1525; translated
AAAPIPIPTPTRHQHPCNNPNPRTETRTKPHGEEDQIQDDGLSITNNGELGKSFRPTESA
ERERRTDRWQGEEVPMPTPSDDARRTESRGILESLWNLTLGMDWLRFSVCLRNSLRNSLR
VPLCVSVSPFTKLGCIRFPVCLCLGPLSESPFRSVPCCPVLRCIAPLRQFRKNGLPAAAA
AFAAASAASASVTLLDEEDVVHAPHYPWRHQGYFSSFDHAALRRGYQVYREVCASCHSLH
YIRFRELVGVTHTKEEVKQMAMDVDVLDGPNDQGEMFERPGKPYDVIPSPYPNKEAGRAA
NGGAYPPDLSLMVKARHNGADYVFALLTGYCDPPAGKEMMPGLHYNPYFPGGAISMPPPL
NDGGVEYEDGTEATISQQARDIAQFLSWTAEPESEERKKNACKYMVVLAFMALSVGYYKR
WKWAPLKTRQIAYVKGVGGAPH
>comp3008_c0_seq1; 128 1 742 LEN=990; translated
XKRIDYVLALVNNCVSILNSYPHSFSTFATVPYFDTNKKQQMQDHTARITTLTALCAFWA
ALFAPSHVTSLQFGHNRIRSDFHGKDSSATQSAAIPKSECAISSRRNILTTAASGLAWMA
CVGGALPAKAATATETTSIETNEITATKKSNFNYDLGSSDCETSCVLNCELQKNGKNGGK
SSEGCLDSCVRSGQRYCHQVASTSDSPSASEAPTSRYTTTTKEPRIQSAKPIPGLKYNSN
RGGAWRD
>comp3011_c0_seq1; 357 438 1823 LEN=2306; translated
MIADSHRVMEGWSTTGFKTSSKENAVAIENLVKRLVDEAEAGNSNANPTTKDYNCMLESW
ARSGEGVHAAERCEEILVQMQAEYESGASSAQIRPNLSSFKIVLQAWKHAGGKKLSSFRA
QRILDWMSSLHESGKNSLAQPDQMCFDTVLQSWSRNNHEEAPVYAERLLAKMETMKLEST
AIVPLVQPRTLSFNAVLGAWNRASLTPSLESPSAFASQPSSTSWQRSCDILEFMEKLYYV
EGNSMVEPDRVSYSLVLRTLARHKNDDRAAPKADKILRFIEKKSRNGELSWRPDTLLFNT
VMGCWSHSHLKGSYRKTRGILDRQIHLFTSFDGKVGSDDCRPDVYGFTTVLSSCALERAN
GEEKAKAFNVARSTYQQLITNKDEYGAPNHVTYGTMLKCVSNLLPADHPQRKKWTKKVFN
QAVANGCVGGMVLSRLSEAASSAKEYKALMRGHSKKKLPAGW
>comp3012_c0_seq2; 90 1 523 LEN=1028; translated
XRSASHSPEGSGAALTPVPARGLARGLALDSTGPAPVPPSRVSGATAAVPLPLXVRVCVQ
PDVGLPPPQLREGGPGAPAGPEGTRREAKGLHGGSGQHARQRREDPAQRGANGDADAAVG
GGGSGSLVKGCRHGGSVSRGLRVCLFVCLCVWFCVCVCLQRVVPVAVSDVLPEG
>comp3013_c0_seq1; 372 1 1611 LEN=1629; translated
AASGLASGLFGDESMPPTTCMAVTVTADPLRSXGVGEGWTGALTRQHIFKSIPGQFDEEG
AGFDGERDPEERPHRTGSDDGTSLEWLSPPASPRPVLIYEHDDVQRAAAGWGVSAFLDTR
GALRVVGRPHDLVSLLRLNRMPGLLQRWINRNHDTSATTPVGTMVSSLIGWATGSLGGVP
GDNERWKVAEQFSLLDDWTRVEVNHSGGTKIGKTAGEFDTTRIKQLACGPGFLAMISECG
SLYTMGVNNRGQCGTGAISNNVWTPQAVRGLSMTSLNIGGGDKISDKMASEQDQPMVQVC
LGFQHGYALSRDGQVYSWGKASRGQLGRTVDSDQDPWARPIQFDNADGNPRVVAIGAGFH
HGALLTEDNEVFVWGKNMVRNAGEEDDANSDGGDEQPVDPSDARTPQKVLGLPDSAHDNQ
SPMKVERISCGSHHTAMLMEDGSIFGIGIASDEPVPILDPVELIPPGVLELPLRHFEAHH
DRTSVIDNSGAVYQVHLWNDETLREYAYFTPSYVDALLDEGHDIQSITAAGAIRS
>comp3014_c0_seq1; 176 69 353 LEN=388; translated
MMRSVFFLLAIIACASAFAPVYNTVRVADTCLSAKHVNDKAAKWAKSKRPRKSRPSDINR
APIDYAIHSYQKPAEYDISSEPAAPMAKPAATEN
>comp3016_c0_seq1; 435 162 1658 LEN=1811; translated
MVSDKASNMEAEKFSRCSGKAMMLCDSEFLDACCPICYEPFLPVDVDPIRPVAAKVGSES
TFCRQEDSQSSTDERNGIYKKADGDCRKEGTSSKDIDILIIHQQDDRHSHTDTEKEHCQP
CHGNCRLDPRQRVTIPECGHEYCRGCIKKHCSYAILIRDMPIQCPGCTSGTSNPCSSLLP
EDIIEESLLLKEKSVEEDGGDCPINTLSSSARKEGSTVNQDLIKFQRLQRMLKDPSLISC
TRCDELIPPDSNIQMIGCVDPNRIECPACHHVFCRIHGDAHLGMDCYTASAKKTDERNER
KSESAIRKRCKPCSHCQSLIERVSGCDHIICPVCHNDFCFRCGTHIYLQGEGMVRDCSKC
QKSYIDHRHIGKYRLCIGLWVPLLLPFYISYITMTIGFAIVTCGFFCCLGCGIRKDKKTD
SIIFMPVMAAREILNFVFLPFVDLSRSCGIPCCFCNNAPGLGKGQYADNDDDDEEDYDDS
VESEDYDEENQMQENSLR
>comp3018_c0_seq1; 200 75 656 LEN=896; translated
MMKGTIRSLPYYGSTKNLFHLALVLFLGISTTHAFQYPRNHQNHQSSGSSSQYNKINSIH
YEGWSSSAVRPIRYDRSIRLSGGFMDDLQGFFKRFTTRASASHILIKGGAEAENKLEDLK
VEIGDSPVAFAKAAAQYSACPSGSSGGDLGSFGPGAMVKEFDAVVFNEPIGVVHGPIKTQ
FGYHLIYIRDRTE
>comp3020_c0_seq1; 711 1 1727 LEN=2065; translated
XEKEMEVMQYYKDHPFKAREIKMHASSSSAAKKLASPRRPLTKPESFHFHSKPSSHRPVK

EDGEKKELFKPFKALPMPKFSSRKVPLGGTKTPGSSRPLTTPEPFKLHKSTAIHKSGNEK
HPEADKEAKSGTFKALPMPDFSASKGSKKLKSPPSRPLTTPEPFRFHKTRSKRSNEKAKE
EKQSAGFKARSMPKFAARNATPSTGTHPKAPGALTKSEKKRTKSTRSKTIPTGASGLLFH
RSTSATNRLSRPKVVQETKQPQSKPFKAKAMPNFIPDVIVTRTPVDEEKRQPSRSPELYA
DEKYNSAYHHCDNSTISSCFNKASLGPKSLTEEPPVFVREEHPTNVTSPDSVKESAEPAL
PREDPVEPAVLNTVQAMEPPSEPPAVVRQRDPNPSPVASPQLNKSTLSMEDAKARLRERL
SKRKSNASKPQQHNGGAAASSASMAFSTLQVQSRLTVQAEKNVKLEEKLRKMTLKKTQER
SSGNGASYLVNDNPTPVKSNNTAPKSTRLQGTVAGDSDDKCTIPEQHEVQQPAGDDNTTS
SILQLAQEVQRAAEDELSFYGSLDTRDRLERELGL
>comp3021_c0_seq1; 151 168 1178 LEN=1415; translated
MLAGMVATRGGIALPLARKLCSQEHGRQRLVSRFSGQAGGGGARRRYSLAIARRVPRSFP
EALGTSQGSGGEIDRERAEAEHERYLAALRKHVPVLCLPALDDLPDSVFVEDTVVAVGNR
AVVTLPGHPSRRNEVGPIRELLAGGLGMDVTDMGSVGGGRALCDGGDVLYTGRHLLVGMS
GRTNRAALEVLEEGLGMEALPIPLARENGRASLHLKSVLTHLDDRTLVVPENDEGRRVFS
AIRDATGGAYTSSDAVWLPAHAGLSCNAVSVGDRGILAQLVRGGDAPTRERLEAAARERG
RAIEFVAQGEAAKCDGALTCCSVLLDLRPAGGLVRY
>comp3025_c0_seq1; 167 1 458 LEN=576; translated
XNDNDNKTPHYLWEDLSPDLQREIQALRGILRSSNRKKVYFSSYHEYLALLAEQHQYYRE
RLDDARHGLKMRLEEDATLRKELELLEQERRYLGGRSSRMDLRIKETTNRLEGLSRGTEY
AISKIEPQARRVDTLKALLVEQKKIFGGGELL
>comp3026_c0_seq1; 371 1 1249 LEN=1316; minus strand; translated
PIQSKPNQPKQQRSANERNNPPSSMFSSLSTAGGLRLASRRILDASRSVAPRSRATPQLH
LPQRQAVSTAAFRAGVARARTAGHRYLSKDTAKTAAEPAKVGWWKSPEFWGFAGSIAAWG
MSGAAIYDSALQGPEVISLTMTPVLMVYSGLFARWAWVVKPRNLSLMYCHVANVLAQTNQ
LRRALEYKMENGEEEQVKEMAQQVGMLGGVAGAAVLGGPAARSALSGANLGIVSTIAAAD
AGPFTATFGELHARDGCGTFCPVRAVLSVVLASRAFDPFLFLTQHDAPVSLRTNATLRMK
LVSCNSRTNAIGANDRSIERTNERTPHAVTNRAPMSKVFISGASMLELDRPTDKISLPQY
AALTATGFFFTRYALLVTPVNYTLCAVNIALFLSSFWHFGRKVKADFVDAKPDAQ
>comp3026_c0_seq2; 355 1 999 LEN=1066; minus strand; translated
PIQSKPNQPKQQRSANERNNPPSSMFSSLSTAGGLRLASRRILDASRSVAPRSRATPQLH
LPQRQAVSTAAFRAGVARARTAGHRYLSKDTAKTAAEPAKVGWWKSPEFWGFAGSIAAWG
MSGAAIYDSALQGPEVISLTMTPVLMVYSGLFARWAWVVKPRNLSLMYCHVANVLAQTNQ
LRRALEYKMENGEEEQVKEMAQQVGMLGGVAGAAVLGGPAARSALSGANLGIVSTIAAAD
AGPFTVHFWAPMSKVFISGASMLELDRPTDKISLPQYAALTATGFFFTRYALLVTPVNYT
LCAVNIALFLSSFWHFGRKVKADFVDAKPDAQ
>comp3027_c1_seq1; 192 1 680 LEN=709; translated
QPRRTTGGGRNERTGVYVTDDMVLSLVRATVRASRLRSRRKRATGDPPPTPEEEEREAAL
RKIPLDSALEILSRLKEDHGLPLAAYYVNPLAAAYQSLGYTAHSKEILETMLSNRTAGED
DLPNVHDLCAKDKXELQPPGPGLGGEGGLGGGGGLAGGNDGRRPLPQPAALQHVERDLGA
ADEAEGRRQLEEEAGRLLDRQHPVTARTMVQLAQEHKQCICLLLES
>comp3028_c0_seq1; 426 1 1382 LEN=1400; translated
KAFGFCARNGLEMRVVXMITAAKGKAEISGLQQSSSSLPMISIQLPETVDFDACEISFNV
YYKHMPFRLDSIRAKQAFNDLAILGSSRARMKILRLVPIDSINASLLFGIPVGIKAAILL
AEDEGEHREKVLMVQSLLKTLADRDCALLLRFVDDGGLCHDTVDPSGEGILDSSREVQHF
FLMPDLVFSKEGPASPRTGVLHRVASADHILEETTASDIFMASNLESASTTQILENSFSE
YVKASLDCLGCSPWNPLLQDVLQKNKISPNPAEIIVKKENRIPKYNSDFRTTLLGRRQKS
PIDQGCTAESAEVWMNEYGVGSLVRQKSLSEHSNEMKGAATWEDSKVSANVANVDGDATS
TDISKGTGYVDENSTNADEEKIIYSFGTSGAGVKTNHAKHKSNAVKSVGSDLDSSDNTSV
GISFDDSDGYSSSSSTTSTCSSSATSGATTFGTFHYGRTR
>comp3029_c0_seq1; 73 521 709 LEN=709; minus strand; translated
MCSRRKARELFDQESVQAWERVWEGVWEVVWEVVWERVWEKGQRPVEGQRPVEGLVEGLV
EQL
>comp3030_c0_seq1; 130 1 978 LEN=1340; translated
PKLKSMSTSVALTHNITPEYGNRNSLVERNNGAAAAATMLPVVCGGLSGMLAKTATNPFE
RIKVLSQTGDHLMKSSSIIDLYTGIIRNEGVIGLWAGNGANLVRIFPAKGILFSTNDVYQ
EFFRKVAKTSKSEKLPLFYTFISGGVAGMTACALTYPLDFAQGRISGKLASHGNRKEYRG
IIRTVLTTVKDEGVLALYKGVTPTTMGALPYEGIKFGTVGFLEQCFPVEQTTPLRKMMFG
GIGGVIAGLVTFPNDTVRRLLQLQGSRGSKTNYCGFLDCIRQTYKSEGITRFYRGFTINI
VRMVPNAAVQFGSYEVLKNLTKNYF
>comp3031_c0_seq1; 280 434 1477 LEN=1477; minus strand; translated

MSSSSPTKNFSNPAASFESQPHSPRSIFENSVVRTEEDAGVIAEDEVNCQTKSKSLVPKV
IIPCGHDGYEKSRSSVGEKDIVLGRRYAVXNVEDALKTDWCMILTRNSLISFPLIFPPIV
VDSDSVQNRSEDNRFRRIVNLYRDEYISSNSVERATIVSKVIALLEKDSYSFLRSGSLRT
PFSNRNSSYPSSLCDGYGINEGDSNGGLESGMFMDGQIRQMVSTSFSRLGRRRSRNDSSE
FKDAIRQQQRQMIDATLNRTPNTLKNPAPLATKDKVSYLKASTLGANQSIAPQQQDGPQD
ELVPPLRASRAPAHSETKLAADKVQKVRPFSSAHESCLLQECQPKVPAX
>comp3032_c0_seq1; 325 1 717 LEN=751; translated
XAKEMVVGTGNRPMRKAIEEHGKNLDDAYLAELVRYLGAPAPAASAPVAGATEDVEMGTG
ADPKEEDAGARAPASSYQSALEAAFLYRYASTLQEQKLLADAYHKHTCAWCLDPSGGASS
AAEDLPRAAAPSSAPTAEAHVPPVVEAPVPDGGEAAAAVAAAAAVDVAVVAAVEDQGAFA
TEXIARRTAREGIEKKSIEQEKQSETNVLDKKHHPFLKRKQPDGTIDSDDNTSNNSHSG
>comp3035_c0_seq1; 137 1 920 LEN=1431; translated
IDQELGAIQHPQRILQNEKVCFCRDHFSYTAASPRSLAVALYMDSICGEKTRRRPTALPF
GSVHARWGSRVGALLGEAVDGGELENVGEPLDPKLVAAASEKLAWELTEDRRQKPILNLS
SRDASPEETQGKEDECENISEWDRGQRWKISLGYLSDLGVDVDATRAAHNGHEEILGAAM
LENCPQLVRLKPEAIRDTAEWIATEFGLGYLKDSVLPQHSVLLSFRSSDASYGLEFMGLM
MMGDARPACSASPAFLVTAIEGGLQERTVGKALGAAGEATSKASRSIASDTMESFRQLRG
ATRHKK
>comp3036_c0_seq2; 141 203 322 LEN=980; translated
MSELAAGKMKRYIMLQIQWFDANILTRLVSHNAGRIQTM
>comp3037_c0_seq1; 375 1 931 LEN=1018; translated
XGTTTTASGPSHSGDQLSWARDTLSEWGEASKSNSVIYTYAFVTFLTLAKTEDGGEGQDD
SELVDRYHKNRGYQGSKLGKLAGTALLLVLVGVAIVIQTVTPIAIVATMELPEGGACPNE
ADGLTKFIGLTLCLYFVFLTISMCQNKLRAMGYLRLFCGHHDAGLGGRMGISCGVLFLDA
GILANMLSMGAAGAAQYLLFVRNANRDVLLLLLLQSLAMQFVLTADQKLMTGAWNALTKK
QIEILLQKDTAGNKPGGAGGAEESGLGTEAIEIDDARLSRILLMHKAETAFLMSVVLIGG
GWSIALTYCM
>comp3038_c0_seq1; 755 299 2575 LEN=2902; translated
MSYHVRLPNRRALRKEDQHQHRRMKENEALNPDLQWTFIGTKRVSNSESMDEEDDSGLHE
NGDYVSVVQKDEAAFMYLPRLNEPLVTYSTRAPNYPIGYSCAHTLVHSSNGIKGIDSSPH
DPMNLSHSEDGESMVLSETFVPIPLEFFIPASLSGDYDKIYSNVEALQWSILNEVAKRSG
LAKGCKVNVQFDDRPLLTALTSLNELHSSATPETTLRHNSDQHTTLSVSSNARSSSQNQG
KNQGNGQRNRRRARTIGTNRRMLSRLPYPMSVYSIESTRPQWTESCSSEMIHSDESESQC
FTAEITLEVRFRGTTGESNLIEEYIQSIVHNQIALDASQLFVEDIFGLKWSSVQEASSLT
QPPSNSSQESNGPISFGPENVEDSSDSIGTTAKDKSNSLFASIIIPIATACIVFVVWYCT
RRYYNRDRKRARGARDHQEKSQAVVRGMKQSTDIEIGIVRTETTQSSHDQLSKSSSNSNG
NPEDVISATASSDTISSSSSSTSLKSISATSDFSDRSITGLPPRPNVVKREGSKQLKKPR
KKKKRRKKKVMSLQRINSRDNISEMPMISESESECDSESGGEDEDYSQDDDGSSYDASGG
CLTPSRRSRTSSRASSASSVSSPPKSPRDDFFQPESATGDQSSYEFVMEAPEFSEDLFAG
LKTKNMFNINANNSNPHTSIETPQSKMTVPETDKMDKHNQQTNILDSVSIGDDIDNDNFE
IETGAELDTSFTNEHGSVMERMMPLPWLTGSGRNQRMK
>comp3039_c0_seq1; 221 1 1103 LEN=1116; translated
XSSSLSESQKGLVATTEPISPIRHAGEGASEGESSWEGASPDTPSSWSVHSGSNDSRDSS
GSYTEDGSQGGPGPSYGEADGILRCLLDLRDRGVLYKGVKPIVSTIFTSQFVFFFLHAYA
KRWLARVTNSGSVSNSPSLSLLSSCLAGVGNVLLTNPLWVTNMAIVSGTTETQNLFREVL
RLSRDHGLGHLWKGTGASLLLVSNPILQFVCYEQFKRKILEGRGKTGPGGKARTTLGAAE
AFAVAALAKTIATVGTYPLQLAQTLLRLADGDRSGERAARYEGTIDCLARLYRTNGAGCA
AWFKGMRAKLLQTVLTAAFTFLTYEQILGAVRRVVESHALRVRTSRRRVGTTGGSSGGNG
LDQLLE
>comp3044_c0_seq1; 718 89 1642 LEN=2177; translated
MSPKVKNDDDNDGTADDSPYISRIETSFQPMDVAFHPHRDNLVAAALVDGSLEVHDFRKD
EDIDPTDSDDEYESLLSSLSLHAPSNYTERSPSKKTPTASCKCVAFSPDGRKLYTGGSPY
GDMCAIDAERACSFSLNSKNEPLWRIQSATASTDSVVTNNDEYNINKNKSKNNPLVALYP
FCKDGEAGTPLLATGDDNGGLRIWDERLCGGSSGASFSSSRPKGCVLEWNEQQDYISGIE
YYPHSNGKNHDGNNILLATSADGSLGVYDLRMNKGTVADTTRTSDHLDDELLSLCVLKSG
RKIVCGTQTGALSIFSWGTWGDISDRFPGHPSSVETLLKVDENTILTGSSDGLIRVVQIH
PDKLLGVLNDDIEGHEGYPVEKIAFNSNRDVVGSVTHDNFVRLWDARILKDDDDDHSDND
SGGDDDDDDDVEDSKKPAGVAKSTGSSQVYMSTSTRRADEKHNSDDEWEDMDDNKKSGSG
SDDDSDDSDDSEPEQETANDRRKKRLKTENEKFFDDL
>comp3044_c0_seq2; 691 214 2640 LEN=3175; translated

MSNRNVSQVKNRAPAPIQISAEQLLRESADRQEVLKVDPVVQIHDAEEYQSYLRDRRKHY
EDNIRYRREHIGNWVKYAKLEEDHKEFERARSIYERALEVEPRSAELWLRYAQFEMRNEF
LNHARNVLDRAVQLLPRTDFLWYKYVYMEELVRDIPKCRSVFERWMKWMPDDNAWMAYAR
FETRCGGSSGGLDRAEAVMRRYVNAYPSPKSFMKFAKWAEYEAKNVDLARTIWEASLSEL
EPEESKQARVFARFAAFEERQGEYVRARVIYKHAIVLLRLEELEKIKAEQSSRTMEQDDD
IPDWELDQRKDLYKAYLTFEKKHGDRQGIERVLLTKQRREYNERVEKDPYDYDAWFEFAK
LEEDSTLLDAGTDNHHRAGDPAAVREVYERAVAQVPPGNTGEKEHWKRYIYLWIYYATYE
ELTNRDLERASEIYQTCLDHVIPHKQFSFSKVWIYAAKLQIRRKNLTAARKLLGRAIGMC
KGKERIFKEYIAIEMSLGEIDRCRTLYNNYLKAMPQNCEAWSKYAELEKSLGESERCRAI
LSLAISQPSLDMPEVLWKSFIDFEIEEGEAGNARKLYERLLEKTSHVKVWISYAQFEAGE
IGEGMPKVRTILEKAYNELKEDGLKEERVLLLDSWRALEQQKGDADSLSAVEKRLPRRVK
RKRMRKDGDGNDLGWEEYFDYQFPDDDDQGASNLKILEMAAKWKRKHDDSDDDSDDDDDD
DVEDSKKPAGVAKSTGSSQVYMSTSTRRADEKHNSDDEWEDMDDNKKSGSGSDDDSDDSD
DSEPEQETANDRRKKRLKTENEKFFDDL
>comp3045_c0_seq1; 435 1 951 LEN=1137; translated
DVDEDVDEDGLLVLDNPCSLEEELDLDSFEQQQFPHTQLDIIQEGSAEESAEEASVRHDH
RVVSEEEEFTAAGAVVEAEDSAAVWRTLYGLVRLLFVSTVVFHCGATVGFWDRVADHLGS
IEGGPLVLEKAEAVVSDLRAAGTSLVSGTREVVSDTVETVQQRIGSIDDPLARLEEAFPG
LEATASHWLEEAASFYHRLEQAASDLAREAASFDIESFETAEEGPPELFGEEDAPMSDLE
VFHQLVEEALSDDEREAAATATASSSASARAPPEDAAVAPLQGGVVSEVVVPATAGPGVE
RGSEEAIEVVAAAGQE
>comp3046_c0_seq1; 176 134 340 LEN=970; translated
MPDDIAIEMTPAPGDVIDDVDDEISFEWTVATIAQSIALFLLAGVAEILGGWMVWMSIRG
NGKDEKKPW
>comp3046_c0_seq2; 182 134 301 LEN=1070; translated
MPDDIAIEMTPAPGDVIDDVDDEISFEWTVATIAQSIALFLLAGVAEILGGWMVW
>comp3048_c0_seq1; 120 184 396 LEN=396; translated
MSGELSAPTTNYKTNSFPTPPRIKKTQNWREAAEKSKKFRDETTTPAQKKKVAIIGGGLS
GLACAKYLSDA
>comp3049_c0_seq1; 113 975 1091 LEN=1183; minus strand; translated
MVEMGKKSSRSLYVMICHVVHAMEYDIKVSLHAEENQY
>comp3050_c0_seq1; 328 30 1345 LEN=1493; translated
MEGLPTEPPPETGKGDSGRREVARDGQEPPSQPRKASKPAPPAFWPLLALEPLLSRWKER
AVSWTEAFASGPRNDLIGILADRALEAAGHHLDPEGPVVRGLLERFLPGGLEPGGDKGGD
NDKHKDKHKDRQRERRESPAKQDLLQKFPFYGQALDAVKYQRDSYLMGPDYANRKLQQQE
QELRQHPKDPREPPEPPWREPQQLREHQPPPKRLLPLRFGGATAPGTTGGLPLPPLGGST
GTSTAYPLLVDTGGRFLESRGARDRLLPIVVSLPSTEPLGSAICSTVSNAIPLAAPPLDE
AVKQAVLGVMGDPFTRELIKSRTQQILRVDDDXALIFASARAGGSRRTRSGAKSRHENNE
CLPLGVDDPSETDGRATGAIDANANIEERKRTKTPQRRGSAWFFPDGIESNRLDSTRIDW
NPSGIDPTEQRKNMVGNR
>comp3053_c0_seq1; 474 117 958 LEN=958; translated
MAGDQSPILFLQEDNRESEVRQDHILSASVFGHRALQSNKLSFGKFLIATLREENGKVFL
CMKTVERAHSPKNLWEKVLLSRNYAKALAQIDEHLQYFPKALLHRNKQRLTKIHQYLLRM
RKLKLKELSGHKAKIVQAPTRKIKQREERREQKALVAAKITNKIEQELVDRLANGTYGDI
YNFEQTSFNKALGQVESDEEEMETEDESEEEESIVEYVEDLDSDEEESMEEDMEDFEYLG
DGQWGRAESDSDESDDYDDDDDDDAESEDGDSKSEKKENRX
>comp3054_c0_seq1; 207 767 1024 LEN=1161; translated
MANDGRGKPNTNRSQWFINTAKTQWLDGRNEIFGMVLDGVDVITAMEEKGTLGGTPTARI
AINDSGSLPLEREDAVPYLVSQKLR
>comp3054_c0_seq2; 299 185 895 LEN=1032; translated
MLFLRSIEILLIGLLRIAPVWSEYDVPTKKAMPPKMDPIPTLPLVTERVFFDILIDDEPI
GRIIFGLFGDVAPKAVENFSALCKCDRGKAPITGKDMCYKHSLIHRVIPNFIIQGGDYTH
GDGTGGESIFEGNTFEDESFAVKHNRKYMLSMANDGRGKPNTNRSQWFINTAKTQWLDGR
NEIFGMVLDGVDVITAMEEKGTLGGTPTARIAINDSGSLPLEREDAVPYLVSQKLR
>comp3057_c0_seq1; 405 166 750 LEN=839; translated
MAEPTEPTDAVAGDRDSDRDVEDKYEYDNDEYDDDDFGDDEAPPALFRMSGVDTSLGDAF
LEECLEAVLLRGQEAAGERGSDEPKLTVTLMEKDFDEKGTLLFLVSDEGGGAGGTAPPTT
PCDVEGAIRDYLEASQPGDGEAHPWVGDPGAASIELVDWELDDAPGAGVDLAELGISEEE
AYRLLVDGQAGDGE
>comp3059_c0_seq1; 210 402 989 LEN=1367; translated

MARDYVENPSPDWWSDEEFVFRGPVIGPLVKKDLVATLTANEDLRTAFPDLQQNAFGFTA
DDPIEPNRVWYFVRPRGTFAGPFPMPGTGKMIQPTNAPYIGPPECRSVIFNREGKIKYQS
VGYVVDRFTGDTTGGRGAIFGQYAVMGQDIDANPGAVSTIFLQKLSEYLPQFPKSYSKRE
DLPKWWTDERMGAEL
>comp3060_c0_seq1; 317 70 1749 LEN=1777; translated
MTKRTKSQSPTRAPQHNGGNIAQPTRARSRSRSPSSGSRAAQSNQPQQLKRGRRPGGSVQ
PSTGNNKNENADRKAEAEAQADPAQGVQIRPKEIEIEAVREAWIRHRGTRNRTDIEAEAV
RKARTHQHRGTGLEAEAXSESMDPASRNKRYAHFDDTQATKEYPSQNRKGLRRGFTARKL
SRGISVRGREMSWCKLAQYIVPGLIIIAATVGAIFATGNGSIITDTLGGLIPILDNSDIE
DPFGNGNAPSWPSNGKGLEITIINALNDDWQNTFAIAVANWNNGDPSAVQITQEPGTYDP
DCEAPEGKIIVCNGDYGETKWRGVTESLSGNDGKLVLATAKLNEYYLLNMGKGAWQYTMC
HELGHSIGLGHTDEVFGNEDLGDCMDYTDNLERNKQPTELNYDMLMSLYGPLGGQRQRRE
RLQLRMLERNEQNFIPEAYTNSSGHHQDLSVQRLRKKVSFRNNIRIHDVSAEGSDDVNAH
VFRTVPDDIKKEKDKAVQVLFDRVRNEDPDDYEIAAGYTHKDGWKLLHRKLYGEEHEMEL
GMGYKVRIQLLLDHKQTIR
>comp3060_c0_seq2; 338 70 1872 LEN=1900; translated
MTKRTKSQSPTRAPQHNGGNIAQPTRARSRSRSPSSGSRAAQSNQPQQLKRGRRPGGSVQ
PSTGNNKNENADRKAEAEAQADPAQGVQIRPKEIEIEAVREAWIRHRGTRNRTDIEAEAV
RKARTHQHRGTGLEAEAXSESMDPASRNKRYAHFDDTQATKEYPSQNRKGLRRGFTARKL
SRGISVRGREMSWCKLAQYIVPGLIIIAATVGAIFATGNGSIITDTLGGLIPILDNSDIE
DPFGNGNAPSWPSNGKGLEITIINALNDDWQNTFAIAVANWNNGDPSAVQITQEPGTYDP
DCEAPEGKIIVCNGDYGETKWRGVTESLSGNDGKLVLATAKLNEYYLLNMGKGAWQYTMC
HELGMSFSFVSFSAVQLVVLKFDLTFTQPTLREFFCVFLTFTLAGHSIGLGHTDEVFGNE
DLGDCMDYTDNLERNKQPTELNYDMLMSLYGPLGGQRQRRERLQLRMLERNEQNFIPEAY
TNSSGHHQDLSVQRLRKKVSFRNNIRIHDVSAEGSDDVNAHVFRTVPDDIKKEKDKAVQV
LFDRVRNEDPDDYEIAAGYTHKDGWKLLHRKLYGEEHEMELGMGYKVRIQLLLDHKQTIR
>comp3061_c0_seq1; 50 1 1107 LEN=1230; translated
RLVLELTGEVVERAEPHIGLLHRGTEKLIEYKNYLQALPYFDRLDYVSMLCQEHTYCLAV
ENLVHCKVPKRAQYIRVLFAEITRVLNHLLAVGCHAMDVGAMTPMLWGFEEREKLMEFYE
RVSGARMHAAYFRPGGVHTDLPKGLLTDIFIFKEQFRLRLLEIEEMLTENRIWKQRLVDI
GVVSLEQAQDWGFSGVMLRGSGLEWDLRKNQPYEIYDELEFDTLAGTNGDCYDRYLIRIF
EMKQSLQIIEQCLDNIPLGPIKTSDNKVTPPSRFDIKQSMEALIHHFKFYTSGVIIPANE
TYIGTEAPKGEFGVYLVSNDTNKPYRCKIKAPGFAHLQGLDMMSQGHMIADVVTIIGTQD
IVFGEVDR
>comp3062_c0_seq1; 366 1 935 LEN=935; translated
PQPQQPQSPTTEEPRGSPTTPSIQAGAAATGTDPTDPGKTTAGPAAGTGAKEDAKEDAGA
NAGTDANANANASVSPISTNSSRRTRSVEVDLEEPKRASVSRGKSARKPSAATKASPSKK
KKASRSKKEDRTPARGRGAGKNAPGKRKDPGSSGGESTVTAETVDVAAVEDTDEEPASVG
RTDAESARAGRAKKRKKKKKKSKGSGASKKKKKKRVYSIVVEGDEDAYKSFDNRLREVE
VFEEKLVRDKLLLQKEREAMAFELESMEIRMDEELGYSEELELRVKELEQLLRSQKISQA
GHRVETEDKVRR
>comp3063_c0_seq1; 234 1 484 LEN=649; translated
NDDDDNNNDDGPVQKNSEVPQRQEQEGSLPGARNHPEMQEQMENQWREVERREREAAANT
RENILLRRQEEEARAPIRREVMAYRKMMEGMGKGEKLAPLDSKEAAQEYDDRLALGKTLE
EQERKMRMNELTLRKMDMEDRKVNADEQSESMCARCCVIS
>comp3064_c0_seq1; 179 1 1009 LEN=1009; translated
XNNNNNNNAFDAHWRSIVLVHQQYGSRMFGAGASWFLWDVSFYGNKLFQSTILEALVGKD
AGVVGITEAALLNASVALAGYFCAALVIDRVERKRLQSAGFLGTGALFVACGRLVSALDD
RGRSFSVVVVVLYLLSSFVGQVGPNCTTFLVPAEIVPTAQRTYCHGVCAAAGKAGALVAS
VLFHALGSRGAGASLFYVSGAASLLAALVTQGFVPETHGLDLLDWTAGGAGSCASAGTSG
EREPEGRRDPTKRAIAIWSASRGGPAATDRPSEMPTRVRTSGTGPGGQRERGFRCGGPPP
GGSPSGTGSGGPPQPQNKTKRNSVDAVVLIDTITAFX
>comp3065_c0_seq1; 521 1343 2230 LEN=2298; minus strand; translated
MTIQGILLLSSVLGSAVATTYPVGISNCGVRSWIEDAPKRAVTMNQGTTEVMLALGLADS
MVGTAYLDDEIWPELAADYARVPVLSEGYPDIDTLMATDPDFLYASYKSAFQAKTEEDSK
RIDYFQVVEDGCDLTLPETNATYCRAELHVEGIQTYLQTPYCELADHRPAEVDLKHLYEE
IWDVALIFDAFEEAQVLVSNIDDHFRRATNLVETHGSSDVQVFWLDGWDDTTPFVGSCCG
SVNKIIEYAGAQNIFSDLGIEEQKSWADANWTDVIERDPDVIVIVDASWDQAGA
>comp3066_c0_seq1; 566 256 1094 LEN=1094; translated
MMEFSRNNLSDEEWMAKMSSSMNQSVDQEFLRRLKASSSAGSSGGAGSNAPSGMGTDGSS

SMMQQNQQQRNLSQQNGGNNTGNGGNDAAEEELLLQLLYQRRRQQQMKEGMGGGAGGAP
GDDGRFGSGVDSSGASGNPTTHLMRSDEEQLKKLRDQQATVVSRRNSLMSATGGGNDRRA
TMINGFSDASGNNLGNGANPLGGNRSPNQTRGSGSISPFNASNGMVGGPGSSLDGVRRSS
MMGAMGNMGAPAGGMFGGMGGLGGIGAMGAMGGMGAMNGX
>comp3066_c0_seq2; 492 201 1039 LEN=1039; translated
MMEFSRNNLSDEEWMAKMSSSMNQSVDQEFLRRLKASSSAGSSGGAGSNAPSGMGTDGSS
SMMQQNQQQRNLSQQNGGNNTGNGGNDAAEEELLLQLLYQRRRQQQMKEGMGGGAGGAP
GDDGRFGSGVDSSGASGNPTTHLMRSDEEQLKKLRDQQATVVSRRNSLMSATGGGNDRRA
TMINGFSDASGNNLGNGANPLGGNRSPNQTRGSGSISPFNASNGMVGGPGSSLDGVRRSS
MMGAMGNMGAPAGGMFGGMGGLGGIGAMGAMGGMGAMNGX
>comp3067_c0_seq1; 191 150 833 LEN=833; translated
MFHRRGGGGFHHNKTVSCSAVKRNISSTARKALVCPPPATTPKNRAGRETNNGSDVGKQK
GEKGFLFFQRHKQRNGSVNSVEETLTNNSSNGIRSIPASSPARTKSAMRSRSRWSPPRAQ
QQQEEREQEPPLSEQLAAHRRRVTFAATATTRVYVKNKDRRRTQQRTRRQAPSETTATEK
NAKTKKNKRNLEKSSSNGPRPTIEGLPPPFLHRNNSEGWSKQKQQQQX
>comp3068_c0_seq1; 379 92 823 LEN=967; translated
MVLSVVKNVQAALKYRGGWKGLFQAMYTNGDYPFKFGTYKGCDAAGNRYYENKVDYPFGQ
HRWVEPGDIHNFDSCQVPPGWHGWLTSMYDATPEEDEEYIDELKKRIRPSAPSDAPYDHN
IGYQNELFNFNHMQQQTQIRSRGYGIGNSIVGLPAFKPEAYYTQPGSPYNPAFQRKLEYE
GDLDEATGGGRPYKNAMWKERLMTAEEKKAMEPVDDAEFAAEATKLTPREEAILARGGTL
PTK
>comp3069_c0_seq1; 293 160 1083 LEN=1629; translated
MTTVPAATQQSEEKNGNANDDVTSRDRRLCEECNEESALYQCPGCLIRTCSLKCCQAHKK
RTKCSGKRPRGAYLPLCRMNDSTLRSDYFFMEEVLDSIPRARKISKLAEEGKSCMNRSMH
GKSSNLQKSIASINKKAKRLVQQAQRRGITVQVMPPILERHKNNTSWYCGPRDLIMWKVE
VVLVPTESTVSFNLSEKEESILDHIAKHITEFHKNDPNMPTKLSPQNYQLLIKRLPSAAN
SPRYARIENNKSLREVLEGFMVVEHPTIYCVPNEKMTDFPIGTKEIIERSCDTHSETVSD
NYLPTSD
>comp3069_c0_seq2; 293 160 1083 LEN=1453; translated
MTTVPAATQQSEEKNGNANDDVTSRDRRLCEECNEESALYQCPGCLIRTCSLKCCQAHKK
RTKCSGKRPRGAYLPLCRMNDSTLRSDYFFMEEVLDSIPRARKISKLAEEGKSCMNRSMH
GKSSNLQKSIASINKKAKRLVQQAQRRGITVQVMPPILERHKNNTSWYCGPRDLIMWKVE
VVLVPTESTVSFNLSEKEESILDHIAKHITEFHKNDPNMPTKLSPQNYQLLIKRLPSAAN
SPRYARIENNKSLREVLEGFMVVEHPTIYCVPNEKMTDFPIGTKEIIERSCDTHSETVSD
NYLPTSD
>comp3070_c0_seq1; 223 146 1084 LEN=1084; translated
MALHESLTVLIQTSPIPSHPSTALLEALFRSFEKADGLLESRIVILADGCEALGHDGGSA
TPTESGKQATENIKHGRCYSRTAENYLRHLELLERAVREGTPPFCPRAGGSIELVRLESR
HGSAPAIEAAMARCVSTPLVMICQHDNFFVNEAPLRGVVAALLEEPRGLGIDANCVHFLS
TATLNYREKIQRRYKLDIGEPRRVKALEDPLVPLVFWYGRSHVAYSDYVRSHCLNRELAR
GSHLEELLGEKQLHDIVEVGMEAHKKYGTYVLDQGFEVLYHMSGRRVRAADPSDATENFG
QKDSSIENKQQQQ
>comp3070_c0_seq2; 74 1 804 LEN=1282; minus strand; translated
LLLLLVLDRGILLAKVLCGIARIGRPHPSPRHVVQYLKPLVQNIRPVLFVGLHPYLDNVV
QLLLSEQFFEVAPPCEFPIQAVRPNIIAVCNVGSPVPEDERDQGILQGFYPPGLSDVQFV
PPLDLFPVIQRRRRQEMDAVCIDPEALGSSRRAATTPRSGASLTKKLSCWQIITSGVETH
RAIAASMAGAXPVPRLQPDQLDAAPGAGTKGRGSLPNGSLQQLQVPQVVLGRPRVAPAVL
DVLGSLFAGFGRGRASPVVTQRLASVR
>comp3071_c0_seq1; 413 83 1471 LEN=1717; translated
MSFCGLANGIIADGFCAVELPLSVSRPRSVRVHGDSDEIFVLERETQSIVHLFDSDGDGI
PDSKAIVASASNLNHGLAIYDGYIYASSDSTVYRWPLYEGDIAGSVSMNYNAFPLVNEEV
VVSNINEDGMGGAPLGHKTRTLEFDKSGMLYISVGSNKNVDGDSYRSRIRRFNISDPSVT
FPQDFQTMEVFADGLRNEVGLAFDKHGILWGVENSADNLVREDLGGDIHEDNPAEELNRF
RPEDAGKHWGYPYCWTEYNLPAPTGIGRGMVWAWPSFFEDGTITDEECRNDYIAPVVPMQ
AHSAPLGITFFNWLPAQEVVSEACNGVKSFPEEMNGFAFIAYHGSWNRDVPTGYKVVYVQ
MDESGDAIGEPIDLLAHSPPNAKWEDGFRPVDVAFDSCGRLIVTSDGTRGRGAKVIRIEY
IGREINGSTEASNPPDISSAAEFSSTQIYAFLFAFLMVAICS
>comp3072_c0_seq1; 186 172 606 LEN=694; translated
MDHSLSDGSGDEDLPLSHPARIGEFGSKNDKNRGGWGESMELAHQREAGRRVTAGAPETV
VAVDLSQFRNTEVGRGYQARHVVRQRSAAIETTKTTTVNDRVHLPRARDSERPVKQEPLD

KEDLLRNEGLRLFRKEIESILASP
>comp3073_c0_seq1; 233 1 999 LEN=999; translated
LIRTIDTLDIIHKMKVTSPQLPTIRDQLPGRRSLSFYYYCSLFLFLSIASAPKTIVVVDA
SPIVKHQERNSNNNQSPYRFEPSYGDQSWIFETINLPGAWKLGYTGRGVKIRINDEDWES
GHKEWKPKRLIKDNKNALDDYSCGATNNTLAEGKFRDSDNKNKFLNHGAAVTSILAADGS
NDFCGVGIAPETELSFCYFRKDTDASVLKYDAVDNMNFFYISVNAFAQEGCKPRTSMFGE
AVNEQTIATGNGDRNRQRRRTKNRHGRQHRQQQDQSDRILNEQEIGGCPFVNFYYDEYNP
GDDPCKVCTTSDFKSVRDSNNENARNNFGSLQD
>comp3074_c0_seq1; 191 373 847 LEN=847; translated
MSSNSKNVVLANIKTEPLPESPPPPLVLPKPLWNLAKGDNVWVTQGKTDHMATFVGDPRM
VCIYPDSDDDHNTSPGEPMQKKQRTDRGTQQLEVRIRWATTGNKGYVQASSVRPVVSGER
RRRQLVSHYDAAPSKRPVYTRKSRGKTGGTTGSTSTSTX
>comp3075_c0_seq1; 161 1 409 LEN=1054; translated
XSSSSSSSAMPTAQGRSSGRWVTKPSRLSRGSIQLSPRWKVRLPKESGGGTVIYKGFIDA
QKMIGRGGKSVSAEMTGVILTGEEVNKEKVIGRFTADFVRQLDDEEVDALKIGGGGGKEI
TATGSSNNGPITLSPK
>comp3076_c0_seq1; 278 1 887 LEN=947; translated
XSSSPCYRARGDAANAFLLVGALGYAFLVLCFARPGSEGVVDEHWKKAGFCIHNKEVPYR
SSFDACLYLDVVLSAVLGAMYLSWSKLPGMEAPSKLVPSLVASTVGHGVAHGVMAKELRE
RIAGGMAEAASAAADSNGGGESGPKPWQLAGFCVGFWFPLLGAALPGLRHGTLFLCAVAA
TCGNAVVRKELAFGYVQTVVNVAFAASQLLLPPDQKNEREYALLPLAGVLPIVVSWAEAL
CCDSLVRSLGGHALYDASIIVSVIVFYSESYLHCTAKRAAPKKTGDALGDKEKTR
>comp3077_c0_seq1; 205 1 289 LEN=702; translated
XQHQHQQQQQQQQHHLQQQQRSQSLNFNNRNANNFLPGRNSTFGTHEPILSSGLNAGEVN
NWRNGNTDPNNSYAMGGNNINMGSNGPFDTRHHHAN
>comp3078_c0_seq1; 389 1 1455 LEN=1668; translated
APGKVLLAGGYLVLEAPNAGFVIAADKRFYTTVDAGSDKGDDHETKHLMRIRVESPQFHS
TWNYTYEKSKCQLVPSPENQSVNPFVEKSLRVCLIYLLQSSKSAGSAIPSSLNILIRADN
DFYSVLPHLPADSKKTPAEVAALPKFLPCPVDPDTGKAIVHKTGLGSSAALTTSLVGALV
HYFSRQSPDETDQLSPMIHNLAQICHCHAQGKVGSGFDVSAACHGTHVYRRFPKCLLPDL
LQLLDQFEKQPDGNNEQHVLLGETITKLVELVPWKEDMVTPIKLPNDLQLILADVRGGSE
SPSMARTVLKWKAGNKNSENDEVPHWSALSRLNPKIVESMQLLATDDTKNVNYDSLAKAP
SSEWSSDADSTLISLRDTFVQIRNELRSMGEAADVPIEPPPQQELCDATSKLPGVVTALV
PGAGGYDAVACLYINRPEVVKSIGELWSSWTSPVVCPLAVRAGDEGLRVENESDFNEKKR
KQIE
>comp3079_c0_seq1; 256 1 1460 LEN=1782; translated
DATMRILDTMKTALLWLAIGRLGPSLAFLPQAVPPATARSRAFVAAEAEPGNSSDRERPR
HPCAGQENPGTEAAAGLETWDRRGFLAGAAAVLASSAAEGSSPARAVVTDETDNFADNYW
SKSPAVQGGTTAPGTASEGAFAAPSDEVTVGVSGADLRSEGGIGLELGEVEFRTNRRVYV
KSVSPGSPADRLGIKKDWIVVSVNGASAERTDVEGVAIMIYKAAREPGRSAKDTVDLRFR
NPVVFRNQLRDLSSSPPGSTVVTQVAPEGDTTQRNPDGSVRQGYGGPTDQADQRLTVTQL
VPPKICRRGATEDDLLEISYVGKVAETGAIFDGSAVRINGEGIAGRGNDVSIFFVLGKQP
FGQFPPGWDAGLEGICVGERRRLLVPPVLAYGSKGLPRRGIPPDATLQYDVTLVSLNGLX
DAAVAGGGGDGTHRKEGGNPGQSNATQRKAVLVRVRVLADTGRYARTTHRTGRNAFAPAS
CNAAAGW
>comp3080_c0_seq1; 370 1 1288 LEN=1332; translated
XEAAGIVQKLAEACGDDFELHRYQRFSDLKVSKKSLGKNPTKTGSYRNVQKGDCVVAFSR
NDIFAIKREIESLTDHRCCVIYGKLPPQTRADQARRFNDPDSGYDVLVASDAIGMGLNLN
IKRIVFNSIFKFNGEKIVRLGHSDVKQIAGRAGRRNSPYPNGEVTCRDVRDLGYLRKCLS
TEIEPLRKAALLPTESHIELFAEAVSAASAGEDPGDQNATDPANHPDLHEILRQFSAMAT
VRGDYFLGRQTEMAMIAKRLRNIPIRLRDAYAMCLSPTTESSARLLEMFATKVSQNEVFG
LPSRSVPRKARSFDDLSYLCNIYSDVDLFMWLQYKFPPGNAVELATAMARKERTMEFIND
ALSATERLRLDHCYLKTAQRHRSVWEQRSGGIGSTANTDESDSDGEDYDDDIVFAFDYSY
DDNIRRRID
>comp3081_c0_seq1; 266 1 943 LEN=1015; translated
XPGGDLRPSEIFWLEDAAVEVARRHYEEVVEREEGVLPPNQRTGFRPPTVGFTHDTPDEF
LLRRSEEIDRFREATAEVTIENQTRIVPAYFPWKARTYTNTSLDNIVGQWHSIQSVFKLM
DYTARAKGFAYGRVGMFRSDALYVTPIDIARLGGTDRYDTTNRCFVTPSFALQPVNDRMV
YGPYGAVKIWATKRFDLLEDRASKQTDPGWILHHERFLNASVFPAMEAAGYEHSLETNGI
CSLRTRADRSAVLSDCCTDGLLPTHRATFWNDPGEAAERARLAAVESIVGRKCSLEDRGS

KWRFAVCGEERPAY
>comp3084_c0_seq1; 138 398 1345 LEN=1364; translated
MPPHVWIPKATAHAASRYASHRREALSLYREILRTARAFHWCDEEGRPWNERLRREARRE
FEAARHETDPLVLARLLVTGRDCVQQVQNKFNEADRAAPGPDPPGFRGPGVMRSGGDRSV
AFRFVAHNAPGPVSTVWHALVWWWFASFRVGVCRSRAHRWPGEDATQSPPRDAVSRTLGG
VDGTARHGTAPDSXGSDGSGGPFGGGRAADPAPGKAFPDRAERIPPEEAPGGLRFGLHPW
ALNNAERVAVPGGRAARSVGAALPPCTALWAVLVDHGPADTDGWTLDSEPKSRHTPFIGW
GVPVVFFEVPYDKKK
>comp3085_c0_seq2; 135 1 980 LEN=1062; translated
XFPEPIGAPGECHRIAAPVTCASCRFEAPRSLGLHGPVPPFAHRQRARTTAPPDAPARCP
WRNAMFHAGRIAGRSRISPTVVKLDITVTTPPLASFLPGQWVDLVAEGQPWVGGFSIASS
PRDLPTLTLAVKRSGDPPAAWVHDADRSAVGSPVRVRVGGGSVLTGPGPLRPSVFCAGGI
GIAPVLSQYREFLFLRGAAAEGSPAAPAMLLYTASRAEELVFGGELADLSREGCTAGGGS
DRVVFALTREPTGWGSERNGDSDSDRTFPPHVELRTGRVLKEFLDEGPADADYYLCGPPS
MIDEAVAHLRGVRSIPHEQIKYEKWW
>comp3086_c0_seq1; 135 1 273 LEN=711; translated
SIPFHSIVTKVVVRRLLEAGVDATANDNAAIQIASENGHIEVVDSLLRAGADATANDNAA
IRLASQNGHPKVVDLLRAHIDAVDRKRKRP
>comp3087_c0_seq1; 445 365 1391 LEN=1391; translated
MGDGNGGHRTKKRPLSAASEEASEATGDNDSEPPPLYLEEGLFAVTKPLGWTSQDVVGKI
RFVLEKDARERGAPDRRTKKRRPWMKVGHGGTLDPLATGVLVCGVGSGTKDLQKYLVGSK
QYRAEVTLGYQTTTLDADPKGEVVEEKSFDHVVSYDEIDAMLQSKFTGTIQQVPPIFSAL
KRDGKKLYELGRKGQTAEEIKIEAREVVIHDLKLIRPDRSGDSDSDAASEPPRIEKFEIE
VECGGGTYIRSLVRDIGIELGTVATMTALERTKQGRFLPEHCIPYQLLGDEPKEQKTQGS
DEKTGTVFIDKKEDCNWTVETITEGIRVCRSNVLGSSDDDDDX
>comp3090_c0_seq1; 271 1 542 LEN=542; translated
QHQHQHEHQHEHQHQREHEHKKFHKDNERHRSYMLYTQKAGLALFQNATPSFANHTKEHN
GIIGNNTNTNTNNHTIDRSTNGGNEDDNNDWKEFFDSAESPLFGKHRELRPIHKERLSSA
SGSVDDATEAEGAEANTDTDTDTEPSVDSYPIIRLSEPDSSTISTSLSEAVYDNNSSSSN
X
>comp3091_c0_seq1; 271 236 658 LEN=863; translated
MAADAYAEEEELSEEDEPADFSSSTLAETTSFPAPPATDVAIAKVAAAAAPPSEITANTE
WKKVRLYQDEMGNSSTEPPKPIQDTRKNWKYFTPVCGCYDIDETWSMLTTSTTTGPKKSP
QGKIMWNDFTQVFLNAGCDG
>comp3093_c0_seq1; 266 1 1001 LEN=1414; translated
GAAVAPALIHGAMEFFAVAPDYLGPLAAPAGALPGTASPSFVELATLPDGSQIVAGGFSW
XTPGLWAGVAPSVVTASFATGYVSSLGVGMAVGRFGWNALSDSLGRKNTYSLFGLGIPLV
GLAPHLAHAAAETASVGGMESEQLVVPYLWAFYGGSVLAITFYGGIFSALPAYIADLFGQ
KHAGAIHGKLLTAWAASAVVGPMGLGYLRSRSVDNAVGDLLEAIGNGGQEADEARKSVFE
ETFGCGLDDTETIEKLIDAKTISIGRLMELVPEGTVDPTPFLYDTTCYASAGFMGIALLA
NLAIRPLEFVKIGARAREPNDENGPRQETESKS
>comp3094_c0_seq1; 211 328 1170 LEN=1516; translated
MLTDYSKWDRLEVSSSDGDSGSEDDAHPAAPRVTRLEAPSRITFGGGSPTIAASPPAPGP
RASPAAAPLAPREAPAPEGTGAAGAPPGGNASAPSAWTERGGLVFVDAAAHGHRARNLYW
SQDRYSVVLRLELEGAEKVGSVTVGGILPYRDRCAAAGTARPVLSAGSRDGTVLLEGELP
HPVHLDEDEEVVDWSVEAAPSEATGRYLCVALRKAVPMQGLSVWWRRPLTEFPEADLGAI
RGAELSKGGSDEFLRAWEEAHKVFREGKPKRLPRNGPGGP
>comp3094_c0_seq2; 211 328 1170 LEN=2157; translated
MLTDYSKWDRLEVSSSDGDSGSEDDAHPAAPRVTRLEAPSRITFGGGSPTIAASPPAPGP
RASPAAAPLAPREAPAPEGTGAAGAPPGGNASAPSAWTERGGLVFVDAAAHGHRARNLYW
SQDRYSVVLRLELEGAEKVGSVTVGGILPYRDRCAAAGTARPVLSAGSRDGTVLLEGELP
HPVHLDEDEEVVDWSVEAAPSEATGRYLCVALRKAVPMQGLSVWWRRPLTEFPEADLGAI
RGAELSKGGSDEFLRAWEEAHKVFREGKPKRLPRNGPGGP
>comp3095_c0_seq1; 151 458 553 LEN=553; translated
MKDSEIEDDAALVDSFFSPGGLFADENNNNNN
>comp3096_c0_seq1; 110 56 1232 LEN=1647; translated
MASRFLKRRFTTNLLGWPTDVTSVDPNPASCHNATVHTIIVWIPGNPGQYDWYNSDFLEI
LSALGHGYAIRSLSHAGHGLMVSIGENCGADHSSSTMDIEGHCHRCRADNEETNGPSPRI
PWTVKGQVLHKIAFVDSLLREITGGDRYSEHERKQTCRFTPNPRFIFIGHSFGCHIIQRM
CVLRPDILERTTGFLFLMPYIRTKPLFVTEKKKLDVGGSYPEHLILVATKLSQLLKFFPE

PIVRGLIRKGLQGDSSGNDDGGIDGESIANVTTKLLCNPLYPRNFFELGTEEIRDIPNEI
DTTALRLLSSRKLLLPQNKTSMGTGSQRDVAQKRRIFILYAGDNDQWCPSFHGEEINALQ
SVKVLPASIERTKMSGLNMTTFAKIQRRDPG
>comp3097_c1_seq1; 158 1 313 LEN=569; translated
GGALRPAPPGLLPLDLQAHEEQAARGLARGEYRAIATTYYGDASFDNAFSENFSDDEEED
GYEEDSSWSNSGNGGRVLEMSGLGKHKQGGHHDDGLSLEEMNG
>comp3098_c0_seq1; 187 672 1361 LEN=1361; minus strand; translated
MKTIVFLILFASLSPPAFVESDPELPCEPVIGDEGHCAPACYCIPDDPGRCNACPGVGNR
QFAYLSGSSEVFFAGLEVEEASVFRLDPAGCNPFPAVASVLNQTLCSGVTTAGKGGGTET
KTKAVGDDGNGDIANGPVRCVFEYKRSKKGKSEGDRRRYSILEEPAPFKTTKKKSKKNPK
AAKRDAVLTHVGSCGVCSSAQDLAVNLSPTLDVDALSCTTKASEELLADT
>comp3099_c0_seq1; 407 1 963 LEN=1136; translated
KEAGEGQGKEAVDSFVLDAEVVAYDHEKGQLVPFQVLSTRKKTVDEGETSKVQVIVQAFD
LMYLNGKSLLGKTLKERRELLKTHFAPVEGRFQFAKYLDAREDGDTTVLEQFLDAAVKGQ
CEGLMVKTMVDIYEPSRRSLNWLKLKKDYLDGVGDSFDLVPLGAYYGRGKRTGVYGAYLL
ACYDPDTEEFQSVCKIGTGFSDEDLQSLSKALGEHELEGGKSSQYVVSESLEPDVWFDAV
QVWEVKAADLSKSSTHKGALGKLEEGRGIGLRFPRFERVRDDKRPDQATSSDQVLDMYYN
QDSVKGQLGGDGYDDGYDGI
>comp3100_c0_seq1; 450 1 1605 LEN=1765; translated
AVSNQSLPAAGAGEETPWIVPDDYEEEEEEKPPPAPRPQKKPRPERKSIHVAMDLPKKLV
EENAEAWDEESAPVFDLSKPQDRIRRHRYRNRLYQRLWKKRKLARLGKTKQAYKLNYRYR
VSAGGDGDDSDDDSDDDTDDDNHDDDHDETGGERSKDTIPKRRRTWLPRPIRGARSDRLG
DVHRILAVVVMACKLVPNWDENHRYVFSRGGDKSHHNSNGGKPSSGITTAENSKNHSARA
MDRFVPFNRDHFGYIGNGKTETDYLNFLEEFIFRGKSYALPKLVESLKEASSEGDGDANV
PDVDDDKASSDRCLVEESDDEEPEDQNDRTCSDITVVVPNDTVVEWRSRRKENNTGCLVG
SKRKFQCWTRPKVDSVPYRLTTVTDTKSLRTLDSPLGSLIEYIAYKTETQPDLILQQLIL
LDKEMSVKYRRSMKKCDRVAPSQIYKFVMGRKKKKKSKVLAVDNECQGTPAKANLLTDDA
NDDKTVDCSNHARLETISLLSPLQFNDVLAQESPEIGEGPDMDDVWSVGERACV
>comp3102_c0_seq1; 152 1448 1654 LEN=1968; translated
MGVIKAIVDSYPEAVLMRTNKGSSAKQCLSLTNAPNKAEVKQMLQATFARVEQKYRPAQA
PKSERVLV
>comp3102_c0_seq2; 188 647 1324 LEN=1638; translated
MITSEDWHKVVQLCQGSPSEAKKWTTRQGFFEGMKDANVLPIHEALVGGAPFQIIEALLN
AYPDSVYCKESSYQRLPLHCACRKNAVPEVVDLLLRRYADAALTPDSLGRLPLHYALSNG
ASPAIVDCILTKRPNSARGFDKRGWTPLHVACGVGAKMGVIKAIVDSYPEAVLMRTNKGS
SAKQCLSLTNAPNKAEVKQMLQATFARVEQKYRPAQAPKSERVLV
>comp3103_c0_seq1; 982 59 2567 LEN=2567; translated
MRYLSSRVLFYWISSLAFEKKSAAFVSPLFFRRAAIQRAATAATTSGTRSSMVAATSASA
SSSAFSSSRSIPKEETRILDLLQGQPAADGRGVKIAVLDTGCDLAAAGLQETSTGLPKYL
DFLDCTGDGDIDTTATATIPEVSNDDADGPIVIKGKSGRDLTLGAWAPPAGCTVRLGSIR
LYDVIPRSVKRRLVRERKEAFVDEHRRLIAQTQQTLDDLQSKISGFTAGNGDDDDAKEKK
ADEKKQLIRQRKDAETLLGQYQAMMESYDDAGPYMDVVLFRDESDGDEYKAVIDLKADGN
LTESVPFSAFAKHRQMGEFGFGSSVTFCIQIYDGGNTLSIVTDAGSHGTHVAGIAAANFG
EEDIKNGVAPGAQVLACKIGDGRLGSAETGTGLIRALIAAKKYGCDLVNLSYGEPSWQPD
SGRVSEVFADATKKWGITVFTSAGNDGPALSSLGSPGALSSCITVGAYVSSDMMVDQYST
LPDGDEGLRDASYYFSSRGPTPDGLLPDICAPGGAISPIPRHALQGKAQYHGTSMSSPNA
CGVAACVLSAVKQRLGIDRVSPPALRKGLSNTAVPVDVADPFAQGYGLVSATTAVDYILE
NRGKLAQDLTLDVSVPERNNARGLYIRDALELEGPMTFGVKVMPDFQHASTRTPDEMDEV
LSMELDLNLVPSASWVKCPERMTLMSAIERNGQQFSIRLAVEDLPAGVHYATVDGIDSND
EARGSLFRLPVTVVKPHSAIVDATNPSASLNEHETITLGDNGIDYSMSYKLEAGAPNRRF
LEVPSIAEWATIKIKSTDPNPSPTSPGRVLIHAVPFVRGDIPNTEIQLKKLIQITEX
>comp3104_c0_seq1; 532 1 1675 LEN=1844; translated
XHTCPQPTSIGWNRSKKVIIANGSTASMERSNENMNNDQSRPMEGAARAXETDYETKVED
GPYLTVEMARQSSIVSDLSSSTSDSKYSQNSFFMDTSNSEDKELKPARATTTEENPRSLK
EVFDRMNCLALQDSTLIEKQALKMSCVDEKDADAPRELSTPGEVIVLGSRQNVYPIVTNL
GGRVKPHIVPTATKQEASRAGSYQTLFVSYDSGFSNSDAAATNAPKRIRIVDFFKCGQPR
FDSAAKSTDGYRGPFSYLVPIISPSKVVASTSPRFLSVSMSMDELSCSMNSIAVEGEGKM
PEKGLGKTKMKQGMFQVPTAKESMFTKETTIEDMPFDLQSSTASTPKQGNKGKARNDSEK
QDIVGEEDGVLENSAPIAASSLALQSVQSDDFIGANIEAIEVNETGTIVNEDINTCETPA
EGHSPQLPFKNVYLSHKIDAGQSEVQDETSLSSPMKDSYEENAKTVSKHFSLLTSFSTPK

LKRRLRALAGSRDKKENGFVSKKPKATDCDSPGANTTATILMDGSSFGEEDFSNSNGNQP
FQLRPRPGGEQQSCFRLF
>comp3106_c0_seq1; 50 1 746 LEN=1468; minus strand; translated
XGQYPGRVPEKNTPTETEVRAHLGEVLEASRRSDATTNGGVCFVSLQSELPPQDDYDSWK
ASGGEIYLEGDARSEWPNPFSHYAPTVEWVRSRMETDGDGDSAVEVQYLHHPIRDLSVPS
NSKHLRLLLWKILEVLDNRESDGSNSDSMVYIHCWGGRGRAGLTACCLLALLHFASEGGT
DGGGNHDDNLSAIFDIVQTGYDSRLGSEHMPLALSRSPQTDSQRAFVAGFYGEVRAAAAE
AGKNGNAK
>comp3107_c0_seq1; 397 258 1348 LEN=1704; translated
MEPRLSSAREACGAKDLRKASETTTTISLMLNSTSDPKSSQGNQKRTHPHSISSAMEEIF
DYKKNKSEGINAHNHKRANPKSRLQLLHDHNIIENVMMFLDEIALFQLENTHMNMIGQLL
FARQWYYLSICDEKNMGFAHKRWRSLNAEDITVALDETESPINLERGNVTNENDHGKNAM
LPDSLSSRELLARKVGRFFAEEAIFVQEREKDASRIYDFDRMPVKDSEIPLSMAHSSATD
IDSSLATSCSSSSQENFWNEWYDYRMNSVNDRYVFVRLSLRDGTGRFWHGFRRLTTEHNR
TFFRMHFGMKELIQDMQWTELESYVQAQDTGYTSAXKSFEDRKIFDAYDAVDSIFGRKII
DGDW
>comp3108_c0_seq1; 250 1 635 LEN=674; translated
XFINDFYHHHYHLGTHSNHYQQHYHHHDEHERESTHQTSATEGFRPWFDRSGLSTGPSSS
AFGGGAESPYHSHHHPPDGPAGLHQQAEDMPFAVEGIGNERSSPRKPQGSGVSASASASL
QASMGMVAPPRRLAMFGGGSAGGQPGSPSSAPKAPTKTQARASSPGADPVVSLADQLSEF
RSFGAGLSSVGSGTPCGTAAALTASVGSGG
>comp3109_c0_seq1; 469 146 2098 LEN=2597; translated
MSNILRRSVKHGIGRLTASADGIPKQAAKNPSSRTRLTQVAHKAPTRLIPVKKTSVQKQG
AAVCALSNYGAGMLQGDSAELSVHVENSAKLALISQGASRIYTQRVPGEVKARMYAKVEK
DGVLVFTPDPCVMFASSSYSQTQEFEIHPEASVALIDWFASGRFKNQERWDFDKLTTRTT
LKWMEDAENKNSAKEETNNQIPFLQDSISIDLRLNSNSDKNNRSVHDTHGVADFNCFASL
IIYGSEMEAVKDKCQVLADLFAAQHTRIRERDGNGEGDTMLRSVKTIDDLKLSGRIVMGV
SKISLPGKPSNAHVIRLAGETNEDLYRVFHECLIPLAPSFGFGFYKDRILAQRSEIPVQG
VTNEKQKIFETPTKKHQTKAAKSYPSSRKPLESFNDFSQTSRSFWSVVMLADSGLPTGSF
AHSAGLEAATQLGMIRNEEDVCSFIHAATRSSIQLLTPFLVRGLRITQCETSNGSDKKTM
EDLWEKLHRQCHAIMVTNEPACSASIDQGKSLIRVAAHWLNGANESHEPTLPNKYILDCL
KSGSAPHIAPALGAIGGLLGLDEVQVCRLFAYCMARDLVSSAVRLSLVGPLASVPMLDSV
QEAAEDAICSAYHDFRSNPEDPLLVASSSAPVIEAVHPSHEVLQVRLFRS
>comp3110_c0_seq1; 321 68 598 LEN=701; translated
MTATPRYYPAEDLPASKPTYRVKQNPPRIRKSITPGTVLILLAGRFRGKRVVCLKALESG
LILVSGPYKINGVPLRRVNQAYVIATSSKVDVSGVDVSSINDEYFARSKDASTEGEEEFF
MGDAPKAAIVSDQRKEDQKKVDAALLKAVASVDMMEAYLAAQFTLNPNDKPHLMKF
>comp3112_c0_seq1; 286 82 972 LEN=1013; translated
MAISSAESNFILDGCQDDCRADGRSRRDFRLYSSVVGDFALSYGSARIFLATKENHVLVS
IKAEMAVPASSAPTEGAVEVHVDCVKGTNTQDDDLESTISNLLVPHLVDKRNLCIAPGYF
AWKLNIDLFVVNSGGGSLIDACSQGIHAALQQTLLPRISVIPATQGGENKPTLQIDSDIK
AAGKILGVGNAPIIVTVTVLKHKRTHLLIVDATTEEEICAFAKVHVVLDRGEPLSSSCRK
RKPTICALHKAGGGALPFEILQDVTSFVLEASGSSCAVESESLPHLLQDNFSIQQ
>comp3114_c0_seq1; 148 1 232 LEN=232; minus strand; translated
XLLVVVVVVDLALDRIGSGGIEGYFCLEEFVAVDEVEEFSEDVDEGDEDGYSFWLSRSLD
RLDRLDVGRWLLLLLLLL
>comp3115_c0_seq1; 348 83 556 LEN=705; translated
MARGRRGGGMGARRPAARPSPPPSRPASTKATPAPAPAASAPAPMQQQSRGGGMLSGIGS
TIAQGMAFGTGSAIAHQAVGAAVGAMSGGSSSGSDHAAAPAAQPQQMAAPADNGVCSNDK
QLFFDCLKSSNGDAQACSFLYEQLQNCQQQSATQTFY
>comp3115_c0_seq2; 372 57 799 LEN=948; translated
MTRTSRLNRSEEPKPLGSSHETSVSGXILVTAHQVFLEETSSLRNDFVAASRLVLILSCF
FVVDAVANTTAPCTRCGYGTNTCAAFHPFDPQRGRRGGGMGARRPAARPSPPPSRPASTK
ATPAPAPAASAPAPMQQQSRGGGMLSGIGSTIAQGMAFGTGSAIAHQAVGAAVGAMSGGS
SSGSDHAAAPAAQPQQMAAPADNGVCSNDKQLFFDCLKSSNGDAQACSFLYEQLQNCQQQ
SATQTFY
>comp3116_c0_seq1; 477 78 1607 LEN=1723; translated
MGGGISKHGGDEAAVRAASAVGDQPGQPPQSQPPPQPPGSEPDAGDEPPASKCPMHKADG
SGYSYDWKQLFRAAAVHGPGGSKPLSEKERAEATGATGGGSACPVVHDPAAAGGGCPVRA
PHPEYDVYSQPIDKTNNMPRGASVQLPNASQRIELSTERVSSTIPKGGADGSSAATWTYP

SPQQFYNALARKGKFDANGGESEVASEEDMASVVALHNNMNEKTWAKVVEWERQTYSEAS
APKLLRFCGRPHDLSPKAALKHYLLGHPLPYDRHDWTVLRDDGSTVRYVIDYYYDESRAG
LTDDSAMPDLHDRSATPSLLVDVRPALDGPGSLLARGVRMPYRMATGQTGYEMLPLRGTP
ELADQVKESVEVWKSIQQTRARQEDEKARKEAAADDDGTAEGFDVSEDRAREIALGFARA
LEDCSGVRERMANCSSEDECSRASMDLTLCFGKHLCSVQHKSLLGALEKDGDDAKAEAAL
ETLTQCVMLKSAERRFAREQHPGLFEERE
>comp3117_c0_seq1; 267 1 940 LEN=1067; translated
XSVPTIVAVQFAVQTTAMQLEMVTTSRLIDGPSTNSGRSVTLANSHPFSQSQPPSLRRLY
ESMVRESSSTDYNELYEERTTLQREWTQFLESVDVGDHNLLDPIAPQTSSRDRNGRNEWP
PSPETPTAGLSASSCTLEEATQISLMTVVTTTTAEDAHLVCSDESSGHCSACQARSQTKT
DPRCAAAGTQLDLEPEDTEEAMVTFRCLNEQVDXIAWDPGVIVADGGRLLPNNNNNNKLT
PSTTDRSSNTKRFSVETASFGSFHGSLMVGGPPVTTQQRSDVQMPLGDTVRRCLYTSDDD
PSPFEKIIVFALD
>comp3118_c0_seq1; 177 1 439 LEN=1134; translated
XAMESMQWKDATIRTEENGDVFEDPGTSHPGQAPGCVETKPPPHCRLFRLAAVSAGASSV
LAACVCLEPNESTARADRNHGWFALTAAKQPTRTLQSAAFPGVNKCSTLVQTFSVGGLPT
GDPWLPSLDPWLFSLLGPSGPKPSQV
>comp3119_c0_seq1; 405 58 1229 LEN=1229; translated
MADLYNLLDELEENEAFDDDAKRDPRRLTVETAATDGLSDDDDDNDDYEEFDAPTPRVPA
ALLDRDEDEDESGDPFYPAKPRADAFSKAGLDLHDDLDETHKQEQHGVPNELYEKLHRHW
LQERHCPELLGYDEAMVGELRERFEDRQQQADDLVDSQDPVEVLMANLAQQDLDRARFVL
SDWLTHRLHKIESHPLHMREKIERMSQNEIDYLAAYGAMVHHHLEQAVTRSIPHAWNQLD
DPGMIDQPDYEGYHFWLVREAIVDRDENEHEPGSCLVAKYLDMKENMNXEQGGTARLRAG
GQRAIGRATTKGTGRSVRFLSMEGAVRCVWSFRTRVRRAMVCSAWLVRHARAAMFGEPEH
ATAGGATDGSGFGLLLGRGESNRCALGGWRV
>comp3121_c0_seq1; 489 474 1847 LEN=2037; translated
MTKPSSLCVLLILSATCSSVWARSRFSPPGSIAVLQQQRRRMQTNGDDNDGIPSPGSALV
FDPPILTTSYVGGDCTGGGGPDTIFPGQLASIKNTAYGEFCIGDLVDGQIEYSLLDEITC
TPWGIFERYSACTDETCNNCTPEAISYFQLEEILNEPNGYCYDLTYSRNPTSFDSDELFR
VGYMFDDNADMSAASDYWSLIGLNSCISTMVVEEAIDDDAALADDKLVNRSVSYNMYCFL
LRSLQHGNDVYWRRAXNELLWYNTNISFEGMVLSFLIEILTACDRLCATSCHFCPSLWPP
LTYLLMYRTADLICNTAGYTVLCDLFTKWAPEVYEAASTDYYTLFAPSDVAFEKISGPLT
DGSLSDEEIGAIFFYHATEGFVMMKDLVCGEKIEMAMAGNSRTKCTDGGATMFQMGGGNR
SNNLFPVIDDADIMACNGIIHLVSEVMLPNFVPEGFV
>comp3121_c0_seq2; 513 474 1613 LEN=1803; translated
MTKPSSLCVLLILSATCSSVWARSRFSPPGSIAVLQQQRRRMQTNGDDNDGIPSPGSALV
FDPPILTTSYVGGDCTGGGGPDTIFPGQLASIKNTAYGEFCIGDLVDGQIEYSLLDEITC
TPWGIFERYSACTDETCNNCTPEAISYFQLEEILNEPNGYCYDLTYSRNPTSFDSDELFR
VGYMFDDNADMSAASDYWSLIGLNSCISTMVVEEAIDDDAALADDITCQSIPDLICNTAG
YTVLCDLFTKWAPEVYEAASTDYYTLFAPSDVAFEKISGPLTDGSLSDEEIGAIFFYHAT
EGFVMMKDLVCGEKIEMAMAGNSRTKCTDGGATMFQMGGGNRSNNLFPVIDDADIMACNG
IIHLVSEVMLPNFVPEGFV
>comp3123_c0_seq1; 950 1 2272 LEN=2363; translated
XFPPCVCGCCLHLHRARFYRTHHRATFVFTFTRYDGPTVKTDGAEGTADGKHAAIENEGG
GREEKDAVDSTTDRSLTTETRLQDGLEAVDGDDSSHPTIAGASILSKNHSLLTTESTAQK
PPIKIAEEGADPVIEDVQSTGAEQEQDKEQGDVSSSKRESQSMEARSEETTEDDPTTNRL
QTSTNQDDDQTDLPDEVTGDKSTPDPDIEDASTNNKSIDIDIDIDITAGETKKADLEATN
PEALDEVKAQRPINDGEIGTPKDGEDDGADSVLESTKKEGAATSAATEDVLIDAENSNSE
VETDQETSPETKDATGNGAETVFPIKDNLEDKPKDKEKEHEGVNDNVFEGSEITATPGDA
RNIHNNDLPIQNLQSDSKNPDDGKPPDAARDEVTESLGDDAGDDADYSKSKTVSSGGPNQ
HNVSSKTATDQSDLEETIENSKDKTTAGMNGIEDAQSGTIDIDEKNTESSHTDNVMDEEL
LSWMTTSTETNIDDQLGNAGGSPPGKSDGDKVSVIDEELMSWMTTPNQDKVEDKSASPVP
TPFSGSNGDKSSAVDDEQLMSWMTTPNQEKEIEDDDSADRNGGNLLGEQIVSALDTAEID
VVGAEGDEVIAPETLADIMLENIEKDAIKTNKVGRSLFGANSLYSIGGETSISNTELEPP
DDAFTDDLLRDIANSKHLEAEDDSVEQTIGEEMVLVADSILNILLPDGEGEPEAIPEDAP
SESTGSAEKGDNGSDGPSTSHRFELKPDGSGGSGLFY
>comp3125_c0_seq1; 213 120 939 LEN=1003; minus strand; translated
MATASGRNLAVGGDSAEPAGACHCLLRLLRVTNGGSRKPAKARRKRGNFTAGEHRPGRRD
RQAKTRARNPTIVGWKINAEAASNVRRMERFDIESNRLVPFHAGPHSRQSSTFNNNSLHS
LWKELSVYAVSMLCIFSTNGAXDFRGLAAGTSLPEKGSGAADRPAAGPGXTKEDSADRWQ

AAAGDGNTRVACKVRGPFVWVGCRVAAWRRSMAVCCLGLFRWRSGVAFVRLESRSDPRGT
RPNSGVVGWCSEKWIGAESSHRGISLISWIAVT
>comp3126_c0_seq1; 303 122 1033 LEN=1174; translated
MIADENKEVLEKALGKLLGFEEGEAEDLLDHLFSIESEEDLSEYLAQLLGGNTPEVSHFV
DSVSRHRKGECLSSRDGGDDKKEAPSKPPAGEDTAALDSFRMMALGKNTVETPKRGTNSG
RKKEAKSRVPPPKKKYSGKQTQEPAKTTTSVAQTSIRSKPQSYNTQPEEKKEEETTVVKK
FHPSRGKAKVVCGCFGSKHKALTNCLYCGRICCEAEGYGFCPFCGFMIEAPVAGGSGKAF
QQKERLLRFDRDFARRTEVFDDQADYQGPATWMTEEEQKEAEANEQKHLETLKRPKQKLS
LVL
>comp3126_c0_seq2; 226 122 803 LEN=1367; translated
MIADENKEVLEKALGKLLGFEEGEAEDLLDHLFSIESEEDLSEYLAQLLGGNTPEVSHFV
DSVSRHRKGECLSSRDGGDDKKEAPSKPPAGEDTAALDSFRMMALGKNTVETPKRGTNSG
RKKEAKSRVPPPKKKYSGKQTQEPAKTTTSVAQTSIRSKPQSYNTQPEEKKEEETTVVKK
FHPSRGKAKVVCGCFGSKHKALQIAFTAAEYVAKPKGMAFVRFADL
>comp3130_c0_seq1; 476 127 1086 LEN=1228; translated
MIRFTPALLSLALMMAMMGPLVKAFVLVLPKIGNSHQHCFQELSLSLSLASDAGSELEMG
MTPELKRVTDAFASIEQEQVRYKQLLYMAQTTEQVNNMPESSKIQENKVPGCLSTVFVDG
EAVYDDEIGDYVINFQGDSDGLMTKGLVALLVRCLSGNTAEAIQKVDPQFIKIARIDQSL
TPGRNNGFLNMLQTMKNKATSLDEAVRTSGASPVDGDDAGEDGAAAVVDGDEANGSSTNH
AAILAALGALKPSSLTVTDNSPDGEADTRFVVEIVAPAFEGLNVIKRQQLIFMILADVMP
EIQDLRIASMFTPEEAENQ
>comp3132_c0_seq1; 429 112 1449 LEN=1535; translated
MEVNKEQAEKCKTIGANALKQGQYDRAIKFFKKSLHLYPLPGVDTLLETAERMARGESSS
NNYNSGSNANSYSQRSSTNGASYSASSSTARPAPAPAPAPSSAPAPAPASNGRSYTPQMV
ETVERIMKAKEGGRGAHYRVLGITQNATENEIKKAYRKTAIKVHPDKNSAPQADEAFKAV
GLAYATLSDPQKRTIYDRYGEEDPDNRGGGMGGMRRGPGGMHFRPGQEVNPEDIFNMFFG
GGMGGMHRGPGGVHFSTNFGGMPGGFRQQQRRQAGGQRQPEPEAPGLANLIQLLPFLVIM
MLSFFNQSNDYAASGGSNSGKSRSPGLNKYFTLHQEVPFINPLHTKLTKVKDIPYFVDDK
FMRTLYRDRYQLAQVERMVQTAYENYLVDACNDQRRHKKNLLKEAETKATEEEMEIARRT
AERFELFRCVELNNFFPLRNTGRRY
>comp3132_c0_seq2; 367 112 1161 LEN=1748; translated
MEVNKEQAEKCKTIGANALKQGQYDRAIKFFKKSLHLYPLPGVDTLLETAERMARGESSS
NNYNSGSNANSYSQRSSTNGASYSASSSTARPAPAPAPAPSSAPAPAPASNGRSYTPQMV
ETVERIMKAKEGGRGAHYRVLGITQNATENEIKKAYRKTAIKVHPDKNSAPQADEAFKAV
GLAYATLSDPQKRTIYDRYGEEDPDNRGGGMGGMRRGPGGMHFRPGQEVNPEDIFNMFFG
GGMGGMHRGPGGVHFSTNFGGMPGGFRQQQRRQAGGQRQPEPEAPGLANLIQLLPFLVIM
MLSFFNQSNDYAASGGSNSGKSRSPGLNKYFTLHVSGWSVLMFRGLFHC
>comp3133_c0_seq1; 262 1 938 LEN=1385; translated
XGPQAKSLAVALTARESLVENGFGGANWPGGTVKGDLIAVIATYRAWKKHRSDQKRRSFC
YQHALDHATLRDMDRLRGQFRELLADAGFVAGSSSRGASGEEQEENRSNAASEDALLTSC
CLVAGLYPNVCSLMRPRRGGPRGGGRLLTNDGDECRPQSSSFQQRRVRQASETGRDAYAV
YHSKHRSIGTSSGTAKERPPEVFLTEVNFVSRFALLLFGGDLEIAGNAIVVDGWLKFKVG
DEKEEGKASSKSGRDTTDNAVLLLALREKLDRMVLEHVVSESGACGPDERAAMEERHRSV
VAVVRKLLAEEG
>comp3134_c0_seq1; -16 526 985 LEN=1042; minus strand; translated
MPSPFPESGPPRTICXTSASTSTSVASDSQLPPPPGEPASGPKSDTGRLRRSYSSASNQS
KTSSSSSVMPDXEQALHAVLVAGVLDDGGKDPARHRVPGVSPGEIGRPVFDGRDPSDQGA
EDLRGHERPIDEAAVTGGDGKQHSVAGGLCIVR
>comp3136_c0_seq1; 333 1 1557 LEN=1909; minus strand; translated
GDDRDEMPDDYYYDEDDQFYYDEQDEEDRRKRARLRRAWICCFVMLCCLLLAIIAFVIFL
ITRSKDRAPEPTPMPTMEPWVPDTDDDYMYDDDIILNPGVVTAPMAPFNRDCEESEGIEY
SGEFRNIMDQCACLGEIVDVPDDVAEMRNLIIERVAPKFYGPNFTIPLNSCDPANMAATW
LATGDNRDGGEARQRFAMALNFYQLNGTVWDFMDGWLGPFNECLWMGIQCNNKGIVNSLA
LDANNLFGPVRITESTVQSDAMLELVISFFKSLQDALYSSHPADTNFYFVTIVGLNNRQI
ICVLFPTEIAKLTGLASIAITRTHLTGTIPTELFSMPKLKELRLYANRMTGSIPPEIVKA
TKLESLRLETNFFHGELISEIGAVSTLTDLNIGFNDFVQTIPTEIGKLKNLKYLRVSNNA
LSGRLPSEIGRIDALEVLAMTNNLFFGSIPSYFGLLWNLRDFRITGSGVGGFLPTELALL
SNLERLDIAISNFHGTLMTELGQLTNLCKSLLEIRMRW
>comp3137_c0_seq1; 297 1 1157 LEN=1314; translated
NENDNDNDNHENQNDVDYDCDSDSEHHQRGAIEASASSRSPPGSPSQLHKTIENEFGRYS

TDSNDTPVTCNKSLVVLPTPPSPHGNGGDGEFLGNSNGSLSGSGTAHAGDPNGLFGKPPK
TILHRSLSLGDALSPTEDDDGSPIRWHRPSGGVEEHKDDVANHPGRPSARQQQIELLQLP
VMEEGPRQGLPAGFVGESGPGGQGSGLHTNAPAHHGLAATRIRDENDDVSALTSSTVSSF
VFXEGEPAERDHVVSPPAAAPAGGKGRPGSRPPRVFLEDQALCHRKVVPVPAAKAPTPAH
AAGAGGCRHGGRGGLVPPREAAAALSVHPRPAPGRPAKNDGGEPAFSGGGDQPGGEPSIT
ADSPPSRLLFLSHARWYLVAKLVAA
>comp3138_c0_seq1; 660 323 1936 LEN=2071; translated
MNDPGPAPDGNIGNDTAVFTGVPTTPEDLYGIMLFFLALVLMGDCVCQRLLKIVPSLVGY
ILVGIAFGPEGFGVLGSNTEETLAVLGNLGLVLLIAQAGLEMDYATLRLVGLRGVAVAVV
GTILPVSIGTAVASRYLGSWRSALSAGCSFGPTSAGIAMNVLGQCTVRPARSGGGLSSPG
GGSNEDGDGGSDNGDTDSGSVLRLPVGQLIVAAAIVDDILALVVLSQLQALTTTTTDAAG
SDGGTNNNIAMDIALPIASAVLWLVVGGGIALWVFPRVLSTIFDRVAKGRPSVEGARESG
GLSLGVLFGLLYTLLPATYLTGASHLLGAFLSGLAFCRDPTGVASLFRSRFGRLVEWLMK
LFFAASIGFQVPVRSFGDTEVVSRGCFFSLALLGKLAVGLMTPTNLDPSGERTSSSDSND
GNDDNGHPDTASPNARSFCRGKHARDCFVVGFSMMGEAEFAFVVAVFGVTEGLVPPDIYA
SIVFAILLSTVISPLLLKTTLAFFPYDYDYEQHDSQSVQREDSEPAIKGAKAAIEAE
>comp3140_c0_seq1; 367 1 1133 LEN=1183; translated
SSRDSIHVHMKTLRIITASTRSKKIPSSLSSSPRSPASFPPGRRRSTRFFSLYTAAATSL
LLFPSLSGVTSALANSNSNQQQRAMSCRKTHVTDRDGSGTITVSPRNEAEQSGLLVISHG
LGDTAEGFADVAEQFASEMPWLKIVLPTAPTQKVTMNMGMAMPSWYDIVGLDERSNENCH
GIDESRARIAGILQKEHDATGLPYSRMVLAGFSQGGALSLFTGLQLESSLAGILVMSGYL
PAASKLKLSQTDTPVWHGHGSADPLVQYQMAEKTKSILTERGVKNYSLNSYPIPHTVSPS
ELGDALAFLKTVLPFDESIKVTLKDPKDMSVKELKAAIRKAGLSAVGLMEKSEFIKMVQD
HRDGKLXTSNFDTFTTV
>comp3140_c0_seq2; 170 259 741 LEN=823; translated
MVLAGFSQGGALSLFTGLQLESSLAGILVMSGYLPAASKLKLSQTDTPVWHGHGSADPLV
QYQMAEKTKSILTERGVKNYSLNSYPIPHTVSPSELGDALAFLKTVLPFDESIKVTLKDP
KDMSVKELKAAIRKAGLSAVGLMEKSEFIKMVQDHRDGKL
>comp3141_c0_seq1; 319 134 1195 LEN=1241; translated
MKLLQRIRALDASRRLDVMNKYIPAVLVENQKDFGESSSGVCVDNTIIIGHIHSDLIDLM
RINLDEGRDDNSFCDSFLFPPLLDDASTSSLTKRFFGLRVNEIPQGESPIARTDARNAIL
EQFTSLLLECKVISKKHTDMYPVKDLRNDCEGEILAEINRSAAPFLGISSVGVHLLCYVR
GDGTSRDDDTVCLWLAQRAANKSHNALRWDPTVAGGQPASLSLFDNLVKEAGEEAGIDPC
VVARNAVSTGCLSQMTCKPNGACMKPSLYYTWDMQVGSNFVPHPADGEVAKFQLFSAKEL
EHEVRNGDRLRPAMILVVTDFLIRHGVITPDNEPDYAQILAAMHRERLILDYQ
>comp3142_c1_seq1; 206 560 1093 LEN=1143; translated
MKTGLFLASLCSLNASTSAWTPASSTSTSSRRGFLDQAAKIVPLVVVAAPAFADEEVAAV
APEAAAAVEAEPETPADPAPATDENEFIARLKAQSEANKDKYLAQSRMNDKLSKRQFNSQ
YDRPSYVGVHTADNSSVKMLLKADFEQMLKEGKIEQKYESKVVKKTGEISDDFSKPIW
>comp3143_c0_seq1; 283 126 853 LEN=1811; translated
MTASGSGKSLLFVTNKMCPFAQKXELKRYYNHPCSFPYYHWIRLGSDNTTNRTYIHSFLK
AWIALEAAKVPFKFEEISLYGGGGKPQWFRKLNPKGTVPVLVVNDDGRKGTENAIVLADS
DLILDEMKLVVDSVGESSASPLTIAPVDDAVRTKIDAFRSSLTEFLPIGKSAVLGGDKER
MWTKLRELDTLIDTANEGQPCFLAGTDKPSVADCAGFPFLWRIDDEFGAGSWESHGCSNI
PVW
>comp3143_c0_seq2; 313 126 743 LEN=1701; translated
MTASGSGKSLLFVTNKMCPFAQKAWIALEAAKVPFKFEEISLYGGGGKPQWFRKLNPKGT
VPVLVVNDDGRKGTENAIVLADSDLILDEMKLVVDSVGESSASPLTIAPVDDAVRTKIDA
FRSSLTEFLPIGKSAVLGGDKERMWTKLRELDTLIDTANEGQPCFLAGTDKPSVADCAGF
PFLWRIDDEFGAGSWESHGCSNIPVW
>comp3145_c0_seq1; 230 982 1485 LEN=1509; minus strand; translated
MPTSSRMIDSTIEDQSMGGGSSSSTNNSKCPDTKHNELPQQKGTKGLSNITETSAHISGG
PSVVNDADTSSKVKEEPLNEVSNTLESEKLGNDHDFSGDTSLQEGDSSKVAATRSSPSRA
PVISRNYLFSPPLVSLLSVSYKIERNTMKMTPWVSRLSTKPYIFPCE
>comp3146_c0_seq1; 309 136 1284 LEN=1323; translated
MATSHPTRKPRRNRWVFYAIAGFAGIAVVDAFVSSGQRGVMGKGPGDGGFSKAAETGAHS
TRITVSRHGGRTNQKHPAALQSTAQAKETPFFAEFKEPSVARPSATASATPKASSPPASA
MFTRGPDPATKPDYANIHGPLGKAVDDVFLWIFRTRLAEFVGVDSSLPKTDYDGLIELTA
ALNARYSDREEIQTIAQKTLRSLFPSWLPGQFAILFARPFPEFSSRMNAWATYMAGTWLM
GECEINDIEIDGTTYKDQGLLVKRCRFLEESGCASVCVNSCKIPTQNFFAEDMGLPLTMT

PDYETHECQFAFGRAPDERDEFDAKNTPCLSRCPTAGSMRSWHSGGNVASVEGPTSFDAT
AGTAGGEPGGKCAFMENAPSER
>comp3147_c0_seq1; 402 1 1272 LEN=1319; translated
VIVTTSMATMGKFALLSRVQYATRGAKAQNRVCCAEKASILATQILGRQRKHFTSSTTTT
MEKETTTEVKDRHLTLLEVEAICSKALSSSGLNPDATAAITEVVTVAERDGCHSHGLFRV
PGYCQALLSGKVDGSVKPKVHDVAPGVVRVDAGGGYAPPAILAGRDLAIQKVRKNGISCL
AIHNSVHFAALWWEVEALANEGLVSLAFVNSRSFVAHHPGGKRKLYGTNPMAFGFPRPIG
TSSENEARGPLVWDQASAAMARGEIQLCERDGHTLSEGVAIDRHGQPTTSPEAALDGAQL
PFGGHKGSLIAMMVELMAAGMTGSQFSFEAHDNDDDPENSNGPTNHGELIILIDPVVTSG
GNDYLQHCEMLFERILEEEGTRLPSTRRYANRLKTPSEGISIPLELYEKIISLGLEDGGP
TPK
>comp3148_c0_seq1; 204 83 1027 LEN=1340; translated
MLARGDGMELCAANSSRLTAVYHRTILGITEDFRHEVAWCVSQKILDRLWDEDGATSAGE
CEKASQNARPAFINASAENSPVRILLCCQGCGFRLHPGWGGTTLRIQRPKIPCSSSAKKT
LRRREQRRRRKATRAEESRSKDHNRRRFLAVAPSSTVESTGLEIGVILRDDSSISRLDRN
HLVLTCGNCQHKTYLKGLRRELENAHAGFAKSALQSSVATQSQKGPGKAPAAACVRPHDL
GQSFELVPKLERNPSACIASASKTSRISANATSSTLSLLEQKGGRKKKRKKSGSNPTNQK
SGNLLSFLSSLNDH
>comp3149_c0_seq1; 344 1 788 LEN=1078; translated
XSSSSSSLNVFNPFAKKEAAPAVVEVVREEPVPGPLETQNYVAGAVWITLIAFAFGAAPG
ELNSAADQEMVTTLVTQPSPRPEQINELWFAIWNCFAVVPALLAALEAPVGRGQRLPAAP
FLWGSAAFGYFSLGPYFATRTMRTEPADTEDLGWASRNIFENRLFGVALSALALSIPFSS
DLLGCDVSATIGGFIELASTSRFVSVASADIVIMSLLAAVLVSEDAKRRGWEDRSVPLLA
ASILLPVITPCLYLAARPSLEE
>comp3150_c0_seq1; 237 1 1169 LEN=1277; translated
XHRHRHRPGAGIGSTALVLPLLLLPLLPPLLLALAPHRSVSAFAFRQPAVPFSASPAAAG
RQGSLPRGTALRLASEPNSSGDSSSGDGKHGPPLFRFRRATPADVAPCFEIESASYPPDE
AASLRALADRQSLAGDYFLVCTEETTGAVAGFVCATRCHAFTEASMSEPHDPAGRLLAVH
SVVVREDHRRKGLATEMLGRYFEGIASSEDGADASEQPPIESVVCIAKEHLLGFYVRCGF
RVNRPSPIVHGRELWYDLERELRTPNKEESSSSSAAAAAAPRTRPGPGESWFCKTERFKP
SLSFPEIKPHLEAHSEWVRSLRSSGEACIVSGYRVDADGRPGGGGLMFLAAASHEEALEL
VRKDPLVANGCVDWELNGWIGQVGGIRAE
>comp3152_c0_seq1; 149 1 778 LEN=831; translated
XRAQEPLYHMTGFCGSPRYMAPEVGLRHPYNAKCDVYSFGVLAWEILTLEVPYGDCNLRD
MTSTIWPGSIGPAEWNRGDRRQPGTGSFRGRNPRWRSKPPCRETPLPAVLGDTWNREVSM
RPTMEEVGRCFRAECERFETRFPSEANSPTEARAALRRWFRDPTLQKLLRKQQPESTVVK
PMAALGTPPPQPHPERRRSSSWMPRRSSAFSLEPMVLRSVTMIRRGSGADRWQSSSVATL
DSTSSRSEEQQEAQGPLRV
>comp3157_c0_seq1; 515 1 1455 LEN=1583; translated
QKITKMKIKSKANQLPCSIISMTFLLLCAIAQIVIIPTNGEMAVESNIGVGKKANDANKD
NMVVLRGGGTNNFSERNFGFVPQTSMTSQLRIRNLKPKEPASSTKKTKKTAPKSKKISKK
NTKKSGNSCAMKNFIGQYDSVRGECELKFRSVITCANENECTFKENEIKDGEMLCTKAGT
FNPSESMIVGSSSCELDYIPLDEGTCEDNSFGVKIRMDGKDELNLFFSINQGEVYYNEFF
PDKAVRVSEKNDRVFQHADEEGGIAQAYSILRGLYKQSIATGVNMCAEENRPDMEYEKVL
APIVKQHNQGREHKINHEFILDKWCPKHPEGMLQPFSRAARTPCSEKVIDFLASAIGLVL
AMIGFPGIVKGALLDIIGFFINGLSGGINEIVKLFEGGQNPINLVERSLQVFILLLNTIG
LGAISDVIWESMAWWEVCLSIAGIGVTLLGIVASGGLAYVAYGLSLLVANVAFVPALIGM
LEAC
>comp3157_c0_seq2; 530 807 2078 LEN=2206; translated
MVVLRGGGTNNFSERNFGFVPQTSMTSQLRIRNLKPKEPASSTKKTKKTAPKSKKISKKN
TKKSGNSCAMKNFIGQYDSVRGECELKFRSVITCANENECTFKENEIKDGEMLCTKAGTF
NPSESMIVGSSSCELDYIPLDEGTCEDNSFGVKIRMDGKDELNLFFSINQGEVYYNEFFP
DKAVRVSEKNDRVFQHADEEGGIAQAYSILRGLYKQSIATGVNMCAEENRPDMEYEKVLA
PIVKQHNQGREHKINHEFILDKWCPKHPEGMLQPFSRAARTPCSEKVIDFLASAIGLVLA
MIGFPGIVKGALLDIIGFFINGLSGGINEIVKLFEGGQNPINLVERSLQVFILLLNTIGL
GAISDVIWESMAWWEVCLSIAGIGVTLLGIVASGGLAYVAYGLSLLVANVAFVPALIGML
EAC
>comp3161_c0_seq1; 276 1 1008 LEN=1008; translated
XRELVVLQTTDEPPIHGPGALGESEGEDRGNGNDNDNENGEAEAFRQLTEQEQEDYASNA
MLLLSGIALRYHRPRAPSLRLCFGAPGKGPLPPPKHPPTGKRAERLPTDTEREAARVLRE

ETGYDALLPDPGSDAERLLAHSGRREDYLSWEDYFMSVALLTAQRSKDPSTQVGACIVDG
QNRIVGLGYNGMPTGLDDDLMPWGRANAQAIFNKYVYVTHAEVNAILNSKEGYGSPRVRG
GTLYVTLFPCEHCTKKVVQSGIRAVVYLEDKYHDTDGCKASRAMLRCAGIEVREHVRTVE
AVTVDFGRGGEEDQEEEDGGEGDXVLIGLEIGLDWIG
>comp3162_c0_seq1; 579 1 1902 LEN=1925; translated
RELYGKGICNRTLVPTVHRSPPTTEYRRLFSRSLSDHKNSNDPTYQPTNQLTDQASKEAS
KQATPSSTAQNRNTNLKXAMKNLGPAFLLFGALAATASAFSPGAHRAREAGAASRTAPGA
PLFVSTSSRPSRASVEEDEALEIFQKAQDFAFSDDEAVQGSSSLTDEEALLAESRYWLRE
MIRVQSGCATGTIAEKGVCENQLGAAEIVGRLRFRIEKHEKRLEKRSKGSDSVVPTIATE
LALGALLVVVAIFWATLDLGQRHDDLPSLNNFQDFLSVVEEKEYSFLVGWANDGGAPPHL
ASRRTKPNQTKPNPIRIKTTMARPKRTVGRATAGFIAAVFLSGAGSLRHASAFVGPTHRP
VAATPRHMFDFFKKDDPEPSPDPSGASPSEDEEPDDFSDDPVDKIFSVFFGKKEATPMGM
KRFGRERFPEQYPAVLDEWAEPVASDDKDMAVLRPMLKNTNLETRGLKLTYSANRDGWDP
VSFHRKVDKLGGGLVVCTTEDGLVCGGYNPKGWVGYGEARGSIAAFLYVFTKGTSELPTK
LRKVGGPSLAQQDLPETGPSFGADSLVIPLEKSSPRVARSKLGSYYERFPGGGNTLFGKR
GSSVRLKDLKVYHGVYAEGEYIPFTDAEPFALY
>comp3162_c0_seq2; 538 1 2089 LEN=2112; translated
RELYGKGICNRTLVPTVHRSPPTTEYRRLFSRSLSDHKNSNDPTYQPTNQLTDQASKEAS
KQATPSSTAQNRNTNLKXAMKNLGPAFLLFGALAATASAFSPGAHRAREAGAASRTAPGA
PLFVSTSSRPSRASVEEDEALEIFQKAQDFAFSDDEAVQGSSSLTDEEALLAESRYWLRE
MIRVQSGCATGTIAEKGVCENQLGAAEIVGRLRFRIEKHEKRLEKRSKGSDSVVPTIATE
LALGALLVVVAIFWATLDLGQRHDDLPSLNNFQDFLSVVEEKEYSFLVGWANDGGAPPHL
ASRRTKPNQTKPNPIRIKTTMARPKRTVGRATAGFIAAVFLSGAGSLRHASAFVGPTHRP
VAATPRHMFDFFKKDDPEPSPDPSGASPSEDEEPDDFSDDPVDKIFSVFFGKKEATPMGM
KRFGRERFPEQYPAVLDEWAEPVASDDKDMAVLRPMLKNTNLETRGLKLTYSANRDGWDP
VSFHRKVDKLGGGLVVCTTEDGLVCGGYNPKGWVGYGEARGSIAAFLYVFTKGTSELPTK
LRKVGGPSLAQQDLPGTNEESEQVHERAGCARGGEARAKNRRVCCCPLTVPCFLFVRLCF
CYWHCCAARSPNTLFHAETGPSFGADSLVIPLEKSSPRVARSKLGSYYERFPGGGNTLFG
KRGSSVRLKDLKVYHGVYAEGEYIPFTDAEPFALY
>comp3166_c0_seq1; 383 1 1248 LEN=1311; translated
SSFLPIWQREANARLEEHTDESVRFLLARGYTLQEIQGLEELETGEQVQPSVLLSRLWKR
LASGTDQPGSSDGCEEDETDLWCRIYESRNEEKDVLEAIYGEDDLARFGWRNDNDESGND
DSDDDSDDDECDDPTRPFSFDCVLPVQGYEPPERYDFPPPLLLEVYVDRSLSYYPYGRSP
SALDPPVLALVGGGLPQSYLREATDRLRASVEEDLGGSDADEGGTPRLFDWLQLAGELAA
EVLEKEEADLAEKARLERVARAKRDREAAKQLREERAREQKEQQEKLAFRSEADRRAYAQ
GVLARGLATEGHGRRPGESGGKAGKSKTRNYDAGADNLSLVNDPLDDAASGASGGNGWVR
PRLATIVHDEHAQQRTDEQTQGTRMVFLLVAGIVAXALVEAATVARKMRLRVAVL
>comp3167_c0_seq1; 501 1 2487 LEN=2758; translated
LRSFEEKKIGRDLDKLRGLLDQHGLRTSRISSPREAVSVYKDSCLGGDDEPANSFLKDGT
DFLLVTFGLHPQQEELEKYFRRSIQNTYCLLLFCKKMREEYHSLLGLSSSESASASKSQH
VSEPTNAIDNEINKQIEMLIGEIEKTTSGLFRESLSSTKIGTTKEHHGGVSSRFAEEQVT
ALIELASAVKGKRRYRAEEWKKEYQRICVANSTISNAYLFLLQLTHEMCIQDILFKYNPK
EPKYERITIYLYDPAIDYRKPGLFSFLPGLRTVLTRGPHELVSDMIESFWSQVLRNGYFP
TAAIQEVLEPQLRSFFLSGLVAADARGTTYLPLPLKASFVPSAPLSIYLHGKAGAGKSSL
AKVLPMALQSTLEEHIDPECMARFVKQNLNKRLETLELEFQLRPNNNDMSVMSIIQGRRM
AMSQSKPGLVVLNLEELPMDSSLGNDPNQEAVAQLISQRFGGRKGKYQSQEKQRNIENKG
RNGHGNNAAPRNSDKRSIGRDFSLVTIFTSNYPLAENSKTALAQLELYKSLAPIEIFSIT
GSDRRQFAQSYLSQCLTDLLEENCDVELSPRNMILDIASISSDGDTRPLVRQLRMFSYYV
GKLLKNACVAKEGPIIVEEIKIKQSDKNCTISVQTKTEGNQNQSSQQKLTMGNLSNWYPS
GEFVFDSRINRVIDKLKTIAGDKKAIELAVILEFWFSSILAPAVILSQDKKIIEHIMDAI
DRMGSGVNCIRRIDTQTYKMIKSLYDPKEMPNLRDDILKFGRGAFVATELSCPTSDSQLC
IREMIEDSPSMTAFSSTKSALHKSGLLFAIHVEGEVTPEVLSRVSFIL
>comp3168_c0_seq1; 234 1581 1955 LEN=1955; minus strand; translated
MAKIRKANESQEDGKPRKKFKKESPHKKKDRKQGKEQGKDGKTDSPAESTEKWSKSKKKR
MRKILGKLKKEQAEGKTSQSAPSATSSTDASSTNSLAASATTETGTKKKNSIQEAFKARL
AGSRF
>comp3173_c0_seq1; 325 71 862 LEN=954; translated
MPSASNRGKLLLLGDSLTQQSFEGWASDLANVYQRRADVLNRGMSGYNTRWYLRYAEDNG
IWTEPGNVVLVTIFFGANDAAILEHDPAKHVPIPEYQTNLETIVDKAKEAYPGAKILLIA
PPPVHREQRLAYQKKRYGNKATGIPERTSEHTAKYAAACRQVAETKQVPCLDLFSLMRND

KENEDADDIGRFFWDGLHFNKTGQKYVYDALANAIHTHFPSLEVRPCPITGQFNNSGSAC
DDVVNSGPYHEKLGGKDWSKAFD
>comp3174_c0_seq1; 81 1 793 LEN=793; minus strand; translated
CNQNTFTFFKGLMRRSETDRSKQRTDFQRVPQNGLPGLSVVRRRPVRPMHACVALGDAVF
GEQAGIDRSIDPVRRSCIAPDPSVDPGPCRETTPGTGHRCPSAGSVPGVSRCGGAFLPWI
LFRSGWEHSRRSPPAVALHRGTNRPTRPAGTRRDDAVVWSVVVRFGSGEQAFSRGRDTYS
YLLGGSFGNPCEVRCGIAVSALYRTRMYVLVRTCSSFRFFRIDGFLGSVQKNFFKIDITQ
PADASSATEAAESSAAIELCRVLP
>comp3175_c0_seq1; 438 756 1745 LEN=2918; minus strand; translated
MTPSASASPNGHSTSSLLLAASSFPEKEAKKLLEVEEKFSFSDRSKLEERLKMEGFALCQ
EVIMVDWYYDRLVANSECLASVLPLVRNDHWLRYRQITSRNCIECVDGEAEWQLKRGTSI
GGGDGGATVYEEIEGTEALKIACSLANRELSKRDEANPTKNETALLLLFEGESFPVPPVD
SCDLQPFAKIVTHRAKWEQRQGQQPTSGTKTTNASLFSKLVVDLDTTPDGFAVGEVEALV
EAYGKTEGTSTAVYQEEAVAMARTDIRELLERILKGDDNNDDRSTPTRSPSMGKLEQFLF
LRQPLIHKVCVESGVIPQPPFSAEEEPSS
>comp3176_c0_seq1; 390 225 1337 LEN=1337; translated
MLPPSMIRNVNNGAIGSMNGASRRIRNGERNNGNGSNKGALQRSIAAGGSDRQQPAVSPT
DKNRLLEQLRLVTAQLQARSSSLSQPAASITQRTPPSTTAPTTTPIPQLEAQPKPSNQQT
MLHPEVPRSNVGIPPTPATVRGGNTGSSISTAINIDSCGSAPSAVATSNSSNPASQASPI
GNDAQEELFPYILHGMLDDVERMGHTNIVSWNADGRSFRIHDPDSFVSLLLEKYLKRYVG
GIASRFTQFRSDLMDWGFEDAVDGNRMGETFTHHCFQRGQANLCRYMRCGRKSEESPAPK
QALVTPTNTIPRRQPVPVSVQSQPRVVVNAQPTTAAVSAVQATNPVTLGSTAQLLLQQVQ
AQAKQQQQQQQ
>comp3177_c0_seq1; 761 95 1828 LEN=1897; translated
MCSIRGPLVTSAIAMLALCQKTSHALVTSARRPGLQCRPSRSTLVALRGGSSSTMAEVPT
ETESSPATASDVYQELLAKLETITHLERASSVLSYDQLVFMPSSAAADRGAQISALAGVI
HEQKTDPALKAVLDRVLELEKKAPELDADAMRLVDLERKDFVENERVPAALAAKAAGLSA
SAYSDWVAAREAKDYEAFAPTLQECFDTAMETARAKRGETDEEATKTLYDQMLDQFEVGM
GQARIDEIFGEVQSKLVPLIARVLSSPSKPSAEPLEGSFDIAKQQQLSEKLVKAIGYDNE
QGRIDVSVHPFSSSMSAKDVRITSRFREDEWYQGLAGSIHEGGHAIYEQNLVPSAISIDK
ALSMGTHESQSLFWERHIGLSKSFWKFAHPSLPDLDTSFETYTPDEVYGAVNSVRNSFIR
VEADELTYPLHVILRYNIEKDVIGGKQDVAGLSERWNKDMKTLLDVDVPSDDLGVLQDVH
WSAMAFGYFPTYLIGAIAAAQLYHYCEKDLPDIESKIENGEFQPIKEWLTAKVHKHGRRY
PSLDSMFQDQLGEPLNAKYYIDYLTSKYEDLYQLDQK
>comp3178_c0_seq1; 70 1 816 LEN=1839; translated
XQRPVGPRGKIVRDDPDDKLLLGLALAGERIRVAERGPAVPFLADGPLHKTDRAAGPTVD
VRQPGASQAAKGKVLVVVQPPYTKDETPTWVSVRLPVASSTGPLCRRGPPESPKQMPFFS
SLAIAFDETKTRETDPVAVRGWLGVAQRIDQRTRSQLTSTNVRGIIVTVDLVHGNGNLLK
KLGGRTGETGRSGEVASKADRNYFFFRRRKHWLFSEPKCYGGVVDIDRGIKLALWRRKEN
KGKPIVLKSGVDSFTSGTIATHTHALGTTVR
>comp3179_c0_seq1; 211 464 853 LEN=1295; translated
MPANEDYGSFQHVDGDRVSNTSMSWKARIDELAERALEAAALLSDNIVVTMSSNEDVNVD
ARWTSRQDLLVERLIKGGASAERITVERLATDAIVPVGMSNNSNMHHSHGSYRTQYCTWR
PKQESVGCT
>comp3179_c0_seq2; 211 312 701 LEN=1143; translated
MPANEDYGSFQHVDGDRVSNTSMSWKARIDELAERALEAAALLSDNIVVTMSSNEDVNVD
ARWTSRQDLLVERLIKGGASAERITVERLATDAIVPVGMSNNSNMHHSHGSYRTQYCTWR
PKQESVGCT
>comp3180_c0_seq1; 462 175 1152 LEN=1267; translated
MARSSPTGVSKRFLAALVVALAAAIRPASSLLQASLAEARAKAKAPAQQRRSPLPLPLPS
LAAAAAAPPRSLPTLALSASPNGSGGGSGRREVLRRAFLAAAAATASFPSGSLAFPNAVP
DARKFADKTKRRGTPPKDLGILPRTTEGIDASVTSPRLRTCDGNPNCFSTTGDALLEDRQ
QYGVDFLVAPWVPPAGETDPIGGIAAVVASYEPGQGGIDGGGFALVKQTDNYLYYQFEAL
KKGFIDDVEFAVVGNDTTAVQVRSASRVGVTDFGVNAVRLNYISRKLRAGGWTIAEITPE
THRDYWITANEAREATFDADRRSMD
>comp3181_c0_seq1; 263 381 1376 LEN=1444; minus strand; translated
MACFLRWGRRASHHHETTKVRGADQRFVAVFFLSGPISRIQQRASVFLGLCIGAXMHLCD
APAAAFEMMHGTEGGGNHCCRVTHSGETARTNRLGQGAVCPVRSQKQGSAMAISMAIQSN
RVQSVAVRSERVSIPLDSRSIAEQSVNDRRGVVWIGLVLRSLGWSWEAASTGVHVSGGFD
RSKAKRDRAGRDEMLRPSPPGLVPSRLAPGVPGGALPTAPRQVFPDLPEKGHDETRNPAE

GPLGSPEPASFRGGRTTAGGGRTGRTRLALGQVLDGPGDLFGPLLVEFLVLLVGPDGKPL
EGPGIGEALDDDLVGDVVDDALDELLAHGCSW
>comp3182_c0_seq1; 180 106 505 LEN=676; minus strand; translated
MSLFDRPEFDSKECAQKRVHHGHLLDVVILLFGQSSTSQILALHISIKVVAVGGAVSDCL
TKITGPFDKPFEEPIDVGEQTAQRLQRHEENPEWSDNGSDWPRNEKKKNDPTERIANISS
PHGFRYYQYNLF
>comp3183_c0_seq1; 495 325 1947 LEN=1947; translated
MDTPAREPEASRLSVLSVQSPPECVLNVDVNLFCPSATPGSGRDNSHLVDDYEPIPYNAN
GNFNGNTNGNTKGNINGKNPSDYNYVSRYNSSRPPPLPDVREAIAKQELLAIQEEMAHLT
LHQQESARRSVLGRSSMQLQAHTTPNSVDNPNNRTPLECPAFLGRKLETMKRELENLETR
FCHTNTCGSDDDSMVSAYRMASMKENNSINRSNSDKENSYVDDPDFRLGFLRAERYDARR
AASRMWRYLSEKRDLFGIECLRRSSGHIFLSDLGHDGLSVLSMGAIQVLPQRDAAGRRVI
FGCPLPGNSHAIHGYTIRGAAKAHFYVYSSLLEDDTDVGRIKGVVGVLWRVHHPEIHPDI
IAATVRVRECAPIKFSAYHMCHGVYNNHAAAPASDQSRHNTNRNYYEAMYDYARRIVTFL
LPNLQRIGQMRYYRAHYGTALECRYSLYSRYGCPSDCFPLLHGSGGDGLVNPRCVEEWMA
YRKRIDDDQEEKQKWRAIATNIGMIQPLAPTQREVTIERDRNSDGWPSTAGDCDGNGNGD
G
>comp3184_c0_seq1; 338 108 1475 LEN=1661; translated
MCRLDSGLKSCDIHAVCQDRNCSVKTIQKFVSKYGVKILKKQDVYGRVSLFYALRKQSSL
SVVKSLFDLCPSALLQKDFCGELPLYMLFRPNADYRILEYVLDRDPSLALYEEKRFSGSQ
SLLQRLCNQWASTIKGQRSASSVRSNSDGNGGNSVVLSHEEIRSDNALCNQWAKLVLTVR
AVHRIAHGTATKVTEFDEEVVSNAMKSDELQRFPPRQLSFEIPELHMAMQLPFLPPGIVC
QFIELYPEQASLPIENVIQSTNNLLDNEDPGEGRESHLVGTPSAKRIAGKMLPLHFFLSD
YSAMKPTPNRPVEGTGAHKLLSQCQKRAGILRGLVGTFPRAATLRQKHQCCDRGVLRSCR
CFPIHVAIARGIPWECGLRELVYANPSGLSIRDECGTKLLPFLQQALAAATCYKPLYEPT
EDGDRYRFVCKEESNLSVFNTIYCLLRENPSVLTP
>comp3185_c0_seq1; 247 194 934 LEN=1268; translated
MNIENEEPREVVASRAMCYYSFDILIHALQRRNTSLPESNRGNELAAEFVSELPDPSIQC
PLFVTWEKSAHNGSGWRLRGCIGCLNPRPLATDVGEYALISAFRDRRFNPISREEVQSLR
VSVSLLVDYEDCEHVYDWTIGVHGIMIKFAVDGKLFSGTFLPEVAKQQRWDHAETISSLI
RKAGYTKAIDQSLLDGIHCRRYQSSKCLVTYADYVNEKCGGIDPVFTPLGQEKESKQPWY
KHFPGL
>comp3186_c0_seq1; 847 172 2790 LEN=3033; translated
MTYAKELSENFFSIPLSTYRDRDVPLRAFPYGMLISAISVLFFRTTIQFPETTSKEPMFL
RWFFGALLAFLTYSNRSYELICAVEIFSYLATFLLFSDCIPSLFSKFADDKNKTNVLRIV
LISISAILCLMVCRMAATGLLFEILKSVTPAFLSKGLMAMFPIEEIQASYDIMNEFVVED
GLLQDQVARLFFITFHIQVGIGYLGIDFLKQEQHRRNELVKMDIDIEGAVDKDGDGAKKA
PLHDAKGGGTAKNKLQRGIEEKKMKRSQKFQRTAAPFIFFTAVPYMVRVIGYGNINAFAF
ACMRDDIHRAVRLYDLFDHDNHLVALADHSAKAPADFAGFMDTIVSTTYDLFNRKLFSLP
KVMLLPVVMSKQPKMIAQIFPIIFISDWMKGRAVSYMTSRIEELEKDVQELNAIRSKVES
YDLKNSELLHRSGPGAMNFTKERWLEMTVKVQMKMAVSNLTSRTKGFFSFIQRNFVFSVL
VDCALAQLMAVGKIVAADIFVFSRAIEDAVDMILMRSRSEAELARMMTQISNLKELSTVW
KSAKERNLLPCSLRSRHVDDSSTPLVVFKNIAYARGTAAVQIVDMELPAGIYALTGANGS
GKSTLFRVIMSCSTNDRPIDIPPSISLSISSESLTPSLDGRRNESHGDISDESHLQPSIT
MPSSHVVEISQTFYWPLYSKPIEWILQQSSAEKLTNKEKENRSRRVAELLHSLEFTQSLD
IEEVSEKDNETRNLTENESNSDSIVASSPTGDAITRIMEDLEEVKEDWFNDLSGGQRSKV
ELVRKVFLMDECPKTLLIDETMAPLDPRSKALVMEQIKSFCIGSVVIVIYHTDVGREQEK
DGGDLVECVPSNNFFDGNIHVQNKTMILRPVC
>comp3186_c0_seq2; 788 172 2863 LEN=3106; translated
MTYAKELSENFFSIPLSTYRDRDVPLRAFPYGMLISAISVLFFRTTIQFPETTSKEPMFL
RWFFGALLAFLTYSNRSYELICAVEIFSYLATFLLFSDCIPSLFSKFADDKNKTNVLRIV
LISISAILCLMVCRMAATGLLFEILKSVTPAFLSKGLMAMFPIEEIQASYDIMNEFVVED
GLLQDQVARLFFITFHIQVGIGYLGIDFLKQEQHRRNELVKMDIDIEGAVDKDGDGAKKA
PLHDAKGGGTAKNKLQRGIEEKKMKRSQKFQRTAAPFIFFTAVPYMVRVIGYGNINAFAF
ACMRDDIHRAVRLYDLFDHDNHLVALADHSAKAPADFAGFMDTIVSTTYDLFNRKLFSLP
KVMLLPVVMSKQPKMIAQIFPIIFISDWMKGRAVSYMTSRIEELEKDVQELNAIRSKVES
YDLKNSELLHRSGPGAMNFTKERWLEMTVKKLIAGTRLSSFYVSSDTLTSCFCRVQMKMA
VSNLTSRTKGFFSFIQRNFVFSVLVDCALAQLMAVGKIVAADIFVFSRAIEDAVDMILMR
SRSEAELARMMTQISNLKELSTVWKSAKERNLLPCSLRSRHVDDSSTPLVVFKNIAYARG
TAAVQIVDMELPAGIYALTGANGSGKSTLFRVIMSCSTNDRPIDIPPSISLSISSESLTP

SLDGRRNESHGDISDESHLQPSITMPSSHVVEISQTFYWPLYSKPIEWILQQSSAEKLTN
KEKENRSRRVAELLHSLEFTQSLDIEEVSEKDNETRNLTENESNSDSIVASSPTGDAITR
IMEDLEEVKEDWFNDLSGGQRSKVELVRKVFLMDECPKTLLIDETMAPLDPRSKALVMEQ
IKSFCIGSVVIVIYHTDVGREQEKDGGDLVECVPSNNFFDGNIHVQNKTMILRPVC
>comp3187_c0_seq1; 451 1 1615 LEN=1862; translated
RTALEKTSADTPVLDKQQKKYEYAFGIRGKGEFDSQPIVAAAPFLHHSSTGASAWSTTQT
PGISLQKDSRRTATARKMTESSAVNAAASKPRLPIPAPLYSDVPGTWAYDTMSRRVDEEI
LQRTFDDNKGAWETEEFSDILERFNALRKELQNAGETKLGLPLPPPSDCSEARRTEWEEW
KQIMEPFVAKGDTWLTAPWMVAEFYVYRRLVVDVIQYWDEYDRNDPEKKNPGYLYDPFID
QKHAGLESSVASAEGVLAKINNLGGNGSSIDQGGRLAASIALWGNKMDLSLWPADAKTAS
KDIFSAILDKATEGLLHDDSSLLVEHFEKLKASGGGNIDIIVDNAGFELITDLVLAQYLT
ESGIAKCVTFQLKSHPTFVSDALEKDLLEHVNHLADFDDEAKAYPNVKISGAKWQEYLND
GRWKCTENNFWVQGFPMWEMTEPLRSDLRDRCDLAFVKGDANYRRLLGDCQWDYSTDSFQ
DVVGHYFPCPVCALRTLKAEIGCGMEANQVDRASKLDPNWQTNGRFGVLQFGTGESQ
>comp3188_c0_seq1; 160 94 441 LEN=803; translated
MDNWIVSQISFWFTVRKVKPAQIVDQIINFWLDVTIPGRGAGENSLLMRGVRSVQQGAER
AEAELNLRSSLRQWDNDFPEIHHEMIVRWGCGLVASVFVSSIYQALKPQPRNRKY
>comp3189_c0_seq1; 350 1 1232 LEN=1262; translated
XLATKKNEKISAQFRRQQSNNTMITFIEIMHYMMMPVTATFAFLWVFYPAWAVLIAERIE
WDVEDLASPGMNKDSFESPLLTLVIPAFNEEDRIPIMIRETFDYLTSQQGKQLLRKLQSS
IKPQTTSSLEYEKLEDASLREESSEQRGAEPKVEWIIVDDGSTDATCDVVRETYKKLMGN
KSNGVGKSTPSATNACDWKWKIVSLTKNAGKGGAVKTGMNLAEGMFHLMVDADGATDFGN
GLENLTRELRAHTQGILASREGHIGSRKGMAGSRIAVFGSRAHLERESTAQRSFVRTLLM
KAFHFFVSLFVSTKVHDTQCGFKLFSKKASNAIFKTLHLRRWAFDTEIVLLCDKQCIDII
EVAVRWKEVDGSKLSTSKLALALVSISMLRDMICVRACYTLGFWRVKMGL
>comp3190_c0_seq1; 437 525 1307 LEN=1626; translated
MPGRSLQRLSSLRLHGTNKDNEMEGERKKPLGPRRIKSLTDSFRIRRFDNLNDGIDNNNK
SRSESVEYQVCTNSVGKKHHKAFDGTSNEYFSPPRMLIRRRPVIKINDDKRNETETNVAR
DNEGADATSSSTTTPSLQRETPIRSNSRRRLSKLKQTLFGSSSSSSKNLFGHRTRNTDGS
GNGIQQNKTNELEQDTLSLAVGDSHGKANIAHNSNDVKYRNDESDLEDSFSIRDVAKDNL
VMLLLCREFNLIDTNESSSE
>comp3191_c0_seq1; 434 215 1339 LEN=2516; minus strand; translated
MPSAADYLVGPTIGEGSFGHVVYAVHKTTQRKVAIKVMEAPVMGATEHKHRQLWKEQKTA
MILNERRILSLPELKSSKWIVNLWAAFCESNSRYLYFVMELATGGDLQGLIDRALTSSRA
KSKRDAWRTDSVPFYASQLIEAIQFLHSTGILHCDLKPENVLLDAATGDLKLADFGCALD
TNRQQQNRNPSSPPIMLPRGTARYSAPELLQAESPLALDAAVDYWSMGCILYAMFRGKSP
FDRGSEALTIRAVFDYLDGAPSDLSPKQKWAEDTNDDESTRYPLYRLSQGLFAMIPGDRT
TFWATSVIPLFDSKSQGESVGGDDAIHGIDNRRSATFATTSNESGVLPSNPDWKDQVESA
TLRNGDLGWVAFQL
>comp3193_c0_seq1; 377 87 1013 LEN=1335; translated
MKSTVTFLFFYAILLTQEANAFSASDSGLFRQAKPSTRSNNGYSNIDLLTTTEQRRNAFS
TLPNKQTSNSNTRLSVAPGAIAGVLTGGVLGGALHAIAGPDHLAALLPRCIGQRWYKAGR
IGILWGMGHGISVTMLGVLGFALKSQFAKAPGARAVLNEVSHLMEFAVGLSLIVIGAMGL
REAKEWGEEMESQPAHSLSSAVSPSEAKNQNRNRAVVFNGLLHGFSWDGAPSLAPALAVA
SWSGNLAFLSAYAAGTVAVMAVATSFIGEGTRRAGEIFNRPDLPQKLSFFTSLLAMAIGG
IWCGLALM
>comp3194_c0_seq1; 280 68 1156 LEN=1372; translated
MKDIQDNPHHPASIDTLERYTLIVSYDGARYSGFQRQSSSSSSSCRNPDSKSDANPLKRR
RPNNQIKKRIKNRGNVPITVQETLEDALEFYTGMDRFQLKLRFAGRTDGGVHARGQVVAV
SLPRAIFSDQKVINNISDENENDREKLWTIRKSINSRLPNDISIEDVSVCQDECFDPRLD
AKRKQYSYMLRYRQMVTFEIKNVKNNECGKTISPICLKGGPQLLRTALDSNRVWICPWAL
DDSKLTHYCELLTGTHDFSAFVHKEARKTRDNKMTVTRLDYERVRFRDDVAPVWEIRFVV
EAKGFGRSQVRNMVGFLVEACRGSFEDLESNQGWLWEEDPDRLAKQIPSAPACGLCLEHV
MY
>comp3196_c0_seq1; 362 79 798 LEN=952; translated
MSGGKIILDKALRSAVNLSSSKLPSEVAKSSAALFSALSGTKFVLPDLAYDYNALEPAIS
AETMELHHSKHHQTYITNLNATMEKIDSAMVAGNVSETIALQGALKFNGGGHINHTLFWE
NLGPDSTFPEGGPLEAAVNARFGSLDEMIAEVSAKTIAVQGSGWGWLVFDPKSKMIDVVA
LPNQDPLEATTGLKPLLGIDVWEHAYYVDYRNVRPSYVSAIWGIINWDVVEERLVAASK
>comp3197_c0_seq1; 204 1 753 LEN=835; translated

XSIRMQNNGKKQLQSNTNNDHDSENIFFSALVPPDHQSGVLSPIATDEVILSQGMALNMA
TTAVTATTILPQAANADPSVVSGLQGGIQDKVAPGHWIGQFIGLNSKTEKWEFPNSTPEE
VSKALVDVLTSLTEDRKAKLYMPEFEIQTADASKLHVLTWTKLEWLDTLDVTFRPKENNG
CTATASFYATGFLPTNIPGAPLFNVAFFWFPFASPGPRGEMLQSFRLRALNGLVTKRLEE
NKVTPDLYFP
>comp3198_c0_seq2; 347 141 1015 LEN=1015; translated
MGDRHLGIRDRTLVLRVLVPGLETRLRDKMKEVEEAADTDPDTKSATERSNTGRTSSSSG
NHNKEILLDLDGVACEPTKDSSTLWNFHCDGATYPARLVNLPCPVELHKTHDHAMYYKCC
DIAQMLIVYEDSMALDDADAYPKKTGYPSYYHSGITLPMKQVVERRFGQRDHQPGIKFPR
NEVSDVERELHELMCKISKEGGKGRKLKIPSLANQNKVLQEVEEEVVIDYEPWMEDGIEF
EATDQIASLHPEIWLSPEKIKEIKQKEEEKEEEERKKRQKEREKKEKKKKKX
>comp3199_c0_seq1; 319 111 1262 LEN=1828; translated
MKHRTAFLAFVTISVGLTFNQLKFFFSVVEFGNQGEKTSLMDKDHPSMGKALECLRLTKP
KLPNLSYPRDAFEHFHHIHYSLARWIQHPDHNPHRAKGYSGPWIENHWISHFEKELKSNN
YSLPDVFGPYVPILIPWTDIWVKNRYKYPGELVGVIKRLLREDVMYITVNQNDDGFVGRN
ADFHDIQNMYHITVLSAGGYGHVPVPLFKQPEALIQKVPLNERKYLVSYVGSDTNAPNDM
RKKMIAHLDQASRYYYKGSEWRDVMAQSKFSLCPRGFGRTSYHVIETLQMGLVPIQVYLD
QPWLPYGNLLKGISFTVRIEEVSDLLLNLSEIPDSKIEKMEAQIELLREDYFTFEGAINQ
IGKFMIDPTNSELICQTLPSTPR
>comp3200_c0_seq1; 495 90 1322 LEN=1418; translated
MELTDPWWKYFLIPLIDGAVGYFTNVLALQMTFAPLEFVGIPLVRFPEQPFGLFGWQGII
PAKARKMATISFDLMTTRLLDLQEIFNQIDPVKFAEAMDEAILLMLDNVISEVAMETMPN
TWGNIPQAVKDDIIVTADNESGKFLTEFMKDMQAHVYDVVDIKEMCVNACVQNKHLIVKI
FQECGDKEFNFIRQSGFYFGAMFGVVHMVIYMFYQANWVMPVFGFVVGTATNWLALKVIF
SPVRPKKFLGMTFHGIFMRRQEEVSAVFARVVCGEILHIKAIWNAIFQGSNSKNFTAMLR
AHTLVFTDKLVVEIEPLAVAAMGSEKFLQMKEDIAQKVTKKIPDVIDSSYEYTQRALNME
NIIREKMSVLPPEEFEGVLHPAFEEDEIQLIILGGALGAIVGALQLLIFV
>comp3200_c0_seq2; 369 90 1177 LEN=1177; translated
MELTDPWWKYFLIPLIDGAVGYFTNVLALQMTFAPLEFVGIPLVRFPEQPFGLFGWQGII
PAKARKMATISFDLMTTRLLDLQEIFNQIDPVKFAEAMDEAILLMLDNVISEVAMETMPN
TWGNIPQAVKDDIIVTADNESGKFLTEFMKDMQAHVYDVVDIKEMCVNACVQNKHLIVKI
FQECGDKEFNFIRQSGFYFGAMFGVVHMVIYMFYQANWVMPVFGFVVGTATNWLALKVIF
SPVRPKKFLGMTFHGIFMRRQEEVSAVFARVVCGEILHIKAIWNAIFQGSNSKNFTAMLR
AHTLVFTDKLVVEIEPLAVAAMGSEKFLQMKEDIAQKVTKKIPDVIDSSYEYTQRVSNIT
SRX
>comp3202_c0_seq1; 582 87 1592 LEN=2007; translated
MRTTPFCKSHTPLLALAYLCAASHTGAHANGVGIVVGVAQNPPVNYVPARIPFAELLPSE
HGTNAGDENDVFWKALREVGLVSITDVPNLGKQAMMADLGECLATTARGAEGLHTEGFAD
VGIDGFHRRRTLAMHTLAGSGEGLFAERDPSPACAALAESSRPFREGVEKVLGAIAERFA
LVPSSLAAAGEGDGAFLSADRLIHEGEHLEHFHSYYPQPQAGKGSGSQTKDKDNANTKTI
EWHTDQGMMLVFAPGQRTDGSSTGGFLVRLSDGSTAELDLDPFVDDLVVMLGDGVHQYIN
QSGNGSIQGLRSVPHAVSLDRNDGAQRLWYGRMVLPPPGAVHPSSAAANTFGELRAATIR
GDPSGLGCSSPTAVVSRGLAGDGHEDPGECDEATSMFCWMRCMDYEEYGASPEICGAGNL
ACANADGEPWAKGVHDPSYKLRCLGSAEADTNATASAGDDGDEPESETMEMDSSGAKDRG
TTAFGFLVAVVAATAGGWIAW
>comp3203_c0_seq1; 414 1 1764 LEN=2592; translated
XLLQKYEQSHQLLVPRAVDSLAPDSSFGSVEGTERGAKEFGPSCKKPQHMKRAIVPSFFI
DTDETIDADKSIVADESMSQQTSRTTKVVVPSFLGHPENILPPLFLIMTCIKVLLFPSYR
STDFDVHRNWKALTRQVDISEWYWNDVSGRTVHTLDYPPAFAFFEYILSNNPITHLVLPQ
GDRCLELLPDTDNEPSEACVVFQRTSVILGDCILWIGSYFACWAYHYKMPVQLTVSSFLL
IVLNPALLWLDHIHFQYNGMMIGVLMSSLGCFMMGNNVFVDQAAKQPIEYDLYHLAGAAI
FALLINLKHLYLPLGPLFFCYLLERYCLTTEIPPGKKRKQFLPGKFLLLALVTGSVLVLP
WIPFLKNQKNPKLQVIQIFKRLFPFGRGLVHDYWAGNVWAIYTFANRILLATIRRLVPLL
MPIMPEIATFLEGLYLPEPSPLVCALLLVLAIIPGLQMASLRLTNKKLIEAVVYVSFSSF
MLAYHVHEKAILTTLMPLTILVEPSSRVEYHNLLFWQSSIWGLLGLFPLLFRPVELVFKL
GSFLCYMGLTSYLLKTPPPWMQDRQHDALAVVSFVILLLECVPIQGKW
>comp3204_c0_seq1; 1129 74 2308 LEN=2339; translated
MSLLEPPATITRLLQESFPDSVTSTSDGAQWNANEDILSYVAFLAAGMADVKEFSQEAWK
EQLSPYLEGIKEVGGDEIDAFRILVAKEVLGEDDESYGDDDDDDEHEEVCNLRFNLAYGG
KILLRESKLRLLRGRRYAIVGGNGVGKTTLMNAINNGKLEGWPQHLKTAYVDSGSNVDPE

YERQNVMKHLIEDTKQSEETIIAKMKELDFTDQMIRGTIGALSGGWQMKLRLIRAILMDP
DIFLLDEPTNHLAEGAVNWLTDYLCGLDHQTVLCVSHDTTFLENVCTDIIHYEQRSVWGP
YRRLVHYKTKMSGFVKLQPQAKHYFELATNDDGLKFDFPQPGRLDGVKTSTQKFLEMEHV
DFRYPGAEKNQLTDVNLKMSLSSRIVILGANGAGKTTLLKMIVGETVPSNSGRFYVHPNL
RIAYVAQHAFSHVERHMEESPVAYLQWRFRDGFDKEKLESEAYRITPEEQQAIDDFKLEG
IWSRRMRGGVLEYEVKKKNVREKDNKYYSRDELLGMGFEHLLKQTDEKIAAKEAGLDLRP
VTTSEIQKHLNGFGLPQEFGTYGKIRGLSGGQKVKLVLAAAFWTVPHLVVLDEPTNFLDR
EALGALSAGLNEWGGAVIMISHNREFYSSVCKEEWHVAGGVVQIQGESEEREMKAFARKK
KFEKELDGNDKLEKAGGNMNSNGDKYKEATVNFWGATVSKKEARAYEKAKKKGNVAAMRK
VLQIPPGKVMPGYEELGDGNDAKQ
>comp3205_c0_seq1; 319 1 899 LEN=1041; translated
XLSLLIAYGSFFTNKNKNRMFQFTAATSIGAVLLTMMAAPIGAFLHRDGRSMTAVTTDRG
GLVSSVPASLSHGAFRLFAETSPESDNEKMRMFESEGWKGIAEDLRSFPLFCIATAEDNP
VAYQITVGEEKTFDVPFFYCDVTEAAAALEKAQASSDNATEGVDLKLLPFPLEQAFKLWC
GDKAVIIPSKASILQAGAPAGTNPIGQRVPMWACLDIAEEQGEGLPPKLPIFMGLEDAND
AVLEAVGGDRAKVDDLEVVCLSLDGAIEQLVTAAPEEAPGFAFIPPSKSMKYIEENVQQ
>comp3206_c0_seq1; 320 159 707 LEN=891; translated
MTALRSTKPLLLASFLSVLALLAGPAASFAPGPLRRDTACQESSSTSVGVGNIIDLLNDG
KKKLVKSLAGDYDAEAVRARTKGLIEANPVLMLSFTTCPFCVKAKGVLDGIGAEYTVVEL
DVDPDGKAIRAELGDLIGRTSVPAVWIGQEFVGGCNDGPMGGVVKLSEEGRLEGLLKEAG
AL
>comp3207_c0_seq1; 158 189 1062 LEN=1128; translated
MAGGGMCLARTTSVSRVLSKRLSFPAVAAAAGRLSSSGAAFGLSLQAPAPGAREGKVLSA
GAPSRQGLPLLRLPPPSCASHLPLRPGPFSKGTHRAQGRWLSSDAEAGTENNGSYTVPIV
IKMPPMDDVGGDESHCFVETWHKREGDRVHPEDILCDVSTPDFVFGMQIDDAEPGILGEI
HVEAGVRVPDHTPLCTIYHRAPPEEAGGERAGRGAAAAGRTARSHAMRCDAARLGLGSIP
RSIRPHATSESRVKGKSEKTERHASSPRHKPMREGSMDVFKSWEHPMRLP
>comp3208_c0_seq1; 421 120 1538 LEN=1955; translated
MPKAEKGSVKDIANRMKARGLQKLRFYCQMCQKQCRDANGFKCHIQSDSHMRQMKIFSEN
AGGFLEQYSKEFEQSFMTNLRMRHGTKKVNANNVYQEVISDKQHIHMNATHWTTLTEFVQ
YLGKTGKCIVEENHSDGTGGWYISYIERDSGILARREAQKQREAADQKAEAALLERMKAQ
RIAAARALDRAGGSVTVQATSIQQNGNTAPAIKLGLGAAKMKTAKKKSTASIGTSAFGDD
DDEENNDDEKDYSKRKSANTNVEKMMKETTMRKQAAATTMNENKNSSSKDSSANPKTTKK
RKLNDDGNRRIEEITKRSRDERSEVSRTKPSEEQQAVKDQNSWLYRDIMVRVVSKRLAGG
KYFRKKAVVDTIVGDGFAAEVEVLGDSEDGVDGDILRLDQDDLETVIPKVSSGGKPKKVR
ILRGEFRGEKATIEHLDKSKYRADLILYKPKRDEDRGRRLRKVPYEDFSQIA
>comp3209_c0_seq1; 177 1 1524 LEN=1625; translated
IQRHERLRKLENSLLEFRLDLERSVSDDEEVEKRVAGERARRMARLEEEEAERKERRNQR
GDSGDGYAARNSNNRDRGYSGLASTSGKSNGRTDRGYRGTAYGRGGGPPRDYGRRPQQYR
RDGHHRGGDRRPFSNTNSHVEATRKEEENHRLADAFGIDKAKHVEGAAFDRDLQEEKKRL
KTEKKERXEKAAEKAQRKLERDQKRKEKALRKEERNQRKKSKPRVDRDRESAARVPIAAA
VVAALEATAGPRLRALDHPLRPTAAARRGRPRTAAARRGRPHRGNPIGEGCVGRRMAERI
AGTQTGASPVKRERGLGAEAQVPTTVGQGAAVAAAAGAVEHGGDRKDKADAKRSKKMDSR
NEREPAENEKNSKGGSEERSTKATEAPSGPGDKKNSEARGGLPERTASPVEAGCGGGRLD
RNRAPGPPAGRGGARPGERKHQRTAAGSRPRPDLALSRRSARAAASEPAGGGPGPDRNRP
RRQEVHSGRRVARRPAAEAPGLGRARPW
>comp3210_c0_seq1; 127 512 1109 LEN=1109; minus strand; translated
MRKPRVSSLSAPPWHSVRSRIAPKPVVGWLVHPPARQSPTGALVLMLPPPPPLPALPPPP
LPPFLPAGQPAGLRLGPGTSGVRNGLPEPVVASHDRQEQGGGDSEQSCPGDPAGTPAGRD
GGGGNHLFSCPASFFIVLQGTSTDGTVCCYGFVTLWFVMVCVPRNAWLGVFPRIVKRSIL
PNAVPYGTAIEKIIPGLVAX
>comp3211_c0_seq1; 227 1 713 LEN=922; translated
XQLHLLCTPVLRAISHANIWPLDYHREHFYDIKRNTASPEQSDGWYSIHRIACFRRQSCG
FSANNPADRMDQMDVSSISSAIDATSTMPEPATYPRPPPTPAQVAAEASSRQSYLAPPAS
SSKKSWLDSFLDLVAYSVARILNHPDVRDAVSSAILEGMVKLCYVEDLHEHLRHVDETLT
EHQVSDAAKKGKDAQKIVRAYLGGMFAGDNDESKTDDADNDSRRNSKHTENNNKKVR
>comp3212_c0_seq1; 233 139 801 LEN=1083; translated
MSTSDVRRRLAPTDCTGASTVANNAKATEQAPIFVFVFAHPDDESMFFLPAIRALVAAEA
TVWFLCLTNGGYDGLGKVRETELEKAGKLIGASKVLIRSDDDNGDERAILDHPTKRYARE
TVANAIRDSLCASLEPSGSANRFVLVTFDELGVSGHVNHIDTYLGVCHLMDEQEERQREQ

RAQTTPKTSRQQSPQELFLLEAWHLESERNLFFKYLPLLSW
>comp3214_c0_seq1; 248 70 1202 LEN=1438; translated
MVNIFGMQTVLFLTILAIVLVTIVNAEIKQDDRQIFSGDGHETARVLKTIQFGEVDGKTL
LAQNAPKEINVDPRTTYNEKFMAQMSSPCRPEYDGFFGATSGDSVQIQYGFQLEVEPLSS
VMDILDLIEDKIVDSVLQSSFPEMCGMRRTRRTESHDQYLARSLNDGHPSGFRFLKFEEV
GKIYNRSNRKIRSEILFLQDFFLITWCDFAQHNVSPEVNDVNFCGIFTGIVYVYGKHQNE
EEIFQAVMPYIDHVFETTNPRGLHSELAMISRVDEFSSTASFDISQTPTENQVKHISRGE
LSGLDTLLIIVSGLIFLSILYYFYIQWDERRRYPDHKVASNSLNVRSGDDYIEDEYGANE
VEFDQYQDNVQSNGILS
>comp3214_c0_seq2; 293 70 1110 LEN=1346; translated
MVNIFGMQTVLFLTILAIVLVTIVNAEIKQDDRQIFSGDGHETARVLKTIQFGEVDGKTL
LAQNAPKEINVDPRTTYNEKFMAQMSSPCRPEYDGFFGATSGDSVQIQYGFQLEVEPLSS
VMDILDLIEDKIVDSVLQSSFPEMCGMRRTRRTESHDQYLARSLNDGHPSGFRFLKFEEV
AQCIPEVNDVNFCGIFTGIVYVYGKHQNEEEIFQAVMPYIDHVFETTNPRGLHSELAMIS
RVDEFSSTASFDISQTPTENQVKHISRGELSGLDTLLIIVSGLIFLSILYYFYIQWDERR
RYPDHKVASNSLNVRSGDDYIEDEYGANEVEFDQYQDNVQSNGILS
>comp3215_c0_seq1; 428 1 1515 LEN=2270; translated
LVLTMTLVLFLGFLFLATRSLGLLSTSTNVFLPRSDLSPNLTTSTSRVYSSRLSSWSKIA
EPDTDFGSFRLVNTEDAEALAASQEGIEFSDSLVFNLASPREWLEFCEARQLYQDQHPND
IRDRAPGCGAYTVLRCDFLLEEKAWRIWGRDFHWNRLRESYRLLLQQKQSIAGQNGSGAN
HCEQAAIDSSIEAMDCLLAEAESEILSKYQRENDETAVVVAMLTLLWDPEPGDATSIRVR
GHGFSTMKPSHNNDTDGTSMGNPNAPIEAVIGFLPVTLAERVADRINNNHHDVTMLPNRY
QNIPQAKLSSWCRRRRLLEKIFKGENTGDVILTKECGLGKETETNKQESSPEAEVPSIEM
LEGLTSNMFVLYPGNVLRTAHSSHVLGGYVRDLIVECASECGYRVKFGPIPLVDSSKWKE
VFLTSSVRLVIPVSRILLPSIASSTRDEECGDSDDLFRLDTVWEMARDDELSDGDRPTRP
ASHVLYEKLMERGRDIPSHTSRSP
>comp3216_c0_seq1; 206 197 712 LEN=1128; translated
MTDPNAKNPLPSDARDNYTPHRSHHPEELLHHDKKKHHTRAEVEYNVFRDSLLRYLGYAN
EVGESFRYQYPRFVVPSYVVSFGYCLADAATSGKKAYDYSTSEGSSTAAIDSVVSTVDTL
LWQSLASVLLPGFAINQIVKFSRFAVTKSPMAAPVALATWLPTVAGLGSIPW
>comp3217_c0_seq1; 270 358 921 LEN=967; translated
MVLTEEQKLRIRKNRERALRIQKERREKYEKQKEEHLKESERSIEDNQTPSKKRKTTDST
LARPTASTTTSQSISPTTNAKQATEDHDYHSSEPLEDFEEGASQWVTKTEAMNTYCLPEG
TMAVCSYEEKQNPRNPSFKPMKLYKRSEIRYRAHKRHGGVEGLVKEREKRRKRKLEKDME
AARDIFK
>comp3218_c0_seq1; 303 47 697 LEN=2266; translated
MKSSIAFLALGVSSASAFAPTQSASKSVTQIELTRDEILSTPNTLELGEIYDPLGLSQLG
GDETISWFRHAEIKHGRVAMAAFVGWCATSGAGLRFPGELSHGLNFADVPGQGLAAWDAV
PGWGKAQMLLLAGLIEFHDELFYARRGTHYMSGGVPGKNMVPGLYDPFNFSAGKSEAQLA
RGRDVEIKNGRLAMVGIAGMYFANVIEGSVPLQPTC
>comp3221_c0_seq1; 364 1 1309 LEN=1385; translated
XERLMIDESDKSCTVQAISFVDHSATRMTRPILPSRILKLVVVVSVALLKFTDVTVDAFA
SFSTRGSILHSSLHHGSDHRCRSTLNKLSARLWAQSQQITIRDWRSNGNEPESIYELLLE
QRREQAQTTNDDFDPEGSLELDVLNRLCLQESYSKEDGGCFLVAVAGDCNDHNDDDENGD
KRKSSIVGTLGMITGTQVVYQSSGSSVSEPEVAAAIRRVCSSWSEDEDDGTAKQGLTKQV
STTTLLEDLIREGEQRAIQSGATRIIGLAYPETTGAAGEKARVAKPSTDLFESLGYRRSD
QQLPGVKTLQYEKKLSRNEALANSNAVLTESSVERWILPAAIVGLVSLGYLVFNLYSNVF
GIEQLWGSSDNAGIGTSLSTQNLQDLIRDEKLGRSGLDDGISSYASTRQWEDLSPEELRE
EQALMKVIQGQSIRFK
>comp3222_c0_seq1; 157 243 899 LEN=1072; translated
MTCLFRNDLTPREKIRTILKLVFEHASNLAAFATIYKVFLAVLKGSSRCLRQGVGLNDYP
NNQPRTTSNAIKSLGRTLIALIVDGPFFSTDTSSVWGSSSSTRRAGEPERPYHSLLAGAA
GGYIVWGRYSSVNHQIVLYLASRIMVGLAKRGWELVYKEPGHFRNDNRGKSILQHPQTYP
MVAATVWGIVMLLFEESPHVLHRSLKKSMDEIYRRGVF
>comp3223_c0_seq1; 229 31 585 LEN=648; translated
MMPLQQKRLRVRVVHFLALATSLIMLSSKLASAFATRSSFITASRAASRSFRSGVVLNMA
PKPGVATLEELKSHVEAAGDKLVVIDVRNPDADKEPGDQKSLAVAPVPDASSGVRPCGLH
LIWDRESDSMPLPSVEKDTPLITHCGGGGRGQKAKDFLESNGFTNVLNGAGPKETELWDV
YGEK
>comp3224_c0_seq1; 377 109 712 LEN=1049; translated

MSEEQANTTPPASEEKESQEESTAEFAPVVELEEVDVVSGEEEENVEYTQRGKLFIYGET
MLDVGSGNKTEGGGIGDIRILKHKQEQRLRVLMRQEKTMKVIVNHALDPRIKLEPNAGSD
RSWVWSAFDFASGELVETVFAIRFADSDIANNFKKVFEEKQAEMQSILDGEDAAPSQEAD
EATEALAGLSTKEEEAAKEE
>comp3226_c0_seq1; 462 58 843 LEN=1222; translated
MSDDKPQDGGRGGFGRGRGGRGRGRGRGGRGGRGGRGGRDENVWVPVTKLGRLVKEGKIG
SLEEIFLFSIPVKEAEIIDYFLKDKLQDEVMKIMPVQKQSSAGQRTRFKAFVAVGDGNGH
IGLGVKCSSEVATAIRGAITAAKLNIVPVRRGYWGRTAGLPHTVPCKVTGKCGSVSVRLI
PAPRGTGLVSSPAGKKLMSMAGITDCYSSSSGHTRTMGNFIKAIYAALRATYGYLSPELW
AENALLPLPFQEHTDFLSKKL
>comp3227_c0_seq1; 190 125 508 LEN=972; translated
MNDPSKSSTGNNHDRNHPGVVRVEATAPIKSSPLSTGGIALAIGTFLALHTPSWLVLVFC
FETDEHKDHHDSLPIRLLSRWLLDFTPWLANALLSFFLGVLCLCLGAAFLSRTERWGAVH
GVRTEPF
>comp3228_c0_seq1; 275 184 1395 LEN=1701; translated
MPLLFRQRASQNSLPRITPSVAALVAYGLCCTVLVALLAGARSFAPREAFSTAASKRTSP
VPMSPSSPATESVRWRPLGPELFPSPVSPDDGCRYAKDEAYLGAVLGSWKTNPAGPRAET
SPWVYRDQDGTALYGHAVRPIERTASTGAPRPGILLFHTAAGPQDVFLFQKAEQLAVSGD
LGPDGCVVLVCDLLSDPDGWAWTPGGDRTRFDRQKELLFRDNARLLRARVEAAVEALSGD
DRLGVDPDRLAGLGWCFGAHPILELSGLRRPLEGGTEEGPTAVALVSYHGVYRRDPPGSP
ATGGDPGDGPRGILPPAPVGDVLICTGGSDPFVSGEDLDAAKAAMEEGSYRVAIAEFEGA
KHGFTNPAQDFNPNPAFGYDEHAATESWKATMELLKRRLGPPG
>comp3229_c0_seq1; 239 249 861 LEN=1057; translated
MYPLLSPTAITHSPNSQNHGAQAVAIAVPQKIQPYGVHVRSKSIAAASAAGRQFVEIFKK
RWLKLQNTIYVIPKCNVSIVRITASAAVAAGGIVVLSRHQLPSMTREKLYKQVWRNEAAE
GQDDATNQNDGSIFDDEDEEDFQPLVDKEESPMTTAMIATIGVYKNFISPLLPPACRFVP
TCSQYGVQAIKEFGPSKGVVLTSW
>comp3230_c0_seq1; 280 176 673 LEN=828; translated
MTDAAIQEVEAGDMIPSVVLQEGLPDFGGKNDIDISELIAGKKVVIFGVPGAFTPGCSKS
HLPSFITAKDELKEKGVDMVICIATNDLFVMEAWGNTSGGAEAGIRFLSDGSGELTKALG
LTMETPMMPRTKRFSLIAEDGVVTEYFSSAEEASNTWAPAVLAKL
>comp3231_c0_seq1; 453 132 1121 LEN=1260; translated
MVFGWGFNANKLKPQLKMAVHRFQMSSNKKAALLKQNMREVAVLLAEDPPKEEKARIKAE
ALIRDDYMIEAYDILSLNCELLSERIKLIQFSKECPPDLISCISTLMYAAPRVDIPELIE
IRKQFIAKYGKKFDEDAMANTGGILNERVVAKLSVQPPAAYLVQTYLEQICEKFEVDWSP
NYRLSAEQMGEPMAPPSGFSVPIGQGTGLGHAAHTGMTVTGDDDTTYDGGQSLPPPRAPG
AGNNNAKVKSNQSEFHIPSAPGTSNAPSLDLPQAPAMTNTSDATNKDDDDEDDNQNNGDS
SGSQSQPSSGKDTASYNDLAARFENLKNL
>comp3231_c0_seq2; 369 62 850 LEN=989; translated
MIEAYDILSLNCELLSERIKLIQFSKECPPDLISCISTLMYAAPRVDIPELIEIRKQFIA
KYGKKFDEDAMANTGGILNERVVAKLSVQPPAAYLVQTYLEQICEKFEVDWSPNYRLSAE
QMGEPMAPPSGFSVPIGQGTGLGHAAHTGMTVTGDDDTTYDGGQSLPPPRAPGAGNNNAK
VKSNQSEFHIPSAPGTSNAPSLDLPQAPAMTNTSDATNKDDDDEDDNQNNGDSSGSQSQP
SSGKDTASYNDLAARFENLKNL
>comp3232_c0_seq1; 155 1 602 LEN=866; translated
XRFSSCKSEKIMVVPDPNVLAAVERGSSVVFFDVSLGEGESAADLGRIRMELFTKDCPKT
CENFRQFCTGEFLRNDQPTGYLNSSFHRVIKGFVLQGGDFINHDGTGKLSIYGTSFPDEN
LTSHKHDNAGLLSMANSGPNTNGCQFFITCAAAQHLDGKHTVFGRVLDDSSMLTVRKCEA
VPVNGTTPRLPLIITQCGEL
>comp3233_c0_seq1; 168 1 364 LEN=559; translated
XAITTTVSTIGTRYIFGVQNIATSFSVASIEMGFSTLFVGGGLGFAAQCTSNAIQKIPIS
RQPWMHVLCTFGGAWAATKWVDAKHALLADVNELRAYKGLPPMVGSKNYMPFVPPVEESK
E
>comp3234_c0_seq1; 266 1 849 LEN=849; translated
QQQQQQHQQHPHQRHGNFSRPVPQRERLRDLRKGRLPPALRPAHARGRLPEGPLCAEDQR
APAIERPGAPPEAPEFQGAAAAEDAAFGSDEADPIAEAVFASMNTGGRLFHSTGHVFSVL
ENCGGPSADPMLVLMVLFHDVIYYSVDANFLEFQLDLLEGVLALVEDEDGDDRRPSEPLA
LSETATREDPLVAAVAAVFGVEEGAVLPGKATNEFLSAVVATRLLSGWLPLSDLVRLAAG
IEATIPFRPVGPSGETALGALHDRLSPLVLLPGNGAAAAAAAA
>comp3235_c0_seq1; 383 589 1479 LEN=1527; translated

MTSSNLQIIRFVLFLQALGSGSIFGEGFQHQQFSILESYAGRRTSRSSLVSFSKSKADEG
SSESTVEEYRNVATKILSNFMQKDKMEISDDENTSSTVDFESPKISPSTSLETLISALDY
ELSEKEWFVTGNVNPSYFSEDFTFQDPDVKVESIEDYSKGVRKIFNQSTARAEIISTALT
EEVSSSGNPVVTVTWRLSGGVNIGFGLDIKPYIVYTDFVIDPNTSLIISQEDRFDIPSWD
ILLSALFPFLIGKVTKPPAAAVPKRKLTMPKIELIGSESRSSDLSGVFKSIFGDKS
>comp3236_c0_seq1; 186 1 717 LEN=871; translated
QSNPIQSKPIRCDMRYAICDAMVEGDSDARQPSAGQQRSAVSPRRSDARCILRIANANAN
EKRKEMTPTDVLSAANAKKDXKVRSKDGEPTTNAVASCTQWLLARSFVCSGAVARGKKKV
AGEPQEKDDQQHDCNGWDGVEAADRPPERTDGAFSCPSIAVSGGNAGIVTERRYGTRREE
RFGTNHRDVVRSKECPGSFVPTARDRSHGIQLFRIRCDVFQYPYTFTLLYFTSLHFTF
>comp3237_c0_seq1; 187 132 764 LEN=924; translated
MYRDERTSSNHMLSIRSILFLALLVVSTTAFAPPSRTSQLKSPLFALEVVEENDPFEAYQ
TTPEQTTVVFKDNQIGTGYLVGEAESQQLKIKYTATFLEPNPGAQFDQSESFVCKTGQNK
ILPGFEEGLKGMKVGGKRTIRIPPNKGYGDQWYKGTIPPNAHLQFECELLSIAQTPQEEF
MEKLNDYGVGRAIGMTVCIGYLAISPFLTQ
>comp3239_c0_seq1; 838 235 2634 LEN=2704; translated
MSGATGSATPNVGLSSNRSNSSGTPSAPPRPASYGAAIPAATSGNFPAIGSTLGPYFSVG
RLGFGTFCSIHKCINLGYHHNKKRKTFENDKRKALRLVAAKVEIGEFKNSGVLGGEASIL
HFLDESLPPNTVPTYMGHYVNSNRDREQNKTVTSAIVMEYLTGQDMHKIREWSTKHQEQQ
QQEKHERAERGDDEDEAQFSPESYGVGKNKGNEHGSPTKGSSATNNRNKGSGASTRRRIA
IKDAVYLTAHVLLPLLRKMHSVGIIHRDVKPSNVVKRNDDGDEDESNSGSNADMFCIVDF
GLSKSIVVPHTNDTADAKQSWKGSTWMGASAPGTAVSSRDPSGFELLSPDSSGPTEGKTN
NRPCYRAERPTADFRGTSMYASVRVHQLREYSPRDDIWSLLYVFCDLASGGLPWMLYAAN
RDRAACKLLKERIHGLEPHADGRIGTDTKRLLLGDEYHRALFRKRNGRVDPPMDHEEGAL
IEDEEDPDLPKPLALSEDVHKVGLLTKAFEHLKGLGYADIPDYDLIQGCLEGFREDGNKP
STNGSEAGNGSCETPIPCIDWGLLAEHSRSTENAVHGNGHGSVPAWGFLDGDKGDNDPLD
ASVFAEAERSILRGNNGEEEGGPLYGEAADMARLPLELRFRMAQMDYNTAHHTTIEPHLA
LRDWLAACLLLLHTPWDSKRYERGGHRSRGDGYKREFFLRLVNKCLGGAARFGGFRARGV
IYRGGGDENENTHNNNNGSSSERPAKRRKVGMRNVHEPPGNETADLLLLSRVLFELRYTK
KLEEKLSPPPPSRLSFGS
>comp3240_c0_seq1; 614 39 1328 LEN=1527; translated
MKFALFTASALYCANFATAFAPATRPAAVRKSVVPVAAEQFDQEQFIAESKEMRLKHLEE
QAMYALKISCENYGNAVFPNAMIAGDVVITHLLHRMGYLKDSSKCTIMCVDTFHLFPETM
EFLKEIEEFYGFKAEVFCAEGIPVGDKDAYDKRYGADLWKENIEEYDRVCKVEPFQRGLK
TLNTDCMINGRTRWQGFERAWIDLFENAPIGGGLAKCNPLAYWTLEDCFDYIAKYEVPHH
PLHASGYPSIGDAKDTIPIPEDGSVIFKDFKFEGDKTQWLDYASERKGRFVGLANADGST
KTECGIHVGGAEKTWDRDLWEEGAVKSIESNDAALEITKAGEASVIAVYAPWCQFCQKME
EDFSTFAEGSDIPVYKFRGDEDREFVSANLNTESFPTVNVVKADGSVVKYESEDRSPEAI
KKFVADTLA
>comp3241_c0_seq1; 514 65 1375 LEN=1400; translated
MTSRFENVRLRKSTKKGKKTKKKAPESSIDDLLKAASAAMESLDVERAYETYSHAATRLQ
GADNEKHGVEDLIHVLEKMGELQVSMGDQDLAKDHFVRCLGLLEERGKKDSAKDMSYYET
RSNLCFYIGQLSLEKEALDAYQNGITSLEKCMQLVADGQEIVAMQDESDETTNHNEEAIL
KQKLSGAYCNIAELYLTDLCEEETAESDCEQYLEKALQLKDSDGEPLVDALQTMASLRLS
QQERQSEAIPFVLRAYEKQRVGSEALATLVGLGEKTTDDDAASKGQAQELLEVEAANNLP
EFEFRCQTAKLLLECAGLDREESGGTDQANECIAAAISVLGSLLAQNDEVIEIWYLTGCA
FAAKQPPLVDAAKFYLERAKEMLTDIQKALKDELQYADDSEKQELQEQLEMNATQMSDVQ
SKLGALPDAVEDMEEG
>comp3242_c0_seq1; 223 53 625 LEN=625; minus strand; translated
MPVLWFLQFLRFLPVLLLVFFSLLAKRRKQRVRLVPGREPPIPWMGGALTVLVLVQFENG
EGLAEDRANGSGTRFEHGFELESGFDPGPPSVENSARGSGRSGAVWHTQAGRREEPNGGG
GCALSLSSFPSSSLPLSSFLSLFRFRSLCLCLGLLSVVSAIVSVFVSVILPGWVGSEAAR
SAFVNDDDDDX
>comp3243_c0_seq1; 201 1 621 LEN=841; translated
QQQQQQRSQEPQQSEDASVLVETLKSQNSILWERILRLQHTVDELEGQAARLVEVNSKLE
IELREKEKMNQQLVQKMISTRSIAMDYTSAKDAEISLLKEHKLLLATKNSRLQSYLRTTD
KFIGSIQSQEQEMMKIQQQQHLESTKSRSKRKLTSIFGDNEEHKTPNKTIVLVESNECKK
EFNTCTENSLGTGSATGKGSDVIELL
>comp3244_c0_seq1; 388 1 1210 LEN=1272; translated
XAKQQEKDRSIERLLQTAFSMCDPLPSSDAGPLIRWAQRVWFDLAEGSFPYPSSYIPFAL

LHKKVDLPPWPTQDACWKSSELHKDWGVRFHGSREDVNYRISYGDSGISLQVDWDSVTLV
GNENGYEKQGPSAIEESADVVGLLTSVRDAVSIWYNITKDVPCYNISQAAPNARTMRRTR
TGTKLPLLSPSPPVEGYARDAYGDPFGLTEAIGSKTVDPERICHEAMVEQGSWGLLCCND
EMNLVITDAQGTGHDFFWPPSHPRSVKTYRDMIRNSTFEPCADPYGIYGYSKEPIDPMSM
RLDAYYGGSNTKGHSNIVFSNGLLDPWSAGGVYRDSVRLEEFFDEDKAKVQKINENDVVA
LIIPFGGHHTDLMYSDDSDPNSVTEGRKIEEEYIERWIRDWKP
>comp3245_c0_seq2; 203 71 347 LEN=347; translated
MKAIIAFLALLSAADAAGSLRGEAEPRGPPIACLVKGGTSQSQCDATTSEDGSTCVWCSL
SSYGACVDESIATQMKQMLPALQCDNEDDDDDX
>comp3246_c0_seq1; 343 151 735 LEN=1235; translated
MSSSRVHHFKLVLLGDTAVGKSCLVVRFVRDEFFEFQEPTIGAAFLTQTVALDDSTVKFE
IWDTAGQERYRSLAPMYYRGAAAAIVVYDITNPDSFTGAKSWVKELQRRGDPNVVIALAG
NKADLESRRKVEFEEANSYAEENGILHLETSAKNANNVKALFVEIAKKLPKNPPQPEREA
FPLAAPQNDNRSCC
>comp3247_c0_seq1; 382 183 1100 LEN=1281; translated
MVGEAFLAPTATTTSRTSSRSMVDPMSTITTAEISEEFGKSVIIALVLGGGLIPATLSAN
SAMFSALSGRKGYIPEGKEPPADLETFENYDPNNTFDPTMGNANNIKYREYVLDSGATGP
ELPSSQLLFASDKIPVADIVAVLGRIEATESIVDWRNLPSATRGTVTPGAEPPMWLPRKA
FKVLIRKSKFLGWPTDPKTGLPVGGEELKQSEEKRISKKDAIIGDAALDAVFDSWTGGAS
IATPDKVAKVLSKVKPAKNEVDLDAFVQAAVLGRANTAFAALSFVVIQIIVFGVLFVQPL
IEELS
>comp3248_c0_seq1; 277 719 1694 LEN=1716; minus strand; translated
MAQYSSAVRASALHCIAFHSASVRACKRFRIRYGGAIGVRIGASTLSVGQLPVAGPRPHP
LSPPNQIQELPVVFFLSVELFSEGGGRRSPAEPEPVAGPVHQPRGAILPAPGFAAALLLE
VRLQPKAMGFRPFLASFGATGSAGGSPYGPALCALVPACRRPRKVAVGVVDAVRDEECVP
VRPQLQGLAILVALVVGRLFAAASPPDCVHRRVVDLGGPARIGPVRKPRIPVDPVGHEED
GTACLAPGGFDKVLSRQQRERFFVEALPLVVNPVSVPKVNDDDGGFPLVVVVARVGSSAA
PFESRGATDQGRMARSSGVGSNLSW
>comp3249_c0_seq1; 147 324 566 LEN=566; translated
MSDDGLWEKPSWARGGLKLKKTGRADTMKSSGNLAAPITFTPFKNTDHSNKVANQQMLKR
SEVGEAVKSGEDLAAPITFTP
>comp3250_c0_seq1; 215 47 295 LEN=295; translated
MAITNLSEITDAQKEELVASLACLVIGEGELTSESVQAVATASGNSVSDSLAALFATVVG
NSKDGMKTFTPPPGGGGGGGGGG
>comp3251_c0_seq1; 476 1 1667 LEN=1706; translated
XPSALIVTKQNCRYKTHTHSTRTVRYVDQYYAGVGAPQHCHDVPSKACGDALGWLCVLLL
HRSTLRRICDRACSGAGPPAAIEPTPPLDCCLETGVFPKRFVEQQKLQRILAVETDLEEN
GVRGEEIEDEESLEEVSILCPPNCYGGANDDESVPDERYLPDGIESGGEEEDDEMFLRSL
PPSGDPFPTAATLAPAENLHGRLLCAAGCAYEIEAPYAQGAGFVAGSKITRLSVGVNSCL
VGRTEDGIVVAFRGTQAGSPLDWLQNAAVALRRVPQGYAPRGSRVHEGFYGALRNKLGRA
VREVLLRLLREDAEAREDGKGSDGSRPPPPPKVYLTGHSKGGSLASLFALVMHNDEELPD
PEGVVTFGAARVGNSEFASHFDRLIDQSTYENNLDIIPFLPPGENSMQDMMAAMEDPEKM
MVMMEGIFWSEEDRKQLKHKSWGWNGRKNKPYAFWRYHPIGNRKYISPDRTVIEPVTSDL
DASRIRDLERKTLLRFSEFRKAHCSACPDDLKGQEGTCDGGYFSALAPEVCRAWRSIEKK
AETATKETKTKNDAQ
>comp3252_c0_seq1; 276 1 1135 LEN=1227; translated
KAKTTGRNNVTIRRQHHWFLSSSRKRARTEHWTSSSSGNNKINSSNNTAIAAGRFETCSI
RCDKVQYRAIRAMPSSTGVTRQRSFFLPLLLVLASCLSPALSLLSLPATTTIASCRTAME
LAAAEPNTAPGEPPRGTAAPSFKVLGVCGGIGSGKSTACKLLVSSCGCLAHIDADKVAHS
VYAPGSRAVEDVAARFGEGVLKNGGKGDVCEPREIDRKQLGAIVFADRSAMADLEKIVWP
HVGAAIGDTIGKLREEWERSGGTADEPGGRLPVVVLEAAVLLDAGWEDLADGVWAVTVPR
EDALRRLIESRGLSEAEAAKRIDAQASCRGIGNVPEEVAAGAVTGVVANDGSLSDLERVL
RESLEDPSFWKPAKEPC
>comp3253_c0_seq1; 299 1 787 LEN=808; translated
XANNNKEQMIAMMETTKSSRRVLIAVVLVAITMTNDVGGFNPAVSVQSNAQSRSKLNYVP
ARVDTFVEADDSAGGVGLAKDSAIKIVGDVKHKPGKAESLPKDLLRYNNLLTVDEATVQS
ALKNSGSTILCMGQGVELYKDPGQTTNKEVTYGPQEAIKDAFTTAASAMESETLVFNFLG
GDDLMLGEVTDACSELVVALDIATKAKISFNSVSHKAIPKGTCTVTVVSVGSPAGDSMIA
QGEVYCRDQTYYTLQESDLNDE
>comp3254_c0_seq1; 372 179 1165 LEN=1628; translated

MAACMPMLMSALLKHMFTAPGQEPWPHGSLRLINCGGIPAQPLNSAERIEALRADFVHKD
EDVWIVSFVKSGTTWTIGILAALYDHPAAVYSGNLQKMTRTFCPQPELPDLGWGDDGFGH
SMEELNSWSSKRRCFKSHWPSQDFVLPNGKSKFIYVMRNAQDQMISHWNQVWGMGFHYGT
ANISFEDGWDTFVQDWLEGNVENGKWFDHVASWYSRSKEIDSDVLLVRYEDLKENTDETI
RKISNFVDTPMSDDIMARVKDLTSFERMKEADEKDIGLKFMRWLGVLRKHHIRQGETGAG
ELKLSEEQKKILEEQYESKLKPLGMPREW
>comp3256_c0_seq1; 170 1 998 LEN=998; translated
XSPFAAHPLCDRSRRGHPNSNKQTQQSSRATIAIPNSSSGVPPAAGEGGPGFEPTEXQKQ
AGRQVVAFRFASFRSLAELRRAPSASTQRNARAARREGGGMSSHGRSRASGGHDRRHRYG
DHETDEHRSETAERYRERNRRADRLHRERERERPGNRESRHRNDDDDDDREAKKRRLAAD
REERMARLRDQLRKEDETLAELDRKQAETEGGAAGSKTSGPTEAVVEVRPGDLEGLDEEE
QMRKLLGFDGFGSTKGEAVEDNHSTAARGGAAKNKARKYRQYMNRKSGFNXAAGKDELSR
PELPVRGSGGARGRHSADAHQSEAALCQYNISAX
>comp3258_c0_seq1; 529 1 1570 LEN=1631; translated
XDXSETTTATLVAREDGRDVLEYNASDTRSKKALQFMGDLTGSHGISFQKTGAGGRPQKP
SVKKRCIIMDEVDGMGGGDRSGISELIQMIKKSKTPIICICNDRQSQKIKSLLPYCMDLR
YSRPTKNSLANRAVRIAEMEGLVVERNAAEAIAESCGNDVRQVLNLLQMWAQKKHVGGEN
DNSLTYKKLKDRGRSIQKDEMLRVSLFDAAKLIVEGPRDLQQADDKAKLDSLFKRLDAFF
VDYSFTGLIVQQNYPKVTNSQYQRIKGDDEAEIQLLERFHKAADSMSDYDMVESQIRGGD
LNWSLLPTTSMLAVKTGYHAGGDTGGFLGGFPEFTAWMGKNSSMGKNYRLLSELSHHMNY
KVSANDQELRQAYVPVLRDRILGLLSRADPDANKEAIDLMDDYGLSRDDVVDKLNVLILG
KKDFSFEDLDSKAKAAFTRQYNAGSHRSQALIREQGGGAGGGAKKKRKRAPITDEDEEAI
GDSGDDQDSSEDELDEEKELARLQAQFKKKGRKKAAPKKKSKK
>comp3259_c0_seq1; 335 228 1073 LEN=1594; translated
MEFPREFISGDKKEFFVPEIPNHTNRSIAIAALAFLLLLPVPASHAAVPAARDESSYNCP
KYFESQTLGIQNVVRQPPASSPPAALLSNARGNTSKTSGSLSSYCPASQDGGKEYNRDEE
EYSVRGLIYLPSNNRMGSSMDNGKSRNQQNPASSSLIVTVRTSENPSSSIPLMGARIPLA
QIESFPISFVLSESSNNFLMADRSWERSVLDASNDLYVTAKICLDDGARGDPPLFEGSGI
SRKILVPSGLSENEEDGFRAVRGAASIRMAGASVPDALYTW
>comp3260_c0_seq1; 279 1 841 LEN=969; translated
XSYGDPTYEDASGEKCIYTSKAKNHFLNISLHTDTVYMKLNSNATIDIRDDPQIGKFFSH
PMNFADVWEIRKLNIDHRFRYTDIPEYFIRALENNPEACPNGLNSILPSRYIANGTNNDG
FVEKYTRKPPTFSNEELMNFKKIWIEQAQSYFDEVAPSPSTNDDEVAPPGSRKKSKEQLC
CHSSNNIKFICCLLFEAIDRDTIERWAIDGDTFLAQVVQSRRYDTHPLCRDLLSNTSYGP
NVEASQKYEESSVDCFSDTKPHISSSGGHFRGAIRDSICHW
>comp3261_c0_seq1; 329 143 1645 LEN=1951; translated
MSSSRVVTTLAGNFGRLWKRGHESFSPDSRRILSVQGKGATAYLQGLVTSDLLTAPVPPR
EEKQDQDTGLETADEESEAETSGVDFSDHLRATCFLDNKGRIITDSLLWKVDENEYYIDV
PADTADPLLSHMKQFILRRTKVKVKDESARSGMASHVIFGTLNARNTPDGFLAGVDPRHP
SLGTRVLSLDLPKGKEFTQMMSRQFPKAPGTYNLIRKLAGVAEGSELKGKVAAEANQEFM
NAVSFQKGCYLGQELTARVQFTGAVRKRIMPLMMTNVNMQCPRPWLIASQVQKEQYQQQE
GIQTDELFTRSDESGNKDEELLPSDVSQLPPLPRLSPSIAGSMIASMSGSPIVAESKGDE
DKENSSNNVGGVDDAKKASDELQAQTEALFEELQSYKVGDKIYDSNDGKAIGQIVALPEE
GTNVLLAQMRLDRVGLLEKPARKPKGDDKATGDADDVPTWKHTNKVSIGEGSSASPLRFL
PYLPLWWPTIDRGSGKAKEE
>comp3262_c0_seq1; 302 451 1197 LEN=1197; translated
MLMRLLLAGSLFMGVAESQYTCQFSEEISIHKGGTTFLSNYVNEDEGTFTMRLRYTGGQA
WVGIGVNTEGKGDMMPAHAVIGDVSRGVKRYSLINDDKKASGVIPLEDVNAHLKAGKFHQ
TDSESILEFTQDLVVREPGSSVVYHEISLSSVWIFAIGLPGNAWEGKHKVHGAFDGLELF
KGCVKDTDPPTLPPTDMPANIPTNMPTLPPTEKLTFEANGVDYAINQEDPYGDEAYTFDG
YNETFRNET
>comp3263_c0_seq1; 537 178 1581 LEN=1736; translated
MKVKEEKENMDQRISLISEQNNSDRDEREDEGLKNQRPIQNERQRGRWRKLAFWRRGQTL
DEGVESEALMPSGFGDDEGLLGGGDISTSDLVYQYKTDNEIKCSTSDYNDDGSDEEEDTE
ENLEAIEEDNLLILTDVPTKNHKVLNAAISPTSVMASLNGESSLNCDSSTRSSESSSSDC
SESRSDSSSESSSEDGSVVRKHNHVDLLLGPLECTSFNSNDNSDDSSLESEIDDSVPAPI
STTGRPALDRRCASTMSFFDKKDHSSKFSQSSVSRSQSMRSGFCDNGKEDDYADIDSDGR
KRLNGRIEGFGSSLSPESKSSSRTLRQRLQSFRNGSSEMKKMSMERSSSWTTTVESRMND
IIDVMFVPPELRPFEEGYMIASRRDRDVIPTSKGVNPTKSNDYIARIERELRQKDLEITC
WKNRVKELEKEINRLKEVIDEETLLNNVCGQKTTEIEWQSEEGDLLS

>comp3264_c0_seq1; 412 62 1192 LEN=1350; translated
MTESSAHFYLLHEEHHCHCCVTSNASKVDTLPMSIDESRNDFMRTILDLNNRGSVSLLDG
DYSVANNLFELAMKRLKDHSESESGVLPPSHLPVQFCDCDCHSSLPLDELDDSTESLPAP
PSRSPDSDEISTVPVPFNSSRSSSNLRIKRMTSTSTLPMNPFYGQQELNQAQPAQCQTDH
KFYQQVYCLPIVLDEGEWNNASTKDKSFVLLFNSALCNHLLGMELLGSRQNLKHQGHEHT
KDDRDKRVLDEMCSRVFITARVLYRLGLENASSFIHGVDKICYLAIFNNMSHVCKTMDGF
NSIEAFAMDKHLLKAIYWWKDTECRRIQETATWARTSPGTANTSYVEEDGEIIDAFLDNT
FYLVGVQQNVAPAPVA
>comp3265_c0_seq1; 542 286 1170 LEN=1361; translated
MPIAGIKTRTDLQNDLFCLKEERDFFESRFLEQVSELKALKQELAKSKKEIRRLRMFLMD
QDTANNPLLLPPDQIDAFPTPKNHNKGSFRGSFGTPRENALCEDKGHGHYEGEDEGRNDS
IHQHIAEKDDASSLTAEEIEELDQQDETLEREQERQRDQERHREDLQIQQSSSSIRTPSR
YESDRGDGEEEEEEEDVRKSAEKLLAWASYRSTMSNRSLNASRTGDLSSVASPSVRTSGS
ARVSLLGKMIETADSHDRDDDNSHCNNDCNEKNLSLEEEKKDEDCSRGNEVQAN
>comp3266_c0_seq1; 613 193 1917 LEN=1972; translated
MFRPLPTSSVASVSSILLCVSFLSQTTQPFTAKPRVATTKNIPSSPPTELFSSAAANPTS
EIPELMLFNSKTRSKEPFQTLSPRKVAMYTCGPTVYDYAHVGNFRAFLTYDLVKRVLLYF
GYDVEHICNLTDVDDKIINRANEQGLAVGAINDLTLKFENYFFDDLKALNVMMADRYPRA
TEHIPEMMEMILQLAEKDLAYETEDGSWYFATSKKEGYGEQLVQLNKDDMESQQDDTAVG
TDMKQDPRDFALWKAFKSPDREDASWEHPDGKIKKGRPGWHLECSAMARKYLGDTLDIHG
GGCDLQFPHHENEIAQSEGVTGKPYCNYWVHNGFVNINDEKMSKSLGNFLTLRTACPKAV
DVRAYRFFVMSSQYQKQLGFTAQAMDASKNSLKRIDRVMQQIEDAVGRNKENADGEPQSS
STLSDAASKALKNFDLALLDDLSMPRASASLFSLVKATEKEFKRVKKDETATLDLKGLAE
VQSALLQMDKVFGIFYDVPLTKEEEEALAASGSSDSEGGATEVSDEVMELVVQRTDAKEA
KDWELADSLRARITELGFAVKDVKGGDPIVTKIE
>comp3267_c0_seq1; 337 719 1918 LEN=2045; translated
MELYSFSLSNYALWPFLAFFFSLLIAVSAQDVSRRYLEEGEEEGEQVRVTISSGLTITSM
VLGLISSLLCILALIFVISFRKRTIVALGQPPFLCLICIGSLMVGSKNLFESPKAVVRMG
TLINMNLDTLCIIRLWLLDVGLIIVYMSFFCKLWRTEKVCQFRKNQIIHVSHVIGPFVFI
LLLQIGLLTAMTILAPPFWTWKVPMKPHDSSNTDVVEKCYQYQFSNRMNMAFNIATLAVV
IICQAITIWMSYKTRKIREDISDSRRIFQTVVFQILICFPYTLVVNGAFTNADVTFVFLY
EAMYPFLMSVSSVGFLIFPKIHCILYQRKFGQLPSYARTIGGGVHVSITSSSLPTNSTPI
TRNATSNKIAESRNNDQTPQVPVRDASATNKSILKEGMA
>comp3268_c0_seq1; 249 1 428 LEN=428; translated
DEEDNSSDEDEENDEEDDEDLIIDNFVRNDDGDGGEGSVAMPKLRECIARAMGFETAPPK
PRDRDQVEEELTLAPTSAPTKKRKKIGKKKRANKGGGGGKKKKKNQKNKKKKKRKKKSEE
FHYEKTDKNETITRHTITSSIGX
>comp3269_c0_seq1; 239 174 550 LEN=550; translated
MFFAKRWLPAVLAVDLVSRSLSLVXQDGGSGLHSRFPQPTQTTPLGASVLDLFRVLEEEE
EEASKNKKNSRSKAGAALEMREPKPFRMEFEEEYGEDEEVFEMTDEDNEDEDSNEGYQEN
ENENEN
>comp3270_c0_seq1; 253 561 971 LEN=1243; translated
MIVGSSSCNSACSHRAGIRQDYRRSLRYTRTLDVILEETSTEQNQQQEQHFYVTPNNSSQ
HLEQDNRSSCDSPTTTVLTTDFWHSMQSMKKSATSVSLNCLVGANDNSNNASEELNSGVL
IEDDEGYDNFLDDWDL
>comp3271_c0_seq1; 483 144 1814 LEN=1996; translated
MDAGEQTKRRILVCCFGSRGDVQPFAALARGLQDYQEGSVRFEVLGITNVNDGVSIFRSL
GVKAEGVHFDMAKMIRENPKMKNAMETGNAVAFGAFMSKQFVRHFPKEFSEQWKIARDFR
PDMILGTTMMWLQASAIGQALKVPVFHADLGPFSFLPISRATQTEMHEPQCLHRFMSFLM
VCSMSMVLNMKNEEGDSLLGTMHKELVAGGPGAGPNAVPESERLLGENGAGQFAREWMNP
LAPNLFAISDSILPKSKFDDLPPIYAERSVWTGNWYVSKAIQEELARTGDPNFSNNTKGV
DVQKTVLEFIEDQQRYGTDLLCSQPAYIGWGSMVAVSPEHMTRLAIESLKLAGLSGIVLG
GFAKLNPDMLDKEQDRDLCDYAEKHVLFLQSAPHEWLFPRCCVTVHHGGAGTTAAAFRSG
VPTIVTPCIADQYDNASLIEDSGCGVGFQKPLRKIKAKELADVLVRCSSSSSEAENGNRN
VDTIRSKCKQVAAELGKEDGILNAIRVIEDFVIKRLDSGTWKQEYEARLQQRIETPWSFF
RALYRMLIYKDPFVEE
>comp3272_c0_seq1; 164 594 683 LEN=683; minus strand; translated
MDHDLAAVAGDAAGCVAGAGMDCVVGVVVV
>comp3272_c0_seq2; 164 766 855 LEN=855; minus strand; translated
MDHDLAAVAGDAAGCVAGAGMDCVVGVVVV

>comp3273_c0_seq1; 110 168 710 LEN=853; translated
MDQHEHRSVSPQNAGAERERLGGGPTATSDDADPVRQSHSIPLQDLPCDAFALVVSYCPD
LESLTNLGSASHPLGRMVFRQNLLNCDRCRIPLFADENLSSRTPHTRAFSCSVCRSTLCG
YQLFDYDRRNCKPQKCDGCGSIECRACMEAHTSDEHADGYGTENYCKACQGEFEFGMGGC
>comp3274_c0_seq1; 166 126 461 LEN=1241; translated
MPEFGDPGYVYDPMDPLPGQKYVEYSKDGEDGETKIKDVDSGLKFEIKGKYIFLKEVKHP
APTHNLHPGDRVIAVNGKTVEKYKKDLTKIKTAVDDHNVVRLVVDPTTLRK
>comp3275_c0_seq1; 350 305 1147 LEN=1519; translated
MKGRTDTKFRSFLSSVLVLAPFLLLRSTSNAFTISSKKLVQPQLSRFISPADGRTNLQRT
TFHEVYAQNARSIKPWYSSSTTSLSMYNLPPGGSGGGGGDKNEISEVAKSVGSLLLVVGF
FISPLGGFVLGIFNSFLLLAFLLPLLATVGFQVWSSLNTVKGECPNCGAPATVMKTKSSG
TNVVDDVMPAQSICFSCGAVLQANDDNTGINNVSGRTSIDDLNSPMGGQSIFDLFSTPPQ
QQQQASTTRTSETRKNTSDSGVDKGAVIDVDILDDDKPFQ
>comp3276_c0_seq1; 312 1 1646 LEN=2135; translated
XQKDNEANIDDSKITNETDSGGSNNFQQKDRDRPPLNIVIFFPDDMNHRSLQDVSGTGYV
KTPFLTELARDGIRFAKNAVTTSICWMSRATLFTGQYTSSHGSMQLKCPRFTLPQYWNDT
WVATLQRLGGYYVGHVGKWQYMKNAKLPHYFNFSNFFEGSHWYSHQRQPMSASDKSVMHA
IEFLQERPKDTPFALTVAYYPPKAVGASKEPGGQWFPKESSKALYANTTYEPPYDVEKAH
ADLPDFLKKKIMRNRYLERWDTTEKYNTGMRNYHALVTEVDLASSQIVEELKRQGLYENT
MIIFTSDNGLMMGSHGLGGKWIAYEESIRVPLIIYDPRMPSEKRGSVDNSMTLNIDLAST
ILGAANIAPPEAMQGRDIADLYLSSNDGTSKLKEKPWRTEYLYEYPIWSLSQNSLIGERY
KYIHWMGFQSEQLFDIQEDPFELNDLIKQVSKDEAEVDSNHIAQMEKLAVGLFETLHGVK
VTISELHDLLKEKGIAVGQNKSESEEATLKELIEALISELKIRYENVRNEVQGPGGKPDW
CDPSLEYP
>comp3277_c0_seq1; 326 1 781 LEN=811; translated
XPASLSPAIPTREPASPGGMALSTPTQKLLGTTTEAATPVTPLDHELEMTSSKNGDGTEE
ELVDLLKHTTTAAPATPNKSFDSTETDVSVLRPIANAEELRLQAKSRTMLGAKTKSRRSS
ILLDRLLRSASKFDPDDANHQSTSTAGGGGTAGCDPERLAGSLMGLDVADLDEFGQMPFH
TKIAYELVEAHKLHIDQVMETLKVEMDALKDFELVLLEQGPVRPTEEEVVEYFESLGLCL
EQRKKAGAILQKKMDRISQG
>comp3278_c0_seq1; 190 1 571 LEN=634; translated
XKQQQTSSSNSKIQATSPFTFVKMPRYQDKFENVLVPFLGNSKAMKDTARESFGQAMKAH
KDVKVIQERRARFVTGFSIVGALLGGNVGCMVAGLSVTIVSFLVSLRDSPYHEIIETLNA
LGYKEKEQLINELERLLQCEGCSKDKFSARLYSAEKIPKGREKIWKLCLESLSYYFTQSE
SESESEWESE
>comp3279_c0_seq1; 384 155 898 LEN=1143; translated
MTNHSRSIVSQVLLAFTLLAFVVLLASPSNAFAQEPKRFQRNNGRESQATATTSNVGSSS
STCLNSTPQSPENQQSQTNKPDLVKQDVFKAAIDRVQDEIEAQYRAQLEIEPSPEGERWL
AERAQAREEDQKYVFLVGKLDVDLPIDTQPELDLTESIGPLVLVTNVWGRTAEATGMQSF
DTITKVSVKGNDDAGASFEYKTKGTPLQETAGALTAAAQHAISTGKTDIQLEVQRLIKGY
YAPEDKI
>comp3280_c0_seq1; 473 322 1410 LEN=1680; translated
MKYSTTAALLAIAMSGGSAFTPSFRRQESTTKTYSSVEVLVEAPLEETVSAEVLEADEED
YEGPLTAELINSRLEKQLAKMRLKDQTSKQLEKEDLVIIHEDKNILVVDKPSGVLTVPGK
NDNPSLAASVHANVKNSLPTGDHMVVHRLGMDTSGLIIMAKNKNAVRELNGVFRSRKVDR
RYEALVAGHIEKDDGLINLPVMRDYRCPPFVRISTDDHQWALVDLTAEDVGKKILEGPKE
SITKYEVVSREFLQGFPVTRVTLTSITGRYHQINVHLAAFGHPIIGDNVYGINGEALPNG
GLTPAELNELIPNPNRADDETQLLLNESVNAKKLPPCIHAKYIKFTHPITKKEYEFSTDS
PF
>comp3281_c0_seq1; 361 54 1235 LEN=1260; translated
MRKIKRTIIPDWMVVALSAAFVFVVVGASSKNNRPHVPSQPRTIGSVEITATVETRTQVE
DSPGNSTIALKKKVTKPKKKRPNGKTSGSNSDKRSYSSGSTTSTSLRRIKREYKDAVDMG
IAYDWAKQRLVTRKSKTKRAPSVDGDADGRIPSIMCLGPITTNLRQWHFSFRGAGDGAYS
KGIYHGQILLPKDYPMSPPSVQMWTPSGRFVPGRDICLSASNYHPESWTPKWSIQGIVNA
LRLHMLTAPNEIGGMQSTHDEEVEFARLSLAWKQRWRDGGSIITVDHRLLLEEGILSDGS
SEEPTNIGELLQDLRGETGVREESEDDESQGPAEVAAEQADHATTEDGLCEYGKESDNRI
RFGARFSLSMSTVTSLVQLACLYVIVLLFFNR
>comp3282_c0_seq1; 317 1 1068 LEN=1385; translated
EFVGKGYHRTLANAGKYGATKPIFRDVTLRYVKLQPSTSRMITSFFKPKRVRASAGKEND
SGEREETTTAVKKLRTHIDPPSKKNATLSPEAKELVSYLNHTNTDSDDSSSWRDALGKHI

SSPSFDRLATFVAKERNQKTVFPPVEDTFAALNLCPLSKVKVVIVGQDPYHGPGQAHGLC
FSVRRGVPAPPSLRNVYKELLNDASIENFRSIPGHGNLERWARQGVLMINNVLTVRRGEA
HSHKKKGWEAVTDEIIRAVDRANRRKQQIAEGGTKRGGVVFLLWGKPATEKATTALAGAA
SRHTIICTSHPSPLGATKTKSPFLGSRCFGRANEALRKMGHEEIDWRVDGPLAQE
>comp3283_c0_seq1; 691 271 2112 LEN=2766; translated
MANSSSTRTAALLSLFFLWTGTHCCSFFAEAWPVLPVSRTEPSSRLFLSFFEKNIDDDAK
KDSNRKNSSNNNHNNKHNHNHNQDPQYNPIRKRRPGHIGSKRNSLNAYDYGFSNRIDSSQ
PPPERKYEPSPSPNRRHEPAEVFYRPGGNGRQALERETPDAKAEEAASGAEETKRLEFGM
EDLRRPGQQWQPPMPQGTIPTPSRGDRGGSRGRNRYHPGSSRPGRTAPSTQSPFSRAYSS
FSSTTTTRRAYSSFSSTTTTRRRTSRQPSGEPFVYFSTTTASSTHRPRSRPYQRRPLTEN
PGMYSRSSSRPEMFDPVYDALQQRRWQQQHEQQRRREQVEQSTSSPVTNADQSGGKNQTA
ASETDANSEFQSVLDQAAIVLRKHLDLGVNEHIAVGPIISQSSSFATTASFGVDSFYANN
ANQRMMVRTLEARLDGCHSPWITATLTVDENDELSEIRLRSNDGRSDRVPIQSSRGRSGM
NSGVANGATDSDINRSGSGASNQNSIDRSFIDVEIIDDDEDEEDYDYDYDYDCDEDQDDG
EDESWNDGEGEESCGDVDDMDESWNSEHGRSWDSDGYEASWGDDGDNGDAPWNPGEGGAS
WGEEAWKSDIDRE
>comp3284_c0_seq1; 264 259 999 LEN=1111; translated
MSRGKYDNLCTSSSWGPRQTPLFAEKQELSVYLDSKLDDERITSLFAWLSRAFAGRDEYN
NLMLAIAAIFGNLPEDSPPVQMAKAALKDVPPEEKTVGDRFSIEERESNSLGAMGAAQWT
GRWKTRPHALLELKNITTIDDWVKTLPRGCKRTIKKALAQNFTVTTKHILADEPAPHSSL
AHFRCVIEHEVRLLRNMYGEDAFFDALAEGVSRYVGTTRMTGDIREYRSEEGKVIAIAHE
VCRTFH
>comp3284_c0_seq2; 333 259 1260 LEN=1601; translated
MSRGKYDNLCTSSSWGPRQTPLFAEKQELSVYLDSKLDDERITSLFAWLSRAFAGRDEYN
NLMLAIAAIFGNLPEDSPPVQMAKAALKDVPPEEKTVGDRFSIEERESNSLGAMGAAQWT
GRWKTRPHALLELKNITTIDDWVKTLPRGCKRTIKKALAQNFTVTTKHILADEPAPHSSL
AHFRCVIEHEVRLLRNMYGEDAFFDALAEGVSRYVGTTRMTGDIREYRSEEGKVIAIAHE
VRKGSTVRGQWFYATDEAAKNYVWFHSVYSLIERSLEAGDVACVDLGPSGSDAFSELKAR
YGFESVDDWTRVADYSGDFYYNGKPRRFSGLFL
>comp3285_c0_seq1; 569 65 1303 LEN=1539; translated
MVPSWILARWILSSILVVAHLGIALAWVPPSRPTIRTASTKCSALGGDNNGHEERESAQK
GRREILTEWVAGTAMAAAASTSVGLPAFAEGGDQPLPPVEVTPLSPDAKKLFNEGRALES
QGNMIAAQRLYGKVVKISPRFIYGWSNLGNTEVALGDLSSADDNYSRAIELCQESSAEAA
AQGFGIKKCNDLYVLLLNRGSVRLNTPGRKVEALQDLRQANNLRERPDAVILQNLARAEE
LNGFYTMADKNYNLAISMTANEVNPFWLRSAMVKLQLGDLQGGRDLLKRVENRFPDAPEV
KAASATFLWEMARPQQANPASALLGATSSPSNNNKNAPDDAPAAAKPSPEDYKVDAQRKF
LEIPDRQRLRFVDPKYLDDTIAWPPIMKEGVTSIARNVGDYKRVEAAAPVPQ
>comp3287_c0_seq1; 320 133 1485 LEN=1951; translated
MPSSTLYLPFKVPVRYDLVQPLANWLDNDDSHVAFHKENIGNSCAGVDAAPAMIVPKPAY
NSVQCRSELLRIAALRNCMSEALQDSHRTALEEHEMLRDCQDYHATLLEFEKRGFPTGGG
GDADEENGIDLTWRGAFSDDDDHGAQKAGCGGNGVPETHHTLLWDRACTLWNIAALRSAS
AALETDDKTKEGLKSAISSLQQAASHLSLCRQILDHSRENEYISTVDLSKAMLQFWEKLL
LAQAQTCIYKLANLGGSSVRNHTTLAYLIQGAAPLYNEALILAQDPRLQSELPTRCREWA
AHCKARSLVCQARAYFHMSIEGRLTKAHGLEIGRLNQTISALDSVLDFCRSTQVLKEAIT
LAGGGKSGKGSAAISPIAEAAATIQPEAEGLLRLASDRRKAAEEDNRTVYMEHIPKPRDL
QEIRAQTMVKTDLPLPETMVKPRTNLFGWE
>comp3288_c0_seq1; 414 131 772 LEN=953; translated
MAPPDTLEALGHLQDYADASEKAHDELKSCLWQLTKSRRNVRSGILGVDSANAYTAGLLR
EELRAQFRVFDAAARGDNDGIADLVNEEEGAAPAKDSGAASARSSAPEWKLCNVRLERKK
PKTDDEQSLDPNPSSSWMVVEEEDLTDGDDERRNEDLDEEERILQTDPLKLFGGYFTARE
LKVAQRNAQRALDGYIQAASDAARLLAMLPAEK
>comp3289_c0_seq1; 308 74 919 LEN=1186; translated
MVKEYELLSFVPSTVPASSHSATYSCTLTNQWSAATHPVLYSSISQNAHWSPPALVAHNE
AYNLWTPNELASPGVENVAETGSTSKLEKEIQKAQKKKKAGDLVVGNGQFNRNDPPQTFE
SITLTPYFPLFSAITMAAPSPDWFTGFYDVAPVDDGETGVVWYKMFEIATFPWDGGTEQG
STYSINNAPEDPHVPIQQLTADTVPDNGILLNPDGDEVLPMAKWTCELVDSSCSNSDNLR
FKNFKKRNCAWVAKRKRKRCAKVFRGIPLSSWCPETCGKCN
>comp3290_c0_seq1; 320 227 918 LEN=918; translated
MIRPSLFLMSLVILAWAAPETAAFFIPSASQRAPLPATSLSPSSSRLSAIYSPGRSASRG
GNKDERSKRQYRVGELVRTELSRIIHSGVIKGRDATYLDEELRTRISVVSVDVSPDLRQA

RVSVSIRNANPKQRQPSGSDEIMYFEDDPAVDKRRAFSWLVDNTKTLRHTLAQKMSHMKT
SSPDLTFHQVDVAAATDVMYLIDKVSKGYKRESIGEYEFLGDIDEEDDDDX
>comp3291_c0_seq1; 397 66 1979 LEN=2120; translated
MELTRMITPWLANTKTEGVTAMSAPTSGEAMNTYSKAKLLVTIFLFRVCYLSGMVVSSHF
LLEHNPGDDVLRFDMRLNDVNGCFCLQGQACDNGATDYTASDCAILSNHSSNGSQRYWEF
LLTPFTKWDAARFLHLATNPSMRDPPRYLLSPPSPSENPWESSEQAHAFLPMFPSILQHF
SLFLYHILPEQILPPTFESLVVLSGILFNNFFCLTVATLGLYHLTFLVISRNKTLNSGGG
QREQQYHDKGRHFVASVVCLVFGIWNPALVFFATNYSETFFATTSILGHLCIKLSKNQGN
IAMWFIGIGCWMVGSYTRSNGSLQSLWLLQDGLSHIILLFRHRNSVCHKGGTNVATNPRT
TNLSYLFRAIVIGIQSTMGAVLVTLPVRYHDLKGWDRHCVGAAVRPSWCSYGNENPSVLS
SSFSLYRYIQDKHWNVGFFRYYEWKQIPNFLLAAPIITLSTIGVYQWIHWSLVTDYGKGK
FPSSRGMLFVGWPLHALAESASAFDGQASSGTVAHGSRNDTTEPVFAPWSLLLENSSLLG
HYAILAILAFVGLFIAHVQISTRMICSASPAITWFIANILMSPPTNDAGTSVSKYNEKEP
KSGSYVDILQEKRRDIVLFYIALYLLLGSVLHVNFLPW
>comp3292_c0_seq1; 427 254 1432 LEN=1744; translated
MPVLRNVDVFVKARADIRTQSASGGLVTLLAASTAFLLFVCQVCVYVFPNATHTLHLSES
THFPMLPSGSGKTNGLSASERRAIDREGRIPLYFKITFLHLPCKHVDVALNGEPIRGDAH
SEWSSSGGSSSKSHGKNHNKPIDRSKLTIKKYPPRSSEIKTIFGSEQSHGARAHRGKGCT
LVGNLRVPLVAGSLTISMTRDSWAEAIQYFMVRSSLPEAEAARQDELHKNDSNTTHFIHE
VRFGSKHGSGGLVEHGYVPPLENKLHSIENDLNGVALQEVHVKLIPTVHSNPGLLEKVFG
GRRGRMYQMSVVDHTLKPETMVAGGVSTLLPGISLGYDVMPLAVHIDELNGEGGFFGFLA
TLVGIVGGCFVTVGLFAGCAVRSVQAVAKKID
>comp3293_c0_seq1; 85 1 722 LEN=973; minus strand; translated
XSFRFVSEAAPGGNREAASAVRPVVLARSDRSFRRVTAGESAGTTASASSKGDPDAKPLG
PDPPEGPPQGRCKEPPPQGPFRVHFGFAPRASGLFPPLRPLPPQPAALFPGLPPQVGPVP
LGTVPCASPAFGLQTQGTGGEAKGKRPASEGCGKGRRHRPPLRKGDRRQNGSGEDPGGAE
PPTHRPRGRKVSVHWNVGIGLDWIENAKDSADRTAPSIYGLSPREFLFLSLSLSAERVSV
W
>comp3294_c0_seq1; 234 359 853 LEN=881; minus strand; translated
MPTTTLTSGTVARLSAALSAVLLLTGAPIIPLASASASGAPFCERVAGGVPLSPPSTWLD
FGRATVEENDLAGALSGGSLRYGSIGTIRGRPVDLLVRTKDDAPDSVRSGDGAAGLVDPI
YEGSFGQIALETVRDEPGSGSGTFEFCFVEPGTTNEVVADFFQW
>comp3295_c0_seq1; 507 205 1449 LEN=1599; translated
MILTAAASAQRSRFARAITAEASHLCRSLSAFGAKLDAPETLAQSPSDSDRRCYSTSPPR
FKIHSFGGDDGKPLSEREMKEIIEAEEAKILAEREAKTYPNWKPGERKRPLIKTHSEEEF
MRELMPEKFLDNPIWTLRDKRCGALAIKVGMMPVWDEVWGVRHPTTVLWLDRNIVLGHKT
VEKHGYSAIRVAAGERKAKNVSKAVLGQYKEDPQLSESPPYLVREFRVTDDEHLLPLNST
IHARHFVPGQNVDVSGISKGKGFQGAMKRHGFRGMPASHGTSLSHRALGSTGQCQDPGKV
FKGKKMAGRMGTDRVTVQNLQILKIDRGRNLIYVKGHVPGQKGAFVEIRDAVKKPLWRTD
KVLGSLERPPLPTFEYDSEIDGTGECYEEFMPLGDEDPLDPDYMDTTIAIKAQA
>comp3296_c0_seq1; 597 314 1945 LEN=1945; translated
MGVFAGHRNVRRGQYLLEDDGDLVVPVYDMTRHVGHAKYHFLWEEYTWRAAGFGGMSEEV
DDVKKVKACSPGMGAAINCMLPLVNVVDDFDNRKVGLSGIEAVPGNNATAWSLSSPGAGA
FSPYYNRRWKAKRYIPAGMELYGNYGEGYFKSRSSIYGMIPFRENYKHADRLLRTFQRIT
RKVATEEVKKDLLISLQSMAAIWDESRNMNAIPQTDPKGDHAVVDWLLKKTGGTGMLHYN
SSIRDLDWLDEHGRCMDNIRDGVSTIPHAGRGAFARRKIRKGAIVAPAPLIQISDRDIFT
IYGKKPEAEDKKGKETYWLATDHDNPIHHQLLLNYCFGHGESTLLLCPYGLMTSHINHDA
VAPNTRVAWAADDQMAHPEWREKPIKKWARQDKAGLSFDFVALRDIEPGEEITIDYGDEW
EEAWQRHLRSYYREEETNGSESENVLRHHLPAFELNKLVDLKLQTLEEGDYELQGLHLFC
RKPYVEWYGASATVPGEDDDTLQVRTESGGTTTIQWNEHYDWDDEVVFPCRIRQRHTSSN
NNNS
>comp3297_c0_seq1; 356 140 1513 LEN=1616; translated
MRKMAGATIKGASRFLRGYRPAASLANSSSLCALQNISGYQISMAPFWSPSCSVSTQRFA
SSESASPPTRQRQPAPQTKPRQRKRLDVAIVGPPNAGKSQLVNVLTQSPIAAVSRKRHTT
RSDLLGARTIGDTQIVFKDTPGFLRIENAKEERLDRDLIATAASEMQDVDFTLLVLDSAR
KLSENYRHALVQLMIGAINSNGRIEDSDDLDDEEDSDRTPKTARNSKDNGSNSNSNVNSN
RYNKGKFAIVLNKVDLVKPKKDLISLAMDIGSMADVCLEDQFKKNGKPFDFEAQLEHSPI
VFYVSALKEDGTDDIMEHLLDLATPSQEWPVEPGRATNMTVKEQVQEVIREKIYRSTHRE
VPHAVQQVNRIFRRIPNKGVVIHQDLVVFTKSHHKLVMGSGGGRTLKRIQESAQKDLQNM
FGCDVALQLHVKFSKSKQRRAQSGAEYNLGEVSQSVL

>comp3298_c0_seq1; 261 1 753 LEN=1005; translated
XKVLNDQGKCFFLKGREVAKARQQDNGIYHPISLPAGIASDFFFNPSCPGFKLGTTTCNS
GIFSRSSIKASCAEDDKVEVSGTVTAPSAFEDAEVTFVPCIRSTGVCFDEYSQRGGNICD
LISASDGSDCGSAGTYTIDQEFDIPEEVTDHSWFMRLVTIRVLINDEEACTQNATTNASS
SAFMATGMASLFAVGSLSMYFMRRRQRPVLVLDENGYADRHLGRFVQMNDLSPSVSSIGL
RGGFSWSGMA
>comp3299_c0_seq1; 317 1 822 LEN=859; translated
GNGNGNNNKDAQGETGTTQNQVSRRLPPASSPTYSWGDSATDVAAVAWELAILETCLSHD
YVSTICGALEDSSTGASAGAELRALVSEVLGETAAQIRPLVERAWNEIVVGHLIDRCSGV
LGAVKGVVATYRMTNRPPPTQASPYVKTILRPLKEFADDLALRTPEHIGERWKIAVLASI
ADRYGTAVEELITTVQRTEVALQSRRTRKATSGGARISDGDKVKLQLFLDYQAFCGDARE
VLGQGGGDLVSVVPGIARLGELTKEGAAMLHPK
>comp3300_c0_seq1; 134 1 640 LEN=753; translated
LSELSNVLEGTETTIIASEIVDASSEKDTKEAREFASAVLTGTVIGRTLQDALRSLSEED
SDDHATDEYATKSDGTQARADGIDEDTEDGGDGDGVQDEKCAVRMDSQSMNRILKSFQEA
VVESRLCHKPDNGKAPRGLLKGRCDYFNRYGQNWMVLMDEVQIKARPQKFTKRRRDDRPS
LWDRDDDKTAKTTKKEIVVKEKMQLLAYGDIS
>comp3301_c0_seq1; 376 88 501 LEN=618; translated
MKFNANVSSSRRKSRKAHFGAHSEARRKLMSANLSKELQARHGVRSMPIRKDDEVLITRG
MYKTREGKVTAVYRKKWVVHVERISREKVNGATVPVGIPASALVITKLKLDKDRKAKLEA
KAGKKGEKKDETMANVD
>comp3303_c0_seq1; 265 688 1527 LEN=1702; translated
MQSVLLTILVLVLSVFYLDAFLPSERFRTRSQILRARQRGSLVSSPESRSAWPSGRSKHA
GNALAGSSVPNNDAGGEGAAAALSHKDIVWKLRPPAGTGRRRRLWLRFAANAIRLDCMVF
RKQLPTALCPKGGQALLEAHTHDENGRLVKIGRFGFTTERGAPAEPIQETIDDLYGVKQL
AGIGAIIYMFVEPEYRGRNVGTLALEVVRCIHAIQNIDFTVLVVDDDGSGKLNDWYRERG
YSEAPKLQNILGSPDAVHGISMIAPTSLTLDEDCRIKWW
>comp3304_c0_seq1; 698 86 1729 LEN=1847; translated
MPNDENEMQTEVWCFREDDIFVNVDDGNGDEDEDADEEEEGNENDATCHDIIVPRNFLCM
DALGLTNEIDDEVLSFLYDFDTTTGLLQDEEEVKEEEGIEQKEEKEQEREEEEEPTKALN
HDKQIISFDASSSSLVMKPERDASYTPIPETHTQRTQRRGGLRSRLRLLRLGRIGRKNSK
KESTTSAVVDCSCSAISSISGASASPNELNPTEEEEEPETTALEEKQQSINNSKKIECNA
THDDTDDDNSKHSPIPLVLSGSSFGGIFFDRKENIEYDFDDDATEIQSNVSSKNRCRSHT
TKSEQFSHFTYNEKSDTSYTDSGSYSDDTRDSASFSASNKSHSNRNLILSSANNAKNDNN
GELLRTTTKVNSFFRNGVWSKKSKSISFSLKSNISGRKSTSSEFKTEGNDDESSKASTAQ
ELWQARNEVRRIKQQKQKHTKYKRTITPTLVHAGNNSQQFNCDDDPITEASPLEPVVNTR
KEENPVDVAALLLNQHKVKIARWKSEQLMKRAVAKLTDSADEMIETVLLVGFDHEDSYAI
QLKSVPL
>comp3305_c0_seq1; 197 149 463 LEN=777; translated
MSSKPFSVIVEAEIQPDRMEDFLKMIQNNAECSRKEPGCIRFDVLRDQSQDNKFWFYEVY
ENKEAVDFHKTCAHYQSWADFKESGGTISSVSHKADGEFIGPKA
>comp3306_c0_seq1; 209 53 481 LEN=1010; translated
MSDPTTTTPASTTAGNSVTGTAATGQSVSSSTAAGTSTRIATAVGSGTTTVTVTDEEPQQ
VLRLTLVDRPTVTWDENVVDNEGMGRKSSKRCCIFHKEKAFGESSTDSSDYDSDGDKNQH
KRIAHKKHNGKKKTPDHLRFHA
>comp3307_c0_seq1; 484 329 2323 LEN=3126; translated
MSSNNIRRNPNGSRRFLYGDQDEDSFSAHSSRSRNTQRSAGFSTRDYIVDQSVNLMDIGI
GDSASSVSLPARSSSIAGMFRSSVETEILNRFDEYGDAAPMADAEEYIDNDKRRRGTAMW
RKCRKALCSMYTLTLLVSLTGIFLVVYTLVLTVNTKGREKTDSVTKADNKSGSGSSNKDA
NHERYGMIRSRIVDAGVTSEGLFDGGSKSPQKLALDWLVLEDPANLKDDHPALLDRYGLA
VFYYSSGNANKTTLVGGWKNSDNWMTGKGICSWHGVECGIREQEATAENKFTPFTKTYDA
DSQVKGIRLEDNNVEGTIPAELSGLMELEILNFDDNKLSHTLPSALGSLSKLKDLLIGGN
ALLGTLPAEYGELTEIHQLHLGKNHFEGPIPNSWLSKLTKLRYLSLERNKITGPFPDLSR
MTRMKGLFLHDNNFEGTLPEWLGRLTGLLDLRIGKNKFEGNIGLLASLQNLETLHINKNS
FTGSIPNMFDHLFRLHELVMNNNMFEGSIPLTLTHLQTLRTLNLGFNQLDGEIPAGLGLL
TDIIIISLDHNRFEGTIPTLLGKLDDITDLTLSHNQFSGPIPSELGRCFRLERLHVQHNT
LLGDIPPELGELIGLIDLRLESNQFGETTMPPQICALRESEELKVLSVDDNVSCECCTDI
PDAF
>comp3308_c0_seq1; 495 43 1326 LEN=2248; translated
MATYQNNHRHYSTDQHHHGTPEEHFSSSYCSSSISNSTHSHNCSQRHDNDDVVSRIPVRM

LNNRACVLVAMGCFAKANHLLEIALSKHKEKMASKLFHCPRASSIHAGPGRDTEYHPTNA
NPNNFTAFEDDIYGFSFDDDSEEDEEDDGHCMLIDDSWTVTNFTNDADNACPLTDATQTT
SLHPLSSPSSSLTYDEDTNIIPPASNQSWGIPRMTRNSQLRTKSYPGQDQRGAERSHKSK
TKYKYHQVYSLPIVMSQLEWDHASMEERSFVLIFNSAVSNQLWGMNITSRVCRQDQQYLP
TNTNPWDLFQASKTLYKLSLEYIQHNIRCVDKICIPAVFNNLSHLCKSLEGPASQEARCY
DELLLKSVYWLIDSLPSTTINSNNNNSSGIYRDNFYDGDNDEIIDAFLENSFYLICESPT
AVLAAAA
>comp3309_c0_seq1; 1081 1 1898 LEN=2032; translated
XQNENNNNNTTTIGSVVDVVPIKRALVSVSDKTGIVDFCTALQKNYGTELLSTGGTAKKL
RESGLEVKDVSEFTGGAECLDGRVKTLHPKIHGGLLAVRGNAQHDADMQAQGMSNIDMTI
LNLYPFEETMKRENVTFEQCVENIDIGGPSMMRSTAKNHVATTIVTNPNQYDALLKELAE
NNGGTTHKTRKLCAAKAFALSAKYDGMIASYFAEQIKKDESDPEAFTKDVVAPRVYVPEF
KLKYGCNPHQKPAAILSRMGQKLPFEVLNGTPGYINLLDAANAWQLVKELKEATGLAAAA
SFKHVSPAGAAVAVPLTDIECKAYEVTEETRASLTPVSLAYLRARNADPMCSFGDFSAVS
DVVDEQTALYLKKEVSDGIVAPGYTPEALAILKSKKGGKFIVLEATKDYDAGINEYREVY
GMTFCQKRNDTVLTSDHMSTVVTDSAKCAPVNDAAVRDLIVASICVKYTQSNSVGFSKDG
MMVGVGAGQQSRVDCVKLAGRKVQTWYLRQHPKVLALPFKEGVKRQARVNARVRYIEGDF
TDEEKIRWNEQFTEVPEALTDEEKQAFLANSTGIAISSDAFFPFRDSIDHASKLGVSYVA
QPGGSVQDGQVTEACNEYGIAMCHTGVRLFHH
>comp3309_c0_seq2; 1115 103 1989 LEN=2123; translated
MAHVLSAVEGSVVDVVPIKRALVSVSDKTGIVDFCTALQKNYGTELLSTGGTAKKLRESG
LEVKDVSEFTGGAECLDGRVKTLHPKIHGGLLAVRGNAQHDADMQAQGMSNIDMTILNLY
PFEETMKRENVTFEQCVENIDIGGPSMMRSTAKNHVATTIVTNPNQYDALLKELAENNGG
TTHKTRKLCAAKAFALSAKYDGMIASYFAEQIKKDESDPEAFTKDVVAPRVYVPEFKLKY
GCNPHQKPAAILSRMGQKLPFEVLNGTPGYINLLDAANAWQLVKELKEATGLAAAASFKH
VSPAGAAVAVPLTDIECKAYEVTEETRASLTPVSLAYLRARNADPMCSFGDFSAVSDVVD
EQTALYLKKEVSDGIVAPGYTPEALAILKSKKGGKFIVLEATKDYDAGINEYREVYGMTF
CQKRNDTVLTSDHMSTVVTDSAKCAPVNDAAVRDLIVASICVKYTQSNSVGFSKDGMMVG
VGAGQQSRVDCVKLAGRKVQTWYLRQHPKVLALPFKEGVKRQARVNARVRYIEGDFTDEE
KIRWNEQFTEVPEALTDEEKQAFLANSTGIAISSDAFFPFRDSIDHASKLGVSYVAQPGG
SVQDGQVTEACNEYGIAMCHTGVRLFHH
>comp3310_c0_seq1; 536 214 1203 LEN=1446; translated
MKLVSPDRNGSRRGFLNNAFFILLILLVALVSGQDEYSQADLEAMTEAELEDICIKRGFQ
LVNEDSDELTKQDYVEAAQRCLAIEQEMNELLTQYPELADELEEEIKRMERENAEKQAIV
DGLENQISDDAEKGRPAPGTAFHREAILEDEENNHAVGSGVGDDELEISEETLVDEVVVD
SMSGEETTLHDTTLTDEEKSSGSQTETNLDEAQDDDFVDTQTVTDATPDAGTQIEAPNNS
AGKEDLTMTNLAIESLRVLVKNAKDDVRRIINLAIPVLQPLLNAGDVAWQQMKSLFLRAR
EAYEAYQATNTAPIEGSEVADTCDDSIPA
>comp3311_c0_seq1; 660 229 1911 LEN=2345; translated
MSATASSSKSAAEQITKKNSKTLSTLLQSNPKNTDWKRIKAILFTYHPEKKNAPNLEGLL
HQALNRACLDPSGVSSPTFDLILSKTRELSLSERVRYTSTAIRCRNRKAVRALIHDDPSV
LFYCTNEEDDSSMITADNANGTTLLHIACEEYGWNDEIAFVLAEILSNNKHHERDGRCYF
HNGLFQEMAESEMPLKLSLQAGSDLEKIICHLKEQHPSYLERNLDCLSQIIAEFCYDMTL
LYDLADAYGIRLLTSVHPKDGSSPLGFACYYQNEEMVRVLLELYHNLNNRTENRESCNNN
NRNSLLQVQKSLLSPNNEGMSPLGHLLLSVGDPDAENAWKCVNTCVRFFTDNDNITNFDV
NDDCNNGGNGISRLQQQRFPVLHLFLLHTWDALIAKKNCMKILDRIIQRFRIDFCVVDDE
TGNTVLSIVVEKLSICSPAYHGKKKRPHAMAMKILDYLTTGHPVVPSDVASNHHRPATTR
DGRGRLPLHSACENSLPWKTGLRSIVDANVPALESYDPLTRLPAFAHHAVGPKSDLDSIY
ELLRLHPGIIDTVIPLANSN
>comp3312_c0_seq1; 288 79 796 LEN=920; translated
MANAAAKKAAAGKLHSSLWRAGFYFLIELCLSKLLNATYFYLLMPENRIISGDLFSALPF
CNFNLLGTVSKRIAKQDAAKVYMPLIVIVNIIYALLLLGTSDSIPYDGTKWGIFGMIVTW
AMQAYAYIGILESSAIAAAKIKSNKDLAGGANLDLLAVTVFVQFSSVLHSTRWFAVTIFG
VPIIAGYKLYRTIYGCGGGGGGGAGTKSQKLSKSENSSDDPAAAKRQRRAEKRRQKWG
>comp3312_c0_seq2; 309 79 609 LEN=733; translated
MANAAAKKAAAAKQDAAKVYMPLIVIVNIIYALLLLGTSDSIPYDGTKWGIFGMIVTWAM
QAYAYIGILESSAIAAAKIKSNKDLAGGANLDLLAVTVFVQFSSVLHSTRWFAVTIFGVP
IIAGYKLYRTIYGCGGGGGGGAGTKSQKLSKSENSSDDPAAAKRQRRAEKRRQKWG
>comp3313_c0_seq1; 505 149 1186 LEN=1308; translated
MSDDDQKKIESTEFIFLVAKSGTGKSFSGDYVDLFGDWKHVDGDTPLKNTHGNPHYENLK

NRAFQLEEYEGKDMEWLVTEGLVPYMEELAKLGLEAAKESDKVCISWASYFRPNRLLLKK
LLLDAGAKDFSMVFLHCDLEAHMDALWKRTCRQAEQAGCTITEFAALFKLEVEGEEYTKE
QFVAFQKSSDWYKMWQDPDESEQPFQIVNVTAKDATVLDSLDAAFGIEDSNRAHLSYDEI
VAKVRAIDEERDQKNYDAYVETLEGSEESKEELEFAQKEPELCKARRSSLLLADKMNSMR
SLSSVEGEEERQSLAIEALSRRSRKSFITTGKLFTSEELKEIDEE
>comp3314_c0_seq1; 327 60 1262 LEN=1497; translated
MVEGTAVNHSYDGGNEDYDYIGRGGFLIECGDNQEVLGITKEQAELVKLNCPFFQKCLHP
GADSNGEDDSDSGGSMMMKEAKSRIIHKPDWSLAIVRHFIEILTKGKTTLSNLELVDGVL
TAGDQSLADLRLSSLVNYIDPCSKENEFMRLVDPSFFRFYFQAVVTSDQWLALLDRGILL
YREETNFVVQLYKDRAFDRDQSVSRRNLDNQRSDFLVHAQRPIEAMFEIQKCLSASGERN
RSARCIDNVTAEAFSIYFETTESIPKEHHQLIDRLAGGEAYIRTCADAAEYQTEGYTVKA
SLDVLSRAIEPISEESVIHCSIRVDNPTPDTLGRFINACQKVNDYPGTLGIDAPINRYFC
RKSMGDIHIVLRYMNDYSTSSKVSEDFRLFSRSSGSQSF
>comp3315_c0_seq1; 593 371 1780 LEN=2063; translated
MKPSNQALRQFFSLGSLAIASASMPPTDAADADGRANDNANANANARAHVSEQLRLQDPI
HGVEVVGNANAHSIADDANQEHKYSNKTDDNTLNDTSKIILDSGYDDSSPNELTNAKDAS
IVHKNIHIKTEKTPKRCLPDLGLLACGPGESCVSGTNDTQQGGDDWHCLPAPAVHGGRSL
QGRGAMTPSRFNTLPTTRDNQEIYTHCAANFVLDENSSPPTDPAAYDVCICKGYDFQLPD
AEYAGYGYCDDSIVSNYCQKYFGSEDAVDVMKYDVCLCTLSPDGQHAGKEPNFCETVHVL
YCAALQADATGSILNCECSLFNVNCPAPTASPTPEVTSSTTSLSTGAPIIGGSMAPTMVD
TTETTVTTTEIISKIPTAVPVPVTAAPVPVPVTAVPVPVAPVVASAPTPDDEVYTASNVR
VPTVTLTPPKEPGHSPATSASSSSFRAGCLLVRSATAVAVLAVGSLFLP
>comp3316_c0_seq1; 102 468 770 LEN=897; translated
MIMRKFHNSMAVGFFMGGVVAMSQMFFFLMLIYIGYLKDQRNDGLPVVTETVMAFFCLLQ
CVLLGTFAAILGAHRSEILESQDEHAPGQDSFGATPYTRS
>comp3316_c0_seq2; 102 653 955 LEN=1082; translated
MIMRKFHNSMAVGFFMGGVVAMSQMFFFLMLIYIGYLKDQRNDGLPVVTETVMAFFCLLQ
CVLLGTFAAILGAHRSEILESQDEHAPGQDSFGATPYTRS
>comp3317_c0_seq1; 242 195 1274 LEN=1730; translated
MFGKLGKQFAAQVADKVEELALDALNERNPGGRGQTQSAKTTEKLHKISGNRKALFIGIN
YIGQKGELRGCINDVKNIKKFFREHYELDDVMVLTDDKRAEPETCFTPTRKNILSAFRWL
VKGAKPGDSLLLHYAGHGGKVKNLDGTEASGYDQTLIPVDFQSAGNIIDDDVHDVLCRKL
PKGVRLTAIFDCCHSESIMDLPYVYNVNGNLDIIENDKNQAVATLVAAGTRFLLDGNKKA
IGSVLKREITNLVQAGGRGGGDSSARARAAEENETSADVIMFSGCKDDQTSADAVIKGKA
SGAMSYALVHTLKKYHLKRGELTYTQLLQDMRTILEGKYTQVPQLSAGRKLVLNQPFAI
>comp3317_c0_seq2; 299 195 1694 LEN=2150; translated
MFGKLGKQFAAQVADKVEELALDALNERNPGGRGQTQSAKTTEKLHKISGNRKALFIGIN
YIGQKGELGECFVLFVASERLLFRWNGWIVPDCDNFDLSMNWLIHLSPCPGSHLDAMRLD
ATQGMHVEGTTGFRGFVPGTSVHIHCSTPLAKRFKSFSCRSEIDSVHLLIAIASDSHPTV
IAMDSLPFASTQNNPSQPVPFCVQMPRSVAASTTXKNIKKFFREHYELDDVMVLTDDKRA
EPETCFTPTRKNILSAFRWLVKGAKPGDSLLLHYAGHGGKVKNLDGTEASGYDQTLIPVD
FQSAGNIIDDDVHDVLCRKLPKGVRLTAIFDCCHSESIMDLPYVYNVNGNLDIIENDKNQ
AVATLVAAGTRFLLDGNKKAIGSVLKREITNLVQAGGRGGGDSSARARAAEENETSADVI
MFSGCKDDQTSADAVIKGKASGAMSYALVHTLKKYHLKRGELTYTQLLQDMRTILEGKYT
QVPQLSAGRKLVLNQPFAI
>comp3319_c0_seq1; 266 194 994 LEN=1219; translated
MALHLYDQCDNREGRNPSDWYPHSIDRRSSLVSPTSTLTTSFRTATSEREREPSSSRIPA
TATATKTNEHRFGSVSPTPSSCCSTSLSSHSSSIATRSETLSHSYGSEEFAMFNLTNRRS
SPKGKAYRHYFARHRDERSESSTDPRPHAVPRPDGTGFDLEIPPPKHLTNAGGSDYNDDV
HPGKRRMKKQRKTKTAAGTIGGMVVGGVALGPIGVLVGALWGGVTAREVARKGEKRAQRR
HEQKSFRRSAFRKGTHWEESEVAVYV
>comp3320_c0_seq1; 497 1 955 LEN=1076; translated
XNNSNNNSFKAGLNANSKKKLYPSIDEKLMGNAAANANNASANQHQQQLKSQPKQEHGLS
TAETPMANNTSAAKNAKNNESSDRFDPVYVSKGSDMEKDTFAPGSVDDQSIGQTVASSTY
GDDRIKVVNKKLLDPYGDKGTYTGVVLRNTGMPHGLGRMIYEEDRRVFEGDWRHGRWHGY
GRASFSNGDSYEGEYKFDQRHGTGVYRWNDGRIYDGQFSEDKRHGKGVFTWPDGAIYDGD
FVNGQRQGHGKYTFADGGQYEGSWKDGRYDGFGTCTWEDGRRYRGEWRNGMAHGRGTETY
PNGNVRHEGEWIDDEPIR
>comp3321_c0_seq1; 239 118 672 LEN=867; translated
MAFILQEALRWPASSFFTEAYKVGDYVDASLSTRSALTIDLLMANQPLFGVPRTVHLPRL

PERFSLSFEEGFHSLPYVEGQTLEQLVVTFVYSKSGDGRIHSVASKAIRLPKNQAFDKKE
DIQVVFDWIEEASLDLEAGAAIMFLAVFVASILFLLQLCTMDNGGEHDTRRKGQDHIDQY
YKGR
>comp3322_c0_seq1; 605 114 1067 LEN=1401; translated
MEETLIPLNDNSAKAAKSPSRSNSFGSDKDISLHEDGEDNTLDYRIHAKHGQNAKTLSLW
HDISLSHIDADTGKQTEYYNFVCEIPKFTRKKYEIATDEEFTPIKQDEKKGKLREFKKGD
IFFNYGCFPQTWEDPTFVHPDAEGCRGDNDPLDVCEIGARIVKCGGIRPVKILGILMMID
EGEADWKLITIDKDDKWAPFMNDINDVDEQMPGMLDAIREWYRTYKIPDGKPPNTFGLDE
KFMNKAYALEIMAECNHAWKELISGEKVRKMGDNNGHTEEVKKLVRKLSTNSLAALGTNL
DEHDFPDTGDDDDAPAF
>comp3323_c0_seq1; 141 91 408 LEN=1013; translated
MVKVWYEPPPGGPRTHLNRGGYYVESARTPAIITKMSKARPITVILPAIAAISAMCYVPL
MGQKGLPSTLNPEHVAAQRAYMRYHNMNPIFGVSSKRARSADPDH
>comp3324_c0_seq1; 170 212 643 LEN=1823; translated
MATKKLFHYPDTPTTVTTAASLEERFDRPNVYGNIKTIAMHHRLDNTVDFTSKSTATSNS
ILEEQASVPSLPSRNDNRDDDNNVENANANANARSFRKSQAALQKSYQKRREAYRKLPLS
VRTNTGINLKNLHPLAVRNGEFC
>comp3325_c0_seq1; 374 108 1037 LEN=1579; translated
MAYRSFLRLAIFCVASLHLHQYCVTRAEDPLQQQQRRRRRQQQQQEEEEEERHASQHSPS
DAATAAKTTKFHNRKIKVDLYYMPQCPGCRQLITSSFNEAFHTPGFSNMADVTFIPYSDG
RIDTGSDSSTKRIFSHTLESCALHFIGIGGADGFETNGTNGLTMHKKHEHQELQFRYIDC
IDHYPSYQYSAATVDVECAKAIGLSEATVTRIESCASSQEGHRLAERYVKQVHSITNPRI
TYFPWTMVDNVHDKDTEERIWNSLFEHVCSIYSGPLRSLHCQAPGDMNGTDDEDDYVYGK
DDDEDEEEL
>comp3326_c0_seq1; 638 1 1880 LEN=2884; translated
XEVQYFVPTVIGTPDSTEAQTKEVGDHYSTVLSFRSPDSLLLYRREQKDDGESHNGEGLG
ASLQAQAFAPCDGAAAAXAASGREPFVPGPQPAGVLGGGPSRLPSPSRAGVALCVLFPAL
RKGDPRPPAPGGPPRLLHLGSARGPPFQHGVAAGLEQGQSKEDGNNNDERTDDEKAAAAA
GPRTTDLLVVAGDISHDPGKIEEALLLLRNHTEHVVFVPGNHEAWLSSSDELKAYDEHQT
EGAEKDAGNPCCGETQTQPKPARYSSIEKLEDIERLCRRLGVFTTGDCVCVGGGALESED
GKLLPAEELWILPMDGWYDGSLSFVDPNPSSGSNGNGTSSSNHHYDLVSDFGRWPWVDFL
RCRWPGLESVGENDENEPPPPPLLRKIPRGLVGHFVKRNQGTIDAFKRATTERESFKRGS
KPPPTKSAFASVLTVSHFLPNRLCLPDWKDLSSADFDFDSWLDHGAGGLSAKFALVAGSD
KLDQQIRSIPLPQYNDDENSEDDSDANDPSSSPRRIHVFGHSHRPKDFVYGGIRYVHNPL
GKPRERELHMVDPEAAPKLVWEAIPSEGEGEGEGSGFSSKRAPQGCHTASSTTTIIRYWE
EKGGGLEMLRKRMEGSKRRRRKGPKN
>comp3327_c0_seq1; 407 45 872 LEN=1106; translated
MTETETPTTTSTTKTVALLGATGKVGGWVLEACLDKGYNVVALARSKGRFDGFLHEHKDT
NLTVVEGDATTVVGVRALLDANAPDVVVSTLGSPSNEILVMKKAAEALVGALQEIESEGD
DFRKIPRIVWMTSTGINKATDQAKSYPLFGAPSRWLFGYGAFGWLQFHLLIPYVIGQELW
DDMGHSEEVIRSCGSESIASRTVLVRPGNMWPVSEHPAFSEAWRMEGGDASGYAWVGACD
PPPGKWITRRAIAAALLELLEDDSKDGAAWSLFQS
>comp3328_c0_seq1; 199 1 1117 LEN=1287; translated
KKKKLKENNALALNGVGNDDGKKVKSGIPSLCQALWVIYLFFHLRCYCDGLQPQLTSAVL
DENKSSSPLTGFVVDGFDATACTTNRRGVLRALFGATFSATSTTLRPSFSWASASAPNSN
APPSPFVYSDSWTGTRLEIKTLSEATTDGAYLINRSSGGGSVTYQWPMGKWPDPALRIPA
TPVPPAMWLEGSAEERRLRLAAAILRDTARAEGAVGLAAQQCGVDARMVYLSSSGRVGSG
DLVLINPRITARSPEPSMRVWTEECLVLPPSFRATVLRDDWIDVEYDRSYEYRAPAKSAT
KHDREPSVRSLSSATARFFGEQSRCLQHELDHDRGILITDHVGLDELENETMRTIEAEGH
DRRQAIAYARE
>comp3329_c0_seq1; 112 1 293 LEN=934; translated
XDESPSGVPSSLDNPTAAPTPLDCPPDKSDPPSEAPIVPAPCKDNAEGSGGRRKGRCEDA
NGLLKEKNATLYTRANLCGKSAESLVGDVKRQIPGTT
>comp3330_c0_seq1; 761 1 2137 LEN=2232; translated
GREEKPVASASENDAAAPESPGRDENGDTQDTPASDEDVRMDEGSGGGGKEKDPPEAGKK
RSRSPTETKSGSNEGSSEPSQTNKRQAILGVPGVKKATCPSVDAGQAPNEKISVASIAAS
SQLSSSMGWNGLAGLLSFSLLTNSGNSVVTASSSSGERTRGGASKTSSAALAGSAASGSG
AGNHGSLPSKEQVLQAAIKAVNDAPPFVHLSKTDSAPQLKITDSEVPPAEGTGDRDESGS
ASDSAAAASSSSAQTAGARTSSNSNSNSNSNSNPSPPPAPAHLTNDRLACKQGEGELRGY
RMTRGTHGVAPGSGHYYYEVVVLEPPTACELVNSLPPNVRLGKKLQREMQEALKAEEDAE

HEAGAANGGGASDAGTTGSKQAFGAHVRLGWSMRTGDLQAPVGYDKWSYGLRDIQGSKIH
CSRREDNWGGEEFGPGDVIGCAISMVSEGPAASQDESSGSAGSGTKSEPQDSQPHPGKGS
ENHIRFFKNGYPMGEFVITKGKREGGVAFVIPDGVYYPAISLYMGATVKVNFGPHFIHPP
RKLPTVLSPKYSRKKQGGSNAFQPVSSLSKPPLTVEEASAKVAKEQKTLFFRKADNSAQQ
RRFSELVETEVNILQEAYKNHRRKHVLDVMRERQKRNLKCDDLEQDDFFVSEDGSAGGVV
DSAAEGGSVSSWATPTTPGMGFVAAQKTKGCGEDVLGGGARMRMRMVRFHH
>comp3331_c0_seq1; 236 310 627 LEN=1038; translated
MTAIVVQLPGCEKSYSDAAKAKRMALSATSSGSDGAASTTASEGGRSDCDPPPQEGVAGR
RRKRDEHHTAAIAAQQEWREEEEARNNSGGQSPLQGARASLPPPL
>comp3331_c0_seq2; 692 1 1241 LEN=1652; translated
XDDDDDDDANQRKDRGKQQHSNTDQHHGTNSNTGSSSSAMAGAAMKAASDHVSAIEDEGD
SGTTSCVVLITPEAIVCANAGDSRAVFARRPPEPVVQKTTEGEGNHDNNPSAVSILGKAI
PLSFDHKPDDEEEERRVRNAGGYVANGRVEGDLAVSRGLGDFRFKQMAVVLNNTQLYPGD
DEEDGRFSSSQNNPGKHKDTVMGTKTDDDDEGHDRGVCRSSSSGAPPPLIFPPGDQKVSP
IPDVTVHARNAERDEYVVVACDGIWDVLSNQQCIAELDAIMYDEGESDLGLVSEEILDTC
LRYGSKDNMTAIVVQLPGCEKSYSDAAKAKRMALSATSSGSDGAASTTASEGGRSDCDPP
PQEGVAGRRRKRDEHHTAAIAAQQEWREEEEARNNSGGQSPLQGARASLPPPL
>comp3332_c0_seq1; 822 70 2271 LEN=2429; translated
MAQPISSPVSPVFLPLQILLIGIDLLISLLTFGWINVLTTLTKPKPLRTVPVADDFSHRV
LPENKGNLTTVPSNGATTLYECFNTTLESHGSSLCMRSRKFKGWKSKKVKEFSTEITELT
YSQVGEKAYKFGAALAATGCVASDPEASLEKVTKPCRIAIFENTCAEWMIAALGAFSQSV
GVATVYATLGIDSVAEAVVDNSISAIVCNKTNVAYLVNNAKNMPCLKTIIYTNDLVGETM
EVDMPTDTPAGLTVIAFDDFVETGDTSKYPVVPPAPESAAVIMYTSGSTGKPKGVVVKHS
SIVAGVAAGESLINLDENVKYLGYLPLAHIFELMYEFCCLSSGASISYADPKSLTATGAY
PTGALETFAPTHMVAVPKIWDTIKKAIVAKVALSSPVAQALVNTALMWRAFAITIGLDTP
LFNVLVFKKFKKFVGGNIQHALSGGGPLNGDVQTFINVAFGFPLVQGYGLTETCAGLTIQ
HIDDMRTGIAGTPIPCVEVRLDSTPDICDKAGVAYLKTDRKDVDGNEVWGRGEIMVKGKN
VTTGYYMMPEKTNEVYIDGWFATGDIGQFMEDGSIRIVDRKKNLVKLKSGEYVALEKMEM
VYGNSEFVDAVTGGICCYGDGDMDRCVALFQIAKPASMQWAQKTGLVGPTEQEGDWEKVK
NSKELYNHVMECFKKEHASSDLSHIEKLIAIAMLDSPWTPENGCLTAANKLQRRKVIEDF
ATEFEEVKKKGIF
>comp3334_c0_seq1; 232 268 614 LEN=614; translated
MNDGGGLMPLPSQPPNHPGASIPHQNQAVYGQYALEQQPQQRAQQYSQQTQQPRQVLQQN
HQQVQQTILGSEGVVMREQQQITQGAPLANSPNPNANLNGAIANPGHFQQQQQQQX
>comp3335_c0_seq1; 173 851 1012 LEN=1274; translated
MNVTNTVKRKEVNAFRASKKRSATSAKFDAHHGVITVLFAIDVFCNSIITVCG
>comp3336_c0_seq1; 214 310 546 LEN=878; translated
MAHATMDFSAYFSPSKSLLRLLAVIGRVFAISADYVIDHTIRPEEMVIQLILMSIAIKEL
VIDKELVVETPANSKHIE
>comp3337_c0_seq1; 436 591 2375 LEN=2611; translated
MSLSTHTDDALGTEGSTQRFHGIVERDKKKVPVASATDLAIKILLLVPVWMVLVLPLTIF
YQVGKSVLNGIGDKQEPEQAAAAAAAQSMKATTASGNEPEDTSQSLPTDFVPRPDRSYDV
VVLGATGFTGGLAVRHLADTYGVGKTVKWAVAGRSASKLDAVLESLADDPAVKNFDASDA
ERVDRIVCDTSDLASLSAMVRNTRSVATTAGPFSLYGGPLVERCARYGTHYADITGETSF
VKKMKAEHQDMAIATGARILSLCGNDCVPWDLSYNILADEFAERCTNNNRNPNVNDNETM
VRIEFGNEFSSSASGGTLKTMCLGISGKLADPSELDPAASTLLRRSFGSSTEHTSPMENN
ISFGIKTGVSKPWEMVVAAEDDRRGGVTAAATDGTDGKGSKLVEAPFVMSGVNYESVGWS
HALRRDKKCSYKEQLLLPDFITALDGVLGLLLFFTSLMNPITGRLLEDYLVTQPGDGPAM
EQMNNDFFLALTGTAYGSKGSVYQSLLYYDKDPGYLETARMVVECALCLALEEEAVSENI
AGATLLAKNVDAKPLPEVVGGFFTPGFALRKILLRRLVDTGCHYEFRVLNDASK
>comp3338_c0_seq1; 208 1 862 LEN=1271; translated
XSNHAIGYGDNDFTADYNRSRFRSAAKYARAVLALASAFVLDLTSAGNTTLVSVAARVSL
TLFAYAVVYTSASRMLLMAIAYLMNHGWYLLAASCALILLLGCGILLAVCEWMWRWQDRA
LSVLDRNNIGYDRLRSVNVLDDDEMEDGPSLRAWVRRIGSAVFACLVWSLFCALSANADA
WIADAYKGARPSAPSIELRLVGCLEGLFLLAGAALLLYVACPSFYRLHLHLSRPGALGTA
AVPLAHDERGLGFGGGLDFDFDDNDNYTITVREDEVTVGRGIQNLIL
>comp3339_c0_seq1; 338 554 1878 LEN=2199; translated
MAPLASASPKKGDGANGANGNKKTRHSPSVASKKEFGEKISIHKPDFQSMEKSSIRRVTL
AIAGDNESDAEHEHDNSRRKGQHFPPSMLIPVLPSHVPTPQTIGTNDEPWAKYKVEDAFH
HIQTIFSRGGGNSEGNTSDGGNANGSNTISHDGPIKYAPSPITPPQSPVSRKATVTLDLT

PTATTTTRWGADFWNSHKKKNTNSPTSTMTNSLDVSQSPSSDKHSKNSAGGNEGNTTTND
NSSNYKNHPNKESIPAENNKIFDQETDDNHFDWAKQDAFLREVMARKRGRRPPSCSNGNK
QQPPARSEEGVVATNTALSQPSKRPHSPPGSNTVGTIGGSRXEKDRGLNENEGGQFEAAA
VIGSALFQPQAATDPPAAPVDARKRSQSTRSLPKKLVRDIEATESNRVRFEESSSQRPGR
FRFREICARGSDLDPRDCDNR
>comp3340_c0_seq1; 489 203 1069 LEN=1457; translated
MTDFAPIVPVGNDILDIGSRKARRQRNADPMAPSAAMLMGAPAQFDVPNVSNPAYYCRNN
FASVCGTGDENQDVKFMKGTTTLAFKFQGGVIVSVDSRSTMGAYISSGTVKKVIEINPFL
LGTMAGGAADCSFWERNLGMECRLYQLRNGRRISVASASKLLANVMRSYRGYGLSMGTMI
AGWDEKGPQLYYVDDDGTRLKGKMFSVGSGSTYAYGVLDNYYREDLTVEEAIDLGKRAIY
HAAHRDAASGGINNLYHMTKDGFVKIHSIDVNDMHYEFAEERANAMSS
>comp3343_c0_seq1; 279 207 806 LEN=914; translated
MTIFDDVADVLEDALSKCTDEPEEKPKIKPEFIKHFPTSAMTNGIEQFYVRLCATISGCM
YNKLSNISQFNNLDQGYNLSDVGLTEPPKVLLYDNHGMFEVTNPPFSVTVAGKKMILAWR
GSTTAMDWDRDFGFYVASSFRWKNVADVVSVQGAYLALVESCMSKHQDFIIEKIKEHGVT
EIMLTGHSLAGKFNHTPFA
>comp3343_c0_seq2; 420 207 1481 LEN=1680; translated
MTIFDDVADVLEDALSKCTDEPEEKPKIKPEFIKHFPTSAMTNGIEQFYVRLCATISGCM
YNKLSNISQFNNLDQGYNLSDVGLTEPPKVLLYDNHGMFEVTNPPFSVTVAGKKMILAWR
GSTTAMDWDRDFGFYVASSFRWKNVADVVSVQGAYLALVESCMSKHQDFIIEKIKEHGVT
EIMLTGHSLAGGVAQVAHLWFSGTMDPAIYPEPNPWRELKDEVGLTVRTMSFEGPMTTVF
IKSGDETLNKKGIDFINKCSANMCTTCYHMDAVPRMYGYNDFGLKVLENFKDDIGNSDGW
IEGSIEKKIVEYLWHKFKASSIKKSIEQSLKDEVNAASQFQHLGHVLYYESQEAEPKVCI
DDPNGILTTSDGKTSTLPQFRDIKYEPVNKGVLQEMYYDHMYIIFGPGLTLNWQPYFEHK
LIIH
>comp3344_c0_seq1; 318 174 1730 LEN=1780; translated
MPEPDNVEPSVTASTPLFRAMAMPNRGPQLMMPTEIATAKYVATPQNFSDEIMPRLPEQK
QERKKQPSELNQQQKQKQQFVLDTESQTYYGWNMSQIQVQKFPMYFPRDMIQWEFPTRDL
PVVLERITKELRESSIQASLIHEPLSAKLQTCQENAEIYLVFFTDRNDTVFMSLQRHKGD
HMVANRWIRHLVDAAKGVLSGNDSAGTKPVTASDVLALERLIERCAAERAVASINNQDLS
PEKHHQHPFLSQTAEQMTESAVRDVHACLSQPRRLDLRRLALEHLLAMTDLKRTLRTTAI
ATSLIVLQGKLPSSSFEMNSQARTIQSILLNVLLTRELPGDRAMLAEAARVDKKNISSTS
DDMDLEMRPYFPEVDEDVTAAAVDLPQYYSEFMNELFNLALRILVQSLEVAACFPESFSS
TGSNMATQLLTVTSQDVGGKDLYHTLLVCVGHAESKLANGYLACKALRLLASDHPGIKDQ
IKLDDNAKQSIGNAYQVGQMRHTLLKEESYHLWHSVSQ
>comp3345_c0_seq1; 296 460 1107 LEN=1823; translated
MIMNSVMFSVFDTVEAKVNNPFVSGIMSGFATAIISTPTDYIKIQMQLSSGQRRSTMISI
LEQEKNVGQYTNVVNKVRKLYRGFTLNLAREGIFTMVYLGIYHWVITNAKNNSEEFGDEL
KSFNRVLIVAGTSAITGAMAWVASYPFDVAKTMIQSGNTFEQVRELWRRHAVNGRLRGIS
AFYKGCLPSTGRAMLVTSSRMIAYDLVIRCFAKTT
>comp3346_c0_seq1; 274 234 1439 LEN=1537; translated
MRTLVLHAHHRRENLPRLSSGLALFPSLLLVLTLLSFSEANPWSASSSLERTTRPTSHSQ
TPPQRRLWRRSLPLTLHVINLDRDTERWETVVTELVAKGVPRKRIRRVSAVSGRDLSPGD
LRANTTRVARTVCTPGTIGCFLSHRKVWQETEDGPEPYRVVLEDDVVVAPGFLEKVRTVV
REIDEACEETRDGNWDVVFLGALGCVHPEGRHGLNRIAAFFAGGGRVPRRTLEGAPHCHV
PRRPLGAHAFVLSKRGAGKLLKGCSRASGHVDVVAWGMPDLGVVSVHPMLAHQNNTGSPS
TIGAVTGGIETRIPNLVVDPYTGLVLEWIYNAPVLSLGPVLLTMGRSVLFVLGGYLASAL
LYNKCPWLIVLHSIVVAILVALTKATTLRHGRGDETSGVEA
>comp3348_c0_seq1; 331 1 1004 LEN=1004; translated
GFGIGPSISVAIAMSAVPEEREATPLHIKTVRNYDTSPNDTNRHHDCGHYEILQKLITSS
PAGGYETESAEIESCLEQFVGRDGLVLWRRVVVRSGPSCPKQIVHASYMLPQDSSYAPPF
PDGTAATTDGVSRPSLLCWTAFPDRPDRPLLCVLANPTLLCIWDVYPSNPETEKEEQSQA
NPSQTEDLEGGEGQNIPLPFEACVIYGVGNGEHGLLLQRCESTEDIIAYHNYQQSINIAD
SMNDLEDQNNDDDGFILKAPPRPKRLRDSTGGGSTAMASLNVSTASTPTMMGGVGEGAAP
NASLSSPLMIPSLFSLHHPQGDILPVSTSTAAAAA
>comp3349_c0_seq1; 483 51 1406 LEN=1582; translated
MASSDTETNHTHEMHPGSEPQSCSGSSGLNINDFIKTHYGAISGMSSNGETAQEDTVTLS
LLAANAKDHARALGVACSETASLNSGYFSENDSNSGDRNKQQWTLRRLALELSLWLNVFI
TLTKAVAYFQTLSLSVLAALLDSVLDVVSQMVLNYTEKHSSLQRSSALYPAGASRLEPIG
VLTCASLMGMASFEVLKESFETLYTKDSSLQDLDWTEGVRSAASMGAIVVLKLLLLWLCN

YSSRTSCRNRQGGGAATSKSLRYSDPTLEAVAQDHWNDALSNGVSAVCLLLALYTSDLWW
CDGVGAILISFYIIYSWYETGKEQIEHLTGKSAPDDFLEELTTLAQHFDDRLLVDTCRAY
HFGPKFLVELEVVMPMDTPLKESHDLGMELQYEIEARGEVERCFVHIDYESREYDEHVVS
KVPELREHLLRHRSSRNNARGTALQTSASSV
>comp3351_c0_seq1; 89 1 236 LEN=236; translated
XKFRWILKYDTERVMLLNTIFIFSLVIAVVQSFSSNNSELCRNNRTESKLHETMSSLDLK
YKQDGEEAKEIAKYLDRKSX
>comp3351_c0_seq2; 283 1 817 LEN=993; translated
XKFRWILKYDTERVMLLNTIFIFSLVIAVVQSFSSNNSELCRNNRTESKLHETMSSLDLK
YKQDGEEAKEIAKYLPYFPFKGIDRFYDIGGFLYEPEVFQKIVDIFVERYQAIGIDSVAG
LDARGFILGPPIALALKKPFIMMRKKGKMPNTIESEEYKTEYGNRSGLTVQKGKIKKGDR
VLVIDDLVATGGTLSSAISLVKQCEGIVVECACVVELKMFIDPPEESGLPSRTKSFQKLG
HQDVPVWGLVSEDLLLNEAELPDGYVDDGEEH
>comp3354_c0_seq1; 238 405 1021 LEN=1021; translated
MQTPQAPPPQMAPAPQAAPQQPMMPAAPVAAPIPMQHTQYAPQQQNNVQGTTFQPQQQQQ
QRYVGAPGTQVGAMPQQQQPVTQQVAAPVAQPQRQQTTQIYPHQGLNGGWQSDQDYNERR
KMIAKIVHLLKLRKPNAPPDWLKKLPQMAKRLEESLYRSAKSFEEYNDASTLKQRLQQLA
HNIGMKTRKIQQAQMQARAQQQQQQX
>comp3354_c0_seq2; 238 482 1098 LEN=1098; translated
MQTPQAPPPQMAPAPQAAPQQPMMPAAPVAAPIPMQHTQYAPQQQNNVQGTTFQPQQQQQ
QRYVGAPGTQVGAMPQQQQPVTQQVAAPVAQPQRQQTTQIYPHQGLNGGWQSDQDYNERR
KMIAKIVHLLKLRKPNAPPDWLKKLPQMAKRLEESLYRSAKSFEEYNDASTLKQRLQQLA
HNIGMKTRKIQQAQMQARAQQQQQQX
>comp3355_c0_seq1; 126 199 843 LEN=1067; translated
MCRFIHANKGTNHSLSTISLILVLSAMLFTVVPVRGDEATLVAPVPGNGTLLSINASEVP
SAAPTISYCKGICGEGGPNSEGYLVDPDFATEYQWNLRVPVCSGASCEKATCAELSMRLR
TKDLSDFECRKHQVELQRTALCTCTDPSEDDDFGMGNDDYWDNRTTRAFKPAARYTLLGV
MSALFIACWVGLYIGIQHDDAEKEANGDNDEDVE
>comp3356_c0_seq1; 469 1 1731 LEN=2038; translated
APSAAPSATPSAAPSATPSATPSAVPSMRPSLNPTESPSVSPTDTPSFSPTDSPSAFPSD
SPSVSPSESPSASPSDRPSVSPSDSPSASPSDSPSGSPSDSPSISVAPSSSVQPSNAPSI
SANPSSSPSSSPSDAPSSSPSASPSSEPSAKPSASPSDSVEPSSEPSSEPSTNPSMHPSE
SPSISTRPSAIPTASPTSSPSSGPTMSSVPSAPPTMIPSSYPSSQPTHKPSKRPSKYPTM
RPTPDPTPNPTPQPTPRPTPNPTPDPTPQPTPRPSLRPSADPTPQPSHSSDQPSQQPSMK
PSRNPTGKPSVSPTPEPTPRPSLRPSRDPSSSPTPNPTPEPTPHPTPRPEKPPSSAPSSS
PSIKATTDNAATPEPTNTKKNCYYNGVWYEHDTWYAGQSQTCKCHDGGWIGCFLNSDTRD
PTETPTQASANPTSAPTPPSKMPVSQLATTSSPTGNTEAPTATTVAPSPIPTLASQSPSD
RSATDLPSESPITAKTGTPSSSPTGSPTAATTILTTKGLSYVPSESPSASPSASPSASPS
TRPSAAKTGTPTSQLPTVSTAPTSSLEPTSFFIFKN
>comp3358_c0_seq1; 765 117 1709 LEN=1827; translated
MTRLRNVLQSRADRFRKKQEKNALHQKEENRLIGSKYYDDETQLTGECIRPQVEPDGVEP
DGVSEGDMYLLQAKGTRGTMAETFSSSEEENRAFSEYNENERFSRNQKITMYSEQDQYYP
ENNRQYDNPAYEEVENEYETSNGVEVVNLARGSARSGRAGVDEVENSEQPKTIEFVDSED
GKVFEIPQQEAGCNDGLLQNVRSLDDTYNDECSKTLNTNDETLQDPYTSNMEGIDHMNSS
DRFGSLQVQVEDDERYEEYDQGDSAPENIGYEQEHYAHHDNTELYDCDDGQMYDPAQVDS
NVGQPYQLERDGTFFEQMRSVQSRGHLEMEPENWESTAHDIPLNNPTSAYLNRPNDVPAS
PQLEDPYFSDGNSSWEEGSFATGASRTLSRVDTACDDDDDDVRNDDDARTFDGTFDGTEY
YSDDDSYAEKQVDRPFVRILKKFRDMNVVDEQSDYESGDEGDEEDYEDSKKRSRRRKPKS
AFDRLGAIGMNILNETIDYAERQDRAPKLEGGTIINSFADLFSCGAPSGY
>comp3360_c0_seq1; 336 1 825 LEN=1055; translated
LTKTKAIKDEAKTTRSRKMVLEAVKVLYCAKCGMPPEYCEYGPDFERLCEPWLKKYHPGM
HVTLKALRGDTSSDNDEEEEPVPEKPAEPWTTEERLTKFYELYVPEKVSDVPSLLEKYAG
KEEKLFIALSKKYGPEPEDPYYMSDNEDEDESEDLAKDMDNLQVGGKKRRGVKAKKSVKS
ETRVLIQKLTRSKRKATTVIVGMETVEGIKLKDASKAFSKRFAGSSSVKDGVKGKEIIIQ
GDHMEDVAAMIVDKFKVPGDCVFLDFDGGVVPYQ
>comp3361_c0_seq1; 399 192 1894 LEN=2105; translated
MVGTAAANGGGGSRERPFGSGTVRFTASLLVGLLGIAVSLLLWPETPGSGNPDPSGLLAF
RPELYHGEGTTRHFFEGWYHKLVRTRTPCPGGNGPSESKNENESGENNISLAVVPGVFYD
HGGGNGKNESHAFVFLVVNGEKQHYYRFDLDTEFGFLAPGATGGASGSDIDTPYVRIGNN
TFSNHGVSLDLVPRDGDDASVSLRGSVRYGPLAPWPIPSARNLGAMGPVGHFLAPFLECH

HDVLSFDHSLEGRLEVSSRDETQGGGGSSTYAEDFYDSECGSGRETGVAVRSRGYVEKDR
GRSFPSLWIWMQTNSFPRHPGTSVFFSVARIPILLPAFLRSLLRGLAEPEFPGFTAAVWL
GGSGETEPRGLVPFATWTGAYFEDLSVEDDRVVAVLDSGGIGGGLAEALWPSESAADAGD
LLVRLGRYSRAWIGSFFVGYDSRALRYRLELEVDRRVPHALLYAPTARRMEQAVREALAA
RIRVRLYEYRTGAGEDALPRLLLDDTGEHAXAGGPPERAVPSGQPVREAHGEEQAGLPVS
EDPHALSHQLDGTMRHAGGAARHSVSA
>comp3362_c0_seq1; 221 72 440 LEN=626; translated
MTKSSQPSGFVGTLLLLVVVLCSSMASVSAFVGAPRCVSQSSYNSALAMSSDDENDPNDI
IARRIVVEGDVQGGYYRSCVRNEAGRFRRLCGTMTPPDDSGTAEIYVEVCSLMDFTNQPI
AV
>comp3362_c0_seq2; 291 72 539 LEN=1198; translated
MTKSSQPSGFVGTLLLLVVVLCSSMASVSAFVGAPRCVSQSSYNSALAMSSDDENDPNDI
IARRIVVEGDVQGGYYRSCVRNEAGRFRRLCGTMTPPDDSGTAEIYVEGKRKMVDGFMRW
CKKADVGLSQQIAVSDVTEEDPTGLYDDFYVKVRD
>comp3363_c0_seq1; 110 1 419 LEN=1170; translated
XSEGETQTIRPGRRNTADQHILTATYTTQQENGTESHSKRKMAYIGRDGTVGGQEQKTIR
QKITDFFAGIIGFVALFFSAITNPPTAIGSNGSNYAQRNNNNPYRVSGGGGRTLGRGGAN
IRGVGNLQGAADAKMGGGG
>comp3364_c0_seq1; 153 300 442 LEN=442; translated
MAAVLSSSRSRNSTAWPVIATLAAVAFAALAASVVAASRTRDSSSSSX
>comp3365_c0_seq1; 319 32 829 LEN=1353; translated
MVSKLFWKEPNGRSAYVLSWVSTIVTAVFAIAGIVLYIATGSVMCLVFGLENCVDLMSSV
VVLWRFWASGSLTKEREKALKRRELRASMAISFILMILGFGVIATSSYDVAMGPETKHEM
DFVLGMSAFSLLVCGSLALVKFRYASVLESESLYKDGVCSLIGTILAVALLSNTLIIRTH
PQIWWLDPCVAMICGFAALFIGVHSVFVAWKHRRVPICHLSWWLMSRGAHEPKQGDGDGI
EKSDPDGGGGDGDPSAPTSLSDGVV
>comp3366_c0_seq1; 205 1 1481 LEN=1661; translated
XTNTTRTPSRHFCSLPNAPCFFLAKFKTKDFAPVAGLYLLLIAGAFEPCRSFQTTSCIHG
NIIFKKYYLAGKDRHSRGTAKKWNIGSREALSSLFVLKSRKEQKHSLSHFSTTNGTNDSS
VGIEEEDCDDLTFTSRPARLYIGDTDAYGVMYHGNYIRSYERALYELHTQRKRKRQQEDQ
LQLANNDDADGHDTNNQKNPAAASLVLDRSDFRLTRCTNHRFKSSPTLGSRYVIRGTLVP
SGGDDDGVSDSQEETWSLEMEEYVPKHANNDCCDGRNPSATIYNTALVTIASASECAEKN
QPHQSLSARVEPFYPSDLDVEKPVKCPSAMYTNRFTIHRDEFDAQGLLPMTTVLDFFERL
RTDVLGGPDELRNLQENDNLLFVVTSLNDLEVLHVLDDFENESKTRKIVRPGDDLEVRLY
GTPKRKGMLFVCEQELWGNLGGGIDYEKKDRSILLAKGICTICTIDSLSGRPTSNIPERV
RASMETTASLTQS
>comp3367_c0_seq1; 186 488 870 LEN=1031; minus strand; translated
MNEYLTRRFDCAXPTPIIAKTIEEAKRIGDGRSMGYEVFPSSGVDGLFADKGSSKASNCC
CWKSSPSGGVEKIFVLFEKGDAIVEDREAAIACSPAKLLLVNAWVPRMKEHATKIMEYRS
IVVAIVV
>comp3367_c0_seq2; 276 184 672 LEN=833; translated
MLRYSIIFVACSFILGTHAFTSNSFAGLQAIAASRSSTIASPFSKRTKIFSTPPEGEDFQ
QQQLDAFDDPLSANKPSTPEEGNTSYPIDLPSPILLASSMVLAIIGVGSGFDIFADEPRF
PLAVNAVISAGGLAVCLGLFYASILKAQAETEEDDKKYMSGR
>comp3368_c0_seq1; 300 1 412 LEN=412; translated
QQQQQQASGAGQALAGINQIPQHIQAQQILQQQAIQAQHKKNTADVTNAVTHLHHKKSSQ
QLPMQPAQNQQQHQHQPQQQFVIKDGTIQAAPAPSQQKLLVLPTAAGPISATAKPTILWP
NTANANGGVVQQQQQQQX
>comp3369_c0_seq1; 204 247 846 LEN=1404; translated
MVFVSGKGRPPLGNAKIGHVSWDEEHSCFVGTNGFYWHGRKYGGWKEGPPDALVANRKRP
APCSRGRRPRGNDHVGIEVTYSVEYDCYQTEKNYFWHGTYNGGWKKHPNGTDFASEKEES
NDSVSGVGGTKYSSENQKINCEKKQKKGNTKGRRPKARSSGEAIEYDTERLCWKTDRDGG
LYWHGTYNGGWKDHAPKKR
>comp3370_c0_seq1; 355 88 1386 LEN=1470; translated
MSDPTIRGKLRVLVITLGGERRRQIEELFDDPVMGEHFERPTFSEGVPSRYIRNRFNFYK
VAHDAGLLPEQEWQAILKATEEKTYDHLPTERFFDCLEEVEVTKGRHGSQEDIKLHYSVE
LWRKAKAINRARSELACVFAHLIAMKTFVEDGNFDLILEDNIRTDPKSCAHKVLEFMRAT
EEWEEDMSEITKSKCHLRFAGWLGSVPNIEWIMQTHIPKRKYSRKKACKDDNDEDERATI
FPLPLLEHLNEDLQTIEESQAKVEPSTNTSTEKDKDEEESEIGEAQQTQKHTKPGGNLLW
GAYAYWISKEGYKSLLETLRSDVGAILWKGKRMRNYAVKPIDKMLPRRTVSKFGPSAVQI

LSQPAFFRAPMLTSKIHTQWDPGFCESTEFQLQKGKLGWSDLWLTDTERAIVDHHKTSGK
WLTIKELELLQL
>comp3371_c0_seq1; 490 35 1129 LEN=1481; translated
MLSSALNFIRVDSLRSVASQNKIDDRDKINAIHEKGCRSNGEVHPTSVNMHKIEERMDAA
IEIVAMSPQYFSKNRSPDLVPLTADYEQMRKRLRTLIGVLKTYQKCTRQVQEARFEIAQK
LTLLSERSPIQDEIGCELDGEAMERLQQLSQRLPSSPSISSPTASTINGENGLSDSESLP
ISAARVAGDYRKRNGLKFISLLGLYRFSAAEAVVNDLQYQKLVVNFATNWMEIVTHRIDV
GLKRVGKLASDRLHYERKVEALRNRANDAETKGKAIPDSATERLSRNEDKLKHAFVFHET
EASKLCALIEAVTQDGYQELYTLVRNYIEWEITRIGRENDMAIQLDATLSSMNEKVNFSA
VIIE
>comp3372_c0_seq1; 416 1 1111 LEN=1237; translated
XRNQTKATKPKIKTSLSHTIIAHIFAKNTTKKKDKDQANSTADSEAVVNNXIMIQCNASS
TGRRTLLLLAQKQRQHQSSLSSSLPLKSRDPLCRLYSSSKACRKKDLPPPLPPKTGGSSS
KQAKGFPSPAQAKKEYLESQKRTGEFLQDPQANILRLQKEMNETKAKLLTVHDKPIWRRV
VDRFKAKRHSVINLLCASMAYILAHKLHLKMKANQELQEEIDSEHAKNSELRALLRSLTT
EEFLRDVLAEATSAGDDNSPESISNANENSSSSWWSSTSSQTTKSASQSVDSDALVDALR
TKLQARIGDEGLEDADRKEKNIQEIWKENEKRIEETEEGLAVALAAVAAEGQPGEASATT
SSSKKRVFDM
>comp3373_c0_seq1; 395 1 1144 LEN=1292; translated
DWKLGLPHRDDDSSTILPYFEEKPELPFPSHDSTAHLPEEVYIPPIRNKAIVFIFSASIQ
TCSSIANSPINNIMSKTEANSSFSKSSWDGSDKDGFASLDHPGYSMECNADFTGGWHVHR
KVWEEQEGDKEDKPSDWIKEILDDITKYDYESMDIGRPPRILVLYGSLRPTSFSRKCAYE
FARLLDLLGCDVRVYNPKGLPVRDPALEDDPKVVELRALTHWSDGHMWVSPEMHGTITGV
FKNQIDWIPLNTGSVRPTQGKTCCVAQVNGGSQSFNAVNALRLLARWMRMPCCTNQSSIA
KAWQEFDDNGRMKDSSFRERVVDVAEEFAKFTAVMAPVKDSLTDRYAERKEKAAKGRLLT
QEEKELEKKKTKMVEESRKG
>comp3373_c0_seq2; 211 146 421 LEN=569; translated
MPCCTNQSSIAKAWQEFDDNGRMKDSSFRERVVDVAEEFAKFTAVMAPVKDSLTDRYAER
KEKAAKGRLLTQEEKELEKKKTKMVEESRKG
>comp3374_c0_seq1; 465 1 1940 LEN=1992; translated
XSIPICSLHSTVVRKEAPDMASKVSICLQSRQGRKVISLASTASTKEFYESASKEFGAPV
ESIKGGFPPRPIPSDGSTPISNFVSNQERVQVEFAQNVKICNTNGSNKKNSHIQSKPVSN
DQSSPSSSSGTRRSKRAASKVATESMPAMIKAQEEYLKNSAPIGKKRGRSNKNFNTTSGV
FPSGKNVKQRPHKTKSTKLSASAGDGRRLVDGATVANPRRAGRQADMRRRRGELGKGEHS
TDMSTALMGALHDKGQMGVILRKGMKNVVLSSYETTRSFSRLAAIQARSYQMTNFSGERN
LSGSGGGDLLKVIYRGSVNKTKEEEIVDRIPRDVLQAVLEGIHASNKEALRPENLSRLSP
RVLWSCVKLFPELSNVPDMYRQLLPDLNWSFLRRRAEQLSEKALENKRQAMDEDEGIDFE
RASNVVAAVEHAMEHLQDHHAEERKARQAQAAEARLRRQQGQKSGQNDGGNDKSWTLTTP
PEPDRDELRECIETSIPEINSNTRGNISNWISRLMKDCGIHNWRELANASDEKIGILVTK
LEVPRPNLQSWIDKAQNESVSEIMVEICDDNIQAVEILTERARSGTPKDLAGWKSIPDIL
LEQLRKKCVDHGYKWMELGTISMWCDRAHDALQDYEWLNWYATPVE
>comp3375_c0_seq1; 168 66 353 LEN=989; translated
MPLPMSRTAPFAIFGCILSAYALYVEHKVAHLSPDGEEFTALCDIEQIGASCSNVFQLPE
GRMLSYFGIVPEGSILDVPNAALGMYRSFCLMHKI
>comp3375_c0_seq2; 235 66 572 LEN=865; translated
MPLPMSRTAPFAIFGCILSAYALYVEHKVAHLSPDGEEFTALCDIEQIGASCSNVFQLPE
GRMLSYFGIVPEGSILDVPNAALGLVYYSYWLLIGPLLPSASTVFVSSIAMASSVFLAIK
LVILKELCILCWSTHVINSRLFWSVVAQTISGGSATSTKKKEKVIKRV
>comp3376_c0_seq1; 685 256 2115 LEN=2115; translated
MARTASVPIGKKSSNDSGKYSVEILNKKIKKVEKMIANSPPGSKECTKLQKKLVEYRAEL
RNQTRIDIEKANAETTSRSTEVEVEESSEVQEESTNNSVANSVPTMAKEGTDDQRMKEKS
VKQDEDKGHSLELLNKKLRKVEKMLLACTPGSKEHQKLQKKRTEYERQIENQKHETTRTS
IGSDKGSKQLNKKNEECQNKVEQESQASWRNELREEAKILREKALEKKRKERESVESAAR
AEEELRREEAEKEERQKHADNEADILRKKAFREEAAKLMRKKALEKKQKEKEARELAERT
KEELKQKEATNQKARSSHEGAVNQGDSKVTEIGKSNGTISEANIFLHYANDEKTRKQAFL
EEARKLKQAAAEKKRKEEETNRKEDTKSDTEKQSIDANDRNHESEEWNPQERIYNIDEKK
RRQAFLEEAQRLKQETMGSKRKENDVTNIYGTSPAESKGQDHEACNEDYTRNSERETEKS
PSNYSDDHDKKLGYPVETQSNVNENEKRAETVPPSSSVFNVSKPKLTFLNEIRLSANKDE
DDSDTHKPDFLDEVQLEANKKETCTPGPNTTVHRTKRNFLDEIRSKVEKIEDGAPAPDLE
CSHRSKPNFLNDIEPKAGKT

>comp3378_c0_seq1; 238 1 674 LEN=806; translated
XENENENGAPQHQHENEHQHEHQHEHEHPQQPEPPEHQLGHQAQQHRSHQQQQDVAQADA
TNNDLDASDELSDGEDGNGSPGKSPQVPPTTAAVTTVGDSWKDWAPDPTSSLFPRTFVGV
LRIMDDPGVLGIPQRYGLLRSGNDDDPSARRSRYYSFEHELFHHDRHGNRKGIKTRTYLR
KLKKRGDPRLSWHQSRRSWSPAERQNLKHFVEVLIEIAMAHFMA
>comp3379_c0_seq1; 349 748 1794 LEN=1897; translated
MNERPICFLLASWMSWVIIGYCDQVFFTLKQQCIETSVPLNVHFCMHRYGEWFMLMFGES
IMSLLIVEGDNESFNHSVKFYCGVLSVIFLAHLHFQSEPHHNSDHAVTRSRHSNYLYTVL
VPIYSMALIATGICYKMFLYDYQKGDNRRALGDSNQEAGYGDTEYQYIEKKQQFTADLFS
GSIATVLICTDLNALLHHGTQSILCQLKTIPKTRLALLTISKYSLVFFLATMSAFQNDPH
FMAPFGLGAILFQEYLHRSFKSWQQYPETIHNADDSSTDSDGTLRTCFTGYSADEDYQYE
IESFADAKMALAKFERRLIVETKPTARSRKKDTEKICEKVLPQTYYAC
>comp3380_c0_seq1; 202 181 390 LEN=390; translated
MTVNPTKSKLLLSAAAVAVALSVSPSPSSLLPCAEAFSPSLFRVAPSHNIASFAASSTEL
AMAKKKKKKK
>comp3381_c0_seq1; 737 136 1887 LEN=2298; translated
MGPRPEERAHQPNAIEEDVLETDTDANGREINIINDAIAVVGNHRTTGDPETTDSTEAWA
RAEGYTPLSDLNTDGGFDGADSYQMFSTGPGAADSNSDSNSDGDSNSNSNGDDQIQGGSA
TGTFFADASATGGSGGAGQDDFEQVAAGALSALEEEYKQTLALSQGGVSEAREKDEDLRI
IGAGFDQRNEELQQIRRQGGFVVHQEWEGPEKKTPPAGIDASGKGTNARSERPGGRPQTS
NVDTDAVRKAIQSLSTKNRNASFQQKFATWQQQQQQTNKNTDHSHVLIPKASRHEFYRPK
PTPASRRATANLTRSATLAEAMVRLSLLAALPDDCLLIDVVGVDHVECQNASTIRDTFRP
FVRWVRDYLFFERGQRRIRIHFRLIGRELMTTTEGVVDLMESAASSISATATCHSGVYHE
FLDERLKLAREEGDQKPSDGLLLGTKIAPDLAVAFNAGIWGYQEWATTIQYLASFPTASL
STAEATAAIADDERTRKIGGIPIVITAYTLDECQEDQEVISEAIASMRTEEANEGTGYKT
TAPFNPCRAEILWESERNPFGSQVTRETKGSTQEYRENSCWQAW
>comp3382_c0_seq1; 141 206 421 LEN=421; translated
MMHPSTKTLLLVIVAVALATTEAKNFRGATSVEEGDMTRRLSAKSMSMKEKSMKEKSMSK
SMKDKSMKSGGK
>comp3383_c0_seq1; 325 469 1584 LEN=2140; translated
MVSSQALLDFAVITIFARETLEGGIIIGEYRTVIMRSNWENSAITQQVALRAVSVSALLA
ALVAIIVCAAIAIPLAILSLDFEPSTAKIIEGVSKIVAAICLLQLSLKMPKFFGLYYSKA
QLKKIKNNEPLDETMDSQGMSLRSIRFNVAWNIWREVAECGVFLIPSFLTGEGILAIPLS
AVVGVLVGGIICVSIYYANHYFKSTKALTIFTVGLFLLLSTGLFTGGCTNFEKVYGKTKT
VYELHGDFWSIDRLPMTIFKPFGYSDTRTVLQVCCFWIWMAFSLALHYFKYKMCMKPSSE
LSDEAAASRSADIENTIEEIKEDGANENLSIHEDTREPSDSGCSGECPNDIEGSKKDEIN
LKGKEFEICEA
>comp3384_c0_seq1; 606 1 1146 LEN=1204; translated
IRFLGSKGNQQSIHPSTAMSSARNANKSIVFWGAGAAVVVAATAYVLFSRNSSSGSSAEG
SDDTKPNENLKSETTVTKPSKVTKEAVASDAVEEPAGKEKSPPTQAETKLTDATDEQAKE
HEEPPPETATDTETTAVVDDVEEETKQEQPPLNSPAEEVVVFDEAKENAREEEPPPTPTP
TAADTEPVADSVKGETEKEDLPSEAPAREVEIGDFELEEALANTITTRRRNKASAKTKEE
PPPTPDNATQEETPSDQESNETAIPSTVTTRNKNRRKKNKKKKWNTNTTPLTEPDRTPAE
TTGDENNSKNSNNGAVTAATNGAKDNMEPGGIDTAAAAAIAARRAKDKKKRNRRKKAAAQ
SAATREIKEFQKTQNGNSGKN
>comp3385_c0_seq1; 438 91 960 LEN=1052; translated
MTKRTRSRAIGSVCAAATGFLLLSACSGPSPSHAFSVVAPSTATTKASSRRWSPPLGMAD
SSNAAAGDDDADEASPEAPAGSFFHPVAPSGEEAPAPAGGATAPPGEKAPAPAGGGDDFD
DAVSKLMQQRRKKPLASEPSTIKGVPTKGFGKQPAGGKKRAAKPISPYVAIGTPDQPVND
PSKPERDDQGYTLYTDTKTGEKSRVFEALVDYPSVFTMKIVGANEGTFVSDMVALVAEAC
DTEPLGIQHTTKVMGKWVSVTVKAPVENAEMLYTLYEKVDLDPRVKFKF
>comp3386_c0_seq1; 550 388 1959 LEN=1959; translated
MMHQRRTVGLDPPSVSSSAASSGTTAANRNNSDGTRRRRVRRTVRSRQHRWWKGVRRILS
DEDALVTLVQIASVIVIIGCMLMLALFFFGPGEIVVGDDDASGIGWTNSILANMNRWIFR
REIRYRLHRDHENTLHTNKKGDDLPDLKPSTIYTIPGSMPHIGDKSDYYAQLRKEYDAMD
LPRVEQRYKFKTMPMRVNDDGDFEEPEHLPAPYDIHNCPEHPPENYPYAWNLLQILDHWN
PDDHEVPEDLRIYQGICVFDYEKDYQKALNYRKQEVPFVVSNDPAVQETAKRWMAPGYMD
RLMSNVKHRTEYSETNHFMYWNAGSQVRRKQHVGMRRGNNQFMGDGHPDGRGVDKDLAHW
QQPTQMMRMTYAEWLTHANLTEGETVGPHDPHWYYRLIGCGEQSPDGKCDKGSSEYLFDE
LPFFQPKENLYIVDPMEQKGIHCRFGMRGVIAENHFDQSRNAIAVMGGERRYILSHPNQC

GVLSLLPKGHPSARHSAVDWCDPDLESFPEFAQAESNEVVLQAG
>comp3387_c0_seq1; 504 68 1550 LEN=1550; translated
MDEEKAIKAAKKFLKKNESGSMKLKDLARLVAEKLGDKENYKNAKKVITKSDKFSLEGKL
VSLTKKTKIDASELDSAAPAFKRPKTEWGMSSEKLPSSIEKWRKDNLIVLKHSKDDEEGK
EETKKINANKPYNPCSSFDECKAIIKDSLIRQCTEANGFTKPSPIQAQAWPILMHQKRDI
VGIAETGSGKTLAFGIPALSCMAKNPVVSKRRLPRMLVLSPTRELAMQVEVVLKEYGAVV
GMRSLCLYGGVPKYMQTSELRKGNIDIVVATPGRLKDLINEGTCDLSKVDHLVLDEADRM
LDMGFEEDVKYIISTCRSKDDGRQTAMFSATWPAIIQEIAMNYMNDPVRLYVGFEGIVGS
NGENCIDDSLSANKRVTQTIEVIEDRPRENRLREILRNNRSKKRILVFALYKKEAERMEY
QLQRDGFSVCSIHGNKHQAARTQALADFKQGNSQIMVATDVAARGLDIPDVELVVNYTFP
LTIEDYVHRIGRTGX
>comp3388_c0_seq1; 282 75 785 LEN=1062; translated
MKLYFLPFLIAAVAEAFNVSPDVSFRRTGSVAMNESNGTGELSRRTIVQSIMLTSVLPAV
VSPQMASARLEAVNRPDLLPSEAGLNVIQTEKFLTPGQAKRLDKMMANLEKDTGFRLKLL
CQNYPNTPGLAIRDYWSLGKEDQKDDKYIVLVVDQFGGRGNILNFNVGDGVKFALPNVFW
TRLSGKYGNTFFVKENGIDVAVENAVEAIVSCLRSEDGFCTSPPDQGQSLRSLGFN
>comp3389_c0_seq1; 742 188 1414 LEN=1446; translated
MARFTLAALSLLGASQYVTAFQPVTPSAVRMASSLNAVSKKDSYTVTLLAGDGIGPEISK
ATKGVLEALCAKQGFSLELKDALIGGAAIDAVNDPFPQETLDQCQSSDSVLLACIGGYKW
DNNPRELRPETGLLKMRSSMGLFANLRPAKVLDQLIDASTLKREVVEGVDVMVVRELTGD
VYFGTPKGIDEVDGERIGYNNMIYKESEIDRIARVAGDVASKRNGKLCSVDKANVLDVSQ
LWREVVTDVISKDFEDVELSHMYVDNAAMQLIRWPKQFDTIVTGNIFGDILSDEASMLVG
SLGMLPSASIGESGPGVFEPCHGSAPDIAGEDKANPLAMILSAAMMLKYDLDRPEEAQML
EDAVESVLEQGFRTPDIKIEGDGCKLVGCVEMGEAVAEAVTAMETISV
>comp3390_c0_seq1; 373 213 938 LEN=1872; translated
MARLPVGRNGDSRTRLLNSLGLFQEQRDERKCMLGKSSASIRYDRDSSPSNNHFENIVGG
SDGESGEKKKFRHVRSWSDIPVRSALHNSVAFETRLNDENIMRNHKDDRGNSQRRSSLSR
KNKEDNSPHSVRFNTVVSGVKIPSRNQYSRRIKQTLWRDRYELSEMVERNTTEFHAENYD
WKQVVLDEEMYVDATSGERIHPCHVELNSFDESDDDDYNDSLVDESGFAPLSRLDSIASS
K
>comp3391_c0_seq1; 573 409 1659 LEN=2121; translated
MLFGSIPDHIWDEAATSLLHKGVATVRLSGRFDEGETFGSTKPSQIHRKAFRSARRVLDQ
ALHPDRHGAEDETVLDIDHNGNADVYGEKLCCPLLPMIAPDADSAHATGFHWAGGMSRYN
VQREGIVFSDENCVGCGDTDQTNPQSFHLHVHHEDDCYLRDLSGMFCSLHSIADMVLLAI
ERKLELPLAEHKIDQSQPISDDDGWFQRHLGPTTTASQWHMKRFVKEQKTKDDEGNDCTE
DSTKTSPLMSTSLLNENGERITLPMHTDPSLISVVIHDIEEENSDGRPGALGLEYYHPKE
HCWIEPEAHGHSVATIFVGSVLAYLTSGQFPAAKHRVVEKESSKLQKQRMAATLFVRPQP
SALLQVPLPSPWLLKRIEEEQEERRRRQGEKEPKNSTLSSKKLKSPSSKTPITFDAW
>comp3393_c0_seq1; 392 209 1375 LEN=1439; translated
MAFVSRELAGKGHRMATATTRLFSVSNGIEADVKSGISRIATLQSMLSSHGAPGSVGCSL
PDDLEPVYVTTDADAEETPELIANIMGANEFSNLHPHLYPLAKSKSTGNFICGLRRAYAE
DASYENSSKAPWPIVEAEIGGPGMRLISLNSEHLMRRIACEVDFGDERKELLELYNEDLG
KGFIQDEGLDRPYEQGSVEKLGYGVDKFVLLRVGPFPDIYSKLAIGHAERGDESSSLIAA
EAANGKISGFASTFLFYSSLLNTFPNRSEETRDAARMCLRMPLSSVGLSAKDFRDVGIYG
QLAEETDSDEEIFAKLQIMYEKMRDHENEDPRAGNADMTPEQKALDDANYLLDITSLTGS
NWSEIRPKLAEIYKSVGRDDMADFVNP
>comp3394_c0_seq1; 460 347 1840 LEN=2266; translated
MTTTMTTRMRYGSSTVSNRSTVAVCSAFVPNPVMGGNHQTKGLFSDSHSRKPTVFASSSL
SASKGFRFPLSATEQAMHTRLRSSGRESTTNRCLAVFSSVPSSPPSSSLATVPPNERAEL
LNLCLEKLLNHRDDACDQTPSSTEDGDIGSPSIEIMDAALRSLRDPSSGYDQRYGRPALR
CYRSFVYPKSKTKAKAVADGGDGLIDNESSRHEDRIQLEAAALRTANQIDFLIKRHTSKR
MDAIRNHDPSGVRGEESGSLEPEPTPEAFPLVLVLDNLRGSFNVGSIFRTAEACGVSEIL
TCGITPHPNGSGAEKVAKSALGADLLVRTRHFATTRKALSALKQPGASSGDEKEIGYLPP
LVVALETTAASELYTRFDYRPYYRKKSGERISQEGANNNPSDNNRQPGHQGIALILGNEV
TGVDAELMAHLLDKDLGDNELVTGQNQGDKARGLVDAIVELPTYGKKNSLNVAAVAPVIL
YEILRQWNEEVEDTGGV
>comp3395_c0_seq1; 299 152 1498 LEN=1594; translated
MTMEDPMIERKMVVAQQENENTGFCLQQVSLDKAVAPLLVRPIAPFDRTERKRAHTSAIF
YSECFLRGIEPKMADTAALQAQGVYDKWWVRSTASSRRFGTFVGDKSESQKGTREEEGTK
NKAKYKKRVADEAFLRNTHDFTGTLVARDPSLSREISIEVVKKLMIEELRSSGGNVDTPE

FLKYQRILEKEFIQRGIKNVNQISQEGNWFTISKPTFTECQGTNDKGENKYSLGRISFDM
FKPTGLVCSFRGAFNYVQAVDPSNPGRPLHVPRVLMQDIKKEKCRLRTYDIAVAITIEAG
QDQKGNFIDEAEENSCYIVPKPIRGILTTLGYSIPDPNVANRLSIWLSGGSLEVQDEERD
VDEWKKIFDTSTAPNRDIREYANSLAARLLLGAHIHDKMEEDGTLRFDLKRPIGGHGSVF
CDIIYMDDNLRIMHGHHDSVYVSVRVPG
>comp3396_c0_seq1; 197 93 902 LEN=1594; translated
MKLPTCNTSVTPSTVTVLGLGSLLSERSSRTTFPDLTNFRLGRISNQYRRVFGHPASIFF
QRGLANVETKEFSSLSAEEIEYCGSNRNDGENLGGFICSVFEVPRDQVYFDETDNDGRIV
ATKPTGAFLEREEEFDIVEVPFLDFSESNSDPDKTAERATKLGTLCTRFTDQGYVERWGK
QRFDENYGRFGIDTIWGWEADSGLLPCRVYLRHCYLAAKSMGSECLDSFLDETVLVDRRT
TIRQYLEEQEPDLIETTIPPPGFEQRYSG
>comp3397_c0_seq1; 491 53 1216 LEN=1382; translated
MKTSQRIALTAIALAYSWSSGSVYAATTSATGNKEGGGEQYLSLIGNAHVQYHPTFFNDV
ERILHQVGVRKNKKKKRASTRKLAKKGKNSKNTSAPTKNPTKAPTKDTTIAPTIAPTPDP
GGRGDDCDPADVKLLDYPQWEAEKGYWIGEYTFLQGDGNPYESSQWNYPYDHYKGFITGE
VVGNAYRQRNVFLYPPQIPSKCNPENENNVIGDGECGVNGNTKVFEADQSATTCSLNPAL
GGIIEGLYVQGPYELPTKTELIGRENALLYQVFIPGTMVGKEEDVLLQSQLTTITKNEDK
VYRTRTAQSFDTFVSPGLTTSASYYRERRVSKEEFYESLNSSIADYNIRTEDLCVWDNSG
FPVEGVTGSIEACEEHLEESFLLGSEE
>comp3398_c0_seq1; 457 484 1880 LEN=1880; translated
MRLLLATHRSSPPFVVSPRKILDFVVVVTAIASAXLFGANHPGAGRPGWRKRPSGRYTNW
PTLGSNKRTGATPELLLGVANNYCAAKRWPWPWRSQPHAFHDAFSAAIQLWGGSTDSVFP
PRPDFENSRRSSKVLRHDEPYQAWDVRFGSNKGDSDSNDEKDPKEDREHVSCKDLQDEAT
FRYGSDPSRDCVWVAEYPIRRCRKIDPIRNQKTRVFCPLTCHKECRVVDKIIDDFVNNNN
VTGGINGTDVDTHSAAPSGTPTVSFFPSEDASLVPTAIDEGVNDVVEEDVFDPSLSPSTL
PTTYMPSPGLFATAAPTVEDDKRNVSLVTSNNREQTFGTTKWILLGVFMVLVLGILVGTA
AAQCFRCSSKRRTRRRRIQPNSNKERAPNFRRRKKKDRADGRQQQQEQSPPVRTTDRGGG
SIGSTDSCSCYLQDDYGVEVDYDVHDCWSVSVVSVLPPPPVEDVDL
>comp3399_c0_seq1; 367 1 789 LEN=1671; minus strand; translated
AQNNLLNKATAPKVYQDFTTKKVFTMERLRGVSMLDEETLTKVTKDPEVGQQAIISALNI
WTQSVMQMPWFHADVHAGNLLLLDDGRIGFIDFGIVGRVSDKTFKAVNELSTSLALADYK
GMAQALCNMGATDEEVDVEKFGRDIEVVMKRIASVQPDITITEMQDGAIGGTLDFDETEI
TNLLLELVEITEDNGLKLPREFGLLVKQSLYFDRYLKILAPELDVMNDARVSGLGEEALK
TTSMPMVNGSGGNTDESIVIDV
>comp3400_c0_seq1; 171 1 542 LEN=732; translated
XIQNTLLLSFFPISLLPNQQELPRTWCILTFNQKKSISNKVTEYDVYHNPSIVEKFVVGL
RPTLLQIDRILEIAPIRWKRDDFENHHQQEKGIPKTSCQARIENENKSSAVRNISGVISS
LRSPLFKGDSKLQLQAKDMFYKRRKECLRRLQLIFKVMSSAGKGEYETTTFGHVQIRRYK
>comp3401_c0_seq1; 184 269 475 LEN=772; translated
MGDGARRKLQLFATNFEDRMKGINHNNGAHTGGLQEARGAAAERRGLLDNDDDDYGEDMI
EFETRKSR
>comp3401_c0_seq2; 265 1 984 LEN=1281; translated
KTSSSTERNQRTVIPFWIHTATPRNTPRYIPLQTMGITYEEALATLEAMFGDPWTRESLD
AVLRHEKGHMENTCERILEHGEKDPVLLVERLRNGGPEPQVAMDEEYARRLAGQEEGSVS
SGRGSSRSGGGHRDGGGNGRGTPTILPSDFLRLPGFRPPPSSTASSSASSSSGANRAAGA
GAGRRQPPHDDETLARMLQDELFSEELARNPDFAHLAGRNPRRSSARPSASNARLPAAPA
GAPLQNPFEGVNVMQKLSTMGDGARRKLQLFATNFEDRMKGINHNNGAHTGGLQEARGAA
AERRGLLDNDDDDYGEDMIEFETRKSR
>comp3402_c0_seq1; 787 47 1897 LEN=2004; translated
MRSCGILSLALVAIINNDSRVSSFVPSSLLARNSPNYGKLFSTVEAVESESSVETDSASA
SEKLPELFPALSTSLTKLGFSTPTPIQSVSAKRALDLENLLMIAPTGSGKTLSYMLPALE
KIVAKKQQQEETAGVLQGGEAGTVLVVAPTRELALQLMRDTTSILSNVDAMSDSGDLAVL
LAVQGVQIPTPEELNSATVLIGTPKELLFVLSEVRGGPEFLAGDVLSSVILDEVDVLLPN
PPKQLRTSLDKSGNAARERKGGKKFNTPQDERRRQEQKRKLMAAKRRGVEFGSDKQIIGP
TESLLKMIANRRLYTADGSTPTPYHVLAGSATASRKTLDRLNKALRDAAMDASQTVDGVW
NGSVSPCRPEISESDQSDDESKKQDHTIRAVTVPSEVKHQYVRLEKDEATKPAAVLQAVA
KATEALKPKRALLFLCGEFRKQKSAAPTRKRTTPQGKTPPRRGRQKTLKQKKAEAAAAAR
AKAPDTGLSARKACAGLAHFGIEAKPLHVALGLEVNAREEDDEDEEEVPPFLVTFEGSAR
GLHLDDIDTVFVVGRPSSAASYLHLAGRVGRSSTSAEGDVVVRPGNVVSFCTAGSSKELN
KWTKQVGGTELEEIAV

>comp3403_c0_seq1; 894 1 1110 LEN=1110; translated
MDMDGDMDGDLEAGDMDGDMEEGGWEEEIEIEEQDFDDISVGDDSVNLQQNEDENDLEAE
EEEDDDDGGWEEDIEFEEQELDADGTDADAAKGKGKGSAKNQQKSQAQEQAAKKGGMKGI
MASIGVAATDLIASQIVGQIFGDGIMPGDLMGGGNDVDLGGIEDAADQAGDVVNDDVADL
AEDTVDEVSDEKKDAKAAALAELERQRGSSSDMYDSTGRKIRAAALPLGAGAAAVGVGAG
LHAAKKKPSSLLGPRTDAAAGGADDDSFASIQSGEEDEFEDEEVSGDEEEEEEETKRKTG
KKTKKLFGAIKNIASATVTAAKETVVSAIVGDDLGELLVEKQEEAAEGSDSEDGSDDEDK
EENDDDDDDD
>comp3404_c0_seq1; 605 1 1165 LEN=1278; translated
XSDETERIGLKANRSSIAPTPLAPTTLAPSQPHRTAPQQHRPLGSLNVTMAPAPLPTAAG
TQEGTAKAAAAGLPAPAADAASAKPASGITKKTTGTKSSSKTVTKASGAGTKAKKPVRKK
KSTKSSSSAGGSSQLSASRAAQLDLASQRSAAAARKSDPLWYQLEDYVLPAVAEETPMTE
GTGAKILPEQVQIVEAALEKNGLTRSDVTPQAFACLLEQARRYAIDILTDSQDYAFVANR
TEITKADLALANDFRPDHPTAVTAQLPKLNLLAQTINRVPLPPIPTQCYSGVLLPSKRFQ
LTARTFDVVTSAQTAQKMVQAVPAFPAKAKTSSTGSSSGTTSKKGTSSSSKKSTKKAPSY
GASRGRQIAINLKGDNNNSNKDKMDTTK
>comp3407_c0_seq1; 470 153 1340 LEN=1386; translated
MRLAAASCSRRNASQRRFCRCLLVFLFALVAPDSFSGFLPMPFSTTASPSNGRLFGLVPA
ASAWEWTDYIAGGENSGGANTLTDSEISDLRVRDIKRRLARSHGYAADELARMLDKKDLM
AALSREETKVRKKHRGQQQRKLFWRGLFVALLCVVAVVGWPLWVQLSEVMAVNWQVYYDR
KRHEISQCVEFRSVKGSFGVLLLGVLDGIRFWLSMTVLLSWVLPSESPYRRYMFPIPSLP
IRPGQFMGDKIANGPMGRYGINAAPMAITWSMGFVRSKIEYWTGKALSASAKRRRKEARS
AETSEERKARKAAKKAAKREAAEERERKQQEAWKAEMERRKEMAQKASEQLYGNGNRQPA
KEEPETENEPPTSNGDYDDAKREFESDMDDINDLD
>comp3413_c0_seq1; 340 221 655 LEN=1069; translated
MADDDSDDSDEWGLEELVIPPKGEKTGGGNTGQGTDADGDADVGDDYWEVKKNTGVAAKG
ASKEQEDDSNGGNNNDDDKGGEPMIIVDMTLLNPDIHSKFDRNSVNQPEEASALRKAIEK
DYETYAHSSDYLSEGIVVPCGSSVW
>comp3416_c0_seq1; 199 1 458 LEN=860; translated
XQTKPNQTLAMGIFKKTRQSLIEDPATVAVDGETIETTLPQKQPQPAQEKQQQQPQQSIA
QWKKARKKERKANAVKRANQKEQRASRIHQLVLHRSANRSGGPQNEYCYSPNIAVQRQKS
AKKAASVGWGTAQIAMKGVFGGLGRKDKIRKN
>comp3417_c0_seq1; 373 76 1389 LEN=1615; translated
MVMLQRQQAMSMRRQSSCPGMLRNQQSNYSHGGSFMSCAMGDDDNGSISSVSSRQSSFSS
MTSDNTEHERRSPSILPSTFGGNMNSSRTNSQNLYHHQQAKTPRWVLLVMCGTIMLSWFR
AVQYRTISYGILSSMDTELESIEFQKRQSTRLWKDATDTKNSFAKQHWKLKKTQKNFQHE
TRMLEEMAELEAASDNDNIEIPEAAREKFQNRRTGDVAKKWIEHRQEALLHKVYSLQAYI
RENSRQRVIEKYGYGPHHVVFDVKSREGRKSGKFTVRMAPLTAVPHAVETFMDMVSNKVW
DNTVFYYHHSQAHVVAAAPVVYGTFQTKDHELESLGHNGVSFPEYSPSFPHKQHTIGFAG
SGPNFYINSIDNEDHHGPGSQGHHELEGDADPCFGEVINGFDTIREMQYARHRGDVPRVW
SDYDLTRIIKATLVPLI
>comp3417_c0_seq2; 373 94 1407 LEN=1633; translated
MVMLQRQQAMSMRRQSSCPGMLRNQQSNYSHGGSFMSCAMGDDDNGSISSVSSRQSSFSS
MTSDNTEHERRSPSILPSTFGGNMNSSRTNSQNLYHHQQAKTPRWVLLVMCGTIMLSWFR
AVQYRTISYGILSSMDTELESIEFQKRQSTRLWKDATDTKNSFAKQHWKLKKTQKNFQHE
TRMLEEMAELEAASDNDNIEIPEAAREKFQNRRTGDVAKKWIEHRQEALLHKVYSLQAYI
RENSRQRVIEKYGYGPHHVVFDVKSREGRKSGKFTVRMAPLTAVPHAVETFMDMVSNKVW
DNTVFYYHHSQAHVVAAAPVVYGTFQTKDHELESLGHNGVSFPEYSPSFPHKQHTIGFAG
SGPNFYINSIDNEDHHGPGSQGHHELEGDADPCFGEVINGFDTIREMQYARHRGDVPRVW
SDYDLTRIIKATLVPLI
>comp3419_c0_seq1; 605 123 1274 LEN=1523; translated
MATQPPPEAKLLDKFPDGISALQYLPSSSNEGGKRLLASSSWDGAVRVHDTSNEASASAS
SSSPLLLSHNMESGPLLSLAVPGATTVVTGGLDGSIKSFDTGTSTVRSIGFHGNKNSGSI
PGGAGVETGNACSCLSSMSNDGASSDVVVSASWNRQLCVWDLRQQNSGNAAARSLLPGKA
FAMDVDASRNRILVATSGRHNCVYDLRMLESPNTFELKSVLERDSSLKFQTRCVRLFPEG
HGFCIGSIEGRVGVEFLDEIGDERMLAVAGKQKKYAFKCHRDSNSGLVYPVNAIAFHPRH
RNTFATGGCDGTVVLWDGQNKKKLTALPAFPTSISAMCFSNDGTELAIASSYTHEEGDRE
QHPQDELYVRKILDSEATPKTAA
>comp3420_c0_seq1; 333 1 1572 LEN=1605; translated
DDTSCLRHFAQICVHHNVHARMETSTLVYSWIARGIYNLDSQRFQKASLDTRTEGWTETW

GNHGEGKSSEESRSEADGIPDGETTNEIDKQSISLQLATPYPQLEIMLTQHRPAWFEQML
LRVADIPHIVRNSNHISNEATGQLPYLTDCTPSKLPVLVGRHQPSNLKRSSNICNNSILA
YLQDCRNVNLDKQAGLATDQEQALSKSFQFMIQSELSKILLHARFEDGDAWEQVYRDQYI
GASTVQCNEGRGPSSLGKRNWLLKLHGRFQASMERATERRRLPGCGCRGQSISIDQLLER
ANEAYFAIDRQLLSAAMKQNKTDSKRKYLLGTDEPALVDVLLWAHLAEALCDVHLVVLLA
SYPRLVEYFEDFYRRYFGQCDAKVDEVCSWKDWNNAQNLENAFQKIPTLSKRDPPQNTAF
KNAVDLMQKSSLQKQDLQEVLDAAKEIRTKECIPEPREPPSYLLYRWCMGETSTADQPVT
GKESENPIRKKLLRDQARNDQMWISGILGISAVAVLVIQGSAT
>comp3421_c0_seq1; 438 536 2021 LEN=2053; translated
MVLSTPELELQSLRENAYRTRDGSLVAAGDQWGNLSALAAVSVASQRLGRSTRVGRLLGA
PVTAMALTFFLASVGVLAQGGTPTSRSLQTMAIRFATPWILLGAEFGTSGGDELVEASSS
SSSTSVAPLVVGFGLASLATLAGGCIGWNLAGGALVSCALGGHDGLAIASALLAKNIGGG
INYVAVCAALEASPEAIAVGLCIDNIGALVYFPISNLLASRYPDVTDSNASQTNSEARND
SGQNKDSGVASAAVEPMTVSSISMAFFAATTLLWMGDYVASSLLKSPSSQIPVVTLLAVV
LAGIVAPRLPTRETDGSISSSTAGGPMSEPKFPLFPSSASKRALRSTCDTLGTFCLYLFF
TTAGAPGIKVAKSMTSALVPLSLFSILLYAVHGGILSALYQLGHEQSRFRRKRQQRREGQ
LRPTRGWLSTVLSPQRLLVASSSAIGGPPTSVALARSVSWDSLVVPALLVGNIGYAVSTF
IGIAFYYCLKSTVL
>comp3422_c0_seq1; 858 140 2020 LEN=2330; translated
MGSPHRQTHKNARLAVAVAVTVSTLLVLVGLQCSNALAPPPRKDASRATATARALTRTRT
RANTCLFNNNGESAATAAAPRSEIDRRRNLAIISHPDSGKTTMTEKLLLYGGALQQAGAV
RQKAEQRATQSDFMEMEKERGISISSTVMNFDYEGFRLNILDTPGHQDFSEDTYRALAAA
DNAIMLLDAAKGLEPQTRKLFEVCRMRGLPLFTFCNKMDRPSLSPYELMDQIQEEFDLES
FPILWPIGDGDRFKGVLDRLEDVVHLYQKPAKRGAKAEVIEVPLSETDRLEELIDDAELF
EKLLEDRELLDELIEPLDMERVLDGEQTPLFFGSAMTNFGVQLFLDKFLKMGTKPTSRPA
ISGDKKEESTEQPVSPEHEEFTGFIFKTQANLDPRHRDRLAYVRVVSGVYEKGMKVGHSR
SKAGKKYNLAQAQALFGSDRSTVELAYPGDVIGINNPGSFAIGDTLFTGAERVAFPGIPS
FSPEKFAYIRSPNPSDYKSFRKGIDQLLAEGAVQALRQRNDDGGGPLMLAAVGQLQFEVV
QARLENEYKVESQMEPLGYTLARWADGGWDAVDKADADGKLFGIMIVQDRWGRPVLLFRN
DWKAASLTEEEKDLQLKPWSQPPDFE
>comp3423_c0_seq1; 238 1 982 LEN=1141; translated
XSDYPSDYPTAGPSDYPSVGPSASKSSDSPTSLPKPPERRTDPPNSLVSSSPTGTPTFEL
PVHCPPSDPILVAVEGETMYPAPPLAITYQNTTHVGFRVENTFGNLISSVFTEYHSGDFG
ETECLEEENVETNQEIEFVAQCMVHAKISIVNVWISDCNDASPFLDELDNAEVPECCHPG
DECKTVQYTFKLPCVDPCPIDLSEVGDAVSTSTGSPKLIDSGTSRPTSSVSSSVSSSPSA
SPSASPKDATYPQGRRKLDEEKLEDEDHFCVVEDYPCGPSSDKVYVCHYSARDGYKTFCV
PEPDSDALRFYPKDYCGPCVGGYSETI
>comp3424_c0_seq2; 82 281 870 LEN=1416; translated
MAKMLPSLIVFDLDDCLWTPEMHELPGCPEIPVHGDLTPKHGNSKQAHQPPREQVGVVGL
QVPRYRDTVTLYDGARSVLYELATNPIYKGIQLATASSSLEPSYSYACLGGIEILPGLCL
GDMMSYNQIGRTGALSPDKTTHFRELHEESGIPYEEMLFFDDCNWGDXLPESHKGVRSGK
PENAAWIAMGRIPKGP
>comp3425_c0_seq1; 247 224 1270 LEN=1508; translated
MPGPPPVVTVFSSIPAIAEALSSCEGLALDVVEDGALTGYGGTVEFDPRNLSPATFKALE
RAVVLVSEPAVVASLLGHEPQCLRNLRWCQSTYAGVDPLFAPGIAHLEKPWILTRFAGVF
GPPIAEWCLARIIGHERSFAASALDQRRREWAGSREAVGHYRYLSSLTLSVLGCGDIGRC
IARAASALGMRTVGFGRIPRAQGDLEGVEKYTLDLGEALRAGDYVVSVLPSTPETRGLLG
GSALAAAAGASGGKSPVLLNVGRGDVIDEESLLSALESGYISAAILDVFEKEPLPRDSPL
WNTPGVVVSPHVSGLTQASDVPGVLLANYQRYVEGNDLLYVVDWDKGY
>comp3426_c0_seq1; 352 139 1122 LEN=1585; translated
MSTSNKSSSKSSSRIARGTALASIALAALSPSASGFSPSLASFLRTTGPLRSMSMDNDSP
SDTPLENDDVVTVDVESAESLETVDKALGSETETPGALVSRIVDLLPSTVGLSPSEENRS
AINEALLGLEALNPTPEPATSALLNGVWELAYAGLYSSAGALDSPTRQAALFLYSGGYSP
GLFCLQLAKQLPRGLVEVTGPLDISISRAAPRVEASVSVKTLLGGTSTVRVKSDLGVVSG
VRLKETYESAEVLSRGFVDLPGVLQYSREIYVTYLDEDLMIVRDGSGVPELLVRKEKSFT
RVWGDEPTTVDDELAPGEEDADEASVL
>comp3427_c0_seq1; 254 87 665 LEN=910; translated
MSNPNHYRHPGHERIVREVYEDRQQLVNENEAEIADLKRQLERATLNQETLLARRNAAKK
NYEAAKPNKVDHLEDDGGSDEVRSNCIGGSDEVSSNHNRDDASTRRHSKRYLRRLMRKTL
TTSTTYATSASDEQGRNTCASNGQRRMSLVNAEGVLVVNNGISSPRKRSERLRERYQSES

LRIARFSDRRQV
>comp3429_c0_seq1; 362 82 1218 LEN=1472; translated
MRQYETTRIRRRQATCRTTAAHGMLCLLAFAFFEANGRWVTTVTPWVQSVGAFAPVVIQP
RIPTSSFPSRDQNCHNSRAVLPCKGMPFPGQAGSLTKTVHASSLSAMPMELAYTLSGAYG
TALLEHPLPTKSLTAGILCGIGDVLAQSRGTNEDSTSNYNLQRTLRFASKGCLGGILWTF
WYDQIDSFLRFQEDIDQIGGDGSYNHNKISLYVLTGSIFSVQANASFLAFCKAHTAAVTT
LMSILMEQFLWCPLVYGTFEIPISTLMNGGSVGSIRGEVTSKLNGLLVSNAKVWTLANLL
IYNAPLEWRLFIGNVIDIFWQSIVADVSADCGGLGQEECGLDGTETTFISAGEEKEIEAA
MMASSTAPVNDRLLQTIE
>comp3430_c0_seq1; 362 134 1388 LEN=1396; translated
MVSTSAQPTATRPGLLVLVLFLLSAPLLAAGFRATTTTGVTSLRGSERTNTSTACFSVRF
RCGRPGDEPVIALTMARELMNPLGISHRNFVVAEDASTGDRVGWAQIRPLGPAGVDPSEL
NSPPGSMKPASIGDAVDEQMWEEFEEDPSAGFGDGRWQSTLPWTKEYKQAFASAAERRER
RAELLAREEDARADRPVPRLWELASVYVVPERRSEGIGSGLVRSVLDQHRELGRASEAVY
ALTLSSTVEWYRSGFGFVEVEEPREVPKPMALEVAAGSILTKLMGNELVCLRMPLEQMTG
ERSGKPRINTEEFSEHANRPIRNASCPSWREMGCQNERENRVDKTPPNPQKERRGTEHER
FENDSSALPFDNYRAGNFSVGIAAIPCRDERIRQAERSFVPPSQIFAPPEVNTAVPY
>comp3431_c0_seq1; 175 1 1079 LEN=1352; translated
XSEENPSKQPKPHSMVDDGAFVSKQPPEALSSTPRSAPARFFRVRPAAGSDDFRAESMHR
SVRCGGAHPLDVDDPSPSGSVPANGNESGKKIDSGEGSSSSSIEARNRTHPGVACSRPVS
IREDLTEEQRNPAVGERWMVDPPTGGNLHLVCCDTTKGSFRILLHERWAPTGVSHLLEML
GGGYFDTEIPLFRCTDACQFGLSANQTLTKAFGGTVADDPLWLPTGPEHRYFRAPGGSGE
IERYPKGVLTYAGGGRNSRGNQFVLTLQPNKYMGGGSPWEVPLGEVVEIESERKNAGGES
SDLSLPDLYTGYGERGPGQGLLHREGASASVRERWPLMDYVLRCSIVDQWISDGGGGRG
>comp3433_c0_seq1; 665 1 1029 LEN=1206; translated
GWYHAVQMLEDKCPSASLDYVVEPWFMGAGADSEAGKEFGEWQAESEAKYGTGFAVSLDD
LPPPAAPRLALISGRTADNPRLLSESIAHGAKCIYLEKPGAPTVVELEAMKDEADAASVE
VLMGYNKNVCKYVRKTREFAETVEGSHVTFVSNNAYENTPESLGECFERNAEGMLKNMAI
HELALLVSFYDVTVDNIESVVADKEFSSLQTLKGPSGDDYTDFDKIKFTITTKTGKEVSV
AADRCGGTDSYATVTDTSGEEVFRYYMPDEEDKVHVKELEAQYPTAMPYFFTQDPDYITV
KERVAKHTVDGKPAEGVATIQIAIDTLKVAEYLTPILQEQLK
>comp3434_c0_seq1; 228 140 658 LEN=855; translated
MRSMSYLSNMTSESNLHRSTTKSLGKKRLVASKSSSKSGPATSYKLSSPVRTILSRELQM
PCFKGSDGPLQPVNSRRRFQRRGSKSASMLRSLSSSHMVAELLKNDSRREERTDTTELLI
GSMKRISTESNRSSSSSYSESSSSMGGSMATLSSRMEDSSNSSLPSREQSSA
>comp3435_c0_seq1; 174 117 613 LEN=845; translated
MSSAPTEEPSTPPEGLRDDELSKRFYYGGLLGLPWLWIVHALHFHGKQRQLEAQRILREE
QEHNVNVRMKLAEWDDNDDNLSVLSSGIFITDVLLFFLASSSHPDTDNQYAEAEKMWVRR
GRNSAVVVSGAWLAWVLFVQLIFPDTLPSSWYVRDPNSYAATGW
>comp3435_c0_seq2; 206 117 515 LEN=747; translated
MSSAPTEEPSTPPEGLRDDELSKRFYYGGLLGLPWLWIVHALHFHGKQRQLEAQRILREE
QEHNSESSSSHPDTDNQYAEAEKMWVRRGRNSAVVVSGAWLAWVLFVQLIFPDTLPSSWY
VRDPNSYAATGW
>comp3436_c0_seq1; 299 133 1533 LEN=1768; translated
MRSILPTTHFVLPFSRQVVLEEVPPNQRLARSPPKHTEGSNRPFPSPRDHAPSNRERQTK
SKLGGLVSSRTVVPCLLLLTLAIVAGTMDLRRPLVATLAVVADADGNTDGNTDGREVLLS
LEHLFHNGGTVGGTTGGATSPGVPFESGLRWNPRPGVPCPHCGQTDDDDGGAVQRFGSGS
ADRDAASLGEGDPPARTPTGGQTRLATAAVSGSPSWGQRVLRRLWSSGGTAGKASPESRH
LRAAPPVATAPGDPLGGSLLPGDSICATIERAFPRAFPERLKRKCRWAAARSRGGGTARR
TALGSNHRASFETGNGGESGSGGKNDVPEDGNNLSEENENNDASEDANDVSKDGNDVPED
GASPSVPYLGSDRDEHGCIVSAGTPGASPWGPATGPGKAPARAPLARTQSPLPLEPTPAA
GAATKATSGAGPPGRATTXWETVCGGGAELPARYNPIGARKRHKFL
>comp3439_c0_seq1; 401 1 829 LEN=837; translated
XQQQHQEREQSTRQQRRQQRRQDKQQRGAEPTRAEVSVDDILASLQQQQQQAPGEPLVSA
GGPPSASEKDAPGSGFRRDGIATQQIAPQELRIAAASNDLASLERYLDVAPQHANRQDKR
GWTALHYASHHGNLGAVRLLLGHGDGESEWEIDAGIKSFDETPQTAFDVALAVHGIDHPV
ARAFVESGWHDGGESDERVRRVLDEKKRRDEKDKRLADKAERERLAIQQKRDRMFGDLSR
YRAINKNGGDLDDDGENESEVELRRKRLDALLGGEL
>comp3440_c0_seq1; 281 1 1613 LEN=1790; translated
RNYREDQILLRFFSVRWNTVTKIPNLEWLIDDFKLMMDNAVEARDQKRTIDPILNLPVNS

LATSLCFVCPAKINAPATKIEPDIETSGNTSYPDEKSAYFCEDDDSESDSDSDDNELLFR
SSSISITKTDSNTSIRGGRHLHRVLNDRFLASCHQDGEALLWDLNRQKNVARIAAPRGGS
GLCVRRTDDPSQIMVQTRDPKGIVSLHSIERCRPIVGSHGIGSDSDTTSNHVRQYETHSQ
TFCRAAPCYGNKHLLALPSDDESTVTVVDNRSNSSVATYSVPGHGMLTSLGLAVTDHERG
YASHNPILACGMENGTAVFFDITSGSAKPVASESSFSLGKDPILTLDLLPSFSRSIVATH
ENCPPSFPITSSSNALLVAAGMAGDAEEVSELSEDEAGRAVLFKAFCRGDSTSSPAWTFK
QRKRFSTCRVDRDGFNGKPGVSSCKFRPGDGRLLAIGGWDRRIRLFERSTGKPMAILKGN
VGSVADLDWAPDSMKSGLLQVHAEEKNRYLFGNAMQSVELSLEKYENALYRFLGLV
>comp3441_c0_seq1; 342 1 1890 LEN=1890; translated
XNNNNNNNTMSSNNPGTAETTGARPMTKEEQEQLFRDAMLALSALAGAVLFLKALAASIT
VYLALLPLAFLYGIQTCPPASSFDAKQQLRSVLRGDSLPPDHPDNPGQKTGFLQQLWADT
KATLTSELVTLPGYEVTIASLAGAALLARVTMPTADLECVWLGCNHRWYYWGARSLSESN
GPGAAVEGVSDNKVGNNVYKAPEHVDLKIGNANFRLDFGKGKQDEXLPDDGLGRCQLAGT
PRPAGENGLLDGQKGPAKQTKTRWARVLVSNSLFPGFLFFFFLLRDSEPESNRLESTINS
TQFSLIPRLCLEVENTMQLLNSLCVDFDRELLLSMLYHIHIQRLIYLVFDRVPKPLFILQ
QWLPWLRREKRAPRTYQLANKLCYAHKLRQNEDKETESTHRTALHRKNNSTGQQHRTTTR
VPAETRSTAIAMCVPMDGLCRVPPHFLPFATERGTGLLYQQHNRPGLQPTDLVEAAMAPA
TESAKETAVATPPLACFVRVAPEEAGGAGGLAPSATAGRKTGDDWLAPPNACPSSPQNKS
PISSQVQKSSSRGSLQTATVPFVPRHAQFSSSSRASSPCQTVPLPSLPSAGSSGPGPSSA
STSNVVLAVEERSGRLEITSCGGDSGRTIGX
>comp3442_c0_seq1; 614 1 1886 LEN=2369; translated
XSDDENKPAEGSKWDGAYSPSFFARFANALFGCDDNDDTDGRFSMKCGGDHREPNTVEAK
LKAFTMPSPRKSAKSEIGSPRRDSGPGPSETSLAGEESSEYQSCTETEYTTTEYTSVGVT
TLGDTTETSNYPGSLSITTREGDGARTVGYEGDDNATFPTYQRGFSATGETQSEPRNGEV
LEFESDPYHEDETLTTYGEAMGVSTLEDTTTFTLGDTTTFTFNDYDDDDDEQREDYDTTN
EDSSYGSNSFRSPLTDSYQTASSRFFGDSQSRSFSLRSMPRFREHFPDTRGNGDRDGAQG
SVVGNQIAFVSHHHEEQKQPLAMGESRSVSTRSPSSLRESLMSSGFRNENDVIVGIMAIA
EDDDNKTGEDSKDSLSYTKSENRNRLINSFILLNENEEAPQSELETSQTTRSRARLRTFG
KKGKTKTAIDRDMAVGSECSSCAEDGEKEGCKENEEPTKKTLARKPGEKGPKSPSEADVS
SKAVRITVLKTVKKADVDGSRSQGSYRTRSSAFQSIVYGRESTSSGKARDEPRDEPEEPK
DSHHQRKANHKVLHRSNGPFLRVDTATVPVSTPEKVPVVSPGGSMASSMHTTSHPKCQGP
SSELLLSGNLSPMARLRNPVSYDVLSHD
>comp3443_c0_seq1; 318 43 777 LEN=1484; translated
MMEGAFFFLLLCLKTLTVPVESFPASSLSHTKISADVTIRRETKIYRAKANDDDVDQSTD
SSSLIPTEAVEFDWKAIANHVFDESVESAETLHSKPIILFDGVCNLCNGGVNYAIDHDKN
GKFRFASLQSNVAKSLLLRDGKDPFTTSDVVLVTAEKNYYSSEAVARIMASLDLPVLKLL
GNLGQITPSFARNFIYKIVSGNRFVLGENDSCRMDFDGEYTWRFVSDPPESISSLTEEDK
NAGE
>comp3444_c0_seq1; 162 1 445 LEN=1232; translated
NNNNTKWKPSLQTIAEEEERVVAGPNTIEDEDEAEAEQGGDCDEEGKPRKASAMAMAMAK
PNTQGKALPLPHPRQQQLDLLVAGGSSAPSPSAPPGTSASSGRSEQNGTDQNTTERNGTK
GNATQGKATAKQRPPNQPNHPRATPRK
>comp3445_c0_seq1; 156 1 364 LEN=525; translated
XRKMIDDVSERPVAMNIATISAGFEKGDFLVREEQGNRVYFDFFEEAFNYISNKGYARMS
REDEKHWVDLMNVIHDSVKGGMKYNTGTLLMVLSRPVNSDGASDKKDKVKAPSKDLKSMN
P
>comp3446_c0_seq1; 920 1 4209 LEN=4240; translated
XFFATRLQSGPRFAFESVPVARTEVDWTNLSRMLRQLSATRRKALPSVAPRRGSHKNVRR
ENYDCGGVVRVSSGNPLCRTVGRFEERQRRYFRHHVQHHHQQRRQQGGGFSFHHNCTDRA
TRCDLSRRDLPSFHRLRACYFSNAAERTLDDENADDDAAVHAGGDQAPDQAPVHANQSTE
HEGDPGLNHIGNLKLWYLDHHGEIGSVVQDAFETKMVQNPSHSILVRCGGDTDAALSGKG
QDAIDAIQQIFTASFTCPVTNERYEAGRLSGYFDIGDQNFYSKKKVALQAVSAVVLRHKM
GASDSNHDNPNTKAVLDNGEEYSYRLPSTNDQNSLRQRLQALYMAKFKVSPTKSQSRMTK
QCFVGKEQGGSWWTAAFVCPVTSRIYEAGDLPTEYAPDSMKMNKAGQIWYRKKSDAVHAS
AYTAIDSIDWDAELSTLEEDKSKTAPAELEQSARSTDVLRALSFWFLEQQGHTDLDLSIE
ENFVVTAGTDDKGREVWTASFLCPLTGERFDSGTINTNDAVPVAEIDHLNWYTNRDTALR
AASLRAYDAFHFRDTGVTDPRFCREDPTDFTTGNVRVGGEDIDSDSENDEDTYTADDLIG
YEEDEEDFIIEVIPQQLNLSRGLESGSSKTLDIIAQAWIDSTVVPSSEESPKHEIQKLQN
IYSERQRTINRAMEWVSKQKHDSVKETNDRTQFDVTGRICNLKIANAILSSLAKNHQRIP
FDSRPAGVEKCAAAILESMWSSESTIPDAQSYALYLKCLEGETAKEVAGRAQEIISAMES

GDEYNGRFLPKPNEAVHSILAELNALAGISPPDTIRGCESLDRNVHLCHLSAMAHNPTMF
DAKLAIECIDQMRTLSETHNCPSLQPDIEAYNAPLRWSGGSLRSRLYSRVIPWDSYSEIF
EDGFVSESNAAALLEQAAKVEEWIEMMKSKTENDDALAPNIETYESLIQAWVRCGSMESL
LRAETIANNLITGDYPCIRPRLQTFYPIIGAWVYSGCDEGPQSVESWIDRILLLQLEPDI
DSRLGFPHLSIMSQLSLQRQILKQSGDTPDSMSRIKESAIKSNEILRSIIEEQNNAAEFR
IGSDMFMLVINAWCNVASTALSQNIMEEVRQCSREIQKVVNLFDEMLISLNMADDSQEEN
QSKYSRLLNEAPSVYGAQIAALNKLQTDFDVSHEGSWSDDGLRQQQIFIEEKIRRLEEFN
FFFSGDNNVKIADSQNCNSEVNIMSPFPNEVYMTDPSCRDWADFIYLSLDSIEKGTNNFS
HGEADFLRLCTLITRVTSSAMPSVLTSESRDQIFHKVIDLLEIYGSQNQDRMTVVSTLVQ
SLKKCVGTQQDKETKSLHGCILRCGTNEQLDEEDANDLTVAXCPNKTKNEEEEVSWERIT
RKLVFSFKSTKPLTVKALSFASC
>comp3447_c0_seq1; 507 1 2185 LEN=2185; translated
XTLLLKVAVIYPETTDVADQSYAGLLHHWISSRVDFGKILTSNRKKSGTSNFTSSDEVRI
VIVKGSSYDICATADIALFVIPPHWSGPEQKGEILGELASSLIDDDIPRVALVLRDSVGA
DEIREINNLIATQLAGNMDAIPIIHPSELSVKAFESALECSFSRIAKLFVHEACVRVSRI
PAMQLATKTILAVLWQSIPSVAGEDIDEDAIVECSRLTVHTLIEELAKQFERNETEWSLW
PAPEFSSRDGVVQSYFSGAVGLPLEWGRCLDRRVLHQTYESLLSAFRGHFRDAYQRIVVD
APITVQDQCASECAQGQYRRCLERALQWMQTSSSFSGCFLYLPEGLLEFVKKKVGEKVGA
SPLLSKSKGSNSTKAEIPHLAAEDRLFCDDVPTKNTSKRSRAAPDFFGKRSAPTNGNHPS
LKKQKQEDPPVAAGEGPPCVTPVGTVGSQARNPVQTPSTKRRTSALRKGDAYATKLERLL
HGDDTIDFVVGQTSSLSEFLRGAPKPKAEGANPSASNGVSIGIKDTMILILQNGKKGTMI
GGHQYHINITSHHTTPHQIIRGRLEILRLLRHFCKNTSQLLNCIILLQQYPYGTVMSGXG
MVRQAARDSVGSQLDRSRLRPCHSVVRSLSTWYDAKSASQRSISDTMARESAFFRCLTRL
PRTLSTEAPAHSGGATTTASTAAXDLVELVADPRQRALDAEGVPVPAGAPSPVGGVVHPD
RVPEPGGEGX
>comp3448_c0_seq2; 301 75 1037 LEN=1184; translated
MAKSKSKMTLVSSNQNESRNSLVAKPMDRSPPNSPTIVNNTKYQIVVFLERGKFFNKCTL
HPGEAVNMTRKQTGGGSLKFSYKVHAMIGDERSLPTKGDSIRNVLKVSAVPAAFAAGCLI
SAASAGTMTGPSAALAPLVRGIVVKGVTVSAADIAAGTLMAERTKKVAKILMENHEETVK
CVTGKYKPKERYLSVEGGLLEGPIVVRDISRRKFKRLSITNTKAPMDPSEVYEKLSAASF
DDSETDADSSRSMRSGSAANGGKENERGGTNIKSRPQMTDVKNVLVKSYKLKKVISESTT
KQNNASTKAKGKNPMYAAIF
>comp3450_c0_seq1; 161 1 700 LEN=1308; translated
EQEATEQKQDNLEQFSKTASFAPYGNYYEELQLNLVTHQQHYYTAMISSSWFMSKTVPLV
ARSILSKSNRATVTAGATLPTRRGYRGDTGEAVNKLTEKEEKLLKIALPKAEKIMKKHIE
LPVDDIATRRKRLIYRAKQRGWLEVDLLLGTWASENVPQMEDGDELDQFEDFVNMETIDI
YNVITLRLDVPEDMKRDGNGVVERIQEWARSHPLGRGDPDAYKAVKMEHKLT
>comp3451_c0_seq1; 666 195 2912 LEN=3153; translated
MLVQLSPWETASTSIRVPKRRRKRVGSKGSDKEGDQKEPPSFASHMITMVSKRSLSKMKM
HSQEFAGCLPLLAITAGHQITVLGKNLSTETEGDSYREFTSLRYSSRPGDAGSFFAGPES
DLPRIPAIPDTKNDRVYALQQGNQRLACWDSWKTGGPDEASAVKVRLENPAVSMSMLPMN
KGIVYGSCKNGAVYVARMVGGALAVEYLRGKHSKNGKLVGTFAEIDMEQAVRGKKRKTSD
ADGNSSVRFYQVFCEGTTITIVRNSAMLSASGSHSNPDKPLIQTGSLVQNAAFVDLLGEA
PANDRDNYELTRLELLVSSSGSAPKISVVFTVTATHSKTKTNGYSRRESAKDPFCGTFCM
AISLATGDVSTSPLRLPPGSNQFGLFTESVLSAASDEMICLYDLETSAFLQSVSLEKPLS
DMDKGGNWILGTNAKHGTLCVFYPRDGLLRVALSTVSGLGSSSERLRSSSKLACSLLANT
TGALELMGNATDETQYKQGLLALTRLDEAVARALATLDEIRRTLVSQGDNAKSSNKPSFR
EAFYVALSNLTKDTEGSASTGGYPHYHCNGETPTKKMKNGKLNGAVHASLAAKGGKIPPC
TPQSFIDGAVQIVLSTIIIGKEDRNGSNSSSFDELGLDARFVLKDLLRSGRVSARLHFEG
LYALQETGKKHPLAIALGCMEYPAAGNPLSPLQIIIEMILNCTDLSERQLVVMLDYMMRH
AKARDIVKIVREKHTHMVSKDLAALTNGDEKTTVLAGVRVVLEMIVGYSECNEAMLRAAL
VEELSSSVEAVLLARLLPGLLLSNPNRNSPRYFIRSTCLWIAALSESFRDDLGWARTSSG
ETYLAFLLNSVADTTKNSQEIMSLKDSIGVAEMIQKYKKVKAKKVSSEALQTEELVGYSI
ERIIF
>comp3452_c0_seq1; 248 1 242 LEN=242; translated
XEEREREEMEKKQLDKKKNTKKEEKSDKKKKKKKEDDDDDGSSGSEKLKSDKKSKKKKN
KKSKDESDDDGDDEEEAKKDK
>comp3453_c0_seq1; 234 1 925 LEN=925; translated
XAKPAGAEPAEDREQETDDKDDGDASASDAEPDRAAPGFSVLSADDSDSDQAETGTSLPE
PEAATGNALLAQLAKERADRQRQREQQAARPSGPGDSKGSSRQAKRKKKKPKGQRLGGGR

NPPPGRGPSKKEKDETLDDDLDDMAFLDAQVARAQNAHGRRVEGSGTAYRTIVHGILNAR
PEPRHQAPRNARASAALRAKLGEAGKARQKKPKKKXGPAKCFSRAARGSASGSQAKNRYE
KTAGWPAAADGGGMMTESLSDSPETPSVVSRTTRMVRNSSSSSATTVAGENCDDSLVEMV
ENQYLDPSWX
>comp3455_c0_seq1; 73 1 395 LEN=395; translated
EDCAKVGCCHFNEGQCLSSVGRDPCHNVLLEGYEGWKDGAVVWSDPPSDGKWEACGKVTV
VFGVAICVTQGAWNNKSKCDHIAHVMYQLLDNNADGIPDDEQVLTFMVQNRYMLFVPENG
EESEELWSKLPX
>comp3457_c0_seq1; 230 255 713 LEN=770; minus strand; translated
MLVNGSMKILTRLVEMYLKRALNPFLNVTKQNRIQDLACPTWPAVHTFALTRGAQIVENP
RFGLGFVLITWPSVLDQPLYGPASSLSSFLLIRRKGMSVVEALNLEQSCEVLNNFNRPRG
RMFDTATIVSRFRASLPSPRGPSQFYSEQLDR
>comp3460_c0_seq1; 316 26 1018 LEN=1018; minus strand; translated
MGSKHESKRKNTGTDKHPIDRLMWLVAALFGFRHLLLLAVSFAAFDRAYRTDVLQANERK
ALLLRGGATGAAPPSSTAQNPALRPPGLLPDREAWHEMTMAEMDENLGCDYDRGLPIHGP
EIWTGMREAYVSVVGRHRSTLASVGRDAFSTGLLVPFEVRYSDGRGRGIFAKEAVDKGRV
LYDFSQSAQFETGTDFAEFLRILAPELACDVLLWSYAQDLGTETGNQTRSERRETTKGAR
LRIVTDLDPGSFCNDGGYEGGNTGWMDQERGGASEGGRHPPHVAVLRREEASSGSGFVRS
APLVALKDIAKGEELSCVYDQFNEGLGLMID
>comp3462_c0_seq1; 601 1 1697 LEN=1722; translated
NEAHARELQALKEAHAKELRELKEQLDSANTISTASASNNSSNNNSANTASRNEAFRQRW
KNRMNNNNNTIQNNAHTPTVAFSGDTNHETNDDYRATEVASTVVSTMSCRELSVARYRAR
LLEQMNRGRGTLVATGAGATRREDSGVDGTNGGSAESRDGNDAEEIQRLLDELQQAERRQ
KLLEKQLQQAGVVLAEDIPYQLAKDKVASISKRMNEIGSSGVVHSDPLVQKQLREEYFRL
EKDMQKYISALMLTDEYAEERRSEEEAWEARNKVANQKALETIRRSMPVDVRRLTPSQLE
DDYGLPGDLVKRFRRINVLQLLRVDPAIVAKWHPSSVEALRVTGLTITERRALHCYLSPV
VCKWNGSETPVVAAGNTGTDAMTQRKLAWFGALRTNFKEALGEYERHLSQRPGPGSCASK
TCGLMGNRCPVRANAAVKYPDLGHPSHGAYEAPFDLVAGGGDQKSPAEVIAXVPQERTDR
GRGGTQGQNACARPGEEGVRAEARRTAEASGRAQGGLGPERPAGGHCRSPDRLSRILDAG
AEREAAGVAGGRYDAMYKKRYNNIP
>comp3468_c0_seq1; 105 743 1699 LEN=2017; minus strand; translated
MPELKYSEGGALPSNAPEWAVAASQRIQERMAEKGRAGLYDNPPPEGISKILLHSCCAPC
SGAMVEEMLSYTEKYGLEVVIFFYNPNIHPRREYMIRKEENKRFAECLGIEFVDCDYDSD
NWYKRMKGMEFDPERGNRCTECFDMRMERTALYAHENGFDAIATTNATSRWKDAKQVDGS
GIRAAARYEGLTYWYRDWQTDVMTLRKYQISAENKFYKQEYCGCSYSLRDSNDWRRANGI
PKIEIGGDRAGLGNRYFSDPIEDAQEESQDVVDEFFAQAQDIAAAGSDRTERKNALEMYK
DRRKNEKEAGIDFGLNNW
>comp3469_c0_seq1; 146 1 1065 LEN=1065; translated
NRGDVMTDKTLTRMDLSEAIFREVGLSRNDAAQLVESVLGHMSDALVRGEQVKISSFGTF
SVRDKTARVGRNPKTGEEVPINPRRVLTFRPSHLMKDRVADGTKXLRISPPMGKAADAFR
TISEVADWLEVPAHVLRFWESKFTQIKPVKRAGGRRYYRPADMALLGGIKVLLHDQGMTI
KGAQKFLRENGIQQVSDMSPPLDEEAQGALSGIALDVPSIPARPADVLAFTRPDGADDPA
ADPEDTAEVAVEPEALEDGAYEADAEAPLSPQTKAPAPKEDLDAPALPPIEDQTPHEAPP
AAPELSETAPSFHTAPEPEPEGDTAAGAEDETPPLEPDAPPTQTAPHPRAPSDLPX
>comp3470_c0_seq1; 219 1 404 LEN=551; translated
XMIMMMMKNNSFLALLVLTIAASALVSGFVQQPAQSIRSAPESTTTAVNGIWDKVVKSME
SGYAGGEESPYAKIKAADAKKEEIRRKKALERQKRGFTELKDVKKKSFVKLKYPGAEEEE
EPPAPEKKKLFGLF
>comp3471_c0_seq1; 1284 93 3638 LEN=3735; translated
MEGGDLIENHRHKQKKEGRGAKERKKDDKAKKEGTRKERHNHRAFSVANIVRTQRTIQRN
LDRSQKKEYVPLTDRRSIDPNESPPALVVVMGPKGVGKSTLIRSLVKLYTNHNLTTVTGP
ITVVTSKHKRITLLECCSDDTSAMLDCAKIADLVLLCVDAKFGFEMETFEFLNILQTHGF
PKVMGVFTHLDQFQTAKNLRKTKKLLKHRFWTEIYDGAKMFYFSGVINGKYLKNECKQLT
LFISRVKFRPLTWRNTHPYVLVDRHEDITHPNETDKNPDCDRSVTFYGWVRGSHMKKGTK
VHLIGVGDYNMAEISPLPDPCPIPDKEQKSLKKKDALLFAPLSNVGAVSFDKDAIYIDIG
RANYTQKADLQGNDESGDEDEEASEPEYDPEEPAGMLKGLQGVDSAVDERMERSTLRLFK
GSKAVEAGSDDDDSDSEDDVPEISTDSMVPYRVRGDSDSDGNRAEEDNDSQSGNDESDSD
NSDSDEDADDGEDDSNSDSSDEDDSEGESDNSDSDEEEGDSIERNGISQNSMSWKTGIAD
RALESFLERKGSIVNLQELIYGVDKAKSVNDDDDGNKSDSDDDEFFKIKKSSKTVSTDSN
SKNILQSKSLLGEEDSSRLISGERENISMEAWLEEGGDSLIESIRDKFVTGNWSKDGEGD

DEEAFDDFADLETGEKYGPNGEVLSSDEDDDDVEEDSDVEDKDPTEEMTDSEIRAYYAEK
KAKQKKGFDDDYDDDKKSKGMGEMKDDEAENEYLEALKRQKEERMKKNHEEFGKDGERSR
LRHEGFRQGVYCRIRIDGIPAAFIDNYNPEQPLVLGGLTPQETKLGFVRCRFKKHRWHKK
ILKCNDPLVFSIGWRRFQSIPTFSTEDDNGRHRYLKYTPEHMHCFATFYGPQCPPNTGIL
AIQRMSENISGFRIAATGVVLELDANFPVVKKLKLVGTPTKIYKNTAFVTGMFNSDLEVS
RFEGAAIRTVSGIRGQVKKAIREGQPGSFRATFEDKIIRSDIVFCRTWMPVKMKEYYNPV
TNHLSSKGTSGWYGMRNKALMQLETGTPIEVNPDSIYKPIERPERKFRKLRVPKKLESNL
PFANKPKDDPKKRKGGYLERRAVVMEKSEKKKFTFLQAVNTIRNEKVKSRKEKKRLSRIE
KDKKNAKKEEAIAAARKVNRKRQYRAEGKREKMQEAKRAKM
>comp3472_c0_seq1; 386 344 1444 LEN=1540; translated
MTFRRRLARTEATGDESEKREQTNRAIQSLMVKHDPILLFASRLLSPQKAEEASALYAWC
RRLDEICDSEENSSKPELVQDQLDVWQERFDDLWSDETKEGARKTTRVEPTATTYYSEDD
FLMDLALKECIERYQSSGMLTRKPFDDMVQGMKSDAVEKRRIRDMAELELYGYRVAGTVG
LMLLPLLLTPEDDANDSSSTSAAEAVKTAREPATCLGTAIQLVNILRDATQDAVLGRIYL
PTDMLRAEGVTEEDEAKWLLEAAVGEGMSPAPDGYKRVVEKVSNRGYELLAHAEEGRSTL
PGPFGPLLVQVIVELYRDYLEELKRRGYDNLQGGSGSNKGTMDRVKISGLRKAQASLRAI
AKVLID
>comp3473_c0_seq1; 113 1 608 LEN=943; translated
XTIIDKRSDAERDEGSIFGQTAGSSQSTWVECDKCKKWRRLRGIVDSNKLPPKWYCTMNK
NDPERARCSAPEEEYESPRTPESKADARTRKHLRVWVRRLQCNEQYESRLPTLTRGKKRS
AGTSPKEPHEWIRCCNPSCGKWRAILRIMDAKQNVMDLTTDGEWYCVMNTWDEKTASCAA
PQENLPAVGCPSWVMQDLEREN
>comp3474_c0_seq1; 235 161 925 LEN=1173; translated
MPLLHRMPSRPHLMVPRGGDQKVEDLDVDEPLVEEPSTEAEAEAEPVAKVASTKVVAAPC
VISKVMATLTSVGACYSKQLESRPILTKSYTAGLIFGLSDYFAQKIESSNDVNGSSSSMN
WTRVITSTLVGLLYFGPAAHYWYEWIFKLLPGTTLASTLYKAFWGQVLFGPSFTCVFFAT
ILMQSGNFSLQSWWQKIRSDLPGAWLAGAGYWPIVDLLSYSIVPVKWIPLFVNMASFIWT
IYLSLVANKKSSSA
>comp3476_c0_seq1; 481 50 1282 LEN=1461; translated
MGLIQRLKCEKKSDGEHTASTATLTNDGEVAKDSGKKKKGKKTKIRSMKVSKKKVEDGDL
LTGLDDTNVPEEYWEEAVVTKIPTEHRDLLAENLFCHIWKTQGKSVALKLLGDYEQENLS
KTANERFRNGCQKWRQDDYEGAGWELERSLIIREAQRNHQTLVNVRYLKQYKEEGDENQV
SKQVSREKEIDEIVQNYANADPTDGKALAKEREDAMDPPPNGNMFRVNTLSPSPTESTAQ
LYYALGMVRMSQEDHVDALMEFRRAIQVAALGLGVAHDLTKAATYMIRAALLTMGYRSHE
IRKYLAQLKSNIYHEIEADVLCESGNFDQALEEYADLNFLHDPDSQVQARIQTKIASIFE
ARGDYIKAIQIWADILVLYDDTPSVGIHHPLARQVVSRIVEGKRLLHTPY
>comp3478_c0_seq1; 239 1 721 LEN=765; translated
XPDIASSKAIMLQLFLQRVVATWLGKLFVGGAAVLTSLRYAGGIYAFLEAEKLERPNYSV
VQRLTDGVEIRRYEPYLIAETTAEDAAGFQKAGKQTFRTLAGYIFGKNKARGGFLSSGKN
EKMAMTAPVRLDGGQKKKTRVSFVIGSDYTLKTVPKPMEKKVTLRQVPAHTLAVKKFSGK
PPSDERVQKQRERLQSALKKADIEVQNGDDTTLVYGYHDPFITPNILRRNEVAVVIDGSV
>comp3479_c0_seq1; 539 34 1173 LEN=1270; translated
MAFAPKILSFVLVALNMSQAAAFTVGGTFLSAAEKAALEKTADAVATAGKGITACDEGPA
TIGGRFEAVGVENSEENRRLYRQMLFEAPGCEDYLSAAILDPETLYQRSDNGVLFPDLLT
KKGIVPGVKPHLKIYTLPGTNGDTVMQGLDSLAVRCKEYYAAGARFAKWRSPLVIDMEKK
CPTDLAIKANMHDLARYALICQSEGLMPIVEPDISLSGSHTLEDAIDINIRVQSELFKAM
IDHGVYMAGSTLKPNIVNPGRDCEKSYTVEEIAEANIFVFEQSFPVAMKGCNYLSGGQDL
PTAAARLSAINKANKKGPWNLSFSWSQALQLPLLDLCKGKGDLQLEEMSKLYVDELKIAG
AAAKGEHEWAKGEGDHEAA
>comp3480_c0_seq1; 355 1 1335 LEN=1431; translated
KRNNHWSKRRKETTSSSHNTFHSTISLAEPKKKVVVMDSFLLLLIAGLLGSTSVVEGFDT
LFGANLNAKSPLVGRVSAGPLEISSMGCGTWSWGNRFLFDYDPSQDEEIYEAYREVRNAG
VTLFDTADSYGTLDLNGRAEILLGEFERRYLDELGISSDGDESGQDTPSSWWEFGSKPPA
SKNKNYRPQQVATKLAPYPWRVTRNNIVRSAEGSVRRLEQPKLSIAQLHWSTANYQPFQE
RALRDGICDVYDKGLCDAVGVSNYGPKQLISVAEKFREREVPLAIAQVQYSLMTYDANGS
TGKTKMTGKDMNEACDEVGCRLISYSPLCLGLLTGKYTLKETNRLPKSKARQQLFRELLP
GAQGLLDTLEVVAQDYGKSQSQVAINWCMAKGTVPIPGGRTLQQARENLGATGWSLRPDA
VEELEIAASKVTKPMVQNVFQRSRW
>comp3482_c0_seq1; 280 1 1130 LEN=1130; translated
XFCTLLPVPSCGILDGTENTDSTGTTANSRDTAAMTPKTNRAENSFVAISNGEYAIDQLE

RCGKWLASIPRDDITVLRHTIEFQEFLASFERLGQAHIRVVVNNQSPNAVGTTQISPAGP
RAFGAHHPAEANKKPSGLGNAPIDAPHNQHAEEGNGSAKPNRKRSAAHATAVDAGNPRRS
NESNDDDDVSMKDPPDGAATRGGAGGLHGNATPTSGNNFLRFTDDVLLRIFEFLRCRCLI
QASRTCSRFHQLAGRSAFQRSSDVLQTRQLGSVMQLLRAREQIHYTDEDAAASGTGRTFH
VPVPLLLPSRRVRVANAGDPEYNGVYYCTECSCNGFVFTKPRGSAMVRAENGEANNGNMV
VPQQYRDLAFHNNNNNNX
>comp3483_c0_seq1; 218 271 773 LEN=869; translated
MGVKRKAPTPVSQAKQNLVITQHPSHSYSFAADKIAAENNTPIATTQQQSNKSVRFSNRI
GILSISAHKSYTNEERQRSWYTTDDYVDFVRRRRGCRCKTQAVRMRRAHSFPTDDEGHRN
RESGIDDIEQQXHGIPPRPAVDRKLWVVVVVVRNSKDQEQRPNDRELQ
>comp3484_c0_seq1; 402 151 1178 LEN=1973; translated
MESLLFFATGASSGVGLEATRQLAVHLSRQQQEEPNRNNSNSDGSNNQVVATIYLLCRSK
EKTQAAIEDIYNSIGENSSNAKNNIKLNFLKFDACDDAETIVNNIQFNNATKIAGVLLNA
GGFGENATGIDKNIKSKTSGSSSNIAKLNIIGHVILVNHLLSVGKTDASTRIVAAGSEAS
FVTQGLSIHAYNAADLRXHLSSSLGSGKMAIGTEYAWTKGILALYWAAFARHHPEVFVTT
VSPGAVSDTQLLSNVPFFLRTLARLSQWSCLGGSHTVQEGAKRYVNALINDIPVGSSGSF
LASRKGFAQDFGDVSSLSKCQFVSDIELQDKAWKAVHNSLER
>comp3485_c0_seq1; 429 54 1034 LEN=1266; translated
MRSSIPASILIISSTATSCAAFSTVSIADQTRRQQQHSSCAVLRSSSSEYDSFWNNDIIN
RRTILSTASAVLASSVAVAGLPLPASALVKGVAPPPKKSVGDKPKCTNVEECQAMADKRA
DEERAKEESGPPPLVTKSGLKYRDLEAGTTILDPETGEDISTTAKDGDGVEVYFKVLKLG
KRSYDGLSGEGTVVFSRGYGLEDDENAAGQKSFQTTVGSFQNIAALNEALVGMKPNGLRR
FAILPQKGWEKATAACDGGPGGGGAGGEIRTDYVVVPTATMVQQEACFDTNKIPFPSTYA
QQRRMAQRFDQSLIMEVQLVSINGKQ
>comp3486_c0_seq1; 126 1 276 LEN=276; translated
XKGGKGYKGYGHGCDCGGPAYPPAYPPAYPSHGEGCYEVCYGSGGYGGKGGKSGYYGYYG
PGYGKKYYGGKGGKGYYGPGPINYGYDGPKYYG
>comp3486_c0_seq2; 59 1 221 LEN=221; translated
GGKGGKSGSYGYEKCEIVCDGGKYGGYYPPAYPSEGCYEVCYGYGGDYYGGKGGKGYYGP
GPINYGYDGPKYYG
>comp3487_c0_seq1; 143 1 574 LEN=574; translated
XAAGDVGNVFCHQVSPWAEFQHTGTDAAVGDCWSGKPLLPGTLVSVGVPRASTSTNTTRP
SDDSKQGDRSTLPAPRPDSKPGTVWDNPSLLAAATATSTTTGGYSYLPPPGTEPPVQTQA
QQMSDAMVLKTMRGTQWRVLEDRSASAFYSGTDSHSPRTCRSLATFAGFAGEFNKGTVSV
EYSCGSSSSSSX
>comp3489_c0_seq1; 567 537 2078 LEN=2251; translated
MRERIVAGNLTDLTAEEVEVSVQTLLKSKKRGPKKRQERKSHNQISFGDLARTIATKWKA
IEPDIKAVYTKFAKAEKSRYTKEVLAWKRKKESELHAAASRPRTGNGSSTHSLSTASLSQ
SWKSLSLQGSFSNGTNQADSSSSSLDEVLKRQRDILVEMGFPEKGLRESDAVARQVPDAE
QDPCLQQRRATGFFRTGDPIGCQNSNTNGCNGDDNISQNCDTLPENIMFGDCGSEEQRRR
RQQRDQKHWHQQHQSQQEHQQHDVEDFQGSEVAESQLQPQTKGQHILLRLQEQQLQQLWE
LQKARDETSRMSNITTTNILSHVRQPRISNCDHSNALHLGQQQQRSRIEKLTDSQNQWLS
MMEQSDLVQLKRLEEITKKLDDLKDQQRRLKQLTKDCHDMDESMFRDAGSNLAKSISKTY
DRISNNDFSASTSSDSPNSPVFVRCQLSTRQPTGGLLPDDIAEGHLLGGNNNGLAADFNQ
ACRLNQALQFESAFLSQPCCESMRTEDGRLGSE
>comp3489_c0_seq2; 576 1 1930 LEN=2103; translated
XNSLSDPQAFKNQILKKVFSSSTMNATMDVDTVDLLQDCVTTDWCQKEVVTVGGRIEKAV
EYVDLSDSVHTSMSTSTNSNATISTSTHTSINTSTHSNDSVTFAKTRRSWKRPKDKPMRP
LSAYNMFFQNMRERIVAGNLTDLTAEEVEVSVQTLLKSKKRGPKKRQERKSHNQISFGDL
ARTIATKWKAIEPDIKAVYTKFAKAEKSRYTKEVLAWKRKKESELHAAASRPRTGNGSST
HSLSTASLSQSWKSLSLQGSFSNGTNQADSSSSSLDEVLKRQRDILVEMGFPEKGLRESD
AVARQVPDAEQDPCLQQRRATGFFRTGDPIGCQNSNTNGCNGDDNISQNCDTLPENIMFG
DCGSEEQRRRRQQRDQKHWHQQHQSQQEHQQHDVEDFQGSEVAESQLQPQTKGQHILLRL
QEQQLQQLWELQKARDETSRMSNITTTNILSHVRQPRISNCDHSNALHLGQQQQRSRIEK
LTDSQNQWLSMMEQSDLVQLKRLEEITKKLDDLKDQQRRLKQLTKDCHDMDESMFRDAGS
NLAKSISKTYDRISNNDFSASTSSDSPNSPVFVRCQLSTRQPTGGLLPDDIAEGHLLGGN
NNGLAADFNQACRLNQALQFESAFLSQPCCESMRTEDGRLGSE
>comp3490_c0_seq1; 371 341 979 LEN=1115; translated
MEYGSSVDLSSSSCPYSVWGQKKYSVGEGWTIKARSEYSKGKYDYDGDSAGTYITVEGSD
ADEETFVWGSGVVGGKNVKALKVGAKKIINTDGGKFMVAPRHSFETSETHVCLGFTKDDT

KAYLTLSEDEKSFLVQQQLDDDNSASVKAGCSGFVAATMTNESDLGSTTVTVTPDDIDVE
IKSDGWVAGIKSALSLDAEPTVRFSKSLTFGV
>comp3490_c0_seq2; 382 94 879 LEN=1015; translated
MKFSAATLFLLAGYASAGSQYSLTLKDGGYDSVRSALSPSLTYSGESNGMEYGSSVDLSS
SSCPYSVWGQKKYSVGEGWTIKARSEYSKGKYDYDGDSAGTYITVEGSDADEETFVWGSG
VVGGKNVKALKVGAKKIINTDGGKFMVAPRHSFETSETHVCLGFTKDDTKAYLTLSEDEK
SFLVQQQLDDDNSASVKAGCSGFVAATMTNESDLGSTTVTVTPDDIDVEIKSDGWVAGIK
SALSLDAEPTVRFSKSLTFGV
>comp3492_c0_seq1; 525 78 1064 LEN=1095; translated
MRVLNICLSALVLSSASAFAPISAPSSRVSSTELYANGGVVITGSAGGVGWAYAGEFMDR
GYDVVICDVKDCATAAAALEAKHKGAKVYHTQCDVSDSDSVEKLAEFAKENLGTVTHWIN
NAGINGGRRALSEVPISQVELVVKVNLMGTLLCTKAAIDLMSKQAGVTGHIFNTVGSGVK
GGGTPGYACYGATKRGLPQLTDSLVKELDEGVQGYEKKSTPGTVQVHNLSPGMVFTKLLL
DNSTPELRKFPFGVLAAQPEEVAEDLVPKILATTTNGGSVEFLTTDRILTKFFERFILQK
KSEYIDDDGNVIKTPGAQYDEDGVRALF
>comp3492_c0_seq2; 260 78 548 LEN=641; translated
MRVLNICLSALVLSSASAFAPISAPSSRVSSTELYANGGVVITGSAGGVGWAYAGEFMDR
GYDVVICDVKDCATAAAALEAKHKGAKVYHTQCDVSDSDSVEKLAEFAKENLGTVTHWIN
NAGINGGRRALSEVPISQVELVVKVSFLGTDGVQAK
>comp3494_c0_seq1; 315 98 733 LEN=1105; translated
MKFAATVLICASAIAGTSAFSPASTAGRRSVSLNANIRDKSEKAEVLANGWDGTTALGGA
VEDAKPSRMLDEIRAAGETIPSECEVFNANVEMDADSLKFEEVMDIIETHYEVGLIEFKN
GDIVNKPGENEGSAKLLSYAALSEFDKDTTLKLWGQYYREVLADPDGDSHQNIRNFMKTG
WEGVPFENGIALTKKCVGDSDWDWDAESWIP
>comp3495_c0_seq1; 191 1 806 LEN=1159; translated
XFTPHKIYNVNNHRSYKTIKSLRLYKTTSIMSAVDAFSSNLSTLSYPRTGKRGVPQQFAR
RLYEMLQSEAKIASDSNNGNAYISWSESGFAFRILDVDGFTTSVLPKYFRTKKFSSFQRN
LNLYGFSKVRRGPDTDMYAHQSFIRGKPELLLNLRKCSSASRRKMSASSSSDSCDSSSSS
ATHRENTCQKESPVTSPRTITDPFLQASKSSSKLLSYDQNTNQTWLSFHHQHLMPKTTYL
PKLPVKQPQASGAGRLDLLALAVEHASF
>comp3496_c0_seq1; 359 74 1010 LEN=1010; translated
MRFHSSILLLLVGLSDAWVTPLTSTNSPLQRSSVTTRTDPTRVAFSSPQQSTTDEYSDND
ITEIPMSSEANTIVGATMAALVCFAAMTASPETASAAMTTNSDVASSSIVTAGGSVKSAL
FAYGHYISLLGVVFILATERWTLESGPNLTDEEEDRLAIADTLYGVFGLLLVYTGYNRFS
DPTLGKGIDFYIHEPIFWLKIAMLGVLGAASFFNTTKIIQRSVGRYTGDKTVEPMSEELC
NRMKSICNAELTGLLFIPMAATFMARGIGYNESIPWQAEAGVAALIFAGLSFKYIKEALT
FESRLAETTSASX
>comp3497_c0_seq1; 240 145 543 LEN=543; translated
MADAKRSYPTTPRNGRHHSIDPPDYYDSPSMRSQRRGRSSQSSSQSFILKAFLGCIILCI
STTSFLHFHEQASERGDLRIHRGLDRYVMDMPDESLEDMMSARKLQRPRPKAGKPQRKII
KLGTDDDDDTANT
>comp3498_c0_seq1; 215 1 151 LEN=226; translated
XGGGGGGGGGAGGAGGEAAEEEEEEEVEEEEAEAPPSGGGLFGDDGDGDY
>comp3499_c0_seq1; 324 278 987 LEN=987; translated
MTKRHGWKHDCFEPLTIDDYLDGCGSNLFPSENHGDASFTGVWQSSPLFLVWLEHNWSKI
VEAATATTPPTSVPFSILELGSGTGFLGIHLAYNLQQTLAGAACQESNPTHRYKVTLSDT
PRSGAYFWTKANLEKALRGQEREQGDGDGGEEKKEAFDCGERRTLAGISSENLCVIPFDW
ACQEQRRAVSTQMDDWDLVIGTDLIYSEEGCHYLVQAMASLLNESPPSPSPAAATAA
>comp3501_c0_seq1; 451 84 986 LEN=1446; translated
MSAACATPQAIAPSEDPGREQSTHDPLSVSSRKSILADDKSSRGEEPGGGNEPSDVPGAK
QRRPSKGSEPAAGANESPSSCSSLHAEPPAQGESSFLEGGTRDDSCRSGSSRTKPSAAQT
QTQHFCSEMLRVGVCQTDFAEQMDIFMRCQHPLDILGMFQDEDDDKTDSINASEHHNGEG
GKVVVASAPRANKTSGYHAIPRSCEYCGSPDIDLCEDYSHNTCERPKSFFPREMPPFFRK
SCGTKLPWGGGGGGDDEKENRFGTVFGVAVAVDPAGKTGDAEAEDVGGAGPARMACSAWV
>comp3502_c0_seq1; 319 135 863 LEN=888; translated
MNSSPSPISLSRLFLLILGLVYALKPTAKVDGLRSVDKSMFVVFGRPGSGKTTVANKAFD
MLSIEAKNRTEKEKCVALDLDVCVTQLMRDNFSKGIYPTLEERKIFAIDACDYVDEQLAT
ICEPKNEPDKINAIISFSFVNTDLREIFISRFPEAEWILIDTSEEECTKRINEREGHFYK
GEVVTDESDTNVNDEADATENEKDNSDWKFAPVTFPHVILNGLDAVEDNAQKVVDKILEK
TT

>comp3503_c0_seq1; 429 338 1366 LEN=1386; translated
MVAENDDVFVTDLARYAIKGFSGDLLDSVLIPGGGVQTFPDDRRYALLKQKNKEKFDEKS
PEWLHKENFLCAFSAPELMASFRSHYEIVEEQEENKSDDGVAPKVYIRRLLTLYRRSGED
DNDAASEEKPVLGPIDLNTQSGREALASFFSYESNEDLVCVTKETDGKVNTFQFGNTRAG
VKNNPGGDTRTVHIVNRATVREFSNKIGLQLNPMRFRPNIVIDGLGPWEEFDWIGRTLRI
VPKEEGDNDENASGVQHAAHLRLKVLTKTVRCEGIGIDPLDPKTGKLDMPKLLSENYPEH
GPYLGVYASIEVIGTEQGGYSGDIDKLLSVGDTLSLLDEDNV
>comp3504_c0_seq1; 168 1 850 LEN=1695; translated
XINPTYAIGNPLHFPLTRPCYLRKVSNILLEKKTKMSCLYSFQIISYLASLLLLISSSGV
IEEVSSHSVNDRRCTRRVWIQISSASIAVAQHPGISYAETRAATNPVEKLMEAQETLNTL
LENYERATTDCTFADVPRELLETKNKELLLEKAATNALFDKSASIETCKTTNRIVRDYLG
VTGKGPLVGIEKRIQSGLKFVDGDFLDDYVSELEGFSQAYSKATSLSYTAGIADLDSVNN
FSKEDADESKRDNNSNLQQTKEAIIEAKRNLDVVILILQKSQL
>comp3505_c0_seq1; 538 110 1711 LEN=1771; translated
MSFSNLPSDSKRRIASFLDYRSALRLSNISRDFHSDLALSLLVPRYELIYSQIVHGDPIT
GDIPIKSVRIPIFGNNPHTILLRCRWKDQGWGERKGRLFIVAHPDKSTDGCVSVRGKAAC
FESGRVVYESPIASHTFESLSITFSPQQHNEYHLFYLVGGGGGHLLIVEDLSIESIVFDD
AENTMKNAYFSLTKNGIICSDDDDNDDRDIVFYTKLLMEILKICASNEMECFDVFFEKYK
LRPTDLRSLSSIQRVCSFFLEYLEVTDNDADQAPSANESICRDKIVSLSPFTLLQYSTWE
EEPGNTSPSKCSRIPFFKRRTKSIRMTCNCHDQGCGTSKGKLYLVAERKVLEDDEPKTQD
PRTPFLSGRRLVWESPIIDHESTFLETSFAPDDNETYFLYQDRGVESEEDEECILHIDSL
CIHLTVFDDEIGSLESCYHELLQRGALDSRKSDCASFQTDLLLASISFLQLVEPSQTTTL
QKFFESTGFGVDANSLRSLKELTQTLPNFQTQQTKKSTWLRNGDADLRMFSCL
>comp3506_c0_seq1; 434 163 969 LEN=1103; translated
MKFRPCIDLHNGQVKQIVGSTLKDATEGDEDKTKEAASALNTNFETDRPAADYAKMYDRD
ALTGGHVIKLGPGNEEAASSALGAYPGGLQIGGGINDTNALSWLEAGASHVIVTSHVFSK
GKIHMERLKKLVEICGKERLVLDLSCRRKPNSTDDSYYVVTDRWQSFTDYKVNPENLREL
AAYCDEFLVHGVDVEGMQCGILTDLVELLGEHSTIPVTYAGGVRSIEDLELVKKLGRDKV
DATVGSALDCFGGSLSYDEVVAWHKAQN
>comp3507_c0_seq1; 211 147 361 LEN=361; translated
MDPYVSSSSDKPKKKKKKSKDPYGPPPVTEDPYGPPPTGTAGTESGGRSFASTTANFGKQ
KTSRGKKSKKAX
>comp3508_c0_seq1; 462 1 1606 LEN=1606; translated
XEPELHVILAPVSYFSKENSPDRKFLTRFNYDYMVVDEAHNLKNAKSTRYKMLDKVRTEH
RLLLTGTPVQNNPQELLNMLSFIIPLFSKAGASFEEKDNYTEQMLKHFVDASVEDEDEQA
AYRKLKQLFAPFVLRRKKDDVIHQMLPKKERKTVFVELSKPIRSVYDSIIRSHFEKNGTT
VNPGTGEHLFTNLRKCAHHPLLLRNRWNTTEERDRLIEDLIRFGAFRGEACTKGRVGEEI
KSWNDFDIHLTALNLIDENEHRRPFLQKYVLDQEDLFDSTKFDHLRGLLPRLVKEGHRIL
IFSSWTSCLDLLGCLVEYLGIQFLRMDGSCPSKERQRLIDLFNSDPSHSVFLLSTKACGL
GINLTSADTCILHDLDFNPFNDLQAEDRCHRIGQKKPVTVYKLITRDSVDEDIFKMQEKK
AKMNAAIMDPASSKKEKNAILKSVLDRASRASNDGGGGDGAVIGGNETDANQPDVLEENA
NGMLWTPVLSHAFYEKHPLRGFQRCEGSRIFLRAAFWQNTANSLLIIQVHKRVTQR
>comp3510_c0_seq1; 279 176 782 LEN=817; translated
MSENAEENDANQSKFYRVVSTEAITDSNELFDALSNFLMKDKKRQLSAGYLDEKDQIASR
LTWNNLRKVANSVLDEDCPQREPIPAWKSKKKLLLDDAEKEPPSEYLGGDTALSTKQEQP
ESTTRTVTKIEPCTEFKAHENTLTPKQEAKKAKKEAKEAKKEAKSARKRERAEKREAKKA
KKEAKKVKKEKKRKSIESGDE
>comp3511_c0_seq1; 287 1 677 LEN=973; translated
XKKQANKMKSLSSTKKQRTNRRSSLSTPSSSFSRKRRTKRELFSRKSNTSGLHFLLRLPI
LDVTFASKISSVTSSVLSVALFSAFIVLLSRQNCFHVHYGLGGSLSNLESLGIEKGNGFV
DYHFTGRGKVVYDQEARDILKANPDINLRDLDLGFVFDADQFGADPMSVEQKEKSTRLAP
TLTSTKGTPKWFVGKRNDDALFYVTGERIRNALKKMEEQMPRPSN
>comp3513_c0_seq1; 282 127 1272 LEN=1343; translated
MNQSSILSNSTASLVNSTALKISSSSNSTASFIDETVAPSAQTSPPTTIPEPDYVFCHRN
CHMRFQELLFVTGLFLLPLLIWCYFAEHRSRNEEDVRESETYEEPSLHATLAGMTREEKR
EYYSELFQRNGNELVLSKDQIVTVEDLDGNHEEGDDPSLYLSLENPSGFASNAASTRTRR
SSFLVFETSDNCGTDRPSLDGISTTTTTTDVRGTCIICFEDFEAGDTIVRSMDTRSCKHV
YHKSCMVDYLSLQKTPKPGMRGSKDEYHPSCPTCRQQFCKLLPPAAKTREQRDNDTGAID
ADAVDLEGGVASAATISTRVSRTTRGMEAQRHESGALTPVVPMIAGVREQDLVMSTNAVS
SPPPATSRRSSSAIFYDIEVV

>comp3514_c0_seq1; 321 62 841 LEN=2668; translated
MAEALPFQPSVEAVGPEHQSIQNYGSLKGGCGEGEDYDGNKSTVLCDILEHKEMKDSFNG
TYEPDSSTEVSDGNGSSGSDNKNGDDSDHNYDDMSSCTYDSDDIEARFSDISGKSFHLGD
HTRMSINNILVNQRRTDSFAQYGEDYQLVPTQLKKNVRVVEDMNMDYGGRGRLYLASYSF
FDNNHETKYALTIQPNIYKQLFTEVHDAYNVPCGLFFCCHGGDGAHTGVSHNDFVDIKLA
WVVFIFVIGCLLGLELING
>comp3516_c0_seq1; 473 346 1335 LEN=1672; translated
MAKKKGNNPAKTEEEEEEKEVVEPTDEELRNHIRRLIPLVNLQNTGVKAFTKLLSKECGG
ADLKSRGKFIKKALSEAINEMDNSSSEEEDEPKKKPPRKTGLSVKKEISLELANFLGVEK
DQLMARTEVVKGMWDYIKKNELQNPDNKKEIFLDKKMQKVFGCKSFTMFTMNKYLGAHIH
PFKPVDLTTSAKKKPAVGSRKRKKKGDSDDVGGKKKRKPKKPGLQPPYRLSNELAEIVGT
QILPRPQVVKAIWAHIKKHDLQNSEDKREILCDEKLKAVMGGEDRVTMFSMNRYITDHLL
EKLDRSEYNHDEIEAELAAREEGSDEESE
>comp3517_c0_seq1; 265 1 774 LEN=912; translated
PSLMNLPNAKFQIRELKKGYSYGGDDLMQVFELSKHFVDHTAEIDRYRVLFRGLSLSVNE
GDFYFVSGPNGSGKSQLLRILSGLSPFQDGTLSLRGKEWKDDFDGYKAVEWRKQIRYISQ
SKVQIPGTPRQFIKKIQSFRSWNTDYDAHSPNSTMKLILQYVFEWGLNVDCLDKDWSFLS
GGESQRVLMAIAMSSRPKVLLFDESTSALDPESKAAVEASVMDFVGNHRGGVVWVSHDEQ
QADRLVHPPAIEECDAN
>comp3518_c0_seq1; 376 41 1501 LEN=1717; translated
MKPKNDNFIKKKIVSVLVLHYVLTCWSLSLSYQTKLLVRQIRSQPNNVTHTLNVIAQIDT
KNDAEHVQKPIGPVNFDKGFSNVIVAALRVCGNANKHELALELYKKYPSESARAMMISVL
GGSGQLAKAVDLLEDNFCPPTAASFNAAIAACGKAKNWELALDIYQNKLPKQHSSTLSTN
ALLTVLAKCRKGVQALEILHEMVPSPTNSSAGCDSVTYSLIISALVRSNMLDEATKILES
LEHPTHNCSAKSIEAMNDMVLSAYSQRSDWSGMERLERIRQKNNGLVHNQKLENSKLVTP
IIQSDYRFHKWEGIRKVGKGKESYWMIGTYLDRIDGMNVTIGLRPHRNPSRNGIQLLFFE
NAFEDETQSWTQQKIGFLLMKNNANDRTSSLLGMFLKPTKRGRGISKVCLSLWIWLCLKA
SIVPVTGIIQKPLLALILQHTFGYTGPTESDAHGGVLVELSKDSEDPDCVVLSPLNGKSL
EGAFGPW
>comp3519_c0_seq1; 381 38 733 LEN=990; translated
MKLTMKSTLFGLLALANSPSMTGAFAPSVRSSPFAPSLLKAAESEVSFGDLDGSDVRIGI
IRTRWNDEHVTNLVDGCKEALKECNVKEENIFETSIAGAYELPLSARFLALSGTVDAIVT
AGVLIKGETMHFEYISDAVSSGLMNVQLQTSCPVVYGVLNCMNEDQVKARSSGDNNHGYD
WGKTAVEMALLRNEALNIKAMGGSENKLADVGFSKKEASEGASEKKAPGFF
>comp3520_c0_seq1; 187 97 1149 LEN=1402; translated
MSLEVLNSPPLVGALTAGCYGLLCAAKNPLVLNSASVPFVALKGINAFSYGLSMYSVSQP
GRYDGAASVNGEEGDSKSKASSRDKDAAQKGMEQMGTSRGRTLVPPAPWAFIIWAPIFLG
ELLMVTVPFLAPGSVSPVVERALREIAGPYLLTQIFQSLWAASFRPKYEKGIYRYISVAN
LAGTAASLSVCHRAFASSIHQHASRSYSAFEYALYFLPLSLHFGWTTAASLVNLNGMFAM
ATDDNRKNATARAVALLGHVSVALATGVGVGITWTRKAPVYGGVICWALTAVAAGLKQRI
LASASNSKKNDQKIANDDKTVGIYGAERQRTLSLFGAAICASTSAAVMLL
>comp3521_c0_seq1; 34 1 747 LEN=1129; translated
PSTATLKKGARDYAERYAGSAIRFPVLTEAEKKSSLGIAMTFLAGLFLTLSQHLTRERAR
AETIGLKLPAPSGAGTPDATTRSATHATQTQAPSGGGPGASVAAPPTGPTREAPSAPMPA
SGSXAVDRGLARTEEDEQGTEPNTAHRAGAKNETGLLERAADGPXDDLREGLEAPAETNT
PPRLSKQMRTSVFSRQEGRVGWRPSVGDAADSDATNSTLLCTGMVSIFFAKHSVRRDRVG
SLPQHGGLW
>comp3522_c0_seq1; 400 134 1093 LEN=1252; translated
MDSIICCKPVDTRDDGDSNVEESSAIDHNGKILECDYDTNCTKLYKMIEGKQWEEIVYFL
ESGKWYFDTTFLTAMIFGAGPDPRFTESRTWVTALDEMGNVRWCQLPLHAAITFQAPFEV
ITKLVEIYPESIRCADDQDMLPLHYAFRFGSEDKVLAHILEEFPQAVKKKALRDRLPLDM
AHYSSKPERGVIIECYLDTSIEEAKSDWESDHRSIISSIKTKANASLSDEVSTRNKKLRS
VERELYEARKKIQEMEEKLDGGRMGPPSGRYSIPRNSSNQRSGKGRRDEISLDPSEVSTD
KKKGFGKMFGRSKKQIVMS
>comp3523_c0_seq1; 186 197 1701 LEN=2158; translated
MRNFTKFLLVFAVLVIVLDLGFVQGFIACDKGSALQRDAMTRGKIKVRTVVAMSTVTPPK
VSNCAKKEVRNDGENIGYGGSDNRHYKASDDGIPLQCPSSYPSKTFGASNIKVSYQRAEK
DDINGDSGAQLLPLQRYHARNQNQYNGCTADQTIRETNDHPFSSVQNNATNSGSNFSRDN
YTTKRKTMLVTHDIPDLDFIHDNLSRFRVSKSSQVLLEEYQNEKQSYRKNYPFDNEMMLD
QDSSERERQKQALLLKGYRLRSKIRAECRASLALSIEETLKIIYCDSHICVVDKPSGVLS

VPGHRRNPSLANLVYDTIDPSSYDTEGRIVNVDQTVVHRLDMATSGIIVYALSKDALQKL
QGDFRDRRVQKTYQALVEGHGLLSSRDRTGDQSQTLRCCGTTEGEIDVALERDPKNPPYM
RIAQTRAEKQYGGDADEQXTQVLAGGPQTEQDDLVGPVLRARQRREATDEARAAAPHGPD
APVAGPHQSGTGDPDRRRRDLW
>comp3524_c0_seq1; 177 116 391 LEN=755; translated
MAKKASEKKGAKIFKTKCSQCHTVEQGGAHKQGPNLYGFYENTSGSMEGYSYSTANKKAG
VTWTDENIFKYLKKPQDRHDIIAYLKKTCKA
>comp3524_c0_seq2; 222 116 442 LEN=806; translated
MAKKASEKKGAKIFKTKCSQCHTVEQGGAHKQGPNLYGFYENTSGSMEGYSYSTANKKAG
VTWTDENIFKYLENPKKFMKGNKMVFAGLKKPQDRHDIIAYLKKTCKA
>comp3526_c0_seq1; 670 421 2490 LEN=2538; translated
MPESTPKNPRPATPARGKSDSASDAPVHDLHRRSRQATLTRMRSSPPQSPPDAAAGSNHH
GDGMAPSLFWPSNSNPTKKPAASVPGEETPVDAEYCNNEGEEYDDYDEGSYYHDCDYYDE
VFDSIVKIYATHNFEPDTTMPWQKQKSYNSYSSGFAINGRRILTNAHSVEYAGQVLVRRR
GSSRKYRATVEVVANECDLAVLTIADETFWTTRTEYDEDEDDEYDDYEEDEEEEDGGSEK
GAVAEASPGDDAIKPLSFGALPELQDEVEVLGYPTGGDSLSVTKGVVSRIEATEYAQCSG
SHLLAIQIDAAINGGNSGGPVVDAATGRVVGVAFQALEHAENIGYVVPVTVVLHFLEGLQ
RHGGTYSGVTCLGVRYSRLENESMRRYLKLPDAVETAKPNGGKKKSRRKKSSNSHAQGGV
VVRSCYPTCPSRNVLRESDVILSIDGIPVASDGNVPFRRNGRERVSFSSYVQTKFSGDTV
ALDLWRDGGSLKVDVPLAVSRDLVPPHWNNQSPPYFIVGGLVFTALSIPYLEDCGAWGEY
VSDSLSYLMNKVYEPLEKPGDQVVVLINVLAHPANTGYDGLTNLHLRSVNGAPVRSLRHL
KELIEAEQQGTHPGASPNGTDSKDTRDTEPRFLRLGFGSKTNEGDGGILVVLETDSIEDI
NREVCEEHSIQHPFYFPPLASNESSESQS
>comp3528_c0_seq1; 220 1 1835 LEN=2478; translated
XQQQQQRIFPPFLLPGKPIGPICLFRGKVSNEILADNLEKISFQWALDQDYAGLKQRMAS
SVTATQIGFVMAEILGRRGPGSLFRLLKKRQWLPEGNRGVPRISFPVDVSGFQIMRLEII
VSLTGFAKRSSVIAAVFDYLGCLRSNPPGRELIAQYCAVAQLYGHVLAPRPPDAIELAFD
GQVYGIDEPNGVGGYDWRLMPMPENLLGVQNLQKTIQTVLNWISDPSNAITIMTASQRAI
LSQHANDLEERFPLFSPASWNISPTTQARYLPQNNVLTGKINEWLITRMSSEGLQPPVLN
PLIPPSLRPPRLTPSLHRSTISNYESSQKGDIVPPIRSLVEDTIDQEKAPLVRDYWALLK
VYSHDDPFPPGISLPRAPPEPSSRSLFVMQLLSSRPPRATAEMAAHAEVWRISLEYALMD
LAELGAPAGLAYDISYNKYGMRVAFLGLSQNIASYARRISRRMVDHTNQLLEGPEQVPEP
IIEAAIRNTNRFRMSPTRKAIVTSTLKQTTAVDAALEGTRFFQSCSGAVCFSQGDLLAAE
TLSLLADLKEIFRKTTGINVRPAPAVPEVSDLLYRANWIPRAASVCTVTGSSLISYPVGG
FRADVKEVRNT
>comp3529_c0_seq1; 376 1 2020 LEN=2310; translated
XKTFDVLAVHSSRYMLETLVTFQNACSHSSSDFRSTMDRTTPLKRPRTADPSPTNYNYYG
NSISNDEAIRRHIQEILTELQQAISSKQRVRALRNLLHFLRYDPSTRLCRCPASTLECFV
HGKCIQLLCLQLGYAIGRHRFDHGNRCGPWGQNHEILLISTVLDIFYRYCPELVTEHSIQ
KNGSEVLRLSQEVFINYQREGVGQDQTQEKSGMRGSGDFLSIVSIWHSCSSCSLGTMLLL
QHPGTLKAINYILSSKMHQSHHDTSRIETIMECLGLLKNLTYFGEDHRQEIVDQTNLLTT
LTRLTDIPNKKTRERLSAVFRNLALSADLRSRLTKQTNVLAAIVRMANFGLIECNNLNGH
DSHHENANCKKNTLRNILSTVANLAIDRSTNHLMVFYGDGILIEELKQFLVQGEDFVLRK
RASRALRLLAKNPSSAPLLIMQNNQLLEILSDLALNDENDSVRVEATEAFAACAELIRAP
MTQHNNILDALTHMLITSTRRISPSRVNLDIVARTLQEQASHQINKRAMIRRKNLLAALA
NVILSEHATLSAKESVSATFVNLSEEEDNQEIISIPIILIALVHILLDRPSVLDANNAVA
NVRASVDRIITSRIRESSVRTILNLAKTPSNRQAMAKQTALIQSLLRFAAASTTNDDLKK
QVKTVILQLAAEL
>comp3530_c0_seq1; 259 230 1330 LEN=1414; translated
MPSVPPQPDTIVHYHNETSFSSSEQESSNNAKGSSALYLFGDPSSPRMALCCAGFPDDHT
VFRPFAKSLSEKGILVGVMCLPGYDDRPADGVPWTEHNPSGYTFDEMVSSIRAAARALRA
ASKAPGDKPAALTGIFHDWGVVPATMWAQRLEEEEKDKADKSQPPTLDRIVFFDVLMPPP
LDRVQGKYNPVEGFTALEAAITVLYKVILGLAFWLRANCSEYLAVVFFLCTVFVLKGLRL
DPLYSFDENSPKALYKNNVGHSNSERMSLSRIIYMSYPYWNLMRKMGMLAGTVHSNWKDT
PILYLYGPNKRTQFHHKQTLRLLEREAKESRSISQAVAISGTGHYLYLQEQERCLELVFR
FMTAKK
>comp3532_c0_seq1; 519 127 846 LEN=1081; translated
MKFTAALVSLSVASVSAFAPLSTTTTSTTLSAKIDGRTVESGTVVPTNNFILVKVAPAVE
ETEGGILLTGKAKVQKTEGSVVSTGPGKTHPDTGVLFEMPISAGDNVVYGTYDGTEIDID
GEKHTLIRDDDVLVRFEGDELTLDSCSTIQDAVLVYVETKEVATEGGILLAASSKTESRP

STGKVVKVGPGRCATNGERMEMEIEEGDFIKFRDYAGNEVEIEGEDYTVVRALDILAKF
>comp3534_c0_seq1; 228 117 881 LEN=1043; translated
MNKKSRRNTGTLLPFSARTFLQHSVLLLAITLSWPRYSHLRADGFASQAVARPHLSSIAT
TPKTPVSSDSVLFNYNNDDNGDNNKNDKSLVDFELLSRRFERADKTLIRDEATLVACYVL
CRFLVYDISSGAKIEPGWEIQDFIWLTGTFSSATVLVIYYTVAGLLSRSFEEQPDYRSWT
SIDDGSDSKDPSSSVVKAVVNVVLCCPIWLATEHLLGFGPADIGGNTLSVAVATGFLGFG
SFMALAKILTAGMR
>comp3535_c0_seq1; 1014 1 2687 LEN=2782; translated
NYHDTHYSNESSSSHEPTEDTTAPLSTTDSPESSATGPADESSFVSMSSSLSALTTQTFV
SGFTGFTTTIRAKPSQATANTGRISGIGNDNDNRYSNNALQQQQQPPTAPPQNSFLGATF
GPASSSGAPPASCTAGERQQRSERILRFASKRLKKSHVEDYYEYKCRKRNPHRSHGSSKH
NNYNHNHNHNDGILDVCHSDIQLDSILGNGSYNVVYSVKRIRTTSGTSRSHSHLQPRSID
PNTVVVKTLRSKLLNDVPMLAACAADLRKEGLVLAALQQQPNEEEDNEGARHVIRILAWA
PTGLEAYSNGCHDAFFIVLEKLEGTLTDTLKEWKALEEFQQRRRRARQRHDRKQQQEQQQ
IESLTEDIECCYNPGRQSISIHNEHNVGYEYEYPHGKGHNKHKRRSRLLGSVKNSFVQKK
RSISKALSQLQQKQLLKQYHRFEEGKEQGFEQREDRERGFDCHYEHIFPSGSNSAHVHRP
DVDADEAYFWNARLGLLIDLCGAVSFLHSQKIIHRDLKPDNVGFDRNGTLKIFDFDVARI
LPPQAEDPDRLFKLTKKVGSPRYMSPEVARGQPYNEKTDVYGLGLLAYEVLSLKRPFESV
PSGRSKNQKSRNPNNHHNNSTGVYEDCVPVVVREEQHRDSQRFPSKRSERRKGRVERLFY
WVRNKDDRANTDGIIQHQKANGNIPKEPINTDDHNNSQQPRRLPKYANLRPLLPVATPQE
LLHEEEEEEQQVNSRLMFQSRVDSMLPSTSKNPSLWTRKFHKHGQVFGKFSVAATTVVXS
SSLNAGNGTKSSSKQQQQQQRQQQHNINVVDFETRSFFWTQSLRTIIDRSWSYDIPTRPS
ASGLKLSLQAEVDRINNAINILGNTNHKSLQSAKSCNSGDDYSSSSLFLDGVSIT
>comp3536_c0_seq1; 204 1 552 LEN=849; minus strand; translated
HKGWSEVLCRLLDKNWFEALSAFGESGLAEQLYGLLKDNVWSSVLKELQKKAPSSNLAKL
LLTVLQNSGWLVILGLVLVYLWSLVRNRKQITANNDSKYSSIVEKKIDLDERIRFMKTVY
VITSETSERTYFVALLSHLSLVIVLDAPDVSKQEQQQEQQQQQPALAAAFVDSSNSNSSC
CIF
>comp3537_c0_seq1; 251 182 577 LEN=1430; translated
MTSKNVEIRTENAPDPLGPYSQAVLAGNTLYCSGNIAIDPKTGDIVGDGDVQKETQQVLK
NMDAVLSEAGAGPANVVRCTVFLDDLQNFSAVNQVYEEYFKNSPVAPSRSCVQAAALPKG
VQVEIDCIAVL
>comp3538_c0_seq1; 268 141 677 LEN=1304; translated
MAEATTFLDIDFTTFLPNGSKDVPTQAGALIQWWKAYQQSGLLRKFPDCSIIENSSSSGV
FLRVTRVEDDDGGLSGVVENKIRSTMKKIRVEDLGNMQATDLLASIPEEDPQGRGLCDLR
DMEKKLSIKIVFDDGGGHVYLVGDAKKLDKKAFAIRNLLSHYHWRLSGTDVSSKNLCC
>comp3539_c0_seq1; 469 1 1266 LEN=1681; translated
RTAFHWDMSGLPRSFHELSDQLVPFVAQQRDQWQDTLAALPSRFDGLSVQVASQTDVLKD
ELVAKLGSVETSLGEAFISGKAAVAHHTGDWQAASHAKIDQWQNDLAQASGRLQGELAQG
YANIRYAGQAKIDQMQQGFHQDYSQLQVKGGEAIARAKDVVLMKASEFQETIQQSTDQIK
DGAVEMNEQARVLANDYSAEMKDLSQQSYGAFREVGEAKMMHAREGLLSEMAKVRETMEI
VVAQKFTDFQQLSKSTMDGLGKDAIQLTESVRTMFANVGADLEATRREIGSTTAPMIQGT
GEFLRDKTAAFRDTSWIEWDDVQHRMGLQGDAAADAIGDNLQKWQENTANGLSALHDSVA
KQVAPMDTIGDNINKFKESTTNQISSFESSVANQAASIVRRDNSLTTESAFLKAMLEEMV
F
>comp3540_c0_seq1; 434 127 1383 LEN=1441; translated
MKEVMADHEFRSDFASGNPFKIAACLNKFRQEHFPEDLRALWPSLESFNPTVVLAGGIST
PKGLLYSFDKTIPYISVDLQVVLPLSDKAPVGLPKLPFGTNKLWVSLLLQKATSAMAARK
KIIKEVLDIDMDGKYEKPAHYTLFTDIPSFPSPHFYGVSTTVITEHPEWPKENFYPCGFF
TIGEQRQEQLMKDESKGYEFGSETCNELTEFLAAGSPPVYLGWGSIMCNSPQWMVSLALE
ALQHVGARGVLLGGWAGLSEAYIPDHLKNFCKKNVIFVSSAPHEWLFPQCSCIVHHGGAG
TTAASIRSGRPTVITPVMGDQFDFAAGVNEVGCGIGLRKMSSVRGNTLGDAIKRCLEEES
IIARAKETGEKLRNEDGCETFCNVFEEWLVHGFASGEWLRKHENLMKKCKESWEAEQRW
>comp3542_c0_seq1; 362 1 1284 LEN=1284; translated
VPSTVPSTIPSMKPTHLLSTSQPSSLPSSLPNSQPSASPSTEPTAQQSSQPSSLPTFQPS
DTPSVRPSISEKPSFTGQTFAPTNVPSKVPSDEPSEKPSDEPSSSPSASPTDVPSTTSVT
NSAFNPGTTAAQVEDAYTKVVTSMVIGSLTRKLEQDSSPSENLDGVDIDVKCEDQSCSFE
SQEDISGKVCFECSCTITASPATQINTADSSSSVETDIASGGFSAKLEDEGLDGAVVALP
PSNEPSSQPSGSPSISVKPSLKPSHMPSIVPSSLPSAEPSSEPSVSSSSIPSDVPSVGPS
KISSTMPSREHSIGPSSMASSMPSSRTSNAPSGVVSSAMPSGIPSMALSSAPSREPSGRP

ISIPSSMPSAEPSTVYSNGPSKLPSGIPSQNPSTSAVPSSKPSVDISNVPSENPSGIPSH
NPSASTVP
>comp3543_c0_seq1; 383 864 2112 LEN=2112; minus strand; translated
MSDVTSPVLRLYSGEHDPLEIMSEICELAKEFDLDWTKFVHVMLEAASTNYSDPGFVDTI
ANSENRIEPMKQTNLLIQTALMPNSRIRENNNSESTAVYVGYDTDEDGMSSPQLFTVALS
AAWVVGNKSADFWYGTDDKTDQLKTYTAPTPVEHSWDTFESFFLYPGNGGKISINGSDIL
AKSEGDQNNAFVGKPLRPPFGGPEATTVAQVASASSAAAGGRSPLAPSVYNQNFSISFYS
IEGSKVRLIWRVLLGISVGLVTTVLVGWLVEYCTRCRKKKSDGDSNNDTKDPSNVESNGQ
DENGQSVDVNTSSVRKPAMASGVCCGILAGCLAGLYFGFGSILLPTVYDVGVGLAYENPH
FDKFAVCSQWPNPCGTQDAQFNDGYYIDNPAVVVNVGYYLQENGVDSNQTIKLIVTX
>comp3544_c0_seq1; 234 203 1187 LEN=1187; translated
MGSRSFSSPSSGSSKIISPYFIPLFPDSEKPKTADNEPPPNCDVVQGPSLLRTPAPRPHP
TLMIMPGMRSLPFWTSDSTPTPSSNPGSSAPPPRRRIAYNDPSVSRAVEYLEQHADIIRE
EYLRVAPSLPSDYENLQDHTQGSGDGKSNDTKLHDGDWDWHTYLSKGNVQGDFVMKFPET
SKILNDGFRGEENNYQLFEGTPFGFVFFSNLGGGAKIAPHNAPTNLRLRIHLPIIVNDES
VNPETGIPNCGIRVATSIRPYKQNNAIVLDDAYDHEVWNETAENRVVLLVDIWHPDVTQA
EKEAVVEMFQKAKRDGCGNDKTKNQYANX
>comp3545_c0_seq1; 332 743 1459 LEN=1642; translated
MCTFHLMEFFVTAFYNPTVVASDSFLVNHSKAYTVASIIAGTEFWLGFWCGGGGGEGIDD
DAPSNATESGWWWVTTIVGIVMVVFSQTMRSRAMMKCGESFNHYIQTERKENHVLVTDGI
YTWFRHPSYTGFYYWSIGTQVVLHNPWSAILFAVAGWKFFSQRIPYEERSLLRLFGDQYY
DYVGRTYVGIPFVPSIPNRKEGAVGIGSASHQKEEHRTEEAIEIEVEAEKADREKKND
>comp3546_c0_seq1; 282 890 1726 LEN=2589; minus strand; translated
MAMTKRLASSALVEAMGDDASVNCNCYNGDDILSRLFGSVEARDSFFANDFGKNVVHIRR
STSGNDIRDLNLPCIDVNEDSNYEKTSKINMKILFDTSEFIALRIRGSMTYLNKAETSYD
DFCEYIGNRGSAVIPVAEENALMPFRNQIEASMRDQLHMRQRNSQEVLHQEQNEVQAGIN
VYHSGPGAVALNRHFDQYDVFVLHLDGHKDWEIGVFDPDHVIDDDAFMDTTSIDKVRRWT
NMTLVPGDVLYIPKGVYHAATTADGYESTTHATIGLEY
>comp3548_c0_seq1; 379 142 1002 LEN=1092; translated
MPMKSNTSNSNNNRGRFLLGRAGASLRRSQNKSNKQGKTAAGGKLGRDRPRLSPLKENVP
SRSMKTNTSLTLREREQHVTFHSNQSSVPIRSNNFFHSTGFSQAEIYDLEESFKLFDIYG
EGNVQVGDLRSILAVLKQEQQQQSSTAGTKYPYLDRLTYHLSELSDEDTLHMDEYIQLM
ASTTITNSIVLDNKGEENQSSQHFSRVFELFDTEGKGYITIDDLERIAIELGEHDMTRGE
LQEMIDRARGGSDSNGNEVVGIEDFTRMMTMSLFPSKDDRGEYEAK
>comp3549_c0_seq1; 276 1 779 LEN=1371; translated
XPNFQRYYYRVVLLPSASIIQRSSHCQGMERRRKKKSQPRTIAQFSQKLFLGYLAFTRYE
YRTTPANPFCHKPKIDPLTKNRDTMKSTARTTQLLLSFLCVMNAANAFVTRGPSQLVGQR
QLQPEYRVLFQEPPTLEKEAEKSEAVDEVKEPEEEMSETQKLMQKVKDSGTAGFISYAAW
EMGFWALSVPVCAVGYYQVTGHWPDLGDPDDQKKLGAEAFAFVNFARFAVPLRIGLALST
TPWIDENIVKRFLKKDDEN
>comp3550_c0_seq1; 461 94 1879 LEN=1912; translated
MCGIVALLLADENAWVNQLIFDGLTVLQHRGQDAAGMVTADCKRLHLRKDNGLVRDVFQQ
EHMTQLRGNIGLGHVRYPTAGSSSCAEAQPLYTNYPHGISVAHNGNLTNTDELYKEMLKK
QRHVNTDSDSEILLNCFAESLNKYQRISADQDDKENAIVEDIFNTCTDVMKECKGGYATT
YMINGVGLVGFRDPHGIRPLVVGCRSKDPEDKGKTWTSESMEQNSGTKKIDFCMASESVA
IDALGFELVRDIAAGEAFFVDMNTGKCHFRQCLSDESVAPCIFEYVYFARPDSIMDGVSV
YESRLKMGEKLANKIKKIHPDHGIDVVIPIPETARTSALQTAHFLDVPFREGFVKNRYIA
RTFIMPGQAKRKKSVRLKLNTIKAEFSGRNVLLVDDSIVRGTTANEIVQMAREAGAKKVF
FCSAAPPIRYPNIYGIDIPTRTELIAHEREEEIAAKIGCDWVMYQDLDDLEASVTELAR
PDQPLTKFDTSCFNGVYYVTGQQIGDEYFQRLYSLRNDDAKQNRDASDRGKIQSNDGCESV
QNDTRKEYMKELVPVKPCRILSVYEIPDFGLKEQHILITSKERCHATEIDKEIV
>comp3552_c0_seq1; 389 177 878 LEN=980; translated
MFTNIFLISLLLLEFVVSLIPATKTLIRHEVFLNTKKNPDNAQSQHSLAVGTFVEFEEKK
HRAHIGKIEEVEHKSNGGTRYGIKDSDGHHFNIPDKNVHFSVYPPNSPGPAGELYRELII
AHEASEEELQSKLEISPEILELAWEEALENEDASGEHAVSPEKLIELVHSHAASAIEKYQ
AWRLLQSDLAHVFFKDLKDHGRITSFKIKGRKAVETAKIAFCNSHEESDLCLI
>comp3555_c0_seq1; 280 202 1005 LEN=1192; translated
MACHRSVARRLAASIPLVLVVAILRSRSCSAFGVLATGRNGVQRGAVSLSEHTLNVEGIR
DGDDFLEADGPKARLLAAIGAPAETACPPRFGFGDDSAAKEEIEQLVGDVSGYVLPLEHP
SEWRLLYTGAPDLLGLEGGPLNRLVSIRQAIPDREKLEIELEYEPSEGILGLAGSFLDGL

GEDRLVQTVRFGYGVGPMNKVDLELSGTSLSSTRLSSLLGQGGSSLPPLSLPSPLPFSGF
SVVFNDGDLRVDRTVQGDFLLIYRREK
>comp3556_c0_seq1; 198 187 354 LEN=354; translated
MLLAAIVNTNGEAIESSDIEEAEEKICVNNAGGDETCSFPAVEYEEDEDYDDDEEE
>comp3557_c0_seq1; 189 610 819 LEN=912; minus strand; translated
MVQSNLTVPEKQNAGILYGIQIVDERNIETGLVTQKAQKFDSIDYIVKRSLIPNVVEAAI
APSVEGKED
>comp3557_c0_seq2; 166 57 305 LEN=667; translated
MSGAASRIVNYNKRLFGPGPVARVPASGIRGYYARLAPLEGFTHAAVHGTVLGFIAAAVY
KFGVGDPEAREIAEYYEENPPR
>comp3558_c0_seq1; 196 615 1051 LEN=1094; translated
MGKSKYSQLSLDDFSDDESVEDGTHHNGNGSANDDHDEYVRNQQVLMQQQDAGLDMLSES
IFRLGEMSNNISEELGQQNKMLDAMETDLDHAGDELDIXDEVDQGADRPERGGLDLLPNR
GAFDRSAGAAGPHYIRLNADRGANY
>comp3561_c0_seq1; 131 156 1049 LEN=1440; translated
MPASGDAASRRRQTRLRREYLYRKSLEGKEKAAYEQKRVIREALASGKPLPTEVRASYDK
LQKQIDAEDITTDTPRSHIDNEYGDAGLLEPRVCVTTSRDPSSRLKQFSKEVKLLIPNST
RINRGNNRVDELIESCRQSEFTDVVVVQETRGEPDGLVVCHLPLGPTAYFSINNAVMRHD
LDPPAAPMSEAYPHIILNNFHSELGKRVGNILKCLFPVPRPDTKRVVTFSNDNDFISFRH
HMYSKPTHDSVTLHEVGPRFELRLYQIRLGTMDQKEADNEYVLRPYQNTAKKRKMLG
>comp3562_c0_seq1; 503 1 750 LEN=786; translated
KKMDALGLEIPRDTGDYSSVDPSGASGIVQPPPMDPLDGLEVIIEGTFTVGILNGIPSRK
SGRFSNMFDADIEVEGIEEESEPPPASVVATPPRTEAPQESYGVTDDVDDEDDEESLERI
ESEMAKAAAEAETAAAEAKRKADKMEALKQRAEAAMARRKQKEDETREAENAAAAEAERK
AEKMALLKQRAEEAMARRKQNKALASEENKEPKEDPAAAEARRKAEKMEMLKKRAEEAMA
RRRQKKKEE
>comp3563_c0_seq1; 497 51 1259 LEN=1275; translated
MKRTFGAWMATIFSSSMNGATAFALKFAAENVHTPVGKDGEGAYTAATKGCFDVIDMATP
LVLQEISEQHINPDRAFHVADYGTADAGTSLGLLTKVVRSVRKRTSGDDKKEVVIHYEDQ
LNNEWKSVFNHALGNIKVTDAYNNEIDTPFNLGGVFVEANGVGFHQQCYASGSIDLGVSF
TAMHWLSSSPSSLKGSKYMHAARCLDGPPEAEKLQAESDWYKILTARSRELSPGGRFICV
NFCVSKEGYFLGQTDVGSSMWDSFAYAWNKLAQDGIINEDEQLGVSFPNYYRTTEEFLAG
IERCPDLKLVSAEEKIVRCPYREQYIDSDSKMSAEDYAKTFVPTTRTWSHSTFKTALSDK
RSDEEKEKILEQFWSNYEELVAQSPETHGMDYVHSYLVIEKV
>comp3564_c0_seq1; 118 578 1016 LEN=1016; minus strand; translated
MAVVTTRARALESSDRSGMGCCSQRTAGLCVCAALRWFRAVPCRTKGWRSVFLPLCFFLG
PADDFAPVHSDPPVGRQGSGLPRFAPPKHPACVTEQIEIGFFESRFLSKQVSGSGCFDGR
QRSVIADKGWLRLCTIRHEMHRRWKFX
>comp3566_c0_seq1; 324 101 922 LEN=936; translated
MSRRRLVEIPFLVCGEAAAKRTIGSARSIAFDPCFRFFRRTTERVGVNRNGNTIGTAIGS
AARFSTGSGGSSYSASGPSQQLKSWESGARMVQNTTVENKYLEHIREVHDPSQHIKTIED
ELKGSIGKALGKQGQKVLMYARLMEEERQRYQDLLLELRGQAREAEPHTPSPAVERTDDV
DRHPELSKIAISHNEYREQCLHARWELIVHRQAVGFIVGNHKYVTDKFPVGDALPVPSED
GEDERATAAASAEKSKKKKVFTDQLDWWQRIGR
>comp3567_c0_seq1; 234 1 824 LEN=1315; translated
XQQQQQQRQREQEPTEETYKLPRTRFVLAGASMGGALAAYASQRILEDSAACSKQPRSDR
PLAQPAAAVAAEQHASFVGTLLLSPAVGVDPSVVPPALLVAALSVLSWLAPGVGLEGVTP
TEDPSHYDCPASTKRNYAGPWPLGTSKLLLDATTVFVPGDVKEGSLNLPLAAAAAGPALP
HATLVVSGVKDPVVPIGAVREFVEHTNSHAAKRGCLGPGIGLAEIENGDHSLLAPQKQKH
SKGQLGATKKKTLATTTGHVEAFLERCGAAEGSS
>comp3568_c0_seq1; 145 1 217 LEN=525; translated
XLCRHCPDDDAMGSQFDIQSLNSNAFDKKAIEVAFCKKIQNSVSKKFATTKSCSIAVESV
IATDVAASKAAW
>comp3569_c0_seq1; 279 1 1395 LEN=1878; translated
LQIGEQETKHNRSVYQDVEGVRGGLLSDNRKMPSYSPSGLRAVCQRLLPIPNSTYPFLLA
FLFIHSFWIYYDIAITSHERHRPRREPVRVTESSPLTPFYPPSPGVKYNGYARSFPVWEH
PFPCGDLEDIGDEKLQRSRKQVNEGFFYVRLLDASPSICSSVTTRIGRNVARRQLHQSHD
HGGNNRTKVCTSRIGSQRAKQFYARSPQKSFLWSVVREPVDRLISKYYHYSRPIEKGGNR
ERAFSEFQHYVLDSERHDYGYYFRSLGVKRTLNPYEKQHEADTQELLESYDFLGISERMD
ESLAVLKLILNLEIEDVLYLATPKAKGTTTPSGSSIDFYQHWQRTCRAIREPDVTMEMKK

WFHSEEFEAYIEADVLFYKAVNASLDKTILEIGRERVETTVRQLKWAQKRIEEECRGARF
PCSSGGEFQKETDCFFADVGCGYNCIDSLSETFSQDPVFQKLAK
>comp3570_c0_seq1; 190 151 810 LEN=810; translated
MALAIFCVSWMAATAPRSTLALSVPSFGVTKTIGGATQHIQSGRPSGIHRIRTGTTGAWS
SPPERSNNEGEAVWSPGLRKTMAGVAGLGAVETGYLTYAKVTGGLPFCGSGEASYSTCDA
VLNGPYAHLPFFEDLPLASLGFVAYASVAALALSPLAAGDNEGGGDATNRVLLAALATSM
ATFSVFLMTLLFGVLEASCPYCVFSACCSIFLAAVALIGG
>comp3570_c1_seq1; 159 1 533 LEN=579; translated
XLPSGGNASAGGRTVLAGFAGAVAGAVLLFAVGGADAASAGSPPGSTLAATISGSSRGEP
QQLLYAPPEITTDSSERALALGRDLASLGGARMYGAHWCSHCYDQKQILGKQVFDKTSGF
VEYVECSKDGINSQSKTCKAKEIPGYPTFEIQGQLYPGQQDLGELEDLVKGLKEASK
>comp3571_c0_seq1; 394 23 1168 LEN=1989; translated
MRIGIIGDGIIGTAIAATLAKNRPSLEITLIDDGRPFKTSRAGQGYLWSIHRFDDAVGLE
TSVMAKNAWRDLLGDGDGDGVDGQPLLKKTGSLLIAPNRDASALNDYYQRARNSPHLPNL
QFLSDASTHNFLLRPGEFHGVYSPDDYTCNPLELIEFLRTKFRLETDHRTVTDLRSELQT
RTAAGGGDPSDSGFDCVICCAGPWINDLEDVNLKPIRGLLLEAGVGDALEDAMPSTSTYK
TDGNEEVVKALEQESKLVPMMEFGYGTQGIHFTLSSRDGALLVGASREAVGFSTTDLEIL
EEAILRHADKFVVSGGIGPVLARRLGFRPACFDQKPKTGRNYWIENSASDDRILLVYGFE
GQGVLYAPLAAMDVLNTFFSF
>comp3572_c0_seq1; 328 1 966 LEN=966; translated
XSNSNSSNTQVFSIMEKKAIEDAIAMAMSTAVSSPMSSEESSAQSAPASAPIAALRPTPI
AQSCTEDTARNDATRTFEGSELESELELESKLELESESESAAAASTIQEQQQEDFKQRNR
QQPPSTHATDVHGILRKSSYRKRTQTSNNFAFRSNLPPHVDTRHGGEFDVLEDEYCEYGC
RRRRSPTGNFNGAASSLFRTTLDRTISDKHFRRRNTSTANNESDSSSSGSLNMLFLKSML
GLVVVLPVVLLTFLGLPYLLLGSLVWTLIFVARWFRTFVEEEFLFSSRKNGMDNSASRGG
GTTPSLLATATRTTDRNRNRNSX
>comp3573_c0_seq1; 658 1 1910 LEN=1978; translated
XDAGVNDIEALKKKMKRELDANRRAMSLASDMEAKRQTEKLMSKIDRMTGDFLRSNEATR
ASTKMAAAASRAMEGRGLEMGTWGVLGGRTVVADDDLARSGAAGLLGSVGNAVREADKAA
NTKTTLDDYNDLGSISSTYREEDPPADVRENRILIVADTQKDKLAKELVPALMAAFEREK
DSIPDLKFDVLSPTSTMPLGGNDSQCVVVFCTGLSQPDSLKKMLDRLLRRTLATGGGKVG
TPPTQLVGVSTLGTERFGTFPYNMQNFLGGKLETRRQIEEVLINTVRNRVVEPPLDYTLL
KVKEGDFLPDESVGSGSPSFSLTPGDVPDDATSVETAARVLLQAIAFQPPARNSTIGLAG
VLPASLLEDPSGSEEVLDEQDDFWRETFACLDGPELWRTVLLEVETTGEGGLPEEELAGT
CDRLLEYLGEWAGVLARSGKGLTTPVKAETEGGLDPASMVTPSNSRTLLVQDGVRLLFLP
TKTGKNYASKEEEEELRREQQQTSGSGNRVPPPPKTAREGGIDVVVEIADLPGEGSSAPR
RRQLRVRARRTNYSEDAVFKEMSEATILKRLEEALEVFKTENNILPPEEKQQAVDWILSI
AGRTRPAGNKTTQTQNKHDHNRVPSPALPGAVPPSR
>comp3574_c0_seq1; 522 54 1652 LEN=1681; translated
MRKELSSRSLLFLLLLEGTVLFCCSGFRLPEQPTTTQRHHRCQQSSVILSVLADTERTAD
SVSMNTDTSERKFLIHWRGEKDPGYSKSFRHLEFESALAAELCSQKLPTSSSLDHARDTT
SDDSKVIPQVNFTNALNYIGNESMTDMNIEHFNEAMQFVSFSSNDSNLLAVSKATVIRAV
ERCSLIHGFYEIVASSDKLDDLADLAIKDGGFSDLYEGGANEKATWCFRARMYNSSVIPE
EHSNKGRGKRYSSRTRSMKIERDGLNALKDLMIRFGGKVNLENPDNKIYIFDGLEEEIDG
VRVGRKVLARRIASGPKIFSIAPAERICITNTPLEPIAAFVLCNIARIKARDRILDPYAG
SCTTLLAAMIAPDAETVGIEIAHNGLVNREHIVEDFTTRNLKLPSQIIHGDSTNVTIRE
TAKEAVGNEAFDAIIADPPYGIRESIGYNENSPLEELFTSIAIDRGATDRERLLKAGGRL
IAFVPVTDEQTLTEMLPHEDLTEKAGLKFEVCREQPLNDKLSRWLVSYVSIK
>comp3575_c0_seq1; 400 1 1486 LEN=1535; translated
XTSLGKVYFMSNMFERAVESYTEALGIEPMYLEAIAARGSSRIILGKYREAGRDLTTVME
RDESGMFLDVYTGLARILQAREDAVPTGWGPIVERLQRMIPLLEEQHASLHDHQRKGRQN
NIAHGLNRLFHVLFMYHDTKTKDTDAAWENLTKSYHYKMGALPPWNAGFENQKISATKQI
FSRSFFPDGVGSQSEVPIFIIGFVRSGSTLLERVLDAHPQIVGTGENSVFNGRLDEIRNK
IVETSLSGDTNQLKRVIASLADGVVDEMRERWKMVSAGTTGGAGPEEEETPLRFTDKMLT
NYYNVGFIHMLFPNALILHVIREPMDTIFSAYKHEFPAGTLDYTSDFPSLAELYHGYRDL
MDHWDRELPGRVTHVRYEDMVHDMPGVARRIVAATGLEWDDSILDFHKKKHAVNTLSTTQ
VRRGVYKDSLKSWKRYEKHLGPLLELIGERTSCGIETTLPGYEKPPVEHAASGDGDPGGA
PVAGGTGAAPSEGEL
>comp3576_c0_seq1; 1194 265 3228 LEN=3381; translated
MMSKATGTLYSILVSLVLVLCCSKCHAFLPQQTKAPNTINTQIGWFGQQKTSAPGHPLSM

GLVDDFVASTDEKTRKSRNEKYLAKLQERVTRINGLEETIEELGDDELQAKTESFKERLK
NGEDINGPLLEEAFAVVREAAWRVLELRHYDVQILGGLVLHDGRLAEMATGEGKTLVSTL
PVYLNALTGETSFVVTVNDYLAKRDMEIMGQVHRFLGLTVGLIQSEMKEEERRVAYENDV
VYVTNSELGFDYLRDHLALTPEQTVLPKGAGEFNGFCVVDEADSVLIDEARTPLIISKQV
ASPATKYQTAFQLAEALKPDVHYTVDLKNKACTLTEVGYRDSERALGIDSLFDIQADGEA
WVPFVEKAVNAKELFEKDVDYALITDDSGNKVGVGIIDSFTGRILDGRRWSDGLHQSIET
KEGIPVSEQSQVIAKVTYQSLFRQFTRLSGMTGTAMSDADELELTYDLKVVPIPTALPIA
RRDYPDVAFKTRKAADEALVKEIKNVGGGQEGGRPCLVGTTSVAQSELIVSKLAEAGIKA
ELLNASPNNAPRESEIVAQAGRTGAVTVATNIAGRGTDILLGGCPKTMARIKLRSVLVEN
GVLSTEEADALPPSPPEDYYPCELNDDVQYMMKDAAASLRNQFGSEMTSLGLNEILTIAI
DTTEAEDDPEHILKLRNSAAVIREIFSEITDADRDLVQEAGGLYVMGTNRHESCRIDNQL
RGRAGRQGDPGTSRFFLSFEDDMFVIFGGDGLQNILKTFRVSDDMPVEAPQVSEALDKVQ
QAVEIKYQEIRGEIFKFDDVLNKQRSIIYARRRKILNMSDEESTAMMRKYNTNVITAIVK
GQTNMGKIDAGAIVDKVGQFFPLVASVVKASDIEGMSESQAIEFLSIAADEVFTKKAEVL
DQQAIAAGKAPNSIGRSARYIILVTMDNAWSDHLQLLENLKEAVVIRKFEGKDPLKEYIA
EAFETFDGLEDDMQNNAVFSLWQSLGA
>comp3577_c0_seq1; 593 1 1929 LEN=1959; translated
KKAEKQILNLWEENCAIRKTLKQKKREKRVLVREVKQLQNTVRDLEDDNQRLSRSARDSD
KPSEEDGTVAMADTMIGSDEERLIIELEEHVASSIRLHNRLVAGSDLDRDAEQSISTNPT
SFAAEGSDLAASVAGWPGLQKGGIEVSQPKLLSLLDDDSDSDESGSEQEDPKDSDSRLEL
GSLLGSVASATDTDHARSIGATGTPASASSVGADGSSSQRANPLLKLDHDDESTEIRHLD
PHFRKPSSPKLVTENGQATSRLVCPLADVVDTMGGSSSGGNGRARGEGNEELRVHHITFY
SRKIGLQFQKASPAPQKPRGLLTDVLAADLAGGVDGSDKTAAELRSVASIASLAGGGTNA
RGEEVTCPLALPEDIVLVCGFEGFDDSGVNQRPRLGARLVAFDGVSVEVGKWTFENISKA
IKSRGRPLTLSFRNDFLTTEQRAILTKAIKDVDAKRDPVRSVIQYERPPSATPSLHSALS
YESGHFLNNEGTQGGHHPNAHGEGGIDLDISIAATEESNCWHRFPGSSSSSLSSFYKENS
RSLPRPPSTSSVSTHRSRNSFTSTAKNYMYSFQEAGSPNRVSSLVNNLAEKVASSSERQQ
RSEIFRGTPSNRKGGSERSRRFGIGINPSGTQQHQDFQSNLL
>comp3578_c0_seq1; 139 47 692 LEN=692; translated
MIRILSAVLWAFLCVVSIPSSEVRNTAAVTAFAPSLSRHHQKLKVPVDLHVGQLRTLSLP
KIPSVRVVAQSPASARTTCTSLPSAKVLPFVYTGFSGALLYKATRLSTDCSETIVLVAVS
ALSFLNLGPSDNARLASAKRAYKKTEPASAGKEKQARQAALTWKKAVRIKIIGQVIGLGR
MVLAKECRGVMKGAAWVMGATLAFLMAGGGSARHDX
>comp3579_c0_seq1; 332 327 1151 LEN=1504; translated
MTLRTSKCSRYNIDQRLTVDAPTETHRPLQHGLNTQGQNGVDCIGHRIVLTNSSNPLPST
PASPRSIFAAYWKTNHRPSSISPRSSPVGEPERIRRQQQRQQQRRQIHQPKTYSYFHNQ
DPFEYFGIAEAQDNSKTQLHDDTDDSSINSYERVLRQEEAASSIAIHERSSTYPCLSAID
IRSMGTLLVSTKKTTQSDTILFAKASSLQRSCLRKGRFSCCNNRKSKRENQLQPSKEATP
RASKVSFESVVEIQWFEIPAEKWAPNGWSSWFGA
>comp3580_c0_seq1; 400 1 1708 LEN=1829; translated
XAGLRQPGRRALSPAPTLLPALAARSDDQGASAFSGLALLLLGAVSAATDCEAASGSDIN
SDINIDDDNDDGPEYTSEDVRANDGMDGRPVWMTYGGRVFDVTSFVRNHPGGDRILLAAG
GPIEPHWHLYRQHFASDLPMRLLEPLAIGTLRESDQDEVDAKMEEIAERERDPYELEPER
RGIVTDKDGAEDRMKVHSDQPMNAEVPERLLTRSYLTPSYLFYMRHHHPVPYLSEKQIEN
YRLEIDLSEYGKGVVRLSLDDLKSLPKEEIVATMQCSGNRRSEYNVFEKTSGTTWGQGAI
STAKWGGVRLGELLKNAGMGDPVEAREKGGIEHVRFGAIDGMKVSIGIEKATNPYGDVMV
CYEMNDEELPRDHGYPIRLIVPGYAGVRSLKWLNKISISREEAEGPWQRGLNYKVLPPNV
HDANEVDVGGMPACNELSVSSGITTVEPVGLGSAPQPGDVVSVRVAGWAFAGGGRNIVRV
DVTGDVANHDWRAAELGEGRDQRYGRGWAWTFWGAEIPARVQADGSVHIFCKGVDMAFNS
QPESAADQWNVRGLMNNSWYHRTILFNKQ
>comp3581_c0_seq1; 247 345 1333 LEN=1642; translated
MAAMDRTRGNSGISSNNRRNSAFLVQREALKAVANNQPVSGSFRSFETVSTTVLSMADEG
SSPVTDGGQNSLSSASKPTPSSAVVVGTNKRNFFASLNSKEILNGATQQRSALLATMIDE
KKVVPVESGNDALSTATETEPIPTVSYARPGSTQTFMKPGSVRSNAKVAEGTWKVIYAPH
MTTAMDIFRGRFDVTYDLFPDQTMVSHAYYDFPVFGKGYLSVSGTYGSVEGDDSGTYSRV
DFDKAWIKRLASEDSNDSNSVPYGSLSEVPDSFLKTAINEIGKRAFIESVAVFPVSFLDD
DTIVFEFQALGDEDLCPQTIALAATLEHL
>comp3582_c0_seq1; 396 1 1021 LEN=1204; translated
XSTSASTSALAATTTEDPWFSSSSSSSSPALATETESAGSVERLKIQLLQLGAALDRGQA
YNPTSGSYYEENMKVARDKIGQLLDAAADAGDADKDDDVADDIADGANQPPTLPRSLADM

DGEWELVLSTVPHGIFRSSPFFLAIQESYEYAESKTFEDGTSKAGLFFKLHELQTCSWGV
SKVGRIAQHIDAQGGYLYSTFDTSLFSLTVVPVLGWFKLLPTFGGCVVTAASCTCSGKEM
LLEPDTFGGRRVSRLDMVVDYTTSRKIEGLDGLPLLGDFIWRIKVPVGAIWKLLPWNKGR
EATCKVYVNYCDGDFRIVQDADGEFFVYTRPVVPRPLEVF
>comp3583_c0_seq1; 213 1 1196 LEN=1279; translated
XCVCAFVGEPTGGTSGRQRLDLDFAALSNRVHFLSLVAPGSLCTSFLPACCARTLTKNYF
LSHFHSDHYGGIASSWDAGTIYCSLPTASLVHQQLGVDKKHLHPLPMNTPTVIESRGRAV
TVTLLDANHCPGAVMFFFEVGRRTILHVGDFRWSRDAMLGDPSSPLGSIASQKRALDELY
LDTTYCDEKYHLPEQSVAIKATIEAFETETAKKGRTLHLFGAYTIGKEKIWASVALRFGR
RVCVDRSRHRVLSALDLPEEITSLLTTNKEESNLWVVPMGHLNMKKLPDYLGAANSKPFS
PHYDRIVGYRPTGWSLSSKPGGSVLTSRTSKDRRLTIHSVPYSEHSSFPELVDCLACLRP
EKIVPTVKASASGEQVKLLMGGLRQRQRTSKLFFGKAV
>comp3584_c0_seq1; 166 276 1190 LEN=1190; minus strand; translated
MSRHHTPHTGCSVPNDGPEELCKWASLPCVLLCELLSGTAIHDPLHSGLACRGSSVPFCS
VLRCLPHHRAAECAVEHDAQHQPVGFNGSAPAHAVPDMPLVVVWHAAQRNANHAAVPRRV
VFWTRHRHWIEAPSARSRRAVPVPSCDEEEARTHPCTNGPFRRGGPFVSRCSLIRARPGR
AEPGQVLFASHHIASSGVSADVVSSTARLLRKKDKPPAALGSRRETNHHHHHHREWIESG
TAHLALVGNATRRNATQGIAPAVADRLLVPPHGSSIEHATRWCDASRRVALCTSVTSIII
IISSX
>comp3585_c0_seq1; 436 153 1364 LEN=1451; translated
MTNETTTIQRNPLVLNETIIPRLWSDDLHSKLFASTYASSITKIISVVPPTVIDAGNATI
RSRPVARRATSCQATAGTTNNSISSTHLGSKLAGDMLVAATVTFGVSPFLTVVDKAIVES
TAGTNSILQSSINSLRSMARNPVEYVRSPTFLLMWGVYSATYTTANAFKTLEEHATYSRD
RSSSSNTTQKSSGMFQMGAFLGTTVVNSGASMLKDRAYARMFSGTTSSTSFPKASYALWA
MRDLSVISSSFLLPDLVSAKLVEHSNGEIDRERALSFCQIGLPVLAQFVAGPFQYLGLDV
YNRNLGHMTTPQAIVDRSRQLTRGVAPVIAARVARIIPGYSIGGVANTKLRTAWRKKLLE
RDVAAMMKSSAANGRQSGGSNEYATRMVALLHGSNQGETKKRS
>comp3586_c0_seq1; 542 139 1368 LEN=1658; translated
MRLSFSSLTAFLLLAVPSATTTAFSTFAASTGPSNPQHSQRHSQRLSPKTATKMSASLKP
YIETLIAGERSLTTDETKEAFSEILQGADEVQVGSLLTLLRQKGETPDEIAGMVQAMNDA
CNAVDLGDRKLLDIVGTGGDGADTINISTASTVLAAACGCTVAKAGNRSVSSACGSADVL
EALGVKIDLDPSQVVECVDKVGVAFMMAPVNHPAMRFVAPVRKKLGVRTCFNILGPMTNA
AGAQRAVIGVFHPELLELMAGALQKVGRVDHAVIIHGVGLDEISPLGPATILEIKNTAAE
GEAKVYETNRFEFDPETVGIPRCELIDLKGGGPKENADKFRDVLLGGDHTDAKRDSIVLN
AGVGCWVYGLADSIEEGCKLARETLNSGKASDLLQDWIAASQEIASASE
>comp3587_c0_seq1; 54 1 326 LEN=350; translated
XIPYHTIPIDQSNGTNSGTLRPNDVPGENETKNAARGTVYFVSTDRPGFRTGWRVSMVET
GHITEEYSPKLRMCSGAHSMPIIPPERYSVVFFCACSKNATKTTTTVR
>comp3588_c0_seq1; 253 1 572 LEN=572; translated
NNNNNNRNNKNTKLRTRQQPCNRRESRFPCVSEERQRQQEQHPEQQEAAADEELWFGYNI
LDGKQPRNSHGVGEISDALATESPEQAPNPTPVPTTEHISKHSSASIYRNIELIEATYAA
LDHNETELESILDEAAGETTNETEIADHPIWEDNAIYYPYEETEVFEVDTTNQYNNNGTV
AIVEDGLYNDX
>comp3589_c0_seq1; 330 74 979 LEN=2544; translated
MGEEAIIRCGTTKGDITLKFIREWSPNGYDKAVELFERHYYDHSHFFRAVPKFLVQFGIS
YSEDQELVSFGKLQIKDDPKHDPPIEFHPGIISYAGSGPNSRTSQLFISYGSAKSLGREL
WETPIGKVIEGMDVAEQFYSYGDMPPWGKGPVQGKIYNGPKYIEDNFPLIDKFLECEVER
TNSGEQSPSGSDDEKAGSTDDGEKSSDADLNEGGNDDDAHGMDDPMNGTGAPKEKKNHYS
DRSEQKIDKFYAVPQGLHEKSLRLRGNFDSNSFAFPAGVIFVMVLLTFILRGRGKISAKE
N
>comp3591_c0_seq1; 106 1 144 LEN=1854; minus strand; translated
VSTGGNSGMPNDCEGCGSLSYTVGDFVGNDDFYYRRQRVAQFDAVVS
>comp3592_c0_seq1; 498 385 1764 LEN=1852; translated
MAVIAEMCPKIRVVVGDISQKQIDAWKTDELPIYEPGLLECVKKTRGKNLFFSTELDKEI
DEADVIFISVNTPTKTKGIGAGSAANIKNCELCARHIAKVATSSKIVVEKSTVPVRTAEA
INRILSANEQKPDVTFQVLSNPEFLAEGTAMSDLHNPDRVLIGGPQTPEGLAAIETVVSV
YANWIPREQILTTNLWSSELSKLVANAFLAQRVSSINSISALCEVTGADVTEISRAVGMD
DRIGKRFLQPSVGFGGSCFQKDILNLVYLCETYGLQPCADYWRQVIQMNDFQKKRFSEEI
VSSMFNTVTGKKIAVLGYAFKKDTGDVRETPSMFVVRDLLEENAKIHVYDPQVSREDMWT
EMSYTCGINHENTKNLDELVTTSTDPYEACKGAHAVCIMTEWDEFKTYDYQKIYDSMQKP

AFLFDGRLHLNHKELRDIGFEVHAIGKPDPIKFPGSTIL
>comp3592_c0_seq2; 414 782 2087 LEN=2175; translated
MEDDELPIYEPGLLECVKKTRGKNLFFSTELDKEIDEADVIFISVNTPTKTKGIGAGSAA
NIKNCELCARHIAKVATSSKIVVEKSTVPVRTAEAINRILSANEQKPDVTFQVLSNPEFL
AEGTAMSDLHNPDRVLIGGPQTPEGLAAIETVVSVYANWIPREQILTTNLWSSELSKLVA
NAFLAQRVSSINSISALCEVTGADVTEISRAVGMDDRIGKRFLQPSVGFGGSCFQKDILN
LVYLCETYGLQPCADYWRQVIQMNDFQKKRFSEEIVSSMFNTVTGKKIAVLGYAFKKDTG
DVRETPSMFVVRDLLEENAKIHVYDPQVSREDMWTEMSYTCGINHENTKNLDELVTTSTD
PYEACKGAHAVCIMTEWDEFKTYDYQKIYDSMQKPAFLFDGRLHLNHKELRDIGFEVHAI
GKPDPIKFPGSTIL
>comp3593_c0_seq1; 299 1 849 LEN=984; translated
RNRNIRIMAPAVATRRLRKELINLKTEPPPGIIAEPDESNILKWHYAIRGPTETPYEGGI
YIGKIIFPPEYPMAAPSVYMLTPSGRFQISTKICMSMTDFHPESWNPMWKVATIIQGIQS
FMASDELTTGGLRASEKDRKTFAKLSMNYNQKHYPKLFEGDIEAALEAADQACGKAEMEN
AKTSCSASNDQKASSSRRSRRMARKAASSGGGNFSQDDENASSQDNGDDNNQTDNNDAPS
TTTEKELTPEEIEKRRKKNAKKRAKQKAKKAAAVTQEKGTEP
>comp3599_c0_seq1; 297 1 1408 LEN=1483; translated
XGSASNTTNPIPGHQSLQSYTAMAFKVQNLDLLASRVASHVLRYNVAQAAAKRIPLASAS
ASASTSQPPPPRHELILYDFEGSPWCRLVREYATILDLTLHIRPCPRETLLFGEGAFSAK
SRFRPEAMEWFKSRCYRCSRETDLCSANSEAIIQSTIEEDEEEVDNRDNLTFPLLVDRT
NAKISTEENSKFSDHGVVVLTESYDILCHLWKHYGASVVQSEPCGGGSGNSRRRPDQTVN
SSSVPFPIRFLSLAGPSYLRPWPRCGLMRFPCSRSSTAAVPLSSPEEPGGRPQQPALTLY
QAEGCPESRLVREVLCSLEIPYRSVPVADGSSNRLPNASDSRNHGATDDARDGGKSLRIP
LLEIAPPNDSVIAATPETAGTSSSIGVIYRVGARECVDYLRDNFYRRSGAPKHHDDHGRD
GDTPTWLDPLPEENLGRGSDGDGSNSLAIGAYTAFLKGSRAFVPARAMK
>comp3602_c0_seq1; 270 108 752 LEN=1083; translated
MAFNNDDEIEEVELYSSSRERQAYDDQAALYAIILATEHLERAYSRDAITQQEYVQECNK
LISQFRLAEKAALPKLNMTTESFMELYQMDCPRATERLLNMGVPEPIRTATGQTADVAVT
VAKTVENFITAMDAVKIETRFVDELQPLLSDLMDSLTKLPDIPDDFEPNHKVKKWLQKLN
GMRAIDEITEEDSRQLSMDLDSAYVEFTRYLKSK
>comp3605_c0_seq1; 247 436 1085 LEN=1188; translated
MRQLLREEKQPGTLPAPNLSTSHDISIISETFDDPSSLLLGSGSFGQQSDSRLINSLIGG
MSRRQKSSGSFSKRGGIRNNTTTSAKVASYRNLKASAALRQTQQQVNVHVPDADDGSDES
GSDDDDTNCEKRLWKLNCFRKPSKVTKRNSIRTLTHETFPMLTSEEEEKRWLAYGMKPIV
EARSLASGSIISMSEPLLLPEKKDNSMRSRQPFRPT
>comp3605_c0_seq2; 518 198 2051 LEN=2154; translated
MPPQSQSTVSPVRRAGGTQRLPPSGARASSGRYGTRNVRSQGELRMAYKAQSRNIGSPSQ
NKNIKTIDGDALPYNNGFFDSSNDHLSTDSAGNSVGTPSSVYMPSSVALSRDMSDLSGVS
GDRHRSSSSHTIPGQIFDSRFLNKPWKQAYTLSNSPPECVNVGTRTTSSNATGKNRGRSD
GNDKSEGNKNVKGTSIWVPQNDVVEIFDSLESSLGLDRFQSMGGKHSRIPIMVLLMNPPN
QSYELMQIWVDRANDSIRDLVHVLQHKLPSQWKQAYDGLFQVRGHRFTQLINIIRLVKYD
IQSHEILVAKPWAMTAKVTIAFAGSAIRHLTRVGVIAVDNNDSGSIRSGRYLRTQHKNED
VPLLLSKRARDRAYFPEGILNHHHALQFVTFSPSFEPTHNNNEAATTRRKAARNASAPNL
STSHDISIISETFDDPSSLLLGSGSFGQQSDSRLINSLIGGMSRRQKSSGSFSKRGGIRN
NTTTSAKVASYRNLKASAALRQTQQQVNVHVPDADDGSDESGSDDDDTNCEKRLWKLNCF
RKPSKVTKRNSIRTLTHETFPMLTSEEEEKRWLAYGMKPIVEARSLASGSIISMSEPLLL
PEKKDNSMRSRQPFRPT
>comp3610_c0_seq1; 128 105 577 LEN=577; minus strand; translated
MGKSCPHDVHAKLSSMPIKSDSTRLDAISFALTQVKKRLKKHTPPYFTTTVWPLSDLAAS
AMVSATSEKFSGAAETDVFETAPGARGARADDGKAKPDATDAVDAATMRTDASDFMFVRL
VFSSFDLVWFGSVLASGGNLALRSSFDRSRLRKASANX
>comp3610_c0_seq2; 505 127 1134 LEN=1392; translated
MKSLASVLIVAASTASVASGFAFPSSALAPRAPGAVSKTSVSAAPENFSEVAETIADAAR
SLRGQTVVVKYGGNAMTSPELKAGFCQDVVTLQNLGVRILVVHGGGPQINAMLEQVGVES
RFEGGMRVSSKEVVDVAEMVLCGSVNKEISSTICSAGGRAIGLSGRDDSLLQCVKKTNKD
GYDLGFVGEVESVNTKLLNDLLDMGICPVVSPIGTGIGDEKDIAYNVNADVAAGRIAGEL
RAARALFLTDIAGVLDKEMKLLPSLSCADVESLIEDETITGGMIPKVSYATSAIELGVGG
AFITDGRVPHALLKEVLAGGAEGSGSGEGGTFVSK
>comp3611_c0_seq1; 252 1 354 LEN=354; translated
ALLKQKITELETSVDRFENQEALLREQIYEEFEAKKEEETIAVNAENTVSSDQYREQEEE

LQETKKKNKEMEQWLTKVVEESTKRETLLKKKVTELETSIDKFENQETLLASKVVEES
>comp3612_c0_seq1; 703 1 1639 LEN=1831; minus strand; translated
XCVLSIASFLGNVGVALTGFGMAIIYLFVYQIIVLAGYDGNLRYAVFIQALALFSAQPLL
LRKAEITKHASWKILHYFIPITIISTPLGQLTGDAISTDLIEIVAGFLVTFVAVFEMYQK
RKLFAKRFRKWCCSVGNEEEKKEPVDEENPKSRDTGEETSTNISRDTSGQNVLLMNDHNG
ETNAENDASLLHTPEGGFFGQYNLGKTLDNGSFSVVKLGTKKKCTRKSFAIKIIDKSHLL
DDEAEQLQEEITHLQQLNHPNILYLQDLYDEPQKYFLVFDLMHGGDLLARLSGVRFFSEA
EARRIAKDVLGAVRYMHDHRIAHRDLKPENILLASGDADCEVKLADFGFSKKEVRPNSFV
TLCGTPGYVAPEILKGTPYGCKVDMWSFGVICWVMLVGYQPFRGESEQEVQIAIVRGKYD
FDEEFWEDINDSPKEFITSLLVSDPETRLSSEEAQCNPWFSDEYKCAERIVDETTPVFFM
VGSQRSGSNWLRTMLNEREDLGGPHPPHIMREFMPIIDKFGDLSDDESFRILLDHVCTFV
ERNQVPW
>comp3615_c0_seq1; 330 1 2203 LEN=2203; translated
XSPIVKATQIIALLSFVVFPEASLLDISSGVEIYPDLWRRNLDESTLLIAFSCTLRIIQG
LLAILAVFLLVITSSNATEIILNFTAVNFISLLDDIAFDLAKDGKYGRKLKVAAKRVENE
ALPRYTVSRNSFLRHKVSVCLVSIILFGMLSYVILCQESSQTWITRIVQIRFGNKDFKAL
SGCYDIIDELTNDGRYNYEISDKLHQTTRMRFGYCKKSRRWVLFSGRHLNGTEFDDPCLA
DEYEIKARSSKTDSFDIMYSLDEPWYSPSGEALDFTLVDLEDNQQKCNSVVGDGLCNEFF
NNEYHRYDDGDCCFMTCNSTNCGKNGYKFTPLFGVTIAAADGYPRCDLKAPDSAGLKYVF
IRLDSISISDRESNPFLVLECDGKMVFSIPVDQEMQNKTEKAIVPKEGKCTLNFELKRSQ
EIREFSWDLNYSIYSAGKDYIYRQEMGEKIFKGHSSSEKSKEYFSSYDLGSEDHLSSNIG
TLLHGNTSLDLYDMQLDGTLPTEIGLLTDLTSINLCGNSFKGGLPSEIGFLTKLTEFDIA
CKDIDGGNANITDGLRGTLPTEIGSLSDLTSIRFGIRSFTGTLPSEIGLTKLSELDFHKS
SFTGSLPSEIGLLTNLAKLDFNQSSFTGDLPSEIGLLTQLSELRFYNTSFTGVLPTEIWS
LTNVSKIWCGNSTFNGSLPSEIGFLTKLTSLVLFNSGFKGTLPSEIGLLTRLTFLRVDNN
HFTGSLPTEIGWLAX
>comp3616_c0_seq1; 505 145 1952 LEN=1952; translated
MTSKEGIDSNPVEREDIEEAIDTAIVKRKDDDDSCKDEDCDRHEVDETNPPYLDFIRAKT
KGWGWIICSAVIVAYSLAAFILDFRRALFLFVVEVLVVTLVAFQYVTDRFCLEQKTRIMD
SIIDWFVEDLEESKIAAGVLLVAMIAIIGVMTRDPMNLVSASGLVVFLGLSWLTSYKPWS
VRWRPVISGVFLQFLLGVLILRVEPIAQAFQWLGDQVAIFLNYTNFGAMFVYGFLADSSL
NETPLATADGGEYYLYPPFYFTVLSVIFFFSSVVSILSYIGVIGYLVKKVGLTLAMLMGT
SGPETFNCVANMFLSMTESPLLIRPLLSEVTESELHAVMTAGFASVAGSVLAAYISFGAS
PSDLLAASIMSAPAALGISKLIYPETTVRRDRESLFDLEMAKSVDPNIVAAASSGASLAV
DMVLQIGGQLISIVALVAMVDGFLSGIGKLIDVSLSFNLICSYIFYPFAFLMGVPMADCL
QVASLIGTKIVVNEFAAYAQLGVMIREGSLSARSITIATYALCGFSNIASIGVSVAVLSS
LTEPRMKPIIVRLVTSAMIAGNTACFMTACVAGLFYTSSIASDPNNIDPLVDQAGNMTAS
EHX
>comp3621_c0_seq1; 239 1 988 LEN=1591; translated
XNSNSNSNTAETDDRDFVQLDLSSCNVLDVAGGKGELAFQLLNLNNAKTCHVIDPRPLSL
KRFRGRLEKGFYHRSQGILTHDVVRPLTEPERPVDQLRCLFSSELWETEPDSNDTNNNND
DNNDDGDDNANPQKIKPKRDLRKEFFQNCYRAQGWVWPPVGASNEHAAMGDDDRDEIVRK
RPSANATAKANGDSDPTPTPSGRGTIDNKENESSFTPTFDHASEVVRKADLIVGMHVDQA
VDAIIDAALAMDISFFVVPCCVYSKEFPFRRVPVLEGGGCGDGNKQRTKPVTTYEELIRY
LKAKSPDIRSHVLPFEGRNICLYRVVGCS
>comp3625_c0_seq1; 657 401 2454 LEN=2454; minus strand; translated
MKWTFNSVMLSTILFSTARHALAFVPGVQRAASRQMATAAGSSFASRYSMPRSSTPARLM
ATVAPEKQAAEDKDTDIFDELPTNDNDNDLLRIRHSSAHVMAMAVQKLFPEAQVTIGPWI
DNGFYYDFFFPETIDPETGETIEARKLNDQDLKKIKKQMDKIIQKDYPITKEEVTREEAE
RRIKELNEPFKLEILEGLQEPITIYHIGDEWWDLCAGPHVESTKQIPKKALALQSVAGAY
WRGDENREMLQRIYATAWKDPTQLKQYKKMMEEAKKRDHRMLGKKLDLFSIQEDAGGGLV
FWHPKGSKVRKIIEDFWKTAHEEDGYDIIYTPHIANLDLWKTSGHFDFYGEDMFDQMDVE
NEQYQIKPMNCPFHCLMYADELRSYRDLPIRWGELGTVYRCVLITCCICCESMLDIIVVL
DSQHKIFTFDFLHNRYERSGTLHGLFRVRGFTQDDAHVFCLPEQLEDEIVKVCDLIETIL
TKFGFDKYEIMLSTRPEKAVGSDEIWDAATIALRGALEKKGWDYGIDEGGGAFYGPKIDL
KIRDAIGRQWQCSTVQCDFNLPERFGLEYTSKEGTKERPIMVHRAIFGSLERFFGILTEN
CAGDFPFWIAPVQLKLLPVTDSVQDYCHEIAAKAKKAGIRVEVDRGTERLAKQIRNAEQQ
RVPIMAVVGNKEVETNKLAIRSRKX
>comp3626_c0_seq1; 220 77 622 LEN=894; translated
MGKELKVEFGRINQDNIEQLRKVNLACFPVQYQEKYYKDVSASVGEGLCKFAYWNGFVVG

AASSRIEPINGSNGNTDEPKNDNESNEKRIYIMTLGVLAAYRGRTIGTQLIQSILDYFEE
NKNESPELKDVIEISLHVQISNDDAIKFYTEKFGFEKGEMVENYYRRIDPPHCYKLYKKV
R
>comp3626_c0_seq2; 203 303 806 LEN=1078; translated
MVCWQLRKVNLACFPVQYQEKYYKDVSASVGEGLCKFAYWNGFVVGAASSRIEPINGSNG
NTDEPKNDNESNEKRIYIMTLGVLAAYRGRTIGTQLIQSILDYFEENKNESPELKDVIEI
SLHVQISNDDAIKFYTEKFGFEKGEMVENYYRRIDPPHCYKLYKKVR
>comp3627_c0_seq1; 486 42 1439 LEN=1598; translated
MVDESQVISRFKDANQSHVLKHLDTLSIEAKSEFLEQLNSIKVDELSDLLGAAMAEQQNQ
DGNDDINPFSKDVGRTSDKDESASAYEEGIGAIGRGEVAALVLAGGQGTRLGFSGPKGMY
DIGLPSGSTLFQLLAERLKRLKKIAECSTLPFYIMTSPINHEETTIFFKENNYFGLPEND
VLFFQQGMLPCMNDDGKIILERADKVAMAPDGNGGIYPSLKNSGMIDDMVNRGTKYIHVF
SIDNALTKIADPVFIGHCVKTQADCGNKAVWKKGPHEKVGVLAEKGGKPCIVEYSEITTE
MAEQTDGEGRLVYGAGNICNHFYTLDFLCDKVVPNLGNMYHIARKKISYYDEESKSTVKP
EANNGIKLESFIFDIFPMSERMAVLEAQREEEFAPVKNAPGSASDSPDTARVYLSKQAQK
WVIEAGGNLTGDLESQCEISPMTSYGGEGLEDLVRGKDIHCPFSL
>comp3627_c0_seq2; 407 42 1346 LEN=1441; translated
MVDESQVISRFKDANQSHVLKHLDTLSIEAKSEFLEQLNSIKVDELSDLLGAAMAEQQNQ
DGNDDINPFSKDVGRTSDKDESASAYEEGIGAIGRGEVAALVLAGGQGTRLGFSGPKGMY
DIGLPSGSTLFQLLAERLKRLKKIAECSTLPFYIMTSPINHEETTIFFKENNYFGLPEND
VLFFQQGMLPCMNDDGKIILERADKVAMAPDGNGGIYPSLKNSGMIDDMVNRGTKYIHVF
SIDNALTKIADPVFIGHCVKTQADCGNKAVWKKGPHEKVGVLAEKGGKPCIVEYSEITTE
MAEQTDGEGRLVYGAGNICNHFYTLDFLCDKVVPNLGNMYHIARKKISYYDEESKSTVKP
EANNGIKLESFIFDIFPMSERMAVLEAQREEEFAPVKNAPGSASDRSVVFYTCIASMFFD
RFKVVVFNILLLCNW
>comp3630_c0_seq1; 366 195 1373 LEN=1466; translated
MTTTGTATQQKWNRWGTSSYTVMPHVSPESKAIAETYQLRPTDVVVLSFPKTGTTWTQTV
CEQLRTRAEGYDGFGDITERQPWLEFAHDCGQDLDDGLYDAYKPRIFKSHQLLSAINPGG
KYLCIVRDPAATLLSWFSFQKAKGRPGYAEYKDANEYIKHRPELFGGHHIFGTNLWQMYA
EIWSARNDPSVKVLVYEALIADGPNDYMDHLPVIHDFLGLRTNHTSPEEDEETKTLYQKV
AFLVSRAEMVRHAEKFKDTFIAEMGKKHGRALRIMEPAAKVRSKSDKRAELDEDTLEWLE
DQWLEKITPHTGHATYDEFATELSDLFFEGVVDEKQVEKAGAEPLPLSHDEIEKKQHRPS
RKSSILVRRKSSIVVQQMPSIRQRRETLAPLD
>comp3630_c0_seq2; 246 195 1484 LEN=1577; translated
MTTTGTATQQKWNRWGTSSYTVMPHVSPESKAIAETYQLRPTDVVVLSFPKTGTTWTQTV
CEQLRTRAEGYDGFGDITERQPWLEFAHDCGQDLDDGLYDAYKPRIFKSHQLLSAINPGG
KYLCIVRDPAATLLSWFSFQKAKGRPGYAEYKDANEYIKHRPELFGGHHIFGTNLWQEVT
KILPFRSELRLCPTLSATSNFLRLTNDEPTRXSQMYAEIWSARNDPSVKVLVYEALIADG
PNDYMDHLPVIHDFLGLRTNHTSPEEDEETKTLYQKVAFLVSRAEMVRHAEKFKDTFIAE
MGKKHGRALRIMEPAAKVRSKSDKRAELDEDTLEWLEDQWLEKITPHTGHATYDEFATEL
SDLFFEGVVDEKQVEKAGAEPLPLSHDEIEKKQHRPSRKSSILVRRKSSIVVQQMPSIRQ
RRETLAPLD
>comp3634_c0_seq1; 112 516 695 LEN=839; translated
MIFKGQDAWRNHHLFQRLWASPFPGFRNAVFIFGAYLVVDSVLEGMKPKSAKIEHGGHH
>comp3639_c0_seq1; 935 90 1793 LEN=1832; translated
MTTAAEFKALGNAALQAKKFSEAIANYSKAIDLDGSNHVYFSNRSAAYLSKGDGNNALED
ANSCIALNPEFTKGYSRKGAALHALKRYNDSIAAYNEGLEKFPNDPALKKGLADVTREKD
APPAGMGGGPPGGGLFSPSMLAQMAMDPRFKQYLDDPTVMGKIKMIQQNPNLLPTMMSDP
KMMEMLGLLMGESPGSSQPSSPPAPAPTPAPPKKKEEPKPKPMEEDWSTLSPEEREKKEI
QKKARLKKEEGNSLYKAKKFEEALAAYDEAIAIDSTCMTFLLNKAAVYFTMKKYDECIEE
CNKSVEVGKENMAPFEDRAKALTRCAKAYQKKGDLANAIEMCKSSQLEAYNKDTQRLLKS
LELEKKKKDKLDYQDEGKAQEAKQRGNDFFREKKFPEAIKEYEEAVKRAPNDAAIRNNLS
AALTKIMDFNGAKVQVEKALELDPKYTKAWARKGDIEMAFKEYHKAMESYKNGLEIDPKN
AACKDGLQKVTMQINYGRSQMTDEQKREQAAHAMADPEIQAILQDPLVQQTLRDFGENPS
AAQQAMNDPHMRGKIQKLIAAGVVETA
>comp3639_c0_seq2; 685 90 1945 LEN=1984; translated
MTTAAEVNRYGHSGEFATFLLVSRCFRRHRRFDGLLADSELKLTQRFLPLVHSFPFXFKA
LGNAALQAKKFSEAIANYSKAIDLDGSNHVYFSNRSAAYLSKGDGNNALEDANSCIALNP
EFTKGYSRKGAALHALKRYNDSIAAYNEGLEKFPNDPALKKGLADVTREKDAPPAGMGGG
PPGGGLFSPSMLAQMAMDPRFKQYLDDPTVMGKIKMIQQNPNLLPTMMSDPKMMEMLGLL

MGESPGSSQPSSPPAPAPTPAPPKKKEEPKPKPMEEDWSTLSPEEREKKEIQKKARLKKE
EGNSLYKAKKFEEALAAYDEAIAIDSTCMTFLLNKAAVYFTMKKYDECIEECNKSVEVGK
ENMAPFEDRAKALTRCAKAYQKKGDLANAIEMCKSSQLEAYNKDTQRLLKSLELEKKKKD
KLDYQDEGKAQEAKQRGNDFFREKKFPEAIKEYEEAVKRAPNDAAIRNNLSAALTKIMDF
NGAKVQVEKALELDPKYTKAWARKGDIEMAFKEYHKAMESYKNGLEIDPKNAACKDGLQK
VTMQINYGRSQMTDEQKREQAAHAMADPEIQAILQDPLVQQTLRDFGENPSAAQQAMNDP
HMRGKIQKLIAAGVVETA
>comp3640_c0_seq1; 662 1 1773 LEN=1804; translated
GSGKTLAFVLPLLSAAKRRPWQRKKVASPRIIITICPSRELAKQVGKEYSKLAGKAFGVAT
VFGGVPIERHVSLLKYKPQIVVTTPGRLRELVREGHMDYSQVSTLVLDEADLLLDPQDSP
DVFSAIEDIEKALEESDDEDPEYQMVLVSATIGKNVKDFAAEMEFPESSFVNIEGIQDSK
KLVDPSSGGAADSAEHQISAASSSNAATVGHWHMSCKSAMRPEITANLISVLSPRLTIVF
VPTKSATESVAAFLSEKAAGTTIRILHGDMSQSARSRCIGMIREDAIRKEQQQSPQSEAG
QILVATDVASRGLDLPNVDLVVQFGLPRIAGKDGTINPELYAHRTGRTGRFRGGSNDRNW
GPAANRYETANAVALYDPAVGEGKLVTELVEAIEDDLGVTIQSMSIPSSAKVVDAAYQRL
SLDVLNNDSDQDTPSLSSSNNKSRSTDLAAYFKQQLKSDKRVDSSDPEELLGHLANAMVR
LSKLDPSLSPYTQSSSLLTGDPTMTTLRLYPRSIGGSSLTPPVVTSFCKQRGSGKLGRVV
ISKKDGSAIFDLPEKRANKLMEIIKAEEEERKDDDGLEYGLEIPISLPEL
>comp3642_c0_seq1; 318 400 1670 LEN=1714; translated
MNHGXAVGVANAGEADGDIIDVEVKEEEVIEPPKPEIKTQFCITGISPPLETALNEAVSK
LTNVTLKEANELVEIGAVWAKIDVMTEDDVLAQYDSSGEELYADLPQGWGNGDSDAGTGT
EEEKLEEYIAKMESQRFRRILSPRKLQPGTDLRIYPNPRRFPACYSIDGKSLLYQDTTFI
VVDKPPMLPTQPDASNYHENCPGCAQDILGPFEDIQGNTIRRPLLCHRVDSVVGGCVVMS
KDRNGQRVFQELQRQRKLKKVYLAVTTKPVPLGMHLHWMWSPQTQRGKAGGPPCQLVSHI
APESRRKARQFWTRCILEVTKSEPITVNADDYDPGDKQHYQNTIRLVTGRKHQVRAQLSS
LGCPIIRDTLYSPMSGITLGDLEVGDEEALENAIAQCRVPEEPIGLQAHAILFGGIKARA
REPW
>comp3645_c0_seq1; 388 57 2204 LEN=2434; translated
MKCLKKRSLVTFWAAFVAMYPQDSQVSATSSCRSINQIVCATDAFSILCQALEETGIDIT
LNNREKDFTFFAPTNDAFQHVLRDLKYNDIYECPKGTLRELLFLHMHKNNVINKSGLKDR
CGERLEMVNEEFTRTVCENGETKIFQKGAGNSESAKPEVIAFNIEACNGIMHIVDEVILP
SHLPGLSSRKPTKKPTKKPTRRPTPKPSPRPSPRPTRFAPLEASSCSKFPRCAALGLTGE
CCPTDEGVKLDCCVEEAIYFRPTQKPTEISAGSCSRHPKCAAKGLTGECCPTDDNAKLSC
CFDIPGSCSANPECEALGLTGECCPTDENVKLDCCFAGVFPSGSCDVYPRCRELGLKGVC
CPNDGTGQKLDCCSEEPGLPILGESYCTNTPDYSCYQFGRPECCLKSTIECPKIQPGCEI
GWPIVGDSYCTYAEDFSCYKNGRPRCCLDDSLYCPDERPECEIGFPIVGDSYCTQSPNFG
CYRSGWPECCGDDRSSNGCPNKKPGCEIGLPEVGDSYCSDPPDFMCYEMGYPSCCLERDL
TSCPNEKPQCNIGRRGCELQDSLPSLYDFACSMRSFEILCYLLGEAELDERFDRDGSYTL
FAPTNQAFKRLGKTTMSDLLVNPREGLREILEYHVSDRIYFEEKLFCDRKIDTLLGDSSD
DFIITKCEEDKDGNQLKSQAGNGNRVWPLIIATDIVTCNGIAHVVDNVILPKEYL
>comp3645_c0_seq2; 380 273 2333 LEN=2563; translated
MVLFCPDQIVCATDAFSILCQALEETGIDITLNNREKDFTFFAPTNDAFQHVLRDLKYND
IYECPKGTLRELLFLHMHKNNVINKSGLKDRCGERLEMVNEEFTRTVCENGETKIFQKGA
GNSESAKPEVIAFNIEACNGIMHIVDEVILPSHLPGLSSRKPTKKPTKKPTRRPTPKPSP
RPSPRPTRFAPLEASSCSKFPRCAALGLTGECCPTDEGVKLDCCVEEAIYFRPTQKPTEI
SAGSCSRHPKCAAKGLTGECCPTDDNAKLSCCFDIPGSCSANPECEALGLTGECCPTDEN
VKLDCCFAGVFPSGSCDVYPRCRELGLKGVCCPNDGTGQKLDCCSEEPGLPILGESYCTN
TPDYSCYQFGRPECCLKSTIECPKIQPGCEIGWPIVGDSYCTYAEDFSCYKNGRPRCCLD
DSLYCPDERPECEIGFPIVGDSYCTQSPNFGCYRSGWPECCGDDRSSNGCPNKKPGCEIG
LPEVGDSYCSDPPDFMCYEMGYPSCCLERDLTSCPNEKPQCNIGRRGCELQDSLPSLYDF
ACSMRSFEILCYLLGEAELDERFDRDGSYTLFAPTNQAFKRLGKTTMSDLLVNPREGLRE
ILEYHVSDRIYFEEKLFCDRKIDTLLGDSSDDFIITKCEEDKDGNQLKSQAGNGNRVWPL
IIATDIVTCNGIAHVVDNVILPKEYL
>comp3652_c0_seq1; 549 326 1971 LEN=2282; translated
MAAVPLAGFTDVADHGPLHSPVYRPWFFFLLFLGGDXFRFSGGKVEAHDTNKFQNFYAHL
SRRKKNNNRRSTSHIIVPRGGANRFLPNDEGDFVDDSEHMKKKRNPIRRWKDNHSRSSTK
RFDGQTTKHSPVVYQYFGKSRNRGGGAHSHHPSHSDETSLNFILLGPNVDHWKTVGQTLA
SRGFNVMACERMIDKDGDGNPTQNGESQYEDAPHLVLEILDALKWNTAVLVGCDDESLLA
IETAMMLAPNRIAGLILCGDLAESNRFAADSGNARTLDTLLHRNLDCPFVIVWDGGLRSI
ASGSGSRGGLSSTFDENDFDGRCAILGGGSAPHRTKPEYFAWVLARFVEEKLEYHPRDYV

GGKMRHANSESSANDNGRIKAVKDRGRILRNLNLPFGIDSVVSPEGRLLLGRAVAAAIFY
TSCMKVLFVQYGAIKAGVISVKNSVDSVEALRRKMCQATGAFVMNFGYIPKFCKFSYKRT
EEKGENGVGSRSAKAGRSTEDKERNETLSDSTPSSPDKDDADHSQKEDEDNDDTLRIKPL
FFLDNIFT
>comp3652_c0_seq2; 488 326 2127 LEN=2438; translated
MAAVPLAGFTDVADHGPLHSPVYRPWFFFLLFLGGDXFRFSGGKVEAHDTNKFQNFYAHL
SRRKKNNNRRSTSHIIVPRGGANRFLPNDEGDFVDDSEHMKKKRNPIRRWKDNHSRSSTK
RFDGQTTKHSPVVYQYFGKSRNRGGGAHSHHPSHSDETSLNFILLGPNVDHWKTVGQTLA
SRGFNVMACERMIDKDGDGNPTQNGESQYEDAPHLVLEILGAVHASADVHHFVCLSICLP
ACLYDLSSDTTRRKNKNLQILIVFLPNCIDAADALKWNTAVLVGCDDESLLAIETAMMLA
PNRIAGLILCGDLAESNRFAADSGNARTLDTLLHRNLDCPFVIVWDGGLRSIASGSGSRG
GLSSTFDENDFDGRCAILGGGSAPHRTKPEYFAWVLARFVEEKLEYHPRDYVGGKMRHAN
SESSANDNGRIKAVKDRGRILRNLNLPFGIDSVVSPEGRLLLGRAVAAAIFYTSCMKVLF
VQYGAIKAGVISVKNSVDSVEALRRKMCQATGAFVMNFGYIPKFCKFSYKRTEEKGENGV
GSRSAKAGRSTEDKERNETLSDSTPSSPDKDDADHSQKEDEDNDDTLRIKPLFFLDNIFT
>comp3660_c0_seq1; 236 1 353 LEN=529; translated
XFLQHNTLHLIMFKSVIALALVASAAAFAPANVGFRSNTALNGENDDKVKEIIVEQVGCD
EDKVVPDAKFIDDLGLDSLDTVELIMAIEEEFDCEIPEEEAAKISTVGEVISFVDSL
>comp3662_c0_seq1; 157 1 646 LEN=690; translated
XPGAELHSIEISCIEQGPRAEGKWPWGNFAKESKEYQNLLDRNLLHCGDASDVDWLYQIW
NEKIYRRNKLLGGSTSGTPPPLKIVVDDGSHLAKHMVHSVFFWFPRIEPGGILIIEDIQP
IEEANLFRTQFLPQAMADLHFCGDPNQNKDEACFPQLYRLLQSIHCEMHICVLERNEVPA
IPDLSIEDSKPPPNALDMTQCYTFANSFGAGMEAI
>comp3663_c0_seq1; 182 1 376 LEN=535; translated
XQQQQQQHQHYDQENTPRGPASPTDSVGAVRPTRGGRSANTEADDKKSHYSAFDYIRAVQ
RQFTQPYKDPPSDKSMATSDSIRAEDAGSALYKKGYLPQKKYENELVKQIVKRRHLALIQ
AGEEP
>comp3667_c0_seq1; 447 114 1019 LEN=1100; translated
MPEKQVNPLINLAAGGISGAVSKTLTAPLEKVKLAIQNQDSDPRVISGEMKRYTGMGDAF
KRHISELGPSSLWRGNFANCIRYVPTAAFNLAFKDTIKRMFPKYDKNTEFSKFAAAQVAS
GSAAGGATNTIVYPLIYVRTVLGADLGKEKKYNGMWDCLKKTVKGNGFTSLYNGIGPSTV
GIVVYRGAQFGLQDILKAFNPYQKDFTAIGLASKFCVAQVAVATSGVVAYPFDTMQRRLQ
IEASKPKGEQMYTGMADCFGKILKTEGPGGFFKGALANVLRGTGAALVLVIYDEIINAVS
K
>comp3675_c0_seq1; 314 526 1644 LEN=1887; translated
MGDREDKLPLPKGWIRTFSNSQKRYYYSHSETKHTQWHFPTASEANDPWMAKRRAETNRS
SQQQQPAKKKQRTEKGAAVASLSDVDALLDVADTTSVAIIVPFRDLHKEQKRSEHLSKFI
PHMIRFLGNLKTTERRISDYHVYIVEQSDDGRKFNRGKLLNIGFDIARKHKGRNGQKHDV
FIFHDVDLLPSKQLNEAYSKFPTVPHHIARCWDRYSNNPKYFGGIVSFSSSDYKRINGYP
NTFWGWGGEDDELQLRCNALGIKWDFPRLSSSASSSSTPPITDLEGMTLKEKLKFLKSHR
EWKCMVKWEALEEHEKNWKTNGLADLQYKVLKTTPLDSKDASKAKATKITVDVKLNPNNH
WANDKCGVEYMG
>comp3675_c0_seq2; 191 526 1792 LEN=2035; translated
MGDREDKLPLPKGWIRTFSNSQKRYYYSHSETKHTQWHFPTASEANDPWMAKRRAETNRS
SQQQQPAKKKQRTEKGAAVASLSDVDALLDVADTTSVAIIVPFRDLHKEQKRSEHLSKFI
PHMIRFLGNLKTTERRISDYHVYIVEQSDDGRKFNRGKLLNIGFDIARKHKGRNGQKHDV
FIFHDVDLLPSKQLNEAYSKFPTVPHHIARCWDRYSNNPKYFGGIVSFSSSDYKALTAIR
TPSGDGEYXLIGPYYVAFNTALWMFRMDPLVIGIETDSHPFLLFMRIFPCHTKQRGGEDD
ELQLRCNALGIKWDFPRLSSSASSSSTPPITDLEGMTLKEKLKFLKSHREWKCMVKWEAL
EEHEKNWKTNGLADLQYKVLKTTPLDSKDASKAKATKITVDVKLNPNNHWANDKCGVEYM
G
>comp3678_c0_seq1; 395 1 1500 LEN=1800; translated
LKSRVVLVTIQAIDCSKPHLEICQLTLQITVRQTQQVNCDSNXAKGLVDFIDLVLSTTQY
PTIDKMSSIGGTSRKTFRLLSFALVVTLVFLFLEGEASSAVDSKRNNDGINVPRFEVYDL
PAIQWACEPFLEAHERFHLNPMKEKTRDLCLKLRGGKLGDAANDVVVLEESTEGAAENTN
RSMDVSSITVLEDEENEVEVDDVEEDLSSPSSPTTGELPMNGSRPKPTTIENEHLDSADD
EHPVPSQESISEPSASPTDYTFSVFQKGDGHETDPDGIPTRYLNMQGGRRDLAKTALMNT
VKWREENDVDTILARPHPKYDVSKAVFPHYFCGRDDTQHVILLQRPGLMNVKLAHKNGLT
GEELLFHYIYVMEYLWRIIDPEPDATMTSIIDLTELNMSILRKREQLRIGSLFLSTMDAH
FPQRSHRTFLINAPRWFGALYKIASPLLRESTKEKIMILSQGKEQDAVLRDLLRDCPIPD

GEKLENVPAGIMEEELAIL
>comp3678_c0_seq2; 435 1 1553 LEN=1628; translated
LKSRVVLVTIQAIDCSKPHLEICQLTLQITVRQTQQVNCDSNXAKGLVDFIDLVLSTTQY
PTIDKMSSIGGTSRKTFRLLSFALVVTLVFLFLEGEASSAVDSKRNNDGINVPRFEVYDL
PAIQWACEPFLEAHERFHLNPMKEKTRDLCLKLRGGKLGDAANDVVVLEESTEGAAENTN
RSMDVSSITVLEDEENEVEVDDVEEDLSSPSSPTTGELPMNGSRPKPTTIENEHLDSADD
EHPVPSQESISEPSASPTDYTFSVFQKGDGHETDPDGIPTRYLNMQGGRRDLAKTALMNT
VKWREENDVDTILARPHPKYDVSKAVFPHYFCGRDDTQHVILLQRPGLMNVKLAHKNGLT
GEELLFHYIYVMEYLWRIIDPEPDATMTSIIDLTELNMSILRKREQLRIGSLFLSTMDAH
FPQRSHRTFLINAPRWFGALYKIASPLLRESTKEKIMILSQGKEQDAVLRDLLRDCPIPD
GEKLENVPAGIMEEELRNFCMARLEEAGAEMQVVEEV
>comp3680_c0_seq1; 357 1 1636 LEN=1848; translated
XDSSIRSNGVAATAAVATPLALWFVSSAALALLLSLPGCWADPSSRGKNSVHTHDAAYAR
LGVRPPDEHNRVLGRGGVLELPIARWSWRIHGRILPLLHLFEGGRDRRPPDVFVNLRVLW
CKLLSSLDPESPAYEGRRSLPGKGSSGVWGGNSGDATDAVLFATYRMLPPCSIRWPLRYL
GLWRFFPRWMHANIELRTAYLKGALRRVLAPSGASTTGPDRCCVVVLGGGYDPRGAVLSA
GKGIERVYELDLPAVVDSKRRLLSRAGFCVEGEDAGGSGRARNGVSGTPRGVRLEGVDLN
DDEAVDRVLDKIRRELADDPSSDPASGKPGVWRVVVVSEAVLMYLLPGKARRVLEGLAER
FRGSDSESGFASASFVFADRLVPRRNTALDAAPTGSVVAAADSTAPSAGASSDAAVIDPR
GTEESEVRQWLETSGWELRELLFKPGXYQTPGHCNCGMTHVIGVEAHAHTNRIDPAPLRI
DGAVLHHCVGFGTNERTNGNQSMTDGASFLPLSMGCRDGFFRGPIGRRDYGAVLGPACVE
CPASD
>comp3686_c0_seq1; 190 444 872 LEN=1295; translated
MNTTGNNILQWTALLRGPSDTPYSGGVFQLAIDCGADYPLAPPSITFVTRVFHPNVHFRT
GDVCLDILKKEWSPAWGVQAACRAILALLSDPDADSPLNCDAGNMIRGGDMLAYNTTARM
FTVENAMFLAWPSTATTTDKEG
>comp3686_c0_seq2; 216 10 685 LEN=1108; translated
MSNTPIEEYGEQSWDQSKAKDNSVQNEQKYTGVHTGNTTSERQDKAIRIKPITMSLNLAA
AKRLRKELQNLKRTKEPDPDIYLIPDGNNILQWTALLRGPSDTPYSGGVFQLAIDCGADY
PLAPPSITFVTRVFHPNVHFRTGDVCLDILKKEWSPAWGVQAACRAILALLSDPDADSPL
NCDAGNMIRGGDMLAYNTTARMFTVENAMFLAWPSTATTTDKEG
>comp3687_c0_seq1; 304 287 811 LEN=1610; translated
MGKGPNKKRNKTTKRGKGCYQSTDDGEEQPPSIGRRCQKSNKRRDNNQQQKNGNYNRGDN
DYDTDDCKKLRKTLNADGLEIVEMSADGNCLFRSLSDQLFGDYGKMHYDVRAVVCDFMEQ
NKADFQVFLVFEDEDDEDQCEEDARDFDHYVGNMREQGQWGGNLEVVAAARLYR
>comp3687_c0_seq2; 454 287 1324 LEN=1479; translated
MGKGPNKKRNKTTKRGKGCYQSTDDGEEQPPSIGRRCQKSNKRRDNNQQQKNGNYNRGDN
DYDTDDCKKLRKTLNADGLEIVEMSADGNCLFRSLSDQLFGDYGKMHYDVRAVVCDFMEQ
NKADFQVFLVFEDEDDEDQCEEDARDFDHYVGNMREQGQWGGNLEVVAAARLYRRSITVY
SETMAAFTIDHGESKSMGPDLLVSYHDNDHYNSVRNKLHPPKLVRNGMLPMNDILSNKIH
TKNTNIYETTHQGSHEIECITTSFSALPLSDKREEETEKNTIKNVKRNAPCPCGSGLRYK
KCCLARQKHATRVERLRVKRQDEEESLPAETEEERSHGMFRVVAI
>comp3691_c0_seq1; 222 816 1133 LEN=1491; translated
MADVFGSEYVSLHVRKSNRAAFHLYSVTLQYEVNDVEKGYYADGEDAYDMRCYFKQRSTP
ANEESSAAVGGTNESSSTEETDGPPTRAMHQLQVSDAAAPVAAGM
>comp3691_c0_seq2; 222 555 872 LEN=1230; translated
MADVFGSEYVSLHVRKSNRAAFHLYSVTLQYEVNDVEKGYYADGEDAYDMRCYFKQRSTP
ANEESSAAVGGTNESSSTEETDGPPTRAMHQLQVSDAAAPVAAGM
>comp3694_c0_seq2; 265 145 1067 LEN=1107; minus strand; translated
MVTQNSDRILSGSSDHPVGCLSAFVGGAVGRSSLLCSAGACAYVCKQTKKERRPMGFVDS
WVELSRVESSRVEPNGTEPMNGGGDPPSSSPPLVFLLPSGLLPPLPVFPEAGVQLVPDLL
AALLLELAVDDPALPPLVVLKGLHPVLLGRLLSRGRVPEVDLHDPRVSEAVGLVVGGTVA
LSRGPALRPPVRGGGIVGEGLGSAAGKGKAPLGPEGGDPAPLEALGGGKRCGGVARIAEA
VLLVAGPLLGQDGPAPFAAFRLSTEGGFRGGRGGSGCVRNHGDGLGWDGSASGGGSGTDG
ARRLFDR
>comp3698_c0_seq1; 799 57 2318 LEN=2361; translated
MAVRGRDADDMIASLMKEMGQNQGYHSEQTLTRDEDVNAHDGKETGQQNNSKFQTPTPRR
RLLPELPEYPDNAFQAGSQSIWNPMAEGKCGSPGRNASDRAQISIVPLMVPTNPRIEVIR
RRVFEKFRQETHTLLQEFQTKNLSFSSRSSASGGSFSKNKSKLPIPSMIDKWHMDAKLEE
RNILFQNMAASSSLSSSSSSKPSNTSLPSAASTTEILREIATRKKHGIHVYDPILLNQHS

PSIFIETILKPQVRKMWQQSNERVESNNNSEFPPKFCKKAQQVQKALFRLVCQARESFET
QLLQDVRQESIRSTTAKKSRRGSNNNRIPKLSRSPTTDGDSSSPSLVRVAFSGVTLKLHP
AYLEKLQRLYDRTKERRLKLEHSPDNFCAADAMHHNLSFEEALFCLLCRYDMIQGAGLQA
GVPGSIMDVLLERFDCRMECFASPLNCRYDCFASAFVDVDAAFGSKGSFFELPLLSDNDD
GDGNDTEDDDSLSSRDGGICYEANPPFCEGLIQKLSETIDDVLSPQRARNHRRPIMFVVF
VPAWRESGCYQALLANERLEGHLLLEQGDHWYAEGTQHRRRDSFRVASFDTSVLFYQNVA
AKRLWNVKGKDAVVLDELAKAFGQDPGNMQKENQTILPQGRRTKPATGAGPSTPAVLPVI
ESNVDNPPLSTEDRKESISTSQTELSSSTRKQKKASTKEKKRKWNQQDEGKAQLNLLASL
GLSSTTKEGTADEKSKPATSHKSKRKKKIRRKK
>comp3699_c0_seq1; 233 141 1409 LEN=1477; translated
MPSSFIVLRLSVCSLLLVAFCFSKTTHSFVLLQRGTIPTSISIPTATPQFEQRDKIRQGF
KSSATGRPQRLHTYLEARDNASKSPVNNATAFKTIAQPKLIRGEQYKNENSTLPSPEVIA
AKLGVRPSKEASKGTWQRAWKLHKRLIPLLHSTDGCKPPDSSLNLACMWWKALSGNDRSS
PVYDKELSYDLLPSGWRKILRLRRFYPRLHHANVELRTAYLDGRVGEIAAQQQNGGNPKK
IRLVCMGAGYDTRGVKMIERTTVDRVIELDLPEVVNAKERLFWRLLKRRPWLKEFECSMP
TLVPSDFNDLLDVEQKLRALLLGDEPDGPDEKAEWHTIFVFEGVMIYLNDGVPSSLLGLT
SKILRENNLEGSICFADRLENVPGGDYDAGVNELKKNGWELKDWSPKPGLARHMGYASLS
LQ
>comp3702_c1_seq2; 130 100 542 LEN=936; translated
MGSCMSSNAGVAEGSASAKSSSSKPRAKAKAKQAFPGAGHRLGSSVPEPALDPTKSERRA
AVLEAAEQRARKQKGGRAKRTQTAKAPSGXAPAGAQQPAPDAVDGLVRLDETRTERNAKG
NETKRTEPQCSARWFEWLVVEPPPAFL
>comp3704_c0_seq1; 389 618 1643 LEN=1643; minus strand; translated
MLSFSSSEQSPLLIKSSKDQGRGLGSVKNKPNKKDFQEPTKEKTVVRDKSTTHNKSADNA
TIYTLAFVVFVQAYLLEGVFPYAGFLTMRLLSCKNNGYENDSSCSTKHAIDEENVGKYAG
FVACCFMVGRTVTAVAWGKIADTYGRVFVLKVSLVASAITSIGFGFAKTYWEALLWRFLM
GFANGITGTIKCLLSEIRASSGSQTKETKTMAIVYGMRGYGFLICPGITGYLSDPIKQYP
STMASVMRPIKSLLRAYPFVLPNILACGLCILACLLVKNHVDETLPNEKCEPFPSFFTAA
QGILCRYLPGCNDNTTGSHKDSVQRELHGTILPDTDKMSGGN
>comp3706_c0_seq1; 320 1 1045 LEN=1540; translated
XEDVFLANGASAAISHLLTALVSDPACGIMIPTPQYPIYSATIDLLGGRRVGYYLDERNK
WNLNLEELERSLKEAREQGTNVVGFVLINPGNPTGAVLSREAVRDVVRFCSRHNLVLLAD
EVYQENVYDEDAEFYSCKRAAYDVGFLGSEPGTEAIELASFHSTSKGVFGECGRRGGYME
LTGFDQTIKDQLYKLASASLCSGLNGQCMTALMCRGPSPGDESYESHEAEKTAIFESLKK
RARIVEEGLDAIPGFSCQPAQGSMYCFPSVELPQGAIDRAREEGKTPDTLYALSLLERTG
ICVVPASGFGQEAGRYGFRTTFLPPEDDMARSVEAMKVHHQEFCERYS
>comp3706_c0_seq2; 356 99 1058 LEN=1553; translated
MIPTPQYPIYSATIDLLGGRRVGYYLDERNKWNLNLEELERSLKEAREQGTNVVGFVLIN
PGNPTGAVLSREAVRDVVRFCSRHNLVLLADEVYQENVYDEDAEFYSCKRAAYDVGFLGS
EPGTEAIELASFHSTSKGVFGECGRRGGYMELTGFDQTIKDQLYKLASASLCSGLNGQCM
TALMCRGPSPGDESYESHEAEKTAIFESLKKRARIVEEGLDAIPGFSCQPAQGSMYCFPS
VELPQGAIDRAREEGKTPDTLYALSLLERTGICVVPASGFGQEAGRYGFRTTFLPPEDDM
ARSVEAMKVHHQEFCERYS
>comp3708_c0_seq1; 334 1 1261 LEN=1691; translated
XLLKYIVHALQSRESDGTGEARVGVCAPTGVAAVIVGGSTLHSFFGIGLGKGSPSSILQK
VRKNSSAMDRIDDTDVLIIDECSMMSSELLETLDMVSRRIRNGGVFRDEPFGGMQVIAFG
DFFQLPPVYRNDGTKDWTWRPFCFESPVWEDLGLSENVVELREVQRQEHGDFVDLLNKVR
IGKVTEQDIRELNRQCLIGPNNPIPTDGILPTRLYVLNKDVDSENINRLAELKGREIVCK
ASDNWRQSMPLGTPAATKKKMKESIAMEIPDEVRLKIGAQVMLTRNKDLQRNLVNGSRGV
VERIVKGPEGNPVPIVRFDSGLVTKVPPVESERFNPDGGPGCLVRKQIPLKLAWAITIHK
SQGSTLTRASLDISSAFEYGQCYVALSRVKSLEGLWLEKPASLRHIKVSPQVVDAFGGLE
>comp3710_c0_seq1; 241 1 1222 LEN=1774; translated
ILREGTEGKLVNYSLFLDEDVPKSESPLSVLIEKRIDMLNCSIVGPELRGFVMLLTLSFV
SHSPRIHLLQSFFHVSEDDITEELFRIDLPAPTKSESNPERAHNGPGSLDSEIAMILYLA
SNPKWIQGDVLELSCDGTTGASGVVGLLSCVASKLTSMGPDELKAFKRERKEVEDEVLSL
HSGQKRRENFSSVFPQGVHRLTLSDESPEFLHGAQEMIQRHFRSSASPPDVSIEQVEWSQ
QGPRRRYNREYRTVVGSDLDIPFPNAKELSKAVANMLLPSNVLAAANMKEERPGVDPTVP
PTFVHLCPDYRDESRYLRQFLEKGHLMTVRTNYVRMERLQFYFQALPQGSPEIDLDLLDG
LEVQQDTSRDYQSMTAIHHPDYAGDGLGEYFFPLETGAYEGGLSPL
>comp3710_c0_seq2; 291 1 1210 LEN=1762; translated

XDDDDETTERAPEDYTGKTIYQRTFYRLSSDSQVQLPNALMIEERLRFLPDPEDLEYMLP
LGPRTLILREGTEEDDITEELFRIDLPAPTKSESNPERAHNGPGSLDSEIAMILYLASNP
KWIQGDVLELSCDGTTGASGVVGLLSCVASKLTSMGPDELKAFKRERKEVEDEVLSLHSG
QKRRENFSSVFPQGVHRLTLSDESPEFLHGAQEMIQRHFRSSASPPDVSIEQVEWSQQGP
RRRYNREYRTVVGSDLDIPFPNAKELSKAVANMLLPSNVLAAANMKEERPGVDPTVPPTF
VHLCPDYRDESRYLRQFLEKGHLMTVRTNYVRMERLQFYFQALPQGSPEIDLDLLDGLEV
QQDTSRDYQSMTAIHHPDYAGDGLGEYFFPLETGAYEGGLSPL
>comp3715_c0_seq1; 253 134 1059 LEN=1059; translated
MGLLLLRIIVTTLVAVTALRFLGRPPWDPFDGSGGAYIRIRNKGGKMSHSMHRVSVYLDV
GTAWNTTASTRTTTLVPPRAWRYLQQEEDDLASIVVPASDNNETKPTTMPVTTTTLAPTT
SSDESESLVPPVDNDQNVTSDDDISDNIQSATPSSTPTITESGPDTNGNETSTLPSMVPT
TVRTTPPPAGVPTIIAEPFTSTANPTVVTTALIPPETIAPVPVEVPSVGTKEPSTSAPSP
AVEAPPVASPIPYDVADKRAHNIYNIGAVVSRPTLTSGVGGGETPTYSDGNTLGNERRKV
DESPKLGIX
>comp3718_c0_seq1; 426 46 954 LEN=1028; translated
MTRQSVKGGYCDWPDTGGCALTKLECDDPANFRSVRQMQGAPVRAHGGSCRWQESIKKTV
LGRCTTGDDGVPTQCASEVEACPETDATRGFLGEIPECTVETTSFGRCDYGMCAWSHKHC
VEDNTWDAFDEGCTCDKVQVGACTRFVDGERESFCAVSEDACDDEQTWLAPHGVKQASGF
DCYLCREKAPPSTAMVTDENDGFEAIAVESDFGKAPGRNSAETTTIIVMAVLGVVISLLI
VGVVGWKTFQARRAIKRSNDGVFSKEEAPPATDIEISKNTGNAGEFGPSTDMDEASVLSD
DD
>comp3720_c0_seq1; 373 1 1347 LEN=3036; translated
TQSKKFVLELNHSNQLIQTIMDPTVQEEETGLPIAHDNTSRGIRCVPYEKKHALGVLLLT
AVEFPFPENVLCSIAALFYCLFFGVVALDENGKVVGYFLGQSPLTIGLSRKKVGILTVVC
VSSKHQKKGIAKTLGKHVMEGKCEEFSPEDFDEIILQDTDTWNSPSWLFSDRNGFRYSPT
HDLIRRFGLVGYVQIILTGNHTLPCQFVKRYAKTEEEHAEADAFETQKGIIACLISGILP
SVYWICFWIMSCDNGVKAKFAGWVFLAVYFLLAVRFALAHLLAQERVVFREYDNWWAPIG
LFWPIATFVGIPLLGWTGGFYIGVPGINYLHDKYKTALAQMYLPINLFTLVLFFFHQLAR
IVSPGVLLWAGGLLDVLGYAAFFCSITDLAMPTVAFPSGAMAIRRWNFLLFVGLWVSWCA
ILVCGILLPNENESSRFMDLSIAACASQ
>comp3720_c0_seq2; 373 1 1347 LEN=2895; translated
TQSKKFVLELNHSNQLIQTIMDPTVQEEETGLPIAHDNTSRGIRCVPYEKKHALGVLLLT
AVEFPFPENVLCSIAALFYCLFFGVVALDENGKVVGYFLGQSPLTIGLSRKKVGILTVVC
VSSKHQKKGIAKTLGKHVMEGKCEEFSPEDFDEIILQDTDTWNSPSWLFSDRNGFRYSPT
HDLIRRFGLVGYVQIILTGNHTLPCQFVKRYAKTEEEHAEADAFETQKGIIACLISGILP
SVYWICFWIMSCDNGVKAKFAGWVFLAVYFLLAVRFALAHLLAQERVVFREYDNWWAPIG
LFWPIATFVGIPLLGWTGGFYIGVPGINYLHDKYKTALAQMYLPINLFTLVLFFFHQLAR
IVSPGVLLWAGGLLDVLGYAAFFCSITDLAMPTVAFPSGAMAIRRWNFLLFVGLWVSWCA
ILVCGILLPNENESSRFMDLSIAACASQ
>comp3724_c0_seq1; 320 219 1067 LEN=1541; translated
MGDAISSLLFQPPPPTYLHPSRHFWLNTVTGSRIPAFYIERPNAQVTILFSHGNAEDLGM
IYDWFNDLARVLRVNIMAYDYTGYGKSNGSPSEENCYADIESAYQYLLEVRGLQPEQIVL
YGRSLGSGPSCYLASKTANEGQSIGGVILQSPLLSAYRVAFDFRFTMAGDKFPNIDKAPS
IQCPVFIVHGTQDEVVPFWHGEDLFMALPQQWRAKPFWVEGAGHNNIEALLRPTGAFVDK
VVEFLDLHIGARRGRVAKPIPVPECVPLAIREMNAARDGISA
>comp3726_c0_seq2; 714 231 2167 LEN=2167; minus strand; translated
MKIRCLLPFLAVVGEILVVTAVLSDYAIGDSNINVESLDDRIHFQDKVSPNISFAVSESG
FPASVDSDWFSVFSNFATLSIRRTIEIDXMITVFVLLIAWYAATYFSTVSRYLKPEKKKK
SKAAKSKKVKKNKSSKSSPAYEVWASDQSNSVPGEERLGVKGSFLWVFDTDGIQGQLSGK
EKATPLPCTPTKTKGPCDAFEIFPPELNQVDAGGAPTGKQLKDISGFGRLHGVLVDPQNK
YVSANFFTPGGGFIGIIDVETKEAISLFRVTGFSTKAERSVHMTLWSTDGSALLSANLHG
KAIERINIGRDSDGKITSAEFDKSASIGLGIGNTIIEEASVFEGVNAYGRQLLGSVVGQY
SDAGMGDLTPNGYCKQDGCTSSALPPTGRTNNVPICPIPSTENNLYTSLGGGGLLVMDLK
TTPISIVGEYGGKIFNGAGCGGTQVDQQMFLNAGISAAASGSDQSTFTVYSMDDTAYSHG
QNVADDPPPTIVYKDNTNTNTIGNVDGVDTPNNTGQIPGVTTRRDSHGIVSTLNKKFVHQ
FDRIQNVVEVFDTKTYERFSYDLTSKNGQLGRPNAPEEAGPCLANSVTDDVNLVLNDPSP
DVLERSPDGKFIFVGFRGPAPVSVGHSAQGSCPGVGVVEITEGGKX
>comp3727_c0_seq1; 31 1 163 LEN=373; minus strand; translated
XSGVPSGVPPGEGWALANKDGAELSPATDGAMLKDGAMLKLGLSVCGPPNVVGS
>comp3732_c0_seq1; 202 971 1506 LEN=1506; minus strand; translated

MGQNTATVEALAKAWTPRLLKLETNSSGSNLDHEQSQHWRACLAAVQRTLPLFQTTGSQN
GGASAASVPFVCRYRADLIHPLATQQVHELQSMVAKHEALQSLRTKLVEHFPCNSSSNNN
EMRNRILTSTSRAELDDLYAPFKPPAKGSILERITSEHPKLVETIDELWNAKDEPDDKR
>comp3737_c0_seq1; 227 481 1268 LEN=1268; minus strand; translated
MGRALVLWACCFCCPRADAFPTARRAGVGRPRALAQRHAEARPGGGTAGGRGAGDANANA
SADADPDDNADAPRTHRIEFVSPLLDYGYPPAARDLEARQAGGTNERHEKPILLYLPGFD
GTYVCPFLQFPELGTEFEVWCMTIGMEDRSTYDELKSVVLDVLRTDLAAGHRAAEAVPAG
ASSESRSEGESETEEAVPGERAPAAGGSGRFSGLFGGNGRKAGAKTTGRPVYLAGESFGG
ILASDVARTLLEEQNAPGGAGGA
>comp3742_c0_seq1; 503 275 1990 LEN=2110; translated
MQSLKMNSSNTYKTTLDLLIVGAGPVGLTAALEARRLGLSARIIDRKKKRSSHDSRALVV
HPRVMELLEPIQDGAVTEKIENAAFNLEGIKIYLEKWYHMNDDSDDCFDCLNANVKRTIW
GDTNYPSLYFLPQYETERILEETLNACGGQVDYGVSLENLSQEGNLVTSILRNSNGDTTE
IVTSTWVLGADGGRSKTRDLVGIKMNRLQSDLYFVVADLVLKGDLPLEDQLSGRGGHVFP
TGKGLIALLPLPTRNSYRFVGKAPVGVTSADQIELNATFFEELLFDRTGRKFHVELGKWQ
NIFHVTHGASDSYRNRNVMLAGDASHVHSPIGGQGMNLGIQDANNLLWKLAWAKRFLDAV
SNEEEEDETTSTVDTILESYHSERHALGQNVIRSVEMATKLMNAKSRFVQFLRGVLMRIG
LRSTRANHNFRKVGQLETAYAPSSSSIIFDGNMKKPCIVSPGQRVPNIRLEDGSNLHSHI
DRVQHTWVFLNPHESLSSRSPNVIGTKVVFLVPAKSEEQVSVPKITEKALTAQQILLVRP
DQFVAGVGRTQEEMLNQLRKVGINEKAIAVM
>comp3742_c0_seq2; 511 363 2077 LEN=2197; translated
MSSPSWHSKLRCFSDTTPPFLVFSELDXTAALEARRLGLSARIIDRKKKRSSHDSRALVV
HPRVMELLEPIQDGAVTEKIENAAFNLEGIKIYLEKWYHMNDDSDDCFDCLNANVKRTIW
GDTNYPSLYFLPQYETERILEETLNACGGQVDYGVSLENLSQEGNLVTSILRNSNGDTTE
IVTSTWVLGADGGRSKTRDLVGIKMNRLQSDLYFVVADLVLKGDLPLEDQLSGRGGHVFP
TGKGLIALLPLPTRNSYRFVGKAPVGVTSADQIELNATFFEELLFDRTGRKFHVELGKWQ
NIFHVTHGASDSYRNRNVMLAGDASHVHSPIGGQGMNLGIQDANNLLWKLAWAKRFLDAV
SNEEEEDETTSTVDTILESYHSERHALGQNVIRSVEMATKLMNAKSRFVQFLRGVLMRIG
LRSTRANHNFRKVGQLETAYAPSSSSIIFDGNMKKPCIVSPGQRVPNIRLEDGSNLHSHI
DRVQHTWVFLNPHESLSSRSPNVIGTKVVFLVPAKSEEQVSVPKITEKALTAQQILLVRP
DQFVAGVGRTQEEMLNQLRKVGINEKAIAVM
>comp3744_c0_seq1; 565 150 1157 LEN=1186; translated
MAPAETDAAPNTEEDPPMFSKWREFNINDEARDGDGGAGGENEEDTAGLFCMFDEEEDVY
EDVRYDYPSFFAEGDGASTTTTVTIRHHGAYPNSTGLAVWRGAEFLARFLCDAQQEADRK
AGREEDGGSGIGVAAVRGKKVLEVGAGAGLPSIVAHSVLGAGRVLVTDGDFDALNNMKHN
VDANRSTNSISNNNNNTIACLQLIWGTTHAEPILETHGKQDVVLMADCVYMVPSLKPLWE
TIDLLLDTDGVVVYAQTAASAVPWEDFRAALDRHGFETIHRVAGTAALSPYEAADRGSES
GSENENENEVVNNPGASKEEEEYERGIYVFRRRRP
>comp3746_c0_seq1; 124 201 579 LEN=1436; translated
MSVTIHTTNGDLKCEIFCDTCPRTAFNFLALAASGRYNGTIFHRNIRGFMMQGGDTAHVT
GKKSSIWGNDVTFPDEFHPNNKHDRRGRLSMANRGPDTNKEVFRTTDQELQVYGFICDAI
DALFI
>comp3746_c0_seq2; 221 201 758 LEN=1133; translated
MSVTIHTTNGDLKCEIFCDTCPRTAFNFLALAASGRYNGTIFHRNIRGFMMQGGDTAHVT
GKKSSIWGNDVTFPDEFHPNNKHDRRGRLSMANRGPDTNKSQFFILYEQQPHLDNMHTVF
GQVLDGWDTLDKWERLPVWGERAQSRSQKTTPLEPPEITGITIHANPLADEGIVYPTPSG
PPEKK
>comp3747_c0_seq1; 357 1 542 LEN=1187; translated
XGSAEESPPKNLQEMETTPPALPFDSRASGMVYQARNQQANDNYEDNDNKNHNNGINNVN
YNVIINNNINNNIINNNNSININNDVNNGIKNNTNNNGNNNVYNGINGISSNIQHTHHGF
QQMSENSFLATAMAANGPPPPAFMATLAGSVATMLGGGTEGGSLLTAADASAARFPPPPR
W
>comp3749_c0_seq1; 364 271 1516 LEN=1533; translated
MSTAVSFNNESLGDDTDDSTASSGISFHSKAAIKHFCDVDVEDSGSVTSSSAERNFRLVD
GSKNLRLRELMGKQVRDLEQQRSKRQRDLPDGGGDGSGNPDAGPKRTVVGIGIGWGEVRR
KNKTHKGKKPRKGDALSLALSPGSAEFGKASSGDLSTTAMPSRVPERASRVVGFSKERFE
RSQRPAVEDALAAPKSDSSGGSLDPCEELEKKCLELEATGRELSERIDETVLRALAKKHL
AEQLEGHAVFLRQRLSVRKETTQPEHRESDGGTDPYRRKLRKLLANAGSTWAFLRKHHGP
LRAELERVRSRNKGLAVRRHRRQNATAELPGTPGHQRPVQNGNPRDRGPPPPVPPPWDWL
VPPNVLPAGKETRLVRRRLPERRAPATPNDSMVVAGIVSGPKRTIDHGNNRTHY

>comp3755_c0_seq1; 205 168 1558 LEN=1558; translated
MAMPLSILLLAETLADPHRVFSISSISVATIPTSTIALPTTQAAVASASSLTIQPVNPAE
YLVESNTRYKKFKARTRDPIGDDDDEKMHSLQLIHADLFPDTMEYLKAMDGKPYAIRSTN
GPNVLYDLLSDTDKGKSVEEIARTYAGLECAVALDPIILSPEEISEANKSQFDLHIPELC
EDKSHICLKLDTSDTSMGGSKFDLEFYLNGGTSGSVAPTTNLETGGSSDPRLLKAMASAT
GCIQEGIKENVKFDLEGHHGLVNYDATPGDQSPAQQTYQVVLSLRANVGFLGHGTTLAET
DGSRQCERRRIRPEVRWKGREKDGFLARLRCRRHKSRLRNRKLQTLQPAKQMGNKILRPV
QLRRRAVHPVEQFEFCPRVLLRTPAPPAPICAGDSNEVPHDGHQGSSSDGDNTFLVNEKT
DGGSCSTGPTGTPSSTGRPVTRGHRLPLEKSRDHPVGRKRTKRX
>comp3763_c0_seq1; 674 40 1749 LEN=1837; translated
MALLCWSSRFVWRWHAQVLMATSLAAILVLSLVSSSNADNVGTFDLVGLIADLPTKQFYI
NGTWVEPAPPSSETFKRFINVVDPSTAETAAKVAIAGPEDVDAAVKAAKEAWLEWSYHTP
LNERKRYVQKLARIYASKIEEMAQLISTEMGSPIEAARESHAWGGLSNIESSLELIDEFE
FEKPLGDDELTTILYESIGVVGMITPWNWPLNQITLKVVPALLVGCTCVLKPSEESPLSA
LLFAEMVHQAGFPPGVFNLVNGDGPFTGDSLTKHPDLDMISFTGSTRAGRQVAVNAALGP
IKTTLELGGKGANIFFADVGDEWMEEVVVNGVESVFYNSGQTCNAPTRMLVQQPFYNMAV
ELATNIAKTNSPVDSAHEDGDEHIGPVVSRNQFERIQEYIQIGIDEGARLVAGGLGRPEH
LANHPSKKNGYYVRPTVFADCKPSMTIMQEEIFGPVLCIVPFTTEDEALQLANDTPYGLT
NYVHSRNFNRRRRMGRLLRSGMVEMNDAAGDHGSPFGGVKGSGYGREGGIFGFEEFCIIK
ALTGFIPGEDDLFFDQELETSAGTEEAEL
>comp3764_c0_seq1; 607 357 1493 LEN=1794; translated
MSSSSETTTTTTTTSRYWNREAFASSSSPSSFEEQAPVREARIVSLSFPGDPNNAPLESE
TSSQLPPGARLLQVGGTLADLDLEALRKEQANVAFVSPGVTRETLAGLIEGIPSLEWIHC
RSAGIDVLTSETLSNSPDELVMTNAKGAFSSTLAEYTMLAISYFAKDLPRLLRNKREGNW
EKYSVLEIRGATLGIVGYGDIGRAAARLAKAYGMRVVALRRNVPAPEAIAADPLCDAVYG
DGSENLNRLFGESDYVLCSLPLTEATHKMIGKDQFDHAKKEGCVFINVGRGPIVDEEALV
EALKDGRLKGAGLDVFTKEPLDEDSELWNLDNVLLSPHNMDQTATFMLESTQFFVNENLP
RFICGQALLNPVDKKAGY
>comp3764_c0_seq2; 551 357 1370 LEN=1940; translated
MSSSSETTTTTTTTSRYWNREAFASSSSPSSFEEQAPVREARIVSLSFPGDPNNAPLESE
TSSQLPPGARLLQVGGTLADLDLEALRKEQANVAFVSPGVTRETLAGLIEGIPSLEWIHC
RSAGIDVLTSETLSNSPDELVMTNAKGAFSSTLAEYTMLAISYFAKDLPRLLRNKREGNW
EKYSVLEIRGATLGIVGYGDIGRAAARLAKAYGMRVVALRRNVPAPEAIAADPLCDAVYG
DGSENLNRLFGESDYVLCSLPLTEATHKMIGKDQFDHAKKEGCVFINVGRGPIVDEEALV
EALKDGRLKGAGLDVFTKEPLDEDSELWNLDNVLLSP
>comp3765_c0_seq1; 191 140 740 LEN=740; translated
MQPCSSVEGSHVTQPLFGGAIACDLPAGWKDLSDVRPIPDNQECFQNSLVDENPIMLVVE
ILERQEQIGDQDAASFFFNELAERNDVLQTKNDIRFFTLAEPVLATALLGDASVVAGTNT
VLTCAGYGYQKVAMGRDYDNAGNSRRENQEVKCIRVDLCVLRLQAQETDLLVTISRPVDD
INLNEASLDSSSAESAILSQX
>comp3767_c0_seq1; 224 607 1459 LEN=1459; minus strand; translated
MAFLNGNDFAGSIDVVFCSSGHRIFEELVSDCKGSAPEVKCSCCTGCCDASGLNCVLHDP
FSAIDTDRPSPTPTRKSVGAQDASTGATASTSSAAQPAGTPSQEEPLKELLSAVSERTLL
EEKHTDQFKAFMWMARMDPSPVDVATTSRHDIMQKYIMVLFYISTGGKNWRHQNSFLTDV
SLCDWEGLTCNDEHKIVAIDLEDNHLNGTLASELGALGPSLQDLRLGGNALFGSVPSELG
VLPSLRTLDLTDNAGISGTIPPSVSLRSLPNLETLELAGTGIEGX
>comp3774_c0_seq1; 175 1 1433 LEN=1559; translated
XSKAKAMTMRPLVLISAFLLFECLLKSAVKSHAFAPHTKSTLAFPKSPRFGTTDCRMVLK
DEGGDDGVDSIACDTDVIIIGGGLVGAATGLALSRRGKSCTIYEQTQSLRKVGAAIGLYP
NGLSALEHIAPDVAKMVRKNCSPCLVFERRDASTDEVVRSTSVPHLNETSPVMYAWFLLQ
QHLVEALPEDCLQLGHSFQSYKVLDDGRVEVYLLDNGKEVTKTCRLLIGADGIHSRVRCQ
LLGEPPTTNYYGKVMYRAVIEKERVDRVLEIPEGSQISWQGTEIGKSFSVRETTEGIITV
TAAALDDSPNGDSRSANATVPHDNFTHKKHLQSLFSGFSPTVGAIIGLLPDTFHRDFLRD
INVPNETGWCDGQQVLLVGDAVHAMTPGMGQGANLGLEDACEVLETIVQKRPLSDFVRAR
IDRVRAIQERSAENTLQSNAYTRDNASTPFQRRKYSQCFKDDLYRWKPSLTKAPTRE
>comp3775_c0_seq1; 287 103 723 LEN=1413; translated
MGNKISLEDEMVNLRIVSKQMLRSSKKCEKNEKAALQKLKKAIQQGNHEGARIYGQDAIR
EKNQALNHLRMASRIDACSSRIETAVRMGQVTDGMKGVVKGMDKGLASMDIEKISSLMDK
FEQQFEDLDVKTQYMEGTMNATTATSTPAEQVDTLIEQVADANNLELGEAFSQAGPVGKK
TPTVKESTVAKPVADDLEARLANLRN

>comp3775_c0_seq2; 198 103 812 LEN=1502; translated
MGNKISLEDEMVNLRIVSKQMLRSSKKCEKNEKAALQKLKKEVEAILILQSIMQYFFVNS
SLMRTAHCTMAIQQGNHEGARIYGQDAIREKNQALNHLRMASRIDACSSRIETAVRMGQV
TDGMKGVVKGMDKGLASMDIEKISSLMDKFEQQFEDLDVKTQYMEGTMNATTATSTPAEQ
VDTLIEQVADANNLELGEAFSQAGPVGKKTPTVKESTVAKPVADDLEARLANLRN
>comp3778_c0_seq1; 285 1 1343 LEN=1595; translated
XAAPAGVAESSVARPPSMTPAIEAAAAMKRNGDEANDSSAAVSTPPSIFVSIASFRDSET
RPTLQALFSRAKHPHRIVVGIVAQLVDGEAYDEAIWESIVRFSQSPEPLAPEPHQIRAMR
MHARDATGPCYARGLCQSLYRGEDYVLQIDSHMRFRDHWDDYLVRTIVDLRARRLVAAAD
VNEAAQADKILLTAYPVGYTLPNEVPSETRGTYLVPWKFDTNGMLRQRGRLLRRDPVAAP
DPTSARVVAHRQYLYAGGFNFGPGRVVREVPYDTNGLHHLFFGEEPSMAVRLFTHGYDLF
APAESACYHLWSRAHRPPPPAGTTDPLRRELRDRSLAAVQRQLLGGESPPTGTTTTTTHG
LGTVRTASEFAERLGVDFRRRAFIREGWEWGSLSPGDFVGGTGEDSGEEPRLPNPTPSHG
GSEWSSLDRTAKDLIGAFLRGLQDAEE
>comp3780_c0_seq1; 322 1558 2526 LEN=3056; translated
MVLPEKFAERLSLWPRQQNEGRQQQTKIETTPIDSIRSADVRLVVETIEDFPSAARQHAA
TYHIGARLILYQSIAYVVGFWMIWLFPTINRQLQYYDITNHLWLVIFQGFFEPLQGAFNI
LVYRYTHYARLKQQHPDWTRRELLRNTLRFSFLGPPKDSQSARAVEIAMPFEFDNDGNSN
LPDEQPRDTSSYGGNNTQIDSIGEFNVSDNNLQCMNSVDFNAKFLDNAYVRNSISSLMGD
LMVDYADDPSLLNGNFVEFGNQDLFSVSLPVQSSFPTFVTRHGLSRQSLDKSKPDLMKNF
QVDELEDDDGNLIPEKNTWDKL
>comp3780_c0_seq2; 420 235 2292 LEN=2822; translated
MAGPLEFGPKVEIFLSILPLFSGFLSLIGSGWIIVEVVTTRAKIQTVYNRLLLGMSVVNA
FTSVAYMFSTIPMPVDSTDAVWAHGNTQTCTAQGFFIQLGIIPPIYHVCLSVYYLLILKF
EVTEYTIASYVEPFMHATGLLFGLGTGVASIILDLFNNGTLWCWIAQLPFNCEESFALPE
GEETTCVRGDNAFIYRFIFFYIPLWLSMILVLAVNVWIYRTVRKRDQKAKKLHAIHHAKH
GRPTLGKQVSLSKSHKKTSIFLRSPKKKTGINFTFGQHPASLFSTLKNSIDKGEKLHIAT
SSGPMLVPISGETELEIIEQQRETSVRVSSDREGEYETKEHDHIEKDVQSSRSSHGDNRR
IPTMVLPEKFAERLSLWPRQQNEGRQQQTKIETTPIDSIRSADVRLVVETIEDFPSAARQ
HAATYHIGARLILYQSIAYVVGFWMIWLFPTINRQLQYYDITNHLWLVIFQGFFEPLQGA
FNILVYRYTHYARLKQQHPDWTRRELLRNTLRFSFLGPPKDSQSARAVEIAMPFEFDNDG
NSNLPDEQPRDTSSYGGNNTQIDSIGEFNVSDNNLQCMNSVDFNAKFLDNAYVRNSISSL
MGDLMVDYADDPSLLNGNFVEFGNQDLFSVSLPVQSSFPTFVTRHGLSRQSLDKSKPDLM
KNFQVDELEDDDGNLIPEKNTWDKL
>comp3785_c0_seq1; 349 1 1538 LEN=1625; translated
XSEEFTAKGLMIEQKNWLEIYHPWERWSTGQGELPHVMVGSRIAPHSFVMKEGQTTPPLP
ISEPELITLMDKSGIGTDATIATHIATIQDRDYATKDGNQRFLPTPLGIALVEGYNSMGY
QLNKPDLRRETEHECNLVANRQKTKEEIVPLILNKMKDVYLTAAREARKLDQSMARRFQR
IGAGNDHTELVQARFSQCGNCQQPMSLKQERRTGGRRRGGGNNNNNDTPRKLLFCNACQE
GYILPRKGIPRPKISEGETNNNNNTPVKCPICNFQVVAIARGDGYEGNGYNVCPKCYNDP
PADHGGGGSGGGGGTHPCFSCSHPACALAGGTQGGDIEVFACPFCREKNVPGGKVTLRKN
SRGYVLSCANYSGRQRCSYTIWLPRASRSIEVPPGEEHVCTRCSTHSPVRKLAFQWKPGG
VLPHLGRETTACVLCDARFRQDVQINLPQMDRVGTTSRRTASLRPNHHGNGRNNHGDRAR
SATTNAGNNNFVCFRCNQPGHYASACPNANNQ
>comp3786_c0_seq1; 427 321 1826 LEN=2155; translated
MAPRSRLQRKRDTSCSAQEEISKTRQHSSCAEDLLDDLCRKHMDLPAQDFAMGCGFLHQV
ALGVPTEEVDRLLSDVPSLVNFRDYDRRTPLHIASSEGHLEICKLLVKRGAKINRSDRWG
LAPLDDAHRHQHLEVLQFLQKSGGKFGSSSLSINLISACVEGDIDEVGSLLEYGKIDINS
GDYDCRTPLHIAASEGQAEIVELLCEKDADVNAKDRWGARPLDAAVFANNAKCISMIEKY
GGTRGSSATCSFGHDAILDLIHTYGKIRDGVLSMDWHDVQDMLTGIGEKPTDEVVQKLFE
VADKDKKGIITTEEFLANHETFLSGRPARIILVVGGPGSGKGLLSERLANECGVVHLSSG
DLLRDEISQGTELGKHVEKIVQKGELVSSAIMVTLMQKRMKDHPGKRILLDGFPRSQENA
KDLVTLCGKPELALHLTCDDTVLLERIMKRGESGDRSDDNFQTAIQRIRTYHKYHNLTID
FLREENVPIVFLDCSKSPGVVW
>comp3786_c0_seq2; 385 321 1442 LEN=2251; translated
MAPRSRLQRKRDTSCSAQEEISKTRQHSSCAEDLLDDLCRKHMDLPAQDFAMGCGFLHQV
ALGVPTEEVDRLLSDVPSLVNFRDYDRRTPLHIASSEGHLEICKLLVKRGAKINRSDRWG
LAPLDDAHRHQHLEVLQFLQKSGGKFGSSSLSINLISACVEGDIDEVGSLLEYGKIDINS
GDYDCRTPLHIAASEGQAEIVELLCEKDADVNAKDRWGARPLDAAVFANNAKCISMIEKY
GGTRGSSATCSFGHDAILDLIHTYGKIRDGVLSMDWHDVQDMLTGIGEKPTDEVVQKLFE

VADKDKKGIITTEEFLANHETFLSGRPARIILVVGGPGSGKGLLSERLANECGVVHLSSG
DLLRDEISQGTGR
>comp3799_c0_seq1; 640 77 1333 LEN=1551; translated
MRFLAFSALLVAADNAKLTAFASSVEPATGGSPPEPGSMIDFEAWEALHRGIGSDRGYGS
ESERSLRKNIFQENIAVVQRHNDAFDAGHTSWKMTMNSPFADLTADEFSSLYLMDAQECS
ATTPPATKAKLVADTLRSDDKALRVDWRTQGIMTPVKDQGQCGSCWTFSTSGCLEAHTCL
AWGKDCTQWDGFAEQQLIDCAGDFNNFGCNGGLPSQAYEYLKYNGGMDYEETYPYKAKDG
AECLAKDGKAGGFVSEVYNITSGDEDDLVTAIAQVGPVSIAYQVSPDFRFYQHGVYDSYN
ATTQSTMCNNTNMDVNHAVVAVGLGTTADGRHNHNDDGGTPYYIVRNSWGTSWGMEGHFW
MKRGENLCGVSDCASFPIVPSKEEMTQIWKEQKKQYFKDKLAKEESKEEDSLRYLRKN
>comp3807_c0_seq1; 364 1 789 LEN=917; translated
HGPSPSSLPSRNRRDNISTTNQDMPGTTSSKGSKKRWFPLESNPSLINSYIQKLGYDTSL
YEFVDVFSTEDWALDMIAQPVAAVLMLYPLTEKQTSNTQDDVVATAEMKDKVWFTKQRIG
NACGTIGLLHALLNAPEPIRIFKKDSWLEKYANDCPISLDPVAKAERLEEDSKIEEMHDE
ATSSEANATDRGSVNDEVLTHFIALVCVDNKLYELDGRKEGPIDHGPTSQATLLKDATTV
VKKFMAKDPTEMRFTITALAPK
>comp3823_c0_seq1; 374 257 1855 LEN=1971; translated
MSLTGEFQEDETERTRLLSDFSESSFLSATGRWATATPLTFSSSSPSSSYGSIFNNSSRV
NNSARTVPNGNVSVDEGPINRFRGDARTDFADIEGGSQQQHEDEEGSLDTMTTSNTNRAV
SGDSTGDSSRVDNNGDEQERQQPSEGRDAEEPRDGAIGNLTRRLRCLFSTVTWPIVPLGT
IVSLALLWVLYAASSLDLEKNCSHPLHSYALVSLVLVAYIPNHSLVRSRMFHYSRERDGP
IRPARVRMYDQFFHTICLLYVYTGVTLIQTCQEDIVDRNSIDVPKNELALPNNGPLNACQ
ATCPNLYQAMSVYVATLELFTFALILPLLFLPCIYLWFLQRATAEADAFARLQDHLQEED
ALLRNGGVTTGEILDSLETVKMVYRGPGSSAHEITNINDNPVAEGESEPQELWILPRDVG
LEDTIGTKSTMRECCICMTNFDFEVEGSPSCEIRCNGNDKFEKRKNLKRVNNDAMDEVGH
EATPTEPSRIVRTRCGHIFHEECLKGWVGGMWSANPNRAGGNNTTGDDSTPDW
>comp3840_c1_seq1; 1277 1 2656 LEN=2721; translated
XASSGGVFGNHEMDISVAGDPNHTVLDRAERLKKQANAKFNKGDIEAARALYSDALACIP
TTGFYSRGVKALLSELYSNRAVTFYRSKQYQESLDDCDKAIERNPEAEKSYIRKARALVG
LERQTDASRCLQDAYKLFPTSKKVKEELSKIIDQRDTLGNLSGKGGSSDSVDFNASVGSL
SNFYPPSHTGSKAAQTLSTGREELTYADDIELSDKLKRNANAKFNRGDVAGARMLYTEAM
AVLPNDRENEVVRTCLASLYANRAVTYYRDQEFGQSVRDCENAIELDPKGEKPYSRKARA
LASMQRYDDAIQCLEEGKRQVPGSDKLEEELVKIKQEVGDAIGTDSKAGGAENANLGHEA
GHAPTVPSATDANDTSNFFVPTVGAAMGSVPEGKSQEFDLPTKIDPEEFERAEKLKKQAN
AKLNKGDVPGARVLYGEGLACMPPGGEHTPEGRELAANLYANRAVTYFREKQFRETVNDC
NKAVEFDPKHEKSYIRKWRALMALGSFDDAYRCLESALKELPESERLNEELENAKEEKKL
ITQVNELIEAKDYQKAHDILHNIVKVSDNVALWLSTARVDTFLGLTDSALSRVEKVLGFN
PKHGEALRVRGYATFLSGEMEQGINLLKESLEGDIEDTSNDNASGILQACQIKMNTFHKG
QARVKRGRSREAVDLFTAIMEDGQEPIPHGAPLYGILLTERAEANLLSDQYEAALADCAE
AISLKQDNLTAWTVKTEVYYALGRLQEARDELAVARKTWGARNETIEDAYKKTDFELRLQ
KVDNELRFLVGTVESGKPLVDESGILHMNHSQRGNRNGLTASDKSNSNSKSSSKSNSNSM
VPPSPMKRSSDINGHVNKRAQSRGKQREGFKLSSVLKSVGRQSNQ
>comp3842_c0_seq1; 624 244 1770 LEN=1856; translated
MSVDNITSPVAAAFPNIQWDALGSLRLTAQETALLRMAETNPIDYTLADSRDASMYARVL
LKLLAEAAAAGAGTGEVSTYIEKCSDERAMRALDVDPLGVVTHYALTKLNEIISTLSKGE
SGVTVAAVFYVGKDGILVDQWKALLRILNKSGKGDIFAQKSASMSLGKILIASCPSKGPD
SKGRTVSYISAKEPMEALIAWIVSQLKNVAGSLVGLCIPALVVLMEALESRLIFAKASGI
KYMSRQLLSASKSGSPSDGQQKFGSGSVQQLYALSFCMWILSYELNSSWEIRADFAKEGV
PVKALCELIAAAPREKVVRVSLAALNNLATCVDTDDAHPAGQTPHEGKFFLSEMIGNGLM
KSISLLRDRQFTDPDIVEDIDNIHKLLVANYKEMSRWEVYKNEVENGHLQWGSTHTEAFF
KENAKMFEGPDGDFRLLKVLITLLSSKDEEVVAIACYDIGEFVRFYPNGRSITKRLGAKE
LVMPLIESENVELQRHALQCVSKLMVQNW
>comp3842_c0_seq2; 171 244 474 LEN=609; translated
MSVDNITSPVAAAFPNIQWDALGSLRLTAQETALLRMAETNPIDYTLADSRDASMYARVL
LKLLAEAAAAGAGTGQ
>comp3847_c0_seq1; 397 470 1414 LEN=1984; translated
MSHKRKNSSAEKMDEKFIFCGSGDRWISVWKIATDDDVGTPQVITRRLKFVQKLGPHTGW
VKALVYDDESQLLHSIGCNCIESWNCSRLDSDHLQDDNEDKLLPIFHSIKRTIENCPAVG
TTLSSDLLCLCLVPPTVDSDEGLSRLLVSGGVDGRIHVWTSDQYVKRKEGNSYVDVRNPL
HIALAHDGRVNAIAFSSAMDVIFTIGNDGVLSLFRVSFGKGFELISRVEIEDSSKETLLR

LTAMEIVVDSREQNECLVAVGTSDGELHFVTTRIGDDGSLVSQLQDDHVKVAQNSMIYSI
SCKEEEGTKSAPRVW
>comp3859_c0_seq1; 276 349 1391 LEN=2255; translated
MSNAVRAFLLLSFVSIVEPFPVALSPKSSATPEHARLFISRDDSIDAENVRNIDTQAEIG
SNSSGSEDTKALSGLLDEMFQAKQEMSREKKELELLSSQDADMPVLGNDGVYRIISQSQL
EKSXEGKLVVKIKCILSTLRQSPYVSLIFLASFRNFKDANSDKLVFLKFSSPICAACRML
KQKFQTLHRSPKFAGVPVVFADIMISNNKKVKDPFRDYVTSQLRVQRVPSIHFYARGNDS
DANHIYCDDGGGACSWPKIQRQMLAFVGEHYTAPEKVKTTHEMSSSNAAVAEATSPXVNY
DDNSDFNNKGYQKDFQTQTDSRAYSILAYVENKKNVEYDLVFGPRKR
>comp3859_c0_seq2; 340 349 1292 LEN=2156; translated
MSNAVRAFLLLSFVSIVEPFPVALSPKSSATPEHARLFISRDDSIDAENVRNIDTQAEIG
SNSSGSEDTKALSGLLDEMFQAKQEMSREKKELELLSSQDADMPVLGNDGVYRIISQSQL
ENFKDANSDKLVFLKFSSPICAACRMLKQKFQTLHRSPKFAGVPVVFADIMISNNKKVKD
PFRDYVTSQLRVQRVPSIHFYARGNDSDANHIYCDDGGGACSWPKIQRQMLAFVGEHYTA
PEKVKTTHEMSSSNAAVAEATSPXVNYDDNSDFNNKGYQKDFQTQTDSRAYSILAYVENK
KNVEYDLVFGPRKR
>comp3862_c0_seq1; 315 317 778 LEN=1108; translated
MTPPPHVKIISGAASTTLLVLGLRNLFAPGAKIPFLPKEHDLQAYFWGTKNPDELVPGQK
AYSKLSGVNLLVLAAAKLAVLFTHAHKGTFLRQNLFLALGASQVAGSILLVSGEEEEKAK
AAGASFWTMAAILGGEGLVLLHDALMRDRPVKP
>comp3862_c0_seq2; 315 197 658 LEN=988; translated
MTPPPHVKIISGAASTTLLVLGLRNLFAPGAKIPFLPKEHDLQAYFWGTKNPDELVPGQK
AYSKLSGVNLLVLAAAKLAVLFTHAHKGTFLRQNLFLALGASQVAGSILLVSGEEEEKAK
AAGASFWTMAAILGGEGLVLLHDALMRDRPVKP
>comp3863_c0_seq1; 269 1 1442 LEN=1442; minus strand; translated
XASPENIGQGKLCGAHDKIETMVDALGSSTPQKSFPNVISRKGSHIKVAQVENGNREILI
QVIDKEKTTICAPGFSRLYDHIPDMVSQDSVKNEIIQVEDSGQYVPLEATDEVERSVAAS
GSSRVHGTIPNVVGRESSEHDVALVEYDSRQDNPFEERSNGLPSVVESNLLQQAVNCQSA
TKDVVATEFDVISTLCTPKFDQESVSEMSFSLVSERAKDCKRLMAPAVASQLDLSSCGSD
PDHIFAKKSEPEDQNKGCSVETPPTMEKDELKSLVRLVASRDKPPGTTQRERPIYSLPKK
MPFHRTQERWRKGSPAEKFRKRIRTPSTPNRVETSRSPFFGRDKEGRLVPTDGSVVGWFI
SPGRYSSIGSPCRRTRCTSQSNAIITGSVPFEKISLSKGSHRSRMRNENDSYSSSSLIGS
ASPKRSTSANIQTLLHSVAGRSRSPAVSLFSRMPHGSQSAIRKLQRNHCDINGKLSGKTL
R
>comp3866_c0_seq1; 498 63 1856 LEN=2109; translated
MVVIRTSIIATLLLLLFSGTERLAQGQNNENRGIYLDRFTYKNRNQKNNVKVNGNDHTVI
DYAPDNWGDISCNEGSKLSECIGYPDKWETGREWGISKNYCKSCPAGENNGCGRHHQSPV
DLQRGVGLDFNVSSKYYSDIANECIDLHWMKYEDSFCSMDQLENSDAFTIERHALRIAQP
ISVYDNIANDKDGVLDGVKLECRLEGRGSRFGRIDYSKGFSEWWHLSHIDIHTPSEHYQD
GVQYDGEIQLQHFYSVPARETQTNVNNEMGTVSVFFMAYEDAAPYRYLDKVICQWRRKEY
EVRKECGLDPVDSTYPGCFPLNKGRRNLRHKTGSEKAESKFKRHPKGFRTIHDVILYNDF
NKEHPNHTDVHVHMEESNWDPPDDSIDWDSFVAEQSQKLKDEEELYQKMMEFENRTSGNG
NTTEEALREEYRRKLIDYEEIPWFNYWPMIGVQTEYYFRYSGTQTIPPCYGDFDSSSRKG
TNHWRVMKDPIRIHPRQLVELRRLIAERIAPKGDPMKSCRADTAAKVNIDSSRDPSDPKK
YVKVDTARPLQSWDRVHYKTFCECKDWVSKWPADRQWCKIQDMNTRFFKKKYNFDAY
>comp3866_c0_seq2; 286 152 1141 LEN=1394; translated
MGTVSVFFMAYEDAAPYRYLDKVICQWRRKEYEVRKECGLDPVDSTYPGCFPLNKGRRNL
RHKTGSEKAESKFKRHPKGFRTIHDVILYNDFNKEHPNHTDVHVHMEESNWDPPDDSIDW
DSFVAEQSQKLKDEEELYQKMMEFENRTSGNGNTTEEALREEYRRKLIDYEEIPWFNYWP
MIGVQTEYYFRYSGTQTIPPCYGDFDSSSRKGTNHWRVMKDPIRIHPRQLVELRRLIAER
IAPKGDPMKSCRADTAAKVNIDSSRDPSDPKKYVKVDTARPLQSWDRVHYKTFCECKDWV
SKWPADRQWCKIQDMNTRFFKKKYNFDAY
>comp3878_c0_seq1; 249 130 419 LEN=419; translated
MTEESTPAPPAPAVGTNTENPAAAAGGPKKKKEKKVPPGLVIPPKRPKLTKAERRALQEK
QRAAKAAAQGGKAGGKQLQPTPKKHVLAAQQQQQQX
>comp3886_c0_seq2; 1047 628 2589 LEN=2626; translated
MDQPASTNENVGSGVGYSVVNDLMRVYENLTQSSTPQARVPDASKALSIAYNTNNNGNSN
SNVPAPPAAAPLYPPQPPMGYPIPPPPGYQQQQHQIASATTTARAPKNPLHTLSKAPSQA
HPTSDNNQEGDGPPNAASYPQQQQQRQGTSAYAPAPMGEAQPGSPPQSGNKDGGSHHSAQ
PPGEETHHGNHNIVRSSSIISNQQQTADASSESDKSGSGGRSRRKRKQQQEGVNSSGGGK

KGTRNKKSKKEDGRWSKRFTWPEDLHRDFVSAIFDVGLKHSSPSAIMEHMPKHEQITTER
IKSHLQKYRLHRVKSKKEFISSYEASLRNFQQNQSNVSTLVSGSGEVAAHLTSTALGTNK
NGSSGTVSKEGADHSNGALAVVHSGDRPKNISQPIDDGNDSLMLPQLTEEEKQSPIGAAM
GYLMGLFFSLKQQLVIQRSLEASGEKAKGNTMFSSSPVAIAPGSTNIIPAAATATAAAVA
AGVEFAANGTMQQSQQLSAPVSIRSKMEENSMMKREMKNQMALQNKMRALKQQELAKYNN
INNQEAVPDPVMNTAAQHGGSTDPHFSETGGGIDRSSNSMLGVHTVRVEQKQHQPIGGNN
SNAVVATQSEMAGTDMSSGQAYSNNNDTSMLDAEDFWHTDVVDEQLFEFLMNN
>comp3889_c0_seq1; 640 206 1813 LEN=1970; translated
MYTNGKALYHCMALTIALWPAANANDSDGWIRHARNLSTRDFMQECDQVLMNAAHIEHHS
YLMSHFADEVTDLCHMIAPNPDQGSCRSTGFESLNGSLKRAFFRQAVQHAGTANFPVDLV
ASWGDAGYIISDKSNVEKDALRNDLCSEVYSSIGEDFIDHIPDSKIPIVSSNEPKRMSPP
VSAQEEPSTFTVVQVEKEDNVLNMGAIISISCAVAALIVVFFYFEARRRKAARVSRSPHG
NKGIDDDGATRVSTFAGGRNNNRFRNFRRSLSSSTDGSFGPVEEVKDAINNADWDRVYKL
ASQLAENDDSLSLPSLDSFNAENRSHLCAEDQERTKTLDELAASGDWTGLAVTAALYAGE
TSASSHDINCGIDSPSSRRQTLSPSQSQDVEEGCASMEQMVSGLGVALNAGNWSQVSKYA
NLIKDQKNACNSFDTDTDVIESSSASVSSAGSNDTEMSKKQTIEKLMRSGKWKGVSIMAN
MYEMESKQQISSTSVRPYPNSPSHPYTERRSIVRTKELQHEDRVQENIVSFRQDP
>comp3893_c0_seq1; 308 317 736 LEN=928; translated
MLPSTTTFAFVLLVCAAVLQAEAFTTTTRKAGCSSALQADRRDFLNAAFSTIAVTSFPLI
AGAEDLADDLAMPSEEEQRKAEADAAAERLRRKAELQKKNSNPTDFRDRLAKEREKQKNL
QMSKEERRNALCEELGRGC
>comp3912_c0_seq1; 647 228 1886 LEN=2078; translated
MSQGVIYQIDFNAVAAGKRVSSSKRKIRWRFGFANKQALGEGLNGTDCRGEEHDVTIVWS
ITSGKRQIMMDGKEVHYSTNRAGVLDHSWQLKGNHVVKVTCHAAPPMSAQPGFRQYDLSI
DGQSFFSMPKVFQIGIKGAVPGQSPGRYRGYQSHTQNFSSDAPLTREQEDAQLHKAIQAS
INESRQHLGEIPKGNPPPSNNTSDLLGFAGPPAPQPGAYAPPSSDARSVASYYSAPTTYD
QSFQSPPPPTYPTPAPAQPSVAQGAIVPAVAPPSYYQAPPPPAAVPTYASPSPVPPQYVS
PPLPPQQAAFTSPPHVPPAPASTPVGDVFGLHQAPVNDPFAPKPPPPVDLATAVMSAYHS
PPTGTATPQTPMNERNEAQSATPQTNGNNGAALSMNSLTIASAAEEKPKSEFERALCNLV
NIDHIDEPAEGEVKLTMMRKDEKKKKAVNGKSVPKPPICLGIAGSNAPLSQIQKDFASPK
SQTTEGVMNAPPPGVFSPHAAQGGALVVHGQGPPPLQQAQGFGVGAMLPNGGFQNQKNAV
PGQYMQQQRLYQ
>comp3918_c0_seq1; 289 63 932 LEN=1048; translated
MVLPAPENPRIIALFDVDGTLTVPRGEVKEDMMDVLKELRKKITVGIVGGSDLPKQQEQL
GEGIVDEVVWNFSQNGLVAYNNGELLESNSISKHLGEDNIKRIVNWVMKYLAELDLPVKV
RNHETPTEKFHLGSDCCPLTITRARSIACLDAIQIFREELLSNSEAAXLNISPIGRNCSR
EERNDYEVFDLANNIRKNMVEAMKKEFSDLDMTYSIGGQISFDVFPTGWDKTYCLQYLKA
EDFDEIHFFGDKTFEGGNDFEIFTSERTIGHTVTSPEDTMAQLNELFLS
>comp3918_c0_seq2; 375 63 818 LEN=934; translated
MVLPAPENPRIIALFDVDGTLTVPRGEVKEDMMDVLKELRKKITVGIVGGSDLPKQQEQL
GEGIVDEVVWNFSQNGLVAYNNGELLESNSISKHLGEDNIKRIVNWVMKYLAELDLPVKR
GTFVEFRSGMLNISPIGRNCSREERNDYEVFDLANNIRKNMVEAMKKEFSDLDMTYSIGG
QISFDVFPTGWDKTYCLQYLKAEDFDEIHFFGDKTFEGGNDFEIFTSERTIGHTVTSPED
TMAQLNELFLS
>comp3922_c0_seq1; 882 258 2651 LEN=2786; translated
MKVPQVALLFVLGVSTTSAFTPATKAFATQQTSLYSSTKAQEEAIFQSLDRNGALANDIE
ELMKAPGSSKSKSPKAIEAELLASGKSDIKNYMKKFEGISGAAVVYKSLLDHGVDVVNGY
SGGAVLPLLDQFHPDNPRHAELGAEPIRWITNSNESSSGHIAEGYAKSYPINGEHKPVGV
VVATSGPGVTNLVTPIQDGMCDGVPMVVLCGQAATTAPADAFQSAPAVQIAEPCTKFSYQ
IKSAAELPFVMDYAFYVATNGRPGPVFIDLPKDLQNQILTSEMIDEYVESKIPDKKHDDA
MGVASMFTAQRDTGEAFKAIRLGFEEKGLSFEINDQNDLVPISSTTDAFNADHHVSNKIF
WTTNALTGEPNPITKEGDLEVNAEITDDILKFIKRAKKPIIIAGQGCNDSAEELKEFAEK
LQIPVCTTLHALGCFDERHDLALNFVGMHGHPTPNFMVQEADLIINVGSRFDDRITGRMS
DFVPGARKAAELGVGGIIHVDVRLTEKAKQIEPNFFVHSTGKNFLRTMNKALSQEEDLVP
ETSEWIERKNELEAKYPITIPKFEPETITMADENGVEKTAIKKPLSAQSVIKVMDQELLK
ADRIDDAIFTTGVGIHQMVAAQLITWTQPRQMLSSGSLGTMGVSLGYCIGAKLANDKKIC
ISVDGDGSFNMTFTELKTVAEQGIPIKILLLDNESQMMVEYWQRLFHNDRKLAVRNKNPD
YQTLASAFGIRSMIAESEDELEEKMREFLFDYPDEPVLFHVRIRRTPCLPLVAPGQPLDD
MILEDINVEVDQSAAPS
>comp3925_c0_seq1; 213 93 917 LEN=941; translated

MPLDSKTHSTLRGAVLIYVVLVKFLLSSPTIGAFAWNGCNLVTHFQSRVKYSDPSHCRMQ
LCASDSGDDSTSTDDPRSISANLVYFDIEVANEPIGRLIFHVSNPSPLPKHAENIIQLAK
GARRGIDPKAHYVGCEFDYSPVAVEDGMGRYRWSHQLKGRGRNGIGRADELIADPKSLSE
CCHSTFGGQYYGDLYTIIDNDPGVLLTVPVVGPGYGSSKFSIVRVGESPKEWKETLLINS
GVIGRLDGSSLEVLHAMARQRRGPPTVVAAGVLQ
>comp3927_c0_seq1; 485 1 1356 LEN=1464; translated
TGMPYAVGELELALEDCVSQAILISRQTSDGGDEGATAAATAAVKNLGILTASNRDEWTS
NRARLIEAIPGMKAALEKLESGLLLLCIDGEDNGRNAQVDSLSQNARSYLMGTQGRWYDK
SIQFIVTPSGNLGLNGEHSMMDGMPSVGLCQYILGQEASPNKRETEPKQKHPTPLFSPKV
APIFANQTILKELTNASLDETKSMVQKATADFDKIRSSLDLEVQSFQGYGSTWIKTNAKC
SPDAYVQMAIQLAIGKLTTSKAFNGNDGSRYVPWATYESTQVRSYRYGRTETTRSVSEES
AKWVGSMLGNNSNAEELKGMFHEATQAHVSYIRNASQGGGVDRHLFGLQMLLAEQEKQNK
QAVDLFEDPLFVESKTWKVSTSTLPVNPGFGPVVVPDGFGVAYHIESNRIFFTCTSYKGL
APALSHYLEESLVDMKEMVLKADNPNDRSKL
>comp3929_c0_seq1; 90 1 251 LEN=251; minus strand; translated
XTNGEELGDPCCTWVAGSSEGANPVPIEAFVPSHPGLASHVPEGPVLDLATFCESEGGSG
EAIVADVEVDAAEDEDSSLSSVVDX
>comp3931_c0_seq1; 309 120 674 LEN=872; translated
MKLSIPTIFVVALFTKDCDCFSTSGKARNSAVFLPAANSEHAENDENGGVVSRRSAVAAV
FYSASASLVAAPMVSFAETSLDFSLPTYDTKMSGFGEGSESYVKKGKIATLGESGDAMMT
DPGSDEKEKQAAAMRKAEEARKAALAKKLAERKALEEEAKRRAIEKKARDKERMKKMWSS
DFSE
>comp3936_c0_seq1; 945 560 3280 LEN=3779; translated
MAPMMMATSGQSGSVVRATTSRCLKIRTLADVLPTVLQSVVSSGKMSILFFLGGHLMLFA
LWLPFWLLSFVVSEVGVYITVVCSIFLVGRAIIRMIAFPGSSYRISSEIEKEFAKYSVRI
ITVSATSLIDLANTIASNDTYYEIPSLWKRAKSYRDRVLGVYLEVLHTLFQDDNNISPNS
ASGLNKYGNNYLVGDIGNLSGLTAEAMTDGRVLLNHLNDVLGQVNHLEDEAKSFLENGIG
NPSESARRIARNIVASATELRDLAESLKPPTTAGSTTALSGGDDGNLSVDEVRRRLEEEQ
ANASIMDTIRTGLSSILPMLDPPPHASIFGFDVLRGCVLSRYRGARQLWVQRPSGGMIDC
LYIPAKPTGTPIAGTSGSRKANAVMYCNPNAGLIEIAAGMSLAGGNVTSEIDNDDNCWTD
FYTQAGFDMYLFNYAGFGRSYGGSFCGMCSRPTDGSYTHGVWDRIKRIFYGLLFSFTPTP
DSLRADGVAVGSFILSQMDVDSLVIHGESIGGLAASHTGRKLSENSHHKDRISLLICDRT
FCNLEAVAQRLVGGWSGYAIRMLAPLWSTDVVGDFLSAKCPKIVANDAADVIIADSSSLK
AGISFWKEIKRSASSTNGIGWMMDAPLHHRMADWENVCVAGSQYVPQPRITRMTAPVWPA
DKHMSLDEAFHFAACAKRIGKVASIEKKRFAAMVSSGILNAEEGVADGCQAPIYLVWKFL
GCCEGLCGSALGVTVKSGFDTTVSWLCSMCVFGGQTVVEGMEERHKCADLNELTQVESSD
FDCRPPGWEHQESDTVVHPKPIPEVVAALQKIVADNPNDEILNSASHEVSYVVGTLNYVT
ARLSAKATIEASWKSRHLHLTGPMAEGCFMNLHCGHNHQYSAVERARLKAVLQQVTEATS
SPFPLV
>comp3936_c0_seq2; 885 560 3043 LEN=3889; translated
MAPMMMATSGQSGSVVRATTSRCLKIRTLADVLPTVLQSVVSSGKMSILFFLGGHLMLFA
LWLPFWLLSFVVSEVGVYITVVCSIFLVGRAIIRMIAFPGSSYRISSEIEKEFAKYSVRI
ITVSATSLIDLANTIASNDTYYEIPSLWKRAKSYRDRVLGVYLEVLHTLFQDDNNISPNS
ASGLNKYGNNYLVGDIGNLSGLTAEAMTDGRVLLNHLNDVLGQVNHLEDEAKSFLENGIG
NPSESARRIARNIVASATELRDLAESLKPPTTAGSTTALSGGDDGNLSVDEVRRRLEEEQ
ANASIMDTIRTGLSSILPMLDPPPHASIFGFDVLRGCVLSRYRGARQLWVQRPSGGMIDC
LYIPAKPTGTPIAGTSGSRKANAVMYCNPNAGLIEIAAGMSLAGGNVTSEIDNDDNCWTD
FYTQAGFDMYLFNYAGFGRSYGGSFCGMCSRPTDGSYTHGVWDRIKRIFYGLLFSFTPTP
DSLRADGVAVGSFILSQMDVDSLVIHGESIGGLAASHTGRKLSENSHHKDRISLLICDRT
FCNLEAVAQRLVGGWSGYAIRMLAPLWSTDVVGDFLSAKCPKIVANDAADVIIADSSSLK
AGISFWKEIKRSASSTNGIGWMMDAPLHHRMADWENVCVAGSQYVPQPRITRMTAPVWPA
DKHMSLDEAFHFAACAKRIGKVASIEKKRFAAMVSSGILNAEEGVADGCQAPIYLVWKFL
GCCEGLCGSALGVTVKSGFDTTVSWLCSMCVFGGQTVVEGMEERHKCADLNELTQVESSD
FDCRPPGWEHQESDTVVHPKPIPEVVAALQKIVADNPNDEILNSGEF
>comp3940_c0_seq1; 227 324 677 LEN=881; translated
MVLPVATSASISRLVSHSASGASITDDFENVILNESSRSIDSEQDYKDELAFCKRLACIL
TKERELPKLCQGFPAVLQTVNKHAEEQQRRETLHQKWQRNVQANNTASFSMPPISDR
>comp3948_c0_seq1; 421 93 1238 LEN=1482; translated
MMLRNCSRALSRTGPAVAPRSCFSSSSAAKGDGPKVTVKDPAPAEPKTKPKKKQRLVVAV
GGNALQRRGDRLTIENMLKAAAQMAPTLAELAKDNELVLTHGNGPQVGELALERAAATFD

VLGAESMGQIGYVLAQAMQSAGCTAVPILTQVVVDQYSEAFKDPTKFVGPIYGSIEAQAL
AQSLGWTVKPDGEYFRRVVPSPPPVEILQIDAIKTLLEHSPGILPIACGGGGVPVARVPA
NPNTLQGVEAVIDKDACGAKLANELDADGFIIFTDGGGIWENFGKPEAREMEQATPEYLR
GTKAGAKFPGSMGPKVNALIDFVEGSNKPGAWAAVGDLKDAAKIVAGEEGTFVREEVENG
VQWRAGKSGPARKESKDPPPF
>comp3948_c0_seq2; 276 1 958 LEN=1202; translated
MRSTLCILRRAMASTDPSLFGFLPRRLASLASLPVLTHGNGPQVGELALERAAATFDVLG
AESMGQIGYVLAQAMQSAGCTAVPILTQVVVDQYSEAFKDPTKFVGPIYGSIEAQALAQS
LGWTVKPDGEYFRRVVPSPPPVEILQIDAIKTLLEHSPGILPIACGGGGVPVARVPANPN
TLQGVEAVIDKDACGAKLANELDADGFIIFTDGGGIWENFGKPEAREMEQATPEYLRGTK
AGAKFPGSMGPKVNALIDFVEGSNKPGAWAAVGDLKDAAKIVAGEEGTFVREEVENGVQW
RAGKSGPARKESKDPPPF
>comp3951_c0_seq1; 372 117 956 LEN=1541; translated
MAARIAVAASGAARRIGATLDTMGANMEVAKYTERLVPSSRIVSVDGVAPALPGANNFIA
PSASVIGNVTIGEHSSVWYGATVRGDVNNVKIGTKTSIGDRAVVHVAKIQGDHPTVIGDN
VTIGAGAIVHACTIEDLVVVGESAQVMDGATVGTNSVVAAASIVTAGTKIPSGELWSGAP
AKKERALTSEEIAEISESAHDTLELAYLHAVENSKDYKQLVEEEEDRLDRKLRKNAWFDP
NIPDPDDVLGQGVPGRIFNSTLTNPEEGLKMHAERKAKS
>comp3957_c0_seq1; 159 379 798 LEN=1031; translated
MIIGARPIIIPAAGLLLLSGILHLSSAFSVQPPLTARDYTTSSTSAIGYTMQMPEFVVTL
PKSTWYDVANPTARRIVYDDGPTDEFMFASVGSDWPELNDIDMEERDNELPESMPNQPRQ
PWSRFNPLRRAKNLIRRIL
>comp3957_c0_seq2; 164 379 621 LEN=1141; translated
MIIGARPIIIPAAGLLLLSGILHLSSAFSVQPPLTARDYTTSSTSAIGYTMQMPEFVVTL
PKSTWYDVANPTARRIVYDE
>comp3958_c0_seq1; 787 68 2869 LEN=3015; translated
MARSSFHLSLCLVAIIACLSPESARAGLSATAEKNSNQLYHERKESSNVEKNFNQLPSIE
NEVESLFDEVSFYSAFQPSPLVDQTEHNNEIEDVFRRALQFQPTVNAPTFQGAQGTRNRG
TSFLGGHLQEVGVFGGVGARRGGVRGSGGQPPLQVQDQVRLNGHGPIPKTGKQKVSSLKM
AKSTKSIKFAKDKHKQFKKDKRYKGEKRDIDHKLTKSGKKDEKFSYDPYDADDDNFPLHD
DWTFDSWSWEWVEEDDYEHHDASAQFKMPDSSTQQSSGFGGALGGMRGWQAGTINSDISE
TMMGRMDLGGFSSISNWKPASEAIGAIPQQTNHMLHQTDEASQSNGVTTAQTNHYGQVTQ
MGGVSSHTGVTTAHANHNSQPSVQTGEASQSIQATTSQSNHNSQPAQTVGSSQSIGATTA
QYDYNSQPTQTGGASQSKGATTAPYNHVTKSNQLNFPTIQHMVFSGPIHLKAHSPVDVST
MDPEIFAILQSILTPYLQETMGPTLHAYTLEVNYSPGHGKNVGKDVVETLIEVNCAFKVI
SDSVESFKSINHVQASRWIHDFFAGPEIYKLLEALRTDNIPVNDITFVDQAFQVSATVAE
ANSQMASGRYPVSKNNEKSSNGAFIGITLSVVIVGMIFFLHHTGRLPSKAQVGAFSLTAR
DSVAKHGATARHSIAKHSKSARKSIAKCIPSSMKRKESRLGKYTDDEVEGGRRRTYSGTF
RRHPPGGLQKAALQKKPARSEEYLGESASTSSSSAGGAARSCRKSSSFTDMLEQDDYSFS
NFEGDYEPSICAPSTPSRRTYNGDEASMTDTLGYDSSAHSRSLLDKVSNTVSQAASLLSP
GNSSYKQQDASRSPIPSSAPRRVTAADIASPNDADNWSIQSYQTKTPSRRSPSNHPLYRG
WNDSGPELRMQKSEINDSEESPGRKRLNIPRFT
>comp3968_c0_seq1; 353 72 1280 LEN=1545; translated
MPNPVLVAAVEGGGTSFVVAVAEINMQSNNTQILHRAEIDSSHDDPNRTLEECASFFRNH
KNISEDGYRALGVAMFGPLGCNAERKEHYGRILPTTPKKSWRGVDILTPLVKACESEGER
GRKIAVKIDTDVNAPAVAEYLAIKADPTATSNPISSLAYVTIGTGVGVGLVIHGQPIHGK
MHPEGGHVPVQPLEGDNFTGYSWGANSKNPFRGKHTVESMTSSVALAERLEQLQGEEGNS
STNQQNLNRSCLADLKDDDHEVWSHAANALANLCVTLALVTSVEKIVLGGGVMNRNGLIE
KVRQQTRFLLNGYLGDLTDADLSEWITSSSYGSDVGLMGALVLAQQAIAEEAEREKATVV
PIKKLKQIAYGAGLWHGFGLGLSAAYIGICLWARSGKSSSRR
>comp3980_c0_seq1; 265 83 796 LEN=924; translated
MTDQSSEFVTTAFTIPLEGYTTPPSYENESQNPYEYMPARPIGGGFLADATNDGDGSSCK
RPFSKRLILDLDNKDIPMFPAPRFTGNHINTIDYNRNYNKRLKAKSSAFSNEEIDIFSTT
SIVRKSLLRMQEAAITLQRLKKEREAEANGTPDETRDSSPTGALSLSPLNISDRRSLIFH
STLQLHPSSSFSSLSDDSGSHCQSEGHTTKLKPRPSQQGVALGREIRRRDSGIARCA
>comp3985_c0_seq1; 498 141 923 LEN=1084; translated
MITRISFRNFLVMLAVFPSSVSSFNPLNTPKSSPSFHIEDGLKAATVDSTNEEDSWNSLT
RMIHSTIEEKGGDSLSEEAKDEIIKTAVAGSVLGTVVGSPLVVGAALGYAGSQMLQGEKG
DKAKQVMGQATREVMDRANSAIVFAKQELENEKDLSNVSAKILLAIKDKAGEIKSEAKDS
PAIMVEKLKQNVMNSVESQDFKTLPKRSFGALKALLESDEVKNVSTSAIDAIKAGLESEE

MKALQNRASKAVKDTLDSKK
>comp3992_c0_seq1; 372 106 1260 LEN=1374; translated
MARRDDFVFYLALPIAVCIICFAAELQALTNTKSYTSINRRSSHSIQQTIQHRRFLLGIR
SKITASATLAGGDNKSEETIRVGDLLSDLPTPTLLIELSLAESALKNTTTSLDDALSLLS
SKSVATGRPDRVSGNNNAIDETVCEQLLDGSIFLHTRVTDTSVRDRINREHGSGKSPVIG
GVDACPDRHVKGGAYLGIGLSNHHVGGYYWARGMGIGASLEAHGVAFRETPTPETETGER
KNTGSKSSEKLASDVGGGDAGRSGGELYWTRRSSDPTKSNSTSGSDAASGALCRGDTTEE
SSNSNDGKRSEWADFLVPGDTVQLIPYSPVRVLRESAFQRLVGVRRVGRPLGADPIVERL
WERETATAEESSSNTTGFGSLVPL
>comp3993_c0_seq1; 327 291 947 LEN=2499; minus strand; translated
MSLRVYMQDLILAHTSCNTDDDYEEENSIKITIVNDNSSSSLSYNYEDESSHKDRGQCDP
SPSSAESMQIVEICSNSFTDPVDDECDKSSSGYGLQSSEHRVAQHQNKSLSDAAINNKVM
LDDFRRRQCFLRAKFHPNRTAQANSQALQISPEPPLVMESQTTMFVTPSASPTKNSDNVA
EDEDMGIGVSEDNTFRCRMASLKSSIQEDQESTILPLF
>comp3998_c0_seq1; 429 204 1760 LEN=1870; translated
MSSSSPTKNFSNPAASFESQPHSPRSIFENSVVRTEEDAGVIAEDEVNCQTKSKSLVPKV
IIPCGHDGYEKSRSSVGEKDIVLGRRYAVXNVEDALKTDWCMILTRNSLISFPLIFPPIV
VDSDSVQNRSEDNRFRRIVNLYRDEYISSNSVERATIVSKVIALLEKDSYSFLRSGSLRT
PFSNRNSSYPSSLCDGYGINEGDSNGGLESGMFMDGQIRQMVSTSFSRLGRRRSRNDSSE
FKDAIRQQQRQMIDATLNRTPNTLKNPAPLATKDKVSYLKASTLGANQSIAPQQQDGPQD
ELVPPLRASRAPAHSETKLAADKVQKVRPFSSAHESCLLQECQPKVPAPRQCGGELREDG
LLWSSGSRTLSLLEGMDSTLVPTTSYSLLPTIEENVFGTKARAGPNSGCSDDGTGRMEFE
TIGYTHQKVGELTIVRPILLLPGVVGGASSPAVKNETNEDRHNRRTRTSHVGNSDAFSTS
NLLVLSDKVHPDGLDENPHAMDDVNSVVSSPTMLFPSQ
>comp3998_c0_seq2; 441 204 1650 LEN=1760; translated
MSSSSPTKNFSNPAASFESQPHSPRSIFENSVVRTEEDAGVIAEDEVNCQTKSKSLVPKV
IIPCGHDGYEKSRSSVGEKDIVLGRSDSVQNRSEDNRFRRIVNLYRDEYISSNSVERATI
VSKVIALLEKDSYSFLRSGSLRTPFSNRNSSYPSSLCDGYGINEGDSNGGLESGMFMDGQ
IRQMVSTSFSRLGRRRSRNDSSEFKDAIRQQQRQMIDATLNRTPNTLKNPAPLATKDKVS
YLKASTLGANQSIAPQQQDGPQDELVPPLRASRAPAHSETKLAADKVQKVRPFSSAHESC
LLQECQPKVPAPRQCGGELREDGLLWSSGSRTLSLLEGMDSTLVPTTSYSLLPTIEENVF
GTKARAGPNSGCSDDGTGRMEFETIGYTHQKVGELTIVRPILLLPGVVGGASSPAVKNET
NEDRHNRRTRTSHVGNSDAFSTSNLLVLSDKVHPDGLDENPHAMDDVNSVVSSPTMLFPS
Q
>comp4006_c0_seq1; 176 60 248 LEN=866; translated
MADRYGIVVVLKAKEGKIDELIELGKGHFVRQHDGREPGATCASIIMPLPDEPNTVRFFE
QV
>comp4006_c0_seq2; 191 60 380 LEN=632; translated
MADRYGIVVVLKAKEGKIDELIELGKGHFVRQHDGREPGATCASIIMPLPDEPNTVRFFE
QWIDKESYELHSAPNENLAVFLEAAGPILDGAPIVLSSPMLHYEKK
>comp4007_c0_seq1; 369 166 1002 LEN=1224; translated
MISKHRTAKPSTNELLVASGMALLVIIASLSDPVAAHRRIESCNHHYRKMYPRHYYYQRS
DCAFRRQKSLYNSAMNALDELVLATTNSLSRQRSRSRQNTAFSKQRQLRYSVEDFGTKGL
ELVIDLPGSNARDLAVEILQKDGTDVITVRGNRASHQNKLVTNLGFSQSFEVNSDDIDID
SITAHFSAGVLRIMMPKIEREGTKKPIAIISTNEYDDIVPIFVAKRDNRNDPSERKGNRQ
VVIEKNLEKSGDLKNSEKENYFTRGDNDLFISEEEDVW
>comp4012_c0_seq1; 407 7 753 LEN=974; translated
MKSFNVALSALMASSAMAFAPSSSTQSSTALNAEMPDRMWDKMVDPTERSASLPWLPRPI
NLDGSMAGDVGFDPFYLSSIPKNFAGFIQPPSWEETEGIPTLYWMREAELKHGRVAMLAW
FGWVATDGGFGVTLRFPGDIYSVENIPNAYNAHNILVDQGSMAFLLFAVAFVEFCTGAAL
VQVSKGELEREAGDFSLDPLQFLKGKSDDEVMKMKTRELLNGRLAMLAFSAVATQTALGP
ETQGFPYF
>comp4024_c0_seq1; 220 1 936 LEN=936; minus strand; translated
FGSTGANIAMSTGGSMSPSLVRDTGESILDHHHLEDYSCEMGNTSENQNPTEDDLIDPID
PKPTEDDPKPTEDNIEISSCLSGSTMLEKEMKGLPKSHSSKVFKADREDNSMIRVDSKDQ
YKAIGEHSIDPKIAEVESKKSPSNSEIEAPKKELTSLEKHICNDGRHRSIGGKVDEAASG
DAKKTHNEKINRGPTRKRKSLAVEKRTESIETLPDTNTKGPKTRRTRNRVSPSDDISAVC
RDADAVYEKRFMKAIERGVVSLVMSGVEPKNMACVTSFCTMKSLNNEAVKVNVSSTIDSD
TTMCVMPTASNN
>comp4040_c0_seq1; 193 294 956 LEN=1222; translated

MSSSSSSPPQCGVDYPELVVFDLDACFWDQEMYEMPALPSKTVSGILGDSGQEGVTGAYS
GRNKISMHPGSMVALQEHARGDVYPGMKVCFASSADTPFAEKIGRATLKLLEVLPGLTVW
DLVLKDWDGKDVNQIGRQPPLSSNKAQTHFPFIRKLTGIPYDKMLFFDDCNWGDHVGMVG
RGCTEESTSRGVVGHRTPSGLTIKDWYRGLELYREAQQQ
>comp4040_c0_seq2; 129 294 713 LEN=1542; translated
MSSSSSSPPQCGVDYPELVVFDLDACFWDQEMYEMPALPSKTVSGILGDSGQEGVTGAYS
GRNKISMHPGSMVALQEHARGDVYPGMKVCFASSADTPFAEKIGRATLKLLEVLPGLTVW
DLVLKDWDGKDVNQVRSRN
>comp4043_c0_seq1; 205 116 1410 LEN=1652; translated
MGRSQREEKKPCCTWPRILVLVVVLIAGGVCLWKFGPWEDTINDVLDSIPIPGGSDGKIP
PPNDDYIPQTLTPVSGATASPTAAPPDYDFIQCNSDGTTTPSNCCNGLEGLCDLRVNEVM
YATLHNAMATFENGFLFGPNHKFQLEGALEAGYRGLNLDICNCGGELQFCHGECRIAYFR
ASCKEFFSSKVGLIDXLIGIIFSGICALGPREIVDVMQSVNTFLDENPTEVIIFIYQVND
DVDQEVDLNKFYDQLLLVDGLVDKLYVHPDIEAPWPTLGEITDPEFNKRIVMFHYGGPNC
SIDPLACPDGLHLYYEYGSDNDWEHSGMESIRDRVNSCELGLNGFNKRDFVGLNNFVSPP
SQSTAAKANEYSEVLDYVNTCSALLETDINFVLIDFWSVGDLPRATQDHNAALIERRRRQ
LQRERGLLRAE
>comp4043_c0_seq2; 311 116 1315 LEN=1557; translated
MGRSQREEKKPCCTWPRILVLVVVLIAGGVCLWKFGPWEDTINDVLDSIPIPGGSDGKIP
PPNDDYIPQTLTPVSGATASPTAAPPDYDFIQCNSDGTTTPSNCCNGLEGLCDLRVNEVM
YATLHNAMATFENGFLFGPNHKFQLEGALEAGYRGLNLDICNCGGELQFCHGICALGPRE
IVDVMQSVNTFLDENPTEVIIFIYQVNDDVDQEVDLNKFYDQLLLVDGLVDKLYVHPDIE
APWPTLGEITDPEFNKRIVMFHYGGPNCSIDPLACPDGLHLYYEYGSDNDWEHSGMESIR
DRVNSCELGLNGFNKRDFVGLNNFVSPPSQSTAAKANEYSEVLDYVNTCSALLETDINFV
LIDFWSVGDLPRATQDHNAALIERRRRQLQRERGLLRAE
>comp4044_c0_seq1; 747 183 2402 LEN=2580; translated
MFIIEKNRAKNGRNGSLNRNVRKSNLASLISVAFMAMFLTAVVLWHDGRKTTPARRNTSN
RFLTDVDADQPTLDDDNARTATCKRFLMNFLNGTTDSHDQCQAFYNAYRSADCAHETHAN
IFGVESADKDNSSTTDDVGIDDFFEHWECCDSIKGYYNNNCEHDSGVHSLQLLGVLLVFL
VCTMIKAMLKKWDVNWLPDACAFILVGTFVGVMLRLLDSRLVPKLSFDNDLFLQIMLPPI
IFEAALSIDKKAFRRDLFPILLFAGIGTAFSSIAIGWMVHRVSNLGTNGSNLPWLDSLVF
GSLISSIDPVATLSILSGVGIAQTDTLYTLIFGESLLNDGVAIVLFEVLKDHLGEGDSLG
NDAYKEMSMHFATVLFGSIAIGAGVGICCTLYFWALREKQSAVTEVGIFFCWSLIPYYIA
DGLHCSGIIAIMVMGFILDFYVIGGVNQEQHAWREVLQQEHENATMQNTRNYNSVSNQLN
RVLKDAFSGKGHILDASRNHVGFLAHVVAMIMETAIFAYLGLFLFKDNEWNSRLIFTAIA
SCVSSRFFMVVSLSFLVNIAVFLDFENRIGSCIKSVNPFQRINLADDDDSVGSHTRKYLD
RKTQLVLLLAGVRGAVSFALVENIPVWDNVSKTGSQFKAELKAMTSSSIVFTLFVFGALT
YMAVKHDPESDSDRARAVLLNRLLNQPMDGTELSETQFERDGRSGGHHQTMSNGQHADGS
RGPQEELNDSDAVYQSSNEW
>comp4047_c0_seq1; 150 114 566 LEN=1232; translated
MVVLEAAAIGAASYGIYKGGEAGIQKGKDCRKEFKREQKRSSQRSVLHQKTRSRSSRIAE
IVQMKNGKNEASAAAVTSARGAGTGSGFLHATSFASRLKSANEASTDIDDRHRAVMETLR
ASRKEESKKEAKTKVGSKLKKALTNPFKKK
>comp4053_c0_seq1; 371 53 1723 LEN=2086; translated
MRRYPSTPAFVFLIAASFSLLPLRQLTCSGSEDASFRRPPPPPPPGSSFNTVSDIGRGYR
NDANKEGPTSNGSAESYGYDDMQHRQKQQQQEREELSSSSHSSRYPFEYSTESVGGPTHT
QSQVDRSSSFREREQKQQTSSLPIHYDFPISDDGATETMGKGSMQRQRIDNTRDGVDDEE
GMTSSARKDLVTRHWSTKRGKVQIQTVIGLVGYGSGRFFLKSLMGDSDMVQWLGCFSAVA
FIVSTWFRSPIGELSRAVGLSLILVLQRTQRIRREYPTWRYVAASVGMIRGGPRDARGKP
RGPRPFPPARNPWKYSPRSPRDPDFNMTFALASMAMVGSAVGGNLPFIPNWIGAIIGAAF
SAFACTWQESHRGDLARTMGMRVVRVVTELWEIQADLQIIPKATVVSSQIIDKAMILDRK
HRVKDRFLSLATKGYAQATRVAEQVQQQQRGAKSSEGRNERVENDRVMPRGEDDESIRGG
QRGGYDRGRDEKGDRYFDGKPENGRRLMPRDKDGRDSYDFRERQGPRRRYEDDDDFDGSR
RRDAYNHDKDSKDYFR
>comp4053_c0_seq2; 203 478 1170 LEN=1533; translated
MTFALASMAMVGSAVGGNLPFIPNWIGAIIGAAFSAFACTWQESHRGDLARTMGMRVVRV
VTELWEIQADLQIIPKATVVSSQIIDKAMILDRKHRVKDRFLSLATKGYAQATRVAEQVQ
QQQRGAKSSEGRNERVENDRVMPRGEDDESIRGGQRGGYDRGRDEKGDRYFDGKPENGRR
LMPRDKDGRDSYDFRERQGPRRRYEDDDDFDGSRRRDAYNHDKDSKDYFR
>comp4056_c0_seq1; 770 1 1237 LEN=1493; translated

XRHQSNSKMKFSTFAFLATAGSAAAFAPSTGFQGRTAPLNMATEAAEETKAFAKLPASVK
PGVVTGQALVDLLDHAKKEGFAIPGVNVVGSSSINACMEAAKKYGGPIMVTFSKGGGYFI
AGKAADDTDDKASIAGAVAAAKHVHEVAELYGVPVVLHTDHCQKAWLPWMDGLIAENEKF
YKENGKPLFSSHMLDLSEESLEENISICKDYMEKFAKLDILLEFELGVTGGEEDGVDNTD
VDASRLYTQPEEVWYAYEQLSQVENAAFTCAASFGNVHGVYAPGNVELRPEILHNTQKYF
AEKLDNGEEKNMKFVFHGGSGSSVEDIQYAIEAGVIKMNIDTDTQWAFWDGIREYEAKYH
DYLQGQIGNPEGEDKPNKKYYDPRAALRAGEEAMAARLCKAAEDLKCVDVLN
>comp4059_c0_seq1; 500 1 1178 LEN=1336; minus strand; translated
XSIVMNEVIRIHPELQKNVVELLNMIEQSRLQIDQQMDHQKAQDLALTASNVTLKNLQNL
LERYSPMQILYDRSRKANRISLRLKLLVSCNYIKTAIAIVTAVDKESSASNIEIPTGDDK
LCDVLGVDARSGLPHPLWTKKHDAILIRSVAKHGWVDVDANLKNIVNDKKIKWGFPFEAS
NNAPVQRIGEQEMKNMRETAGRAASILNEKPKILEILTGFNKKLGRCATAIDIFSFNYSV
FLPKVAHMLCALVFLFFKVIDSYGLTQQSEDDENDEMDDGEKKWRVDNRMLHQASKKSEP
VKEAVDLPAKKDFVKRAKIVIQKTFAKLRSEGTNSKSISSTKAIDEDVKSNESHGYIVID
QGSRCNILLAEMIRALVKGSTKVSVQIRHMVR
>comp4062_c0_seq1; 306 113 861 LEN=861; translated
MVDICLNDKQCSNGGVCSGNKCICPSGTYGARCDAQNVDSDTDISLCTEDGNQCQNGGVC
IKDTTEINNVTVDANFCKCPDGYFGGRCHGHSGGGGSAAVPDGTTWISVCTQDGGECKNG
GTCVLETETHNGQVVDANYCRCPDGGLGSSCDFDHSSGPDGTGDEDEDKPEPEDNTEDEV
KPVVPSRSVCTKDGGQCKGGTCVLATEKVNGQTVDANYCICPDGGLGSHCDPAKSSGQGG
ANDEVKSDEV
>comp4065_c0_seq1; 184 60 733 LEN=733; minus strand; translated
MSSNNIRRNPNGSRRFLYGDQDEDSFSAHSSRSRNTQRSAGFSTRDYIVDQSVNLMDIGI
GDSASSVSLPARSSSIAGMFRSSVETEILNRFDEYGDAAPMADAEEYIDNDKRRRGTAMW
RKCRKALCSMYTLTLLVSLTGIFLVVYTLVLTVNTKGREKTDSVTKADNKSGSGSSNKDA
NHERYGMIRSRIVDAGVTSEGLFDGGSKSPQKLALDWLVLEDPAX
>comp4069_c0_seq1; 258 159 842 LEN=903; translated
MDANTGTPGQSASAMGTLVGVMNTAKEKWDSSGANDAVSKFSASIPDSTKDYISQTTGQF
FSRERLRTVSVCFGIGEERPFYVEKTPSLLLVRLKHNFQFFYLNYLLMLAVLFGLTLFVT
PSAIIGIALLAMAWVYVIRASQSGSLKIGSFSVTQTQATIVMGVVSSLTLIWILSSVFWW
ALGSSGFLTVLHAGLRDASMHQDGEDQVTMVGEVEPAGEQAAFLGQV
>comp4074_c0_seq1; 269 229 672 LEN=730; translated
MARVVRRNINPTGNGAAMAIPGYGVAEQVFVGGFANFLSIYNIVITARVLLSWFPQAQGV
GVLQPIFQITDPYLNLFRGLIPPVFGLDLSPILAFVLLNVMTNVTAAVGAEITADDRKKL
QESSPFAVKPYHKKNSQKKYSPLMMQV
>comp4077_c0_seq1; 319 253 1785 LEN=2348; translated
MHSAIQICRSSVLLLKLISVLQAPTWTNGYVFSNRNVPICEAGCHCYGSEGNCPSFPTVT
EDMLKTFRELNLVNPMKVWCDPLQLTECISTLEEGEACVVDLIAPDSIQEAPCPSGYSYR
LTTVPSLEDAIAEGQYITHTGACGTCSSLQDLALMIEYPNLPYKAQQCFFRSSALKNIEE
GIACYQEIGFTQTCSATLAYHQKRIETKVVDINAQLGHMMGIWGDVSDIIKDYVSSWLHA
KNFSLILQLLLIHPFPISTDSSCEDVTGCGPCVDNFGITAQLELISGRTFANSGYPSQKA
QQCVNIAPIDVIGGIDICAYAPIEEPPAMETEAPTMSPTALKTAVPTPTSIVQTSQYPML
STTQEPVEPPTGLPSVSLVEVTTAPAPVENVAPVEVTTAPVETTEKPVTTAPVEPTEKPI
LLITAFPTPDPAPLPTADLYFQQCLNSAAIEVRMGALSGGSGVVCDCVEAEKGVTNNQDA
SRVPVDLLRQNAKSSMIFVRLMRIAVVLA
>comp4077_c0_seq2; 365 253 1678 LEN=2241; translated
MHSAIQICRSSVLLLKLISVLQAPTWTNGYVFSNRNVPICEAGCHCYGSEGNCPSFPTVT
EDMLKTFRELNLVNPMKVWCDPLQLTECISTLEEGEACVVDLIAPDSIQEAPCPSGYSYR
LTTVPSLEDAIAEGQYITHTGACGTCSSLQDLALMIEYPNLPYKAQQCFFRSSALKNIEE
GIACYQEIGFTQTCSATLAYHQKRIVDKGCGYQCATWAYDGDLGRPSCEDVTGCGPCVDN
FGITAQLELISGRTFANSGYPSQKAQQCVNIAPIDVIGGIDICAYAPIEEPPAMETEAPT
MSPTALKTAVPTPTSIVQTSQYPMLSTTQEPVEPPTGLPSVSLVEVTTAPAPVENVAPVE
VTTAPVETTEKPVTTAPVEPTEKPILLITAFPTPDPAPLPTADLYFQQCLNSAAIEVRMG
ALSGGSGVVCDCVEAEKGVTNNQDASRVPVDLLRQNAKSSMIFVRLMRIAVVLA
>comp4083_c0_seq1; 508 97 1052 LEN=1052; translated
MMKSWADHCSSDEDTDDNFSVGDEEQQSDDDACEETSIEDDFSDKVRLQEQQQERTYDFP
DRPPFTAFIGNLSYDIQEADQLQKALVDVVFRHLGERITPLGGHISYDRDNNRQHRGFGY
VELNTLEELKIVMKLNDDGQAKLAGRKVQVNTANNHNRSNNNFNNRRGSSHNHHQNHSRG
SFQNDHRRNSNSSGNNSTDQKPYEKVDGSKFRGGIFSNKNNNNGSHNNGSHNNRNSNRSQ
PTRGAAVPRERPSLKLAPRSKPIEGEGRSSASNIFGGAKARDEQAWEKNRHEREEKQKQE

KQANEKKSTAKKHNNNNNX
>comp4092_c0_seq1; 212 236 886 LEN=1568; translated
MAFVPAFLLRGVDSFALHLSAAVYTRCRSTNASTDLRLWLSPEEITASLGPGRTIASEPF
SWNELKEIVVNGDPTMHSRSMEVQESYLLHSRRIKSEWNSMNDYILHSKFGFEKITKNEK
FCSSPSLEQAKLEGRRESKLLRNEYPYFVCHGIEHWCLWKLGSKVEPYDLDWAKNKLVEM
DRGNQGEIMSWVNPPNLQSIPDIDHAHILCLRSDDI
>comp4114_c0_seq1; 246 1 540 LEN=540; minus strand; translated
DAVADDDEDADMVTGPGSSSDSNVIDAMFRVAEFMSSPPTDGGVTFTGFYSLPTIQLLSG
DREFDLSMSELKECWADGSLGATRVDRAGFERVWIEVQEYFEQDIMTEARKEARKKTTKK
KVRGGAKSVSATPTTTIGDNLSDKELEEVNQRVKNLSDDEVGSVLDMMEQMDPAQEARLK
>comp4116_c0_seq1; 650 137 1634 LEN=1736; translated
MNTSKANVEEPKKVSTTEESDEAIQNGKDDGKNAENVEPSREDKAPITGDKAKRFLPAYK
KANAALTFPEKEDHCARELCSVLSVEQVMNLTFSPHARLTKMMNLMKYVDEKSKSEKDFC
ISWLPEGKAFVIYNIKEFTSNVIPKFFKASKFCSFTRKLYRWGFRQLNRGIGPDEPIIFG
NEYFQRDNADLMVNMRSITAASIRKRESDLLRHMLASKKRALLSDHTSSYVPHQDGKQFN
MAAQFDQKQSVSRMANVLASQQRFGGGGVGSSDGGFSTNLSHLNNQELQMMYQSVIDQSR
KSSNGPDSGIAPIISDAMSQMSNPYGGFNTSGSSHQQEFNQMASMHMNSSSSNCSAMMGG
STSNFMNSQNCNSSSFDAVGGNGCNGRMNNMGNGYGNNAMGLNGAFNNNMFSSDSMYGNS
SSRGNGNFNLSQQFDGDLNGGRKSLMNHGGKHNFENGNYNNFNSAPMNMNINQQLFNLSS
SAGPGAGSDDLYFGGGQY
>comp4116_c0_seq2; 679 137 1543 LEN=1645; translated
MNTSKANVEEPKKVSTTEESDEAIQNGKDDGKNAENVEPSREDKAPITGDKAKRFLPAYK
KANAALTFPEKMMNLMKYVDEKSKSEKDFCISWLPEGKAFVIYNIKEFTSNVIPKFFKAS
KFCSFTRKLYRWGFRQLNRGIGPDEPIIFGNEYFQRDNADLMVNMRSITAASIRKRESDL
LRHMLASKKRALLSDHTSSYVPHQDGKQFNMAAQFDQKQSVSRMANVLASQQRFGGGGVG
SSDGGFSTNLSHLNNQELQMMYQSVIDQSRKSSNGPDSGIAPIISDAMSQMSNPYGGFNT
SGSSHQQEFNQMASMHMNSSSSNCSAMMGGSTSNFMNSQNCNSSSFDAVGGNGCNGRMNN
MGNGYGNNAMGLNGAFNNNMFSSDSMYGNSSSRGNGNFNLSQQFDGDLNGGRKSLMNHGG
KHNFENGNYNNFNSAPMNMNINQQLFNLSSSAGPGAGSDDLYFGGGQY
>comp4126_c0_seq1; 325 129 1421 LEN=2047; translated
MPKHQRDNRKSRRSAPYESGEVEEGEERERNRSSRNRSSGNRRSKSASSSIHTPCATFDD
MNLKPDLLKGVYAYGFEKPSAIQQRAIKPIVLGRDVIAQSQSGTGKTAVFSIASLQLLDE
TSRDVQVMVLSPTRELAEQSQRVMQSLGDFMNVNCHACIGGKSLGNDLKELERGGVQVVS
GTPGRVYDLIKRNALHMSRLKAMVLDEADEMLSRGFKEQIYDIYRFLPPSCQVVLMSATL
PSTVLEMTRKFMNDPIKILVKRDELTLEGIQQFFVAVEKEEWKFDTLCDLYDTMTVTQSV
IFCNTRQKVDWLAEKMRESNFTVSAIHGDMDQTERDAVMNEFRSGSSRVLIATDLWGRGI
DVQQVSLVICYDLPTNRELYIHRIGRSGRFGRKGIAINFVKEDDVRTLKDIEQFYSTQID
EMPANVGDLL
>comp4126_c0_seq2; 204 1 1092 LEN=1718; translated
ILCKMANKNTTLGLTGLEPLTCRYKNRVTSTTLRCSAASSRFSFVDVLLPIGWKTSHKFS
TLCVLKYDDPSHLFLNNFMMVFFNKVNCHACIGGKSLGNDLKELERGGVQVVSGTPGRVY
DLIKRNALHMSRLKAMVLDEADEMLSRGFKEQIYDIYRFLPPSCQVVLMSATLPSTVLEM
TRKFMNDPIKILVKRDELTLEGIQQFFVAVEKEEWKFDTLCDLYDTMTVTQSVIFCNTRQ
KVDWLAEKMRESNFTVSAIHGDMDQTERDAVMNEFRSGSSRVLIATDLWGRGIDVQQVSL
VICYDLPTNRELYIHRIGRSGRFGRKGIAINFVKEDDVRTLKDIEQFYSTQIDEMPANVG
DLL
>comp4127_c0_seq1; 337 133 894 LEN=1206; translated
MISLASIKSDTFLLVWNIASVVTFLLPLFAFTTARLTTYGNEDENQNGNQNNQYQSNYEN
PDYYDEYGNYVGPNHWWEFWKKTNNRRNGNEENEDELSAPWWYIWGEREGREPEEESRAV
LFVYLWSLALLACIAYVGNTCGTNVIKLEKLRVILLGFANCCFVTIILLVGSEDTIRTEG
REIEETGFYGQRAVLLFLTCIFGLLQSIVFVSWTSKRIKKLKIDSLTGKSDEYVNVEFDN
APPAGSSGPSVPV
>comp4131_c0_seq1; 196 64 351 LEN=499; translated
MQIFVGSNAYEVDPSTSVEALKLQIENAEFVPAHQICLMNGADELEEGTLETNGVEGDDA
LEMRMAVTSGMRKKWKKKRMRRLRRKRRKMRQRAR
>comp4143_c0_seq1; 407 74 1024 LEN=1291; translated
MGRISSAIRVVALFSAVCSSLSFVAPALDSFNQRQCGDNNLHRHQSTRPAFGLDDTTDDR
LVDIRNALEKMSKLSQSKDESHSMQTFETRIRKVISSGESSIPGAGRGLFATKNIKKGTV
IGFFPVDGIGVEFDDGSSICCGLTPEDQTYFDNTPKSNYLLNLFGSRRLGTADFGEDAQL
FIDVNPGRTSDASTPWNGHLVNDGATVLENSEQGMLDYYAASQRRKNCVNIPFGPSPLLA

VMTTKKVKKGEELFTTYGCLYWMEYILKPDEEVADVTEAVQLQARETAQDLFDAMKDAQV
TYAKQQAEIEESFAQM
>comp4144_c0_seq1; 75 1 223 LEN=223; minus strand; translated
XREVDPTVFHMQASEEDGGVSFADIGGLNEQIRELREVIELPLTNPELFTRVGIKAPKGV
LLYGPPGTGKTLLAR
>comp4145_c0_seq1; 69 1 205 LEN=205; minus strand; translated
XGVGDGSESTDNVTTRGVVDNPQVALQPSIAQTNLLTQNLRANFNAAQRQAAATTTTNLP
NFQPLIALP
>comp4151_c0_seq1; 821 153 2752 LEN=2752; minus strand; translated
MKSIFLFSALWSGASSVLAACDPETTVSLAGGVSKLTVTGFVDPIINYFSSVPGTVSKWN
VLHSVSHENKCSACVTGLGFSHRDRLTPAACTVFRASIPYVKGDSVVLKGYDLFPEVLST
GDELVITAGEPCGSVSSPAAPSISDASGSLAVFGATSYFFGGHAFLTAASIFLAMVPFSA
AAEEAVPSITVEIYVDVDKLLHEDSGNSDCPPETTLWKHHDSVFGGYEGCVSEKYLYPCA
QYVRPEDEWNVDRVPIDFVDGECIESGYTQXEQNLLDLVGGSAGRKSMASSVSLFSMVLF
DLSKLXRNNLTMMSPLPCFHPNPFAERIAMKYIPRNAPQGDTLMEKTGLKPTAKFPLRRG
PYPSYKTAEGFEEALDDSNDAKAVALDFLVYLGAIEESAKDDWYVTINEGAEQGISTLWA
AKALEIAATTCNRDIYVLMEAPAYGYDTGTIASWVNQNAEDFYSGEECLCYPLCNEPTVT
VNNVGFFPGNIPEALGGDNVTYVADSTSPTSPWFESMVFPEEESHRIAFPGIPNRPSRQD
ASHDASRIARDQPTQPNPTQSHPTQRVFLSPVFLGSRFWHSLPIQNPSGVIKTPQLPANR
RVCDGVYVWPMYFGMRGFEIPLDEMPDCDGWSYAITKGYSASVRAGFITYKKGSEEFAGY
VSKITSPFHSLSYGTLSQWTWEGQMQIQKMMMSRRLDDPTSWVGAYSEIMKEKWEVVTEA
FRDCPVLELTNSLAGAYAFFVYKDPYLGLQSGTYPSFFMDVLGIRATTYFWGFRGADPSE
YFGEGVSTTDFTRMQLYRDVEVYHEVARRARIVCSDTSASIGDLVSIDDWQAAGLASSRH
LEEGHDPETRRRLLREELPHLHERKLA
>comp4158_c0_seq1; 366 1 656 LEN=1037; translated
XDDDDDDDDIPLAALKSKSPAKKKKTEAKTKTTKAKAKAKTPAKKKKATPKASASKSTAS
GSSTKDDYSYPSFALYGTESLKGKLIQNLLCRWWYAIAWPDPSAIPKEPPTDYDAMDGFP
GVFICTQGEEVGTIKDFRDKENTPCFNNFARKSSEELKELLVEAINEQKRQLVQAEGTGT
ATEKELDTMLKWAKKVNAKKADTEAAKILKANKLSIPA
>comp4164_c0_seq1; 216 139 915 LEN=1219; translated
MVEITDIPFVKPLAEWIWGEEGSSSCLSQVPFLSGPCLSQLLTKALGVLIILASMLNKIP
IMVNMLKAQSAAGISRNSLYGEAIVYANCALYGFLSGHPFTAYGENASLLLQNSVLIVLA
WNFLSKTNTPVGSDEKIMAILFFALYVAAVLNFLPEDYWYMLMSTTWPVMLYARGSQVYE
TFCVKQTGNLSIVTTTMNLVGAVIRILTTINETGDVVVMSGYLLSGSLSLMMFVQHWMYL
KNTREIAKKAEEAKKKTD
>comp4164_c0_seq2; 195 820 1185 LEN=1489; translated
MAILFFALYVAAVLNFLPEDYWYMLMSTTWPVMLYARGSQVYETFCVKQTGNLSIVTTTM
NLVGAVIRILTTINETGDVVVMSGYLLSGSLSLMMFVQHWMYLKNTREIAKKAEEAKKKT
D
>comp4181_c0_seq1; 153 1 442 LEN=700; translated
XFLADQAFACIPLARRAKTSALESIRDTTSLFATQAPSIARWVKTRTMNGLRITFAFFSD
QLKHYDYSNGVILPYLKDRWEYAFTDRAEQEQRIREEEKLKELLAAAAAAAAKEAAEHQQ
MLWTQTMVAGACAFAGSVATTILWSVL
>comp4186_c0_seq1; 239 137 990 LEN=990; translated
MAKPSPLQPETGTLSLSPPSPRGKKRPRGWGKQPAGIETGSELASGKQEKQHRHPERQQI
QHQQKQQRPNLRAQRIIVLGAGISGLACARELKQRGYEVLVVEARSRVGGRLKGEVLELG
ADYPVTLPELSATRTKTKRKRKTEGSSASKARTKQRKTTGDGNDSRDSNKENHTATTRKH
PIDVGGALIHGIENNPIHQITSQMGVPIHSVSEYCLLTDENGWPFDPKTDERTSNFFNDC
LDVTFARAKLQRDSGESFGSLFERVCREKTGTVAYGSKQKQKQKX
>comp4192_c0_seq1; 364 93 932 LEN=1133; translated
MESEATTTAISALNWANGLAFFFNVLATFGSQSAKGMQDNATLSAKYQTLVTPAGYAFSI
WGIIFTTQAAWTIMQFGEGMRSSSFVTETVGYGFVSACLAQALWSVVFGLEQITASIVPM
VAILVSLLPIAGRILTGSSNDDESQNFWVTAFPMLIHAGWILAATLVNTNVVLVARGAST
SVQTLAAWASLAVLVSCGVGLLALPMASSGMTTSVSTPTVVVIPCVLAWASYAVSRELGK
PKASIATNFSPEVVARTGMVARNGAFLIMVLVVLVALLR
>comp4196_c0_seq1; 72 1 103 LEN=404; translated
XIQSVVEAENGESGALCNSLHSVVEVESGESVSE
>comp4200_c0_seq1; 269 39 653 LEN=697; translated
MAEETHPLQNEWVMWEHKANQGPKDPDKWKENMIQLCKISTVEDFWRYFQHLPKPSQVFF
DGECRKTVGPESKTIEEYSFFKQGIEPEWGDPQNVTGGEWFCRQCLASEVLDLYWQNLVM

SCVGETINDAVAPEGGESIEQYVNGVRVVDKSRAYPMYKLELWLNTRDPAIKDKIKNKLV
EIITDGQSSTKKLQPKFDWRDHSA
>comp4214_c0_seq1; 1383 187 4047 LEN=4641; translated
MLTKFESKSARVKGLAFHPVRPWVCASLHNGVIQLWDYRVGTVIDRFEEHDGPVRGVDFH
KTDSLIVSGGDDYKIKVWDYKLRRCLFTLLGHLDYIRTVEFHPDPAKFPWILSASDDQTL
RLWDFQKRSCLSVLTGHNHYVMCAAFHPSEDLIVSASLDQTVRVWDTTGLRKKQTGQDVM
DPMRGPQVSATTNAAMNVQAELFGTNDVVVKYVLEGHDRGVNWACFHPTLPLLASAADDR
QVKLWRMSETKAWEVDTLRGHANNVSSCLFHPKHDLIVSNSEDRSIRVWDVSKRVGVQTF
RREGDRFWILAAHSNQNLLAAGHDSGMIVFKLERERPAMCYNAPRSKLYYVRGRELFARD
YSRGDGSGNDIPIASLRRVGQQAQTDGIGSAPRTLVYNAHNPSEGNVLVCSDIDGGSYEL
LTFNGANVTDGKRGACLGPAVFLGRNRFAVLDRHQRQIIVKNLDNETTKRVQPPVANVDA
LLDGGASGRVLLRADDRAILFEVQSRRILGELTAPKIKSVVWSPDGSKVAIVCKYGVVIA
DRQLEQLCSVSDNVRIKSGAWDCSPSSDGTASNLFVYTTLNHIKYCLPSGDSGTIRTLET
PIYAVRVVKDQLHCLDREARARVIAIDTTEARFKLALANKKYGRVMHMVNHSRLCGRAIV
AYLQQRGFPEVALHFVREPKTRFKLALACGDIEAAMESAFALENQTQQNSEIEGGSSTDE
TREIWSELGAEALRQGNYEMVEMSYQRTKDFDRLSFLYLMTGDTEKLRKMLKISNMRGDI
MGRYHNALFLGDAAERLAVLEASGNLPLAFMCAKLHGLVEDADRIKVAIETNEGEEKMQT
VLEQTLAIETQNDRSGGRLLQPPTPIFRDNNWPTLEVQKSTLADLTAAVPEEEEVETQAA
LNAPVSELADDWQDAVDDSDLAADLAAADDDFGDDDWGDDDGLDDLGDFGNDEDLDVDGD
DFDNIADDSGFQLPPSGRPPAACWVANSSHAADHVAAGGMSSAMQLLNRQIAASEFSVLQ
QNMMGCYAGSMMSVPGIAGSGSMALPLMRNDSDGHPGNASLPRTYITPKNLVDGVKSGYR
FFQGGKFNDSKASFMAVLTQIPLVVTNNPKEQAQIKHMVNVCREYITGIRIKGAMAEAGK
DPVRATELSAYFTHCELIPAHMVLALRSAMGTAFKHKNYILAASCARRLLELPEMSSERN
ADLKNKAMKVRQKSEQIARNEHELNYDETKSFKIDCATLVPIYSGASYKSCSYCGSQYAG
DDMVNKLCSTCGLSQVGIKTLGLVTG
>comp4226_c0_seq1; 80 1 226 LEN=226; minus strand; translated
GDVKAVYIECLDYQKRGGKGTRSSLPEDFFRYEYKVNNEIASCIATTPIISLWDGLVMGG
GVGISIHGKYRVATEX
>comp4233_c0_seq1; 297 1 549 LEN=719; translated
QQQIPRSLPVDQTTSFQQIEIMKITAIIASMAIGSASAFAPNPTIASSATQLSLFGGGGE
GGEKKAGGGMMDQLAMFKKAQEMAQKKNKLDQELAAETFEGKGADGKVTATCTFSPSKNP
MDPQPDYKVSEFDFDEDWYESASPEDISAGVKEALMDGIAVTNEAVNAKYSQLEEDIKNL
QA
>comp4234_c0_seq1; 956 1 1845 LEN=1845; minus strand; translated
XALGSSNGDGFQPDLEKQMKPEETVVATGTANEEDDEFGEFETGPTFHDGGVDATDHSKD
EFENREKKVENSLITSVDFPMVTDLGKTMSEPMVAVAPQMIPATSAPSSLSITDAFSDMP
VQEDATLPSMQHNMATNTILEDKNLDEVDGVFGNFEKVEAAPSEPKRNDDQTKDDGDSDD
EFGDFGGVSSSDRGGNNELSSGSDVMGSDCVGINMEGDLPPSMTDIGRTVSEPIRVDETL
VPAKGMMGTGHLRSISIDDAFGELASTEDRPLPSLEMMTANSNVGCLSASEENKKKRNES
NDDFGDFTAVRPQPEHLAGISSKSDDGDGDEFEDFDAAPLENDERPPEKSHDKSEPFEST
GTMAVSDADRQIPIFDALGTEDAPLPSLEMLAATPDPNVESKDLVGVVDTHNNDSFGGFK
TADTHPSGSMDGTNERNEQGELRYVSDDMKNESTSDDYLLQASTVLSTPAITETGPSLSI
ADPFGNLEVPDAPLPGLMSFGGTSDTRMSASKGTTASIVDLYGSSTTQNVGDSGDDFTSS
SGIERRGTVLSEADDFGDFGSAKLTIPADCNENSPGMAEDEFGDFGSAEVVPPIDNEIQD
GPSEEDDFGDFGSAEX
>comp4237_c0_seq1; 267 292 624 LEN=895; translated
MVLDEASERRFHSSDSNDNDEHQQPKVTYFTDIPLGLYIVRGDSVVLLGQINPEEEDETI
QQGTVIMKKLDEEAFEAMVQGDDEGDEDESADEAAKGSHLEWDFDKDLLA
>comp4237_c0_seq2; 268 108 902 LEN=1173; translated
MEENSEQQPATPMPPPPQVPPQKQPPGLVGEVPPASREAMSPVQRGRGRGRNSGDGRGGS
GRGGRSGRGRTGAGGRTIHDPNARGRGRGRNQPQNNQGTTQKIAPNTGIPFGHVPAYLPG
SSSLVEELDQRSMVVLRDGRHIVGKLRTFDQFSNMVLDEASERRFHSSDSNDNDEHQQPK
VTYFTDIPLGLYIVRGDSVVLLGQINPEEEDETIQQGTVIMKKLDEEAFEAMVQGDDEGD
EDESADEAAKGSHLEWDFDKDLLA
>comp4243_c0_seq1; 314 680 1971 LEN=2078; translated
MIQPLANLTNYCNLGCISWLTREFQGWEFIXMIFGACNVVGTAAVPKGSTNAVGNHGTVT
TCTVQGFLIYVSLMTAFSYYCSFSVYSYVGTLKNFDKSAFQNGMETYIHVLVHLYPLATA
LYITSQQLFNNTGFGYCFVEGDPIGCGGSPRATNEAIVPCDRGGQTYQTIQLVELLWDIP
LFIMLLFPTAVMITLYLKVKKRQDSILILSCEVAQQSLYYLLVIYAGVIPSAVVHTLDWF
QFLTTELATNLTLFSNVMATLCGLWTVIMYLYFSNPKTESVQKYRGGEEDGDGRENNNDS

ADELDPSLSDGVRMNSGESGANIFSFCPETETEHNSTNDNNQSNHLSLVAAGTKTTNTDP
TNASTRRRNSSTKSRNRSGSDRVSRKFSFNIFDGTNASSAYSDFIFDGDSEDEREDHRET
QQWSSVQDQI
>comp4243_c0_seq2; 404 328 1836 LEN=1943; translated
MEGHDLPLPENYYGGSSRTPLFLFEFGMRRMADSSETNEMAEPQEAEAVPPIEFTHHQLI
AGMWTTRITGVISLVSSLVVVRRALKRRRKVFHRLILGMGVHLMIFGACNVVGTAAVPKG
STNAVGNHGTVTTCTVQGFLIYVSLMTAFSYYCSFSVYSYVGTLKNFDKSAFQNGMETYI
HVLVHLYPLATALYITSQQLFNNTGFGYCFVEGDPIGCGGSPRATNEAIVPCDRGGQTYQ
TIQLVELLWDIPLFIMLLFPTAVMITLYLKVKKRQDSILILSCEVAQQSLYYLLVIYAGV
IPSAVVHTLDWFQFLTTELATNLTLFSNVMATLCGLWTVIMYLYFSNPKTESVQKYRGGE
EDGDGRENNNDSADELDPSLSDGVRMNSGESGANIFSFCPETETEHNSTNDNNQSNHLSL
VAAGTKTTNTDPTNASTRRRNSSTKSRNRSGSDRVSRKFSFNIFDGTNASSAYSDFIFDG
DSEDEREDHRETQQWSSVQDQI
>comp4251_c0_seq1; 152 136 447 LEN=632; translated
MVVRLRLQRFGRTHAPFYRMVAANARAPRDGKFLEIVGTYNPIANRDGMKEIRLKEDRLK
YWLGVGAQPSDRVAWILGKFGILPPPPSRFNPEKSRVKKAEEE
>comp4257_c0_seq1; 367 343 1320 LEN=1651; translated
MSRTPSLCGGFLTDKRSMVAFTWSVTTVLTIFAFATALVLTVKVHTHYKYLERYYDGDDW
YEQNYAYYNYDNSQDEENDNENEDSHDEKYRDQIRESYMLLSSMSAKSVTFVAFYTMVLA
TALSMYGTMAIVGFTSLRGVYIAPCFSIGSNKLKVGIFGGAIVIFANLLLVCAVVLGEVK
VAGNYNQEKEEGEGDGESSEPYEVERIATILAVTFMFLSVLYTIFAVLLFLCHAGEEQSI
LRIEQGTGGVGIGRHVNAIGTSIPPSSLTRPSAKTTPLVGLGVGVVDGNVDRSYDSALGV
GQIQSSTLGDGRGFITMDNSSQGTE
>comp4261_c0_seq1; 433 706 2207 LEN=2385; translated
MTLFWDIFFWLIPVWVFLMIPFSTFFYEADDGMLMAGTSVDPNPVKKSRILSALGYTTGV
VVFITVLYVVAYMTASEASISVQDYMGNDLVSASALGNDRRGVVITKTSIGASGNFTTAQ
LAEMEGNPYDQAYASTVENMGSDTLVMQVSISTFFAALMAWLGWFLFAVFGGIGMASMPL
DLILVFKNRPRNMDAVEYAEAQKSLRDRVNELVDIGELIKIERANNPNMGTVGGMGGYFS
AEKRKEARIERQALLEFKQGVYLLEQDVEDFKACTSDYDNYNPLRPYIALFLGICSIVIS
LVWMVHIAVYILPTPPLTPFLNSYFAWFDKWFSLFGVLSVALFTVYLLFAAVTGCFKFGL
RMACIQLHPMVLGKTYMSSFLFNTGLVLLCALPVVQFSAQAFSDYARNSTINQIFNVQIE
NLTFFGWWFTQKVFVYIFFSFAGLTMLYLLVKPKETGPSGVXPPGPVAIEEGLSKHFKQS
SASKPTIRENEEKFRKETKK
>comp4261_c0_seq2; 782 37 1787 LEN=1965; translated
MADIFLIVATSVAFLLMLVIAIYLLVYYSHPDDRNDAYFPKLVVIIGIMLTGATCLLLPL
DVANNEGYAGCDGYNTSLCGGLNMTLFWDIFFWLIPVWVFLMIPFSTFFYEADDGMLMAG
TSVDPNPVKKSRILSALGYTTGVVVFITVLYVVAYMTASEASISVQDYMGNDLVSASALG
NDRRGVVITKTSIGASGNFTTAQLAEMEGNPYDQAYASTVENMGSDTLVMQVSISTFFAA
LMAWLGWFLFAVFGGIGMASMPLDLILVFKNRPRNMDAVEYAEAQKSLRDRVNELVDIGE
LIKIERANNPNMGTVGGMGGYFSAEKRKEARIERQALLEFKQGVYLLEQDVEDFKACTSD
YDNYNPLRPYIALFLGICSIVISLVWMVHIAVYILPTPPLTPFLNSYFAWFDKWFSLFGV
LSVALFTVYLLFAAVTGCFKFGLRMACIQLHPMVLGKTYMSSFLFNTGLVLLCALPVVQF
SAQAFSDYARNSTINQIFNVQIENLTFFGWWFTQKVFVYIFFSFAGLTMLYLLVKPKETG
PSGVDXPGPVAIEEGLSKHFKQSSASKPTIRENEEKFRKETKK
>comp4274_c0_seq1; 318 126 1358 LEN=1461; translated
MLGRTAAARLAQRGARCSPAAHRRAFGSTTSLGATPPGDDKPDLFLPKITDRRLNEGGPG
GRQSNADLRVAIFGATGFIGKHLCHQLGHNGAYAYIGNRGDEAEHRELKPMFDLGHTRFV
FYSPNDVDSMREVIGDADIVINLISKTYASGQPVQTDKFPFIGWQTNHSLEDVNVRIAKQ
IAELCVEMQVDNLIHVSALGISEDHPSEWARTKWAGEQAVRETYPWATIVRPTQLFGSED
KFLHYFANMSKMYGFIPVCGDGSVLTQPVWAPDVAKTISRITDAPEKFQGRTVDCFGPSD
YTYEELGKFVLDLTEQDKKIMHIPEKQYIMMAKALGYQSFVSFNEDMAKLWSRDIVPPMT
QEEYRNQPEEGKILTMEDIGITATPIEKEAFSYLHRFRTAGHFGRVDGYH
>comp4274_c0_seq2; 169 126 470 LEN=739; translated
MLGRTAAARLAQRGARCSPAAHRRAFGSTTSLGATPPGDDKPDLFLPKITDRRLNEGGPG
GRQSNADLRVAIFGATGFIGKHLCHQLGTYRLSRPARLRHTHHSWNPINRQTDR
>comp4275_c0_seq1; 103 151 464 LEN=464; minus strand; translated
MGFHGAKFPLPFGPAQGEEGMRKNIEMVKKWREAVGEDYPLMIDCYMSLTVPYTIELARR
CEPYNVKWIEETLPPDEYDGYAKIKEKVSTTLLTTGEHEYTRWGX
>comp4300_c0_seq1; 456 191 934 LEN=1046; translated
MTIALSAMPVTIRGPANVDECIMSSYLEYQSFWPSIPPLALTTSSSTSTTVSGTSISTRQ

RPSHSDLVAKASDISGRKLCLPPVKSSRKRPSLIDDEPSPAEENHVNSGYCNSRKRIKLC
SRTFTLALNPDAATRNIPSTTLIMLPSFESKGNDKLTISDDEDADGDDCCHDSKPPPVGF
SKKETITPLSSPPSSRFFEEEEYVDGLHQRDNFFPQPPTFRRRLTIRNSSDLDESIEEFT
HRRLFSE
>comp4301_c0_seq1; 459 1 1534 LEN=1665; translated
XDDEEEAEYLSDTDADDDWATEEAPFSEREWPRRAGRFLARTWGLARGCLSFVLNVDNVW
DSPAFATERGAGGASKRKKCVVFFWFAVLAGSYTIERVSFKLLVDRSGPFRLVAVSAVVA
FHALLAGGSLLLSAVWSGRRGEREGPWGSSGAASAPALRALGIPVVDAGLMALLDTVHMM
LVFLTGYHVAPTQTVILVQWTLPLTALLSQFFHGDGCVAACLPHQGPSERGHPSAGAGTA
AGRPLPGWGGLSIEHVVGSVVISLAVLLALAPSIYTTLVDNEYFRYASPTKIPTQTAYNT
ILFVSSCLPAAASQLYKEHVFHKHKQPVHQDHLNFVLSVFQLVFAAIVSPLAYVLLGFAA
SDDWTELYPSSGFGKNLAEGLECFAGVLDEEEAENGFYDEANCRNSFSLVALYAFSVIGV
GVAVDKIVNGGATKVMYRGVSAGIILSVTSLYLYDLHIPDFNYGPAIDSLNLVCLILLVV
GSEIYHRVSLQDASFETVYPEIESFFDDITE
>comp4301_c0_seq2; 430 1 1649 LEN=1780; translated
XDDEEEAEYLSDTDADDDWATEEAPFSEREWPRRAGRFLARTWGLARGCLSFVLNVDNVW
DSPAFATERGAGGASKRKKCVVFFWFAVLAGSYTIERVSFKLLVDRSGPFRLVAVSAVVA
FHALLAGGSLLLSAVWSGRRGEREGPWGSSGAASAPALRALGIPVVDAGLMALLDTVHMM
LVFLTGYHVAPTQTVILVQWTLPLTALLSQFFHGDGCVAACLPHQGPSERGHPSAGAGTA
AGRPLPGWGGLSIEHVVGSVVISLAVLLALAPSIYTTLVDNEYFRYASPTKIPTQTAYNT
ILFVSSCLPAAASQLYKEHVFHKHKQPVHQDHLNFVLSVFQLVFAAIVSPLAYVLLGFAA
SDDWTELYPSSGFGKNLAEGLECFAGVLDEEEAENGFYDEANCRNSFSLVALYAFSVIGV
GVAVDKIVNGGGESQATKRSSAGHGKFGSHFLFCFFYWTFSFCLGSRLTATKVMYRGVSA
GIILSVTSLYLYDLHIPDFNYGPAIDSLNLVCLILLVVGSEIYHRVSLQDASFETVYPEI
ESFFDDITE
>comp4303_c0_seq1; 250 1 639 LEN=639; translated
KKKKKKKKDDSDSDSSGSSSSDSDDSHKKSKKERRRRNDDDRTYFSDRKTIDTRSTAEMT
DWSRMPPPSYAQPVQTMPIIRPVIYSEYDYHGLRNSRDIFEDEQALLLSAKRAAKLARFE
SRMKTSKLSSLKGVHKETAARLIKAKQKAKKIEKKAVSEIEQTEKEKMKMSRSMRKLKRE
KEKMAEKLAVMEEERSESKSQQQHNLQQQQQQQ
>comp4306_c0_seq1; 331 41 694 LEN=1211; translated
MKISSSIFSVAAGLLAVACNKSSTLVSAIEVGDSLPDGLTLHHGFPPERIALADRTKGKN
VLLVGLPGAFTPTDPVAXVPGYLANQKALKELGVDDIMVYCVNDGAVMAAWAEDQGVKCM
ADAEDDEDTILHLYADPYGEVTAALDMEMTAAGPKMVGLINRSKRFALYIVDGVVQIARV
AEADDDPAGDERPDITLAEAMIDAIGDYNKKAAGGEL
>comp4306_c0_seq2; 315 41 895 LEN=1412; translated
MKISSSIFSVAAGLLAVACNKSSTLVSAIEVGDSLPDGLTLHHGFPPERIALADRTKGKN
VLLVGLPGAFXADLIQSPGSRXVVAAALASLITRVVLFHSLHFNGPFVSVSVSISLRFGL
FLTTRSCLRDVLYQSRLPAFHELXVAPQGYLANQKALKELGVDDIMVYCVNDGAVMAAWA
EDQGVKCMADAEDDEDTILHLYADPYGEVTAALDMEMTAAGPKMVGLINRSKRFALYIVD
GVVQIARVAEADDDPAGDERPDITLAEAMIDAIGDYNKKAAGGEL
>comp4307_c0_seq1; 232 1 406 LEN=406; translated
NNNNNNNNEDYCLNSCLLEDGLDYCIEGEEEGQMDIQEFAECRALEVEGQNNNNNQNYYA
NGNSYVQYYTGAYCTSSGVYAGIFTDPACTKHAPKGTYEKINYGYSLPTTPLVSSTCLSC
KANQYDNDNNNNYNYX
>comp4312_c0_seq1; 482 1 1672 LEN=1672; translated
XRATQGNFIKRESPSADNGHNISNRXGPNPIRDERRPIMNDPEAKNHIINDTNDVDILQR
KAHIMRSPVTVAAPSIAPSTDADDEDNGPTVQRKRRSLECEKQHRRQNRNRCDANKRALF
LLATTAAAAATICLSSTSSMNMIFVESFSFNPFRGGLRWRGRTGGGQTISSASSSPAEEE
IEKMSKSLQNSIEKNYNSAKSTRNATPSLVDWKVQSDGKITGRSMNHPLVPDGEIITTSL
VDPNCLEGGVKAGDVISTVRGTKYELLKTPPVGSSYFSSASNSQTFSEDVFSYTTTLPYD
QQLAFLEELTDMIKTKQAAVEGTSSATVSQPASSSIAGSTVAPVAAKTLDATPSTAAPEV
DTSTKIQEQPEQPTQGAIQRGEEPPKRPTYSISNIQQNQEQSKSKDKSSSTSNNNSVLGG
VATLAAATTAAATVTLGGQAGSEFLDGAKRQVEAQVPPQAKELIASVNTQVQKQSKVVLP
YLEEQIKAAEAKLEEAHLEEKTKTAWNGLADYLNDQEIAKRETVSTTPITSSEPGEAITS
ARISVGEAGTRMAAVEEGX
>comp4319_c0_seq1; 401 98 925 LEN=948; translated
MRSYRQKVLSIFLFVTIFWYDGFTRAYVFRGCPSRSTYSFHGSYENLSSMRLNFSKDSGR
LSDLSDFGASSFDELEEEVLASTRAKMDFQKVKMTITEQPPPLSNEPQRTAIMAAQWKIS
LAAASFVSTFVTIATHSNFLITSVVFVSVFLVANQDPLEEEGAAGALARLLGRLTIQSVE

TTKPKLRAMAKAAITSEEESQEQVIRLLQQRIEVLENENADLKQWKKQRNSADTLLSQYA
LEELKAMARANNLPVGGTKIQLLMRLLENRVLIPM
>comp4319_c0_seq2; 200 253 516 LEN=539; translated
MAKAAITSEEESQEQVIRLLQQRIEVLENENADLKQWKKQRNSADTLLSQYALEELKAMA
RANNLPVGGTKIQLLMRLLENRVLIPM
>comp4320_c0_seq1; 299 39 695 LEN=805; minus strand; translated
MHAGGSSLKLLDFFGDDKLFKSGAERLVIAGGLKYLFPIFIGTRIPKPDVRVSRSAKAKR
KWLAELKAQIIRIFYALSFQLDAQSPKDAMSRFVAKFVEDDLKYCDRLVELLLVYDERKR
KAEYSFFRSDIEDVLDANQVTLASFEAKLNGGGDICNRLAAITAFVCANSKRCHERISLQ
LRMQQSGVSLIKDALNEFAASLESGRQKRHIESLSEQI
>comp4326_c0_seq1; 138 1 450 LEN=450; translated
QHQHQQKSKSKSTNAVSLSRLCSTIETIECVLGIRGQSMNGEKTNQNSIVGSTNKIQISK
QDPSIPCVGTGDKDRVDSGDGIAPTAANTTASTAAGEKQEGNGKTNANSREINEDAIGVN
NILTEVDNAVIAAQTIAFQPTNANTNTNTN
>comp4332_c0_seq1; 169 1 494 LEN=494; translated
DELDVTFHEASRSQKLSQSNGPITGILXRKNNSNNQAEAHTTEPIMSDNDRRGSSLFSNS
GAGPSGSAPEGQPPPGAQRRSSSVLQPGALGGIAFGIDFDDSDYEDSDDEDNEPAAAAPG
AGTGGPNHRPYVGGFAAAAYEAARMHHTQNTLKQQQKQKLQQQQQ
>comp4334_c0_seq1; 74 1 381 LEN=1034; translated
AITYPKRMRKLFQEAEFQAARDNKIQAVLPNLPVFRNNFLTCNTAEGIPWTPPENNDDID
QRISVGQGTENDGEDNAQPSNNSNRQITNSNRVSEHQNPHWLTTCSPVSLGSTLAIGIPA
VIQVFTW
>comp4334_c0_seq2; 91 1 465 LEN=1118; translated
AITYPKRMRKLFQEAEFQAARDNKIQAVLPNLPGEYPLVLGTIRFLDCSNKLTSGFFCRA
QVFRNNFLTCNTAEGIPWTPPENNDDIDQRISVGQGTENDGEDNAQPSNNSNRQITNSNR
VSEHQNPHWLTTCSPVSLGSTLAIGIPAVIQVFTW
>comp4336_c0_seq2; 147 1 602 LEN=906; translated
XPIHFRQSSAKIKRQNRNQTHEAIPQKTSETTKMKNQSLSLALLATLTSFLVGRVVSVDV
DYEALNAAAEKNPAFRRSLENRHRRSLQNKDKPAFDFFDPDEDTCDNSKNPTEDRALLSA
CDFSRFPTCGDNEEICYNRKPSRDHFHRDNHQHKFYIQYDRVFCYPDSWGGCSSCTPGRY
CKSEKRCILEEVGYPCQEWF
>comp4337_c0_seq1; 508 546 2449 LEN=2893; translated
MTRIQSMTIDPGAGLSVTPQDSDEEDDNDEEQQGDHQLDLHHVNNDDRDGSKPFMTSSYL
AAVSPKDHQRRKKRKNNKAMKDISFLRKRTSTLLALTEPESYLSGQQEAPHQQAHLSRGM
KINLNTFNFLIDGWAFSGESDACDKAMILLERMEEMYYRYGEDSPVCPDVRSYTKVINAL
SRSKHRDSANLAEQLLYKMEAMAESGENPSAKPNTFTYTAAIEAQSYSGIKGSPEKAEEL
LEKMIQKYQSGDPDVVPNARCFNAAISSYAKSALPGAAQHAEYLFERMDGLYMSGVEEAK
PNSFNYNSLITAWANCRDQEDENGVCSARKAQEILERMEQCYAAGDESCKPTTVSYNAVI
DAYAKSKQEEAAERAEQVLRRMGALYKEGADIQPNTRSFNTVINAWAKSGRADAAEKAQD
LLDFMTNLYEDGNAAVRPDAHSFCTVINAFARSNQLTDKAERANNLFRSMMDAYEKDPKM
SHLKPNVVAVNAVMNACAYTIQGDINEQNRAMEIAHNLLKILEDSDYGSADQITYGTFLK
VCANQMPDCATRQQIMEVIFQNSARDGQVGNLVIQQLRAMGPPELYYRLTGHYIEEDIRM
EDLPKEWWVNVVEXTMEEAQARRPPQQLDQHPVE
>comp4337_c0_seq2; 491 546 2366 LEN=2772; translated
MTRIQSMTIDPGAGLSVTPQDSDEEDDNDEEQQGDHQLDLHHVNNDDRDGSKPFMTSSYL
AAVSPKDHQRRKKRKNNKAMKDISFLRKRTSTLLALTEPESYLSGQQEAPHQQAHLSRGM
KINLNTFNFLIDGWAFSGESDACDKAMILLERMEEMYYRYGEDSPVCPDVRSYTKVINAL
SRSKHRDSANLAEQLLYKMEAMAESGENPSAKPNTFTYTAAIEAQSYSGIKGSPEKAEEL
LEKMIQKYQSGDPDVVPNARCFNAAISSYAKSALPGAAQHAEYLFERMDGLYMSGVEEAK
PNSFNYNSLITAWANCRDQEDENGVCSARKAQEILERMEQCYAAGDESCKPTTVSYNAVI
DAYAKSKQEEAAERAEQVLRRMGALYKEGADIQPNTRSFNTVINAWAKSGRADAAEKAQD
LLDFMTNLYEDGNAAVRPDAHSFCTVINAFARSNQLTDKAERANNLFRSMMDAYEKDPKM
SHLKPNVVAVNAVMNACAYTIQGDINEQNRAMEIAHNLLKILEDSDYGSADQITYGTFLK
VCANQMPDCATRQQIMEVIFQNSARDGQVGNLVIQQLRAMGPPELYYRLTGHYIEEDIRM
EDLPKEW
>comp4344_c0_seq1; 283 283 1401 LEN=1651; translated
MILFHTTPKHKCFMWFLLATVAFAGENKNFPNLRGKLSGVDDPQAQAGARLLRWQESSTT
TAVTTATATTTTLNIANLAIGDMECEEAAGGSVVCAFRTVLPTSASNLNTGSVLVDCLRQ
NGSNSQYCVTTEVYRSSYQSIPEPKPQPKPEPKPKPPINPSGGPGSGINPNIPRPEPIQN
VPDVPNVPSDPAPPQVPPVTPAVMLRADGCPEKTQLTGVRCDDFVPPGASETYCNYFSSG

SSTILRCGCTLRVGGVPARWVCGAVEEAEEEPPLAVSLQPPAPIGGISANVQSVVVPPPD
PPGQILPTINNPAYCADTEAECDGASCYMHQRCTYYVLDEETGDPTGANECGCDPMNGIY
KCYKSPNPIFNP
>comp4344_c0_seq2; 216 283 1587 LEN=1837; translated
MILFHTTPKHKCFMWFLLATVAFAGENKNFPNLRGKLSGVDDPQAQAGARLLRWQESSTT
TAVTTATATTTTLNIANLAIGDMECEEAAGGSVVCAFRTVLPTSASNLNTGSVLVDCLRQ
NGSNSQYCVTTEVYRSSYQSIPEPKPQPKPEPKPKPPINPSGGPGSGINPNIPRPEPIQN
VPDVPNVPSDPAPPQVPPVTPAVMLRADGCPEKTQLTGVRCDDFVPPGASETYCNYFSSG
SSTILRCGCTLRVGGVPARWVCGAVEEAEEEPPLAVSLQPPAPIGGISANVEVLQSTSAL
YYTGNRSHVCLHCFDSVVSYPYFSPLFDPLLKYAWSHNNTLQFTTMNRKSKRSKAVVVPP
PDPPGQILPTINNPAYCADTEAECDGASCYMHQRCTYYVLDEETGDPTGANECGCDPMNG
IYKCYKSPNPIFNP
>comp4349_c0_seq1; 218 293 682 LEN=898; translated
MSSMNFSTSKQYVRPPQRGIFPLDHDSECRPFMEKYLECLNDSSQVHHKCKPLSKDYLQC
RMDRQLMSKENLDDMGYAKNKDVVGAKEYDKAKEKAGFIAGKHIEKSSKWWFQSYKAKWV
GDDKEGEAK
>comp4349_c0_seq2; 195 306 473 LEN=689; translated
MGYAKNKDVVGAKEYDKAKEKAGFIAGKHIEKSSKWWFQSYKAKWVGDDKEGEAK
>comp4355_c1_seq1; 463 156 1184 LEN=1422; translated
MMMRITTVVGLSTAWFLCYVANIGSSVSATRAFHGQSHSFGVLSSSMGKIIHPPFLRACT
SRALLNTRGGADDSDDEEEYSEEDSYDEEDESDDEDLFDGFDLDEHSSDDFKEDNKFDQI
VEEYHRTPPITKAYLTSSFAVTALGYVMTNNEFPPILTLDWNKVFKGGQIWRPFTAFLNL
GSLGLIGYPMTIHFVHQYMSFLERLAHNKPYDFWIMILFGMTSMLVGYPIMKMSPRFLGH
NLSTFLVYIWSRMFEGVDVGVFEFIHIKAELLPWFLLAQTFLLEGEPPTLDFLGIVFGHV
YYHFRKVGMLSAPQWLIDWYDNSPKAEILRKKYKTISSDFGV
>comp4359_c0_seq1; 243 1 572 LEN=601; translated
XRHNQPGSDFDCRPISSQIFVDRILSAVAVVADPKTPLVVCSKEFDSSQPAHRRSFRIDI
IETFMMEARKRGGTSRESVGDYDIETNQGVGAVDMASASPYSRMAKDPVEVVGGAPDGIL
APGPSPAEDSHIRSLAKGFTWRFVASGTTIAIAKIVTGETTIAFQIGFIEFFAKIMIYYL
HERIWAKIRL
>comp4361_c0_seq1; 470 98 1600 LEN=1600; translated
MPPVMRYFVLFICFGLKGGVAIDCGVSYSNSKSAGKQDDVQFTECEPVQQRNESGIHFQG
PSRAPVSPPVDIKIPIGSAFPPTPENTIAGRVPVLEPKEAASSPHSIPSFRPTLSATLVS
NVIRSATDAPPGDTNWCPQAVVADEYACASILPSGASWGQCTYLVVLKMNGSTSTESQTC
TCTRSNPFWSCQVDNFSLTPPSGSAPSTLNTAFPSSTPTTRSFDNVVIPTITSSTQISSE
SPSAIQTVDRTDIPTISPSVAYSSEPTGSPTAGPSLLSSDSPSLQPTSTSSIGPSNIPSD
GPSLKPSDGPSSRPSIAPTASPSLLPSDAPSLVPSEGPSVHPTRMPTIAPSATPSDGPSL
RPSVGPTAGPSVLPSDGPSFVPSDDPSLQPTRTPTIAPSFFPSDGPSLRPSSAPTTSPSM
LPSDGPSLMPSDGPSLSPSVAPSTKPTGVPTIAPSSVPSDGPSLIAGGSRLRPGKAARRT
PFPTDTPSLVPSDGPSLMPSD
>comp4363_c0_seq1; 91 1 534 LEN=761; minus strand; translated
LVYWNFFLDFGPLNLGQLWRFTKKLNRLLALAEKDGSTVLFYSTTHPNKRACAIFLICAW
QVLELHRTPDDAMFGFSATAYNANHSTAKTNGSNASDVNRSNPPLLPLSGLGSQTINPLP
PFHDASPYKCTYQLTLRDCLGALVKARQFQFFDWGDRFDIKEYEYFEQVEVRRTNQV
>comp4365_c0_seq1; 427 187 1416 LEN=1734; translated
MASDHCSDLKPAKEGIEFEALPWNLNHPEEHSYVHLTTKTGEWTKEHYDPTTDTGALFDS
VYKYDETDLPLSPACCSLNYGTTIWEGLKCYRDKDGNAIVFRPDRNFHRFSNGAKNMVLP
PPSRELFMRGIQHALQKNSHLIPPHGEGMKLYVRPMLMGSGEQLGLYPSKQFSLIFYVSP
TGNYFKSATGGLNLHLETRRCRAARGGVGNVKCSGNYAVALKPLMDAKEQGFHDNLFLEL
ETYREKGSLDSAILQEMSAANIFFVLKTGEIVTPSLDRGTILPGVTRDSVITIVNEFADD
LKEAMMKSTGQDANITVSSRDVTVGEVKNATEAFCTGTAAEVVPIARLATGTDEEPFEAV
FEHGKTEAGPITSAVLSMLREIMVGDRSSKGTEGWLRNPFAPPEEFCKE
>comp4365_c0_seq2; 253 123 867 LEN=1185; translated
MKCTAFAQLLVCIEFKLEQGNYFKSATGGLNLHLETRRCRAARGGVGNVKCSGNYAVALK
PLMDAKEQGFHDNLFLELETYREKGSLDSAILQEMSAANIFFVLKTGEIVTPSLDRGTIL
PGVTRDSVITIVNEFADDLKEAMMKSTGQDANITVSSRDVTVGEVKNATEAFCTGTAAEV
VPIARLATGTDEEPFEAVFEHGKTEAGPITSAVLSMLREIMVGDRSSKGTEGWLRNPFAP
PEEFCKE
>comp4366_c0_seq1; 233 34 1226 LEN=1226; translated
METRYYLHQHCRNICLAILIVLLGLLVEPTSSFLPLQRLEHKCRITKDVSASVLDDNTIP

ESSPPSIHSTGEGSIDRRPHEQRTTGPRAARRMNHAFRYLYRTTSHLYENVTALEYLTTF
YSEEQVMEMNRTFPPLLDLDVSRHVHPKIRFLQETILQSNRDEMPLDGSPRQNKNADTLS
IMNLSDYEIPAQYFGARLEKTIAPSHAFLEYMDLPHGLPLLMRKDDNDERNETHRGGKRT
TTLWQDFLISCRRTKQFCALCNQWQREKEHKLGNSDIPRAQITAKQIEAFHFIFGRGLMA
ACRGELVQASNPWPLEHVNITSKEVLELLIKHGANPLEKDIRGTTLLHWAAGSGNAEAFE
TLLPYFPDRLLEKTERDGATPLHWACAGATSGEFGTGG
>comp4368_c1_seq1; 190 1 1087 LEN=1159; minus strand; translated
QLQELLVSQWGQYRPTAINGLRVVEVVGLALLTGALFYDIGNDTTATGFVKVNSLLFFSV
HYGEFLEVFFCDVRCRILRSCKIESYTFQAIVLLRLLTPTLFAPIFHYRTFTRMYTSVGS
TYLWYIAVEDDVVNKRNYAVGPLYLSRLIVVMLAESFWPFLYVFVCYPLASVVGDWTVVI
KIGMLLCLNNAAYISLGSCLGVLFPSIPQGMIASTLFSQTSLVAAGFYTTLPVYLSWIRY
SSPVFWTFSGIVKAAYTWSDTYKCIKGSNLAGNNRCFIETSPVIDDMKERGINAAVYGDA
QSDNIWLQVCMLILLLFLLQTTIYITVNVRCSRRKKAKEEGIDDSERSAGSAESKQCKMS
G
>comp4371_c0_seq1; 589 167 1360 LEN=1488; translated
MAPKEKTKTDPAPRGQKARQASAGALYHDLLRDLTDHNLFFDELVDMIPSKLYIEGQSGD
DYNPKYFKGQAKESKEARRARNKQSKRAKFDPASAETTTQIKQRLEGGAGVPQAPTAPKR
PSSGAAKATAGTPDARASRIEALREKLHAKLAEKRGNRPVDPGSVSKRAARRAEKKKRQE
EAIRRNKKKAASSAEAGSHKRPRLSVGASGSAGDAASDLAMVDFGRLSGLNPVGNGNYTE
VNKALKNLSKSKNLEKMLADAESKKKRLEELKQSDKQEDKEKAASIEWGDTLREATGERV
KDDPAKIKKALKRRAAKKLKSSKAWKTRMESAQSKMDERQKIRNHNLTKRKLGGSAGANL
SSKRIETEAEADGKKKTGRRLSRAGFEGKKQDFLNKN
>comp4389_c0_seq1; 304 20 595 LEN=647; translated
MGVNLRLQKRLAASVLGCGKRKIWLDPNETNEIALANSRRNIARLQRDGLIIKKPSVVHS
RARVQLRNEAKRKGRHTGIGKRRGTANARLPFKVLWMRRQRVLRRMLQKLRNAGKIDKHI
YHSLYMLAKGNQFKNKSVLLEKIHELKAVVVQEKALAEQATARKTRAKNRLERRAAKEAK
KAADAEAASNL
>comp4392_c0_seq1; 499 121 1494 LEN=1699; translated
MNAQTEKMGDPNHRSAGATASASMELHQQHQQAPMGSSGPPHMGLGGHPRYSQHAEQELR
PGGPPPPMGYGGPSALSHGSFGNSYSSYPPAPYGGDGGYGGAPPPGSHPPGYPRGAAAAA
AAAAYSMPPHSFPYGAAGYGPGGYPQSAPHPAYPYGGGYEGHYGSQGANLHRPPGSFRGP
SPVNPSAAPGRGNGRMQNGLLPGAVAPLSDLNSLVSGGSSALTKKGGSSTAGTKGLPGSA
LSDANGKRSGPQQSLKPDQGEVERLRAAAATEITTEEVKPIQTDFHFFVRENIEQYRSLA
EEEVKRSMEDQSATPDPMLVNSNLNTRLMSAWECLTKETRDTFMTHEETDRRRFMEDDEI
ASRHCATLTARGKSPRAVGGDSLKELAALQRQKHQEQEKKKQESGTDGGTPNTVAAKTKT
DGEEKKAEESAKEESKRPGDSGGDVHESPAKKNKVSQ
>comp4395_c0_seq1; 374 1 603 LEN=906; translated
VNMYAQYFIGAKCSHKDGKSIHLGVFTDNGCTNSAGSGVYEAFNYGSSLPYSSEPIIAVN
ECLSCLQVDEDANDNNNNNNNGNNNNNQNYNQNYETTELCQQSYEMSAKCETKLSNYLGQ
YFYADTSACDFINNILPNLHNAARKISGGSTSSVGGSSAATAFAVIFGLTTALLGAYSFF
LYRKIHRAKVNLAQAEMGIA
>comp4401_c0_seq1; 1473 64 2193 LEN=2767; translated
MPAESESYAFSADINQLLSLIINTFYSNKEIFLRELISNASDALDKVRYQSLTDSSVLDA
DSELQIKLIADKGNNTLTIEDSGIGMTKADLINNLGTIAKSGTKGFMEALSAGADISMIG
QFGVGFYSAYLVADKVEVVSKNNDDSVHTWISEAGGSFTVTDTEDSGLKRGTRIILHLKE
DMSEYLEEKRIKDLVKKHSEFIGFPVMLYTEKTTEKEVTDDDDDDEDEDGDDDKPKVEEV
DDEEEAKKEKKTKKIKEVSHEWEHLNSMKPIWMRKADEVTHDEYVAFYKSISNDWEDYAA
VKHFSVEGQLEFKAILFAPKRAPFDMFDGGAKKKHNKIKLYVRRVFIMDNCEELMPEWLQ
FVQGVVDSEDLPLNISRETLQQNKILRVIKKNLVKKCIEMFNDLTENEDTYAKFYEAFSK
NLKLGIHEDSTNRAKIAKLLRYKSTKSGEEMTSLEDYVGRMDDNQPGIYYITGESHQAVK
TSPFLEKLQKKGYEVLYMVDPIDEYAVQQLKEFDGKKLLSATKEGLDLADDEDEKKAFEE
AKEKTEPLCKLMKEVLGDKVEKVVVSNRLADSPCVLVTGEYGWSANMERIMKAQALRDAS
QSQYMTSKKTMEVNPTNSIISALREKAEADQSDKTVKDLIWLMYDTSLLTSGFSLDEPAT
FASRIHRLVKLGLSIDDDDDADGEDDMDDLPPLDDDDDDNEESAMEQVD
>comp4411_c0_seq1; 33 1 925 LEN=1181; translated
RIQYKPNTDRTEHDEDTKSKSQQNKAARMNKLQNSRNIAEWIIIASAIAIVRCHVSNLAS
GIVGAAAYDGTADRDPGDSAKNIGWDPKRSHPCTVETISMEDFLKKYRKQGGLPPLYPRP
LVIKSRSGNPQRNQRFQELTREDTIRDSFPANFTVTLSSSNSFSEHRRTIPLSQYLEEVA
TGPPLDADTKSNETWYLFGETYSDEWKSLLSEYQVPPCQACYDESLVALSFGIGNSGSGV
QWHIHGPGFSEAIHGRKHWVLYPFEDQPAFHPDQTSLNWMHYNYPSFEQLEDRPLECTLD

PGDLIYL
>comp4411_c0_seq2; 54 1 1039 LEN=1111; translated
RIQYKPNTDRTEHDEDTKSKSQQNKAARMNKLQNSRNIAEWIIIASAIAIVRCHVSNLAS
GIVGAAAYDGTADRDPGDSAKNIGWDPKRSHPCTVETISMEDFLKKYRKQGGLPPLYPRP
LVIKSRSGNPQRNQRFQELTREDTIRDSFPANFTVTLSSSNSFSEHRRTIPLSQYLEEVA
TGPPLDADTKSNETWYLFGETYSDEWKSLLSEYQVPPCQACYDESLVALSFGIGNSGSGV
QWHIHGPGFSEAIHGRKHWVLYPFEDQPAFHPDQTSLNWMHYNYPSFEQLEDRPLECTLD
PGDLIYFPDMWWHATINVDDYTAFVSTFTQEHLFLDRSETKKAWA
>comp4416_c0_seq1; 909 1 1330 LEN=1511; translated
XSHSFTSIIKMQFSKAVLCLLASAASVNAFAPAQSFRGLPTALEAKKSIEDLSDDELKGK
KVLVRCDVNVPLDGKTITDDTRIRSSIPTIEYLKSKGAVVTVCSHLGRPDGKEDKFSLAP
CAERMSELLGQDVTLAPDCIGEEVSKVVDAASEGDVIMLENTRYYKEETKNEPEFVEKLA
APFDLFVNDAFGTAHRAHASTEGVTKFLQPSVSGFLLAKELEYLDGAVANGEKPMVAIVG
GSKVSTKITVLEALLDKCDTVIIGGGMVFTFLKAKGLEVGTSLVEDDFVDTAKEVMAKAD
KLGKTILLPTDIVVADEFSADANSKVVTADAIPNGWMGLDNGPETTAAQKECLSTAKTVI
MNGPMGVFEIEKFASGTFDLVNILADITKENGCVTIIGGGDSVAATEQSGRAGDMSHIST
GGGASLELLEGKVLPGVAALNEA
>comp4417_c0_seq1; 231 194 633 LEN=1177; translated
MARQVARIRKSSFKKLTFENDRREGETKSVQFGTIEIRDYSQCLGDHPDVNRGAPVSLDW
DYQSEQSYDIEEYEQRYFRKKRTHQQMIRLAFERKHILKTLGYSEEEIETRSKSVKKDRK
KRXTNEADDDFRAFHNDIGENATMDD
>comp4423_c0_seq1; 336 384 1196 LEN=1384; translated
MFFSRLSFHYNDDKSAIHGTNTSFKAVDKVACEDDLTELFPSNRLKALWDWRDRKKKLGI
LVSKYDHCLWELLLRHKARELDADIHVVISNHDALRPIVETFDIPFHVTPIEKPTSKAGA
TSEDDYTRSDKSTLPLHELRQLELLKEVDVVILARYMQILSPEFLNHFPPDGIINIHHSF
LPAFIGGSPYHQAHERGVKLIGATAHYTTEELDDGPIIEQDVTTVSHRDSVKQLMKKGRV
LERNVLLKAIEAHLDDRVIVHGNRCIVFGD
>comp4433_c0_seq2; 318 276 878 LEN=1466; translated
MKYLLFSLFFMLIGSGYSFSTPSRVFVGTPTKTRTRLVQDCMTPNPKTLKSTDTIEDAIV
ALLSAGFNGAPVIDPITNNLVGCVSAFDFIQREETGVLLPFDMEDPENQREMSINARKIV
ATTVGELATEPALTIHMDTNMKDAAEMLTQKQKHRLCVVDDNNNLVGILSTSDVMRNIVE
VLKALPETSSAEMSNSELAP
>comp4437_c0_seq1; 665 1 1739 LEN=1739; translated
XQGSGSLSNTERPSSPIKSIKKSIRNRLQRIKDGKESRQRHSQRSGLRSRPSKEEKQEKT
QEKKQLQLQQQKPQQKQHPFNATLSKQKKSPQNAVGSSGKKQKVPAASSAKQVSSKSNNI
SESNAAIANRNTKPLVKNFISPPSQTLQASTRTEERREEKSPVRNRREKRNKSTKKHPSQ
DAKTKQRNSFRSFSESSDDYDYSDHTGLGANRTYDSVGDEEDDYLDRRYSRSYDTRGWSY
STRTGSRSSSSSSSDGSYDSSSSFDDDESTISATKRTMRMAVHRNPSDDASSGRGRSRNP
RRRPPPSSPRAREDHISSPRRNPRMQTNRFVDDDYYYEGTNTNTDDESQSCYSGISSGNE
YSRSCSSGVTSGGTFETNDDDDYSSSSSNDADERDFRNPGGGRRGSNPHRKHPVSDDEDQ
SECWTEAPVRTYDSEEESDRSSGSYSKKNHGQCVLDPVTTYDCEEGYGNGSGLGLDLTRT
QTDDQTYTSRDIDPEEFAAFVQEQEAAEQRAERKKTDKSEGKDAAVGSGVFSWIFGGTAE
TTEPEERTAPASPARRGRSPAPVRSPRAHQGDTDRRAFFD
>comp4443_c0_seq1; 429 1 950 LEN=1044; translated
XGGASSFHHDKVSLTGEELAALPTLLIQMAGDEALNRAVAEEHGGPAGVPGLAGDLDPEH
PLDVIVAVPPSHYMEHLSTGRYQNRIYATEKYPDGSVLGANTMMGHDILFDPTQNRIGWA
ESSCDYHELATKHGFPDVLGDELQTAETKEAIATEEEEEEDQVEAKEAFDVDHVEAEIQE
EEAVAVAAGAEHQQHEQHNNDDEQGNDGPAGDTPEEPTAPSLRPADASKSSTAYIAEGPE
GPQTALLALGLLLAVCAVCGCLYRRCCPRGPPARAAYHRAPEIELNGRSSYKDGFGDEDL
EEEDDDVADEYGDKYE
>comp4443_c0_seq2; 267 212 838 LEN=932; translated
QFDPTQNRIGWAESSCDYHELATKHGFPDVLGDELQTAETKEAIATEEEEEEDQVEAKEA
FDVDHVEAEIQEEEAVAVAAGAEHQQHEQHNNDDEQGNDGPAGDTPEEPTAPSLRPADAS
KSSTAYIAEGPEGPQTALLALGLLLAVCAVCGCLYRRCCPRGPPARAAYHRAPEIELNGR
SSYKDGFGDEDLEEEDDDVADEYGDKYE
>comp4469_c0_seq1; 468 1 922 LEN=1184; translated
XGGWGNLGGGVTQLIMGSALFPLFKSFYGGDASKAWRVVSVVPAVVAFGTGIMIYKMSDD
CPKGNYSDLKKHGQMAEVSAGASFRQGAFNINTWFLFIQYACCFGVELTMNNAAALYFKD
KFELSTESAAAIASIFGFMNLFARGLGGYFSDKGNQYMGMRGRLWAQTICLIGEGVLVLV
FAQTNSLGAAITVMVFFSLFVQAAEGTSYGIVPYVDPPATGSISGIVGAGGNTGAVCFGL

GFRNLEYKTAFMLMGFTILGSSALSLVIFIKGHAGLVCGEDAPKEATTATLAVPAKDAEA
SEDVNEE
>comp4472_c0_seq1; 681 89 1684 LEN=1889; translated
MEGTTTLADALLGDLDDLMDSDDEDQNEVAESEEVDASKDANNVGESMDGMIPPSPFKSS
SMIESEKTRTRKRAQSQFLDNRSLKNHLKIIREQNDGNSTVNDNSNTNSSVGAVRRRKEE
EEENHQMVVQSNKYLASLAEEMQRAHSKLADTYRPKFPELEDLLPNPLDYKNAIRIIGNE
TDLTKVNDELNEILSSNQIITLSMASSTTSGRVLTEKELEEVDAAATYTEELLLVQKDLM
KFVEDSMESLCPSICALIGAPTAARLLGLSGGLQELTKIPSCNLQVLGQTKVNSESRAGL
SGGSIRQHTGLLADCDLIQSLAKAYKKKALKVVAAKLALVARFDFSNVDSGRQRSASAGI
SYRKQLEEKFEKWQEPDRAPVAKALPKPNLEAKKRRGGRRMRRMKERFEETAMMKQANTR
AFSTQQGEYGDDAMGITMGLLDTADNGGAIRKVTEKRKMRQANTKASRKRAMQMAQQAKN
KDGLASSVVFTQTTGMELINPEAQKERVKAANDKWFNSNAGFQSALPKKGT
>comp4473_c0_seq1; 193 151 759 LEN=1007; translated
MGTTIIFHITLDYLAILVAFSFLFTPVQSMETPNGYLCNLCYSRENEYHYPPPSIEQKVV
RFTSPISSTAAKSEQQERESTTVTSINVAFGMPWDIGTTCIDVWNKVLDYKNPNIVDESS
CRSMAALFAPQCCNQHEHEREREEVSVKEASFTGLEENKTQTELRESIGSDSHNLQRRGR
RAAATSYNLRGYSLTGYEIKRR
>comp4476_c0_seq1; 261 226 909 LEN=1167; translated
MSVMKKRILLVGLCLSTSMVGLAHAMENVGLEIMEAESSQNTPASPRTFVRSFLRTKSEK
SNKKSKQLLGSKKSKKDKKEKKSKNDSNKPTPSPIPSPTNNPTKSPVQPTMAPTKKPTIS
PTTQNSETTSESSSQSNSGPTGSPTIPTPTLSPPTSSPTTFSPTTFTPTDTDQGVNARLE
VAALSAVGQSDTQAKSQRKRMHNVEREPRIFNRMGLIDQDNSGIGGD
>comp4477_c0_seq1; 375 111 824 LEN=1345; translated
MTTAGTLRLSSAGNSSNGSGTSTSIDAYLERITGDASLTTAQKNNCLKTLAVVLKNLIDP
SKGAAPGDAGIKYRTLKLDNPKLADRLFCSASVRDLLEDDRVGMVEASRTLVLEEAPCPS
ARETLAREVLPAIARAQSALQPRASVHKKARTDADRPAAALPVQQPLSEKQKARLLLEEK
KRLEKERDRLHKQTVRAQIAADKRVRLHDENWKPAVSAAADKTGNGLQSFRDRHGEQ
>comp4477_c0_seq2; 149 1 784 LEN=1305; translated
XFVLFGFVASSRLVVPYLTSPHLPLFPSERQHFLWFRCNRIRCRRNENYAPWTRQHDTTQ
QTKPNPMRQQLKTLAVVLKNLIDPSKGAAPGDAGIKYRTLKLDNPKLADRLFCSASVRDL
LEDDRVGMVEASRTLVLEEAPCPSARETLAREVLPAIARAQSALQPRASVHKKARTDADR
PAAALPVQQPLSEKQKARLLLEEKKRLEKERDRLHKQTVRAQIAADKRVRLHDENWKPAV
SAAADKTGNGLQSFRDRHGEQ
>comp4479_c0_seq1; 1016 94 2328 LEN=2430; translated
MATKGDSFPSYVTYGLLHKSNDYIDWIRVERDCVVPNETDSSPNVDAESTEPIDVTSCES
EGERTEATALDVRAFEIFRSLQQKEGTDDGEQNKIEDPILKNNDENESDDDLIGIGDENH
ISPGLFGKGTKLQMELERGMNKFSSNIAAKIMSAETSSFGVFDANWNPLRFSPRHSNSDV
AILLYAAAKEQTRPDEESVTEASAWKRRTSIEKPGALSVDAASDTVSATACAKDGFSCSL
QDIETEIERGTLPVVPVRRSSQQQFLVKQSVNMFFRKESVQFFACFPLRNSPRTIYRFDM
DGSDGTNGDQSRASAKWKGKLARISSVSLASLKKQSIAATGESNELSVGGDNPPAPKLSL
NERLSKMKVSSATKTDDIRRSLRSSLSNTKEKFENSSFRSTVSNTKKKFSEKFRKMPSLS
SPNAEATEEDIVDYLEEEDIVIFDSSDIVDAEDQKMGDDSTNNDSIVAAANGANNDTAAT
VGTEHETVSEESESQNAQGPAGYVALSIDDVPGRFNFADKSLSLLPKVLDLFDKYDAKAT
FMVISSFLSPFHEPDMIRLLQEGHELANHGIRDEPMDKTLATSVDAFLEALDECNDKIKD
LQQKANASNHDEFAFTSTDNSHATIPIGVKWFRAPQSRYTKVMEEGLAKRDMYNVMCDAY
AACPIIEDGAWIAASLSKQIKNGSIALLHMPEALGFRGYCLTALELLLEDLVVRRNFKVV
TVSELQRISEQQKASATTTGSSAE
>comp4480_c0_seq1; 539 192 1637 LEN=1868; translated
MAKVEPANTTGEAALARKHRIAMRKQRAVSSCSLSDEDSLCERARSKRQRVALDAEVEPM
VISSATDSDSVKKTTPNDILESIKKKKPHITGIKKQSRYDPGVEMTKEELKAWRKEARRV
RNRESAAASRKKNREAIEQLESQVKGMQSKYDTALGYIIHLQEQLRQIGMSPLSCPSSVL
LQDLEDLNKDSSECLPRQTVSPPQSPTPIEQSSVEVDPLHLTPQAEHRQQDRQRHLVLRN
DPDKNFLSQPSSRHSPSHPAIPNSQKHIIDNMIIRPIASSRSIKPLIIHSAASAAVHSAA
ALCYYINNFQQRRSSPGANEKTVNDCGGIDHELSKAWAHTVRDTVVSDDDAANAFKTVTN
GDSPSRHLAVSQTTTMETDHSSFSGSDDDTVIESEQPMFLDGLPPVTVNDGCIDEIDEAI
LGCDVGTEHDDVMAVFLQDTFEPNASRSQVEPSLESVVDSNDIDALLIAENEALMLFPDR
S
>comp4480_c0_seq2; 624 192 1538 LEN=1769; translated
MAKVEPANTTGEAALARKHRIAMRKQRAVSSCSLSDEDSLCERARSKRQRVALDAEVEPM
VISSATDSDSVKKTTPNDILESIKKKKPHITGIKKQSRYDPGVEMTKEELKAWRKEARRV

RNRESAAASRKKNREAIEQLESQVKGMQSKYDTALGYIIHLQEQLRQIGMSPLSCPSSVL
LQDLEDLNKDSSECLPRQTVSPPQSPTPIEQSSVEVDPLHLTPQAEHRQQDRQRHLVLRN
DPDKNFLSQPSSRHSPSHPAIPNSQKHIIDNMIIRPIAAKISPGANEKTVNDCGGIDHEL
SKAWAHTVRDTVVSDDDAANAFKTVTNGDSPSRHLAVSQTTTMETDHSSFSGSDDDTVIE
SEQPMFLDGLPPVTVNDGCIDEIDEAILGCDVGTEHDDVMAVFLQDTFEPNASRSQVEPS
LESVVDSNDIDALLIAENEALMLFPDRS
>comp4483_c0_seq1; 354 150 1447 LEN=1447; translated
MVLPFLITSSFDEDDSISVMSLNDRDDKNNTCNNSVTSDNLSPTSAGGGTVINLVNRNKR
QRFTSESPFTEESENEGRQESKKPAKRARITMTIDEPIAAEATAAMKNFPSEATQGSFGV
SISNQNDQHGIARPSFEDLSAELCKIVDAAHYSRHEAIAALTNLCQWGYTDDPVFLEEML
ELGGIQRVLLYLKNNEGDPACVAMAAKAIMTCTVRSPKHQSFEVSKDIVRALTKREGIEI
LLQASNECGRESMASQVEALRWIWSALFNITDKSSAYEVGDLEEQKERLLGVFENGVNTM
AKLGGNIIAASTAQKGLTSPTVSKGMNDSHVKLVHVVVGISSRMMYXGGADYQLRAVQWI
FSSICGGVGSRKRTAMEMSTNRRKRRIVRPSRTVKAKTKNTTKLKTITENSNSVLASQVM
GLVFITLSNINNN
>comp4484_c0_seq1; 360 413 1537 LEN=1727; translated
MPSAADYLVGPTIGEGSFGHVVYAVHKTTQRKVAIKVMEAPVMGATEHKHRQLWKEQKTA
MILNERRILSLPELKSSKWIVNLWAAFCESNSRYLYFVMELATGGDLQGLIDRALTSSRA
KSKRDAWRTDSVPFYASQLIEAIQFLHSTGILHCDLKPENVLLDAATGDLKLADFGCALD
TNRQQQNRNPSSPPIMLPRGTARYSAPELLQAESPLALDAAVDYWSMGCILYAMFRGKSP
FDRGSEALTIRAVFDYLDGAPSDLSPKQKWAEDTNDDESTRYPLYRLSQGLFAMIPGDRT
TFWATSVIPLFDSKSQGESVGGDDAIHGIDNRRSATFATTSNESGVLPSNPDWKDQVESA
TLRNGDLGWVAFQL
>comp4484_c0_seq2; 360 413 1537 LEN=1904; translated
MPSAADYLVGPTIGEGSFGHVVYAVHKTTQRKVAIKVMEAPVMGATEHKHRQLWKEQKTA
MILNERRILSLPELKSSKWIVNLWAAFCESNSRYLYFVMELATGGDLQGLIDRALTSSRA
KSKRDAWRTDSVPFYASQLIEAIQFLHSTGILHCDLKPENVLLDAATGDLKLADFGCALD
TNRQQQNRNPSSPPIMLPRGTARYSAPELLQAESPLALDAAVDYWSMGCILYAMFRGKSP
FDRGSEALTIRAVFDYLDGAPSDLSPKQKWAEDTNDDESTRYPLYRLSQGLFAMIPGDRT
TFWATSVIPLFDSKSQGESVGGDDAIHGIDNRRSATFATTSNESGVLPSNPDWKDQVESA
TLRNGDLGWVAFQL
>comp4485_c0_seq1; 237 1 2390 LEN=2596; translated
XKIISNTIAITNTNTIDILATKTSSMSDTYLNNGPPDPIAFFRQASDANVGIVDSSLADI
EDGGDSRKPCSGVTCPPSPRRNQEAPARERPPPGHDWPYRENLSFSRSLMFYGAGAVTVE
NVKACKNIEKCRSYRKKYFGSHSSIVQSEILMDAKSNDQSALSYRMGSDGVMEIYHSEDM
EKTNNLCVVPTIEDFERDYRDMTKMVNEGFMRSYCFQRLQLLSSAFRSHTVMNASMEAHE
QGNLLGTDFFRTLKIDNHIHAAGAPTAKQFVNFVMNKLQTESDTVVSSDGRTLGQVFEQA
GLDKDHLTIDAFNVLADYSVYQRFDNFNAKYSPFRMADMRRIFLKSNNHIGGRYFAELLK
IVLNRREESKGHASGMELRLSIYGMERREWDDLAEWMLRDWKGGDFPGPLVSECNRWMIQ
IPRLWRIYRSKPGKDATTGFNEMLENIFAPMFEATLHPERKPKLAEALTHIVGIDSVDDE
GAPEVRVVQTWFTECWQAMAAFCRWISWLIVFNVLKLFPLLFPACFLPLFSSKAACGCQR
PHEWTSPGNPSYWWQLYFLWANLEVLNSLRKARGLNTIAFRPHAGETGNPLNLACTYMLS
ESIAHGINLHQQVSLQYLYYLDQVGISSSPLSNNFLFRKIRESPVGKLFRRGLNITLSTD
DPLLFHMSDDPLLEEYSVARATFDLSMTDMMEMARNSVIQSGFEEEFKQKYLGKDFRKGV
TFCDERLTHVPLIRAKFRAEHLALEHMLCALTAAGKGPSVLAEMKTQFGLARDAHRAILF
DNLDEIDASFPELGQL
>comp4485_c0_seq2; 441 1 2242 LEN=2448; translated
XKIISNTIAITNTNTIDILATKTSSMSDTYLNNGPPDPIAFFRQASDANVGIVDSSLADI
EDGGDSRKPCSGVTCPPSPRRNQEAPARERPPPGHDWPYRENLSFSRSLMFYGAGAVTVE
NVKACKNIEKCRSYRKKYFGSHSSIVQSEILMDAKSNDQSALSYRMGSDGVMEIYHSEDM
EKTNNLCVVPTIEDFERDYRDMTKMVNEGFMRSYCFQRLQLLSSAFRSHTVMNASMEAHE
QGNLLGTDFFRTLKIDNHIHAAGAPTAKQFVNFVMNKLQTESDTVVSSDGRTLGQVFEQA
GLDKDHLTIDAFNVLADYSVYQRFDNFNAKYSPFRMADMRRIFLKSNNHIGGRYFAELLK
IVLNRREESKGHASGMELRLSIYGMERREWDDLAEWMLRDWKGGDFPGPLVSECNRWMIQ
IPRLWRIYRSKPGKDATTGFNEMLENIFAPMFEATLHPERKPKLAEALTHIVGIDSVDDE
GAPEAACGCQRPHEWTSPGNPSYWWQLYFLWANLEVLNSLRKARGLNTIAFRPHAGETGN
PLNLACTYMLSESIAHGINLHQQVSLQYLYYLDQVGISSSPLSNNFLFRKIRESPVGKLF
RRGLNITLSTDDPLLFHMSDDPLLEEYSVARATFDLSMTDMMEMARNSVIQSGFEEEFKQ
KYLGKDFRKGVTFCDERLTHVPLIRAKFRAEHLALEHMLCALTAAGKGPSVLAEMKTQFG
LARDAHRAILFDNLDEIDASFPELGQL

>comp4487_c0_seq1; 236 443 1064 LEN=1236; translated
MTLFFNFGGDRPVYYGALDVSLSPLGEAEAKAAAMYLEQFDLDLVVSSPLSRAVYGAEQV
LSLQKDLDTPVVRMEGFKELDRGSWCGKTKEEIGLEAMAKFDACDESVTPEGGESYPFLK
TRVLEDRDAVLDGLPAGGTAAIVSHLQVTRSMLSDALGIPTAKMAGLGIATASVTCIDYN
PDGSQTVHFQSFKPDVGLEKSVDGAN
>comp4487_c0_seq2; 236 443 1064 LEN=1141; translated
MTLFFNFGGDRPVYYGALDVSLSPLGEAEAKAAAMYLEQFDLDLVVSSPLSRAVYGAEQV
LSLQKDLDTPVVRMEGFKELDRGSWCGKTKEEIGLEAMAKFDACDESVTPEGGESYPFLK
TRVLEDRDAVLDGLPAGGTAAIVSHLQVTRSMLSDALGIPTAKMAGLGIATASVTCIDYN
PDGSQTVHFQSFKPDVGLEKSVDGAN
>comp4487_c0_seq3; 194 1 907 LEN=1079; translated
XNEPLNSSPRPLRRLKMERTARALFIFQILICVTVRNFLASSFAPIANTVGARRYRLFRM
KSSLSAKDLKNLSSGKIIGPEIPSVEVSSKRLFLVRHGEVINPGGDRPVYYGALDVSLSP
LGEAEAKAAAMYLEQFDLDLVVSSPLSRAVYGAEQVLSLQKDLDTPVVRMEGFKELDRGS
WCGKTKEEIGLEAMAKFDACDESVTPEGGESYPFLKTRVLEDRDAVLDGLPAGGTAAIVS
HLQVTRSMLSDALGIPTAKMAGLGIATASVTCIDYNPDGSQTVHFQSFKPDVGLEKSVDG
AN
>comp4487_c0_seq4; 194 1 907 LEN=984; translated
XNEPLNSSPRPLRRLKMERTARALFIFQILICVTVRNFLASSFAPIANTVGARRYRLFRM
KSSLSAKDLKNLSSGKIIGPEIPSVEVSSKRLFLVRHGEVINPGGDRPVYYGALDVSLSP
LGEAEAKAAAMYLEQFDLDLVVSSPLSRAVYGAEQVLSLQKDLDTPVVRMEGFKELDRGS
WCGKTKEEIGLEAMAKFDACDESVTPEGGESYPFLKTRVLEDRDAVLDGLPAGGTAAIVS
HLQVTRSMLSDALGIPTAKMAGLGIATASVTCIDYNPDGSQTVHFQSFKPDVGLEKSVDG
AN
>comp4489_c0_seq1; 450 404 1444 LEN=2037; translated
MGLNWIFHDLLEISESIVRRGANFALDQLVLDDLLRAIYHPINGLWACCVGTYLGASSMY
GLQMTDDDKTELLQSSLYLKDKNQAQAVLLKAGGCKALLPESVQNWLQQSSSSPTLEKTI
DISVGLESDKSTSDSDVTEVDEHDQIFRGSTVVDEDFEKIGELSDVSQPSNMVHSFKDER
MKRLSGSESQQEAKDPTGWEDVSQTDPVEVFFRILRNMAMDRIRSYAEALPRSQIENVGI
VALMALGIRLSLRRASYDRAPLLCAGVAALSFGTIISREAILGNIYNKQSMHIVCKDIAS
SILDKLKKRKYSSKKTFFAMVTMLIFCRRKQGKMGAPRNSQSFKTR
>comp4492_c0_seq1; 615 91 2184 LEN=2778; translated
MTMAIGIQSLRRRSLLVLQISLLLATANIRCIRAFQRIRSESVKNLAGRTSMSSSSCLSG
SANPLLEAWTQPFSLPPFDRIQTEHYRPALEVGMEEHLRDLQAIVDNPEPPSFENVMVAY
DRAGRTLNKVGGVFGNMCSSQNTPELQAVQTEMSTILSRHRSSTYTLPGLFDKVEQVHRE
QQAENGGGRTREDNRLTERIYLDFTRSGAHFDTDKQAENADIQAKLASLITEFMQNVLKD
EEQYELVLKRDDLAGCPDSLVEAAKNAAEERKKADDEYVITLSRSLVEPFLTFSSRRDLR
ETAWKAWTSRGEMDPDRDNIKIATDILKLRQKQAKLHGYKNFAEYQCVDRMAKTPEKVIE
LLENVWEKAKVSANAERKELEDYVKQQGSLGEDEKDIIENGVQAWDWRYYAEQVRIAKYD
FDESLLKPYLSLESVTNAVMAVSNKLFGLKYIPRDDIKTYHESVKAYEVRNEREDDKLVA
IFLHDNYARPFKSGGAWMSEFRSQTKNLSEEMKEEYQGIPIVTNNNNFARGKNTLLSFDD
ATTLFHEMGHGHHGMLSDCTYGRLSSTNVLRDFVELPSQLMEHWFEEPEVLKEYARHYET
GEPVPDELIEKMNKARSFNQGFATIEYTACALLDMLLHQVDDYENFDMSTFEREELDRLG
MPQGVVMRHRPAHFSHLFASSGYAAGYYGAYKLAALL
>comp4492_c0_seq2; 645 91 2370 LEN=2648; translated
MTMAIGIQSLRRRSLLVLQISLLLATANIRCIRAFQRIRSESVKNLAGRTSMSSSSCLSG
SANPLLEAWTQPFSLPPFDRIQTEHYRPALEVGMEEHLRDLQAIVDNPEPPSFENVMVAY
DRAGRTLNKVGGVFGNMCSSQNTPELQAVQTEMSTILSRHRSSTYTLPGLFDKVEQVHRE
QQAENGGGRTREDNRLTERIYLDFTRSGAHFDTDKQAENADIQAKLASLITEFMQNVLKD
EEQYELVLKRDDLAGCPDSLVEAAKNAAEERKKADDEYVITLSRSLVEPFLTFSSRRDLR
ETAWKAWTSRGEMDPDRDNIKIATDILKLRQKQAKLHGYKNFAEYQCVDRMAKTPEKVIE
LLENVWEKAKVSANAERKELEDYVKQQGSLGEDEKDIIENGVQAWDWRYYAEQVRIAKYD
FDESLLKPYLSLESVTNAVMAVSNKLFGLKYIPRDDIKTYHESVKAYEVRNEREDDKLVA
IFLHDNYARPFKSGGAWMSEFRSQTKNLSEEMKEEYQGIPIVTNNNNFARGKNTLLSFDD
ATTLFHEMGHGHHGMLSDCTYGRLSSTNVLRDFVELPSQLMEHWFEEPEVLKEYARHYET
GEPVPDELIEKMNKARSFNQGFATIEYTACALLDMLLHQVDDYENFDMSTFEREELDRLG
MPQGVVMRHRPAHFSHLFASSGYAAGYYVYLWAEVLDADVYAAFKETGNVFDPDMAERVR
KYVYSAGNTEAPDELFRKFRGRDPDIKYMLEKKSLVSAS
>comp4493_c0_seq1; 165 1 858 LEN=1077; minus strand; translated
TGIDWYSIAGYNNTHPSDTLRDIFSSGKDVLVRVDADGFYYSTYIGYFYLPSKDSDGDPF

TSNSVFRMEVAPNCQGVWIRAPRHDGTSRVIRMNSGDSVTLTYADTGFGTGYWFTAGENE
EWNGSTNSFKIERGDGTFDPYDMNVNCVTFTSEKCSYYSYYGSMGGFRNRELGKYAGEAD
EPAIAFREQESGARRLQTDSDEFALAGGEGTIILDFGSRRLEGSNARLLQATQDPLSSTI
SVDATLSTAFDEIGELRTASGNRLQRATGVIGLLLAWATAMVAFAW
>comp4494_c0_seq1; 478 188 1690 LEN=1982; translated
MPSSSSRDVERRFLLAVNDVCFFFLGGSAWILVNGIYVQIPFIILFFDGDYGVVSKITLC
VTLSALLPIFGSVSSFGSLTSANHVCSKLNVCNINAICASMGGTESTSGIDEHERSIHTG
ITLVLSLGFVTSLVLTIWTEQILSRLAPMLVASFAAGIVGTTSSILYYPHAASAVSSKTN
TAKRQTTAMLFGTAVSNLFVALVAILQQQEQRQQISERAQATASMRSYFRLILVLMWFAI
VGFLGTLVIRRFGDTTPKILESENNNKTQSALESLPLLEENYLQPGNTETILHRRSFQKA
ADVSLSFWEVSSLNSTLNTAQCALNFLVFFLPGIVPYSVRDSSKDENYNTTSLRYLVVSQ
LVAQTLGVLASSGAGGSSGARASFCYRRPDSDNNHSAAPYQLGFIFFFLWIPLVFLSFEN
DLACGAGIPIVLNALLNGAYGYCNTAWFHLLNDNHCGSQNGDTSNGTPTSAVRVMGTWHQ
IGATMGSAIAFELVERGVFG
>comp4495_c0_seq1; 553 1 1749 LEN=1902; translated
RKTTIGHQGMEIVAISSDGAAVTVSKPSVTEVGVNDSFSTDSTCSVPNDDDESDAAGNAN
PPPRGPFSGFRQRRAERIAARTSLRCERDEADAARKKAIEEERRRAQEQQRKAEEAIPER
LIEPDLGSLRAGRISVTVSKTESNSYGFGLAQVPRKKNMVKVDALVNEGDKHLLSQSPLR
EGDILKMVNNQEVTDYRTVMLLLMGMEGRVTMVVETPATQGNPAMVQAFCRKPLPETTIG
IEFQVVEHNTTQNSLCRDPNGTMEVTTTKLLQIKTVNPDGYLAHSALRQGDFVLAINDKP
CTDTTAEEAAAMILESESTVNILALNPELAQKHCSPTRAQRWMRRARRTGVGVAGGTMLG
LGLIMIPLPFPPPFGEVLVVGGVSVLGTEFEAPKRVARSARNALQNAVGNGEKTVSAEVL
KQMESVSSNDDGNIAGGSVGPGDDNVGESEHDCKDENPTESEKVVLRDSFLTVIGHHQTG
MPIVSGDETEPSDEATNVVDCWSQANIDANIENMYSGNEHDMPSSPTGTRHFLKNVGRNV
VLPFLDHVVGDGKQGESQGKLLESKPATAKEELNITAATTEN
>comp4496_c0_seq1; 273 335 793 LEN=1104; translated
MSSGCITKYDYKNINFSYPSRFCLKGLTLRFSSTSRIVRLTKRFNHFIDDANSGSIENSN
DVNSYHDTSGTRSSSTTDAVMVEKVKIAACGDIDSDDEDDDEMEQEEMFVEAHVSPAFEH
GATEWGGPRRGGRFPEPTRFGDWERKGRCSDF
>comp4496_c0_seq2; 193 28 489 LEN=737; translated
MLVPSPSLIRLIALRVRSNALPSLQSNSTSTSTARTIRSCYPVQSSAFSSWFGNKSEENS
NDVNSYHDTSGTRSSSTTDAVMVEKVKIAACGDIDSDDEDDDEMEQEEMFVEAHVSPAFE
HGATEWGGPRRGGRFPEPTRFGDWERKGRCSDF
>comp4496_c0_seq3; 193 28 489 LEN=800; translated
MLVPSPSLIRLIALRVRSNALPSLQSNSTSTSTARTIRSCYPVQSSAFSSWFGNKSEENS
NDVNSYHDTSGTRSSSTTDAVMVEKVKIAACGDIDSDDEDDDEMEQEEMFVEAHVSPAFE
HGATEWGGPRRGGRFPEPTRFGDWERKGRCSDF
>comp4496_c0_seq4; 273 335 793 LEN=1041; translated
MSSGCITKYDYKNINFSYPSRFCLKGLTLRFSSTSRIVRLTKRFNHFIDDANSGSIENSN
DVNSYHDTSGTRSSSTTDAVMVEKVKIAACGDIDSDDEDDDEMEQEEMFVEAHVSPAFEH
GATEWGGPRRGGRFPEPTRFGDWERKGRCSDF
>comp4497_c0_seq1; 541 181 1989 LEN=3121; translated
MATSNNCSSSDSPTSTSSRDRASVQTGERNQRNERQQRNQRRLLLRFVLEHRHKFPGRLS
RAIERARCTSAGRANGVPPTTPEAPLVPSPGTTSDSDSDFLSRIRRDFEEWLFSEPSKSN
YRPPAQRNSSRVGEEQRQASRLELRSNANANADENDSESETATASDIAVSGLDDRVDTQD
QVELALRCFPEVLTARRFGLYPIFWLAKSIHSVSFIPLFAKIGNESGLFQDFERGGLVFG
GCGKYINVFSQLAAGVADAGHRNNASNNDHDQAYHRLVDEKFLASIKDLRESRLMEKKDI
ANHDLLGALHRQSIFPEQRFRYLVDWDPRNTLLKKKAYGTTLSWDLVSRYIINNIGENHL
VTGHSHHTVHNQSNHNRNSKNSHNHNKSNHNGPQQCATAGVEMLFELALKYFPIETGFLF
HKCDVRRRKRHTRGVTDYYFLYCDDATLKRRCCRKHTLGSSRRSLSPSSSSPQPGVAREN
HEHGFFEQRSASSSLPTTNTATTKGTTKTRATVTKTMTPYRIACEIYGIECANKILETAL
AGVIDGTSNVASSSTANSSSNDDDVMAMALINAASDPNVDLDCVYILLQRDPGLCKPNGK
RN
>comp4498_c0_seq1; 334 1 1106 LEN=1106; translated
XETLKRLQDRRQLSELPGTDRHQPVPSEEKDGGGDASADATLPSENELLLMIGHCMRGLA
ILGRGKEVENIFARVAIMPLIRSKISMGRLDEGGSRGECAGLKSLLEDMVLSVSSTFGRV
LELSEAMFDLETRVEVDLITDGVWVPIATALMADAGIKMAIFSPGLAGILQSNYVTLDKF
LSKLAGSLLRESGAADVGAASTSTSASAAAVPSHLATAQSESESLAEALYYTPRLSAETI
RRAQERIYLHPKTAEFSKKWNLPIYYQLRFGDCCARLNKAIESTTREGWTAEVFTGDKAS
ETLVRNDVGFELSFFVELYDTLLGLWKPDVILQPLTNRFLRGAVQLIGRSVAFVKAGMDG

ELKFGDDGN
>comp4499_c0_seq1; 329 334 1332 LEN=1413; translated
MSLLTEEWWTDFVAGWVSGAVGVATCQPLDTVLTRFQAGRVLSAPGLEVAVQGGVTASAV
RSSVRGLVGEFGLRSLWRGSSPMIGAVPVQNALLMGGYGFGKRYSEGARGADTGACDALL
PVFVGGCAGGVLQSFLMSPVEWIKVQQQTHASSGAAATASSVSVLRRFLTHRHALWNRGL
TATLLRDGIPHGVWFATYEFCKLRMLAGPAEAASGGTETPGAGAEDGPRSSLYKTVAVPV
VSGAVAATTAWAVGYPFDIIKTRIQASADGVRVPPGVWETGIKLVEEANGNLLRGLYRGF
GLKLLRSVPASMVGFFTYELVSGAIAGAGSNP
>comp4499_c0_seq2; 270 334 1340 LEN=1340; translated
MSLLTEEWWTDFVAGWVSGAVGVATCQPLDTVLTRFQAGRVLSAPGLEVAVQGGVTASAV
RSSVRGLVGEFGLRSLWRGSSPMIGAVPVQNALLMGGYGFGKRYSEGARGADTGACDALL
PVFVGGCAGGVLQSFLMSPVEWIKVQQQTHASSGAAATASSVSVLRRFLTHRHALWNRGL
TATLLRDGIPHGVWFATYEFCKLRMLAGPAEAASGGTETPGAGAEDGPRSSLYKTVAVPV
VSGAVAATTAWVSSANLGPFCFDTAVRLLVVWISRATHGLCSFFLLSIPNSHTQAVGYPF
DIIKTRIQASADGVRVPPGVWETGIKLVEEANGNLX
>comp4501_c0_seq1; 172 167 976 LEN=1154; translated
MKSDGNHGARNRKVIGIIGGSGPEAGADLFGKVVSMNRKKMGETYKSDRDAPNILLISVS
ELGGPRTAIDVDPRNPDGTYEQSRTALLDTIRKIVPLVDVFCVACNTLHMFEAQIRETVA
ALGRNPSMFVSIISSTIKACKDELNGDESAKISILGGPVTMDLDGNSSYRRLVEEVGKDY
FYRPPSSCTSILQRVIWKVKQEGTAHTSGDALTEYEGLLRDVASQSVKVCVLACTELPLI
NAKHITDLPMKFIDPAEVVADALLSVAHR
>comp4502_c0_seq1; 260 235 1002 LEN=1093; translated
MTARTNCDTTSSYPLSKMSNNAMTVNHRDAKSSLRKQKNRAWATTHGNNKYFRDHNRRSI
ATSSSRKGMPASSRTGALAKPRHISNAKRAVSLPADLRNTTALATKDLFWFLPKRKHRQT
HCSESDDPYQAPRSQQRHRRHCSESHYPCKATDATVVVTDTRLVECGRTELHRSHFRAFD
EFNGMTAHCREKTSSSGNKFRLSPLKNLNWSGDYFVDTADEHDFPLLGLEIMESVTENEI
EKENQTKFIRHQIPK
>comp4504_c0_seq1; 377 665 1816 LEN=2144; translated
MDSTHCSVRGTKSIIGYHHLGSTTTTSTSSGPEMSAPASRQRKIPKIMHQHGRSRCVPSR
LYNLNQQWKQHLLMDDRDGNGDNEGDEWSIYFHTEDAMARLFRAAIAVRQGAAGPNTRRH
GSPLIISDDFPHLGQILRTCIKNRSFIRGLLWRFVCLYIYGGMFVDLEYAPPPTESYGDN
MNTIIDDNRNDAVFFIASDKDKGDSNNLRNSWGGINTNIMAVSPEHPILYYAIQHLLFQI
TTDGYADQDDSNKTIQHYIVSDILERSLADFMDKQSVSLFEFTLGKHSTESRTKTYSGTG
KRTVTIIDSSSGDTSSIGTSNHRHFFQMIANFQRKPRASPIVDGSSFDHKVFSPSNQSLR
KHPPSCLHKILADISKSSSEVGS
>comp4505_c0_seq1; 254 72 671 LEN=958; translated
MESKTTTDPMPFERVTRTLDEFGRNCFIDEDGIAATNMCTIHGFFNKCPAGSSLTYAGTK
NSIDAKLACPLDSVAQSDWREASQKGLCDCEASLLDGECEVIEEQMDCQCFGCPDGTLLG
FAYSCTTPIVGPCLSFDCYGVCNGKFDPGNLTGEKETFAPTEMAQDEDEDTSSARTIGDP
NNHVMGTAVMVLALLRMIM
>comp4506_c0_seq1; 407 363 1864 LEN=1948; translated
MTVISVASIPIGRVERLHTAAVTEATAKEDRTDGILRTNTSTTTTTKALVTTIRDPNPHR
SPTQTAKQAQAGANSYTPTSLQRDRLGRTAVGAATAGAFPSTGTFTLETTQXSAAHLLNG
DPAHQFPPSLYQPLADAPKSDTATVCSGSMLEDIEDVGSHYDGESTTSGQLSASKQPQQQ
HHHNYPPPTGAIRRSNSKEGTRLVDFAERSQEALRAVFPKQSKSAAYASLSQESSQSHAG
ASTSAVATASGSNDEDAVQHKKGMGPPLSRHWLLQQHGNSPFRTGSVKSIGSNTGSDVVV
NVGSDCGGSLTDAQSVLSQEDTALLSTVNSEISSDVPPAAASAASAGKSDAIDEGVPHPL
LCDRSDGKTNGRTSPGGTIYRGRGIRRYKGRFYALPLKRFHQDGVHLHSDSEPESGVDRQ
QKQHDRWDDRSGSDLRFGGQRGGERHNYHGHAPSRRDRSRSRSRSRSPPAEGRGRETRSR
KMDQARGYALEKNRDDDGSS
>comp4507_c0_seq2; 593 177 1076 LEN=1249; translated
MSAKRYPRSVVPLALLLALFSMAVSVLASTEGSQRIAAGRSRAEERGGIRGLAGAGQAAG
SSSDDDDDDNANEDTTAAPKKDDRSSPAVSPKTSGQKATAPTEPKTSSPSKATTVSIASR
TSSVASATKEPLSASMQFETPQTASPQTSPGVSKSSPSSGGSKPEKDAAASSKPSSASSS
DTTESPVASGSTTTTTSSSVGVGVGTEGNQTATGDDDLYALASPTADNGDDNFASVIFST
SNNSTKPLVLPKEKEQPKQWPEIVGGIFVAGGISFFLATYCKNYQKRRQYDQVPASLTV
>comp4508_c0_seq1; 616 117 1388 LEN=1643; translated
MKFQLAVLSLFCAASPVHVQSFAPASTTPSSTRSASAASPSVARSVPLFSTAAPAEEQTE
ETLELNADYQAALDQATAAIKAGLQGASPEMLSSVVHFCTEYVSAHQAANAKQPTEENSA
TAALKRISEAIQFGKKFGMGEDKFQFGVTHDALRGNDPISEEIGNTLDFYQWGCDFFRHF

IDVEDSIVLGEDNLKKAMEQASKGENVVFYANHQSEADPQVMSILLEKAGYTEEAAKVIY
VAGHKVTTDSLAIPFSMGRNLICIHSKKHIDTDPDTKSQKSRQNMKAMGGMLSKLKQGGC
ILWVAPSGGRDRRDTSTGKTPIAPFDSKTIDMFRLMGTKSKVKTNYYPLSMVSYELCPPP
DFIEAGTGEQRNFRFVPVGIKCGDQIDAESGSEDAFHPGAFEQTLADYHELRERIFPGTA
PEL
>comp4508_c0_seq2; 224 1 720 LEN=975; translated
YPPRKGEDNLKKAMEQASKGENVVFYANHQSEADPQVMSILLEKAGYTEEAAKVIYVAGH
KVTTDSLAIPFSMGRNLICIHSKKHIDTDPDTKSQKSRQNMKAMGGMLSKLKQGGCILWV
APSGGRDRRDTSTGKTPIAPFDSKTIDMFRLMGTKSKVKTNYYPLSMVSYELCPPPDFIE
AGTGEQRNFRFVPVGIKCGDQIDAESGSEDAFHPGAFEQTLADYHELRERIFPGTAPEL
>comp4509_c0_seq1; 614 63 845 LEN=1194; translated
MADNWDDDSDDDWDVDDDALDAKLGLNKSAPAPVNNFDDEEDLALKEKAEQDRLDKINLK
KKGSALAAKKQAEKDRQDDLEIARKALELEAEMEANMTLDERKLMQRKREEESELDMMGD
AFGGGIGDGGVGRKGVQQAGDKVVMKDMKDHMKHARKVADCMKGHGKIHLTSIFIKEVIQ
QSKDLLDDAAISDIIKTCNVIKNEKVQASKRKVKGQAQKSKKQEKIEKAKAQAIQNELFG
DSNQYDEYDEYGEEYEDAFF
>comp4511_c0_seq1; 234 29 721 LEN=1031; translated
MAKRNNMHNTSHANINTVRFSLAHVPEHHQDLCMGTKRSAWNSDEDYHSSKNRDRVALPP
RRPSRIRKNPNGLNEEVFVSSGIDGQRMLPYREARQLSKPSTERKSILKKSKFSHRDSTI
SKIPSTSKCPESPNCSIASDDSSSAFDRGATSLCSSLLRNHRDSVLDLHRLARHRSGVNC
PRKPSIADESITDSSILNDESDDDRSMSTLGQSNHTFGSNPPLTDLLLEM
>comp4512_c0_seq1; 246 553 1098 LEN=2502; minus strand; translated
MHPPCPFSLRLCRVVSLRFVASLLLRRASSVLQNNTKLHGAAKALDCDDLLSSWNSEGSP
NGKKESFLVVDFSGGGKHPNRTVRALELGLQFQAGFAAEEITVYAKAQQQEGETGGARWR
KAEEIEAEDDHELQSFALPAEEPTTALKIVFGETTDFYGRVIIYQIQVWGKNLGNDCDKS
S
>comp4512_c0_seq2; 246 461 1006 LEN=2410; minus strand; translated
MHPPCPFSLRLCRVVSLRFVASLLLRRASSVLQNNTKLHGAAKALDCDDLLSSWNSEGSP
NGKKESFLVVDFSGGGKHPNRTVRALELGLQFQAGFAAEEITVYAKAQQQEGETGGARWR
KAEEIEAEDDHELQSFALPAEEPTTALKIVFGETTDFYGRVIIYQIQVWGKNLGNDCDKS
S
>comp4513_c0_seq1; 228 198 1502 LEN=1621; translated
MAAEEEGEGNDAVEGDIIRDKVDLWVPRLIGSVTFLCSIFILGLSWRRRAYVFHRLVLGI
SIHLIVSGVCLIWGAAAIPSDASDAVGNYGTVATCTVQGVLLYVSFHSVFFYYVCLSLYS
LLGVPNNFCDKRRLLVAEKWIHAGVHVFPIASAGYLVGIEAFNNVGLGYCLTVSDPLACN
NPESGVPCERGPSVEEWKALNMFWIVPQLLILLCPTAIMIALYFQTRKHQATVRIASADV
AKQSALYLLSVYWIVLPFLVQQLWSSLSTTGYSTEPIAFFVFAVLSTSNAGLFGLWILLF
YLCFSFDISISSMSATRKMNTVAEDKGPQRPGRSGRPTVATSPKTHNIFRSQDIVGAGEP
GSPLATSHSSKKSLGSRTISFNIFDGTNAAGAFADFVFDGDSDDEKADATETAYCIGLQG
PAFQEDPQRVPPST
>comp4513_c0_seq2; 223 198 1346 LEN=1465; translated
MAAEEEGISIHLIVSGVCLIWGAAAIPSDASDAVGNYGTVATCTVQGVLLYVSFHSVFFY
YVCLSLYSLLGVPNNFCDKRRLLVAEKWIHAGVHVFPIASAGYLVGIEAFNNVGLGYCLT
VSDPLACNNPESGVPCERGPSVEEWKALNMFWIVPQLLILLCPTAIMIALYFQTRKHQAT
VRIASADVAKQSALYLLSVYWIVLPFLVQQLWSSLSTTGYSTEPIAFFVFAVLSTSNAGL
FGLWILLFYLCFSFDISISSMSATRKMNTVAEDKGPQRPGRSGRPTVATSPKTHNIFRSQ
DIVGAGEPGSPLATSHSSKKSLGSRTISFNIFDGTNAAGAFADFVFDGDSDDEKADATET
AYCIGLQGPAFQEDPQRVPPST
>comp4513_c0_seq3; 197 498 1646 LEN=1765; translated
MAFQCLGISIHLIVSGVCLIWGAAAIPSDASDAVGNYGTVATCTVQGVLLYVSFHSVFFY
YVCLSLYSLLGVPNNFCDKRRLLVAEKWIHAGVHVFPIASAGYLVGIEAFNNVGLGYCLT
VSDPLACNNPESGVPCERGPSVEEWKALNMFWIVPQLLILLCPTAIMIALYFQTRKHQAT
VRIASADVAKQSALYLLSVYWIVLPFLVQQLWSSLSTTGYSTEPIAFFVFAVLSTSNAGL
FGLWILLFYLCFSFDISISSMSATRKMNTVAEDKGPQRPGRSGRPTVATSPKTHNIFRSQ
DIVGAGEPGSPLATSHSSKKSLGSRTISFNIFDGTNAAGAFADFVFDGDSDDEKADATET
AYCIGLQGPAFQEDPQRVPPST
>comp4514_c0_seq1; 238 238 858 LEN=1151; translated
MTNSSIRTRSRSNIFVNSFTWNNKRIIRSENAMKNYKRHDGKIHRKHRLQSIPSMEGLVL
RTAKVDISTNECDENKAPGGGLINQDEKDVFFLASPSSLLIPPCNPTRSLPELPYKRQQR
SQFQYEDKVYIPKFDPSLLQFPILPSPPPSPSKSRAAGSKYSKKKQVGKRYSAPKHNKPW

DPSHIPIPSGIRLLPKTRFVLPRDNC
>comp4515_c0_seq1; 425 331 1512 LEN=1792; translated
MCGKRIPAASSLVSSAPTGNVRCRGRTQTIPYFYVLGASLLMLQIVVAATSSNIGNFQGI
SFAKSHKRPSFAVDRSIPRGGALFGTATEDFDDEEDEEEEDEENDDIRQHPEYDKLLAYR
MKQQVLLQLRATYLSETLAKRGLPIATIQDVATPEGTSPPQKVDWDCAMSTEEDPGHCLI
SYEPEPGAKLVVPIELAHTDKWITLAALNRLRRDDPSKVEPMWNDKYAVLTSWFSPNSRY
SLLQHMGPKGVLLSTLLDGNRLPLVVGILVLLVTIQVLPIIEAVMNRLLVSGFVWERWPS
WYRYVRVGLPFKLLILQVAFGQVSKAFSALVVFIKDKLVDMECRILEETIPLTVGVPDGP
YDTDDDEDDVDQEETDDDEHDDESSSDDDMDE
>comp4516_c0_seq1; 410 136 1437 LEN=1844; translated
MSYAYQNPTRTTTLLLLWIGLLGCSTRTIHSFVHIQARASNPRQSGSAGNHNHRIGSAKH
HRPSAGPTPPATGFDGSSELYGSEGSREEDIDEDASYERAQRIMYNQLSRKRESVRYQII
AAEACEPLAMRMAEKYPDRFHFHPTTWAKFPDGTDNIEIGGFSNPRNVVSGEKVLFLGSF
HSNDTTLSQFQVMICLLQSFIESLTVVLPFSPVGTMERVTNEGQVATAATYAHMFSSLPN
CGRPTRLMVYDLHTLQNRFYLHGNSVASLHTAIPLLKEKIEATGINCVAFPDDGAAKRFS
GMFLDMDLEIIVCGKTRGEGDARSVTIQDGNADGKHIVIVDDLVQTGGTLYETGRVLEEA
GAASVNAFVTHGVFPNESWKRFNKGGDRACFEKFWVTNSIPTVTNDLPEEDGIFEVLDLL
DLIIDDLDHYNSI
>comp4516_c0_seq2; 362 136 1590 LEN=1997; translated
MSYAYQNPTRTTTLLLLWIGLLGCSTRTIHSFVHIQARASNPRQSGSAGNHNHRIGSAKH
HRPSAGPTPPATGFDGSSELYGSEGSREEDIDEDASYERAQRIMYNQLSRKRESVRYQII
AAEACEPLAMRMAEKYPDRFHFHPTTWAKFPDGTDNIEIGGFSNPRNVVSGEKVLFLGSF
HSNDTTLSQFQVMICLLQSFIESLTVVLPFSPVGTMERVTNEGQVATAATYAHSEYSXSV
TIAVCAVPQDLASDTHTRTHTHTLVPTLFLVVAVAHSSLVSRSHSVLVPPQLRPADAAMV
YDLHTLQNRFYLHGNSVASLHTAIPLLKEKIEATGINCVAFPDDGAAKRFSGMFLDMDLE
IIVCGKTRGEGDARSVTIQDGNADGKHIVIVDDLVQTGGTLYETGRVLEEAGAASVNAFV
THGVFPNESWKRFNKGGDRACFEKFWVTNSIPTVTNDLPEEDGIFEVLDLLDLIIDDLDH
YNSI
>comp4517_c0_seq1; 292 1 1051 LEN=1197; translated
XDAAMFRKRTKKAAAKSNLRRKRRDDEDPVGENVSGNDESEGDAAATSARIRSTLKKQKI
LATLPMTSGGGSGRFRASAKQNRSGDDHHQQQDGSQAAELSVLDTKHKNNMESFIEERMA
SSGAGREGGTGPAGQPPEASASASASASTEDDLYRSLAREVAGETEPLAPGNEGPPEQDG
DQGAGGAALLGGTGIAEVILPSTRHEPVALFTGQRGHRHFSEGRSSAVPEGERHGRSLPE
TTPKPLVRFSGKASANLLETKGSSEGKHGSRREPSGGEGGDLGPEDGKADADRKGFDAFR
GRAAASSSLSDQDPGGGDDRTRHNRWKTGQNRDDVVYRQFLKKALNPRNI
>comp4518_c0_seq1; 517 227 1612 LEN=1640; translated
MTSLSATKRFSVPSLSELVRFVVGLSFAFLLVGIDVLMSVQSLSSSPMTMASSKIPETPS
TSTIQQRSTFVRPSLEESSNTSIQLSNVDDVVDESVELKTRRVFVGLYAAGIASTLLGSS
PDMASANEPITSVDSTVEETLLQVPTLEISDAAAVDWNSIFQKASKRALGGGKAGASAAV
IQVLSLMWLRTSMNRQYRYGGDLKSSLKELWEEGGIPRLYQGLPFALVQGPLTRFGDTAA
NVGILALLESIPETQTLPLPVKTGLGSVSAGLWRIFLMPIDSSKTAMQVEGKEGLKDLLE
LVRKDGPSQLYNGALAQAAATAAGHFPWFLTYNYVDELLPAVSKADDLLLSLVRSAVLGL
CASSVSDVTSNSLRVIKTTKQTARLGGTNGSGVDGTAVSVEKKENDDQDNSNDPSYAEIV
QMIIEKDGLAGLFGRGLKTRLLTNALQGALFSVLFKYFQQA
>comp4519_c0_seq1; 389 163 1359 LEN=1454; translated
MNNFIFDADRSYNQTTANIRRACIDTNLTYASNANYHNQQKKPPRMTPRRASIAYFADEP
TRRSSIAYFADEQSQKTDLAFDSNEIFRPEHVFEIDDREDKSIQYARDQRVERSTKPTQD
SLTSISFPSSKNYTNKLFQPTRTSVRKDEDQPSKSSNKIGTSMSQFFDVFSTPTAPATDR
EQYQQHNHHQQQYQQQHHHHHHQQCGPNLTPTLSPPKEHNTRKRKATDETPAPKKSFKLR
FRSHQAQGWMEKYEELLDYRLANGHCLVPNQFPENPPLAEWVKRQRYQYKLKGLGKHSSM
SDDRVVALEKLGFVWNSHDAVWEERLKELKQFKSIFGTTNVPSKYKQNPQLAIWVKRQRR
QYKFLTEGKQSTMTPHRMEKLREIDFSWSGRKCKTSSV
>comp4520_c0_seq1; 363 1904 2953 LEN=3489; translated
MTKRARIIMESSTTVTPVARSIKSSCHSEDSGHVWLVTIDYDGSPSEGCSDCGHSTSCVF
GSEAERDRFLASATMIPDSTDSSSGGAKSKPSIVGYSVQRIKTSDATKREGFTGSETAAG
AGNGQASPNERTALLNPTQHQQRHSATKHNDNFNQIDHPSSLIQGDDEEECYNDFNELSK
GFDALVKRFELFRRVIEHEDDLLNQRMSWIILAQSFLMAAFISTGSDNDNNNNRRFLSIT
AAVGLLTCLVTLPALVAAGKNIEVQQHIYFAGIRSDSLCQRLHGHGRDRSWGGDTEHKLR
SSSSHLLPNTAFRSRFGVPILTTVVLLSIVQVAGWCGLLVALIMADDDR
>comp4520_c0_seq2; 363 1653 2702 LEN=3238; translated

MTKRARIIMESSTTVTPVARSIKSSCHSEDSGHVWLVTIDYDGSPSEGCSDCGHSTSCVF
GSEAERDRFLASATMIPDSTDSSSGGAKSKPSIVGYSVQRIKTSDATKREGFTGSETAAG
AGNGQASPNERTALLNPTQHQQRHSATKHNDNFNQIDHPSSLIQGDDEEECYNDFNELSK
GFDALVKRFELFRRVIEHEDDLLNQRMSWIILAQSFLMAAFISTGSDNDNNNNRRFLSIT
AAVGLLTCLVTLPALVAAGKNIEVQQHIYFAGIRSDSLCQRLHGHGRDRSWGGDTEHKLR
SSSSHLLPNTAFRSRFGVPILTTVVLLSIVQVAGWCGLLVALIMADDDR
>comp4521_c0_seq1; 104 1 498 LEN=560; minus strand; translated
XTPTTSNSYYLRDNDIVHTETMDQKSPILGNGTIHSSPPQSKIADQSXLHRSALLGDRPL
DVALDVHPPPDGPNAGGKVGAAHGLVAARGRNVVDAHPEIGLGVGRGGLQKDPEAVVPGT
ELVGGPAGGTLRREPDELLVALLDVLDRPQDRCAAAGWQPRNCNEK
>comp4521_c0_seq2; 150 1 792 LEN=906; translated
ENESESENDGPPGEEDRGPSRGPVRPSCGWRLGAACFGTASFLLSCLFLYLTVMNLGATY
QQNIVRLRLNDSFAILYPDDYHTGEMCAWSDRSPEGEFRTFASLREVEETNGAFTVVHCG
ACGACSNWEDLSLQWTTRTFLAQASKQCCRTSILGSVEDVQECNEELIGFTPECAACWTT
DELCARDNCFWIFLQATTTNAETDFRVGVDDITAASCDEAMCGPDFAPCVGAIRRRMNIK
SDIERPISQQCTPVQEDWSAIFD
>comp4521_c0_seq3; 231 90 1079 LEN=1079; minus strand; translated
MELFTRLLLNQKSRTNPLAPECILKRNAIQWSERKRNETKQEKNRVRRGNSIALAASPWK
AQPTNHKTQGVSTQTPFVPLTLGDRPLDVALDVHPPPDGPNAGGKVGAAHGLVAARGRNV
VDAHPEIGLGVGRGGLQKDPEAVVPGTELVGGPAGGTLRREPDELLVALLDVLDRPQDRC
AAALLGGLGQKGPGGPLEREVLPVAACPTGPAVDHRKGPVRLLDLSQRCKGPELPLRGPV
APGAHLSRVVVVGVQDRKGIVEAEADDVLLVGRPEVHHRQVEKQAAQEEGGGPEAGGSQP
PPARRPDRPPGRASVLLAGWTIVFAFAFVL
>comp4522_c0_seq1; 154 36 131 LEN=836; translated
MASAAIQAQANALNAKMEGEASKMLDEVERVW
>comp4522_c0_seq2; 198 36 494 LEN=591; translated
MASAAIQAQANALNAKMEGEASKMLDEVERVWMRKVAKQSFACAVKCYDKAGTSGPAESL
EACTRSCQAPYQQSSNLVQQEVAQFQNRLNRNMQECQEKARDMIRPGTENDSYAMSKVET
ALITCMEKQVNEHIKLLNPMKERITSVLKNYQ
>comp4523_c0_seq1; 714 213 2075 LEN=2976; translated
METRILLLSLLLSAVVATRSVVEAKTTVSRIEFSSSAIKYKASERLLENDGQSAVEAFLS
EHPQNDESVTNSIINDSGYDPYPDGTAVRYQDQDGTWMNGRIDTYNSDTKVYTVKWDEDN
VLEDFSDLNKVDQLVANAEEQVQITNTEEQVTETNDDEVDEQFDWDDNEKLGQAPSSNGS
PYRGSVPEDFDFGYTYQYDNLADYEPWPIGTVTLLEFSDGWYEGKITSFSLSEDKTNATY
VVTWSDGTGDRFVNELEWMDLMVANAQHYEPWSIGTQTYGYPNPDENDPDSEKFYLDGEI
TLFADGAYTITWSNGEAVVYSDFDEVDEFVNNAAAYFDPDQFNGYNPWPINTPVSWDFDD
GWWDGTITGFEDGVYEVTWSDGTNKYYSNFEKIDQMVAFGAGEGFLEGPSESYSSGDDYY
AISGYYELGTLVYAKFEDGWWAGYIDSYDDDYYIVLWSDESSDRFLPGPDMDEMVNNAQY
IQYDFNLYPEGTQVYAEFNGYWYWGTIEHSESGFYEILWEDGERTVHVAGAYLDDMVSNA
YYAGNSGNVLSTFGIVALLMIVLGGSGGLAFFYMRRGRKQRRSAELTEQVQENELDLDED
VASGSEYTDEPNGRIRANVV
>comp4524_c1_seq1; 205 635 904 LEN=904; translated
MADSDDYGTDNYDIGTASYGETVGSSRQSYEDEQSPSESGGSSSSSSSSSNIDSDSVSQQ
NALFSGESCSNSSFRDANTTSDYRHDDDDD
>comp4525_c0_seq1; 405 118 1236 LEN=1424; translated
MGSASGKAKNFNTAADKDNINEMSGGYYRRASHAGSWYSDDESALDQTLSRYLAAAAEEG
GDERQEDSESSSLSSLRAIICPHAGFSYSGPTAAYGYRALEEELSKHESPVRRILVFHPS
HHVYLNGCAVSGASAIETPLGDLPVDAELRDEILSLKKSGSLGGEAAFTVMDRDTDEAEH
SGEMQYPYIAKVLRNAKQRKGGASLPIPILPIMCGSISNVKELEYGKLLTKIVARKDVVS
IVSTDFCHWGSRFRYQPTEATAPTAAAGTKTTTMEIFEYIQNLDREGMRHIEMKEPGAFA
EYLKNTRNTICGRHAIQTWLNAVVHSETASSVTNTNAALDIEFNKYAQSSQVRSMRESSV
SYASAVARVSRV
>comp4527_c0_seq1; 493 156 1429 LEN=1429; translated
MEQPNEQYRSDSHRENTTMMVAGHSPSPPNVPSMEKKNISDAIVEHQSRILDREGIYLTD
RMFDPPSKICGGSMILKLKSMGNGNNKKKKKKNKEQSLEDKSDPSSAMMTMLRSKMQKKK
RNDKKLNENKKSEKQQVLVVPASTTMSSISEPTASVSFLPSVPPTNTKENADEEMLSFRD
VQLLLKEIKTANGTRKEKSTSEEKPKKANEKDIPSSITSYNRRFRDGEKEKLRIKDIENW
EEESLDSVYIDDSVGDKENDTTIDRLKLERKFYRSEAKTLRSELGNIKEELSELRKFLQR
LPNNKDENHEPSEGHQVQHESDAASSMTLKSFDTIYTEHGDLFSNLEDAKNKVLRTKQQR
ERNSTKLERPVPLYAANLAQSLPRDILSVKRDTNNNMDIRSGSDYQEQRTSPKVRRELRS

SERFS
>comp4527_c0_seq2; 812 156 2330 LEN=2848; translated
MEQPNEQYRSDSHRENTTMMVAGHSPSPPNVPSMEKKNISDAIVEHQSRILDREGIYLTD
RMFDPPSKICGGSMILKLKSMGNGNNKKKKKKNKEQSLEDKSDPSSAMMTMLRSKMQKKK
RNDKKLNENKKSEKQQVLVVPASTTMSSISEPTASVSFLPSVPPTNTKENADEEMLSFRD
VQLLLKEIKTANGTRKEKSTSEEKPKKANEKDIPSSITSYNRRFRDGEKEKLRIKDIENW
EEESLDSVYIDDSVGDKENDTTIDRLKLERKFYRSEAKTLRSELGNIKEELSELRKFLQR
LPNNKDENHEPSEGHQVQHESDAASSMTLKSFDTIYTEHGDLFSNLEDAKNKVLRTKQQR
ERNSTKLERPVPLYAANLAQSLPRDILSVKRDTNNNMDIRSGSDYQEQRTSPKVRRELRS
SERFYAMISSPAPKVIVENQRVGTGQRTSNNSQRNITICKSEHKNDPEEKGDQVAVKNVS
PYRTRSYRSRSMSPMRRRNKLNPPSPSVGKTRGDNRKSERSVSRPGHSHEAALKDEMKDN
ERLVMQNDGVVNSRRSINHAPEEKNSGMEFNYDDAKNFYRSYQQSSEPDAAKELGNISSK
TESSQSDSLPNGRNATSFTFNEPKSPLFDLKAADRKYREMRRSMQQVGSNNVGHHLPECE
SEDSDSFLGYCTTDDSQSENDDSSRLSFQHQRPISRNFSLKKMCDEEEESSDSILLRPPL
PPSR
>comp4528_c0_seq1; 461 260 1666 LEN=2406; translated
MMRSIAVCPISLTILALALTAAPGVSGYAIPKSKFLPLGVGNQSKMQTKAPVLSNRRGSL
LRMTKDDDSNKKDSSDVSPSNNFPSDAPTTKASSSKREMLKFALPALGIFLANPLLSNID
NAFVGKTVGTEGLAALSPATVCTDQLLYLFLFLSRATTGLVSRAQGSKTDFEEKKKAVAE
AGSAPLTVALMCGLLLTIFYAYCTPNMLSMLSVTPALRGPASSYIYWRGAISWAALAQSV
SLSILLATKDAITPLKIVALAAGVNVIGDFLFCVFPLRLGVSGAAAATTFATLLSSAFMV
KGLKKKGILPKIRWPSRKELLSLMEFTGPLLGITLTRMFGFVKMQTTAMTLGVKQTAAYQ
VCLNLMVFFLLFAEPLSQLSQTQLPELIEAGDGEQVKSNFKSVLSLGAMSSIAIGGVAGL
ATYFGSFLFTSDPAVQALAKGAAPSLFLTVATAIFASEYYSEIGKAIT
>comp4528_c0_seq2; 535 260 1906 LEN=2270; translated
MMRSIAVCPISLTILALALTAAPGVSGYAIPKSKFLPLGVGNQSKMQTKAPVLSNRRGSL
LRMTKDDDSNKKDSSDVSPSNNFPSDAPTTKASSSKREMLKFALPALGIFLANPLLSNID
NAFVGKTVGTEGLAALSPATVCTDQLLYLFLFLSRATTGLVSRAQGSKTDFEEKKKAVAE
AGSAPLTVALMCGLLLTIFYAYCTPNMLSMLSVTPALRGPASSYIYWRGAISWAALAQSV
SLSILLATKDAITPLKIVALAAGVNVIGDFLFCVFPLRLGVSGAAAATTFATLLSSAFMV
KGLKKKGILPKIRWPSRKELLSLMEFTGPLLGITLTRMFGFVKMQTTAMTLGVKQTAAYQ
VCLNLMVFFLLFAEPLSQLSQTQLPELIEAGDGEQVKSNFKSVLSLGAMSSIAIGGVAGL
ATYFGSFLFTSDPAVQALAKGAAPSLFLTVATAIFAITVDGAMLASKDFGFMLSQGLVMM
LLQLFLLKTWCSSISTIFATFTLRLGLYALCCLLRAGLGSGNLGRAMFKSGRVAFFKRKP
INGLASGN
>comp4529_c0_seq1; 283 907 1464 LEN=1852; translated
MVSITGCFLSVCVLPALVFLKNGDLPSARQVTLGTFATIGATGSTVALHFVFGAYVLEMK
PVTNSNDDTNGDDQQNPLLLEATTRSIFGFWNDIHVFDPKNDVTPYVGMRPFANFCANEI
PLYVHPERLDSTTRQLLLHNAVGSSSSEKCDDSMSSHGDSIDIKDYRDINEGASSKRKKE
DDELF
>comp4531_c0_seq1; 685 137 1870 LEN=1936; translated
MTTGAIITKAELPRDLPGPSEERFLVIVCEPDGIKWSLHDGVSFHYAPTVFWTTPHLQDE
DRSASPCKSLTRFLCQHALAHIFGASDYHKATKQNATKTAAKRMAPSTTIYVLSSNKQHE
TDLLFLFSEVPSRILRFSASDFFDAQALSSVKATIGQLAAMHAIKKDYGSPVLLMECSVA
IEYFGLDGNSKFLGGGVCAGMPIRCRSLFDYCNNQDFPSIDFERYTKLTEKAKKSGTPMS
LFSTDPFQRVAASATAEMAGQLRNIVRQFLGLVGPTDAPVTVVLCGDDTHLTRELLTDNC
SSIVAVEPDVEFPGPSKVTFVVRKNMAAYGVQHLLSASKEQKVPLDADDELREDLVGLRA
AFIKEPGNTKIHRGSITRVVRGAVFEEDSVILLLDEGDNVTLDLIKLYDCLSLYQEVGEE
SKIENKEVWVGEKQQASTKVQVELEKKNEVFATRKTILSEAKEKEGSIVKMVAPPENDKR
GAKRPRQSPSPKPKKASKESPEKYVGHRIAKLFDSPNPDDEGDMVLYFGSIDKYNGETKL
WHVTYDDDDEEEFDFDEVRDGILLWAKNKDDDKNAKS
>comp4531_c0_seq2; 523 137 2053 LEN=2119; translated
MTTGAIITKAELPRDLPGPSEERFLVIVCEPDGIKWSLHDGVSFHYAPTVFWTTPHLQDE
DRSASPCKSLTRFLCQHALAHIFGASDYHKATKQNATKTAAKRMAPSTTIYVLSSNKQHE
TDLLFLFSEVPSRILRFSASDFFDAQALSSVKATIGQLAAMHAIKKDYGSPVLLMECSVA
IEYFGLDGNSKFLGGGVCAGMPIRCRSLFDYCNNQDFPSIDFERYTKLTEKAKKSGTPMS
LFSTDPFQRVAASATAEMAGQLRNIVRQFLGLVGPTDAPVTVVLCGDDTHLTRELLTDNC
SSIVAVEPDVEFPGPSKVTFVVRKNMAAYGVQHLLSASKEQKVPLDADDELREDLVGLRA
AFIKEPGNTKIHRGSITRVVRGAVFEEDSVILLLDEGDNVTLDLIKLYGTHMHICDVLKQ
ARYFAHRVSFRCFLYPLRLGGVLVARSVFLSALACSNLCSLSLSLILADCLSLYQEVGEE

SKIENKEVWVGEKQQASTKVQVELEKKNEVFATRKTILSEAKEKEGSIVKMVAPPENDKR
GAKRPRQSPSPKPKKASKESPEKYVGHRIAKLFDSPNPDDEGDMVLYFGSIDKYNGETKL
WHVTYDDDDEEEFDFDEVRDGILLWAKNKDDDKNAKS
>comp4532_c0_seq1; 440 278 2110 LEN=2341; translated
MAQGANGDDDDDSSFPALDERRVSFNDRFDSYYSRTAAFYGNLLKVSQREHWQLASPMTK
HVFPSEHSAQSAGSFKMPRTRSRSFNDYIESRDNDDGYGSARTFKTTAMPTWKTNSNRGA
TSPVRTKESDPERGGHQGRDVRKDDGAMPFSLSVLYGAINCTIVLPVIMSFGNIIYRDDA
FIAYMPVLIKLTLFSGMVHQLCFSLFSTLDFAVGSVQDAGLIFLSRMAHDMVQYCRARNY
DDDTMLATVTVGLGCAAAALGVGLIIIGKLGLASYVQMLPTCVIAGYLAYIGWFVGYSGL
GIMAGESSLTPALLVEKSLYILPGVAGGALIYVSVRKFRHAAVLPSCITLLLVAFYVILL
VTGTSVETATENGWIRQTQDEPAWYQTWDYLQVDKVDWAALPQLWLTWLGMLFVVALSSS
LDVAAIELEMNDPLDYNKELRMVGISNFVSGLTGGYTGSYIFSQTIFSLRIGVRSRISGF
SIAFFTLVVIVLPFPILSYVPNFFYGSLLSMICIDLVYEWLWEFRHKATTAEYLLGLSTF
GLIQVLGVEYGIAAGVVLYVACRQLEIDVGELKTVYVEEDENTTRSCEICAEPDETTKLV
VNGDDSQPHF
>comp4534_c0_seq1; 302 1 1593 LEN=1593; translated
XSTTLNLSSSESSAVVSLQDLSGNLITTTSTTPQANRVIIETELSNLTESPSVKESDRET
IGGRNATICGELILLPDQIAASMIRLYQLELKKNVLRYRRFILEQRLHNIQRVSVFPSPE
GDPHPKRKMVVNQKFEVTLNHTNGDTGSSDVGERMSLSKAFLYTGWLDAAGQPHDDKGIL
RFEDGQEYKGNVYKGQRHGSGINRWPDGQKYDGEWRHDSRYGRGAHTWNDGRTVAGSWKD
GHGRLFYRWPNGAVFDGNANMGKKEGRGVSTRSDGSVYNGNYKNGKENGFGTVIRPDGVK
FRGHFRDGKKEGYGVMLWQTRTYEGEWSNDKPHGRGRVVWSNGAVFSGRFQQGSFSGMGV
YTWPSGEKYVGMWENGAKSGHGTHTWPCGKKYDGHYTNGRKEGYGRMSWPDGSAYCGGFR
HNKRYGRGIQTDRDGAVVHCGLWKDNIPLDEAGGTTKLPPVVEGGTPEFRLGMKNARLWS
GSSTDTGGLESFDHVQRGENPLLSESRQDADAVHHRSSICQSSGPAIIERVX
>comp4535_c0_seq1; 284 772 2204 LEN=2204; minus strand; translated
MWATPDAKELCALYVSAGVPIYPHSLGHSYHTLIWRLSIEVKQLQEQQTKNSINSVDIST
PPNAPGAPRAADETIPVDPFYNERDDMRMREELILGSGRVGLTYLEEKAMHKLNSKYTES
HADRIRSVNWRRRSTNARGLPQVPFMPPPFPPTASGRRETHADWHAAGRCASSRGQLSRS
NSFSGSRFRTPSTILKVHRHLLDQHLLGRSGVDDGEGSMPTPPVSLSADFFHPFFSFLST
EETMRNRSNGSDSMGRTTTGNTSLLPAYYQPVSGRNLESDEDTNDARRAGSMGSNYTDDS
DSDFEAEMMHPRPDIMEPLRLRNADEIVAAGAAMQPPAMVPPVPPMVSLGTRPTSASASI
ISEMRKSSHQLTVQSGILSNIELDVYSTSSKSLKIGDDSKAGRQLSHYLKGRFAAYHRCL
ERHISKNDNDGFEECILDFWDEFLPQTANIHYYDKHTAVPRISRLDEFLTKPCPKAVX
>comp4538_c0_seq1; 383 99 1265 LEN=1314; translated
MTNFLMAKRLGLNGMSAATMRVGKYLVVASTPASSLAATYSSKAFCSSPQSECGGAGIGN
VEDRILPSTFTDSRLFKHCRHPMTASIIGAPMTYGQPFVGTDYGPKKLRERGLRSNLTKL
GWRVEDTQDLNFDSVNQSHVPDPPNARNCSLVGKGCSLLSEVVETKINDGHFPLVLGGDH
SIAIGSLAGILRARPDTGIIWVDAHADLNTPDTSGSGNFHGMPLGLLMDHPDGQYDFSKL
PGFEFLVDGPRISPEQLAYVGLRDVDPMERVWIKKLGIKTFTMTDVDFLGIGEVMKQVLE
HLEGRPLHLSYDIDSVDPVLAPATGTKVRGGLTYREAHFVAEFVAQSGDLASAEVVEVNP
NLSDDDGANETVELGMQLITSLMGKSII
>comp4538_c0_seq2; 383 97 1263 LEN=1312; translated
MTNFLMAKRLGLNGMSAATMRVGKYLVVASTPASSLAATYSSKAFCSSPQSECGGAGIGN
VEDRILPSTFTDSRLFKHCRHPMTASIIGAPMTYGQPFVGTDYGPKKLRERGLRSNLTKL
GWRVEDTQDLNFDSVNQSHVPDPPNARNCSLVGKGCSLLSEVVETKINDGHFPLVLGGDH
SIAIGSLAGILRARPDTGIIWVDAHADLNTPDTSGSGNFHGMPLGLLMDHPDGQYDFSKL
PGFEFLVDGPRISPEQLAYVGLRDVDPMERVWIKKLGIKTFTMTDVDFLGIGEVMKQVLE
HLEGRPLHLSYDIDSVDPVLAPATGTKVRGGLTYREAHFVAEFVAQSGDLASAEVVEVNP
NLSDDDGANETVELGMQLITSLMGKSII
>comp4539_c0_seq1; 551 372 1472 LEN=2044; translated
MSAPAPATVNGTADGEAPQPMFQMSNPAATTNGDGATPSIGGMLDIGNLDLEELFVDCYY
DVPGMADMANDQHANAPAPAAPVPTPVSAMHPAVAGDPAASAGMVAPPAPPAPQPAAVPS
AAAPAAESSKKRPASEIADGPSSGGQLTAKQKLERRERNREHAKRSRLRKKFLLESLQEQ
IHGLQEQLDKFKKAIRTEIPAPRAEEVIDQICGHENKYTPLPMPSGFGPVKTLMEPDFRL
MSALSGSQQNFAISDPTLPDNPIVYVSQGFLDLTGYQLDQVLGRNCRFLQGPGTDQSAVD
VIRKGISEGIDTSVCLLNYKADGTPFWNQFFVAALRDAENNVVNYVGVQCEVSKAVVEKH
MGKEKK
>comp4539_c0_seq2; 458 1 1074 LEN=1646; translated
ASPTASTLLPDGFSFVFQAATTNGDGATPSIGGMLDIGNLDLEELFVDCYYDVPGMADMA

NDQHANAPAPAAPVPTPVSAMHPAVAGDPAASAGMVAPPAPPAPQPAAVPSAAAPAAESS
KKRPASEIADGPSSGGQLTAKQKLERRERNREHAKRSRLRKKFLLESLQEQIHGLQEQLD
KFKKAIRTEIPAPRAEEVIDQICGHENKYTPLPMPSGFGPVKTLMEPDFRLMSALSGSQQ
NFAISDPTLPDNPIVYVSQGFLDLTGYQLDQVLGRNCRFLQGPGTDQSAVDVIRKGISEG
IDTSVCLLNYKADGTPFWNQFFVAALRDAENNVVNYVGVQCEVSKAVVEKHMGKEKK
>comp4540_c0_seq1; 445 1 661 LEN=955; translated
XLGLDEVLHACSDTLDAHYIENPVLYSTMKSAILFAMVGSAAAFAPSQQQQKTTSLSAWK
DENVIGITAPMGFFDPIGLSKNKDDATMNMYREAELKHGRVAMAACLGWYLNAAGVHPAF
NSSLSNNPLEAAKELPAVGWLQFVLGCGAIEWLAEKIKDRPGYVPGDLLGAAYWVDNTDE
GWVDYQNKEINNGRLAMVAFMGIITQDIYFGNYGDMIFKN
>comp4541_c0_seq1; 886 128 1873 LEN=2055; translated
MATEKDDVVLVEHTDVTVDDATATTTTTTTVPTVSDDESSLDGGNKVGAKSEPCKPEPLQ
IVSIGTEEDKFAFQFHEERLNSIMRKIPPGWKVSVVSVVGAFRTGKSFLLTWFLAYLKEL
KKSGSTGDLIEGDKKWFETIESIENDAFHWRGGSERNTTGIWMWSQPHFVKRGDDTMAIL
IVDSQGMFDNETTMSLTASIFGLSTLLSSYQIYNVDKRIQEDNLQQLALFSEYARLAVEG
TNGDEAKKEETGDVKPFQTMEFLVRDWQHFEDEEDYDRMEEEMNEYLQKVISEREAKDLQ
ETREQIFACFSKTSCFGLSHPGMAVIKKNFKGEVSKMDETFLNLLDRYCRRVFSVENLTP
KVIQGREVTASELGSFIKAYAEMFASGASFPEAATMLEATATANNSNALNLAVGEYKDYM
NRISGPNCSSYIKPKELEDDHRHLLMKALNKFDSMATFGSKHSIEEARQSVLEMINNEFA
VYASLNEGRNPLAGLETYIVPLTVATASYILRAVADMTCSNVSQTCRNTSELLSHIYAVV
VIFMLIIMATKAQQVKEFLGRVKGAVELVANSGGDTKMKKD
>comp4542_c0_seq1; 206 168 578 LEN=1155; translated
MKRSSSIAALDDTIDVKLGNKPHYDEKLLVSSMMLVAIAEEDRQGRRFLRTQRSNGSIGS
MSARSGCGSYGSLRGWGSNASRKSYKVDLCSLSDENSGDIANRKISTKIQKKCSTLTLQK
GGANGRSSTENAELETW
>comp4543_c0_seq1; 471 147 1505 LEN=1849; translated
MIVMRKAKIFFVLLGCHNLFGAYGVNSDIIEMNSISRNNDEKHGRNLVNMMVPGDRVSSI
DKSSAICTADSGDGQEVEIVRTVVDYYYAIESTDKITTKDSIGRTMIRLLENKLFRSIRP
AVLWCYFDEPQNTRRNLLRDVADAFDKGDHLRKLTLEEARRRLSIVSVSNNPDDEETTIE
CNFPTNHTGNCTVMHGKVTVMHHATSDVSLAVASIFDSTQKAMDYNEIFIALDLDENAAN
ITNIEWLGETEEEAINGGPIIVSDVPIISEVNIDEIEQKENKQSLLAYALTIPVLLLIAF
ALLVTKKKTKRKIKTREQVRAIRSFDDVLIGTGDPPGSFHEGMYHYTCQGVRYLSTNCAD
CIETKRNGFFTALDLETISEKSLEDDEDFSTHRKKHFVNASQSALGKKHSSIDVHVCNSA
RCPICTYKPRDVCFVSRSENFLGALREGELEV
>comp4544_c0_seq1; 226 57 560 LEN=615; translated
MASDDDVTTGNVFDLDKLSLEELNQAKQQEEGRLQALTSRYAQLRAAAARLTASSNAISE
ISPSSEGKEVMIPLTESVYAPGKLREPNKLLVEIGTGFYVEKKPSETNEFLNRKLKIVDA
NSENITKAVQATRQNLDSIAMSMQGKMIEIRARQEGQRHRAAAEGSK
>comp4545_c0_seq1; 742 271 2439 LEN=2463; translated
MQKTLSNPALSQQLDKSSWLWTPPGSTRKKYEPHDFPGYGSYDVNISPDKSDMEFIDSSF
ESKEHEEKKLGELEATAISGNDILSSTFYVSGLVTLSAGKLAPVCLALVGFVLYLFRGIY
HETVMALPCNGGTYNILLNCTSKQTASIAAVFAIIAYITTGVVSALEAVAYLQTILPESV
EIDQQAATISLLFFFCLLTNLGISESAVVAKTIFVIHVTVLSSLTIMGIIFMISHSDLLY
ENWQTPYPSVDAAGDILTGSLFTAMFYGFSSAMLGVSGFETSSQFVEEQAPGVFPKTLKN
MWMGVVVFNPLLSLISFAAMPLNDILENKDTVLARMAGIVGRWLQSRLRISEEHVSIGSM
FSLIISVDAFIVLAAALLTGYVGINGLIRRMSMDRCLPQFFLISNPWTGTDSFILVGFFL
LCVSQVILLDSDVEALGGVYCYAFLTVMTIFALGNVLLKIKRPSLPREITTSWFQSILGL
IAVMTALLGNVLGKPELLTYFFVYFLIVGTLVFIMFQRVQIVRLLYKVFKSFHKPQDCDV
EESSDSEGTEDESLLSRKLRTVSSRINEEEEGMMSGLVPEPPGSYESCLERIANTVKKFQ
DTPFIFFMKHDDLHMANKAVLYIQKNEQTNKIIFVHCAFTTEGGLKKNTEGLQEHVKLLD
LLYPRVKISLLVVNSNFNATTVQWLSRTLEIPTNAMFISCPDENFSIKKSQLQGIRIITS
SD
>comp4546_c0_seq1; 490 1 1414 LEN=1825; translated
XVQCPRSRVETNTILRRNYLPNQTMSALALPSAFFLLLIGARSVDGFLQEHNRRTGSIWD
GRTTEASGRAGRRCFLEALPVRSDETDPSSSNPAAGATDRRRALKATGMAVFSLWLGDTL
GPDSIPSKWAASAAEEAASSEGASKTILITGCNSGIGLEAARILARRNPADRLVLACRTQ
AKAEGAARQILAESPSSNLVAAECDLASLDSIQKFAASLDKQPLDVVCYNAGIALNTKGE
IERTTNGFEKTVGTNHLGHFYLHHLLQPKLNTSTAKVVVTASSVHDPESPGGKQGVPATL
GDLVGFARDGKNFQMVDGNPYNGDKAYKDSKLCNILFCKELQRRLGSDEATKNIVVNAFS
PGLITSSGFFRYQNPVFSSIFNVAASSVFRVAETPEYGGAALAYMTTVEGTSGKFFDSKP

GTSFKYAADGNDYQGAFGKEFAPVATSTESMDDEKAKRLWALSEKLVGIST
>comp4547_c0_seq1; 472 1 1177 LEN=1292; translated
XTTNFILANQEGSKQLKEINKHTRQNHHIMEVDSTGDSTGVVGETRIAVHPLAIVHMSDQ
YTRITSGGSPMDKTDPVVGLLFGLSEDQTTATTGNLNNSNVHEGPLIQVLDADDIPVEVS
ETSTMQVDLHKAVFPKHKVVGWYRAVASHSDDEEIEPTSEDLGITKLLKNHYTSSDLESF
CFCLLKVQKENKGDCQEPGDDAAMKTEDATTDTLNKELPINLYELQKFNNNTVLVGLSNW
QLDTSPAERIAVERVMKDQPLDGLESESSQSPSQNPFILETKAIGHSLTSMNDRVEFLVA
YLKDIQEGKTPVDHGILRQIQQIVGCLGPLSTLATCTNEGEEKDLQLLTHLAIVARTVNS
MQSYTEKFRVMNEHKTASFERQQASTFPSWNF
>comp4549_c0_seq1; 184 1 433 LEN=433; translated
RRLGDSGRQPIPISIHRRDIDSLLVPLPNHPDRLHASKQATTKAEQGSPQLEPRIAAASK
GTLSDSKQASAENKGKPRALGSEQIMSDTPSTASSTDPSSDPIVADYTPLHETSAPASSS
VNGSGNSHNANANSNSSSNSNSNSX
>comp4550_c0_seq1; 248 187 1175 LEN=1175; translated
MIASYSMHSNHTVHRPTFICESPRQVYTARNDVGPLSQKQTIPPVAILRNLPIRKEPSLN
DIICARGRGYWDHPGNKMYRQCIDLAKAQYCRAPNRLGKSLIVSEIIDAIHNSGXGIFHG
KENEWVECNENSIREKVTQSLRDGLSFKYSSSITRKREKKARVQEAFYGDIDVIVHSNKA
VSQKIKDLEKKVEWANRNYGIENDARVSDEAIMKIFDEGNLNILETMKKDRSILDQLHQI
GRKKTKSHVHRVSLPLSPPKMGSRESFSLEFPLPFSTANNNEPGKISPSASVDLEAVTVQ
QHDEYVFDSNGKMDIDLESTSTMFLDNIVH
>comp4552_c0_seq1; 432 312 1451 LEN=1808; translated
MNQEEEKEEVIEGSVFLPDPSKPNFGMRASRWARDIPMKDDKGKADSNGVPPNMVCISED
PDELFDVTIGFWHSSQPRHMELDIRIHRAADDNADDEGKRDLLKDSVYVLDAYMGGDFSR
FHWYHPKPNSLWAMYGTRVNDYLNEHFWNGERHSKKTRAPFAIAVDVKERWFGFDATHYF
FDEKTHRMTGSTFRLLLKRFLRWGYTLGKAQLFKFQPFFFVNNHSQDLTNSDRTEENTLR
FINCQASVSLCALWWMREFDELSKEPEHDATNTDLICDKLSPLLFPRIRMENYVSVSPLE
WKDVSKEESRKQMDEFLHGAAVKRALSDEFSEENVTKLLVLINNSAGTTAPTLQTIEGTE
AGVIGDSSIQQNGRFVCRR
>comp4554_c0_seq1; 114 180 848 LEN=984; translated
MCSIIRYLKLLSCILYLASDCLGLSHQDPSISRRHAFAQTASVAAAGLVTAGASSTKIAG
AASPDLKFQTSQSGIQWADARVGTGQPLKPGATATIDYVMSTTGARYGTKIYSTTDVDRP
YRWNLGDGSTIAGIEQAILGDGESLTPMRPGGIRRIVIPQSLGYAKLAADSRTLCVENGA
PGPIPPPKEAFEEYQRFKNIYCNNERQYQPDIVMDVKLYGQR
>comp4555_c0_seq1; 519 392 1831 LEN=2283; translated
MDQSAGTVDFDDDWEEWDGTSPFWVHCVAGSMAGVAEHILVYPLDTVRTHIQVCAACHFN
PATHNASTQAATVAAHKHAPGNAGTPSARHLPPGSVLQQAAASATSSSKSSIAQAVCKTK
AANLTASPRTGGMHHLGGSGSPGTYNLSILQAIRQLVSQRAAVSQTGSAAAALEATATTT
ASPPGVPAFAPRDVTRLWRGVQTILVGCIPAHALYFSTYEIVKASTLDKDGNVTAYGSAL
AGGAAVFGHDLVLGPLDTVKQRLQLGHYNGLTDALTQMIRTEGAVSLLRSFPVTLATNIP
YGMIMVGTNEFLKDQWTRIKVANDGGDSDGNSDENYHIDHSELTMGVTLGASSLAGLVAA
ATTTPLDRIKTYLQTQQLAPKCLLQNNHHSASSPEHCPLNNGSGGSGPSAGSTNPKAPRF
KPIVADWRQAALRIYQQEGYAGFFRGVTPRILSHTPAVAISWTTYETFKRYLKQHFVEE
>comp4556_c0_seq1; 311 197 1204 LEN=1235; translated
MNSTNPSPHHQLPKSHSYFDEWFNIPSKNSSSSIHPITSLIQCLDPKAHINEYWEQLTRP
DGRLFTQGRTTQAVYGMLKHSAGSALVSQNESGGHISGTKILAATTFNIGQPSPEQPDEG
EVVVQVSGTGGRNVAGDSKSQQHYWESLQSWLQRTIEQDEMMAKSLNLLTGKSCLQLVIT
VMVLEDNGNVKDTALLACMAAWKDTRLPKVGKDLTEVQGKLWLRPHPELPVNINEAVRCA
SNEETPNDQRVQRHYRVSLTMGIWVHPRDQTIHLIADPSNLEEPFFEGSITIVVSLANEK
LQVDYTGKVPLTGHIVALAAKLALARAREVTTILS
>comp4558_c0_seq1; 1375 141 2186 LEN=2292; translated
MLSSSVLRRSLSSLAAKKATTPGVSIGAFDGIARRFKSDDAGDVIGIDLGTTNSCVSIME
GRTARVIENSEGARTTPSVVAFVHGEGGAEAERLVGMAAKRQAVTNPENTMYAVKRLIGR
GYNDKEVNTIQGLVPYKIVKSDHNDDAWVADTQGTKYSPSQIGSMVLGKMKETAEGFLGR
TVSKAVVTVPAYFNDSQRQATKDAGKIAGLDVLRIINEPTAASLAYGMDKADGKTIAVFD
LGGGTFDVSVLEIQGGVFEVKATNGDTMLGGEDFDEELLSHLLKEFQKDSGIDLAGDNLA
MQRLREAAEKAKRELDGLAQTDVSLPFITADATGPKHMNAKISRAQFENMVQPLVDRSIG
PCKSCMKDADVDKDDIHEIILVGGMTRMPKVQETVEDYFGKAPSRGVNPDEVVAMGAAIQ
GGVLKGDVKDILLLDVTPLSLGIETLGGVMTKLIPRNTTIPTKKQQTFSTAADNQPQVQI
KVMQGEREMAADNKSLGEFDLVGIPPAPRGVPQIEVSFDIDADGILNVSAKDKGTGKEQN
IIIKSSGGLSEDDIEQMVRDAESNAEADAKKRQLIESKNEIDSLIYSTEKSVADNGDKLD

AETKTEVEKAIAEAKEVKDGEDLELINSKKEALNQASMKIGQAMYANKGEGEGAEDKKED
ENTQDAEFTEKKEEKKDDEKK
>comp4558_c0_seq2; 1375 242 2287 LEN=2393; translated
MLSSSVLRRSLSSLAAKKATTPGVSIGAFDGIARRFKSDDAGDVIGIDLGTTNSCVSIME
GRTARVIENSEGARTTPSVVAFVHGEGGAEAERLVGMAAKRQAVTNPENTMYAVKRLIGR
GYNDKEVNTIQGLVPYKIVKSDHNDDAWVADTQGTKYSPSQIGSMVLGKMKETAEGFLGR
TVSKAVVTVPAYFNDSQRQATKDAGKIAGLDVLRIINEPTAASLAYGMDKADGKTIAVFD
LGGGTFDVSVLEIQGGVFEVKATNGDTMLGGEDFDEELLSHLLKEFQKDSGIDLAGDNLA
MQRLREAAEKAKRELDGLAQTDVSLPFITADATGPKHMNAKISRAQFENMVQPLVDRSIG
PCKSCMKDADVDKDDIHEIILVGGMTRMPKVQETVEDYFGKAPSRGVNPDEVVAMGAAIQ
GGVLKGDVKDILLLDVTPLSLGIETLGGVMTKLIPRNTTIPTKKQQTFSTAADNQPQVQI
KVMQGEREMAADNKSLGEFDLVGIPPAPRGVPQIEVSFDIDADGILNVSAKDKGTGKEQN
IIIKSSGGLSEDDIEQMVRDAESNAEADAKKRQLIESKNEIDSLIYSTEKSVADNGDKLD
AETKTEVEKAIAEAKEVKDGEDLELINSKKEALNQASMKIGQAMYANKGEGEGAEDKKED
ENTQDAEFTEKKEEKKDDEKK
>comp4559_c0_seq1; 524 1 1231 LEN=1231; translated
ELTNDHIRGMLRSTDDIVRPMVHPASIWDEEGTGKDYRALVLDRIAEQRGASRKRKKGVA
FRDVPGPASSGGVPSLTRPFLADASESGGPGLHPVLQRLWEDNYWRANERPCPYRRLDPG
GDGNDNDKDDKVVDDVEHVRRGTAASDDESTVGGRSELGVGAEKDDDDARSRLSTGQEMP
RDDEMDFPVANDEEDEEDDEMDAPVPDFGDDEEDPTNLAAGGDDEDLLDLGMVNDMVLDS
DEDDRQDDGEDDEDREAIGDAASSSTKWHKHTVRVFRHLRKCLRDPNADTDEDGGIANEG
LPDSVGFADLTKNVVSRRNASSVFFEMLQLKTWDFIELEQERAYGDIAISAGLRFGEDAP
EHWRTKRKRKRNESKRKAWKVGHVFRKQRCQNKNYNDSNSNSDDNAFDRG
>comp4560_c0_seq1; 322 153 1546 LEN=1601; translated
MFGSVTCGALEPFLSVDGVDEIFNGVEKKKKKVRVLRWSSNDGDRDRDCDSDCDSDCDWA
IVVATSEFDSKTYHVHRSVLGAGPRKSVFFSKLFRKGNKAGSNSTRIQLDEQDAESFPLF
LDFVYGAAPGADESNSKSLKLNVREDFGAPNAVSLRHLAKVFDCEGLTLAVNTFIQKDLS
LRTGPVYLVEAHHYEDNRLIESSKRLCIENFALLDTRAIVGLPPELFQSVMSGVIRRAKS
AHESTPEEENALGYHVGEVVCQYFEKNPHELSAPLLLELTDEEVIKEIAPEPAIGITALI
RDLDAKEIRPNSGEWRGVVGLSQRCARAIVREYGWKDFNVKSALEEYLYGPCADGDSSST
KLDSLLFATSFAATLEGAQNETRTPFKKQGVRLQQQNDELQRQNQKLKEEVDQYKSMAAS
TKEEYLLLKXASEESKAAAKFLIRQTLLHRMRRALSTTSSRRGG
>comp4561_c0_seq1; 527 314 2215 LEN=2532; translated
MKVLSIFWGVHIVVVAAVLFELGPPFFVVEAAKDTEQNPNKSRSSSSSSATMKESSPSRL
RRTTQVLQVAIIEESDRKKQQQLKEISSNNHYDNGKIVHNKPGSNSDERRETSRHLLQAS
KITAMNHYQAVKEENVLMNVNEMVRMFERDDRGDRGYYYSMSMEPAKRPTQKPTRQPTYR
PTNRPTNRPTNRPTEAPYFENISRPPTRSPTRRPTRPPTRPPTGSPTRSPTRSPIQVDPN
PIPTPVVAPVVAPVNFDVPTPNGDTICGELTPQERETLILGQLATYSGGDVTSPGTPQNE
AFRWLIEKDSAMVCPEDFLNVQQRYILAVLYFSTDGNNWFNCNALSSPDLRQCPGGDSQR
FLSSSDVCFWYGISCNRLRVSGISIDENNLRGDLPDELGFMEQIEDIDFDGEIEEGLNFL
SGTIPSTFGLLEKLVTLDLDSNQLTGPIPEEIFEAKRLTIMDLDSNELTGTLSPNFANLE
ELQILFLDNNGFNGSIPSEMGAMQRLRFLRLDRNELTGSIPDDWVAMQNLRVMFLNNNGG
ITGTIPEFLGEFVDMQQLSLFDTSISGTVPSSLGQLTGLVNLFLHFTDLTGTMPQEICNL
RTTGALNFLTADCAGTIRNSSVPSPIVVPFVFR
>comp4562_c0_seq1; 1066 332 2344 LEN=2742; translated
MFHNGGGGGAGRSPVYQIDFTAVASGKRIASSKRRVRWRFGFANADALAAGQTGTDCRGE
EHDVTIVWSITSGKRLVLMDGKETHFSSSRNNLFEFSWTMKGNHVLKIAAHAQPPMSAAP
GFRQYDFFVDGQSFFTFPKVFRLGLAPNDPRGAGGSSSGRRRDASNYGNRSNNGYDNYGG
RGGGDMVPYANGAGGGDVRSMNSNNIASIEAPHNPEEEDAYLRQAIENSLKEGPSPNRRP
APAPAPAADNLLLDFMTPAAPAPVAALPPSTSQYPGDMYGAPTPQQQPAYAALPPSNTAP
AGPPTQQQQQMVPTGASSDPWGMASMANSLPSTQGAVAQPAPNPYGAPAPRPPQQPAQQP
PAAAVPGQQQYGFAAAPPPFGSPPPTSISATPKETPVTQASSLGFASPLAQPFGPPKEQP
QLQPQQLQLGVEQEEQPIPQEPVVEPAPAPAPPSDPALLSMNVLSGQPGPLVTEAMTNGT
AGQAAGSMADQAYAKLVNLDAFDLVQDKSADSRQNPFDMAGTTSTANANLSLADMKKTKK
PSEPKKSVMNSPAPAPGALVLSGNQQGNFGGYGGMGTMGQQPPATGMMGQQQPPPLQQPG
YGGMQQPQMQGYGQPLPLQQQQYGMQAPAPPMQQQYGMQPQQAQYGQQQPTQQQQAYGQP
PPLQQQPYGF
>comp4563_c0_seq1; 944 502 3018 LEN=3234; minus strand; translated
MAWTMIRRSCRQWFPSRNQMRRTGTNHHQQRAHKHATTSPGLWTSAAPSAAGSSSARTFL
DSRQHRHRSWGMHAAHKPSHDGGDHYSSSVSRKNRRRPRRLSSSLAATTSASSSSSRLYR

YRDFLEKGEHDRIVLSYLTDIEGDKFYLDRYVDNSKILTWIPSDDDAEPPGNDTEQSPFS
LSYYSCKEYPPARFPYNRRMDFVDSNSMLVFGGDLWDKGGFDLYVTRQLMDLKRRYPDRV
VWVLGNRDINKLRMMQELGLPPAESSTDKSNTGAAAASNLSVPYHPGLTWFRGSGRVGDP
SGPLPSMVPGDRLRWILGNTMGSPDAMEHRRQELAWEAGGCRETGGTSAASSAEGISDCD
VVRSYQESCHPRGEMGRFLAEGLLAAKVGPILFVHGSLPLTKDTMERGLRDGGDGSNIWD
DLTFCMPWINKTHHEYSEDHRFESASDLGVETIEDWLDAVNRFASRNVEAWERDIARLER
EAGQQATNATHNASDGVEEESKDSIWAYRAGYGNGPSHSGIYYSDLVQYGMGMIPGGKKN
PTVVYNSFTPEGMPTFFLPPEESGGTNGDCEEALGTEHASERDSGNNPSSSESDVAICTR
QFFERSSMRLILTGHKPQGDMPSPIRVDRSSWVICADTSYSGDTKWFHDERNATNPSKME
RRKRQEEEQHEKRKRSNLGRGNSISFRGETAVSEVLVELSGKDLSPESVSYHGVLSDGTE
YESVNLLDDGEDESGAVSADDAGSNSNGSGSCGSLDSMPATAAILGRVAPTNMVPDPLNS
PHEGRWWTKGICRDGSHLYYAGEGYNVWNYIVSPSDIDGNRSNTKYIDNTASESDAKK
>comp4563_c0_seq2; 944 425 2941 LEN=3157; minus strand; translated
MAWTMIRRSCRQWFPSRNQMRRTGTNHHQQRAHKHATTSPGLWTSAAPSAAGSSSARTFL
DSRQHRHRSWGMHAAHKPSHDGGDHYSSSVSRKNRRRPRRLSSSLAATTSASSSSSRLYR
YRDFLEKGEHDRIVLSYLTDIEGDKFYLDRYVDNSKILTWIPSDDDAEPPGNDTEQSPFS
LSYYSCKEYPPARFPYNRRMDFVDSNSMLVFGGDLWDKGGFDLYVTRQLMDLKRRYPDRV
VWVLGNRDINKLRMMQELGLPPAESSTDKSNTGAAAASNLSVPYHPGLTWFRGSGRVGDP
SGPLPSMVPGDRLRWILGNTMGSPDAMEHRRQELAWEAGGCRETGGTSAASSAEGISDCD
VVRSYQESCHPRGEMGRFLAEGLLAAKVGPILFVHGSLPLTKDTMERGLRDGGDGSNIWD
DLTFCMPWINKTHHEYSEDHRFESASDLGVETIEDWLDAVNRFASRNVEAWERDIARLER
EAGQQATNATHNASDGVEEESKDSIWAYRAGYGNGPSHSGIYYSDLVQYGMGMIPGGKKN
PTVVYNSFTPEGMPTFFLPPEESGGTNGDCEEALGTEHASERDSGNNPSSSESDVAICTR
QFFERSSMRLILTGHKPQGDMPSPIRVDRSSWVICADTSYSGDTKWFHDERNATNPSKME
RRKRQEEEQHEKRKRSNLGRGNSISFRGETAVSEVLVELSGKDLSPESVSYHGVLSDGTE
YESVNLLDDGEDESGAVSADDAGSNSNGSGSCGSLDSMPATAAILGRVAPTNMVPDPLNS
PHEGRWWTKGICRDGSHLYYAGEGYNVWNYIVSPSDIDGNRSNTKYIDNTASESDAKK
>comp4566_c0_seq1; 196 781 1452 LEN=1642; translated
MPSVGLYFGIYSFCKKTFQHSDPDNYEQRRALYIGLSAAIGNTIASASRVPYEVVKQKLQ
TKAYASFGEAMRDLSYRTLFPKGSIASQTLRDVPYAIVTLLTYEHLKSVWKTRAQNRFPK
VPVRSWDLLVGGLSGGLGSYVTNPMDVVKTRLQTDSQLYNGSIRTCAMMTLEEGGPAAFL
RGSIPRLLHKVPANSFFFLFYEFFRSILRVQDDANFSMAKDEK
>comp4566_c0_seq2; 250 246 1334 LEN=1524; translated
MVQLTVLVAIATLLLSTPVVSSKSPPLLPQNTHHRRGQIASSSRNSDTIRYASSVTTSSR
KSQEAPWVSGFKNSLASGLAAGCSKLLLAPFDTIKTLQQAASVSGTTSLSVSQAAQKILS
RPKGFLEFYAGVGVAVIGSMPSVGLYFGIYSFCKKTFQHSDPDNYEQRRALYIGLSAAIG
NTIASASRVPYEVVKQKLQTKAYASFGEAMRDLSYRTLFPKGSIASQTLRDVPYAIVTLL
TYEHLKSVWKTRAQNRFPKVPVRSWDLLVGGLSGGLGSYVTNPMDVVKTRLQTDSQLYNG
SIRTCAMMTLEEGGPAAFLRGSIPRLLHKVPANSFFFLFYEFFRSILRVQDDANFSMAKD
EK
>comp4569_c0_seq1; 266 38 544 LEN=756; translated
MNTINFKVTTTPLGENTITSETTVFVPKFKGNKGPNDVVCFVGSGTGLQPDIKSFYGAGG
TNCQAKNGCGVHVHSGLSCKNTETQGGHWYNKDVLSVDPWAITGYEITSAAGTADFASCV
YTGFDVATNPDLLEGHAFIVHNEDGSRASCGIIKKGGKHNKSTKKTRL
>comp4570_c0_seq1; 224 91 525 LEN=691; translated
MTSDSKQVRFGDLTITEYPIILGDNPSCSGAPITIDWKPMGSYTRNLELYEYTRMQRRHG
RKKLVIPVQKRSQMLLDAGYTEEQIIKRALEVAQIKTQRAETLNEPKQNGFDKFSSSLVK
SFSKLKIDKSGNKPPKTTVAARSA
>comp4571_c0_seq1; 195 322 1225 LEN=1225; translated
MDQKHSRQQILWLLIMATVTCSLCLSSVVTAFAPPPHYCSKRTNSNSLRHFPPTKQKTAQ
AVTAARIAPALHVKKQSTADFEYQELQIQLDAMKKQNVKPSQLDPSKRAELRSYVEEILS
RRLPNMKTVDSSVPLPLRLRSALPGTKWRMAFTTQSLMAEALPKDATITMDFASGSGSAA
TGSNRVDYGLDFFKTLALKRLVARSTYSVMEPRTDGILGSAIVQITYDKIVTDVMGFSNL
GIGMFGMLKGRNTYIQTVFFDNKLWIESGVDPANGETYYNVYTREDKDSDSAGNDDDDDD
DX
>comp4572_c0_seq1; 872 139 2772 LEN=3074; translated
MLSSSLGNWEELRRNESANILRRERDQVLDVLQNYINEEDEGPAKNIAFCHLVSLMPFNA
IQNQTSDGYNDFAYEASVAIAMAISHLNEGNGILVKEVEGLNERCNIRFTAEFIDTQLDP
GITMNRILELTDTSGTRHPSAFLGAFRSAVSGPSSIITGLQGYPQISGASTSALLDDKNQ
YPKFARTIPSDDGVATSIIQYFSTFLGLEHLAVININDSYGNSFVRGLKVAAAKHAPNMK

IAQIPIEENDESIKQAIESLKQTNYRFVFCITFKPWFDVIMEEAARMEAAGDGKYNWMFP
DSFSGALSHRKFERGSLLQKAYQGTGMLTSSAKSGSKFQSFSSSMQALKNEEDFQYLKTL
IPRFDQTPFFELNSTFMHPINYDYTVFFYDAAILLGLAVCEATGDDLRITGNTLYDQIAK
FSSFQGVSGTVALDPNTGTREPNSALYTLTNFVERGGIDEENGMDMVEFETITTDQYSNG
TWSSILNYTFNDGTSNRPLGIPSPSTDKNLIESSIRSVMCTLCALTMLLSIGFALWTWYF
RKTKVVRASQPFFLLLICLGSFIMGSTIISLQFDHKIADLDGNNIACNAVAWQAFIGFGI
VFSSIVCKTHRINIVMRNALRFSRITVTVEDTIKPVLLLTSLNILLLSLMTALNPITYET
QIVEADEFDQPLETYSYCSWNNSLRYVIPLAVVNLISVIVAIVEVWKARQIATEFSESQY
IFRALIYMVVVFFIGGPIIFIASDNPNALAFVSSGIIFVATCGILLLMFIPKIIYQRKRY
LMKKKGTNKNRYSFSLGPSLTDSNALESDYYNTGGEKILTTKTQKELSEEVKMLKRLLKE
ARGTKKNESERTPCDQFQVVEGENGNSGDDIVESEDI
>comp4573_c0_seq1; 203 437 1168 LEN=1751; translated
MSRATSTPGGKPPLYKIVMLGDSGVGKTSLVARLTNPDRPLNHDISATMGIEFDTQMLDT
PQGKVKAQIWDTAGQERFARVLLPTYFRKAKGVILVYDITNAKSFESLSERWMAQLNDHS
NADDLAKLLVGNKSDLESSREVTSEKASAFCQEYGMEFLETSAKSGDNVLQAFEKLIGIV
HDRALNASKAKGGVGGAGVGGNGSGAPKGASDSGKSDAAGGDGTVKLEDSGAAGGADANA
CGC
>comp4574_c0_seq1; 250 992 1894 LEN=2479; translated
MANKKRKNAQKKATVASSGRSSRQSSKPGGSDCGKGERSSHHQTQRSFQSFPLHLVPNNE
YAGKDNARELRELCRGRVWVVPSFFSHKECRAWVDFCESFGNAGGNGFQYTAHPASKYIT
NRECYRMQQENASELSDRIFQRLKGMTITPNKNCGNGKGDANSSKPKETNACPTILSRIQ
NDTADLYPPPKSYGPIGCNPNLRVYRYDEGHAFGRHVDETNAVCGGATEMTMLVYLSACR
GGATRFHIGSGRRSSFLAFEPEVGALLLHVHGKHCLEHEADAVLAGQKYVLRTDLVYGRR
>comp4575_c0_seq1; 450 1 1582 LEN=1794; translated
XFLAAMFRMIFVCVAYEQIEGHALAFLGFQVVLLNVAITNAMYIWLTGQEYTRVTLLPCI
PKISLSAPVVAFVTKIYLICTVAISVVKIYGTLLFVLGGDISWYLIPVPFTGKVLGQIID
LIWMTFNAILPFYFAYVRAENEDPFIIEIDAPKHIYRSRDDGGHNLGDANGEDEPQNGDS
GPATETTGLLTGGGSDDQPPDEETPDEAPAPPPVAPTGPRIIQGQENLDSIKGSEGNNDN
LANIFKTEGYTEAEAILVDGGWISDIFLPSGCPEGSKLTVRCNSTWNVKIGNLKGVGSKV
VATKQRLVVVVIDGVWTIQEWADEKGPFVVKGQQNLDRFKGTGGKYFVVVANKRVVYHNA
DLEACKNFLMTKFGHGASRPSRMVVDVSGGKVSEDPHSCGGQNQGGGMKAGFNKWWQGWP
DIRSMNTVAKTFLKTNKVGGPPKNDNLLHLFNPKDYKDVELHTYDGSWIRELYLPKANGI
LSGSSLTIKCNSTWGVKVLYPGNWQKDVATKTTLALIVKEGKWIVKK
>comp4577_c0_seq1; 193 104 639 LEN=639; translated
METPEKTPEPAPPARTRRAGKRSGDTKIAATMAVAILVVTGGLMALTSPSADGGSSPLAK
VFAQAQCTDPCVPGSEDIMKPKAHGTSEYPVVSVGXTNSATERTSGPAWKRSDAVPPAVF
VATVEPTVTRTLFVSVVAVSCNNSSHSRENRPIFVSQELVVCCVLLLAFFSTRSNKICV
>comp4577_c0_seq2; 328 104 802 LEN=1100; translated
METPEKTPEPAPPARTRRAGKRSGDTKIAATMAVAILVVTGGLMALTSPSADGGSSPLAK
VFAQAQCTDPCVPGSEDIMKPKAHGTSEYPVQQNLRWGVDWDLADRINNFNRHYAEYSGY
WESTSFLSDVRENIETESGTVTFYDSGWKGSPLFTAPRDRTWADFVDESRNHGWPSFRDS
EVDWNYARVLPGGEMVSVDGSHLGHNLPDRKGNRYCINLVSIAGSLPKDDDE
>comp4577_c0_seq3; 128 238 558 LEN=558; minus strand; translated
MFVRHSVHFSDKGLCGIVLGAIRHRPLEDFRQSKPDLRYGRLPQNKTSGKRISINRNNLF
HERTADANATILTTIQKKNQRNVQHSTKRENTNGSHAVSKLTDAIPI
>comp4578_c0_seq1; 287 1 798 LEN=839; translated
MLEAERNLFGNESTEMSKSPIHNDKQWTQPPVRRSKKFHIMPFKMILNICLLKLLLLMST
MMSHVPRGSAFRARSVNSYGLQHRIRIKNLSMQSPVTRESGDASLLEPAVVSSRVFDTDK
RPIILFDGVCNLCNNAVNLALDWDPNAKLRFSALQSNVGKSLLENNGRKADDISSIVLVT
KDGAFVKSDAVLKITEELTPLSLLPLKPAAMAGRFLLPKLLRDIIYDSVADNRYRLMGMR
NECRFDADGEFEDRFVDDDIALKKP
>comp4579_c0_seq1; 596 117 1331 LEN=1429; translated
MKFSAAILLSLTLGSIEAFSPNASPSRTATVVRSAVDEAVDTKDSSKGGETKDPLLIRAA
KGEKVERTPVWMMRQAGRHIQEYRDLCKKYPTFRQRSEIPEVAVEVSLQPWRNYKTDGCI
LFSDILTPLPGVGCEFTIDEKLGPVMDPIKNWDDVKKMHPIDPSAAAPFVAETLQILRKE
VGPETAVLGFVGCPYTLATYMIEGKTSKEYLEIKKMAFNEPKLLHTILQNLADSIGDYAL
YQIENGAQLIQIFDSWAGHLSARDYEEFAMPYQKQILDKIKKHHPEIPTVVYIKHSGALL
ERMAGTGADVVSLDWTVDMAEGRARVNKGRELAGLSGPGGVQGNLDPGVLLSNHDTIRER
TEEILRKAGPTGHVMNLGHGIEACTPEENAACFIETVRNFRHEE
>comp4580_c0_seq1; 167 1 388 LEN=508; translated

XTNSTFTMVFTVAAATARRRIAVMATRHAAQGQKRSMGGGPAPEWEGIDKVVRGVFPGDH
QLAMAIMGGYTSLILLFKMKSAVSKKAPEEKIAAAPASSSSDGGVPSIESPEFGEWLGSP
SFEAFLEKA
>comp4581_c0_seq1; 413 76 849 LEN=1317; translated
MFEAQMQDGKIFKAIIEAIKDLVQDCNLDCSEEEVAIQSMDSAHVSLVAVRLSAGGFSNY
RCDRPNSLGINTANMSKILKMLGKDDSITFKAEDESPDNLTMLFEGGKTTIADFDLKLMD
IEAEQLGIPDTPYQCTVQMNADEFQRIVRDLQVLGDTCTISCGKEGIRFSVTGNIGNGNI
LIRSNDEQNEKDRVTITMDEPVELNFALRYLNFFTKATPLSSSVIISMSPDVPVVVEYPI
EDFGHIKFYLAPKIEED
>comp4582_c0_seq1; 371 117 1670 LEN=2183; translated
MCINTQVERNPDQMVDGTTSLPTLEAEVETTNTTRPSKRMSREEVSACADSDSLCLMIIG
DNVYDCTRWQKYHPGGHLTVRALCGKDATDAFNVTHPRWVTERMINKFLYATLEPDDEGT
ISNDSSNETSDVTKDARLTISTRPHGVKDDEATLAFRLLTEKLEEAGLFETDYTFYFRKF
AVYTCLFLLILGGVLLSESLMVHTFSGILLGIWWQQLAFIGHDLGHNAVTHNRVHDSYLG
LFVGNFCTGISIGWWKRSHNVHHIATNSCTYDPDIQHLPVFAIDPVLVTTKLFSTYANIY
MPLSDVTHVLVKFQHFLYYPVMAFARFNLYIQSLNHALRLSYYATAGDLIWGQKLQIASM
GGFWAWLLALTFSLPTWESRVLFFLSAHIVAGILHVQITLSHFPMETYTGVTYDDSSNGF
LRTQLATTMDIDCPTFMDWFHGGLQFQVEHHLWPRLPRHNLRRVKGILQSFCKEHKLIYH
QATFFKANCMVLDTLKQTAKSTKTFSELFHESANLMG
>comp4583_c0_seq1; 371 51 995 LEN=1115; translated
MATAKENANPKLFSVDNPGRYSRARQTPLDWIHLGDFRRERRGASPGGASPFVVVPTTAG
DKAVLRALADASRSDPRGVGLLRKAGLAIPVRTSASSPGKTAGSLLVPSCLADFSRPVFR
IIPRDDDDDEEKGDVEEPRPGGEAKQNDVDNNDDDNDETDHGGHRPTGFSETEVSAEEIF
DIIRNVQDPEHPNSLEQLGVVSLEQIELSLARDRDRGGTTHRNKDEIRIRFTPTIPHCSM
ATLIGLCLRVKLHRSLPPARFGVDVSIEPGTHVSEKAINKQLRDKERVRAALENKHLAGV
VDKCIRNGMEAATD
>comp4583_c0_seq2; 286 51 942 LEN=942; translated
MATAKENANPKLFSVDNPGRYSRARQTPLDWIHLGDFRRERRGASPGGASPFVVVPTTAG
DKAVLRALADASRSDPRGVGLLRKAGLAIPVRTSASSPGKTAGSLLVPSCLADFSRPVFR
IIPRDDDDDEEKGDVEEPRPGGEAKQNDVDNNDDDNDETDHGGHRPTGFSETEVSAEEIF
DIIRNVQDPEHPNSLEQLGVVSLEQIELSLARDRDRGGTTHRNKDEIRIRFTPTIPVRNA
MQRNAMQRKNNHRSDTRRGSVGIFIDLRCPHQIHQPLVRIEIPCNARNLSWLPAAPHX
>comp4585_c0_seq1; 289 302 1230 LEN=1230; minus strand; translated
MRVSFCKRWHVPARAAAQGNHGPGVAWFHLSCLCGLDWSTELERHVPRRPQGNADAQGPF
VPGGGVPIRRATPVGGLGFVSFRLVLHRFSPPQALSGPVALVALVPTAGTSPSGVVTAGW
ASLVPFVAAPAPVVSGCGPSGVAAHQEVGIDPEVVAQALGRQGPRQNLPVEADPLFLLRP
AAPGLRVVPGVLVPKHAAAQAPVAALGHKALGVPGGSKEFQTLHRCVGAGARSAFASVRI
ANEQAPIVLGQEAVLVVRRKIGKGVGAGRVVEEAHHRFPKNRAGAAFVAGGGVPKIADQV
RQLGVPVPAX
>comp4586_c0_seq1; 722 253 2028 LEN=2212; translated
MEIEEDDQPYWYLLNCVAGLEMDLLRQVKQLAQDLPQADVVKFVVPTVTKTRSHGANRMV
RETKVKYQGYVFAKLRLTRETYEPIRNMDLCRSWMGTINMKGYKKLPPAPLPLSDEEIES
FDLENPKWEIDEQDKSTGNDSDGIILDTHENDMKEMEEEEAIEQEVERVYKGLKVEDMIK
VTGRNKFKGEDGIVKRLKEEMVLIRFFTYGQTFDEWLDPSDVRKLTDDEVLKGLGGPGKP
ITQQDLEGPRDRREDKPQRKFDSRNSVGAFGGGPSRERRQDRNERRFGGEMDHKQERENW
SQFKENEKRGRRGRSYDDGNGVEFRPASEAEGDVDSQWGRRRRSNQQEDDWSSFVSQGPS
DYNKKNKSSPSKEDTDDFFASLMTDLSKDIDGHSGKSRDQPQESSGSDSDDDFFASLMSE
INDSPSSQKQKSSTNGGDDDDDFFASLEREIRGSKSATSKPKRAEKETKPDISSAPTADE
DDFFASLEMELETDLGESKSQGSEQSADAAEDDFFSSLESELSTDLSSSETIDAPASAKQ
TRAPQVTSSSEFDASDLKKRTVPELKAILKDRGLKVSGKKADLIERLTSGV
>comp4589_c0_seq1; 441 83 1618 LEN=2301; translated
MTSTSRSISVVVIQLLLICTCAVLSAANANNNDENGKNRFERKRINGNGDVTNEMINNRD
DTAREYLESLWDNFEDKQDQKVQGRRLAYLESMMDRLLMSMSMPTKSPSSEPPNSKTPTA
PITPTEFPTSKEPTDMRPTRSPLPGPTLPTRVPIPTKAPINAPSEPFTYNPVEVPTLPNP
PTPAPFSAPTRPCIAADKERNLYQAVSSTTDRSVFLNINTPQGMAYSFLLEEEPSFICSP
TLMQRYGLSTFYFATNGDSWTNNLGWLTGVQECNWFGVKCNEGLFATSLNLALNNANGII
PNEISAVSTLQKVDLFSNSITGKLPNRLSRLSSLVTLDLQDNLLTGDAFPRGITSLERLV
SYRISNNQLTGSISADITSLRRLKELWAGNNMISGSIPSNIGDLRDLKTVYVNNNALTGR
IPSELGLIPLEGLVMNDNSFFDVIPSQLFNVASLDTIRLDNNFLVGTLPDSIGQLTDLEI
FRVENNNLSGKIPESIGFMTSLGEYGTSRKF

>comp4589_c0_seq2; 549 83 2083 LEN=2192; translated
MTSTSRSISVVVIQLLLICTCAVLSAANANNNDENGKNRFERKRINGNGDVTNEMINNRD
DTAREYLESLWDNFEDKQDQKVQGRRLAYLESMMDRLLMSMSMPTKSPSSEPPNSKTPTA
PITPTEFPTSKEPTDMRPTRSPLPGPTLPTRVPIPTKAPINAPSEPFTYNPVEVPTLPNP
PTPAPFSAPTRPCIAADKERNLYQAVSSTTDRSVFLNINTPQGMAYSFLLEEEPSFICSP
TLMQRYGLSTFYFATNGDSWTNNLGWLTGVQECNWFGVKCNEGLFATSLNLALNNANGII
PNEISAVSTLQKVDLFSNSITGKLPNRLSRLSSLVTLDLQDNLLTGDAFPRGITSLERLV
SYRISNNQLTGSISADITSLRRLKELWAGNNMISGSIPSNIGDLRDLKTVYVNNNALTGR
IPSELGLIPLEGLVMNDNSFFDVIPSQLFNVASLDTIRLDNNFLVGTLPDSIGQLTDLEI
FRVENNNLSGKIPESIGFMTSLVSLRLNDNFWTGTIPDVFRNYKQLDYFDVSNNMWEGSI
PKTIFSIETLRLAYMSNCNLKGTIPSRFARPPDLRDLYLDGNNLTGTIPPIKDGQLKKLN
ELLLQNNKIIGTMPDSICNLRSKFLLDDLWSDCGGPSPEIECEFPECCNRCFEAEALSMT
RRKDRN
>comp4590_c0_seq1; 488 134 1498 LEN=1630; translated
MASNAGTKKLISGLTTAIRNRPFVVLGAAGVVGVTGYKQYKAEKRLAEDAKKKRVLVLPF
HRMKIVEEKKSPASALFSNLTGSVENGGVGTEKTIEMQSDELITLIKEAAEDPSIVSMYG
VFGNGGTISTGGWAHLEEIRNALETFSKPSSTVDEDDESARTNETKKTGKAMHAYSNTFG
GQKSMQEYYLASAFHKIHLQPQGDLNLYGLHTTNLFIRDFLKKYGITVHVWKHGVYKNMA
NLFTHSYYSKEHLENAEGVLLPIHKHVCNAIYSSRHEQLKKYGYDFDQFWSMVENAGSLP
ANVAHQIGFVDHLPLKNPLDALLKNNKREMRVRKEGVGGVDTDVDASSIMTDTTSPPSTS
GDSDDDSATEKVDTKREKENTAVAKSGDENSSSAAVGDNWKLGTDSGSFQADSQIFIDAY
SRQRSKLRKKRASSGNLLESLLSAFGIGDEVSCK
>comp4590_c0_seq2; 871 134 2497 LEN=2779; translated
MASNAGTKKLISGLTTAIRNRPFVVLGAAGVVGVTGYKQYKAEKRLAEDAKKKRVLVLPF
HRMKIVEEKKSPASALFSNLTGSVENGGVGTEKTIEMQSDELITLIKEAAEDPSIVSMYG
VFGNGGTISTGGWAHLEEIRNALETFSKPSSTVDEDDESARTNETKKTGKAMHAYSNTFG
GQKSMQEYYLASAFHKIHLQPQGDLNLYGLHTTNLFIRDFLKKYGITVHVWKHGVYKNMA
NLFTHSYYSKEHLENAEGVLLPIHKHVCNAIYSSRHEQLKKYGYDFDQFWSMVENAGSLP
ANVAHQIGFVDHLPLKNPLDALLKNNKREMRVRKEGVGGVDTDVDASSIMTDTTSPPSTS
GDSDDDSATEKVDTKREKENTAVAKSGDENSSSAAVGDNWKLGTDSGSFQADSQIFIDAY
SRQRSKLRKKRASSGNLLESLLSAFGIGDEDEKSKEVKEKIAVIKINGAIGEMTARKAEK
ALRKVKEQKDIKCVVLRVDSPGGSINACESIHQEILDLPQKVIVSFGNVSASGGYYISSS
AERIFALPSTVTGSIGVVMMRMNFKDLAKQYGITFDSIPTSDLSGSNDPFYPVNKKMNEN
FGNQADRSYHRFKSLVSSGRNMSMKDVEKVAKGRVFTGEQAKKVGLVDELGGLDEAIDYA
QQNCTSSGAARVVNWPPKKSFWQFLSMLGSDEEGANNMDEYDVPDVVHVALSRFVNLNNS
WTPSSRNIELGGIYSIFGNGCFACPSLPVTSGVMLTVDENAAIQCMLEDANVLKGGFASH
DGTNNGM
>comp4591_c0_seq1; 248 1 961 LEN=2170; minus strand; translated
XFRRDIEREGPSVFSDLYEAYKVRYDKYKNMIENVDFNPVQDLRNLVAETAQQAAKMSHS
AEESALKSAQNMYETIVQPGSQMEKHLSAFGHATSLQLTEAWKSVKGMKERLDTANFLSE
RRKALMKQVRGNRAMLTRMREMSYAVNSKQMAALMRTITEAHEALESIEAAAKDGFISAA
GKLADNSLFKRQEPRRYARYSSDPILGIASSPLCITLFLLISTESILRTLLKRRGFERRN
IGPVTYYYHPGSNYASESMNSENIDHNGLPPRDGSKTIPVVFIHGIGVGLIAYIPVIDAL
MESGRPLFLPELPYVSAFRPW
>comp4592_c0_seq1; 1112 256 2664 LEN=2772; translated
MKFSSLAIAVVLAVTTSQLQSTEAFTSVLNPQAHGTSSLLKASTQERDAENFQRRLLATR
LKQNSSGENVRSDGSDDKKNDGVLLEDADGLGVASESTASAAQEPAPVEAEDESTAESVV
LLNGSVADADEVEKLMSDIEIESEKIVAEIVEEECQIDEETLGPVDPLCADEEQAAGFRA
GLKNRISNTLQSLRGISKGDKAFDEEEEEEEIDGDMLEEGWFKRGESSALRRNAEVWKFA
LKCVFRSLKPRSMKKKGASEEEIKQAQIDAATFIRDGLLKLGPTFVKLGQVVSTRTDVLP
KTFTDVLKTLQDDVPPFSGARAKEIIAKELGAPTDEIFTDFSETPLKAASLGQVHTAYYK
GKKVAIKVQRAGLKELFDVDLKNLRKLAVLLDKFDPKSDGADRDWVSIYEESERLLYLEI
DYLNEADNGERFAKDFKNIDYVRVPEFYRDLSTPRVLTMEFVESLKLTDIEKIESLGLDR
ELLAKRTADAFLRQIVETSYFHCDPHPGNLCVDEKGNLVYYDFGMMDELKPNVREGFRKF
CTALFAGGPMVNDATLAKNAKQLIDGVEEAGVLARGSDRLAVEKLARYFMRAFKNNQLGK
SSGNIKETLGVDLQTLTENDSFRFPSTFTFIFRAFASVEGIGKGLDEKYDIGKLAQPFIE
KFTETQKGYENETQKQFSIFQKATGLNTKDIETAVTSPKKVAYIEETLRSMEEGTLKIRV
RSLENEKALERMALTQERTENVLLATMLLNVAGFASKQLVSGAGYAGFAFFLFQAFMANT
KVKKFDKTQAKFVQTKFNGEEE
>comp4594_c0_seq1; 224 113 313 LEN=523; translated

MAGKKKANDAANQAKEGLNALTAGKTDEQKANENRAKDRNAAYEQKKREREERKKKLQSQ
WADNKK
>comp4595_c0_seq1; 234 130 1614 LEN=1803; translated
MTDGSVASDPVYDLVVVGGGVVGLACLRAATLRGWKCALVEGEVDLLSHASGGNSGIVCT
GVDTRPGTLERALIRDSVSEFRTYCQSHNVPMRPCGSLVCLFPWDVTSNEKDGADNIAND
PDCTNGETGTHHLFPLEKVLKESHVAGDSGAALFGNGQKVLLEKEKNVSPSLMGAVHIPG
EIVVDSWVYSISLAAHALENGAQIYTNFRVTSIKREEIGDQDDGNDRTWKVSRECGNEPS
DVPTVIRGKAILNATGNWSDATEKVAFGSSEWVAKPRRGQYRVYNSNDQTQIIHPLQPIP
SQRTKGIFVFSTLYDQLVVGPTALDQSSKTDRSIDPKVARELDGHIRRVIPNIDTEKSYV
GDYVGIRPGTNHRDYQIHLKPEKRWIAVAGIRSTGLTASLGIGNYVVRLLQSILRDPSDA
LREDGESQVHDQASSERVIKTTPMPKVEKLAEDFVTNGDGCCTIHGLRYRVTHPLTKLGF
KSLMSNKLPSKSCR
>comp4596_c0_seq1; 248 20 793 LEN=1830; translated
MNDGEHVDDGTTVRVSVLPNPPSEFDVNESLSGALMNLELKDRIAIQEEIHGVRCGAVDE
TPELLRQSLARFDELLNARKEKDPGNRLLRNITRICPSDLATTEAGPAGGNGSSKEPVIK
PKKPSCYLNDPDIRLRFLRCECFAVEDAVQRMINFLEFTAILFGDYVAERQIELTDFNTK
EEENALQNSRNQYLPFRDRSGRRVLVGVGNCNFHLEYRLRYKILMYLFWVVSEDVETQRR
GIVIIHLIFDEDGVENMW
>comp4596_c0_seq2; 392 20 1660 LEN=1727; translated
MNDGEHVDDGTTVRVSVLPNPPSEFDVNESLSGALMNLELKDRIAIQEEIHGVRCGAVDE
TPELLRQSLARFDELLNARKEKDPGNRLLRNITRICPSDLATTEAGPAGGNGSSKEPVIK
PKKPSCYLNDPDIRLRFLRCECFAVEDAVQRMINFLEFTAILFGDYVAERQIELTDFNTK
EEENALQNSRNQYLPFRDRSGRRVLVGVGNCNFHLEYRLRYKILMYLFWVVSEDVETQRR
GIVIIHLIFDEDGVENMWESQLRPGIRKKIRELHKLQNGSLPVREAVIHQCLRNTPFFRT
LAAMYVFGLDAHHRSIYQQHFGELTELLYRMASFGITPDLIPVSSTGKVKFGAHISWLKV
QRIKLQTKNDSDIVECPRLVDVVFKHGPAYKNNPGNMYFRELIEISLGDHLDGTKDEKCK
ITMRILKQIEARKGRILEWSKRESTWRVVRNRNVMRKKIAATFKQQHRQRKTTTQMKKTI
ENALYIAADNETSREQANPKRYYDSDQLYHERKRVRTGCFDCDGDAEDPDFSCFGRSFHP
TWGGND
>comp4598_c0_seq1; 222 1 1236 LEN=1283; translated
XNTTRTKQNETTVEVVCFDPELRMTLACGLLREYNLEDRLSSSGSVTFFAPTDEAFARLG
DEHPDVFDTVLPGVDESILLYHLANGGFSLSDLECTGLVPTLLEGRSSRTKCVGDDRFQR
GPGNGTGGSAGEGLPRIIDPDLPACGGSLVHVVDHVLLPRLPAAATATGNEPPVGGGTEE
RATGGPTVEPTAXGIARADAASNIAADPEAKHGANGCRTNPGADHPAHHNAKHGENRGAY
LAADLGANNHSKNLGANNHATNLGAEHSRRCGTTGTRGPPVAGNGHSGSRGPRRRHPVPN
AGVDLVRDVGSHHDPFPDVLGDVGSHIHPLPDVRVGRGLRHRRSAAFRAIDRCIPLFRVF
FSVDSVVAPLTLCQRLLRSVLWGKCGSSFGRENVVIDNNISSTTTAFFGMNQ
>comp4599_c0_seq1; 265 127 660 LEN=1465; translated
MPRKFGLKVDLNLKENHEGYIALSKCPPIVGQSQLQKAAHLSLNPSSAPSKNTAALVAKR
KQKAMAIAMKPGSQVAMNAFMMYMSGSNLNIFTINTVSSAILTPITSLFMIERTFGPLDV
DAQMAKSIYVAINLAWLGIGLYKMSAMRLLPTTSADFADAIVWKDMMETSSIPSIIS
>comp4600_c0_seq1; 468 170 1372 LEN=1481; translated
MKRVVSAPQLHPRFGTQSFQMRVEKINDLGPLLEHKEYGTESPQAVRTPSNESDKYGTEP
TDDEDYGFYDAIDGNQEDSERSSTTSSLQDEIQNSTPSAEIASGRPSRRRSSGKKSSLKQ
RSSYGEDFIPLDFERKEFNRVLPKPDLSQRTRVPEGASTEKANGMIRSSSSGLFRVVSEP
ILFRPIYQEQFDIDDLSSNNEGGRLENSDASNSDGNALDNSDSSFVEGAMKKRISFGTIQ
IREHSVTIGDNPSCSYGAPIQLDWKHEDMEDLKVEDYEAYRPKTRTKDKLYLSHFKRMDL
LKQSGHSTSEIKESRRKTKKHRNQREWTKFVVMNYPTVSTMEDAIESGIRKMKRSLSKSS
LGSKEDIPRVSSKDDMNVIRDKALLLDMIDNDGSNATAPF
>comp4601_c0_seq1; 217 111 743 LEN=762; translated
MTSIVELFPKCRKLAYDSRQQLAQVQNGTLHASELFISLDELSRQLELMNKLVMRERPAQ
REVWKRKIHELRVEADGVRRAGEHYDRLVNTNVRQQKERDELLTRRRQRKENFSSVSERD
MNNLADESKSWSQSQYMVNDLIANGEASLSALVEQRQRMTGVSRFLGQIDDRLGISNSTM
KIIERRDITDAYFVLGGCVITCIVIYFTWL
>comp4603_c0_seq1; 190 269 1231 LEN=1231; minus strand; translated
MLSNIVFAVLACFVLNASATHAFVATAGRSGNKTRRGRLLGGPQRRPPAETATSLSDMKR
PVLDRVASFLFRLENDRVESSSVVDDKGRKGEPMEWSESDSLANRFSAIVATNGLGYRFK
QFVADIVAGDDYDVEEVNNRIDAFISEHPVAMFSFTTCPFCRRAKDLLDEKGIPYGVIEL
DELDGNAGNEXTGKPREKDRQNLGAVDLYRGKLHRGLQRRAGAPAPRGIRRSGPDAAGGF
IGAHGSIGGLPTELRTVRIDAWYVLHTMLNAQTHGSAAAWDCRVASSTDATVRPKQLGHR

SPQLRHSRVSESSNTVPKSKNX
>comp4604_c0_seq1; 577 134 1834 LEN=1870; translated
MSNPNASAASGLFIQGSRESGEDVRVGNVTAVVALANIMKSSLGPVGLDKMLVDDIGDVT
ITNDGATILEQLEVEHPAARLLVDLARLQDKEVGDGTTSVVLLSAELLKRGLGDLVQKQG
LHPTTILAGYRTALKAAIAYIKESILTPISLLDSKHLLEAAKTSMSSKIIGKEGNFFAEL
AVDAVKSVAHTKEVSGKNGIRKKKTIYPLSAIHILKVHGKSSLESYLMKGGFVLNATRAS
QGMPTFLGDPDDETSEVKIALIDMNLQRHRMAMGVNIQIKDPKEIERIKQRELDLTKEKI
QKILDTGAKVVLTTKGIDDTCMKYFVEAGVYCARRCPKEDLKRLAKATGGQVVITMADME
GDESFDESALGKCRAIQETRVGDGELLKIFGCSGSGASTIVLRGANEYLLDEIDRSLHDS
LCVIKRMLESNSLVPGGGAVETALNVYLQEYALKTLDTREQLAVEAFADALLVIPKTLAV
NAAKDGAELVSKLKSIHSKSQRPDCENDDLKNYGLDLVNGEIRDSLAAGVVEPAISKIKS
LRFATEAAITILRIDDRIQINPTEQQ
>comp4606_c0_seq1; 836 127 1949 LEN=1949; translated
MNFIKDLSIDKIQNLAEDAFNQAKPKTDVEARVYECLSHKNWGASGSLMNEIARDTYDME
RFQVVTNLMWDGMENQRPAAWKIVFKALNLLDHLVKNGAERCVDDARNHGHILRSLGQFN
YYEGTIDRGLGVREKSKQILEMLSDDERVREERQKARKLREKFGKFNKGASGIGGGGGSS
GGSGYGNQDSWNTSSGGDGGYGEGGIYDNEKAYSGRYGNSSSTAAPTFASIPGEKKKSKK
KKKKQQAAPAPAPEPVGDLLGFDDAPAPAADTGGSSDFGAFQAPSAPAPAPAATNASDDF
AAFDQIRSKNAQQGPDPFAAAPVQQFDAFANNNSGVGVNNNTMNNNMMNNNMMNSNMMNN
NNNNMAAMGNAFNNLSVNTGAPTGGVQQPSASPSNDDDFGDFADSKLSPSLSKPVTKSSD
PMAGLINLDSLTKNASKKMSMNSPVAVEAAAAQYQRDMQNGVQGQALMQGQAPGVVSVSA
GGSDAISSMMGPVPTQPQRGSNFSMSTQAGDGNPQMQHQQNFNQGMQNQGMQVGGMNSQM
QGGMMYGNQMGGMQGGMNNTNMNQMGMMNQMGGQQQQMNPQMMGSMGMQGGMNQMGGMQG
GMNQMGGX
>comp4607_c0_seq1; 242 163 696 LEN=1634; translated
MSSFGSWYDEQKAAENGGASNSNWFSIDTDEVLPLFNAENLQGFSFENMKQTMEGQMPKK
ILGMGYQQRFKVFCALLFLSGLFFALAFFVGVPMLALKPQKFALSFTCGSITFMGSFGIM
KGPYEHLISMCTMERMPFTIIYLGSMMTTLYLTCTKGGMKGYALVLISSGVQLVSLLW
>comp4608_c0_seq1; 209 1 1128 LEN=1349; translated
XGLLYGTVWHVRHPKPRTARSFHAASTMLSNIVFAVLACFVLNASATHAFVATAGRSGNK
TRRGRLLGGPQRRPPAETATSLSDMKRPVLDRVASFLFRLENDRVESSSVVDDKGRKGEP
MEWSESDSLANRFSAIVATNGLGYRFKQFVADIVAGDDYDVEEVNNRIDAFISEHPVAMF
SFTTCPFCRRAKDLLDEKGIPYGVIELDELDGNAGNEXTGKPREKDRQNLGAVDLYRGKL
HRGLQRRAGAPAPRGIRRSGPDAAGGFIGAHGSIGGLPTELRTVRIDAWYVLHTMLNAQT
HGSAAAWDCRVASSTDATVRPKQLGHRSPQLRHSRVSESSNTVPKSKNVPQIFVRSTRQY
EGKQDSCRKNNKHACL
>comp4609_c0_seq1; 287 57 422 LEN=813; translated
MTKSSFLGGALSFKGDKKKSKKKSKKSKVKHKLKDEEATAKISRETEIPHHNRDDVLLSD
DEDELTDAEKKALKFKKERQQEENKKIAAQSHRERVEALNEKLGNMTELNDIPRVSAAGN
G
>comp4610_c0_seq1; 942 27 2111 LEN=3001; translated
MSAATSNPAEAVPTPAPMTGAAPVPGQAGPGGDEGPAPFHSASLYVGDLVPDANEGLLFE
IFNAVGPVASIRVCRDAVTRRSLGYAYVNYHQMSDAERALDTMNFSMIKGKPCRIMWSQR
DPSLRRSGVGNIFVKNLHESIDNKQLYDTFSLFGNILSCKVVTDRETGLSKGYGYIHYET
AEAADAAIDKLDGMLIDGQEVQVGHFIGRNNRPNQDDFTNCYVKNIPLDWDDDRLNKEFS
KFGTVVSAAVSRGIRRKKKPFKKEETKAKESDEEKEVEKKDEAKEESKEEEGEKKIESLG
FGFVNFMDHDSAVAAVEGLNGKTHKFMQDGEEVERDLYVGRAQKKSEREREMRAKFEAEK
MDRISKFQGVNLYVKNLDDTVTDDMLRDEFSVIGTITSARIMKDMGKEGRSRGFGFVCFS
TPEEATRAVNEMNGKLIVNKPIFVALAQRREVRRAQLEAQHASRGPPPPAGQAGMMRGPM
GGPIGYPGMPAMYMGRTGPGGGMQPAYPMAMPQMMGPGGRGGQRPGAYPMMGQPGGRGAY
PMPYGGVMPGRGGRGPMQPGPNPRGGRGRGGAMQGRGPQGNQQIKFNQQARNAGSVPTGP
AQPPVQAAQQMPPAEGALTASALASASPEMQKNMIGERLYPLIHQSQPDLAGKITGMLLE
MDNSELLHLLESPEALKAKIDEAIQVLEAHQSSE
>comp4611_c0_seq1; 298 1 530 LEN=769; translated
XFEQTNEIPNKIGKMKSTTTKRQLSSSTPNTTTMSFRHESKTMKTLNLLRGEQQPYEVTP
TNSEDEDDDSTDQSNRDDEEFKTFHGIQSQNDHDHHSDLYRPLTPEEADEDQLAEFFTAL
DFDISFGALPLTQVQCETDATIRNYSKTLEEQHTSKRRRLLSPRIHVKRKAMMEET
>comp4612_c0_seq1; 306 296 1313 LEN=1467; translated
MTILRVASWNLLAQDYVKEYKYPWTRKTPGCLEWDHRKSLIVDKLLDKKAQMDIVCLQEA
QMDSFSDLLKDVSPVFDGVIQNVTRGHNVGGAPWPELTSFLKQYFTQRIFSPSRYLGRLL

LGTAVLIRNSCKLKLKRVESRSRALIATLEDKDTENNLYICSVHLDADKSTDPKKREHHQ
SQRRSQLKSLLKRINYACKQEKQIIKEAPILIAGDFNMLRENPLNELLVEGSLTTSPSAY
VSLRDAYLEAERHDRFSIPVYHQSVLEGGNDIGETASNEKHLVKTYKGGAILDYIWTSDE
VKVVDTLLYHPDSSTVGTEKWPCHDHPSDHLPIGIDIDW
>comp4612_c0_seq2; 249 671 1426 LEN=1580; translated
MLVSAPWPELTSFLKQYFTQRIFSPSRYLGRLLLGTAVLIRNSCKLKLKRVESRSRALIA
TLEDKDTENNLYICSVHLDADKSTDPKKREHHQSQRRSQLKSLLKRINYACKQEKQIIKE
APILIAGDFNMLRENPLNELLVEGSLTTSPSAYVSLRDAYLEAERHDRFSIPVYHQSVLE
GGNDIGETASNEKHLVKTYKGGAILDYIWTSDEVKVVDTLLYHPDSSTVGTEKWPCHDHP
SDHLPIGIDIDW
>comp4612_c0_seq3; 359 296 1216 LEN=1370; translated
MTILRVASWNLLAQDYVKEYKYPWTRKTPGCLEWDHRKSLIVDKLLDKKAQMDIVCLQEA
QMDSFSDLLKDVSPVFDGVIQNVTRGHNVGTAVLIRNSCKLKLKRVESRSRALIATLEDK
DTENNLYICSVHLDADKSTDPKKREHHQSQRRSQLKSLLKRINYACKQEKQIIKEAPILI
AGDFNMLRENPLNELLVEGSLTTSPSAYVSLRDAYLEAERHDRFSIPVYHQSVLEGGNDI
GETASNEKHLVKTYKGGAILDYIWTSDEVKVVDTLLYHPDSSTVGTEKWPCHDHPSDHLP
IGIDIDW
>comp4612_c0_seq4; 327 212 1329 LEN=1483; translated
MSKAKLSVGWGCKLYVIIAVIXPALYLQYDNTSSGQLGKFVSFRLSTTRTKSVLNRFYTD
AFVPNGCLVSQHNKKNLLAQDYVKEYKYPWTRKTPGCLEWDHRKSLIVDKLLDKKAQMDI
VCLQEAQMDSFSDLLKDVSPVFDGVIQNVTRGHNVGTAVLIRNSCKLKLKRVESRSRALI
ATLEDKDTENNLYICSVHLDADKSTDPKKREHHQSQRRSQLKSLLKRINYACKQEKQIIK
EAPILIAGDFNMLRENPLNELLVEGSLTTSPSAYVSLRDAYLEAERHDRFSIPVYHQSVL
EGGNDIGETASNEKHLVKTYKGGAILDYIWTSDEVKVVDTLLYHPDSSTVGTEKWPCHDH
PSDHLPIGIDIDW
>comp4615_c0_seq1; 244 37 1059 LEN=1270; translated
MIAWNISIALLLISLESATAFVAYTDAHRTGCFLVLSSSSSHSNEIEESEDPSSSLSNNR
SKNRRHFFSDVFSQSASMAAMCSCCSSPLLLPQPAHGLAQITTPSREAIGAFDPPRNAFM
DAAFAQGMAVGMVDYEREAYPKKKEIFKALFSSLPKNINSNEEPVIVEIGMGSFPNALYY
KGMKGLDIIGIDPNDWMEGYAKDNANRAGLLNEDGDNLRIIHGVSEALPLLDASCDAVVC
TLTLCSVLDPAKSVSEIKRVLKPGGKFLFWEHVLSQTDPDFARQQILNTPNQVKRADGCH
LDRRTGETIREAGFSKLDLQYIELKRFGFLNPTVCGIATA
>comp4616_c0_seq1; 118 1 378 LEN=1299; minus strand; translated
ATFERQRNELVTMQKSTQVIGQSVYNRMLQSARAREWISDEQSRLGIMHSTPMVATAKQA
PAAAITSPVDAASVPEPSSVSAMLSENDRDILTIKLTSKAISKQKKKMARKRKRAAMMDW
GDDDE
>comp4616_c0_seq2; 118 1 378 LEN=1043; minus strand; translated
ATFERQRNELVTMQKSTQVIGQSVYNRMLQSARAREWISDEQSRLGIMHSTPMVATAKQA
PAAAITSPVDAASVPEPSSVSAMLSENDRDILTIKLTSKAISKQKKKMARKRKRAAMMDW
GDDDE
>comp4617_c0_seq1; 553 973 2793 LEN=3153; translated
MEQRSGRKERSQTFKWFSSTLLYYFCDRMAMYMNHRYYTKLVSSSVVWGKKGSKLIILKL
QRPVLFEFKAGQYAYLRISTINNEWHPFSIASRPDSSNLEFYIEVHEGKTWTKHLWNLFD
DEVTSGSFDVASLKIEVMGPYGSSLSRGQNYSHTLAIGAGTGVVPILSMYKEHMHKLFRL
DPKKFFIELNSSERRQRRIEQAEQAQTGSLAQKIFRVLNGKYSLLRNHKTILTSGSSYDA
DNLQEDDHETIYKSATAATLPIYGSVVLSSLPVLGVALFALMISWSTIPIEIYPSMAMIL
KYFTIAFQTCFALVSIFFWNADGILAYVDVVFVVISPFADWYWIEVCSDFQKLLPSDITL
FSILIWYMIGRFWVKAVSPNQSVRYRVTKHVQSSQVDQLKFLWITRSASQVSEILPDILM
LWDLLVVKWGIENAQKVCKITIHVTDPNELSCALLRKEYENSEFFRNGGIIFRRPNLRRV
IEDHTIELINSRVNSHSLLAFCGSPSLSNEIEYNKISNDVITAMTGHSQSHMMDFVSESY
GGKKAASSNKKRKKGDTLSLEIDHDDEDGVEVQLLTSRKNLSYYSNESCLDCLSFMDIQK
SFEISV
>comp4619_c0_seq1; 117 1 189 LEN=580; translated
QQQQQQQQSRRPQIQQQPQQQRRQQAQPRSQMSRPPYVDPNTNIIYETDNAEYEGFLTKQ
SMW
>comp4619_c0_seq2; 362 1 782 LEN=1130; translated
QQQQQQQQSRRPQIQQQPQQQRRQQAQPRSQMSRPPYVDPNTNIIYETDNAEYEGFLTKQ
SMWLKDWRRRFFLLKGSKLFYCSNEYSTPHGMIDLALCTTVKSADLKSRKRNSFEISTPE
ITYLLYADTEQEKDDWIGRVGKAIVRCSSTYYSQSASGSGVRRQPQPQGYAGNYYQQQQQ
LHQHSAMDSDDDDIYYEDPNNENPYFNDXSGGTNQESGPGDGRCPGNVAQRRTEETTQRG

MVQQHNATQHDTTRHNVAET
>comp4620_c0_seq1; 258 1 1083 LEN=1514; translated
ITPPLRPYFILLPNFLPQMNSNTEKKTTKTAKAGGYQVPWIEKYRPRKLQDVVGNEETLV
RLQAIAKDGNLPNLILCGPPGTGKTTSVHALAHELLGDSYKDGVLELNASDARGIDVVRN
RIKSFAMNKVTLPKGRHKIIILDEADSMTTAAQQALRRTMEIYSNTTRFCLACNVSTKII
EAIQSRAAILRYSRLSHEQILANLLKVCQAEEIPYTEDGLEAILFTAEGDMRHALNNLQA
ASSLAKGAGDMVNQKSVFQVCDQPHPTIVRDIIDSCGRGNLHEAIEQLDELWSSGYAASD
IIGTVFKVTKAHDSLPEGEKLEFLREIGFTHMRISNGVNSYLQLLGLIARLCHMVEVTQQ
>comp4620_c0_seq2; 94 1 487 LEN=918; translated
XQTLLCHSNLGIFYFSPHLDGLEAILFTAEGDMRHALNNLQAASSLAKGAGDMVNQKSVF
QVCDQPHPTIVRDIIDSCGRGNLHEAIEQLDELWSSGYAASDIIGTVFKVTKAHDSLPEG
EKLEFLREIGFTHMRISNGVNSYLQLLGLIARLCHMVEVTQQ
>comp4621_c0_seq1; 310 130 1137 LEN=1313; translated
MSTATETGEAGNHSGCNSSCDKDPTYSVPICSPASSPSTPASARVPSLAFGLYKIPDSDE
GVGIMADAILEAGYRHLDSASIYGNERSLGRALALALATTAAPSNGSKRIERSRLFVASK
VWNDAQRGGRDAVRKSVLQSLRDLGLDYLDLCYVHWPVPGHFVETYRELMDLRREGKILH
LGISNFRIEDYEELEAAILPPEEFVPPLVHQLEVSPFLYRPETLRYFKEKGVLLAASKAL
HRAATLDDPVVEAVAESHSHHRGGAGVTPPQVLLRWGLQKGLVVLAKTSRLSRMVENRDL
LDFELSLAEVERLDALTTPGAIETREALEAQRREG
>comp4622_c0_seq1; 416 250 1281 LEN=1467; translated
MGKKAGRDPGAPKRNMSAYLLYQNAMREDFKSINPGMTFGQLAKYTSAMYSELPPAEKET
WVQRAEADKARYLHELANYVAPPGYDPKGDAIVSTLQKTGRKGKPEKDPNAPKRNMSAYL
LYQNAMREQFKRENPGMTFGQLAKYTSHMYKNLTPDEKATWESRAAQDKARYDAEIAAYQ
PPPGHDARGVLIEEHRPRKRSKRGPRDPAAPKRASGAYVFFTNYMRPKVNEEFPGIKFVE
LGKLLGERWRALTPVQKKEYEDMAAEDKVRFQIEMQQYTANQAAAAPPSQEAASSTEAVS
QEHGTSSYYDPTVGHNALAGHEYNAHHAQSYHHDPYQQQYHAA
>comp4623_c0_seq1; 362 1 989 LEN=1194; translated
XPHQHANKKERNKPKMVASIPRTGSAASTAAARPFVPRACLLLLLAGTSATGFTLAPSLV
ARCRSVPPRSPVVRYDYADDGSPSDYDSSDLPSEFKQVAVDEREEDVAIRAALKQELLLL
ASVTDRGAYCSREEKDIVIDLVAQLEALNPTKDPAANCGGEWDLGYSSTQLFRSSPFFQA
IRVAVGEENKDVAENGFALHAQATSGSRVGRVRQSITSETLTSEVDLEVGMLPGLPFSIQ
GTVVSTASLDVVSDKRWELRIQNTRVKGSNIPLFDQFVDNLQMELPVGDFYNTVQGTTPV
IPMTTFYVDDTLRITRDVDENFFVFIRES
>comp4623_c0_seq2; 327 1 1310 LEN=1515; translated
XPHQHANKKERNKPKMVASIPRTGSAASTAAARPFVPRACLLLLLAGTSATGFTLAPSLV
ARCRSVPPRSPVVRYDYADDGSPSDYDSSDLPSEFKQVAVDEREEDVAIRAALKQELLLL
ASVTDRGAYCSREEKDIVIDLVAQLEALNPTKDPAANCGGEWDLGYSSTQLFRSSPFFQA
IRVAVGEENKDVAENGFALHAQATSGSRVGRVRQSITSETLTSEGXVCVVCGVDGVFLHW
VVVEGAARILPTGAGAESGGPGWSQHSKPPRIVKLATVNDRLSFPPGITAGFEYRPSASV
FWRSDHVRNPAHTGFLFCATQSHSSLLVYGAVDLEVGMLPGLPFSIQGTVVSTASLDVVS
DKRWELRIQNTRVKGSNIPLFDQFVDNLQMELPVGDFYNTVQGTTPVIPMTTFYVDDTLR
ITRDVDENFFVFIRES
>comp4624_c0_seq1; 313 200 1528 LEN=1543; translated
MSSACMYSSVMQSLLQNSDIDWNQFLETIWERKCHRFVFNEDKLSSKSCSENNVWTEMVH
RGWAVLTQLMDGSCLVGDGDRSQQKASIATGKGDCDSPLLFRDLQPMDDPDEIHRLYNNS
LYAAYLAGTSIVWNHVDLKSPRVAALCEDLQGSRCKPEQEHERRTYSYKSLKLDRAFFPH
AYANAYLTPPKSQTVPPHADDRDVLVFQLVGRKRWRVYQQIPIHYPFTHEQVGKSGIAVP
ESVLKGPLAFDDSLQPGDVLYIPRGMVHEAKTDTGNDDVPELSFHITVALATYDWTLVGN
LSRMLQTKLMNIGELLPSSTDASSSARTSNTTLSPMNLRRSILPTTRENFRRKHRDEGKG
DDCKSVTDANGAASPARLDCGGFLVDTGAIQKGIDSVFKKLRTEITAESVAEAMDHRIKA
HNLRASGKRNALIMIGEKDESQ
>comp4624_c0_seq2; 465 200 1951 LEN=2341; translated
MSSACMYSSVMQSLLQNSDIDWNQFLETIWERKCHRFVFNEDKLSSKSCSENNVWTEMVH
RGWAVLTQLMDGSCLVGDGDRSQQKASIATGKGDCDSPLLFRDLQPMDDPDEIHRLYNNS
LYAAYLAGTSIVWNHVDLKSPRVAALCEDLQGSRCKPEQEHERRTYSYKSLKLDRAFFPH
AYANAYLTPPKSQTVPPHADDRDVLVFQLVGRKRWRVYQQIPIHYPFTHEQVGKSGIAVP
ESVLKGPLAFDDSLQPGDVLYIPRGMVHEAKTDTGNDDVPELSFHITVALATYDWTLVGN
LSRMLQTKLMNIGELLPSSTDASSSARTSNTTLSPMNLRRSILPTTRENFRRKHRDEGKG
DDCKSVTDANGAASPARLDCGGFLVDTGAIQKGIDSVFKKLRTEITAESVAEAMDHRIKA
HNLRASGKRNALIMIGEKDESQCEPKKKAIADPMDLVVGPVAAKNVSLETHIRSSTPIER

DYAQKKSSASSSSAAGLNVRDEIGDDVAEIVTKIKIQQPQQNSTFRVGDFHKLLEAYSHS
LVCKLTSLSLAKRAVELGAFAVCDWDGTEVPRATKRRKIDVNI
>comp4625_c0_seq1; 343 109 1203 LEN=1896; translated
MISTRSSLCLRAAAAASRMGRALTPSRSPPSSVGLPAAARSFVSPFVGEERLRIRSLLSV
PGCDGRKIQKSRALGADAVVLDLEDGVAHDRKEEARKLVRETLLDEGRSFGASELCVRVN
GLHIEHAIRDLEAVLPCPRLRYIVLPKVESAGDLLFVERMIEEHRVGRVGASTSTSTSTS
AEETPRRDVRVLAAIESARGLWNLREIVETAAPERYGRRFPLLEGLVFASEDYNADVEAI
RTPGAEELLYARSKLVVAAKAHGLQAIDMVHINFRDLDELATECEAGRRLGFTGKQAIHP
SQVATIHEKFSPSLEDVDFAARAMAAFEETEAAGSGTCIVDGIAVDLPVYKWAVKICRQS
ERPA
>comp4626_c0_seq1; 231 141 1571 LEN=1571; translated
MTDATQTLLVVLGVTWLAMVATLLIPSLSVQTHSFSARSDLEMDAHHLLRSSKKDSAKSR
KRRGSRAITIENLPAVAAAALNVPTSEKILSNDDVDYQSIIEKWQMPKEAISLLRDVRSR
PVGFDYNRETDILALRHPLKTGGTSFSMMMKEIFGDGVMPGSGKSQWFKRQDFEKALQEH
PPEIDPNYWRNITAFYTHSFLRETDENKNLLNELRARVPVLEEKKFRLMTIVRRPLASGF
EFLGNKMSHRNILRVERDQGCRNLSHSESDSDLGTNEEEKQECLAGTREKRCGFLLKKTE
RFFEFCGSLDTMLERGGAHNQLYQSLMGFLPEPPEFERSMTPTLKDVAIYTIQDLGGLMD
VHKKYKEDIIMFLITERMKESLCLFHYHFKIEPVKERHALYKHCRPLRIWEERHKRIVEE
REQFDYTVWRAANSVMDVRMAAMRLEIKGRIDAGESLDEIPFLGQGCYDDTTISSTX
>comp4626_c0_seq2; 228 141 1344 LEN=1685; translated
MTDATQTLLVVLGVTWLAMVATLLIPSLSVQTHSFSARSDLEMDAHHLLRSSKKDSAKSR
KRRGSRAITIENLPAVAAAALNVPTSEKILSNDDVDYQSIIEKWQMPKEAISLLRDVRSR
PVGFDYNRETDILALRHPLKTGGTSFSMMMKEIFGDGVMPGSGKSQWFKRQDFEKALQEH
PPEIDPNYWRNITAFYTHSFLRETDENKNLLNELRARVPVLEEKKFRLMTIVRRPLASGF
EFLGNKMSHRNILRVERDQGCRNLSHSESDSDLGTNEEEKQECLAGTREKRCGFLLKKTE
RFFEFCGSLDTMLERGGAHNQLYQSLMGFLPEPPEFERSMTPTLKDVAIYTIQDLGGLMD
VHKKYKEDIIMFLITERMKESLCLFHYHFKIEPVKERHAL
>comp4628_c0_seq1; 297 53 895 LEN=1124; translated
MASCLQKFHSSSAYRLFVLWMLYYPLACVIFSLFWAWPIALADKTAYLNAFFFEMSLFTG
SVIPLTDWAPPTDGTGSFAIVNLLAVAHQVILACFIGVSAGPMIEGLLDIKGGMLMKKID
PEGTLLIPRTKSGLFLKMIVFYIIIVVLSIIWAAYWGGFMSLAEGWPFLDGFVTALGVIT
SGLTVLQPDKFPVTGWGLFCGFYTGVMGAAMLGLTIAVASVPLLGVDLAYDDSPAVRIFP
MLLLSAEQREKLGLKGGCCGAKGGDSKEESADNGGVEEAA
>comp4629_c0_seq1; 304 153 2294 LEN=2303; translated
MPLNLSYRLFGPTTVHLRHETYLIISLALLEYASIRDLRFAAFVTVWLSRNLVVRWLLTA
HDTRTSVSEWSLPFVIKYYRFQFNPLENVINFITTTVLAGQTFDRPNPHVVAKFIGTNHM
LNDLMAENNLGNLNSNNPSRNNNRRGGNSSSSHHSHYYGNSSKKNHYDLVCDWCKRTFYK
IKELSPSSLAVASSEAYWTNLMRSCGPSPQLVLQLFTLLFLYFCWNFPLSENGGGIVRYF
TTIYTDVAQVLRDIYNIAVNRNVIYYHYGRHDDSNGPLVTNHALIMETLKDSGSAMGYSG
DIAIDNTKAIGQYQHSFSPTWIDLWYYIIVCGTCLSMALYGRILLPIPDLVAGSCVLSSF
RLRDGAGGKILTNVGGGGGGNKGSRNNNSQRGGGSSSIGGLATGSGGGDPNFDQPWLEQY
QSIITEHRLKLILNVAFVRVVENLFLIAILPRTDFACKTTGQCPNKSGLGELAKVLFYAG
ITEPLRSDSQYYQNDDGKNPFSVGDNPFRPPVASALMIGISAVVITVSLLLAQAATLNRS
HLGITGYLAGGWVADDQQTHPSVTDKKASKSNSRNKKSTPESLAPEWVPRKRYKKGELVT
YERTVYRATTNNPEGCPYDFYLRATHKIFRNELGHPATSRVVAFVSTVQFGLISLLILMV
LVHQLILGEDEPGERTTCLLWTLAANLVAVFGTVSVAVPNYSEFDRLAADLMN
>comp4630_c0_seq1; 239 767 1339 LEN=1578; translated
MAISKNTPKQNNMLFMLASLACNGQNKARDSIPAAVAVSDASDDDRSVTALPQVKEVVVA
RRRRRPIKKRIPGNDYQCSPLNSPPGSSLAIPKKSLLESSIKILRCPRKTNKIESISLTT
YNKKKAASVHNQRQKKTRCCIRTSVGSGTLALAHDDEKLKQVHSPLDLPSFLPCPAIALR
NVESISLNLG
>comp4631_c0_seq1; 732 1 1707 LEN=1866; translated
PTGGSTGGSTEGSNEGSNEGPTGGSTEGSTEGSTGGSTGRPQGENSTEGSSNSSSSDGSN
QHGDEGKRKPSEQDGNDVVQDNQSDSSQQTGSNKSNDENEANNDSQTTGSSSIENNGSTE
NQTGGEQVIFNSDNIFDKEVLEKTDSATAAFCPDVSPDREVLVNWLYAVEFIPNLTEEQK
EQAVTIMELKLVSEVLKCNTAVSVLPGKRAVRQRNLVEERVENNGIAGLNYMPLDAKNED
IVCFSNSTAAGVCETYFGRMEMYLEEDAVVQVAINEILHSIRDTMERKNEFPAVEGIVQV
WYLEPDLSGVSKISGADDTIGDQKPPIDGSFSTGTLIAFAALGVFVFAALIMGAFRMRRN
NHDGISTLEPGSSLGCSAFTTGNDSYVKQSNNFTTMLPNSYNLDDGDNMSAIPEGDSSDA
DSRYECSVIVSDGGYTSDGDSRAGDALHPSHFDTVLGAPHGDEDNLDTDRDLLFENDENY

EDISSMGNRDFSNHRKHNLVSADDVGLGMRHSTVDVHQCTSANCKICNYKPRDVDFIHNS
PHTPDVSLGNFFNEQPPSPPETPDEVEL
>comp4633_c0_seq1; 113 629 1309 LEN=1900; translated
MVPKPHSCDGHSHDHEHSDNDLGLSLRPQIDFDGVKCLNEELPNMGRDVLKLHEERLTSD
PFLRSQDDDPELLLYIPFTEAVTITHLSVRSEPRVMEDFPSAAPRTVRVFSDRDDIDFDT
ARELAPQCTVDLVPPDHFVEGTVDYPLRPAGRFQNVSSITLFFEDNFASLRLDDEDEVAT
IVTYVGVKGKGSRQKRMAVDAVYETRGMKKDHQVPGAEFGAQNEIG
>comp4634_c0_seq1; 118 1109 1644 LEN=1729; translated
MAXIVNIDVTTFNRGGYHGDLNETFLVGNVDDDGKRLVKTSFDCLAAALDKVKPGTLYRD
LGTAIHKTASKNKCSIVRTYCGHGIGSLFHTIPNVPHYHKNKAKGTMKAGHVFTIEPMIN
LGNPGDFTWGDNWTAVTTDGKRSSQFEHTILVTDTGYELLTGRENEPVMTWDAALNSR
>comp4634_c0_seq2; 191 549 1517 LEN=1602; translated
MSEPSWAKYFSFSGSLRPALQSPKRVVPRTIRRPDYADHIAGVSESEQRDKRNHNNIRVY
TSEELDGEDGLRHACRMGREVLNEGAKAIRVGVTTDEIDRVVHEACIERDCYPSPLNYYN
FPKSVCTSVNEVICHGIPDFREVQDGDIVNIDVTTFNRGGYHGDLNETFLVGNVDDDGKR
LVKTSFDCLAAALDKVKPGTLYRDLGTAIHKTASKNKCSIVRTYCGHGIGSLFHTIPNVP
HYHKNKAKGTMKAGHVFTIEPMINLGNPGDFTWGDNWTAVTTDGKRSSQFEHTILVTDTG
YELLTGRENEPVMTWDAALNSR
>comp4634_c0_seq3; 285 132 1355 LEN=1440; translated
MALTCSSPGCSNAVTGNLACPNCKKLGLNNYFCTQECFKKNYAAHKQVHVIAKQVLAASG
GQSATCSTAPQDGISCALDAPAETKMSEPSWAKYFSFSGSLRPALQSPKRVVPRTIRRPD
YADHIAGVSESEQRDKRNHNNIRVYTSEELDGEDGLRHACRMGREVLNEGAKAIRVGVTT
DEIDRVVHEACIERDCYPSPLNYYNFPKSVCTSVNEVICHGIPDFREVQDGDIVNIDVTT
FNRGGYHGDLNETFLVGNVDDDGKRLVKTSFDCLAAALDKVKPGTLYRDLGTAIHKTASK
NKCSIVRTYCGHGIGSLFHTIPNVPHYHKNKAKGTMKAGHVFTIEPMINLGNPGDFTWGD
NWTAVTTDGKRSSQFEHTILVTDTGYELLTGRENEPVMTWDAALNSR
>comp4634_c0_seq4; 171 132 1482 LEN=1567; translated
MALTCSSPGCSNAVTGNLACPNCKKLGLNNYFCTQECFKKNYAAHKQVHVIAKQVLAASG
GQSATCSTAPQDGISCALDAPAETKMSEPSWAKYFSFSGSLRPALQSPKRVVPRTIRRPD
YADHIAGVSESEQRDKRNHNNIRVYTSEELDGEDGLRHACRMGREVLNEGAKAIRVGVTT
DEIDRVVHEACIERDCYPSPLNYYNELRVLLVWFRFRPVGKLRCYDKILEGDSDGLGLFL
FLTSCNSFPKSVCTSVNEVICHGIPDFREVQDGDIVNIDVTTFNRGGYHGDLNETFLVGN
VDDDGKRLVKTSFDCLAAALDKVKPGTLYRDLGTAIHKTASKNKCSIVRTYCGHGIGSLF
HTIPNVPHYHKNKAKGTMKAGHVFTIEPMINLGNPGDFTWGDNWTAVTTDGKRSSQFEHT
ILVTDTGYELLTGRENEPVMTWDAALNSR
>comp4635_c0_seq1; 98 1 232 LEN=232; translated
XEGIPPDQQRLIFAGKQLEDGRTLSDYNIQKESTLHLVLRLRGGMQIFVKTLTGKTITLD
VEPSDTIDNVKTKIQDKE
>comp4635_c0_seq2; 108 1 136 LEN=221; translated
XEGIPPDQQRLIFAGKQLEDGRTLSDYNIQKESTLHLVLRLRGGF
>comp4636_c0_seq1; 269 81 674 LEN=1040; translated
MSTRSLITPTSKDVGTEKFKVVILGDESSGKTSIIRRAVDDKFSKEYEATLGVDLFTKTI
CTPGQKDIRLQVWDTSGQERFKSSIPSYIRDCHIAIIAFDVTTRGQYDSIEKWVTEVSEL
REQDEVWIFIVGTKCDLPLSQRCFTQQEIDEKLNELQRKEAPNNNILQYIETSGKAGFNI
RRMFYNITTKLIDQSSQ
>comp4636_c0_seq2; 245 81 602 LEN=1147; translated
MSTRSLITPTSKDVGTEKFKVVILGDESSGKTSIIRRAVDDKFSKEYEATLGVDLFTKTI
CTPGQKDIRLQVWDTSGQERFKSSIPSYIRDCHIAIIAFDVTTRGQYDSIEKWVTEVSEL
REQDEVWIFIVGTKCDLPLSQRCFTQQEIDEKLNELQRKEAPNNNIVSLSFFI
>comp4637_c0_seq1; 301 142 930 LEN=1335; translated
MRTFQFLIFLVLSGIVVSSEQKIAAESTKEKVKYRKDASGDNDSNDVELEVNPERKGGGF
YRDDLEKGVMDDIKLDNSKIKKNRVNQRNKNLRKETEKVNFVYGLDGELEPVLSKDDLER
HITENLLEEKEANALRRAGPLPKGIFDERRSGTDGHGGVVLTEEELEDIIKENNIDDQEA
EVFRALRPYSYYNYRAPSYSYRSSSYYRGYYNSKGGKGKGYGKGYGKGSKSYYQPTTYSY
TSYNNGYSYGHRKRSYQYYYFY
>comp4638_c0_seq1; 487 1 901 LEN=901; translated
DDAILERAQQREYIASMLESARGQPSSMAVVREAPTSKPKTNTGAPSKRQAPPAETELDR
RVKQVLQVLPHFGEGFVEVALGLYKGDVEATVATLLQHPADGDSADPGYPTALRMLDPKL
PRRKREFVAYQTAEEEAASAREARADVKERLALEEKQKREEYEALVVVSASASADPPPPK
PVDGGGGSPTDQPKPGAARKLTRKELRQQKLREQHRRVLADEYNDDYDDQYDEVDIKLGA

ADDGFTTDHTSAAGSANNMTYEQIKLYNKIVQEDMSDASFWESNRNTNKKGSNNAGNNNN
X
>comp4638_c1_seq1; 415 96 921 LEN=921; translated
MDTASPVDVAQQLVSDLEGLAVSATAPSSPQLLPSLQQFYRLGLVLFRILDDNNGDGHPV
AIAYRKLIESLSEEMLEPVFRDLRRASGRLLASGTVRVETDLARAARSVAGLVGAFSDGG
APDPTCGDGKYRSGRSRLCRLAYDHEWTSTLAAFYDRFVVAEKRQQRDALPDPDSSASEM
ETNKTAVLSCLSRMLLDGLILPVPFHRKRKNAPNTPPVEESLLEAIRSMEEESIDCLRDL
QEWQLLGGDPFRRTLEESLSHIGGNRHRRGDDDDDX
>comp4639_c0_seq1; 354 98 1807 LEN=2221; translated
MARGHIERTLLLLAGVLVIGTQLHPAEGQENTNVLTGNGLYLDRFNYDATVQRPDGFTDY
GPSDWENIECDERDALQSCLAYPDKWETGRAWAVNRNYCRWCPASDPDRCSRHHQSPIDL
QRAVGKEPGTDWLANECIDIHWMKYEDSTCTMEQLIEADAFTIERHALRISQPIAVYDDF
RDDPDGVPDGVKLECRKEGVGSRFGRIDFSKGFSQWWHLSHTDLHTPSEHTQDGVRYDAE
IQLHHFYSVTAREAGINNEVATVAVFAQAYDDVAPYPFLDKVICQWRRREYEVRTACGLE
PIRLSYPGCFPLKRKERSLRKGAKPGSATPRFQTAHDVILHNDRHRTDPNHTHTRLRMEE
ANWGPAEDKDWDTWIGEQSKKMEADEALYHSMREEHHGGNHTEALHRDFRALFQFEELEW
FNYWPMLGVRTEYYFRYSGSQTVPPCYGNFVSESRTGTNHWRVLKDPIRIHTRQLAEIRR
LTAERIAPPGSAVGSCTPDTASTVTYDEADPTKIVSVENARPLQSWARPHFKTFCECKDW
PSKWPEDRRWCEIEDEDERFYDKPYNFGW
>comp4639_c0_seq2; 304 160 1416 LEN=1830; translated
MEQLIEADAFTIERHALRISQPIAVYDDFRDDPDGVPDGVKLECRKEGVGSRFGRIDFSK
GFSQWWHLSHTDLHTPSEHTQDGVRYDAEIQLHHFYSVTAREAGINNEVATVAVFAQAYD
DVAPYPFLDKVICQWRRREYEVRTACGLEPIRLSYPGCFPLKRKERSLRKGAKPGSATPR
FQTAHDVILHNDRHRTDPNHTHTRLRMEEANWGPAEDKDWDTWIGEQSKKMEADEALYHS
MREEHHGGNHTEALHRDFRALFQFEELEWFNYWPMLGVRTEYYFRYSGSQTVPPCYGNFV
SESRTGTNHWRVLKDPIRIHTRQLAEIRRLTAERIAPPGSAVGSCTPDTASTVTYDEADP
TKIVSVENARPLQSWARPHFKTFCECKDWPSKWPEDRRWCEIEDEDERFYDKPYNFGW
>comp4641_c0_seq1; 571 153 2210 LEN=2262; translated
MNSPINATGKQDGGSCIGDEERQGQDSVYAAAIRLCCSKCCSYLKPNYDEIRADKVHVTP
LKCWLCLGLWQKEEPEEENSTDEEKKCLEEFKRSGIFCLRRAIEEACSPYGGIGRKSGNR
FSRMPNQTHTVSLSGDLCLRYKWLASFSSNSHVPFSVYLQDLKQHLHSRIKSIIDKDDEN
GEKDCNREKEKNAQSWKRENHQVFEVEEQGCLSIHLLCLPPKDYPMALALNKYMKTNAVG
KKTKNQRNRYNKRQKCGPFTTQGGDPRQNLEVRLRSKGYEWISQSTAEQLSASLSSSQAS
PSLAMLFLNDSAPNNSMTMTKMEYQVAVFRRPMYLYGYYTKSRRDVSQTPFVVMREKLSS
PAVESGAKSVANEEYDAIKSNNDGGVTAPLPKKEAQTLGATSVEEQICGPIKDLLGVSTR
NNITLPDSNDSLTMVGNTGGGGGVLYGMIKFHASGREDMDVRMLVRSNSPTCHGRPFCVQ
IIDALKGIASPIELKGLIETINHSSAESKTTTALPASRNDSGRNEGVSYYGKNPMGVGIS
PDIFRVVPAATFSGLQGDTESKTKHYGCYCWSKNTLPFDDNGGNELTQIIFKDLIFPLAI
QQKTPVRVMHRRSNSVRDRNVLDAKATRVDDHHFRLELSTQAGTYVKEFVYGDFGRTIPS
VSSLMGCKTNLLELDCEGIEIPFSS
>comp4643_c1_seq1; 144 1 812 LEN=812; minus strand; translated
ALLAGRVPDGELDPLPAHLEVLDLKVDADGRLDVLVEGVVREPEEHRRLLGGASWQGTEN
GEACNGVGSSARAFPREKKKRDNTVVRGPAQRGAKRNKREEGKPRRELLWCGVCQTRTAT
WGGEGDKSRGSVPGCQKSIRPFDPSIRSVQPNRDHGTGEERRTFPTEASPMMSTLKRKSY
GNVFGGGATGGADMVAGVYVLVFVVVRQWDLHFLVRAGVGVAIAVADAIVPKLGVLAVVS
EREEGKSSGIVSYRLVSCSTKYGRIPVGYKX
>comp4643_c1_seq2; 153 232 468 LEN=468; translated
MSAPPVAPPPKTFPYDFLFKVLIIGDASVGKSSMLLRFTDDSFDEHIQSTIGVDFKVKHL
EMSGKRVKLTIWDTAGQER
>comp4644_c0_seq1; 27 1 1792 LEN=2321; translated
RAAKQGRMCVIVPERMRGRARGIRKPRPDPGRADGGSLRCGASASRLGGSPSPVPRPLRW
LLFAVVLALVGVAPAEGQRQRRGGHPGKVLRHDAPRPDPPGTATDLTGSVLGRRDKKRLE
RRIRGGRPLPFSLQWPRPSSRKRRSLADLDLEVVVASGVEHGYTPREMAADLLHKGWGMA
DQTETHSGGMIVLVAIEDHRIEIVVGDSLAHVFGADWCHSVLYGRGVTEAFRAGRYGPGL
ERLLDEVEDRWGEAGAETATETHAGPHSGRRHRSRTGTAVAYLAALAGGYAGLPFWQRGF
GGGGGDPRYGHGHGEGHHGHDHGHGHGHLPTGDPRHARRRGGLAHGPGRGLDMGMGMGLP
LPLLSGLGGALLGAKRGARLAGVARRHRPFVTFGERDCGAQRIEGSPPSAAAAGANTKRP
FRSPPLSPLRSEEQRQRLRDAAALGGTALGDSAGGPPGGPRANTTRPFERRGPGPRPDRS
GAGGPPGRSALPEPAGTGTRRNPTRVGPAAGVLPAVERRGSKHSTEQQKLSISPWWHRAP
PQICTVRVPAFPMKLCIVEQNYCTTSFFPNFRIKIKTDCDFCFRVWTEDLTILNHE

>comp4645_c0_seq1; 943 182 2569 LEN=2609; translated
MSHVIGMTNRISLCLQLLVIVSVATIPPAYSFTSATVSPRSNHRSQKSAIAMSSSDAKQD
VPYGAWESPISSKSITAGSVGIGGLHVSPDGNGGDIVYWTEGRPQEGGRNVLCRYDPTSE
EKSERDAVDVTPKDTNVRSRVHEYGGGAALFAATTEGSDKEDTLFFIDFASQQQFKLALD
GGSGEKEAATTDDTGRYRYADAIYDKQRKLIICVREDHGVDGKSSPKEVVNEIVALDPNE
PAREPKVLVTGTDFVASPRLSPDGNTLVSIVWDHPNMPWDATKLVSVALPEGDLLSSSTE
VGEPTVIAGQDGDTSVMQPLFHPSTGQLFYISDESGYYNMYIQDGGEAHSLLPMNVDFGG
SSPGWMLGQMGYTILKDGRLAAVIKRDGSSVLIVADILNYTPGTPVASVEYTSDDGLPMQ
FGAVKGGTSEDTKDVLYFMGGDPSTPASVYRWSLEKKSDATILATSSTLKFDDSVISIPR
QIEFPTTLGTAFGYYYPPKNGAYHCTTESAPPLLVKAQEGEFFENVCELFEQEFLSSAFR
KDCLALCSAGDQETIDXDLMYFFLSSPTGATGTMFNAGIQYWTSRGFGIFDVDYGGSTGY
GRDYRRRLRGSWGIVDIDDVCNGAEHLVNEGLADGDRLCIDGGSAGGYTTLGALAFRDVF
KAGCSLYGIGDLTALAADTHKFESRYLDGLIGRYPEDEAVYKERSPIESVDKLSCPILLL
QGEEDKVVPPNQAEMMYEALKQKGIPACLKIYEGEQHGFRKAENIEDALDSELSFFGKVF
GIDVPDSISLQVDNM
>comp4645_c0_seq2; 921 136 2370 LEN=2410; translated
MSSSDAKQDVPYGAWESPISSKSITAGSVGIGGLHVSPDGNGGDIVYWTEGRPQEGGRNV
LCRYDPTSEEKSERDAVDVTPKDTNVRSRVHEYGGGAALFAATTEGSDKEDTLFFIDFAS
QQQFKLALDGGSGEKEAATTDDTGRYRYADAIYDKQRKLIICVREDHGVDGKSSPKEVVN
EIVALDPNEPAREPKVLVTGTDFVASPRLSPDGNTLVSIVWDHPNMPWDATKLVSVALPE
GDLLSSSTEVGEPTVIAGQDGDTSVMQPLFHPSTGQLFYISDESGYYNMYIQDGGEAHSL
LPMNVDFGGSSPGWMLGQMGYTILKDGRLAAVIKRDGSSVLIVADILNYTPGTPVASVEY
TSDDGLPMQFGAVKGGTSEDTKDVLYFMGGDPSTPASVYRWSLEKKSDATILATSSTLKF
DDSVISIPRQIEFPTTLGTAFGYYYPPKNGAYHCTTESAPPLLVKAQEGEFFENVCELFE
QEFLSSAFRKDCLALCSAGDQETIDXDLMYFFLSSPTGATGTMFNAGIQYWTSRGFGIFD
VDYGGSTGYGRDYRRRLRGSWGIVDIDDVCNGAEHLVNEGLADGDRLCIDGGSAGGYTTL
GALAFRDVFKAGCSLYGIGDLTALAADTHKFESRYLDGLIGRYPEDEAVYKERSPIESVD
KLSCPILLLQGEEDKVVPPNQAEMMYEALKQKGIPACLKIYEGEQHGFRKAENIEDALDS
ELSFFGKVFGIDVPDSISLQVDNM
>comp4645_c0_seq3; 944 136 2232 LEN=2272; translated
MSSSDAKQDVPYGAWESPISSKSITAGSVGIGGLHVSPDGNGGDIVYWTEGRPQEGGRNV
LCRYDPTSEEKSERDAVDVTPKDTNVRSRVHEYGGGAALFAATTEGSDKEDTLFFIDFAS
QQQFKLALDGGSGEKEAATTDDTGRYRYADAIYDKQRKLIICVREDHGVDGKSSPKEVVN
EIVALDPNEPAREPKVLVTGTDFVASPRLSPDGNTLVSIVWDHPNMPWDATKLVSVALPE
GDLLSSSTEVGEPTVIAGQDGDTSVMQPLFHPSTGQLFYISDESGYYNMYIQDGGEAHSL
LPMNVDFGGSSPGWMLGQMGYTILKDGRLAAVIKRDGSSVLIVADILNYTPGTPVASVEY
TSDDGLPMQFGAVKGGTSEDTKDVLYFMGGDPSTPASVYRWSLEKKSDATILATSSTLKF
DDSVISIPRQIEFPTTLGTAFGYYYPPKNGAYHCTTESAPPLLVKAHGGPTGATGTMFNA
GIQYWTSRGFGIFDVDYGGSTGYGRDYRRRLRGSWGIVDIDDVCNGAEHLVNEGLADGDR
LCIDGGSAGGYTTLGALAFRDVFKAGCSLYGIGDLTALAADTHKFESRYLDGLIGRYPED
EAVYKERSPIESVDKLSCPILLLQGEEDKVVPPNQAEMMYEALKQKGIPACLKIYEGEQH
GFRKAENIEDALDSELSFFGKVFGIDVPDSISLQVDNM
>comp4645_c0_seq4; 966 182 2431 LEN=2471; translated
MSHVIGMTNRISLCLQLLVIVSVATIPPAYSFTSATVSPRSNHRSQKSAIAMSSSDAKQD
VPYGAWESPISSKSITAGSVGIGGLHVSPDGNGGDIVYWTEGRPQEGGRNVLCRYDPTSE
EKSERDAVDVTPKDTNVRSRVHEYGGGAALFAATTEGSDKEDTLFFIDFASQQQFKLALD
GGSGEKEAATTDDTGRYRYADAIYDKQRKLIICVREDHGVDGKSSPKEVVNEIVALDPNE
PAREPKVLVTGTDFVASPRLSPDGNTLVSIVWDHPNMPWDATKLVSVALPEGDLLSSSTE
VGEPTVIAGQDGDTSVMQPLFHPSTGQLFYISDESGYYNMYIQDGGEAHSLLPMNVDFGG
SSPGWMLGQMGYTILKDGRLAAVIKRDGSSVLIVADILNYTPGTPVASVEYTSDDGLPMQ
FGAVKGGTSEDTKDVLYFMGGDPSTPASVYRWSLEKKSDATILATSSTLKFDDSVISIPR
QIEFPTTLGTAFGYYYPPKNGAYHCTTESAPPLLVKAHGGPTGATGTMFNAGIQYWTSRG
FGIFDVDYGGSTGYGRDYRRRLRGSWGIVDIDDVCNGAEHLVNEGLADGDRLCIDGGSAG
GYTTLGALAFRDVFKAGCSLYGIGDLTALAADTHKFESRYLDGLIGRYPEDEAVYKERSP
IESVDKLSCPILLLQGEEDKVVPPNQAEMMYEALKQKGIPACLKIYEGEQHGFRKAENIE
DALDSELSFFGKVFGIDVPDSISLQVDNM
>comp4646_c0_seq1; 367 111 452 LEN=503; translated
MKELATYLMLQIGGNASPSKEDVTGALSAVGVEADSASLDKLFADIEGKEIAELIEAGKD
QLAKFGGGGGGGGGAAAGGAAAEEEEEEEEEEEEAEAPAGGGGLFGDDGDGDY
>comp4648_c0_seq1; 545 1 1358 LEN=1425; translated

XADPQSFFLVEFLGSHEFIWVREADIVENFNPQEDPNKKDGHQRSKKISRKSLANILGSK
IYANALEEAQWALEEFELQLQDIGGSTEGMEDDEHEEGYSYSVLSQSDDEADDDDDLATE
DLDQDECNELLATNGLLDFSVAGRKRRAQILKQQKLNAEKKQKTVKAKKARADKAKKAKD
EKQKEKERRQEKRDLEKKRRKRMREREKALKGVQHQKKKRKLSDPAPGRRHLIASKRDRA
EAIVSGYVSRAAKAGVYKPLGLGGEQRWIQSAFIDSTNLSGMALAFRASAGLIPMPKQDT
GAPGKIVLMPWDKIRLRGKKTSAERSEVLLKQIDLLEKEIRLRKASRTRRENRLKGVLQE
VIKMEAGIAEDDKLSRENPLKKSKKPHPLAGKKRKRSGASAADPKAAADAKKEGGSGSPS
AKAAKPEKPAASAAASPGGPAAEAPAEPAGAE
>comp4649_c0_seq1; 745 175 2121 LEN=2478; translated
MMMQPVPVNVAPEPGVIVDKLEGHPVHVSDSGTIDEKKESPAEEEHAEYNMSMQQEQRLI
QQHYIHNPYAQHQHQYPPHMMAGMVPPQQVYIQQHQQQHHQLPLESQFQSMGLGDTNGGG
AGQGGNDSSNNNNSSEDANNGDSRNNVNDGSNDRNDESNDGNGDRIANEDGGDRNGNEDE
PIKLFVGQVPKTMNEEDIFPTFNDFGPLKDVSIIRDKHTQLHRGCAFVTYLSASDAEQAQ
EALHGRHFFPGARRPAQVKPAEPSVQENKLFIGMLSRRAGEDEVRELFAPFGEIREIYMI
RNGDGTSKCAAFLRYVGRDAAVNAIEALHNNLVMEGAARPLIVKFADTKHQRHVRQMRSV
RRHEMFIGGPAGGYPGPYPGHHMPMGPPPVGGPPHQYAAHMGAPPHQMQHYGGPYPGPGA
GGQMYNPMGYPPAPHYPSPHPYGGFSERQGQGPDGGHQHQSTHPQRMKDGIRGNPRHHNQ
STNPRPREGPAGANLFVYHLPHDLTDADLATAFNPFGNVISAKVYVDRYTGDSKGFGFVS
YDSVISAESAIEQMNGFQIGNKRLKVQHKRVHNNGGGNRGGGQHGAGIGHHEQQQPKPQQ
QQQQPPPQGIPLDTAPSNAALDSGKPVPDPAEGSAAPSSGGETSGILP
>comp4650_c0_seq1; 238 77 433 LEN=692; translated
MSNPLKSFFQGFQETEDSRIGRLSERVAEGSVDSQKHSDEYNRPNMFTHLYYLQDEPRQR
GYNVYSNMSSFFKGRDGGAASSFHSVRYYNEEAKKKRAALVEKYNVESAVATHTGTTE
>comp4651_c0_seq1; 709 341 2001 LEN=2001; translated
MPSIVHARHELKYLMVQEQEVIVNQQHPKKSMSVEMSNSIGELDTYCNKPLLLAPVTATD
AECGDTSDDATKTTSGPEDDEDGSRIECETDDRDANNEENNSLSTLDSNSISSAAVANAL
ATEDAAAAIPNFPECNKKATENKRSVQFEPMSTMYVFDDDESSEGCEDEVEGDEVRAQEQ
SIDRDVFSCYSTNAIASPKPSQSDPSDSLSCNKYYIENAWYSANDIKAFRADAWLTVNWM
VLNGFAGENMAYITGKHLEEAVALDSGDHPEQDELKLDSDTESAAMLSARVQQEKDQKDP
QPRYSFCSRGVECRTPLGRIVKNKRRLDAMRAVLLYQQMQKNSRRVQRRKAIKEQQRSNS
NKGYDNCNETDYDHLERLRRIEIEIDTEALANVYRSYCEESCSVALALGRQDALDAGIPK
PPPIENRNSNPVVDSSPPGLTLVASSSDVSSSSSLSNSTSATSVTASVAPPKLPPIEPEF
LTHRRSRSLSIEDPQTIGKVIVDCPSLCDDDDDDEEDLATLVSLSYVRHPIVEEYGCGEQ
DEEEEEHQKQGLSX
>comp4652_c0_seq1; 275 82 921 LEN=1575; translated
MRRTTAVFSVSPLLAVSLALVALAICGAGTTSAFTAPAGSVSDRGPSGSSLASTVAQAPP
AEQQHWMDFMKFGGTSPGFDVIEQTRAFSECKTYDDAEGYFDEDYVFRGPIVGPITAKDV
RETQERFNVMDAYPDLVMEKFGYTVDPENPYRCYWFERWKGTNREAVQVGPLALPPTGQV
AVVPTHVMSANWTPSGKIIYLCLSGPLDRFEGNTMGQGAVFGLLKTAGVPLPVPSVGNPS
LSFNQKYVAPLISQKAFSADADVPGWWKSTAKGADANDV
>comp4653_c0_seq1; 356 154 798 LEN=1027; translated
MNIVLSTTVLASVLAPAYSFSYLDSLGGGVAVAPPASSNGASYLDALNGAPVAAAPVAAE
PVAAAPVAAEPYVPAATAPVAPAANYHDSLSTGATEISGPGIMTHADTLNTGANSLLGGA
GIITHASSLLTPSALAGGAGIQTHCDSLSPSSGSSVSYNPFGSASVASASFSGSTSADGV
SFTLETGDISGLVADIKAGAKLRLTGSIDSISYN
>comp4655_c0_seq1; 312 370 1299 LEN=2658; translated
MGGDGGVIASNRKFMRGAGTADCVGDLHRKAAQKFNPKEAMSTCSLTKTPLLVRASSGSS
SSSSSQGAIVADPYGVLYHKEAAVRALLKRAQKRGYGDDAGNDASIGPQVRRLADLYEVR
FHLDGETPVCPISGKALNGSIPALLLVPGKQGAPNVVSESTLSRLSQEELEAEFGAIAKK
VRLAPDPLLLEQIKEEVRTEQEKDEEERFRAKKEKRSKKDKSSKRKRGSKESGETKKKRD
KGSGHKECPPLPSATHKISSSSSSSLGQQVQQKVNSAIQQNSVLSSLFTNKNASSKVTEK
EKKDNLFAR
>comp4658_c0_seq1; 641 87 1975 LEN=1975; translated
MLVSFTIFHKGGRVLFQHSDEASGEDDDSRSRSINTWLEEIYFNPTVSKTLHKNQILVDG
TTRQVVEWEETSELIALAVYPDLRHEDSPWIRSLLAASLQEYQVFARARSAGTGEDKENS
NATNVFQGPAAASPESQSLFGKTFEILVQKHRSESKLSSSAAAASGARSNKASGASGGNS
NKGGKEKRQWHDGNAKVTEKAMKELDKSKAATAEAIVKSEEQALAEARAAYLPTDADLAD
FDIEDTTQLADDENDGDGDEQGGSGWGKSLQGLFEQMTGQKVLTKQDLEAPLTEINKLLT
SKNVAGDIAEQICNKVRDSLVGKKLNSMYRVKTAVRQALEQVIGELLLPSTELNLLRKVV
SKRDQASSFFAKSKTQRPFVIAVVGINGVGKSTSLAKLAYYLKSNGCRPLLAACDTFRSG

AVEQLGVHARCLGLPIYSKGYAKDPSAVAAAAIEQATKEGNDVVLVDTAGRMQNNLPLMK
ALGKLAAENQPDMVVFVGEALVGNDGVDQVRMFDKAVSFQNRNQRVSAILLTKFDTVSDK
VGAALTMTHVTNAPILFVGTXAEIPPPAKTVDGVGDSVPLPLACQHWWASGGLWLRTESG
ARVAQTEKRTVAGRNRLKRPERVLRIVTKW
>comp4659_c0_seq1; 983 263 3523 LEN=3620; translated
MASASSPDLQGLAQKLLSGDFDINLLDQIVGAAYDPISPHRAEANKALMQLQESPDLWQM
ADGIIEQSQNPNSRFFGLQVLDDAIKIRWKVIPKDQKEGIKNFVVGKVIKMSSDETTMKQ
EKVFIGKLNLTLVEILKQEWPQNWPSFISDLVGSSKTSEVLCENNMQILKLLSEEVFDFS
RDQMVTEKVKRMKESLNGEFSQIFQLCQFVLEHSQKPSLLRVTLKTLQRFLTWIPLGFIF
QTNLIETLLNKFFPVPLFRNHALDCLTEIGTLQDLEPQYDPLFRGLFTTFLQRLRDIFAP
DMDLRKPFQEGSEEDCFFIQKLALFLSGFFKAHFRVLETPESEQALISGMFYLVRISEIQ
DVEIFRICLEAWHMLAEDLYKADNNQSSFYDASATSRVLNLNGNASSRSSRKFVYSSVLT
GVRQVMIANMAKPEEVLVVEDENGDIVRETTKDTDVIAQYKTMRDTLVYLTHLNCEDTES
IMLSKLSDQVRVESKFDFNVLNTLCWAIGSISGAMSEDEEKRFLVTVIKDLLGMCEAKRG
KDNKAVVASNIMYVVGQYPRFLRAHWKFLKTVVNKLFEFMHEVHPGVQDMACDTFLKIAT
KCKRKFVTLQQDEQVPFISELVESLPSIISDLEPHQVQSFYEAVGCMLNDRGPNVAIDRQ
VLQSKLMEMPNRTWEVIMDQAKLSVENLVDPATIKEIIKILKTNNRLCRSVGGLFTYQLQ
TFFMDMLNVYRVYSERISAAVAQQGAIATQMSLVRTMRSAKKEVLRLLITFIDKSGPPEA
EPMTVAKGFIPPVLDPILGDYKRNIAGARDPEVLTLFTTVVEKLKSNVVDEVPKIMEAVF
ECTLEMINKNFEDFPEHRIRFYEFLRAINQHCFPALFNIPPEHQKLVVHAIVWAMKHKER
NISDTGLEILHELLLNVGRTPNVAQAFYQQYLLSLIQDVFAVMTDRFHKSGFKIHATLLR
HMFHLVQANQVTVPLFEPSTQPPGQTNPAFLREYIGNLLITSFPNLSKTQVSKFVDGMFD
IKMDLSTFKTHLRDFLIELKEFSAEDNSALFSEEQEAERRRQEKAMMAQRSAVPGMIKPS
EIDNDL
>comp4660_c0_seq1; 259 1 673 LEN=696; translated
DDDDESEEAVKASLDVGTGTTTNAAAAAATTTSTHMSEAYSDPVLEASIMEDVEKMRFVE
IKEALRIRGISVTGSKDTLKERLFISLMDDAGTNRGLLRNNSSNNDDNAVRCDATPFDSI
QFDWRWDPARAAKEYRSNRTFETKPFPPFHCGIECPTVGSCDGTNGSVAVRFGVVLHGDR
GASGPNLNLIERNTRKLRHETKRIESKQNERSRNEGIANNPHY
>comp4662_c0_seq1; 939 95 2251 LEN=2843; translated
MKTVSVFVFASLLANAESSSLFGVRHFGKALGATSTKQQIGDKTVETQDAVQSQLCDAIK
LRGGGDGSISGPCIGIDLGTTYSCVGVWRNDRVDICANEQGNRITPSYVAFPKDGSSSGG
RLVGDAAKNQASQNPTGTFFDVKRLIGRNYDDATVQKDKKLFPFEVAKDKNAKPIVRLPK
EIGSKFPKSDFTPEEISGMILRKMKETAEAYLGTEVKHAVVTVPAYFNDAQRQATKDAGT
IAGMKIERVINEPTAAAIAYGLDKQDHEENILVFDLGGGTFDVTLLSIDNGVFEVLATSG
NTHLGGEDFDYRLMEYCISQFKRKSGIDISNDKRAVQRLRKQCENAKRTLSTQTSTMIDC
EALSNGEDFSLTLSRAKFEELNLDLFKKTLTPVTQVLKDSGLSKNQVDQIILVGGSTRIP
KVQTLLSEFFGGKKLNKSINPDEAVAYGAAVQGGILSGDAAEATKDVLLLDVAPLSLGIE
TAGGVMTTLIKRGTTIPVKKSQIFSTYADNQPGVNIQVFEGERGMTKSNRLLGQFELSGI
PPAPRGVPQVEVSFDVDANGILSIGAHDKGTGKQETLTITSEKGRLSDEEIERMVQEAEE
FAEEDAKEKAKIQARNELEAYLYNLKNSVNDSLEGKLSEDEKDQLNTAINDGLDWLEDHP
AAEKEECDSKQKEVESIANPILKRAYESSAGAAGGADDDFLGEDLDGVDDGPTVEEMD
>comp4662_c0_seq2; 527 95 1504 LEN=1698; translated
MKTVSVFVFASLLANAESSSLFGVRHFGKALGATSTKQQIGDKTVETQDAVQSQLCDAIK
LRGGGDGSISGPCIGIDLGTTYSCVGVWRNDRVDICANEQGNRITPSYVAFPKDGSSSGG
RLVGDAAKNQASQNPTGTFFDVKRLIGRNYDDATVQKDKKLFPFEVAKDKNAKPIVRLPK
EIGSKFPKSDFTPEEISGMILRKMKETAEAYLGTEVKHAVVTVPAYFNDAQRQATKDAGT
IAGMKIERVINEPTAAAIAYGLDKQDHEENILVFDLGGGTFDVTLLSIDNGVFEVLATSG
NTHLGGEDFDYRLMEYCISQFKRKSGIDISNDKRAVQRLRKQCENAKRTLSTQTSTMIDC
EALSNGEDFSLTLSRAKFEELNLDLFKKTLTPVTQVLKDSGLSKNQVDQIILVGGSTRIP
KVQTLLSEFFGGKKLNKSINPDEAVVRKRSIFTSCLYFDYNDIASDDTK
>comp4663_c0_seq1; 390 269 1042 LEN=1078; translated
MAEESSSSSSAGEAIREAALSAENAVEKCTANVAIEVPPAANAAEGAPAANVSAGEKRKL
DEGDGEDVVDTPSTSIKEVSTDDAPENKPKKAKISMPPPVSSSLTDVEKYTLECPPPSDN
TNEADAKTKITTPNLMLFGLHPLIQELPLQKFLEDYGKVLAITVRSAFASRYGHVTFETV
EEARKCYTSINGAKLLHKAFLVQPSMASAAPKASTSMDSKSKESKDTETTEVNTISTDSA
GAAAATAAATATATTPT
>comp4665_c0_seq1; 177 231 422 LEN=502; translated
MEAMSVLLKSNNETSAELDGTDTMDVDGSDGEIDGLEASGAAAAWSSLVVLFTSFMGASF
LGS

>comp4667_c0_seq1; 113 1 697 LEN=1205; translated
XNKNNRFLREGMILRTKESGGSNSDYTSRELVERLRSGPYPLEIGFQNLAAGGDAISDLG
TSIVTPKDALDLAQKTETPSAGGNSGKARGVEYSITTTQQKPPSECAIKSRRNDVLEIEY
EASYVADENAKKVIYDASAFRGTGNRPYQMVLGSGDMIPGVDQGLYDMCPGEQRVLKIPP
VLGHGPNSRRLYRIPDKYVNLEWKVGLVTIDSTIREDNNVVSRDERESRFAY
>comp4668_c0_seq1; 268 409 1143 LEN=1321; translated
MAEATQKRRLEQGKEEPAGLDSEWNKRMKERLERIKNKKMRDTSITVMQRGLSFSNTKDN
KQTTNFDEEGNIVTSGAGRIREELSQENGRIEFLTGKDAGTVEICVQSISASPKRPARLH
VMVKDEASDNEFDDDDAYYDDDDHVSDSKANTKKVKDPDHLEHRELTQTMTRLERDLQSL
QNRIKACINNADFNKDQEHKFHEQSVSMNRATKYWPIIQLIVLILTGFTQANHIVGYLKS
HRIV
>comp4669_c0_seq1; 149 214 369 LEN=1194; translated
MAAVPDAEAMARERRKESFEYTRVFLLGKRIKCTLTDGRTLTGTFICIDRL
>comp4669_c0_seq2; 128 214 753 LEN=781; translated
MAAVPDAEAMARERRKESFEYTRVFLLGKRIKCTLTDGRTLTGTFICIDRLKNLILTDVL
EERSIDPSDYKHRVGSSEPSTEPPTARCFRRVERKISQAMVPGXAAGAGRAAPRVGSYGS
VVPPAAVLETTDAPVMMGGGDETGGTQRGAALCDEWHATQCDATQGMREKQSRAPPCTV
>comp4669_c0_seq3; 113 69 418 LEN=871; minus strand; translated
MPCVASHCVACHSSHNAAPRCVPPVSSPPPIITGASVVCEHRGRRHDAAVAAYXGEQLDP
HQPLAGDHGLGDLALDPPEAAGRGGLGARLGRPDPVLVVRGVDGSLLEDVGQDQVF
>comp4670_c0_seq1; 600 75 1121 LEN=1291; translated
MYSAFFFVVAILFGVIVDAAVAAESAPLLSIYKTPALMEIQFASENEFSADDIYHIEESA
ALYLQELLTNDESLRFVEEQDSMIDVSVNVKTQQLNGNEISLNSEVDVIHYGEVGIADLA
ALLTFLVNRNNTDSSLIYLDQVVNADASVELISFDQVSESIPMATDLTRMSSYSELANAK
ESKKRTLSFVTTLLSITLFGMSAILIWVGGGWLVLRKKVKALLDREEEFTRMTMNIESKP
TAETDSGDDESPSKEDMNKKKQGPLAVYGIAVKTTPPKMGSESNPDDMATPMSVMSDYSD
TNRAPIGIMSMRKLVPNIQRDDNESESEGDEEEQNVERGFGSMKKLQY
>comp4671_c0_seq1; 197 951 1875 LEN=2601; minus strand; translated
MPISNARAIPPPTPVPDPIHVSLLCSARSVPVLPPLPLGVFKRRARSGRGGRTAARRLPP
CSDSGSDSAAPGGPACTAASATAAGVLARAHGPRPPRRVVRAAAGCPVGRGGTGSGPAGS
HAKASHRALDLGRVLPELVEDVLVLLVLVQDGVLRRYRVAGDRAPPAVPDAALHVAAASA
VAAVAAVAADPVPVAGSARVDADGIDRGDAEVAAVGRVGGEEAAGPPPRVPPLVVEVARV
WVRLWIVRLGDAGGPSARVAFAVVVAVAVAVGNGGSGLSVAQLPMGAGLVFKATVPAATA
GGTESLG
>comp4672_c0_seq1; 295 295 519 LEN=519; translated
MGKKKDDDSSSEEESSDDDSDDESGDDSSDDDDSDDGSGSDDDSDDGSGSDDGSDDGSDD
ESGSDDGSDDGSDDG
>comp4673_c0_seq1; 365 1 846 LEN=941; translated
ATAAEPAASIHPVATLADGCRFPLASFGLQIYDDSTAYKLTLTALECGYRNFFASVLAGN
QKGFARAIKDSNVPREDLYICGSVVSNRVSGFENARQATTKGWKRNMEAFGVGGIDYLDQ
IMLDYPGPDCESIQGQWTAFEEMHAQKLTRSISVSNFSARQLDCVLEKARVKPVVNQLPY
SVAYHPGDAVADNASRGILVQAWAPLGGSLGGRFDKNVKKACGKIGKKYDKSFAQVALRW
IVQSGGSFTTQSKNKDHFSEDLNIFDFELTEEEMKTLNSLA
>comp4673_c1_seq1; 140 141 403 LEN=403; translated
MIFSKRATAALALCSVLLVSNSPLGRALSTKSQNQSQNQIQTHGSGVDRRTAIQRAGIAA
AGLGTVTGGGLFLPNASPAVATAAEPAA
>comp4674_c0_seq1; 179 720 1172 LEN=1585; translated
METGSELSKTVATFIVQKILLEETGLNYVCATAERFYAVSTVLANMVHLLQVHPSVRLLK
HVVRCYLRLADHPRAREALKTCLPASLKDGTFGPHLHNDLTVQKWLSQLLTIVGSDDGDP
SGDNGNNGNDNTNPLLTQPSSTGYLQKDSK
>comp4674_c0_seq2; 268 120 1109 LEN=1522; translated
MALTSSSNSSSHSLTSGPGNSASGGGGMDELLPLVLQLTNAESREVVLLELSKKRETFAD
LAPILWHSTGTVAALLQEIVSIYPLLTPPTLTAHASNRVCNALALLQCVASHADTRQPFL
AAQIPLYLYPFLNTQSKSRPFEYLRLTSLGVIGALVKVDDTEVISFLLSTEIIPLCLRIM
ETGSELSKTVATFIVQKILLEETGLNYVCATAERFYAVSTVLANMVHLLQVHPSVRLLKH
VVRCYLRLADHPRAREALKTCLPASLKDGTFGPHLHNDLTVQKWLSQLLTIVGSDDGDPS
GDNGNNGNDNTNPLLTQPSSTGYLQKDSK
>comp4675_c0_seq1; 430 115 1662 LEN=1759; translated
MLSRVLPAARSRWHSAYSHARRCASSGSGTTTAPSSVSFEKMETDKRYKPTTDVPESLL
SSPEGSPSPFSIRGQFREGRAAYLDMSATTPMDPRVLDAMGPYMVGSYGNPHSKTHAFGW

EAEAAVEKARAQIAALISGDPKDSENENENQTESGAPKKKRGRRKGVDPKEIIFTSGATE
SNNLSIKGAAHFYKKRGKHVITTQTEHKCVLDSCRSLEMEGYEVTYLPVQASTGRVDMKD
LEAAVRDDTILVSVMAVNNEIGTLQPLREIGELCRSRKIVFHCDAAQMLGKMPLDVDEYK
IDLMSMSSHKIYGPKGIGALYVRRRPRVRLEPIFSGGGQERGLRSGTLPHALCVGFGHAC
ELAARERPFDEEWIGYLSEKLRRGIEESIPDVVRNGDPDHNYPGCLNYSFAYVEGESLLM
ALKNIAVSSGSACTSASLEPSYVLRALGVDDELAHSSLRFGIGRFTTEEEVDFVLELLRK
HVVRLREMSPLWEMVQEGIDLKEIQWSQDEHHHHH
>comp4677_c0_seq1; 557 1 1629 LEN=1975; translated
XHRANWMATRVLGNLEEQFDLVLFCLPPGTGRDWIGFASVGRFDSYYNNQWCGRVSIQLH
EVGHSLNLAHSGEKKAGTSDYDDQSGMMGFSYPRDDWPRMCFNPAKSYQLGWYKRQQLAI
DPTKMLLADHQGYGVEGEGEGESESYGIGYDTDDDTDDDTVSTAKPISTTTTETSATSVF
VFNGVADYRATDDYYDDYIGDDDDDSNHHNATAVFPASADADADAGDNDHTDDRAVVVLR
LRETGSGTDYFVGFNRASGANQETQEDANEIVVVEKPSGGSYGYGQSWKVAALGTKGQSI
VVPFRGSLVSAFVEIKLLADTTTGYSGSNTNTNQAPINAPVAITSYRRADPCGNGERTLL
FEARGMTDGLGGREASWSLRVDPDKKASGNNADENAASASGARGGYLGFGSGYGPRESFR
RDFEVCQNTCYIFAISETGGDDPSGSEGEAAWGGVVEGFLNGEKVFDTRDEPKQNQKQNH
EPPEHDLXLALAFVLLLHRPPPAVPGLADLQDPDRKGQEQKDPEGLQVGPREPQEAVPEA
VER
>comp4678_c0_seq1; 204 308 2023 LEN=3265; translated
MNALRKAGGCHEYSSIALSSRARNAAASIGPRTYPSCEESKIRDINDNALDDGGIVDGHD
SFAVVDIEEAIDRLGMGMFQYQVVLACGLCFASDAMEILLLSFLTVILQGEWGLSEAEAD
SIVSVVFAGALLGSLVLTPLGDRWGRKPVFFVVAATISGFGILTAFCTSYSQVLVVRFLV
GVGVGGLTVPYDALGEFMPNTFRGPNMLCTSFFWTASSLAVPLFAWLTLRTPDHPDGDGS
TQESPGGGSWRAFVVVCALPGVLSTVLSLLWVPESPRWLLTRGKHDQALAVLRRAAARNG
RDPYGSFPEHTRLADHRLPSARTARDACSPLPQHRAVDHHPRQNAARSNQGDAPDREEER
REFAGSAEPNDCTNVSMVPESGCRWVLCSSPQWRKISLLLGGQWYGLAFMYYGAIIAVSI
VFSSTHYGSANDNDDGDSNADDTGGGFDFDYQALCITSFAEMIGLTLAIKCVNRMGRVST
QAWAYSLGGFCLLLLSLLDYFVGGGNEKGGFEEGENDVSRRWLLIGFAFLSRMFVMTATS
VTWLHTAELLPTEIRATGHGLGKAIRCVVNA
>comp4678_c0_seq2; 252 308 2341 LEN=3134; translated
MNALRKAGGCHEYSSIALSSRARNAAASIGPRTYPSCEESKIRDINDNALDDGGIVDGHD
SFAVVDIEEAIDRLGMGMFQYQVVLACGLCFASDAMEILLLSFLTVILQGEWGLSEAEAD
SIVSVVFAGALLGSLVLTPLGDRWGRKPVFFVVAATISGFGILTAFCTSYSQVLVVRFLV
GVGVGGLTVPYDALGEFMPNTFRGPNMLCTSFFWTASSLAVPLFAWLTLRTPDHPDGDGS
TQESPGGGSWRAFVVVCALPGVLSTVLSLLWVPESPRWLLTRGKHDQALAVLRRAAARNG
RDPYGSFPEHTRLADHRLPSARTARDACSPLPQHRAVDHHPRQNAARSNQGDAPDREEER
REFAGSAEPNDCTNVSMVPESGCRWVLCSSPQWRKISLLLGGQWYGLAFMYYGAIIAVSI
VFSSTHYGSANDNDDGDSNADDTGGGFDFDYQALCITSFAEMIGLTLAIKCVNRMGRVST
QAWAYSLGGFCLLLLSLLDYFVGGGNEKGGFEEGENDVSRRWLLIGFAFLSRMFVMTATS
VTWLHTAELLPTEIRATGHGLANALGRIGGITCPFVISRDTSLRTIGLVMFFVSILTSMV
VKSLPETTGKALGNFAVSLKDQVQGPSILPTTPEAHVVDSRSTHELSSNEDSTNKSTREL
QGDTAAHGKAESFSQIV
>comp4679_c0_seq1; 633 256 2045 LEN=2188; translated
MSPVPSDGPSTPPTDFPTESPAPSPSPSEIPSTSPSMSPAPSMKPTGSPSEIPTSFPSDS
PTGPLLITVVGVELKLRNVTGEREMNLSDLKYFEEKTTLYLQNEVDQVPGVFKIEVENTL
VTNQTLIQVNGNGTIMEKDSMLRKRSQLEQNQTDLIITMDVVVSAVLNRSESANFDVFFE
DFFKLEDNQNKLRSMLEQENTFAKGIFFNGEIVSSERLSKKGSNGTAVTGIVLAIVALVI
GSGLLWIWIQTRRIADRESTDPRKLTNLGTISKSYDSDDGNATGAEKPRRIRITTSDELR
LIQYPKKLNENDALSSASSSQLMYIPTIMPTESNIEVPDTPGTAFGINEFGTPSNSNGFA
TPANANGFATPASANGIPTPVSTSGLVTPASVLSRGGNSTHTGASSKLLVPTMKPETAPA
LDISSAQILKDISSATARKQKIPLPPNILGSIKSPFRREKKTAKSEDDYDYDRSSMFGAG
VVAGMNNVSPSDXKEKKKRKTWQQYLYDMPPIEIGGPGVTTVAVVPHGKRKSTSRSLNSR
SHPMVIGGPPDKSNSDARSTRTKNRDKHGVVDIVDEIAYLYSTNADPPPRNSDASI
>comp4680_c0_seq1; 653 1 1629 LEN=1629; translated
AASPREQIPPQYGRWSSSPTQAIQAAAAALQPVAARNRNQNHDQTVRQEISIVNALFFSA
FAVAVAVVVAAVFLFAFELVLRSAIAAKHIAPVWAHRRNAAEGNXKESSPARQKACAVAN
KNKPTGSVAWMGICRAIAVAVAVALAVAVALAWIVLGLLPTDSTTGFENKPHTGFERPAG
PTKTGLHPETPGAPRTSGRKKKNKPPNIVVVLTDDLDWTLGGATASTLKKTRRLLGGGDG
NASGNGSGNSNSNGNGNGNSNGNYENESPRRQQHRSPGHQADLIRDFLARYDEKRRNEPP
RGRSGSPPRRTATATTTATTARIRGRTKSPGRLPLWNPEKTVSEKEEQEQEQQHQEQHQQ

QEQEQEQEQQQPQEQQQEEDRKPQHQDDDEHDAADGESQNQQLHGGDRKLSGSLSTYLQS
TSEPVCEKGSHNKNDKENDSNDGSFDSSELTDQFPEIPVRLRTVKELETCNGYNAQDTDE
DDKENPQQQDDGADPGDQDSKIYIDALIRGRDLQHAKELERHAEELEKLHKLFEEASEKH
RTE
>comp4680_c0_seq2; 668 1 1608 LEN=1608; translated
AASPREQIPPQYGRWSSSPTQAIQAAAAALQPVAARNRNQNHDQTVRQEISIVNALFFSA
FAVAVAVVVAAVFLFAFELVLRSAIAAKHIAPVWAHRRNAAEGNXKESSPARQKACAVAN
KNKPTGSVAWMGICRAIAVAVAVALAVAVALAWIVLGLLPTDSTTGFENKPHTGFERPAG
PTKAGAPRTSGRKKKNKPPNIVVVLTDDLDWTLGGATASTLKKTRRLLGGGDGNASGNGS
GNSNSNGNGNGNSNGNYENESPRRQQHRSPGHQADLIRDFLARYDEKRRNEPPRGRSGSP
PRRTATATTTATTARIRGRTKSPGRLPLWNPEKTVSEKEEQEQEQQHQEQHQQQEQEQEQ
EQQQPQEQQQEEDRKPQHQDDDEHDAADGESQNQQLHGGDRKLSGSLSTYLQSTSEPVCE
KGSHNKNDKENDSNDGSFDSSELTDQFPEIPVRLRTVKELETCNGYNAQDTDEDDKENPQ
QQDDGADPGDQDSKIYIDALIRGRDLQHAKELERHAEELEKLHKLFEEASEKHRTE
>comp4681_c0_seq1; 152 370 1662 LEN=1737; translated
MISVRSTAAYVRRSVFATAGVKRNIRHSKKSTISSTPSKVEQQAASPPSSSASGEGSGGV
PVLASLAAAASGVGVVGAAALAVEQSTADTCPPYTSRGGAQRFDQETFGGRFARMLLACD
PTLLLCGDETILAARERLTEAAERESNSNNANNLFSTKESRSLWESKRVVESALHPDTGE
FIPRPFRMSGYVPYNGPICVSMVASTSTIPLLFWSWVNQSQNALVNYYNRNASSPMTNET
LMKSYTAAVGSALVVAFGLSTFVQKRYPPAKAKELMKYVAFPSAVVASSLNCYVVRSPEI
DTGVPLTDQEGNKVVLPDKSENTSQIAARRGVESTTASRALLQAPVYFFPSLLMGSLPPL
KRFLEQHPRTRVPVTTFLVLVSFGLGLPATIAIFPQVAEIKSSDVEEKYRHLIDPKTQKP
YEVFYYNKGL
>comp4681_c0_seq2; 136 370 1104 LEN=1852; translated
MISVRSTAAYVRRSVFATAGVKRNIRHSKKSTISSTPSKVEQQAASPPSSSASGEGSGGV
PVLASLAAAASGVGVVGAAALAVEQSTADTCPPYTSRGGAQRFDQETFGGRFARMLLACD
PTLLLCGDETILAARERLTEAAERESNSNNANNLFSTKESRSLWESKRVVESALHPDTGE
FIPRPFRMSGYVPYNGPICVSMVASTSTIPLLFWSWVNQSQNALVNYYVSCVQYGIVDGS
MFPW
>comp4682_c0_seq1; 322 189 1004 LEN=1079; translated
MEIIIVVTCSIVNLVHHIFRHWNEGRVLRRELGIGSADIEGNSNNITVAANDIIEIASRR
VGRILDAPPVDDNTRKRRRDELIYRNLILHHVMAVPSSSNQSQEQQKGDDTSNTNNEPFS
DSDISGFAQQQFDVNTETKESFPPSSMLPSLVFCNRANTPKLDEEEKRAAAEVLTHQKCC
GICLEDFAVGDIVVHKWRRRDQGTTKPKKVHNTDYRGRLDIPAGKGGDEKYKRNKDKRNA
SCNHCFHENCMIQWLQNHHDCPLCRANIIQE
>comp4683_c0_seq1; 326 218 1433 LEN=2252; translated
MTEAEAEYPLPEQILPEIHDAICNPQIWIVPSVPPDVDPRSSLLRRNHPSLQPVYRQVFL
CRQQDAAQAYQIIQPPAFQPVELSHGGPTWGYLFMGVILPRRKDDMLYEEPMEHEAQRVA
IKRLDLNVVTNELRNGSKENPFKEIWRMQTIGDHHHVCAIIEALQAENYLYIITPWCGGG
SLSDNLPLSPNDRYSVEDQARILFQQILEDLHYLHKVHGICHRDIKTANFLVSESGRVLI
GDLAMSFVMPFMVPETLLNRPFDGAKCDFWAATVTLYTLVTGLPYLYRTPRPDDILFRYC
IMAKGLSRDRHNELVKEIISEVDNPQDVSLLSTVVEHICNMSYDILELFENSLSLIPEER
WGSNKHANPDGCRTEEATMAIYFDDALAHDEFEHAKDTEINGMK
>comp4683_c0_seq2; 332 218 1478 LEN=2297; translated
MTEAEAEYPLPEQILPEIHDAICNPQIWIVPSVPPDVDPRSSLLRRNHPSLQPVYRQVFL
CRQQDAAQAYQIIQPPAFQPVELSHGGPTWGYLFMGVILPRRKDDMLYEEPMEHEAQRVA
IKRLDLNVVTNELRNGSKENPFKEIWRMQTIGDHHHVCAIIEALQAENYLYIITPWCGGG
SLSDNLPLSPNDRYSVEDQARILFQQILEDLHYLHKVHGICHRDIKTANFLVSESGRVLI
GDLAMSFVMPKGGIVKDIGTFGTPPFMVPETLLNRPFDGAKCDFWAATVTLYTLVTGLPY
LYRTPRPDDILFRYCIMAKGLSRDRHNELVKEIISEVDNPQDVSLLSTVVEHICNMSYDI
LELFENSLSLIPEERWGSNKHANPDGCRTEEATMAIYFDDALAHDEFEHAKDTEINGMK
>comp4684_c0_seq1; 246 175 1239 LEN=1606; translated
MVRYNRVSMRTESEDHGDSQDTIAVESSHSPANDQKLHPSINPLARIESNDRSLSSKSYK
SRGSDAQDYLNSSGSHRFGDSGDSDDIDEEERSPLTQNAEYASNDGLLENMPMPGDDRSV
TPLSLFPSMAAISFDLDDDDDDEVEANLQRYHLDFTSTENGDYLARVDIDEGDGSDQGAA
SGSSNWCSSGAKYVWFSVQSARQKARQRRAQMLLDQTERNWKQSLKICVLTSCDNTDLGI
FLFAIIALVWIVVLLFIRNPVARRKGILAGIILVAVRMGARPFFHWLHKHWQRRSSLRQR
VCGSAQDSSLDGMASGQRGLELHSIRDDEKRDYNHADGLMSPVSAGSDPTVAAI
>comp4685_c0_seq1; 464 338 1441 LEN=1717; translated
MKSSIVLQSNKDDGKIIATRMKQRHGLANCTIFRGQCPRTDQMMQKWHKNIDLMMEKLTI

TDDYSSAKVMAPTLVLSSENQMDPDFTNACRKNYPKHFDLITWAEDFLHSSDQEKKLPEK
KHPVCEETAKIKETQAEIFLEEAAISDSIGNPKKRIRDETTFMSTRGKKNRTETSLRFHA
SDRERQNNSPRCIFSPIKEKPLKESKSVMPIAPLPSPILAIDNSITVALAPHPFNTSKLP
KDNVSQSLEVVAGESSLLSERTCRRQRSKIAFGQNLLSASVATARTLQFKSQNQDNSKAD
KSKTANLIGGKRCESSIYRPTLVPIKEETGNNKNSSQAPDTHEDSDKENSDESTNVPPRS
ILSVTFL
>comp4686_c0_seq1; 259 252 671 LEN=927; translated
MSSGVQVDDEIPKLFEEFKIQKKLRFYTYKIEDKKIIKIDVVGPKESTYDDFCSAFPENE
PRYGVIDLEFETKDGRPTSKLIFISWNPDSGKIRDKMIYSSSKESIKAALNGVGIFINAT
DSAELDLEESILPVVKKFA
>comp4687_c0_seq1; 414 190 1029 LEN=1221; translated
MKQTCLHDRLHSFQWIGIGWNVVSVLMVGSTAILAEGGGDVDSSESAASDSDGSALVGII
LVLFGAFVQALQYVFEEKVLTMDIPAPPLLLIGMEGLWGTIFCVTVAYPLVYYLPGDDHG
SYEDPFNTWYMLNNSPTIQIAFWVYFFSIFFYNFFAVMVTFLLNSVWHAILDNFRPITVW
VTDLVIFYCLTPTLGEEWTIFSWVQVGGMIVLLYGTAIYNAPNAGSISLKGEWYSFGFNF
SEEYKAVEEEMMLDKQFESRKLSSRKLSSFRGDLAASLR
>comp4687_c0_seq2; 559 294 1526 LEN=1718; translated
MMGLHGKGIDGDIEVFEKPIFQTFGMFLGMTFALLMHFVVMALKIPFPGYVHDGASQESP
DDEERGEKSGLLQKSKGENAATTTPTWMYFFLAIPAIFDLAATALCMMGLRYISVSIYQL
LRGSGIIFVAVMKQTCLHDRLHSFQWIGIGWNVVSVLMVGSTAILAEGGGDVDSSESAAS
DSDGSALVGIILVLFGAFVQALQYVFEEKVLTMDIPAPPLLLIGMEGLWGTIFCVTVAYP
LVYYLPGDDHGSYEDPFNTWYMLNNSPTIQIAFWVYFFSIFFYNFFAVMVTFLLNSVWHA
ILDNFRPITVWVTDLVIFYCLTPTLGEEWTIFSWVQVGGMIVLLYGTAIYNAPNAGSISL
KGEWYSFGFNFSEEYKAVEEEMMLDKQFESRKLSSRKLSSFRGDLAASLR
>comp4688_c0_seq1; 489 310 1260 LEN=1371; translated
MGLVRQRLAMGLASPPSLDRCGAFGFKAFSSGDSEPTAPKTPSSFAVVDHSRAYEASMEG
RHGQQLALARLEGIGKDDLPFDPFEEEERKFLEEEEERAKLLEAADGEGGDVEEADEFGL
EEEDDDEEEEDNDDETTGRYNRDGSVRLKKSVLATLRAGYPAGGLFAVVELAGSQHKVTT
DDLLVVNKLKPIENYPIGSVHTLTDVLLVGSSHRTLVGMPRVAGAEVDVMVEEITQDAKV
VIFKKRRRKHSQRKNGFRRDLMLLRVLDVRLPEEHRDHAHVGRDESLEDLDTVNQRIGGD
DEVSRFDNGGRSMASA
>comp4689_c0_seq1; 401 277 1197 LEN=1281; translated
MKRARCASSGGGTGGREASDAVSLRNCLVLAATAAVLLVSEPVSALLCASEARVQQPCLL
ASLPKHRNGNRHRAPGIGRRFRGENGPCGLVHRSHRQQNRNQELWYVARSGNDNSSDDAA
DKDRSKTGMSIKKQREVEFNEFVEAHGDDLDSLRAQAAKLRAEADSLRTNLDEAREARLQ
KETDKVDGWIEDLLIRARVGGGDGDTEPIELLNTVDQVTEVLRDDRYSAEQVLKIFRRLS
QLRDESRSNCSPLMELLVDATGKLDGQERADNPNKRWNHKVERVLRKKLFARDWNIQYVS
EDEENW
>comp4691_c0_seq1; 303 1 1311 LEN=1839; translated
PNKTTSKTSKQTSTVKEIKPAIQKPSINDEEIDTRAIDQTDKPSPTTEEHANKTTSKTSK
QTSSVKEIENPPLSNHTNATIPGDEEPIEFKRYEKVVIATKIHGPHQWTLVEQSLCLLHF
AYNHKVLYDIVVFSAEPIPNEKIEEFRKVVAPAKFDVVMDNIGLQEEIAALTPEKRELFL
ERCQVKSPGNLTWFSNCRDPKRRKGSRLAYNWQAEFRSVRVWEHPALDKYKYMLWIDSDG
FSSRPWKKDPVEYFIKNKGVIMFDNFPQGSTKQFTKEIVDSFNATVCDLELDKQKGQLKR
NLITHEEYGILVNGNKNKTVNCSDANISMIHGFMHITDLDFYRQPKVINGLKGLLGNCFL
CRRPDDQLAVTIPAAIYAPEKSWDMRMHGFNLGVVHNFFVDGHEKNKPPGFSRWWYEGGK
KQLPRADKVCKVIHRE
>comp4691_c0_seq2; 348 1 1659 LEN=2187; translated
XLSSSSFNMTERKEKEHLLNDLLYSTKATESISMYSXALLIRESSTLKKEVYRMKLRIER
SPVPGSPRSESQGAWSMSMFFWTFLAFSVGFNATKSISYNAEYLATLQKNFSPLSTSPTS
VVSSATNSTTTGDATEPAIQKPSINDEEIDTRAIDQTDKPSPTTEEHANKTTSKTSKQTS
SVKEIENPPLSNHTNATIPGDEEPIEFKRYEKVVIATKIHGPHQWTLVEQSLCLLHFAYN
HKVLYDIVVFSAEPIPNEKIEEFRKVVAPAKFDVVMDNIGLQEEIAALTPEKRELFLERC
QVKSPGNLTWFSNCRDPKRRKGSRLAYNWQAEFRSVRVWEHPALDKYKYMLWIDSDGFSS
RPWKKDPVEYFIKNKGVIMFDNFPQGSTKQFTKEIVDSFNATVCDLELDKQKGQLKRNLI
THEEYGILVNGNKNKTVNCSDANISMIHGFMHITDLDFYRQPKVINGLKGLLGNCFLCRR
PDDQLAVTIPAAIYAPEKSWDMRMHGFNLGVVHNFFVDGHEKNKPPGFSRWWYEGGKKQL
PRADKVCKVIHRE
>comp4692_c0_seq1; 801 1 2445 LEN=2997; translated
KSSEPANDSNVSKSKPNFLDEIKSIAEKQKETPSPAPEKNEPKKKTNFLNSIRSRIGAKG

GKQKEEKTAQIDDSNMRETKLNFLEELRSKAESHKEAKEASHSVASLGIDVDGTHSTTDS
VEAKILQSQQQLDPKSTGNVSNLPTDQSPSDQGKEPSKDYLDLRTLPEPYTDNTKKLLRE
LETNDERRKKLERSLMQNGIPISEEISYEEAKDKIAEITESMKSLTVAGMDSFAMEKKYF
QLEEELSKYTAALMLTDEYAKETRRLEQEWEDSIEGDNIIAIRKLRSHMPVTIRHMTEDQ
LATAETPNGMTLPKAFARKFKRTNILQLIRVDPGNIEMMHPSLLEGMRTAGLTLTERRAL
HHHLKDVGERWAERRADPSIEKKWQWYQILRSKFKETLNAYLRCVQQHGPPENHKYAKRN
DTDGSGCRLIGNQCPIKADATFDYSQDYGYTKDATYESSSDGNQSTIDFNKGKPASKDKF
VDGQKASEAEILEEYRHRLRMDAHETEVDKKLLRELYHSNKRLQRLEKQLTNAGLSLPKE
DVPYSVAKTRIAELTADLQKVGIEMGSTNDSQQMAKLEYTFGQLSQELDKYTNALMLTKE
WAQEQKEKERQWEVDVSCGNYEALQKLRRHMPVGVRNMSESSLATEPTPNGKILPKAMAK
KFKRTNILMILRTDPSAIEQMHPSSLEGMRTTGLTLTERRAIHEHLKEVAPKWKATAGDK
MCERKWMWHASLQSKLREMLERYDKHIEEYGPPGSHECPLIGNQCPVKADLVMDYNGDYG
YPDGAEYEQQTVAKSNLLSMEELEKRRREDQFGY
>comp4693_c0_seq1; 249 387 1049 LEN=1179; translated
MSDPVDRATALEDADDTLRSATGTKRSFKMEIRLVQDQNLRNKYTKRLKRLENELRTLKA
DLKALQAEEHRGELFISGGAGDPDGNGGDPTRAGSNMLNEASALQDKTQDALSNTKNMIA
QSKEVGVATLEELQRQREVIANIDKEADRMGDNLARAEKLVKEFGKRMSGDKFIQCFAVV
NCLLLCGVVVYAIVKRRGGSLFGNEPAPENPLGNRMLRGY
>comp4693_c0_seq2; 285 384 1127 LEN=1257; translated
MADIQYWEDTLVEGVEQIRSILDGVQAMSDPVDRATALEDADDTLRSATGTKRSFKMEIR
LVQDQNLRNKYTKRLKRLENELRTLKADLKALQAEEHRGELFISGGAGDPDGNGGDPTRA
GSNMLNEASALQDKTQDALSNTKNMIAQSKEVGVATLEELQRQREVIANIDKEADRMGDN
LARAEKLVKEFGKRMSGDKFIQCFAVVNCLLLCGVVVYAIVKRRGGSLFGNEPAPENPLG
NRMLRGY
>comp4695_c0_seq1; 203 145 621 LEN=1045; translated
MNSTFFGLVLPLLLALGTPSPFVCDAWTAVTRLGRRYRSAPTPTLAGGPPEAGVIHNPPT
RLHMVGTGFSFEDGEQILVSVQKPFGIVLEQGGGDEDDQIVVTEVDPGGAAGRAGVRTGD
VLVAVQNASTVSVDLAEVLGFIAEKCPRVVNLRFRRPG
>comp4696_c0_seq1; 220 9 377 LEN=907; translated
MNMMNMNMMNFFSRPMRGMMMMMMMRGMRRPGFFMNVANNPNIMVNMRGMTTVITIGRNI
LKERSALKEVGAAQPDNERRLKIAAKNSSKNNFVKKFVPASGATKGSSPRGSNRASERRN
EQ
>comp4699_c0_seq1; 234 253 993 LEN=1011; translated
MTITINDVALRNVSELEGRRMKANDVKHRKEALFHCCEIVLGKLLGTGAFCEVHELEDVR
LLNVKELPGCIHSKCRIIDEENERRKREHLRLACHDAKGKLRYVVKHLRPDLAADRGMKV
FSRAAVDCLRELNILSRLCHKNVVRVRGSALSGRNVINKSDNSNKDFMKIASQNDPKAFF
IVLERVEETLSQRIRQWRLSEKPKAIDSSISDADTMLPLFYPDKLRFGLHIASAIDHVHS
HGMIFR
>comp4699_c0_seq2; 334 253 1680 LEN=1721; translated
MTITINDVALRNVSELEGRRMKANDVKHRKEALFHCCEIVLGKLLGTGAFCEVHELEDVR
LLNVKELPGCIHSKCRIIDEENERRKREHLRLACHDAKGKLRYVVKHLRPDLAADRGMKV
FSRAAVDCLRELNILSRLCHKNVVRVRGSALSGRNVINKSDNSNKDFMKIASQNDPKAFF
IVLERVEETLSQRIRQWRLSEKPKAIDSSISDADTMLPLFYPDKLRFGLHIASAIDHVHS
HGMIFRDLKPDNVGIASGGIAKLFDFGLCRSMPLEDDKTLSYKNNAKREPVYRMSTVGTR
RYMSPEMISGDGYNQKTDCYSWSMVFYEMLSLQKPYARYNREAHKVFVCDNHGRPCVSAG
NVPWSSRDLLERTWAHDISDRPCMKAVCKELELMIDTVEKQALPLVERSLRAVFEMAKLL
AFDENEAVPCLGSTTCPFNDDDQHPGKRYSSHSPQKSSGTETSISVPDLSIVAAE
>comp4701_c0_seq1; 971 404 3040 LEN=3229; translated
MASCTDSDSDEEDEADLQNSITTRNTVGEGALSHEEAWMINLGRSDNNEWLTGPRDPDEW
FTGKQPKICPGVDGKEVVRSLPLPRLDAVTRESAMEYFDNSWTLYETLFAGLNGEEYFYR
PPPHGLRHPQIFYYGHTACLYINKLRVSGVLDKPCNAYFESIFEVGVDEMLWDDMHKNDM
LWPTVREVHEYRQQVYEVVKDAIMNHPALDDSNGPITVDQSHPMWALFMGYEHERIHLET
SSVLFREAPKHLVQAPANWPPVHPSAYNETTSSNPVEGVDYPSNEMISVKSDKVDLGKPA
NFPSYGWDNEYGERTVDVPDFYASEHMITNGEYYEFVKEGGYRSKEYWCDDGWAWRTHRN
LKWPFFWQQSGPAGSHEYNLRTIFDVVPMKWDWPVDCTYYEAKAFCDWKTKKYGSPTSKP
YRILTEAEHHVIRHRDNNLEAARKDVSADKVMVTSGEDFPAGSHGANLNLAFSSQSPVGQ
FDPSHTGHRDTTGNAWEWTEDHFNPLKGFEVHHVYDDFSTPCFDGKHSMIVGGSFASTGD
EASVFARFHFRPHFLQHSGFRLVASDEDAPATHLFAGNFGGQAAARDAANAEAAESNPSN
ESINANDTNVYETDDSLHMYLGLHYPSSGAKEGVSPILPHNQSPVHCTHFPQRVARLLTS
LNPERTTNKALDMGCSVGGSSFELAKSFDHVDAFDFSQKFIDAAKSMQAGEKMDFKVPTE

AELFETVEAVLEPGVTPEVSSKVNFFVGDACKIDELSSVKGNGGKTTYDGIMMSNLLCRL
PDPVACLNGLSSIVNKGGVVVMVTPFSWLTEFTPRGKWLGGFLDPVTNEEIRSKDELQAI
MEANGFEKIHEEEMPLMIREHARKYQYIISEATAWKKK
>comp4702_c0_seq1; 577 571 2439 LEN=2793; translated
MANMNALITILLAAIIGILTAPRSSTTCVSAMPEVQHTYFRTNIRNAAKVRVNEIDIGNG
NGNDNYNNNGFSTPDADLREQQQENGDATEVFLKMLGGSMPSLSPSQPPSEAPTLSTTKP
SLFPSFSPSNAPSLNTDFPSASPSESIEGRDDPLSFVPTESNAPSTVPSTSPSTSPSIGP
SSITSMIPSISVEPSSKPSEIPTSSTDRPSASPSVSIQPSGMPSQAPSLSPSPSLSPTKE
PSTSPSGSPTKLHSFAPSNLPSFSPSLGPSNLPTDSPSFFPSKLPTDFPSFASSDSPTEE
PSVLPTPAPSLLPTDACGLTPNDRRDGIRAALVGATNATLVDDPFTPQGKAMFWIIAGDD
LKLCPDDPKIVQRWTLAVMYYSTNGDSWDQCFDGDDSCATEMDDVGNSLVNLLSPVDECE
WFGIRCITLDDGRRCVERIVYEDNNLSGTIPTELGLLEELKVLGMEQGTTGGRIPSELGR
LDNMIFIDLDFNELTGEIPSEIYQLTDLQTLDLNVNFLNGTLDTRIGNLKSLYFFQLQNN
LFSGTIPTEAGGLVDLETFNVHRNDFSGVIPSGICDMREADPVKFKSLIADCQCKGRWGK
KNPEKVECPCCSGCGCKNEKEG
>comp4703_c0_seq1; 225 71 478 LEN=789; translated
MGKGSNVQKAQQARERNQKKLGKTDEERRAASNKAKQDACAKKCIVCLQTFMVNSRPPLL
YQHVVAKHPDMLADPTRCFPELVGYDPSDPKGEKAAAKAKQEAAAKKKKKKGAGGGMDDL
DSLLNAGLKKGKKKK
>comp4703_c0_seq2; 120 71 337 LEN=1176; translated
MGKGSNVQKAQQARERNQKKLGKTDEERRAASNKAKQDACAKKCIVCLQTFMVNSRPPLL
YQHVVAKHPDMLADPTRCFPELVGYDPR
>comp4704_c0_seq1; 236 89 625 LEN=910; translated
MVKHGRNKKRRAGRIGKTKIKNSGNFKRWDPDLHKKIKNPTVREHWDPSKSFTANLAAIG
LVGNPNSDVRNTNPLQKPKAVTDVSAIELFDIPDSDELAEQKEKRCPINEEDQKYIAKCM
SKYGVDYGRAFRDIKINYMQHTENQLRKLGARFLLLSSDQRIVDVPEKVKSLVSTDGA
>comp4705_c0_seq1; 242 501 1217 LEN=1899; translated
MGLTQTKLHHRVLQQNWSAIRRRLQTSDGQRWVKTKNPCGDLPLHLACYGGQTPPFVVRA
LLQAYPEGAHVRNDGGFTPLQLARVNYRTDHPFREEVLEFLEVYTNGNANTPEEQEEDLI
SLSQYDDAKPIVSYQTSATCVICLENEADHVVIPCGHQCLCGDCAKKVLAVGRSGNSNSN
NNNGNDHHETTITKQRFCPVGRCKIAAIVKVSAPPACVEASAPPLCVEASAPPLCASE
>comp4706_c0_seq1; 1484 242 3274 LEN=3575; translated
MMILKKQRPAVLAILSAATAFSGLYSSTTDAFTPVAPRQSTSIGPRNSSFCAQQRLVTGR
QLSKTNKVRSTATTALRMSSDDFNEQKYTEAGWAVVAALTKAADFYQTATVESPILLDLL
LNPAKHKAGDDAESARKAVERVLKDVKCDVKALRQELETHLAQQPKMQGDTSSQQKTMGY
SLPRVLEAARSAMSTLGDSFVSAEALLLGLVKEDDRFTTKAFLKQDIKYTDVSDAVVKMR
EKAGPIISRSAENNYDALLKYGIDFTERAEEGKLDPVIGRDDEIRRAIQILSRRTKNNPV
LIGDPGVGKTAIAEGIAQRMVAGDVPDTLQGCKLIGLDLAALVAGASMRGEFEERLKSVL
EEVQNSDGEIILFIDEMHTVVGAGAAAGSMDASNLLKPALARGQLRCIGATTINEYRQYI
EKDKALERRFQQVTIDQPSPEDTVSILRGLKPRYETHHGVRIRDEALLAAAKLSHRYIPD
RFLPDKAIDLVDEACAKLKNELTSKPTILDEIDRRIIQMEMERLSLQSDYESGEESQEAN
KDAGKRINVIDKELEELKNEQQALNLKWMAEKGGVDRIKDIKTEIAQVMLEIEKCEREFD
LNQAAELKYAKLPPLQEELEKLELAGDETELDEGDRMLRDEVIADDIADVVAVWTGIPPT
KLLESERARILSMGDRLRDRVIGQDEAVEVVTEAVQRSRAGLNDPTKPIASLIFLGPTGV
GKTELCKALSDFMFDTEDAIIRIDMSEYMEKHTVSRLLGAPPGYVGYDEGGQLTDAVRRK
PYSVLLFDEMEKAHPDVFNIMLQLLDDGRLTDSKGNSVNFRNTIIVFTSNVGSQDILDLH
GDDSMQKEMMKERVMDAMRDKFKPEFLNRIDEHVIFNRLDKPALREIVKLEIRRLEKRLA
EKEITISVSDAALDFLTDIGFDPVYGARPLKRTIQKELETVVARGVLAGEYGDGDGIAVD
AVGDRLEVYKTYDGGYNSNNTGGGYEADDYGNPAGYAGYQEDFSETPPFN
>comp4706_c0_seq2; 432 242 1168 LEN=1266; translated
MMILKKQRPAVLAILSAATAFSGLYSSTTDAFTPVAPRQSTSIGPRNSSFCAQQRLVTGR
QLSKTNKVRSTATTALRMSSDDFNEQKYTEAGWAVVAALTKAADFYQTATVESPILLDLL
LNPAKHKAGDDAESARKAVERVLKDVKCDVKALRQELETHLAQQPKMQGDTSSQQKTMGY
SLPRVLEAARSAMSTLGDSFVSAEALLLGLVKEDDRFTTKAFLKQDIKYTDVSDAVVKMR
EKAGPIISRSAENNYDALLKYGIDFTERAEEGKLDPVIGRDDEIRRAIQILSRRTKNNPV
LIGDPGGE
>comp4707_c0_seq1; 434 336 1072 LEN=1902; translated
MTSPVAHQIHLALVAXPRLGETKEKPKPPKQKSNSATCTRSNKLQTAKPFAMFDIIQNSE
SCASSFEARLIKKYWSKQNEHSLPSFLGKIHLEDNSKQIECTVARTQENANNTTPKEEMK
NDIMAGYSKIAKDNPWWEEQETCDDHHAVPFHSKEDEYHPPAVEQRDEKDWKVCEDEETK

TLSTVQEVFDANVNHDGSGLSEDGDSSTITSSSAGAEFKLFDEMSVVTDESSMGDDDYNK
EDKDE
>comp4707_c0_seq2; 456 458 1042 LEN=1872; translated
MFDIIQNSESCASSFEARLIKKYWSKQNEHSLPSFLGKIHLEDNSKQIECTVARTQENAN
NTTPKEEMKNDIMAGYSKIAKDNPWWEEQETCDDHHAVPFHSKEDEYHPPAVEQRDEKDW
KVCEDEETKTLSTVQEVFDANVNHDGSGLSEDGDSSTITSSSAGAEFKLFDEMSVVTDES
SMGDDDYNKEDKDE
>comp4708_c0_seq1; 291 268 1164 LEN=1515; translated
MTALKIIRDFFPCDLSRDAPRTKRRKKSESSLRHVMEGGSEVQKNQQQQNEILSKVAPSL
FVAHALAENGLPDRIDPLSIEYLMEFDYDDGSFHHDMPTEVLMAVKSGNVDALTRHYVTR
DRLLAQRNSHGETLLHLACRLGSVALVKSLLCDAQMSALVLDRKGRTPLHSLCMAMDAKN
VRDVTNYPNCDQLESMRLLLREKSTLVVFQDKEGKVPLQYIRQSECMKTNDDHQRTLLYS
VINEMLASEQVVERVVEEISSRAKSLRLGQRLSTIEKINTLLDFSGIEAAIMETGLSI
>comp4709_c0_seq1; 552 131 1312 LEN=1484; translated
MRVCTSSLSVRSIGTGEIAPSPTESKKEDTSSNDSSAKPFVVRNFRGFTSDWLHVNKNNT
KYGTAKENTKTLTKQRTGSTDSNSSESELEGEETDKSNFEASYDTTTDLSYHNASEQEGA
KLVHQALTADEQSRMPDAYMPLRHYRAEKGDIAKAIDAIKFTLKWRKEFRVDNLVSSLED
DEAKKKGTYSSNSVEIDDQDDEVDFAAILRKENETGKIYVRGYDKDGRAIMYMRPGRENT
MHEDNNMRHLVFQIEKAVRCSEKNGHGKICLVIDYEGFHMSKTPPMSTTRRTLDILQRHF
CERMYRTYICNPPFVFRSFYAMIKPFVDPVTKQKVCWVVGKKGMDQVVEDVGGPEKAAAQ
LEKCCGGSGNDVRDFDSEEYMRLPLKIAFDEEC
>comp4709_c0_seq2; 305 175 865 LEN=1037; translated
MEEEFRVDNLVSSLEDDEAKKKGTYSSNSVEIDDQDDEVDFAAILRKENETGKIYVRGYD
KDGRAIMYMRPGRENTMHEDNNMRHLVFQIEKAVRCSEKNGHGKICLVIDYEGFHMSKTP
PMSTTRRTLDILQRHFCERMYRTYICNPPFVFRSFYAMIKPFVDPVTKQKVCWVVGKKGM
DQVVEDVGGPEKAAAQLEKCCGGSGNDVRDFDSEEYMRLPLKIAFDEEC
>comp4710_c0_seq1; 255 286 1035 LEN=1066; translated
MDKDRLWMPSSGLQKRREERTILSPHVFANYHSPTPTDSTVEKDGACIQDYGFQSSPHPS
DSFEVSLHRLPRTLNSEIELVFPEIASQAKREKREQAKHKKDECTKENTKRNDHKREYLV
IPTFHVTRFSILERNDRTEDERIRVFLRFRDFATEFKHRLSQKDPFSRLDIPDIDGASTF
SDSVHTNSAYDEVASTRSILGYSTFLYMGIQIVQHPRIGYEKLAIHTIVAYANCRHAAET
YLELLKKYQ
>comp4711_c0_seq1; 161 1 956 LEN=1275; translated
DGKAKDGKAKDGKKKRRVLDEEEIGKGRIYLRSPYESSPVTVSSSPRNARRPETSVPIVG
TSKAKTASISSLESTLDSIFSLASFVRHAVXACHIFQHGGGSDGRVAESLSSTASISGSS
FAPSNPNHNAVRRLKKFECTREFFGCEDGSHKCEIKEGGCGKDVPPGYYPDLSSVFSYCY
CPPQRDSKAAQNATVSSPYSESVRVGHTRGSRTPLTREMRFHDGRNHTSSNVTDIPTRAS
ADSSIVPHTNPAVVARYERCSFGLVWDTHSVPEGSNRTDQVAYLPGKYGNGGLWGTSGGA
CVLGNNISERGRVRPTRN
>comp4712_c0_seq1; 432 254 1264 LEN=1457; translated
MEEIDDQAMLRYVHEVLIEQFVGREEENEDLKFELMMLVLRDPSVVKDFSVTTLSQRATS
FAMEIDDAATPREVHKQLIERFVGREEENKALKRKIKELYHRFLPSEYWEKYGFLPVDPM
VVQDVSIRASNKSFNRKLKERVMSIDYLFVFDQPEAEFDGDCNSNYDYNRRVSIKSNRAK
NESGTKEYFCGEAAFVDTPRLLPDKCTINQLLREDTVIEVFDDETSDTPCPASNVSRLLD
WHYDPRLAEASDSNSPGGGWTCSIFLEKNIDDFDDCCCNLRLSVEIKFEAGMKLLHKRDG
FSYENLSDVQTLGDIRSGFDGEVVDISLETYWLTP
>comp4714_c0_seq1; 312 146 787 LEN=948; translated
MSHQTNDGAPPPPFEASGSETTAPVANKLEEDLKKSAPRVMKHMNDDHGDSLVAYVLAFG
VDDPSRTSDNQQPHQGAGLLAAVQNGSLAIASARLTDVRTDGFVLEVLVEASAPGSGEPG
PSVLQGVLVPYGRPLSGARDLHAVAVELHHSAHHRLGVVYKARSGYYKGSVAALARRVLG
KPKQRSAGWALVAAATAAIAAVAWRARRPAAAA
>comp4715_c0_seq1; 356 450 1689 LEN=1689; minus strand; translated
MTELFFPASKRAARLLTIGALLTSQVASAADSSQGVSLYDFVEYTTLQREDITGLYLTMG
GTAESENNCIFGGQEYKGSMHNVVHTIEPVYFNTATDTTPLITVFTYPEGLIQEIVFGEG
ESGKFINLQYNLDLLKRKDTEPTKTDGPDSGVATTFPKTQSYGVWIGFPFRQLEQVYLAG
DIVVRIKEGFTNMASLVATDRVAVEAIARPPILDTLESENSNVPDEAFANRTLVVTAARN
ASVTLEAGHERPKPIEAVASGSADLSIAGSVEHIQCTDNAVCIVDGKVSYGNCTDSFVAG
NATLETDDCSCVAILVTAANASSPINGVSVDPGTPKIQRSSAFGDEFFIRTADTANCLDT
TYVPPVRAVTDGPSTTHAVFSCPALQGMLEDETGFSKTKENPHGGMSPPAPFSX
>comp4716_c0_seq1; 514 1 1636 LEN=1867; translated

XQVSQTELWSKISKSRYEPNHVRNVYEIDDPSKVVRFFCGFDPYRCRVPVGSKTEGGEGS
DKTAMTPVPGGNQVMGGNNSALKVYLYSRQSGRLIKVRNDPRNELGLTGGSTDFCQGMTV
IIDDYNGTLPLNPTKQDTAYGHSKHGQIHASNVSEWTAAIAHFYWNYYYYRLGDSKTAIR
EAVLETKPSLEEAYRHYQDREQIEEDNDFVSIVPLCKGTFISYDNVDFTLRNYRGVATIR
CNKKWRETAVPKIARHEVVRFSEEFVASATKRRAATAKKKRDTLDLAAGHRVSQRVTSLE
VVGPPPAKRLRSSHHDEARIDLLRDDNNDDGGGGENSDHPQQPPNGDQAYGRVSDGRTES
FEERGRQDGGSHPQAAVGFQVQHLHQQLQQAWYDNSRLEQECSRWRRQDAVLQQECQNLR
LAKAANERELYDLKTFQLKARGDVERLVAEVEHLRNRGAPPQHQPNQFSPGGPAAEHAQV
DKLRREVQVYKSRAEFYKKETESKKAQIENLVQERRRFEERVQELEDAQLDDNGGSNPLE
SIVTI
>comp4717_c0_seq1; 108 128 430 LEN=547; translated
MTMIRAFARTRALKGPARNDVDLAMTKWWGKKRPAEGQITRSISAHEQYVVGPWLKTFPQ
KVVRRAPTYIIYPGGALALTYGSIKWASDATAAEDYSHRP
>comp4717_c0_seq2; 135 128 256 LEN=683; translated
MTMIRAFARTRALKGPARNDVDLAMTKWWGKKRPAEGQVSKH
>comp4718_c0_seq1; 285 330 1118 LEN=1491; translated
MKSNIPIFSSADEDILSGSSGGRINSAFSRNEFSRVIYIDRIFQQRGRNNQQRDHEVKIR
ASDEECQALADRFELKKIETLEARLSIRPATEALAAGTSGGGLPVEAEGTIEAHLTQTCV
RTNEEFEVDVEFPVYLIVKPVTSNNKDDIELMIAQQQEEEEAEGGKKKKKKKFKPKKDD
VFHQNKKTYNLSDIFDLQSAIQEADFGGAGDGLADTVEDEAIYSLSSDQLDVGELIAQTF
CLELDPYPKKPGTDPVSWEITG
>comp4719_c0_seq1; 128 1 534 LEN=605; translated
IKVNETKVIKISGVTKAPIRRYNRNKIMLPLSMLNVAAETSTPLLVELKGGDTYNGRLVS
CDSWMNMNLTDVVCTSKHGDKFWKMPSCYIRGSAIKYLRLPPDLLDSTKQKVQEDERRNK
IMREQNNGGRGGRGGRGRGGRGRGGRGRGRGGRGGGRGRGNNNNYGGGRGYNNNYQK
>comp4719_c0_seq2; 177 555 890 LEN=961; translated
MNLTDVVCTSKHGDKFWKMPSCYIRGSAIKYLRLPPDLLDSTKQKVQEDERRNKIMREQN
NGGRGGRGGRGRGGRGRGGRGRGRGGRGGGRGRGNNNNYGGGRGYNNNYQK
>comp4720_c0_seq1; 280 1 688 LEN=999; translated
XAAFFPSNDWGIAHTTKLSNLISIMSILEYNGAAIIAMQGDGCVGIAADRRLGAQLATIA
TDFTKVFRMSPKLYVGLGGLATDVQTLHQLLEFRMNLYRLKEDREMTPKVFSHLLGQLLY
EKRFGPYFVEPIVAGLDEKTGEPFLSGMDLIGAPVHTKDFVVTGTCTGNMYGMCEALFKP
NMNPDELFETLGQALLSSVDRDALSGWGGIVHIITKDAVITKELKGRMD
>comp4721_c0_seq1; 824 711 4179 LEN=4739; translated
MHVYDRYVHRITLSQQTKLRRSISLANSIGDHRVTAEQYLLKINECLRDIDQPLIPSTIN
NFLNRRFVLKKEKEIIDIFLDCDATTLNYLVCHLKLALLVYKIKDHGFGGQHRTELIQIL
AVERLAALTVYSRVIVLHALQILKLRANPRAEYWVRNILLNSTGDDLSDIKTLTDAKGDY
YCMSKLIYDDIKSETVRHDILIHMRKEGAMQVTRRHPGTRRGPRRRYLMAWRKILSDVDD
TLLSSGGHYPAGIDKRYPKKVVYPGVLAFYRELDLGISGPDDWPENRVGNLVFLSARPHV
YKDMSEKSNFAKFEKLRAVGEDGRRGMHTTPSLLAGDLASGSQYITTNDFEPLAQKKFEN
FKRYVSIYPEYQHIFVCDNGQGDVKAGEMMHDNFPYEYQATYVHLVQARERTYGYNPERW
KETEFRPCFFRTYPEAALHAATQDPPLIRMNGLNRVCSDAVKDFNAVPNKKWQSPKQKAE
RRAELNQSIWKANKTLANTDAGVVCLIKAERLWEDGAKVRTPYGNGTIRGFDPEFDLYDV
ELDWRPLDVQVREHVRNIEDETIRPKQGKETEKRSSSVLETVVETDEVTIITEGGDETGF
EGERTNGDENDRSALEQGLTVTCSTTPEDYAAPENDKILNSISIDESESTTSETESDNHA
LGTNSSLPAVKTAIAGKASRRGKEPDDSFKATAKICGRSISKFIPPTLPTFNDKKGTPLF
SFWVPNPRKRTGEKCSTPYGPGVVVDHRIDQKMVVVDMVGWTARAYLNEDDVKVTKESLL
STLFRRQAISSDIPPSPKYDHFPYIKGSIIRTPYGNAEVIVPLPIPDNDSIDSNDTENKI
ICLGVTSWTLADGSHPKLYSTVKACQTWKDAHEAKGIFSAFGTLVSSINREIKDRLTTTA
GTQKMKDDGLATPKFKQYYRDSAAVSTAFGNGRVMKFRKSDGFYCVCLSSWKLANGRNPT
AWLQENDVSYQIARGCKEGYPVLTKMGVSGILESVQPTTGIHIVSAPSARMVLYLQPEAI
CRGLKAAVGEDVLTAYGEGIVERYDKVNDTYVIKLNGWNARIYAKAETFDRLRDSMRDRD
GAFGMDWLLRFFFSPSDSIHQGTRSRSNSVVSGTSTSVLSLKTIRGSSDDHHLELDKPQE
WDDFLIISDCFNAFM
>comp4722_c0_seq2; 224 267 779 LEN=1075; translated
MDKAMEKLEPVIQKVDNFIAQYPSVTQYEKFKDLEEKTGYPKALFFVGMCSLIMFMVIVT
GGMKLLSNLIGFLYPAYMSFKALDGGKGVDGDATQWMMYWITFCSINLMDETFSFLSSTI
PMYYFIKIGLVVWLYHPKTTGAQIIYSSGVRPYLMPMMEGSSVTTGTKKD
>comp4723_c0_seq1; 225 115 618 LEN=865; translated
MNRSIPSTTILARAACLLVACSVLSDSLVSALSSPSSLKIRVCQGSGCLGKCRGAFDPLE

SFEKLAAGTGDSDGIRLEETFCMNQCKRGPNVRMINADNGNVLVFDDAMNETEAGRKAFQ
GVSNDGRVEHLWGIANQLLAKGASGSPMEGVAEKGSADKLNDIMPTK
>comp4724_c0_seq2; 375 1 1229 LEN=1319; translated
XTCKTKGTTMAASIASARVISPSRAEGQDQDHNGIHGLHLLSNVVDTFLHHDNAKTEQSQ
YLIPIESRHYGVPRVSACPSPTEDVLGRRINAFVSSQDRSWSIEHLKSALQLPVSHPNEF
RFRNAAIASFSEMVSNCPPPIAIGIDGLKFIRSNLVPPSPPISPQDASLHEKFPIRHSQE
ILTQQAQGLAEDPTLDASLKRKQNFNANRGPLRKRIKSAYKNCSVREVKKTSVPALKTNC
DGDGTKISIAELNYIKHKAVPMREKWMARFSELAEFKKKFGHTKVPHNFPDSPKLAEWQK
FQRQQRKLFVKGKHSQLTPERVAMLEKIGFVWEARIDAWESHYQNLKRFKGSNGHLYVPV
ANTVLSQWVKRQRKQYKKYQKSQESSLTTDRVDRLNQLGFIWDGRNVTD
>comp4725_c0_seq1; 213 1 572 LEN=752; translated
XDNDNDNDNDNDNDYDEEEDSNSNDDIDEAKDLSSLIADAEETNRKLRDRIGRLRQAIRS
IRSMEAIKKGKGMVEEYGLRVEADALGEDLRFLEAKQEGLGETLFELHIQRLDLLRLEED
LQKERVREEAKERCAPTPYGKKNERVAAGAEKTTEAGRETETETGARRSAGPCPMSASEP
SESGWQAWCV
>comp4726_c0_seq1; 151 236 919 LEN=1063; translated
MNQVECIDRRAINDNNSLERCKLTEILDRVLRMVHPENDPTESAPLRSFCHAEQQPRACP
LCQNCTGGERFCNASSNPCGANPQPHRSNGHDGRDPGNGTERERPPVSARKVRFCHRVEM
MTRQSESVTIDSERLKPPMATVKSNLEIVLPNTTRENPEGAAIDRLFRPPAVARGRISAG
AGHNRWASEEGNPHIDHVPSMPSRRGRNGRRKIGKDAGTGTRTNRRR
>comp4727_c0_seq1; 228 1 754 LEN=987; translated
XQYQHQYQHQFQQHQHHEHDFGFTQNNVQDSSVAFYASSPYSHSDPTHSKAHNYGVPLAL
DGSYHPIAPTTEPSVASQNNPIRSSSHYHNQQPQCHSHPHHHHQNQWHGRDPTCGAPFAQ
IDTNHPVVSAGFPSSDTPAKATASHRSAPQEPGAEPSSESHATERRSATKMLRTVFGSGT
GGTDGDAPRKHDRSETSSAGPHSGAASKRGLIDAGKHAFRLGRGRQPQQGSTTTRTRLTH
ADWCASEYVV
>comp4728_c0_seq1; 225 1419 1820 LEN=2088; translated
MQSSRSQAVPATNDPSNATGTKENPSALSTPSKPATDHSTDTAESSVDETGRGMQQQQQS
SPAAAVTRSATSSGSGTTRELRRARILVTVKRTESYKRWLEENPIQRQAIIAGTATVTAE
EIVGKESSLPSSK
>comp4728_c0_seq2; 342 684 1682 LEN=1950; translated
MSTEAGQTKRRLSRNTIESEERLADTSTNKPNKGADTETQTGEPLRSRQQQHGTDGDKTK
NVGEQPGTIGEGSADTGAPDALGKPVPDDTSHQNVVPGDGTRQIIAVSANKNPTAFFQLA
RKFLMTNEMCDLSALEGAIVSAVDAAHLLERSKLATIVSINTSYVNVEPKRRRQQKSEEP
TSEAIKKEGVPSIQTSRNTMQSSRSQAVPATNDPSNATGTKENPSALSTPSKPATDHSTD
TAESSVDETGRGMQQQQQSSPAAAVTRSATSSGSGTTRELRRARILVTVKRTESYKRWLE
ENPIQRQAIIAGTATVTAEEIVGKESSLPSSK
>comp4729_c0_seq1; 513 167 1621 LEN=1851; translated
MENLNRNASFTRESFGSHSVKVLSMDLSGKDRDKETVPVRMFEDQHIIPKLEQRRKKGSL
RRLKPFDSEKDERPSECDAIDFSSNDYLGLAQSTQQQHKVEELYTTLPSRYLGSTGSRLL
TGDSEFAQNLEKRLAKWHKRPSAMLCNSGYDANLSVMSCLSLNSTVIMDELCHNSIQMGL
RLSKGYSLRKFEHSSLRDLKRILNEEHKNVDQPGKPAIIVVESVYSMDGDAAPLKDILDM
ALSYNACVIVDEAHGLGVLGENGLGLLEEHGLENHPSLLCSVHTFGKAAGCHGAVVCGSL
NLKTFLLNYGRPIVYSTSLPLHSLASIACSYESMTGPAGRRLRRDMRDRARLFRDLFAEK
ILKQGIRHTPGDLVPIGLVPSASPINALIIPGNSRCLEFCDNLWIKSNRSIRLYPIRSPT
VPKGQERVRIILHAHNTEEEIVYLIDLIKSTLCDMGIMKHHATGKVSPPQRPKRIHRPNI
MSKL
>comp4730_c0_seq1; 560 154 1326 LEN=1369; translated
MAFVMLSAQIVAFQTTSVTISYGRTSSKAATTVSYATTNTDSNSSDKSISDAQRLLEKAA
QLRAEIATAEGKTVEQVEEEASQARIQREKQEQKNREAREKIRQEQLEQKSQQQPTLQVP
VTFDDMVRQGARAVERAFHDGKTRQVVRFNLVGEEDGASSATEENQWPGGAKQMYRESGK
PLTNALLREVRAPTKRLDSFPEERRTNPQLKVQDIWDFDGTALHTAEAAEGPSADIQAMV
FPNTDTKYLNDIDEISQAMGKRLFLLVNPFWRNIDSWGFNILAPGAKKKAENVIYGGTGD
DDAVPFEETYVLNVFSVRGEQCVALKAYPYDWQLFAFREDDYYPNNEEYSTCIRLGSCEE
DPTSAIITELLNERPEFKETKTMRQMKKLG
>comp4731_c0_seq1; 228 117 681 LEN=681; translated
MADAFPAEDQGGPLLLYTDLAFAEERPKNLDRNQNPKRDRSNSNAEIVGEACESDGGGRP
GGDSRLVRAVARSPFVFRERGIGTAVSLNAEFFGDVSWPRMVCFVPGVCESAETWTVQHL
ARICASKEWRLAVLEPEGHGLSDGVPRGLLRPGKWERYVGQVTAFCGHALEVDRMQKEQL
QFQRQQQQX

>comp4732_c0_seq1; 153 1 544 LEN=1156; translated
XKTTTKSPISLFVTPVMFLPSTVTVVKRRSYVTLGRLSAAWSPVRNSRGGRLAAATESLA
HPGKRAMGTTIVEGVDFDTIAREWRCKWSAENDKQSLAELQKILNEAKAEIKAVKGVKSV
QRVVCGGCLDFKVVVALEDSKFGEWEESGFAPEESFLEKIKKIDGVSVVETQTYTLMPVE
F
>comp4732_c0_seq2; 151 396 960 LEN=960; minus strand; translated
MDTKTVNKTQDDGFRPTTVVVIRHGERLDYVQRAQGKNWCQANPEQPWNPPLTDEGVEMA
RALGTALKEKILPEMRMAPIAAVYTSPFLRCRQTAAGIIGGMSSVVITDENNSGSDGNGT
AKSSLKVRVELGLSESMNENWFRSWSLPGADGTWGYKKEVPLNQFEVDPRALQPAETLLN
WKDSTADTX
>comp4732_c0_seq3; 153 1 544 LEN=1303; translated
XKTTTKSPISLFVTPVMFLPSTVTVVKRRSYVTLGRLSAAWSPVRNSRGGRLAAATESLA
HPGKRAMGTTIVEGVDFDTIAREWRCKWSAENDKQSLAELQKILNEAKAEIKAVKGVKSV
QRVVCGGCLDFKVVVALEDSKFGEWEESGFAPEESFLEKIKKIDGVSVVETQTYTLMPVE
F
>comp4733_c0_seq1; 431 317 1543 LEN=1670; translated
MNMLHNDDYDDDNYFLLINSFATPEPTNVTLLSDIEDIDEYLPEQELIVDDSSSLPSTLS
HGEVGATIPDNEPVKNDGKSFVGATKNDTKESVQGNALSQEAIIGISVGGSILLIAIAFI
MCCFCFVKKRRVEEGNDVKSTKEIRSLDQSKNENNDPSDDVESTDLASTDDVESQAMYSY
NQSYGDSGSVYTNGSHIKIHLINSYNTSGNDSASYAYSLEPGIEASVVGSLSANNAGGIS
KHSGKMPIREIPQVSMAASEGRENHSNNNSERQKQNTIPDHFGNTFIESAPSDLKLTASE
LQMLPSNLRSSDDEDESDRHEGNQKVTRKVLAPAGKLGLVIDTTVEGPIVFKVNEGSELS
GKVFPGDIIIAIDEVDTRAMSASAITAVMVRTAKQKRTLTILGVDFMS
>comp4734_c0_seq1; 298 129 1862 LEN=2213; translated
MSEASSNGTTNGKSNSYSDLASSSKNVVVRTTENWLELFLTTPEKYRFGQWKEKIADKSL
SGRILRPLWDFLSRKIPNTVAPNVLCLAGLGLLGQAWYVIYHYGGAPTGPGYPFFAAMVI
TVFFAVNSMVLVHADRIRQRTPLGDFFKYATDSAATVFLCVLTIYCLLGGRIKDDEKGNS
VDTMLEIQWYAVQTTQLVLFVKHLSAFDRQAGLRYRFFSGPGEVIFTAVSLLFFRATIGF
DWLWEYTRLAFESSLLKVENHIGEYVGVDDITKTYNSITPFLLVKYAYYSMYLLAVTRTL
LLKDPHGWSRFGLSASLLMRLIPALLYFHSDDVIRVGGDIGGLDTESLGGDVPVVGMADV
ICDGLFMAVLTSDLTLAKMGGREIHPWVVLMSLAAVFSHSTILALVVTYYVAVFADLCAY
LNLPLLTTCRNVYCDGIYDLCHVGHKMLFQNALTYGNRLYVGVVGDKDANAYKRPPIMSS
AERCAEVEACKAVTKVIPDAPCWGLTKEFLDLHQIHVVAYGQEYLERFPDPKDDPYYRVP
REMGIARPLPRTQGLSTSDLIRRIQNARPADEKNSPT
>comp4735_c0_seq1; 506 1 1064 LEN=1241; translated
XSSEDIPSQMSTDTNATLSEDITSIEPSVNDEKNLEDIENPVSHTVSQPSRSNITLEEDE
EIDSMAPAVDFSLYLEEDNTDEELLSLSQVSASKQSCTGILPTEIDIENESDLSTSSDDF
VYHQQQYQEDDLISTSSHNSYLRAISDITASASSKTDRVPTKAVDDIQQIEIPYGHEAEV
EDYEEYDEDYRVSFSPSSKVDNEGFRVSFHPSNELILQAMEGASLRAKVDIEQATARALS
KINTVTSKSRQETGSERSRQRLERSKNRVLGYSFLLLLLYVINGNSLIQSLSSSIGIDDF
QIPAKGHSDLTLWVDDKVLHPSVQDSTSEISKKYENAHNVACVSKVGCLALGSI
>comp4736_c0_seq1; 472 1199 2842 LEN=2860; minus strand; translated
MTAVEWAKKKPPQNGPKPSCIAGKEARHDRNNDNTSSNEILFEEKVFFCEGDVDGAKHDK
SDPRFASGVVPLPLAVQHCWPTLFPTISSSKKSIRKGVFWVSSSSSNSDGSVRTMGNDRD
AWCNTSLQSSRSPFRKGKCDTQIHQFDCIRRKVTRTKEQHFRKRGTSTKDLIRRSIGGKL
RIAFIDKYLAVVVKPYGMPVHKVVDSPIAARPAANIIGTNGVADINDHSRSGNCGTDADK
EKDRCDNDIAAKTGEKIVSMHELLLHCGLLIVARTRPAARFLSHAFENRSEFLVKRYRAI
VHGHLEGGDEHRDSNAAGFVRSPLSGKECCSEYRVVRRFEVDPAAENSSPRDSNYENDDH
KKKASTAPRVPLTLVDLFPHTGRKHQLRRHMASIGHPIAGDTKYGNQSDDDARLLLHVPT
MLPQHNGGASSLDTSRTRTSDNFQRPRDGPALPLMLAAVEIIFPHPASTADKSLQSEVEA
PRAALESSIWEPNRDNGSHRTSDATEDTDHYVMAEDAEHSYNVSNGTQRVQIDMPDIMKC
ISRHSSI
>comp4736_c0_seq2; 488 1199 2851 LEN=2869; minus strand; translated
MTAVEWAKKKPPQNGPKPSCIAGKEARHDRNNDNTSSNEILFEEKVFFCEGDVDGAKHDK
SDPRFASGVVPLPLAVQHCWPTLFPTISSSKKSIRKGVFWVSSSSSNSDGSVRTMGNDRD
AWCNTSLQSSRSPFRKGKCDTQIHQFDCIRRKVTRTKEQHFRKRGTSTKDLIRRSIGGKL
RIAFIDKYLAVVVKPYGMPVHKVVDSPIAARPAANIIGTNGVADINDHSRSGNCGTQLPM
EVATSKNENRLDNDPLRRPAAVHRLDQPTYGLLIVARTRPAARFLSHAFENRSEFLVKRY
RAIVHGHLEGGDEHRDSNAAGFVRSPLSGKECCSEYRVVRRFEVDPAAENSSPRDSNYEN
DDHKKKASTAPRVPLTLVDLFPHTGRKHQLRRHMASIGHPIAGDTKYGNQSDDDARLLLH

VPTMLPQHNGGASSLDTSRTRTSDNFQRPRDGPALPLMLAAVEIIFPHPASTADKSLQSE
VEAPRAALESSIWEPNRDNGSHRTSDATEDTDHYVMAEDAEHSYNVSNGTQRVQIDMPDI
MKCISRHSSI
>comp4736_c0_seq3; 519 1199 2950 LEN=2968; minus strand; translated
MTAVEWAKKKPPQNGPKPSCIAGKEARHDRNNDNTSSNEILFEEKVFFCEGDVDGAKHDK
SDPRFASGVVPLPLAVQHCWPTLFPTISSSKKSIRKGVFWVSSSSSNSDGSVRTMGNDRD
AWCNTSLQSSRSPFRKGKCDTQIHQFDCIRRKVTRTKEQHFRKRGTSTKDLIRRSIGGKL
RIAFIDKYLAVVVKPYGMPVHKVVDSPIAARPAANIIGTNGVADINDHSRSGNCGTDADK
EKDRCDNDIAAKTGEKIVSMHELLLHCLPQLPMEVATSKNENRLDNDPLRRPAAVHRLDQ
PTYGLLIVARTRPAARFLSHAFENRSEFLVKRYRAIVHGHLEGGDEHRDSNAAGFVRSPL
SGKECCSEYRVVRRFEVDPAAENSSPRDSNYENDDHKKKASTAPRVPLTLVDLFPHTGRK
HQLRRHMASIGHPIAGDTKYGNQSDDDARLLLHVPTMLPQHNGGASSLDTSRTRTSDNFQ
RPRDGPALPLMLAAVEIIFPHPASTADKSLQSEVEAPRAALESSIWEPNRDNGSHRTSDA
TEDTDHYVMAEDAEHSYNVSNGTQRVQIDMPDIMKCISRHSSI
>comp4737_c0_seq1; 518 280 2177 LEN=2215; translated
MIGGADHPPNCVKQEGRTRKHKPPRVWVGIVLFVLLVANRWWWKELPSAGKLVGHHHYHH
PIQSAIAILEDPKDHSGSNQSTTTLDSLESKLSTLSLRDRTSRNDTEPTGNPLLNEMLGG
SQPTSGFFPSSNNRHNNLRLQFETGTRVTLLHIGKTGGSALRQLVRSASEYCRSHNHNGT
VRDNPSSQSTAIENHGEGTAAEILHHHMCALARVTDSRASDDGEGGDGTQYQTNGKGEPP
GRGLVHLDRNLHKTLTDSHFLVPIRNPIDRLVSWFHYERHFQAVLGKRHSHALHDLVDPH
RCNYRTVEELVLGHGGAPGAGTNSSNSHNNSSNSIHKDIRKRGNRFFDDFFPSGIASEYC
SRLARQCLEGSIPCYAHNFFNYEYYLEDVLVRILMGRSRAANTTANATSNATTDSNMDQA
PVPRIDVIRAEDSAGDLNRTLLEWTGLPVTPEMDSFYTVRKPFGVDTHYSSNEITTATTS
DNDDNDDQSHSSNDIVAQQKPPPLSDAAASTLCGWICPELLAYAKIVSHASNLSQGDKQR
TLDRLDESCGGGRGEXSEALRGDLLLPAGPQPAPAEVLRARHRHYQVRPVVKIGGGDGCP
TSEAVRDIPRADWRGHTPASNDTRSALCCNRV
>comp4738_c0_seq1; 651 491 2359 LEN=2693; translated
MFWFRVVSIQSRNLGTFGTGKRSRGNALCKTCFYFLDHNYGHSRQCSIRNAPSFAPDDPK
FTSFKGTVSFQDEYDNRIGTFLTIITRSYSSASKPEASATMKVTKMGMSGKARIPFVIQR
GGTSKGIFFHKEDLPKCEELRDIVIQDAFGSPDVRQIDGLGGADVLTSKVAIVSKRMDSS
GCTDNVLADVDYLFGQVEFGRPSSSPSDATNLVRKRILYDVNCGNISAGVGPYAIEEGLI
EVNDDTVAAIDIGSNDSNGIRCPAYRQTVRIYNVNTGKVIIAEVPVTKVLEKDEADGCGD
SWNDWTVVEEGGYEIDGVPGASAYLPLDFSNCVGTLTASDGSSNSQTADTRDQGSSRLLP
TGNVRDAVVVDGQSYDVSLLDVANFVVHVDGRQLGLTGKEPSLEILKNSKFWNTVRRIRF
EAAKLVMEEDEITLTKPFTAVVFPYLPDHGYESIRGDLICDAVDQEKDEPPSGENHDGRT
NHAGIDFRSFVLFCDHPHKAYPGTASNATAAAALIDGTVVNDVLNNPIETTTLEPNKRTR
EKIVTIGHPSGRMEVSSRIKETLGDVHGAIEPIDRDDGKSHNSSKEYFLERSVIGRTFRR
LADGFLYLKPDTVRRWKEAKTE
>comp4741_c0_seq1; 381 121 1515 LEN=1562; translated
MNRFGYLIPTLIILSICCVSFVFRYSLTGYAINEQRHHHFQEVATNRLRKVRNGESERLG
SGQVNLQDGVDENEEYDVCIAGAGLSGAVIAERYASQLNKKVVVIEKRDHLGGNCYDYID
DETGIRVSQYGAHLFHTVNKRVWNYVQRFSDWTNYEHEVVGVVEGKHVPIPVNIETVNTL
FGLNITTTEEMDNWLSQEQVKYEQPPRNSEEMALSRVGKRLYELIFHPYTIKQWNKEPKD
LGPEVTARIPVRNNFDNRYFGDPYQALPTNGYTSIFENLLCHPKIDVRLNTNYFEIRDNL
RCKHTYFTGPIDAYFAHMGYKKLEYRSLEFERKVVKNTGDRGTFQPKFVVNHPSSDTNFT
RIVEYKHLLNQTSKDTILFVERSKDGGEPYYPVPNEENKALYQKYQELAEKEPNVTFVGR
LANYKYFNMDQAIINALELFDKDTGLSEENDDSISLCVSTSNGD
>comp4742_c0_seq1; 164 1 372 LEN=810; translated
XNDNGNDNGDGNGDGNGEPKSNSNWKMIVTIVGAILGFIVVVLGIWFAHQRWKRGNNQKY
LEIKEKGGXHAKDELGMSCADLDTKTGISDNEFGQLSTREQRAHLDAIDNLFDQQGVKKS
ITSS
>comp4743_c0_seq1; 506 249 1481 LEN=1791; translated
MAFRELREVNGRCRGGVKHELMALTGTTSIDDVHKALQDLCNEALAEATEAVNDETWNRL
QEEPYNIDLNEFFNGYGHLNLETGNFRQYPSSFEKRGGSERFLYIGEDPRLNDHYATTEA
SYLGGEGVNKFYNEEAKKFFLQAPTSNFEQGCRSNTAMCCWQRDRQYLDRNGNCGFNDCS
NQNPMDNTDLCWTEEDGTIFPYPGDETENDLHCHGFAWGNDDGGVNTEAKWNNLFFVSMY
DHLYQRGYVESITNDPKIAGDQVMCGCVEDMAPVARADCTEAIGLLNYTSYFDPEKKRLA
IKHVPDSFELNFRSCRGYDYMPFYQPKDYKANQNDLDLENSNNDLAAFVFRQYLEGKMTD
DQVDLVEKTIIGYRNPKVNENDEEREKACANAYKERFGNDNYVEREIEIA
>comp4744_c0_seq1; 686 78 1409 LEN=1737; translated

MGDKKDKKKKSKKDDDKDKKKKKSKSDDASAASGGTEDKKAKKGKKEKEDKKGKKDKKGK
KEEAIPEESAHDESVASEKVEEPPPAEEEPPPPPKKKEKKEKKKKKERQRSVEEVEAAQA
IEIQLFQPDEPDLPTKGAKKYGDVKTDAQKKQCKKTYKFLNFDPWNEATRYDDDDLCDYV
IEYPDGCKVKYEFEGFSGCIYPFLMCFALKASTDTVEVVYDAYPKALKETDFWIGNAYHY
AALYDAPIPIVNFLLTKFPEGIKTTNYYGRTALHMGALYGCDENLIELLAGKYSKACQIV
DKDGYTPLHHACERGAGSTVVKALFGKHPAIVFHKAVNDMMPLHLACNSTSNVRVIRALL
LAAGDNSICRAVDVMGNTPLHLALTSVAPYESIELLVASCPETVWTKTTNGELPLEIAQR
KRAPQDVLQLLEKMMERLLVERD
>comp4745_c0_seq1; 290 349 1131 LEN=3016; minus strand; translated
MAGTYSTLLSFAIPGMIAFGQLSQYFSAQKIMYPEVYSSAVGLFANLAFGLIFVLGWPIP
GFAGFGFEACPIVTTSVTYVQLLFMVIVFVYIQKLHEPCWPGFKASEITWDRIKTFVELY
FPAALSSSSDFWRVAVIGSMAASLGEADVAVFNTAYRIMWIALIFVVSIAGAAAINMSIR
LGKRDPYGARQAGHVSIGMVVIMLCILGFFILLKNRWFGLIFTNDEEFLSLFQQCSVPFT
ISLIFMNLSVAIERIPYSMG
>comp4746_c0_seq1; 359 227 1183 LEN=1413; translated
MVALPARKRRICCTFVVCLSWESFSIGFLGPQATCGRRGLAKGKMHSIPVLNAFAKNSGE
DNGEEPGGIADPSAGSDDDDDDGDDTVRLPTGKSPRSPGVLLPRQLPRGVLVSAEESRRR
ATERSLLEALRDGDDAVGELRDFWRAQPRNYRDGDKHETEHERGERFREAALGIGDPSSW
ERSREVLESLCGEDPTFLQAFALLSKLYCLEGKLEDSRAVALEVLKLKPWHFLAIETMVA
TSYALNKINASVFWASRRLPPPGRAEQRSEWIDRAIGESLELEARIATLEVTGGDVTGGI
SFDWSSESDRTGDDDPWQ
>comp4747_c0_seq1; 257 135 680 LEN=966; translated
MSEHDTNEIENNEIQEQYRMMAQMEAAMRVKDNTGFDSVEYKRQRRLLEEQRAQEEKEAT
MPWYTKPTPRLPQAKTAGGGENGSSGSRAQEPPLPFTRANRRFGRNSRNRDGRVRVPDLC
LGVVVAVHKNGSVEEGSSCRESNGNSDTVATTCWGCDRVLRVHVLSTLVQCPECWTVGPV
T
>comp4748_c0_seq1; 501 59 2054 LEN=2259; translated
MTAERSQGAQETLACTSDNHSARIQSPENATVTSGVESVGYENNPLSCHDDWRIALYYIY
IDLTPLEVDAHIGIQRNYCEEHGLKGRIRVSTEGVNGVLSGTHETLRAYERAISTALQAP
TRHQICENEHQHRNILPVMNARASTTDTCNPADGTICILDNNEKTKLEEEAQNNVKDDNY
TTTIDLDVKYCRLRKDLPIEKQLFDRLVVKRTTNVIGLFDQSLCGQQEQQKQKRNKSERY
RRRRERKREEQEARRCSNLPNQQQQLYSAENKVKSTKNEASSPKSNHARNDNDIKKQQDG
EETSSTLENPSPLLDMSSLRRTVMEEPLNPARHLSASEWNEKLDETSSSSNKKSALLLDV
RNVYEMKVGHFAHPSTPTLLTNTRKYSDLPMMLASNRDMQERDQIFMYCTGGVRCERVSM
LVHELYPDKEIFQLQGGIQRYLEACSEQPQQQEDNQEPHSNQQEAVDRKDGAIGKKNPGY
FIGKNFVSSLKSLFGYLLLELIVRKICSDENFWRIRGFHFFNSCLCYSVKQMIVSTDFFR
PVHESKVFDPRRTDPVHFGETVGRCLVCAKPHDDYDNGHSPSDNKEARCNNCRMLILICN
DCRPKYRCHGEEEIDHGIKDEKTAAREEKDSSNEHSVRPLLYCNLDQCIHEGSSPDPELR
VFTK
>comp4748_c0_seq2; 578 59 1873 LEN=2078; translated
MTAERSQGAQETLACTSDNHSARIQSPENATVTSGVESVGYENNPLSCHDDWRIALYYIY
IDLTPLEVDAHIGIQRNYCEEHGLKGRIRVSTEGVNGVLSGTHETLRAYERAISTALQAP
TRHQICENEHQHRNILPVMNARASTTDTCNPADGTICILDNNEKTKLEEEAQNNVKDDNY
TTTIDLDVKYCRLRKDLPIEKQLFDRLVVKRTTNVIGLFDQSLCGQQEQQKQKRNKSERY
RRRRERKREEQEARRCSNLPNQQQQLYSAENKVKSTKNEASSPKSNHARNDNDIKKQQDG
EETSSTLENPSPLLDMSSLRRTVMEEPLNPARHLSASEWNEKLDETSSSSNKKSALLLDV
RNVYEMKVGHFAHPSTPTLLTNTRKYSDLPMMLASNRDMQERDQIFMYCTGGVRCERVSM
LVHELYPDKEIFQLQGGIQRYLEACSEQPQQQEDNQEPHSNQQEAVDRKDGAIGKKNPGY
FIGKNFVFDPRRTDPVHFGETVGRCLVCAKPHDDYDNGHSPSDNKEARCNNCRMLILICN
DCRPKYRCHGEEEIDHGIKDEKTAAREEKDSSNEHSVRPLLYCNLDQCIHEGSSPDPELR
VFTK
>comp4749_c0_seq1; 1026 614 2986 LEN=3353; translated
MAMATAFAVLVTCGDSAVFSPKMDSLEPTRTTTTALATAAAATNTRPSINHRKGRSASDR
PVTRAKKRSRMLSIQSSNENKKPNRWLEQEEAEKYDGNNDDSNDDGSNGGGSDDDDYSLY
SAYATGYISALSCRSYRIALSEDYNDYYTYTGGQYQREQRHDEPGEKRSPTIIGQETVLW
FDYVLDNSSDNTDDDDNDYDDYYSYNGEEENEQGEKEENNNRSRRKLEDEGDGVQGDDEE
EVDQYDYNYNSNYNSHRGNIVLAKDWLAAHSGGTLTSGCQQLLDPLDVFEDYVPSMDDGR
LVFSNLYYGPMCSDSNSNTTVATVEMGIFLDSSCHAHLPGLTYLLNHKVLSDWMTGVPVG
NSGTRSSGSSNGSSNENDEDNAMDGSSNEFNDRNPYDYMYEEANDDFNDIDDDSDSSSKY
QNDLKRLNSYRTLVHQLNYYYNRDPTDCSSHQDVCGQILQKAIDLNSCRSVDQELETGAY

AYYGDQNGDDDSTAYYDDAYNNDGQSKSQSGDEKSADYIDDNAVQEQAQNYGAFVEVDDG
IENSNDEENICYQIFQNVLEQYSSRFSSNEGDDDANRNENGDEGDYDEQEDAEYYYEGIC
YGILEMEIDSSSQGCSLENYYDLSSSNKDDDETETTNTAWTLLQQELEEVYGIKDPNSID
LLALFGIALGVVCLLGLLAWGVDMATKRRGSGTGQSRRQSAHKTKSRKSRSRTKVTKESS
AANKRAAPLLGDEDRVGDTDPDFVEDPAVLALASSSSSKGFLRAVESIEAVFRPEDRELK
SMTLSEGEFA
>comp4750_c0_seq1; 401 606 2501 LEN=2769; translated
MEFDELKALAKYQKSVRLHRRGIRDTIAAFNCTAEVNSLREAVFEEEEDVLKLKQSAEKK
KEEARADGRESAMHSMASFRDSRAFANSKVAGQNLAREAERENELSETIMNKTLEQEDAA
KDRLKKAQATLELANAAQHHTEIDTGICKWAPAVCNLINSGSSAATVTDEAIQANKDIQE
ALLQIRNAEIERAKAIKLHNLASTHANLSTSMFADAEDFKTQSQEQWQEAKQSRSSAAKE
ESEAEKDMEDAKLEESDIATKEQEIRDDLNSSRIIFDRVVEDRRKESIAREQMTHYLKLV
RERRTQWREKKTEATHHVARAGWEALLASVAASCLLVVVSSRILATFRYRQPLRWIVREM
PYVPQDLLYLNCHAWIFLLAMGYGGELLTAFHDRGFVARAAITILIALVAAILQVTLLHL
IPSVCALLRESRLDSGAIQPLVQEVVIKRGIIIALTSALELLLCWCWMGNIVFQRVHLFN
TFVVWIAVLCMSTFYWIVVRREHVHTRIDALSRFSNSGLLVQGGSNGESERYSLLTASDL
ERSQGSSMSGYSEGSFYGATRKSLGTNLSSIGSPTSMQSIPIGRSTEPSEQEGDFGSLAD
MYQAQAFTTPWHSELEKVRLLFEIYIASVAIW
>comp4750_c0_seq2; 401 399 2294 LEN=2562; translated
MEFDELKALAKYQKSVRLHRRGIRDTIAAFNCTAEVNSLREAVFEEEEDVLKLKQSAEKK
KEEARADGRESAMHSMASFRDSRAFANSKVAGQNLAREAERENELSETIMNKTLEQEDAA
KDRLKKAQATLELANAAQHHTEIDTGICKWAPAVCNLINSGSSAATVTDEAIQANKDIQE
ALLQIRNAEIERAKAIKLHNLASTHANLSTSMFADAEDFKTQSQEQWQEAKQSRSSAAKE
ESEAEKDMEDAKLEESDIATKEQEIRDDLNSSRIIFDRVVEDRRKESIAREQMTHYLKLV
RERRTQWREKKTEATHHVARAGWEALLASVAASCLLVVVSSRILATFRYRQPLRWIVREM
PYVPQDLLYLNCHAWIFLLAMGYGGELLTAFHDRGFVARAAITILIALVAAILQVTLLHL
IPSVCALLRESRLDSGAIQPLVQEVVIKRGIIIALTSALELLLCWCWMGNIVFQRVHLFN
TFVVWIAVLCMSTFYWIVVRREHVHTRIDALSRFSNSGLLVQGGSNGESERYSLLTASDL
ERSQGSSMSGYSEGSFYGATRKSLGTNLSSIGSPTSMQSIPIGRSTEPSEQEGDFGSLAD
MYQAQAFTTPWHSELEKVRLLFEIYIASVAIW
>comp4752_c0_seq1; 251 56 766 LEN=919; translated
MNAVNFPLIVTLLLFIGERAPVAKAFVVPSEQHNAYVSAIRRTPASSQSTKLGIVWDPRN
PDNDTGLVEFPTASQKSEIKREAKKRKARKQMPSFSFSDDETNGPWSDETIQAIWQQLTE
SEMMELRGICRESRKDVYQTALWFCEDLEDLIAPSSSEDEDEGQGDEEEGGIHLPVALVA
VKGHTALIYCPTLPIDHPKKLILRTSVGQKNVWKARPKPPRDIRGQIIREPKSEYE
>comp4753_c0_seq1; 276 194 1288 LEN=1523; translated
MVSSTSFSLPGAAVGAILAGFVSVSGAALTPQEYEEAKNGGWLNDIDIESGNDDDFFNTS
GIISSQHNSLRGNASATEDPVISVGSALCSNEQFSIMCGLYNETVKNNTIAGRIQGLDWG
LSYYERFTLFAPVNTAFLTPVVTTLPPALSVRDFLDTHIINRALSFEDLSRTCFYELETR
NLGETTTTYCEIDGTPRYQVGEGNVASDRPEFLSRSFVNIRAGNGYIHAIDAVIKPSQFL
NPETAAPTSKPTTMEPTRTPTKSPTRSPTRAPSRRPTSQPTSRPTFRDSEETSETTTEES
SNNPNALISPGSPHGKLDVEGRKVVREERGKFVPSSLPSDGPSMLPSDGPSLRPSDGPSF
LPSS
>comp4755_c0_seq1; 619 1 1291 LEN=1867; translated
XLACLRACCGELPLNGRLWVALLDKAATGLVVDQSLVGRRRARGDADAGAGAGAGVLLQR
THKERCMLWCPHVLPIGDLSRIPPAKTTSGGGGEEEEKEAVPTLQQLLEADCAKRPLSTT
ARRYYDFDTKPYDFEVEIPAGLFSFGGGSGKNGSSRIETDTENDTKQKQPWKTTRSMRFS
ALTGYLFLGIDRVLPDPTTTTGGSNAHESRRLDRSELAVPPTLDLTRLSEGQSHPGPVLY
DLVGGALCDEGDYVAVLKDHAAAAALAERQVASNKNSNNEKDNDPKNGEGSDSDDDEEDE
TWKLIEAEETIPMSELDVLEFLKGEGNDDDSEEEDDDSDSDDEGGPCGTLAVYRLRTEAV
SSSSFSLQRHLPQNETVFDEMNRLLSDIVLSQVTGSLDSLSTDYYIEEEIYEEEIIEDTD
DDDEAEGEDA
>comp4756_c0_seq1; 437 88 1128 LEN=1415; translated
MTDYDVDHHSRNGEDERSIKVCQGLTEDQRREIRKRQRLLRKEIDDLDVDQARDKNNEIF
KQVKYVRECVLDSENVEEITKKFGNKIDQLIQIPRYDADRVVSMLVKKCGITRGGYSYFD
WRGLGDQVGVCFNAIPSQVSFLNGPLVDGKEVKVKQRAKRVRSTQPESDAEEERPEDIKG
HTARGKNQLSAVKENIQNVERALVKKVDSTYRARSRSLTETYGGKDDSSGKIKKMTRLKK
KIKRHRDVNAIELLFNPNSFTQTVENLYHYSFLVKEGKASLDVRQNKVLDEESGLKIDGG
PVVKYVRDKNPPPPPRQAIVNLTMEDWKNLCEAYEVTSSCVPHREG
>comp4757_c0_seq1; 485 363 1004 LEN=1303; translated

MKFVATTILALAGSAAAFSPFGKKAAAPAVAEPVPVVDTLVGALEPVGFWDPLGFAAKAD
DNTLRRYREAELTHGRVAMLAAVGFLVGEAVEGSSFLFDAQVSGPAITHLSQVPVGFWLA
LTISIGAAEQKRATIGWVEPENVPVDQPGLLREGYLPGDIGFDPLGLKPEDPEEFRLMQT
KELQNGRLAMIATAGFMAQELADGKGIIEHFTA
>comp4758_c0_seq1; 454 197 1741 LEN=2322; translated
MSSTSGRPSRRRSVISGLDMYKKVPIDLMEGTKRGSVLSWIALLAMVTLFLYETNEYWTT
KLVKDLRLDQKDHTGDKRLRLNFNITMLDLRCEWAVVDVVSVLGTDQNVTAHVTKWDLDA
DGVRSNFQGRNRNQKDIDLFDETVTETIDELHENGEDALSLDKESLHFAKENFDFLFVDF
FASWCSHCRDLAPTWEKLAEIMNDHHYDNLDDDYTTKPEKAKTAKDHIEDSQNNRDDDGN
FQQVVVAKVDCVEHNDVCNEHNIQAYPTLRLFVDGTPWMDMFAENKNEGTRKLSGSSDYN
GHRTVLEMIEWLHFVEEQVREDDGARTLHLAHQAARNRLDGDKKAEEDKKWNDPGIQKKK
RHHHRVRKNWKDAEHPGCQIAGHLLVDRAPGNFHIMARSKHHDLVPEMTNSSHMVNSLYV
GDPTALHWIQERRSKIPTEIEPAITPLDGNVYTTDELHESYHHYMKLVATKIDGMKVGSR
ELVFYQMLVNSQLAYYEKDVAPEAKFAYDLSPMP
>comp4759_c0_seq1; 668 1 1758 LEN=2128; translated
ETDALNTTLGDPVFFADNLAQLCEWGMRFSVDVFEAPSASPTTFPSEEPTIAPTLSPSST
PTTSPSEQPTTSTPSSTPTETLSEQPTTSSPSSTPTETHSEQPTTSTPSSTPSLVPSSTP
SDHPSGVPSGQPTPAPTDLASKAPTTAPVLTRAPTGAPTATPTKSPTTAPVAPDVPTEPP
SGGEVEPPTSSEGERVSNTIEGLRMGMAGITEMSPSAETEWQELTASFSSSFVFNDLKDK
VSNFVTTYEVTGVSPITVQQGRQLQLRANGNSRHQRSLQEQGIVVEYTQTVEYNTVDLSE
FTPEFLVTAPFATQAERTAYVTLLKTTSTTDLANIKGVSETQLPVTPPPTSAPAQKDAIL
TLPAIIGIACGGAGLLILILLFAIYCRKGSDVGGDAKSADEPPLHVDVKEDEVSTLAGPN
GPPTYGDQSVATMDYDYSKAYGGAGDTSVSSAGGTFGSNTQNLSLPANAAATGAALGVMG
DDDESYNTKYDDPRAAQGMEEVIHIFAPPGKLGVVIDTPDDGAPVVHAVKDTSVIADRII
VGDKLVAVDDEDVRSMTAIKVSKMISRKGANPSRKLTVVRTVPLE
>comp4760_c0_seq1; 430 736 1374 LEN=1476; translated
MAFLAKYDLGKQEELQTKVDEKISRKKEMKAVSKAMKGREATLQSLPLEITVGADGSTQT
ARVADLQKGAKQHMIIVAGPRKVCKDALIGANLLKMDFALSNVLVVPYETDADDSAARPS
GGFGDRPSYETQPYIAKANGEDWEDYIQQEISDAVEQSGENVKTEGIAIVVANNGNIIRR
GVGTVPWRLMVEQLEETVNPTTEEKGLFGFLE
>comp4760_c0_seq2; 545 83 1141 LEN=1243; translated
MKKQLLFSVGVLSAIGGASAFVPAQTAPNAIGSRKSSSHGRDGLVSVSDANSPQSLSSRT
ALLSDKKKGGLDESMRNKLVTESIAPWRTLRLFLYGSFGSGAFVGGLLNGSEAIANSNSP
DFNLQVELVNLGIDFGVVVLMAFLAKYDLGKQEELQTKVDEKISRKKEMKAVSKAMKGRE
ATLQSLPLEITVGADGSTQTARVADLQKGAKQHMIIVAGPRKVCKDALIGANLLKMDFAL
SNVLVVPYETDADDSAARPSGGFGDRPSYETQPYIAKANGEDWEDYIQQEISDAVEQSGE
NVKTEGIAIVVANNGNIIRRGVGTVPWRLMVEQLEETVNPTTEEKGLFGFLE
>comp4761_c0_seq1; 270 96 350 LEN=967; translated
MTSSENKTPTDAKNANANESSAPSAGATKQQEAQEAIEEDDEFEEFQGANWGTGEEDAED
TQQWQDNWDVDDMDDDFTKNLGMN
>comp4762_c1_seq1; 663 1 2427 LEN=2519; translated
PDVESHAVPNPQFFVDNLKPPNRNHSQSYDSLLHRASSGELSSKEVAQGGQEQDDQDLER
RNSNVVEDCSSYSVSSHTSDDSIENERNIHHLHDNSCHDNRVIRHHHLDNPQAKFDRRQF
LPKTSSIYGNIGGESKCPTFVCPNCNTRQRQFFTVSSAPKNNESAGLFISLGFATYVFAS
LYIFGLQEGWGKLDCIYFAVITLTTAGLGDLVPTSNGAKIICSIYIYFGVAFIGLLLGSY
IASMLDERSYREAIANQIRACPNCTRIRNIRYASERRAAFGPVSNNKVSMERVAQMHAS
TLHRDPKPVRRAEEAPNLLPPGRQHPMERNKVDLSTTPKSPSGFDSMSPSFRRQLLGSPM
TSQILLRQSHTRHGSMDLRKDNNFGSASHLKGGLNRSFHDSPNSSVSPQSGLSVGAGRFR
NNSADIHIPATVNEGAQSNYGTNNTASPMPPTPEPPLEAKEDNNGNTNPEDPPPPLSEIP
LKNHQVMENDDLTTEDSDSYDSDSLDSEYGVRRRRKKYGARSADAAYVIRTLREALVNSM
VIIAFGCLGFYLIEGLSMIDSWYFTTVLLTSTGYGDIAPKSDGGKLFATVYLLVAGTILL
NNMSMISMIPLELRKKRTEQAVLSQFGDSLDDDALRELATGPLVQRINLERQDPRGLDEC
TREMFALAMMIRLGKVTEQDINQTFAAFRKLDVNNEGVLNSKSIIAGMIHKRRRTMNKHN
NGNGRTRYSGMSQGYGSWMYPVAPPYVDGNRGANSTNNGNIYVSHAVPNSQGHSVHYSDH
TPLLSLSERGPPLHSNRRRSSTSYSMDE
>comp4763_c0_seq1; 265 277 1864 LEN=2265; translated
MAVLCWEALHLGSNLEIMVMEQQLIATANEGRIAALERIKRLQSASGSGDGPTGRRDYLV
PIVEQRAQQSEEHLPGSCGAFDHSVRRPRHGCQVNKDTETVFCNFENLRVDPRKIHMVAR
GGEVLSTVMGREESDEFPLYQRGAFSTPVKPHFEVPNEFRQNMHYLGDVLNALRYPTSRN
KHRLDLSCKKIYKGTTLFLTRYEYVNLFHTVTDWWNAFLVSSSAQNHRDEKPDRVVFLDG

HAEGLLDSTWKSLFGNFRFVKHMDEGSGDGGLCFERAVFVPAGYKSELFNEWRIPRCPSK
GMTDAFSEFVLARHGLLQQPEVARGRIVLIDRQPFVSHPRSSTSNFLRQFRASDLDELQR
RLERIPGTTVDLVRLETLSVREQLELVRRAHVLVGIHGAGLSHLLFMAESRSHVIEFLPD
DQVDFFEYLSRWKGIDYTSIPFLDTLSGGPIDDAVESSKTTWEQSDSESRQHSMGCMDCF
DALWAGLAACLSNQLAPWYRLEGRNSEANHETLQNSLHKKLQRTTIFL
>comp4764_c0_seq1; 658 139 1452 LEN=1719; translated
MNVATRGVLRAASKKGAAVAGRQGKLLSTLVDATEEFPGVPALIPEPAKASKASVSTLPS
GLTVVTEDACSTSTVTMTYPNAGSSSESLDEQGAALINKCLAFCSGSGLSTTLVNRTIEN
EGATPFAKADRTSATLGYTVEPDNALGLVPLLALDCSLEKWDVRDAKKLANYQIDEANKS
LEVVLTEQIYAAAYGASSSMGRPFYSAGASAYEIASFRTRAYGINGAVLTATGIRDHEAF
CTEVESLLSEAPTGDASGSPKPKLLGGESRLSSPSMGYAHVALAFDAKGTAPMRNVLKHC
FGLLGKEAGVTSFVAGEMVGVYASAPTAGAGALDASITDALTASLTPEVIEKAKTLAKAE
AAFDLDSGSVGLAAAMTSAVLESGSALDAATVATAYDSISESDVAAAASAMLGSNPALAA
VGDIGVVPYQGTFASRF
>comp4765_c0_seq1; 576 111 1310 LEN=1486; translated
MASEETTGDASGSSLLLNQPVVIDNGTGTLKAGFAGKTKPKVVIGTKVGRTKHTRIMPGG
ALETEQSLSSSGPSSIFVGKKLDEHRGAFLLEYPMDKGHIVDGGWDGMEALWEHIYSKPG
INVKAEDHPVLLTEAPLNPCQNRDRMAEIFFETFRAPALFFAPPAVLSLYASGRTTGVVL
DVGEGVTHAVPVYEGFALPHSVTRSDVAGRDVTRQMQLLLRRSGLSFTTTAEADLVKTMK
EESCFVTRTPVADESMEKDSRTQYTLPDGQAVTLSKERYEAPNILFDPSLIGSEEAGVAD
ILVDSIMKSDIDLRSTLFSQVVLAGGSTLLPGFGDRMLYEVRSRSPSHTKIRISAPPERV
NSAFVGGSILGSLATFKSMWTSKTDYEEYGSNILHRNKL
>comp4765_c0_seq2; 523 615 1646 LEN=1822; translated
MPGGALETEQSLSSSGPSSIFVGKKLDEHRGAFLLEYPMDKGHIVDGGWDGMEALWEHIY
SKPGINVKAEDHPVLLTEAPLNPCQNRDRMAEIFFETFRAPALFFAPPAVLSLYASGRTT
GVVLDVGEGVTHAVPVYEGFALPHSVTRSDVAGRDVTRQMQLLLRRSGLSFTTTAEADLV
KTMKEESCFVTRTPVADESMEKDSRTQYTLPDGQAVTLSKERYEAPNILFDPSLIGSEEA
GVADILVDSIMKSDIDLRSTLFSQVVLAGGSTLLPGFGDRMLYEVRSRSPSHTKIRISAP
PERVNSAFVGGSILGSLATFKSMWTSKTDYEEYGSNILHRNKL
>comp4765_c0_seq3; 409 322 1086 LEN=1262; translated
MAEIFFETFRAPALFFAPPAVLSLYASGRTTGVVLDVGEGVTHAVPVYEGFALPHSVTRS
DVAGRDVTRQMQLLLRRSGLSFTTTAEADLVKTMKEESCFVTRTPVADESMEKDSRTQYT
LPDGQAVTLSKERYEAPNILFDPSLIGSEEAGVADILVDSIMKSDIDLRSTLFSQVVLAG
GSTLLPGFGDRMLYEVRSRSPSHTKIRISAPPERVNSAFVGGSILGSLATFKSMWTSKTD
YEEYGSNILHRNKL
>comp4766_c0_seq1; 283 121 651 LEN=1102; translated
MSDTEMKSPKSAKKSKKSDRTSSASAADAATSGKTYEERVAAVSVIAKPLACKKSTKKAH
KLVKKAATAKRIRRGVKEVVKGLRKGEGGLCVLAGDIYPLDVYSFLPCLLEEKNVPYIFV
PSKQDLGAAACTKRPTSVVLVKDKADKKAGDKKFDGQDLMDTLLKEAREYNPVDGN
>comp4767_c0_seq1; 547 1 1760 LEN=1760; translated
DESTTHDRSDEDDRYRYEEYRMARRSQESNISSANGDRAARYRASGNRPSSPGARALDRG
EKRGGAAGTLDPCERMAVRRIGGGAVSKEQHEELIRINRKVFDKLHARIDRLKQSNVQLA
NELRRARALEPEKEEQPDPNHPSETSWEEGVYKSQLELLQSRLEITTIQADKRQRELSAS
NELARSEHEFAVARLRKRISELEISNRRLALQQLEHCKPKQPKPQADTPNQRDPCDSARG
GKDKVAAELQRWKDRNAFLEQKVASLEEKLSEIQKPEKQDQQFQHAGEGVEFPYEDTSFF
QRKHGEGLPGGIGEGSAGNRESNSAVAAEIGESNSHNVDANESPEDLSESSRGNSGNRSN
EEHSNKNLLLLDSSRESSNGENPGGEHDCKNLLDDTNDTESVELVCSGGIGIGRVPDEIA
KRSSGGTSLRLVYEHDFEPTPLATAEASNSVCGEHKTGSGIDHPHDASFESATSMVLIER
ILELESVLHERRDMTDPEKAGFVKALQYRIEKLTERGYERVDSNXVTRDSFLWCMIGFVC
QQMKGAASKEELGGLTSTGEGYFGHVQQICSSSMAIISTNVMINDVR
>comp4768_c0_seq1; 456 1 1087 LEN=1112; translated
NVNVNVNVDGNAGDPVSVMASASFPGDTAKDSNLGGGDPLRPSSNDEPFDEAFDEAFDEV
FDEAHGLSRPNQQTTVEPSPTSDKSGSGDKSDKTSNNNSDNNTADNNNNNTADETRDDGF
FRMDLFETAQAAAEQMVERTTSLGLGFRTPSRPATTPEASPASNEKEQPGYSQRLDVDVS
GEPNPWDLLPQARTAGEPCTQDAKANADAKAKANPSADANANNHPFRACGRSVEELVETG
RECVGTVGRATEKAALDLLESSSCKGKTAMDYPAAGSSEAHRRSEPLAALHPLLEKTLEE
LGDFVEYHRALLEERGVVVPWCGRGKRKGSRHDPTEKCDEHENENEPVLNVAIEVEMKEK
A
>comp4769_c0_seq1; 504 1 1470 LEN=1722; translated
XGAAAIEDAVGKRMELGDAGLEAVFGKGEVAFVPHMFDREALNASADKGLGLLGRNHVAL

VDAELTGMPPILDRARPEALEAGGGLFETDGENRPDPRFRFVVLLQGGNYDKLDRKGWDT
SLQAFARFYHSLDDPSGIHLLIHSMESYLIASDSNNEEDAPPMVLPQGYNIRLALHELGL
PKTAYTVDQAKHAPEVVAAYKKRADVCLHPSKVEGFGMNVMECQIVGTPVVTTKYTAMED
YTKFGRSVPPLQTIRNPDQLYSMAMPDVEGIAKALGELYLEHKALAAGDEGYKRRREQEI
ASFNDWLETTCSPDAVADKFGELLPRAIDEFSARETARGLLLTGAVPTEGAYEVSSGYHA
TIADWDAPWTLMAPDGTVVTAPEELNKMAWMMLLADNSSSSPAMAIVVPPTYEDGTKADE
RLFMEGKLPLLVRTYVLCSLQGLSTRFSTLMAQAINVSGNSVKRMPDGVAMIDRNTIKGA
TTAPEGRSEL
>comp4770_c0_seq1; 229 187 894 LEN=1119; translated
MILKGTLISSLRNCASRGLVVPSQSRDFVSRRFASSNSSNTPPKKTNNLVWQVGTVAAVV
GAYAIGLNFTQVISDRAKENEDDDIGDEALREITSRVFFDMSIDGNEVGRIVVGLYGNAV
PKTTLNFETLAKGDTTHPRGRKLSYEGSTFHRIIPGFMIQGGDSRTTMVPEVYRSTATNL
LTNETGLHVFDTQDPEFCLWPIPVKIPMALNSSSPQKRHPGWTHDMSFLVKFWME
>comp4771_c0_seq1; 424 1 1072 LEN=1525; translated
XNLSKQGRKIESNWNTAMSNRLIDGETYLEGLIESLSTLPAEMRRNLELMKDMDASCSSL
VEETMKLQQNYLLRVEGKMGRLEVVDGKGVRVLTASRDETDSKTKDDDGDEKMAGPDDER
PVVIPTTEELMAYIHEPETRSKIENLQSDALQQAEEKVAIANQTFELIDNLCRKLDTDLE
EMERSLLARNGADFHNHSSGGVNGAVSGTSSSGTSGGLSSTTTGGRGRGAPKKDDLAAVQ
EVPGSPDWILAKVITLDPQTGMYKLSDEDMESNKVFNLPESQVIILERMKNLRIGDVVFA
VYPDTTSFYQGTIAQPPRKAAGGGMFVMVTFVDDEDAATGKTLDKAVLLKHVMPPPF
>comp4772_c0_seq1; 681 324 1985 LEN=2566; translated
MSSANSYGEYEPYGGPDDFAEFDEDYSTTNDVRDPGSYDGEDYNYGDEDEDDEQTKTNDS
ADPYKEYGNEYGTDDDDDSDSESSYSRDEYYGDDRDEMPDDYYYDEDDQFYYDEQDEEDR
RKRARLRRAWICCFVMLCCLLLAIIAFVIFLITRSKDRAPEPTPMPTMEPWVPDTDDDYM
YDDDIILNPGVVTAPMAPFNRDCEESEGIEYSGEFRNIMDQCACLGEIVDVPDDVAEMRN
LIIERVAPKFYGPNFTIPLNSCDPANMAATWLATGDNRDGGEARQRFAMALNFYQLNGTV
WDFMDGWLGPFNECLWMGIQCNNKGIVNSLALDANNLFGPFPTEIAKLTGLASIAITRTH
LTGTIPTELFSMPKLKELRLYANRMTGSIPPEIVKATKLESLRLETNFFHGELISEIGAV
STLTDLNIGFNDFVQTIPTEIGKLKNLKYLRVSNNALSGRLPSEIGRIDALEVLAMTNNL
FFGSIPSYFGLLWNLRDFRITGSGVGGFLPTELALLSNLERLDIAISNFHGTLMTELGQL
TNLCKSLLEIRMRW
>comp4772_c0_seq2; 650 324 2435 LEN=2528; translated
MSSANSYGEYEPYGGPDDFAEFDEDYSTTNDVRDPGSYDGEDYNYGDEDEDDEQTKTNDS
ADPYKEYGNEYGTDDDDDSDSESSYSRDEYYGDDRDEMPDDYYYDEDDQFYYDEQDEEDR
RKRARLRRAWICCFVMLCCLLLAIIAFVIFLITRSKDRAPEPTPMPTMEPWVPDTDDDYM
YDDDIILNPGVVTAPMAPFNRDCEESEGIEYSGEFRNIMDQCACLGEIVDVPDDVAEMRN
LIIERVAPKFYGPNFTIPLNSCDPANMAATWLATGDNRDGGEARQRFAMALNFYQLNGTV
WDFMDGWLGPFNECLWMGIQCNNKGIVNSLALDANNLFGPVRITESTVQSDAMLELVISF
FKSLQDALYSSHPADTNFYFVTIVGLNNRQIICVLFPTEIAKLTGLASIAITRTHLTGTI
PTELFSMPKLKELRLYANRMTGSIPPEIVKATKLESLRLETNFFHGELISEIGAVSTLTD
LNIGFNDFVQTIPTEIGKLKNLKYLRVSNNALSGRLPSEIGRIDALEVLAMTNNLFFGSI
PSYFGLLWNLRDFRITGSGVGGFLPTELALLSNLERLDIAISNFHGTLMTELGQLTNLFK
FSLHDCDFSGPLPSELGYLWNMTRLQMKNNQFTGSVPRHWGRMAELEQLTLEGNQLSGTV
PIEVCDLLNDKLGQFVVDCYNRRTGFGFDCEPDCCTLCRDTS
>comp4772_c0_seq3; 771 324 2267 LEN=2360; translated
MSSANSYGEYEPYGGPDDFAEFDEDYSTTNDVRDPGSYDGEDYNYGDEDEDDEQTKTNDS
ADPYKEYGNEYGTDDDDDSDSESSYSRDEYYGDDRDEMPDDYYYDEDDQFYYDEQDEEDR
RKRARLRRAWICCFVMLCCLLLAIIAFVIFLITRSKDRAPEPTPMPTMEPWVPDTDDDYM
YDDDIILNPGVVTAPMAPFNRDCEESEGIEYSGEFRNIMDQCACLGEIVDVPDDVAEMRN
LIIERVAPKFYGPNFTIPLNSCDPANMAATWLATGDNRDGGEARQRFAMALNFYQLNGTV
WDFMDGWLGPFNECLWMGIQCNNKGIVNSLALDANNLFGPFPTEIAKLTGLASIAITRTH
LTGTIPTELFSMPKLKELRLYANRMTGSIPPEIVKATKLESLRLETNFFHGELISEIGAV
STLTDLNIGFNDFVQTIPTEIGKLKNLKYLRVSNNALSGRLPSEIGRIDALEVLAMTNNL
FFGSIPSYFGLLWNLRDFRITGSGVGGFLPTELALLSNLERLDIAISNFHGTLMTELGQL
TNLFKFSLHDCDFSGPLPSELGYLWNMTRLQMKNNQFTGSVPRHWGRMAELEQLTLEGNQ
LSGTVPIEVCDLLNDKLGQFVVDCYNRRTGFGFDCEPDCCTLCRDTS
>comp4772_c0_seq4; 560 324 2153 LEN=2734; translated
MSSANSYGEYEPYGGPDDFAEFDEDYSTTNDVRDPGSYDGEDYNYGDEDEDDEQTKTNDS
ADPYKEYGNEYGTDDDDDSDSESSYSRDEYYGDDRDEMPDDYYYDEDDQFYYDEQDEEDR
RKRARLRRAWICCFVMLCCLLLAIIAFVIFLITRSKDRAPEPTPMPTMEPWVPDTDDDYM

YDDDIILNPGVVTAPMAPFNRDCEESEGIEYSGEFRNIMDQCACLGEIVDVPDDVAEMRN
LIIERVAPKFYGPNFTIPLNSCDPANMAATWLATGDNRDGGEARQRFAMALNFYQLNGTV
WDFMDGWLGPFNECLWMGIQCNNKGIVNSLALDANNLFGPVRITESTVQSDAMLELVISF
FKSLQDALYSSHPADTNFYFVTIVGLNNRQIICVLFPTEIAKLTGLASIAITRTHLTGTI
PTELFSMPKLKELRLYANRMTGSIPPEIVKATKLESLRLETNFFHGELISEIGAVSTLTD
LNIGFNDFVQTIPTEIGKLKNLKYLRVSNNALSGRLPSEIGRIDALEVLAMTNNLFFGSI
PSYFGLLWNLRDFRITGSGVGGFLPTELALLSNLERLDIAISNFHGTLMTELGQLTNLCK
SLLEIRMRW
>comp4773_c0_seq1; 1052 253 4101 LEN=4243; translated
MGSNAAPAPAPAATPVKDSEGKTKPLQRAKPAHEKRTWPEDYDRDAPEGFALSFGRWMTR
IWNNWTYSYMNVVLSKGAAINGASAKQKHNRNSNEPNTNIAEDSHDPSSSSDTDKNDDDG
AVEDIHLTSDDLWPVPRKMQSTRLVNMFESNWKNASRTRTSPNATTTKRQLMKTLWKIAT
PTFLPAGIFQFVSTVLVSVMPLVVRRFLSILEKETTDGSSILREGLSWAILLTVMTLLNG
FATQRYRHQSVKTGIALRSAVVNMVYRHVLMLSPRGKRGLTSGEINNLVAVDAQKLYEVT
QEGHLIWALPLSVVLVTWFLCLVMGPSTLVGVVVLVGFLPLIKLVTKRMTEARAQRLKYS
DDRVEITCSMLLGMRTTKLNGYESKYEARIKEARSKELRWLAKEQAWWATTLLMTVSSPI
LAMASTFATYVLTSTESEPRVLTAADTFGVLLLFGALRFPINFAGLLIGSAAQALSATRR
ICAFLDRPLREGGSRGEEQDPTGSLRLLESDGDKDNKDIPLILTNGRFRVGVAPEDLTES
SSIVNDNETDSNVDNNSDAAAIKNITDQSTSLGENYNNNIENGNRDNLSFTVSEFNFELR
RGEVMVVCGPVGSGKSTLLNGILDEAEALGPNWSESATASTYSTPMVQKNGRISYAPQDP
FILNLSLRENILFGSDFDVERYNRVLDACALRPDIEQLGGSDLVQIGERGVTLSGGQRQR
VSLARAAYKQPDSSCILLDDPFSALDSGTGKIVFEQLIAGPDALLRDSAVLLVTHASHFI
SHRAVDKVLLMVNGRNEFLGTWEELTHFNDEEHDDQTRRAVDHIQSQIRENTDGHNHEST
ATKEGDSSSSNLDNKNGKSSHHFQQQPEESSSINDNKIMQQELREHGLSSMKTWILWFKR
AGGAWFGMTIFFFLFLDRFAYVAVEWFLASWTSGAYEPVTILGVDFDAQSDGLQAQAQYL
KVFASIILVSLLATAVRSEFAVTGGVRATKNVFNSMLESVLSAPMSYFETVPMGRILNRF
TYDTDVNDVTLTQKISMFIISCSWYVASIAVQVAIIPWSALALFPVSMLYLLFMHYYRLT
GPDLQRIDALSRSPIQSMISECLEGSTSIRIFHQKVNFVRKFEEIVDVNSSALLNYISVQ
RWLSLRMEILGSVVVMIATVLLVCLNETMRLSAGLAGLLITWSANFTITLNFLVQTFSET
EAAITAIERVDAMADLPSEKSRETAKEFLPPTSWPEQGLLEFKNVSLRYREGLPLALDNL
SFAIPAGKTCGIVGRTGAVRLL
>comp4773_c0_seq2; 1210 253 4761 LEN=4862; translated
MGSNAAPAPAPAATPVKDSEGKTKPLQRAKPAHEKRTWPEDYDRDAPEGFALSFGRWMTR
IWNNWTYSYMNVVLSKGAAINGASAKQKHNRNSNEPNTNIAEDSHDPSSSSDTDKNDDDG
AVEDIHLTSDDLWPVPRKMQSTRLVNMFESNWKNASRTRTSPNATTTKRQLMKTLWKIAT
PTFLPAGIFQFVSTVLVSVMPLVVRRFLSILEKETTDGSSILREGLSWAILLTVMTLLNG
FATQRYRHQSVKTGIALRSAVVNMVYRHVLMLSPRGKRGLTSGEINNLVAVDAQKLYEVT
QEGHLIWALPLSVVLVTWFLCLVMGPSTLVGVVVLVGFLPLIKLVTKRMTEARAQRLKYS
DDRVEITCSMLLGMRTTKLNGYESKYEARIKEARSKELRWLAKEQAWWATTLLMTVSSPI
LAMASTFATYVLTSTESEPRVLTAADTFGVLLLFGALRFPINFAGLLIGSAAQALSATRR
ICAFLDRPLREGGSRGEEQDPTGSLRLLESDGDKDNKDIPLILTNGRFRVGVAPEDLTES
SSIVNDNETDSNVDNNSDAAAIKNITDQSTSLGENYNNNIENGNRDNLSFTVSEFNFELR
RGEVMVVCGPVGSGKSTLLNGILDEAEALGPNWSESATASTYSTPMVQKNGRISYAPQDP
FILNLSLRENILFGSDFDVERYNRVLDACALRPDIEQLGGSDLVQIGERGVTLSGGQRQR
VSLARAAYKQPDSSCILLDDPFSALDSGTGKIVFEQLIAGPDALLRDSAVLLVTHASHFI
SHRAVDKVLLMVNGRNEFLGTWEELTHFNDEEHDDQTRRAVDHIQSQIRENTDGHNHEST
ATKEGDSSSSNLDNKNGKSSHHFQQQPEESSSINDNKIMQQELREHGLSSMKTWILWFKR
AGGAWFGMTIFFFLFLDRFAYVAVEWFLASWTSGAYEPVTILGVDFDAQSDGLQAQAQYL
KVFASIILVSLLATAVRSEFAVTGGVRATKNVFNSMLESVLSAPMSYFETVPMGRILNRF
TYDTDVNDVTLTQKISMFIISCSWYVASIAVQVAIIPWSALALFPVSMLYLLFMHYYRLT
GPDLQRIDALSRSPIQSMISECLEGSTSIRIFHQKVNFVRKFEEIVDVNSSALLNYISVQ
RWLSLRMEILGSVVVMIATVLLVCLNETMRLSAGLAGLLITWSANFTITLNFLVQTFSET
EAAITAIERVDAMADLPSEKSRETAKEFLPPTSWPEQGLLEFKNVSLRYREGLPLALDNL
SFAIPAGKTCGIVGRTGAGKSSITVALFRLVEVESGSMLLDDVDLTTLGLSDVRGRGMSI
IPQDPFLAGANLRECLDPFGQRTDDEIMEALESVRMGRSSYSGVAPPSVEVVLSTKLEEG
GSNYSVGERQLLNLARALLSQPKVLVLDEATASIDGETDAFIQKMLRTRFPNTTLVTIAH
RLNTIMDYDYVLVMDAGKAAEFDAPAKLLGGGDGDEKGIFSQLVDATGAESSKALRQMAK
ESW
>comp4774_c0_seq1; 537 286 2475 LEN=3671; minus strand; translated
MSSSFNATTRNGEAAKSYTSSQQNQKQEQTSRGAELIPKAALTKYPPAPRDESEAEASFR

RQAIDKAHESSAISWLQHIENHDADARYSRKLHRRTSSKDYSASFHSICAGTTLQKRRSC
NSVISTTPKRITKREGNRPSASFVVSTSDNRGSNDVEMVSDVAAFDGLPSKRGSGKTDRM
PKHKREWNQGASWEKYCTRHIQLLVSLAFFSYLGEFSRYSIDILFGGGCHSPGTIGWYRV
GWHICTTDPGTTESTGGAFFVDLPTNVIGCFLMGLLVSGDGESIAVNLPVAALGRTCFFQ
NWVVTHVGLRTGFCGSLTTFASWNTQMVKMVVGNKGTALGYSQWVSALWGYIVGLYAALQ
SYQFGAAIAFALSRCFNPHLAREADKIIDKKAIGVLINRDLPDFERRFLHSIFLEESERH
HHNDTNTCDEPRENLAKYGSSGYESFYGDHAHHLRTWKETTDYYRKGAGMQEESFVAELQ
EIEKMLLVDRIEPRQELLNVARDAGWDVGALRNWAEALDEEEAKLAMRNTGLEGNNDGDD
GNFDIETKLEDAYFLASHSSPIEFALTLSLFLLTTGLLLLGFVYYSNPTESDSVSWRSYR
TQCLAALLSPFGTYSRWYLSRLNGRIRHERWEWLPIGTFLANAMASVVSALMAAFLVGLN
PEANPLAVVVCKAIQGGFAGSLSTVSTFATETVGLLQALPRAFWGYYYSFGSMLCAFVLG
VASYAWFVV
>comp4775_c0_seq1; 276 252 1073 LEN=1073; translated
MFFHKILLSVVAGLSLLASSVSSQQIRGHPQPAARNLQVPEVCVPEPIRTLANGTDVFAE
KKFLVVFLAEPQNRTNFIDVLALQKTAPTAYDDSVDCSLSEGSIRSLKNCTVVTRNIIYE
EASLLMECLEYSSNSLDGSSFVVTKNSYTQDPYCGCQCAGDDGVSFQAFKQPNGTCTGSC
PCSGPRPECNCPAPNNATLVENWNTLYQNASDALGVNVTIVNATDVRVLQNCVNTFNYTY
NATSVXPSRAGDEPAHPRSHPVAYETAHQETDEAX
>comp4777_c0_seq1; 500 1 1094 LEN=1135; translated
XNLNFVVTKSTIKNIENMSAPIESGSILGMGNPLLDISANIGQDVLDKYGVEMNSAILAE
EKHQPLFKELVEKYDPQYIAGGATQNSIRVAQWMLTAANKPKHTAFFGCVGDDETGKTLE
KCAQSDGVHVHYMKDAETPTGACAALIKDGERSLVTNLDAANNFKPSHLESDEAKQIIDL
AKIYYSAGFFLTVSVPSLVEVAEHAAANDKTFCLNLSAPFIVDFFGDQMATAMPYADFLF
GNESEAAAYGKKHGMGEDLKEIALKVAALPKKNESKPRTVVFTQGSESTIVAHNGTVTEY
AVEPLAKELLVDTNGAGDAFVGGFLSQLAQGKDMEACVKAGHWGAKYIIQTSGTQLAKAC
DYSP
>comp4778_c0_seq1; 434 97 1419 LEN=1877; translated
MPKIRRFSRSRQNGRPEDGGVDDIFADPSQPLPDAPIDRRYRRNQLYKRGPNNQDQERSS
ATRQGTESTKGPPRKGDRNQSQRRKHLGRNQHRRNANGNSSANHLHQPTGENVTDRKNPL
YQPAIDGDGRSDPLQRKQPQPHPNRKKQPSNRMPRRKAQSRQNQQPTVTTDRGENDDHAK
TLLWDSPLPCIPLWQEEKMIEFMFQRNGKKERGMMKDKPNFLVTKYKKAVRNLAPSKDER
MSLPQVLSLRRHHIKLLNPPKSMPQLGLGTYDVIRESARIFEVVIEEFLKRSSVDYLTED
DQKRKARAEGASLAVTPDFLLRTPVLLRKIRREKPRGKQIVLPGERTIHWIEAKMYYGAS
SIPHGSTGAVGSVLAQARKYVDRLGEGAFLFMMGCGERLAADLNDVGVSVLDCFGNTVSL
ERVHEHQRTWCANGEGVILP
>comp4779_c0_seq1; 131 1 1325 LEN=1325; translated
TQHTMLLVECARARAIRNLPAVGTTEALKEYLANIKAVEAAERQKTVERTLGILVCAPHL
DLPQAMHCAGFREKECSDPGLLEKVRSAREESGPEPLSPRKRRRVEIYRQRVAALEALAE
KPDPWKLTYGDQLTKKKVVAHIKATLLYEAGGLGAPSVCHLVYREHGALPSEDSIRKSCR
EGRAGQVPRLWARKWSHLAPPEIEDGDAPCPKKARIEADERETRHAAAHIEATLLLESEK
KRPNGMGAKKVVAQINEEYGTSLNAGTIASYVQQGLAGHPPKNTKAKGFPIPPPRPLVRP
EEQAAEERARKDLEETRIAAHIKATLLFEAEKKLPCGMSSKQVVDQVNREFGTKLNARTI
ARYVREGLAGKPPKKNGRPSTIFLPEIAQHVLLRRLNELSEQASQGGGSTHLVRSAETQL
AVGFIPQCHHFYVIQYSDFGSX
>comp4780_c0_seq1; 515 126 3019 LEN=3225; translated
MTQLRNLLLGASVLVGIDKSDATFDKRGASLRGKARKHNGRKLVSEDANLGEFPFFTQWG
GCGATLIWGDILLTSASCDEVKSKTVLVGAHRSNEEQGGSVKRKIVSRHIHPEYNQENLL
NNFMILKLNETVDVQTALINATIAIPTDPELYGVGFGFTPSLVHVNEIYSRSFHHSVLDT
SELLQSGNDQGSVLQKAKMTIIPHAVCNAYDQYAGFIDDGSMICVSDDDATCLGDNGGPL
CAMIDNKCLQIGIVSFGDSCSTRLRPTVYSSISAAYGWIEKMICHVSSKPPSSCQTVVKI
REPYPGLSESFTGRSAHKNSKELPLPSTHLESKVPTDYQSFIPSNKPSPVSSLMPSDGPS
LAPSEKPSFHNNKPSSADQDNRSLRKQAIRYKMPRYPRTDLDVTVRKEPDGIHLFFWRFG
SPHSKLGLCEGDCNIDDDCAEGLYCYHKDPSVTSVPGCVGTDTSRTDFCTIKSDQNSSVH
DESSRIDETAKLPVLSLKKLGQCEGDCNSDEDCAEGLYCFMRDGNVESVPGCSGVDNSRT
DFCTTKTVSGTNTSTNGPRISNQVLIGDGAMPMLFVYPENPPDISNLPLLACQGDCDNDS
DCADGLICYSRHGEETTVPGCSGESTQXTDFCVDPAYIMSPTFEPSRKASGPPAETAGRS
ENEPLEPTPSPSRTWNAIDDLFANAAATQPPTLTPTSIPTQFIEETLAPTIPSPSPSLIP
TSTIASPSQSQSSVSITVAIFFDPWPEEVSWKIESEEGDLITAVTAGSYTSTIDQTFELV
TVKPGKMYRFTIADAGNDGIAGIGNLYEIVMTENPDIMLLDGDGVFENGRTKMFYVPNTE
EYPTSAPTEPTISPAPTFNTVNVYLTIVFDNWHQETSWEITAADDPTVVFAEAAYDTYRG

GEKITNEIPLPPGQEYTFTINDFFEDGIKDGEYLLKSADGIILFEGNGDFGASRNHTFTV
PENP
>comp4781_c0_seq1; 763 1 1078 LEN=1228; translated
XEVEGMMMTDDESSIEVEPTKMTEEEAAAKAEAKAKRKAEKKAKKKAKKEKKKKKKAKKE
AKRIAKEKAAAEAEAKTSGNEDPPAEAKVLATAETNTIPEEESANKSNVTAEVDDKMAVG
EEAAGAEAEKNTDEEAAERTQADNAAIAEAEVLLAKQLDEVEAEVIDAERLAAKEMAEVE
AKLAAAEKAFAEAQRSEKKNLVNTNDALKRDDVHPTIERPKLAAFAERAKSFKNRISQEL
EDRKERIGNNESAEQEPNGRSWRGRLSQGASVESKNEQTRGDESAEQQLKTSTFANRAKN
WRSRFGRGANNNSTDSTVASDGNSEAATAGKNAINAFPKVLFNDGGESNSVKEPEIGSI
>comp4782_c0_seq1; 462 163 1845 LEN=2137; translated
MSSSNVVKVKRKRCRREMEDASDEQSNNERNVQGDEANRDNQPILPLEGVIACLSGFAQD
RKDHLHNIILSLGGRYTRELNLDKNTHLITEAAQGAKYNLAVSSDPKSIHIITSSWLSST
MESGQRASEMAHVLASTMDSSEGNQTQTRSSSKSSVLSLMDNALDEVVAPCDRLSRSFLF
EKFQFFLIGFESSPELKMKTSVLIRRGGGTIHWDMNEEISILVLSDACDDAVQKAAKIVT
SHHPNIPPAVSPFWIIESYKGITLQPTANYPPIRTRQPKLRSNEGQIRSSKRASEVTSRT
TSLASTASNVSIFRGCLFSSVRTSPTVELGSSLAMNPIIEFNQKEIDNLIKAHGGKILCT
KLLDALRIDAEKSTSEIKRKCHVVCWGGSPPRLDTNPLVSQLKRHNLCELILVTPIWVQT
CVSVKRRLRPEYMPVVFVPQSWSMKSVKLRSEEVGGKRRQHSDAVTKLQISLTGFQGSEK
AMLINLIRAIGGVYHGNMSSATTHLVFKNTATGLKQKKAIEWGLRVVSVQWLYHILEHGY
RGVHKDEVGCENRFSMGIDS
>comp4782_c0_seq2; 408 163 1961 LEN=2253; translated
MSSSNVVKVKRKRCRREMEDASDEQSNNERNVQGDEANRDNQPILPLEGVIACLSGFAQD
RKDHLHNIILSLGGXVRITIIFIKFDIKPTAPTQIVACQTLALYSCAIQKLGKRYTRELN
LDKNTHLITEAAQGAKYNLAVSSDPKSIHIITSSWLSSTMESGQRASEMAHVLASTMDSS
EGNQTQTRSSSKSSVLSLMDNALDEVVAPCDRLSRSFLFEKFQFFLIGFESSPELKMKTS
VLIRRGGGTIHWDMNEEISILVLSDACDDAVQKAAKIVTSHHPNIPPAVSPFWIIESYKG
ITLQPTANYPPIRTRQPKLRSNEGQIRSSKRASEVTSRTTSLASTASNVSIFRGCLFSSV
RTSPTVELGSSLAMNPIIEFNQKEIDNLIKAHGGKILCTKLLDALRIDAEKSTSEIKRKC
HVVCWGGSPPRLDTNPLVSQLKRHNLCELILVTPIWVQTCVSVKRRLRPEYMPVVFVPQS
WSMKSVKLRSEEVGGKRRQHSDAVTKLQISLTGFQGSEKAMLINLIRAIGGVYHGNMSSA
TTHLVFKNTATGLKQKKAIEWGLRVVSVQWLYHILEHGYRGVHKDEVGCENRFSMGIDS
>comp4784_c0_seq1; 544 1 1871 LEN=2162; translated
XKNTNSCQNGRSAFSYTSKIMRPSTSSSEASEQAVIGSDNSSLGSDSDSEEGWGFLLGGL
STESMASANWEKKEEEGISNTSGRINEPIESKNVVSVLPMGSSSREVPSYEVTGGESKQC
GATAATSDNIVEQKISQRAFCLSPNGQILKPPVQILDRSCEQKGNITIATSDIDKGQVIF
TEKALLGIQVVPSQTTSLADTKSGMYAVRSCQLCFRSLESASCLSEEGLPFSELWPIPEY
DDPTTMECVAIGDPNEPAVATHEYTRTKSGILTCRDCGVMFCNRFCASNHSKVVGDCCRC
TRAIKGVVKSIVSGERERRQLTEQYNNNLDTSNSDNSGICNCGAEQEDFGDSEALVDIDP
VLILAAKMFVAHVHVCRKNGDGFRGNDGDNEVASDTLFYGLCGQAEDVASLSLGSASIPS
SFSDDGDQNSKSPLPLLKEYNAIADAIELTEMERRSSSLCSLQQFQKIAAIAQRNSISLA
TGSPFRTYYQGMIRKTGGRGSSRQQQVVSDVARLLGAKDGKLTKDMDRIVEEKCVVKMGG
IFTLTARMNHSCDPNAEIRAQEYVDCNVDIVAKKNIRRGEEICISYVYLGQNPSNSVIAR
NRRQRELRSRYLFQCQCSWCSRC
>comp4784_c0_seq2; 547 1 1829 LEN=2352; translated
XKNTNSCQNGRSAFSYTSKIMRPSTSSSEASEQAVIGSDNSSLGSDSDSEEGWGFLLGGL
STESMASANWEKKEEEGISNTSGRINEPIESKNVVSVLPMGSSSREVPSYEVTGGESKQC
GATAATSDNIVEQKISQRAFCLSPNGQILKPPVQILDRSCEQKGNITIATSDIDKGQVIF
TEKALLGIQVVPSQTTSLADTKSGMYAVRSCQLCFRSLESASCLSEEGLPFSELWPIPEY
DDPTTMECVAIGDPNEPAVATHEYTRTKSGILTCRDCGVMFCNRFCASNHSKVVGDCCRC
TRAIKGVVKSIVSGERERRQLTEQYNNNLDTSNSDNSGICNCGAEQEDFGDSEALVDIDP
VLILAAKMFVAHVHVCRKNGDGFRGNDGDNEVASDTLFYGLCGQAEDVASLSLGSASIPS
SFSDDGDQNSKSPLPLLKEYNAIADAIELTEMERRSSSLCSLQQFQKIAAIAQRNSISLA
TGSPFRTYYQGMIRKTGGRGSSRQQQVVSDVARLLGAKDGKLTKDMDRIVEEKVRYRLQT
SIADAIHCAGGPTGVREDLKRLEPVIDSSSFLTIIFLNLVLQYLLALWCHKIMRSAVRCQ
NGRDIHADR
>comp4786_c0_seq2; 311 132 1703 LEN=1888; translated
MAMATTFNSVEQRVNDLLAPPVFNEDDTISKELLKLSLKERNALEEEIHGVRSLNPDETP
HLVDRSLRDFDQKLLLVAEERQRTREKKILTLHGFDNSECFKSNDVLRNVRRIYQNCNGQ
KSKITIPPLCDCYVNDPAIRLVFLRGELFDVEKAVRRFINFLELTSELFGDFVAHREIRI
TDFRTPEERTALRNSRQQMLPFRDRSGRQVFVCIGDCGTGLPAKLRFKIRMMLFWTTTKD

IQTQRKGLVFICWPSSEILSKLSKSDLLYRQKFMDARPVRFVSWHICCQDTPMCRAIGTL
WVVSSRPDVRVCTKVHIGGLIETQYMLHGFGIPLDIIPLSHTGTIKTANHIQWINALHYK
LQEAEGGEEIVICPGFHDVLSRKGQRYASHPGNMKFRELVQSMSDLHERVPRTQKFQVTQ
SVLFKVEILGGRFLEWKSPSNVWIVTKDRKSIHAKIAFAFREIKRRKRLAAQKTRTSNGN
FNDDSPDLNREEVAGSAVDYSMQGYQDQKCFLFCRESAVHSNN
>comp4788_c0_seq1; 419 1 1246 LEN=1390; translated
XPTFEDQQEDAMDSANNNNDNDNNNNNGDEIAEPAMHASNGYTSGDVVKKLKAVLSLMAS
PLTDEVGSTRSVLSGLATIFLAGASIGLVTPGNPVLSPPYRSVSAAIGYVYFLAWSVSFY
PQVISNFKRKSVEGLSPDFVVLNVIGFACYTAYNASFFWNTTIQEEYKRRFGPDAEITVQ
SNDVAFAIHALVLSFVTFCQIIYYNYFYCPGPTGEIDRRTRTVIQLSRPVLAVIVGIVAI
CVGYPALVLLGKGSSSSSKNDNLDVLLGDDDALARFNWLDFLYLLSYIKIFISLIKYIPQ
VVLNVRRKSTVGWSIWNILLDFTGGTLSDLQLVLDCANAKDFSGITHNKAKLALGSLSIV
FDIIFLVQHYCLYSSAEPTIRNEQNEPLLPTTDSDPEGVQEVSEEEQSQPETIFV
>comp4789_c0_seq1; 573 249 1574 LEN=1766; translated
MNAEGSELRSNDPSPMSNAGDGGVRTPTIDFDDWAVDPFSLSKEIDVFSSFAGVEEDAPN
ADSNRASFPPSSGAVVGKNSTLTGTEDKKTLVANQKSRKRNHAQQSQGKQATTVSSKNAS
SAKKRSDCIPISRRKKKPKGMPKRPLSAYNLYFQAERTNIIASQQAENGPRIGFEGLGKI
VGKKWRDLSSADKKEYEKLAEKDSERYRREMDAYQKKKAKRFEEEAASRTPVVSSIPSST
TSAQAAPFDLRFPKRQGSIQIVTIGGEGVGNSVAFAHPPESMVSSVSSLPPGTTTIPYRP
AHSAHVEQPQSRHALQQNSILEALNIASAVGGAVTRVSPPNYYPHQTVPDNGDLPPYTVP
HRNDNYPMQPGMEVVLADRNGIDRKYRVQYTCYSMTRQNANKYIESLTRSSNSSSTTPAP
PPPPPVATTMGANTEYGVWGV
>comp4791_c0_seq1; 269 63 455 LEN=740; translated
MIRATPFLLLAALAASANAASCPIPAVMPVTGDTCSSSGQMCHYAQKPDGTVCGDCDFWT
CNCVGGSFFCSCSGVGCDNLKATDEDSDMDLFNNVTSDLVEDFEMDSGGFVSSLGLAASG
VVLAATVAGL
>comp4793_c0_seq1; 243 1 833 LEN=1182; translated
XEPNRTEPTETKQNKQSMVSHCFQHRAIAGIALLLLGRLSASSGLATPRSFPAKLPSDLG
PWEEAAAPLPGESRRGFLGAALGVCLPLALGGAPSASLADEYGRETEAPTLATGESFEIC
TKRGPLGACQKTEVRTASNDNDKAEKYFRQPTELVRRKDTEARTAEATEGNVLIERLKKQ
SEENSEKNELLVYQRTLLNDSSASFGPFDGQVLILNEDGRGFTLLANPQAMRLKKAGLIE
NNQKKFVRQPTKEELDAALAPEPAGPGFLDRFLGGGP
>comp4793_c0_seq2; 163 316 459 LEN=808; translated
MRLKKAGLIENNQKKFVRQPTKEELDAALAPEPAGPGFLDRFLGGGP
>comp4794_c0_seq1; 581 1 2270 LEN=2636; translated
XRNREKRDTKRGSMKASAGKSRCKYLLILLCCGSYQLWMGFHGLGTNVPTNQNDIYSDQT
EVSPFWRQQRPQEQHDRNRISRVISNQSARGYNQGFDIGTKTVHSGSINGTSSAIQLRPT
KVKDTAPLNDTATKLSKEDHEKNNNSTDIETRITLDLEPPVDTSELSVEKKGEQEKTSNE
FGNESNNEDTRKSNDEPAAPASDQSIDDKFYDGFFFSDYTNAWTEQEEHPTAGFIGQDAF
SGSWWQSKEQFLRYEEVENENPPSPEGASFDVMDINDDNMRVTRDWIDLNVEHMSRWFRG
LLETRHNSNPQPTLERILEIFQNYVHSEDRLFMWERLEHESDRAKHQHHHNHIHNFDAQD
WLEVSQSTIAVIAYMPYGQYGGAEITKWSLAATMMSLVQLGVGRIIVSGNQDSYDQQSFV
QEAWQEVLEVTKSWTTSKQKRMTTHSFCSCDKEILTDKANATEGENKTTATATTASLVES
NIPYAALRRLRYILLHRDDEFAKGKDKAEAIRRCWAGSSKGQQNKTDSDDSDPMDQWKYV
FLGEPDLILNTRPLALPKLAGALTGGGVLAPHRLQPLPHASDFGVNGLDLDSLKLLIPNL
PPHFDNIAQIDQYERISNDDTHSSGNHGGDIHGHGTKTRLSNHACCDAGNQKPYRQFETC
DSWWWNCAFTALQYYDDNHYEGALEALVESNNETYVLDLHKRHSVYPMVRLKRGTGVVFA
GGESGRMCNPSQLGTCKRQDKTERNEMKENQSKKNR
>comp4795_c0_seq1; 87 201 641 LEN=923; minus strand; translated
MLSEQGGSRWDEEEEEEGTPVPFGTGLGSPKRSPPAPFSVGFRSPHGARSRSPHAGHHPS
RDRSDSSERLGATPSSTTTGTPWASVPRVAEGSRCSGEPFPGRCLREPHPWGGGQLLEEG
PDLRELVARADLVQELAAPAPVGPSA
>comp4797_c0_seq1; 230 22 534 LEN=775; translated
MTITYNTAQVQIENQTGQSIRSVKVLHKYSNVYRNSLEFTGRLQPGKKILMNETVEYNTG
FLTTGCDWWFVSFQMEDGSVYQSNPHNFRGIFDWVEQLGQKKIRDRPTGNNGLEKAACNI
ALSILNSESTVGFKKHILRAKDSVNTIVIKKDKIIFQSQSGNSVTGVMRV
>comp4799_c0_seq1; 204 119 847 LEN=1327; translated
MGKKRADGRGSDSTLRPLACELSCLHRSDGSALWKAGSTHVLAAVYGPIAPQNMNNEGDV
GKVSVVIKSGKADNGTYENEMGKFLTETISSCVDVSRFPRCIIEVVLQIIQSDGSLLGCL
LHGAVAALMDAGVDLLYLPVATTFLVRSIQSGANATTASKVRLDPTSAEEEEERDSSIIV

LVNEQRRPERILGSHTVGSGIDLDELLSCVQIASKACSAIPTFWRLAIEQKLKRESQTLW
SR
>comp4800_c0_seq1; 245 1 1375 LEN=2080; translated
XASQALPLPQTRDIALGTTIAFGLAPSKAAKDRVTRYREEQNRPAFDFDRATELPDVLRR
ESNRTESIDSNPIQFIPIQFIPIASDETESNPSGFLLAAAISGLRTAEGRGGTAAAIPEV
LASSSPGGTLYSCFLATDPGGPPEREACCGAIAGARDPGGGPSDHARCLAEMAEVPSGVS
LGGDPGDTARYAWAVPGDGSGSAEQRLGRETVRTISPYHKTAVPVPGIETGLVVSRLGAE
GGMHRLFHHEITLPGTPAGDSGANPRYYLYVTVPSGMFIDLDDPFEAPQGGRIEPAAAAP
GGDASRDAGFEATCTTGPSAGTETGPAVSFRARLHAAAVCDIEQPSFASGQHLLVWELEV
LGASKPGATERIGFATKLHLRYPRPSPDGERWIDLPAPVLVDPVGGGRIAGWEDPVRVAA
GRDGDHDWIVAATVGACLVGVAAMVRDISGVSLWDDA
>comp4802_c0_seq1; 684 192 2015 LEN=2301; translated
MASLSLPPPRNSTGVALSSTKASDVAESTEMVVSSADNDNERTEDFARPAAKPKSNSAAN
NKAPTYHQRCKAAADLSRLPPSERKKKKLFVPRSLKDFDDGGAFPEIHVAQYPRHMGNPH
LKKKKGQVASSGAGGSISTSRAIVNVEVDKNGEISYDAIVKGGTNSDKTVYSRYEDLRGG
EAKPEDVALPSAAEEAETANRTQLALQKLLVDKTALNKPSGSAIANANTSKDIEKNTQFI
KYTPRPDAPGYNPAAAQRVIQMVPAQVDPMMPPKHQHKKAPRGPAEDPVPVLHAPPRRLT
KEERDQWNIPACISNWKNTRGYTIPLDKRLAADGRGLREHTINSNFATLSESLYVAERQA
RQEVRLRAQVQKKLTMQEKDRREEELRRLANQARLERSAALGEASSAKEAVSDETGGDRR
PQQSSISDEEHSDDDRSQRQSNQKSNKVDEDDDVVAARQRERLRKERRREREREL RQENN
AELKKRKLEKERDVSEKIALGVHTGSGGAGEADSRLYNQSAGMDSGFGAEDEYNTYSKPL
FDRQGVTSASIYRPTRGETEHNADESYDKLVKGATSKFQPSKGFAGAEGGADIQAGPRTA
PVQFEKS
>comp4803_c0_seq1; 303 359 1448 LEN=1540; translated
MDYGMSESFVSDNIRAEIFTEDCEYPVLVDGIISQAIDDQGKHLFQIDPRTLSNNTKVFS
LLDDPYKAKMKFCLRHTFFSGPESDNGSIPINYLESILTVFFDLRAAVYYVGFSVEQKELD
FGSDRNNVTEKEGYEADGYICHPETYERMETPKEGYGPGSVVCICAELNQEAIDEGGGET
LYSTPYDVVGNLPSLTTANLTIYAFFPGLYLVGLTEFVWIRERYLGDELIRTVQYSVKDG
VAAVGGLTEYECITDYFCMFKTFLLADFYILPGVISGYGSVAFAFGSRSDNEVGRRVLET
HGLRSLQQEAEDPNSDLFVALPVTNRRPELKSVETAAATNISYKRSTLWFIACSATGALL
SF
>comp4803_c0_seq2; 311 359 1351 LEN=1443; translated
MDYGMSESFVSDNIRAEIFTEDCEYPVLVDGIISQAIDDQGKHLFQIDPRTLSNNTKVFS
LLDDPYKAKMKFCLRHTFFSGPESDNGSIPINYLESILTVFFDLRAAVYYVGFSVEQKELD
FGSDRNNVTEKEGYEADGYICHPETYERMETPKEGYGPGSVVCICAELNQEAIDEGLYLV
GLTEFVWIRERYLGDELIRTVQYSVKDGVAAVGGLTEYECITDYFCMFKTFLLADFYILP
GVISGYGSVAFAFGSRSDNEVGRRVLETHGLRSLQQEAEDPNSDLFVALPVTNRRPELKS
VETAAATNISYKRSTLWFIACSATGALLSF
>comp4804_c0_seq1; 642 151 1554 LEN=1801; translated
MFGWINDCTECLVLTKFGEETWQQIKEKAGCEVEDGGFLRYKYYPDSDTVQLVVAASEVL
GIEVDDVLFAFGDYFIDYVQENGYSNVLKCLGSNLRDWLSNLNSLHDHLQASYPKGFVAP
VFWSEDDEASSKCAILVHYYSHRGSLLVPLVVGIIKKVARVYFDIEIEMDQLQLQDENEA
KHTSWRITTVDPLKTAILRGEKIRTSAEKRRGHETFDGEETISTSTSTASQNRYLNAFRT
GGNVASDLRAEEFVKRHFYNASCELYHALTLEQYIYLSKYWKVNENEEEKFCYEVWSLQD
DDPSTWASLEDLPSKLLPEMITKNKYFGGMVPKTGKYPPGEDGKPQSVPPLISVVNGITG
KSKNLVVKKDESLSLEDGIYNHPDMEGAGVKEFPPGWQQRIVAGEVEIQCDVWNEETDDS
YHSFSLEDLKTASTKQLYDLVPMSPDPIKIVLQCQEAVEVDEEWEDI
>comp4805_c0_seq1; 398 139 1233 LEN=1434; translated
MSFQHVLIPAIESEPIARQEASKAGGLTNDALSKNAKAYFFEKSGGSKRAELLEKASAPE
KKQIADRIRKQYTGADESSKRVQAMGDDEIVNLLKMQEQASNCEITCLTIPTPLNGQTAV
SMYGDDQARTKNHPYNPRATKLMLACGHAFPPNSTSGDSDGKPNGIYGDVFVGRAIDDEG
KDIWERVDLTPAEVEGDLYKQQWCKIARRKGGGGGQGGSASSLSKTLQNFQNQQQQMCPS
AAVAASSGTPAIAADPANENQAKYTWSQTDDEVELKFVVPKETRAKNVTAQFGCKSLRVS
LSGVDGCETNELLLCDGVTWDQIDVDGSTFTLQDEPGAKPTGRELCVSLEKASGGQTWNY
AIEE
>comp4806_c0_seq1; 305 182 1262 LEN=1283; translated
MDKKISSEAEFALKTAKERAVAILIDSDVTLQESLQELERARELAGKAGLSIDSSIVKPV
DTTLSDAGVPSTTSNWANETTCGHSNTDHIVVTTGDPRLDRIAELKRNLLRRHRAMQPED
RGDDRWDKPKLPGERRRRIVREQDKDEYPSEPPISGWCVFINQMTVKMRHDRPNERHDQT
KVVQQLAKIWRVGMSKDEREYFTKFGTCPFSVTSFKCWQRLCSFRFARTVNYQNRQFFLP

HVAADDARKEYEQQVIEYRATGSYKPSKHFKRLGNSSVWVRRDKPSPLEEEICSYESIQF
PKRPPELDQEYEEREIRGILKRKLKLKGLLNDDGETWKEGVDFEAELELERKRRKKLKI
>comp4806_c0_seq2; 357 182 1147 LEN=1168; translated
MDKKISSEAEFALKTAKERAVAILIDSDVTLQESLQELERARELAGKAGLSIDSSIVKPV
DTTLSDAGVPSTTSNWANETTCGHSNTDHIVVTTGDPRLDRIAELKRNLLRRHRAMQPED
RGDDRWDKPKLPGERRRRIVREQDKDEYPSEPPISGWCVFINQMTVKMRHDRPNERHDQT
KVVQQLAKIWRVGMSKDEREYFTKFADDARKEYEQQVIEYRATGSYKPSKHFKRLGNSSV
WVRRDKPSPLEEEICSYESIQFPKRPPELDQEYEEREIRGILKRKLKLKGLLNDDGETWK
EGVDFEAELELERKRRKKLKI
>comp4807_c0_seq1; 836 118 2750 LEN=2843; translated
MSDGDSVDDLRQKGNHEFQKGNLDNAIAFYTSAIGIASETDTAAYIVNLCNRSACHFQMD
DLEKAKVDAELAWRASEESNVKSAYRLAKTLIGLKEFESAIKVLKKALDIPDLKETEKKS
LQELGRNVLTKMDQPSGEDETSIRFAQRPLSIREFKKGKSLGVGNFSEIVVVEHKVTKEE
FALKILEKKTAADLAKRQHPNVYNEIAMERRVLLQRVPHHPNIINMYHAFRDFNNLYYLM
DLHNVNPDLWAQLRYNGKMVGAPGSQVRRWMMQLVDALEHLHLHGIVHRDLKPENVLLNK
RNHVVVIDFGTAKDLIETDLNGPEFVGSTLHRTLRFSVLYCSLLFLLFRAFLIRMDTQIA
QIEPEQSCSNQCPSLSVASLHNAIQFQLHLMQAPDFMAPEAVTGFSGMPNQPAAQAMEHG
AAPSTAATDLWALGAMAYILHTGSTPFWSPSPYLTFLRIKRGLLPRNTWGIPDEDAWDFI
SNLMRVEQNDRLGADCFEVESGKVHVKGKGYDVLRSHPYFAKTDPGDESSVLPSLQDLCV
RACAELAKQDAMDLDVCDRHPPGDGSSHDLERLSVRQRSLVCHVLDKSKAFSRGDETRIF
QRFFASETDSLRARVRPASRDFVGLTQMNDDEYKPLTGRGSDDPYATKIEPEPTKIAVLT
NPMLLGAGGVSPEQEKAYLKGWKNCIAAINKKRPRAVVVCAKAIPPKFWKFLARIRDSIH
VLWNDGSVHYSFWLNGFQGLVLQSSGLTEESSPQMLWLREQMEQARMAKQQLFCFCDCDP
GDLSPIVQKRLARGRVLCLSGLSNQTAPFEREVCYSANETLDDDTSVKSTDSTEDEDDGS
TMRVYGTSENGLRWITVHERDEWHSEFEAIDVPSAV
>comp4807_c0_seq2; 882 118 2553 LEN=2646; translated
MSDGDSVDDLRQKGNHEFQKGNLDNAIAFYTSAIGIASETDTAAYIVNLCNRSACHFQMD
DLEKAKVDAELAWRASEESNVKSAYRLAKTLIGLKEFESAIKVLKKALDIPDLKETEKKS
LQELGRNVLTKMDQPSGEDETSIRFAQRPLSIREFKKGKSLGVGNFSEIVVVEHKVTKEE
FALKILEKKTAADLAKRQHPNVYNEIAMERRVLLQRVPHHPNIINMYHAFRDFNNLYYLM
DLHNVNPDLWAQLRYNGKMVGAPGSQVRRWMMQLVDALEHLHLHGIVHRDLKPENVLLNK
RNHVVVIDFGTAKDLIETDLNGPEFVGTPDFMAPEAVTGFSGMPNQPAAQAMEHGAAPST
AATDLWALGAMAYILHTGSTPFWSPSPYLTFLRIKRGLLPRNTWGIPDEDAWDFISNLMR
VEQNDRLGADCFEVESGKVHVKGKGYDVLRSHPYFAKTDPGDESSVLPSLQDLCVRACAE
LAKQDAMDLDVCDRHPPGDGSSHDLERLSVRQRSLVCHVLDKSKAFSRGDETRIFQRFFA
SETDSLRARVRPASRDFVGLTQMNDDEYKPLTGRGSDDPYATKIEPEPTKIAVLTNPMLL
GAGGVSPEQEKAYLKGWKNCIAAINKKRPRAVVVCAKAIPPKFWKFLARIRDSIHVLWND
GSVHYSFWLNGFQGLVLQSSGLTEESSPQMLWLREQMEQARMAKQQLFCFCDCDPGDLSP
IVQKRLARGRVLCLSGLSNQTAPFEREVCYSANETLDDDTSVKSTDSTEDEDDGSTMRVY
GTSENGLRWITVHERDEWHSEFEAIDVPSAV
>comp4808_c0_seq1; 581 298 1781 LEN=1913; translated
MAKPQPEKPPRKKNVEKYGNNNSSSSSHVSSESNKLRGHDVSIYPSKSSKNPSRDGSRDE
YDHDHHTFATATTASATISFSNSEAHSIASEAGMEVVMSSNQSEMERASPVEEYKTHSFP
AGKXHFYSRQAIFIRVVGSGAYGVVISAKDSKNTDGENVAIKMVPKAFNDEIDAKRILRE
IKLLNHFQHENIISLVDMMPPTARYLEDFHDVYMVTDLMETDLHRIIYSKQKLSIDHAQY
FIYQVLRGLKYIHSADVIHRDLKPSNLLVNSNCDLKICDFGLARGIREKNTEGQGGTMLL
TEYVVTRWYRAPEIMLACHEYSKAIDLWSVGCIFAELIQRKPYFPGEDYIDQLTIICEKL
GKLQDKDLDFVTSEKAKRFMRKLPMKTPAPLREQFPSAPADALDLLRKLLQIHPTRRIDV
EDALSHSFLSQLHNEEDEPVAEESFDYSFENEKLHRLRLQELIWREAGHFRPSCLPVAPS
RNGGSTRSNRRRDP
>comp4809_c0_seq1; 361 20 1162 LEN=2024; translated
MVGFRPLGWMGISTAVTLVFLLVSINALGNGSGDLSSSYVSVRALDNYGNAVQLSHAKEA
AQRYGRSVVVAVVDIVHDQNNNSTGKQPEISRANEHKEHQTESVEEFHSSSFLMVISLGN
SPILHPIQLPLESDPTSLLAICFTGVKGDANWLLQQIQKYAADVWERYGTTVMSVPAVSH
VVARLLGRFAAQPEDREWQSSLGLPGKHDSDDDHQSSWSRPLGVQTMILSTGVQDVQKPK
LLIVEPSGRVLNPLARSASGCVSLAAMGKESDKIQARLSRLISGKEQERKTGIGPFSFSS
STRDETQTSSWEDIPPTYERCRDSLIRVLLEETTGSNTNNIGSQQNNGGGEIMVEGYSLD
RGQIERQIFRYENANTFVPL
>comp4810_c0_seq1; 706 149 2231 LEN=2307; translated
MLVSSKVMAAFLLGAVAPSTTNAFVTKASSASIQRQNDVSLNVLPPLIIGPMLRKMKEEK

AKKNAPMATEADLQGQAPGLRVGGSTWKWPAVWPYDQNFFTPTEDIPKPAAGQNAMAQMM
TGGAPVPPQPQVIEVEKLDPIEYWQVEKADTKTEMDEEAIAXIETVSIILDSMLVVYDYD
ATRIISIDGLVKTHRTDXTPVHVQLSVTSHYGFYLKDGMSVLELGAAEESYLPDGIKLSR
HVGVGLSDKLMGENPSLTDKLIVDLNKVIKDGDVDSDELRKLAEEPFDAIIMANTAEFLM
HPREVFKTAWFLLKPGGECFNAFGTKTAFGDKFERAQTRMWRDYNDDQHMWVAGSFYQFS
AGDGWQNVRGFDISPESAKENFGETGPLDFLNNQGKDNNIYVVQATKGYQDDSIDESDPA
KSINSKMWMLPTLEDRDKKLVVPRLGRSYTVLKNPKRKDAIRDHIQYLPKIYEALIKMDA
FAFTFEMQSQLAADLVLDPDFDANDEQIKALKEGLGLRTPSKEFWEPVGQLTGAMDINDK
INLLSYIVPRFGSGDEKQEAALLAYASGLKPTFDTLRSKCPDMPESDIQLLGTELLNADI
LIPDRSTKEEFAAWLGAMNADEMKSILSVRKGLNEESDTQLKAFKKQQKEDEEKRAEIRK
EYEQQVKDARKNRTIIFNPATGNFQELEKQKKLF
>comp4810_c0_seq2; 797 149 2101 LEN=2177; translated
MLVSSKVMAAFLLGAVAPSTTNAFVTKASSASIQRQNDVSLNVLPPLIIGPMLRKMKEEK
AKKNAPMATEADLQGQAPGLRVGGSTWKWPAVWPYDQNFFTPTEDIPKPAAGQNAMAQMM
TGGAPVPPQPQVIEVEKLDPIEYWQVEKADTKTEMDEEAIAELKRHYGFYLKDGMSVLEL
GAAEESYLPDGIKLSRHVGVGLSDKLMGENPSLTDKLIVDLNKVIKDGDVDSDELRKLAE
EPFDAIIMANTAEFLMHPREVFKTAWFLLKPGGECFNAFGTKTAFGDKFERAQTRMWRDY
NDDQHMWVAGSFYQFSAGDGWQNVRGFDISPESAKENFGETGPLDFLNNQGKDNNIYVVQ
ATKGYQDDSIDESDPAKSINSKMWMLPTLEDRDKKLVVPRLGRSYTVLKNPKRKDAIRDH
IQYLPKIYEALIKMDAFAFTFEMQSQLAADLVLDPDFDANDEQIKALKEGLGLRTPSKEF
WEPVGQLTGAMDINDKINLLSYIVPRFGSGDEKQEAALLAYASGLKPTFDTLRSKCPDMP
ESDIQLLGTELLNADILIPDRSTKEEFAAWLGAMNADEMKSILSVRKGLNEESDTQLKAF
KKQQKEDEEKRAEIRKEYEQQVKDARKNRTIIFNPATGNFQELEKQKKLF
>comp4811_c0_seq1; 357 1 1607 LEN=1607; translated
XFGFENKYVRQIHWKLTDSGREPLPTSQNTIESKDVLGRTNAMIVLPVETIGGSGDSREH
WTADATGLLRRRLANETIAPEVALNLEGADSHHVGSWIAIFAFSLTAIGSAVSLASVFFI
YHYRSNQIISVCQPPFLYLVCSGVLLLSISTCLNTTLGHHSSERSQTSLDASCISWIWCV
SMGDLIIYLALVCKLWRVEKVSHFRKGQKILVRDVIWPLVFAVVIAVAILVAWTVSSPPY
YEREIVVGNDGKIYSVGICYYEDGGIYQLVLTTMFLASATVVYWLSLRTRKLPETLSDSR
RITQLLLSHLIMGLFVLVTNLVMTLEMTTEVFGAQSDAESNARNLVRAGAFLTNVFYQFL
FGMTSVGFLVVPKIWYVRYEHVHGRLPSGIHKVAGGRVTVRVNHQRVGETVGRPSSDGIG
RPPAVRKVSAFGDTRGDLRDRVPSHDDPPCPDHGGIEGDGGDSEGVDALEYVMEVDSSTS
SAGDDEKSSTDVENNSAVFDLVDKIAFEQDGLNLARVLYITIMYFQLMRKCDNRMT
>comp4812_c0_seq1; 317 210 1144 LEN=1508; translated
MSDEIPYAQPFPWTEEQPPRAKSIRSLRKRKFVGSGLAVLREKSLGSTSKMGNVAKNILM
NLFHQHGIEIDGDMESDANKFNGIGLSDGSLQKETTISNEESNTASKMESGYQSIFVPLL
KAVTESGTSQVSTRQILGKVCLLLHPGTLPGIMDSKDMVLAALHFLSSTNDTKSKYILKL
PLIRPLQAYSDLERRNYEKIGDWKLKDIEEKLLKAEEIFLSSPSSWEWQKRESLSLRLSE
ADEQSLFMRGTVPLCALASXKKVKTEGVKKRKPAIKKTKTESLASAVETFTDENATSDAL
EGDVSYENPF
>comp4813_c0_seq1; 401 157 909 LEN=1125; translated
MTSTRYSSLALLAIIACNSVASFGGVAHAFSLGPFPATTSTMAPSVRRFSVLHASEADET
AAEKPKKQAPPPPPPIDVSKLDIRVGLIEKCWEHEEADKLFCEEINIGEDEPRSIASGLK
AHYTSEEMTGKKVLVLANLKSRKMVGFPSHGMVLCAVKEGESEDGSEDIVELLSPPEGAV
PGDRIVCAGFEGEPATENQVIKKKMLNTIFPDLKVNSEGVAVYKDVPLAIGEGDEASVVT
SGSLKDVIIS
>comp4814_c0_seq1; 141 1 893 LEN=977; translated
GIFVCPLKNNCILFXAASVIVTTPQELSFADVVRGVEMFDTVDVPCVAVVENMAYYDSDP
ASQVNNKDELEEDVRKMLAEKGLLNNGDGDKFAEELVQTVLKNAKTDPVQIFGPGHKKRL
SDQWGIEHTYSMPLMDRIAANGDSGTPYVLENPESSQTQIYRELAKSVVSEVAKAKFASR
RPEIEYIEEFNIIQVDDEPLDSATLRRACRCAACVEELTGKQILKASDVSDDIKPLRMSG
TGNYALSVDWSDGHRSLYPYRQIRSLIGQDEDNTALAPKTTQESESTESDQKSCSCC
>comp4814_c0_seq2; 403 202 1983 LEN=2067; translated
MENKSSLRIVLTIAIGASILFDCNAFVTRTTTTSATAKTYEQAQPEHLYLNQNQYYSGSS
STLLSSVTDDDSTTTTSENIPIPNEWQGEVLKTLKSIIDPDLGQDIVTLGFVQNLKLDEA
ERKVSFDVELTTPACPVKETFRSDCERLVSALPFVGSVDVTMTAQEPSNTETDTSGMAGI
GAIIAVSSCKGGVGKSTTSVNLAYALQSQGASVGIFDCDVYGPSLPTMVEPDDDMVRFVG
RQIAPLQRNGVRLMSFGYINDGSAIMRGPMVSQLLDQFLSVTQWGSLDYLILDMPPGTGD
IQLTLTQRLNITAAVIVTTPQELSFADVVRGVEMFDTVDVPCVAVVENMAYYDSDPASQV
NNKDELEEDVRKMLAEKGLLNNGDGDKFAEELVQTVLKNAKTDPVQIFGPGHKKRLSDQW

GIEHTYSMPLMDRIAANGDSGTPYVLENPESSQTQIYRELAKSVVSEVAKAKFASRRPEI
EYIEEFNIIQVDDEPLDSATLRRACRCAACVEELTGKQILKASDVSDDIKPLRMSGTGNY
ALSVDWSDGHRSLYPYRQIRSLIGQDEDNTALAPKTTQESESTESDQKSCSCC
>comp4814_c0_seq3; 247 202 1146 LEN=1198; translated
MENKSSLRIVLTIAIGASILFDCNAFVTRTTTTSATAKTYEQAQPEHLYLNQNQYYSGSS
STLLSSVTDDDSTTTTSENIPIPNEWQGEVLKTLKSIIDPDLGQDIVTLGFVQNLKLDEA
ERKVSFDVELTTPACPVKETFRSDCERLVSALPFVGSVDVTMTAQEPSNTETDTSGMAGI
GAIIAVSSCKGGVGKSTTSVNLAYALQSQGASVGIFDCDVYGPSLPTMVEPDDDMVRFVG
RQIAPLQRNGVRLMSFGYINDGSAIMRGPMVSQLLDQFLSVTQWGSLDYLILDMPPGTGD
IQLTLTQRLNITAA
>comp4815_c0_seq1; 545 246 1463 LEN=1538; translated
MNSKQKVMVLLQVIAAVYTIVGHDRFNGLFASAFLPPRGNGNTGATASTRAYLKRHKNDL
VPFSETFSAATAASRNRILGLNPSKRIHGNASDSLFALRATLLPCWSVANRLTRTDAQAL
LDQDDLLPSSAYGDRKTLGLKAAEQECGGDLGGNDEDAGVILADDPRLARTYAEFPLASL
DVLLDAAVGHLSSGAGAGGSAIQMVDIGSGLGRIVLYSALTRHGRGAGETDTGEESWRVC
GIEISSVLHNGALELVDAGMGRELFRSCGGDPGREDERKGDPSFLSKSNKLSFHLGSATE
PEGSALLGSADIVFSYSTVFKAKYFDPDVGALLLDAEEWSRPLGEACKPGCVAVTTDRAL
DPRFGWKLVERIDVPNPDVLGTTGFVHVLETNEQNESEAMGTSQL
>comp4817_c0_seq1; 1630 158 3319 LEN=3756; translated
MAEEQKVVVDPPPWHTMSKEDVISELDLPADIRKVGLTSKQAAERLEKYGENKLTEKEKE
TLLQKIWNQVNNILVLILVIVAVISLISAFVIPNEVNPRYTNWIQIAIIMGVIIINTIIG
LIQEGSAEKSAEALKNMLSSDAVLIRDGVETKVPAALIVPGDAVVLSLGDRIPADLRVIE
VNNMASAEAALTGESVPIEKVVEKIDLKDGQLPKQVPLGDRKNMCFSATLIAQGSGVGIA
VATGDETEIGTINKLVNSQEKTKTAVLEQIDTISLYLAGFIMSVALITFLYEWLHIKTSP
IMALNTSLVCAVAMIPEGLEAIVTVTYAWSTNKMAGLNAIVRALPAVETLGSVTVICSDK
TGTLTTNVMSMTAFVTSNAHYKNNVHATERTPDNLVRDDTFFGEKADIAKVIRRNEAKKN
GGEVAVEEGEVEAESDVKIDPNDKTPVGQGGSASSEFWRQALIGGVMCSKCTLGKDGTRE
GEIGNPTELSILRAAYWSGVDVNKAKDASPIIAEVPFSSEYKFMATVHDSCPENDGPGMD
DKLIIHVKGAPDRMVKLCNTQAVAGQLGKTEPINADFWQEQIAILSSHGLRVLALLRGTL
KKGSVSQGDALKPEFVRDREPWLTMVGLCAIVDPPRPECIVAIEEAHHASVRVAMITGDH
KDTALAIGASLGLVDTEHPAAVTGPELDAMSDDEIKKAVMTYNVFARASPENKIRIVKAL
QSQGEVASMTGDGVNDAPALKAANMGVAMGKEGTDVAREAAEMILADDNFATILSAVKEG
RVVWDNLRKVLMINTPINNAQGLSVLFGIICGFNRGTAQNDKVILTAIQVLYCNLICAVT
LGFVAAVEPAEDGIMDVPPRRVGKRLIGRFLFLRIALGTVVLVFITVASAFWAEDILVNQ
YGLDKFSRECIDRVHSQASNSLTFGACFVTVSARFSFSNAVHPRAFKDNPAVLWSLLIVT
LLQLAITYIPVLNSVVFAMAPMKAFQWGIVLLGGLIVFISLELEKALRRYLKAHGSDVDD
SEYGIFDNKVPLPNQDIRLPKGASHLRLTHVKD
>comp4819_c0_seq1; 384 960 1685 LEN=1782; minus strand; translated
MPSRQYAAYIIGLFPSVYDWLVNVSARSPLIDDTFSYDISSVQSPTWMGVLNWKNGSLLV
SLLWTSILVNVIDRQWVSAAIWSVIASLFAVFGIIHSFSAGFSNFNSPSWQYCWDADGTC
WEYSEQWMEFTAYLMVAATFVILHFVSKHDEQMKDPIDDESRHAFDDWFKDAYKYKDSEG
NTRDSRIDQSGEEMKEAMANPGNEGADAMKLVYGEGHPEEVQDNPGNEAAKSLSKAVEMA
A
>comp4820_c0_seq1; 189 1 1149 LEN=1205; translated
XVTSNEKGSKDRSAPRWESPGARRQARRRASSCEENRRLKPPPLGHREVLDVFGCCSSDD
EDRTQTRNAPKNGAEKRTTPDHVGARGDRASNAPATHGNQRPRAPPSNTGAGDERKTAAG
PQSDARTTGLGTTRSQPAPAWSTGSNGSPNERPVLSQQTPSGTNGGETPRAAESGARSLP
KRTRKRKSADTVRILYRPRPEDVPPERWLNSSIGRTREELNSIRDMVQYMGSCKFGQSSV
CSKFGRVHAIVMSDEWNDLAQRNDVLQNWYVGLGPGRKRLARLVGACESTIPIFQAPKKE
HRTPEIFYVGHYKVLGLQELDPTVTIKGIERDIKLSFAFDRFDGRLDAIIQGGPVRSGST
PGSGSVESGQVLAKGHEWVQTP
>comp4821_c0_seq1; 591 134 1843 LEN=2301; translated
MATNSNDVPKNDSILSDEHDCTSWHAGGDYNPLPHVLPEGDNAAPLEVMGSSASDKLVII
MVGLPATGKTHIAKRICRFLSFFHDIPSQIFNVGDYRRQLCGAQQPASFYNPENADGLAQ
RKVACDAALADLMEYMSEDGVRVAALDATNSTKERRRHIKSVLKKEKNNIKTIFVESICD
DEAILLSNIHKVKLSTPDYKGVEESKAVADFMARRANYERYYEPVEEDDGAFCKIVNSHR
YVVHNIRGYLSLKVVHFVMNLHTMPRTFYLTRHGQSEYNLLGKIGGDSGLTPAGLEYARR
LAIFAKEEIATPRPEDHMHNGEVKSGGSDNEGRIQRPCRLWTSTLRRTRETAQFIEHTNF
RHTFDNGDNSDWCQFRPKPRRNLDELYAGTCDGMTYKEISEVFPDEFSRRQADKLSYRYP
RGESYMDVTLRLEPLAQEMERIREPLLIVGHQGILRILYAYFMGLDRKEAPYVSIPLNNV

IELTPHAYGCHEKRYCLMMKEEMLADGQDEPVTSMPVAQKGSQDVGLYDTAKKKWTVPRA
ELLKKRSSVQEIDAKYEEDDSIMNAPSC
>comp4821_c0_seq2; 150 569 1486 LEN=1947; translated
MPRTFYLTRHGQSEYNLLGKIGGDSGLTPAGLEYARRLAIFAKEEIATPRPEDHMHNGEV
KSGGSDNEGRIQRPCRLWTSTLRRTRETAQFIEHTNFRHTFDNGDNSDWCQFRPKPRRNL
DELYAGTCDGMTYKEISEVFPDEFSRRQADKLSYRYPRGESYMDVTLRLEPLAQEMERIR
EPLLIVGHQGILRILYAYFMGLDRKEAPYVSIPLNNVIELTPHAYGCHEKRYCLMMKEEM
LADGQDEPVTSMPVAQKGSQDVGLYDTAKKKWTVPRAELLKKRSSVQEIDAKYEEDDSIM
NAPSC
>comp4823_c0_seq1; 461 862 2319 LEN=3844; minus strand; translated
MCSAACSIHWLLVSSALVSVFSFSWLAPRTICQDGFSHHSITSNWRIRRNKICISGRRGQ
MREHPLFGASVDDDDLFSYDHDVNLPKLISLFGGSPSDLLRLDSNNGIRGVYANKSLKEG
EILLKMPLSSCLRDDQPPLWLLEHQQQDQSEEESSESALLIQDWVTRLAACVLEKQHEQK
IKKEADCLDRATQAWLKLLPSRSLLRQFLPIHWPESILSETNYMPLELAVDSAYFARAAP
MGDLMGAIPSSYCNNGGEIQDTIEWALDLVQTRSCRCALRFSGESTGGDVKDLRVLVPVF
DMINHSPEPNAEFFRKDDYMMVQALRDIAADDQVCISYGGSTVPAWRRLFSYGFCDTSGG
DGDGFAVYEDDTVEIVLEDRFRFEVSPTEIPGELIQYQAQQAAGIDSDEPLDIELTPEIG
LAIVDHLTNAAANSKRKRDEAINAATNSSEESLSVPLRLVGDLHESHRRTLLTCAGGLRE
YLGGQ
>comp4824_c0_seq1; 571 71 1285 LEN=1521; translated
MMVFKKVFLKVLLMVCLIAATGIVVADDEASPPPKDDAKAQSETKTDNRQARPLAKEIPQ
DTFDVKSHFNWGTYYDPKNVFCGKYDCYGILGFDYESFDKEKPTQKMITKRYRALSRHWH
PDKSKHPEAKERFQKIARAYEVLTSSKSRKEYDDLRYDQDSYLKKYGSDVVFTFAPQTDT
AFVIILIMGLINGFVYFAQFNRWKKVCDRLAKAAIEDWNSSQGGSPESKDLRERALEIYA
KEKGDSKDDTVVTKKGGKKGSKVSNKEKKELENAALRPIVEKLAYEITDFGAGFHKPTWK
DLAILKLAVWPYHLAVGTAWQMKFWIRRIQKLDLNDEEKNVLTERAVGRVIWEFSTDDER
ETMVKRELWDLSNLKEWNEEREFAQLSKTEQKQYKLWKKHEKMN
>comp4825_c0_seq1; 150 81 584 LEN=1112; translated
MTKDDHGDGNSVGDDXMSVDSNYYGRLADSSRNRKDVLTLRNSTQDSQELEIVIDERKKE
LMTKTRSVAYRLAASTPTPTAREQEMEANGDAGGNHDGHEEQRSKRQKTSGTATSARATE
RGFLEVTQKGTPILNPSEETVRGPIRLRLLRLPPQSTELGREAARE
>comp4826_c0_seq1; 368 1240 1818 LEN=2221; minus strand; translated
MPKEGKCVKKAATAKHKITASKSTKGLTSSQQKMIDTIAAISLQMQNNVVSRSIVAMQLK
LTLKSVNNALSKLKQKGLVTMDTGVSVSITQDGVEQANLDSAKICKNNREHHLNTMDTFK
LTPKQRQLFEELSDGSRKTKKNVASSLGYNRMKSFQNLLMPLKKFGILCYDKDTLWLSDA
MFIFEARKESGP
>comp4827_c0_seq1; 306 229 1239 LEN=1863; translated
MATTQGQLRLWILLSLALQLLFNLSRYETPHPQNAASRETKVVGDATNYRGVPHDTTTVG
ETIVYGHLHMQKTAGSNINGMMATKYDNVCGNKGSSYNAFVVNQERQEAITNMENGNRAE
VSFGRKGVPYNEVRDTIGFANCDYISVETKWKNWKQDIVDALDFEKRNMRLELHVPCRDH
LDHLLSLCNHRNKKFQCPDISDEDAIASEIKKCLWAGPKKEAKEDKVSNILINNARFDPK
LRDESHSKGIDLKCFSTFPVENYINYMGQFMRKRRIEAPFVDRKTNKDREKTTECLLNQD
DNYKATVKRLVLQLDFMGYYQFCEDCMGSSDQLKLS
>comp4829_c0_seq1; 258 298 1146 LEN=1430; translated
MIKEVSGMRVSELKAELGERGVDFSDCFDKESMVAKLASARVNGVGEGDNDHEHQSGESG
ENTGDAAAVDANTPQDFDRDAALRELKGMRVRELREELGRRRIPRAGLFEKEDLVAALLE
ARALASVFCATGSLVAGEVTEVSEDVARREIGFGGGPPLLLDVYATWCGPCQMIAPFLKE
IAAEKGTGLRIAKMDSDQNPGLSSELRVQGLPTLVLFRSGEEVDRIEGAPTKEQLLAWID
XPPVTDGETAGSGFDRVGHDHKKRTTGYQEARGKTGAIVLSF
>comp4830_c0_seq1; 432 8 973 LEN=1675; translated
MIRSLRVTSVIAWKSCRKRRWMPPLSATSSLVPPKQTSFQASFSGIVSSYLNAAIPGNYY
VSRNNDVPSQPIGLFFFSTNCKRDPNNNESEHDAPTQKPQTIRDQLKSIPNILTISRLAS
TPLLCYWIINDEPYLAIVGCFFAGASDVLDGYIAKNYGQKTVLGTYLDPFADKFFINSLA
ISLCYSGILPMPLILLWASKDVLLLGGMGWYLYQEQNTFNFFSNSIHSKPLTVTPSTLGK
ANTGLQFATLAAGILTPVVPPDVLQPTILESLCWMTGASSIVTTISYARGGKGFTYTAKN
NQNVKKRDAADANSANGNNDT
>comp4831_c0_seq1; 598 130 1485 LEN=1742; translated
MPSLIDNVTNDDDESELEAGADPQPGKKEDEAETLGERASKDGTKADEDPDAEKSGCFAS
FLTKFCAFYWQNEFLILIVCAILLARAYPPLGATYLAPQYTATWIAVMFIFLLAGLGLKT
EEFAKAFQRIYFNTYVMLFSFGVDSSIVFGISRLVAHYNIINQGLADGMVIAACLPLTIN

MCVVLTKASGGDEASSIFNSAFGNLVGVFLSPALILGYIGVSGEVKVLNVFFKLAIRVLL
PIIVGQLLKYFCPPVNDFVKTYKPKFKKLQMYSLVFIVYCVFCKTFGSDEASIPIGDIFL
MILFIGISLTILMLLAWYSLRLFFREKPKLRIMGLYGCTHKTVAMGVPLINAIYEGDPNI
AMYTLPLLIWHPMQLIVGTFLSPRLLAWVESYPVAGDKAGSGDDSNESGETFAEKKVVVG
VASADGDDDVVDDAVGDANSIKSGKSIEMDV
>comp4832_c0_seq1; 572 299 2485 LEN=2810; translated
MAAKAEKAEKKKVELKKRLARLLRNSDNKICADCPEKRPGWISFIQPQQSFALGSKVLAS
FVCLECAECHKKLGTDICYVRSIKEDEFKKDEIEHAQHSGNGIINEIFEGHLRKSTTDEI
GIKPLMDADVSKKERYIRSKYVDLYFYRKSAHYKQIADMNEVLAESRKTKTKKKEKKSSK
PAGKKRRKSKELGAFLGEKMNDSKESRCSLTVGNTVSTPISNDDGNHFSGSINVENSEDE
LTLVTDQGSSMGEPQDARTIKRGRSRRKLGKKSISELDIGKRQIERVEVRGGPKETDRVE
DGEGAGAGVVHQKTMKRIRLSKTTTEIGNGASQGDDRFLRVHLERARASQGDGHFRRVPL
QKSGVRQGGDRFLRVQLQKFGKRVTGVQSFAVSVRDRGRNRELGGKVIRAKLKMFLNGFT
ASPADELLASLSITDLVLDNLGSPTRDTKKKARSKGRRTSEGLSSSMDYLETPARDENKK
AKIRQRNTSEDAKTTRDNANLEQNSEESIDDNGFKLIDSSTFCTKRSSFGNSWNSSGSSF
TKPYKKKGVGLFHNRQGQSVEFEGSTTIRHNRRERKSAIFASLERESSCEHFEALNDHNS
DSFEWETLQRSSNSDNSGSFVRAAPRRSRSSDTLMRVAPRRTRSSDAIGMQSSSSKMRGV
SMKSRSVPIRSRSSATVPKTTLKMPEWKTYKSRTSIKNETKLLKEEWEKLKKENEDVFVS
FEKSFSKF
>comp4834_c0_seq1; 274 266 1237 LEN=1844; translated
MRRTPIASALSGKSRTGNGKKGSRSIDNKNGSAASQTSVCKFCARNPPALLVQLPVLGRK
KRAATPYCLSCYYTTSAVRQDPEKYVTIFDEEEKAKQLPAIQNLFSECFLELQHELSEES
VRAFKKQKSDPLAMLSFSSGSSSSSFASKKRRKLASNKGQAPDPRTKQEGRANDGGFLRE
IDLPERLKRTQKQQEELQNQQLARMNQAASAARRASRIASPLTKSTMNKRRKGSGKSIWN
LAMDKGKDGKLSTSSESYQAAAMDPATMNVSCTCGSKDVRSFGNITSRNQDVRKGEIWGT
DRGDEVINRFQCNICGRTWNEAE
>comp4835_c0_seq1; 212 254 736 LEN=1189; translated
MSSDPYLNSFRPGEGFPSGVEYRWSDGNSGEAMSVSAPVYIEKVLGWIADQINDETKFPD
DDDETEALRVFQTPQFAALCGQIFRRLFRVYGIIYSSFFGTLEALDMAPHLNSCFKHFMY
FCTEFGLLPEREIEPLEVLVKPIRKKYYAAKQGAVTAPGR
>comp4835_c0_seq2; 272 275 967 LEN=1420; translated
MAPASTGTFQPLRHRSGTMRERLHSYTKRTLGAGGSYNDAVALPQGESCAAWVAVHAIDF
YNDVSTIWAVMSSDPYLNSFRPGEGFPSGVEYRWSDGNSGEAMSVSAPVYIEKVLGWIAD
QINDETKFPDDDDETEALRVFQTPQFAALCGQIFRRLFRVYGIIYSSFFGTLEALDMAPH
LNSCFKHFMYFCTEFGLLPEREIEPLEVLVKPIRKKYYAAKQGAVTAPGR
>comp4836_c0_seq1; 563 121 1785 LEN=1999; translated
MSIDDEARRAMQGPPTTQSEFLARSMQQSTSGGSNPRQTSMASSGPPSAQYRVSQTPEGI
DLESSRIAPEIQSKEGADESTARMRYWVIIFGLLIVICVGAAAIVVPLYVKPQYESDPIV
IIQTGAPAPSASVVPNSSPTQAPTPKDVPTLPPTTAAFATFVNQFALEISGSEAFENPDS
PQYKAADFIANDAEYAPRLSDKAMLGDLYAASVFYFSTAGEYWFECSQGSDNCPDGISWM
SSNVTYCHWSWIGCNDAGRIVDIVFSENKGNNLTGTLTSELGLLTELEQFVAVNDDIKGA
LPDELGSLTHMTHLILANNSLSGRISDTFLENSPVEVFMLSDNKFKGNIPKSLTLMPSIL
QIFLRNNRFSGPIPEELGALPHLATLDLAGNRFEGEIPDSIFSPTMENLYLGGNSEITGT
LSPAVGSAANLVRLYISGTRISGSLPDDLFTLGHLSELVLSNNTLTGELPESVANLGGTL
RLMKLDGNKFSGDMPSAAINSLVVSNVLTFDRNSFTGAISPTTCRRRGDGYHDLQILSVD
CAEVTCSCCNNCAL
>comp4837_c0_seq1; 419 1 1335 LEN=1335; translated
FKITESSLGRLCLFPIIPIERTGSNCDLSSPATQLTVQADSQRNNIHTITXHHYQQQQQQ
QLALQHHHQQQQQTALQQQQQPQEQVYREPVQMQVQDHSIQIDVRDHQQQRQLIQASTET
QQRHQNLSASQPAVQQQQVAHLNVQPPPSQSEREAQAEAHLQAQSHAITQHMSDTAMQVP
APPIQTEPQVQAQSQSFPQQTTYAYPPHQQVHQQEQQGQQHDHNMAPMAIPAPAPMVGQQ
QDHNLAPVPMPAPQPLSVPAPPLAQPTQFSHAQTPVTQQQQQVVRMPYSQENQEHMLPTG
SHQVANVSYEQRQPQHQQNYDAAKIPANLHTQQSGAVPGQIIPAPLQQQQQQQHPQLQYT
QPSMPSAPETLQLPPPQQVDTQQQPAYSQQDHQQAYSQAQPQTQDATSISAPPEPCISAS
AQHSVQHSYPQEQPHDPHMQQQQQQX
>comp4838_c0_seq1; 454 69 1340 LEN=1561; translated
MTSDYKHGSYRTHRNKSNQYAAAFGELHKDDAYSKRSRQSRMSEASGVNYLSHQLKQTAS
FDFASIDENSENIRNGANSRFNSMGTTKTTNTLEDSYSRAMQKMNRGDFDPNRKVTSDAE
KGRFDDKNRSSRGSSKAESQSRHSNTSSKHTQMDNHSAHRRASKSKNSATTGKKKRSVSK
KDPSEETIALFGANGVTGHYFLQQAMEAGYNIQALVTPGMPLEDFSNNERLNIVTGSLEE

IEKIQAVVHNATYVVCLLQDNDQETMKPPVGNIEDEAANESYSFFNLNFVHNLVPILEQS
KTCRVLLYEATSLAKDSKGNAPFLTTMTKKLAFGKNWRWNKKEQDRIVQYLAAQTENAAF
NYIVTRPSATLIWDRPSRKKLAASKSKPGPFPITNIDLAEFTLNALRMEKVYNSCPYVVQ
DGI
>comp4840_c0_seq1; 245 127 279 LEN=279; translated
MTDVEDFEDMEDSMDMGSMEFASGDDGGDDVDAEVVVDDNDGEEEEEEEEE
>comp4840_c0_seq2; 245 1092 1244 LEN=1244; translated
MTDVEDFEDMEDSMDMGSMEFASGDDGGDDVDAEVVVDDNDGEEEEEEEEE
>comp4841_c0_seq1; 428 273 1643 LEN=1834; translated
MTSEDDKFSPRCTASQAAYEKTQILSQQSKLSVLKECIQPVRWESVQQKSLLGKGAFSEV
YRVDINTPELKDKKCALKYLSPKITNIKEGSFDLAAIDLATEANLLSRLNHENIIHLHGV
FGGELRTSYVDYEKGYFLLLDLLHDTLPKRLDQARAKERRRILSGSISFPKMLERIEDVA
LGVSRGLEYLHQNGVVFRDLKPDNIGFTKDGKPVIFDFGFARELHTVKAGDIAGSLRYMS
PEMAFGQDPKLPSDVYSFGVLLFEVCTLEKPFKQFNQQAEFKEKVLIEDYRPPLTSIPST
ELRDLITRTWNPDPKNRPDMTSVVKLLRVEVALSERQVQKGKLGLHRPKSCSSNISGTLK
RGSTMAALTSNAMFRRGSFHAENKTIVSTPVSEMSDPISDLSLVDMGRSSTRKVSISSCS
SASDISQCNSSHSLQKSEFTLGRLRKPFRRRPCAKS
>comp4842_c0_seq1; 274 17 499 LEN=653; translated
MKQFSTLIAVVLLVYNSIGTEAFVANTRSSRSAVITSSRLAAGPLQKFSNPKEYNKVVEN
LMFTRGITREEAVKEYDAYLNNPNDYALNKGEAYYKSLGYKSLMDGVIGEAEKEGRGDEV
RARIEKFNQESKAKGLATISFFIALGFYLKITHPYVPPLH
>comp4843_c0_seq1; 156 145 639 LEN=1485; translated
MPTGTNQAHVQDWGSVNVGRASRPTAKKSVGVEKRYGAGGNASAHSGGIMSARKLEEATD
VGTIAKVDMSLRKAIMQARTAKKMTQKELATAINEKPQVVAEYESGKAIPNPQIISKLER
KLGTKLPRPGKSKLAKDTKTTARKPGQSAAAAKGLTRGGPAKRR
>comp4846_c0_seq1; 335 225 1517 LEN=1690; translated
MNVVLKVRNSLQRLPCAAKAVSCFTCGTDTTLSSDSCAEIRTGRVIDASFCMDLPNKSVT
ATQLYYDRVASESDCYSISNPSYCPYDGEDEREDHNNNDNDSLATQASRTKSGLQKSKHE
LSGIFEYPGNSFMDDSSYETFSPYGIPDDDTRSDAENSTRALPDNINKNNTCDKPEMPET
YRNDRRDMTFNKIGLGIVGLVTDVVTSKASRTAGGAGGGASRARTIPTGRMNTSSGFRSN
INDWNGPSQIIESREGSGLAKKKNCDGSGESQTRELPSFGKSREFRSFGSNRSQRSSLDD
GSLYSLKPTFVTDDESLLGQSMQYSYITPTRSAEETEMETASAERAEFSSIYQSAIFFDG
SHGLRSRDSSFDAAEYPTTTIASKPRDREGAPSSVRPRGFKGKRPPFPFLDNAGTVPGVS
KQQHLRLVPP
>comp4849_c0_seq1; 604 216 2387 LEN=2554; translated
MKVETPQILWNNEADKGINAPLLSIDILESGLATPNQSNCAQSQSQTKSENRSKYSHVLV
TAGNAASINLWKICFGSHRHKAQQSTDLEKDDASVGLSECNSSIFQKQTRPLFNKIDYMM
SLNRHDATVNCVAFSPDGLHLATAGDSGNIIIWSVPVNKRGNNNGRHFWSTVTKENDLIV
RIVSTHCEGIVDLSWSADSKRFTVGTIDSCVLIYEDKHYGINACSPDTHRKESDWQPVFR
NSDQHGSFVQGVAYDPLDVYIASMGGDRTVRVFPRKTPQKSKKKVLRPANASTSVRSNAS
RSLVTPPPHQHQRMVAQILTEAKIDIGKPKRIKQRLVMVEDSTNNGTGSDKNHNNTNEHH
VKQIKHKLFVDESNCESFFRRLNWTVDGAFLVTPAALWHENNDIDAGKTTQTSASTSPLF
STYIFARHRFEEPYRVLTGLDKPSVVVRPNPILFELPPSVLEESKENQYPLTSKSQNASK
NLMHEQLPSTNPCGLPYRSLFAVLTLDSILIYDTHHAAPLSVLQGLHYSGLTDCCWSSDG
MNLMVCSSDGYISIINFAPGELGNVYVNHSCSDDAASTTVQPHSVDKTEENVETPAPVEF
EPLPPCDQDPMPAVLVAPAAKRVKKTRITPTLVSNSISCHSLRTQDEAIEAASTTNVAAT
KRAISETEIVGEAVTKLSLDNNNIGKTISTETKNTAEEGNTSSCEKPKKKKKRVQPLLIS
NNI
>comp4851_c0_seq1; 251 135 707 LEN=739; translated
MATIVEFETTEGNFKAELFTEKMPITCGNFLDLIERKFYDGLYIHRITPQFCVQFGCPYA
LNPHVYYGTLDDDAGKGSGPPDTSYKSCNGKEHTRDEYGNIEDEIVKGISNEEGTLSMAN
SGDPETGGSQVFINVNDNKHLDWWNSSSDSAHPVFGKIIEGYDIVKKIEAVETLSEQPVE
PIQLVSVRKI
>comp4851_c0_seq2; 235 135 793 LEN=825; translated
MATIVEFETTEGNFKAEXIYGENADYMVSSEITWSFENRSLTLTKDSILWMKKSSSGNFL
DLIERKFYDGLYIHRITPQFCVQFGCPYALNPHVYYGTLDDDAGKGSGPPDTSYKSCNGK
EHTRDEYGNIEDEIVKGISNEEGTLSMANSGDPETGGSQVFINVNDNKHLDWWNSSSDSA
HPVFGKIIEGYDIVKKIEAVETLSEQPVEPIQLVSVRKI
>comp4852_c0_seq1; 291 1 795 LEN=795; translated
NNNKIKGPDGDADTNSISPWVFGHLFTPNEHGSAKATSNKKNLFKKKGDSSAKKASSEGG

SAAPKQNGKRGNKDSKRNRLLNWKDSGTNRRGNSPLSSTAVLGTLMYVRDYRRLRDGRIL
ALVHAAETFVVEKVHRSLPYAIVDARILPDVEELEFDPPATDGSESLPSPSIYSCGDHPD
EQFLVDSLRADEDASAGPARARAVEDSIHVYHRYECDSEQRLDGIPNKSDLGILDITHNA
ISRAHLPYCPFAASSFLNATTAATT
>comp4852_c1_seq1; 208 1 727 LEN=1314; translated
NNNSSSSNDDARNHPDTKDETGASGSSPSEKDPARAAPSLEFELLRRGITKIPPSDRRFS
YNAEPTYASSSSEETKESTTHSWTTDELEYELWLVLDYFLKATKKEISPVLLRLLPSEPE
MPRAWPPGFSLKAAHDTKHGNSQGDPGRSSGGGGGYPHRRRQRRLSYSAAYLLEAVLPIG
RAVGSGSGRSVRCSGGSGVDEAEIQELRALLLSVPSTRQRLRVVLERFHRWRLYEECDEF
A
>comp4853_c0_seq1; 83 1 225 LEN=225; translated
XDNKDNKDNTPKDIDSNDSHLAYGNTHHTRQQINQEPIALILKSIDSSRNKRIKRNKPNQ
QYETQLGPKNTTTTTX
>comp4854_c0_seq1; 487 115 1410 LEN=1903; translated
MKFGIFGSKKKDGHEADPTAGVLSDPEEAESGVNMKWNDMYVEIDDDANETIANESYNST
TEKRCGGRTIPGSMSEPTMDGSRMNSAFVNKSFANNEEKQDDGPDSLEVPSNADSGTITN
STTEDCTINVDDLPDHLPQGVETNSKDFSITNNGKYLSNNYFANAQLMRWKHAAEEGPIF
IQVLAALAAIGAMTTTIYPLVTFYTVWTAPLCICALHTTVLCSLIVTFEMRAWCVRNPMS
LRARIRNLLVRYLNVLRFLWGRGLLYIFAGSMNITIYYKPYTSYSGFTLIALGILAIMVG
GSSALNLEHLRLSLTDRSFLWSKFVEADSDSDNMINLTDFSNLIWSLGLELDDFYTYRAF
SDVDKRSHSHITFDEFQYWWMASQDGDETVLTMAVSKISAFPSVAPSHYAVANSNYVPKH
IKDHIPAHIKV
>comp4855_c0_seq1; 313 1 1130 LEN=1195; translated
LFSLQGPNFFAPPTIGSPQHRISDELPYVSLHXSIRVQASLHLLEMKASEALGLCKLADS
GVASVTSQARVANLWAGMGSIIRLECSGGSQKSIIAKRIRCPCDPSSIGDQRKAASYRVE
ASFYASKYCRELSEEGICCEGLHTEDDGKGSITILMRPLPNETLSYMEGKVAEAAVRSVA
KLHAYFWGSERAREAVEETGLAEQGTYWYLDTRPDEYDRMDGNRGLSARLKRAAHGIDAA
LKEHEYQTICHGDLKACNMSLSRNPDFVTLVDFQYLGKACPAKDLAYLFVCGIDVDDDFE
ARREDELLRLYIEELAANGVGDDADAPLPTMEKLKEALDLSYCDLYRWMLGWGVWGNGFL
EGRVEACMNNYGSMYN
>comp4855_c0_seq2; 279 1 1073 LEN=1360; translated
LFSLQGPNFFAPPTIGSPQHRISDELPYVSLHXSIRVQASLHLLEMKASEALGLCKLADS
GVASVTSQARVANLWAGMGSIIRLECSGGSQKSIIAKRIRCPCDPSSIGDQRKAASYRVE
ASFYASKYCRELSEEGICCEGLHTEDDGKGSITILMRPLPNETLSYMEGKVAEAAVRSVA
KLHAYFWGSERAREAVEETGLAEQGTYWYLDTRPDEYDRMDGNRGLSARLKRAAHGIDAA
LKEHEYQTICHGDLKACNMSLSRNPDFVTLVDFQYLGKACPAKDLAYLFVCGIDVDDDFE
ARREDELLRLYIEELAANGVGDDADAPLPTMEKLKEALYVSARCCARFRKLEPIPFS
>comp4856_c0_seq2; 330 177 1517 LEN=1702; translated
MATTRATTKEKNQNEHSSPCYYKLSAAELPDDLFRKDVPVTEWIGGKEEDNKNADDARPW
IATSSSPSGFPPGDLKKVHPIDRMVDIERCIGISLYMVGNVTPFALPSLAFAWLALGMEA
AKYLFWFVVVYHGGLYALWRLWMVPIFLRRYNRGSRLKDDLNPVDPGYSQYLYTERNTTK
YCSTSYVWPASLHRPALEKGPNNDRPVIYCIIPHGLAPYGIVGYPYFSKVWNSKLCSWTC
APVLLSLPFVGYFLKAIGYIPAKSKNILEALTKKDRNVGVILDGIEGMFHNANATNTDTE
VGAILKRKGIVKIALKANAALVPVYGFGHAEVYDILVDPFGIMKYLSITFGVSLTPFFGR
YKWFLGPPKRNVPITVCLGDPIYPPESSSSSGDETTPDSPSNTITQEDIDEHHARLLKGF
EKVFDTHKRGYYGEQTGARKKLVFCK
>comp4857_c0_seq1; 150 148 452 LEN=452; translated
MTQNKDHHGTHVDVRGAKSKIIGLISGSVVSRREKQSTAKIDNPLSSDDLAPPPPSDRLN
YALLIVLYTLQGIPMGLSASIPFLLQEKIQKMTAASAAAAAG
>comp4857_c1_seq1; 586 1 1691 LEN=1999; translated
XAAGTDVAMGATAASAVAAEAARASYNANAIFALCSWPFSLKLLWAPIVDAVYFKRFGRR
KSWLVPIQTLAGLIMVGGSDFVERQLGLGNVAAVTESGVDAAAAAAASMNVRGVTAFFFV
LYFLMATQDIAVDGWALTMLSKKNRGRGPICNSIGQNIGYFLSFVGFLALNDVESANTLW
RPLLRLPQNPNKGLVSLKGFLRFMGSFMLVTTAVVAIFKREIKEPPKVVDSNNIIAKIGR
ANSNVSDEDEDDDNELDASEIGLRETYHRLWAVSQLPAVRWLFIVLVSYRLPVALSDNVK
FLKAVEYGLSKSTTAILSPTIILPLGILVPLVASKIWHMAPLTQFMRAYEWRVTIIPLLD
ILMLRILKSGQPHNTSFFWGAVIASTAGQAICNSLQFNAQMTFFASRVDPAIGGSYMTLL
NTMANLGGTWPASFVMKLLGWMTPTVASTSAESPTKASSSWFGEDPYEFVQTILSVLGIA
WIIVLGPVVRRLAVLPDDSWRTQLLDDDGDDDKITKGGKKAEKSSKLSSVARKRGGASKP
RMSAGTKRINAMESGGVEDTKTK

>comp4858_c0_seq1; 495 1 1376 LEN=1615; translated
GLPGTFIFWACSTIKQKECRFSSQHNIDTFFLLCNPGSYWIERNVNPIIFVLFSVAVVVK
KGMCSFAQKAEFASRNIHPKGVVKILIIDGEIRINDADDEGYAFGSEIEDDKGGEDDLGE
NNHLSSFEFPSYYNTDDEKKTTLRREHEDDISVALLHVSYRTGYELIETILDEDIDVRNA
GGTRVTVDAVSPPLTGAVIIVWTAVSIVICLILCCCMAAALEDLLENQEPEPAPLRRPRR
QRLTTDQVRELHVGIFDGNKLVYDEENPTANGVGEYCCDHILQHSNDKNFPQPTINSMDT
CTICLDDYQIGDKLCCLPCGHVFHAECISKWLVERSATCPLCNLDLYEEEVDDNSEGEED
EYTNNERQARNGSAPTTQVEPDMNEDASTIDHSIGSWLRAIFRSPYWRNRTTGGSSGLEA
LAEPLLQQDQPEHPTSAGIDVEATPSNEESSTGPTEID
>comp4858_c0_seq2; 584 317 1639 LEN=1878; translated
MKEGLEYEARLQRVNGADHFCGKGSWNVTVPSDGLPVAVVVKKGMCSFAQKAEFASRNIH
PKGVVKILIIDGEIRINDADDEGYAFGSEIEDDKGGEDDLGENNHLSSFEFPSYYNTDDE
KKTTLRREHEDDISVALLHVSYRTGYELIETILDEDIDVRNAGGTRVTVDAVSPPLTGAV
IIVWTAVSIVICLILCCCMAAALEDLLENQEPEPAPLRRPRRQRLTTDQVRELHVGIFDG
NKLVYDEENPTANGVGEYCCDHILQHSNDKNFPQPTINSMDTCTICLDDYQIGDKLCCLP
CGHVFHAECISKWLVERSATCPLCNLDLYEEEVDDNSEGEEDEYTNNERQARNGSAPTTQ
VEPDMNEDASTIDHSIGSWLRAIFRSPYWRNRTTGGSSGLEALAEPLLQQDQPEHPTSAG
IDVEATPSNEESSTGPTEID
>comp4859_c0_seq1; 328 212 823 LEN=1627; translated
MTKRSSLIKDLKKDCVPYFERDEIMPYLGDKLGKGGFNSVYELEKIELDETSPVNDDQRK
QRSFVTQNIDQKLLAVKFLNESAMSNSNEFCNGAADLLLEAKYLSAISNHPHPSIISLHG
VAAAGAAGFATGQMGGYFLVVDRLYDTLDKRIDIWKELKRRKLRHPSPSNVKLLQAMFLQ
RLHVATDICGAIRHLHSLKIVFR
>comp4859_c0_seq2; 383 212 1105 LEN=1407; translated
MTKRSSLIKDLKKDCVPYFERDEIMPYLGDKLGKGGFNSVYELEKIELDETSPVNDDQRK
QRSFVTQNIDQKLLAVKFLNESAMSNSNEFCNGAADLLLEAKYLSAISNHPHPSIISLHG
VAAAGAAGFATGQMGGYFLVVDRLYDTLDKRIDIWKELKRRKLRHPSPSNVKLLQAMFLQ
RLHVATDICGAIRHLHSLKIVFRDLKPDNVGFNFEGRVKLFDFGLAKELDPLQKTGDGMY
EMSGGTGSRRFMAPEVALGQPYNLSADLFIFCFVLGISGFRESLWEDEPRRASRESN
>comp4860_c0_seq1; 309 106 1621 LEN=1873; translated
MRKNDSSHQNSPLINKEGDENISVEERLQDWAFLPERDSSSADRKLLDNSWSDLYRMIHD
DKNSKKTILDYIYSSPHEVEAPIANGDTPFRLALRTGCDFDVISAMIEVAPNASKDKGYP
TPFMEALCPEPTICTDPMRVDVDMEVIETLIHFRPEAVRNPLALGYLCSKGNPSVTSLEL
VKLLISHCPNAVQTTNGATNRNMLPLHSACESGMSIDVVRYLLNLFPAASSTKCEPCNSM
ITSKGPYVLDVEARLPIELACVSKSPSVDVVKLLTERQTKNMNQMNTDGYDANFGKLLLL
IAWATRKSYLEHGKDGTVQRPNYKEIIEYLLPMTNSKERLRVLNAIITENAWDDAQSFEY
TKLLCDCTSVNGTMVLHAAVERGLINISKLICDISPSLARKRDRNGCLPLHIASTVEKHH
STQKTPMAEFLKSANRRKVVIQRILIAKYPRALVLKNHEGRIPCEMAGRYASGTVAINAK
SSILREDFSAFCTKKHDVYGVGKG
>comp4861_c0_seq1; 408 668 2234 LEN=2601; translated
MAASSSSPLSSFIFSGDNRHNTASYASQGTTTSSFSSVVSRSNVTGDEKFSVMKINTETG
AIRRIQMCQSEIMVQTTLSPRDLVSLRLTSKKERRENRVGKSLVEQMRHPPTIMARNKFI
LLSMGPVRAVAEPDAVYVFDVHSKAAKLFATDLAKVYEERSEAIKNKTLLNHEEPTELVF
LETVLTDAVDSFTGRIRIFEPIVNDLLLRISLDVEDVSNTNTIHQMAPLKEQLQSFEIFV
TQAYDCVTQLLNDDEAMLQLLLTEQEGGDIGAVQLSHPSSFICNGSQIVSPLVCCYHIAL
ISNVSEARRTNTTVDFERHQHVEHILGIYARRFSSVIQEVNYLLGRIESRQEYIELELDL
YRNRLIRMNVDLAILATATGVTTALSGTFGMNMVNGFEESHTAFLVTSATSALAALSVGH
FFRRMVSGNVIQQRAQERIGEIRTMSNALSDMSALDYAVKQMIRGENMNKTEFKEQLAGA
RHSKTCTDEEVDLLFRVLNTHKDDVLDKEDFVEKRDSGESP
>comp4861_c0_seq2; 443 668 2116 LEN=2483; translated
MAASSSSPLSSFIFSGDNRHNTASYASQGTTTSSFSSVVSRSNVTGDEKFSVMKINTETG
AIRRIQMCQSEIMVQTTLSPRDLVSLRLTSKKERRENRVGKSLVEQMRHPPTIMARNKFI
LLSMGPVRAVAEPDAVYVFDVHSKAAKLFATDLAKVYEERSEAIKNKTLLNHEEPTELVF
LETVLTDAVDSFTGRIRIFEPIVNDLLLRISLDVEDVSNTNTIHQMAPLKEQLQSFEIFV
TQAYDCVTQLLNDDEAMLQLLLTEQEEARRTNTTVDFERHQHVEHILGIYARRFSSVIQE
VNYLLGRIESRQEYIELELDLYRNRLIRMNVDLAILATATGVTTALSGTFGMNMVNGFEE
SHTAFLVTSATSALAALSVGHFFRRMVSGNVIQQRAQERIGEIRTMSNALSDMSALDYAV
KQMIRGENMNKTEFKEQLAGARHSKTCTDEEVDLLFRVLNTHKDDVLDKEDFVEKRDSGE
SP
>comp4862_c0_seq1; 407 185 1589 LEN=1589; translated

MASISRRKNSLVLGGVGLVAASIATVWALQKWRQSRRGNDKRSSTSTILTAHEQLIEGGT
PLIELKRVTKLIGRSVFVKMESMNPGGTGKDRAALNMIRMAENEGALPPPDAVPTNSTDY
DSSLFLRPSVPSGSSHGNGTSATAEHTHDTADVPTSAAENGLETMILQALERSRTGGLVV
EGTSGSTGIALATLCAARGHACLVVLPDDQAVEKQRILRSLGAVVYEVPTASISNPKHYV
NLGRKAAKLARDKFGISAVFVDQFENLSNFDIHYQQTGRELLRQLYDQNRSLDAFVMSSG
TGGTIAGIATYLKEQRIHNNNDGEVRVVLVDPPGSALYHKVEHGVAFAVEQRERYLRKHR
YDTLAEGIGLDRITRNFSSGLSCIDRSIRVTDQEAVDMAHWILRTEGLWIGSSSAMNLVG
AMKTALSLPEDSSVVTMICDGGQRHATRFWNPKFIKEWGLEWPSPDCLX
>comp4863_c0_seq1; 411 26 829 LEN=886; translated
MRLAPTLVLAARASSLLLSPPTTLPTARAFSAQHSNRSSSNRSSNHKRNMSTESDQDGKP
ARQLVVDPFCFRQFGEHPQSGSYGGTVFSSLTVAAFEEVANARFREEDLRDGYAPFCKHV
FLENDFTGDDARVNVLPVEGNEDKIRTEYAARNDKELPVLQRFIPLEAVGGAEELPRAKY
LDLILYSRDQIRKENESMGNENVEGDEEAPWGIVSIKAQMEDFELPMNPITQMRNALGKD
QGGSGIPLTREEYMKSVEYWTANVNVS
>comp4864_c0_seq1; 380 81 743 LEN=907; translated
MASDQIRQMVNFILQEAHEKANEIRVKTEHDFNLEKQTLVHEAKLSIQDDFAKKEKQHEI
QERIARSSEIGACRVKKMQLRDELLNKLLAEGGAKCAVVARGANYPQLLQKLIVQCLIKI
EENNTEIYCRTEDVDTVKGVLPDAIKEYVEIMKRESGVTLSPNVTMNADADKNLPESSHG
GIVMTALNGKIVCDNTMSARLELVYEELLPSIRSIMFPDE
>comp4864_c0_seq2; 259 444 961 LEN=1125; translated
MTXAKKEKQHEIQERIARSSEIGACRVKKMQLRDELLNKLLAEGGAKCAVVARGANYPQL
LQKLIVQCLIKIEENNTEIYCRTEDVDTVKGVLPDAIKEYVEIMKRESGVTLSPNVTMNA
DADKNLPESSHGGIVMTALNGKIVCDNTMSARLELVYEELLPSIRSIMFPDE
>comp4866_c0_seq1; 375 1 936 LEN=975; translated
RKAQSEKKDSHIKNLLLRGQSIILRKRGYIGMKPLSVQAPLFRSGLLIVVSVLSFCSKNG
LFVVESFTLKRDAARCKVDREQSLQAFEATWINARRKSTSLFQQRQQARRWNGNYKHKSK
KLSDVPKSKKLMLNLTANGVTKTLEKKRMETLQHVLTKAMIWKMFMNDYPNLEIELDIGD
PDYLPDVIGATSKGYDKASIVFWGESGRMKVHKALDLMRRYPNAHIVHCRWAIDIESFMG
PFVEYLETELFDEDTSWWKGKFSFASLPLEVWDFINEDTGTILIEKSDLSWRELDLSQLD
FNRTSLDEDNI
>comp4867_c0_seq1; 313 45 947 LEN=1195; translated
MRSSHLCVPIILLSAPRAVTGLQTNPHHQASRREILGGLVSACGAGLVVGGAPQPARSAA
AVQDSLDVDAFLRKGVDTGGTMGVSSQAGKSRPETGVFLRDGSDVSRDKKTGDVSAEIVV
NGSNGEKMAVFVSYSSPWPLAEGPFYDIECRNQKTSDGAFVQVTTDVGGKTVANIDNAFI
LENLLAPSGRFSFYGQPTDVKIKKSTTVGDYRIIDLSFATLSQATQTEIPRKAQLVATIP
KGSSQAIVLVGSAPAARWNKGSDQQVFSTIESFRAIAAPKSSLKLRRKERNTFSIDENGE
>comp4867_c0_seq2; 275 45 1105 LEN=1353; translated
MRSSHLCVPIILLSAPRAVTGLQTNPHHQASRREILGGLVSACGAGLVVGGAPQPARSAA
AVQDSLDVDAFLRKGVDTGGTMGVSSQAGKSRPETGVFLRDGSDVSRDKKTGDVSAEIVV
NGSNGEKMAVFVSYSSPWPLGKNERECAAWSCKLNDNICYAPLPLYDPERAFIXESKEVS
QFSPSSTCFSTHPAEGPFYDIECRNQKTSDGAFVQVTTDVGGKTVANIDNAFILENLLAP
SGRFSFYGQPTDVKIKKSTTVGDYRIIDLSFATLSQATQTEIPRKAQLVATIPKGSSQAI
VLVGSAPAARWNKGSDQQVFSTIESFRAIAAPKSSLKLRRKERNTFSIDENGE
>comp4868_c0_seq1; 12 1 212 LEN=212; translated
XQNSDAPPITATVKLATPKRKKSRRPRPQEREQEHRYMEDTTNNNIRVGSGRNTNREPHI
GTQQCQPQPQP
>comp4868_c1_seq1; 105 229 495 LEN=495; translated
MTKNKNAKRQCPSCGITTHKKGGLLGKKSIPITVEGKVSNGRCLACDPFPPCLTPAGQAS
VAIEEVCQSPVRQAPLNNNSDNRSGGGGE
>comp4869_c0_seq1; 1075 266 3208 LEN=3963; translated
MSSEDEFGDSSFLDDFDVDAAVAAGSATPEKRPHRKSGSDGFGDTSFLDDFDVDAAVAAR
GGLPANKKRPRVTPEPHQGQRHCRHPSTAFPTEELEACAKHYYGYSSYKSGQLDAIRAVL
RGQDATVFWATGSGKSLCYQIPALYSKKTAVVISPLISLMKDQVLRLNGLSSEALATYLG
SAQTDGAEEQRALRGDYRLVYITPEKLMTGGFLDRLAALDLCCVAVDEAHCVSQWGHDFR
KDYLHAGEALRNHPRLRTVPIIALTATAVPRIQNDIAKNLRLRSPHVNKQSFDRTNLSIR
VTLKERNHPLPSAMEPLIRSLLREEKGSGGASSTIVYAATRNATEEVASYLQQRLCNKNS
AVDVRAYHAGMTTDQRNETHVRFLTGKTAVVVATVAFGMGIDKPDTRRVIHWGPPKSVEE
YYQQIGRAGRDGFPAECVLYASPSEFDKYMDDFYIGDLDGPARQASIQSTRALKSFALDK
EGCRRKGLLDFFGETPAFGERCGTCDTCLAAKQFGDDALRDFGPEARLVLSAVSGLREAS
IGTIEKVLKGNIVEGYRYAYRTSPEALQTMMTETRKALPRARASIQFLREMVSTLSQKGY

LTEATKSANLGGGNYKRSWTVYNINETGRRCLGGTEKVVLPVPESIREAERQEKARKDRV
LKNLEEKGVSLDKLPSEELETGDGEVIRAYTKWNNYVALQEKLGKEGRNAELEELLSLVQ
EWRSNAAVEHTMAPASVLPEHIMLSISYAVATYPAGVKVSRSDLIAAGARTRELESLADI
LNSWIDRYRAADNPSGSSSASDQAKDVPMRFASGGPTQGKKWEFAVYKPQKKTGMATWES
SYERFAAGESPQAISMSPANGRPIQVMTVVGHIQDAFLHGRPVDLQRLSSISKPPTKNQW
MELEQAKESTGMDPVGNPSCSGLDGGSFTLTEFLRPVMGDDFMETPREDRSESDKARFGE
WCGVLKWYLFMKRAGVEPVF
>comp4870_c0_seq1; 248 74 676 LEN=1036; translated
MKVLSLATVRTGNELEDPIVCSMSSELSSFGFFQRPTIKEMLGFLTRTFMKRTEPGQRQS
ITHENYVVHCYRRSDGLGGTVTTDLEYPSRVAFVLLTQLLDDFTAAVGDTWKTCTTPESM
AFPAMEEYLQKYQDPSAADKITKIQNDLDETTQILHKTIDSVLERGVKLDNLVERSNDLS
AQSKMFYKQAKKTNSCCVIS
>comp4872_c0_seq1; 815 110 2689 LEN=2914; translated
MGGGQRSSIVAESSASEDENNVLIIRISKRFIILTFVVSCLVAFAVGHVSRIVLIDNPRN
EFMKAYQREHVSIDGAMGTKPFGSKDEGQFTLPDPVLQSGKTPPITTYTSKNFNTALSST
THSRWMVTQDGEDLDNGLLENESNGDQCRSSTNDSCVEDEQTEEEEHLPAGQHLLIDIEH
VDALFLNSEDRLATAMLDLVNECGLTLLSYHCHGLHPSGVSCAGVLLESHVSFHTWPEEG
VITLDLFTCGPNSLLPIVPLAEKLFAVASDALRAMEPKMIWAHKFRGFGRENGSASEVTD
MFRFPVGQMNDFKKEIASIQTDYQTMDVYDVFRPGRHDMESYEKSLSNDGSYEALHPELF
EPDRLVYLDGVVQSCKTGDAAYHEALVHPAMFAHENPKRVAIVGGGEGATLREVLKHNTV
EKVIMIDIDEQMVELSKKVLPYWSDCSMLQDSTKSCFDDPRVETYFLDAFKWFIDRFSDE
KSDDFDELPFDVIIMDALDPQIQKEFVDALYDGGPFLRSLPNALRESGILVAQVGEANSI
QSPSGEHSIDRNRINFIETLVSLGFPTIRDYEESHSQFDDPWEFVAAFKDFDYKVDWYAE
SPVIDLKIRERGMITTAGGSPFLHFDGATMKSYRYPSKGSETAFCRLKPNEVDCANGHGF
DLERDDVMVSSLEIKPSSLGVKAGRGVFAKTDIPKDSYVGLKEIGSSVHINPHVFEVMKE
MKEIPWAYSNYHGKELEAYTNLYGHFFVSQGETEVFIDSTIQCMINHGCMGTSNIGYNIN
VTEASADPLSMSEEVTSSYIGRQYIYNPAKERQVRFYSSSTPRRDIKAGEELLGNYLGMA
GADNPHWKDVVLALRDHCV
>comp4873_c0_seq1; 433 235 873 LEN=1069; translated
MTYEEMIQRVDCYEITVPKPLGVIFGENPDPYLGLVVDDVSEGMNGGKAGLRMGDQLLAV
NEQVVIGRDFDTVMGKLQEQPGRLNLVLYRGPVGQLFTVLSNQLGEGQNMYEDEDEYEES
EEVMMDENYESPVRIEVKEEKPLTPGDFVKAFGKLGSMLGETLTAPVEQEDTGAPPPPKK
KTGFFGIGGESVQLDGEDARGYRREKIEPDDF
>comp4874_c0_seq1; 640 836 1762 LEN=2325; translated
MTYYSDTEIISSDDDDVSISDCSSSFHFSDNVVLNGLDNNCNNCYNNTKGKYEEANRASF
PLLTNHSQRTQQSFFSLKDLVSMGTVSSLSDNETPDDDSDGGDTKDTNTDGEEEEKQYFN
NDDTERRIEITFASSNISMDQRPQEQRQLRIITGRRFINEDTILSDGEEDLCELTEEVMG
NNDEEELTIKESETEMEERFDDDLEEEEEYRVLLNDVDDETGTRKRDVRGSKHRNDEYSI
ESVDDETVEEEEIIEIEEVILDDNEQSEEEIEEVIINNNEHGEEVFICHNGEAGKEAIEE
VALEQYEL
>comp4875_c0_seq1; 479 209 1231 LEN=1942; translated
MICVPTARLLFFCAFCFDRAVSFTPKAIWAHQTRKPCQQFAYRDSNEEDSFESLVEEATF
GATGLYGGDFAGLAATFNPGDGSFIPIPDYLIPEDLKEWGQEPKCLQVLVSEDVHDDGSG
ESCLMSRVTTKILPETGCGVDNMETMIASDEIDLESQWKSDDDNDGLEDVVGLQYPLGEE
KLRVETIFGLEEEYGVDRIRVAIDLIPSEENFAIVSPMVLTLERRTSSISSGGTIAEGGG
LDGRTVYMLLGERLRSETTFVDEPSMSNENYYDTGNDIRYVGLPANISIAFGWLNEGRDT
WMLQVSHITSNGSRRVVSRQFTVASNNELDFEIRSWVEDL
>comp4877_c0_seq1; 50 125 482 LEN=482; translated
MRHHKIQAAVRRILRLSFLLGLAFAGTTAAFSAAPPRAPARSGARPSPARLCSTRSGRVA
EESFLMKPFQTASGETVNPYRVLKVPRSAERKEIREAYRRISKRYHPDIVRHKDVLPGNX
>comp4877_c0_seq2; 77 215 662 LEN=731; translated
PVTHHTTSHQIAPRPNSNSHDDVEAEWERIQLSYDILSERKRRMQYDRHELIADPGAAVQ
RAAFEAAAAGVVGLGKGIFRAGSSALDLLGGAGKRDAGGERWGRWRRVALNRIESSKTEP
NGMVVGDAAGGETEWSSCDGPVDSYCKQ
>comp4877_c0_seq3; 154 566 1112 LEN=1181; translated
MLPFASRNPYRVLKVPRSAERKEIREAYRRISKRYHPDIVRHKDVLPGNCNSHDDVEAEW
ERIQLSYDILSERKRRMQYDRHELIADPGAAVQRAAFEAAAAGVVGLGKGIFRAGSSALD
LLGGAGKRDAGGERWGRWRRVALNRIESSKTEPNGMVVGDAAGGETEWSSCDGPVDSYCK
Q
>comp4877_c0_seq4; 77 344 791 LEN=860; translated

PVTHHTTSHQIAPRPNSNSHDDVEAEWERIQLSYDILSERKRRMQYDRHELIADPGAAVQ
RAAFEAAAAGVVGLGKGIFRAGSSALDLLGGAGKRDAGGERWGRWRRVALNRIESSKTEP
NGMVVGDAAGGETEWSSCDGPVDSYCKQ
>comp4878_c0_seq1; 164 1 546 LEN=781; translated
QGLKQGTARDRGDTKQRYNTPPSPSPRDDKTTMVWGAIKNAIGLGNPLDSLPEGPTRLPD
YFPLEPKGCEKPSQKLFKCLSREATEKARDMERVGFHKSHFPDVVVGPLDPKAAEAVAND
PENPEFPKPGDNPLDECRLFIANYMKCCDRNLKQKRNRLLQETVRVQEEYRYQGPTASAE
E
>comp4880_c0_seq1; 374 163 1416 LEN=1587; translated
MRNVARCPPNRPRGISRAVLGLAFVAWVAAAVSADAPPGRGPCAFNEGEDREDLYGSHPS
LCAMLDRLEEDPESVASELASFARAHGRLVGETIGDPGAPLSEHERDPAQLPVVFAHGMG
DSCFNDGMVRIGEHTAGLLGGVYVTCVPTGDTKTEDTTNGYFLNMDASVDAFAARIAADP
ELRGGFHAVGFSQGNNVIRGYVARYNTPTVHTFLSVNGVNAGEGTVPGCFPSGGAAAATL
GSVCDLLLEQASRRAYTEFAQKHSFQSNYWRDPRASEREAYQKFSQLARWNNEGPELHNA
TLNENFARTSRFVWVRATKDSMVWPSEGEHWGSPDPDDPFGKPVLPMNETEWYTEDLFGL
RTAQEAGKNVFETFEGDHLRFSMEDFDRWVRTYLVGGGETERAGADAIAEAATAATE
>comp4881_c0_seq1; 539 1 1438 LEN=1959; translated
XSVWKAEGFHASQQQLQWHRSPLLQRAPVEGAAFVRGGAERSATVEEAAETETETLPTWE
DLQSALEDLRSGAPEEKPVLTLYRDTNGWCPFCERVWVAVRAKGLPYQETLVPLQGKPEW
YKQMVPTGLVPAVLFHGETEDKTDEGGSEPTSRRELVWESDAILQALDDKFPDTVRLTRK
GDEDYEKALDLQNRVQAEGFRFAYGNRNGTATEAELEEIRSGFEAVLDELDEALGAQTGG
NFRLGADFSGIDAIMVPTLERWRYQLPLTEAFDILEGRPHLREWFEAMDAFPAYSGRVAG
DEYSWTATASMFLRYFGGGEDKPKVAEGIARADAAAEDLSGGFADGLGSIATPREAALEA
AAKLVSNHDAVLRDCLGAVEPKSQGHIPRADEEKGPTVDALLRHVASLLLDAARGGERAT
PLAPPSIRGGRSTGEKTQGALALRTVAKRLCVPRDMGAPSAALLRGILATVADDLSPVE
>comp4881_c0_seq2; 625 67 1461 LEN=1982; translated
MLFLSDAFLDFVACNLLFVCLFVITNQWIRLPKPIRFHSFLGGSHFILTPSSTPRWFALL
SRTKKLKPSDTNGWCPFCERVWVAVRAKGLPYQETLVPLQGKPEWYKQMVPTGLVPAVLF
HGETEDKTDEGGSEPTSRRELVWESDAILQALDDKFPDTVRLTRKGDEDYEKALDLQNRV
QAEGFRFAYGNRNGTATEAELEEIRSGFEAVLDELDEALGAQTGGNFRLGADFSGIDAIM
VPTLERWRYQLPLTEAFDILEGRPHLREWFEAMDAFPAYSGRVAGDEYSWTATASMFLRY
FGGGEDKPKVAEGIARADAAAEDLSGGFADGLGSIATPREAALEAAAKLVSNHDAVLRDC
LGAVEPKSQGHIPRADEEKGPTVDALLRHVASLLLDAARGGERATPLAPPSIRGGRSTGE
KTQGALALRTVAKRLCVPRDMGAPSAALLRGILATVADDLSPVE
>comp4882_c0_seq1; 879 86 2443 LEN=2497; translated
MFAGKFLKQTGSLFSSRGSTDTSAVDERELHDLEGRELQKKTNSREIRSNSSASDYSKEY
FEAPSMSIDSDIDRLEDLLQMGCMDALFLPQDPKPAAKKSLVKVNSQGHRELNLNVKGAL
ALGLEAQMREDAKNRKEGNHSFYLEDGTKVPEFHKNDHFRSLMSLSHESASSGDFDDGRS
DDSDIRNANFNQPFQLPPHPMQQPHPASWQHQHQHQHYNPHMFHDNQNDYYQQQQYHQMP
MMMAQPNSPYAMVAHGISPPFHQQAPPQERKSGIMEVKSEVSAPGRMPLQIVLENTSSYE
EDSCLSYENAAAPDPPQSSLERIPEDDYSSCASSEKESDTSIVACGSMKSDNGKNSRGSH
NRASKGDVPKEINPNEILPKEIVPKSGKRLTNLERWKKANIQRLYLKKNQMALTGIGSPE
ESPAAQQSRRGEVNLPVVNKDKPEDTVSVGSDEAAQSTVSGEEAVEEASEEATQPVERSG
SDNTTDTKTTTEVSNHRSAHTVSTDDHAKPFDEPETVSEAMTFDSILSRRIEKRRATKLK
MGTRSDLASLDHDHHSMESSGWDRDDTENHLPSHGHHDSSKPRKQERRKNPFEDDDFLCS
DDGEDEDEDEDEDYHPLLTKSSSDDDGIDIPSPRHYSKPSRPMPLPRAHQGSSPRYTKTT
VASPQRAVLSPRSNGGNRSGGGFSPSSPRSSYHQKSSPRVSGRVAAAIQKFEKKTPTAKH
QPQGYQPPQKQRSVRANEWHGSIENVSEKMASASIGGKVPQYSPDPVGYYGIDEVCSYDT
PFDES
>comp4883_c0_seq1; 1028 203 1969 LEN=1999; translated
MRFSTCAVLLLVALVAGVDNIAAAKKAPRSASSRRSSSSGRPSRRSPSSSRRPPVSKRRA
EIYEDDEDDYDVGFDDPFDDEEDEYEDAVDDAESEEEEEEYRPKRKSSGSSSRRPPPRKS
ASRRSSKYDDEDDYPPPRRQSGRRPPPGRRPGPPPRGNRRPGRGGRVVPYTNRRQQPSTF
TRGLSAIRNSMPDASSVKEAAMKSLSVARETTSGLSANLYREVKGLTSSELEQVMLKATR
PDDTPVKGKHAERLVGVTYQISARYDIYDAVLRKLWAKMAEKDWRTTIKALYILHRFSAD
GSPEHAPALKARVRELRRTRDPKRKGKFFSSKLLLAGDTSEETAKYRAFLGRYAHYVLLR
AQCFGGMFDEIAAPSAKDTKKKKAAPKPITSTSLKADHLDAAKLLLETGLACVLKKGEEN
ENTALAVERVASDLLGLTTAVTGALTRALKDDDLKGADPVLIKKWCEFYSESLLPQTRSM
VKKTAPTLDAYGLFLPSRMGPTLSQDLLQKGLKLDESSSAGDAEESETSKKEASSDEEKE
SDHADEEAAGESDAPLEEDEDEVEEELDDMDEYEYDEEEEYYDDEDEL

>comp4885_c0_seq1; 256 1 995 LEN=1031; translated
XPVLPSLSDRLGARVALGHDAVGFGFTDRPKDPFWYTAGQSSRIARAVLAKETQGTVEPK
EPVCLVGHSMGSLSVLRLALLLPEETPKLVVLSSPALGLLKRRAEPKTPPAARSILRAIG
AGLAKKMLLPPSRYLLRRAIGAEGAWKKGLEAAWGDPSALKEDSDVLRYSWPSIGAGWED
GILRFVRAQALPAEDDLEDDAVLLEKVLELPSTRVLVVLGERDRVVQGSALRAFLESVEK
APRAQARFSPVVVELDRLGHNAFEEDREVFCDLLERLVEDHWDGVVADDDYDGWQDGIES
NPIHTIRGRVATQYCYPAASKSRTVVRAQYF
>comp4886_c0_seq1; 513 106 1938 LEN=2938; translated
MKGNRRGRVCNGVALRWMRDPQMRRRIILFTIAASGILYCIVDRIQMTSLLLSPLDDTYS
NSYYLRSSLPSSTTDIHRSTLSGSAEPEHQNNVLCDFYLAESAIPRGGLGVFTTLPLNKG
DQAQGWPDICIYLTDARQSKGTEIDTHTWQSFRFGAQWLGGRREVRAACMGLVTIFNSMG
MKEYASARPDAKAGLIHTNGGLDRSRDPGAGAITHYYGASSVAIRDLLPGSELMLWDSGH
GGDQYALDEVKNDGRSELGIELVPPPIRSPEWLQKHGMCADNIQVKLATDPSMGRGAFAK
RFLEKGSVIAPAPLQMYPNRSKFMAPTDQERKRREKALDIKYENDQLLVNYAFQPKGSTL
LLYPYGPGVGLINHANKKSGKINAVLRWSNHHMNHGTQWLDSSFSLDQFWKMQYPGSLIL
DFVALRDILEGEEIFIDYGTEWEEAWKQHVKNWKPYVSSKNHYVYPADEMSRHRDPIKPY
RTELEQETKPYADNLMMVCNTDISDNRKARRKWKPTKFWAENLAECSVLTRSYDESLKTF
LYQVQLWPDEEETDSEDGKQIKYVDFDVPHSAIRFVDKPFQSDQHIPNAFRHPIGFPEEL
TPSFWKNDSD
>comp4887_c0_seq1; 431 82 1308 LEN=1490; translated
MTSSSKKSSRQLTSAILVALAISSNPQFADAFVPPTNSVTTSESRSTPALLAGFGSSASP
SPKNKKKGKRGKTKNTSETPDQLKQRWANKMKKVYGGTSKEAIAAGTEKRIAHLMKNSLD
ESVQKALELREIVQMFERRITGMGPEDIRQRYSQEELETAIQNRRTFEEHMETNDLTEED
LQVAMQKLTWDASADAKACRSVTGNMPATFKNRVTKAAQVAYEAATDKESLVLDVGCGYG
VLVPFLKKAGFKPSQIHGVDLSGEMIGHAKSFYPDVASKGSFEARDFFGDTEDDQKNKYS
SVLFCSALHDLPDPKRALLKARDDLLDGKPGSRLVIVHAQGAFHVLNQNKQNPLLVPRGL
PTSAELKEWLCEEGEDSGKSSMKLVHEPAEPKSQQEVREGYLAVLETV
>comp4888_c0_seq1; 326 329 1549 LEN=1846; translated
MATESLFDPQKQFLSKPLRQRMQTKTTFPCFIVVIVVAIAASNLSLSRALSNQRCIHRSR
RSVVSSFSTVGFHPAIGSMKAEHANGVKTFRSGVQLYASVKSSNKKTRKRKQKRGSKKVS
GEEKKRGSPAPAPGHSVSEDELHQHLDAQFNYGNSSPVGPRSRLRGIIEKDGGEKDGIED
ENADTKMREQQFFLRQLNNRPTLVLNANYLPLGYMPLSLWSWQDAVKAIFKGSVTVVDVY
PDITVRASNIKVPLPSVIALNDXRPPEADQARVYAQERLSEGRVHMPVLREPVPHDGPLP
RPRDPEIDGRPAHVGEHRDLLQEMQRQEVEHTAGRTGFDRDEAEPASDLSHPGGTGGEVG
PDDPQAGPSDVEALPGHPGTAAPSSASGASGTKGDEEFIDDRYFEE
>comp4888_c0_seq2; 298 329 1669 LEN=1966; translated
MATESLFDPQKQFLSKPLRQRMQTKTTFPCFIVVIVVAIAASNLSLSRALSNQRCIHRSR
RSVVSSFSTVGFHPAIGSMKAEHANGVKTFRSGVQLYASVKSSNKKTRKRKQKRGSKKVS
GEEKKRGSPAPAPGHSVSEDELHQHLDAQFNYGNSSPVGPRSRLRGIIEKDGGEKDGIED
ENADTKMREQQFXSEAIEQSSNVGIERKLPGKFATNHCGAFACWRISMVSGKVLPSPRLQ
SKPLSPVFSIATGLYATESLVVAGCRQGDFQGQRHRGRCLSRHYRSGIKHQGAPAVRHCP
ERFRPPEADQARVYAQERLSEGRVHMPVLREPVPHDGPLPRPRDPEIDGRPAHVGEHRDL
LQEMQRQEVEHTAGRTGFDRDEAEPASDLSHPGGTGGEVGPDDPQAGPSDVEALPGHPGT
AAPSSASGASGTKGDEEFIDDRYFEE
>comp4889_c0_seq1; 943 259 2715 LEN=3004; translated
MRIFAAALLLLNTDGNSVGAQMMGGGYGARREEKHHRGEYHEEPEHILTKEEIGDRDRFW
KRNLVEESIVHEGVNHYHNIKNCIQERLTEKEFSELKTVNNHSVRSIGRTIDDPNNHGIP
MLVTVENAMHPTHVRAVQTLASCIRSFLPHLYESRAMYQEMNLDEDDGSGGNCPTHLAPL
VGVFLPEVDREMAATLEVAYRGTPGWIATVKEDQADPHMPRDKKLYPPAEVGVRASEHLT
YSDFPRLAEHHDGDTCYTMNFAFSAPGDYEGGEFFILSYDDDGENEFRDTIKPNQYDALV
FLGGRYLHGVNEIHGGHREMFSTELWAYPDTPFGSSLWSNFPNNMEDYVERCNKEQAKTG
SDTCTLPYDLITPFADSREEVRKKYEGGGDDDEDDDDEDDAKQYDYDRSERDGHGDPLPP
PLPGIDRQHDNKIRPNRVRPNSLRATRVVFTDGYGKEMELDDFYPHEDEPDFLVPKKLGV
AEMEPIRWREDLSPVDGPDGESFVIGFPPELHEEFKKYVARSGMMRIAHQILYQEEELEP
DEQRIYTLDDGEKWTAMVQGSWDTDMVWLDPGDESCFESLLGVLRRGGFDKVLDKVGKTF
DLNGLMIQGVGAIFLSEYTKTENMHVDIPGSRGSFYNIIVPVHIPADDVAIFKISDKGDD
FQGRIKLDPNVGVVLGGESRHGTGECNYRKREDFRLSFAVYVADINDENIEMIAGDSTSL
WPTQGDTFFFESQKGRLWTRDGKNSLARDRGRAPMNIQDSLPECATMDRDLCTRDVQGMR
LACPKTCELYLEDDVYYSRYFPNRTAEPPTCDNPACDA
>comp4890_c0_seq1; 544 1 1736 LEN=1919; translated

XGDTPTDVVKESIEGSREVVDQIYKAEHENLKTHMAHMENDETNYVDEDSDDEAPDLIAI
APDRVEKRPDGGGDGDAPASNSPDGSNGVETPPPCPVTILSGFLGSGKTTLIQYILKSPD
HGRRIAVIENEFGEGLAIESLIARDGIDPGSGSLQDLIELPNGCVCCTVKDSLVETLENL
IAKRSDLDYILIEASGMADPGPIASVFWLDDALESRLKLDGIVTLVDGRHILDQLEATEE
AARQIAYADRILLNKVDLLEGNAPGESVTASSVRSRSVSEVEQVLRKIHPTAPILRTSFS
KVPNLDWILDADCFSGFDHLEEVEHGLAHASSSAGKTHGNSERGHGHDDHNHHHEHGDGH
DCSECCEHDGTDHHTEHHHHHADHHEHTHTNSISTVAFEQPGTVDVARLNAWLADILWPN
QDETDKVLTAMLHRQEEDGGKHDEGRGDPPMGNTRNGRHESGSKEGTQQQIFRIKGILSV
EASDDYFWDESGAGDVCDSEAGGRGSSSSSTTTTTLVLDPRRFIVQAVHDLWDIQPVTSA
NLHWNTGEERVGKIVVIGKSLREADLRAGFEACFLQDA
>comp4891_c0_seq1; 167 82 444 LEN=1335; translated
MPAPTAHRHMSAAHASTEGNSNSGNHRQNHHHRRHLQSHQSAANSQARLEQLFDEQDHKR
CSEGNRKALLAENLDQLRGLAKVLEADDWKYHSNNTNSNNTPFPWSTMLSSAGGGSNHA
>comp4892_c0_seq1; 123 1 869 LEN=1011; translated
XSHSNSHSSSQFRSNSHLRSNSFPSIHDQDRRWVPALPGPGKTHHLSQLPPLRVRDGVRD
GVRVRLWALPAPAPPLSVPRCWVPEANAAAEAETEAGAAPTXTIVRWSSGPIGSGVSFPD
PPERNAYDAWLSGGPLPESLHLDEETAGEVLLELVGSFYGSSVFGCHERAASIGITGSLS
LEEVCGPEVVLGLEGSFWHKRSTVLGRAAVWLNARIPEVASVVVSDLEDLEDFEEIRDEE
SGEVLFRRDKRSEDFNGDRATMEYQGLDPDDRGPFPQSAMGGGGSMINPA
>comp4893_c0_seq1; 169 1 839 LEN=973; translated
XENEFLCLNEKGTAVQGKCFLSHSKEIDRAMGFPTDTFYEAFPWARKVHTLLNQWTSHIP
PPPLPDPGTKRKILLITAHPLPESFSMAIAGTVEDTARKEGHEVQRIDLGLERFSPSLTG
DERAGYFLDRDQETRKTLPHQIRGYIRKLRWCDTLFVVYPTWWMGTPAVLKGFFDRTLRL
HHTWGFPEAASPASILPAGLAPGLTNIRRVVGISTYGASRAAVLAAGDNGRSMISRPITA
VFHPDCTVLWHGLYDMDFTTLPQRQAFLREVEATVRDRV
>comp4895_c0_seq1; 503 186 1715 LEN=1910; translated
MTANAEARALLDQLMGVERDALLPAGAALPRKNKRPLGDGGMILPGAKRSKSCYDRDIDP
LYCAWGVDIYELFVNTKSDIGPNPYTADEAARKEYVSLPREEKDRLGYESRLFVKLQELV
QHCDRTVNRNHEKLKRELQRQAQKRGGNDYVVDVSEEKVEELVRAECQLEDMKEDLVKAL
KILDEVQIKEEAILDEQRKELMLKKAEKPNETNGETTTVKAEEGGDEPEKAVTKEQETPD
AAVKTEDEQGANPTIVVDRQGVESSDSTNNEAIKTNSDGKLEVQKDDGDRKELDKNDANG
DPSGSATLEELGKLALEKQSLLCQIATIISQLGPQEESIEILHAKLNFVRSDTTTDKTVC
EVSGNFMSARDADERIAAHYAGKQYVGWKLVRDKYQEMVKKYGRYGPPRPGGRGGGAPLM
SHQKYQQMQQGGGHGDRDRYRGRGDDRRGGSSYGRDGGRWERGGYGDRGGGYRGDSRGGY
DDRRRGSGYGDRDRDRGGYRGGGGYGSRR
>comp4895_c0_seq2; 485 539 1945 LEN=2140; translated
MILPGAKRSKSCYDRDIDPLYCAWGVDIYELFVNTKSDIGPNPYTADEAARKEYVSLPRE
EKDRLGYESRLFVKLQELVQHCDRTVNRNHEKLKRELQRQAQKRGGNDYVVDVSEEKVEE
LVRAECQLEDMKEDLVKALKILDEVQIKEEAILDEQRKELMLKKAEKPNETNGETTTVKA
EEGGDEPEKAVTKEQETPDAAVKTEDEQGANPTIVVDRQGVESSDSTNNEAIKTNSDGKL
EVQKDDGDRKELDKNDANGDPSGSATLEELGKLALEKQSLLCQIATIISQLGPQEESIEI
LHAKLNFVRSDTTTDKTVCEVSGNFMSARDADERIAAHYAGKQYVGWKLVRDKYQEMVKK
YGRYGPPRPGGRGGGAPLMSHQKYQQMQQGGGHGDRDRYRGRGDDRRGGSSYGRDGGRWE
RGGYGDRGGGYRGDSRGGYDDRRRGSGYGDRDRDRGGYRGGGGYGSRR
>comp4897_c0_seq1; 1103 175 2520 LEN=2817; translated
MMALDCGSLEFLSPFPLQPVSVDNSDFDELPVEERLEITRRNLQEVQANLRIKNSEYEDL
QQEMKFHAIKNEELMDVVNAFRSSSVDRSRDIMKAKAEQNSELTLQNHALRDLLTKSGEE
IAALQQEIREKTAEGESLSSQKRSHERLQAQLNDLVKTLEKVEVSNVDIPSEWINFKWIG
IGGKKGDASDTYEHSKAIELISGKITAMNADRERLLKESKLYSQSDGAKEARIASLERQL
RQTEHERDELKETTDALHQRMAVREGKIGALEELFQNINSNRTLEARTRRKSKVSMDRSQ
RSLLEDDDDDEDYDCVDIDSTAGDKSVAQTFEEMFVNIWTSVTGGSRGEDDNDDDSSRGN
SSQASTCLSTNSYHTKQIEDELQAAARQEFEELQESHQLLNEDYESAQFKISDLSARLEE
TTIKANSFEKKAGLREVMLKDVIQQYKELQLEHSSSLDRVEQLKQKVKVFVQREQEREQE
RIASEAVTANGNVKGSSTVLLVEETPTFDMSERTERTSNDETVGEDSPSSPSHGLEKNFA
MEDYERLETECDRLQHEFDVAIEKINELEKALETAKAEVRDSQATQVEQARKIALLEGEK
TDLQERLIEASAKIMDSQSTHTQRTVQVEDELRLAELREQEARAKQVQREKDLWEVIEQY
KKLADEKESTEGKETEAEDQMVLTDKVKIQKRDLVYEYRKMEKALEEAVATEQQLASDME
NAKNEAAKCREETKAIRKRLAGCHSHYKQLREEYTLLQQENEELDYRLSEAQTNGRVESL
S
>comp4898_c1_seq1; 521 10 939 LEN=1029; translated

MTNILLVLLSSFVCAAAFSELPVLPSPSPSVAGRSPARYRMRNPTLSAGPKPDLEELEPG
VSDSNFGRMAYWDESYAPQSSESGPSEEPNVFSWYCGWEDELGPFFEELVPDKEALVLVP
GIGNDVAIRDMFDVGGYQRLMAFDYAPQGVECARAMFGEDRLRTISERLGRGRQPPQTED
TDGNGVLRVCDARDLSSEYGDNTFDAVLDKGTFDSIYLSGGKDKEKARANLSMAVAEMKR
VVKEDTGIIFSVTAACVDAVREAFDSDEDWHQLRDGSMHITEDGYASNNVDATMLAWQLR
RGSTGAAAT
>comp4899_c0_seq1; 251 231 1193 LEN=1758; translated
MGTSKDVKSKNKDTGSSKTTSFSAAAKFLLIPTTATFILSAMGFSYTIAALPYVVIKSRM
LNADKGESTAQFWTKFLIMEVFYCAMLSAFAFVVLLPMFGIQFTGFHTLAFYLPMFTITL
HDRRHQYGPFGTFVVQTFFDVPLVVAGYICRELFGLRLLAPWADILDDTIVQGSLPFPSD
IAVLAAEPYNVGLIINMCREYGGAKDEMKRCGIVQCYLPHQDTTAPSYESLVKGCAHIKQ
FKKHNPEKRVYVHCKGGIARASTMSLAHYVSNEDQDPSVAIRAMKTKRPVVFAGVKDFPA
IIKLNDERLSKEQQEPKKAK
>comp4900_c0_seq1; 427 370 1605 LEN=1947; translated
MVIQAQTGSGKTLTYLLPLLSKLENRASVQAMIVVPTRELGLQVATVAKRLVARRFMVMS
LLQGSQLKRQRAWAWAETPQVVIGTPTELLDMVQYAGLPRISSIKTVVVDEVDACLVHQS
GTSLQGSNIKGNSDARTLSSSLHVLLSKFLSPTYADDGDIDEDDVIVIADDSNKKRKRKF
RHRQTIFCSATIPQHRYFLKQCKSNQWTLEEPVYVCTSPGEALPPTLKHAYMVCQSKTKK
MGTLRNLLKKLNSPTNKVMIFVDSTRPLDSIGKNLSDNLDEGILYEDRKTILDDIESKPV
VSILHYDDSLSQRAGATKAFTEGSDFRVMVTTDLAARGLDILGVSHIIHFDLPPDADTYL
HRSGRAGRLGKKGQVISILSDDQEFVLKRLMNALNVDAKCVGRQKAKKNSS
>comp4901_c0_seq1; 1079 266 2884 LEN=3156; translated
MARWFRSEPMEYVSLILNEDAAHDCLADLGKLGVLQFTDLNPELTPFQRRYVSYVKRCDE
LERILRYIHGEIDRFEDLELAPAGSVDSFLEESVGTQKTDRSGNSKLDVLETDLGGYERQ
LRELGLYSEKLTHQYNEKVELQQVLEKARDFFRFDRNESTTTPRLDIMRFQSIAGVISTE
EKVRFERMIFRTTRGNSLVSFAPIEEPITDPETGKEADKSVFLVFFKSKAIEVKLKRICD
AFMAHRYTLPDMDDLATVEKLLEENARELEDSETVLKKNRDTRYKLCKELAAKCEKWTWI
VLKEKAIYHSLNMLKADVSGMLRGEGWVVSENLDDVARAVDRAHSSLDMAMPSLVKPVDQ
PWPTPPTHFATNKFTYAYQEFVNTYGIPRYREANPALFTAATFPFLFGVMYGDIGHGLFL
FLAGCSLVWNEQAVENGPKPDELTAGLHTGRYMMLMMGFFAVYAGFVYNDCFSLGMNLFG
TRYSFDGMDDGTTEEGDVAVLNAPYGSDESVYPFGLDPIWHVASNELLFFNSFKMKLSVI
LGIIQMSFGILLKGSNAVYFKQPLDLFLEVGPMVVFATSLFLYMVVMIFMKWSIDWNHRM
SLATCYDPTGTEWQSDWTVCDGGTQDANGLCTPWGHSCTDSDTTADKCPLDYGGSGDGCQ
PPSLITSLINIALAPGSVDEPLYAGQAALQNLLLLVAVVSVPVLLLAKPIVLHQRAQEAH
AKEVVHAVDGCGGDEEEHGHGGDDHGESDEEHGFGEIIIHQAIETIEFVLGMVSNTASYL
RLWALSLAHSELAQVFWQKCLLTTLPLGWLPTYLGFGLFAAITLGVLLMMDVLECFLHAL
RLHWVEFQQKFFKGDGVRFVPYSFKQIITDAM
>comp4902_c0_seq1; 108 1 381 LEN=413; translated
KKSVGRPRSTRGRLHDSEMPPPPPVPNTNSLVSGPMKSVEGWVIFVTGVHEEAHEDDIRE
AFSDYGRIKSIVMNADRKSGLAKGYALVEFIERNEAQDAINALHGKPLLGQKIGVHWAFC
KSNSTR
>comp4904_c0_seq1; 286 207 1097 LEN=1280; translated
MTEVIFSSHFARSHHPCNHHHSLQKSSNSDFVDTKKKTLMTTEATDFIFLNQALPIDFEP
GEFDVICSQGKKAKVHTGNIYFRAVVQKRAKEYSKTFGKKDKSKIVRDIIQHIQTKSPSG
GFVQKIAVTETIKGRCSWKLIDAERAREKVSQCLRNILVGQYRSSQDAKKRSRKESNLKK
IIDFQEIVASNHFVSERIRQLAMMIRKNCLSEPQFLRLMSKTNLEILLELKNDQTVQRKV
QSRTPSTVRNHEEEVEFANESFPSPTCGVDEILKKFQSDEMYILLQQPDTKRRKTK
>comp4905_c0_seq1; 487 333 2514 LEN=2646; translated
MKIISSFTVCFALSTVLFAAPAIHGMPANPLGSFIETQPDGTNITLRLHGDPHDAWMTDE
HDYTVVKNSATGFFVYAQPDGNGNLEPSSEIVRDSNDDEREVFFEEEEGHQKLPPSSNSD
SVVDTQSSPSTTVNGTVITTISSFNKQKNIRPTKRDCRDKICGEHDERGDNHQENILQGL
RGTNSVPSTNTATSFVASTYAKRHGDGPGKKVRLRNLVLLLKWSDHNDRVLPRREDYEVL
MNNRGPNARFCPTGSVRDVFLENSYGALEVDSYVTDWIPMDNTEAFYSNGDKGTTTRIQD
AIRYALNYVDGRIVDGVSVYNKVDFDFFDEDRDGYIDSITFIHSGYAAELGGTDFYGRFY
EERIWSHKWSLYHEQFQSKKGRNVRVSTYHISSGLWGLDGSRIGRIGVLAHEMAHFLGLP
DLYDHDSSSRGVGNYGLMANSWGSNGQQLHPPYMSAWAKMILGWVTPIEPTPGTNIIEAA
AEQNPFQPQVYIIQEGFPEGEFLLIENRQPVGYDAYLPQGGLAIWHIDYGSTKSYNIWDV
YMRQSREGYPAQEGWPENGNHYAVALLQADGYYDLEMGFNFGDYEDLFHASDVDELVPCR
RKHDCQYPNTDSYQGGNIVRSNVHITDISESGRIMTFDYWVGEFGTIPTNSPTGYPTYFP
TSFPSVPPTIAPTTEKERKGGEKAGSAGRIANALVAFAERADGGYSKTNTGSLAGVERKN

EHSTKA
>comp4906_c0_seq1; 144 1 898 LEN=974; translated
RKNVKLTLQPLSADVEFFPEHDPMFSYDGMRKVGGRAQFATSNYFWSSSSPSPTPQQSTV
SPNSFFGSDNEHGKYGNQHGAPLRRKAVDYSKKCPQYPARKFYVALDCEMVGVGINSSRS
SLARVVIIDWKGQILFDEYIKQSEPVTDYRTFVSGITEQDLSQATMSLETCRNRVSAILF
NHILVGHGLENDLNVLGINHPWWMIRDTACYEPFMNNRFNVLMPRKLKDLVHENLGKEIQ
EFGKPHSPYEDARWAMNLYKSVRSDWESSVRYLVQRSVIESSIGRLHIECASRSQMIY
>comp4907_c0_seq1; 780 255 2114 LEN=2245; translated
MINFIVLSIIFCIISSKNGSFKNFIDAFVIREQNTVIRQCQDQSAAMFGSDAFNICRSRR
GLNTGKGTDLRNIRPRKVVASQGTMLFESVVTDDDVVTGREQNQILSKEPTNSSLPTSSQ
SKASSTSSQIQVVMPMDEKSAEPSTNTATTNISGKANDMTTHDKTALLGKMKEKSAEKDN
ADLPHPTANGGYSHTKASRAKISAANKGKTPWNKGKARSPEVRARIAAGVRARNRQVFLK
KLEDIGMTEEEYEAKKKEERRIKEAERRSRRTANGGYRPTEETKKKISRILKEKHAKGEV
KKRAKPDPNRVRRGFTHSEETRKKISQSLRKRWQNDKQFQKKMKDSFTNREDVRKKISDS
LKKKWQDPEFRNGMMDKMSKRKSGKDAMSYDDNHRKKISEAMKRKWQDSSYREKTLTSIK
KSAQTRKVSSPRAKQKLPHSKPSPTRTKSPGIEEVKPMKAGDIPKRKTTRKRVTRKKTIT
KKAVKVEDGINDSSDGNSKVTAMTAVKNVRGKRSKIKGVTSSEKSPSVVSSIGDKGPIAS
KEKKKTKKKKKKEKDGSVTRLREERRDLFDLLYGDEDEMNDDEEQNLGKLLSDSSSDKMD
ILFGDEDLDAFDPYGLDDY
>comp4907_c0_seq2; 338 1 778 LEN=909; translated
XCAWTSYPSSRYEILDKMSKRKSGKDAMSYDDNHRKKISEAMKRKWQDSSYREKTLTSIK
KSAQTRKVSSPRAKQKLPHSKPSPTRTKSPGIEEVKPMKAGDIPKRKTTRKRVTRKKTIT
KKAVKVEDGINDSSDGNSKVTAMTAVKNVRGKRSKIKGVTSSEKSPSVVSSIGDKGPIAS
KEKKKTKKKKKKEKDGSVTRLREERRDLFDLLYGDEDEMNDDEEQNLGKLLSDSSSDKMD
ILFGDEDLDAFDPYGLDDY
>comp4908_c0_seq1; 193 122 1291 LEN=1809; minus strand; translated
MPWVRNKSRARRWTDGQDQRAPAWPRGAPPPREPLPVGSGIAAGSARNAVHTHRWRVHSI
RGRPVVSAVRCALPPTGARRWFLPEEVHQRRRPFPGNHVRRSRNNNQIAHRPVRDVPRSV
PQVVGSLDDHEFLGARLPDGFELVRERAVLAQAGIEGPHRGPGLPAELPLGVVQPGFDVA
VLASAAFPVLCEEEEPGEKLDERALLENRKGQPAQDPGPEQAVVPFQLGSRNGGHHVPPG
RRGGQDQLLDGGRVPCRQQESHGGQPPLRHHRPRRGRPGGFSTVPVVDLPHEGLAAPGPP
EGEGEAPLVRVPGVRSLGDPAADQVGGVGPEGGAATLCRLSLVSVLDAGQEGIPEPGAKP
DPDGVNEKNVVVVTAAAGFVFAIATVAAG
>comp4909_c0_seq1; 298 1 544 LEN=1014; translated
SNLSTAVFNDEHIDAELKRINQKEVIMSSNSNNTQGNIFLTTENLAFSMPLILVCSTYAY
FKIVPLKYGVPILVGVFVILQIRGQVKTLREKKIENMDESDMNDLALELEDDAEDKKKKE
EEARKLEKKKARLAERLAAEERKAEKAAKRNQGKKKSGDDDDEGDVTVFAKGSRNQKKKN
>comp4909_c0_seq2; 303 417 671 LEN=1141; translated
MDESDMNDLALELEDDAEDKKKKEEEARKLEKKKARLAERLAAEERKAEKAAKRNQGKKK
SGDDDDEGDVTVFAKGSRNQKKKN
>comp4909_c0_seq3; 303 437 691 LEN=1161; translated
MDESDMNDLALELEDDAEDKKKKEEEARKLEKKKARLAERLAAEERKAEKAAKRNQGKKK
SGDDDDEGDVTVFAKGSRNQKKKN
>comp4910_c0_seq1; 412 111 1908 LEN=2029; translated
MFVRDVVVSLYFCALCTLIMDGSVATEHRRRNLRHLTSQFKKRQRIGNDNHHGMQNSKRS
LIVGGKDATQYPGSIVFLSDRIDDLSCGGTLISPTIVLTSGHCEVSLVTHAVFGRYDHPE
DRRDSTGKFANSDREIRIPIKQEILHPDYDDISLTFDVMLLVLEISPNDHSFAEVTEKIP
YMRLHSPSDISIDHIADSLASPNGGGIWNGAGPEQTNLPLKAVGWGYNVDPTDDDILPTP
KSTETLQEVTLNYVRNEVCRNVEDKVDNSQKYRYRITDDMMCTYAKDRDTCNGDSGGPIM
IPNPNPNPSPGEHRFIQVGIISWGEDCADHAYPGVGSRIASSYDWIRDRVCSIDGASAPN
EFGCVLGDEHTFSPVPSASPTTDEDKLVASVNLDALSMLDFRSGDPSDVQVKVAIEIFLD
VFAKETSWSLVETNTGISVMRISEKTYQHEDRTKHEVVLTEGISYTFNIKDAFGDGITQL
GFYRIYVIDETKENDIGRLLVKGLGKDFEFERSHTFVVNSPATPNPASSRPSPSPSTPTP
TATFDENTPIESGSISKRQFHERFGILKDVPSYLKTPFAISKHCETYCTNALVKEQYL
>comp4911_c0_seq1; 578 241 1991 LEN=1991; translated
MEGGNAAAPSATGEQGVFNNAGDPSTTTGGSGNSGANGGNDGEDGSGRGGSRRLSNVEQQ
LLAEDNAATAGGFYPRGPAPGLQGVTAAGMGMYGGANDAAAQGVRAQQINSIYGAAGLGG
TAGLGGPGGFRGVPGLLPGQFEDPSGGLAGLQGAGGVGGPPGLGGLNAMNGLGVRGPASA
LQQQQQQAMVAQQQVLAAQQQAGGADFQGLMPTGFEGADASGLPGNLAAMTGYPMFNPAA
AGLGVGADGSAGGGGMMNPNAGPLGGGAPSAATMQAVRNATAGAPSLFDQGYQFARSEMA

LVRHQAAFNNNLMGAGVGLAGLDRQGAAGLRALGPNPYGSYPTDPMGMGAAGPVGTDPYL
ARGGAEGGANAQHLATIPAIRLAQHRSALQRRMMANNLPERKDGSFPVVLYQESDEHKLT
AYQCLLRKQLELFEADEDDVRCSTRQGRTAPIKLGQVGLRCRHCAGVQLAARTKGAAYYS
QTVEGIYQIAQNMSKVHLCERCYRIPRDVRRQLIVLRSDCRRAIGGKEYWSENIRALGVY
VDEGILRVKKAPKEESKEESKEESKDEAKDEPMEESKEESKGEV
>comp4912_c0_seq1; 425 1 1414 LEN=1564; translated
XFVVSALLSKMGFQTVLTEFQRKASYLHVLFGILIASRLHILAKAECGSFGCSLTAVDII
YDESAREALSILRQTDPKKNSHDEGTNGEYIDSLKLLDQRSEALSKLEIAGDESKTTLTL
RGYKGGRLQDQINQDRAMVVSPMDILTNSSKSTPIVQLLGVFDGHGSGGEKTSQYAVEQI
PSLLATKLGAIFRNEDDRESRKQENLIAESDSPVVEALKETFDEVDKNDPTRGDAGCTAT
VVLRVGKKIFIANAGDSVSFIGIYITGESISTHLQKQVQIVYQSREDKPDLPDEKARIEA
AGGYVSIPPDDNGDVSRAYYVDEHRRLRYGLAMSRSLGDWKVQGVIAEPIVDVLTLDEVV
EQALSTYAKACGSKIEESEEFGDESNGSCHVVDESDIHAFAASISDGMMDYLNTNDIGQA
LAEAFFDSENSSHPHSAAENLITSAAMGWEGEFNGQYRDDIAIASFIVPLR
>comp4912_c0_seq2; 256 1 1122 LEN=1771; translated
XFVVSALLSKMGFQTVLTEFQRKASYLHVLFGILIASRLHILAKAECGSFGCSLTAVDII
YDESAREALSILRQTDPKKNSHDEGTNGEYIDSLKLLDQRSEALSKLEIAGDESKTTLTL
RGYKGGRLQDQINQDRAMVVSPMDILTNSSKSTPIVQLLGVFDGHGSGGEKTSQYAVEQI
PSLLATKLGAIFRNEDDRESRKQENLIAESDSPVVEALKETFDEVDKNDPTRGDAGCTAT
VVLRVGKKIFIANAGDRYVWFVGLSFLANGXMTQRGPQPVYQRNSVSFIGIYITGESIST
HLQKQVQIVYQSREDKPDLPDEKARIEAAGGYVSIPPDDNGDVSRAYYVDEHRRLRYGLA
MSRSLGDWKVQGVM
>comp4912_c0_seq3; 287 1 1039 LEN=1688; translated
XFVVSALLSKMGFQTVLTEFQRKASYLHVLFGILIASRLHILAKAECGSFGCSLTAVDII
YDESAREALSILRQTDPKKNSHDEGTNGEYIDSLKLLDQRSEALSKLEIAGDESKTTLTL
RGYKGGRLQDQINQDRAMVVSPMDILTNSSKSTPIVQLLGVFDGHGSGGEKTSQYAVEQI
PSLLATKLGAIFRNEDDRESRKQENLIAESDSPVVEALKETFDEVDKNDPTRGDAGCTAT
VVLRVGKKIFIANAGDSVSFIGIYITGESISTHLQKQVQIVYQSREDKPDLPDEKARIEA
AGGYVSIPPDDNGDVSRAYYVDEHRRLRYGLAMSRSLGDWKVQGVM
>comp4912_c0_seq4; 394 1 1497 LEN=1647; translated
XFVVSALLSKMGFQTVLTEFQRKASYLHVLFGILIASRLHILAKAECGSFGCSLTAVDII
YDESAREALSILRQTDPKKNSHDEGTNGEYIDSLKLLDQRSEALSKLEIAGDESKTTLTL
RGYKGGRLQDQINQDRAMVVSPMDILTNSSKSTPIVQLLGVFDGHGSGGEKTSQYAVEQI
PSLLATKLGAIFRNEDDRESRKQENLIAESDSPVVEALKETFDEVDKNDPTRGDAGCTAT
VVLRVGKKIFIANAGDRYVWFVGLSFLANGXMTQRGPQPVYQRNSVSFIGIYITGESIST
HLQKQVQIVYQSREDKPDLPDEKARIEAAGGYVSIPPDDNGDVSRAYYVDEHRRLRYGLA
MSRSLGDWKVQGVIAEPIVDVLTLDEVVEQALSTYAKACGSKIEESEEFGDESNGSCHVV
DESDIHAFAASISDGMMDYLNTNDIGQALAEAFFDSENSSHPHSAAENLITSAAMGWEGE
FNGQYRDDIAIASFIVPLR
>comp4913_c0_seq1; 305 1 1291 LEN=1347; translated
XQDQSYPSTKKRYMIQRNHRRKQQNTKPNDRGLGSNDIPTEQYYGSGRSDLIMGHKGRPF
GRCRQFHIAASAISVVGAATSVPMIVARGGGAPLPPPLPEPAGDGASRIREALRKDFPEI
DALCSDAYLESVASVPGRSLDYAIHRKVRESLEWRRCYGVDDLRSAFLHVRGEGGGSETH
PATNGGGVFVPAHAQEPSDPEGGVREPKEVNATTPTTSDDSPVSTNSENGTIRESHFVPT
PELVEVCASGAFVLRDEEMIGDLRDPAVQRDEDGSFRRENCRRLVVHADTSRLNWWRTGV
TAGLQYHVLVLEEALERIRTENQNRERNLRESVGEEAVGPLSESLVILVDTTNLPRLPPP
LSAPRGMIQLMRKAYPDRIYRIYVGPVHPWLRKLYNYLSSSLRPRTREKIVLLDEAPSLA
RFGSVRTSLQ
>comp4914_c0_seq1; 452 365 1703 LEN=1983; translated
MKSSTFLPAAVSAFLFITAQPSEAYIAGTKTKPKAPLIKKRIARSVDTTKIGSITVPTVG
TGTISWSSNKIFSTENKELDNVVAEAYRSNAAFFDTAERYGTHFKTAFGMGYGETERMTS
KYLEKAKLAEGPSLVEPVVATKFTPIPWRTTVQSVVDACEQSCKNLGVDQIDLYQIHMPD
IVQPLRAFGKVETKDRIYWEGLAECYNRGLVKNVGVCNYGPTLVEQCHEALAEKGVPLAS
NQISYSLLGRHNGSQETIDKCNQLGVKVLAYYPFAMGLLTGKYSNAFISENDSLNGRKKS
NLELADLERYAYGDGESIPEGGIQPLLKTMATIAKRRKKTLAQIALNYIISKGAVPIPGC
RTVEQLEDNLGAMGWRLSPTEVKMLELEADKLGFGFDGAGFKRTNEKFVVTVLKNGHWIE
NNILDEEVMFKSISSAKQNKIVAMV
>comp4916_c0_seq1; 464 68 1642 LEN=1771; translated
MKRYLTAKALAFALASQQKSTKAFSSIARAVPNSFRTSRNGNSFPSIKSGYNSVVRRNMV
QGVVIDNENCLVNTNPATGEIISRVKCTSPDELEGIIETARASQRAWRMKSLEVRVGLLK

ECIEELAAVSDPMIKLIVQEMGKPIGEAKEEVDYAVEDQDEYFDILLDAIKPKAYGKSTV
VRHPYGVVAIMSPWNFPLGEIMLLALPSLASGNTVIVKPSEVVPETGALMVKTMQKILPE
GVIQLAQGSGSVGAALVAHPGIDLVAMTGSSATGKKILEAAAPQLKRVILEMGGKDPMVV
CADADLEKAARDAVMYSLSNTGQVCCSIERIYVADSVYDEFQELARVAAMDYRVGNGMDA
GNNVGPMVSKMQKDQVHEQVEDALAKGAKLLHQSEIPETESNKGGSFYPVTVLADVDETM
KLFTKETFGPVVAIAKFDGTEEEAVRLANDTEYGLGSSVYTKDTDKATRIASLIDAGQVG
INCYPLDNMGVQCPWVGHKASGFGFHSGKDGCLQFSIPKTIVSG
>comp4916_c0_seq2; 252 68 646 LEN=784; translated
MKRYLTAKALAFALASQQKSTKAFSSIARAVPNSFRTSRNGNSFPSIKSGYNSVVRRNMV
QGVVIDNENCLVNTNPATGEIISRVKCTSPDELEGIIETARASQRAWRMKSLEVRVGLLK
ECIEELAAVSDPMIKLIVQEMGKPIGEAKEEVDYAVEDQDEYFDILLDAIKPKAYGKSTV
VRHPYGVVAIMR
>comp4918_c0_seq1; 412 157 1792 LEN=1856; translated
MASTNSTENAPALDPLPCEILADPTSADGIGDDRLREGLHSFLDKLGKRDDVFLLPPDFT
RFHSKAGKITQMIARYYGCIPPSPGSPAPPPSGFPPPQEFKIMPALGTHAPMTREQIETM
YGPELAGLSDAQGGGPFLVHDWRKDVVTIGHVPAAMVREATNGMVDRPWPAQLNRAVWEK
RLALHRGGEKEAPPPLVVSIGQVVPHEVMGMANFNKNLFVGVGGLEAINLSHFIGAVYGM
ENMMGRADNPLRAILNRASEDFLQSKLDLWYVLTVVAPNGSLRGVFVGNTIECYRRACKL
SMAANFTMLDRSPSKMVVYLDPDEFHSTWLGNKAIYRTRMAIADGGELVVLAPGVKRFGE
DETVDRLIRKHGYVGTKKIMDCLNTGDDGDGDETASSGIGRNRELAENLGAVAHLIHGSS
EGRFRVTYCPGPHLSREDVEGVGFGYADLGETLREYGLDPKDLPGDGWRGGSGSGEFFFI
GNPALGLWAVPDRFEDDGRRGDPASTTSSSGKQQPNHMLFLVSGNDAQQAKAWHDTTTTA
LDHY
>comp4920_c0_seq1; 187 221 865 LEN=3825; translated
MTRSISASTIRTLDSTVHSDDSTCSFTPSEDEQAFYGRNEAAQDITSSCSHSPCAPLGPS
SPESPASPVEATATPTSTSSSGKKRVRFGSLVIHEHAVQLGGSGVPGSGPSISLGWREEN
CYTVDSLEEYEDSRPCLPRKSIEMLQPKSQRVDLLLNAGYSMNQIRVCSNECDEIRKLRS
LVVRGRQGVDDHRIKRVYFQHGQLFPNEDCHQEQ
>comp4920_c0_seq2; 171 221 853 LEN=946; translated
MTRSISASTIRTLDSTVHSDDSTCSFTPSEDEQAFYGRNEAAQDITSSCSHSPCAPLGPS
SPESPASPVEATATPTSTSSSGKKRVRFGSLVIHEHAVQLGGSGVPGSGPSISLGWREEN
CYTVDSLEEYEDSRPCLPRKSIEMLQPKSQRVDLLLNAGYSMNQIRVCSNECDEIRKLRS
RTVSRVQFGYRTKSNLKKLLTWKKEQPKNE
>comp4921_c0_seq1; 880 106 2346 LEN=2589; translated
MLKLRPFRGTAPQQYRNGTRGSKERPQRRASVVTKLLATSEKAKEAISNRSALRKSKEKN
VGGSVYRPQPNEKKDHESTKTVKKWMRRVLPKEFSSPRPDGKPGLFPRIRKTTSAKKSDG
KLSPTVIACCNTVTDESSSKYSRSKNDDSPREMILAGPLSSFGDILRSIENNGATLSTMR
ADSISKRHNENIFFEDDDTCESAEVAAQTVAVNIKKRELKSSCSTRSRNEGLWDMHSKDS
KHGKEEEPPVIREEQKNLSTSKAEILSKLFPLRNQNTCETEETPINTLMQTTKHDEKAVD
KENDDREIHFSQFRRADIISSLFPFGHPDDRKKEWPVAESNTETYAERNETNCSDSSVMA
TSPLHQTESAMTSREIDEKLRDETNENVNDSLRPTESGEQNEDKFIGLVITFDPTWDLLV
PDESNFSTIMPRSRLKKFHEIENYTDSLLGNFAEDEFSEESIDLIAGLDESFDTEPSFSK
DFVAPKSLSGSFELGFHFDRERFSKVDPLHEEEICFYSDDESSIESRTAKCQMINLETMS
QLQNGIEICFPAGKRTEVIFRLVESDNAGQDEDVCCSTTTSEEYTDCIEYEQQVPVNNHT
NSPIHSSTQSPIPIFESTPELHSPVLIKELTAANLYLPRIVNKDASDESSPTNEAEIATP
IFQTVPNKHLKTPPHVYPVLSPRSGTSTYYSILSKDTPDSRYEGKDACKIQFYDAVMEKS
PAIPSNYRDVQNIDSFHSPMGVCNST
>comp4923_c0_seq1; 248 63 995 LEN=1083; translated
MSPLLPHSQDPALLESFRYVNSMPGKNVRGKMIDCFQLWLAIDESSELKVLDSVKEIIGD
LHNASLMIDDIEDNSKLRRGVPVAHSIFGVATTINTANYAYFLALEKCHALQNPQAMTVF
VGELLNLHRGQGHDIMWRENGTCPTEEEYLKMVKDKTGGLFRLAVGLMQAFATTNLRTDY
TALVNNLAVYFQIRDDFINLVDEEYMKSKSFCEDLTEGKFSFPVIHCIWSFPEDHRLSSI
LKQRTDDTDVKRYAQQIMVESGSLQYTREKCTSLKREITTEIERLGGNPPLMQLVELLDI
QIHEMNSVKL
>comp4924_c0_seq1; 1089 133 3015 LEN=3100; translated
MKINSLALRAVLLLAAGINTNAFAPPKTGTTPAKMLSFSSSQQNAVVKSKSALFTATVSS
AAQVGSDLGLEYAPIAPFGKGAADANLGKDAKRILGGKGANLAEMSELGLSVPPGFTITT
ECCDRYCFDEEWNKSLPKPLMDEILDSIDAVEDEMQSKFGSPENPLLLSVRSGAAISMPG
MMDTVLNLGMNDEVVEGLAKKTGNPRFAWDSYRRFLEMFGNVVLEIPRSHFEDELDDIKY
EKGVNEDSDLSAEDLQELVGRFKDVYGTMDLSFPQDVHEQLKLSIGAVFNGWMGPRAIKY

RQVENIRDLLGTAVNVQAMVFGNMGDTSGTGVCFTRDPNVGTPELFGEFLINAQGEDVVA
GVRTPRPIAEMQDVMPEVYEEFLKNTKILEEKFGDMQDIEFTIQEGKLFMLQTRNGKRGG
EAAVKIAVDLVGEGLIGKDEAVAKVLPEHLDQLLHPRFPDVELQEYKDAVISRGLPASPG
AAVGRIAMSNEKVVENKENGIPSIMVRDETSPDDVEGMFAAEGILTARGGMTSHAAVVAR
GWGKPCICGCTSLSVDEANGKIILTMDDGSQKTFKEGDFISLNGQSGEVLEGSQTVAPPA
ITGNLKTFMEWVDDAREIDVLTNADTPADAKEARKNGANGIGLCRSEHMFFKPERIAKVR
QLILGNQEQEKEALKELLKFQRDDYEGIFTEMDGLPVTVRLLDPPLHEFLPSLKEDSVLS
ECAEQLGITVDELKEKVEAMEEVNPMLGLRGCRLGITRPSIIEMQTRAILEAALNAIDAG
VDAQPDIMVPLVGKIEEFRHQKALIQSVAAKVFEEKGKSCNYKIGTMIEIPRAALTAHQI
AEEAEFFSFGSNDLTQMTFGYSRDDVGSFVPNYMSQGILQDDPFVTLDTEGVGQLIKMTV
ERGREVRPNLKVGVCGEHGGDPRSVHFFSNEAGLTYVSCSPFRVPIARLAAAQASVERKK
>comp4925_c0_seq1; 369 264 1382 LEN=2237; translated
MSQPGLTRGVPRMYGSEILPASANRSKTRLHDDGNGSLNTKQSRYYPYSVLTGGKFSNKQ
RQNNDWEVGDWCWEEVEEQKYEDDGICFDGWSRGSWCWLDSNHSSSSNDEQQYEDNTRRS
SRLASIRTTVNSGNNLLECEICCVRKPVQEISHCSNVTCRTNVCAMCQLRFISCRRGVYD
SNHIDEFSWIDTVQSKRLCCQTPLDVRVFAKKFFARDIEKLLTSDNSGIEIADRSALISL
FKYLRGIRKATESARADGFYNEDGPDIDIEGLFDEDENNEYHTSNSNDEYGNDDRIVNDN
HYGVRNDDNDNNTMVNGSPSGSRQVSGRSRRLIRRIAKANKRHRIQTRNSDDAWVPGSWC
FTEDQSTEETEA
>comp4926_c0_seq1; 275 406 1350 LEN=1624; translated
MVKVLDRAKFLSASAATFASGTAGFVAANTNPEQCEAKAVAAGSQNPRYIDKELQMSLGD
DGSGNPRTRGILVRRMTGDSTPYEFPVQPVRLVQTWPEEWPFKEQDFSRSDPSNDKGFYT
IPKLVYHIDEPAVASLTQYYRKNIPAKSDILDICSSWVSHYPLEFPETMKSISATGISGL
ELGFNDQLTGGFEARDLNEDPTLPYKDNSFDVVTCVVSIDYLIEPVKVLKEVHRVLRPGG
KAIISQSNRCFPTKAIAMWLQMNDRQHLELINGYFQYAGGFEPRKAFDITATLPDNGYRD
PMFIVEAVKESKSD
>comp4926_c0_seq2; 237 406 1481 LEN=1755; translated
MVKVLDRAKFLSASAATFASGTAGFVAANTNPEQCEAKAVAAGSQNPRYIDKELQMSLGD
DGSGNPRTRGILVRRMTGDSTPYEFPVQPVRLVQTWPEEWPFKEQDFSRSDPSNDKGFYT
IPKLVYHIDEPAVASLTQYYRKNIPAKSDILDICSSWVSHYPLEFPETMKSISATGISGL
ELGFNDQLTGGFEARDLNEDPTLPYKDNSFDVVTCVVSIDYLIEPVKVLKEVHRVLRPGG
KAIISQSNRCFPTKAIAMVRTLLLFLFFHILLQAKLLSVALGCTSDFSPFVVFLTAHALT
AVAQMNDRQHLELINGYFQYAGGFEPRKAFDITATLPDNGYRDPMFIVEAVKESKSD
>comp4928_c0_seq1; 623 117 2552 LEN=2966; translated
MEFDGVFELLETAKRLEKNDQRIEAATKYYESCYLMRRILSRFPRSEQDSPTCRLLEENI
RFYNQAAQALYFDDGSLEPNSAPTPTVVTIRHSLSNDSPDDVSVLTPPMIALPAISHEAR
QSEKYDKSRFTQSDDFNRMISLANTRLGQAIDLDENNQADAALKVYISAAEMYLGVIKLT
EDSPLMVSITNVVKRRLSSALDRIEALKNPARSQPPNRQQQERPGSLRSDVSNRESSHSL
SYLSEEEIAVLKKSSLIASGLFLPFSEEEARSLSRQVAAQKSTFKPWIDPDGDLSLSEKQ
KKRFYAWSRPHEILQRRSQMGFKQRPPSMISNISPYTIRQQYVTDCSFIASLCVCALFEK
RFRKRLITPIVYPQDKDGIPVYNPSGKYIVKLWLNGVARCVVVDDRLPVDRQSNLLCSQT
SGDHNHLELFCPIIEKAYMKLCGGYDFPGSNSGVDLFSLTGWIPERIFFPSQHGETRDFE
TPPDRAWERLFSANSFGDCLITVSTTRDISEQKADDLGLVTGHAYAVLDVFQSKNGTKLL
QLKNPWARKGWTGKYSSHDKMSWRDQSLRSEVGYDPVEAAKQDDGVFWISWEDVLRFFRN
LQLSWNPKLFSYRVTTHGFWPVEQGPKIDTFNVGENPQYILVLSDQAVLKKATIWVLISR
HVSKQEQEGAEVHDFLTVHLLRNSGKLERVWYPQSKKSIVNGCYTNNPHVLLRHDVTGPE
DKYISLVLSQYEKKKDLAYTLSCYSTEPFELRRPPKPLPRKLNLSSEWTKHSAGGAFGSN
KFFVNPMFELLLTEKTTIQMRCTTSNALAGM
>comp4928_c0_seq2; 535 117 2692 LEN=2713; translated
MEFDGVFELLETAKRLEKNDQRIEAATKYYESCYLMRRILSRFPRSEQDSPTCRLLEENI
RFYNQAAQALYFDDGSLEPNSAPTPTVVTIRHSLSNDSPDDVSVLTPPMIALPAISHEAR
QSEKYDKSRFTQSDDFNRMISLANTRLGQAIDLDENNQADAALKVYISAAEMYLGVIKLT
EDSPLMVSITNVVKRRLSSALDRIEALKNPARSQPPNRQQQERPGSLRSDKKKPDXLSRQ
VAAQKSTFKPWIDPDGDLSLSEKQKKRFYAWSRPHEILQRRSQMGFKQRPPSMISNISPY
TIRQQYVTDCSFIASLCVCALFEKRFRKRLITPIVYPQDKDGIPVYNPSGKYIVKLWLNG
VARCVVVDDRLPVDRQSNLLCSQTSGDHNHLELFCPIIEKAYMKLCGGYDFPGSNSGVDL
FSLTGWIPERIFFPSQHGETRDFETPPDRAWERLFSANSFGDCLITVSTTRDISEQKADD
LGLVTGHAYAVLDVFQSKNGTKLLQLKNPWARKGWTGKYSSHDKMSWRDQSLRSEVGYDP
VEAAKQDDGVFWISWEDVLRFFRNLQLSWNPKLFSYRVTTHGFWPVEQGPKIDTFNVGEN
PQYILVLSDQAVLKKATIWVLISRHVSKQEQEGAEVHDFLTVHLLRNSGKLERVWYPQSK

KSIVNGCYTNNPHVLLRHDVTGPEDKYISLVLSQYEKKKDLAYTLSCYSTEPFELRRPPK
PLPRKLNLSSEWTKHSAGGAFGSNKFFVNPMFELLLTEKTTIQMRCTTSNALAVNIMLIS
LPDTKITRDKRNLRELVNYSKKPILDSGNYRHGFTITEMKLMDPGHYALIVSTYDGDQLG
KFMVTIETSEKITTNYIA
>comp4928_c0_seq3; 602 117 2801 LEN=2822; translated
MEFDGVFELLETAKRLEKNDQRIEAATKYYESCYLMRRILSRFPRSEQDSPTCRLLEENI
RFYNQAAQALYFDDGSLEPNSAPTPTVVTIRHSLSNDSPDDVSVLTPPMIALPAISHEAR
QSEKYDKSRFTQSDDFNRMISLANTRLGQAIDLDENNQADAALKVYISAAEMYLGVIKLT
EDSPLMVSITNVVKRRLSSALDRIEALKNPARSQPPNRQQQERPGSLRSDVSNRESSHSL
SYLSEEEIAVLKKSSLIASGLFLPFSEEEARSLSRQVAAQKSTFKPWIDPDGDLSLSEKQ
KKRFYAWSRPHEILQRRSQMGFKQRPPSMISNISPYTIRQQYVTDCSFIASLCVCALFEK
RFRKRLITPIVYPQDKDGIPVYNPSGKYIVKLWLNGVARCVVVDDRLPVDRQSNLLCSQT
SGDHNHLELFCPIIEKAYMKLCGGYDFPGSNSGVDLFSLTGWIPERIFFPSQHGETRDFE
TPPDRAWERLFSANSFGDCLITVSTTRDISEQKADDLGLVTGHAYAVLDVFQSKNGTKLL
QLKNPWARKGWTGKYSSHDKMSWRDQSLRSEVGYDPVEAAKQDDGVFWISWEDVLRFFRN
LQLSWNPKLFSYRVTTHGFWPVEQGPKIDTFNVGENPQYILVLSDQAVLKKATIWVLISR
HVSKQEQEGAEVHDFLTVHLLRNSGKLERVWYPQSKKSIVNGCYTNNPHVLLRHDVTGPE
DKYISLVLSQYEKKKDLAYTLSCYSTEPFELRRPPKPLPRKLNLSSEWTKHSAGGAFGSN
KFFVNPMFELLLTEKTTIQMRCTTSNALAVNIMLISLPDTKITRDKRNLRELVNYSKKPI
LDSGNYRHGFTITEMKLMDPGHYALIVSTYDGDQLGKFMVTIETSEKITTNYIA
>comp4928_c0_seq4; 556 117 2443 LEN=2857; translated
MEFDGVFELLETAKRLEKNDQRIEAATKYYESCYLMRRILSRFPRSEQDSPTCRLLEENI
RFYNQAAQALYFDDGSLEPNSAPTPTVVTIRHSLSNDSPDDVSVLTPPMIALPAISHEAR
QSEKYDKSRFTQSDDFNRMISLANTRLGQAIDLDENNQADAALKVYISAAEMYLGVIKLT
EDSPLMVSITNVVKRRLSSALDRIEALKNPARSQPPNRQQQERPGSLRSDKKKPDXLSRQ
VAAQKSTFKPWIDPDGDLSLSEKQKKRFYAWSRPHEILQRRSQMGFKQRPPSMISNISPY
TIRQQYVTDCSFIASLCVCALFEKRFRKRLITPIVYPQDKDGIPVYNPSGKYIVKLWLNG
VARCVVVDDRLPVDRQSNLLCSQTSGDHNHLELFCPIIEKAYMKLCGGYDFPGSNSGVDL
FSLTGWIPERIFFPSQHGETRDFETPPDRAWERLFSANSFGDCLITVSTTRDISEQKADD
LGLVTGHAYAVLDVFQSKNGTKLLQLKNPWARKGWTGKYSSHDKMSWRDQSLRSEVGYDP
VEAAKQDDGVFWISWEDVLRFFRNLQLSWNPKLFSYRVTTHGFWPVEQGPKIDTFNVGEN
PQYILVLSDQAVLKKATIWVLISRHVSKQEQEGAEVHDFLTVHLLRNSGKLERVWYPQSK
KSIVNGCYTNNPHVLLRHDVTGPEDKYISLVLSQYEKKKDLAYTLSCYSTEPFELRRPPK
PLPRKLNLSSEWTKHSAGGAFGSNKFFVNPMFELLLTEKTTIQMRCTTSNALAGM
>comp4929_c0_seq1; 487 79 1392 LEN=1447; translated
MSSRKDMESTIRAVRCHRFACVEKKNDPDEENGRKGPSRSTYQRRSPKALRLKDCLSLDR
IAFIESTNPSETRNDTRVLIRTEYAGIQYPDALQALGLYQVKPELPYVPGMDVVGLVLKV
IPSPSRSNSNVAARIQPGDRVLATMMDFGGTGGLSEIVLAPLKHVYKLPKLSPSRCPVQS
AAGFANVGRNYFAAYHALVEVGGIRLNTAYHPNTNHDKIILVTGASGGVGMATIELAKAM
GCTVIAGVGSNSKREGPISVGADAVLCYGRSKAERKQFRKQVLEVSKTMAMARKANKGVD
LIVDVVMGNLFEEALVGCLKPLGTIALVGFAAGQKPIRPGILLVKEVRVAGSIWGSIALE
ATEERSDNKYQTMVETILGFFATGAIKGRVDRIVPLNRFIEAFEVFEVNKGRGNTVISFV
DHHDDSRAESNNARSRL
>comp4930_c0_seq1; 359 1 1321 LEN=1524; translated
XASASASAFAFDPSPSPSPAAPPLPITESFTEDEVLAREAARAWSRDVLAPSVRSMDEAS
AIDPLVLEDLFDQGFMGMEVPQDLGGSGLSFTAACLAIEEIARVDPSVAVVVDIQNTLVN
TALRFWGTPSQREEWLPRLAGGGCLSAFCLSEQAAGSDAFALATTATASSSTDGSSFVLN
GSKLWISNAREARLLLVFATVDRSLGHRGVTAFLVDRERHGNGNGSDDDDGNGTITVGQP
ERKLGLRASSTCPVHLENVRVPASSVLGTVGEGYKICIGVLNEGRIGIASQQLGIAKACL
YDLALPHMMDREQFGRPIAEFQGMEHQYAEMATEVHAAELLAYNACRLRERSGDPSGGRS
SGRSFAKEASMAKLACSRVAEKAASKTIEWLGGIGFTRDLPAEKFYRDCKVGSIYEGTTN
MQLQTIARILREEQASGKQR
>comp4931_c0_seq1; 289 271 650 LEN=650; translated
MQMRCNDNHGNIGNSKMSRRQTSRRSRRHRIISHFPPSLMRDASQEGDGVNRNHIDCLTD
AINVLQEGRRSMSSFLQDGDQTHSSGGSGDGSIRDSLHQRVSIQQQQQQQRHQHQHIHHQ
QQQQQQX
>comp4931_c0_seq2; 289 422 801 LEN=801; translated
MQMRCNDNHGNIGNSKMSRRQTSRRSRRHRIISHFPPSLMRDASQEGDGVNRNHIDCLTD
AINVLQEGRRSMSSFLQDGDQTHSSGGSGDGSIRDSLHQRVSIQQQQQQQRHQHQHIHHQ
QQQQQQX

>comp4932_c0_seq1; 1439 366 4438 LEN=4454; translated
MSTYMKRFASSTKKTDTNANIKKERTVAPALETAKQKSYRRHINRLVPPSETPAEMAPST
ESRQATKSNNGTRPGTKSKSPTAGITKKLSTRRLRRVRNPSISSASDVIVSSGHQSEKLD
TSNTEPPTTDSRVSPTKESKTERHSDNEGDDEVLEDVEKPNSLEPESSAPTSALWDEGKV
GNNSMRASSTRSMLLEKENMQRELTEVQLDRDILRLQLLEAQTELETSRTSLRDQISVLE
NQLRTFQQRERRQSKISGKKMDDGKSKESSHPPPDNPIVNEDSAKQSAEDNFRDHNNGFD
NGLLEQIRTLERDRESREEVERALRQQLKDMTEESKEAKSQAAIAEDRVRALQASIDASK
SASSTRENAENEDISVQVNTNGNEKDQVNDIDTEGVSEVASLKKSYDTLKEDNNHVDSEQ
DRRKRDVDSSEKLFARSNNKNEVVHNLHLKLDEEMAKLKLRKERRRSRAVSATASFSTHR
FRALGDSSSTTSFRRNEHKRDDVRIAIAPDISPRSLSEETSITGLPTNTSTRQPDQLFDG
IKPTQSNPVMIGQTLSGSIPEEAPNCVVNYEKELKILNTECKKLKQELIDSRMEKETFET
ALEESTSEILKLRSLVSEKAISARSNEEDEALNLESNVKDEEKGALGKLEEKNIQLAKSL
ETVNNVKVQLEVQLQNAMQQVNESRDEVDKLNSEVTSYRSSLEQHVSEEKRLTELIESTK
NETALQAHVTRGRSEQSSYERPTEMKEESNDKDGLERKILSPENDSKVAIESLLNERNSA
LEERKAVELKLRDTSKELDESKDRESVLEKLVQEISDKLEATQKELSGKQNRLEQASTQL
SDLEKELVGRGISKTSENENVDFQRQIEEKGICITNLQNELSNKDRRLQQAAEQLYELEK
ELFGRDEEIDTLNAKRQYIEDYANRQLTEMQRALEQGVVQIDRLEEEKLKLQDSIQKYEE
QIEKQGGRLAEASLQLSELESELFAKDDDIEEIELSVIAMHALEIQRLEALEDSENKRRD
LEKQLASAKKRMEYLESLGGEIKRGDETSTTEARRLFIEDERKSKELQSSKNQLNRQLQN
VSKLTELNDKWQLQLESASDEKQELIRTLTDATNRVDLLQRELEEASNVIKDLNVTNAGL
KDDLDCATQEGDKNRRRISQMEEQKEALSEKLESAMDNLADLKKNYKESQEREEALGNDV
IEKLQANLRSAQAVDSETEIRVRNLTEQLESERRSLQMTEVRRNELETENESLKNRIRLL
IEFEFEKGDNAAGDDQGELREQRIEELAVAVSRRRESETKRLGTKAAELEEHMNPSEKGE
KLCNEPILASLEDKSXAGPPVQHLTQKGSNETGALIT
>comp4933_c0_seq1; 844 158 3085 LEN=3274; translated
MSSSSANESAWVKRQRDRQIVVLVDPYSTGCVVAQELQKRGYLIVAMWTIGFNEEMKSHV
PVSCGKMEYFEQIDQLETNEATMSKLLEVAKGHEVIACLAGGEAGVDYADAFSEYLASVS
PSSSKILTNGTSIPNRRDKYIQQELCRKMGLRSVRQAGSDKFDENIQSFLKSEPFPVVLK
PTESAGSDGVKLCYTFEEAKDHFETLMNGQLVNGGDCPSVLCQEFLNGTEYVVDHVSRDG
VHKTCMIWVYDKRVANGANFVYFGMKPVDPSTPLASILINYTRRVLDALEIKHGPTHGEI
MMTEDGPCLVEMNCRAHGGDGTFAPLAKALTGAYSQVEMAADAFVDPFAFAQTPDRPSYP
FKAAGQEVIFVSRSKGIVKSTPGYKIIKMMPSFLHMDGMKTEGSVVDYTIDLVTGIGSVI
LMHKDPEVVKRDIDFVRYLEDINGFFVYETKLENLARPRGDAIVFSKTKTSPSKPPRAPH
RRLKSTDTSHHQHVFSSDGPNLVRIMSNDRPELGGFGGMPMLTKRMTTVDSSREAVVLID
PYSTGCCIAEEIMKRGFSVIALWTRGFSPEMKTHVPLSCGNIKYLAEVEEGETLAQTSQK
MYEAAGSLRVVACLAGGEAGVDLADALSEHIKVRTNGTVIPNRRDKKIQQEIIRKHGLRS
IRQAGGSDFGEVKHFLETEPYPVVLKPVESAGSDGVKLCHSFEEAEEHFHVLMNSQMVNG
GNCPSVLCQEFLRGKEYVVDHVSRDGEHKTVMVWVYDKRPCHGAEFVYFGCVPVDSESRE
AKIVIPYVQGVLDALEIRNGPSHAEVMMTPDGPCLVEMNCRAHGGDGNWRPLCRHLTGGY
SQVEATVDAFLDKQQFSVLPSKPPSPFKASGQEVILVSYGRGVVKATPGFDIIKEMPSFV
YLETGVRPGSRVDYTIDLFTGIGSVILMHKDPAILEGDIQRIRDMEKNNELFTFEQQSRY
MKSPSIIGMDKLASA
>comp4934_c0_seq1; 113 1 655 LEN=908; translated
LFFANLEHRVHPPKYTQGPSSVTLLEMASGRLKKELAEVGKVDQSSGVSARATSDDMRRL
KGKINGPESTCYEGGIFEIDISIPKQYPFEPPKMKFITKIWHPNISSQTGAICLDILKDQ
WSPALTIKTALLSLRALLSSPEPSDPQDAEVAKMYLNDRKKFEKTAKFWTESYAKEADYE
AMIKGLEDMGFDRASAKKALEKHDWDESRAVNELLGM
>comp4935_c0_seq1; 345 484 1452 LEN=1678; translated
MKHKCTHGADANKSDRLASNINEERMETVSEESIDNSIANTVNPGVLKSKEDLSSILLEN
ENECVNHKNSKNYSSSIEVPSHSLLSSSISESSYSSLYISIHNDKRNFEDNDSASGSFSD
SNSDSDPENMMKEYDDGLDINFSIARSRIADDAANEALKRLGNCECWTLISVGCGSSAAD
AIHERDSRPIWQQGLNQITDSRALAPFGECGLQFSVTNVPDELQLLDLPYSKTKNPERIA
MVNSXQKFDSNGSEDERDAIYSVKISDDAIERCNGNEGGRFCDDEDVNSVETFVSLATDG
ANKEISYDERGHKKIAYETYEG
>comp4937_c0_seq1; 806 78 3415 LEN=3523; translated
MKRGTGISTQRLNTLSTGSIYSDDKAPEWNLKRIYGREKESKIIQNCVDKGSNGVIILKG
CSGSGKSFFVDGQHFEEDGWIYATGKHDMRRKQEPYSALIDALDGLVAKWMANNSAAQLC
RMNSFNNLLVDDMEFLETIVPKMFREVKAECYKSRPKGLQTTSSQFGTEATVDSKTINAA
FWRIMSFLSESKPIIFFLDDLQWADQASLDAIQALSTTGNIEGFWLILSYRDEEVSEGDP
VSTCLDFIKEENECVETIHITNLDEENVNNIVCSLLEQDPEQTSELSKVIHNKTAGNPFF

VIQFMKMLRHESFLNYNAMILQWEWGDVGKMEQIVHVSDNVADIIAASMSKLTESSLFAL
QMASCLGKVVPMQVLIEFIGTCVVEEASNDEELAICGALVGIQLQGLKDVFDEAVKFGIL
VRLEDDETYVWAHDKLQFVAYSMIRRKNLNKVHKSLGMILRNMYESNPENCWALYMAADQ
LNRLTNVCDDNLNADIALSSLQASKISISKSAXFPALDMLRSAAEHLTAIADPWKTNYQL
SLEVLSSLAEMAMRFGSHKEAINAAIQVDRNAKILEDRSRAQFIFILHKLEGGNRDYKGA
VESIKSILLDYGVKFPTKIMPGQLILEYRKLRTRLGGAGEAFLNLRHLNDAHPKDRRTKI
IIILLARLIEYLDHGEKTTDLQSYAAMRILNISSKEGTCAATALGVACFGSNMIQDGREY
DAREFGELAIKLVDSFPSKVGSCHALVHSWVAYGVLSQAVPFHDLLDPLLDLNRISLRGG
GISEGAMAWIGYAYTYMCVGLPLHPLDLDLLSFSKNARQFRLPATFKVLFPIIRQAIQNL
QELKSNPTLLKGDAFDQEKELKNFKDLGHKMTLRDINSFRLMLACIYQDWKIAEELVVAL
EPYLHGDKLFIRGHILLVYMGYACIVLSRRANMITRLHHRQLGRKIIKTFKDQLKNGSPN
ALPVVMMLEAIESPSKERFDEAIRVTARLGMVQHAAVVYENAGLFFTEQGNEGLAEYYFS
EASKLYTDWGATGKTKDMIEKHNFLGSSSLNRNQSNGFIKGRTRFSPETMLSMQEIKIGP
GSSERISDITGGISKGSNLSSSVASTLFSTSK
>comp4938_c0_seq1; 633 1 1685 LEN=1889; translated
XATLIGDYQANSIRGILAIFHNQLVAKLIVKITARQSLWLPIENLYHHPMTSTSKFLLVA
VVWSSCFTFSSGFQGHLNIHGLNAGKRTTTTLHTIPESLAEEGTWQAYLDEDTTGLIYYF
DTSTGESRWEPPTSTFPEVRLPRRKQRLADNLRREYRKERIEAMETEDEGEGIVGGLFSA
MTSVLDQEEAVVEETESVDDSQTEDEEEEQPSGGFFGNLFGGNTADQDTIEDTASVEEIT
EEESEQPTEKNFFSSIFGSGGSASAEVEDTGVIEDEPAEPDEKKSGMFSAPFFSTSSTDR
AQKEVQEKIIDDSPKPIKIEIGSHILPHPAKVRWGGEDATFIKGRTFGVFDGVSGAEKLD
GVPLYSRTLASEMKRMCGMNGETVQDMTAYMSSAASYADGAATGASTAVVASIGEDGFLQ
ALNVGDSACMVVRDGKVTAKTREISHYWECPYQLSEDSPDQPKDGTKLNVELIPGDIVLM
GSDGVFDNMSDDQLLELVAKSPSVKATTLAKRVCDASRKISLDKTAVTPYSKQAQRRGDR
DYRDGLGGKVDDISCIVVACK
>comp4939_c0_seq1; 175 1 318 LEN=758; translated
IGEAVKSGEDLAAPITFTPFKNKDHSNYVANQGRLRRSEVGDAAKSGEDLAAPITFTPYK
NEDHSNKVANPNILVATEKGKQMKTAGNLAKPITHIRDAMKNVSM
>comp4941_c0_seq1; 522 220 1740 LEN=1740; translated
MVLVCRSGFRARNSAVVAAFSGRHHPAAGGSCARDGNLRTPSTTSRLFSSKPQKMSSKSS
SSSSSSSKRKTPPPPPPLLGPEDFPEWAYEPRDFFRFEILHESTKSQARVGRIHTPHGII
DTPGYVAVATNAALKGVDLRDADKAGQQLIFSNTYHLMLHPGADLIAEAGGIHNFTNRRD
RPFITDSGGFQVFSLAYGSVQEELSSKGELKRASKSKKNHNGNGMGKDAVKVSEDGVVFR
SYRDGSKFLLTPESTVEAQKKIGADIIIPLDELPPYHIDRKVLEESVDRSHRWEARSLRA
HIENVREQAMYCVVHGGIDRELRTKSVDFLTSLPFDGYAIGGSLGNVKQELKDLLDWMMP
MFDDDSDGKNRKGKPRHLLGIADEENIRHAVARGIDTLDSCYPTRIARHGTVLTKDGPLR
LKSGKHSKEFGTKIDGACTCPTCQHYDRAYLWHLFKANEPLAVTLAAQHNIHYMNDMMRT
IREDIMEKEYDVPSSTKTCSIYFSTAX
>comp4945_c0_seq1; 316 434 1183 LEN=1424; translated
MPLGDEESAIILTSSPKPDGLEERVTSLTSLSANGQPQHPQQIPMATNHTPSARGRHSLE
MKKNQAPPMPKLLHPILHSLPLHFRFGLNGFLSNVLFMVAYNAAVVQFGQNASSSTIYAT
VYLIFIPISHAFISLLVFGWPERYVRSLMSNAPVGLTAIVLGAALTAYLDEIEFNGWVAD
AVTEYWAMMGYKIETTDATTEPTTVQDEESRGGGEFYSSLFVLIVTGIWTFVLSVMVNAP
QESQEKKDL
>comp4945_c0_seq2; 245 306 611 LEN=852; translated
MSNAPVGLTAIVLGAALTAYLDEIEFNGWVADAVTEYWAMMGYKIETTDATTEPTTVQDE
ESRGGGEFYSSLFVLIVTGIWTFVLSVMVNAPQESQEKKDL
>comp4945_c0_seq3; 162 434 661 LEN=877; translated
MPLGDEESAIILTSSPKPDGLEERVTSLTSLSANGQPQHPQQIPMATNHTPSARGRHSLE
MKKNQAPPMPKLLHP
>comp4948_c0_seq1; 231 484 945 LEN=1721; translated
MDSSNHEHTASAARPPRASPLAEPKNGTPTAVKDTNIASTAVKRPLASATLSPCAHEAPA
AQVAAADNQTRENTTNSKAIQNTKKKKPFQAQKQLPFKAKKTLKKTKKKKKFSSILSGMM
KPKEKKAADLVKDREEALRKHLGGGAFAKVDKI
>comp4949_c0_seq1; 279 1 647 LEN=666; translated
QQQQQQEEEPPNGESKSSSGDTTAETALSDARIPRAELARRMGQLLGRLHNIGIVHGDLT
TSNMVLLPAESKPEPRDDDTPSDGNGDAFELALIDFGLSKSTDSSEEQAVDLYVLERALL
STHPDLPGGFFETVLEEYKATTEGDQAPGGDAGKSKKRKNHCQSALVRLEQXADAGEETG
VFWLIRENEEPSKALRRAAPPRPALRYTTPRYNRK
>comp4950_c0_seq1; 365 143 1518 LEN=1622; translated

MAQRDLNQHRRTQASLPKFSKSRDGEVEGKGGRVSYGNDDIGTWEWKGKGKGDKKSKKSS
KKGKSGKKEKKEKKYGYYERSWGKGTPSQYQTKLPTLNPVLSPTFLPTLVPTPTKNLGSG
SDSEDARPSTETSNDCPPERPPTIVPPSPSFVSTKNPTRAPSPTIATDKENTMSVNLPIY
AIDYTFSQTSRTPIKVDFLELQTLTQSFFKNYMLTAYEVSAQVTLVDFSTSFVTAHFTPG
EPIHIQYNSTAYFDSKSINIPTAQNLFVVLENSLVSTQAYVDDLQSQLEEVNPFSSTTEA
IFTDPKDLPVTQSSAKTEVGAASASVAAALYPYSSSECPFFTHEKSTDDNVYDERVKKEI
KGDTTVAGETYVMENDHFSQGISLSDSLSMRSSEHDQLPNSTSLLGETIHQNIPKSRTIK
ETGNVHDVSNGHSPAVKNKPRTIEDIERLLSLGDCDII
>comp4951_c0_seq1; 212 386 1444 LEN=1634; translated
MCKPEQASKESDPQSKDAKDEVIGTNPKRQMSKNKSQQKDVASMSSSVLSGVLHGVVWNV
FAANLPFFLSGADVYHKLYQHASFRYDASISLFDNTVLTYGTDYAIAAMTLAIAFWILRT
SNRAETQVAAKLSKLSALMLVLYATSTFAGGLGHHFFLTVDSRNTNLFRVLWTVCVGGVY
LAPMTMGMIGNECLRIFQPRSNCPPLLKSMPLLTDTYWCLYGSIGTIAWILGFSSFQRPV
CDIFLAGITQTPCTFYFMAFLYLVEHPVITNAVKNRGLVGFIMNAFLLPLYPVLVFNFGL
SLGVANAIQHANLFVAWGLQGMTMQTVVKALVEEKTEASRSPSSSDQKTKDQ
>comp4952_c0_seq1; 434 1 1373 LEN=2027; translated
XTKPNSIRETPFKALTMMRLMVSALLFLGHCPSGLVDANTGIEALVSKAKRKFKSYQSDP
LNLLRGIQAGEYDRIYIQYHQCVWSELDSYSDNYEESGCSGDENGGDKPWYMGNTQCYRA
NVAYSLYGVRAGDESPQDACRRRYYINTFFTSNGIEEFGDLVGLENHEDATTQCTLVGDE
ENGDDDADGADYDSFQHNTEIYPNAQSYTTFCADGRFVNTLFGGAYCTGKSDLEVLDPLD
DLNSELMELECALVYTAENENNGGGNDVGDENENARKLEDGEQERDEGDNDQQENSGDEE
DNEDDNGDQDNEKQENEGNGNDKGGTLWNVLSHSSTCSILEYPNACPDPYRAKKHFDMNP
RTSSGFSRRLNPVDWFSLVLFVLGSVLLAVTLLIEDRQPEEKKRSEKASFLLDLVLDPAL
DPGDLGGDLGDDREEDLGDDRESTQQEEIIEAMVVNT
>comp4953_c0_seq1; 163 413 852 LEN=1459; translated
MTPLKDLVEKVVTKAICHKXQPPPLVLVTDDTPYTKNESMETTIDSPCVLTTSKSSASQG
ENEKDSLAPAFLCRDDYPEGWLIYHSVLGVISLTEAERYDEQQLLDKGKSDKNQDYRKKN
TLHFQSATNETGIRLAKTIATGSDGS
>comp4954_c0_seq1; 336 170 1204 LEN=2075; translated
MRIFGAVSAFLVLVSPILLCEGFANAGKRRGLRSTFDSIEGLLRVSREDTDDVVDNPNLS
ESPTPIPSLESDQFRHPLDQRLTSLVRNAPFYDLAEETLRRAFPLIEQGVQLDMLASSVK
VSPQQLPELHDLLIEACTVLNFPSTFVPELYVQSNPQANAYTLALQSRNNSKKDKDGNPY
SSSVIVVTSALVDRCTPRELQSVLGHELGHLKCEHALYLTLGNLASSPLSRLPVMGPRVD
STLQDWRLAAEYSCDRAALLVAQDSNIVASALMKLFAGTSRYDLNTEAFIEQSLDYERQL
ESANPLVRMSIQRQQRTHPLPVRRVAELEKWFQSDDYQKLVPSK
>comp4956_c0_seq1; 627 158 1705 LEN=2045; translated
MPFGAKYIGIALGITSEAWGNTGLGNDPATAPSDDVDETATRITALLLIRICAWIMPRGM
FTDLSTTTMCRSMLVNDTLWPEQVSEEVVVQLRDYVKSILLGYNDVPYHNFKHCYHVTIS
TNKLVDMIVHKHPDEKPSNTFGFRDDPLMQLALIFSALIHDVDHRGIPNRQLALEDEELA
MKYNDQSIAENESLFIGFSEILKKEYDKLRSVIFPQREDYRRFRAACVNLVLTTDIASPE
RTQLGKSKWKAAFGDPYETVERKVMKKAQGHREDPTQILKDMHVEKSANEDESSVSSSYN
SYEDEDNNLGREARRIQKKSDKELSGKALKFHRRLSQAGTSTPSSPRKSSRLGFRRTMDL
TGESIETYPGKKRVSASGDGLFDVPQIDLPPPEKIDDLRESVIMETIIKSADVAHNLQGF
DQMAKWSDCLYLELRKGFVENRGDDPSNGWFNNQIGFLDFYLLPLARKLDDTGAFGDARG
GIFASSIENNRARWTEEGMDVTMNIIKQGAKDYPN
>comp4958_c0_seq1; 535 261 1865 LEN=2320; translated
MKYFLVPAVVCLVLLPKGVSSFSPPASRATSRLPPPAVATSAATTTSTRLFGYNPIEDET
PEERAARMELVRKLQQTFYKEGIDDDDHQEHTEETQTGTGDADTRVELEAEAAATTTPST
TVLRDLPLWRVQWRELPGSQNVLNVHVPHYTNMFQKILKGRTKPWGGFGRNATTGDSESG
GASTMDGDHYFGHVFLDKGSENLDNPDHALQVGDEGVLMRISDARQIMDDGRLTLLVQAL
EKFRITRVVRSHSPYAVADVELVVDEEQVASAAGSEASPGHEAAARALAWHPFEVRRVAM
DECELARDASGRMGLSVSPLSNYDAGQAGGCAGSNNPPGEDADLVAIAERDAWLKLDELL
RLLGVAARGEVGLPVPTQILGLLPKTSCALAPVAEREAGKSETNPIAIALEPRPWPGGFS
LVGTADRMEAAETATVGTHSESPFVRIDAASYRSYPPLRRAQRFSYAVWTLIDSVDLDSA
GGDGGEDPYGRARILEIGSTRERLALATEKMDRICALLQRIIRMHDGRGSGVRG
>comp4960_c0_seq1; 440 1 1525 LEN=1622; translated
XAPLSPPIESCANSGGGASEDSPSLWYSLVGSGKGIRASVCRDPTNFDAVISVYAASSCD
DLVCVSGSASEESTGETGSSCDTHWVAEEGTTYYIRVHGSSSAETGSFNLFLETLTNDVT
ELCSDENSEGTVDEACVACSKERANRIAMFDDPNDVDCRCIENDGTGGFHLTCVYVSCLK
CNPRQDRCGFDTFELDIESEGGVSSLGSYESFYFMNGNGEENQANEIAIIKDQECLEMRD

PYQQCMMAKEEMTGGEDSKVFCDCRGISEEGDYMLLCSIYDAHDYCSTGSSGGDEICATI
LFGQTISQYGAVASDFRTYNFRSGGEDRELVVDRSTDECTVTVNDQQCSSCRVIECDTGP
GREDALDVSARVGAGTRAFHDLSIDCSNVFGGETATYECGSAGVGELSLLAGELSAASEA
EAPSPTPPQTLPPNAFPPTLPPVTKPTLAPETPASAPPVEPNTTTLVYDEGAGGEGESSG
AFGPRWSHAQTTMASAAATAVLAISLVL
>comp4961_c0_seq1; 281 307 1764 LEN=1907; translated
MVLLVAGVFVLIWVGAIFSMGFSHHQNAKHNEHHLAADYDLSNVRKPRHLTNDQKRQQFE
SMKTHKTGTKKHGNGSTIKNPYEGWQPVIDPNQDMGNKKCDSWRTCFIKDHSCPAKCRDS
KSDMGEAPPRPGFTPDPYGDAEDNNEKAQAVEWIPDVTVLHRMLQAGKDEHGNPWPPPLV
TDTNRELCEQIGVFGGQNDDNRVALNAIPMRGKDLLGKGIDYSSTRMKRAPRVLCMVYTM
ENNHHTNIRAIRETWGPGCDGFLAFSTRDDPRLPAISLPHDGEEAYGNMWQKVRSIWRFV
GTHYLEDFDFFFQGGEDLYVLPQNLRSYLADAIDDPNTQDFFGGRRFQQGSQNIFFNSGG
AGYALSRATLRKLIDTGLDHPGCFPDKKSSMEDVFIARCLKDVFEIGLVDTRDEKGRERF
HPFSPGSHYTWEPPTNGKRDWYEAYNRLWPPKLKEECCAPDSVSFHYMKKPAMVRHVHAL
LYNCD
>comp4962_c0_seq1; 1174 341 2674 LEN=2732; translated
MDRQLLARATENSEAPTPGYMYLDIAKNAAGNPQTSADIAKYLTGRLASKNNPNIKFKCL
KVIAKTAESPYSRGQFKRCLSQDPRAMAAIKEATQFRGRPDPVRGDQPFEKVRQAAKEAL
DAIYSDTPAVAPSGGGGSFASSVSSSYGNPSGGYGGAPPPGAGAGPRRMEGIGNPMFSDP
RLEPQPQGISNMTVNEVVSEAKDTIIGMIKDPLARNASVQISNRQGQMPRPGSYSGPGGS
FNSGPPGRSNLMNQTNGQWTMASNRGPGAVAPPPNYANDSAYYKSREAAGGNAYSWAQAN
AGGAAGGVGGSWGAAATAHQATPSITVNTIPGMGAGVSGGAGTGVSDGSYEKQLVMELCP
PGGMKPEPPPDKLAQFARVVSSLNPDFVCPVLLDCMEEGQPWIIRAKALCAMECCIKNGQ
KPGAPSNAFADFLHACNGEFAPLANHSRAAIRDPARRVLNLLGVATPVGGAVMPPVSHAA
APVAAPPAAAPPAPVANLLDFDSDVPVAAPAPVAPPPPPQQSPPKAPNAAAGGGSLFGGL
NMASTPSTASAAAPPAAPPAVAPPAPAAENGLLDLMSGGAASPAAASSGTAEPKSAFGFM
NTETAAPAPTPPVAVAPAADMFSNLSVKGADEPKSSEPAPAVTTPSATTGSAFGFMNTAA
GTPTAGAATAPTASPSTDSFDPLKNAASPSTQTKMMSMSPEQMQAMMYQQMMMQQQMQMA
QMQMAMQQQRGATFMTMPGHVMQNPAASKSTFAFMDKPQKKDDKSFDFVKDAMKSEK
>comp4962_c0_seq2; 926 205 1758 LEN=1816; translated
MASNRGPGAVAPPPNYANDSAYYKSREAAGGNAYSWAQANAGGAAGGVGGSWGAAATAHQ
ATPSITVNTIPGMGAGVSGGAGTGVSDGSYEKQLVMELCPPGGMKPEPPPDKLAQFARVV
SSLNPDFVCPVLLDCMEEGQPWIIRAKALCAMECCIKNGQKPGAPSNAFADFLHACNGEF
APLANHSRAAIRDPARRVLNLLGVATPVGGAVMPPVSHAAAPVAAPPAAAPPAPVANLLD
FDSDVPVAAPAPVAPPPPPQQSPPKAPNAAAGGGSLFGGLNMASTPSTASAAAPPAAPPA
VAPPAPAAENGLLDLMSGGAASPAAASSGTAEPKSAFGFMNTETAAPAPTPPVAVAPAAD
MFSNLSVKGADEPKSSEPAPAVTTPSATTGSAFGFMNTAAGTPTAGAATAPTASPSTDSF
DPLKNAASPSTQTKMMSMSPEQMQAMMYQQMMMQQQMQMAQMQMAMQQQRGATFMTMPGH
VMQNPAASKSTFAFMDKPQKKDDKSFDFVKDAMKSEK
>comp4963_c0_seq1; 1518 392 4918 LEN=4918; translated
MNSSSGMMHRISQWGRSRPSEHFHSSVCQSETFRRHPMKRARNRMSSSKEKATSFLCGQS
ILIRKFTPAAITALAIFSMFLPETIAAIPLSTRRPTIIGVETHLSDIDYRNLQRQSELQQ
CKVALGVGDGNRDEMLGQEEYVLFVNLLASKQNSDYDAHSSFDKLPQGLKDNYNNFADVS
KGGIDVAGFRPGSAATEEQDEQLSGVCQSTIASILDSNSKPKTPVVAPTSPPGDSNSVVV
VDVSEVDCSKMIERAQCNVDLSISDSYQNDLLDESDYVKFVNRLSDNKYARYRFEELPSN
IQENHKKFVTVDGQVDISGSKPGQRVSDKQDEFLDFFCCETDWNIQNPGATLTQAPAGQP
ANTLPPTFDLFFCQRSMASSDLERNDNLNEEEYVIFLNRLTNNEFIGQSFEELDSFLKKN
FSELAGEDGQISIYGSKPDQKSISVKEKEHLENICIKTGNALNGISDPDPTPTPTPVEVE
VGIPSVEPSGQMSLPPTFDESTCLIAIASSDPDDDDYMNQDEYTEFVVRLTGKAFEGLTF
EELPSPLPETYNSLVSEDGNGIYTYGAKPDESPEQDQEEFLQQICLNVAVAYSQTEGWTE
APVIVTEGTFPPGISEGYNSFIVSNRGGLTADDLVSGLNRDGLDQAYTIFIERAIEDSDI
VIGTLVEENRLRRRKLGVDLAPNSPEIYILDDIDCPEGLSSVEKCQIAFAKFQLTIDEED
PQTVSRVYTNYTQSLISNRFLEIVLKEVDPRTTLRIVDASYPVLPEIEITDPPTTAPEVE
EEKEEEEKKKSAAGPVVGALFAIVIICAIIYVLVKGVPFDLPFGLKLPAFLDGRRRRVGI
KGEDGAEGGLVQGDDERSASGPFGLKSSVFGDDEEDNDEEGQRQNLFGFVGKKKKKNDDF
GGFGLESRDGTQSYGTSVNHHKYDYEEPSEAESEEDSEDDQRSGVDEDNAFGGSTASPTW
GADNVFGSDPSSSTPGWGADADADGDEDFFGEGSALGDKKEGEEGSGEGSEESFSSDDDT
YESGELDPSEENSEPISENSAIDAVEQESFSVSSADFSDNSKSLSEKLMGDELKKKNDDM
NDAIDKGDWDAVVNATKSFGKSDVDSTMSKTPLGDSEEMDFDDDDSYTDYSGSDEGSVYS
ETSATTTSEILEQRAEYRTQVEELVRIVLPNETEKVDAMMDQFKGREAELVSTLQTMEER

SGNQRARAAVHKSKPPAPQQNAGYSAANGAGIQGGEGSTAGTAAIAAASLPIPAEEMFDE
DEDGDDTFGGGSFGTFGGGGVEPIAEEGEEDYEEEGIEREYSGDFEDGSRSYYSDEGGSR
SVGSRSFYSQEEGSGGSRSFYDDEEEGPQNGDEEQGQVPPYDDEGSKSFYSQDGEGSRSF
YSQEGSVPRSYHSQEGSGVPGAGTAGKDPGARGAGSAGRDRLVRGAFTARMYPVVPGAST
ARREAGAIIPKSEATIAARKETPRRAAFRARREAGTTTRKSHQIEQIKTTGMVDSVEHLN
IIEYSILAV
>comp4964_c0_seq1; 214 160 1197 LEN=1471; translated
MTKAPAALGAILCVALRLGILATLGERAVGFPMAAFLPRACRHGFGGRQASFYRAGTVAA
RMASNTTDVGAGVVGGGEILASKRPGSLPADLATWFDVSLPEGRCIGVKTANETACFPQS
PPGTELEPDHWIRSFFHSDEIEFGMTLKQTRNSFWLGRLAMRMALGYPGYPIVRDYYGRP
RLREGAFGSISHKKGRGVALASHSVADPGLPGAIFSGVGVDLEMTSRPGRPSLAKRVLTA
SERESLGGIPGIAADEEVLLRFSLKEAIYKAAHPLLCQYVGFQEAEVTPLPDGTASCSWL
LESGADDRIAGLTAHWRTIDDGAYFLTSASVFARPDFEGCPLADE
>comp4965_c0_seq1; 147 310 495 LEN=851; minus strand; translated
MAIVHENTIDGSEQVRVYAHPCGMQHDAQTQRRHGDVPVVGVIGCTHTNHPTSILFGKND
P
>comp4966_c0_seq1; 458 228 1631 LEN=1834; translated
MTLLHHGIANTLPRLSTKYATAIRRLIASSSRVAAIGSGEHLSPVWGHLTTMQPVKGEGI
YLWEADGTKHTDFTSGIGVTNLGHTHPKITEAIREQATKLHFGQMNVVMLPQFVELSEKL
DEITPDGINNFFFANSGAEATEAAVKLARHASGKKNIVVFKGSFHGRTAQCMAMTTSKYI
YRENYAPLPGGIHVTPFPYSYFYGWGQEATVDFCMKELDRVVMGQTSPDETAAIFVEPVL
GEGGYVPAPNAFIERLREFCDRHDILMVADEIQCGFGRTGKMFAIEHSGVRPDILIMAKG
LGNGMPISAVGASHELMSRWKTGTHGGTYGGGNTIVARAALATIKTMRDEKIPEKAAAMG
EYLMDKLRALRDRIPIIGDVRGLGLMVGTEFTDPSSGKPAGDVAGAVRSACIEKNLLLLS
CGTYDNVIRWVPPLIVTENQIDDAMDIFEQAVMNATGGMSSKQKNIA
>comp4968_c0_seq1; 390 568 1410 LEN=1410; translated
MISLSKEKAAAISRQNTALYDTSRRFGRNNEAERLKRKRDELEAKALQQELLTEVREAEQ
ARKRIERDIREAEERRRALDEQSSTGRDYIDNLRSGRAGAVNDAKVFLDTGISGKKRGRP
SKLDIERERLELELEQLKSKQKAEENTKLLTTYGGLATVLGVAANVLNANGDLGDVGTAS
GLRQLFPSTSKYLTDLSAENDNQRITAAAAKKASGNIEMPYLDAKIAELEKKVKEERNTV
KEEARELDRLAREKEEARKKGLIEAEQKAEREAKEKAEKEA
>comp4969_c0_seq1; 252 1 955 LEN=1167; minus strand; translated
TDRPTDRPNQPAKQPRLPVVSEHDRDETEQNVPTEPNPPVVPPAAGVLADRRGPGLCFSF
RPPAPASSSRPSTRLLGGGFGKDSPNYKAPSKSYGKKELEPIKDLIDEESGMREFFESNE
PWQPLFRSVAPDDSVPAMAFLGGAAGEGLEFTETSSPWRKLPAVPTDEADIAVLGRFLDA
MQTSLIEDIPVDESTKDDENDLHFLEEGRRMLVCSRYHVVQGIEKGSVETFDRLFSICWS
ELTELREAGEEDSGSLIVAPGFDYDELRRFVDMNLQRPLQWLGVEGFEVASLEKGGLGVI
RIIYKLSDIPTELPDRG
>comp4970_c0_seq1; 245 1 968 LEN=1227; translated
XNRSCVENYEAITMDYSKGQRHFSLSLMLLGGLFFIGGSSILGVGVNAFVPRSSCLSSKS
IQTATAIAASNKPIVVTLTREDGKNTKLRRKIEENTQLMEQIDILEMPCIAHADGPDYER
LSSTLLSERWDFVAVTSPEAAKVLASAWDAVRDNPLSVVAVGKATEKSLKDSKIPVSFVP
SIATAKTLAKELNPVHETKKTSVLYPASAKAKKTLQVDLMARGFDVTRLNTYDTVTATWG
DEEKESSRRVEVACFASPSAVEGWLKNTEGNTDVFAACIGITSATACREHGWDEKQIFFP
EAPGLEGWIDAIQDATEASRNT
>comp4971_c0_seq1; 549 1 2043 LEN=2187; translated
QEAPHREPTTDGTYKGSFVLFQRTYNTAMPDLTSLSKPEEAIVTHLDWEISVDFEKERLC
SVATYSVRVLESNATTLNLDTAHLDIASVTCGDGNPLKFTLHPAAEDKSYLGRRLEIDLP
RRDSNDKCRIAVSYSTTKFCSALQWLPPPQTAGKKHPYLFTQCQAIHARSLVPCQDCCGV
KMTYTAKASVPSWATCVMSALLKKEEEDKASGGKTYTWDQPVPICSYLLALAVGDLTKRT
ISERCAVWSEPGIVESAAYEFSETEMFLSTAEAITGMDYAWGRYDLLCLPPSFPYGGMEN
PCLTFVTPTLLAGDKSLADVVAHEIAHSWTGNLVTNATWDHFWLNEGWTTWLQRKIMARM
KNNPKFLDFDAIEGRKDMADTVEEFLVSAPENTSMVLNNGDGDPDDSYSTLAYEKGFTFL
LYLERLVGTREFEKFFQAYVARFASKTLTSTDFKDFFLQHFAGNSKVGEIDWDAWFYSQG
MPPVLPPLDQSMAKASTDLADAWCAFDRGTASSPPATNETSAWASGQITCFLDALQSLTA
EKALKVSTLRAMNALYRLAESRNSEILFRYCQLAIASEDESIIHVAVRFITSQGRMKYIR
PLYKSLYRSKMGKNIALSTFLKHKDIYHPIASKMIAIDLKDAMEGSTTSNLSMPTNHILM
TGAAIAVAGIAVALVRKSRK
>comp4972_c0_seq1; 721 232 2025 LEN=2223; translated
MFCQPKIEPPRLSLLGQNNNNQLTSSQKKKRKSNTIAGPSSLDEENRLQRSTNLTINTTD

CDLSPQKSYQHQINKSPRLEKSVKNNEVEDNIVVDKMKDSTNLAIDTLNDDLKSSNIHSK
NPSPNASECASEVSSVSQRREWLKSFEQKQSSNIFRSLTKDERPEMSQSRQVDNRNKKET
RDGNTEKDPRQSPPASPAKSNVSFNRKISISHYPTGVTNSATKPKQRRPAINRRASSAVM
MRSWKDPRVHEGVKATDEGYASVASLSKWLENDPTSVKKKRHIRRGRNIISKSRQFERGL
EDVIVLENKISRGAVGDKKKWLENAFQSVQEEHEDDTSSIYSGYVQSDVGRSFRSHPNFN
RQGAQTEIITDDAASSMSVSDKQTWLKNAFSKSSDERKRPAYTRARTDIVHNRGQNRDET
ASRAKMMFKERSARKLSSAQSRSSLMPTDPKKSVALEAPVSSSEDFPEDEAASQPEEVIV
AKSEEEAPIEPKKTVQISESVEEDEAPVDFRAARQALIERSKKNGHNTQVVNKVFLRKKK
FEKLEAESRRKSMGVGLMLKSSWDLVDTSSGRPSNVYEKRYVPTQNIAPKKSFEELP
>comp4973_c0_seq1; 413 136 1689 LEN=2127; translated
MKSRSCFEAIGLLFSRLRVKNKGKDHEKIGNGRVRCDFSGNRSCGNATCDLVSDTEDVFA
RRPGHDNDESTFYYFNHASQAPLSPEVQRIGIEQIQASPWHDPNPNEMAHTSQARIRELF
TKLIDGGCCDDIDEPIENGEVLKATTDGSRIAILPSTAFAITLAARNIIEGQRRQQRKYG
NGDSYNPVSGRILVLQDQFDSAIYPWQQMCDESKGKLRLDIVEHPDENACPSVTSNGVRS
NNGQGVIDGIGAPNGGWTQAVLQKLKSDDDHSIIAACLPPLHWSDGTLIDLEAIGAVCRH
RNIPLVVDATQAVGIMACSVRKIRPVMLACSTHKWLRGPSGCCLAYISPEVQDDWIPLDF
HGRGRDFEAGADSWDVSKNEMGPKGYPERYYADSRKFDSGGKANPFLLPMLRTAMEEVCK
IDTGKAQIRLKTLMQPLFDWAVRNGHSIKLDPRAYHLIGIVLKDKTPEEMIEISKRLAEE
KKIILAVRCGGFRVSSYLTNTEDDVRKLIEGLQDTVS
>comp4973_c0_seq2; 230 186 707 LEN=1145; translated
MDPFLQVQDDWIPLDFHGRGRDFEAGADSWDVSKNEMGPKGYPERYYADSRKFDSGGKAN
PFLLPMLRTAMEEVCKIDTGKAQIRLKTLMQPLFDWAVRNGHSIKLDPRAYHLIGIVLKD
KTPEEMIEISKRLAEEKKIILAVRCGGFRVSSYLTNTEDDVRKLIEGLQDTVS
>comp4973_c0_seq3; 189 200 808 LEN=1246; translated
MACSVRKIRPVMLACSTHKWLRGPSGCCLAYISPEVQDDWIPLDFHGRGRDFEAGADSWD
VSKNEMGPKGYPERYYADSRKFDSGGKANPFLLPMLRTAMEEVCKIDTGKAQIRLKTLMQ
PLFDWAVRNGHSIKLDPRAYHLIGIVLKDKTPEEMIEISKRLAEEKKIILAVRCGGFRVS
SYLTNTEDDVRKLIEGLQDTVS
>comp4974_c0_seq1; 297 1 433 LEN=700; translated
XAKAKDEAEARAKEEAAAKAEAEAKAKEEKFERAKKEALAWAKEEAAAKAKAEAEKKIKI
NIICWSGDMVVIDDVCGNTTIDEVKERLVTLKSVAKEHQTLLEGGIELSVDDKSLLDYGI
GDDDALMLDNPDKKKKQGGGGWFGW
>comp4975_c0_seq1; 583 1 3017 LEN=3272; translated
XQPNPTQPKPTQPSQIQSKTILTPTQEMRLMGKSSVRSFQGCNSDNTIDSQRQRQRQPPL
SSVATARTARAKLCTFPTKSSGSWISGGIWAALFWCLYAVAQCGLLHVYHSAALRRSGVE
IEIERERVVPGNARYTNGNSKSSNKSKSTNEQSRYKDKPPAPPTPDGIFNGYPIYKLRGA
GSNFTGDDTSHSGDSPAPNHKNNANANANSTPANKRSSTSRPLYXPVPLRWGDLETTRFP
SKVEHLARGELEVPFLPLPVFVFDTEQQEYVLFLDPNEHPDTNHEHIEHISRQQQQQQQR
NPRSESGTKTETMPHHPNPSLQKILRKQREQVEAQERKAKRVNANAKANSADIQGKKKPL
LPLSFKQPSLFDDTSTVYRNLTSIVDGKGDVTVGDVINGKHYGVSIGSMNGKWGLVDGQR
LKWFPRIEWGGVPLDVDVYTLPSSVVVVPFHSLSASNPGHLVWDDFLPMYTLLDMFGLLH
TIGGDDDETGGANSDKANNTPPDLLPIRYILPPGPTGDARGLWAGCDWLDSRAQECHAML
QKFGTLLGTRRSYKTHGFSQQDTEKTRDGVDASKSERSNTPVLTASKMPKIPITTNRDVE
LRLRKDYDDNDWQQISQRNTNTNGDSPPLSPTQKRALVCARNGLAGFGAISDHGDGSRSH
GWEENDYKVSYNQGRGGKLWDFRNFMIRNLYGENNANSNGSNFVENENATAIVRTTFVPA
ATASTTGIHERDPDRRRVEGITQGGKEEQRPYYIADSVFRGIEEIGGHQREQQQQNEWYP
PASITEWGPDEPLVVLFSSLSSKTRRRDFVKEANDLRRVIAERSGPDDPQIIVETHKFSD
YTLEKQIQMASRAAVFVTFCGGGAITASFLPKGASVQIYYNEKGGIRHNIDTQLPARLDW
DFFNNAGYLHVQWLPVISIDKTRRQPHKTEPNTTTIPGSSGAAKNVPSDLIFGELRRIHR
ERKAFFLSAASHPEDGGHERISVQKEPNKPNHAILLLSPFVYRIY
>comp4976_c0_seq1; 326 1 439 LEN=439; translated
EPSTVEKQSDDGFGDFDSARTQSGTLDTNQADDDGFRGSPQAEASNIQNQGDIDDDDNFG
DFDSAPKNVELSGHGDQANDGCDDDGFRDFNATSPNAEISNDGNESHNDDRDNTFRDFGV
VTSQTEASTVEQLADDDDNDAFGDFGX
>comp4977_c0_seq1; 930 106 2880 LEN=2903; translated
MAEQRTVTDASTKAIEKALVLARDNGHSTADPMHLAAVLFNGDDSIGARVCSKAHGASDV
TLIRRNLQKLLLKRPSQSPAPLEATPSHALAQLLNRAKKAAAANGDALIALDHLLIQIFE
ERELKKAIETESGLTKKAAQAAVDEIRGKKKVTSASAEDQYEALQKYGIDLVKSAEEGKL
DPVIGRDEEIRRMVQILSRRTKNNPVLVGEPGTGKTAIVEGLARRILEGDVPETLKGVSV
RTLDFGALVAGAKFRGEFEERLRAVLDEVKSAEGKMLVFVDEIHLVLGAGKTDGAMDAAN

LLKPMLARGELRMIGATTNDEYRKHIEKDTAFERRFQKVIVNEPNVENTISMLRGLVGRY
EAHHGVRIADASLVTAAELSNKYITSRFNPDKSIDLMDEAAATKRTALDSRPEQIDQLQR
RILQLEIETTALKREKDKKSRKREKELQEEISNLKEEVAPLNAKWEAERGRADELKESKT
KLDALEAKLLQAERVGDYVKAADLKHGAIPDLKDHLANIEREEAERRKNRNKDDDDGSIM
DEVVTPEDIAGVISRWTGIPVSKLSQTDRERLLRLDQRLQQRVVGQNPAIKEVTDAILRS
KAGLSREGQPTGSFLFLGPTGVGKTELSKALFSELYDGDERHLIRIDMSEYQEQHSVARL
IGAPPGYVGHDDGGQLTEAVRKKPYTVVLLDEVEKAHPKVLTILLQVLDEGRLTDSKGRT
VDFTNTVIILTSNIGAESLLVLTENSQEHEVEAAHDKVMEKVRYSFAPEFLNRLSAIVMF
NSLGIKQLEKVLHKSMKGVKRRLASRGIKIELERSGVQAILEASYTPSFGARPVERYLEA
TVVTKLSRMLISGELTSGTIVHIEGKEMTQSEESEHGRIAKRSRNGHHLYYRLENADYDN
EVKNDFEMIDANDSEMELMSTQLN
>comp4978_c0_seq1; 1330 210 3830 LEN=3894; translated
MRISAAGISFVAALCFTTASVSAFTTPSQRAISIQQQPSVQGSQATILNEKDDKKGNFFS
NMFNGSGESNDGTTALRGKLPPHPSVRPHISPLNLLGPDPQEKPSSDPLPVHPDVRSGTL
PNGLPYVILPNKSPPGRFEAHLQVFSGSADELEPQQGIAHLTEHVAYMGSRKRELLFGTG
SQTNAYTDFHHTVFYAVCPVKTPRGDMPMLPMALDALVDVMEARTEGSRLEKERAAVLSE
MTMVNTIEYRVECQILSTLHRENRLAKRFPIGKESLIRSWNTDDVKSWHRQHYRPDNVLL
YLVGDINPDEAEKVIAQKFGRLSAEKQGSEIRLEEIKEMASDLSEAVVEGSVKAAQSWHY
PPVRHDWCAPIDQEVPPELIIPANSLEYDLQLQESYPLDEKTNFLESEEVAPGKQIRPHI
FRHELLQSFSLHFFSKRPVEPIVDIDSFRKSLARRVALAALQIRLNVGGRSDDPAFTFVE
FNQLDSAREGCAVCSLDMTAEPHKWKEAIHKSLSEIRKLGVHGVTMGEMERYASSLMTDG
EQLAAQGDRISHGDQLSYLMETVANGHTFMSPEQSYHMTAKALSSLTIEDVNAAAAELCS
HVTALKDGELPLSGPVIAVACTPKGVSEDDPAYCDEHSLVQAIYDACQIDVEPEEDVVVP
FTLLPEDKLAQAMEENPPVWLGGQFSDGTPNTAPDTITRPFTLRRLGNGIRVGVSYNSHE
SQRGHLRLVAPGGRDAEKRLGFKKGSMSIGARTMQEGGAFGPWTREQVELFCVDHLLMVE
INCSEESITVDFVFPTNNVGNVGFGDDVQLGITGTESVLQIVREIIVGFKWEDDALGRAK
SSFRTTHESLLKNLENFSTERIMEAMTDNDDRFLSIDVDAVNDISLADAKNAVMSQMSPS
NLEISASGDFDVTEVLEMIYKYIGTIPADANSEFLKEDVGLEQGAAVGSVPALPKPGKFL
ELELADSDPRAVAYVAGSAPNAWGYLSDGTSMADYIMSQDKRATDYDRQRRKHPLFGHVA
LLLLSEIANRRLFSYVRERKQLTYDANFSFTGFERLLGGFFLVTVTASKENAAKALQACK
ETLFNLKKGQRITPDNVESAKRVVLNRHDFELRTTAYWAQLMSGIQEESVPLKGPLSFTD
FAKVVEAITPTDLQLALETMGIEDDELFTAIGRTVQTNVPTDDEDETPLVRQAPAIGMRR
GGALRD
>comp4978_c0_seq2; 1285 210 3961 LEN=4025; translated
MRISAAGISFVAALCFTTASVSAFTTPSQRAISIQQQPSVQGSQATILNEKDDKKGNFFS
NMFNGSGESNDGTTALRGKLPPHPSVRPHISPLNLLGPDPQEKPSSDPLPVHPDVRSGTL
PNGLPYVILPNKSPPGRFEAHLQVFSGSADELEPQQGIAHLTEHVAYMGSRKRELLFGTG
SQTNAYTDFHHTVFYAVCPVKTPRGDMPMLPMALDALVDVMEARTEGSRLEKERAAVLSE
MTMVNTIEYRVECQILSTLHRENRLAKRFPIGKESLIRSWNTDDVKSWHRQHYRPDNVLL
YLVGDINPDEAEKVIAQKFGRLSAEKQGSEIRLEEIKEMASDLSEAVVEGSVKAAQSWHY
PPVRHDWCAPIDQEVPPELIIPANSLEYDLQLQESYPLDEKTNFLESEEVAPGKQIRPHI
FRHELLQSFSLHFFSKRPVEPIVDIDSFRKSLARRVALAALQIRLNVGGRSDDPAFTFVE
FNQLDSAREGCAVCSLDMTAEPHKWKEAIHKSLSEIRKLGVHGVTMGEMERYASSLMTDG
EQLAAQGDRISHGDQLSYLMETVANGHTFMSPEQSYHMTAKALSSLTIEDVNAAAAELCS
HVTALKDGELPLSGPVIAVACTPKGVSEDDPAYCDEHSLVQAIYDACQIDVEPEEDVVVP
FTLLPEDKLAQAMEENPPVWLGGQFSDGTPNTAPDTITRPFTLRRLGNGIRVGVSYNSHE
SQRGHLRLVAPGGRDAEKRLGFKKGSMSIGARTMQEGGAFGPWTREQVELFCVDHLLMVE
INCSEESITVDFVFPTNNVGNVGFGDDVQLGITGTESVLQIVREIIVGFKWEDDALGRAK
SSFRTTHESLLKNLENFSTERIMEAMTDNDDRYXTDKIVCVSALLVFALPYCHASRLTHA
HAHIHILPLVLDLCHSFLSIDVDAVNDISLADAKNAVMSQMSPSNLEISASGDFDVTEVL
EMIYKYIGTIPADANSEFLKEDVGLEQGAAVGSVPALPKPGKFLELELADSDPRAVAYVA
GSAPNAWGYLSDGTSMADYIMSQDKRATDYDRQRRKHPLFGHVALLLLSEIANRRLFSYV
RERKQLTYDANFSFTGFERLLGGFFLVTVTASKENAAKALQACKETLFNLKKGQRITPDN
VESAKRVVLNRHDFELRTTAYWAQLMSGIQEESVPLKGPLSFTDFAKVVEAITPTDLQLA
LETMGIEDDELFTAIGRTVQTNVPTDDEDETPLVRQAPAIGMRRGGALRD
>comp4979_c0_seq1; 311 1 1098 LEN=1764; minus strand; translated
HMTRQLPNKFLPKSATSKGKDRRIRGTTKLESFDHENRRAGSTVYSVSANEHPADPSRTR
SKTSVSRTNWMEEAAAATKKRGPLHMGGGRRGFSLSQFQEKRRTSTAGNASFVTPANRNR
KTASINTVGLGYDGTVFVPKENKILFQQSASIQEIPAKRMVTPINESDRTKQLRQQQAEV
AQRLQESTTKEKSSGLSSALVNSSNVKQSSRCRKGVTSEKGRKSDNCEVDAFLAAMGGAV

DEDKVRSAKSRFANEVAADEYVKNRRKLLELEKLEASNESRNKKKHQNAEQRMTKNWYCF
NCRQTYNQRPGRCISSNHNVKNRFGLKESTSKDEERNALHKKSVQDGGLHLGSGIEWRDS
GRFRT
>comp4980_c0_seq1; 523 248 1303 LEN=1531; translated
MTINAFLLALLASVFAPIAFGQDSNSTCASIADIVCAEDNLNAVCEALSISGLDDDLAEE
TWTVFVPTDEAFEELGRDNLDYLVFGNDTVPLTDLLLFHVVPGVALTSDLLPCVAGENLI
EMANGDDSRTKCRRGIPVFQKGKFNTQEDSPQFIETDIMACNGVIHILDKVMLYESLPHP
IPVADAEEEPEEEPVADAEEEPEEEPEEAIEEAVEKCQTIVELACSNPRFTTVCALVNEF
GLADDLSDGMWTVFAPSNQAFDNAPPFPADVDLNFVLQGHVVANEAIRAEDLVCTEKIEM
LNGKLTRTVCKNGTTYQNGVDNDDDNRPEIVLADIEACNGIIHAVSQVILT
>comp4982_c0_seq1; 209 1 338 LEN=338; translated
GDGDGDGDQDEGTKKPDNSAAEGMDGGLDSQNNDDWKEPAGKVSGSDLDSGATGGAVGDM
VSPNMDEATKKPNHDDQDEATKTPEANAEDDDNTGATGDGEGDTVSPKVDEAT
>comp4983_c0_seq1; 384 331 1453 LEN=1453; translated
MMQRYFLIIASTILSALICVDIAVVDARELFESFNGRIGPDSSSWFEPSLWNGKGKVPTQ
NDAVVFDESDTYAAIDKKDIVASVSELIIGRSASSDSIGSVQLDLLSQTKLAVQQDMTIG
ELEGSDGSVYVDDNASIAVEGSLTVGSGGNGLLVLAKKASIRTGNLSVRGDNSGGGSKIV
MGVASTLTVAGDKRTMVNEMIENGLIVKETMDSTATLKVGTTDGNTVVTVATEPPEPPIS
CENRGLKYRNQGRKNCKWVAKKKNKRCKLSWKKEKLSAWCPGSCNKSCIDDGDGNNPCAD
GTLKYKNIKKKNCRWVKRLTRSRCKLQVSDGVKVSEICKKLAANANFYSAKAKFTTHGKK
SINEFNKISIYVRQX
>comp4984_c0_seq1; 249 1149 1496 LEN=1868; translated
MQEGRAKLFDLVYVSSDNDSDQMKGEIEGGWDSIDYEKKEDRSMLKKCFGICAQKEMEAL
GITSEQRKGGIPTLLLIDKGNSVVISPDGIPDLMGDNKVEDPLAKWKALLLPKES
>comp4984_c0_seq2; 308 752 1249 LEN=1621; translated
MSSTSLSKCLTLVKNAVDESSLAPIIALYFAASWCPDCVESKPYVSNVFKMQEGRAKLFD
LVYVSSDNDSDQMKGEIEGGWDSIDYEKKEDRSMLKKCFGICAQKEMEALGITSEQRKGG
IPTLLLIDKGNSVVISPDGIPDLMGDNKVEDPLAKWKALLLPKES
>comp4985_c0_seq1; 310 390 1529 LEN=1976; translated
MGAKALLASSASLDDTSSGLGSTSQGAPMQKAIAYRTISLPIPEFDVNVPVSIWYPFQNG
ANGSQPAEIKYSYRISLRRIGQLLARWDFIPDFISKNFALNPSITTASTVRVVDGENIPI
SVTSTGTNVVFLAHGFLGSRSDLAYLAEDLTAQGFVCVASEYPESLEASYPRLEGLDRVV
INQLLIPYVQDMVPQPILSYSAIGHSLGCGTVLQMGDDSWNRVLMGSGKAPELPSLGNVK
PEVSTTTKYQTPVVGGRLLFISSVNDGPVTSWGGGIKIPQGYTVLEESQLDTATKFDRAA
LVFDREDAPNHISYLSENVNDAMLSFLSPLLPVTKALNIPVLDFDKYANSRDSVPTGNVL
KPLISKFLLTAPELASANH
>comp4987_c0_seq1; 156 160 696 LEN=1056; translated
MIRAARIAPPSLSSLASRAVVRGNSSVGVGLPGRGTRARFLPLPARSHSTTTALYSPVAA
ASRSGGGSIRGTPHGFLSSLRGFSSLLWPPAAVPNTAIFPGLFAPARTAAAGEAASLSCR
SNSSSILSWAIWLIKRTYQPSLLKKKRQCGFMKKRRQSVGGRRIFKRRRQKGRKRLFGA
>comp4988_c0_seq1; 359 349 1710 LEN=1842; translated
EGTEALADGEYDAIVLGTGLKECVISGLLSVKGKRVLQVDRNGYYGGDGASLNLTNLFEK
FGAGQAPTNLGENRDYNVDLVPKFIMACGNLTKMLLHTKVTRYLDFKSIAGSFVYKGGKI
HKIPTSGVQEVLASPLMTLFEKRRFRQFIIYVDQYKEGDSTTWEGRDLNTMTMSELYKDF
GLQPDTAQFISHAMCLKLDEAHMNEPALSTLKELQTYMYSLMRYGTSPYIYPCYGLGGLP
EGFSRICAIHGGTFMLNRDVDEIMFDDNGKATGIISSEDGKKMMAKAPMIIGDPSYFSQD
KIRPTSQVVRCICLLSQPIPNTNDSESAQIVIPGPQVGRVNDVFVCSMGHALQVTSPGVY
VAIVSTISEKHDPDQDLEPGLKLLGRILKRFTSVSQQYEPVSDGSVDNCFISKSFDATSH
FENDCDDLLSLYKRVTGEELDMNINADSVEGDY
>comp4988_c0_seq2; 281 349 1455 LEN=2008; translated
EGTEALADGEYDAIVLGTGLKECVISGLLSVKGKRVLQVDRNGYYGGDGASLNLTNLFEK
FGAGQAPTNLGENRDYNVDLVPKFIMACGNLTKMLLHTKVTRYLDFKSIAGSFVYKGGKI
HKIPTSGVQEVLASPLMTLFEKRRFRQFIIYVDQYKEGDSTTWEGRDLNTMTMSELYKDF
GLQPDTAQFISHAMCLKLDEAHMNEPALSTLKELQTYMYSLMRYGTSPYIYPCYGLGGLP
EGFSRICAIHGGTFMLNRDVDEIMFDDNGKATGIISSEDGKKMMAKAPMIIGDPSYFSQD
KIRPTSQVVRCICLLSQPIPNTNDSESAQIVIPGPQVGRVNDVFVCSMGHALQVTSPGVY
VAIVSTIR
>comp4990_c0_seq1; 199 148 680 LEN=790; translated
MPPQHLHDSVSNXVLDIIALRKDGLRGQAFVVFDDVQAATAALQTEQGFTFFGKDLKLAF
AKEKSDRIAKRDGTYVPRAKRRKREVNSAADGGEELTEAAGKNTEQANLDSAGAPPPPSS

GAPTAKVATTSATQQQPTHILFAENLPSECNEMMLAMLFRQVRLSYETIWGSNGWTV
>comp4990_c0_seq2; 321 194 910 LEN=1270; translated
MTSSIAQPNNTLYVSNIDWKVKKPLLRRALYSLMTRHGKVLDIIALRKDGLRGQAFVVFD
DVQAATAALQTEQGFTFFGKDLKLAFAKEKSDRIAKRDGTYVPRAKRRKREVNSAADGGE
ELTEAAGKNTEQANLDSAGAPPPPSSGAPTAKVATTSATQQQPTHILFAENLPSECNEMM
LAMLFRQYSGFKEVRIPRPGLAFIEFDTEPHATLALERLNGFKLTTADALTLTYGKA
>comp4990_c0_seq3; 269 194 805 LEN=915; translated
MTSSIAQPNNTLYVSNIDWKVKKPLLRRALYSLMTRHGKVLDIIALRKDGLRGQAFVVFD
DVQAATAALQTEQGFTFFGKDLKLAFAKEKSDRIAKRDGTYVPRAKRRKREVNSAADGGE
ELTEAAGKNTEQANLDSAGAPPPPSSGAPTAKVATTSATQQQPTHILFAENLPSECNEMM
LAMLFRQVRLSYETIWGSNGWTV
>comp4990_c0_seq4; 251 148 785 LEN=1145; translated
MPPQHLHDSVSNXVLDIIALRKDGLRGQAFVVFDDVQAATAALQTEQGFTFFGKDLKLAF
AKEKSDRIAKRDGTYVPRAKRRKREVNSAADGGEELTEAAGKNTEQANLDSAGAPPPPSS
GAPTAKVATTSATQQQPTHILFAENLPSECNEMMLAMLFRQYSGFKEVRIPRPGLAFIEF
DTEPHATLALERLNGFKLTTADALTLTYGKA
>comp4991_c0_seq1; 477 100 966 LEN=1020; translated
MSEEELKNSHKKALKSLEGEKRAAVKKAKGMKGKKGKEALAAVEADFATKLKALEASYQE
SLASLSLESLNISADNNPGNAQEVSASDDKNDDYDDEAAARERKRAKAKKKRDRQKEKEA
ELEKQIEEENANAGPGLRKIELEQIQAVLTPLKLKVSEVDADGHCLYRAVAAQSDRNHMA
IRSLCADTLAKHQEEFAPFCEYTNEISTFDEYVEKVRNSAEWGGHLELRALGIALGRPIY
VYSVQSGAKPLAIHEDTDNETKMREPILLSYHLHYYALGEHYNQVVKD
>comp4991_c0_seq2; 427 100 1118 LEN=1172; translated
MSEEELKNSHKKALKSLEGEKRAAVKKAKGMKGKKGKEALAAVEADFATKLKALEASYQE
SLASLSLESLNISADNNPGNAQEVSASDDKNDDYDDEAAARERKRAKAKKKRDRQKEKEA
ELEKQIEEENANAGPGLRKIELEQIQAVLTPLKLKVSEVDADGHCLYRAVAAQSDRNHMA
IRKSRGFQAMGPLSFIFAXMSSDLCLIAFCLLIYDHRNFIACCSMVVAINNIGSLCADTL
AKHQEEFAPFCEYTNEISTFDEYVEKVRNSAEWGGHLELRALGIALGRPIYVYSVQSGAK
PLAIHEDTDNETKMREPILLSYHLHYYALGEHYNQVVKD
>comp4992_c0_seq1; 399 160 1299 LEN=1656; translated
MRPLKKIALLGVFVNFYCSLSRTDAFVSNAVVGSITKQGLVITGQAPFEPILEAARRQQF
PQHFVTVNDSELTAVEEKTDLEETLLSRQDPTPKESEYSAEQKVINFLFLSATFGYALYT
ILNIDKGMTRGWSASEIAMRIPLDNWGAYESYLDSKPIVTKTLINVIIYLLGDWLSQTAF
RGENLLEFDISRTLRNGFIGLCFGPLVHQYYEFSDAILPPENGLITRLQKILMDQTIYLT
VKCSVYISAVGLLAGEDLSSVTQSVKDKIGGIVITAWKFWPIVHCITYSVIPAQHRILWV
NSVDLVWNAILSSAAQRSSVFEEVDDEEEEVSGLNIGSREFVVAVDQSDVSTAAVDEVEH
DKFIDQFNTTTSSGVLVAT
>comp4993_c0_seq1; 430 636 2318 LEN=2764; translated
MALFRKGRQNDAPPRVIHITANVNSEEPRGGSGNGNGNRHKGILQTPTKTSGVPCGILKP
SSYHRRGASSDKNKSNSDSSNTPSTVHTTWTNDSNSSSTSTSSDSTETRSLDRIICNLHA
KHGSTDGSSQSSSSARGNSSHLPPPSATNTITATTPREQKLREARIRHIEDLNERIKAAK
ARKLVERKAYRREDRLLLKLARELKVASKKLHSRALDIKQVEESNRFLEERLARTKQELC
ELRASRPELEHRLKSESMVRVANERSKHQFQLAVQDEKLESVKTTHQLRCQELCESVLDA
NFELDRLRGCLGGDNNKATSTPPSSPSRRRRAGTAKTTIGSTPERAQWWRTTGGTVLLVC
GLAMVIAMALFVDGNGHRAAGKMRSHSVPSAGLSMEFVPSSESASLRESIDSAPKALPYS
RILALEDSAAISNDLLAPIAVHAIGVGSTASPDETTNKSQGFDTIVRKELSVNRSISSRG
TSSREVIEDGPVVATRNPVDVVSTDCPLPIALGTEGRLGNTCLGPTVERKRRFPGPNEFA
ASVARLVLDGTTLGTINRRE
>comp4993_c0_seq2; 430 814 2496 LEN=2942; translated
MALFRKGRQNDAPPRVIHITANVNSEEPRGGSGNGNGNRHKGILQTPTKTSGVPCGILKP
SSYHRRGASSDKNKSNSDSSNTPSTVHTTWTNDSNSSSTSTSSDSTETRSLDRIICNLHA
KHGSTDGSSQSSSSARGNSSHLPPPSATNTITATTPREQKLREARIRHIEDLNERIKAAK
ARKLVERKAYRREDRLLLKLARELKVASKKLHSRALDIKQVEESNRFLEERLARTKQELC
ELRASRPELEHRLKSESMVRVANERSKHQFQLAVQDEKLESVKTTHQLRCQELCESVLDA
NFELDRLRGCLGGDNNKATSTPPSSPSRRRRAGTAKTTIGSTPERAQWWRTTGGTVLLVC
GLAMVIAMALFVDGNGHRAAGKMRSHSVPSAGLSMEFVPSSESASLRESIDSAPKALPYS
RILALEDSAAISNDLLAPIAVHAIGVGSTASPDETTNKSQGFDTIVRKELSVNRSISSRG
TSSREVIEDGPVVATRNPVDVVSTDCPLPIALGTEGRLGNTCLGPTVERKRRFPGPNEFA
ASVARLVLDGTTLGTINRRE
>comp4994_c0_seq1; 276 173 676 LEN=922; translated

MAGDQYNLGLLDDDDYKVSVLRTKLLGIAECFMYRLPPGSTSPFRADGWPLTKPLQCVSL
RVERRGDVLLLIFTYTHGDEGAIGQQKLFALCPIDIVNQTDVAKQKIEHFVEAVLDSTRY
FVVRVKDEKADREALIGLGFRDRSEAGDFRAALSKYENDIKKEQRKR
>comp4994_c0_seq2; 167 191 596 LEN=842; translated
MLNVDLPIQNISADGWPLTKPLQCVSLRVERRGDVLLLIFTYTHGDEGAIGQQKLFALCP
IDIVNQTDVAKQKIEHFVEAVLDSTRYFVVRVKDEKADREALIGLGFRDRSEAGDFRAAL
SKYENDIKKEQRKR
>comp4995_c0_seq1; 834 235 1899 LEN=2016; translated
MVEDTKMEDTAGIAPTKEEGPDKQVNPPSVPPLEAAGRRLERLLGGGSAAASSSGPSDNA
SGRPLARLYTYGTPVKVTRRWLGTASGAAGEAISSDIVSAATTLLDPSHEDCRTRSLLLA
IDESSSMEVEGEDNDNGNDGGEAPSTQAKDEPEYLSASRREVESWLLSLQVRILWKAGKH
PEAMELVEKGIAILLEQVTIAAKKLTSLPGGSVSSLFPLLARLYRYRSLVADSMKDPSVT
AGLRNVMAKAHNMACLRRDVDSQATLLNLMLRDLLEHSQVEQANKLLSNATFPESASNNE
LCRYLYYSGRIQALRLEYTTAFSNLSQCLRKAPTNTGLGFRITVQRLLVVVQLLMGEIPK
RHVFFSKGMKTEMAPYLAITQAVRRGDLAVYTKVVSEQSSQLRRDDTFTLISRLAQQVVK
AGLRKLHVSYSRLSLEDVANRLGLPSATSAEFVVAKAVRDGVLDATIHHEEGYVQSHDLV
DVYATKEPAEAFHRRIAYCLTTHNDAVRGMRYPPDAYKKQLEASRGNRRGKRGSDDKTDE
EKAQELEDEFDEDY
>comp4995_c0_seq2; 458 235 1076 LEN=1266; translated
MVEDTKMEDTAGIAPTKEEGPDKQVNPPSVPPLEAAGRRLERLLGGGSAAASSSGPSDNA
SGRPLARLYTYGTPVKVTRRWLGTASGAAGEAISSDIVSAATTLLDPSHEDCRTRSLLLA
IDESSSMEVEGEDNDNGNDGGEAPSTQAKDEPEYLSASRREVESWLLSLQVRILWKAGKH
PEAMELVEKGIAILLEQVTIAAKKLTSLPGGSVSSLFPLLARLYRYRSLVADSMKDPSVT
AGLRNVMAKAHNMACLRRDVDSQATLLNLMLRDLLXTLPR
>comp4996_c0_seq1; 503 174 1964 LEN=2071; translated
MNGSENGNHRRRRASATGSMPDVKVFLSRPTYPLGGTVVGTILIQQKGPLPLPLPSEPSG
EGEDGEDEADLHPLRAILESVTVYVAGFCKIDSRWHDSRDYKKIYGKMHPYLELLQQDEL
TFDADLLEQSEDTVCIWATNGMEALDLPERTEGRRWGGSDADFQRGLGYDGDEDSKSMGD
SGNDDESDDCGAASAIGRNVLAFTFRVDIPSDLPHSVHATTCRYYYMANVLIKTKTQQQI
LKRGFKVVTGSSGPDDGDEQNKRQIQRARVRFGECVGVAHSNGLPCHLSSTEIHRPRGQL
VVEQDHCLVQQYQPNADVRTMRVTNATGRPVCILTVIGSQSLSPGSRVHLQWDFPKVLLS
NREVAMRTRRRKKNSLDGNSSDINDYWIPCHQVCACLQGEEYAVFEDDTKTRTQSFLFDA
CHERVEPGVTDRITKTLWLSSAGTNSEDGNGSDNTSFFEGGTPPCDLKTDVMEVSTWCKV
DITIPDQQNRRDRYSNLSLTIPCRVRHHRSSESELEDRDALEQPVAPLHELLFGGEDSLP
ATAAAFPKADIRSDLKVLSLALEEAVAGGSTSLSASRSGHGGLEQRTEAPIGRIDE
>comp4997_c0_seq1; 896 1 2705 LEN=2963; translated
XLLEIQFLQYTSNKSSIKPADVGRMKQLQVLQSNETNPSILEALVAQEEDISYSMSAAQF
SSPTSRGSSIAMNTKPTTSSKSNVSYELFAPRATAQVSRLSRIAALTAGQTALGSAKSKN
NQPSAVDSDEATILKAMSPPCASSDSISLSKENESSTVKTALKEATESSDHLTRLLKHTT
SLRLALRASINIAEDISNRHADLIRHSGELSAAADRLQGEQEMLCRHAEEIGMPLKHYDA
VDNIGILVGVLFKGRTVVRGLAKIKVDNDDFPSILDQIDDAVEFFARESGGREALDAELK
RQRRSSAKEQFETSGSIEYYRRSLALQEAALFLIREAVVDRISNTTAQVASGLDIPKTPI
AADKLEASLVYTRFHGISSRSNRLLTLVKKRLHCGEAYHELLQLCRTTYCNSRESLLKTT
IRAHMDKLKDQHGLVGMTRLASVFLIRLCTVETALYLDFFGEEKVSEEGKDPDDSDEIKT
KQPNQSKRAMALPSRTAKEDATYYDREFQAYLASLTSALHRTIRRGLVTLLDLDTLCQIV
SVLREERSMASSSPTTLAAARSISSVIEDAQERLIFCATNTLTKEVIRFKASPSDLDYPN
KLTKNTKQSQEGGEDDAIKKQLQVYESWFPPMRSVLRILSKIFRVVEPKVFEDIALQSVQ
SCTRSLRDGAKYIEKRSGTIHADLFKVKHLLILREQLSPFDIDLRSVERQLDFSDAGRAV
SRFLANRNRRLFSMSAENALVTLLREGVSVNEESVDSKRDLEEGLRNACNDFIDHTCDSV
AEDVMKLVAQLNSATPESLKGASFFEASKVRDILSKTQENFEPKAKDIRNKMNLYLENPT
TQSILLKPVSKKISKGLEEIRKTAYGMANDTGGWDENVRAEILGMIDDLERVVKRIGRAA
K
>comp4997_c0_seq2; 598 1 2111 LEN=2111; translated
XLLEIQFLQYTSNKSSIKPADVGRMKQLQVLQSNETNPSILEALVAQEEDISYSMSAAQF
SSPTSRGSSIAMNTKPTTSSKSNVSYELFAPRATAQVSRLSRIAALTAGQTALGSAKSKN
NQPSAVDSDEATILKAMSPPCASSDSISLSKENESSTVKTALKEATESSDHLTRLLKHTT
SLRLALRASINIAEDISNRHADLIRHSGELSAAADRLQGEQEMLCRHAEEIGMPLKHYDA
VDNIGILVGVLFKGRTVVRGLAKIKVDNDDFPSILDQIDDAVEFFARESGGREALDAELK
RQRRSSAKEQFETSGSIEYYRRSLALQEAALFLIREAVVDRISNTTAQVASGLDIPKTPI
AADKLEASLVYTRFHGISSRSNRLLTLVKKRLHCGEAYHELLQLCRTTYCNSRESLLKTT

IRAHMDKLKDQHGLVGMTRLASVFLIRLCTVETALYLDFFGEEKVSEEGKDPDDSDEIKT
KQPNQSKRAMALPSRTAKEDATYYDREFQAYLASLTSALHRTIRRGLVTLLDLDTLCQIV
SVLREERSMASSSPTTLAAARSISSVIEDAQERLIFCATNTLTKEVIRFKASPSDLDYPN
KLTKNTKQSQEGGEDDAIKKQLQVYESWFPPMRSVLRILSKIFRVVEPKVFEDIALQSVQ
SCTRSLRDGAKYIEKRSGTIHADLFKVKHLLVRVIHYSILFETA
>comp4998_c0_seq1; 436 156 1160 LEN=1721; translated
MVPSTLKRKLATIEAKNRKGSKKKRGLTTTKKKQNKTTKKASSARSTSKKKDPTRSSISK
PRATSNSSNAPCPANGNSRNVQGDARLQGNNRRKVERSPNGRARCQKCRQIIKRGEIRVG
IPISSASAKTTAYQYYHNRCLSDVHKPGCVKETHSQIREKILSQRANLLWNLQQYWSLLG
TEKIHKLVIALPQNERDLFKVLGATSSSFPYDHSRLKYLLDIIRKARQKNEAVSTAAALG
GSTAQRQMAANSSRRTNDPSEVIVIDDTDDEDEVDMGNKSDSAVPEGLEKDDRLGDDDDD
DDGEVLVGGTLSCAQVIQQKFDQAAANGEILTID
>comp4999_c0_seq1; 320 1 1303 LEN=1371; translated
DRRRNEMKHETGFTNPVLDTNPGIFPTSNLFQGNSFIKELTFSFKFTKTIEDKNGRSNFN
DSVLFAGSKELYPVSEIVRRVQPSFRRSLIALELIEIYVDLTHTKPYTIPNHPFSSTRTG
YLKHIEKYKSPVQKTPYIRVGQEGADGVDLKNKVVIVTGANSGIGKDLATYAAAKGAKLY
MFCRSKERAEKAKKEIVDATSSESVDIVLVDLGELASIRKAVQDLQSKENKVDVMVCNGG
VLLNEKQTTKEGNEVTFASHLLGGSYLLSQLLIPQFKAAEDPRIIFVSSGGMYNVPFPDW
ATATNSKGSTHEYSGNLAYAYAKRGQVLLAEEYAKMYPGIKTVSCHPGWTLTPAVDQAYG
DNKKYLEPMRTTWQGSEGIAWLMGEKGSELESGAFYLDRSPQRKHLGPVTFYTKNKQKDI
DDMMANLKQTAGC
>comp4999_c0_seq2; 395 161 1168 LEN=1236; translated
MGGAISTTQFFLQGRKNFTQTGYLKHIEKYKSPVQKTPYIRVGQEGADGVDLKNKVVIVT
GANSGIGKDLATYAAAKGAKLYMFCRSKERAEKAKKEIVDATSSESVDIVLVDLGELASI
RKAVQDLQSKENKVDVMVCNGGVLLNEKQTTKEGNEVTFASHLLGGSYLLSQLLIPQFKA
AEDPRIIFVSSGGMYNVPFPDWATATNSKGSTHEYSGNLAYAYAKRGQVLLAEEYAKMYP
GIKTVSCHPGWTLTPAVDQAYGDNKKYLEPMRTTWQGSEGIAWLMGEKGSELESGAFYLD
RSPQRKHLGPVTFYTKNKQKDIDDMMANLKQTAGC
>comp4999_c0_seq3; 396 161 1306 LEN=1374; translated
MGGAISTTQFFLQGRKNFTQTGYLKHIEKYKSPVQKTPYIRVGQEGADGVDLKNKVVIVT
GANSGIGKDLATYAAAKGAKLYMFCRSKERAEKAKKEIVDATSSESVDIVLVDLVSEPRI
GESKNEGFLLQLRSFIILYFNGSTRKLTLRSRKSLHINHQGELASIRKAVQDLQSKENKV
DVMVCNGGVLLNEKQTTKEGNEVTFASHLLGGSYLLSQLLIPQFKAAEDPRIIFVSSGGM
YNVPFPDWATATNSKGSTHEYSGNLAYAYAKRGQVLLAEEYAKMYPGIKTVSCHPGWTLT
PAVDQAYGDNKKYLEPMRTTWQGSEGIAWLMGEKGSELESGAFYLDRSPQRKHLGPVTFY
TKNKQKDIDDMMANLKQTAGC
>comp4999_c0_seq4; 321 1 1441 LEN=1509; translated
DRRRNEMKHETGFTNPVLDTNPGIFPTSNLFQGNSFIKELTFSFKFTKTIEDKNGRSNFN
DSVLFAGSKELYPVSEIVRRVQPSFRRSLIALELIEIYVDLTHTKPYTIPNHPFSSTRTG
YLKHIEKYKSPVQKTPYIRVGQEGADGVDLKNKVVIVTGANSGIGKDLATYAAAKGAKLY
MFCRSKERAEKAKKEIVDATSSESVDIVLVDLVSEPRIGESKNEGFLLQLRSFIILYFNG
STRKLTLRSRKSLHINHQGELASIRKAVQDLQSKENKVDVMVCNGGVLLNEKQTTKEGNE
VTFASHLLGGSYLLSQLLIPQFKAAEDPRIIFVSSGGMYNVPFPDWATATNSKGSTHEYS
GNLAYAYAKRGQVLLAEEYAKMYPGIKTVSCHPGWTLTPAVDQAYGDNKKYLEPMRTTWQ
GSEGIAWLMGEKGSELESGAFYLDRSPQRKHLGPVTFYTKNKQKDIDDMMANLKQTAGC
>comp5000_c0_seq1; 238 164 958 LEN=1004; translated
MAESGTRATESGGSSPRKRPRKEAPPPLVEGVGEHKKARGQRAAIEDPKKTSKIWLNHKL
VARPNYITSYGILMYRESTASDDPGSRRNSESADEPAKDNGCRNSHGNVPHHRKEFEYLL
GLIPQGNAWTVFKGLPEPNETPEETAVREFEEETSLRFPYPFPNTGKNVFTTKLYGVTST
KKLLQIYLVPSPPELDTSRFDVDKVVTIDGSGRFSGLPEIIEIRFLTKDKAIEGVRGKGN
HRIAKIYKSQISILERADSILHQE
>comp5002_c0_seq1; 304 112 884 LEN=1441; translated
MSTAGVADASVGDDKANVDNMSLLSITEGENDRGIPHVKFIDDVDAFAASFSPPSSAELL
IGAYSELHSKFKTFETSLTQKSTLNFKIGLALILRSMTHRLKGLQVTLLQNTIEKHSDLR
TFFILLEQNYNLKIPDIEKSLALVKNLQSKQKAGETVTTRYNLADNVYGKADVDTSVGIV
NLWLGANVMLEYTYQEAIDFLSKNLETCRRESKIVKEDLAFVRDQIVTSEVSMTRIFNWD
VRKKRAEAAVQAPSSXW
>comp5002_c0_seq2; 370 112 747 LEN=1304; translated
MSTAGVADASVGDDKANVDNMSLLSITEGENDRGIPHVKFIDDVDAFAASFSPPSSAELL
IGAYSELHSKFKTFETSLTQKKQNYNLKIPDIEKSLALVKNLQSKQKAGETVTTRYNLAD

NVYGKADVDTSVGIVNLWLGANVMLEYTYQEAIDFLSKNLETCRRESKIVKEDLAFVRDQ
IVTSEVSMTRIFNWDVRKKRAEAAVQAPSSXW
>comp5003_c0_seq1; 271 64 813 LEN=919; translated
MAPQSQSTMLDSFFGSKSNILCSNGGGLGGVDHGQYHCYQSSMAAIPSMPSMPSIPSCSK
ENGGDHRNAVRHSKHYKQPQLINSTIPSCTPKYVPQRQNTPPIKKKKNHTKRVTFITQEK
IREIPSLSSYTRGEIDSLYLSRDEMNRSHEECWRLVDLMNTGIEYEDHEQFSKRGLIDLK
DDSVDRRRKMRDQTYQIVLGVQQFHRDNKRAQATMDVVEVTANLYRKASVQAKEEALEAA
WFDAVAVRV
>comp5004_c0_seq1; 648 139 1884 LEN=2305; translated
MAAVGQQEPTPTSARPGKAGRCNYDQWDKVTRTLVNEAEQEEEQEIEESKKALGLDGKYA
DSQAHAEEREKAKDVLKAKKVLEKYQKRESAVRTELKGLLGPPPSKNGDGEEEESKATES
KTVRVTRDMIDAGKRVVVIADTSGASQSDTLVLTSDLSLLESKMKANTTTKPKQYAEDAE
NDVDESKEKGVERKIYGLIKCFVTNVHNCTILIKCKVISGSLEINHCSNVVVKIERDATV
ATIQADLCENISIIFNDAPSGKNGATYAGGIPMDTSTDSKNRIYWGQDKEDRIFHAGVKN
MRVQINRDGFMETERICDYTFDGAKAIGNATEEEFQFVTSCIDEELVTEAVVREGSTTGQ
NARPLTKREIEEEKVKREKAGNMATQMAENMFKFKEVGKDGAKKVSKTEKPKPEEVPVVE
DAEEEIEEIYAGMSKEDIDAVVKECEQNKARGNEAFGTGEYAQAVLLYTLALDKADELPD
DKDKTKLFARDVTLSNRAACFLKLGQHEKAEADAKLAHEYNNQNVKAFFRHGLALHAMKD
YKAAISILAQAYKLEPKNKSIKEALRFAEVRLNQEMRKRME
>comp5006_c0_seq1; 316 82 1194 LEN=1272; translated
MSDGPYGRHGFSANSNAGGYGGQAGGTSYGSQQGGGGYAAPSYGQSYGGGASYGASGGQD
YGSQYGSYGGGAPDNYSAGSDNDDGKKGAKKSIAVQALNMYVLMWAGFITASLLVYFLLS
GGDFSFLMTYGGMARMFGFGILNLKTFKSQRATGVSKKTLQLYCCVFFFRLTSIIRHEGY
LPYDKSGDWLYHFVEGMSLLLCTVSLYACTSPFKHTYQADLDKFGEFMVPPGTGAVYLAV
PILILAVIVHPNLNADFLSDTAWTYAMYLESAALLPQLYMFQKQSSGVVELLTAHFVFAL
GFGRVMEFLFWIYSYHELADASGSKLPGYLALFSQAVQLIMMLDFFWYYYLAVKNATPLV
LPSHGGAGIV
>comp5006_c0_seq2; 214 421 1236 LEN=1314; translated
MYVLMWAGFITASLLVYFLLSGGDFSFLMTYGGMARMFGFGILNLKTFKSQRATGVSKKT
LQLYCCVFFFRLTSIIRHEGYLPYDKSGDWLYHFVEGMSLLLCTVSLYACTSPFKHTYQA
DLDKFGEFMVPPGTGAVYLAVPILILAVIVHPNLNADFLSDTAWTYAMYLESAALLPQLY
MFQKQSSGVVELLTAHFVFALGFGRVMEFLFWIYSYHELADASGSKLPGYLALFSQAVQL
IMMLDFFWYYYLAVKNATPLVLPSHGGAGIV
>comp5007_c0_seq1; 421 247 1503 LEN=1941; translated
MGQCSTLPSEGRQSRRGGREEESTYRDSRSRKSLQVDSRTAQAAQYSQHQQVTTGHNEGM
DSTEPMDTREDIPLPPAIATRTRCYKLNLDSDLYPHHSVFLGPYVDVPPPLTCSLSSDSA
ESYSPLQVAINTAKIFRGIQVGKDGTILSQNARATRSNRGSKTKVGEKSRQATKIDKAKD
LVEETILTGKAPDSEEPAKMQSIVVMGEYDDMKYLVRDGAKKLREGSEVADESLRSLARG
RDENSSNNIMAKVSSPRKRGSMSGPRTPNKSRQFSQSGVPPKLKSHPRDRLGISRKEASS
ARRDNRDSNADRASSNGDSRSGRQEGDWGNTLPVSFSRGFQNIWNCGATGERSDDRMHSP
TQQVASPRDGKSPMNDSSGMHHNQAKVATQPYQHKAVYEGRDSQQYGTRESGVTTRAN
>comp5008_c0_seq1; 414 1 1009 LEN=1405; translated
XNNNSNNNSNSNKKRSGNRCDPHPEHDRELFHEWEGPSTGSPWAARQEAFLPATEPEQVA
LSVTASYSTSPSSSFGIPPWSSPMVPPPSPPPEEAHPPLVLFSRSSRSQPSTKDLPVLRP
RNALRNAPPAVCPGQRIRRSSPTEKIRSPPGGLSRDENGNNQQFPQQQQQQRQEEQQARA
KSSCPETSLSLSLSLGTLAQEYEKDTLRMLERIQKSRSFEQTAKQKKQDLRPGNCPAAKT
RTRTPTRTAPTSFLQHPQPPVESIASEAAPWKENRGVDSQKPGDFYIGDEEAGDGFLAGG
HAPAQQPQETTLLLGPGGVGHEHERETGEDIFELDL
>comp5008_c0_seq2; 459 1 1390 LEN=1603; translated
XQQQQQQRQAHQHEHEQKRDENSTIDIDMSDRIHIRKVSVSSPSSHKGPSSGTIDNAQHQ
IHEHPLTVSALSQREEYSFRDKNSSSPAASSCASCSSWSGSEDDYDEEDHHIRVREREQT
PSHSRAATEPAAITNTSDAVTAHVTPRSQSSLHHHQEQSPNTREHLEALSLDRPGSDTKD
IIRNNTKVDVPPPTMIHPMTKRISVPSAAPSPPAVARVVTMPPTNGLQSFLQTANSNANT
NTQCGQSVSSKPPPALPFHDQHRRHQHQQQHSMHSHSHSHSHPQAYPHEVQCHSQYAPHY
GLPPPQRQHFHAQPAYSQAPNPMGAPVFFPPPGAVAPAFLPHYEQKPPRMLQPGMEVILR
DPSTGIERSFCVQYMANSMTSEQAEHFKNANAHCMRSMPMTMFDTHANMSQRGTTNTPKS
HGNSSSLSTSTNVCAAPHTRNSSIEQRSVVEAGKGTAIQIARV
>comp5009_c0_seq1; 394 206 1579 LEN=2475; translated
MASTGTDHLVETTVDSVSLLSIIQKSFPEATSYSVRDNIKQANAEGFRVVLEGIEKENDS
STSLFIKKVKAREYSHKPWGDLRRTLLYSRTEARFYTDILPLMREKTKTANSKWSIAPNC

FYAETILDGLVGEDESTSASKGESASDPFYNETDTAILDHKGGLLVLESLGGDFYQKSPL
TPEEATECLRAVAGFHATAYEDAAMLKVVSDRLCEYGGSYHLRNRNPKELKNIQTTWAEF
CKRVGSAAPEVFERPSVVNLAKRVQDMAESISEELSPAYNDKYATIVHGDYKAMNVFLAA
DKTEEGKECAAVAASPLPIIIDFASTGVGLGISDVAMHITHAVPPEHLVDGGEEKLVETY
LDALNKALPDHSRPCYPRDVAMRHYRLAVIDYFRFIMGRLWRGASLETFEKRKDSMNSVY
VNRNIDAAINLIERADSYLTIFEEESRMNTGRSNMTQ
>comp5010_c0_seq1; 575 102 1790 LEN=1928; translated
MTVEPSSTTDQSERKKLVKARWGILRNALMRGAQEEQQQKHSIHCFPGFNLLKGIPVNL
TSSLSGSQSESCQIEKWLTEYRWCDAENHTLEQNIDNMEIAILALAACFPKGKCMRIIRT
IDTTSGDGDSEIENDFSSNWLESIKQRCQPSVRVWKASEEEEVLEQKCAAKNVVTTLLVQ
ETSTSTKYQTLRYNLDNTKCNTVSTIVTVESLCDILWTREPRETRLSLDDLISHRKNKGV
DNTGNICIWDSERTLAYLLYHYFDDFFSFTKVNSDDDSMSIVCSCHNDSGDPDSCIVFQQ
QEKDHSKVRVLELGTGMAGLSAVSLGLRLAMRQRKNGDETNESNDNDDESEGKNKKDIHI
TLTDGNSNGVKNNAINQYLTELNSRTMLKERGIHSNYLDLSVNCATLLWTTDISGEKNTK
KEYSYLDQDVILVSDCVHFQNFHAALAITTLRCLRVGGSAIFCQPRRGPSLNNFFNLLST
ATSETGFTRSTSLLSISRFSHPIIDQKHDESLVQHSDIYDENLHRPEILAITKLREITEN
DRMRFISFQQRYNPVKSKITEE
>comp5012_c0_seq2; 250 82 618 LEN=1138; translated
MSDQSQTATNKQTEPSRCAKGCGFFGNPATGGCCSKCWADMQSALKTKAAVAAAVVPPPS
PVCKTASTTAPPSLETASAPVAAKTETPRSASASNLSSSASTPSVAGASGTALETPAAVA
PSAATAAAPAKKKKKKKSSYKNMMASMMKESSANKNDAKDAAIRKVTGGGTFSKIDKI
>comp5013_c0_seq1; 483 1 1375 LEN=1755; minus strand; translated
XNSNSSNNGNSTTAAIQRRHRHSSPREKERSNCAIGLFGLPRSFKKYVLPSLEANVIRPN
AAYGCDYFVHYYKQDSEEAGRSGRGGSVRPDDVLLLRGVVETVHNEHWENRHQQQRPATD
TINDGRITTTTTTAAFPLPTMPPPMPTVAFVSDTNETFWDARFEHYLKRYRMARDKDTGNY
LFYPYKEPTYEYPGTLDNIIRQWHSVQAVWEAMEGHQQQNQQHQHQRKKHQQTNKRYDRV
AMLRNDVVYITPIDIYRVPELGTSTSSKGGGNEIYSTSVVPGFAKYPVNDRMIYGPYEAV
EIWASKRFENIELYATDPNRNKGMVLHSETFLNASIFEAIKGLGSQRGETETETATTPAT
VEKSSSNSSSNATTLARQARNVYRVVEDPWICFLRVRADGAVWIEDCDPKKSGFGGGYPG
GEANYTDLLTKFLPNHGKRCRRRLVRDKQRRIVELACS
>comp5015_c0_seq1; 292 1 511 LEN=529; translated
XDVGFDPFGLAGEDKAKLVYMREAEIKHSRLAMLATVGWPLSELWDKPLADAVGLPSLLT
SSGDAPSLLNGGLDKIASAYWVAIISLAGIIEIENMKMKEEKGKAYIPGDCNFDPLNLLP
QDKNGQMEMMTKEIKHGRIAMMAILGFAVQEALYKAPVVAETPFFFHPIF
>comp5016_c0_seq1; 797 55 1431 LEN=1701; translated
MADNNSNNNTNTNTNDGPQPAMTAEEVVVTPCGLLLGCFTCLLGCMCLPFLCCCAVTATA
ADTAANRAQGKRFDNVQGKWVIDDLVEEEKTLNGIPDDDDDLIKLSQESGDEKPEESSAT
SGKKVKDSKYYDVLGVPTDASASKIKKAYYIQARKWHPDKNPSDEAKKKFQEIGEAYQVL
GDEKLRGIYDRDGEEGLSADKTDANVENIDPSFIFTFLFGNDCFNDIVGRLQLVTQQLVA
GADGQPKCTAEEMREMERRRVLRLALALRKRINAYVSGGNTEAAKAEWKKEGERLVEVRY
GQELLNTVGKLYVLIVTQITGTFGEGLTAKKEGFDMKFDAAKNAQNAAKKTQGGATGAEQ
DALPSMIQMMWNLNVIDITSTIREVVMKLCKDKSVSSDIQQKRAEAIKVLGSIWESIESK
NKEEKDARTMYMSATQAAMESHLDKVSKDEAAKSGASN
>comp5017_c0_seq1; 356 219 1388 LEN=1782; translated
MAPFISSIRYNSISFLASLAVLAISSAEESVEFSVVTPDAHASLRRRLGGNENNTAQPRR
QPRIIGGSNVNDPSEYPYYALMNGRALCGAVLIAPTFVLTAAHCVGADDDFEIGAIIDDS
NSIGSWWNSLIGGSSASGTGTEYAIKSRIVHPDYDDLNMFNDIALYELQQAVPDTVPYAK
LRREPLTEAGTQLTVIGFGDTDASEWIQDTSTALQKTTVNYVTESTCQSRLRSHLIGPDM
LCAFAEGRDSCEGDSGGPLLLEGPWGASSDELVGLVSWGLGCADDNYPGVYTRISHFYDW
IIENMCEMNPSGVPDGVACYSMATTPEETIAPTIFPTDGYGDDDLYSNWFYTDDGGVYAH
LDDYVTDDNFFGNFWDDVTDWVSGIFGL
>comp5017_c0_seq2; 288 521 1551 LEN=1945; translated
MHTINFVRYSNRDGNLGIIGGSNVNDPSEYPYYALMNGRALCGAVLIAPTFVLTAAHCVG
ADDDFEIGAIIDDSNSIGSWWNSLIGGSSASGTGTEYAIKSRIVHPDYDDLNMFNDIALY
ELQQAVPDTVPYAKLRREPLTEAGTQLTVIGFGDTDASEWIQDTSTALQKTTVNYVTEST
CQSRLRSHLIGPDMLCAFAEGRDSCEGDSGGPLLLEGPWGASSDELVGLVSWGLGCADDN
YPGVYTRISHFYDWIIENMCEMNPSGVPDGVACYSMATTPEETIAPTIFPTDGYGDDDLY
SNWFYTDDGGVYAHLDDYVTDDNFFGNFWDDVTDWVSGIFGL
>comp5019_c0_seq1; 259 491 1282 LEN=1703; translated
MTRLSTPPKSKNRLGIADSNVRPIEGNRRSQNIPLPMSHMHRTQSEVQLCEDMETAEQRD

LNMFYRVVNGIRDRQMQLVRENKSSVAAAAFYNPAHGLTEAERSLAHIIHTRNAPLDRKN
GRDNSSNNARRTNLNLIGASNPQQIHTGLNSGVPILEDHSECDLMEHEKESYERTGAEDN
EWSVAGFDNDSPNTKMSLDSRQNEHYQYHQNLYQQESTVYHDQGVSAAAPMYEKPTNTLS
TATLNPSRSSSNIVDEGMFDFEL
>comp5019_c0_seq2; 259 374 1165 LEN=1586; translated
MTRLSTPPKSKNRLGIADSNVRPIEGNRRSQNIPLPMSHMHRTQSEVQLCEDMETAEQRD
LNMFYRVVNGIRDRQMQLVRENKSSVAAAAFYNPAHGLTEAERSLAHIIHTRNAPLDRKN
GRDNSSNNARRTNLNLIGASNPQQIHTGLNSGVPILEDHSECDLMEHEKESYERTGAEDN
EWSVAGFDNDSPNTKMSLDSRQNEHYQYHQNLYQQESTVYHDQGVSAAAPMYEKPTNTLS
TATLNPSRSSSNIVDEGMFDFEL
>comp5020_c0_seq1; 305 1 1232 LEN=1417; translated
GLWIAGKFEHVQVNAGIDTXLHSNLFEFNVLQTLIKVNITRPLLLATILCMLVNPKAAQA
KLEIIVSTFRYLVLSKDKKWKKANDDPASFFSKDSDGELIVLRKKTVVFLRHGESTWNDT
FNKGDRKMSSFVLGFIPGIFHSLATEWYFLVRGMSDESWFFDSPLSAKGIGQAEGVARFL
RETDAQYATPREAKFLSLVKGEKSEKNEAGEMCVLVSSNLRRAISTCAIAMKDRLDQKIP
GDNIIILQELQEASINPDAQSITPAFEKLVTSFTDSEAVKSIYANQSDTSINKGNKGIKS
NGLQRMEAFCNLLFATDGYESVKGNDGDDSNNAATILGNANNVMCTGHSYWFRAFFQTYL
PRDFQHICKTKKLVNGGLVGFTLCHTKVKETGEEKFMIDPASLTVLYAGF
>comp5020_c0_seq2; 320 1 1405 LEN=1590; translated
DQTVIFENSNELTPDHMDGQFLSQSNINIEIAAPVVIFILLINFSGVRAKYQEPPLQKRT
VTQHTQVAEKYNGTIRSDSGPAIFGLMLRALDSSQHHSALAFGYHSVHAGQSKAAQAKLE
IIVSTFRYLVLSKDKKWKKANDDPASFFSKDSDGELIVLRKKTVVFLRHGESTWNDTFNK
GDRKMSSFVLGFIPGIFHSLATEWYFLVRGMSDESWFFDSPLSAKGIGQAEGVARFLRET
DAQYATPREAKFLSLVKGEKSEKNEAGEMCVLVSSNLRRAISTCAIAMKDRLDQKIPGDN
IIILQELQEASINPDAQSITPAFEKLVTSFTDSEAVKSIYANQSDTSINKGNKGIKSNGL
QRMEAFCNLLFATDGYESVKGNDGDDSNNAATILGNANNVMCTGHSYWFRAFFQTYLPRD
FQHICKTKKLVNGGLVGFTLCHTKVKETGEEKFMIDPASLTVLYAGF
>comp5022_c0_seq1; 944 329 2845 LEN=2866; translated
MAWTMIRRSCRQWFPSRNQMRRTGTNHHQQRAHKHATTSPGLWTSAAPSAAGSSSARTFL
DSRQHRHRSWGMHAAHKPSHDGGDHYSSSVSRKNRRRPRRLSSSLAATTSASSSSSRLYR
YRDFLEKGEHDRIVLSYLTDIEGDKFYLDRYVDNSKILTWIPSDDDAEPPGNDTEQSPFS
LSYYSCKEYPPARFPYNRRMDFVDSNSMLVFGGDLWDKGGFDLYVTRQLMDLKRRYPDRV
VWVLGNRDINKLRMMQELGLPPAESSTDKSNTGAAAASNLSVPYHPGLTWFRGSGRVGDP
SGPLPSMVPGDRLRWILGNTMGSPDAMEHRRQELAWEAGGCRETGGTSAASSAEGISDCD
VVRSYQESCHPRGEMGRFLAEGLLAAKVGPILFVHGSLPLTKDTMERGLRDGGDGSNIWD
DLTFCMPWINKTHHEYSEDHRFESASDLGVETIEDWLDAVNRFASRNVEAWERDIARLER
EAGQQATNATHNASDGVEEESKDSIWAYRAGYGNGPSHSGIYYSDLVQYGMGMIPGGKKN
PTVVYNSFTPEGMPTFFLPPEESGGTNGDCEEALGTEHASERDSGNNPSSSESDVAICTR
QFFERSSMRLILTGHKPQGDMPSPIRVDRSSWVICADTSYSGDTKWFHDERNATNPSKME
RRKRQEEEQHEKRKRSNLGRGNSISFRGETAVSEVLVELSGKDLSPESVSYHGVLSDGTE
YESVNLLDDGEDESGAVSADDAGSNSNGSGSCGSLDSMPATAAILGRVAPTNMVPDPLNS
PHEGRWWTKGICRDGSHLYYAGEGYNVWNYIVSPSDIDGNRSNTKYIDNTASESDAKK
>comp5023_c0_seq1; 392 468 1844 LEN=3300; translated
MAQSVNQHMVTGFGRTVRRIRNPNPQDVLSGRGGGINSHAGNKAFRSWVEERKPAYNLAE
NKQAKTEVAMQVVQQVHNQRPVAGRFLTKDKSMEGTGGTTWWVEIDEAKALAKTTQALRE
GAPKIREALQNQTGSPLLGPNSRPKPPKKRKRKPSGKAANRDGSITTATGQPRVGINLGA
PLLDDDSEKQILQEFKQDVCHLKRYKSEQLLLPTSNSNLKQVYDQALVQLHANAEKAKKV
AAERTGGTQQNVQKPQTFFPATGSRIAPLTSNKVFKEKYSQQQQGNVPPAMPAIDPNAAT
PPLMAAPEPNNSIPYLSLNAQPAPKRARLRRNHSLALSDYDENALDPATDEVPAFVDPFA
NETDVLLNNNGVLPSHATSFVPDQLSKSGDRLAETSGQSSRLNYNRSVHRYLDRILSFSS
NPLHLWEDGLKGNNEQVETRVVDIRDDYFLCDALPQDE
>comp5023_c0_seq2; 344 468 1709 LEN=3351; translated
MAQSVNQHMVTGFGRTVRRIRNPNPQDVLSGRGGGINSHAGNKAFRSWVEERKPAYNLAE
NKQAKTEVAMQVVQQVHNQRPVAGRFLTKDKSMEGTGGTTWWVEIDEAKALAKTTQALRE
GAPKIREALQNQTGSPLLGPNSRPKPPKKRKRKPSGKAANRDGSITTATGQPRVGINLGA
PLLDDDSEKQILQEFKQDVCHLKRYKSEQLLLPTSNSNLKQVYDQALVQLHANAEKAKKV
AAERTGGTQQNVQKPQTFFPATGSRIAPLTSNKVFKEKYSQQQQGNVPPAMPAIDPNAAT
PPLMAAPEPNNSIPYLSLNAQPAPKRARLRRNHSLALSDYDENALDPATDEVPAFVDPFA
NETDVLLNNNGVLPSHATSFVPDQLSKSGDRLAETSGQSSPAYFFYENDRFNA
>comp5024_c0_seq1; 104 1 529 LEN=684; translated

RRAWKIACSSSVNEKAIFASLGNRKIGIQSFAMDFNSFVFISRISIDLKVIANFSDQLFA
THVVIKIPVPKNTAKAKIKHSFGRAKYEPEQQAIVWRVKRFPGKAECHIHADVDLMPTTR
PKPWSRPPINAEFQVPMFTSSGVHVRFLRVYDKSGYHTSRWVRYITKAGNYQIRI
>comp5024_c0_seq2; 480 176 1450 LEN=1605; translated
MISMIMILNQKGDIMISRQYRDDVSRAGADSFRLQVVATKNTGTEAPVARIENNSFLYTR
HLNMFFVAVTRSNVNPALVFEYLYQLVRILKSYLGDEFDENAMRNNMTLIYELMDETMDF
GYPQNCAVDVLRLYINLGNVKAKEEQEPSQLTSQITGAIDWRREGIRHKKNEVYIDVLES
VNLLLSSNGNVLRNEVTGAVQMNTKLTGMPECKFGLNDKLVIEKEGQRKPGVEIDDCTFH
RCVRLGKFDADRTITFIPPDGEFELMKYRVTDNINLPFRMIPAVQEEQSLTKISIDLKVI
ANFSDQLFATHVVIKIPVPKNTAKAKIKHSFGRAKYEPEQQAIVWRVKRFPGKAECHIHA
DVDLMPTTRPKPWSRPPINAEFQVPMFTSSGVHVRFLRVYDKSGYHTSRWVRYITKAGNY
QIRI
>comp5025_c1_seq1; 99 1 773 LEN=773; translated
DDDDENHGDHSEKQKNQTETDATNEPPETEPEEEPRRKRHDPATAASPAAKDPPDDRRRK
RCWKDLLESLFAVAEAVGSDLASIWGGLENGRLRWLAAVAAAHPLKAVLKEALALEEDAS
QGEALMVWIYALCVHLSANVDAGPEPGDPSGLAGAVEDWVVAVEMKDPVRPLEGFRSELW
DPARDEWRPLDVGAQEAAEKGGADLLEAWGAEEVGLAAGASGSDRGGAGETAARVPKRTN
ERTTAGDALNHPNTTTAX
>comp5026_c0_seq1; 500 88 1671 LEN=2341; translated
MTNDSAIQNIQRQAELKSLTLEAIVKECSNKSSSVHADLRAIADGLSTGRELDLTILTGG
LCNYSYMVHFKDHEKNKDVALFAKMTFGQPVLFPGMNCPLNRTNCEYQMMEWYSKVSPFP
ETTVTPYFCIDVEGEEENMKVLATEFAPSNLEEQGGHAFIEGGIIGKGFACNLAAGLGAL
HNSEVTDPDFNGEMKPFFLALAGMISTIFDGYDGSDEDDGDSKNNNEDDPVEELDMALEL
GKSLGKHNLDKIVESYRECLQRTDCYIHGDFHPFNILVEGKPKTPGDLIEENGDDGKYSI
IDWEFTHCGPMGKDIGFFSAFPIACLFAHAAQGDVMSAESIYQFLGTLWKEYSQVIKLDQ
KENLSLTDVYRQVLGFSGCFLMGYGAIGFHMEYLPIEEGDATEDILNKVQASLRLLGLKV
LNWGFGEEGDTLTIENLQGRFNEAIREEMSRLTPPADRKSSKRRSSILRETGRRVSDSHT
LFGLSKNFERISISSTPSGMSPRNSLSEKRTSILQAEKLIADMDYEF
>comp5027_c0_seq1; 390 181 1791 LEN=2409; translated
MEELQDAIEDAQYVNAIATQVQGPRPVLSWHFPETEELERWEAKKIEEGANASNGLEVFG
IDWCLQRAIGFFLFSEFLKDTCNDWLRINFIEDVIHWRKMRGRQRIERAKRIAQKFLKSS
PVDKVTGERIYPEKREIVTHDMFRQVPKLTETEVKELISANKVVDYDSESPPVPNSIGLT
GPVLDEIFTAISEAENKYNNESSGSERSINIESAELREATRNLPDRRSVREKYSSMKQLT
ESLKQVDPKNLNDLFAKADVLVIESLRKKYWQQFTESEKFVRMKNFLWFYDRPVEPDDFF
SMRVLGRGGFGSVTACKKGTSGKLYAMKVMNKKRIKVKKSELLALNERKVLAAVESPFVV
NLKYSFQSTDDIYLILDLMTGGDLSYHLQQKGSFPPRECRYYAARVMLGLQALHDKGYVY
RDLKPENLLLADDGRVKITDLGLATKITPKLHGAAGTRGYWAPEMLRRDEDGRRMPYNQM
VDWFSFGCCVAEFITGSNPFRSQTALKFGLDAGKETKVRQSNTLGFDTLIHNDFKM
>comp5027_c0_seq2; 511 181 2184 LEN=2295; translated
MEELQDAIEDAQYVNAIATQVQGPRPVLSWHFPETEELERWEAKKIEEGANASNGLEVFG
IDWCLQRAIGFFLFSEFLKDTCNDWLRINFIEDVIHWRKMRGRQRIERAKRIAQKFLKSS
PVDKVTGERIYPEKREIVTHDMFRQVPKLTETEVKELISANKVVDYDSESPPVPNSIGLT
GPVLDEIFTAISEAENKYNNESSGSERSINIESAELREATRNLPDRRSVREKYSSMKQLT
ESLKQVDPKNLNDLFAKADVLVIESLRKKYWQQFTESEKFVRMKNFLWFYDRPVEPDDFF
SMRVLGRGGFGSVTACKKGTSGKLYAMKVMNKKRIKVKKSELLALNERKVLAAVESPFVV
NLKYSFQSTDDIYLILDLMTGGDLSYHLQQKGSFPPRECRYYAARVMLGLQALHDKGYVY
RDLKPENLLLADDGRVKITDLGLATKITPKLHGAAGTRGYWAPEMLRRDEDGRRMPYNQM
VDWFSFGCCVAEFITGSNPFRSQTALKFGLDAGKETKEKAIDYATLEMIPEFPETLFEPD
AADLCRRLLDKNEHARLGSKGCEQIMAHPWFKTCNWEAILSDKKTPPYVPPKDVNAASQS
EIGTFREDEKYLECVIDEKDGKYYSDWNWTNPHAYAAEVIDFLIYERETGKPLLPMDENS
SCCCSVM
>comp5030_c0_seq1; 1142 337 3249 LEN=3551; translated
MMDHSRLAQQANNSTASSDSSTTPGDTRTSNDGVGSVNTNASSGHSDGTGGSSNSNNRVA
VDKSTNSLLLDNYVDNTFEKVELDLDRTLTLDSWAGSGLDVGPTQSVSKIGLSSKLGDIG
DDFEEPWKKIADSYFKESTTNSFDDQDAQTQTQTQTQSALHNQQAPELMEGTGDSNSSMS
VLLGLRPSGKSGSSKGSSNISDNSIERLLRGADAPFDKSNYSFEKVLGGSDNSLNSLDKI
ISMDFDTTDTDRTNTHLSDYNDHASNDMDSEMLTLLPTDVDQVVSANDFKINNGRNFSAT
TNNKSASVDPSLTLNQQQNVDNAGDSGKSYNDDELLASLRNAVSDSDHTTSVDGAAAKEN
EDDNHSSTVTSNSASSTTHPSLKNNDRQHKFDGAFDSESSLPSIEVGNIPSSTSEQQTGP
RSNSPSVHPFLPSNLSLSDHARRDSQRRRQQQQQQREPYLKDQQQQQQGKRNNSNAHSSI

VNIVNNQLPQLSPQTANNSTSNQRAQMDYAGLQQVKMKLIRRLQEIERAGMGKNHNRGQQ
FFAQQQQMILQQQQQQEIQRQFGMGNSLKQQPQNGLNSLQAKAGQQQQQRQLPLSNQFQQ
RLQQQQMGLSQMAQMPIGFAPTPIAIDPTPSPFSTSITNDNSSNTNNALRKQQQQPSSIF
AETAANSNSNISSVIGSGRKETPLQSFLRNKRSSIGSVSGNITAPQLQPQSKFSAGSTPF
GGNNNMVTTASILDATPMDFGAQSNNPFLRKQMMASMDRSVSGCNTSARGRRSSAGRNSS
SSLSSSIRQSMMLQMAPGGSRHQRSNKANNLMNSRSEHGTTSTSSSASNLSSGGADIYQS
TGITQRHASADHLMLGSSRHSMSVGTAKAQNRRFNAVSRSSSSSFGTLTKSKSNGGLKKD
FSAGSLIPVKRGNGVGGRGGFGAKHKLSQNSTISLRSQSVPFMIQRGGGSASERQQQRMG
EGNNHNDGWV
>comp5030_c0_seq2; 1142 385 3297 LEN=3599; translated
MMDHSRLAQQANNSTASSDSSTTPGDTRTSNDGVGSVNTNASSGHSDGTGGSSNSNNRVA
VDKSTNSLLLDNYVDNTFEKVELDLDRTLTLDSWAGSGLDVGPTQSVSKIGLSSKLGDIG
DDFEEPWKKIADSYFKESTTNSFDDQDAQTQTQTQTQSALHNQQAPELMEGTGDSNSSMS
VLLGLRPSGKSGSSKGSSNISDNSIERLLRGADAPFDKSNYSFEKVLGGSDNSLNSLDKI
ISMDFDTTDTDRTNTHLSDYNDHASNDMDSEMLTLLPTDVDQVVSANDFKINNGRNFSAT
TNNKSASVDPSLTLNQQQNVDNAGDSGKSYNDDELLASLRNAVSDSDHTTSVDGAAAKEN
EDDNHSSTVTSNSASSTTHPSLKNNDRQHKFDGAFDSESSLPSIEVGNIPSSTSEQQTGP
RSNSPSVHPFLPSNLSLSDHARRDSQRRRQQQQQQREPYLKDQQQQQQGKRNNSNAHSSI
VNIVNNQLPQLSPQTANNSTSNQRAQMDYAGLQQVKMKLIRRLQEIERAGMGKNHNRGQQ
FFAQQQQMILQQQQQQEIQRQFGMGNSLKQQPQNGLNSLQAKAGQQQQQRQLPLSNQFQQ
RLQQQQMGLSQMAQMPIGFAPTPIAIDPTPSPFSTSITNDNSSNTNNALRKQQQQPSSIF
AETAANSNSNISSVIGSGRKETPLQSFLRNKRSSIGSVSGNITAPQLQPQSKFSAGSTPF
GGNNNMVTTASILDATPMDFGAQSNNPFLRKQMMASMDRSVSGCNTSARGRRSSAGRNSS
SSLSSSIRQSMMLQMAPGGSRHQRSNKANNLMNSRSEHGTTSTSSSASNLSSGGADIYQS
TGITQRHASADHLMLGSSRHSMSVGTAKAQNRRFNAVSRSSSSSFGTLTKSKSNGGLKKD
FSAGSLIPVKRGNGVGGRGGFGAKHKLSQNSTISLRSQSVPFMIQRGGGSASERQQQRMG
EGNNHNDGWV
>comp5033_c0_seq1; 819 151 2637 LEN=2839; translated
MASFSGGGGSSVSERKKFFENCISEQKQRESLYAPPSVASTTSRTTDSCMSNGSRTSEFL
EERSDSESLFHNISDRTDPDESYGTFGTFFTKSGSTSDSDFHHFTRSESDSEPDSDVNFP
LLFEQENIFYESNEKNTSLGITSINCDEDVKGDSVPQSHSYLQKAQSLDEKLLPFLDEEL
EEEPGSQAFVEVPNHSSDLTSTYGRVNTFEEFDAWVSNHITSCSEKKDKAIEQENEIQRG
VEESEIQRVVKIQVTTTPAPTKDFQGVDDFAAKGKKNIDVMIAEFQKVSSELSKELSFTP
PTSRSNSDDYSNSQSYHASNQINEIPKDSTTHPTNSAEESLSYNSLPALSIDDINILKEI
HEKQLPTDVCISKDYVCASESTDCSIDSLGNAETTKSVCSRSGKSLGSLRYLRRNKGEGM
GKMLKRAKKGMGKSAKNISSLMKKTVKSKTAKELSANEKQASKASADHNQYRDFFNEKLA
ELLMRRHKGISKASQYLTTGKNLLNNGAQYKKNHDFVRSKEMIQKAHTYAFVGRQLAKQY
LLEHQDEIYDSTTMMTQSDLTSSENEDAINAFEDFFFAFLHCVGPCYGYEDNHGEIQVHD
TLEILSKVSEEETDAINRRLPNNGTVNDGKEKIRAAIERALQQTDSFDQKDEHETAQDRN
LAIRQFLSDLQSFYQVDTFLNLSSVALSRKCTSESTREGNSSKSQIEQDGTDETEAAQCS
GSQEEGSQESKFSMISKSLSDNCSGRSSFLACSVSTTRSMRPEKTVKRSRSKEREGRKEE
SQVKGIEPGTTVLKIPMKKRGRLKNKTKPTNDNKKKKWLSAIRIKRKA
>comp5034_c0_seq1; 150 105 500 LEN=500; translated
MATXIKSRKEANEFLSCAHLLSDEEILEFLSLDGGDESRGDEKVNTDGTGRGIAKKKMPS
WKSRKEYCCERTLWRRAFRRRTAEIRALASELRDGSGRARIDEDGRLVPVFEPPQPPRAD
LPPPGRAAETNAX
>comp5035_c0_seq1; 282 340 1086 LEN=1277; translated
MSNDSKFSASALGELKETIRSQTKEIERLKAQLAKSGGGGKSKAASHGGGHGGDVELTVE
ELNKYLHDPFYRVGLERVGWLGIFLGSLSATALIMNVFEETLEKHIELALFVPLLAGHGG
NTGGQTVGTILSALSAGTIKVSDAPKVVWKESCAGILSGSILGAIVAPIAYKVLGVSFHV
AVVLFFTLPLVSMIASTLGSIIPFICVLVRLDPSVIAAPAMTSLVDVAGLMGYFMIANYI
FKLYGLEL
>comp5036_c0_seq1; 179 89 388 LEN=783; translated
MSLATSRIVATRVMARRAPVQQQRRGIVDYLTNYPDKVNEIKKIQKRGGTQQGEANPTWL
KQDSDKMVAGFVFAVAGMGLVRLGSGYYKLATGTGKIPV
>comp5038_c0_seq1; 363 49 1332 LEN=1660; translated
MGKKRRLPQPVTSSVDDDSSKVGFDRPNDDNERKGGIPPPSSSSDNEPPTRNTEQLYASH
VTYDCTMNRECIPCAYKIGCFDVALDKEFCRRRPNQRPDQCPRSKVDYNTAANNNTDTDD
GVLGFRRGMSILTVGDGDFSFSLGIAQRLLQQKKFSSDGKDQKTFIVATSYETEATLRKV
YPDFDKTLRRLDELGVIVAYSVDATRIFDTLSVSAKGRIKTIKGRFHRICWNFPCTAISN

GQDGQNKAMEENKNLVRKFVQNARSILTGGGDGELCLCHKTKPPYNQWNLEEVALSSQKD
DCDSTTKSPPLLFYSGRIVLDRFLLPPYTPRKALDRKSFPCHDACFYIFAQKSSDEPAVK
RKNLFLPTIPHKEQPAAGNKESRRENRTSQMLIKVTTELLQLIRERHINATPRKKRKRNT
KHTNRSF
>comp5039_c0_seq1; 486 1 1984 LEN=2040; translated
XVSKMRTSLVISPVLAADNSSQSCRVSGRKFDVKTLPADLNRVYKRLQEKFGEEYALTVV
FVIRTAVELHRAWHMTLLTSRLLQQIKDRFQLRMSWNLGVTDLVFRVEDGLYELQRRVPY
DYDSEYQDMYEKAAVALIEGHIGIHDALIFQSELKDGKHTSRSGLFLRENPGRLVLYPLQ
AATCCVIFFGGDWVDAGIAAVCGLTAGLIEGVLSSKRVFANVNDSKMLIDFLIGVSTGVI
GGLFYYFMSSQDDLCLKGVFLGTLYWFFYGTAFVIGLLEIIAGELQTGVTRFLAVSVKTF
VLSVGSAVGMTLVLAETVYEEWTNQFDPEANKCNSIDLSEKWWRLPLFILCSVSVLGQYR
FIIMNYWAGLLVQVAAYEAQYRVMGYYDKAHEFDGMDTVFADLAGAMAAVFAASMVCFFI
DNLRHYSNLSVVGKSAEETKLSRSGQLTNKIYECLVFVGSSLGLGRGLFRRHANCKKKLS
EIAKAEGKNKMEVRLSVEEEATMVEAAVEAQETNVWSLLMPAVYQLVPGSKLAQFWYKLI
FPSEESTGIVDSPETALWLTSVALALGLILGLTAVRLLGFSLITIASCVRKKIESEDDEI
GKSTRQRERRGVTAQSNDFDPSDLVWEKEFDLKYGVNASTDSNDENKPSDSSEEIVMVCS
A
>comp5040_c0_seq1; 560 180 1958 LEN=2463; translated
MGSLYDDNCFRRIGTRSLVVLWVAWIANTLGYKSLRGLNGERLFNTPLAQAFSSSRKNRN
HHSSIQGKRFRHAATIFPTTGSSHYKDNDNSTTSVIPSLDGKKVRIPPPISDTTPCQVLD
IFHDGEEFYFPSPEQYSVLPKGDQGGFHIVKEYTVPMPGTVIGDDVLKVIDTYQKRNKVN
CITLQNALLALDPERYPTLTSARKACRKGKILVKQHKMNLTGLNIDRHDCDNNSSNNNWS
VNDGNSCVFRRGRVGDLVFPGDLIGSQLFLGTYRKKQCYPNIDYPRPQFELEVVYEDDHL
AVVNKPSGIAVYSDTRMDSTGTSKGNRRTIQFLLPFVLTPPQRGTQGGLLRRPGIVHRLD
KATSGLLVVAKTKVAMESLKEQFRTRQIQKLYTAIVNGDLLQEEIMQRKLQADRLEDGCD
DESEHQSSSEKTSESSSWNVIDYPLGGKYAVTAWKVLRRSPSLNARDGMLTKILIRLHTG
RYHQIRRHFGWWCRKPLVGDPLYAGLLQAHHFRKRGLYLCSSGISFFHPFPGNNGDGCNK
TNDKEKNNKDVGMNTQRRRVEITIESPKRFDKLMNAEEAWKRSKSSKENIDK
>comp5043_c0_seq1; 180 1 1042 LEN=1377; translated
XAPSHSRAGFPMPCAPEIVVDGILPETARVFRSAVYPALIEFHALRVVDKAAFAQDPRPS
SRSGKRGAAPPLPKPPPPTLKRPGSYRVLMKTGDDLRQDQLVMMMIKLMDRLLKRASLDL
CITPYSIIATSPSSGMIEFVEDSVPLSAILANHNNSIARFFRSVAPQAGARHGIRPDVLS
SYVRSVAGGCVLTYLLGVGDRHLDNLMITKTGRFFHIDFGFLFGRDPKPLPPAFRLTREM
VEGMGGSESAEYRQFCSLACQAFNALRKSAGLVLNLLHLMSDAGIEDLSNNPSADADGVI
AKVEERFKLDLTDEQAERFFLGLIHESQSALAPRLMDVFHSIAVARR
>comp5044_c0_seq1; 305 148 993 LEN=1051; translated
MATSAYMVLFPPDEGKTDGSSSRCLSPSTSFATSGSRIANNPFWLGVGVGVILSRVASYE
IKRRRFWQRTRLKFLSWLYDGVSSATATGGEPKTRQTPLLQKLRKPSLISELNGNPLVRR
SESDENKDNSVCGQHVELVSPDWALSSDLYCDIVRLPSGTELMSKNAPGVEFYYVLEGNG
QYVDQNGEKHQISAGFGFIVDPECARGFLVGGMSNLTLFRATDVAMSDGHNHVVRLQASS
LSSAAAIVRAGLRKIHRLIDDNPLAAASDQTNRLPPSPCED
>comp5046_c0_seq1; 359 1 697 LEN=784; translated
XGLSHRHPIVEEYDCGEHDEEEEEHQKQGLSHRHPIVEEYEDEEDDDERNERSRDTTEEE
GSQQSRASHATNHSSSFSVAISVSSDGCTNKDNSNNKGTIDVSVVEVTLHYKNPTTSLPS
TVQTDDDHASNHQEIPHIESIPPSHNRDASLPAPKVWKSPSELLSCSSSKPCASPDATAN
ELDLPSVANKPVAIAHESSSKVAHDLPSSPPSSPPRSSSFDRLLDVVSGGLYW
>comp5047_c0_seq1; 350 56 1759 LEN=1937; translated
MALQTGVFHTAGAGVLRFLILLVTSLVVALAFFLGGAAAEHGSPRGCSLCPDGGEIEFPD
APIPFFALPTADNPTCGDLAFLASMVPHDSIRCTEDLQPHAGYCGCPNVEQSNICSFCPD
GDAPDRPDLTLLMGDTCESLYRYVSFFNQDQCNSLQYSKIIGNAYECGCDISVAKLRQAD
LAPLQKQHNCNLCADGSMPPDPDFYLELGQMTCGEYVDYAHSLNPRQCEIQSRRGTFALF
AFQCMCPGAEPPACVKQENPELCTVSLLNSVDHDEKCECYSFCDGEFVGCDKYPGHFLGD
KCSGTKISGCNYASAIVDVAPEPKPEPEPIDPDPNSCSICPDSSNNIGNPEAVLPPFSGI
TIPGLLEEPTCRDLIDHLGKKSKGDTECKVAQSRLSHLCGCDGAEPSCTLCPGGVPPSFA
SAMATVDETCEQFASTVLTWEADTCEGGESYLSVMAARCGCVTAEWPVCPVLRQPVLCTV
NLLRSTDEVCECYNFCGSEFHSCADYPGRLLDESDCPEGAEAIAGCNMALATSQRCSRGS
IGPPCDGYYEEVAQTKLRHNLRHNLRH
>comp5048_c0_seq1; 449 202 1206 LEN=1206; translated
MRHANSNSNQHHYDRSRQDHYEDEARLGNDQTENHPYPHAIASPYHQQNVIFEQQNRRVM
QQQMQSSGYMNGMPQYPEEVMQYNFQMNQNGFNKTGHINGNNIMNPQMTYGHRQTLPFED

EQDNIRFREERNGHRYSELYEESYAPRSSTPSGMMTSTERTRGTRAHTSTSRSTSGYGSS
MVSSSSDSDTDDSNWEDNIFSALNDMAGNLKKMGGIHCNQAGPDTTEVQLKINLPLPIHS
LDAVAKDAMATLSPKKCSNMQYDNNFDMEEQFLGAMDKLQGNMMPVYKSLFESQSDSSSF
EDDDDDQSESTYEDPRFLPTNANIEQQQQQQLQQQ
>comp5049_c0_seq1; 135 1 740 LEN=871; translated
XRSLFDHCSRNNLTNTPVQHSDMSRAPANILDRPLGGRGSRADVQISLSSFAYLYSELVQ
YHQNRVDSISELERRLESSGYGVGLKMLELISYRNRXDLNSNNDAMICIGFTKTYAAQTR
NQTHERIAFCLDPGVEALFGKPADSLERSVENADEFMIIDNDPLTSTFCSVPADFGQLSV
DAYMSGIVAGVLTGAGFSARVTAHSVALEEGERSNLNSSLPPRKDKAVFLVKFAPEVLER
DAAMDR
>comp5049_c0_seq3; 185 1 683 LEN=814; translated
XRSLFDHCSRNNLTNTPVQHSDMSRAPANILDRPLGGRGSRADVQISLSSFAYLYSELVQ
YHQNRVDSISELERRLESSGYGVGLKMLELISYRNREHKRETRLMNVLHFVSTQVWRALF
GKPADSLERSVENADEFMIIDNDPLTSTFCSVPADFGQLSVDAYMSGIVAGVLTGAGFSA
RVTAHSVALEEGERSNLNSSLPPRKDKAVFLVKFAPEVLERDAAMDR
>comp5050_c0_seq1; 146 1 559 LEN=706; translated
XCKKNTFNDFVDVGRWLINDQKITTSDQLSCEGRSAGGLLIGASMNQAPGLFKAALLGVP
FVDVVPTMTDASIPLTVVEWEEWGNPNEEKYHKYMMEYSPINNVQSGARYPSCLLTGGLH
DPRVQYWEPSKFAAEIRHRSDSANSGPCCLRIDMAAGHFSASDRYKYLRELAFDYSFLLD
QLGLVE
>comp5051_c1_seq1; 759 218 2302 LEN=2797; translated
MVSLSGAPSLVRRRRTTTLAAVLPFVLLVLTAATVWFRPSSYYHDFFHRDGIGIGEVAQT
DPLASASAPKSAPGSNPNNHEDDGPAKMVPAELLELGVYDESRDFVNERGFSPTGWEEPN
ADPTVWGPCRFTPVRPGSSFSMAADVSWADRRRWTRSNETANATALLSRSDDYYRNADPD
DFEYRVAVALSQLTGHNKSNEKHPPFKGGWCRPGFLIIGAGKCGTSSLYHYLMGHPRIVP
AIEKQIHYFKYHANTRPLAWYYGHFPTPQSFLDHGALMTGEASPGYLPYPEVAKLVHQTY
GGTGQDGAGTDVGMSLQYLLGDPAYRKRAPAVPPRIIAVGREPLDRMYSSYRYNYVVPTI
EHLRSRGHPAIRKHRRYGTKAKKKSSHKEHASEEFVFELNHPDDEYYMPYLFTLEEFVRA
ELKQLRGCFYDWGPRKTYMQWRRDSIYGKTIKLRNGAFVDNESSIAAGKNATDSSAPPPL
IALDDVCYGKSVNKTVYREQWAEMQMNNPEKKVLLNKNLHLTQALIGRSLYTFPLEWWYL
DVHKSPQPPIFVCTDELTDSETLSELTAQLGLPAFDGFDQVLAEGAYNVGGHRGYDTATS
WEELELEKQQKEQQQQNNDESSSRYSSSGSGGPNRTRHRYAAAEDVPTAPKGAAPGTLTT
NGIPLPEDLYRELKDFVDPLNERLFALTGKRCNW
>comp5052_c0_seq1; 228 204 1160 LEN=1262; translated
MISNTESGSEDAYSLRSGVARLDICSVLVLCLVGFYSLLANTYLFQQHESSLLLHDENDA
TLQQCLDSAGGLWTPQENNAIYQYDGDLHRKNISTDFRILCTSLNKRITWGSYRLRCNDL
KRWTDVCAPNVDITVGVSIEQIHSMWGKKAHDIKNNSISRIGPNNNDSMNVDDIFYNATI
FVKSLSRKEFPQFGNKFIDLVDEYRWKAENIPLDMHLIFQTSWQGPTLFPNHSSSVVEHW
YNSYPSDMTNLGDPEYLPSIEQKSPRHLNIATIWNTRRSLDPTEGGCPRLKTPNVKYSCI
DKVSLVSNHEYGSQNNEA
>comp5052_c0_seq2; 341 204 1604 LEN=1685; translated
MISNTESGSEDAYSLRSGVARLDICSVLVLCLVGFYSLLANTYLFQQHESSLLLHDENDA
TLQQCLDSAGGLWTPQENNAIYQYDGDLHRKNISTDFRILCTSLNKRITWGSYRLRCNDL
KRWTDVCAPNVDITVGVSIEQIHSMWGKKAHDIKNNSISRIGPNNNDSMNVDDIFYNATI
FVKSLSRKEFPQFGNKFIDLVDEYRWKAENIPLDMHLIFQTSWQGPTLFPNHSSSVVEHW
YNSYPSDMTNLGDPEYLPSIEQKSPRHLNIATIWNTRRSLDPTEGGCPRLKTPNVKYSCI
DKEFDITSWYLDLFNTKSDECDMARTMASSQLGTGMLYYNLFRKFDALVVLAKNHTDKLE
YGNVQRAISQMRSGVPILLEVRGRVFEDFMDKYNYSCAFRRYNDGRTSQKTRPLMSFDEA
VEKMKSPEIRRQCQVQGLEIVKDYSPSKIGQKFLKAVGYHGEFQC
>comp5053_c0_seq1; 579 670 1812 LEN=2660; translated
MDAAIHNFRMRQQRRPSNLLHCMDRDGNIDAFRYIEYSKRRRVDFLKRANFICTMKSSLQ
QQRQQHQQRQQQLLRQQRPSSLSGMNTVMQPAMSIPPVVPSVLTKLPADLAAFGNAAAPS
SAFGGVGMSGLAFSKVQGSAARRVSNAGHRQDHQFVSSRASSMPLPFASSFAINSTNYNN
ARSATTTTANKRNIGSRIVSVESDCSSSNAKLGSADDKKKKTNKRMASSSIPKVISSPPS
PSLYQGQRQENRQQGLSSKPHRRLEKEELEAAEALLFGMGRDCKVNKTPENAAAAAQGRS
LGIDKRNVASVSEYEQQAGEEVSKKRKIDHCHHGLCTTAATDDDAESSTIVSAEEETSLD
NSINSMTQKCAKCIAREFGR
>comp5054_c0_seq1; 261 187 1095 LEN=1586; translated
MSSHPLIQVLFLLSLVDATDAFVAQSPVASRSSLSNGSYRKTGSSDSRCFNFLKDALDKA
FSNDGSLDKDKRAGQYDDIYAGEEYIESDPSADDKLTDVQRKWRQSTAAFDTVVPEMIAG

KSFTLDLFLSGVPDRDPSNDLYGSRVNISSRDKSTGLSLPSAPSASVQLDFLENGECRCS
ESDFTSGESNGEWKLSDDGKVLRFSMDAIGYTRTVQTKGSIQNVYWTDEEEKSIQTSTTY
SIPPGMVYGDVQVSLGRKPGTFEVEKEGVLRLEKSTGMFGIGTQLVACGKFVPKLKIDDA
VA
>comp5055_c0_seq1; 790 1 2174 LEN=2204; translated
XNGNGADGNPEDGRNGENAHLEPYATQATTYRYALFEYGDLDSVAHEFSKRRLGMTDMEV
TENEYDEVVRSRKRLLKEEHNVWTFDDWFEHVRSGKGNKNDKESTDRKNNKNDPAVHLVL
EASVPPWEVEMHRPSIRNDDGVSDGDSKNATAEDFRLHPAAECVRCLSEDSEASFEEDFD
PSDDGIGSYIDFVYRRMMAETLATTEEKPGGEVVPGEQPQNATPCNADTVDGKPLDNSGV
GSCSDHAMENQWFHCVDTRDLGCEAACHAESLKQHWYDLLTQEESKLLSNPEEVQKDACP
VDESGSRMRFLPAYKLNPELIELERLKSEGLLVQHEEEDDNFEPEDHDNSLDFTFPSFEL
FFGNNTDNIYYTPHVKMAYSPFLAHCVKSLDNWEAFFSDIFFGGTIPDALGRLSLREEKS
RGSVHVRSPILQQWDPESNSYVWSKRDEEDYITCPFFPFAFHLVAKGSSPPRTWSSQLFD
NLHNSRDHEEKNRTSRRRVADSIKSWILDSIRRHSSDPKSSDDQECGGEWFASFLRAVHR
EIYDDIDVSDAAVLLRKNYMKSLSGNRFAKHNIGKIAMPSAKESLTKSSHALXEQTDLSP
EEHLVTPRVEVLGKILIDVWMSSLVDFSLLLKIASICSNEPRDNVVVVCYVGSAHAKAVR
DFFCGPLGFKRKASVGKFTWDDCEAQTLDLPSKLWNLSELFXLMQRRTEYYCKRVLPAQV
VSYHA
>comp5056_c0_seq1; 374 197 904 LEN=1015; translated
MITPGIQSFFLCSWAVLLLFSTRSEGFLPTGTPRKNSVSSPFLPTNGSLPGCSVATKSTS
TELQNLFGNLFGQQSKQEGGGDTESAGSSAPQPVVEIMDIPARVIKIKPLKFYLQLFLVS
QQNTPFKGAWLLNANEEVEEYLEVYFSDGTGMVSVQFDDLYGLKIQRKGQRPSLQYLLQE
SILLHALLDELEAIAFGQGDDDGEAVDEEKRLLQFAEGNKDVLSRAREALPARAA
>comp5057_c0_seq1; 198 172 1035 LEN=1073; translated
MGRMEPKVPSRRIAPCLPQRSMASWPKVRPKRIRMITFDITGTLVSFRGSLEEHYLGAAT
KHGVKNVDSSQFASAFGRAYKETSKLHPCFGGEDMTAKDWWRICVLKSFKYAGATMDDYT
SEMVFQRIYSTFGSSNAYEIFPDALPFLNWAHRNGLVCGVLSNADERYGDSILPMLGLTH
DELQFQCYSKDLGIEKPDPRAFVAAMHRGEPLLPDSREPLEPSNVFHIGNDFQKDFQGAR
RVGMHAALLDRYQETELASEWRRRGVPVFTDLLDVVEFLGRSDCILG
>comp5057_c0_seq2; 140 1 1015 LEN=1053; translated
XYEYEYEYNTSNNTGTDVVFSSYGMNNVFRRNKAVVTNASTCCSLHTIRASMGRMEPKVP
SRRIAPCLPQRSMASWPKVRPKRIRMITFDITGTLVSFRGSLEEHYLGAATKHGVKNVDS
SQFASAFGRAYKETSKLHPCFGGEDMTAKDWWRICVLKSFKYAGATMDDYTSEMVFQRIY
STFGSSNAYEIFPDALPFLNWAHRNGLVCGVLSNADERYGDSILPMLGLTHDELQFQCYS
KDLGIEKPDPRAFVAAMHRGEPLLPDSREPLEPSNVFHIGNDFQKDFQGARRVGMHAALL
DRYQETELASEWRRRGVPVFTDLLDVVEFLGRSDCILG
>comp5058_c0_seq1; 128 76 673 LEN=975; minus strand; translated
MCTWCGDGILCCIGRYDVGVAATVVGSTCLRNVVVPPHETAIRLEADLRCGLICSDLQSF
QCSELACPVNRTDTPSSRRLQDALKDLVSGTLVEVRDNRLHVVMGNLVSKDANVLQERLD
SRDGGVVIRRNRLQRRGPVALDDELLLEAGRNLLLIVLGAVLLQDILQSSHGFLSLGGNA
TSAGGVAEARNRSQKCSG
>comp5059_c0_seq1; 411 94 987 LEN=1719; translated
MSTSYRIARKALARTSNLRCLSQWSLPSATTVDLSRTKGFPVAAKHMSTSSAGGSLLTTL
AKENPYKDVVRYEFKNRKWNLHHVDFYSEALAIGLLENGLQPGDVVLSWLPAHFSESMVL
QFACSKAGLVLYNLDPTLATKDTEAAKEALAQALTVTKANVLISQEAGSDVNYVRLCESV
IPEIRFFDFAEGKPFITPRFPHLRLPIHAGFDQDDKWGMLLLKQFIVPADNLSDHLHGFT
VDGSTPFLGELIVDAKGIPTGTGKIQTNDEVAKSGALETYSKILSRDFHVVEGEGVVW
>comp5060_c0_seq1; 268 171 854 LEN=1259; translated
MASTDTQTEITLKGSVDIVSEFFFTAINSILYQRGIYQPETFKRQSKYGLTVLTTTDDGL
LKYLGQVMAQVDSWLLNGDVQRLVVVVSGVDSGETLERWQFNVDLDGGSEGGDENRAPGT
NPNNTAKNAASSDSKKSNKSLKEIHGEIQAIIRQITASVTFLPLLNEPCSFDLLVYTKKD
AAVPTKWEDSDPCYIMNSQEVKLRSFTTSVHKVDSMVAYREAEDWEL
>comp5061_c0_seq1; 115 1 1171 LEN=1171; translated
XLPFGVAVQRERKKKSKKETIDCRNRTTAHRANTTPTTMECVPDHHRFRASHPWPSPFGL
GASSPAFQPDRTSARGTPRAGSAKRQRGWELDHPRHERVEEPGHCPPEHHVILSSSQEEE
EHDESPMHRGDDDFFYGEFRPSAGKRQRLAPPPGEIADVPPRSSDADLFSPEEPRLVPME
DWFPVSGEAFGDTGASERFESEHECEHECEHEHEHSEWWKNRPPPAGPDSSVSRRCVVEQ
EARASGTRGLSPGGTTEASCHVCHRAFRLPPPAPLASVMPANALLNYFAPLRPQKGRDAP
MAKGAPLPASKGICAXLRPAGLQGVPQAVRGLRQALLLLLHHHRGRARGRGPRDLLPGLL
LVPAAGIFGSVGRLWVAPSSEPKYNTLCMGR

>comp5063_c0_seq1; 269 1 441 LEN=1884; minus strand; translated
XTVTQMMKAVDFEKIANASPETWNKKKEEKAEAETEKAPPLTPSVSNTHIGNVNPADTAG
CKVIPGTTPGSNKEVTAKDNVQMHTPTVQTKESSMKTLQEHTSKQQHFYKEAKDNPKGKG
PSASAAIVKMVNSEFGAVLNHKTVLR
>comp5063_c0_seq2; 253 36 623 LEN=1800; translated
MTECTTSQQQAWFLYAKYPLFSQWEFALKGGKSVEGEIYCIDPEADLIVVQDVENGITMI
SLESIEAESPSSSCAGKNEENTNASCGTTIVDEPSIDVVHAKKTLEDREKRAIRLAQESL
KHLNPKALPKGQLVFDRLLKACNEVVWKSNSIVVLDSIVVDPPYTQGDCKILAKAPGKQG
SLDRVQKIVSSISRT
>comp5064_c0_seq1; 459 109 1662 LEN=1865; translated
MLSVLAAAPFLLSLSTVTATTPDHVSSRLMIHVPHKLFVAEGYDHREALFGIPPYGGSIT
QNVYYADDQLCDPVVDTTKGYPRREEDANGKMEEWKAPYILMVDRGDCTFVKKVRNAQRA
GAAGVIIADNTCLCSDARCIELSDNPTCETSEPIMADDGSGSDISIPSMLMFKHDADMVK
DQLIEDQPVQLEMAWSLPNPDDRVEYDLWTTPSEKTSKKFLEEFKPVAKALGDSAYFTPH
QYIYDGIKTNCRGNDGENFCYNLCTNNGRYCATDPDNDLDHGISGADVVKESLRRLCIWK
HYGESDGIGEVWWDYIIQFMDRCNTPDYFMNEDCVNDCYKRAKVDRDKIERCMSDSGGLR
KDNSNALFDSELELQEQRGVVVLPTTFVNTAAMRGQLTVGTVFGAICAGYAEGTTPAICE
TCSKCSDVVACATKGKCQDSGSSSGQVSAHTFGFTIVFMAMAFAGAGYWHYQKTRDDMRE
QVRGILAEYMPLEDNDADGGPGPMEFAMRGGNTPLFA
>comp5064_c0_seq2; 383 286 1377 LEN=1580; translated
MADDGSGSDISIPSMLMFKHDADMVKDQLIEDQPVQLEMAWSLPNPDDRVEYDLWTTPSE
KTSKKFLEEFKPVAKALGDSAYFTPHQYIYDGIKTNCRGNDGENFCYNLCTNNGRYCATD
PDNDLDHGISGADVVKESLRRLCIWKHYGESDGIGEVWWDYIIQFMDRCNTPDYFMNEDC
VNDCYKRAKVDRDKIERCMSDSGGLRKDNSNALFDSELELQEQRGVVVLPTTFVNTAAMR
GQLTVGTVFGAICAGYAEGTTPAICETCSKCSDVVACATKGKCQDSGSSSGQVSAHTFGF
TIVFMAMAFAGAGYWHYQKTRDDMREQVRGILAEYMPLEDNDADGGPGPMEFAMRGGNTP
LFA
>comp5064_c0_seq3; 424 909 2189 LEN=2392; translated
MEEWKAPYILMVDRGDCTFVKKVRNAQRAGAAGVIIADNTCLCSDARCIELSDNPTCETS
EPIMADDGSGSDISIPSMLMFKHDADMVKDQLIEDQPVQLEMAWSLPNPDDRVEYDLWTT
PSEKTSKKFLEEFKPVAKALGDSAYFTPHQYIYDGIKTNCRGNDGENFCYNLCTNNGRYC
ATDPDNDLDHGISGADVVKESLRRLCIWKHYGESDGIGEVWWDYIIQFMDRCNTPDYFMN
EDCVNDCYKRAKVDRDKIERCMSDSGGLRKDNSNALFDSELELQEQRGVVVLPTTFVNTA
AMRGQLTVGTVFGAICAGYAEGTTPAICETCSKCSDVVACATKGKCQDSGSSSGQVSAHT
FGFTIVFMAMAFAGAGYWHYQKTRDDMREQVRGILAEYMPLEDNDADGGPGPMEFAMRGG
NTPLFA
>comp5065_c0_seq1; 412 210 1319 LEN=1442; translated
MTIPRHLRQRSEFLTSLEKRRRRWSFRHSVVLGILSLALVGCLRYLAVPLAYDYSPLHST
RMQTNTDSYSGERLFDSPGDVPMDLIRSFDAILVLGGGKPRAIDRPPVYYVERRCDDAMEI
VRTRSESYANDTKHKHKHKHHGAKEVDHQLPILCLSAGTAHMPQLLSADGLPIWESTACA
AYILRESHRTNNGTGASLGTTPSLIPPESIFVETSSYDTIGNAYFARTSHTDQNGWRKLL
VITNEFHMARTRAIFDWIFSIPPAVAMGVAAGGFGEGFPAYDLYYLESPNVGMAREAIAA
RKEREASSARSVDALSKKYSHSLGDVYRFLTHDHALYAASPLVDRGKATAADILEDSVND
AVKKSYGLS
>comp5066_c0_seq1; 605 43 1431 LEN=2184; translated
MVSTRLAFLLVSFSLGQLGDGLNIFQGVFLAGVGWAQGAVGTALSLMGLTSLVMQPFAGN
WVDTTSVDRRLFLVLASVITACSASTILFVHPSSLNHVLIYASKFVEGIASSFINPCLAA
LTLACFGPTQFDEVMASNVYWGHVGSVAAAVLAGIVAFVSYPRIEYCFLVIGASALVAIV
FIRYLPQGNPLWGRGLDASSIPSLEYSDTDTEIGSSQSEDSTENEISSESTPLAPTNCSG
ENTPQVASYWEVLSEFRTLILCMTGFFFHFANANVLLVLGELMGQGDEEQEGVTRTAIPL
TAGAIVVAQTTMAFATHWADGRTEQGWGRKPLFLIGIATLPIRCALIILLKDAGHQYLLA
TQLFDGLGGGLFGLIHPFIVADITFGTGRFNAVMGLTASAFGLGATLSNYLGQRVAEHFG
YVHSLMASFVISFIPIILFAIFMPETLGRRGTTTNTKSVSLH
>comp5066_c0_seq2; 605 43 1431 LEN=1833; translated
MVSTRLAFLLVSFSLGQLGDGLNIFQGVFLAGVGWAQGAVGTALSLMGLTSLVMQPFAGN
WVDTTSVDRRLFLVLASVITACSASTILFVHPSSLNHVLIYASKFVEGIASSFINPCLAA
LTLACFGPTQFDEVMASNVYWGHVGSVAAAVLAGIVAFVSYPRIEYCFLVIGASALVAIV
FIRYLPQGNPLWGRGLDASSIPSLEYSDTDTEIGSSQSEDSTENEISSESTPLAPTNCSG
ENTPQVASYWEVLSEFRTLILCMTGFFFHFANANVLLVLGELMGQGDEEQEGVTRTAIPL
TAGAIVVAQTTMAFATHWADGRTEQGWGRKPLFLIGIATLPIRCALIILLKDAGHQYLLA

TQLFDGLGGGLFGLIHPFIVADITFGTGRFNAVMGLTASAFGLGATLSNYLGQRVAEHFG
YVHSLMASFVISFIPIILFAIFMPETLGRRGTTTNTKSVSLH
>comp5067_c0_seq1; 506 24 1208 LEN=1266; translated
MKMKSLCNLSAFVTLTTFGASTALFVPSGWRQAHALKNNESRFLSQRGASSSSLPDTGTR
NAERNNEFVEGLMGNLSKLCDRYIMNGSPKIREQIFNVLDQIATEAVDRDLVNQSIRIVK
RAGVPVHKTYLEKYSSELGASDADRRRSEADKRSKWENEKKAAGKSALSRRIAGSDGRPD
LLTPSLLDPDSIATIAGEKKELQKEIQNGSKDRTQPNSMSTKAAEEAACRVSEIIAKAGS
GDSFDGETLGVGGLDDVLVQIKRRIWTPLAAPPRLLQELGIHPARGLLLYGKPGCGKSLL
ASTLGSLLSPFRPITVVSGPEILDKFVGSSEKNLRDIFDNPPDIYEKFRVNEEDGGDSLA
SAAIHVVVMDEFDAVARSRGGSDGKGGQGDAGVA
>comp5067_c0_seq2; 703 24 1751 LEN=1944; translated
MKMKSLCNLSAFVTLTTFGASTALFVPSGWRQAHALKNNESRFLSQRGASSSSLPDTGTR
NAERNNEFVEGLMGNLSKLCDRYIMNGSPKIREQIFNVLDQIATEAVDRDLVNQSIRIVK
RAGVPVHKTYLEKYSSELGASDADRRRSEADKRSKWENEKKAAGKSALSRRIAGSDGRPD
LLTPSLLDPDSIATIAGEKKELQKEIQNGSKDRTQPNSMSTKAAEEAACRVSEIIAKAGS
GDSFDGETLGVGGLDDVLVQIKRRIWTPLAAPPRLLQELGIHPARGLLLYGKPGCGKSLL
ASTLGSLLSPFRPITVVSGPEILDKFVGSSEKNLRDIFDNPPDIYEKFRVNEEDGGDSLA
SAAIHVVVMDEFDAVARSRGGSDGKGGQGDAGVARDSVVNQLLAKMDGVQPLAVPTLVIG
LTNKRSLIEPALLRPGRFEVQIEIPPPRTNEQRFSILKVHTQHMFTAGRLQVNDPPPDSP
AAQQIENADTSTLLAYEDLLMKLATLTDEFSGAAIAGVARAAASRALERAVSQFSDEIES
RETLSIMDCLVTQDDFYEAVNDVRESMGTRDHSED
>comp5067_c0_seq3; 141 1 449 LEN=459; minus strand; translated
XRNDIFLNDCRSTYVVIIAHTGTPARFTIRIDWFTKSRSTASVAIWSSTLKICSLIFGDP
FMIYLSQSFERFPIKPSTNSLFRSAFLVPVSGKDEDDAPLWDRKRDSLFLRACACRHPDG
TNNAVLAPNVVNVTNADKLHKLFIFITVD
>comp5068_c0_seq1; 719 1 2541 LEN=2789; translated
XSNNTTHTNPIAIPEGLTSSTEHRSSSKNYHHGILSAAAANELEESFPAPPRACFNPNAA
TENGKASAPTSLVPDRQGEAPLPVRVRGVARLLVDAASLPALVHAGFTAYVLPREDPYKV
LKIKRKDRNNRDAIKKAYREMAKSAHPDKNRHLDPDVANKRFHRIQEAWEILGDKTNKRK
YDQHLRDKEARERRNRRPTKQKEQERRQREKEARETRERFQREQERMKKEQQRKKQSEMH
AQEAQEALLRISTLEQLHERSNLFDKNTNTFKKHFLCVFVGNKKAEQTAEKDYQFPYPFG
PRGTNSFDWTEIMQTAKVRFNKATPLTRAFRVSARASDPIIVFAKKGSRLDLFEFETYTG
GNLEDWVLRMMQTSVSVVNRYASGGPTIKIFFDDGDTLRSAGKAVPPGFMLQIPLKISDR
LLVVDAGTDDFTGGVGIGEKRQQDLEKLRDEKILDRIALDRMYAREEGERFDIGRGHGTT
RVCYDLSTSCHRDATREKCEGRSRFFHAMCAKSCGVCIESNYWNGIYYYLLHKPLDTIPD
VPCVRRILSWLRDHGEFGSVFWHDLSHMWEKRRNIMFGFVFTGLVLGIQIVLLARMFMMG
NRRSSGRGRAGECTFFSSPAVVGFLLVANSAAIGARFWLSATPRHKVPPFLRGFRADLKG
IEKSSYQLLRILFCLGAVSSYVSIAGVRRLRLRPILRETATFATIVLATALLLLGIDHYF
HREFTGDKLWKRHRYMQWESIWDLRKNAAIAIIVSGILSGALIAGLGRFMKQYIRPLYLV
LSTANIGLWSGLLLFAVHFDRFFYEDLKHTTEMRMSAAIPCLILGLIWGMSGAHLVSTYR
VKVKVD
>comp5069_c0_seq1; 477 1 1449 LEN=1538; translated
XAFQVMAAHSAMELPIKELKPVNMRKFVYLQLILVSSAADVATPDPIPPPLGDMPPALVE
SAPAIEVIETYSLPNPDAIAVDPTGTFFVSGTKDGYVYKTDIESGDIETIFSPKDFRPDY
FVNQTDEEVRGLCDGSTLANENTCGRVLGVKFDLDGETGDCNGLWMANSYFGIYEGTCGA
PWEIVKRVAIEGGFVNDIQPLSDGEGNFLYYTITHQSKTRNELPYVVLDTEMPSGSFHRL
ETSNYTTEQLGDGIYFANGLATSSDEASIFISETTAARILRYDIMSGEIEPFLENLPILP
DNIVVQDGELLVPGYTRNEDMETFLKDPAALIAFSHQFPDYVQHRFKSMIAPQGNLVIYD
ESTGRMKEQIFQADGGLFSLASSATRLDNGDYVVGSIFVPAATRFRILPHISQLPSSSET
TNEDNGNFVEWVFDPNLVSNKELGPESGTDSEPELEQSSSSSTEKTIIQVLVVLTIISFA
NA
>comp5070_c0_seq1; 443 1 863 LEN=1016; translated
XSTSQSSLVPPPPSANADHPSFSMIFEGLVAPSPQMKMVQKCKDSIEKTSANVCKPSLSF
FSLPLETEKTYSKNELNATNAIDLVVPDFVPDKSFAGMDALQNHDKGGRTFSVPLSKENQ
VSEGTGSEDSVEEEIGDVNHILDLLLVNSPKSEGHYVNSPETNSLPGEDISETNPGDCWI
PSQSTNKKSTTDEAKGPSKVCNFEINSVKNIAHGNNQCNNLLLLNSPALQSRAQTEETAS
ATFMSPDVYHEQKPESETPETLLLSYNEKDSFVENSTSFEFSEPQTT
>comp5071_c0_seq1; 231 81 1412 LEN=1504; translated
MDPTTDKSRAPRSDFYSEIDVKTKHPDNEVDHRPNGGPLLKLLWSEEPLTGQTLFLGGEV
GSDGKIYCIPGHSPRVLVIDPETDDVTLIGSKELVSNGTKFKWLRGIPIGDVIYGLPCHA

SEVLRIHVPTQTITKIPIPYETFYEDPAVAKQQRKMIWKYHGGNICHDGCIYAVPQSALH
VLKIDPETDTCSLVGPELPGKYKWYGGVESGGSIYCVPQNSPHVLRISPEAITLHGDYGE
GGHKWHGAAAADDGTIVCIPNNVDTVLLVKPGEGDDGPTLTEIGDGSFIQTGRHRSDGKY
KYLGAMTGTNGKVYIFPSGSEQVLEVDTARNIARNVGPNLRDSGMERIFQNKWQNGLTNP
HDKCVYAIPLSGETLLRIDCSNDRDAGGDGDVEVTTWPLPSPFETLDKWEGGVICANGLM
YTMPNNCKAVLKIQPCNWKPDNE
>comp5072_c0_seq1; 683 161 1822 LEN=1851; translated
MRXLFVPLGLVIGFAVAYEQRIKHTSHKLDDVSGGQATSDDRFDEIPPPIWLHSVRSAAD
AFVGYPAAIDRSFDTANIFPSALHDAAYPRRKLVFFSNSTHKHDSVLCYSTREPKPDTTN
DSGESSLAESISRSYAEKETDGVVELATTSSLLTDLSVDIEDDLIPAILEASGGRKGIVA
SIMNAMIGSGCHFIDETKTKAGTTPGLVSDRVLSLMEGLFDAADVTPDIVTYSLAYEALS
KDDPDDTDGLADSILETAERSSKKIAGGKRRKLLASSRRKVTSSFSDAEDDLRELLGADF
EVLLETDGFAVVNKPSGVPCFHARTTTAGKIKRGKGKKKKGKKGQQQNENKANDRLPSDV
SLEDALVSCNVPLSTLNPEALGLVHRLDRGSSGCLILAKTNELHARLVSEFFLRRTTKQY
VALVRETQASSISKKGSIDHAVGGRSARSDYQLLERYRSDGDDAAMVGFEIFTGRKHQIR
VHAAEVLGSPVWGDPLYGAGDGGPNTKPPQQERIFLHASRLKIPVLGVDVESPVPSWWES
TLSTFGEKNETKD
>comp5073_c0_seq1; 459 53 673 LEN=870; translated
MGITREGRHKRRETGGRRAQFCKKRKYDLGRPAAMTKIGEPRIHKVRCRGGNSKFRALRL
DSGNFSWGSEVCTRKVRVLDVAYNSTNNELVRTKTLVKGAIVVIDAHAFKAWYESHYGVK
IGVKKNNKEGETSGQSIDLSSKSAQVAKKLKARQADRTLDEALDAQFASGRVLAKISSRP
GQSGRCDGYILEGKELAFYQRMMHKK
>comp5074_c0_seq1; 1074 1 2602 LEN=2730; translated
XPPRSTAAETPSPAEEEEDEGSSFLYCCLSPNHSFDKLKDGTGESGRLPPSSGALRDEFE
QTAVIEEPSFGKQLGYREAEQAFAPEYDEATLASTIFTEANQGSEPRNPRSPSGSWHPRS
TSGFDPEGASPCPSTHRSWDGNDAGEMGDARYPRNEAESWTGPVGNTEEYGRGQDCDAEQ
IEMELGSYHPDPDHISLSSNDTDSLTYNGEEEAAITEQVKTTVKAECLEDYDVSNVLHGK
RSFKSKTTEDGSSILPLHSPSERRERRKNAMKAHRDFLYNMSTSASACNTEDHDRNDADN
AEGAVDSGEENGSDQHEGEVAVETPGIAGSSSSSGSKPNSIVHDNAGRSSDADKPDDIIG
MVRNAFTVMISSKAEDEPDPEASVLNESKPDLQVDQETAGLPLAQEEASTSGPQSPVEEA
LNAAYRFFAPSQADEPLINEPVHGEKQILQCTNVEPNPVSRPEVTVEDPVQEFPPEETIE
EKNSIHSLPSIHSVSSTPSDEAVTNKLELPKKPKAIKDVGYNVEGIPLELRPQKIWRVPP
STMIAEEENYMSPTCAKESYTAQIAEAPASPPESIRDAGREQAEDASIAEQQHEPSPPSD
PVPSQMESDIDTNDKKLGRHAQRFRRGRDLRRFNRRLQQGDGEKRPVPTKEPSLSADPPE
KQYSDTSNPRVQRPHRDVVPSPIVAGGGETIRDTAAGALAAPSMPDPVSPRSNKADFVVG
SRRSNLSEIRSRIKQRRKSKLQSRHQAEQLSPLNGRRDAASPQRIVSKHNTADAPTKRSG
IEAAKTRNATVRSAENTPVVNAATDRRDDSASCSSKDGDEFFDTITFDEDNMEGMIDAMT
PPRVQRYLEASSPMQSPNTIGAAISLD
>comp5075_c0_seq1; 562 107 1234 LEN=1408; translated
MKNQLKALSLGDGNDLTPDLTVDMKKLQTFKTEVVNDGKKAEAMIKDCEEIYQDWKKILA
SASYLTSEYPTILDAFHQTELVESDFKSGDILKGVEEMEKVYEDIEEVYQSRAQLMHSFI
AIERWTGNIDLIVKQAIDFCEKHDQKAKEKAEEAEKSLKLRLRKMESRILHRMHLDHLLV
KMGKGLDKSTIESSEPEPTTVVKEETKTIVVKHISMREEAEKFASSVEEDTKALVVEAEN
IEDFMKKNKTTIDAFVNDSKEIKKDWGASLKAIANVQSIIPLVTKALDEVDAIGNLIQDA
CGAASLNPTAIIAVIKDGKKAWDDFEAIYQLGQKIKQVFEHLVVLEAFIKATVQLISTGR
VLVKAYQASCGPIKL
>comp5076_c0_seq1; 193 1 922 LEN=1220; translated
XIELLTSSAPLYQLHKSKNMATTDTMKRPYRHSSTTSRFPPTSLQDITEMPRHLEGPYNQ
DGPHERMEQRNTYGSGSNSTNNKKTTTTSGSSYDDQLLTKGERVGAIIGLVVVLFVVLYF
IMLNIRVRRPRSRRKHRRRNTGRRKRTYEEDHDSTTDDDSSTAELEMAEQQSVTPYALTK
NEDKQVDKPQVSQARSWLWNLFKRTVQEEDKNQLHQAPKHTVGSSDCYYRHDSDEITFPF
DEKSSRRVANGTTENLYVSNDDSVITLRDNDERDTNGDDVFDGIEDRDVLASVRLSSAST
AYTTNQI
>comp5077_c0_seq1; 236 53 541 LEN=1234; translated
MDVSKNETSHGEDEFFGSQDDFERGSSLRIGEFGNMNEHEIDAKRNELKHVGFLDAYDEF
KESRMQEGFERGFLDSFAAGKIIGKIIGEATTLEKIYEQQSEDQSANSHCASCKPVRSGK
VELIRDYFTNKFQKDLDSNNGKQGLRNLISKTEEYPTTGDFT
>comp5078_c0_seq1; 106 1 484 LEN=670; translated
XKYRTIQYVLVLLNSFNWASSHKLISDNLVAFQLPCTQNKNEEKNMKAKSTCIAAAATTC
VLGHSVLMNTPGAEAFSQAKAPIARASSPYAVAVSTSRMAAAVADSDVGCSCPCPKCQAA

KSQSAGCPCPNCKGAAAACDCPDCSGAQRGQGCPCSACQGA
>comp5080_c0_seq1; 1025 278 3082 LEN=3424; translated
MVSALQSKQKENRCSNSSTEIYEDESTSSDGSTKCADSSNSDMTTEPPSRSTSGTKISFL
ATLVSILGVAVFGFLAGRIYATGSVDGSLALLVHPLLAETLGSSKEEAQCVEEDLNDTRV
TLQFNYAEIEEDDETTSKIVDDDEDEEEEEDDDDDEVEGVGNLLQLYLNAYGLEDHFADY
DGTNGKEIITEFMEDMVEASYDCEEMFPLYGHFAHDTSKHGGKSDTLQALGMFRYGMGQV
SLKAWPKEGKIMADFLYIPPRSTEKETYAADEDDEEVSEETLLACVDSLARFFYPDDVIE
PSGVVGDSLNVGHVSQLGPFESLSKHGKDPFRFRWWVDNKVRGMKPVDLNDADLLEQMGS
SDDWVNRVTSVQSPFQRIDIVDSEDDYETMPWLEYYTSDSEKFSEYRAYMESHPHLFRAD
RLLYLDGVVQSTLQGLAAYHEALVQPAMFTHPNPKRVAIVGGGECATLRETLKHNTVEKV
VMVEIDPVIVEVSKLYLPEWNDCSMFKGSSRYCMDDPRVEMHHLDALKWFRDRFSDEARL
EGHELYGTEEKFDVVILDALDPQNAVDFVEALYGDGAFLNSLYNSLTDTGVLLTQVGESA
YIGDISETYPDNFNYQRAMYEKGLKNQGFKMVFHYEEPRAGFNGIWSFYAAFKDDSSRAR
WQMNEAQLDLEIRKRAIQMVDDRDVEEGELKGPFDVFDGPTMATYRFPSKHAQVIYCLRD
PQPYGCTTNIEDHHSRRRPIWEPTGFDPEIPNFNADNFALVGSNGKGLVPRVTIPEKSYL
MLEQSIHDVRVAPSTSSILERASAKSVLGTTIGPVARFVSGNANGLTMPRRRDIHSAYRV
DSGLLSFIKHGCNGNFNTETYHSFSLTEDSADPDVVPTHGLLGRLYSNTVMKEVVFNPSH
TRNTIRPEFLMSSRTINEGEEIFSNFVPWYNKAGW
>comp5080_c0_seq2; 1014 278 2872 LEN=3537; translated
MVSALQSKQKENRCSNSSTEIYEDESTSSDGSTKCADSSNSDMTTEPPSRSTSGTKISFL
ATLVSILGVAVFGFLAGRIYATGSVDGSLALLVHPLLAETLGSSKEEAQCVEEDLNDTRV
TLQFNYAEIEEDDETTSKIVDDDEDEEEEEDDDDDEVEGVGNLLQLYLNAYGLEDHFADY
DGTNGKEIITEFMEDMVEASYDCEEMFPLYGHFAHDTSKHGGKSDTLQALGMFRYGMGQV
SLKAWPKEGKIMADFLYIPPRSTEKETYAADEDDEEVSEETLLACVDSLARFFYPDDVIE
PSGVVGDSLNVGHVSQLGPFESLSKHGKDPFRFRWWVDNKVRGMKPVDLNDADLLEQMGS
SDDWVNRVTSVQSPFQRIDIVDSEDDYETMPWLEYYTSDSEKFSEYRAYMESHPHLFRAD
RLLYLDGVVQSTLQGLAAYHEALVQPAMFTHPNPKRVAIVGGGECATLRETLKHNTVEKV
VMVEIDPVIVEVSKLYLPEWNDCSMFKGSSRYCMDDPRVEMHHLDALKWFRDRFSDEARL
EGHELYGTEEKFDVVILDALDPQNAVDFVEALYGDGAFLNSLYNSLTDTGVLLTQVGESA
YIGDISETYPDNFNYQRAMYEKGLKNQGFKMVFHYEEPRAGFNGIWSFYAAFKDDSSRAR
WQMNEAQLDLEIRKRAIQMVDDRDVEEGELKGPFDVFDGPTMATYRFPSKHAQVIYCLRD
PQPYGCTTNIEDHHSRRRPIWEPTGFDPEIPNFNADNFALVGSNGKGLVPRVTIPEKSYL
MLEQSIHDVRVAPSTSSILERASAKSVLGTTIGPVARFVSGNANGLTMPRRVRTKTIVFW
VIGFVFLSVVLVLTLFLVHAFRSQ
>comp5081_c0_seq1; 174 1 1113 LEN=1280; translated
XSVDHIETDWKVDANNITEYNKYESLRTSALSISAQFDNETTNIMKLAGLILLCCLLMQG
GHGYNIIRKNTVPTTKDTASSEKDTASATSRRNLLRSAAALSAGALLGIANAEIAVADDD
VDTPLYFGVGCFWHIQHEFVVQGEKGILGRGPHQFTSAAGYAGGKSTDKEGRVCYHNFSG
VADYGKLGHGEVVGMNIPQSKIGDFSELYFSLYNPKTKXYVGLQFRELLLCVRFNFRAAH
PTIIGFCIKTKCIFVISIIDRVDPMDRGAEYRSLIGLPGGMKHTSYPLVESAAEKAGFKL
VEGKGNDPDTLGKQIVYVYDSTKFPFYQGEVYHQYHNDFQSPAYGKEYNNLVEVALEDGR
IKGTGCPDRW
>comp5081_c0_seq2; 274 1 990 LEN=1157; translated
XSVDHIETDWKVDANNITEYNKYESLRTSALSISAQFDNETTNIMKLAGLILLCCLLMQG
GHGYNIIRKNTVPTTKDTASSEKDTASATSRRNLLRSAAALSAGALLGIANAEIAVADDD
VDTPLYFGVGCFWHIQHEFVVQGEKGILGRGPHQFTSAAGYAGGKSTDKEGRVCYHNFSG
VADYGKLGHGEVVGMNIPQSKIGDFSELYFSLYNPKTKDRVDPMDRGAEYRSLIGLPGGM
KHTSYPLVESAAEKAGFKLVEGKGNDPDTLGKQIVYVYDSTKFPFYQGEVYHQYHNDFQS
PAYGKEYNNLVEVALEDGRIKGTGCPDRW
>comp5082_c0_seq1; 382 1 796 LEN=1438; translated
XTNMGKENSTTTNNNASLVSSSSSSNSSKSLGKEGNPGQNDGLKPVAEKSNGEGIVSLVS
SSSVRKEGRPDQNANPLKETRNEEGVASCSSKVSSLRKEGSPDQNTRATPEKKMAFEKEA
IAGDSVRVGGSDGNKSPGSRPSGEHQQQQHQPDKHFDGTNGLQQQEQEQQPDQKSDGSLE
TAADPEPPTLGTEEAVGETNGTSPGTSDNESRPQGGDRSGADADGSTIDNDDDSDVVFVE
VVRPYGITTSFGHDLVVTKSEVFDF
>comp5083_c0_seq1; 436 49 918 LEN=970; translated
MKFSTALTVLSLGGSTNAFVNNQFSSPPVTALDARKPFITGNWKLNPQTKAEAVDLARGI
ADSITEDSPADVGLFVPFPFLETVQDIVGDKLIVGAEMVTPEMKGAFTGGVSPAMLKSIG
TEWALAGHSERRVLNGESDEYINAQCKLLVENDMNVVLCIGETLEEFEKSLVDAVCEVQL
KKGLEGISADQMSAVTIAYEPVWAIGTGKVATPEIAQSVHKSIRQILDKMYGSDVAESTR

ILYGGSVSPDSVDGLLAQPDIDGGLVGGASLDATSFGRIINFESADVMA
>comp5084_c0_seq1; 1000 369 2804 LEN=3449; translated
MSSSARVSTNQSVPSAPIATATTTASTFDTTTKKKFGKNLNKLTAPPVAPVAQGNTKTN
SSSSKNGFLLLSTKRPSSSAAGTGSGGILSSKSAQNSGKPIPSLGLHTEFSSSTHDALMG
VVVGASRLENHQQPDAWGVAEKQQQEQVQQKEISVTAASTETNRNNPDTKTREQVHKNSI
EEKSAFEVKQKERPPKSEADQRNVDGNQGGYRTSNWDEYGGRDMQTGGGKDSNESSKRKE
TTDDQAAIMTKLARQRAEQKRNEEENRMMEQKERAAQRLRELETKMALKKKEDLASKKTA
RNAKGSDGVNNDLESRSRSRIVLEKLKQKESGTSPASTTPARTQRALYDPNTPSKSYSSL
VAGSSSMKQDRNLKNPEPNTKHLSSSITSSKKNAVQLQQGISTYAADPESFDRKPVIQLA
SYEDRDRGDRNASAAPRMLFDPKSGSMIEVTSRDEGAANARNRKAGKKNKNSRDKQSKKD
LAVDNGGNADSKGRRKVKGNKESSSINQRGKGMAGESSNSKKDIISKPKKLNISDPRKLP
RTCGVLYARDKKGGFVCSDGCEGDLGYGVHSVPGGRVKNAEAYSNYVNTQKQMKQGTMLN
YDGINHDELVLETGFRISEAKEPKHEWIKPNEKIELITGDEDSPTLQATAREWAPTHTTF
TLSEHGKMARSSTGSFEDEDDEEEDVDDDEVAPLGLGFDPALNMDSVMQSPSIDPSDGLG
AVDLTTLSLEPALQGVAKNSHIFAFESGATWGASNSEGNNDWGMHSGSAYGNGQANNNGG
VSTSFLSLSTANTWGGFGSPLNGDNSKQSGE
>comp5085_c0_seq1; 226 37 474 LEN=593; translated
MKAITVVLLLFVSAQEILGFVRPVSVRSAASRPLSADQSTTDSIEFDGGYVGTPFEHPSS
LAGIATGILGFVGKAIAEDEYEIAELPPVWVPAIFGVVLLIGVGVLTTSLGNVMDEEASL
GMQSGARAKKEIERSRSSYFGKRPK
>comp5086_c0_seq1; 270 1 940 LEN=1045; translated
XWLPLNFQSLVLFVAPVRNPNCFDPTITNVTFLLPSEMKIAHSLFRRLAASSRPAAAVQT
TLSPFRGAMGSAASSAASAFFCSGTPVDMPLSAISNHPSAGSRSFSSKSNLLEILSREEQ
EEIETGNTEMPPELSDLKATIEKSWKIVEDGATTNFFRIESSSSNKIQVSFHCQDTLEVE
EPEYDDSMEEDSDEEEDEASAPVRFTVTVTKAGKSLNFACFSEYGQVKVEGVSTTSYSTA
EYVHENQGTLPKHEYQGPDFTELAEDLQEGLAEYLDEECGVDSDMAAFIAMFADYREELQ
YVDFLKQAQSIVS
>comp5087_c0_seq1; 563 233 1495 LEN=1605; translated
MDVELGGIGRSESKAYPRNHGSFFAKIQLYMRQHTLATVATVGFLGFLLIVSSDQLNDSG
IGLRTTSSSMGSAFSGSLHGGYFSVADSKIGENQFRFAAVTDLDQLSFIKDAKKPSYRSL
LLPGTITYHKNSRKYTMVMEDDATRTLVTKHNEGGRGAEFSELTVFDNRLLTFDDRTGEV
FEIMNTKDGKDSFVAPRYVITEGEGDTDKGMKWEWSTIKDGLLYIGSMGKEYTRPDGSIE
NVNNLWVTIMDNHGEFRRINWTDNYNFVRKVLGCPSPGYVIHEAIRWSPHMRKWVFMPRR
ISKDVYDDVVDEKRGSNKVVIVDEQFRNAEVVEVKMADKDGLHGFSSFAFVPGTNDRHAI
ALRSVEEDCAINLDDCKQRSYIVIFDVVTGDVLMDEVKIKEDMKFEGIEFVNMYTKPHLS
>comp5088_c0_seq1; 158 219 493 LEN=493; translated
MVLLLLNKKTLVVLTAILSVLVTSASANTEVEYDGQKNVGSKLLRSGNEGNERGEHRELN
LANMLNQINSVIDSTPAPQPEKKSKAAKAEKX
>comp5089_c0_seq1; 188 451 1077 LEN=1246; translated
MIDPEYPGTAVERMLAVRARVTSLTTEELSGPWDEVRRKILWAGGLRDLPNARPGQGYTG
HSFNDYNHVDLTCMRDDVSDNENDGSVKMIAIGNQLGPGIRQASLPELGSGGSWSTCAIG
CNQDPPQDVAHIQFRSRIAFKLVWCPTETFDTFVLVDDDGKKLAQGTPSGSLPSLRERQV
NYKISAGSKYAIVADQIAKGLSTSTETM
>comp5090_c0_seq1; 1148 136 4261 LEN=4499; translated
MNSDASIKSNKNNSNNQNNKKNHSRDGIHAKLSAYPTVDSSDVGSNVNPDTTGGNIAESR
RSNTRFRSNSVFESNTRSSDVKIMSRQRQEPMAIPKSYDGARSSPFIFRDGIQPAPQLNR
VHSSTIPGMSSISYCGYLYKRSNKPQRTVDALQQEQRDHYGHMHRPIHEVYSASGVPEFP
VMPEISLNSGNDTSVDHTNSERIALDHDQDGKISAFNVHSLPARNDSITGLAQSLQPLMH
QQQQQCTLSPPRDESKKTIAKNPIQIGLEMGAAFFGIDLKDSNHKDGDDNHSEEIDFDPI
SPPTEQTIGTMPGTDNNTEARVGFPKTTTVPIKFRPSNSRNVPGEIPSARQPIPMITNEK
SSSSTSPFHESLHRLCSESSQPLSDVSITNGNDGIPHDFVDPEDGHLWRAKYCVLEDGIL
YFYRNAHDGNHAEAAAERRKNTSTLLGANINQTNDSTFSTSYSDLIGNDGNIRANNRSNK
KQRPLTSDTTTSRGQLISGISALKISVPSAATTSRRRSSAHDLSKSPMVRPGRNLLHHID
PSGFSLPDIDPDIDNRIYWEKRVVMDCVRGVRTAERQYGLNSFELLAMDDEDSDEDSSID
TLVLKGQNPTEMKEWIFQFHRTLATFMRNFIDNVGSTSTRTSGTFFENRRPSSDMLSSSL
SASDLKEERGALPQSLSEKHLTRLISTSPSLNQTVLSHGHGRISLKRRMDLKRSSSGSAS
LSSTPDAGDMDYSTIPFAITDPSPGNIRTSPSSPTTSRHLIPPPTQRPFLNKAFMSTDSK
NSRDPDLPPTKSSPVDPDIYRPKQSSNTNKYVPPHQRSKSKYLPPHLRRQQNQQSQGSFM
GSSSALNNGQNNSQKQEEIIPSTETVSPPTQDKLDIVNQSTTGFVLGGCADPLLLKGSIL
DAAYIPKKASRLEKTISEAYGSFGGGFSKEKKASNSSLRWEVGAISECGVRESNEDSYIL

TNDLLNAFDSASYGSEAQTAWKEDDTDHSVGLFGIFDGHCGNQGARFAVEQLGRFIHSEL
QNEIRNIDENPFSPSNMESVLHEAIVKMDDEFCKLCQQGGREWESGATALVALLANENLV
IASLGDCRGFFCRFVENNESYESDGGWEELEIGADDFFSSSVEKGSESEPLQRCFWREVT
TVHSPSAEQERARIEQANGWITTETEIPIGQFGRMDFHDEDVIGILKRCMRYPSSTGESF
TDSERSTKECKAAPQRIIHISRVCGELAVSRALGDRDFKAGFNSSSTQRNDDIDKLGRPT
FDHTSAWESPLWLTFPENHDCRFRGDLVINTPDFHRVRLGKEGVSMEFLLLACDGLWDVM
DGDDAIRVVRDLLYRKNVTAKKAAARLAELAIHLGSSDNITVVLVRLFSRNDIE
>comp5091_c0_seq1; 254 1 1103 LEN=1230; translated
XNQTFRTSDVRCALLLFFFLSSSVQGRQWQSIVNHDIVDKSNIDSSRYELAIEIAPLFNP
EEKSPTEIPTEAPKVTTTTVWTAGVPTSSPTKTLPTNGPTLVPTAFSPQNNGGCQEGLRP
YELHMNDQWGDGWDGTTITIEGISDLDPMAADLPTASMTNTNTNAEGETVVSITQTVDFD
TFDSNKQTDPLGKIFEGTLSQGYHDFANICLLPNRCYRVVTSGGDFLGEVSWDIRLGASS
EPILSGRAPTGCTFSIPDEHGHHFCENTCSDTMPASGAANPSELTDHPQLNEDAAGAEEE
TEFIQPAGTEEPTEAAPETVFMKSGTLDGVSASVTPVTQSMLSGGTSAATRNTLKNFQSL
NAEDSSN
>comp5092_c0_seq1; 668 331 2232 LEN=2509; translated
MASLGGSVHSAGSTSNNPITKDLQLNILVHDKLDKIKKYTPFHRWKSKFLTRFDTFLTEG
GKKPAREASGRIQNHLQNCLNRADVLIELIERGDLSPITGRKTVRAFSALKEFCQDLDGT
RVELQELVPGNSMDERRLGYTKFHVGASLIEECFPQYVAMRELEDKVQLISDSTRDIDRD
EDVLDKTQRELFRNYKKQVTRFCDVMASLDFYEIMLKCVEFLNPPEHEESGDEELTIFLR
PFVRNTGTNGSKNRGSGASLFRSKKGFEPTHAKVINPVKIDAEETETIASVTILALDELG
FNLKKHGDVEILDQLRVRYNNDNNVVKNPKTSTLKDLNIENGDVLTIEQALVPIQVRRTT
SEGEAVFVELMIDPHTPLKELKATLDSEKNRHEGMERVSADEQILLLDGTELTEDEKTCF
DCGIASGSIVDLKSLAKIALAEEAMKEQIDKILIVDTKYGTMFGVGRQEAIQKAVVTPKT
VRVDDEFLEATNSDIDKNHMLQSMMSSPNLNVKPQIVIAKLKIEDYEIEDAAAVHNLWGV
NLKKTSQRQRSTAIFFVDLKTNAVGFLDRKKLMDMNFITVVKVNDPSVLGDNKKHEVNET
LEQAEVDQQKYDFFVARIRRIFGIESLGLQYTD
>comp5093_c0_seq1; 275 205 1020 LEN=1261; translated
MSVQDIRREYRPVMFYFVFDGDVMVSDLELLRKQVSLVVNLAKPGHDRCVVSITSPGGAV
SQYGLAASQLVRIRKAGIHLTCCIDTVAASGGYMMASVADTICAAPFAVVGSIGVVTHIP
NFQRFLNEHKVDAYLMTAGEHKRTIDVIGEVTEEGKAKMTEQLDDIHMAFKDHVSLARPQ
LKDRMEKVGTGEHWLAVQAKEKGLVDQIMTSDEYLESVCEEFDIIEILEKIYKPKGLSGL
LGDNLTSAGKVVKSVVDRFQPSVEPQPMAIV
>comp5093_c0_seq2; 464 187 1767 LEN=2008; translated
MGRYSTADFGYDFPAKAEETKSLVVPAAINEDHGGNAKNGAVANFVGKPEQSEDGAGYEL
YDDHQRRKQEQKQAYRYQPAKINRGGENFFADSKESTGVTINPVECIGAFCCCITVTFII
FGGILVLFVKYRDSDPLLLVGSVLLVIAAGSCLCGCTAICIGTTADDLIDAKGTNGSKAD
PHFHEVQVRFRRLNDRFQKGCMNAEDGLLDVRLDVVGHMKEVKEALNKEAKEKKEKEKKN
REELERRVKHDLKAGMSVQDIRREYRPVMFYFVFDGDVMVSDLELLRKQVSLVVNLAKPG
HDRCVVSITSPGGAVSQYGLAASQLVRIRKAGIHLTCCIDTVAASGGYMMASVADTICAA
PFAVVGSIGVVTHIPNFQRFLNEHKVDAYLMTAGEHKRTIDVIGEVTEEGKAKMTEQLDD
IHMAFKDHVSLARPQLKDRMEKVGTGEHWLAVQAKEKGLVDQIMTSDEYLESVCEEFDII
EILEKIYKPKGLSGLLGDNLTSAGKVVKSVVDRFQPSVEPQPMAIV
>comp5095_c0_seq1; 420 508 1641 LEN=1881; translated
MAVATRKRKATRAATATAAASSTRSGTKIKAEVDNEQGTERTTSITNDQKNSNNKYKLLM
ILSPAKTLNLKDDVPDGIGLTKPLDGLKSRRTEVVAAMKSHASSASKLGALLKTSQKITS
AAQGYWQQMSGDASGGTTTKPSIFLFNGAAYSGLDIGTMATKANLEYLQGHLRIVDPLYG
WLRPMDEVEAYRLEMATKGVFAKSKTKGIDPKVKLEDYWKAPIRSCIEEEERGQADANAT
TNPAVIIVNLASDEYSAAVDVPAGRIVKVVFKHGGRTIAVHAKRARGLMVRFMAMNAVES
LDELLGFDLEGYTFRPGESTYKKGDHENFSNLAALGSRQPKQEKEESASLAKKQKTKKAR
EQPLTLVFDRPGTWKRS
>comp5096_c0_seq1; 413 257 1288 LEN=1623; translated
MTSLFTVVVLFALLLPASNGFVVLKPSRLSVSTRTSSIHQKAYAPAIFLSASASSAAETQ
ETDLSPEVLAEVLEVATRAAKEAGDIILGNAGGAEVTERKANSRDLLTLIDPLCESTIRA
TVLEQFPDHDFLGEEDVDPGAEASAAAIDNKLSGDDKSDWLWIVDPIDGTTNFVHGMPLN
MPSIAAAYKGEVVVGVIYDPHRDEIFCATRGGGSTLNGEPIKVGEQNELGDAVVAMGSPP
GEESMKMSLLGAQTLMPKVRTIRMLGSAAIMLAWIANGRLTAYWEYDLSSWDVAAGALII
QEAGGNFTDLAGDPFSLRTRKTVASNGKIHEELLAALNEAGVV
>comp5098_c0_seq1; 655 73 1899 LEN=1963; translated
MRIIVAATTLFFTCELAGLGHAFTANLSTPRSFIGGTADTTPSRPGGFVSAVHGAPTPLK

AASGNGEEFDDSCDVLVLGSGPAGCAIASVLGASGNLDVVVANKDFDADWVPNYGVWKDE
WQTILDRYGSVGIDLNDGEVNGVIDREWPVTDCFFGGSYDIPTENRMRLDRPYCRVDRFA
LKKSLTKNFRVVKANHISEAIGINMFSPAGSLVHDEDGTTIKLQPQDADASPLTMRTKIV
IDTTGHETKITLKDSIEPSTPPGFQIAYGALVEVDESQSPNLSMVGPYDKEAMTLFDYRT
DHFDGDEYTSIKATKAPTFMYAMPLEGNKIFFEETSLVARPAVSFQECKDRCFTRLEHLG
IKVANVEEEEYCYIPMGGALPARDQRILAFGGAAAMVHPSTGYHLCRALMGATDAAEAIE
EELASETPNLDRAVASAYNALWSRENIRQRNFANFGGEFLMKQDVVGLRGFFDGFFKLPL
ELWGGFLAGWPGLANNEYHESWAKRIWFGLNFIVKLPVPVALDMASSIATYSIFNEGWSL
IQSVTPFLDPPPSYEYKQNNYRNGDIAAKREAVQMIRETKAFAEMPTEVAIIASSSSELN
CNEPVEQT
>comp5099_c0_seq1; 848 313 2652 LEN=2945; translated
MSEKTEIEFGSDNVCQKRNYCLRNCRQPLVIETPFGVTLRGKGQLRAAVQALAFGALILG
CSAYHAYSTAFSEDDFSFDSDDELMRLLQDASNATNLDQSIPGESLVACDDLKKADSWWM
TVFYIIGILYMFLALAIICDEFFVPALEELSGPRRLNMSMDVAGATLMAAGGSAPELATS
IIGTFRQSEIGFGTIVGSAVFNILFVIGMCSLLAKDVLTLTWWPLFRDTTYYTLGLVVLA
VFTGFHTKNNIQLWESCVLFGMYLGYCLLMWQNKNLYKAITGKVLVYPDEDDEDGDDGRT
ASNGSGGSGDQNGNTDNKDKKEEGADEEKDVKSAGGMRSSLQKQSSKSSVHSVASHISQA
SFRGSDKSTASHPHFLWQGTFRAGILKLLKDADSWADTAGAGMVSKIMGDADYVFEQVDK
DGNGEIDREELRQLFDLLECYATHEEMEEVFVQLDANKDGVISRKEFTEWYFRSEERMLS
QVRHVFDQIDVDKSNTLDKDELKTLLSTLDPHVSDTDVQSALSEMYKHGSPDEITFDEFS
EWYKQSIMYEHQLQKIEENMVGVCDNLYPPKDGGARDWAWYIICLPLVFLLTFTVPDVQR
PGYEKWCYASFFLSIAWIGGFSYFMVDWAEIVGNTFGIPDELMGLTVLAAGTSVPDLLSS
VIVARRGQGDMAVSSSVGSNIFDILVGLPLPWILYSAANKGTPVVVSQEIVDVKNCCRSL
LLFLSASTIKPSQFLTNLFISLTDWLGRTCGIIAYFGRHDCLGDYFYSLSRVEVDEGVRW
>comp5099_c0_seq2; 757 313 2770 LEN=3063; translated
MSEKTEIEFGSDNVCQKRNYCLRNCRQPLVIETPFGVTLRGKGQLRAAVQALAFGALILG
CSAYHAYSTAFSEDDFSFDSDDELMRLLQDASNATNLDQSIPGESLVACDDLKKADSWWM
TVFYIIGILYMFLALAIICDEFFVPALEELSGPRRLNMSMDVAGATLMAAGGSAPELATS
IIGTFRQSEIGFGTIVGSAVFNILFVIGMCSLLAKDVLTLTWWPLFRDTTYYTLGLVVLA
VFTGFHTKNNIQLWESCVLFGMYLGYCLLMWQNKNLYKAITGKVLVYPDEDDEDGDDGRT
ASNGSGGSGDQNGNTDNKDKKEEGADEEKDVKSAGGMRSSLQKQSSKSSVHSVASHISQA
SFRGSDKSTASHPHFLWQGTFRAGILKLLKDADSWADTAGAGMVSKIMGDADYVFEQVDK
DGNGEIDREELRQLFDLLECYATHEEMEEVFVQLDANKDGVXEFISAVIRVYPACASVIA
SFFIVHLSTLTFTFTFFRGCXISRKEFTEWYFRSEERMLSQVRHVFDQIDVDKSNTLDKD
ELKTLLSTLDPHVSDTDVQSALSEMYKHGSPDEITFDEFSEWYKQSIMYEHQLQKIEENM
VGVCDNLYPPKDGGARDWAWYIICLPLVFLLTFTVPDVQRPGYEKWCYASFFLSIAWIGG
FSYFMVDWAEIVGNTFGIPDELMGLTVLAAGTSVPDLLSSVIVARRGQGDMAVSSSVGSN
IFDILVGLPLPWILYSAANKGTPVVVSQEIVDVKNCCRSLLLFLSASTIKPSQFLTNLFI
SLTDWLGRTCGIIAYFGRHDCLGDYFYSLSRVEVDEGVRW
>comp5099_c0_seq3; 793 313 2734 LEN=2948; translated
MSEKTEIEFGSDNVCQKRNYCLRNCRQPLVIETPFGVTLRGKGQLRAAVQALAFGALILG
CSAYHAYSTAFSEDDFSFDSDDELMRLLQDASNATNLDQSIPGESLVACDDLKKADSWWM
TVFYIIGILYMFLALAIICDEFFVPALEELSGPRRLNMSMDVAGATLMAAGGSAPELATS
IIGTFRQSEIGFGTIVGSAVFNILFVIGMCSLLAKDVLTLTWWPLFRDTTYYTLGLVVLA
VFTGFHTKNNIQLWESCVLFGMYLGYCLLMWQNKNLYKAITGKVLVYPDEDDEDGDDGRT
ASNGSGGSGDQNGNTDNKDKKEEGADEEKDVKSAGGMRSSLQKQSSKSSVHSVASHISQA
SFRGSDKSTASHPHFLWQGTFRAGILKLLKDADSWADTAGAGMVSKIMGDADYVFEQVDK
DGNGEIDREELRQLFDLLECYATHEEMEEVFVQLDANKDGVXEFISAVIRVYPACASVIA
SFFIVHLSTLTFTFTFFRGCXISRKEFTEWYFRSEERMLSQVRHVFDQIDVDKSNTLDKD
ELKTLLSTLDPHVSDTDVQSALSEMYKHGSPDEITFDEFSEWYKQSIMYEHQLQKIEENM
VGVCDNLYPPKDGGARDWAWYIICLPLVFLLTFTVPDVQRPGYEKWCYASFFLSIAWIGG
FSYFMVDWAEIVGNTFGIPDELMGLTVLAAGTSVPDLLSSVIVARRGQGDMAVSSSVGSN
IFDILVGLPLPWILYSAANKGTPVVIGSDGLVVSLLILVGMIVLVIISIHCQGWKLTKVL
GGIMFLLYIVFLVQAAVLGLPFEVCVE
>comp5099_c0_seq4; 884 313 2616 LEN=2830; translated
MSEKTEIEFGSDNVCQKRNYCLRNCRQPLVIETPFGVTLRGKGQLRAAVQALAFGALILG
CSAYHAYSTAFSEDDFSFDSDDELMRLLQDASNATNLDQSIPGESLVACDDLKKADSWWM
TVFYIIGILYMFLALAIICDEFFVPALEELSGPRRLNMSMDVAGATLMAAGGSAPELATS
IIGTFRQSEIGFGTIVGSAVFNILFVIGMCSLLAKDVLTLTWWPLFRDTTYYTLGLVVLA
VFTGFHTKNNIQLWESCVLFGMYLGYCLLMWQNKNLYKAITGKVLVYPDEDDEDGDDGRT

ASNGSGGSGDQNGNTDNKDKKEEGADEEKDVKSAGGMRSSLQKQSSKSSVHSVASHISQA
SFRGSDKSTASHPHFLWQGTFRAGILKLLKDADSWADTAGAGMVSKIMGDADYVFEQVDK
DGNGEIDREELRQLFDLLECYATHEEMEEVFVQLDANKDGVISRKEFTEWYFRSEERMLS
QVRHVFDQIDVDKSNTLDKDELKTLLSTLDPHVSDTDVQSALSEMYKHGSPDEITFDEFS
EWYKQSIMYEHQLQKIEENMVGVCDNLYPPKDGGARDWAWYIICLPLVFLLTFTVPDVQR
PGYEKWCYASFFLSIAWIGGFSYFMVDWAEIVGNTFGIPDELMGLTVLAAGTSVPDLLSS
VIVARRGQGDMAVSSSVGSNIFDILVGLPLPWILYSAANKGTPVVIGSDGLVVSLLILVG
MIVLVIISIHCQGWKLTKVLGGIMFLLYIVFLVQAAVLGLPFEVCVE
>comp5100_c0_seq1; 241 35 478 LEN=542; translated
MDAAKVAELRKKRQFRKFTYRGIELDQLLDLTNDELMDLVCARPRRRMSRGLKRKPMALI
KRLRQAKKEAPPGEKPRGIKTHLRNMIIVPEMIGSIIGIYNGKMFCGVEMKPEMIGMYLA
EFAITYKPVRHGRPGVSAAHQTRFIPL
>comp5101_c0_seq1; 188 411 875 LEN=1352; translated
MLAVRRRRRGSSRKVESSADESSEEGDAGSTLQLMHAPVTDPVEFSEAASRLLCKIERSV
EPMKTRNETFITKRSDGEIGEMFTIDLGPQLGIFQIETSTDEQVFEYSSPISGKLLYCLS
SATGEWINMEDGHQFEGILVRDLLRSNCIGLPDL
>comp5102_c0_seq1; 631 96 1928 LEN=2128; translated
MKLSLLSFLLSAVTSTAFLSPTKVSSVSKSTASTSIWSSLKEDETTTTASSETDAEAEGL
PWWWDMVWKLDMMKTGEDGEDIIFGDSANVLRSNIEQIYGGYPSLDGCPLAEGALDDIAD
GTMFIGLQRYYKNYGSPYKLCFGPKSFLVISDPIQARHLLRDGNKSYDKGMLGELLEPIM
GKGLIPADPQTWATRRPQIVPAFHRKWLEYMIGQFGYCNTPLIESLNKLADTTGKVEMEE
KFCSVALDIIGKSVFNYEFGSVTKESPVIKAVYSALVEVEHRSMTPAPYWDLPFANQLVP
RLRKFNGDLELLNNVLDELIAGAKETRSEADIEQLENRNYAEAKDPSMLRFLVDMRGADI
DNKQLRDDLMTMLVAGHETTAAVLTWSLFEVSKNPEIKAKLLEEIDRVVGDRHPTYEDIK
EMKYTRLVISESLRMYPQPPLLIRRCRAENTMPKGAGREARVIRGMDIFLPLYNIHRDER
FWPNPDTFDPERFTRPYKNPDVPDWKGFDPVKWSKMLYPNEIASDYAFMPFGAGSRKCVG
DEFAVMEAVVTLAMTLRRFEFDFDEDKLVGKDDIHDEPSGLNHPVGMRTGATIHTRNGLH
MNIRRRQHSQ
>comp5102_c0_seq2; 540 96 2075 LEN=2275; translated
MKLSLLSFLLSAVTSTAFLSPTKVSSVSKSTASTSIWSSLKEDETTTTASSETDAEAEGL
PWWWDMVWKLDMMKTGEDGEDIIFGDSANVLRSNIEQIYGGYPSLDGCPLAEGALDDIAD
GTMFIGLQRYYKNYGSPYKLCFGPKSFLVISDPIQARHLLRDGNKSYDKVSDSMLFFLIQ
EYGSEFDSVLESSSVSGCQYSSLTEIVYMCRLYVSINXGMLGELLEPIMGKGLIPADPQT
WATRRPQIVPAFHRKWLEYMIGQFGYCNTPLIESLNKLADTTGKVEMEEKFCSVALDIIG
KSVFNYEFGSVTKESPVIKAVYSALVEVEHRSMTPAPYWDLPFANQLVPRLRKFNGDLEL
LNNVLDELIAGAKETRSEADIEQLENRNYAEAKDPSMLRFLVDMRGADIDNKQLRDDLMT
MLVAGHETTAAVLTWSLFEVSKNPEIKAKLLEEIDRVVGDRHPTYEDIKEMKYTRLVISE
SLRMYPQPPLLIRRCRAENTMPKGAGREARVIRGMDIFLPLYNIHRDERFWPNPDTFDPE
RFTRPYKNPDVPDWKGFDPVKWSKMLYPNEIASDYAFMPFGAGSRKCVGDEFAVMEAVVT
LAMTLRRFEFDFDEDKLVGKDDIHDEPSGLNHPVGMRTGATIHTRNGLHMNIRRRQHSQ
>comp5104_c1_seq1; 555 219 1469 LEN=1597; translated
MTSGLHNFPKIELHAHLNGSIRETTLFDLAEERNIQLNESFFNASPSEITHGIGDFGSND
RTGLIHNKQHRSLVDCFNIFAEIGKVVIDLEAIERITREALEDFAKESVAYLELRSTPKR
LLPARQWCDETFSLASVAIEDLATKRQYCERVINTIINFQNEEEERYRKELETSNSLKDL
NSQRLPMMACFIVSVDRSNPVDQGFENIDLAIELAKEYPGVVVGVDLGGNPLKRNFLEFR
DCFQKARQSGLLSVTLHCAEVPCTDDDQGSAAYEDALSILKFGPDRVGHALILPPSLQQI
LAKSGIPVETCPTSNVMTTELVEKSSGGSLAEGVKRHPQLRQWLKNKHPICICTDDPGVF
NTNATQEFLLLQEAFLLENDHFKEIVMNTVDYSFAKEETKATLRKRIRDKLLELKL
>comp5105_c0_seq1; 802 103 1707 LEN=1707; translated
MVLAELGGKLRESLRKLHTSSGHVTATKLNQVLTEVTRALIESDVNARLVMTLRNNVKKK
VERLIEDDEDSETEEDSDAKKEQRASNVSKMVQKAVVDELVGLLEPSRKPYGMKRGKPNV
ILFVGLQGAGKTTTIAKFANYYQRRGWKTAMVCADTFRAGAFDQLKQNATKLRVPFYGSY
TQADPVAIAEEGVDQFVKEHYEVIIVDTSGRHRQEAGLLEEMQEISAAVAPDNVVLVMDA
TQGQAVFDQAQAFSNAVDVGSVVITKLDGHAKGGGALSAVAATESPIIFLGSGEHFDDLE
PFNAQSFISKLLGFGDVRGLMEAMNGSPDDKEELMEKMSKGEFTLRDMYKQFQRVMNMGP
LNKMMGMVPGMPEYLVPKGGDSDSNNRLKKFLYMMDSMTDKELDGKVDFSDHEEPQTMSR
IRRIAAGSGNHPKEVQMLLQVHKQFEGMVSKMGKSGMVGKQAQAQQQALAAQMRRNPNFI
NQRLNQMDPKLLEQMGGREQVKSHMENMARGGGGGMPGMGMPGMGGMPGMGGIPG
>comp5106_c0_seq1; 917 85 2631 LEN=2711; translated
MDSSLWQDEAQLANMFLEEDSNRSSSESNSNDFYFNSTAHLKKNLATTFDKELSNDLVAK

QVDHLVPSKPASSSSPVRSGGQLPSIRPPLPTGTDRVEHQRKSRKKFAVFLRIRPLQIYK
STLEQDDDVNSKVENASTIEVLEPSDKSNDETIFPTTIRTYPPMVSNVHKVNMNRNARSN
FAYAKEFHFDRVMGPKISQKDVYFAAAAPIIHDLLNGRDKRTELSRVRRESALLFSYGIT
NAGKTHTVLGDINSTNKANWGIIPRAISDVFQRNRVKLNVTSTTEASESSMGNCKHQPPC
DLYISFFEIYNENIYDLMPEKAANSSKHKSNSLPALKIRECRGQTFVRGLKKLKVRNEEH
GISLVKEAYNKRHTSCNNLNMDSSRSHFVCQMEIVPLRQQSTPFPVRDFSTETDGGKKAS
NIDLMSGYSTDEEAVARSRLSASTIWIVDLAGSERSKRTRVGSIRQKESTKINNSLMTLM
RCLNAMKENGDRRRGNNVIPFRESKLTHIFMSHLTSKSAARTAMMVNVNPSVEDFDETQH
VLKYSRKAKLIEMNIDEFGAKRKQFFGEEYDRNGRKKFKCENSKNCDLKKPKHPLLSRMA
KKLSPKKALEKIVVKNTVKEERIVSSRSYDHAKEIEALKNVVQIAEERVYALKNKNTQLQ
VELESKEDQIRAEVAMEMEARLRETRTKQKEKYDHLRSVMQNQMSKNDVSLSISRAGNQI
EELLDKIDECEQEMLRMSKEHRQEVICLNAQIDDLNRTKEILAKEKVEHTIEISNLKQAL
KKSHDEVIHLKGFKKCDNIEMDEENFLAGSIKAIKKRATIKQKIQPRKALGNATNQSSSS
RVLQMSIN
>comp5107_c0_seq1; 337 43 1033 LEN=1471; translated
MNLKSSYSCSFQLLLLLLLQNADNAASFSVVSTPYRSSSSKNHRVAGTSSLHSRTTRDVV
LYSTNEGKEPVSEEQKAEAVGNLVENDEWEGLTMELSEVIKMAVVEDIKKNTADFIGKDN
YQVGDITKEIDNRVKSEIASMRGNDEYQLGDLIVXDRWDGKWNKGWKVSRIDFLSVTRIF
SRQVRDLVLISSVSSLAKDMTEEMTGKPYEAGDLSKEIDSRVKGTVASFCGKDEYEFGDL
TQEIDRRVKDRVSDYIGKDEYEFGDITREVEKRRREWVKGYLGEDAAKNYKFGDLTMQAL
KNISGDDDYQFGDLTKKFVGNIFGKKKLK
>comp5107_c0_seq3; 435 43 921 LEN=1359; translated
MNLKSSYSCSFQLLLLLLLQNADNAASFSVVSTPYRSSSSKNHRVAGTSSLHSRTTRDVV
LYSTNEGKEPVSEEQKAEAVGNLVENDEWEGLTMELSEVIKMAVVEDIKKNTADFIGKDN
YQVGDITKEIDNRVKSEIASMRGNDEYQLGDLIVVIDGMAKDMTEEMTGKPYEAGDLSKE
IDSRVKGTVASFCGKDEYEFGDLTQEIDRRVKDRVSDYIGKDEYEFGDITREVEKRRREW
VKGYLGEDAAKNYKFGDLTMQALKNISGDDDYQFGDLTKKFVGNIFGKKKLK
>comp5108_c0_seq1; 486 128 1912 LEN=1989; translated
MAEGKKKASLKRPKRVRLQDFTCIEDPHPYGCLPGGNRFFGTPSSSSPKSTDVSKRKAED
VFDDKVWQQIISFCDGCSFGRLVQSSRYLYVVGHQPELWRDQVLRKCDAEKLVISRVGPS
WKDTYVRMFHDKNHRNNNSDNNSSKNETETDGASAYRPHRPMRMPNVYSDDIYRSHLCRS
FAIPPAWLKSQDASTEGTQQHWHREVDNIAVDDLNAETFFANYEEKNQPVVVSGAANGSA
VEKWKEWDYLTSKNTKDSYRSTSGAAPLPGNFSLEAYRDYTVSTRYLEESPLYLFDRTAF
ASNREWGDDFFPDFYEKCPYWDPSGEFGHDMLQYLGEKERPDHTWVIMGPKRSGSVFHID
PNATHAWNACIRGRKRWIFYPPGQPPPGVFPSADGDEVALPLSVGEWIIQYWKEHTEQYR
KRPVDQRPMECTTHPGDVVFVPHGWWHSVVNLDDSNIAITHNYVSPSNLGNALKFFVEKQ
DQISGCRDRKESIKPEYIHGELVKALAIKEPDHLNRAMKQTGWTCRAWEETPKSDETVIT
DDDEHSSSSNKNRNKRKIDSVQGGNEQGTNKAEELPTSVMSKTEKVPAFSFSFL
>comp5109_c0_seq1; 325 173 1783 LEN=1930; translated
MSFDNNGYAANSYAAAATAGYGSYSGGRGIGSSAGVGGQNAGDVDSGLRRRGNTHLNGGY
SNHHYNGDYYNGDYDDERNKGKKSSTVEKLDFMFPKVAEEFTVKTQGGGMATLFAYALIL
ILVTTEIFTWREQNRLETSTAVVDTSLGKKMRVNMNITFPSLCCDDLHLDSIDVAGDSQI
NVEDTLVKKRLHMNGKLFSQEEIKAELNMHHQEQLRKLEILKSKLPADYCGPCYGAHERE
DQCCDTCDDVIEAYKNKKWKTDLLQYTSEQCVREGRDNAEPKKMTKGQGCNLSGYMTLNR
VAGNFHIAMGEGIERDGRHIHTYNPEDSINFNASHIIHHLSFGVEDGHEPLNGATKIVTT
DTGSTGLFQYFIKIVPTTYVGHGVNFPEFDQNLPSLFEEAEEFASSDHRKIETNRYFFTE
RFRPLMTELLDDEDYMDDDSGKAAADTGYTGGHHNKKHHSKQNSVLPGVFFIYEIYPFAM
EIKQNEVPFTHLLIRLMATIGGVFTLARWIDSVLSERMSGNTRGDMRTPTRFSVNT
>comp5110_c0_seq2; 376 40 1057 LEN=1057; translated
MAVRTRRSRGGKNEEELEAIPQKAKPDQTSSVPVEEGDQENDEAEEEARNTDSSDAGEDN
GNDDDDADNDSDNDDGENTNDSDSDHDGSGTGTRGAIPSTPAAPGQRAGTPGGNGPAKQK
KNALTHLVPGYTARMKLDSSSLDRYRCGIGELGRRAEKGDASTRGFVREATASKAGSAMS
SRRGGFLPSSYEASYSHFKKGTRAAPDTSAGRGWFGMKPAAMTEELRTDLAIIRNRTYLD
PKRFYKSSDKHHSVVQVGTVVEGASEFYSSRLAKKDRRANLTEELMADAATASYAKDRFR
KMAREKAYQAXAAENQAQEEVPQVLLGGGAARLGAEVGGG
>comp5111_c0_seq1; 418 214 1200 LEN=2511; translated
MATEGFVPDADTADLQREEVQALEAIFEDAFELRSIRKQDDEDGMDTFDFPIVYCIKLND
MDTADDDGSGNSGYDGTKNWPTRPLTVEVRYPKNYLSDEDGDDENTARGATATDPTFRLL
HENSVLEFPSSVSEGLLAVLAETAESERGMPCVLSCLYAARDYLDRDREWDDRSNQPARN
EGNDLDGAFAKNCSIDAISHDNQTTHTNDSCHYACLSTHHLLDHKPDNLLKTGFKKCRLR

GFYKFGTPGIAIAWGDKDQIEDFLDVLKQAMPQKKFDLVFCRTWENIQEIPNVWESVDPP
TLKQELDKLGLPEEDYYTALGLEKRGK
>comp5112_c0_seq1; 981 137 3397 LEN=3724; translated
MPKTTSSCGVLLWLTLLAGCLPTTALSVDQSKFRTCEQTSFCRRHRHQHSASLYQYRIDP
DSVHFHIPSENKHGSDGPKHQSERDADQTMDNVEKPDVDTDTDNGLLKSLTERIFGKSPH
EDDANQNKKDPYVRGPPPTLTGTMVNTATETSSGKKEYLNFSVHCISDGMVRMRITEVYR
NRNDDDHGKTPHDKARTTYDELVLTTENMEPAEHVNWVRPGEDYLVELLGSQDEANRYMG
LRYGDHPTTKEKSGMLFLMRLDSFATYLYRESDLQQGPVIAMGDKHMTHFEIRRNKNVVD
GPDNGNLDDMVAEESSGDAEQPEKEIVGYWEDGLAIYADGTREERKEQKEATDHRQLSEL
ELDKEGMWEENFGPHTDSKPHGPMSVGSDITFPGSKFLYGLPEHASSTALKQTTGNKAEY
KNPYRLYNLDVFEYDLDVPMSLYGAVPLIVSQSAKTGTSGVFWFNPTETFVDINDDESGS
KTSHWMSESGIIDVFFIPGPTPKDLYRQYAKLTGFTPLPPIFGLGYHQCRWNYKDEQDVY
QVHDKFEELDYPYDVLWLDIEHTDGKRYFTWDNHLFPHPKPMQEKLWSQGRRMVTIVDPH
VKRDNGYYIHKEATSKGLYIKDKDGKNDFDGWCWPGSSSYLDFTNEKARSWWADQFSYDR
YHGSTPSLYTWNDMNEPSVFNGPEVTMQKDLLNLNGDEHREWHNIYGMLFQRSTMEGLIR
RNKEENTRPFVLSRSFFAGSQKYGSIWTGDNAAQWSHLEIAAPMLLSLNVAALSFVGADV
GGFFGNTDAELMTRWMQAGAYQPFFRGHAHHDAKRREPWLFGDETMMRLRKAAMARYALL
PFWYTLFHEASVTGMPVMRTMWMNYPHIESLYAIDDQYLIGSDLLVKPVTAPHVTESIIK
FPGDDIWYDAESLVMVSKQGMQGAVQEIKVPSDINTVPVFQRGGSVIPRKLRLRRSTHTM
TRDPYTLYVALDKDGRASGSLYMDDENTFNYSTKNEFASANILVEINGSRGRIRNAPTVG
DGWKGTSEESNKMIERIVVMGVETHPSSVDEVGESLGFTHDSNAHVLVIRKPELSALVDW
EVNITF
>comp5112_c0_seq2; 671 137 2092 LEN=2327; translated
MPKTTSSCGVLLWLTLLAGCLPTTALSVDQSKFRTCEQTSFCRRHRHQHSASLYQYRIDP
DSVHFHIPSENKHGSDGPKHQSERDADQTMDNVEKPDVDTDTDNGLLKSLTERIFGKSPH
EDDANQNKKDPYVRGPPPTLTGTMVNTATETSSGKKEYLNFSVHCISDGMVRMRITEVYR
NRNDDDHGKTPHDKARTTYDELVLTTENMEPAEHVNWVRPGEDYLVELLGSQDEANRYMG
LRYGDHPTTKEKSGMLFLMRLDSFATYLYRESDLQQGPVIAMGDKHMTHFEIRRNKNVVD
GPDNGNLDDMVAEESSGDAEQPEKEIVGYWEDGLAIYADGTREERKEQKEATDHRQLSEL
ELDKEGMWEENFGPHTDSKPHGPMSVGSDITFPGSKFLYGLPEHASSTALKQTTGNKAEY
KNPYRLYNLDVFEYDLDVPMSLYGAVPLIVSQSAKTGTSGVFWFNPTETFVDINDDESGS
KTSHWMSESGIIDVFFIPGPTPKDLYRQYAKLTGFTPLPPIFGLGYHQCRWNYKDEQDVY
QVHDKFEELDYPYDVLWLDIEHTDGKRYFTWDNHLFPHPKPMQEKLWSQGRRMVTIVDPH
VKRDNGYYIHKEATSKGLYIKDKDGKNDFDGWCWPGSSSYLDFTNEKARSWW
>comp5113_c0_seq1; 241 1 1527 LEN=1785; translated
LSAPLSMPVYPGLVQGHLVCAGKEFPLTGRFNASAILTVEEKQADGLLSDEELFGSKEVY
LEALENSTSKGSFSMEKVAVFGGIFIAVFKTHPSIKNPWRDVSIRSIETVNYDIVDLSHP
MQGNRMDGNHHEGAIMDNIPYSRVFYHAFPGAIITHRGRRYKIVSMTRPPAFGVGFRSTV
TLGAYAKPSAHRYCTRPLSNLKITVVKQTERVDLMDKVTTTKESDQTPDPSTIFADDFDP
SSGSFAGCGTITVKRNVHGYKKISLVTRDELSRAELSLPDMEYDTYAFWIDCDATGLGKL
MTPQSFGYGVHALSHALCNVAPRFVPCVLNDVQCDHSFYSPTRVVIFDSRAGGSGITAQL
WKSIFRPNGLVEAAIDLLESCPSCVDDAGYTGGCPACIQAGECIKFNDFLCRSSGLIIAR
HMLRRLEKTERYKLAMEEHAESDVEMVEDDRKKRKENRPFASPRREKRRRAMRAAMDIDA
TKPRQLVIGRPSWPMDCCDSIPRQQQEG
>comp5114_c0_seq1; 382 99 548 LEN=1062; translated
MADEYTEEQIAEFKEAFSLFDKDGDGTIDNDELGTVLRSLGLQPTEEEVEDMIREADKDG
NGTIDFMEFVEMMPSLERDDNAEEEMLEAFRVFDTDGNGSISAEELRQIFINLGEKLTDE
EISDMINEADKDGDGEIDYKEFVSMMFSG
>comp5114_c0_seq2; 239 481 732 LEN=1246; translated
MEFVEMMPSLERDDNAEEEMLEAFRVFDTDGNGSISAEELRQIFINLGEKLTDEEISDMI
NEADKDGDGEIDYKEFVSMMFSG
>comp5115_c0_seq2; 158 731 1714 LEN=2440; translated
MNDSNESDDSNRGRGHGSVRGSIVVVAKCPIPGKSKTRLIPLMGEEGSVRMAKAMLSDVL
KTIDGCAELDSVRKILLYAPGTDEGLKRMQTLVAELGLVTEAEPGCESTGGGKESWNLLA
MASSPSDLKSHVLGTKLEDALVRVRALDRQLRPPNGVVFLGMDAPILPLNDIVEGLERAA
GETAAATLCPALDGGYAMLCVPGSADPARTFSSVCWSHPMTGMSQIKALTDRGIPTVVGK
AVRDIDEAGDVEELCRLLQKESRAPDSPPHGSDRRTKNLEEPGKWSSRCTAGDNGVSSSH
PVCHYTRRALVEAGLVAEHETGGSLDE
>comp5116_c0_seq1; 252 71 814 LEN=1350; minus strand; translated
MIDKKKDNPGLYTVKKVFICCQHSNTTGTKPVILFSSVSFLAQEIRTNLSPLTPPLSSIL

GSGATETLRTSLLPSAALSLSPAVPTFPSGRIIRLSFGSPPSTPSSLMPPLNEGVPSVIT
GPAERTVVGGVAVFPTLGGGDAAAALLAVSSFFSRYSTLASKASSLGIRDAIAVSEYFPS
TSTSRLTFSPIALLGITIFFWVYSTSMKPKVRFSTSTSTTVSDVPSIAINPFGTTYLTIA
SANSSSTW
>comp5116_c0_seq2; 469 138 1298 LEN=1493; translated
MTNFSFVFALAASLLSTTTHAFAPKSTLVATTAATAATMNHQNSAQGMFTGIVEEMGTVV
KMEDRNDMTLWDGSIGSGTELTVRGSVVMDGAYLGCSICVSGVCLTATVLDDQHGTFTVG
LAPETLDKTYFRSLQPGQAVNLERASEIGGRNSGHFVQGHVDGTGTIVDKRVDGDSLRIT
VQVDEEFADAIVRYVVPKGFIAIDGTSLTVVDVDVENRTFGFMLVEYTQKKIVIPNKAIG
EKVNLEVDVLGKYSETAMASLMPRLEALEARVEYLEKKLLTASNAAAASPPPKVGNTATP
PTTVRSAGPVITEGTPSFKGGISDDGVLGGDPNDNRMMRPEGNVGTAGDSDNAADGNNEV
RRVSVAPDPKMDDNGGVSGDKFVRIS
>comp5117_c0_seq1; 514 116 890 LEN=890; translated
MADKWMHKLTEVAGEYQNDKGNAIQMAMKLAEEMTDDLETDEQKKASKKAMLKQAEALTG
QVFGTQSKPEEQQTAEDDDADYVPPKASKFNSGSSSTKKPAGKPSYLISKKEDEKQTTES
SEGGSSNDEDKQNNNEEDEEEYTPPKASKFQSSSKKNSGKPTYLISKKKEDSESPEGSND
RDETAADQIEEEASSVEDDKAEEEEYTPPKATKFQSSSKNNSGKPTYLIAKKKEDPEPLE
GSNNGEETAADRIDDTALX
>comp5118_c0_seq1; 331 85 858 LEN=1078; translated
MAVQQLMKTFLLVLSLSGLCQTADALASTRTSQKIFAVEDRITEDRRIQPFESAGAEFSR
RTALRNFGVASAVLFGSSAGFTKPLPAVAADDSLDAYLYKIVRVREATQQERRLITTGKF
KDQQRANVKLAVKFMVQNYRLSDSVVGASGYLTGGNALQMRAIDAGQGAVQNLQTILEYF
DSSDVENIKVGKNSMAGKEEIVVKGLEATQVKLDEFLGFFDDETVNKVKAKVQEENELNV
KEFDRSLGDIINLAPPS
>comp5119_c0_seq1; 360 604 1741 LEN=1741; translated
MVNEIEYDPDNDYFAILGVSPVVTQAELKKRYKKLVLKLHPDKHPLGARKYFHVPFTRMQ
KAYDILKDHSEHMMYYHYRMRYYTKMFFQSKSNGWSEYLCELSWKHKHKIDTETKKIDAL
ISISEKWKQEEKKRRDTQRAQEEEHRKEHERQKEIQAEKRRQEEEEAERQKRKREEQAKE
REKRATEDEAEDNAELKAELKAELKEVQLEFEIWELRQKVKRAKEVENRESELRLLDEKR
DKAIQELNEALNDKMENLRNEFRRKTQTREEQHEREVQNKKAFHETERETQKIDRKRVRD
SYRSWDNEMRSIVEGDGPPPAKAPKRPDFYNPALAGKRTARTVGSARKPEGFAPCTRTRN
GEPRNRDSVGQLHNANACE
>comp5120_c0_seq1; 257 1 861 LEN=1135; translated
GKKSKKAKQKPKPASSYSYEAVDTGPNPVLEASGWKELQDEHKAGGVLSGTIDGSGGVFE
YAITGDESQIVTVAVPPGESCQGEPGSMMYLTSPVSMKVSCGADWFGRCCGGESCCVVNF
NNHTGEVGYAALTTNQPLAKVVPVSLSSSEVGGTLIVQQGAYMASYGHVELSYDCDCNLV
RCCCGGMGLVRQKITGSGTAFLGATGTIVEKVLAEGEVMLMDTNCILAYAESCTFDLKKT
GGIVNWIGGGEGFFNSSLKGPGLVVAQSMNMCLLLESLAAEKMYRR
>comp5121_c0_seq1; 877 164 4378 LEN=4732; translated
MGKVVDVCVLSIASFLGNVGVALTGFGMAIIYLFVYQIIVLAGYDGNLRYAVFIQALALF
SAQPLLLRKAEITKHASWKILHYFIPITIISTPLGQLTGDAISTDLIEIVAGFLVTFVAV
FEMYQKRKLFAKRFRKWCCSVGNEEEKKEPVDEENPKSRDTGEETSTNISRDTSGQNVLL
MNDHNGETNAENDASLLHTPEGGFFGQYNLGKTLDNGSFSVVKLGTKKKCTRKSFAIKII
DKSHLLDDEAEQLQEEITHLQQLNHPNILYLQDLYDEPQKYFLVFDLMHGGDLLARLSGV
RFFSEAEARRIAKDVLGAVRYMHDHRIAHRDLKPENILLASGDADCEVKLADFGFSKKEV
RPNSFVTLCGTPGYVAPEILKGTPYGCKVDMWSFGVICWVMLVGYQPFRGESEQEVQIAI
VRGKYDFDEEFWEDINDSPKEFITSLLVSDPETRLSSEEAQCNPWFSDEYKCAERIVDET
TPVFFMVGSQRSGSNWLRTMLNEREDLGGPHPPHIMREFMPIIDKFGDLSDDESFRILLD
HVCTFVERNQVPWTDKHGCNINFLRPIVYKCALESCNRVKDARHSLRNETPICNGIYLLS
IFDAIMTYFTKVNGKRLWVCKSMGMSQYHDILLEFYGSKRLRYIYLVRDPRDVAMSFVKT
PVGDCHWHAIVTKWTKLQACALRIVKECPELIYKLHYEDILEDREREMKNLYDFIGERRF
GGVKRTASVMYMESVENVVALAKEGRESLKAHRLSSQFMNLSRGISFFASQFEKWRHPDT
GLQEDAIQILETVAHETMKTLGYEPHLVGVTTKPSVWNDADLAVFERLNQEGISKMNEDL
GRENPDDLARRIYQAAALAMVPVYKPEVWVETDASFDEIVSTGIQKILNEDELLKRRDIT
AEKTCTTKSGLRFRWACGTQRGYYPDNHDKPNQDDWIAQTASTEEGDVHWFSVYDGHGTQ
GHKCSEFARKEIPNEFQKRIATGQSFPLALEKAHVTVHNRLIANPQIETELSGTTAGTLM
LMDDQCIVSNVGDSSFILGWEESCGEHPPKITAKTLCTPHTPNRADERSRIERAGGVVMT
ADQRDGLAPMLRTDRSRDDDHEPLRIWSDESAKVPGCAFTRTIGDGMAHKLGVVARPELF
EHSFNQNDRVVVLASDGVTEFMDQDTCVRIACAEACPSRAAEALIREASKRWKSNSDYMD
DITAIVVFLDPSKVAFREIDPLATAGETAGNNHLHHHHGDGNLHLIVPTGSDSGDGSLEE

GPRAIGPWASFWTLLAGGFSGFLGGICGIRGPPVILYFLHPPYPVTFDKKSQRATGAVIT
ATNVAMRVAYYVVETLVLETKESYFRESDLVLYLCVVVSSILGVLVGTKLFDLMKDSRNN
IRLILSFFLLMCGISLLISSFHF
>comp5122_c0_seq1; 530 1 1536 LEN=1631; translated
QRNATQRNTTQRNTPDPRAPFLSQTAGHRSRGLPETAQKARRPAQNPMFSSSVLRSPAFR
RSTTLLTVAISANAWVANNAFAISARRAIAPATRTTLAEAAPSFSSSAEFGANEGARHGA
CGRAFYPRDVYYDAEAKSKLEELRALRRDEIADGGGAASGGGNKTNTNSTTLGVLKESGG
KYSGTLTLIGYKGGPLESQINQDRAVVLSPFVVGNKKDTDGAGGSDNDSDSVDNNSNKLI
GCFDGHARLGEVVSDYVVKELPKLLASKLDKIQPKSNYTDQVSQAITETFVEMDRTAPAE
ISGGCTATIVLQKGSRLYVANAGDSRSFVVVYRAKAKTAEVVYISREDKPSLPDERERVE
KAGGKVLLPPTGTSRVLYTDPESGLTSGLAMSRSIGDWEVGKFGVIPDPIIDTIDIDEVV
EQHSGGSEDDVCIFAVSATDGCMDYTNPEGVAAVLARSMYEDESPHLLTAMEQIIYTAAA
GWERAKRGTYRDDIAISVCAIRIPPSLLQGR
>comp5122_c1_seq1; 298 222 884 LEN=957; translated
MPTLPGREPRKGRRARRCAKAEGKKNLLVVDRGETVPEEEWIRRMLEHLSRDGHRLVAGK
RIVLVASSRSAPPSSRKGQKHSTASLWVSRLASRAKSLCLVVSEQEEEDEEQQQQQHGLR
DGSENGGSSAGGAGPCSILWRNTEVVGSTELEDLLRKAGEGGDKAIDAIVLSTTSAEVVH
NDPFVNDFLRRTDAPIHMSSDCADGFCYYSRLQEGANIVLW
>comp5123_c0_seq1; 309 52 1642 LEN=1642; minus strand; translated
MMNRIITTKSLKAKSNHTVFKDEQKIMWLYKNDPFAVAPDRLDAMRCDAVQGPRRAAQRL
AGWAGSCLRPQKRLKDARIDAGLGFVPGVPLDVPLVVARKDAAGVPPERGRQGAHVPAPH
PHRVRGGVFGPLSASGRGPPQGPPVREAVDHVEPNDLSLVPETPQLEYGHDVLVEEQGGP
QFLPPGPERRQLGHQHPQVVPVEDVPPADRHELVPLHRSLFDEGLVGVVGDQKGVPAQPQ
ALAYGRQDAGGLDVDDLRDRPFRKAGVGIAIAATAIERRFRRKVPGAEQRGNHGATVRGP
VLSPLARRDVGHVYPSAVHQGEKVVPEARVAASRCREAPAPVGAAVLGRVVTGAPSPVEW
HRDALDDNGLPPLARPAAVAAIVAAAAAANVVAVLIDGVAAAAAAMRVVSGRCHVVAFFS
LLAAAAVVAGMGNAVGALVEPHPLPDRGHPGGLEFALVQGFLELRIVALLLPACRLEIGE
MVVAKVFGFGSRKVAVQGAPARAGTRRESLLLLKHGREAPLSQRSVRCCVX
>comp5125_c0_seq1; 371 223 1536 LEN=1932; translated
MNGLWQHHHLGESVSGDDCQLEEFGTAEHDRHATLRYRDVANAGPPARPLDFPSPKVFRG
ELVDLRARDHKSGTIHYLKNVLIRSTVNSNNNGNRYFSNISICDTAYLVTKKVSKTVYGC
VSLCIVLKRIKNDPNDSTNSSDATKEEDRQDNNMDCSLIEWESTDDQAAVKISEWKKIHA
LRGKHLEDPIKEVSVMQLLGNYHQHISGALEVLQDDKCLYTVMPYCGGGDLYGRLVEFMG
FRSVRSDTANSVVATGFDEHSARIWFRQLLQAANLLQKKGVCHRDICLENILLDEDDNIC
LIDPGMSLRVPYTDSETGGVGDVSAGTSRRLMIAQGQGGKLMYAAPEIISKTTDVDAFAI
DLWSLGVVLFVMVVGLAPFRWAHASDKRYARISSGDLNGMLKELGISISPEVCNLLQGFF
WADPQKRLNLAEVIQHPW
>comp5126_c0_seq1; 278 92 1214 LEN=1214; translated
MKTSSSTLRLLLCVLGSATIRRCYGGKGTPRSIGRFLQIRGGEASYPDYRGANLPPDLPE
YGDGDGVQNGRSPQQQGGHNRYHYDSQQQQYPAGDHGGDPRYNSQQPPYYPQQSDPYYHQ
HPPSHPSGPYGENPNQGPPPLPSGHGQEQQYPSLPGDPSLYQEEEEPEMDMNSAFGGGIG
DENSGDGSSGMDLSSFDKEYILKGLAKLYKKKILPLETSSGYGHFHSPPLSPADFVAPPQ
VLLLGQYSVGKTSFIKYLVGRDFPGIRVGPEPTTDRFTAILWGPNDKVVPGAALCSQQNL
PFTGLSPYGNNFLSRLEGVEADSAILRNVTLIDTPGILSGQKQRSRNYDYESVMKWFGER
ADLVIIMFDAHKLDX
>comp5126_c0_seq2; 150 137 527 LEN=527; translated
MKCSVGKTSFIKYLVGRDFPGIRVGPEPTTDRFTAILWGPNDKVVPGAALCSQQNLPFTG
LSPYGNNFLSRLEGVEADSAILRNVTLIDTPGILSGQKQRSRNYDYESVMKWFGERADLV
IIMFDAHKLDX
>comp5127_c0_seq1; 296 64 1185 LEN=1529; translated
MGLNLATRYGFVYNQVARSTSLRRSQIPSFPWRQHPWQNKCQHIASQTAYQDPALSWAQA
NPRLAQQCLNPPPVDGEDHFPLAERSLQSFEAYLEWRKWTFPNEIFHESEGVDSGDQREN
AIAIISHLLSAPLTLASQFRKLMEYFSQSSPNENGASERVGFDLCCVGARAEASIPAVYW
REFLRLSMASMSIRSEFPCDGRVLLERKSLELYLDFVGPDIPSKVSKQSVCVPEEKRNHT
ASSASISLRNYYRGYFHDVPAVNASNQKGKLWDAYVFFNPGFGHPNLQQSWEPTLKLLLD
QGRSGFHKKKSILLLTAHSEKDASRDFSILRDVYGLKTVEYKENPFASRVTYEDPFEKEH
FVRPNHYVATILL
>comp5128_c0_seq1; 290 110 826 LEN=1015; translated
MGNAAGKAIDEAVLVEGTLNKDEGRFISKPFVSNEGGITKIKNKGKTWVTTDGESLLWTS
KSSGVFKCHSVITDAGGSQVAIIVTEKKAMTSSTNWILKTEPSFDGQDPVSEEDLQKAGI

AKKDEPSGILLYKFSKIEVNRKLTTGVCTYGIVKGQEDFLPLYVGERLSSMGFKALFKEV
SSTNEDGILVAKAYMPGMSMSPHVEVAAGVDMLAIVSIGYALAGDDSSAGALAGAGVV
>comp5129_c0_seq1; 472 90 956 LEN=1166; translated
MSSEPADQFIQMTDSEAIFEEECDKLEKRGRRLRRAALQTGILTIVSILCTIYTTIAVRW
VLIEWQQPEKTIYDIAESLTMFKIGIFFDIVVTICDIVIGITLGLIMVGAGVNPATAVTI
CTFKIVQQAVSAANIIFLIAAHLLLDGASPIYEVIQKYFYSDNMPPIGTQISYMFLLINQ
YGYYFQQIFGGVYMFLLGFTIVLWGVFPRYMGYSMCLAGVGYGVNSCLYLFFPGYDGVVS
WLLLFPALATHFWLAGWLLVNTPHPSKNRDLWGSRPAVPQYPGKQVEP
>comp5130_c0_seq1; 305 565 1794 LEN=1898; translated
MKILLCGGGNAVHVLTSFIASQPRHEVCILSLYPGEAERLKDAISEKGIRCTHDLGDDAY
GRPVRISSSAEDVADETTKLVVLALPSFAHESYLRALKTCLKPGIMVGAMPGEGGFDLLA
RHVLGDAFVDASTVFALETLPWACRILEYGTSVEILGTKKEVDAVVASKRGEPPHMAIEV
LQSLVGRLPVMKPACNFLAVTLMNINSIWHPTISYGYYRDRDVTKPFDEAPLFYEGADEY
TGEMLAAISDEVLELKRVLVQKYPELDLRSLHHVRDWMLRSYGDDIGDTSNLHTMLHTNK
GYKGLRHPVRPVVDDESGKKQYLPDFGYRYFSEDVPMGLIVTRGIAELVEVPTPKMDRVI
LWCQKQMGKEYLVEGGTLSGKDLGTTRAPQSFGFTDLDTFMKTNHYVKA
>comp5131_c0_seq1; 497 56 1762 LEN=1901; translated
MATSPRLPVLIAGAGPCGLVAATTLKKEGIPFVILEKVSRQRICSNAGSGFELAPTAVAI
LEEGLCLDLEKTRLVSRYRGMCLATMEGRPLRTDRLGEGYVGGSLNRAELQRFLLGVLFS
SPKDEEGVLVCGSGVESYRETKNRTVVVELASGKEFEGCALLACDGIRSRCRDRMMGDLS
REDSLHYCKAVCYWGKTLAREGTALEKEFLSLQSEAPRTKKAKARTKTKTETQTLSAKAH
AQEEPGLALALEDHRRSSFVLSLATPRVPAVLFVIPSQHTEGGETTSTSNTKSKTALMLN
WGITVASKNEPAARGDSTRRGGGVLSEDEKKQLLSLGGKKETPKKRWGGATKTKAKTTNR
SLLANIEDFPLLEELLAATPAKDITEAGFYDRKDLDLPYTSEGKLVALLGDAAHPQTPYL
GQGVNMAITDAYIYSSAIADALQKSRNKSSTTTTTETEAASVADAIRWCDPDFRRRDNKS
AVRWARFLCDFSSSSNRIVCWLFHAYCKHIVSPEEFLSTIEKTDRSNARYWKYFVERYGT
RFFSGIKNPDQKKIANGEIKPLREEAEC
>comp5131_c0_seq2; 348 56 1319 LEN=1319; translated
MATSPRLPVLIAGAGPCGLVAATTLKKEGIPFVILEKVSRQRICSNAGSGFELAPTAVAI
LEEGLCLDLEKTRLVSRYRGMCLATMEGRPLRTDRLGEGYVGGSLNRAELQRFLLGVLFS
SPKDEEGVLVCGSGVESYRETKNRTVVVELASGKEFEGCALLACDGIRSRCRDRMMGDLS
REDSLHYCKAVCYWGKTLAREGTALEKEFLSLQSEAPRTKKAKARTKTKTETQTLSAKAH
AQEEPGLALALEDHRRSSFVLSLATPRVPAVLFVIPSQHTEGGETTSTSNTKSKTALMLN
WGITVASKNEPAARGDSTRRGGGVLSEDEKKQLLSLGGKKETPKKRWGGATKTKAKTTNR
SLLANIEDFPLLEELLAATPAKDITEAGFYDRKDLDLPYTSEGKLVALLGGTQWNGTEYN
GX
>comp5132_c0_seq1; 182 1 454 LEN=1100; translated
XQKVFVSKTILNNSQKFDLKQPPTNTMSNVMEKVKRASNSVVNAGAKQMLKMDVLFLERE
INTRKQSFGIEIYDLMAELEKSEGMSDQDKEAKIRAAFDNARKDIAVIEAKKECKKEEIG
VLAATQNAGAVPEPAIPPSSGAVLTTGHPEL
>comp5132_c0_seq2; 163 1 487 LEN=1133; translated
XQKVFVSKTILNNSQKFDLKQPPTNTMSNVMEKVKRASNSVVNAGAKQMLKVRKYGRLSR
LRMDVLFLEREINTRKQSFGIEIYDLMAELEKSEGMSDQDKEAKIRAAFDNARKDIAVIE
AKKECKKEEIGVLAATQNAGAVPEPAIPPSSGAVLTTGHPEL
>comp5133_c0_seq1; 294 1 990 LEN=1131; translated
QHSTALHCTLLPQPLAIESKRSDTNPAAEKNSPTRTAPTKDNTPTTIRYDTMRCDTMPRT
HPAAPAPGSPHPLRVLAAMGLLAVAQRAHPASAFSPCPAAGGARPGSLRMADAPADTTWT
NLVPADESAPVRKTVLEDGKGGLPKKGQTATIQYTGRLVLGGDSGGLAVASWTPGAVVDC
WLAEQQGLSGILEGPFAAQGVDGAFLLDGDRFTEDFLLDPLGLENKLQRKKAVMAARRLR
TSLEGYEDGAVFDSSSDKEGGVFRVAVGAGKTIKAVELLVASMAVGERARVRSRCDYGYG
SEGYRTSRGDVVVPPFCGLEFEVELVSLE
>comp5136_c0_seq1; 597 132 1688 LEN=1884; translated
MTTIPDTIDEETLNSGAEGFMATQVFVKNPGCVGMTYDDLIVMPGHIGFGVGDVDISSDL
TRNIRLQVPLVSSPMDTVTEHQMAITMALQGGIGIIHSNMKAEDQAAEVLRVKRFKNGFI
LDPVCLSPENTLEDILELKTKLKFSSFPVTDTGKMGGKLLGIISGRDTAFIDDISVKVSE
FMTPRSVLTCAKDGISLEEANEILKKSKKGKLPVVDEDDQLVGLVARADIEKNKENPRAS
KDKKNKQLLCGASIGTRPDDKIRAKLLADAGVNVIVVDSSQGDSMYQIEMIQHLKATHPQ
VDVIGGNVVTPSQAHNLIKAGADGLRVGMGIGSICTTQEVCAVGRAQASAVYHVSKYARK
FGIPTIADGGIKSTGHITKALSLGAGCVMMGSMLAGTDEAPGEYFYQDGVRLKRYRGMGS
IEAMKSGSEKRYVWDTASTHVKVAQGVSGAVTDKGTLRRYIPYLMQGIRHGLQDAGIQSI

DIARERLYNGKLRFEIRSPAAQKEGGVHGLHSYQKRLY
>comp5137_c0_seq1; 454 106 1815 LEN=2597; translated
MKRRSLEDVSKSDAAKDSIEDPYNRDAAADGWYMCSLGCDGGQQQEIQTNISPTSTYDAP
GADIFLNQTSQTFNKVRRRRSRTKNKDQHDNVGVSSSKHTDLSSLIASELSRLSIEDREK
ALEEIHGVVEVSDEDPMEIQNLLDQVKEELKRIRYKQAYEKAAFLSSAYVTDPDFVIYFL
RTDNYNPRKAAIRLVEHFKYKLELFGEHTLVRDIMYDDLSEVEKNILNSGFVYCLPKPDR
AGRHVMICNLSEFLKTGTLMDMNRAVWYISLRYIQMFPSLSKLGVVGVYFAHGMCNVFDN
LSREHFVPKTAFLGNALPYKFASVHFCFEKNILASALQTTLMMSLGKYARIRLRSHCGSR
MESTYTLKSFGIDLEEFESDGLMGHAASAHSFLQQCQKFDQYYQQKQNTRLLPRPNDVLL
GRGRPFQLYPGNLALTAIIDQYRDEYTTSKKMHKKAITAKVVNDILRSGGRFLKKAENGK
GVDWDKVDFETARLKVSHSFRTMSKWHADNEDEENENRGDGQKKGGGEDESLKSISPINT
DTIESLGLIIEPISKNETLSNKRRKGQNY
>comp5137_c0_seq2; 420 106 1940 LEN=2722; translated
MKRRSLEDVSKSDAAKDSIEDPYNRDAAADGWYMCSLGCDGGQQQEIQTNISPTSTYDAP
GADIFLNQTSQTFNKVRRRRSRTKNKDQHDNVGVSSSKHTDLSSLIASELSRLSIEDREK
ALEEIHGVVEVSDEDPMEIQNLLDQVKEELKRIRYKQAYEKAAFLSSAYVTDPDFVIYFL
RTDNYNPRKAAIRLVEHFKYKLELFGEHTLVRDIMYDDLSEVEKNILNSGFVYCLPKPDR
AGRHVMICNLSEFLKTGTLMDMVRSPLLKCFLDNEKTRNIFKFSEFDCFLSHQFHLLFWS
TFYRIEPYGIFRLDXIQMFPSLSKLGVVGVYFAHGMCNVFDNLSREHFVPKTAFLGNALP
YKFASVHFCFEKNILASALQTTLMMSLGKYARIRLRSHCGSRMESTYTLKSFGIDLEEFE
SDGLMGHAASAHSFLQQCQKFDQYYQQKQNTRLLPRPNDVLLGRGRPFQLYPGNLALTAI
IDQYRDEYTTSKKMHKKAITAKVVNDILRSGGRFLKKAENGKGVDWDKVDFETARLKVSH
SFRTMSKWHADNEDEENENRGDGQKKGGGEDESLKSISPINTDTIESLGLIIEPISKNET
LSNKRRKGQNY
>comp5138_c0_seq1; 197 149 667 LEN=1045; translated
MTDVESSVPRRANKPVNPYSDGPNQNSRWNPINFVPRILWMSFSFYVLFDRFNIYHTIMH
SPDVSHEWFKIGLAASIALFSIKAYVELYTGKIQKKEVSYKAVPQLTHAAILLIFVSGIA
FHIALWPVYGGKSMVIMFLVGAFLLNFCLMFPTSVQNLVAIVLFTFFLQEYQ
>comp5139_c0_seq1; 452 270 1190 LEN=1417; translated
MTFEKGCSVEAFGFILAVASAASPQEKVVVEYHRSSKSTKRLARRRRKIPLALIQYNDGG
GAEKKPSYKKNSETSLALGIHMDALPQKVQPQNPTNNVGKAIKDKDASVIFIVQSENYDT
IDQDIKPPPPPPSSIVSTTSLEDYIINSDKNDNDKQDKTREYPVIQKVNARIRKNQLAAM
DETNALIKMLRTADDSTMSMSMGFGPSKISMAPMTSPTEMTQTSGYVPVLTPTKNDGPPK
RPSVSTNANSNSIVLPTESIPPFPDETTSSSVADVIEDGASTVFLASAHFFTTVIVLGCI
FSCLVS
>comp5140_c0_seq1; 430 1 1416 LEN=1496; translated
XCRLQRWVGGTGNFQPILQHSVNREKSSQQHDTQQPITTTSQDQEDEQXRSKMGVMEKIK
EIEAEMARTQKNKATNYHLGTLKAKLARLRNELFVEQSGGSGGSSGEGFDVARNGDARVA
LIGFPSVGKSTLLGALTATESEAAAYEFTTLTCIPGVMRYKGSKVQVLDLPGIIEGAAHG
AGRGKEVIAVARNADAILIVLDAGKEGLNRHREILERELETVGIRLNQRPPDVKISKRKM
GGGIRFSSTVPQTKLGPEPEKVVTQILREYKIISADVLAREEITVDQLVDVALGNREYKP
ALYLYNKIDTITIEEVDQLARQPHSIVGSVNKKWNIGQPLEDDALKQKMWEYLGLTRIYT
KRKGSPPDLEEPVVLSDIRKGTTVRSLCANISTQMLRDFNYALVWGKSAVHAPQRCGLTH
RLDDEDVVQIVTKTIKQQQNSSSYAMLAQEYQDKHSKKRLEAKKQKQKRLRG
>comp5141_c0_seq1; 11 1 475 LEN=781; translated
RSSGGRARSSSREEVEASLEDGRQVPVVPGVEVVPGSGAAVGPEVEASLEEGLRVPVLVE
IEQLVPVALVAEVVLGQRATTELGVAVVQDSLAAVEIGVAVDLRIGLTALQETHQKAVAE
DGVEANLELDPVEAGPGVVVVRENQGTPVQEAEVGQE
>comp5141_c0_seq2; -41 1 448 LEN=448; translated
XEKHLEAAAGLGAGVDQEIAERDLEVAAVLEAKVDQEKLTVPAEIDLGAAVELGAKVDPE
IEPIGLQALSVPGVEVGLEMLPVLVETGQEVVVEHGAAVDPEIDSRALALVEVGQEVVAV
LGAKADHEIDPGLPVEHGVIADLEIDPRVP
>comp5143_c0_seq1; 143 208 839 LEN=1126; translated
MSIRGTASVSRRRDSSISKSAFFSSMPGSPENEEELFEEEGAESLLDSFDSYIVVSVLTA
TASFAALFEANLEDKFWGFHNAWVFICAICSLSGIYXDGRIFPQLDLRTYRGGDRKGPHL
RDLLRAHGGVSDAGLLDVPSEPGPFHFAADLYRGGQGREEVPDTLSWIPPGALDFHVPRL
DDNHEGGGPHFCASRREETKEDLEVYEKVRW
>comp5143_c0_seq2; 143 363 994 LEN=1281; translated
MSIRGTASVSRRRDSSISKSAFFSSMPGSPENEEELFEEEGAESLLDSFDSYIVVSVLTA
TASFAALFEANLEDKFWGFHNAWVFICAICSLSGIYXDGRIFPQLDLRTYRGGDRKGPHL

RDLLRAHGGVSDAGLLDVPSEPGPFHFAADLYRGGQGREEVPDTLSWIPPGALDFHVPRL
DDNHEGGGPHFCASRREETKEDLEVYEKVRW
>comp5144_c0_seq1; 299 224 1192 LEN=1309; translated
MQNVFSRGRILIPGSSRLYAKEHAVTMANIASHRQLPVSVHPKKKTLHGFGRRLFSANAD
AAGSSSASGKSFRFSFRSGFDWYSKKLDTHPITTKCISAALISSLGNALAQKITHEQDRQ
QRRDEWERKHADKPTQHEEHEPRGEPFELNLAQIGRFALLNATFVAPVLHQWYQFINRAV
PGRSMSRVLQRTFWDEFVFSPVYIPAFLAMLWKLEGVANDRILEMIKSECPSIIVAEWAL
WVPTMIVTFRYAPVKFQVLVINVVGVVWQTFLAYSANNAHSSSGAEDDSVDQEQKGKATD
TGIVIPEDKKPLVLESIVQNKQ
>comp5144_c0_seq2; 229 224 937 LEN=1132; translated
MQNVFSRGRILIPGSSRLYAKEHAVTMANIASHRQLPVSVHPKKKTLHGFGRRLFSANAD
AAGSSSASGKSFRFSFRSGFDWYSKKLDTHPITTKCISAALISSLGNALAQKITHEQDRQ
QRRDEWERKHADKPTQHEEHEPRGEPFELNLAQIGRFALLNATFVAPVLHQWYQFINRAV
PGRSMSRVLQRTFWDEFVFSPVYIPAFLAMLWKLEGVANDRILEMIKSECPSIIVAEW
>comp5145_c0_seq1; 197 162 647 LEN=793; translated
MASTVDLLPSNLQQTLQLIIERSNASGGGIRVILLSTAEGVPLGRVYSSADHASPLDEEV
LSNIESTWAPASKQFPLLHMGKEVKVVKVVTAIYDNRTIFHIYQAPVVVTLLVGMNANLG
AVRSTAIPLLKEILEPLCSTLLNSLAPAQTDHGYPQQGYYQ
>comp5146_c0_seq1; 639 368 2800 LEN=2939; translated
MVASSNSVTVARGGVGASRDDTVLDDLLLSRCGTSPHIMRRRMNSPSSSTANRNPQKESS
LTASNTRKRLQVELERQRLFPPLNDGKNSTSSGTVMNKNNTNQHMRRVRSDNLAQSKRDD
PSVRRSIFGPYFQEKPRSNSVPHLVRSQLPPPPPPLTPPLEIDSPVPAREEPQAPIRSFR
RSSSDFVVASKKNGDKNDGRHRRKSQEQLHRHNLPVDYRVFAPSQHQAATSAICYRYREL
NRDHEKEYDSVLERQVSVEHSLPPFPSPLIRFCSETTTTVAGNDSFSLNTTHPNGIRRSS
SNISSHDREIHYHGVYSLLTPISILRPSRYAGSKRESSSDSNVAVDTGVISQDSAGVTSY
TRSNNKRNATIEEEAEEGHHHLSASMSASFNFPRSYIDSSPILKAFVASPTDDGSFSGIH
LVEEKKESDIAPQVPLGAASVASSLKPTKFLAPDISRSDEALDGRPSSGITAAATDGKFE
KDTFSDKDTTDTVVLKQPLQQEQQQKLRFDPRVTVTEFEDPIPRKWYDENELDQHKREAI
AIAQNYLRQHRAVAEWYRRSTLDPVTNTYRKKALFSLPVFSSMYSDNRTENSQESPRGAI
VPSEPRIRKILIVEPNPAILSLFHKSMKSMFESAEIVAARSSEKALRLVEESIAPTRHDA
AAFDIIVIEQALAIRGNHGAKRSDTCLPGLLGMFLKKNHEPIISAVPSTKIHRGSELIEQ
LRELSLEQQTQFGNADPPRSSLLIGVSLRPERDGAAMRQAGADLVWGIPIPSVGRNLRND
LVSKLQDKRSATARPTSTPLLPDQSNPETK
>comp5147_c0_seq1; 399 19 2006 LEN=2128; translated
MWFLWIIILVAYGTLTWQSLLEASAPAVPCFDEGNGDDSSNPNSWRCYRPAVRVGESLTL
QLELFSSPSGSGAAGGTIPRWTPVETCRIDLAVPPVGKLPGLVTDSKKGSGIGSSSDGSA
MPSNGSDNTNEYRDEKSSTKPQEGNQRCEVIFSKEARHRSSIQSEVQPLKARFVVLAPLG
GDNNGRVKIETMAPXFLTRVEKRDPNSIMRLLMSNPNPGAPDKDSGTQVETCRSDSTESN
NCTIGSNTGDNDSNKIMWIPFVKFGRAPVRIRFVAEERGYAFLQRADGIRLKALNSTTYT
PLLYVDELSLQRGAQIELAPFEANKPAFELQIKLGSISPMVDTLYRQVQDAFETIEGSFF
QGSELDELRYFLQDENLYRFALTNIISTLHVWLDYLAFRDEVRFYRGKQNLSGVSPSTAV
TKMICSMIILLYLIDGGGTSWIVLFSLILSFVVDAWKVLKLLRPTPTRSFPFVKIREFAS
KQEEEIAEYDRIACRRLSMLIYPVVVCWSIYALKNYEYRSLYSWFISNAANGVYTFGFIS
MCPQLYVNYRLKSVAHLPWKVFLYKIFSTFVDDAFAWLIEMPLKHRLMTLRDDVVFLCFL
GQVYMYRVDKTRSNEFGYSYGEDEDDTSQELNTTEGTDKASPSDSTRKHRNEVPSSSKLK
DE
>comp5149_c0_seq1; 502 262 1822 LEN=1943; translated
MSPKSETCDAGKCTPKEPSHEVLRMPSFYQRQLPDTCVAFASRQGKKLFRSALENNGLKS
FYNLIEQHHTQTEPAYCGVSTREYRVILVVCSNFSVKSFCVSKSGTTHGQSFDYYCPRLS
VVMTLNALAVDPGQQWKGVWRWYTESMLNCCVDLEEIKKDGITLRDFQCLAHCQGLSVES
RYCDETFSLNEFRRVVERACIEQSVEEEDELEHNGEDENQLLECLIVSYSRKTLGQTGDG
HFSPLAAYDKESDSVLILDTARFKYGAHWTKLPLIYEAMKPLDKSSGKPRGYALLSFVPE
HKTDNIAIDTMATTHGDNKSSGPTNTNQLSTQPASILFRSKMNQKGRRQLYKEYIKSLRE
ETYEQGDIPYSAVRSYWCKDTHGPISVWEIIVPTRLHNEKEKELLRQIRRLLAELKDRTM
NEAIPESIEGNNDNNRRKEGCTAYDLCDNAPVRSTNHTAYVSAEETLYLIYLATLSEERR
YNYIMNFESEASDIARKQIITEADMIASAIKVSDQLTSL
>comp5149_c0_seq2; 539 262 1704 LEN=1825; translated
MSPKSETCDAGKCTPKEPSHEVLRMPSFYQRQLPDTCVAFASRQGKKLFRSALENNGLKS
FYNLIEQHHTQTEPAYCGVSTLVMTLNALAVDPGQQWKGVWRWYTESMLNCCVDLEEIKK
DGITLRDFQCLAHCQGLSVESRYCDETFSLNEFRRVVERACIEQSVEEEDELEHNGEDEN

QLLECLIVSYSRKTLGQTGDGHFSPLAAYDKESDSVLILDTARFKYGAHWTKLPLIYEAM
KPLDKSSGKPRGYALLSFVPEHKTDNIAIDTMATTHGDNKSSGPTNTNQLSTQPASILFR
SKMNQKGRRQLYKEYIKSLREETYEQGDIPYSAVRSYWCKDTHGPISVWEIIVPTRLHNE
KEKELLRQIRRLLAELKDRTMNEAIPESIEGNNDNNRRKEGCTAYDLCDNAPVRSTNHTA
YVSAEETLYLIYLATLSEERRYNYIMNFESEASDIARKQIITEADMIASAIKVSDQLTSL
>comp5151_c0_seq1; 924 222 3347 LEN=3387; translated
MPSTIRDRINSFEAASSLQGGVPSPSRPIASISPRGGALGTKRDAFKLPHYEGEHRHQQN
EKEENKPQSATKQPKPHRFRNRNRETQSRSKFYRDMKNANNNAETALSDVNTNSSGNNNK
NNPTSASNRERGNRMSHNEGNQPFEETSSTTATASENRRSRIRQSQQQIRENSISATTTN
FTDLRRQRRMLQNQRRTYQAPSNDSSTAAAKSQDIDQSEASTTMFGRENLASTVPDSPGR
TSTASSSLNIASSSPGGSRWSKLSRLQRRPNGINERQSSIATNTTRNSGYSAVTSVVRNT
TSDNATSKHLSPVKLENVSAASPNTKSMTISKLMSATAGLSTKTRRQNEEVSSPSISTSR
HSNRNRKESDDNNDNNGTNLRSPRSHRDDSSDIGGAASPQQINASLEAPTDDDATLTSVH
RIMEGSNNSNHHRTTIDNSNNHHPNSHQNSVNYTYEPLQSNHNPSTSGRDSGSVGPSWKP
NRGVHSNSNRNTIWKEDEKSDKIGYGLGGRVLRTEYSSDYDMDDISKADRSRHSVHSVDG
PSTSQATDANDFLISSRYNNSNAINSGLNNNNNSAATMGTKRIDDDDRTFDYGDRDDDSN
GGTSFARRRREADQRRARLAAQNDLNSNNNGSIVHNNQLGQSIHQGGGSIIGNNGEPKLL
SKEEMEHFTAKDNPALRLSAGVAAVATVGLAVAGPAGLLIGVAAGGLGFGFMQIPEDERI
KIQDRAEKAMNTLQEKACDASEAVTSSCLSTYEDSGVAEHVPHCLSSNIADQLVPNCPTG
QTAKSENLFNPAGATAPAGSQMTGPHSAGTNHQEHPSASPAGPMMEQPKAAPMSHNERMR
NKKVACLRNVRILPIAQIHGLDPTSQPRAWLDVVASANTSNDQKNEAMEEILLLAKDKRR
AKIFLDEGILDYIIWTISRYLEKLEAADKKTDWAFPEITANEKSAANLAALCCVTLGKAH
CAAIHTEGDLLLMSMYERGTVPEERQVAQMLHEVPHHARVTKISDPTIVEPSKEVFAPRQ
LTLAQAEELARSIKAVADGQM
>comp5152_c0_seq1; 1229 271 1764 LEN=1949; translated
MKFSSIVVLAAVAPGATGFAPFQRQFTTPRTVLRKVEYDLDLGIEEAPKKKGGKKEAPAP
VPAPVPAPEPAPAPKSKKRASKKAPEPVPEPTPVPAPAPAPKAKAKKVKPQPVKTPPPPP
LPVTKPAPKSASASTKASGVALGVAPLVLAPLAILGAGRSVLSGTKERREKIQKEIEEFE
QAKKKKQLQAEVDGGGLVQAAGFLGAAGLSLGLIVANPFGQTSNSPAPVKAPAATKQAPK
KDVSAKTSSAPPAAVKKVEKKKINAQRKAELKSGYDLSLDAEVKADEKAAVKAIKEEKAL
EAAVKKDEEAAQAAAKKEEAAQEAEKKAAEELKKDEAEAKALKEAEKKAEKAANEAKAKE
LAKKEKEAEAKANAAAEKEKAAKAAEAKAAAEEEKAEAKARADEEKAKIAADKAKAAEAK
AKEDEAAQKKVKEEEAKAAASAPAPAAPAPAPKPAPAPKPAPSGDAALGKVLGGESGGRI
LPKEELDPGVLEFLKKY
>comp5153_c0_seq1; 398 68 1316 LEN=1853; translated
MASSNQNENVGVALALVFGAGASTGIGAAVVFIPSLVKIASRRVLASSLGFSAGVMTYVS
FIEIFNKSADSFENAGHPPDKAYIYATLCFFGGVLAMLVLNLLVGVLLGKRGHHHDELPK
KAKKSTRKSVDKQLVDGVNDCPTCCSADPVKQLNDVHRMASVIEHEIEDAAKQAAEVSNA
EEEQEKVVSVDLEEKSKMETVDEENIPAAELVEPAGDSAENKRLHKMGLNTALAIGLHNF
PEGLATFVAALQDPKVGAVLAIAIAIHNIPEGRKRRTLFIARLPTPMAGVGDYSKESLLR
TKLIKTLASLPFTGLCVALPIYYSSGNRMKGFLWALLSGASEFIAALLGWAVLANSFSES
AYAILFGLVAGMMIMISTKELLPTAHLYDDKDTVVSYSFIIGMVVMALSMCLFQL
>comp5153_c0_seq2; 362 68 1387 LEN=1924; translated
MASSNQNENVGVALALVFGAGASTGIGAAVVFIPSLVKIASRRVLASSLGFSAGVMTYVS
FIEIFNKSADSFENAGHPPDKAYIYATLCFFGGVLAMLVRTEHEFLKKRRREPGGFVESI
VAIVVENEVCXAIYFFFRQTVTHASQNNCGADCAWGLVFGKQSKVLNLLVGVLLGKRGHH
HDELPKKAKKSTRKSVDKQLVDGVNDCPTCCSADPVKQLNDVHRMASVIEHEIEDAAKQA
AEVSNAEEEQEKVVSVDLEEKSKMETVDEENIPAAELVEPAGDSAENKRLHKMGLNTALA
IGLHNFPEGLATFVAALQDPKVGAVLAIAIAIHNIPEGLCVALPIYYSSGNRMKGFLWAL
LSGASEFIAALLGWAVLANSFSESAYAILFGLVAGMMIMISTKELLPTAHLYDDKDTVVS
YSFIIGMVVMALSMCLFQL
>comp5153_c0_seq3; 412 68 1189 LEN=1726; translated
MASSNQNENVGVALALVFGAGASTGIGAAVVFIPSLVKIASRRVLASSLGFSAGVMTYVS
FIEIFNKSADSFENAGHPPDKAYIYATLCFFGGVLAMLVLNLLVGVLLGKRGHHHDELPK
KAKKSTRKSVDKQLVDGVNDCPTCCSADPVKQLNDVHRMASVIEHEIEDAAKQAAEVSNA
EEEQEKVVSVDLEEKSKMETVDEENIPAAELVEPAGDSAENKRLHKMGLNTALAIGLHNF
PEGLATFVAALQDPKVGAVLAIAIAIHNIPEGLCVALPIYYSSGNRMKGFLWALLSGASE
FIAALLGWAVLANSFSESAYAILFGLVAGMMIMISTKELLPTAHLYDDKDTVVSYSFIIG
MVVMALSMCLFQL
>comp5153_c0_seq4; 348 68 1514 LEN=2051; translated

MASSNQNENVGVALALVFGAGASTGIGAAVVFIPSLVKIASRRVLASSLGFSAGVMTYVS
FIEIFNKSADSFENAGHPPDKAYIYATLCFFGGVLAMLVRTEHEFLKKRRREPGGFVESI
VAIVVENEVCXAIYFFFRQTVTHASQNNCGADCAWGLVFGKQSKVLNLLVGVLLGKRGHH
HDELPKKAKKSTRKSVDKQLVDGVNDCPTCCSADPVKQLNDVHRMASVIEHEIEDAAKQA
AEVSNAEEEQEKVVSVDLEEKSKMETVDEENIPAAELVEPAGDSAENKRLHKMGLNTALA
IGLHNFPEGLATFVAALQDPKVGAVLAIAIAIHNIPEGRKRRTLFIARLPTPMAGVGDYS
KESLLRTKLIKTLASLPFTGLCVALPIYYSSGNRMKGFLWALLSGASEFIAALLGWAVLA
NSFSESAYAILFGLVAGMMIMISTKELLPTAHLYDDKDTVVSYSFIIGMVVMALSMCLFQ
L
>comp5154_c0_seq1; 297 1 1540 LEN=2188; translated
RSLTNVLSRGHKKRYYSWWYGMNTSIVETHQEAPKHHRSARVDDRVARGDQQGIPNKKVR
KKGVKMVMSSTRACTACHAAKVKCVKVEGSTKCKRCERLGLKCIEHISRQGQGTRRRKKA
KTKVQKNDVQECKFSAPICTPLNCNITNDKGSVSKKSTTENGASTCYAIPMGYTSTVICD
GSGGNNMRNNDGSLKQKQPDPSTICAAMDALEVEDKIICSSITSGLGKDHFGLNHIIRQW
IALAFSRRSFSLLARASFIAEKMKIPMDDIISNQSPFAAATDSEPMDFLAADILLSKKER
KTLGYPIDLREIPWDLLESVQIDPNRLEETTRNRCIAIRWASQGSTRYWTSPVFCREFAS
TAEIGQVWEENKEGYDTIDLFIEKSEKGRFAQNLFHIIFVNSKPNMPCYVTKDRHKVQRR
NNAPPIEANVIQTIKLLDLDASIHYCEIQFLDDDISNANNLVGDGLVRANKRDYIDDHSL
DNIDPFLGDGIEFTDIPVTDEMIEFMKLISGE
>comp5154_c0_seq2; 324 539 1845 LEN=2493; translated
MQLKSSALRLKAVRNANGANGSXLKCIEHISRQGQGTRRRKKAKTKVQKNDVQECKFSAP
ICTPLNCNITNDKGSVSKKSTTENGASTCYAIPMGYTSTVICDGSGGNNMRNNDGSLKQK
QPDPSTICAAMDALEVEDKIICSSITSGLGKDHFGLNHIIRQWIALAFSRRSFSLLARAS
FIAEKMKIPMDDIISNQSPFAAATDSEPMDFLAADILLSKKERKTLGYPIDLREIPWDLL
ESVQIDPNRLEETTRNRCIAIRWASQGSTRYWTSPVFCREFASTAEIGQVWEENKEGYDT
IDLFIEKSEKGRFAQNLFHIIFVNSKPNMPCYVTKDRHKVQRRNNAPPIEANVIQTIKLL
DLDASIHYCEIQFLDDDISNANNLVGDGLVRANKRDYIDDHSLDNIDPFLGDGIEFTDIP
VTDEMIEFMKLISGE
>comp5157_c0_seq1; 102 1 202 LEN=202; minus strand; translated
RKKKKKKVLRKDSKEIPPVEITDSSATEESKAENEEIVTRKASRSTRKIVETNNARPTQI
TKTPSPQX
>comp5158_c0_seq1; 629 117 1993 LEN=2254; translated
MEEVQQLAELEETKESRKIVSRFWNNGNSDDDDGESDGNEVPLPVMGAVKIFLLEKKTXN
KSFHRVEQNQDGSGDSDGDEEMRRSQDPDYAWEQIRDEQIFLEPPTKWIPSQNASTSTSD
IRVRQAIVEDLKQKEQDSFVRFVFVSDTHANHRDLAHLPRGDVLVHAGDFSKTGETGSIE
DLALYFHENKQQNENHQTSMIQEIICVAGNHDLTLDQDYYHRNWSKFHRKAFDTTVAEKA
IRRHSIYLRDEGCDVVVKQKEEEDGTAPIHIWGSPYSPEFFDWAFNKDRGPSIRKIWDQI
PSPHHPSTSTVTPCTVSFDDNGGIGGEHDSAVGHESEPASSSDSMQQQRQHQAQPISKQV
DVLITHGPPLGRGDLVFLDETESKTTRAGCYDLLRVVQGRVKPRVHVFGHIHEGYGVTFD
GTTMYINASSTSQDYEVINPPIVVDVRVPDAITKGKSMNYERNSKCASISQSNNKPPSAW
VVRPKSLFRNGKTNICSVEQLIDWCLCNQHEFIANELRSKFVTVKIDNSTNEEMSSSSSI
CRSNTCNYDDTLQSRRKRLDCLIASNGRGSNAGHEDAISITAEQVFELLAGVLGYHFGFR
GIRDARRKLAKMVLHLYAESFESER
>comp5159_c0_seq1; 273 1 1195 LEN=1583; translated
XMACSKRTRPVETSPASGRSARLSILTALLTSLCVTVCHGLSPQLHGVADAYNNGIVDRQ
AFLVDGFWSCSSIALATTTATTTSEIICDNGALMGELAVPGAYSSVCMGLPERAVPLPRY
RERMSKKQSPDAESTIVVQQNTGGSGNTGMTVWNSGLLLTRLLDTMVDELHRQGDGNGGE
SIFWRNQDVIELGCGTGLCSIAAHRLGAKSVLATDGNPRVLQLAEANIRKNCYNNSDADD
GATTNISASSSPSPTIQAQPLQWGFLDAMDYSERASFMIGADLTYNPGSWRALAETIETV
LKIENDSIDPVTGTRVKNDRCYFLYLSVGHDGFNVNAEMDGFLSVAKEVGLVNLDGMVEG
IDVSRLLLARLSDSEKKILAQGGGVRVSVLGRKNSSRK
>comp5160_c0_seq1; 190 81 665 LEN=865; translated
MVGATSDSLTKPRQKRTHIEKSPVTSEEFMAGAPLTNEESISSSRNHSSSDESGARNFEG
WKNTGKLSYNTDAAFAGLVAGMLQIKKRRREQEAVPISPALNERARDKKTISLWLRSNKA
TRNIRRCNGCYYEDQKNNDDVFCAQSQCSCCSSTSSSICSWGLVRNEKKPKLWRENNKRK
ASPSLHKAKPSTTH
>comp5162_c0_seq1; 1024 1 2535 LEN=2552; translated
ASYGKMTAVDVAPGIVAVGTDTGSLHIFTYGGGRRVLRPYLSIPSPPSNDMAIVVCKISV
GKEKVSVFVAYQRGRKTHRGAKTDGSPTPRTSSGVSCYDMPLPKGNSPAAVTAPSARHDL
DGRNVSSSSLCDAVFNENENELQLCVARPDGLYTYSTTQKVDVSPIDGSKLAICLVPSPK

AAGTSREMVSSGKTGSGFVLVASTDSKSRRDAVDLYDPHNKLVAFHLLLSPGHTAIRAAG
VTTPPTRSADGSLQNGRSSAVVFTSGGSLVTLTEKETAQKVNLLVQKNLYSAAIFVAYAD
PSYETEDITLLYRRHAEYLYRKGEYSSAIEQYIHTIGSLEPSHVIFRFLDAPKIPLLVKY
LEELRSRDLTTPVHNELLRTCYLKLNDTESAEAIAAFSSRSMDSESLSTMVAKSPKDAFA
TICSFQAPEAAEALAVYGATLARLLPRETAGLVVSLCLGTFSPQKLADSQAAVMANAKKL
LEQPVDDRDRACDPYPVHVFASAFLENPKILRLILTHCNRNKCYLSPSLRRTLLELTLQE
WNQAKRTGDMEAERMRRKEAIAALTDSHSREIGDYDALVIVQLAGFEDGELLLYERLQMG
PMLLSRYAKDGGVKARRQMLAMCRNDPEISADVLGYFVNMVTERMALAEKEGVDDGEESD
DEVNDILEDIQDTLALAKRQGVLPPVRIARILAGEGTGQFSGSSAEAASDRSPVRTVPLS
VALDYVGDILDESRKEISRLKTEVEDYNQLCNSMEAQIDALLNTSASASQQRIATTQSSE
EPSKQLSPRIHVEDMYTKVRLHLDKNDKQDNKADLSREAFWRDMDQSEDSFQTLARFFAQ
NVIQ
>comp5163_c0_seq1; 756 135 1769 LEN=2201; translated
MVAKSTSVPLLEDKSPSTKINDDDDSGNEGFPSDEDHLIHEILKNRSWMKEDIGSEDLED
DELEKEMEMLSVAEQEVSQEISDAIRSFQNQLGAVNDRSRRKSDSRASRRTVIRTEGKNW
ETHYTSQEKPARINQEILQIRSKRKIHTSISNIRQVEIDAIERDTRDSVNESHPSDEACH
GVYDICNRDPPEEMLFIDDDESYDEEDDSSNVISDVIPHMSTNVKENENENEPESVDIYS
LKDFEGFDGSPFAQWHREEEGLSRQLPDLETGSNNFLEEEFFVGDIIHENTNKTAGENTK
DSGTNNSKKQQDANGSSNLEWHSEEDGLDTWDQRAKQISDLEWGKAELFEEEAFWKEGFE
TIPVPNSKIKTSSSSIREDNRNASSGKSHIPSASESRRSIIEILALPTLEHHVKDIQDVG
AKFDRQPIQSASDESVTQSVHAPAESTLQIDFENNESIFQKSTQLFHSVIEDSPLICIEE
SKNDCSFAPEPTVLIEEAAVRGNEKPRKKQNHSCDLRTIILAVHVVLAIVGIIVFLAFLL
RTDL
>comp5163_c0_seq2; 756 135 1769 LEN=2335; translated
MVAKSTSVPLLEDKSPSTKINDDDDSGNEGFPSDEDHLIHEILKNRSWMKEDIGSEDLED
DELEKEMEMLSVAEQEVSQEISDAIRSFQNQLGAVNDRSRRKSDSRASRRTVIRTEGKNW
ETHYTSQEKPARINQEILQIRSKRKIHTSISNIRQVEIDAIERDTRDSVNESHPSDEACH
GVYDICNRDPPEEMLFIDDDESYDEEDDSSNVISDVIPHMSTNVKENENENEPESVDIYS
LKDFEGFDGSPFAQWHREEEGLSRQLPDLETGSNNFLEEEFFVGDIIHENTNKTAGENTK
DSGTNNSKKQQDANGSSNLEWHSEEDGLDTWDQRAKQISDLEWGKAELFEEEAFWKEGFE
TIPVPNSKIKTSSSSIREDNRNASSGKSHIPSASESRRSIIEILALPTLEHHVKDIQDVG
AKFDRQPIQSASDESVTQSVHAPAESTLQIDFENNESIFQKSTQLFHSVIEDSPLICIEE
SKNDCSFAPEPTVLIEEAAVRGNEKPRKKQNHSCDLRTIILAVHVVLAIVGIIVFLAFLL
RTDL
>comp5165_c0_seq1; 682 142 1899 LEN=2256; translated
MAIRKGGSSSGETKVKPAQVAKQIKTLLKELPEIRADAARLDELRRVFTRLEAAGELTFT
VGAQSDSSSANNSVTAKWCSFLRKSHDDMVSQLCERVKLGRHASIRCLWGVIAGSPITSV
SKVTNNTNRKGYKNSSSSSSYKYVNADILKNWMRAMALQESSVMDKGMRHMIEAEFFSPY
RDIQYFSLGAISKIASSVYDGDDDDDEKTKVGGKLLELLMMIPVPSSDDDLDGSKQFLFP
PPSNVTPVDETEVESEESEEETDEESDMDSEDESSSDTERLKEETLESRPKKRQKKESTH
RFSFQQTRMFRREYKKAWLAVLRLPLPVASLKKALHVLPQNVLNYVAQPLRFADFFMQAY
SDHSSGIIGVLALDGLFLLITKYGLEYENFYTQLYKLVSSRLMYAKYRARFYSLMTKCLV
LNTMLPAHLIAAFIKRLCRCALSAPPPSALFALALVSNLLKKHPECYCLVQRKEGMEIED
CFVADVDDPVECRALQSSLWELAVFERHYYPAVATLAKSIGRAEEDQAPLYNIDEFASHT
YASLFDQEKKKKTKTALTFKKPTSLFTNDDIFFGCVETGDTEKEN
>comp5166_c0_seq1; 406 1 683 LEN=683; translated
XNIVQRIFCLHNDRNDWQNTRAARATAKMKFYSFLISASYLGFVTEASAGSGATRNPGVR
GATRIPDDINRSLKSDKRIAKVGTNLVNGSVMPRQKFRGNNAMILDEGKEDDEGGSRRND
NMILDEAKEDNGGGFGSSIINKKKENEMKNKKDNNDEEDKKNNNPKIINEDEDTSEENEK
NNNKEEDKKNNNQEKNNNKEEEKKNNNGNKNNEEEGMSKEDEKKNNNQ
>comp5167_c0_seq1; 706 190 2364 LEN=2852; translated
MITYNRSAFGWRLIFRAHGSAVYRSVLPSMISVLAYFLYHKYNTEYAGFAADDVLHPYAI
GVLVSSSTFLIIFKLSQSYNRYWEACSSTYQFMSKWLDATSHTCCYHMQCDHYNDIKPPS
FYDYPELNHLNMTRDRERSHTHSIHDVDDEETKDDNNVGLRNRKQSQTQTSAPTENYPPM
PSRKMFSARSLSGSIRSDASSGEYGRNAPKDLKGRSTEEKSKEYKARAVAKSINKTSSHR
SLPRAESRRAWAKEYSKMSTFSESIRTLYNLDVSRDMMDSESDEIYEEGIFDEKSLEPIP
LVGKPRMDGNWGEYYSKKKKKKLSTFMDPNDPNKIDSKGFASMQGGRTPPLFIQELAHLS
SLMNAVALATLRNDKEESESPLSIYEPGSPWPEVDPNNEELLKQTGFKAFFTALKTFLGV
GLSKEERSEHNAAQPLPVIGGVSDAEIHFLQLARGASAKTQLCFNWLTEFIIREHLAGTL
GDVGPPIISRIVQFLGDGMIYYNHSRKIMFIPFPFNHAQLSVIFVLFIIIVVPFLMHQYT

DEPAVGALLTFLTVLCLQGINEVARDLENPFRNFPNELPLVTFQAQYNEALITMYAGYHP
DFFWNGDQVLRMTQSKSKKQGLSKPNGSKSLSGSNGSLQSIDSGFSFAGENTPPTSVNAP
APVTNSLGNDSTEVAFMKRELEMQAKLIEQLFAKVGSTGSSFDELLPRAGATGLHEKDGA
PAKK
>comp5169_c0_seq1; 223 283 942 LEN=1069; translated
MYQPHRCTEGVHVWLLSSMADEDGVSSSKCISISEKNNRNRRDVLCQACIAATCISIAFV
FSPKPAVADVTNKIASSTALRALKRAQDQLPTKLLPDAQANNFVGIKARLREAPFDEFRK
NALILVRGGEDGPKASELLRAYKKLIASLEAIDATASLGMKGRKVDPFQLGLQYEDVAKA
LNSFLEVGAAAYAIPLQEAPSMQESLRYGSIDTKVLKSE
>comp5172_c1_seq1; 467 1 1763 LEN=2447; translated
XGLRSAEGPPRAASTTASTAASTATSSLTVVAHSMGGIVARRVLVEQQQQQKQKQLQKGY
DSGDGSDFRVHQMITLATPHSNPLYAFDKSVADYYQKWLRFPPRHGESENENDNDNERAP
LMVSIVGGLRDEMIEPAACYLDGPNSETILATKLLSSGPRPSDANQETTTAATPTKGLGM
DHRAIVWCHQLLEEVREILWSFIDTGGTGSRDRLHGSLERFSGGSNNDQQSSVRFARDLE
ELNQDLVELFGSLASVCMQASMLYNLPYLLALYAVLVVLSCAVAAFPSGSVVFGTPALPP
TELLPLGGTVVLGWVFRGGDLLGSTTLILALVADSVRLLLVHLLGKLCGRDHKPRSRNPF
RTLGRALLSTVLAVSFLSTGAFCGVQRFGSEGLTEFLALWAPRDLVAFVVYCCVATSICT
LSLAGMGCACDDRGGAFAFEAHLVALVVLVVPFLAAGPLALAAWERGTRPSSWWTLLSLQ
VPVGARALLRAASSAARAGNGKPRDRGARTTGSPGGRPRANPGTPRDFRNRKLSLARSAA
LVLAACWMCCREPGLLSRGTGYLVPQMAEVLVWVDAVYVVAAAGNAS
>comp5174_c0_seq1; 600 219 1543 LEN=1614; translated
MASGLAASIRGSTESDTFDLAGQTPATVTTNITQAFKDPFPIDEMIRITFVTGAGKLGRQ
KYDEGCAKAVTSSLRALGFDEDRAASCVRECAGLFKLQHDTGKNLKTVVVFPRINDASDP
GGGDDNEGGGASGSDDSLLLQRGSXGGDDCPVPDQHVSKPLEESLSFVEPEKGCNAAISD
IQRLVSSLEERLMEGVILDDNEQAFYDTMSVDDLKEKHNIVKEAMQKQVETDGNITPEEH
KQLLTQVSDRLDTINADLEAAKIENKPKKAEKLSNMKTKVQQRQAMLRKITPKNPAPLRY
QSEIENLNVELIPLLKLEDSTKGRLLSVKETKTLARKDEILEEITQLENDSRGWFEDDAS
FALRVKKSKAAAKQASKASAKKSSTSAKKSSSTNTSAWMSSSSLGRRPVAKARGKVRGAK
KKTSGGSGGVFAAMMDDSDSD
>comp5175_c0_seq1; 121 1 485 LEN=730; translated
EXEAAACPLPAPREAPSRRTALHRREGNASARGIDDLSPSRRRDLKRPRHGEAHADATLS
SAVSKSLTLDERVVGREREQQQQQQHQQQQRQQHPGSASPGGSGSPNLISPSSMTQSGLH
ARRHGTDAASLDKKVDMSALSGLAALSTAAFLKLDEDDGSP
>comp5176_c0_seq1; 929 329 2893 LEN=2971; translated
MTERRASALRPSTRGSGGLVNGNKIQNVSFSEDPMDHIPKKAQELRLSSPSNGSSPRRTS
FLKRNKKSSKDNGDKDTSIATNGKENNHNKAGGNSIEIKLKNPTGSSGASSPRKWGSRRF
KGFSLFGGSGDQRNGYGTLNDSNNYENDGEVTTAVEVDPVMTIFESRNTQRVRFTLAGDS
NESVISGGGLSRNSRDHQKNTKRGESSKNANSGSGSTGNVKQFMSRALFWSKSSSKKLSE
EEMKREAKELGCGPLIWESAATPTGPEVIHTKNNRNNNQKRNQKTPELTVSFTEDPKARA
AVSKILAKASSAEHVHFRYEYAVKCYLKALTILQKQAKYPDDHPTVVKTVRLMNNAHNVL
ISYKNSANIVKMGIKNEDAGELVKALKMYTIAYRIRRDNLSRNHPSLVVLLNMLGSIQIK
RGELLEAMQIYELALSDDSTVVPEAPTESAAPRHQQQQITTKDRNLLAKSVTFREMGTIY
EMWGNVDEALKFFHKSLECVAEWRESVRSSYKSRNRIVKNTDGGCDNGNDDEKSGSISLR
TSSTESQHYSIDIVQTVSSNIGDSEMELVFGSKGSPKNCSESNSKSRNKYERFFPNEIDL
KAKDGVKNSDENSHADMDLSLTLHQIARLYCSQGKFDRALDAYEAALRGMKTALGKHHPN
VAAVLGNIGNLQKEMGDLDAAYKTYQEVLGIESYRLGLSHPDVAITLHNIATIDAARGNY
QHSLQLYQKVIGLQKQLFGEDNISVAVTAACMGDVYEKIGELKSSMTSFDEAVRIKSSVL
GRHSLEVGRLLHKLGKLSFLSTDYVEAESYISRTVLIYRLNKLDEHHEWFVDANRDSADI
DGALALGVADQCEI
>comp5177_c0_seq1; 221 181 882 LEN=1364; translated
MTDTVLEEVWTEVQQRKADATGEGDGNSDSESPPLMNHLKNGYTTDEASQVRESVHEDCF
NVVPPPVDCPAWLCIVLPCINHLKSMKAHKNCIPEDAEVLRNGRWIRYDAASLVLGDVIR
LEEGDVVPADCVIAADEGELLVDLRVVTGQSRPKQVSGINAKRRDQRTLLFGGTVVQGHA
TALVTAIGTETTLGQLIKNGRFPVPEKESDDMEETIMLTEDSTMRTMEMGTMS
>comp5178_c0_seq1; 546 99 1565 LEN=1736; translated
MGKRSLEEMTTSNNDDTTKMKDTSNKSSDGDGDPMDVDDQKAPPPASKLTIPPRGSLDTL
LNRPSPFANEEGALPIGEFEPTENKASALLSNARVLVVGAGGLGCELLKDLAMSGRFRDV
HVIDLDTIDVTNLNRQFLFRQKDVGSSKAEVAAAFINERCPWMNVQAHHGMIQDKDPSFY
AGFNVILSGLDNVEARRWLNSTVVGLVEVDEDGDPTDPESIVPIIDGGTEGFSGQARVIL
PRITSCFECSLDAFPPQKNFPLCTIAETPRKPEHCIAYASILLWPKEFPDKKLDKDSPED

MKWVYERALERATRYNIEGVTYMLTMGVVKNIIPAVASTNAIVSAACVLEAVKILTFCSQ
TLNTYMMYMGSEGVYSHSFVYERKDDCPVCTSKVRTVTVSRRTTLNELLQKLCENNGSGS
LNLRLKAPSMTTAGKLLYMQKPPALNASTKPNLDKALSELVAEGEEIAVTDPVLQHTHLS
LAIAFEPE
>comp5179_c0_seq1; 335 1 1020 LEN=1094; translated
VLNHNVVPFRPVYVHFLSANTQLRKYTNGSRRHYFCSTTSNHNPRESPGVSIMMVPPHNA
HEDRSAIRQALAILEQEGVRLDTSELGYELDSFVMDDEIQSRQRQKQRSRSKSPSRTRNL
KNKKSLREPTPQSRYVEDGSVSSTSYSSSSTSSSSASSNREVGGKAKRSRSKVRSRRTSP
KRPELRETPEETGEKSVRFSNYDHTINVPHILDFTPEEIQRCWMDEEDYRSIRTRSLALI
EMMEDEKKYPISADYMIVNKHLVCVRGLGEKTTQCTRELERVQRKLYKSVFRVQEELREE
GIIDDEAIRKASRKYSKKSIKTARFVGISDEVNVASKFG
>comp5180_c0_seq1; 194 434 1169 LEN=1169; translated
MSVSVNAIDARDEMLKTIAEANNLCIHAQYHYQRAASADEDFYRIEFSALFDDKSLKLNS
AVEEATIGTAKLQRAFSLIHQNSYLNHRFPVLMSPAGSIHTEELERLSSAVFLGNNTCDQ
KESLYSSMMQFVRKREQLSIHQQSLLAAVDVQIRSGPAYMTYNTRGAGRRREHPVNYFVP
DSPQQVQPTACTNIEPIPIPIKITHALIISQKHMYLSIPIRDESQVGEDKLEETNTVQSR
SHPFA
>comp5181_c0_seq1; 656 153 2000 LEN=2647; translated
MMKRCLLSRSFRRHRQGSFASKGFDLLRFLSSSGQPTSDQEGGEKVFIKYSSSGLPDFIE
EWDRHKFRKVGYGLAAITAASVCNTVMIFEEQTVFFTAGFGALTASYWIIGKRDMEQRSQ
TIRRNFPVLGNMRYLLEMIRPEIRQYFVEGDDEKSVPFDRNHRAIAYERSKGAPATIAFG
TRRDTEAIGYEWVNHSLYPTSLKDKEQRVLIGGNNPDCIQPYSSSLLNISAMSFGALSDR
AILALSSGAKLGGFSHNSGEGGISEHHKKGGADIVWNIGTAYFGCGKFSENGVRMFDPEL
FAENSEHAKMIEIKLSQGAKPAHGGMLPKEKISPSIAEARNLKYPVEQDCHSPSRHNAFN
NSIELCEFIKELRDLSNGKPVGIKLCLGQPTEFCALVKAFLETGIHPDFITCDGGEGGTG
AAPPEFSNSVGTPLTEALSFVHAVLVGTGLRDPTEKSKSKIALIASGKVLTGMSMYKNFA
LGADVCNAARSFLFSLGCIQALKCQANTCPTGITTQDPELSWGLDPSFKQVRVANFHKAT
VEACIELMEATGIESWKKIQPMHVTRRVALGKSKSYEEIWEHLKVPKGGLLEGIGPDSLL
QVWKNSDVVSQAASI
>comp5183_c0_seq1; 747 1 1478 LEN=1520; translated
XTFVSKLRVDSGKSTSSKLLFIDLGGGKKRLGGSALATVYSQLGNESADLEDFATLKKAM
EVTQDLIDKRSILAGHDRSDGGLVTTLLEMAFAGNCSIDVTIPSSTGTEIDTLFNEEAGL
VIEVAASDVESIMKAYSDASVPVEEIGTVAAGDSIKIAVGSSAPCIESKMTVLRDVWEAT
SFKLEHRQRNPKCVVQEEEGLKSRQAPQWKLTYEPKPTDDSIMKSETKHKVAILRQEGSN
GDREMISAFLSAGFDAWDVHVKDLLDGTVSLEQFRGIVFVGGFSFADVLDSGKGWAGVIK
FNESVYKQFQDFRNRPDTFSLGVCNGCQLMALLGWIPEQKGLPEERQPRLLHNESGKFES
RFSSVAIGKSSAIMFKGMEGSSLGVWVAHGEGRFFFPDESVHDAVKKNNQIPLRYVNDTN
EPTEEYPFNPNGSPGGMAALCSEDGRHLAMMPHPERVFTTWQWPWTPSDWKDLKAGPWLQ
MFQNAREFCDNN
>comp5183_c0_seq2; 1739 101 4054 LEN=4096; translated
MTEKIIHYYRKIDTSHSLLPSCKEQLASLGLGEDADKITDVERESCFNVQLSIDGDLEPT
QKDRLEWLLAETFEKNHLKLEKSFFDEESSPGKIWQVEFGPRMTFTSAFSSNATSICKAC
GVPVERLELSRRYRFLLSDDISDTAIKELKKMLHDRMTEEEYDKPLTTFDNGAQPVPVET
VPIMTEGRAALEKINAEKGLGFDDFDLDYYTNLFKEKLGRDPTDVECFDMGQSNSEHSRH
WFFGGKMVIDGEEKEDTLFKMVKATLPKDVPNNSIIAFHDNSSSIEGYECDNLRPSSTTQ
AGSVKVGKQTLHPILTAETHNFPSGVAPFAGAETGTGGRLRDVMATGRGAYPVAGISSYC
VGNLQIPGYELPWEDKSFVYPSNLAAPLDIEIKASDGASDYGNKYGEPVIHGFTRSFGQR
LPNGERFEWVKPIMFSAGIGQMDGRFSEKGEPEKGMLVVKIGGPCYRIGIGGGAASSRVQ
SSENADLDFDAVQRGDAEMENRMNRLMRACCDLGDDNPIVSVHDQGAGGNGNILKELVEP
IGASYDIRNVNIGDDTLSVLEIWGAEYQENNGLLLRPESRELFEKMAERENCPIAIMGEV
TGDGKVVVHDSMDGSTPVDLPLELVLGKMPQKTFVDNRVKMDLEPLKLPEDTTVMSALDR
VLRLLSVGSKRFLVHKVDRSVTGLCAQQQCVGPLQLPLANVGVTAHTHFGITGTATACGE
QPIKGLIDSAAMARMTVAEAMTNIVWAKMSKIEDIKASGNWMYAAKLPGEGAKMWDACVA
LRDCVLELGIGIDGGKDSLSMAASCGDEVTKAPGELTMTCYVTCPDVTKTVTPDLKCPAS
GSKLLFIDLGGGKKRLGGSALATVYSQLGNESADLEDFATLKKAMEVTQDLIDKRSILAG
HDRSDGGLVTTLLEMAFAGNCSIDVTIPSSTGTEIDTLFNEEAGLVIEVAASDVESIMKA
YSDASVPVEEIGTVAAGDSIKIAVGSSAPCIESKMTVLRDVWEATSFKLEHRQRNPKCVV
QEEEGLKSRQAPQWKLTYEPKPTDDSIMKSETKHKVAILRQEGSNGDREMISAFLSAGFD
AWDVHVKDLLDGTVSLEQFRGIVFVGGFSFADVLDSGKGWAGVIKFNESVYKQFQDFRNR
PDTFSLGVCNGCQLMALLGWIPEQKGLPEERQPRLLHNESGKFESRFSSVAIGKSSAIMF

KGMEGSSLGVWVAHGEGRFFFPDESVHDAVKKNNQIPLRYVNDTNEPTEEYPFNPNGSPG
GMAALCSEDGRHLAMMPHPERVFTTWQWPWTPSDWKDLKAGPWLQMFQNAREFCDNN
>comp5184_c0_seq1; 445 117 929 LEN=1026; translated
MKIYIPISFLAVASAFSTTKRCPSSSFRVATESNTFLFAETSPSDATADKKKPMTPKEVE
AQLKARRELGEASTHPKIFSDDLLDDMKEILLILEKRIQEGPGSIGAAEVDNFVGMSKNV
LDEMKQKEYERLEDAKSPPAEPSPAATAAVSTPAPVIPTSEAIAASGKNESPEDDEFFNP
DDMSEGPGYSPDGGQGSIPKDTTNTYVIPGMDEMSPEEYQKALQKSIIDRQSVRKSSGTY
GNRNTWDYLNGLSGGTDPVVLKQDEDNLEN
>comp5185_c0_seq1; 498 67 1002 LEN=1390; translated
MSNDPELSKKLLDDNEYEDVEIQNKKTPRVVKGRVISTNPSPTSANGNAKKSASRCRLPC
WCTCLVITIGIFVLFALVVLGFAYAGLRDVVQHLTIETDSPKKFPIVDMSPLELRIVKDR
ITLFADQVAEGATSNLDDLVVTQDEINGFISNSEYLQGHMMITLEKNLYYEEYSLPMDVF
GFGGRYFEGNEYLALDGATTDKKENTIEMKTEIAANPSDWFDGPLFFAQLQYLVTKNEDG
GQGVLELFLDKGTFFGQELTVDDHENLLEGLYNTHFDDDGVEYIAEVIKGIGSVSIEEGQ
IIVKPRRTTKN
>comp5186_c0_seq1; 451 225 1424 LEN=1441; translated
MISFLLSIAGIIHICSAFSVHTPTSRSLSSRPLVGFETIERHYHQPTELRSSALSSNGDL
LPGISVIDDTNGVIFQQMEILQDSLYFRLFCVDILASCEYMPQELFECYSETCEVYPVDD
EEVPENIREVDFREHDFELDGWARWDMPSNDYYDLEEFPEGYTGYDGSEVWQFIHDKIGF
TGYDYDDNHWKADFNKAVSGIHSLVSAQVTKGIQDKIDAGEEFTDDEVWRDPKVEFDRRL
GRGGEQPMAMENLYFAYMLFLSAAAKAKDKLLADCDNGSIDSESSAILKGFLELPILCDP
AVEVAAKKLHDHAMDSQNTLWEARMRTRDLLRIMNCVQCNKCRLHGKVAMLGLSTALQIH
LGRTGEGGDPNRIHRVELAALLCTIQKFSKAVEFCKESR
>comp5186_c0_seq2; 223 1 970 LEN=987; translated
XTRRYVWSNQXFIKLSLRPCIFVVCRVIHIAAPGDWSSHFFAIFSQVPENIREVDFREHD
FELDGWARWDMPSNDYYDLEEFPEGYTGYDGSEVWQFIHDKIGFTGYDYDDNHWKADFNK
AVSGIHSLVSAQVTKGIQDKIDAGEEFTDDEVWRDPKVEFDRRLGRGGEQPMAMENLYFA
YMLFLSAAAKAKDKLLADCDNGSIDSESSAILKGFLELPILCDPAVEVAAKKLHDHAMDS
QNTLWEARMRTRDLLRIMNCVQCNKCRLHGKVAMLGLSTALQIHLGRTGEGGDPNRIHRV
ELAALLCTIQKFSKAVEFCKESR
>comp5187_c0_seq1; 572 1 1276 LEN=1276; translated
XGSSRLECSADLKPSPKIQEKLITPLIKIPILYIDTGILVERKVLHGMTDDGRGDIVHDW
SAFRDDNGEIFYHNSKTDETSWDPPPDNAPFNPVETKEGEDKEDKNKEEEEKTTATARDW
VEYMDDEKGEPYYFNTVTQETVLERPPGFSSGEEKNQEKPELKIEAEEAVPAEENKASVP
ATGDWAEFQDDEGRTYYYNEKTEETSWDRPAGFKEPKGEKDVDGNQNLISPGYSQSPALS
KSDEDEKKTTKGDWVATQDEEGRTYYYNEKTDETSWDRPENFKETDSKKCSDKTNANGTS
PMRNVPGNEEDGTDEENKTAEDWSEVQDDEGRTYYYNDKTEETSWDRPAEFRVNAKNGND
QARGEHEKEGEVAEKKEETDISAKSKAHASDWVATKDGEGRTYYYNEKTEETLWERPAGF
NPENEX
>comp5188_c0_seq1; 538 268 1293 LEN=1329; translated
MSEQFDVNSPTLNRFVMASTHDHDLVILMNAIATSCKLITSAVQRAGIANLYGLAGEQNS
TGDDQKKLDVLANDMMINALVNSGVCSVLVSEENEEPIIVPPGKAGKYCVAFDPLDGSSN
IDCNVSVGTIFSVYERRPGSNGTVEDILRSGADCICAGYVAYSSAVEMVFTFKGGDVHGF
CLDSTIGEFVHTRVKMLFPETGGKKIYSCNEGNSSRWDQAIKDAVEIFKDESNPYAARYV
GSMVADIHRTILYGGIYLYPADVKSPKGKLRLLYEGIPMAMIIEQAGGIASTGMFEGKIQ
KVLDLVPDEIHAKCPIIMGGKRDVQVVYDQFAKHGADVPTL
>comp5189_c0_seq1; 595 98 1135 LEN=1308; translated
MSSWFQSSGLTSAFESLNETVQAVTESVQEAIPTEHKEFLSKLTLNTDEMISERKNYKDE
EFRKAAAKDRLNRILPWETLDAEREILVEECRDAILELSETEETFFGPFEMPLLNVDLSV
QETLEIGDETNGDEEEAQESAAGEEDSGFVDGTSFVGEENNRTGVSEESQEMLAKLEPLP
PLLEDFDLDEHVGLIQRVLKEDPKLVMMQAKLSGGGARERIFWRNYFFHCAFTRYKCGLS
LDEIWSHQEESSTTKNPSDTTSTEYGDADVVSTPVPDDVEEEAIIFDNTNVQDETKTSES
SSDGDLDGTGNTECSFQLIDEDLDEAGGDPELDELEAEIARELED
>comp5189_c0_seq2; 588 98 1297 LEN=1470; translated
MSSWFQSSGLTSAFESLNETVQAVTESVQEAIPTEHKEFLSKLTLNTDEMISERKNYKDE
EFRKAAAKDRLNRILPWETLDAEREILVEECRDAILELSETEETFFGPFEMPLLNVDLSV
QETLEIGDETNGDEEEAQESAAGEEDSGFVDGTSFVGEENNRTGVSEESQEMLAKLEPLP
PLLEDFDLDEHVGLIQRVLKEDPKLVMMQAKLSGKYQYGVFFITTCFVCIDLLYNFVSRC
FFSRTKIHLMLVNYFSSIDGYLFFDSSGGGARERIFWRNYFFHCAFTRYKCGLSLDEIWS
HQEESSTTKNPSDTTSTEYGDADVVSTPVPDDVEEEAIIFDNTNVQDETKTSESSSDGDL

DGTGNTECSFQLIDEDLDEAGGDPELDELEAEIARELED
>comp5191_c0_seq1; 598 314 2549 LEN=3224; translated
MQPTEESHQENILEISAVEVEYSNASNQKGTITPSNSSDEDEDGNTKPKNDYKDYERDPF
FAFVPQLARWGCGLLANEGRIVQKGVLSAPGFTKDFLADTYRYYRENPRILYKEIVSGFT
VAIMQVPESIAFSFVAGVPPLSGLQATWWMAFITGILGGKPGMISGAAGALAVVVTKLTA
SDGVLSYLTVEERLNVLYMTMFVCGLFQIGFAVFRLAKLVRLIPETGMIGFMNGLAIIIF
MAQLPAFQYCTSEPLFIECEVDERQWLTFGGQPVELSLVLVHVAMCMAIMHFFPKIPKIG
KLIPASLVGLLVGTLIEWTLFRKVIGQGTRTEALGILYGSRKSAILSRQKKDVANSLFFP
FIQVAETAPIAGAPPKFDWPTIPTDGQTIQTMLLFAIQLAAIGAVESVLTLQACNEITDT
VPKISDSNQECFAQGLANLVCGLFRAMGGDAMIGQSTINIMNGARHRVSSTMSGVFMLLF
TLVLSPFINLLPIATLTGVLFIVVISTFQWKTFVILRYGRLSDSVAILLVTIVAVFTNLA
IAIAAGIILSALVHAWDSGAHVDADVELKDMIINGKRVNGVKYVHIRGAIFFSSTRKFVN
MFHIGEDPDTVILDLKDALVIDHSAVAAIQGLTHRYGLAGKRVIILNIGGKCVGRMRRTH
GDHDKFHEQISHKVQDIECQMKADNGSESSDFESETASIKNKVRDLQDLKMFQTNLDGID
KEIEKLGREENRYSVQSLEDKKSS
>comp5191_c0_seq2; 614 314 2452 LEN=3127; translated
MQPTEESHQENILEISAVEVEYSNASNQKGTITPSNSSDEDEDGNTKPKNDYKDYERDPF
FAFVPQLARWGCGLLANEGRIVQKGVLSAPGFTKDFLADTYRYYRENPRILYKEIVSGFT
VAIMQVPESIAFSFVAGVPPLSGLQATWWMAFITGILGGKPGMISGAAGALAVVVTKLTA
SDGVLSYLTVEERLNVLYMTMFVCGLFQIGFAVFRLAKLVRLIPETGMIGFMNGLAIIIF
MAQLPAFQYCTSEPLFIECEVDERQWLTFGGQPVELSLVLVHVAMCMAIMHFFPKIPKIG
KLIPASLVGLLVGTLIEWTLFRKVIGQGTRTVAETAPIAGAPPKFDWPTIPTDGQTIQTM
LLFAIQLAAIGAVESVLTLQACNEITDTVPKISDSNQECFAQGLANLVCGLFRAMGGDAM
IGQSTINIMNGARHRVSSTMSGVFMLLFTLVLSPFINLLPIATLTGVLFIVVISTFQWKT
FVILRYGRLSDSVAILLVTIVAVFTNLAIAIAAGIILSALVHAWDSGAHVDADVELKDMI
INGKRVNGVKYVHIRGAIFFSSTRKFVNMFHIGEDPDTVILDLKDALVIDHSAVAAIQGL
THRYGLAGKRVIILNIGGKCVGRMRRTHGDHDKFHEQISHKVQDIECQMKADNGSESSDF
ESETASIKNKVRDLQDLKMFQTNLDGIDKEIEKLGREENRYSVQSLEDKKSS
>comp5191_c0_seq3; 544 314 2658 LEN=3333; translated
MQPTEESHQENILEISAVEVEYSNASNQKGTITPSNSSDEDEDGNTKPKNDYKDYERDPF
FAFVPQLARWGCGLLANEGRIVQKGVLSAPGFTKDFLADTYRYYRENPRILYKEIVSGFT
VAIMQVPESIAFSFVAGVPPLSGLQATWWMAFITGILGGKPGMISGAAGALAVVVTKLTA
SDGVLSYLTVEERLNVLYMTMFVCGLFQIGFAVFRLAKLVRLIPETGMIGFMNGLAIIIF
MAQLPAFQYCTSEPLFIECEVDERQWLTFGGQPVELSLVLVHVAMCMAIMHFFPKIPKIG
KLIPASLVGLLVGTLIEWTLFRKVIGQGTRTEALGILYGSRKSAILSRQKKDVANSLFFP
FIQVAETAPIAGAPPKFDWPTIPTDGQTIQTMLLFAIQLAAIGAVESVLTLQACNEITDT
VPKISDSNQECFAQGLANLVCGLFRGKLGASRAVQRDSLADGFARMLRHNTKSNXAVVTS
YSAMGGDAMIGQSTINIMNGARHRVSSTMSGVFMLLFTLVLSPFINLLPIATLTGVLFIV
VISTFQWKTFVILRYGRLSDSVAILLVTIVAVFTNLAIAIAAGIILSALVHAWDSGAHVD
ADVELKDMIINGKRVNGVKYVHIRGAIFFSSTRKFVNMFHIGEDPDTVILDLKDALVIDH
SAVAAIQGLTHRYGLAGKRVIILNIGGKCVGRMRRTHGDHDKFHEQISHKVQDIECQMKA
DNGSESSDFESETASIKNKVRDLQDLKMFQTNLDGIDKEIEKLGREENRYSVQSLEDKKS
S
>comp5191_c0_seq4; 560 314 2561 LEN=3236; translated
MQPTEESHQENILEISAVEVEYSNASNQKGTITPSNSSDEDEDGNTKPKNDYKDYERDPF
FAFVPQLARWGCGLLANEGRIVQKGVLSAPGFTKDFLADTYRYYRENPRILYKEIVSGFT
VAIMQVPESIAFSFVAGVPPLSGLQATWWMAFITGILGGKPGMISGAAGALAVVVTKLTA
SDGVLSYLTVEERLNVLYMTMFVCGLFQIGFAVFRLAKLVRLIPETGMIGFMNGLAIIIF
MAQLPAFQYCTSEPLFIECEVDERQWLTFGGQPVELSLVLVHVAMCMAIMHFFPKIPKIG
KLIPASLVGLLVGTLIEWTLFRKVIGQGTRTVAETAPIAGAPPKFDWPTIPTDGQTIQTM
LLFAIQLAAIGAVESVLTLQACNEITDTVPKISDSNQECFAQGLANLVCGLFRGKLGASR
AVQRDSLADGFARMLRHNTKSNXAVVTSYSAMGGDAMIGQSTINIMNGARHRVSSTMSGV
FMLLFTLVLSPFINLLPIATLTGVLFIVVISTFQWKTFVILRYGRLSDSVAILLVTIVAV
FTNLAIAIAAGIILSALVHAWDSGAHVDADVELKDMIINGKRVNGVKYVHIRGAIFFSST
RKFVNMFHIGEDPDTVILDLKDALVIDHSAVAAIQGLTHRYGLAGKRVIILNIGGKCVGR
MRRTHGDHDKFHEQISHKVQDIECQMKADNGSESSDFESETASIKNKVRDLQDLKMFQTN
LDGIDKEIEKLGREENRYSVQSLEDKKSS
>comp5192_c0_seq1; 214 1201 1803 LEN=2148; translated
MQSNNQSRLNRLHQSQINAYRKAANQAIKDWALSVPLQPMEETDTKTVFVERCIAAISTA
APGTNALPNWHLSTAKATATTEIRRATAIYEKYHPVEEADEGKNNGSGTANASEAIAVE

NGAETEDGEATSDGISDDDGQAQTSKLAAGKSNQPNRNKKGSGQKIIATRKKGTNAKGGP
RGIRGGRGIRQGGRGRGNGK
>comp5193_c0_seq1; 294 88 1002 LEN=1172; translated
MTIAKSLEPYVCGGTAATFGSCCIHPIDLAKVRMQIFGQINPGKPVPGFPTIIKNMIASD
GVTSLYRGLDAAVGRQMVYGTARIGLHTKFSGMLVEQNNGQPISFVQKAASGMASGAIAV
SIGSPFDIALVRLQADGMAKPEDRRNYKNVFDALIRTAKEEGFGALYKGVLPNILRGMSM
NVGMMSCNDQFQEMYAGLLGDPMVGGPQMPTRILASLSAGFTAALFSMPFDLIKSRLMAQ
RPDPITGEMPYKGVADCVMKIIRTEGPLGLYNGFSAYYGRCAPHAMIVLLSKDSITKAYR
NAFD
>comp5194_c0_seq1; 434 92 808 LEN=1462; translated
MSNKRKAWNEAMLSAAGSAALPKALQPRTQKRRSDRRKKQDRRDKARKTSVLHSSESKEF
RAATWMDALEDVPLTGADGEGADGGDGDDSYDELDELEGGNKKKARGGRAKSKSKSSKKQ
QENSLPKRFKARSMANILLEEVNRDDGVAYAWLADEARDLPQTNSSSGRRAKIPRLPARK
FCSVTGMEGIYTEPKSGLPFANLKALEQVRERPPPWMTLAGSIAYHEAVKSIRDEGEP
>comp5195_c0_seq1; 331 70 717 LEN=961; translated
MRMPVPSYSTSPHHSYNAPQRKLNRSTFCKDINQSICNIDRTASENSINPRLQFGPRVTY
NPSKSSTIDRSVSENNISPRGRFGPRVTYKPRKPVEETRPECLRMFHETMPETKYLDSEE
ECRLAASVYGFGEALESEESSPEDEKKEKKKSSSKKSKKNDRGNKEGIKKEKSDKSKKKS
SSKNSKKKDDRKSKSAKKSSSKKSKKKDRKDSEKQ
>comp5196_c0_seq1; 218 74 728 LEN=838; translated
MTAIAPPPPTTTTNTASAKSNPSPHKRIHHETIIDAPIELAWAALVDVEDWSWNKWTRLE
LPEGEGPPSPGKTGTLRACYEGDDRSWESFGFEFAAVDDGSGSGKHKGNRRTLAWKGSVG
AFGPFCLFRGYHSVSLEEAADGSGRTLLVHKEDFGGLLPALRMGLPYKTLDRNYRLMNES
LRDFVEARHRETAARPSKRAAAATRSGAEGCEYYHYW
>comp5197_c0_seq1; 358 933 1550 LEN=1993; translated
MTYFDRATGEKVNASKFLIFQYMFARRSKEIGVTLVMFLMGIALTCFLGYHIYITSMGQT
TNENSKWGDVKKWYKAKQKMHRAAVEEAKVNPFETSTNSERSTDIGNAHGDSEKQESQES
FDPGPIPKNLYNRGFVENWKEVFFPLSLRKDALERGGYTKAAILQQKQQQQERISKEAEC
PTGSLCLADGATNSSSPRSDKPKDV
>comp5198_c0_seq2; 215 1 1065 LEN=1205; translated
TRVIVKRVVRDDFNCLQFMKARRWDGASNIPYNDRNTNSAPFLLSAKRFPTKMGGIVSTL
AFPSPEKEWSKADLLQRKRLDQLVFLKTRSGYRIPAVHIRRDDETKFTIIYSHGNAEDVG
LSLSYLDNLSRCCDCNVLAYEYCGYSISEGEASEKNCYECIDAAYRYLVEGKREPFEGNK
RVSAQTSIPVDPSRIVLFGRSLGTGPTVDLAARLLSRTGESRCIAGVVLQSPLESAGRCV
LGEFASCALYPLDLFRSYEKIEKLAPVPVFIMHGVDDKVVPCASGRALYTSLTNERKRRQ
ALKRKNTTKNDIEGKPSVEYSPKWIAGVGHNDMPEFECMNDVAKFLNFLEQRRR
>comp5200_c0_seq1; 301 1 778 LEN=928; translated
XPLPSSLPVRNTHCNEIRIRARTVLDMSPTPSNSSAWVALTAFAAGAATALVASKILSGN
KTDKRNAGATIEGEDLSLFYGDVEELTTITPSRKFLPEELYGNLVRDCIVFCVDIVLVRT
SEGGKKECLLVKRSSEPVKGAWWWPGGRMLKGETFFDAAERKARQETGLSDVTCVQVLGI
WNTFFPTSNWDTDADKGTQTVNAIVLVELNSGGDVELDDTSEECRWIPLDPEVAEANGED
KYVLQGLLRLKAWNPSYAK
>comp5201_c0_seq1; 732 63 1841 LEN=2102; translated
MGLGTKKRWRIPRRSKSKSKAKKESDDDDAFPTSSESSDEDPSVLEEKALPASMSDVEDS
DSDDDLYLDNDIDKSLDKIADLPKPPPTIPASDFGDPGNNVKGTNSNTKNDENHDIHIIV
HSKQQSDHNLYNIVRSNQQRDIIDDDDFSDSDSDDEEFSNYEEDMERHRQETRSSPFMSD
VLHKIEEESHDDIDRRNSEDSKGQSNEEQSKSPNNDNTDEAGPPQNPKQADKTAKKQKHK
TAKKRVYKDRAGNPLILPSHRELSKELCKIVATDHYSGAEFLRALERLAEWAHTQDTNLL
KHFLTYGGVVKVVDFLREQIEDPRCEGEFLMECIHKACDVICNVCFVGKFGINEDIAVVN
ATVVVKYEGIETLLMASNEYNEANKDHPLALKAAEGVWNAIMNVYCNAETAMTKKLSMLV
LDAGLQMLARIGSVDHPVAAETIANVFNTFYRITYHGYVTNEEFQKLDILTHCLDVFKRD
VTSSDGDEELLEEAISFFYGCHEKTLFGKSSDYERVLPLCVMGLREFAYDNANIREWATK
LLDGACSNIQKKETIMMAEGAIEALAPLLTAKDVDTAEKDEVRKLIRKIIAA
>comp5202_c0_seq1; 306 403 921 LEN=1348; translated
MSQSRAKKKNPPPAQPVPVLPVNSDVLQEDLGQEAPNSTRVQEKVNYAECSAGSSGEDCS
CASNSDANSIISLDEYMSTSEEAGAVSEKNIRIAIAVHFEYVLGAPPPSEWKERKTITTI
AKAFNQNQRKAFRNTIRSVIENVCRCRSDGKKYTGDRARIASGRKPILKSDS
>comp5203_c1_seq1; 365 1 1355 LEN=1775; translated
XGDAQTATSVLERVLARSISTSSRCTGFAVAFEKLKLSDPGDGTTTVSAEFVLTIDHHES
MGFAEVKKKCNEINSSLAQAMDDGDMQLAMAAAAKQEGAWPEDFRNRIVEEFLFEDDDGD

GPLDETGPPPPERGGGAKPNLESTIDSLLSDLNYEVQGFQNEDRRDVPFGMPGSPVYSSD
DIFLGGGNDGVFWNYAGDNADGAPYKGSLGLRMVDAVVEKAKQRHPRVIAIGDVHGCIDE
LQDLLRECDYRPGDLVVFLGDLVSKGPDSMAVVQMAREIGAIGVRGNHDFEVVRWHQAIM
SGVDPPVVGSEHFHIANCLTKADMKWMYSLPWYISSPDLGALFVHAGFVSGIRLAKQNPR
LMMNMRSILPDGTVTSKFFNNWPWARLWDGPQTVLFGHDADRGLQQYEHAIGLDTGCVYG
GRLTACILPEKRLVSVGARREYFKYRRKHYD
>comp5204_c0_seq1; 344 97 1239 LEN=1239; translated
MRALNVFRLLSLTGILATAESTNHISEGLDKVSDETAGDSFESKDRDLQYYYNNAFTFFT
TGAPTERPTHKPSPYPTPRPTPLPTHKPSPMPSPFPSPRPTPHPTHKPTMMPTKFPTPPP
VPGPTRLPTPNPTPEPTPRPTLHPTPRPTKMPTMEPTSPPTEPPTPRPTAFPTARPTRRP
TPFPTVDPTPRPTNNPTRRPTAPPTPRPTNFPSREPSDEPTNSEEPSENPTNSEEPSEEP
SYGSSFSSSFSSSLGNSGCGETIAEIVCAPRNNNILGRLCTLLRDTGLYNTLDDCNGRYT
VFAPNNQGVQRFLDFADDFDNLVNNIIGSDDIGFIIVDPNNPIQRRKLESQKSSKASSST
SSSAKLSSSRRLQNMNMNMNM
>comp5205_c0_seq1; 146 67 657 LEN=1244; translated
MESAPTNLATTSSGGSLPLFETDSVAHLETNLDDATGESLAFAIEVLLDHGAIDAWIAPI
VMKKGRPAHTLHCLCKDTINGGEDNPTITKLLELMFLHTPTLGVRIQRGIQRAKLKRSVV
SVATPFPNTVRKGRVDVKVSSFKDGRVVRTKAEFDHCREIAIEEGVGIQTVAEAAIKAYH
ENHIERLESSVSGKGL
>comp5208_c0_seq1; 593 145 1719 LEN=1976; translated
MKTSQKTIATSALVILLSLSTGMLSMPAVGATFAVEKINKQRGDASHTHRNTLRQKQSNK
AKLESIVRSQNLLPPLLHDEDLPIENAQQKWVSLNSDVEFLPADNIDRKIVHRFLEQGNY
DEEEEEADGGMTGMESIYNVEPFAYGVDEYDEYQQAWRLMGFIVDCYPMVDDDYYQNGGS
GSGDQGTEDGCSRYVLWAAYVDLDYEGGGIGEYQYYNVTQNSWDTSACDASGSSRCQKMD
CHLENTDFSLLGFFKHKSYDDWMEQLFKHEGICVWNDEEYAFMKNARKAWPQGCIDSYTM
TDDGTEIYYDIKPIQGGDIRVGLYTDTQCIKEYVSSGSNDPITVENILGNFFEGGGSGSG
DNYNYDFSSDTLEESMARWDSAFGKFRICQPCVAYDLENTDGTKFYDDDSGNSGDMFDCY
DDADYTNVNQCMKFMAKTTMNTATFRDMSLGRYQGTLTATQMNGFSGSKNWRQEAWENAL
TYLFLSFSLIVFIYGSVYFYKAKVATNYQPTFGWINFKEPLVMT
>comp5209_c0_seq1; 664 69 1319 LEN=1520; translated
MTDAPVYMATVSAPTNIACIKYWGKADAKFNTPTNDSVSVTLDQSDLRAVTTAAASPKFE
KDRLWLNGEEEEGASTSKRFRACVDGVKLLAADPEKWKDLKVHVSSYNTFPTAAGLASSA
AGYAALVAALAELMDAKEAYPGQLTTLARQGSGSACRSLYGGFVAWRAGTPEGRSDDKEP
TPWSDSMAVQIADETHWPELRAVIMVVSDAKKDTSSTAGMSTSVATSTLLPHRIAKVVPE
RMVAIEKAFLARDFAAFGELTMRDSNQFHSVCADTYPPIFYMNDVSRQIVRIVHAYNQWA
GETRVAYTYDAGPNAVLYTLEKHSVEVGALMAHYFPPGEASVESFYNDASFASDLGGFSL
DPSLLAATDKCGRTPASGDVKMVYHTKAGPGPQVLDNETDGNLDPVTGLNRYTKPN
>comp5210_c0_seq1; 218 1 930 LEN=930; translated
XSVQNIATRRNYDFVFDKSSDLVMLYSNKKHDISELVLNSIVRDRKVKQKKEKRAKNGGL
TEKQKKAQEAKEAARKKKEADRLARQKKIEEARKKRLADIEAKRKLLREKKEAARKKKEG
NKSKEDDKTTEQDNNKLNRNEKIEEFTIVAFLALGMTGVANAQKVGHVNLDRVIANMPET
RSLLAEIEKISKTYKDDLEASAKKLDGKIKKYTAEQATQTQQANEQRAQEVQGERARLGQ
AEQAALQDIQEKQQKGLIPIFAKAEKAINDIASSKGLVYIMDASAGKGLLVAKGVDIYED
LKAKLGLLPD
>comp5211_c0_seq1; 266 35 1063 LEN=1558; translated
MNHILLKKSFTLIAFTVHLTSSLQPARKIANITRNHLSINSSLETATPSSPSKMSTEFIG
KDGENGSHSKKDVITFSSSFLPDPRGDDRQQQPEATRTALIVLNYKIPNKSVLLEHLWKT
SGTRIAADGGANRLYQYSKDLIPDRIRGDLDSLDPDVRAYYEGRDVSVEQDFCQDTNDLD
KALQALVDADGNDSSSPSSINRVVIYGAFGGRFDQEMASFAALYKWAPLFDGQIYLYSDE
TFAFLVPAKKECQILLPFYDSHVLGDGEQPEMGEGPTCGLIPLGCPCESIITSGLKWDLD
GSMPLEFGGLVSTSNHVMKPIVTVCASHPLVFTAELASKRTL
>comp5212_c0_seq1; 676 1 1665 LEN=1690; translated
GAGVEFLCGPRNEVTSLDLQDGRCNGFWLGSESGRYVSAKAVLVANGAWMRNLLPVPLEP
HKGQSLSLRMPKDRPPILKRVLFGGDSYIVPKADGRIVIGATVEAGSFDSAVTPAGIMHV
LSYALELVPGLADLPIEETWAGLRPTTPDKGPILGETPWENLYLAGGYWRNGVLLAPKTG
HLLATLIASQNKGEGNTLLSRDDEAMLEAFAWDRFTSPEGGKRMAANARYAASMYPVHKR
KSGTGVAAAVGTELGSYSTARSAGEERQKDRKSLFGSDGFDGIDSETEDLFEKAAMMGRE
DAEVFEGLDGESSSSSSESTSDHTGGSPSSSGGDGDDKSLKFYYSEQETALGELRVLDSD
ESSPAAPSPEEIETTVPYDGSADAITVGSADNDAPEGDEDAGDDASRLELLYESIRENKA
KQKVDLPDQDIKDTRPDPGFRISHVCKKTGKVREMPPYVQPGEFFESLEQQEQKEEAEVE

TPPSNGIPTDEVEGSGSAPSSGSGENYDETTYDGYQEIFKANASSSRKEELEKMKLARIS
NRSSAPPREVRKKE
>comp5213_c0_seq1; 377 1 1178 LEN=1223; translated
XPYEKESEQYERIIQIPMPCLVIVGHPSAGKTTIAYLLKERALLHDAIDDVVLLNEETEC
GCIQTDDNTNERLETSKNERDNKVTTTKQELYENSLAEKQTRGALKASFDRAVKKSGGTN
SSNRRLVILDSLNYIKGFRYELHCISKAAGEKHGILWVLNRLSVVEEWNKDYSPPLLQEL
ISRFEPPDNRNRWDKPLFTIDVEPTSVSGCSTGTESNLQTRKTHSSKNEVLEHSVYNMHS
LGEAFGTSRTKEQTDNNTETQRSKKSAFSAASNASKAPKKSAFRRRKKASPPSASEKNEK
KIASVYNQNNKIIFDTSMMDSSAQDGIIASEKPSDVCTSKSALSPGVPQQSIPNEGQTSD
GKSLEEKLDEILDIFLLQTKNLKEGMSTRQYD
>comp5214_c0_seq1; 166 76 309 LEN=622; translated
MALRRPPTRVELKADDIDEYTKIIREKEMSMEDPMAMQHGEAGRSSGADGGRMKRSNMTP
GAKKSSAAQRIGIGRQR
>comp5216_c0_seq1; 373 1 1427 LEN=1679; translated
XQQSSTMINQQLHHRGREGVSDAAKNGMLPRTTASAAEPVNGKTRRKNFNAFVGSSGLVS
KQSFLILLFGTMMGYVILPMIVLRTNSIAQSGSVQLKKVLERESKSKYRNLNDAAMARLV
ENKNLMATQSLPTDRTPAIMETASLSDHRRKKILVTGGAGFVGSHLVDKLMMEGHEVTVV
DNFFTGQKKNVAHWMHHPNFSLVVHDVTEPLMLEVDQIYHLACPASPPHYQFNPIKTIKT
STEGTLNMLGLAKRVKARILLTSTSEIYGDPKVHPQAENYWGNVNPIGPRSCYDEGKRVA
ETMMYSYRDQGDVEVRVARIFNTFGPRMHPNDGRVVSNFIIQALQEKPLTVYGEGEQTRS
FQYVDDLVNGLHALMNGDFDGPVNLGNPDEFTMKDFAQYVKKLTKSESEIRFLDKTQDDP
SKRKPDISRAKEKLDWEPKVTVEDGLTQTIKYFAQVLEEAGEIIPTGPQASKPQN
>comp5217_c0_seq1; 575 151 1374 LEN=1420; translated
MNLSSAVAGLSATLALGAHSFTPTSSIRPAAASWRNTNTALGALDEGMMGRLENIKRSYN
ALTERLGDPDVLGDSKLLQKVMSDRASSEEVVLAYDEYTGLLEELEGAKELFQDAGDDPE
MKEMARAEMKEIEPKLEPIEDKIKILLLPKDPNDDRNVMLEIRAGTGGSEASIFVGDLLD
VYRKYISSQGWSANIIDESTGDDGGYKNIVMEVKGDKVYSKLKWEAGVHRVQRVPATESA
GRVHTSTATVAIMPECDEVDINIDKKDIEISTMRSGGAGGQNVNKVETAVDLLHKPTGIR
IKCTQERSQLKNKELAMKMLMSKLYDLENEKRDMEERARRGSQVGTGGRSEKIRTYNWKD
SRCSDHRIQQNYPLNQFLGGDIDPIIGAMIMKDQEEKLKELAEQNAV
>comp5219_c0_seq1; 369 325 1599 LEN=2313; translated
MDDMMSGSAAHCEDGKPLTRTTSWQKDLNSTTAEVTFHVVGDAYVDFFSFLDGEWPESGG
DSRLGEPVKCYAGGSSVNTATHLKALIRNFTDEPKPPVVLHTVLNPDDQYGQILMDHAKS
HEIPIDNRRREGDASTTGHCIAIVSGGERSFMTHQGVVGNFSADDLDVEEIGSTPTHLHL
HIAGYYNIPGFWNGKLKQKLEAVRNMRKRMFPDKNTTISLVTQHDATKEWDGGLSDLFPL
LDFVLMNDLEARSIIRCAKGGKRIGYEHEHLEWADYFGGLDRSSKTIITRGEKGSVALQH
KRILSKQKPIVMNSIDPTGAGDSFTAGFLFGLWSWKLGRREETTETKPGEREGMDGMNGG
DGAWPTEAIEEGMRWGVSLASAAVMIRGASVPPDGSEIQKILEQAKEPEYPVFDNGYSQL
SSCY
>comp5220_c0_seq1; 548 1 2050 LEN=2407; translated
XFFAKEEHQLKNLFPMRSNNNRKRYSRFNEAVISAAQKDSNTPQIIFGENGCPEFSSTGC
ALLSLSQLVRGSDPSALCDQILSSSSIRDVSDLIILAFVTRNIRGGKGEKDLSYAMFLRI
WYHYPETAKKLLPLFANHYGYWKDFLLLTEKSQTKKQSQELVEPILEESINLMRAQFQAD
LDALAKYEAKQKDGDTEGMDRGDMEHRSEIDADHADVVGKIISTGPKISLLAKWLPRENT
YFDKKLNFVQRFLNEGPIERRNGSKIWKSHSKKSFRKEVKRLTSFLSLPEVYLSAQRADE
IKFHMLSSKATLKLSRALLNEKVDGSVRRPNDAKRQRCAELFVDHLTQKGLKGSTLMPDE
IVAKIMKGKVSPNQEKVLDAQWKDLWNTAIEEVQAKSKLEGVDFDPSKMVPLSDVSGSMS
GKPMEVAIAMGIGISEITHPAFRDMVLTFESTPCWHKLNPEDSIVKKVRSLMCAGWGGST
NFERAYDQIMKVCVEHRLAKKDVPSLIVFSDMQFDLAQGSFRRYGACYDRSRCVTMHETI
RSKFATTAQILGWKDSDPTPIVYWNLRDTCGHPVEKDTEGTVLLSGFSPSMLKMVMTGEA
LEDKEVEVVDADGAVRTKKVRVKPAEILRKILDDSIYDPVREILVASKEGLLKHYDYVKP
QWACMAAGSTCHADCLDEDFTLL
>comp5221_c0_seq1; 623 181 1603 LEN=1603; translated
MSGQSATIAVGDRLDLDDSFLLTHDPEGLPSPATDVENCSYHDSFYDSVRMVEMESDMNG
ALRDSKNDTSLSEYLDDGDDSTPPPCDAGTDTNTYTNTVTTINTNATTDGEVSDEGNVVL
SPPPSAPTMLHDQRKLEEGLIPLPESSTSASSSSSSSSSAPSGGKVDSPASSIAVTPATV
LPPRNTRNDEFVSSGSSVIPDDSSRREEESGEMMAVERGEHDEATTRPLFEDQRDENKLI
RFAPHIYSVLGEPNGRMDELLPDLAENDERPLFGRTSTERQLDIFDQDNSPSYASMYGGN
GDYGGDDDASFVSCISGDDGDDESKIKNKILLSGAFVGAAVVMEFVFGTFRRKKEGNEVL
DEIGALGKDSDAVNNNGAIFSDNGALASNNVTVGSNGTSIAPAEFAKASFSSGSSNNISL

AMGQPVTTTTTGSAATNSASTAAATSAAKSSATTSAAATGSATSSATSSAASSAX
>comp5222_c0_seq1; 1220 186 3176 LEN=3474; translated
MTVVQGLPHHEDALSGDPYNVKAWNYYLEEMDDLIRGKISDLSSSGGMKVKQTQAQNTTN
YSLPELIQLRDMVGRRSVQLLPRSYKLWKLHWEFVVSRQQYLGGSSAVLGCFERAMVTLH
KFPRAWIAYLEFAHTQQKEEINGTDNENSIIIHPTQLRHLVNRALEAIPVTQHDKIWPVL
LKYYRSNSSKTAAAYIPKETKLSVLRRYVQYNPAATKEVADFLADDLGQWGEAALMYVDL
LNHDDVRDGGGSGFSGSKDMVITSATERKDLWMSLAKICTQHPVEDEIGLDFEAIVRAIL
TNSNNSNKTFQGGALRYLPREMEGVLWSQLADAWIRRGEFQLARSVYEEAIESVSRVRDF
TILMDAYLQFEEGLLEATMESQAEMMDDDEALEEDKQDGVDKDENDWDILLPHQNRDDNK
NSKSASMSDLELALARAEDLTARRPILLNGVLLRQNPDDVGEWLKRARMYKDVGQTHQAA
AALEDGLKTVKARRAIGGNSNQMVIQLAKIYEEDCKDVAKARNLFDRICNQWVYEFKNVD
DLAECFVNWVELELRQESWDDALDIIRASVIIPPFAPKWTRGLPKSLRLWDLMLDLEESL
GTTQTTKDAYSRAIDQKIATPLHVLNFATFLTEQKYFEESFSAYERGIELFQFPGVKIVW
KAYIDAFLLRYEGTKVERTRDLFQRCLESCPPEESTDFFLMNGQFEEKHGLTKRALGVYK
KMCETVPTEDKYTAYQLFIAKTGKYLGQTATRDIYQNAVQELEDEVASKLCVEFANMETS
LQEIDRARAVLTYGAQTADPRRNEEYWRAWNEFEISHGNEETFREMLRIKRSVEASFSTV
NYNAAEMSGTDSKATETLSNEDAMKMIAEREGVQLNPNTSTISGFVASSSSGKRSAQAAS
LDDMEERVAKLRKATAMAGQTNEGGDEDEIDIDELVDELEGDADEIDIEDEDEDEDDDND
KEAEKEAGNRASNNAIQDISTKSVPAAVFGGLVKNS
>comp5223_c0_seq1; 379 248 1504 LEN=1621; translated
MIRMRTNGEPSQAVRGPVSKALLGFAVVAAMLAGHASSFQLSSVGTISSRHNRTTRKGRT
IVARSMGMDGNLDREETTKTKDRDLLRQERSSGLLRLAKLSLQDYEWRIGVFKDRDADRK
VEESLARMMGDEASYVRPMDASEKSIGPLGLWEKESVEWLQQVIDEEARRAREIVRLGGI
LVRPMDASKVPGEVEELGPLGAIERRVVDFVESIKRSEQERTKNMVLRPKDVEESKRGPL
GQAELRASEAIREILQSERMRVQESRRREGLVRPIDIPGPLGELEMAVLEIVRAEEQRKL
EIEENNSAYLVRPMDAKTKGPLGELEAQATEAVKRLTDEERERVRNIQRYLDENRPMQQS
ERTALGVLETIVVGIVRAPILFYQIFNRVKDLLESEPMEGRDAEILKENEANANAKKK
>comp5224_c0_seq1; 324 1 581 LEN=1205; translated
XITNCQEITFDISPTNQQQSAASMVVIEAREINSSIDMAMEVDRSNEDTHGTTGQTKPVP
EAMADDDENDTKSETSDNAITCPLFMTGLPKNFATNPQLAAIASLLDDDEATDDNNDEDS
DAIQKDERPLDRQDDKIVSADACSKRNLRSIRTQRRRRQLRASPYPRPANDGNAKRKNGS
VGEITLFMNMWKP
>comp5225_c0_seq1; 257 306 977 LEN=1176; minus strand; translated
MTKNCWMSFPFVMAVYVQRGCNQNRVHFIQTNEFFAIRFCFSYGYAETQEHLDPSEIISL
PFPLVNSTIFAILPVFVLMIVWTVYNPSTVGWKILSLIKVTGFIFVIFGLCGNDLIATWE
HSVSAALFLAALLCTNLTEKRTSKLLEELPFYDHSDLLSTIRLYTTLAFIIPFSILSVLD
HGDQKQRWPCPVFMGGTYGFAFGSVGGIFLNYFKAKKLEKVKN
>comp5225_c0_seq2; 257 306 977 LEN=1478; minus strand; translated
MTKNCWMSFPFVMAVYVQRGCNQNRVHFIQTNEFFAIRFCFSYGYAETQEHLDPSEIISL
PFPLVNSTIFAILPVFVLMIVWTVYNPSTVGWKILSLIKVTGFIFVIFGLCGNDLIATWE
HSVSAALFLAALLCTNLTEKRTSKLLEELPFYDHSDLLSTIRLYTTLAFIIPFSILSVLD
HGDQKQRWPCPVFMGGTYGFAFGSVGGIFLNYFKAKKLEKVKN
>comp5226_c0_seq2; 657 172 1935 LEN=2079; translated
MNRAHAVEAKAVEDGYDYIIAGGGLAGCLLADRLSADGKKVLVLEAGTPDYNSMIVRIPA
GILRLFRNTKYDWQHETGGEKACNGRNVFLQRGKVLGGSSCTNVCLHHRGSAQDYDDWGI
PGWKASDVLPFFKASQKDMTDRSPEFHGKDGDWVMDDVKYQNPLSKRFLEVGEKAGLGTN
DDFNNWSRPQEGVGRFQVSEVNGERCSGAKAFISKALKRKNCEVRTGTMVRRIDFDGSKT
ATGVTYDLMGDDTMTPFNAKLNDGGEVIVTSGAIASPQLLMCSGIGPADHLKDHGIPVVH
DNPSVGKNLQDHPAAVVSFKTPQKGVSVTSKLRLFGLTNPFPIIQWLLTKTGLLTSTGCD
HGAFVRTSASSRGQPDLQIRFLAALALGPDGMTTFTQFRNKKNHADGYSFQSVACRAKSK
GCVRLASSNTHVKPKIDGGYLSDPADLKTLREGIKLGRQLGNRPEWGEYLGEEVYPGIDV
QTDAQIDEYIKNTIHTANALTGTCKMGTGKDAVVSPDLKVIGVNGVRVADSSTLPNIPGG
QTATPTVMIAERAAAFLRSPQQSVTTTTEVIMEEKAVEEPAPELAAA
>comp5227_c0_seq1; 305 105 752 LEN=945; translated
MTGKESTPLLQKNGDGNNFYFKKLQKEGSSYDSTTDADGGHVVETLPHGSSEEDFAPRAL
GESIKKQRRPSEPTGIFGKWFGGSKKNDANVSETRMIKPRKAPIKVEPKVFFANERTFLA
WMHVSIILAGASVAILTLSDYENIEKQLYGLIMLPVAVAFLVYAMYQYVKRAHMLRNKLP
GPYDDTVGPTVLSIMLMVSITAQFAIKMISSANRV
>comp5227_c0_seq2; 273 105 919 LEN=1112; translated
MTGKESTPLLQKNGDGNNFYFKKLQKEGSSYDSTTDADGGHVVETLPHGSSEEDFAPRAL

GESIKKQRRPSEPTGIFGKWFGGSKKNDANVSETRMIKPRKAPIKVEPKVFFANERTFLA
WMHVSIILAGASVAILTLSDYENIEKQLYGLIMLPVAVAFLVYAMYQCKFSKNAFVWRYE
RVVNMRNILRSMLILLLRHACLTTIHYTHDDNNQYQCVEXKPKDVKRAHMLRNKLPGPYD
DTVGPTVLSIMLMVSITAQFAIKMISSANRV
>comp5228_c0_seq1; 1821 116 4063 LEN=4142; translated
MGEGADVYIRDDEHAWVPARVVESDGTTAKVAVPQYACEEEITSDGGKAATGFKTAEVKL
KDYHSGVLPLQNVNESGTLIEVNDMVDLPYLHEAAILYNLKSRHVRALPYARVADIVIAV
NPYQWLTHLYEDEVRMHYAQRIVWDDNENDPRIGLDPHIYEISSLCYKGLALEKQNQSIL
VSGESGAGKTETVKICMNHIATVQQDPNEEKMEGMSPVVQRILDSNPLLEAFGNAKTRRN
DNSSRFGKYIMLQFEHLAPEGGAKPRSALAGSACEVYLLEKSRIVTHDSVERTYHIFYQL
LSSSDEIKSEIWEGLTGKVNEDFKCVGTSPIKGIDGKSDADNFTDTKSALELVGIKGEPY
IELFRAISAVMQLAELVFAPDPSDDEKSVITSKKEFDDLQKLLGVEPTTLELAFTEKTIK
TRGEEYKVPLKADIATESAGAFAKEIYAKTFLWLVRALNDATCAENNWPAGDTVEYGLIG
LLDIFGFESFPVNGFEQLCINYCNEKLQQKFTQDIFRTVVEEYKQEGLDLEEIKYDDNSD
VLALIEGKGGFIRALNEECVRPSGNDPTFVSKALKANKTVACLIQKRTFSPLEFGVHHFA
GSVIYCAEGFVGRNTDTLPVDLAACAKMCKNEIISKHMDNNSASNQKKKEDPDKAPPKRA
KSSMTGDTVMTQFKTQLTSLMKGLASTSSRYIRCVKPNTRKAKCVMMHNTTLDQLRSAGV
VAAVTISRSAFPSKLDFPDVLYRYGILNPEKDKAYPDDKAEIDEMLTTVLKRFEKTRKDG
SISKAFAVGKTKCYFKGGCLEFLESEKAKIWFKWAVKIQKVIRGRLTRDRLERERIAREE
ARLAAEKAERERIEAEERAKREAEEKARKEEEERLAAIEWEKNREAREEAERLEKIRLAE
EAEKEKARLAEEARLEKIRLEKEKARLEEEARLEKIRLEEEAKARAEAERIAKLKAEAEE
KKRIAEEKARIEAERIAAAKRAEEERIAAELRRKEEERMRAQRELENKSAITIQCMFYQY
TARQRFRDRKKEKKLQKRMGKKWKKAATKIQAIARGFLQRPKYRQALLDKAEEDNLKNQL
GNMEAKLEEAEQARKQELEDVKFKFEQEMEDMKEKLKEQLEEEMANAGNSAQHQTLIDES
GKIIEYLRKENAKLRKQSDSTKREYKTLKEQNTRLKEANDSASKSFNELNNHAKGLNATN
ARLIKNVDAFKKQLVKMKEDLKNRQAFYLAEAHARVAYQKTLARIVSQVQNQSRDAQLVE
DVVIWALECEAEAKAERAALENAGQKAPPGVINAAPKRAARPMDSDSDDSDSDSD
>comp5229_c0_seq1; 212 238 633 LEN=1010; translated
MNPIRLLLVFLSTTMVHSFTGSLLARPHDGALFSTTGKEVMSDIDIMCLENAADLCSYYD
ECDIEEREAILNRFAAETEIMVNRIATMNGLVKHLKTGDHKHLEDEEVESLGAKILDLLD
TEVRSGEGLSP
>comp5230_c0_seq1; 389 191 1288 LEN=1618; translated
MRNTIRDALSRSRCSSSNTYGISNVRTRGSACMEETPLPTVRRFQQPFLFCLLLFSICSA
TDSFQQSDWRNNRRQQHRISLASSLQATSIANGPPLIGVYGGDIFDPCSDSNWYSRQRQR
TRKKRKQRRAKTACNDAEYQKRKQEWADRYTTLEGLRETFGSNRNKLYGDLDVATTRRLY
KSLLPTAVCELVLDLEVKPEELAQLAYDARKAAKLYARERCHLPARVFANCYDGVRSFRR
YGRFQVEGMSYDQIFNKYYERSARERSMRKISSQKKGEQGIDIGDVGGTDVSHGWDEEDI
VTQTCMKILESACRTNPFIDQLALSSSNESNNGRNKKRMDRKELEQIAQKLEQDVRKLLD
PYNQL
>comp5231_c0_seq1; 450 814 3102 LEN=3241; translated
MSTETVDEENSDKAMRKAEIGARIRAIREETQGQHQQLTRKRPRYTPYLPSLAGKKKPKP
LQVETVEELRHAILDQQLRLSDTTIVEAKSQSNVDAAQTTAGKGVPPVLDHAVRDLIKER
FVNGTTPGHRLPEDKATLALAIEGGGMRGCVSAGMVAALTALGLSDTVDSIYGSSAGSVV
GAYMVSRQVCLDVYVDILPASKKLFVCKKRMVANLASLGLTQMLGKRNSRKTGNGKQKID
FISSSTLSPPSPLPKTLRERLLQTQPGMNISFVLDGIMGDDHGLRPLDVQAFRENSEKQK
LRVVSSCVDPATGKLLSKCFGSKEFFCKDESLTSPDRKRQGLFACLQASMTVPVSLIDFF
PVXFWLECDVVWTIVCYCFESMKGSIKIKRRRGSSHXIVEKDFQMTSIALALHMQITLTM
IFGMLSTNXGATGPPINLVRKNEASEDPLPCFDAFCFEPIPYRSAVEEGATHVLVLTTRP
EDFVPLTQPTVYESGVAPLYFHSHGQKGVAEFFEKGGQQYIYAEDLMLLQEARHKGSEGI
LVPPPEILYGVPRTNEISKNIRDRDNSWNKAHLFPLRVPKGYKELEALSQDKDEVLEAVR
DGFMTAFDSLKDIVGLENLEGSKVAEMLFPSSGDDEIKTEETGKLSSLSPVEEEILRTKV
NVPGAPIPESRLAMNKESLKLSRGIFRRPRSIFRRGGAGGLFGHGRHDEAEGNGIEEKHE
MHQWREEEFSATTLLECLPGFQDGKYSHLAKGLIEEQQRRTTI
>comp5231_c0_seq2; 759 574 2877 LEN=3016; translated
MFSPSVLSLSRRSISSSSSSSLSPSPSPVMMEAVPSGRKRRKFQQVRKRIFSRTRRGYRN
DGDGIVILVDEDVSSEPETPMSTETVDEENSDKAMRKAEIGARIRAIREETQGQHQQLTR
KRPRYTPYLPSLAGKKKPKPLQVETVEELRHAILDQQLRLSDTTIVEAKSQSNVDAAQTT
AGKGVPPVLDHAVRDLIKERFVNGTTPGHRLPEDKATLALAIEGGGMRGCVSAGMVAALT
ALGLSDTVDSIYGSSAGSVVGAYMVSRQVCLDVYVDILPASKKLFVCKKRMVANLASLGL
TQMLGKRNSRKTGNGKQKIDFISSSTLSPPSPLPKTLRERLLQTQPGMNISFVLDGIMGD

DHGLRPLDVQAFRENSEKQKLRVVSSCVDPATGKLLSKCFGSKEFFCKDESLTSPDRKRQ
GLFACLQASMTVPGATGPPINLVRKNEASEDPLPCFDAFCFEPIPYRSAVEEGATHVLVL
TTRPEDFVPLTQPTVYESGVAPLYFHSHGQKGVAEFFEKGGQQYIYAEDLMLLQEARHKG
SEGILVPPPEILYGVPRTNEISKNIRDRDNSWNKAHLFPLRVPKGYKELEALSQDKDEVL
EAVRDGFMTAFDSLKDIVGLENLEGSKVAEMLFPSSGDDEIKTEETGKLSSLSPVEEEIL
RTKVNVPGAPIPESRLAMNKESLKLSRGIFRRPRSIFRRGGAGGLFGHGRHDEAEGNGIE
EKHEMHQWREEEFSATTLLECLPGFQDGKYSHLAKGLIEEQQRRTTI
>comp5232_c0_seq1; 899 100 1506 LEN=1521; translated
MKFSAGVTAMMAATTVGGAFAFAPAKVTGTASALSSTATETYTFTKSEEIFKEALDLMPG
GVSSPVRAFKSVGGNPVVFDHVKGAYAWDVDDNKYIDYVGTWGPAILGHADDDVLEAVSK
TMAKGTSFGAPCPLENELAKAVIDAVPSVEMVRFTNSGTEACMGMIRLVRAHTGREKVIK
FEGCYHGHADAFLVQAGSGVATLGLPDSPGVPSAATKATFCAEYNNIDSVKAIFEENKDE
IAAVILEPVVGNSGFIKPTKEFLQGIREMCDANGSLLVFDEVMTGFRVSYGGAQEYFGVT
PDVTTMGKVIGGGLPVGAYGGRKDIMQQVAPAGPMYQAGTLSGNPLAMTAGIETMKKLSQ
DGVYDELERKSKMLVEGIMEAGKKHGHSIQGDWAGGMFGWYFSEEKVKNFAEAAKSDGEK
FGKWHRAMLERGVYLAPSMYEAGFMSMSHTDEDIEKTIAIADEVLSQL
>comp5233_c0_seq1; 260 1 775 LEN=775; translated
XAKYIDTVPYCTGTWWCNLLNKTESDPGQADRTMRIVSIVQGVLMLLAAVRSEVPPAEQA
ESSLRGGQENDSVGAEESLRQLRSRLGYSGIWRGRSYRTTRTYVDDYDDDDDDDDDNNDD
DGSIAGRYNFKSMKRSYQKGKGVGKGYEFKSMKKGYGKGDGKGYNFKKEKSYGKGKGASS
YSKSSKKHYGSAKGGGKGKGTSLHYYDSKKSSKKSNGKGKGKGISYYSKSTKKGYGKGKG
ASSYSYSSKSAKKSYGKGK
>comp5234_c0_seq1; 345 103 1464 LEN=1731; translated
MDFGGVDVDVGVEVGSGYGLFLWDAANLWIGRILVTIAIVVLWDSYVSILERKQKYEQQP
QKKQQRYRGTPGPRNTDGQLNEDVSVLETPRKEASDRSVPKTTMHDQENESVDASNVSTP
VPASYSSTESEKRLESPTATAAAATSVSAAKTTEKKSTSEKAAIVARTQAVRKMKGTSNE
HPGMEGFSNWYEVETSLFRIYTLAPRDPSVVTIPPYVPHSRRGDVSIELEVTNHTNYNIH
VFWVDYHGNLVRKGNIPRNRGVWKQSTYIDHPWVFQDADTAQIYLYYVPFRAIPTTPEVS
TIGSDGTTALHKFALINDWESAFYIGIQDDVLPFPGTARLQNPLDAINWTLKHMSRANTT
FNLLPDLDTLQAYLINVLQHPDRVKYRQLRIASPRFRTIWASPLRGVLLAVGFVEAGPYA
ELGCDRPLSSQRLQDVGLLSYLLNEWKEKESAA
>comp5234_c0_seq2; 296 103 936 LEN=2083; translated
MDFGGVDVDVGVEVGSGYGLFLWDAANLWIGRILVTIAIVVLWDSYVSILERKQKYEQQP
QKKQQRYRGTPGPRNTDGQLNEDVSVLETPRKEASDRSVPKTTMHDQENESVDASNVSTP
VPASYSSTESEKRLESPTATAAAATSVSAAKTTEKKSTSEKAAIVARTQAVRKMKGTSNE
HPGMEGFSNWYEVETSLFRIYTLAPRDPSVVTIPPYVPHSRRGDVSIELEVTNHTNYNIH
VFWVDYHGNLVRKGNIPRNRGVWKQSTYIDHRKCRRE
>comp5237_c0_seq1; 462 318 1376 LEN=1417; translated
MTRQNVDTELIDEQEETPQEAQRKMRNDKKLYALPAGNLSRNFNATLYKELINAPFEIYE
SAFGVGKEEREESFPVFIVPDWRSDNTGIFSAFSRTATPLTFQNMQTLLSWDEIVDASTG
AEEELLSFMSLNNFHASLFIGKTLGDAASHTPFLGLIVSYHQHSQKNILVPFAKLDHFFH
DSSEKHLLVESQVKVDHDLLHEILDSERAFNTTNSFFHPEIIAETHKNNKREQLEDVEAL
HFRRLFQLLIRRTFLFSYGYADCLMVEPHNLRTCLDDTFSLVVQMEASIRDAIDDQLLKD
LEAVTQIENSSRYREACVLVDMYGTRACSDSPNSNVRGLGRTAYSSLSSTFL
>comp5238_c0_seq1; 117 1 1016 LEN=1285; translated
XGHMYRTNFGIGHNMKEILDAHRPPGGRLGAGHVGLFETITNSLHMQLGLALASLGVATS
LTAQHIYALTPYAYLSKDFTTEAALYTHHQYIAGFLMVGAFAHGAIFFVRDYDPELNKNN
VLARMLEHKEAIISHLSWASLFLGFHTLGLYIHNDTVVAFGQPEKQILFEPVFAEYIQAA
SGKAIYQFNVLLSSSTSPATIAGNQVWLPGWLEAINNDKNDLFLTIGPGDFLVHHAIALG
LHVTALILVKGALDARGSKLMPDKKDFGYSFPCDGPGRGGTCDISAWDAFYLAMFWMLNT
IGWVTFYWHWKHMTIWGGNPGQFNESSNYIMGWLRDYLW
>comp5239_c0_seq1; 544 1 1219 LEN=1270; translated
XAVVEKKYPRFEMKTPKASLLAVLAVVPSVTTAFQQPVAHRYNAVKVAPFTKSAVSSISS
PMRPSSTSLVMAVEAASEDAGAGGEKSLGKKIFDTYVRVADVATTLFPLWTVLFSVAAIK
TPQSFAWFTTEYFTAALAALMLSMGITLTPQDFLDVMKEPISVAMQFSLCYAMMPALALG
LGKAFALDPAMLAGMVLVGSINGGQASNLCTYIARGNVALSVLMTTATTLGAIFMTPLIC
KAVLGTVVPVDAVGIAMSTIQVVLAPIAIGMTTNKFFPNFVKQILPITPVVGVLSTCLLV
ASAVAQVSEPIMNAGLQLQTPVFLLHLLGGLVGYFLPKLTGFSEVKSRTMAIETSMKSSA
FGFLLAKLHFGQYAARVPSAVSVVWMALTGSLLAVVWRYIPVGDEE
>comp5241_c0_seq1; 802 33 4402 LEN=4402; translated

MSVNIAADDDACSTRRTRSCISKLRGRDPLVEIADFLNQFQSHFLSLSAKKRINGGCGDR
FRFGEITAVDLKDALKFQSLMERIQLLEQETFIAGEYLRNAEGSESMAGRIAVRKKTELE
RIQKNICGVPDRTKLVLSMLWNCKVWTCKVVAQDHLVSPARLMPCTGRKEAGLCCFSPFL
NIPCSKYFAKLLPPVPVEETRPMAESEASIAEDSATSGKQPAAVTEADNATPASSRIPAF
HRKKKFRRCCSLALPPNKYTWEAYARFAIMATLVGDQEFATDVDGSDSAWKNHKESSLIQ
SFKSPLCVYELNTYKTGKNSSHSKQFSFSPSTDLVPLWIRAHRNTQSLRQLIQLFEVKRR
DKYDDDDASCSTNAIVRGINRSGEGKELIVNDSEDADTDAKQDTETAFERKFEATFRGNP
LDTKSQLMKVGDYVDQLVEWAVVNQSIFTYQYMKNNILTAHLRKQEDGQRRKRKRQRSNV
CRNIRCNENCEQQRFKLYRACQSYILRWFARRNRRAVAILFSMRLKHLKLDIGCKMDKNS
LEMANALEKAGFIVKAQENMRKLPTDRIHHEDLNFSRIDRRLSISGRYDGSVSITCQSQV
SNLLKTVLKTCPTNDKAYKHLITTAIDDLDLLFGTLSPGPFDVYDKACELIDSSNKYTLS
YLADLLESVPKPWVEKCCRCNKVCSSDLRTCFNCEQVFKETCAASASGTLPTAISLKDLI
RSFPPLHDIFKLKHPKNIDRPDFRMSDNEWVEEVLKIERKSNIDENQARLGISFISTEDC
SEQFDILHSDAMSLVELMPSETKNGGANNLLVPLRTSHKGCLVVTVNQELPGGRAGLQIG
DIIIGMEIEKNSSHSKKRNVEAKKRFTMSDLSKRDRTELLKNGSTKIKLIIRRPPINVVE
LATNWYSVIKEENEKLFNILQRMDESELWYCGSCCEPSTHDDSRSVFLEASYCRAVIRRV
GMESYAKPFLEDNKTSSSHFCLRRLDSILTDIMMVESNEESYDDAAKSFLRPSLANSTRQ
RLLRAATSLEKQPMTLLCRAMKGLIESSVLGANDRNTHQIAFITHFLVVFSSWCVGGTVK
SSSFKRLAGPPEIFQLCRVPWLGSAYSACVSRPSQKMAICNAQVCLNHSLDSEEKEIERT
ENAIKEYTMRASLVGTSFLVFRCDPLVECVSSAFPIDTKSQLIEFLVASYLPSDYHDAVV
KGRRKDRYDQISKNDGIYHLIPVVSCDQQQFLLERCKMRNEHQNNIISEISWTSLDVLNL
DGVTRYSSAALRKKIKESNSIRLAIDDAIIRDVCNSSTVLSDFSFYFQKSIDSEITMGVS
SLRARNQDLTLEITNKLIESLLKGKGSTAVVRQLSSSSSDVNPISQVEEQKTPSKWKIIK
HDENSDDLEFGLARLQPGLIPINPIKPSCQILTLPHISEDQYMLFYSDLIFYDERDKEIL
ENKLSPPNFFPCQDLRS
>comp5242_c0_seq1; 665 106 1734 LEN=1884; translated
MIKIAAPAFALLMSGFAAAQNGSDQDQSATLVGNLVDDCVVDFDPNAGVDYFPNKYSKPI
IKSYGDRDIFGEKFVPHNTTDFLDIEYFNTYKIVTNLHQDPPKKYLLYQCGTQIPEDVDV
DSFDLVTSVPHKGGLALTETPQIPYVELLGLRESIVAYIGDSFYVTSPCMTHMMEGETPM
IETVYNYSIQEELIDDFRERNPDAIIVSGPSNNVVGEQVIVSSATQERTNVATFDWISFF
AAFYNMEGEANRITSNMQESYDCSSDVASFMAAEQRELEAEEGPTIMWANYLTYQNLGWS
VGECPTWDHAYYCEYATHCGATVLTRPEGVGVNKTWGSPTVYWYLNDEEMLDMGMDADIL
IYSGSDWNEIYALKNETLNKMKAVQNGQVYDVLGQGSSAWYEQRYAEYDVVGLDMCNIVG
YAPASGHQNRWFRNVFTDPIGELEECDVLGGEISEPYVPPQQECVRPLIAPSDDAGSEDT
LMPEPSASADAGSEDAPMLEPAAPTSDEPFATVPSTTGLESAATRPVTIGTATILAAVVF
FL
>comp5243_c0_seq1; 220 327 1055 LEN=1229; translated
MDRGDAAEGMKHSLGATNSMEIQHYRRGWTQEFFCCITKSDFEYMNEEGETVAKSKEDFS
FVCRCCFAPCHAFDMTITDTKTNSEFLEVNRPFRCCMGNLKCCCRQEATVFSGDEHLGDI
QESCWCFVPSFRVLDETEKEVYVVRPPTSCGNMCIDCFPDGKWCCPHGCCMIPCDVYAIE
NGVEGTKPIGRMAKIPKETFKETFNEINYYKIDFPENATTNEKGLLLGSSILVNALYFEN
SE
>comp5244_c0_seq1; 160 496 1306 LEN=2915; minus strand; translated
MSAVDEVHENETDEDKKPSGLDEALPMEVDDQDFFDQKSSSATAANSAMKPESCVSAQSS
SDEDQSNNESPIDSIHTDWSESDYLDAFQRKLSRGALGLTHSDRNSIIEEIHGVNTHIQE
ESPDLIRSSLLSLSTELRALVQDNAANGNTNSKEPSLSTAYILSQCIGSSSKRTYVNTPE
FQLRFLRCDNFDXEGSGDPDDEMAEPGPRSLWGLCSLQAHPDYGFHKKRAQNTSERMAAS
PPVSGPERATCLDLAGGDGVQLRSGSXGYAW
>comp5245_c0_seq1; 617 171 1421 LEN=1421; translated
MGGGKKRALGGSGPSSSSSMSVVEKLQILRGLSCGGFSESDLSNCLRQSGYRVDVAAERL
VTGQFQPSKKANRNKGFFGRSSASTAPTSQTNDGTAPCPHKVATTPRQKPTLHLQSRPAM
PQLPSAAIDLSIDDDPTSLKVMGASHSPMPPSMEGTSPSSSSSSILVTPKGTTPVAKKAK
GLAPVPRGGKTTTTKPGHGDEEWLLCHRWVGDGVNLSRHGACDYQEEFRVLMEGSSSSSA
TTKTSSCPPKALRFRSKSHRMDGSFPRHLSFLGTLLKHNLIRLKATALMEEKQLPVGAQV
AFGIRVWIVDPVRFFAVFRDGNNKNGGGSRSSYSKDFLYSGRNARMGQAATAAVSRSLLA
SCRECAFLLLQWAQHGKQETRNDDADGNGTGGTSSENESSNNDRGVDGSDDDDDDDD
>comp5246_c0_seq1; 1277 101 2875 LEN=2900; translated
MRFTAASAFLALVSTTDAASFFKSTPSIAQKYGLDVGSTTRSLTRQDGWFAGVATRGGAT
EEDPTKKAKKKKKKTVSASTKKSSGKQKSNADSDESDLSVAILGGSTDRNAMHVFKYSG
EEGHSVVGMTEAAMMEMGLFEGDTVSIKGKRGKKTMASVAVLSEADVAALSDGSSDGAAI

GMSTDAMKNAGVRAGDKVTVTAAPNVKFGKAVLISPMLNSLKLSGIDEETMTDENGDEVK
LFEDYLAPYFSDKFRTLYRGDTFVVDGPKGELEFQCVEIDSVEKDGDNACIVVEETVLEC
DGEAIEEEVDDLENAGYDQIGGASKHLAAVRELVELPLKHSELWRKLGINTPRGVLLTGP
SGCGKTAMARAVAAETGAYFFVINGPEVISKRAGESETNLRRAFEDAEANAEDYNGAIIF
IDEIDSIAPRREKAGGEVEKRIVSQLLTLMDGLKSTSKVVVMAATNRASTIEPALRRPGR
FDRELDMGIPDEQGRLEILQIKTRDMRLGDDVDLEFLARNTHGYVGADLQQLCMEAALEC
IRGKMGLIDFDKDQVDKKILDSIVVEGKHFDHAVGIVAPSSLRENQVEVPDVKWEDVGGL
EDVKRELHETVQYPVEHADKYIKFGMSPSKGVLFYGPPGCGKTLLAKAIANEAGANFISI
KGPELLTQWFGESEANVRELFDKARAASPCILMFDEMDSIAKTRGSGGGGSSEAGDRVIN
QILTEIDGVGARKNVFVIGATNRPDILDPAVIRPGRLDQLIYIPLPDFKSRLAIFQASLR
KAPLDPAVDMEVLARSTHGFSGADISEICTTASKLAIREAILAAEERKEEAENDDDEEDS
SSSSSSKEKTQAVGEQMLITKRHFNFAMSRARRSVTEKDLVLFEEFAEKQKAGRGEAANN
FKFGESNSSGAAGEEAKEDEDLYS
>comp5247_c0_seq1; 402 77 835 LEN=1211; translated
MALRLAASRFAKNGVHFATSARSLSTGADHMRSKENYEAVLDKFAEIRACAVLRTPTSES
CPKAMQAAIDGGFKIVEFTLTTPDCLQHLSDFRAKYDGDVMVGCGTILTPEDAADAMAAG
SEFIITPVMLPDVIEWCRDRNIVCVPGCQTPTEMVNAYRHGAPLQKLFPGVAGGPGWVKA
VSSALPFLSINPTSGVDLDNAGDYLKMGAASVGLVAPLFDQEAIAAGDFDQIAQNAEKVM
ANVREAGPYQRK
>comp5249_c0_seq1; 519 581 2257 LEN=3480; translated
MAVYDSSYDFLGDALSFEHDGDALLTGSDYRFLLDASNNETMSPSEDIIPVATTDGFTKN
QDIFLVYMGRVFGFLSILAGFYIFYWAWRRKEHVYHRLMMGISVYLILTRPMFVYGVSAV
PEGTPGVWGAKGTTATCTAQGFIIHIGGMAAPTYFIGLSVYAWAVVAYANFDAKKYQWIE
KYIHILANGYAWGSAIYILSIQGFNHSGFHICWIASIPFGCGDETDIECTRGPQNISQVM
SIFVGIPAVTFLVVPTIAMLALVLFLYRQQQRQQHQQDQNRQRDDERQEQVPKNQMEQQE
PSDSSKNDASNNTEKAISMKSIRSLLSARSEHNRKRRGISALPYHGLTPKLVAKQSFIYV
GVIYFNYLPGILQHMVGAFLNHKSFFLSLLASSITAGSGIWYALAYRYFSTTGVVLDGTK
VEVGFGIRDNAKLQGGNIDDQNNAGNARSVTASESHGFSSMQLDSVIEEGDCNESDNSLQ
RTEEVKGSNDKPLPRASSSSSNREQRRQAEHKQFTFNIFDGTAPVNSRYSDFIFDGDDED
QQGDDEESRFWSSCQDLR
>comp5249_c0_seq2; 519 581 2257 LEN=3363; translated
MAVYDSSYDFLGDALSFEHDGDALLTGSDYRFLLDASNNETMSPSEDIIPVATTDGFTKN
QDIFLVYMGRVFGFLSILAGFYIFYWAWRRKEHVYHRLMMGISVYLILTRPMFVYGVSAV
PEGTPGVWGAKGTTATCTAQGFIIHIGGMAAPTYFIGLSVYAWAVVAYANFDAKKYQWIE
KYIHILANGYAWGSAIYILSIQGFNHSGFHICWIASIPFGCGDETDIECTRGPQNISQVM
SIFVGIPAVTFLVVPTIAMLALVLFLYRQQQRQQHQQDQNRQRDDERQEQVPKNQMEQQE
PSDSSKNDASNNTEKAISMKSIRSLLSARSEHNRKRRGISALPYHGLTPKLVAKQSFIYV
GVIYFNYLPGILQHMVGAFLNHKSFFLSLLASSITAGSGIWYALAYRYFSTTGVVLDGTK
VEVGFGIRDNAKLQGGNIDDQNNAGNARSVTASESHGFSSMQLDSVIEEGDCNESDNSLQ
RTEEVKGSNDKPLPRASSSSSNREQRRQAEHKQFTFNIFDGTAPVNSRYSDFIFDGDDED
QQGDDEESRFWSSCQDLR
>comp5250_c0_seq1; 278 1 908 LEN=908; translated
XSRERTTVVIMDLIIDFLSGASPYHAELRTFGQAFMGALAVRSGVNKNKDLNWFHAFALT
TIAAFGGGWFGFVWMGKPTSLITNGDVTLTLTAIAFVLLTYSPMDIGYRFGTLLPVKILL
NSWAQLFRGLGMIGFINACAAEVAPSKYYPTPILGPVVYGTLLGNMGAFFLKGFHKHLEK
DIPWAFQNGLVVGLFYHLYTHDKVGSVGVALRSFVQSTGIQGTMTDKMFATIVVTAFMQV
SGMMMLPEFYGPSFNLLLLPKRMLMGTPKTIDSAAIEKDLIAEEEKEQRNQATKSKKKKK
KKT
>comp5251_c0_seq1; 735 1 2374 LEN=2779; translated
XPGSLSIMSLTNGSNSSSWPRSVPQEWSIPLWVLAKSGTDLGHEEELLPFVNDMMDNEPG
FLERFKPQELSNSVWAAATIISKRPQAAMGAASDAALGILRHTSRELIRREGEGYKTQEL
ANSAWAMATLGFGVKKQTTSATSKDCHLTHTYTYLVSDDPEGDRALMEEAMKITLEKTKQ
NLRRFTSQELNNLCWAMARLEEKDEGLIHMIGRELANPRKSVNSQDLSTSLWAMATMEYY
DEDLYRSVVARFPGIGPHGFNPQEFSNTVWALATAGVVPEYSNAFDDKLLPSDLRPTKEE
ALRDPVTSIFAAAASELIRRPNEFKTQEIKDVLWSFTRVGMRHPELFRSMAEYLVGKEDN
LETTGRGLDEFNSQGLANLAYSFARHTQLGGETMNKYERRCRIPFTGGKLAYYTISYLDV
GEGLLRKLFAAIAKAGLENYDNLSKCSAQDVSNTVWAFGILGLKHERFLNAVEGTLRKRM
ESYVRGDKSSFIQGQEMSNALWALATLNYIPSGLLSLVERYFLELLHHGLTVSNISRLIT
RQELANIAWVAAVFGEYPSKLIKMLYMGILGVGEKPDPAYIQNVYNDSGIQATHSNSMLY
LQIMMDFDLGRKKNTFALPNDFPSAWASSMAVMNDGSSVDSSNGIMEISTTTSGIQNRIS

MAFDRIDFGHVDEHLFTMKDLATEFDIQMPPFPVEVLSVDIANIESKIGIEVDGPGHWVS
NIDPATNHNILASVGEYRGPAPNESKKGSDHNRIFEYNFIWNSEDQMINGATSLKQRMFQ
HMGWDIINIPFW
>comp5251_c0_seq2; 1021 293 3632 LEN=4037; translated
MAYEIDDVSKVSREERPNLIYRYIRCLRSNKRILRTEKELERITKASTTITKMVVTKTKA
TTTDTSDARGWSKRIWSAEPEDHKEETAQGVLSLLASTKGALSSVAGGGTMSTVNFSTSI
HRIARHMTLYTTKNLPGNERAKILSDPRFALLICSTSEALLDGAEKYESQYKSNNRKMNP
SSSGRVCFGARELSNIVWAIAKLNIAPPDSVLKVDIDNAEKLLREKSKDVRSIIFEVAKH
RASPGSSSSSPSASSWIPALSELCGVLVDAVSSKALDLDPKRFQQQELSNLMWASATTKR
PSENVFKYVVSSIIASAEQRKHIKLCNENNPNNKDSVKDEKGAVPQEWSIPLWVLAKSGT
DLGHEEELLPFVNDMMDNEPGFLERFKPQELSNSVWAAATIISKRPQAAMGAASDAALGI
LRHTSRELIRREGEGYKTQELANSAWAMATLGFGVKKQTTSATSKDCHLTHTYTYLVSDD
PEGDRALMEEAMKITLEKTKQNLRRFTSQELNNLCWAMARLEEKDEGLIHMIGRELANPR
KSVNSQDLSTSLWAMATMEYYDEDLYRSVVARFPGIGPHGFNPQEFSNTVWALATAGVVP
EYSNAFDDKLLPSDLRPTKEEALRDPVTSIFAAAASELIRRPNEFKTQEIKDVLWSFTRV
GMRHPELFRSMAEYLVGKEDNLETTGRGLDEFNSQGLANLAYSFARHTQLGGETMNKYER
RCRIPFTGGKLAYYTISYLDVGEGLLRKLFAAIAKAGLENYDNLSKCSAQDVSNTVWAFG
ILGLKHERFLNAVEGTLRKRMESYVRGDKSSFIQGQEMSNALWALATLNYIPSGLLSLVE
RYFLELLHHGLTVSNISRLITRQELANIAWVAAVFGEYPSKLIKMLYMGILGVGEKPDPA
YIQNVYNDSGIQATHSNSMLYLQIMMDFDLGRKKNTFALPNDFPSAWASSMAVMNDGSSV
DSSNGIMEISTTTSGIQNRISMAFDRIDFGHVDEHLFTMKDLATEFDIQMPPFPVEVLSV
DIANIESKIGIEVDGPGHWVSNIDPATNHNILASVGEYRGPAPNESKKGSDHNRIFEYNF
IWNSEDQMINGATSLKQRMFQHMGWDIINIPFW
>comp5252_c0_seq1; 236 150 695 LEN=791; translated
MSILRRSTDARPYWSLGKIANIVFVNALFFIAIQSVTSKDTCGATATPPQKLLNSAFVFV
KPHANTEKVRELVAEKLESAGISILFEDDIGGEEIDRNGLIDQHYYSIASKATILPAEKI
PVPPKVFRETFGEEWSKVLEENRAANALDACKRFGCSPEELNEAWNKVSTVKFGGGFYCG
E
>comp5252_c0_seq2; 349 150 1208 LEN=1233; translated
MSILRRSTDARPYWSLGKIANIVFVNALFFIAIQSVTSKDTCGATATPPQKLLNSAFVFV
KPHANTEKVRELVAEKLESAGISILFEDDIGGEEIDRNGLIDQHYYSIASKATILPAEKI
PVPPKVFRETFGEEWSKVLEENRAANALDACKRFGCSPEELNEAWNKVSTVKFGGGFYCG
KVSVNGKPELYVFNAFFMTMRSKFVGKDNGIHCFTVEWDSDTLSWSSFRHEILGPTDPKK
APSNSIRRSILDQYKNLGLSSVPNHSDNGVHASASPFEALAEKLNWLSLNLESDSLGKKL
LDAGISETRIKEWCLDPQIKTSDSSTGSLFDELEDLNVDDCVSKLLELNRLN
>comp5253_c0_seq1; 242 482 841 LEN=841; translated
MNDQFGFDLGFGLDCFSPQQIQQPQAQQMQHQQQQVQHQQHGYLNNNNDNNNAGNNGPCM
DGYNPNQVSNVLGGRAQHGHSAYNNSINASGALHSRNNSVGSNHNRRNNLHHHQQQQQQQ
>comp5255_c0_seq1; 305 1 351 LEN=600; translated
NNNNSSIDDEEFTYVPLAEQRRIEEKAKETDAGQLQRAPEDKPEMPMAIPTDGSFLEVMK
RKLEEEQRAAKERTDNENGNGNNEEQKQGPEPPVPTDNNNDDDDDSEGPAPPPAV
>comp5256_c0_seq1; 368 50 1465 LEN=1912; translated
MVRCDGHNQKYSTIDEEYAGIDFSNWKRGDWCWLSPPPSPPRKPKPKPIPSVSSHPPQKR
RLSSEDGTDTPSNPLRFHKQMKVKPDFPVSSSGGIHISHHRHESTTPTEPMLMEKRGSSC
KTKAEPSGDRGNDTNTVPAIATTVSTSKQWFLPKQEKRERTEHKLRAAREKAYEKYADRT
LLHRFRGIGLLGGNLQMTFPSPRKGCPKTFDENNNIRQGYPRPRRALNHPNVALATGEKF
NMEAFTIATFSGFWQPNGPNFAAQPTAAVTSLPGCMFEDRSLAVPFFIKRSLTKRGAKRE
GRILGWEYVGNYRYASDPDLTLWGSAGSVPDACKRGLEDRILRSAASGDDDFGRRILDHW
RNKLNVALEQQSDSGNDGENDTPNSMIQRAIAMGYGPATNDKDLLRLVLELDEYHEQYII
EFVEYDEAIYKYCSKGPTSKNSRGEVQATRGGALATAEDWYNFSNENMVML
>comp5257_c0_seq1; 371 375 1475 LEN=1580; translated
MNNSANSIPNNRAATEKTNHFPWKLYDMLHTAEKRNEEHIISWIRDGKAFKVHKRDQFIE
EYMKKIFNQTKFKSFQRQLNLWGFQRVQIGPDKGSYFHPLFVRGRRDCCQHLSRAKLKGT
AEKLTHAGILEKQEPQNSPYSMATTTAESLSTTGAAILPLAVATPSIVEGNCNTVSPASL
LGAFVNFSRPASATAIEHTVQGHSLQTQLSEFLHYHTSERRKEIIRKAAISLSPELSVTV
VGSPVFAIATDTSKQNPRSEYQQKYHNDYTHEQQEKQSRIPPRQPKIEVSTGDHQQKDEK
ESLNFQQGRTKFDRQSKIECQGQKSRKRVTSSGLEALLAVTAQRRREEEMALLLGIPDGE
DTPRTK
>comp5258_c0_seq1; 618 224 1882 LEN=2489; translated
MTQRGRYPIGDEDVVTIKGRYLYKGDGDTRFFVKGIAFPIPPPAPVKIDIDGWITVLEQL

ANDTDINAVRIYDMDCYHVDDTYDPFLRRAAELGIYVIVPLTSSEGDGNLSRDTSPPDCY
PQKLFHYGKTCIDKYWDRPNVLMGVIGNEVMNSFTTYKSAPCVKSYLQDLVKYSRSLSSQ
SSSSRKAFPLIYATQHDSPTSELHPDEAIKLTLDYLSCASGTERSDGALDLVFGINIESW
CSSSESFDVEDDGITESSYHSLWRTFSGKNKTVKIVDAVTGDFTLEQVLPVSPIPVAVPV
VFSEMGCSRRDFNRDNPLIPEDLKNIRDWKQIPLVSSEDGKMADLWSGFIAYAYDGGGFD
VFRMMGGNSTKWNGKESLPPSTEYNNFRYQLSKVNNRSLTGESSFSLSEIAEIDQSTLSC
KDATAKIRKFFGVKLYSLSEMPSYFPEVRDNVKRLTTFDPRAPPSAGTPTESSITNRVAS
YGELHLLISVLLFVLLAVGFLFLGRRRGERPNIRFGWLEQRFGLNVDSNNNKRHFEDENI
PLKSADMNYGSM
>comp5259_c0_seq1; 310 211 1116 LEN=1862; translated
MEDQFSVGFQASLPCDPLASFSCLVVNGQSDRTFTLAANVLPVTANCNSNLERWHRNDLI
LGRFWQPEDGIDEALFESKSEEADHLKSQPHYLTYGEVTALGVRQLAYEMGIANCDGGED
RRNRFDDANAEYNYNEDDGGGIVFYDLGSGVGRLVTQMYIDQPDRVEKSVGIELSEGRHE
IGINALKGIIGEDLVERFDGLYSGGDAMPHFPIELIHGDATEVVLDTRTTHVYISSLCFP
KNVLLTIQEILLKLPKIRVIAALNRLDTILQLEGEEWQERNVPIQMSWGPSTAKLYRKVK
S
>comp5260_c0_seq1; 155 695 960 LEN=960; minus strand; translated
MAAPEIAPIAPVNPRKKKKKPKADVDSSDLFDDEKSVKSTRSSRSGRSTRSKKSRKIKRI
PKRDSSSDYLAQDSFVLDDSSLTISTKSA
>comp5261_c0_seq1; 377 627 1803 LEN=2351; translated
MAFAEINPQLGHRLAKSTNIAVENLEKCQVTANFTFMLFVSQIASLNAKIENTLQSKSDG
TLVAAAKEVTLAPIRGPPSKVKCRRTLKGHFGKITALDWSTDGTTVVSASQDGNLLLWDA
LTTSKKMDIRLKSAYVMSVCMDKSGQYVAAGGLDNACSIYSVGGTDQAQLKTELVSHEGY
LADCKFFHSPSKMLTASADATALLWDVEKGQIIDTFQEHKSNLTEIQLVEQDMYLSCSTD
MTIKLWDVRCAKEGSVQTFTGHAGDVNGITMLPNTEGAFSSCSEDGTARIWDIRSYGEVA
SFGQLVQQSERDPFSDDDAGFTSISASYSGRLVFCGHSEGSVWCYDIFSGNKNPAYILGN
VHEEHISCVKVSPVGEVLCTGSWDFNLKLFA
>comp5261_c0_seq2; 369 59 1153 LEN=1701; translated
MAVTLQQRLDAAKKEIASLNAKIENTLQSKSDGTLVAAAKEVTLAPIRGPPSKVKCRRTL
KGHFGKITALDWSTDGTTVVSASQDGNLLLWDALTTSKKMDIRLKSAYVMSVCMDKSGQY
VAAGGLDNACSIYSVGGTDQAQLKTELVSHEGYLADCKFFHSPSKMLTASADATALLWDV
EKGQIIDTFQEHKSNLTEIQLVEQDMYLSCSTDMTIKLWDVRCAKEGSVQTFTGHAGDVN
GITMLPNTEGAFSSCSEDGTARIWDIRSYGEVASFGQLVQQSERDPFSDDDAGFTSISAS
YSGRLVFCGHSEGSVWCYDIFSGNKNPAYILGNVHEEHISCVKVSPVGEVLCTGSWDFNL
KLFA
>comp5262_c0_seq1; 644 155 1666 LEN=1842; translated
MPTTAATTTPSEQTDEENSGSVAPPTGCSLLPNDGNGNPNDRCAENSGKGTDKAAKKARK
RLLNKHLKQRNKLRKYETRVQQGILRKDKSLEEEARKDWENYKQLLQNDPNSVLNYTGEE
EAPGTTASVCQTGSHCCQLNQSSRYRHSHQSLRSSEDRETKRVREGGAWLLKFWMKMAPL
LALSSPPSNNDNDSPGSKPITSKTTKEQQTIRAAKLKANMDRGTQTESMFDDDMALLGYT
RQKFYERALLAFASLDRLDPCHRENEGDAAVDGTEGGKDGNAGGIGTPRGKSAAERKLVD
GMWGRLIGTKQVVSIGCGPGCDATGVLAFFESHGAQLENGILLMDFVVDRWKQIVLDQLI
ERIVPRYVPFVRAVGCDVRDSLLPTEQHPNKQDNKNHRALLELRKESPSRNRLIVVSYLL
TETRHKWKSFFHHLLSMLQGTESLLLLTEPTAWQLHEFLACFSSSDADLVRAHVWLDSSR
DLPHMQSLDKRNGPAILMVCTH
>comp5263_c0_seq1; 969 282 2939 LEN=3057; translated
MTTKSGMATMEHQISFSHEHSAEAQHRSHSPVSSVSYLAREEPTKLSWSKLHSPSSVLYS
SKDDKKLEFYRKQRLCEIGGYMYREGETVRTFELKCGSVRDFPCVCAPDLDPPLWCPYCG
IYLSPKWASSAASSDSNIERLRISNAPGFFCLKDGETSDPFVGIESGIRQVCSCALTDEG
KPVSKCDVASPEFGHNSEKKEGGYDTHTKGNESGTGDSGNDTVSSAIAEGNTSAHSNMSS
NASASANAYGNGNGNGYGTGVNTAGETDLNTDPGGTQRLANPNNKDNNNGKKSTPGFNDN
DSFPKMKTTSSSAFNDKTSKDDTSKESNGNTEEKTTYKNGSGVNDGDDDVCTLELPGESG
ETMTFRRGESYGPFLRNRCQAPSKHPCYCDPDAYRQMDCPYCGFDLGENSLLCARHNETV
SFFAPDGPHGEVRTCSCSIQGTPITNCETSNHPYVVRSDDGNASPGASSHQETITTGGQK
GCPIKDPSSGRVVAVVPDGASFGEYASTVMGTCGDGKQWPAFCDASSDAEPTSAQVKSSV
TTSQQGLHNTLSVDIRDRADVLYPYCVYLDVESGSPVCARDKEEVVYTNDEGIQVKCSCV
IDEKDSRVDGSFTCGPYTGNNETKTEVGLSKEQGGGNNTGTPNTAFANENNTKTEEFPNE
VEEAKEKKEEEEEDYDDDDDVGTAYNVGDEKDVKLVDENKNNHVPSTTGIGSSLDDNNVN
KNIDKDGNALSSGNNLGSENNNNDIPSMPGNGSNLGDNIMNKNMNDKNSDILISDLQGEN
YNKNLPSMTAIGSNLGGNGINKNIINNNGGGVIEDSNLPGIGSNFEGGALANNRPTLRPT

MSPIYVPQSGTIATYGGTSGNSIIVQILLTTIGWAMGTMLILKTI
>comp5264_c0_seq1; 473 49 882 LEN=971; translated
MNTAAELQANRPDGSAAEAGVEGPRKYLIGGNWKCNGTWDENLDRVKMFNECGSIPANVE
VALCVPDINIPQLLTGLRKDIAVGAQNAGVNKGNGAFTGETGSHQLKAIGVTWVIIGHSE
RREGFGMAGETEELCAEKCKVAIDNGLKVMFAIGEKKEERESGVTMDVCAKQLEPLAKIL
TAEDWANVAIAYEPVWAIGTGLTATPEMAQDTHASIRKWVAENVSEPIATATRIQYGGSM
KGANAEALLAQPDIDGGLIGGASLKDDFFNVINGIPK
>comp5266_c0_seq1; 308 518 2851 LEN=3017; translated
MDVDERGTEDGIPTTPTDEGSALSPLAPPTPSRPLEVGGGSVFQGSRRPRFGGVMSDGDA
DVNEETENDINDENHEGDFLDAQDGFATPRGTDIDVAEAAQAFRGFLRGFRGLRGHDDSD
NEIDDNSSLPPPVYSSELESMVQRTGPASLDVDVMHLCFFDAACQKLYHQLLHYPSEIVP
LLDLVVRREMEELRAGHQEQHESHLPHEPPVVQIRPFNLREVQGLRCLDPITVDTLVAIQ
GMVVRCSPVVPDLRIAHFSCCVCGHAHNVAVDRGRISEPAVCPSCGTRDSHRLEHNRCSF
ANKQLVRLQETPEAVPAGRTPASLAVYCFDDLVDAAVPGDRIEATGILRAQSVRVNPRAT
RVRSVYKTYLDVLHFRRITGMAGDSSEPPRLQSQRRELKIQDPQRRQPLPRRVGTASGRC
KLLTSERKEELVALSRRPDIYDILTRSLAPSIWGLDDVKKGVLCMLFGGTQVRRVNDGEN
HDHDDTRPRKRGDINVLLCGDPGTSKSQLLAYVHKLTPRGIYTSGKGSSAVGLTASVVRD
PETRDPVLEAGALVLSDKGICCIDEFDKMSDATRSVLHEAMEQQTVSVAKAGIVSSLRAR
ASILASANPSESRYNPRRSVVENLRLSPTLLSRFDLVYLVLDNASPDADRRLARHLVGLY
QETPTAGEPPLDTALLRDYIDYARATVHPRLGDAAEEELLERYLKLRKIGTGNNGTLAAT
PRQLESLIRTSEALARMRHAPLISGADAREAARLLSVATLASSTDPKTGRIEMVEVP
>comp5267_c0_seq1; 524 370 1680 LEN=1746; translated
MYTLHPIPPIRIESETTTTGIGGGGDNKSFVGSVRYQQQVAAPSESYNRGNANTSKNTAT
NPQHHRSTESPIFRCCNDTPLSSPSSSRHLSAPCCRLNFNPSADFSETTRTTTTTATATT
ICTTDKSLSSSSLSTSPMVVRGSRPSDCGAAFSSSSSNLAASSASSPYFMRFQQQLPLRP
RSNTTISDESDMKEIITAGEEAKQTNEPNFEVVAGVDGHADSDSERERGEHFTSADEAGP
LEPGISSSIGSSLFRTTRYNSSESDDSSDGNAYNTAVFDPGLDFLRKNYNYRTACELSRD
PSREEVLPGRESASKSTAVVFCEQDGSKHPRQAPTSGSTRQSLGSIGDDGTSCKHPEALA
MKLHRKHTRATYVMGGAAVGTMLLPVVGTVVGGALSGYSVNLYLKRKEKHAQRKWDRDNF
QTNADASPVAKYAVFV
>comp5268_c0_seq1; 208 212 712 LEN=1306; translated
MAPRQRNISPPSPATSEDEDSSSFDDGLKAPLIQGEHPAPAMTANKVDATTRAPPKSLRI
TEADIARDNHDWFNVVALPVVVIACALNYEYPSFHYSGKYFWSMWAATTMYFFLDFSWVT
IVPTCVKSPVVIQKHHVVAMIYLLGPIYYPQYRWLMGAILSVEVNTW
>comp5269_c0_seq1; 628 1 2026 LEN=2669; translated
XGGNGWILSVLATMILLAAGVNVYVSVSQDFSATDWIAGASTRDMPHIREMTSTTHILST
AENPVSVPQNTAAATEPTGLERNERTATKSLATTHPKELKTNSPTASGSSSGSSASKEPA
PEPAPKQTPTTKTVAQTVDEGQDEQPDDRTGSNNSILDEIIANFTEPKIKLLKSQKEMDW
FRKRAILFRDEIIVAKKIRSFYSKATDDPLSRSGDPDHYYAIDQGNNTVAAKRIYDENKL
AEFNKMNNISGRPDPSEGPYLDFMVAGNPKCGTTTLMANLRRIAPMPVKDVCTGATHTLR
FAYLNWPNDPNCNALVTEHLGPDQTRWMRGSKCPRLLNSNLLLGHSALLPSTKLIIGIRH
PVLWFSSFSSMREDAQSKLQTTYDNIELCKCPVNPLTGVRPRSCDSHPDENCFGECRCGA
LMCLHRSRLHLTLARLGKTALSKEELKLLAPNDNDGGENLFNAKIQNPVFLYDLTQMRND
TYWEDLAGYLGVSNVPNHDYRGSHGVRNRHKNLIRSSAKDYKKENLLCDPHLDFFRSKIM
TYSYELGMWIQNYWLPVAADPDRPDVVVAGGTSNTSATDEFWAIAETYKVDPCGRLVRRP
SDGLYVLRGDVGDETNSTAVEFPTGTAPLHPVYGHLNTTAVPQCRSHKKAQQKRTKDEKS
FGTTGLAPSSAAGRT
>comp5270_c0_seq1; 475 139 1020 LEN=1174; translated
MVLYIIGLGLSDEKDITVRGLELVKQADSIFLEAYTSILGGVPKEKLEAFYGKPVTVADR
GMCENDADELIIDPAKEKNIAFLVVGDPVCATTHTDLVLRAKEVGVKVEMVHNASIMGAA
GACGLQLYNYGQTVSIPFFDDKWRPTSFYPKIKYNRRGGMHTLCLLDIKVKEPDFDAMMK
GRTEFLPPRYMSVNVAAEQLLEAEEKLGEGVYDGKKTLCVGLARMGQPTQKIVAGTLEEL
TQADLGGPLHSLIICGELHDLELDVVKEYLIEGSTYQREFEDKSQDNENGAEN
>comp5272_c0_seq1; 1194 1 3274 LEN=3359; translated
XTCKAVPSSESVLKTNIASTSPNKFSLLARRFWTSKALELAIGIGMRIIGEAVMAFLLSA
LALRGIGVSSFSLRQAVSRTSTTLAAKGGGGGGRKKRPKNPFASTIILPDTGFNQRANAV
KREPEIQEFWKDYGLYDKLSETASAEGKERFVLHDGPPYANGSLHCGHALNKILKDFINR
KQALNGKQVHYIPGWDCHGLPIELKVLQTMKSKERQGLTPVTLREKAASFAKATVEDQSK
SFQRYGIFGDFEKPYLTLQPKFEAAQIRVFGEMFQKGFIFRGRKPVHWSPSSRTALAEAE
LEYPENHVSKSIYVGFEVVEPSDGLKEYHSEDEPVKVAIWTTTPWTMPANLAVAINPELS

YSVVNHASTGKLVVATDLIETIANKVGLPEGESFEIVSTMPGSDLVGTKYTHPLYDRNSP
VLEGGDYITTESGTGLVHTAPGHGQEDYLTGLKNGLELLSPVDDAGKFTIEAGEEFKGLN
VLGEGNQAMIDALTTAGALLKVEDYGHKYPYDWRTKKPTIFRATDQWFASVEGFRDSALK
AVDTVQWVPEIGKNRINSFVVGRGDWCISRQRSWGVPIPVFYDRETGNEVLLNDSTLEHI
EKIFAEQGSDAWWKLDEVDLLPEELKAEADKWKKGTDTMDVWFDSGSSWAGVASERDELA
YPADMYLEGSDQHRGWFQSSLLTSVAVNDKAPYKTVLTHGFVLDEKGFKMSKSLGNVIDP
LYVIEGGNNKKLEPAYGADVLRLWVASVDYSGDVGIGPNIIKQTFESYRKLRNTARYLIG
NLADFVPEGQPDSTAVPYDELPSLDKWMLGRLSASLKEVDEAMDAFEFQRATQELLRFAT
AEMSNFYLDVAKDRLYISASDDFRRRSCQTVLHALIEGFAKGIASILPHMAEDIWQNLPY
EKTTESVFEGGWPAHLAEFEQFESEKWDLVRAVRDDVNKMLEGARNDKLVGASLDAAAYL
YMADEEKRKILESLAGDVSLVVPPVKTNGVDELRTTLMLSQIHIVDSPEKISESCDEKYI
STADTISGCVVGVAKAKGTKCGRCWFYDEQIGKHGLPHDDACQRCNDAIAVFEKTTGEPF
VRPPVEEPVEA
>comp5273_c0_seq1; 134 1 555 LEN=1066; translated
XTAKHKTENCQQQRNQQNTKVTIATLYGIFQDEHKCKKTTFKDFKRLQNDPPSGVTGAPM
DTNIMLWQAVIFGPDDTPWEGGTFKLVLEFTEDYPNKAPQVRFLTKMFHPNIYNDGQICL
DILQNQWSPIYDISAILTSIQSLLCDPNPASPANSEASRLYNENRREYNRRVREIVEQSW
QDES
>comp5274_c0_seq1; 473 561 2489 LEN=3004; translated
MAEKNHRVSDSSLLLEEKSMNSMRIESNVFRMQSSIADLSIDLSMTNSGSGMRCGINDPS
KVSLEIHKQTRSDPNDPYDSPQDYCFPRGLPFPNQSTEERRSDTAEYQHCHGLHQQDVHT
FPHAPNLHAPSPTFCQRPMIGPQLTSQQTTGCTAQSSFIFASSRINFAASPIVKSIRQDV
RSMTEGTRLVPVHEGQPNDRSIRFLNTTEDTLQTRVILGTGSFTQVTSVTINPRRNNDSF
RQGSQTNHEYRYYACKALKQELISTGGDEFRHAASQLAYEAHVLSCLDHPNIIKIRGLDD
KGVLGFERTDRGFFLLMDVLCETLDQRIDRWGKSSTTYAMNMTGSNFHGNHYGKGNNNVS
ALLRRHLDKVVICLELASALEYIHANHIIHRDLKPANIGFSSTPCPNHPEGEEIYTRLQL
FDFGLCHEFTPSSRTSNTLTKMVGTMRYMAPEVCLEMTYDYSCDIYSYAVVCWELWTHRT
PFEAMTSDLYRELVCRRGFRPYPDQSKEEYDSLRFGWSDPRTKRESSMRETLNNKNLPIP
REISVLLSQAWTPDPQSRISCSRIQNQLKLYKTLVELQLEAQDLSESANNTDFDEGNNDH
DNHDVHEYCKNYDEEDAQQAFQTFVTDNVNYISDRASWKCCD
>comp5274_c0_seq2; 473 435 2363 LEN=2878; translated
MAEKNHRVSDSSLLLEEKSMNSMRIESNVFRMQSSIADLSIDLSMTNSGSGMRCGINDPS
KVSLEIHKQTRSDPNDPYDSPQDYCFPRGLPFPNQSTEERRSDTAEYQHCHGLHQQDVHT
FPHAPNLHAPSPTFCQRPMIGPQLTSQQTTGCTAQSSFIFASSRINFAASPIVKSIRQDV
RSMTEGTRLVPVHEGQPNDRSIRFLNTTEDTLQTRVILGTGSFTQVTSVTINPRRNNDSF
RQGSQTNHEYRYYACKALKQELISTGGDEFRHAASQLAYEAHVLSCLDHPNIIKIRGLDD
KGVLGFERTDRGFFLLMDVLCETLDQRIDRWGKSSTTYAMNMTGSNFHGNHYGKGNNNVS
ALLRRHLDKVVICLELASALEYIHANHIIHRDLKPANIGFSSTPCPNHPEGEEIYTRLQL
FDFGLCHEFTPSSRTSNTLTKMVGTMRYMAPEVCLEMTYDYSCDIYSYAVVCWELWTHRT
PFEAMTSDLYRELVCRRGFRPYPDQSKEEYDSLRFGWSDPRTKRESSMRETLNNKNLPIP
REISVLLSQAWTPDPQSRISCSRIQNQLKLYKTLVELQLEAQDLSESANNTDFDEGNNDH
DNHDVHEYCKNYDEEDAQQAFQTFVTDNVNYISDRASWKCCD
>comp5275_c0_seq1; 794 525 3659 LEN=4141; translated
MPSQSRGFFACDYCGLQHDTLEEAIEHEQTQCPQALQAAARTRPIGYLNAQPQQGLPQQY
PSYYLQQQQQQQQQQQQQQQQQHRHGFTTGMGPLVSSTTQGAETYPYARSAHYGEYGRRI
SQAPAVPGMEAAYSQKSGEGILASTQTTSRPRCISLFDSPTDPGERLNRPDVTLIQNIEL
FEATPEVIAEYERTSGDTNRIEPNQVGIRCRNCGSGSNNSSGSSRSTISGAILFPIDLET
IGECARSISDRHLISCEMTNPDAQRELGVAASARGNNENDDQSSKKLLVDYCVQFCREVG
ISKRGNNKLGIEFIGSSATPHGESFVPPSQQQQQYLTTMQTRPLGVGTGGHGTRQRSQSD
TPPANFPFYQENDRTWHCRYCSYIPHHLRDPKSIWSSPSGVHPPNSFVDQHLSLCRAYRQ
QSISSMPPGRGLLPSEQSPFGVLPSYANPPSMQLGMQGQISYPPVSWEATTALGGMGGHM
QYPPVGLLPPHQDPSNYRYGYLPPGLVQPHSLDNSLGGRSGGRFLPMGQGDNRGGYSERG
GAPIMPSMQAAGAMGPSYALGSGDNTADSMKDAMSFLASYDADYYARDPASAEIPKLVLD
EDHLLLTDYFFWLMKQLRLVRFTENDRKTRGGKREKIKVGYGGLQCIHCADLPMSRKFFW
SNVDRLANSFAEIPGHVLKCRRCPQANKDALMRLKKGHPDQMSKLPRGSQKIFFRRMWRR
LHEGDPQDDDVVVESTARAHAGSESKPPVPTIKTSPEKSIEDLSHSGSITVTSDETILVI
QRSAKEAAKALAESTLNKSGPLSPSSRILVAIPEDKEWLSDIDSYVRKQLEVFCATEADV
AAAKADRKYPISVGQVGIRCIHCAISQGNDAVGHAIAYPYSISGIYESVKEFHRLHLDSC
PNLPATSKAKLKSMKGASSLSSVLRKYYTLAAKALGLVDTKEGIRTGGESTPINSVAAFT
FSEVDDAEPLTKTKEGGEKETEPNLHGTNTQAIRRNETGESTNEHFEEAAASTSVGMKRS

GSFTSPEMESQPKMAKTDGGPTEV
>comp5276_c0_seq1; 553 82 1692 LEN=1948; translated
MTNKLHLLFGCYSFVAVLSALSSNGFIVKCTESRSGRIIHTNHIAESVVRLKVLQSPELD
SSNEKEADGSEKKWWEDFTFENGDVAARCRPDFEILSSTTIGDDNSIEPNGNGNTDSSDE
TGKINKKRLIYLDSGATSQKPAQVIQDTAEYYEHTNSNVHRGAHTLSRKATAAFEDGRDT
VASFINANSRNEIVFTSGATEALNLVASSYSRAIKLKEGDEILLTEMEHHSNLVPWQIIA
QETGAVLRFVKIDFENTGCLDLDHMETLLNENTKIVGFQHVSNVMACINPVKKMVAMVRA
KASPNAIIVLDACQSLPHMKVDVQSLGVDFLAASGHKMCGPTGIGFLWGKEELLNSMPPF
MGGGEMIDQVTLEGCTFAPAPARFEAGTPPIAEVVGLASAIRYLNSVGMDKVEAFEHEIA
AYLYDRLSEVDRVTVLGPPKGVERAALCAFYVDGVHPSDLSTFLDMEGVAIRAGHHCCQP
LHQACGISHSARASLYIYNTKHDVDCFIEHLENTIKFFTGLQNESEGQDDDFVPFI
>comp5277_c0_seq1; 430 103 1176 LEN=1956; translated
MKIPTPSIFLALTTCRVVDGLARGPIRSQLSKRSLVDTTPIRRSITPEVSSENRVRNEIE
QTSKPAIQSRKQALRSIFVASTTCVLSSTLATSPSVAYDSSFPVELSDADDKSKFVTIGK
RINSQQRKENAEMAKVKMDQNLASFNLKNDFLPSLTWGLALFFASGSRSNPLANPLANII
YDEKEEKWLEDRNAGLFSPLPLPFLFLLGFVFFLLGTVTQYIFLQLAEGDSGVCAQLAGV
ALINGGFFEFGRIASGEKRMTRDEKDRAVELNEEFGEFAEKRLQSGGSCHRSDVVKSFRR
YFAKYRQADSQQYPLTDLEIEKLLRFWNETQNERKAEMTSSGFYYGIQINSDADVFA
>comp5277_c0_seq2; 248 270 566 LEN=1346; translated
MTRDEKDRAVELNEEFGEFAEKRLQSGGSCHRSDVVKSFRRYFAKYRQADSQQYPLTDLE
IEKLLRFWNETQNERKAEMTSSGFYYGIQINSDADVFA
>comp5278_c0_seq1; 400 1 754 LEN=1160; minus strand; translated
XVVAAVAVAVVVATVVAAFLPTRGRPLRLGRHDHPRLGKEVDDESVPVELVLPERLVVVG
EHLSAKDESLLGRGDLVDPGRPPLEGSDRPSGRAGDRESLLGRVEDGDLHVRGGIGFPAL
AFNVAAAAAAAFERMGFGSAGGSQRLVVKGTVVRGGSVHRVVAGDAGVVPAAVAPIGGGG
RHGGSVVVAAPSDRPVIAVGVIRVRVRGDVNVDVVVDVDVSVDVNAGVAGVLFSWTKHKD
GCRVVLVSDFG
>comp5281_c0_seq1; 291 300 1328 LEN=2484; translated
MQAATQKALPLAIRHVNATDTNTPQKSNYYPSPAKTTTIHNMTTADTSPVESSSQTCPRQ
VLLHENAQQLNNHAANFMEVGKLDEAIDALALLLESLRDFEAEPAGDETSRVYCLDECIK
QPLETGHTTPIASILRLNRHRNDPPDRKRPRGFNFIDRALPCIDIHYRPIRVITGGQSMG
GNFHAIVTYNTALAFHLRASFMTTTIAKYEVSTQRQLQRLQQLELRRALALYELAYEIQR
DQNTRGPASMVQTNADESKGSVYFQSILLNNVGHIHLLMNNPVNYKRCVEKSISIEATKL
VRAETENDYGDKKKNNDSFLRKRSSPATVSFDEERNSSTIAM
>comp5282_c0_seq1; 252 1076 2221 LEN=2379; translated
MEGNRAYVRLQCYDIDSAHPLVEAHARMYVLDHKLKMHPLRLMDPNDDLGGVLYPSVPAD
IVHHIDCHSPLSPQRLPFVERSNGLVLRTLDSSTGNRDQIICPVCGEAYGTYERLKKHVE
YNAMIEAKEGDYPTERSHVGFQFPVIKPLTLDDVRSHIEETLSEIIVVVEAIDPQLSGTF
QSLQSYKYEDIVFGSEFAKCMSVNKERTEFLVDMQQFHKTYKNNPSIKHNIFDSIKRTKS
DKTDPSTEHRVFDSMKRLKSDKSIENRRFLLSHISEDTFEDDVSEYHEQDIDLDDDEVSL
ENGGTYFSRSYNTFSSLHNPSGNLASIFTGRFHSSQSTRRSSTSSILSTNSSITVSEEEI
NTSMSQKRPTYDRLPSYSSHV
>comp5282_c0_seq2; 252 1195 2340 LEN=2498; translated
MEGNRAYVRLQCYDIDSAHPLVEAHARMYVLDHKLKMHPLRLMDPNDDLGGVLYPSVPAD
IVHHIDCHSPLSPQRLPFVERSNGLVLRTLDSSTGNRDQIICPVCGEAYGTYERLKKHVE
YNAMIEAKEGDYPTERSHVGFQFPVIKPLTLDDVRSHIEETLSEIIVVVEAIDPQLSGTF
QSLQSYKYEDIVFGSEFAKCMSVNKERTEFLVDMQQFHKTYKNNPSIKHNIFDSIKRTKS
DKTDPSTEHRVFDSMKRLKSDKSIENRRFLLSHISEDTFEDDVSEYHEQDIDLDDDEVSL
ENGGTYFSRSYNTFSSLHNPSGNLASIFTGRFHSSQSTRRSSTSSILSTNSSITVSEEEI
NTSMSQKRPTYDRLPSYSSHV
>comp5283_c0_seq1; 207 125 1153 LEN=1233; translated
MKSRESIPNQSILLRYFISAVSVQVVLQLIYIDFRISSTLTHNIFHSAPDETSANHTSLR
DVSGQVGVSHHDTGAIPTIEALASTTTFSSTLGNLYMKYGKEIADYRPIMRKWCEETKSC
KFGDYEVEMLYMLVREHKPQNVFEMAPNRGYSSHWILHALHLNDATSKLHSFDIHDTSTK
FMSPDYQARWVFTLGDYSELYDEGKLEMDGYDFLFVDALHEPEFARGYCQRLFANHIRPN
TVVAIHDIVADSSGRGRESEEVYKYLAMANNARNVFTYSRFAMPNSLYAPQTDIVLPKIN
KIRSDLGIIPDCGTPENCAKHDQGFMYFENNDAPTIFFTLN
>comp5285_c0_seq1; 486 25 1179 LEN=1516; translated
MTLLRERETTIRELKEGSEELEEKLQRLQKSLSVYESTIESLKGKETETEVELEKVTEAL
EKHDARMGELEETDHIRQKATEEMANATATAAASSAKLILELQEALKERDEEIAELEADL

EEFESEIERRNNSQHQREQPQSGELRTLLEEKEKRIQQLEKERMARAEQSRSLQEKEISE
ESMEIIRDLQNLLCERDMKIESLKADHAKEVHQLRGELEETARNHAKDMKVLVNRTNALQ
VSLEEKMNRVAALEETNRELSGESYGSDPMANAVEEAARNSQRMERTLSWFSPSTEKKAQ
VSCVPESLDSSTAFLTTPCTSTSSSDDSTTEDCTSSDEEKEPDRRMVGAPRLPPLHSTEQ
PEILDDQGAVTGRRSSFWSMMRNQ
>comp5286_c0_seq1; 1489 308 2941 LEN=3210; translated
MPNNKKNGQVKMSLGDFIGGEPTGMAALPTRPKERGPDDDGSFKRRSDYDNNRRDQEISR
SDGDDNWRRGGGGDSFRGGGGGGFDDRRSGGYNRGGGGGYDRGGGRGYDRGGGGGYDRGG
GGGFDRYDNNRGGGFDRGSGYDRGGGSGFDRFDRNDRRGGYDNNNDRSGFDSFRRGGADR
GAPPGDRRGFSSSQIGGVGSAGMRPRMQLKKRTVPLPEPQKEEEKKEVAPVEDKTAKEEA
VVEATQAPVTESASEKDETPVADVAAPEPEEDGWETVEGVKNKSSKDASKAEKKSSEGGK
KNSEENAEKRKKKREPEVVNSRAAMLESAPTIKRDSNDANNNRRDGGRNRGPPPVVNKRF
EQLADEERGKQQEREARRGPPPVADSRFAAAAEADEDMRRSRDEFTRSEGPPAVVNSRFS
AAVEADKDYNSNRNDDFDNGERGPPQETNSRFAAAAEADRDRNYNRENNYDRADRGPPPV
ANSRFAAAAEADRDRNYNRENNYDRMDDRDRGPPPVANSRFAAAAEADRDYSRDRGDYDG
GRGDRGPPPQASSRFADAMENDRDRSYDRQNNGRMDSRDRGPPPVANSRFAAAAEADRDY
SRDRDDYDGGRGNRYHDDRRGFDGGRGDRYDEQPRYQSRVQEPPKSSVADLLKPKARPME
ENILKVPTKEQASNFLAPPSKAQSENVLKPPTKAPAASPKAAKSKAPVAAPAPAVDHTAL
LAEFASGKKLGDDLKAWIESQAVIPSVEKLVFHLLTETQPHSPDVECEWAAPEKYGAALL
SLVEDDLLKQMGVLFAVQKYCDKIGMPKLDDEYVVQSMFRSMYKYDLADDETFAMWKEDE
SPENEAGKLKAVIQTVDWFNWLEEDDDDDEEDYEDEE
>comp5287_c0_seq1; 574 84 1445 LEN=1494; translated
MRFTSSIVCAIAAFAASSTAFAPQARQGVSTARFSTAEEVGVSQVERNENFAKLAGGYLF
PEIGRRRNAYLEENPEMADRIISLGIGDTTMPIPPHILNGLVEGAKKLGTAEGYSGYGAE
QGQGLLREKIAKVLYNDLIDASEVFVSDGAKCDIMRVQQMFGSKVVSAVQDPSYPVYVDT
SVMMGQTGVQNPETMQYDNIVYMKCGPENGFFPDYANLPRADVVYLCSPNNPTGAAATKE
QLTEMVKLCKERGSILVFDAAYAPFIRSENVPKSIFEIEGAKDVAIEVNSFSKYAGFTGV
RLGWTVVPSGLKFKDGSLVRDDFNRVMTTAFNGASNIVQAGGLACLDEEGQKEINTLIDY
YLENAKILRDTFEEIGYVVHGGTDAPYVFVELPEEIGGSWDAFSMILEKAQVVTIPGAGF
GPGGEGFLRLSAFAPRDSVIEACARLREVMKKD
>comp5288_c0_seq1; 306 252 943 LEN=943; translated
MAFSLCGSVSSLLFDFCFIWCLAMASGMTKSAPITKEPSSQRTTTRFKLQFDGCLRPPRD
PGFPTIPYRMAVCSACVWVVHSRNNSDWVDFDRNDVDDNDETLITPLAVGARALPVSTET
TSQHAEYEGLLLGLQWMVDFFSLPDWNIQYTFAENSRALENISGIDSYTSTMRQPQRVLI
SIEGDCKTVIDQLSGKAIPRKLESLHVRAQSLLDELTNRNVVVINDDDDDX
>comp5289_c0_seq1; 117 1 292 LEN=363; translated
XEAKSRFPENLRIDYALSREQTNSKGGKMYIQDKVEEYADEVFSKLENGAHIYFCGLKGM
MPGIQDMLKGVADKKGLNYDEWLKKLKKNKQWHVEVY
>comp5291_c0_seq1; 207 82 501 LEN=633; translated
MINRIPVLSAIISSAVIFVSLATDFTEARLGNDPGRGLLPSKFTSSSASEVRDADLQAGN
SLDLRQQEINRHEEVPINEGVAVPNENEGCDVPCSDGSGRITVCHPRAGGFVQICTERES
FDDHKAKGDVCGLCISADM
>comp5292_c0_seq1; 508 196 1728 LEN=3188; translated
MRFFLLLVGILCSLSTPSWLVEGFGQQQRLPQTTLARSSVGNTQVFLGPPKIDEWEILRS
GAVRGTVSNHPELPDGYEITTSPLETKPVAIKDNTVVVTLSGSKYRLLKGIKTAASKDAK
AAPATPRSTMSLFGGTAKKEPKPAPAAAKAKPKPKPRAAKPAPVAKAKPKPTPKAKPAPK
APVKQAPKAKAAPAVDYNLNGKVVGGKGKCAYLLVGTQIRSSSKRSQIFYAYEADSDGVT
PTGPRLTVKLANSRERLAAESRNYDRVFSRGRVAFSLQGSASCFVKKVALIENADDSPLT
KGIPRGSSALVLESGDQNLRTRALESGGGLKGGDLRKAAVNVCRCVESMHSSGLVWTDLK
AENFVVLAGSGEVRGIDLESAVDVRSAPADYSPEACPPEFAAAEKANRGYEFECLKNYDT
WSLGMLLYELATGNNYFMGKSEDLITRNLATLELVDESTGRVKGLDAIPDKNLQDLVDQC
LSVDPGKRPSIGQILLHPYFLTTGFGPITF
>comp5293_c0_seq1; 309 335 1300 LEN=1636; translated
MKFFGTPQAAMAAALSVASSTAFAPMPALVHGPVPLSAPHQQRRRRGARGTTTGLASSRQ
EDAEDRIVSLKKSWSDQSGVCLPSEETEAPPALAMLGNLKKKYWKLPPAPEDQFIMTGDI
FTLFLYAFTSHSINDAIVGGLLEDPSLTIPEAVRELDPLQTVVKLQTPVWVDLASNPNVP
FGNPSVDHALEVTARESLMNHWGILLSTEGSACVALCTCWLVAGYLHRSFSFDNSTYCGG
DKALTKTVETWATAALLLAWCAAGAEGLAGHFPALQALVCGQCHPPSQVVLTQSDLLFVV
DSLTVLIAWRWSAHRILNTFR
>comp5294_c0_seq1; 757 188 2323 LEN=2423; translated

MKTPTLLLSTLLSSSRAFNNGRYLVQHSLASQSRLYSQAEKKNNPVSVETKDDWISDSLS
DADDLQGAGHLSPSTSASQLSQFVQGPKNCLIFDTTLRDGTQGELVSASCDDKLKIATRL
AAFDVDYIEAGWPGSNPKGKKSNRRNFEALLDPMDGTHGXYDFLTLGAMQTDLEFFKRAK
AELTEHARSKLVAFGSSRRKNTAVEKDPQIQALVESDVSTVCMVVKAHSWQVTEILRATR
EENLQMIYDSVEYLTNLGKTVLVDLEHFFDGYKDDPEYAIACCESAVDAGARCLVMCDTN
GGSMPWEVTDTTTAMVKHFEPFCTIGIHCHNDSGMAVANSIASCHAGVGLIQGTINGIGE
RTGNADLCSIVPSLALHVETDMTCKDKVPELTKLSRFVDEVLNRTPNTAAPYVGRSAFAH
KGGLHVAAMERSPLSYQHVEPESVGNEMRVLISELSGRQNIMGKMQDMFGSEMDVGQKSA
RSLAVLNRVKELENMGYQFEGADASVHLMILYSTQGYCPPFQVLDYSAQCYDQTMDSASR
LMTTRADSSTARATIKVRTVSDDPDLEGSGLSFVENLEVSDGAGPVDALAKAMLKALTPS
HPSLESIELTDYKVRILNPNAATKAVTRVLIDFRDAETDQKWTTVSVDSNIVSASLNALV
DGFEYALIEHSDTCMLCDDFFAYTKNPSVPSWWLASTLHLPKQRNEFATAI
>comp5294_c0_seq2; 760 188 2225 LEN=2325; translated
MKTPTLLLSTLLSSSRAFNNGRYLVQHSLASQSRLYSQAEKKNNPVSVETKDDWISDSLS
DADDLQGAGHLSPSTSASQLSQFVQGPKNCLIFDTTLRDGTQGELVSASCDDKLKIATRL
AAFDVDYIEAGWPGSNPKDLEFFKRAKAELTEHARSKLVAFGSSRRKNTAVEKDPQIQAL
VESDVSTVCMVVKAHSWQVTEILRATREENLQMIYDSVEYLTNLGKTVLVDLEHFFDGYK
DDPEYAIACCESAVDAGARCLVMCDTNGGSMPWEVTDTTTAMVKHFEPFCTIGIHCHNDS
GMAVANSIASCHAGVGLIQGTINGIGERTGNADLCSIVPSLALHVETDMTCKDKVPELTK
LSRFVDEVLNRTPNTAAPYVGRSAFAHKGGLHVAAMERSPLSYQHVEPESVGNEMRVLIS
ELSGRQNIMGKMQDMFGSEMDVGQKSARSLAVLNRVKELENMGYQFEGADASVHLMILYS
TQGYCPPFQVLDYSAQCYDQTMDSASRLMTTRADSSTARATIKVRTVSDDPDLEGSGLSF
VENLEVSDGAGPVDALAKAMLKALTPSHPSLESIELTDYKVRILNPNAATKAVTRVLIDF
RDAETDQKWTTVSVDSNIVSASLNALVDGFEYALIEHSDTCMLCDDFFAYTKNPSVPSWW
LASTLHLPKQRNEFATAI
>comp5295_c0_seq1; 263 77 745 LEN=866; translated
MKAKELKILRKALVKANRCEELLKGFGTETVLSVNNNANYENKLSECSGHNRKYCISIIF
SLSRQMTENRFKECLGLFEKNMAEMYINSSWGLNMEEKSAELKDDKARFLLALDDDKKLS
GFVHFRFEYDDEESPTCGVLYIYEIQIESIYRRCGVGRRLMRMTQRIAKEQSMTKILLTV
FKKNRQAMTFYRDKLGYGIDESSPSKFGDHDVDYEILSLKIS
>comp5296_c0_seq1; 560 1 1900 LEN=1978; translated
XQSLGHATPLTRHFLSNRFRSDLNASNPLGTGGKLAHAYEAFVKDAWMRAGRGAVSPTAL
KRAIALFAPRFANRNQHDAQEFLAYLLDGLHEDLNRIRRAPYVEMPDVTDGQNTTIAAAE
AWNAHRRRNDSLVMDAFYGQFQSTCVCPRCNHVSVSFDAFNHVSLEIPSSGAAWATVAVP
VLVHFSDGNRKPVRYGVTLRKQASVLDLKRAVASLSKIPPEKLIVTDVYENSIYKLLDDK
QSILSIKSTEDTIVAYEVTPYTVKDTLHMIVTHSLSTDPSRSGEAYADQTTSGAVGGTDG
GKGAGSDGNGDGPEENYFGLPFMTSIDAKNSSCRDLWQLLRGLVRRMVPEFTGPIAGEGG
SRPEKVLVVHVVDAQGRPRRIVPKMGASGATSVLPECSKTLLSSILGEDCAENFLFLNLV
WKPLASNKKNGSRIDPKRFLDFDIDPTLTEAIRRQQSTSRSSTKQGVTLDQCFHSFSRPE
RLDRNNMWYCSRCKDHVQALKTMKLWRLPNILVVHLKRFEFKHALRRDKLTTYVDFPLAG
LDMNAHCARSNTTTDKNDFANSYVPADYDLFAVVNHYGRLGFGHYTSFAMQWDETGISKE
WNAFDDSSVHPVEPSEVQSSAAYILFYRRRVFH
>comp5297_c0_seq1; 897 235 2604 LEN=2740; translated
MGHSRKLIRAISPTISDHMRQNAAQRRTILGISKALDKRLYRMAKGVMPPISQTEQVALG
CGTIGFDRDIFTGNPSLQHLVDTYDPKLTDEEQAFLDNEVNTLCELLNDHDVSMEKDFNK
ECWDYMRDEGFFALKIPKEWGGKGFSTHAVSLILCKLSTQSFDANATVAVPNSLGPGELL
ARYGTDDQKEYFLPRLADGTLIPCFGLTGPHSGSDATSLIQSDCVVEERNGELGVVASFK
KRYITLAPVAGVIGLGLNLSDPQGLLEGKGTEGFTVALLERDHPGLEMGKRHMPLNAAFM
NGTVEGDNVWIPMSNILGGQERCGFGWHMFVECLAEGRGVSLPAGSAGCGRTVVSAIGAY
SRVRKQFRVPIAEFGGIQEALSQAGSDALITIAGTDLMNAIVDNHEAPMVVSSIMKQNCT
ERGRRIVEHGMDIAGGAAICRGNNNFMGNAYMSLPIAITVEGANIMTRSFQIIGQGLTRC
HPHMLELIYSLQAPIEEEDKAIKTFTSQFYKVVNHGATNFVRSVTRGVGSTISSMTRSKK
EFRDGDKLLAHHEKQLLRLSANFALTADLCFTLGGRLKFEELLMGRLADAMGAIFLGYST
LHHYSRNRGVDGLEAITEHAMLRLEKDAQDALKEASDNFPGPLGTVASVVMKMGCFPLGS
ITRPYNAPNDDLTKEVSRLLTTPSGLRDLFSEHVYMAPEGHIHQPSDLMRALPLCVEADK
IASSLRREKREPTQEETDIIAKAEALRDVLIQVDVFDHLTDAEGEEGYVRPALLGTEERL
ASLDQKRFG
>comp5297_c0_seq2; 897 445 2814 LEN=2950; translated
MGHSRKLIRAISPTISDHMRQNAAQRRTILGISKALDKRLYRMAKGVMPPISQTEQVALG
CGTIGFDRDIFTGNPSLQHLVDTYDPKLTDEEQAFLDNEVNTLCELLNDHDVSMEKDFNK

ECWDYMRDEGFFALKIPKEWGGKGFSTHAVSLILCKLSTQSFDANATVAVPNSLGPGELL
ARYGTDDQKEYFLPRLADGTLIPCFGLTGPHSGSDATSLIQSDCVVEERNGELGVVASFK
KRYITLAPVAGVIGLGLNLSDPQGLLEGKGTEGFTVALLERDHPGLEMGKRHMPLNAAFM
NGTVEGDNVWIPMSNILGGQERCGFGWHMFVECLAEGRGVSLPAGSAGCGRTVVSAIGAY
SRVRKQFRVPIAEFGGIQEALSQAGSDALITIAGTDLMNAIVDNHEAPMVVSSIMKQNCT
ERGRRIVEHGMDIAGGAAICRGNNNFMGNAYMSLPIAITVEGANIMTRSFQIIGQGLTRC
HPHMLELIYSLQAPIEEEDKAIKTFTSQFYKVVNHGATNFVRSVTRGVGSTISSMTRSKK
EFRDGDKLLAHHEKQLLRLSANFALTADLCFTLGGRLKFEELLMGRLADAMGAIFLGYST
LHHYSRNRGVDGLEAITEHAMLRLEKDAQDALKEASDNFPGPLGTVASVVMKMGCFPLGS
ITRPYNAPNDDLTKEVSRLLTTPSGLRDLFSEHVYMAPEGHIHQPSDLMRALPLCVEADK
IASSLRREKREPTQEETDIIAKAEALRDVLIQVDVFDHLTDAEGEEGYVRPALLGTEERL
ASLDQKRFG
>comp5298_c0_seq1; 594 137 1972 LEN=2434; translated
MGPRERPRGDEPETTESTPLISNATPAAIVSVSSEATPGGDSDPSSEARKHKEWLTTTSA
LSFSLLVQSYLLVSVFPYSGFLSMHLIEGLTEETAGSYAGLIASSFMAGRTFSSFEWGKA
ADRYGRVSVIKASLLLSAVFSLLFGLAGSFPMALALRFLLGVCNGLIGPIKTLVSEYAHG
DQTKETKTMGIVLGMWGYGFLINPAISGYLSDPAKQYPEAELVEAFRPWLEEYPFFLPNL
VGCGFCLVGYALTHAFVTETLPEEKRQPFRLVPSIGLWSWIEAKTSQNPKILRTVSSWGL
FKHLHSEDGEISENNWSVVVSPTGGQGATDGGGDTNESRDDGGNTATIASLLQRNSTRQH
LLVYWVYSFLIITLDEIFPLFCISKTSGLGVTEKIIGNILSGTGLVYVAMQYFLLTGLVN
RFGFYTALTVSAALSVPTGIWIPLSLVTNEGAAEGALNGASLAYLSIVYAFIRAFSSVVF
STITMLLNRTVPAHQRGTMNGLSMLGGSLAKACGPLFGGVLFSRSVNSVTPPFGSVLVYG
IMSALGGLLVLQTTFLRMHGDVDDEPAQMSSSPPPPKRFPEDDPETEFCITMDCNDQEDS
SREETDSPPAF
>comp5299_c0_seq2; 220 1 466 LEN=490; translated
XHNNNNNNNINNNFIINNNIDRNMNMNMNVDDWFQPQRQHQHDPHQQIATMPDDRSDSDC
IEETPLRCIISKAYSGNDLLWYMSKEVEVTDGGSRRRKTFSFYAPLLLDAPPDLPVYPSQ
TSMLVRQHQRWRGLAGSTTIQPPTVEVIDADDLDL
>comp5300_c0_seq1; 232 1 640 LEN=933; translated
XESSTIRRRKQQYNTILAGPHHCTNTGTVIKKNQAKMISSTRFCLLASIACLWMFSATAF
VAGPAVARRSALFMAEGEEVKSAPKVSGEELEVMLQDWDTPLVIDAYATWCGPCLLMSPE
FEQAAKELDGKVRFVKLDTDENEEMAARLAINGLPTLLFLDKYTPPEGSDEEASAKEAGA
QAVLKGRIEGALQKQNILDFCNFHFFDGPEPKL
>comp5300_c0_seq2; 122 1 351 LEN=644; translated
RKPTHNHRFDRERCGPCLLMSPEFEQAAKELDGKVRFVKLDTDENEEMAARLAINGLPTL
LFLDKYTPPEGSDEEASAKEAGAQAVLKGRIEGALQKQNILDFCNFHFFDGPEPKL
>comp5301_c0_seq1; 441 72 1574 LEN=1636; translated
MKFGKVSQTIKANISGVGGSGHKLNDGGNRDAVQNDQPREAIVVGPLQSEGNHAFISVRS
PWEWVMDGVCTQNGAGLVAITRVGASGDLPSGKLASIAAVAVASHPRQSSTSRRRQQRGI
NTAATDANCTLRVEIVSNRSTGHNSNHARPWSLEVFHCTSQAFDGLDDKDELARTLKRCR
CEDMNISPPSQLINWDVTKEECRNVVGNSLPHLSKESNGDSSTAIAVLKEPMGSRGTGVF
FVRDADEIHSIIEQHRQRAVSESGFLDNLIAQKGRIPSWVLQAEVKPCLLIRDRRKFHIR
TYVVCVETNVMLDEPLDGEEEEDLVKLFLYNRHEIRIASEPMPSSPCDAAGSSERSRGAH
ITETFDRGLLQDEPELVARNLHHKVENFVANAMEALYPDMERRVARSASALDGENEASRG
CMVLPHKFAIAGLDLLVTEQERIYLLEANINPAAPPPETVTEEFQSHLIGFFRDTMELVT
SGSKTSDRNNFFSTKSILRR
>comp5302_c0_seq2; 179 401 907 LEN=1396; translated
MTKEVIDAGGNVGASQAARLGKYFSLMMLVEVPASAVEPLEESLRTMKDLSASVCVAKDD
DDSDKVAETDSSGNTIGYQGALSLEGADHPGIVNKVTKILSSNGLNIDSLETTDELAPGG
GTVLFRMIGITHAYEPLSAGFDVEKIKQELEDLGNDLNCDIELIDLEE
>comp5303_c0_seq1; 350 1 1525 LEN=1795; translated
XGADVGADVGADVDVDVDVESEPTSASKSIAKKVNGNSREQSSAFCLLVKDDNDILPEWI
AYHYHVFKMRRLIVALDPDSETSPLEILQSFASETSFEGRFHLDFTIWNDKDYTPEFFQG
DKKDYSKIPITVKEEIHFKKKITDPKRLTAADIENRHILSTAWHRNETYNKRHQTVVKRV
AHKMNNHRFRQRIFVRECYRKIKEEQMDAYQVGQGEQQDSGSGLISWTVHIDTDEFLVPN
PWISAYVQSNSSEHLTNHSSVNAYEHKEDYEKLLNLTGYKPHDLVALYPKRPSDVSLWNF
FSDYISKLPRYKECIVIPRVLFGDKEEDDGTSISTTFANFSGMSASWNHAKFQSLRWKYH
ARFNSSIELSKTIVRTNTLDLTSNRGSEPVISIHRPLDSCPPQELSLQIYPKVRRVHKPL
VAYHYLGSQERYLSRKDPRRNILSFRKLNSHANDAIGDENYDADMTNTTQDHPSIWLEGW
LDAFIETHGADAVFTVLGNPYATVHMQQ

>comp5304_c0_seq1; 322 1 1202 LEN=2205; translated
XPFSIMRQTPSMIVVLPRAMRITFLLLLQQFYGAAFQREPHLHLRGVQYHSSNLGRSSCV
PSLVQINTRTCPVAISMLPTLPPSYEYDDSVSFFQRATGTRIRGRKELERSVQRWEKDFR
EAVDDELVRTILNDGGGNGMKLQLQPRLDTVSATTTSPTTLLVRWNLTYVDPSVQWMVSL
AESVPGWTVDLSTYTDKVEEERRFSYSAVGRLFARAIFTGCFRVPMACIEGVSTCEFVQT
ESGDTSSKRRKIRSIVEDLAYAQDLNRGVMANRLCARDLQFFLETVRKPPEYYNELNSAE
AKRSGGSGTNGVKQDDEYWEDRLASLLPWRSVPGMMDPLYIEAQAEEDIAVNLPVLFGVF
SIAVVVLFASWIAPNLIGQSLFGQPSYIVPPSELNEIIAF
>comp5305_c0_seq1; 330 13 889 LEN=1145; translated
MSFDDNGGKFAGGTFVSIVLSSVATNFVTNLATERITKAKCHEGDPRRIDEDQDCPEGDV
DKAKSKKPKGGNATKKSVRKKTSTSAAKKKNASVKKTGRKKQMITDTDKKTDDSRQSDSS
DDDPEWEAYHLHSEKSESDAFWNENYRRLQEFHSTHGHSGVPIHWRVEPQFADWVSRQRQ
FFREIQSGYRRPSIRDEGRWKRLQILNFPLDYEKWHWQQKYNELIEVLHGEKYDENNTNI
PETLKVWANHQIYLNQSDVHSRIDSERRRGLEILGVLKKDNRVEDCASYSD
>comp5306_c0_seq1; 635 144 2159 LEN=2248; translated
MKIAAATSVLLVAHAQRTEASGRVAETDKVQTATTREAVEPSGLLRSSARATLRGRHNEN
VLGEYKSKSERRNLERNKTALNKLLGNLEELKNRRKTLQRTSKNYEEEIAVPTSHEDQQL
VDLGILGKGRLLESSERTDKESIFDQNSVRDPSVATTADEETKDQGTSTSAPIDVFDGAA
EENDFFLANAHYLCEYENCNCGSFSFESMQGIIQCSSEVVATGGYCATTMNYCGEPVDVC
YRETSSLEAVGPQDYTYTVCSTFSKPYQQHICVTFDASAEDDNNINTRSEVLSDNNGISE
VVNYSQSSAPCKVEFNNERCSSCSTELRTYERIFVDQSIGDRHVLSSYQRRCFQVDCSNA
AAQDHVFNTCDTNDLSTLRENVVFGDDCTRCPPCGMGFHMTAGTAMGSFPVIGDYQCSGL
ELGAITGFFSRDVCGDIQSKAKEYCGCEPNFYDPNLVLPELLTEDIEAEKSRAIGGDGDE
LEISNPDNMFVLPGDTIGAESCHLCGANSAKIANPYNLVYLPNGKQTTCAALEGAGRLGM
MTVDYCKFIAMPLAFAECGGCLTWGKGEEDEEVDAGHAGPWTTSDEILRLNGNPVNETTS
PIPRLYRENPQELNVTSPLNATSPPKTSSPTASPPKAVSALSTDEESSAPIGSTMLSCFT
MVPISLLVYFS
>comp5307_c0_seq1; 652 1 1731 LEN=1873; translated
PTASPTASPTASPTADPTVTPPFAGIVSYEFPATETTDFLHGLCVKGAASPKKIAVTSND
ETLSYTKIPKFKEGTKLWSKFKQFVGPLGKDMCLGGTYLQPNEIRLIPKGTKISLEAPAG
FDKPVTICAFVEANGPKRDGGWPAPLKDDGFKVSEPFDWASGNEKDNLARFYCKEFTVEE
PEDELDRFELFAVGDGNKKPYGKLEATSPLTAKVFFEAHPQQFIKKNSVVDVALYAKVPK
GKSCDKGTLLSSADGYDVEVGELFEYPEYNKLYGGKGPKFPSTSNGLIGAAEFIISDFTA
PSGDSQIYFPGVGDGDGEINLCVQVSTQIDYRGNGKKDIVSRIDTHYAVSVDLTGDFEKF
EQTVKIQSTDASDLKDQIQSAVDVTAFLCKKGNKPIEEITFREGEDFRICVQPVVEKDTR
LDYSVVKFDEVVCSNKAEIRDVITKKGPDALTLVDKAPDKKGTLGFASVVTNGYLSKNED
SFLCAGKVTLRSKQKPQILPGRALRSEETVAEFLIQRRAEEEAADDENSEGGVFGLGIKI
APPTDEDAAYSAPASVASPVSMFALAVASMAMTLWF
>comp5308_c0_seq1; 365 1 1080 LEN=1173; translated
DDKSDKDDKDNRSKEDKDEKNDDKSNNDKSDKEDKVDKDKRSKENKDDSPTQSPTAFLTS
SFSSTSSWVSTDPDNDSPIGSPTQRPTQRPTRKRRNPTRNPTKNPTRNPTQSPTEAGTSS
STSTTTEIENAEIENDNNSNQINSQNDKDNNNTSNSSSNNDGGSNNGSSSSNNSAPFSFL
DLPRNLPDRWRAGYDFGGAASDPRDRSGGERDACVEDAPPYLLSLGPEPDRELQGPPPRP
FGEGLPPTELCCGSLAKYFAVASGKACGFLQVPSPSSSLSVAKSWDHAPGVLCVLESGGD
VRIVEGNGVGGGSLPAFCGREFPMGGGIVCVARESSPAVRERLFGSLRRGREEARGWPG
>comp5308_c0_seq2; 346 1 1519 LEN=1612; translated
EDADADPKEPKQRLLRGDTRLPVVAFLVGTALAAFFRAKGSLSRSPSPSRSLLLPAAAAL
AFRPMPGTARSRAFRRNGTGRAEAGSESELQAQAQASSPAAWGGDLHEEAGVAAELVANA
LRLCLELRPRCQEFTSESKSEGAFRRDKADGTPLTALDLAIQGYVLSNLHRSFGGDSFLA
EEDASLLRGAPDHKGGLSERSHGLASRLDPGLGFGEFLDAIDLGTNGVCGGGNRKGESDT
NGRCWILDPIDGTKGFLKGGHYTIGLALCIDGLPVVGVLGNPCSPSIESSTMVAVKGHGL
RYYRGGPCSGNNGSSSSNNSAPFSFLDLPRNLPDRWRAGYDFGGAASDPRDRSGGERDAC
VEDAPPYLLSLGPEPDRELQGPPPRPFGEGLPPTELCCGSLAKYFAVASGKACGFLQVPS
PSSSLSVAKSWDHAPGVLCVLESGGDVRIVEGNGVGGGSLPAFCGREFPMGGGIVCVARE
SSPAVRERLFGSLRRGREEARGWPG
>comp5309_c0_seq1; 384 1 1535 LEN=1572; translated
XQIRIHSTCVATVRQSLRAGQGILSSTDHPASTRALPDRLSEGIKAEEHGECTTDGGVIL
FADTQGIMFSFCERQRPRRLRILAASFSVVACAKATPPTGDAPASVSTGDFLFEEVPHSE
FSDCELGCANGGYCRFVHGTEEDLARAAMSGALVESCRCPEGFTGKTCQFLAGADVPPER
RCQGDETNAETQASFHGCDCAFADAVSDFAGAMCRSPVTEYCGGGFGVQSRASVSFCTNG

GRCRKDVIEATFLPKDPEGGRSSDGTPGCLCPRNFYGPHCEFLRMDPSDGNLVFAEVEVA
PDDDTRLSGGTDPSSTSPSASSLRSTVDYETATKSRGAATLVFLLAPVTIGLLALSVCVS
RTFLARHPGAAHRTGMLQRARLFLRRSNGSKDHKTGALSSSLSSKPWDGRSDTSSDCSDN
GYRHCCSYADDENKVFDQVNFNEEDELGIASMASRLDLGPIDEDTERPDAFXATNHEARR
VSMYRLHRVSKVWRLALIWTDLDGGLLQSSV
>comp5310_c0_seq1; 333 1 1426 LEN=1574; translated
XYACLLKFVIKPRKLSAVFLLLHSNSLYMNTNSSLFRLLKMRITTAYLHWLLFFPSLVSA
EAVSPLVFRGGSANRLDQGSNALVPDLGQDILQEKKLNTNSAVDEKYLERIVQSQWEKHR
RWRAVAKKRKFKVDASRISKLVLVSFGAVAQVGSTQLDKYASLSKYKSTASLLGSVCIAA
AGLIKNKILTDKEIHHMVTSYATSQAIKAEVYKFRANVKPYSQFKMNPDEALNLLRSRCS
SISESTSDEKFYLTRQDSSPPPGWLNTVDQYMEKRIDDMINRFYVKRGRRLKQRAAFCSR
CENALLGLSGAASFCSAQQLSGPLQKVFSTVGGWGGAFTTVSAAFANHIAKEKFNDIAVE
YFEAAAKLQDVKDSWPPTAAMSGSPDWEEQVAKSENVILTTIEEWAKAKTGNEDLHLKAA
PPVKKDPVWNPDVICGTDESGFYPASDRAEWLVTNKNMTQADAQKKVMVEFPERF
>comp5311_c0_seq1; 474 350 2221 LEN=2510; translated
MMELDSYIDRYTGENRLRRLLIIAAAGPRENCNLEQRKRALNLAEQQLRLRDDNVLLYRE
IFGGGSEARRADPELFEGFPTYDANWANSTESRNNETRFVLEGRFTTAKSRLHKDAIRTA
CLALAAHDKRTGQDTFKSLADLLGKSRGDCGNENKNKYINNKAKVNKALYLAMDVCTTRV
QTAQLGLLIIESALNAGDYFMVREYTGRVESTLHSLKEKVGVSSTISRNNRIDKNQDPNQ
DAILRDIGVKVQLAKGIERMVSGDYQTAANVLVALVMKESDGGIGSNNVSNENNHHYQEN
SDSDACHTLHWPGVTAPEDVALYAGLMCLVTRDRSKMIALADHPDALELVPSVKELLVHW
SRANYAPCMRAFSMSSAVGLSQSSQSSLAPLLSQPLLPMGMVVDLYLTPCRWDALVQTIR
ETCLIEYIRPYQRVKLDELRNLFFPVSSPTTNGNESTLDALIDMLADLIDRDLLPTTTRL
DCREGVLFQTKALTDPAPNLQAMEEKILDDTHAMLVRLACLEHDLGLASNDASSLGGKRG
NMGGRQHLRSQKSSRPGCNQSQRSGILDSSEDEECVAGNVYETFAPAASRANPRGDVGDM
NDTHMIDAESSHANSAMNPEDMY
>comp5312_c0_seq1; 346 1 1748 LEN=1968; translated
LTLGYAISAAAVARSWAVYVSGFFHGWLQGYIVRESVSVAIATAADNFLQWFVNLPISNA
FGEQEYQCCPLAAVVILLCTSLLVTGAKESARFNTVMTILNISVLGFVVIVGWTTGNVKI
QENLLTPSFFPEGFDGVGRAAGLVFFSYLGFDMVSCLSEEVRNPEKNMPIGIVGSLVAST
IIYCVVSLTVVGMVPIKLLGDDVPVVNALLANACCTHNEQLQDIAIHSDTDYECLSYPSC
KGSSFIFALLFNGSRLISFAAIFGLTTATFACLMGQPRIFFSMAQDGLLFKIYGRVNPET
GVPTAGTIITGITTAFVACFVDLESLANTISLGTLQVFTFVNAGVIVLRMTPSLIQIEID
TKNDDEESDAGQGSNKKTEADAGWNLSSENTAQNERSYLIPCKTNGRERTNNICSSSCTA
TCCAERRSSLLPDEGGAVLVREASREFRSSLRSISSSIVDNGESFESQHLLVELNGSKPY
WLTLIFTVSAVLFSASMSXLMAHRNKVGIGDHYATFGSYVVATTAEQLARDFQLPVRSGC
AIVRHIFQFLYDGFNALIHMVNNHPLASSGRLFLLLLRNAPF
>comp5313_c0_seq1; 314 101 1219 LEN=1326; translated
MGNRSSSSVRSAGPPPLLSAAVVGDAAKFRDFWMGNDSVDLNDAQDNNVLHALFSCRLEN
GRCSEILGFINESVPRSQLIKAHRARNKIGCTPLWILVAYGNVALLTEVLRDFEEKGQMD
LFSELIEQSNNQGDSPFLATCSQGNTGMMRYLKEEIYTPSQFKAALTRKNKKGTTPLQIT
VANNHESLLEYMLLVGEVVVRKQLLECNSIGLSLFHICSERNAHEILQTILKFMDDDKSS
SDRSDTFDKIISLKDKNGANPIHVAAFCGNVEVVEVWIDFVKKACSHVDNDSKKSKIVAY
LDQLDGQGRTPYWLGVVQGRSRIGELLAGEGVDTENPKMVLEIEEAQEKRQKASAARQHE
NQTIDGSALLNR
>comp5314_c0_seq1; 272 101 772 LEN=1084; translated
MAASPVNVYTEEKAQRLIRTFSASLAFCLLIGWFEIMILRPAQDMAQEMEHLDPSEIISL
PFPLVNSTIFAILPVFVLMIVWTVYNPSTVGWKILSLIKVTGFIFVIFGLCGNDLIATWE
HSVSAALFLAALLCTNLTEKRTSKLLEELPFYDHSDLLSTIRLYTTLAFIIPFSILSVLD
HGDQKQRWPCPVFMGGTYGFAFGSVGGIFLNYFKAKKLEKVKN
>comp5314_c0_seq2; 257 349 1020 LEN=1332; translated
MTKNCWMSFPFVMAVYVQRGCNQNRVHFIQTNEFFAIRFCFSYGYAETQEHLDPSEIISL
PFPLVNSTIFAILPVFVLMIVWTVYNPSTVGWKILSLIKVTGFIFVIFGLCGNDLIATWE
HSVSAALFLAALLCTNLTEKRTSKLLEELPFYDHSDLLSTIRLYTTLAFIIPFSILSVLD
HGDQKQRWPCPVFMGGTYGFAFGSVGGIFLNYFKAKKLEKVKN
>comp5314_c0_seq3; 255 101 640 LEN=952; translated
MAASPEHLDPSEIISLPFPLVNSTIFAILPVFVLMIVWTVYNPSTVGWKILSLIKVTGFI
FVIFGLCGNDLIATWEHSVSAALFLAALLCTNLTEKRTSKLLEELPFYDHSDLLSTIRLY
TTLAFIIPFSILSVLDHGDQKQRWPCPVFMGGTYGFAFGSVGGIFLNYFKAKKLEKVKN
>comp5315_c0_seq1; 405 279 1467 LEN=1631; translated

MRLATPLSNLDVAKPININTKSYYPSEKQQLKAYGFGLTENQSVSNHLREADVTYISNDE
CFGRGINFNNVLNSNEVMCTDPFAGKTATCLGDSGGPLTDASGTTLIGVISFGSGCEADL
IPDGHVRLSQVSEWVQDQICLLSANRPASCGPSAEYRDPQAFEVVVDFTHDFYPEHTTFA
VRSKSSQKTVYAGPEYIPTRNGNHKESIFLVPGEYTFEVYDLEGNGLLSERGNGLWKISV
LFDGCTETEVASGGPSFKNEQGTKFVISADKATHNSRCISKNDEIAIEMNECLTKKQAEL
SAGVAFSTSCKCAPDEATGKIELACFNKLGIHALSTTKPAQTQQIAVVGVGVLMGSADRV
LRQHQVETAAELVAKASGALQVEQGEITGISVEDE
>comp5315_c0_seq2; 498 285 1869 LEN=2033; translated
MMLSYLIFLLTLTASILEGSTIVADELEDPFAYMLRKPRIVNGDKSNPSNRPFFARAGYD
EYYFTDDILCGATLIWSDIAITAAHCQGAFNNGLLILDQQTNDYTKLTPINRQIRHPGWS
ISRQNLNYDVLVMRLATPLSNLDVAKPININTKSYYPSEKQQLKAYGFGLTENQSVSNHL
READVTYISNDECFGRGINFNNVLNSNEVMCTDPFAGKTATCLGDSGGPLTDASGTTLIG
VISFGSGCEADLIPDGHVRLSQVSEWVQDQICLLSANRPASCGPSAEYRDPQAFEVVVDF
THDFYPEHTTFAVRSKSSQKTVYAGPEYIPTRNGNHKESIFLVPGEYTFEVYDLEGNGLL
SERGNGLWKISVLFDGCTETEVASGGPSFKNEQGTKFVISADKATHNSRCISKNDEIAIE
MNECLTKKQAELSAGVAFSTSCKCAPDEATGKIELACFNKLGIHALSTTKPAQTQQIAVV
GVGVLMGSADRVLRQHQVETAAELVAKASGALQVEQGEITGISVEDE
>comp5316_c0_seq1; 340 1 933 LEN=1010; translated
XTAIDIADQFDEPGRSSGHTIASAEFNNTTRKMAAGGSFFFRGAVLLAAGVALMASGGSD
SLRAAAFSAPSPARTGRIDNPLSALEPKTEATACGQETTTTTTAAANKAPAAAVSLGLAL
AVGASALAGEPANAYIPSDYASETVQTSIQDLKKASGNVDETFKVYESIAGIITEGKGVG
GQINYKGIQLERGYVADEDTTIYNPGLSLLTESEKERLVEAVIDARKAGLEGNQWSENNE
YAFEFLRQKLDPFHITELRGFLGIVPFYGAAIYLAVLAVQQLFRDGFQIAYLVGVAAFFL
PIVGLVLAGP
>comp5317_c0_seq1; 875 53 1912 LEN=2076; translated
MDAESGECLANATLLSKVDATFSNPVFVAAVVVLAAYLLVEKYAIIYHTKLWIEYLTTSI
PTLEVSMTPEESEDDGVKGDPVTEMPLLNADDKSKLNCYDPSTKQFIGHAKNMTAEEVNE
ILVKAKAAQAEWAKTTYAQRRMVLRTIQKYIVNHVEDICRVSARESGKPKVDAVMGEILT
TCEKIRTICEWGEFWLRPDSRPTGPMMVHKSAWVEYVPLGVIVAIAPWNYPFHNSINHVI
SGLMAGNAVVGKVSEHASWSAAYFGRILHQALVVNGHNPDLVATVTGLAESGVALCTSPL
VDKIIFTGSTPIGRRVMESAANNLTPLVLELGGKDVMVFRGDVKVPTIVPFVMRGCFQNS
GQNCVGVERVVVYESILDEFLEAVVPRVKALRQGNPLPSCGADGKVDCGSMIMPRQLEIV
KALVDDAVKKGAVLHCGGKPNPNLNGQFFEPTVLSGVTPEMDIFNQEVFGPVMTIVKVPG
DDDEECIRLVNHCEFGLGSSIFTADDARGLAMGKRFKTGMLTVNDYASNYLVQSLPFGGV
KESGFGRFAGIEGLRALCCERAICVDKSSWIRTSIPPVIDYPINAVKGFPFTESLVQLFY
NESILGKIKGIIGLIKNGA
>comp5317_c0_seq2; 875 141 2000 LEN=2164; translated
MDAESGECLANATLLSKVDATFSNPVFVAAVVVLAAYLLVEKYAIIYHTKLWIEYLTTSI
PTLEVSMTPEESEDDGVKGDPVTEMPLLNADDKSKLNCYDPSTKQFIGHAKNMTAEEVNE
ILVKAKAAQAEWAKTTYAQRRMVLRTIQKYIVNHVEDICRVSARESGKPKVDAVMGEILT
TCEKIRTICEWGEFWLRPDSRPTGPMMVHKSAWVEYVPLGVIVAIAPWNYPFHNSINHVI
SGLMAGNAVVGKVSEHASWSAAYFGRILHQALVVNGHNPDLVATVTGLAESGVALCTSPL
VDKIIFTGSTPIGRRVMESAANNLTPLVLELGGKDVMVFRGDVKVPTIVPFVMRGCFQNS
GQNCVGVERVVVYESILDEFLEAVVPRVKALRQGNPLPSCGADGKVDCGSMIMPRQLEIV
KALVDDAVKKGAVLHCGGKPNPNLNGQFFEPTVLSGVTPEMDIFNQEVFGPVMTIVKVPG
DDDEECIRLVNHCEFGLGSSIFTADDARGLAMGKRFKTGMLTVNDYASNYLVQSLPFGGV
KESGFGRFAGIEGLRALCCERAICVDKSSWIRTSIPPVIDYPINAVKGFPFTESLVQLFY
NESILGKIKGIIGLIKNGA
>comp5318_c0_seq1; 444 734 1991 LEN=2073; translated
MKRVVVPEXENPPVQMPDKDAELRLADLQATITEEYKRGNFTNALATSQDLLKQTQQHFG
YNVDHPATASAMNNVGLMHKLLGNFTEARQHYVAAXAVYASVCGRDHASYAMTLHNLGNL
NKSQVHFDASLKATDRLSLVESALEYLEEAYAVRIAELGVEHPHTIATRSAIGSTLAAQV
LYQHKLVENNTEGSEKIRKQYVALNPQQITEQQWRAAEEHLRESLQTAIDNPRGKQIKNN
QHRKNSNKFRKNGGGVKHIKQLQNKIPGNPGSSFSITTLSAASAAQNLAVFLKSLAMTMD
EDSPGRKYNLYEAKDLYENVQQVRTQLLPSDHPDLYATKYSLAELLEVLAAVPSSGEGTD
KEQEERQRHQESADALRQEIIDTYDPPNKKLEEIDSLVPNDESQRIEIVVEKTKGTQNH
>comp5319_c0_seq2; 411 52 1467 LEN=1667; translated
MGEPIVIQGTAVSAPAQTTQAVTYGAEFETPSSNNPEPAKTGCKDPLFAILFYCNVAALL
IVVVLYGIPAMRESDSSTNYLRYVTAALVFGVTSFVAGLAGLFMLITCPALMIKVGLIFS
VAVAIAYCVYAFLYLNLVFGILAVVFAALMACYARGVWSRIPFAAINMLTAATAVKANLG

VTIFAIFFTILEVAWLVLWAIAMVGVYDKTTKCDANSQCDINYGILFGLFVCLFFTQQVL
QSCVHVNVAGTVATWWVAPNESGCCSKGVCNSFIRTVTTSFGSICFGSLIVAIIQALRAL
ASAARGNDDAGFLACIAECILACLQSIVEYFNKWAFIYVGVYGFGYIEAGKNVMQLFRDR
GWEAIIADDLVGGAIFMLCCVLGLIIGVVGVVYASINSEFKTLAQDSLGAAFGIGFVAGL
FVASVILSTVASGVNTVIVMFADAPQEFNQNHPELSRKMRETWTQFYPGSV
>comp5319_c0_seq4; 368 52 1595 LEN=1795; translated
MGEPIVIQGTAVSAPAQTTQAVTYGAEFETPSSNNPEPAKTGCKDPLFAILFYCNVAALL
IVVVLYGIPAMRESDSSTNYLRYVTAALVFGVTSFVAGLAGLFMLITCPALMIKVGLIFS
VAVAIAYCVYAFLYLNLVFGILAVVFAALMACYARGVWSRIPFAAINMLTAATAVKANLG
VTIFAIFFTILEVAWLVLWAIAMVGVYDKTTKCDANSQCDINYGILFGLFVCLFFTQQVL
QSCVHVNVAGTVATWWVAPNESGCCSKGVCNSFIRTVTTSFGSICFGSLIVAIIQALRAL
ASAARGNDDAGFLACIAECILAXPPEYCGVFQQVGLHLCRYVKSLGFEIDYAYGVSVALK
ASRTLVLIDSVLFYLHFSFCFTGVYGFGYIEAGKNVMQLFRDRGWEAIIADDLVGGAIFM
LCCVLGLIIGVVGVVYASINSEFKTLAQDSLGAAFGIGFVAGLFVASVILSTVASGVNTV
IVMFADAPQEFNQNHPELSRKMRETWTQFYPGSV
>comp5320_c0_seq1; 174 1 589 LEN=716; translated
XEVVQPSDAGTLQFFLEAKARTNTDTKRTASFPTIYLTTQYITILRANQKTHLAMVITMP
CRSLTGVLSLSLLLLLCSPPAASFLAPHRSHHPTTHRGSLPPLCSSFPPGPGHPGGFPPQ
QPPPGGEAEATDDAAAGSEAEGQANTRFSKFAPDPSLDTDEFRLQLRENMKADLERRRSE
DPNRGNQVAKNYLDSL
>comp5320_c0_seq2; 174 1 589 LEN=920; translated
XEVVQPSDAGTLQFFLEAKARTNTDTKRTASFPTIYLTTQYITILRANQKTHLAMVITMP
CRSLTGVLSLSLLLLLCSPPAASFLAPHRSHHPTTHRGSLPPLCSSFPPGPGHPGGFPPQ
QPPPGGEAEATDDAAAGSEAEGQANTRFSKFAPDPSLDTDEFRLQLRENMKADLERRRSE
DPNRGNQVAKNYLDSL
>comp5321_c0_seq1; 478 179 1546 LEN=1794; translated
MSDKTEDQNGSASSPNSNWTKEQLTGKSVVSTAGLAEATASKLTVSPPKRKSIGKAPKDE
GVDPSLWGRPGHLTDAQADTYLRFKEIVDLRGGDFRDTIFCFGEEEGEVFALCRWLRARK
FVFNDVMVMVEGATKCEASAKKAGFYADPAVALGCAEAVYNRQYPQCYSGFAKNGSPVFY
SKVGELDIDAVECVTTISNIVKYHWYVMMHDFANRLRAHKKKNPQFNEFECVCILDLANL
TMGKLNSKTLAIIKEQSAIDSLCFPETLNKMYLVNSPRFFSATWTVIKGWLDARTANKVE
VISSRKAMEKALLEYIDVDQLASDYGGKGPATQFSMEKDCFTGNLKRLHTETVYLRTTAS
VTCDIQAGEELEIAVYTRSTIGAKFTVSDAKGGPTWATDVPIKHDSEDLEKMPSNVTITK
ENIKGPATVEVKAKSMGGRFAGSTNYLVVFSVRSP
>comp5321_c0_seq2; 358 1360 2349 LEN=2597; translated
MVMVEGATKCEASAKKAGFYADPAVALGCAEAVYNRQYPQCYSGFAKNGSPVFYSKVGEL
DIDAVECVTTISNIVKYHWYVMMHDFANRLRAHKKKNPQFNEFECVCILDLANLTMGKLN
SKTLAIIKEQSAIDSLCFPETLNKMYLVNSPRFFSATWTVIKGWLDARTANKVEVISSRK
AMEKALLEYIDVDQLASDYGGKGPATQFSMEKDCFTGNLKRLHTETVYLRTTASVTCDIQ
AGEELEIAVYTRSTIGAKFTVSDAKGGPTWATDVPIKHDSEDLEKMPSNVTITKENIKGP
ATVEVKAKSMGGRFAGSTNYLVVFSVRSP
>comp5322_c0_seq1; 669 325 2513 LEN=2513; translated
MVSLSSVPRFLVSAIFGLAAASQLLTARADVNFSITPSPTFSPTSFGCDQNNKAIFQESA
GSDNYPYDEDTIEILHQDIDSVTIRVHQLWHRDGAPMMAVAYRAEVGEQETCSMDVPEEG
LISYGSYHQYTAKCMHGYAEVSVYGFVGPAADFDVDECEACTVSDSNYIGYYVVVPCVPI
CDPKIPDCFEGTQVSLADIGDEQKCIYNSAPVTVVSENLPPAGDVPENVMFRVADTWAFD
DFSDGGRAQLSVYFQDPNGEDRCETAEVTPNFADFVFEAKCDYDATATVVVSLLSSDLHH
FPELRPEICGPDDGLGGCAYEFVIPCMDGLICPESFTEPPTTFPTFSPTAGDENLLPTPF
GEVDCVDSATATFEGAVGNVKSVFLQEPPVRIDSQDIDSVTLSIVQEFSPVAFPMVSVSY
RDPITLEQQCLERTDFGPGQDLGLMAACTKGFAEIELFFYVGFGDDFDPDECEACSVPDE
NNYAAYNIAVPCAPRCGPKVPNCVDGPIVVDADIDDADDRPMCLYEFMPVVVEREDTPDD
MVVFRVENTWNPALEFTGYSVGYVHPGYDFPICDGEFFDGRPPPLPEKPFARGSEKLAXD
PPPLDAKRAFQAPYFEFHQSPLLEAKCENGFASVFVSVYSQMIRHSAARLANDSENGCGL
RYDSGSCTYHVLVPCDESVRCGRQGTETPTLQSPTGVPTKSPTGSPTPGPTKSPTDAPTE
APTKAPTEAP
>comp5323_c0_seq1; 341 1 1038 LEN=1266; translated
PKQNKCVQNQNQNNAQSAEAAKLGVSFHYDASSFFRGLDVVVLSVPLIRLQEAVDSLPIH
ELKGKLVVDVGPLNDAPKSILLDAFASYPDIDVLLTNPILGMLPREEDQPSSGKRPSSEA
GVTVMTPKHHNNPLMGGPTGSIWEGRQMVVERARVANVPRCDRYLSIFERARCEVVEETS
NEHDSAIGDAQFVTHLIGRLLSRDHLLTPTPIMSEEYKDLSKIAEMAAAGSFDRFYGMYK

YNHKTATNHIRKLRENLAALECELAARGAYLDAKEELARGDRQKLLSETRLLLQELAKTE
AFDESPAASTASSESESDVYEAITDGYEAITDEIDDEGEPLFVLK
>comp5324_c0_seq1; 234 801 980 LEN=1301; minus strand; translated
MFTEFVLDDAVDENSEAADLEELLHDAKWDEEDAMQEFVDDVRNRIQHERLMVFRLEEE
>comp5325_c0_seq1; 425 192 1064 LEN=1505; translated
MSSNDAVSSNNNSEEEILRKKLKPDPTADQVIDAVRTYFYGGKEDVEIKLKKELESYDDR
NFWITVDGTDYLAKVHNGVESRDLIQYLQNDEKEDNSYKKSAIHLQNAIMKHLNNCGIST
NIPQEPIATENTSSSTPVAVVSLPVHSKDDSPTPLVLRLLGWVSGRPMSDHHMLPIECLA
DAGRFLGNLSVALSSIPDADHLQAAKRYHQWDGKNTADLRDFVKYVTDERRRSIVNSVID
AFQKDLIDSKVAETSFKKSLIHADFNDANFLLGDDFCVSGVIDFGDSVER
>comp5325_c0_seq2; 404 192 1526 LEN=1586; translated
MSSNDAVSSNNNSEEEILRKKLKPDPTADQVIDAVRTYFYGGKEDVEIKLKKELESYDDR
NFWITVDGTDYLAKVHNGVESRDLIQYLQNDEKEDNSYKKSAIHLQNAIMKHLNNCGIST
NIPQEPIATENTSSSTPVAVVSLPVHSKDDSPTPLVLRLLGWVSGRPMSDHHMLPIECLA
DAGRFLGNLSVALSSIPDADHLQAAKRYHQWDGKNTADLRDFVKYVTDERRRSIVNSVID
AFQKDLIDSKVAETSFKKSLIHADFNDANFLLGDDFCVSGVIDFGDSVESWNILDLSVAM
AYAMLNAFGKHQRSLAAAAAVLRGYNSVRPIGPEERRYLPLLIACRLSCSATLGAYSYAQ
NPGNEYLLLHATPAWNALELIWGTDPERRSSVLETVQEVFDLACSSCETDKKTRVIGCTD
LNFPDPSVPDPLAGARSRKRKEME
>comp5326_c0_seq1; 944 236 2356 LEN=2612; translated
MGGNNKQHESNNDGENRELSVIVRRRLALYEEETTRLLRRAHGLAKQKDDEERRLYQRSI
GDNTVPVLPKARAKPTTPGTGASNGDEAKDIVSDNSSSDGGDGFGLATIESEDESESENS
SSDEEDEDDEGSDNESGIVGTPSSAIESTKTATTKPQKTGKLSGRKQKRLGQDINRHADN
LIRVDLPLPPGDSDVDEGDGNSTSLSLSPHLHLFPAAALLPNNKLLGPFCASDQAGIVRD
LSETMSSESCEDGLVVVLLIQSGRFAGGVFRRGKCLAHRATTRYTIRKGQGKAQSVQDGS
RRPKSIGSQLRRAGEEQLNEDVKLALTEWTKKRYIADASLLFLACPKTMRSTVFSPSDAN
SNRAGGNKSKHKTDDCMLSKDDDRIRKIPFDVGRATFHNVEVVYKVLMTVDLCHVQELGS
TTSERHAIESHGVSVTTETADKDESAHDDLEEEKRKRKEKLAADLPMTPLHVAAREGNLT
VLLDLVHKINHGADDCPGVDQPAGYSCSTPLHFAAAASKNVDPVTASAIISALLIQARAD
PTVLDASGRPPYFLAGHDKIREGFRKARAALGEDYCDWDSAAKVGPPLTESDLEAKKKRE
AEKKRRKKARQKEKKEKDRAAAREAEERRAAEEEARHTAEEAKRIRDQLPPKPTAANACD
FCGKICKGRNASKNMFRRLDYKYCSTECVNKHKRELMAAAAMARFG
>comp5327_c0_seq1; 279 204 1205 LEN=1429; translated
MSQSESRRENLRQRRSQILQDKKEDARIQLVIQDSIGKNQFSKHLMKTFLVIITALFCTI
AAVLNHTRPRFLERFWKQPVRPFHLATRYPKNIILDRDLPRFFGHYSIATPENLLARQAV
QKVLRSRKKLRSQAGPIKSTLKAWDDSNVEQLLKQGICGDEFELAYRRESTHQGENGLLM
WCLLASQVTEGFFTESVEILDSILELTRNRGIVVRKQPARGIVDGYDALSTTFYLHPRTN
HDKVNWIPTKMLSMFISSSNDERVDDNNELAQRMLYELVVNQGNEEEYLILEEICQETPP
KRSIAFDTTEDPNGCYFVVPNKYGGIFGPEEDE
>comp5328_c0_seq1; 533 237 1649 LEN=1676; translated
MYPISVILTLVGMITAGAARIVVVKIYYQIELEESRPVTPLFVTLLYLVGQALSLLVHFV
CVRFDYSKRERSDNGIGYDTIEMAAMKLDDDDDNNNEPFVAAVPPKAIEDEEQADRPAGT
DATEETDTECGSGDGSDDETDVPGDEEGSECERAETGSDPRCGGSPLHAGGPVRVDPGDG
LLLEGNSDGRRGHGKERPPPRGRMRRRGSKTGLTSDSREAVAWIHRIPWYLKPGIPGFFN
LCNSALRWACLVYVAASTAEILISGLELVLSSFAARVLRKRIISRRRWIGVSVVTAGIVL
VGFAKLEETRDSDSETHNFLVGNLLIVGQCVFSVLQDLSEEVFMQEANFPATLLLGMEGL
FGLVFGLPIYALFFDEEISFTRFNIEYQIGLVVLVLLTGILNIRTTEATSSMTRNVWKQF
RIMLVWILGLLVVYYLGSGGRLGEAWVFPDSLFVLGGFGVILTGVFFYYSG
>comp5329_c0_seq1; 1251 1 3651 LEN=3689; translated
SNSNNNSSNSSNSNINNIDDIRASTSRAQLQTPRSTSGHKQKGSNRYRHRRTEGNRGRSK
RHKNGFQRREDGNSNSTSTKSNSTAGGDSSSWTDPSVILYAMGSWWWIFASTLVYLSPLI
VWTGERVLGCLGSMCGARCQRTGRPQGRGGSLVESDGGGAADEEPSSETTAIDDSVASPP
PPSRLAALCRRCRVPFPNLRMGSVVPWRIVFWALVFLPSVVVFSYGTHQGMDRARQNLSE
RLGDGGEAAGSQETESSLLDWIGALVLASAMPAGTAGMVSMGVYIFGTVAKHSPLWSAEV
ILPYTTNKRGGSDESIGTTHHSDTGQNETSQKGDRNPSHGHENLMRDALRLHQWAGYMTF
FWFLLHSVLYCVIYGYLGVVSHRQEDIAAQAASDIAAAGNATDDGVEASATAMGSASTTR
SLVEIFFASVWNAIYPPPECRPILGRKDGAGSDGEETGNRRLDIVRLLDEDKQQESTASG
QSFPCYGYYRNFNGAIAILAFSVLVGTSLELVRRKNYRFFYASHIVCAVVFVLTMVFHMK
RISVYLMPSLLCYLSTTIPTVIQAMVSAVRDGGVRIEDFRTLENDGGSSDSNNLQGCAEL
TFSVDDTGVFSGAYDRKKCGAWTTYRPVSVKICVPSISLLWYPFTVIETNSIKNAHVGDK

IVNGTDPSGAGDSGSDVSNGDGTTKGDAGGSIQCTILFGTVGYFTKSLWKRMRDAQEAKN
GFRISLANENGDEFIDEEPITDHPLEAMNQTPKALPLILVDGFYSGASDWIESALGHDSV
VIAAGGTGVTPFLAFLPKLIRVLTDDLSTVLSGTADEESPDGRDALREGSQETVASLKTI
SFLWCCRDESLIRHVLATYLLPLFRGREHELWQTKSMLPENANDPPFRDLAINTNGVRFR
LMIYNTSRDRGDKSPFELSASDDGVGSIRCGDSRNSASISARGLAMRPSKFQFSPSSSRS
GATIGVVGFLSITLVGMLLHHRASDYVQEERLRFSIRAYGIYAVVVWTIAAGFATEFLWR
CRCLCSPTAWHRRRGYAVADGDGPDSSSSNLSVDPNHETGGGEELAIANENEFSERGKAR
TSCAEARSCIGTASLLPGRIYHEELLPRTDSFLELGISNGRPAFHGDDDDGLHDPCSNES
SPSSPAAWADEVRRELIDADRPGVFYCGPDGLLEAIRSGVKAGRSERRRLSGGRDARNFN
RDRVAECVFYEESFQM
>comp5330_c0_seq1; 369 305 1681 LEN=1848; translated
MRFFQRRKDGMGVEQQGSATVAFHPAQNVDSKHSPPQYHHGDGIHSLRKDPFAPLTQEEL
RQCSNTNLHDIDDKTESTIVENEYPKNRQSIGRLVRKLNCSASRKSIHGVDQDPIAFTDR
TGSLFSNEGGMNRATRSDGLTLERLILPSKSIESAASSDITRPVLITVKDRDTKQPSTKD
VRDRSNCVTPEMKRSFVVGEFPVRTSSFRSRSSARKRLHDVDYHNNNLTRSASVSGRWVN
NGGKWEHSSGGINSSSNSSRSNPSNSPIRTSSNLSKKNTERDLEAIRVAQDLARRERWKR
GLENELYKKSSTAAGRSNTKSKGPIAAAKRYIEGESSNNTEDDQSLSDSTQGSHTTGTSG
YDDTTGESSSLDEWTDGSTTDNDSRVYRYHQDYPQHQQRQYRKTKSGRRCINNEQLARSV
AEDVGIFADLILSDGFACFGTAAAITKETVASCRGNDV
>comp5331_c0_seq1; 270 1 1019 LEN=1492; translated
XEYANEIENIANEHAKEIEKITNEHAKEIEKITIEKESKSVEPQTIEEQACLDLGDGNNM
DELLSAHKQVFVAMPAKAAGSSMKSFTSKCMKRGWSGNYTDRWVENVLAKPDVLKELLHG
PLEVPPLIASHMYEDADFMRLMQAATKETLIVYIHREETSRLTSAIRYVMDHRKCPEFLK
EHRANNDKKTCTVEEEQLTQIIQDRKTNSMGEIEYGAVELLTCRTYSAIRSNAPKLVFMH
YKQASKLQKLLAKRFCPRQGDSLKNVRSEDAKIQVELREGGSVVTLDQWLDAKQHFLELA
LGMKETMTCQHETKSMEETLFACPDETFYWTPEMNLVSR
>comp5332_c0_seq1; 282 169 642 LEN=1501; translated
MKKHQQECSEIHTIQIFREASSCIPRSSLRQCNQQCPGRTVSIQSTTCVTPTSQKQDGTS
YAIGCDWAIEMDEDLVEEPSTLIEMKESNSDGQSSLYSFYFETPLISIGQDQSFDEYTIT
ANDTDSFHDSNDFDSDSMDDIWEQGLTRSVSCFEMEK
>comp5334_c0_seq1; 834 69 1262 LEN=1424; translated
MIVVSVLLCFVLCIARTEFRLIQPDATDSATPGVVVALKWNKQGALSDPLETAHERDMGF
LRDKLYDAAGGDRDVPWFYKNSSLSHIRRSGGQTGDASPATPHAKTWHKYESAHQNTNRA
ASATATKPKAVALKAKPTTAKSFFGNHKASAANKAKPTPKPTPAAAKAKPDNKAAPKPTS
QKRSEASKPADEAPDEAKEGSSPSNEHGDTNATNPTVAIGNADDFVGDEDEDSEDEQDMK
TRRAKSIEIVRKLKERDDMLDALAKEEDGDTEREKPTLVKGAMDAFCHATSGPSTDVSVA
PAQANKKKRRKVFRETTKMNAQGFLVTTTEAVWEEIDEDDEAAAPASSAAGNHTKAAPAA
TTTKSKPRPKKAAPPKKKASDMKQGSLKGFFAVKKKK
>comp5334_c0_seq2; 916 128 1633 LEN=1795; translated
MSAATAIQPLPNGTSAVLASEQVLSWLHDEESLVTSQRIQHHCLRDENENDGEGEGNEDE
SSSLLLSRREASFLLRAIYEKDPCRYKATLCTRETFRSVPSMNHKKEAGRPACSAEEYKT
TEFRLIQPDATDSATPGVVVALKWNKQGALSDPLETAHERDMGFLRDKLYDAAGGDRDVP
WFYKNSSLSHIRRSGGQTGDASPATPHAKTWHKYESAHQNTNRAASATATKPKAVALKAK
PTTAKSFFGNHKASAANKAKPTPKPTPAAAKAKPDNKAAPKPTSQKRSEASKPADEAPDE
AKEGSSPSNEHGDTNATNPTVAIGNADDFVGDEDEDSEDEQDMKTRRAKSIEIVRKLKER
DDMLDALAKEEDGDTEREKPTLVKGAMDAFCHATSGPSTDVSVAPAQANKKKRRKVFRET
TKMNAQGFLVTTTEAVWEEIDEDDEAAAPASSAAGNHTKAAPAATTTKSKPRPKKAAPPK
KKASDMKQGSLKGFFAVKKKK
>comp5335_c0_seq1; 430 320 1363 LEN=2915; minus strand; translated
MEPARIRQFAYATASFLLGAWAFVAVNAISGPAFEPIIAACTNPDFASVEDFVENTGYHE
YEPMVGLGAFNLLVCLITQFLFELRTTHPAGVLTWGGVVLVSLPSVGLGTFEGARRGARG
PIRYPVVLGLLYQLFGISVVFPLIFVPAFVFGEGERGAPLTRFRVAVAHAMPVPIILLTL
AVFLAPTDSALWTTSAGILGGPILALMGLLFFFDKSSELATTRKAAEATSRGLQAFYAYL
AVVGFLGWYALVGIAYQSYGTSFGDLWRDIWVEAKPSVAFMTIDTGVFYLAIVLLVAYRS
GERMAAKAILRTSFLGPATALCLAMIEIEKETVCVMDEADKSTKKED
>comp5336_c0_seq1; 111 108 410 LEN=854; translated
MKIRTLFQLFALFILAVASAQSSAIDDEHTERRLRGPHKAGRGFNKRPFKGGRGKKGPYK
GPYKGGRPAKKGFNKGPYRKGGPGRKGPYKRGRGGPYKKGW
>comp5336_c0_seq3; 111 108 410 LEN=863; translated
MKIRTLFQLFALFILAVASAQSSAIDDEHTERRLRGPHKAGRGFNKRPFKGGRGKKGPYK

GPYKGGRPAKKGFNKGPYRKGGPGRKGPYKRGRGGPYKKGW
>comp5337_c0_seq1; 656 294 2123 LEN=2561; translated
MLTYAKQAINFARVYLSVTRKDMQFYASLDECEVNPNPNLKNLTSEDLQFENPRVVSFKT
IDNKPAPETETEKVSTVIPKSLVLKNDDKRAVPVEPSPANNDLVLDTNNELVLAANDELV
LAANNERVRSAGDAFGLAPSELATLTRERVRERFREAVFSYHPDRNPSSTTVQMQVIRDA
KDILLAACDGNIPEIENDTTPVALIANDVSPACTKANAIVPQITSGIAIKNEAAIQIAQT
NVPQITSDIALSCPKTATGTKLPASLEALQSLQLQKRETSLGFYEKKSTKRPLPFPVGYE
FKDSGKLLRKGIKRNVFLGNSTIEPCKFPTIALIGYTSNSNNKSVFYEFKDSGKRLRKGI
KKKNILKPQPPSNSDSHPNSEGNLVTDAEKKNCIVNASKQRLLHRSLSTNSSHGTGTLPG
TNLPTVEYVREITDKDITTHEQPCGRKKRAKRIHEEKLDTDADEINEDDNRSSTGFKKNA
LLQETDTNSDQSNQPSCAIIGDTNDEISEEESASQKAFHTNQNPHGSLVPFPLSIVLPRQ
GDLVWVMQGKTEHMAIYVGELRNSTEGVGYEEVKVRWSTNRLAQYVPLRSVRVFEMGTKR
GARKRRVRS
>comp5339_c0_seq1; 441 108 1696 LEN=1696; translated
MMAIALVALLTALSLTTAYAHPDNPKVLRSRSARRLNSPVPVPEPNYVINLLSERDDATQ
NDYGDATFDGNPTSIKLGLDFEVVYVGTTDSPSTSLEYRVGVYKYGDCSGDEVFGATNAL
ADAFADNKVSQSIEISLNSVDSSEGGETYESLFWVNDADGSTRYVDGSMRVQFCFRVSIP
SDLDTVPFEADAWVNFPETYDASFRLSNSVQNLANQDTWRSSITVNLQYTVRSCLCNYVP
ADNQEPERYECLTESKLFRPGTTIQMCIEPGHLVKSEEEEVDDVNTGSLEIERVTEFHII
QEPAETSGMTVTRKAIDGSDADGLTVVEYKSMAITPQDGSSPRKFVGGNAPGAIQIAVVS
TPLNAAFFLQVEQPVDVHGQVLYRDSNRRSLLKANSMYLSSSTLAEGSIESNTTEGLVLP
DVQVNLIGEEKTVSHSINTNPGGDFDVKLSLDESFKLNPQFEHSSSTLILGIMICGILLG
LIAIHGILYVLGFRMTSPGVRRVGYRSRGTYGKDDISFCSSINTFGAIPA
>comp5340_c0_seq1; -81 1 408 LEN=795; translated
PGMPFKVDVHGKRFTVTCPQNAGPGQRIQVRLPISAPRAKTPPRHTTPPRHTITPPRTPP
QTPPRHGGTKTELFDVTVPPGVRPGGRFKIRAHGQEFIVTCPPNASPGRTIRVPVKVQVE
SPMVQVFKQQGESYC
>comp5340_c0_seq2; 538 1 1300 LEN=1300; translated
XKFQSSSKKNSGKPSYLIAKKNEDPTTSVESAVAAGAVVTAAATSVAATSALETNDASAA
ETERLEAVARKKAALQAKLQAEEAEIERLEREAAERAAARERLKAEAEASERRRNNKKAT
AAAIGAVGVAAAAAGTAAFVASKKSDDEESDNDDDEEALAAKQRRRQEEKAKARRQQKEK
QHPKPSAPPAPADQTMTTYEVTVPQGVSEGTDFKVQVEGQQFEVTCPIGASAGQTIRISL
PTTDNFSSSTASHNSHNSGKVPTNASIEESTSTTMHFVTVPAGIFGGMPFQVDVDGERFN
VNCPQNSGPGDRIKIALPTKSGGGTGLSSSHKTHDPESFTSSHTSHSSTKAPETKMHHVT
VPAGIYPGMPFKVDVHGKRFTVTCPQNAGPGQRIKIAIPTTTGGAGAGGRGRSKSPQPPT
GTSLHHVTVPAGVY
>comp5341_c0_seq1; 237 400 943 LEN=1675; translated
MALFDDQNNLCLVPMMCLDNGNATEHRIEITTELEVRNPDDDHLLVSADVVRDDDRDYKG
LHSNSEVEEIEVIDEDECTRTREASNSGDHAADETQSRIIDGSISSGPIVTFSTLFDDTL
QGPESEVDNETEARQKLAEKHRVKSQAKRIGPPESIQIAAVTEASDVASVLGRYKFTFMK
>comp5343_c0_seq1; 261 181 618 LEN=927; translated
MPLTENQEAQMKTVFTKYAKNGSIGSKEIGAACRAGGLNPSEADLALWKGETKSGLDLNG
FQRFMGGKFDKTNDTVDEIIETFQAFDTNGTGTIPVGELKVMLTSMGEELSEPEFATLMD
ECEVENGEVSYLQLAHMIFGTTGDE
>comp5344_c0_seq1; 373 139 1533 LEN=2185; translated
MKTPFYNANGIPVVQGMSCENKRTSRSSLNHSINDAQISDAIARKQQDEIDQRLSYYAAT
DHHKSSLTSMNNTVMIGDRNRNGGSRQQEGSIPRGEIQPTAFRDVGWAILFCAHVIAIGY
ATAHFAPLIASDIANEYAGGAHRWLEQELQQIGDGFYNDSDHKYEYDDKSFKNYLGIFPD
DTDRSESIIYWEQQNRVLNNGDQDSELSMNTLLTIMAISGAVGFLISSLAMSLMMAIPTA
MIKIALTFNLIVTTGVLVLSIYIGAILVAVMSGLGLILTIYYTYIVWRRIPYAASTLVTA
LTAVKCNMGLAFFAYNNLVVTFLWSLWWSTAFVATFYVVGDCDAEGYCENEINGFLVFLF
LVSFFWTTQVIKNVVHVTVAGTVGTWWFVPGEARGCCSSAVRNSYWRSVTTSFGSICFGS
LIVAFIEATREIVNSARSGENGLIICIIDCILHILEQIAMYFNKW
>comp5344_c0_seq2; 456 139 1941 LEN=2001; translated
MKTPFYNANGIPVVQGMSCENKRTSRSSLNHSINDAQISDAIARKQQDEIDQRLSYYAAT
DHHKSSLTSMNNTVMIGDRNRNGGSRQQEGSIPRGEIQPTAFRDVGWAILFCAHVIAIGY
ATAHFAPLIASDIANEYAGGAHRWLEQELQQIGDGFYNDSDHKYEYDDKSFKNYLGIFPD
DTDRSESIIYWEQQNRVLNNGDQDSELSMNTLLTIMAISGAVGFLISSLAMSLMMAIPTA
MIKIALTFNLIVTTGVLVLSIYIGAILVAVMSGLGLILTIYYTYIVWRRIPYAASTLVTA
LTAVKCNMGLAFFAYNNLVVTFLWSLWWSTAFVATFYVVGDCDAEGYCENEINGFLVFLF

LVSFFWTTQVIKNVVHVTVAGTVGTWWFVPGEARGCCSSAVRNSYWRSVTTSFGSICFGS
LIVAFIEATREIVNSARSGENGLIICIIDCILHILEQIAMYFNKWGYVYVGLYGYSFVEA
SVNVLSLFQARGWSLIIADYLVDTVLLMVSICVGVITGIVGALLGILMEQNGAVVVGAFL
IGAAIGFVLCSTLFGLVSSAVNAVIVCFAEAPAEFQSNHPVLSHQMITAWREAYPTEFGY
>comp5345_c0_seq1; 438 110 1816 LEN=2044; translated
MITTTCAPPKKVEVVDKSKKNKVEMAPSDQLIMYMSPKEVMNDPLGALASAALAAEASEA
SCSDASKDATKKEPTTVKTDSRKAAEMEGENGTTKTSQTTGKKTEEETADSETIAKQTIP
PTITIRRELSLTDSNRPSKSAHPQHIYPQVPVAGYFGPLPGHPPHAPVPHAPPHAGYYQA
AYGPWVGYGEHPSAPVPYGSPVHWKHPHAPPPPPPHAHAHAHPHPRPHPHPHHVVYHDGH
YYPPPHPPPAPQFGFARSITADSPARSITSPAPDHEGEGSRKRKPSTKEYSPSNSPPSLV
RTSPPLPEISLRKQTAHVDSIRSKEVKSCSDNRAVFKRRASMGKWTEEEDELLREAVADY
GGKSWKKIAGRLTGRTDVQCLHRWQKVLKPGLIKGPWTPEEDGKVIELVKVHGNKKWSFI
ARQLKGRLGKQCRERWYNHLNPDINKGEWTNEEDKILIQAHSELGNRWAEIAKRLSGRTD
NAIKNRWNSTLKRIMSKDASPESKKKRKVLVDGLADSQPVKKDREGTEFRPKAKKQKLQT
RHELAAHTLSDLSSPSIKRHFRGRFASKW
>comp5346_c0_seq1; 134 810 1055 LEN=2150; translated
MGFRFVGGGRACWYSLGGSNADGHHRPRPGGQTVLVRGGKGDRGLSAGKSPLLLPELLLL
PPGETVLESEKRRHALTLERE
>comp5347_c0_seq1; 299 609 998 LEN=1386; translated
MSIVGGNGGSFRKVNLQISQTIESLQLICNSINQSKDNWWLSPAMLSSVEGMRTSRILKD
QQEALLVEEEKDDIDDFAGQEGQTNDNGDETEEMIDLQADEFEKDFADTDSANCEQGTDA
NISDDETIF
>comp5347_c0_seq2; 299 707 1096 LEN=1484; translated
MSIVGGNGGSFRKVNLQISQTIESLQLICNSINQSKDNWWLSPAMLSSVEGMRTSRILKD
QQEALLVEEEKDDIDDFAGQEGQTNDNGDETEEMIDLQADEFEKDFADTDSANCEQGTDA
NISDDETIF
>comp5347_c0_seq3; 392 229 873 LEN=1261; translated
METSHETILNNTLSAAGLLVEENKATKEMRRSISKFARLLMCPLCNKIFDRPATLAACAH
TFCMNCIDRHSAKHSVCPVEGCGMPMSIVGGNGGSFRKVNLQISQTIESLQLICNSINQS
KDNWWLSPAMLSSVEGMRTSRILKDQQEALLVEEEKDDIDDFAGQEGQTNDNGDETEEMI
DLQADEFEKDFADTDSANCEQGTDANISDDETIF
>comp5348_c0_seq1; 165 1 813 LEN=928; translated
IRITFNLSLAIECTRPNIQFDLMENNGVKNPIFVYVAEGTQHSIGITANGAAFSWGRSTS
MGQLGRDNTDMPFKRPGRVNLPNSVRVTKAFASNNSDPESGHSAIIDDKGNLWMAGCDRW
QQLGLGSSNSGSTGYTWQGGKQWQDRFQISQSVEELMKKDNYSGIRDVALGGDHTLVLSS
NRRFVYAYGKGGDGQLGFVGKPFVSAPKRSPILSGKNIAAVCAVESCSVTLDDVGATIAK
VGKCRSSKVSEGISRCVENARRQGLLDSNK
>comp5349_c0_seq1; 223 1 360 LEN=1468; translated
GQDSPIARKNKISDSTKKDKMMNTDNDETTIIKLKSMPDEEGNVDVFEISMAAAKISELV
RDSFPEDEDEEYSDDVLEISRVKGPCLAKVVDFMKHHHESPMKEIPTPLGGSSFNDVSS
>comp5349_c0_seq2; 339 1 609 LEN=1282; translated
GQDSPIARKNKISDSTKKDKMMNTDNDETTIIKLKSMPDEEGNVDVFEISMAAAKISELV
RDSFPEDEDEEYSDDVLEISRVKGPCLAKVVDFMKHHHESPMKEIPTPLGGSSFNDVMDQ
EWYQNFVSEENLGGDNTMLFDLLTAANFMGIKELLDLSCLKVTFQLTGKSADDIRKILRL
PELTPEEEAQARTEHKWIFEDS
>comp5350_c0_seq1; 250 100 756 LEN=1021; translated
MLALKKKREAEKKKAEEEAKAAVAAPSVSAPSESTEEGGGQVSLFGIGGQKKTKKGESSA
TKKTPGEIRIQKDIGELDGGKVAEVTFPKPNDLTSFNVSITPDTGYWKGARYEFEFKVPA
HYPHSPPKVECKTSIYHPNIDLQGKVCLNILREDWRPVLDINAVIYGLIYLFYEPNPDDP
LNHEAADLFRKDIQSFERLVNRTLRGGTLDGVTFERLV
>comp5351_c0_seq1; 163 346 744 LEN=962; translated
MPELFQGKKVAIFGVPAPFTGTCTTAHYPPYQKLQDKFVAKGVDKLVCYSVADPYAHYNW
GKTMGNDFDKISFLADVDGAWARSNELEQDYTVVSLGHRSARFSMIVDNGIVTAFNLVED
ADGDAELLLEQA
>comp5352_c0_seq1; 280 543 1295 LEN=1445; translated
MYAPPFGVSCLMVQHVHPCCLPSSSGLAVEPNTADANPNANGPPSTSDSIFDLAEAAGSA
SGGGDGEVRRTITMYRDGFVVDDGPYRRLDDPANKEFLKSLARGQTPSELTGDSSSGNIT
VGLIDKRGEDYVEAFRSFSGAGQSLGAGSGASAGASSSGVFDPGALGEAPPPPPSGASTT
AIAVRGLDGRRKVVRLSRTATVAELAASLDPRPQQAFRMVAGFPPKPLVEEGATLEEAGL
VGAQVQLQKA

>comp5352_c0_seq2; 350 123 902 LEN=1052; translated
MSNIRGLYDDKNDESSDDENNRYVGGIGARGGGSGLAVEPNTADANPNANGPPSTSDSIF
DLAEAAGSASGGGDGEVRRTITMYRDGFVVDDGPYRRLDDPANKEFLKSLARGQTPSELT
GDSSSGNITVGLIDKRGEDYVEAFRSFSGAGQSLGAGSGASAGASSSGVFDPGALGEAPP
PPPSGASTTAIAVRGLDGRRKVVRLSRTATVAELAASLDPRPQQAFRMVAGFPPKPLVEE
GATLEEAGLVGAQVQLQKA
>comp5353_c0_seq1; 601 445 2298 LEN=2339; translated
MVSGKMQIDNNADTSTNNINTQPGHPYTPAGKKIVFWESLYRRKSNFHEHLAGPMSDAIE
EDEEDEFTSFDDFENRSYNDNENAHDNRNLKSSSDSRSSDENFWKQAWKEGWGNSANYDK
YINGDDDWSIYIGDENPTLLPLSTNDMFGFMLASLGATLGSLGGIGGGGLVIPCYVVVTK
LDLKQAIPLGSVTVLGGSIAALMLNMKRRHPLADRPIIDWDLILVMEPLVLVGTIFGSIF
HRVMPGKVLSVLLVLLLSIVAHTTLTKARRMYDAEKRYIEHLKSARSDYLTRVASFRTAF
RMSTGAWSDDALHGAGMDPESKASYLPPEPVRTESLDSQSLATTPISPKMDMHERQRILI
LNPDFVTLRSDMLEQEKITPRGKVMALIAKFSVLMFLNITLGGGAFRSPWGIQCGGVAFW
VVHIIMIAFLISSAWAAQTYLVNRHELKEIIRFDYVHGDIKWDQRKAIIYPCFFILAGIF
AGMFGIGGGMITVPLMLAMGVHPAICTATSSTMVLFTVMLSSSSYAVFNLILWDYAVVCF
CIGFIGSLIGNGIMQRARKTDTGAVNFERNSFIAYCTGYVIMLCALCMTLQYILQIVLFH
DDMSNSEVGLCAGYRIN
>comp5354_c0_seq1; 836 102 2168 LEN=2209; translated
MSKRYNEKRAALAQKAVDRELDQGYGREDFESNVAKDVRGAAMGEGEEELFEKKMSKAEK
KAAAKAAREAKRKAKGLTKKKKGGDDDKEKAAAEEEKKNAAKILDPNASAEEKREAALEN
LSREEIIVTYESRKGKMHANARNINVSGVTVTFHGKPLIEETDLVINYGNRYGFIGPNGS
GKSTVMKAIAARAIPIPENLDIYFLNSEYPARDDITALEAVMESNDEVQELEAKAEHLND
AMAEADEDEQAEIQQTLEGIYSRLDQLDASSAEARATQILFGLGFTQKMQHMKTKEFSGG
WRMRVALARALFIEPEFLLLDEPTNHLDMEAVLWLEEYLSNWNRILFFVCHSQDFMNSVC
THIVRLDATYKKLRYFSGNYDMYVQTRRDRDMVQMRQYEAEQRDIAEIKDFIARFGHGTV
KMVRQAQAREKLLEKKLEAGLTQLPEQDPDWDWSFPDAGELPVPVLSVENLSFAYPGGVE
LYSNVDFGVDLQTRVALVGPNGAGKTTLIKLMTGQLNPTKGAVKRNTHLRISRFTQHFEE
KLDLTMTPLDFFKQKVMPEQPIEKIRPLLGRYGCTGDQQSQIMDQLSAGQKARIVFAIIA
HERPHLLMLDEPTNPLDMESIDALARCINKFKGGVLMVSHDMRLISQCAEQIYVCDHKKV
TLYKGDIMKFKLHQKKENSKNLAQHMNG
>comp5354_c0_seq2; 761 1 2072 LEN=2113; translated
XGCDGRSNEKRAALAQKAVDRELDQGYGREDFESNVAKDVRGAAMGEGEEELFEKKMSKA
EKKAAAKAAREAKRKAKGLTKKKKGGDDDKEKAAAEEEKKNAAKILDPNASAEEKREAAL
ENLSREEIIVTYESRKGKMHANARNINVSGVTVTFHGKPLIEETDLVINYGNRYGFIGPN
GSGKSTVMKAIAARAIPIPENLDIYFLNSEYPARDDITALEAVMESNDEVQELEAKAEHL
NDAMAEADEDEQAEIQQTLEGIYSRLDQLDASSAEARATQILFGLGFTQKMQHMKTKEFS
GGWRMRVALARALFIEPEFLLLDEPTNHLDMEAVLWLEEYLSNWNRILFFVCHSQDFMNS
VCTHIVRLDATYKKLRYFSGNYDMYVQTRRDRDMVQMRQYEAEQRDIAEIKDFIARFGHG
TVKMVRQAQAREKLLEKKLEAGLTQLPEQDPDWDWSFPDAGELPVPVLSVENLSFAYPGG
VELYSNVDFGVDLQTRVALVGPNGAGKTTLIKLMTGQLNPTKGAVKRNTHLRISRFTQHF
EEKLDLTMTPLDFFKQKVMPEQPIEKIRPLLGRYGCTGDQQSQIMDQLSAGQKARIVFAI
IAHERPHLLMLDEPTNPLDMESIDALARCINKFKGGVLMVSHDMRLISQCAEQIYVCDHK
KVTLYKGDIMKFKLHQKKENSKNLAQHMNG
>comp5355_c0_seq1; 533 385 1473 LEN=1884; translated
MALTIPPELKKITPYVRRAEELDRDQGNPESRLVAYYCRQFAVHTGIPLATSPAGKLCLG
ELLGVLEGEKEAMDSFTRDESKFLCEKFAEKIFDKADTEDQSGEANRNTARTFYAAASFL
EILQQFYNDDDDSAEVAEQKKKSVYCKWKSTEILKAIKEGRTPTPGGYGEEETNNADAEE
KEEDTPVAIAVEDESPVMPETVEEDSHDDNGDEEDEEEEEIKPNVPVDEDSGDDSGDEVG
AQVPLGPPPAYPIGIEPVDLPLPPPAPPVKPPLVFDLPPPAPMSFPSPIVPTPTPTPTPP
MEKPKMTTLFGLANKKKPKKKVGRAEIADATELTRFALAALEDKDIEIAAERLEQALQCL
GR
>comp5356_c0_seq1; 1010 176 1882 LEN=2243; translated
MSDPVSEQYDSCGLPEKIVGTENDSEEAESLEFEKLELMECEDGATKEIDMPTTPSQKKD
PNSSDGEGTEGAEQEHGTSFLEQGEDAFGGDDGRNEKKDDDDNDDAEEEEYNDDYSPEAD
DRKPAAKQPPNRKRSKKNPGDDDGCSPRRNTKGTRTSRRTRNKIPKYFDDEDDEDAEEFS
EEGAGEDPCDSKPPAAGASASNGGEDSAEEEASAEENATEEEYKSNSEAQDEAQDKGKKR
KADFQSPDKTTDRVEKSSETSQSSDIEDGSEEPAVDNDDVVKSPKKKKKKHADQEDAKA
FTNPKKKIDGESVKEEDEEASMNVKKDNVKNDNVKKEKRSSERKTRKSHAPNDRMRDLLE
RKKTWTKKKETKLLTVKDVRKAYETEGTNKDDALLSMLMAYYIAGVKAVTFDKLFEGLGH

RPKNKAIEKAWKEVRAENLVEEAPATAGGGSKKAKKVFRLTQKGFDRAAPEGYEEETLSP
AKTSDELHDIIKGNAKNNYSILVFEILLEKKDEGVSLTTRELADECGVDKDSHGFFYGFK
WLKDNGYAVSDGRRKNAKYSLSEKCFVD
>comp5356_c0_seq2; 1010 703 2409 LEN=2770; translated
MSDPVSEQYDSCGLPEKIVGTENDSEEAESLEFEKLELMECEDGATKEIDMPTTPSQKKD
PNSSDGEGTEGAEQEHGTSFLEQGEDAFGGDDGRNEKKDDDDNDDAEEEEYNDDYSPEAD
DRKPAAKQPPNRKRSKKNPGDDDGCSPRRNTKGTRTSRRTRNKIPKYFDDEDDEDAEEFS
EEGAGEDPCDSKPPAAGASASNGGEDSAEEEASAEENATEEEYKSNSEAQDEAQDKGKKR
KADFQSPDKTTDRVEKSSETSQSSDIEDGSEEPAVDNDDVVKSPKKKKKKHADQEDAKA
FTNPKKKIDGESVKEEDEEASMNVKKDNVKNDNVKKEKRSSERKTRKSHAPNDRMRDLLE
RKKTWTKKKETKLLTVKDVRKAYETEGTNKDDALLSMLMAYYIAGVKAVTFDKLFEGLGH
RPKNKAIEKAWKEVRAENLVEEAPATAGGGSKKAKKVFRLTQKGFDRAAPEGYEEETLSP
AKTSDELHDIIKGNAKNNYSILVFEILLEKKDEGVSLTTRELADECGVDKDSHGFFYGFK
WLKDNGYAVSDGRRKNAKYSLSEKCFVD
>comp5360_c0_seq1; 291 261 1304 LEN=1356; translated
MKVFLNYEDNENKDFHKALKITLPKSWKTGPVTNLLNQFAESYNAKFQETNPLDASEIHL
AIREKKEQAYGSMAGIPETRMVPVCNDAVVIETIPDRSDVYICHGAGRTKAEVEAEEKAE
QREQEELRKNTVIALTLAAKTGSRRVDLTRHVDTTSSLRYFMKQPSIGLAVPRRKLMVND
DSSQALELKLLFYVDVFDVSSLAFPHVYFDPDFLVSMKTSDWDEFQMIPGCMTGRCSEVK
DDGKLFLGGMDLREQAQGEKLKSIDDFNKAQSAGGAEAAPVLERLRNAMFEIGIDKELFD
QVVDGMKKKYTQSAANESELLAAVSQELGKELKTSMKAIAVDQLRIK
>comp5360_c0_seq2; 293 261 1175 LEN=1227; translated
MKVFLNYEDNENKDFHKALKITLPKSWKTGPVTNLLNQFAESYNAKFQETNPLDASEIHL
AIREKKEQAYGSMAGIPETRMVPVCNDAVVIETIPDRSDVYICHGAGRTKAEVEAEEKAE
QREQEELRKNTVTCTHFGCQNRFPKGGPYPACRYHKLPPVFHETAKYWACCPQKKAYDWD
EFQMIPGCMTGRCSEVKDDGKLFLGGMDLREQAQGEKLKSIDDFNKAQSAGGAEAAPVLE
RLRNAMFEIGIDKELFDQVVDGMKKKYTQSAANESELLAAVSQELGKELKTSMKAIAVDQ
LRIK
>comp5360_c0_seq3; 258 261 1130 LEN=1492; translated
MKVFLNYEDNENKDFHKALKITLPKSWKTGPVTNLLNQFAESYNAKFQETNPLDASEIHL
AIREKKEQAYGSMAGIPETRMVPVCNDAVVIETIPDRSDVYICHGAGRTKAEVEAEEKAE
QREQEELRKNTVTCTHFGCQNRFPKGGPYPACRYHKLPPVFHETAKYWACCPQKKAYDWD
EFQMIPGCMTGRCSEVKDDGKLFLGGMDLREQAQGEKLKSIDDFNKAQSAGGAEAAPVLE
RLRNAMFEIGIDKELFDQVVDGMKKKYTQSAANESELLAAVSQELGELKW
>comp5360_c0_seq4; 256 261 1259 LEN=1621; translated
MKVFLNYEDNENKDFHKALKITLPKSWKTGPVTNLLNQFAESYNAKFQETNPLDASEIHL
AIREKKEQAYGSMAGIPETRMVPVCNDAVVIETIPDRSDVYICHGAGRTKAEVEAEEKAE
QREQEELRKNTVIALTLAAKTGSRRVDLTRHVDTTSSLRYFMKQPSIGLAVPRRKLMVND
DSSQALELKLLFYVDVFDVSSLAFPHVYFDPDFLVSMKTSDWDEFQMIPGCMTGRCSEVK
DDGKLFLGGMDLREQAQGEKLKSIDDFNKAQSAGGAEAAPVLERLRNAMFEIGIDKELFD
QVVDGMKKKYTQSAANESELLAAVSQELGELKW
>comp5361_c0_seq1; 620 176 1801 LEN=1995; translated
MACGYEIEDITDPSQISQAKAIIFPGVGSYGSAMKVLKEKGFEQPMREYLSARDRPFLGI
CLGMQTLFESSDEHEEGADAIPGLGVIPGKVIKFDETKMVVPHIGWNGRKSHKESSVLKY
VNPEDSAYFVHSYYAPITDENKEWILTSTTYCGQPFISGVQRGSVVATQFHPEKSGKTGL
NILRGFLEAVENGTLNNAKSIPVDPKAETMLAKRIVVALDVRTNDHGDLVVTKGDQYDVR
ENESDGAAVTGRGGVRNLGKPVGLAGRYYDEGADEIAFLNITSFREGVVEDMPMLQVLEQ
ASKSIFVPLTVGGGIRTYTDQKSGQTWSALEVAARYFRAGADKVSIGSDSVHASEALIAR
NGEKTGESSIETISHVYGAQAVVISIDPKRVYVASKDDADKKHVVVELDDGSSGPNGEKF
CWFQCTVKGGREERDICAVTVAKACQTLGAGEIMLNCIDMDGQGNGFDLPLMRAVSGEVS
IPVIASSGAGNEDHFVEVFSKTNVQAALAAGMFHRKEVEIEACKSAMRENKIPARNVCTS
Q
>comp5361_c0_seq2; 257 176 841 LEN=864; translated
MACGYEIEDITDPSQISQAKAIIFPGVGSYGSAMKVLKEKGFEQPMREYLSARDRPFLGI
CLGMQTLFESSDEHEEGADAIPGLGVIPGKVIKFDETKMVVPHIGWNGRKSHKESSVLKY
VNPEDSAYFVHSYYAPITDENKEWILTSTTYCGQPFISGVQRGSVVATQFHPEKSGKTGL
NILRGFLEVSYFLSFFLVANQFCSMTQIFSVQLPSDLTLCF
>comp5363_c0_seq1; 625 144 1811 LEN=1883; translated
MDDEDEEDPFDVFGDDDSDSDNDNDAEQVVTSKESMEFARSLVDATNEKISHPKSESGRS
TAIVSTPVSSSERNDNNDCQDRQLEDLSYLKAWENKWPDPMYKGEILLVSPLPVGGGRGY

VATTPIAPGTLVLVESPMMTWPDEQIGKKLGMVSVKHLIEHPNASKIVDDLEDFYPTKEQ
VDRYNGNEDGTMEIGSDEYEQIPKMIQFLKSELASSAKGEESGEPNSKEKDQELLDLVEL
SKTLHIRSRDNTELTEIDIIRLLLVLRYNGLESGVYRHVAMLNHDDYPNCAKFLPTGGKT
FSEVRTTRRVQPGESLTISYLPRVVSHASRRKVLWEQHRFDIGVKHLKGERYKMETIASS
LPPSPIQGVADETLTDRIELATEELEKMQAEIEASLHLTSAATSAQTFETVKALEQTALE
LYKEAGEQLQNLNHILLIPILILHMDICALVLRDSSLSNSVQLGVISRQVVSAYHLLPLQ
KLLLGADHFDIARTTLDLADSVSQLLSRSAKTLYNLNFPSMDSFAAWSTFEHETRKEHNR
IKALYPHDVEKHINV
>comp5364_c0_seq1; 456 224 1876 LEN=1970; translated
MSEEKKGQESSGQDGKILMTEEDFDVESSGDRFDTGAECPTDKVEGVGPKGSRTDGNTNG
AALVPGAFPVSNPLNSSGFLAHITGSDAEALVTVSTTNGSKEQYLAEAMRVDRDKIVVAT
EVNDDEEPKSKSYTKRLIAIAVVGVIAIVIVLIINFTIGKKQKPNNEPTDPTISDSITTP
EESNLSIEKYSERRSSLIEILTPLVSDPEVFNPESPQASPDRIEALEWLVTSNPARFSIL
GLKEHSSNTTYNHSLEEQSETNSDTVVQQIRQHFVLALFYFATNGPNWEEQYRFLSDLDP
CLWNTFRSTATEEDEFYVPKEVDARGIICNTQGRVSFMHMWWNDLSGTLPPELSYFKDSL
REINLAGGSISGSIPDFTDLEKLEILAVNDNCLTGRIPEGFSDLPNLYIFNTYGNAGLVG
SLNEFCNGTAYQYKNEVTAFVSADYCGNYGGLSSSGVECNCCVCCNPNNFECYSQEWGLA
STVFSQIKRKNKYAKSFEKSCLTFEHKQWVEEECPCLVESHTLGNDDSPYYACSNCTVDG
VRPSHPNWDD
>comp5366_c0_seq1; 248 1 523 LEN=523; translated
XSLMPSDGPSLSPSVLSSSKPTRAIIAGKSLPSSDDISLFPSDGPSLVPSDGPSLTPSDG
PSLTPSDGPSLSPTIPPSSGTSLLPSDGPSLIAPNHVSSKPTYAQMKPSSTSISQRLPPG
IFTRRTPPPTGVPSLAPSDGPSLQPSDGPSLITEDPIDSPTASQSLLPSDAPSLV
>comp5367_c0_seq1; 425 285 1457 LEN=1500; translated
MGAASQTTRRITFHWNVRVNPKGFSSSPPPRPRRDTRLLSSLLPSPLPSSSNSNSPNASL
PIVLSPLDENYFLNQVETTIARVFRQQQQHGDPNDVNILDLPKEQREAFGIGRNLDKRLK
SLRKNNDCPRCWMQRKHCICSACPSVVNDGSEIHGSNDYNSGSLKRIFLVMHHKEIGLKV
DTAKLILSSFPFQSKLVVGGIGPEHQHSMQEMLESIHDSKRTTLLLFPDETAKTLAEIVN
PQKSTREERQPSKDCEKCKEKTQVNERSMDTYDIIVLDGTWAQARKFHSRYFPSDESPPS
LQRVQLSEGSVDILNKGSTETGHQLRRHDIAWRQVGTFEAFRLFMRDWSREFAFTARNDE
FELWKQIESYQRISNDAALRELGPPRFESK
>comp5368_c0_seq1; 424 175 1386 LEN=1710; translated
MAAMVQDDNMVPEERVSSPINSSGDAQLMARLSIADGSEGKTKLSSASNISSNNNNGSNN
VRRNSKHTVNEEISPSDSLKAQAPQARDIKSSDLMRGPPARTSSGKKRSNNHNKQTTNSK
AQEASKALHSLQASAANNDRIIRATPKGSSAGAQTRASPPEEEPYIEEDEESSEMSASDE
DGSWITWFCSLRGNEFFCEVDEDYIQDDFNLTGLSSLVPYYEYALDMVLDVEMPMEDSLT
EEQQEIVESAAEMLYGLIHARYIVTNRGMHAMYEKYRTVAFGRCPSVFCQGQPVLPVGLS
DLPRNYTVNVFCPRCHGLFFPKSTRQANIDGAYFGTTFPHLYLMTHPDMIPAKPSQTYVP
RVYGFKVNESSLFYKNHEEGPPKKSIDGSGRHSRRSSGRSRRK
>comp5368_c0_seq2; 423 175 969 LEN=1839; translated
MAAMVQDDNMVPEERVSSPINSSGDAQLMARLSIADGSEGKTKLSSASNISSNNNNGSNN
VRRNSKHTVNEEISPSDSLKAQAPQARDIKSSDLMRGPPARTSSGKKRSNNHNKQTTNSK
AQEASKALHSLQASAANNDRIIRATPKGSSAGAQTRASPPEEEPYIEEDEESSEMSASDE
DGSWITWFCSLRGNEFFCEVDEDYIQDDFNLTGLSSLVPYYEYALDMVLDVEMPMEDSLT
EEQQEIVESAAEMLYGLIHARYVR
>comp5369_c0_seq1; 461 1 2119 LEN=2447; translated
XVLQPLIDSKEYCSGAAPGQNRSEKEAKATDAQGTHVKKTMAERAISISSTSMPLSRSKK
RQRLPRIRGALRFVPLIFVAVFQQEAATKAFVATAFRPPGTTAKFPFHRHCTGSHVLSIP
EADALDDDCKERIAGHIIESGIWKGKTDDPSSSDQLLLLARDFVDRPEVFSSLLMSDFGF
PALVAHQTRALAMAAIRREQAEVRREAELVEPQNTQDSGDASRTSNDSEAVANPGVTDHR
DGVVGARHSIGGEPAADEKAGVAAKRFSHSELQSSVVNERARRRMRSLEDYNYGLPSDYA
KVYPKLASELDEYYTFMTQPSTYSQESPIRDATAKVYMNHAKLFLGWCCRKTEGNRDETE
ASDASLFALIPNKDKESADHIIRFVLWLRSREISVSYEANLLRGIIKLLKFRFARESNSD
PLEGQNTFDDIPLIREVRKLHRDANQRQRLAPRSSDETKKWITWKDYLEVVRKVKNEVLE
LIEGYENLPASERTYPGGEKEGLYSRQQKKVAEAYQRYLILAILASIPDRQRTLRELEVG
RTLAKDPETGCWTVKHGPGDYKTGKHYGERPAMQLAEELTPAIDDFLARWRPALEPSTGL
LLVQARTGKPMTSNSVFKRVVRCCFQHTGKKANPHLLRDILVTHVRETEASEKELEALAL
YMGHSIQMQRTSYDRRTLGAKVAPAVELMRSVNGSPGGRAPPRLAG
>comp5370_c0_seq1; 940 151 2025 LEN=2155; translated
MRFYASSRGGSRSLATMALATTQLIPSQSYLSSRVMVTAFSSSLAVKQVNSLKEARGYSS

SSTDLNMSSKMNQSFETWSFDEPCKTMAWSPLSAVELTASGSGLDNWTDDADLVFVGIFA
PSKEDSEEEEDDDKEEEVVEPTLSGSAGDLDASLGGAISNLMIENFKDFKNGAEAGSTLP
TMRFYEDGKTKRYIVVGLGSEPKDSDSSESIGSAIGKALATKCDSEKKAVSAKFLLPESI
ASDSKNLKDLSSAFYSTLYADNRHRTGKKVVTPAEDLKSLTLLSDGGSDSDTMQDSLDIG
KKLATGVYMTKDIVNAPHNVLNSESLADTARRLAEESGGSLTCKVLGKKECEERGMGAYL
GVARGSETEPQFIHITYTPSEGDIKKRVGVIGKGLLFDTGGYNIKTQMMELMKFDCGGAA
AVFGAARSIGALKPPGVECHFVVAACENMINDKAIVPSDILTASNGKTIEVLNTDAEGRL
TLADALVYCDKELDCESIIELSTLTGACMVALGQSVCGVFTDNDDLAEELKDVSKVTEDK
AWRMPMEKSYNKQLKSKVADMTNLGGRYGGSITAALFLQNFVNKKKPFAHIDIAGPVWDD
QIGATGFGAKLATEWIRRQGESDE
>comp5371_c0_seq1; 223 369 1436 LEN=1891; translated
MTTSGDSSKSTTAHTALSEIDDDGAFRRTASSFTSTIEVGGRFEPEANRYHLYVALACPW
AAGTLAALKHKGLDSIVGYSVVHPTWRRTRPDIEGDEHCGWHFANPGDPPVQNSNGYGSF
DCDDALIPDRVNGAKTIRDLYEMAGDTSGKYSTPLLWDRKEKTIVNNESMLILKIFNEAF
CNLLGSEGEDTDRPNLFPPDLQSQAEEWNELIYNAVNNGVYKCGFATSQSAYETAHSELF
AALDKLEAHFGESGHTFLMGDTLTWLDIRLYCTLVRFDPVYVIYFKTSHKRIGSDYPNLL
KFVRACYREAAIRDTTNIKHIKMHYFTSHPALNAYGIIPVSNGPPLETSTEGEHQ
>comp5371_c0_seq2; 223 195 1262 LEN=1717; translated
MTTSGDSSKSTTAHTALSEIDDDGAFRRTASSFTSTIEVGGRFEPEANRYHLYVALACPW
AAGTLAALKHKGLDSIVGYSVVHPTWRRTRPDIEGDEHCGWHFANPGDPPVQNSNGYGSF
DCDDALIPDRVNGAKTIRDLYEMAGDTSGKYSTPLLWDRKEKTIVNNESMLILKIFNEAF
CNLLGSEGEDTDRPNLFPPDLQSQAEEWNELIYNAVNNGVYKCGFATSQSAYETAHSELF
AALDKLEAHFGESGHTFLMGDTLTWLDIRLYCTLVRFDPVYVIYFKTSHKRIGSDYPNLL
KFVRACYREAAIRDTTNIKHIKMHYFTSHPALNAYGIIPVSNGPPLETSTEGEHQ
>comp5372_c0_seq1; 201 1 895 LEN=950; translated
XGSKTDEELLQSSVAALDLLDQYTMSSWDLSSKSRRFGSISRSARSFFRSSSRSSSRSGS
RNSSVRSAVSSDDGIDHAYDTATKKTIYLIRHAESEENVHMHGMQELGTSLVGGRVPTSE
NLSSSLKFIGGVAKGDIDSKLSEGGREQVKELHSVLKSDGMENMSSVINDVDIIGHSPLV
RARDTCYGGFGLHTNGVCENVVELKCLEEATPWETAVQGRKKTVHKRIDELQRWIDNQTD
ATTIALVGHSEYFMIMLGISRAEKFWNCDVWKVEYAAGKWTGLTLKHRLTSSKPMGVF
>comp5373_c0_seq1; 340 296 1630 LEN=2044; translated
MTRHKSQRVVLFGLASCALILSFVTYSRQNRKKRKSEQKSKPGACSCRDGQRITKATKTT
AAATNGLRCKPMFRWQDPELTRTILESADQRAKRWVSFSLHGAHHMVGQVGGRSHDPILI
LDTVVMKPVRRKHTGIREVAFYEAIQASSKRSGFDRYCSLFSEDPGNCDRATVAEETKLL
YRLEPYTPRYFGTVEYHEGPEGTTNRAVPPPDAEREPALLGIGPSSYILLNNATKTFSRP
CVLDIKMGTQTFEPGAPEEKRLAELGKYSPQQETFGFRLTAMRIYDPSHNDADADGYVYY
PKQFGRSLATRGEVKEALLRFLGGAGLPEPVRSHRTSAIQRILSKLKLIKGWFRDNRSFS
FYGSSILVAYEGDTEPDDVSGVQPDMAKAKMIDFGRVRRQAGGDPGYSKGVATLVSILEE
ILRESFLEESRYISPKRNGHAMNR
>comp5374_c0_seq1; 183 108 584 LEN=1374; translated
MSSGIARGRLVEERKAWRRDHPFGFYARPTSKGDGSSDIMKWEAGIPGKEGTDWENGVFK
VSMEFSEDYPSKPPKCKFVPPLFHPNVYPSGTICLSILNEEEGWRPAITLKQVLIGIQDL
LDDPNPNSPAQSEAYQLFIHNKTEYKRRVQAEARKNTP
>comp5375_c0_seq1; 206 340 979 LEN=979; minus strand; translated
MTLFEIYGDFGDSTRDGRKDEPIHPSAPSRNRLYPEDLLDVFFRLDSVLDGGKQSIEARF
ADLDVRSEATLGTFGDALGGFYRDAQITGQLLEVDQGFLSAVSGAGRPGAHHHGRSFFEG
GKRVASRDTCRREEAPWESPNASVPTKAETKRRRDEILGIVFGLSLIKIYSGNENGLNGS
ASLQEVWPVLLISCTAHRYVVGCRWSVGGKEVV
>comp5376_c0_seq1; 374 81 899 LEN=1237; translated
MKSYSPITLLLALSLALRQEHAAGFSRPNPEGGCSRAVSSSAAQQSAGNRRDWLKQAAGVV
VATTAAATPRVARAEEKQKMFAPGGTLVDYQVGVRVGNERASASRRSDNSNVVFGQDYYY
KFGTAPSFIPEGDTSFPVKMPFVLSQQRYDGLKKYRERVQRGIDKVASLKSSISAGDFAS
IPPADDPEYSIRPMGLLANAFLASENTGTTNELFLARWYINEIYLDLADIKQAPSKEAAE
TAHANAILALNSYLGMLNRSITAKVGDPFVLV
>comp5377_c0_seq1; 548 95 1744 LEN=2375; translated
MDNEVADATAAKQAKAAAAKARKEARRALRRKLEAETAQTLKDLGLSASAVDVGASKSNA
NRPLPAPPVNLPADSCTICLFYQYKEPAWKSKDHKAALNKVTEFAKAHNVTGRGRCAPEG
LNCTLTASANDIRAFCYALRDWDPAFNETDFKLEDGVENKHRFRTFTLRKVDELVGYTLD
GIKAPSLNRHAGKHLEATEYHKQMQQKDTVIIDVRNAYESDIGHFQPPEGGAELLDPKMR
TSQDFPKWLNAPETKEKLKNKTVMMYCTGGIRCERATALLNQITEAEPEFDTKGVVMVRG

GIERYLKTFKQGGYWKGKNYLFDKRMEQAPPEKSQEALDADVESKCCLCPKPYAKYRGQH
KCARIIPSSGLPCAVPVIVCDECQKTATDEPQCLACPLCVKGYQTPETAPDLIGQKRKLG
LIEKGGTDLVTGQKVARKNDVASQRLFVGKLPLTTTASKLRKAFGDAKIETIQWIVDHDT
KAFYGSAFCKMASIEDAIKAQSQGTIPLLQSKKIKRQKQRKARVCFAPLRENELWPLESY
TETEYPPLG
>comp5378_c0_seq1; 829 319 2349 LEN=2486; translated
MSEYAALHATSLLLVGKNCKTSESSAATSRQVTASAAAVTTITSRSAHGDTVFTTSDLFP
DDEQDFGFKQPSAKNFRKSAANDNGIETIMQLSSNNSNATHQSNDVLVLPADDLSDGPDE
SRWRSHGITRRRKLLEEDKRIGGRDFSMSFDCSVQRYFSVAHWALEQFHQLYQKSSSSST
DSAKELEELYLMGHRVVAFLTECLPRHPGFARSPFVRQRCKQELDHLMKCLEDVAIRIDE
QVCNQFVDDGDFFLDSMLAAEMANEDEDQEQRKSPRRFRTPSKAKQNNSAFAEWEVDFPD
DEFGPLKGEATKCVSFKADFERTTAESPTAETVGTNGTESLEPSDTSYTNFDSSDNSKGS
EEALSRSGSEVTDRGGSCDGSNDYRPARDDDQQDVFDSIEFSPEDEGALDLPSQPVSILK
GVRLDFLKTIACEPVLYETDSEAADSWANTADDAKSRPCLPSSSGVTPTCDPARIAFRDL
MNKLPHKSILQRNNQSKLLGGFLSRTPLNPPDRSPASSPTLIEILGERNTNKPAGVAHTD
FDDVIENEIKEYLDSSQDEEDPKQFVESCHAKMSTQSKSMDMGTIRHRKPRDDRSSVSSY
VSSSSSSTVASTTSSQSLKSLTNKTGSQTSAFSSFGAHRKEQQMQGSEASKTIFEQDDWI
SFDNSSGDSFFADSSF
>comp5380_c0_seq1; 348 1 1092 LEN=1174; translated
XDDLTTSTAVVAAVVAVYIQSSLYLKPQHEAEQSKSTMVRSHEARGFGMLISAMKIPPHI
RILCPEQIGHGEDLKAALDGNPSNQFYYKEAGRETANTMLESTSEFLDVVQAGPNCNAFG
ISLGGALVYSLRYKRPEIIKKTVLVCPAIGFCLDDAFVDSIVKGTNNFVDFQSRDDVKFL
FRDLLSTGPYEDTDATNPAAPAPAATTATKQRRKKYPIPPFFMEAIYRQSQKTTPEGHYR
KMMLALLETTGRVNNCNDEDFFGDGNECNKRDDDNARIFRATSDVDPKSPRLLLWPENDQ
ISNCAKGMLYFEHSIVPSSSSGADTGTIFETIPDCGHCFTGDGRMIYDVIRPRVKEYLLD
FHTH
>comp5380_c0_seq2; 419 83 1288 LEN=1370; translated
MKRNSRSRRWFEATVGSHLLKKAMELGQKHGKLVGPKSVFLPKCGIRMRYYEREAQPIAD
DQHQPQHLPTLLFCHGISEEARGFGMLISAMKIPPHIRILCPEQIGHGEDLKAALDGNPS
NQFYYKEAGRETANTMLESTSEFLDVVQAGPNCNAFGISLGGALVYSLRYKRPEIIKKTV
LVCPAIGFCLDDAFVDSIVKGTNNFVDFQSRDDVKFLFRDLLSTGPYEDTDATNPAAPAP
AATTATKQRRKKYPIPPFFMEAIYRQSQKTTPEGHYRKMMLALLETTGRVNNCNDEDFFG
DGNECNKRDDDNARIFRATSDVDPKSPRLLLWPENDQISNCAKGMLYFEHSIVPSSSSGA
DTGTIFETIPDCGHCFTGDGRMIYDVIRPRVKEYLLDFHTH
>comp5382_c0_seq1; 190 227 721 LEN=890; translated
MSDRKVQFNGIDVILPSLERVEYTRQEHAATWVSPRELKMTRKDIKATVMLVLKGDERAH
LDSESHNTRGLEALTREAAATKKKRKSQTRKVVIVEQHDPESLSKKYMEVTEASRNEARA
IALQDAKYARSNRDISDDPTQPSDIEKRDDLEQIVGTKAGDLGR
>comp5383_c0_seq1; 229 142 707 LEN=707; minus strand; translated
MTAIPMDHEEAASQAPEASSKSTEYASVPVPVPVSPVGGPTEPSEPVSAQPEQASALEET
QGQAQAPPDAYNIFFKHTRSRIVRGLPEEGTVAETVLSIEHIVVNSTMPKQSRKSHGQIS
FGDLARRIADRWKTIGTNQRALFDHYASLDMQRYRREVALWKAKKMRELAGSDVICGNVI
SSNSINSSX
>comp5383_c0_seq2; 321 142 898 LEN=898; minus strand; translated
MTAIPMDHEEAASQAPEASSKSTEYASVPVPVPVSPVGGPTEPSEPVSAQPEQASALEKQ
KEKQKQKQAPKPKQSEAQAQNAAKPPEEAPKQNPNSKAKAQAKAKAKQTPKEKPKQKRRT
WKKPKDKPKRPLSAYNIFFKHTRSRIVRGLPEEGTVAETVLSIEHIVVNSTMPKQSRKSH
GQISFGDLARRIADRWKTIGTNQRALFDHYASLDMQRYRREVALWKAKKMRELAGSDVIC
GNVISSNSINSSX
>comp5383_c0_seq3; 190 79 702 LEN=880; translated
MTNQRQILKIVLCSLLAASTVAMKDPATGISFDSESRARPIFGVGARKKGPIKVYSVGMY
GSEDLKEELSSISRSESKGKKAVDALRKGASNSSTAFVLKMNFKVGSEKMAQAIADSVKP
RHSSDEEVGQLKDIIFEGVQGKGAAVKGTVIEFDCSDTDGVEVTVDGKGCGKVSSPGLAK
ALCDVYLDDKAVSPKLKESILENCCAE
>comp5383_c0_seq4; 190 148 771 LEN=949; translated
MTNQRQILKIVLCSLLAASTVAMKDPATGISFDSESRARPIFGVGARKKGPIKVYSVGMY
GSEDLKEELSSISRSESKGKKAVDALRKGASNSSTAFVLKMNFKVGSEKMAQAIADSVKP
RHSSDEEVGQLKDIIFEGVQGKGAAVKGTVIEFDCSDTDGVEVTVDGKGCGKVSSPGLAK
ALCDVYLDDKAVSPKLKESILENCCAE
>comp5384_c0_seq1; 137 1 266 LEN=568; translated

XSSSNTNSSNSNSTNSLENLSRFELMFLPQERRVMKAVKAKAASRRLQRERSRRLHYCLL
ASVVTGIVLVVAWYVVVVQERNDGPGTD
>comp5385_c0_seq1; 318 1 1190 LEN=1728; translated
XHSTTASNNSSLTSRESAKHHTVAKQYFDRTRFVPSATGFSTIDPGTPLTEHAVAAVRNE
TKRNQTKPMSPIASASASSGTGPGDAAAWASPHRRRIRPRPRDPSRSGSRGPGLVVSVVL
FLLALSRLQGAAGEQPSPRSRHPSTGCISSGTELLRAVTAYYDPEGQAVDSYGTPIEDWC
LSPSVLDALYREGSDLETRFSATWEGIVDSHGETFAGGLTEGSLALAWLGASTNENHVCA
DLCAPAGEDVRVPVPVPVPFDPEEDGIRDEDEAVGNDPHEDAASDNDRDQEEPPSLSASA
TTRSTTTTSMLVRLIPVKLVAVLSLMCLALGLRQMYFSVDSDSRCWCCCKWFSSVFGSRY
ASYEGIDPSDVSEHQKNVEFELLELAAELDAEVVIS
>comp5386_c0_seq1; 425 540 1271 LEN=1521; translated
MATGTTIGDLHSHDDVKDLQSEGLQGSPAYSLRRLQDTRPKKDGHQRGLNPLEGSELKSL
VMDDRYDYTGNGYYDRKGGKKGADYFGGKGGKKGGYYYGGKGGKKGVDYQGGKGGKKGDG
KGGKKGGYGGKGKGGYENPTIPPETEIPSLNSSSTPSPSDSPSKSPSDSPSNTPSHSPSN
SPSYSPSNPPSDSPSNSPSFFSSSLSSQSSTDTTSANDADEVGEGSNDESLTSDAADRIG
IGS
>comp5387_c0_seq1; 420 103 1080 LEN=1304; translated
MAVANGWAFRPHLTKTIRAAGLVAATKGQAYQKHNRCVLQMGGYYVEAEEEEAKNIIDRS
KTHMTFGIRCKEEKLQISIPSPSSSSGNSAITKTVVGLVPLVDDAIDKEAEEESFVFSFD
DVKDDENVEPTNARTVSRLMNLLFHSEYGDDEESSPSWTLVEIGADPIFPLAVAEWMEAV
GSEQKIVTNVIVKHSDEQRLRVLEHAHLFFNQRKPKSSSTLTFSTKPLTDDSNPWIETLQ
KGTNNKIVVLISNEKFATQLLDKMKENANPTANPAGDFVYRTIIPSTIMSREKLLLDGFQ
CQSVGFGDENGDDGLDFWEIVPIRQ
>comp5388_c0_seq1; 223 184 1005 LEN=1253; translated
MSNSKPSIDNKTKPHPSEMGTGLRRLMICGGTDGIGFSLLASELLRSKYTEFYILGRDFT
RVRRLCKRRDDDEMPEKSSQKVIEIPCDITHLNEISTAVAKIGDDSIHDFVLTIGTFTSG
KIADIIPISSEENDCKNVVAEHFHLNCVSTIHLVRSILPKLVAGDSQILVCTASLALKAR
SPYGLQSATKAALRSVVDTLRIELAGKTRVMNVMPPSVDTKIFEKAGDSRNTDGYPPASR
IATIIQFMLDCPPDICVPEMLIEQHHFDRVHEK
>comp5389_c0_seq1; 410 123 881 LEN=1435; translated
MTLSSSLTTDSAMFNPINLEASIGRIEDHTINERACEAEQVSRSSPSSYFSSSQHLKNAI
HIPTTKSRTGITRTSSRTPFQTEAAANTSARSDSSNSLLPQPYPISETDHEIKERMRLTE
SVDWSNSLVIANSPPKNTESVIIEATENADQQSMMEVTVDDFVLSAEEANSLTDLDTATI
QDDPNGFWDTPAQVASTMSVDRDVVDMTTAVPFSVTISSQENAFMMDENEDGILVEGEDA
DASPIRSSDMEW
>comp5390_c0_seq1; 584 353 1654 LEN=1690; translated
MKFAACSTMVLLAASSSGVRANSAAIADLPGKLVDGVRRHLNLNGILGSPTRPTNSASHS
PYEKIMGHPVFQVTTSWGSAYMNMEELSQDDEDASAGAESEGSMLGVAKSGDEAAGIRKD
RYRTISLYYMDPDDAIGAHAEFKQMDGMKDADVRITSVSLAKAVRSACNLGNGLLTGQPI
DFHKGTVLPTKEGGSLRHKILPPKKQLYYAAFCRGKERIGLFSDSSDEKESQKDHASAAV
IGNAALAYRNAQRKQAIRDRKLAYKPKNQLEADYAHMDGNLGIPVFYVPGMERRRFRIKS
LVSGGVRKEIPLFFNYEDLQEAWQKTKPIGGDKDTYLPNEPQGVEVFNLWDVLTSMEKEQ
DKMAQKRAKESPQNRLVQSLSQPFRNRFRNLVDSDDEAAMLDGIVFIPSSDACTYKEGIT
ARGNGKARLRPMR
>comp5391_c0_seq1; 395 1 1201 LEN=1897; translated
XLPKDECKHQNTAMNPEHITGTVAAEVIGNNANCSGSVCMMSLSNHHEKTTGIDDSSSCY
LGNSKNRANEDHRAVITEVKENDDKEGQYPPKTKGMKKHRVSGTDAMMEKTNKVASSLKD
AMEELDRIWDVDDDDNSCASSLSSFDEDHEKQMQNVESLPSSPSRNRVRQTAALLVRVFS
NELYDPAVQALAEHEELRSEAINLTHQLEISQREVKRLKGSELRSKESIQNLLKAVDRST
EEAKDSSQAKLVEAKLRADLLQTRKERDDLHHESMVSKRSLELVTNEANSLKQEKSRLQK
ENIRLEREARSARVLAESLSRSKNANLNNGNDEHNAEFYKRKTRDLETHLQGMTARLAEK
NQELMELRHCRDRNLSQNRLEALRAGKKISNKKIRVSNAR
>comp5391_c0_seq2; 328 1 1338 LEN=2034; translated
XLPKDECKHQNTAMNPEHITGTVAAEVIGNNANCSGSVCMMSLSNHHEKTTGIDDSSSCY
LGNSKNRANEDHRAVITEVKENDDKEGQYPPKTKGMKKHRVSGTDAMMEKTNKVASSLKD
AMEELDRIWDVDDDDNSCASSLSSFDEDHEKQMQNVESLPSSPSRNRVRQTAALLVRVFS
NELYDPAVQALAEHEELRSEAINLTHQLEISQREVKRLKGSELRSKESIQVSIVSDVVLI
RFASQSIISQRFFVFSHSNYXIHFNRHKTNNNECSQNLLKAVDRSTEEAKDSSQAKLVEA
KLRADLLQTRKERDDLHHESMVSKRSLELVTNEANSLKQEKSRLQKENIRLEREARSARV
LAESLSRSKNANLNNGNDEHNAEFYKRKTRDLETHLQGMTARLAEKNQELMELRHCRDRN

LSQNRLEALRAGKKISNKKIRVSNAR
>comp5392_c0_seq1; 160 259 1158 LEN=1688; translated
MGGITEEPTTKIINSDSDSDCNNDDDAGCTKRSDEIFKKTLAHSLHRRKVALAKQHNGKN
TVRFSTVRIREYMLCLGDNPSVTRGAPISLDWVYIRDNCYELEDYEQGKHSLDRNSDSVA
LATSTSIPAKLLPLERIHILKERGYSRSEIKDAMDRAHKIREQRLWTKRHCERVDRFQTF
VRAMSCFSLSQDERIKCVDVETCSSTALSSSDLSLVHETDKSLREHKARWKRQPRNSLDL
MTSQRKKLQRNHLYKPRNLLKMLRSWHEKPQETDSIDTARHMSVRLEIDGSTLVILGGE
>comp5393_c0_seq1; 903 35 1465 LEN=1519; translated
MSEEAAKRIKVAAEERNFLFSSESVNEGHPDKLADQVSDAVLDACLKADPFSKVACETAT
KDNMVMVFGEITTSGKIDYEAIVREQVREIGFDSFEDDLGSVNSKGLSCDSCEVLVRINK
QSPDIAGGVHVGKSDLDVGAGDQGIMFGYASDETEQLMPLTHMMATQLGKKLTDVRKDGS
LWWLRPDGKTQVTIEYKQHTDGSVTPIKIHTVVISTQHAEPLKAKRTEEQSGKDNRGKYE
GEEQIAPSMDEMNELIHDKVIKATLESIKLKSGESALSIYDRETCNLHINPSGKFIIGGP
QGDAGLTGRKIIIDTYGGWGAHGGGAFSGKDPTKVDRSAAYACRWMAKSVVKAGLCKRAL
VQLSYAIGVAKPLSIFVETYGTEKEGLTAAQITELVKIEFDCRPGALARDLALREPKYNK
TAAYCHFGREPFEKDGMKFFSWEDPIDMSKYASMSTEDIDKECAAQKEAILKKWVD
>comp5394_c0_seq1; 273 105 1709 LEN=2042; translated
MDNNYTHEEDASPAVAGMMMAQDVDTCVSTGGHGDENSKSIPSTSATMYSKCKSPYSTSE
FFFRLWENRWILLLGQVLSLLLASGGAAQATLHLQCGLSAPTFAMSLVYLGLFAIHCPLL
WFRRWRESKSDGLSTVESEEEVVVFDQHAYSNSNVDANGHAIASYCRPERSEERLPITVE
HGEKDAVHPIHSQTTNDNISSMRVSKPLLKKTYFWYLLLAFCDTQANALTILAFRYTTLT
SVTLFDSLAIPSAMILSSLFFQSTRRYQALHYLGVFLCMVGVVFNVLQDYKSDNEIIETT
DSSSYTHKLRGDVCAIVGGLLYGLNDVLTEVTLSETRDTTEYLGMIGACGSIIAILQAFV
LERVEIQGFLSETVGISVDGDELRACSTAEAMWLLVAFVGVTICSYLGASRFLMVSEAAL
FNLSLLTGDLWSVVFSVFGEKIVPRPLFFCALAAVLSGVVVYEMAPSPALEKRHGGKHSN
NIVKECTEGTPIRKDGIFRETTDGGDAEVISSDSLEECGGDSNTLEIELKGIVS
>comp5395_c0_seq1; 279 201 518 LEN=747; translated
MTQKITTNMLLGLLVSSVAINSNNCSVHAFTGMKSLSRNCRGSNLARSPSVVFMSDDRSK
DDEIAKLEEQLRKLKEEKTSEETAVAATTTTTIEEEEEDEDRGGG
>comp5396_c0_seq1; 235 1 905 LEN=1324; translated
XNSPKSSLTTTQSCGHYGVVSIVSWCLVSHVNFTINFNTTRNRRNTSLSFFQKLPYAARV
AIVLEECGEPYETIAIDLQNKPTEFCELYVRANPIPNARAKVPVLEVLGKDGAHRSIVLA
ESLVVADYLADRYPDVGLTPSTSEDRAVMRLFSELCGASVFSYWEILRARGDDEKFESAV
QKFKQGLINTNAFLEKKGKSGGPFLFGNQFTLAECNVAPFLQRACNVLPAFTGARNAEMR
DYDSAGVDPIKICEEEGLIRLKSWVCAVLARPSVKYVELSDEEMNQSVSKMLKRFEEMEK
K
>comp5397_c0_seq1; 381 150 1208 LEN=1404; translated
MAEKPISQNGKTDISMTDPSKFRPSPPAFQCGEELQEGGGTIGKSGVSFYCSLPQDEKRL
TFSCRDRSVGVLESKESAITNDEFDATQNACAMDPFFFDEGYTLAGRTGFQVWSGTRLMI
ESLLFPLPDDKDRLVAIQKEINSADGKNILELGAGVGVVGTALSAATRCRVLLTDLPTLV
ENSLLPNLKQNRDESRVEEATNETSWLYKASSNIEFEEEEDNEDVVFSLGNRGGWASIAT
IDWTKTLNKEVSSVSSLDWIVASDCVWLVSMLDSLLDTVDSLFGLNPRARLILSFQRRDA
GDNVRFTSVPSILGSVRRRGWSIDCLAWRHVRQEGMTEPQEVFLFEIVPGSK
>comp5398_c0_seq1; 180 78 461 LEN=696; translated
MTSAQNHNQSRVVHCTTEREYWEAIESAGSNLVVVDCFAEWCPPCRQIAPFFDALSLEHP
EVVFVKVDVDKVPGIGRILGVWAMPSFFFLKDGETVGSFTGAREGLLRQGIANGGRVGGV
CSSCAVQ
>comp5398_c0_seq2; 131 78 729 LEN=896; translated
MTSAQNHNQSRVVHCTTEREYWEAIESAGSNLVVVDFLPSGEWFVWMQRSAGQRRARPST
ERRKTRGGGFVRCFRFVSFRSFLTTSCVRAAKPSSRRGVATPRTIAQVSPLPADRPVLRR
PVAGAPGGRLCQGGRRQGPGDRPDPGRLGDALVFLSQGRRNGGVLHGGEGGPPAAGDRQR
RPGRGSVLVLRGAVSTAYSIGDLAAPPWRAVRFVPR
>comp5400_c0_seq1; 466 487 1564 LEN=1721; translated
MISQLSELSTMHPQLLRKLSKPSKVASILDWKKASDNTVLALNIHKNRVGIAVASHPSLG
IPCIELDPLRFDDSHGNRTNISRSIDRHCLERFSDIIEDYKVRRQLSWLDQTRARVDSLP
AFLRFCASDTLSFFLWFRLQVCGVVVNWPLQHDTGRMGAACGRVIFALEQLVERSNESTV
DIAEERLVGGNAAANDAKDHDGVGILSRPFCLWDAGHIDVKQRADPSKRVDAFGRCASYG
REAREPPVARGQERRDNENENTYFASKEQYYEDELTVVLGVWDDFCREHWPDLYETRGGN
AEGILGKADGAHQAPASPSNDYGDSEPATPEKLPIRVSSPRLQNMAKMKQKKCLITMR
>comp5400_c0_seq2; 485 487 1446 LEN=1603; translated

MISQLSELSTMHPQLLRKLSKPSKVASILDWKKASDNTVLALNIHKNRVGIAVASHPSLG
IPCIELDPLRFDDSHGNRTNISRSIDRHCLERFSDIIEDYKVCGVVVNWPLQHDTGRMGA
ACGRVIFALEQLVERSNESTVDIAEERLVGGNAAANDAKDHDGVGILSRPFCLWDAGHID
VKQRADPSKRVDAFGRCASYGREAREPPVARGQERRDNENENTYFASKEQYYEDELTVVL
GVWDDFCREHWPDLYETRGGNAEGILGKADGAHQAPASPSNDYGDSEPATPEKLPIRVSS
PRLQNMAKMKQKKCLITMR
>comp5401_c0_seq1; 662 56 1666 LEN=1750; translated
MEQNQQQEDIGVQASATEAKNSHSNRSPVRAKRSGRMLYESSFATSFTSARGQKRPPGFV
ASSLDYKSTGVNAASSPSLTTKPGRNSLHNKQKKQKEQLEVEQQQLRQYRSGQSQPTRAQ
GNFGPPPHSITTIEDLESMSEEQIYKLFMEDPELYESFIKSTETERGSTPTGARKARRST
SKKPSSESKSKRRSAKAKTIKSESQDREIPYFQWIFLLVLVGVAIYQGYKTYSSSTSTKD
KNNSIVSRRIKGGKQKNKKGGKSDKKVRVKTKEVSQRRSEASSLMEKKHDVETANTAGRP
SSSKKKKKMSKSKSKAKTNTFTGDSKEEEQNKPDLDSSDVSSEDQALETSSKLDSTAPSN
PSTIPNREEDNDGEWQTVTKSSKGSKKEKALPPQNFHEDKEVIAIPVVESKQDNPSEKAK
DDPESPEERNTFEDSDFIEVKSKKKKRRNLSNSGKEKTNNAQSAMSVETTSIEKPSVTKN
ENLSEEKQNGESVASTEEDAALALKLHEEMNLSSKTDMSNSQEEGWEEVTSKKKKA
>comp5403_c0_seq1; 323 107 1699 LEN=1809; translated
MMHRAPSRGDSEQARQAQAPPWGYVDNPAEVNNFLSQEHLLELSYTDIRNLSSIEESDSN
RNKREKNAHDKVEDLLSKKMLQTSLEDRNAIVGEIHGIQNLAPEESSEMIDAALSELDQE
ISNVPPRSRQSFDRSQDIAPNGYINSSEFRLRFLRCELFDAKRAANRMVQFLDVLSELFG
DVVLKRPVMMKDFSKEEMQAFRVGHQQLLPYRDRSGRRVFACVGGVGLDVPLLTRVKILI
YIILAASEDVETQRKGIISVIWPGTNTPSADKKSNVKLDRIMFMKRIYGNLPLRTCSIHF
CSPSNELNTPFLRVFRTLTILTMPHYLGRLKFHAGQPIELLYSLKTFGIPAELIPITDTG
NIKTIYLKQWMKLRRIIDNAQELSDHAKMALNFIECPGLNDVIFRTGTSLYCHPGNVRFR
CLMEQKHEIPSYIIQKTRSELAAQIIEETEAEGGKFLKWDQRNMYWTPIEQSQVQNKVLL
AIRDFKYKSKIKRAHLEANQDKSFMFLNGEEHNANEMCFGTTFSELSER
>comp5403_c0_seq2; 196 107 1844 LEN=1954; translated
MMHRAPSRGDSEQARQAQAPPWGYVDNPAEVNNFLSQEHLLELSYTDIRNLSSIEESDSN
RNKREKNAHDKVEDLLSKKMLQTSLEDRNAIVGEIHGIQNLAPEESSEMIDAALSELDQE
ISNVPPRSRQSFDRSQDIAPNGYINSSEFRLRFLRCELFDAKRAANRMVQFLDVLSELFG
DVVLKRPVMMKDFSKEEMQAFRVGHQQLLPYRDRSGRRVFACVGGVGLDVPLLTRVSNFN
VYSXSLKWNKTDVRQDIIELXIILRQLIPCNFRLMPPNLPPLKKVKILIYIILAASEDVE
TQRKGIISVIWPGTNTPSADKKSNVKLDRIMFMKRIYGNLPLRTCSIHFCSPSNELNTPF
LRVFRTLTILTMPHYLGRLKFHAGQPIELLYSLKTFGIPAELIPITDTGNIKTIYLKQWM
KLRRIIDNAQELSDHAKMALNFIECPGLNDVIFRTGTSLYCHPGNVRFRCLMEQKHEIPS
YIIQKTRSELAAQIIEETEAEGGKFLKWDQRNMYWTPIEQSQVQNKVLLAIRDFKYKSKI
KRAHLEANQDKSFMFLNGEEHNANEMCFGTTFSELSER
>comp5405_c0_seq1; 854 148 2361 LEN=2468; translated
MDQLIPIVSKLQDVLGAVGQNTQLDLPQIVVVGGQSSGKSSVLEGIVGRSFLPRGTGIVT
RRPLILTLVNTRTPGSEQPEDENRDPNSNEDGEGVEWGEFVHCPGRRFHSFQQIRQEIVR
ETDRLTGSNKGISSDPISLKIFSPYVLALTLVDLPGITKVPVGDQPENIEEQIQEMILEF
ISNPNAIILAVTAANTDVANSDALQMAKAVDPYGNRTIGVLTKLDLMDPGTDAADVLLNR
VYALRRGYIATVNRGQKDINEDLSIRSGLQKEEEFFNNHPVYSTDRNLRGKCGTKILAKS
LNNILMHHIRDVLPELKNRITIMMADVQQELDALGGPGQQSRNTLGGTLLGLLSKFASNF
ASIIDGKGGELGSSEANTKNLALVELFGGARINYIFNDIFATSLMTAGPFDGLSDEEVRT
TITNAMGTRRALFVPEIAFDILIRRQIARLESPGIECVDLVYEELQRIAAQSEPKELTRF
PFLRERLCDTVSTLLKRCMAPTQMMVENLVKVELAYINTSHPDFIGGARAVASVTNRAAN
MEPMRNQELHIHTGSTGVPETPASQADSQYFNADASNGGEQTPYHDKDGGIMNFIFRGKP
TPIADGRSHSGSLPAVVHLPSVPANMKQTDIPPSDRELSEMHVIKSLIESYFKVVRKTFI
DMVPKTIMFFLVNHVKDAMQNELVSELYRESEVETLMKEADDVAERREQSEEMRDLLGKA
LEIVNEARDFNTISQVE
>comp5405_c0_seq2; 790 349 2549 LEN=2656; translated
MGHGITRAARALIPFFSNPISKKCVNRSAVNLQESLVYWKASSDEVSFPEEPVSLPVDXL
ILTLVNTRTPGSEQPEDENRDPNSNEDGEGVEWGEFVHCPGRRFHSFQQIRQEIVRETDR
LTGSNKGISSDPISLKIFSPYVLALTLVDLPGITKVPVGDQPENIEEQIQEMILEFISNP
NAIILAVTAANTDVANSDALQMAKAVDPYGNRTIGVLTKLDLMDPGTDAADVLLNRVYAL
RRGYIATVNRGQKDINEDLSIRSGLQKEEEFFNNHPVYSTDRNLRGKCGTKILAKSLNNI
LMHHIRDVLPELKNRITIMMADVQQELDALGGPGQQSRNTLGGTLLGLLSKFASNFASII
DGKGGELGSSEANTKNLALVELFGGARINYIFNDIFATSLMTAGPFDGLSDEEVRTTITN
AMGTRRALFVPEIAFDILIRRQIARLESPGIECVDLVYEELQRIAAQSEPKELTRFPFLR

ERLCDTVSTLLKRCMAPTQMMVENLVKVELAYINTSHPDFIGGARAVASVTNRAANMEPM
RNQELHIHTGSTGVPETPASQADSQYFNADASNGGEQTPYHDKDGGIMNFIFRGKPTPIA
DGRSHSGSLPAVVHLPSVPANMKQTDIPPSDRELSEMHVIKSLIESYFKVVRKTFIDMVP
KTIMFFLVNHVKDAMQNELVSELYRESEVETLMKEADDVAERREQSEEMRDLLGKALEIV
NEARDFNTISQVE
>comp5405_c0_seq3; 808 148 2469 LEN=2576; translated
MDQLIPIVSKLQDVLGAVGQNTQLDLPQIVVVGGQSSGKSSVLEGIVGRSFLPRGTGIVT
RRPLILTLVNTRTPGSEQPEDENRDPNSNEDGEGVEWGEFVHCPGRRFHSFQQIRQEIVR
ETDRLTGSNKGISSDPISLKIFSPYVLALTLVDLPGITKVPVGDQPENIEEQIQEMILEF
ISNPNAIILAVTAANTDVANSDALQMAKAVDPYGNRTIGVLTKLDLMDPGTDAADVLLNR
VYALRRGYIATVNRGQKDINEDLSIRSGLQKEEEFFNNHPVYSTDRNLRGKCGTKILAKS
LNNILMHHIRDVLPELKNRITIMMADVQQELDALGGPGQQSRNTLGGTLLGLLSKFASNF
ASIIDGKGGELGSSEANTKNLALVELFGGARINYIFNDIFATSLMTAGPFDGLSDEEVRT
TITNAMGTRRALFVPEIAFDILIRRQIARLESPGIECVDLVYEELQRIAAQSEPKELTRF
PFLRERLCDTVSTLLKRCMAPTQMMVENLVKVELAVSFNDAFEIQRKSCQFLVIHFLVVT
DSFHFVHLFGSTSIPAILISLAEQXAVASVTNRAANMEPMRNQELHIHTGSTGVPETPAS
QADSQYFNADASNGGEQTPYHDKDGGIMNFIFRGKPTPIADGRSHSGSLPAVVHLPSVPA
NMKQTDIPPSDRELSEMHVIKSLIESYFKVVRKTFIDMVPKTIMFFLVNHVKDAMQNELV
SELYRESEVETLMKEADDVAERREQSEEMRDLLGKALEIVNEARDFNTISQVE
>comp5405_c0_seq4; 744 349 2657 LEN=2764; translated
MGHGITRAARALIPFFSNPISKKCVNRSAVNLQESLVYWKASSDEVSFPEEPVSLPVDXL
ILTLVNTRTPGSEQPEDENRDPNSNEDGEGVEWGEFVHCPGRRFHSFQQIRQEIVRETDR
LTGSNKGISSDPISLKIFSPYVLALTLVDLPGITKVPVGDQPENIEEQIQEMILEFISNP
NAIILAVTAANTDVANSDALQMAKAVDPYGNRTIGVLTKLDLMDPGTDAADVLLNRVYAL
RRGYIATVNRGQKDINEDLSIRSGLQKEEEFFNNHPVYSTDRNLRGKCGTKILAKSLNNI
LMHHIRDVLPELKNRITIMMADVQQELDALGGPGQQSRNTLGGTLLGLLSKFASNFASII
DGKGGELGSSEANTKNLALVELFGGARINYIFNDIFATSLMTAGPFDGLSDEEVRTTITN
AMGTRRALFVPEIAFDILIRRQIARLESPGIECVDLVYEELQRIAAQSEPKELTRFPFLR
ERLCDTVSTLLKRCMAPTQMMVENLVKVELAVSFNDAFEIQRKSCQFLVIHFLVVTDSFH
FVHLFGSTSIPAILISLAEQXAVASVTNRAANMEPMRNQELHIHTGSTGVPETPASQADS
QYFNADASNGGEQTPYHDKDGGIMNFIFRGKPTPIADGRSHSGSLPAVVHLPSVPANMKQ
TDIPPSDRELSEMHVIKSLIESYFKVVRKTFIDMVPKTIMFFLVNHVKDAMQNELVSELY
RESEVETLMKEADDVAERREQSEEMRDLLGKALEIVNEARDFNTISQVE
>comp5406_c0_seq1; 211 35 466 LEN=716; translated
MPSVIYNTRVFKIGLLFILSWGFVSAVEHVLGVSETTTTATTNHIAKSASGDTAPLRFFA
KDHTDALPLDLQIEPNATEAESAIASRRATPASFGIEGKVVPEEDDRPPLKADTASGNQN
AYSATTKTTMLLLLGCTAQQLLL
>comp5407_c0_seq1; 229 277 795 LEN=1171; translated
MSIIASSSPEIKALLLPTTSMPAADKTDDYSGNDSSSTYGFPISCERNIIDDCSGEISPS
LPRDIHCQHQRSIGSLPHYSLEEDANHDIAEAESNKCIPTFLSTSASVDERRRGKFDGSN
NWFIEHRDQHNGPSMNRFGPTEDRDVSTNNKENSILGDETDELTLYLERYCM
>comp5408_c0_seq1; 117 367 1227 LEN=1227; translated
MAAIAIPARTRSVPDDPRXPPPIPEPDAGQARGQAQDGGGPFLDTEKLADRYALCQDRYE
AFRFRYYHLGADLDANAKRTTDSADRGTKTNGEWIAETFWPGLELAEAILLDLPEAGGWD
DGSCHTARAVAAGHASEALRLLLRILRWLEEEDRDSFHGVSAVVFRRRKRAWAEASKGGG
HNDSLARCREYLEALRGLAREQSRGRWSTYEPMRRAVAGEWGWEPEPGRELEREPVLGSG
LHKGRPHLAVGSSGKRGGENARASQGTSGFFRLDDCHAELCTGRSSVX
>comp5410_c0_seq1; 276 99 1268 LEN=1408; translated
MVRVARRKAGHRLPPARHLAVAALAVVLVCKLAFGGDGDSYYKYKYNADTSTKDASASPA
RGGSPEVSPVSFRTPLAYGTKSLKERTGELVATAISEGFRHIVTGGHHSSHNETGVGHGW
KASGLGRSELFLQTCFVPFGNRADFAPDPLDPEQAPESIEEQVRLSVSISLKNLQTDYLD
ALVFHNNKAKRWADDEIRRAWKVFEELVDEGTVKHLGMTSVHDPEWFETFWNQSRIKPEI
VQNRFHSNRQYDVNMQEVFRRHNTQVQRFWLLNGSSRGGRRSKDEAEARGVTPAQLMLGF
VMSMGSQTCLVGTKTQQHMRDDVEVARCYPSLFWEAGSAGDTERAGSDEARVSYADKLGM
KQPGDRPLPGPDEAARGAMDYRPCRSAAR
>comp5411_c0_seq1; 557 282 1727 LEN=1842; translated
MSTEPGNDPSMTPAPPSTTYYIRHCDPNSTGEGLGSHARRTLYPLLQVALHFGWTVIWEY
DQQPCWKNNEYREYYSQECMELGPWLGFDTKLSDLDRDRTINVPLHIDGDDARFGNLRGA
PELINFCLEEFIRQHGMDHFVGRQDRNRDYPFVLVTLYGRFHHQDDPSPPVLQWMHRRCR
QWKDIVHQRQHESLLLERSSIDDDGDANDRNCSSNHHVINTTKMTRVSDECSITTFVPLN

IVVHVRVPEDICTQEWKDANSVSHVLTALEQFLRQWNNIRHEQQQKSKKSGLEVVEKYTT
KLQLDVYTEESFTQAKEDEFTAALSEMRLLDHNGNDDSADPTASPAIISQLRFHRQTPLL
PCVQSMAVSDVFFPASSYLSSMAAFFHTSLIVLPNESTRHDPYFATHLKYRRKYHRKGVL
SRVDVDTETSDASICSANTSGGNESSTSTTASSSHVCPIVRVEEEEELKEATLRLWQGKT
R
>comp5412_c0_seq1; 170 1 766 LEN=1004; translated
XDKPKHDRIKQEAQNSSRILAQLQYWVLQWYSSLKDVDLDPDPDPPPRATELLPQTMPAA
TSENSTCLMMISAPCRAVSPSPLPEAVAETETEMSVSSKRQRCDRVSFDPRTSIVYIEPL
SEFTDEEKETLWYDAFEMAWFKESARNLFRNRYSSTSKRNNNRKTHDRESKERSEGHGAC
VADVGCVATAATTVAAYYGNENIVTDGEEEDSTRGMEVFVPARQKYNKKFVRNVLEAYHV
RCTGNDEHVALLSERW
>comp5413_c0_seq1; 1094 105 2333 LEN=2504; translated
MKFSFALPALLVSYVAASGSNKSAFTPVRVHYDDVLNEHFSLLDALEDSSGIISMTNLPQ
NFAKVKKEVMGSLHSCLLDQQEGGSIVGEESFGDGTVRRSFATATTAEEGPLRLELEDTL
TPSTACQAFEGNLQLFRSIAMKATNKFTAALSQEMEPHLMKPLLTKKGDNNSSYDTIQDL
VENGEQLEHFHSYQKIERISEEYHDDDHEEAMETIDVHADQGFFIAFTPGIMISHGKKDG
EASASDGFYIVEEDSNGAPIRVEFDVETDDLVFMLGDGVNQYINNRLIDGENIHRRHLRA
TPHAVSLKLHDPSQARVWYGRMVLPPNDALVAAETVQTTLTYGDVRRRLVETPDAYIPAG
LGCSSPNMRALQHSSNHNEPSACEKGTLFCWARCMGLSDYEITEEMCEEQNLGLKCINPR
GQFQTGERHGDFWPACSNTTQEVTPYPVIAQKNEVICTNATWDAFVADTDGITYDHSFDL
TTEKNSGTILQWSVVDEEQGIVNGRLVFNDVFGWLALGFADPDGKHNGMNGGNILMALPG
TNYTPQFGLEVPGLTSAGSDEISIEIAKQKSALLATKTDPEGSIVSEYKISPHGSAFRHW
SEPTGINTAQNSMEVHTNECATSMTFEVVSINGQKFNMDGMDSMIWAGNSNDYHVGYHGR
ANRARFNVNWKTGEAEFWTDKEDEKVAPAEDDHGDDHKHKDHGNDHDHDHDHMEDTEMDD
SGVGRLSGYFTFFVAAVVPFLF
>comp5415_c0_seq1; 282 46 1117 LEN=1235; translated
MAHNPIFRKKYDGKRQKLIEYFFSKALFGIPDLVFPVVGGCVAFGTTLAVSTAAQRFIGI
STGTKFVPSFLGFATVCGASLVSEQAAILSYELKRDPRKRSFEYVQKKIRHQFLETSRNV
MGSSSLLRGEKGHQFKLPMHEIRVCLLGLLAFKSLGGRFWGIAPSSYTHLGSFTRWSIPC
TENYANTKERVMIEQMGRKWGCHTCGSKMYFSSAKSGITGKSYRFVGDHMPPKSVAKHMN
NQWFRKIGLWPKVKFRFYPQCVSCSNVQGSILSKTGSGLGLLPKHSWASTMHGAGGGTSA
HFHGFTPRINHLAGGVLAAAIVVGADDDEIAKGNPKRLKQWQKQIQKRFTNELRNF
>comp5416_c0_seq1; 344 2065 3135 LEN=4039; translated
MYSSKKRKFISDGLDVLESFSALEGDPLGVHTLILGTLPSDKSYGQNLSKKEILLRGGDG
HQNYGHSRNSFWNIVGSAFGFHRHKIAFNDQVNILTDQGYAVWDVLSQAKRKGSLDSNLV
KGSLVPTDLPGFILDHPNLNRFVFAANSAEVFCKKEVWADWLATGKANASSATTTRDPDG
IDNDDNFSCDATIETKFWIQGEVLNPETFARTNSIFGKKKTVLIVDDAAIARSYSKTTTY
VEHDGTLRRMIELIVMPSTSPANASNRPPEKEKKWHVACYRLREPPEYYICPGCEFHRKR
HGSSATGNDEYEYGKHWFHDCPYREDWKAMKKLKSKKSLKKQDADFDAIDPFGWYL
>comp5417_c0_seq1; 250 208 612 LEN=1217; translated
MPQSNKGLNAARLKANRAAGIGDEMGRLPARVKAEAKMICCLVCKAELKCTKTNTEMKLH
AESKHGKTLEECFPGATAEAKAINAKLDAAKSGGKGGKGGGDGMTKAERKKKNAAMADNL
LLAGLSAGKKKGKK
>comp5419_c0_seq1; 369 366 1634 LEN=1902; translated
MGLPRSLLLLAAASLVSETFANDPFLKEILMGRCYERWSGPSPVVPPPTTCGELVGSFLN
LLESRTESGLTERAFAEAYTSKADFSSPRDAGLVRVALGGGSQDGTEDETEERTTSPFGL
LEGGPWVTPETTPGGSLLDGLVFCANGNQENCTRESSGAYWKFWASAYASFARSAEGHLS
ILLEDERVDGAFLERAFLSHLGASGVEAVGIWGVDCASPLARKVSSFVAETVGLPCSCGG
PDVVELALCGSASTSGAAAEACAAYRRGKEAANATDNANEDAGGAPGEPDSGTEPPLATA
DGDAIIDQKDHERKHGGWFFPWFFLAIGGYLWHKKKHPLSPCEPGRLHRWCEDRTYDCIK
GDLFLPSTSPSFRTQGSRDEPPTSSSGSGITDSSSSMKYSSFANSYHGSGASGSTGGVAS
SQ
>comp5420_c0_seq1; 390 93 2747 LEN=2747; translated
MLPRSAAVASLGALLCLQLIYDAMGQQSETERLMLLGEKGHVYERYDRDDKTPYSSVSSP
NTGHSSVRGRRRSGSSSNKIKNNSRNSNAMRTDNITRQIPAGQGRLRRHLTTEETTCRLW
KVEIRYEEYEEESHWECEFTNPDLLEATQAQTRDHHIIGTNHFDVDAFLDEQGAESGTTTL
ILSTGVELAMTESENQELHLGRNQVVAMEDNAIEVTENDDLMVRRGLADSIGNLRTLVVR
VSASDTEPPSAEKLYDDMFGDEYCLKSQYKRCSFDKLNITEYLPDEEVTVPVAEGVAGGI
VDVQININAVESSSYNLQRLANSAARDLFGVSMWSGGLNGIFDLVLFCLPPGSGSWVAYA
YLNRWDSYYNGPYCGSLSATLHEVGHNLNLEHSGVYYGDAITQDYGDASGMMGFSYLNDD

GPIQCFNPAKNWQLGWYDDKLLSVEPESLRNGPSTYILNGVVDYQDGNANGYVVIRIGDF
YLGFNRKASFNRGTLASGDQVTVTKMTIPYRYTTGKSKLLVAMDAGSEYAIFEENVLVKF
VENANVEDAVIELSLFDPSLPEPPPCETQIIVDVVTDNYPQETYWGITDPGGAIIAGQDG
YTDPNSSSRTVVEGLCDNVQYTFVAVDRYRDGICCEQGNGSIVGTYDGNIIFEASEFEFR
LAEPFIINRRTLSPSNSPSVQPSAGPSYIESASPTIAPSASPSASPSTSLVPSSMPSKSP
SEKPSTSTAPSELPSASPSMVPSETHSEEPTGLPSLFPSSLPSEGPSDFPSTSPSHFPSS
SPTGLPSSSPTDFPTQHPSETPTVFPSEFPSSIPTIAPSVSPSRSPTEVPSNFPTISPTK
SPTKNPTSAPSNVPTPSPTDLPSTFPSRNPTNSPTHVPTNVPTNV
>comp5420_c0_seq2; 79 90 892 LEN=892; translated
MYSTHLLQLIDTETEFVXEQGNGSIVGTYDGNIIFEASEFEFRLAEPFIINRRTLSPSNS
PSVQPSAGPSYIESASPTIAPSASPSASPSTSLVPSSMPSKSPSEKPSTSTAPSELPSAS
PSMVPSETHSEEPTGLPSLFPSSLPSEGPSDFPSTSPSHFPSSSPTGLPSSSPTDFPTQH
PSETPTVFPSEFPSSIPTIAPSVSPSRSPTEVPSNFPTISPTKSPTKNPTSAPSNVPTPS
PTDLPSTFPSRNPTNSPTHVPTNVPTNV
>comp5421_c0_seq1; 372 174 884 LEN=1014; translated
MAEVCLLEPPPSNDEESTGMDSSELWWTSNDYENNKRKHKSLARRMLSSSLSALPDPNDV
DEDADGSYRGLERLLDGGYCKARMRRVIRVVRGEQARHFLDGVEGEASDLAIAQVYGALC
VKSVNQAKRLGRFDAMEARKALEDNWEDDDATASTSTSASVEDSSASLLLEESEESPAID
CDRGQVEEPINNENDNATPIVAVNSKTKGKLWSGRLSLRTNYKFSMSNVMNRRPHAW
>comp5422_c0_seq1; 444 157 1380 LEN=1651; translated
MSSEEKTSVTPRRSLFPKSYSKKESKKEKFVTFSPYARCLKLEPQNLSEDERACLWWQKN
DYEDFARLGKIISKAMLEGGSEVWLRSKCTPFCASPTSSCSVSEYGNAATVPTTTRDDTM
VESHSSSTRANPPLHLHNHVSETEPQEFYEMQGKWWHKFGHSRRGLEHIASNSEGRQRHA
NARYAIRSVLEEQQRQEMFLPKGYFDVDKFRSVYLKQTHWARLLALAAGESDTDAVQTNF
DESRRKPREHYLKKHFDNNIDNAATNNDVDIPNFMKTIVSTARPTKKLNLDANTISQICF
RNSQIDPRSDNSKLKGHNNKKKNKVTGKTQLSSKPAAPESPSSPDEEGKAKETVVNDDAS
TSTEDSSTSNLAKMAAGWGGDESSSQEDMSFVLIGMGIAEKPNITVG
>comp5423_c0_seq2; 598 231 1871 LEN=2208; translated
MLMHSFVLDALATTGADTFSHFEELINEHRKMCLANGDNNWNSSSSDHEPKQSRLKRLVP
TVGVFHTPLPLRRAYEAYNAKYKLDRRRHLAISFNEIRHILNLAQVMAFSEAMEPAGHRS
LSTASGNNAGRCIFSRNPLDSPAFTPLNGKEMGAMPPLSLDAPAETPTMTVTASKQTTDD
SSSDDSSDSGPFRGPSMITFDGDQTLYSDGANFESNPKLATYLYLLLRNGVTVAVVTAAG
YEYQTAKYEMRLSGLLQFFRQKKPLLTEEQLNRFYLFGGECNYLLRLGSDYGLHPVREHG
AGGWMTATKYLGLDSPANWPQEDVTRLLDLAEESLRGSMLDQNLQSRGRFLRKKRSVGMV
PIGGDAAPIPREALDETVLRAQDHLDRHNLRGNSRASTIPYCAFNGGRDVWVDVGNKRVG
VQILQSYLGVPAAETLHIGDQFLNTGNDYAARQTCPCIWIINPDETTYVLKSILRLAGID
LLTVEHGDADNHETTTPPTATMRRGSNVDFAELERRTNAVATMDVFTGELITKGVGTGAA
SSDAKQ
>comp5423_c0_seq3; 618 65 1714 LEN=2051; translated
MKSMLMHSFVLDALATTGADTFSHFEELINEHRKMCLANGDNNWNSSSSDHEPKQSRLKR
LVPTVGVFHTPLPLRRAYEAYNAKYKLDRRRHLAISFNEIRHILNLAQVMAFSEAMEPAG
HRSLSTASGNNAGRCIFSRNPLDSPAFTPLNGKEMGAMPPLSLDAPAETPTMTVTASKQT
TDDSSSDDSSDSGPFRGPSMITFDGDQTLYSDGANFESNPKLATYLYLLLRNGVTVAVVT
AAGYEYQTAKYEMRLSGLLQFFRQKKPLLTEEQLNRFYLFGGECNYLLRLGSDYGLHPVR
EHGAGGWMTATKYLGLDSPANWPQEDVTRLLDLAEESLRGSMLDQNLQSRGRFLRKKRSV
GMVPIGGDAAPIPREALDETVLRAQDHLDRHNLRGNSRASTIPYCAFNGGRDVWVDVGNK
RVGVQILQSYLGVPAAETLHIGDQFLNTGNDYAARQTCPCIWIINPDETTYVLKSILRLA
GIDLLTVEHGDADNHETTTPPTATMRRGSNVDFAELERRTNAVATMDVFTGELITKGVGT
GAASSDAKQ
>comp5424_c0_seq1; 235 1 904 LEN=1035; translated
XDVLTIISLSNNSCVLFRITSRFSNHPDISTTKMSARKFFAIFSLGLCFCASVTEGRLFA
RASSTSSSNDVAGPAEHESETTGLSRRPASVEIATRKFHDFGEPSKNTSMCARIHPEQLP
EECSCSEPHPYSLVVECHKVFSSTYFNDTIGMKIDLDPCDEEGAKISLDVTEEDHNIDFP
LAGIRAGERKDVPIPGLAMIVPGVGNVGLDVSVFIAGNPTLLRLKVGLNACAQLSTDHQV
CASSIPGLSTIFPWYVLKGTYNFGGICESKSGAGVDGNVEVDGTNNSDDTKEKKAIVIEQ
K
>comp5425_c0_seq1; 606 36 1583 LEN=1923; translated
MKYSAAALSLASALLAAPVAAQPAISVHGSGTTNPSKCYWTIMDQMQIQSKNPIRMTYRA
VGSSTGQAEFIGDGVLSDNMFGSGDIPLSQEKFDSLAQGSFFHLPVLLGAITFFHSAPVG
DQKLNLSACTLSKIFKREITDWNDSEIILANPNLDLPRPAPITVAHRVKGSSSTASITQY

LNQACPEEWPEELVGKTIDWDVDTIGCEGSGGMSACIKDIPGTIGYIDMGHGHSEGLAEI
ELQNAAQKYISTKEAAELGGIMAAAENADLPDTLQGSFANVNLLNQGGSNTWPITAMTYI
YVKKDLTFIEDPAAQSLVKAFLRAVYSDDYIPQCEEEFGFVRIGGELREKALAAIETITT
SPGAPEWSFEIDTQKNAGQSDYVISKKRKSYSELEQDKLVEMMNKMSFRIDQLEAQNAAL
MSQETVSATPVSSSPASSKPASSDFMKTSSSTGAFGTFIDEELDEDQQVKTALIMSSVSI
ALWVVAVLGMIVKYASGSTASKASKPEEMMEAGMN
>comp5426_c0_seq1; 252 460 1572 LEN=1679; translated
MRQNYAYDLYPRELVGLEEKWNLTQNGADFQGMKYRLKYIVSDFILESMLRTVAYTEECE
EGGVEIPSSHMTYEVTFDDTPPGDGGLERTFNIQTEVNPGVEDSLVYKTVIDPETGAKLA
EVRFCMLTVLRTNTPNPIGVNFLETVVGFTANLTSGFGVSANVGPKEKIVTTAAESYNVN
AYLCDEYNEELTGSALTNARTQGQIVRACVTPDQDARDDGVFMKAIQDFTWFRDYGGGLG
MVSQVAVQDSQAASNLLTILYCTPGSITCAFESILFASMFLTPGDVSGTGTALLQLGDSS
EERKRRGLESSSLSRSLQDAGDLPATDFGLEFDLRLGEEYTGTLKTASSPKLELMHSIAT
MGTIALINLL
>comp5427_c0_seq1; 233 195 569 LEN=659; translated
MISSLGARAFSTARALAGRNAMARPFGPASSTARFLAAGAGDETSVVTVCTNKIREALET
TEVTVTGAYDDPNGSHVSIEVVSGKFEGKRPLQRQQMVYKAIWEELQGPVHAVDSMVCKT
PDEA
>comp5428_c0_seq1; 900 113 2665 LEN=3205; translated
MSATANGGSGNASKAPAVSTGTPTGPSRLSNYSFVIGTTLICVFVVTLSSMETLTSHFGN
LAGMSSPGSSSSTIEIKEIPGNKIDTGSSLRKKSKSSKRSKKGSATLVKSGADGAVNPAI
VNWDVVNEPKKEGKPKEEKESKTDESGSKKKQGIENGNGNNDSSSPTPFTSALDSVIDAR
KDMIKKLKKQYGKDNFDTIFRYDKEADDASNNEKYDGGVPTHKKLQMKYRPIRPLAPKDL
YRTNHWGKESDELKEYFLKSDAKYWNSVENLQRKMMIKLLLQKAPDAKGKSSNKYVWATG
GHSAAAGHGNLFKESYTKVMEATAVSVFDAAGLELEARNFAMGATSSAAEMSMCFKEVYG
DDVDIFSWDFGMLEARYYLSGRLLHYATRGFLSNQQDPQSIVPAFVGLQELDESRRKSMV
ELHDAFRGTGSYAEDSLAENNGLALFLTDTSYWKIMTEAVPETSGLTSAEIEELPELVRY
FKCNGGIEKGDPFCGEKKYSEEVCPKRDGQVSWHPGWKYHALLGNSMALFLTEMLLGSVT
TLEKEVREIKNGKGISDDDALAELLGRLLEEEKTLQNFIHSATTKQLPKSHDKVYWYNNT
EDNWEMYPEVLEYLSVNATDDFRDRFNALDRQALFVGPSICHTARLPSKTRFTGTLTNQP
EMTGEQAIFGRETYFVGIMEEEFHDQNVAKQQEGIVSDDNHEITLVSILKEREKDCGEEL
AAPDYHDYFMTHSSEGWTSLTFPNDAEQAAYNYKQNADRYKGMLIIVPRFCGFGQCEEGF
LGYREYNEGKWEMVVNGVPVTALTMMGHEAILLEHESGIVFDRNSEGRYDLEFRVNEPNS
FIKISAFIMY
>comp5429_c0_seq1; 111 155 787 LEN=1223; translated
MSSVGFFPNLSEELLLKVASYLPPRDLLRFQCVSKDLSNLDTDSIWEWHCEERWKPWPRY
RLTDQKRVELNSGGTETFGMTWKRRYMFIEKDATRTTLVSRDLQNLKWYLSFVLSGIRGE
GRSDHLPIEFAFGEVLLVPGYPPLSYQIVNKTPPSNGDRIRKNLRGDQPFSRKQYLKISN
FPPHLISRKKSDAEWLIGNENVIMVSRGEL
>comp5430_c0_seq1; 223 279 1730 LEN=2140; translated
MVSSSSPEPSLTYLRPEVLKLKESLDQFIQQEVIPAEAEYHEHMEGRHSNGKGRNDSRWS
LDAMPPCLSRLQSRARELGFWNLFLPPRLLKYPHWDPKLLGPVDPNNVLCPSITLTYREY
GILAESMGRSIELGSMACNCSAPDTGNMEVLLEFGTPAQQKQYLKPLLEGKIRSTFLMTE
PDVASSDPTNLETVLINHKQKQNGNQSYTLKGRKWWSTGAMDPRCRIGICVAKIVDEKEG
EGNDADNSSVGKANLHGQHTIVLVPLPNPNVRMIRALQVFGYDDAPFGHAEVGLENVPLT
QEHLIGGLGSGFKVSQARLGPGRIHHCMRSIGLAQRCYELMLERSTQRKTFGKFLCQHGS
VQKDIAASFHDLQQARLLTLDCAHRMDTSPNGPRGARQHISSIKVAVPALCLNVIDRALQ
VHGGLGVCQDTILASAWAGMRTLRIADGPDEVHRRSVARIEIKKWVMAKQEAQEQDQLKR
SRL
>comp5432_c0_seq1; 198 698 1318 LEN=1985; translated
MPKIDAILVLGSDGDRLAGKYYGDFLKTADDGHSREETRHNFEKEVQQKIGGIVARPDAA
EVVSVRGKTVVFCGGSSQGGNGEVRIVHVGPSNESELVLAHLCEGMHDALAQLMGGTTDR
TMVLDNLELVFLLIDEHCDGGIILETDGSKLASAVLLRDEDAQPGEGGTGGQPQTPGLPP
GVTAGDMTLGQAFRQARDQFLSGGM
>comp5432_c0_seq2; 185 146 781 LEN=1448; translated
MLAPLMPKIDAILVLGSDGDRLAGKYYGDFLKTADDGHSREETRHNFEKEVQQKIGGIVA
RPDAAEVVSVRGKTVVFCGGSSQGGNGEVRIVHVGPSNESELVLAHLCEGMHDALAQLMG
GTTDRTMVLDNLELVFLLIDEHCDGGIILETDGSKLASAVLLRDEDAQPGEGGTGGQPQT
PGLPPGVTAGDMTLGQAFRQARDQFLSGGM
>comp5433_c0_seq1; 580 205 1491 LEN=1491; translated

MGSGRRDLLLQPVPALRKSTIPFTPARTKVYAIAPSASGYSSSSRVLGNANGDDDDDSSE
STGGERLPHGDEGFPYEGNDASGDGRNHRDREEDQELSSYPSKEHIYSLQDENSDLQKRI
QRLEIVLRDNGFDRDGKRIIISPGARATDGAAIDGRCIEMMSMMGMDPERPTVLTKYMEF
KVLELQDELEAMELDRDRLRSMIRDLKASGSGTKPRMPKGAETGEGKELENDDESSSSSD
SDNESDSDSDSDNEHARNKKAKSRSMKLRRDGGVSDDETDDDETDDDDTADDETDGDESE
SSVSDDAGSQEESTRAATPGNRATPDESRKQEEKMEELRLKKSKLEELLTKVVDESTKRE
TLLKQKVNELEAHIDETEHKEELLREQMRSEFETKEKEENQATIAPPENTISSDQYREQE
EELQETKKK
>comp5434_c0_seq1; 177 1 365 LEN=773; translated
XNSNSNSNGAIGLINEYKGRLFFVCELRDDDFPSDEEHPPPPPSPGDFENKNNGRDLSSC
RQQTGIRLSLEHSGFRWFKDTADGKSRPGEELSRLLRFHSGGKVRKAIEMAYRVVKEEVN
G
>comp5435_c0_seq1; 389 108 731 LEN=944; translated
MKVVAFLLTTLSCAGAFTSGSTRSGRKSFGLFAEEADVAEPETMGAEAISALTSDVKTHF
TSVDVDSILPHRYPFALIDKVVDVVPNKKVIGVKAVSRNEDFFNGHFPGQPVMPGVMQLE
AMAQLAGVILNTADGIEEGTIGFFGSADGVKWKKPVVPGDLLVMEVEIKKMNLRFGIVKA
SGKGYTDGDLAVEVGEMTLMLAKPEKK
>comp5437_c0_seq1; 692 213 2882 LEN=3275; translated
MKARELSSLTALLFICSIYRYEAFVNTAPSSSTRPNSSTETSPAILFYSTRRNFDADCSS
RAGHNKRRSGYRRQHQNRRFVRKPRNDAAIRHKNDEAYNERRKWLQQATQDVLEKEAGSL
VKGKWHELVSMIKGWHKFAKMDPEAPIRIELLIKRLHDERLAGNDEAKADISMYNSLLDT
WCCVALFGTNYREQSGKSVNRLASPLAASQRAREILMGMQGNYDLAVEAAGSNTTALDWA
QPNEESFGLVFDVVLKVEGVAPARRLVAWMEHVSKTGRNDLAKPARKYYMRLLNAYANSH
DTNAGNLAEGILRHMIKIGEIPDTVCYNIAIKAWSKARRGRESAEHADRILEEMQAPKDV
ITYSSVISAWSASGMQSHAVVRAEELLREMEETPEVEPNTVVLNSIMSAWVKSKNPAAIN
RTTELLEYMEQAQHAPADLVSYNTHLHALSIHSHREPDYAQRANDLLTSLEARYERGEIR
FRPNLFSYNVVIDAWCRSQASDAAWNAVKLLRNFINHDSKYQPDTFSFNNVFSALSRTNR
PGTTLLAEHLLDYMEIAYKNRIFKNAKADIVSYTCVIVTLARSGEADAAERGEKLLERMK
EQYKSGKTYMKPTRVVYNALIDAWAKSGRGVLGSRKAEALLKEMEEMCAMGDASVAPNVI
TYNNVMTSWARSGTRCAGNMAVKYLDRMTMINGTEGKIEIRPNDKTFHTVINAIAKSPNE
MKAQKSLQILRRMDKLYQSGYTGAKPNAVTYNNVLNAAASSGTSTDFKTKRKALDTAIFT
LQELQSSQYAQPTESTYSTFIEACYNLLSTDDEVELRDIIENTFEECKEDGQIGDMFLSR
LREAAPKDLYEDLLSEVIVANRDEVKVDDLPQSWRCNVRRGGRKSRQPP
>comp5438_c0_seq1; 338 138 1103 LEN=1495; translated
MNGETLGFSLTMSSTSLDKGSAAKPLEKKKVAVFGTGGYLGGCAYGFLQRAGSLYGTGID
GVGAPRAIVATSFGSANLNSVLSKNFVLAQADESYIRLTDMTSSESIQSKLRGFDAAVLA
TRYSFKTLPITMGSYGKGPNDKTREFFMDQPRSATTKLMQDPEYSDQVFLDTLEACKNSK
KLKKLIVVETDAEFDAEESFVGDKYIQALEESEIPYTYIRPIGRMENIENFTFKKGVQTN
LNIGRANSVEELWPVENKPIYREHIAAVCVQSLMTLGWEENQVIQVDQSPDVLDVDVKKV
NTGKEWCVNSSIIQNSLAALS
>comp5441_c0_seq1; 131 111 535 LEN=1050; translated
MEAIHGRPSLWHNILSGGTIGFLGVATGRLGIPLVSPAHAYRYGVSPPVLAFGVYGSISG
VMAXDAGLEALLRSNEELSEPERKANGRPNSWTGAKSRKRAKPNTTRQNTAQHDGLGGCR
LAFWSIEGNEAGKQALSPTIR
>comp5441_c0_seq2; 84 103 728 LEN=1243; translated
MYFYVDTKSWAYQKRCLTYPIQSSAVATILFSAIDIFQGVPLQNVMNPRSLGTYFGGIYC
YHAIQCPMEAIHGRPSLWHNILSGGTIGFLGVATXPAGDPPGLPGPRLPVRGVPAGPGLW
RVRVDLGCDGRDAGLEALLRSNEELSEPERKANGRPNSWTGAKSRKRAKPNTTRQNTAQH
DGLGGCRLAFWSIEGNEAGKQALSPTIR
>comp5442_c0_seq1; 419 114 914 LEN=1089; translated
MDFGKRHEQQQPLVSSQYDLLTPSRRAQLMFGSETLLMDSKKQNELLWARVFRQEKNIAN
LSEENGRQEKTLAHMAEENRKLADMTARLREQLEVQAELNKDLANRNDELANRNTKLASY
LRAKDTQISVLKENKKSLIEKNSNIVSYLKEMDKFIGSIQIKQEELFAQQQQLRFQHSVT
DGGSTKRRRDEQKSNHGTGRNTKARGDVEEENDKLVRVVSIGSARDDKTPNARVYVIEDS
ATDVISTSSSKDGEDGDDTTCGDSRA
>comp5443_c0_seq1; 428 434 2026 LEN=2072; translated
MTRMISMDSTVTPIKNGSTRNWDLDSNNPEIPGGKASSGNYPEYVENHSYSNGDHGDHHH
QSPLNEKVEVIKAIDQLQGTATATITNNHTSESQSGKPSDLLSKYNPLNAPVILDGWNRT
VQVHLIVDVTGGEDINPASKLLLSAVQRSKYTQLKAVTFVRPSIETIDFFSRKPGLPLLF
LVDWGSMDRDCHRLQLTLENIREQEKNHSKRKREKEQPYFLLIDSSGSTRRTGCSYLFVS

NSDDRRDSETAISYARANDNSRIRLAKRSIVQNRFYDRRNNKVHLGDVSPNQWDGAPSDY
SKPILHSPFALRESFVMSIQNITKGKRVSMNDSKRTVDVGFFWKSGDYSHYGFYRRDIGK
VVKTLHHSSINKEAGTRMENLVQISYTDEKGMEAGNVQYKYTMELLRCKIVVIAQRDEWE
DHFRLMESLASGALVMTDRMVALPAGLEDKVNIVIYDNPKMLKDLIKYYTKPKNKNKRKR
IALKGYNLVMGRHRSWHRLEELLFGRPLTNVDQPDAPAPKKGALPTFKDR
>comp5444_c0_seq1; 372 277 1452 LEN=1856; translated
MQEQRRSVRYALLAAILIMSAKRALCLHSVPSQEGGSEEEYSSRSTDDMVNNDTSSNSSG
AGTTGKIPFVGPLHSFQYQDPFAKGFEITTRVYTDPQDKLAHFDDVLSNDETSSSTPESS
LPPTVPIVLPYWECGVSGSTTVPVPLSSLTIRHLLGGSKPGSFEEGCGRGDTVGVGGGIG
RGGGSNHDDTHPRLVVPLTSLEIVLNSGQMRIFQPGDVILLENCLTGGHILQGHEGNDMI
VMLLTLPHAYHHAGKDRNSLSSIFENNFWKQSPCKTGLVGNNNDRTESRKNIVVPWSRSS
RIRRRLGLGIIGAGVSLAMADFLGKVAPLALALAAGGGLVAVGGTYSIIRLGEYGLDELD
FWHERRLLKLQGGSSTPVNDGNEKEDKDQDS
>comp5445_c0_seq1; 412 126 1616 LEN=2019; translated
MVKRSIFRRRRREGDDIEHIRTLELEGYATAMGAVLIVGVGFAMAHMCVFLLQPACIYQQ
DNSTLNPPTATMDTAPFNATPPEPGVTPAAVPKTAAAAGVAITKSAHTSDSHATTTANTK
TESMPGFPPTCTDEQFAVLHKQLPPEGCEENAKKPWKTKMCSFAKETHCGNGNPHWFYDF
IHLPQSLSSDDDPTFRGIIVGCNKGYEAVEMLRIASPPNEKYNLSQWKEEFLKMGSSEGE
IDTHFDTSVDCKADGMATSNRSGRIQNAEVYCLEATPKTFAKLVETRDALGYSDEELHLS
NLVVGANFDEDDMDLGKLVTTDERIGSMGVGLHHWAQTCERHKHKNVNKNCVRVPTTTID
GWIEKHPKLSSTLSTTKRTPLINILSITAETSDYEILMGSAKSLKNIQYIDFNYHWYANW
ADHSLNDLIMRLKKKGFACYFTSTNENMWRITDCWQDHYEIKYQAAVGCVNVNLPDAKPL
LAKMEEMFLSTLQDTE
>comp5446_c0_seq1; 601 148 1263 LEN=1452; translated
MSQDGKPAKVAAAASDDDSSSSSYSDEEDEDLVLEGVLVRNPDASDSSSDEDSCEEDPDE
TTKPPAKKQKKNDTNESLKSTTRDPAIERKDDKQSTTKNQKQLQKKNKKKKKKEKNSGPE
IVDMEFTFCDMDEKFFHGIKNLLASSSPAYGATASGLTDLMIENAAVGTVISTDYSGYYQ
PGQQKDPDYEGVVYGFASVLNVSTHGDSSSIQALKKLCLSKCPGDRKKELETVLSGTTKR
PAGFYFQSRMVNLPLEIVEVLHQQLVMDMDWAVENAAGQGKTKEETERLRKSLDFGAFVR
IAPTYREKSGTASYYKYFDDELLANHAEFAYEIELPKTFGMEETPYCTVIVATKTGHRAA
VQQLREMVGGG
>comp5447_c0_seq1; 405 106 1311 LEN=1582; translated
MGKKSVAQLRRLEKRAAERGEKYVPPIEIPPSDCVQDKSTSKETIGDKDKSTTGIKNSCR
LAAAQKLLKILKNIENNDELKSKDRRSAKRKAEAICSEETGIPVEELLDWYEKNKSLHDN
KKGEATVSGRRRDPLIAFVGQLSYETTRDDLMDHIQSHLGGEFPASQINKITKIRLLSDP
QTKKSRGMAFVEVDDPEFLYALLKLHQTYLKGRRINIERSAGGKRNSQARKSKLEHYRKE
QGAYFAEVVENILAEYKQNGELREDELDEGVVSVCKRHSGPVVQAAVAEYIEKGGRDMNN
PSAYLTFLVTKFAKEGIHDVEDTGKSKTLKPTRKRKEAPPTGDQDGRQKKEWKGASGFAR
LGVDMSMSVKNPSREGPRDVSKIFPSSQRGRGRGYMSNVRR
>comp5448_c0_seq1; 280 827 1471 LEN=1612; translated
MPISQNIFDAESQARQSLSSEFSVATRGPLHTASSSPFQSSNAVLRQQDHFSSSSNSRHG
PTNVTHPQQTINNQQLESSVADTCRWLIDAGVPMSAFDPVSIGDISRSAPLAVPQLNTSA
TSSFSPTISQKTVKQEIKTGNSSATNIMASVDNIGDLAPQMNSSGGNDNNYDLTSLFTMS
LPSNNSSASIAFDNDHLLTDLLPGENLPLVDDSLW
>comp5448_c0_seq2; 328 129 1340 LEN=1481; translated
MIQDLWRSKEQRNLAPLSVPGASSAGSVRVVSGSGFRYVIPFPFRLHEMLSNVDTLHDSS
IVSWLPDGRHFKVHDPRRFVETVIPSAFKQKSLKSFQRQLHLYGFQRVHEGPDKGAYYHE
KFLRDDRDLCLSITRAKAPKRNRTASTKTTTNTKKNTKKVPSLYANTKTTETRGSEKSSL
SAMPTPTINMPISQNIFDAESQARQSLSSEFSVATRGPLHTASSSPFQSSNAVLRQQDHF
SSSSNSRHGPTNVTHPQQTINNQQLESSVADTCRWLIDAGVPMSAFDPVSIGDISRSAPL
AVPQLNTSATSSFSPTISQKTVKQEIKTGNSSATNIMASVDNIGDLAPQMNSSGGNDNNY
DLTSLFTMSLPSNNSSASIAFDNDHLLTDLLPGENLPLVDDSLW
>comp5449_c0_seq1; 671 57 1517 LEN=1656; translated
MVTTSDLLFRFVAAAITFGVVNVNGVDANWLPWSSSSEAQVGSVSLNQFQSFWIEATDII
SDLDSYDKLWIKPHSCVWSECNVDDTDDGYTGDNRDGDEQWYQYRTQGFCANAAYSLYGR
KKEDGRFPSFSGCSRRHFINSFFTYGGADNLLKSIGESPVVYNYNGEDNGSNDNANNEDG
NGSSANEICVEIEYGSGYDDNVNEDQSGSGSGSNDNNEFSGTLGCGPDGEYVIAAFESSS
CDGNYFAGIVNNFDEYNEQHSAIGCHNIYQYGNEASVEDVTMLLSNSWSCDRRLYPNGCP
DPYGKKQSYDFAIRTVAHGGNAERAYRNMMLKLPLHIVSWVLLALTVTVFILTYLVKNES
RAIESKGGRNFVGYLRCIGEDIVTGWGCFCTLIVNWCRSSCEDGQSSTEGSAAKDDDDNV

STDDSDNSYVKVEEKQPKKKNRVGVALKRSVSAFVKLKGKTRGSADKEKSKELQKEKDFS
RSPTWH
>comp5450_c0_seq1; 525 1 1628 LEN=1815; translated
XANARSYQNSCARDFSKRTTTMSPSAKTTLLAILAGIVAKASIAAGQDPANSTFPSELPY
IGDLIEEAFPTWCIDEDEQRIYDVETCEDGSPNGRWSPLYFTKEFSGADPALGGYPTDID
VQYAFYYGAPFLGQACAGGPHHCSEDFDGENLNCAACPKVKTDEDNGPNGPGHIPPHIAL
AAVQRFYNDMAGDQDMDVADIYADAFDYEANACRILPDVLLRMIRTYFPRDPETGEAYYP
PPFTELGGGYPLEFVNLVGETCEKEKAKHSEAGEMECFEQHSGDASLYPEYLELGHGSPH
YCTKEGKEADVYNDWCPYIYFGPNRGKYRHPHVAFAAVETYLANLVMPDKCGTTWDDSNY
PPEVDTTPAFPYMEDMTDIDAMAPTQPVINDGHFVWPGPAGTKKKPAKGKFNFDLYVTAS
DAIDGYCGLPSDKTCYALGWPTCCLASDSECPEEKPACDGQEPPEDDEPTSVPGASYCTW
SPETDCYASGWPACCDEDAETACPDERPLCEIDPPTEPSTDPPLTGTSYCTYAPDYDCYE
NGW
>comp5450_c0_seq2; 210 211 723 LEN=910; translated
MEDMTDIDAMAPTQPVINDGHFVWPGPAGTKKKPAKGKFNFDLYVTASDAIDGYCGLPSD
KTCYALGWPTCCLASDSECPEEKPACDGQEPPEDDEPTSVPGASYCTWSPETDCYASGWP
ACCDEDAETACPDERPLCEIDPPTEPSTDPPLTGTSYCTYAPDYDCYENGW
>comp5450_c0_seq3; 595 1 2126 LEN=3477; translated
XANARSYQNSCARDFSKRTTTMSPSAKTTLLAILAGIVAKASIAAGQDPANSTFPSELPY
IGDLIEEAFPTWCIDEDEQRIYDVETCEDGSPNGRWSPLYFTKEFSGADPALGGYPTDID
VQYAFYYGAPFLGQACAGGPHHCSEDFDGENLNCAACPKVKTDEDNGPNGPGHIPPHIAL
AAVQRFYNDMAGDQDMDVADIYADAFDYEANACRILPDVLLRMIRTYFPRDPETGEAYYP
PPFTELGGGYPLEFVNLVGETCEKEKAKHSEAGEMECFEQHSGDASLYPEYLELGHGSPH
YCTKEGKEADVYNDWCPYIYFGPNRGKYRHPHVAFAAVETYLANLVMPDKCGTTWDDSNY
PPEVDTTPAFPYMEDMTDIDAMAPTQPVINDGHFVWPGPAGTKKKPAKGKFNFDLYVTAS
DAIDGYCGLPSDKTCYALGWPTCCLASDSECPEEKPACDGQEPPEDDEPTSVPGASYCTW
SPETDCYASGWPACCDEDAETACPDERPLCEIDPPTEPSTDPPLTGTSYCTYAPDYDCYE
NGWPACCDGGADSCPEEKPVCEVACDTLVGMVCSVDDLSQLCSLIQRAGLVDALSTGSWT
VFAPQNSAFERLNVGDWSKDRLRRLLLFHVVADEVLYSSDLKCVSPENLITMANGFDSRT
LCRGGVPAFQKGRSNDSDDIPAIVLDDAVACNGVAHIIDEVLLYKEYD
>comp5450_c0_seq4; 226 220 1221 LEN=2572; translated
MTDIDAMAPTQPVINDGHFVWPGPAGTKKKPAKGKFNFDLYVTASDAIDGYCGLPSDKTC
YALGWPTCCLASDSECPEEKPACDGQEPPEDDEPTSVPGASYCTWSPETDCYASGWPACC
DEDAETACPDERPLCEIDPPTEPSTDPPLTGTSYCTYAPDYDCYENGWPACCDGGADSCP
EEKPVCEVACDTLVGMVCSVDDLSQLCSLIQRAGLVDALSTGSWTVFAPQNSAFERLNVG
DWSKDRLRRLLLFHVVADEVLYSSDLKCVSPENLITMANGFDSRTLCRGGVPAFQKGRSN
DSDDIPAIVLDDAVACNGVAHIIDEVLLYKEYD
>comp5451_c0_seq1; 431 608 2053 LEN=2363; translated
MGFSFRPKISPSYNTNKNGVLLFESSKLKKKPSVGGNERSSGLPTASTSKRRSSSRSASS
GASSLRIRTSSLMSSLGTGSLGRDGKVVSFVPSPKARSAYGFSSFFPSSAPTRDAEISGT
RLDFDQAQPVSTTSMAPSTKSRIIGRCCSFLRVSDLAMDDESVAATSVASEISYINEMQE
HTHRPVVNPPPGIEIDPKERWIALCTTAGETANDVSNNGSNSEHTPIAPKAVESLANYGM
TTALDESMWVPDSKTDKLIKKASGGSEWMKHTFHEGLVKASSFSDISAKDVLVWQGSYKH
GLFGSEIPAIRSAGIVNSSAKKMVELLVDSSRVKEYNKISLGRTDIITFDGNFDEEGPFG
KSITKVMKSESKPPMMSPLSLTSVLHAKKIPNGSGYLIVNRSVHRPEDETVASSMKTEIV
MGVNLFVDFDDNAHESKSGGIERCLMINVNHMRSPMVPMYIAKKVAVTSAHKFINDIRAL
A
>comp5451_c0_seq2; 385 462 1505 LEN=1815; translated
MAPSTKSRIIGRCCSFLRVSDLAMDDESVAATSVASEISYINEMQEHTHRPVVNPPPGIE
IDPKERWIALCTTAGETANDVSNNGSNSEHTPIAPKAVESLANYGMTTALDESMWVPDSK
TDKLIKKASGGSEWMKHTFHEGLVKASSFSDISAKDVLVWQGSYKHGLFGSEIPAIRSAG
IVNSSAKKMVELLVDSSRVKEYNKISLGRTDIITFDGNFDEEGPFGKSITKVMKSESKPP
MMSPLSLTSVLHAKKIPNGSGYLIVNRSVHRPEDETVASSMKTEIVMGVNLFVDFDDNAH
ESKSGGIERCLMINVNHMRSPMVPMYIAKKVAVTSAHKFINDIRALA
>comp5452_c0_seq1; 358 1 1516 LEN=1680; translated
XTRDYTAQHSTARYNTTRGAERTDQTRPDQTKPPHAPRTHTQNTAMFSNQAAELSVNRPV
AAVDAVRGDRVNSKFLAGTSSVRESEDNHLLLLRFHTDANQLGIDARLSHPTGPVGCIRT
SPADPSYVLTTAENNPSEATLWKIPPESIDSVPADLDSDGGDDNDHDGYNYGHDESPSAN
YGGAGSDGASAMEERAVLRGSASSGASARLTDLAWRGAAWDDGDPSTESTMGDVATLCSD
GTLTQWDVAFGSAESTRSCAADLSSPLRPGLPPRMAWDPHHPDLTAVSSGEAVTLVDWRE

PPSAPSLGSSGRPHHRYGITDLDFNPNKPNLLVTSGRDGLLKFWDLRNTGSGFFDADSGG
GQEIVAGGSAGRTKKQRPLLVARGGHRHWATRVRYNPFHDQLVLSAGTDSLVNLWRMSTI
SSAPLLTFDDGSAEDGESLPQGQHQNQHQNTQEGPNVRVSRYEHMDSIHALAWGASDAWI
YLSASYDGKVVLNHVPSKEKYKILL
>comp5453_c0_seq1; 196 862 1068 LEN=1424; translated
MPKALPALILVHDNKILDSWKGLISPAQLQDMLAKHVKNGTKTKDNNPVATKPRPFRGIS
FFNESMAL
>comp5453_c0_seq2; 255 294 920 LEN=1276; translated
MVSSRPYIRYHGSPLFFMIKNILFFVLVSNGIEKAQSFSVRPLHPPSTRSYTGNDSLLNA
VNSKSIVAVTDSNYKELFSGEKFLLLDACAQWCGPCKLIEPLLEQCARDWKDTLVVGKFD
VDGANSESRDLKVELILQGAMPKALPALILVHDNKILDSWKGLISPAQLQDMLAKHVKNG
TKTKDNNPVATKPRPFRGISFFNESMAL
>comp5454_c0_seq1; 148 1 1051 LEN=1225; translated
GTPNTPASKQTTSNRFVQSQCLPRRPSSCNWLPVVFDEYGQNDVPAEVPPTHHREKRKGH
RPCLPAPLREGPPRRIGQAVHYSYAEGPGRFAFSDEASAERHARRLQGWRERGFTVGIGG
PVGSGKTALVLALIKRLAAPGAGHPPLAVVTNDIFTQEDAQFLWRNQDVLPADYIKAVET
GGCPHAAIREDVSANLTACEDLTESLLGSDAGGACATPPPLILCESGGDNLAANFSGELA
DLTLYVIDVAGGDKVPRKGGPGITQSDLLVINKIDLAPAVGADLGVMRRDADRMRSTGGH
AHDHGHDHDYNHSDPASGPTVFASVRDGVSLDEIEAFILEKYRKATAEA
>comp5455_c0_seq1; 247 75 653 LEN=870; translated
MSPIASILFFCIIYCASTVDARSVGSLVAPKPSSSKPRIFRSSINRFVDKLFQETDANND
GQISFEEAYIGCLMLYVQLNQSAPIPPPDRKKFRRIFSQAAAEGNKWNNKKNALDLEQYE
NMLKKLVARAVLRLTSHKIVTIAGAPLLAEMIVRSLAKRKKGFEELLRSIIPLQFQDATI
PTLTSKAFHRGLW
>comp5456_c0_seq1; 260 1 949 LEN=1158; translated
KKKKTQDRNHFFSMKPPPHQFDCFNHVDSRFNDGTHYAKTGEMESLSGCPMKEIVGDVFS
IVKRETSRIKESFTQKRSKRSHDLNDFLFKGFENKPKKQRFLPPDENTSHPATNVPPNGI
QRVISSRSSSVSLETGELEMLNKWKQSVASNSRQGSDATITQKVRALQTNFLVSPIRYCL
ASHPSIVSSNWELKGTVDSNYLKEKCRVTLAPSSYSAVMHSSRDLSINGKLLELIEMITA
RRKELAALKRPINSRFDVDNVYLHGLSLKIKNGLRLTDFEVDWLERMKKNNTKYGSIEKD
PSFNQELESFDLQVA
>comp5460_c0_seq1; 713 408 2528 LEN=2761; translated
MGVMDEDSPMSFLAENLVAVVVTTGCVTLGLLGALLAVKSESKSRAATSNKQAASSGAGK
SNTVDRAKYPGGKLTVYFATQTGTAESFAKELEREAKDHGFLVKIEDAEELESPEHMADF
ESRSPFGADSKPDETPRAVVLAATYGEGEPTDNATELFNQMEDILAANEEEEEPQKPLLG
LEYAVFGLGNTEYEIFNAMGKFFDSAMEKLGGTRVYNLGLGDDSDDLEADFEKWKDGLWA
TLKKRYVNEGALLPTSAEDGKLSLPDCEYTVSWEEEASTGPDSDANRASVPLDTVYGSSK
HYFTAVDCPVTVVKELRSPSDGGSTVHVEIDISDAKNLDYKTADNLGVLPCNAPEVVEEV
AEALGFGQDLDRVFSLGKGVNQDGEEHEWHGLPFPSPITLRECLTRYLDLTSAPRRSDLK
LLSSYAKDPVDRKALQRLSSKDGKAEYKEKIVEGKIGLAQILKLCPSLEIPLEHLIGNVC
RFQLPRFYTIASCPKVHPNSIHLTVAVTRENRPDGSLFEGVCSTHIAQGLNQSLRVFVRP
STFRLPSTLSTPVLMIGPGTGIAPMRALLQDRKHQSEQEGAKNMYNALYFGCKREDLDYI
YRDELEGYKSGGILNDLYLAFSRKDPSKKEYVQHLLKKNASSTYTLLKEQGAYVFVCGGV
KMGHDVTETLKKILVEESNGSMSQDDAANFLSKLSSEGRFVQELWS
>comp5461_c0_seq1; 216 250 699 LEN=1697; translated
MNDRELGSLPAAKSALEAPLDDDRSLRVPCYCEENVWRLAYRKTYLQKHQNKIDTPNKSC
SYHVAFVSNPKGCVPMFEQVAAADRENPVFWDYHVILLMCEEDDSSTARKGHVFDIDSHL
PYPCPLDEYIERAFPNHTEWKDEYLPFFR
>comp5461_c0_seq2; 340 250 1017 LEN=1586; translated
MNDRELGSLPAAKSALEAPLDDDRSLRVPCYCEENVWRLAYRKTYLQKHQNKIDTPNKSC
SYHVAFVSNPKGCVPMFEQVAAADRENPVFWDYHVILLMCEEDDSSTARKGHVFDIDSHL
PYPCPLDEYIERAFPNHTEWKDEYLPFFRVVDASVYLRHFSSDRSHMFDNEKKKWNAPPP
LYDCIVPSETKKRIDLNGTNNNTSTTTFHQYMTISKNDVVDSRQQHQQQNSKYEDSPFGR
IYSLSQLIQRFGIHK
>comp5462_c0_seq1; 906 57 2405 LEN=2738; translated
MVFVASTNEVGLDLRKANARTKLSKEKNGDNFGGSNYRYGGKAEEIRSKRASDHMKYLTM
KASKMGEDVESIADTKTNSEALTVPGGVSDIAWYDGVFSSLFGGNTCEDRNDMKNDVGSI
LICGSPGNQTDVSLVGPVFGQMGDCQAQTQYDEERQDIDNESASEGTVSTLGKDLGDDLK
SLPSVNGGILPGEDDTHIDVDIDNLPMSPRMGTRKSFGNKTKEVSKNENFESLVEKKIKN
LKSERSSMDVSKNVPYLRQKVFPMTAGRNDSEYSSNGHRNLDPAVLETRRKFLVRELRSA

ISKHGRFDVRCANITAALGDLYDENKEYRQSLRLHKDVVSVYSVNLGDDDKKTLDAKLRL
AKVQENAGDIDGAILNYNFVMNMMKDLKGEKNPDVVEVLTRIAGCLKKKGKYESSIKLLK
RALKTYREVLGDTHPTVSVTVDSIASLYITIGEYEKASAILEEVAKLKAATLGVTSKEVA
SSLSELATSYECAEQYGKAMKNLKKAYKIYADIVGEHGEKAIIILERIALIYQATGQYKK
AAIAYLGVLRGKKRALGGSHPTIADTYFHLGVSLRQSNQFDKAFKCIKQALNIYVGEGKE
IHDVQMIAEVMHELAIIHKANHNTSDAIKTFKQEIAVRRKLGQPEYPLIAQALNHLGVAE
FELKSHNRALNYFMEALAIYEKNRVETASKAADGTVVAGTDMAEVSYNTGLVFESLHNKK
RARNAFIEAARIFKENGYSTSHPHYSKTIGKLKRLGYNFQEIDDKKSNSNPSERSNNSRR
RG
>comp5463_c0_seq2; 187 1 1119 LEN=1287; translated
QINKPRKKMDEAKVTNTLCFRDGNCRFLMALAVGWLCLFVPSLVQASRGPPYTEEVRVQR
HHERGYEFPFHSYNPDTPGWRRLMDQRFDQVMALTDAQMKWDGWIQTCNAAVTAPNFTEY
GWGLTQAPDLLTLDIRQTIYDNLSNARSEGGIDVIGGPNTPLFIDAPHLNERALTELQPI
LEAWSGVELVPAIAYGFRLYRNESNLWMHTDRAQTHVISCIYHIASSDDSEPWPIVIEDY
EGNTQMAYLEPGDMLLYESSKNFHGRPTTFNGSWYTSLFVHWYPKGEWNRKDHDIESHYA
VPEEWNELTPTSREHPDLKVIGTSMLEPNCPDNWCNLAKAKKVYGPGQYGTVLTTGGKTI
SLNLEDEDAEEL
>comp5464_c0_seq1; 228 1 1166 LEN=1508; translated
XTFSPAISIGPKAKPQPFRTPPFQPSGYNKKHIMIPCRLVDRRKRRGRKVFQGTLPIDLL
LAVLLAVLGVANTTKHIFVDGFDLQDHRLSYGSLLKSRTVGLEYNSSSQKLHNRVPQTAA
EDDQPSKEKVTNYAFSVRGGSRTVTRTAIAVSQPKRRSQIVPIMVSLVIGSFSVLEIIES
LHKEVESVVGHAHGMFLLSLIRLTRSVAILQTQTEELAEAVEELTEGKETKAGSSKKSFL
SFWKQALLRFVVSRHVAIAACVMASIASIVEVVEDLKPGAHHGAVFLALSELNYQVNRFS
HIVDHSNGGKKKSEPKGLLFSFRSIVLGPLLFLAAAAFAAFEVYEDTRPGAHHAVAVLAL
AELIENLDRSKVLRRKRRFILFGPKMAS
>comp5466_c0_seq1; 677 84 2372 LEN=2795; translated
MAVLSQTHVSSADVTVSSPGISAEQGGSGAKVQSGGSNAPILSPSTVSQVSQEDNNTGGD
DDSENDNNWKGQSWSQEDDKKLKSIVSARKKALRKKKNGQPADSSDLMINWQGIGSTMER
DGEKCKERYDFLRQSQGGRGPVPWTRQEDEQILALVAQHGAKKWSSIANSIAGRSGKQCR
ERWHNHLNPNINKSKTWTMEEDRIIIDSHIRFGNRWAEISKMLNGRTDNAIKNHWNSSMK
KKIEKYLERKRVDPTAPVVDESGRFLLRREDIEGCLQALQQSGSNSKSIKNQAKSYAGRP
LGSTRLNHMHPNRMVPLATPMSAAQNPAAYNSMMKRQYDAMMVGAYPGMGYTPKSVKRVK
SSSEYNKNSKLAEMSPVNKKILEEFLSELKGGYIKGVYHSALERRRIVEKAVKSGSIGEL
NALGLSQSEYSRLQKAVPMNQSRRHLDGPKWATPQQQLYPHHHRYSNGYMVPPHPMHWGH
PSPLYPMAQLFPGYPPVRPPPTTPLPTNNLPTPSSTKDSTQLQQASSKIETLSNSLSLKH
SPILRTKDNTVQKDTKSKDNFELGESSDLGYTLDEYALLGSPCAKGSKSNCFSPFLIPTP
NKSSTPGMCTSWGGDDAKLLHDTFSKGYVSSSKSSSANTPRVFFKDDLTEQSILLDSGTK
VKHTNEETPFQSDFTMTPGRPKSRIGVVTGSGRERIRGAMVDERDNLLSTAILATPKSPK
SGNVQEIDHSLHHIDAYSVIKSPLHFGSPIVKAAARSPMRLL
>comp5467_c0_seq2; 764 252 2270 LEN=2515; translated
MSSGGALSMSGRGTRKRPALSSSSPASALILEQDNEPPTRRARRDVLSCELETLQAELEH
EQSLRALDSKRFVQIKQRLEKRCDFAMEEAKDCKALMEEMREENERHMDQLKRAMARTKA
ELREVQEELEEERFVGVRKGSDGDPRVDMLKEGLEEKEAENEQLRGTIDQLRCDMKELLE
EQQSVDRQSRMQPGGEETEGLISEARPKALRELNRVRIRLAETERKNRQYKRIAEDAQRK
TKQFLQEKEQARVAGKRIEQLEQELQRAVKAEESTSEQLRHWQSLGVEMVRVFAPGAAKG
SRDPDIAVVQKHLNDAKTRASASEKHESELKKELEKANEAIQILQAEKSSFGRKEAAWEE
QRLEQEKRQQLYQKDMKVLRSQEGVYKREVESLRSIVKTFDELPLKASQGGSVGTNSGGN
SHPANVRALEASLEAADQENAVLKEATNELRGDLKTSMAEKKELQNKHATVLEKFGKLRD
ALYAERAKAEKAEERANRAEILAGKGSFNPERTRVLHMGANPLTMALKEEIGVLKKQIEL
LSSAETNKKTKSAFASDVDPNKLHQRLKQSFKEQISRYREGVYLMTGYRVEMIPDNDRYT
FKVRSMFAERERDYLVFQWPEGKEVTSLDLLNTEHAKMLTKSPSYEYMTKFHSLPAFLSS
VQLSLFEKQTMI
>comp5469_c0_seq1; 502 180 1364 LEN=1595; translated
MPPSSSDGTPWVERYRPKTLDDVSHQTEVVSTLKNAVKTGRLPHLLLYGPPGSGKTSVAL
ALCKELWHPTQWRRRVLELNASDERGISVVREKIKQFASLSVGKAGSHTAPQARKDFFGT
GSHGSADATAGEASEQTGNTNDSEAKSYPNPPFKVIILDEADTVTKDAQAALRRIIEAHS
RITRFILICNYVTRIIEPLASRCAKFRFQALPPSTMKERVTAIAREEGRKQSGGRNEAAV
ITDRQIDAVLAVSGGDMRRAVTSLQSVQALLAGQQPGTAADIDEEAIYELAGLPPGAVVE
VLYQSLLSNSFDTMQAAVKNVLHDGYSVQTLLQKLLDRFLETDDLDELGRARIAIRIAEA
EDKMNDGADEYLQLMTVCGLALRCLQESANKMKQ

>comp5471_c0_seq1; 358 26 379 LEN=643; translated
MKSVIALFVTLLATASAFAPVQQTVARAATTSLSMAAQSPELKKGMIAAMGAAVPTVFSS
LATVATEGTSEWFGVDDTRLLAVLFVVHWAILTLWLQQYGDADEDEDFFGEIDYTGN
>comp5472_c0_seq1; 627 365 2182 LEN=2416; translated
MYHTKPHFYRQLLLLLVVAAATFSCRISNGLVSSFAPVVSIVPRLLLSSSVSCESGRSIC
LRARSSSSNDNESDEEDEDKMVSDFFFNISKSNLDSSNKNGRTSMDAGIQEMVSGGTSSD
YFYSFDGSTDFPLDDSSGDQAHSENSNSDVVMVVDWECLLDALPYHVELGIHAARTVWPH
LNDLCNFDTDRQWLENKLYAMSHVLGCPSDGPPRHEGHRHVACEFALATRLILEEQALDR
NESTGKNGKYSRKFHPRSEINGSDKFQEESRSRRPLTAGELAVNWREFIRSTLIVKYSRG
PDGKLKKTEPTLELEQAIEEYLTTPSSSESKPSFASKPFLPLFPQTKFALRNSSTKIVIR
ISHRFDNELVARKLNKQLQLGPHERIAKTYKPENAIRRLFLNEDKNLVLLLSPEHYNDDG
NGIGNSKKYDNGSHQNHATEESCLRSIMKFAHQCSNRYQEKGEVEDVTTRTATTITRSPC
IVWVDSSWHRLQTLVPVFGDAIPSLKNGKKNTAECTVIAAEDRSPGTGEEDDGDEDRTIA
PSSSVWLSLYLAEWPSIDSSTSASETARATILSTTFSENHQAAMVYPWTDCLSWDDAEDI
LLPADW
>comp5473_c0_seq1; 104 1 255 LEN=856; minus strand; translated
ENESEGLDHVIGEMCTDLTRMTSINNTRISFIERLTSDSDIYFNKRVEEEQAVIVAKCQN
LMKRTKEKAKKAKQKKDESLNLPW
>comp5473_c0_seq2; 212 176 1564 LEN=2022; translated
MKITNSAAATVLGSTSVVFALDHGSSSSSSRDSSGSYFVSSGCLSIDDLCETNDYSSFCS
LLKKHDFYDRVSTGTWTMFVPTNQAFDRVSNGLASLDDHSLSKVLKSHLVEGDLVSTLNS
VYESGDYISVGRTDPENLHSSESLCGGGVYYILNEVILPPSIFSDMSQFVQSKEDKSVTS
TDNLPTSCYVSPMCNDIPTKGYVDPGSGFTNSTGFDTVQTDQCCNICADGGVTNIGPYDY
LLLDLMWNPTFCNALEDGHDFTLTHMPSMRCSPSLSERLSIHGLWPSWLKTFGTCCNATG
SNKPLDPHEVTNEWDNSLRLRMLEDWYDPVLYNGRFNEDNGCQICYVQNHEWQKHGLCYT
DSPEEYFQAGLDMYDIVEEQRKQIDSMHGTIITVKDIEILFPKKVNVVCDPQASTSTDKY
QTFSELQICFGDELTIIDCPAATYESNWTTACKHSIMIPSFL
>comp5475_c0_seq1; 540 37 3144 LEN=4973; translated
MTDESLSMDADTVLERQNDNRLEVFEDGLSANCEIVGLLQFGRGNPSSDEYFYNPSQEDI
EHVVSTLLAIEHLNTANTSLVPELSGIHECPITFSAAFAEASIIPSYAFEVVDRLTRPET
DSSARNAERPLPCAFLGAKSSTISSATAMVTGMRGFPQISPSSTSVSLNNKVEYPLFGRT
IPDDGFMAEVFLQFLHDTLGIRHFFVFFDSDSYPRSVYESLRRAILDLGWGSLGGRETEE
ESKDTMYLQSYLLERDADGRDVENILVDAIDAFNRSEFRYAFVITDVDTTDSLMKLAYDS
ELAGYGDYQWFFADTFSLSSRKFKKDSVLVKAYNGVGSIKANAISDMEIHDAYVKQFRDS
KRDLHKKYAKLVREAVSQKETDDDDINVSNATIVSEPVYKIMGGTEDSLFLDDVPFLNES
DWIGDDMNSKYTPFTYDATILLGLSACAAVRRIGDSLVLEGNEFFEKIKQTRFAGVTGDV
VLRDLDGTREGHSAHYSITNWRAFDGDDDDMVFFNETTAYAYHPGDDQNWDDVSVFVYQG
GNVSIPPDLPPPVVSINEVNAWIRVAVYALVATVLAATVGFAIWTYRHRQCRVVKASQPF
FLLLLCVGVALMGCSMIPLVVKENEVEDLDLNCNSFAWLVTMGFGITFSTLFAKTYRINX
NRTQRKEMPASKTYGSGHGLPRRLDMRMYVVFSIVVSPSAGSSILALYPHVLMEPLNVSF
ILCVSTLFVSYEVNVMMLALMSANVPITHEEFSRKPDRFGRDTEIYSRCMFEDSLPYIGT
TAAVNLCVVCLAMYQAWRARDLSTEFAESMYIAGALFVTLSATILAVPLLLIVKENLDAL
IFIICALVFTLTTSIMCCIFIPKITFHLKDKRSGQSASRTRFSSVYSRASSVSANSAGEV
ILTSKPQKALISEISDLKRKLGRAEKRERILDLRNAQLEKQLSGSGMLNSKEESALTFRT
GPALSGVTGSTKKYVSFQEDTIISSSNTKTPRVSELSLEMEREEKQELQVNDESDHASHG
GDEDPLSNGLDNSAV
>comp5475_c0_seq2; 588 37 3009 LEN=4838; translated
MTDESLSMDADTVLERQNDNRLEVFEDGLSANCEIVGLLQFGRGNPSSDEYFYNPSQEDI
EHVVSTLLAIEHLNTANTSLVPELSGIHECPITFSAAFAEASIIPSYAFEVVDRLTRPET
DSSARNAERPLPCAFLGAKSSTISSATAMVTGMRGFPQISPSSTSVSLNNKVEYPLFGRT
IPDDGFMAEVFLQFLHDTLGIRHFFVFFDSDSYPRSVYESLRRAILDLGWGSLGGRETEE
ESKDTMYLQSYLLERDADGRDVENILVDAIDAFNRSEFRYAFVITDVDTTDSLMKLAYDS
ELAGYGDYQWFFADTFSLSSRKFKKDSVLVKAYNGVGSIKANAISDMEIHDAYVKQFRDS
KRDLHKKYAKLVREAVSQKETDDDDINVSNATIVSEPVYKIMGGTEDSLFLDDVPFLNES
DWIGDDMNSKYTPFTYDATILLGLSACAAVRRIGDSLVLEGNEFFEKIKQTRFAGVTGDV
VLRDLDGTREGHSAHYSITNWRAFDGDDDDMVFFNETTAYAYHPGDDQNWDDVSVFVYQG
GNVSIPPDLPPPVVSINEVNAWIRVAVYALVATVLAATVGFAIWTYRHRQCRVVKASQPF
FLLLLCVGVALMGCSMIPLVVKENEVEDLDLNCNSFAWLVTMGFGITFSTLFAKTYRINT
IVRNAKKCRRVKLTVRGTVYPVALICGFNVMMLALMSANVPITHEEFSRKPDRFGRDTEI
YSRCMFEDSLPYIGTTAAVNLCVVCLAMYQAWRARDLSTEFAESMYIAGALFVTLSATIL

AVPLLLIVKENLDALIFIICALVFTLTTSIMCCIFIPKITFHLKDKRSGQSASRTRFSSV
YSRASSVSANSAGEVILTSKPQKALISEISDLKRKLGRAEKRERILDLRNAQLEKQLSGS
GMLNSKEESALTFRTGPALSGVTGSTKKYVSFQEDTIISSSNTKTPRVSELSLEMEREEK
QELQVNDESDHASHGGDEDPLSNGLDNSAV
>comp5476_c0_seq1; 724 97 1407 LEN=1545; translated
MSVPSMSGFAMMSPHRAVLEANPDDDAGQVRLLEELQSRGRAAVGAKSWMDAKLLYEKCL
TMTGCSLVDKNKEAIFHSNLSLVENSMGEFEKARVAAEAATQDDPKYVKGWWRLGQALLA
LHRPEEALDAFSRAKVLDPTNKALTKLVAKTKKQVEEEKALMMETEGDKDSGKDATKTTV
SMPAPPPKSVKSTKPAAAKSSSSSAANATGDDDDDDSKLFTKSEPVRGYKVVNGKKTSYF
HNELDEKSRELIGDIAPKKIENPAPTPAAANASKQGTSVWNTGGTWEERNVTEWAKKTLS
DFLLQTTYTLPPSSPAPGAVARVEKVAKLDGHASVAVVRGKTRFIYEFCCKLEWELDKPD
DELSCAGSLSIPDIDGTIALGEGYEIHDFGIESVSNPSVRPLVDRFVHRGGFHEALNEQI
DDWVRHFKAEHGPKDA
>comp5477_c0_seq1; 610 96 2305 LEN=2346; translated
MNHQSKTSNFHREDEQPATEELGSLFSRLKCKNTGEEIISSRKSLKSKTSSKKVHKILTP
WLVNDRAAKISGESISKGIQVDDRDINLPMRTEHVIYPSVEGNSVEIQSTLAPASNSKTQ
VQNKNSIGSVLFGRRRRSSSKSNAIKSSEYNESKSYPQFGSFPENESDEDKTRNNKLRGS
EEKIVSTNPVNGSDNLSTSVAEKAGIKPTRTNSYDQRFSYSNGTNSILDNDDDCGLPSMM
TSSTISTKYDVGDTSENKTGVRCSQRDIVTPRRRNSRSPSPSRMRREPNPMNPSDSPKNG
KVQHHQKVKFKNDWNHEMLMAHEMNKQLSQLSSSFDCGTMNSNSHEAGKAHSKKKATIQN
DWDHEMLMAVEMKKQLSQMSSSFDCGTMNGKNRETSFSGSTRESLRQENEKISVLSSSAM
SCLNPTSQLSSLGKPNSTMHGTDSGNENNQNLINISGTRQMSTKPATNTLCGAVSSALLL
DVKDDDALWKYYERNKMPQLPLYQDQISKYREHITKQDPPPGSTAKAPVSNGEGRIIQDA
PRFAGISNSNRIRPDKVDLQXDIDAACCNKLLTKSTQACPSKADGYDHRRPYDNETTGFQ
INQGTNNNVSDRELQLRLQYESLMGYQNQASYSHEPVKPSKEQLLDQLRLAVRKASAELD
SLYLQKGIRSDFRERYNMRTMETEDCSEESSFMPEYYGRNTQQRHLDHEEEALDYIISTG
SIHSPTNRKDKYDFSY
>comp5478_c0_seq1; 1334 505 4686 LEN=5362; translated
MPPSATPSSLSKSTQLGSGSDKTSETTLAFNSTEAEAEASQSLALLLLLLEINEGDETLS
NLDDDEDALDIPISTETTDHIPMSLVNGNNLNGIDAKAKPSFLVICTVDGYIMVLNANDG
SLVYAFSSGIPLAGPSEPLDEDIFADGTRGSPNNNNQDYERRIVPFPDGQLYDIGDGGMS
VKPLGISVQDILANPAKTCWKSGKSTRNTEASHGKWDRDVGNDGYDDASYWDDNINNNEL
DHIEDKEECGIVTATKSISLFALDSFTGNLVWHQYPNGTTLKMDDHETAHGDNDHTNPNA
SSSGRKKKDQTQSTVLLQREDVVVQQISTDNGEPVWNVTWATLRGLEFGESDDQGGRPRS
PRGHGSGDLLPPSEQGLLPTGEETTDGNGFGKDDCDSVFSTRLPDILFSEDGTSITAVES
SRSNCPPPEIMWTREFPKTVASVFGLNGKSWEPLTVLDDVGAGPSGSWNGEKHLLPQSSN
NYDDNNPTDLGKELAVIKQQPSHITSHEFVNNYRTDRLYHGRDFLFRNGIRFPFHTQRVA
PTLQSFTPITGGRDDKDNRNSFVGSNYLQLPSPEGYSDDKTQQKELSVRLEWHIWLLAAI
GVVSLMKSCYRRIYEQKEQEATDTNAVRLQAMPGMNEQKQQISVTQPTTSDSKKEERTAT
TKTSTTNGSPKVDDSTPHYGVGLIDGSIPLIQYTRYASEFEEIGALGKGGFGSVFQCRNP
LDKRDYAIKKVLIRSDSKQPQSDFTNRLKRTLREVKSLASLDHSNIVRYYTAWLEREQLN
GGNDSENPSDGTPGASDYYMMSYTRTSHVKGFGSGQFSESVGSPMWKSNNNFLNPFGSGG
GLPPVFDHSFSTSSGQNHSISEVPEALDDYGFVFDRSNTEGEQCDETARNERTETQVISR
PQSKSTTVPSSGGGLPPVFQNSATSSSCQSHNIPGVPDALDDYGFVFDRNSTEDEHSVTS
ATEESLKNQAVPELKSAIKNGTILPRNKSRMNNAISFQSLRSSRSSIEESTSEWSQESKN
QSDATQEGNESNTIEEKVAEPTVSQKYILYIQMQFCSQKTLADFLSNEEARRGPSGSSIG
GIDIPYALSLFLQTCQGVKHVHSKGLIHRDLKPNNIFIDDDGAVKVGDFGLSRASSDSNK
GDGEADSSVVVGSTNNPSNADITAGVGTRSYASPEQMKGGSDYDSSTDIYSLGIILFELC
YPMYTGMERNIVLSNLRNHILPNLWTETVAVDFPRLNSLVLSMISNKPKDRPTAQAVVEQ
IQSVLEGFTISSLDKRDYEGSILLRVEIMFCENGLHKTMNLLREAALPNLIDIVQYGLRS
STSKGQMKSIMEFAIVPRPQQPEDEPEDAPECSPASIGALMVKKLSEKTLSEKTPCDNPQ
VLLIRQVSATKYA
>comp5478_c0_seq2; 1231 505 4806 LEN=5482; translated
MPPSATPSSLSKSTQLGSGSDKTSETTLAFNSTEAEAEASQSLALLLLLLEINEGDETLS
NLDDDEDALDIPISTETTDHIPMSLVNGNNLNGIDAKAKPSFLVICTVDGYIMVLNANDG
SLVYAFSSGIPLAGPSEPLDEDIFADGTRGSPNNNNQDYERRIVPFPDGQLYDIGDGGMS
VKPLGISVQDILANPAKTCWKSGKSTRNTEASHGKWDRDVGNDGYDDASYWDDNINNNEL
DHIEDKEECGIVTATKSISLFALDSFTGNLVWHQYPNGTTLKMDDHETAHGDNDHTNPNA
SSSGRKKKDQTQSTVLLQREDVVVQQISTDNGEPVWNVTWATLRGLEFGESDDQGGRPRS
PRGHGSGDLLPPSEQGLLPTGEETTDGNGFGKDDCDSVFSTRLPDILFSEDGTSITAVES

SRSNCPPPEIMWTREFPKTVASVFGLNGKSWEPLTVLDDVGAGPSGSWNGEKHLLPQSSN
NYDDNNPTDLGKELAVIKQQPSHITSHEFVNNYRTDRLYHGRDFLFRNGIRFPFHTQRVA
PTLQSFTPITGGRDDKDNRNSFVGSNYLQLPSPEGYSDDKTQQKELSVRLEWHIWLLAAI
GVVSLMKSCYRRIYEQKEQEATDTNAVRLQAMPGMNEQKQQISVTQPTTSDSKKEERTAT
TKTSTTNGSPKVDDSTPHYGVGLIDGSIPLIQYTRYASEFEEIGALGKGGFGSVFQCRNP
LDKRDYAIKKVLIRSDSKQPQSDFTNRLKRTLREVKSLASLDHSNIVRYYTAWLEREQLN
GGNDSENPSDGTPGASDYYMMSYTRTSHVKGFGSGQFSESVGSPMWKSNNNFLNPFGSGG
GLPPVFDHSFSTSSGQNHSISEVPEALDDYGFVFDRSNTEGEQCDETARNERTETQVISR
PQSKSTTVPSSGGGLPPVFQNSATSSSCQSHNIPGVPDALDDYGFVFDRNSTEDEHSVTS
ATEESLKNQAVPELKSAIKNGTILPRNKSRMNNAISFQSLRSSRSSIEESTSEWSQESKN
QSDATQEGNESNTIEEKVAEPTVSQKYILYIQMQFCSQKTLADFLSNEEARRGPSGSSIG
GIDIPYALSLFLQTCQGVKHVHSKGLIHRDLKPNNIFIDDDGAVKVGDFGLSRASSDSNK
GDGEADSSVVVGSTNNPSNADITAGVGTRSYASPEQMKGGSDYDSSTDIYSLGIILFELC
YPMYTEVCNTKLTKLKQRVKLNGSLQLYPVSFGELHIDFLSYLTGDGAQYSFEQPSQSHS
SESMDGDVAVDFPRLNSLVLSMISNKPKDRPTAQAVVEQIQSVLEGFTISSLDKRDYEGS
ILLRVEIMFCENGLHKTMNLLREAALPNLIDIVQYGLRSSTSKGQMKSIMEFAIVPRPQQ
PEDEPEDAPECSPASIGALMVKKLSEKTLSEKTPCDNPQVLLIRQVSATKYA
>comp5479_c0_seq1; 369 206 805 LEN=1013; translated
MNNHKGILALALVFVAPDATLGFLIHRQAPAPSSRFDGNNPFRDLDVDIDLAEDCASNFG
KYSFEEIKQCRDEIHAHRVQNVALGDSTTPDLIKERFLETELTMQLERLQNDMPESYLFP
DEGSFDEGIDTDVGLDGILLDNGLIAMDLPTPRDAASSENRAGTKQVNVWKDLVAAEGVL
ESIAICGMLGFMMMAPNLN
>comp5480_c0_seq1; 275 1 1145 LEN=1252; translated
XFIRSRMKSFQSERDRKYKNNSSTRSFLWDIVRWVTCLHHALFLFNFVVDRCDCLVTKPR
PRQKSKLSSARTALPASREELYPPLVLLGGIAQTKSSWDHHLGALAEHRQVLVYECIGQG
EKAIDDNNLDASLPAQARRLLEQLDAEFTTGGRSNDDDNQLVVDIAGFSFGGRVAMAAAC
LQTDGVSQKIPDHARVRIRRLHLTGVGCDRSDYGHVAMRSFSELIKSDPSLRSFAWSILL
ATYSPAYLRGLPERTLERLVDFIASSNNADGLLAILDQAEVSDVEDPWHVVNMADRLVSS
GETPEFSEGTGSKGAVTGKLCVGEFDRMAPVEGVALLQKKLGWIRHGVDVLPDCGHAVVL
EQPRAWRESVLSFLNANGAVS
>comp5481_c0_seq1; 452 200 1777 LEN=1910; translated
MVQIASSLQNPPVGGRGPAARPLRHRSRRFTAWIRPCLATSFCGLVAVWWGLYYHVLLLH
KSEHQLVSGFEKQQQQQQQQNRPHQQKQKERQEQQRALLLSPEAYPVYGTHMELEWNGS
NKNGNNEQNNGNGNSISMVYAVPSRSTPGRILATNKPPSATGTADPLGRIRGVVVLLHAC
THSALKFFAPSEDSCPSCVGLSEEMRLARIVLERGYAALAVTCSNAKSGCWGGHEDVRRI
QHALREFWRASVPMEAGRDPGALPAETSAPVYAIGASSGGAMAAKLVAEGVARSATVMVM
ALRDQLLDRLLALPHEHKLYLAPMVRDKGTTNRVRKNYQYITKQQEQRSREDPKSTAGNN
HHDFQVVLDETSCKPLPVTSHYLWNRVPGMTLEAAQIIVDVLLRHGHLDPSTHLFLIDPT
RSNWRDHLLETRDGGKDNSSNHPGEHNNKARPPSLELPQRDAGPVQQRPKNDGESQLLLW
GTFDLTPGMSPLAKALHRAWAFHEYCSEVVEKSLDFFEGTRKGTQ
>comp5482_c0_seq1; 450 140 1366 LEN=1519; translated
MEGLGGPVDLERYSADGIDPSLEDCCRREVVGNRRHNALTTTLRRHDVAALAERRRRHLV
SMPNNADDEACRCSCDPNSDGGEYKALTEFKERKQREHSVSEQYEEEQQEQIAREEAAYE
RKAAVGSGNDDSDSDDEFDYLLDEDFGVEDETIRLLEEERRAELEYQVLVRQVAGHHGYG
VHRQLHPTRVLRIGGLGRDAAKSKGVPPPPQAVVLHLVDPESITSASLDYYIEKELAPQC
PGTMFLRSGGRSVLSMDSDLARKSLPPNVLNPDRDLPALVAIRDGVAIHACPRLLGLGAE
GGEIEPGAVRHWLDRARVLRTDPPSDDVCLIRPEEEAHMDFMLSQAQPPAPAAREEEEDH
YNCGVEGCHKYFRHEHVGVKTEEQSGLVVKEDKILGMGDFEYCQFLEE
>comp5482_c0_seq2; 349 397 1514 LEN=1667; translated
MLSQRPFGDXDVAALAERRRRHLVSMPNNADDEACRCSCDPNSDGGEYKALTEFKERKQR
EHSVSEQYEEEQQEQIAREEAAYERKAAVGSGNDDSDSDDEFDYLLDEDFGVEDETIRLL
EEERRAELEYQVLVRQVAGHHGYGVHRQLHPTRVLRIGGLGRDAAKSKGVPPPPQAVVLH
LVDPESITSASLDYYIEKELAPQCPGTMFLRSGGRSVLSMDSDLARKSLPPNVLNPDRDL
PALVAIRDGVAIHACPRLLGLGAEGGEIEPGAVRHWLDRARVLRTDPPSDDVCLIRPEEE
AHMDFMLSQAQPPAPAAREEEEDHYNCGVEGCHKYFRHEHVGVKTEEQSGLVVKEDKILG
MGDFEYCQFLEE
>comp5483_c0_seq1; 408 1 1715 LEN=1837; translated
QALVCTGTIARGLPKGFSEARAQTSIRSVSSLPKRKKDIPTKQRKMYEDGIDGDSLREVV
ATAIKRIDIGTAVLPEPLFCSDTDQADSGPGSEGYELLGGETSSGRNSHRDSNREGRAPD
ELHATSSSTLYARTLRSEFNALVVEHHLKWAPMCVSEPGPFVDGSLSTRLGRYDFHHADA

IVDWGIRYNMVVKGHVLVWHVXIVSIAMQCNPCIVSQLNAVQCLQRNGILFVLGRQTMHG
DHLVCFWEKNVIKCTQLSLLFLSFFACIRSIDPTPHRPQLLEDLEPEEVREQLRRHIFTT
MGHFRGRIRVWDVVNEALAPDGSLAENVFFRKLGPGYIEECFRWAHEADPDAVLLYNDNK
VEGMNGPNKQKADGFYRLLAGLVKKGVPVHGCGIQAHFNAAGTGRNRVPTPRMVKNQIHR
LGKLGLKVNISEMDVRVSKLAPELRQIAQRQIYHDIIAAALTEPAFDGLWLWGFTDRHTW
VTQFYSEDEPLVFDESYGRKEAYYGLRDALSSIARGGTVGAHVALDSDEDEDGNRWGHAW
MPPLLEEETRSSFLDQYGDDITGDQRPDWEV
>comp5483_c0_seq2; 303 1 1870 LEN=1992; translated
QALVCTGTIARGLPKGFSEARAQTSIRSVSSLPKRKKDIPTKQRKMYEDGIDGDSLREVV
ATAIKRIDIGTAVLPEPLFCSDTDQADSGPGSEGYELLGGETSSGRNSHRDSNREGRAPD
ELHATSSSTLYARTLRSEFNALVVEHHLKWAPMCVSEPGPFVDGSLSTRLGRYDFHHADA
IVDWGIRYNMVVKGHVLVWHVXIVSIAMQCNPCIVSQLNAVQCLQRNGILFVLGRQTMHG
DHLVCFWEKNVIKCTQLSLLFLSFFACIRSIDPTPHRPQLLEDLEPEEVREQLRRHIFTT
MGHFRGRIRVWDVVNEALAPDGSNVKLHSNPVWNRLQEVQYDAYKRYVQXDAELILSIEX
YSDLISIACLTFLGSLAENVFFRKLGPGYIEECFRWAHEADPDAVLLYNDNKVEGMNGPN
KQKADGFYRLLAGLVKKGVPVHGCGIQAHFNAAGTGRNRVPTPRMVKNQIHRLGKLGLKV
NISEMDVRVSKLAPELRQIAQRQIYHDIIAAALTEPAFDGLWLWGFTDRHTWVTQFYSED
EPLVFDESYGRKEAYYGLRDALSSIARGGTVGAHVALDSDEDEDGNRWGHAWMPPLLEEE
TRSSFLDQYGDDITGDQRPDWEV
>comp5483_c0_seq3; 434 1 1494 LEN=1616; translated
QALVCTGTIARGLPKGFSEARAQTSIRSVSSLPKRKKDIPTKQRKMYEDGIDGDSLREVV
ATAIKRIDIGTAVLPEPLFCSDTDQADSGPGSEGYELLGGETSSGRNSHRDSNREGRAPD
ELHATSSSTLYARTLRSEFNALVVEHHLKWAPMCVSEPGPFVDGSLSTRLGRYDFHHADA
IVDWGIRYNMVVKGHVLVWHVTSPQLLEDLEPEEVREQLRRHIFTTMGHFRGRIRVWDVV
NEALAPDGSLAENVFFRKLGPGYIEECFRWAHEADPDAVLLYNDNKVEGMNGPNKQKADG
FYRLLAGLVKKGVPVHGCGIQAHFNAAGTGRNRVPTPRMVKNQIHRLGKLGLKVNISEMD
VRVSKLAPELRQIAQRQIYHDIIAAALTEPAFDGLWLWGFTDRHTWVTQFYSEDEPLVFD
ESYGRKEAYYGLRDALSSIARGGTVGAHVALDSDEDEDGNRWGHAWMPPLLEEETRSSFL
DQYGDDITGDQRPDWEV
>comp5483_c0_seq4; 329 1 1649 LEN=1771; translated
QALVCTGTIARGLPKGFSEARAQTSIRSVSSLPKRKKDIPTKQRKMYEDGIDGDSLREVV
ATAIKRIDIGTAVLPEPLFCSDTDQADSGPGSEGYELLGGETSSGRNSHRDSNREGRAPD
ELHATSSSTLYARTLRSEFNALVVEHHLKWAPMCVSEPGPFVDGSLSTRLGRYDFHHADA
IVDWGIRYNMVVKGHVLVWHVTSPQLLEDLEPEEVREQLRRHIFTTMGHFRGRIRVWDVV
NEALAPDGSNVKLHSNPVWNRLQEVQYDAYKRYVQXDAELILSIEXYSDLISIACLTFLG
SLAENVFFRKLGPGYIEECFRWAHEADPDAVLLYNDNKVEGMNGPNKQKADGFYRLLAGL
VKKGVPVHGCGIQAHFNAAGTGRNRVPTPRMVKNQIHRLGKLGLKVNISEMDVRVSKLAP
ELRQIAQRQIYHDIIAAALTEPAFDGLWLWGFTDRHTWVTQFYSEDEPLVFDESYGRKEA
YYGLRDALSSIARGGTVGAHVALDSDEDEDGNRWGHAWMPPLLEEETRSSFLDQYGDDIT
GDQRPDWEV
>comp5484_c0_seq1; 919 186 2105 LEN=2704; translated
MLQYDNSAFYFFALSFITIYLVPSWIYIAKSFKKVLFADDASIGAVSRTSAEKAKADALK
KDSGISKLFKSRKFLYNFVITIACTILFFWLLFSVKEDGIVNSFDPFNILEIDSGADTKT
IKKAYRTLSLKYHPDKNPGNREAEAKFMMVSKAYESLTNEESRENWEKYGNPDGKQSLEV
SIGLPNFLLQTQYRNLVLVVYLIFMVGIIPFCVWTYYSDSSKFGEKDIMYDTYSWFHNTL
NEHTLVKWLPEVMAASAEFRNHNVPKTAEDKKDVSQVMGLVKTNMQKPKHSLPAIVKSNV
LLHAHLMRKSDQIESEQLQDDLNRMLRLSTSLNDAMIAVCKHQDHMQTAMNCIEFGQYLT
QAMWVKDSSLLQLPHFTEEEVKHAEKGKGKAANIVEYRDQPDDQKKGMATFTEEQKSDVL
NYLKMIPDIDVQTKVYVDDDEDSNVYEGDLCTVNVMLTRKNLEKGEKAGLIHAPYFPFPK
QEAWWIILGTKEGKIIHIEKVVDPSRVVEHEIKFLAPRVGEYEFDLFVKSNAYVGLDQHQ
KIKLTTLDNSVLPEFKVHPDDAELDDEPTLFEEMLNANVEDDSDDDSDDSDDDSDDDDEP
DEAIAPKSEADMKKDRLKNARNQDDDSDSDDDAEEVYAD
>comp5486_c0_seq1; 293 66 419 LEN=724; translated
MKFIATIAASLLVATQGFIAAPTASMTTSLRMSDDDQTATGVTGKVKFFSDKGFGFIAPD
NGGEDVFVHFSAINKDGFKSLNDEETVTFDIEYDDERGKYRASNVNGNGDGIQKERW
>comp5487_c0_seq1; 327 175 1005 LEN=1089; translated
MSGQSKSRRKLCFELLCSLWFVSVLSHTRPIHGFAAVANRPRPGSSSSSLSAYQKVFVAG
GSRGVGHAVVEKLSERGTEVVALVRREESAKELSSLPGVKAVLGDALVQKDVENAMDGCD
AAITTLGGTTDDRRVDYEGNNNVIESAGILGVQRILLVTSVGCGSSKAAAPPHVFEILRD
VLSAKEKAENVLIEWYTNSNWSIIRPGGLVSEPATGEAVLTEDTTAIGSIHREDVADLVV

RAMDSPRTERRVLTAVDPSIASQVAVEGKEIPAFEL
>comp5487_c0_seq2; 338 175 1290 LEN=1374; translated
MSGQSKSRRKLCFELLCSLWFVSVLSHTRPIHGFAAVANRPRPGSSSSSLSAYQKVFVAG
GSRGVGHAVVEKLSERGTEVVALVRREESAKELSSLPGVKAVLGDALVQKDVENAMDGCD
AAITTLGGTTDDRRVDYEGNNNVIESAGILGVQRILLVTSVGWQVTAARMRANRVVLLFW
AAQRGVVVWCGAVWRWPNAGYRESGTIPLLFFFLTTFFFSFCGLSNRWIRPRWSARNAIQ
SNAIQSNTMPCHARSRHSGSSKAAAPPHVFEILRDVLSAKEKAENVLIEWYTNSNWSIIR
PGGLVSEPATGEAVLTEDTTAIGSIHREDVADLVVRAMDSPRTERRVLTAVDPSIASQVA
VEGKEIPAFEL
>comp5488_c0_seq1; 330 1 2000 LEN=2361; translated
GDYNIPKIRINIKRIYLIHDTNXEIHSEGNILTMEKDNNKNCSHLEITDGMVDVELAKEL
LGMSVDDRNKLHEEIHGVRCQAVKETPELIERSLLEFDVELNKIAKKNQKKSSSDENAYM
IMLSRRRELLQDQDGNVQKNHYALDDPAFRMRFLRCELFMARKAAERFWKYLNFVYEQWG
EIALDRPVCMEDLMNNKEHRKYLKRGHMQILPFRDRSGRRVLVHLGNALRGVDPIVLSKV
IFFLYDSCTRDCIESQQAGIVVVNEFQSWDINVAKFFSMEALGMMTRSLECLPTRLVTQH
MCVSNTPGSRMIVDLFFLHGYRDKRTKVNMLFHRGSVQETRYSVRSYGIPTQLFPMTATG
NIKLVYLNQWIKTRLILEQQEHIQRLKLGTESYLKGKDKSGQQEQSTLANHVDNTSIDGN
EDPNNIPMEIEQKKSAREDRQVIVECPCLNDVVFRQGTPSWGNPGNATFRQLMLDHFAKQ
ELLKRNTNEGCTSESDDCVLKNANSNAHANDNLVFCDWLMDEIEVKRGGRFLGWDKERVA
WVQMKTDQEKQLAKIRQKVFIAIYNCGRNFNSALHLPVQNQNVIGQLRSSSRNDNLNTTT
HTTKANRPAKHLTMDNSYGNRGDSGDESLQYQFLNEGKRASPPNGTFCLSVCKRASKMPK
KSGNHP
>comp5488_c0_seq2; 330 1 2000 LEN=2177; translated
GDYNIPKIRINIKRIYLIHDTNXEIHSEGNILTMEKDNNKNCSHLEITDGMVDVELAKEL
LGMSVDDRNKLHEEIHGVRCQAVKETPELIERSLLEFDVELNKIAKKNQKKSSSDENAYM
IMLSRRRELLQDQDGNVQKNHYALDDPAFRMRFLRCELFMARKAAERFWKYLNFVYEQWG
EIALDRPVCMEDLMNNKEHRKYLKRGHMQILPFRDRSGRRVLVHLGNALRGVDPIVLSKV
IFFLYDSCTRDCIESQQAGIVVVNEFQSWDINVAKFFSMEALGMMTRSLECLPTRLVTQH
MCVSNTPGSRMIVDLFFLHGYRDKRTKVNMLFHRGSVQETRYSVRSYGIPTQLFPMTATG
NIKLVYLNQWIKTRLILEQQEHIQRLKLGTESYLKGKDKSGQQEQSTLANHVDNTSIDGN
EDPNNIPMEIEQKKSAREDRQVIVECPCLNDVVFRQGTPSWGNPGNATFRQLMLDHFAKQ
ELLKRNTNEGCTSESDDCVLKNANSNAHANDNLVFCDWLMDEIEVKRGGRFLGWDKERVA
WVQMKTDQEKQLAKIRQKVFIAIYNCGRNFNSALHLPVQNQNVIGQLRSSSRNDNLNTTT
HTTKANRPAKHLTMDNSYGNRGDSGDESLQYQFLNEGKRASPPNGTFCLSVCKRASKMPK
KSGNHP
>comp5490_c0_seq1; 285 123 833 LEN=985; translated
MLYSVILPTYNERENLPIIYYLIDKSFNDAKLSYEVVIVDDSSPDDTLGVARAIQKSYGE
DHVQIVSRKGKLGLGTAYIAGLKAAKGDRIVLMDADLSHHPKFIPQMVDLMDKTKVEIVT
GTRYARGGGVYGWDMKRKLTSKGANFLADFLLNPGVSDLTGSFRLYERKVLEQILPEIRC
KGYAFQMEIIVRSKKAGFRVDEVPITFVDRIYGESKLGTKEIIMYIQGLMQLFFTT
>comp5490_c0_seq2; 222 123 563 LEN=684; translated
MLYSVILPTYNERENLPIIYYLIDKSFNDAKLSYEVVIVDDSSPDDTLGVARAIQKSYGE
DHVQIVSRKGKLGLGTAYIAGLKAAKGDRIVLMDADLSHHPKFIPQMVDLMDKTKVEIVT
GTRYARGGGVYGWDMKRKLTRYVELCW
>comp5491_c0_seq1; 94 1 638 LEN=638; minus strand; translated
XAGLGSGIKSVTKGTVAGIASLIAQPMVGAKQGGVAGFFTGLATGVASCVALPATGVCVG
AYQVGRGVYNSAEAISSSNKGMVWDEKKENGITTIVKDRTYYDLMGVPTNASQGEIKKAY
YVKARKCHPDKNPGDPTAANRFQELGHAYQVLANDQSRAAYDRDGLSINDDKNQKLHMSD
IDPYIFFAVMFGADSVKPYIGELWIADKAEMFL
>comp5492_c0_seq1; 275 48 1280 LEN=2508; translated
MSNNTNRHNTKLLFLALFLVLSSLGVLSQSIPHTSEQAVASPQPNENNTSTENTQLVYLE
REQTFAAPPRRDVDTRLPGIFPRGARSLYKTDLDWIALRGISPWKRVRSGLKFVFGGGLS
QLLRVTRAFYFRSIPATIYDARAKEAEEGLTKAEFFERYGFVVLNHTSAMEASDWSESDR
NIAALLGAYGNRSKDGGSSYQAIIDDFRNGDTPVKRIYAKETTDLLRSILPRAKNVMGPS
RGIRRYPTNNLNKGFAKQVHNDYGLDFDAVVERNPFFDFGSQKALYEEKQADEYMLVNLW
RPIKPMSDSSELRSFPLCFLDPTTLQEGDFVEVDSASLGVVTALKENPEHRFYYYPDMTV
NEVVVFKQFHQLRIETSARMPVFHTAFADPAADETTEDRVSFEYRVGILL
>comp5493_c0_seq1; 394 146 1000 LEN=1109; translated
MPKAKRNASLNRVARRRGVALENLPSQETRKKNESGGGVSNDKKFEKATPSVDQKKLIVD
KSNAVQEGIQKNTANKDDSINQPDIPPKQQLSRGQRKRLAKREQFLKKEKMILSSLMLQR

QEEQKKRIDGLDAIKQALMDTSFNGRNQSSISIKPPTQHVSTNKAKRKLVAKEVEHMNLI
LQHPAFKNNPFETMQEHLRNTLAEERKQQEILSRKRSEEEKQKNEAKKSIKKKSQKKNTN
HGELDECGHFILQAVYGVDTFSATKILINFRIIQHSFVIGYTKF
>comp5494_c0_seq1; 389 79 1749 LEN=2388; translated
MKNPYHGGNFRGDIQHNNGSASASKSVAFLRWTFVGLSVACFVQISYQQHLRVSSGYYGD
LSQSPLRNSAGADPDVPVRGNARSIYKNDGSATKNLPPIPRFVVDPLFKAEQDRIDSAAD
EETRCKQYGVPPLPRDSKSRQRRIFFGSMLANENPEVIVAHALEAYNKYDVVALVESNTT
HFATPRTMNFGPGSISVRTLQESELFGFRNTTKVVVDYWLEDEPDLKMMSREVEQRNTIW
KIWLKEGMAPWDVGIMADLDEVTSRDFLNALRVCDFPKLRYDADHRPSCQTPKMALSTMQ
FESTPLCVKEWEWFHPDLIAGHCILGVGDPQGRATPERDTTVGGDRRIPAGNRKGWGSND
YKKYPEDVLSNDRFPLWDGRDIREINGNEDNLVNFDRPFREGMGDTAVYGTAYHLHNWFK
DLTVLRHKYATYGHANPDAGKKTLSNIQGDLDMLVRCTRGLGNNPALEIGKLPDDGYPYY
TSNRLLSLEESQKRREVGSNDSAIRTNANHMFSLGGNRPIFFQNRTYVLERHALVQKLIR
DDEQRFGSLYKDPHNK
>comp5496_c0_seq1; 416 156 1358 LEN=1456; translated
MTAFLSTLTLSRCLTVQSWVLLPPQRALKSRLGGTMKPRMALNGNIKKPTPSILSMVSTS
ATSINGGNGVSKEDRALLARALEHAKLGFGHTFPNPAVGCLLVREDTGGIIGSGFHPRAG
SPHAEIFALLEAAGHVESGIAAAKNVLHGDVGEENLQELIDTYKGTSEDDAISDGPEKLF
GNAFADIPVTAYVTLEPCCHYGKTPPCAASLALAKVNRVVVGFRDPNPRVDGGGVKVLQD
AGIDVDMAKDKGCARIVDSFVKRILPKNYDSENYSHVTGQMRRALRKMAGQQKMENTLQQ
VGWSAKSKATTEDAVDELVLPPEWIEHLDALLWQEELVNIRLNKAVGKKKLAKQLGNRIA
STLGAHVAQVVGHTVLLYRPGIRPVLDLEELVRTQNVDDE
>comp5497_c0_seq2; 460 58 1801 LEN=1801; translated
MSFATATTMLQLLIVAALSLSIVEGDEATDASVGDLTTVVSASQVRDWNERSYEHFRTEY
RETGCVEFLGDFPVLTAEEDSFGAHNSILTRGRSLQMSMDDTSGGMMCSCYMLYDLGRLA
ARKKSSGVAAGKAQATAGADCVGGYAGIYPCENVDLVGFVPVDSLPLPRSGEPANAANDV
WGWTSSTDREFAIWGTTEGTFFVEIMDSPSNPYFVWGFMESEDGSALQRDIKVIGDTAYV
GAEKDNHGIQIFDLNRLLNMVRSDCSSPACQSLAPDHVYRGFGSDVIGNSHNIVANEESG
YLYFVGSRQGCNGGLHIVSINDNPLDPKFVGCFGDDGYVHDAHCVKYRGPDDRYQGRDIC
FCFNESVVTIVDVTEGSNFEILSRADYKQTGYTHQGWLSSDHKHVVFGDETDELQAVWGG
REPRVRTMILNVENLSSPKNYRERFGKATIDHNQYIRKANAAGLNYNNENDLIFQANYEA
GLTILQAINYDEGDLREVGSFDTFLYGGEDPYFSGAWSTFPYFSSGLILISSLREGLFIV
KPNLGDALVTSEQPIESPSKPPSKPPTKQPSQPPTQSPVTVX
>comp5498_c0_seq1; 163 1 328 LEN=555; translated
XEEEEEDEEMISLEEASMDMFLSEGWKEARSNYDPSNTATKKRQEEEQGNLIGTVAKLAG
GILALVLFSQIPVGQEDLSKYSSIKSGPATTTIDLGDTNKVKGQTMGDL
>comp5499_c0_seq1; 308 97 1143 LEN=1873; translated
MKGGIILLAFSLTAFFVEKGGAFVFVAPQSIRSPLPSGEGMPTSRHFAILGDGDTDNGAD
FPDDDSGLAMFSMASLNSRLKEVQEEESKLPLVVLDSMLPRQVLELQINNDLLIELVRDC
LKRESPFFGMLGIARLAGGQQVHLTRGVEVEILEDKLQFLPEGQGVKMALRATDRRFEIQ
KGGIESIGGEDGYTVAKVDFLDSAQEEAEEIQNAIRRDENYVADTEEYDRMSVARAIMKA
KEFTSPNVNMAKNLSLVDRWIELARERERSSGQIDQILNDLGKIPPADQPSERAFWVGAL
INPIPAMGVSLEIRPALLTAKKAEQRVEVALNGLLRSIKYMEGSASLV
>comp5500_c0_seq1; 213 177 1061 LEN=1193; translated
MPKAHHRYQHTIGGRSPCSGPIPSETSNDGSLPFSSSPRKVSFEDNRSDSKSSICSDDTV
LMENNKGKQISEGTESGREQLPSTRRKRSLLVRPITSLSLVDLARSMVEEDDGSMTSGQD
GTDRLEERKSKRIRSFSLSPRSVISNSDISNAHQDHRLWSPSASVVEESESIKKSPWGHF
IDMVPNEDSNYRTSTHPNFSNMLKSYRRIPSQNLMGGLQHRSLRRSIPYCESEDYRPRRT
QHATPSFIGLKTDSHLVNIMSETKNNFRLTPRSPNTKGKQQADRLIGVFSDLQL
>comp5501_c0_seq1; 198 1 677 LEN=1055; translated
FLQPKNESYRIGNRNRNRADSSTIRIKMSNSRIALFGXFPGLLSTVLATGIEALSFSDLP
VRESTKNHLHSRRHFVGMASAFVAAAPLVSVPAVSYAVQEGNAKERFVAARKELRSLIDD
YAEISKGGGDAVRARLGTQGISSDLFGIQKVLKTLSEEADDLVEYTEMMEEFDAYYFQAE
GAAYQSMFVEHSSAKGTPESFLKTAKTDILQMEKYMDQLAVQLKL
>comp5502_c0_seq1; 636 441 2165 LEN=2423; translated
MGNALHRATDRCSIALEAATSTPTGTVAVAAGALVSAGMVAKNRKRICKSVCEDLPLYFL
TYHRSTVVTVFGLPLSLLWNFAMNARSKYIHTFHSAPHLHNDRVATIQKKVRNRPNKNAP
ICTARPGWQSVSLSYRNYKSKWNAIEMPLYDILEVDEGSMIVHVEPFVSIGQLTHALNPK
GLTLPVVPEMDDLTVGGLINGTGIESSSHKYGLFHEIITELELMLGDGSVVVATADNEYS
DLFHAIPWSYGTLALLLSAKLRLIPCKQYVRLTYTPHRTREGYVEHMRELSGEYNKEVEN

GRKNTPVPMFVEGLAYSLESAVVMAGDFVDAKDVPRGSANRISLWWKPWFYKHVEKILNE
GEETVEYIPLLDYYHRHTQSLFWEMELMIPIGNHPVFRWLLGWLMPPKVSFLKISQTELT
RKLTERTHVAQDFLVPTRKMFDFLAICDEDFDRIYPLWLCLHDHAPMPGSLLKDPAVPKP
SNGHEMYIDIGVYGLPRCVHEKREEDFDMKRSMRAVLKKLVDMNGFQMLYADVFFTREEF
EGMFDHTGYKKLRAKYKADGGAFKEVYDKMNILL
>comp5503_c0_seq1; 365 171 1187 LEN=1413; translated
MSYNTSGNLRNDGTFTAAGKGKVCIPTKDKNLQFKKLKAIQSNQVCFDCPAPRPTWASVT
YGVFLCLDCSATHRNMGVHTTFVRSVDLDEWTQRQIDAMRLGGNANARKYFRSHGLTDMH
GKIEKKYTGKAARAYRAELDKQLDIAAAKRGEETASGAVASTGGLLENLSLQDQQKAAAV
SQTAVVKPVAPAVPTAKKASELAGAKGKLVVTPPSSGNLLRKPTTSLKTGSLGMLRKPVG
KTMNTKMLLKKPSGGTTSNVKLRLNKLPVDDKMTGFDDMEEKPAIPAPATAPKPIVAPVV
APVAPVEVKKEPPKPKLSAMQSGVSRMQSMNSDFFSGI
>comp5503_c0_seq2; 365 159 1175 LEN=1401; translated
MSYNTSGNLRNDGTFTAAGKGKVCIPTKDKNLQFKKLKAIQSNQVCFDCPAPRPTWASVT
YGVFLCLDCSATHRNMGVHTTFVRSVDLDEWTQRQIDAMRLGGNANARKYFRSHGLTDMH
GKIEKKYTGKAARAYRAELDKQLDIAAAKRGEETASGAVASTGGLLENLSLQDQQKAAAV
SQTAVVKPVAPAVPTAKKASELAGAKGKLVVTPPSSGNLLRKPTTSLKTGSLGMLRKPVG
KTMNTKMLLKKPSGGTTSNVKLRLNKLPVDDKMTGFDDMEEKPAIPAPATAPKPIVAPVV
APVAPVEVKKEPPKPKLSAMQSGVSRMQSMNSDFFSGI
>comp5504_c0_seq1; 320 90 1139 LEN=1449; translated
MTTRITLFFALTIGRCLSFGNFQMPNFPWSGNGGEGKGLLKTTNGGILQPGDTVAVVGAS
GNVGKLVALRLSDTYKVNGIVRNADSVKPFFEGREDSISLFETDLLEEVTSTSKPETLSC
PDSLYPALCDANAIVICTGTTAFPTKAWSRSGEFSVTSEVINALLDSKFDVREALNQLDA
KGLNTPRNVDNLSNKLVLDAWKASSKVSRKRAIMLSSIGVTRRESMPFPILNACGVLDAK
AAGEQALMDSASEDGFSYTIVRPGQLFGGPYDNNYYLGTLFQLDKDSETQDVNLGKGDIL
LGDTLRSTLAEVTAQLMEGNSARDVDFAVVNVKGDAPTADSVNEKLVAL
>comp5508_c0_seq1; 251 1 892 LEN=1340; translated
XEELSSVPDTSDKHQQPTAEVQRRGQEELPPLSADAISGIKRRLWEETANGLGKVVRNLR
VAWYKGDERVFYKREEQQLEKYLPLFQYGLALFVFSFFNFRATKTPRFQKWRTGFLERIR
PSPPESLSKASQQRQGLRQVSSIKSPPTVGYLERKRKQNVDEALNSMKYFTDFLVSLSIG
TSATLFLLERKCGNNLRTDFEEAPLVAGRSLIADKICPGMLELCFSDLQVRKAFEDNQNQ
KDRPWEASSFDPNLHTFFIFINNCQKRKDYESSLRQQMQQATTLKSGAILIPHTGVQ
>comp5509_c0_seq1; 561 209 1711 LEN=1939; translated
MMKLKTGNPERLFLFCFFWSFCSVNIFYVAIAQTNLFRGFKTGKEDATGAIDDVPLTTTL
SNGVVVPLAGLGVGNLQRNLVETSIHEALKDDHRIRLFDTSHAAGNEREVAKGITTGVKA
LKDERESLGEEFDRKKKIQVHVVTKIWYTHLGYQRTKLAVEEILENFEEAINDPNVDLKL
TLLIHWPKCFDEIPWMKCKEEEDRLPERIKNAGPPPHLDDNAWKESWWALEDLYDAHNSV
DNNKNDSSSSVIAAIGISNFDRASFETLLSFARIRPHVNQINVWGLLHNKWMIDLMNEAN
VHMQVFNVMDGMIRHVYENPKAHHHLLMIANRVHQKYIQDSVSVAAEENSGRTLEKVFLV
QLMLKWFVQSGISVIPRTSKFKHLDQNSAVSLSMIPDLDDEETEVVSRAMVALIENEDFE
DDVLLQVRFHATVADMFLYWVPDEGDVEKQVAFIEKGSSYEESAHPGHVFKVYQAYDPDQ
VRTYSIGGHYGDTQDIHVEL
>comp5510_c0_seq1; 318 959 2164 LEN=2472; translated
MEANGLTRCSLPYSFESPVVCDEMITDDGASASTPPSRDPAAMTTDAMPPAPISPCGSPK
RYSPSFVSSLNDQVARYCSNECVQRRCGTAAMRTVCNNVAVAQTRVRQIEDHLVAGQQQG
HGSPPSLRDQLFAEKIRLIDALVVMGWEHYRCFLEESECFEKAEAAVRHIYEEALELSVE
LFCSAPPATGTSIANNGRLMMLLLTLDFDEYCESLLGYQLARESTSASLTADSNGADAAA
GVRGADGRLAEILKPAAQGNAYLQQVHELWKTGAFERFDVAASHEEEEHLAKIHAAGLCL
VLFRKLEYKQTDLKATERQFRYLFDEYLPRQLAQPSMNNIIATKHARILFQDGTPTVYWS
LLADSYRKEWAEHLEGYGSGAAEELFDMDMDEDEDDEDDGL
>comp5510_c0_seq2; 318 591 1796 LEN=2104; translated
MEANGLTRCSLPYSFESPVVCDEMITDDGASASTPPSRDPAAMTTDAMPPAPISPCGSPK
RYSPSFVSSLNDQVARYCSNECVQRRCGTAAMRTVCNNVAVAQTRVRQIEDHLVAGQQQG
HGSPPSLRDQLFAEKIRLIDALVVMGWEHYRCFLEESECFEKAEAAVRHIYEEALELSVE
LFCSAPPATGTSIANNGRLMMLLLTLDFDEYCESLLGYQLARESTSASLTADSNGADAAA
GVRGADGRLAEILKPAAQGNAYLQQVHELWKTGAFERFDVAASHEEEEHLAKIHAAGLCL
VLFRKLEYKQTDLKATERQFRYLFDEYLPRQLAQPSMNNIIATKHARILFQDGTPTVYWS
LLADSYRKEWAEHLEGYGSGAAEELFDMDMDEDEDDEDDGL
>comp5511_c0_seq1; 550 40 1239 LEN=1897; translated
MAATIPPTCDNDGDTVLVHLVLTGASGYLGQHLLSQWITKGLGLEEEESPGAPNRKYKTK

ITALYNRSEGFPGAVESFCSKSQNKGSGRGVSDVAVRSIDLTNADDVRSFVESSLLAEDS
SPGHKTTVFNVVVHSAALSSPRVCQEDPDRARAVNVPRDFFDAVLGTASASRRTAIVALS
TDQVYDGTRTEAFYTETETKGLEPVNAYGQTKLEMEKLLLRGRDDSPMGDCFALRSSIIL
GPPAPIDPGCAHGTFLDFCRSRGETETPTTFFTNEYRSVVRVDRVLDTVRGIVVRRVLLG
RSGDRGEKAAARVVYNMGGPCRVHRLDMARAVFDKFGYDPKLLLATDQTSPHSPLDISMD
SSLLFRHGILAGSDDKTQGLATESYLKALVDYAFPDGEK
>comp5513_c0_seq1; 448 170 1525 LEN=1715; translated
MKIALTSYAIPTLLLLLWGTTILGFVSAEETCTTSNDCESEDSYCASSGTCLSVGACAEV
SDCSDPTNQPFALVMCVGTMECREGMCGMKCGDMGDDRMACDANSTDPSVCGERAYCSSE
GICTKIGSCAIADDCFHQNNVGFGVALCFGNMECTSEKKCEMDCDGGSDAIMGCDTSEDC
LGDDDYCSTEGICMENGSCNVVDDCSNLNNTYAMIECVGTLTCESNMCGKVCGDFDSASA
GNASAVEAPAAFCTRDEDCLSLAPRERTGLFAPDETPEFEYYCADGTCRQMGSCATDSDC
SNPANRLYQDKKCLGYLSCDVGTCDRICGQECLRGRPGRCDANPCAADPATACPGAVSCI
TDTCNGSCEAVYYGEAGNILAGCGSGETSVDQSAAAPESGETSVDRSATAPDANDRVPTE
SVASSLESGAAVLAGSLGPALAVAAFVLLAC
>comp5514_c0_seq1; 382 163 804 LEN=1062; translated
MAKSKNAKKKNHVSKHILKARQDDTATTDDVDGKSSKSMKKKKGKTNSHIKHPDEISSYL
TQWKASKSEDSSVRASSQWKFNKNTQSWMIRHMYEADKMPKSSFMLLLEYLEGLEGKTIK
DRMRSDASRRCRRHKEYCKHNPDENKKGGETEKGTEDKEGDDGQAEEKSSRAAVVFTGDA
ELEYEEAMWKKLNDHEKRRQYKRARKILDLMKN
>comp5515_c0_seq1; 197 94 387 LEN=519; translated
MTAEAEAKQLDSVTDVVKEAEIDTAKAQEAIGLIRSKTNDDQQAAALAAVTISRGDVELI
VSELEVTEEVAERTLREVSLDAKGGNVVEAALRVLIA
>comp5516_c0_seq1; 254 1 791 LEN=791; translated
XECQQLCFDRRRFTVRSRTILEFFGGIHPPPDCPHCSQDQESEMDYQHHNLRSFCHRPGE
QLQQQSQVRNGMKRARDCGGFPLVDAAFASDPSCEQASKRFKRTGHGGALGKRPHCDAGD
RSGPLLHSKSDDEVSLTEEAQASPSAFPHHSTFIPREQHRHDQRDCQRGNAPVGPNDSSS
SIRSTTATAASTNGVAHATDYQPMNSLLGNLHMMRQQQQRRRQMQGARVQTQAHNATTVH
PQLQQRHHQQHHNYNHHYQQQQQQ
>comp5517_c0_seq1; 327 112 1113 LEN=1212; translated
MGRFLSLGALYPFLAFPSIRALSTAAESAASKRLVQEPTPWSDVAIKAAKDFTISQRQKL
ADLGALDIQRPMKIVGTPLENNDNIDSESKINTNSKVTKTIHFQRHGQGFHNLICDVWRE
RGLPIDFDSTDPELNPLIREEFLDPPLTETGRRQSMDRRIQCAALTPELVIVSPLLRCIQ
TAKLSFVDHLPSRNGKIDPRSADYVPWVSHEGCREELGLLVGNKRRPVSDIVVDHPDIDF
SAIEHDDDVLWETYGVRRETLLQKSERIYAFLTEYVMNRTEDNIAIVCHSAYLFTLLNAV
MDIESEELRYWFLTSEVRSMKVTFETNDDYRIQ
>comp5518_c0_seq1; 423 258 2024 LEN=2135; translated
MGNPRSKQPKKIALVGSTGGGTATLGHTDVTGLVEMIRHHLGCIHAGADSSTITSTGNDE
KALPPHPLVTLGTVLFVSLDSGAGFDSATGAEEATLLFLADGGREMIVRDRLDRINDTMR
DFEKGIARDFQNNELHGIIGVSCKPSLVSSTLQAAAEMGAPLTGTGGSSLSEAASAFEGL
RLIGNAGGSVGTTPETKAVSFASAFASEWGLGYDPAAAGAAMRKRTQTASQTNPSSTATA
TPTSTTADRPPTWKSVLNSCLPGFWGVMLLKRILRTTPVGDWLPEGDRRALEYAMEFHVL
PMVCAVVMATSRRKVESVQMAALLAAAACRGSILGGLLSGWCAAILEERLLYASILRWKL
PATFTNLLTGGLVGVVTATLMAPLSPYLGAATGKFRNLTAVYLWGSAGSSNDNNDIHESL
RLVFSSLLGCLFCYGSKVGWYHSIFLPLILIEMELGDPSMLGALDVLALVLVCAGVCLGL
LVTGNGEDKGLARRGMTTNLLYGDFVEAYYPHAEKYPLVNAAGYAASALSSAILTTDCRS
SAYLPFPAAVWLADDREAFLAASLIAFFIPFLGTVVNHTLFVRKRKSE
>comp5518_c0_seq2; 217 147 557 LEN=668; translated
MELGDPSMLGALDVLALVLVCAGVCLGLLVTGNGEDKGLARRGMTTNLLYGDFVEAYYPH
AEKYPLVNAAGYAASALSSAILTTDCRSSAYLPFPAAVWLADDREAFLAASLIAFFIPFL
GTVVNHTLFVRKRKSE
>comp5519_c0_seq2; 340 347 1042 LEN=1201; translated
MITTTATRSQPFVVPATPNGMRPGNPNCSQRSVCLGSSSMIRSGPPSLSSSSLLSGIQTT
EMDTSMFLLPDNPLLSPLLIATVALVLLISAQSFINKMLEGDQGLGAFLSDGSGFNRSGF
RPSNRGDDGGKDKDPLPWLKLPRLDFVEVAGQNELPPPDAAANEMDEEERQRAYEELEGL
RLRLNRELENVRKRRAQDGETSTGDDIAEARRLQTKLEGLMKLYGIEYETD
>comp5520_c0_seq2; 304 73 1233 LEN=1961; translated
MAGRNATLDLLSVESSDSIARIDENPIPNAAIGIGNRPNRRRVSSFDISLLSDQSIRKRD
LHEFSVHYSVSTSSWITRIVTFRGAAEENERRCLSFSFSKENEARKFGKAYSPPKMMVGS
IRCFCCSAKFTSKCTSNYCKNCGVQICEKCSRQWGIRMIPKTYLGGLFNATSKTVRVCKS

CDWLTNAFCMALLQGQFNNAVIIHEKGNINLRSTFADIHQEAMFPIHCAVMGGNLDIVKW
LVDAHGCPLSVKSKTKTGMAQSIQTSRSRTLMDLVMTGRPKIDILEFLVSKNMSVMDTND
PTLASKTLQTLMSKDNNLLRTSGYANTCCDASASTIEDACIICCEQSMNCVLAPCGHQVC
CSDCGNHLSECPVCKQSCSVMRIFKS
>comp5521_c0_seq3; 132 84 329 LEN=1197; translated
MAVKKKASVSENYCKKLLDDNKDIPDACKRFASIKASLDAKKSDPYAGMTPGAQATVRNA
QIKRRMSTRHNQGKEGKKSSWW
>comp5521_c0_seq4; 132 84 329 LEN=1106; translated
MAVKKKASVSENYCKKLLDDNKDIPDACKRFASIKASLDAKKSDPYAGMTPGAQATVRNA
QIKRRMSTRHNQGKEGKKSSWW
>comp5522_c0_seq1; 456 278 1974 LEN=2107; translated
MNTAYLXLILEEILDIFGAQTGNGEGTGAGGDRDTDGCNGWKLDDIQKVKSAQQKVFILT
FSVCKRKNGRDLHQICQVPCQSNHNRRIDPMASQTFTKLIDDVQSGRNQNLQLVLRIWKG
GSRWWNLNRNESPQILARAEIDGYRVASRSIASEQEEESANRAAGLPRIPQVLYVSECFR
NDVLVQEHPCWALLEYVGPDKNDRLIFGRAGLGDHPQNAKEGEDNGNYCANANGIENLEK
LRTTTEFGAIDRSYLNGMIKSRMEFGFDEPHPRWGRVPVDQALDYAKIILNEVLLPLHQS
SRRLFRDQPRVQQDASNSTKSFSGMVEIYCNAWKDMFTSNCCLATGGEALLQKELQTESE
DNPLGGGCGSDRIQTCLQRLKLALDLLSQNADATILPPLDPVLVHMDLQPQNLIFCRDVA
SSYHHNRHPSPPLLPYVFSVLDWEDAAWADPRFDLLLLCRKVCANRDQADALWLNYERTV
LSSKKSMKRSIDKLASFGGGEEEEARKKEFLGPIEPWLRLETVHSITTMLLQSMDLVNGG
RNPWETKQDLWQKLQREFARLEDWK
>comp5523_c0_seq1; 498 93 1889 LEN=2360; translated
MTLYLMVTQRKHTSMKTYVTPNAMRRKNNGSDFRQSACRSEWHRHRHGARNDLRRRRSFA
FMALPAIFIAFCASFGHLCAEAFRHNSCTFDVGKQFQFADCYLPDIADDLALSDASFLMS
HDAATGYLKKTSGLVTNGATNLYTKTQIGDVYQQLDDGARALDVRPKLLANGTVVLHHGA
VPIAVTLERLVEDARQWALDNPSELVLILHSNFAYASSSSNNDDDDVDNGGSEDSDDDFV
PSADTTVEALSQVYANLGVNYVECSDLYGLTVGETKRLAALSSLTTDDDDNDNANDNSGN
AYYNQDEGESWRGGYLLAMDRHDQYAGSCAKQNWVEDSLVTCYSSDGDVLPCTDRKSVQH
KKLKEYALASANNKPTDNSNELGPPESTYYYPFNEIQALWQVDGISAALGVRHVSSIIDD
NTKSHLNARVVDWIYDDEFDSINLLVADHVSLNGNALTSVLRNRCGQSELSYDQFHGDGQ
DAREYNHEYFDIPCGTAVGKPHLRKGKPLSTLSFFATALVCAVLGLWIAVFLAHYRKHHD
HAKEIEILEKDLQIVIHRCGCSSAEAATMAVDDLDSMVGDGNSQEGKKTPLIFLPNID
>comp5524_c0_seq1; 394 39 1400 LEN=1876; translated
MIFVFLAITALTSPRLSPFRLPRHSRLSGRLIANESSKATHINQRSTSATTIHLTWSPQD
LTADNDGFRPIPDDDYLKKYQEIPDLWPVEFFLVPYRRRRKATNCKDNDDDNNNSETQIL
VRRSANGTSKYGLGTGVPATRWMVSTGKLPRGYEWTDPPVVFEASDFPEFPKGTGKEEPT
RSWAYRKIHLRDDAFHDSERDNDDVAEFKDAALEEYAKEIREHLKTELLKISREGNENSS
SRWESERNSVVQTILESDNSIAAIQGTLRMSGLFEKRKSDTSARFVAFSDGPDPAGLARS
VKIYTMFPQMPDPMPLPSAPPAGLKEEIMSRPSRMAKSGRDPHKDDYGRTYTHISTSNVS
NTIHGVYLSFDATNCLPADASGVDTTEEVPPALDLFGTTAVEREWKSLQELKVLDQGGNN
ICTEDPKSTFISGFIARQLVKEGAIDIHGSTNQ
>comp5525_c0_seq1; 1115 68 2281 LEN=2546; translated
MVAETKIENLPSEMGEETTVENGDDGTISSKVLGSTFMKTLFAPFESKEEDKKESETEKE
SEPETEAETETEAEAEAETEAEAETEAETETEESTEETSEEQEKSETVSESETEEREFDK
NPTVLYALVQKKLWKETIARAKSNPKEARAFICRREKDGKIRWRLLPLHAAVVFKAPEEV
IETLLEAFPKASEEKDDQGMLPLHLAFRNGASKGVVNLLLMAFPQSVESPDRKGRVPLTL
AKAAASPNREMYIEALEKGPSHYAVTAMACARERILAEQTALFEAKMVQTCQIHESTLAE
MKASAEKQQKELQDKISEKEQELTKIHENSQVLVDHVGSLEAQMNTRSDTERFLATKIAK
LEQNIKQNGIDKEEREQFWQKTLAAKEEEMEAAKKVQGERELEIEGEKTKFTTEKETIET
KMNQTEKDLASTRDELAQAIETMKNKQYDWDVKEKETAEKISELERELANSNANGAILDS
QLKKRMETEHFLASQVSSLATRLAESSESQMKFKKRIEELEVGELSYTTNTDALKKRLAD
VTAVMESTRKHQMSILDDAITQEEMMVRTMENHAQNVSKSLEHEKQIEETKAEMMALIEK
SFAAISEKHSANFAAVSEQGNFLTNMNNSRGSMLSSVKTVTTDVMEALTVEMNLNAAEME
QAAKEARAEAELAAKEEQATEEEPAKEETAEVVVAPTKIVAVAPTVETEAVVAEEGRADQ
DEVRPEEPVNTSRITAE
>comp5526_c0_seq1; 866 486 3491 LEN=3744; translated
MEEYASRYLQAASLPYETEYLDGAAQASAEFDASTSPPTDWNDDWYHFYDGYVPTTVDPD
WWFTIYIILGCFFLNLTLPLWIYLGNRFGFHEKSVRKANKQMFMESPRTESPKTGEDVLR
KQLDEARSMISGYSFAPEEDGGGQAPPGSAVSGSIALSRAGTHLGFHHRPNNLMNEDTAS
IFSGTSGFTDAILGARPKRAPHARRRAAKTKKIVGSKSCVMGEESDHTRGGTKKRLDVRM

AAEFTKAEMDLLKERNGQQPEKDTYEDITPKTSASVSDVDYGDADDSRSVSTHSMAPSIL
SKLDADAISVRDAVDARDGGPPLMEDDVKLVNYVSPGGTAHGNGYLEAVWSRLMAIADWD
KEMKKYIGLAAHYSAQGILDEILGVVEIAAIGHFMGVPQANAYIIVGTVTGFTGTISTGF
YECTGVLIPQAHGARNNLMIGRYMQLGIFFYMVTMLPGAIFWCFFTDRAVMFYGFDEATA
EMGRKYFLATLPGYIEYGIDAVLFELLNTVGYEKYCTWFTVISSLLHSGIVVGMLWWGVD
DLFILGAVETVSGIICLIVNLAILVNRGWLDPYWEGLFKTNGLRDWRAVKNVINTALPLS
VAWILTYGEWEIMTFYCRYMSDTGAEVAAWGLMGYLWSTFETLTDGFGDAAEVRVGFRMG
AGQVRLARLSTDKALYVSFIVAVYATGILFVMAKYIPGWMTPDPTLQRMIFDIIPLIGFG
QVLMVWGMVAWAILGAQGRIRIATALEFFISWGIGAPIAAIFVFLFNYNIEGIIGALSIS
YTISTNVYLYMLHTSDWENLSAVVVARSAAEGKTYDEFYWDELPDNIQDAAAELGYNKWI
WESDDMEPVSDDKDWDELTPAERKAASLLGYTRKTWDCEDNTEDRDDSSSSSESSDGESW
KNLSKEAQSAAKTLGYTQAIWDNDGSPPTEEKDWDELSPTEQAAAKTLGYSEKKWNADDD
DESDLSSDTPKPRKQTGNGNVLEQAFGSIRNACTFDSNSTC
>comp5526_c0_seq2; 288 102 607 LEN=860; translated
MSSTIGFGESDDMEPVSDDKDWDELTPAERKAASLLGYTRKTWDCEDNTEDRDDSSSSSE
SSDGESWKNLSKEAQSAAKTLGYTQAIWDNDGSPPTEEKDWDELSPTEQAAAKTLGYSEK
KWNADDDDESDLSSDTPKPRKQTGNGNVLEQAFGSIRNACTFDSNSTC
>comp5527_c0_seq1; 411 391 1467 LEN=1769; translated
MAGGKATTAPKNTLVLSLALLLVQCCGKVAAGWGNYNNNNNQDYSIYGNVASRDWLYDGS
SLSIQVLGCVWGVVYDSEEAGCLEDESEDGTYNWYMMANCRRPQVAYSVYASDSGSASCN
SGTFVGSFMTTTGVSEFLGNLEAYDQNFNYDDDNFNYDEIPECYGGDNGYVGLDCSDGAF
SLNYYNDQYCMSRTGETYDELKGINNIMNNYKSCSSAGGGNNNGDGNSLIASLVYYSTPC
QSYDYQLCTDDASFTARSNGVSSASGIPKSWSSVNGGGHHSWVTKLKYVIGGGFLLASFI
MFTGILFTNRRRRRAMMMRKYRQAKKAKRDKGNRKSRSGSKRRSKSKGRDKSGDGVFT
>comp5528_c0_seq1; 705 273 3087 LEN=3600; translated
MKPFDVYLRRAQDPEWSPHYVDYASLKKTLRMFGKRRRKLQRGDLKWSTVLEEEPHAGGN
QSYNHPDANGSDNDNNIIFGYTLQDISSMGMNGNDTNESRLRGCGGVFGGETKTERLSRI
EHDDFKKALQAELDQASRWFQTCWIELQSSAAFTESNDETEEENTILKLYGFAVVNICVL
RQLLLRYNAHVRMYDGSMFLSEYEILQPPQSGNESSSLMVSEAEYERATDDFSLKTVVPS
LLASLEPLQDRMMTLPSYWMDDSSVLKNVHQGGTPSSAKPAGTDPSSSTFRARADELEAL
LDKTSWKQQTRQSPNKTERILQTIRYFYTMGTAQMGISMEPRFLFSQVRGFKKEMKALAL
WRERPLDVADGIAYGDEGDEAIGAGMDPANVWPLVLNLISCYLFMMNNYIIEPSSAYYAN
ALGSSDALSGIMMGMAPWFALTSAVGYSIWTNTSYKQPILFSGVLMVVGNTLYGAAYSQQ
SMVMCLVGRGISGFGAPRIINRRYVADATPFSLRTMSSAAFALTTALGAASGPGFAILLD
MVPEFEFSLPFLGVQTFNGMTGPGFFMAVFWFFFTVLIVLTFREPNRSGLEELRQREAAA
AKKKAAEDDASAALRESFDGEGDRSFRDGAVTDDDDDDASSVGSLSVGSAMDESGVSKHS
PLYCVRNMTRATALCMALIFMKRIALEVSKQRFTIHFFTPRKLCERFCVVRTTELNEKPT
PLVFCLYFSTILSLGFSFPNLMASSELQSIVGSTSVITKNRYAWSIRNVGVLHLVNGIIV
IPVSIFAGWLSQYREDRYLAMWFMGITSLGLLAMVDLTDLVDTETSDSYNDHIPLSVGPV
QYIAGSLVAFSGIEACESFVASLMSKCVPSALAVGTFNSGLLATLVGTGGRAVGDLFITA
MGLISIRNLLNLLIIPGLALMILSMFLLWRNYTIVAV
>comp5528_c0_seq2; 726 273 2903 LEN=3416; translated
MKPFDVYLRRAQDPEWSPHYVDYASLKKTLRMFGKRRRKLQRGDLKWSTVLEEEPHAGGN
QSYNHPDANGSDNDNNIIFGYTLQDISSMGMNGNDTNESRLRGCGGVFGGETKTERLSRI
EHDDFKKALQAELDQASRWFQTCWIELQSSAAFTESNDETEEENTILKLYGFAVVNICVL
RQLLLRYNAHVRMYDGSMFLSEYEILQPPQSGNESSSLMVSEAEYERATDDFSLKTVVPS
LLASLEPLQDRMMTLPSYWMDDSSVLKNVHQGGTPSSAKPAGTDPSSSTFRARADELEAL
LDKTSWKQQTRQSPNKTERILQTIRYFYTMGTAQMGISMEPRFLFSQVRGFKKEMKALAL
WRERPLDVADGIAYGDEGDEAIGAGMDPANVWPLVLNLISCYLFMMNNYIIEPSSAYYAN
ALGSSDALSGIMMGMAPWFALTSAVGYSIWTNTSYKQPILFSGVLMVVGNTLYGAAYSQQ
SMVMCLVGRGISGFGAPRIINRRYVADATPFSLRTMSSAAFALTTALGAASGPGFAILLD
MVPEFEFSLPFLGVQTFNGMTGPGFFMAVFWFFFTVLIVLTFREPNRSGLEELRQREAAA
AKKKAAEDDASAALRESFDGEGDRSFRDGAVTDDDDDDASSVGSLSVGSAMDESGVSKHS
PLYCVRNMTRATALCMALIFMKRIALESIVGSTSVITKNRYAWSIRNVGVLHLVNGIIVI
PVSIFAGWLSQYREDRYLAMWFMGITSLGLLAMVDLTDLVDTETSDSYNDHIPLSVGPVQ
YIAGSLVAFSGIEACESFVASLMSKCVPSALAVGTFNSGLLATLVGTGGRAVGDLFITAM
GLISIRNLLNLLIIPGLALMILSMFLLWRNYTIVAV
>comp5529_c0_seq1; 142 348 858 LEN=915; minus strand; translated
MNPERCAYCNIVNTNCIVQRLPPVGGIVPKVLVAVLLFSQFRVPVHEELDVADRLLDVVG
LADADHVLGRAPTLGGGHVDLDVVVPADMLDFRSAGPDDTVEEFRWDVDLLMNEPRMGDL

VVVVLAEVSDDLLGGLHVGGDVPGDSNSARRFHVSDLVCQVVVVTNGVT
>comp5529_c0_seq2; 760 151 1557 LEN=1965; translated
MSDFTTHIAAVAVGVAITYFLLGGFSSSDKASATVDPPAAASTSNGAKSAAPKKKNRKKN
KKKAAAPKKAEAPPPAPAPAPKPAPAPAPAPAPKPAPEPTPVSEPAPAPAPAAGKKKKNK
KKKAANGANKAPAPAPAPAPTPTPAAPAVVPLQPRIVVEEEWETIPKKTRKKRPKKQAAP
TPEEAVAAPALTTTVTIDASRVGIIIGPKGATMNSIQEKTGTKLDVNAPDKPETGGSQFR
PVVGGAKAKATVVISEGTAEDQATAKKAILELASRGYAALLQAEGFGESSVSVHPRFLSE
IVGSGGKTIKAIQTALDVKLTIPKTEWNPKTLQIGNVEPSCRVGIAGDVPANVKAAKQVV
RDLCQYHHHEITHPGLIHQEVYVPTEFFHCVIGTRGSEIKHIRGNYNVEVYMPSTESWCT
TENVICVGKSNDVEKAISYIKLLMDRDTELREQKYSDEHFGDDSADGW
>comp5530_c0_seq1; 216 23 478 LEN=992; translated
MGRLHAPGKGISKSAKPYKRTPPSWLKISAADVEEHICKLAKKGLTPSQIGVILRDSNGI
AQVKSVTGQKIVRILKANGLAPEIPEDLYMLIKKAVQVRKHLERNRKDKDSKFRLILIES
RIHRLARYYRTTRKLPANWRYESATASTMVA
>comp5530_c0_seq2; 216 145 600 LEN=1114; translated
MGRLHAPGKGISKSAKPYKRTPPSWLKISAADVEEHICKLAKKGLTPSQIGVILRDSNGI
AQVKSVTGQKIVRILKANGLAPEIPEDLYMLIKKAVQVRKHLERNRKDKDSKFRLILIES
RIHRLARYYRTTRKLPANWRYESATASTMVA
>comp5531_c0_seq1; 234 147 1265 LEN=1589; translated
MNRHGSRIACLAARRKLHRRAPTASILRNLGIRAPSPPVAPGRSPPRTEAQRWSSLSLET
ASSELPGTRPRKPPCLSGRNDVSLARYPNLRTAGGMLRGADYAGTVTFALTGTITAAQSG
LDVFGCCLVGILTSVGGGTIRDSILLNKRPFWTEEPEYIWMGLATGLVAFFAWPTSVLEW
KRREQQKRDEETRQRLRDGTSAGSREEPSPTDRGGHRGSGFDAIDVFLESMDAIGLSAFC
IIGAQNGIRAGMPPVVSAICGMATATFGGVLRDVLCGRPVRIVHSNTEVYAPPALAGAAV
YLAAGSGRIAGLRIPVASPLAKIGLATATCLGGRYVAIANDVKLHTWDTAHDNLGVTIRK
KPTQNRNYRNSD
>comp5531_c0_seq2; 204 147 1747 LEN=1747; translated
MNRHGSRIACLAARRKLHRRAPTASILRNLGIRAPSPPVAPGRSPPRTEAQRWSSLSLET
ASSELPGTRPRKPPCLSGRNDVSLARYPNLRTAGGMLRGADYAGTVTFALTGTITAAQSG
LDVFGCCLVGILTSVGGGTIRDSILLNKRPFWTEEPEYIWMGLATGLVAFFAWPTSVLEW
KRREQQKRDEETRQRLRDGTSAGSREEPSPTDRGGHRGSGFDAIDVFLESMDAIGLSAFC
IIGAQNGIRAGMPPVVSAICGMATATFGGVLRDVLCGRPVRIVHSNTEVYAPPALAGAAV
YLAAGSGRIAGLRIPVASPLAKIGLATATCLGGRYVAIANDVKVSAGRRFDSNRSLPGSH
SWKVSAHDDPGFLDSHRSLLLIFLFSKPIGAASITSCIHGTRHTTTSVSPYGRSPPKTGI
IETATNSARGVVPRAFKKQGDDGPWSCNTQFKVQSICVRALVGQGHPNGSLAGKKNIGGP
ALLRVNAMQCDAANQPARNTLPCAKNPTACRKLGKANQCNACPSTEQATIQAAR
>comp5532_c0_seq1; 201 1 623 LEN=766; translated
XSFIVGLTPSRVEASTTMPFTNHYHDWFAPERKSLLIISLLLIQNVAFASSMSSCKPIDS
ADLAHEYQNIVSEYLTNVDDIAVLTDDPDGWYYMHIEPMQSNGIQWNIVAGPRVVVADGE
DEVQALWESFSNRNFSFHQWSHFSSCTKRNGDVQTDLRYHGIVREDEDGNIQDVFGTATV
VVKTDGNRAGLVTEVTATRSETLERPY
>comp5533_c0_seq1; 1287 144 3299 LEN=3391; translated
MASDEAIPMEASATTEVDEKLYSRQLYVMGHEAQRRMMASKALLVGCSGLGAEVAKNCIL
AGIHSMVLVDPLPPNTYDLGGNFYLKPEDVNSGISRADICRPLLAELNPYVNVSVASEVT
ALTAEQLLPLLDTGITCLVITIPLSKELLVALNEKCRLVNASFIYSLSCGAFGKVFCDFG
ASFVCSDKDGNPPATSQIESVFEEDNKVVVKVLEDQGRHGLETGDLVTLARLKGLPGLET
NKNYKVKVTGPYMFEIECDAGKAITIPGEPQQGYITQVKQPVTIAFETYEKRLADMGECM
MSDFAKFDRPPLLHKSFQALEEYRSENAGEYPTPGDVDACKKVVDIVNAALAAAGEDALV
AAQERIVLQLASGAKAVLSPMCAALGGIVGQEVLKACSGKFSPINGFFFLDADETLPDEL
LPADEVAAQNDGSRYDSQIACYGKEIQQKILDLNYFLVGAGAIGCEMLKNWALMGVGCGP
NGHITVTDMDRIEKSNLSRQFLFRSKDINHFKSSSAARAVQEMNPNIKITALQEKVAPDT
EHIFSDKFYDKLSGVCTALDNVEARLYVDQRCVFYRLPMLESGTLGTKGNTQVVVPGVTE
NYGATRDPPEKSIPVCTLKNFPNQIQHTLQWARDYFEGEFKQAAEDVNAYLSQSPEDYLA
SLQPNNRNETMQSIRRTLVDERPVTFEDCVTWARLKFEELFNNQIRQLLHNFPKDQVTST
GTKFWSGSKRCPVAINFDLNSQCEDAQMTNHLDFIVAAANLRATMYGIKGRTDKDYFEST
IKDIIVPDFTPKSGVKIAANDEEAKANQDSMVTDDEEATTILESLPKQADLAGFQLTPID
FDKDLDDHMLFVTACSNLRALNYSIPTEDTHRSRAIAGRIIPAIATTTALVTGLICMELY
KIIGSPRKATTIESYKNGFLNLAVPFMTLSEPTGPKATKAVLKGKEWNWTAWDSLDVNVG
NITLAEFMDYFENEYNLEISMLSYGVSILYSFFANKKKVEERKAMKMTDVITSITKKEFP
EDQLFLILEVIANDKETDEEVDLPYIKFRFR

>comp5534_c0_seq1; 739 244 2021 LEN=2486; translated
MTTKRLRASVLVVATTLFSDAFVHYSSSQTRCNGAGPAASDSAPLSSLELWPLGRGGFHD
SGADSDSDSDGATQRTAVKELTAMASTAALAAALVFSPLAASAENELADKYGGKGFDSSL
VDQNCLVDRCSVQAKACLADDPSCRKGLTCTAKCLGDNSCITGCFARFGNKNLDNLLKCT
IEDHDCIKIAILPGGADKPGEEPRAPKATVANFDYKTMEGTWYKVVGFNPNYDCYACQRN
TFALPATTKAEGASASASDTTLVAAYNNEPYMNMGVEFSMPHLLPDGSPPPPKQVRESLA
VFGKEDLRKKDSANAGLLSIGLNEYNTNEVMVFDDKGSETNIVLNKGKENEARYSRTAHS
EGEMFGLSXVSFDLCVFSKWLDVGSDCFACDSLYGAIPNASCLSITFPPTPTEFWENWYV
IGENDPGMPEFKFIYYNGKTRQNTYDGAFVYSRSKTLEPESMSRVYAIAKEAGMNPDQFC
RIRNGCFDQQKEEPLFGPDKIGAPTSEPLRGLIASTKVSELLGVEAVSARDFNVQGLGLS
QQLQPEPKAYDSGTNSNRPWWYEVADYLENPHRHFEVMDSLRKPMDWSESPR
>comp5534_c0_seq2; 767 244 1887 LEN=2352; translated
MTTKRLRASVLVVATTLFSDAFVHYSSSQTRCNGAGPAASDSAPLSSLELWPLGRGGFHD
SGADSDSDSDGATQRTAVKELTAMASTAALAAALVFSPLAASAENELADKYGGKGFDSSL
VDQNCLVDRCSVQAKACLADDPSCRKGLTCTAKCLGDNSCITGCFARFGNKNLDNLLKCT
IEDHDCIKIAILPGGADKPGEEPRAPKATVANFDYKTMEGTWYKVVGFNPNYDCYACQRN
TFALPATTKAEGASASASDTTLVAAYNNEPYMNMGVEFSMPHLLPDGSPPPPKQVRESLA
VFGKEDLRKKDSANAGLLSIGLNEYNTNEVMVFDDKGSETNIVLNKGKENEARYSRTAHS
EGEMFGLKFWENWYVIGENDPGMPEFKFIYYNGKTRQNTYDGAFVYSRSKTLEPESMSRV
YAIAKEAGMNPDQFCRIRNGCFDQQKEEPLFGPDKIGAPTSEPLRGLIASTKVSELLGVE
AVSARDFNVQGLGLSQQLQPEPKAYDSGTNSNRPWWYEVADYLENPHRHFEVMDSLRKPM
DWSESPR
>comp5535_c0_seq1; 580 374 2308 LEN=2695; translated
MMAIDHSPNRKSKHHNGNLRHNSGPNEEEMVPGMLISPTGSCSTNSSSEENNGRWSQNLY
SSSLRMQEKFASALSAKRRTSREEKQKSNLHNEHQHQRNNHYQASQISRSQKEVQNVTNS
SILMGAEIIKRQLSQDSPVGAARFDHRGSSIARRRGGDDWVRDSGGGTRLDRDDWDPWRT
DSNDETCNDHSNGTGNERGVKNDDYDDTLRVLQQAHFIIDKYNKPRSEEPSRALPSPATK
KSETQNEDATRTTPRSYNCLVQRVSEMDDSTFASSSIGQGYLPKSLMRNLTCGQLNTTSD
ATTKVEIKNEREDFANCNLDFLANLHVEEKNLNSIMKKFTEKQQLLPDESQCINYNPEAS
KKGLPTGNPSRGTPGTSRSQPCRVTAAAIPFEKMISPRTAIHDELMEDPAYRHALKAGTL
WQSLCSQHVRFPALWWDGQEPIGPPLGSTKKRKKPWSYLGRHRVQGDYKLNSLIGNRGSS
GRILLHLVVRDVVSGEPIEDIACGCYHPNARGIRTTPDFNPRIEDCRDVWIAHRRRVLTR
SLLDADFDDNSLCDSDNDEYSWTTIESLLRHQNKGKVDASPLGAQGGKSCVTNTNLKAVF
GSKPPVYTVFVPEGELFELFQNRLDGSIPASVALLRYYLRYRIG
>comp5536_c0_seq1; 262 167 622 LEN=1301; translated
MVAVSDQLVWEVIKSNNSFLKKRNGKTSRSGKIEFSSEKGNVKSLNRFKYSGLANTKVCD
VVCTDDNKAELIVKTASKCATQPAKGKASIALNKSDFRKVESTIKKSTSDVFYRRDLEAA
MLGKWTKVYQANKRANGVKKGVPPKMGRGTL
>comp5537_c0_seq1; 563 1 2236 LEN=2450; translated
XNAMSFIASIKRFTVDIPLTVLNGATAIGVITTTTALLLYWRSIEKHKEEFKSKLARSLV
YRDPDSKSTKAIKILELHPDFVREHSSSWQLKSLGQYLSTLRFITEKTDDKSRSDGDKNE
ATEKESNSRLVQALERELYVLMAALLMKTLGETMGAVFLPVMGSGQAESIIGAVSSKIVH
YIMAQILVDSRHTEDWDPTEDRAAMPLNVSEVIXVSELASVALMLDHETKPLGKLKQLET
MKAAQIILPTTAHCLNDPINRLIRPNNDLSTNPQICLTTIWCLRKXFVNLNQKLTDGNQM
SLSPLQWMQKGEIGYNPTYDTPITSGDEGTEQPPVLVPNPFVVEEHFEKAIAGMEDRIRG
SGATYDPSDRSHPEPVPINNTILPDLHMGWGDAKVTHTKREVLCNRLFAVLLTKLSYNYE
LRKTKKTDNIDDDSGEPYFVVQMDGKNCVFPDQFVEVLYDTGHSIEVCPRSTITTFGLAA
CVKEKDGSWTNIPLAFFFRTGYESSHRRPAYFHPLHGGIDLSIEGALVGNNKDTGEPRKC
DIQFYMAIEGMCGWQSNHNPDVPWIERVSTCDPYTREQALAAVRMSGIMGCTFNRIGTDM
DLPFGGYGVLGVCNDTAALIDRAVRGSTNMYPLLSTGRYLMHITRFLMAFHDTIAGADEH
GAGAEGDGDCCKTHQIASDTLRLVSAACSMESDIHCAPHGMAGAARRYRSNYPRPFFQIT
DDSQSVMDEIAREYEDLETTVQTNIP
>comp5537_c0_seq2; 634 1 2023 LEN=2237; translated
XNAMSFIASIKRFTVDIPLTVLNGATAIGVITTTTALLLYWRSIEKHKEEFKSKLARSLV
YRDPDSKSTKAIKILELHPDFVREHSSSWQLKSLGQYLSTLRFITEKTDDKSRSDGDKNE
ATEKESNSRLVQALERELYVLMAALLMKTLGETMGAVFLPVMGSGQAESIIGAVSSKIVH
YIMAQILVDSRHTEDWDPTEDRAAMPLNVSEVISFVNLNQKLTDGNQMSLSPLQWMQKGE
IGYNPTYDTPITSGDEGTEQPPVLVPNPFVVEEHFEKAIAGMEDRIRGSGATYDPSDRSH
PEPVPINNTILPDLHMGWGDAKVTHTKREVLCNRLFAVLLTKLSYNYELRKTKKTDNIDD
DSGEPYFVVQMDGKNCVFPDQFVEVLYDTGHSIEVCPRSTITTFGLAACVKEKDGSWTNI

PLAFFFRTGYESSHRRPAYFHPLHGGIDLSIEGALVGNNKDTGEPRKCDIQFYMAIEGMC
GWQSNHNPDVPWIERVSTCDPYTREQALAAVRMSGIMGCTFNRIGTDMDLPFGGYGVLGV
CNDTAALIDRAVRGSTNMYPLLSTGRYLMHITRFLMAFHDTIAGADEHGAGAEGDGDCCK
THQIASDTLRLVSAACSMESDIHCAPHGMAGAARRYRSNYPRPFFQITDDSQSVMDEIAR
EYEDLETTVQTNIP
>comp5538_c0_seq1; 880 335 2008 LEN=2700; translated
MSSPNITTIDATTTVMATVVVPPSPIEDVTEEAATKTHEETPEEREQRILTLTMLARQLE
YYFSAANLSRDTYLSTLRDLNDSYVPVSIIANFGKVQALVPYESALEAVVVAATDHSDLL
EIVELDEVGKKIKSSQEEKKDESETTTIIVAVGSISQQPIPMSQIQPTEKTKQVSNEESP
TGSSPSSTANSQLASTVASAGVQNTIILRDVAEDMEEKTLRDLFNFEGCPSVETVREDLH
NCWFVTLDTESKDDMFNVMMKLRTTKYPSGDSVKARLKSSASANSSSTPTPNNIVFNPNI
STMFSPQFSNNGNMTANSNSNGNNHSRKKRSSNNKGRSSSSAGTNTNNKPKSNQQSMSGT
SKRRNGKDESSLGRPPFPGTAGVKVAGNNSKKIQSKPPSMGESNFPSLPLSTDGNSKPCQ
VEKVPTDHEMMRGCEKERNASGFSDSSSTATTSTASTPTEAPPSGVVSGGYAAALLKPAA
PTPVSVKQKETKVVSQQQSVDKGKESSKKKDNKKSKDVKLSTKNVPSSESISVDLPAVSV
QPPAWGKGRSFADIVSA
>comp5538_c0_seq2; 413 335 1075 LEN=1101; translated
MSSPNITTIDATTTVMATVVVPPSPIEDVTEEAATKTHEETPEEREQRILTLTMLARQLE
YYFSAANLSRDTYLSTLRDLNDSYVPVSIIANFGKVQALVPYESALEAVVVAATDHSDLL
EIVELDEVGKKIKSSQEEKKDESETTTIIVAVGSISQQPIPMSQIQPTEKTKQVSNEESP
TGSSPSSTANSQLASTVASAGVQNTIILRDVAEDMEEKTLRDLFNFEGCPSVETVREDLH
NCWYVNW
>comp5539_c0_seq1; 634 69 1358 LEN=1389; translated
MADSSSCGGQEAATNLVPENDTLPPETPTKEDWEVQRDDHKQSADQAFRARGFKTAIDQY
TKALSLDPEFVVLYSNRSAAYLANGESSKALKDARKCLELDASFTKGHSRLAAALMALKR
YEQAKASYQHVLEKDPKNAAATRGIELCAKEITKRMTLEEQKRQEEEKREKEEAKKAEAA
AAAKEEDDDDLLNGFFDEVEEVVAKKKEVTVPSNNAIRNDRETLGSTQQQIERLLQPRYE
WRNLNPYYVLQLPAATSTDDDISRRYKALSLLLHPDKNRSTLSTEADRDRAQLAYDQVQK
AKTILADASKKRYTQSLVEEGMKNGEARWKREQHQNHKGKGSGVSSGNETLESIQEKEVM
RIFAQVEAKRKDMEERERKFEQREQQQEDDQLEKERRERKFNKQWKNQDRVDKRVGNWRD
FDAKKRKKL
>comp5540_c0_seq1; 370 261 1304 LEN=1397; translated
MSDFKRAKLDDSVSTKVIGTHSGSFQADEAMGCFLLRQLPEYRLSKIVRSRDPKVLETCD
IVIDVGGVYDHEKKRYDHHQRDYDERFDSGKEGLTDGRCTKLSASGLVYRHYGKEVIKAY
YPNLSGEHLDLVYTKIYDSLLEALDAIDTGVEMSTTELVYRDTTGLSSRVARLNPRWNEV
NENGEPPEYDERFMKASNLCGEDFLAVMTKIVESDLPAREVVEKAVLARLETDPSGEIIC
FPTGGLPWKGHLYELEKLHKVEPLIKFVLYTDQGGMWRVQAVTVEGKAFENRLSLPEEWR
GVRDEDLEKLTNIKGCRFVHAAGFIGGNTYFEGALEMARIALQGSSK
>comp5541_c0_seq1; 1402 234 5003 LEN=5187; translated
MASNVLCGQAGLLQLPPVRKSKSGDKPVCSMLADAKLHPYLRVDRKKLKSDSRTGIEVFK
ISKNGKLQARRLGISPDNRCLLVTTNRVNSFKSFLKVATGNTHDPSSTKVIDVSRIDRLI
KGQLSKRFLMENHHSDDLAAGGGTHSQSNSEAFLEQHEATSITIVYRQTRAERLSSLSLS
SATEAGDASYGGRIGSSISGGLNKTVNMNASVSGDESTIASRSIKSKGQSKGHGKAPVES
CAYYGGYECLDLCITDPKDFQQLVETLEELMELYKKERRWMERSVLLYQHHRIDMGRTDV
SDPISPGDWISLCDRLNAPLRRPEIKSLYKDMQNELKRACKELGTELDFLDSGLPPWAVA
ELLKDLHFQSTTAMGLKYTKQDPTLRLWHEILDSDPVPILNKDGSKNNMDTKMINQSAST
DTTISPVALLSFIRSQQKEFKASLEDATEIIKAVKAQKSFKHLAAETGDAGNGNNPGGGA
GDGRLTKDEFMNFLLSDTNDLMDPRKGKVNADDMTHPLSSYWIMSSHDTYLDCWNPDHIV
LDEQMYLAALYRGVKCLELDIFDDCSLAPKPVIAREKPTSVDDPCISVRVALRAIRQYLV
TNENSFPIIVNIENHCSYEAQEKLADYIYEDLGSIGLIVVPDNSGSIDSSDLLPSPASMK
GRVLIMGKRPSIVEDGAKVINDDFDDENDVSIDEADFAIPNVKSEDGEDSERGIVVGFDA
KGPIRVYNEREQKNTIEHSPGELLYIAKQELETAELQAAQVEMKAIALEEASLRVEKYAD
ALIRNAGLSDEAVAELLQRLNGEDIDPDRHLDSLPRRDEGVEVEDFFADAVNSAKLTFAK
ADQFALQAAEETNRALQQLNQATERLHEAEKLRETSFVKGKRVVSKFQKAAKIAREKQEN
AAHAHRRVEKLRQMLEECETGANSANNVVNTAMTEAKISEKRAAETEARAARAAAIAQKD
RARAEEETRKEEILEQEAGLLHEKLLEIARKEKEVRMEAQRCAATYEKLSKQIKLIENSR
QFIEEEKMEGELSEENKEFLTKGGKVHSKHAAKVEERNTISLRMDQMATEISEAAKLRTI
SQDAFEKKAHEWKSQADTAAKFRRVVDKSSHAAEDLAEHAEEEREAANLRKVARDRAQSH
VTQKDSYKSSLEAQLAEAERHTVVADGEAVASRKEADRLAKANESYENSGFEDISAIIEG
RKLTRTTLLAEYEKKKENQKAAEEKAAELKKEYETSDNVFSGAKQNSALHEKVLKNQRQE

DKNALLAVNSAKATRMKAQLALEEARYAQSVVSEKLVVVKRAEEYKLKTERVVEIPTQLA
NMTFLHTLKQKYWERSLQLPSTHVHSFADCILDKLALKNKDSFVDNLKNFTSSHLCRTFR
SWKDAEDSSQLNTDPLSQWSLGCQLVSLNFSTYDEHLQKADGRFRRNGSCGYTLKPEFLR
SYDSIPERPESWKIHVLSGSYLPTPDSKQVGGNAMSYINPFVKINVYGGDLGQRKGEHVT
STVMMNGLNPVFDDKMGFIFKATSPSLAILTFTVWDKAENGTEEFIGGSAMPLSCIREGY
RSVPLFDKQNTRGGAFAYACLLVKAKRMT
>comp5542_c0_seq1; 185 358 645 LEN=680; translated
MNSRLKLVMKSGKATLGYKHSMKCVRKGKAKLILVASNCPHLRKSEVEYMAMLAKVPVHH
YSGDNIALGTACGKFFNCSVLTIIDGGDSDILSQN
>comp5542_c0_seq2; 217 85 408 LEN=443; translated
MVSKKAKKQQDNMNSRLKLVMKSGKATLGYKHSMKCVRKGKAKLILVASNCPHLRKSEVE
YMAMLAKVPVHHYSGDNIALGTACGKFFNCSVLTIIDGGDSDILSQN
>comp5543_c0_seq1; 700 219 2789 LEN=3131; translated
MPMITKKFTRGSLKRASSSGISLANDSEDPSKAEIIQLKTARARSKSMQNRNAMNQQHRR
HRSLSRDPQSRQPSADIGGQTIRNYSHPNDTYNQAFNEAELQTAMSSLTEPTWAPSDLGR
SDVKPTHHRQNQQMQRSRTGNYNNVRYTSRPQYKKKLNRATVTISTGKDQKETKILLLDN
NDTRKSQEGIEASPIDPDEERNDEADDKTTTTGLDHLPTKPKAIRSSDLRDGVSSRDDAP
STPKHDASSSRARSKTRRAGDQSSVSKTRGRSGRPSSDRKSLRRSSSVASRRSAHSTRTG
STAPTVPTSSTASSQSRISRTKSKIRGVSTRKKVNDNKSKSSTSRSSSVPPTMSDSKRKQ
RTLSVPPLDQKKSDRKNDNNEFMDAILDTSEIVEDPAVMSVRSKSQFHPPSFRSLVESPF
DCRPTPSSAVEAARMSLRPRLRRSSSNRSVSSIQSESIISMMSTASRRQTEISGGNLQES
HRSPQIIPASPSNSRYGSRSHKLAKDVHSKRIHERRQKKREEHEKERKRRQQKNEEKKQR
KEARASALTNETDTEEPQYTTINTLLYDFLVFNLLLVLSFPQYLVSFLWSRTFSKQWSAR
RKTVVVTGARSPLAAETARQYAAQGANLILISHSSTSVEDDLDWLVEECHQLGSSNVRSY
SADLSKADSAEQTLQQAAEDFNETFDVVILNGENKSHGCLFEEIIDVHEIEDMVKGNTIG
CLTTLHYALKHIPKTSDSRIVILSSTSGLIASPYKSVYGATQHALKGFCDSIRMELNSNY
TERRAPKICYASFPELIGKNIHHQNCNIHISRMGAENPPMKTHSWAAIPLQKAVHDLLEA
VALGERDYGSPRHVKAW
>comp5544_c0_seq1; 255 58 762 LEN=1461; translated
MSAKKYVAAVLGGSGSVGRHVVNSILADPSCDQVLLVSRRELEDIKSMDPERVTVHVCDP
LDDIATKDYLAGTNIAFCTLGHGSSRKSTKDDLVRVDATIPGEFAKACKKAGVTHYCIMT
AVGSDENARWSSLTKTAAGGGWYNHVKGLAERLTIEAGIPYTYVAQPGALLGSPHTPKIV
EMIPNFILPAIYSSAHVKDIAKGMVTTTANAYQEDKTGVVKITGGIPISEGRAQ
>comp5544_c0_seq2; 255 58 762 LEN=1224; translated
MSAKKYVAAVLGGSGSVGRHVVNSILADPSCDQVLLVSRRELEDIKSMDPERVTVHVCDP
LDDIATKDYLAGTNIAFCTLGHGSSRKSTKDDLVRVDATIPGEFAKACKKAGVTHYCIMT
AVGSDENARWSSLTKTAAGGGWYNHVKGLAERLTIEAGIPYTYVAQPGALLGSPHTPKIV
EMIPNFILPAIYSSAHVKDIAKGMVTTTANAYQEDKTGVVKITGGIPISEGRAQ
>comp5545_c0_seq1; 305 187 1281 LEN=1363; translated
MASSSRISTTRSAVVFAAFALAVAKLLLFRNNTGDGTAQGVAKVRHIESMYDESVRLYTD
PYAKSMYQGSSVVEWMGISMIDRLLDVSFPGILELLTLRTKWIDDQIAIHVVGSDDETDS
SADATAIPSCRQLIILGAGYDTRGFRLDFFPEGFEVIEVDQSPVQASKKRILEDIATNDH
KVASRLKNGMVTFVPIDFNNSDDDVGGHSIGKKLGSFFSTEGGDQTMTVVLLEGVTQYIP
KSATARTLKQLHELLPRGSVLLVSYVPQTVFDDDTDRVSRLVLKGASLVGEPWISVWTRQ
GFASFLLSECGGYQVVSDSGVRELHEQYLAPLATREKDLPHNRRSRNMRNQVPPIERYVV
ARVV
>comp5546_c0_seq1; 215 155 988 LEN=1149; translated
MAVKKTPIALESLYAVPLMMIFLFLQIFLIPTTICALSGSHGSPSSVNTGSKPSPSDRIL
TPEYYRSVYKGLKIDQWKEQPVQNGGNTPWDIGNHRPQPAIVRAYEEGKLRGHVLDAGCG
VGENCVYLAHKFGISSVTGFDLSEDAVKIASDLVALEDERETTAATPFWTKPRFLAASCT
EVADKHGSELLEGLTGEEDDKIGGPKLFDVVIDSGLLHCLSEEAASSYVQQISKLLKPTT
GRFYIGCFSTENPDPWDNPRRISEEYLRNDLFSCRETW
>comp5547_c0_seq1; 999 1 1980 LEN=2035; translated
VGFVLCFLTLHPDLTLELRRAKFSATASLGVIHAGHVTEAMHLLQPYLPSNPSVGEDGTG
GSAVSAEGGYAEGGSLYALGLIHGSHSGSSAEKRKETSDFIRTHLRASHANEVISHGAAL
GVGLTCMGSADFLVVNELKELLDTDSAVAGEAAGMAIGMVLVGTGAGNVHNSLPAEGKEE
ITEIVSELKNYARETQHEKIIRGISIGLALTQYQQEENADALIEDMRTDRDPVLRYGAMY
ALALAYCGTGSNKAIRILLHTAVSDVSDDVRMAAVIGLAFVLFKTPERVPQLVKLLMESF
NPHVRYASCMAVGIAMAGTGDPESIAMLEPMLDDMTDYVRQGALLGTAMIYMQQSDTSNN
RKVKAFREKLSSIVSEKHQSTLTKMGAVLSIGIIDAGGRNCSISLGSRNGFTQMTSAVGL

VLWLQHWHWYPMLHTFSLALTPTYTIGLNKDFKFPKKFEVVCNSKPSVFAYPKKLEEKKE
EKKKRVETVTLSTTAKEKARQARKRAKEEGAADSSDAMDVESEEKTPDKTETEDSDAMEV
EKKEEPEKKGKKKREPEPTSFRVTNPSRVTKAQSQFCAFDLSQRYRPIRVEEKPFGVVIL
TDSTPGEEEDLGAVKAPSLEPEGECAPPEPFEWTPPEDPVAPKEEEPKSKEDDTKPSAA
>comp5547_c0_seq2; 1424 72 3215 LEN=3270; translated
MATRPQSTPLAPDAVATASGYIALLQEEDPALKSHALKKLLDCVDHLWHEVAESLPDLES
LSEDFDLPLETRRTAAAVASRVFFHLEEPSQALRLALEAGETHFDLRNKHRTPYVESLVA
AALDAYIRERRKLIRQQEEASDATSTSTNADAEASADDTGISIEQLRGMVYRLLDACCQE
GRCDHALGIAFEAQESEKLREILQASGPDEGLLKYALDASIRVVASKSFRQEALAVIADC
LCQLETPERKRASVDTLIRVYQLLGKPDAVAGVLADLLKQSSEEDALLCYQLCFDLVDTG
DQAFVASVASELEPLLGEEHKDRWASVSRVLTGGFSSELALSFLHKQSQADRLVMENLKK
SLEERGSGGRSSLLHNAAVMTHAYLYAGTTNDSFLRDYLDWMKKASNWAKFSATASLGVI
HAGHVTEAMHLLQPYLPSNPSVGEDGTGGSAVSAEGGYAEGGSLYALGLIHGSHSGSSAE
KRKETSDFIRTHLRASHANEVISHGAALGVGLTCMGSADFLVVNELKELLDTDSAVAGEA
AGMAIGMVLVGTGAGNVHNSLPAEGKEEITEIVSELKNYARETQHEKIIRGISIGLALTQ
YQQEENADALIEDMRTDRDPVLRYGAMYALALAYCGTGSNKAIRILLHTAVSDVSDDVRM
AAVIGLAFVLFKTPERVPQLVKLLMESFNPHVRYASCMAVGIAMAGTGDPESIAMLEPML
DDMTDYVRQGALLGTAMIYMQQSDTSNNRKVKAFREKLSSIVSEKHQSTLTKMGAVLSIG
IIDAGGRNCSISLGSRNGFTQMTSAVGLVLWLQHWHWYPMLHTFSLALTPTYTIGLNKDF
KFPKKFEVVCNSKPSVFAYPKKLEEKKEEKKKRVETVTLSTTAKEKARQARKRAKEEGAA
DSSDAMDVESEEKTPDKTETEDSDAMEVEKKEEPEKKGKKKREPEPTSFRVTNPSRVTKA
QSQFCAFDLSQRYRPIRVEEKPFGVVILTDSTPGEEEDLGAVKAPSLEPEGECAPPEPFE
WTPPEDPVAPKEEEPKSKEDDTKPSAA
>comp5549_c0_seq1; 300 60 1058 LEN=1248; translated
MSSSTSNEKTSAVLTEDPKSWIGKSLVSVTQITPKGFDFLVTIAQQMRDIVRSEGGDDRL
KHKLLATIFYEASTRTSCSFQAAMMRLGGKFLHVDGNGNSSAAKKKESLEDTIRCLECYV
DAIVLRHPLTGSVPKVVQATTKPVLNAGDGIGEHPTQALLDSFTIWDELKEDPKVVVFLG
DLKHGRTVHSLAKLLSQLRPQSDLVLRFCSPSSLVVPEYIIEYCKNNGVATELFENPTEA
CKGAQVLYVTRVQKERFETEEAYESVKGSYVIDKAFMELAPKNMIVLHPLPRVDEIATEV
DTDPRAAYFRQMENGMYVRMALLALVMGKSSP
>comp5550_c0_seq1; 211 283 699 LEN=699; translated
MDTPVPTSEPTAFSSSDLTTELSSDDAPKAPKPTLTDVLEDVPLPNATPVAPPTVAPVPV
PIPVPTNPPVPPPTVVPIPVPVPTNAPIPLPVPTAAPIPLPVPTNAPIAVIPDTPPPAPQ
VLVDVEVTEPPTHTSSTFD
>comp5551_c0_seq1; 148 1 705 LEN=819; translated
XFLSSRVFQNENELKNELKNDPIDPTEPANXTAGPTALPTPAPTKPPTVAPSGVPSGEPS
GEPTLPPSAAPSPAPTPPPTKAASKATPRPRPVAQVGHSGQQEPQPQLVPAPRPVPVGSP
TKAPIDQSIPVMKPRPAGHATKNTSEGVATIPVMKPRPPPRPAEVESGGSGPSKQGTQEE
PRPQQENEEEEEDGSSHPHQQLLNLELHRTTQISGLRRQPKIDERPGKALRLPAS
>comp5551_c0_seq2; 142 1 850 LEN=964; translated
XYFPNGDLVGEAECLCESFGQQDMCDFVPPEGWVPPTPAPPVAPVVPTEFPTRTPTKEPT
PAPSVSPTQRPTGSPTQKPTAGPTALPTPAPTKPPTVAPSGVPSGEPSGEPTLPPSAAPS
PAPTPPPTKAASKATPRPRPVAQVGHSGQQEPQPQLVPAPRPVPVGSPTKAPIDQSIPVM
KPRPAGHATKNTSEGVATIPVMKPRPPPRPAEVESGGSGPSKQGTQEEPRPQQENEEEEE
DGSSHPHQQLLNLELHRTTQISGLRRQPKIDERPGKALRLPAS
>comp5552_c0_seq1; 411 1 1236 LEN=1999; translated
KQIAMASTAFESDLDPPNVEQDSNYGPTGNNNSHNNHRGINGRNSKRRQSQNSDNLMLQR
NSTKLNGTNLEPQNYRYGNLEPQNSYGQSYSPFPTSIDDADPSSLFNSLTAGFCSTAAAA
SGSFFSSISSTDLENDPELTPGRGNSNNDNSNSNSNSNIEACPPHASLPLKIEINGKIVVVG
GGECPSHLNWKTGVVTDENGVRTLLTNQQLESLRRERNRMHAKMTRDRKKSFIATVEKTI
EELESSNKRMAAALTDVIHFQRSPPCSPAATTLVSFRNENDHDHDHNREHNHGNDNANHT
EPERNSDGSNRHGQNPPGSVTPCGSRSPAPAAGERDRYTSVALSSVPSLIPAPPDSFVTF
VDGDRDGDGNNHGDGDRNGNNNGIVNNNTKLDSEQLPPPKRFCHGFSIAPY
>comp5552_c0_seq2; 375 1 1404 LEN=2167; translated
KQIAMASTAFESDLDPPNVEQDSNYGPTGNNNSHNNHRGINGRNSKRRQSQNSDNLMLQR
NSTKLNGTNLEPQNYRYGNLEPQNSYGQSYSPFPTSIDDADPSSLFNSLTAGFCSTAAAA
SGSFFSSISSTDLENDPELTPGRGNSNNDNSNSNSNSNIEACPPHASLPLKIEINGKIVVVG
GGECPSHLNWKTGVVTDENGVRTLLTNQQLESLRYVFLCCTAFTSYCYFWKLGGPLLLVS
CLVFLSSSSDHNHSLPFLSSMFDCFLDVSGXERNRMHAKMTRDRKKSFIATVEKTIEELE
SSNKRMAAALTDVIHFQRSPPCSPAATTLVSFRNENDHDHDHNREHNHGNDNANHTEPER

NSDGSNRHGQNPPGSVTPCGSRSPAPAAGERDRYTSVALSSVPSLIPAPPDSFVTFVDGD
RDGDGNNHGDGDRNGNNNGIVNNNTKLDSEQLPPPKRFCHGFSIAPY
>comp5553_c0_seq1; 250 686 1385 LEN=1442; translated
MKLFYDSPTSVLGLNQVDELSKFFESARDLDESDPVAKHVAVLMAKKDAPPSKLVSSSLR
RAVTTVAGGFKDRLARRPQEKILILPSLQEISRNPDTLSITPAQTQIQASWIDKGSKLCD
FQSTFMNQVDMSLHMGNKPVGTNGLKRMRKFCDVIFSSSVTEENVIVGGHSIWFRSFFRT
FLPFEENHVGKKKKVVNCGVIAFTLMKVVRESGRSTYMIDPKSVDVVYGGFH
>comp5553_c0_seq2; 387 342 1272 LEN=1329; translated
MPYITFDPILEAEKEGTLVAEVVERKTIYFVRHGESTWNDTFNKGSHRSQAAFAIGFIPG
LIKSVVFELYLILLGKMDSWFYDSPTSVLGLNQVDELSKFFESARDLDESDPVAKHVAVL
MAKKDAPPSKLVSSSLRRAVTTVAGGFKDRLARRPQEKILILPSLQEISRNPDTLSITPA
QTQIQASWIDKGSKLCDFQSTFMNQVDMSLHMGNKPVGTNGLKRMRKFCDVIFSSSVTEE
NVIVGGHSIWFRSFFRTFLPFEENHVGKKKKVVNCGVIAFTLMKVVRESGRSTYMIDPKS
VDVVYGGFH
>comp5556_c0_seq1; 51 1069 1451 LEN=1451; minus strand; translated
MEVIKNIVFVENGKLVRTKLSVPEEERAGILEAGLEKIRENSIKIDEVLPGTTPTGVWHS
FEADRAAFATVASSGPCKIWLMPSEKFRAVISRPEFSMVVMAAMANRIRVGSKSIRNLLE
KVKDGASX
>comp5556_c0_seq2; 51 952 1334 LEN=1334; minus strand; translated
MEVIKNIVFVENGKLVRTKLSVPEEERAGILEAGLEKIRENSIKIDEVLPGTTPTGVWHS
FEADRAAFATVASSGPCKIWLMPSEKFRAVISRPEFSMVVMAAMANRIRVGSKSIRNLLE
KVKDGASX
>comp5557_c0_seq1; 260 1 343 LEN=448; translated
XQQQQQQQRARATYNDPSVEMKYQVALQESYTMKVEDLQQELNSRGISTQYCMVFKDFCV
EYAKAIAENRQKVVANDEVPGEGEGPGFFGDDEGDYDPTYRDVVMQKYDPSMWI
>comp5557_c0_seq2; 260 1 343 LEN=451; translated
XQQQQQQQRARATYNDPSVEMKYQVALQESYTMKVEDLQQELNSRGISTQYCMVFKDFCV
EYAKAIAENRQKVVANDEVPGEGEGPGFFGDDEGDYDPTYRDVVMQKYDPSMWI
>comp5558_c0_seq1; 1051 1 3289 LEN=3289; translated
XDNANIGEYGSGKEQEGDGNPSDKGNQPGDEIKENTEEDVEEKTDRMTSKVEANMRDKAS
IRDDDSGKERKGDGDSTSNKSDQPVDEITGVTEEGVEEKPDRTMSKMEASTQDDASVVDN
DSGIEREGDGNLAERSNKPRDDIKEKSIPCQKDSDEILDAVPEPKVRTRSGRVVKPVRRL
IDGCETSIGGENQYGRAHSVDFEIDETEDHALDDSSKTVTQAASTDVQETTNLQLGSNTE
ASLESDRTTDEVTTQTKPNEKFSPPSMDDEKEEPSNGTTDNSWTEMKPIGDEEVIINSSI
MNGSTKMSPMFGLSEDALYQNEEVHTLGDGDGDGDEENSTNNAVATILNVTSDHRPFVQR
FDRHALKDKKSFVKFVATRLTGFDIEVKEKSNKHDLFDPSSDYPIPDSVFEKRIPPIIES
STSSSVSSPPEIDMALHIEEREDGSCKNDDENDLVPLFDLIPSKCEISRESCVGEESRKL
ESNLSEEESQRRKRLKQGSIGSQNGSLSSFSTHQRSSKSMSFSNKRSSRRLRRQSSRLTL
SLSNTQHTKSPYMTPVENRSLVDDKVQIEAQQGDPAVQLVGWDTDIEANFPSVPDTEEMT
EAQERSVIFHETLAAFSSLENIDFLRDLPLSRSEEVPKPTSSLMWRQLIANWKHSESCKA
MLARPSSIQLSKLTKDVGFFGVDDDFSSSMSTIQFQLDSGNVFFSNDFVSSRAYSLLRFT
NADNYCEADEGFLVLTRYLCDIGPGTSSSEEKKKSTAEEELLQHSLSKTLENNEHGCTIG
ITEILREAKAQLPNLDKIINCVVNYASQNDPSSIDLECYDITSSSGVKDYTSIRRKARRK
YSGDVLQVKDVLRGEITFPDEASLICGLHCLQSMNGRNKTVPPFQIVRLKNLFRTTSVGK
EIYTALPTGYRHILINLKLECGLIIELQFQIAQLFDIMGEEGYQLHHDIMSSETDMKESF
ASSTSGQSTTKIPTEENPEATSTHFVRSILTNPTDLAIISRQMGLKKMNHCRIDASNSPV
ISSTNHSVNSESVGGIGALAAQAALRKSQKHDIKSCLPKLPVGGIAAIAAEAALKKSQKQ
ESEHGAQKPPVVGIAAL
>comp5559_c0_seq1; 705 1 2799 LEN=3012; translated
LLSRHHSLLTPVAEALLSAIFPFRWVGLYIPVAPLKMMDILDAPIPYFIGLSSDYFKAVD
VADRPADAIFVDLDLDTIHIGRNPLPRMPEPDAQKLAASLEEAGGSVYVIPNSGIKGCIM
AGSDKDLLVPNEQRPRYAHMTTMTGLGADAFVREALFAASELAYGGGEADRTASGGGSSS
NRGNRTGDGSDGKKKPRPLFRASPGKRPPVASPIKKPRFAKNKKADIILSRSHYAMNQGN
LLDMAEPEGFSVAGIRGAFLRFVVATFAHYQDSLLENSGRRLFDEEKFVGDVASKGRPMA
FLRNVLKTQLFQRFLEERRENPNLPEIRFFDESIAAKRNRSKKASLAMKGKQPTPFLDDK
TSWKIREVYASPPPNNFGIPDTEATYRYETFPVLDKTRFGRIRPPPKWPQHTNLDSVRYS
KKSDLKRRKKQSELVTRAMKPVLAAPKAVAARRTAKDLESTLVALSMDPTGSEAKETIGR
GAERKTKRTPSRGRVKTLSTADTIMMDARRKQAILLDVIVAFQALCRGYLVRRYHHEGVE
WEQDESLDRKLAYRHYHVRKTRATMIQSVVRMFVVXEESRAHDSGNRSFSKPPASPERTA
EVRSDEMQRDRGCQMRSGKANESDLPRTEADDCTRSEPSEGRAGSVENEDRDEGGMDAYA

NQIFVLWARLHVSLFFRTRLWPEISGCRTFVGLRVAESELLRLWDIAGVSNRSNGHSRTN
TKLLFRSAVGFGNDTCPRYKECTNKILGDKSIASKFDNARRMEETERLQIHERLGSSVSG
DDLAELYSGFGIQPKKKLKKVALSRKLWTDLPLANLSASSMHRIFPELHNSLGVRFVVPS
SKGRRRFRNAPKASVPLVDHALWEEISTQGRTKRCVQEVGMLFINKVPSPMKKVDSTNNT
FEIHSFLRATMEVNGFESIEEARRSVITQYLG
>comp5560_c0_seq1; 336 176 907 LEN=1228; translated
MLITIHYFYFDSGNICRDPPTGERVLTNPGKTFTQSNGLTVSCGEVQEWVQDVRLSGGSL
EETRYCELTQYLAWKHCDCDGPDIPSPNDDVKDLNPVCNLCGNPDFDFEFVPQPNKNKLT
DTGCCGRYNCEILYYGASQGVLSANLCSVIKEKSGPDCCNLDIIEAPPVNENNDNEQNDE
QNNEVDNENENEDYNNIEVEEENEQSDEDPEEDNNEIEDNNDQIDCQDDPSFRFKGRESK
NCDW
>comp5560_c0_seq2; 302 145 954 LEN=1275; translated
MLFYGHPNCWLLGCVYLIIYFRQVYHCEGTPLEGNDYACNICRDPPTGERVLTNPGKTFT
QSNGLTVSCGEVQEWVQDVRLSGGSLEETRYCELTQYLAWKHCDCDGPDIPSPNDDVKDL
NPVCNLCGNPDFDFEFVPQPNKNKLTDTGCCGRYNCEILYYGASQGVLSANLCSVIKEKS
GPDCCNLDIIEAPPVNENNDNEQNDEQNNEVDNENENEDYNNIEVEEENEQSDEDPEEDN
NEIEDNNDQIDCQDDPSFRFKGRESKNCDW
>comp5562_c0_seq1; 178 509 1552 LEN=2005; translated
MGITSAFKGLRRSRYAIANSPGILTPSRHASKSMRAASSGSSSASSTSVVPLPTREQLSI
LAIRSAIPMVGFGIMDNVVMITAGGAIDSTIGVSLGISTMAAAGLGQCCSDVAGITSGGI
VDAAVSRLNLRHHGLSPQQLDTRVARVCGTIGACVGVVTGCLLGMTVLFCMDTDKADRAR
RAKELKSIFESVMNDGHKLVGAERATLFLIDEDSNELWSQVATGMEGENIIKVPSTKGCV
GTSVTNNIVCNVPDAYASEDFNKEIDVSTGYRTVSILVVPIRSESGKVLGAIQMINKKQQ
PQEGFDGDIADNSNDATIIPFSENDEKVVSVLASHVASFIRVVESSE
>comp5562_c0_seq2; 211 1169 1702 LEN=2155; translated
MDTDKADRARRAKELKSIFESVMNDGHKLVGAERATLFLIDEDSNELWSQVATGMEGENI
IKVPSTKGCVGTSVTNNIVCNVPDAYASEDFNKEIDVSTGYRTVSILVVPIRSESGKVLG
AIQMINKKQQPQEGFDGDIADNSNDATIIPFSENDEKVVSVLASHVASFIRVVESSE
>comp5563_c0_seq1; 220 1 805 LEN=1087; translated
XPISAFSRTLFCMEQFSFLVERGETETTEKQHRERQSEPRNYKSKKQAMITPVSPSSRIF
FLAVPILLVLHMHGTSIHAVCPQEYNADRSSHHTSRMVEEDDSGNWGELLKSCPLKIALF
APCIDTESDLSECTVCVTDFVANSGTLSSGTGDDNNSSNTNTTSTECDKVQTEVCSAVSV
CSEKCGLSATEGNNGKIASFFQFGSDCDTLFTDLLACSLEAEDLIPTECSLDTCGSVDGS
SSSASSFFLSRAVAATLIMVAPGVASLL
>comp5564_c0_seq1; 570 341 3255 LEN=3455; translated
MEIRHRKNARASRISNPVAMGALFVAVCCLLGFNAVIRGNTGDTAAVSRKSSVVASAPNG
DINNNNNNENTSNSNRNTQTIGNNSNNVVYSRFGVTVFYPIYNYAKDKDGEAPPASPVTE
KLLEDLRHSPSVTQVVGLDTDHPPSFLESCGTGHGIAVVDAAGGEQTTGAVSERFRQLRA
SRQPHLAMELLKYCALDHYQGGLYVDSHSLLTSTVENLLSRVEAGGNGNLAVLNDPKISP
ESIHGALLYIADDSKXFGAEETEQGPIDLRRRGHDPLAAVDRRVGPGILPALFAQVALRP
GGKGRRGRRTVREPGDDRGEGHPLVFAAAHLQPICAGTEAAHGTDFSLRAEQPPVSRARS
DGAHRIAVSCRVVPCGMVWHGMAWYGGWKRIRFGFVLAWACAIRLRFPVVGRAALDGPKA
RLPGRNSLFLCAXDGALPPLPTGRDLTFLPPLSWSFSYVKTFPFLFLNDRATISGDNRCS
RRLTQNCPEPNGFCCSIYDSITHIPTMMTKHLLLPYQVLPPTSDLPQPLNADKGEFDEAD
LPYISTISERVHARPEGHALLTPNFFETLLQSDCLPSDKKCSDCLRNKQGSNCKTCAHAC
PCYCKALCHVPVEEKFVSKSLVISPPIYAKEPSRLVPRIVHQTYYETVTSEKYPNMSRLI
ESFKQSGWEYKFYTDEMSLNFLSTHFPPEVREAYETLRPGAFKADLFRYCVLLIHGGVYA
DMDIMLETNLDLAIGPDIGFMIPHDEPGTPVNRRMCLWNGFIAAAPGHPFLAKTIETVVN
NIRNRFTGVDTDAMFCPNPEMSMLHSFDTLFTAGPCILGAMVNKVLGRHGQTSFEAGEID
LWERSKQDALQNGTKFVIGVDDKPSKKIPGRTIILSQNKWDMGSHRFTFIEKNLVVAATD
LPGADDRANQETTKEHYSKTHVKAGIYGLDRLYTDNVRANENLRFYVDAQWSRYVQRGTR
DPGGTASGSKT
>comp5564_c0_seq2; 612 341 2872 LEN=3072; translated
MEIRHRKNARASRISNPVAMGALFVAVCCLLGFNAVIRGNTGDTAAVSRKSSVVASAPNG
DINNNNNNENTSNSNRNTQTIGNNSNNVVYSRFGVTVFYPIYNYAKDKDGEAPPASPVTE
KLLEDLRHSPSVTQVVGLDTDHPPSFLESCGTGHGIAVVDAAGGEQTTGAVSERFRQLRA
SRQPHLAMELLKYCALDHYQGGLYVDSHSLLTSTVENLLSRVEAGGNGNLAVLNDPKISP
ESIHGALLYIADDSKGSARKKQNKAQSTSVVGGMIRLLLSTDVSVLVSSPLFLPKSLYGL
VAKDEGVGELSASRETTAGKGTHWYLLQHTCSLFALGQRPLTAPISPYALNSHRLTQNCP
EPNGFCCSIYDSITHIPTMMTKHLLLPYQVLPPTSDLPQPLNADKGEFDEADLPYISTIS

ERVHARPEGHALLTPNFFETLLQSDCLPSDKKCSDCLRNKQGSNCKTCAHACPCYCKALC
HVPVEEKFVSKSLVISPPIYAKEPSRLVPRIVHQTYYETVTSEKYPNMSRLIESFKQSGW
EYKFYTDEMSLNFLSTHFPPEVREAYETLRPGAFKADLFRYCVLLIHGGVYADMDIMLET
NLDLAIGPDIGFMIPHDEPGTPVNRRMCLWNGFIAAAPGHPFLAKTIETVVNNIRNRFTG
VDTDAMFCPNPEMSMLHSFDTLFTAGPCILGAMVNKVLGRHGQTSFEAGEIDLWERSKQD
ALQNGTKFVIGVDDKPSKKIPGRTIILSQNKWDMGSHRFTFIEKNLVVAATDLPGADDRA
NQETTKEHYSKTHVKAGIYGLDRLYTDNVRANENLRFYVDAQWSRYVQRGTRDPGGTASG
SKT
>comp5565_c0_seq1; 817 284 2359 LEN=2421; translated
MKWTFNSVMLSTILFSTARHALAFVPGVQRAASRQMATAAGSSFASRYSMPRSSTPARLM
ATVAPEKQAAEDKDTDIFDELPTNDNDNDLLRIRHSSAHVMAMAVQKLFPEAQVTIGPWI
DNGFYYDFFFPETIDPETGETIEARKLNDQDLKKIKKQMDKIIQKDYPITKEEVTREEAE
RRIKELNEPFKLEILEGLQEPITIYHIGDEWWDLCAGPHVESTKQIPKKALALQSVAGAY
WRGDENREMLQRIYATAWKDPTQLKQYKKMMEEAKKRDHRMLGKKLDLFSIQEDAGGGLV
FWHPKGSKVRKIIEDFWKTAHEEDGYDIIYTPHIANLDLWKTSGHFDFYGEDMFDQMDVE
NEQYQIKPMNCPFHCLMYADELRSYRDLPIRWGELGTVYRYERSGTLHGLFRVRGFTQDD
AHVFCLPEQLEDEIVKVCDLIETILTKFGFDKYEIMLSTRPEKAVGSDEIWDAATIALRG
ALEKKGWDYGIDEGGGAFYGPKIDLKIRDAIGRQWQCSTVQCDFNLPERFGLEYTSKEGT
KERPIMVHRAIFGSLERFFGILTENCAGDFPFWIAPVQLKLLPVTDSVQDYCHEIAAKAK
KAGIRVEVDRGTERLAKQIRNAEQQRVPIMAVVGNKEVETNKLAIRSRKLGDLGSFDVDE
LLEELTTCVENTVEMTKMGEVEEKEEPASKK
>comp5566_c0_seq1; 235 269 716 LEN=716; translated
MTSDMPIAHSSSMSSTISANNGAQQQPAEDAVIGNAEPHDGTALTDSTNASNNERPEPTT
APGLPKPVAIRAESRDTSMCSRNSRDSLRRSVSMRSSTFTELERLFLEQLTVEGTDAQVE
AALRTVEDEELFFADTPVIGNNNSSSSSSX
>comp5567_c0_seq1; 250 368 1672 LEN=1884; translated
MNFSAAEFNELNASAFPYSERSLEQFRMEVSDDEISKADAITEAAVSFTAAENSSSFTNI
VGMDAQHEPHRITPQSSLVYREASSSSRTTTATENNLNKNSLPLLPPIAGSTGDRQTPLT
IEKRNSRALNSLVGLVDEYAVNSTARTYIGCQVRTPRVRDAGLDLDESPGHYFNACRDGN
PTAGTYSSTDREKGIHKNQELMVNLIPHNFGPLDRERNQCHDRPTSSSNSITITYGITDC
NKKVRTRRRESHKTKSDADKDSSNISNTHSKQNDFITTTHSKKNKTRKTSPTRNSNTNTS
TMFSKVGECLPRQRSRAIAINRSQPVGYNRSEGQANASEKVGCDAELYDYATWRMYNRII
DHRRKNLLRYPQAQPEGQPSAKEAATNQTVDESAVIHHPSPVVIGGQQYFLHQSQLPYPA
DEYQEEEDIFDFEL
>comp5568_c0_seq1; 91 141 452 LEN=452; minus strand; translated
MPAARWSRIVNGAYSTAQNNSTALPHSFRCAGCRSPPVVGSPVSFAAEHGGPRGTRERPE
AAAAAAAAAAASREKGPLHHQARQRELSLRRFRPRKGRRIAIAI
>comp5568_c1_seq1; -16 1 657 LEN=657; translated
QRATAFESSTKQPRGSARATQHRTSTQRDKTTIMKEHRTKLRPSQRQTHKLQRIEIMDET
AIPSIWNTPPSHGHGGSGNTARGSGRKGRTGEQEXRKSDPPTEGPRPWPVPRRLGPEARP
RKVQGRTPKTESATGAATPKEPPPRTVSGHGQRGPARPGSSTGTEGHRTGLLAPTQGPAL
AEAAGTPQAGAGRGRVSPLAEALLLFFVLGARRRWRWRWX
>comp5571_c0_seq1; 286 309 734 LEN=1039; translated
MNELTEENDKSLKEENKKNYDKIQTALVTETAFFSALIFSLLWLASDNPFAPLSFLFGSI
FGTMYTYGLGKYVSTVGGSIDDAGAVEGAGVGQARFAFLIVLFIFLGKFRPYGLQEIPSI
MGFFTYQLASLSQGLKEEKID
>comp5572_c0_seq1; 203 248 655 LEN=1915; translated
MTSSKDDATTSSGNAAKSNTRRIQALEAKAVAEATAEADAKYIPSVALMNSSDPLALVRQ
VDGLSDLSEYEEKESVYVGRSVPISAGGSLEIPIQVAMPGSVVEYAVENKQYDFGFSITA
ERDNSVTIVKVSSPV
>comp5572_c0_seq2; 308 248 1108 LEN=1587; translated
MTSSKDDATTSSGNAAKSNTRRIQALEAKAVAEATAEADAKYIPSVALMNSSDPLALVRQ
VDGLSDLSEYEEKESVYVGRSVPISAGGSLEIPIQVAMPGSVVEYAVENKQYDFGFSITA
ERDNSVTIVKESSRVDASVNGPITGKFLVGSVPCWVRFKFANDFSWMREKVISYKVTVKP
PSLETLYSGRRRRAKACLKAIEEDLTAADKRRKAAGDQLTLLETELEELRREVTQKAKSL
QAARDEEKWCMDRVALRTEQKKLLDSRLQDGWEDEENAAGDESDEE
>comp5574_c0_seq1; 187 204 794 LEN=901; translated
MERFSEMRRLFPYRAIVSRNPTFHDTWSHRNQAYQFRFKVTTSRAKSMLGFSTDSKPTVI
ELRQAYYEAAKLCHPDVKEDDHKLDFRELKDAYEHLIKGGHVTDHISDEISLDEEKEYRR
ACMDVLGLKAEIVEESKQNPMFLHWLSGKTDGAQYWRNFFTAHGGLAQKLRPPAGYLASN

AKHVAKASESRRRRRR
>comp5575_c0_seq1; 361 183 1518 LEN=1777; translated
MFRDNFNFNRIVLRAAGIASFLLLVSVSASPEGPPAKGVPLDREHLPKQDAPTKFIGVNA
TGDPVYMPLLGAGTWQYDDDEAYESLCKAFGAGYTFVDTAYGYKNQKGVGRAIRDCWKGN
REDLFVLTKIPGGNTEQNRARFRPNKSRFGAKPTGPGLRRDPPVRGLFSRTGSAHAVLLL
YRSHGTNAFGRDHTYRAVPNATRCNATQHNAGGRNASEVQRLHETNLEELGLDYVDHLMT
HFPADWEETIVGAEARQEEWLALETIYHTGEAQTIGISHYCSRHILDVLWVATVTPALNQ
VEYHVGSGDVDGVMETCSDFGITFMSFSPLCGPCSYEPEDSLVNGDLVQEVAGAYQHGNA
TTPGAGKPVVGSQVALRYIVQQGIPVVPKSNTMAHVLSNREIFDFELSEAHMERLGRARA
PSAEAGDCGVEAPALASASALATA
>comp5575_c0_seq2; 387 183 1277 LEN=1536; translated
MFRDNFNFNRIVLRAAGIASFLLLVSVSASPEGPPAKGVPLDREHLPKQDAPTKFIGVNA
TGDPVYMPLLGAGTWQYDDDEAYESLCKAFGAGYTFVDTAYGYKNQKGVGRAIRDCWKGN
REDLFVLTKIPGGRNASEVQRLHETNLEELGLDYVDHLMTHFPADWEETIVGAEARQEEW
LALETIYHTGEAQTIGISHYCSRHILDVLWVATVTPALNQVEYHVGSGDVDGVMETCSDF
GITFMSFSPLCGPCSYEPEDSLVNGDLVQEVAGAYQHGNATTPGAGKPVVGSQVALRYIV
QQGIPVVPKSNTMAHVLSNREIFDFELSEAHMERLGRARAPSAEAGDCGVEAPALASASA
LATA
>comp5576_c0_seq1; 532 82 1161 LEN=1278; translated
MTTILFVSLLFVWPGVNAFLPPSSITSSQSAPLRLIRGNNCPVATIGAVKIDANSMTSTA
LAANRDGKSDKKKPSKAQGVYVRPSGAIEQGSGFFVPGLEGPKVRLVIGLVLLAATGINH
FVLGTYTGEEGISFSEGTAIVYSVLLLFQFAIEYAKEAFLPPPQSSVSSSSATRKKIQDS
LSAVAAASEVLEQKWSGILALDDGQEEKYRSRVQWAAASYLAMTPTTQIMLLASSGEDNR
GDIRYRLGGKEANQADDGTTIDFGVKAALDELRGSKGGRIALPLNHPAVEALLPMASLPA
DSESTSADGEDLRPLRTVILQRIADDSCWMASSDQLLAGYTSGDLKWLGQMAEYVGDRH
>comp5577_c0_seq1; 854 338 1915 LEN=2135; translated
MAAKATTARKRRSTGRFAVGLLLLLLLLLVPGETAAASRRASGTKKKSHDKKQFASDNYY
TVLGLTKKAKEKEIKSAYRKLALRFHPDKVKDGEDPEEAEDIFVRVSEAYSVLSDKEKRK
IYDRYGKNGLDAYEKGQDPASAGFGGFGGSGGGGGGFNGFGGFGQQGGGFRQSSGGGFGG
GFGGFSGGFGRGGNHGGGGFDPFSMFEEMFSGQAGGGGGFGGGFGGGRRGGKGNRDRPPA
LFPKGQSHVARLGSPKFPDQDSKHMWMVLFYANSNQASRAVSEKYEHLASQTNLPYKVGA
VDCKLSPKEEKFCAEKGIEPGHDLPSFGMVVDGELLRYEDFDSRSPPSSKALHEFCMDHM
PKRYVRNINNVPQIKERLLAPEEEETASSSWFQYSSSSKPKAKPPRLPAVLLLTDKYETS
SMFYGLAYYFRRDFVFGESRAKNLKLSQTFQVKKYPTLVAFVPSPVSGSLVGKTVPYDDN
YHTIRYTGPPKKEKITAWLTKLNTVVRESKHDKSNTGRRSGRTEF
>comp5578_c0_seq1; 683 1 2889 LEN=3062; translated
XGDIDLHKDRPRGEPQGKLEELKGKKNRGDRARMIGQSRRRIFNGRAVPGQNFFHGTMGE
NXDESEAANKNRQREPLDQDSACALRRVCGSKRNVVSLAQRRSAQEIRKSKSRLGFSEVA
FDDERRVFVEPDEMEAKNENKATVKKRGSIALFFFPDNLFEVDGITPMDLTYPFFPATNQ
VIGDDICGSENERENGYNSRDRIIITGIFSHPVAAELALTLAERCGVRHILGMSDHLLDA
EESSRLDFLMRHIPSLQLRVRRGSLGDRATEDLFRDYSPSHIFYFQSDSFKTPDEDDNDN
EATDFKTHSGTNQLQQICNAMVKLQCSNTTDANGVKTNLLYVTSTVSEMNDLDNGNRLSV
ATISTFNQITLDAYRMQYQLNVRQLALPNIFGPFKEGAAWLLSEKSIQRARNEENLRKTQ
WYPYSSSNPNPMMSQPTISIIDAVQSILVSGRLVHVHGEKSVPILEAARSQTATFVELSK
ALVPLLSTKSIQTDKKNSVIEALLPILSWNYKRYKPHQDPSNFDVSPVENDKIATLGLIN
TRQNLLMDNTMDSAAVSQLERRQHDIFPCIATCTSYVTCKKSAWDTKVNVTKQATKGCTF
LLYTADFSSALESLPEMRESVNDAPWPRDSFCQVAFVSSRSTIVNNALAENDRSTIVKND
LEDDDESKISSRESIDEWNGKVSNNGWKLIWIDADEESISQMDSIMPKIVPETLLDQSVQ
FVFYVEPQHFEILPPLQLMWFLMGRQLTAAARTKKRDSGTNEEWLIPEQHIAIFTHTYRN
SDIEHLDQKKADYMAKAAQFILDQSGKTNLSKGEDQVPLKNRRQVQAYAKSLLWRSEEDV
KFELIDTPLLIYPIHNRLGRQLRCEWYEEHLFWSTDDNINLEGLSLSYVLYRWHRQKRLL
PNTDDDKWGDMIMLGEDGKSLSPSSVKFRTLVDKVEDRQEILPESLPPAFRKIAQSLNSE
EDV
>comp5579_c0_seq1; 520 389 1270 LEN=1554; translated
MVATSDEAEKDLNLIFDYDDDDESSIQEMIRSTSGLFATLDKLTNEIEEDLVSAEGTPSV
SGSNESPPCNDEEDDDESYSDDEEAAYSDEEDPDSGETKLFSMMDDLVAELQIQLEEDSD
EEDNTSVNDEGKGNKNEDPVQNTDQACRAETPEDVEKENHRVESQYQLRQSVNILLEQVI
EMKILFNLDQTNHVRNKNHDKISANIQKKIEQGEYDADRLEHLMGAISSYANMNSRKSRN
YVKTKKKKRKKRTNKHKQRKPTASVQTTQSLHILHDSLLALEEKFAREDQPRH
>comp5580_c0_seq1; 540 1 2406 LEN=3091; translated

DDDDGHNHRLGEAERVRSFMHSVDPSAPRGCGGDRPASAPSPPPRSGSGRGLPFLPAPSE
MADRNETETVVPSLKPSPLFSPSVFSAASSYASSPSSSRVPSFRAIYSVNYNNGNRSGSL
GIASSPLVRARLPLFDRRSETPRTLLDPKRASITPGTDKGGRCSTGSSNSNSYSNRNSNK
NHNYHRGAEISRLLEGNELDPLQWERFVSAKISSSGMLPRVPTFWDGSPTRKTCGGTWST
PSAAPKAGAEVSPPAAAPCVATPSTVHNPTSSRRACRTPPSPPGLAGLGPGNHGTPSRGP
PGEERERPGDGLGQILRAKDDDEEEERESPAPPVVVGKAWAPPKTLLRPQASVATSMAAS
VLLSFMVTTASPPESSSSSPSPGPPQRVLPWKRQHPATSAANTVLGYDADDDASACTVLF
GPATKRRRKTAPRRISNFLEQNEDDPATTDGTGDPGRRIAAEPLCEIRSGNHGNARSTFK
SGKRRKSRTVSIPTFEEETAIVSKAIEWPPLLAGSCAAPSSPGDYGPVGSTLASPGRSNK
ANTPAVSPSSAFKRIPRRYLDDPTLPILRDGMRLSAPDDKDHLNSLHCFVRKELLEVFVL
DDTIQLKAEEDSEVLSADKNSIPKRTIGRPRKSRQREQRQRYQQIHRVGIRCVHCGRKPK
SERGGVTMSTFFPKSLEDVYRGVCTWQRIHFKACRHMPDHFRVLYKHHKDSDKSRGKKAH
WIRTALEMGFRNVDEHRSGVVWTGKRPADATNLSMVRTHGKNDPLPRLRSPVAVALDLTR
PSPERYQSDAPGHSGEHHASV
>comp5581_c0_seq1; 484 205 1326 LEN=1808; translated
MTSGDGDPEVAAVGGSEKAEVERQNSAVVPLIDIPIVEDKSSAHLLSLETTSKSLVSALR
DSGFALVRSPLLTKELQSEALRAASQFLAKETKSKVASENGSASKNEDGNETKPGSIDVI
PHPTDPKVYAMLSSQAQYELVESDTHVISAYISALRSIKKEVLRLIAIGLGMNDDVDFFA
KLHDEDNDTLRIITYFPTFSETTGNRCKEHSDYGTITLLSTDGVSGLELLSSKEEGEAKW
LPVPYIEGALVVNVGSLLAGWTNGALKATLHRVAGPASLNSGSDRRVLIEATKHSRTSIA
FFADPNANVRESLETNENNQNFKGALGGMSVADYIRWRSGGNGFERSGVSFTPEERKIIA
KGETAASVENCNR
>comp5582_c0_seq1; 229 379 2079 LEN=2097; translated
MVDSLALRMPPLVDKKKANAEISCSECKGPVFSLGLDTYRVPMALHELNRARLVQSMTKA
LTETEQEPSISSSPENPSLPRGVILLEGGKQTTRYDTDHEPVFRQESYFHWAFGVPEADT
YGAISLPSGDATIFIPQYGLDYEIYCGSSPDLEQTRSKYGVENVLPIDQLDEFIASELAP
SDDGGDDARLFLLSGRNTDSGNYAIPAHYKGIAKHDERRNTESLFPCMVESRVFKTKAEI
ELMRYTNWVSSMAHVEVMRSCRVGMMEYQLESLFQHFTYTHGGCRHMSYTCICACGPNPS
ILHYGHAGRPNDRELVDGDIALLDMGAEYHCYASDITCSFPVNGKFSADQLMVYNAVLSA
QIDVITAMRPGVSWTDMHRAAERAILEGLKEGGLLKGDTDAMIDADLGAIFMPHGLGHLI
GLDTHDVGGYTEGIAPPRSDRPGLKNLRTARNLEAGMVVTVEPGCYFIDILLNFALANPA
QSRFLNADRVNEFRGSGGVRLEDDVVVTEDGCENLTVCPRAAEEVLDVMAGGVWPPEKDV
LPELRRNWVRNERGTLVRFELPSATN
>comp5584_c0_seq1; 471 1 1413 LEN=1869; translated
LEEKVSAVYQRLSEVLSSRAQALQNEPIDAAEQQAKPPIDLSDPEGRPYKIHMWDDTTLE
SPREDILVGEGWFEYAGKRNENEGGGSPSPWFVPTDDPASADLIVWITTMGQHELEKPPL
DPVDHARKVVVLDYSDGCTVHHKRDEIRGNNTELAYFKRSFVFKDDFNVFLENCTELDSG
ILPYSYSGSRVMMVPNDSNIATTKHRAELFSRGIGTMYNYHPPSDWNSTRRVTTSNTTKP
VDQGYFRDERYDNFLVPFAERRWTLTNVLVLNDEYHSHNIVRTKIIGWTHEFCRAKAHHP
GHDPNGTDVVVDGANNANGTSDALEFLDSSSEDPTFAAYVGLVRRGCDGHCFGQNYLRHL
RDAKIIVTCNPSGYEGDFRLWESFLSGAMVMVDEMLIPDWMPNPLVHGVHFVTYEANNRT
DFLTKLAYYSHPDHLHEAEAIAKNGYRFVLEHHMATNRVEYILDNIHRTD
>comp5585_c0_seq1; 244 1 1392 LEN=1533; translated
XRIESNQITQQRTTRPPAPRNQLRIFSRLVSSRRKAGQQTQPQRDDHETPREDAVRPISR
RAPAESVFPASARPVAVLRSAFLPLWLVVLVLAARPPSGAEALSSLSMAAPSSGAGRVAV
VTGASRGIGRGIALELGSAGYTVYCLGRSSREAEPTGVGYQRPVAKGLDLTVESAAEAVT
ERGGRGIPVPCDLGRDGAIEDVLASRVDENEGGRLDVLVCSAYATPPTGIRGDFWEQDLE
MWDCVNGLGLRQVYAACRAAAPRMIQTARNNGGGASSPPPLVCLVSSFGGKSYTFNVAYG
VGKAAIDRLAVDMGYQLAKHGVATTALYPGLVRTEANLQMEADGTWDEQSGGLDLSMGET
PSFSGKALVELAALPREAMMERSGSVEVVAELASEFGFCEADGSRPFSIRSLGYLLPNFV
FPAIEKELGEGKELPGWIKNNVPNVLLPWSTFSSGPPPEMDDR
>comp5586_c0_seq1; 759 305 2320 LEN=2601; translated
MELPFAVESPDVGTEDNGARNNSLQQSESDSLVTASSQGSFIHSTENNLHIIPDHCGPLI
PNTPQEILLPSHHHAQSQQHQQQHQHQQGQLVVIPGASEEHTGGMADGGYGDPEYDFTYV
NVTEIEAGEDGVLLEGEDFGIPQDQVRGDVLAVLIGGMQENDGCSILNKTESANEMALKA
SEAKKNGDLLSALEYHTKAATLYKDIAKVIRDRNPPLASSLLLLSQTQAKSAIALKGIVK
LNPAELRQILSPSSRDSGDNPVPAAASHKERLRAAVRGALSSHPHEADLSDSQFLGSAEN
ETTGKLATTKNRLTSVLNDSKGARKKKNDDIRNDHEGTLLVAEEQNISNPIDVMMELERE
LRDIDMAPELGNSISSLDTRMQNRMKGSMMGDGSFMVVPPGSSSYMASSMWGTPNNPRQI
ANNQQGTAGVRARANRVQAPASRPVHHPSQIGAAPVPSNKYAQGLESSWWGNTNTASQIL

TSSVMSLGVRGIEGGPINEISSGQSTQNTKQIMRLMDSLKTLGDENAALLREVEELQAAR
AEAKAARETMKRFKSEYGQRFNSLKKALEKFRQENPTGKGNGSGPVNTSEFMKNASVSDQ
LQRKDQLIEKLTRDLKKEKEESKKKDSVLRKYESFYRDIKARSAQKAAQRQTQQQRQNSK
QTRTATSRVQR
>comp5586_c0_seq2; 333 305 868 LEN=1159; translated
MELPFAVESPDVGTEDNGARNNSLQQSESDSLVTASSQGSFIHSTENNLHIIPDHCGPLI
PNTPQEILLPSHHHAQSQQHQQQHQHQQGQLVVIPGASEEHTGGMADGGYGDPEYDFTYV
NVTEIEAGEDGVLLEGEDFGIPQDQVRGDVLAVLIGGMQENDGCSILNKTESANEMALKA
SEAKKNG
>comp5586_c0_seq3; 384 305 955 LEN=1087; translated
MELPFAVESPDVGTEDNGARNNSLQQSESDSLVTASSQGSFIHSTENNLHIIPDHCGPLI
PNTPQEILLPSHHHAQSQQHQQQHQHQQGQLVVIPGASEEHTGGMADGGYGDPEYDFTYV
NVTEIEAGEDGVLLEGEDFGIPQDQVRGDVLAVLIGGMQENDGCSILNKTESANEMALKA
SEAKKNGDLLSALEYHTKAATLYKDIAKVIRDRNRE
>comp5587_c0_seq1; 289 99 809 LEN=1509; translated
MGENSVVLNVYELPMSIPDDAPCASITSFFSRILPSTGFGAYHTSIDVGDNTYSYAVGGI
SKTSVANKHRGLPPEANFKESIVLGCVSSSTTDSQSDIERNIQKCLDYLRKNFFTPTGYH
LAYRNCNHFSETLATALVVPAADLLRNRALKSYPGYINRLARTSRMVLDESADKTEGTTG
PCDVMKEAKASMGVNENLSSKSKKAVSGEKEKQRKQKKELTEAQKKILAKIKSPKK
>comp5589_c0_seq1; 402 66 755 LEN=1825; translated
MSKLNSALLEKAVNDILAYSAGEDVKINGQDKTGKKRNFVETIEAQISLKNYDPVRDKRF
SGTFKLPTVPRPNLKCCVLGNAAHCEQADRIGVDHMSVEDLKKLNKNKKLVKKLAKRYDF
FMASDNMIKQIPRLLGPGLTKAGKFPTLISGGDDMQEKINEVKATIKFQMKKVMCLNVAV
GNVSMDAKQIIVNTQLSANFLASLLKKQWQNIGVIYIKSTMGPAIQIYF
>comp5590_c0_seq1; 267 130 1251 LEN=1364; translated
MYGLVTTKIFIAVGTFFISFTSAAAPLRVIRIDDHLFSIGNMVAAGVLLAGGLVHQLPDS
SESLEDKFGDYPLASFIAGLTFTFFLLLEEYLHLQMKDNELFAQIHGPRSISDSEQANQQ
RPHERSSLISHDTPGNSGCCIIASENDCAAYLTSSTRSDAERNEECRTIPKKKIDIHHRR
STSGTNSVLDSMRDYSFSYQHPVHYHESHLVNHAKGSLLSSVLLLTALSFHSLFEALAIG
VATDTASITSTTSAVLAHKAFAGYALGSAMVASHMRELHVLVLSFVFGLCSIAGVFLGIF
LLPAIDADNSVTVIGIIQAMVSGTFLYVSIVEIAMKELMTHREGKSDLPIQTWQIRKLLG
FLVGYLMMSVLAIW
>comp5591_c0_seq1; 282 169 1086 LEN=1329; translated
MTSTAVILRSVVILASVASLPSSLGFQLPNDSRLPLPSAAQASGSRRHSPFLLRQRPVVG
LPLSGLWGGIQGSKENEESDNGDEVNAEGGTEEEDLIPTENTSPVEIVATDSSDVAALNS
SSIGSEKSLWVLLNEIGNNFKAMAQRATVKGSESDSQSKKIVYATKACVYYTLFILYRAY
RGFFVLLPATFLQVYRKMEGAMNSGNLSLEQVGFTESGQDPANKTTTWRTKLTVSILTTV
ITASYIIGGTLKMATRFLRTIAKTSDVSKSFEAAADEALDFETRISSVGKVNGGEDAGSP
SGLAP
>comp5592_c0_seq1; 130 1 290 LEN=526; translated
XNVPDFETIKTTNFIGIEILSLANTNGFSAQGISTSKEGSGGTQEAGENREQKQENHGTE
SKFDRLSPHFVRVIHGCHRATGICAAQRTETPRRFL
>comp5592_c0_seq2; 130 1 290 LEN=801; translated
XNVPDFETIKTTNFIGIEILSLANTNGFSAQGISTSKEGSGGTQEAGENREQKQENHGTE
SKFDRLSPHFVRVIHGCHRATGICAAQRTETPRRFL
>comp5593_c0_seq1; 282 54 1160 LEN=1478; translated
MALMDHSMGATAIGACVVVGGAVIFKQTLSVAFADIAHLINQLFFKVFVAYAGMVPKSVE
MRVNGQVVRAHYLERNLRKTSRSRGNSTCQDILVVPGFTAEATFMAQLIPAMNLSPETRV
CIMEFPLHGKNVENFDGKTFPKPQQTVDYALAFVEAVGLGKTSETKIPLNLFGYSMGGAI
CFQMLAQKSPGTYRSAVLLAPALENTMDDDFNALSTKDPRKVHGWETLPECKEFFTTSAG
GFYPTKLPYVVLAGVHRQRELTYAPHDRSFFTNFFRRLHGGGFELEPDEAKQKLLRAHRK
TPVLVVCGDRDDCISLDKCQRFFGDSDNECFLFRELLDTGHVGGPADRRAMFTILDMAGP
IAAEFLFG
>comp5594_c0_seq1; 144 266 1192 LEN=1415; translated
MAANLCDYRILKSEFLGEVEVSRTYTGSNGETVTTVHSIRHHIHNRMNLSSQQGAPVLVL
HGGPGLPSDYLLPLRDVIPYRSIVFYDQLGCGRSPGPETNEAYSIESSLDDLEVLIKKIG
LTRFHLYGQSFGGILAFEYMKRVAERGSDDAPRCLSAILSSAPSDVDQMDANWDARVASL
LEEDPNSSTVMNRFLARHACRSISADGEKPKPLADAYENAATRRVVWNRQHKRMEGGQTL
GIFSAHAILYGDEGRNDFVTAECLEAWKDAFNHKFVRVKELENASHHGLLEDGKTYGRIV
DSFFSEYD

>comp5595_c0_seq1; 575 100 1182 LEN=1927; translated
MKFAAALATIASLGMAQAFTLPSSTSTTASTTALGAERPKIAENVLDLIGGTPLVKLSKL
NEGCEAEIVLKLESFNPANSVKDRIALSMITQAEERGDIKPGETVLVEPTSGNTGIGLAM
VAASKGYDLILTMPESMSMERRVLLKAFGAKLVLTPAAKGMGGAIAKAEEIAATQGGFLL
QQFNNADNPKIHRETTGPEIWADTEGKVDILVGGVGTGGTITGCGQYLKPLNPSLQIVAV
EPTESAVLSGGKPGPHKIQGIGAGFIPGNADTSLLDEVIQIAGDDAMEMARNMATQEGIF
CGISSGASVLAATQVAKRPENKGKRIVAIIPSFGERYLSTALFQNLWDEASAMKAESVEA
>comp5596_c0_seq1; 564 210 1535 LEN=1869; translated
MPSAASSSSSSSLSSSSSSSSPKSITDDNSNNNCSDTKRSSSNYYLTEEAKKNLKAYTYR
GADNSLIYQYVLSPLAGYLVTTIIPKSMAPNTVTSLGLAWMVSAYLSHWWFAPDLVFEGC
ESDSRDPSCDPPRWIFLWNAISLLVYQTLDNMDGKQARRTGSSSPLGLLFDHGCDSINSI
FGSANVIIAMGLVPSESLLSIFLLIFVPMVVFFIATWEQYFTGELIMPIVNGPTEGLLGT
AAVSFLSFWFGIGVWEETHAYDLLSAATGFGGGQDSTPWKNSEFLVGAISVLSVQEILLK
LAFGLQKRTAAALVPFFAMAASFFVIGWIGPEIWLSIPRTGMHLSMLLFVEICTELMLAH
VTEGSFRPWRWQIAPLVGIAAWVGLSGSAGGNHGWVAPALVAYTWCLGAYLVTKCTLVIR
EICDALEIWCFDIVTPHCRRL
>comp5597_c0_seq1; 396 215 1212 LEN=1297; translated
MSPAVKWDQIXEPISLSPRRLSASPQDSQPNYPEGGDETDLLQSPEGESTPGGGDATGTS
KEDFVGLVEEVMKSSKSEHIPRLLANNIEVIIGLQGQDGAQIITSILEDAKADGEKDGDD
SVYTQTLQTIDMILGFAEDFVRQAQEMDAANKQLLGKIIKAMVTSENDQSVAGGEGRIIS
KEDSASKREQLLDTIMKEEKKNFSSGFLRHIDGECDRISNQEKLTPESTRLLDILRMIQT
RVLEELGQDLGEAALVLGQLMGYEKEEELLGVLEAGLTVRGNEFALEMASLTEEALDGFS
RVPGGADPELVDKVRFIDRRVQEFLNETNEFQ
>comp5598_c0_seq1; 175 541 1134 LEN=1622; translated
MKRDLTKERSSIAQNRLGATLSSDNAHETICILEKGSIHTLVQRLASSSGTKIELAREVP
VEVRTYEQVGASMAWHKDDILYDPPQVEVVFTLENDSDCVTMWVPDKGEGTAGETNNDII
QSQETHPNSMLILLAGGPDHCVTSLKRGRRVILKCAYVCEGATYVGEEDLHQFGSVKSNT
NANRTSKPKRRKQKRRR
>comp5599_c0_seq1; 438 475 1533 LEN=1973; translated
MNSIIENEAFDESNSMFNNNAKSTIEDNCEDQGSLVSALSGEDTDVSLLLGEEVSHCNDH
YAENGETKQPRRDDSEEDTGTTIDATTLVSTTSSNASNYFLPHHKHNDSLCYSTTSSTCD
TEHQRVAPSSYLTNSNLGSFQQAFPSSMFPGQTLLPPHHSKRTEQPEGKSSNSNSHLPSY
YYYSSSYQNSTLNDIEVGNNLNRTPAVGSNVYQKRTKTSDRWVLFYDDGETSSLLRAPSA
NSWRGALKCFFSPRVVLTVLLCSVILFNLMRYGTHFKHQFETYQHGSNSLGGSSKVDTSV
DYIKLETIGGAANSPYSESWKDDDSLSLLSKLESKIHAVGGRENENGGGERR
>comp5600_c0_seq1; 883 79 2433 LEN=2614; translated
MTSISQNARTLKMSTSPRNHYQHKINQSYIGANDMNIYVSSPVPSVDVHNGSITNIKTEA
SSMDSNPHNVSNTSSSMKFSSSHHHQYHHGTRPSLAGFDPFSSEQQTPPPTKSIAPDTRG
NSSPSGEVDLAFEGADSGYSKMEKHRPHRLGLGNVENFDNEQVDTNGVASAGANPSKKTT
SAKGPMKKVNKFLRKSILAKSLTMARQISAERNPSTQKTPSAVSPVSVVSLNDNVSNPGN
NDEHLPGKQQMTASTITTNPKGMPSSFIHADQSPSLPSQPLVLSKGPTSLTQDLLELKEG
FQNHATLEIPLLTTPHKQRNKPNYAPTSFLTGTQSKGIDYEAPVLTSCLFNDATPFQLEI
PSLLEVVTMARLNEFVENYRKQDQNLDLRQFVGLSRLQLEHKLQTSCATTSNHSSNKNIA
TSNNILVKEHIPIVQSLLDCSEHDHDEITATTNTKGGEISIQGFTTESGGENHSADDRTE
AVIFQGQRNFTVVFRGTTEQQAKGLQGGGNNSKSKKRAVDLNDDENGGSEFPDLNKNNPN
SRCTIEVYSGFKESYSRIEEECFRQVDKLVDENPFCDVFFSGYSFGGALATLAAFRYAKA
RPMMRVGCLTLGSPKAGFAGFRQAANALPNLKVMRLELGKDARCQGPTAGGSHAGHTVVL
NHTGSGGPTGSSGSNPHNKSNAASKSTGGGNAATPPVSQGPFPSSPSDHRKPSVSVYKFA
TPKHKNGFFKSSNPGLKKYISTLEDLATLNKTSGRSASSLTWATDFANNAGEGVVVNNEK
RQMV
>comp5603_c0_seq1; 160 1 398 LEN=760; translated
XDSKYHRYSSTTNESTMSALAKTLEPLMKRAARTYQGVLATELNKMGLRAEDLMSRDEPE
VNEALELASPDVVEGRYRRLKRASDLAFKQKELQDYAPDMILDPMKKEISADVDKIMKRD
LEFDLLNNHKKG
>comp5604_c0_seq1; 398 51 1650 LEN=1676; translated
MNGNLDALTTIFTEFYYWVTVVFMFLIHVGFCMYEVGASRRRNHMHTLMKNVMVIPLVTV
TFFFFGWWIYWAFPMFPFFGGLNHEMASANLPWSPNMATNLDDRITGVFWAAFLLFSWTA
ASIVSGSVIERIRSSALWVHAVLIGSVWWIIDAAWGWHYDGWMVKYLGYHDAYASGVIHA
IAGGYALGVIMVLGPRIGKFAADGTPRDIPPHNPWMLTIGIFLIYTGFWGFYAACNVPLI
SPEVIGGQITGTTWTATNIYLAPTSLSAITFNFLMSLSGGLMAAYVVSKGDAFWTFSGGL

AGIITASAGNDLYHPVQAMLVGAVGVVIVYKLHFWVERRFKLDDAVGAVAVHGYSGVVGL
IIAGFLLWGAPSSPFEGYATINPLGQTLGAVIMFFCLGFLPAFIVAKMQAAAGVLRIPVE
VELQGLDYQNXHAYEDAIVQVIEADKAYLNTKPTPAEXRIRHMSTIGYESWAVDLAEVGP
IYPFQGYEIPMVVAGVIFWLAWHRIQFVREGEHLEQARKMGDPEKIRKHLERY
>comp5606_c0_seq1; 458 292 1722 LEN=1989; translated
MAFALVMGTASADLQDYIDDETGEVKSFDPNEQFSIHVVNKSKYRADVYWDDGWQGVSIA
TVDPGETMGVTTSQGHAFFITRHGVRENLYPEAVEGEEDKPLRIVAQKPNEKLVIPEGAA
PLRGERAEKNRCVDRYSMCAGEGETSLSSDSSVVDTSGARNRVFRLQCTLLDSVVNLFRC
LPFPATKGLCSEAPGWMIVNCCKSCDEELNASELLDADIRCSRENLGMGGPAWEPGDLNK
LFEKWVTDEDFQQYTPNVLSSPGAKFGGRDGPWIITFDTFLTDFESDQIWKGGELAGFDR
STDQGQVNQFGEMEQVVSKTRTSSNAWCTDKCEMLPGVISATEKIQKVTGVPKVNYESFQ
ILEYQKNQFYRMHHDSSDGKDDTAAGHRILTFFLYLSDVEEGGATRFTKLGLDVTPKKGR
ALVWPSVLDENPEISDRRMYHEATDVIRGEKRAANHWIHLYDYETPNLWGCTGAFS
>comp5606_c0_seq2; 458 292 1596 LEN=1863; translated
MAFALVMGTASADLQDYIDDETGEVKSFDPNEQFSIHVVNKSKYRADVYWDDGWQGVSIA
TVDPGETMGVTTSQGHAFFITRHGVRENLYPEAVEGEEDKPLRIVAQKPNEKLVIPEGAA
PLRGERAEKNRCVDRYSMCAGEATKGLCSEAPGWMIVNCCKSCDEELNASELLDADIRCS
RENLGMGGPAWEPGDLNKLFEKWVTDEDFQQYTPNVLSSPGAKFGGRDGPWIITFDTFLT
DFESDQIWKGGELAGFDRSTDQGQVNQFGEMEQVVSKTRTSSNAWCTDKCEMLPGVISAT
EKIQKVTGVPKVNYESFQILEYQKNQFYRMHHDSSDGKDDTAAGHRILTFFLYLSDVEEG
GATRFTKLGLDVTPKKGRALVWPSVLDENPEISDRRMYHEATDVIRGEKRAANHWIHLYD
YETPNLWGCTGAFS
>comp5607_c0_seq1; 138 1 892 LEN=1022; translated
XVKRDVFWFDGVDVDSSSTNDNPEIVYVSSPAPLKSLANLELYYEFATGPVQHSKTPNRN
HRDVSLPPSSFVAGQDPFVTALKIAVVTGSTRTSGPPSINGPRIASFVRHELEERGNTIV
AEIDPRDFPLLEKPEFAHHRRTVPKPLREARAVLEEADAYVCITPEYNHAPSPGLVNVLN
HFGSSVFSFKPSAIVSYSAGQWGGTRAAIALRPILSELGALPVSAMIHIPNAQDAIDDDG
TPTTDPKRWKEYCGRCFSQLEWWADAAKNHKEIADPFEESKPFRLTPSQRNAPKKQ
>comp5608_c0_seq1; 182 1 1007 LEN=1196; translated
XNTSGRYDTTHKTNKNLHLASSISRSLQYINQSLNFSFSSGPKYRELMGEGGWVQHRGTL
ERIDLEMLSRYQQNSLSGDDVTIPAFTEESNTDDNWYSITPTILRWEETFSAQVGNTSGA
QKALEKKLENIHRELLFVNKPSGLLSVPSRSLNDCLATKLQSSYPGSKPCHRLDRDTSGI
VLFGLTYDAHRDISMQFAARTTSKTYVALVAGRPEKDHGTVNLPIGKTRTEGGFNRWTIG
GYKPRDSVTEWSIDKVFNDATTGAVFTRVRLKPITGRGHQLRLHMKAIGCPILGDTIHGE
GSIAMCSSRLCLHAQTLEVDWDGLRLKAESVPPF
>comp5608_c0_seq2; 285 185 1318 LEN=1507; translated
MGFQRLTGTVTRTLVAIMGIVYGAWRRKINAFHPNQSSRVVAGVGYIARHGQMLLRCEKT
RTESSSVQTLLNPISKRRIHLGGPKYRELMGEGGWVQHRGTLERIDLEMLSRYQQNSLSG
DDVTIPAFTEESNTDDNWYSITPTILRWEETFSAQVGNTSGAQKALEKKLENIHRELLFV
NKPSGLLSVPSRSLNDCLATKLQSSYPGSKPCHRLDRDTSGIVLFGLTYDAHRDISMQFA
ARTTSKTYVALVAGRPEKDHGTVNLPIGKTRTEGGFNRWTIGGYKPRDSVTEWSIDKVFN
DATTGAVFTRVRLKPITGRGHQLRLHMKAIGCPILGDTIHGEGSIAMCSSRLCLHAQTLE
VDWDGLRLKAESVPPF
>comp5609_c0_seq1; 570 1 1962 LEN=2182; translated
RMDTAENSSTEGEVISLLQAHESRKCQKRGNVTFDRMEVSSESTRFSASSLLGNVSTGGD
TIEVDNSNVRDLGGNEKPLNEKNILTDVKGITADSATLVMANESHAQKLNRYQDDESCTR
DCPFEVLGESDVAATRRKSLSSNSFTLNRVDHVQNLVNDITIRQTSSNIDKSVSFSDTTE
FIKTINVNSGPSDDLLDKPTNVDIASFTSMIFEGLHPVRIQEDGLSDSFTRSGKSNDAVG
KIVCDRWRQFIEAVCGEVERRIDQEGTAATSLRSLVEEDPGLYYSIQHRFQSSGEHSFIR
ESLKRLVEAGQILIGYEWNSWLATVLESFHGPLTDIPQAFTDDISKIDEALQQSKILETD
VSFMNDRRTKRARRKSLARHRTMVTALEREIESIESQLCAIKAELEQMEREKVDLLKATT
SCYDFRHNAELHDGLKVEAEISQRSFSSLRGLHSWSVSTLSESDIEISIIGSCKQTYLEL
MYKGTKCGTANTNVLSKIDSSQKMEKSLYVYHGSIATFLEIATKRLIYMAQKSDIKGEFR
INDHLLRYAWLAGRLDIVAKEFQVVQRRYRGKLYRDSKGSFSFIVEFESNKSKMVADFAI
ESEYPSFPVEVRLDLISGKQDLERIRKALVKNAKPGFGSLSRACDIIHSIMNG
>comp5610_c0_seq1; 631 106 2310 LEN=2388; translated
MVRSAPIVGKAETERDGREMNENMHRIVEDFNYSTFSRSLNRTLIAEKYGVSWRSETSNE
IGVKIIYSGADPNANAASALDATLSETGVCQNLDCRDFMCIGWCDITALGKNGVIESLAE
VSHIIALMMPETNSFTGSGSIQTQAIAAMQINTMLRKYPELTGKGVKIGIISDSFNARAG
TLAGRNVAVNGTSEEADIESGDLPGGDQRVKVLTEVEPDFPFPVSDEGRASAQLIYDLVP

DAEIVFSSGFLNAGGFFASAIEELVEYGCDVIVDDVSESDTILLLTYEDDFVANFFRLTL
FLNLYGLFVFQSADLKXADGFEPIFQLGPTPKAADNAATVDGVAYFTVARNLASRSWENR
GFKDTACPNNPIFDDYISCHDFGNGNPLQRIAKFAPNPLTFYWDDPWTSVSGLPGPESDL
DIFAVDIETGDILKRSATVNSGGDAVEQLVFPPQFFNLVIAKSSGPSPSLIKWIDRGLTS
SDPPAESATLTGTGLAPGVAAVGAASEKQVFGQLELEPFSSQGGIPLIFNDDGQRFPQEI
VLKQPRFVASDGSYNTFFGYLEPYEFSIGEQALRVPPRFYGTSCSVVNAAAVGVILIQAM
SLFPGERRLLNSKKTKKTGEEGGVNSTSAKNESEEDQDVRKGFSLPFDVYKIMEDTAIDM
NEGGFDFLSGFGFINAFDAVEMVVQKARKGTKSKKASKKSGRIESVSTECPVESFLGPIG
SYFDDSYVANATLS
>comp5610_c0_seq2; 598 106 2454 LEN=2532; translated
MVRSAPIVGKAETERDGREMNENMHRIVEDFNYSTFSRSLNRTLIAEKYGVSWRSETSNE
IGVKIIYSGADPNANAASALDATLSETGVCQNLDCRDFMCIGWCDITALGKNGVIESLAE
VSHIIALMMPETNSFTGSGSIQTQAIAAMQINTMLRKYPELTGKGVKIGIISDSFNARAG
TLAGRNVAVNGTSEEADIESGDLPGGDQRVKVLTEVEPDFPFPVSDEGRASAQLIYDLVP
DAEIVFSSGFLNAGGFFASAIEELVEYGCDVIVDDVSESDTILLLTYEDDFVANFFRLTL
FLNLYGLFVFQSADLKXADGFEPIFQLGPTPKAADNAATVDGVAYFTVARNLASRSWENR
GFKDTACPNNPIFDDYISCHDFGNGNPLQRIAKFAPNPLTFYWDDPWTSVSGLPGPESDL
DIFAVDIETGDILKRSATVNSGGDAVEQLVFPPQFFNLVIAKSSGPSPSLIKWIDRGLTS
SDPPAESATLTGTGLAPGVAAVGAASEKQVFGQLELEPFSSQGGIPLIFNDDGQRFPQEI
VLKQPRFVASDGKDHDSNKPHDEFISWMTSPQLVVVAFNDTHYIFARRTFFQSLNDNIXS
SYNTFFGYLEPYEFSIGEQALRVPPRFYGTSCSVVNAAAVGVILIQAMSLFPGERRLLNS
KKTKKTGEEGGVNSTSAKNESEEDQDVRKGFSLPFDVYKIMEDTAIDMNEGGFDFLSGFG
FINAFDAVEMVVQKARKGTKSKKASKKSGRIESVSTECPVESFLGPIGSYFDDSYVANAT
LS
>comp5610_c0_seq3; 659 106 2190 LEN=2268; translated
MVRSAPIVGKAETERDGREMNENMHRIVEDFNYSTFSRSLNRTLIAEKYGVSWRSETSNE
IGVKIIYSGADPNANAASALDATLSETGVCQNLDCRDFMCIGWCDITALGKNGVIESLAE
VSHIIALMMPETNSFTGSGSIQTQAIAAMQINTMLRKYPELTGKGVKIGIISDSFNARAG
TLAGRNVAVNGTSEEADIESGDLPGGDQRVKVLTEVEPDFPFPVSDEGRASAQLIYDLVP
DAEIVFSSGFLNAGGFFASAIEELVEYGCDVIVDDVRADGFEPIFQLGPTPKAADNAATV
DGVAYFTVARNLASRSWENRGFKDTACPNNPIFDDYISCHDFGNGNPLQRIAKFAPNPLT
FYWDDPWTSVSGLPGPESDLDIFAVDIETGDILKRSATVNSGGDAVEQLVFPPQFFNLVI
AKSSGPSPSLIKWIDRGLTSSDPPAESATLTGTGLAPGVAAVGAASEKQVFGQLELEPFS
SQGGIPLIFNDDGQRFPQEIVLKQPRFVASDGSYNTFFGYLEPYEFSIGEQALRVPPRFY
GTSCSVVNAAAVGVILIQAMSLFPGERRLLNSKKTKKTGEEGGVNSTSAKNESEEDQDVR
KGFSLPFDVYKIMEDTAIDMNEGGFDFLSGFGFINAFDAVEMVVQKARKGTKSKKASKKS
GRIESVSTECPVESFLGPIGSYFDDSYVANATLS
>comp5610_c0_seq4; 626 106 2334 LEN=2412; translated
MVRSAPIVGKAETERDGREMNENMHRIVEDFNYSTFSRSLNRTLIAEKYGVSWRSETSNE
IGVKIIYSGADPNANAASALDATLSETGVCQNLDCRDFMCIGWCDITALGKNGVIESLAE
VSHIIALMMPETNSFTGSGSIQTQAIAAMQINTMLRKYPELTGKGVKIGIISDSFNARAG
TLAGRNVAVNGTSEEADIESGDLPGGDQRVKVLTEVEPDFPFPVSDEGRASAQLIYDLVP
DAEIVFSSGFLNAGGFFASAIEELVEYGCDVIVDDVRADGFEPIFQLGPTPKAADNAATV
DGVAYFTVARNLASRSWENRGFKDTACPNNPIFDDYISCHDFGNGNPLQRIAKFAPNPLT
FYWDDPWTSVSGLPGPESDLDIFAVDIETGDILKRSATVNSGGDAVEQLVFPPQFFNLVI
AKSSGPSPSLIKWIDRGLTSSDPPAESATLTGTGLAPGVAAVGAASEKQVFGQLELEPFS
SQGGIPLIFNDDGQRFPQEIVLKQPRFVASDGKDHDSNKPHDEFISWMTSPQLVVVAFND
THYIFARRTFFQSLNDNIXSSYNTFFGYLEPYEFSIGEQALRVPPRFYGTSCSVVNAAAV
GVILIQAMSLFPGERRLLNSKKTKKTGEEGGVNSTSAKNESEEDQDVRKGFSLPFDVYKI
MEDTAIDMNEGGFDFLSGFGFINAFDAVEMVVQKARKGTKSKKASKKSGRIESVSTECPV
ESFLGPIGSYFDDSYVANATLS
>comp5611_c0_seq1; 1234 698 4423 LEN=4927; translated
MQGVPTKNVSSAGKKNNLQQQSQPSPTSVKDLTIDVNSPPIDRGLPSPIDSKSRRNRRRR
KPKARGNQGEPVASPARNPNKTPNSTKNKSSHNNSPNNNSNHTSSPFCSPIVSWRSSRPR
NGNNNSRHNTKNNKQWNHPSPRFSKNVSSPACPASTGFPTSKKQHDAPMKKRDLYFALHV
ERVGIAPLPENETQQQQKAVARVTLTNWENEIVLDTFVAIPVPVTDFYETGIRPEHVKPN
TKSTNAIGSAVAEGVASDKNIAPAHSSQCFSEVRAKVEQMLRGKILIGHELDEGLQALGL
VHPTTDMRDCSVYFSDERKPGHSSLKSLEELSRKELNRWSVKASKARVAKDISNKEAIIF
SSKDHSISKTPVLVCVTSMDIYKKHRNAWETSLIVQARERERQQQEHLLKISEQRQQEQE
RARREAQQRCQQQYRDAITTVSMNCETVRTGRTKTLTRVTIVDGPSRNVVLDEFCQMVFP

VTDFTALDAKIPGMHVPRHQTDNSCSSKPISVVRALVEQSLRGRLLVGYKVEEGLKALGL
THPWAQTRDIAYYPPFLRNASVADTSMVTVRPLDDLSEDFLRQQLRPVGDRCRPLDVCRT
TLCLYEAVRDQWEGQQRDSYPHHVHPQHSQNLQPQQPGRMMTCSPSASSVGMLHSPHPNH
YNHQPNHCYGQQTDLTSYGGPTMILSPTQRQQYQPHVMEEQQQRPDMQSRSNPSSWFPWG
KNQPLQENQVGGASQMLSPQAYQVLQEVSGVQYASFSPSKKSFFQDNSSVDSYYGNSSYI
EGTSRYEESTVGGRSGLISETVATESLISSLPDESSSVISSDQPLSTVGSPRLCLKNDSP
SSSSWFRFGSKKSKYPTSNDVKTNCETMAAVQEMEVLSDDGMLPTPTTLFSSKGLEKDNS
KSQPKTDNAETITEGTAMSPSLFSRKLFGFRKPSISTGGKERSHSPSSCSSESTIDELSI
AAEPEGFPDTATEASIEITLSIPNSTETEGGDTCSLLVEKAESTTLASRPSSSSWFGFRR
SSKSLGSKCKNFPSDSLTKVSDPTVMPYHLGLPSAPTERTAAMEDDWLQEVMTGTTDDLS
PWLNENPQDKGGEETGKSSATSRGQASWFGFKRGKVTSLNKVTPLTNGFDSIGGMEMNQD
PRGDSAWRSDSPKDDYWSAATNTPMSGSDEETLSTILMNANSENNNDDSDIFRPRARLPT
ESTVPSVATEVASEEEKCVSDDCYSKELDFGAVESFNFLKI
>comp5612_c0_seq1; 349 117 965 LEN=1051; translated
MASSSSRLNLCHQSSFSAENTLVTIVPSFGYSGSFPLLSCTQSVNVGPFTAGMPSDVPLW
VAKALYRRNFCQIELPEWLSAPFLTSILKEERESVLLSTRLPFHYYEIARALDCCLTDSK
NPSGKATKVVLQDLVAVRSDKLRMHFHELSRSDLVHPPAGYDRVDENGNYTQDSLGKGSP
APELPIISVTGICSYELNKVGPFLQRAFSDYGFLTRRPQAAAGGGGTSEPTKGPASSSSS
AHSSEYDKDGSGLLEGDAGNGGMDATSRRVSVARSRLRRFRD
>comp5613_c0_seq1; 108 339 804 LEN=1057; translated
MVIDGNNFLGQRGVTGLPRDGDKLAEKLVAISGTQGGSSTPNDVLLVFDGRRDVDLERTD
SKLTDSFTKVQLQHGTIADDFLVEEIEQIRKEDPSRRVKLVTADKRLRARALGIRPTVKQ
VVNPKVFWKKYVPRMSGQKKNPTPHMPRGNSGEE
>comp5617_c0_seq1; 627 197 1942 LEN=2572; translated
MATAMATGMATATARLRSAASRAVGRTALRFGPAGPVLNGHRTVPGILLPTAAATTVSTV
PWPSFRRHPSFSTTPDRGEPIPGEEGSEGQRQGQGQGQRQHVRDPPAASWMKAYRLLEKH
HREHGTTMVSTTAAADDGSGPSIAAWASLQRKRHKSLSPEQKELLNKIGFVWDYQEARWL
EQYERLCDYERRKLPSKLRATENGASPIDLESVFEKRYHGDPVLKRWVDAQRLRYRRESI
SAEKVRLLEEVEGFFWDVKEARWAEMLRRLSVYAEAHGGSCSVPQNYADDPQLACWVANQ
RQRRHQLTSDRIQRLDALGFVWDVRELRWLETYEAYKAAFFGGGTCGARGDDEQARQLGD
WAIKQRNCFRRGTLSARRVQKLEELEDPSDGSRFVWDPHEENWMDMYRELEDFRSVHHHV
VVPTRSSSVGGNETAREGDASPEDEPPRSSSTSSSTNPYKQLGTWAANQRRAYKMGTLSD
KRVELLERLGFVWDVPRAQWEDSYRRLLEYQREQLEAGREASQSTMVPQHYDKDPQLATW
VKEQRQCRKNKSLPPDRERLLEKIGFVWTVAGTGSSPPPGD
>comp5618_c0_seq1; 166 120 386 LEN=386; translated
MNIQFIPFICLLALLVVSDAQPGQPDDKDDKMDTPTDGGGSENCPAADQIVDGDPCGDFI
PAGSQEASCFVGDTGEQCNCPGQETPDPV
>comp5618_c0_seq2; 166 307 573 LEN=573; translated
MNIQFIPFICLLALLVVSDAQPGQPDDKDDKMDTPTDGGGSENCPAADQIVDGDPCGDFI
PAGSQEASCFVGDTGEQCNCPGQETPDPV
>comp5618_c0_seq3; 166 65 331 LEN=331; translated
MNIQFIPFICLLALLVVSDAQPGQPDDKDDKMDTPTDGGGSENCPAADQIVDGDPCGDFI
PAGSQEASCFVGDTGEQCNCPGQETPDPV
>comp5619_c0_seq1; 271 133 1044 LEN=1287; translated
MSSDKNFVQEYYYHLSLSSTWTGIFGATFTVLITTLDDSIWLMPFVATGGCEKRSRFVHA
GVFLGTLMTLSFFCCAIALGFVSFWGQNTDNDTQSSIRRVEIQLECISVILCWAIASGFF
IKKQLKHRRRLREDHEGRDRRQHGTRDESNAVLRTEKKKDDVKGGAETSHADDDSEKFRR
ALYGSISGPPVRKYSHDNDLCMPTSNEKEPDSTDTDGLAPMTVASLTALGFLDEISYFPT
LIVGNVFSVSELILGTLFAGLTVLAIQVFLFGRCKPFIDFLDERVPLYAIIACFAAIMTC
RLVW
>comp5620_c0_seq1; 370 192 890 LEN=910; translated
MTKNRSIRSFVTFRSTLTAIVFVWCCAHSEAFQATPNQKPALSSLAPAATAATTLAVLLT
LGTPLEPAFAASKESPTAGRITINQIPPKTVRIEARDLPVVGKLVSGTYARVDESSVAGP
PSVIVSSPRDTIGAIKKATTGGHVEFDVGGSVGLRTHLDVDIAAEKSGVASVRVASDLIP
QLPFRNAASSLYGQPGRKESQWNIVTNMGSGESYYFNTKTGVTQYEQPSKF
>comp5621_c0_seq1; 622 130 1534 LEN=1534; translated
MSSEITTVSASIADTHIDPSINDTNNDANSDEVGTEKITPIAVAKTEEELSGKDDSAPSP
LVIAAKPPNSAMDTAKSLNESNNKIIKELDDETAKTFPQILMEILTNDENSDTIAWLPHG
RSFIIYKKKKFAAQVLVKYFKATKFTSFTRKLNRWGFTRVTRGTEMGSYFHKMFLRDNPE
LCLRMSSHTSSKYHHSHHHDPANPIQNAPHMHGHLVMNPGGVSGFMPPGALGMPVVPFPF

YGMPGMAPGATTAGMVSPPNGIPPPAPMQANMPAPPTTAAEFSQQNQYINQQLQQLQWQQ
FQLQQYQHQQMQAAAAAAANHPNNDGAPYQQSPPGTLPLYGGNPYPPQPLQPQAISDLPD
NTPPIALTAPEPPVVGQHPPSTGQAENGQHPSPARPVQQLQQQPQQQDQATSLMHPSTHG
GAHASQQHPHYQNQQLHPSPTQQPPNNNPYGHNPHYQYGQQQSSLSIRX
>comp5621_c0_seq2; 646 130 1836 LEN=1836; translated
MSSEITTVSASIADTHIDPSINDTNNDANSDEVGTEKITPIAVAKTEEELSGKDDSAPSP
LVIAAKPPNSAMDTAKSLNESNNKIIKELDDETAKTFPQILMEILTNDENSDTIAWLPHG
RSFIIYKKKKFAAQVLVKYFKVGSPGSFLCRFNKYYDTYITILMPFFDVVXGNDLTFLQS
LKATKFTSFTRKLNRWGFTRVTRGTEMGSYFHKMFLRDNPELCLRMSSHTSSKYHHSHHH
DPANPIQNAPHMHGHLVMNPGGVSGFMPPGALGMPVVPFPFYGMPGMAPGATTAGMVSPP
NGIPPPAPMQANMPAPPTTAAEFSQQNQYINQQLQQLQWQQFQLQQYQHQQMQAAAAAAA
NHPNNDGAPYQQSPPGTLPLYGGNPYPPQPLQPQAISDLPDNTPPIALTAPEPPVVGQHP
PSTGQAENGQHPSPARPVQQLQQQPQQQDQATSLMHPSTHGGAHASQQHPHYQNQQLHPS
PTQQPPNNNPYGHNPHYQYGQQQSHVPHGPPHSHQGVSNGPNPPPVGHLGGEHYGGQQPS
QVQLQPYPYQYPQQQPYHHHNPQQQQQQQ
>comp5621_c0_seq3; 580 130 1657 LEN=1657; translated
MSSEITTVSASIADTHIDPSINDTNNDANSDEVGTEKITPIAVAKTEEELSGKDDSAPSP
LVIAAKPPNSAMDTAKSLNESNNKIIKELDDETAKTFPQILMEILTNDENSDTIAWLPHG
RSFIIYKKKKFAAQVLVKYFKVGSPGSFLCRFNKYYDTYITILMPFFDVVXGNDLTFLQS
LKATKFTSFTRKLNRWGFTRVTRGTEMGSYFHKMFLRDNPELCLRMSSHTSSKYHHSHHH
DPANPIQNAPHMHGHLVMNPGGVSGFMPPGALGMPVVPFPFYGMPGMAPGATTAGMVSPP
NGIPPPAPMQANMPAPPTTAAEFSQQNQYINQQLQQLQWQQFQLQQYQHQQMQAAAAAAA
NHPNNDGAPYQQSPPGTLPLYGGNPYPPQPLQPQAISDLPDNTPPIALTAPEPPVVGQHP
PSTGQAENGQHPSPARPVQQLQQQPQQQDQATSLMHPSTHGGAHASQQHPHYQNQQLHPS
PTQQPPNNNPYGHNPHYQYGQQQSSLSIRX
>comp5621_c0_seq4; 688 130 1713 LEN=1713; translated
MSSEITTVSASIADTHIDPSINDTNNDANSDEVGTEKITPIAVAKTEEELSGKDDSAPSP
LVIAAKPPNSAMDTAKSLNESNNKIIKELDDETAKTFPQILMEILTNDENSDTIAWLPHG
RSFIIYKKKKFAAQVLVKYFKATKFTSFTRKLNRWGFTRVTRGTEMGSYFHKMFLRDNPE
LCLRMSSHTSSKYHHSHHHDPANPIQNAPHMHGHLVMNPGGVSGFMPPGALGMPVVPFPF
YGMPGMAPGATTAGMVSPPNGIPPPAPMQANMPAPPTTAAEFSQQNQYINQQLQQLQWQQ
FQLQQYQHQQMQAAAAAAANHPNNDGAPYQQSPPGTLPLYGGNPYPPQPLQPQAISDLPD
NTPPIALTAPEPPVVGQHPPSTGQAENGQHPSPARPVQQLQQQPQQQDQATSLMHPSTHG
GAHASQQHPHYQNQQLHPSPTQQPPNNNPYGHNPHYQYGQQQSHVPHGPPHSHQGVSNGP
NPPPVGHLGGEHYGGQQPSQVQLQPYPYQYPQQQPYHHHNPQQQQQQQ
>comp5622_c0_seq1; 149 1 554 LEN=554; translated
RYGAYCSVTGRTKHRRRLNSNIVCLCTYNFFMVASLKRKRKSEPKQKLETGWKQRWLTPS
RSLFVLFFVSLLRIIHSDLFSIFDSANVQVETSSNNGGNETKETKSSRPASIQMEMSSNS
GRNETKENNAAKELGKPKSSRPASIQMETGSGSGRNETKESNDAKELGKPNSSRPASIQM
DTASX
>comp5624_c0_seq1; 288 77 556 LEN=688; translated
MSNFTTNEEDQREAAKLQARLRNAASDDDGNGNNGEIDSAATYHPIPNVTMAEGKHKYVL
MSAVLPNSSERQNFVVSSAFASYHRDAAEPMVAALESSGYKSVAITGGGRISLDTNKRSI
SVYGFSYGFGLADHALSVSVIKNDERYKDFDVTWSNEGY
>comp5625_c0_seq1; 515 59 1330 LEN=1584; translated
MAPNDSSYVRSHSSIAGKHVNVGMMTSGGLAPCLSSSVAQLSKYWIEALAQGKISGLTLR
MYVDGYSGILTGHSFVVPESEWEGMDKLNFLGGSPIGNSRVKLTNLADCEKRGFIKAGEN
PLEVASQQLLKDEIHVLHTIGGDDTNTQAAVLSKYLMDEHNGSVIVIGMPKTIDNDVIPI
AQTFGADTAANQGAIFFQNVVNESTASPRMLVLHEVMGRDCGYLTAKTAQYYRDILSKQN
LASSIFPSNQSSRDIHAIWIPELKLDLIAEGARLKKVMDENGCVNVFFGEGTGVEEIVRD
MESNGEEVPRDAFGHVTLNKINPGQYFSKRLANYVNAEKTIVQKSGYFARSAAANEFDRN
LIGECAKVGVASAIEGVSGCMGQDEEKEGLPIRPIEFERVKGGKAFDLKQEWFQIMLKEI
GQI
>comp5625_c0_seq2; 344 262 1065 LEN=1319; translated
MDEHNGSVIVIGMPKTIDNDVIPIAQTFGADTAANQGAIFFQNVVNESTASPRMLVLHEV
MGRDCGYLTAKTAQYYRDILSKQNLASSIFPSNQSSRDIHAIWIPELKLDLIAEGARLKK
VMDENGCVNVFFGEGTGVEEIVRDMESNGEEVPRDAFGHVTLNKINPGQYFSKRLANYVN
AEKTIVQKSGYFARSAAANEFDRNLIGECAKVGVASAIEGVSGCMGQDEEKEGLPIRPIE
FERVKGGKAFDLKQEWFQIMLKEIGQI
>comp5626_c0_seq1; 229 326 883 LEN=1466; translated

MNSLAAANGGGATETSASTKKIIDLSDKLEKTACYARNEHSRFPWTNLLIGDTRLGCKSD
ADEQLILHFEFSEFVRVHSIKLTEFNNGVEPEENPTRVLLFVNRNNLGFEDIDDVDPTTT
LELTAEDLKENADNLLLKFVQYQRVKSITLFIEDNNGGEVSALGGLKFYGLPVATTNMAD
FKKQG
>comp5627_c0_seq1; 461 80 1378 LEN=1462; translated
MADLDLDAIIKQLFALSSEKPGTVAELEEDAIISLCSAAKELFLSKPMVLRLNAPIKICG
DLHGQYFDLLRLMDHSGRPPNTSYLFLGDYCDRGKQSIEVLCLLFALKLKDPERVHMIRG
NHESRKLSRMYGFYDECKRRYNPELWEKFVEVFDCMPAVAIISDKIMCVHGGLSPELEDL
EQINAIERPCDLPEQGLLCDLLWADPEPGIEGWDENDRGVSYVFGEDVVEEFVKKHDLDV
IVRAHQVVEEGYDFFAGRKLITLFSAPNYTGEFDNSGAIMIVDSDMNCRFQMLKPSQKTD
RPKKGQELTPLQRLRMRSNQVKNALTAVKAFGKPSSEDILKKMNEKRPCKEASSDYRSMN
RKKSETKINEADFSNVRFTDNALILETENLTRRKTSWGDVFEDLYYTEEQIAEFRYEAFN
SEMDDDEDDWFN
>comp5628_c0_seq1; 226 1 1102 LEN=1248; translated
NPMATTPHSDLAQIHLREGDLQRTNGNYTSAVGDYSRCLEILQEHNRTPDEKGTPRNLDR
KIADTQFNLGLTYLTSSSDLQKALAGRTGKTEDPASEARKKKEAETLARDHCERGVLQQV
DCAGTFCGILAGLCGADPETVLSATRGGGSPGPKTTGLDDGGAPLLSEAATAASRRLEAW
RTAAAALVGDRFPEVAPSFGGEDHRKVLDLLQLLDEIQETIDEAERSLEGVRQAAEIRVR
AQEAAEGKASEEQKHADGSTTTIGFGPATAVAATVASGASAKPVMVVKKKKKKRTGAGAT
EDGSPGKPAAENEASGGGGKRARTDWAVEPPPESRDWNPCGTQAVLVGRMPRFWSVETPG
ARQPTE
>comp5629_c0_seq1; 511 1 1606 LEN=1727; translated
XNGQNNMISSSNSVSMSNRGNMTNQMHQIYNQMDPPIPRVIHSAPVVSGPLGGPLTSPRS
VNAGSNGFTPYNRGGVHPMPYSNMKAIEEEEFISDPTAYTSCMDTLFGSSIENQRDKLRT
KKAGFSACNPLNVSTSDEVEVDIDLQSPSARKQRSRNENGFLCTGIRGPGKKFDYSCGKV
PNHFGPHNDSFSVVSDANEKEEVSSSKSSSKSVDSKLILDVSENNNPKNESENEENTQDR
ASPKFSYEKIASSATEPDEKEAMGSHSSDGVLSVCNESYDEQSYLVDTMSVMNFKKTQCT
VNTMEDTDFPRVANAFTEGPIDFQKSHVVSKDDDHSQDNGRKPDSKTSRDGSVNGDHPND
EVDSFKTKNIGDLADSTNSTRANANSVPTLHEVGTAQSTEEARTEEGVVCEVCTEESLPG
VCTEESIGEMDTERSIHPTSCTGAEAEIDYLADDVPSFDCAYPSFDEPAEPRKGSNTTFP
LTRSSSGGENKQVNANSSPLKPCHMREETLKPIGALIAKNTKLVANSDSFSTAVQ
>comp5630_c0_seq1; 630 69 1989 LEN=2558; translated
MSPPSTQPPLFSSLASIFRDVGLYHRQREMVLDLKESLGDIWQAEDAACAFDEGKIRSLS
WPVEDGEESIDGDEDEEGDENTDDESKTSGSGQSQNDSDNDNGSDSDSDSESNTDGLEVV
FRNESPAPLVLCWVSESGKLHHFYRLEPSSLTQTVTQDARASFRRLKTDHLEHTHPGHAF
CLGYAGSDTEAEQVRSSRSFFRRDWGDKRSGSGRGCNGEGQIGADEDTSASSSIAVGGYR
PFAPSSSSSSSSSLSSVLSRRIQLVTISHVATNEGQPGARCLRRRLRGSLSCLKARAWCL
DPWRRSAMHGKRRDSDSEDDGDIETGTETHRRSLDPRGWRVEARWVRTESKPLDTTAKVY
EEVALGGWPCCLEPNWSGGDTVSARKLERDIRAAAALLPPQARAHLRKHCRIWVNRSLEW
GPSDCPVKGRGCCYHPGRAWLTRNGLSAKKHMCVEVNDAPNYRKDCDLWGIGGVMLHELS
HAYHHGMVPDGYKNKEIQECYELAMKEGLYDRVKYHSNCSSSSSSSSNGNKRSWNQYNGG
NKSHNGVIFPNSTMHCYDSGGVSPSPPAYSCVSPTRPSLPSLGYTYSSSHNGTNHEGETN
HYYGRNNSSENLFRREQQIHLEQSAEKAATKVRIPVATS
>comp5630_c0_seq2; 630 171 2091 LEN=2660; translated
MSPPSTQPPLFSSLASIFRDVGLYHRQREMVLDLKESLGDIWQAEDAACAFDEGKIRSLS
WPVEDGEESIDGDEDEEGDENTDDESKTSGSGQSQNDSDNDNGSDSDSDSESNTDGLEVV
FRNESPAPLVLCWVSESGKLHHFYRLEPSSLTQTVTQDARASFRRLKTDHLEHTHPGHAF
CLGYAGSDTEAEQVRSSRSFFRRDWGDKRSGSGRGCNGEGQIGADEDTSASSSIAVGGYR
PFAPSSSSSSSSSLSSVLSRRIQLVTISHVATNEGQPGARCLRRRLRGSLSCLKARAWCL
DPWRRSAMHGKRRDSDSEDDGDIETGTETHRRSLDPRGWRVEARWVRTESKPLDTTAKVY
EEVALGGWPCCLEPNWSGGDTVSARKLERDIRAAAALLPPQARAHLRKHCRIWVNRSLEW
GPSDCPVKGRGCCYHPGRAWLTRNGLSAKKHMCVEVNDAPNYRKDCDLWGIGGVMLHELS
HAYHHGMVPDGYKNKEIQECYELAMKEGLYDRVKYHSNCSSSSSSSSNGNKRSWNQYNGG
NKSHNGVIFPNSTMHCYDSGGVSPSPPAYSCVSPTRPSLPSLGYTYSSSHNGTNHEGETN
HYYGRNNSSENLFRREQQIHLEQSAEKAATKVRIPVATS
>comp5631_c0_seq1; 423 1 1276 LEN=1276; translated
QTITPLQFGESRPRSDSLGCEAPVRSESDERGTGRHWSNETSHRGMYPLQQVLDRHGFRG
IPGPVFPEHERSDGEVEEAKARQAGGKAPSGGGHPALVGPDLFLLLQGRLFEKNVSLVVC
GESHNDAIDVTRRSPPGRFDPTEGWIPIADSSSPEGDLDLRSGLPRVLFVSGGDGEDRPS
GPAVEEAEREAAAVTIQTEREAETTAAVIPYTETEAAAEAEASTTETTTGPTTTAEMAAT

TTTTPTTAAAAAGPTSPSLETIAIRFLYPINGKKNRPVTLGKAKELAKEFVDKYEIEEEM
FHCHLVLLWIATTATPGSSHQRVEKGQAFLLEIETEENIDYNLYERYIDAEDEEEEVYDD
WVVFPSNAAVYESEIQRRNRLALPDLVAQWMDDVMMVTNRESDFYDYDDDITVGTAEESD
DEDGG
>comp5632_c0_seq1; 342 299 1762 LEN=2255; translated
MKSNRDAKASNRRHVPHGKPLARRVTFTVKLSMAMCIVYVVWLGTLSSLHNYGYHNLHSF
RKSFGFQQSALQEFRKTKRPIAEVHVVSLLESKFEVFRHRNFDLANGGNEVDDDDDDGYN
NDIPTEQRRPKVSVRWFEALDGMDERVLDEWHQRTQLYRLNATEFAGPDAAGRKHEYASP
HAVGCYLSHWKLLERAWNSWVGNKKYQRRNSKDRYSNDGEATGGTETLSDGKRRRLRLRP
DMLFVFEDDAHCVSNLIERTWGVVQQLPKDWDILYIGGKPFSYHTMGKSLSDYVAANEAP
NNATNGIADKKRPSDRELLQRKCNGEFGTSLSGPFPSEGAQWRNAGKFTTNDSLPPYYQT
KTTLNTNAYVVNPKRIRRVLRVLSQPMYEYLPIDVVLAKDMFREFYDPVGTNSSSNSAPL
KAFLTPSMYCDQNSKRLIVDRDKPPPWEGYYWLPWRKKMKGFPDVSAFVWGTIAGLPNMC
SSLASKQ
>comp5634_c0_seq1; 522 85 1293 LEN=2064; translated
MVFSVPPPPLDDPNVSDPLECPPLKWGILGCGRVSNDFCQAMKHLPTQSTVACAARRTES
AEAFAKKHSIGKYYGSYDEMLQDPEVEIVYVGNVHSFRRSTGEKCLLAGKHVVLEKPFAC
SVEDGEYLISLAKEKDLFIMEGMWSRFFPAVQKARDLALGGEKILGEIATVFSDFNFNAA
DSEEYPSSFVYQRKLGGGANLLVGPYPIAAASLFFDGRMPEAVKAMGQVDQETGVELQAA
VTLSFSPTDTVKPLMGTADDDGLGNVYPKLPGSGVALLSFGFLCETPEETVVVGSCGRMK
IESPGHCPTKLTVEVKAQGRGQVGKNLTFEYPLPEDTEEIINSGGYFYPNSAGFCYEAAA
VARCIASGKRETPQFTLDETMLNLRVIEQIRNQLGVKAVNDE
>comp5636_c0_seq1; 251 61 576 LEN=639; translated
MNIYTITVFIIALQSAQAMVSPAVTCCEENGGTYEILDGSDGQSGFCRKDGVNTEEMTFM
YQNCPPAPMPHPSNEPTTRYSVYAVNNCDATVTANFALRLSSNGGNRNSRVQVQQGQCKY
VGPTNLDTVTYTSQGSYVSRGNDSCLSIGNSNNSDCNLMGMAENACVINLC
>comp5637_c0_seq1; 134 123 251 LEN=251; translated
MPRIDDSSDEAEYEEEEVETPSRKRRASRGVVTYNEDYDDEEE
>comp5637_c1_seq1; 375 1 291 LEN=1035; translated
EANNTEDENNDDDVDEDETDNDEENDSDSDGGDDDDDEELGTKALLGPEIRDDEDAAVDF
VPEEVDDEDEDMDDDDEDDEADEEGKKLTMTICLLK
>comp5637_c1_seq2; 438 1 315 LEN=948; translated
EANNTEDENNDDDVDEDETDNDEENDSDSDGGDDDDDEELGTKALLGPEIRDDEDAAVDF
VPEEVDDEDEDMDDDDEDDEADEEDDDASDDDQDGPQLKKQKTS
>comp5637_c1_seq3; 582 1 432 LEN=432; translated
EEEEEEEEEVDLDEYEDQDENGDQEEDAEAADEEEDADTADNAEDEEDDDEVEAEEGESE
AEGEPEEEVEAEEEDAETAENAEEEADDEVEVEAEEDDDEVEVEEEDPDTADNEEDEDDE
EVEAEEEANNTEDENNDDEDENDE
>comp5638_c0_seq1; 218 177 839 LEN=1047; translated
MEPDNSLPLQLLYEFDGKGVGSSQEIGWTNEDVMNPYNVTSNSNVEVVQFGDHDSIFDIQ
SSTSPRQITHDRISESAHIRENLPTEESRCEDSTSSPSQPKPSTKRKRSRSLVSSSWNER
FQELIRFREANDHCFVPHNYPANPKLSQWVRKQRHQRKRKEKRLHSTLSDERQELLTNAG
FIWDSHRAMWQERYQSLELFQMTNGHCNVPSNFHDSSLSNW
>comp5639_c0_seq1; 360 118 2243 LEN=2351; translated
MMQQKQRRRRRNEENAMNARSYDACDESKCVSRGLSHLYNYSFTCYGDNKDFHMMCADGY
LPQVVDDEDLVLTEGLNDGAPLLLNVFGIWGAVGSDDSNFQYFTCCPPDDLSAETKNVTR
HCSDPILGETEGDSNATFTCDEYDIQPYPRMMKQPRLYVHPDDCFTNKFEGNPYMCCDAS
GAENAEKILEDTECVPYRTESYDEAKAMNRYGGIIPITCDFPDGGFKFPRFIEDDGSTHP
SQWFHYQCCRSGPAIPPFIHDSPFKLTVYPILVLSALAAIGSLLVILGLTIPLVIQLRTE
AHRKLNQVRDTGDSGQSVIIPRLPGRSRASLYSTTICSQSSGQQTARQSQHRTTSSYSRQ
SQFSRSQYSRPSYSSYNLYLIYLAIPDLAMALYQLSMSASMINQKFNMKFHSVVVFPLLR
IFSHEIAINLAYMISAILYINALMSYEVLVLLRSFTQVRRVQPPSLTKVTLQALSVYCFA
IIMVVTDFFVRRTAGREFLDGNLHRSQSLLAWGGRYGITILVMPILFVMCVCIIIFWKGY
LPAIKGNSPKERALRELVWYFIRIVAVIFGIWLPFILLGGWTLMNQGGIGDSWYLALVQL
LGPIGSLFTTSVILTKSDVRAHTLNLITLSYLRDCFHSRCRENREHAEGGKKKEENDRSQ
XKSTVTVFKNMRTYTYEDPCSLESTDMVTLQAVAEGSDPNKTEDYNA
>comp5640_c0_seq1; 394 48 1387 LEN=1387; translated
MSSKHSGCPPTEFGGPIGAFGTVVALPLLVLILAHWARFGCLDLDFAAIFRGHRDLIFGG
EDAMELLAKCTLGLLGWFLGLVLLWKILPGKWVEGAPVIDPEADHSAASKDKAPLRLKYK
MNGHATFWTVMILLVATGFPLNDYLYRHYERLAFVDILLCFGLSAWLYRGSFQKDPLTGR

NKILSHTGRSGNAVYDFFMGRELNPRSLGGTFDWKEFCELRPGLILWVLLNIACAQEQYK
VTGSVSGSMVLLNVFHAIYVWDSMFMEKCILTTMDITTDGFGFMLVFGDLAWVPFTYNHP
AKFLVYHDPRLSNPVLACILGLYVLGFAVFRLSNRQKDIFRNDPSDQRVAHLTYLPTKRG
TRLLTSGWWGLARKINYTGDYMMGLSYSLLCGFDSIVPYYYAVYFLILLVHRSIRDDELC
SEKYGDDWNXVQAAGAVPVHPRDYLDR
>comp5641_c0_seq1; 731 113 1414 LEN=2201; translated
MPPKKYTKINGIMKLNPEYKRWKDAQNAAAGSPAPATTVVNSAQALPIVSSMDDHMTMND
DLKQEIPLAESTNATIEMLQEPEIAAEAGMAPDEMIDELGAILAKYEIPIGLTNKLMLLS
EYQSLEFIVDDSGSMQLPTDSNDPTTRRPMSRWKEAEIRVKEMIEIIAYVPFEQIGIEFL
NRKDRISLKREGRDPKSFLEDANRSIDAVFARKPAGTTPALEKLQESFLRGQGNNIARYF
FGDGRPNGGQRAIDEIIKILRHRSDPESNPMTFLSCTNEDEAVEWMKDAEEIAAFCSESD
DFGDEAREVYKDQGAALPYSKGFHLICQLVAAMCPEDLDAMDESVPFTKYTLDNLLGVVS
NDESYKHYFDNFLKAQRERVVEIDERTGQPSRLDTIRKNTRWDFNQFRSTNGPATKIAQV
GDFKRQLQQASQF
>comp5643_c0_seq1; 375 1 664 LEN=744; translated
XGMAPSPSPFAGGTAPSPSPFGGGAGPSPSPFGRGNTNANPSPFGGGGMAPSPSPFGSGG
MAPSPSPFGSSNRAPSPSPFGGNTSSSPFGRGNQMQQNSPFGGRNAKKKGPCRFFAQGKC
KKGDNCEYSHESGGGNSGFGGGFQQNNHGSSGQGFQQNYNNNNKVGKKGPCRFFAMGKCK
KGKNCPYLHETGGDSGNNGFGGGNQGFQSNNNWNSFGRRRR
>comp5646_c0_seq1; 505 129 2014 LEN=2058; translated
MMPSSLVRSAKRQKLDSMRGNPYEATGALNTNGYHYNTLLSLVDMLVSVQSSDTEDFIEI
IEKIRSHPFLLEHDSLRRNKVAAIHDYHKTVMISKLHGNLILRLGFLLASFKEVSLRVFS
SLVFCLRDLYTNDDISMENLNTEEEFGELFILIPKVVRIIDDKIDESNVXEAIKMRDTVL
GCYQIMKSWLRAPRLLKKVLAYSSTNTLEDFLLSLIIVIDQRIIKSGSGEEEIQNVLACL
GSICNVEAEFRILLDVTKSAVQLRQASPHVNLSPPLVIFFWRCISRVHSQRQTKSERKIQ
LDFADRAVEAIMPYCASEDHLLSNKATVGITELIGETDYNTRIRLITFIVESVKKKIVVD
NDGSYGKVEENEIDGNLVALLECFGLCINTENVCDYFFQIDEWEKVFDALVSISVDQKNI
YASEKSAAVVLPILNSLVTKVEQPLAPLFHKAIEICLELTHSESISIVKRTLDLLFDLVE
NPGVERRIRSASLAPDMINALALLSPKAFLLEESTKSKLAQTFALLVEGVGNIHFLARQT
SNLAFLVRLASGSYCETLQSRAQQISVSVLMKLARNPCNRRILANQPGLLSSLIRYTRMT
PEGTDLFVESKLSRKEMKDQILLVADAL
>comp5647_c0_seq1; 92 189 851 LEN=1130; translated
MAGGEEVDHFTPGAGLSAPAMIKATPLHHGPVLKLHVPVLYSILPAFFQRLILALPFLRS
FAPSWKERHLILGGSYLYKFKDPHSQIPKGSPFDVETLTTEIVGHDTSLQFPEIGNLPAG
CTGFFAVSTLRRQHFYAVSTREEALLWFRSLHDARQDSITRNMGHASNVPYPSSWKYFDS
LGKSLVKTKARIKDRMERSTFDSMEMTSFTDGPIARGYHG
>comp5649_c0_seq1; 507 92 1232 LEN=1232; translated
MDNDAINDNKNDTPPTSMIPCPDCEQFIPQQNLELHRLRSCRAGRSNGTGNDRGQDRART
AAAVTTLRTTTSIAVGEEKQGDHSDCNEDAQKRVVVTAIDSASRRHRKTRRGNTDDSNSM
AQSPPVSRAEERTGSLDQSNEIIDLMDDSESSSRGSDSLDRRMDVSYGSTPMNDHGDSMA
VNLTGIDWEDQGEDDGDRKMPARDASAHEAPSEDEALEDDAGESHHHDSGADRKPPAIDP
GDDENSSEPAENDARDQWSCPRCTLLNENTSPVCDACGYHNLEIENALIRRNNYDMLHTP
EEHPHQRPQQPLPASPSSPSHLGLIGSGALIGAAVGMAGNWLQGRGTLSGALNGGSTGAM
GGVLLREVLLSNNRDNNNNNX
>comp5650_c0_seq1; 823 283 2025 LEN=2244; translated
MARTLAGILGISGVISSSRAFSPTANTNLGHSRQRHQREAVVLQSIFYDDFEGFSDFSLN
QNSPDTESNNNNNEATPSLSDTDIFASLRARQDSLKEEQQRIEEQTEKAVEVDLIERERL
NYNWNEANCVSTVRLTIDDWIRRMAVDLYPMVVCGSSRGNLYLGNLDDGDQLDCLEGVHS
EEIHEDIPDDVAQACIQALYDGYDGNGPFAIAMKKDLVVSSGREGGIYACTIIGEEVDVA
SGSRGGSVRQTKLRLQREGKFRGLEGGTDSNGKSIPPPLITSLVFDNRGTLWAGGYDGKL
RGYNHEEFDLDDRPLMLRQKAPDFDIDVGAPILDMNVEEETGIIVVTTHTKGVFLYSLHD
GRFLFHCNPFAPSKLRQNASISQRQFARTAMAVLTNNSTTALNESDSDAVLIIGGSYGHM
YQIQLSIATDEKNPSRTLLVTSEGTTKITPKHMGPVVCLESPSPGLFVSASHDGTMRVWD
CSGRAEDDDDDNDIDDDDETRAVAEESKGPKPKMPKVLYALSGYKVWLGSVFANERKLVS
DGADNTVIVHSFDEDEDAILRSQQEDDEDNNLDDDFSSLA
>comp5651_c0_seq3; 422 83 977 LEN=1095; translated
MSESSPAVLGDAPIDESREQPSGVPTEAVPREGEENTAAFSEGANSASLAPDDDGSGGQE
HDVVDDVDGEGEDEDAKSMASASHAMDDEEIEDEEMEEDSEETGSVADSGAAEPEAENAN
AGVNAKAPNEAPAVDAPIKKKRGKPQIPASARKGRAPAVKGLNIPFRTVKKAMKLDPDIP
IVQNEAAIMTTLAAELFLKRLANQSQKICKSRGKNTIRYEDVADARSNDPALAFLERSFR

DPSKAVGFGGGTGSNTLGPEKNWHRAIPTRTNERTNRMPLFLRWCVRGMACDTEAML
>comp5652_c0_seq1; 402 119 1423 LEN=1613; translated
MKKKDKSSTNKISNNVTVGTLADAQKPDMQKWETGRGALGKGSPKDRYVYCDFAQIKEEE
YDVDYEIDQEGLLQALDESTTMAAAQSAQHQSISYLQQPPPVLRASGWVRSQRFPTKLYA
LLSQPQLSNIITWMPHGRSWKVLETRIFETSVLPVFFESDNYHSFNRVINAWSFRRKSSG
PDRGSYFHELFLRGKPHLQKYMRRLPRTHKKLAMSKSEEPDFFELEKTSPLPTLDQARAS
LESRKYLRLRDHFDKETVVQGGMADHSRSQLPAPPLDKNGNDQAKRQGSEQHDAAALQCR
TMLATKGAPPKGTGTLNNVDQFTLGVQHNVNARNGLKVPESLFRLNHQDKQLKDQLELQS
RITGQHQQVTIRQQQEQQMIARHHRAQILHQFEQEQYLRAQSVHKAELTQQQSQMNLLRQ
MDLLQQQHHQRNGF
>comp5653_c0_seq1; 585 143 2200 LEN=2640; translated
MGWFDDSDDDEDQQPREIGSLGEKLGKGTDAHNVEKKDDEEDPLDAYMKTLDKTAIRSKS
GGNTNTDDDDYNFREGDTNAASMSGGRLDVENEDEATGHWVQKKKVGNKKNSSSNNGKSV
VSDRVTDNPRGASAKHALHGTFHRASDQQLQEDQNGNVDIRLEQVQHDRMKYTHFRKCLL
PLSPQVSIHENGSNESRADPIIRNTPEGGRWREENNVTCQPPTLDPIYDFLELRDILPVE
VMTWNAMKHLTSPTPVQSQTLGVALCGKDAIVTAPTGSGKTLTYLWPIVSHLMVNHRSGD
TGSSSRALILVPTRELALQVEQIARSMFAKLPLTALAITGGNMGRYQLSQKLASTKPHCI
IATPGRLLDVLSAQQKSRQQWLLPNITILVLDEADKMIQMGFSNQVTQVLQNLRPDRQSL
LVSATFSSKLQRQCQKWMHEPFRISVGKTGESSQHVTQHVVCLPNTQAKIEFLKELLPTF
AGVGRTLIFSATRQRVEDLAADLSTIVPMETLHGDRHLSDRKAALKAFRKGDTKVLVATD
VAGRGLDIPQISTVITFDPAKNWDTHVHRIGRAGRLSAEEQQQHGSAYTLLLPSNVDFAQ
SMIRAYEKEGRPIPREVEELVAKKNNRFTWQDRISNNSQGGRMFSDSSGANRSKGNHGID
RGGRLHHNDPSENYHGLETKRRRFS
>comp5654_c0_seq1; 842 128 2341 LEN=2439; translated
MNKTSGSGFGGSASDKQKRNKGKKEARKRENGTWTEWHNELEKFLAQHRDSRTNASIDRS
VLNTLKKVLRCQAPDWWWKGLQQKMYLLAIKVSIVLTEQYPQDWGDPDDTESAIGALEEL
AEMSKILVASSTASASDASVSSAKGHSSESKTLSTKKYENNTHDNGSINAYNWIRDQTSK
KRSVKHKFTVKDMLLPKAVLQLKEEAMAASKKFIEQPECSMVGTHDYYRQKMRPLAFSMV
EGSSFESPKHYYAPGGGCKRYRQSRLPNSKGSSPSKSQSSAAILWKELSAYPTALPVEYG
SSIFVRALETELDKLRVLIIGPEDTPYANGCFFFDVIMGDDYPNKPPKVQFLTTTSFLPT
ACGNKRVRFNPNLYADGKVCMSLLGTWTGPSWTPKESTLLQVLVSLQSLILGTANPFFNE
PGYERMEGTPSGDRNTKAYNVMIRQQTLKVAMLPFLYNQLGGNVNECSGMNDCKAAPVPR
ANASIRGNHKRKSFCNASFPPNHYFSEFKEVIQQHFRIKKRSIQTQLFQWLKEDVAGLEA
LISEYWSVFNQLEEQRKKLLSASKRSRTITGTFVTASPAIPRSKGRGNKDEAIELLPDSE
DENSNSDNNRKMSVNTVELLVDSDDDNENNCKAKHVDYRKPPAATIELLVDSDDDNNYGV
DAQSVSEKKSAATVDLQINEKQKSVNNEDPDEAIELLTDSEDSSQRDGEAQSLNSKKPTE
TIEILMDSDDENPAQNK
>comp5655_c0_seq1; 608 527 2374 LEN=2506; translated
MMYCSDGADLKQQKPSKDQPIRGSGRSLDKYERFENWLRKNGAQFDLLELREYDDSTGSI
VDHGNEEHDEAEEKKEQQMPIDNDHGSCEGDSEMRGVHARTRLPPNTVCVAIPRRCLITV
EMGQETPIGRSILRSDLDLDAPKHIFLMIYLLWDRKVNGKNSFFHPYYEILPQTLSNMPI
FWSPQELEYLTGSYLLKQIEDRNEAIADDYYAICQIAPLDKICTLQEFKWARMCVCSRNF
GLQIDGHRTSALVPHADMLNHYRPRETKWTFDEDRQAFTITTLQPIQAGAQVYDSYGQKC
NHRFLLNYGFAVEDNRELDGFCPNEVPLELNLSPEDPLYQHKLEFWIRGEMNLVGSAALV
ASSRPHSAHAKRVRICVSNNENTRLLFSLLRAMNCSADELRAITSPVASDMRSLFMTEHR
SSLTSYYRSCRDIRHPLSLKNERQSMVNLLQIISVALAQYPTSLAVDKADLLDEHRYPPF
SNQRHAKIQVRGEKEVLYHFATWARTALEVLEIIEQELREEQQRGSVEVQLGKGVIVNDD
APSRRGSFDAFVRKMEQDEAEDGVHHTIVRYCADVLGSLRREELKSMRRQRATLAATSLK
SSGDAGKGGGNGYC
>comp5658_c0_seq1; 275 216 788 LEN=1007; translated
MAADPEAKKETTWDRITGPKLFKTVTNWNGIHSVPLVPLRILTGMLMIHHGSEGGIGPAN
FGTAEFQGFVDYVMAPHFGFLPGPLELWAALHDYAEFFGGIFFALGFLTRPAALSLLVTM
MSAVYFHLSAVGAQGFPLGHVSNYSYDFEEPLLYALIFLVFWFNGAGPLSIDSIIYKNIA
PEDLSDTNSQ
>comp5659_c0_seq3; 531 123 1610 LEN=1610; translated
MKRPQLRAHASLPLSVGTSALSSARKQQQGRAGRFSSLWSPVGLVLCCACSVVSFYAGAS
IGYHAGAASAGVRRPPGNDAVSPEEPGAPVYDAGELEWLRKTNKEHTHQKEKLERRIKTV
ERDLMRCHKANRDGDKDKDKDKDEDEPTNDGGNNPGPFHSGDFSEAHTGQFASGMHLVDR
EDFAGAFDTGVPLDPSGFGNDRVLLLYSDPSAFPSGGEKTSKSPVVSARDATANCNNLHI
VYTQTGRKKQCVALMGQYESYHLHRFMRVDKTEKRGAVDPSLPLVQVSRGYQQNGRKSHK

VPTVENSETHWDNLVAYLQSLRGKDNFLEATLDPILKKVASHNDHNTVVVMVCNFGQSEL
LWNFVCNARAKGFGDVLNNILLFATDKETHELATEYMGITSIYIESIFGDMPRGAARAYA
DRTFRQMMAAKVYCVQMVSVLGYDLLFQDVDIVWYQNPLEWFHDTNNPHYHFDMYFQDDG
NHSLFYAPYSANTGFY
>comp5660_c0_seq1; 397 1 1404 LEN=1609; translated
XSSDVHVGAGGLPVVANSQLQLGCVGNFDVVQKMSSVKKKSKQRGSLEKSGASKDEDSSS
PLPRIAESEPLQLPTAEMASTRIGDKSEAIVADSDEFAKEYFNIERSPIWSGSNKGWQLT
WGIWHRLPRDERKKIALEHGMKSIGEFEEYVSLQQAVDDSEYSMVELVENSSHTTTTISN
VASHDNTVHQTKGFEENREEESDEDDGDDDLLHNEATELATELTSEELVAVGGNILILPS
EILHQIVAWLPADAYGTLGLVSPHWKFFTRTEVVYKRICERLYLNQSKRRQLHVSRFGGS
YRRMLEIRPRVRAAGGCYVLKYSKYKKIERDMWCEIPIGAVLETVYYRYVYFHEDGRCLY
ALSNSPPKVMFPRILNVLLHKEETDPAIVTGTFQVQKYNCTIIAKQPWHTVKFEITIVPE
SVHGRFAALTIDRHVTSVSGEIDDWSYDLVEHEVPDKRFRFMKDGRL
>comp5661_c0_seq2; 261 176 712 LEN=1032; translated
MLARTTRSITRVAGRRLQAPSLAATRGFSDKKKDDESGATIPFGVGKWLGEKSDHLIPTD
KDHDTRPKHMPEFFPEDIKKEMKEMGLEGIEDLDELEQKNIDGYNDLGLVPPEGTGTFAS
PILIPSRRTSRVVGYQDPDSHAMFWFTIHADDNTYYIKDLGLFFKMLPIPAEEDAHAH
>comp5661_c0_seq3; 175 1 583 LEN=903; translated
FFKLTHNASKLIQIDETNQQTPSITRVAGRRLQAPSLAATRGFSDKKKDDESGATIPFGV
GKWLGEKSDHLIPTDKDHDTRPKHMPEFFPEDIKKEMKEMGLEGIEDLDELEQKNIDGYN
DLGLVPPEGTGTFASPILIPSRRTSRVVGYQDPDSHAMFWFTIHADDNTYYIKDLGLFFK
MLPIPAEEDAHAH
>comp5662_c0_seq1; 257 624 989 LEN=1985; translated
MDNSNREAKELYAENMGLRDSIQKLEEENERLKKFVEQQHQRGAGTVSAKKNNNDKTIVL
ERFEGEHLFDFDTEPSTSSLSSQAVATNEMKEELWCDVLDGDQCPVEPSVSFGEALRDRA
YW
>comp5662_c0_seq2; 334 624 1643 LEN=1848; translated
MDNSNREAKELYAENMGLRDSIQKLEEENERLKKFVEQQHQRGAGTVSAKKNNNDKTIVL
ERFEGEHLFDFDTEPSTSSLSSQAVATNEMKEELWCDVLDGDQCPVEPSVSFGEALRDRA
YWLVGMFCLRDWKYIFLRIEPVPISVLWFITGNSPLLIRIVWHRFHTGLLIMQSLSGIIL
SRNELLLANHPVIIYYLTMMVGAGGNAGNQASVRVIRGIALGTLNEQTRGQFLIRELKMA
GALSLILSAAGFFRTIFFKTPFPEAVTVTSALSLIVFTSICLGAILPLILERIGIDPAHS
STTIQVIMDILGVALAVFVSGLILDGPLGAFLLPKLGFS
>comp5662_c0_seq3; 343 624 1514 LEN=1719; translated
MDNSNREAKELYAENMGLRDSIQKLEEENERLKKFVEQQHQRGAGTVSAKKNNNDKTIVL
ERFEGEHLFDFDTEPSTSSLSSQAVATNEMKEELWCDVLDGDQCPVEPSVSFGEALRDRA
YWLVGLLIMQSLSGIILSRNELLLANHPVIIYYLTMMVGAGGNAGNQASVRVIRGIALGT
LNEQTRGQFLIRELKMAGALSLILSAAGFFRTIFFKTPFPEAVTVTSALSLIVFTSICLG
AILPLILERIGIDPAHSSTTIQVIMDILGVALAVFVSGLILDGPLGAFLLPKLGFS
>comp5662_c0_seq4; 307 624 1651 LEN=1856; translated
MDNSNREAKELYAENMGLRDSIQKLEEENERLKKFVEQQHQRGAGTVSAKKNNNDKTIVL
ERFEGEHLFDFDTEPSTSSLSSQAVATNEMKEELWCDVLDGDQCPVEPSVSFGEALRDRA
YWLVGLLIMQSLSGIILSRNELLLANHPVIIYYLTMMVGAGGNAGNQASVRGMFAVSTGR
GASGIISTYXVSLKSTKPLCFFGVLFAIHILKKYLVQVIRGIALGTLNEQTRGQFLIREL
KMAGALSLILSAAGFFRTIFFKTPFPEAVTVTSALSLIVFTSICLGAILPLILERIGIDP
AHSSTTIQVIMDILGVALAVFVSGLILDGPLGAFLLPKLGFS
>comp5664_c0_seq2; 267 92 715 LEN=753; translated
MPISNCFGSKVAVTSVLLVLGIIFDTSQSLLHEYWHHSKPLHSSYQRLDWGDSKHFTFLS
NRLETSLGIASDGYDSVEAGSRVEGDPHVETVLFVECGFGNDSHGQSATKAAVRACRNSI
EFNSIPSVSRLIPNGYQGLKLDVLLAVPPKYKDSLDLSEVQKVFPYGSVRFQIQDGGMVA
PSGIAIEKLGDQNEDMVVVCAAVTVGY
>comp5665_c0_seq1; 176 593 985 LEN=1683; minus strand; translated
MNGSRVAGHMAFWLADSLPEPNRVLFRFAIVRSLDLSEGAPFLALVGQPPNLLQGSVVVV
GLVLPLDPASVDLWWCWSLALALLELPPLLLGACAGTSARVAVPAAYVGPRQGGSRRTGG
GNLGAVAVPF
>comp5667_c0_seq1; 556 363 1790 LEN=1824; translated
MPDTLLVKKLSENATLPVRGSEWSAGFDLAASRPQTIPAGGRGIVHTDLSIACPPGTYAR
IAPRSGLAVKKGIDCGAGVVDADYRGPVGVVLFNLGTEDFEVQKGDRIAQLILEKISMAA
VEEVADLDETGRGAGGFGSTGVSDPPAGSHKKPKTGDIHRAARPVSGNRNTRRVGTRAAM
DEIKAAFGASRITNKPAKKRRTIRRPDALVLNHIVDGFADHLGRTDEPDFEFALETFDDD

FMEDNYETFDFIKDYSGYESEEDGLGRRFKARVMCDYDINNRTVVSEYFAIDNIYFEALS
YTKEGFQARNHGISWMKIYLSKQGLDQFVSKFEEEVGWGIKDSLFYTDDEQGLVSLPVSF
DEEDPPVWTNLVLPDGNGNENDEEPGFGLEEWGTVPEVMRPPIDYRSRDRRGIYLGTGIF
TMSFRVVADGATVETLEAIRDDREYDWGSPLLGRIDLRLVGTRVIREDTRVLPLT
>comp5667_c0_seq2; 457 363 2040 LEN=2074; translated
MPDTLLVKKLSENATLPVRGSEWSAGFDLAASRPQTIPAGGRGIVHTDLSIACPPGTYAR
IAPRSGLAVKKGIDCGAGVVDADYRGPVGVVLFNLGTEDFEVQKGDRIAQLILEKISMAA
VEEVADLDETGRGAGGFGSTGVSDPPAGSHKKPKTGXVSRVEKSRVDFDETRRVEFRREK
WNRHRHQIPRRILDRTVSKLGKNSNTNKLFHVTLSFFYRFLRLHLTPVQPNAMPASFSDS
IHRAARPVSGNRNTRRVGTRAAMDEIKAAFGASRITNKPAKKRRTIRRPDALVLNHIVDG
FADHLGRTDEPDFEFALETFDDDFMEDNYETFDFIKDYSGYESEEDGLGRRFKARVMCDY
DINNRTVVSEYFAIDNIYFEALSYTKEGFQARNHGISWMKIYLSKQGLDQFVSKFEEEVG
WGIKDSLFYTDDEQGLVSLPVSFDEEDPPVWTNLVLPDGNGNENDEEPGFGLEEWGTVPE
VMRPPIDYRSRDRRGIYLGTGIFTMSFRVVADGATVETLEAIRDDREYDWGSPLLGRIDL
RLVGTRVIREDTRVLPLT
>comp5668_c0_seq1; 286 110 1006 LEN=1395; translated
MDRYQKIEKPDSALGEGTYGVVYKARDKQTDEIVALKRIRLEVEDEGIPSTALREISLLR
ELTHENVVELKDCVQELGKLYLVFEFLDKDLKKYMESCSGLLSPLLVKSYLFQCVRGLAF
CHARGVMHRDLKPQNLLVTRDGRLKLADFGLARAFCPPIRPLTHEVVTLWYRPPEILLGS
QTYAPPVDVWAIGTIFVEMVTKRPLFPGDSEIDQIYKIFRLLGTPDEDVWPGVTHLQDWN
AAFPRWQKRPYSSAVTKHLDNAGMDLLERFLFYDPKDRITAKESLNHPYFDDLDKENI
>comp5668_c0_seq2; 185 1 1250 LEN=1639; translated
XYVHGIGPRKPEKNKNDCIACFLMDGPIALENGSPYKSFTAEATGPVVAMYWDPSVTYVL
ILILTISFVTSLTLTLQLIDLGFDGITMTYWVAQLMLYSCIAVASRPGNIASKNNQSTLH
SYQKIEKPDSALGEGTYGVVYKARDKQTDEIVALKRIRLEVEDEGIPSTALREISLLREL
THENVVELKDCVQELGKLYLVFEFLDKDLKKYMESCSGLLSPLLVKSYLFQCVRGLAFCH
ARGVMHRDLKPQNLLVTRDGRLKLADFGLARAFCPPIRPLTHEVVTLWYRPPEILLGSQT
YAPPVDVWAIGTIFVEMVTKRPLFPGDSEIDQIYKIFRLLGTPDEDVWPGVTHLQDWNAA
FPRWQKRPYSSAVTKHLDNAGMDLLERFLFYDPKDRITAKESLNHPYFDDLDKENI
>comp5669_c0_seq1; 314 1 740 LEN=881; translated
DQTGGDEPFPPSLPLRLPHGDSSSVRADIVLPSGACLRINRTVPAWALPAQEAAVLYLCR
KHSWPAGAVAGGRPFRGGGGGKGIDAGGVGIDDGIGVDEDDGVDEDDDDDDDNGVDNGVD
EDNGVGVGVDDIDCNDDDEDDIDRNDDDDDIDPPAFPVPDPKRWKPLGAGRFRRPEAPLG
SAMQPVGGRDPRGSGSSDGDEDGDGRFGDAAEGTSDSECECECDKGFLVDMIAAAWNKHT
TLQQYK
>comp5671_c0_seq1; 480 135 1379 LEN=1426; translated
MIAAATILCSTSGRYATAFSSRSTFAGSVIRQGTLMSSRRTYSSGTMTMKLQTAIVGLPN
VGKSTLFNALTESQGAEAANYPFCTIDSNVGLVKVPDEKLENLSELNKSVKCVPTALEFV
DVAGLIKGASTGEGLGNKFLASIRQCDAIVHVVRCFDDEDVIHVDGSVDPIRDAELINLE
LAFADLAQVEERLSRIRKEQRAGKADPAEQAALEKVCAVLETDKPARFADLLEEEELAIK
SLGLLTRKPMIYAANVADADLAEGNEMVDRLREMSVTEGAKIVVVSAQVEAELVDLESED
RTEFLESLGVSLEDCGLRALIREAYDLLQLQTYYTSGPEESRAWTIRKGWTAPKAAGVIH
TDFERGFIRAETVSYDDLIECGSEAAAKSKGLMRSEGKDYVVQEGDVMLFRFNV
>comp5671_c0_seq2; 257 1 861 LEN=908; translated
LFLFFYSFYDKTDPAGKTDFVGMDFSHLWINFFRISNFVVDPIRDAELINLELAFADLAQ
VEERLSRIRKEQRAGKADPAEQAALEKVCAVLETDKPARFADLLEEEELAIKSLGLLTRK
PMIYAANVADADLAEGNEMVDRLREMSVTEGAKIVVVSAQVEAELVDLESEDRTEFLESL
GVSLEDCGLRALIREAYDLLQLQTYYTSGPEESRAWTIRKGWTAPKAAGVIHTDFERGFI
RAETVSYDDLIECGSEAAAKSKGLMRSEGKDYVVQEGDVMLFRFNV
>comp5671_c1_seq1; 836 1 1825 LEN=2132; translated
XDSRPSLKDLPIKILLRIIRLDSYHKRESAQRETNIAGAQIRHRKQNREQQFLIMSMVYD
EYGRPFVILKEQQAKNRVKGVEATKANILAARSISNILRTSLGPKGMDKLLVSPDGDVTI
TNDGATILSQMEIHQQVARLMVELSSSQDDEIGDGTTGVVVLAGSLLEQAEILLNKGIHP
VRIARGFEAASQVAIQHLEKISEKVEFSKDYLDPLLTTAKTTLNSKILQPYKEEMSKIAV
QAILDVADLERHDVNFDMIRMEGKTGGSLSDTELVNGLVLDKEFSHPQMPTIVDNAKLCI
LTCPLEPPKPKTKHKLQIDSAEAYDELYNQEQEYFRDMIQKIKDSGANLVICQWGFDDEA
NHLLLQQELPAVRWVGGVEIEHIAIATGGRIVARVEELTPDKLGTAGKVKQLSMGTNNSR
QQDRILVLEDCANQKAVTVLVRGGNKMIVDEAKRSLHDAMCVVRNLVKDNRVVYGGGSAE
VSCSLAIKKHADTVTGVDQYAIRAFADALDDVPLALAENAGLAPIEEVTAIKSRQVKEGN
PTIGLGLLPLDRADLNVGSKTGDHTMDMKELGVFETLIGKQQQMQLAAQVVKMILKIDDV

ISHGNYQ
>comp5672_c0_seq1; 345 119 1248 LEN=1248; translated
MPEDGNANDRKTTPANDSRPGNNSEVAVVLLSGLPASGKSTLAKSLEARYNRCAGDADEP
GRRLIHIEYDELEDSLLPPNGDAEESNDTASRRRDAWNRARESALERMERELRTLGNERG
LPPSPLPPDGSGGALASHVVLMDDNFHLRGMRKQIHRLLLGYRPIRFGILHLDTPLEVCL
ERNRNRSGRRRVPDDVIVKMATVLEPPRAAWEAGSAMTIVGGSGEDPGEGKTLEDIVRFL
EQCPAIVEVPPEAPIDPEQLAADRARTRESRVHNLDRLLRACVGTVARFDKRRAGAANAA
RKKLMEDFRAGCIDFATVDSGDGGVADAFLDLVVPMEPPDANTNANTADADSTNPEEYGS
DAHAPSTKRSQLGELLX
>comp5673_c0_seq1; 212 83 466 LEN=966; minus strand; translated
MNRNRPCNVCYLRGVSIKIDGAGDGNSVKFPVDAIVGASDVWASVGAADGAADGATDGAD
VGDCVTSDGLKEGSIVGDGPKVPISFLIASSTVLQIVMRLSSMNLDCDESKSILAAPPTQ
QKGACDM
>comp5673_c0_seq2; 347 1 1479 LEN=1618; translated
XKTPIDGNIVSCPNEHRGSGVSRLALSKLPNRSSHKRTNGSPDSQVAQTCXNIMMRKFNQ
QKCCFLWLIASLLLPDLVDCMNGSITDSDKGLQRRKAQSYQLSRAYSHHQGCVDDMYSSD
LDGDMKLSEDEYVLFISKRSRGEIGADGYTELPFSLISTFIYGSCFCSYMSHAPFCCVGG
AARIDLDSSQSKFIEDNLITICRTVDEAIKNEIGTFGPSPTIEPSFKPSDVTQSPTSAPS
VAPSAAPSAAPTEAQTSEAPTIASTGNLTEFPSPAPSIFIDTPPPEPDALACVNFQYGIR
NDEGLTSADIENEIGNSYKSDLIVATRFVTIQALNETFPEDAEERALRKASTDRSPSKTH
KNRLTIVNLGRFGLDEYYSPQGERISSILAVKDEGKKFRRRTAYLPVTTENRISNMDSRR
LVFYTDSNPPVISSIFDNSHCPELRIDGIVCSIVDTKVCVTLEEGDDEDDVKEALLDGIE
KSFDDGSFASAIP
>comp5674_c0_seq1; 277 402 899 LEN=899; translated
MASQRMACLLALVAALFVVIPRAPLASAFAPSIPLKASTKALVPSGPSSDSDSALGVYNS
YGYGRDWDYDYGGGLAFGTSQYRQRYNDGYSGGVNLSHNGYSQQTNVARGGYGGLGYYDD
YGGYGGYGGRMGGYGGRYGGYGGRMGRRGGYGYDDWDDYGGYGRRG
>comp5675_c0_seq1; 488 68 1388 LEN=1388; translated
MKFASSALSLVAFAASATAFAPISRDSVAVPSTTSLSETDAMSFDFVPVRPKRVARKVEV
EKAIPKPAPKTEDPPFFLDESRTATSAEDISPEEVRALFKLWNNALATGDSRLVASRYST
KSGAVLLPTVSDTPRTDYDSIKDYFDGFLKKQPQGEILSGNIRIGNGWCQDAGIYEFTMG
AENGAKVKGRYTYTYVMEDGVWKIAHHHSSVMPEGIDIATPITKEEVQGLFHLWNDALAT
GDSSLVASRYASKGVLLPTVSDVPRDDFASIKDYFDAFLLKEPQGKILESFVTIGTNWAQ
DVGIYEFTMGATGAKVKARYSFVYTYEDGQWKIAHHHSSQMPEEVVPGPASITNDQVRGL
FHLWNDALATLDPDEVASRYSVATAPCLLPTVSDVPRTDYDSIKAYFVDFCKKEPQGKIL
ESYVTVGHNWAMDDGIYEFTX
>comp5676_c0_seq1; 187 124 654 LEN=1147; translated
MVFVTIIQDPFDVGCDEDSQLDQNVMGNEHPRDKYTKGLGDRYEIGSYNSNAASRTGGMN
QLRNDSDYNATKCYKSKTAHKTNGLREDRYSNLCDANECSRVIMTNNGMPRASLDPYDMR
ISATGHTTNNNKYSRMDRTFVDNNHCMGEYDNFKENEEVAASHIDRYECRMDKYSC
>comp5678_c0_seq1; 468 100 1419 LEN=1534; translated
MLSIGNLTASPGQKVSGNVPIQRSPSATGGATNSSNNATTPPTTAQGESENNSISLFIPM
TIVRGLNDGPTLVVLSGVHGSEYVPIRATQKLARELDPSAIRGTLVLVHIVNLPAYLGRT
VYTSPNDGKNLNRVFPGSADGSPTERIADFLVNEIYPLADGLLDMHSGDANEQLGPSYTA
YYGKAGSEDIIQKSKDMAFSFGLGLVVEFQWELQGNSSKVSTFSGAIWAGSAAVVRGIPS
IDVEVAPGMGAAPSGSINDVYQGVQNVMGHLGMFLEENATTSNDTVEDDHPCLVTNRRFI
DAPTEGSWVALVNTGSFVKEGTLLGTITDFSGRNTIFEAKAPCDGLLLIRFETPPVLAGD
TVAVIAVLDGDSCDQLRSGGQFRLDPSKVGALVLWQWSAAIGWLVALGLSIIFRRSRRNG
LYREQPAHQAVPVEDGSMT
>comp5679_c0_seq1; 300 268 927 LEN=1858; translated
MPVDIISPDPGKTSPATVAIDVTASYPNGVRELKQLLQYNEQLYMPEELMMANDSIKVWN
LDDDDDEDENPAEKVQPKMFRNDPFRVSQEARRMLINRVSIEESTDGIHQDHRLAAAAAA
AEMVVEETKEDLMSPLYIPSEPIAPAPEIPRRVSSTLPPRRPSQSPVVLDRGTTRTVVLD
NDDLDIGRHAQPGAEEPNPRPSHRRRGHRRNHSHFDFQF
>comp5681_c0_seq1; 306 653 1708 LEN=2130; translated
MWEECSWKVSSLSEIPLDYPLERTRRGICNASITEVVNRISKCLRLLSIEAKFENEYGTA
KCITSDMVSFHIQLYAGGFKNQESITVELQRRSGEPRYFLRVCNKILDAADGGQIQEETI
PARKKVPLCIMKMPVAKMKCIQGVNKPDHSQEPRRCMNRSLQILRSKEKDFDVEDFEVLC
FLTDPLKTRADIVTTICKGILQDHHYSFIRDAIENALETDVVLLEDYESRGRLDAGGKLR
RLATILMSNVLAQTSSDGCLANAIQNEKWFADFLIPSLSDEVKNFEVSSNNAYEAISGLN

HMAACSEIARHLIKEECSLDDLRSANDFAVHNHKLLASETKLSLGILGYLI
>comp5681_c0_seq2; 403 149 1471 LEN=1893; translated
MDFNDTNTNDVTETGGTANVLFGMKREGLASMEETQPPLRAVSLLDRTTTGLQCSTDSKN
EGKEYTKNSKRMSLESKSRLTSNRNGAKDMWEECSWKVSSLSEIPLDYPLERTRRGICNA
SITEVVNRISKCLRLLSIEAKFENEYGTAKCITSDMVSFHIQLYAGGFKNQESITVELQR
RSGEPRYFLRVCNKILDAADGGQIQEETIPARKKVPLCIMKMPVAKMKCIQGVNKPDHSQ
EPRRCMNRSLQILRSKEKDFDVEDFEVLCFLTDPLKTRADIVTTICKGILQDHHYSFIRD
AIENALETDVVLLEDYESRGRLDAGGKLRRLATILMSNVLAQTSSDGCLANAIQNEKWFA
DFLIPSLSDEVKNFEVSSNNAYEAISGLNHMAACSEIARHLIKEECSLDDLRSANDFAVH
NHKLLASETKLSLGILGYLI
>comp5682_c0_seq1; 654 98 2176 LEN=2357; translated
MEIYDDDDVDRRIRPQQVRKTELKLTNGDDETNDTKMNKRRRLSKKERKDRKNLQKKNKM
EVKSLQTNGHDNNQEDVEGEEERIRVECMETYSPIDIPEEPPMELASQGEKSKVNNDKKI
ANDEGECRTLGKWFPNAILIKSISYTNTGQFITNKNCQKENRQMKANEEDLIVDNPRSSL
VLFYQYTTNDGSSDVTNNERNKKWDRRQLQLLITYLSTIAHRRNLGGRIRVATEGVNATI
SAVDTTECTAKAALRHFAEDLRRFDPRVFSHTDFKYLDDLPADRQFKEFKIFPVQELVFY
DIGETDAPLDSDKLVKKYGNNSNSESEVSGGGIHLDASSFHEMLQKDNTVVIDVRNNYET
ILGRFDGQQRQKEGPTEKIDSRGCSSSGTNGGAEYIDPKMRKSTDFKSWLAKPTTQEKLG
NKTVLMYCTGGIRCERASAYLRSEMKDKVKGVYQLKGGIERYLKQFKDGGFWRGKNFVFD
KREAVGVDNPHGDGGILRKVERRQRKMHEKKTEGSPLTAKCCVCGDSWDRYVGKKKCLTC
GVPVLMCEKCMSLKPDKTPGMELKVRCPLCVEEGVTVLAKELVFTANGIKNKIVSSAKAA
ISGNSVEKVSGDYRNKCEIVDKEYESKKNLGGTGKAADSVLKWGGGHAINKKKLKKMKRR
VCQFGAECVRKDCFFYHPQRESSFKATTEGQK
>comp5682_c0_seq2; 281 81 1378 LEN=1559; translated
METXPLGSFNLHNNRVTDDLPADRQFKEFKIFPVQELVFYDIGETDAPLDSDKLVKKYGN
NSNSESEVSGGGIHLDASSFHEMLQKDNTVVIDVRNNYETILGRFDGQQRQKEGPTEKID
SRGCSSSGTNGGAEYIDPKMRKSTDFKSWLAKPTTQEKLGNKTVLMYCTGGIRCERASAY
LRSEMKDKVKGVYQLKGGIERYLKQFKDGGFWRGKNFVFDKREAVGVDNPHGDGGILRKV
ERRQRKMHEKKTEGSPLTAKCCVCGDSWDRYVGKKKCLTCGVPVLMCEKCMSLKPDKTPG
MELKVRCPLCVEEGVTVLAKELVFTANGIKNKIVSSAKAAISGNSVEKVSGDYRNKCEIV
DKEYESKKNLGGTGKAADSVLKWGGGHAINKKKLKKMKRRVCQFGAECVRKDCFFYHPQR
ESSFKATTEGQK
>comp5682_c0_seq3; 531 98 2261 LEN=2442; translated
MEIYDDDDVDRRIRPQQVRKTELKLTNGDDETNDTKMNKRRRLSKKERKDRKNLQKKNKM
EVKSLQTNGHDNNQEDVEGEEERIRVECMETYSPIDIPEEPPMELASQGEKSKVNNDKKI
ANDEGECRTLGKWFPNAILIKSISYTNTGQFITNKNCQKENRQMKANEEDLIVDNPRSSL
VLFYQYTTNDGSSDVTNNERNKKWDRRQLQLLITYLSTIAHRRNLGGRIRVATEGVNATI
SAVDTTECTAKAALRHFAEDLRRFGSICFELKTIVIFIGILEIXPMNAFPSLDPRVFSHT
DFKYLDDLPADRQFKEFKIFPVQELVFYDIGETDAPLDSDKLVKKYGNNSNSESEVSGGG
IHLDASSFHEMLQKDNTVVIDVRNNYETILGRFDGQQRQKEGPTEKIDSRGCSSSGTNGG
AEYIDPKMRKSTDFKSWLAKPTTQEKLGNKTVLMYCTGGIRCERASAYLRSEMKDKVKGV
YQLKGGIERYLKQFKDGGFWRGKNFVFDKREAVGVDNPHGDGGILRKVERRQRKMHEKKT
EGSPLTAKCCVCGDSWDRYVGKKKCLTCGVPVLMCEKCMSLKPDKTPGMELKVRCPLCVE
EGVTVLAKELVFTANGIKNKIVSSAKAAISGNSVEKVSGDYRNKCEIVDKEYESKKNLGG
TGKAADSVLKWGGGHAINKKKLKKMKRRVCQFGAECVRKDCFFYHPQRESSFKATTEGQK
>comp5683_c0_seq1; 173 379 1101 LEN=1500; translated
MSKAGVDGALIVQPINHKYDHRYVTDALKGHPDKFKGILLFDPSESDPDAALARFEELVL
QGYVGVRFNPYLFEGKMCENEAAVKIFKRCAELKFPVGIMCFKGIDLHYEDIESLCDTSP
ETVVVLDHFGFASVENQDQFEKVLSLKNRNTVVKISALFRLGDPGAPKYERVRTERFEPL
LEAYGPDRLMFGTDFPFVTQEEIGYQGAVDTVLSWMSSPEEKSAVMGGTAERLFGVWGVN
>comp5684_c0_seq1; 226 1 871 LEN=2028; minus strand; translated
XMAPGAELTHAPMEPDMVPGTAYFEVMKDIAHTLDFLMPQYYNGYVRSYVDFAGALSHFT
TLANELFEGDASKIVYGFCINDCGSFNLDGFQSAEVMKWLAGEYPCNGGAFFWVANDDFN
GNWSTEVNNQIASDADLCLDQSPVDPVPAPVPNPTLSPATFSTQNPTVSPSPKPTSLPTQ
SSTPYPTSAPVVNPTNQPIPSPPVSIGSAGCCSNDYKTCASWCNESRQKCGSPMCASMVW
LENGSLSSDTCVSRWGACTNDVSSCCGGLVCKGESRWYKQCLAPNDPAPN
>comp5685_c0_seq1; 221 144 1220 LEN=1809; translated
MPESSTAGNEMPRPLRIVSLIPSATDICVHLGLGDNVVGITHCCDAGDVPSSALVVTEDQ
VHASSNSQGDIHAKVVENGQKAVETMETNTRDASDPAACSLTTEEIPTLYPINKELLKKA
DPTLIVTQDLCHVCAPSSATVFRALKEAGIDARVVLLTPMNLGDVVENMQQVADAAGISR

RGKELCDELRANLRLLGDTVRKHRKPEQANKKVLVMEWVDPPFCGGHWIPDMARLIGTEL
AIDPNPDGRSRRVTWDEIERADPDVVLVACCGFDLERNLDDASSSGKHFGRLRSNREGNL
YAANGDQYFARPSPKLLTGAVVMALGAYNERDEPDVVRAIRDLPFSSRAFDSIEAMEF
>comp5686_c0_seq1; 793 271 2039 LEN=2194; translated
MTGKTEGVKDVXKKDRKLNRRSDNSDDERRHRRRTSSRTEKKMKKRRKKSSKEKRKKDRK
RHPDTDESSLYSSNNSDSDYSRRRRHRRSKKERKRRRREDAEDNHKTKKSRIDTKEKSRK
ESSSSTTSSAQTVSLALHNLLIEKPDFGSELPLILIRLAGGTTFDLGQVTDPSIATGLRS
VFEALEIYGVKKQQSSGMWMFQNPPCATRRDELVLLKVIRSLLDEIGLTMEELAKFENKQ
RVEKEEQKQADETLKPDERKKMKEFTLKILEKFLLKDATLGSQLATLCMTIVEGESVSVD
GIPDENLKTALESLFEKCGLEKSEIEEDDEDDDDDSDESPLMGYGLPDSSDTDNDIVQM
KLAIMMSACREGPPKRKSIGPTRRPMTAEEEQALKKMYPENNRKEEEDNDEGPLLPGEVR
KAPGPAMSLEMIKAQAEHRELQLKATASGVPMPTNAGGREEWMIIPGEHDFLSGIKSGQA
IKSRGFQNKKSRDADKKSQPIHPAVQAEMDSIMQAHQDARGPSLMDLHRSKKSQEKELAS
RANNGKKEWKWNRDKDLDAGRRVDKDALGMILGGAAGDLKSKFAGGFNR
>comp5687_c0_seq1; 1006 84 2600 LEN=2732; translated
MLVDQGFSPAYDSSLNGSKSMPSREERKKLRNKRRSKGAHNINGKDNRGSADNVGSSFSP
SMDTEDRYIALLNRNEDELLKFVAQVNKVYQDLLKRPAPFMTFVFCGMQSAGKSTIMERF
LNSVLNIVQQGTGTRCPLDATCIHDTTCNTAVCELYGEELPEDERGDQLTVNDVFERITN
HNKKLGDEDRFSTEPLYLNYRSANVQNMRFVDTPGIISNQSTGKDNREDIKSILRSEMRK
PNTKLCVLLEPKEFATNPILDFCDDTLGGRKKWIDDATFLMTKFDKQLEDARTSTKANDF
FDEFFLNKCRPHLVITPTLDREDLPPDKLFEERTKLIQKADVYENDKFEQWREGHERYCI
ENGGVEDPLHIDIETKIGFPSAKKVMREIMLKDTIERLPEVISSLRNDLDKYNAEYKNLE
DKMKFNDPQNLRNVVTRMLFQVQDRILNYLDGDLELSLKFPDKLQTLDDELDEEEDSEWS
TRSLNHHSEKEDHWRGRVSNLDEFPDEIYAASRFLGGKQYQRALEFFRVIMIDALPDPFQ
LRDKVANLTGFLSGGLQQENWERAMVEITRVCLKDVSHPGLNYVIKHIGSIFRRLFPIAL
QDVKQGERFSAEFKQIPAAVERFLQSEFDSVLWNLLKNASNEVHSSMEPMYSSIDPNLPT
FHCKRLVNHQQKDLFVKKDQKFVSVDEDREKQFENSWLSSFKHRAVALINNDSNNSAKLF
LKDENRKRATTKKSFLPDERAAMITKEETEMILQRSFEYIVGLMEFNLIVFRFQLNYHLF
EGFKKAIKETLLTKVNDADWDELVRPDSTIGERLEIVEKQRQGLQKSLDMVLRMQRII
>comp5688_c0_seq1; 55 1 322 LEN=965; translated
XNKQSFLDKTQIDNRRIRSIKPNRIVGNRNRNRMNSRSLGFLPRGGPAVAMGIASLTFVG
AVFYSHDSQVRDRETMKAGVERDKERIKQKRRAKQQQQRPQQSLDHE
>comp5689_c0_seq1; 185 83 394 LEN=787; translated
MIPKINKIFSDKIPELDEKQEDLLHQVKNVRTTPRDARFPAQNQALHCWNRYNEWLVCLK
QSGDDADDKCQVVYRQYSTSICPDAWIENWDEAREENRFQGIKI
>comp5689_c0_seq2; 185 180 491 LEN=884; translated
MIPKINKIFSDKIPELDEKQEDLLHQVKNVRTTPRDARFPAQNQALHCWNRYNEWLVCLK
QSGDDADDKCQVVYRQYSTSICPDAWIENWDEAREENRFQGIKI
>comp5690_c0_seq1; 697 1 4075 LEN=4181; translated
XEEEKAPFAFSLQSDFHGNAGMEHSTGIATVQKLLPAGSKVLLLEDFVKEYAPACVVSSS
FPEAAPARTRAETIPNRTEEMTTSPVPVPRIPSIPEQLRRMIIAPLPTCKVLRTNGPRSS
KAEKISLCRESVRTLVYDVVYSLVHRRERLQRRRKYQRKRMHSRRNKPPPPPSVGAAEMQ
VVNGNCTRSETNGKNENTSCRVSSSTASKVQSDISNGSDRFESDDGRNVLARGFVVSREG
DGPPPTGMKAGISYLHPNENVTFCRSSPVIRRLHELVGDEVLRTILLHTRCFLPIVGDCH
ADGMAKSAVARKGKRNRKRRREGRDVQDRDNGDSRNYILLCGPPLPSGTNHPLCKTSRDN
SRCLQNDLPKRKRKRRRCFPSNALSLRNGTPGLHLHANDIISRYSLMYCDAYTPHVALPK
SHPFSQRIENQPSHPHPNGDTDYRFVLNVVFDLHLENKNKQRRIRKRLEGNGRAVCEKLW
KNYTRCRNYGRLLERYCGLPNDYLEETTASRTSSSSPPSSRGSDNERLARLARAFTPKDG
VVSFLGSVLRQVFPAGFWGSERNFQSVMASVRCFVRLRCNERLANKNILHGISITELEWL
YPTTKHQEPRAAGENPLGGANESNDRQTHSRCRGKNKSGRKQRTNHEAATELALKALRWL
FRGFIIPLLRSSFYVTETEFSAKEVQYYRKPVWSLFRALSVRKLTTAPGTSGHSRHFSIL
PKGQAHLNLRGQTLGLSRLRFLPKPTGMRPIAQLSRNVPIRLSLPTTAEATAVVTEGRFY
RKRSLASDSQGNDSRPGKRANTGSSTITATGNNVMGPDPVHPSTEPKPSEFSPSELSHVF
SRLPTNVILERVLHVLTLECGRRKRPYGNGMDNLQEFYTRYRNYVSHIKQQFLQEGSGSN
KMQLFFAKVDIEKCYDNINQHYLLNLAQDLILNNTYVIQRIKLDSANFGGAENATRFQTL
VDSVDDYQSFHSNKHALARRSRSTVFDLTKCTLTERKKILDLLREHLLQHTVVTSGRYGR
KLLLQRSGISQGSTLSMLLCNLYYGQVEKLMFSEDGGHLPWSNSTANAKSTPTEPAMHLI
SRFVDDFLFISPDKAAFQGFLDRTHEGITELGARVNSKKTLVNVRSFVQRNADDGRTQSI
SLSVSSKLNRSGSVLFPWCGFLFDTETGEVFCDYERFQGGKLRDGITIDYDGNEGRKMEF
RVRTFVYPRCLPILYDSSINTFETSVGNFYQMMLFAACKTAEYLRGHITHNVDHVVRCIR

GLPQYAVWSIRCRFEKSPGTTQDFGAVFAINGHLASVLSWKAFCDVFSYLQGFAVITREL
REEASSSSRTMTVRKRQQVQRIVSRAFEDFQIRHMIEK
>comp5691_c0_seq1; 663 146 1972 LEN=2272; translated
MAESRDTAGSSSAGKQRRRVKGSGSKSSSSSSSSSSGCSLCPGEIVCTDRELRKSSRSTE
LALLLGAAIVGLYLFGFAELMQSMPEISSRISHYRLGGNLNLAKMEIDSGMAGGEAWEED
EDQDGTKKHKYKRKKKKNKDKKNKNKDNNDNNASIPTGVWPVSVRDEEFETMIHVGDLET
VMKVPRFWSPPVHNQQRFTREQARKIGTCAVPDPSNGSSARGEDCPPDERTIFVGIASYR
DFECRLTLESVFHRAKNPRRVRVGVVDQIVEGEDVRCDEPVKPCGEDPQQALCKYRDQVD
VYTMDAPLSVGPVFARHLGYRLYRGEYYATQSDAHVTFTTDWDADIIEQLEATNNDMAVL
STYLTDVQGSIGPNGNSLRNTRPIMCNTYYEGGPQGLHLRHGSQPERTPAIHGTPQLQPW
WAAGYSFSRGHFLVNVPYDYLQSMIFQGEEMSIGIRGFTVGYDFYAPERSVCFHHYATGA
NTKVRKKVKHFWENGSRYAGTGKKAMKRLLGIVHMNPEVDPSEWDHTDVDRYGLGAVRTP
EQFYSVFGIDVVKKKTQGHLCEFVSERARMHKMFTPKLRSDGMGIDYSTIDFRWVDPSPE
TRETRDES
>comp5694_c0_seq1; 765 141 2091 LEN=2492; translated
MRTSTAVDIDTTCTEDNEKNAIMAIQCRQNNEVFLRRKFGRVAEAVIVAAGAAIFLTCSL
NCCTGIHAFSLRSAAKGSTARVASTFRNDDDHLLPGIHSLPFATTTSLQTYYTTNSRLWA
KEEEKEDDRGDAELYIVSSASKNVTEDPSVPFETPMVDDDGIVTNRKPTSGPARTGERDV
TIRGDRDEVGRNPVPGAAIKRAKNEDLDYVTIAQENPSLETDLDKTVNAVFGAIAFCQKI
PSKVEEAIEKTQQLPGKVTKSVQETKKSVEETIETTQKVVKTVQEFPDKVTKSVQETKES
VEETIETTQKAVKTVEEFPGKVKESIEDTAHDVKVLFGLERPSLKKRMQQQKIPMTAKEE
ALDLAGKATVATAKVAIWAGKGVGSMAWKGTKLAYNNTIGPAVEETWNEQVASINNAINA
TTKAIQDVPSKITSPSSEVLDKRKDPKRPSFTRKRERKETTIVPDSVSKIITKSPAQLQK
EINDAQNLAKEISAALDAAERAMKMNPNRELEDPMIEIGEVSPAESTSKAAVRPSTARNN
GPFWNRRQKQNKTPSIDVLARLNVAKTQDQLDAELKEEQALEKEIADALAMAEQALMTSA
PANSGTSDGGSGDSKKNTGDHHRPIPTHQEQQNDPIKSLPSASATARMV
>comp5695_c0_seq1; 281 62 1192 LEN=2163; translated
MDENNEPEWESASNDQAFREQVREVHARETRGGAPQASEEPVSPAVTSVRWDLGEDNSNN
QNGNDVSFLTSETQNIPETNANASGRRRGLASSLCSNCRSKCSLRYFLHVWDIAFTSIWI
LFGAYHLFFNKNNESSRSTHMILLLVTCVLLAVLNALRGFLWIWTSVPSLYTICFCGCCY
YSEDELADIASRLATNLTLSLGILYGGIAGVAWFGPDAWLPWCDFFGSWCHKLIPTHTGI
PIALTAVSIIELCRWIFLQGQLSSSSPVRGADYYNDDVASSHSQSSRHRPWWFGRQSNPD
GDGLHEPLLEQDMAVNGQPSWAVTSWFRSPRNTNSNTSNAEDGVINGDEEDVESVLDSLG
EDWASRAESDPYWWTR
>comp5696_c0_seq1; 461 1 1729 LEN=3399; translated
XKRISGETIPPSHYMLPISSKQQYQHAKRRMPGPLGPAAILAGPGAIAAAKHLAVRAATS
PGVTSTTLTAIVFYWSFQKLPDWVKKDICFQNLLKNRENIATEELEGLYSVVDKLQNVAS
NIQELDTNIPQLHAALLAYIQLSGQIKIQQVRDYCSVPDDDNTCTSQVTTAITRDNLYET
AGRPVDLFTLRSLEIRGALQFATFAYYEELETLSEKLHKKDYTVLHHKLVDIRPGRVAHY
IAVSPAERNVVIGLRGTSTLGDLLTDCCGHAVPLVDDDDPNADKLRVEIQAEVPNIVLAR
SDEQTMEVISGHERIVLENDDDDGDNYTRCHEGILISARNVLDEIESYLKPLIECNYKVI
LCGHSLGGGTAVIVATLLRSKYPDLCLDPENKIHAYAFGSPPVLDHDSAIAAVSYCTSII
NNADLISRLNITNLAVSLACLRKIQSKLVENDLSPTGPLTSVAFFNKIAEGPSGTPLMTL
LELNETIDEAHNNIALRKPEHLFVAGRVLLAFNPWLDGPEGCPDDSTTPNIATNLQCVET
TGTSPVFQRLEIDGLRCFTNHLTSSYYEILGMKYEF
>comp5697_c0_seq1; 426 797 1756 LEN=1926; translated
MVDLAGCGTAATENWRNQKLPLAKIKKIMKSEDLILQELEKDRLEKKAAEEGQPMPSSNE
KTTKFMISGEAPVLMSKACELMIKDLSFRAWRHTERNRRRTLQKQDLHAAVGESEVYDFL
IDIVPRVTTTTPKMQHAHTTHLSSSSSVPGTATMIPPTMGMQVATGMPVGLPAPAMPQQT
TQSTAVIDHANAGMMQLIGTAHPLQAQQHAAQQQQTTQFHHDPSQQIVVGTQQQPQTQIP
QPMHAQVVQHHHHHQQMQQQFAGMPQMPSAASQSPAGAPITLPQTGGVNTLNSTAPSASS
ATAAAQMQAQHWPGGVDPP
>comp5697_c0_seq2; 455 545 1570 LEN=1740; translated
MAGLPVLAGQQMDEGLENMWHGSADCIRAIDAATENWRNQKLPLAKIKKIMKSEDLILQE
LEKDRLEKKAAEEGQPMPSSNEKTTKFMISGEAPVLMSKACELMIKDLSFRAWRHTERNR
RRTLQKQDLHAAVGESEVYDFLIDIVPRVTTTTPKMQHAHTTHLSSSSSVPGTATMIPPT
MGMQVATGMPVGLPAPAMPQQTTQSTAVIDHANAGMMQLIGTAHPLQAQQHAAQQQQTTQ
FHHDPSQQIVVGTQQQPQTQIPQPMHAQVVQHHHHHQQMQQQFAGMPQMPSAASQSPAGA
PITLPQTGGVNTLNSTAPSASSATAAAQMQAQHWPGGVDPP
>comp5698_c0_seq1; 328 1681 2562 LEN=3093; translated

MKFQFVGSFPTLVVIVAALFLGGNPLNNVFAQIESATNSAAYDKNACQPGSHDVPNPYPA
ADLVAPDHFYVKMPSTVSDGIPIIFEVNRTWSPFGADRFFALAKDNYFDCAAFFRVIPSF
VVQWGIASDPVETVKWGTAIPDDPVMESNTKGMVSFATSGPGTRTTQIFVNTADNSFLDT
QGFSPFARVVQGMEEVFLGNNMYVADPVPNQFTYESEGNAWILEDYPAIDIVKGLPQGDS
VTNTTTEVMLTLEEKPDEEQEELEENPFESDSSSGSSSSDISSDASSSASPSLW
>comp5699_c0_seq1; 389 145 1008 LEN=1377; translated
MSTNLNYKQVANVSRRTWDVEAYEKRAKDRAKNTDANSKEKRKVFDSTSKQVERDGNVQE
EFVPAAAGAAGPQLSERAFLKARTSKVSILDAKIGSVEIVNPEAVATTKSMVENGECIDK
DSAVTKSGIGWHCKVCDCFLKDSLAYLDHINGRKHQRNLGYSMRVERSTKDQIVSRLAAL
VKAKERNKNSGLKKPMDAEEKDFNEIVNTKDEELRRNIEERKRARKERKKKKKERLKAEA
EMLGHKSEVSTPEDETLAVEERTGDGVDPNIAMMMGFSGFGASNNIR
>comp5700_c0_seq1; 477 32 1000 LEN=1081; translated
MTFFKKAAILSTLCSSSVAFTTLGMSKQSATSLKSAVVDFSKYEGLGNDFILVDDRDKDA
PSLSPEQSEKLCNRNFGIGGDGVIFALKPPSDDYDFSMRIYNSDGSEPEMCGNGIRCFAQ
FLKDLGADAESYQVNTLAGRIIPVMNDDGTITVDMGEPILKAADVPTTLAANFEDDISVV
EQDLECNGKNWKVSAVSMGNPHCIIFVDDLENDIDFESDGPALESCEAFPAKTNVEFVQV
MSDTHLKMKVWERGAGPTLACGTGTCALVIAAIQAGKIPSGNKVRVTLPGGDLFIEWNKD
TDNKVFMTGPATYTFSGKAALK
>comp5701_c0_seq1; 789 1 2249 LEN=2614; translated
XQTATPHHITTQHTTPLQSTNRITHQPEPRVILFEAYNPKQNTKQSTAQRERGGTPIILH
DCTGRNTRAPPHHPAMVSLSHSPGGILRVLLAASWLATVVLGQDRTLYTGWTMKFDNTPY
PTQTAFVGDKLTFIWTIDNLQNVFIHPTNDCTQTGRIAIGNQSPTEYVFQPEDAGKSLFF
ACDIDDRCEEYGMRLIVNVISLDNNNDEDNTPAVSLTQEPTAAPEFFKFRTQSPTFAPIA
PSEPARTPEPTIAATDFPTWAPPVVPTDVPTVSPTPDPTGFPTARPTASPTDSPTVSPTL
TPTTPPNPLPTFPPTDRPTTLEPTVAPATPGPTVSPTIADNGSDGTTRVAMRGLQMTLTG
ADAFSDETQSAWRGETEAFVTSHYESDHRGISFVTSIAVTNHEALSPSRFRQLVRGRGDG
TPNTRGGPNDRRLQESSVVVTYDQVVSYRDASGKVFSEAYLATSAFAFSEQRANYVEGLR
ATGDPVLEAVTDSSPVTIGSKTTPTEAPVSTPVPRPSPTPKSGGLSTTAIIGIACGAAAV
LVLAALVAIYSRGGSGDPEPDEDGDSPPKKISIKEDEVSTLAGPTITNPNTGAGERSIGT
VDYDYSKAYGNGEHSLSSAGGTFGSNTQNISFPMNADATGAALGPYGEDGTYENQFRPGA
KVKEEILTIFAPPGKLGVVIDTPDDGAPLVHAVKDTSVVVDKVKVGDKLVAVDDEDVRTL
SAIKVSKLISRKSANPSRKLTILRTTQIG
>comp5701_c0_seq2; 282 1 499 LEN=864; translated
XHHFCLPLACRSRSIGTVDYDYSKAYGNGEHSLSSAGGTFGSNTQNISFPMNADATGAAL
GPYGEDGTYENQFRPGAKVKEEILTIFAPPGKLGVVIDTPDDGAPLVHAVKDTSVVVDKV
KVGDKLVAVDDEDVRTLSAIKVSKLISRKSANPSRKLTILRTTQIG
>comp5702_c0_seq1; 200 1 1136 LEN=1328; translated
RQSEPTPRKRRKQNPGQPKQNSSSRTYRTEQTMPPRLRSRETKPPRFRNTDASIRAILTE
SRTVALVGASNKKHRDSYEIMSLLLERGYDVYPVNPFLAEAGETIHGRTVYASLGELVGK
SADKEHPGGRARRGRTRTIDLVDVFRKSSHAGAVVDEAIAIGARSVWLQEGVVDEDAALR
ALSAGLRVAMDVCPYHELPRLGIHGPASAGERSFXRQREQNKTKQNPPTHATTNHGAASR
ATANAADPEREPGKGTSSVRGTDPANANEKKENNKRKRTGTWLRCRQLTGTETQPTTPGR
NATGDCCLQTPQEHLLRQRDAQNGNVTEIECCAMASALVPARRQQCGREGGFSHGTDLVW
FRNTRREAQCNAFELNAK
>comp5703_c0_seq1; 328 377 1279 LEN=1512; translated
MISFQESVETLFTGIFGCGRSCNYPDFSDYSDCKENQNDVQIRFSSKPEKYLNSSIMITN
EKNCSILNETATTCQDSFLNESTSSLDNNGSVVIARLPLQPQQEPPRVRLHKRELVAIPP
GGSMKSWSPVMPHSVLADPVFVSKKEHQRARMPIISPMAPPKIQRLTLDDAQAFSGHPRR
QRHDNRTPSTATSRNNSEGMMYDIDGLPVEVPEAFSRSRSLDFLFSFPSLSHKENGPLGD
LCIVEECDRHRDADRASYPSSWHYQKRGMVEDTSAYPYPHKTRKQDESILSHLNSTHITL
>comp5704_c0_seq1; 241 95 867 LEN=1192; translated
MVSLLQKAVLLVAVSWRSATAFVGPSTALKSKSSASFDTACHETLTVPGMWGSGLNFGKG
DFKFYRSFDSFMSPFTDEDKAEYPEVFNLPKGVFEVSMTKPLGIVFEEIEAGEGVFVQDI
VEGGLADVQGKIQPGDVLIGVTAIKVVGGKNVIPRDRDDNEMGRMRWLMNXLFSFYLLLI
SVSCWAILKAKWERRMIPARKFDFDTVVGAIGSNEPKWSCSDVVLMFERPSECDSAEVDA
FMEFFEPPFDNPWKQQQ
>comp5704_c0_seq3; 303 95 745 LEN=1070; translated
MVSLLQKAVLLVAVSWRSATAFVGPSTALKSKSSASFDTACHETLTVPGMWGSGLNFGKG
DFKFYRSFDSFMSPFTDEDKAEYPEVFNLPKGVFEVSMTKPLGIVFEEIEAGEGVFVQDI
VEGGLADVQGKIQPGDVLIGVTAIKVVGAKWERRMIPARKFDFDTVVGAIGSNEPKWSCS

DVVLMFERPSECDSAEVDAFMEFFEPPFDNPWKQQQ
>comp5705_c0_seq1; 241 203 1135 LEN=1885; translated
MSKKIVTSAASLCRNQCQLSRLFSDALSISTSRSPSLSISSARFSSGLPPPVATTNNTRS
LLSRAAFGANTTDNNDWTTTIRRSMATNRKVIDAKNARRLKIQQKKKKNANKPGAAVATA
EDASGETADKNSPFLQHQEWVKFQQSITVEGFQTGQTTTATALKKSRGGKQARRKREKEL
ARLGALSDPSEENISAVAQKFPAIRYSPEETEELLKQAYATLPERAGKRGNRNLRRQAER
WRRVRKIRSDYKAHILEAHHRRMEARSYKRERTKAAREEAAHLRQKDVEYQGAVLRRWAE
TMGGSGNASA
>comp5706_c0_seq1; 574 167 2192 LEN=2741; translated
MISEKRLFLAAAAAVLAANNRCLHTSAAPLDDLITELPGFGRTPTPQFSGYLDASKGCDT
KVNGPVCKIHYWLAMAESEGDSEGASDKDNNKDKDKDPPRTVLWLNGGPGSSSLLGWLQE
VGPLMLNASGGLMENPWGWTKAGVNLMAIEAPMGVGFSYCSRELDEKQPKPCVNTDTNTA
EASRAALVDFFTTKFPELARGDFYIAGESYAGVYIPTLAKELLTHKDSKDGVVPLKGILV
GDPCTDNAAQNDSMDSIWYSHKYGLMDNEVYDVLASDKCSDYYRTKLTEELMIGRRSRGK
DRSNARGTHSSRSHQSVGKRWSSHVTAADLNQELEAIEDLTERRLAAEALYRSRVLGIEE
EEDTKEHKLLRSAASATKGNGNGNGNANVAITNIINNNLSFAIMGEQNSCSNSLTNSCCT
MDGEGVVVVENLPFQHIQQSAVGTTKISGLCSSTINAAIQQLCRTVEHRKQQSGIKPSTS
SGIEGEAIKQNSLEYDTSSLFLTVGSSTSADLENSDKIIIGNQNSVIYDGEYELKRESFL
KQEEASAATRTKTSWNRKSKRRIQLNGNEGNHLDAARSPPTSLSSSLSSTLSSSISSSSL
RSSERPCMRKTLPFDGPIMRPYRQVSLGSLKIGEEEEGEPKDGFELDEADSDISKKCSGN
NSISSLASVTSFAE
>comp5707_c0_seq1; 362 349 1284 LEN=1584; translated
MSPLTRFLNGRASAHAPLHIPHCTNTSEDEFLKSEVLLLRLENNIKDDKLVAVTQSLHEK
EQEVIDLRANMSVVNTSIHRLKRNIATNTELERENNKKQKVIDDLLLKICNYDQRLKEQS
AIIAQQHQLLQKQTSQDVDLNARVIPFLMNSVSILRPSVTNITAIYQKGGYQQQEHQQRL
QYSKLLESTKAHLSSNTTTTNNSACSKTSTKKSTSQALSSVESTSKKASKYAHISMADRW
MNRYNELIEYKKEHGSTLVPQRYKKNPKLGKWVTHQRSSCLTEERKALLDEIGFVWDATR
KGPLPVSILKS
>comp5708_c0_seq1; 362 192 1400 LEN=1526; translated
MAVSTICLLFLASGPAPANSLLPQVRIARNSDAFRGIALCPTSPSRIGSAPSSSALKGSL
TPLPRDISPFDKSASKGRDVQGELRKLATVALERALSSKDGPPTRLELEFPPLIGGDQSK
TQFDDFDNVQELNQNQAWCVQWLPTLAQSLNQKSADDKRVWFVLPDTKEVELCKEEWAGE
RYRQSAVFSCIEAVAQHYATSNGGDENEAGYSKPWGATFASAVNRLVKGGDDEEGSGTGL
LGDERALDSLSDAGSPSLHLVCQPGNGGPVEDWINVKKFHDNAAKLSSGVGPTTIVVNGA
LDKVRDGYYAPFIFPALAKTFDFYKSFQPILFLKPVSDKGLYGWLFKVYGEPWQVFLQKP
RTVTKASGTTIEVEDIQALVSDTRPTYQECIQALLACNVEAN
>comp5708_c0_seq2; 273 192 1573 LEN=1699; translated
MAVSTICLLFLASGPAPANSLLPQVRIARNSDAFRGIALCPTSPSRIGSAPSSSALKGSL
TPLPRDISPFDKSASKGRDVQGELRKLATVALERALSSKDGPPTRLELEFPPLIGGDQSK
TQFDDFDSXVFAVLVIFVFNYESTTSPRKISGSFQKTNKTTTDIVRVVFLGFDDTNPYRP
DLASQTSKNSTRTKPGASSGCPXLAQSLNQKSADDKRVWFVLPDTKEVELCKEEWAGERY
RQSAVFSCIEAVAQHYATSNGGDENEAGYSKPWGATFASAVNRLVKGGDDEEGSGTGLLG
DERALDSLSDAGSPSLHLVCQPGNGGPVEDWINVKKFHDNAAKLSSGVGPTTIVVNGALD
KVRDGYYAPFIFPALAKTFDFYKSFQPILFLKPVSDKGLYGWLFKVYGEPWQVFLQKPRT
VTKASGTTIEVEDIQALVSDTRPTYQECIQALLACNVEAN
>comp5709_c0_seq1; 386 184 1152 LEN=1152; translated
MTMAEQSYYGGANYGYSSTIYGSNAYLDSTETEYDAYAQAWRYLGWYVDCNGGSSRYYDR
SGSHSHSGSGDQQSFGNNYCQRYLMWAAYVDETYSGGGIGEYAIFDTTKGSYDSTACDTN
GNGSCKLMDCHNPASTNWKLIGVFKEASYFGNDAFFEQLFKHEGVCVWNDSSLYEFMSET
RESSFSSGCLDTGISYGDYGLYIDLKPSFNGNMTYQLYEDYVCSTEYEGNDYNLESVAAS
MGLLYGDYMQAWNDAMEPFKVCQPCRAYNLQQTSSSSSSKKTYYSYSSSSSSSSYGGYWR
YTGNRDLEDEKAESNKVEENQED
>comp5710_c0_seq1; 2296 307 3963 LEN=4011; translated
MTSYQHDTTKDGYMPIGGSETARPDSKKKSKWILAGVVVAVLAVFCGKSLYKPAGKSTQI
AMKKSGLTINEDGTVMLFDKLKRFVMEDYDAKSNFASFLPGVGGYLGKPVWAFYVNRGQI
VSTFGIESKDYPLLEFNAANKAYQVTSYIGFRTFVRGTRKGASVLGNSFQVEPFSPARSR
NLDDPGDDPDKPKRVLYVGTNEVEIEEIDGVNGLNTGVKYYVLPEENFPALVRRTTFTNT
GTTDLELEVLDGLAKMEPSGGKLDGMLKSMGRTLEGWFGVYHADDTLNMPFYKLSMEPGD
TAKVTIEEKGNYCLSFIESEDETAELLPIVFDKDKVFGKSTSMEYPRGLEASSVGKILDN
KQYGESKTSSSFAAVRSVTLKPGEDITIASVYGRADHIDEVPEIAEIVTAPGFIAKKFAR

AREMINEMTVGVETNTTNPLFDTTVRQMFLDNSLRGGIPTILGNVDDDTNYDEDPGVKVY
HSFSRIHGDLERDYNWFSIEPAYYSQGPGNYRDIAQNRRNDVTFFPRMGGFDVKQFLSFI
QADGYEPLTVEAQVYVFSDHEKADEIAREVTVDAKSAKVLGEILKGGQFRPGQLFQLADQ
LNINRIDDDKLFLNTIMAASEDHAMALFGQGYWADHWDYYIDLVEAYLAIYPDGEEELMY
DNELRYFFSTATVRPRADKYVLDYTFDGTQQHVLQLEATYYDMDKAQEQEDFRSSKTGLL
GIEASWQRTEDGVTFKSTPIAKLFLLGSLKFAMRDAWGMGVEYEGGRPGWLDSMNGLPGM
VGSGMPETYELLLLLKYVKKVVDAYDRDVEVPSELGTMITTVEAALDKLEGASYKDTFPL
PHDVPEPLFEYWDTVATAREEYREAVEYYFSGSTTTYTGKEVSKMLKRWIEEIELGIERS
HDFGTKGFGDDGTSGVPACFFSYDVTEWELTGEHNEVGLPLVDAKAMKVGVFPMFLEGPV
RYMKTIQDDKKKMTDTYERVLTSGLRDQKLGMYFLSASLTGQSYDMGRQIAFAPGWLENQ
SIWMHMSYKYYLQLLRGKLYDQFFDEYKGGGILPFMDPDVYGRPLTECSSFIASSVFPDP
SIHGMGFLARLSGSTAEFMDIYQLMFYGPTMFYLNEEGDVEFQLKPAIPSWLFHDDDSHG
DSMMDEDGQYIVSFKLFGAIPVTYHNPDGGNIYGIPPNKYVIVMKDGSTEEINEDFIPNK
YALKIRRAKEVASIDAYF
>comp5711_c0_seq1; 508 67 1692 LEN=1692; translated
MKFLQITVATISSFSFLVEEVHGHGYMFEPISRNYYSNQFGLTWGTEPNIPMKEYCFHCL
NTKGVGSVCGTSEQGVNYDSWLDSLQQPISWNSNGNVYQEGGIITVETFLTAHHTGHMEL
RACPMGRASTQECFDQHVLEFVEDVNNFDVKMPKDENYPERGYYYGSQAFNNNRFAMNFR
LPQGLTGEEVLLQWLYVTANSCSPEGYAEYYSLNSHLPQEFWNRQLSTCTPEQFPPEFYT
GDSPERFVNCAEISILPNDNNIHIDEPAPTPTPTPEISTQSPVQVISVPDVSPPTPIATP
ITNESESRVIAYVGNWQACPTDEQIAQYTHVVIAFAVSYQWSPGKNICSETCEIAMPPVC
ENVNRPDLIAKWKGMGKKVILSFGGAGMGGSWAGDNNDCWDYCFGRETKVIDRLTTIVNE
MNLDGIDIDYEYFYSDNQNGSGFSKGAEARNFLTEVTVGLRNSMAPGAELTHAPMEPDMV
PGTAYFEVMKDIAHTLDFLMPQYYNGYVRSYVDFAGALSHFTTLANELFEGDASKIVYGF
CI
>comp5712_c0_seq1; 200 1 558 LEN=741; translated
PPYRTYRTVKYGTVCFFRFSHDDRRCPSGDKHHDHPAEDSAPLQLGSASRPLGQWRTKQD
LPDGPAGGMQTNKAGETGGGGDKDGTHKSRGARSSSMDGSVETVSSMSSIPSISSIPSID
VSPDENEWESGLPSLGGAEGVGRAPEREPEAGQPPDTKPDARQFWLLFYLGHLCALHDPT
PRTFA
>comp5712_c0_seq2; 767 1 2559 LEN=2688; translated
PPYRTYRTVKYGTVCFFRFSHDDRRCPSGDKHHDHPAEDSAPLQLGSASRPLGQWRTKQD
LPDGPAGGMQTNKAGETGGGGDKDGTHKSRGARSSSMDGSVETVSSMSSIPSISSIPSID
VSPDENEWESGLPSLGGAEGVGRAPEREPEAGQPPDTKPDARQFWLLFYLGHLCALHDPT
PRTFAVHVLDFYEQGIFDRKAIRFLSDLGLFPASEVDSQGLVAIAPGSATAANANARANG
TSTENRVSVVAVGDQRESLGVLVDRHGDREVVAIHASSGLAAIGDTTEQALSPTTNVLHH
IGSAVPEVTTLHSPQTLRSLEADRIRNKLKQHDNAYDNSTKKKHPTTRGTATDGDRLPID
HGTRDKPKSWDVKHYPLSMSHYQREFKEIALLNSGAFGSVYRAIRKLDGCEYAVKKITFD
IMGYSHESIERVIREVQCLAVVNDHPNIVSYYTSWWEPTWMTSNASDSFGARETLAIEAP
SVKDLSLPQNDDSSSWSPDSGYDDYYWDSTNDAESSRNARKDPRNRAQNRNKISLYIQME
LCLPKTLQDWIRERNLQIPESDHEKRMGPALEIFRQICSGLAHIHKSDVIHRDLKPANIF
SSSDGKVIKLGDFGLSKQLHEIVHQQQKQAGSSKPTSPRPQSPSKNYSGLSNRSPSRNDA
IVPLRGETRPAAQALVRCNTVGDNLTAGIGTASYAAPEQVQLNTYGTAVDVFSLGLILLE
LVCCFETEHERLHNFQQCRNQRVPGWLEENHPDIASTILACTHPNPSKRPTASALVDAVE
LKTPRAHKEIQVLRGQLAEKNAEIAEKDRTIEAMRLEIERMQKSLLASKKWNRPEEGIAV
TDVETFDEECVH
>comp5713_c0_seq1; 210 51 401 LEN=566; translated
MGSSQSSATSSSSANMKDFINGQISANKIVVFSKSYCPYCTKTKQLFAQPEYKALNPVVH
ELDKMGDGPAIQAELLAMSGQRTVPSVWIGGTFLGGNSETQAAHKDGSLQSKLGLN
>comp5714_c0_seq1; 505 190 2508 LEN=2576; translated
MSGDNSPVTENDHAREVSPTNSDGHGTSKSTSRRKGDGRENSGDKLSSHAMHPYRSDQPQ
STSGLTKKVGNRRRISRSTRSPDDESTISNGSSELSKRRSVSRSESEKPQGRESDRAPLP
PSRMRERHDSDGPPKPKSVSRRKEPRSSDSLDTELRAPREHKEKQRVTLRGGEDENNVGQ
PSFNSSADSVGTSRTNRSRKSSSSTSKTEDPTTDESSQLSDKMRRRNSNSSGGSSKQGSS
TRSRRSKSEREQGNRLSRSRSNRSPQSLKGGTDERSSSRKQLTSDDRSSSRKKLTSDDRS
SSRKKITVSEDEIMALLGSTYDGPLNTGDEEVRSISPKLEKRISRSRGTTSPRRNNSPRR
QNSPHRRSSPHRQSDKKRMIRRASNDPDLTTDKKERKQRIPPRRSRSDELEPNNGLNHFL
EKDLGSRERRKGAIGSRSVASATLSGSRSILSTRSSQSRRRRQSSSTGRILKHQVSPDVV
EEENLSDDMNNSDMINGFNHDDYDDDQESIDLDLATARENFAQQNANAKLQLHLNKTDEL
LYSVFPKHVANALRNGQKVEPENHELVTIFFSDIVGFTDISAKLDPLKISDMLDRLYNSF

DALSDYHDVFKVETIGDAYMAVTNLTKEQPDHCKRITEFAIDAIRVANQTVVDEENPSMG
FVNIRVGFHSGSVVSNVVGARNPRYCLFGDTVNTASRMESNSQKNRIHCSEISANLLRRQ
CPRMRIYPRGTIEVKGKGEMKTFWVHTEGSVAHKDKKNGTIRGFMKSIGKRG
>comp5714_c0_seq2; 443 190 2616 LEN=2684; translated
MSGDNSPVTENDHAREVSPTNSDGHGTSKSTSRRKGDGRENSGDKLSSHAMHPYRSDQPQ
STSGLTKKVGNRRRISRSTRSPDDESTISNGSSELSKRRSVSRSESEKPQGRESDRAPLP
PSRMRERHDSDGPPKPKSVSRRKEPRSSDSLDTELRAPREHKEKQRVTLRGGEDENNVGQ
PSFNSSADSVGTSRTNRSRKSSSSTSKTEDPTTDESSQLSDKMRRRNSNSSGGSSKQGSS
TRSRRSKSEREQGNRLSRSRSNRSPQSLKGGTDERSSSRKQLTSDDRSSSRKKLTSDDRS
SSRKKITVSEDEIMALLGSTYDGPLNTGDEEVRSISPKLEKRISRSRGTTSPRRNNSPRR
QNSPHRRSSPHRQSDKKRMIRRASNDPDLTTDKKERKQRIPPRRSRSDELEPNNGLNHFL
EKDLGSRERRKGAIGSRSVASATLSGSRSILSTRSSQSRRRRQSSSTGRILKHQVSPDVV
EEENLSDDMNNSDMINGFNHDDYDDDQESIDLDLATARENFAQQNANAKLQLHLNKTDEL
LYSVFPKHVANALRNGQKVEPENHELVTIFFSDIVGFTDISAKLDPLKISDMLDRLYNSF
DALSDYHDDLKLKRKLFMPQQIGMCSDXKSIEGDFLTIFVFDFFKSCFESIGDAYMAVTN
LTKEQPDHCKRITEFAIDAIRVANQTVVDEENPSMGFVNIRVGFHSGSVVSNVVGARNPR
YCLFGDTVNTASRMESNSQKNRIHCSEISANLLRRQCPRMRIYPRGTIEVKGKGEMKTFW
VHTEGSVAHKDKKNGTIRGFMKSIGKRG
>comp5715_c0_seq1; 199 377 820 LEN=946; translated
MSLKRINKELVDLGKDPPANCSAGPVGDDMFHWQATIMGPDDSPYAGGVFFLDIHFPADY
PFKPPKVHFTTRIYHCNINSNGGICLDILKDQWSPALTISKVLLSICSLLTDPNPDDPLV
PDIAQLLKTDRARHDSTAREWTSKYAM
>comp5716_c0_seq1; 520 301 2019 LEN=2119; translated
MSAPTPVLVSGSSPGPSSPSPKTEKDGEMIPNATSNNRSSCIRKTSSTIKKILSMALILF
SVIVISAAIITKQTVATGENNVHPILALAVFWLSLFWLSVMEGGLNCMVGLQPISQCLYK
KSHPKTYRCTSVAHKSQNIERFIVGRQYLDLMIVFITSFMVSAIDGASVLGLPKIVNVIF
LGSDLAVILCTIVFGQLIAQINCAHSMLDFINNWVMVASTYIALCVEASGILHAVYFVQI
IFTKIVDYKHSKEQELNASKNNSSGKSSTKTMNTSVDSRDSSSEEESDESSSDEKFEIKC
DHYESSLSPSKKSLYQKIFFWIRVVFSLAISIFAIVVFSTALVNGDTTVRDNIPVAVSFV
SLLVLLVLGGFMEALQIALFAVKHLPSECIEANPTAKRNCEYILGKAGSSKRDNDKNSRL
QSFLVGRQISQTVIMFMIARIITVEMKVEGETLFGVTPQIQSVFFDSGLLNALVSTIFAS
LSWRVTANFFPMLYIGCPLSIWIIRLCLVVEGTGICDAAWTLAKIPAYLVGYKSDDEWIG
DSIYTNEKDLETGGLENTILTKERSTVSEETEW
>comp5717_c0_seq1; 770 1 3724 LEN=4178; translated
RRSRPKERQQFAASLLGRDKKGRRKLQLFNDANSPKHGALGFGICNDLFADVSVRRGSLA
GGFRQRNSDPRTKAVTGIFVPNLRRTDHRPMMLLISKKGRVRVLENPENLDTDDDEYINK
EDEIDNDDNTESNLILDLSGEGTLCIDGERGLTSIVAHPNFLENGWVYLYYTRWSQWCLE
DPVLGPQNILVRYTMNSTTLLLENEQLLVEFGVLGERVHNGGGMMFGTDGYLYLATGDGG
VRQHAQNRQNLHGSLLRLNDDGSVPDDNPYARNSTLGVPCGKSDGVVPEIFASKEGGENV
FCSEIYAWGFRNPIRMALNHKGSNSEKVLFSISDVGAQLFEELSWGGTDYKEKDYGWPNL
EGPCITDSIDDCPLPSPDSNRIDPFHYYVHKSYENGGCVAGSTFVPDGIWPDEYKYLFID
FIFLKIYNLIEDPESECRESCSPPTSGYSNETFFESIHEEGENINEARIIDMFFGPYKDT
EALYVIKYGNNNSILRIRYTGVLGVPPVAKIKIENEQENIRKDTTSSLPTSSASNIGTGQ
VLPLQKTTVVGKGQVINFDGSGSFDPEGNGLSFTWDFGDGNNSTANATNLSYAYEVAGEY
NVSLVVTDSKNKEHQAFFMVLVRKTPTVDMLSPKEGDLFSMGDILHLDGEAYDNQGNRIP
DDQLIWEVRVHQDDQLDSLLNKRSGNDFHLDPALGPEDLNGFLSIMLQAMDDDGLTTTIV
RKINLDVVDVKLTTDPEGLNMTIDGVAVISPYHIQSWVNFELPMEVKTEQSYYRFKNWSD
DNTNSSHIFVTTPRENATIDATQAKERAVSITAIFCLDSNAPCGDVTTTCCSGICDEMGF
CKDELQDSVSVETKPTAENVSMTDPDLLPPYPHGTIVASQIMPHGYDTEPDQKPGSPGID
SSIFDEPELDVINSSTILDEQSNLGEQNAYYGLRPYGKWLLSIFLTIFSIAPCYLGWKYL
QLKRQMEAETKISTDKEVSDEPLTPKTVVAISSEQDTPVPLFESNTSNISFQRSGTIWDR
IAEETERRAKLRAKYCCKDTPKTPDTAEYRGSPSSFDSFSSPSSVLTNNIDQTLVRLDDL
LSKTFSRKLSRISFCQKQEEHNDLSDGGDRKSGDLESGDFIEKIEDSEQSTLAPLWLSSN
QNSVPGSPMRDDSNCSKTAGENLTLGSLYALGSSKGDLNTSQVVRLSEMCDNSVKVFQDN
NLSAATSNTSEGNLRANDAANQQNRSDAAGDGGGNHISNI
>comp5718_c0_seq1; 127 1 1259 LEN=1259; translated
KTRGRTKTGRGDRAVTSRHTRWARPIALLLMASLVAGCGLPRVGPTKREIYAGSVQKQGD
SFVVTVNDRVTRATAVQPALGFTEQFQNAGLLGSDLISPGDTLGLTIWENVDDGLLAGEA
TNQTTLGEVQVDGSGFIFVPYAGRIKAAGNTPEGVRRVITEKLEQQTPDPQVEVRRLAGN
GSTVAISGAVSGQGVYPIEAPTRTLSAMIASAGGIAIPPEVAQITVIRGDQRSKVWFQDL

FKYPQFDIALRGGDRILVEADTRAYTALGATGAQSRVTFETQTLSALEAIAQVGGLSTAA
ADPGVFVLRNEPMEIANQVLGRTDLQGAQRLVYVLDLTKPNGLFLARDFSIRDDDTIYVT
EAPFTQWSKVIGAFTGTLGXGWIGINGVLNALGRLLSQPSDGQPAGEARASRRVFYFNGG
>comp5719_c0_seq1; 502 112 1185 LEN=1221; translated
MARINARCLLRSFHSLSVQSKQGPRRLSPNAMLVPAALPGPLALAHRAFATDSNPPRRRP
RRKKAKYEQFSDAKLATGGGVPTTSALEDPFQFSGDSIEEYREKTELSPWVPVPDSVARK
IFDRALPEEEESSDTSKADEIHVELGCGDGRVNFHAIEYGVKESIGIDVDEEVVKRADDR
LNRIHPKPKLSFVVSDLMDPESPAWTEHVPRATILTMYFATDGLEKIRPLLEQALRGKRC
KVFACGYAIPGWESQIVETVLDIPIHFYDWGNDEVNMMTIQPSDSLADKLTANGMGMPGA
PSDNMDQYMGRKNKKSTFVQDPLPGYHPDDQIDYGWMDDFEEEKTKKENGDLKEKST
>comp5719_c0_seq2; 332 112 1332 LEN=1368; translated
MARINARCLLRSFHSLSVQSKQGPRRLSPNAMLVPAALPGPLALAHRAFATDSNPPRRRP
RRKKAKYEQFSDAKLATGGGVPTTSALEDPFQFSGDSIEEYREKTELSPWVPVPDSVARK
IFDRALPEEEESSDTSKADEVSGFVGAVGLSIHSVSFVHXNVRGRGLLFVWFDSHSPICF
LDHSINVLLIHVELGCGDGRVNFHAIEYGVKESIGIDVDEEVVKRADDRLNRIHPKPKLS
FVVSDLMDPESPAWTEHVPRATILTMYFATDGLEKIRPLLEQALRGKRCKVFACGYAIPG
WESQIVETVLDIPIHFYDWGNDEVNMMTIQPSDSLADKLTANGMGMPGAPSDNMDQYMGR
KNKKSTFVQDPLPGYHPDDQIDYGWMDDFEEEKTKKENGDLKEKST
>comp5720_c0_seq1; 178 1 495 LEN=609; translated
RTATRTLAIFSEARQVQYSINQPIKMISDNAKIGTGLLAGGVACLFLGIMFFFDPALLAI
GDVLFLIGLTLTIGPWRTLRFFSRSDRIRGIISFFGGVILVMMRWGMIGMICQLYGLVYL
FGQFFPIISQSMQNTPIIGDILKIQAVEDFFSNFGGGSSRRAPV
>comp5723_c0_seq1; 109 233 405 LEN=405; translated
MMSCTKSFVSFVLCLSLLASPVLARTSIATGGGAGGGASRDSIKKATNTGSSSKPTNX
>comp5724_c0_seq1; 260 233 932 LEN=932; translated
MALARHVTFVHQNEGLEAQPEDLDSDDDDDSGRAAEENSDFNEEKGIVSPRLLREYISRA
RRHDPVVPPEVAPYIVEAYVSLRMQDRPGKSNNGNKVGDQTVMTARQLLSILRLGQALAR
LRFSDYVAREDVDEAIRLTHMSKASLSEETGEDGKIVRREDVMSRIFNIIRDYSATSNST
TVEMKLAEAMVIRKGFTVQQMQSCLEEYEALEIIQINQNRTQIHFISLYWVPDX
>comp5724_c0_seq2; 477 1 1707 LEN=1707; translated
XNPGGGGGRRVVGVLKLETRGSKFTKFQELKLQELPSQVPMGHIPRSLSVYCRGELTRLA
SPGDVVTLDGIFLPQKVNDGHGAAGRRAGLMSTLYVDAQNLSVHKKSFDDAATKRAGEEK
AGGLDADVRRVASAEDPVGVLSRSIAPEIFGHADIKRALLLQLTGGVFRKMADGMKIRGD
INICLMGDPGVAKSQLLKHVVSVAPRGVYTTGKGSSGVGLTASVTKDNTTGELSLEGGAL
VLADRGICAIDEFDKMDEADRSSIHEVMEQQTVSIAKAGIVATLNARASVLAAANPLYSR
YNRHKSLSENINLPNSLLSRFDLMFLILDTPDMDRDMALARHVTFVHQNEGLEAQPEDLD
SDDDDDSGRAAEENSDFNEEKGIVSPRLLREYISRARRHDPVVPPEVAPYIVEAYVSLRM
QDRPGKSNNGNKVGDQTVMTARQLLSILRLGQALARLRFSDYVAREDVDEAIRLTHMSKA
SLSEETGEDGKIVRREDVMSRIFNIIRDYSATSNSTTVEMKLAEAMVIRKGFTVQQMQSC
LEEYEALEIIQINQNRTQIHFISLYWVPDX
>comp5725_c0_seq1; 389 104 1267 LEN=1405; translated
MPITNPHFKLGQDKIDEYLENGVLVVENILADDELEEAKKGLLESLRRRGIKSIDVEDEE
SALAFQKLSSTNGSGGVLDIFHDDWKLKLIATHPKLFSITQQLWKAGYNCGGRDKDSLPE
NQRFQWHPYGGDIDFENKGYCYIDRVGYRLPTELAQEWGDRIYKRNNGVTSVTSKKKKKL
RSIQRSLTPHLDCCPDTLYENTNKWRPIQCFVSLTNNLEANTGGFEAVLGGFHRTFDAWT
KIRNGDKTAIGQSKATINKAAPCLGEYTHIRPKEDADVMKRVNHISVKAGSAVFWDNRIP
HANAYRNDNKSVPRAVVYASFLPDIELNRRYVENQLLAWKLRKPIRDQWNHIPEEQSPCS
NEDQIASSSDEGIFTEIGKRLMGIEPW
>comp5726_c0_seq1; 153 1 353 LEN=609; translated
XEETDEAGPDTTTDSEPEEETDEADPDIEVDSSNSNSTSTDDELDGEDEDVPDGEVPEGP
PPPGPAQVPEPNPYKPFLHVDNLRYAATNLIRPSRVWQSRIDRQNELNANPSTIAAF
>comp5728_c0_seq1; 445 116 1435 LEN=1528; translated
MLAIRFKEALVFSGLIALACHIHADNPEESSSKASPRSSASSPTRPLVFGILSQPFYDDE
SDHDPGDNGYSHTYIAASYVRWLESGGARSIPIPFDAPPFLVEDILGQVDGVLFPGGGSS
LPSSAITIWELLHSEKYYYPDEKEGRQGDRIPLWGTCLGMEFIVQLAADVVGSGRKNCEM
DRGCAYDRNILEKGYDSTNVSWPLLEVRKSGLYKPDSVYEIATTYNVTLNNHHLGISPDR
FRNNSGLSRRFDITSINYDHKGKAFVSTIEPAMEARNTTDLSKPSPFPPPVYGVQYHPEK
NSHEYGLYPGTAIPYEAIDHSQEGVDFSIYEARFLVDLARKNVLNRIHDGKKDNKYNKPD
LYPMVFTYPRRVGHKFEEKYILPPAKHWTDKIEALDNSDFRDRNPEDDGESEAANSWYRD
NWKEQRRAEKLPSLRGRHD

>comp5729_c0_seq1; 558 340 1617 LEN=1830; translated
MSLLDQKTQQVFGTQCHTQLLMSTSATSDRWGISETSRWDKEMDEQTAGNQEIDTDGNES
TVLLQEHQAWSNAVDSALKTLKKKRTSLQSELKKAEDVEAIKARAQLLVSNLYLFQDGVK
SAIVNDWENDGKEVELTLDSTGKYSSASDEADALFSIVRKLKRGSRIVADLLKETDTAWE
VLTEAELDLQSACGGDGETGIDEGRLAIVKERLVRTARATKFQAPTIEDDNSKTKSKGSS
QNRRERKPALGTPASNVRKLISPGGCIVLVGRNRRGNEHLSMTVARGNDIWMHSRGCPGA
HVIVQNRRGGPTVTEECLQFGADLAIFYSDLRNEEKAEVTAAEPKHIQKPRGAPLGAVKL
REEWKTFVGRPGQVPDELKEAREESGQTDEYHMFDKAKHRRRTKQVREEDRAKKRRQQRE
KKNQR
>comp5730_c0_seq1; 485 68 1219 LEN=1590; translated
MGENEDSNTKSGDKAPSMSLNDRLDAARAMAESSPSECLPLLDGLQKDVQAVGLFSKNET
IDDISTKSIPFLAIDHLLAVVLGNLPVQPGAMARRKENLQRSFTLWTQFLERLELLEILS
KEETNEFHRLLETQQQVESSGTSTTISSPVNREEKIARFKAKQQQRKEIERLQSLKDRRS
RCDVAPEDEFDGHDLDSLERSMTLKELHLFKLEALESWSQSLRELPMIEQMIKMDEERQQ
RQKFSGKGNDAVDPNRRPPSSSNKGLKVTHVSKDAASGQLRFKREEIRSKVFQPGWSQPT
MTLEELGEREYQEAVEREARQNAAEAERVKQPRRYEQLANDGLEDNADLVDASAKLDREW
DDWKDENPKGSGNKHANRGDKNF
>comp5731_c0_seq1; 1081 161 1963 LEN=2351; translated
MRNFLFALLFCLAAGTTVVVQAAGGGKCSSEQLKCPTEECESGYSYVTDCLDCEGNLNTF
NGEDKCFTRKLLSSKNPSPQYLWRDIIGMLVWFCAAGVATACGVGGGGIYVPLGILLLNF
APKPASGLSQASIFGASLGGLLLNLRNMHPFTAKIETTAVAGGNGSPAEERAVADVPTND
DGEPNTETTKYYTRPLIDYDMALFLAPMEMAGAVLGVLIQKILPNWLYLFTAAVILGFTA
KKTFHKWWDTRAKEMAKAALEAADSEEQAALDPSANAKKDTATDNDNENSEGPSSDGNGE
VEVTASASMAAEDNDENNEAEIVMDSEKIAKRTWFLERDARQYPVEKMFCFGVLWIGLTL
LTFFKGGKGVESLVGIDCASPWYGVLIGIQFLWTLGFATFFAIKLMRETTQKKAVNYPFH
EQDVLWDFQKTRFYAFFTFVAGIVAGLIGIGGGMVLGPLMLVMGIYPRVSTATTATMIVL
TSSSVAILYITSGMVPWQYAVTFFCTCFTGALIGKTYIDGYVKRTGKASVLIFLLAMIIA
LATLGALVIALLRLNKADWCFAGFNKFCTLDADAVEVVCVPTETERMLGSSFYGDVVRGY
>comp5731_c0_seq2; 1081 257 2059 LEN=2447; translated
MRNFLFALLFCLAAGTTVVVQAAGGGKCSSEQLKCPTEECESGYSYVTDCLDCEGNLNTF
NGEDKCFTRKLLSSKNPSPQYLWRDIIGMLVWFCAAGVATACGVGGGGIYVPLGILLLNF
APKPASGLSQASIFGASLGGLLLNLRNMHPFTAKIETTAVAGGNGSPAEERAVADVPTND
DGEPNTETTKYYTRPLIDYDMALFLAPMEMAGAVLGVLIQKILPNWLYLFTAAVILGFTA
KKTFHKWWDTRAKEMAKAALEAADSEEQAALDPSANAKKDTATDNDNENSEGPSSDGNGE
VEVTASASMAAEDNDENNEAEIVMDSEKIAKRTWFLERDARQYPVEKMFCFGVLWIGLTL
LTFFKGGKGVESLVGIDCASPWYGVLIGIQFLWTLGFATFFAIKLMRETTQKKAVNYPFH
EQDVLWDFQKTRFYAFFTFVAGIVAGLIGIGGGMVLGPLMLVMGIYPRVSTATTATMIVL
TSSSVAILYITSGMVPWQYAVTFFCTCFTGALIGKTYIDGYVKRTGKASVLIFLLAMIIA
LATLGALVIALLRLNKADWCFAGFNKFCTLDADAVEVVCVPTETERMLGSSFYGDVVRGY
>comp5732_c0_seq1; 511 350 1798 LEN=2062; translated
MSLLATAQSSRRSALSLGKRALSAMAASESSPASSSFPSYVLKAPTTDVTTLPNGLRVGS
ETTVGAETATVGVWIDSGSRYETEANNGAAHFLEHMAFKGTSKRTQRQLEEQIENMGGHL
NAYTSREQTAYFAKVFKKDVPVAMEIISDILLNSNLEPQAIEREREVILREMEEVNKNQE
ELVLDHLHATAFQGTPLGRTILGSEHNIRTLTRDQLVDYITTQYTAPRMVVAGAGAVDHD
ELCGLASECFGALPTESPNALSSDSSYEPATFTGSDYRVKFNSEDVGYMAVAFPTDSWTS
EYAFPLMLMQLLLGSYDRASGMGHNYASNLCQDVAENELCYSLNTFNTHYKDTGLFGVYA
VCPDNKIDDASWYIMNNLIRLVHSVGDEEVERAKVSLKAAMLSNLDGHNNVAEEIGRSLL
NYGRRMTPAETFCRIDAVTTDDIKATAYKFFNDKDHALAAVGGIHELPDYTWFRRHSYWL
RY
>comp5734_c0_seq1; 311 352 1587 LEN=1888; translated
MESSLNARLTFEQVRASGEPCRPLEFPPPQVGKGIRVRALVIDSNTGERRGWSIIHREDF
GISSKIANGHCEPQFVAYAPSRQLQTAIYGSVWACVVLKRHYGVAANNDAAIAVGIEPGS
PCAPIVWETTNRFVAIKMVEWAKVQQSRGALLEDPIKEIAAMQYIGNTSPHLLGPVEALQ
DNDCLYNIMPYCNGGDLFGVVVNYAQESGGERGMPEPVARYWFRQILKGLFHLQSVGVCH
RDLSLENILVDNENCLIIDMGMCLHVPYNDPAIPGNVVDARRGSTRRLMRPQGVCGKHNY
MSPEVYANTGPFDGFAIDLWSTAVILYIMFTGFPPYEMADMRNKRFEIIAKGRLVQQLEE
WDVHLSEDAGDLLQNMMKVDPRERLTLAEVMSHPWVVNGDVQPPPPRQSFH
>comp5735_c0_seq1; 264 83 1003 LEN=1191; translated
MGRCFIQIVMGPAGSGKSTYCQAMQYHASTLGGARKRTIHVANLDPAAEDFGYQLAFDIR
DLISVEEVMEELDLGPNGGLVYCMEYLLSNLDWLQDHLDSFDDDEYLILDCPGQLELYTH

IPLMRNLIDRMRMWGYAHTMAAVFLLDATFVGDTSKFLSGSLLSLSAMIALELPHTNVLT
KCDLLTDEEVEDILDHGSATQMWDREQDRQSLIMPTPFSVTAASSNQGDEVTEEQRRLER
RRRHRQRLTDSISQLLDDYSMISFIPLNIRDEESIDALLTCVDGSIQYGEDLEVREYDGP
EANDDT
>comp5736_c0_seq1; 335 240 1220 LEN=1309; translated
MSRHGNSRAYVVDHGENFVPGSEDAPNLAPQPLDHFKRGYLERANQRERDVANMDNPGGN
GENGGGPQRDNSRYSNKSERGRGGEGPMPTRYSGGNNGPFNGSGRGSGGRDQRDSHYGGG
GVSRDDGGRRSSSSYQGGGRGRGGRGRARRDTPYRGDPGSSFRGARGDRRPSYYGDRDDR
GGGRGGQQVIMGLDIQALVDRLVRPGCDIYRELGITRESNMAVFQSGKAVTAIISNLARR
RNLRIANCVWDWIDHIGIEKNTFHYNAMISACEKVRDYNKALRLLDEMKEKKVSKNEVTF
SSAISACEKCGQWRIALDLLDQMVRH
>comp5736_c0_seq2; 303 952 1773 LEN=1862; translated
MDNPGGNGENGGGPQRDNSRYSNKSERGRGGEGPMPTRYSGGNNGPFNGSGRGSGGRDQR
DSHYGGGGVSRDDGGRRSSSSYQGGGRGRGGRGRARRDTPYRGDPGSSFRGARGDRRPSY
YGDRDDRGGGRGGQQVIMGLDIQALVDRLVRPGCDIYRELGITRESNMAVFQSGKAVTAI
ISNLARRRNLRIANCVWDWIDHIGIEKNTFHYNAMISACEKVRDYNKALRLLDEMKEKKV
SKNEVTFSSAISACEKCGQWRIALDLLDQMVRH
>comp5736_c0_seq3; 705 952 4047 LEN=4047; translated
MDNPGGNGENGGGPQRDNSRYSNKSERGRGGEGPMPTRYSGGNNGPFNGSGRGSGGRDQR
DSHYGGGGVSRDDGGRRSSSSYQGGGRGRGGRGRARRDTPYRGDPGSSFRGARGDRRPSY
YGDRDDRGGGRGGQQVIMGLDIQALVDRLVRPGCDIYRELGITRESNMAVFQSGKAVTAI
ISNLARRRNLRIANCVWDWIDHIGIEKNTFHYNAMISACEKVRDYNKALRLLDEMKEKKV
SKNEVTFSSAISACEKCGQWRIALDLLDQMKKEGAGQTAIAYNAAISACEKGLVPHKALE
IFEQMKREGVRPTVVTYSALISAAEKGQQWKLALEVLEEMKAAGHGANVIAYSAAISALS
KGQMWHKALELFREIESSGGKPSIVTYNATMTALEKGLQWERALDLFDEMKSKNMPVTVV
SYGSAISACEKGLQYRQCLEYLDEMTEMGIKKNVIIFGAAMSCMEKSCRADIGFQLMERM
RLEDVAPNVHIYNSAISACARCNLWEKGYELFVEMDRIGVNKDVVTYNAVLDAVSSQVEL
GRRLFKEGVERGFYARVSRLGTQWLELDLHFLSLGGGEIALGWWFEECLVPYLINTSKLE
AVQSISIVTGYGKTRSRGARMNDDGMRLRVRAMLKYMEIHETPQPNKGRIHINKSALIEV
VKKNGGRIRFDLEGYTRFKEEETTANKFPDVVQQVRPRFRPARPGQGPPGTFIRDGDPVP
PYRSPDDKRRELDHPVDRRSFSFRKSRDDRFPDRGPDSARPSGDDRMPDRASSNYGRTGE
SPRNNQDQRYGDEWRGGQRDAPRRGAFGDQEGHGDRGSRGDRPENGIDHRRSQRGYGYEG
GRPGIGNDGDRRGGERQHEYDERRGGVRNWDDSGRDNMRSTNRDYDPNLRGQAGMDRRGR
DERDGRSSMSRPDQSYYSDDRRPSMDGRGGPRAPRGPPGQNMHFQDRHSDRGSRSSGPNG
GGGRNGPNDMPADFRGARNTDLNAQASYRNEELELASTRKRPYDDYQKQQPSSRGYDIEP
SYAKAAAHSITX
>comp5736_c0_seq4; 737 240 3494 LEN=3494; translated
MSRHGNSRAYVVDHGENFVPGSEDAPNLAPQPLDHFKRGYLERANQRERDVANMDNPGGN
GENGGGPQRDNSRYSNKSERGRGGEGPMPTRYSGGNNGPFNGSGRGSGGRDQRDSHYGGG
GVSRDDGGRRSSSSYQGGGRGRGGRGRARRDTPYRGDPGSSFRGARGDRRPSYYGDRDDR
GGGRGGQQVIMGLDIQALVDRLVRPGCDIYRELGITRESNMAVFQSGKAVTAIISNLARR
RNLRIANCVWDWIDHIGIEKNTFHYNAMISACEKVRDYNKALRLLDEMKEKKVSKNEVTF
SSAISACEKCGQWRIALDLLDQMKKEGAGQTAIAYNAAISACEKGLVPHKALEIFEQMKR
EGVRPTVVTYSALISAAEKGQQWKLALEVLEEMKAAGHGANVIAYSAAISALSKGQMWHK
ALELFREIESSGGKPSIVTYNATMTALEKGLQWERALDLFDEMKSKNMPVTVVSYGSAIS
ACEKGLQYRQCLEYLDEMTEMGIKKNVIIFGAAMSCMEKSCRADIGFQLMERMRLEDVAP
NVHIYNSAISACARCNLWEKGYELFVEMDRIGVNKDVVTYNAVLDAVSSQVELGRRLFKE
GVERGFYARVSRLGTQWLELDLHFLSLGGGEIALGWWFEECLVPYLINTSKLEAVQSISI
VTGYGKTRSRGARMNDDGMRLRVRAMLKYMEIHETPQPNKGRIHINKSALIEVVKKNGGR
IRFDLEGYTRFKEEETTANKFPDVVQQVRPRFRPARPGQGPPGTFIRDGDPVPPYRSPDD
KRRELDHPVDRRSFSFRKSRDDRFPDRGPDSARPSGDDRMPDRASSNYGRTGESPRNNQD
QRYGDEWRGGQRDAPRRGAFGDQEGHGDRGSRGDRPENGIDHRRSQRGYGYEGGRPGIGN
DGDRRGGERQHEYDERRGGVRNWDDSGRDNMRSTNRDYDPNLRGQAGMDRRGRDERDGRS
SMSRPDQSYYSDDRRPSMDGRGGPRAPRGPPGQNMHFQDRHSDRGSRSSGPNGGGGRNGP
NDMPADFRGARNTDLNAQASYRNEELELASTRKRPYDDYQKQQPSSRGYDIEPSYAKAAA
HSITX
>comp5737_c0_seq1; 508 110 1429 LEN=1634; translated
MMFKAACTILLCTLAAVNAAKDDEYYRAGMGNPNVDLKMYWADAHNVIQDLDEFSYLYIQ
YHNCAWTQNRNTNEEQESGSGDETDYWYMGATPSYAANVAFSLYGSLKGEKFKGCNENTF
INSFTTNQGFEGFASAIYYAGATSTDYSGVYSSECQGGAGIACDYDVGFASVTYSTDTCN

PAYATGITDTLSYMNSDFKSAQCVKIYDYQQSYNSGNRELKKTESKNEAEQDGKTEIDEN
TDKEDRNLQNNYGYGYNNYGYDSYYGYNNNYDYSGTPLSILYYSHACFIQNYWDPDGGCP
DPYGKLEYYQENFNKGVRMSMKVDTYVNYRANMKIGQRYVGIGVFLFMTAVAMLATEQYM
AYKAKKRTNKSKTSDSLMKPGSHRKKTVVELVSTTGNKIKKSVRGSSKGRKKKSSKKKPT
PNKVTTEDYDAPTLEMTDA
>comp5739_c0_seq1; 192 98 670 LEN=1107; translated
MGEEEKQPKRPSTVDFKIDESAESTKKRPSVKRGMSKRKSLRASFLRLKSLSFRRSGGER
GADIDTLRGTHGPEFEGYAKINRSGGAGISCGCFGGSGDTKEKIILIKGAYCFVFVNESD
PAPKYAIACAHMKAKIHSPSHGVHPVTIETALGDVEWDLTFQQKQIAKQFADAFKKQAAV
GESDEVRKVI
>comp5739_c0_seq2; 234 98 814 LEN=984; translated
MGEEEKQPKRPSTVDFKIDESAESTKKRPSVKRGMSKRKSLRASFLRLKSLSFRRSGGER
GADIDTLRGTHGPEFEGYAKINRSGGAGISCGCFGGSGDTKEKIILIKGAYCFVFVNESD
PAPKYAIACAHMKAKIHSPSHGVHPVTIETALGDVEWDLTFQQKQIAKQFADAFKKQAAV
GESDEVRKRLGHDKLLNKRGSVRYAEAIAEKKLEEQPEKKENVLLEDVNRIEPMMAGC
>comp5740_c0_seq1; 473 327 1490 LEN=2071; translated
MGGGEISASGWAKGILLSILASVIGGISKLAIRKSWLLQAGGDRNSGNNDNLLPGEITNN
HIAAENTTGGNGDEQMSGQYERQRKELIPYVLRYSGMFGMSVLNPICCVLAMNFASPSIL
APFSGLTLVWVILGSPFVNSEKPSRRQIMACCLIILGEVVVAMFGDHTNDEGVTVEAVRK
SYKKPSFISYFVGLALYIALLMYWINFSESFIFRRFAWGCSGGAITGCQNFLKDSLTIIK
ATEPDQKLPLIFYPLSLFAAGTAFVGLLLLTACMKRYDATYSSASFVGSFVVSASIMAAV
HYNTFEQLDGVINYILYPFGILILMVGVYALVKESGESDGYDDIPEQQRGLDADQVGFND
SEASMFTKTVESVFLFRSVFSQVMNLQ
>comp5740_c0_seq2; 481 327 1493 LEN=1924; translated
MGGGEISASGWAKGILLSILASVIGGISKLAIRKSWLLQAGGDRNSGNNDNLLPGEITNN
HIAAENTTGGNGDEQMSGQYERQRKELIPYVLRYSGMFGMSVLNPICCVLAMNFASPSIL
APFSGLTLVWVILGSPFVNSEKPSRRQIMACCLIILGEVVVAMFGDHTNDEGVTVEAVRK
SYKKPSFISYFVGLALYIALLMYWINFSESFIFRRFAWGCSGGAITGCQNFLKDSLTIIK
ATEPDQKLPLIFYPLSLFAAGTAFVGLLLLTACMKRYDATYSSASFVGSFVVSASIMAAV
HYNTFEQLDGVINYILYPFGILILMVGVYALVKESGESDGYDDIPEQQRGLDADQVGFND
SEDIFVYTEVSELGREETLSRNVCSEDG
>comp5741_c0_seq1; 502 1 1516 LEN=1570; translated
XNSLPQVNCLSSACDFQSAKNSKKELEFAPAARNGSMLSGIRTGKAKKKKSKAKTENVPS
EPNSDATSIRDPHPRIVNSKVNSSNNTVANANHKNEANLSVAERLKQALASGVPFQKSSK
PSTATEEAPASSGSYMERVVGKGRVEGTSAGASMIVLDAPGTSASYSKKREEDMTATELA
AKERATGGENSMSWNEHMARNIMRVGKKRKMKARTAAGEDSDEEVERMKRHLPDYHLNES
GSGSSAKRAAKQLEKEEQRDRRRQIDRYQKQEKITSMCSWWINSSSFAKHRLLAFGKHVS
LMMAPPNSSLLPGHQFYLVPLKHAPSMAECDDPDVREEMKLFRSSLENMYAKERKGLVLC
ETVLPNKNFWQTRVEVIPIPFSQLQDAPIYFKSALVEQAEEFGTHTKLFRTSHKKPLRSV
VPKRFPYFHIDWGNIATSDSTGYAQIIESSSFPRDFGLDTLAGMMELDPVRFQRKVKFPK
DTESKLVAEFSAKWKSFDWTQRLED
>comp5742_c0_seq1; 237 86 268 LEN=268; translated
MDLEKGAEALREPLLHDDSDSGADGNENENRKNDKQEERSNIGASFWGDEAEGDDDGDDD
G
>comp5743_c0_seq1; 485 247 1152 LEN=1577; translated
MTIEEGDSDDNSLRRSEATHDSFPIAFLFILTFGMSALILGCIQNFGCSTVTFPQVSTAV
DGATLGSFNSGVFGYEFVGVDFNNPFEESSTSSDTTTNYDESVNSMEMWEVCRRYGTLGE
DMKLAGVETEFSYKGADYMKAAQGMAIIAFSLGLVMMLIVCSVPNGGILCVMQWKCYGLF
FFLTSVFQGLSLLIFSSSVCLDNPILGVIDDNEKIFGRLGDTFEETCEMGIGAECGIAST
VLWFVTAALVCCEKPPGRYKEECLTHMLRAHKQELMARRMKKAAALEEYQQRREQTNNNE
Q
>comp5744_c0_seq1; 251 214 792 LEN=934; translated
MGTISQLRTVYLVSVVVLSTLLLQSSQAFMLAPTAASTRYSTSFSLPASAAAENEVESTL
TPKDKKVYQFLQELSESNLTFRIVVIGNGAILESTNLLGPTFKLGQSPKTGNHIVTFAAE
DQSFEFHVMPGQVASVALVEKESPIGNKTMRLMRLLNADGGSICSLILADDSQAASDWYG
GMTETYGSDFEF
>comp5745_c0_seq1; 200 1 958 LEN=1315; translated
XSTQTASPESNPTLKNPSKPSNMSTASDAPLPKSVIFGTAGLGGCMGWVVVHPFNTAAVR
ANLASASGQTFSISKAIKKDGFRSFYDGLSAGVARQVFYATSRFGLFETFRDKLHEYRGK
TDFASRVGVGAVTGGIAAYISCPMEVCVIRMSNDSSLPVKERRNYKSLPDTFTRILKEEG

VSAFWRGSNPFVARAMMVGVFQVATLDQFKDFYAKALDQKRNSISNVFCSAMTSGLIYSL
ITMPLEAAKNRMASQKPDPKTGKLPYTGTLQTVTKVASDSGFLALYNGFAPYYMRCGGHT
VTMFIFVQMLRDQYKGFMM
>comp5748_c0_seq2; 219 1 533 LEN=714; translated
XALTADLSSCTLPATKVLLFFVPSPDSSRRVNPGHKYLVENSWIITPATTIASTPSPTRH
KTSRNNMEKASATNDLDERNSSGSVNEDKAKGVTDLVSDMLLEMENDFKKSGNSIRDRMR
EMGSKLDGLEQSINGLMHDAGLIDGEEESPSDSNPSPRRPPTNSSPPRTTMATSDSV
>comp5749_c0_seq1; 430 182 1534 LEN=1592; translated
MALSTKTKPSVDRCPTMRSIATTLTDFSSSSSTELSSTSIDERIKQMAERAKNGTPFTED
EIRDIANSLQNLAPVVDNKKVASSCKEIDFSGLKSLLGGVAHLSHKDWAVTSENSDKLCE
TLAVESSSPSDSASTDFPLSAPARQLLERIIKEGNWDGAVRNANRSDRPADEKSWAVLVT
GVNGIRKTTSMYQPWFGDLLSEAIICPATCGASETKQKAPKGLPTGSNSFFRQLDHMICT
LCNEEFTRLYAWSTSQLPKVEDDGKPIPSDEVVDQYSKYKAAIFSRYRTISELLGALLLK
QAQKVDINCMMETSGRDVAMFHYVDHFFGENTSYNKLALHFTINDLTFAKQSVDRRMIDE
IQRGSQALGNSNVFDIVYTNQGGPYGSKVLDGVQADSDRVWESEVLSGNVGEDWYKATIA
INAHETEPWTAQAVKPDGSLGTKFAFGPKK
>comp5750_c0_seq1; 434 108 1664 LEN=1853; translated
MTSLCSASGEDIDVPKDLECLICGDLLENAVSLQACAHTFCSVCFRIHYREAKKKRCPLC
KVSVGNDLRKALLVNGAVQKRVREFKSKHSILQPSLPFHDKLPNAQEAPIREQMQRRNYS
MLLKHGKKGLQKVCKEYSLPTSGNEQELVDRLRYFQCMWDAELDTIYTPLRPSELVAKFK
KKERLQREEKSRAMMTGSINDSKHMKNLTSSLLNDNTRKDQKVRNERKVSATSSGNAQFD
IKFRSNFEALIAQGRKHMKKGPGCPRRVYDDDNIHDNPAPTTNSLGSDNEKISPNIDGSC
AQIVANINVRISNRTHNNGVTSVGSSVEHGSSLNRKKRPLVSPCTLERKKNTLCYTAKES
LGASESNIFNGHSSKIASNQTTSSGQLRPSKSQKGTVINNPYRKKQRRSPSKAKADIQDK
GGNSSCAILPFQKRKFQTCNTLGDGGKRSDSTVLAQENITNGNTIGSNNRIHQNKTDSSL
SIGKGSQDDSSIKTETIPTRKKYVIRNPYLKSSGKKDR
>comp5752_c0_seq1; 422 39 488 LEN=657; translated
MADQLTEEQIAEFKEAFSLFDKDGDGTITTKELGTVMRSLGQNPTEAELMDMIQEIDADG
SGNIDFPEFLTMMARKMKDTDSEEEILEAFKVFDKDGNGFISAAELRHIMTNLGEKLTDE
EVDEMIREADIDGDGQINYEEFVKMMMSK
>comp5753_c0_seq1; 214 213 782 LEN=905; translated
MKTIEIIRSSPRPETSLRFSPSEDGDEKSINVLGTRMECCCSNVGNSGIGTGFYRNGYCS
TGPQDFGRHTVCVQVTEDFLEFSKSVGNDLSTPIPEYHFPGLKEGDQWCLCAQRWAQAYA
FGKAPKLYLRATHEKTLDYVEFAVLRQYALDGKEADAEKGDLDEKRNILENIFGQESSPS
SPPNSESEE
>comp5754_c0_seq1; 293 106 1161 LEN=1161; translated
MYISEKNCSALRSDSVSSHKRIVSETKTIDKNLGKNGNDISNNAKDTCTNSKTKSLDRFP
LTKDKQQKCLRTTNVTVEVSGSQFCIVPTLFQHIENLPWKKQKERPRGKKRTTLRLNANP
DVFECVLRFFLHGNLPDPKELSTRRAKTLIDFVSPLDPVAVKPLVHHLNVFVAGQSSSSV
NSKRSLMLKKSISNFSSWTKLVHSHRGASTASNVDRNNAATVSGGTTTSTNPPRECGDSA
ANNAAKAALPTPMGSSSSAVTTVPTQIEQPIPGSITTAAEVSVCSMDEASSVSKLSLQSS
WTQPVPDENSLSVSGSASAHAASSSRKPAPDPVPAPVPAPVPVPAPVPAPVPX
>comp5756_c0_seq1; 447 261 1667 LEN=2251; translated
MSERDIGAGYHGRKSSSGRGRGRGRGGGRGRGSGSRTRGAANEIAASTNAAKGANAEDLH
PNDKSIKDAPIRNHRFQPKKGRGTWGETSNRNHNKKNNHTRNNSYNNQHKKAYDKGGKRG
QLPLKVYYTGDGEEDRANGFAGMHRDCKALRYKVHKPMCDCPKLLRMVRQSEATIEGTTI
TEDGKIDALLVVPDTKQRYLYVCSDTSLDRFEEDPRGAERKLRAEIFGGSEGGTTPVDES
SFKLSCAGCLLFGHEPNGFCLVASPSFEAIDAVREESLRSKNRRADGPLHHDHAHFVAGL
PRSSLWDLHRAKTRAQNAGAVETILCHLSGQGVSAGTEPTADRLSGGIELGHHLTSALSL
TQNGTKHGSHRKPPAVAPGDVGDEPSWWFVIGYDDGSSLELDLPGGKRHLGETSLEGAIR
ETEEEASLVWDPSWVVECLESKKASERGNRYYLLRPPQSFLEQRGGDG
>comp5757_c0_seq1; 1148 337 3945 LEN=4023; translated
MIRSESGTDLLLHSSLSVPFHIIDFEVNGIGGGFVVVNHYGDYGLSAARIDIGVLGDDWR
DIDSALLYLHSTASLTSTKCTTTGLNLGGAGVLLYFCSRNQFTDSNRKTLLAALVEELKG
FTKSNILCLPRNLSNEDLTHMYKKNEIVTMRHDNEGIDNAFVPLAKTKRKGSFAIATESF
DYPIPKALITQEENLVAKSAAYSSFGSILTLMKAKEWTSARIVLLGVTHLALELYQLLKR
ETDTVFLSNPHPSKEDLQSKIPSDAFISWDEAHKSKEEWDIMVFCSQSCPSLDESVISSI
RTKAVISVSDDLLPMDEKRREEVLLTLVKRDIFEAADGISDLGEIAKVFSLAGGTTFSFN
DAFGMGCRVMEKKLHLHGIVHSEDVTAKRKFHDLMLHALEKDENARLFGLGTVMHQSSDR
MTAWLWSRARSMCPSYRAMSSKSATVKPEKVSYLDMGSGNGAAARWICKQGKKIHITCID

VCAKQSYENRNISDEEGLGSQIDVVQGSYERLNSDYSNFFDGCMSQDAFIHAFTKRSAFL
EAFRVTKGGGWLLISDLMRGDGKDGNEEMEAFVKDHNITSWATPNECVQLATEAGWSEVR
FIDCTTEINVSLHGLLKKIKSMIKSGEYEGRNLQLLKTHRVRLSNRIGQADRGIFKWGII
SGRKPYDVVFMSEPPVSPESRSMINYSVNSLDGDLKFGTDVLVVNIGEKLDYEKIMELPS
TTRLIVTLSAGLDHICVKAAEERGIRIRRAAREAIVKSVADYLLSNVIFGLRNGFQNVGV
PFPGASWDLTWNSDGVDLDCSKIGFIGMGAIAIETAKRIRSLSKSCELVYHVPSDIRCRF
EEGTHHMYHVGIADLLSTCDVIIPMVPLTTSTTGLINYSSFSMMKRTAIFINMARGKVVE
TAGMLRALDEGLVRHAILDTTDPEPLPPDHDLWNLKNCTITPHFATNTTYVRKELVEDIP
NQVEDTLEERGILRLEEQRMRHELSDAYRITREFGLDELVWNHISVLLSDGSFLITPGSR
MFDDVGPEDLVKSSGNITADIIHEAVYKRRSDVKAIVHLHTPASVAVSCLEMGFVPLAQE
AAPFIGRVSNHPWHGVSNDREEQALLGEAVKNPKTNTLLMENHGFCTFGKTLGEAWVLAY
YFDKACQTQLSCLQTGQKIRYPSEDVLAHAAEQSVLPEFLPGACEWDALRKMLTRKQGRF
RR
>comp5759_c0_seq1; 1018 161 2122 LEN=2312; translated
MARVTSKALLLVALLGSLQTAASIHRAGGVRELSFQHPIEPHLPHQGVSKGTGSSLPVNK
SGKSARDDSGDSPYYYEYQKHGKKEKKGKSSKSGKKEKHYYKHNKYEVEMEVVVDYSKST
KGVPKFEFIEIGHKSWKSSKDSKSGGAGNEGDGTMYAKDAKSSKDGKKPKSYGSNGNTNK
QTKAPSTVSSYPVAPTSSAAPYSSPSTPVPTMLGSFGVKIPEYSLAYNLLTTLEPTNKEL
SELEVATTSYLDDFFVDEFDKDDFTVFEVFTTEIVDSSTQGKVVVVSFGKSVARFDGLSL
IAPTSEQLDSAVVEAFTGLHMIQYEDWLKQMLPSNNIFIGSKVQYLQDDEGIADKARRGI
GATGIAASAVAFTLLVAGAVLYKSKSGDREGQTDKLNKASGDMTVAGETFAGETYDGTFA
GDTYDGTASVSAASIDYVRRFDGDEEDGMKKDNLGAIPEKPWGDDDSDGENGEPYSGGGI
PANKTGTAFRKGVTSAPRTASFEDVALQAPAYGSGFRHNIMPDPSSSEDDQSQMSDSELS
QFVASTRQVGTHISGDNTLEIKSLLSMDSMDDHSSSGLSVRDNSSRRLRTVAEIEALLSS
ELNDDNGASNTDGKEPETSRPRTVEEIESLLTADDDESIVESPFSDEDESIVE
>comp5760_c0_seq1; 166 889 1101 LEN=1543; translated
MDMSDLCGRTIKVSLAQQNQLNKLMSSMGGGKGQAIWSSDEWFQEHVVGGSEEDRQKAKE
AAQDRETLKD
>comp5760_c0_seq2; 280 258 713 LEN=1155; translated
MSMESSSNTSGDAGIIVTSKRAVYVGGLGEDVTPQILRSAMIPFGTIKSLDIPMDYKIGK
TRGFAFVEFDDPEDASECIFNMDMSDLCGRTIKVSLAQQNQLNKLMSSMGGGKGQAIWSS
DEWFQEHVVGGSEEDRQKAKEAAQDRETLKD
>comp5762_c0_seq1; 221 103 570 LEN=1438; translated
MTRAEYGSNSVNVEHNETTNLIDNEGFMKSMSETDSNGGASNNNSSDDLYESETWREYLL
SWRFLELVLCVVPVLLMGMYFEFVNPEPMTDRPIPFQYAKGGSTAMVEEGIDASSDFHRS
EGGDDEYKSYTNIIWRSSNLEENTGETVSHVAYIVW
>comp5763_c0_seq1; 1030 107 2740 LEN=2740; translated
MDGPPEGGSSEQEQPPGIAGPGIVARNDDGIPFAFDASIGRSGFRIGIVAPESPVLFYRP
EHAPVPIPQQHDAGNATTSSARLGGFWSGDRNRNRHSNSNAFSRYRGGSLSCFSTTTGDE
KSEATSSDSGPETGETAEASPSPPPLKNQKTMEFQAETKQLLDIVTHSLYTDKEVFLREL
VSNASDACEKLRHLQSASDSASDLTFVDKERPLEIHIELDEVESSLTIRDTGIGMTPADI
VSNLGTIARSGSKQFMNQLEQARAESSDDNGALDAAKGIIGKFGVGFYSAFMVADSVAVT
SKPATVTDGEDHTVSVWTSEGIGSYELAEIDDSDEGGNTQRFDRGTTVKLFLKEDYWGLL
SESKIKDVLNKYSNFVRFPIYVNGDRVNTVEAVWARDPREVDEETYSEFYKYIANAIDEP
LETLHFRADAPLELRALLFVPSFHSEKYGMERMEPGVSLYSRKVLIEHKSPDILPDWMRF
VKGVVDSEDLPLSISREKPQDTALIGKLKKALTRRFLSQLEKMAKKQPEKYVDEFYREYS
HFLKEGICHDYEFQPVLSKLLRFETNKNTTRDLISLDTYVGNLRPEQDKIYYLTAPSREA
AAMSPYLEAFEAANVEVIFIYSAIDDFVMANIGSYEGRQLVSVEKSDIDLSKFEKKKDSN
DDKADSDDDKDKTSDDDNDSETKLSATEAIDVCTWMKKTLGDKKIVSCRPSSRLVNSPAI
VTDNESGMMRRMMRMVDNSDGRDGMPLPAQNVEINPSHPIMVGLHQLKETEPVLARVLAE
QVYDNCLVAAGLMDDSRTMLPRLNDLLVSLVKGAKDSKKSRERGAHLWMHRRVLLQESAR
SPSLIQELGNGGVIRENISFVHFLDAGPTAAITSLYVX
>comp5765_c0_seq1; 1010 132 1904 LEN=2026; translated
MGADDVARQHAMVEFLGTFPTLSGSPPEQLSDLSDGVALFEVLSEIAPNHFDPTTIARHL
GDNWALKSSNLRKLIRNLEYYCHEDLQKDAAESFQKISSNVGKISRTGDADAIETLVELV
AAVAVTCPQKSEVVGRIMSMSSESQIQMKGIIESSLELLEDYVIEENDDFNVEDDPEMIF
SEEAGEFGVHETGKANHEASLFHANHLKQSSDADPGELEQALAEAKRELAAQKSVMTDIR
EDADKAQSKLRALVEDLQDRLAKRQDELITSEEELRDATNVLDDLKSRVGELEEEKAQLA
DELDLASAKANQLHKAEATVMAYKKKLEGVGVMNQQMTDLEDQAAGYLRQIMDLEGEVKK
TATLQRQVDSLQKRLGDLEKQKNDSVDSLKSSTTEISELKDTLAEAEKAKKLYMDELKEL

RAQQAADSEDVDIVGFETPAQVSSTAQRERTMRLEIENTKLKSEVENLKKASSSGEPVTT
VSNAEVENLHNQINALKEQLEKKNAENKKIVSDKDKLEAYTKRTLAKFQDKYLVALQECK
AKLKEKQDKIEVLESRSATEKSAQKREERLLSSTIFELGLSIMQNRLKDR
>comp5766_c0_seq1; 125 1 698 LEN=1618; translated
XSGDEEYLSTGDTDNETDTETDTETNAVDRTGGRVDDFWRYDVLHAYEREFTVNGGMSWL
RATNSTVAFVELLVEKCGTMCDDQIVLNELLGLDDDNAEAHRGTGGLRMEWGASHPSTGR
SAVTGHSVKIWERDFVFRGVVAADSNVNGETKPPKCPPKERNWISMPRIDDHNVVVNFLD
QLPPIHTSNRLRKPKVRKRKHRLTRASSHMYKLALYDVWDTIAAIVLLTHWP
>comp5767_c0_seq1; 456 1 978 LEN=978; minus strand; translated
XRESLKKLEILKRQLCGGDAFDKDRVAIDVHLCARCGVGDVQRRKEADKTIAAVGDILNP
GVGVKPADEKTVHPIFEIGDHIHLAIEKGREHELIRAGTAAHAITARAAVDRVIASAAVD
RVIATAPGEKIVAVFAKDAVIALAAAHAVIACACVDGVLTGAAADVIRAIIAKDQITAKA
ARYAVIACAARERVIVRPPCDHVIAVAAQDAIRPKAAANNVIALAAVDGVIARTTADIIV
SGATQYEVIAIRGGNPVIACAPIETVIAQAPRNYIIAVAADQSIVRVCAEQHFARVTRHN
EIARKPEHRDAHLSHRHSLIPEFHPFX
>comp5768_c0_seq1; 748 342 2117 LEN=3023; translated
MLLSRSIVNAVRSQNPPGRFLQKNGKTKLWNDVGDQRAQEKTSQALREGAPDIRKKVAAA
ITASTSGDSNSGFMNETVTSAQIPVPPSAPQHQQTDATDPSKSLPSNGRMTARESQQLYQ
HQQQQHALDNSNNNMYGRNGMSSGGHGHMNLNERGRMVQNAVMMANNGMNVNGQHCEQSN
NNIQNFQIHQHPYQQQQPSYNRYQQHQHQNLHHQEQYQPQNQQQQLAYYNKQNKQQESNN
TTTVPTPTVDELSAATPVELETNGLSFGSIAMTDDEMHLLQQAATISRNNPADQQYSIEN
MSKNGRHNQRHLVSREYENNNIDSNVNKNNDIINSRSAVPAAPTGETLEPTGTSIGDISM
MSGGTNFSNFQMKLDPNAGGTSFGTIMSYNMLNNSDPNMVDGGLMDVVGTSFGSMSLDKN
SRDNLFRTLEIAGGGREVPPMFRNETKAFGNLLDCSDTESESSRDKDQLTKQKSQAWEIM
KTTLEKQTSKDLMPPPLGRAPHENNAPQPSSVTSGNPHPVFDNVEIALPTTTMEPNLSTL
SAWSAAEDDDSAAVVAKNQNALSTNREDDEEGDAAATPAPPPPQLTKTDSF
>comp5768_c0_seq2; 733 1 2012 LEN=2918; translated
XKIISQQQDIENDSTLATAQTPPMNMYANHPLKDIQKPHEHDVLCGRGGGTNNHIGNSHW
RMLVAANKQLYITLPKRQKMLLSRSIVNAVRSQNPPGRFLQKNGKTKLWNDVGDQRAQEK
TSQALREGAPDIRKKVAAAITASTSGDSNSGFMNETVTSAQIPVPPSAPQHQQTDATDPS
KSLPSNGRMTARESQQLYQHQQQQHALDNSNNNMYGRNGMSSGGHGHMNLNERGRMVQNA
VMMANNGMNVNGQHCEQSNNNIQNFQIHQHPYQQQQPSYNRYQQHQHQNLHHQEQYQPQN
QQQQLAYYNKQNKQQESNNTTTVPTPTVDELSAATPVELETNGLSFGSIAMTDDEMHLLQ
QAATISRNNPADQQYSIENMSKNGRHNQRHLVSREYENNNIDSNVNKNNDIINSRSAVPA
APTGETLEPTGTSIGDISMMSGGTNFSNFQMKLDPNAGGTSFGTIMSYNMLNNSDPNMVD
GGLMDVVGTSFGSMSLDKNSRDNLFRTLEIAGGGREVPPMFRNETKAFGNLLDCSDTESE
SSRDKDQLTKQKSQAWEIMKTTLEKQTSKDLMPPPLGRAPHENNAPQPSSVTSGNPHPVF
DNVEIALPTTTMEPNLSTLSAWSAAEDDDSAAVVAKNQNALSTNREDDEEGDAAATPAPP
PPQLTKTDSF
>comp5769_c0_seq1; 301 184 975 LEN=1241; translated
MHGNGHTNTHTGQFVSCSLPRGTTLPVQHAQSAMRSAIGEMELDEILHNRSRLNALIKGG
LQEAALPWGLEVRRYEITEITPDHQIRIAMDKQAAAERDRRENVLRAEGDKRRAQLTSEG
VKISLTNESEGNLVKVQNEAEATKQRILLEAEGQAEAIRLTAKAQAEALELISTQINKKG
GSEAAKLALAKEYVSMYADMGSKSNTIMFNERPADVNALLSQAVVAVQSATQAATTAPPL
EADDQKPPAEAIDMSHSKVETPE
>comp5769_c0_seq2; 574 132 1373 LEN=1639; translated
MTLTTRSLRVCRRAGAVRLPGFSGTSTNTTTNTTTNTTTNTNAFVGDFYSSGPKRQFSSY
VLDGFREGSGVGGGIPSRWPVTRPNTIFNIVPQGFKYVVERFGKLHSIEESGWFLAIPLV
DQIAYVIDVRERAIDIEPQAAITRDNVSVEVSGNLFVKFQDPEKAAYGALNPLYSVTQHA
QSAMRSAIGEMELDEILHNRSRLNALIKGGLQEAALPWGLEVRRYEITEITPDHQIRIAM
DKQAAAERDRRENVLRAEGDKRRAQLTSEGVKISLTNESEGNLVKVQNEAEATKQRILLE
AEGQAEAIRLTAKAQAEALELISTQINKKGGSEAAKLALAKEYVSMYADMGSKSNTIMFN
ERPADVNALLSQAVVAVQSATQAATTAPPLEADDQKPPAEAIDMSHSKVETPE
>comp5770_c0_seq1; 263 39 1184 LEN=1337; translated
MQSDKTIQFWDDYHTENNSLEWISKPGKELLAMISDQLEIEKNEDDNGCGDNDDARGRNK
VVSMLEIGCGTSTLVRDVKSYVEERHSGISVVACGSDVSRVCIDTNRKRDCESVDGDYSN
KTPGKGESSIWYEVLNILEGEPSRRNWDVIIDKGCLDTFLFRSRQRGAQNRVYPESLRLV
LDKIHGWLAATTPVGGSCGGGLDSNRTNSSGTGGKSRFIFITPRSKIKAVRDYSGFSSVR
RLPLPPMYRATLESKQCCRINDNTEAGAAFRNDINSSKTIPGKESVLSPGYMFVCTRNDA
YQPGVSQVFVSNGSTRGSVPTDDNRCPCCLLTFRDFRKAENVEFRGEAYWTRRWKAHGLH

CRGQPPCMHEKVVVMLPLSTD
>comp5771_c0_seq1; 361 285 1355 LEN=1640; translated
MPSPSEIVLEYVCPAMGVVLANLMFLAPLRDLQKAVSLGLGLGDLNPTPWAFMVGNCLGW
TAYGIIINNWFVFWANYPGFLIACWLNLGAVKLLYSSHHQEETRRSMVQYLSDRKFLVNG
QLESTEEDGEVEQQPQSLVHIDDDENNKDKYTNWDTSNQSNGCDQEHDEDPETNAAATTS
TAEDWAKIIWDVTSQTTPAKTPHERLIMGILILWTVVVTVVGFHAHYHTPSDTDTDSGAD
SFAQTVVGYVVNFNLVFFYGAPLSSILAVLRTKQSDSLHGPTMVMNTSNAAFWTAYALAP
QINDPFIYVPNGLGVVLGVTQLVLWVVFPKTPKATAKDDARNNNMQLAASDESASA
>comp5771_c0_seq2; 338 789 1541 LEN=1826; translated
MVQYLSDRKFLVNGQLESTEEDGEVEQQPQSLVHIDDDENNKDKYTNWDTSNQSNGCDQE
HDEDPETNAAATTSTAEDWAKIIWDVTSQTTPAKTPHERLIMGILILWTVVVTVVGFHAH
YHTPSDTDTDSGADSFAQTVVGYVVNFNLVFFYGAPLSSILAVLRTKQSDSLHGPTMVMN
TSNAAFWTAYALAPQINDPFIYVPNGLGVVLGVTQLVLWVVFPKTPKATAKDDARNNNMQ
LAASDESASA
>comp5772_c0_seq1; 583 190 2093 LEN=2178; translated
MILSSSRFSLFLASGSLVLFRSEAFVPTSISSSRSTQQWMAGGEGGESEWVKALLESTAP
VPGAFEDEMKMKGLLGKKMDGNPKLSANARLINWLSEEGDMYLSEESSWGEAPHPMAIST
ETKDEITNESSGRGLLARRDINDGDSLLKVPLKLCLTKASARKALGKDVLPREINEYLAI
ACQLIHERYVLGEKSFWKPYIDVLPETEEVNPTFTWSDDDLSFLNGSPIIAATKSLQMKL
QREYNDLLGGEDGLISKHPDRFPKEXTFLTQSVLALVEGGKKSLAQHSSSVSCLLHKPIT
QKPCLXIDHDSYDPIQIAFHDENWVWAFTMLFSRAIRLRSLRDGEALSMVPYADLINHSP
FSQAFIDAREGGDWLFSSGEEEVILYADRGYRRMEQIYISYGQKSNAELLLLYGFAVERN
PYNSVDVTVAISPLTKSFVKESGDENLIVDPLADEKLAFLESVGRESLVDFPCYADRYPV
ELLEYLRLMQMTPEDTRGKPLKEFDYARSISMANEAAVLTSVINAVTRQLDKYPNTEEED
AALIKDKAMFRLLSYNQRMAIRHRRNEKRLLKRTIAALENQIRKSGLDEVDLKRAEGSTL
GQLLPGDERRYGMKQRTALEDRLEKMGLPVDIRK
>comp5772_c0_seq2; 703 190 1938 LEN=2023; translated
MILSSSRFSLFLASGSLVLFRSEAFVPTSISSSRSTQQWMAGGEGGESEWVKALLESTAP
VPGAFEDEMKMKGLLGKKMDGNPKLSANARLINWLSEEGDMYLSEESSWGEAPHPMAIST
ETKDEITNESSGRGLLARRDINDGDSLLKVPLKLCLTKASARKALGKDVLPREINEYLAI
ACQLIHERYVLGEKSFWKPYIDVLPETEEVNPTFTWSDDDLSFLNGSPIIAATKSLQMKL
QREYNDLLGGEDGLISKHPDRFPKEHFTYENWVWAFTMLFSRAIRLRSLRDGEALSMVPY
ADLINHSPFSQAFIDAREGGDWLFSSGEEEVILYADRGYRRMEQIYISYGQKSNAELLLL
YGFAVERNPYNSVDVTVAISPLTKSFVKESGDENLIVDPLADEKLAFLESVGRESLVDFP
CYADRYPVELLEYLRLMQMTPEDTRGKPLKEFDYARSISMANEAAVLTSVINAVTRQLDK
YPNTEEEDAALIKDKAMFRLLSYNQRMAIRHRRNEKRLLKRTIAALENQIRKSGLDEVDL
KRAEGSTLGQLLPGDERRYGMKQRTALEDRLEKMGLPVDIRK
>comp5773_c0_seq1; 55 1 359 LEN=969; translated
XENKTIRHADAGLESAIESESIRLQPPNRTGRDRTGQNGTPKQGTIFHDKHSIVSLRVDP
RTEPWRLQEQPQSQQQPPPGPPGPCSPHRVAVSLAGPRCRASRVPRPGAENPRSTFSPV
>comp5773_c0_seq2; 172 191 505 LEN=737; translated
MAFARATAIAAATATGAARSVLTASRGRLVGRPPMQGIAGAAAWSRESAIDVLSGDERLR
NDFAYALSMASPSLHDDCGTGGNSSVFLSQRQREQLAISKDQLR
>comp5774_c0_seq1; 112 1 631 LEN=1033; translated
LLSNKRQTNNPFPDLTTHSSKNDATNESFPFSHDLSPVDTGIRFVPHNVCEGLQVAPAAA
AASGAWTWYKSMLIAKPLVTKSITSSCIAGVSDVMCQKVVISATPKEERTSKLDSTRVLH
TAITGAVWSGPITHFWYIVLEKMYKAIAEAANIQDPAIGLVIKLILDSTIFSSVTLTGYF
TVRSILEGTGLKGASEKVRTRFLSSLFGAW
>comp5775_c0_seq1; 109 310 597 LEN=1152; translated
MEGHRGGFERCDRRRGTKKALSAPAERRNPSPGRLCVDPRGCCAKRGTIGRWGPPQWSRP
AARGVFRPGDRRDAGFSGGGDVPAERVCLVVKVSV
>comp5776_c0_seq1; 178 203 367 LEN=1269; translated
MSTQVIDWDEAMQQCGEDEEFLRELLGDLRDETNTQMAKMEEVIRVCRAYRVSL
>comp5776_c0_seq2; 261 203 586 LEN=983; translated
MSTQVIDWDEAMQQCGEDEEFLRELLGDLRDETNTQMAKMEEVIRNPTGSPFVELHRAAH
VIKGAASNLMCSQLRDTALALESAATSAANAPDGATNPAVIDTVKAKFEELKNAVCLYNA
HLTSLNV
>comp5777_c0_seq1; 200 160 639 LEN=809; translated
MAILCLSLAYTTTAFVGRPALSAARSTVRIQADGGKSDDAVVPSAESAAAITDFMAKAHE
EKVAAMARVEDKYKGQIAELQQKIAELESVNKQTTPTSGNSFAFPATNKDLTEKVQAYRA

FISDYIVKAQEEKAEAVKAAESKLTAKYEAIIDGLKSAA
>comp5778_c0_seq1; 339 70 600 LEN=765; translated
MAESKQNVMKEIKVDKLILNCCVGESGDRLTRAARVLEQLTGQQPVYSKARYTLRSFGIR
RNEKIAVHVTVRGEKALEILERGLKVKEYELLQQNFSETGNFGFGINEHIDLGIKYDPTT
GIYGMDFFVCLTRKGARVAKRKIRRSKVGFSHKIQKEDAIKWFQDKFDGVVLAKPI
>comp5779_c0_seq1; 405 158 1568 LEN=1568; translated
MRKLLTSVAAIGLLAAPALAQVSDDKVKIGILNDQSGVYANFGGRYSYEAALMAVEDFGG
TVLGAPVEVTTADHQNKADIASNIARQWYDTEQVDSIMELTTSSVALAVQALSKEANKIT
ITTGAATTELTGAQCSPYGFHWAYDTHALAVGTGGALVEQGGDTWYFLTADYAFGYSLEE
NTGNFVKANGGEVVGAVRHPLASTDFSSFLLQAQASGAKVIGLANAGADTQNAIKQAAEF
GIVQGGQRLAALLFTLSEVNGLGAEAAQGLTLTESFYWNRTDASREFGKRFLERTQAMPN
MIHAATYSAVTSYLKAIEAAGTDETEAVAAKLKELPVQDLFAENGVVAANGRMIKDVYLM
EVKAPDQITEPWDYYNVLSTIPGDVAYIKPAXERLPARDAVRHLRLMTAHPVSKQDQPRV
VLSARGLGKDFSGFTAVNNVDLDVQHARIHALIGPNGAGKTTVFNLLTKFL
>comp5781_c0_seq1; 259 1 1336 LEN=1511; translated
XDVAYYVYISRASLVSNNLVGTLPAEISLLTSLTYLESIENELGGTIPYEWHNLQNLRTL
IMGFSQLTGTLPSWMPLAWPNLEYLYLSNNELSGFIPDEFFGFQKLSILALDDNLFTGRT
DVIWNSLHSLEHLYIEDNGFTGTLPNLIAKSHPALVTLDISSNRLNGSLPDDLFRLPKLE
ILDVHSNHFISSIPEDVPENNQHLKFLALYRNDLKGSIPSAIANLRHLSHLDLSSNYLTG
TIPAELEKLSDTLTYLFLGANDYNEGPIPSFVYDMTNLRELSLKSTHRTGTISGVIGALE
SLLVLDLDDNKLSGSIPDQIGMLTNLQFLLLNRNQLTSSVPSHALAKLEKLRVLLLDNNR
IFGDLNPICGLDDLTTVFVDCGEVACLEGCCFCCLDGQVCHNYDHIASHDPLWEGDYKRQ
FFDFSSNEENGNGGRYYPNRDDDYW
>comp5781_c0_seq2; 678 1 2448 LEN=2623; translated
XSLPGTLKGCSSQCMVKTYSSLEEEKDVYCDDDNNLRWQQQQRKXQKPTPPMAAFPPDPF
LSPENGGTTEFSGLPTIDSSASGDADTTDDSLPHSAISPVARKILLGADDDLFDDLEFGD
ESNNEHKSPLAEEFPFEMPFDDREVNGTTNLNYRRERQDSFPDVEELKLSVAAAGKSSVS
SSLLSERKRQIGCMLFSLVMGIIALIAFVAIAIDARKNRHQTTSIDSQNHNKNSVENLDD
SGSSTLVFDGKGTSVGKYDDREVHLKNYFARYGIVEEINYQGSWTAAMNTPQSEAIAWLA
NEDTQFPVLPTHNEGPDTPQGYKLLMRYAMAVLYFATDGENWKNDLHFLDAEKPTCDWFQ
VFPPPMGQVGVLCNQNTQEIVGLSLISNNLVGTLPAEISLLTSLTYLESIENELGGTIPY
EWHNLQNLRTLIMGFSQLTGTLPSWMPLAWPNLEYLYLSNNELSGFIPDEFFGFQKLSIL
ALDDNLFTGRTDVIWNSLHSLEHLYIEDNGFTGTLPNLIAKSHPALVTLDISSNRLNGSL
PDDLFRLPKLEILDVHSNHFISSIPEDVPENNQHLKFLALYRNDLKGSIPSAIANLRHLS
HLDLSSNYLTGTIPAELEKLSDTLTYLFLGANDYNEGPIPSFVYDMTNLRELSLKSTHRT
GTISGVIGALESLLVLDLDDNKLSGSIPDQIGMLTNLQFLLLNRNQLTSSVPSHALAKLE
KLRVLLLDNNRIFGDLNPICGLDDLTTVFVDCGEVACLEGCCFCCLDGQVCHNYDHIASH
DPLWEGDYKRQFFDFSSNEENGNGGRYYPNRDDDYW
>comp5782_c0_seq1; 716 1 1791 LEN=1866; translated
XTIRLMVHGSSSVNPEEFPFPSLSSLSTSTYKSKEFGTVGVSSLGLVYKVSGFDQLAKSS
EQQPERGVQPVVSAESSNRRRPLLPVATESVSLGKEGSYRAFGINKNVTNCINITKSNIV
INNNNLRSDFCQMPAGGDSCGGSNNLRRSHREQGLKKSPSDTATVTPFSVSSLPLSLVNA
AWSVLILLVLGTQHGGSGLVPRYGTRNAFRAGRGRSCRGGQGLHLPGNWGGLLLLPTMSG
YSALFADAFHPEASHPSADKTSENENNNKSNNSAKSRDHGNESTCEADASNNSGTPHPEE
DQLLLFHYDSTTSTQDEAKRIAKRLSENESSSSVSTFCVTAASQSSGRGTTGRQWLGAPG
NVFVTIGFPMQTYMTTMLRERRIPLTLLPLKIGDLTASLVQSELDDENCAAVVGDASGPA
RVTVKWPNDVLVDGKKISGTLIESSSDWFLVGIGINVAHAPTVPTTGKDYGRPSVSLHEY
CGPQQQNQQQQQSDDDAITEAAQIEHARDLGVGLALGIHRWIQNDPSEKDGTAESIVEGW
KRWLDWDAELTLRNDNDADQSRRKTVRLVDVLPDGRIRVRHKGSGKEEVLVSDYFV
>comp5783_c0_seq1; 108 185 691 LEN=1193; translated
MMWKRTPMNREREAAILVPLVSCEGTISLLFTTRSSRLPTHASEVAFPGGHRDKMNDVSL
EATAIREAQEELLGKYPWEQVEIIGRASPLPSPSGMPVTPVIAVLPHDIDPDTFPGCPSE
VEDVFCVSLEELLEVETSEWSKRFQNSAPVFHALNGKKIWGLTAIVTR
>comp5783_c0_seq2; 123 185 604 LEN=1067; translated
MMWKRTPMNREREAAILVPLVSCEGTISLLFTTRSSRLPTHASEVAFPGGHRDKMNDVSL
EATAIREAQEELLGKYPWEQVEIIGRASPLPSPSGMPVTPVIAVLPHDIDPDTFPGCPSE
VEDVFCVSLEELLEVETSEW
>comp5784_c0_seq1; 242 37 609 LEN=875; translated
MMRISTLATIALASFLCAKTQAFSMRMGPEKQTSMESRRGFLSTATAAVVTAAALNPIQN
AAAAPTIYNLSNGIKYAITQDVTKGSYPEQGDIIAIEYTGYLSNGAIFDATHSQGKKNAL

LFKLGSSAVNEGINSMVSEMKVGQKVQAIIPPALAFGDKGICLENGECLVKPGATLVYDI
FLKKASIPPP
>comp5784_c0_seq2; 221 37 417 LEN=1000; translated
MMRISTLATIALASFLCAKTQAFSMRMGPEKQTSMESRRGFLSTATAAVVTAAALNPIQN
AAAAPTIYNLSNGIKYAITQDVTKGSYPEQGDIIAIEYTGYLSNGAIFDATHSQGKKNAL
LFKLGR
>comp5785_c0_seq1; 527 135 1593 LEN=1593; translated
MAYLNRIAILAATYVAANWIQLAFARRQREVVANEHNHRRQLIEAYDSFVLYLKMTEYEN
HDEETWCCEIPDSIAESLEIGSLVCNFEGVSQEFINMSGAISGHYIMKVQGAIIEQSLTT
SEVTMVVPSEATVIVEKIAEKDLRHRRRLASSLGTQNVLVVRVIDSNGAAQSSVTQLQND
IFLDNQCLKTRYEECSFNQVTIQPAGVGTGGVVDVTLDIPVTSGLNAVLYATERAAQRQF
RASQYDLVLYCLPPGTGSWLAFAAVYGYQSYYNDNFCSSMSALVHEVGHNMGLGHSGRGG
DEYGDTSGMMGYSYFYDDYPRQCFNAAKNYQLGWYNNQRIAINPLALSNNRETYILNGIN
YYSTDGSSPNKIVTMRLIEYGETSGTDYYIGYNQAVGINSGTTTSINEVTVLQKESGDPN
SYGRSWQVASLGIGETHTITNFRNSNTDVFITYLSLSGNDAAIEISTLNPTPQPTAPPPP
PPPPPTX
>comp5786_c0_seq1; 285 175 645 LEN=1257; translated
MNILNLTNDTPLEEIEQLQMDLEAPLLGKNNNSYTADDSCVMSFHIADNSSNNSNQVVSK
RKKSIAKNKKKPKQLMIHVNEDEDLDQSSQTTECQVLNCSFYGGLALGIILQALSYYVNV
FVMPMLQANDASGAGSGAQDALRIPLILGLGFLARYW
>comp5787_c0_seq1; 184 713 1333 LEN=1741; translated
VVLDNEDGTGHFLNKTDPIEEILKSPLAKDLFKIDGVKAVYFGRDFVTVTKRAEAKWQFM
RPEIFSALMDFFENPNNKVLLDKPQITDTTILEDDDEIVAMIKELIEDRIRPAVQEDGGD
IHYRNFEEDTGIVTVQLAGSCVGCPSSSITLKQGVENMLMHYIPEVTAVVAQEEEEDEGE
APFGEEPKKQKTYEERLAAAGIPFSD
>comp5787_c0_seq2; 351 1 1035 LEN=1443; translated
HNDSLRTAPSIHHTTHNKIRFLQEVLDIMRSPGGCRSFLARRWRRVSASAVSNKGQGERA
TTAPLGVATRGFHHLSMGDAFGSQPRTGQTPSQHFQNHTNLLFNYNRNGNSSVQIRTVFV
QTENTPNPESIKFLPSNTVVLDNEDGTGHFLNKTDPIEEILKSPLAKDLFKIDGVKAVYF
GRDFVTVTKRAEAKWQFMRPEIFSALMDFFENPNNKVLLDKPQITDTTILEDDDEIVAMI
KELIEDRIRPAVQEDGGDIHYRNFEEDTGIVTVQLAGSCVGCPSSSITLKQGVENMLMHY
IPEVTAVVAQEEEEDEGEAPFGEEPKKQKTYEERLAAAGIPFSD
>comp5788_c0_seq1; 353 211 777 LEN=893; translated
MTNMIKDVFDKQVLELKKLWANKRQLLFQLLNLAMIVFSALMIWKALMCYTKSESPVVVV
LSGSMEPAFQRGDILFLNNSVEQVGVGDVVVFKIKDRDIPIVHRILKVHQDGPDGRVELL
TKGDNNRVDDRGLYAPGQLWLEREDILGKAVGTLRYVGMVTIALNDYPVLKYVLVGMMGL
FVLTSKEG
>comp5789_c0_seq1; 426 103 993 LEN=1062; translated
MKTKLSTLLVLQLVFAHATTPTWAFSPQLEQRRASPSAVFASTEDVEEMFCGPLVSSRRN
WFHGIMVTAASSILVSLPVSAEDAVVAASEAPTPLAMKEFVDPAGLFSLRVPQGFYTLRR
TQKGDLPDAKTGKGRRGSSIFTAGNMAKAEVIAVERFPSRVLLEENGIEATGDLSTFPGI
GDPKAVAALIIVRREKDNQTQGTKTLDSVELTPDGKTLTFRLKTEIDVQKPELLLEQTGL
SRLFRITVAKATLDSNDGNIMAVFASGLEQDYTNGVDGPALEDSVNSFQVTNQSQS
>comp5790_c0_seq1; 483 132 1653 LEN=1653; translated
MEDSGVEVAGGPVNAKRQRKRIEKKKKMKFLFRKVGRQIGKRTGRGKQKVEYFSHSADKS
MIEYSLDQYQQEIAERGHQEEHRQGRDDDEWIRSPDLAGGIRTNTANGVNNLEGQSIEVD
SYFNPFNWMHSLTSESTPENEERKEINEDEREGIEIEENDGYLADQEKEKTPERIGRKHA
NKIESAALENMERYSVHPDTSSIQDAEARQVRKIKKHSKSRSSMPFASRVKSSGRIAKHP
PKVLTKETSGSPGDASIVLVSSVKRMVAANQKKAAATLVTSVRSMLLRSSEYKSPKLLLN
SVRALIHDAESKSARKRRFAWRERGRTIRESKAKMETEKAKEFEDTPPMGVVLLKSPSFD
EESEASLHSDASNHGERSKDGNGTDEYGTETEWDESAGVIIHDGGTSSETSEDYVGESDW
DGLTRDSVYSTGTSFDHECTRDATYSTGTSFDHECTRESAYSTGTSFDESDFDDDESSLE
EYCATVEVVRDVAEESDDESGDDDDDDX
>comp5790_c0_seq2; 269 137 656 LEN=656; translated
METEKAKEFEDTPPMGVVLLKSPSFDEESEASLHSDASNHGERSKDGNGTDEYGTETEWD
ESAGVIIHDGGTSSETSEDYVGESDWDGLTRDSVYSTGTSFDHECTRDATYSTGTSFDHE
CTRESAYSTGTSFDESDFDDDESSLEEYCATVEVVRDVAEESDDESGDDDDDDX
>comp5791_c0_seq1; 105 154 648 LEN=1480; translated
MKPRRRSPFRPLPAACPAPPLLRMTCLLLTLTLLTVLSMAPGALGFGFGFAATGKCGRTS
TPFVVPGHPLSLFGRNRTPCAKRPLRGILPLPSRSGDPFREPGEPEEEEEGAAPVPSGGT

DPGVPPQDGFDGEGFAKYLLPYAVALVGSVLATAALFRFVLLDY
>comp5793_c0_seq1; 487 1 1031 LEN=1186; translated
XAGNDSAGNDSTDNDSTDNDNADNDSTGNDNTGNSGTDNNDTNSDTDDKNNKDSENETDN
DEDTGSNKDSGNKDGGNGNSNASSSSRPGNPDDESLAKALGLDKSDLACMANSTGFIDRS
ETLQGAEKEYQAMAQLSPSIHQPKKVGYPAEASEEMERVCKAEGGAWFIVPRGDVTCESK
GCIHVYNFGNCVADTDSCAYMDPMVLVRVFFKEVLGLNCHAGCDDQEHNDVYNKKYDQKP
SGTAKPPAASPKPQTRAPHATPSGSGNLFHGASQAQTQAQTSASGTSMITAAFAVCAVLG
LIGFGAVYHKKWSGRSGRQQLRQGDIEMMGNDNEHDLWFRPIS
>comp5794_c0_seq1; 439 204 1670 LEN=1867; translated
MPQTKEHLCSTIGTSTTLAESTLASPDSSSNRSEFITENTVCGGSPDDSRDESGSRVSDS
KNELVSQLEKYNDTLASIFTPNHSGPGNDTDIGTGSSIVRVAPDELRKIQLLGTGKHCQV
HLVTASLSQTGDRVGKAKYACKSIDPERVISVKDFLDAASDLASEAKMLSELDHRHIIKI
RGLCSKPFSASFTDTVIPSVTRQGSYPKGTSTNGIDGFFLILDALKETLGDRLKRQRRAE
AIEKASRKRAKNYNPFKKVPPCDMTAAVTNADRLALYERIKHVVVGIVSAMQYLHSRKIV
LRDLKPANVGFADNEDVQLFDFGMTRPLAECSPDEACGSQLYMAPEIMNDGRYTLKGDVY
SFGVLLFELCSLQAPYAKEKQMMIKKYQPPQPKSGLSVLFRKKYRSPDDGSNIRQKSARE
VSMLVFDELSEKVGKHHLRPAEDFEELKALIPCERLCVLIQDCWSGEPEARPDFDQIQQR
LNEIFAKQ
>comp5795_c0_seq1; 249 843 1364 LEN=1605; translated
MTSSPEGGSDAKEQPAEPTPSRQQSPTNNKPKPATKSAPLRSTNASAKVTSTRGGPNRNK
PPVRMEATGGSSFEAMNAAAIAEEENAVVPATSMLAGLLPTLVAGLVFTTAGSMVGYVWN
SEWTETASSVFSYFWGDETDEASSPYGSNTNSATAEEYLGDHFYNAAPVTPDR
>comp5795_c0_seq2; 249 711 1232 LEN=1473; translated
MTSSPEGGSDAKEQPAEPTPSRQQSPTNNKPKPATKSAPLRSTNASAKVTSTRGGPNRNK
PPVRMEATGGSSFEAMNAAAIAEEENAVVPATSMLAGLLPTLVAGLVFTTAGSMVGYVWN
SEWTETASSVFSYFWGDETDEASSPYGSNTNSATAEEYLGDHFYNAAPVTPDR
>comp5796_c0_seq1; 244 182 625 LEN=942; translated
MFALRRTTISATVGSTGRKSSLSSLVSVTAPDETRSEQYEDRKNVFQTLFTTRSFFAVPS
PPQSSVDVLSIATATAANQWSYCWKSNFSSTTAPAFSLLDQLQQPMLLETLYRKGIYLIS
TLKRRRKMMNKHKLRKRRKKNRMKNKK
>comp5797_c0_seq1; 309 387 1001 LEN=1308; translated
MGIGRRLMAEVLEAFPKEEDEAAGISAQERKKMIEDKVYHIYLDNPEDVGGAHTRWMSGV
VATDDVEKYCDPLFNKNPKIKREKELHKNEPESEKKASELFEQALYQSIDLPVVDSIAIK
FPFSNGFLSGLVFSYKIIPEMRQLAAERGEPGNMSVVVSRCSVEGAECTHYIPLSKGIGF
HAGQPSTEDYQEVFLTKGLALLKL
>comp5798_c0_seq1; 505 176 1660 LEN=2069; translated
MDDQTLESSGSAITPRMSICLKASRDDPDMALFLTEIEDDGFSLEDNNAENDNPPSKDEP
SSTSNHRSKTRRSLLSSSSILLTGTPHSILSPSMSYDSVTTNTTTKMPTEDETDTQFGKS
RQRGKRRKPGNDRVRFSESVHFRNYMHVTEITSEERAAAWFCGSEYRTIFRNNMRIIRSI
AKREKELKKTIAKRKKEEQKLKKKLKKNKGKHSNNSNELGSCSDCGLCGDKSDLYWEEII
SIERDEEQGYSVRGIENEIPKKRKKRDQVYLQAKYTILSLQADLDAHMEAMQDEYEERMQ
KLIEGFSRTKNGKKKKHDRRNSFSMSSSSAQQMERDERTEKEIRDITATNKFQFAQYAQS
QYNNMIEAIAERYSEICKQHAKNALEAGCGDERTARAIDWLEESSRRNSVAVESASKGVL
VDTAVLSDDADDKRRPSTSTTVTDNETIIPPLSMRSIDEARNSDEVESLKETISKNQAAS
RMKRARKFLRRLTM
>comp5799_c0_seq1; 302 648 1253 LEN=1327; translated
MGKDNVYDTGVDNVGTVPASLATFLAVEKMAAEGSPVQLILNGGTCGGFKRKGGAIGDVF
LTTGVANHDRRIGIPGFDKYGIGTLETTIDPTKIAEELGYKTGICTTGNSLDKTDKDDEY
MMENDASVKDMEAAAIAWVAKMQNLPYMGVKVITDIVDGGVPSHEEFMENLMSAAKSLQD
ALPKVVDYICNHNQMDVKEEL
>comp5799_c0_seq2; 376 186 953 LEN=1027; translated
MTIKHVLVPIVMEAEATPFVEHLELTQVEDFFPSQTPFLAFSGTHSETKVTVITMGKDNV
YDTGVDNVGTVPASLATFLAVEKMAAEGSPVQLILNGGTCGGFKRKGGAIGDVFLTTGVA
NHDRRIGIPGFDKYGIGTLETTIDPTKIAEELGYKTGICTTGNSLDKTDKDDEYMMENDA
SVKDMEAAAIAWVAKMQNLPYMGVKVITDIVDGGVPSHEEFMENLMSAAKSLQDALPKVV
DYICNHNQMDVKEEL
>comp5800_c0_seq1; 324 1 686 LEN=998; translated
XKKQKKATKATIASMYSSSSLFLILATAIALCSVDAWTATSSASSFGGRSLTSTTKQVVV
LGNGSNMDMKKGKANVPPQMRGQYARQQEMAAMQQQMRASSVPGDDGLPVFNLFVRTTSQ
KIWYPCGSFKGDDRSAALAKSWSEGGMLSGISKKQLDGGIAGSLYSDLAKLKDTVMRAYP

QLRKKSNEMEFGYKLAFDGLPEDKAKQTFPVEPKEPKGALDGLRNIFS
>comp5801_c0_seq1; 277 1 635 LEN=667; translated
XVNVNVNVDVDVNVHTTAARGSICIQKRSFSSMQELPPDDPQAQFKPTPPPELNMEMARG
IQSANHLILKHGVGKQRLELLAREPESKTPLVVKWQRMMEIYLGAQLHVVAALGYETSEQ
GIMLYTQQLAQFVGTKCDPSVQEDFRAKGRDTWREMLVVAFGLDREAIEEKYGNELSIVD
ARNIVHRVATALIEPEILEEVATRVSLVGVP
>comp5801_c0_seq2; 172 1 488 LEN=923; translated
XVCLFCWLAGWLADGLHRPGSNVSGGLSTTPELNSPRPKRLFPDPFPAPRLMRCAFATPP
QVGQLPPQTDPQTEMGLKHSIIQDVVVNNVYLGGETPLTEELGFGTGSKGYALMQYVMAY
HESDPLCAQYTSSSMTKIWKAAGLDLGEANPVASKLPMSGPQ
>comp5801_c0_seq3; 376 1 917 LEN=1352; translated
XVNVNVNVDVDVNVHTTAARGSICIQKRSFSSMQELPPDDPQAQFKPTPPPELNMEMARG
IQSANHLILKHGVGKQRLELLAREPESKTPLVVKWQRMMEIYLGAQLHVVAALGYETSEQ
GIMLYTQQLAQFVGTKCDPSVQEDFRAKGRDTWREMLVVAFGLDREAIEEKYGNELSIVD
ARNIVHRVATALIEPEILEEVATRVGQLPPQTDPQTEMGLKHSIIQDVVVNNVYLGGETP
LTEELGFGTGSKGYALMQYVMAYHESDPLCAQYTSSSMTKIWKAAGLDLGEANPVASKLP
MSGPQ
>comp5802_c0_seq1; 1786 73 4935 LEN=5040; translated
MGKKQPLVQLIPTTREVKPPRDSSDAKETSSDQLPQHFLDASRNPFGTTSSEPTSDGKYS
DTRLIWKGKQPPPRNSRNNETNVTRMNRSTTNTSEGSSKTNKSTSSCEESSDGLCSFVHS
TGSISSLSAPNLSSSSPARSKHDGLEWKIETEANSTSPTGDCGSPISKRNSVQIAIQNFE
EIIELTREASDEVERKSKTAAARSKNRTRRSRQLSQKAISVSEGSPESSFFSTSFVSAMD
EHLASRRPNRLPTGDSIEVRKATSQDHQTGVLSTEAQVLVPEGQPILQPETPQRPEQMQF
ISFRSPSTINGLRRSRSPAEAATWFVTPPSPTWGVPEREEPNRTKEQIDKYESVPTFSEE
EANNESQEESFEVVHKSEPKEEFSRHRNSKNTRQKHQRKRTHTQRINKKKQQQKQEPYQL
VRVEESNAIEVLRQSNIEPRHSPKALSTAKKVLKLMSLRRHTHKKSPSHSASEAPSNKLV
NIDEDNSTGAIELLRSNSTIVPTTAPISITRGTTASANYLSDNDDDQSREDNILVDNTAA
PSSPLEENGKGNTSNTCTVDNDHKLSYKIAGTCVSTPTLVEEKMSIEIKVAKTLLPINND
SRDDSSSSDLVEEAFVDDNMPLPDAAMGQSAASLFETSENRPSSCLDDPHSLDEKASNVV
APLENEVRKSVNDNSKHASSLQQHENGPTTKSPAIPVPKIPAPKKDTVANYPTNYSNTTR
LVTNRDQSSEDSDNASVSTGKVRQVKIQVHTGSGDGNNINNIEVSPSVASLSDSKLAVQE
PDVKQGTLAIAKPTNNESTPEKGSGPVVKDKKYTTMVVKPLQGAVSTKPVSTKAVAKHVV
RVNVLGVAGIVVDRNRCRDVTGYKLSPAPPEQMKALVGISEPGRSVDNVSAFSGSLIHAP
MVGKERNRDDTSQRHIAVWASSHLGETPGSIVESNVLETDIADSASRVLNLNVALAKSDH
DWNHHAVVIGAAQLEITNEMMSDGDNRTIDLPVQTVAKDGPLSSNSNTNSLIKLRSTEER
KGIRKLLCEKVSDWEDANQGRVSHLTAAYAIDPTGDSMIRIQVRVEEVHTSTDQEKIKRF
GSFANRTAATEETDDSSLEGFKNAFDPADFIGGTPSNSMDEGLYSKPVELPSDFDEDEQR
AFQTVSWKEKLLKNQIEDIDEDPGCRVFGRNISIPSCTSTSRLSVVKHFDDGLENIADQV
FDTNQCAGIRSDEDLFSLDRNETVTTMENYTEYADSGVTLTTREEETVTAQKLLEEMKEA
LGPVPSMTEMKSFAKELALTSGEYFFSKKVRKSENGVDGDNASVGSATPSAVLTSEESLR
KKQDDDNKTHSTVSYESEANRFSETKFDIDDNDAIITAEEEKTLDSYQAVPVARNDGYKG
DDEESTLRDSTALMNEVVEEDENEDARHAGNRGDDKPSLPSVVENIAEVLSIQIQPCSFF
GTKEPAEVAFPNGTWDQNSVPRNVPQTVDPSDELSIGELTATSLEKDEIYERNRKFLMSK
LRLPTDIFSRRNASPTPDDEQLGNHRQLVHRSSSVSNQDQSTSENYFSDYEDGASGVTSI
YDNSRSTGSNSTKQTKNEQRDLMRRRSDEVTKTSTTGETGASETNASLQRIEEEEDVKSA
>comp5803_c0_seq1; 500 116 1818 LEN=2092; translated
MASFRSSXALALLAAALAQPILSGRSEAEAEPGHPEGTAARGHFEAAPPVPRLTREAFDN
LPVPGKYLLQLVPKSMPLDERESFEEFWDLLATRAPESQPFLMSRVDCGREPGFCRALVG
PHVLEHMESTKTFRLLSTEFSDDGRIGINVFHQDSLDPNTVNTWVRYTISRRQESLLMGG
DDLPESRSGGRKNIALLMLSVDGLGVRSDIWCRFLGLAAEQDVDVHLFLHDKSNSIGDGP
LCPMGTNIGTNVLHALANRRIFAIPGNMVLGTQWGSASLLKAIVYLLRYVVYHPEFRGAD
RYFLVSENSVPLVDPKTLLRIVSGPGYNGIDDNGNRQTLSVFQELLPGQPSPTERIDRYR
RLRPRYEREREPEEGSRGKGPRGEQTTDRARTTTGHETPPHRLDLALSDFSIVETWMVLD
RAAGAWFAAHDYFAEFELSYSAEESYFVSLCRRFGLSHRLASAAHHTDAAPAAGSEHERP
ERPEESHPMYTQWSTDFGTRHPDTLTEITQETLQRWGDGRDKKHSFLFARRISEQTRIDL
PWIKDGADAASGVATTGTNHHVTSNEL
>comp5805_c0_seq1; 756 231 2255 LEN=2367; translated
MASSADDYFGTLGGSLMKDLLADLQVDDNDWSLEQLEKELAYLDQEPSLVEQQSLPSLNA
ASLVVSHAQERQSSILPILPLEKNAVPLPPPGMGNSNAADAWSLSLQNFTSLSLQDDFLA
ADSARKQTRPPPPPPGLASLVGAEDYDVKEKQVVGPPPGLGGGAPKPPLPVQKVPVSAEM

SKTEPIVDPQPSSTTANDIPKEATTLPASNVPTMMPVQGTVVPPPQGKPPSMMPLQGAVM
PPPQMNPPNMMPPQGTVMPPSQMQGRMTMPPPPGQLLPPPPMGVPVQMPMQMPPAIGTPV
AAPVTGVVVGSAVSTPGPAWQTPSQVPMPVLHQPTAFAAKAYCNPYPRAPPIPATALASS
CMSGRDIAYVVHAIMKPLLLAGTSENDYYIQYIKSRVGGPGSDPINPRRIKDATHDVASR
TTKSKEWASEKSVLGHITKTNVARPRALIATPQPSTDQENKEQRQRANLWKARVYSDQAY
QAYQNVIDIWRAAPSKNGIPPQVQIHLVKLMKCMGVVLDNEKKVYTVSTDALKLILKLDK
GRTLICRVLEQALLPPNAVQAMLPDLLDIIIAMPTSSGKEMSNSAIVEEKTVGRLFQAIA
GIVPKLNITADTLVKCLEVAMVHGKSATRSIVRMGCMHSLLQKGAQVVPQDPSEDVKQAW
GNAERKFMSGLQGA
>comp5806_c0_seq1; 244 1 706 LEN=1116; translated
KEIIEAETQLQPNNTSSSARMQQHHRFFLVAFAVLVSTLSQNQSLALLIPQQPLSPTTHG
RAGHRLQMSAAATPSAVSSLSPVTDASNGAVVDDLAERLKGKRVALYFSAGWCPMCTSFE
PALASFRQAASDSGKDVEIVYVPSDRSDADGLERARAMGMLCVSKERAADYKKEFKIWAG
SETLAFGFGRRSGVPALVVLDNSDGKELAFLPAESQGARCLGDWPLDDESNGVW
>comp5808_c0_seq1; 619 49 1767 LEN=1920; translated
MLVPFKYLVNPIAILLLAGSVASATIRRKQSSIDNQNQSDSKHERCLLQSDHPDRMTSFP
GWDQPLPSAWYSGYLDYELEGQMIHTHYVLVQAEDHEGNDDELPLIYWTNGGPGASSLFG
LLTEIGPLMLSDESLKTDAYKETGIPTPIYNPYTWTRLGSILMIDQPAPVGFSYCNDDDT
KNATSHSCGGIAWTDELTSLNAYTALQTFYKTKFPCLAKKELYLTGESYGGIYIPTLARR
IVEGNGKEDNSTIPLDLKGFAVGDGCLGTETSMCGELGSVGFVDYWHLWFMAGHHQIPLS
DFRLVMKACAHSDKPGFLTTPNSKNDELCRAAVSKVKKEIGGFFEYALYDECTYENGLKW
KRTLGLLDSMSTSSSSSKDNSFVGGALNDYPCGAGPVLEAYLKLGPVVFDAFHVRSTFFE
VDNAEGDFDYTPTEPDLQPFYKEMNGKLKILVYNGDTDPAITSFATQNWTSHLGFDETLD
GHWRPWTIDGCQKMGGYVERYEGMFDFLTIRGAGHMVPTYKPEASFAFLKAWLRDEDYPT
FDANCTDPESFSSAKSNRGAASSSSFENIEIE
>comp5809_c0_seq1; 318 466 1662 LEN=2540; translated
MIQGRNLWIVVVLATFLGQESTGFSAPVTTNSRSRSVSNQRASTTHLELIAAGGNFFDDF
RSERRDGSGGEDSGNGKDHHNRVRNERSSDSTKFVTGDDLHRLRHQVLAMRLELQEARRS
GDIGRVDDLERAIMKTQQVDAEFVYTVSLERQELALQAGDVIAAQRFHEKAMDARSSLPQ
FQLEGLWVGKYGEEQGYEMINVTYHGDTLVAHKVTSGAKHVPRGAETFRVDLAPQFPTES
TLSNRRNNHNRQTGSEPLLHNQQSQNQDLTPIELGESAAKQWGCKYLQRFGGQGQVASEG
YKDSQWMDGQLILVNEYFSFAWLPIGHQVFFGRPTAELVVKLMKDESKRESAVSSPARSF
LEKCWEETEHIEDELEADRSQQSSWNTNIYYDQDGCFE
>comp5810_c0_seq1; 375 682 1593 LEN=1593; translated
MSSTILYKFRASSTFEALPLPGAAARLFDVKKAIVKANKLDTGTMEFDLAVRNADTNEEY
GDESMLLPRGTRIIVQRLPAGRGQGFLARMARNEHGGGMGNSYQQQQHPSGYYTIDNQTR
ENEEDEFVESSSGAEKELAALQAVTSMQAEGPTMVGGQAAAMGAVRGGRGGPPRPAGMGG
PPTKPGFHQSIAGSNASKPGGSSHFRPNADPELREQEKKLQPKKRATGIPRTFLNLSAPP
QTDGNKEGADGEQQAPLLQPNKQGFKELVSRGGGQSESTAGTRRDLDYVLKLTSTTVPEH
LQCA
>comp5811_c0_seq1; 238 267 1748 LEN=1952; translated
MNDNRAFSSPGQSASASRRFRTAQFTSVAALALLAATSTNRVESFSAKIHKNHRPGFEPS
QHEQPGAGLTVISHMRLSHRRTVSSSSLQSTSETKSGIDSSIAATNTTVANQASVAKAER
AWTLAPISAENDMSLMDAMADSELQSDETEISLMSKLTSADVLGPLFTIAFVIVFLCNTL
LNSGEISDTFLAMDLHQKLNVLSKISWFPGQFQEAQLVVALVMAFSAFAQALTGFGFAVV
AVGAMSSMPWLLHSELFGVITPVAATLGALVGFILLIPYAFTETVTEEEPGLEWDQILPL
LIPCTVLTPVGIQLNNMVDPLLATRVLAALIMGFVGYKLVPTIQDALSSDESMADNEITS
LVATEPEPTDGFLQSQTAAILFGSAAGIFGGAFDVQGPPLCVYGDAKGWSPARFRNNVLA
VVCLNSALVVAIDAAQGSLGGFYYSYFCLTSLPGVLLGIVAGQWASERIDPVLFKNLVLV
MCLGLGLQLLTVS
>comp5811_c0_seq2; 375 289 1770 LEN=1974; translated
MNDNRAFSSPGQSASASRRFRTAQFTSVAALALLAATSTNRVESFSAKIHKNHRPGFEPS
QHEQPGAGLTVISHMRLSHRRTVSSSSLQSTSETKSGIDSSIAATNTTVANQASVAKAER
AWTLAPISAENDMSLMDAMADSELQSDETEISLMSKLTSADVLGPLFTIAFVIVFLCNTL
LNSGEISDTFLAMDLHQKLNVLSKISWFPGQFQEAQLVVALVMAFSAFAQALTGFGFAVV
AVGAMSSMPWLLHSELFGVITPVAATLGALVGFILLIPYAFTETVTEEEPGLEWDQILPL
LIPCTVLTPVGIQLNNMVDPLLATRVLAALIMGFVGYKLVPTIQDALSSDESMADNEITS
LVATEPEPTDGFLQSQTAAILFGSAAGIFGGAFDVQGPPLCVYGDAKGWSPARFRNNVLA
VVCLNSALVVAIDAAQGSLGGFYYSYFCLTSLPGVLLGIVAGQWASERIDPVLFKNLVLV
MCLGLGLQLLTVS

>comp5812_c0_seq1; 640 1 1949 LEN=2326; translated
AEPPIYPPEFYRVQQRQRDRNDATKPWRHAPNHTAVADASSWTHAERSWYRSEFEALNRD
SVFSLLLLEGEGSASTDTVLQWSDDQDEDFGLIKEHGRKRHQQGLYEYHDVQSAGENGSN
QLLVLEAAWGNDAHVGNPSGKTGSTTNKSGDSASPGHRGRPEPYRPRLIVEPSLCHAFRH
AGAIANFSTPHVLVTHMNENWGALSSEVAGRTAEWGDIVRNWRALGGSGGCDPLELEELY
LDSPHTLAVFTTQHQALFDHPKVHSIPIGVANQRGFAEDFLARLRSQKKRSDQRARVGSM
RVSPPGSETPHAPDPRPQLLMINAFPSDTRAPQIEAVVRSFASSGHRVANTYKAGGTGYY
NELSRSKFVLCPSGMGYDTYRIWEALSMGAVPVLERHTYRYEAVGSGRGRATILRTVGDG
EGNTTTTAIGSSIGNTTNNTTTPFLLEYSDGWQKTLDGLPVVWTERGKDGGGGFEGLTPE
FLERAYDDLAARSFAGGFRYEKLTSLYWIRFLESFLLLPPDQARREPPQEAPLRRQSQTQ
KWNLAMESLSSTXQPPPPQQPLTAGAQHFAPWTTNNAGALHRRPDNRQHPGSRIVAKQER
ESAARAQLLAGRRAPRHKPLPFQQEEGTFKVQCDCCCRGERRWMWDDEAW
>comp5813_c0_seq1; 381 68 958 LEN=1437; translated
MKPAILFLSMLSAASAFAPSFLGTKLSSGLSQSASTTCLEMKYTLVLVRHGESTWNDENR
FTGWYDCPLSEKGLKEANDGANLLKEAGYTFDVAYTSTLQRAIKTLWIILEEMHLMYIPI
VNTWRLNERHYGGLTGLNKQETVDKHGKDQVLVWRRSYDIPPPDCDDSSEYYPANDPMYA
NVSKEDLPKSESLKLTEERFMVEWDANLVPLIKSGKKVIIAAHGNTLRALVKHLDGISKD
DITGLNIPTGVPLVYELDDDMNPIAQEGAIAPLTGKYLGDLDKVRERIGAVAAQTK
>comp5813_c0_seq2; 353 341 1111 LEN=1590; translated
MKYTLVLVRHGESTWNDENRFTGWYDCPLSEKGLKEANDGANLLKEAGYTFDVAYTSTLQ
RAIKTLWIILEEMHLMYIPIVNTWRLNERHYGGLTGLNKQETVDKHGKDQVLVWRRSYDI
PPPDCDDSSEYYPANDPMYANVSKEDLPKSESLKLTEERFMVEWDANLVPLIKSGKKVII
AAHGNTLRALVKHLDGISKDDITGLNIPTGVPLVYELDDDMNPIAQEGAIAPLTGKYLGD
LDKVRERIGAVAAQTK
>comp5814_c0_seq1; 401 68 1789 LEN=1900; translated
MNKEANDATSKDDAFDYVIVGAGPSAMGILRGLLEEISEGFSRNDCLSPFSIAIVERGQG
PPHDEATISPHRWYEAANPNSSSENVKLFSSEILGRQMDIPCGIGLGGTSNVNACLCLPP
LQQDLESWPEPYRSCIRSNASYLKTIMERHNAIHYASMENMCTPFQQENSLLEFGTRVPT
MVEWNKTTAKPVRRNYYDALVKPFLKQHPYLEKYLHWFRGYEAQRLLLTEDTNHVTGVEC
IFVGNECTSIYRQIYATRRVILCTGAIGTPALLMVSDLGNKESLCGVGQNLQDQALLARV
FIKAPSRKVEVASSNGIVALGHLNIPEKDCKGNCAVFQVAITDSVADASIIPSVVAMALR
WKFQNIFLMKLVENAFRCVRAFVRLAILCTPLGSILHYMTTTTMIFLMHPLSKGSVAISP
KDEESIREPKRLQNMSIQVDPKYLYDPRDMRSLKTAWDALERATHPSSFEIFPRPIFFAL
SPFKRQWFQIYCHCFLLPYYHYAGTCAMQSGIDSEDKTDWVVDTCLKVRGNNGLYICDAS
VFPSMVSNPPALTCAALGYEFSRMISTELSKKE
>comp5815_c0_seq1; 170 201 512 LEN=970; translated
MNRLSSNQRTETLSSRGQEAIRSELLDSLGIARTMKPSASQDSFGKAMEARTLTSTIPQR
FQSRNYKWELSFSSARNHSVLSNDEVAKLSLNGTNNFRWEQHR
>comp5817_c0_seq1; 423 375 1385 LEN=1458; translated
MYTMRRMEITCDNEYKARNIRGFCHLYDGQEAVATGIQEALDKEDSWVTSYRCHVLSLMR
GGSVAGVVGELFGKANGVTKGKGGSMHMYSKEHNFFGGHGIVGAQVPIGAGLAFAHKYNA
KPGQPMNVAIGCYGDGAANQGQIWEAANMSNLWDLPMIFCIENNHYGMGTSIDRHSSISD
YYKMGNAIPGLRIDGMNVLAVTEGMKFAKDYVGNGNGPMYVEMMTYRYHGHSMSDPGTTY
RNREEIEFTRTNRDPIEHVKSCLQEAGFMTAEEIKETEKRIRKEVQKEVKKAKEWAVPEA
NEVVEDIYAKSLTPHDSTHPPFVRMPDMAKSFKFDV
>comp5817_c0_seq2; 465 204 1358 LEN=1431; translated
MVRSASRRAMSTSHTFDITGSFEIHNFESGPSETAEVSKDQLVAMFETMYTMRRMEITCD
NEYKARNIRGFCHLYDGQEAVATGIQEALDKEDSWVTSYRCHVLSLMRGGSVAGVVGELF
GKANGVTKGKGGSMHMYSKEHNFFGGHGIVGAQVPIGAGLAFAHKYNAKPGQPMNVAIGC
YGDGAANQGQIWEAANMSNLWDLPMIFCIENNHYGMGTSIDRHSSISDYYKMGNAIPGLR
IDGMNVLAVTEGMKFAKDYVGNGNGPMYVEMMTYRYHGHSMSDPGTTYRNREEIEFTRTN
RDPIEHVKSCLQEAGFMTAEEIKETEKRIRKEVQKEVKKAKEWAVPEANEVVEDIYAKSL
TPHDSTHPPFVRMPDMAKSFKFDV
>comp5818_c0_seq1; 490 528 2003 LEN=2120; translated
MSLQIRRSSRIRGMKSDNGTGEQVRRQSVRLFQGKSVDAGTKRKRKRGNNNKDVSLSHDG
IIPVVPSEKRVTRSISGGLKSCPTSESTPDAGVDGKRETAKRGRKVKATTVASTPIRGTN
AIPVTPEPPISCNIDVSSEKNDSKTKTIAKTKSKAKTKTPTKTAAETKKKSGGKKKKAKT
TTKKKKATTGTAIWQAPAGWRETYTLVEELRKDKTAPCDYMGAEALVMPMRDETENGDED
LLKTRRFQTLIALMLSSQTKDAMVGQAMKNLRDTSDGSGGLTVASILAMDPKVLNSKIYS
VGFRNNKTKYIKQVAQILQEEYSGDIPSTADEMIKNLPGIGPKMAYIIENICWDRQSGIG

VDTHMQRLFPKLGWVSPDTKTPEQTRKQLESWLPREFWGDVNLLWVGFGQEVQQERQKIL
GKALQSSRPLEALRLLKAVEFDVGKEWDVMLSSSLSATEGEKKELPWTDNNEMEKMNAMI
EGALLEGKLTK
>comp5819_c0_seq1; 442 227 1507 LEN=1629; translated
MDEIDRSYSKAPSFSDGPSTITDQIGLVFFFGATLLSVVVVLVCSFRQYVASLSDAGDET
CATTFFAKICCHCRERTASVREDETEQYDLDRLLALALHRQLNEEGREKEREKERELKRK
ERRMWYEYYMKPYSVVISESNLLRASGNSSPSDDLPVNHTETETQAPSIDSDEDEAGENA
DKRRDFACTTACEEATLGGTTAFDEENLGCTIACDQADEHPTLCLELCDVAEGRRCFDGT
CALCIEEYCAGDTVVWSDLHCSHAFHKECLMQWLTKGKKRCPVCRHWFVPGTKIDDQKTA
HGGAWQCALSEMQKREEQAEQEAGESEPGNGESTETVATETSDSDAASRSESSGLEILHA
DSSDSEQHDTGYTIGFDASQMVDIESLTVIPTNTQNETDKFCANDSQDGDLKHSGEPQRQ
CNEDTV
>comp5819_c0_seq2; 217 750 1647 LEN=1769; translated
MYIEIESVISESNLLRASGNSSPSDDLPVNHTETETQAPSIDSDEDEAGENADKRRDFAC
TTACEEATLGGTTAFDEENLGCTIACDQADEHPTLCLELCDVAEGRRCFDGTCALCIEEY
CAGDTVVWSDLHCSHAFHKECLMQWLTKGKKRCPVCRHWFVPGTKIDDQKTAHGGAWQCA
LSEMQKREEQAEQEAGESEPGNGESTETVATETSDSDAASRSESSGLEILHADSSDSEQH
DTGYTIGFDASQMVDIESLTVIPTNTQNETDKFCANDSQDGDLKHSGEPQRQCNEDTV
>comp5820_c0_seq1; 325 348 1204 LEN=1355; translated
MSTRNAFLIALLLAKSGSGLSPGRVGPSARATTASVESSXRNEAVSIDATPDRRGFLASA
LGVVGAPTMIALTSIPSPADARDELFRPNPLTNPLLEQVRIMEQAEKDNIKYGGELERGD
AGNKGKVDAYPSLLVPILKIDKELNEIRTLVRSGGENKPSNEAMSQALAILQQPTYEKVN
FKKTFNKYGDNIYYSDPDRANLYLGGGATPKTEQSIAYLLRNDVLTNMENLRAELEYLIK
SEENEIEDLYSYADICTSAMKKYLAQVPPQQLKEAERLLSSEQSS
>comp5822_c0_seq1; 603 1 2007 LEN=2476; translated
PYISSFALKSCGVGIALKTNGTKNEEGIGLDAVKDSRLHCCNSTMAGNKERNVREDSVRR
TNLKLNRRKGTKKKSGSGINKITIHGAAVLAVAIVLGLVLNKRNTPTTSKSVLNPDLAID
PSFADFLEIACDKNGTSSSVDPSTNKAWCGNGKIEATRRTFQASVAGTRPIRRGDTLVEV
PREFQIWEIDALRSDFVESENLLKARHEQTGNPLAGGAFLAAYLAHERKRLTERSNRQQN
LEMAASANASKDELRESYLQSLPSWEELSAYHPILVSQSDLKSMLTPHSLNFAVIIMYKV
MVESEYKAMAIVSPKGFGQQITLEEYQVARIHVLSRSFSPGPDACKAEVEKYFRPDDLER
VKSTWGMRSSKSTFEDGCHAMVPILDTLDSHPRPNVLYTYDSEKQAFVISAKSKISPQYE
LINSYGKYSDSHLFAKFGFVNGDGSGYTQASMALFHRPFDILMGEDFSLMPNVVMNGKDD
ESETQSLMDETLNHQRKEMKRYLTMDDGYDDCIQKDLHPEAFKLKRLKLMHLIRMANDPS
SWIATLQPRKPRSRPRESINLLISEEPPKFNPSNVQVNLIKLMNTCRLIVLNEEDYDGRA
IQILEDNLENKDFVVPEMEGNKALEYRSLKYLGRMTASALMQYTPVKMKQEFETVRQLNK
ENAFGNSTW
>comp5822_c0_seq2; 653 1 2190 LEN=2311; translated
PYISSFALKSCGVGIALKTNGTKNEEGIGLDAVKDSRLHCCNSTMAGNKERNVREDSVRR
TNLKLNRRKGTKKKSGSGINKITIHGAAVLAVAIVLGLVLNKRNTPTTSKSVLNPDLAID
PSFADFLEIACDKNGTSSSVDPSTNKAWCGNGKIEATRRTFQASVAGTRPIRRGDTLVEV
PREFQIWEIDALRSDFVESENLLKARHEQTGNPLAGGAFLAAYLAHERKRLTERSNRQQN
LEMAASANASKDELRESYLQSLPSWEELSAYHPILVSQSDLKSMLTPHSLNFAVIIMYKV
MVESEYKAMAIVSPKGFGQQITLEEYQVARIHVLSRSFSPGPDACKAEVEKYFRPDDLER
VKSTWGMRSSKSTFEDGCHAMVPILDTLDSHPRPNVLYTYDSEKQAFVISAKSKISPQYE
LINSYGKYSDSHLFAKFGFVNGDGSGYTQASMALFHRPFDILMGEDFSLMPNVVMNGKDD
ESETQSLMDETLNHQRKEMKRYLTMDDGYDDCIQKDLHPEAFKLKRLKLMHLIRMANDPS
SWIATLQPRKPRSRPRESINLLISEEPPKFNPSNVQVNLIKLMNTCRLIVLNEEDYDGRA
IQILEDNLENKDFVVPEMEGNKALEYRSLKYLGRMTASALMQYTPVKMKQEFETVRQLNK
ENAFGNSTWTAAQLRLGEMQSLHAVSGIAMTFARKLLEGMEEEQIGVQFKIRESSCPKAY
TDILDKDLN
>comp5823_c0_seq1; 306 1 1052 LEN=1504; translated
XPRADRLGRSEAFSESLQARQDHPDHHDHHVCEPNVRRERTLRVRGIPEGNPPRRDRRPK
EAIRQGPVEGDPDELRKVRDLDEYNDEPISINTNTNTIGSHHSNAVGHGGNRTSHGNTVV
PSLSLSGSAFPEPDPENNFGSTSNNCGARSHNAPCRERGTLATGIDPVYNAIPGLHLEPS
PLPPPTQEHTDNDDLSAAEFEFDPGVIAAAPGDRSRSRSRPPPGSVVIDVDAESSCDDLS
LAEFELDPLPFFGSHDKNQSNQPVRFPGLPTAVLPAGGGANNYHNNKNKNTPMPQRTKQN
SELLFGLGLAAPPGGLRKTTKNNPFSLQAMQRSSDWKEREFIDLTGPTDV
>comp5825_c0_seq1; 838 200 1819 LEN=1979; translated
MAPFEDGVEAMTLPENRFDVTIICTTDDHQAEFWMRKLKTPTPIGGAASVFPLVVAVSED

WSGSGGAGNGLGTLYAWEKACAYASEHVEGGPDLRALLAEGSISAALYHTAGKGTRMAPL
PASENNNKPGVKLPFPKQITVLEAVVRQTGIYASSRKGRLSVFWGDQVFLPPQKENYLYE
PSHHIDIMCTLLGDTAPTAKEWTDQGLDKYGVIAVLKESGDGKVAEAAQVEKVSHETATK
MLGELGTIAQVGPSLGSFSVSAAILAGLCEEYSSELTQKQGKMDTDPHFWMPLTLPEDSY
VQLMSQKGVDSSTSKVHYQRMKAFSEKFKAANAGSAFGLFGAVDVGKDACWWDYGQLKLY
SKNSLLLLDDKNPESKLLRKFLGIKEGPQFGTFAPSDVMNHHSYAFDCNIATGKMRNSLL
SHVTAREVNAEGAIIVNCVAPKITAGKGAIIYNIIGEREIVAEEGKVMVAVSSEDGTSME
ICSRMDIDGGKVWKEVVEGNPMSFEQVRNNNMDANISQVDLKRKENYTIVTERIFQTMK
>comp5826_c0_seq1; 754 96 1727 LEN=2323; translated
MALRLAATRLSSKTAAKRTWLDRSITSRFTPLRCYLSTEAGEAKIESVSLGENIPAPPMV
YIAGEEMTHYASDLIVDKWIKPYFDTSAWETYDLSCKSRDETDDKVLHDAVEAGKRIGAI
FKEPTITPSAVQVEEMGLSKAFGSPNGAMRKGWNGITISRDTIHIKGIELGYKNQVLFER
HAVGGEYGAGWDKVGEGTLLTTYLPEDGSPPFVVDKRDLTDENNVVVVYHNPYDNVKDLA
HLFFKRCLEHKVTPYVVTKKTVFKWQEGFWSTMKDVFEQDYKETFLEEGLLERSGGELVH
LISDAATMQLIRWTDGGFGMAAHNYDGDMLTDQIAQVHRSPGFITSNLVGVNDDGSLIKE
FEASHGTVSDLWHDHLAGKETSLNPLGLVEAMIGAMQHAAMLDAERNPDDDTKLATKLKI
MNFTETLREAMHNTFRYGQGTRDMSGPGGFTTEDFIGKVAWRLQRYLAMQDEEAPPPQLV
EPPRKYRRNLHIDTEAVNKMFHKFDKDGDGFIDYTEFEAMMIKLNLAPLKKTKEEEEKEP
WGK
>comp5827_c0_seq1; 225 297 908 LEN=1335; translated
MRNVAGDGDCMFLAVALASATSMGLGGSDKLLRAISIETRDIVAQVLSSTGKLYIGKDNI
VDASRLLESATMQEPSTNTTEDYLAALRKEGRSGGLYGGGPELVALSNILRRPISTYEVD
QDYLNQQESMGDDTSSEVFFPIVCKGSFGDGVFEDPCITSIPDSVIVSNIQPGAYSWRLH
ILVLDASPTEKHACVLLPQQIYD
>comp5828_c0_seq2; 526 68 1414 LEN=1504; translated
MSTRDPFKMSLRLKAVTGALVAVWLLSLTTVVSFLPPQNGRATRTSLSIGLKLSSQTAAI
CEITKDSPISKQSTRSTWLDKGLLLSSFTDGLKTNPIVVDWLMSALVESLWKEEQAQAQL
ALKESNLASPCNGPDPVFLDQLEETDRALENMRGGDWKKNLQLYCETKRARNGESNPLSV
DLRFLYIPTAMYALRPESTSTPGKQRGRNRADGKKRRNEIMGLLQRQLEEFASSSEGNAD
VLYSIRTVTMDFDDCSVKQPEIVTNSNSLSETDDEFPETGKQAIRQWQPHFIYVQGGNTF
WLHYCMEKGDWTKDLIDACCYNPNNDNEIESPFSAVYCGVSAGAILAGESMQTACWKEWD
NPSVVPGKETYEDWADVRGLDVAGRTSIFPHMTEEWQDVAERKTEELLESTLNAGNDDDL
LSSSVCCIRDDQAYIVNGRRQTIEMLS
>comp5829_c0_seq1; 513 1 1268 LEN=1407; translated
XLNERKAYEAEVAELSHTVNALREEIGVKDHTILDGNRTLQSRIDVLEEENAALSSELEH
TTEQLESAPSQSLVDSMKRELRILKRLEYNAEDPDMPESDPEIAGRNEDEGKDLESVLVA
KLRRVEADLVKERNKNTDLQKAQEALNEEVKTLEQARVDAETVVATLEQDLDRAIKSSTA
TKAQGSDAETTTAAAMPMAPSDPSALQSVLDPNAPPPPPQPVPPTPTSAQEKASDDRSVA
NIIMSQRDRLRTRCDALEAERDSFKRELQIQVEASESLRSDNTKLFEKMRYLQSYNKSGG
GRQGGSNAYARQNSRDLDLEALEQRYEASVDPFRQFGKSERQRKLQEMSPMERTVFVVAK
TMLGTKEMRTFLFFYVSALHLLVFSTTMHWSHSDSGHDCLLLQEQHSHLPPLHDHSGETA
GS
>comp5830_c0_seq1; 340 258 1352 LEN=1562; translated
MVASDEANLHPTNLSRGRGAESRLRLYLVRHGESEANHRGIFAGQLDSPLTKGGASDARM
LGESSVLLPTSSGARSQTKGQKEGSERTCPFDCVYSSDLSRAHDTCKLMLEGMHKRQLDG
RNSYNNVNEDQETTAPGKVSGLCDIRLDERLRERSYGTLQGMPWNSDRSETNKIWRERGD
SDTMPKWESDDDIWIRVKSFLSELVEEELLSQSDKSIQQPDRLDVVETTVTIGNDEYMSG
DSCREDTASDIVNNNPKRILITSHGGVLRQILLRLVGVSKLKEMGATFDAKRKNKLITPN
TSLTILDFSIRSDEHDATAENRCAIDGECLKEDHDSEGFRGVQIEIKVFANTDHLNGDVR
IHDD
>comp5831_c0_seq1; 182 172 423 LEN=423; translated
MTRRNPTSHFIIAVITFACALSIIFVAREKDLLNALQETAVPEKGLQVVDPAKESPRETP
KISIDASKSGSQETAASEKGSQVV
>comp5831_c0_seq2; 305 1 1203 LEN=1530; translated
DPAKESPRETPKIPTDASKSVSQELILQKTPIGSIENFKIGSKIHVPSRFWTCHRNVDDR
EQKDRKLFFVHVFKTAGTTFRQFFRNYAHICNRRIITIRCTGVLSESLANQTSVWMLKGS
DSAVRGMGYNAGPCIPYDFVDGESNILERQSITSIEEIQAYNVRQKTRLFEEVSYSKLRE
YDIDIISGHTPIGLHSNWFSEDEKVVTPLYITFFRDPIDRYVSSHLQKNKEMVQQGREKS
WDFEGALAFIRNSIQKKRKDSLDPYLMTSAQKVGIKNMTEARRTNLMKSNLAELPVVIGV
VEQMKDSLNLIRHAINSDKNALVENMIADLDIHRQSKRRLLEMNKSKLSTSALVREIKKD

SDLFSKLKNLLRYEYEVYDYAVELHKQQVKRLSEVVESMP
>comp5832_c0_seq1; 145 1 897 LEN=1281; translated
XNGNSNSNRNVFLYEKTSLGTTVSLCRCDMSNGPTSRGSRSVWFLTELDEEQPNTDCDRD
YYYAPPPSTSAPPPEPHRGPRRQRLPPDEGWTTCPGGHGIFPAPGVSKELHFHGGPGADP
HRRRLAEWITDHGVVAIGLSAPGSKFAGSGSRSRDGWNQSLSETEDCVGNRRHRENGIAS
CLEILMELYDEEDTNRNDQLPGHNGSRARVSSSLGTEFLVKFAAWCLWHRGILAQTKTRQ
QIHRHGVETAAGSAKXLTNDCRDTGAGIGNELSVSAIDFQIATLKCETVGSEAIEFRED
>comp5833_c0_seq2; 116 189 644 LEN=1256; translated
MAQQLPPPQMNSGISGPAWANPHGIPQHQTSKSTATGPIGVDQHESEFFAPLSTLDEPIR
ETIMRDVRSVARKLKVVLLPMERSLPYGGYSGVSQSESIDLTERDKLIIESLRDWDLWGP
LVVCLTLAVLLSIKAPVGQASLVFAAVFCAVW
>comp5834_c0_seq1; 156 99 230 LEN=484; translated
MTQQIALFRRLLREAKQFNDYNFRAYAVRRIRGGFEKNRHLEG
>comp5834_c0_seq2; 169 99 353 LEN=892; translated
MTQQIALFRRLLREAKQFNDYNFRAYAVRRIRGGFEKNRHLEGDAAQIALKEGQEQFSVL
ARQSTISRLYPSAQSVMEAPTAVV
>comp5835_c0_seq1; 611 1 1586 LEN=1603; translated
XGGEDLLISVLSTIACHGILPELNFEEYKDEQHQPPENGGKHENENENKEHDEIPSVAAS
KGSGTHKIPGFRALQQLWEARSQPRAGTVEKKNTGTGRDHESISGDNDSDSDCASFATAG
DDSLRSFLTAACHEANNDYYDSHQRTRRGNNGNNHNDNDSNGIIGMNVDVDVDADAEDAR
SLLSGLSGRSVAEGLYGWLARMPWPLSALLLPEQHLPSLAKAPEEARGGRGLPAPPSAPL
SDGDVYFRGGGETDCGEKLCIEILLDHGGNECLPSESSSVTGGSSFADRFRVVAYSVGEG
PLGNKAGGPDRWFCCPFLSASFGTEAPGSEPEPAKETNTPKKKEEKKKKTKKKKKQKPRP
WYEMPQSWEDIAEALEGLAGQSTAAPTALESSTGGSSHGNSHGPGCPQNGPSXGGLFPAD
QARAHPAGAEPGPDPARREGQARLGTDDGNGAFPDLAAERLPAAKPLRRQKRTKETSEET
NERTRVAAGARGMDPHPARDALDVGMESSRYESVLNVAPAPTKLESIM
>comp5836_c0_seq1; 383 246 809 LEN=1168; translated
MKLCSQTAILCTVLVAAAAIDVAEASDRKQHIRTSKETLKERKRNLIKVEDSVNVDTEMV
RVLMGSDDMSMSMPSYSGGGGGGGGSSTSNSNSSSNNNSGGGSNGTSSGGSGSGTNNGSAS
EDGDASGNVPSASPPGEGMQPTVAGDVEPPEPFDGSAGTGAEDSSASRRKVGVAAITTAA
AIVPLLL
>comp5838_c0_seq1; 807 1 2314 LEN=2997; translated
XKMGYRDDEQLIPKSKGSWQDGDHGYNSIHNAEEGSTNNAGGKDRGAEGVATSWLASITE
MISTIPAVMVLASWNIMLAIPFGTMFFSNDVPLSHEVKEKLGLRMSLLALGICQTLIGGK
MTSFEALTLWAMAEVAPFYQAFAEIAANNCGDDPSRIYPTTAYLISLSTISVGLAFLMCG
AFRIGKILSFFPSYVVLGVIGGVGVWAAKQSVTVSVSPHVFDGADEDGLWNYMITPHFFL
SAFFGLAIRPLRVFIPPERFLLLDPTYYLSIPIVFYCVITVFGVPFDEVVEAGYLFKAPT
ANTTDDQPSVFAEAAELWTKFDFSKVQWDVAFQAVPQIFATSILAVMVTAPFVPPIATLL
DMDDFDLDREFIAHGLPNIISGLLVPGGIPACACYSSTALYVNSGGKGKFANSLLAGTSF
ISFVYGPALGGYLPRCMASAMLIDVGINLFLEGVADQNSKCDPLEYTSIWVIITVTTVFD
QTSGLIAALFMALFTSTFSSSNENPVRRMCDAATFPSSRQRNDLELAVLDNPITGRKKIV
IIQLQGSVFFGNIAKVQDQVFGYLGKREIHDSIVIFDCTLVSSLDSSACIFFDKIQATLK
KDHRVATIVFVASPPKLELEKAPERRSSLVFRSQNPRRHRANSILFHVDEINPAVAFGER
NNESHPSHKRRSSALTVRGSFDNDNEQKHLTQICGTCFDYLDKALAMCEDKLLSMANIPI
SKMTIPKPFTFIGEDEEVDDPDRAMAINMLKEYCNVKDTGLIKIIVDGMKR
>comp5838_c0_seq2; 896 1 2686 LEN=2889; translated
XKMGYRDDEQLIPKSKGSWQDGDHGYNSIHNAEEGSTNNAGGKDRGAEGVATSWLASITE
MISTIPAVMVLASWNIMLAIPFGTMFFSNDVPLSHEVKEKLGLRMSLLALGICQTLIGGK
MTSFEALTLWAMAEVAPFYQAFAEIAANNCGDDPSRIYPTTAYLISLSTISVGLAFLMCG
AFRIGKILSFFPSYVVLGVIGGVGVWAAKQSVTVSVSPHVFDGADEDGLWNYMITPHFFL
SAFFGLAIRPLRVFIPPERFLLLDPTYYLSIPIVFYCVITVFGVPFDEVVEAGYLFKAPT
ANTTDDQPSVFAEAAELWTKFDFSKVQWDVAFQAVPQIFATSILAVMVTAPFVPPIATLL
DMDDFDLDREFIAHGLPNIISGLLVPGGIPACACYSSTALYVNSGGKGKFANSLLAGTSF
ISFVYGPALGGYLPRCMASAMLIDVGINLFLEGVADQNSKCDPLEYTSIWVIITVTTVFD
QTSGLIAALFMALFTSTFSSSNENPVRRMCDAATFPSSRQRNDLELAVLDNPITGRKKIV
IIQLQGSVFFGNIAKVQDQVFGYLGKREIHDSIVIFDCTLVSSLDSSACIFFDKIQATLK
KDHRVATIVFVASPPKLELEKAPERRSSLVFRSQNPRRHRANSILFHVDEINPAVAFGER
NNESHPSHKRRSSALTVRGSFDNDNEQKHLTQICGTCFDYLDKALAMCEDKLLSMANIPI
SKMTIPKPFTFIGEDEEVDDPDRAMAINMLKEYCNVKDTGLIKIIVDGMKRETLPKGKII
WNLGDRGDSAKLFVQGAVYAFVEDEDEDDDFRFQSKMTLGSFLGLRSLILDECHLATARC

DDECTFYSLDRAAYKRLFQESPEAASLLEVSLARYLSHKLRHVSNRIYQARSLPV
>comp5839_c0_seq1; 114 121 254 LEN=254; translated
MVNGGKPLAKRPLLTRAEEIAEAYLTEQVVSKIITSKEDGSKEDX
>comp5841_c0_seq1; 272 1 1002 LEN=1002; translated
AAKRVSSSSVLSSALSDANERIKESGVASVALDPPHIAHAFVAVSNAHARQSRSARAKIA
SWKSRASRGLWAESFGFEAEAMRRRVLSGFDAETLLAAGLPLVAPYRLEMRNQLASLVDS
SIVELFGAQVANLEKRTSKRLGSQLLKKLDSTPPEEVASENAAAIRAAMVSFEATMGDLE
VPSLSLTKAKPCFDMESKLTDEAINFYDSPAAQIKRTKKVAEVVSKEKKPGQRSVDLGLD
LVAVLRPDGFGSLQGFAGYQLGGNSITLGIHNDADDPAXDFPVRGGPAAAPAHPAQAARG
RGDVMEWKGRCGGATPNVLKKPLARDNILDNYLKX
>comp5841_c0_seq2; 718 257 1963 LEN=1963; translated
MSRVSLFLASPLLALVVSAVLLASGAHAVTSPQYSTLMGSSGGLRLNTIASSDVSTSEGV
VSVITSPGTESFLVGGSSDADDVLSFDGKQLLVCRGATLRGTSPSERSALAITSLVSDAI
VVDGITEGDVRAAGFGKNTRHARTLTALFRARLASSSGDAVEKQALVLCVKSAPSADGDS
LDEILAKEVRDLFEATAAEAKETKASFDDLYDVSVVSVTSKEDASETLATAKSLAASLSS
SSSSVLSSALSDANERIKESGVASVALDPPHIAHAFVAVSNAHARQSRSARAKIASWKSR
ASRGLWAESFGFEAEAMRRRVLSGFDAETLLAAGLPLVAPYRLEMRNQLASLVDSSIVEL
FGAQVANLEKRTSKRLGSQLLKKLDSTPPEEVASENAAAIRAAMVSFEATMGDLEVPSLS
LTKAKPCFDMESKLTDEAINFYDSPAAQIKRTKKVAEVVSKEKKPGQRSVDLGLDLVAVL
RPDGFGSLQGFAGYQLGGNSITLGIHNDADDPAXDFPVRGGPAAAPAHPAQAARGRGDVM
EWKGRCGGATPNVLKKPLARDNILDNYLKX
>comp5845_c0_seq1; 350 928 2067 LEN=2169; translated
MDQMDQKPIEDTLCASVKNENDKPDILIREPEESMNDAFEVFEVSKGRRDDDEQVSVNDN
LTLGATTIEDTYSCTPSMYEIEKFHRELELELFGEVNEVPSMIGQILELPISAENRSSAT
RSSRGSRDYVQNDVLNARDSDLKRVLIQSDEYPCDAFTETSRSGSTACDAMKNSQTSSTV
IPAIPTRTTTHQFPSSQNCHHSTTFHHDALPAPGTGWEQHMNYDGFRREYNDSLCDPFPP
SMANGMRHSMQCHRHEIHSKHSQHIRDSTEETDSIRKHSQCRQGSSLDSSHGTDSKRAIS
NGHIVTSSDFKDLPFCHRRLCDHYRSSSSRSFRSTSLHGPKVCHVPLSGHISASQLVNGT
NGEHCFSSNATITITEENL
>comp5845_c0_seq2; 350 1107 2246 LEN=2348; translated
MDQMDQKPIEDTLCASVKNENDKPDILIREPEESMNDAFEVFEVSKGRRDDDEQVSVNDN
LTLGATTIEDTYSCTPSMYEIEKFHRELELELFGEVNEVPSMIGQILELPISAENRSSAT
RSSRGSRDYVQNDVLNARDSDLKRVLIQSDEYPCDAFTETSRSGSTACDAMKNSQTSSTV
IPAIPTRTTTHQFPSSQNCHHSTTFHHDALPAPGTGWEQHMNYDGFRREYNDSLCDPFPP
SMANGMRHSMQCHRHEIHSKHSQHIRDSTEETDSIRKHSQCRQGSSLDSSHGTDSKRAIS
NGHIVTSSDFKDLPFCHRRLCDHYRSSSSRSFRSTSLHGPKVCHVPLSGHISASQLVNGT
NGEHCFSSNATITITEENL
>comp5846_c0_seq1; 411 134 1060 LEN=1497; translated
MATWNPKRNNFTLCCFFLALLLEPQTILSSGYKGRVLRGSIDGQEKKVSGNRTLKQKMVA
NKKRNSKKRQISKIATSTRPVPKPSPVQSSTIPVVLPAQPTPSPIPALGSTTPGVLPSPS
ATNAIPVAGSVTPVVPPTPPTPSPFANLFTAAPAPVAPNLLTASPIPFAIGPRIPVVSRT
ETEPSGGEGPSGTMTDSSTSEESSSGSTSSTTWGFAWDSACPEEASQLEKCIGSGKITID
DCKSCIVSASFMSSTDYMMKACKTQFCNGCDGGQYLDCAQAANIQKFPVVNGGATLIREK
ELPEDGSD
>comp5847_c0_seq1; 197 81 569 LEN=793; translated
MIRSTTLAALMSWALVARTTCAFTATSCSVAAPAKSAASTTARFMSFEDFGQETPEDTKE
RIQELVDNHPVLLFMKGSKLFPQCGFSNTACQILQSFDIDFHSVDVLSDEAVRQGVKDFS
QWPTIPQLYVCGEFLGGSDIMIEMYQSGELGELIEKAKADMI
>comp5849_c0_seq1; 464 1 1585 LEN=1804; translated
XESILDEENRSNNQSIRRSAVIDQFSSKERLEYSMRQQTLSMADSTSSVSSVYERPVRVP
AVMFFIVFIIFHITLQLKTAFQIDIARSLTTNDLASDQISDALADQQERQEKKKKDSDHS
ATSLIHNVESIKQALGGGESGETVTLFNNDNNLFPEKVVGVQEHRDSDDHHDISSPSEDS
DHTNNLLVTTRMQKYELDLDDFFDTEGEGNQAISEAKKTQKKKKKKRKTRAFFGIMTYDS
ESERKRRNLIRKTFLSYFTNHTNRTLVSEDEYNEKKHWICSLNDLDYGRLEHPEKCRMAY
AFVQGANPNGTTMLLNFNESYPLSLPPPKFETKKEQKDASDSVYLNIQENGRFGKSPTWF
RYAIDVLERHGWMKDFDYIFKSDGDNLIYTPNFFRFMDKLPQKRAVRVYGGRPLNYNKCG
GDSHDHCSQMVGPHFMAGGCYFLSLDLAKFISDETTFDHMAVKLPHEDMTTGNFVYSHPK
KIRVVKEPRKKGVIRTHPVKKDRAFKHRWNKMLKEEQVRLVAAQVNDE
>comp5850_c0_seq1; 191 232 810 LEN=861; translated
MGIDLKAGGRRTGHKSSMKVVKTKNPYNRLLIKLYKFLARRTDSKFCATVLKRLHMSKVN

QPPIGLHRLSKYMSKKEGKTAVIVGKVTDDVRMLECPKMSVCALGFTENARKRIIAAGGE
CLTFDQLALKAPKGANTVLLRGPKSREALAHFGHRVTVNNPHTHDGVKPYVRSNGRKFER
ARGRRRSRGFKV
>comp5852_c0_seq1; 1232 147 2681 LEN=2809; translated
MVRAFIKGGVWKNSEDEILKAAVQKYGKQQWARVASLLNRKTAKQCKARWHEWLDPSVRK
VEWSREEEEKLLHLAKLMPAQWKTIGPLVGRTATQCQEHYEKLLDEAAAASGKVSGEDET
AGATADIRQQPLRVGQIDSHPETKPARPDPIDMDEDEIEMLQEARARLANTQGKKAKRKA
REKMLAQAKRMADLQKRRELKQAGLISGQAKKKARRRLKGGDIDLGIEIPFHKQAPAGFH
DVSQEVVRSEVLRAKQAKRVDFQKLNEQQYRSRDEEQQKAKKREESRLRALEKSNMDYVV
AQVSKTNDPLNIRKRGPLSLPAPSLSDRELQTLAKMEEAATTPAAIASTSKGATSTLLAD
YSDRPLPTPMRTPAVASGVASSGESKQKSIMREASNLRMLERGQTPLLGGENPLLEERLG
EADDGTAMDGKPRARPMTPGGPSSAAAVTRDHFGLNQRPQSESRGGFADDGASVGASTFA
TSTTAGMLTAASSIRDLARAERRAAKRARRELEEALAALPAPQFEYELEAPTTIGDDDHE
GKVETVMEEDMADVDAEEKRRLQEEADKLYEARSSVVKRRDLPRPVGVPPDIKSLSETGV
SDQSVAEALIDEERLTLLKHDAFAFPVETPLPFLGSGEKKGKKKKKQDKTESFVLEATH
LEPISEAALDAAKELIQLEYDRDLNERSQSLLPSGNTMDQAMRLVLAEMSNSAKENPSSR
DVFVSNKWKDSREDNALLAESLAQEFETIEEATSALHKRNEKLESKLALLTGGFAKRAKN
TSAGILNLHRDMLNACIELGVYKKLSEQEELGAVSRIDRLQLSISKLKEDEERLKSLHNS
LLAH
>comp5854_c0_seq1; 690 73 960 LEN=1447; translated
MKIGSSLKFCTGAVVALLLGGASAAPPTPVSNVHVTLKGKKYQIDDVTTLAELQERLEEH
SGIPPSKQGKILHNGKRLSESDDGLSEAGVQEGDQLNCVPAKKSSSKKKSASTTSAPDSS
ASPSEGGSADSLADMMKGLEGSMGGGAGGLDDMMSKLMSGGMGGGAGGMPDMAESMKMMK
ELTDSPMFAEYMNDPAKLEESRQMILNNPMMKAMMASMPGMSEILEDKDAWAQTMMAAAG
IMKSMDPEDMMKMMEAGGAPGGGMPPGMGMPGGMGGGLFDGGASSALDELSEDDE
>comp5854_c0_seq2; 624 223 1107 LEN=1594; translated
MXCVVLLQCTGAVVALLLGGASAAPPTPVSNVHVTLKGKKYQIDDVTTLAELQERLEEHS
GIPPSKQGKILHNGKRLSESDDGLSEAGVQEGDQLNCVPAKKSSSKKKSASTTSAPDSSA
SPSEGGSADSLADMMKGLEGSMGGGAGGLDDMMSKLMSGGMGGGAGGMPDMAESMKMMKE
LTDSPMFAEYMNDPAKLEESRQMILNNPMMKAMMASMPGMSEILEDKDAWAQTMMAAAGI
MKSMDPEDMMKMMEAGGAPGGGMPPGMGMPGGMGGGLFDGGASSALDELSEDDE
>comp5855_c0_seq1; 430 406 1764 LEN=1972; translated
MSNTAVLQRLSPSEVTLEIKDPVNPRALEQAKAILSELVPEGSTDNGRVDPSKLLEVAKR
LGDVEDGTTVGDLIVSKEACEAAYLALGETERVALTNMHRRIKAFADLQRASIRDTEMDI
PGGKAGHTVSPCRAAGCYAPGGRYPLPSSVLMTAVTARAAGCPTVVLSSPKPAPITLAAA
HVAGADVFLRVGGAQAIAAMAFGMEIQIAGSDGDDADATRTVSVPSCDVICGPGNMWVTA
AKSIVSGKCGIDMLAGPSEVLVICDETAEASVVASDLIAQAEHDVVARAILLSTCGDLIN
AIDAEVEKQVNALPEPNRSTAMAALKQSFAVHCEDIAQCISISDDIAPEHLEVQTKDSNG
DAFKCANYGGLFIGVGAAEVLGDYGAGPNHTLPTGGTGRYTGGLSVFNFLRIRTWMRVDD
KEASQGMVDDSIVMARLEGLEGHARAAEQRKL
>comp5857_c0_seq1; 346 1 1286 LEN=1756; translated
XGRNETKENNAAKGLGKPKNSKPGAKQFKYFILAPPNATRSLLTKITETSASSFYAETLN
EDSAEMWLHRAFENLSYEEGHTLDPSEADVFLLPGYFHLNRKVGFITGPALAQNYKDIII
EPSKPHLLLVPTTNPTTSRQIGILAVTDILRRNKVNLWSVGFERNKFWQRIDLDRIVSIP
YVLRPKEHQNKTDDFQTSKKVSNSVFYAGDGRQNAIMWAGCNRTGIVSSLHSSLNVPNAS
EKFGIDVNATYVQIMSKRKRLAQDEYNSRMSTSEYCLILCGDTPTSRSLVSAMVAGCIPI
FMGSRLRGLCEPPCKKGWGWDISGPENPHLPFGDYIPWDQFPEVSEQGFIDNGAETLSEL
FRRFPLSKKETIRSIMKQAQPGWIYGWGDPVTSNDFGDVHKYLWETFQRLLFHETSQPST
FDLLPVLS
>comp5858_c0_seq1; 488 368 1719 LEN=1868; translated
MFSSGIFWTPVAEASHFGSRVTAFAYGGETSTAEWTFIGSENFFLMGEINHAYWFFQFAF
ACALSSIVAGTIAERTQMKGYLFYSFFLVGFAYPIVAHAFWSTNGFLANVNAEPLWGSGA
IDFAGSGPVHMMGGLTALMAALVLGPRMGRFYDDDGSPLETPADIPGHSTPLAVLGTFAL
WFGWYGFNPGSTFMVSNGNAGEVAALVAVNTTLAACSGALSAMFTSTWLSYRSIGLHTYD
LAYTMNGLLTGLVAITAGCAAVESWAAVVIGIVSGWVYLIASKVLIKLRIDDAVDAIPVH
LVGGAWGLIAAGLFSTPDLMDKAYGQSDHVGWFYEWGRGSGNFTLIGIQLIGILFIIGWT
TALMYPFFYVLNYFGALRVDPLEEATGMDISRHKGPGYNVDQSKHENEAAEELQKSRRSL
LGNSSGRRSTGPETSPERANPTAVEVEESA
>comp5858_c0_seq2; 554 41 1621 LEN=1770; translated
MSNLYETCSTQFSTDQLDSLLSCLSEGHGAALDGATLDIAKGLDSFYLIFAGALVFFMQT

GFAMLCAGSIRSKNVKNVLLWNLLDSCGGGIAFWITGYAFAYGGETSTAEWTFIGSENFF
LMGEINHAYWFFQFAFACALSSIVAGTIAERTQMKGYLFYSFFLVGFAYPIVAHAFWSTN
GFLANVNAEPLWGSGAIDFAGSGPVHMMGGLTALMAALVLGPRMGRFYDDDGSPLETPAD
IPGHSTPLAVLGTFALWFGWYGFNPGSTFMVSNGNAGEVAALVAVNTTLAACSGALSAMF
TSTWLSYRSIGLHTYDLAYTMNGLLTGLVAITAGCAAVESWAAVVIGIVSGWVYLIASKV
LIKLRIDDAVDAIPVHLVGGAWGLIAAGLFSTPDLMDKAYGQSDHVGWFYEWGRGSGNFT
LIGIQLIGILFIIGWTTALMYPFFYVLNYFGALRVDPLEEATGMDISRHKGPGYNVDQSK
HENEAAEELQKSRRSLLGNSSGRRSTGPETSPERANPTAVEVEESA
>comp5859_c0_seq1; 244 235 930 LEN=1202; translated
MTIASQEKSHYRYEQAQLRRRHSIAKIHRSSSLVGNPAFIIGLSLALMGAIGFFAAMIHL
TSKVEAFRPVYASHHRRHGSSDTRVFLEDWVADMIDKELYRQDHRKEYEGEWMKKNKGAV
LSRLSDSETSPSGYPSSMLELDGEDRAAFADHKRDERMAFLKPEKYCADRCVATGNCDIF
EDFYELGPEDVLKFCEECVLFSEEAESDGGEYSSACEVPDAFYEKEPPLRP
>comp5860_c0_seq1; 771 1 2382 LEN=2618; translated
XTEGTQKSYTLGAAAAVPPAAPAAIQPSKSFGMEGPPPLGSGAMGGVSISDAFEGLSTEP
TTTTTLPSYVPQEPAAAPVVVEPPTPQPEPSYVVPPQPVTAETVEPPTPQPQPSYVQQPA
PVAEPSMAASTTPPAPTPAATYEVSNSHQEELEKLKVVLQKLKAENISLKSQLGNMTEEE
KNVQQELGQTVAEITALSSDLGAQRQQVLEAKNRLLEARAELKAQQGXEGVSLAAWITLY
DVVLTKLNSGFRLISDXALKNWRIQPQRLLSAPKTNAFIIAPLPLFPLWLTLLFELCLHR
VLTELISEAEQTKEAIESATETLQSNAAAPPPTSQPVAGPEVDLFGTSAPATAGVQDSQE
PEGFMNEHTIPNETKNLPAGVPSMPAPSSAEIAAPGTPTRPPQPMEQQQQIAQAPPPAMY
SNYAAPAPPQAPQPAPPPMPSPMPERSHAREQQYLSPTNLNRPPVPGHFRQSSGFDSGFL
MGGSPTVVYPDSGAPDADNVSQAAFSHASTGDYGYDDQAFEVVEGMKKRAKAADGVARDA
EAASSKLAAEADELRADADRAEANYRSLMAAAEEKKKGRFGGGKKKKMMDGAEQAGKDAA
EIKKHFMEIQGRALEAASVAAQTREEADKLLVEAEAAELKMAAVASAQQKQPAPAPVPAP
APPQTNDYYGGNMAAPPKTGAMPMPQYGQAMPAPTPMVGYNGYNGYNANGGAVPGDGYGQ
QQHQNYGQASQPPGPMGANGGYDMPPPAAFQQMQPATAAPTAGGGALREPFLIFTDGNGA
LDLVNLETCAANGI
>comp5860_c0_seq2; 950 1 2172 LEN=2408; translated
XTEGTQKSYTLGAAAAVPPAAPAAIQPSKSFGMEGPPPLGSGAMGGVSISDAFEGLSTEP
TTTTTLPSYVPQEPAAAPVVVEPPTPQPEPSYVVPPQPVTAETVEPPTPQPQPSYVQQPA
PVAEPSMAASTTPPAPTPAATYEVSNSHQEELEKLKVVLQKLKAENISLKSQLGNMTEEE
KNVQQELGQTVAEITALSSDLGAQRQQVLEAKNRLLEARAELKAQQGTKGVLTELISEAE
QTKEAIESATETLQSNAAAPPPTSQPVAGPEVDLFGTSAPATAGVQDSQEPEGFMNEHTI
PNETKNLPAGVPSMPAPSSAEIAAPGTPTRPPQPMEQQQQIAQAPPPAMYSNYAAPAPPQ
APQPAPPPMPSPMPERSHAREQQYLSPTNLNRPPVPGHFRQSSGFDSGFLMGGSPTVVYP
DSGAPDADNVSQAAFSHASTGDYGYDDQAFEVVEGMKKRAKAADGVARDAEAASSKLAAE
ADELRADADRAEANYRSLMAAAEEKKKGRFGGGKKKKMMDGAEQAGKDAAEIKKHFMEIQ
GRALEAASVAAQTREEADKLLVEAEAAELKMAAVASAQQKQPAPAPVPAPAPPQTNDYYG
GNMAAPPKTGAMPMPQYGQAMPAPTPMVGYNGYNGYNANGGAVPGDGYGQQQHQNYGQAS
QPPGPMGANGGYDMPPPAAFQQMQPATAAPTAGGGALREPFLIFTDGNGALDLVNLETCA
ANGI
>comp5861_c0_seq1; 165 949 1269 LEN=1853; minus strand; translated
MPLKSAYVQGSTFPNGFQIAAFASSCLCLARKALAAPVIDAENARRVVDCPTFFCMKPLW
RSVGRGTAIEWLPEMRQKKIWDSIRIERILRDVIVEWIQSSQGRMV
>comp5864_c0_seq3; 192 176 790 LEN=1630; translated
MAHQSSNVFWKQQPLPNINAIENDDDKNIERVNCMVQISDPDRREMMPPPEKKTRNHSAS
RTMATAKLKVGSHKRDRYRCRVGDPKGLLLLSFPLKLHLILESYEFARDRRKVNKRLKSI
GGNWNTKHNDTHETATCRFCYKNNGASVKKDEIIIGWLPRGNAFKIYDKGRFVSEIMPHY
FLGHGKNAFSFETFLRCLDLWYV
>comp5865_c0_seq1; 488 64 951 LEN=1401; translated
MPTKKKNEAALKKLEDTMVDDDGEEDEVEDVGGGSGRGKRKRRESKSYEPDDFTMASHNH
TLKLSSVPKGRGKKLGDIPAVKSSMNKYKLNTEELLFAYKFVFSNRGTANKKAMKSKLLE
FSGYLPPLPKGKYNKEQQDEKDEVFETKYATKAFRMNLNQIKTLCTFFGVDYNDENGKPL
KKDDVIDRLLDFLGEPDESLLKKTISSEKQKKAVPKQQKRAGGNLPEDPFSLIRDYKKGK
FPSDSAMRQWVKAYIVCIDMETATTKDAVHTATAKFGVDMVSKKARIKELLAEEI
>comp5865_c0_seq2; 416 64 1057 LEN=1507; translated
MPTKKKNEAALKKLEDTMVDDDGEEDEVEDVGGGSGRGKRKRRESKSYEPDDFTMASHNH
TLKLSSVPKGRGKKLGDIPAVKSSMNKYKLNTEELLFAYKFVFSNRGTANKKAMKSKLLE
FSGYLPPLPKGKYNKEQQDEKDEVFEVSVPASFSGGRHLTLSEIHFQSARLSFSKPARKL

MKTKYATKAFRMNLNQIKTLCTFFGVDYNDENGKPLKKDDVIDRLLDFLGEPDESLLKKT
ISSEKQKKAVPKQQKRAGGNLPEDPFSLIRDYKKGKFPSDSAMRQWVKAYIVCIDMETAT
TKDAVHTATAKFGVDMVSKKARIKELLAEEI
>comp5866_c0_seq1; 287 1 1403 LEN=1509; translated
XEDAQHRIYYPIMVKPTSSAASAANSRRGQRTIRSLMEFLRFQARLEANKPTFGQFLGLS
VSGNDADETNKEDYFSTVREDRFWFLDASPSNSIVRSGERRRQEQSLVFRYRFPRNRLQG
LHKGTRKTSISESGMNCGPVGLSTYTSILDEVTSFSIISATGPSYPRPGVSVTMQSQWGP
AYDATSSLSFLEEVDIVTTITKKGRNLAFVRAEVRDPSNYDGIICYFDHVKYLTVGWGMG
LLLTPVGMWFLNLYSRYILPYLYRRKQRNHSNNNNIESNRHTIHSDILDSFLLTSDTTAT
FQFGPPHTNGFGGLHGGVQAILMERLGELVARRELLKVLQSTTNSGADDVGGAAKISCTS
NSGDWMEAECQRLVISYQSSASRVLELEAHIINPPRLDHPSVTLRIEILRGNGQPQANNQ
SSSSNNESINSIGNRGSQKPTSTEFKRVVVSEGILTFVSTSPKTKVQ
>comp5867_c0_seq1; 435 143 1132 LEN=2173; translated
MTSETDKKETCSSTDAKKEGIVKTTREFAEKLEKLSKPALEFLIVLIPMVVETGNKLYAI
WIKLDDAIVKSIIGFAFCFFGGMYPTVFAGLQAAEQGGRALVVKSIGELSEEATRIINES
KKDDKDSGTDKLSNKDYMKQKTLFVLQKMNPEKVNTALCNIYSVWLAVISVLVVHFARTI
QMANSIAEFLAKPADRYFKPIVATALPPEYQQWVPIIIPWTCRTVGMSLAWTLTSIQVAF
ASSLQGGLMLARSAYQVLRKRNITLGGLIVDDQSNSTVDEYLAYGLAALGLSFQLYFRLN
PPFPLNIILAPFKLFEWALRYGMMKATAY
>comp5867_c0_seq2; 456 143 1352 LEN=2393; translated
MTSETDKKETCSSTDAKKEGIVKTTREFAEKLEKLSKPALEFLIVLIPMVVETGNKLYAI
WIKLDDAIVKSIIGFAFCFFGGMYPTVFAGLQAAEQGGRALVVKSIGELSEEATRIINES
KKDDKDSGTDKLSNKDYMKQKTLFVLQKMNPEKVNTALCNIYSVWLAVISVLVVHFARTI
QMANSIAEFLAKPADRYFKPIVATALPPEYQQWVPIIIPWYVQYCRHSIFVACIMAPLVL
IPVENQTLLWDVSVKISSLIVPKSIQSLSPPTCYICSPKSFFCLAFIRACFYRTCRTVGM
SLAWTLTSIQVAFASSLQGGLMLARSAYQVLRKRNITLGGLIVDDQSNSTVDEYLAYGLA
ALGLSFQLYFRLNPPFPLNIILAPFKLFEWALRYGMMKATAY
>comp5868_c0_seq1; 330 186 956 LEN=1245; translated
MKVLVGIKRVVDYAVKVQVQKGSVSLNNVKMSLNPFCEIAVEEAVRLKEAKAAEEVVAVS
IGPKQCTETLRTALAMGCDRAIHISTDLRTDYMELQPYAIAKMFEKIVDQEKSDLVLLGK
QGIDSDCGQTGPMLAGFLGWPQVTFAANLTPNDGGKGITVERETDSGTETIKIPSLPAVV
TCDLRLNEPRYATLPNIMKAKKKKVDTINADDLGVDLTTYNEVVEVFAPPPRQQGIMVES
VDDLVDKLRNEAGVIS
>comp5868_c0_seq2; 330 424 1194 LEN=1483; translated
MKVLVGIKRVVDYAVKVQVQKGSVSLNNVKMSLNPFCEIAVEEAVRLKEAKAAEEVVAVS
IGPKQCTETLRTALAMGCDRAIHISTDLRTDYMELQPYAIAKMFEKIVDQEKSDLVLLGK
QGIDSDCGQTGPMLAGFLGWPQVTFAANLTPNDGGKGITVERETDSGTETIKIPSLPAVV
TCDLRLNEPRYATLPNIMKAKKKKVDTINADDLGVDLTTYNEVVEVFAPPPRQQGIMVES
VDDLVDKLRNEAGVIS
>comp5869_c0_seq1; 187 84 566 LEN=1135; translated
MRSFMDDVDHAAQRVGAMTLAGFLGGAAHAAFKGFPRRAAALGAASSCALVGTSLFALER
VASVAMRENNRNDKSDEELDARDDWRSTLSSHAFGGVFGGALNGYLYHRQPLRGMIAFTP
FMLFIGMIEVEAKKRKQQRLDELLDQRQNQTQAATTTTPE
>comp5870_c0_seq1; 354 1 2159 LEN=2849; translated
XMTSRFLEEGSILRAFFSFYLVQLFFAAIAALCVYIEPISGGSGIPEVKCYLNGIDLPRV
LDVKTLFCKVIGVTCSVSAGLPVGKEGPMIHSGAVVAKMVSSGNVKNDRQLRDLVTCGAA
AGVCTAFSAPIGGILFALEEGASYWSPSVTWRTFTCSMIALTALMMLESIGITFGRVGFD
KLFSFGNFIFEKGQSSYAIYELGIFVAIGAAGGLIGAIFNDVNERITHWRMKHVNTSKKR
RFMEVMLISTLMSCVMFFSSLAWPMCASISDIIEEESDRQNLYEKLVRFRCEEGEYNELA
TLIFAEPGDAIRLLFHLHKNAFSTYALLAFFLLYIGVAVVTYGIAIPSGLFVPSLLSGAA
FGRFFGNQLRKLYPHLAFSNTYSLIGAAAVLGGMARMTISLTVILLECTGNEQFVLPLML
VLMTARIVGSVYNDDLYHIHIHLKKGVEFLEAELTSITLHHNLVAGQIMGANVIFIRPVE
KVGVVYDILMSAKHSNFPVVDTDDRNVLDGTIGRNTLCILLKERAFGYPMNDTHVGPGNH
TAISSNFLQVYERKYHPLVQWDVLYRSYPRYPDAKDLRITDEDREQLVDLRPYINNAAIT
VQETSSVEDVGDSWRACSAPDHFQFNLVQYCAIPALVSHSCFSISIRPMLCNNAEIVMRA
QRTYSMFRNLGLRFLPVVNKYNQVVGTITRSDLTPDALAETMLQKGKKHEPVNNGSFMR
>comp5870_c0_seq2; 389 1 1999 LEN=2689; translated
XMTSRFLEEGSILRAFFSFYLVQLFFAAIAALCVYIEPISGGSGIPEVKCYLNGIDLPRV
LDVKTLFCKVIGVTCSVSAGLPVGKEGPMIHSGAVVAKMVSSGNVKNDRQLRDLVTCGAA
AGVCTAFSAPIGGILFALEEGASYWSPSVTWRTFTCSMIALTALMMLESIGITFGRVGFD

KLFSFGNFIFEKGQSSYAIYELGIFVAIGAAGGLIGAIFNDVNERITHWRMKHVNTSKKR
RFMEVMLISTLMSCVMFFSSLAWPMCASISDIIEEESDRQNLYEKLVRFRCEEGEYNELA
TLIFAEPGDAIRLLFHLHKNAFSTYALLAFFLLYIGVAVVTYGIAIPSGLFVPSLLSGAA
FGRFFGNQLRKLYPHLAFSNTYSLIGAAAVLGGMARMTISLTVILLECTGNEQFVLPLML
VLMTARIVGSVYNDDLYHIHIHLKKGVEFLEAELTSITLHHNLVAGQIMGANVIFIRPVE
KVGVVYDILMSAKHSNFPVVDTDDRNVLDGTIGRNTLCILLKERAFGYPMNDTHVGPGNH
TAISSNFLQVYERKYHPLVQWDVLYRSYPRYPDAKDLRITDEDREQLVDLRPYINNAAIT
VQETSSVERTYSMFRNLGLRFLPVVNKYNQVVGTITRSDLTPDALAETMLQKGKKHEPVN
NGSFMR
>comp5871_c0_seq1; 253 417 875 LEN=1233; translated
MLAGVVVRTSIETSIQIIYREIIDKAIDHNNLLLIGFSWGGAVVAEMIARGMLGGQDQPS
ALLIAPTTSLVAKVAFQRDAATRIKERASLSLSMNSTPKVIVVHGEFDDVFCPNQDRWNR
IRGVNVHILPDSHIFHRSSSQHALIELMKRQL
>comp5872_c0_seq1; 510 126 1259 LEN=1733; translated
MNPAVGMNIARSSRYRLPSRCRRVRHPQSASLMKTRSLAFSSTTTKHEKVKYLLPKRIIL
LRHGESLGNVDDTAYSTIPDWKIPLTRRGERQALRAAKELVDLIDGEALFAYCSPYKRAI
GTWDIIEDYIESNNKKKKNDEEQSSNDKDRVGVEIIGMREEPRVAEQQFGNFQNPHKVRT
AKSERRTFGRFFFRFPEGESGMDVYSRCSSFLATLSRDIKQIDLRYSYLRNQETNNKANE
NETEAGNPLCAMADMNILIVCHGLTLRLLLMRYFQLTVEEFESSYNSQNAKLVVMNRFVY
DDSDADTDPGNRKYYCSITDTHQFREYYKLDEVSKEALNLLGDISNKKPSYWRSSTRMDD
EFNGGLLLVDGDDDLEE
>comp5872_c0_seq2; 437 126 1386 LEN=1860; translated
MNPAVGMNIARSSRYRLPSRCRRVRHPQSASLMKTRSLAFSSTTTKHEKVKYLLPKRIIL
LRHGESLGNVDDTAYSTIPDWXNTVDEAVSKSLRLISRIIFSIEXQYLLLRPIEIQEAHI
QFDLSILDRLRGERQALRAAKELVDLIDGEALFAYCSPYKRAIGTWDIIEDYIESNNKKK
KNDEEQSSNDKDRVGVEIIGMREEPRVAEQQFGNFQNPHKVRTAKSERRTFGRFFFRFPE
GESGMDVYSRCSSFLATLSRDIKQIDLRYSYLRNQETNNKANENETEAGNPLCAMADMNI
LIVCHGLTLRLLLMRYFQLTVEEFESSYNSQNAKLVVMNRFVYDDSDADTDPGNRKYYCS
ITDTHQFREYYKLDEVSKEALNLLGDISNKKPSYWRSSTRMDDEFNGGLLLVDGDDDLEE
>comp5873_c0_seq1; 150 203 587 LEN=811; translated
MAQRDPNSTDSLHPGVVTAEHVRQKLSKQLRIDLDDSEPVHILRTASPPSSPSEETTPSS
TTTTTNPLNFAELDDSKIQALADDLVPVVDEEGRCLIRINRLGEYLAVIGLKGGYAVPLR
VLVRQRV
>comp5873_c0_seq2; 334 328 984 LEN=1208; translated
MEDLPHGKAYKGDVVTVKAGYARNHLVPQKLALYATPQNFSKLGIVDPDFETEEERIARL
QRESNMTAAEDRYLKDSDLLKKYLRNKVLQIWRNVDPNSTDSLHPGVVTAEHVRQKLSKQ
LRIDLDDSEPVHILRTASPPSSPSEETTPSSTTTTTNPLNFAELDDSKIQALADDLVPVV
DEEGRCLIRINRLGEYLAVIGLKGGYAVPLRVLVRQRV
>comp5874_c0_seq1; 282 154 933 LEN=972; translated
MAPSDDEHSGSETSTGEPDSGSSVESERNEESETIKKPKKIHRLSLTETEDFNKKLKRRG
VIYVARVPPRMTPTKIKSLLSDFAEVTRVFLQEEDASVRKRRKKEYGNSGGKRYIEGWVE
FASKRKAKHIAESLNLTPISNHKRNPHFGTLWNLKYLHKFKWSHLTEKVAYERRVREQKL
RVEMMQARRENAAYVEMVETGKKLDKIEERKRKRGDESFTVSSTKDKKTGGEKRHKHRQR
KPVQEGTRATKSAILGGLL
>comp5876_c0_seq1; 459 130 1641 LEN=1950; translated
MPPPPPPSKSYLLVLDSDSSSDESEDESYFQQQYNRTFENINTVAPPQVLDTKNNINGRV
NNDFSSLSLVDESKNDQNQQDSSSTKLTKNQKKNQKKRLQRKKKKKAAKQKEANTLKGVS
FSDVSIREFPRAFSTDTVPAHGGWPLGMKLEGHEDLDQIPLDEYETSKQKRLHERWTTML
SARDKKVPGSPKSPSKKSSPSSKKKSDPVAPFDEKVIQMVGAGAACGESCSEENCNVVYE
TRQWDYKSRIKNPIFRSVSEDERHAIFLDASEFTCSEIQKHVNNSNSVRRARSNSIGNHN
HNSNNNRRSRSNSVGNHDLGNSMKGKFNEEYNQAMVHHVRNELEQIRFERNKSGATGCNC
RKLVVYLPPSDGSGGKKAQHRRLKPSKLVQELKKRNLYDSKVASSSREQMEKILHKAVEK
EPCCKDADCFCVRNGINCQADACSCWHDSHVHPKHSSGSDGRGLTNDDIRTRCGNPLGTY
LVETEAIEAYRTNILKAQKLQME
>comp5877_c0_seq1; 317 94 1020 LEN=1586; translated
MRAGSEASSAPGNPGFSFSSQLSRTSIATLSFLAMVFVCGTTFFSSAMIHHNVRAGTKAS
SATSGALSSSFPRRVFCFGDSLTAGTSPPNHDLFPYAKHLEESLNKHKLNPSDNVVLDNI
ERTVQVRWKGYPGWTAPSLLRNGGLSDIIEKAAQQQQQQETEGSVSSSEEEGQHTAIDLV
VVLAGTNDLAHSKDGQSIFESIKSIHEQAFSQGHVHRTIALGIPPSGWQVHSESARSLAL
EVNSKLESWSATTSTSDAKTVFVPFPIQEYDRNSDLWSPDGLHFSPKGYQSIGESLAPIV

ADILWNNK
>comp5878_c0_seq1; 911 118 1413 LEN=1467; translated
MAKKGKSPSKKQSRKTGKDSAEKKKGVDVDKITWDDIAAMSDSDDDAGGEKAIELSKKAK
DLRQNIANNLSGLLASINQKDGDDEEEFEEAVLDESSDEEDDAQSEDGNDEDEGQGGMEE
EEEEEEEEDSDNNESFIDKIKIKSNDAEDDGDDSDSESSDDEMDEETKKKQEKYARLENN
NNINSKALSVVAAELNSVHSKMPWAETFVIVPPTPLPFGENGDPESNPLDIHDDLKREVA
FYNTALEAVNLARPKCQEAGIPFTREGDFFAEMVKTDDHMANVKDRLIFENKKIEAVAQR
KSNKEQKLRAKESQANRLAEKAKRKRDHFRDVEDWANSAAKNRGGGLRDDVDDHFLNNRY
DGSGPSKKRQNADKKYGYGGKRGRFKQNDRKSMNDMSGYNPRGNFAGGMKKTGSGANRAG
KRARDAKRSRR
>comp5879_c0_seq1; 603 1269 2393 LEN=2940; translated
MTTASVSASSSCSNVSEPTANTKKKSTASVAPHTPPTTTSKFGSELEKDERFKRFKKRSE
SFASTEGSSTPKKDREIMESIAMTTEERQRLEEEMKAQHCHPLVLKIEAEAEERRLENDR
AYRRSTSGNNSRTQATGNRRLLRVRSGSARNVDQLANFLEGDDNDDLSALERAIIHSIGQ
GERGSRGSANSNNDSSTQNDLDQQEGFPLLRSLLTGRLDIHNGNASSISAAASRRSTWYR
RQRHPHSAFASSSLMALSEEEQIAMAIAASMQDQEQQSAQNDDSNEDGSDNDETAEEESN
ETDPGNNSENEDSNTITTPDNEDPDADGVSVASSAVASQQCRHQGNGDEASTITELARVV
TDSRSPNVDTGITA
>comp5879_c0_seq2; 638 208 1788 LEN=2335; translated
MGNQSSSSSSPIDIERLRHFSRNGGSLGLSRTELDKRCQPSGLYGSCNWDDKVVRRLISD
GKIAARLKGEEYPDPSSGCNMEECPICFLSYSAVNITRCCNAVICTECYLQVRPQKEKDS
ACPFCNCENFSVTISKKKSLSLVPGTISSASSMTTASVSASSSCSNVSEPTANTKKKSTA
SVAPHTPPTTTSKFGSELEKDERFKRFKKRSESFASTEGSSTPKKDREIMESIAMTTEER
QRLEEEMKAQHCHPLVLKIEAEAEERRLENDRAYRRSTSGNNSRTQATGNRRLLRVRSGS
ARNVDQLANFLEGDDNDDLSALERAIIHSIGQGERGSRGSANSNNDSSTQNDLDQQEGFP
LLRSLLTGRLDIHNGNASSISAAASRRSTWYRRQRHPHSAFASSSLMALSEEEQIAMAIA
ASMQDQEQQSAQNDDSNEDGSDNDETAEEESNETDPGNNSENEDSNTITTPDNEDPDADG
VSVASSAVASQQCRHQGNGDEASTITELARVVTDSRSPNVDTGITA
>comp5880_c0_seq1; 341 451 1917 LEN=2105; translated
MTAASVVLFSSSTAMMAPALPAFADTTDTDFTIVATTTSEKEQESMLLKGVSQKEIARRA
AEQRNGDFSSSVIDEVWTLLSNYYIDQSFNSQDWNKVREKVESKGVKVNFNDNKSMKIVT
EMVQSLGDKYTRVLDKDQYAAIQKFDLIGVGVTLMPNSQKDIIIGAPPIAGSASDKAGLK
MGDYVTAVNGIPTRGRNAFDIIDQIGENPNAKQVSFSILRDKGPSTIDSIGNPQSADNFV
VTLERQTMEVKDPVQFKISETRSDGTKVGYVRVSEFNSLVNSSLQHALSELNKEGANAYV
MDLRGNTGGAFQSAIEISGLFLRDRVATYVVDSNQVELPFRTPKLQDLSIDPETPMVVWI
DGLSASASEVLAGSLHDNCRAVTMGDKSFGKGLIQAVYGLKNGAGLVVTVAKYITPSGSE
IQGLGITPDVLPLGKNMPAPVYVPVLSTDTSKIDWRDVRERLSSKFCSVPEDRVPAKTPT
GAVAEAAL
>comp5882_c0_seq1; 956 134 2392 LEN=2446; translated
MVKFTSGALSACLLATGATAFAPKSFSGKHASSLSMSTNTAPNSSDVLWTPSWKSGERDE
VYGEIGKYCEDLSFVTRRKTRTVHCGPIKFGSEHPIVRQTMATTNTADVDATIEQVMRCA
DEGFDLVRITVQGRREADAAAKIREGLFKKGYDIPLCADMHFQPKVALMVADCLEKIRIN
PGNFADGRKDFEEKIYETEEDYISERQYLLDAMYPLVERCKELNRCMRIGTNHGSLSSRV
LSFYGDTPRGMVESALEFADICRSQDYHNFVFSMKASNPLVMVQSYRLLAAEQYRMGWDY
PLHLGVTEAGEGEDGRMKSAVGIGTLLADGLGDTIRVSLTEDPEFEFEPCNRLAEIAESR
LAINEDARERQAAVKPYKDTRDVTSYQRRTGSLPEQKEGDVVDARGFLHRDGSVLSVVTP
EMLSKENVQYLYQELGCKTAVGMPFKDIATSDSIYMPTIPPSSDTDARTALRRLIEVSMG
VIVPAEELEKDPLPNAVALVSLKDAIANDGKLPEGAIRLALALDGDETDEEILKVKDLEP
IMCILKPKKGISHLHSSRRVFEIFNDNEIETSVVHHFTTDTDESNELALQIGTQIGSLLT
DGNGDGIMVEQVGEKTPFSVDFLRKTSFSLLQGARLRNTKTEFVSCPSCGRTLFDLQETT
ANIQEATGHLPGVTIAVMGCIVNGPGEMADADFGYVGTLPGKVDLYYGKEVVRKGIPNDD
AVDALVDLIKEYDMWKDKDEEEETEEAEAVTA
>comp5883_c0_seq1; 316 191 766 LEN=911; translated
MKTYGTLEAESIKCAFSFTFSRDFPALVTCYAALGFTTPCATLWAHFAATNGSKCAAECI
PGKGGITELNGPAPECAPSACMECQDEFRDDFDDFAGLEMQNAGITERITRDCDDFYRVI
HDPCIGLDPDYDVGGAVDNSGDDTGGTTPASTPEETTATQTEEPEPSSGTTINGKTTYAM
AAWLAFSFAAV
>comp5883_c0_seq2; 271 626 958 LEN=1103; translated
MECQDEFRDDFDDFAGLEMQNAGITERITRDCDDFYRVIHDPCIGLDPDYDVGGAVDNSG
DDTGGTTPASTPEETTATQTEEPEPSSGTTINGKTTYAMAAWLAFSFAAV

>comp5883_c0_seq3; 467 77 1147 LEN=1292; translated
MTRVLLLVVVASTASLATAADFLGTARCGDCWCITDDGTTCPSDTVGISDSFSDTDLLFG
TFELTNSPNFLKLQSATGGDCFPFKDSVGAIDRYEESNLDQCIASDEDDDVVCGYVYDAS
STTCEGRKYSIQNFASSNDAMASNAHIVHKGSCGVCSSAQDFSTRMKTYGTLEAESIKCA
FSFTFSRDFPALVTCYAALGFTTPCATLWAHFAATNGSKCAAECIPGKGGITELNGPAPE
CAPSACMECQDEFRDDFDDFAGLEMQNAGITERITRDCDDFYRVIHDPCIGLDPDYDVGG
AVDNSGDDTGGTTPASTPEETTATQTEEPEPSSGTTINGKTTYAMAAWLAFSFAAV
>comp5883_c0_seq4; 297 77 823 LEN=1484; translated
MTRVLLLVVVASTASLATAADFLGTARCGDCWCITDDGTTCPSDTVGISDSFSDTDLLFG
TFELTNSPNFLKLQSATGGDCFPFKDSVGAIDRYEESNLDQCIASDEDDDVVCGYVYDAS
STTCEGRKYSIQNFASSNDAMASNAHIVHKGSCGVCSSAQDFSTRMKTYGTLEAESIKCA
FSFTFSRDFPALVTCYAALGFTTPCATLWAHFAATNGSKCAAECIPGKGGITEVRTRSTR
NIVPTFVQ
>comp5884_c0_seq1; 498 110 871 LEN=1048; translated
MSDNKLRCALIYRIGSGGTAVCVAKYDHAGQYETHGGADSSLYGGHDKSYAEAVAGVVTN
DPPSGLSQSGTLGGFKVVQSDQHQIVYGADIDGLCCATITGTQYPSRVAIGMLQDLYTDF
SAKYGDAAKIAAENSLSKKAKSILQAVSKKFEDPSAVDKTSKILGQVDAVKGQMQDNIAD
MLKNTEKAEDMAAKSEQLNEQASVFKKRSTDLKKQMQWKNLKMTLLLGGIVLVIVLSITI
PLVNKAKQVASSF
>comp5884_c0_seq2; 216 110 400 LEN=652; translated
MSDNKLRCALIYRIGSGGTAVCVAKYDHAGQYETHGGADSSLYGGHDKSYAEAVAGVVTN
DPPSGLSQSGTLGGFKVVQSDQHQIVYGADIDGLCK
>comp5885_c0_seq1; 269 106 1125 LEN=1376; translated
MSGSVCAAGGAVAETDPPAMSLPGGDEARGGFGFRFRRYREELALRQRKQGKVRLQDQPD
GFPDAPNGTAPGEKASNDGPGENDRKDDDNDDGGGTVLRDLSGARLVVDENGSVSVSFAD
TEGGPVHGIPSMVPEGTTLLLRNLRDCRVAIHLPLPAVHLVGIRGTEVDFFNGGSRVPSG
GSLPTNTTIHATDCHGGSVLRFERPAQQLRIHGSIGLGIGVAERPGAHRGAASTTPASSP
DRQWKPGSIILEASRDLCFTVPPIDHRALEADTRAYWHGVVVRDFHWLRKGVPSPNFRVE
VLGGNPPRGEGPLPGEERGPSGETNRGEDSSDDSDDDEL
>comp5885_c0_seq2; 82 1 626 LEN=877; translated
XLCLELPCRIAFGVSLLAARHLPLPAVHLVGIRGTEVDFFNGGSRVPSGGSLPTNTTIHA
TDCHGGSVLRFERPAQQLRIHGSIGLGIGVAERPGAHRGAASTTPASSPDRQWKPGSIIL
EASRDLCFTVPPIDHRALEADTRAYWHGVVVRDFHWLRKGVPSPNFRVEVLGGNPPRGEG
PLPGEERGPSGETNRGEDSSDDSDDDEL
>comp5886_c0_seq1; 467 181 1209 LEN=1287; translated
MVVEARKLSDSIQKKAADGEEDNEDIVRALDAMGYDEAAQYVYGMAYPDWKKRHAQKASD
DQMEKFNESKPLWAAHDKKVLAKRSEELPPPLPVDSNGKNTADAPVCAINPSKDNNNGTT
TKPSLLSNVCCEDIDSIDHSKPKTATENTLSSAVQSTASSAFAPPDLPADLSFSLGILTV
SDRAFSGGYETGDLSGPAVRQAIESILESSSAGATLEGVETAIVPDESEAIQQKLTGWSS
SAAPLDLVITTGGTGFAPRDVTPEATAAVIERSCSGLMAFCGMHTRSQQPLAALSRGTAG
VRGKTLIANLPGNPEAVRELLPVLLPLALRAVADMKTTMGEF
>comp5886_c0_seq2; 536 55 1266 LEN=1344; translated
MATEIDNPNYVETHLPPSVIKRLESGAFEQLCTHLRERSDEVQNIDLMTVSGFCRNCLSK
WMVVEARKLSDSIQKKAADGEEDNEDIVRALDAMGYDEAAQYVYGMAYPDWKKRHAQKAS
DDQMEKFNESKPLWAAHDKKVLAKRSEELPPPLPVDSNGKNTADAPVCAINPSKDNNNGT
TTKPSLLSNVCCEDIDSIDHSKPKTATENTLSSAVQSTASSAFAPPDLPADLSFSLGILT
VSDRAFSGGYETGDLSGPAVRQAIESILESSSAGATLEGVETAIVPDESEAIQQKLTGWS
SSAAPLDLVITTGGTGFAPRDVTPEATAAVIERSCSGLMAFCGMHTRSQQPLAALSRGTA
GVRGKTLIANLPGNPEAVRELLPVLLPLALRAVADMKTTMGEF
>comp5887_c0_seq1; 325 291 971 LEN=1419; translated
MTRGLNPATTFSAEDVQTLRNVRRDIYYQGIIGGGLGLCGGLVTYTGAQWARKLGFIKGK
SLNRNTGMLVVLSSFAFGQFFMASTTGKELVHLLHPIYDGWKGDPSSTNDERANHNASSG
LVDPKRLREMRYARRKSLMETLHHQKGGISDSHSGHWVEERSSEEGAHDVGSESPAQVDP
QRLREMRLARRKSLMDTMFHQNGGISDSHSGRWVEENSETKFKKEY
>comp5890_c0_seq2; 440 314 1619 LEN=2293; translated
MTASVLPSHLSQAHVKVNVRLCGSIPRIALSMMQQEATRRPQSAPHLPMFTSSDRQDMFL
KSNIAVSEKDENCVAKQLCSQLSTVKQQQLEQTFESQKLTTQPQHSLFSGTIPSTLRRTP
PTYEAPSWAVPATGESRLEXEHGLSNFCTSESSMPIVLQNQTNTNAXFLCFVSSQPVFDS
VERQAPVDLTSRAVFRVGRSPQSDVQLMHVTSSRRHAMVFHHTNGSCYLVDCGSAHGTFI
NGVRISSTPNDSNVVVPTRVRRGAIVRFGGPGAPSYMLKSFSFGLEQMKETLIPDMNEYA

ANLPPTPMLTAMVEHNTRFNSLGKSAKEELMVQRHFSSKRSFDSMETVLDCQSDDEMDEN
RHTAPVRLSSPPMSPEQPLRLVSPELNCSTSSQTPNFKRRRVTFSDETLLAVYPTLVTPD
VSSDEQDQDIECCS
>comp5890_c0_seq3; 492 314 1513 LEN=2187; translated
MTASVLPSHLSQAHVKVNVRLCGSIPRIALSMMQQEATRRPQSAPHLPMFTSSDRQDMFL
KSNIAVSEKDENCVAKQLCSQLSTVKQQQLEQTFESQKLTTQPQHSLFSGTIPSTLRRTP
PTYEAPSWAVPATGESRLEPVFDSVERQAPVDLTSRAVFRVGRSPQSDVQLMHVTSSRRH
AMVFHHTNGSCYLVDCGSAHGTFINGVRISSTPNDSNVVVPTRVRRGAIVRFGGPGAPSY
MLKSFSFGLEQMKETLIPDMNEYAANLPPTPMLTAMVEHNTRFNSLGKSAKEELMVQRHF
SSKRSFDSMETVLDCQSDDEMDENRHTAPVRLSSPPMSPEQPLRLVSPELNCSTSSQTPN
FKRRRVTFSDETLLAVYPTLVTPDVSSDEQDQDIECCS
>comp5891_c0_seq1; 281 249 665 LEN=852; translated
MREFQHVFGDKYLRNDDGDDEMRGIGDVSSASASTSTLSAELLAIPTNQHARHDLVAIGD
HIEAEKDRLLNCFLEFGEEVCSKIQQAGYWADYIDPCSGLPMLTKNCNKVYSEVDGMECL
LNYRAYNAGFCKVLTHPRW
>comp5892_c0_seq1; 371 94 1431 LEN=1535; translated
MISRTCHIASFILIAVSFLIGESQGFTTSPSGSNHRQGRTQHTLTYSAVDEFSLAETELS
LLKKAKKGQKVTASPQSMYGKNNADELLDAATQKATIENEFPKRRITAKVRETGTDSMKH
YIKSMCNHELLNKNEEIILAREIQILLKWEKQREKLEEELLRPPTYAEWATSIRTNMTVP
ELKKQIRRSLRAKTALTESNIRLVVSITKRYQNRGMAFQDLAQEGILGLTRACEKFDPER
GFRFSTYATWWIKQSIMRAIADQGRTIRLPVHIHDQLSALRKAERELKSDFGRQPTTAEV
TERTGFTSERIDFLNRVSQQSISMEQELTSSKVKGSGAGVGGGGGNSLTIGDMMGDPDQK
PVDQASYQMLKDDLSRLICTLNSREQAVIRMRFGLDDGRPKTLEEIGKRFSVTRERIRQI
EARALHKLRQPYRNHAVKCYVKEYS
>comp5894_c0_seq2; 240 1 618 LEN=850; translated
YDRLRGKVETIDNRQSTTLSSTTVYHVLSNNPTGTSTHFENPKSPAIRQQNRHENMFSSR
SILLLIALVVAVSAGTSKEGLEFLAKKATEEGVVKLESGLMYKEISPGNNSGKKAKVNTP
CECHYAGTLIDGTEFDSSYKRGQPLTFAPNQVIKGWTEALQIMDEGAKWELYIPSELGYG
DRGAGASIPGGAVLVFTLELIDIKL
>comp5895_c0_seq1; 535 493 2370 LEN=2544; translated
MRGISVETKDTELFEIKGGKGNSAVANGGGGSRKKRKSAGGNNSKGGTASSTTKARKKAS
QEGYFRLKVKDNGCGMRHDKIPDLLGRVLSGSKYGVRQTRGKFGLGAKMALIWSKKSTGV
PIKITTAHLLVPKSKSKAYNIDTSSLEPPKKVSTCVLDIDITNNCPNIIEHRLRKNEDGW
VGTELELVIAGNWTTYRSRIVSYFQQLAIITPYARLELVYENRSEPHRNVTVRHDRRSEQ
MPPLPKEIKHHPSSVNNIIVSQLIERSNHKTIIELLTKELSCVSRALAVRIVKECGWDKD
DIIEPSDISGRDLTKLVQVLRTVKMFKAPDGSCLSPLGEYNLNLGIRKVLEPEFVATSRD
KPSAYEGHPFVVEAAVSLGGKEAKEGLNVVRFANRIPLLFEAGADVCTRVATSKINWSLY
KIDQKRDKIGVFVSIVSTKIPYKGTGKEYIGDDITEISVSVKKALQLCCQQLRVHLVKRN
ALRDQRDRKSKLVKYIPDVSRSLFALLEGMRKRRDDIAEGVNPMSSPRKRPRREQHWQEK
HVDTVVEELEKGDITISIIRKSLEETVDAQNLNEEQEEAKKSKTKDRIPIFMKPMFDMNN
SNFDIDHPLFTFRPMVCPVMPPVDK
>comp5895_c0_seq2; 600 114 2264 LEN=2438; translated
MARVNSSDSGSSTKTKKTTKSEGQGEIQRQKSPAEFFADNQAIAGFDNHGKSLYTSIREL
MENSLDACESIGVLPSIQVQIEELTQDEFNIMRGISVETKDTELFEIKGGKGNSAVANGG
GGSRKKRKSAGGNNSKGGTASSTTKARKKASQEGYFRLKVKDNGCGMRHDKIPDLLGRVL
SGSKYGVRQTRGKFGLGAKMALIWSKKSTGVPIKITTAHLLVPKSKSKAYNIDTSSLEPP
KKVSTCVLDIDITNNCPNIIEHRLRKNEDGWVGTELELVIAGNWTTYRSRIVSYFQQLAI
ITPYARLELVYENRSEPHRNVTVRHDRRSEQMPPLPKEIKHHPSSVNNIIVSQLIERSNH
KTIIELLTKELSCVSRALAVRIVKECGWDKDDIIEPSDISGRDLTKLVQVLRTVKMFKAP
DGSCLSPLGEYNLNLGIRKVLEPEFVATSRDKPSAYEGHPFVVEAAVSLGGKEAKEGLNV
VRFANRIPLLFEAGADVCTRVATSKINWSLYKIDQKRDKIGVFVSIVSTKIPYKGTGKEY
IGDDITEISVSVKKALQLCCQQLRVHLVKRNALRDQRDRKSKLVKYIPDVSRSLFALLEG
MRKRRDDIAEGVNPMSSPRKRPRREQHWQEKHVDTVVEELEKGDITISIIRKSLEETVDA
QNLNEEQEEAKKSKTKDRIPIFMKPMFDMNNSNFDIDHPLFTFRPMVCPVMPPVDK
>comp5896_c0_seq1; 233 132 1042 LEN=1042; translated
MAGVGCNNILLVKVGGSSITRKAELETLDAESLAWFSTTIRSIVNDRFLDDAAGNVVETS
TEFDCEKPSIVVVHGAGSFGHHTAKKHGLGGKSSPPPDGMNLTRDLVTGIARTRQSVKKL
NGAVVSHLIENGVNAVGISPCLNVPGLMTHGGDEYNGVTVLVQSIRETLSAGLVPVIHGD
AGLYGTYRDPSARDRFQSMSAGILGGDTLVEIIATHPLIRRKISKAIFLTDVNGVYTKDP
KVFQDAVLVKNIFIDPVSGDVMNEVSASGSQHEHDVTGGLEGKLCAATNVAKTGIPVVVA

KCGS
>comp5896_c0_seq2; 179 132 1144 LEN=1144; translated
MAGVGCNNILLVKVGGSSITRKAELETLDAESLAWFSTTIRSIVNDRFLDDAAGNVVETS
TEFDCEKPSIVVVHGAGSFGHHTAKKHGLGGKSSPPPDGMNLTRDLVTGRKKSGGCSTGF
VGCGMERKKINEGSSDTLHCRKVLRGHAKAVKKLNGAVVSHLIENGVNAVGISPCLNVPG
LMTHGGDEYNGVTVLVQSIRETLSAGLVPVIHGDAGLYGTYRDPSARDRFQSMSAGILGG
DTLVEIIATHPLIRRKISKAIFLTDVNGVYTKDPKVFQDAVLVKNIFIDPVSGDVMNEVS
ASGSQHEHDVTGGLEGKLCAATNVAKTGIPVVVAKCGS
>comp5897_c0_seq1; 460 1 1192 LEN=1296; translated
XPFEPHCTGHTPRNSDRSEQQHRRRKMWGAITAKAQMLGDSAANKAKQAKLTADILMIDN
SMDSRKRAFGIAMYDYLSPLTKSADFYAASDKLTELVRPNIILAQKEIQALAARRVKQKE
ALAKAEADRAAAFPTKAENYGQKLFNIGKAGLYHGGEAKIKTELTVTDRLIKGHKEDFGV
ALFNAFAEAEDKEGFLPTDRQIRNVYDTCRQDLTTLEQQKKAKEDEIVELGGRRVSSETP
SIVQKPSAVPANATMNSTGANSTFVAASTFMETNDAPFSATNQNTGYGATTTPQQSAPFP
TTTQQSPAMVQVAGSGIFSHIVPSNASAPASGGYSDNAGPFHSVGTQQGFQNQAPNNAFA
PQPAAAPVDPFSAFTNQPPPSSSQQQGQSADPLVGFF
>comp5897_c0_seq2; 469 309 1334 LEN=1438; translated
MIDNSMDSRKRAFGIAMYDYLSPLTKSADFYAASDKLTELVRPNIILAQKEIQALAARRV
KQKEALAKAEADRAAAFPTKAENYGQKLFNIGKAGLYHGGEAKIKTELTVTDRLIKGHKE
DFGVALFNAFAEAEDKEGFLPTDRQIRNVYDTCRQDLTTLEQQKKAKEDEIVELGGRRVS
SETPSIVQKPSAVPANATMNSTGANSTFVAASTFMETNDAPFSATNQNTGYGATTTPQQS
APFPTTTQQSPAMVQVAGSGIFSHIVPSNASAPASGGYSDNAGPFHSVGTQQGFQNQAPN
NAFAPQPAAAPVDPFSAFTNQPPPSSSQQQGQSADPLVGFF
>comp5898_c0_seq1; 229 1 900 LEN=1135; translated
XSSFTWVSNAMFYIMLCRMLSYNDVVCFRCAVSRFWEQKRTTSIHFNLLRDSIQFKSNTI
ELTPRPPFAVHFLLFETNRNTVGEGVELQYRLKGYGIPVDLTPLTDSGNIKTMYLKQWLR
LRKIIETGITPSGVKTSTVSIIECPGSNDVIFRPGTSMLCHPGNVAFRGLIEAKQNRITV
KRAEKEEVALEIIQEVRSTGGRFLMWDNGGWWSELNDIPLISVKISISYRDFKTKVKTLV
QQHQQEQQRKQQNLASATNDFLPGGDKKRKRSMIPTFCGTFEXATNNDYHDNDDNQIAID
>comp5898_c0_seq2; 462 110 2067 LEN=2302; translated
MPPNSQQQSNANAIFDEMVDSSDENQAYATSHSSKTASDFDTRIHSMGCETYNGKGNINA
NANVNVNVNETRDRGRRSTKPRRTKESDDLDPSAIIKKYEIQCSNMGSSDQRMLGSHHQL
HPQSQQQAAPNTQGLVGEEDADTDSTTSSSTAASAIATATLSEAGSPFASGMDVDEMLSR
ELLKLSFQDRIANDEEIHGVRCLAPEENPRLLSLALASLEREIERIPYKPAYDKAQAYAL
VPRFAKTSYVNTDDFRLRFLRCELFDVRKAAVRLIGYLELMVELYGLFALQRRVKLQDFS
KEEMQILRSGGFQVFPFRDRSGRRVMCTVGNMGMMFDPTLRLKANFYFWMTAAESVESQQ
KGVVFLVWPGPNDTLRAIPNQTDRALHCKCNAAAPIRIAAVHFCLPNKPFFHLLRNIMAA
TLGLAYRLRLKFHVGEGVELQYRLKGYGIPVDLTPLTDSGNIKTMYLKQWLRLRKIIETG
ITPSGVKTSTVSIIECPGSNDVIFRPGTSMLCHPGNVAFRGLIEAKQNRITVKRAEKEEV
ALEIIQEVRSTGGRFLMWDNGGWWSELNDIPLISVKISISYRDFKTKVKTLVQQHQQEQQ
RKQQNLASATNDFLPGGDKKRKRSMIPTFCGTFXIATNNDYHDNDDNQIAID
>comp5900_c0_seq1; 95 1 283 LEN=1256; translated
XNEDLGVELLDKPTCILNSNNDTDSIMNNNVEDTFVDYAEVEIDGVMGDTDSSPLYRTIV
VTLSATMFGTVLASFQGMLCGECGASITREYAG
>comp5901_c0_seq1; 337 217 858 LEN=858; minus strand; translated
MVRLADARCPVPFSSAPAPSYLRRVLDFRAIRRALAVPLATTGALPALKPPALLPPKPGA
PPAPMEKPPFIPRGGGLPMFDGWDFMKPFDGAGAWFSLVLPNENCEFVGLEGWLPNDIGF
ACWGAGAPNDGAVAAPKDGAGAAAPKAGTGAPPNDGAGAEDAGAAAPKAGAGVAPKDGAW
ADGAGADAPKAGAGADAPKVGADGAGAGAVVPKA
>comp5902_c0_seq1; 585 139 2967 LEN=3163; translated
MARFSRNVGARTEALIYRNRVFWSLLVFATAWLCVMMSFVPKLLIGRVGEETKAWSLSLK
GGDGARIFSSSYPFQSLPGGDAVSGVVFRGEKTRSYEGERIFRSLPSSPPSKDTTTTTPK
ALPLMDPAIRCKNWNVVTTIFDPSEAVRRASWVEGWCTVIVADTKTPEGYMEKLRNMGGI
VGSDTESRFIRTGRRRSEKQEGKDEDEGIVVFLSIDDQKEWMKLERGSSSATSDSTAVGK
FLEAIPFRSFARKNIGYLYAIAHGAQKIFDFDDDNLLPVSLGKDGEERVFAPFHNRDALE
NPAMVVTGPTAFNHHNIMGISVNGTSWPRGFPLTQITNPATRGRIALESKAFRDVPMSEI
GILQFVADNNPDVDAIHRLVQGQMNPTTGETVHFKRHVKYDSNSSREEAANYYNTVSEIS
TEGSLLVPSHAYVSFNAQATVFTHQAMFGLLLPHTVMGRVADIWRAYFSQRIFREVRTPK
RESSFGNDGLKVVILPPDIVQERNEHNLLGDLQSEEHLYCRTEALLSFLDKWEYEGDKDG
DNRSIPAMMEKLWIGLYERDFIEKQDIEVLQLWLSALVEVGYEFPKVQSNSARQIRDVVV

MGQFNYGSSKVADNQDELMKTNLLFWYQKWRQRFRKVVIRGPFSADLAKEFSEQHEIDVE
STRDRLSHDKGFVSPMDNLLSTLKKYEDDPTISGVLYVHDDMIVNVTNVFDGVNRTTTVL
GTRFLTAPQPYRIHPSWASNSKPNSTDKTLIFTAPDGFRTEKWYELLGHLENWDHNPECI
HQFSNIANDPRIKPYLEHDETIENGNPYLSVPYRMQADFLYIPTRLSRKYEEIANLLIEH
NAFLECGVPKIVQHVTTASSNVIFKFVDLCTSFLNRRTRGSLNMITRCEPEYANSQPNTP
LPSPHGAIHPIKINARGYKTWGMLFDWATSGTTRLNVADYEN
>comp5903_c0_seq1; 631 288 2645 LEN=2919; translated
MASSVAAKMMQVFARSKTAPMPIMKRARNRLISPSSITIRQASVFSHFITENTNTLTAKQ
SSVNAANKDWHSPFFMSDTTIAEKEAWLEDLLTETNNSSNTNIISTTEHHQRNRESESSI
DVEAFLVVLKALASPDAVEDAGAPRRADVWMNRLLKLNHYNIRPTSECYQYAIQAWANST
KEQSMVIRNRSQRWFNEIVGKSGDDLLSSIIQPTYGNARTGQDSFNVEPTIECFNAFLDG
LSRGRQGKSKRDRQILLENAKMGEVILRRLHSLYTHRSKRENNSKNEMSHKNRDIKRQTE
TSIAIRPNTETFNFVIRGWTRCKHEPFIHERVLAILRLMESYQRGNPAAFIVDRDAPRPN
TKSYCMAMDALISEAKRKARNYNMQRRRKNHVEFGDKYEETENTHLNGIDEINEAASILK
YLHDLYDAGVEGVVPHRVPYNILISGWAAIASFSQHKHYYQTQNSNQGEEFKAEEILRTM
ISHRENGFIEASPDVISYERVMLAWANSGHPNAGKRALWWLKQLWKDYDLYPDSTSESSH
KSSLLPTVRTYNVVMKALASSDGALAAENMLLDLGEKYRGSTDHPGLCPNSESFSIVIRA
WLESAKQTRNVDERISSLRRAYEWLSSLRGIENENNLSTSPELFIGLLTMSKSCAKQRPH
VLDLAMQIFEDYRQSRHRLDCISYATLLQVGLRAHSRRSGEIREAFVEKLFAECCDDGLV
SNVFIRTLVDDPSNECKNLVNRVSQDWPLPSSWSRNLKNNNNRCTLIDTKSSSERRVLRR
RRNTN
>comp5904_c0_seq1; 530 226 1422 LEN=1853; translated
MKTSSTLLLGFVLLILLCMISIIMQSIHLMEHHDVHVTSEESIGNIPIDAMVSIGTEPLE
HEKMFEDTLKDCMPFVFEDGDRKKNKKECKTFVPEGSKERIGVLAPPGKMTSALVKFINI
VLAKAKKEGGDEIAATGAEVISTTHMAPYGYGKTHGLTRMIRVVPQPLLLGTTDTLKAAV
QSSPTPMSLNDITINDLKAALRQQIRYHCRLNHIAAHTAMWTIGVEDFAEIGREVLVETA
QEFFSLDMDQDDLLNLVINEYDKLGDDDDENQGVVEKDDDDNSLAQLNYMYADGAKLMGL
MQTKAKSEVRKAKAKEKTNDILKMLDDVLLDEMKISNNLTNWPCESFWTVGEPDNRLELS
PIIRSISAAMSPNCTAPYTTCFVKKDKCEAKGDGVCN
>comp5905_c0_seq1; 1272 46 3129 LEN=3164; translated
MSKSTKAPKAGPDPLLLSRKEVDVLYDVFDVVRKALALLNVEYIVTGGSLLGAIRQHSIL
FCDDDIDIAVIDHRCGEEDGGASAPSIYESVVVPRLQSALDEVVADESNDCRDRCVYQIK
PWEGGDRIRSKRYNSVFVDLFVLRKYPTLQSFRSVVGVKRNGRAQPEAYVDSILDTVQAS
IGARRAGNRDSHGSVAVVEDADADARARESVFPFWQFATRKAIEMWPKEVYCAAGASSTE
PSNHRESPSQSMNTVDELFPLQTNLKLGPLTGVCGPNMPVTLLKRAFGDDCFDVYYRSLS
HHQQKQSQNKSQAQSETQQQQQQGTEIKGTEGNKKDADLPPLVAPGGTWEDSPKVPLED
EHYLPMQPLARKRRRPTLHCKERLFEFLDVQAKKEAEERERFLSLRQPSGSDAIPPSGGA
KTVYMDGVFDLFHVGHLEAIRQCAELGTRVILGVTGDEDATGYKRKPVVPQAERVAIVSA
LEQVDAVVCPCPLVVTKEFMDAQKIDLVVHGFANDADAERQKEFFEVPMALGKFQRIGYY
RGLSTTDRIRTIQERDRQDQHRLSGDGENQAKDGSDEQLTIQTKGGEEEKEDSVAMSVPS
DVAEPRIQNGDGAGRDSLANETAPTKTASQPNPQWFGAALAAATNDAPLLPTDPFPLSLR
TVIEPHIEKARRRRREALGAIRAATGEPLYDSVMKNFLRQQEQLRVGTTREPLEGNNSID
RKLSEGLLSSLLLSAGLPPDTDLSRLHEIPLLDGRPAKDRLLHSLTQNPGPFQTSYDAYV
RTVCIPRMASAIEASERRNGNESFCCEEIYYQAFPCLRIVQPHEFSIGPHADVAYGHHPC
SINCYVSLTDPTYSGISSDINGDEMGSSPSSSLFLESRPGAEDWHALFSGNDEGFPRLEL
FAGAMNLHWTTENKTPRTRVSLDFRLLDGRVFQSLKCGGHLEGGQMDVYRATPGYYHRCL
RSKEPVTKPVAQNKTKGGTWDSWIRDKDYLNNGSGGEFPHPDFRVGFPWTVKNWDKFWRK
NTKPDDK
>comp5907_c0_seq1; 473 577 1779 LEN=2123; translated
MTVVFGAAVCVTEEAWKEKTKCDHIVNVFYQYLDNDANGVPDDPRVHAEIVDKNYLLFVP
ATEEEWEDAWSKMGSSFHDQLGYIQMVGTWETNLNSCVVPSNRGASDTDRSTWSAFIDTD
DKDCQGGRPDYGDASIEEILHLITVGAAAVYPSKWGGSFESEVGVAIEEANGNCGWGHDG
NYVNPSSNKCSGQYAFEGVYNKNCRSCEVWVLVMEGIYWAVVSYIGGLYTIGRAEFAMNE
WLMSTPDSNMEITPSGVTNAISLEAGSPALYAMVSDSTSEGHLWLPTIMPNGNYKGFDDN
PPRPTLPPAPDKSPTSSPTYDGTSLITSTTTSGCIDDKDFRFKNKEKKSCEHWVAKGADK
LHKKRNVRAIKKRCRKKYRNKRVWEYCPATCALVDLGDCA
>comp5909_c0_seq1; 474 357 1490 LEN=1701; translated
MKFSFTALSLSQLLAFACPASAMFQIDNSPNLSGNGRGLRKLLSKARRLEDNDEGNNDDG
GNNGGEQNQEEMEAFLMDFSMKFMECIPDQVLTDADYNDHFGVVIFRLCPANSCSDNSGC
KSGYADFAVDVGTYVDAFLTDQADNMNWDDKFDGDEMGECTQFKAEGGNDDGVSYYVGPG

CTSDGKGVKLGLFEDQYCYQESETSFETISNGWGLPYSNGGLVSTQCTSCTEDGGLREMC
SDLYENSPHRCEKEFDFKHYYYDTNFEMYRYGQDTTGCTKIDVMLNPKGVFSQGAVWTDA
ILAVMLLVVTGAGYAWYSSWWEEQKLNLEEIDDDEDSQAYHLGGDEDEDDHSEFSQHGAE
LNDSTLTEPVVAEGTMA
>comp5910_c0_seq1; 574 238 2376 LEN=2459; translated
MTAAIMAQQKPINLFMQDLVRKNIHSNDPKDDSDSTTIATFDFAKKEALHRNDSISNSIS
NSINNSDSSVLTSCTGTSGSYFLSDDTTITDTTASSRSVVVISDNARVSARARPKQTAVL
NPVRSSPHIRKKLTASNRWGADCISQMNKNLNHYTLSNNHSRDGLVVSSSHSSESRNRWY
PDELKLPSRGNSSSSNNKNGKSNDTNSTDPSIRRGVSDSCLTMPRRSWKQQLEPQLEIEE
DSTEELVEQLLNAKTNANTNTKRIVSCNNLSAYIEPNHTTATPSSDIVQAKKALAKSFCS
QKSSSEDLLKAASKAVQRDLHRVDSMELPNSRCNHKFPTQQQPRRKQQRKSKRPVAPGGP
SSTALLLNRNRLLDIISSPDGQESLRRLTSAATAPGECVPKPAVKDKEEISNRLLDKTVS
TSTLKLLQKRRQTSSKTNRITVYKSIDNSSHSIYSSVGEGESAFSSTRSKRRNNSMKKIT
RSSSSTVSILEKALGEMAPKVKKTTSKTKTSKTIATSNSKSTSPKVSKTSSMPSRSGKKL
ERSHGVPSRSGRHTSSRRERNSIDEEYSRSIQSRNDSSSKKMLLETTIGDDKPLPRIKKV
TLGAAVGTDTTCTNSNTPPRIPGMGSLGGSSHRGARSRSSSGKKGVSTKLTSLERVLGGM
APRSRTSSGSCTSGDTSNRERRRRAASRSMEDAPPRPTPRKNSLQISDGTTD
>comp5911_c0_seq1; 832 1 846 LEN=846; translated
QQQQQQQQFPMSNAVPPPPPSLSQDAIGNDNSNGGGIAMGGGMFMGRLTQFLDVVAPQQE
DDAGDGFGHGDGWDDDDVDVDDDVNEDGWGDDDAQELLSMLDDNDDAAGGASSLAPNDSS
TVQTNQAVVAPIPNHPPPPPPPPPPQCQANDRVDESFGGETLLSSAAVGTASTLGAANDV
LDTDWETNDPNNSNNDVGGGGWDDDDDDGFSFGDDAMEPVAMGAEEADENKPLQSPEPVH
VVPPPPPPPPQSNFANNNDVHVSVVENDDDFGLDQDGDGWGDD
>comp5912_c0_seq1; 541 1 1551 LEN=1683; translated
XVLFGNNISIRRLLPIQIRFDLKPVAFLSCRQDFGWLRDENLRCHRTTTCRFLHGLGGVS
ARCWNPSICLFVIGVGVTIKSVEKTIESINASAKGRKPRGTAENGGETSGNDEECGYYDH
VPWLGSVSSTNGQGIDEAARYEELPPSVFSPTGRLHPVEAAVRAGKVATPLSNLLVAIKC
RDGLLVVSTYPVSPNVDARIASLAGSNQTNTISSASIQQGNDDNSGHSDDGIESNQTTTN
ETLDVSGSNNSNPHTGEESHTYPSLFLFEETCASTSTGPIFDIHPCMVGATAGNAVDNRI
LGEKLLDLGLATTNFEEDDPLAGAADRVAKDLANQLQVVTQDITATQKQRLGRMLASSAV
VLGNKKLWRIDPTGQFWDCQATVLGHDADRAEEAFCCRLFEQCERRHGDDKNGIHDPREL
LESMTHDEAFEFAKEFLRHQTATQRQPKSNTALRSSDTKASIDASATKEGGIELPVTELS
SQTYWQAVILDYASLTTRGTPRRILRRGSFGIQNKE
>comp5912_c0_seq2; 355 115 1092 LEN=1232; translated
MRICGAIARQHAVSCTVLVEFLLVVGILLSASLSSASVSPSSRSIKTIESINASAKGRKP
RGTAENGGETSGNDEECGYYDHVPWLGSVSSTNGQGIDEAARYEELPPSVFSPTGRLHPV
EAAVRAGKVATPLSNLLVAIKCRDGLLVVSTYPVSPNVDARIASLAGSNQTNTISSASIQ
QGNDDNSGHSDDGIESNQTTTNETLDVSGSNNSNPHTGEESHTYPSLFLFEETCASTSTG
PIFDIHPCMVGATAGNAVDNRILGEKLLDLGLATTNFEEDDPLAGAADRVAKDLANQLQV
VTQDITATQKQRLGRMLAVRCDCDVW
>comp5913_c0_seq1; 635 228 2207 LEN=2263; translated
MTTDRKKEENTTAKVMEMGERENGDHQSNQADGTQAEDGSEGSRSSRSGIWSNLEEDVET
VSSGVSPTTNQGSQLSHQTGMSKPESEESLNIARKENMAVMTWRLVMFGVLIVTTVAVAV
LVFLFVSNTENQEFVKSFDVDSNKIEESLLSTMDTKLEAVDSLAMFMVTNAREKNQTWPY
TTLNDFGSKAAKMRILSHAIALQQYVYVTEEQRPEWEAYAKANEGWVQETIEIQREDTTF
SSPTYIPDYDTNHSTSIRYAGPVPNGTGPYTPTWQTYPMLPTGTMSAYNWNAIQHRLLGP
GIEKVVADHKVVIGPVLNFDDSNEGSGTNRMVVWAGRHSSSPDVNPKEPIIRVLYPILDT
ATGAITVDEEDSKVVGIIASSFYWKSYLENILPNDENGLVVVFENACNQTFTYQINGHEA
EWMGTGDLHEPEFDGMGKTLTFEEIGMNSANSGGRYGGLPIDMDYCAYTVKTYPSTVMKD
SYQTNEPVLYTIIAVGIFVFTALVFIGYDKLVSMRQKKVMSTAVQSTTIVSSLFPSNVRD
RLLEGNGDTKAANNVTGSIFQPTKSRLRTFLNDGDSSTDSTKPIADLFTDTTVLFADIAG
FTAWSSVREPTQVFTLLETIYGAFDNIASRRGVFKVETIGDSYVAVAGLPDPRKDHAGKW
>comp5913_c0_seq2; 1044 228 3788 LEN=3837; translated
MTTDRKKEENTTAKVMEMGERENGDHQSNQADGTQAEDGSEGSRSSRSGIWSNLEEDVET
VSSGVSPTTNQGSQLSHQTGMSKPESEESLNIARKENMAVMTWRLVMFGVLIVTTVAVAV
LVFLFVSNTENQEFVKSFDVDSNKIEESLLSTMDTKLEAVDSLAMFMVTNAREKNQTWPY
TTLNDFGSKAAKMRILSHAIALQQYVYVTEEQRPEWEAYAKANEGWVQETIEIQREDTTF
SSPTYIPDYDTNHSTSIRYAGPVPNGTGPYTPTWQTYPMLPTGTMSAYNWNAIQHRLLGP
GIEKVVADHKVVIGPVLNFDDSNEGSGTNRMVVWAGRHSSSPDVNPKEPIIRVLYPILDT
ATGAITVDEEDSKVVGIIASSFYWKSYLENILPNDENGLVVVFENACNQTFTYQINGHEA

EWMGTGDLHEPEFDGMGKTLTFEEIGMNSANSGGRYGGLPIDMDYCAYTVKTYPSTVMKD
SYQTNEPVLYTIIAVGIFVFTALVFIGYDKLVSMRQKKVMSTAVQSTTIVSSLFPSNVRD
RLLEGNGDTKAANNVTGSIFQPTKSRLRTFLNDGDSSTDSTKPIADLFTDTTVLFADIAG
FTAWSSVREPTQVFTLLETIYGAFDNIASRRGVFKVETIGDSYVAVAGLPDPRKDHAVVM
AKFARDCRQQFSELCSILESSLGPETGDLNLRIGLHSGPVTAGVLRGQKSRFQLFGDTVN
TAARMESTGTVNKIQVSQATADLLRAAKKSHWLQARTEKVEAKGKGLMTTYWVEPKNYAS
SQASTASTALSHEVQFSSTRVSSQTQRLISWNIDVLERLLKKIIARRIALKRKATPVNWS
KPHKNMIVLNEVKEVIKLPEYDSKYYHAENPDSIILEPRVKFQLSQFVTEIALTYHDNPF
HNFPHASHVGMSVAKLLSRIVAPDQDFEEHEQLHDHTYGITSDPLTQFACVFSALIHDAD
HPGIPNSQLVKEGTEMAKLYEGKSIAEQNSINLAWNILIEEKKYPELRKTICETQQEEAR
FRQLVVNAVCATDIIDKDLKSARNKRWEKAFSTTNPNLKLDQDLINRRATIVIEHIIQAS
DVAHTMQHWHIYIKWNERLYAEMYKAYVEGRADKDPTDTWYTGEIGFFDFYIIPLAQKLS
ECGVFGVSSDEYLNYAIMNRNEWEKKGKDVVASYEKKYKKDDKALA
>comp5913_c0_seq3; 575 228 2299 LEN=2299; translated
MTTDRKKEENTTAKVMEMGERENGDHQSNQADGTQAEDGSEGSRSSRSGIWSNLEEDVET
VSSGVSPTTNQGSQLSHQTGMSKPESEESLNIARKENMAVMTWRLVMFGVLIVTTVAVAV
LVFLFVSNTENQEFVKSFDVDSNKIEESLLSTMDTKLEAVDSLAMFMVTNAREKNQTWPY
TTLNDFGSKAAKMRILSHAIALQQYVYVTEEQRPEWEAYAKANEGWVQETIEIQREDTTF
SSPTYIPDYDTNHSTSIRYAGPVPNGTGPYTPTWQTYPMLPTGTMSAYNWNAIQHRLLGP
GIEKVVADHKVVIGPVLNFDDSNEGSGTNRMVVWAGRHSSSPDVNPKEPIIRVLYPILDT
ATGAITVDEEDSKVVGIIASSFYWKSYLENILPNDENGLVVVFENACNQTFTYQINGHEA
EWMGTGDLHEPEFDGMGKTLTFEEIGMNSANSGGRYGGLPIDMDYCAYTVKTYPSTVMKD
SYQTNEPVLYTIIAVGIFVFTALVFIGYDKLVSMRQKKVMSTAVQSTTIVSSLFPSNVRD
RLLEGNGDTKAANNVTGSIFQPTKSRLRTFLNDGDSSTDSTKPIADLFTDTTVLFADIAG
FTAWSSVREPTQVFTLLETIYGAFDNIASRRGVFKVEVSESNFVIIPDYFAVRRVLGSYI
VVSASASFCQTIGDSYVAVAGLPDPRKDHAX
>comp5916_c0_seq1; 303 1 1601 LEN=1701; translated
XSSSSSSSPGHATAIRRWWEDPKGNIRRLYQWHETNLSTISSSTENQLAANKRRLALVGR
ILIAPEGINGTVSGNRAFVEAYVERLREYDSVFGATETGGSNTDGADLRIFRDVDYKMSY
DSSPEPLFPDLKISIVKEIVSTSGLVDVHDVPLETGKHLTPEEFHQVLEDSKNQEQSKEI
ALIDVRNTFEHDIGYFVDPRTKSKAIDPETTTFSSFDKFCERNAEELRNKKVMMYCTGGI
RCEKASVLLKRRGVEDVNQLSGGIHRYLEKYGNTGHFKGLNFTFDKRVAMKPNFATEFCN
DTGKDLESDDHKAPSCDDTKDDSYDIVGRCVECDDPFDELCGSRVCTVCRDLVLVCEKCR
SRSSLREYHCKRHSEWKNCYFSFLEIFDLNELEAQLNALRALRDEDSMAKSVRRTLTRQI
NKVESRIRAVNDGSQSVDKNAPRRCRSCQEVLGQACDGKCWGFWRKVEVLPTLTTNENKT
QGVGICDDSKNTSNDSTKRAKIAAKRGRRAKQKLEAEMEFLPRPPAGSTLASE
>comp5918_c0_seq1; 275 1 1583 LEN=1752; translated
XVGAPVAPPTEKPVAPPSEKPEPADPTPRPTPAPTLSNFRIMFRQDLFEDVVKDDNSITF
SLRFEHFSDPCTGCSLVARFYSYTDENRCVTSNDSETPIPQIAYDTEMPKNAYHEISLFP
SARVDIELPLQEWEDNNDDDIKLCIRFGIEYPVTNEVVSHIDLPMTINAHNGEYVGSFSA
SGQLADDSDNVVTSLTDIQVSDKVDVQAFLCGVPGVQGITGLGTPSATHYKVGQKFRICV
DIMEVDHTYYVDNIKKMVCKVEGQYEKTIIEDGMATDGLTDVKWSGDVSEGWIQGIGVER
QKVSGGVTGSVVTSTVVSGYVGADAGTIQCEGTVEARLWAEANALYSLDNDVEVHYIHTL
PIGVAFKECYDLCELESYWYYGLQCLNIDEGVIDCVCSNPNYVSGGSVDVFHYPELPRRI
EQTDQFTKNRNSGECSDIEIYSGFFLGGFNYHPFVYETTYKRRLQESNAETESEEFGFSI
TIGIQNEVDDLSRLQDLAAPGADPKVWTIADLVAGLATAVALAMAAI
>comp5918_c0_seq3; 315 1 1567 LEN=1736; translated
XVGAPVAPPTEKPVAPPSEKPEPADPTPRPTPAPTLSNFRIMFRQDLFEDVVKDDNSITF
SLRFEHFSDPCTGCSLVARFYSYTDENRCVTSNDSETPIPQIAYDTEMPKNAYHEITRVD
IELPLQEWEDNNDDDIKLCIRFGIEYPVTNEVVSHIDLPMTINAHNGEYVGSFSASGQLA
DDSDNVVTSLTDIQVSDKVDVQAFLCGVPGVQGITGLGTPSATHYKVGQKFRICVDIMEV
DHTYYVDNIKKMVCKVEGQYEKTIIEDGMATDGLTDVKWSGDVSEGWIQGIGVERQKVSG
GVTGSVVTSTVVSGYVGADAGTIQCEGTVEARLWAEANALYSLDNDVEVHYIHTLPIGVA
FKECYDLCELESYWYYGLQCLNIDEGVIDCVCSNPNYVSGGSVDVFHYPELPRRIEQTDQ
FTKNRNSGECSDIEIYSGFFLGGFNYHPFVYETTYKRRLQESNAETESEEFGFSITIGIQ
NEVDDLSRLQDLAAPGADPKVWTIADLVAGLATAVALAMAAI
>comp5921_c0_seq1; 284 72 868 LEN=938; translated
MENSLFLALPLSVLVTQVSSFASPSTAVALSAGARSWQEGPLALLNDNNQDNHYAPSRAE
FLSKTSSAITAAASLSLVPIDPAFARGRATLEQAYDRYSSRIIDGGNFYKTQMKTMVAKD
DWAGIKTALQEPPKKTKADNAKIDGGIQERAAQAGQFSDARVLVALDLLAAQFSDNSISA

KTKAMKKDVEEIRSVVVEMQSICKQALGEESAKGGFFGVGKKQASKKELSNRMKELYIIG
GTAWNRYAYEANAGIPKTVQQLPFL
>comp5924_c0_seq1; 525 1 1906 LEN=1906; minus strand; translated
XVYTRKFMVIESINLLREPSSVGSLRINNQTKFFHSSTGFEESTLLEKYGDKMPGSNLNS
SRSTGGAAAILSRKRLPASGAGEESNGVRTTNNESRTTTIGRGDGNSIRDANTGIENNNS
SSARSSTSASTNSNNGSTRKMRRGSFRRTLMLQRNKRSNNGSSSSHSKREGNAVDGGATP
ATAVTNQLPVSKRNSTYHDQSSAPVPLKGSVGASVAFALEERETGACSPTLTSSNVTTPV
RGSQQRHERLPRGHIETIRKHYGSNREGVLDIYEDVLKISPDASDREIRIAYFRRGREIL
GDNASSIKINKKNQKAIKQLDPAIKSRFQAVSMAYEILSTPAWKEAYRKQGGILNTPGAR
ASPLVPKVVVEQDGVGLSHPRITVGKKKQQEQEPFDPFVSYPLASRASFWENTNANTGNP
SAMFPTTFSSDPVLPAPSTASRSPKRLVGALRKSKFLGNSGDVDNSNSSRSRRQRPSSAS
VRWKDHVEELIFVNHPDEHGSSDEESSEEADYYSSDDENLRGDDSTGNDAGNSRASSRPA
LPVAVDLYSEPADPGYNAQENNSNNSLSYSSRGSGSTRRRKKNKARIVIDSEELESHLKL
MDSEAEKHFVQDFWDNFEESMDGILTLVDSIGGGGX
>comp5924_c0_seq2; 582 601 2362 LEN=2362; minus strand; translated
MVNKMPGSNLNSSRSTGGAAAILSRKRLPASGAGEESNGVRTTNNESRTTTIGRGDGNSI
RDANTGIENNNSSSARSSTSASTNSNNGSTRKMRRGSFRRTLMLQRNKRSNNGSSSSHSK
REGNAVDGGATPATAVTNQLPVSKRNSTYHDQSSAPVPLKGSVGASVAFALEERETGACS
PTLTSSNVTTPVRGSQQRHERLPRGHIETIRKHYGSNREGVLDIYEDVLKISPDASDREI
RIAYFRRGREILGDNASSIKINKKNQKAIKQLDPAIKSRFQAVSMAYEILSTPAWKEAYR
KQGGILNTPGARASPLVPKVVVEQDGVGLSHPRITVGKKKQQEQEPFDPFVSYPLASRAS
FWENTNANTGNPSAMFPTTFSSDPVLPAPSTASRSPKRLVGALRKSKFLGNSGDVDNSNS
SRSRRQRPSSASVRWKDHVEELIFVNHPDEHGSSDEESSEEADYYSSDDENLRGDDSTGN
DAGNSRASSRPALPVAVDLYSEPADPGYNAQENNSNNSLSYSSRGSGSTRRRKKNKARIV
IDSEELESHLKLMDSEAEKHFVQDFWDNFEESMDGILTLVDSIGGGGX
>comp5925_c0_seq1; 346 134 1123 LEN=1446; translated
MQHQELIHDTCTIDATPPLRRIEIYPPHLLAQKLNNRAGYLITTGDYDGSIALLTKALKL
TEENVAADKSMQACCCKYCGLESILGMSTDSRVSILESEKEDCTGVTADDFQQSACHACE
RDEDMNYSLGEDFNSFGLHGHPQKSYPASPSAFEERVGFVYDRPFLVNKNSIEEGHFMGI
TLSLIILVNLALAHHLKAVSSSYANSRSILLSSSNMQVLRQALQLYEIAYQLHNDYIQKP
TKTLSTAQPEDCNRNIGSLRFTMILSNNLGEIHRIAGNAIKHKMCLEHLLSAIMYMVDCN
IIIMNSAEMDGFYHNVIPIMLTDNCAKAA
>comp5926_c0_seq2; 153 486 848 LEN=1135; minus strand; translated
MKRHNYATGSNKGRQPFPDAAHLPTKTPKTGSIAVDWFGWITWLSYHVVLSYHMWERAVQ
QPIDFDKIGHEYDKNALPGHIVIRQGRYEYHGIIAAFVLDKNDHIRFTIVCTTGSHLELN
W
>comp5927_c0_seq1; 129 296 1066 LEN=1066; translated
MKSSSFFFTAFFSSFLSGVVHAEYDQGSLVSDGSFLRRGLQAPLPTCTPMGVSPYEYTVY
TKISYEGVDFDFTNLTVLTQLEDSFKTSYNSFIGCQTVSANAFKILGLTLVLGSNTTEKS
FLIGSSIRSNAGEFECTPAAPELFSNATHPQVLSSPNDCSCKGPTSTIMKNGYNIAIHTG
DCPQNITGVKQLCMGACTENTTDVTYTNELVSIPCNLGTDVQSTPSKSPTKRPTKRPTKR
PTKRPTKRPTKRPTKRP
>comp5927_c0_seq2; 93 1 387 LEN=749; minus strand; translated
GSLGGSLGRTFRWPLRGTLGGSLRGTLRWTFGWSALDIGSQIARDANQLIGIGNVGGIFG
TRAHAELFHTGDILRTIASVNRNVVSVLHDGGSGALARTIVGTRKYLRMRRIREQFRGGG
RTLKLSSV
>comp5929_c0_seq1; 802 107 2045 LEN=2063; translated
MTAARLLLVVFSLAATTPTPVASAGSGSHDEDSCPLWIAPSYITEQAATIPKFGLFAGRN
YEQNSTLPLSELAIPLVDFFEDFTRQRAYGAQVLALMENFMWTQEKIGSMWEGYFSSPAV
VPGVGILANYHSTYSNADFLHASMLLRETGDEFPKAGEASPSRGAVTPYFNATLKATQNI
PAGMEIFADYGEVWDGNFTENVFQDKIHRYDYHLADEMVKKLVDLYEKHPDMSLDMQEDV
MDFLIAKVLTTVAGPNAKTIESLIPANPRKLKQIQDMGGTFMYRYRDMVRSQEWLETNGF
CLDTIRQEVSKIPDAGRGAIANRDIKKGETITITPLLHIADKTLLTMYPVDEEEEAFDEE
NGPIGKQLLLNYAFGHPESNMVLVPTGPLVTLINHGGSKASNAKLEWAGEGDVLSNPGEF
FSYDVDQMAEVEDTVLVMKVVAKADIKEGEEITINYGTSWQTEWDAYEERWKQNRRGKPH
PLKALDLRNSYKNKPLETGESLAANPYPENVVPACFLYVRQFPDGRQMVHQEFGTEMNQF
ESPTRYEDYDTSVLYIVEVLDRREAPGFFYNYTVRASLGPETNDFADIHDVPHAACTFFD
REYSSDIHLEGAFRHPIGLPDNMVPLAWRNIVGSHTNQCGAMQSN
>comp5930_c0_seq1; 1076 119 3104 LEN=3104; translated
MGAPVEKAAAGNGKTSNKVRVVCRIRPLAKYEINNGSKSIVTRVPGLTVDGPETLQIEES

HDGQDSTRFYELDAVLDGESTQKDVYEKSGARKAISEDLFSGYNATILAYGQTGSGKTYT
MGTAQNTVDSTRDEENDGVIPRACHDLFNTIRERCDGQAKVDCSYMEVYNEEIRDLLIDS
EKNGGPKQDLRIRETLDGQIYVRGLTERTVTSPLDVYKIMEEANKRRVVAFTKMNASSSR
SHAICVLRIKGVIVVADENGNPNAGDDGVSRTFEAKLTLVDLAGSERLKKTQASGGRAKE
GISINKGLFVLGQVVSALAERGRILSEIEEQKKLNKPRKRKVLPPRKPPYRDSKLTRLLQ
DSLGGNSRTIMVACCSPADFNVEETVNTLRYATQARNISNTATANLVETISQEEARKLKR
DNTLLKQQIADLESTISKLTQQVTPDDLERSMSILQQEQNFHQQHQQAAAERAKRRMTVA
IVEEEEEEESSSDESDANSTDAAGLDRDTSHRSNTSSKDGKKKLKNKINLEEHFERSARL
NDERSDDEGSLLADDIFDNRDKPLYERPKRSPKRSGGGAESVHSVKTIDEVEEDNVELEA
RLLLAERDVRATAFDSAVVLPALKKRVKQLEDSLDESRWIQSEAEKLKEELVQTKSEKES
AQKAAKQLSEFMKKQKQDLGFQGNEVMMNQLYFCRHKLNEDWVYLVVIIVKYFKEEMRLL
GDYFQLVSKIVDSPDVLGLLGTLNGKMRNDKLQNAGKGWWKSKEQKAKELETVEAEKRLR
NDMLRTHLQFFNERLEEVEDHVDYRSQSLEDILEVLTLERAVLQNEFESDEEVHDIQDLF
LRKGRKLLAELRVLMTEKPFIKDNGNAEEKEEEEEEASGIDEISRSAIGSTKKKKKVLNQ
EGKEEKVQPREDDEKETEPAVAEVVEKAKEVAEEVIDRSAPLTESLTEPSLSGSASDSDS
VSDTDSEESQLPAIAEEKESESVEEEEEEEPEPVVX
>comp5931_c0_seq1; 692 238 1939 LEN=2613; translated
MVGPRCSAKPFSSVLERGQFGFPWNSSVREREKNQQQDKKFLVDESGTVGETPPPPPETV
DALSSLAQTILALLSGDEDSAKALTGTAAEKRELIEQIFESYDACGSGTLSVEEAHSLFI
DLSRSIVEEIADSPSNDTAAAHSTGNGSDDSNEGSDDSNEDQHRGRIQQGEVARSHARRV
LDADENGTHTIERVATKLLLLADEDHDGKISLTELASIFDTVQKAQSGDIDTFPQPLRAL
AGSLQLLPPTEGTEVAEAAGKALSWHIGVPGDDHTLRRVELERGLSIVGLGRSADASTYF
VPELGIVFDAGFHVKSLRPKTVLLSHGHRDHIAALPVHAADKAKIFAPGKLCSLIERFLL
AEAQLNYGDETQTDEETIAALGGFDVNPVSDGDEVLLPRGSYTGSPKPLGVQVFSAPHKN
GVPAVAYGLYKAKARLKEEYASLPKSELGELIRRDVQITESYNQGLLFYTGDTTIDLLRE
RYQEILPKYKHIIHEVTFLGQPSSELDESTRRKGHTHYSQLHPWICAFPETTFVCVHWSL
RYSREDVLDFFDHQYGGVPKNVVLWV
>comp5932_c0_seq1; 287 114 782 LEN=939; translated
MKLLSCLLVGFKWQQRSQLTPNTFYVGGQKGKTSTGFQSQLASSSVSVEDRNEKLSKVLS
TAENIGQVGYLASEEDQLLMEEVAKEAIPFSLDKPGRFQLKGEHQLVYSAAEGASSGRVF
GNVVGKVSQLFEDDETFYNRVRFGPLEISLRAKREIKNDSTIKVTFLETSFNLFGKTLKK
GEVSGGGVWKVKFVGEVEDEKGGKKLVRIMETPSLFILEQDL
>comp5934_c0_seq1; 242 200 793 LEN=1180; translated
MIVMVGAFTTSLQQICLRNVVSQVNGFNPMYMTEESRCNVVIRQGCSEVAVEISSQTTRE
ANAASNQFLLDRRSYLTFGLAASAAAATWSAFAQPSSAKSYSENAKNLERINNGDYSGGA
VFDNNPSTERGKKRRAMTGCKTTLSREEASNTILKRSKMLSEKECNQMVMAGESEFMLQA
LRNLDCPTCANGIGRTN
>comp5935_c0_seq1; 378 158 1168 LEN=1566; translated
MRLQFSLLAVGALSAILDHSVVSALSVDSKSLRTSQNRLFQPHAGIAPSTLSQKAVTKAS
GSSSQFMNYATVREPIRMPSTTPMVPWKPPNSDRAQMVDINSAMYRDGTLMISRFIDEEA
ANEIIAILLWMRSESSIPSISLYFNVPGGLLRPALAIYDLIQQTKEQGVEIETCNLGLCT
GMGSFLCAAGTKGMRRAMPNSRFLLQRMGLDKPVRGQATDIVLEVKNIKMWNDRCEKELA
KLTGQPVERIQEDLKRDFYLSSDEAVQYGLIDEVLMPAPARRAASKEFAALGAFEGEEEQ
KYQGREAAEGGGWGSQQQASPNKKDDDDDGPKIAKG
>comp5935_c0_seq2; 286 1 782 LEN=1180; translated
XNANGVANLPLQPPNSDRAQMVDINSAMYRDGTLMISRFIDEEAANEIIAILLWMRSESS
IPSISLYFNVPGGLLRPALAIYDLIQQTKEQGVEIETCNLGLCTGMGSFLCAAGTKGMRR
AMPNSRFLLQRMGLDKPVRGQATDIVLEVKNIKMWNDRCEKELAKLTGQPVERIQEDLKR
DFYLSSDEAVQYGLIDEVLMPAPARRAASKEFAALGAFEGEEEQKYQGREAAEGGGWGSQ
QQASPNKKDDDDDGPKIAKG
>comp5936_c0_seq1; 298 1 656 LEN=656; translated
XQEQEPEQEPTQSGSNAKANAKANAKTKTKDGAEDHQVSVPTQTFSKWSFAERLSEAFCE
GIDHRFSCGVGRGMEVYHDYDDKGHAQDDSILGNDGDAKGGSKEQEGDHGISGFRLAKQL
SSDSSAAHGDDRPSPAEPYSTNQIHRHKAFVSEYHKTRRATIHIRERERKETDAACASDS
KESHLIRTQNDTTQGNHRGWGKASTTIASNSTSESASNT
>comp5937_c0_seq1; 361 61 642 LEN=1975; translated
MSEANAAETKKAPEPVAPAPAPAPVVNTPAPAPAVNTPAPAPAVNTPTPAPAVNAPAPEK
PADDAPAPDGKPAEDAPAADKPVEDAAPVSDIKVGSRVAKKFKKRNYYGEITEKWTDDAA
KAPRWHVKYDDGDEEDFNEKELARAVRLYEKVKKNDQKINPRKPRSTPTKRKSFDGKPPP
RSNKYPKRKSTGK

>comp5938_c0_seq1; 380 429 1793 LEN=2351; translated
MLRYPFQNQNSTYSDRIPSLPHSAIKTKNFNHNLQAKTNMPGLHFYFHDNHDYHFSNNGS
KMSLGMKRFCKTCDDDAGRHITKSHSSVFLRNRIGTRLSDPVQEFALPNNSHRRGLFDSI
SCDRKLNDILATVSNDDNDPMFFPLTDLDKDSRLNVESSQTWIAGGRSPEFVDLDSRSFP
PASSHLRLFSKDSTRIDSTTSSSTQLKPKPSTYTSARINTDETTNPSLRKGNNIVVLFAS
DTKKNHPNVKNKATRARFSHPSSNVSRKPATSSTRHKHWTREEDEILRHAKAKESRGKIF
WRKISEQYFHGTRSAAQCRVRWNNHVQPGIVRGKWDSHEDQIILQMVSKGCSWKRIADKL
PGRTNENIRYRFINKLDPKLKKTAWTKNEDDILFTMQRRIGNKWAGIAKFLPGRSDNGVK
NRYYNKRNAFLRKTRLPEKERNGSDETYSSWEPV
>comp5938_c0_seq2; 176 1783 1962 LEN=2520; translated
MQRRIGNKWAGIAKFLPGRSDNGVKNRYYNKRNAFLRKTRLPEKERNGSDETYSSWEPV
>comp5938_c0_seq3; 331 429 1877 LEN=2435; translated
MLRYPFQNQNSTYSDRIPSLPHSAIKTKNFNHNLQAKTNMPGLHFYFHDNHDYHFSNNGS
KMSLGMKRFCKTCDDDAGRHITKSHSSVFLRNRIGTRLSDPVQEFALPNNSHRRGLFDSI
SCDRKLNDILATVSNDDNDPMFFPLTDLDKDSRLNVESSQTWIAGGRSPEFVDLDSRSFP
PASSHLRLFSKDSTRIDSTTSSSTQLKPKPSTYTSARINTDETTNPSLRKGNNIVVLFAS
DTKKNHPNVKNKATRARFSHPSSNVSRKPATSSTRHKHWTREEDEILRHAKAKESRGKIF
WRKISEQYFHGTRSAAQCRVRWNNVRSTFDXLSSSGEEGSKLTIFLLFFLLKALQPGIVR
GKWDSHEDQIILQMVSKGCSWKRIADKLPGRTNENIRYRFINKLDPKLKKTAWTKNEDDI
LFTMQRRIGNKWAGIAKFLPGRSDNGVKNRYYNKRNAFLRKTRLPEKERNGSDETYSSWE
PV
>comp5938_c0_seq4; 303 737 1878 LEN=2436; translated
MTLEDTSLKSHSSVFLRNRIGTRLSDPVQEFALPNNSHRRGLFDSISCDRKLNDILATVS
NDDNDPMFFPLTDLDKDSRLNVESSQTWIAGGRSPEFVDLDSRSFPPASSHLRLFSKDST
RIDSTTSSSTQLKPKPSTYTSARINTDETTNPSLRKGNNIVVLFASDTKKNHPNVKNKAT
RARFSHPSSNVSRKPATSSTRHKHWTREEDEILRHAKAKESRGKIFWRKISEQYFHGTRS
AAQCRVRWNNHVQPGIVRGKWDSHEDQIILQMVSKGCSWKRIADKLPGRTNENIRYRFIN
KLDPKLKKTAWTKNEDDILFTMQRRIGNKWAGIAKFLPGRSDNGVKNRYYNKRNAFLRKT
RLPEKERNGSDETYSSWEPV
>comp5940_c0_seq1; 229 141 752 LEN=752; translated
MPRSPLPGTQRGKDLVCSLVGSIEKANTTVIHGRRGRSPYGRVAMEDHDNDEDDKNTNGT
VEADEQGQHGIARTPRHDPEDYRNFCIDECDDIDYGIDASDDNDEMLRPALQAPFPSDDG
CDCFARHCIHYHPQSPTPPQNYICPLTLQIMEDPVRDRCGHVFERRAILEWLETTSASND
GQPAICPISRKPMIQAHAYSSSSS
>comp5941_c0_seq1; 212 106 315 LEN=665; translated
MDEQAVINAFKVFDADETGKVISEEIVEALTTYGEMSTDEAEKMIKDAGGKSSFDYTKFV
KEMNAKAKG
>comp5942_c0_seq1; 291 271 1368 LEN=1809; translated
MSTVITLCATGVAMGVVHVLTGPDHLSAIATLSVNAESRWRAAGYGIRWGIGHSIGLVLV
ASIFIGLENSHPGRPDSSGSENDDVNDDDGTRSIEVPERIESMAECFVGFFMLGLGCYSL
YQAHRKRRDSIFGHHHHHYHAHFLPRENESTSQERSDHPFGLGEGVDEASHNVLPPNAAG
EEVDDEERSFNSRARDQPHRPMEAMGIDDSHASFYDQHDHESHSPSLSKQFLALCVGIVH
GVAGPGGVLGVVPAVKLHNLWHSIAYLGSFCASSIATMGGFAACYGAVSTRLSNAGSSPG
LYDSVEDSDDGDASTDNNSNDDSGNREKFFTTAYRIEVFSASLSVLVGCTWLVLLYFGIL
HDVFP
>comp5943_c0_seq1; 219 411 1661 LEN=1792; translated
MASSDDSVGNIGPRRRRISRNPSYEVHRSSCDRDTNVTIDTLGSVSSNSKSETDFALAPS
LAQRLVKRTEARVSQVIASSEYVRKNCTLNHIPRFERDDFVLGNLIAFGGFSNVYAITHF
NVESELTEKPEEGIKKFVIKHLNPKLALNPKKLVVGAKDLVMEAHFLSAFDHEHIIKLKG
WSAAGIPGFSAAGRADGFFLILERLSITLSKRISGWREEEKVRKTLTKGRQAAKNKLLNV
RLKAAIDIASAVAYLHERRILFRDLKPANVGFDKQGTLKIFDFGLAVEIPYHEDPEATFK
LAGNTGTSRYMAVEVIRKETYNLKADVFSYSILLWEIMALTKPYDGLLGPQVKETVSLFG
ERPSVPKSWPTAIRRLMRRGWSETISNRPTMDVVLETLTKVHDASSKTSGKILLRT
>comp5944_c0_seq2; 140 1 216 LEN=398; translated
AVNNAMGTANDAVSNAMGGVGDILSNLKDTNETDTEADAIMDEAEADESSSAAYGMLAGA
VFSVLATVVSL
>comp5944_c0_seq3; 116 396 536 LEN=536; translated
MFRLTISKVFIAILAAIASYSGANAAVEEALGTASEAVNNAMGTAND
>comp5945_c0_seq1; 380 103 1484 LEN=1721; translated
MNFSKLSLVCLALDSGSRFVQVQGVANKNPVGKWELHSLSDAEIEVSDSDTPNTAAVETE

IFYEGLFADGQDPIVEIYAGVETGAPCVTGTPATPLGETTPSLSVSFSTADTNDHPDLVE
NMVTSNGDIGSVHIHQTLTGEDNPFWTPYKDDRPDVGTFRMCECXDIKHSTTVPYPCRLL
ILYCSFCLSFTFATSYRFVRGPNTHLVLFLLLGVRVCKKLETRYISYVDSLITVRYDHEG
VFQSFAASIDVEEISSTNEYVRDEFNSVPVEAFPCFTTGSPIQETYKPGQRFSICVRPGE
DDLQNGFGVSGFQEVTCAKESQIVKEGSYVAAITSVSSDTDAVDFLDKEEVAVGPGTAVL
TTTVLPTMVSGSGSMTCTGVVELTFTAPGSRDLSPVGRRLQATDAGVGESTPFSVSIKVS
EEKDWSDAFASPAAGNFEGSDGLVVAGAATTAAVVMAAAL
>comp5945_c0_seq2; 433 103 1332 LEN=1569; translated
MNFSKLSLVCLALDSGSRFVQVQGVANKNPVGKWELHSLSDAEIEVSDSDTPNTAAVETE
IFYEGLFADGQDPIVEIYAGVETGAPCVTGTPATPLGETTPSLSVSFSTADTNDHPDLVE
NMVTSNGDIGSVHIHQTLTGEDNPFWTPYKDDRPDVGTFRMCVRVCKKLETRYISYVDSL
ITVRYDHEGVFQSFAASIDVEEISSTNEYVRDEFNSVPVEAFPCFTTGSPIQETYKPGQR
FSICVRPGEDDLQNGFGVSGFQEVTCAKESQIVKEGSYVAAITSVSSDTDAVDFLDKEEV
AVGPGTAVLTTTVLPTMVSGSGSMTCTGVVELTFTAPGSRDLSPVGRRLQATDAGVGEST
PFSVSIKVSEEKDWSDAFASPAAGNFEGSDGLVVAGAATTAAVVMAAAL
>comp5946_c0_seq1; 266 1 1368 LEN=1474; translated
HLLGALVLLSLKASVASRQSPAASNKGFTFYRHISIQTMKTLITAVLIFLACHHLTGAFL
PSQSAIQAPRKVTRQSNYGPPTTAAFNFRWDPSVQLGSQSTFVSTQLLSSSASDNTGDGA
KQENEAENNRLDTILATLTSAFPFFVLGGALLAFFRPSLFLWTNKGDLITLMLALVMWGT
GLTLTKDDFTVVLKNDLVSVPVGVLCQFVIMPMTAFTIGRTLLLNASASHGSALFLGLCL
VGCSPGGTASNLVSLIAKADVALSVMLTSCSTILASIATPLLVKLLVGSTIAISGWTLCA
ATARVVLLPVLLGMLVNAQAPKLASRISRFTPFASVVLVALICGGVVAENASLLASTSGR
LIPLIVSSVLGLHSIGFLAGYLVPRRGAGFGERTSRTISIETGMQNSALAVVLARSIGAP
SIASLPGALSATAHSCMGSILAAYWRGVDKRKKGN
>comp5947_c0_seq1; 389 91 1728 LEN=2024; translated
MHQRKPLEAEAGPRTAGLSLSGSSSGSFASETNGSSSARQRHAHSSSGSLSRSPGERARK
QRILALHSGRTQWSRLFFLGLWISIGAWGLVGTWMVLEVRRELLDGGADSNLRSRPIQGT
KTALRWPGRKPAEAFQRISEAAPECYDLEDPSEITLTLVTQVSHDRLWMMEHHCDRYRVT
GPEDQRKQQHRMSIAVYSNATKGEIVDELEAMGCGVITNPESDDATYNEVGVEVSVLSAR
THGAWNNYPVNELRNLALSRVRTTHILYLDVDFWPSDSLYEMLLGDDDEGNGPPSPVQQA
LLADPRQALVIPAFQLWRQCAEWADCRGENLPHMEAARTLHGLAHEVKTYKSVSIFDPTN
VGGHGSTSYPSWFRQAPGSVLPIDCLQSNRYEPFVVVRYCRDLPPFQKAFAGYGKNKVTW
VMHLVAAGYVFRQVGGTYLIHYPHLDSASRQHWNNSGKKKEPGEEEKPPGESSGSNSERN
SRSGGNSKRSRVDHLFVEFKDWLADTIPSADRRLPLCDDAQDDDGKLWVDHHSPKRRQHR
SGDDG
>comp5949_c0_seq1; 683 128 1426 LEN=1451; translated
MTKRSSIVACLLFLLGTSTAAAWTPPVVVPSVTHHTGKAAAQHQSQFQHPHDALSEHVQE
WVSSRGQTVNEWFGVPPANAAEASGPPTKNEILTLQKAFAAFYGTQRDPEAALPLLDESV
KAFERQPADERAGLYRVRGDCKMALKDPVAAVEDYSMAIDLLKLPEAKEKADPAELPAST
LGRARAIRSLGARATPEQAKSAVGDYKEALILNSRDDWDTEAEQIEDGAKTNPYAAWEYG
MALRRNGQYEEAKAMHTFTSDLFDSIGDRPRSVISLLDAGVDAASAEAAGSASKGGEDAK
SLLKRAISYTTTTESRDVSLLQRVVAKEGEGRIVLAAIEWADKDKSLRADAEKELSTACE
RLDQLEQDALSRGRKAPSSTSTALAVRFNIDDYPGALETSCSRITKNRDFQRDRLELPDL
LQEKIQKLNQLK
>comp5949_c0_seq2; 342 1 909 LEN=934; translated
XFLLMRLDLVPRKKTLMREKIIALQCPEFTTRADTGSFFFMYKSMNGIRKTGRARAIRSL
GARATPEQAKSAVGDYKEALILNSRDDWDTEAEQIEDGAKTNPYAAWEYGMALRRNGQYE
EAKAMHTFTSDLFDSIGDRPRSVISLLDAGVDAASAEAAGSASKGGEDAKSLLKRAISYT
TTTESRDVSLLQRVVAKEGEGRIVLAAIEWADKDKSLRADAEKELSTACERLDQLEQDAL
SRGRKAPSSTSTALAVRFNIDDYPGALETSCSRITKNRDFQRDRLELPDLLQEKIQKLNQ
LK
>comp5950_c0_seq1; 189 1 1223 LEN=2016; translated
XMTDPRKGNDIVLRNQKILLVRRPNGLPQPEDFRLEQENIVVSDAKLNDSDDRNSLIVLE
TLYVSIDPAMRGWMSTQRSYLPPVPLGSVMRASTVSRVIAAAPRALERAFPVGSLVRSEI
VGVQSYFVMEGYSEARARKRLTRLEMASIPRSTGLSPASHLGVLGTTGMTAYFGLLRVGE
PKPSDVVLVSGAAGATGSIVAQIAKHVVGCKTVIGTAGTDEKCAWLEANGVVDVAINYKT
AGSSLSRAIREASSPGKHNAGRAKRMQGGVDVVFDNVGSEFLEAALGNLAEKGGARVVLC
GAISRYNQTGASAGASNGPRNYMNLLVRRASMKGFVVFDYKKEYPTAIRDLVKWISDGKI
RCYGEDMRHVGIGQFYPALLSLFRGTNTGKVVLKVNEENAVASRSRL
>comp5951_c0_seq1; 224 389 760 LEN=1239; translated

MGGFVEVNETAEDAVKRELAEEMSITLTRSPTLVGIYSDPRRDNRRRTASVAYAVHVAAD
IRPHAGDDAKEVVKIPINEIVDYEYFTDHRTILLDYRSSLRGEVVEDHSTEGDFAADIQR
STC
>comp5951_c0_seq2; 224 389 760 LEN=1102; translated
MGGFVEVNETAEDAVKRELAEEMSITLTRSPTLVGIYSDPRRDNRRRTASVAYAVHVAAD
IRPHAGDDAKEVVKIPINEIVDYEYFTDHRTILLDYRSSLRGEVVEDHSTEGDFAADIQR
STC
>comp5952_c0_seq1; 450 178 1617 LEN=1662; translated
MKFSGSFVLAACIFATVADTSFVDGFVPLSGVKTPSLSTRLHYDIQRDPQPNDNVWAILS
NTEKWIANTLADAQTGNSNPLSRKEVSYVCETSNDPAMILANIFRKVKEARQLGESHAQD
QEALIDEKGEDKHNRITLRQTQVLVIPANEELNQSFHVFDAVINAVNAARKAARDLVTDH
SLEKLDEQMYGEAEDRDWVVSVNCAHLHPEYGKKTPQQELKEMQEEEEEGEVDLNLKEYK
RQRILARRSPYPSVVIEVRSMAPPEFAPPPPSGPVSPKSIDEIDVESEEGESSSGAADDK
IDADVVNKLEALFSKSSLDSDRTEEGDFYESIGSHIETFSSVTPLMVAQNWINKNDASFE
ITQCAFTVSDATYVDEAYEFVFTNLGMQTSQFLESTGSTGGAASKAQAKDVQKRQYMVLP
HFLSSSATSLEKFTTQATEIIRTLPSISDKVELTCFHPEAVDEGKRCPVPVIVLQWKGD
>comp5952_c0_seq2; 367 178 1782 LEN=1827; translated
MKFSGSFVLAACIFATVADTSFVDGFVPLSGVKTPSLSTRLHYDIQRDPQPNDNVWAILS
NTEKWIANTLADAQTGNSNPLSRKEVSYVCETSNDPAMILANIFRKVKEARQLGESHAQD
QEALIDEKGEDKHNRITLRQTQVLVIPANEELNQSFHVFDAVINAVNAARKAARDLVTDH
SLEKLDEQMYGEAEDRDWVKFLNFCGYELGEIDRXVMMQNSVRKGRGIYSAERSRTLTFR
SRSFPTPQHPSYDRVSVNCAHLHPEYGKKTPQQELKEMQEEEEEGEVDLNLKEYKRQRIL
ARRSPYPSVVIEVRSMAPPEFAPPPPSGPVSPKSIDEIDVESEEGESSSGAADDKIDADV
VNKLEALFSKSSLDSDRTEEGDFYESIGSHIETFSSVTPLMVAQNWINKNDASFEITQCA
FTVSDATYVDEAYEFVFTNLGMQTSQFLESTGSTGGAASKAQAKDVQKRQYMVLPHFLSS
SATSLEKFTTQATEIIRTLPSISDKVELTCFHPEAVDEGKRCPVPVIVLQWKGD
>comp5953_c0_seq1; 215 91 534 LEN=1446; translated
MAEQFFQKVADDVDAAAEQLWSIFGAGSAEQEDKADATGGPGADSGSFAHQFDFSDTDID
GLSDEEIQRLLGEEMMRNNPLQDIANDVTQNIVAGQVRNTKRKSQDGGKRSLEGEVTMRG
IILQQKHERNRSPRLKSGLHLSNDRCC
>comp5953_c0_seq2; 369 91 846 LEN=1238; translated
MAEQFFQKVADDVDAAAEQLWSIFGAGSAEQEDKADATGGPGADSGSFAHQFDFSDTDID
GLSDEEIQRLLGEEMMRNNPLQDIANDVTQNIVAGQAQPQSAREHFDAFRSAINWSEKFV
IGLVCFQIVMFLLCLYFSKKDRGLAPRISLLVFIGIVVRAAEWLNSIGADHWESFATQDY
FDKKGIFVGIMLCAPLLLDSLMMLLFFMKEASTLLVQVKRNEIKQKQKKKSKSDDGKKEK
VGGRTKTKKQD
>comp5955_c0_seq1; 771 1 1702 LEN=2171; translated
XQQQQPSQLQTNNGGTDANGVPKTIAAHTHLLELSDPDGSRLRAFYRLSVDELFGFPPTP
TDEEYCARLNIPGMTPRMIPGTHLAALSAARFAEVALGAIVHNEAPLAMELCNAVVHCLK
ESVQEPVQPPYMFEVARSYFLLAVFRAFRGDTVRYFKYRRVCLTYVSKLENATSATTLVA
AVSFLDAWAYMIYNADEKKVPRIDTSIPPVERPPQSMLARPTAVEMEYNVRCNPACIASD
PRNQNWIQGAPPVFLNNEAPLRARSLDALACAVRTCCDQANGRFAAISKEAKADNMEAIP
QESIITPTTTAVLAHENELCSRNMVLSGFSLLEQYEQVTPNSHKNQGMHLVMSAMDAFLD
NGDDGGEGGFTDSQIQSLLSVANIVIENPLLLHHAGPTYHMVSNAAVMLCHLLNSMYVMK
GGAEGIRKDQELGGGMEAAMFEEILDTFTALRKLLVIHRRKLPIKLRCHSIPRPSLVAFE
NGKPFVDLGETLLCACRGCQGFVLMACSPVVAAQKAQAAATKRSVEAAREAQVEAADELD
KTLDDLSHDFNLDDDALLGMLSQLISG
>comp5956_c0_seq1; 408 100 582 LEN=683; translated
MFSRSSAFLRSQVGKRMLRNAQQQTHQSQKRSMGGGGAYMQTNEFGERLGEAFGTIAWLW
VFWRFKQDGKVLLGMEHPWDHGHSGGDIYGSELRVQGLTHQFQVKKQTEEEMIANWDKFN
EKSIVPGEDDDDDDDEDDDDDDDDDEDDDEEDDDDDDDE
>comp5957_c0_seq2; 598 149 2005 LEN=2402; translated
MRHQHHSLNSRREYRGGWSTSIGALLDDPNRKSNCCALTCCGIFLYDRTNYLVTGNRPNW
KRRRTTHIRIPLLILITIAISGMVSTCDDFDDNCTSGGLIVFFLELFFVGYLIFLCCLGG
KNHGVLRQAVHQKMRDQQLGGSEEQQTPTDISTNRKPLGCCSCYPYDVVLSVNEGDDPDD
ADDSRGSKGMASPDLSTCLFRAWSSFCCGALCQCWCQCCGMCAIGQEDRELELILEKDGN
SAALHIDYITFQPYSEYFPAIRDLQNSREGSFAKHRLALSDLSKRILRYAAFIVLLYCAL
FIVSQEHYGIKIGVALGVWAQPCVVLYFVWWRWNRFDISLDAVIKYFASGYLIGMFQALI
VETIIFIPYAAFMAIGIAIEIRGEVGQDTVAPATYLTYFSDGKNTAQMMKDYWGLNAIFM
FVLAFVIAALVEEIVKYYCYWTLETPEQVGDRQKSKLSQANYITIGMVSAALGFACKENM

QYVFQSKNVRDEILTLVLRSILAVHPVCAAIQSLGVVKRDIQGDPSSSLGRILFPAILLH
GLFDFVELLLSYFFAIDMLNTKGTLDSNEGVVTTVGVGVIFMIVGLTYYFCRARQQRNEL
ALSDFSEGTSSLELNPIL
>comp5961_c0_seq1; 202 183 884 LEN=1616; translated
MRKVSSFPLAILGSFVFFLLWLAVVALLGNQLCGLSVHSFAWTAPLRVGAVVGIPRTTAE
AATPTTKNRTLLGVPPALSATTDSIDYQSDAPTFGRGEYHLSAVVEEGDTVVYRTGSWYV
DGVLVGDAGEGAAPSYELCRIDTIQVVWTHNCEHGVLRGLAVGADTDAGENCGSSVLFLK
EPLEDVEFGPEQLIARIRGVEWRKSVGEGEFGTCPVSLHESMWKENDANAIYD
>comp5962_c0_seq1; 264 1 1239 LEN=1770; translated
HDDTTGHFEGRDGCVVADRYRILREVGVGTFGRVVECLDTKRRRGTRRGRGDGDRGCRPR
QHCGGGYSCNSDRDRDESPGTIVAIKIVRNIQRYYDSAKIEADIVEDVNRKGKRGVTHCA
VLYDTFSFRGHYCMVFESLGPSLYDFLKGQNYHPFPIHCVFDFARQLLETLEFLHSFRLI
HTDLKPENILLVDGRTISQRGGHSIPQSTRIKVIDFGGATYDDEKKGSIVNTRQYRAPEV
ILGVGWSMPSDLWSIGCILAELYQGELLFATHDNTEHLALIEKVVGPFPRAMLKKATQRE
VVGEAFGANGAHKMERVLPADQLSYVNKTPPLESIVATHYEEDAWLPRLLRRILVIDPNE
RSTARDACGISGSRSGRPGTGEQSTSLSNAAILVGSGWDVGNEEHARKMSRH
>comp5963_c0_seq1; 197 1 460 LEN=639; translated
XVHQGVHQGVKQEHHHTRRRSKNAAEARASSLRKARLLYELLSAHTASLEEHEHERERND
PSSNYNRSPRFRMALSNNLARIYGLIGDTVLERRCLGDLLSTILVEHARTANGAGATGNT
TNNSNTNSSVGCLEGFLSSASVLWVPDQPAGAA
>comp5964_c0_seq1; 319 1 699 LEN=2428; minus strand; translated
AVTMEAFERKRAILQALERCATDEDNDTSKHATWLLANLDQINRTLKAASMHLQVLYGSL
YGSPVSGTETPEIKREKMALRTELPKTNEFREFMASLASATDKIGEFAFSFLGDGNGDSS
NKRLQSDFLDSAKLLLIANYLAETSSTLLASGIEKTIYSGMLNPALKSLLDNFVNNCLPS
TSETLLVGKRLKEESGIEFELKQLGLAVGMLRAEVAIAADESRAFELNNAMA
>comp5964_c0_seq2; 280 1 699 LEN=756; minus strand; translated
AVTMEAFERKRAILQALERCATDEDNDTSKHATWLLANLDQINRTLKAASMHLQVLYGSL
YGSPVSGTETPEIKREKMALRTELPKTNEFREFMASLASATDKIGEFAFSFLGDGNGDSS
NKRLQSDFLDSAKLLLIANYLAETSSTLLASGIEKTIYSGMLNPALKSLLDNFVNNCLPS
TSETLLVGKRLKEESGIEFELKQLGLAVGMLRAEVAIAADESRAFELNNAMA
>comp5965_c0_seq1; 555 168 1199 LEN=1286; translated
MSGKKFFRMGKQLYRPPVEDDEDEDVDEDSEDESSDDDEDTKPASASKASANANLSDSDD
DDDDDEEEDEESEDEEETKKRRRSASKGSSKRPKVSSFIDEEAEDDDDEEDDEPYGTHHD
PDDVVRKHYTEEDIRKEQMDDEALELMRQQDRRRAQAGGRYVHERSVEEMAQEIEDRHRL
SRTAADRRSLSSHQRREPSSTKMSSSRPGASSSARRHHSSVSDDRPGGPEATNAVSQQSL
VPSVNDPSLWMISCSTGKEQELVLQLMNKCVAYARQGRPLGICGVISGQSKGRIYIESFN
EPAVVEAITGVRGLLQYTMRLIPIQDMTTVMTVVPKKKPGEFM
>comp5965_c0_seq2; 1036 168 2810 LEN=2810; translated
MSGKKFFRMGKQLYRPPVEDDEDEDVDEDSEDESSDDDEDTKPASASKASANANLSDSDD
DDDDDEEEDEESEDEEETKKRRRSASKGSSKRPKVSSFIDEEAEDDDDEEDDEPYGTHHD
PDDVVRKHYTEEDIRKEQMDDEALELMRQQDRRRAQAGGRYVHERSVEEMAQEIEDRHRL
SRTAADRRSLSSHQRREPSSTKMSSSRPGASSSARRHHSSVSDDRPGGPEATNAVSQQSL
VPSVNDPSLWMISCSTGKEQELVLQLMNKCVAYARQGRPLGICGVISGQSKGRIYIESFN
EPAVVEAITGVRGLLQYTMRLIPIQDMTTVMTVVPKKKPVKKNEWVRMTRGHYKGDLALV
VAVRESGLKCIVQCVPRIDLAALGLPPEEARIRRKTVRPPQKFYNEQEVAALGYADRRHR
QRFPNMDYMCDYFENNYYYDGYMLKEVTVGSMVKPCAENKPTLEELQQFRKRNKSANDDG
NDENEGSKMAASLLDELSELQGKTGFVSSSSNGGIMTGDKIEVVEGDLIGMKGKVMSMDG
TTIKIKPLDTSLDIGGTGEIEFLASQVRKHIAVGAHVKVVDGRYTNETGTVVAVEEVEGE
KDFTAVVLTDVTNKEISLRVSQLRESSEQYSGQDKLAGYELYDLVMLSGGGSANEVGIIV
RVGREDFTVVNNHGISRDVRPEELRGKRNSTSNRAVALDVQGNQMRCGDQVNIAEGPHKG
KTATVKRMSRSQLFLYSQTRSEHAGIFVVRSRKCLLAGSRSQQRGAQVGDASPFATPQSQ
SSRGGPGGGRGKGQDSLIGKTVRIQAGQWKGYLGAVCDATATHVQVELHSRLKKVMVVRE
RVAVVGDKYGAVEDVNQNDMQNGIPTSFTTATPMHGGATPM
>comp5966_c0_seq1; 920 118 3168 LEN=3300; translated
MKVSSVALAALCLLANHNGCVESSSSPSAGIPAATARRSKFLPWTKARVYNPTGGQTVLG
QDQEASESLGVSIPLSQSSTLSANDAPLMRDIEMLTDILSDLVLSENPKVHELCREFINY
GQQRSENPNDGAILETMVERAKSLSASECLGMNRYISTALSLVNSAEVQHRLRAIRQHEL
ENKQQLPGPLYHMEDSIKGTIHHLLDTDGVSKEEIFQKLCNQKVEFVLTAHPTEVNRKSV
LRKYRKCSELLAHLERPDLLPHEQLTGTIDLQRIISSVWGMDEIRRTKPTVQREAAGGLA
IVDTILWKAVPNYLRKLNAQCQASLGKKLPLEVCPIKFASWIGGDRDGNPNVTPKVTREV

VLHQRLRAARLYLQDFAELESQLAISSKFSPKMIELAKSVPGTFHQRELYRRVISHLRKR
LVKTADDIEMKLENENLQESDESTASKTLVGDGEVDEEDIELITKSEDLLQPLRVMHESL
METGYDLVADGLLSDIFRRLKVFGITLVPLDIREESTKHTNALDAITRWLGIGSYAEWDE
DARVNWLSSELSSRRPLFPSERLNKMDFDAGVKKTLQVFQTASELEPEALGAYVISQCQT
ASDVLAVMLLQKQFGMTSENGNMMRVAPLFETLNDLNNAPERLKTLFSVPAYVGAINGKQ
EVMVGYSDSAKDAGRLAACWAQYLSQERMVEVAKNAGVELCFFHGKGGTVGRGGNPALYR
AVLSHPPNTINGRFRVTEQGEMITQNYGSIPIAEHTLDIYTAAVLRETFTKHVEPKDEWR
QQMERVSDVSCQDYRHLVREEPRFVPYFRQATPELELQSLNIGSRPSKRNPKGGIESLRA
IPWTFAWTQTRTHLSAWLGVGAGLDASEEDLACLQDMYNEWPWFRELIDLIAMIVSKTDF
SVSKNYDDQLVNSEESKKLGVEVREKMVQTRQSILDVTKSENVAGHHVALQRASNSIRAP
YLDPLNVIQAELLKRFRALDQREDDLTTEEEMEKQTLQDALIVSINGISSGLRNSG
>comp5967_c0_seq1; 327 328 1131 LEN=1187; translated
MTTLVVMLLALPSFPASVIPSVSALTTTTTGTAAHLPASTRHSPPLRPFVDSYQTRATAV
DDASTSERWTLQEMEDFADSVGVVVSLTTFGPGYRAVARAKHDESIIMGYVEGFLRPTQP
ELLHIDKMEIFQPVMDRVRRARPGSLEFGGISIGLGLLMGYRCLLHATAEEESRGRKPRT
IAEFLAIDDEEFQHKRLVRYYRYAGFKVVKYVGDEFESIPDRLVWGGCGTLMEQDISVLM
DKWSSLFDLMKTRAENGTNSTSDEKTK
>comp5968_c0_seq1; 332 159 1638 LEN=1774; translated
MPGDDNERDCIVATILALGKGEWSDGDVIMEGESETQTQRQTQTQTGAEKYQGKSHRSHR
RGSRPVLPGVVIRDGILSYRPFRSLKVVLVRGSAARSKPDQGGGCDPFRKGCGFPAGQNY
HGDASSLHSCYALTFSTNRGPLRFEVFEMPYLPPRRRNHESPVFPGERAPGSRFLRGNHP
ATKDGIGNGIGIGCDYDYDYDAVYRDADGVVVSCFIARFFEFCDRYGYDYASSSILADVV
SRAGRTPPPGGRTSGSGTRTRPHPHPNPYPNPYPRPNPPKNTTVVLCGNTTDVTLAEARL
SCVVVRDQARAAEADRANPTGSFIDGPGHPFASLARAVTGDGGLSFGVFGSGLGGEPATP
EGRSRSSFCSGENDDRIQHEHALHWNRLRARVDSYQSASRPGVAHRGSERNAIQPNATQR
YPTPSNATEPIAIQRNGTMHHATPRHIKKWFLAACCCVLPASAALDSIPHNFWVHESIDR
GGAEGIPVGVVAW
>comp5968_c0_seq2; 342 42 1518 LEN=1654; translated
MSDDNERDCIVATILALGKGEWSDGDVIMEGESETQTQRQTQTQTGAEKYQGKSHRSHRR
GSRPVLPGVVIRDGILSYRPFRSLKVVLVRGSAARSKPDQGGGCDPFRKGCGFPAGQNYH
GDASSLHSCYALTFSTNRGPLRFEVFEMPYLPPRRRNHESPVFPGERAPGSRFLRGNHPA
TKDGIGNGIGIGCDYDYDYDAVYRDADGVVVSCFIARFFEFCDRYGYDYASSSILADVVS
RAGRTPPPGGRTSGSGTRTRPHPHPNPYPNPYPRPNPPKNTTVVLCGNTTDVTLAEARLS
CVVVRDQARAAEADRANPTGSFIDGPGHPFASLARAVTGDGGLSFGVFGSGLGGEPATPE
GRSRSSFCSGENDDRIQHEHALHWNRLRARVDSYQSASRPGVAHRGSERNAIQPNATQRY
PTPSNATEPIAIQRNGTMHHATPRHIKKWFLAACCCVLPASAALDSIPHNFWVHESIDRG
GAEGIPVGVVAW
>comp5969_c0_seq1; 500 191 2801 LEN=2801; translated
MAAEIGEPRHERFLAGDDSSTSSAIDTAGAGTSVEQHPSLSQSFSFPSPVDRERKPPPTE
YDFNILQSTSCDSFVYDSYSSSTADFDNFDLDLPYRWYKRAWSFIMCITGLFDILAYMEP
NLRHWYNDGSICVPANFTNTSDFIEFGDGVSNLLVNLDTGPPSPWYCSRYMTFLNRLHDY
NVVIAFVFSLLWFKQSHTKAKEEYHYNATVKEDRIQLLSPTTIETRSSSSNDSNSNQKSR
WSQVKCMKMYNPSFVYYRRIILRMALLPVGFYIILFQLIRGLMNGKWLYRELLIDQPANE
TAYLLLQDPKEYVTIEVSRAHAKMSTIFAIFLYLKYHFLSVTAVAKAELDTHYIPQLRRK
LVIRAFRNPRNFIRQLKTVLKYVRWIKYIIPLVVKLNKLRGNTMAALRKRRQFTKAKKEA
KRQRLLQKSIVKTTSQLEDSAAILIQRVWRAYRNYMHHRVLKHFQSNKKLNAAMKIQLVF
RRASLRAQIKLARKRTELQRLIWLNQRKIKKLDDDERRRLYELQDEFMTEATNTINKRLL
MRPNTWFSVSWNSLFLFCILVEISHNALRPWLEIPKGEQINNQKYKSMRMFFAESLIPIP
IEETPACKEFLKKKSAIRRLFFRKDHCERPTRKEVMSAFIDEIIDPEYDLEQLRGDTNNT
SKPVVPWQCSDPVLTWRNNWRTIVRLVFSPNPISESPSCQQPEKTLVDIVITRFHQKIDR
PTPWYCTKPYSSIHDFYRSTWNFVIDQIQIVISVICFLDVFVKFFTGEIDPNTGELRPRP
FFRRWIFPGLLLQLLVNPAIETFSVGFFRCMGLGMEIGPVRVFRWCIAVAVPIAYASKKI
TIAALHEAEAEYDRELAQYGMMFSDYSFNG
>comp5970_c0_seq1; 1071 94 3126 LEN=4096; translated
MEDQLSFLSGCLSIVVIGASGDLAKKKTYPSLLNLFADGLLPKNVEIWGYARSDIGDNGL
RERIRPYLLQSGRHSTEVVENFLSIICYQQGSSYGDLEAWEKLKNSIEIFESSKSSLKAF
NRLFYFAIPPSVFAETALAIKTTSMQDEEKGFTRLIVEKPFGRDLDTFEELNKTLSSQFT
EDHMYRIDHYLGKEMVQNLTVIRFSNVWTERIWNADNVKMVILTFKEPFGTDGRGGYFDQ
NGIIRDILQNHLLQVLTLLCCEPPTVAHGPEAGNAIRDAKVHVLNSIPPIKLEDCILGQY
EGYEDDPTITNKDTNTPTFACIRMRINNPRWAGVPIILKAGKALNERKAEMRIQLKDAPA

AEYLFEGHDCPRNEIVLRLQPKEAIYVKTNVKRPGFTSQPLQTEMELNYDTRFFANNKMC
NPDAYTRLILDVLQGNQGAFVRDDELRRAWKIFTPILHQIEETNVRPIKYLQGSRGPIEA
DEFIATLGFTRNDAYVYNDSSGKAKAVSGAHGKSKFDFSDDEKSEIGLWGLAVMGQNFAL
NMASHGFKVCVGNRSPAKVDTTVARAKLEGDFPLVGAMSAEEFIARLSKPRKVIILVQAG
KPVDETIKKLSAFMEPGDILIDGGNEWYPNSVRRSTELLNKGIQFIGMGISGGEEGARNG
PALMPGGPKQAYELVAPIFEKCAAQVERTGACVGYLGPVGSGNYVKMVHNGIEYGDMQLI
AEVYDVMKTLLSMNNDEIADVFAEWNKGELDSYLIEISEICLRKKDDTTNGYLVDSILDK
TGMKGTGLWTAKESAERAVAAPTIVAALDSRVLSARKDERVAASKIFGSPMINAVDKDQV
IADLRSALFASKICSYAQGLSLIKAASNEFQWGVDLAECARLWMGGCIIRAKLLDNIQMA
FSTNPDLQNLMIDPGFAKEIIDAEPAWRRTVALCATSGIACPSLCGSLMYFDTYRRERLP
ASLTQAQRDFFGGHTYERTDMPGRFHTAWTDAHKDIGDVAGRTAGENLKT
>comp5971_c0_seq1; 383 328 1569 LEN=2813; translated
MVDDNDNTPLHVACQLDPPVNIVISLKEAIAQTNSWGATPLHVAASHRCNAEALKKLIDF
SPGSLSRLSRMNRTPIHYACMSYRGLDLIAFRVLLEETLIESRRRKEEQHLHYDREKENS
ECDSKVSVFIDIIKETNENVKKTEMLDDEEISLLITDNVSRRTMAASPSDSLSTSSSDMG
KMNTTDQEQDYSIDNGLYKNNSNIVTWKDTTGKTPLGLLFCRYRERVKRVIEILEQMKSS
GTRSPQSHTSLQTDLGHLWGKARLIVVRLSEECNQQQLQQRNKEFQGKRDSDSVDEAHNY
CSSLGQQWSLAASWSKERLSCYSRVKEEGSDFSTDNEFVEMKSSDNDERQFRIVHASVGL
TGYGCPPEMIRLAMSIYPYQVREMDEDGNLVRKLFPLTYCLFFSLLVNIDFIF
>comp5971_c0_seq2; 418 328 1938 LEN=2707; translated
MVDDNDNTPLHVACQLDPPVNIVISLKEAIAQTNSWGATPLHVAASHRCNAEALKKLIDF
SPGSLSRLSRMNRTPIHYACMSYRGLDLIAFRVLLEETLIESRRRKEEQHLHYDREKENS
ECDSKVSVFIDIIKETNENVKKTEMLDDEEISLLITDNVSRRTMAASPSDSLSTSSSDMG
KMNTTDQEQDYSIDNGLYKNNSNIVTWKDTTGKTPLGLLFCRYRERVKRVIEILEQMKSS
GTRSPQSHTSLQTDLGHLWGKARLIVVRLSEECNQQQLQQRNKEFQGKRDSDSVDEAHNY
CSSLGQQWSLAASWSKERLSCYSRVKEEGSDFSTDNEFVEMKSSDNDERQFRIVHASVGL
TGYGCPPEMIRLAMSIYPYQVREMDEDGNLPLHIAASASSLKSNTSSGNAVSEEDSSVFS
DSIVSLFSAMSLNNGEEDSSIASFDKVIRLLLKQYPHAAQTPHGRSGRLPLVLADHAGER
TWNDGMKTLLRAYPPALFSGSKGIIPINLYPHVLSLIGGGDPSESPQRSNLAQLIL
>comp5972_c0_seq1; 214 230 887 LEN=1214; translated
MPGFCRNGTFVYTGPSTSRASNESTGNEGILVDQPDAATSPSVDSVEEATRILSEWDKIF
NPESQGQKQDTEANEILTSSRDALKASVLLLSAAATEERNRDSVMGRCMLGICAPSAEDG
VAALKAWVTALELPRGLLHGMDKDGVPIDLSGSVYIKYNSGGVYTFADIRKSGLGFDALW
KPGDALLESYDGTYRGVYFQVELQDGEFRQYLVPLDIF
>comp5974_c0_seq1; 394 65 1593 LEN=1950; translated
MARLRRKVERKQTDDRVVGRPLQLLLVGAFLCFMAMNSLDTVMLEIKTAVLDSYQTVAKV
TYSVTNSSFLQQQQQQQRRQQQHTLPPLEELVDSITNSTHPEKSYPCPNGTIVFPDTPLP
PTENGTHHKIPKIVHITAKSRCVPPNIYKHLKQWEFKGYGLYLHDDTAVHKLLRYAILDN
DGKGLVPDLEAVLSCVTSGATLSDIWRYTFIVSYXRSAVMVGVLMKREKIIDLCVLFVSF
PSYALLCLGAQCIPLSVLIMHVDTRPSSNYKSSSGYYGGIYTDLDNSPTGFSENTISPSD
DAFFPLEGLGIVAQYFFAVSPRHPVMLQSLNSGISNLKKIKHVMVNNPAINTGPKAIKIG
FVEFQKAVGISTDGYIDAGTYMGTLDEPLRSYQTASGGNSKIDDELYQNRTILVVGKKQE
SQEYVDRGGLQHHAKLNYMEAHGMPHYHGRWKKYAGVRDQISCKQHIEMQEERIRAQNLT
LPPAYAPGMEHNEWYPRYHRANYTYGKRL
>comp5974_c0_seq2; 489 65 1405 LEN=1762; translated
MARLRRKVERKQTDDRVVGRPLQLLLVGAFLCFMAMNSLDTVMLEIKTAVLDSYQTVAKV
TYSVTNSSFLQQQQQQQRRQQQHTLPPLEELVDSITNSTHPEKSYPCPNGTIVFPDTPLP
PTENGTHHKIPKIVHITAKSRCVPPNIYKHLKQWEFKGYGLYLHDDTAVHKLLRYAILDN
DGKGLVPDLEAVLSCVTSGATLSDIWRYTFIWYYGGIYTDLDNSPTGFSENTISPSDDAF
FPLEGLGIVAQYFFAVSPRHPVMLQSLNSGISNLKKIKHVMVNNPAINTGPKAIKIGFVE
FQKAVGISTDGYIDAGTYMGTLDEPLRSYQTASGGNSKIDDELYQNRTILVVGKKQESQE
YVDRGGLQHHAKLNYMEAHGMPHYHGRWKKYAGVRDQISCKQHIEMQEERIRAQNLTLPP
AYAPGMEHNEWYPRYHRANYTYGKRL
>comp5975_c0_seq1; 82 1 834 LEN=1178; translated
XQNTTQTQPTLPHPNHTEDPRQSAWNPDEDPVGEAPDPGARDALASRSCGPLACNAYALV
CGSTGEAAVVDPSFSTPCDLGALESFLEAGGATAVTRILLTHGHPDHVAGVSELTKAWPG
ASLSLHPLEAANYEGAKRMGRDLFGMDFPNDGPLPAPTHALEDGQVLAIGSSIELSVVHT
PGHSPGHVAFADRRTSRGANQGGSESDKGRGAAGSVLLSGDLLFQGSVGRTDFHNSSLDD
LYASIRRLYELFDDDSIVLSGHTTPTYLKKNESQILS
>comp5976_c0_seq1; 426 110 1234 LEN=1896; translated

MKRTSEHLCCVLYGLLFSTFYICISYNLGFLWTPKHSIECGIKKNEDLIPPQRSNVLHNA
NKETNKKYRNPYDETDSEALSFYNDLELIKMIGEGDVSYAFEARYKNQTVVAKIAADDEL
YFSDIEIDFFRELNAPPSISNIPKLYYAVRSMPNPFFAGNQTYLKKDLKIPSNSARRLTK
KRRISVLVIDLLENRVDPKGLDDVRRLMKSMLETMQFVHSRNIMHCDLHGNNYFWDGNKV
SFFDWNAAFKYEPNRVPIHYDRAPDQLFPPEAEHNTSSVHTSVYAFDVYTIGRLLKMLLE
KNCGVTLKKLRKSLKKLDTSNKSEEARSRLLDSALAYELAEYMMIPDPYKRPDTAAALMH
PFFEKLTAEEQISR
>comp5977_c1_seq1; 511 1 1057 LEN=1408; translated
XEHDEGSSREGEPPKPPPVPSLGVLGNVAAQEKEGAELLLGDPTRFFVFSGGEGGAGAGD
ESSKAPYAGSSLGVPVAIRDPLRTGQPQGRVFPVPVGKLPKPDLRTEKEKKRQEREERLW
AAAAGKASGESDEDLMLIAEEDREIGEAIDYEEAHANWDSDDEDWAKGLDKDLDRDLLNL
PREFRYNQEDIEAVIERLESRAEIYQSHVKNTIRTMEQEARSRLDRSDTGDDEPGKQEAA
DSSSSGEDKAPFFDASTAEKLRSVGADVEKYERLMASLTDDQLLTMFALEANSSSSGTSK
GETEGSEPTATESARTQVFEGIPGLSPQQIAELTELESFIKVAETESSSATN
>comp5978_c0_seq1; 389 675 1622 LEN=1730; translated
MAQKLRSEAASLEAEQAEERANIAKMAFEKFDDNSDGKISKEELKAGLENSLKTEISEER
VQKLMDKFDISGDGYLQLEEMVSVDQFRNQLEAFAREEKQLARDAIVEAQKEEEAARMAE
ARLALLNDKEPTQTDKIVSVLPYLFPLLDSLQFGRFLILDNPENPLFFALALLYAAYRTI
PFSGFLAFIALNTLSNNPGLNKLVRFNMQQAIFIDIALFFPGLIAALVGGLGGMAGIQIP
AAVNQAGSTAIFGALLLVVTYASISSLLGIKPDKIPLISKAVEDRMPTIDMFDDAGRFIP
RNERQEKDDEGEKKN
>comp5979_c0_seq1; 173 144 696 LEN=696; translated
MNLLNFGPRSLIRILQSAIVFSVFSSMWLLQRSETSRTLVNDVDVEVWRVGLRKGKDFAL
AETIQKRNKVLLSRRLDITETPIQNVIGFPSKNRSELSAPQSAHLQDVLANPEKEKRQEG
SYAAVTKDQSVDSENEEKQMEDIAGNSFRDGKRKDDVGSESHIESYEGSSAGANNDRNEL
VTTRX
>comp5981_c0_seq1; 466 1 1339 LEN=1546; translated
XMASTVTEDDYSKYSAFNETDSGMGDSSSFAIRSKAGTGASNSLADGDFDNSLVVKQRNQ
QERVFTRDEFLYALSSPSSIGAMAWILSAFVAVLYGSSVVVLINFCIPLLLGPFVINEQM
YAQLGLVSLVDTDHSLAMSDLMLQALSAELSIFLLIXIFVSLIAIIFVFVKAVRRNYHKI
RDEANKLAMINVRLKGNLSRLHRQEYRLSAVEVRFEHLCQRSNKDITQMKHLAKRNATLR
QKLKANLAAKKIHNLLTTMLGLDYNGNCTISEKQIDEIAMLMKVFAGKNAYSKFDFHEIH
EAIVNSLVKNKKVILIPPSIDDTDSETKESEREYNDERSNGNFTEEPDSVQPSFEGDVDD
STKEEQSLNAPTDEYEDPSVRSDPSGLLAPEEYARDEVSDEVCNDDFSGMVQKTVTNGTM
EEPIDIEKLVFSDHDGMAESNNMYQK
>comp5981_c0_seq2; 405 1 1321 LEN=1528; translated
XMASTVTEDDYSKYSAFNETDSGMGDSSSFAIRSKAGTGASNSLADGDFDNSLVVKQRNQ
QERVFTRDEFLYALSSPSSIGAMAWILSAFVAVLYGSSVVVLINFCIPLLLGPFVINEQM
YAQLAPSVRRNYHKIRDEANKLAMINVRLKGNLSNSIAKNTGFPPLKYVLNIFANVEQGY
YTNEAFGKEECHPSTKIEGKKNLRRIFEGILCALRRSNIGYITLGQTLKFDSVAQFQANL
AAKKIHNLLTTMLGLDYNGNCTISEKQIDEIAMLMKVFAGKNAYSKFDFHEIHEAIVNSL
VKNKKVILIPPSIDDTDSETKESEREYNDERSNGNFTEEPDSVQPSFEGDVDDSTKEEQS
LNAPTDEYEDPSVRSDPSGLLAPEEYARDEVSDEVCNDDFSGMVQKTVTNGTMEEPIDIE
KLVFSDHDGMAESNNMYQK
>comp5981_c0_seq3; 334 890 1456 LEN=1663; translated
MLGLDYNGNCTISEKQIDEIAMLMKVFAGKNAYSKFDFHEIHEAIVNSLVKNKKVILIPP
SIDDTDSETKESEREYNDERSNGNFTEEPDSVQPSFEGDVDDSTKEEQSLNAPTDEYEDP
SVRSDPSGLLAPEEYARDEVSDEVCNDDFSGMVQKTVTNGTMEEPIDIEKLVFSDHDGMA
ESNNMYQK
>comp5981_c0_seq4; 498 1 1204 LEN=1411; translated
XMASTVTEDDYSKYSAFNETDSGMGDSSSFAIRSKAGTGASNSLADGDFDNSLVVKQRNQ
QERVFTRDEFLYALSSPSSIGAMAWILSAFVAVLYGSSVVVLINFCIPLLLGPFVINEQM
YAQLAPSVRRNYHKIRDEANKLAMINVRLKGNLSRLHRQEYRLSAVEVRFEHLCQRSNKD
ITQMKHLAKRNATLRQKLKANLAAKKIHNLLTTMLGLDYNGNCTISEKQIDEIAMLMKVF
AGKNAYSKFDFHEIHEAIVNSLVKNKKVILIPPSIDDTDSETKESEREYNDERSNGNFTE
EPDSVQPSFEGDVDDSTKEEQSLNAPTDEYEDPSVRSDPSGLLAPEEYARDEVSDEVCND
DFSGMVQKTVTNGTMEEPIDIEKLVFSDHDGMAESNNMYQK
>comp5982_c0_seq1; 388 154 1965 LEN=2511; translated
MSEAITDTSKGGGTVVEIVDDSSSSSGFHIDTLVKLTEDDPDYSSTSGQIKKNDATEQQL
DLERGIDANFASDSTVAKESETNKQDCSSTTLNSNHDSSAQQQEILRTTPGAFAIERLIG

ESGVVSGPSTAILRTTPELRQSMSLGFRVRAPDETLPTLAQSETACALLSSQDIYRESGE
EEQTNVIRAVLVKNNTHIYEASPVESVAEDNMKNKWSSKTLFLCAASAILFVLVTTMAFV
VAGKTKGEKSMKMNPGSTPDPLKEKRRDYLLSLLAPLSGPAEEQVFNRGNNTAMSMDRIN
ALSWLVEDTRNKISLTTERNDPITNKSVAEPVPLWKLRQRYVLALFYFATDGVNWDDQYF
FLSTQDECQWTKRFPLANGEKQMGVSCNDEGRVTGISSRWNGLAGTLPHELCFLEDTFEH
LNLAGDLMTGTIPEEYERFTKLRTLVLDDNCIDGTIPQSLLRLPTLTRVSFVNTPWLSAP
MNEFCNRQDYKEGTISIAGDCSGETNGCECCTCCNPDTFRCTDRESGNSWNSYNLNAFTE
RGFVKSFRKNTCRTENSKHWIANECPYIINSNTNLDTNRFEGECTKNGAQKGAQISHDAF
LWF
>comp5982_c0_seq2; 432 154 1380 LEN=2626; translated
MSEAITDTSKGGGTVVEIVDDSSSSSGFHIDTLVKLTEDDPDYSSTSGQIKKNDATEQQL
DLERGIDANFASDSTVAKESETNKQDCSSTTLNSNHDSSAQQQEILRTTPGAFAIERLIG
ESGVVSGPSTAILRTTPELRQSMSLGFRVRAPDETLPTLAQSETACALLSSQDIYRESGE
EEQTNVIRAVLVKNNTHIYEASPVESVAEDNMKNKWSSKTLFLCAASAILFVLVTTMAFV
VAGKTKGEKSMKMNPGSTPDPLKEKRRDYLLSLLAPLSGPAEEQVFNRGNNTAMSMDRIN
ALSWLVEDTRNKISLTTERNDPITNKSVAEPVPLWKLRQRYVLALFYFATDGVNWDDQYF
FLSTQDECQWTKRFPLANGEKQMGVSCNDEGRVTGISSSEYMLNATIN
>comp5983_c0_seq1; 782 166 1971 LEN=2049; translated
MADFQSKRKATDITGGDDKPDDSSAIVACPYLDTIQRSVLDFDFEPSCSVTLESGPHVYS
CLVCGKFFRGKGKHTPAYTHAVDEGHFVFIHLNRGTFHCLPDDYEIKDPSLADVSAALHP
TFTEEEIQRLDSNTGLARDLFGRRYLPGFVGLNNLHKTDYINSSIQALAHVRPLRDYFLR
LSHRNTFVLTSASDKKKSKKNKPPKAPRRMSDLVQCFGELIRKMWSNKRFKSNVDPHMLI
QAISRASKKKFRVGHQAEVGEFMAWFLNQLHVGLGGTRKPGSSIIYRTFQGTIELTTRQR
KTAAALEAAEAGDEDGNGDAKNGGGGTGMEADNGHDETTEPLTTDKTTDPDIEETTIETR
FLQLTLDIPEKPLFRDDDGGLVIPQEPLVSVMKKFDGITFSEAISKSGVAQRKRYRLLGL
PPYLILHLARFKTNKYSREKNPTIVAFPVKNLDLGSYTFPKNGKEVPPTEAEVRAMTVAE
LRALLNEHGREDLSAHALEKSELVEAAVDFVTKSLPDLLADKYDLVANITHNSPADVGRE
GKHDPLQEGSYKCHIEHKSSKQWFETQDLHVEETMPQLIGVSESYLLIFERKAAQQMDLS
R
>comp5984_c0_seq1; 265 72 551 LEN=922; translated
MVSSRIERLFLVAAALAAPTAIEALAGAPQKEFTGAGIGRLNMDELAGKAGTWNCHYDMI
LVERLQKQKGPQDVEDDGLFVPESDMPRFHLCRVLSKGNGREEENGMVAPDINGLQVGDL
VVAKNPWGIGPKDEELSDGKKLSFMREIDIAAKVTGELN
>comp5985_c0_seq1; 715 379 2892 LEN=2936; translated
MVSGKKNVSHKINKSKIVSRILIVVVVTCILRQLTALTAVSRDLLQVSTYDDAASKLVEI
NSVPKSIRKTNLSKGLEIEHKAKEESEIKSITTKATKKILSNEMNDGGDREYEDNIERSF
VIICMGDSAIDSNYVERFVWSARKIGKFTGWIVLLTDAPEQRYAHLSRNWADVDNDKFTI
LRPEEKHYIRHYDHAPAMVFKQFKTYVLQYLSQDSRLDNVKLVYYLDVDIVFGNPVGPLF
NDLEQKYQIGTNSTTTAQTNTGGNVAAKMWMFKGNSKKSKIQGGQMILDRSTSQPCLDRF
RSLMDPKTSTIDQVHLMQMLSDQKLARKTEDYTSLKCEIVIMPQEEKHILFPSKTLIKDI
ADGKINDDDPIPVLNHIKNTGGDLKKSSPEDIEAYLRYLFRFETDQKDTLGITTKTYLDG
GQREKGKKEQHQEKSEVKSAEEKKSDVPRSNHDEETTQESEDSSNVLFRSSQNDDNWKNV
SPNELVTTLADLSNSTEIVSSRAKRRRRRGYRDKKGKKEKETYEDDIERAMFVISMGEKA
AKMNTVERFVYSAREIGKFSGWIVVLTDAPSDRYANMNNWTEKVIFMDPKKEDTKTHYKV
SNMVYKRFKTYAIEYMNRDMRLEQVELVYYLDVDIVFGSDLGPAFHGIEKTYGIGRLGVN
TTANVTDSIARGKMWMFKGNSGKWQIQGGQMVLDRSLSQPCLERWREGFDQKKVTDYAKD
QYLLMAMKSEQEQAMNRTMNSTDPDLDVDLACEIVTMEQAPYIDFPLVTTIKQRSKVLRK
HPKRKFHYSPMVHVRNDGGTANMRDGNIRPYMRSLLRFKKDQPDPLKILKKVHMDTT
>comp5986_c0_seq1; 567 80 1087 LEN=1291; translated
MTGTNGNGNGNGSAVVGTSTVKQGLAQMLKGGVIMDVMNVEQAKIAEAAGAVAVMALERI
PADIRRDGGVARMSDPSLIQEIKNAVTIPVMAKARIGHFVEAQILEACEVDYIDESEVLT
MADDVHHIDKTQFSVPFVCGCRNLGEALRRIAEGASMLRTKGEAGTGNVVEAVRHARQVQ
GDIRRLQTMSKDELFVAAKELRAPYDLVKQIAEDGKLPIVNFAAGGVATPADAALMMQLG
VDGVFVGSGIFKSANPEARARAIVQATTHYKDPKVLMDVSTGLGPAMVGICDIKGDPVNF
RDREGGEYTYSGKVPPKKELSGYTETQMYGSSWNN
>comp5989_c0_seq1; 455 62 862 LEN=909; translated
MATKFADLAKASKDLLNDDYTTKQSLKCKKNAGPVAVTIETGRGEGGALTSKVGTKFNYG
KLNVDKAQATADGGQVLETSLAVAPGVKVSFKSGKGADLGVDYTKGNLYATGVLDVVEMS
KFTTSACVSLPSGIKAGADATYGLSGKTGITGLNVGASYATGSLLGAVTSVNKFSSVNLG
LLYSVNSKVTLASMTSHSGDSAFNLTAVGGSYKADFGTVKAKYTGDGAVSAALIKELAPK

VSLTVSGTASVSAPSESFKYGMGIVM
>comp5990_c0_seq1; 370 289 864 LEN=1204; translated
MNRFQYIIFFLGALMMINTPTVLGSSSTLEVADTKRASLEQIDVKKRILSSPHPHPSVPS
SGGSSGGSSGSSSSGGSSGGGSSSGGSSGGSSSGGGSSGGGAAAANASNLASHNAQKGVG
LMLGIAAVAGMVIAAVVIPRRKTATEAKHPLKGSLNKRIDLFANLAKHTSSAERPPRRDE
EGRYINADSIV
>comp5991_c0_seq1; 281 215 991 LEN=1611; translated
MPHSSSSYVFEPPPISSLPVVGTSKQFPVRRVYCVGKNYVEHVIEMGGDVKRNKPVFFTK
PNIDGVVYAGCLGNSSDKPTSIDYPPDTKNLHYEVELVVAIGKEPNDCVPNDVLSPNILD
CVFGYAVGIDLTRRDLQDEAKSKQWPWDTAKYFDQSAPIGPITPRKEGNEDYFDDNTGKL
EKRTMALTVNGATRQSVDLEKMIWKVPEILSELSKSFRLKPGDLVMTGTPSGVGPVVVGD
RLIGTIDGLVEPVDITLV
>comp5992_c0_seq1; 393 288 1970 LEN=2166; translated
MPIPEMPGTSKNPLRGAVEVIPNRLYYCALKNKETLQNQKILIPNPHTNSTSPDQKETVF
CFHIDDELVYWNFFLDFGPLNLGQLWRFTKKLNRLLALAEKDGSTVLFYSTTHPNKRACA
IFLICAWQVLELHRTPDDAMFGFSATAYNANHSTAKTNGSNASDVNRSNPPLLPLSGLGS
QTINPLPPFHDASPYKCTYQLTLRDCLGALVKARQFQFFDWGDRFDIKEYEYFEQVENGD
LNWIVKDKILAFAGPSYVRTVSPEGYCTLSPDDYIPYFKRKNIGLIVRLNEKKYDETQFI
NAGMDHFEEVYTDGSCPRMEVLQRVLAAFEKLPNDKGFAVHCKAGLGRTGTCIGAYLMKH
YRMTAKEVIGWMRICRPGMVIGPQQEFLEYIQSIMWQQGDAMRTKSSQLTSPLPLLAPRH
KHITNLHSAIHSSSPSSRSTSTATTVPESPSSLVLDKTSSHEIGQEIILGGSTGYDHLEG
ELETAYERERLEGRPGQAEGLLAARQNRNREPIPPQSPQPRGHEQSNSNDSKTAIPVTPD
TRATSTTSSSSTSLWSRAFA
>comp5992_c0_seq2; 308 288 2122 LEN=2318; translated
MPIPEMPGTSKNPLRGAVEVIPNRLYYCALKNKETLQNQKILIPNPHTNSTSPDQKETVF
CFHIDDELVYWNFFLDFGPLNLGQLWRFTKKLNRLLALAEKDGSTVLFYSTTHPNKRACA
IFLICAWQVLELHRTPDDAMFGFSATAYNANHSTAKTNGSNASDVNRSNPPLLPLSGLGS
QTINPLPPFHDASPYKCTYQLTLRDCLGALVKARQFQFFDWGDRFDIKEYEYFEQVEVVE
QIKCESVEVPKRTHQSIPEGSISERILLSHCANNFFCVFLFNKNFPKNGDLNWIVKDKIL
AFAGPSYVRTVSPEGYCTLSPDDYIPYFKRKNIGLIVRLNEKKYDETQFINAGMDHFEEV
YTDGSCPRMEVLQRVLAAFEKLPNDKGFAVHCKAGLGRTGTCIGAYLMKHYRMTAKEVIG
WMRICRPGMVIGPQQEFLEYIQSIMWQQGDAMRTKSSQLTSPLPLLAPRHKHITNLHSAI
HSSSPSSRSTSTATTVPESPSSLVLDKTSSHEIGQEIILGGSTGYDHLEGELETAYERER
LEGRPGQAEGLLAARQNRNREPIPPQSPQPRGHEQSNSNDSKTAIPVTPDTRATSTTSSS
STSLWSRAFA
>comp5994_c0_seq1; 670 80 1702 LEN=1913; translated
MVAKKRNLASSGNGASRGMQRGNSAAGGGGGGGVSSKNAHRDTLPGIVWSVVLCIVKCSI
WFAIAFPFAVATHSLSLVFGRPPNLVYLQQTLRYLGFVWKSLTFATPEQERHMQSFSVTE
KIRLTTTILVHTICSPLSAFGWILDEILYGRRLNGLEPKEKAAVENPVFVVSAFRSASTQ
LARGLVSETRYTCEKGGTSTTEPCFVAPNAMMCAYPYLWLWKLVYAIVGDIPDTLVDAER
AQTEGGLTKEDVREKFNAGFTPDSMARHANDPFQTDTFDGTFLNCHLNGFVWQLCRGLPA
SAIEQEFNYACQKEGDALNRRIWQTDMVRHIERLARKTILFHNEGGGETAAGGVRPPQRF
LLKGHFLSVCPQLREAYGGRARFVTILRDPCERLRSGINYMAVNPTLYASDPRQQIPWAA
FAEALGETEAQYCERELQWFGGTGGDESDRLAVTFDSFVADRGKAMGEIVNWLGLVPEDD
PGEVAIASTSQAKKSKSPRKKKTPSASSAATKKAYRIDRSLAELGVDEKAYRERLSDYLA
W
>comp5995_c0_seq1; 222 231 570 LEN=762; minus strand; translated
MKSTKDAPNIVADRVVPSIKNFPFAAFDEFFSTSLWPAIRIPSMKPKTHPVMDPMHPPKA
AFLKSSPHLKGILAWEIDDVDAGVDVVEDKSPAEEDAGASCGDDLRRRCSNNW
>comp5996_c0_seq1; 717 317 2224 LEN=2306; translated
MDVAKMEKVETPKSPAKMSFFRKWGPALVALVGVFALNASLFGPTLSDMPDMSEKMLNRR
ALAEKRTADVLAMAPTQESTKYTPRSRGFGSRNLSAEDGGVMEYTRRRHRYLGEYVHVDV
SDQFTKFGKWTVSIIYFAYWVFLMFPNWFLPIGRPGIALGGGLSMVVWRYILMVTEQGPF
FDAERVIILEPLFLLFGLMLTTIYLEKMERGGLFDKLRDSLDDPVNWKRSAKIMLMSTIG
SAAVMNDSVVLIFSGVVVDLCVRHKVANSLPYLLSLATTANTGSALTLTGNPQNILIVSL
MYDDISWLDFAANMLLPVVAASVINASMMFLYYGAELFPGSSGMMENIGIMFFGNRTPEM
LAQEHAYYARQAATEGESDGNGWTFWSKLQVLIIVLFLIFFACGFDVCVVSITTGAILMV
IAAYKRQHYDPKPDHTQYDVEGNPLPPKKTYDMDGNEIEPEEEELVTESETTLTEVDYGL
LMLFIGQFLLIGSFDDTGVPQAFFGLSMGKCADQMTEGACVYWFVTIVTVLSNIASNVPV
CQMLAATFPYATPYDWLQVSFSATIAGNLTMLGSAANMIVAFQAAKVGDRTFTSERHAPF

GIPSTILSLYAGTFLLATFHFAPECSERLGECDAQ
>comp5996_c0_seq2; 655 1 1951 LEN=2033; translated
XREDILVSSSKTSTIMDVAKMEKVETPKSPAKMSFFRKWGPALVALVGVFALNASLFGPT
LSDMPDMSEKMLNRRALAEKRTADVLAMAPTQESTKYTPRSRGFGSRNLSAEDGGVMEYT
RRRHRYLGEYVHVDVSDQFTKFGKWTVSIIYFAYWVFLMFPNWFLPIGRPGIALGGGLSM
VVWRYILMVTEQGPFFDAERVIILEPLFLLFGLMLTTIYLEKMERGGLFDKLRDSLDDPV
NWKRSAKIMLMSTIGSAAVMNDSVVLIFSGVVVDLCVRHKVANSLPYLLSLATTANTGSA
LTLTGNPQNILIVSLMYDDISWLDFAANMLLPVVAASVINASMMFLYYGAELFPGSSGMM
ENIGIMFFGNRTPEMLAQEHAYYARQAATEGESDGNGWTFWSKLQVLIIVLFLIFFACGF
DVCVVSITTGAILMVIAAYKRQHYDPKPDHTQYDVEGNPLPPKKTYDMDGNEIEPEEEEL
VTESETTLTEVDYGLLMLFIGQFLLIGSFDDTGVPQAFFGLSMGKCADQMTEGACVYWFV
TIVTVLSNIASNVPVCQMLAATFPYATPYDWLQVSFSATIAGNLTMLGSAANMIVAFQAA
KVGDRTFTSERHAPFGIPSTILSLYAGTFLLATFHFAPECSERLGECDAQ
>comp5997_c0_seq1; 206 294 626 LEN=626; minus strand; translated
MVDMWRKHFVLAENMLEMECTNLTPEVVLKTSGHVDRFTDLMVKDPVTSECFRADKLLED
AIDDLLEKNPTLDAETREEHLRVQRQADAYSPEELDKLLLKYECKGPSGEA
>comp5998_c0_seq1; 749 86 1996 LEN=2307; translated
MIHKTQSFCSLAFLFLSAGTTLLLLDSNAHQVFGCTVFLVGKNASADGSVMCSHSNDGEF
DTDPRLVKVPAADYPPDSLRPVYFSPENYPRYVGYDRGIPEYYPREGEPHEPFQPIGFIP
QVPHTYAYLEETYGAVNEMQVGIGESTCSGVFGARPIHQHPNGTALFSIDELSRIAMERA
STAKEAVQMMGDLAVAHGFYGAGEFEGTSESLAVTDPEEAWIFHILPDPTGKSAIWAAEK
LDDDKFAVIANMFVIREVDPTDTERFLMSKSVHDVAIDYGWWTSVEADGLLDFTKIYSDG
EYSHKFYSGRRVWASYRLAAPSQPFPTDYTDLQSDPVYPLAVTPDRPVSERDLFRFHRDT
LQGTKFDLGAEGNLAGGPFGTPDRWKAGPNEETKVQGNWERAIGLYRTSDTYVVQSKKEE
RQQPDGSGGMGSGGPGAILWFGPSSPLATIFTPFVVGMSDIPKSFRSGHQSVFSRESAFW
AACYAHNIANLKWSYMIQDITKRQDELEGASLDLVASIQEEYRRHNNMGLVEDAFAKNAE
AIVASLWSLSDELMFRYADGFVNSNESEVDQGGEITKRVGYPAWWLEAVGYKNGPPPPPT
TPKCCDPPKGRKKNAGEPSPHLLRGAESSGTAIEID
>comp5998_c0_seq2; 702 86 2106 LEN=2417; translated
MIHKTQSFCSLAFLFLSAGTTLLLLDSNAHQVFGCTVFLVGKNASADGSVMCSHSNDGEF
DTDPRLVKVPAADYPPDSLRPVYFSPENYPRYVGYDRGIPEYYPREGEPHEPFQPIGFIP
QVPHTYAYLEETYGAVNEMQVGIGESTCSGVFGARPIHQHPNGTALFSIDELSRIAMERA
STAKEAVQMMGDLAVAMDSMERENSRERVSSSLVPGFHEVVVVDLICDHPKLSFYRVFLV
HNTTAESLAVTDPEEAWIFHILPDPTGKSAIWAAEKLDDDKFAVIANMFVIREVDPTDTE
RFLMSKSVHDVAIDYGWWTSVEADGLLDFTKIYSDGEYSHKFYSGRRVWASYRLAAPSQP
FPTDYTDLQSDPVYPLAVTPDRPVSERDLFRFHRDTLQGTKFDLGAEGNLAGGPFGTPDR
WKAGPNEETKVQGNWERAIGLYRTSDTYVVQSKKEERQQPDGSGGMGSGGPGAILWFGPS
SPLATIFTPFVVGMSDIPKSFRSGHQSVFSRESAFWAACYAHNIANLKWSYMIQDITKRQ
DELEGASLDLVASIQEEYRRHNNMGLVEDAFAKNAEAIVASLWSLSDELMFRYADGFVNS
NESEVDQGGEITKRVGYPAWWLEAVGYKNGPPPPPTTPKCCDPPKGRKKNAGEPSPHLLR
GAESSGTAIEID
>comp5999_c0_seq1; 369 564 1504 LEN=1775; translated
MEQDYGESGKYDVKFHMRGENYKVSTRDGFSVNSIDGDIFISPEDLRAFENPEDVGPVDW
VIVCIKSSSLDDIPGLIHPLLDAKRTRVLAIMNGLIEDDLVRLLKERNGEEFCAALYGGM
ALVCSNRTGPGKIEHSYAGLLSGGVAMSRRSFTAEDNQQAFRDLWAPTSIDIAYEPSLLG
GRWRKCLWNLPFNGISVAMNGITVDKIVNDSGLRRLAFKIMDETVAAANADLEANGFGEE
FFLGDVDKKKMMDLSDNMGAYRTSTMIDFVEGRPLEVHYLFTKPVQKARKLGVPTPYLET
LVTQIEAFQRLRY
>comp5999_c0_seq2; 407 206 1399 LEN=1670; translated
MATAFRSISRSSLSTSRRGAISKRVAPHRFGKHSSLKASTKSFRFLSSSPSLSRLFLTEN
GIGEKGGSERQSVAIIGAGAVGGYYGARLWESGKYDVKFHMRGENYKVSTRDGFSVNSID
GDIFISPEDLRAFENPEDVGPVDWVIVCIKSSSLDDIPGLIHPLLDAKRTRVLAIMNGLI
EDDLVRLLKERNGEEFCAALYGGMALVCSNRTGPGKIEHSYAGLLSGGVAMSRRSFTAED
NQQAFRDLWAPTSIDIAYEPSLLGGRWRKCLWNLPFNGISVAMNGITVDKIVNDSGLRRL
AFKIMDETVAAANADLEANGFGEEFFLGDVDKKKMMDLSDNMGAYRTSTMIDFVEGRPLE
VHYLFTKPVQKARKLGVPTPYLETLVTQIEAFQRLRY
>comp6001_c0_seq1; 140 38 409 LEN=521; translated
MIKSILILTLAIASGDAFLSSPLHPTRPMAKKLGASSSDSNSFVDTLQTSFRIARESNAE
GNGFKQVLADVLAGDDYDKEAVSATIEDTISSAPCGKNVLCIVIDRTVQLIPSTASYGHF
ILT

>comp6001_c0_seq2; 162 38 616 LEN=1145; translated
MIKSILILTLAIASGDAFLSSPLHPTRPMAKKLGASSSDSNSFVDTLQTSFRIARESNAE
GNGFKQVLADVLAGDDYDKEAVSATIEDTISSAPCVMYTWENSPSCKKAVEAFETIGATV
KIVRLDDPWEEGNPVRAVLGRKVGRTSVPFVFVGGEYIGGFDGGVSTSAPGMVDMAFQGK
LREALSEAGAMK
>comp6002_c0_seq1; 565 320 3412 LEN=3963; translated
MDDGSAGNDGARRDELVLLFELVGARNLVLRDDYEICQGIDPNSLQPFCVVKYNGVRIHK
TKPSEELGCNPIWTPHSSRSIFLVRTTAIELSRSIMNISVYIKKESALPSMLTTVTSVFM
GQIHVDSKTILSHCDEERFELNIEDELGEQTRNLGKLAVRCRVATPSDIEILSVLNKDLS
ASHMERIGDMMLDSSTRPMETTSTLFHKRSDRPLATMMTEKDEAEVAQSSIFQSVGNIFV
SQTSTCPESGQVMKRVKPYPDPDRQEKTEFLRPYDLNVETRLPSTKWTEAGSGSIGKLFV
EVLSCDDLPNLDSGGEMMGNFTDSFCTLVYEDTCAMTDVIDDELSPRWMPWTNRAFSFNI
MHPASILYLGVFDFDVLGTHDPIGRVAVNVCNLQRDTVHTLTYNLYPSSNVTERKAAGSI
TIRVRLEWFDPRAALLAAMKSRPNMHVNVLKEKTFRVVRYTCYGEYGDKGEFDITLMRSY
INEIFEYKAGVSYAISDTLRSLILWKGQVEIFSIMLPLHSLILFVFSTRLVEKPQLIVPY
SLLAIAWMMLANLTIRRQHPSPWQSRLSFFDYLNILRTGKSSIPVKCIKEYEGAAEARAY
ELAWEKRIEKDRKIAERKAELLQEINDISDVNIHTEVSNQALIPVDLLNRLARYQGIVGR
ICKKLRFVKIILTWEESIVSFLVTATFLSAGIVALALPWGFILTWVGRLIVWGFLGPHMK
LVDLFLRANEKRDGKLNDLIKNFDIQSHQARLSREEALKIKDMKEVAFGTYSVHVPSFNL
SRHHDRPLPQSSSRVYRKPPPHRKVGNNARVSVTDLGSRCTLIPGQQLYGAMIPRPEHEE
EIHKHQRILAEKRMKSVKDFLRRLNEVEGLSEYETKQLKKMDIDTATPMAIGYEVVPFEE
CESVSSDTESSEGSTNKSAALCSLRNFSIKLMSDRLGYVDCREARDLILRSDGVTTYCIS
ENCEEEDEGMEVIGTGRFQSFDYGDEMRSSIDSKDPNVEERLTCALNSTEGSQMVYYRPP
ENGEVKLKEE
>comp6002_c0_seq2; 495 320 3567 LEN=4118; translated
MDDGSAGNDGARRDELVLLFELVGARNLVLRDDYEICQGIDPNSLQPFCVVKYNGVRIHK
TKPSEELGCNPIWTPHSSRSIFLVRTTAIELSRSIMNISVYIKKESALPSMLTTVTSVFM
GQIHVDSKTILSHCDEERFELNIEDELGEQTRNLGKLAVRCRVATPSDIEILSVLNKDLS
ASHMERIGDMMLDSSTRPMETTSTLFHKRSDRPLATMMTEKDEAEVAQSSIFQSVGNIFV
SQTSTCPESGQVMKRVKPYPDPDRQEKTEFLRPYDLNVETRLPSTKWTEAGSGSIGKLFV
EVLSCDDLPNLDSGGEMMGNFTDSFCTLVYEDTCAMTDVIDDELSPRWMPWTNRAFSFNI
MHPASILYLGVFDFDVLGTHDPIGRVAVNVCNLQRDTVHTLTYNLYPSSNVTERKAAGSI
TIRVRLEWFDPRAALLAAMKSRPNMHVNVLKEKTFRVVRYTCYGEYGDKGEFDITLMRSY
INEIFEYKAGVSYAISDTLRSLILWKGQVEIFSIMLPLHSLILFVFSTRLVEKPQLIVPY
SLLAIAWMMLANLTIRRQHPSPWQSRLSFFDYLNILRTGKSSIPVKCIKEYEGAAEARAY
ELAWEKRIEKDRKIAERKAELLQEINDISDVNIHTEVSNQALIPVDLLNRLARYQGIVGR
ICKKLRFVKIILTWEESIVSFLGSTDPDTLSQSSIVDNVSSYIPFVVTATFLSAGIVALA
LPWGFILTWVGRLIVWGFLGPHMKLVDLFLRANEKRDGKLNDLIKNFDIQSHQARLSREE
ALKIKDMKEVAFGTYSVHVPSFNLCKFEMIFIIHYPEKGYLIHILIVEIDPARHHDRPLP
QSSSRVYRKPPPHRKVGNNARVSVTDLGSRCTLIPGQQLYGAMIPRPEHEEEIHKHQRIL
AEKRMKSVKDFLRRLNEVEGLSEYETKQLKKMDIDTATPMAIGYEVVPFEECESVSSDTE
SSEGSTNKSAALCSLRNFSIKLMSDRLGYVDCREARDLILRSDGVTTYCISENCEEEDEG
MEVIGTGRFQSFDYGDEMRSSIDSKDPNVEERLTCALNSTEGSQMVYYRPPENGEVKLKE
E
>comp6002_c0_seq3; 572 320 3493 LEN=4044; translated
MDDGSAGNDGARRDELVLLFELVGARNLVLRDDYEICQGIDPNSLQPFCVVKYNGVRIHK
TKPSEELGCNPIWTPHSSRSIFLVRTTAIELSRSIMNISVYIKKESALPSMLTTVTSVFM
GQIHVDSKTILSHCDEERFELNIEDELGEQTRNLGKLAVRCRVATPSDIEILSVLNKDLS
ASHMERIGDMMLDSSTRPMETTSTLFHKRSDRPLATMMTEKDEAEVAQSSIFQSVGNIFV
SQTSTCPESGQVMKRVKPYPDPDRQEKTEFLRPYDLNVETRLPSTKWTEAGSGSIGKLFV
EVLSCDDLPNLDSGGEMMGNFTDSFCTLVYEDTCAMTDVIDDELSPRWMPWTNRAFSFNI
MHPASILYLGVFDFDVLGTHDPIGRVAVNVCNLQRDTVHTLTYNLYPSSNVTERKAAGSI
TIRVRLEWFDPRAALLAAMKSRPNMHVNVLKEKTFRVVRYTCYGEYGDKGEFDITLMRSY
INEIFEYKAGVSYAISDTLRSLILWKGQVEIFSIMLPLHSLILFVFSTRLVEKPQLIVPY
SLLAIAWMMLANLTIRRQHPSPWQSRLSFFDYLNILRTGKSSIPVKCIKEYEGAAEARAY
ELAWEKRIEKDRKIAERKAELLQEINDISDVNIHTEVSNQALIPVDLLNRLARYQGIVGR
ICKKLRFVKIILTWEESIVSFLVTATFLSAGIVALALPWGFILTWVGRLIVWGFLGPHMK
LVDLFLRANEKRDGKLNDLIKNFDIQSHQARLSREEALKIKDMKEVAFGTYSVHVPSFNL
CKFEMIFIIHYPEKGYLIHILIVEIDPARHHDRPLPQSSSRVYRKPPPHRKVGNNARVSV
TDLGSRCTLIPGQQLYGAMIPRPEHEEEIHKHQRILAEKRMKSVKDFLRRLNEVEGLSEY

ETKQLKKMDIDTATPMAIGYEVVPFEECESVSSDTESSEGSTNKSAALCSLRNFSIKLMS
DRLGYVDCREARDLILRSDGVTTYCISENCEEEDEGMEVIGTGRFQSFDYGDEMRSSIDS
KDPNVEERLTCALNSTEGSQMVYYRPPENGEVKLKEE
>comp6002_c0_seq4; 488 320 3486 LEN=4037; translated
MDDGSAGNDGARRDELVLLFELVGARNLVLRDDYEICQGIDPNSLQPFCVVKYNGVRIHK
TKPSEELGCNPIWTPHSSRSIFLVRTTAIELSRSIMNISVYIKKESALPSMLTTVTSVFM
GQIHVDSKTILSHCDEERFELNIEDELGEQTRNLGKLAVRCRVATPSDIEILSVLNKDLS
ASHMERIGDMMLDSSTRPMETTSTLFHKRSDRPLATMMTEKDEAEVAQSSIFQSVGNIFV
SQTSTCPESGQVMKRVKPYPDPDRQEKTEFLRPYDLNVETRLPSTKWTEAGSGSIGKLFV
EVLSCDDLPNLDSGGEMMGNFTDSFCTLVYEDTCAMTDVIDDELSPRWMPWTNRAFSFNI
MHPASILYLGVFDFDVLGTHDPIGRVAVNVCNLQRDTVHTLTYNLYPSSNVTERKAAGSI
TIRVRLEWFDPRAALLAAMKSRPNMHVNVLKEKTFRVVRYTCYGEYGDKGEFDITLMRSY
INEIFEYKAGVSYAISDTLRSLILWKGQVEIFSIMLPLHSLILFVFSTRLVEKPQLIVPY
SLLAIAWMMLANLTIRRQHPSPWQSRLSFFDYLNILRTGKSSIPVKCIKEYEGAAEARAY
ELAWEKRIEKDRKIAERKAELLQEINDISDVNIHTEVSNQALIPVDLLNRLARYQGIVGR
ICKKLRFVKIILTWEESIVSFLGSTDPDTLSQSSIVDNVSSYIPFVVTATFLSAGIVALA
LPWGFILTWVGRLIVWGFLGPHMKLVDLFLRANEKRDGKLNDLIKNFDIQSHQARLSREE
ALKIKDMKEVAFGTYSVHVPSFNLSRHHDRPLPQSSSRVYRKPPPHRKVGNNARVSVTDL
GSRCTLIPGQQLYGAMIPRPEHEEEIHKHQRILAEKRMKSVKDFLRRLNEVEGLSEYETK
QLKKMDIDTATPMAIGYEVVPFEECESVSSDTESSEGSTNKSAALCSLRNFSIKLMSDRL
GYVDCREARDLILRSDGVTTYCISENCEEEDEGMEVIGTGRFQSFDYGDEMRSSIDSKDP
NVEERLTCALNSTEGSQMVYYRPPENGEVKLKEE
>comp6003_c0_seq1; 195 1 676 LEN=676; translated
XNNQQNPKHVRIRTANTPTNNVRTSHSTNTIISSQRIAMFPSLVPVPGMNSYYDDAGRAD
DDDDLGLSAMMAKTVSAATSRPARGRSERDRGASRSRSRSRSSSILRRMVESVRAEKETK
TTATVNHHSRDGPSIPRRGRSRDRIGGESAAGDENKNENDTSTIATNTGRSRSRSVVKSI
KSRDEKTKTSSKEKEKTKTKRKSKKIRRISLPVTPSEEKSETASSS
>comp6007_c0_seq1; 339 1216 2337 LEN=2792; translated
MVKRLPKTKPDVRDVLEANATPDSDTQIAFRKLRDQLEKDGWWKRDLKHESKLLGIWGSL
VAFGAVAARTGHPVIGTVLLALSMTAAGWLGHDYIHGVDPFSDKLRNFAAMAAGLLPVWW
SDKHNKHHALTNEQGVDEDIATDPFLFQWAPSPENDHPLRKIQHLIFYIPFSFLFALWRI
DSVKVAISSVRQKRAGSKPGLYSILFHYTWLFAIFPIKVWLPAIFMSGLLSALIVTPTHQ
SEEMFSEYQPDWVTAQFQSTRNAVATNPFSEWLWGGMQYQLEHHLFPSMPRMNYPKLRPI
LQKFAKDNNVPGGYRESGEFEILKMNWDLYRRVAKADPVPGAPSSRGTIGQQGGILDSDN
SPAAALARASQIA
>comp6007_c0_seq2; 452 491 1990 LEN=2445; translated
MKIIANSKKTMPAAAAALAMLPAISTDAFVMPGRKAAVASVTSSFSPTSLVASTLETPNV
VVGDNELSKDSNSKPRTSLDVRIHGEWYDLTGWRKAHPAGEHWIDWYDGRDATEVMDGFH
SEKGRKMVKRLPKTKPDVRDVLEANATPDSDTQIAFRKLRDQLEKDGWWKRDLKHESKLL
GIWGSLVAFGAVAARTGHPVIGTVLLALSMTAAGWLGHDYIHGVDPFSDKLRNFAAMAAG
LLPVWWSDKHNKHHALTNEQGVDEDIATDPFLFQWAPSPENDHPLRKIQHLIFYIPFSFL
FALWRIDSVKVAISSVRQKRAGSKPGLYSILFHYTWLFAIFPIKVWLPAIFMSGLLSALI
VTPTHQSEEMFSEYQPDWVTAQFQSTRNAVATNPFSEWLWGGMQYQLEHHLFPSMPRMNY
PKLRPILQKFAKDNNVPGGYRESGEFEILKMNWDLYRRVAKADPVPGAPSSRGTIGQQGG
ILDSDNSPAAALARASQIA
>comp6008_c0_seq1; 580 1 1046 LEN=1185; translated
XLLESKNEPGELLASVSEDSDEDSDSDSDDDYLPTSNQRRNVFAGFAAAGFSSSDDDSSE
EDDDDDEGDRDGDQKLSPDRLKDPPPGSRGGKKQPPQQDDGEDDRIDFDELIAQNEILRR
ELDERRKREESEEAEAQAPRALPFHETYDPDDYDANEQRIASVQHRLRKTLASRGVAPRD
RSSETFDPLDAIAMGKTLLQEVLQANIEDLQNKLDAYKRHKEDCQLMARQFALQGGNSKA
LPAQYVRKQDAADNARRRANVHHAGSHYHMQCSRSMQFGKKYKGLMARHSSQGSRLQASR
MAAKEMQLQQRSHAKQVTSKKKSQQKKRGEAGGSKKNGGKKGKGGGDD
>comp6009_c0_seq1; 377 213 1538 LEN=1762; translated
MKMEKMKSFRDKEYVPSTSQNLIESSWDSGILSHEWDGVEFTKSNKLSACSSFFDSLLHD
HNIADIDFVPDNAKPRSNVPRSVYFHSPEDQEKKSANRWNSCITRSNPRTLRGKNLKVSL
SRSNVAPSIETGQGAASRYDESLSPPMRSKHPKPGDGLESTHRVSTPRRADKSPSMQSDC
DEPMPTPAAAPSSLKDTGFLPPLWERLNNGGNATHQLPRSIPLSAKQVKGRSPSYHCSSP
PLLISTPPYPLRGEDLQRRTMSKKTTAQNSNLWHDVGSLKPRRAPRNDLIAAETQSKPRF
PVLKETSSSRSTSRVDGADCSNCRTSLRSLRGKHWGGDDTVAESTISSFKIVKLATDLLN
QNPPLVSSSTPRNSLRRRSIRVSHALDNRRHSRLHPIRQHSDNSNMERCPKPPVRRRSVS

FDSPIPPVRRRSVSFGSDEEV
>comp6010_c0_seq1; 148 88 888 LEN=999; translated
MMQTGTCAPMDASSPPRPPSPRHPESPSAAPGPADSLGCGNDRSPDAPLPSPLVRFEARY
YLTSPRHRSLFVRQRCYVHYRATYGRWPAPGARRGLPPARNDGDNSDGDNSDVFLGHNPW
KRSPPRSPLVRQDAFRSGGPPGSPDYRSAYRSACRSAHRSAPSDAYSRSASGARRRRDPG
QNGQRRDGGTRLLAEGVRKQPALGACGRARLRLPAGNALSGVSPLRHQSGARGGSPNGPP
GEGPAQKRWFFDPLISKTSAMNNFLE
>comp6011_c0_seq1; 555 54 1907 LEN=2397; translated
MMKFPLLLSSVFICIVLLQFPLRVQGSSEEEERWQRTTRSRPRPSRRQRRHGFDANVEPD
LQDHAYSEFVTDYFSKLQDDRQKPRDIVEGTISAFQSLLVGGLASLSTFLGIPIVTVISL
VASNAVVNENEEINGGAVLPAIFGLVVGSIIGTTFTFFISIFSLWRAGVSFLDGLLETPR
ALKSWILEGKIWNPYEREWKSKTQSLENERRELLLMMEAQEAEAARKRRGLVVVDTKLYD
LLEVSPDASTSTIKKAYFSKARELHPDKNKDDPQAHERFVEVHEAYSVLSDEKRRSQYDR
WGSSSEGSDDGIISPNDFDANVFVAVLFSNGSGIVSTPTIQKYVGDLGIASFIDKGYKLT
ALAQAIALLKEQQGDGEPLNRVLGTLADELFGLENSEMRRKTRSIEIATHLVSRAELLSD
GQQCNNSGDGRTYNQKSISEETFRHVVRDEAQIMLRESGFYGPVYLEIIGSSLLFESSLH
LIPHRAQTFYQKWTSRKQFVQTLYDLVKNIDPSVEAKSHVLEIFSTGNLQGILKLITLYN
RMDISTALREAIWRVLNDPSASRSERKNRNRAIRIIGEEFSILANTEMALDNDSEEAVNQ
FESNLVLAIQVASKLTT
>comp6012_c0_seq1; 257 1 1276 LEN=1645; translated
XATAIMITSAGTHCYHWLRNKMKYVAETSNKWGGRRTQASIDSRNEIPAEVEAGDNDYEI
NDDYDDENRDENDGQLASSRAIQNDIISKFPQDHLVHCFGYGSGVFTQSLSREKGAANAG
MLDLILVVDDTQKFHNENLQRFPHHYAPWLRFGGPNLATRMQRQGLPWLPDAHVLFHVVD
ADESSGLPNMKYGVVDKKDLVGDLVGWYSLYLAGRMHKPTLNVVSRDDDLVRAQTKNLRA
ATSMALLLSSSANDTATSAVSWPSLYRQIAALSYTGDFRMQVGGEDPLKLDKLIQAPGQL
QRFHDMYRPIFKSYEMSGFLSTSYPSSGGGGSGGVEWNPNDPSVINQLVRQLPKSLREGT
ATGTATTKSTKHLRVNHDVLAKALSAIVAPAARNQSFKGVFTLGFRKSIRYASAKLSKGL
FARKK
>comp6013_c0_seq1; 443 403 2127 LEN=2173; translated
MSSKLGRKGDPRMHRAVAARMANPNLSLYEALCIGGFDYSENDDKSVLDSKQVTLGQRKN
QLSRRLRLARKSNSGGGKNPPRSPSAVGNNGGATSTSTTENRNITNIVSSKEPQPRENNA
FSKAKDHHSTIYRQKVDFKNELATGSFNGNNKSIGIGDAVGDHQAKRTRFTCPPAPVTQP
KLNWNHSLHQGWMYVTDPYMNSQVTTSQSVSQYQPGASAVALSSLTSTAQAVGLTLEQLA
LTLSSNTTSLTKLVAEARSGESETKQELMALNLYQNDIKPLYTKCLLVAGVDASLAEQNT
PTYVKFATKAWEFEGERLQTLRNAMNIGKETKHTVAFAESSFQQNYTKSSSRTPTTTNSK
ANKDNSNNDNNTITSSDWNSTNRIECNQDNCQADKEHNQHSHEHEHSHTTTKNANNDSDR
HDSSECESRHFHRVGECGHKAVIHKPKDGPPHIDFIVNDQIECYQGQDSMSLAGTSLAGR
SEKSLDSYWPSKLKCKEVDEAGPKSCAKSVGAQFDSNWGHLEPLVQEPKTFKLSEISAQD
PEWTFDATDGAGIDNVAMGLFELGREVSSDVANC
>comp6013_c0_seq2; 438 159 1823 LEN=1869; translated
MANPNLSLYEALCIGGFDYSENDDKSVLDSKQVTLGQRKNQLSRRLRLARKSNSGGGKNP
PRSPSAVGNNGGATSTSTTENRNITNIVSSKEPQPRENNAFSKAKDHHSTIYRQKVDFKN
ELATGSFNGNNKSIGIGDAVGDHQAKRTRFTCPPAPVTQPKLNWNHSLHQGWMYVTDPYM
NSQVTTSQSVSQYQPGASAVALSSLTSTAQAVGLTLEQLALTLSSNTTSLTKLVAEARSG
ESETKQELMALNLYQNDIKPLYTKCLLVAGVDASLAEQNTPTYVKFATKAWEFEGERLQT
LRNAMNIGKETKHTVAFAESSFQQNYTKSSSRTPTTTNSKANKDNSNNDNNTITSSDWNS
TNRIECNQDNCQADKEHNQHSHEHEHSHTTTKNANNDSDRHDSSECESRHFHRVGECGHK
AVIHKPKDGPPHIDFIVNDQIECYQGQDSMSLAGTSLAGRSEKSLDSYWPSKLKCKEVDE
AGPKSCAKSVGAQFDSNWGHLEPLVQEPKTFKLSEISAQDPEWTFDATDGAGIDNVAMGL
FELGREVSSDVANC
>comp6013_c0_seq3; 443 215 1939 LEN=1985; translated
MSSKLGRKGDPRMHRAVAARMANPNLSLYEALCIGGFDYSENDDKSVLDSKQVTLGQRKN
QLSRRLRLARKSNSGGGKNPPRSPSAVGNNGGATSTSTTENRNITNIVSSKEPQPRENNA
FSKAKDHHSTIYRQKVDFKNELATGSFNGNNKSIGIGDAVGDHQAKRTRFTCPPAPVTQP
KLNWNHSLHQGWMYVTDPYMNSQVTTSQSVSQYQPGASAVALSSLTSTAQAVGLTLEQLA
LTLSSNTTSLTKLVAEARSGESETKQELMALNLYQNDIKPLYTKCLLVAGVDASLAEQNT
PTYVKFATKAWEFEGERLQTLRNAMNIGKETKHTVAFAESSFQQNYTKSSSRTPTTTNSK
ANKDNSNNDNNTITSSDWNSTNRIECNQDNCQADKEHNQHSHEHEHSHTTTKNANNDSDR
HDSSECESRHFHRVGECGHKAVIHKPKDGPPHIDFIVNDQIECYQGQDSMSLAGTSLAGR
SEKSLDSYWPSKLKCKEVDEAGPKSCAKSVGAQFDSNWGHLEPLVQEPKTFKLSEISAQD

PEWTFDATDGAGIDNVAMGLFELGREVSSDVANC
>comp6014_c0_seq1; 250 1 1001 LEN=1337; translated
QVHRSGITSELKDWKKGHRVRPYVLLNSQILRLGFALVVYYRLNRKNSTVVRVSWVAHKS
STIEPRAEAGTALEEEGGVAAMKEVQATVDAMAPTRDTAIIGNNSIMXHPISSGNVSNSK
NSGGDSNPKGKDYENEYELLLAGRDTFLRSKDHQNSSAFEEEEEIYQCAALGASICIPSM
AATLNSMSVSHRLRMPLHVVASAFPSRFKADQVRDEGQKEGEEEVHADSENGDASVGKAV
VVEDDADDSSANLEDWLDDACGNTQEPQPQTSIPHRTVSPKASDGSIHNSDNGDDVSPSA
ANLGNEKTAEAAVDNKIEDGGDGDLDDWLDSVI
>comp6015_c0_seq1; 443 152 2292 LEN=2533; translated
MPANDGGLGTALLGEKMVMGNLNLTITKLLGEGGFSYVYLAKNSDGGGLLGSQNTNNGRA
VSPHRGGSNSESNDEIPPGPLVLKVTSIHSRSQRDIAEKEAKLLSRLSHPSIVKMYVASY
RTVTNQHSGKRGKESMHSGKERPQHCILMEFCEGGHALDVCNKLRMEGKRFDRSTLIIAF
GQICNAVSYLHAQRPPIVHRDLKPVNFLVKNGAYKLCDFGSAVFGHVDLRTQQARVEADE
VVQKTTTQMFRAPEMVDLYVAKXIDAIVSTVPCFDIIFIQFMMSSNATSGQFCDDASNKW
NNFYPAPTFGLWGAALYSMAFLQNCFEEGSNLAILSRNYTIPDDNPYGDGLVELIDRMLT
VDYKARADMTEVILCLSAVYSGRPLPPRKKSTRSSKSDEKEQNKNPEETTKKDTQNDSRV
GKFRTDSQGYRDENIYDPTKAKEGKKLASNSVAARRKRAAASSAAVSAGQHSPLSDPLTF
SSIQQSGAELDTDAAFSSFGDLDRKXLISLTSVAILMPNITEDSXALDLITVSHIILTRQ
TGFENSKADNYFFEAFDGVKGIQQGSGANDGFDPDVFGTTGWGDSIQDNMENFQRMKLDA
IDGDKSKEDNAQRVKSRRKSGMKFRSNRDIDAKNPGTDGEKNERRSRKEGDQKGEDDKEY
RQRRRSSSRSRRKGRTPRQGSGKAFDEKEGFSGDSEKFSKLGSFRAVFNRKQGT
>comp6015_c0_seq2; 543 152 2065 LEN=2306; translated
MPANDGGLGTALLGEKMVMGNLNLTITKLLGEGGFSYVYLAKNSDGGGLLGSQNTNNGRA
VSPHRGGSNSESNDEIPPGPLVLKVTSIHSRSQRDIAEKEAKLLSRLSHPSIVKMYVASY
RTVTNQHSGKRGKESMHSGKERPQHCILMEFCEGGHALDVCNKLRMEGKRFDRSTLIIAF
GQICNAVSYLHAQRPPIVHRDLKPVNFLVKNGAYKLCDFGSAVFGHVDLRTQQARVEADE
VVQKTTTQMFRAPEMVDLYVAKKLTQSTDVWALGCCLYSMAFLQNCFEEGSNLAILSRNY
TIPDDNPYGDGLVELIDRMLTVDYKARADMTEVILCLSAVYSGRPLPPRKKSTRSSKSDE
KEQNKNPEETTKKDTQNDSRVGKFRTDSQGYRDENIYDPTKAKEGKKLASNSVAARRKRA
AASSAAVSAGQHSPLSDPLTFSSIQQSGAELDTDAAFSSFGDLDRFENSKADNYFFEAFD
GVKGIQQGSGANDGFDPDVFGTTGWGDSIQDNMENFQRMKLDAIDGDKSKEDNAQRVKSR
RKSGMKFRSNRDIDAKNPGTDGEKNERRSRKEGDQKGEDDKEYRQRRRSSSRSRRKGRTP
RQGSGKAFDEKEGFSGDSEKFSKLGSFRAVFNRKQGT
>comp6015_c0_seq3; 488 152 2180 LEN=2421; translated
MPANDGGLGTALLGEKMVMGNLNLTITKLLGEGGFSYVYLAKNSDGGGLLGSQNTNNGRA
VSPHRGGSNSESNDEIPPGPLVLKVTSIHSRSQRDIAEKEAKLLSRLSHPSIVKMYVASY
RTVTNQHSGKRGKESMHSGKERPQHCILMEFCEGGHALDVCNKLRMEGKRFDRSTLIIAF
GQICNAVSYLHAQRPPIVHRDLKPVNFLVKNGAYKLCDFGSAVFGHVDLRTQQARVEADE
VVQKTTTQMFRAPEMVDLYVAKXIDAIVSTVPCFDIIFIQFMMSSNATSGQFCDDASNKW
NNFYPAPTFGLWGAALYSMAFLQNCFEEGSNLAILSRNYTIPDDNPYGDGLVELIDRMLT
VDYKARADMTEVILCLSAVYSGRPLPPRKKSTRSSKSDEKEQNKNPEETTKKDTQNDSRV
GKFRTDSQGYRDENIYDPTKAKEGKKLASNSVAARRKRAAASSAAVSAGQHSPLSDPLTF
SSIQQSGAELDTDAAFSSFGDLDRFENSKADNYFFEAFDGVKGIQQGSGANDGFDPDVFG
TTGWGDSIQDNMENFQRMKLDAIDGDKSKEDNAQRVKSRRKSGMKFRSNRDIDAKNPGTD
GEKNERRSRKEGDQKGEDDKEYRQRRRSSSRSRRKGRTPRQGSGKAFDEKEGFSGDSEKF
SKLGSFRAVFNRKQGT
>comp6015_c0_seq4; 498 152 2177 LEN=2418; translated
MPANDGGLGTALLGEKMVMGNLNLTITKLLGEGGFSYVYLAKNSDGGGLLGSQNTNNGRA
VSPHRGGSNSESNDEIPPGPLVLKVTSIHSRSQRDIAEKEAKLLSRLSHPSIVKMYVASY
RTVTNQHSGKRGKESMHSGKERPQHCILMEFCEGGHALDVCNKLRMEGKRFDRSTLIIAF
GQICNAVSYLHAQRPPIVHRDLKPVNFLVKNGAYKLCDFGSAVFGHVDLRTQQARVEADE
VVQKTTTQMFRAPEMVDLYVAKKLTQSTDVWALGCCLYSMAFLQNCFEEGSNLAILSRNY
TIPDDNPYGDGLVELIDRMLTVDYKARADMTEVILCLSAVYSGRPLPPRKKSTRSSKSDE
KEQNKNPEETTKKDTQNDSRVGKFRTDSQGYRDENIYDPTKAKEGKKLASNSVAARRKRA
AASSAAVSAGQHSPLSDPLTFSSIQQSGAELDTDAAFSSFGDLDRKXLISLTSVAILMPN
ITEDSXALDLITVSHIILTRQTGFENSKADNYFFEAFDGVKGIQQGSGANDGFDPDVFGT
TGWGDSIQDNMENFQRMKLDAIDGDKSKEDNAQRVKSRRKSGMKFRSNRDIDAKNPGTDG
EKNERRSRKEGDQKGEDDKEYRQRRRSSSRSRRKGRTPRQGSGKAFDEKEGFSGDSEKFS
KLGSFRAVFNRKQGT
>comp6016_c0_seq1; 454 201 1655 LEN=2114; translated

MKNSSLAFQRRRRVAIALVPPFVSLLCMDAIPASSAFLQSTKQIHHSSSLLYSSSLILSK
ESEISPKTCNDGSSDCLVSSVILDSESSPKEPEQRRYDQSKHSHNPINGWLKDQGLPRGL
RHALLDNIQEVSSKRIWIVDNSGSMKMMDGHENLESRDRDGRDEYKDVNKEDDKILVPRI
GVVSNDFADGDISRWTELRQTVNYHATLNSVLGAPTEFRFLNKPSNGGPRNFRVGYENQH
IHRQSNNNENKIRIFGGGGKNRNVGRKRDSWRAHKIMNRNQPKGQTPLHEAVLDVKRDII
KMRPQLLADGMRVTLVICTDGCSEDMETVLESLKGLPVNVVIRLCTDFGNVLDFYNALDE
RHDDPSGSFLDVLDDHEGEAAEVYRHNPWLNYALVLHQMRELGLQGSNGLLDVLDQRALS
IDEIRDFCALLFGTTADLLPDPLCDWENFVTEVDHLQQREQCHWNPSLGEVTPWVDVDEL
LAME
>comp6017_c0_seq1; 776 183 1604 LEN=1903; translated
MSESKPLIPNDDDAEMLRRLKAYQPMAFFVTFGGYFMSHFSRKSYSTVKQQLELKAGYTA
SILGAMDTVFMLTYGAGNVINGKLGDTFDPVTILAIGLWGSGACLLVMTAAVLADLVSVN
VLLANAVILGTLFLFGFFQATGGPVGTAVMGNWFCDKDAVKNRGSIFGFWTCHQYVGDVS
AGLVTAWILGSGAHYTWALFVPAVANILFAFITIQLVADPYNMGIITPEVKIRQAKFEAK
KKEMAEKGETIEADTGLQAISFVDAFRIPMVANYAFAFGFFKLTNYCLFFWLPYFLGKHF
DPVEANLIASLYSVGMMPGGIIVGYVSDIFGGRRALVIGVFMGLLIVFLSIFAKFSEAPD
LNPTVLLIMLFCMGILVGGPNNIITSAVAADLASHPSVRGSSKSLGTVTGLINGCGSFTS
AVGLLAIGPLQTAFGWSAVWIYLIGCTAAGTSLMGSKIYAEVFPSNEVRSTDV
>comp6018_c0_seq1; 455 1 878 LEN=1292; translated
XQQQQQQQHHHHHHQQRQHHQRQDQQQRLLLTLQRYEPQGKQQTQFAQPSLSLHQSTQMY
PNSTHYEPVAPVESSSNDDVLLGSGPPVTPDCSYHQSPKSTALYIHSAPPSDFNLSFSTT
EFQVDYGVKGDDSKFSTLKAPTPIEGDGTCVTTSAPSFSAAIATNAYQTGELTSSDVLAT
GGYSAMLHLTTAKSLEPSQKKHTVAPTSFSHSNNQRQQQYHEQEYKLHQQQQYQPPEQQQ
QYYQQEQQQQHCEKDDFGVLPLTTLSRIESGTVDSHLRKGEGMEEWDLDLLL
>comp6019_c0_seq1; 423 265 1311 LEN=1460; translated
MGRVAKYKKVKSFDPYSKKNRGNVDLSAVGVWGLGDNGRKKKKRSLKAQKLHAKKHRNSS
KKKGTGGFDDDGFDVPPDEEDEFTMADLVGSIKKKSMKTNEELLGDSSSSLATTTSSFLG
ASSSVSNDKTEPKNNKTADESYDTIVTSSGNVANIPKTDQDEKKIERLLRVEEQMEQKSK
LEKQQTQGRMEGESKRAYAKRTRAETREIIKQSTTTVRNLEKLQKKKEFLKNKKNKKRKG
GAAYYDDEVDYDKRRSTRTAIKANQEPEAPVRFNEQAERPPIFRQIPRGAKSKKPKTATS
MASLSKTKGMTDHQIEGEKDAMELMRRRIQAQYKAIKQKRRDTGDFHL
>comp6020_c0_seq1; 257 78 953 LEN=1245; translated
MKHRQLYLYLLFCAVVPRSVSGHFFWAETDQDADHLTVAFSEHAGVPDKVVERFADRVET
MAYHNGDTTKNVALALNEENNLLEGNLPGLYPPGPAFVSGYLDNGPYERFKDVQFSFGAQ
IYDSEADYDGFFRPLLRDVGYVPTIVMRNCGGSPGKGSSYQFDVAGLPSQGPFGVCLYRK
GGILLDCGTFSPPQVQELRGEDAHGATPTSVDRFRRLRGRDVANGDRGNSYTMNLSMDSM
SIESLDEPSLASPNLIYALANQTVVDDASGDISIYIASTSVYFQGPCQADF
>comp6021_c0_seq1; 109 1 208 LEN=1351; translated
XRAEQHDRERGEKQTVSSTKANLKDVNVYDTVEYDVRKEQHMREKEWKETGKQNKKNNTN
FDMARALFN
>comp6022_c0_seq1; 222 158 403 LEN=1115; translated
MTNGQRMLRVRFGAAVLLAATRASTHATIAVMIHDRRDVAIPSANTSSGSASVFVPRHSN
PISGWNNSTEESPVSMYLYYQ
>comp6023_c0_seq1; 760 1 2875 LEN=2991; translated
XKEQQQRERERRVLKNKLLRARDSILYFGEGSENEPGSGPSEELKSDILPTLTRSYSSVI
LRAPSPSSSSLDLFDPWDIRDVILQESRPVQERFLGLLAVLMQVTTHIQEEYTVYNNTAA
TRKAKQKSVAEITDLIRAVQSQLWRTCRLILEDMLAKTTNSAETMNQVTHVLSPIPITAL
ATSLRALSAVSGENGNVYAGSGVEEIKELIQLVNEARIAGSETHTHESIDDDKLDRALLT
AWNCYLSSLCDRALITNNPYDSGLASAAALLRMKSNETGLIDPDITSYNTVMGNAAKLGN
ATLVDVLWEDLLVKRGELPEDRRLSLQPNSRTYNARLMVTNDWAKRLEILDNEIHSDTHD
NTANGATAPTEMTNIDGFTIDLLLIPLLQAHRKKELFKLIDDWMITVDRHHHKPPRKGEA
TDEKQRKQNKNKAQRAFQNAMSAFFITLVQRNGDVCTARELWNRYMLVEIDNNKEVSNSG
DDSGEETASCSNATNIVFPERRHYNILLDGYARLVDEVRDRQGPNLDIAVKKIGFSKKKT
STNSKKIWQPNGDAGSDLDVVSNSKFYREVQEKAISDGQNLFAIMMAQAQQQNPRNKVGS
DVYTKSTMIRLCRSGREVRNLLEQASDATSARAGRSPSDKTWLPRSVVRAAITTCGRLGD
PSMACEIFEDFMFPENDSDRRKFFSNYRAFNNLLGALANGAKMENPKLNVMPTEFEGGTR
PQTLLGHVHGLTCTEAVVCILDLMTSKNAQTYCVAASALQYAPLDEQSRYTPSSDGVDEK
GTNTPLGLQIFHNATKNGIHADGRLVNAIFRCYGDDIAGALDGWKTHIRRATDQYENRTR
LSTRKNMLAAYNGLLYVCGRAERPDIAVRIVYAMKKEGLEINENPYNCYRSGKTTRESLL
SSRPDEQEDVSKEGKWRRMRLPKLGMVGQYENILYVECKKYDTRDRRTQNDKRVRIIV

>comp6025_c0_seq1; 631 181 2391 LEN=2431; translated
MSSLSDNSVCSTKTSQAWAILRRHARDEISNLRLQELCRDNDRVSSLVSVYNATTPSANS
MLMIDLSRQRMTLETLNHMLCLGTARGLPKFIRQLSWGQNDPENPIVPTRFLPDQDNPYA
DGSTPNAKNKSNRHRHGERDRRNIEQNQKQNDCSRCSIPSYHMALRAPSGRNLEMLTRDG
LNVLVGIHSDWNRIKRISESLRRGKLPGVTGGMIKDVVVVGHGLTAMALRFVYTALCEDE
AATIGRRLGMDGRNPKKGSELGHRRMKFLTTVDPVQAAAVVTDLDPSATLVISVAMTGKE
NRDTTMLATQTLQSWLLSKLGGHGHRPEFILSKHCMLVTSNEDLASKHKPESVFLIPDHS
RCEPFTTCTAATLLPLSIVFGWPAVEAFLSGAHDIDKHFVETNPRHNLPVLLALTDVWND
CLLSPNNNNRSAPYSANSGRILTPYTEAFVAYPAFVAALEAQVCGRKRKERNNNSSWMSS
SSMSSSLNEMHPQSMSMPPPATVQSSACSSLVIDGGLHGIYDHSLYQSPKIIPSELVMTL
DSQLATNGCLLGEGENKKVFEAQDTLVCSLFAHADGLAFGFDQSNDDHYADDKSPVGTGF
GGDESNTFVDEQEPAEHNRSGGNRPSTLLLCGRLDAFTCGQLVAMAEHRAVIKAWIWEID
PFPVNREGSSTTLPRSKRTDLLKERLGKMMSDGYSEEDDEDGDDIEHAPGLNLSTKTILR
HYANTMKGQRRSAMNN
>comp6027_c0_seq2; 209 160 540 LEN=742; translated
MSGTNDPLDVVQQLIDKNKDSFGQMGFGGFMGYCSGMAFRKAGRAVGVVIGLGFMGLQTA
KSMGYIDVDWTKIKDDAIKPLDTNADGKLDGQDLEVWWKKSQAIMKDSVPEAGGFSAGFL
LGARRG
>comp6027_c0_seq3; 178 61 279 LEN=481; translated
MGLQTAKSMGYIDVDWTKIKDDAIKPLDTNADGKLDGQDLEVWWKKSQAIMKDSVPEAGG
FSAGFLLGARRG
>comp6028_c0_seq1; 496 1 2694 LEN=2859; translated
XSSSSSKAEPSSSTASTSSSSPYSSMVTILGQTTGMSPVQAERAVGWSTITGTGLVAAKL
ALYAYRSLKGEGRIEDEISDCTTEVSKEGKVPKDVVTKPQQRRTASYSILGIFRRTLRRM
LLDESEYIARAPPPTAEEDAIAEQSEGPLVTYQGSCHCESIQFTALAPRELKAQDGPGKI
QFRHTQVRASNFRIYAGYEHLKTYYVFYHSSGNKGAHAFCQRCGVHVLYAPSKSTPFLSI
NTRCLRDEDNRKIKLTSKKDGISEGIPVAGQFDNTNNVNFSDQLSTISEVTQPFHFQMNH
SNNANAQYKNPVTDLRRRPSDISSMESSIISGDGAIEVFYPTPYDRKNRPKQQLQRPMTP
TTSSVTTGGEADSSGSLYKGHLNQLQMICPTSPPRSGGASIGGIDEFSFGESVSLMDDNL
SVASPKTATISIRRNQHTLRVKAGGENQSLHSNHPPRHPTVTSPETRNKMKFFMSKYKKQ
PSSGNKMRERTEIVYVGRKKPKTLAFSAMGFVSHYVKAIQNRDVPRNTTEQLFEMYNRNN
TTARRITPLELLDRHLFQHLEXFVLELVKSTTFFEKTEGFLKSERMLRSMRFSVKKXTEA
SKEISSVRSLPSPSMDDKSGTDFTFENKNDLVGAALAAIRLIGGRIRGMKGKGKAERKKT
TEATPDAALRGFGSGAMQGIAQPYXNEANNCLTSTLDVNASTTRGQSFAFITSNMLHCHE
MPPLKEATVVTTPSPCGMPMVHWEVYLKTSPTLSPPAPSAVHMARLFFGSTFSPKGSLYR
TSTSSLGPEKTEGNLSLATTLELEGGMLSSISSRSLSVSSAAANSGAETVICSRATGAPG
DGDTKARAVDDSDATRRVKTSVANWFMVKNYCLRMFVSRTEGINGFVATESVLDQRR
>comp6029_c0_seq1; 301 1 857 LEN=1016; translated
XLHGNKIFVKTKQTTPNTLLLWHPSKRKTMRKMNSDDDDHQISPTHTPGPTMDAEKDTNI
RSERPGMRRTNASKPLTHIHFDSSDDVVATIDMPREEFTERWYTNSEYYVFRREATRACK
KRQRVKQKDKLKDDNVSCYTGLDMVCDEALEARKLLVEKALDTVLRTQEVHGKTDGPSAR
TSGESHGSNRRSNNDNDNDINNDNEGRPPETKPPMDEAIARKYIEISCDTKARALKAAKV
NENEAKEYCRALRADLVDIGVIARTKTTSLDLSRALHRLLRLKSE
>comp6031_c0_seq1; 319 223 861 LEN=927; translated
MSALNLKTAASLMVHLLLVPTTVEGFQHYHRVSICHLASSSSTAERGNTVLFNTQGDDND
DDDWSSSLPLSKSPTPFSQQLEQGKFNPLDYRGKSKTNRSSGSAQAQVNLRSMRMSSMTD
DLLNSLGNKAAMRAVLEENRDFLLEPLESEETLAGSGSIYTPDMSRSERYQAYRKNVNKR
LESSNNAKAKAVLTAMRDFVLEFENEGPLQNE
>comp6031_c0_seq2; 248 223 693 LEN=1450; translated
MSALNLKTAASLMVHLLLVPTTVEGFQHYHRVSICHLASSSSTAERGNTVLFNTQGDDND
DDDWSSSLPLSKSPTPFSQQLEQGKFNPLDYRGKSKTNRSSGSAQAQVNLRSMRMSSMTD
DLLNSLGNKAAMRAVLEENRDFLLEPLESEETLAVR
>comp6032_c0_seq1; 158 124 431 LEN=431; translated
MNQGQSVPAAQPGARHVSQNVVDTSRLPQPLAKVAPSVDDDAQSLGVIAAPAADETQAHT
QTQTQSQTHQSSEIGTNNTTNSVAPAHIPVVEETTNSSSSSSX
>comp6032_c0_seq2; 149 124 362 LEN=362; translated
MNQGQSVPAAQPGARHVSQNVVDTSRLPQPLAKVAPSVDDDAQSLGVIAAPAADETQAHT
QTQTQPVVEETTNSSSSSSX
>comp6033_c0_seq1; 477 1 2297 LEN=2411; translated
ERSTHWTIIHPTYSCCIRIVLFQLLIVITAELHNXEKTVKYEVASSLFISLLYRANANDS

SASLRGSAGIQRRALRVQHPCTLVQIESEYADELGEAELKYGSRERMYNGSGHSNRRELH
DEDYEVKCELQAEDRAAVGTYFVPINGIERGQLDNIISGDTTLIAEGAMIMDGEMWLPAG
AAIEFGSNGRRKLQQASRTKGTKKVLVVRANAQDTVTSSSKEILSDKIFGTGNDLANLSS
QYSACSHGQLEFEPFRGMTEGGFYVDNGVIDVNINMNAIDTSRFVVEDALEEAADKIVGS
LSDQFDHVMLCLPPGSRVGSTNKNWVAYAYVNSWLSVFNDSFCESLTTQVHEIGHNLGLA
HSGKDGVSYGDKSGMMGFSYKLDDGPLQCFNPAKSYQLGWYDEKVVEIDPNDGTWVGSLI
GVADYENASVSQNSKVVVKILSPDNKNLFVGYNRKKDMNSGVLMAANRITVVEQAPNYSA
SDFLANISIDEPTHTFFNFGGSNKNLVVEFVKRQSFFNEVTVAIYYDTCVFPSCCNGSTC
NDPNFWTPTDAPIPAPTLAPQPAPTAAPVPAPTFAPQPVPTPVVSPTRLPTTPTPVPTRG
RLNVLTGGQINRPKPLLTEIFKDGLGAFHGGGNKVEQNEFQYILTAKFQMKDENKLPYIE
TDLDLDGSNVVQVNFWYIIEKMDFTDGFKLQYSTSHGDSWTDFKEYRYGINIDEKQRWNF
VNAASFEVEKGTSTTKVRIVGDTTSKNGSNAVFYVAGVSIYGIEK
>comp6034_c0_seq1; 513 1 2635 LEN=2779; translated
XQSSKAKASLSSSDFGVHRSGSYVFLPMVVRITGKMMCGRWQLHSCPKRIAAGIGRRRST
LRELHSVRSPSRIRPRPRSLLEELTTVQSFARHNTGNRTAATVTDAPSHERGIRNRNSLG
LRRDENRVLIVGGGPTGLFLAHLLRSYNVPFRLVEAQTPEGRFQHPQAHFLNTRTMEILK
HGLSYCNDHNGNPNEPNDIYRRLRGAMPPVEEWKSFRFGPDMTCNTLDGKILAEVVHPVD
RPLAATEDANGRLVPESAAGDHRTPSTARERGSGTALSPESVGHLAQHTFCRILYDALVD
SPDDEVMYERRATGFDWNEETRLWNVQTDRGDLFDDVDVIVAADGAASSIRKNLFPGDGT
RGGNDSTMLGTHTLQRLINVHFTVSEETTSIPPAMLYTIFNTEVLAMCVRHGPGEFVLQI
PYFEPYQTPEDDFGIESVRKMIGSILGNSNGFTIHSIRPWTMGSLVARKYHTDNGVFLAG
DAAHVFPPAGGFGMNTGLQDVYSLAWQIAFNRQTKQNPKDRTSNNEDGNSTFSSLSEIGR
TYERERQPVARRNAALSVRNYNRVLNVMRACYLDDRHPAFLIQALDATASFVPLEARRQT
FRTLLKTALSPLSQLESSPDGIYAQTIKGNLRSLLGSGQGLPLLFPRYELDFSYGAPGEH
GTSVDGEDEWSQDSVASFPRLVVGGLFPHIVASVEPDTLLSFPRLQPIDNGEDNANGRNR
FIVTGTTADELRTISTRDLPAQLATDETPCAFCIVEIVSSTGVKPDDTLPSLQKSLQGKL
GIPILMSRLTVYSNSDGDQATLNGASQIDVIDTSICDMHMDAHQWEDLNLIPGLSEPNSS
FKMFVVVRPDGHVAAISSEVPHLENLVLGMKAFLQYDK
>comp6036_c0_seq1; 842 292 2745 LEN=2844; translated
MATCNGCGESFPSRNAIFRHLKETNGACLSKEDYKQFVKYARKTTKAPKVIIMYGYLPYD
GSSCSKSIMIRNGEDAGHILMETINELENEINGFSDDEDDNGDGENPNSSSSINNINRSY
GYSARSAECVKQDEDTAAITEVMTARLYPLHKDMSMENWLDLVQKKLDDKFDEGHDNTMD
DGNSFRVTPVRIIGRQDMPNLRFNAEMDITYRRVEYVLPVDFFSWSPRNAEFKEKIHETP
IFKENPKHDIDHVKETTNPLDPATKAFMMELKNFFKLLTTRVVKLDVTDEASVLEKGFSA
KKLMKRKRDKKQRDAEDNRDKCAGCSTSTNAQQKGLEGASSSVDFKDSRPTKKVKPTKKP
KKTNVLKRKRYHNFTEKLMAHEYLTYRRLDRIYHRSTLKFPMESESKMKTTSYMILSMSG
DMFLTGQTSRLIGVIVALGNGLIDREFIDCVFDENYPHLVPTPPAPHFGVVSSEVQYANQ
EGKTRRILSARMSDRYTEGWNNEATIRRVKDWQNEVYKYVDSKWKKDGRDEQGRLRSEQE
WTEKVLLPWAEKAKEHLEDYKRWKKKQHQSSHDLSESSAKHDSHTESSYAPESSRIPQIE
PIDPTVPEVYKDVLHHLRKLDASGEWPMTSSKRQLVMVSSSDGVKENGKGTESLALACLK
AKRNELTGSSAYSFAEGQGGASGSFSVGIMPGGDKKQPKANSMFPDLVKAAFLLETKLFP
DREPSSTIAVNRNAQFRPHTDSGAGAGQSTSLIVGLGTYGGGELMVEGDKHDIRYKAIEF
DGWKQRHWTMPFKGERYSLVWFTPKGCEGMKGIELDL
>comp6036_c0_seq2; 842 457 2910 LEN=3009; translated
MATCNGCGESFPSRNAIFRHLKETNGACLSKEDYKQFVKYARKTTKAPKVIIMYGYLPYD
GSSCSKSIMIRNGEDAGHILMETINELENEINGFSDDEDDNGDGENPNSSSSINNINRSY
GYSARSAECVKQDEDTAAITEVMTARLYPLHKDMSMENWLDLVQKKLDDKFDEGHDNTMD
DGNSFRVTPVRIIGRQDMPNLRFNAEMDITYRRVEYVLPVDFFSWSPRNAEFKEKIHETP
IFKENPKHDIDHVKETTNPLDPATKAFMMELKNFFKLLTTRVVKLDVTDEASVLEKGFSA
KKLMKRKRDKKQRDAEDNRDKCAGCSTSTNAQQKGLEGASSSVDFKDSRPTKKVKPTKKP
KKTNVLKRKRYHNFTEKLMAHEYLTYRRLDRIYHRSTLKFPMESESKMKTTSYMILSMSG
DMFLTGQTSRLIGVIVALGNGLIDREFIDCVFDENYPHLVPTPPAPHFGVVSSEVQYANQ
EGKTRRILSARMSDRYTEGWNNEATIRRVKDWQNEVYKYVDSKWKKDGRDEQGRLRSEQE
WTEKVLLPWAEKAKEHLEDYKRWKKKQHQSSHDLSESSAKHDSHTESSYAPESSRIPQIE
PIDPTVPEVYKDVLHHLRKLDASGEWPMTSSKRQLVMVSSSDGVKENGKGTESLALACLK
AKRNELTGSSAYSFAEGQGGASGSFSVGIMPGGDKKQPKANSMFPDLVKAAFLLETKLFP
DREPSSTIAVNRNAQFRPHTDSGAGAGQSTSLIVGLGTYGGGELMVEGDKHDIRYKAIEF
DGWKQRHWTMPFKGERYSLVWFTPKGCEGMKGIELDL
>comp6037_c0_seq1; 50 1 498 LEN=691; translated
AKAANGGGLPSGVPESPHGFPSDGPWHASCHMSVVLPDTACPDALAAALEVIEANVDTGT

EFKGTMSVFDRGSDWIWSSRLTYDRRYTDDQLFGFAGAPGDPSSCFLAMRSRSRSRSVLD
YGVNYCNMWNVVSRLPGFEGAGAPLVSECYSRNVPSDPVATCARY
>comp6037_c1_seq1; 156 1 514 LEN=514; translated
EWETTSNTKMQQLRYVTYACHRHADYSNPSILAIQRTVQRDLATKVAERRPTNHSSTTKS
VQQEPQQTATGTAAPNMLQQHRSSYYDDQLGTARVAAATVDAPDAKASPSAPGRARSAVL
VFLAAAWAIAVAVAVVVAPRSLRPGNSKGNAKGNSRSGVREATAKAANGGG
>comp6039_c0_seq1; 482 279 1835 LEN=1988; translated
MRVAAICLSALLAVMIPTRSSLALATAKSSGVFGVPHGGSTATTTRASTQIASAATMDAS
TEVPSTDDRRPMHILFLSADTGGGHRASAEALAKQFLIQFPGSTYELVDIWSDHGIFPYT
NHVELYKQWSAKPWQWRLFYHLSNTKVNEVVSNLHSRFLCGDSIRERLKSDKPDVIVSVH
PAMNHTPLKSTHKNSKELGKHIPFFTVVTDLGSAHCMWFERKVDKLYVASDRLYKLARRR
GGTAPEDIVRTGLPIRHGFADQAEKLGQDRSAPQGIQYQAQVRKELGIAPDKQMVLVMGG
GEGVGSLSDIVNNIYAELSSKGVDATICVVCGRNEKLKNELATRNFDEVIEEIKAKAEKR
RNGVRGVLGKVRRKNRIAARQAAEASTATMSQKGNIDVMPLGFLTNIPEYMVAADVLVSK
AGPGTIAEAAAVGLPVMMTSFLPGQEAGNVDVVLEQNFGEFEKRPEKIGERVTAWLQDKT
LLHTMSQAAMVAGNPYAANEIVEDIGRQTVAWMNLNEK
>comp6040_c0_seq1; 282 148 529 LEN=529; translated
MDPSASEARGNNANRGSEDGANSSSNNDDSNGTTFRASRPTPLTPYSFIDPLGLGMDSPY
ILPNPQMAANYFAFMNGLNGSAASPATAAAGAGTAPAPASSKRVTVHIVGTNHKNKNNNR
TSNSNSNX
>comp6041_c0_seq1; 344 614 1609 LEN=1697; translated
MGSKKDQDTTTIGTNESLVVLHETLSRALVTPNSSSNNLHEKALQEKQEETQRKALSPRP
LKKRKISIQGTMATQVSATQVSEAVDAGEDEALIRRKQLAAKLRAKGVCSKLVLKPSSFA
RTIFVKNTRQYSSSPIIELPPFYDPTPADVDIHQNHSHKLYNYVRKGTDMEGFKEFVREL
RRKYNSSNTSMQGNQPFRCCNKFGESLIHLTCRRGRTEMTRFLVEELVENNAVAARQTLT
VRDDCLKTPLHDACWTPTPNFELVELILKHCPEQVLMADIRGNTPFDYIRKEDTNLWLKF
LWQRKHLLRGISTSTTMTRDATPSNVAIPSN
>comp6042_c0_seq1; 116 1 694 LEN=1055; minus strand; translated
XVTTDAIKGNGAVESPGEQICNLAVKCAPVAQGVAMWAREITRGGEFVSSASYVSISPSI
MTLARIVYMHHPFTRDDVVEVAFQFLKHSNSEVVYHTMNTLKEQSLRLLLFLCTRGEAPV
VFSQLMPLLKDPGRSGLDASLIRYFVSGLLEIASPPFSIPFVRSLMILLKAPGSVDALKT
SYFDKDSKSRLKSVMNYLRTRLSGKLHTRPLAKEDISLINSVLSIYSPTSG
>comp6043_c0_seq1; 324 339 1604 LEN=2380; translated
MPAKQELDVFIHDMLAMSAFCYHFHSIRTIVRSKDNTTVKTGFLGFGKPREIKFFRPELI
ITRNSGIERDPNQQLLHDITPAAIQEFIENNRSLFQDDDMDGSLEFDPSDTKKDEPFLVE
GVLKEQMEDNLKIIDYDDRFSTDKGGLVYMIVTNLTQKTITVCFRGTIGTTDMHTDRDFR
LNTEAFFGSEDDFSVPKSESHNPATHNGFTTYLTSNRETDSIERPYAQRILACLNAEFGN
NKDVKGKGFKLFVTGHSLGGGLANLFAFRAAQLKARGDKAVEHLPDMIKALTFAAPCVGN
EGYEAEFQALEKKGFLRHIRVANEEDVVPTNCISLPFSLAIKGDTSLYRQNGVNLQFHPD
KTCDIVYGNTKSMLSQFSPTRSLNTHYLTEYRVRVGKPENEKVYDQTIEDIYKEATNNFA
A
>comp6044_c0_seq1; 86 232 615 LEN=1684; translated
MAGRHLGAFSRTLPAKMAVGPFARLIAQAIIPVVAVLARAIPAAYGQALNNAKKAGVDAS
EAAAPVFGKTISRSEALMVLNLTEDEVKADPKAVQKVSIDHRSAARRCVFSFVALVVVGI
AVQFDRVW
>comp6044_c0_seq2; 130 232 675 LEN=1237; translated
MAGRHLGAFSRTLPAKMAVGPFARLIAQAIIPVVAVLARAIPAAYGQALNNAKKAGVDAS
EAAAPVFGKTISRSEALMVLNLTEDEVKADPKAVQKQFEKYFEANDVSKGGSFYLQSKIY
RANELLQEFQAEKQKEASSTTAGDKKQ
>comp6045_c0_seq1; 415 119 916 LEN=1003; translated
MFRNQYDTDVTVWSPEGRLLQVEYAMESVKQGSACVGVKSGTHVVLGALKRSVSELSSHQ
KKLLEIDDHICMGIAGLTADARSLAKYMRNECLNHKYVYGAPITPAVIMGDLADKHQRTT
QTYVRRPFGVGLLVAGVDPQFQTPHLYQTCPSGNMYEFYASAVGARSQSARTYLEKHYES
FPEASLDDLIVHALQALVGCVSGDDELTKENGSIAYVGKDQKYVLLEGDALQPYLDKLEV
KNAQEDEDDDDDDEEEKSEDETMET
>comp6046_c0_seq1; 601 122 1510 LEN=1649; translated
MVGSVKLLIASLCLGQVNSQREQLRGRNAKESLDERERNLQVAPTFTFEEQEGYDDYFEL
PTDVAFSGGQAASDYFTETGDGMEYHWESASGGRVESGGGYFEDATDPATDPPKIGKFTK
AVKSAKSKEGRYFAKSGKSGKAFKSAKKGKSMKGQHMNTRAYDDKHDGYYEGDVVVEAEH
DDYFAGTSFGAAFVDTGPSYADYFAYDPTTTGRTESDDYFNPAPKPRSEVEGRSSFQHRS

SANQQQQQQQRQRQPTTFDDDFFNEEEVVIFRPRPVVTVGGSVFSVNPQTLIPPLVPDGS
GNTLTVGTQYLFSEELEDAANVNSQLVPVEVDNARVNFLVNLDGYCDRIGPQDQNSVQGY
CFFTYTFIDPQTRFISGAFTAQGIIVNSQVPGQLTITGGTGMMTGASGLVEVLPAAIDNG
RNPPLLIQPQAGADPFNDVSGWAHFFEFDVDVLFFLPDLYAQ
>comp6048_c0_seq1; 189 1 1123 LEN=1916; translated
XSVLLGTLRTARFQGIQTKATFRSKTKATRMTEASPTSTAMPIWITDSRIDPSWIEENMP
GDFPSVSGCTAEDISNDGRRGNNPRNGSTLLLRVTRRDDGIETNTATKIKTLVAKQIPPS
GLALSQRLGLAREAMFYNQLAPRIKFPVRCCTPKIYCSSGSMDDGSKIIVMEDLSGGFVD
SGILFGPGNPNNWSRDLWAAIAGAYPTLSRVPTPFEVANQSFVAMASVHAAFWRDSALLG
DEYSWLRGSSWTSGSDEASWRASQGVVQASWARYTASEIENDNDGGNNNDEQRIQWDPLV
REILETAMKGISWESQRQRLNAGSNFCLVHGDFWPGNVMISKDQGPATSVIENDGRKGCR
LRQLRLLDWEMVGE
>comp6048_c0_seq2; 220 1 1447 LEN=1784; translated
XSVLLGTLRTARFQGIQTKATFRSKTKATRMTEASPTSTAMPIWITDSRIDPSWIEENMP
GDFPSVSGCTAEDISNDGRRGNNPRNGSTLLLRVTRRDDGIETNTATKIKTLVAKQIPPS
GLALSQRLGLAREAMFYNQLAPRIKFPVRCCTPKIYCSSGSMDDGSKIIVMEDLSGGFVD
SGILFGPGNPNNWSRDLWAAIAGAYPTLSRVPTPFEVANQSFVAMASVHAAFWRDSALLG
DEYSWLRGSSWTSGSDEASWRASQGVVQASWARYTASEIENDNDGGNNNDEQRIQWDPLV
REILETAMKGISWESQRQRLNAGSNFCLVHGDFWPGNVMISKDQGPATSVIENDGRKGCR
LRQLRLLDWEMVGLGSGPQELGQYIISNMDPSERLVCEETLVRNYYQQLVHELGSNGSSC
NYSWEECWSEYKIGGLERWLWFLAYFSGQPSMVEWTRFFHDQVKAFVHDHKIGPANVTQP
RP
>comp6049_c0_seq1; 436 98 1387 LEN=1458; minus strand; translated
MMGTFNGNDDAPQLCYNAAKSWYLGWYEEENGHEEVPWNGYWEGKLVAVPDYLEKIKGIG
NPNDEYRAIVKISDLYLQFNRKKAPTLDFQPEDVDRVAVVSQLTQVEGTQSWKLGNSLDV
GEMVESGIWAVKVLSIDTEATIPYAKVAVYNSKHFDEPMVDEARSQMPFPTTAPLPITKR
RATKNPTPLPTRPTTPIHNTHPIHSPTSPPSPNPTKDPTFLPTSNPTNGSTPFPTKDPTH
LPTPDPTKVPTSLPTNGPTSSPSVTSSDLVIYEVFPNPKRENDKDAEYIKLFNPSTVATV
SLSEYQVYDSTSNRYSVLTAKPTLGPREFFILCRNKRWFDDVESVIPSGASDGCDQQGHF
LLHDRLSTIILQRQEGGEHEDIDSVQTVDARDHGDKIYARLSDNVNAETECATCFDWFPA
YDGPKAAQG
>comp6049_c0_seq2; 426 116 1753 LEN=1824; minus strand; translated
MEHLLLRTHTSAGDGKWMFNVDRTSSRDSMKTTRIFLKLIMKRGRHSQRRFANVGRNLLF
FSESLPPLMKHLRSVYKDPYGSHVWVQIHEVGHNNGLGHSGLKRGHTTNSYGDKTCMMGT
FNGNDDAPQLCYNAAKSWYLGWYEEENGHEEVPWNGYWEGKLVAVPDYLEKIKGIGNPND
EYRAIVKISDLYLQFNRKKAPTLDFQPEDVDRVAVVSQLTQVEGTQSWKLGNSLDVGEMV
ESGIWAVKVLSIDTEATIPYAKVAVYNSKHFDEPMVDEARSQMPFPTTAPLPITKRRATK
NPTPLPTRPTTPIHNTHPIHSPTSPPSPNPTKDPTFLPTSNPTNGSTPFPTKDPTHLPTP
DPTKVPTSLPTNGPTSSPSVTSSDLVIYEVFPNPKRENDKDAEYIKLFNPSTVATVSLSE
YQVYDSTSNRYSVLTAKPTLGPREFFILCRNKRWFDDVESVIPSGASDGCDQQGHFLLHD
RLSTIILQRQEGGEHEDIDSVQTVDARDHGDKIYARLSDNVNAETECATCFDWFPAYDGP
KAAQG
>comp6057_c0_seq1; 233 727 1394 LEN=1555; minus strand; translated
MKHGVLVLARQCFHSVRCYYEGVLELRRTLSVNGRGRPVVWPVQNLVAPLADHRFDRKCV
PFRHLSVGFVVAVVQDVGSGVEDAANSVTTKVSDGRKAKRNNVVFHDGPQFFVVPAGLNK
LAGFDPAIVCCLQQSLPLLVDVAYNKHFTAVSVVAVQVYRDIDIDNIXHPLVSDRLEFRG
KSPRLHSYSNSLEIRGSSVELLVKQNRFTRTTNKNTDKEESE
>comp6057_c0_seq2; 444 179 1417 LEN=2035; translated
MPLAAAFRPSGLSPRMVPSTFSQTNRVENDRTSSSPTALFMGKGKGKKKGKGGPPPAGKG
FGGAIATDSPIDKFPYAGTVRPGIQSPQRKVILSSEEAKKILPDYALDGRPKKGSNSPLL
PWVIEVKKPEEIEKMRVSGKLAREILDLAGRAVKPGITTDEIDTIVHEAILAAGAYPSPL
NYHGFPKSCCTSVNEVICHGIPDDRKLKDGDVVNIDITVYLNGYHGDCSEMFVVGDIDQQ
GKALLQATYDCWIKACEFVQPGRDYKELGAIMEDHVVPLGFSSVRNFCGHGIGSVFHTTP
NILHYRNNEPNGQMAKGHTFTIEPMICEGSNKVLNWPDDWTATTVDGKRSAQFEHTLVVT
SDGVEALTCKNENSMLHFWEKESEVYKGFWLGSSEAAKERAEALNTKILGDL
>comp6064_c0_seq1; 242 512 1105 LEN=1238; translated
MPGKHWPWVSFTGGLRELRVVEDGSFISMRGNQIVKPLKGCWELVWRKGDPDGTLTFGLE
LDQVYKRNDATLPKGMIYISLTTWSSEGLKNAQSFKKRSTERADMALKKRDEEIFKMNHT
TNIFQKGLHYYKALCAFDEYLMEPNLKMRRIPSEDEVVHLEGDLYICKSGKVWTHDSPMG
KQATIGTVSLELLSKKM

>comp6075_c0_seq1; 182 1 543 LEN=671; translated
CPSISFILFGSFSIFPAGIDLVHDGVDVPTFLSVLFFRLKNSPSADFDRTKGRFSEEEFP
SSALPLSIFSLKLARRNNSPREFLDLLRLKEGFSIGGLSRTWSSTLWLINSSNPVRSSAS
FSTPSSWMEGSTSDLARRCCEILGRTSMEGGDKDGVALLWLRDRAILGDLERFFLNEILF
>comp6078_c0_seq1; 104 1 238 LEN=238; minus strand; translated
XGDYMASIGLSDRTEAIAGSFLEEFPAELSKCDAFIMRYVIHDWGDEENVKILENIRAVA
SKTEKKKVVVVMDQILDTGA
>comp6088_c0_seq1; 275 188 910 LEN=1378; translated
MAKESSKGNNRNSHVNGHALLLYPSDGALPLQMYLENALSTVDNEEDPAVTENDVQTAIT
SAKNDGNSRNSNILVVVDGTWAQTQSMVQNSNDLLWKLPRVMFDETTNSLFDSLRQEPAP
HCTSTLEAVSRAIRLLSGSPASAADNDCTGSRGADALEKSLEAMVDGQLRFALDDENARP
RYYRKNETGEGTKPVSKREAGRARKLVSKRRQTSLSLPKAKSKEEIELDRIRFVYIAHMG
>comp6088_c0_seq2; 293 1 977 LEN=1445; translated
XSTAGSRGYCPRCKRPNVVCICHALPDEPIDCGTRILILQHPREARKRKRTSTVPLIGLS
IQNVEICVGTKFERGSHPLLDEALDMAKESSKGNNRNSHVNGHALLLYPSDGALPLQMYL
ENALSTVDNEEDPAVTENDVQTAITSAKNDGNSRNSNILVVVDGTWAQTQSMVQNSNDLL
WKLPRVMFDETTNSLFDSLRQEPAPHCTSTLEAVSRAIRLLSGSPASAADNDCTGSRGAD
ALEKSLEAMVDGQLRFALDDENARPRYYRKNETGEGTKPVSKREAGRARKLVSKRRQTSL
SLPKAKSKEEIELDRIRFVYIAHMG
>comp6089_c0_seq1; 502 194 1609 LEN=1909; translated
MSFCIQSLNMLQLLLVLLLVDVSYSSHTWGGKSSSASSKKSVSFHPSSRSRLEEKYGFRR
DRRNANSFKLHNVLSKNIRGGDTNNNNDIQPLRGGSLKTEKGQISENISLKNKKVHNIAL
NAAAAEAASTWSRKSMSYADGFVEPICRQDDDGTRFSLFPIEHPDLWSMYKQHVASFWTA
DEIDLSADLTDWQENLTKNEKHFISMVLAFFAGADGIVVENLAERFCRDVTVPEARCFYG
FQMAMESIHQETYCLLIDTYITNPKDRELLFSAHTRVPSVEKKAKWAQRYIGSDASFAER
LVAFAAVEGIFFSGAFCSIYWLKKRGLMPGLTFSNELISRDEGLHCSFACQLYSKLERRL
SEKDIHQLIGEAVEVEKEFVCDALPVSLIGMNKSLMAQYIEFVADRLLGDLGYRPMFGSK
NPFDWMDMISLEGKTNFFEKRVGEYQKSGVMANLDQTTAGTRGNLNFDADF
>comp6089_c0_seq2; 316 194 1049 LEN=1349; translated
MSFCIQSLNMLQLLLVLLLVDVSYSSHTWGGKSSSASSKKSVSFHPPLAADLKKNTDFAV
IEGMQTCLLIDTYITNPKDRELLFSAHTRVPSVEKKAKWAQRYIGSDASFAERLVAFAAV
EGIFFSGAFCSIYWLKKRGLMPGLTFSNELISRDEGLHCSFACQLYSKLERRLSEKDIHQ
LIGEAVEVEKEFVCDALPVSLIGMNKSLMAQYIEFVADRLLGDLGYRPMFGSKNPFDWMD
MISLEGKTNFFEKRVGEYQKSGVMANLDQTTAGTRGNLNFDADF
>comp6093_c0_seq1; 561 490 2043 LEN=2958; translated
MRPLLIKKRPRRQVIGEDGKYMDIELHNHREFPFSERSEYPKFSAADAIASAPTTNKKPK
GKNKDKGAVPIDISSRRQLQTARRLSMYIAPADLQQDETDEESDESHGADDDEEDNILDE
AEYGYENMEDSDEEEDEDIEPSPYKKRRGRSLETGKANKHRRIRVGPQQNNDKSGAKKQP
PQVHLTKVENMLHRVSKNISVEHVHFPPPQLQNQLGKEVNAKGPAGDVLTAKEKKHYDEF
DKLLGYRLKNPNPIVKITSSFLGPLMRIIRIAIYGSRISFNVWTWRDPYLTFWIFSFLWT
LMFVLLIFPWRTFFFFCSFILLGPQNIGVRKYLERRAKQREQEEKEDEEREALKAQLKIN
DPQRLAEQQLQQSRTTSPDENKKSEEKDGRKKRSWGLSRKKEEEIHQQQDEIHEADMFLS
PRPAFYAHCKPNKRTSLVPRDVAVPYFRFRKDRFYDWPPDPTVSRATPMFITSSLGEGEG
GQYSTINYGPRVGSPHQGPHEQVGLRNRGRINHQKDH
>comp6093_c0_seq2; 733 389 3310 LEN=4225; translated
MGSNFKGSTGSLSSYDGRYEGNNSGDLFGEYRGYGGFSSDSQIQGVTLRAAESNSGSYTS
GMNYVSSDFVVGTNHTSSLIASGSQMRRIESDHDQKRKKVRVKPHSAFQSATFMNETDLY
QCMMAGSSNFVFLTSYLTPSTKATRHAKVSQAVQRHFGSPKEDGRIGSLRLEILGCVGLD
RFKPEVSVYAVCGDCAFTTDIIEGTRSPMWPNSSKRATVFPLHHAYARAFIGVFDVKQQK
EKEADYLCGRVTIDIASLRPDTEYDITFPLRVSSFIYDRRPRGVVRVRFSLHWFSERSAL
LSYLKRPRNPLAFSRQAKKFPTIPCGDPKTFRNVAVTVHGQDFPGKYTRAAFRSTMREFN
LSQQNIRFLLKVIILDCILYENPLMSIYLFLTSMYCVSVNSIRLAPPFFVGWLIYLLLEN
NFYFTRTQEGNLGYQGLTIQELFFSLVFDGKKENRKMRPLLIKKRPRRQVIGEDGKYMDI
ELHNHREFPFSERSEYPKFSAADAIASAPTTNKKPKGKNKDKGAVPIDISSRRQLQTARR
LSMYIAPADLQQDETDEESDESHGADDDEEDNILDEAEYGYENMEDSDEEEDEDIEPSPY
KKRRGRSLETGKANKHRRIRVGPQQNNDKSGAKKQPPQVHLTKVENMLHRVSKNISVEHV
HFPPPQLQNQLGKEVNAKGPAGDVLTAKEKKHYDEFDKLLGYRLKNPNPIVKITSSFLGP
LMRIIRIAIYGSRISFNVWTWRDPYLTFWIFSFLWTLMFVLLIFPWRTFFFFCSFILLGP
QNIGVRKYLERRAKQREQEEKEDEEREALKAQLKINDPQRLAEQQLQQSRTTSPDENKKS
EEKDGRKKRSWGLSRKKEEEIHQQQDEIHEADMFLSPRPAFYAHCKPNKRTSLVPRDVAV

PYFRFRKDRFYDWPPDPTVSRATPMFITSSLGEGEGGQYSTINYGPRVGSPHQGPHEQVG
LRNRGRINHQKDH
>comp6095_c0_seq1; 448 1 1851 LEN=2238; translated
GTFCIQFPISCNDPRRKTFAFFSRAGKTPETTYFKEPNTASKSNGSQTMPLGDSNGGGET
ACSSLWWCAAVSRAISNDSGDGLVETALVVGATTVVLVLWLLSGGRGKKSSNNGGTGHHH
YADIPWAPGGLPLLGHALSYQRDPSGFLLGAREACGGSSLFWVNLAGRRMVLVCGPEEQR
QLAGLPDSVLSLREAIAEIGFEQSLGRKNVYEGTQIHKGIVKAIWSRAANEKNSDSGNEQ
RGPADRQLVAWQDSIRGAMEKELAVASSAATAGSGVKVEFLGFLRRVMLRASVEMFLGKA
FLAGWTGFDFLEEFVGFQDTLEDVTAKAAVLPRWFALVALLWPLGRKRETLQRVIEKRLE
AVLGMDGDNDAGFWLEAVLAQGVPVSDVAEFVVGLLFAAHKNPAIGAAQTYLLLREHGSE
EVLERCRSEAEALLSETDPVPWSGLGEALPTLRRACLESLRLTAHSIGGIRKAQRDITVT
VSAEKGDQLLRNEAAAAAKTITYQISRGTTVGFAHTTSSLDPSIWGADAARFDAGLDRYP
EDKYQDDYSFTTFSNGTHRCPGRVLAMVQLQLANALLLAEYEVELPETLPALDFERATLA
QREGPVLVSITKRTRT
>comp6098_c0_seq1; 204 1 574 LEN=741; translated
XPTLGMSIGLTVRMGHIIAYDIVKAKLLAIWCMLFTVVFGAILSTSLYIFRVEIAMLFTD
DIEVVEGCKAIWGKLSCYVFVLHIFGINSAILRVLGLQWRMAIIIFGFLWFVTLPSIIFF
AVHRGGGLYAVWSILPIFYTVMQVLLAFSYLTADWESIGKEIHDRAHGEQSQKVPTTEDE
TKALLPSEQKR
>comp6099_c0_seq1; 499 311 2368 LEN=2483; minus strand; translated
MSPVAISQFNHSRRARSPSSGRNESITSSATTAAATEPERMVWRGRAQDTVVSSGTNPNT
LTRKPKTINRDNRDSNNNSRVQKDNANTNDGDSISRIKNNNNTICGNRRRQCCPGSTAWI
VAWIVLVLCRQLMTETGLLRTALASYGAPCHYFDRGGWTEQGDECGYGRGNGRNRPSRSA
HGSSVDASSNQTLGSDNDKNSSSINITVPSLPSALDLLNPPFKKNYTIRVLGGDSIGDIY
NSNCNGSDTAVTTTSRKNKNSNNRNIIVTAYYKIPSKHTPERYRDWMKNFLGRFRDPVVA
FTSPDMVDSLAALRCPLSNSSSSNSTSDKDLNRSLSQSPACFPTVLVALPLEELPLAELY
PKSFWVDQLDRDPEGKIHAPLRGDPRVGGRFESGELYWIWLSKAWFVHEAVGLLMQEQHQ
GANEEKKSVEEEVAATTRLTSTATAAEGEKEHCPERDKRGDVFAWIDIGSFRQSENETET
TPTTITSKARHKVLIRHPEVVPDDEVLFWSHSPAHKISSGNDKNKQKKHDYSPVPPNLSP
YSDDKLSSNGRKHFFHAGAHFAGRPESIEKLYFRFLETVDVFVHRNMLLADDQAVMQSAC
LGYFDGQGYNRPSGGSVNTSGNNNGNLCAYATRDMVDGRGGSKWFGLAGLLQYGPAKKAY
RSDRSKLLGAYWRPPSREHYSEYSA
>comp6110_c0_seq1; 412 1 1648 LEN=1787; translated
XCIEEEIATTKKKVEDAEAMRIVHPSTQNERNLTKAKAAHERAKMGLCVMAREHHRTGHI
LCSRAFGTFRGAGPKSCASLENAVAAQIEASTADLAEHTSIIHRETKTIQSIEGKIAMNT
NRTTVVNLNTFEVLQALKKGDTEKTNCPICYDVLGNGDGSHGKVYLTRCGHLSCKCCLNH
WINQKERQGSTISCIECRKRITRNQLICVDPKKVDQDHLMDSKQKARALVKQAAKMLEEN
FGKLKPHLWEALYLVIDLPPGIDQDLDESFTAIPGLFLAHLRHFLRGSMPLGAPPNFPAG
AVSKGSNECNLSSKFRALLSDLPRNELSVVFASSKSIVLHLKRVLETKGFTSKTLFVGQS
EEQSECAISDWEDSNGSKNPMVLIVQAGAAACGLTLTAASKMFILEPFRKHEEEKQAYAR
LHRYGQTREVHCKVYYTPVSVESRLLEWRRRATAHSPEEEKTVYAPLRQNDECKDEDDGT
DGKSTDQSQSNERSADPEENASGIEDENQIRFLLGIDPPLATTTSTSTVESTTLATTAES
SSSTNSSSY
>comp6111_c0_seq1; 497 1 1347 LEN=1899; translated
EAAGLEAALDGSSNNELFSSLTVFAPTNVAIENYDLLGFSDSMFNNETDAGAIRDILLFH
TIAGSVIESSAIDCSNSSSLSLTTANGGSAEISCALDGFVVTGDANANTTAAEAVVRITT
PDVAACNGILHGIDGVVPSLPGTISPPGLDGPPPDVLPDDEDCQSFGDAVCSRADLKDFC
RLVGLSRNDPEGVELLNALDTTAATVFAPANAATVFSPPNEGLFAYTSEGSFDLTNPGDV
TKILRNHVVLGTKLLLEDLVCDQTLEMSNNSTTTTECGMNQAGTEVVKRFQVGDGNSPGN
PEVIDYNLVTCTGVVHILNGAILPLFSEATATPTSSITVEMSVGATPGDTDGGTDGASAG
ATPGVSGEVSSNSSVDVTGGASADTSASANGTPGPSTDASASSNGTPGLPADGTPGASAD
ASASSNDTPGVSAGDTPDASSGVPPPWA
>comp6111_c0_seq2; 181 1 600 LEN=600; translated
EAAGLEAALDGSSNNELFSSLTVFAPTNVAIENYDLLGFSDSMFNNETDAGAIRDILLFH
TIAGSVIESSAIDCSNSSSLSLTTANGGSAEISCALDGFVVTGDANANTTAAEAVVRITT
PDVAACNGILHGIDGVVPSLPGTISPPGLDGPPPDVLPDDEDCQSFGTNETIIFFMGQXG
FRLFHAVRSETLKSSLFLTNX
>comp6113_c0_seq1; 165 621 1200 LEN=1200; minus strand; translated
MEERVYSKAVNKIGLGNRVIDGKELHRFFSTDEIDSLARDNDWVECIRCKQWRMFPPDHT
ANVAKLPKEWVCEMMNEYDARMELTCSFKEKDSEWYYHHFKKPNKKVLSRTSHTAEAVIR

ETTNVISAAQSEIKVDRDVILKKLVTLFTRGKNAARIVTKHYFHEALFSDASTRTNASRT
NREHPASDTEIFKX
>comp6113_c0_seq2; 165 714 1293 LEN=1293; minus strand; translated
MEERVYSKAVNKIGLGNRVIDGKELHRFFSTDEIDSLARDNDWVECIRCKQWRMFPPDHT
ANVAKLPKEWVCEMMNEYDARMELTCSFKEKDSEWYYHHFKKPNKKVLSRTSHTAEAVIR
ETTNVISAAQSEIKVDRDVILKKLVTLFTRGKNAARIVTKHYFHEALFSDASTRTNASRT
NREHPASDTEIFKX
>comp6118_c0_seq1; 365 258 2154 LEN=2246; translated
MQGFKSTTLFVVILLVSTSLPFLEGARSRDDCRCLTASELNAGDFVLKENLEEDIESLVT
QVGLANGIADGNATESFQNEYGIGCKAHDAGLSECGAAGKPGEWCDYEWCYVDNGCKLDF
EPSRIFKKRSYSYAACGFIDESDRGPTLLNGMVLQGVIHDNHRGVEGTECEDKDNPTQTT
CHGTTMDLARYLVEEPTLNITINKDRAESYGPFVENLENMDHGACVYAASLGVVDICIGA
FTKNRQRTMASSFLEMGVTAEYLVVHEHTEQLSVMDKVKSIFEPFSPALWGMTFLVFSFL
GFLLMLQEVSWETLREKPSANVKMSLYGSVMAFFARRPGPYCSDKVTSWGGRLTTLAICI
QILLSGAAYGAQLTTLLVRSGFSVTIESIDDVMLYKFPICVLRNKIKVLDNAYGDSRFNY
AINPEDGLPGFNNRTELLLAMDKGLCDCLLADEHVMAAHYSMGSHCDKIIVGKPVIELPW
GLPVSTNYAAPLNTAVTRMMQRGKWRDIMEDAEPENICPEKDINTKTTPFEIHNMLGAYV
LSGVIAATGIVISLWSYFRKRSRGRSDLKELPSEGNGDEGEQTLAHMEYGSTSNTDYHHL
QSAMSGLMAEIAGLKATMMEAKEEAAKKKDV
>comp6124_c0_seq1; 339 1 516 LEN=550; translated
XSFCFVLFCLPPFCSLDEAMATTDPANLLIALADLLACRFLALIAIPGRAEGTPVTDETF
AEWRDKFNAEMEELKAQQKAEASADTRKKGGAKGAVKEVDKSGRLTGYQHFTDKTGGTLN
IEAMEAAAENAQEDINEDLFDVSDDDFDDLDDMDFDSSDEEDEDEDEEPDI
>comp6124_c0_seq2; 334 266 784 LEN=1199; translated
MSEELREEQEMEAEALEAIFDDCLEIISSEQPFEWAVDLYPEQLAADEEDEDAPQNHVGV
KVVAKIPLDYPEESLPEFNIKVLKGLTEEHEQELLELANEEAENNRGMPVVYAVCERLRE
WLLENNQKGMDDRSMYTLMLKRQKEEKRDEVRSESNESVWIQRKLDERFQNDW
>comp6124_c0_seq3; 685 266 1174 LEN=1208; translated
MSEELREEQEMEAEALEAIFDDCLEIISSEQPFEWAVDLYPEQLAADEEDEDAPQNHVGV
KVVAKIPLDYPEESLPEFNIKVLKGLTEEHEQELLELANEEAENNRGMPVVYAVCERLRE
WLLENNQKGMDDRSMYTLMLKRQKEEKRDEEKAKQVYEAQKKKEEMTQAESEELAVRRRR
AEGTPVTDETFAEWRDKFNAEMEELKAQQKAEASADTRKKGGAKGAVKEVDKSGRLTGYQ
HFTDKTGGTLNIEAMEAAAENAQEDINEDLFDVSDDDFDDLDDMDFDSSDEEDEDEDEEP
DI
>comp6134_c0_seq1; 123 894 1690 LEN=1690; minus strand; translated
MLQNWEYMASQLHLQWAVLALDNELYEELGPSRAIPAKEEFTVSGPQEFRKGSFQGLTCN
KVRMALEVASACKMNVVFTDVDNVFFQNPFEHDLGRLIRSQRYDYVYQPNQPAWAPLEDQ
CLKGNPRKEANTGFYYFNHKSEIYRNIAESMLEVCVRPENKIDDQSLFWKEFWKAEKRIT
SRNQTEGSDASFHHCDLSEYVNPWSQQNNFSKTSGSNKRIFQYCCLDPYFYPIGKHNARD
GPSNKNPVTYHANYASKYGRKVEKLV
>comp6136_c0_seq1; 256 1 880 LEN=1087; translated
XGIINRDGINDSAPTSVGLRPDPKRPARFSDAMKDDLVVAVTEQASKRRKLAMSAQPPPS
SAALALDWEEIAESVGHGVSAEDCRHNFVTADLGEDDPAEAATPGSGVADGNPTAGVSNQ
KAFVQSLIDSSDPKVVEAMFEAAVRATGGGSPADTRAAAILGIHATGAVANARGHEIDLA
LRLSKLLDARMQKLENRMAMLDDVEGILEAEKVALEMERRDLYTAXVPALVRGRLTGSMQ
PVPPIDFGGALSFLSVACNTGTICNSNNIVETCPVPRNETFVELFRSKRNDAEW
>comp6144_c0_seq1; 147 172 540 LEN=815; translated
MKTKRISSKDDSSSASQDTNSTVSLYSGNNSVLGKRAADVLASSDLSAFGEPKPDSKTLR
EIRESAERLAYELNDFLTKNEVPTTKLLKYNEGNGSPDFSHMTDEAIADLQLATARKLRE
VR
>comp6144_c0_seq2; 969 172 3072 LEN=3114; translated
MKTKRISSKDDSSSASQDTNSTVSLYSGNNSVLGKRAADVLASSDLSAFGEPKPDSKTLR
EIRESAERLAYELNDFLTKNEVPTTKLLKYNEGNGSPDFSHMTDEAIADLQLATARKLRE
LLPRNRDLLFTARENQWIPILLNWLTFHDRPAVQVESLLALTNIAELCAQQSYYQHTQAQ
SSAVAAAAQAAAAAVKGDSSSSSFSMFGYKDGQATYPYPSLVAPAPGSVPFPPLSFSQTL
SPEAVASFDASLTATIQNSKVFDKSSKDGKSSFSLPPAPLFPSSAQSSHGLFQNAKASQA
AGINFTSLAPPPGGAHSAVPNNTFLPPPTPEPSLTCFPQQLSPSSQHLLLRHADAIPTLI
SLLSSSNKEVYEQSMWILGSIAVGDSPGTSSSGGSSSSSSHELSREKSVSARDVILAAGV
MNQLLRCLEAHPGNLSLQRIGSWTLSNLVEGIFQQQSHGKGGGKASDYSSSDEIEMPILL
PVLRRLLRMADAEVLSYTCWTLSHLCDGPSSHIAEVVTTRDESNAPKGGLVPRLVELLLH

PSWRVTKPALRTIGNIVCAECSDDTNQIGTVTDYTEVILECGAVPRLKELVTHTNREIQK
EACWTLSNIAAGTVDQIQAVIDSGAIPPLVKLVRNKKTDQEVRSEACWVVLNATSCGSDS
QIEVLVDEGCVSVLGVLLSEASMVMMALEGLERVLQVEETREMIRREQIENGEIKDVQKT
RNPSLVNSSLIEKARESHNSTAVTKRAERIWKQHFVSCALCKKSFSKHRPSDASFCPECK
CYVCCNCNCKVYHLDYQEELWAETEKTEQKKSSKKNKNKKKKEKAKQKKAKQEMNLVTFT
EGVKEGTRVPQQAAKGAVGDQSRSTVSSESVDKELKSTDDIRRTTDSSKNSEPIEVDHSE
GQDEKQHQQPPIDFVLYLQQTGSIIALAKLMDALEYGEDLDEGLDDSELQLIREQQVLNQ
QQATT
>comp6158_c0_seq2; 190 99 545 LEN=768; translated
MRLTAELIASSEQRTNPLGEREIVLRGLAIPVVEHLGVTRDAYDAMDLTDNRLTRLENFP
RLLRLSSLSLSGNIIEAIDSSNLSKNLPNLQYLDLSHNHISKLHEISNLGKAYHNSNAKK
SESHSSKGNLECLNLHGNPVQRCMRYHK
>comp6158_c0_seq3; 245 99 992 LEN=1113; translated
MRLTAELIASSEQRTNPLGEREIVLRGLAIPVVEHLGVTRDAYDAMDLTDNRLTRLENFP
RLLRLSSLSLSGNIIEAIDSSNLSKNLPNLQYLDLSHNHISKLHEISNLGKAYHNSNAKK
SESHSSKGNLECLNLHGNPVQRRQHYRLYTIHSIPSLKVLDHQRITKSERDRARRLAVSA
AGAALESDVQDEAGKSKTFTPGEGASAEQSFVVNFTVEEKELIREMVANAESPADIEEIE
RSVQRGVLPIKYLKKTDAIQRKDVDENETNSRKRSLPVPESGENQSNESEKKIKHGQ
>comp6162_c0_seq1; 329 1 931 LEN=1020; minus strand; translated
XVSVEKSDSSENPGIRMGMKETSSGRAVCISEISPSSLFARTPLRMGDIILSINNVSLHD
NADVFDAYAAMGKSEKRITLVAKKGGESLNDFLLAARSQPVMKGKLNHSGHDSFDSQLSS
KTAGTVGTTASGESGRGGSNDSFESFDGKSEDGVIGFKSTEEWKSNFRHDAVNNTSKAVS
SPSRRRNEFEFDEESYGASLFGYNASSNVRIIKSYCDEDIGFKMLEITTEWGRLLVVSNV
TPRSLASCTDLKVGDAILSINGISFRKKASAARASSIIRDAHREVYIEYQKLSSFSPAVP
VDLKEDRILR
>comp6173_c0_seq1; 773 30 2242 LEN=2359; minus strand; translated
MVSLSGAPSLVRRRRTTTLAAVLPFVLLVLTAATVWFRPSSYYHDFFHRDGIGIGEVAQT
DPLASASAPKSAPGSNPNNHEDDGPAKMVPAELLELGVYDESRDFVNERGFSPTGWEEPN
ADPTVWGPCRFTPVRPGSSFSMAADVSWADRRRWTRSNETANATALLSRSDDYYRNADPD
DFEYRVAVALSQLTGHNKSNEKHPPFKGGWCRPGFLIIGAGKCGTSSLYHYLMGHPRIVP
AIEKQIHYFKYHANTRPLAWYYGHFPTPQSFLDHGALMTGEASPGYLPYPEVAKLVHQTY
GGTGQDGAGTDVGMSLQYLLGDPAYRKRAPAVPPRIIAVGREPLDRMYSSYRYNYVVPTI
EHLRSRGHPAIRKHRRYGTKAKKKSSHKEHASEEFVFELNHPDDEYYMPYLFTLEEFVRA
ELKQLRGCFYDWGKNLLNAFLGFVRLFRLTECTLXYLFELTRGLFAYRSTPFSPQGPRKT
YMQWRRDSIYGKTIKLRNGAFVDNESSIAAGKNATDSSAPPPLIALDDVCYGKSVNKTVY
REQWAEMQMNNPEKKVLLNKNLHLTQALIGRSLYTFPLEWWYLDVHKSPQPPIFVCTDEL
TDSETLSELTAQLGLPAFDGFDQVLAEGAYNVGGHRGYDTATSWEELELEKQQKEQQQQN
NDESSSRYSSSGSGGPNRTRHRYAAAEDVPTAPKGAAPGTLTTNGIPLPEDLYRELKDFV
DPLNERLFALTGKRCNW
>comp6173_c0_seq2; 691 1 2156 LEN=2273; minus strand; translated
LLRCGFVLLVLTAATVWFRPSSYYHDFFHRDGIGIGEVAQTDPLASASAPKSAPGSNPNN
HEDDGPAKMVPAELLELGVYDESRDFVNERGFSPTGWEEPNADPTVWGPCRFTPVRPGSS
FSMAADVSWADRRRWTRSNETANATALLSRSDDYYRNADPDDFEYRVAVALSQLTGHNKS
NEKHPPFKGGWCRPGFLIIGAGKCGTSSLYHYLMGHPRIVPAIEKQIHYFKYHANTRPLA
WYYGHFPTPQSFLDHGALMTGEASPGYLPYPEVAKLVHQTYGGTGQDGAGTDVGMSLQYL
LGDPAYRKRAPAVPPRIIAVGREPLDRMYSSYRYNYVVPTIEHLRSRGHPAIRKHRRYGT
KAKKKSSHKEHASEEFVFELNHPDDEYYMPYLFTLEEFVRAELKQLRGCFYDWGKNLLNA
FLGFVRLFRLTECTLXYLFELTRGLFAYRSTPFSPQGPRKTYMQWRRDSIYGKTIKLRNG
AFVDNESSIAAGKNATDSSAPPPLIALDDVCYGKSVNKTVYREQWAEMQMNNPEKKVLLN
KNLHLTQALIGRSLYTFPLEWWYLDVHKSPQPPIFVCTDELTDSETLSELTAQLGLPAFD
GFDQVLAEGAYNVGGHRGYDTATSWEELELEKQQKEQQQQNNDESSSRYSSSGSGGPNRT
RHRYAAAEDVPTAPKGAAPGTLTTNGIPLPEDLYRELKDFVDPLNERLFALTGKRCNW
>comp6177_c0_seq1; 524 1 1334 LEN=2111; translated
XRAGTGTGRPSLGSVLEEHRSPVAAASGSDAAKALVARPPTPLPPPPMAVPVAPPPSRAE
PPRKALSSDGVKDMGLSLAFARIDRMAIQQREAEARDTPRRVPNRKLCKTRSGTVALSAK
EVDRRVMALSLDGILNETKKGRPAPVVRKMVSSKSGTLHKARKKNSKKTGTDKKKDAKSK
TSKKEKEKATTRASKTGKKTKVPMEITDDEGGSSPKARSSSRTSRSKSRDKDKKPKDSKA
GRKSVTRQKSESSRTTTCVSDDDDEEFLQFLEDFASPSDEESVFSSDVSISTCYDSDSSF
EHRMPLRERLAARAPRAPVRRRSPSKKKSSGKKAASSRSGSDTDETKAKKKKKKKKGAGS
DYDSDRKDPEKKKKKKKRRESAADVATRESLARRGKNQEPATRATKTSKIRRIDHQKRPI

NHQKPMPQRQDVQRLDQRGNTCPL
>comp6197_c0_seq1; 306 66 959 LEN=1143; translated
MGVARLVVLLSVVLCFVPSNGFSAAPASSGTSQLSIEDLVRDQLGYFPPNYVKVSAWKKK
SNESTPLAIQTYPLNGGAKRRQNKAKLPQDQSKEKSEQQPIVQSPFPTLFWLTCPDISRA
VANLERRGFVQIFEDELNSRSDLAIRLFRCHEEYARLRWKSLTEEDRNVLTSSSSSSPLT
PSLQRMRNMMEHSGISGTNFTDISSWDKDYDSNFESDCGSIVEAYVGKRSVQQKKECGES
SLSSTEAGLVKVPPIKCLHAHYAHYRSTVSEKSTTYVNPVGELIHRQLEKDFPDLDL
>comp6198_c0_seq1; 380 1 1856 LEN=1856; translated
GGSITRNIQEMPQAENTKVWNEDLVTALRCREEKCRQQFSQRQYLWRDGAAAIASVRKDI
YQYKNGRIVALPRTLTNVVEEECRRIIEGERTILPEGYLPTTLDEQNMSRGPVARKSVNP
YHKDPYLQRIKMRGGSYAILMALHFSEAKVMTQKQICNAAQKYCDEEMESNFYSGRSYGA
WSSKKTLVKHGLIKESRTTQMGRHGQVCNGVFEYSLTEDGKLFIDALLVKFPQSSEERST
FRANDSNATIHHKNDLYQTKEKSTINIKLGKKFIDKENFGRNGIKVPNRLNANEKIEDHH
CLLLSDSDEDDHDDYDDHGMLNKSGNHRYDEQEIMSSKSQCHVIDLLYSSDEESVSELFE
YHQIKRNETFDSQVRGLQKSHENILNPIPSSSVIKKEVNLSSKLVIVIDDRERNKNRTPR
YMRIELSRHLSHASGSLCQIWPSNLPIGEVEEDQLNYGDFAFLIKSKSAFNSKAPVVIER
KRISDLAQRSSRAHHWQQLHRMRDCCDHAILLIEGNVKMTSNFSTYENIEGESWNPDHHT
IDDEHAFYRFLGRAILSSPIVKILQTPHEQASYRSIGATGLVASLLSWDGNAPKKLPNTK
VRLNHLYNKLKSRGIPWQX
>comp6203_c0_seq1; 230 649 1669 LEN=1669; minus strand; translated
MTNLMTREGLGVFLALMAVFVCDHYAASFLLNQATRAYQPAPFDRSVVKTFSTPFSASSG
RNNERDNKNNSRKPKKSVTDRSQEEAISLIQDIIRAAVDAGPQAGPVRTLQAYKAFSETA
REFLPRFGQSPPVFSAPKATRTLFEKLGATYVKLGQVRLLHGVMCFRLTWKFARFLKALS
TCCCCFNPFGSXVASSPTIFPKEYVLEFQKLLDKTEPVEWRIIKRVIEKDLGPISKTFDF
VDEKPLASASIAQVHAARLKTGEDVVIKVQKPGIDASLKADLSFVFVASRVLEFLQPDWE
RTSLSGVVGDIRSSMLEELDFTKEAKNTEEFRRYLAQNNLX
>comp6204_c0_seq1; 396 171 839 LEN=1222; translated
MIASLTACLSLLVIQAYAFQEASNAAIFSSRSQLGSIYTHVSLHESSQPFDSQERNLQER
RSFLSKLALIPAVVLPSLATLPTSAQASGGATAGGAYLLSAKKRYYERVKESVTGLLKAE
DGLRNGDSKVAKEYFSSEDGGSWKDLTAAGYLLSNAFRRSSSTAPDSLPAVKKYKAFAKE
VEAFQKTLKKKGAAAASEAFPTVEAALDEWLSEIELPAAREL
>comp6207_c0_seq1; 261 1 827 LEN=1044; translated
XLNVDNIDSIRQNISHWIFTKMKFSKILLLSCYILTFSVKVNECTGNLRSSLDDEEVTGD
LRSVVSNLVRRIEALEEASWNDQEKIFHMENHIITLENKIIQVEGALDAGLEKIQSLEQN
IRTVEQRNLQLSNGPLTNRPVVSLLGNSKKDTDNDSSPCMPVFENNRCIFGNTDTIAFRF
ESPTFFNDDVEFNENVGFDQDADCMPVYNSTSGQCLMNSNFVYQSGNHYYNNPSKVFFRS
KEVWMRPKKVFFRNTQTSFQGGKLEIKKDVNYTNA
>comp6207_c0_seq2; 313 315 1079 LEN=1296; translated
MKFSKILLLSCYILTFSVKVNECTGNLRSSLDDEEVTGDLRSVVSNLVRRIEALEEASWN
DQEKIFHMENHIITLENKIIQVEGALDAGLEKIQSLEQNIRTVEQRNLQLSNGPLTNRPV
VSLLGNSKKDTDNDSSPCMPVFENNRCIFGNTDTIAFRFESPTFFNDDVEFNENVGFDQD
ADCMPVYNSTSGQCLMNSNFVYQSGNHYYNNPSKVFFRSKEVWMRPKKVFFRNTQTSFQG
GKLEIKKDVNYTNA
>comp6209_c1_seq1; 530 1 1433 LEN=1531; translated
XQPLLSLVDTAFVGLSAKVPAESAAQLASLGPATTFIDGATYLFAFLNVATTNLYSTALA
QKGGQSDEAEAVVCTASRVALRCGIGIMFFLFAFARPLLALYIGHGADPALIDSAVDYVK
IRAVSMPTSLLLGVVQAALLGAKDSVTPLIAILYSTVVNVVGDFLLVRVASMGLKGAAIA
TLLAQLAATAALLGPARRRLVRDRSLGLWKKKKPAEGSQGEEAVTSKAFLGFAAPVLTLI
LGKLAAFGFMTHSAAAVPGQPTPLASHQIILSLFFFVSPFMEVISQTAQTFVPSYLAPVN
EYVEAKEERTLADPLVKPWLDRAFDLGSYLLKLGFISATIVASLASLIPAFFGGMLTSDP
LVKNGVKPLAKYLWAGAFLTAPVAVSEGILLARRELGYLAGVYYVLTTALLPSALLRVKSI
GGSVDQVWACFAVFQLCRAGFFAGRIWSGSVLRKIAGLFGGKEAPAAAAEGSAPAAS
>comp6219_c0_seq1; 410 1 1465 LEN=1577; translated
XDHTTAEEETQCFLSVVEGDGECDKRKSIPSARPVLMALSSRTDQRQNYQTCAAASIEKT
EKYSKSKTQTIDNIVPDIQENRNMGLVQVEERRHEPVKQNYDFENTIPVGESSEGDTSIS
ERKMNLSPQNMGLDENVGPDLGIVLDKDFDTALNNRDRKESSEIINYNSLDFEALMQDSI
THASSDQTATEESIQPDSISDDGCESNFDIELEKKLDLMILENPLWRKAMQDTYTKEDIS
ATQKVGESKTSTPTDLIEGSGGSSKIFDESNVVNDKQKKEMKKEPYTAVQYLQSNQWSCV
RPDAGDLETPLASNVRKPDQDGVGVTKGFSKYPSRSQPTSLPTLHSKDDGNGNATNVDEE
YSGNNSANPLDLPRFPAISTDFTVDSIALTSTVQTNVESLGDESLVTRNMINAGTDPLSR

DSEDTGRVVDENSTFSTKAIQGFCADDIKKFFAKPSILFQCTGPVAIHSVSSPSHGIFGD
TGSETEKR
>comp6226_c0_seq1; 378 253 1329 LEN=1368; translated
MVQSAGTRNGVYGAWGNQKTNILLLLVLMPNAVAFHTMHTPSSPVRGFANNPSLSHKASE
LNMMSWEDAMFNAQSTASSMASISLDDPSNLLSSVPIMYGAGLLTAFSPCVWGLLPLTIS
YISTAAGEREDKEIVLPTLSFAFGLASVFCSLGLIAASVGGAVYGNSGSTNIILPIFSNA
ICCLMAFQLLELIDIPLPALKTQTTTNSQSSNEQILIDGSGKILSSTKDSSSKTKGEQGS
LFRTFLLGGSSALVASPCATPVLTSILAFVASSSNPLLGGFLLLFYTLGYSTPLLVTAAT
GGKALVGLKELGGKDETSIYASIAPWVTPFTAGILLWYGTNGLLTAVLGDPSLAGLLS
>comp6226_c0_seq2; 307 253 1425 LEN=1464; translated
MVQSAGTRNGVYGAWGNQKTNILLLLVLMPNAVAFHTMHTPSSPVRGFANNPSLSHKASE
LNMMSWEDAMFNAQSTASSMASISLDDPSNLLSSVPIMYGAGLLTAFSPCVWGLLPLTIS
YISTAAGEREDKEIVLPTLSFAFGLASVFCSLGLIAASVGGAVYGNSGSTNIILPIFSNA
ICCLMAFQLLELIDIPLPALKTQTTTNSQSSNEQILIDGSGKILSSTKDSSSKTKGEQGS
LFRTFLLGGSSALVASPCATPVLTSILAFVASSSNPLLGGXPAAILYPWVRQCYTSMFFV
FKIKQRVITFYALFYHLIFLQIFDTLTVTAATGGKALVGLKELGGKDETSIYASIAPWVT
PFTAGILLWYGTNGLLTAVLGDPSLAGLLS
>comp6235_c0_seq1; 291 72 1022 LEN=1669; translated
MTREEQTIAGGSGANGPPPEKCCISWTGGKDCNLALLECWRDPSLNVTDLVVFRPESRAD
FEAHPMELMKAQADSLGLALRVMVLSASQSYRDAYVDAIRRLRDDLGIRVIATGDMDLVE
IENSSDNYIKECCEMLPGGGIRAYLPLWKAEREDCLRTLLVDNGCKVVFSCVKSPWFDAS
WCGRTLDETAVREMEAIRRGEVDRVSFTEAERSGWGTKDPRRIPLDLGGENGEYHSMVLD
GPLYRYGIELRAAPGGDAGGIDSRPTASVPLRSVVSGPAKNGEDRWWTYTGQTRWSLVGF
EVGGVPENGSPEARER
>comp6242_c1_seq1; 230 1 806 LEN=897; translated
XRFSTYAVYWVRAAVLRCIAERDDVVRVPGHVSQAIVKINKAAKRLGWVLQESGGILVTE
HGKGSKSVVSGAACESPSWRAARAAKCLAEEAGLSEQHFEEAMRARRRRYSGGYVPVEQW
MESSGRSGAGGYHTDENEDQDRTRMESEAANEDIRTVLSRHLRPKEMEALSWRYGLFGDE
QPAERANRQLLEMEDRLFGPSAVEAAPGAKPASAAPAKGGRWGEAMTFVEVGKKMQVSAE
YTRRLCHGALDKLRRAAEEGTLEPAFLC
>comp6247_c1_seq1; 339 92 842 LEN=1118; translated
MSEETAAAATATPPAAADEDNEEEDLEKLQKEIERMEAEAARITRETDDLEKKKEPGEAS
ASTPGANGGGGDTGGSEADKGEGGSREATSIYVGQVDYSTTPEELLGHFEACGTVERVTI
VCNKYTGKPKGYAYLEFQTEAAVENATKLDGSEFKGRNLKVMPKRVNVAGYHYQQGGDDG
GAGEEDVATPAGEATGAAAEDGAGADTAEATGEDGAAGGVATIPTTRHCFIRTNRMESNR
IRRHLLGTR
>comp6247_c1_seq2; 284 92 511 LEN=1593; translated
MSEETAAAATATPPAAADEDNEEEDLEKLQKEIERMEAEAARITRETDDLEKKKEPGEAS
ASTPGANGGGGDTGGSEADKGEGGSREATSIYVGQVDYSTTPEELLGHFEACGTVERVTI
VCNKYTGKPKGYAYLEFQV
>comp6259_c0_seq1; 200 1 1322 LEN=1322; translated
XFSNSSMIARRSYLSTPMLEASNFEESTKRKYLKPILVLAIYGAIFLFVEHRVNSSILSG
DQVSERLNATNKIPTTTTGKEPGYEDESIELDPTDGSAKHSIAIKPKFCSRERIRKGEWK
AVRREKPLYALDEDAWENKCYQKKQTEKPAIIDSWEWIANENGGDDKADCSFAPFEVHRF
CQLNSNRTIAFLGDSISWQQFNSLNYLLGATDEYRTTATIKINGVMTGSINNNACSGNTK
LLWLRDNRAKVSRLHHIIGLYDPDVIVLNRGAHYVESSVLRMELNLTIGRALEWQRDCDK
RGGERDCLLVWRTTAPGFPDCENTPGPIGIDRQAMAKDTVSDPSHPWYQTRLNGHKFHWW
DFAGQNAIVEDLFQSRIRQGLRVSFLDFYEMAILRPDLHIGKGDCLHYCLPGPHDAVNAV
LLHEMEVXKINSEVTLSPLAAX
>comp6266_c0_seq1; 540 1 1877 LEN=2480; translated
XDDDDEEEVIEGEARLEIQHYGCSEKNLLKTSVYLEALLIGHEEMVTSVAWHPNPKALYD
EEMILVSSSMDRSIFIWSVTDDGVWTPISRVGSPCGILGGPVGASLLGYLNIQIEPHFGR
WIMGHAYGGALHFFSCERNTEDRNLNVESSEQSHMSIEERAALVKWKAQPCITGHFDEVT
DLCWEAIKGEYLITVSNDATCRQWAPITKLLDAWIEISRPQVHGYTLSAVTSLSTPDHEY
QLVTGADEKELRAFDGTKSFLRMLRLISEARGETNCGATRNDISRVDRAYMPSLGLSNKD
SAADGADEDTAGASTTSTLLPIEKDLGSTSLWPEVRKLYGHNSEIARLTSTLSARTCLST
QYSTSYFNQVLVASTTKARDVNTAAIRLWDINANRCLQNLQGGHRSTVTALSFSPDGTYL
ASSGKDRRLCVWKRRTDDNTSAQNELFFLASAVNSSHKRIVWSVHFCPHNPTTLASGSRD
GTIKLWKITEGAGQGDQNTTVVEYSKFVPSVDVDTGHASKDKPEAVTSLAFSPCKFNNQN
NGVIAIGLENGFIQLWSVPIIPSLDGNVVERPSTLALFDAHFCHIGAIWKLAWRPRRDDE

IGPFVLASCSSDNGCRIFSVWLHNE
>comp6268_c0_seq1; 130 1 819 LEN=951; minus strand; translated
LFSLASKSWLPIPLQRNQYAVAHYRAKYPGEPFRETQNRTVLRETTIHAVECAKTRVGHS
RTQDNSQNNRHSSSVASERVSAVYMASDTGXVNILQRFRRSTSRMQALILRMVRKNSIVS
FPLPIFEQTKSTIRLLTSPGIEAAREAYVSLNDSGSKDGDHEKDSLNVWTYLDLQSYDTD
YNIAASDGIGQIKTKNTIALAEDPPHLNFAKPDHLSTFYSIFVDLFLMSYSNCVVYGAGG
FGRLGSLVSYRPRCGMAFTENQGVLQQCDPYD
>comp6272_c0_seq1; 409 79 1550 LEN=1748; translated
MTSKIIRNRISQAALYILLGVAFVASLPSTIEAANGVRGQPLRRLSESRVRGARQHLGTI
KKGTEGSGDKQTSKQKAGRNTPLLKNVQRPVAKAFDKLNIKGALQKPAKPEKQLPEPEVT
KINVKKGSGSSRRPSKNPDDLVATTEIVENGTEHFTEVKVEVYQRELPPLSIKFEIDHSS
TSGSPTMGDYNELSDVAEEYLDDFLTSVFEDVPVRHDGTKLFITVSDEDQYTVEFKLVLD
FLIPGEVPTIKYVLFLVLLKDXAYQNTGIKSLLTSNVSIRVLPWFGLHYYQFRDSFLIDQ
LREGLETETSQLFFISKLETMSETNPFSGTEKYNVVSRPPMSAAEIAGEGRKPQVNTVEH
TGKHYILASVLAGMGCVVLVGAGLLWRNKHSAESSTPVSGQKFSLFDKSRKNDDSSGANS
TGLYGFDTETMSYLKSLRLRYSKDRDDKTPSLSPKSNIAAVEQTGSYDDGEESLCDSVNS
GSGLERVDIM
>comp6272_c0_seq2; 448 79 1419 LEN=1617; translated
MTSKIIRNRISQAALYILLGVAFVASLPSTIEAANGVRGQPLRRLSESRVRGARQHLGTI
KKGTEGSGDKQTSKQKAGRNTPLLKNVQRPVAKAFDKLNIKGALQKPAKPEKQLPEPEVT
KINVKKGSGSSRRPSKNPDDLVATTEIVENGTEHFTEVKVEVYQRELPPLSIKFEIDHSS
TSGSPTMGDYNELSDVAEEYLDDFLTSVFEDVPVRHDGTKLFITVSDEDQYTVEFKLVLD
FLIPGEVPTINFLIDQLREGLETETSQLFFISKLETMSETNPFSGTEKYNVVSRPPMSAA
EIAGEGRKPQVNTVEHTGKHYILASVLAGMGCVVLVGAGLLWRNKHSAESSTPVSGQKFS
LFDKSRKNDDSSGANSTGLYGFDTETMSYLKSLRLRYSKDRDDKTPSLSPKSNIAAVEQT
GSYDDGEESLCDSVNSGSGLERVDIM
>comp6279_c0_seq1; 255 1 1162 LEN=1162; translated
XDAVDPSSGRRRSSGEPLIARVDLWHGPDSTPQNLAIYLEEEADEDGDDYDEDWGASGSA
AQGHSPRERSSSNGNGNAHTSSVDTPFSTIIETPQGHNTIAIRNIAETSDLLACVEGEER
DYDYGHQESWFTPSAPPASDSGHSPLQSVIQRLKATTTPQRIDSTIANPKHTQGEGYQDY
DDYDEDDEANQPVVGSCAVELPSNIASVQVLLQTQFMQPLQARIELELREGDSENTPTGG
YRVVKRTIVEVFSEDGMHRPFFAVLEAPRKAGWFGSESGAAPPSRYTTSMRVVNLSTTEF
PLFATVEPHAVDDHEPRRRPAPSGDQRGEEAGDGPGASQPEWTHFGGXEQLRRRVQLHDP
GRGDLVGRRSKKSGLAGWLALSSKTGGDX
>comp6282_c0_seq1; 503 131 1380 LEN=1380; translated
MVGARATMMRSSMTTRRLVTLFILSSILVSLRHAGMFYTRQLGNVDDGYYSDYSNGEERP
QISVITLDGLTEPPDWNNTTAKSTSGSGNRHTATIDSRSEKNHISGNTTDIDKDFEKRAI
VLISMGQKAENMSMVERFVWSARNIGKYKGWIILITDASYDRYADLKAAVIPSEERGQEM
DGLQNNPTTLSPSLAMKGEGDNLDADMMVTTKEDSDQNAMENKFLVFRPKEKRFAKITKH
GRFTQSKSMNSKIFKTYILQYAKKEPLLDSVELFYYLDVDIVFGNNISPLFKGLEGIYDF
GLHTTSSEYNQSTNAQIHFFEGNGSQEIQGGQIVLNRYRSQPCLERWRELMQATRRERYL
KDQLSLTVMLDEQRARNSKNASAASNHNHSAIIGGTPSTSQSHIGASTDDKDKKKKX
>comp6284_c0_seq1; 587 1 2652 LEN=2769; translated
HSSANESVEESGTEVTHSGNAELHMHPAHAVLYPPLILTLGVIVYYCLSRYLHWLPYTAL
MFLFGVIMGTVSASEVFFRPNGSETYMHDTLTAWQDINSEVLLLVFLPGLIFKDALGQNA
YLFAMGIGQLLIFAFPLVLAGTALTACVAYYIFPFGWSFFLCLTFGSILSATDPVAVAAL
LEEVGAPPRLSTHIAGESLLNDGAAIVFFYIFSRIFYTEFRVPVGDSIGWSEGIKIFAQM
AFGGTAIGMVAGGTIIFVLSKLSRRFGKEENIVQVTAVVGLVYLTYFIADLICHTSGVIA
TVAAGLSVKFFGRSSINDIHLMDDFMGVLEHILNTVLFSLGGLVWGEVLIETHMEGYWGA
KEWGYLILLYVLLHIIRFVLFAAAYPITVRIGLRTNWQETAFQIYGGLRGAVGIALSIAL
DNQVLEYGNAQDLSEERRSVKQLYAMVGGIAFLTLLINGSTAGPLLKKLGLADSTEERGK
IIEAYKVRLRSEQIDNCVKLLTFKMFKSVDFQFIQDHMPYLRDLTIEQLAEAVESLKDST
PSSKYSPPHLENVLSVLKRQDRDANVEAHTILTEDSARFLRMQRIESRKKGRKGVCRSSM
HHMMTGDPLSAKELRLLFISMLQAQYEKLIDEGFLTSQHGLTIAFEQSLEMAESDVNKGG
ALNDLTHLTEFYTIALNCTRVAFKATAWLRRDKISSFNDFELHHFILLELAFVTAHERAQ
DFFQQELGDSDAQLSEAGKIVIRESKRQVSKVEEDLNSPELTKFVIEVATYKVCDVLLSQ
SIHGVERLVRYGLLKESEAEHMIEEMIRVQKEVRRGKCNMNAHASKRHVSILDVNRETRR
NSFLGGTGKKKPPATVAENARMASVSADLDGLMESNSIHLEEA
>comp6288_c0_seq2; 190 400 1032 LEN=1577; translated
MIQTSSRFLGRLLGFTSLLILLESSGAFQPRKQNQASTPSKSSSDEKKTVMSDEMNILFE

RHDRVANDLIDKAQEFASHFGKYSYGEVENMRDNLHKHRVQNMAIGNDEYSSDMLFLERV
LEDDLNFQLAALEADMPEPYLFRYPDKDPVATASVEAVADPIIFHESLEEDKVANYEKSN
LYGELVQEGVIESIAICAILGFLMVTPAHVW
>comp6290_c0_seq1; 588 299 2028 LEN=2028; minus strand; translated
MAPSPMKTPGKASGPKKVYLFNCPYKEKASAKKAGAKWDASAGCWYVLSSQNLEAFNKWH
PNENKYLVCSYDEKDDAKRAGAKWDATCKQWYIAANQDESRFARWLPSSAGNRATKATKL
IKGKKSEIKTTKTPTKLSTKSQLATIPRINGDMTVAQLQTECKARDPSIKGITTKRKDWF
LEHLKVGTPWISAEVPYLDYEPESPPXKAKQKLTTSALKASKSNSDTKKNNSAVARISAD
MTVAQLQIECRARDSGIIGISNKNKNWLLEHLDVGSKWVSQGWKEKDLQNLISANKKKAS
SQTKQQSSSKHKDSVDCRDIEGMARQTSSKNNSKKRLGDFPVNNITIPQQVGKKAKKDST
SAKKPSVKLVAESVHKNYQQNDFVKLKKEAIARNVEIAKQKSLHVHKFPRVHRCAMAKSN
RLMMNGNARDGECDLCYQKRERHFFVVGRSSTAIYTCEKCDFDICQKCFDEENRSESEKE
KIRKKRQREREEETRRIQEREEQRAQRWDSNLRFKNTITKVKGKHLDAESGLKFTVWCSD
GYDYDGRHSYEGEPTKEFDSVWKTKEEANSRAEYYSS
>comp6290_c0_seq2; 584 299 1602 LEN=1742; minus strand; translated
MAPSPMKTPGKASGPKKVYLFNCPYKEKASAKKAGAKWDASAGCWYVLSSQNLEAFNKWH
PNENKYLVCSYDEKDDAKRAGAKWDATCKQWYIAANQDESRFARWLPSSAGNRATKATKL
IKGKKSEIKTTKTPTKLSTKSQLATIPRINGDMTVAQLQTECKARDPSIKGITTKRKDWF
LEHLKVGTPWISAEVPYLDYEPESPPXKAKQKLTTSALKASKSNSDTKKNNSAVARISAD
MTVAQLQIECRARDSGIIGISNKNKNWLLEHLDVGSKWVSQGWKEKDLQNLISANKKKAS
SQTKQQSSSKHKDSVDCRDIEGMARQTSSKNNSKKRLGDFPVNNITIPQQVGKKAKKDST
SAKKPSVKLVAESVPDASLQRKRPAKKEQNKNYQQNDFVNAQERSYSAKRRDCKAEIVAC
TQISQGPSLCHGQV
>comp6290_c0_seq3; 632 299 2073 LEN=2073; minus strand; translated
MAPSPMKTPGKASGPKKVYLFNCPYKEKASAKKAGAKWDASAGCWYVLSSQNLEAFNKWH
PNENKYLVCSYDEKDDAKRAGAKWDATCKQWYIAANQDESRFARWLPSSAGNRATKATKL
IKGKKSEIKTTKTPTKLSTKSQLATIPRINGDMTVAQLQTECKARDPSIKGITTKRKDWF
LEHLKVGTPWISAEVPYLDYEPESPPXKAKQKLTTSALKASKSNSDTKKNNSAVARISAD
MTVAQLQIECRARDSGIIGISNKNKNWLLEHLDVGSKWVSQGWKEKDLQNLISANKKKAS
SQTKQQSSSKHKDSVDCRDIEGMARQTSSKNNSKKRLGDFPVNNITIPQQVGKKAKKDST
SAKKPSVKLVAESVPDASLQRKRPAKKEQNKNYQQNDFVKLKKEAIARNVEIAKQKSLHV
HKFPRVHRCAMAKSNRLMMNGNARDGECDLCYQKRERHFFVVGRSSTAIYTCEKCDFDIC
QKCFDEENRSESEKEKIRKKRQREREEETRRIQEREEQRAQRWDSNLRFKNTITKVKGKH
LDAESGLKFTVWCSDGYDYDGRHSYEGEPTKEFDSVWKTKEEANSRAEYYSS
>comp6290_c0_seq4; 540 299 1557 LEN=1697; minus strand; translated
MAPSPMKTPGKASGPKKVYLFNCPYKEKASAKKAGAKWDASAGCWYVLSSQNLEAFNKWH
PNENKYLVCSYDEKDDAKRAGAKWDATCKQWYIAANQDESRFARWLPSSAGNRATKATKL
IKGKKSEIKTTKTPTKLSTKSQLATIPRINGDMTVAQLQTECKARDPSIKGITTKRKDWF
LEHLKVGTPWISAEVPYLDYEPESPPXKAKQKLTTSALKASKSNSDTKKNNSAVARISAD
MTVAQLQIECRARDSGIIGISNKNKNWLLEHLDVGSKWVSQGWKEKDLQNLISANKKKAS
SQTKQQSSSKHKDSVDCRDIEGMARQTSSKNNSKKRLGDFPVNNITIPQQVGKKAKKDST
SAKKPSVKLVAESVHKNYQQNDFVNAQERSYSAKRRDCKAEIVACTQISQGPSLCHGQV
>comp6301_c0_seq2; 39 1 498 LEN=555; translated
XTIVVGKNSDRDRREHTARQNGLESTHTRTAPGCSATSPARGEPKNYVRYGATNGQNLFL
KVRRNLGRPIGQRGTEMPRLVPGPIPGDPKPGAASPGTTAGKQHDVAPVTPHMANXTQQY
AQNASNERAAGGMRCDNGKEANEPMGSIGACMHAVHNKSPQSTKMN
>comp6308_c0_seq1; 636 90 1780 LEN=1780; minus strand; translated
MSFSAPRRPLDLAFVVVQSPQVPHPMGDNNSNSSNNYADSDSDETFDDMPPLTDMDTSTS
IHANIDADNDANTGGTGTNVVAMTGIPSERDIYEETNLRTEYIVSEVDEDEDEDEYEDED
YFDEDEDDADTAAEVMMAHYLQRVLEDIPEYDTGDVFEDEDYEESHAYDDFDEDLEGSDP
CNCPQCVLHRIVGRHSSCYEATDSCSDEGDEHDNDVPLDPLASPCAICMEPETAARRFAC
LPCCSSGSRMATRQDSHGHHLPGTLPPTTTDTSSTRFCYKCLVTCLVKNGCGVHRTLERG
DHDHGSSDKTNGNTDAQLAGECPRCKKLLVLEELPLEDNKDASKPKWQHPLPGGLPKQCL
AKPPLARKASEEIAYHKRTVAAIPSTEAMFWYVASRNHLRIYLVVLATCPNPRYIPEELL
LTIDPSSTERIRLLCQWGLIAKRQNSPACASTKSRTSLRKRLGSIMQQLWQRGPLGTAAA
AAEMPDRSDAFWNKHYQAILGSVRTWRISRACCSLAEASRNLPNRSGTVYQIDPTVQHTL
HRLSAMYLQLGDLDDPAIDRVGEX
>comp6312_c2_seq1; 201 1 1144 LEN=1144; translated
XPGGTPDAGARRLPDRVVRSLRYERVLGAGSYKRVYLVSATSRPPEGGDPIAGATAPRTF
RYAMAVERLRNKRDVKNAFRGVRIPDLIREGPEGGTPVDAGDRELFETIVDWWVQASDVP

EFAGGRPVFPQAQRGDGPLPGLDRAIGRTRSAPRKNFVGSRWMLSFKPVYETDLKRFVRN
SPALYPVGGSSETLRGSPVRDEYWSEPVLMGFVLEVLRAGKLMHDAGVVHRDIKPKNIMV
SLSPSASDGSLRRRPVIIDYGFSEIGSPTVLDEGGKGPPGRTAEKASDVCVVRPGQLKGE
IGYVLVEDLANYRGCQRGDTYAMGKTLYEFIFGSAGLQEARTEQEDRDQEEISVEAARVQ
ANEFHSLLFGNPTAGNESRFRL
>comp6320_c0_seq3; 165 1 709 LEN=1157; translated
XLLSSNSNIPCNSDKRKMISMNRKIRKRNFRRHLPISMTQFIRSSYVVVCILYPCFCLAI
IGPNWGHRQIISSVLTSKSSASTVKKNASSKVETLSEPKEEERLQSFRDTANELLTFVSR
KPPRYVQHVLYWVTATDAILSVILNKYCSFSMSSPLIAPQAEAQSVVREVYSKLFYFARM
RPRLLYSIGALLRALSLCTPLRHLFDPTVGVGAGIHFFASLASSRWGEYCLLLRSS
>comp6320_c0_seq4; 197 1 853 LEN=1047; translated
XLLSSNSNIPCNSDKRKMISMNRKIRKRNFRRHLPISMTQFIRSSYVVVCILYPCFCLAI
IGPNWGHRQIISSVLTSKSSASTVKKNASSKVETLSEPKEEERLQSFRDTANELLTFVSR
KPPRYVQHVLYWVTATDAILSVILNKYCSFSMSSPLIAPQAEAQSVVREVYSKLFYFARM
RPRLLYSIGALLRALSLCTPLRHLFDPTVGVGAGIHFFASLASSRWVKPLILGWATTKSF
WTWLGARPVDRAYVPITLSIHEWEEKKRKRAKIENTKSKKDDAS
>comp6341_c0_seq1; 1092 413 2421 LEN=2612; translated
MYGDNTTSNVPKDSFSENNRKLSPAIPRPRPTLGAMASASSLSSSSFRFGNTEADSTTNS
SRNYSDDSDIPWGDRKPAAKVFPFSDYGNDYQHFQMEDIDDDDDDDYXNGPEDYDDPDMD
LVEYPSKEKFESSIATGSDEEFELNTPTKDGVLLPRNSPSQRIIKRLASSSSVVAKPSPS
PDKKGEATLKTDHSSHTDDEENGRCDCREDDDGHDDGHDDSSALLAAKNGPRTTKTTVFG
DGTAISNRELLWAASIFFLVVGISVLTAALVLIKGQSSSILDANSEGRIPSTPSASPPFG
EPPTPPPVVVAPRLSVQNEWKLVYAAIRDNPLTQSLLVSGDGEEVLPADPTFYEGLVEGM
VFSEDHRDWIPAVMPLSDDNDDLVNLYGMTTTDQQQIAGNNNGNSINTNNHSGGQHIGMP
PSAAKKNPMTPNQKATAWLLYHDRRKDPNESVWRWALASIYFKMGGHGWTIFRRDQEADR
SSPKWFTDAPLCDWERLEGCSGYFKGNGNRNPQAQTHLHHAEHHRELPVDLDFDDSNLSG
SIATEFSLLRLYNTGSDDEDTVLRAITLTDNRLTGRIPGRVFEFLFPSLGKLYLDGNELT
GTIPYELGGLDALYVQNNALTGDWPEEFCADGENPAGSESENGLTVVDDFGMDCDAIECS
CCGILQCYY
>comp6342_c0_seq1; 622 15 1819 LEN=1819; minus strand; translated
MTESDAELIERCNGTSRTYEKTSIVDFQHSSPDDVALVVDGSCVQEVTFQEFQGFISNVS
SFLLHHESLRHQERVGLLIKSSANAMAVIYGIINIACAIVVLDPEKTPIDRCKMIFDDAN
ANVVIVDDEYHSAFQELASDVIGRKLCLISEVVSFNSSDSSTAKVEALPEDIFGIFYTSG
TTGTPKGVVIKHSNVLNLVCWWKEFFDVTHKDSILLFSSLSFIMSLRQYIPTLHAGATVV
IPETALDFESAIVAGKVNKLVCTPSALAALDMERVANRIEAIQLAGEAPRKNTLDVWSTK
VSKVFIGLGPTELCAHAVCSQYDGETLCIGYPAANVKLYIVDPNTGLQCPVNVPGELWIA
GENVSFGYLNREEATKKSSHTWLKNGSXLGDLAKRLPCGRIQFIGRNDQQIKVNGFRMEA
GEIYNAMPKTVEKSHVMVQNNALTLFASPTSINVNELKSHLLKILPTYMVPTVIHTLDDF
PLNKNRKLDVQSLLLMVGTSQPEEEEAQEDADFGLSNRTETTLKLIWCQILNKDVKSIGK
DDNFFSLGGTSLSAVVVSRMINQELQMELSVHDVFIHQTIHSLAQLIREKEKNVEDSTSD
PH
>comp6345_c0_seq1; 708 241 2624 LEN=3072; translated
MTTSDQGEEEKTFPSIRELRNELRKELKNTSPQGAIKAKGILDLMFLRYGLDKNTENTTV
SRNERRRIQKTRPMVDAIDCTQVINAWSKSRRRDAPQQALATLRRMYTTFQKDGNPCIRP
DNVAYNSVINAFAKQGDFNGACSVFALQTEDFKEGGNTKAKPDAYTFSTMINACSRSNRE
DLSETAEKLLTTMNDWHARGDLEIGPSTMAYNSVINCFSKSDALDAPERARRILETMVSR
FESIGSDSNDGHDIARPNAITYNSVLNAHARRGDVEGATEVFERMKKDFESGNNGAKPQV
QTYNILIDGWSKSNNKDAPENAESLLQEIIRMESEGKFERGEGPDCITYSAVINCFSKSN
AYDAPTRARTILETMISEYENSSEKNSVRPDTVVYGSLMNAYARQGDTKGASEVFDMMKK
DYHSGNQDAEPNVPSYNILIDAWSKSNNEDAPNQVELLLKEMIQMCSDGDIKKGPDAITY
GAVINCYSKSNLQDAPQRARDLLESMIAQCKGNDTKEDIRPNTITYAAVMDAYARQGDAE
GATEIFEMLKRDKNCKPNVQTFTILIDAWSKSNSEDAPLEAALLLQEMMGMSSSNKKQPK
TGTLQKDSLGRARDVLAEVFARYSYRNMYYRPDTVTYNAIMNAFAKRGDVQGATAIFNAM
DSDYSYSYNKESRTHEKTGPDVRTYNILLDAWARSSEDNASAESERILKEMNHRCDKGEA
HMKPDSITYRTVIRCLELFEGTEKRIEELKSIPSSAAXMTRRQGSRKQLGNGRNYGMNSN
GLRWAFERQLHQCG
>comp6346_c0_seq1; 93 1 221 LEN=221; minus strand; translated
XFMMESLTDELYNKAKCIMDDVESKGGMTAFVNDGHAKLLMEESAAKKQGRIDSGVDVVV
GVNKYRLSDEEEQE
>comp6353_c0_seq1; 438 1 1384 LEN=1458; translated

KKNPNDDIGWEPLDRSTSFNRLHPQNKNRRNANNRYSYAYAAQKIISSAHRTMSPAISGL
VLGLLATYQQVPSAAAFMAPNTNQAMAAAAFLKEPSVAASPTSTLRMVGEEFDLSGASGD
ETNEVRVEPDTHDELMYALGVNLARQLGDIRPLVESGEELAQVAKGLLDTVVGRLNDDGQ
RLLLSKRGRELDQLIAGRANALREKLELRGREMLQNMADTEEVIALDSGVCVHVLEHGPE
GPGQGARATAGSVVVIHYHGTLSDGTVFDSTLGGEPVKFPLAQVIPGWREGVLAMHKGET
AMLGIPPEEAYGAEGTPDGRIPGGSTLFFKIQLVDILSGAVGGAPTLFGADGQPVSSNDD
SGGSGLIGVDGQALEPRRPTTAEPALPAPGRVRRRRSGAPASVPRFPGEATTRKKPCPL
GIKKPRNTVELFEPILDCSCSQNLAMGLVSLASNNKKQK
>comp6353_c0_seq2; 196 388 1123 LEN=1197; translated
MADTEEVIALDSGVCVHVLEHGPEGPGQGARATAGSVVVIHYHGTLSDGTVFDSTLGGEP
VKFPLAQVIPGWREGVLAMHKGETAMLGIPPEEAYGAEGTPDGRIPGGSTLFFKIQLVDI
LSGAVGGAPTLFGADGQPVSSNDDSGGSGLIGVDGQALEPRRPTTAEPALPAPGRVRRRR
RSGAPASVPRFPGEATTRKKPCPLGIKKPRNTVELFEPILDCSCSQNLAMGLVSLASNNK
KQK
>comp6360_c0_seq1; 341 44 1195 LEN=1311; translated
MFGSNQSGKQKRLTSYFGGGTKDDKAKNRQSSGKKRPRPVSGTSFRSHNGGSGFGVCPLC
QLSFAWHILENHASECTGIAINHKNSAKRSTATAVESKSPEEKSTPKQPTKSEERDEIIP
LENVIEQNSSFTKTPNAKRKRQSCIVTPRLAPIFQAKAGERGNNQALHSKQHQQLRPHSY
EPIPGLFVYEDFITEEEEALILKACVNVDRDVLPAWKQCRFNGKHIGKRWGVHCNLRDRR
VDSPENPLPEVLSEIVFPKLRQLNLPATKNFWPNEANAIDYRRRQGHWLKAHVDDRKLSK
EAIANLSLLGDCYMTFCNVSKHRNLAVSEQRVLLKRRCLQVLTGKARYDFSHGIANCDLL
TDRRVSITMRESPLTRTSPTIDN
>comp6392_c0_seq1; 134 1 855 LEN=855; minus strand; translated
XEIERVKLGSKNSMKGRFFPTYEYRLFIRHRPADPMSDLFDSQGLIDGIERRDTVLMMAK
NRGRKHTESSMQASKKGSNNYYLSLPLQDDLDLHHRSVNRLEETKKPSPNGAGLRTCSSD
LSGQLGRLQSNFVGTEFQIFTSHLSMMRKKPDSLLSKSMHSPTMSDDEALSDSGRQGESS
RRRSCFGRLSLRGKSNNDGRWTDFSEPPQPHSSPLALLRPRSGDATPGRNRKVSAAESFE
SKQYIAETQPLFYEQEDGAITYTANLLGSRPRIMDVCIPRVNHDGX
>comp6398_c0_seq1; 326 138 1142 LEN=1142; translated
MRSVSLFVALAFVALGTTVGFTQYRRVEFDGARKTASAALRNRRLVACAMSSLDYYSIRN
TTLEDEREALKSRMPPPVNEDMTKFQVEFRELLEGILYTPEELSTVLNPRMRAILEGITA
SYYEFPVYHAFEILYEDYIPLRLAGRVVYRELRKVVDESKNYKESQINAAMEGTGMPRSE
VEACWSTFAEIVSDRRLPLEDLESYVGTMTLKYVLDSGSESIGNRAREEGGVSFEELLVC
LNNYRIRYSGNDGEETNATAFMGKPATGNLLQQALNPSGDAAAQARKGHELSPKRQKYNQ
KYDDMLVQFSKWKPLIPSGEGRRLQILKGCFVGSE
>comp6399_c0_seq1; 282 103 795 LEN=968; translated
MMKQTLLSSLLVTLFAAPSLSFVPQSQRGYIGQNAGRMNRILPSQRAAGSSDDDISTATT
ESTTDSDENDTGADAGKRSSVFSESVQEEAREVLEKVGWAGVAPDMSDPEQPMTSDDPFV
QNIDAGIREDFGVGLDDLLNPAKVVNLERELYQLREELEGTTDESTIQSITKSIEKKEGK
LAIERRSVFRGWLKNIFVGQAVISFGISYVMATNPSSLFGGFDWYYYNSM
>comp6399_c0_seq2; 381 103 1128 LEN=1575; translated
MMKQTLLSSLLVTLFAAPSLSFVPQSQRGYIGQNAGRMNRILPSQRAAGSSDDDISTATT
ESTTDSDENDTGADAGKRSSVFSESVQEEAREVLEKVGWAGVAPDMSDPEQPMTSDDPFV
QNIDAGIREDFGVGLDDLLNPAKVVNLERELYQLREELEGTTDESTIQSITKSIEKKEGK
LAIERRSVFRGWLKNIFVGQAVISFGISYVMATNPSSLFGGFDWYYYNSMDISIQVLGYW
FWWLFIVPSLRSRRPRGFEKEALDIAFLGTPLVSLAAPVLTKDTGLIWFANFVIVAGAYG
FAYLTDGDDDSDDSDSNKPAWLKFVYKSLDFGSGRERGARQ
>comp6401_c0_seq1; 654 70 1278 LEN=1494; translated
MKFLLNFLTFGLATAALPIYGNPKNKAPVKKFDSLNDVKADSALGMSLLSKARKLEDENE
IDFTWVSNFSIKFQGCHHISQWNDEADGEEDVRIETKRLIRFRLCPAYECSPDKAGGCDS
NYGDYVIDMNTFLESYYEALENYNQYKCEYTANMLCDCEDGDDKGDDFDAEICEYDCYVE
HGIADTCADRNPYNDDEQEQEEEFNLAEYVECKQAEFQNEERRRLDEEEIEYFIGPYCAE
QGGAIFMGLFTDDTCTSFADDYGGQETYSSMAGKSLPYGSESLISMDCVSCKEPEDFNND
GNDAQDEDQVIEMCEQIYQGAGKCEQGLESTGYVSSANNNACNYIQGIKVVRKDGIITQV
GSKANKTASIFIGVFVVAFVLLAAYVYYLKTKLDRASINLSD
>comp6401_c0_seq2; 654 199 1407 LEN=1623; translated
MKFLLNFLTFGLATAALPIYGNPKNKAPVKKFDSLNDVKADSALGMSLLSKARKLEDENE
IDFTWVSNFSIKFQGCHHISQWNDEADGEEDVRIETKRLIRFRLCPAYECSPDKAGGCDS
NYGDYVIDMNTFLESYYEALENYNQYKCEYTANMLCDCEDGDDKGDDFDAEICEYDCYVE
HGIADTCADRNPYNDDEQEQEEEFNLAEYVECKQAEFQNEERRRLDEEEIEYFIGPYCAE

QGGAIFMGLFTDDTCTSFADDYGGQETYSSMAGKSLPYGSESLISMDCVSCKEPEDFNND
GNDAQDEDQVIEMCEQIYQGAGKCEQGLESTGYVSSANNNACNYIQGIKVVRKDGIITQV
GSKANKTASIFIGVFVVAFVLLAAYVYYLKTKLDRASINLSD
>comp6403_c0_seq1; 48 1 94 LEN=472; translated
XGRGGRGGMGGAMGGGGMGGAMGGGGMGMSF
>comp6405_c0_seq1; 651 92 1729 LEN=1802; translated
MTGLNKKSNGDMSDDDIDFDEDDEYDAEKVEKKAIDQQEKLKLLLQNLEVYCNDESEYDD
LRDLGKAVKSGAKLAGTILSGGYTNYSYKIHLDSDGSDNDEGNYGKKEIAVFAKIAFPYA
LWNPDTSVPYDLDRVTAEFDLMKRFSEELKTTSAIDGSEKSPIPRPYVLIDVPAAVDGTS
PRMKIFVAEWVAPTDEQWGNQYIEGEIDLRVVDQCAKTLAVINLADCDDDINQGYVETFA
KIAKGFDGPVLAVLDREDEDKAVRYVRDVVGKEKMIAIMKEWHIRNRKKEALVHGDAHVF
NMLVERKPNPFQLSSSFGANGDFILCDWEMAHKGHKGRDISNFFSFPTMSACFMAARGQK
DKADNIIASLKQFWTTYKTALAEGLDTKQAGSANGDDVLDVDRYMEEVFNSAISFFGYFS
FVAFYIVGVMTEYNETEGLTEKEEKHVMGVLGWIGIRSMEVGFLEPAAEEAFGDHDNQRL
SRMESFFFGMIEQEINELFEARKTSHRRSRRRSSMLRESSRRVSDSESGFGRMVRRMSQQ
VTDEN
>comp6405_c0_seq2; 265 141 875 LEN=948; translated
MLVERKPNPFQLSSSFGANGDFILCDWEMAHKGHKGRDISNFFSFPTMSACFMAARGQKD
KADNIIASLKQFWTTYKTALAEGLDTKQAGSANGDDVLDVDRYMEEVFNSAISFFGYFSF
VAFYIVGVMTEYNETEGLTEKEEKHVMGVLGWIGIRSMEVGFLEPAAEEAFGDHDNQRLS
RMESFFFGMIEQEINELFEARKTSHRRSRRRSSMLRESSRRVSDSESGFGRMVRRMSQQV
TDEN
>comp6413_c0_seq1; 191 1 561 LEN=657; translated
LLWICLWYPLFTPPNSNRCLLQEWEEKLTEAKEQAETFKRKYNNVKEGKDLSLMEDARSM
MSQKFSQSVDTNEGESSTLRSSSFRSTTSRPEFKRATESVASMGSNLAAHAKTLVGSFAC
AGNNERTGQVLSSERATEEWRDRRNANGVDHSRHPSAPYQQGGHPGLEMSHSTSSRSYRD
TRRVDV
>comp6413_c0_seq2; 696 1 1852 LEN=1948; translated
XNNNSNNYNQSASTPSSAARSYRSNSVGRISTGSGKSGPSYRQSLSIRERAMASVEKESN
SNLNTSYRSTGSNSISPTKMQQQQPQSKSQQQQYSSPSPQQSEGNRSRRKSPAHSVRSRA
RSLSRGRRSSTGEDKPAPAVQPPPQEEGQAAQGEAVVSPELLVDALSGHEDGLLAIAERL
MEHYDSGYDAMGEAIIDAFADVQKLFQHVVEAAHMEGAAFESSRREEEIRELKRRVASGD
VDHGMADDNDGANDLASPSSSGPVRHDEFIDQDVKDCLNEAIRNGVTFKEEQKHAQCFEL
YERACQAASALLPVDSDHRGRLQLSIARAESMTPDRGCAILRYAMDDVLRSGLRAGKIPK
NYPLSDPSKRADVVLSKPSMGNTQQSSEEQLNSLVEEMKDVINAPVYENTPLQDVARRFW
EALQENQKIRSKNEDRLEHNLGKLKGDYLLARAEWEEKLTEAKEQAETFKRKYNNVKEGK
DLSLMEDARSMMSQKFSQSVDTNEGESSTLRSSSFRSTTSRPEFKRATESVASMGSNLAA
HAKTLVGSFACAGNNERTGQVLSSERATEEWRDRRNANGVDHSRHPSAPYQQGGHPGLEM
SHSTSSRSYRDTRRVDV
>comp6421_c0_seq1; 510 1 2390 LEN=2390; minus strand; translated
VEWIETIRNASLKASNSTDVHVCSQFSLLHNRPPKTRNLRDQRKEHLLETLKKGYFGAAD
EILLVSGSGTKPLSSSPCSETPWNTADGIDAVNDYYENRGDNDVCQTTFPHISVSYNPYL
SDPHLQDQENSRLERQLATGCLSKIYLPFGTDLDRLRTGLEFCADAIHTAKRKHQRAGDV
SLVGSFVLPTPRWMAHQRKFFRSSQKRVMLDPRFFKSDENQELTAIVVQVIRMYEKHGFG
ELLLEASPSANTLSCTKDDLSFLFDILKRVSASRDDGLGPNRCNDSERSSSMGMNNFRNN
SNGVDFSSNSKGTKTGAESSQSPEILKKSLPSSLRQDWRSRSSSPCILLFGSHDVRLQDN
KALEAAARSHKKLLPVFLWTKEERDGDQWGCPRNTAVAVCLEEALKSLQDSLQSFGLPLI
YCNCCGPSPRHDSREEQLHQQENSHGIRELKHVVEAVGATAVYWNKDPTPEGRDRDAYRR
RILEQHRLPRRIDVFEAQSSLLYDVSTIELPTGFRGGHFGTLMPFLNHCRKQFGVPSRPT
PYFETFRILEESSPPEAFDTIFDMHGSTMSTHLRDLKIIDTFKLSHKWDQPIRARFPMSE
KIAQSTVDSFVRNGMKRYETDRSRADISDATSRLSPHLRIGTMSPNQLYWRIEDSELPYK
DTKTLGRRLIWRDLAYFQLHCFPSMKKKCIRSHYEAMEWVETIEEDRRFRAWKTGTTGYP
IVDAAMRELYATGWMTQSIRMVAASFLTEYLRINWTKGAEWFHHTLVDADSAINAMMWQN
AGRSGIDQWNFVLSPTT
>comp6428_c0_seq1; 452 533 1518 LEN=1518; minus strand; translated
MNDSKYMKPVGERTSRGKTTAEAKGLEWTKNVPVTKEYEFVRRDDSHMAKNETGFLEIGK
ADPTYAYDVGGTWKLLYRKPDEKPIGPGIKPKNEQPMKIFVRSWNDEIFPLDNVKPQDSI
DEIRFRLEEEHCIPREQQRLRLGSGQPLVKDRKSLEEYGIKDEDILDLEPMSITVRLLGG
QTVHLEVEPQDAIKDIKWLVGNKLDIPIDDCILLFKDKSLPETSTLDENDIHHGDVIDLQ
QMEIYVIDLDGQKLEYLVSPSDTIESIKNVLEEDTGLKREDQRLCFQDNLLKNDKMTLKD

AGIKHQDTLALEPMQVTVRSTTGGTVDLL
>comp6429_c0_seq1; 105 434 988 LEN=1165; translated
MVLAPRQLAVAVLFLPGSLSLTNPRCQLQRRRHLASTASGPPRPLLALETAPSSHHGQAS
DNADADSKPDAIAEQEKKFKIVACSKKRQSLGLDPLSTFGALYSRAAATGVRVEEGPCIG
SCKRAPCAAVEHDEYFGSVALEGMTSEEFESDAFLNIVTEEDADRVWSCVENAVKIMAAA
EEDD
>comp6429_c0_seq2; 17 576 1037 LEN=1520; translated
MVLAPRQLAVAVLFLPGSLSLTNPRCQLQRRRHLASTASGPPRPLLALETAPSSHHGQAS
DNADADSKPDAIAEQEKKFKIVACSKKRQSLGLDPLSTFGALYSRAAATGVRVEEGPCIG
SCKRAPCAAVEHDEYFGSVALEGMTSEEFESDA
>comp6429_c0_seq3; 67 434 895 LEN=1378; translated
MVLAPRQLAVAVLFLPGSLSLTNPRCQLQRRRHLASTASGPPRPLLALETAPSSHHGQAS
DNADADSKPDAIAEQEKKFKIVACSKKRQSLGLDPLSTFGALYSRAAATGVRVEEGPCIG
SCKRAPCAAVEHDEYFGSVALEGMTSEEFESDA
>comp6429_c0_seq4; 105 576 1130 LEN=1307; translated
MVLAPRQLAVAVLFLPGSLSLTNPRCQLQRRRHLASTASGPPRPLLALETAPSSHHGQAS
DNADADSKPDAIAEQEKKFKIVACSKKRQSLGLDPLSTFGALYSRAAATGVRVEEGPCIG
SCKRAPCAAVEHDEYFGSVALEGMTSEEFESDAFLNIVTEEDADRVWSCVENAVKIMAAA
EEDD
>comp6430_c0_seq1; 290 235 1140 LEN=1615; translated
MIVVCKTTHKLGLLADRQILIKSKTKQNHHAKLQKAVPILQESYSKTFNDRTEYQAGAPF
DTDGSKQAGVLGIVNELFGMYFRLNILRLCKNLIRPVETKKLSEKGSMAEMVTYTYYVGR
LSMFEDDHARAETNLEYALAHCHRDATNNKKRILKYLVPVKLYRGKFPTTRLLDKYGLEE
FKPLVEGMRKGDLRTFQDGLVKYQDRFIGQGTYLLLEKCKTVVYRNLFKRVWKIMDSYQI
KLEQISKAFKWLGMPIDLDEMECILANLIFKRYIKGYLSHAKRTLVLSKRDPFPTSAVIS
N
>comp6430_c0_seq2; 345 106 1338 LEN=1813; translated
MVKIREIKQTLENALINKDPHRLAQVFEPLVGVASSAIATNGGRQSQKHNPQSFTVNGKN
CGGLLTSLLDAYAAAEVGDNEAAFEAQVKVHPKFNQLFSSSEGNWMVPAMIVVCKTTHKL
GLLADRQILIKSKTKQNHHAKLQKAVPILQESYSKTFNDRTEYQAGAPFDTDGSKQAGVL
GIVNELFGMYFRLNILRLCKNLIRPVETKKLSEKGSMAEMVTYTYYVGRLSMFEDDHARA
ETNLEYALAHCHRDATNNKKRILKYLVPVKLYRGKFPTTRLLDKYGLEEFKPLVEGMRKG
DLRTFQDGLVKYQDRFIGQGTYLLLEKCKTVVYRNLFKRVWKIMDSYQIKLEQISKAFKW
LGMPIDLDEMECILANLIFKRYIKGYLSHAKRTLVLSKRDPFPTSAVISN
>comp6431_c0_seq1; 1221 1 2264 LEN=2264; translated
XEISKRLFLDIESMGKQYQALHEAQKASQRGDGERDRNLDPLSSSSVLSGLQELYIPPDV
DVTDTDLPETIFGQVDLQNVALAKLVKKSTKRLNAASTDGDDAIRLLDLSIVDSDGDDDD
SDSDSDSNSDDNSDDGENEASEANSDDEDEDDEAERIRRRMERAMEDMEEEDGDSDGEST
EGPGTRKQAEPKKPEEELYDPVAEDMNDGFFDLNEMEEFADEEEDFLPNEAFGQPSDDGP
AKKQNKKSFHQRQREGIHMDDDDDSDNELDSDDDSDGGGLYEEVKRDVKRKKYRDQEDID
ALYELYQETNPEDDDEDDLDDVVNMTAADYFGAPRRNYMERYKKNHPPAKPSPRTNDGDD
DSWGEYDFENEDQEGWRDENNDDGDDDDDELEDMKDPAPGEGESEELEAPRRSNADTSRK
LSTEDEKLRKQTDELEKELINEKPWQMRGESKSGSRPVNSLLEGTPEFEMASKQAPIITV
EHTMNLEDVIKARILAEDWDDVVPRELPDVGWNQKKGEAPEVSQEKSKLGLGELYEREYL
KKAVGYDKDAEEKQTEEDKAKDEMKMLFANLCSKLDALSNYHFAPRPIADEAEARPVTVP
AIAMEEVLPLHVSDARGVAPEEVYATKRGREGILRADSEMDRDDRKRQRQSKKAARRKKR
KEKEADDKLISRLQPGLGLNNPYEKRKAREELALARSRGKVTEGEREMDGKYGSSGTFFK
RLQAEAQQTIQDRSSGQDDAPQKXNKPQIQCLQAVX
>comp6440_c0_seq1; 315 256 1044 LEN=1559; translated
MLEGFELWVPPTVPNYKDEMPYYDILGTDAQIVQFPVRAGRQIMCFSGAMAYMSEGMKME
CKLAGLGKTFGRLAGGGSLFQITYTNDSTEDGYIGMTPDYPGVIVPINMAKAGKVIALRD
SFLCATVGMGETMTDVGAGFNPASSALAMCCSGMDFIVQTVENGEWAFLMAMGTVIKKTL
NQGETLLVDGKSILCFESSVSVDVRPVGGIAAMCCGGEGIFNTELTGPGTVHLQSMSIDK
LRMLFPPNVQSNNPDDGGDGGE
>comp6440_c0_seq2; 283 256 942 LEN=1704; translated
MLEGFELWVPPTVPNYKDEMPYYDILGTDAQIVQFPVRAGRQIMCFSGAMAYMSEGMKME
CKLAGLGKTFGRLAGGGSLFQITYTNDSTEDGYIGMTPDYPGVIVPINMAKAGKVIALRD
SFLCATVGMGETMTDVGAGFNPASSALAMCCSGMDFIVQTVENGEWAFLMAMGTVIKKTL
NQGETLLVDGKSILCFESSVSVDVRPVGGIAAMCCGGEGIFNTELTGE
>comp6446_c0_seq1; 290 473 1524 LEN=1982; translated

MVASREXNILPEKRVARPDAFVPVSRQPNSYQHSNRQGVVMEQRNPEDEQREAQTLQHVM
HRSVSMNNNPSVAVNPSSYQFGPADNIDERDAEDPLCATEYVQDMYDHFRAKEGITSVRP
LYMENQTHINERMRSILVDWLVEVHLKFKLVPETLYLTINLIDRYLERQEVSRPRLQLVG
VTSLLIASKYEEIYPPELRDLVYICDRAYTRKEIIEMEEQILKTLEYNITIPSAHAFLVR
FLKAAHADKRIVQLSCFILDGTLQSYNLLHYLPSQLAAAAVFIARRTVGRNSWSPTLLRY
AIYREEEIAPIARAVMAEKSSAAHELRAVNKKYTSSRYGAVANLALSCDF
>comp6446_c0_seq2; 347 175 1335 LEN=1793; translated
MFHRSSNIAQPSQSVTGNPRRGAFRDLTNANQQENNNGGKSGKNILPEKRVARPDAFVPV
SRQPNSYQHSNRQGVVMEQRNPEDEQREAQTLQHVMHRSVSMNNNPSVAVNPSSYQFGPA
DNIDERDAEDPLCATEYVQDMYDHFRAKEGITSVRPLYMENQTHINERMRSILVDWLVEV
HLKFKLVPETLYLTINLIDRYLERQEVSRPRLQLVGVTSLLIASKYEEIYPPELRDLVYI
CDRAYTRKEIIEMEEQILKTLEYNITIPSAHAFLVRFLKAAHADKRIVQLSCFILDGTLQ
SYNLLHYLPSQLAAAAVFIARRTVGRNSWSPTLLRYAIYREEEIAPIARAVMAEKSSAAH
ELRAVNKKYTSSRYGAVANLALSCDF
>comp6459_c0_seq1; 245 1 938 LEN=938; minus strand; translated
XGSTYKSNKGNGGFSSSNNDPMSIKGNGVCLVEVARVEWNPIQPWQRSPQSKSSGTAFVI
EGEQLLTNAHVVKSAIDIRVRPHGSTRRFPAQVVCYAPDVDLALLELVGESEKADFFGHP
SAGEADGDEEEERIAKRRRTTKSLEFATELPGLQETVHVVGFPTGGRTICVTEGVVSRID
LYNRIVAIQVDSAINPGNSGGPAFNSKGQVTGVAFRKRISTKSQKVDNIGYLIPAVLVRA
FLGRVDREKGTYQLSGTIPYRCHSLENHSLRLAHGVPDFVHGILITSVADAVTESTEDGT
SAEESPLQLQPGD
>comp6459_c0_seq2; 187 1 638 LEN=1195; minus strand; translated
XGSTYKSNKGNGGFSSSNNDPMSIKGNGVCLVEVARVEWNPIQPWQRSPQSKSSGTAFVI
EGEQLLTNAHVVKSAIDIRVRPHGSTRRFPAQVVCYAPDVDLALLELVGESEKADFFGHP
SAGEADGDEEEERIAKRRRTTKSLEFATELPGLQETVHVVGFPTGGRTICVTEGVVSRID
LYNRIVAIQVDSAINPGNSGGPAFNSKGQVTG
>comp6466_c0_seq1; 224 464 838 LEN=1276; minus strand; translated
MVMTFFHFTTLSAFHDLSSIDLSSSTAVSVLNHVNAMQHMIKQEEPSPQQPKPALAIQKL
AANHNNLVHPVAGLDCTEHDGPQEMEDAQEMAYWRDIKADNSWTSPFLKKDTRQYLTFEP
DGGGW
>comp6475_c0_seq1; 137 1 286 LEN=286; minus strand; translated
XTVITDDLADRVLLMMSIGVGVLTGLVGLLVGSVNPGLLEDFGVEDGGGVATVAFVVGFF
VGLGFCSILMSVVGSAVNTVIVCFAESPAEFEANHP
>comp6476_c0_seq1; 101 1 515 LEN=515; minus strand; translated
AALDRPAGDHLVAQITELMNENNELMDRMTALKLVARQQKDKKDEELRFLKSELDRLRAE
RDQQNLEKLRPEKPSSMQLVDKIWNQKRDATEQSYRRQIQDLRDRVRGLEASNCKLKREL
GRVTVEIKSDDDEETRKAKEMARAVSRGRLTRTRSAQRVRDRCERQLERSKX
>comp6481_c0_seq1; 486 102 1613 LEN=1800; translated
MNRYVLEEQIGQGTFGIVYRARKKETEIHELYAVKKLKQVFPSWKDVVVEREFKAIEMFM
RKREKERATNDESKENKECFDCCGCHNGSDHLVSVFEVLRERDSCLYIVMEYMKDGCLQD
YIAAKAKSKAESPQCIQKISQNGHTFRDSETRSILCQIFHGLRHIHSLGYVHRDIKPENI
LLHGRTAKLADFSLARPFGMDRKLKKEAPRRSCNQASSAFHCFKAPVLDDTGCTMTNYIG
TRWYRAPELLRTHSATTHPIGNGDESKHDVAFYTEAIDTFAMGCVAAELYRCRPLFEGRD
EPEQLRLIENLLLTDPPHYRTPTSFFGDDRAKCCDFLDDYGQDERNEGMVRNRLASKIET
TDQETVRFIHALLHLCPERRPTAWEALRHSYFKVECKNRKSCETQSVLTTALARASSLQR
TSESMVTPPLASDRCGIYEPRSTAYPRSIGNTGAKSSRKITPTPMIAPTTDCNNTGHKMR
LETDAVEQYIGIPKQSPWAFHESW
>comp6482_c0_seq1; 706 1 2299 LEN=2299; translated
XKAAPKLSCFAPDLERTHRAVTRARQEWKKREQIQRSKPGDAVLGLPLLNVGMPKSGSST
LLRFVRHDLKWKATHNVVERAASERDGDQRLSPTRTLTPIPTSPPTPPPRRSYVGKLMKQ
AIDVGHPSPFSLLSQGYRAHTQMDYTVVSSSGQEDDETNFFPQISLLDEIHASEPNATFV
LLFRPIDDWIRSVRSWHRLPFRWMEASKPYYGAATQIPGLVLTEDQRTRRTERPEEPLAA
TESQLRDWWCHHVRHIRRFVDEYPSHTLVELDLYDNHNRDKHEAGDDSSLSTTETLRELF
FPPGERNRTITWGMANANPAAAFSIGSRPGGPIARKTKYYEQWNHETQRLELERELEAST
RTKGHQNSSNSSATNSTLPKTSLEILETVLREREESKEEGRDWDEECFLPRPFSVEKDVS
HGSMMNSNSAALSNPTNNNSDDEVTKGSKAAGTVLLPRVPYPILNVGLPMDPWSDALRDF
FSCLGLKVVHRKALGSNGTRRIGSVGKKMREAVRDAHVGPISGFFGTNRQVFSALDYTPS
LDSASAASSSSSSSSSAAIFPQIQLLDELHEEDPSFTFLLPVPPIDEWVKWAQTFHNFSE
RWARMEMPGLVLTDEQRSARHRPCPLVEKSTDGEQQQQNNNSNNGNHTATLKEDTGNHRD
TEEQPEETRRCLRLTDQQLRDWWCGHVRHVRSFAEAHGDSHALLELDLSPASRGPTSSAL

NKVFGANQTCIAKLFXETDARGGRILAVQEATHPQDPLDLWYTLQEA
>comp6491_c0_seq1; 52 1 215 LEN=215; minus strand; translated
XLHEPKTLDEVKSLLLRAGSAGQLVVIDFTATWCGPCQMIAPLFKEMSSTHDDVMFLKVD
VDENPDTAQQYSX
>comp6501_c0_seq1; 623 1 1775 LEN=1880; translated
XEELRARAVQLIAERSGSLYLGKSETVLFVDMIPHLLQLLGETTGNFLQQSVFAAIDSIG
RNSCLSSDSVINDRNLEIFSSAVFKASSVISTASQEISEGLFLNIPPESRQLVSSAALCA
STAIKICGPRALPVLPKLIKPLLNFQSAATAFIKSSISGDDSIDKKELSQARLMQLAILR
TFTAIIDRMPMLLKPYLVDILKSFSQVSESINIDQSHSIQTELIALQNMTTSRIPARLLI
PAASKSIITTPNLDLNLSTLSIMTDSITKSKSSQVSGMVSIIIKTATYVFDQETNQDGDS
LVMQATDKLLLSIVMKLSEIQLRSLYRKIRDWRGDFDATNPEQSSQRRSAFWRFSSALSD
QLKSIYLSCLSSVFSDAVDELEMAAYYLSKKDSVKKSDGKKRQRISSNEESNGLEISSLN
ALKSLLLCLEVSLRSDAHEGGLWIREMESQRYDKLLTPLGKLLQCRLPCDASNCSFDAIV
LGGDNHSGSVVECLVALATAAGDEQLWKPLNHSVLQACSDENRPEVRKAGVSCLLALINS
IGEEYMVLIPECLPILSELLEDPDEDVAGIAQECISQSEELLGESLQDSLR
>comp6501_c0_seq2; 553 1 1851 LEN=1956; translated
XEELRARAVQLIAERSGSLYLGKSETVLFVDMIPHLLQLLGETTGNFLQQSVFAAIDSIG
RNSCLSSDSVINDRNLEIFSSAVFKASSVISTASQEISEGLFLNIPPESRQLVSSAALCA
STAIKICGPRALPVLPKLIKPLLNFQSAATAFIKSSISGDDSIDKKELSQARLMQLAILR
TFTAIIDRMPMLLKPYLVDILKSFSQVSESINIDQSHSIQTELIALQNMTTSRIPARLLI
PAASKSIITTPNLDLNLSTLSIMTDSITKSKSSQVSGMVSIIIKTATYVFDQETNQDGDS
LVMQATDKLLLSIVMKLSEIQLRSLYRKIRDWRGDFDATNPEQSSQRRSAFWRFSSALSD
QLKSIYLSCLSSVFSDAVDELVSPCLFHHFSLISLHALTINFICELEMAAYYLSKKDSVK
KSDGKKRQRISSNEESNGLEISSLNALKSLLLCLEVSLRSDAHEGGLWIREMESQRYDKL
LTPLGKLLQCRLPCDASNCSFDAIVLGGDNHSGSVVECLVALATAAGDEQLWKPLNHSVL
QACSDENRPEVRKAGVSCLLALINSIGEEYMVLIPECLPILSELLEDPDEDVAGIAQECI
SQSEELLGESLQDSLR
>comp6509_c0_seq1; 701 244 2478 LEN=2577; translated
MMERNKGGRSLHRGRRMNATNALSASTLFQSPDALCLHVAIIFLFVGSVPIGQAQTQANT
PSTPNTPPPVQSSNVASPVDSALPTQPPLSPPIPTNAVTPPIPMPPIPAPPSPFPTSIPG
PTVLPTNFPTGTPSVLPSFSSQPSSLPSKSFEPTISDSPTAQPSAAPTGQPSWLEPSFKE
NKFRQNIQVGNGRVFTDDEQNIIETIYRLQTREFAPVPETELGKIMTVCTVDRQKIYTPS
EESTEGERWLSYHSNVNESEALLLRRRRRRRVEDALDVENNDVTVVVDFTMQYESRYFNV
TQYPKEFQIWTNDHLDEILIQMQALKLNVTKVEKAQRIVVNTQAPSMPISSSPSIAPSLF
PTVSHQPSPVPLPSLSPTGENEDPGGVETTSLIAISASVIVAVGILLVGVFFWYRKRQIS
LQREESAKLDYQTNAMRLSELDSDGRWTTTANAGSNLQFRNSDSKAYGSSFAKQPKTPTG
LSGAGVILSPNGSLRSNPSLVSEGILIGGDDSGDEADATQILADEFDQYQDQNLEKMRAD
VEGNLKGCDGMMSQAVARALIDDVDYNPYSGAYWGGDGEDASAAEIEASALGSVLDWLKR
HGNASGREKREFFQETLNKMTASVRYGFVGPEDGARTIHECAGLLGLQLAVEIPVTTIVI
SGMRKSTTEEDIRDTLGVFGDIAVAAVAPKQKGYGILRFTSDSAAERVMNRFRTEEIVLD
DVAVQLIVIKPGSSLEAKEPNGVI
>comp6509_c0_seq2; 255 176 553 LEN=652; translated
MTASVRYGFVGPEDGARTIHECAGLLGLQLAVEIPVTTIVISGMRKSTTEEDIRDTLGVF
GDIAVAAVAPKQKGYGILRFTSDSAAERVMNRFRTEEIVLDDVAVQLIVIKPGSSLEAKE
PNGVI
>comp6521_c0_seq2; 356 1 1000 LEN=1565; translated
XPIQSKPNQPKQQRSANERNNPPSSMFSSLSTAGGLRLASRRILDASRSVAPRSRATPQL
HLPQRQAVSTAAFRAGVARARTAGHRYLSKDTAKTAAEPAKVGWWKSPEFWGFAGSIAAW
GMSGAAIYDSALQGPEVISLTMTPVLMVYSGLFARWAWVVKPRNLSLMYCHVANVLAQTN
QLRRALEYKMENGEEEQVKEMAQQVGMLGGVAGAAVLGGPAARSALSGANLGIVSTIAAA
DAGPFTVHFWAPMSKVFISGASMLELDRPTDKISLPQYAALTATGFFFTRYALLVTPVNY
TLCAVNIALFLSSFWHFGRKVKADFVDAKPDAQ
>comp6541_c0_seq1; 106 1 496 LEN=943; translated
XGDVGNSHCDGNEFVSYYLYVTYSIGCILMKKVLNISLHDDVIPLNFFGRHRLRMGTKPV
MAGHCFFGTFMVVMLPSYYFCYRRREHQEQVIEMMMKYNQFGHATDLPEEPPLEEHPFWE
QNNEGDTSNAETKHDREFRGLIKERKEWQKPQDPSFDEIFQERK
>comp6541_c0_seq2; 178 147 734 LEN=1181; translated
MSGSGTSQTVETSVDGILTSVGGAAPNPNTNRSDPLETAKWTRFLPEAFGIRESVRKSSY
RWCVREGGMWGIATATAMSLHRLRMGTKPVMAGHCFFGTFMVVMLPSYYFCYRRREHQEQ
VIEMMMKYNQFGHATDLPEEPPLEEHPFWEQNNEGDTSNAETKHDREFRGLIKERKEWQK

PQDPSFDEIFQERK
>comp6541_c0_seq3; 190 645 863 LEN=1310; translated
MMMKYNQFGHATDLPEEPPLEEHPFWEQNNEGDTSNAETKHDREFRGLIKERKEWQKPQD
PSFDEIFQERK
>comp6559_c0_seq1; 540 206 1480 LEN=1982; translated
MIHIPVQSSMSLLLVMLLQLDSKNSLVGATTASQSSQTHTIPSSHQIFKAHFIDDTYLPS
HSSKSLLWVARILGDLRGGGAGDEYDEDESYDVYDDYDEYDSDEEDLEEETPIQRGDRNR
KGGYDHSMIDPSRPRPRQRPPSGYHANDFRRGKKGSSSLSSATNAASAATKLATKSLSVT
GSLAWNALVKQPGKLAFHVIRPKHVDLVETFGLWRLDQQVVEKSNKGGGDQRTIASVATI
ELARPPPPSRGSTGQSKRGSRHRSGPPPPPIVILRRSKSGENDDNEGKDNDIVYRAPFTF
VRKNKYLKGASLSSFQTSFVAPAFLVGENQTRLYGYKGSWQRKLADKSVIKLKGKIYQVH
KQKFGKKRGSYVYGPAVGTFVMRRRISMSEEEEEDYDDDLDSDEFEYDNDSDEEYSEAEG
DEYD
>comp6559_c0_seq2; 540 206 1480 LEN=1946; translated
MIHIPVQSSMSLLLVMLLQLDSKNSLVGATTASQSSQTHTIPSSHQIFKAHFIDDTYLPS
HSSKSLLWVARILGDLRGGGAGDEYDEDESYDVYDDYDEYDSDEEDLEEETPIQRGDRNR
KGGYDHSMIDPSRPRPRQRPPSGYHANDFRRGKKGSSSLSSATNAASAATKLATKSLSVT
GSLAWNALVKQPGKLAFHVIRPKHVDLVETFGLWRLDQQVVEKSNKGGGDQRTIASVATI
ELARPPPPSRGSTGQSKRGSRHRSGPPPPPIVILRRSKSGENDDNEGKDNDIVYRAPFTF
VRKNKYLKGASLSSFQTSFVAPAFLVGENQTRLYGYKGSWQRKLADKSVIKLKGKIYQVH
KQKFGKKRGSYVYGPAVGTFVMRRRISMSEEEEEDYDDDLDSDEFEYDNDSDEEYSEAEG
DEYD
>comp6559_c0_seq3; 540 206 1480 LEN=1779; translated
MIHIPVQSSMSLLLVMLLQLDSKNSLVGATTASQSSQTHTIPSSHQIFKAHFIDDTYLPS
HSSKSLLWVARILGDLRGGGAGDEYDEDESYDVYDDYDEYDSDEEDLEEETPIQRGDRNR
KGGYDHSMIDPSRPRPRQRPPSGYHANDFRRGKKGSSSLSSATNAASAATKLATKSLSVT
GSLAWNALVKQPGKLAFHVIRPKHVDLVETFGLWRLDQQVVEKSNKGGGDQRTIASVATI
ELARPPPPSRGSTGQSKRGSRHRSGPPPPPIVILRRSKSGENDDNEGKDNDIVYRAPFTF
VRKNKYLKGASLSSFQTSFVAPAFLVGENQTRLYGYKGSWQRKLADKSVIKLKGKIYQVH
KQKFGKKRGSYVYGPAVGTFVMRRRISMSEEEEEDYDDDLDSDEFEYDNDSDEEYSEAEG
DEYD
>comp6560_c0_seq1; 275 254 980 LEN=1148; translated
MTQNIINKNAVSGGKPPKFLIQFRDSDHVTSKNNPRVFFDIEIGGKKAGRIQMELFKTIN
PKTAENFRALCTGEKGVGKSGKKLHYKGSVFHRIIPGFMCQGGDITNGNGTGGESIYGGK
FEDEWYENELFITHNTPGLLSMANSGKNTNGSQFFFTTARCKWLDCRHVVFGQVEDGWEV
VKAIEAVGSASGKASDEVIIADCGELKDFKLPKKVVNGDYNNEWKTDHLDEYQAHEAKKN
K
>comp6560_c0_seq3; 436 94 987 LEN=1155; translated
MLNLKLQKQTAVAFVALSLLCIVPMVSAFDPTAFVADAIAYWNSLNLFYKFLVGAPIFFL
FAAATGLDGGKPPKFLIQFRDSDHVTSKNNPRVFFDIEIGGKKAGRIQMELFKTINPKTA
ENFRALCTGEKGVGKSGKKLHYKGSVFHRIIPGFMCQGGDITNGNGTGGESIYGGKFEDE
WYENELFITHNTPGLLSMANSGKNTNGSQFFFTTARCKWLDCRHVVFGQVEDGWEVVKAI
EAVGSASGKASDEVIIADCGELKDFKLPKKVVNGDYNNEWKTDHLDEYQAHEAKKNK
>comp6571_c0_seq2; 140 112 804 LEN=1580; translated
MITARSTLTRNLFRSRKDAQNALAAFVQTPLPSPTSDEQQKPGFARHMTSPAYRRYMQKQ
KGDVAIKGNQPSIGAAQDGIQIPTNEVARSMGWKFSEMENEALVVIAEMKNHGARVEVLR
RHIMATDDIDYEAAGEVIEKIAAKNQDGMTRAALPYKVAIATAVVTGFGAIPMVFSIDVA
KWFNLAFVTMEIPEPSDLETALETGAWTWNWMEPVLGTASYTLLCLQVAR
>comp6571_c0_seq3; 187 112 945 LEN=1416; translated
MITARSTLTRNLFRSRKDAQNALAAFVQTPLPSPTSDEQQKPGFARHMTSPAYRRYMQKQ
KGDVAIKGNQPSIGAAQDGIQIPTNEVARSMGWKFSEMENEALVVIAEMKNHGARVEVLR
RHIMATDDIDYEAAGEVIEKIAAKNQDGMTRAALPYKVAIATAVVTGFGAIPMVFSIDVA
KWFNLAFVTMEIPEPSDLETALETGAWTWNWMEPVLGTASYTLLCLQVARQNMQNLGIKP
YTSRLMEKRAAALYEAFPQYNRRMLHSFVETDAMVKV
>comp6582_c0_seq1; 559 1 1797 LEN=1970; translated
DEMDVELGREVGYTIRFEDVSDPRSTVLKFCTDGMLLREAMHDPLLSRYSAIVLDECHER
TLATDVIMGLLMEILPKRKKGSEHGELKVVIMSATLDAQKFQEYFHGAPLMKVPGRTFPV
DIFYTAKPERNYVEAAVRTTLHIHQCEEPGDILVFLTGEAEIEQACEEIREKGRAMDSEL
VVYPLYSSLPPEQQRKIFSAAPGPRTPGGKPGRKVVVSTNIAETSLTIDGIVYVVDPGFS
KQKVFNPRIRVESLLVSPISRASAKQRAGRAGRTRPGKCFRLYTEKSFYDDLQETTYPEI

LRSKMSSVVLTLKKLGIDDLVHFDFLDPPAPETLMRALELLNYLGALDDEGEMTEIGYQM
SELPLDPQLAKLILVSPDYGCSAEIVTIVACLSVPQIFLRPRESAKQADEAKAQFSHHES
DHLTLLRAFNEYDQIPDNEKRQWCWDNFISERSMISVMNVRRQLIGIMKRLDIPLVSSDV
KGDGSFASADVRKALTAGMFMQVAFRQRTGDYLTIKDNQHVAIHPSSVVNNRPEWAIFEE
FALTTKNYIRTVTVTNVDWLVELAPHYFDLENFPKCEAKGELEKAYVRLAHSRKKRGR
>comp6583_c0_seq1; 164 1 454 LEN=553; translated
XDKVSIIQNKKQHAGQNTTTVEIGMLNMRKFLWAFAVAEWSFLARDTTAFSVDHRAASPS
LGHRTASTSEASPAPLQFPASPHRQLRRPSTQLFSLQPLIEDITQSAMSPASGKKTIFVG
GKGGYVGGRKRREHHSRTLNTIRSNHAFTSV
>comp6583_c0_seq2; 841 1 2479 LEN=2494; translated
XDKVSIIQNKKQHAGQNTTTVEIGMLNMRKFLWAFAVAEWSFLARDTTAFSVDHRAASPS
LGHRTASTSEASPAPLQFPASPHRQLRRPSTQLFSLQPLIEDITQSAMSPASGKKTIFVG
GKGGVGKTTVSSSLAVELATKHDLKVLVVSTDPAHSLGDALDADLRRGRGKPVVMDDPLT
RGNLYAGEVDAEEALGDFRESLASFDVDRLAASMGVPMDLLEGLGLREFSGLLNNPPPGL
DELVALGNVMDENKVDDYDVVIVDTAPTGHTLRLLALPQFLDGLLGKLIKLRMKLSGVTS
TLQAFFGDSGAQERAQTIDNALDRLDQFRIKIKNMETALRDPERTSFLVVTIPTKLAVDE
SKRLVSDLTSQGIAVSDIVMNQCVGDLTETTESNGLLNYYQRRQEGQSQWVDKLKRAASE
VSATEEYQSNGSPSPIAVSEFPFFDVELVGVPALGYVGSQHIMTNPSFEYLMNDGNEPDG
EPRVVICGGKGGVGKTTTSSSLAVSMAAQGHRVALISTDPAHSLGDAIDMDLSGGDLVSV
PLIGVPGSTGEGSLSAMEIDPSSALSQFKGIVDKLIGTSADTSTGGGSDLSATLRDLESI
FDTLPAGTDEVVALAKVINLVKKGDFDRIVLDTAPTGHTLRMLGTPGFLAELIDRILDIS
SKINSNAAVKMFITNAASQQGATGEQLDSAATAAKSQLIGFQLQMYDLEDLFANPDQTEF
LIVTVPTELAVRESVRLLNDLTFEDPDMPIKVRNIVANQVLKNDGSDVYTFLSHLGKTQA
SSIASLEQSMQGMPHAPKITKVQYLDTEPRGVFGLKVLGDALVQDE
>comp6589_c0_seq1; 192 1 366 LEN=986; translated
LLDGELAPVDGFVVVLAVALAAAATAAAPLRMGIATDRLAVERNIDVLAGKVQEGDLEIE
RDPSLAVDRSSLRGREAVGFAAAARQHGKMRRFRKVQRVGSVRKDVDTDLAHKEQKGRRG
G
>comp6595_c0_seq1; 323 236 807 LEN=807; translated
MDHHNYNEAFATPLKRKLPQQRNTDTDVLVTPTLAWEDSDAETLSVGSTSQSSSPPATPV
SCPPTPVSFTKKRRLADDALVYSTPPCNVPGPLRSGLSRRPPPAVPRLAMDLSRLGEDGR
RDEEDLSFLAFPALYEDAGIPAPSPEDGLPSRAFALEPRPNNGFPALPKRLFQDGTNTIA
SDPSSSSSNNX
>comp6595_c0_seq2; 323 346 917 LEN=917; translated
MDHHNYNEAFATPLKRKLPQQRNTDTDVLVTPTLAWEDSDAETLSVGSTSQSSSPPATPV
SCPPTPVSFTKKRRLADDALVYSTPPCNVPGPLRSGLSRRPPPAVPRLAMDLSRLGEDGR
RDEEDLSFLAFPALYEDAGIPAPSPEDGLPSRAFALEPRPNNGFPALPKRLFQDGTNTIA
SDPSSSSSNNX
>comp6595_c0_seq3; 323 165 736 LEN=736; translated
MDHHNYNEAFATPLKRKLPQQRNTDTDVLVTPTLAWEDSDAETLSVGSTSQSSSPPATPV
SCPPTPVSFTKKRRLADDALVYSTPPCNVPGPLRSGLSRRPPPAVPRLAMDLSRLGEDGR
RDEEDLSFLAFPALYEDAGIPAPSPEDGLPSRAFALEPRPNNGFPALPKRLFQDGTNTIA
SDPSSSSSNNX
>comp6600_c0_seq1; 473 1 1521 LEN=1868; translated
CLSKSWKGKKERNMNLLAFQIITFLFFGLFIFKGLYGNLAHAFSLSTAARRGRNNPDHGN
NANQKYKPVVVVGKIIIDEYGDPYPSSGEKNTINKHGDQSSYEPRETEASPLSISIGGGG
PQAAFGAAAALAVWDSYHVNGIGDKNSSVSSMKLTPSCQQPIPPVLFLAPVGKDWTKSET
IALLSALNTTTSRSSSGGDCKECITPASITSQGQNNNIVTHLIQSEDSITPRIRLWHDQD
QVVHWYAINDSFGPKGADGLWRNRPSAEDLVSVLSTENCSECDGIILHVIAEAGKDAAGG
QLDCLPLITDPTLVESHLSFLGIEPVASGESIEEEDALSAGTILEGSWKKTSSLDSVFWC
PDYALDQAMRKHNIYDTFHKNGGKDKESTSILVSTRDGPRGSQIWYPFGDGSSRDITIPA
AALSTKNGEPIDPTGAGNAYSAAMTALLGNSVPLNIAASIATGVGAVVCEYEGLPTAMIN
KATWSKIVERIHEAATEVRAKLKEEH
>comp6601_c0_seq2; 261 90 779 LEN=1114; translated
MTQFLRNSLALALVAIASLTAVRGFSVSNGETRRGFASSVAGIVTGSSALLIDPSRVFAE
DAAATPLARYENSNCKFSIDLPSEWTMTEQSLPDRRKIILYIKPDSNQKTLFFIAFTPTR
DDFTSLGSFGSVDEVGQATILPKAELAAKQGIESEMLSAVSKKQAYFFDYVQSVPSQPKT
HFRTIFSLATGATGGAGNVLVTITAQTPESDYAAMEPLFNKVLDSYKNA
>comp6606_c0_seq1; 656 273 2372 LEN=2690; translated
MSGSSSSSREAAVVRMFEMVTASHWTARSSESNVVDDLRPLLLMMDIGSCASAEKHTRVN

CVTASMGDVVIEHYPRIGELLELRAAPVSVGRTSLDISVLVVAHGCNESNGSAETKQVCE
AFFTYVTTRGPDGKKRFAPPLAAREGTGSYACEKTWERTMAQFRKQLIRSEQTLVKRHNN
HLLEEQRSGGNDAAPSALAIFECSEVVLPRHQNHMEHLFGGIVMGWMCKSALAGCVLACR
KPPSAFRIRSIQRVDFPKGADVQDHVVLRPRITAIFDGGRHAEVEVRVTKRSITREQPEP
SSGEALMNVGHFYVTGVSSYEQAGSKRRSDIGNTDGGPEEKLSCAVAPFADLLNKRSDLY
GSNPKDKPSLDLEMARERLNALLTRMNLLAGIGDSIRWDASMELQAPILTILSVLRLCDD
YIEESTSTYSSNGRGVDENHSGPIWTVPDLLLSHNPPNTTVEWARGEESLFGHSNTFLLR
GKTVVSRRRPVEPNLSGSGFLEAVYATIRDKRPEWDPACMAIECLERSSDKNTEAENGKE
ALEWAVVEYQMKSAIADFRLCLLQAGAYLRRDGSIAGSGEDDDGDGDSTQEENTSAVLAS
RSVRHQRSKGTHRVLPSGWVLKTMEDDGEDNDEAKTTDERLEITYVAEFDYDTVRQSGAL
KGHEDYATEELIAGYVGLFVGQWFENLASFYHYRSNDGD
>comp6614_c0_seq1; 362 302 1237 LEN=1499; minus strand; translated
MSPLTRFLNGRASAHAPLHIPHCTNTSEDEFLKSEVLLLRLENNIKDDKLVAVTQSLHEK
EQEVIDLRANMSVVNTSIHRLKRNIATNTELERENNKKQKVIDDLLLKICNYDQRLKEQS
AIIAQQHQLLQKQTSQDVDLNARVIPFLMNSVSILRPSVTNITAIYQKGGYQQQEHQQRL
QYSKLLESTKAHLSSNTTTTNNSACSKTSTKKSTSQALSSVESTSKKASKYAHISMADRW
MNRYNELIEYKKEHGSTLVPQRYKKNPKLGKWVTHQRSSCLTEERKALLDEIGFVWDATR
KGPLPVSILKS
>comp6614_c0_seq2; 238 441 1118 LEN=1380; minus strand; translated
MINSLPSPSRCTKRSKRSSILKICNYDQRLKEQSAIIAQQHQLLQKQTSQDVDLNARVIP
FLMNSVSILRPSVTNITAIYQKGGYQQQEHQQRLQYSKLLESTKAHLSSNTTTTNNSACS
KTSTKKSTSQALSSVESTSKKASKYAHISMADRWMNRYNELIEYKKEHGSTLVPQRYKKN
PKLGKWVTHQRSSCLTEERKALLDEIGFVWDATRKGPLPVSILKS
>comp6614_c0_seq3; 124 859 1089 LEN=1351; minus strand; translated
MADRWMNRYNELIEYKKEHGSTLVPQRYKKNPKLGKWVTHQRSSCLTEERKALLDEIGFV
WDATRKGPLPVSILKS
>comp6615_c0_seq1; 307 115 558 LEN=717; translated
MKTAAILLATVSGVSAFTQAVSQNRVATALSAEPVPLANGAMSFNRVCREWRCKYEGDKA
TSASLEAIAKVVDESLPEIKKASSEITVNRLVCGGCLDFKLMMTVPLEDFGPWEEAGFPP
ESDFLAKIKAIEGVSVVETQTITNMEI
>comp6642_c0_seq1; 263 1 1403 LEN=1475; translated
RADVSQVLRIYQNKASKSSSSSCGSNLLESIAETVLLKEMKRAIRGRTKQARNAKIAASF
LALNGEHSSNNNSDNHYNDDDETTRNERHMRLARVLLGIGTPEDTEEILSQQSLLDDNEH
LLGIIRHNAFGPDFHHYHRMERELFDANHADTTTTGGNNTTSIASLYSRVLGHYPLAAMI
NHSCGPTNAARVFYGETMVASATMDIPRGTELVWPYCPPILPFPERHSRLERGYGFSCSC
QRCLLEGAALEGGIPPPLPLMGATGHGGAEARLGPDEWSSVLESVEAWYRAVTAGEPPKH
SNNNSEPRSPLNLGECLRLGHTQLYIDYFNQSLAEKGAVEVLLDQATELHRSFARVHNAC
TEHLSLLHLSYELEAIGGSTSKETGNGXADLDRAPQAGTPLQVFERDSREGPGNTTDGPQ
AYPDRPKDEEWMESQQNTGGREFFFMRMERANAHACRRLAQVVLQQW
>comp6670_c0_seq2; 429 149 958 LEN=1054; translated
MTCATTTTTTAFASLFFCLALIGSISEQKVDAFTLGGLSPGRFGSVAVTKMPQPVTSIMS
MAEEDEAAEEVEESSEAEEEETPEDPEVTALKESIAELEGNLADKKSKLQYELEKCEEYS
KSGYARKVADMENMKRVRSNIASTSKASATAAVLKDFLPIYDTLNSLRETYSEDEFGRKY
SELNLEQTFATLGVTSFDIQAGDKINNFRMKVLESENSNDFEKDTVLRMVEPGMELEGNV
VRAAICVGSLGAEGADDESSEGEDTTEEA
>comp6670_c0_seq3; 385 149 1250 LEN=1346; translated
MTCATTTTTTAFASLFFCLALIGSISEQKVDAFTLGGLSPGRFGSVAVTKMPQPVTSIMS
MAEEDEAAEEVEESSEAEEEETPEDPEVTALKESIAELEGNLADKKSKLQYELEKCEEYS
KSGYARKVADMENMKRVRSVSRTTIEIGKRQEGRSGNWRHAQILCHLKMAIHKFGSTTRL
PATVILSLTVSFLVSLFANVFGLPEHFVAGSTRVFTNTIRSCSITIPQRAFLFNLKNIAS
TSKASATAAVLKDFLPIYDTLNSLRETYSEDEFGRKYSELNLEQTFATLGVTSFDIQAGD
KINNFRMKVLESENSNDFEKDTVLRMVEPGMELEGNVVRAAICVGSLGAEGADDESSEGE
DTTEEA
>comp6673_c0_seq1; 378 372 1160 LEN=1655; translated
MSYNYNNTVNANPYNNNPSYPSSGNNSYKKTLKIVIVGDSGVGKTSLMNRFSTGKFTGQY
KATIGADFLSKDNVIVNDNFMQQRHLVTLQIWDTAGQERFQSLGVGFYRGADACLLVYDV
TDPHSLDNLDHWRKEFLEQVGGGIQGLGDASTQFPFVVVGNKIDKANDRLVTREQAEDWC
RTAASATIMGSQPLPYFECSAKTSESVEDAFQEAARRAIVYEEYKKRSQPQLFVPPPMGS
SNAPINLRGQYDQSNKQPDMCC
>comp6673_c0_seq2; 113 1 121 LEN=910; minus strand; translated

XSAMKMETSVPATSSGVVKRVLVNVGDKVDGDDLLVEIEV
>comp6688_c0_seq1; 75 1 801 LEN=914; minus strand; translated
VGRTWKSTSEQITGKTNNVRIKWRSFIFSVLPAPILGALVPGATLATKSVIVTALAAAQV
AYTLAEAEFCLARATDAVAIKARSAAVCDTYANQGARSAAILPFTSALSGLCAAATAAIV
ELPFLETLSASGTVASLTGEMAIVGVLPLFSTLFAAAASVSKARCEVDAEAAVQAASTLA
LEYSSNEDEDPILRPFRGVAELIRLVSTSTLEPYQRFYRRFRFSMSRSWNKFKNIFRLFS
RRKRGGSDETSNASDSGEEGPVPALA
>comp6692_c0_seq1; 275 1 1063 LEN=1063; minus strand; translated
XAVYFPTSIDAGVIEAKDSGVNSIRVLIHHLLDDEIIELSATLDVRSYRPEMASIPIALS
FGLFLVFNIHMVHSLFLAIFIGSWIIEGSMTRGFKATFDTFLLEAATDNGXRVNVSDLKA
SSSQSHFSTGRISNNWFSLLSMLLLPCYDSILFIVGIFGLMAMVNRSGGTSAVIKSLTQH
SSKTRLAQFVIFLMGLAFFFNPYVSIMIVGNIIGSIFKSFPLSSEKVSFLIDTTAAPVAS
IFPTSSWVVFSTDLIEREIEKIKALGYEEVSGVSGHSLIISSIMYQFYPIFVLGLAVLQI
LTGREMGLVLDAENKARLSYNSIDPRKTSFSRLKERSFNWYIPVVTLNVFLWYAF
>comp6695_c0_seq1; 339 81 1163 LEN=1840; translated
MLATKVPSLLPKFLPFRRELTQSLTALSGNGNGRTSLTSPSTPTSTFSSSSSSLLQTQQQ
STNSQKRSYVSRAHPRPIPEFSVPIGLQMVLDGVEERKKARAAKWERNKEKRRSRGIEDV
GEYRNQNETIDLSINLNVDPRRPGQALRGSVVLPHGTGKTVSCLVFTSDPSTIEEAKSQG
HEAGGEELIDSILAGEASIDAYQRALATKETMPLVQKQLARLLGPRGLMPNAKTNTVFDE
GKVLLEALQEQSSTITYRTESGGILHFPIGKGDFTSKQLIDNLQVICQAVQDAKPEQYGR
GKKPKNKGGGAGTKGNSKKQSKNVKYWLKAHLTATQGKGSVRLDLRTVDPASPFFMKEPE
>comp6695_c0_seq2; 226 682 1409 LEN=2086; translated
MSENIGIXNETIDLSINLNVDPRRPGQALRGSVVLPHGTGKTVSCLVFTSDPSTIEEAKS
QGHEAGGEELIDSILAGEASIDAYQRALATKETMPLVQKQLARLLGPRGLMPNAKTNTVF
DEGKVLLEALQEQSSTITYRTESGGILHFPIGKGDFTSKQLIDNLQVICQAVQDAKPEQY
GRGKKPKNKGGGAGTKGNSKKQSKNVKYWLKAHLTATQGKGSVRLDLRTVDPASPFFMKE
PE
>comp6701_c0_seq1; 163 1 519 LEN=1060; translated
VRLASDVLDQQDPSATLAIVSEFMNPSGDFADRLSSWWKTDVGGGSARALLLTNEKALDA
AEYAKRRADNDKEAKQWEGHLGNNAIEFISPGLMFLRRNQRKSNLTGDNTTTAVDLKHVV
VPKTREGSIWTPTNIVGRNFTSRHIQKFASEWNTVPPDTVHDGDGRQAITKH
>comp6704_c0_seq1; 643 284 1948 LEN=2353; translated
MEPTKDDATMETPEVSVSAPAPGGPDPEEGSFDSPQPKNKTTPIPPGPAQAVMLAPTTGK
QPDRDRTLVEPSGALASPETAVTGRSSDEADERLELMYSKDVAHKKSSDGPGGDKERRKR
WLTIGLLAFLAVCTIAAASAVVVLRRRKGPLASSAATAGLAEPDTVSPAPTRTPVPTADP
SEGPTTGPTESPSAGPTGEPSVVPTGAPTTSEPSSAPSPMPTRDHVALLQEFLFGSYAVD
YNETSNMLAIEWLAEEAARIDSFEPDERLVQRFALLSMGFALRGANSTLDAPVNVVPGVD
HCDWDGVGCDGGDEEGSVTEVRWAYQNRTESGDGRISPEVRLLAEDLVHLDLSNNDLVGT
IPEELYDATNLEKLYLFRNELEGTISTKIGNLDSITHFHLSHNNLSGSIPAEVKSGASGI
RPLEYFNLYRNRLTGTLPESMRFRDMIYFDVGRNELSGTLPDDIGEKFVSLRHAHFDHNY
FWGTLPESYNTVGNGRLESFSIDHNYLSGTVPGKRDLTNKLVQYTLHSNKFDAMDKGNCK
MEVPQGEMIEFRCV
>comp6704_c0_seq2; 591 284 2217 LEN=2298; translated
MEPTKDDATMETPEVSVSAPAPGGPDPEEGSFDSPQPKNKTTPIPPGPAQAVMLAPTTGK
QPDRDRTLVEPSGALASPETAVTGRSSDEADERLELMYSKDVAHKKSSDGPGGDKERRKR
WLTIGLLAFLAVCTIAAASAVVVLRRRKGPLASSAATAGLAEPDTVSPAPTRTPVPTADP
SEGPTTGPTESPSAGPTGEPSVVPTGAPTTSEPSSAPSPMPTRDHVALLQEFLFGSYAVD
YNETSNMLAIEWLAEEAARIDSFEPDERLVQRFALLSMGFALRGANSTLDAPVNVVPGVD
HCDWDGVGCDGGDEEGSVTEVRWAYQNRTESGDGRISPEVRLLAEDLVHLDLSNNDLVGT
IPEELYDATNLEKLYLFRNELEGTISTKIGNLDSITHFHLSHNNLSGSIPAEVKSARAES
APSVSYVFACGEXVRERTLDPAHVLLCGVSVPEYFNLYRNRLTGTLPESMRFRDMIYFDV
GRNELSGTLPDDIGEKFVSLRHAHFDHNYFWGTLPESYNTVGNGRLESFSIDHNYLSGTV
PGKRDLTNKLVQYTLHSNKFDAMDKGNCKMEVPQGEMIEFRVDCWICECNGFFDLCDXAM
LLKQKAPADSETYFGDWRYSHEHNQGLGRWEKKMILIGSTFLDP
>comp6704_c0_seq3; 646 284 2130 LEN=2211; translated
MEPTKDDATMETPEVSVSAPAPGGPDPEEGSFDSPQPKNKTTPIPPGPAQAVMLAPTTGK
QPDRDRTLVEPSGALASPETAVTGRSSDEADERLELMYSKDVAHKKSSDGPGGDKERRKR
WLTIGLLAFLAVCTIAAASAVVVLRRRKGPLASSAATAGLAEPDTVSPAPTRTPVPTADP
SEGPTTGPTESPSAGPTGEPSVVPTGAPTTSEPSSAPSPMPTRDHVALLQEFLFGSYAVD
YNETSNMLAIEWLAEEAARIDSFEPDERLVQRFALLSMGFALRGANSTLDAPVNVVPGVD

HCDWDGVGCDGGDEEGSVTEVRWAYQNRTESGDGRISPEVRLLAEDLVHLDLSNNDLVGT
IPEELYDATNLEKLYLFRNELEGTISTKIGNLDSITHFHLSHNNLSGSIPAEVKSGASGI
RPLEYFNLYRNRLTGTLPESMRFRDMIYFDVGRNELSGTLPDDIGEKFVSLRHAHFDHNY
FWGTLPESYNTVGNGRLESFSIDHNYLSGTVPGKRDLTNKLVQYTLHSNKFDAMDKGNCK
MEVPQGEMIEFRVDCWICECNGFFDLCDXAMLLKQKAPADSETYFGDWRYSHEHNQGLGR
WEKKMILIGSTFLDP
>comp6704_c0_seq4; 588 284 2035 LEN=2440; translated
MEPTKDDATMETPEVSVSAPAPGGPDPEEGSFDSPQPKNKTTPIPPGPAQAVMLAPTTGK
QPDRDRTLVEPSGALASPETAVTGRSSDEADERLELMYSKDVAHKKSSDGPGGDKERRKR
WLTIGLLAFLAVCTIAAASAVVVLRRRKGPLASSAATAGLAEPDTVSPAPTRTPVPTADP
SEGPTTGPTESPSAGPTGEPSVVPTGAPTTSEPSSAPSPMPTRDHVALLQEFLFGSYAVD
YNETSNMLAIEWLAEEAARIDSFEPDERLVQRFALLSMGFALRGANSTLDAPVNVVPGVD
HCDWDGVGCDGGDEEGSVTEVRWAYQNRTESGDGRISPEVRLLAEDLVHLDLSNNDLVGT
IPEELYDATNLEKLYLFRNELEGTISTKIGNLDSITHFHLSHNNLSGSIPAEVKSARAES
APSVSYVFACGEXVRERTLDPAHVLLCGVSVPEYFNLYRNRLTGTLPESMRFRDMIYFDV
GRNELSGTLPDDIGEKFVSLRHAHFDHNYFWGTLPESYNTVGNGRLESFSIDHNYLSGTV
PGKRDLTNKLVQYTLHSNKFDAMDKGNCKMEVPQGEMIEFRCV
>comp6709_c0_seq1; 111 451 1105 LEN=1232; translated
MSPSAVQLRGVWYDPWVQPSCKWAHSTPTVIRLYIFYNPFGPDAMPRGFCRAPPHEGIGH
RSRDTVHSRRFLAWCRGCCAPVRAASRQGTDRTDPKVPRRQARPRRRFGFLQDEDDSRNG
AIVCARAAEARRPHHRELVAPPMLALLCARRGPTPPGARFPAAPPGHGGSIRFPRAPPLA
GRGARREHPKRHREPRARLGPVRAAIPLERTQDEEHL
>comp6709_c0_seq2; 111 470 1124 LEN=1251; translated
MSPSAVQLRGVWYDPWVQPSCKWAHSTPTVIRLYIFYNPFGPDAMPRGFCRAPPHEGIGH
RSRDTVHSRRFLAWCRGCCAPVRAASRQGTDRTDPKVPRRQARPRRRFGFLQDEDDSRNG
AIVCARAAEARRPHHRELVAPPMLALLCARRGPTPPGARFPAAPPGHGGSIRFPRAPPLA
GRGARREHPKRHREPRARLGPVRAAIPLERTQDEEHL
>comp6710_c0_seq1; 316 1 1121 LEN=1237; translated
XIASHPQGVFSSYRTNHIPIDGNFCTESIFQSKPIRSNTIQYDPIQFGTAMAPGSNERSG
ETGQQHQQQAPPLPPPRPAPSDGPALSFEADPMVSDIPLCKDGDYAGLVLVTNWPPSPLE
GVGREYSEFLAAVRSCFDPCDTSVQMAPGEAPTTVPAVYFQPISALHVTIATFSRIHKID
DDGDDRGEKRQRRDRERKRALALVEAAARLPGWPKKPFRLVVESAQIGKKAGILLWRDLS
GGIEAARACLREAAAAAEQGAAPANGNANANTNKNSNSNTTTKAYPTIPGIVHSTVLRFS
RVPATPGETVQERFRSGVLQRLGTDLFAAGSAIASDTVRLADETTPYMHFAPDEDHVLWS
CGLGGGSGSGGAE
>comp6712_c0_seq1; 574 61 1539 LEN=1617; translated
MNTILRVSRITNIHPFFAIVASVGTTTAGAAWYKNTTRCEVALKADVNEADEQTFRIESH
LMDRIQQKKYLSNAKDSIPSTLRVLAIDVPEMRTEAFTGECVISHDKVFLDDIAAPKVVN
VVLDNQGDKERNDKKEKLKIVQKSLARSIIYCSSPQSQQHVGVELLEASFSNLNRFKVRK
KQSFGNYTYDPGKYYDSSNSSNENDSDDDNEIDEVDEGELPMDKETLDEIEAPWNQYAWS
EELRLRINGHIEFDGPVEKSSRYQRVLFGKNYKSTVPAVSRVMDLFIPFSFARSDPDGID
GRNQSNVRACNKPHAVVANGAALQLVPSSLRLLQKLCKKADVPLFVVHDPRQWGGNTQAS
LQEALIAMRSTVKNRIIGKALEQQGSSAFTRGRMLGQVETEAKWQLKDKTKRAKELLGLE
RGRRRKVEKEDWSLLDADTIKSKLIERKAIRVEKDGSDSKREVCTEELMKIARECCDGSR
EQTAQKANPDQF
>comp6726_c0_seq1; 655 242 1618 LEN=1793; translated
MGVSEAEKIKEVEEGPEALAAAMDLAEDDGGERDGGDDASTSSTLDLRKLIHAEVVPPKS
LVLIWILFAAELGFDLITTAIAFFSTIGKQECCGYTVYLGPLPMTTTIPFFFLIVTEIVF
LGRAILLTLWPSVFEARRLELHGGLDEDDDNIGNLGQVGFEVTLATTEDDEESGSRGESD
SDGSKTPNGEEPETEGSVTQADASDAEKHESPSPESREEEATEIADEPEAAEEASHPCTS
ETAREEGGDPKDRAPEFIDTSAAPPGKEAFWKRACCCLLRWNARVILMVLNFLTLLNPFF
GCLIAYILLYQSDKTECFVVLGIESLSIVLHFVSVRMEGGLRTVWSRALHSLVLLPFFVT
VILVLVFLREGGMCYSAESRIFLFSGCEICPDTEEPPIDGMCGNSTLEGAGGLYEDLQDL
GSSFASFSDLQKIASIAKRGAEQGDYCSEQTNFCFFEF
>comp6726_c0_seq2; 420 594 1828 LEN=2145; translated
MGVSEAEKIKEVEEGPEALAAAMDLAEDDGGERDGGDDASTSSTLDLRKLIHAEVVPPKS
LVLIWILFAAELGFDLITTAIAFFSTIGKQECCGYTVYLGPLPMTTTIPFFFLIVTEIVF
LGRAILLTLWPSVFEARRLELHGGLDEDDDNIGNLGQVGFEVTLATTEDDEESGSRGESD
SDGSKTPNGEEPETEGSVTQADASDAEKHESPSPESREEEATEIADEPEAAEEASHPCTS
ETAREEGGDPKDRAPEFIDTSAAPPGKEXFLEEGLLLLAPVERPRDPDGAELSHPAESVF

RVSHCLHPAVPIRQDGVLRGPGHRVALDRPALCFGPHGGGPPHRMVQGPALPGSPPLFRH
GDTGPGLFAGGGHVLLGREPDLFVFRVRDLPRHGGAADRRDVREFHAGGSRW
>comp6743_c0_seq1; 102 146 595 LEN=1791; translated
MLQEQEDGRNRAQKTSATTASEPTTTGEGAPDPKRTPNPGNTRKVFYTTCREGMANEYIR
RHNDIPPDVCAGLRTAGIAFLSIHKLPGASNVLVLTIETAGSNLNLGRATGPGSVYRQNP
VCQKWEVDMETHYHNGWTEMEEIHSSDVEW
>comp6745_c0_seq1; 347 1 902 LEN=902; translated
XTKLDYWTITARNHCYQQTLQVARKKMVFYYTIRGGQCKNSHSHITLTSEDITCYVGRDK
HENEYLIKYGWPGDIWFHVEGLSSAHVYFRLDCFGNTKNTSNTSSIKGLLGQIPIDDLPD
ESVEDMCQIVKHNSISGCKLASCKIVYTPHSNLKKTFGMESGAVTYHNPKLQRFRRCDKD
RQRIKLLEKTKSSDLQIDYYEEMKANERRIIEWKRFLRKQGGGGSGVEIYDPVLDDLRGA
KAKANRQGDESSGLDSGLAALEELGLGGGGAVQHQHDRDPTRPVAPSTGKTASGNDDDDD
D
>comp6746_c0_seq1; 297 504 1130 LEN=1130; minus strand; translated
MNDIFGFVKSVGKSIFSPGSNSSENSKTPSSPRDEGNNSSDNLQAETTSPMRGPAHIPGI
TVPLSSQKVLKTNESPVIGLVSTKDQVIGGDGRCDGKEDTGRALRIHGRSLEANLKTIRK
ESSAERCNVTDLKPTPSPSTKSPSLATSDTSNVYFDEEGGNGEGKENAAATTSVTTTSVT
IISKPFEDPLETSTVTFGNTTDPKSSLLA
>comp6748_c0_seq1; 460 1 1078 LEN=1240; translated
XDLLVEVAAIAADTSFDYLLIESTGVSEPMPVAETFTFEDSTGLRLGDIAEIDTLVTVVD
GSRFMSEMHSLENLQARNWHADPEDQRTISHLLCDQVEFANVVILNKCDLIGDDERVQVK
SLIRKMNPDARILESKFGNVPLDCVLGTRLFSMSDAEKHEKWLQEARYGEHTPETEEYGI
GSFTYRARRPFLPDMLERVLEDLLGGTEVPFDSSKIIRAKGFVWLAGCSQLQGDFSLAGN
HYSLLPGNPWWAEIDKSHWPEHLESSIAPLWHEPYGDRQQEIVFIGQGLDREAVCKAFDT
CLVPPDSMEAGQETWDAMVHEYGDPFAETWDAAIALAQKEGEGHSHDHDHSHEAATAAQ
>comp6750_c0_seq1; 708 186 1376 LEN=1506; translated
MKTPMTMGILNSMAAIAVIASISNTMDISIEAFLPSSHYPRPAALGKLTGSRGVETRICM
FDWVNDLFGGDAKKKEEEAAAAAKAKAKEEAEAKKKAEEEAKAAAEQAAKERAEAEAIAK
QKAQEDAAMLAKAKAEAEAKFAEFEAQQKAEQEAIASISARTKAKAEAKTAVESETEKVE
DETTTKAEEETEIKDAAVETMKTEKKVVAKIKEEAEATVATEAQAEKVKDAANSTAEVED
VAAAQKDTEEKTTSETAENEDDGELARFNAYLDEVIKKGDRIKGVVQWYNPKKGFGFIKP
FQTADEEFEISKAIRKERAVKEHEKKVVTYSPGIYVHHNSLYTPDETYFRKLYPRESVEL
EVDYDNEGRPTAKDVTGPDRTDIQAILKEKLRREAS
>comp6752_c0_seq1; 397 164 1942 LEN=1979; translated
MAATPNGNRDPSPNKNKTRNGSGDGGAKQQGGVAPSPRSGGSSAASLNNASKAAAQMQKM
RDANSKYKNLLKMAKERIEQQEVELKELRGEYQNEFSILLFVSFAKQLWLERSEMCWIRI
MKVIQDGHVRESMNIXSYHAYAVLFFNSVSLIEYAHFYKFSTMMLVCSGYGETEAERKRM
EEEVASGNSREITANAKPLEDANDDLNSSDWNLTRVSQLIRVDLESHELNTRGLNHEIWA
LCHFELEETDEQKFLINNNNSDSTNKKKQWKRFDAESDLTDFIRCDRGEPLTLPPYSLTP
RQSSRTVSEADKKVSDITEEYRRFRVKSEMTRKQMDAQIRDLQSSQVEATTRQIEGQEHA
EQGMESSRGTEHSQLERMRAEAARQEDQWREAYDILLKENEALKSSGSEALLASQWRQRY
EACLKERDEAVNKLQSAQQRHEQHKQQQHSASFSSPPGIRDGKISKGVGNEGKYEMKYRD
LKESFRLYRKKAKEIFESQAAAAATNNSAGGKKNAADMMVLQMSQQHSNAEEESKLDYLK
NLMVNYLTADQETRDHMEMAIGTVLKFTPTDIAAIKKKKEDDASWGAYIGYT
>comp6752_c0_seq2; 494 164 1690 LEN=1727; translated
MAATPNGNRDPSPNKNKTRNGSGDGGAKQQGGVAPSPRSGGSSAASLNNASKAAAQMQKM
RDANSKYKNLLKMAKERIEQQEVELKELREAERKRMEEEVASGNSREITANAKPLEDAND
DLNSSDWNLTRVSQLIRVDLESHELNTRGLNHEIWALCHFELEETDEQKFLINNNNSDST
NKKKQWKRFDAESDLTDFIRCDRGEPLTLPPYSLTPRQSSRTVSEADKKVSDITEEYRRF
RVKSEMTRKQMDAQIRDLQSSQVEATTRQIEGQEHAEQGMESSRGTEHSQLERMRAEAAR
QEDQWREAYDILLKENEALKSSGSEALLASQWRQRYEACLKERDEAVNKLQSAQQRHEQH
KQQQHSASFSSPPGIRDGKISKGVGNEGKYEMKYRDLKESFRLYRKKAKEIFESQAAAAA
TNNSAGGKKNAADMMVLQMSQQHSNAEEESKLDYLKNLMVNYLTADQETRDHMEMAIGTV
LKFTPTDIAAIKKKKEDDASWGAYIGYT
>comp6752_c0_seq3; 117 164 566 LEN=566; translated
MAATPNGNRDPSPNKNKTRNGSGDGGAKQQGGVAPSPRSGGSSAASLNNASKAAAQMQKM
RDANSKYKNLLKMAKERIEQQEVELKELRGEYQNEFSILLFVSFAKQLWLERSEMCWIRI
MKVIQDGHKRNENGX
>comp6754_c0_seq1; 122 81 515 LEN=708; translated
MQRNAVDTSARLGPCQIPHTDPNFLSFLVPLFSASPLPPPPIPRKDCHWVYQKNKCFGYQ

HGVHIGENYCRKSCGYCNDLPPTPLPTPDPTVSPTKSPTRDPATAEPTRFFVFRPPPTAA
TPAPTPKETESSSLVSTLGESIGG
>comp6754_c0_seq2; 479 74 1585 LEN=1778; translated
MASTCSRFSVLLLFLGLLLRQAEATDRYVPFVANALDYNDAVEKDNKVKGNCKAGQPPLD
EPDAQTTKDSVCRETGPCQISYTEEGESVTYKFKTSGSDGKRYADITARVSSKNPKKFEM
KLFNGRNADETEQFRTKGKGFDDFYDVTWRRVPIDMLYDHHRVVVTFTDGNINMCSIRVD
WSDKNPTPAPHKKPTRKPTPKPTPSPHHPEPTRSTPAKWSAFEYSVAYDNSYAYEGNCHK
GDRDDGVDGSYTSDKVCINRDDSECHIGWTRPGEWLQYDFFAEEEGRYNVRARVSTNLYG
KYIRFRLTADDRAPFGKEIDVPAYGWRDFSDVVWKNVYLYRGHYELQVYFSTGYVNLCSA
AVYRSASKPTRKPTRHPTRHPTHEDNYNDCEDDEGYRYFGYHWKDCHWVYQKNKCFGYQH
GVHIGENYCRKSCGYCNDLPPTPLPTPDPTVSPTKSPTRDPATAEPTRFFVFRPPPTAAT
PAPTPKETESSSLVSTLGESIGG
>comp6761_c0_seq1; 125 738 1097 LEN=1475; translated
MAQFCKDFNEKSGEIYETNTPLRVELRAMSDRSYTFSIRSPPTAWLVKRAAGIDKGPNKP
NMSEQPFGYITPEMVHEIALIKQGDDNTWHLPLEGIARQVVGTARSIGIKVAEEEDLPE
>comp6761_c0_seq2; 125 402 761 LEN=1139; translated
MAQFCKDFNEKSGEIYETNTPLRVELRAMSDRSYTFSIRSPPTAWLVKRAAGIDKGPNKP
NMSEQPFGYITPEMVHEIALIKQGDDNTWHLPLEGIARQVVGTARSIGIKVAEEEDLPE
>comp6764_c0_seq1; 443 48 2024 LEN=2147; translated
MQLFAAVLALSSTFFGTSFVNADQGCLGGVYGNKTTTKLDIMDSVVRENSLHQIGGQLRY
ENVGTVRDRPVSLVVTVVDGDYADIAQVWIDRKRGSPYEMNGKKEGGKFANINLQTVEGK
PKSGEGNFRMCFVDTETNELTTVEKFSWTVYDLDERGNDKINKPVIKEKLIIDTNQARAF
QLWPNEAQSEVKLSCEDGSQLPCNAGVRTVFHSSTDGTGDDNPTDPNILTDLQKSRSVTF
TFIDTDCWEFTYDHYCPVEQPEYDGPSPRKTCKKYTGGNFLFAGESEEIINGGECLTPPP
SPTASPTPVPTAEPTTSPTPSPTANPTASPTVSPTVSPTAGPSSRPTVPPTVSPTTSPTA
GPTSGPTTSPSNSPTAAVDLPTTLSPTESPTSFDTAFPTKFENSDFPTASTTSFDTASPT
SLDTVAPTVFPTILEMPTTSPTTSPTKSPTPLPTVGATESPTASPTASPTEPPTAKATES
PTTSSPTDAPTFTEDETSCPGDVTLIKKNGITDIPLENAIKIVKQDKSTVTVELHQSWTS
GTKRESVDSIFYSFRTSIFDQKCYESRDVGSQRYDTIELTCEVLKPIAFVEICVVDDATH
GILVPGQGNDNATVPKCCHPEEISPEVPVVCYTVEIKCQTECVEEIERGRRGLRGSYP
>comp6764_c0_seq2; 426 48 2188 LEN=2311; translated
MQLFAAVLALSSTFFGTSFVNADQGCLGGVYGNKTTTKLDIMDSVVRENSLHQIGGQLRY
ESKTIESMQLKSLILFLNSNQTVLREAGTLTTPTLSFSDATQSDICYPSNIFMTSDVGTV
RDRPVSLVVTVVDGDYADIAQVWIDRKRGSPYEMNGKKEGGKFANINLQTVEGKPKSGEG
NFRMCFVDTETNELTTVEKFSWTVYDLDERGNDKINKPVIKEKLIIDTNQARAFQLWPNE
AQSEVKLSCEDGSQLPCNAGVRTVFHSSTDGTGDDNPTDPNILTDLQKSRSVTFTFIDTD
CWEFTYDHYCPVEQPEYDGPSPRKTCKKYTGGNFLFAGESEEIINGGECLTPPPSPTASP
TPVPTAEPTTSPTPSPTANPTASPTVSPTVSPTAGPSSRPTVPPTVSPTTSPTAGPTSGP
TTSPSNSPTAAVDLPTTLSPTESPTSFDTAFPTKFENSDFPTASTTSFDTASPTSLDTVA
PTVFPTILEMPTTSPTTSPTKSPTPLPTVGATESPTASPTASPTEPPTAKATESPTTSSP
TDAPTFTEDETSCPGDVTLIKKNGITDIPLENAIKIVKQDKSTVTVELHQSWTSGTKRES
VDSIFYSFRTSIFDQKCYESRDVGSQRYDTIELTCEVLKPIAFVEICVVDDATHGILVPG
QGNDNATVPKCCHPEEISPEVPVVCYTVEIKCQTECVEEIERGRRGLRGSYP
>comp6767_c0_seq1; 309 1 1327 LEN=1327; minus strand; translated
XDAKKQSLPLHQSAMDERLDTSQKVMALKESEQDIITGPPWRENSFMRLSACSQIRNGSL
ETGDYERNSISSNCEASIQDDFMGPKAAQTTHQAGSINIWSNKNKSHLTRDLQDHLEAPA
CFRQNQKMCSVGRSMREFLEGCKETVAMADCSSNFDSPVSLTPRGQEILTQFSNIQDYGL
DSAPFTHPGKKNQRTSSTGSNNKIQVQHSENTADNQLQTTTATTPFGEKLKQNWSPSTTK
KTATLKKFKNLNGSIDNASGGGEESYVSTLAPDSDYNSDNDQFQFQPENSVLKCEDLVWE
TKERNFENDQKERKSRGRIQTSSHDDGTSTKIRCRKRRKFFRSSDYFRNRAPAGEPENNI
AWSSEQGSKETDTDSVELIHVTMPDPLKKGDSTRPYRKSNYVTDWVPSNGLQSLSEDFLK
EEIWDIAAPDLQTSSTMPKSISL
>comp6770_c0_seq1; 206 338 895 LEN=1369; translated
MTEQQQQMATSASHIETAKNGLFNKMFASCAPDPRGSVHRAWGISLLFVVVYFSLSIFEM
VSLHQSGDASIALVIAVVWTGLIHLALGVLGTFVLKRFPTSFSVGFFLGVLVVLANQNIL
LYASFHKYTQGSARANQVFASLGFVLFGVLAFMSALLVHFKNDVVISPMETMGDNDEEQV
NNTSN
>comp6771_c0_seq1; 298 387 950 LEN=1177; translated
MQLVIPIEDTSSGTKCSNHCVEQQQELESASGRWEPRNNGSILSKDLRDQFETMQSVWRQ
ARRDGEDVDSSSSNNSNRERGGNMRSELKRKERETAVETAIKVDREIAFWQEGIADLEAL

LAAEDEQVTNEEGASAANNQHAVEENGPPGIREISFVRRNHLQAPVRPAQIQFPFLPPAI
PTEEESD
>comp6773_c1_seq1; 119 1 1329 LEN=1329; minus strand; translated
XTIVLYRKQQQQRHAQTKHRIVFXESFLILNSSNRVVPCRAVPADRSIAKARNGPPAPPT
LPTNSTRLEKRVAGVLQLLHDLPHRPVQLLRDPRLLLRRQRVEEGVAQLAVDHVHHRRIV
RPVGLRVPARRLGGLRPEVLLQLGVELDVLPGPEGDQRPDPPGHRGIDQGLLHARHQGRP
GQARHDLPDVVLAQRLEPGSTAAPLMGPRPGGGLPEGPLAARGVHGGGVGPPGEPEDAHV
PELVPDRDRVEIEELLADDRTGGRVVRDQHQPVGLGLVPDNVQGPHHVAEVGPIPRRLER
LDKGREALQLALSQQLPLPHLVQAERPSDPHQQPALKVRHGQHRAVSLRAVHGRLVQELQ
PFPVDDPVQDHVARGLLQRVHRHPRHGPHRQEGFPPAGVEAHRRAAQGRLVPAHLDAIQA
LARVGGIDQADAGSPRGPAGGNRP
>comp6777_c0_seq2; 42 1 887 LEN=1264; translated
XFETRAKVVVYLCFGVLAEDVTTQRNGVVSLGFFSYDNNDLLFVERESFLRFFDAVPLRW
SGFHLCIPNNPAYSIMKALLLTVIPKEMRRITRIHSGTNLECDYSLRCFGVPTEDIPRTC
TGKVKTKHLAKWIKVQTSIDDHRRMMARNNYNMYYLTPATERIFPGIECPEVNCVLFRSG
RLASRHPGNVEFSIFLQEKEAEREHMKTLQQTDDYLYRIIQQTVNRGFRFLAFDETKFWY
REINNYEVLRKHIFQAQRDVLKRTKAREALQLNKSNTTVFAGLDAKILQTDRRRE
>comp6780_c0_seq1; 667 110 1498 LEN=1939; translated
MAQPTSSKSFKKKKKNTRAPMPLVKGTTAASLAKKRAAADREEASGTSRLSSASRKRKRK
DRASENNGNGNNNKNVFSSTAESLAVISEYHTLNKRLEQAENDESLTPEERQKIILDLKK
QQDAMGGLDRYQKASIYGAKSSKFVCADWVVPLLKKELEKEEEEERHAGAKTMTAKSQSD
KQSESNPRRLRILDVGAIDNQYFGKGYDDWLDAVPIDLHGGQHDTVLQVDFFDYAHEYCA
GKDLAVAKKEEGKTKKQNNAKVHRDASSVSPSPPQPFDAIVMSLVLNFQGDPRKRGDMLA
LASDSRLLKSSSGDNHGSSSNRTRKGGGMLFVVLPSASLDNSRYCDLERFIEVTTNPLFQ
LELVEKKRSSKLILLAFRRTTKASRQQKLDDEQDAANQYGEQQKSNGKKQKRDQNAHCYD
VDKKMFDYGNHEMKRLQPPKPGAKRNNFAVILKSCHNRTSNK
>comp6781_c0_seq2; 189 1 1066 LEN=1066; minus strand; translated
XSAPSQIELGGDQRQLLTGGSQQGRDSIGIIRMAVSLPRSRLIVFFLNICNAVAFSRKMG
NPKSPNSISIGGNEGPKWCEASQIYIGGVPENEEVQKLIEESNGSVKIFGYGSLCWNPGA
DGGLSKASQTLGRVKGYKRVWSQRSTDHRGTVQFPGIVCTLLTKDEYHSVVESVAKGSGS
DEGALSPIDGTHDNETKDYAESMVTEGVIFSVPPELVQETLAELDFREKGGYARDVCEVV
EEGSDKTYRALLYRGTPDNPAFSSRCLVDLPFSAAIMSASVGPSGPNDIYLNNLDGFLDR
IKNSAAQQDDTKTLANMVRSFQKGYQLYFLTGGGSNQHNQLMLNRPGIPSLIAAED
>comp6785_c0_seq1; 691 1 2062 LEN=2153; translated
XGFRVSSHHPFTPSSPPSSLQRSGKMNTKKGDATDGGAKKIGFSLATNKKAQNRATSAAL
SGSAFSDDDEDQSLDEGNDVNTDIPKKPLVIPCQKDSRQSLQEQARARRQKEAGHPDVVE
SGDVGNHDAARRAESKSAGVEVPVKREENTQEADGNSSDGATTDRDRAAIEALEREAAAA
QGGGHDGTVSGSSQKRVIAVSGDTFQRQRERERGIDPTKTEDQKKLDEDIEDLPPDISVK
SQVYKKVPIGEFGAAMLRGMGWTGGDNNSSNKRKNNDATTMPRPSRLGLGATPKMFATGD
APDTHSRRRPRRQDQVQREERLKQQQQEMEKERQRQLALDKQRVLQIGSIVYVQRDGDRS
RKEQLPRARIKQLAGVPGLNMILVQYEGDAAPTKTKKGSIELVDRSHLNERPFQEAEEDR
RKKSREGDRGERERDPKRSSRGDCSDGRRRDKMGSRDRSYRASDDYEDGRDKDRERDRDR
TRDRDRRDRERDRERNRDRDRDRDRDRDRDRDKRDRDRRYRDGNCDRDRDRLKRKRDDEI
RRGDDSSKRHQSSRPSGKTNSGSLAFSESWLTPNIRVRIVSSKYGRSMYKEKGVVVDVTR
NGVATLKMGSGQVLTVPERHLETALPKAGGNAIILSGHQRFSKGQLLERDGKKNRGVVQI
FEDMSVVTTSLDDMAEWCGPLDDDLAH
>comp6786_c0_seq1; 403 155 1480 LEN=1582; translated
MTIKTLFFGSLLFATAVPIAGFSPPCSEVSTYRDRKQQSPLFPTELKMSTTENKVTATSP
RAPPRTGLAQTLLNFALESPLWKLILVPQARANIVQTAEANEIPWTAAKEWIQSQTSLEE
LDTALNEKVSSSWSSSIPPYYQKAFHAYETGNLSWEAAFEVEIASAAVGARNFPEYGAKG
EDAFRGAFESALTEAGANVPNSEGGDVVRIIDFGCGTGMSTRQLAQNFPQADEIIGVDLS
PYFTTIGKRLLELTPSSFLEGGPWVSTIQSDPRIEYFVGDAAKASQLEELVGAEGTADVV
NIQFVLHELPPDASRDMVDEAFRLLKTGGQLWICEMDFESPGYAAQRANPLLFSLIRSTE
PYLDDYAESIADLFSYIQSKSQFLTVVPATGRHFALVATKGEESMDRIEGVMKDLRFDKD
GNYRVEDTHLPTFKRKSETKS
>comp6790_c0_seq2; 60 1 461 LEN=745; translated
XMNMDMNMNMMNMMNMMGPNNNPDNDDNRTRVLRALLFYHVAPNVYRTQDLFCNGPNRYV
RMLRLGTSRTRCEGNRRTAQLGTCNGRGNIPYFTQRDILASNGIIHVISNVMIASPDGTL
QGCGLLRSPRPTPNPTRKPTRNPTRNPTRSPSF
>comp6790_c0_seq3; 3 1 518 LEN=518; translated

XMNMDMNMNMMNMMNMMGPNNNPDNDDNRTRVLRALLFYHVAPNVYRTQDLFCNGPNRYV
RMLRLGTSRTRCEGNRRTAQLGTCNGRGNIPYFTQRDILASNGIIHVISNVMIASPDGTL
QGCGLLXKPATDPKPNQEAYEKPHQEPYQKSIGKSNANADGLPVRNADGCSIVX
>comp6791_c0_seq1; 189 1 458 LEN=1133; translated
XQTPARKTTRLFNLVQNYSADPFTADNSFKNMFSVGRVLASRVAVRAVNTSLFTGVRQAQ
AVRTFAASTFLDPAEVSGRVTDVIKNFDKVDANKVTDTSKFTDDLGLDSLDAVEVVMAIE
DEFAIEIPDAEADKILSVADAVEYISSHPMAK
>comp6791_c0_seq2; 231 137 502 LEN=1177; translated
MFSVGRVLASRVAVRAVNTSLFTGVRQAQAVRTFAASTFLDPAEVSGRVTDVIKNFDKVD
ANKVTDTSKFTDDLGLDSLDAVEVVMAIEDEFAIEIPDAEADKILSVADAVEYISSHPMA
K
>comp6792_c0_seq1; 457 114 1037 LEN=1398; translated
MKLSLVSFFAALTLADAASHGASRTTAPWGIPRGGASSKYASKLDAVKEQVLASTLPSIE
ELRKSIVNESTTVEDLGLKADTIVNKALEEFASSAPEGDDSDVYEAKLEQIEAALDAPLQ
VLYLKQLALLREQALQNYRAASKASGGGTSEYEAMLQADAFFAREAERSTRDEVSDASEA
DGGWDFTGERAALQTVMNRLAESGQKATEVQMKSAQQQQTAMQFLQSQQQMIQQLQMQLY
GQSSPWNVGVAYRIPDTNFNLQGSYQQGRANVQLSCVPDEYAPFLGPNGFTNGVGPGNLG
LSLNLSV
>comp6792_c0_seq2; 188 114 359 LEN=610; translated
MKLSLVSFFAALTLADAASHGASRTTAPWGIPRGGASSKYASKLDAVKEQVLASTLPSVR
TICNESHPKMLETESDAVGAI
>comp6793_c0_seq1; 377 1 2236 LEN=2403; translated
XGDAASLDWHPRWPFVVATGGSGDRFVKVWDLESSLDPIVSSQNDRHSKRNNPRKEVLHH
HQENSNTHGSTKSSSSSTYSASSGESLHSSAGGASLSSYGPPNKWDLTSRSTASAWSMGG
RHHKSTSSGFSKYVLSISASVTQVRWRPPSNDFGPNDKNSDDPNQNETMVDRHDSMLAVA
TARLTSAGGSGTLSLWSFHRPFMSLSIVEGHEESAVSDFVWVRTPQTVPHRKHASEQQEH
DRMGFKHLASESSLSALTSSQRLRKKSWDFSDLDYRGDEQATEVKSVSYIWQNVLSVGRD
GQCIMQSFARGERRIRHVPSSCFAMANLSPFQEGYGSLQCFSVYQDVPNRAEDDFMLTGL
RQDEHTAKAPGVFFEEDLQKHLVNKKKEVEGVGKRLPVLVPKLRFSVLDQGSLDANGMPI
ENEEETLCIAPEVLHLSRFARLYKMYPDNQCQTRVELCLYNSTIAEQLKCRPLARMWKTV
ATLLDASGLDELPAHDSASPANAFQFVIFPTVKSLLFERAEAGDVQTNVALCEVLQVVDP
GGEKTRIPGLGIRLVREWYLSYIDLLHQMCLFSEAAFLIKHSKDPAIGALSQQSTTFHES
CPRCGKPLDSTADTSGANGVSNAGSSGSSRQVCKNCRRRVGICFICHEPVNGVFVWCPGC
GHGGHLEHALQWFGGLDGRAVRTVCPTGCGHKCNFVQFASTFPRTMSMALSDSSPIDHTT
LAIPGHQARCMPATTSSLYNLDKTF
>comp6795_c0_seq1; 471 68 1402 LEN=1414; translated
MDLKSGVSPSPSTSRIETVEREENTVARHVPIKGRRRNLGRQARKRRKKYLEIEAAASAF
VAAAAAVTACTIENQSSNPESTVLESATNPQNEKQKSGSSNIASTNCILPTDRSYILDDV
RRRTMLSENDRRDLIQQLGYLPGNALQVMARVEDAFSTEDLQHIIAATVAGSKTSSKNSP
NATATMESSTNCKEIKTNRKDPGMTARMVQTMMKEPLVIKLYPLALRDESSSTKRRRKRR
REEKEDLSTSSDKELDVICQGTSKTVREDQSIHPAASAPTHTIPLMEPFPTIYWVTHPLI
KALISKLELDKVNVRFEQRLLDEIRERKAPEKCKYKDNSSSSSLESMKCAHKSYGRERRE
LLSEQHREYVKKRRWYEDSISSNNHDSKKMTPSKAPFGDGCGVAGIRNPRAVKCLHAHAA
HYWSGNRDNLVGYWVEQELKKRRT
>comp6796_c0_seq1; 331 178 1314 LEN=1971; translated
MDDFNDSFVKDDVVFDESDAWNAEEGVFDPFQIGAACAEKLQSKKLTADPTVSTDNQSTR
SENGSTVKSNTSSALPPRLMINFQVHEEISSTAHLSNEQEGASDVQIQGTLMEVFFGRMK
VSLSLFDGFQDDSDRIGTLPNPFPSQFQNENXAQVVSSDALKNIPFLLESKTRKQNGSFE
FIPNNVYAKGVGQQDENNFKNMSVVTIPKEVLGFVNVGTYRIAQSMEHMPLLLEQKVLHS
KSTIKVAIQVRSKLSNPDDLSEFSIVVFIPSAVDKDSVTIASGNGEFDSFKRCITWGMEM
LPKGESFMVSAKCALDKIFVASQGAKVLEELNFPVMLRCRSKDRLGSVRFETMEADGHPA
TISSSVVGKSYRIVHRLN
>comp6796_c0_seq2; 391 178 1194 LEN=1851; translated
MDDFNDSFVKDDVVFDESDAWNAEEGVFDPFQIGAACAEKLQSKKLTADPTVSTDNQSTR
SENGSTVKSNTSSALPPRLMINFQVHEEISSTAHLSNEQEGASDVQIQGTLMAQVVSSDA
LKNIPFLLESKTRKQNGSFEFIPNNVYAKGVGQQDENNFKNMSVVTIPKEVLGFVNVGTY
RIAQSMEHMPLLLEQKVLHSKSTIKVAIQVRSKLSNPDDLSEFSIVVFIPSAVDKDSVTI
ASGNGEFDSFKRCITWGMEMLPKGESFMVSAKCALDKIFVASQGAKVLEELNFPVMLRCR
SKDRLGSVRFETMEADGHPATISSSVVGKSYRIVHRLN
>comp6798_c0_seq2; 241 917 1551 LEN=1551; minus strand; translated

MVMLQRQQAMSMRRQSSCPGMLRNQQSNYSHGGSFMSCAMGDDDNGSISSVSSRQSSFSS
MTSDNTEHERRSPSILPSTFGGNMNSSRTNSQNLYHHQQAKTPRWVLLVMCGTIMLSWFR
AVQYRTISYGILSSMDTELESIEFQKRQSTRLWKDATDTKNSFAKQHWKLKKTQKNFQHE
TRMLEEMAELEAASDNDNIEIPEAAREKFQNR
>comp6798_c0_seq3; 241 502 1136 LEN=1136; minus strand; translated
MVMLQRQQAMSMRRQSSCPGMLRNQQSNYSHGGSFMSCAMGDDDNGSISSVSSRQSSFSS
MTSDNTEHERRSPSILPSTFGGNMNSSRTNSQNLYHHQQAKTPRWVLLVMCGTIMLSWFR
AVQYRTISYGILSSMDTELESIEFQKRQSTRLWKDATDTKNSFAKQHWKLKKTQKNFQHE
TRMLEEMAELEAASDNDNIEIPEAAREKFQNR
>comp6803_c0_seq1; 265 1 767 LEN=940; translated
XNSRHQLNQNVLTNHVLAREKERDNDDHGIYYMDPHDALPAVVVLFSNANESLGFTARSV
ARRPPFRGGAAQTTRLRASTDSQEAFPVLKKIAGCNWEGSCRYVSEDLKPASFELTGGIR
FDLPGPSDDNDIVEMNSFIVFPNGKRRDIQMRGSRGSLQRSSMRLESTAGDGPIYTIITE
LGPDTVLVNEVSKETGMVVMTSSLSLVVQDGDGGAATELIQVSHEVGAPAAANKSAMIEG
HQVWRFSKQDESSWE
>comp6806_c0_seq1; 149 339 605 LEN=818; translated
MVRWEGHEIGVLSTLVGCVGILQGSMPKGWARGFIHRHPIPAMSCFLFTVGVTLPVTVVP
LRRALGMPTNQYDSDYPGVKYPKYETLE
>comp6806_c0_seq2; 149 122 388 LEN=601; translated
MVRWEGHEIGVLSTLVGCVGILQGSMPKGWARGFIHRHPIPAMSCFLFTVGVTLPVTVVP
LRRALGMPTNQYDSDYPGVKYPKYETLE
>comp6808_c1_seq1; 233 1 1222 LEN=1409; translated
XLYLTRLEALYLNDCSHLDLSAALGPGPSHQTGSDNGFEKTSSHNDDKNNRNARWTNLKT
LKLLDCENVTPQTFRWLTGLETLAMIRWNPHNPNTRGLGSRWIRQLAAPNHGRAAAPPLP
AGNPAFGPTPLLFGFRTTLRSLSFRDANLTNRDLQTIFWEILPGFPALEKLSVARNPAID
SFRQATARPRTAPGRESASPDAVVAAAERGRPSNLKSLEILHTKITWTKAEVDSLVWFLE
TIGASVGTLDVFSPWTSPRFDHPRNPRISHLVERTRTIEYLLARNATNTCSMLTGGSAFE
SGAGENANSTGTGGTEAIARPRNVGGPPPRALWSLALARAYARGYSTFSESNGTFSVQAS
SSMPFAIAYGFLRHGSVFSAGQTESWCRRKRRFLPRTVKKLVKYPE
>comp6812_c0_seq1; 313 1 620 LEN=620; translated
XPPFSRASIQGGREASDYGEDDAPPSTPRFGDGKEPQAQEHEGSPATPETPLTPEEQRAR
DVERQREIDELQMEFDMARDQILEIERQQQQKREEREFLGGLGGEHGAAAKSNRFLLSPS
YSTDEDDYYGYDSEELFPVADSPESAVEMLHAFSGARAAKGSPAPAAQEATAEDRRKQDA
DDSRMEEIRLVLQKVAALHEQQQQQQQ
>comp6814_c0_seq1; 913 287 3544 LEN=3781; translated
MTTTFLSVAMTDLDIHKSRCHSQPCIAEQFSTISQDDAARSFLVETNADADDASSISISL
SSGVFPVEKLYGREIQKASLFDAYDRIVRKRTATDVGNEMNNEQDFILVSGSAGTGKTSL
VELLRQKIQHEGGYFCTGTVDQFNQRGPIAPLADAFADYVQQLFNQGDPIEIYETQCRIR
TTIDETELERLQGIVPSLKRLLGNDTIKSIQYDSEEEDYVDIKHESDIQDENMEKRGRCF
FAMAKLMRTIARSKQSVVIVIDDLHYAQEHVFEYLKGMVLDDRNKSMVFLGICRSDILPS
DAPITKFLRGIEDSSVRITNIELENLDFHDVEDMVKDCFLRSHEQITSLSNFMFKTSFGN
PRSLVTIVESIQVEPNFLFYDNAINTWSINTHIAGNLLNHCPYPLLQKKLDSYPSKEKEV
LMVMACLGSCSTLNMIQEALQEYVSDAITQLVKRGNLKHDANMNTYTIRSNAVQEACYSL
IGEDIQPGFHLEIGLRLMRYLPAEELDDNLFCVVRQMQLGVNLLQAQDQETKYKVAILFV
RAAEKATVSFSFREAHDYLSAAHSLLGPSHWIDAYKLSLSLYNNLAETEFILHDGDRVDS
LLSDIDKFGRNLSDKLRAYTTMVHVLGVRGQKIAAIDKGISILADLNVHLPRHRSAAKFV
LSLAKIERGLKRKTNEMLLRLPRMEDEKQSRAMKILNLLFVYSFEYCPDLFPYVVFKMMK
ITLTQGLSAMSSVAFAGYGALLCLLGKVDHGQRYGDLALDITNRYSTILFNSRCIAMVYG
IIRPSIAPFQYSFEILREGHRVGLLSGDIDCAMLNAHLQFGLMMVMTDGIKVVDIIGSMK
KLIDLTERNGHAHHLRLLYNLISLLEIGSNPDEDLLNCLTPIEDGLKHALDTRSAFALTT
MCNLKSFFLLMAGQFQLGLDATAFSKTVLNSITMKTDQSILNGFLLYVQGLLTFGAARQQ
TNQTIRNKLIRRGKRTVNVMKVFTSKNPTRFGGQLALLEAEFAALSHNYTLAKQKYTEAI
EFADAHERLVEVGLANFFKGLHLSNDMRNPTDGIVYFENAIRNYDEFGLCVAGKYCREIV
NNMKE
>comp6814_c0_seq2; 672 287 2875 LEN=3873; translated
MTTTFLSVAMTDLDIHKSRCHSQPCIAEQFSTISQDDAARSFLVETNADADDASSISISL
SSGVFPVEKLYGREIQKASLFDAYDRIVRKRTATDVGNEMNNEQDFILVSGSAGTGKTSL
VELLRQKIQHEGGYFCTGTVDQFNQRGPIAPLADAFADYVQQLFNQGDPIEIYETQCRIR
TTIDETELERLQGIVPSLKRLLGNDTIKSIQYDSEEEDYVDIKHESDIQDENMEKRGRCF
FAMAKLMRTIARSKQSVVIVIDDLHYAQEHVFEYLKGMVLDDRNKSMVFLGICRSDILPS

DAPITKFLRGIEDSSVRITNIELENLDFHDVEDMVKDCFLRSHEQITSLSNFMFKTSFGN
PRSLVTIVESIQVEPNFLFYDNAINTWSINTHIAGNLLNHCPYPLLQKKLDSYPSKEKEV
LMVMACLGSCSTLNMIQEALQEYVSDAITQLVKRGNLKHDANMNTYTIRSNAVQEACYSL
IGEDIQPGFHLEIGLRLMRYLPAEELDDNLFCVVRQMQLGVNLLQAQDQETKYKVAILFV
RAAEKATVSFSFREAHDYLSAAHSLLGPSHWIDAYKLSLSLYNNLAETEFILHDGDRVDS
LLSDIDKFGRNLSDKLRAYTTMVHVLGVRGQKIAAIDKGISILADLNVHLPRHRSAAKFV
LSLAKIERGLKRKTNEMLLRLPRMEDEKQSRAMKILNLLFVYSFEYCPDLFPYVVFKMMK
ITLTQGLSAMSSVAFAGYGALLCLLGKVDHGQRYGDLALDITNRYSTILFNSRCIAMVYG
IIRPSIAPFQYSFEILREGHRVGLLSGDIDCAMLNAHLQFGLMMVMTDGIKVVDIIGSMK
KLIDLTERNGHAHHVSYARRCA
>comp6815_c0_seq1; 633 1 1807 LEN=1905; translated
VEVRKLQRNSGGNTESVTGGTGRSDINEQVGVTSTSPGDQNPENPLIQILPFQLGTVTYW
QKGSNSINLFSLSDHLQHRFSAIMKVVLQSQRRPQTPYKRPRRKKSMEELVIYLKRGYSF
RCVRSEMKNNLTVVLAAVSADGMALKYAPKEMKNNLQVVTAATKQDIRAIKFASDELKNN
LERVFPGVLLSKCALESASEELRNHPLVMEAVVNWGGKWYFPSEEVMEELEILTKNPMEL
ETASEEMQHNFQVASLAVAKNGEALLFGSLKIRSTPEVVTTAVTQNGAALRHANPVLRNN
VQIVTAAVTQIGFALDYASEEMQGNKEVVTTAVKNSGDALKFASDAMKKDEEVVIAAVTQ
KGGALRFASNELRNNLKVVTAAVKSQAQALMFVSNELTSNMDVIMSAVTKHGHALAYVPE
KWGNNAEIVTAAVKTCNWVLGQASEELRNDLVLVTTAVENHGKALEFASKELRSNLKLVT
TAVENYGKALEFASEELRSNLKLVTAAVKQDGNALEYASKELRTNKQVVMIAVTQHGCAL
QFACEELRSNMSVVTAAVKQDGNALQYASKELQNDTRIVTATVKQDGNALQYASKELQNE
A
>comp6816_c0_seq1; 772 1 2151 LEN=2240; translated
XEEAILTSIEGRQWRLLDRPIFVFFDTESEQPQQHRQGPALEEREHKRLXASTPIKASMS
TSTSTIAPWPTKMVESHGDYRDEAWLEELAHNKDTTYKNQSSLPQLPVPAIEDTLSRFLP
TALPLAESEEEKRALENAVQKFPAQAQKLQERLLERAASVQKNSSWLQHWWNTLGYLQVR
DSVCINVSYYFQFADDRTLAGKGSKGNSLQVQRAASILYATGQYRNIVASGRLAPETLGR
KNIPLCMTAYKYMFHACRIPLPQQDSYRIYDPHHYKHAVVARKGRFYKIPLIDESTNEVL
SVESLEGHLSQILELADEEDSKTEAPGLGYLTGDHRDAWATARAALLELPGMAEAMEVLE
SGCVLVNLDDASPSSRAQCGKQLLCGNGDASNRWFDKSVQLMVCPNGKAGLLGEHSMMDG
MPVVGLANHIVSTTYQQARDESTTGNSGLETFGGVADIFGSIEMTPKVLEMIETSKGNFR
EWTTGKHDLEVQSFWGYGSGFMKKSGFSPDAYVQMAMQVATARLFGKQVGTYEASQTRVF
RHGRTETTRTVSPASAAFVQSLLGRNGGGQPLSATDKIALLNKACKSHARYIGNAAKGLG
VDRHFLGLSMLVRDGEAAPDLYSDPVFVRSKKWRVSTSNLTHPKLDNWGYGEVVPDGVGL
SYSVHKDRVIFGITALKEHGGWPERLSQYLEDALVEMRLLVEEAAASDAAPPTRSKM
>comp6817_c0_seq1; 333 1 1084 LEN=1084; minus strand; translated
XAFARAGGKEVYVASDSHRSLEYIIQHFPPDVQRMIRTQGSFVVRSSWKWPAHMLEKHHR
VNSEALVDVLAMSKCQFLLHGNSAISEAAIYLNMNLHNQSVNWEDPHRLSPVEFYSMVEQ
VLATSSGKVSKNANANEVRAEKTSVKIDAKISKGSQNHICKRNAIVYLGQKNHSSYDRNS
FNSLLESLELMNTNYLSLNNHQNNTDVIVFHTADFTDNDMNLIEGRLGSKFREALYFIDL
YNTSYWRRPSWHSQDNPHDWYAYPLFSEGYRQMMHWYAIDIWRFFEDYRKTHGCLYEYIM
RFDEDSFLRSPVKYDVFDFMKENDFNYGFRLCSYEMQVTRRIWKIWRNLKGSPAPIRDID
LD
>comp6821_c0_seq1; 414 368 2878 LEN=3159; translated
MDLTCVICTENFDLKEHIPVVLPCGHTYICRVCVKKIKICHQCREPLYWTPPRPQPPPHS
ITNHPHRSPATGRYSRYNNRYSHTHSPSTPPHPGTGVTKPEEIPLPCPKNVVLMEVIEAK
QRQERLMAEQKARKDQKRKEQRDEKERKRRERREEQKRLRASRQADTTPGSYRPGTHCDG
QLNEIEVVDIEGDQVKNHVVCGNDGQSEEEEDYDYDDEISSSSSSTLPLGDPELNSGYAA
LSGTCGTYAVREPEGLVVLPYDPTRPKHHITTSTQQQRERQEPMSDEKKEDEFEDISRKF
SSLFTDNAKSSDAAIALNDNKGGSCAGSRTREPFTIEQGQKVQVVGVLESNDQGVYQLAR
GRGYVVATTKQLVKGAVNENFIRHCITTHCFLGLLLIIPFWFVLGSCGLFALVFSTISHV
FGFFSIQLDGTTCIYTRVHTHMILAVGGPMETSCKLEGMLQSVMGKQQELHKKLNQVTDL
SMMLRKEIILEQDKTEEVPVITPSIQKKSASGTGVDEKNGDAHPTTPTKRLTAAHSHSPA
EGSLGVTSIASADSTAVELGMENPRTPCPPSTTASGSYVEEHPLAAAYNRQQYHPPQTNH
YAYSPAHSCPTPTNHVEAAHLEQPFLDDDSTYGSGVLRYRVNNDDEMSGMGWAGVLGCGS
SLFGERLLQPTQREADAAGTIFHSTANVNREFLGMAENSLIQNSNVQRRAEALAQVAAAG
ATTSLRQHRTLESSGSADSPLRRGGSFDAGVGGGSVNFRGGYSNHGGLGRTRRNLYDQQG
THNRNASMTNAHHSGESHEPRRQLMMSQMSQHRGVGVASIQRRGLNADLVQMSSSR
>comp6821_c0_seq2; 668 368 2665 LEN=2946; translated
MDLTCVICTENFDLKEHIPVVLPCGHTYICRVCVKKIKICHQCREPLYWTPPRPQPPPHS

ITNHPHRSPATGRYSRYNNRYSHTHSPSTPPHPGTGVTKPEEIPLPCPKNVVLMEVIEAK
QRQERLMAEQKARKDQKRKEQRDEKERKRRERREEQKRLRASRQADTTPGSYRPGTHCDG
QLNEIEVVDIEGDQVKNHVVCGNDGQSEEEEDYDYDDEISSSSSSTLPLGDPELNSGYAA
LSGTCGTYAVREPEGLVVLPYDPTRPKHHITTSTQQQRERQEPMSDEKKEDEFEDISRKF
SSLFTDNAKSSDAAIALNDNKGGSCAGSRTREPFTIEQGQKVQVVGVLESNDQGVYQLAR
GRGYVVATTKQLVKVGGPMETSCKLEGMLQSVMGKQQELHKKLNQVTDLSMMLRKEIILE
QDKTEEVPVITPSIQKKSASGTGVDEKNGDAHPTTPTKRLTAAHSHSPAEGSLGVTSIAS
ADSTAVELGMENPRTPCPPSTTASGSYVEEHPLAAAYNRQQYHPPQTNHYAYSPAHSCPT
PTNHVEAAHLEQPFLDDDSTYGSGVLRYRVNNDDEMSGMGWAGVLGCGSSLFGERLLQPT
QREADAAGTIFHSTANVNREFLGMAENSLIQNSNVQRRAEALAQVAAAGATTSLRQHRTL
ESSGSADSPLRRGGSFDAGVGGGSVNFRGGYSNHGGLGRTRRNLYDQQGTHNRNASMTNA
HHSGESHEPRRQLMMSQMSQHRGVGVASIQRRGLNADLVQMSSSR
>comp6822_c0_seq1; 447 65 1417 LEN=1450; translated
MQRTKRNCPCCSFDLDLFLPDSEKKGVKEEDKCDEDDIDSPLLSSPPNISETFDRLVSAS
SGVENEGGSQIIIVDTHGHAQLDRGRNNVYELQIARNENSVNHEERKNDFGSRNENFHVK
TLACSVEPADFGITLKYAATSDSILPALGVHPWYIENLPNFASMTSRNIDGSHGDDPENI
DDFDEIDSEWLLPMEKLLIEHPNCLVGEIGLCKIAKWVRTHPDGKARAMGIQKRIFKKQM
ILAAKLRRPVSVHCVNSHGIFVAAIKELVEMQKNEEDTARAADNERHSGSRRFLLPTVIA
MHSFTGTAHHVKELLELEDQIDFPNDRFRTAMGKKKVPKDKEEKCSRIMKAPLTSTRPPL
FYFGFSHTVNYAMCTSEKSRKKGVEAVQAVPLNRLLVESDVHSDADVAGGTIGAISYVSW
ARDIHPREIASATSKNGSVFLGSIGSSNGF
>comp6825_c0_seq1; 208 1 773 LEN=773; translated
DDDDDTPKTTRKQLLLTVLSNTSSPAKTTLDRLHNRLGFDRSRFIGAVTSGEEAARYIRE
TFGGPPAAQAERQTERQTKRFFVWCTWDRDNQNVPDPLEFLELCGPGIEPTLDVDRADFV
VAHGSGAIRGVRDGGGGDRGLEVRSMGTFLDDGDLGEGTPIHGLLSRCADANLPMVCANP
DEVVMYHDGSLKHMPGKLAGAYERILRERSTAEGGEGTGERTPTIRIFGKPHREHFEACL
RDLDLAHRRRNQQQQQQX
>comp6827_c0_seq1; 733 1 1581 LEN=1728; translated
LATKHTDERVHDHSPLGDKDLPMFVTVEEDFLLKDNISTCLSASENRSVVCKPDPLLYSV
SQKAMALMASKTSKETITNDSNQKKATTQNFILKRQESLRKMSKEGCRREPNASKEVAGS
NNERTVTDDKAPQHQLYNRVARKKVSSQFHRHSIAQKETDDNKSTEDTSGNEPMPKNESL
EKIQRYNNFAKTKDSNGENEEESNISNQPSFHRFKYSRFKKIGTKPTSPIEPKREQPSTL
RKEGSHNAGAEDEKGPLIKPSNENKDDNEVGKAAPAHRFKFNRFTKIDVAPTSQTEPKRE
DVGNTDTEDDKDVSIKASTGNERKVAKSKVAPSHRFKYSRFTKNDTALASGVQSIREHPS
RSEIVLDEKERADSEALPLSEKKERNQMNKTNDSHRFKYNRFTKKDAPLDQTVEPKEESE
LEDDDTAQFNAKQKQNKTDASLQSLGLKCNRFNKKPLNVDSKEIQDGLDRTDDENNEALT
ETQEAQTSNSAEQPEASSQPMARSMSIRERRKFFESQSKNYQSSRF
>comp6828_c0_seq1; 141 1 863 LEN=982; translated
XRVWPLPEARRFDSVAFLTFADGLRFLRALSREPRGMPACVRLLDNAHFRLGQALRPDEA
SWLGRTKDALSKSLAHAWLGKDFGPDSIVCATISYEGSASEVREQMAAVSRLSRAGGGVL
LGPGVGRAGYELTYMIAYLRDFAMTYHVLGESFETFVPWSRIEPLVEATKARIAREHSSR
LLPGAPFVGCRVTQVYHEGACLYFYLCMGFEGVGSGASRVYSELERAARDEILAQGGSLS
HHHGLGKLRASFAKTRSSAAFRSALVSIKDALDEDNVFGARNGLFAA
>comp6834_c0_seq1; 226 684 1544 LEN=1544; minus strand; translated
MGVGGMVVVAATAAPVVVDRPLEVGHEVVVALDAPLAGLALDAPAELLHHGPMDPAVDQV
LVLGPVQEVPHLGLDPVALVGRPVEHDRRVLLAADLERARVLPPGLPVDLDRDLALDDDG
GLPALAELLGGAQADPDLAQVEGPPDRHDVEEAVLAAAGPVAGGHAGVLFVVRQRQVGQL
LEAVGPGERRGPGPGPGALRGRGHARDVVEALGPLLDGGPRQLDLAVHAGRVGVQDRVPG
RPEGDSPGVRVDVQHDDPLRIRRQGDLDAVGDEGNVVAEVDRGSLDN
>comp6835_c0_seq1; 190 1 378 LEN=566; translated
QMNGQMNNGNTKNQTNRQMNNGNSNNQMNGQMNNGNTNNQMNVQMGNGNNEKQTSGQMGN
GNNNKQTSGQMSNGKSDKDVQMSRRRRPSKIPTKMPVQSPTFHFGNSSVRGHAVGVSLTA
PPTSQ
>comp6835_c0_seq2; 326 258 693 LEN=693; minus strand; translated
MKSWALNWHLCWYFGRPPPARHLDVFIAFAITHLSTRLLVIVSIAHLSTRLLLVVPIAHL
HIHLIVGVAVVHLPVHLITVVPIVHMSVRLVIAMSMNCRSIHLVILLTGAHMSVHLIAIF
AAAVVAAAPLATADVAAAAVVVVVVX
>comp6837_c0_seq1; 353 1 1755 LEN=2516; translated
XEEEEEKQQQEHQPACRRLLVLFCVVLIGVVVRKANSAFNPSKEPSTCETARNSTLPHQY
FFHSSPHENGRVVVVVLGGSNRLWKEQGTSASIAREDRDPSDPFAPGVPAVPRTASPGAP

FPSGRASQIHGFGGAQQTLRGALAEFSPADGSLLPVPLRLVPEELVAWGQAPKTLEILVS
EAIEAETETETKTLTGPAPEKCGAPSRFFRRRTVTVLPAVGCGLDSLDTIRSEEYHPCRE
SNSNSNDDDAFEWNTSDPIVHIWNDETNGVGIIDRVVGPAATPAATKPAASAAAATTTTT
ILLETTFALPGSHRLRVAFGLDAASDRNGNCRYGLSSSSPIRVHLERWYDTDPDPSVQGA
ARNAEDVNGHLDAATVACLLGADKGQRQRLRAIAAEPPIREAAGRGETVDLALFGRLGIS
GRDGSDGGWSLDVALGLPFPPSEEEGGGKRAANRHRNDGAARVPGTVLHAVLFRNETASG
GEGGAISSEQTARGIGAKASGAXTSSSTATKFCSIVCFTGDVSGGKNTKWGSRKPIPPRD
PEAIRNIPRGNLKNNCRDFSTLSHRGQGNPEARPKCEASFTLAWK
>comp6841_c0_seq1; 261 29 598 LEN=1118; translated
MASDEDKNRNEGEQPGTEAAAETGKTTAMVDEPLPRKVYQDDFRPGRGNALQAAVASVFG
SDLADVPNFVERPEGYESAIEMFCRNRNYSCEKRKLPAEGSGVGGTQGPPPSGQRRRLCL
LRGKSPRGPFGHVVIARTSVSGPECVHRGAVFEMVHDPHPDESFLDESEPFGWCMFFTPI
EEQATQLTS
>comp6842_c0_seq1; 459 379 2163 LEN=2330; translated
MRATQTPDFVGIKRAGDECYEMARETSGLCKTTIDKANEMVAFGQELQTTLDDVCKSKYK
SRGASGAHNSKGIIDASKFKIIQELVEGEKMQSATLLAKELSALSLECVDKSKAMMLSME
KGIDALPDIVEPFVEQGLKASAARNKGHRKGDPELPDIEASVQDLKSLVSGVETVGLFTV
VERGSAAFEGLRTNGELSKNMFLSIQEFAESIESVSGSVRGLKRESMTAKSYLKLMGPMK
TVAKDAWRCLRLSGLMKDFAEQVGRLIHWIIALFQTASAKLGAIWGALANAKDAMAGCLV
YVTESIRLCDDSKTKSLLLRDTSRNIHHHLSNILKFRKTGAAKAVDSLRELADGDEILLC
IDLGTNIDDMFTDCVEQVVATIDQINGAIVKMPDVLTRDVPELACATNCVEYDDEIKQCV
RGTTNPPGIRATASSLNVDDDVRQLKSIQSSIEEASPLTVIHRSADGFASVGSTIETCGN
MISTSRGFAKTCQTSIESFTNAEWDLESARGHILELVAVRDAGVQMKEFVHGVLELVRAN
LALMKAVRSKSKGVAGGAESTNIGSLVGSLATEVDIDDLQAIGQGIQKLGSLFK
>comp6842_c0_seq2; 554 42 1937 LEN=2104; translated
MPNRFASKFANFGDSLKGAVGDAIDKIDDAIDKIDDKIDMPIIIDFVGIKRAGDECYEMA
RETSGLCKTTIDKANEMVAFGQELQTTLDDVCKSKYKSRGASGAHNSKGIIDASKFKIIQ
ELVEGEKMQSATLLAKELSALSLECVDKSKAMMLSMEKGIDALPDIVEPFVEQGLKASAA
RNKGHRKGDPELPDIEASVQDLKSLVSGVETVGLFTVVERGSAAFEGLRTNGELSKNMFL
SIQEFAESIESVSGSVRGLKRESMTAKSYLKLMGPMKTVAKDAWRCLRLSGLMKDFAEQV
GRLIHWIIALFQTASAKLGAIWGALANAKDAMAGCLVYVTESIRLCDDSKTKSLLLRDTS
RNIHHHLSNILKFRKTGAAKAVDSLRELADGDEILLCIDLGTNIDDMFTDCVEQVVATID
QINGAIVKMPDVLTRDVPELACATNCVEYDDEIKQCVRGTTNPPGIRATASSLNVDDDVR
QLKSIQSSIEEASPLTVIHRSADGFASVGSTIETCGNMISTSRGFAKTCQTSIESFTNAE
WDLESARGHILELVAVRDAGVQMKEFVHGVLELVRANLALMKAVRSKSKGVAGGAESTNI
GSLVGSLATEVDIDDLQAIGQGIQKLGSLFK
>comp6848_c0_seq1; 344 102 1046 LEN=1832; translated
MTTNDGNSRSPKAIMARLLSRQPSEYETASPSPSPYFSSVSPRVAKMFEILELRANFPQK
LRKDMDEALDGAAGNFLFHLESSVADLLYASANTSNNHSQEDQPQIGLNSDVDTENQVET
AIRCFPNVICRRSSTISRSEYNAPIEAQITLQSVAFLPLLARLGIELGDGTTFATEQRGG
LVPPKATWCLDNQGSSPFDGLNYHLRHPDSRHQLRNNQTSTNSLVRLLAPHLSRCTNRHS
SCNVLFELATTTTELHDPELSERIDKAFLAVIQKLRAENLFFQHDICGSSKSIYNRNNTH
SDWNMVTTLCATNPW
>comp6850_c0_seq1; 172 361 1332 LEN=1332; minus strand; translated
MTVFRPQRHNELFGTRFWSSQFIRYCGYKDRDSEEIMGDPACVDLTSYLIEKKLWTPPER
RTAFDILPLVLKIPGNDIPFIYQLPKSVTHEVHLEHPEFPEVKDLGYKWAAVPAISNFMM
NLGGIKYSAAPFNGWFVSIEVARNLLERYHVTEPLAIAMNMNLSDKFLAQKVSAELESAI
IHSFEKSDFTIVDPDTVGHSFITHCKRERSAGRECPAQWSSCFFSGPTNPVWHLEMRDFR
VVPQFEYCCDPWRVLDIDGVSGTKAIDTTGHILSEDDFSQKVSMPEVLIAYGSETGKAEA
VANSLARTLKFCRPTVLTLNGALE
>comp6850_c0_seq2; 120 361 1102 LEN=1102; minus strand; translated
MTVFRPQRHNELFGTRFWSSQFIRYCGYKDRDSEEIMGDPACVDLTSYLIEKKLWTPPER
RTAFDILPLVLKIPGNDIPFIYQLPKSVTHEVHLEHPEFPEVKDLGYKWAAVPAISNFMM
NLGGIKYSAAPFNGWFVSIEVARNLLERYHVTEPLAIAMNMNLSDKFLAQKVSAELESAI
IHSFEKSDFTIVDPDTVGHSFITHCKRERSAGRECPAQWSWIGGLVGKTGTNLDHFLFDH
SSRSQSVX
>comp6851_c0_seq1; 341 1 1397 LEN=1615; translated
XNGCKLIRSSLGLVFQVLDSWWIYHDGVRFLPIPSLILPLFYFRYCVDGGKVDITDTTKE
LHIQFSYSVEWVDEPDLVWRRRMDRYVDSRFLPSSFEIHWLSIINSIVLVLLLTAFLTII
LLRVLKNDFSRYMELDDEAIEEEESGWKLIHGDVFRFPENPGLFCAAVGVGNQLIVTTFC

HLGLALTGLISTTRRGSLLAGAVVLYCLTSFVAGYSSIRMYRQMNGKNWVRCILLTASLF
PAPTVAIFMWVNSVALAHGSTSALPFTTILTVTCLYGFIAFPTTVFGGIVAKNYVPAEFN
APTRTTKVAREIPTEIPWYRSRPFQILIAGFLPFSAIYIELHYIFASMWGHQIYTLFGIL
LLAFILLIIVTSFITVALLYFQLAREDHRWWWAAYINGGMTGVFIYIYSFYYYFQRSGMS
GILQSSFYFGYMAVVSFGFFLMLGAAGFQFSLVFVKYIYSRVKCD
>comp6851_c0_seq2; 742 123 2009 LEN=2227; translated
MLGKQTILLLLGLFSSIAIVDGAKKIRRVKSGKIYKEHENVHIVVNKVGPFNNPTETYRY
YSLPFCHEHSTEEEESDAALEEEVELHKFQGIKQVGAQRHKQRLGESIVGDRRESSPYEI
SFDDSVEWRLLCKKHMLPVDIKKLKEAIHNNYFFEMFVEDLPMWGYIGDIDNEDMIVGEM
QDSKTFLFPHLNFILGRNNNQIVSAKVTTDTERKVDITDTTKELHIQFSYSVEWVDEPDL
VWRRRMDRYVDSRFLPSSFEIHWLSIINSIVLVLLLTAFLTIILLRVLKNDFSRYMELDD
EAIEEEESGWKLIHGDVFRFPENPGLFCAAVGVGNQLIVTTFCHLGLALTGLISTTRRGS
LLAGAVVLYCLTSFVAGYSSIRMYRQMNGKNWVRCILLTASLFPAPTVAIFMWVNSVALA
HGSTSALPFTTILTVTCLYGFIAFPTTVFGGIVAKNYVPAEFNAPTRTTKVAREIPTEIP
WYRSRPFQILIAGFLPFSAIYIELHYIFASMWGHQIYTLFGILLLAFILLIIVTSFITVA
LLYFQLAREDHRWWWAAYINGGMTGVFIYIYSFYYYFQRSGMSGILQSSFYFGYMAVVSF
GFFLMLGAAGFQFSLVFVKYIYSRVKCD
>comp6851_c0_seq3; 657 82 2013 LEN=2231; translated
MTFSEKIMVKTQEGIPRIEQHEPLVSNSWMCDEPIDDNNASPNNXNDIQPELTIVNFFIT
KNRRPFNNPTETYRYYSLPFCHEHSTEEEESDAALEEEVELHKFQGIKQVGAQRHKQRLG
ESIVGDRRESSPYEISFDDSVEWRLLCKKHMLPVDIKKLKEAIHNNYFFEMFVEDLPMWG
YIGDIDNEDMIVGEMQDSKTFLFPHLNFILGRNNNQIVSAKVTTDTERKVDITDTTKELH
IQFSYSVEWVDEPDLVWRRRMDRYVDSRFLPSSFEIHWLSIINSIVLVLLLTAFLTIILL
RVLKNDFSRYMELDDEAIEEEESGWKLIHGDVFRFPENPGLFCAAVGVGNQLIVTTFCHL
GLALTGLISTTRRGSLLAGAVVLYCLTSFVAGYSSIRMYRQMNGKNWVRCILLTASLFPA
PTVAIFMWVNSVALAHGSTSALPFTTILTVTCLYGFIAFPTTVFGGIVAKNYVPAEFNAP
TRTTKVAREIPTEIPWYRSRPFQILIAGFLPFSAIYIELHYIFASMWGHQIYTLFGILLL
AFILLIIVTSFITVALLYFQLAREDHRWWWAAYINGGMTGVFIYIYSFYYYFQRSGMSGI
LQSSFYFGYMAVVSFGFFLMLGAAGFQFSLVFVKYIYSRVKCD
>comp6852_c0_seq1; 183 1 1119 LEN=1316; translated
SDDVLGGRSRILASPASACLVTPSASLKLVSIGTSNALVVWKADTDTDTDAFGNETRASP
AKRARTSDPSGTLPAAVVAPCRLVQPGGSGSSFLVGQPHDPDQGDLARFFEQRGPSLPPV
STAELCNLFQAAPGPLWKALEGVHAVVSHCAPGGGEAWFRVEGEEVLKGQRALVEFLCEE
DEDFEAEGGSDASLALGAVADRVGKRLAPLLADKGGPAMGREERSLAIARKTVLLASAPG
TTGGSKAWRDVRDRRFRPDASKIAFCVLRDLFSKYPSYAWSDLSEQWSSRLPLGERYERI
TSTTEWIEDPDLGGHGGLVPDQLVFSPSAAGDAGGGEGADRTTGKKGSGAGNNPKGVLGL
VDPHAVLVWRGG
>comp6854_c0_seq1; 791 325 2699 LEN=2982; translated
MPPSSISNAACSSENDNLLTLLSLPKKWIDFDTKKDLVHRVANIRHFETLLDESTEVSWD
NPTKENRNSTSTKDSPFDTVVEVDNLDPNGLFVSLDLDLSWEETKKAEQKSREKYSRDLS
IHLNREAMESAHRAIQRLEISVSKKIEPKSKKKRKVKAPASALRDGTNPLPATETIVSPS
RLIQYTDTIENGNNDDNHIEDIDTKEITTIELCRRLQSLPESKIGLDLSIPLRDEMSAAC
IDEAPSHPARFRCRIRSNPPVILATSTFESFRSKLFVGIPIVVNTILLHATRAEISWFVL
NGVNENGKEAEELVLRDSPSFIPKAHHVGKRLLVVVRPLRESKTKGDSYGKADVYIFHNV
IQDFPRMPIVSPLRDGFIKGEKESDQKSMDTLRVCTYNILADLYVSRQGNGQDSPTTYSH
VKYEHVEKARRIPMIVGELMAYQADIICLQEVDGSVYDAFLQPVLLTLGYDGYYSNKASS
QREGCALFWSRNVLELSEASSFQVRDLFRPSSSNNEKDRGEWKSMREIYQLLNSNRQLKT
VVVEKLGQILQIATLRLKNSSQEHAKEIVIANTHLFWHPMADHIRAMQVYVVAKKIDEVR
RKTNFDNSLKKNHPPRPFLLCGDLNSDPLSGASQLLCSRSLAPDQHECWKYLHRYKWDID
DCEVDEMMEGGTSGEERRAEHRDGRCEQSISFAKSCNASPPHIDLPDCFPTIVSGCKESP
PFTNFSLDFVDTLDYILASKLLKLSYLDSCQCGXAAMPSVLDVKKFISMPNEFMPSDHVS
IVCDFDLIRYD
>comp6856_c0_seq1; 492 95 1321 LEN=1405; translated
MVSLRGIGLAFIAIHMHENGPTFSSSLVHKSLASRRVALSNHDAHLKMTLSALSSPATND
VTLPDTSQPLNPQTYAGMIEEGLKKKFNETEIERVLTSWRLVDQGYYRKEYVGDKIDASE
IASSNMIQECHSYVPGLSIKPFWDTKEFDWTQTLESRYTEIREEFDAVMGNMEKLQREGN
NVWAGALTEDASSYGEGWKTLVLMDRGRWDPTNANLFPKTAQAIHASGAPATEIFFASMK
GPSSIKTHSDFTNFVLTSHLALDIPYSGENRCRLTVGDETREWINGKSYVFDTSLLHDAE
NESDKMRYILMMRVWHPDLTAIEKEALQFTFDCLEYDQLLSSNEEERFKAEKITEASRSF
PLIPESGTGFRAQIKSPEISKKGFGKNQKKNKRKQKGNGGGKKRGFGK

>comp6859_c0_seq1; 361 1 1652 LEN=2030; translated
XLEDRFRGIAGGSPAAPLEKDGAGGGPGTEGSPGQAGPRFDRDRLRSYLSQRIRAEEREV
FSDPSAVEPRGDRTEHLRSAYIEAETRRLWNFLQGGEDPCPDRTREQTAKTKTATTMAVE
TACDRVLVSARAVDGQRLWPLTLSMVMIGLSVGVTTPAFPFVVQNLGLSTGEYGMVVSAF
ALTKLFGNIPFAVLVETNGRKPYLVYTMIAISTGVGCIGLASGFEELYACRLVTGLGVAG
LSCAATMTVTDISTPLNRASSFAPISAGFAAGTALGPALGGVLIDQIGVNPTFYLVGGSF
LMLGGVNSFLLDETLRKPNQSFPWREETKTESKTDDDEGSIKNAFHKALGQWVPLLSEAP
VRSVCIINGFYWVALAGSQMTLLPLVLTDPHGLAMTATQVGQVYMGMSFVQVFGNPLFAR
IVDRAGTVPGIVGGCSLISGAMFALPHATASGDLGQMAAVLGVWSAGSSLLSTAPIAHIS
DRVDDARRAQAIALLRTSGDVGFLVGASTMGLLSDWTGGLDTAMQSSAAILATATSWYCV
RSAMTSRQKQ
>comp6863_c0_seq1; 338 587 2137 LEN=2254; translated
MEPRTISNAEDASGIVGNAPRARVLSLALRSLPVSGCWAVPVTLSSDDDEFFDYLAVVDT
GSPFLTAPPGAAGALRQTKQRSVTSKRGGNLGPKKTTMLFRSKESFRLGDSHHDDDTSYE
QYGNTVGGVQWRIAPHLTLVGNGKVADIVARGIKDYACDENGKICCSRSDSGVDTPKGVR
LLGESEIEPIVIQDESVFTVGVPSEDVITETGGIFFGLMTVDGARPTTLQQLGYDAFVMR
FGENEASLSSGSRKKDKSNTDPATLVLWNGKQNDSGKTNPATPLVDPFDPCSMKLFDLTP
FGPNLHHYAVLCDRFECRWVGNDSISFDCVQENGSNICTSPLKGENGVSLSRPLIAVFDT
GLSGCIFSDTLWEEVQRERRRKHKNEAGGQRRGRGTPENPEESYPVGCTVRLPTISNAAE
KSGSTPTVLPPKSSSSHSTSIVELSSISEHWRFQSFRLPWWYADSVEEKNGNASQAQERD
NNFPHVIVLGSTFWRNPNIQELAVDTISQRAKIETT
>comp6869_c1_seq1; 553 1 1514 LEN=1675; translated
XDGDGNGNGNGAAARSSVSDQIVKRYLPAYLLAVFSDWAKGPCGYELYSSGEYYNYDSHH
AYLLCSVVPFVSSMLLGPVVCGPLADMGGSKRAAVVFAIVAALGNVAKHFRDFRALVVGS
ILEGVSASLLFTAFDSWLVRSHHRGAPPETKPPATTISPWTSKAAPSLRLSRAFATAEYR
NNMVAIAASFVPSVATGAMPFQPLALPNEKGSTAVVYAGGVLNAFDLSTLALLLCGTFIG
LVWDEIPTEPAEERRETRPGSHAEGAGADAGRRLPLWRRHRSLLGGASFELLRSEEVFLG
GAVCSLFETAMFVFAVSWTKAIASRVDDDENIPFGLVFATFMLGCMVGTSVYALLIDETK
VRNETIGEGLLAVATCTFIAMASTRSATPAVFLVWFFLFEATVGVYFPMMGTLKSEIVPD
CHRTTVCNLYRVPFNLFMVAFLWVLEHRGMCPESCFALCACMAGAAFFFQVALGRCRETR
LLSRLSKQHEASFRDRGMRQKKAA
>comp6874_c0_seq1; 551 137 1732 LEN=1831; translated
MFAKLLVTALVLWINLSVSYGEDTADDNIFVGNNGNSTEDEFPIDWDYEAIIVVRWDIES
PPIVEYDNLQFDIQFSVSDYIEAEKHVRYNVYKTPECGNPDDIITASDGYMETWVSEDKT
PIGPGLDENVKKVVTVSNKLNSQTISQSEIYSEDSLETGTDASITYCVLFGLWNGDGPSD
PDAMMINDMAVTVILSLDLGDDFKITGQNVAAKARDVEISDDRFFVEAFFCDETGKPPTL
VAPKRQGSIVRVCVQPTKQAADVGFRMQSIDEFDFAQGSTSQEAISDGKESSNALTRLSC
KEGAKQCFFDTVLLASFYESGPEGAPGQINGDGVATLQWGGEGVDRRLQDIFLDQSLEFA
TVESNSEKDYTISEQLDFDFERLLRDRTSQKSIQLPNFAVQIEEGSQRPPLKADVVAKSG
KLNPLLIVSAVVLCALVVLSLIYIHYTSPKVRSKEVEEGEDEENNQRNNSQTLPLPSPSD
EDASECECECEDNSNAFDHEEQRGTNQLRLSVIDENPTGVFSARSVLSRGF
>comp6880_c0_seq1; 396 163 675 LEN=1354; translated
MKPDWDKLMDAFADSPTALVADVDCTAEGKDLCSAHGVKGYPTIKWGDPSALEDYNGGRD
YASLETFAKENLKPMCSPANIDLCDDAKKAEIEKFLGMAGEELDKLIAEGNKKIEDNETN
FKNEVSKLQAQYQKLMDDKDLEEIKIKASGLGLMKACAAHAAKSKGSDEL
>comp6880_c0_seq2; 396 314 826 LEN=1505; translated
MKPDWDKLMDAFADSPTALVADVDCTAEGKDLCSAHGVKGYPTIKWGDPSALEDYNGGRD
YASLETFAKENLKPMCSPANIDLCDDAKKAEIEKFLGMAGEELDKLIAEGNKKIEDNETN
FKNEVSKLQAQYQKLMDDKDLEEIKIKASGLGLMKACAAHAAKSKGSDEL
>comp6880_c0_seq3; 343 1 518 LEN=1197; translated
XAASIPNNSFPPLRDSFSFFLVSILSHDAILRTHTIYLLFRRQPTIKWGDPSALEDYNGG
RDYASLETFAKENLKPMCSPANIDLCDDAKKAEIEKFLGMAGEELDKLIAEGNKKIEDNE
TNFKNEVSKLQAQYQKLMDDKDLEEIKIKASGLGLMKACAAHAAKSKGSDEL
>comp6882_c0_seq2; 363 138 818 LEN=900; translated
MAAALPITKLAALVVKTLAKPLSKSIKNNFRENQAAKKVLISIGQTNHTVTSYMTIWASG
YRVRSIKPIEAEKALKDGAEIAGESFVVAVTAATVIFEYNRSSKSAARKAEEKRERIRED
QKKLNAKLNALDIRLKAIEDLVRQQQRIEDKKSLLTQIVPVGGTEKAKYVEPPKEQLVPI
IDNDEDVSATGINEESDSVKERQVAIDSSRTPSEKENDQKQSHSSWW
>comp6882_c0_seq3; 321 426 947 LEN=1029; translated
MTIWASGYRVRSIKPIEAEKALKDGAEIAGESFVVAVTAATVIFEYNRSSKSAARKAEEK

RERIREDQKKLNAKLNALDIRLKAIEDLVRQQQRIEDKKSLLTQIVPVGGTEKAKYVEPP
KEQLVPIIDNDEDVSATGINEESDSVKERQVAIDSSRTPSEKENDQKQSHSSWW
>comp6883_c0_seq1; 137 1 263 LEN=263; translated
XTKAARKSAPATGGVKKPHRYRPGTVALREIRRYQKSTDLLIRKLPFQRLVREIAQDFKS
DLRFQGSAVLALQEAAEAYLVGLFEDTN
>comp6887_c0_seq1; 476 115 1215 LEN=1253; translated
MNPTEPPGTASTGEASSAAEPKTDTKTNPKTATPSESDVYDRQIRLWGAEAQKKMQNSRV
LYLHVTGSSSEVLKNLCLAGIRAEICDPRPASALDASPCFFTHPNHKSKSQGDGESESEP
ASKKPKYGSVALAVRPLVEELNPLLGSCPIVDKAISELTREDLKGYTVVVASQVPLSEVV
RLSQLLAEGSVLYTVDCFGMRGASMIEFGKKDFCYRPEQGKKLLDPTPIKDYVSLPDMVR
VPLHKATNRFHKKGPPPTWIMYRVLMEHQRQTGKWLGDPSIDPWEAKKASEGFLAGEGVA
LTETELDDLVTAGVAQVPPVCAVLGGMVGNEVIKIISGKGEPANNTLLLDGGACKAWTFM
VKAKET
>comp6888_c0_seq1; 407 118 945 LEN=1177; translated
MSDRNLFSNLRSPLAKNDEYGVTELLREVSKRNSSLSDEERKEVSEMVKRVEQHNAKPSP
MDKMKKKFAELSVVNDMGSNNRTTKNSTVGESDDNGDDPRADKSVKKFANDLFQKFSNMD
TISAGQPKDPVFDGNRGKEFFRSLSMKGIQSPLKMTNSASTEQQTPSRAEGDRRPEILKA
FEQSSQSGKELLRNLSKKIQSPLSSVPNKDPPPSAAETAAAVSEAFRDIQKIHSPNIADL
PQKEAIDISNEDQKKNATNLNSIQTPTIVDFPEEE
>comp6889_c0_seq1; 295 257 1255 LEN=2042; translated
MHRSLLGFTLLSSAIGSSAFVTLNVAPRCTTNLFEGGNGGDEENASFDVENARRRLENML
SDPEKIASGNNSSNDGDSDLAAAATPFSFSKFLADNDEPFSLSALPPPPPLSTIERSRRL
VEIKLIECLNDGDDVISELWDHWYSERGSAAKQKLQQSSGMFMDESKWEECEQNLIELVV
EHGIYFAEPVNLLATLYFIQKRFDLSYKLCQVVLAIKPHHIGALSGIVEVALGSGDMGAA
RYWAAKRLPKHVPASVSGSDGAASVYDENEDKKSVDTPRRVEWVQKAVARAQDTLDQAER
RTKQKFFGEADIHFDDSGGGDTLLDGDDDGDAW
>comp6889_c0_seq2; 295 257 1255 LEN=2204; translated
MHRSLLGFTLLSSAIGSSAFVTLNVAPRCTTNLFEGGNGGDEENASFDVENARRRLENML
SDPEKIASGNNSSNDGDSDLAAAATPFSFSKFLADNDEPFSLSALPPPPPLSTIERSRRL
VEIKLIECLNDGDDVISELWDHWYSERGSAAKQKLQQSSGMFMDESKWEECEQNLIELVV
EHGIYFAEPVNLLATLYFIQKRFDLSYKLCQVVLAIKPHHIGALSGIVEVALGSGDMGAA
RYWAAKRLPKHVPASVSGSDGAASVYDENEDKKSVDTPRRVEWVQKAVARAQDTLDQAER
RTKQKFFGEADIHFDDSGGGDTLLDGDDDGDAW
>comp6891_c0_seq1; 171 227 439 LEN=778; translated
MTVSAVNWFKRVLGFGPYFRRRSNGLEGMIVASFLGVTSGIYIWKPILEEMEINRARREL
EERSASKERT
>comp6892_c0_seq1; 413 583 2115 LEN=2333; translated
MNCPVGLGSLGNNNNNIKCDGGLAGGNMSSDSSSSQSSSDDDSMALDDDSVDSSSWSPHI
QNDANEIKHAYWIQRTIREAIYGRVLFAVILKRRPRSLVLSDGAEWEVTRDHCAVKEMSL
EHIRREKDRLAEDPIKEVASMQYLNRWWRYSQQCSIQQHQLHQLHHDHMVIPPLQQADNN
SHADVTQSFFAMRDTNIMMPLDLLLDERNLYSIMPYCDGGELFERLDLNERFSEDEAAVL
DASGAQRKSDIREGIFPYGTVTCLALNASSMVAFPFSHAMDNLPLPSVKGIYTKRVFYVL
SYYLLHFCVQGLDTLQKVGICHRDMSLENLLVHKDGALIIDMGMCLLVPFCDEDPSSLAA
PLGNMKMNGESKLNQSTLPGRLRRRSLIRPQGTCGKWIYMAPEICRNEEPFDGFAVDMWA
AGVILFLMLTGFPPWERACHTDERFHYMTAGYLVQMLTEWDIGLTSDAMDLLQRMLFLDP
KDRLSLEQVRAHPWMIIRRQEQPLGLQRNM
>comp6892_c0_seq2; 492 583 1923 LEN=2141; translated
MNCPVGLGSLGNNNNNIKCDGGLAGGNMSSDSSSSQSSSDDDSMALDDDSVDSSSWSPHI
QNDANEIKHAYWIQRTIREAIYGRVLFAVILKRRPRSLVLSDGAEWEVTRDHCAVKEMSL
EHIRREKDRLAEDPIKEVASMQYLNRWWRYSQQCSIQQHQLHQLHHDHMVIPPLQQADNN
SHADVTQSFFAMRDTNIMMPLDLLLDERNLYSIMPYCDGGELFERLDLNERFSEDEARYW
MHQVLNGLDTLQKVGICHRDMSLENLLVHKDGALIIDMGMCLLVPFCDEDPSSLAAPLGN
MKMNGESKLNQSTLPGRLRRRSLIRPQGTCGKWIYMAPEICRNEEPFDGFAVDMWAAGVI
LFLMLTGFPPWERACHTDERFHYMTAGYLVQMLTEWDIGLTSDAMDLLQRMLFLDPKDRL
SLEQVRAHPWMIIRRQEQPLGLQRNM
>comp6897_c0_seq1; 179 1 715 LEN=829; translated
XNSNSNSNFTNGHGYENSGSRVETFVDIAVFEVPDHCTSRNCDLSKFGVGDLSHFNGGSY
SNLCKAGRLNLDHSVFKGHHTQLMVPIEGPMQDRIKQRKTARFSGTKERHYEVVLANCHR
HGRNVRVNGQVVFDLVETESIANNLTRGSAAILSSVALVVFLALSVVAVRVRLGTRSDFE
RELYGLVPSTDDDGDVAGDGEPREPTEVEDQYEDDNDERGAEPGAGSPDLALANTRVV

>comp6897_c0_seq2; 120 597 1043 LEN=1157; translated
MQDRIKQRKTARFSGTKERHYEVVLANCHRHGRNVRVNGQVVFDLVETESIANNLTRGSA
AILSSVALVVFLALSVVAVRVRLGTRSDFERELYGLVPSTDDDGDVAGDGEPREPTEVED
QYEDDNDERGAEPGAGSPDLALANTRVV
>comp6899_c0_seq1; 508 303 1391 LEN=2443; translated
MAGPANPPGQPKGGFNKLFVMLPVMLAARKLDAEDPQTVQYLRMAYGSMQSLCVLVVLYT
FMKASSIDDKKASNIVYIPAPPTPFADPEAKKKYTSSEYRAHVLSTARSLVGSTLFGICM
TVGLHIYKGMAMGLAIQTIMGPFNLFENPLVKAILMGNGFRPEDKIFEEKDLQELTESDE
VVDETGNVIPKHQLLASSKTQKAKKSQSPEDAFEELLLDTWDAGNGADVSKFVSAVTKKN
CNQRSKGNEWTPLMILSGLTNKEDTASAIRKVIGMGGNPGVTDKDGWNCLHWAAFHGSLS
AAKALYDYDASLLDVKDNEGKLPLEMAQQEDNKEVAKYLEEVTATAAASKTDGGDEGIRK
RK
>comp6899_c0_seq2; 508 303 1391 LEN=2340; translated
MAGPANPPGQPKGGFNKLFVMLPVMLAARKLDAEDPQTVQYLRMAYGSMQSLCVLVVLYT
FMKASSIDDKKASNIVYIPAPPTPFADPEAKKKYTSSEYRAHVLSTARSLVGSTLFGICM
TVGLHIYKGMAMGLAIQTIMGPFNLFENPLVKAILMGNGFRPEDKIFEEKDLQELTESDE
VVDETGNVIPKHQLLASSKTQKAKKSQSPEDAFEELLLDTWDAGNGADVSKFVSAVTKKN
CNQRSKGNEWTPLMILSGLTNKEDTASAIRKVIGMGGNPGVTDKDGWNCLHWAAFHGSLS
AAKALYDYDASLLDVKDNEGKLPLEMAQQEDNKEVAKYLEEVTATAAASKTDGGDEGIRK
RK
>comp6901_c0_seq1; 333 148 1422 LEN=1481; translated
MFIKKDLRKIPTILSDAVDCRTYDEGNDENSNNGTKIKRTKREEPLRILKLGRRKQEFNG
CVRVLCQPEHFPKLGHLSVVNLYECDIHDLTGFGPVFGASAPNLETLNLGRNPLSKGIPD
DFSEVKSLKHLWLDDCKLAGALPRALLKLPNLESLRLPNNRITHLDIGEADMTNGSGSNI
GNSGKNRDDGEEFEYEWDDTAMKDASTTTNEVTPLKNLKILCLDRNLLGTTDADGSRDGA
GEGGDTGNANPNTSKDAGDDPREAEQSGTSPARAVLPPNLAEWFPALEELLLRHNHVRRL
GVRDWPEHLRILHLSSNRLESLSELSSGGLRSLAHLSANGNLLGALPEGILSDHPGLRRL
VVSHNPPLRDLPVEVWERMGLLPRAAAGAVDEAPLPPCDIVWQPNPNLSAPAAGGGREDG
AMEE
>comp6903_c0_seq1; 392 1 1340 LEN=1546; translated
XFKTLTTPTPCQFLSLAVDPAGEIVVAGAMDPFHVYAWNLRNGRLLDVYTGHTGPVAQLA
FSGGGGLLASASWDGTVKLWDMYKANVATESLSHNSDVVCVACRPDGKELVSGTIRGLLS
FWDTETGKLKHELDGRRDIAGGRKENDRMASDNNASGRYFTSVCYSADGSCVLAGGNSKY
VCIYEAQQRILLKKFQVTFNRNLDGVLDELSSKNMTDFGPVDEHAGDSEDETTYNALRLP
GAKRGDDGSRSSKVEVLTRQVSFSSTGREWAAVSGEGLHVYSLDGDMVFDPISLEEAITP
SAVKENLSLKNYSLALTMALHLNEDALLEQALEEIPHASIGSVVRTIGPEHLERLLAAIG
TAMESSPHVQYYLSWCLEMLRTHGVHMDRHRSSFMRAFRSVHKAVQTKHDELKTICNQNR
YALEFIESQASLTTAATAAALAVGKD
>comp6905_c0_seq1; 226 1 622 LEN=897; translated
XMGTGEGGAPLASANLTGASFAGYTNVDGLNWDLFATHQQTGMGVDLTSAFMGTAYAVDE
GKKFNTLAGLVLVVAGALALLLAVFFVAARRRKNRKAFLEHARCIDGLALDAEDDPSLDR
ARCVVDDASLFGQESLPAGYRVRTEGPGHDYGTCANPSCKLCGSARDRPGPVFVKTDPNH
SFTEHRSTLESRRDKEEAERRYESVML
>comp6907_c0_seq1; 203 113 889 LEN=1402; translated
MSSASSSVARGSLYRAYSRSSSTGSISSVENLPPRTIARVSKEVRDLMKTPPGGINLVVD
PETGMPSSLGEIVAEVEGPQGTPYENHYFRLKLVLGVEFPASPPRGFFLTKIYHPNVDMT
TGAICVNTLKKDWTPDTTFAHVLSVIRCLLINPFPESSLNDEAGKLFMESYDEYSKRARL
MADVHGRPNSSAMSEGDDKRRKADLKDDEHDSPQRRKSRIEPGMNPLGSKNSNSISSKSI
GAKQVSGRLKKKKSLKRL
>comp6907_c0_seq2; 246 741 1163 LEN=1676; translated
MTTGAICVNTLKKDWTPDTTFAHVLSVIRCLLINPFPESSLNDEAGKLFMESYDEYSKRA
RLMADVHGRPNSSAMSEGDDKRRKADLKDDEHDSPQRRKSRIEPGMNPLGSKNSNSISSK
SIGAKQVSGRLKKKKSLKRL
>comp6910_c0_seq1; 384 1091 2059 LEN=2080; translated
MSQSPVFITVNHAIQDDGEYDDDSSMANSAIYQDRVIERLEQIRDIFSASDDKGDLLPAE
LLPVGSIRIPDNLGGVVSKWPLYDEGAWWVQDVSATLPAIALFNSLSSKHEEQHVQDMHV
VDLCSAPGGKTAQLCSMGFGSVDAIELSAKRSRSLRQNLKRLGMEEICNVVVEDGREYEP
DAMSGPVRGILVDAPCSATGVGSRRPDVLRKSIDITELSAIQRELLVHAADNILEVGGVL
VYATCSLLKQEGEDQINWLLSEVGSNASNAQLETIPFAPGEIPGFDDCIDENGWLRVIPG
VLPGSLKFCDGFFVARLVKVSK

>comp6910_c0_seq2; 460 674 1882 LEN=1903; translated
MDVPKYAAIYETVESLRLHPKIKVPKSQISFANAVLRNIDREGIPVLKTTSSINNLDLWL
AKQWVKQYGKDTTETIVEAAMSQSPVFITVNHAIQDDGEYDDDSSMANSAIYQDRVIERL
EQIRDIFSASDDKGDLLPAELLPVGSIRIPDNLGGVVSKWPLYDEGAWWVQDVSATLPAI
ALFNSLSSKHEEQHVQDMHVVDLCSAPGGKTAQLCSMGFGSVDAIELSAKRSRSLRQNLK
RLGMEEICNVVVEDGREYEPDAMSGPVRGILVDAPCSATGVGSRRPDVLRKSIDITELSA
IQRELLVHAADNILEVGGVLVYATCSLLKQEGEDQINWLLSEVGSNASNAQLETIPFAPG
EIPGFDDCIDENGWLRVIPGVLPGSLKFCDGFFVARLVKVSK
>comp6910_c0_seq3; 539 1 1713 LEN=1734; translated
FCSKAKGTTKKQKLFDRSTSRKHFVKKMKDYVACLPTHRLLIFIAVFCFVILDQCNGLSG
KTNLNATPEASRQITHRASAIFALMESMKRKNKFAIRVLEQDPSFLDLDQKDRAFARLLL
STAERRLGQIDIVIESFQRKEKKKNSKPRLSEILVEAALRIGAAQLLFMDVPKYAAIYET
VESLRLHPKIKVPKSQISFANAVLRNIDREGIPVLKTTSSINNLDLWLAKQWVKQYGKDT
TETIVEAAMSQSPVFITVNHAIQDDGEYDDDSSMANSAIYQDRVIERLEQIRDIFSASDD
KGDLLPAELLPVGSIRIPDNLGGVVSKWPLYDEGAWWVQDVSATLPAIALFNSLSSKHEE
QHVQDMHVVDLCSAPGGKTAQLCSMGFGSVDAIELSAKRSRSLRQNLKRLGMEEICNVVV
EDGREYEPDAMSGPVRGILVDAPCSATGVGSRRPDVLRKSIDITELSAIQRELLVHAADN
ILEVGGVLVYATCSLLKQEGEDQINWLLSEVGSNASNAQLETIPFAPGEIPGFDDCIDEN
GWLRVIPGVLPGSLKFCDGFFVARLVKVSK
>comp6910_c0_seq4; 384 922 1890 LEN=1911; translated
MSQSPVFITVNHAIQDDGEYDDDSSMANSAIYQDRVIERLEQIRDIFSASDDKGDLLPAE
LLPVGSIRIPDNLGGVVSKWPLYDEGAWWVQDVSATLPAIALFNSLSSKHEEQHVQDMHV
VDLCSAPGGKTAQLCSMGFGSVDAIELSAKRSRSLRQNLKRLGMEEICNVVVEDGREYEP
DAMSGPVRGILVDAPCSATGVGSRRPDVLRKSIDITELSAIQRELLVHAADNILEVGGVL
VYATCSLLKQEGEDQINWLLSEVGSNASNAQLETIPFAPGEIPGFDDCIDENGWLRVIPG
VLPGSLKFCDGFFVARLVKVSK
>comp6913_c0_seq1; 534 1 1088 LEN=1126; translated
XDVLDTNVGFSWGAAALGSNSSGAGAKEGDSSDDSDSDSDSDDDEGEFGNEKSNSRRSRK
RQAKKRLEEQETSRRETALADGTADSNPETAGDFERLLSGEPNNSELWIRYMAFHLSLAD
VPAARKVAETALERIEFREEKEKLNVWSALLTLEHKFGNDETFQGVIDRACQQNNPKQVY
MRACEILANDAEQSSNDPVSVKKADALFATMCKKHKSKKTVWLAHMRYLLKQSRDKDAHA
LMKRAMMSLGSHKHAETMSRFAQMEFELGSPERGRTIFDGLLLKYAKRLDLFFVYLDKEC
KFGNLEHARSMLERKVEERKLSDRQMKSLFKKWYRMEEEHGTEETQEHVKESARAYVTTS
TK
>comp6917_c0_seq1; 245 149 1179 LEN=1244; translated
MPNKKRGRXIPLAAPGLLRPMTSRKRARRVTTMFHKLTKERDQALKEGDADKARSCERSL
EEMGGRPEYQRASQLSTSFHSTSRWVLGHLANNGWLYGIKKQALDESGGSTRRERRKQSA
RRRTTRILEVGAINTELLDAAGIPAAAATARPSPRKAADASGATPRSTGAAAKNIHVRAI
DIHSMEERIEEADFLELPLVHPDREERYDVIVCSMVLNCVTTAVDRGKMLALLYHQLRPG
GLCFFTVPKFCLTKSAFLTHGLFKKMLGEKGVGFRVVSTRESPRVAFFVLERPPEEKDGG
GEESRPARQLDPVFTREIIRNKGKKFPNQFSVVLRSEHVFGTD
>comp6918_c0_seq2; 199 150 542 LEN=942; translated
MLRFISAIILLSTTSGFHFSNRKVRSLGTSLQSDSSSSSERYETYTDGTVNPCWQDIYDA
DCTMDSIFTARFVASEWIKELPCGSGLEDCDFPEGTVVPEMRPESHNHPEVDVMDFLNIK
RVDDDSKLSP
>comp6923_c2_seq1; 327 1 762 LEN=1126; translated
XSNSSNAHLLLVPHGKKGLDLALLRKERKRLRKKDEDSGAANANANADANAPTTKPPSNA
DGVRSGIPSMHEAHRILRECLESVDGNGNSNSSDDPVSGKAFPSAPMVEYLRELLRWKAL
DPAAWDGASTQTAAGGLAGRTSYALAIDGDPRDTERAWEVPRALAARGGTIEAHKGRSWL
PVGEQRAVEAVFARRTSVRXEAPVVAPGTRGGGVPNERPGPKKAGNKVDDYFNDESDSND
DGEDDDDMFGGLDD
>comp6927_c0_seq1; 503 165 1505 LEN=1586; translated
MFPRRIIIAAATASRGPASLARNVRLKERVAVASSPPPGLWRWLGSSSQTASLIQSRSNN
ETETTDEPQNDEPIQQIQPQHQMKLKDKHDTEAKRRDDSPRSLPQAKKWRAPFWLPPSSS
QGKRTPHHRRNPKHQQQQHRPKPRRFRNERLPCRQHLDPNGSAGLPESDWVQLFGAFPMT
SLDEILESVEGTLRKELFDPAANRNNRGGGVVDLDADWNPTEEDPPLVSEITYCTPDAPR
DDRPEYALDNDVRGGIPDDGDGNDPNPGNSSDDETDDTRVESFRVVKAHVVLSPFGRPTG
WNLKLASPSMVNALLSVANNARVRGSIRIGWRFATVKEHVPTPIKMDPRDTRTMLVVNDT
MVRFENCPSDLTEDFLRHLLSRYELTAKGPTIIKWKGRTDDGKAPPLTYVVRFASAAYAR
AAIREMQGKLLKGQAIKLIQYPKQLI

>comp6929_c0_seq1; 201 1 1285 LEN=1445; translated
XHPGRNKTHRVHQPQREMAEGEEAPVRNANEIHGKGVPETERVPPAERRSCLALVEAVAT
TGGGGGLPGGRRKVRLELPPRLPPGQRGEDDDRDWLEGVDRTLAGALGITGCGTIELYDP
GTQAFRRLDRIDEIPSSCCRLRVAETVPEGPISSTGDGGFLALPWREFSGDIDQDGRFVL
GDHSIVVQEESNSGLGTGTNLWDGAVALASFLGHHQGTARSNKDKDNDRDPSLSVEGKRV
LELGAGTGLVGIAAHLALGASRVLVTDLEYSMANLEANLSANRCHRTSAERRDSTIDARV
LDWCDPSSCETALSIWNESGGAHSHSPPPPDVVLAADVVWVDFLVPPLVETLRRVCTWGG
TGSDTDPPLECWPPVVLLSYQRRSQVVEDLLFQSLREASFRTEDVTIPELASSKIRIYRI
TYQHQGVL
>comp6930_c0_seq1; 468 132 1217 LEN=1652; translated
MDIQSHYESNGSGPEEVADRLADNGRRYSQFLAQSLNNKASIQISNGNYDEAISDLSYAL
DLSTSDLNENGETSMCPFQEFSLGSFLMVALEKEEEQHFCLDVDRPADIQQPERSSFCSE
NTDRNYEDLDDCVTMEDNDESTFVGFDAATSNMIESGGSHRNQRSSPLSRDKAQELISLK
SNEDNEHKEGFIYSCPLCVNSRCIAEGHNLGLVLPVMILFNIAIAHHLKAISLSKIATAG
STTAMESFEMMQQASKFYHLAYELQRKKTLRRQQHTKRNSDCWNEEHVSMRFMMLITNNL
GQIHRFTGNPHKQTMCLQHLLGAMLYMFHNCDRTVLTQKELDRFFDNLAPVLGTKRCAPA
A
>comp6931_c0_seq1; 1180 516 2945 LEN=3698; minus strand; translated
MAQKKSRKEEETPSLSPVDSGSEDENTDDEDSVYDSDVPYYEDGVDESDEEEFEDQGSSD
EDEDLLEKQEEDDESDDGMEKLEDNMSNAYETAPRKWNTGKRTISGQKSEPSESNNTEKS
AKWMHTDDFSSDDEDEDGTGNRIGRVPLHWYDEYDHIGYDAHGKKVIKTASIGGNRVDQA
LSNADSKGKDRFMVHDALNDKNVALTPRQLELIRRIQGGAYAHPEFDGNAEYIDYFSGVD
PELSGINSNRTLPKSKFQPSKWEKMQVDELLRKLDKGDINMDYLTGKIRDMNDANKSSKD
KDKPFLMWTGGEEDELNMRKGPQHIAAPKLPPPGHAESYIPPDEYIPTEDEIKEWEQMEV
KDRPHGLLVPKKFPNLRSVGAYEHSVRETFERCLDLYLCPRQMKRRLNIDPESLVPKLPK
ASDLRPFPTAKCIQYKTPHEGDEPPMIRCVTPSPDGKFMASGASDGYVRLWEVETGRLLK
SWNVSKVALSADDEWEESTGSESVKPVVALEWNPNRAHHCLLAAVGKCAIVIATGTEGQR
NAEITGALLSSAKRGGNVSSAKASKAVQWVSVSEKETPISMFASASGPVCALCTKSNMTK
IRWHGKGDYFVTISPKAGASSVLIHQLSKGNSQQPFSKAKGEVQAACFHPNKPFLFVANQ
QHVRIYHLVKQAMVKRLISGCRWISSLDIHSSGDHLIVGSLDRRMVWFDLDLSHTPYKTL
KYHERAVRSVGFHHRYPLMASASDDGAVHIFHSMVYSDLMRNPLIVPVKILRGHEIKNKH
GVLSLSFHPTQPWIFTAGADGKIFLYQDI
>comp6933_c0_seq1; 111 141 502 LEN=502; translated
MAEEASASADVPMEDAAADPAPVSVXPFVIAIKGMTDQNTGNPPKVASDCPISCAFLSYF
THPLFLFAVIFLIGNAMSNTHHAYIFQAKPAAKAAPGAAPRFEIKKWNAVAMWSWDICAD
T
>comp6933_c0_seq2; 235 141 539 LEN=861; translated
MAEEASASADVPMEDAAADPAPAKPAAKAAPGAAPRFEIKKWNAVAMWSWDICADTCAIC
RNSLNEPSIEYQANPSPTNDNGLSIAFGNCGHVFHLDCIQRWLKTRSVCPLCNKEWDFAK
IERIPGYGQLGI
>comp6933_c0_seq3; 75 1 159 LEN=841; translated
AKPAAKAAPGAAPRFEIKKWNAVAMWSWDICADTVSENIKHPKKPYDVILS
>comp6935_c0_seq1; 188 311 518 LEN=518; translated
MSSETNDDAIVRGTRNDIGIDTGVHPIHNSNNDNDDIDQVSTNSSLAPPDADQILSNDIG
IDTVAHPIHX
>comp6935_c0_seq2; 188 438 645 LEN=645; translated
MSSETNDDAIVRGTRNDIGIDTGVHPIHNSNNDNDDIDQVSTNSSLAPPDADQILSNDIG
IDTVAHPIHX
>comp6936_c0_seq1; 991 1 2173 LEN=2293; translated
XCSQNQKLNPIDLALSYNQEELMQMEPSKERIYIGGLNPPRLSARDIFRRLKSINQIEIE
SASIVDKGVNGDSSDEDEGTRPYLHITASSKHDSDSALSIISKQYHNVKWKGCKLIVEKA
KPHFLDRLEEERRQRRERTTGERANEIRDSIEVKEQPEESEGIPSRIPRRLRVRKKFGDT
ALHVDTKPWTVESWSRFHKARGKLQKQEEKHKINVIAYNSASKDEHISQPGPMMHRAVHI
RFSGGETGLNGGIGSNKKDNNESRMVISSSASSSDSSTSDSDSDTEASNESINKKEQSER
YTWSEEDSDSSHSEEEMEQNPDDGSQSTTEILLNQKRKASSNDQKDDGKYQWSTEEESSD
DEEDGTNRWSSTSPRNNEPLRAVHGTDEFAAGFEDTSSFDRYENDDNFEGYDHGKSGNLE
SDLTGDVTSNLNILSSIFPDMVDAKPINPNNGDVTKSTMGSTESSNVPQSKNTTAYGIMP
RFDPTAKSSKQFEVEEEPEEEEPNSGDEESASETRSSIEDADLSDNKENSNHTGDIREND
SGAEVSPAPDIYEQEKLENVFRDARDAWGTEDTGAAAPRDTSATDEDPDKSSGKSSFSFG
FNLDDNDASKKQKSKSATEAFSFSFSIPEQDQATEHDKPNTIQKEDDEHMETATQCDEER

DSKNVNDKVDMITTDENVATRLKGLTLPEGDLKTYVDNFFALNDGARIMKDPEGFRRDEK
EKTEW
>comp6940_c0_seq1; 235 118 606 LEN=846; translated
MRSVTIATTRRCTSMDRNVFRRRKRTQHDQHCHHRQQHSSIRPAARAPLAPSVPNRHNSS
VLYRRNPDVNVSCLGREAAVVQQHSGDTFGGGYILPLPHDEKVGTDTTATASTVAFAATN
NKDSDLLPSWSAAETVMKMMKASYYHASHTSGVAAAGGGSRE
>comp6941_c0_seq1; 577 279 1702 LEN=1702; translated
MNVSPSSQETSIKPFSESLGSGEVLHRMQGNLPFSNTKPRLDIDMKIGKPGGVSIDGVTT
KIDKEVTKSDLVLPSEGLKQLVTVATAEKEAHDSFYADSNPRGDSREDTGRGSTGDEKRE
TECQLSGTASAQMTEDISTEIGADNDDDKEMKNTDEKASSYVDAAEKIIDEPKTEEAKSE
QPKLEEGTKDELKEEQPKKKPRKRWKKPADKPPRPLSAYNLFFKRERSIMLGDAAEKDDQ
EQGKKRVHRKTHGKIGFAEMARIIGAKWKKLPDEDKEEFMVVAKAEKEKYAELLANWREE
KKKQSIANSVEAGKKRKDLDDDTDELIKTINEDREQLLKRHSAFRMQMKMYEEMALSRLG
REGRPMPSLDYMRNMPDDRPYFGERGQYPNAAEDSGRNILQHMLSLSGHEGPGPMSRMSD
GPMGSSMGNTMGSMGSNMRGSMGSSGNQINKPIGNSMGNQHQTGHPMGHPMGHPX
>comp6942_c0_seq1; 703 214 1974 LEN=2152; translated
MSTRVVTVTTPSFDVAGSPTTKDCNNGETTPSYRPNSSHSYLLCDDPSNHSPFVNPNATA
GGSTGGGSFDAKKDRPGGRPHRQRRNWPSRSTTPVWILQSIAFADKIFVSWWLMWVLDVW
HKHVPLFLKRKLCFWAWGWYFSVHTLVVCGWMGLYSDDRFYRLSPPSPHSDGKGKSGSDF
ISNEYKAYSTLCYPSQFFVVDVPRMRFALSQLNVISSRDIPCEECVERVADGTNSRGVDQ
TGTNHRSERPFVVPDAQRDHQTVRGLYVTNPNKRSNDNTLSHTRSILFWFFGGAYLAGDA
EGNLSLANEFMEDCNADAVFVPEYRLAPEAKIDDVLWDAAWAYRYLVKRLQKESSQQNQH
TDTEIVFVGISSGGAIALRLLQLLRDRSLTTKEGSSLPVTPSFLEPVLDDIVSRTIKSAE
PSSSKTKSIRVAGAVLFAPYVDYRDPQPPSGSFVQNSEFDWVVTQAVQHYGLPYLNEFVP
PLGDDLLRTNDDHSGYNTNGRIQYSPLTHDMNGLPPLCLIASEHEACYDMAIAIVNKARQ
GVERGDAAACPPTEVTIGVWKYMCHVFSMMQAFLPEGKASIDFAKGW
>comp6944_c0_seq1; 407 1 1082 LEN=1162; translated
XFFMSPVAVSGTSSAFLSCALNVYGSVVLKGIDRLQLELYILKFVPSCRGLDATGLTGSG
KGGRVTKGDVLAALRDGTPMPALVKVEEKGPELATAPAPAAAAVPKAPTVPAEDLPLPEV
DTYGSYEDVPNNNMRKVVAKRLTASKQEVPTCYTSMEVELDNVLKLRKTLLNDHDVKVSV
NDIVVRCSSLALRDVPEVNGKYDPKTDSVSLQDSVDISIAVAIPNGLITPIIPSTDKLGL
SEISDKVKDLAGRAREGKLSPEEYQGGTFCISNLGMFGIDEFTAIVNPPQAAILAVGGGV
RRVVPTPYVDGAEEQAKPAIKTIMTARLSSDRRVVDEATAALFMSAFRHYINKPELLLL
>comp6944_c0_seq2; 840 273 1892 LEN=1972; translated
MSPMSALRRLSTSSVLRGRRTVATAIVGSHRGASSSTAATIRLGLKGNNNRYSLPETNGL
NCTSSIGRRQFHQSVAVLDYPDHVVIPFPALSPTMEIGTIAKWEIAEGEEFSAGSVIASI
ETDKATMDFEAQDDGILAKILKDGSEATDLPVGTPIAIVVEEPEDVAAFADYVVSEEEST
GVAGADITMDEAPSSSLEELSTATAPVIGGSAGTSLLLPSARFLAESKGLDATGLTGSGK
GGRVTKGDVLAALRDGTPMPALVKVEEKGPELATAPAPAAAAVPKAPTVPAEDLPLPEVD
TYGSYEDVPNNNMRKVVAKRLTASKQEVPTCYTSMEVELDNVLKLRKTLLNDHDVKVSVN
DIVVRCSSLALRDVPEVNGKYDPKTDSVSLQDSVDISIAVAIPNGLITPIIPSTDKLGLS
EISDKVKDLAGRAREGKLSPEEYQGGTFCISNLGMFGIDEFTAIVNPPQAAILAVGGGVR
RVVPTPYVDGAEEQAKPAIKTIMTARLSSDRRVVDEATAALFMSAFRHYINKPELLLL
>comp6951_c0_seq1; 681 1 2696 LEN=2696; translated
SSCDDQRGYVHLQGAFEPKRKASPNTQQSEPSPYWKSSEALEEAILLALRDHYKRLQLPF
PVLSVRVSDGKNLSRLRLEYNCPYEALQIQYAFRDQRISPQTILAPIVNSRGDCVTSKSD
GCALFGSRPCQATMITTTPLPKDTSSFWPRSNPPQFRRLLHNRGDDEQERSQTRFVYVTG
LIDNNNDSEVTTGRTLSDWWGKPYAVYRALRQVFGTDIEIFLPKKQNRKQQLIQSCHLGL
RSASDAQRIVREYQGRVVAWNDGTKKTSDQQQSNEGEIHSGQLFLDYATITQKSKAKEKG
EYEKGKGDASRPECTSTTSHVHVPGLVVVENFLTEPEEKILLAILTGPQAPWAPQQTNKS
QTGAVKRLVQHYGYVFDYQTADVLRRDENTPFSANCPPMPAIETTAENNDPISMDDLIRQ
GRGWEVLAQIVEKTRRHEFETEPPSSDEEKSDGPNSQESMTRKMTFPHLNQMTVNQYKPG
EGIGSHIDTPSAFGDGLISISLNSGIVMEFTKVGAKTEGEARVKKLVYLPRRSLVLMAGP
ARYEWEHQIVTRRTDTHDGTTSPRGLRVSLTLRTALSLDGSPLDFFESSVFPPVWGGSDD
GKRGPPGSNGNALVTPSTERNHVHAVYDAIATQWHHTRGKRGVLWPSATQFVQNLPEGSI
VADVGCGDGKYFPAIWEAGSYVIGTDISLPLLESSFDESSGSTVPGTRTVSGYRQSLQKR
PAVAVADAMTVPLRSDSCDAAICIAVMHHLSTTDRRLRCLEELERIVGKGGRIHVQAWAM
EQASNSKRRFAAPDVFVPFNAQPKYLDRVSGGAGGPEPESHGSGGNDSVAPHGPSKSVAE
LYSEAYEKADFDERKGLVVFQRYCHMYREGELEDLVRRVPGLRLVESGYESGNHYVILX
>comp6952_c0_seq1; 590 372 1605 LEN=1605; translated

MPSLKQLFGSPLGKSKHKKKPNKDRYNGMDILINDHKKKYTTTPKFLATENSEVTVSLFK
EEEETERRGTLIESTACHSEEVRDEEITIDFRNYKDCVGDAELKKSGMKTKATTKEIQDH
LHEESEKDETIYFEQRDHYDNRDCGDDEDFRTAAMPSKSKESPTTKNFWADKELHLPNCK
DINCENADIWCDNDDNFRIIVVPATASLSKPEEEQEKREKAKKHESLAESHHREQQQNE
FSMDEDGIEYDKVDDFRTTIMPTPRISSFAREEKEKSSSAKESESNKEIYVPQQQQQLQH
ELLVHQNDDFQTTVPDLQGMGESEPRSKESGNLKRVARDDNRYQVSPVNSVEFENHIHTT
LNANEGATNSRSSRKEYSNKNDKTSSAIYPIPSHLNTTGYHLDLDDGDDEKX
>comp6958_c0_seq1; 274 33 686 LEN=929; translated
MSTSTLVKGYELHGWQPSKSLCKDENFMDLCMIITRSSKLKQGSMACILVKEATTQREEP
MPATAATETELAGDMCGAIISVANNRPLFSETTSDVHAEIAALGEASRNGVSTENATAYI
TMPPCKNCFGALTVAGIRKIVTRYGPNQKIQEAASKNNIEWITISNHAEMMARVNAIVCP
GSDGKKRKRDHHSDNESVDETGAKNRKSDCTSSLTKC
>comp6960_c0_seq1; 126 1 990 LEN=1066; translated
RVLFRSGNRGGGPQWTFRWNTGGGSRNHRYYKPRLKDRFDVKQAQSRILHIVSLEQLETI
ITATIDGEAVLERDIMICFYTPPLETHLMDEMVYPWPFAAKSSQGIWWEDLLQTSVVRYH
NSNDLTEFFGIPRGRSLEKPVFIFGKKGQKFWDKAAWDRRLSTDERQVFDQWTWKQLEVE
VVFVNHHDHPVELFWIHDRGGHAKGIIDANGGRAVHVTTLASEWWVRDARTDTLEGAPGR
WKMTDGTCLYNVKILSDEHRREYVIPKRLCYDLSGHCPFWNRSGKECDKNPNFMHQWCRK
ECRRCTPDVDPKANEKRHDGTDYPGSDEL
>comp6960_c0_seq3; 359 66 1409 LEN=1485; translated
MSVLSAILSLFFIFFACSSNNSINHHFTWFFPPTVANAAASEITDLYEILGVSRTASVQE
IKKAYRKKALDTHPDKNKGVPAEEAAAAFHKVVHAFEILSDKSSRNRYDRTGQTSGNGNT
SNGGGNRGGGPQWTFRWNTGGGSRNHRYYKPRLKDRFDVKQAQSRILHIVSLEQLETIIT
ATIDGEAVLERDIMICFYTPPLETHLMDEMVYPWPFAAKSSQGIWWEDLLQTSVVRYHNS
NDLTEFFGIPRGRSLEKPVFIFGKKGQKFWDKAAWDRRLSTDERQVFDQWTWKQLEVEVV
FVNHHDHPVELFWIHDRGGHAKGIIDANGGRAVHVTTLASEWWVRDARTDTLEGAPGRWK
MTDGTCLYNVKILSDEHRREYVIPKRLCYDLSGHCPFWNRSGKECDKNPNFMHQWCRKEC
RRCTPDVDPKANEKRHDGTDYPGSDEL
>comp6967_c0_seq1; 421 1 1403 LEN=1468; translated
XKISDDAHFSGSDRTIPEQNAMGKQSKSKTVEPANDDKDGNSVVEMEISDEEYQELLSQA
KELTLEEAQEEWMESCRYGEVDVVRALAENFPSSQETLIGFVHPDTGNTGLHMASANGHT
NVVKLLVRQYGHSFTKNASGNTPLHWAAANGKAEVVKFWTSQTIVEVDVLEKNAHGRSAL
TEGFSSQNEDVVAAILEHDSASEEKLLSTGSQKGTGGNEDGEEELLDSHVHSFFDKERPL
KIRELAMKNADNPFADTDRPDQDTTGLSIWSASLVMARWMQAVGMTGYWKDSSVLELGSG
CGVPGLMVATSDASRPRRMFLTDLNPQTVDNLRYNIELNEVQDFVEAKCMDWEDKSTWPE
EKVDHVIGSDLIYIKSLVPLLTSVIFGTIKPGGKFLYVCPDTGRDGLDAFVEAMKQKCPG
WVEQVAPKEYHSNPLTNEDDEECFLHFQELSSLTYMLYEFPIPNNN
>comp6968_c0_seq1; 213 86 928 LEN=1213; translated
MIGIWIPDLRESTDGLPLATKISDLFLGFPYDRVPENWILQLVLHPGSVFAMLAFYLVSK
PLFQQIAKLVDPKATWFIVSIAEHNFLLAVFSLVVVINSWPIVIDHYQRYGAFDTYCDPE
GRLWDKNGSGFGPWALIFYISKYWEFVDTWILVLKGKKPSFLQVYHHTGIAFCMWVGVLS
QSSWLKAVVLLNSIIHTLMYTYFLIKTISPATQIRSARYLTKAQIGQFFTGIFYSAAVLF
MGDKCDTQSSRFGLACLQVYGYGLIALFMAFSKRKYQKKD
>comp6969_c0_seq1; 211 385 1158 LEN=1335; translated
MVRTNNLLVSSLALSTCCVSPTYGFTPESAASRRSHVRLDLDLGFDATEASSSVTIRNKP
LASIDVVDDDQINAVESSELPPVLQQITDERRNFQMNLGKAMDTLRKDMPYILKDTPDYS
IYHSDITVVDPSGVQLTGIENYKSSIKFLQQFVAFWFQPERNGLQYRLVYDFARSSIRVS
WHAVLVPKMPLGKPLHVDGISMYRLETDTGKIIEHKLENLSINNTPIVPPYGVFSLVQQE
WGLAGTGVPGGQIPMGV
>comp6971_c0_seq1; 324 196 765 LEN=955; translated
MVATKKSSSKSTVSKAKTGAAKKSKKGSKAAMPSTDRIVLAIASRHGLGEEKPSRDMVMG
LALITNKKSFNTTLLNIRKKEGWIEYDKDTVWLTEEGRNYVGPNAVAVPKTNDAMQARLR
EDMVKGLKPRQIFDIMLDGRWYTREELADAMGIPNNKSFGTYVSSLRKVVVRDGKKIRLS
DICFPCGRP
>comp6973_c0_seq1; 342 38 571 LEN=571; translated
MKHCISTGMSEEECQTLVDTCKEVHNQIEAQCGSIKEQCKVDELSTEECKEKVEDCKRGL
EMEECKVLFPSKENNQIEQCKSTGMSEEACQALLAECREVHHQIKEQCKPIKEQCKENEL
NPEECKGKVEECKEGLEKEECEVFHKPNNQIEQCVSTGMSEEACQALLAECKEIGEMI
>comp6973_c0_seq2; 312 38 501 LEN=501; translated
MKHCISTGMSEEECQTLVDTCKEVHNQIEAQCGSIKEQCKVDELSTEECKEKVEDCKRGL

EMEECKVLFPSKENNQIEQCKSTGMSEEACQALLAECREVHHQIKEQCKPIKEQCKENEL
NPEECKGKVEECKEGLEKEECEVFHKPNNQIEQCX
>comp6974_c0_seq1; 794 1 2046 LEN=2162; translated
FSRDGVCLVLLHQNPLSRRQDKREYRIQSQTTPHRTAQHRTAPPENPYDTKHTTKQPPPK
HSPGPQTNMTLLEFQTAQELKTFVESNPVCVVAFSAHWCGPCKASRPGLEQLARDSQKVP
VSLVHEDDNDIGDYLHTFRVRAFPTYVLFASGTEAERVEGANLPGLKAMIERHEAKAGPS
MPSSGGNTLGGSHSEADARALRLARLGGGGGPAEAAATVAEAQPETKEEQAVPMETEDSK
PAAEETEAVAPQNSNSNSASNSNSNSNSDEPKQSPVDSLDPEALRNLTEEMGFSLVRAQKGL
LYSSGNTVESAIEWLMEHQDDADIDDPIPEDPSGGGGSGAGSGAPKAVSYKCNATGKIFA
TLADLELHANRTGHTDFEESTETVKPRTPEEKAAKILELKALLKKKRAEREEAEKVDHTE
REKQRRFMGKEMAQTREQMEAEQRKRDARARKKEKIEFKKERERIRAELEKDKRERMANK
GKLKGRLGVDGYAPSAIQYENGSGEEPAAQRPKTSGAGAARIDECISKVASYRAGGDGGK
CLKVLGLLVGNAADHPEEEKFKSINMETKTYKNKVKPFVGAKSLLLAVGFGLPEKNNDGT
HLVLREDADLELLKATKAKLQEALAKYGSSSRTDDAAWVVMAGAKPTENPTFAREDCPTG
FSSAVGGDPTNSRKRETMLQL
>comp6974_c0_seq2; 550 1 1445 LEN=2169; translated
XQLKQEQLRNQQNRASTPIGADSSEATPVSSVADPAGNKNPAANPDAAANANETATNTKP
ASALASAFSPVRTNGNGSNAANTTTTATSNSNSNSEGTVQAFAGLEEMARHSRIRQKAME
SEVFHDRGGDEEEEDDDLNGNVYVNVNVNDNDNNHTAAGDATKNQQRQNHDRHVPTSTNR
RAPPMLAPTKKRLRWHALVDSGMGRLGFKTDPVTTEDHGKRRDTVEILKELADLEVTPDC
PVEFYGMCTHMADASNSTSTFTDSQMNRFKSLLKRVRGAGISVPTISTDNSAALLTTNLT
HFSANARVLLAQNDADSRGFVRTGGAIYGQRPSFEQLKAVSTLVASVRHVATLRAGESVG
YDRAYVAPTSVRIATLTIGFADGYPRELGNGVGSVSIRGHLFSVAGNVCMDMLMVELGPA
GETNGPGAAVAVGDVALLWGPDEGEEGEGHVRLRDVAEALGTTQSALTCGLNKERVLRKY
A
>comp6975_c0_seq1; 440 254 1549 LEN=1587; translated
MMEMMRLKKGGNGNVRSLSSRRERVSLLGDVQIRSSPTSRSNSGLRGREQRRRTRVRAHP
QDDDAEERISLLNDANSPGKLKRYNRYESIINDGVPWTRSRLMKYVAILVVSSSMSFFLL
RRESKVIRWEEYHSILEPEGSKKARCFETSRYSLDERCSCPDPNVALKNDDAKLWDSNHD
HMVSQAKNAPGDLDIVFFGDGIIEQLSGTRGLGAEIVEGMEEYFERTFVKKNGGTFNAIA
LGSSGDTGPNLLWHWENGIRQANLRPKLWFIMVGSNDLFVHKCTDEFVMANALNVAKRIF
EDQPEAKIVIHGIIPRKDNPESTSNALGHLWNRAQGLNLQVRKWIKKHSSRIFFMNVGQV
LMQNGGMKGRKFLDPNLIDEKMNPTAKGMEKWGDLVVKKLIPILKGFDMAKHRKQTKPPN
KEEKSQTKGTR
>comp6975_c0_seq2; 359 254 1694 LEN=1732; translated
MMEMMRLKKGGNGNVRSLSSRRERVSLLGDVQIRSSPTSRSNSGLRGREQRRRTRVRAHP
QDDDAEERISLLNDANSPGKLKRYNRYESIINDGVPWTRSRLMKYVAILVVSSSMSFFLL
RRESKVIRWEEYHSILEPEGSKKARCFETSRYSLDERCSCPDPNVALKNDDAKLWDSNHD
HMVSQAKNAPGDLDIVFFGDGIIEQLSGTRGLGAEIVEGMEEYFERTFVKKNGGTFNAIA
LGSSGDTVCYVCFVNNNISXKIDLFENIVWSCNAQMCLFPGSRNXLNASTFLHQKKGPNL
LWHWENGIRQANLRPKLWFIMVGSNDLFVHKCTDEFVMANALNVAKRIFEDQPEAKIVIH
GIIPRKDNPESTSNALGHLWNRAQGLNLQVRKWIKKHSSRIFFMNVGQVLMQNGGMKGRK
FLDPNLIDEKMNPTAKGMEKWGDLVVKKLIPILKGFDMAKHRKQTKPPNKEEKSQTKGTR
>comp6976_c0_seq1; 123 111 404 LEN=737; translated
MDNVGNLPPYQQQEFMKYLEQMQLKDSLTMYNRLVERCAEGCVTSFRSKTLDKHEISCLE
NCAGRYIKMTQRVGLRFAEHQAMQQKRAAEAAAQGSP
>comp6977_c0_seq1; 571 276 2664 LEN=2715; translated
MAELFNGPVSFSDPQGDPDRSRPFVHEAMDPEDGVEDFSRKDLEEKSLQLMAELLRPLFP
LGKGVGSEEETEGCALDDKVYAEANQMSNELAKGRFVDFCCESEGETRVESFFLQAADTK
VGREIIKSGMSEDLILFRKVVEGAIVSLQSLLVFGMTYGLTLHPTGVSRAVAHMKDDAEA
LDVTWPTNGQQWKPAHSLRLKYLSFLDHDADEKVPGLQLLSTLKWKRTPLRAYELLTQIG
VWGKHENLALLRSGVPLRFTESEEEAAEKVAEESKKDLGDDADTLLEIRRDFRGQKVFTI
DSASTKEIDDGLGVEVIEGEDGQKRYRYWIHIADADRWATRDSDLFEMARRRATSIYLPG
RSIPMMPASVSEQVMSLRAQSDVCALSLGVELNDDGSIVEDNIVVTPSTVRVTYRLTYDD
ADEMLEDGVGYSEEWQLGRLYTAAQQRRQFRVDNGSAEDFIPTQIPQYTISTFPDQGAPD
DVGIKIDLQVSHNGGKNQSSIVAESGDGNSDKEELPVSSSFLLVTEMMILAGEAIGKWAV
RETQTPCGNHRKNTLELPFRSQEKPDYRTRERERKIMMDLLDCNIGGGYCHAWYARRFFS
PAKVTPEALPHSGLGLECYAQWTSPIRRFQDLQVHAAVKRFLRRQRVLELLETGEPVPNG
LSPSDLGCGLPTQNEDGTLELPLEDADGDIDYGDGVKLLKPVQFVMRNTNKFWMLEHIRR
LKEADPQLSTEVLVLGCVNPSKRQYSVYLYEFGLEWRYNSPAGIQAGDRFMVRFGNVLPQ

NGQMTLVREPNSSKP
>comp6981_c0_seq1; 409 1 2546 LEN=2621; translated
XLLGRYQFRQSRGSGQNPTGGLVSAVGSIDNTFPRPQREYIRCLSRLQESSSLRGFLDVI
GRPRELLTAYNHLIECYAGEGGMLQAHCRKLYSYIHNNVQSSTSGTNHLASKGDAGDKSK
SEPPSGPGCPMAASASNKTSSPGQQSGCPVALSSLSNEKPPSERTGCPMATSSSEGKEPP
SQQSGCPMTMSSNDSLNAIDNHKFAITMFKHMREAANDRWRLRLPPLMNEVTKIIYSTSE
SGGFTTVALDLSGTGLLYEYGDVVKVILPNDDRQTRSWLHSLKSMNQEYFKLEDMRNLQK
STGNGWGWDGLWEALGWGRFEEHGGSGVPLEMIARYIEQGNIRDESSKKFNWVHSPLDLS
VGGASQMFASPPPIPTERIGSLEPVSPRIYSVSGVEPDRVFLLVSKPDDLARHHGYAAMS
DPKITKVHCSFSPATFFLVPPKDVNLVCVASGTGISPFVGLVDSIRSRTGSYTIVHQCKS
SDMFMCNSQTWLDFTAMNPGAIVMGYISGDRSRRNCPMRYVIRNGAFEETTVLRRRRISA
YYFECESFQSRLLETYKSNGLNLAYCCGGVKSAIMPLRTFTESCGMQFEFTCESYGVSPT
LTKESNLLSQIGGTIIDLNHVSPIHPGGDQILHQATDIMTAMAKMKNSENTNTPDHSIAF
YELHPHAYNLHRCLRTPVDADAKAFAQFLERQAMSKSVLGNMAMKYAQAVLSSPESSKIV
KIATELQSSAISAQLSSGDTESAQKSASYLKELLNHVPESDADSERWGESLSLIPPVSRK
SSSVFRSSTFSNKTTMSGGEALKRVSFLQEVSLKSAAYEDSDDESWGESLSLMLPSRRKS
SSVLRRQE
>comp6982_c0_seq1; 447 66 1130 LEN=1597; translated
MNMVAAKVQNLAKNFTKRCRVIVCGVGGRMGAIRTGLVYANPRFELCGVCDVNMEAADRI
ASKYSTRAFGDFEGLLDEFGSKDSTATLDGLVISTPTPTHGDLIKNAAENGLGMFVEKPI
AEIPQEIEELYQVCEQHNVPLCCGFQRRFDESYARAAKSVQQGLIGKVSNAHIFFGDSPG
PSFEFLLEGGNIFYDLSCHDVDFIRWALNDEIESVYACGSSSREELAKCNIQDNATMMMT
TTRGCVVTLTMSRRAAYGYDQRCEMFGDFGKVSIGNEYETAAIVSDQNGDHLSRLKNSFD
SRFHDAFGNEMNAFADTLMEKTPWMVKKEDVISVQKVAAAAKKSLETNQIIYVD
>comp6982_c0_seq2; 347 380 1272 LEN=1739; translated
MHFTFCYRXRAFGDFEGLLDEFGSKDSTATLDGLVISTPTPTHGDLIKNAAENGLGMFVE
KPIAEIPQEIEELYQVCEQHNVPLCCGFQRRFDESYARAAKSVQQGLIGKVSNAHIFFGD
SPGPSFEFLLEGGNIFYDLSCHDVDFIRWALNDEIESVYACGSSSREELAKCNIQDNATM
MMTTTRGCVVTLTMSRRAAYGYDQRCEMFGDFGKVSIGNEYETAAIVSDQNGDHLSRLKN
SFDSRFHDAFGNEMNAFADTLMEKTPWMVKKEDVISVQKVAAAAKKSLETNQIIYVD
>comp6985_c0_seq1; 292 62 670 LEN=817; translated
MNLSGMKLCGCIALLPSSTLAFFLKNLARSEPRNSALLPELERLIAASDSGIDGSYEESI
KDTMVEISKSREGDQRESLPGRWELIFTTEKEVNFFKTSWPFAEVSLITQRLDITDEKFV
NNVIGFKGGAEFAVTGSAGPVDGDDEYDRVAFEFTSAKAVVWGVEISLPPLGTGWFDTMY
CDDTYRLSRDSRGDWSVFKRIG
>comp6987_c0_seq2; 509 1 1611 LEN=1677; minus strand; translated
XAGELTFTVGAQSDSSSANNSVTAKWCSFLRKSHDDMVSQLCERVKLGRHASIRCLWGVI
AGSPITSVSKVTNNTNRKGYKNSSSSSSYKYVNADILKNWMRAMALQESSVMDKGMRHMI
EAEFFSPYRDIQYFSLGAISKIASSVYDGDDDDDEKTKVGGKLLELLMMIPVPSSDDDLD
GSKQFLFPPPSNVTPVDETEVESEESEEETDEESDMDSEDESSSDTERLKEETLESRPKK
RQKKESTHRFSFQQTRMFRREYKKAWLAVLRLPLPVASLKKALHVLPQNVLNYVAQPLRF
ADFFMQAYSDHSSGIIGVLALDGLKSLIIDCVKDGLEYENFYTQLYKLVSSRLMYAKYRA
RFYSLMTKCLVLNTMLPAHLIAAFIKRLCRCALSAPPPSALFALALVSNLLKKHPECYCL
VQRKEGMEIEDCFVADVDDPVECRALQSSLWELAVFERHYYPAVATLAKSIGRAEEDQAP
LYNIDEFASHTYASLFDQEKKKKTKTALTFKKPTSLFTNDDIFFGCVETGDTEKEN
>comp6987_c0_seq4; 410 1 1408 LEN=1474; minus strand; translated
XAGELTFTVGAQSDSSSANNSVTAKWCSFLRKSHDDMVSQLCERVKLGRHASIRCLWGVI
GDIQYFSLGAISKIASSVYDGDDDDDEKTKVGGKLLELLMMIPVPSSDDDLDGSKQFLFP
PPSNVTPVDETEVESEESEEETDEESDMDSEDESSSDTERLKEETLESRPKKRQKKESTH
RFSFQQTRMFRREYKKAWLAVLRLPLPVASLKKALHVLPQNVLNYVAQPLRFADFFMQAY
SDHSSGIIGVLALDGLKSLIIDCVKDGLEYENFYTQLYKLVSSRLMYAKYRARFYSLMTK
CLVLNTMLPAHLIAAFIKRLCRCALSAPPPSALFALALVSNLLKKHPECYCLVQRKEGME
IEDCFVADVDDPVECRALQSSLWELAVFERHYYPAVATLAKSIGRAEEDQAPLYNIDEFA
SHTYASLFDQEKKKKTKTALTFKKPTSLFTNDDIFFGCVETGDTEKEN
>comp6987_c0_seq5; 385 1 781 LEN=1041; minus strand; translated
XAGELTFTVGAQSDSSSANNSVTAKWCSFLRKSHDDMVSQLCERVKLGRHASIRCLWGVI
AGSPITSVSKVTNNTNRKGYKNSSSSSSYKYVNADILKNWMRAMALQESSVMDKGMRHMI
EAEFFSPYRDIQYFSLGAISKIASSVYDGDDDDDEKTKVGGKLLELLMMIPVPSSDDDLD
GSKQFLFPPPSNVTPVDETEVESEESEEETDEESDMDSEDESSSDTERLKEETLESRPKK
RQKKESXTQVFFPTNSDVST

>comp6988_c0_seq1; 478 152 1822 LEN=1862; translated
MASCANDHNPPRKITNAALSKTLAEFQPRPSQVASCALNLYRGKLPKKGKPKPGEEWTVY
AAIVAFHRVDNRMWVVSCATGTKCTAQRHNECVLHDSHAEVLTRRGLIRVILSEIMEKKK
GIENNEKKKNTDSRSLLVVSKGYHSDSEERQYRFNPNIDLHFYISDSPCGDASIYSVPCD
DATRNNDGILYTGAKVIVSEATKVDATDCGGNHQLLSTKGTNASNKVMSEVARNEKDLND
ISEKAGPVFAREAIQVLGKLRIKSGRSNLPAHMRSHSHSCSDKLASWSVLGLQGGLLTKF
LDPPIVPLASVVVSRDSRLSGNDGDFDNGRAERHQSQRMALQRAIPGRVRSVWEHLSIHQ
EHELPTWKPSIPKVHIVPETFESGKAQMIAPRNSSNFSGKKRKQSNDNGENMISNKTKEI
EHGTIGKKAASPCGMSFNWNQNEDIEVTVGVRGVKHGKKPKTPQDIEKHASRLSRARFLR
EYSFLWESKDLASSASSIPMYCKRYHQFKEENASHEWTDLKNRILTQNGSPLAGWIRYVP
ANDDDLIDSKGRKADR
>comp6989_c0_seq1; 575 161 2142 LEN=2283; translated
MSFQLHHRFNEDGSSTYTVMCGLDYIEDHLGNITENPIRRLRWSEEANLYEVIEEGDEVN
DPGRTLYKQSSSLDEDGLLKTGLSGVDSILYQNRTKDNIVLARECWCAAIFQGTLNEPGQ
PVDSTSVVRNPPVWCPVDTNGDFNDGKPSFGDPNVCRAQAIVGQDGVVHTAPGSYLAACY
VEDRLAEFVRSAWPLCCFWVAMLIYLWFATNYGRRCRGYVWRTTMLFMHGRCCVMCGGSP
LRQIEAPPPPDVEEGRSPVAARGEGGEIAVGTTLDQSTPTETNNSSNENEDQPNNTSMEL
VVPTTRNTEDSVQNGDDLPPPYIDPWFGSYENYEMLDHDMRDQAERTNNEFISWLWYSAV
QMEERRVKRERLRRRREERRRRRGEDPDEGSMLSTEQENLRAMLAPVYWWPPAYPDGGR
WEHLVNLTSLDARVRRERRRRRMLRTGDNNEDDDSNDDSQPQQECLFLRTKVFCPSKPE
TIADHEHFEAGKRDRDDNQDVSVEDTNKIRTIGGADSTPSEKKDDSPPQTTSTIAVENSN
SDDSETNDPDTTIANSYSNLEEEDISENTCSICLCEIEEGELVGGHPLWTLFPQGLPQGM
ADEEQPLPVVPASRHCVVPKNTAMSERTNSIQSGGTLSYVMVRIEYAGGDDTSSKHCKDV
>comp6990_c0_seq1; 674 332 2707 LEN=2751; translated
MVDIYLKAPGMKRKIRETRGRLYQRIVARKRPRYLTSWEESFRRMPCSKGTFHSKNDSCD
STKQHWFGLVDLFGGNRWACPPGEASSFEKNTIHKTNFAIGKSKRAGQPSNNGGHPSPKT
NMPSKFFTLRMEHRQGGAAAKHVGEERFLLFRSDQAGAIAKVVFSSSPVTIGDSEDASTK
MPKAQTCKNPDEEIFTMANAKIHVLYVKDTYRGFNLGGFLFVLCTTYLRERYCNSFNPQL
SNRPILRGISSVRCLLDAEEDVRRHDKLVQFYEHLGLRRRTRAKTVFINNNDGETYRKIP
MKIDLRVKNREIERSKWKINHGDHHFAAEYSSFLPAVLKSSSGESARLNDKRIDSWLIME
SGGGNIQLRTTDGRKLEMDQWGNCRLVPVHEGSSGCSTNTGFDSFKLLRVSDMLDKVLYD
KEEEYEEERFCLRDKLLREHHHQQKYHWLAKKKELWMIRSSVFGTFMRLTQSNDLFFSEE
ASFWQADDNFCLTHTYDSPARRQHHRRMWMKQSVDYVTHMRHRYSEFNLQRMTIEEALNI
TKNLQANPFSISMRREQKDFIERLNTPSPPSFRTYLFHTAELARREGHPDWVQFVALIHG
LASALTCVRSSSGSIFQSEDSVTHDDQDDFDWTIYVDTRIMGCKASINSTFAEFRHLNPD
KGDTRYSTRTGLYQEHIGLERVLLSWTGSDYMYWMLKHNGVKLPREAYAILKLFPLVDWH
SRGEYTALSNDDDEELKPFVADFYDLRYRAHILVLSNPAVEEMGDEECRALWANHYSLIA
NKYGAGSMLRW
>comp6991_c0_seq1; 206 445 1104 LEN=1334; translated
MDSFVFTVANAATGLAVDASMDNTIVNGNLYYFSWAGFVTSILLIIEYLRGVFGVDVYGT
VRNRAARLTLWAGLLACQLVVLGASANIFDTDCTDFNGYAPESAYCKRTKYGLSLGVIGT
LFSLVIVAMKMLTTVAPVVVEGVLAFVLCIMNGCGVAFLTSAEGPGSPIGNLYYFSWFSW
LVSFVLLASVYEDYRGMRTPENEEVKVDGEIPVEAIDDV
>comp6991_c0_seq2; 280 47 994 LEN=1224; translated
MAVDPSTGELINNGQSPRFKLWLAFFAFSTIVMGSAVGVKKRENQVSSNSVWAVFCSAFS
FTVTGVVTMMHLSPMLSDYIVGTKWEGIITVVLAAFWSATVAVVANAATGLAVDASMDNT
IVNGNLYYFSWAGFVTSILLIIEYLRGVFGVDVYGTVRNRAARLTLWAGLLACQLVVLGA
SANIFDTDCTDFNGYAPESAYCKRTKYGLSLGVIGTLFSLVIVAMKMLTTVAPVVVEGVL
AFVLCIMNGCGVAFLTSAEGPGSPIGNLYYFSWFSWLVSFVLLASVYEDYRGMRTPENEE
VKVDGEIPVEAIDDV
>comp6991_c0_seq3; 206 466 1125 LEN=1355; translated
MDSFVFTVANAATGLAVDASMDNTIVNGNLYYFSWAGFVTSILLIIEYLRGVFGVDVYGT
VRNRAARLTLWAGLLACQLVVLGASANIFDTDCTDFNGYAPESAYCKRTKYGLSLGVIGT
LFSLVIVAMKMLTTVAPVVVEGVLAFVLCIMNGCGVAFLTSAEGPGSPIGNLYYFSWFSW
LVSFVLLASVYEDYRGMRTPENEEVKVDGEIPVEAIDDV
>comp6991_c0_seq4; 208 79 747 LEN=977; translated
MVHNFQCFVYSTHNTAYESYFIFPLYVHDHDWQNWQSWAGFVTSILLIIEYLRGVFGVDV
YGTVRNRAARLTLWAGLLACQLVVLGASANIFDTDCTDFNGYAPESAYCKRTKYGLSLGV
IGTLFSLVIVAMKMLTTVAPVVVEGVLAFVLCIMNGCGVAFLTSAEGPGSPIGNLYYFSW
FSWLVSFVLLASVYEDYRGMRTPENEEVKVDGEIPVEAIDDV

>comp6991_c0_seq5; 239 272 1015 LEN=1245; translated
MMHLSPMLSDYIVGTKWEGIITVVLAAFWSATVAVVANAATGLAVDASMDNTIVNGNLYY
FSWAGFVTSILLIIEYLRGVFGVDVYGTVRNRAARLTLWAGLLACQLVVLGASANIFDTD
CTDFNGYAPESAYCKRTKYGLSLGVIGTLFSLVIVAMKMLTTVAPVVVEGVLAFVLCIMN
GCGVAFLTSAEGPGSPIGNLYYFSWFSWLVSFVLLASVYEDYRGMRTPENEEVKVDGEIP
VEAIDDV
>comp6992_c0_seq1; 300 199 1134 LEN=1327; translated
MTVDSAPEPEPAVEVCLTPPASPRTATPHSHLLEAYETLKTEKESLTALVLLKTADEANV
QLELDETKRLLEEKTKKMEEMDDRLKLKSVSIECQLSEAEGSAMDQMCATIKERDLWKDK
ASELESKLEQLQMKEQSLSQEVETLTSRDRLQTATIETSRQEIARLTKDLLSFEDRFSSL
ADKAVEAIVSGSGTSTGDNRQEPPGQESQGNEQDERVRTLEALVKELRSSAEDSNSALLT
KLSEQKKQWDTERAALQHKIAGLVEEKESIEKEVAEIAELLEKDQKQQRYSGATEIEVNV
LNPTKPSSVVE
>comp6993_c0_seq1; 287 121 852 LEN=1006; translated
MSSLTPEHRREEVTASAWNDGMVNGAMMFVPTLGGLWLAMKNPRFRKLTNAQSRTAIAIM
PPLFTFAVTSEQKLTHRMHEVASESEHNLETVAWAERAKNKGTTSEEVQLRQLYRQSILD
TGVRLVDTPTLSAYQSTANFVQSNPFKCIAAIGMPSVAYIFYGQGSKNGVAESFQMRLLH
TRVFGQFAVICTLLGVMSLKEVMDRYGRYVTEDEIEARVREMEQTRTRLLTKQEYQNALR
RPS
>comp6993_c0_seq2; 257 275 802 LEN=956; translated
MSETHNDKTTRAAQPKTHRQRQTVAWAERAKNKGTTSEEVQLRQLYRQSILDTGVRLVDT
PTLSAYQSTANFVQSNPFKCIAAIGMPSVAYIFYGQGSKNGVAESFQMRLLHTRVFGQFA
VICTLLGVMSLKEVMDRYGRYVTEDEIEARVREMEQTRTRLLTKQEYQNALRRPS
>comp6996_c0_seq1; 169 1 1373 LEN=1477; translated
XHQRTKRLDTTLHDETATTRYFRAQTMVDLVPGGLSRTCGHRQRWVPAFLWWVLLLSSSG
RLPQRHHTQEGPPRGKGRPPAVSVSTPGTGPCSVLGFSATPVAPARRNRASGGDRRRGGR
SGGSLSRVHGSFRPPPTVLPGSPAGEDPGVEPERATDTRIEEVSIEFCPGCRWGPRAFWT
ATELLSVHGSEGGSTDTDAGNACAPGRIGGVSVVPADPGVYRITARVGGSEGWVLLWDRA
TEGGFPPMDGRALAERVDRCLAGDPSFPPLAPPEDGETSAPVAVVSYCPGGLPGSGLRRA
SYYGQELLATFATGELAGVSLRPHAGDPGNLRVGLEGRDDGDPNRWLWDARLHGGAFPQP
KDLKRLVRDVVDPRKDLGHSDVSRGAGGAAASGPAMEADGGECRSCETSDAGSNSNGENE
NDDGDDEHNDDDGNSNDGFGFMDDDDAEEARRYFGVM
>comp6998_c0_seq1; 628 577 2382 LEN=2602; translated
MPIPFLQDPNNHDNSYQIGFSLSKDVKAKLNNFQPTIVHVTVPDVTCLHLIQYAREKQLP
LMGTYHSNIPEYFTHYQGIGWLKHIFAGYERHQYNFLQRLFVPTPFIRRHLMTEDHYRLQ
NVTELKVWGRGVDLERFNPSHRSQDYRSRYGFAPNDVVVTWVGRLVPEKRPDIFAYVVRR
LAREGIPFKALIVGAGPSENEMKSLPNTVFAGWASGDELAVAYASSDVFLFPSAVETFGN
VTLEAAASGLPVVVDGGCSGHLVQHGVNGFACKDGDAEAYYKGTLSLVLDKSRREAMSKD
GRKLSLKYEKRVVCRKMIDNYSTITEEFYGLYQGDHANRDRKYEQKPDSFLSGCYVRPTS
LILVEFVFILIFRIMYRIAQCLNKIQRLFVSIGTRKVSTAKGEAVVASLSKELQSQPFSP
SSVITAPTDDSSSNATENEEDLTSYSDMEEPSDDQDTSSIASSSCCSVSSTTEKNRLPIS
HVIAILFVKAVRAQGLIECRIRKWYCRTMLPSSSSFSEDIQCKQRKRKCSEELTPDDLYD
SDMIALQLKESLLPNSCCNSVASTRSSENSLEEDKDAIVKPTTADKALDPRKATSSSNLL
V
>comp6999_c0_seq1; 247 492 1082 LEN=1202; translated
MRFLIIRHKSLPLIGPPPGCRSTIATSTKHKNLELEDLVEIIRSDWKPDRQHKGYYITGR
LNTTIYRDDCWFDGPDPDMPVRGVRKYLNAASQLFDTSKSTAELLAIEIGNDAERNHVPK
TKSTIVAYWKLQGVLHLPWHPSLPTWTGRTVYHLDEENMIYRHEEHWDISVLQAFTQTLW
PEVGNVLWETTTPDAK
>comp7001_c0_seq1; 431 1 1564 LEN=1860; translated
XLNNQGLKAQAFSPLAVGSRVASMKMIGGSFLSLLPQGLFNFAVDGGVCGKMSEMSEDKV
RKRRRDNVGGSRTPLLVVPPTGVFACGNRVLGHENSDENDRPRKRMRGESKIDDFVASSP
STPSPVVEETCDCCCHKPVQCHEDLKKSQNSDLIKKLPGDAVAHCLSFLGSTEDRHALQN
TCKLFQAISNSDPMLSKVDVFGNLETGKGGIIQENDNPATASAALAPFARAGNLQALYML
GIVKCYCYQDLKNGILMLKIASTRGFVRSSYTLGIILRDALADEAAKFMEIAAAKKYFPA
LQEILPAREMKERFGEPNADELRRHLDPVGLNRLLLRDYVNSAELRGMNTSHCWNPLCGK
WAYKASSNSGCANNSKVNLRMRRSAAAQVQPTYDVDENMLDTLSPSNNNEEGTLSPHRGG
GIQRLLGTNNSNSLGFGRLHRRPHYHAFDSVAVYLSKDRRVGLSPSPKVTQALAWNENGV
EVDRVARMKMCSRCCRAKYCSKLCQVYDWRSSQHKMECQFI
>comp7001_c0_seq2; 367 1 1287 LEN=1583; translated

VCSSDLDRPRKRMRGESKIDDFVASSPSTPSPVVEETCDCCCHKPVQCHEDLKKSQNSDL
IKKLPGDAVAHCLSFLGSTEDRHALQNTCKLFQAISNSDPMLSKVDVFGNLETGKGGIIQ
ENDNPATASAALAPFARAGNLQALYMLGIVKCYCYQDLKNGILMLKIASTRGFVRSSYTL
GIILRDALADEAAKFMEIAAAKKYFPALQEILPAREMKERFGEPNADELRRHLDPVGLNR
LLLRDYVNSAELRGMNTSHCWNPLCGKWAYKASSNSGCANNSKVNLRMRRSAAAQVQPTY
DVDENMLDTLSPSNNNEEGTLSPHRGGGIQRLLGTNNSNSLGFGRLHRRPHYHAFDSVAV
YLSKDRRVGLSPSPKVTQALAWNENGVEVDRVARMKMCSRCCRAKYCSKLCQVYDWRSSQ
HKMECQFI
>comp7002_c0_seq1; 643 564 2804 LEN=5040; translated
MTTFSHIPSLVLDSNTTWYSPDLFTNAFNKSTAGVTLRMLTTVIVCFIIYTYTCVLIWEE
WIQNLALRRVYYLEYNHYERRKSELEYLQKLEDPEDINTTDRPIFVVHPEFRDTVPNIGL
YSVLFRLPGVNLEDDESRASNSDSGVNRQLKVAIEFFDKIVPNQPGFSSSIAALTILPDT
KRLSQAWKKWFECAGKVRRLKFIRQRLKELENDKNIRSVDDADHQQNISYTEVSPSHGIQ
RLYNLDSDTHDALPERNTGPRTRVSRRAYVGSELETARRTASIEIREETNMEMYLNDDET
EQMAVYYREFARSSASCGPRGCDEHSIRQADYTTLKDFEQEAAEEVHLAIQELHTARKQI
KGEETSLPEIPEIHSTPSDNLVIKDEDDHELDFPDDVGELSSHADVERGDFSDTGNMFPN
VTLSLWEEAEKLVGTEQYMVKLGNQVLKRKLNTGRWYNPFAKANASEDSRSRSRRVMEKF
IPSGVLSKIAKSESYAVVTFTSRQAAIAARQCLSDGSGLEGWREVDKIPIPPLADAVPWN
IFDCRGCCRPVTVTIPPQQRRFRFKIVVLFLIVFSTIWTYPFYLFTSATTPEALVKAFKE
VPNIEFYAQYLSPWLNSGIMILFFAILPQIFKMLANSDGASTSIQEAERHALVYYWWFML
VFVFAGKSLGNIVWSAFRNNQANLESATSILQDLAKIVPTTQSFYWITWMITQTGIILPF
MYFLQFNNFMFTALRWHCCARATAGG
>comp7002_c0_seq2; 838 564 3698 LEN=4953; translated
MTTFSHIPSLVLDSNTTWYSPDLFTNAFNKSTAGVTLRMLTTVIVCFIIYTYTCVLIWEE
WIQNLALRRVYYLEYNHYERRKSELEYLQKLEDPEDINTTDRPIFVVHPEFRDTVPNIGL
YSVLFRLPGVNLEDDESRASNSDSGVNRQLKVAIEFFDKIVPNQPGFSSSIAALTILPDT
KRLSQAWKKWFECAGKVRRLKFIRQRLKELENDKNIRSVDDADHQQNISYTEVSPSHGIQ
RLYNLDSDTHDALPERNTGPRTRVSRRAYVGSELETARRTASIEIREETNMEMYLNDDET
EQMAVYYREFARSSASCGPRGCDEHSIRQADYTTLKDFEQEAAEEVHLAIQELHTARKQI
KGEETSLPEIPEIHSTPSDNLVIKDEDDHELDFPDDVGELSSHADVERGDFSDTGNMFPN
VTLSLWEEAEKLVGTEQYMVKLGNQVLKRKLNTGRWYNPFAKANASEDSRSRSRRVMEKF
IPSGVLSKIAKSESYAVVTFTSRQAAIAARQCLSDGSGLEGWREVDKIPIPPLADAVPWN
IFDCRGCCRPVTVTIPPQQRRFRFKIVVLFLIVFSTIWTYPFYLFTSATTPEALVKAFKE
VPNIEFYAQYLSPWLNSGIMILFFAILPQIFKMLANSDGASTSIQEAERHALVYYWWFML
VFVFAGKSLGNIVWSAFRNNQANLESATSILQDLAKIVPTTQSFYWITWMITQTGIILPF
MYFLQFNNFMFTALRWHCCARATAGGGPGGVVPYRVYVNSGVIFLCVVALAPLCPMVAPF
STLFFLFITPLLKWGFIFVYRPTFDAGGMRWPLLHRILMISVIVSQILLALSLFLKKAYL
LVLICVISIIPTWTFKTVCEDTFLQSFDDAALLQTSELDGWNVNEQMSFDERERYRKWIV
DCHKAAYVPVCVNAEDNYLTSEPAVVVDMDGEGIRSEDNLFQPGETHDSREQSSTSSVMT
NGRQRTSTIDSYNSLRSQKNYGNRQPGAIYRRMSTRFIHLNSTGENNTIISEECESKGSP
NFSPRQFEEADLSTFLNEDTDKSL
>comp7003_c0_seq1; 656 41 1426 LEN=1560; translated
MPKAKKLRAPALVSQQVRHEPLGQVIEGDSQRGSKYAPSTSRRKRRKDQVGEGEFLDEKT
SRKILDMSREQQIEEEIEEQRSWHQKNRRQQEQQAIGKKMLDDSDDEEEEEIEEIILEEG
EEDYIENDQGYVNLKDGALGLSAEDEAIVAAMNSGGGIEERKTLADIIMEKIAEKEGEKH
GDMDEDDEEGLPPLPEKVVEVYTDIGKILSRYTSGKLPKAFKVIPSLSNWEQVLYLTRPD
LWTPHAMYAATRIFASNLSPKLAQRFYYLVLLDAVRADIYQNKKLNYHYYISLKKAVYKP
AAFFKGILLPLCKEECTLREAAIVASVLQRVSIPVHHSAVAIHKLAQMKEYNGAASIFIR
TLLNKKYALPVPVIGSLVRHFASFLHDERDLPVLWHQALLTFCQRYKNEIQDQARESLRL
VMKAHFHHKITPEIRRELFGASAWKDERAAASTQGGTSMVM
>comp7004_c0_seq1; 300 33 509 LEN=744; translated
MSDEDYAIESTDAGASLTIPMEAGQIKKGGYMMIKGKPCKVLSISVSKTGKHGHAKCNFT
ATDIFTGKKLEDMIPSTHGTTVPIVNRADWEIIDIDGDELTLMDEGGNQKTDLNLPTYPD
SMAEDIRTAWADGENTVQVTVQGAVGIEQVIAWKKVDN
>comp7006_c0_seq1; 167 94 456 LEN=1059; translated
MFFLFFSTRSSKAYGGYQHSGYHQHQQQPYGYQAQGGYQQQPQYGQGGYGQNSGYGGGPP
IQPYQQQGQYQQGPPQGAYGAPPSAPPVSEWKTATAPDGQTYYYNERSGETSWQKPPGMP
>comp7006_c0_seq2; 697 140 1843 LEN=2446; translated
MSTDDTAENATEAVKRERPEDDNGTQEPPSKLAKNAEADDPSETKTEANGASEIKTEADA
VAVPTAQAEEAEESKEVATGQNEIIQTEGSKETTESAPKETAGAGAGETATPTGLPSGLP

TSEGQPVQAAEIPNIEATLEEKGEVSAVYVGRVIGKGGEMIRDLQARSSCRIDVDQNVPP
GQPRLITYRGTRKTIDFAKQLVRMLCQENVNEADLPLGDAKREFLVVPATSVGKIIGRGG
EMIRELQSRSQAKIQVDHKGMSGLDSSEKQVTMTGTEIAVVKAKEMVLFLVANPMMDAMQ
SLTMLVDDKIQRNGVWGSGPPYPNLPQGGQNMQPTGGGGGYGYDQPYGGGGGGHYGGGPQ
SYQQPPSYGSGSGGMESEIFFAAKHYMGRIIGTKGVTINDLQKRSGCDIQINQDVPPGRD
CEITLRGMRQGIEMAKNMLREVIDTGPNHPYAGGSGGGNSYGGYQHSGYHQHQQQPYGYQ
AQGGYQQQPQYGQGGYGQNSGYGGGPPIQPYQQQGQYQQGPPQGAYGAPPSAPPVSEWKT
ATAPDGQTYYYNERSGETSWQKPPGMP
>comp7007_c0_seq1; 281 249 965 LEN=1026; translated
MTLKKYILTLIKVFGLCFSSTAAEDESSHVLGTCAATTKPSGCSTEPSSPTYTNLPSGAQ
KFQVRNVYDGDTFTLIDERRVRLLGVDTPEMKPPQPFAKEAKEYTKLRCNKKEVWLLIDG
KDHYDRMLAHVFVEDTSGNFLCINEGLVHEGFANAYIPKKDKKTFNWKKLLDLQRGAREN
KRGIWKSFVDATVYKTTNGSAYHKRTCSHLSNIRKLIEIKISEATDQGLHPCRSCMVG
>comp7008_c0_seq1; 511 1 1704 LEN=1837; translated
VSDFASIKCDKDTGIATASIVILVFEVSISERKHQQEKEVANMERQVILSVIVLVCINVM
DSIMGAVVGPTLIFYVFELGGNKDDYGMMKSVVSLSAMIAIPLFGNWVDSNGNKYLVPYL
TSFFLGIISFVMYFLVILLPKGPIAIYSLMFSRFLDGAAIAGRTLSYSWVASSIPPNKQR
TIFTLLSSSRTLGMVLGPLTNYLVSEINTEITIFGTTIKIDPNNSIGLLMVGGEIILIVI
TLLFFQEPPVKDDKLKKSTTETSVTESKGVFYALTHFDIFFPVFTMFVVGCNFSLLGTAF
SPVARNMGWTPVNISEVTAYGSIIMAFGMVASMVVTTRDVSDAQMLLFGFGTMFFSGFFM
YEWWIDGVGYWRFTLPMYLVYFSYPFIGPANRSRYTQAVHSNKELEGSHGIMMSLINQAG
AAAGLVAPTLVASFVLRTQEDIDASPDKHQLTPGGLYVPIFCAVIITGVIYQHFTEIRTK
NEPVGDTGADSESTKLLSPRENRTPRASVVEISETFSRASEVNRRMSVDCMGISNPVETK
YEQELATKLLEDKKIWEELQELDEIED
>comp7008_c0_seq2; 292 1445 2230 LEN=2363; translated
MGWTPVNISEVTAYGSIIMAFGMVASMVVTTRDVSDAQMLLFGFGTMFFSGFFMYEWWID
GVGYWRFTLPMYLVYFSYPFIGPANRSRYTQAVHSNKELEGSHGIMMSLINQAGAAAGLV
APTLVASFVLRTQEDIDASPDKHQLTPGGLYVPIFCAVIITGVIYQHFTEIRTKNEPVGD
TGADSESTKLLSPRENRTPRASVVEISETFSRASEVNRRMSVDCMGISNPVETKYEQELA
TKLLEDKKIWEELQELDEIED
>comp7008_c0_seq3; 616 151 2100 LEN=2233; translated
MLPVPIEWRVMCNSDTHCFPELLCVYVVQESFGPSFVIGMSFDSYVSTRHAGEMLGDNSF
RLLFVSGSAYDGLQEKSPLYFLTLDNSFRILIKDTGIATASIVILVFEVSISERKHQQEK
EVANMERQVILSVIVLVCINVMDSIMGAVVGPTLIFYVFELGGNKDDYGMMKSVVSLSAM
IAIPLFGNWVDSNGNKYLVPYLTSFFLGIISFVMYFLVILLPKGPIAIYSLMFSRFLDGA
AIAGRTLSYSWVASSIPPNKQRTIFTLLSSSRTLGMVLGPLTNYLVSEINTEITIFGTTI
KIDPNNSIGLLMVGGEIILIVITLLFFQEPPVKDDKLKKSTTETSVTESKGVFYALTHFD
IFFPVFTMFVVGCNFSLLGTAFSPVARNMGWTPVNISEVTAYGSIIMAFGMVASMVVTTR
DVSDAQMLLFGFGTMFFSGFFMYEWWIDGVGYWRFTLPMYLVYFSYPFIGPANRSRYTQA
VHSNKELEGSHGIMMSLINQAGAAAGLVAPTLVASFVLRTQEDIDASPDKHQLTPGGLYV
PIFCAVIITGVIYQHFTEIRTKNEPVGDTGADSESTKLLSPRENRTPRASVVEISETFSR
ASEVNRRMSVDCMGISNPVETKYEQELATKLLEDKKIWEELQELDEIED
>comp7008_c0_seq4; 467 1 1834 LEN=1967; translated
VSDFASIKCDKDTGIATASIVILVFEVSISERKHQQEKEVANMERQVILSVIVLVCINVM
DSIMGAVVGPTLIFYVFELGGNKDDYGMMKSVVSLSAMIAIPLFGNWVDSNGNKYLVPYL
TSFFLGIISFVMYFLVILLPKGPIAIYSLMFSRFLDGAAIAGRTLSYSWVASSIPPNKQR
TIFTLLSSSRTLGMVLGPLTNYLVSEINTEITIFGTTIKIDPNNSIGLLMVGGEIILIVI
TLLFFQEPPVKDDKLKKSTTETSVTESKGVFYALTHFDIFFPVFTMSSWDAILVCKLFLC
FCCLFQDFEAPDKLVFETFLKELLFLFIYFHILQWTCRLGTAFSPVARNMGWTPVNISEV
TAYGSIIMAFGMVASMVVTTRDVSDAQMLLFGFGTMFFSGFFMYEWWIDGVGYWRFTLPM
YLVYFSYPFIGPANRSRYTQAVHSNKELEGSHGIMMSLINQAGAAAGLVAPTLVASFVLR
TQEDIDASPDKHQLTPGGLYVPIFCAVIITGVIYQHFTEIRTKNEPVGDTGADSESTKLL
SPRENRTPRASVVEISETFSRASEVNRRMSVDCMGISNPVETKYEQELATKLLEDKKIWE
ELQELDEIED
>comp7009_c0_seq1; 171 364 727 LEN=727; translated
MERAHTLSDCRAPIDFINRFAHNSVSKKVKTKSNPIQFLKIQTMFSSKILFAFLLLAASV
QADLRGASSSVLDNTRELRRAMKMGMKGNKPNRECRTLMKACIESGSTKEECRALYPDCK
RX
>comp7011_c0_seq1; 243 1 714 LEN=1550; translated
XICFSSIETLCFGEHVERNERNNTNGLQVSISTASTKLNDLKRILADEECFGPEEAPVRR

QRIFHLGREFKSGGRSLSNLGLGRFNNRIIHLSIRPCEEECERNGNAFGQQQSPVAAVGA
RKRRRTDGNAGGEEGVDASSLLPLPVSSPLQSSFLPPFPFMNQNQALGMNNDNDPDNRLN
NAIHASNALCEDRGMPATYTYTNTNTNSNNNLTANNNSAIDLMDSSDDEEVEVIEIS
>comp7011_c0_seq2; 326 91 783 LEN=1619; translated
MNQEEPVTFTVVRTSDAATKEVSISTASTKLNDLKRILADEECFGPEEAPVRRQRIFHLG
REFKSGGRSLSNLGLGRFNNRIIHLSIRPCEEECERNGNAFGQQQSPVAAVGARKRRRTD
GNAGGEEGVDASSLLPLPVSSPLQSSFLPPFPFMNQNQALGMNNDNDPDNRLNNAIHASN
ALCEDRGMPATYTYTNTNTNSNNNLTANNNSAIDLMDSSDDEEVEVIEIS
>comp7012_c0_seq1; 366 89 1069 LEN=1110; translated
MPKRKSSSVKDALSALTDVLSEREKALEEGEEKLRREREALEALEAEKASFYGETKPSDV
LHLNVGGTKTSVLRRTLTSVPGSMLAARFSGRWDDSLEKDKDGHFFIEQNFETFEIMLKY
LRNKANGTEKYSIASPSLRREQKQDFYRMLHYFGMTDGIYPTKLMMKTVPKIEDAVEIIG
TKKVIAKEFTTFQMAPDGHSRRVKGFEIRLGTTQRIQVGWSYKNDDVMEKSVGHYEGSFA
LDLTGSSFLVGGGAKRLSYIEHPEGTVVRSENFGRTWYVNGELLTVNASNDQVEKIWRRY
WSNLESLRPLISIEGEVEVTSVELVD
>comp7013_c0_seq1; 148 1 1178 LEN=1178; translated
XENKDSTMVERNEKFYGIPLVEDIWDFPALQLSTLCILVFVIIFQPDGLCKTKSRSLTFW
VTIGAVAIATPETTLNLILSTIIHTRSTYAYIIVIPHSFIAAATYRSKHGNNVHYLQSFL
LAFFLYGFGGSIVSDLLMGLPVTALSHARIVPCYIIGWSLVWLFPFDLLFKKYSNTSSSL
HYFLNACEAIDSVTTPMGRISRSARELGNKTSAPIVAGLFAGFGGAGVRHAVGESASIAA
LEAGFWKTLSYSLLWWWLAVHKCQDEGGNIPNEQERDIMRENNNCDSYSGSDLLRLIMVG
TAYTLDDTSWNGPGEWAPTCMVLQGGHDQKNRIRIRPIFSDSPGIRSETFAEKGEGQQKE
EYRKNQKTGLGHHHKEKLDLSHKLATTKRKSFRX
>comp7014_c0_seq1; 467 172 1293 LEN=1450; translated
MNRAGSFTSSSNSWILPFMLGASTSLSLLWISKKLEILIATSSTSRSDEKTKPREMKQDI
LDHEELSFRMLRKAEAVIQWRTSRLILVIERCTNDHNYSAILRTAEALGIQTVYMIDPPA
VMEEDGGRRKGTQKEISRTPEEIEQRRLHHIFAQNATEWLTVRDFDSTEDCIACCRKEGY
KLWVTDLSQEAEALDIYNPDSNEIPEKIALVFGTEAVGASTYMLEQADKRIYLPLRGYAD
SLNLSVATALITQALFFMDPSLIGAMNEGERKRLRTAWFTKLAQQRILSSKQKKNRKKLA
SLVKKCETIDKRLKEEPNYHIQPSEQKRLDEWGSYNRELQALDALIDPVKVQKGESSLLE
IRLVCDLPINEIF
>comp7014_c0_seq2; 506 172 1479 LEN=1595; translated
MNRAGSFTSSSNSWILPFMLGASTSLSLLWISKKLEILIATSSTSRSDEKTKPREMKQDI
LDHEELSFRMLRKAEAVIQWRTSRLILVIERCTNDHNYSAILRTAEALGIQTVYMIDPPA
VMEEDGGRRKGTQKEISRTPEEIEQRRLHHIFAQNATEWLTVRDFDSTEDCIACCRKEGY
KLWVTDLSQEAEALDIYNPDSNEIPEKIALVFGTEAVGASTYMLEQADKRIYLPLRGYAD
SLNLSVATALITQALFFMDPSLIGAMNEGERKRLRTAWFTKLAQQRILSSKQKKNRKKLA
SLVKKCETIDKRLKEEPNYHIQPSEQKRLDEWGSYNRELQALDALIDPVKVQKAVQEWID
NPPDALTDVRRADTHRVCFVGKNTRDFHKEHWKDMVATSNKGTVQGITATAFREKMGIES
MGDTSKLHKSGQDSA
>comp7014_c0_seq3; 390 1 1041 LEN=1157; translated
KTFFQDHNYSAILRTAEALGIQTVYMIDPPAVMEEDGGRRKGTQKEISRTPEEIEQRRLH
HIFAQNATEWLTVRDFDSTEDCIACCRKEGYKLWVTDLSQEAEALDIYNPDSNEIPEKIA
LVFGTEAVGASTYMLEQADKRIYLPLRGYADSLNLSVATALITQALFFMDPSLIGAMNEG
ERKRLRTAWFTKLAQQRILSSKQKKNRKKLASLVKKCETIDKRLKEEPNYHIQPSEQKRL
DEWGSYNRELQALDALIDPVKVQKAVQEWIDNPPDALTDVRRADTHRVCFVGKNTRDFHK
EHWKDMVATSNKGTVQGITATAFREKMGIESMGDTSKLHKSGQDSA
>comp7014_c0_seq4; 351 1 855 LEN=1012; translated
KTFFQDHNYSAILRTAEALGIQTVYMIDPPAVMEEDGGRRKGTQKEISRTPEEIEQRRLH
HIFAQNATEWLTVRDFDSTEDCIACCRKEGYKLWVTDLSQEAEALDIYNPDSNEIPEKIA
LVFGTEAVGASTYMLEQADKRIYLPLRGYADSLNLSVATALITQALFFMDPSLIGAMNEG
ERKRLRTAWFTKLAQQRILSSKQKKNRKKLASLVKKCETIDKRLKEEPNYHIQPSEQKRL
DEWGSYNRELQALDALIDPVKVQKGESSLLEIRLVCDLPINEIF
>comp7016_c0_seq1; 189 235 1038 LEN=1755; translated
MEESILQTNNDGVSLLRAGNNKGAMVRLTRSLAIMKKCPLEGERQVRYRFLQSSQHTQTN
TDPETSRRIPRYIFGSPIEVIACAQQEGTSRSTSFSETSFLTDTEGRTCKLLSIVLFNLS
LAHHLWAQEVSEKIYHNVRDELSRSHEVCSALTKTLRLYELCHSTLIFSSSGLLCSDLPI
AVVVKSNVGEVHKELDYQLGQNEQGLYLLNPHQYDRNKSAIACSEQLVQIFMFFTANGEA
ERLEDFSDIFSNAMATLNGRVVFAPAA
>comp7019_c0_seq1; 140 291 560 LEN=769; translated

MESQQQPKSVTASSDQYVPETIREGFEKIIGQGAAGFVLGGMAGIVLSRGGSSSARRVLA
GFGAGVGMGSAWTRTSMDIEEFTSTFKKS
>comp7019_c0_seq2; 140 211 480 LEN=689; translated
MESQQQPKSVTASSDQYVPETIREGFEKIIGQGAAGFVLGGMAGIVLSRGGSSSARRVLA
GFGAGVGMGSAWTRTSMDIEEFTSTFKKS
>comp7019_c0_seq3; 140 363 632 LEN=841; translated
MESQQQPKSVTASSDQYVPETIREGFEKIIGQGAAGFVLGGMAGIVLSRGGSSSARRVLA
GFGAGVGMGSAWTRTSMDIEEFTSTFKKS
>comp7021_c0_seq1; 296 97 1077 LEN=1335; translated
MSPLTDVSNSADPAKKKSGKTTKKSREVLSTEEKILNRFTQTVDNAPPSMNRSPELIPLY
MKWEDTKKKLRLLVSLATDYAETARTMHESRSKLVNQMAVLSENSPIFDSVGETTLSGKS
LKAIQSIREEPPVGKRATISQIVKSSIESESRSSLSSLQEIVTTQGIIQERDFRAQVIDY
ATEWEKVVSEKVESELKKVRALQGTRSHYEKKLEKLRQWADDLQKKGKSIPPAQAEKLER
NEAKLLEAFEAHEQEAGRLCVLIEEATTNGWRELYHLVKYYCKWESFRVEQESNIYSRLL
PTTLESMKAAFKENPSSLSTNKNAKP
>comp7021_c0_seq2; 296 97 1077 LEN=1335; translated
MSPLTDVSNSADPAKKKSGKTTKKSREVLSTEEKILNRFTQTVDNAPPSMNRSPELIPLY
MKWEDTKKKLRLLVSLATDYAETARTMHESRSKLVNQMAVLSENSPIFDSVGETTLSGKS
LKAIQSIREEPPVGKRATISQIVKSSIESESRSSLSSLQEIVTTQGIIQERDFRAQVIDY
ATEWEKVVSEKVESELKKVRALQGTRSHYEKKLEKLRQWADDLQKKGKSIPPAQAEKLER
NEAKLLEAFEAHEQEAGRLCVLIEEATTNGWRELYHLVKYYCKWESFRVEQESNIYSRLL
PTTLESMKAAFKENPSSLSTNKNAKP
>comp7024_c0_seq1; 181 1 605 LEN=658; translated
XGIDHSFIHHKSLLLILYLEKMLMRKNNKNGVRSTAVFLVMFLFSLTSVVTSRAHSSFST
SFETASSPFNTNGKTYFLHDKDTARRPLEGNLARELALLSTTYGIDESWKLVPRGGWLIP
AGYNPFGYQITELGQKFLEFDGSLDSDIGRFLASVKSRKRFEAIKSQWLEILRVSKKGQS
MRIYKSLEELISFCLKAGFLD
>comp7025_c0_seq1; 901 149 3595 LEN=3684; translated
MTSDSQENINISTANSSYDYDDERSAEILSRIQNETNRIRRKRRNRDFVEGAPGTPGGNS
SYDDISLGGSLASSSQQGSPQKLNWNDIVDSSKRSNQRSSEKGSNFLSQETTKDHRTHLT
VELKRTSPHSKSYNNRHESEPSVSSSDLIYSGIELKGSSGESTSSKSGNSVVQSNQRKSS
FFPLRFSKNMSTTAAEIHKENEAFRKKLIDEVLGKESGEKVPMEKVMMIIWQFRDDKEVI
KFMTRFVSKFAGHRDVFEGIEFYLPQLAHMIIHLETEWDDAILERFSLVIAQQSIHFALQ
LNWILAGAIEDYQPELPDGTPNPTHNPLYYVRCVKLLTNIERCVVYRKPRSRELQRLYEK
GNITKAEMHSLEQADRRFTAMQITANTPEVEDEEIFGGELMYKRAVRTSCFKFKRWKKRY
FAIEERMLNCYNVEGGTLVRSMPLEGATVQVMKKSKYDNMFRVTNRSFEFRIRASSLEER
NKWTKMLGTEASTRRVFGHTDASLALDDNEDAQRVIEELTPSQRDRYHFYRQERDFVRRL
TDVAEDLRFLDPADRKKEAPGLMKKLEIPSYSYSPLMSSTDIFRRIHSALWKDTRVFNTK
ARCPTVMYFLGVRGELEYKGKRHANMDIAEYIHLKFDSSLDDRDTKVTTIFENGQEMLEV
KMDTFPSLSDEKETLVVKKVVPTIEEGDENEKSSNDLTELDRPTDSKSPDFFSTPQKEDR
SSMVWSGSFTENAVEATPNKRQSGKGSPVRANRQLRKFLRESMVSMPRKLAHHLERKQGR
QSHNGGSSRSTISDVPIAMGRRSSETHGIGEAVYVGKSSVREQNKILKGKLRAGDIDQES
MARAKEIVCGGETWAEKSFRMLEGIKDKPEDKKVHLEIVCLLSKSNDDLRQEVFVMQMIH
YYKSVFIAADLPLWLKTYKILSTSASTGLLEFNQDSTSLDGLKKSEHYPAEGGLLKYYEN
TYGGPDSKSFKAAQRNFMQSLAGYSVVCYLLGLKDRHNGNIMIDMYGHLIHIDYGFCMGM
CVGHEFTMERAPFKLTEEYVEVMGGSDSECYKEFVHLFVEGMKEARSKCQIALGLVEIMM
FKSNYPCFSGRRYGGGQALRGFRERLMLDVPDKMVERRAKALIESARNHWGTSAYDKFQK
WSNGYAI
>comp7026_c0_seq1; 159 393 617 LEN=2413; translated
MIMGGSRSRSNRGIGLMKCFPIPKKGRDSEHWDVFKQKYPTDSFAKTSLYGKGYVYASRR
NKCMGQSLIKNVFG
>comp7026_c0_seq2; 159 523 747 LEN=2543; translated
MIMGGSRSRSNRGIGLMKCFPIPKKGRDSEHWDVFKQKYPTDSFAKTSLYGKGYVYASRR
NKCMGQSLIKNVFG
>comp7027_c0_seq1; 263 386 736 LEN=1014; translated
MSGSGDYNPPSADGGNMGGQSRMTVAMQQQDAMIDELAVGVGRLRDQTQVIGDEARMHVD
LLNDMEQNLDAAHGGLDSETRRAARLKEDQSVWRLQLMVAGLCVLFVLLFFMGMSP
>comp7028_c0_seq2; 56 1 375 LEN=375; translated
XPEAGPEADPEAGPEACPEAGAXSRSSSFSSTETFFEIARHGVRLIDGVAEYERRRVGRN
LGGGANVAQQDGLAIRDERHVVVANLRQRINADQEPIQDTMDDMALAAEIGLNGGRLIGE

VGNLFP
>comp7030_c0_seq1; 223 527 721 LEN=1076; translated
MPYENLNATINDTITFFWDDDENHTVYINPTGNCDDTGSLLIGDSSPASYTFTETDALNT
TNYN
>comp7030_c0_seq2; 618 100 2097 LEN=2452; translated
MAATRTRASAMMLKSMMDSAKMKHLARSHLFLSPQAGLKRNSFWRETMESDVLVYDFQDG
CPPGERGNVMKGLHQAHEIYPNTPLSVRVTELERDKKTNEGTSTIYDELSTSLKQKQVYW
LMLPMCNDTRDIQEYIDMINHIDEDWLKSHGGLQIICETPLGLHNLDDMLANYPQVKGVI
AGSGDYFRFAQCRDDTLLPKFRWEIINACLRHGVFPIDAPPLVLGIEDGAAIDHFQGGAN
AGYRAGLILHPKQVRLSNEIFSPCPSYLEECERLIDPWLQERETGYVRASGDHFRGPPHL
KQMHWSLKYDHQIRGKKTSHIQAVDPDLFADVASLLSKAHRVTDKASENGVTSENETSVE
RFLNTSMFLALSSSCHDRHEDVLTNLGYFNVEISATEDGTPRSYKEGAFVASQIKGRRLT
SAGKLIVTTDVHVLDKDFKTICKMEKRLMEKKLNFDDHAEEGAYTHVDPRATPRYTRFTT
EIVRSITDGAVCINEAKDKTVSVDVHDDYCSILNLDAPLHHTHKAVSDSKIKNKEDNLAK
PVVPSTLHISHHDIQGIMNEGYDCCRNVTFHSPMRPDEDFTNRKYYVEEIGSLFDRNSST
TTKHESAAMGPGFLSVIWNANGELLSSILLSRNSINDTGSLLIGDSSPVSYTFTETDALN
TTNYN
>comp7031_c0_seq1; 427 885 2526 LEN=2526; minus strand; translated
MSYHVRLPRLNEPLVTYSTRAPNYPIGYSCAHTLVHSSNGIKGIDSSPHDPMNLSHSEDG
ESMVLSETFVPIPLEFFIPASLSGDYDKIYSNVEALQWSILNEVAKRSGLAKGCKVNVQF
DDRPLLTALTSLNELHSSATPETTLRHNSDQHTTLSVSSNARSSSQNQGKNQGNGQRNRR
RARTIGTNRRMLSRLPYPMSVYSIESTRPQWTGKQNRHSKLFTVLDYVFHPVPLTDHFFP
HHKIESCSSEMIHSDESESQCFTAEITLEVRFRGTTGESNLIEEYIQSIVHNQIALDASQ
LFVEDIFGLKWSSVQEASSLTQPPSNSSQESNGPISFGPENVEDSSDSIGTTAKDKSNSL
FASIIIPIATACIVFVVWYCTRRYYNRDRKRARGARDHQEKSQAVVRGMKQSTDIEIGIV
RTETTQSSHDQLSKSSSNSNGNPEDVISATASSDTISSSSSSTSLKSISATSDFSDRSIT
GLPPRPNVVKREGSKQLKKPRKKKKRRKKKVMSLQRINSRDNISEMPMISESESECDSES
GGEDEDYX
>comp7031_c0_seq2; 524 885 2739 LEN=2739; minus strand; translated
MSYHVRLPNRRALRKEDQHQHRRMKENEALNPDLQWTFIGTKRVSNSESMDEEDDSGLHE
NGDYVSVVQKDEAAFMYLPRLNEPLVTYSTRAPNYPIGYSCAHTLVHSSNGIKGIDSSPH
DPMNLSHSEDGESMVLSETFVPIPLEFFIPASLSGDYDKIYSNVEALQWSILNEVAKRSG
LAKGCKVNVQFDDRPLLTALTSLNELHSSATPETTLRHNSDQHTTLSVSSNARSSSQNQG
KNQGNGQRNRRRARTIGTNRRMLSRLPYPMSVYSIESTRPQWTGKQNRHSKLFTVLDYVF
HPVPLTDHFFPHHKIESCSSEMIHSDESESQCFTAEITLEVRFRGTTGESNLIEEYIQSI
VHNQIALDASQLFVEDIFGLKWSSVQEASSLTQPPSNSSQESNGPISFGPENVEDSSDSI
GTTAKDKSNSLFASIIIPIATACIVFVVWYCTRRYYNRDRKRARGARDHQEKSQAVVRGM
KQSTDIEIGIVRTETTQSSHDQLSKSSSNSNGNPEDVISATASSDTISSSSSSTSLKSIS
ATSDFSDRSITGLPPRPNVVKREGSKQLKKPRKKKKRRKKKVMSLQRINSRDNISEMPMI
SESESECDSESGGEDEDYX
>comp7033_c0_seq1; 360 132 1028 LEN=1291; translated
MYGRQQAHQRGGGMQGRQIVLPEPGDVGQMVLRGASAFARRHKVVTGGYLLGIVVILLVG
SGTKLNYDQRREYNNIMNTIDVDAEYDASSDFWRANQAYKATKGWFSCDGLCQRNKRKME
DAKYRLDIIRAEGNQRMSDAKAVAGLFSEVGVGEVQDSFWSYFSAGKQFAKRQSMWDAMF
MGMRSMGRDESMIEYGLKVLMQVLMNFSMGLIIALFTFVIGLWSIVRSYQPNPIVAVLFF
ISAACAAFSFVATFLLAMYGTAAGGVYGVLKVAESNLRLQQGHPGQQPRHMHNRPHYD
>comp7034_c0_seq1; 310 293 1033 LEN=1138; translated
MKVPTLSLLLCYLSTASSLSIQPALSAVNAAFEYNPYAAGAIVAGFKAGTADILAQKRQI
RNAAKKVGDAVQESVEAEKTAGKELDVQRTLAFLMYGAIYQGFCQEFTYNHLYPFLFGSE
TTTITVLKKVFCDMCIQTPLLTLPIAYYSKALLAKNTFKEAMAHYVDDVKNKGLLFKFWG
LWTPVQCLTFSIIPEHFRVSFIALVSFFWVIILSSLSSSNDELADTTSSTAKIAGVNGQG
ATKEAY
>comp7038_c0_seq1; 459 248 1666 LEN=1933; translated
MVANSDSITERMPSPSKDFRAAQPSQLPIAEATTKSTASDVLSSVDSNGPSVMAVSRSGL
MKQSTIYRRHEARLARSYALRDDPRPAFLRVLSGLPGFRIIIPPYQKERNQEAGKQDPKN
QQKETHAPRTVIDTYSQENSFAPYTNVADNAVGSTFGDANTNDNIRAVKWEETMQAMLAT
GVCSAIGEYVFAHHSNISTAKRAVRNSNTLGPMMMTTTTAAPQTPHSMPYTGGFGALRGG
SLDMPRETNKNHRALAATKSRPSNVALSKAMSASFSTSLLFGTKVFLDATISERQFGNDA
NRSNSTTVMGSLVSSATAGGVIGLSQLASLQIKHRRDPSSFSVAQNHYLTSDGSSKLIGR
NVLASLLYFSIYEAVSFFASTQTSTSIHPRDSISLSNGKAGGEKGTLSIVMGGAFAGVAQ

AGTMNYHRYARYGSTIWWTRVMFPAASRSAPIHALVFYGYEKMKEGIKTAHR
>comp7039_c0_seq1; 253 65 1167 LEN=1191; translated
MDYVRKQFYRSKREPGGDNDYYDDHDQVEERAIVIHPGQVEEATHIISFPGRISLEELRF
WAYVPNLHENTFEPTGYTNNNIKLADGPSSLQNTQAKTEQKQKERRRQTNNSYVDIAVIE
IPLEFAQVNSHHXDLTSKGIGKIEHRDDTSFLSLCDETTGRLLIDHEVFRGYHTVLHIPP
TGMMMKDRIIDPKAKSIPSFPSQDSKYDVLIANCDQQAYGTGRNIGLSGQIIFESDPLLE
KVICGDENKNSEQGTRFWSTTPDSSRAEHYYNHVGTKHQQEEYGGVPLDSVSQMRLLAMG
LSICFIFSLLSLRIHNGTRADYFTARLNRRNRILENLSGSDDEDTDDDGNGERSSSNYRD
NVNIPLV
>comp7040_c0_seq1; 308 75 749 LEN=1217; translated
MKLLFSILVFAICNDTVESYITGSSPIGGANWMKGNNAINHISTKKAPSTETTAAADVED
LFRKEYNEWATRYGKTTTDERRFEIFKRNYMMQMQHNKKTGSFRLLNEFGDMTEKEFELG
EYETQKSENSPKSADNVVEVELVMDDPVPKVRMLDSETIPRVSDFEIPHNRWRHEITQQQ
QRQRQQAPVGFEPKVNPIRYPSMEEPRRRKRRVVRLQVGPDRIL
>comp7041_c0_seq2; 314 313 1710 LEN=2492; translated
MHSVSQAMELIFSFYGVALLLASNIDAFSGQPQSSKNPVRVLVTGAAGKTGRIVLSKLEE
DHRYEPKGLFRTEKSARRIVKNVDTKCPLEHVVIADVTSPTFLDDLSSSNDSSSNSNDHH
SETTVLCGKTDQSEKCNGLAKMDAMIICTSAVPRIRKRSLAAMLVLAPWRALRGKPIMNV
RSMRFAWKHGDYPEKVDYKGQKAQIDLAKRLGIPHVVLISSMGGTNPDNFLNSVGKHHHQ
QHQEEDKDREKQRRYKQGEGHGDILLWKRRAEKYLVESGLDYTILHPGGLVDSPGGKGEF
VFGVDDHLLELNHNEEQPAVRTRISRSDIADLCVAALSAGKGKKVSLDCITVPSSTRSTL
MGHGHSFRMVSPGMGIPEAKGSESGGRKSKSKRKPVNLGSDTRSRSSTTAATPTATLSAD
ASTTAEARLAIETEKQGRQKSAEESLSEFVDLSITANYDLGVDKL
>comp7043_c0_seq1; 520 1 1540 LEN=1588; translated
XVWNSPEEEAETGALSVSEPQVVLHPSCVWSAMGLPGGDFATCCQDGTLRIFTRTSDRMA
PRAEREAFAQAVQEAHAKKQSGPSPEEVAKLPAWEARLQKRGTSEGQVQLFNKAGVAIAA
QWSASSQTWIEVGQVMGSSDSGTVDGVRYDHVLPIEVDQTGGGVAKLQIGYNDGENQFVA
AQRFIDAHMLPQHHLNEIANYIEQRAKSEGRTLGGPPAGATSSAPAAAASGMPMATYEHL
PAPAYKSFELSAKSAATTLEKMKKKIAEHGGVSESQLAAISSLSDTLAATNRYHSSRIQP
SELETIARMLETLPRAEVFPALDLARLVVAHPSASGPASAGYWTAVVDRALALCTGDDNL
AGPAAVAIPMLSLRLFANAFRGGPGSLEAVGGRLTDVLACSQRFLGSANKNVRLSAATLL
YNTSFYVFSNPDNAPQGAALAVVSQAGTVLRLGAFEREALVRTLVALGTVAMASPGAREA
ARSASLSSLVEPAASLHGDLARALAREVYSVLS
>comp7044_c0_seq1; 497 360 1358 LEN=1656; translated
MKFQIADIALILSIACLSSAAAFSPSSLAPRQSAGASLTNRISKTTTSSNTKLFSQWDEE
EETSVKRSSFEEAAKAATDEDAAKELEEMGDFDATSTYNSDDIGRYRDAIKKRAESIGLE
KRSAEEIAADQAAAMQKAIEQGEGLVGAAARSNPDQLSDMLDESQVQRLDLSKISNDAPR
GPDEDVPAIMYDPTNDMTQEEMEEADPIGFKPINEQVAWVLEKSEFPSPLAVLGETIVTV
VTVVVTAVIFTQWDELCREAAFNFNFVPRPEEVQKALEGMLAPGDTILGMGANSMSGQEM
LQMLQDGTKETIKAVQDGSIKDILAGDVPAEL
>comp7044_c0_seq2; 279 1 772 LEN=1070; translated
XNPTIITXIAFTLENRNNNAMQYNSDDIGRYRDAIKKRAESIGLEKRSAEEIAADQAAAM
QKAIEQGEGLVGAAARSNPDQLSDMLDESQVQRLDLSKISNDAPRGPDEDVPAIMYDPTN
DMTQEEMEEADPIGFKPINEQVAWVLEKSEFPSPLAVLGETIVTVVTVVVTAVIFTQWDE
LCREAAFNFNFVPRPEEVQKALEGMLAPGDTILGMGANSMSGQEMLQMLQDGTKETIKAV
QDGSIKDILAGDVPAEL
>comp7048_c0_seq1; 761 344 2668 LEN=3187; translated
MSPRRLSTLSFPLSLAVGLCCLLGVDGYNGNHSPMIIPTNGWTAIEIITEGDTIGPTGTW
AIPDQMDGLGAFLVSNRWSSGPGDSDSGDTTLRLHVNHEDEEEATITEIDLHKGRLKQAI
SHAVLTSRAGGASVSNNYNFVQSARVAYDRWSPDGGTTWKYIGGAISDPAGAGGFCRFCS
SQFYPADAFGTGRGFQDAIYMTGEECWDSNGRLFALDANDSARDLYLVSGVTGSMPLVRP
MEYTAAGAWLSAPVPQTAFEEGHGGMPFDSWENAALIDTGETNHVALLLSADGGSETLKL
YIGKKFTNAYGYTDRFGFLARNGLAYGRWFYLGGLEWTMNDATDLWESTTKGRFVTDEDD
AWSESKFEDIDTNPVAPNQAVLAESNSGVYVLDFDLRFGVGDGSFRVDQSGFSVRMVTSS
DHKDMQYPDNVDWTKNNLIFVNKDNDSGGIWYMNPDGTGKVQVGETRSSGESTGILDVSA
LLDYPPASAMLTSNQGSPSSITLLLNPKLRSVLKEESGSSLFPPPPTAVATPVPATIPKP
VAPPVPAPTRKPLAPLVPAPTRNPVALPPLQQSDSTFETTLDCPKSDSRGRYQVLQAEDA
ELYSALIKTKARNYCGVGYVDFDDLDAGFVVPPSSGTDGGYSASSGKSPVFSKKDGIFPS
GNREPVVASRQGNVVGISFEVEITFPGDYEVTVRYANGGPLSSRRSGMFMVDGNFPNNNY
EYRFGNTYGWNIWFRESKTVVFETEGTHTLDFFWTEKRDRPNIDWVSVKLLAEY

>comp7048_c0_seq2; 121 344 838 LEN=838; translated
MSPRRLSTLSFPLSLAVGLCCLLGVDGYNGNHSPMIIPTNGWTAIEIITEGDTIGPTGTW
AIPDQMDGLGAFLVSNRWSSGPGDSDSGDTTLRLHVNHEDEEEATITEIDLHKGRLKQAI
SHAVLTSRAGGASVSNNYNFVQSARGTYAGDGCGIVAPRTVPRSP
>comp7048_c0_seq3; 677 344 2855 LEN=3374; translated
MSPRRLSTLSFPLSLAVGLCCLLGVDGYNGNHSPMIIPTNGWTAIEIITEGDTIGPTGTW
AIPDQMDGLGAFLVSNRWSSGPGDSDSGDTTLRLHVNHEDEEEATITEIDLHKGRLKQAI
SHAVLTSRAGGASVSNNYNFVQSARVAYDRWSPDGGTTWKYIGGAISDPAGAGGFCRFCS
SQFYPADAFGTGRGFQDAIYMTGEECWDSNGRLFALDANDSARDLYLVSGVTGSMPLVRP
MEYTAAGAWLSAPVPQTAFEEGHGGMPFDSWENAALIDTGETNHVALLLSADGGSETLKL
YIGKKFTNAYGYTDRFGFLARNGLAYGRWFYLGGLEWTMNDATDLWESTTKGRFVTDEDD
AWSESKFEDIDTNPVAPNQAVLAESNSGVYVLDFDLRFGVGDGSFRVDQSGFSVRMVTSS
DHKDMQYPDNVDWTKNNLIFGKXETANLYASVERTPCVLSSFADPYIKQIVVRIYFVSFR
LPRFDMDGFAHMVLSPSFLHVRRNKDNDSGGIWYMNPDGTGKVQVGETRSSGESTGILDV
SALLDYPPASAMLTSNQGSPSSITLLLNPKLRSVLKEESGSSLFPPPPTAVATPVPATIP
KPVAPPVPAPTRKPLAPLVPAPTRNPVALPPLQQSDSTFETTLDCPKSDSRGRYQVLQAE
DAELYSALIKTKARNYCGVGYVDFDDLDAGFVVPPSSGTDGGYSASSGKSPVFSKKDGIF
PSGNREPVVASRQGNVVGISFEVEITFPGDYEVTVRYANGGPLSSRRSGMFMVDGNFPNN
NYEYRFGNTYGWNIWFRESKTVVFETEGTHTLDFFWTEKRDRPNIDWVSVKLLAEY
>comp7049_c0_seq1; 594 1 2246 LEN=2374; translated
XDDDDDDANGRRQLGGSPGDRKQQKHCVNGIFKIRDMKESIWEPAIDFLSDEWYRPYQEQ
QVERQRQNRRQNHEISGHNHWMFRRDLENYADDNYNTNDDFSSLSATSSSRDVASDLRLS
FVFAFLIVLGVLGRRRRMRTRYYIVRARAQEDHLFYASSGSGIKRVGFQDSREDQYEGAC
SHTLCGCYPTDPPREGDEIEDEVQVNDDGVSQRKKKPHNEDFVGRGFNCILGMCCGVVCR
CWFQCLSICALAQEAREMRLLVPTRFQRVDYITHQPFHDYQKEVIDLRRGWMGKTRKKSG
IMPHFNALSRLSRHILVLSISLTVIIGATLFLNPLASFSWPDVIILSATFLQSFLVIWIV
HWIFHKSDLSLDAVIKFFAAGFVIAVPSAFFFEGLLINITLSTAYFGYGLGQFMGGDTFV
EWVSQQYQVFWILVEIFNAYIVAAVTEELCKYYTFRCVEHPDLIFLTGLVSNTQDERALE
GGVVKYPFSSHQVQQTNAKNPYDDDASHYSRSSHRSKSSGRNTGLDPNEDDFEEDDQDVR
THRQKAAAVTTAMISVAVGLACAENFLYVFLLGGTGAGVSDDGSSAMDGYLQEWIVLLFR
SIFPVHALAAAMQSVNMIRKFVEAQDVNGHRIGVGRIILPAVIMHGTFDAILLGINIFIE
TSWDEYLEANGGNIDDENATPYSPFVVNVVAWLSITLVMLSGFLWYYKENRSQRLRLIEL
EEQEKANLVGTSGYTSPRAPHVSEVELV
>comp7053_c0_seq1; 570 130 1743 LEN=2341; translated
MSLTFPRQLSDSRLGEKHETLDDFHRCTSRTNVQESKEYTLVVVTETFPKAETQAPTTND
KIDKEIPQPTISSQAENTRILLGMKNRGFGCGYYNSFGGKFLNDEETAEACACRELEEET
NLKVGLDEMAKSKVGIQRYTFENHPTEMVMHVFWIHLEETLAYKGGCHDVRECDEITPMW
FDNINAIPFDNMFADDSLWLTTLLSSPLPIEINGSYHFQENCQDTNTILHYHMDVQKKKP
KFSMEQRLFHTLHKNHSNLLSIKEFKECYAFCNGVRTTFGRNKNKKISFDIVIDVAGGHG
ALGALFLICTSASKAIVLDPANVGGGRIQQAWGKFMGQDKQLSYRRECLRTGLPDELKQA
LQMTTRYRILVVACHACQHLSEEILEIACRFGVHAAVMPCCQKDRSVGSTWKAASKNLSI
PVAKMMDILQCGKMMALGSHNVRLKCIDSKITPQNRIIVCRALDENEIIESRRHSQTEVD
RAHSKLELVYQKAHTVPGSVHESKKMPNFKNFAAQSPVWYMAAGFVAGFLASSVRKK
>comp7053_c0_seq2; 573 130 1626 LEN=2438; translated
MSLTFPRQLSDSRLGEKHETLDDFHRCTSRTNVQESKEYTLVVVTETFPKAETQAPTTND
KIDKEIPQPTISSQAENTRILLGMKNRGFGCGYYNSFGGKFLNDEETAEACACRELEEET
NLKVGLDEMAKSKVGIQRYTFENHPTEMVMHVFWIHLEETLAYKGGCHDVRECDEITPMW
FDNINAIPFDNMFADDSLWLTTLLSSPLPIEINGSYHFQENCQDTNTILHYHMDVQKKKP
KFSMEQRLFHTLHKNHSNLLSIKEFKECYAFCNGVRTTFGRNKNKKISFDIVIDVAGGHG
ALGALFLICTSASKAIVLDPANVGGGRIQQAWGKFMGQDKQLSYRRECLRTGLPDELKQA
LQMTTRYRILVVACHACQHLSEEILEIACRFGVHAAVMPCCQKDRSVGSTWKAASKNLSI
PVAKMMDILQCGKMMALGSHNVRLKCIDSKITPQNRIIGKFRSSLCFLAHISDIEFFALN
QQHAWGFSQLSLQSSRRE
>comp7054_c0_seq1; 378 98 1369 LEN=1615; translated
MINLKRLGKKHLDKNSSATSFSTIVKKDPTVRQTLGELDDCLEQIGLRKLIQTRSRSQTA
RTVVTDVFSECENDLRDDESSKQASLASKTCVERVHRHDFKTISYLGKGAFSGVQKVKNH
SGETFALKALDSSRIKTPDELTTAAIDLGMEAKILSELNHKNIIKLRGVCSSTFSYSYSE
CTDEGYFLVLDLLEDVLSQKLIRWKKHSRLRLALENRRAILPTANVKMMYKRMQSVALGI
VEGMVYLHKKGIVIRDLKPANIGFDRNGNVRLFDFGMARRLEDCDKGEICGSLRYMAPEV
ANSEGYFFKTDVYSFGIILCELCSLTVPYDQMMKKPCGSKEFSRRVAKDGMRPSLDQIAC

DATKELIKDCWKPSPSQRPSFEEIHERIVRITSCDRSETRGDLTRNYRKKFCSRLERQGD
RNV
>comp7056_c0_seq1; 600 396 1391 LEN=1949; translated
MGTGSSAKSTEQDNGVRDRIDSGEFQKVSPPGSPSFESNTKTDEMSYSGILVEKPDETGG
KEQPPTAKTGVPSSEGAIFAADLTSPPISNRSKGRTKTGSGAADSSRAQAQPRRIVKATP
PPRVAPQAAQESGTGASSVSVHSGKSNHSNHNAAPLSSPIGNNANAANELLEESIEKNDP
KTKQRERHTSRQKNDRERLLKEKRSQRTAEANASGTGEKQKPRVEANPFSRFLSAFSVDA
NPKHKRKEQSTADAENKRLKYGFQDTSGTHGGAASDDTPAAAAAVEDSSSSSTREEKEAA
LPSLLSSPWMAAAAAAVALLVVAIAKGSRKK
>comp7057_c0_seq1; 513 269 1401 LEN=1401; translated
MTNLRSDYTRDGDDAPSQRNGSRRSGNGIRDKWRLAIPIRKVGRIGSNGNSNSNSINNAK
AGNDDDRQCPPVSAAKVDPKKQTVVLCASCDRCRARKTRCDGKRPCGNCASRYMKKHKLT
SIEGVDVALFECNYSPAKRRGPVPGRAGQTRKAAEAFGNSIGGRGRFGAPNFLSHAGVNS
DALQTLGGVAAHRSSGMMSATTQSALEAIQMRQMMAMKMGNGMGTVVSRGGGGSIQGIMG
RTLGGAGASGHGSMPGGLAAMNSGSQQRQHIQQQLNLIQQLQAQQQMGSGGGVADGGLGA
IAQRGDSSSGIASAAMGLPSIDGVEAARRMKLNDQTNQSLSQLQLQQERQLLQQQVQQQQ
HQQQQQHRRRRQQQQQQX
>comp7057_c0_seq2; 512 269 1564 LEN=1794; translated
MTNLRSDYTRDGDDAPSQRNGSRRSGNGIRDKWRLAIPIRKVGRIGSNGNSNSNGKSNAL
NKDYCEYEADSEEDVYADLPPIEVKNDKESRSTNRRRPLKSALLARTKKQNPAASASRTT
SMMTPDMLETLGLTLVEQEAALLSREDGFNFEYTHASATNGGRFGTGNENGFPDATTKTT
KNTSTSTITIRKAVRIDENRNTTFQGSLPGTPVQTRQQVQDASSNGNGHSENKDADAYRG
VYGSPYGCPSTDSTSSSSSNSNSYEPIPRDPEDWTVEDDLSTIEDNDNRTLLQYYKRGAG
KDCDSLSCLSLACALPLACGLLCLDSLADTKLLEEAMPGSRARRSNKTEVLAPWGRRRTP
AKTATRSRNTKKNQNHAQRRRGRSRYNGDEDGDEGSSILDGNNCEFVREGNCDVFPSSIE
LPVNKIIAPCW
>comp7058_c0_seq1; 265 84 677 LEN=846; translated
MNVIAYAFLLSLLFSTVDGFSFVLMGRRGKGDLKRALSGNDGQSKRRGKNQDPTAAMNDG
KGQEITGVSLPEEGTIKGWEFGDKKVVACANVRGEFYALQGDCPRCSFDLYRGDLIVDDP
GFEDLPRVACPTCSTTFSLKTGLYGPPLKRTGLAGFVSGLAKTATANQERNADAYTITVD
PETGRVYSKKKVLRKVY
>comp7059_c0_seq1; 625 49 1947 LEN=2528; translated
MEVPSIVKRKSTIFLAHETKAMILIDEKKKMKSKIGVKHENDDSSLFSSVSSVKPDDDEN
CSSSTALMVSSAISTECSVSISSSLSLSSDFGKARGNEEENDSFSFPRHSFPSFPSLRAN
YLLVILIIMLADGLQGGNRVILMYSISFGNNXTEGWYFSGTDFSAFFLIPGTHLYVLYEG
YGFNVASLYALGFIAGGVTTPITGPLIDKFGRKRSAILYCALEIGINMLEQYPFLAGLLV
SRIVGGVTTNLLSSVFETWLDTEFHNRCRAAKPGVDAFARSSDDTTTVSESTNEWNVVED
DEDDIAKNEYELIMRDSVIISNLASIASGYLAHMLAERYGAVGPFRGAVSCTAIAFVVIS
LLWKENYGGPSTLSCADKQTEDTLLGLETDIDNEGHSPNNQGQNSSSSSKSIRAYIMKGF
STLRADPKILYLGVIQGLSAGSLQLFIFLWSPTLQNFAPGQEATFTTNNLSLFGLFRWAV
DRNGEPNYGLIFGAFMAAGVAGGFCAPYIRHAVALLFLQSDESSLLQTVHVGTKRVSVRP
MELEFLLGVNYISAAVLMLVPSILQKSSPESFSISLAAFLFYEFLVGVSMTYEGVVRSLH
LPSNARATMMMVPRMIVNLAVSLGVLLTKYIA
>comp7059_c0_seq2; 690 49 1842 LEN=2423; translated
MEVPSIVKRKSTIFLAHETKAMILIDEKKKMKSKIGVKHENDDSSLFSSVSSVKPDDDEN
CSSSTALMVSSAISTECSVSISSSLSLSSDFGKARGNEEENDSFSFPRHSFPSFPSLRAN
YLLVILIIMLADGLQGTHLYVLYEGYGFNVASLYALGFIAGGVTTPITGPLIDKFGRKRS
AILYCALEIGINMLEQYPFLAGLLVSRIVGGVTTNLLSSVFETWLDTEFHNRCRAAKPGV
DAFARSSDDTTTVSESTNEWNVVEDDEDDIAKNEYELIMRDSVIISNLASIASGYLAHML
AERYGAVGPFRGAVSCTAIAFVVISLLWKENYGGPSTLSCADKQTEDTLLGLETDIDNEG
HSPNNQGQNSSSSSKSIRAYIMKGFSTLRADPKILYLGVIQGLSAGSLQLFIFLWSPTLQ
NFAPGQEATFTTNNLSLFGLFRWAVDRNGEPNYGLIFGAFMAAGVAGGFCAPYIRHAVAL
LFLQSDESSLLQTVHVGTKRVSVRPMELEFLLGVNYISAAVLMLVPSILQKSSPESFSIS
LAAFLFYEFLVGVSMTYEGVVRSLHLPSNARATMMMVPRMIVNLAVSLGVLLTKYIA
>comp7059_c0_seq3; 712 49 2127 LEN=2429; translated
MEVPSIVKRKSTIFLAHETKAMILIDEKKKMKSKIGVKHENDDSSLFSSVSSVKPDDDEN
CSSSTALMVSSAISTECSVSISSSLSLSSDFGKARGNEEENDSFSFPRHSFPSFPSLRAN
YLLVILIIMLADGLQGGNRVILMYSISFGNNXTEGWYFSGTDFSAFFLIPGTHLYVLYEG
YGFNVASLYALGFIAGGVTTPITGPLIDKFGRKRSAILYCALEIGINMLEQYPFLAGLLV
SRIVGGVTTNLLSSVFETWLDTEFHNRCRAAKPGVDAFARSSDDTTTVSESTNEWNVVED

DEDDIAKNEYELIMRDSVIISNLASIASGYLAHMLAERYGAVGPFRGAVSCTAIAFVVIS
LLWKENYGGPSTLSCADKQTEDTLLGLETDIDNEGHSPNNQGQNSSSSSKSIRAYIMKGF
STLRADPKILYLGVIQGLSAGSLQLFIFLWSPTLQNFAPGQEATFTTNNLSLFGLFRWAV
DRNGEPNYGLIFGAFMAAGVAGGFCAPYIRHAVALLFLQSDESSLLQTVHVGTKRVSVRP
MELEFLLGVNYISAAVLMLVPSILQKSSPESFSISLAAFLFYEFLVGVSMTYEGVVRSLH
LPSNARATMMMVPRMIVNLAVSLGVLLTKYIATQSAFVVVALLMTCSGILQLSFVSSQEW
NTIRRKSSHRARSLSSSAAKLVWSSAFKPKDD
>comp7059_c0_seq4; 777 49 2022 LEN=2324; translated
MEVPSIVKRKSTIFLAHETKAMILIDEKKKMKSKIGVKHENDDSSLFSSVSSVKPDDDEN
CSSSTALMVSSAISTECSVSISSSLSLSSDFGKARGNEEENDSFSFPRHSFPSFPSLRAN
YLLVILIIMLADGLQGTHLYVLYEGYGFNVASLYALGFIAGGVTTPITGPLIDKFGRKRS
AILYCALEIGINMLEQYPFLAGLLVSRIVGGVTTNLLSSVFETWLDTEFHNRCRAAKPGV
DAFARSSDDTTTVSESTNEWNVVEDDEDDIAKNEYELIMRDSVIISNLASIASGYLAHML
AERYGAVGPFRGAVSCTAIAFVVISLLWKENYGGPSTLSCADKQTEDTLLGLETDIDNEG
HSPNNQGQNSSSSSKSIRAYIMKGFSTLRADPKILYLGVIQGLSAGSLQLFIFLWSPTLQ
NFAPGQEATFTTNNLSLFGLFRWAVDRNGEPNYGLIFGAFMAAGVAGGFCAPYIRHAVAL
LFLQSDESSLLQTVHVGTKRVSVRPMELEFLLGVNYISAAVLMLVPSILQKSSPESFSIS
LAAFLFYEFLVGVSMTYEGVVRSLHLPSNARATMMMVPRMIVNLAVSLGVLLTKYIATQS
AFVVVALLMTCSGILQLSFVSSQEWNTIRRKSSHRARSLSSSAAKLVWSSAFKPKDD
>comp7060_c0_seq1; 484 59 1351 LEN=1505; translated
MKFSTVAILGLIVSTASAFTSVSRPNRAPFSTSLGLVETGEYVDRDVYTFDEWATSCGVQ
KCAGFQITTQDGSDFFAVTDQDLPAGTPIVYVPPELVLSSNRAQEEFGQSLQYAEDRIKE
GGVANQIPIFRLGVKILAEWEKGEQSPWYYWLNSMPRIFMNGVSMTSACFDALPPYAGWL
AMKERANFVNFKKALLSAPLNEQTVTNEELVKWAYNVALTRSYSLSSSCRRIAPMVDYFN
HASEPNLDIQYDDYACTVYASRDIAAGEALTISLGTGTNPSPLFATYGFLDESSPGTFCK
LMDLQDDMPDMKLGFKDLLFYKNGEIAPEVYDLVLYSILKTDPNFELGPFFDACMAGDED
TKNAYHSQYFSYTLEYLKNHVDNTLVELDRLSNMAASYDPNAHPRAPLILQHNAFVKQTF
EAVKYNLDSM
>comp7061_c0_seq1; 224 294 584 LEN=662; translated
MKRLIDANKVVVFSKSYCPFCVATKNLFKSLDVVEIEVLELDRMGKEGEELQVALFQETG
QKSVPNVFVNGKHMGGNDDTQAAARNGTLQEMLKSA
>comp7062_c0_seq1; 372 1097 1840 LEN=1952; translated
METPSPPLILEEFMEVQFLRQHARQQAIEVSHLRKPYANLTFCEKGSESFPGNGESKAAP
REEGDIAKHHEIMLIVARGEKGVKGGARWLGHLFVPHAAHEAPVPQIVGDLFGNGECKSK
AAVLLVVHRGPDGVVKDSGLHASAGLRQPPDFRDAKEATADQGGEERSEKKDAGPVVLLP
GCFPPLLLSWLLVGHVSGSEFGGGYGFCSGDPDLDLLEYTDSVGEPYSNAWDDRLLVDGT
RVGSVRR
>comp7062_c0_seq2; 372 843 1586 LEN=1698; translated
METPSPPLILEEFMEVQFLRQHARQQAIEVSHLRKPYANLTFCEKGSESFPGNGESKAAP
REEGDIAKHHEIMLIVARGEKGVKGGARWLGHLFVPHAAHEAPVPQIVGDLFGNGECKSK
AAVLLVVHRGPDGVVKDSGLHASAGLRQPPDFRDAKEATADQGGEERSEKKDAGPVVLLP
GCFPPLLLSWLLVGHVSGSEFGGGYGFCSGDPDLDLLEYTDSVGEPYSNAWDDRLLVDGT
RVGSVRR
>comp7063_c0_seq1; 633 119 2105 LEN=2144; translated
MSSSGTENSQQIFHLDIHAWLDSQQTSHGVRHDDYAQYHAYCTRRLSRLSHKPSGAKAYL
VNSSKYASSNPSKPKTPGGRHAFCGRSHDTFALTKEVEVEADAAEPVNAAAAETKTTKVV
QVPVSHENILWYLLVNAERSWAHANEIQKANQIKRRQPILKKLKRANKWAPAPSGKSKFS
TDEETQKECEAYAAWMSANYALEQMQYQIASEGYARAMSLCHELSGENDTDNSANDRTND
VQRLERHDLFITRADTILRPLFRYCQYELKQAGQPTMEEPRLQSSGQEQEHEDEDSIVFR
DRELILDNKDLRVLLLKLQSVEQEQKEEAEKGSSATLKETQFLTALSILDDAMEVVQLLE
QGLSKASSSSGPAVQAKLQQYSLWRGYLQYSKTRKVMEHTENLLTTDSGESMGPAEKVHI
YDAVLQHAKSLLNLPHPDDGNNSGGSANEEDEFILQVQANILRLRALKAYYMGWCYYTQL
FKYGPALAXLEHSAKLSERAEEEIAACDEDMPHADKYLQQLEDLPLESATGAVRAAMALQ
QRQHARKLQKAAGAAAANGSSPSCWQEPVTTDRPLLLRLYEYDGGAPDVPIADLRPMPLP
CKPVFYDLVYGHAFDTAKSVDALEDFLYQHTAAPVSSVVDKEEKDLGNGGSSSGGMFGWL
RG
>comp7064_c0_seq1; 283 137 580 LEN=791; translated
MTPTQSCSKSGVSNTQRFMFGGAGAGSPTEDDTEYDPEAEKGIANAAAAMGMSAEEYKLA
MRAREQLVKSMDAKMVKGGNADTVLVERDVNNPPKQLDITITEAGKELGRDALSKKLVDA
INKGSEEARKGRQECQQEMLKWVQSQG

>comp7065_c0_seq1; 251 96 695 LEN=1104; translated
MRFESQDFIMSQREWQENRRAKANDHSATKQDGSLSRDGTQYNASGSGLIRKQYGYSSQT
KNIARDDSRSSTQKKNAVAQHEVRHDRRDEADSDSDSDVDSRDESTPNVPSDFSKKELQE
LVRSRRRQKQDQSTQVGQSQEQRQTSTYSYHGYADKFRQESLDSFVAARNKVKESNRDLG
VENTRGMDCLLLCMLPTNVW
>comp7067_c0_seq1; 129 1 1280 LEN=1673; translated
XSRSLCPTSRSIRRHYRILRTMVSGCENGEFDETDCVDERYRQLSGSPGPGDEEDRLVGL
LAPFYLYLVENWNGISMTETQLGEIVEMLVPGNDDGGSSTEETPEEKLDDPAQRQTPPCH
HQGTVETDERYVCVGDFAGPHCKVETGMATVLDPLLRSLGDGVLRRNRKVVSVTDKEDRV
RIETEGGETFEAGVCVSTLPLGCLQEHLRRPQGEPGFFRPPLSRAKEEAVSSLWSGSYKK
VFLTFDRVFWPVDPPMIGLVRSTPPETGAKDPLPGRHLVLYNLWAGRGIPSVEAILCGDL
GKWAFGRTDEAIRDAVLGFLAAAMGIPGADLSSSCMGCHVTRWEEDPFTRGSYSSVCLDT
SDKHLSELSAPEWGGRLLFAGEATEPDHMGSVHAALMSGDRVAAEVLAAVRHRCSSNDRD
VPTTRC
>comp7068_c0_seq1; 261 137 631 LEN=1782; translated
MMTENKIDGKVDANLSGGISQFAKGAFTSENFEAAGRYTKEKVAELKAQAQEGDRSLRFL
AFVGGIGCIVVGMIELTSRIVRLQLVDAIIDVYIIMLGTVVVILEGKDMFLSRRLVQNVN
KYALFLKFLWGRGFLFFLCGTLQLTQIDLFNLIAGKFKSKWNRI
>comp7068_c0_seq2; 387 137 943 LEN=1646; translated
MMTENKIDGKVDANLSGGISQFAKGAFTSENFEAAGRYTKEKVAELKAQAQEGDRSLRFL
AFVGGIGCIVVGMIELTSRIVRLQLVDAIIDVYIIMLGTVVVILEGKDMFLSRRLVQNVN
KYALFLKFLWGRGFLFFLCGTLQLTQIDLFNLIAGGYMCAVGILYIIVGNNTAKKLKAIR
KSLYSEGALRTKFQAANVEGDGLDVKQFRFLCDDLGLDFTARETEAAYGHIRKMGAETND
KLSYDDFKSWWDNVEGGDEIHDCAFNFV
>comp7070_c0_seq1; 221 674 1782 LEN=1840; translated
MGYLCSATESDLQAGTLRERAYESSPPTPRASAPLALHRKKRTRAEAFSCSLTRTHARRP
TRPLSPNPVSSDDSSSDEDDTERETVEGTHACCTLAKRRLGRPRKKRVGFGDVAATTTYL
ASDLTRAEVKASKRLLWSTKQDRLDAQAECQRVLKAFRASHAREVRSFSSVCFTSMQVPL
TLEASDLLERARISVPLAIRGMEWGIAPRLRKRRKEHVSSVLSAQAAISESLLREKVVAS
RSLRSSRSARIMARIIGEGDVGIASTTALTVTTADGGEPVAKAEARATANATAGAKEDGP
AGRDASXRAPGKPGARSAAPEAGALEKQSGKEAGHHSRINERGFWSSSVPLCPGMARLCT
DRMTMAPNL
>comp7072_c0_seq1; 428 1 946 LEN=1313; translated
XRHWAMVLNPDEAVAGSNLMEVARHSVRDAIPNDQDAEPGGGWDEEMRRLVGFAEASEEQ
ANHALGIGDSDDYEALTLKNVGAYYNRGDAPSNESCDNQQEQQRAAEAKRCAMFANLMNQ
RMNKLLEDHRARASSGGRAASSSPSNGSESSSRLGMAFPPPRHGHQLMQALTKRMQLDSR
TDADTLEVVNALPVGFKKRLHSYFRRASELLRHFFGLQRLAQETDANGSGGGGADRNAYK
QKLEKIKNGLRTLHGDIDAMRNEQEMTSEEGKTMAGMCKQIMDQLNSAFKSSEEATNTGG
FVPSGSGGFTTVETF
>comp7073_c0_seq1; 171 1 1098 LEN=2246; minus strand; translated
XGSENDWFIGYNEAVGAQADTAEAANMVAVSQTEQKFDDDYYDSSKLSWRHAALPVGNVY
GINNFGGTEYGVYVKFVSLVGTDATVEVITSTTRVPPSCGGVGRFRIGLLTDDYSYDTSW
VFRDSSGAVIDQDDGYAKYSYNTQYNFPSAEGYYCLSPGCYQFEIRDTHGDGLTEGDGRY
TGTLDGVEIFSGSEFGYSEVKDFCIGAAPVPVLECEDDGTFSFRGNSKKKCGTKWLQRFA
GNINKKNRXEAAKTVQEEVGGKTGLRILQGNLRPGRSGTLRLGAEGLYARPGPCCWSGGN
REDDDPGFPCPGRTVAVPAGGLAPXIVRSDGAFGSETAATEVACLGSFEPIGIESGPEHR
HVLAN
>comp7074_c0_seq1; 656 204 1811 LEN=2025; translated
MVIDRRGLALFVTTALATTCNAFSFGVAPGATRRQQSSLKAFASDRGENAAQFDHFTVSD
DSSSPSVSTGTFGQESTTIPLSPPPMARPAESSNLARAQSAPPPADMEGEPEPAQPQLSV
WERQKAAEVQGGSLRTWSFADQHKIDMIQVHMKTDGRPMNANVELWQGPDNVPQKVAIYC
EDGSTRPFRCFLATPYTGNSISIRNTGSLEYPLQAILEGGKVGMNDPETKEKMERVQTAK
PKICQGGAVITERFEPGVQSAQIFLQSEGRPLQCRIELIQGPNNVKQVMEVYTEEGTTRP
FYAIIQTPGYGTGVIRIVNTSTLEFPFSVTVQPWDVGFADDDYDARPTGGSPFFEISGGA
EGRGIGNSNLRSVGSPTDSMGGMFGSFGPSSSSTTSPAPSPAKAKVKRNMIAPKEQPPSL
EPEPEPSPIFLQNEEFAVSSSKSSLSQDPILTTVVPAPRRKAAPKASPPPPEPELETEVE
AQLEVEADPTPIANDPPPSPPRRKGPVQKRYVPPPSPPPPPKKSMVFLDIDPAYK
>comp7074_c0_seq2; 463 395 1306 LEN=1520; translated
MERVQTAKPKICQGGAVITERFEPGVQSAQIFLQSEGRPLQCRIELIQGPNNVKQVMEVY
TEEGTTRPFYAIIQTPGYGTGVIRIVNTSTLEFPFSVTVQPWDVGFADDDYDARPTGGSP

FFEISGGAEGRGIGNSNLRSVGSPTDSMGGMFGSFGPSSSSTTSPAPSPAKAKVKRNMIA
PKEQPPSLEPEPEPSPIFLQNEEFAVSSSKSSLSQDPILTTVVPAPRRKAAPKASPPPPE
PELETEVEAQLEVEADPTPIANDPPPSPPRRKGPVQKRYVPPPSPPPPPKKSMVFLDIDP
AYK
>comp7075_c0_seq1; 413 1 1159 LEN=1418; translated
XMIVTFMRQQKHTPGKHPKPEQKDNDEVNLANMSSNEDSKKSGSEKGAGGILGKQFQKMR
RRLTVTGSKLNIDGAFDPSAFTSGEFRIKKKDEGMKVKTAVTEFHGVSKKGHAPYNPRKK
NQDSMIMKEETKTNSVIFCTLDGHGEHGDHVSAWFRDKLVKEMSSHPSWANDMNVAVADS
IAKVEKDLLKNYRIDAEFSGTTLSMAIVRDNHLTGVNIGDSRVILGKETNGKIVPVEITH
DHKPDTPKEKERILASGGRVFAVEYDDGIDGPPRVWLGHMDVPGLAMSRSLGDVVAHSAG
VISEPEFTEFDLDPEADKFLVIATDGLWEFITNEETVDYVVGQSGPAAAVDTLVTEAGTR
WMAEEQVIDDTTVIVANLFGWKPSSA
>comp7075_c0_seq2; 361 778 1764 LEN=2023; translated
MRRRLTVTGSKLNIDGAFDPSAFTSGEFRIKKKDEGMKVKTAVTEFHGVSKKGHAPYNPR
KKNQDSMIMKEETKTNSVIFCTLDGHGEHGDHVSAWFRDKLVKEMSSHPSWANDMNVAVA
DSIAKVEKDLLKNYRIDAEFSGTTLSMAIVRDNHLTGVNIGDSRVILGKETNGKIVPVEI
THDHKPDTPKEKERILASGGRVFAVEYDDGIDGPPRVWLGHMDVPGLAMSRSLGDVVAHS
AGVISEPEFTEFDLDPEADKFLVIATDGLWEFITNEETVDYVVGQSGPAAAVDTLVTEAG
TRWMAEEQVIDDTTVIVANLFGWKPSSA
>comp7076_c0_seq1; 209 25 739 LEN=739; translated
MSSFTRTITHTASGASCQVHDFGACLLSYKTGAGRECVFLSRDAKLDGSKAVRGGIPLVF
PQFGQPDKSMPQHGFFRTNFWKVDESSVFDNEESAGITYTLELKDVKNSRGGLWDENTEF
DCSCSYHVEITGKTFSTRIEIKNTGGKEIPFQTLLHTYYLVDGKAALDGSNCYVTGLEGY
AVSDKVSGAEYTMGSDPIVIEGLTDRVYTPAVGKDVVDVVVGVGEGKAIKVTASGKCSX
>comp7076_c0_seq2; 211 63 738 LEN=738; translated
MFLQKVHDFGACLLSYKTGAGRECVFLSRDAKLDGSKAVRGGIPLVFPQFGQPDKSMPQH
GFFRTNFWKVDESSVFDNEESAGITYTLELKDVKNSRGGLWDENTEFDCSCSYHVEITGK
TFSTRIEIKNTGGKEIPFQTLLHTYYLVDGKAALDGSNCYVTGLEGYAVSDKVSGAEYTM
GSDPIVIEGLTDRVYTPAVGKDVVDVVVGVGEGKAIKVTASGKCSX
>comp7077_c0_seq1; 894 143 3076 LEN=3107; translated
MHPFRTLALVAITFPLLWHTDEAASSRDASGGSRLILLDVDNTIYREEYAGIESQIVHGT
HSYCRDVLGMDKETADDLYKQYGSTIEGLRHTTWKDLSEAELREKLWGFYHAVYDVVDPS
SVLRPETDAGGSGSTGYSHDSREENKMARQLLKSSPLPLAWASNSPSWHVRRVLRALGLA
RLSSPGDRVMFTPDRLPWYPTKNQPEAFFASSNDEDGDDYSSCLDLEKSISFLDDSLFNL
NRVREAFPHIVDRVHHINRKHENERGESSKLVGNGEENVVQALLQDFGLIDPGFRLSQTK
YLESKNKVDIKSLNVGIWNKVVGELEDKLLKGVPNDGNQLTIVDLGAGLLSMLDLLLHGD
DENGLAALVSAADSKVNTIRYTAYESNQELYESCNQRLLSWGFGVVEDGTSDAVDYQKEH
NGKMFRVRLVLRNFVEGDSGDLAEGTTHPDLIIGCCFADLMDPEQLVPDLMRSFGLVNLP
SSLSTRGTLIYFPITFTGTTQFLPPGPFGFRSDGKTIPSDTVGFRSYSRALDTVLGHNLD
PHLLQDIMEDYGAKLIDFGASDWKIDPEKDAYLHKTMLYFFGSTGGPQLLKEGWDAPGWI
QRARNNRPAIRVSNRDLLFRVLPDGERDPDHNIDTEAEKEAQLSEISFTAPYQVTTVQKD
IPSQLGPRQVLVRASYSLISSGTELKIFKGNFDDDAALDVNIKDMEDERMAYPLSYGYCL
VGVVVDCGSDVSRKDFVGKNVFTFSPHSTHVVTDADAVQILPSGIDPKDAIFMPSVETAL
SLVHDANVRIGENVAVYGQGLIGLLVTALLSQGRSDEPGGWFNSITTFDMFPDRLAASSK
MGASQALVPGNKDHEGFDVSIEVSGNFRALQSAIDQTLNGGRVIVGSWYGSKDVSLKLGI
DFHRSHKTIQTSQVSEIPANLSRLWSKERRFAYTWDLVKQIRPSRLLTKIISLDEAQDAY
EALDNGSEIAIAFDYNR
>comp7079_c0_seq1; 315 1 614 LEN=692; translated
XSANTGFYYVRNNPRTQFFFNSLLMAGDLILSTSSHQIALIALLNEHVSLYGLTVKIWER
NKREFPGGFLYHNRKAAGFMKELMAFGDNHNDRDEPAGDNNDNNISNSNENKTYIFHMSW
TKNKNDKVKFFQQMGEWYLDGSCKSTEPKDLAKKHKPKISDENNNNNNGCCLAMPKIQCH
YRDKPSKIPCNDSPTIDKKGKPFW
>comp7079_c1_seq1; 75 1 499 LEN=656; translated
XRIESSRVESAPRRLGRTRFDATQCDGFPCHGMRRKKSGMHGAKQASTTLRCPFDLPGHR
AGKPAAFRGRTGIPPRRVTPLCAGTPPRRRLLARSLPPADTACVHSVAACLQTATRAALF
VSFGWTATGTVLWQTANQTRAAEVVRSRDILQAMTNDKMLRKVTKY
>comp7080_c0_seq1; 633 55 1521 LEN=1632; translated
MKIETVSSSANGSAAAGSRISAHTHISGLGLDENGDAIVPMAEDAQGSGAVAVNTSGLIG
QVAAREALGVVSDLVHSQRLAGRALLLVGPPGTGKTALAMALAQEVGSNQSNKLPFCHMV
ASQVFSREVKKTEVIMEHFRKAIGLRIQERKEVYEGEVTELTVEETEDPLGGYGRTISHV

ILGLKTAKGSKTLKLDPTIHDSLSKEGVAVGDVIYIEANSGAVKRVGRSDSFATEFDLEA
EEYVPIPKGDVHKQKTVIQDVTLHDLDMANAHPSSGGGSSSQRDVLGLLRSMGKPKKTEI
TDKLRAEINKIVEQYINQGIAELVPGVLFVDEVHMLDMECFTYLNRSLESPIAPIVVFAT
NRGVTTIRGTEPSGIKAPHGIPLDVLDRMLIVPTLPYSVQEMISICQIRARTEKLDLTPD
AFRLLGAIGEKASLRYAIQLLTPAKLVAETQGRTQIVPEDISQVDELFLDGKASAQMLLS
NDSIGLMS
>comp7081_c0_seq1; 597 447 2165 LEN=2295; translated
MFPMAALLTTCLLLSLLADNTDGGGPEMDDMNLGLGRRLRFRNPSTSRLWQITEPMKNDF
DENAPELVGGLRYLTLGGPSTFGRGLGEKRTSEAYPYLLSSEGTIGQERVRNVAQSQYET
GDLAFASLCTQSLVEGESARERAKEGSSLESEIDSHLDSSSWSSSSVAYDVITLEYTKDG
TSDAASAAYTSSMNLLTRRLRKRYPLSTIVVVQLWSPLDLVYYDPVANRNVSFAEWRNRN
EQNNQQYQDKNRLKMKKQKKLSVEAMKEHVWTFRDLSPAEQQRSNDLENMVVIEEGEIYR
MSRPRNINDSLDIIMDWFLEEEIKEEEEDNTSLSLHYTLSRRGHSVVAAGLRDVLALSAV
PRTRQNDSGAFQVQSAPRQYLGSWGSGDSCQLWYRTGKDRIPDSLEYSIGLKSEEFYPKQ
YALEVVSSKGGTLTVYNHFDEDRIVYLTYLTTSARAAANKVYPKTKVSMVKEPMQTGHTT
IVLDPAHDDNSDVMHRTRTSAVGLVKARSKGILEFTPLEEYTVHHFRIVGISFLTKEKTP
HDVPFEFAISPRKLLISKSDDAIANEDMEGEE
>comp7082_c0_seq1; 541 198 1448 LEN=1623; translated
MKFSHVATCIAAAATTVNAFVPATSYRSTRSQLMSTATDASAYADVDRYVSDHPNNNIPP
HIAALVGRGLHTKEDHPIGIVRAKIQDYFNSLDEEYKIFDAEDPIVTCKQCFDDLRIEPD
HPGRSPSDTYYIDDETLLRTHTSAHQSMHLKDGYERFLCAGDVYRRDEIDASHYPAFHQL
EGVKLFDKDAVSGADGLTKEEWLASPECKMIADDLKNVLEGLMDHLFGPVEKKWSEDFFP
FTEPSMECEILYEGEWLEVLGCGVIHTEVLEMAGREDRRGWAFGLGLERLAMILFKIPDI
RLFWTDDKRFHKQFKAGTITEFKPYSKYPPCFKDIAFWLPEDFVENDFFELARGIAGDLV
EKMELTDEFTNPKTGRTSKCYRITYRSMDRSLTDDEINALQMELREQVGTLGVEVR
>comp7083_c0_seq1; 197 88 492 LEN=1479; translated
MTMEDGILYVKRKEGMPETATKATYEMEYDKHRDDQLNTSVVSALVGGFSLTNSWELERS
GSFLDTITYVLAIVAVHACTCSALTSALLYRVLTCSDPEKATVWMQKHSFLATLPYGKFV
FGTLCYLASVVLVAW
>comp7084_c0_seq1; 420 464 1573 LEN=2307; translated
MPKNKSRQQRVFSSDSSNISCASEKIQKRQKRRASSPALLHNTTCTRKMFCMAAATVLAT
STTSMTITEAWAFNGIANNNRNKPALLYSHSNPNPLRCGPSASLYLATRKGTSPLSKTST
RLYSSFRTRKRFQKEKEHEDARKEIFGFRRALRGVARKILPTKWFGTAKEKEALERKQQV
KNRVQSELDQMLKGAPLPFRMLGKLVAAPLMSKVASTVAEASYQQAETMEAILDETRSYL
INDPTTVDLIGTPIQIGQPHSQRSGTTIINGKRQMRMEFTTEISGPKGSAISRVSATNEG
IGQLLVEGNSRVYQVDISSKAKSSSRFGRSSSSSPPRSNGGNSSFGGTKGNTDDDNIIEA
EIIEKKTNR
>comp7085_c0_seq1; 1098 675 4526 LEN=4849; translated
MATTSPTNGSTKICTGKSGHDRTNSSNSTPLPVANIYVDSTIPLTRSRTVPNGKVTLGQQ
QLHPHPQILETSPSRQPERCYNSSGINNTINHSEGMIPASDSNDWHQQQIISNSPSRQHQ
HFSQAFPDSQEQALFEQRLCEDIYGVAVRKINQNGKSNLRYVKCLYVDSSEILHSSSDDP
STTNLNGPQNFSSSRSVSSRHSRGSLGALSRFLSGGGNHSSAAGTPSDHDAHRSLLSSGS
HHQIPTHNQSKNKTRVLVWGKKKDVVVPLDHFTAVRKGKVNERSRRNPCETSRILSLVTV
DIHSEDRKNAVSLDIEAPTRLDRDKFATAFSRFLNVPLLEEGESMKSQQFTPRTPNTSFP
HQQYSHHSFSQQQQQQLLPLNQGAGGGDSSRSIARSIKSDLTAQSWKGDPHNNSIPDIAL
TATNSQTTNGNGGSVRSSSNKRQQQQNAPLPVVTARVGGAPLTNGIISSKPPRNNMRRST
PPPIHPGTNLTALQNNSNSNSSKHQPQQSTANGGSSRTGSEVDDPPLASLPSSTRSRGAN
NCNNDNAHDTYLNTISSSAATKRGKMVLEIPMATKSKLVSKTSLNNQNPAVSTPTTTATG
AHVNSSNTTLHTPISTTLSTANAKNHYHNQGEAAMSDMDDMDGSIVSSITGVGFDQDLVE
ELHLALERMRVELDESRAEASRAVKVAEQAIQSAENSNSKDWNNTVTHKAAEAAATAQKR
SAEAMAKARIAEERLEMERKTSALRKKQIKEAEQMVAHWQTRAVAAEVQRFAVAEALETE
RKRIREFVLQQQQEDLINQQPPNTFVSSELNTSAGNVGTTEEVDRLRSNLAMESARRRKL
LDELQDLRGTIRVYCRLERASCHDSKHSATLEMVSNEILVLHQNSSEKGHKISDTNQSQL
SFEFDGILSSDMSHEDVYAEFEAACASVVEGFKICVMTYGQVNSGKTQTMLGDIRLNAVD
RSVSIENEGIHLQAMRQLFSILEQRRDRFQDIVTMNLVEVHDERLTDLLAGTKFGEEHGK
VEESRKSKRQQQDLKPTGTAEEKQQHPMGANSVSSSRPKLEIKTNRDGETVVRGILSVTV
ASYQDVLRVWTESLTRRTRRLTEQDLVDHRAYELDSHVVASLKIISKNICSGVTTTGRMQ
FVDLASSEIVTNRPFGSIDGVSSSATSKRNSSSSPSPEQLREDWKFSNKSLNTFREVVLA
RNQYQRAVPYRNSTITHILSDSLEADTKVVVIACVNSRDEEPSTTACTLQFAQEMRKVVV
GKATRHATIAPVGTNISNIKPK

>comp7086_c0_seq1; 353 1 791 LEN=833; translated
XPPKVDLLVQRLNDSIGAALKTSEEWASAAAVAEPPGDGSVPDGMAPACIVIDEETDRQA
KDIRYQESQVEGGWIEDHALIDEDGRARCAFHFCRKLFKDSSFLKKHLIKKHSEFLRAEI
AKCHDSYMMTSWDAQEQRPVPPILVDCGHRFGLKPSPVLGAAEPIAEDPEPELWKREEER
REFEEKEAELRRERFQNSQNYHNNNHSGESMDATLSEGRSQGRKNNFEDVDDMKVEKVEI
AFDSLEIPLQPPKKKKKKKKLL
>comp7087_c0_seq1; 74 1 257 LEN=363; minus strand; translated
XRNSKKVLERIKMKSMVLASSSTATVKELLKVLGVRISPAVPLVFKDGFTAVVFRFEFRI
GKDLVSFGNVLEFLFSFFNVVCGDV
>comp7087_c0_seq2; 744 154 1716 LEN=1736; translated
MTVNLLYVGRQCALATLVYFASTARTIAANPSNTNARDDINNWSAGKLRSRGEEAFSLRQ
FDEALRLYQKATQVEPSNGTNFYKLFRVHNRMRKFSSALSDIEKALELEPSNNAWRMQKA
KLLQALGQCDRSFVEYQQVMQNQNANGEGASASTDNIEELLRQAGECDRVITTAQTALMN
QNYPEAAQYFQMALKYVDATQATDLLLQKATATLETGDYYSVISDTGLILKSYPKHLEAF
RVRGMAYFWLNEHEHAVRHFREGLKLDPEHKGCKEGHRLVKKIDKKKKGDTAMEAKNFE
EAIEHYKGAIAIEPGHKNFNRLTTFLVIQAYSKNKQHDEAIQLGREISATDGSTEAFWAL
GDALTEAEKFEEALRIFRDALQAAPEGGDNETQQKAQRKVKEAEVALKQSKEKNYYKILG
LPRTATPKEIKKAYRALALEWHPDKNADNVEEAEKKFQDISEANEVLSDPELKAKYDRGE
PVFENQGNGGGNPHAQHFQQFFHRGGGGGGQHHRFHFNSF
>comp7088_c0_seq1; 590 21 872 LEN=986; translated
MTIVQTEDTRNSEWRKSNVGSRMLEKMGWTEGKGIGKRNVNTTALRAVKRQEGLGIGAKR
QTEGGPSESTGTFAAVLANLKAHHGTGDQGSSSKKKKSKKEKKEAKKKAKKAKNALSLPQ
NKVLAGHARKMRAAKFGAKSAEDLACIFGNTEVVATATNGMVPYGALPVIGAGSGGAKKR
SRAGETETKAKPTGSSDDDGESSSTSSSSGDDQPANAAQTTAATGDSEPEPTSKERKRAE
KKRKKEDKENKSKEASSSSESKKKAKKEKKSKKPKKKSKSEI
>comp7089_c0_seq1; 262 141 815 LEN=878; translated
MLIASTTFVSTRRHLSRHYNFGAIEFKYFINHVNKRYDGTKTSAKLTFHEDEHQKPENHR
ACNRKYRGILKIRESSSGKGLGLFATKDFAIGDLVMSSRPVVTTKERSSHTVQIDWDRHI
LMDLPATLVNHSCDANLGIQNNSNGVRDEDDGEEYFGAYDFYAIHPIREGEELTWDYETS
EWELSTFFQCTCGSTKCRGFLRGFKQNADMIRQQNGEYHAAYLK
>comp7091_c0_seq1; 112 1 664 LEN=1193; translated
XPRETARAICCAFSFAIDTSPEALRVATPGGGIKTERDSGSGNGSLRSRLQSHSQSHSYY
YPPGLRAIEVTANLRTRWFSLPYGDQCLCLTARVFEHVGGFPDQCLMEDYXAGPVPADAV
HREPPPVVCGDAKRRGGPFEATERIAVLSGHSAVCSPRRWQKHGVLFVTYTNSRCVGRYN
DGSVSPGELFCEYYGTERPPARSGGEESPWEKGIPPPAGFA
>comp7092_c0_seq1; 432 215 1571 LEN=1732; translated
MTKNKFSTSAVALILLGFHHLYQQQPWARVYGFQFDPNIIHRTPFHRGNHRCQEIIKQRD
ALAFKGRAESNIDPYISPARVGARYPLFMVTEGDEQPPSFDEGSTSINYQLVLQNVANQA
LIGYQIWTGGTGFRLLSDQAHFGPGAIILALPXLIPLLQLSRAIETSESPFVSGLNLSTN
MAVLRIFGPRKKPIMAFLVSLLLATSTGIVEETVFRGQGGVFLVKENTYFSDLALSLTIN
IYLLLKSIALPGFANAYGNGDIFTGALLSTLLFAVLHTNPLGFFKGKDSFIDNFSLLILQ
IINGGTFCTLYLLTGNLAVPIITHALYDFYTFYKTHLVDVAGQMTYAEEEALMPICSSNG
VEDKWIQKRGEDWLKDAKQSFYLMDTNRDGELSRKELRIALYSYGITLSKIQSEEVEQAA
DIDQSGTIDFDEYLEFIGPTGSRYKAIRNTLL
>comp7092_c0_seq2; 473 215 1482 LEN=1643; translated
MTKNKFSTSAVALILLGFHHLYQQQPWARVYGFQFDPNIIHRTPFHRGNHRCQEIIKQRD
ALAFKGRAESNIDPYISPARVGARYPLFMVTEGDEQPPSFDEGSTSINYQLVLQNVANQA
LIGYQIWTGGTGFRLLSDQAHFGPGAIILALPXLIPLLQLSRAIETSESPFVSGLNLSTN
MAVLRIFGPRKKPIMAFLVSLLLATSTGIVEETVFRGQALPGFANAYGNGDIFTGALLST
LLFAVLHTNPLGFFKGKDSFIDNFSLLILQIINGGTFCTLYLLTGNLAVPIITHALYDFY
TFYKTHLVDVAGQMTYAEEEALMPICSSNGVEDKWIQKRGEDWLKDAKQSFYLMDTNRDG
ELSRKELRIALYSYGITLSKIQSEEVEQAADIDQSGTIDFDEYLEFIGPTGSRYKAIRNT
LL
>comp7093_c0_seq1; 531 606 2591 LEN=2942; translated
MESEVLIERQDEDEEQSFMEEIGEETSEQNEKTFSEEEDQEQLLIDESHMRLAIEYAKSQ
TAGNNSAYPMPTVGCAIIASDGQVLGLGCSSYKNDAIQDALEQTGLDVMPLREWCVTWPA
QAAACWKLRDDLASATLYVTLEPLASRRGETLPPLTQLIELSGVSRVVIGTPDPVPERAG
MGSQSLHRQSGLDVSMGSVLESECRELVEPYAERTNSKLQILARKHRARTGRPHGLLHCS
VVDSDDIEAFAQLGNAFGKLSGGQTLSFRDFGSYEMAPPPEQIWAEGEGEEEENLFPELE
VEDDIQVDGDDDDIDSFFRDSAETKRSNSGNVIMPWYSQVDAVIGTFPREGNNGPMKENN

TIKSRLNGLKWLSTFGRELPPGVERILVLDATDLENLPLSNDDPNLPDGVDVEQFWRGTG
RKPTRVLLRKGRSSQAEGAARAAAVAAEAAAKAALATKEAIETGEAESAAEAAMEYHSAA
LKATEMIQEELSKTLERKQQLQSFGVVVETLNGRNPIDVMEHLGKRNGYGSVVWRAGCWG
QRGVQSILDGAFQSVSAHLAVDANGGRFWQQMLAERAVQGACGPESRVKVFCSDDDVSLE
FCDSPNADGDCVLTKDGRPVRHVRLDCRVALVDRERSREFVIAPTKRLDRKVIEEEAPWF
L
>comp7093_c0_seq2; 531 606 2591 LEN=2795; translated
MESEVLIERQDEDEEQSFMEEIGEETSEQNEKTFSEEEDQEQLLIDESHMRLAIEYAKSQ
TAGNNSAYPMPTVGCAIIASDGQVLGLGCSSYKNDAIQDALEQTGLDVMPLREWCVTWPA
QAAACWKLRDDLASATLYVTLEPLASRRGETLPPLTQLIELSGVSRVVIGTPDPVPERAG
MGSQSLHRQSGLDVSMGSVLESECRELVEPYAERTNSKLQILARKHRARTGRPHGLLHCS
VVDSDDIEAFAQLGNAFGKLSGGQTLSFRDFGSYEMAPPPEQIWAEGEGEEEENLFPELE
VEDDIQVDGDDDDIDSFFRDSAETKRSNSGNVIMPWYSQVDAVIGTFPREGNNGPMKENN
TIKSRLNGLKWLSTFGRELPPGVERILVLDATDLENLPLSNDDPNLPDGVDVEQFWRGTG
RKPTRVLLRKGRSSQAEGAARAAAVAAEAAAKAALATKEAIETGEAESAAEAAMEYHSAA
LKATEMIQEELSKTLERKQQLQSFGVVVETLNGRNPIDVMEHLGKRNGYGSVVWRAGCWG
QRGVQSILDGAFQSVSAHLAVDANGGRFWQQMLAERAVQGACGPESRVKVFCSDDDVSLE
FCDSPNADGDCVLTKDGRPVRHVRLDCRVALVDRERSREFVIAPTKRLDRKVIEEEAPWF
L
>comp7099_c0_seq1; 570 157 1668 LEN=1833; translated
MAPLSTTRNQQPQPGAPASPASSETSLEEHGCLEANVLSVYDLPYPDVIPKAVTLSVRGM
FVSSGPPLARQKDRNSFRFAPVMPDKTKTQGGSNSIGASKAKTSAASAAPIKLVAPLREL
YQETLKIRVVYEDPEKCLETELDLRQLHIHEQKWLILNLAPTPRPGTTHGSSNNGSNGNA
NTSESAIVKSTATIPSVAEEDMSPPPTIRIKFKLSGPYRPEIAALVNLARTWFGVVDAIE
GKISRSSSAMGNSLPNIGINFNKNLLLVPAAPFLAVVVAAVPLLAGVAALALPFLLPIVL
VCAGLLATVALSGGLLYSSTSTGRSKLGSLLTPLVETLVVSRPGQALVYDTGPRPTPVSV
CRQIMPDTMWAKLWISLLIDAIGSSSYLLPLAGEGLDLAWAPAQTVLIMAMYESTSPNLK
YLSFIEEALPFTDIVPSACIGWACEFLPKLWKDHAERTNTHVDPEVTRAVTQLVTTAANV
VRTRNSRSSNPTAVPPATATSVY
>comp7099_c0_seq2; 492 157 1818 LEN=1983; translated
MAPLSTTRNQQPQPGAPASPASSETSLEEHGCLEANVLSVYDLPYPDVIPKAVTLSVRGM
FVSSGPPLARQKDRNSFRFAPVMPDKTKTQGGSNSIGASKAKTSAASAAPIKLVAPLREL
YQETLKIRVVYEDPEKCLETELDLRQLHIHEQKWLILNLAPTPRPGTTHGSSNNGSNGNA
NTSESAIVKSTATIPSVAEEDMSPPPTIRIKFKLSGXVSIAAAFVSHRIAFLERAVWFCF
DALPQNANFHSRVCDSFSSCTHASHHRYRPEIAALVNLARTWFGVVDAIEGKISRSSSAM
GNSLPNIGINFNKNLLLVPAAPFLAVVVAAVPLLAGVAALALPFLLPIVLVCAGLLATVA
LSGGLLYSSTSTGRSKLGSLLTPLVETLVVSRPGQALVYDTGPRPTPVSVCRQIMPDTMW
AKLWISLLIDAIGSSSYLLPLAGEGLDLAWAPAQTVLIMAMYESTSPNLKYLSFIEEALP
FTDIVPSACIGWACEFLPKLWKDHAERTNTHVDPEVTRAVTQLVTTAANVVRTRNSRSSN
PTAVPPATATSVY
>comp7100_c0_seq2; 437 207 1370 LEN=1785; translated
MRTMASTVCILFAVVLLSLSLTAVDAFSSSNRLVHNSVRQCDGRASVSSATGTRRHRLGM
ISFGGSGDESKEELPRDVKEAVSNCRQATQTALQKRISRMDIEFPVGTDFGVEKKAKPKG
GGRRRRQAGGDTDTSGGPTQSMLDRSNRELARLFVEMFQPVGGDNIVVSFADSELADLAK
ANWKNDAGASCRVLAMNRSGAAKKKKKKKGKKVLTSARGFAAKMAAEVEDNDEGTGGQGG
GSGPFQLPPNTEVALFVAPGPKELVVIDKICETVGMGTLVVLLNARLDKVTNFGTEAAEK
LFTEDFESVFSLAAAPQQEAPGCLLYRAFPGDWVLARKPSVGRPKTILSQPEKPTNEECR
ETFDNLEISDLEKNVEGFVDNVAGWFR
>comp7102_c0_seq1; 316 121 933 LEN=1288; translated
MSSSEPTKQEPNDSNKERPRSASSAWSFSSLFSSSSSFKDRFQEFDDKQRIERLSACQEL
DKILSDCRERHQQREDPSKLGINEDNGDHHLDPIESVSIGLRNMKYFGWRGILLQRKREA
NSPTDGNVEVQMVENNDDGPDLSAFHDKIRSSCAREQHAVWACRAVATGCGKDISELKRC
FESVDIFDEDGIDNSSMEPARLPQHIRVLTTPYTNYEGIIEKVADSTDGNNNDVASLRNR
IPCHDIQRRLGSCVTANGRALLERKQQRER
>comp7103_c0_seq1; 324 523 1681 LEN=1681; translated
MEGQPWASSTHGPRSALNGMDLRHQNGGDHNFLGSQVYKSNEPPFFVSQPPDTPVDRLKL
STKKNYSLRVKGYTTPNCGGAGIGLILEDSCRKETVWSARMYVPGDRTVFEAEYSAIILG
MDYSHNVLGIQRLTVFSSNHIIVNQFQGIFTVTKSNLALLLDTIXGVSTVLAKFCNARNQ
PGKKLKAFAQAVKALATRKSLNVRDRYWEVECHDPIRKIRRNPLKMGRWRKPDRPAQSAI
IDPSRIYRLQFDGGAKECLGVGMVLYDDRGTEIWCGWHFHPDAASNNVAEYMGLVCGLRC

ARSLGVAKLIVEGDSLLIVRQINERYRKRKGELVMFRREAFEVIKDLSYFQIRKISRAEN
KRADWLASHAMNIRQSDGFIRMSFSGG
>comp7105_c0_seq1; 272 1 1146 LEN=1160; translated
QQAETEQNRSPKSHQTRILIIQFIIQPATTTKMVVSREGFMFFFAVLLGTVWSTTGTTVF
GTDVLVPPATFGTRTSPSRSRFSLSTVRSDKSYGKLLLGNLSSRDTDTAVFCLRGGEISL
PDDEEEGSGIIATKLRNMIRSILDISDNKIPIFSGFLRTCFKTLETLTGMSILPEQKSKK
KDKAKSKPAKNESKKKKKASSEDGVKTKKPSKKKGAASKKSSAATQKHLSTSIKSSNPNY
RIQKELKSFIKSPPPNLSVQVGTNIRIWIVTLKGAKDTIYEGEVFKLRVEFPKDYPTVPP
SVYFLKKHMPVHEHVYTNGDICLSLLGNGWRPTMSAQSIAVSILSILSSAQSKSLPMDNA
QHATAKPGQFQKDWVYHDDRC
>comp7106_c0_seq1; 413 1 1126 LEN=1335; translated
CYLPERLDEDGGAKRDSDSYTTEKDIENALAIHDEIDGVECAEVSNLSLLAPPSELFQDE
LKAILQVESQHYKPPLNYFEFDGCRSGVNENWRRRLCEWMFEVTDHYDFDREVVSFAFDY
LDRSISLAYGPTSNKNLSKKDYQLYAVTSLYLAMKVHGEMDSDVDGKRIKLRISAFQELS
RGCFTTETIAAKELELLTLFKWHINPPTCARCIFSFLRLLPTWSSMEWDIPREDVVCQIF
DVAKYLTELSIFVSDFSFSYSPSMISYSAILCALDYVEESIKPMPFETKIRFLVSIRQIS
NAFFPESEDVRKLQVMLRKLAPKMFLKLHKYHQLPRTVSLNDVDPANSVLGKTSRSISPI
VLGGDEPKTKRQKV
>comp7107_c0_seq1; 217 127 663 LEN=714; translated
MEPATVTIRVAGLGHKLTLEGLPASTTTVDDLRARIHEATGVAPRYQKLVGPRRLVLDYR
DGESEQHHCALEGGTSLSGAGIVGRTKLLLLHSGLYAAEKDAREALEAIEGEIDALEASI
RGGSAATKKAGFVAEMVTRLCCKLDAVDPRGSGAMRAKRKELIQRAEGLEALADASKC
>comp7108_c0_seq1; 285 158 1530 LEN=1554; translated
MPSRSCFSRTIFVPYRSSIPEQRAAQPGRTTQKRKDLSIGKEFTKEIAEMTRILSFATPL
HLALGLALIDDVNANHPSTGLREEHAVASEFYSELLENDHVESDYFSPLPVEYIDTDELP
ESFDWKNVSGISYLTKSLNQHIPQYCGSCWAHAALSALADRIKIARSGAGTDINLSIQYI
LNCGTESVGSCSGGSTMKLYRFVKDGSGYVPYDTCMPYIACSAESLEGFCPFVDTTCSPL
NTCRTCDTFVENGGTCREIDIMPNATIAEYGYYRPQDYGYGDEPNTNIGTPDTIDHNNST
NLIRMVKAVKAEIFTRGPVVASINGHGLAEYVGGIYNNATASRDTTHAVSITGWGREEQT
QQDYWIVRNSWGEYWGEMGFFRIAMGQNILGIEKKIIWATPNNFTEINYPCHENGGNCGP
TMHTYSDPSLLSSDSRXAAATPEEILISAETIAKIEI
>comp7109_c0_seq1; 520 1 742 LEN=1008; translated
KDEPMEESEKACKEQSKDDDKKGTEDESNKESKIKSEPKDDSMGEADEDDKKEDKDEATE
KSKEESKDAVTNDRDKKDDAASKEKETDNAKKGAMEVEAAEKGDDKETKETQDGATPVND
DEEETEKPTSKGDAADAEEDHKNSEEKSVGGGVGEKAEQKNPTKEKEDEKKTNENVMEAD
KNEDSKKQENDEDDEEGTTEIDAKESKNDDQTKAQSQSTSDGRFEDCCDPLCNGTLQQQI
LDSNED
>comp7110_c0_seq1; 417 108 1756 LEN=1838; minus strand; translated
MVNDGGKGVTYNDRDVFFLPENLGPRLMGLSSSVGDDSSSLAYLSPALAISSDPSAVAHG
RGLIATSDISAGELLLVHPPTVRADVSQVLRIYQNKASKSSSSSCGSNLLESIAETVLLK
EMKRAIRGRTKQARNAKIAASFLALNGEHSSNNNSDNHYNDDDETTRNERHMRLARVLLG
IGTPEDTEEILSQQSLLDDNEHLLGIIRHNAFGPDFHHYHRMERELFDANHADTTTTGGN
NTTSIASLYSRVLGHYPLAAMINHSCGPTNAARVFYGETMVASATMDIPRGTELVWPYCP
PILPFPERHSRLERGYGFSCSCQRCLLEGAALEGGIPPPLPLMGATGHGGAEARLGPDEW
SSVLESVEAWYRAVTAGEPPKHSNNNSEPRSPLNLGECLRLGHTQLYIDYFNQSLAEKGA
VEVLLDQATELHRSFARVHNACTEHLSLLHLSYELEAIGGSTSKETGNGXADLDRAPQAG
TPLQVFERDSREGPGNTTDGPQAYPDRPKDEEWMESQQNTGGREFFFMRMERANAHACRR
LAQVVLQQW
>comp7111_c0_seq1; 386 177 893 LEN=1228; translated
MTLQMFQLQMTGYMFKNAEYRLSMSQSLGMSSSLSSNFMLKGVEEELPDKEDDPLVSGKI
KGKLKIRYRKRDADTDTEEVAKTTASDDVEAEDGMEIEVDAAAYMSELRDEVSRLRDELT
EKKETKDEALKQDLLLYIRTLPAQELKSLTNTMSQDVLVCMKGLVQAVLAGIGDGKIGPD
TVTEQSGEAMAQLCMWQLAVGYNLRELEVREEMKKNMMSLMGDSSADDSGLSDLGAFE
>comp7111_c0_seq2; 717 160 1647 LEN=1982; translated
MMANTPSTRHLPLCAAGRGVIPVTLVVGLLLRITTTDAFSVTAFRSASRHQWRSVWTQQT
NVVLFSSVGDGNGGGGGSNDGDDLGDFLDPMKKPDSENMKRAREYMSETSLPISFDSIIA
DDGTDAIDETVDEIADKANDVDAESKDVDLMSDQPSSSSLISSSSSSSSGSENATSSTLF
GGGGDGDGELSPPSPSLLAKNPYMQVVSKISPSDLISKFTAESDPRVQEAVRTTILGLIG
SLPKMAFETTSITTGQRLASLMFQLQMTGYMFKNAEYRLSMSQSLGMSSSLSSNFMLKGV
EEELPDKEDDPLVSGKIKGKLKIRYRKRDADTDTEEVAKTTASDDVEAEDGMEIEVDAAA

YMSELRDEVSRLRDELTEKKETKDEALKQDLLLYIRTLPAQELKSLTNTMSQDVLVCMKG
LVQAVLAGIGDGKIGPDTVTEQSGEAMAQLCMWQLAVGYNLRELEVREEMKKNMMSLMGD
SSADDSGLSDLGAFE
>comp7112_c0_seq1; 1006 82 2406 LEN=2466; translated
MSEVAALMLCALGGKSGSAADITAVLEASGAEVDADKVAAIAADMDGKDINELLAAGMEK
LKDVPMGGGGGGGGGGGGGAVAAEEEEEEEEEEVVEEYPKKKKYSRDGENPDPWQVEAL
SVPFTEDHGKWKLKKGGSDIKSWQGYFYPASQFSVKNYLFSKGVNLKFPDYLYMSKNYYD
TSWSSRTHRRLKNVIVTMDFIPSRGAVKEVAAAGKGLTKSQEKMLKRAFTSADNNKSGTI
SFAELEEVLKAVDVDVDGEEGDKFFQQMPDFANRPVSFEELKRLLEQRLYNRVQAGRYYV
ALSLFEAECMRAVMHHQLSLPLIPGKDCAVALRTDRTLLDSTYGYQRARSFQDSTAKACY
RFIDSSLNYQPREVSLLFRAIQENNKEMRYNYFVEVMSNRRRKQKDPATSALSKVFINAD
EHHVLNYKIAAGRITAMLKARGMYPRDCFNAIDRDRDGLLNYDDVKRGLAWLGLPLDQQL
LLDFMKEVDQDKDGYINLDEFKAAVGFEDDGTMASNAAAFNNGMPLPPMPSDNKEKRVVN
IPPPVLAAVKVKVKKVTKFHMIWNSQGSMSRKKISIWEPVIQSGAFRQNKESFSLGHFAG
SGYDNPSRDGKDRLCIEVTDTTGSWVGGSSWLPHVLDKFMPHPARYRLAWSVTHGSNPFY
GWEAVPPGDEFVALGFIGTKQDKQPDVKRMRCVPRDWCKPSGYLAKIWDDSGSGGRQGSI
WVFNTLNLVGFVSGSDPPAKKGHDLKSSRFFLKEYSDTNAKNVAPAGGYVNPKA
>comp7113_c0_seq2; 281 313 780 LEN=1089; translated
MVLLRLSPLIPYNALDYISGVTSISLKHYSIALLAMLPGIIMLCSIGASASSLSDRTAST
SNATVKIVTIVSGLLFGGCGVFMASYYSKLELDRILIDQSTGSSALYYVAHELSQNEAEE
IAIELGRDEESDEEVVDDNESENEEGRDSEQIHDSW
>comp7113_c0_seq3; 289 339 1256 LEN=1565; translated
MQRDRDHRRKRVVRFLVGLILLSCILCVVVDFARDRKIESALVGFLEWTREHTYEGMIAV
ILCYIVATVCFIPGSLLSFGAGFAIGSSVDTTFMGIALSSGCVFLGASIGSICSFLLGRY
LFRDCVLQLAFSYPIFQAIERALETKGLKIMVLLRLSPLIPYNALDYISGVTSISLKHYS
IALLAMLPGIIMLCSIGASASSLSDRTASTSNATVKIVTIVSGLLFGGCGVFMASYYSKL
ELDRILIDQSTGSSALYYVAHELSQNEAEEIAIELGRDEESDEEVVDDNESENEEGRDSE
QIHDSW
>comp7114_c0_seq1; 441 176 1687 LEN=1977; translated
MFNRNNRSGIDNAVRHPKSARLSKHGETSDNDFDKQAEYLIPAEVRMISGCHSLETSADV
ANIYSIAEPGKLPSPEGRAGGACTTVLLSILYDAQKKGNSGADSVTFQRLLLELRRRLAQ
TGMSQIPQLTSSRPLELKETPFSLRSKGPIFGNGRYQQQQRQGESGTQRALLVGINYYGQ
SGQLSGCINDVLNVKKYICKYQGFLEKHVLLLIDDGRHHHPTRENIIRALRRLVEHSKPG
DSVYFHYSGHGGLLDPNYWDRFKSRVSNKKYDETLYPVDHVTSGQIKDFNLFHHFVKPMA
AGVTVTCVMDCCHSGSVLDLPYSYRPTNDGTIRMRQSMDSLTNLTYLYILAGGMLPSHGF
ESIAENLETKTGEALDSIQGIGVEELSSDIDGFENVYTNSGNIFSDTTGDNIDIVGTAGD
DGNIPVYSSDNGLVCEAGSVPGQGITDGDGGNAGVFGNQNSDGSAQDFFVADDYTSADCD
CGGEDCDGCDVVGELLNTMFENS
>comp7115_c0_seq1; 1325 374 3922 LEN=4457; translated
MKSSITIFTLWAALASRDCAAWAPVGLSASRATSIDRRRNRFSSFHTKIQPSSTTSMYAT
IEDEEQEQANKPSEKDSVSEAFNTETVNEFFSPIPYSELTIGVIKETFKGENRVSQTPDS
VRGLVKAGFTVIVESGAGDNASFNDSTYIDAGAIVLSDAEQVFQDADIITKIRPPSDEEV
PRLQGKTLFSMIQPAINTELYASLTQQGTNIFALDCVPRMLSRGQSFDTLSSQANIAGYR
AIIEAADAFPRFFAGQMTAAGKVPPAKVLVMGAGVAGLAAIQTAKNMGAIVRAFDVRPVC
KEQVESMGATFLEVDIDEDGSGAGGYAKEMSDEYKAAQRKMMLEQAEDVDIIVTTALIPG
KKAPILVDQDMLSLMKAGSVCVDLAAANGGNVAQTQPDEIITTSNGVKIIGYTDLPSRLA
STASNLFANNIAKFILSIGPQTTKEKGVFQIDLEDDAVQNMLISYGGNARWPDKITPFTP
PPPPQQEVAEVVELTPEQEKALVDQRSKDAFVKNTLIASAAAALLVGFGLTADSSSSVNL
MATFGLAGLAGYQVVWGVAPALHSPLMAVTNAISGCTALGGMLLLASGDHTGASLIPDSP
AHWMGAIATVLSFVNIAGGFLVSGKMLDLFRRPEDPKEFFELYGIPVGVIVAGLAAASFS
DVGDIGLMSGTASIAASLLCISAIAGTYALNFLFSFVGPKRMPSSLNNYSFFGXELFLLI
LHIKTALANQETARTGNFLGMAGVTLGLAATTSDMAIAGASPVVFQQAGLLAGTGAAVGT
AVASKVGPTELPQTVAAFHSLVGIAAMAGAAGEYLGNSGALPAGTLSSIYLATFIGGVTF
SGSLVAFGKLSGMMDSAPLKLPARDQINLAMLGACALGMGAFLGPDIAASLLSMDADTVG
LASLGMAAVVSSVLGWHLTASIGGADMPVVITVLNSYSGWALCAEGFLLNNPLLAQVGAL
IGFSGAILTWIMCEAMGRDVVSVILGGAGSDTVASAGEAVEYDGEVTTVTVEAVTEALTE
ANNIVITPGYGLAVAQAQFAIADIAKNLKAAGKNVRFAIHPVAGRMPGQLNVLLAEAGVP
YDIVFEMEEINDDFDDTDVTLVIGASDTVSSAAEDDPNCSIYGMPVLRVWKSGQVFVLKR
SIGNTGYAGMMNPILFKENIDVLLGDAKDTCDALKSAVSAKL
>comp7115_c0_seq2; 1407 374 3802 LEN=4337; translated

MKSSITIFTLWAALASRDCAAWAPVGLSASRATSIDRRRNRFSSFHTKIQPSSTTSMYAT
IEDEEQEQANKPSEKDSVSEAFNTETVNEFFSPIPYSELTIGVIKETFKGENRVSQTPDS
VRGLVKAGFTVIVESGAGDNASFNDSTYIDAGAIVLSDAEQVFQDADIITKIRPPSDEEV
PRLQGKTLFSMIQPAINTELYASLTQQGTNIFALDCVPRMLSRGQSFDTLSSQANIAGYR
AIIEAADAFPRFFAGQMTAAGKVPPAKVLVMGAGVAGLAAIQTAKNMGAIVRAFDVRPVC
KEQVESMGATFLEVDIDEDGSGAGGYAKEMSDEYKAAQRKMMLEQAEDVDIIVTTALIPG
KKAPILVDQDMLSLMKAGSVCVDLAAANGGNVAQTQPDEIITTSNGVKIIGYTDLPSRLA
STASNLFANNIAKFILSIGPQTTKEKGVFQIDLEDDAVQNMLISYGGNARWPDKITPFTP
PPPPQQEVAEVVELTPEQEKALVDQRSKDAFVKNTLIASAAAALLVGFGLTADSSSSVNL
MATFGLAGLAGYQVVWGVAPALHSPLMAVTNAISGCTALGGMLLLASGDHTGASLIPDSP
AHWMGAIATVLSFVNIAGGFLVSGKMLDLFRRPEDPKEFFELYGIPVGVIVAGLAAASFS
DVGDIGLMSGTASIAASLLCISAIAALANQETARTGNFLGMAGVTLGLAATTSDMAIAGA
SPVVFQQAGLLAGTGAAVGTAVASKVGPTELPQTVAAFHSLVGIAAMAGAAGEYLGNSGA
LPAGTLSSIYLATFIGGVTFSGSLVAFGKLSGMMDSAPLKLPARDQINLAMLGACALGMG
AFLGPDIAASLLSMDADTVGLASLGMAAVVSSVLGWHLTASIGGADMPVVITVLNSYSGW
ALCAEGFLLNNPLLAQVGALIGFSGAILTWIMCEAMGRDVVSVILGGAGSDTVASAGEAV
EYDGEVTTVTVEAVTEALTEANNIVITPGYGLAVAQAQFAIADIAKNLKAAGKNVRFAIH
PVAGRMPGQLNVLLAEAGVPYDIVFEMEEINDDFDDTDVTLVIGASDTVSSAAEDDPNCS
IYGMPVLRVWKSGQVFVLKRSIGNTGYAGMMNPILFKENIDVLLGDAKDTCDALKSAVSA
KL
>comp7117_c0_seq1; 133 173 853 LEN=1336; translated
MRKQVQPSSSCERDQQPARRPSSPVSPQRPLPPWPVVSPAPYASPAPHDRRACPGAPVQQ
KKSRSRIFAFGERDYGEHEASPLRPRRLFFGDDGDGCGGQERSRPSDREATGEDVDCGNF
FVRRFEASIESPRSNTGTLGSSLAPTSASTPSATSASPSRPTTTSRRIISPQRGSNRENT
IDERNTNAEYALGQEPGTTLAGQGITGRSRSISNTSTNTSDARKRT
>comp7118_c0_seq1; 399 328 2073 LEN=2529; translated
MAGAFRHSTLTIAKIPRRSQSSLALSMTNNTCSKQIHGNSFRNSLPRTHNAYGIGHNNFH
STSTCRSPENKPSAAFPHLRSPTSEHARELFLLMNSRPGSLLTTLLDCTKESGDANAESL
EKYNVDWTGHYRGFSSIVVRPNSTDEVSRILKYCNEHLVGIVPQGGNTGLVGGSIPISSK
EIVLSLEKMNTIEDDGRESNVLRAQAGCILQDLQDHANKNGSLVPVDLGAKGTCQIGGNV
STNAGGVYYYRYGSLHANVLGLEVVTPTGDVLNLGYSPVSHLKDNTGYDLKHMFIGSEGT
LGVVTKVALRCQPSPVSRGAIWLSCNSLQDVVKVLNIARTERLSEILAAFEFMDGDVLEL
VRQTHPFIRFPLLNAGDTDAPMQPYSILVETHGSHDQHDREKLESFLECIFEEGLVTDGV
VAQNLGQIEDFWKIRELCNPSSAASGYVYKYDVSLKARDFEGFIRDMKLRLGDVSHASSS
ADHHLLCVNWGHIIDGNLHCNIVSGGKFEKDTGLEKFINECVFQEVMKRNGSISAEHGLG
QYKHNYMPQIKDPPTLQAMYDVRSLFDPNGIMNPGKYLPST
>comp7118_c0_seq2; 48 1 212 LEN=1046; minus strand; translated
XGKKIDPDEIFPEVHSCDLEAIFGRRKSKNYRSRASSGNWANDQVTAAEKLVYKRQMGFA
TDHAMDSTGV
>comp7119_c0_seq1; 656 138 2189 LEN=2313; translated
MAPFVKLRKTKLPEDQHHPSEYRGIFGEKGDSDDWNEAVRDFPNKVPRFESQDDCLQFMK
EYVRHHGGSNPGFPHLKHIFTTLITWDQIEQILIPAITKARKEPSQAKKVPQDYGRTPDD
NSRNIYERSGGKEVVDAINFRLNLPFHKATTPVSVLNTLKYLFFHMKCGIYVMIRDGKVR
IFCPFSNRDYRNTWSGRLKIEGGGSLEEYYAKKKGICRKENIEEDLSVWWANGNIICNEL
SSNKNETKYQIQVWGDQFLSPLKDMLAEACRERQIPDCEFFLNKRDYPQLKVNVERGIPV
EPYGFIFDKDDRDPAQDVDLVDEHKFKTYAPIVSFYAAAEDRFSDIPWPSSEDWEGACGL
VFPQTFLYKEVDEEGYPVFKPPRDLFTEQNFQKFKRGWYEGRVATAFFRGTATGGGTTVD
NNQRLKVASLSHEWKDDEEKGGDEPFLDAAIVGWNLRDKKEAAHPMTFLRPSDFKFTAGR
HHFTPIYEQSRYKYLVYVDGHCAACRYAFMMRLGSVILKVEPRQVADRMWYFPLLQPYVD
HIPIKSDLSDLEEKIRWCREHDEECREIAENCLKLYDKYVARAGLLDYVEMVTKHIAKRH
IEAPGWWEAPPPALPPPKLNKPTTLCYEHKGTGESRLCVRCQEDADKEQKELEATQKLAA
SEKKSKKQRKQALRERMLKRSRK
>comp7121_c0_seq1; 585 1 1103 LEN=1351; translated
XLSAGVVEASCSACRGRQVLHSCGRRSLPIDYEEIARAEREKKEKEEEEKKRVRAEKRRL
NDQKRREAKRQKQREAEEQRRREEEQRKEREEQARLQEAFASQDLHRHKRKEALVSYQAA
HQSSTDSSREAVLSSPPQSVSNGHKQGAVGAAAAAAAATAFRHYLPSHQKPAAAFPEEYL
SGYAAPVQSQAAAGANFQATYQAGNHLLAQQQQQPPETTAYPATDHPVARVSDAPSVASS
EQAAFSRPTHSSEADLLLVFAGHNTAKTPTAATATPAALQTATAGLGAPNHGMEGGPAST
AANQTYPSLSLFSASHGFDAVSTTIEDDQALAHVRKGIPSYASLRGQGDGTGGFWAASAP
GANGTGR

>comp7122_c0_seq1; 744 363 2423 LEN=2866; translated
MTSTEWSQVDPSIQALAPHASEELFGGDFLGDELMDIYNAAVVGGGDENDDDLSKEMSSL
LDTPTDAKKPEAIDDGFGAFRPSTSFNNLSTALLDPNPESATLSIVPTVEPSNDVPPGSV
STRVISSSSLEHSLVISPPQPLVAPTSSMVTYVTTPGGTKRKKTVAANAGPDLKRKSVSG
SKQKKNVQKGAATKKVTKKTSCNPRKVTISSPDLATTSVVPRTTVLAPTKPISPSVSVHA
PAASIKQESGPIATSAQSNSASQSKASAAPQAVSSSAPVAAPAASVLSNPPTEADFKSVA
QAAVNNLIMNAGSNNTATKSKNEKTTETRPNSVPVPSATSIPVVTPTIRVDTSTEHVKAL
TGSNWVAVCGGGITSTDSNNQNNSDKLNRARRSNLTPDERARQNRDRNREHARNTRLRKK
AYVEELKATLSALVTQRDAAELEKRHASQRELEQREVRFRVLEEFLKLRGRNEANAARWA
AILEENFTLTMPFVNFRDMVHSNDGSPRYEQVLTGVGEVMNDSTLFSNFLQNFCKLGNNE
RITCVYNCDRSNFFMDNCVAMMDWTAATHGVSGQRVELLLKGQIRAKFSPASNKLISVIM
SFDTGAINSQLNLIAQAKQSSEADAAAAAAQVAANEADALLDSLEMPHFEPPNSGFISSR
SNVVPPSSGSSASSNDDKAVISSDDE
>comp7122_c0_seq2; 744 589 2649 LEN=3092; translated
MTSTEWSQVDPSIQALAPHASEELFGGDFLGDELMDIYNAAVVGGGDENDDDLSKEMSSL
LDTPTDAKKPEAIDDGFGAFRPSTSFNNLSTALLDPNPESATLSIVPTVEPSNDVPPGSV
STRVISSSSLEHSLVISPPQPLVAPTSSMVTYVTTPGGTKRKKTVAANAGPDLKRKSVSG
SKQKKNVQKGAATKKVTKKTSCNPRKVTISSPDLATTSVVPRTTVLAPTKPISPSVSVHA
PAASIKQESGPIATSAQSNSASQSKASAAPQAVSSSAPVAAPAASVLSNPPTEADFKSVA
QAAVNNLIMNAGSNNTATKSKNEKTTETRPNSVPVPSATSIPVVTPTIRVDTSTEHVKAL
TGSNWVAVCGGGITSTDSNNQNNSDKLNRARRSNLTPDERARQNRDRNREHARNTRLRKK
AYVEELKATLSALVTQRDAAELEKRHASQRELEQREVRFRVLEEFLKLRGRNEANAARWA
AILEENFTLTMPFVNFRDMVHSNDGSPRYEQVLTGVGEVMNDSTLFSNFLQNFCKLGNNE
RITCVYNCDRSNFFMDNCVAMMDWTAATHGVSGQRVELLLKGQIRAKFSPASNKLISVIM
SFDTGAINSQLNLIAQAKQSSEADAAAAAAQVAANEADALLDSLEMPHFEPPNSGFISSR
SNVVPPSSGSSASSNDDKAVISSDDE
>comp7122_c0_seq3; 744 325 2385 LEN=2828; translated
MTSTEWSQVDPSIQALAPHASEELFGGDFLGDELMDIYNAAVVGGGDENDDDLSKEMSSL
LDTPTDAKKPEAIDDGFGAFRPSTSFNNLSTALLDPNPESATLSIVPTVEPSNDVPPGSV
STRVISSSSLEHSLVISPPQPLVAPTSSMVTYVTTPGGTKRKKTVAANAGPDLKRKSVSG
SKQKKNVQKGAATKKVTKKTSCNPRKVTISSPDLATTSVVPRTTVLAPTKPISPSVSVHA
PAASIKQESGPIATSAQSNSASQSKASAAPQAVSSSAPVAAPAASVLSNPPTEADFKSVA
QAAVNNLIMNAGSNNTATKSKNEKTTETRPNSVPVPSATSIPVVTPTIRVDTSTEHVKAL
TGSNWVAVCGGGITSTDSNNQNNSDKLNRARRSNLTPDERARQNRDRNREHARNTRLRKK
AYVEELKATLSALVTQRDAAELEKRHASQRELEQREVRFRVLEEFLKLRGRNEANAARWA
AILEENFTLTMPFVNFRDMVHSNDGSPRYEQVLTGVGEVMNDSTLFSNFLQNFCKLGNNE
RITCVYNCDRSNFFMDNCVAMMDWTAATHGVSGQRVELLLKGQIRAKFSPASNKLISVIM
SFDTGAINSQLNLIAQAKQSSEADAAAAAAQVAANEADALLDSLEMPHFEPPNSGFISSR
SNVVPPSSGSSASSNDDKAVISSDDE
>comp7122_c0_seq4; 598 65 1957 LEN=2400; translated
MSSLLDTPTDAKKPEAIDDGFGAFRPSTSFNNLSTALLDPNPESATLSIVPTVEPSNDVP
PGSVSTRVISSSSLEHSLVISPPQPLVAPTSSMVTYVTTPGGTKRKKTVAANAGPDLKRK
SVSGSKQKKNVQKGAATKKVTKKTSCNPRKVTISSPDLATTSVVPRTTVLAPTKPISPSV
SVHAPAASIKQESGPIATSAQSNSASQSKASAAPQAVSSSAPVAAPAASVLSNPPTEADF
KSVAQAAVNNLIMNAGSNNTATKSKNEKTTETRPNSVPVPSATSIPVVTPTIRVDTSTEH
VKALTGSNWVAVCGGGITSTDSNNQNNSDKLNRARRSNLTPDERARQNRDRNREHARNTR
LRKKAYVEELKATLSALVTQRDAAELEKRHASQRELEQREVRFRVLEEFLKLRGRNEANA
ARWAAILEENFTLTMPFVNFRDMVHSNDGSPRYEQVLTGVGEVMNDSTLFSNFLQNFCKL
GNNERITCVYNCDRSNFFMDNCVAMMDWTAATHGVSGQRVELLLKGQIRAKFSPASNKLI
SVIMSFDTGAINSQLNLIAQAKQSSEADAAAAAAQVAANEADALLDSLEMPHFEPPNSGF
ISSRSNVVPPSSGSSASSNDDKAVISSDDE
>comp7125_c0_seq1; 718 107 1396 LEN=1553; translated
MNAATTYVVKATVLLCIGRHVCEGFTTTPPMTHRQQHQNEAISGEISRRVNTVALSTTPP
PTATRMALRSSFYDDFEDFEMNKDGEDDDDDDEDEYAEIDERSIADFKSKMSNLFDDSGG
DGDKSAEEGMGTIDDLIDFARKQQAEEDSASASAVPAAAPGDWAKATTEIGPGTVLVANP
SKFCDEGDYGRMSTDANEPSGGGKDFFASALGRTGPAPSLLAKFGLTQPPPRHLGADRQA
DLLPVVVVVVSTEGGQVQGVLLNRRTGYLLGDLEQPSADNGDSNEDMAPILEKFCIQPLWF
GGIDSLSVGLDMLHLCPTVKDATKITEDNVYWGGDPAQAQDAMEDPSLDRAYSGFDFKFF
VQSTIWSNAGSLQSEVDDKIWFPAKVSQNIIFKSRDRMGTKKAQPLWTEIMGLMGDEYGE
IMKNFYATE

>comp7125_c0_seq2; 346 144 548 LEN=548; translated
MGRKKGGRGKKNRGDDDWSDQEEIPVGGTPNATEEPAAPKLSRKQQKLAKQQAKKAAAQK
QQQQKDSDSDSDDLAALMGGKMNLGKKKSGNYSDDDDDDEDSSDDEMLPPMPSKRNNDRG
RGRNNRGPPPPMDDS
>comp7127_c0_seq1; 440 169 1638 LEN=1862; translated
MPSSTTDSNNNTTEGSLRSNTVRSFKVMDVLRGANELQQRLDREYASAVASGKSPEAPEK
VLHLEVGQPESGAPKTVADAAVEALTMPRAKTNVMGYTDAFGLLDLRQRIRQHYVEKYSN
SDDRTEQQQQSLCVPDVSQIVVTTGSSGAFLLAFTGCFDPSDTVAIASSGYPCYRNILQA
LGCEIATVPINDEYKITATELSQEIRDRLADGRPSIKGLILSSPSNPTGAMLTPKELKEL
CDLCNKEKIVFMSDEIYHGISYGNVPEATALQYAAADTEHPVNENIVVINSFSKYYSMPG
WRLGWMVVPPRFVQAINKLQQNMFINAPTVSQTAALRCWDEETIQELETHVTKYRANREV
ILARFESSSRSPGGEKQQVLKGLTLEKNVAPADGGFYVYVDLGEDNICINENANLDSVSM
CEELLFRYYVAFTPGIDFEDPNCPKNMGRRRFRISYAGGTENIRKAMDKFTEFWNSFWLV
EIANAKNLV
>comp7128_c0_seq1; 625 30 1574 LEN=1988; translated
MAPDHSDRESTTWIKKVQYVVSLTLLGLCVALCTAGIVANQTPVAAASNSAVALVLMWVL
ILWLGILEGGQGSLVGLQPVNSEKFKFSHPYAFACTELAHKGDNLNRFIVGRQFLVVLVV
FLLNMACTVVDDFDVPGVPHALMSSMVASGLAVMIITVVVGQLAAEVNATNCMLDFIDNR
LVWVTTLICLMIETSGLLHSVYLVQCFFSNESMSKESTDKTFQQKVWYWGRVAMSVSLLT
VAMAVTCAALWHDETTMYPGLTTMASFALFFGLVCFLGLLEGIQIAAFAVVKLPTHIVRA
SPAAGANCDLVFRGKNFKAVLIGRQICVTTSMFLLARITTTNVDPNDLIDTSATDSTVLG
MPACIQNFFNTGLPGALITTVVASLWWRILAAAFPIAFMGNSVVYWTIRLCLCLEATGVF
SSAWLMADGIKWMLRFQTDDVYFSDGDKDIDLELNKYGVDDSQTLLSVSTGKNDSESTFL
TSSSSNGNFSTYGSNSSQEFDTFSNPSPSPSLEF
>comp7128_c0_seq2; 284 1 675 LEN=1089; translated
IRIHIRCCDACQQEHANCDLVFRGKNFKAVLIGRQICVTTSMFLLARITTTNVDPNDLID
TSATDSTVLGMPACIQNFFNTGLPGALITTVVASLWWRILAAAFPIAFMGNSVVYWTIRL
CLCLEATGVFSSAWLMADGIKWMLRFQTDDVYFSDGDKDIDLELNKYGVDDSQTLLSVST
GKNDSESTFLTSSSSNGNFSTYGSNSSQEFDTFSNPSPSPSLEF
>comp7132_c0_seq1; 1049 147 2819 LEN=2830; translated
MANIAPTSAANYRSVTPAMNNVRRGVGASSGGTPNSMNQMRYTDSFNPIDTPQQQSFNQS
QSNHGNLTPSSTGLLRLTLRKPMGIVFEPMYDPNTPSIQRGVRICDLPRTGAAALSRKLE
VGDELLSINDKTMSRLTFDEIMDFIIEADPEEVNLLFRRPRKETLHARQGINLNKASNPN
GPGVKWIDDEERNVSGGKGAKPETSTMSSRSGGRKDRKSSRRSGNGSSSRNHDDETLQSE
DNDYSRSGTGRSSRGRKGRRRKDPYESESFLDILIDTICANPDSVCRDRRRGDDDYYSDD
DETFASRDDSTYVTYESEDRRELKKKMKSKQSKGSKNRDDDSSEYGDETEEDTLDDKRGS
ANSSSKREKGSARLSPPSEGLKSPSDMMKKKKSSGKYEDDGTLETVDSVERNKAMEAALA
PDPTPPLGLAPNPEPAAGPVLPSDTDDDGNPAPIKTVEYDDMDNGADVSVMESLGGPSLL
IEKQRQAAAVVATKVAVPPVPADIIENFGKDYPLDFGFSREQTIQNDPLRFYTFVVKGLL
QEHEPEKVRLLEKLLAKYRGREDHLVQKLSVRYNKEEEEEQDQHEHGLGAARSFEDAVNQ
GKGSAHANATIQTARERLQNDGFQTDQWPEKETKEQDDEDDKQPEMQSESGTEFSGDSID
GTSPAVIAQVSELLNYVYGKTSVPGQIDRVSTIMRAYEGREAVLLELLETKALIKANREK
ENAGQLPSFLRNSANAQRNAGNEMSAADGDVPPVTPMATQAAINDDISSMSGVSSPAEGQ
QHAVNAGVLKQINGHDIAPHSAPGTDGNAGHEVEFPRATSSPQKLKGARAPVPEKGDAKK
KKKGIFGGLFGGKKGKKSKDADKSERTASRTRGLRSPMGRKLKNPTETSA
>comp7134_c0_seq1; 421 34 1089 LEN=1257; translated
MVRFSQLCLALLAVDCSVEAFNVLVIGGTRFSGAYLWKSLYDEGHDVTVFNRGKTDPRPV
ARESDVDFQARLAASTAIKGDRKDPESLKSSIDPSKYDYVFDMNAREVTDTKPLAELFVN
QPNFKQYVFMSSAGVYLKSDEMPHLERDPVDPNSRHKGKLDSEDCLRKLNIPWCSFRPTY
ICGPQNYNPVERYFFERADGDRPVCIPGHGQHLTGLGHVEDLATAMTNVIGREEKTTGKV
YNLQNSQAISFDGVAKAAGKVLGKDVEIIHYDPASISFPEGKKAFPMRPQHFFTNIHQAM
TDLDWSPKYDTVESIMKDSYENDFVHIKAAGGLKNDFICDDIVAEHAKAMA
>comp7136_c0_seq1; 860 1 1952 LEN=2422; translated
XKNKLEDKKYSSRKAQFCLRIIHSYHSHNLTTATMRFNQSVVSVLGLFLASAGCANAFTP
LNLVSHAGRFATSLEASKMEKLPESCVRVTLEVPGPATKAAYEKVCTELSKTVALPGFRK
GSKIPPQVLEQAMSAKGGRNALKTQAINTLLSELIESALKEEHGLEPIGQPSLETAAEEL
ASGFVPGEDFEMAVKCDVWPDIQWKKVEGAVDPEKPYMGLKGTYTRKPFDKSKMDKALTD
LKERYATLDKIEDGEYELQMGDACTVNMEGYMANAEGGKGEKLPDAASGDNVEVILGEGR
YMTGLVEGLLGAKVSQTREITVTFPDKLRDKTLAGKKAVFDVTVLEAQKRNIPEITDEFA
EKVRAGLTAESLIEELQTAVDQEDAKEFTPARNSALAKALSQVMDVEVPDTLVTNQARDK

FALMMSDMRNNGVADEEIKKQISPENFLKYKKIVKDDIVADFKVSMATDEIARIEGIEIE
DYMVEEQMESIKQEMQESDDDFDEAGIRARVEATLQRQSVMDFLAENADLDVKFVEGEGD
FDSSLMEKLAEESLAREQEMESQAPKETGESPATPPDVEAVAEEEVPAAEEVVEEAPVDE
AETETVEEESDPASDSDSVAEERDVSSMSLQDQAFYALMDSGALNRDDTK
>comp7136_c0_seq2; 448 122 1105 LEN=1575; translated
ESQKLRDKTLAGKKAVFDVTVLEAQKRNIPEITDEFAEKVRAGLTAESLIEELQTAVDQE
DAKEFTPARNSALAKALSQVMDVEVPDTLVTNQARDKFALMMSDMRNNGVADEEIKKQIS
PENFLKYKKIVKDDIVADFKVSMATDEIARIEGIEIEDYMVEEQMESIKQEMQESDDDFD
EAGIRARVEATLQRQSVMDFLAENADLDVKFVEGEGDFDSSLMEKLAEESLAREQEMESQ
APKETGESPATPPDVEAVAEEEVPAAEEVVEEAPVDEAETETVEEESDPASDSDSVAEER
DVSSMSLQDQAFYALMDSGALNRDDTK
>comp7139_c0_seq1; 461 1 2124 LEN=2224; translated
RPEESSRVILLSGPPGVGKTTLAHIVARHAGYRPVEVNASDERSSGVLKDRIQRAMESTT
INFKPGNTGADGNNAADYGKPNCLILDEIDGADAKGAIQGLVDIVKAEIPSKSSKQKSGT
TYLRRPIICICNHKYAPTLRPLLPYATHFSVEPPSPARLVVRLKAILNQEKMAMMGGGPL
LHQLVVSSGGDIRSCLFTLQFAAAEAKNSTDLSQALAKSLSGTGLKDDRNDITSTISTVF
RKTKPKRARFSSSKLLVGSSSSSRDSENRASVSRVMAAVEGLGDNSAAINALFLNVPRVS
YIDPAFDRCSAALELISFAVDANQQQSGYALPVVTAGIHLLCRVEVKPDLTFSSRELADM
KYSRDSNLGLVQRFSEGLPPGAKNLKCRALLSEEFIPLTMWILSAGEGACSLSRPASSID
ILTSPEQEAAKRHVSSLCALGLTYVVDREKEETKERVSSFRASVREMRMEPPIHRLVEYS
SQIPPGRASRNEIPAQLKELLAHQVLLEGFRNRESPRASGSPVKKKAGIAAAESSKTPVT
NGAEPTTTTEETSVVTPAKVTPRKLDQWSRPTGLTLTESKEVKVCQAFLAVLXKHGSGEF
FGVKNPGGKSYFFDFSFIFVTLLPSPHFFQASPSSTPGNFLGIAARKGKAAKSARRAARL
GLNRASSKKQKLAHSGSGFLLSHVVRMKYVKGFTQAVRTPCRLSDLE
>comp7139_c0_seq2; 485 1 1983 LEN=2083; translated
RPEESSRVILLSGPPGVGKTTLAHIVARHAGYRPVEVNASDERSSGVLKDRIQRAMESTT
INFKPGNTGADGNNAADYGKPNCLILDEIDGADAKGAIQGLVDIVKAEIPSKSSKQKSGT
TYLRRPIICICNHKYAPTLRPLLPYATHFSVEPPSPARLVVRLKAILNQEKMAMMGGGPL
LHQLVVSSGGDIRSCLFTLQFAAAEAKNSTDLSQALAKSLSGTGLKDDRNDITSTISTVF
RKTKPKRARFSSSKLLVGSSSSSRDSENRASVSRVMAAVEGLGDNSAAINALFLNVPRVS
YIDPAFDRCSAALELISFAVDANQQQSGYALPVVTAGIHLLCRVEVKPDLTFSSRELADM
KYSRDSNLGLVQRFSEGLPPGAKNLKCRALLSEEFIPLTMWILSAGEGACSLSRPASSID
ILTSPEQEAAKRHVSSLCALGLTYVVDREKEETKERVSSFRASVREMRMEPPIHRLVEYS
SQIPPGRASRNEIPAQLKELLAHQVLLEGFRNRESPRASGSPVKKKAGIAAAESSKTPVT
NGAEPTTTTEETSVVTPAKVTPRKLDQWSRPTGLTLTESKEVKASPSSTPGNFLGIAARK
GKAAKSARRAARLGLNRASSKKQKLAHSGSGFLLSHVVRMKYVKGFTQAVRTPCRLSDLE
>comp7140_c0_seq1; 265 898 1503 LEN=1603; translated
MAIANQTRISDQSIVHTDDSTKTDSVSNKTDRKSYKRPLRCSGSSPFKCTNMPYLPHSSR
DLSLDTITEFPYLEDKFSMSLPCSLFSSSFDEGNSQKVRKSHHNKTRSLDFVMDAISIVG
IDEREELQSTVSVSSPTPFTRSRSPLSPPSLASVSDDESVDSSRKDEERSILRESRWDDC
QSRDFVVRKCDSAPTCPRRAQ
>comp7141_c0_seq1; 265 1 1715 LEN=2039; translated
RYFTNPNKHSLAGAVLQFKLEFFLGTIFPAKYSSVARKAHWCVENAPMDHYDSGGKXKVK
RLIVIEDQNGSAKPKNGWDSHNMPPWLATFLFAVTLGLFELFRNLHQMEYISFHGEDGMS
TLLDQPLVTSNRNNTAKTTIAYAVSITHFEVDQNKNEPHNVFDRAAVLHQSIKVAMMKSP
RYDYHIYAFVHPDAVGAKPLLQRLGYRVQIRDTPFNISKVSNPELVEAQGNGCCSEREYL
KLYSYLLSDYPVAVHMDLDTIVLRSMDDVFDMMMLKTGEGPSTSQQSELFSRKSTMWTSN
PQNNIKSTHSSKRVLENPENINFMFTRDYNMVDPSDPPYKQPYQIGVQGGFLVIRPNQRD
FDRMIEIILSGGEEYTIGDGWGGSKLGYGGYYGAATIQGLASFYYDYHEKAQRSIELNKC
RYNTMVDNPYXFDKGSNKTLCRTLESECEDCRKTKLEDVYSAHFTVCGKPEWCDTLDPDK
DRLCSQLMKKWHQTRLSLELEWMNRFSSSKNGNDKANLMYVPQLNGVDPSESRGKLLESN
KLGHCDGENYIPLQYPAMATTSLHSDEAPLI
>comp7142_c0_seq1; 152 139 861 LEN=1175; translated
MTVGSESPLIGGASLSDTPSRTLFTPQTARVPKTSYAGFETPTRQPRTPFVFLSDRKSVP
TELKNLQERTIKRRRDFVARMHELDCHTAKLMSKYAEERMDLDLATCDTFERTIIHPLLS
STERLSLDRQASMNRSVGIPALERRVGAIDAQMTHHINVIMSDAKMDKLDSIHDELQRSV
KPEIRIENNKSNKIEAGIVQRFESVVGTINKSYRSEWASRYVFLYFRALPFDFSSPLLTI
>comp7142_c0_seq2; 204 139 1041 LEN=1088; translated
MTVGSESPLIGGASLSDTPSRTLFTPQTARVPKTSYAGFETPTRQPRTPFVFLSDRKSVP
TELKNLQERTIKRRRDFVARMHELDCHTAKLMSKYAEERMDLDLATCDTFERTIIHPLLS

STERLSLDRQASMNRSVGIPALERRVGAIDAQMTHHINVIMSDAKMDKLDSIHDELQRSV
KPEIRIENNKSNKIEAGIVQRFESVVGTINKSYRSEWASSRAEIEVLQRKTETGVPNRKE
RVEVTLAKISELRARVREERAKRIAADQAIYEDIVRKTSAMKRAMIAMASDGDSTSASSS
>comp7142_c0_seq3; 192 430 1064 LEN=1378; translated
MXNTLASIQILVANEVYEDGDVKXTWDLFLERMNILLSCHRSSHYVQSAFLHDRKDCHTA
KLMSKYAEERMDLDLATCDTFERTIIHPLLSSTERLSLDRQASMNRSVGIPALERRVGAI
DAQMTHHINVIMSDAKMDKLDSIHDELQRSVKPEIRIENNKSNKIEAGIVQRFESVVGTI
NKSYRSEWASRYVFLYFRALPFDFSSPLLTI
>comp7142_c0_seq4; 244 430 1244 LEN=1291; translated
MXNTLASIQILVANEVYEDGDVKXTWDLFLERMNILLSCHRSSHYVQSAFLHDRKDCHTA
KLMSKYAEERMDLDLATCDTFERTIIHPLLSSTERLSLDRQASMNRSVGIPALERRVGAI
DAQMTHHINVIMSDAKMDKLDSIHDELQRSVKPEIRIENNKSNKIEAGIVQRFESVVGTI
NKSYRSEWASSRAEIEVLQRKTETGVPNRKERVEVTLAKISELRARVREERAKRIAADQA
IYEDIVRKTSAMKRAMIAMASDGDSTSASSS
>comp7144_c0_seq1; 517 192 1592 LEN=1920; translated
MQLRRNSEPHTRNRDRKGVPLAPIYENEHTRDLDEMRFVPLRNFMSETILMQARQWNSSI
SNDAASEDSYHFNGNSADADSGVDDRNKDNTSKKAELPLRIIVDNAKSYIQPKRWQRCAS
AHSVMMLADNRWEDSDSSSNHGPLTSSSSLSSSSLASSMQPRSDSSSVSPISKTPLKSCL
KKSSKFSSNPASSSSSLSPRFSSLIPASTSESSTNFSINNSNRSVPHSPPPPKAEKRWGN
GLSSSSLSNRFKTNLKCKSSAKKSGDFEIPLNDDGERSDDSECSVQSFCSTDEKRTTTEI
ITQAEAVVGSTNYTRYNYPETNYPPSLDPNFSVSKSIDRKEGAPVRPERVPSADTFCNEG
REVRAKQRKAKNPLGRRNFQWDTPTTAGVPVNTSANRCSSTTISARRNLLRGTGLSPGIR
RVMKTSFDDRNSRKQPMADVVKTNTTHTMQSMRSPGNSASASSTRR
>comp7145_c0_seq1; 523 178 1665 LEN=2130; translated
MDIEWVIYHLTHNDDEDFTSLVDLDIDLCRSDQDQWDRLLIGLSRNRTVTSVELLRGYQG
SVVNEDDLRRLFEALRLIPRLSKVKLDCFTTFDLEHSELLFRNNSTIEELWVENCHFHRD
NTGSSSDEDNDIDNDHDGKDDDEGYNQYERFLGHLASMHRLRQLRIEMPMQLSRDAPLAH
LLRCSSELRSVVLETSTARTVQMIRNATRRQNFADTMVALRSNETLETFDMDFGISFSEF
KEVAAMLKHNNTLKELCLRLDPSTAIINSIGEDSGAYLSDFHNDFNDSVQSLFEALKTNT
NSSLRKFRQYNMEDRNLEFTLSKEGDNNSNRKKIVQNLIEIGLDMLECNLSLEFFSFFLF
DLDFYRGGGSLREKKQLFLRLNQRGRKSVLHPSIRERVSKVSFIEQLAKHSIDDLDGLFY
YISMNPSICKPPTEDAVCSAITSVISRQNGCDPKITGDWASSISLESGLVEDQQVVPQTK
RCRFDRISQDQVTKP
>comp7146_c0_seq1; 292 165 1145 LEN=1464; translated
MDKENSRFHQPTTASRPWNAHHQKHTHSFDKPSPLSGISDGFSPDDEMAQRARQGKKWAL
NDFEIGKPLGRGKFGSVYLAREKQSKYIVAIKVLYKSQLLKAGVEHQLRREIEIQSHLRH
SNILRMYGYFYDSKRIYLILEYSPGGELYKRLVQKVHFSERSSARYISDLATALDFCHTK
HVIHRDIKPENLLLGAHGEIKIADFGWSVHAPTSRRNTLCGTLDYLPPEMVEGREHDKFV
DVWSLGVLLFEFLYGGPPFEAEGHSATYRRISRVDLRFPAKPEVSEDAKDLIRKFLVKDP
HLRIPLKDVPKHPWVVRMLRETKTSY
>comp7147_c0_seq1; 230 1 583 LEN=885; translated
XTRSAPTIPCFRFDFDTRTVRTLPITAESTPQERQQQHAMTRYSCGLVCALAMVTPDVAS
CFAPQPASLSRPTAMVAGTTLFAEGESESEGQFNWLEEWAMQGKDAVALMKVQERTQRVM
LAQMTEDRIYEITKLLDAMVDEATGEISESDLPKAKELAVQTRNLQKEYKDLVTGEPSTL
LDTFQNLKLGEEDE
>comp7149_c0_seq1; 554 1 1976 LEN=2169; translated
XECAALTLFQRFDTPDIIHAIQLLTATKASRRNPATSPSPATAQRNTTPRKQDLDVDPNE
LVDVATAARRGVAVGWCGFDRGGVRAAACTGVCLRSKSTRARSPRTNGETHLVGALNGIA
RRKRIAGGGEAPSRAKPQQPRKGLGPETVTKPIAVGSPAEATTVLAMEPEIQHSSDVNEF
VSPSELFFAGDTPIPVTSRLHIVTPKEDTPRGTWPIFRIMDEDGSFRDGGNDGYMHDGDA
DNLVLGTSSPANKANGSNVMEPSASKNHAPGLSKLRDSLSKLYPNHSLAIEQNGLFQPSK
FEYADTNSRDTLQRLLRHMVRLREMDSIFQNAQRQGRISFYLTCRGEEGIHFGTASALEP
QDIILGQYREQGPLMWRGFTVQQFADQCFGNASDLGKGRQMPIHYGSRALNYQTISSCLG
TQIPQAVGVALRLKMMAAADSEAKKAVSLAFFGDGCTSTTDFHSGLNFAATLECPVIFFC
RNNGYAISTSTREQFAGDGIVSRAPGYGMAGIRVDGNDVFAVHAAVREARTYALENNAPV
LIEAMSYRQGHHSTSDDSLQYRSAEEVDHFACQSDPFDRLRKFFERHEHCGVTDDAIQSV
IEEEKRAVLDALRTAERKPKPPLEDLFNDVYHEMPPSLKAQQAKLNEHVSKYPDAYAF
>comp7149_c0_seq2; 428 1 2095 LEN=2288; translated
XECAALTLFQRFDTPDIIHAIQLLTATKASRRNPATSPSPATAQRNTTPRKQDLDVDPNE
LVDVATAARRGVAVGWCGFDRGGVRAAACTGVCLRSKSTRARSPRTNGETHLVGALNGIA

RRKRIAGGGEAPSRAKPQQPRKGLGPETVTKPIAVGSPAEATTVLAMEPEIQHSSDVNEF
VSPSELFFAGDTPIPVTSRLHIVTPKEDTPRGTWPIFRIMDEDGSFRDGGNDGYMHDGDA
DNLVLGTSSPANKANGSNVMEPSASKNHAPGLSKLRDSLSKLYPNHSLAIEQNGLFQPSK
FEYADTNSRDTLQRLLRHMVRLREMDSIFQNAQRQGRISFYLTCRGEEGIHFGTASALEP
QDIILGQYREQGPLMWRGFTVQQFADQCFGNASDLGKGRQMPIHYGSRALNYQTISSCLG
TQIPQAVGVALRLKMMAAADSEAKKAVSLAFFGDGCTSTTDFHSGLNFAATLECPVIFFC
RNNGYAISTSTREQFAGDGIVSRAPGYGMAGIRVDGNDVFAVHAAVREARTYALENNAPV
LIEAMSYRQXPSQVSEWDIENHKAAYVSKTLFVFEILSTNNRFCYLQTIDNKARVMIPSS
IEAAEEVDHFACQSDPFDRLRKFFERHEHCGVTDDAIQSVIEEEKRAVLDALRTAERKPK
PPLEDLFNDVYHEMPPSLKAQQAKLNEHVSKYPDAYAF
>comp7150_c0_seq1; 622 124 1311 LEN=1518; translated
MISRTSFAAVLLAATTASAFVAPTHNNNNHHHRIASASTKTSLSATDRLEQGDPVLLVGP
GFQQLVLAKHMARAGLKPIVVASQTKLDSFFKSFLKTGDAEDHVGDEDPEGLHKQIRDDS
TIGMPEAGDPYFGELKGVVFCAEEAVLPKEFVERVIDFEDSGMSPFADGKPARTICLLPV
SNKQTKEKANNWIPIFNMDSKKDDMWSNFEKAFVAHPSYKEADGNASIVRFGSLLGGSTD
GPPVLRDYGLDEGMYKMTLEQYRDMRERAFDRYKLSAQVLKGNTINARPADQDTKEKDAL
AKYPGNVREMFPILGDYPEIDRTNRHTLASGLVQALMQPVSAIGKEITILSKASSEIPTE
EEWLEMFANPAAASWPDPFAFEPEKYGMFANDDLN
>comp7151_c0_seq1; 1024 156 2798 LEN=2824; translated
MGRKWRRKNKNKHSGEGKPRDSNKNPDWGKERDPYSIVEAGNFRMEAFYAYQGIHGFRMS
TDPDGNAKGDFVECSASGEKEAERQRWLKALKSSLPISFRIGNNVDPDLRKHLILELEEF
VGKKMKIEVEPKGGKRRQIREQQYREQEEQKNEGESEDMPRPELEKEVRWISAAERISYI
PHAYQVSIDKQTLRRNSALKPFHDWIKIQTEAGFITRQEAVSMIPPIVLDPKPNHVVLDM
AAAPGSKTSQLLEIVNLPTDKDCHEPKGCVVANDSDVKRAYMLTHQTRRINSPAVFITNC
DARFFPLLRNNDDIKNNESPEGIFDRVLADVPCTGDGTARKNPGIWKSWSALNGYALHPL
QLAIALRGAQATKVGGYLCYSTCSMNPIENEAVVAELLRASEGSLELVERKSELENLWSR
PGMTTWTNLAESRSAREIKNKKKKNNPKMIARRKAWEEKNGEKKPESTSTGAGENNEKEI
EKVETVSTELEEPKIISNKTVFKPDSMDDKEELKKLVESAGMVEYLSHDDVPALMRKRIR
SSCFPPTPEEISKFNLHYCMRILPQDMNTGGFFVALLKKVAPLNARVRKKFEKLEKDIET
NNTNSGISTGDEKGGKDDGDSEPKTKKVKVDDPAKDEGAKEDDMIVETDEKFSENATKTI
ERGAGGEKDKQRKEEKNIGKDDFLPVPDNIFNPLKEFYGLDDESFEESNFMVRSGGDGKI
LYFVSKTIRSLLIDRGIQDKLNVINSGLKGFTRNNKECVVDYRVAQEGVHFVAPHMKKRK
FAANLKDFEMCLSGPTVQIKEFSDEFASKIRELSMGSFVVTLEGYENDYIKKLIIVLWKT
RGDAVNYLVTQTEIDGIRCKLRAISKIQELEKEAPSVAEP
>comp7152_c0_seq2; 166 86 307 LEN=798; translated
MPETKFDIGMTCDGCANAVKRILMKMDGVTDIVTDVEAKSAVVTHDESVPPSLMLEKLLK
WSSASGKSVALAA
>comp7153_c0_seq1; 287 1 1120 LEN=1230; translated
XGMNELIASSTQSSANNAIKPAKISNLLLLLSDKLSVPTHSIPEEVTASPLFIEKQKNQI
FEHTKMSDRKYTLGYIAYGAIAISSFCLDGGVSAFSLPSTTTRLSNTSPSLQLSVDDSKV
VEGLDSRRELLGRAAGAFASASLGALCMPSSGFAAPNPPKSLSEEYRQGTAALADMDEMA
PVPREAYKKLPSGTIIADLRPGKSEDGXGKDREPRQSAMGEFARNQSAVSWYFFVKHKFH
TGRGSLFEFLTTSLSFPSNSLNTIRRTKVLRKSNGYFVDSSQVAYDGVPFIFTVGDGTAI
AGVDEGVQGMASGGVRRLLIPPSLAYVDGLEDGKPGPMPVGFGPRQQMRRVQNVRKDVPG
EYIYLEVQVTRVR
>comp7153_c0_seq2; 294 1 1105 LEN=1323; translated
XGMNELIASSTQSSANNAIKPAKISNLLLLLSDKLSVPTHSIPEEVTASPLFIEKQKNQI
FEHTKMSDRKYTLGYIAYGAIAISSFCLDGGVSAFSLPSTTTRLSNTSPSLQLSVDDSKV
VEGLDSRRELLGRAAGAFASASLGALCMPSSGFAAPNPPKSLSEEYRQGTAALADMDEMA
PVPREAYKKLPSGTIIADLRPGKSEDGXGKDREPRQSAMGEFARNQSAVSWYFFVKHKFH
TGRGSLFEFLTTSLSFPSNSLNTIRRTKVLRKSNGYFVDSSQVAYDGVPFIFTVGDGTAI
AGVDEGVQGMASGGVRRLLIPPSLAYVDGLEDGKPGPMPVGFGPRQQMRRVQNVRKDVPG
EYIYLEVS
>comp7153_c0_seq3; 325 1 959 LEN=1177; translated
XGMNELIASSTQSSANNAIKPAKISNLLLLLSDKLSVPTHSIPEEVTASPLFIEKQKNQI
FEHTKMSDRKYTLGYIAYGAIAISSFCLDGGVSAFSLPSTTTRLSNTSPSLQLSVDDSKV
VEGLDSRRELLGRAAGAFASASLGALCMPSSGFAAPNPPKSLSEEYRQGTAALADMDEMA
PVPREAYKKLPSGTIIADLRPGKSEDGVVKTGSRVNLQWVLRKSNGYFVDSSQVAYDGVP
FIFTVGDGTAIAGVDEGVQGMASGGVRRLLIPPSLAYVDGLEDGKPGPMPVGFGPRQQMR
RVQNVRKDVPGEYIYLEVS

>comp7153_c0_seq4; 318 1 974 LEN=1084; translated
XGMNELIASSTQSSANNAIKPAKISNLLLLLSDKLSVPTHSIPEEVTASPLFIEKQKNQI
FEHTKMSDRKYTLGYIAYGAIAISSFCLDGGVSAFSLPSTTTRLSNTSPSLQLSVDDSKV
VEGLDSRRELLGRAAGAFASASLGALCMPSSGFAAPNPPKSLSEEYRQGTAALADMDEMA
PVPREAYKKLPSGTIIADLRPGKSEDGVVKTGSRVNLQWVLRKSNGYFVDSSQVAYDGVP
FIFTVGDGTAIAGVDEGVQGMASGGVRRLLIPPSLAYVDGLEDGKPGPMPVGFGPRQQMR
RVQNVRKDVPGEYIYLEVQVTRVR
>comp7156_c0_seq1; 198 177 1797 LEN=2306; translated
MAPKTSETLLTPAEEATIDSCANHHLTRNPVSATSVADFDFDRDLTLEESKTPEACKNVA
PRGPFADHYTNLASASGGARVLFATDDWFATAENLLEDGPPRFDPEAFCEQGKVLDGWET
RRRRQAGHDWCLIQLPKRSEISALELDTAHFTGNNVPRISLEICDLPAAELSGMVAKLPG
AYERLLHGGVRGTGRLPGEVAAASEAVERSGAWRELLPETPLSPGFEPTRMHYVRLETPA
TGNLVRVNYYPDGGVARLRCWGTSLGDVKPATTPLYMPVQTCDACTVVAHSSVSDPSELP
SRQPTEHRELSSLDQGGIGGACSNKHYGEPWRLIQPTLGRDMGDGWETARHPSRPGILRR
QRGSTLLDTPLSDWAVVRLGGSGSRTGVAKIIVDTKHFRGNYPESVRIEGCHSLPCGETE
APEAAGGYDPSERGSGEWFSLVPRSRTSPDAEHVFERSRGQLENADRPVTHVRVSIFPDG
GSPASGSTAERSSPWSGRAGRTRQTPASGGLSQRQSEPRVPVRLRNKQQQQQCLEQFTI
>comp7157_c0_seq1; 407 106 1391 LEN=1391; translated
MRLKKLTSWSGTRGSKVRRQVEKEKSSKGSRPSSPNSPNSPNNNLGLDSSPIRSLKHSPG
FGTSPNKGEGEKPATSETDRGVLRKHEDDRPCLLFEDVEDEDDEDMGYAKNIVAKEIEAN
NSAVKDKKLNSTENIPTKKISNTNPNQKSLSLCTPKKTSLPPQEIMIDHLAVPPSPMREP
NVDIMASPSMNKRLLDDQGLYLNFNDSSPRTPCLGDPVGDIAMHRQLKDAERLVRIVLGR
PSKGDQNFLEPNTILQAIRSYALMKAELIELRAKQETEDGDPPAILQALGSPTATTPSKS
IPSLYSPRRSVQNSHFFSDDVNGGRSRKHGIKSSVVDAKASPAASHEASIPSSSCAELTH
AKEMIERLQKELKKANETILELKKKINTEENEENQYPIKSPNTIEIQNENADEKQLMKKQ
NHDFREEPX
>comp7157_c0_seq2; 387 1 760 LEN=913; translated
XKTTNKVENEENQCPITSPDTIESQNENADEKQSMKKQQGDIHEEPKQPENINLLLNDTI
HCPEFVPAENMVAISGEKQQEMEQTNTTDEEITSSTTTVEGENRREAKQSVMIEKEIIST
TTPAKGEEEMQISQEELNLQLETNQKKQIQTPILDEENLLGTTMAQAGEETQTSRGESDQ
KVRKVENGLNRYDENDKKKSQAATIDKEHILESTTTSNKNNTSILMEVSHNTANSKSNSN
LASSSSLSNTPQS
>comp7158_c0_seq1; 188 57 335 LEN=1479; translated
MTPTRSAAACLVLLLAGTTTNAFSPVAKTTPVASATSTTSLRFGFLKELGLEKPSWLPDF
GGKKGEEVPAPAAVEAEDADAPADEAADTPEE
>comp7160_c0_seq1; 696 448 2835 LEN=3313; translated
MAALKLVPPNATGSAATTNANHQHHTFLPDPPSNFSIDNSLSTELLRLSFEDRVAMEEEI
HGVRCSAPTETPELLECSLREFDALLMARKLNHGSGGNPFGIDGSGKNGTNGIHAADGNG
TTPVYIRRNLLRNVRSITCSSDCGNEQSWYSQLPHSENQTPTLHQQQTNSNSPTTTYCYL
NDPEVRLRFLRVDLFVASKAVERLIHFIELTCELIGEVVAHRPIGLQDFTNRREEVALQN
SRSQYLPFRDRAGRRVFMGVGSCGMHLDPVLLHKIFAFMHWTVSGDVETQQKGVVVVVWP
TEEGFAINENTNKNEPPDSEGWWERSIRPKLDKKMRKMFQKMVKGFPLRITSRLGCYKDT
PFFRALSALYYIGFDSQNRRMYKAHFGEDTELRYALASYGIPEHLLPLSNSGTVKTANHS
RWINALKAKQNREQRLEKKKQEQQLQKQQQNITMNQHGTNNIHNYFYYAEQQMQPMMASL
SSSENSYNNNTTNGFTIGNTNDNNAESELDEEEIVDCPGSRDVIFRKGPTYKNNPGNMVF
RSLIEATHEEHSKATRKEKCRITWEIVSKIEEQGGRFLDWSKSKEMWVVAKDREKIRTKV
AACYKQFHRSILAFQEQRLKLSQRQQKQQAKHHKNATNLTSEPQSPPQSSLPLPQQVQHN
YVDGCDHPGGPEFAQERNGDALQQAVAITGMSPKPHVLHLYGSHSAKDGGYSASIFSKKV
GRPQRGVSRFAVVAAAGAIIRVIVRPPTPRNTRQERALMKALPILGKKKTRSHNRKKAID
VLERMKELMVMYEPA
>comp7160_c0_seq2; 569 448 3166 LEN=3644; translated
MAALKLVPPNATGSAATTNANHQHHTFLPDPPSNFSIDNSLSTELLRLSFEDRVAMEEEI
HGVRCSAPTETPELLECSLREFDALLMARKLNHGSGGNPFGIDGSGKNGTNGIHAADGNG
TTPVYIRRNLLRNVRSITCSSDCGNEQSWYSQLPHSENQTPTLHQQQTNSNSPTTTYCYL
NDPEVRLRFLRVDLFVASKAVERLIHFIELTCELIGEVVAHRPIGLQDFTNRREEVALQN
SRSQYLPFRDRAGRRVFMGVGSCGMHLDPVLLHKIFAFMHWTVSGDVETQQKGVVVVVWP
TEEGFAINENTNKNEPPDSEGWWERSIRPKLDKKMRKMFQKMVKGFPLRITSRLGCYKDT
PFFRALSALYYIGFDSQNRRMYKAHFGXVMVWDTILEIARSTSRCCRIGLVVLIHGNVIY
ALLYLAGVAQGLIRVAKKLFCCLGKTMVDCFIILSCQNTHQKYSVGVVFHFFIILLTAIA
FAVAEILYKCIIIVSKGEDTELRYALASYGIPEHLLPLSNSGTVKTANHSRWINALKAKQ

NREQRLEKKKQEQQLQKQQQNITMNQHGTNNIHNYFYYAEQQMQPMMASLSSSENSYNNN
TTNGFTIGNTNDNNAESELDEEEIVDCPGSRDVIFRKGPTYKNNPGNMVFRSLIEATHEE
HSKATRKEKCRITWEIVSKIEEQGGRFLDWSKSKEMWVVAKDREKIRTKVAACYKQFHRS
ILAFQEQRLKLSQRQQKQQAKHHKNATNLTSEPQSPPQSSLPLPQQVQHNYVDGCDHPGG
PEFAQERNGDALQQAVAITGMSPKPHVLHLYGSHSAKDGGYSASIFSKKVGRPQRGVSRF
AVVAAAGAIIRVIVRPPTPRNTRQERALMKALPILGKKKTRSHNRKKAIDVLERMKELMV
MYEPA
>comp7162_c0_seq1; 541 100 1470 LEN=1470; translated
MTTTIPRYELLHHGAAANSSPKAIACLPNTGGDILQGRDEECGAGSEVGTTELIYASHLM
LNVAKPMKLFVGNAKDSGDETNAVKNHEKIWNVEQTLRTSTSLTAEEVETAAANVSTRTA
KRVITCVVQLKWLSHQDSAKNRNRDDVLVCGFSDGSLTSWYRPCGSKDWEEYVLLSHSEG
SKHKHFSSDENSNKDDVTEMKGRSITDIDGYVIYGNHDDEEKQTLNLSIVTCSSGGAQYF
QFSLLLSINGRNDGASTGRQRKEPQYAAIKDTKNVIGTPSNTVKFHTIVSSEGEKSTRHT
IDGKQNIRGIFLVGTAAPRHNKIHVLVLPPTVQENDIPAAPKYSGSLTGHEDWITCFDWN
TNFELTFVHKANDGESGAIPTVDCCYLASGSQDAKIRLWKWVTTTCKVEDEIAFSDRQIG
DSQASIKEQGISSIDESGDGDRNVDVADDDDDDDEEE
>comp7163_c0_seq1; 353 52 720 LEN=808; translated
MNTCNLKMKVPIAVAVFAVSCSSVMGFAPVHHSNQICAISADPQAPTATCLYAGGFGGGG
MGKKSGSNKKKKNSSKEIKLKPKQQWDRYSEFKREPKIQVAVRIKEGGSDEWLEVGRVKS
KDNKFIEAAVFRQRAIIAEHAKRLYPVQLSSKKSLEWGYKSSEEAEETTEWKAVDKSSMN
EVVELDGFEKVIGFEGRPDPSSGFYCVYDGGRLKIGEETSFV
>comp7164_c0_seq1; 473 113 1315 LEN=1431; translated
MATNATTRPSKIEKDTSSSDRCISRFFRPKRKPSRDEENDLKLQRENDVIILGSDDDTDD
ETDDPSCDKNDSVDVANEEKRGPEKPGARCELNTNIEILDDDDPVDACDVISQKEKVESN
RLATAKEEDESLGHSDRNFNGSNLDVDTDGDTITTKRPRIASPQKPSVVAGSVKENPFAR
FAYSASDASEDNVKTTNGLDSLCSSWQRHSFASKSKLRKSNIANQSNDIKNVSSSRIRVS
LKQKQKDFVKIKDISPEEQSKIVRKWQSMADSSAPLEVRRYQVLLAARLHARCQEVTVRK
AMKTLRDYFSSLAEPKMVTVSVMANIDPGLLASHISNLQFYNAKAKQIVKAAQELLERHR
GIVPEDECSLLKITGIGKTFADLLAFVNTREAHKKFGECK
>comp7165_c0_seq1; 689 585 2515 LEN=2836; translated
MTDGQLWDRYIWEQYVGEVEAFEDLDRTNERVACFIPGVGCTVNSMLELGNIHSAQGSQF
DEVVDRTSPGAGAFTPYHSTPTIINAPEGFNDGVEPGQELFATYGDEWIPWIPGAAVTFS
KNFKKADKLMEEFEDWIVEHEENPATKDDVTDELLEDMWKVMIDFPNVGPRQWEELSVLP
REWDRGRLKELKHVKEQQKRNQETTSSPLPLPNPPSTSTRYWADKGKVSLEFLREEGKCQ
DHIRPDISEIPHAGRGAFASRDLPKGTIVGYAPLIHVGTHGKDLFEIVYNGESKHGMERF
EYRDIEDEDEVDGDLEEMAESASNRQNRYTKQDLVLNYSFGHKNSTLLLTPYGAMVNYIN
HKSAKDGDGPNVRVQWPTREFVAHKPEWLSEDLNFFRDSVDKIGLSFDYVALRDIKEGEE
ITMDYGDEWVGIXLFLRSLRARIESVLFLVMLLFFASLAVGLRTRCCFLAISHSSRCLSI
ARKDQAWKDHVASWTPPENAEEYVHSSKFETDYLRTPDELLEEPYPWNLHTLCTEYYTKK
PGDLENSYSSTFVSDRAEQLLPCRVLDRISLEDDDEEEDYLYTVEIQTSENSVVVVHDYP
RSDNGIQLYDKAYSPMWHMKEAFRHKIAVPDDVFPENWMTKQD
>comp7165_c0_seq2; 823 60 2159 LEN=2480; translated
MMLSSAARSRGLVLTAAAALFLANAADAAQGGACPAGPSSADGTCLNVNTNLGDADDVDC
GVYMAPSTIGDHSNLGIFTAKAMKNGEKVPYPEIIVPLLWRIFGEHPAKSMTDGQLWDRY
IWEQYVGEVEAFEDLDRTNERVACFIPGVGCTVNSMLELGNIHSAQGSQFDEVVDRTSPG
AGAFTPYHSTPTIINAPEGFNDGVEPGQELFATYGDEWIPWIPGAAVTFSKNFKKADKLM
EEFEDWIVEHEENPATKDDVTDELLEDMWKVMIDFPNVGPRQWEELSVLPREWDRGRLKE
LKHVKEQQKRNQETTSSPLPLPNPPSTSTRYWADKGKVSLEFLREEGKCQDHIRPDISEI
PHAGRGAFASRDLPKGTIVGYAPLIHVGTHGKDLFEIVYNGESKHGMERFEYRDIEDEDE
VDGDLEEMAESASNRQNRYTKQDLVLNYSFGHKNSTLLLTPYGAMVNYINHKSAKDGDGP
NVRVQWPTREFVAHKPEWLSEDLNFFRDSVDKIGLSFDYVALRDIKEGEEITMDYGDEWD
QAWKDHVASWTPPENAEEYVHSSKFETDYLRTPDELLEEPYPWNLHTLCTEYYTKKPGDL
ENSYSSTFVSDRAEQLLPCRVLDRISLEDDDEEEDYLYTVEIQTSENSVVVVHDYPRSDN
GIQLYDKAYSPMWHMKEAFRHKIAVPDDVFPENWMTKQD
>comp7165_c0_seq3; 302 1 731 LEN=881; translated
XSRNRSSQIFFDEVKETNTTMMLSSAARSRGLVLTAAAALFLANAADAAQGGACPAGPSS
ADGTCLNVNTNLGDADDVDCGVYMAPSTIGDHSNLGIFTAKAMKNGEKVPYPEIIVPLLW
RIFGEHPAKSMTDGQLWDRYIWEQYVGEVEAFEDLDRTNERVACFIPGVGCTVNSMLELG
NIHSAQGSQFDEVVDRTSPGAGAFTPYHSTPTIINAPEGFNDGVEPGQELFATYGDEWIP
WSK

>comp7165_c0_seq4; 712 585 2354 LEN=2675; translated
MTDGQLWDRYIWEQYVGEVEAFEDLDRTNERVACFIPGVGCTVNSMLELGNIHSAQGSQF
DEVVDRTSPGAGAFTPYHSTPTIINAPEGFNDGVEPGQELFATYGDEWIPWIPGAAVTFS
KNFKKADKLMEEFEDWIVEHEENPATKDDVTDELLEDMWKVMIDFPNVGPRQWEELSVLP
REWDRGRLKELKHVKEQQKRNQETTSSPLPLPNPPSTSTRYWADKGKVSLEFLREEGKCQ
DHIRPDISEIPHAGRGAFASRDLPKGTIVGYAPLIHVGTHGKDLFEIVYNGESKHGMERF
EYRDIEDEDEVDGDLEEMAESASNRQNRYTKQDLVLNYSFGHKNSTLLLTPYGAMVNYIN
HKSAKDGDGPNVRVQWPTREFVAHKPEWLSEDLNFFRDSVDKIGLSFDYVALRDIKEGEE
ITMDYGDEWDQAWKDHVASWTPPENAEEYVHSSKFETDYLRTPDELLEEPYPWNLHTLCT
EYYTKKPGDLENSYSSTFVSDRAEQLLPCRVLDRISLEDDDEEEDYLYTVEIQTSENSVV
VVHDYPRSDNGIQLYDKAYSPMWHMKEAFRHKIAVPDDVFPENWMTKQD
>comp7165_c0_seq5; 173 585 926 LEN=1076; translated
MTDGQLWDRYIWEQYVGEVEAFEDLDRTNERVACFIPGVGCTVNSMLELGNIHSAQGSQF
DEVVDRTSPGAGAFTPYHSTPTIINAPEGFNDGVEPGQELFATYGDEWIPWSK
>comp7165_c0_seq6; 800 60 2320 LEN=2641; translated
MMLSSAARSRGLVLTAAAALFLANAADAAQGGACPAGPSSADGTCLNVNTNLGDADDVDC
GVYMAPSTIGDHSNLGIFTAKAMKNGEKVPYPEIIVPLLWRIFGEHPAKSMTDGQLWDRY
IWEQYVGEVEAFEDLDRTNERVACFIPGVGCTVNSMLELGNIHSAQGSQFDEVVDRTSPG
AGAFTPYHSTPTIINAPEGFNDGVEPGQELFATYGDEWIPWIPGAAVTFSKNFKKADKLM
EEFEDWIVEHEENPATKDDVTDELLEDMWKVMIDFPNVGPRQWEELSVLPREWDRGRLKE
LKHVKEQQKRNQETTSSPLPLPNPPSTSTRYWADKGKVSLEFLREEGKCQDHIRPDISEI
PHAGRGAFASRDLPKGTIVGYAPLIHVGTHGKDLFEIVYNGESKHGMERFEYRDIEDEDE
VDGDLEEMAESASNRQNRYTKQDLVLNYSFGHKNSTLLLTPYGAMVNYINHKSAKDGDGP
NVRVQWPTREFVAHKPEWLSEDLNFFRDSVDKIGLSFDYVALRDIKEGEEITMDYGDEWV
GIXLFLRSLRARIESVLFLVMLLFFASLAVGLRTRCCFLAISHSSRCLSIARKDQAWKDH
VASWTPPENAEEYVHSSKFETDYLRTPDELLEEPYPWNLHTLCTEYYTKKPGDLENSYSS
TFVSDRAEQLLPCRVLDRISLEDDDEEEDYLYTVEIQTSENSVVVVHDYPRSDNGIQLYD
KAYSPMWHMKEAFRHKIAVPDDVFPENWMTKQD
>comp7167_c0_seq1; 304 536 1885 LEN=2033; translated
MHIFSVGFGLLTAVACAFMGMYHNANLWCWIAAAPACPYDQEPHQIDNWQHYEEKADKCE
ESKYDDDVDVYRLAFYFGPLWLCMVFITVTMGMLICAVSRIGCSKKSIASCLNRHKNSRQ
KANTDSRKHENDSVEEQSEGDEQSNSSISGAGDAFKRQKANSNSRNHENNSDEEQNGGDE
QSNISISGARDAFKSDRRSSFFIPAKKKKRKTPRNENTTHVLKVLESYLTASQQAKNSQS
YGSRVVYKQAIYYTLAFYATFTFATINRVFQHFGVSFFPFILLHSFTIPLQGFFNLVIYR
YFHFVRLRQRNPYVPFRILLQWTFRWTFLGPPPGARRDSSQKFQSFELPNNGKSTKSRSF
GPSGAFDSHNCEYSEGSDSVVDDDAELERMPRIEDEFENSFWFDYTPSAELAYEYHTTRA
LNNPAQYPSTLNNVPTQYPTMVTDFDSDD
>comp7168_c0_seq1; 317 1 1249 LEN=1408; translated
XGGQELGRYEQLNCITKVMYDMAQDSPESAGAVWSRRIGILRNAHDKRLRDSSFIRGDEE
MEDGGFWSAWPTTGVFLLMRSLGHIFPVTDRRHHVVTPAILLLAQYTAQTPVLSTYDLTM
GILCSGLLMEYSKEAKRVVPEALSFIASAIRLFSPDDAGNLTNPALQTAYNQPFCESLRK
NISRYEDASLPSLKIENDFIMSEESPEIAVTLLSLCIGLAEKYVEALNGSFSVSAETEML
YDITNAILSLQPKSYPITLQKKISKTAALIAKVCPAQRIPLRRRGKPTASEMAIKTLAPR
MEDPTRYSMSRDKGKKSAQAAIDRTRREYKREHKAISRELRLDGQFIESERRKEKERKDG
KARAKRQKNFNWLEGEVAAMNEQVRQGGGLLKGGGTGLARAKAATAKMGIKKGGKF
>comp7169_c0_seq1; 353 1 987 LEN=1518; translated
RNLEAQLHHYANHNKSVIISDEAFSRMGIIVGDSVDNRIVLYRLLNKYYPGRVRVVVMYR
RYYEWMQSMWNEGNKPHKNFYDPVSPYKEKFQKWPSEGGKTCKTFLEYITRKHKKVARDK
LKWSEYQKLAEIDNIHVAEYLRRLWKNHSSNVVMLNMHEMEHKNDGEDITTLLLRENLSL
VAGDVLRMAKANASYVGRPNPSKNFDYDRLAVKAHERGLLVNQSATLRFEIAQIAEKYLL
QKLNKTKDSLPFVCLNQTHLESLLQKSLQYEREIYPDLSEEATKHHEEAFFKAFERKKFC
NFDTDKLIEDNAVQNFFTKDIPQLLDIR
>comp7170_c0_seq1; 285 60 863 LEN=925; translated
MVQSNNTDKTSRSPIVSLNRFERKKNIGLLIRAYAYVQVRDGGAGLPPLVIAGGYDARNA
ENVEYRGELGTLAKELKVDVDFRLDISDAERASLFQTALCVVYTPDREHFGIVPLEAMHA
GTPVLAVNSGGPVETVRDGITGYLREPTPSAFGTALLEWTRNPERATAMGRAGKKHVEEV
FGTERFRDEWGVLVRETKAAASRRKAGGRPSTGLVLWAGVPGYAVELAVAYAAVLLATGI
LRWFGALGASQSIPGALRAAVVGREEL
>comp7171_c0_seq1; 877 1 2089 LEN=2283; translated
XVKSEKAKRNFWNYRTRTFTDHRVQSRDICNTQPHAFPFGSIATMLFPAVHQLGFQAGQA

VSNFISNTAKMMDTNNVNSGTILDVLTDSELLVKTASAFLRYVLTLPSLYKTWWFNLVAK
DPLHVAIETIMLMSIVYMMLSRSTEGYKDSSKDKLTKREQEELLWEWKHKTRAPLAPPLK
EKGNGNGNDDDDGKKSNKDNNSKNRGVHFDNDDDLQIVVQKQEGKWLTVTVPDHWITDPK
QRKSTNATTGNKRGPKNVSSKNKTAKKSKADKSKTTTPPQRSEVELRVLNFCNFDFLGFQ
TSEALRQASTEALNKYGCGSCGPRGFYGTIDVHLQIEEEISRFCRTDQAILYSDGASACS
STVAAFAKRGDLLVVDEGVYEPLSTGVYLSRANVHWFRHNDMEDLERVLKELQAIDKKLG
RKPGAQRRFIVVEGLYKNLGTIAPLDEIVALKHKYKYRLILDESFSFGTLGKTGRGTLEE
HDKRPMYDAEITTIALENAMGSIGGVTVGNEEVVDHQRLSGAGYCFSASAPPFTSSASIA
SLRVLEERPELVERLQSNVRYTYQRLLQKVDQHNTTNKTKCYNLVVTSDSRSPIVVLQLE
RESVYESAIMSGIMRECLQRGVGIVASTTPLEETLGEAGGTAASSSCATELAPCIRMTVS
SLHTEEDANTAIDALIESAALVTSRYAASSPVSRTT
>comp7172_c0_seq1; 186 374 529 LEN=854; translated
MEDPTNGKIKAQNVTGPMGGYVQGAPRRNDFRNDDGYGSSSSDGGYRNDGF
>comp7174_c0_seq1; 200 1 838 LEN=975; translated
XDDDEDSSSSSPFIRISKSTFRSGMQVIGQFNLGFILARCPQNHLWIMDQHACDEKYNFE
QLCKKTVMHVQPLIRPLPLELNPSEEACVLDHMDVFASNGFRFRFDETAPIRHRLSLTSL
PHSGAHEGRKAVAFGPSDVSALCAILTEGSSYEAGSGGTGTDGTGLYGNNAVRRHAGGSQ
SETPDRLLARLPKAIAMFASRACRTSIMIGTALSRKEMEAVVQKLSDTDSPWNCPHGRPT
MRHVANVLPFLEEDERRAETYASLPAISVVPAEEAERES
>comp7175_c0_seq1; 200 1 821 LEN=1283; translated
XRKNKEVTAITYNSRDASRIKMDRNIVMGRNILNLLLLLFSVVQISRVTGFVTTKASSVA
RKYAIHRLSSATLDSVSSDEKMEETREISNALTEPVDEAFTPYRGEQPKEALSEIFVPQV
FSHSLDGVENIEEEKLWVPQTDSVSFRPLCFCVSGGYYVNLLRVRGAGLLSRHRHPGPVH
GHVLKGKWSYLEHDWVAEAGSYVFEPPGEIHTLVVPEGVDEMITLFHVTGSLLYCDPDGN
VVGAEDVFTKLELAKKHYEKVGLGEDFVQQFVR
>comp7175_c0_seq2; 77 1 324 LEN=786; translated
XEGVDEMEEANDLQPNEMAGHDNTKKVLALSYTMDCKHTALSSHHNKTRYFFSARKITLF
HVTGSLLYCDPDGNVVGAEDVFTKLELAKKHYEKVGLGEDFVQQFVR
>comp7177_c0_seq1; 353 1 1921 LEN=2502; translated
XVATHSIFSLSHLFGIPLSEATATGMNRSWKNSDSGSNASTKSKETLVKGQNKSISVKHQ
RAVRDYNSSPMRQPYSPCNDTVDLSYISSDEDDSFLHHRADKSSANSRVRLNRRRARFEE
KEEGYSLLKIVVSVFYVAVTLSSMHIGFFLRAPASPLPAPTHQIPSRASRKPMVPIGRRD
RSPKPQREVDFLEVGFAWSDSFEIANKTKKEFISQDAFSASWWLGKEKFLEVETLETMSA
SPEGASFQVFMKVGAKQMRVTRDWMDLSIEHMSKWWKIILDPWEYTPNGGTRKRNDVEGY
LNRIIDIFRTYVFSEDRRMFWDDITTDKYRTEWLEVSQSTIAVIAFLNTDRQNGADELMK
WSLAATLMSLRQLGVGRIVVSGSEERTETEEATVRAAFKIVAEATKDWDPSRKLVSEMEF
CVTADARTGDKDTALNIPIAALKQLRHVFLKQTPASVTECWLGGSFEKRAKKWNYVFLGE
PDLLLTTRPSSTAALARELRRGNVLAPHRLQPMPHSADFAEVRPEAEDDGVYMLPRTLPG
FDRVEDADHIDTEWSCCDAGNLKPKYDYESCGIFWWQCGYKYERLHLQHEEGRPNPAEAH
KIINSYPLVRLTKGTGVVFSGSEIGRKCTPQLGPCTKDTL
>comp7178_c0_seq1; 302 334 1389 LEN=1653; translated
MAQSLTRIDDSRDILQLMIRQEESYYKCPHYLSLEERSTQTLVFTPVPSVLQVVEEMASL
VTDIRAEPSMNCKGDDHCASPGSSTCDLYGDLRAPLRAFLPNSRSEQFQEVLSLSSWRHR
MLEWGNAVCVTFGIDASVLESAFNILDRYIAVEISHSNDAFENLSLTREDFQLFSMVSIY
IAVKMFSRNQNLRVHDLLEMAQGFYTADHITTTERDILEALNWHVNPPTVMDYCDVYLTL
FPQHLYGCYGNDAYSPASQWSSSKRVGEKQRRKCRCRCQSMVGIVLQDLFFVDKPNFLTA
LAAVLLAADAFRGCARDNSFVLEHFLRNIQGVVNIQKEEFDSIIRRLECYC
>comp7180_c0_seq1; 398 1 1122 LEN=1122; minus strand; translated
XGNYQINKAPLPATTSTIPSIQQQQQQQLLLLALLLQQQSNSNNNTGAAMVDLNALSMAL
SSPEGIRLLNSFAIGQAGVSGTQQNPHVNRQGQQPQHDTPSIAQTNRMAVHNIMPLATTM
IASSQQGDALANNQPSLFLSPQSIQQPQLLLQAPPTLPSAQSQPAFQLHENANATGGASA
GMFPATAAAAINQHLATRAAPPPLHPSSAVSTAATNPSCSSTSRNSGAMGSMLTVNNVNS
QTGCGKNVDRNVRNLLKKRDKNSSTMKRVNTSNSSYKPATKSRFPRILYNEADDGILGEY
QTLLRQQLELFEADGQDVINGTFRQGRTTPIKLGQIGLRCRHCASTPVSIRSKGSVYFSQ
TIKGMYQIAQNMSKX
>comp7181_c0_seq2; 58 1 902 LEN=1091; translated
HTQGADPKRPHERAKPATEAPARXATGAKTPPPPDQEPPAGPLSVQALPVAGLPGPWLRG
QPRAARPPPRTPPREESPPGAVSQGRSRGSIARPIAARKGGGRNAAAPGPVQEPLLPGAR
DPPPGAAGPGDLPPPGPGPRGEGAGGSAGARCFSRNRCRGRKDHLRLGGSRPKGVVVLEA
LPGRDGADPAEPERQRGIRPGAERERHRRAGQPRGELPEFCVRGPPGGDGGRGAAGTAEA

HRRAEKRGRSEDGARPREKKAAGKEKMALVAEQSTPPIHSTPLNTAIPPTERNGHFSHDA
>comp7182_c0_seq1; 424 108 1175 LEN=1693; translated
MASTSTIRLGPTDDNPSAHLLQKAITAFFENKNADHSSNGDTDSGFVIRNKYFSADISLK
GIGMETNGAQDETAASTIEDGVILVFDAVQSNPDCCRDQPNAGAIATFDSLHATHQRAIS
DHTCGDLLRLCVGVSLSELSPSELRGKDHEKEYSRRILWCLDNGYEYIEADLSEEGQQKG
RDDRDKDGFARVVEAIEGTVWSSAAMSASKSSTLKTTYADGKSAVDKSAPPAATKSCKGE
ETIETKPEGSGYEPPVAPPVAAAKSDNAASVFGLKEEMPALDPEDQVTEKLFVEMESVLK
EASEIRQASKSGVLTDDERRERAGNAALALVNLMGQFGLDEEDDDSYGSDGSMVE
>comp7184_c0_seq1; 229 346 999 LEN=1112; translated
MKLHVGEDTEVRYALGEYGIPVDLIPVTGTGNIKTNHHKPWIKIRVMVDMSAHDDDKNGA
NGSLGRIVECPGSTDVIFKPGKRLMCHPGNVLFEELVVSRIQGSDFTASKRETHRWLANE
ITKKRGGRFLKWNDEGYWEKMEDEDEISVKISTACKTVLKKVKQKGSGSRIVVESSTFAF
QENNLGRNVGDNGTDQGRSRKRPFGDCFCGDTLPDSK
>comp7184_c0_seq2; 154 1 768 LEN=843; translated
RCCCSRIIRANEPISDLSASYREGIERRHAQHDEANLPTTATMKALNQENEAISKGEKTN
DDEVSPLIDVMNAALPQNYQQEKSRAVDECIAEDFLKMSIADRNSVADEIAGKSCMAREE
TPEMLAASLARLDVELNAIPLKKKIGYLQSQKFSKTYINDCNFRLRFLRAELFEAREAAV
RLAKYCDFVLELFGSVALQRKIWLSDFDRKEIKWIQMGYIQMLPFRDSFGRRVILQIIDA
SVLREKRVRVSNNVN
>comp7184_c0_seq3; 345 1 1680 LEN=1793; translated
RCCCSRIIRANEPISDLSASYREGIERRHAQHDEANLPTTATMKALNQENEAISKGEKTN
DDEVSPLIDVMNAALPQNYQQEKSRAVDECIAEDFLKMSIADRNSVADEIAGKSCMAREE
TPEMLAASLARLDVELNAIPLKKKIGYLQSQKFSKTYINDCNFRLRFLRAELFEAREAAV
RLAKYCDFVLELFGSVALQRKIWLSDFDRKEIKWIQMGYIQMLPFRDSFGRRVILQIIDA
SVLREKRVRLKTFIFLALVASEDLETQRKGVVLIVWPGGPCKDWRRREPSDGRLSHLITQ
RHEVVPIRYAAFHYCMPDNPFFRIVRSFAVLYGGIGSGTKSRMKLHVGEDTEVRYALGEY
GIPVDLIPVTGTGNIKTNHHKPWIKIRVMVDMSAHDDDKNGANGSLGRIVECPGSTDVIF
KPGKRLMCHPGNVLFEELVVSRIQGSDFTASKRETHRWLANEITKKRGGRFLKWNDEGYW
EKMEDEDEISVKISTACKTVLKKVKQKGSGSRIVVESSTFAFQENNLGRNVGDNGTDQGR
SRKRPFGDCFCGDTLPDSK
>comp7185_c0_seq1; 281 345 749 LEN=1149; translated
MNHQLRQDPSITSSLRSGSMEKHSQYQEQNQSFALGNSASDARKPWTSILQLNNRGPFSR
EGLEFDKMLLKAVFWWRDSHEQQQRQRQQQQLREHDVDNDELYDDDDGEIVDMFLDNVFY
LIGMPEQVAPAAAA
>comp7185_c0_seq2; 571 345 1697 LEN=2097; translated
MNHQLRQDPSITSSLRSGSMEKHSQYQEQNQSFALGNSASDARKPWTSILQLNNRGCVNI
TLGNYSEASRIFEAALSRQKASADSFSAVVRYGCNHTGLGHPDGAYINSAADATPVDNSS
SRAHERPNVEDTEEEFDYDDCSIDSTSCFDDDEDNYDDYMEENDELYKPQPSLVSLDATY
STGSYTTELEAFTSSQSSGQIQHLQRIRRMTASSQLQTFSFTGKDRYLEEQLQQKNRDPS
HSAIQRKSMHEIYCLPIVMDEHEWLSASAEDQTFVLIFNTAVCNHLWGMALQQQLKEHSQ
NQRIHLSQQTERVFLVAFKLYRLALNNASTQQHASPASPDASANGNNNSIDYRLSVLALL
NNLSHVSKTLLGPFSREGLEFDKMLLKAVFWWRDSHEQQQRQRQQQQLREHDVDNDELYD
DDDGEIVDMFLDNVFYLIGMPEQVAPAAAA
>comp7186_c0_seq1; 382 87 1043 LEN=1256; translated
MADPAEGTPDAGTEANHRKRRKREPLRIGLLGCGAINSTVAKMIANQQAGPSVLAAILVR
RERSEEEVRSVLTDDSSPPIAPPKSQEPRAAAAAAVRCTTDADEFFAAGSDWNICIEAAG
QAAVREHGKRCLEMGRDLMITSIGALTDETLYRDLRSAAESSGARLVLCTGSMPALDWMG
AAALAGCGEVLVTQIKPPGAWLGTPAENDHPDLLSLSEPRVLFEGSSREAASTFPKNANV
AAMLALATAGLDATRASLVADPRAGGNRVLLSFRGAAGTIAVEVEAAPSKTNPRTSAIVA
LSVVKAIRKLCCPVVVGL
>comp7187_c0_seq1; 333 41 619 LEN=851; translated
MKLAILLSAIAGASAFAPASHQATSTTKLNAADLETLRGVGPETGGKIFDPLELSQWAPI
DHLRKAELANGRSAMLATVGWVFPTFWTFEGPVTTTDPVEAFWAADNQWWAQFIVFCAVI
EGIKYRGEMEGKSFTGEIDKEACLDWTGNWAKMSDTQKEDMAMKELKNGRLAMIGIAGFI
ASVVVPGSVPAL
>comp7187_c0_seq2; 333 41 619 LEN=944; translated
MKLAILLSAIAGASAFAPASHQATSTTKLNAADLETLRGVGPETGGKIFDPLELSQWAPI
DHLRKAELANGRSAMLATVGWVFPTFWTFEGPVTTTDPVEAFWAADNQWWAQFIVFCAVI
EGIKYRGEMEGKSFTGEIDKEACLDWTGNWAKMSDTQKEDMAMKELKNGRLAMIGIAGFI
ASVVVPGSVPAL

>comp7188_c0_seq1; 331 80 991 LEN=1576; translated
MALKKLLIPTTFCLVLLGIVSDTALAFVPSKSTGTATQTRLPSQLGVGVVPSTAEAIQEM
SNARFAFGMCFYGALGIGSIGRELIPIVFGRYQTTSSLESDGSIAVRSGSAVANSDEDLR
IWGYPEKIYKKDVEMILNNKLTPLAIAKKYPIARDEDTDPSYEYTHLTEAVPFLGYEGFV
KANPGANPVALRAVFDSFSNSIGGSNAVSPITAQRNIETYLDNKKVTGALAKKLNGGKSL
GIAAFVFLLILLGVGDALAIFHLWKGWFPEWQGFSDIPASLFDQNIGIAVLPNFFVGDVP
PIP
>comp7189_c0_seq1; 606 591 2975 LEN=3022; translated
MSPRLLKAKILLDQLTAMEDQEPSLADAAAKNNGSDSQDDESTVVVPDTVWSNGLLDGSD
FVTRYAFRQGVKIAEPLVKYDPVAAEKNLFQQPNRWFVRNVQIAGPLGLWAVGVVQDYLI
SQSSSSSGTSAAAKKQADASRRRKRAIQLRKAISNLGPAIIKAGKALASRPDLLPSEYLE
ELQKLQDDVPRFSNELAFRTVEEELGVENFSDVFDLEEDEPIAAASIGQVYKGRLKANGD
LVAIKIQRPNCEEIIALDLYILRWWSGIYNKIFQLLNRDINVQSIIDDFGELIYREIDYV
AEAANAQRFNELYASVAEQVFVPKVYSDLTTRKVLVMEWVDGCRLTDSDRMQQFGLDKSX
AGGYTGSVVDQADSGKRYELMSLSQSYRFVNNQVEDMSSKTKFEIVETRKMFLPFNSSVW
FCFDLFXLSIGFFHADPHAGNLLACPDGRLCYLDFGMMSYVDRAQRNGFLLAVVHIVNRD
WPELVRVYRSLGFIPNDTDAKPIEVALERALPDVLNADISELNFKNVVNKLGDIFYTFPF
SLPPFYISIIRCMGVLEGLAIQVDPTSRIISEAYPYVASRVLTDPQQDLQEALKRLALTS
DGRVRWNRLEGLLDEAKGSSGYDVTSALNLLTDYLISEEGDAVLDDLADGIVHEADNLGT
EGISYILAAGRALAIRDEISAVNAFRSLQSVITREKNLNEEVKGVLPEPTESMTRFGKIL
SLLDIGAETDYSKLVPIVRKLALEPKVQKTANDVTARLGERALSRTLRSLFGLPPPVFDG
KGTSASTVFEESMS
>comp7189_c0_seq2; 739 591 2813 LEN=2860; translated
MSPRLLKAKILLDQLTAMEDQEPSLADAAAKNNGSDSQDDESTVVVPDTVWSNGLLDGSD
FVTRYAFRQGVKIAEPLVKYDPVAAEKNLFQQPNRWFVRNVQIAGPLGLWAVGVVQDYLI
SQSSSSSGTSAAAKKQADASRRRKRAIQLRKAISNLGPAIIKAGKALASRPDLLPSEYLE
ELQKLQDDVPRFSNELAFRTVEEELGVENFSDVFDLEEDEPIAAASIGQVYKGRLKANGD
LVAIKIQRPNCEEIIALDLYILRWWSGIYNKIFQLLNRDINVQSIIDDFGELIYREIDYV
AEAANAQRFNELYASVAEQVFVPKVYSDLTTRKVLVMEWVDGCRLTDSDRMQQFGLDKSK
LVDTLVQSSIRQILENGFFHADPHAGNLLACPDGRLCYLDFGMMSYVDRAQRNGFLLAVV
HIVNRDWPELVRVYRSLGFIPNDTDAKPIEVALERALPDVLNADISELNFKNVVNKLGDI
FYTFPFSLPPFYISIIRCMGVLEGLAIQVDPTSRIISEAYPYVASRVLTDPQQDLQEALK
RLALTSDGRVRWNRLEGLLDEAKGSSGYDVTSALNLLTDYLISEEGDAVLDDLADGIVHE
ADNLGTEGISYILAAGRALAIRDEISAVNAFRSLQSVITREKNLNEEVKGVLPEPTESMT
RFGKILSLLDIGAETDYSKLVPIVRKLALEPKVQKTANDVTARLGERALSRTLRSLFGLP
PPVFDGKGTSASTVFEESMS
>comp7190_c0_seq1; 192 1 845 LEN=1491; translated
XRFQYDELPVTIVDDDDNLDDNTNSSESIILFLLSFIKSRKMHSIAGFRRKCSRNKSNFS
RGSSNGATIQSPRNSNPRSSSPEIAPKSPIWSPLLFSGSSPQYKTGKKSAGDSDKQSAAS
TSLGGGSIRSLSSSPFAGRHRRQPSLDLCTIAEDRRISSNSLNKMAITTPAKTIARTPTI
MLTTHFSEPLGLQQLPRLEQQPRRDPYNDDTDDDYTNSNDSFDCDNCWAYNKSRLPNDRG
CCFFGFSGQLGVSDSEKWNGARGSSSFHSRSRRQLQRRISL
>comp7191_c0_seq1; 590 430 2318 LEN=2618; translated
MVVVXFQAIPKQEGKNSNKKSIRKRKTTTSLSTISSISSSLFERPSWLQCDPFPYAQLDV
QGQQPYVKPFEDYHDQEENYLHNQQQQPSERIVSYDTGGDSYGQPIDPPELSVGRGRSNK
NKQSKHYPNIRPSGELGQPEQKRKHREPDLQRIQSYTTDGKSFAQPIRPPKSSVGRAKTN
NNNNNRKRKYFGTGGEWTQLQQQNQEEKEQRQSYASGGSSFKQPTQEPEPSVGQRNAKTR
YSQLEKQQLDGSTTTQAKKNASLHSYGTDTVQPIVMDHQLPLSALSSDVRSPFLSGSKNY
YKAKHYYAPKIERSLTMANQEGLKPYSAIRSNSSNSSVIKQEITFPPAESPPPQRLATEM
GRDIATAESISPYPRVQTQSPMLLAKGLTAGRSRFNSSYKTQQIYPKVRIPSPFSFQRRT
QGNGSLNPYNEGGVSGKEPNDPPIQKKGRIPRFKGVPRRTQISPFVKPKYSDSEKTSSSG
DMYTNDNLSEASLDYSTNSDLMYTEESSNTYDNGVNGNFYGQGTGDGVFRSVESYNDVYQ
GFGGENAILDYGQRNDGYDHNEEAREQYLQGLYDGDADDDWYSQSHSFQIPITINICGRE
EMNQGNDSTEQTINININVNTNEGEGYYEW
>comp7192_c0_seq1; 179 1 1608 LEN=1693; translated
XSSSSNNAGAARVSRRCRNALSPATPYLASFLRGLAYACSPDDAPDGYVLLCMAENKLLI
DLLSQRLQTPSTTVAAFSNPMVYCYNSFLGLPVARQAVSYFLANRFLYAPAESGSGAGEG
RHFRGPSPIMAGTTAGTARVSLPQALQSINPAHIGIGSGAAGILNGKMLRKNRQXPKRNA
APVDQNESKKAFSGVDYLFFFGANGSFXIVSLAYLVLALFFLLGDEGDACLIPAPYYAAF
ESDLRIVARVVPFAVHMADPASGPSASELDLAFIEAKSQRLNPRFLLITNPNNPLGVVYS

STIMRNAVAWARKRKVHTIVDEIYALSTHRRHGHGFESIISILDNRLGNDVHFVWSVSKD
FGASGLRFGVVYSQNETLLEGLANLNVFSGVSNPIQMVVSELLTDDDFLDTYLDESRARL
RRSYHICIEKLEEMVLPFVPAVAGQFVYVDFSSLLPQKTTEWEQKLSALLIDHARLVLTP
GESQRERTPGMFRICYAWVTPEVLEIGMERLSRIVAKIRRMDWSDLNESTLSGVII
>comp7192_c0_seq2; 260 1 1453 LEN=1538; translated
XSSSSNNAGAARVSRRCRNALSPATPYLASFLRGLAYACSPDDAPDGYVLLCMAENKLLI
DLLSQRLQTPSTTVAAFSNPMVYCYNSFLGLPVARQAVSYFLANRFLYAPAESGSGAGEG
RHFRGPSPIMAGTTAGTARVSLPQALQSINPAHIGIGSGAAGILNALFFLLGDEGDACLI
PAPYYAAFESDLRIVARVVPFAVHMADPASGPSASELDLAFIEAKSQRLNPRFLLITNPN
NPLGVVYSSTIMRNAVAWARKRKVHTIVDEIYALSTHRRHGHGFESIISILDNRLGNDVH
FVWSVSKDFGASGLRFGVVYSQNETLLEGLANLNVFSGVSNPIQMVVSELLTDDDFLDTY
LDESRARLRRSYHICIEKLEEMVLPFVPAVAGQFVYVDFSSLLPQKTTEWEQKLSALLID
HARLVLTPGESQRERTPGMFRICYAWVTPEVLEIGMERLSRIVAKIRRMDWSDLNESTLS
GVII
>comp7192_c0_seq3; 201 1 1799 LEN=1884; translated
XSSSSNNAGAARVSRRCRNALSPATPYLASFLRGLAYACSPDDAPDGYVLLCMAENKLLI
DLLSQRLQTPSTTVAAFSNPMVYCYNSFLGLPVARQAVSYFLANRFLYAPAESGSGAGEG
RHFRGPSPIMAGTTAGTARVSLPQALQSINPAHIGIGSGAAGILNGKMLRKNRQXPKRNA
APVDQNESKKAFSGVDYLFFFGANGSFXIVSLAYLVLALFFLLGDEGDACLIPAPYYAAF
ESDLRIVARVVPFAVHMADPASGPSASELDLAFIEAKSEVASSHFRASCFQDSSFDAVVF
RGFFVPNPGASCLIHILCCLLLSINPSGVLLFGTKSLGRITATAQSQILVDHEPKQPAGR
RLQQHDLRNAVAWARKRKVHTIVDEIYALSTHRRHGHGFESIISILDNRLGNDVHFVWSV
SKDFGASGLRFGVVYSQNETLLEGLANLNVFSGVSNPIQMVVSELLTDDDFLDTYLDESR
ARLRRSYHICIEKLEEMVLPFVPAVAGQFVYVDFSSLLPQKTTEWEQKLSALLIDHARLV
LTPGESQRERTPGMFRICYAWVTPEVLEIGMERLSRIVAKIRRMDWSDLNESTLSGVII
>comp7192_c0_seq4; 282 1 1644 LEN=1729; translated
XSSSSNNAGAARVSRRCRNALSPATPYLASFLRGLAYACSPDDAPDGYVLLCMAENKLLI
DLLSQRLQTPSTTVAAFSNPMVYCYNSFLGLPVARQAVSYFLANRFLYAPAESGSGAGEG
RHFRGPSPIMAGTTAGTARVSLPQALQSINPAHIGIGSGAAGILNALFFLLGDEGDACLI
PAPYYAAFESDLRIVARVVPFAVHMADPASGPSASELDLAFIEAKSEVASSHFRASCFQD
SSFDAVVFRGFFVPNPGASCLIHILCCLLLSINPSGVLLFGTKSLGRITATAQSQILVDH
EPKQPAGRRLQQHDLRNAVAWARKRKVHTIVDEIYALSTHRRHGHGFESIISILDNRLGN
DVHFVWSVSKDFGASGLRFGVVYSQNETLLEGLANLNVFSGVSNPIQMVVSELLTDDDFL
DTYLDESRARLRRSYHICIEKLEEMVLPFVPAVAGQFVYVDFSSLLPQKTTEWEQKLSAL
LIDHARLVLTPGESQRERTPGMFRICYAWVTPEVLEIGMERLSRIVAKIRRMDWSDLNES
TLSGVII
>comp7194_c0_seq1; 553 73 2067 LEN=2254; translated
MKIFPLYTLLLLTEKLNFATAEGGCGFCEGGSLSNPDALIALTSPFQVHSCFEWQQQSSL
TEQFSEDACNFIGPFYEAICGCSDIPQTFDQCDLCGSPEKVFSNPSKGLLIPKSNSEEIL
CSAVYNGVRLGAVPPNDCQVLRGITNFCGGCIPGPTTGLEETDRVSTLPPSIEAFVSNVE
SPEPTASPTNAPSAGPTTEPTSAPTGVASAEPSYLATTNSSSAVTEISGRRQIDPPTIEN
FVNLSEIPVTCDLCPSTGIVSTPDKLITMIKSDNKIVSKTCGALLWDVRAGRDHIDETAC
SFLQPFFGAVCGCSTFTPLNDEEAQTDTKKTTNIETNQCKMCENPAHAFQKPYNTLQIPG
SKTTEITCGGLFSGAMKGAVSGEECEILSSLAYTCGGCGTFQSRFYEEATPEEQCIDCFD
DNESSMYIYKDFGASGTTFHKAFSAPLHCDKKNKKQPGRSKNDNCSKLLYPELEGCVLAP
ITSFDDLLTIQSIIPPGKAAYTAVHKDGAVLKEDAETCYTSTENDLDSVQISRNRVSERL
SSENCRVENWMEGLEFGYSHDQYENSGESTRSSSAINGALNTCELLKMGWANYNTGTNVP
SSTWENGQLSYCNLGPVQEVGAVWAVEPEGQNYGDLRDVSRNWLLPGAVYKCCKKIYSCF
GEKD
>comp7195_c0_seq1; 302 1 1487 LEN=2087; translated
XPIDLLSPAPPFRTIVRTGIEDPVELGETRLNKSPRQPCKPLSRHKSTQLDTTQLNSTQL
NSNRSMETPDQTDRRHRRAPRTPSSFSFRRTVVLLLAASGLSAVRSLSPPSSPRPPNTNT
NTKTETNCLSDHHPIGGHPQKNKNPSSSSNSNSNHQRTLPSRRQLLDETARITVAAIAAG
SGAPWMAGIQPAAAVAAPSDAKPRGPVVAVVGASGRTGRLCVDACLRRGIRVRALTRTGE
WTAPATPEGAPRPFGGDTTGLLSVARCDVANDSDQSIGEALSGCGAVIYAASASKAGGTA
LAVDHTGVTRVASACLGVGVNRCVLISSTATTRPKSLGYVFTNLGVGGNIMGEKRNGERA
LIEAFEAAGERTLAQGAALPSYAILRPGGLEEPKRNKVLGPPSLELSQGDVFSGIISRAD
VAELTVELALSEAAGTRNTALELYYTDSVVPVEARFGGLLKSESASAGSKLRLHGGTYEE
LLGGLAPGIDFAAVS
>comp7195_c0_seq2; 353 1 1781 LEN=2381; translated

XPIDLLSPAPPFRTIVRTGIEDPVELGETRLNKSPRQPCKPLSRHKSTQLDTTQLNSTQL
NSNRSMETPDQTDRRHRRAPRTPSSFSFRRTVVLLLAASGLSAVRSLSPPSSPRPPNTNT
NTKTETNCLSDHHPIGGHPQKNKNPSSSSNSNSNHQRTLPSRRQLLDETARITVAAIAAG
SGAPWMAGIQPAAAVAAPSDAKPRGPVVAVVGASGRTGRLCVDAVSKNLPRVHRIESNGM
AWRGIGVHFVSQCLGLARNFVVSGSNPQRVSERLFLFSLPLRDTKQIHPATALFGSVLFS
LFSFFFTPLHSSCFPPSAGPRQCLRRGIRVRALTRTGEWTAPATPEGAPRPFGGDTTGLL
SVARCDVANDSDQSIGEALSGCGAVIYAASASKAGGTALAVDHTGVTRVASACLGVGVNR
CVLISSTATTRPKSLGYVFTNLGVGGNIMGEKRNGERALIEAFEAAGERTLAQGAALPSY
AILRPGGLEEPKRNKVLGPPSLELSQGDVFSGIISRADVAELTVELALSEAAGTRNTALE
LYYTDSVVPVEARFGGLLKSESASAGSKLRLHGGTYEELLGGLAPGIDFAAVS
>comp7196_c0_seq1; 267 125 458 LEN=458; translated
MSNAGSEIGSEINADHEENIGSNFLSRLEVSLLVDADGDEDLDIEEEVIDIEEVMELNMM
KEKDRREVKKNREKGKKKGKTLHEGVDPRLFPSYYIKEEEEEKNGDDGDNGX
>comp7197_c0_seq1; 656 1 2578 LEN=2715; translated
XRITTKEASTAISGQTITGRVTTKNVMRILDRVRASGKCPPSLTCEDTNEDFDAIAVEVV
DANELCLSSSQTVSDDFAKLLEMSSSTVSGSKEISNQNAVFISRRSVILTVVGATPTENS
TLAAVLQNDYLGVVKQWMDDILRGTVGGIDLLLHLLTNIAQLPVTKGIVKESGMGKAIGS
IEKHRICVDSPNKVAIVERVNAIKEAWKVSVKVRKDKTLSSSSTPLALKRDLEASSESSL
LRAKKPKLDDTKKSSLSSLLTKVAPSSILSTDDKHGVANVDSSKKVGSILAKKTGKRLKW
KDHFGGTLEASKILEDDHTLRDENPDEDISGFGDRKSRDRNRERELLAKAKKAKLSDDDD
EGLVLTDVNLSIQVQPTIAWNIPPSLPERSDATCNNSKEKLAQTTRMATVTPVEYSSEYN
VPMNPAPLSDVEQALDMTSQSSTVTQIIPFFVLQQPIAPDLAPKVIPTSTPAYSPLLAQP
PSGIANVETVQSLGLPPFLAGQNLQSLRTLANTPELLRSFVDSNGMYDQIRLMNLVETLS
QNSPSSSQQHQGEKTGFQQQKAIGQNTSASGLYGQASGHGGMYGMPSNENKYGGQSAYGS
NNNWQGNGMKTGGYRGAQNSSEGNLHLSGYGPGTTQSEIIALFSPYVQVSEVVMKATFCF
VNTNDPAGAKQAREALNGALLGGQPVRINMAQRKNRDNNMQQNGVSNTGSYYGRNTGMQA
SNTGYSGMTNSGFVQPPPPPMGISPSQTGSDVRDDRGNPATKNLFVAGYGQGTSENQLRE
IFSAYAQISGIISKGTFSFVNTTDKNAAIHAREVLSGTMVNGGVLRINFAKETGRLGTSF
DLTYGGVGGQHQNRSHYGR
>comp7197_c0_seq2; 667 1346 3679 LEN=3816; translated
MSSSTVSGSKEISNQNAVFISRRSVILTVVGATPTENSTLAAVLQNDYLGVVKQWMDDIL
RGTVGGIDLLLHLLTNIAQLPVTKGIVKESGMGKAIGSIEKHRICVDSPNKVAIVERVNA
IKEAWKVSVKVRKDKTLSSSSTPLALKRDLEASSESSLLRAKKPKLDDTKKSSLSSLLTK
VAPSSILSTDDKHGVANVDSSKKVGSILAKKTGKRLKWKDHFGGTLEASKILEDDHTLRD
ENPDEDISGFGDRKSRDRNRERELLAKAKKAKLSDDDDEGLVLTDVNLSIQVQPTIAWNI
PPSLPERSDATCNNSKEKLAQTTRMATVTPVEYSSEYNVPMNPAPLSDVEQALDMTSQSS
TVTQIIPFFVLQQPIAPDLAPKVIPTSTPAYSPLLAQPPSGIANVETVQSLGLPPFLAGQ
NLQSLRTLANTPELLRSFVDSNGMYDQIRLMNLVETLSQNSPSSSQQHQGEKTGFQQQKA
IGQNTSASGLYGQASGHGGMYGMPSNENKYGGQSAYGSNNNWQGNGMKTGGYRGAQNSSE
GNLHLSGYGPGTTQSEIIALFSPYVQVSEVVMKATFCFVNTNDPAGAKQAREALNGALLG
GQPVRINMAQRKNRDNNMQQNGVSNTGSYYGRNTGMQASNTGYSGMTNSGFVQPPPPPMG
ISPSQTGSDVRDDRGNPATKNLFVAGYGQGTSENQLREIFSAYAQISGIISKGTFSFVNT
TDKNAAIHAREVLSGTMVNGGVLRINFAKETGRLGTSFDLTYGGVGGQHQNRSHYGR
>comp7197_c0_seq3; 378 1 1590 LEN=1727; translated
XRLLFRNRLDQILIPVFFHERKAKLSDDDDEGLVLTDVNLSIQVQPTIAWNIPPSLPERS
DATCNNSKEKLAQTTRMATVTPVEYSSEYNVPMNPAPLSDVEQALDMTSQSSTVTQIIPF
FVLQQPIAPDLAPKVIPTSTPAYSPLLAQPPSGIANVETVQSLGLPPFLAGQNLQSLRTL
ANTPELLRSFVDSNGMYDQIRLMNLVETLSQNSPSSSQQHQGEKTGFQQQKAIGQNTSAS
GLYGQASGHGGMYGMPSNENKYGGQSAYGSNNNWQGNGMKTGGYRGAQNSSEGNLHLSGY
GPGTTQSEIIALFSPYVQVSEVVMKATFCFVNTNDPAGAKQAREALNGALLGGQPVRINM
AQRKNRDNNMQQNGVSNTGSYYGRNTGMQASNTGYSGMTNSGFVQPPPPPMGISPSQTGS
DVRDDRGNPATKNLFVAGYGQGTSENQLREIFSAYAQISGIISKGTFSFVNTTDKNAAIH
AREVLSGTMVNGGVLRINFAKETGRLGTSFDLTYGGVGGQHQNRSHYGR
>comp7198_c0_seq1; 566 125 2086 LEN=2951; translated
MNVLVVEESPPPSSPRGGDRAGVEARNRLESRGRIGVPKNRGQSRQQRQRQRRVGTSTSL
AFPIVIVALCGCILFPGVNFVGAFSPSARNGIRGRPRGTGTPRVAPAAQSVELQWQPFEG
SPSPTILPQVRRRTLRPTRSRSSFPTTTRLTTASGDAETQEPSKNTKPGVFANAAAKFRA
RPGTYLMIPVIAGFVGWLTNYLAVQMIFYPVQFKGIPIWRKAEVPLGLLGWQGIVPCKTA
TMSRALVEMVETQLLTVPEAFARLNPGKVAGHLARAVPQLGEEVIRDVVGTAGAAGEPFP
LGLWQKVFGGGGWYSGILRFCNARVLTGVVKDLIRNSERIFSLENCVVDQMILDRAKLGQ

LFQKVGRVELDFLTNSGLWFGFLLGLIQMAVALVWENPWALSIGGTIVGLATNWLALKWI
FEPVNPTKIGPFVVQGMFLTRQKEVAAEFSKFFADNIVTSKQIWNSILTDASTRPALSQV
LGGHVKRLLSIASFGLFKGLPSPETIGKVTESAITNLPGHLSGKLHTYVDGALGLETTLR
TQMEAMSPAKFERVLHPIFEEDELTLIIAGGVLGFIAGLIQQGLETGKIKVPPLPKPLQE
LMARCKTSCSSAVQRLKCVLKKNNNKEKGSSQKNGQNDNVGYDIDNGDGVSGE
>comp7199_c0_seq1; 549 192 2378 LEN=2684; translated
MSGAPRLPSICVGVLVRSLLLLLFLSLSIFRVDGRRRNPTLESSNPSETAGRRLTESNDE
SEGATTTSALLRHVSNDDGSSRSLIGTNVDVPKATTPDDHLVTNLPLLKDFEAKHWAGLL
PVNDKGDGYLFYWLFAPDPKVLQEEKDRGRVKQDESNVPLVLWLNGGPACSSMDGLWLEN
GPFRLDTGNGNGEWGNIKLDPHSWHHAPAYVLYVDQPVGTGLAFTTSGNYPNNDQRVNED
FYYFLTEFMKLHGPNLNVQKNSDGLLTLGRPFFFSGESHAGHYIPSMMNYIRKQNANPKT
LKQRQNGGVVMPLSGALIGNGWLDPVYQYSAHEAAYGYGLIGKAQKRSLALAEEKCQADL
RKGRYTSGTCFGLLEKVVSNSLGKGNDFVVSQYDQRVWEKRRFARDFPPGHKEVEAFLGR
THIVANAPIESVLSAIHAMPSWESGQRYRECTDPPYNALKHQDGLGVVGDIVEVLEDDTQ
KTRLLFFNGVHDLICNHVGNENALEALPWKHRAEYINAERYGWNAASTNKLGGYMKEFQN
LGFLKVLDSGHMVPMDVPSIALDMVRAFVYHEPFNTFRQDINPVSESEVTQNSNGCKDCP
VCEECTMQTCEERFQVSKGSSMLSGIGWGVTAFCAMAGCYCLYFYGSKLLCRRRRQKYYS
DASGAVELSASSAASNGGYSDVDNDVASESDGLFYGDEEFEDQKIDLSDPSFGGSDDDST
RRRVRGLS
>comp7201_c0_seq1; 620 118 2208 LEN=3663; translated
MTRSIHRYYSQAVFAKKKTWPRGVAIQSWGLLSSESPAIAAPSRDFASFHRNKIEGEFGA
YAKQYDNSIQSPEIFWKEAADSLHWFEEPKTSLERNEDNPYLHDWFPDGKINTSFNCLDV
HVNNGRGDQKALVYDSPMTGKKEEYTYNELLEKVSLFAGALQNDLGVKPGDRVVIYMPLI
PQAAIAMLACARIGAVHSVVFGGFASHELSNRIDHSTPKVIITASCGVEPTRIIPYRPIL
DDALRLSKHEVESVVVVQRSNVEECELKSADVDYDELMKHAEPVKALPLPSTHPHYILYT
SGTTGLPKGVVRDTGGYATALKYSMDTFYSSRPGDVFWAASDIGWVVGHSYLIYGPLLHG
CTTVMYEGKPVGTPDEGAFWRIIEEYKVKTMFTAPTAFRAIKQLDPSSEFSKKYDLSSLE
NVFLAGERSDPEIIRWLERTLPHIPPPIDHWWQTELGWPAVGNATGLGRVPIRYGSCSLP
VTGFKISIVDDEGNELAPNELGNMVIKTPLPPGSMKTLYKDDARFVEKYMQKYPGYYDSG
DAAFIDEDGYISIMGRTDDTINIAGHRLSTGAMEEVLLEHPSVADCAVFRVKDDIKGHLP
FGIVVCTKGTDPADHEQICDDLVAMVREKVGPVAAFKNVSVAECGLPKTRSGKILRGMMS
KIANGETYTISPTIEDKSVVEILEPKILEMVGKRVR
>comp7202_c0_seq1; 676 127 1746 LEN=1811; translated
MSSPFTPEHVAEWISFGPKLKGKPTPEQQVAAQERKKKCTEMKVRLAKELNDPAWTEKKI
EKELDRLVAAGESAANEIPRPAYLDPPSGTRDFYPAEMRVQNWLYGKFRESARLFGFQEY
DAPVLEHVQLYERKAGEEIVDQMYNFVDKEGDHVTLRPEMTPTLARMVLNKTNLQTGEVR
VPLPLKWFSIPQCWRFETTQRGRKREHYQWNMDIVGVDHVHAELELLAAAAYFFKSLGIG
SDLVGIKVNSRAVLEAVLAKHGVKKGQKNPTDGGDLFASACVIIDKLDKIGGCAVTEQLA
TIGVSSESSAEILKALEAPTIEELAAQIGDDCEAIHEVKRLFELADEYGIGDYLVFDASV
VRGLAYYTGVVWEAFDRSGELRAIMGGGRYDRLLELYGGEKCQIPCVGFGFGDCVIMELL
KDHNKLPNFVGSGVDYVVCSFSPDLYGQAANISLKLRMTGASVDLMPTPKKKVAASFKHA
DNAGARRMVFVAPDEIAKGVVRIKDLRTKDKNGNAIQVDCPLDALDTVDDLVAKAVEAA
>comp7204_c0_seq1; 449 169 1365 LEN=1628; translated
MVQIHAVSAILFLALIASSKPITVDAELPDAKASFSSSNHKPSNIFRGDPFDTDWDDFDT
VTKRNKSAKTPQFSSSRRGSSFFGFGREFSFFESAPCRVYQSSNNDDGEDRFGGYAYDDS
DADGYDLSNHEDDDRNDEDDDDDFFSRMERYRQRRKSISSISSGGFNDNEGYSFSRGGRP
PFNPWQDKQQRRRREQRQHQKPRIIQGVQDWWMERVDPKLQSLPKIVCRIEPNTTLKLRK
TFRPLKTIVRLGADYNTQQGIWQFKSSWEDSIIGGKITLSGNKELQLTKSWQMSVGGVED
LVTRLRLRATINLQTFQAYAKVGFRTERLTPIDVIEGFTILKRLPLDGSGGNLKLEVKAN
VALPEPEIEYRTESQARSLTGMGNVEVSIDEMNLLLDY
>comp7205_c0_seq1; 621 76 2079 LEN=2079; translated
MDSSTTVTADTRSEAISNRISFKQVVVIFVGILFLAKQFESSLKFETGETLWWQNTAEKH
SHNITQQEIIAPLDEINSTPRNTTRIENDKDKLQRLPPRDDKEGREGIIREGKVTVRSKR
NSRKNSKKNAKHDTWRKGDEDPDLIIPLHIDTEKLEKNQFGNDYDVAKAEANDPLLDILP
STYRKSETLPTWMKEYFDWHRIQTASMNETNWMGSDDYYNRTKDEGMVNEEEYIANNRKY
LIVHCLTKEKCGGLSDRLKPLLMSVATAAHQPTGLNGKEHRRILLIRWQRPYRLEEFLVP
NEVNWTVPSYMEKALSRKEENRSPEQRFEGSSIKRVSVRKKIELFSEEHEDTTVMNIKLQ
EQDGGRGFYQYIVDVAYGSNETATDPLHIKLERYQGVKKTDKSQREERAFQSWNKIYHSF
FRTIFKPSEPVRALIDDKLRVLNLTPGEFLGVHYRADYGMGEAERSGPELIGNTASTIVG
KCASLLTDGSPIYFASDTTSAIERVQEESTVLSTKNITVTTPLAADLAMNKSLAENPKNP

LHIEWDMGPVEDFYPTFVDLLVMGSSKCMIYGQGGFGSFASLLSYNASCWYNFATKCDLP
IERQKFVSPVASTLPSECCIPHKIDRKQSKIARKERLQEEKIKKAQLAEGSANDGSGSDD
GTDDGTDD
>comp7208_c0_seq1; 986 1 3357 LEN=3532; translated
XKQNHYNNQYHEPRTTFVFLSTHEQSLRTNYHGNDEPCTAFATGVIPLDAFIIGSDRDPS
SLLAAKRGEKGLAPKLKNTIHLSTPNSGIVAGLSSSATSALPIPPTRSTRPFRSIVDNGT
NWVTREIAALEFLQTGIPMAKEGSIVHEGWIRQNNGSQHYSHHHHRRFIDHFSETNFDST
NDLKKNEQDQNASKTDRRIEEEEDSIPRISPSLLPVGSIQNISRPPDLVRQGRWWEKWIV
GQNLKESQKQQEDVDVIEDLEEPDVQQQSSSQPQLNQNRQPKISATAAAPIHAPGRRLEG
DYAIKVQIPLANVRNNNKSEVGVVGGGGFGPDHQHNVRQNSYFASLQSTSHQSIARSSIL
REWELRVAHGLEERHLHGQYNSNHFQHQQRHSNPAMLDGRMYMSAKESYPMMMFSLLRYE
PKKEEAVRRRQKLEERGGGGTQFFIMPSRDWRGISYRALLQPVPTHKDDESLPPDESKHA
QVVTSTATTPTRRNKNNQRVLFDRFAPRRNGSKGYDASDPTADSKESDGETTPQESNELN
VDSDEEDERMGDDTYVAGLLDDPEMVQGRHRNVMIGDRGVGPIVSSTIQFVKPKLLKADL
NKQFRERFDGYEPPESQRKFIKAKVVDGIYTLMDPTQELGGEEDDCSSMKDDDNDTIETN
TGGTLNTPTTRATSVSSQSKSNNQRFHRRRLGSTSSASLSTGGGVDEAGSEGDRETIRMP
PSLTLSKIRSLKQQALAAAVRAKLEISTVALAIIYFERLCLDCRVDKSNRRLSFAACLLL
AIKINEAHVGIIITKAIEDSSTSKKSKTSQRIKSLIRPTKKSSNMFASLLVFFTEDWNIS
LKHLFAAEWGVFAALQFRLTAKPSDVAFHFKRLMKTLGSDPRRYLGSKVYRSWQRTLAEE
EFRRQEWEERVEARRQRKEEKKLQQLQREIEAADRRGSSSSHGGSVGEKQDEKDIEDARE
VSSKTLEEPKGLPPLGTTNTGRRRRYFGEILKSRLGGGGITGPPKRSVSTERRRQGNGES
MMATPSAISKPSSAATGGPAKQRLRPLSQSKSMLSLSARGREMIGKNFVIDEVPKSQVVL
RMMKEENIDTKIELNTSVEDPGGGKNGILIDGEGGIII
>comp7209_c0_seq1; 808 225 2498 LEN=2535; translated
MDCVSLEQELASAKSALAKLEEKQRQQQENEEEDGWGEDFNDVQDTPSSVLERLLLLQIT
TDSLDLSQDYLSFLETTRGILGESCDDKDGNDVFIEHDALLAHAQLCEELAHQVILKHAT
TIMDACPALYEEAYLPLFGYVHECLVTLFRDELQSSRYPTAQGCKNLLNSKTGIEHLASI
CQHVVRLEKIHEETVNAVEGVEVATNGNTAMLELFHPLLDRVYFHFINTASNNGDNDELP
LTVTRIDRLPEWLLNYVNSNFLQEQGPFEVVVSVMGPQSASQFCEELVRLIEWVLVEQRK
FFDDPAIVGPKSNPQALLQSIEQFLEFDSALLELLADTTSTSDNMNSAAVKKSTINLPVT
PFSGLMDLLVASSDDLFDWWIQRERESVFSTLFSDESEDDNTSKTQLASHISPRAELFCA
LIRSVQLKASTLTVPGKYLREVAVPICSQFVDALHETSVDLRNRLVQQHNGQQASPQTSG
IVDSEKQLLVSNINEWIEIINGTQLASQLLLSKERAVSSQSDHDLGRFGRSLERLVEVMM
DEFAVTFVETTLMEQAKFANYLMLASHILASPEFAEETKTWKSGITVELEDTKTVLQYFQ
ETCDSILQPEKSLDDDLGIVEEHLSSFAPLGMRARVADRIVDKLLEVALDSNQVTPDIWL
EGAKIFAHDVQVLVGSFSDIPVVNRLLDVTKFMTQDYDSFFGLFSAIGGLMGSDAFLDIE
ELNADTTLHEEATSMLKAKNINCRLEYAVSILNRRRS
>comp7210_c0_seq1; 425 603 1706 LEN=1946; translated
MFESILSALLNRYGEEHHRVGSALHNVAVANLRAGLLNDARDAIEEAVRIRKLTLGEDDP
KVADSLVEYGIILLSMKKYKKSINVFEDALELREIECCEAEDGESKREAKLKKAKIHHNI
GCVNFEIKNYDEARKKYMQAIKEQKEIFGSMWLSAFRLVSNTSKPGHLTMASTMCNKGYI
DLEQEKYRDAIETFTESLKIQKVLLEADNKLILSTMTNIAFCHCKLEETERARAVYKDLV
KLQSESYTEQSQRGWSQSLKKLIYCQVVSFQFEDAFDNLRRLEEYLTTKARDKHKNAASE
LRKTHQLMGEVNYQIFKFPTLSDYTSRLTCTNTCGCADDRANVDVSNWFPKKPVNGSKMS
GHRMTYA
>comp7210_c0_seq2; 542 151 1584 LEN=1824; translated
MGRKSKDVGRSGNQDDREQDGFFGRLFSPGTVDTSVSENSVEEFDSDFDESIDSGTYYTD
IDDSQSEESDEFTEESSHGASLVRFDKELRAKHRAACKSMTIGKYGRAIKMFESILSALL
NRYGEEHHRVGSALHNVAVANLRAGLLNDARDAIEEAVRIRKLTLGEDDPKVADSLVEYG
IILLSMKKYKKSINVFEDALELREIECCEAEDGESKREAKLKKAKIHHNIGCVNFEIKNY
DEARKKYMQAIKEQKEIFGSMWLSAFRLVSNTSKPGHLTMASTMCNKGYIDLEQEKYRDA
IETFTESLKIQKVLLEADNKLILSTMTNIAFCHCKLEETERARAVYKDLVKLQSESYTEQ
SQRGWSQSLKKLIYCQVVSFQFEDAFDNLRRLEEYLTTKARDKHKNAASELRKTHQLMGE
VNYQIFKFPTLSDYTSRLTCTNTCGCADDRANVDVSNWFPKKPVNGSKMSGHRMTYA
>comp7211_c0_seq1; 290 74 775 LEN=1288; translated
MAVLQTKHATLILLLLVACVLGLASAGTNEAGLAFLEENRGKPGVVELPSGLQYKVLRRG
RGTAHPTSGSPCSCHYEGTLIDGTVFDSSYERGEPASFAPNQVIKGWTEAMQMMVAGDKW
EMYIPSELAYGDRGSPPNIGPGETLIFRMEILAIQGSSTPALQCKIASPDDDCNEREKTY
IGKVSGYDASKASKEIRRLRSILGGPMKDDLRDWARRRVNILEQVVEGQEAEL
>comp7211_c0_seq2; 286 74 957 LEN=1470; translated

MAVLQTKHATLILLLLVACVLGLASAGTNEAGLAFLEENRGKPGVVELPSGLQYKVLRRG
RGTAHPTSGSPCSCHYEGTLIDGTVFDSSYERGEPASFAPNQVIKGWTEAMQMMVAGDKV
RVAMNSWRWVSSFALCRLELGEVLWVRLCSLFVCWIAGLLTLALPHNVSHQPPRLFYSVS
XEMYIPSELAYGDRGSPPNIGPGETLIFRMEILAIQGSSTPALQCKIASPDDDCNEREKT
YIGKVSGYDASKASKEIRRLRSILGGPMKDDLRDWARRRVNILEQVVEGQEAEL
>comp7212_c0_seq1; 562 57 1166 LEN=1636; translated
MTMAANENGNKVALITGITGQDGSYLADLLLKKGYIVHGIVRRSSSFNTGRIDHIYRDRH
DSDVKLFLHYGDLCDGTNLITLLLKIKPTEIYNLGAMSHVKVSFDMPEYTADVDGIGVLR
MLDAIRAAGMENDVRFYQASTSELYGKVVETPQSETTPFYPRSPYAVAKQYAFWILVNYR
EAYGMHLTNGILFNHESPRRGRTFVTRKITAGIARIVCGLDNCIYLGNIDSKRDWGHARD
YVEGMWRMLQQDEPDDYVLATGETHSVREFLEKAFGHAGITLKWKGPTGTVDEVGVEEGN
EDRVLVKIDPAYFRPTEVDLLLGDPTKAKAKIGWEATTTFEELVKEMVEHDIAMMKGEVE
DTDNVKAAN
>comp7212_c0_seq2; 442 423 1244 LEN=1714; translated
MSHVKVSFDMPEYTADVDGIGVLRMLDAIRAAGMENDVRFYQASTSELYGKVVETPQSET
TPFYPRSPYAVAKQYAFWILVNYREAYGMHLTNGILFNHESPRRGRTFVTRKITAGIARI
VCGLDNCIYLGNIDSKRDWGHARDYVEGMWRMLQQDEPDDYVLATGETHSVREFLEKAFG
HAGITLKWKGPTGTVDEVGVEEGNEDRVLVKIDPAYFRPTEVDLLLGDPTKAKAKIGWEA
TTTFEELVKEMVEHDIAMMKGEVEDTDNVKAAN
>comp7212_c0_seq3; 442 523 1344 LEN=1814; translated
MSHVKVSFDMPEYTADVDGIGVLRMLDAIRAAGMENDVRFYQASTSELYGKVVETPQSET
TPFYPRSPYAVAKQYAFWILVNYREAYGMHLTNGILFNHESPRRGRTFVTRKITAGIARI
VCGLDNCIYLGNIDSKRDWGHARDYVEGMWRMLQQDEPDDYVLATGETHSVREFLEKAFG
HAGITLKWKGPTGTVDEVGVEEGNEDRVLVKIDPAYFRPTEVDLLLGDPTKAKAKIGWEA
TTTFEELVKEMVEHDIAMMKGEVEDTDNVKAAN
>comp7213_c0_seq1; 590 240 1691 LEN=2065; translated
MTILARSRRLFGSGTHPKAHVHSLSSFKAAPLATGASSRTRLQQQQHSQTTNRRFGSVAA
ASSNAWTRSRSSNGSRTNGSTTYFGVMASAAAATSLLLAPGSLDWNSPSTTLCEEAAPAG
EGNSIPEESEHDPYDNLPEEDEPTHCSICLTYRQGPCRPYWRKVEACTKDHEKENKNKAE
EGKSPSDKSKGDDDSSDEGDPSSPSERCFKYMMPWIDCASKYRNLYTLIEMDTNYTEGIQ
ELEATSRHFCWMTGKEPAIDWTKWQEYISSKEQEDEDQTSAETTKNSSKDQGTVSDADPS
VAMWKTLDTALGDPELVTIETKVPPTIQSPDGDSSGLGILECAYVTDQDNNVIGFAYGTK
PSELMSDSKDTNKGTEENTTEDNKTTARSNEKSGDGTSEDGGEKMVPLNIRLVVSRTREF
VLSASYTHRRIAEGTAAAAKTNGSDSSKKEDAKNFYLESHLYRSKPFSLADAPKAPQQKP
TEQ
>comp7213_c0_seq2; 501 240 1887 LEN=2261; translated
MTILARSRRLFGSGTHPKAHVHSLSSFKAAPLATGASSRTRLQQQQHSQTTNRRFGSVAA
ASSNAWTRSRSSNGSRTNGSTTYFGVMASAAAATSLLLAPGSLDWNSPSTTLCEEAAPAG
EGNSIPEESEHDPYDNLPEEDEPTHCSICLTYRQVRRRVALRANDRGKSDXEVLIHLIVG
FPEHILSEATNSKNSQRFDSITVLFELFHSATSFSIKFMGPCRPYWRKVEACTKDHEKEN
KNKAEEGKSPSDKSKGDDDSSDEGDPSSPSERCFKYMMPWIDCASKYRNLYTLIEMDTNY
TEGIQELEATSRHFCWMTGKEPAIDWTKWQEYISSKEQEDEDQTSAETTKNSSKDQGTVS
DADPSVAMWKTLDTALGDPELVTIETKVPPTIQSPDGDSSGLGILECAYVTDQDNNVIGF
AYGTKPSELMSDSKDTNKGTEENTTEDNKTTARSNEKSGDGTSEDGGEKMVPLNIRLVVS
RTREFVLSASYTHRRIAEGTAAAAKTNGSDSSKKEDAKNFYLESHLYRSKPFSLADAPKA
PQQKPTEQ
>comp7215_c0_seq1; 674 1 1870 LEN=1870; translated
XSTDVRGILNLLRDGSEESSLDPFGMIQELQASLSIKKDQSLEERSLDIANTSIRIGHGG
STLYDRRDSEELSTPPAPFSVFYRSPEQRSKDLSDATNWLREKNSDNYLDPTGDFFKLDS
ILPRKRDQSFEDRAKVIEEGLDWIRGQGMVKDDANDELTGFQELSSLGISKSSPEKRLED
LENAIIWIRNGKVKPGKNDPSRDFYKLDKLLPKKRGQTSEERGRAIESTLDFLRSHNASP
DDDDVIDKYSVIGSIPVSPRTPEQRYKDLNDILNWIRHQGENDGIYDPNGYFQKLDDVLP
QKRGQKVKDRSRQIEKFLDWVKGSEISSDEIETPLLDEIKAVRHIYRSPEQRIEDIEKVM
NWITRRGKKDKIYDPSGDFRKLDKLLPKKKNQPLNDRARAIEGALDYIRNTGISLKDDGI
VEKLSKVGVIPISKRTPEQRVQDEHDVLEWLRNKGKNNNFLDPTGDYQKINSFLPKTSGQ
SREDRAKEIEMALDWIEHKAFGGTDYLLNSFDQKTIDSDEGDNETKSKTYVEILNWLKNA
EKENKTYDPTGDFKKIDKMIPKKRGQSFEDRAHEIEEALEWMRQKEDNNKSKGKRNEKDS
VRTIQLTVRKSPEDRLKDVDDILN
>comp7217_c0_seq1; 194 401 877 LEN=1070; translated
MPVNAAPPISVIAEELGYFPINTKDGDVMYVPKRVSRKSSPQAIELANALKEKGITMYGA

YWCPHCSRQKELFGAEAWSVMNYVECSPKGYGFKGQKMCQGIDGYPTFKDKTGRIVNVSG
ERPLEFLAQQVRFSNFDPLLEEDLPSAIGTACKLPARQ
>comp7220_c0_seq1; 411 104 1078 LEN=1104; translated
MPHQRFPKIKILHMAPHEMRFILSETDTSVANTLRRIMIGEVPTLAIDLVEFHENSSVLN
DEYIAHRLGLIPIRYQPVDSMKGQDCNGAFLPHRECVCYERCPRCSVEFELDVAFDEANT
FRSEEELMAPLTITSKDLKSNNENVIPAHFLSQDEQDESQDAGVAIVKIGPGQRLKLKAI
ARMGIAKEHAKWCPVAIATYRFWPNITINEEQIATLTMEQKQEMVDVCPDKILEIDDVTG
SIKAVDDAWDRCTYTEDLQEFQQSIKKRKEDDDFVTVEAAEDKFIFTVESTGVMDADEIV
ISALRVLKDRLNYLAQEVENIKDM
>comp7221_c0_seq1; 55 1 485 LEN=780; translated
XTMKKASTMATTGFVFLASRTISIIPTARAFTLVTPKHAAVTARNRFLSTSMNASGPRQT
AIEERLTEAFAPTFLDVQNTSHGQKSDESHFKVVVVSEAFEGTRLVGRHRAINKAVSESD
GSLGFHSLEIGAAKTPEEWNQNSNVAPSPKCQGGDGRGMKN
>comp7222_c0_seq1; 1521 90 4145 LEN=4203; translated
MSEETRFPIPASLQDLEREPYNLLPYPNDFDDDNEAARADSFSRLVTSIEQGNQTLLTDG
ICLFQKQDPHLNEVHYTWMDGERTQALYTLVRKSTSLAPGTRWALIQALCQSVKILSSLL
EGDVNSNPDDREAIQGDKAMAMPQSSQKTSRQNNVFSQEFRDAMACHLYMLFSIMFFMES
EARIGNSSGNKKSSSRGGVKKGGKSTGDSYLDGEDTIKMRATCADAMLIAAQSMGANRFK
LWKRGVADESVIVLPCRIAYQMLESASGVIARKAASGDAALAMIAATVDSCDSLMGTIIA
ALMDLMHSFEHLAPLCAELCTLVSTNKLGVELIREVGRLDTSRSGGSDSANKASGIKFVA
PFVCELALCLPQLVLSNFSHLLHHLETEPYYLRSAILTALGHIIEFIGKSLQPNSSESSS
SQSIECETSPGNLQKSRSALLDILQERSLDISSYTRSAVLKSWIRLADSGSIPVERVLPV
TRMAIDRLQDKTVIVRKQSLQLLTSLLENNPFMGDLDPKPYRNKLAEMYSFVKDNLPENI
KEAYEASLAEARANEESENSILQLEQATLAAAIAEANTMDGVDELNVRENEFRSKVQALK
FAQSALDFIDHFEDASSNLHGMLLSANVSDVTEALRFFVKARHFNLPCAVTGMKQALTLM
WSTEQNIRDEVLKAFLDVFIAKPGTDGKDFLPSNQIANNLLILMSDANVSELASIEEAIG
SLVKDEKIPAEVFSSLWSATVIARDRDTRASALHILAMAANTDRAIVDSKSRLKIISDHA
LGDRTEENRDWKMAQAGALALQRVDRAQIDTSCAKYLVLEYIIEQLCTIARGDWCIDDVE
KDTLQWFSTAEQVIGALFVISPEPEVCCAEIVNGMHNQTLGGSSKVEECHPLRLARFFHV
LGHIAIKLLVYTEALSGSVRRANAKKCLKKQEEADKAKQERMSESCRSIDEDIEAELGME
AELEAENERQVAEIAEKEIVGRGLLSVFGPLLVRVVANDGEKFNSEILRQTSALALCKFM
CVSSSFCDQHLPVIFTALNNAPEDDITLRANTVIALGDLAFRFPNEVEPYTPRIYSCLRD
KSTKVRRHTLMVLTHLILNNMIKVKGQVCEIAILLRDPDQRIRDTARLLFHELSKRSNNP
VYNLLPDIISQLSLVELAKEDFRYILAFLLGYIKKDKQNEMLIEKLCQRFSKCRTISQKA
DISYCMAQLKVNERSIKFLVDNFKLYKDALHDDDVKRNFASILSKAKKVAKPELKQGLDE
FESKINQEAEVGMENQLAGEKAAKAKARASKRYGRRLPKAIAEEPILESDNDSDNESEIE
SEDDVPSKPLTPVNPNKRSGRRACRKTRNLM
>comp7223_c0_seq1; 314 422 1534 LEN=1665; translated
MYLSDVQDEHVWTRYMAVDKMRHYKQVLYSTHDLKRFSINGETFSRQAVEDENIFDIVDA
LVLYQGGKDSTIQELVLESFVLSPVGWKSLSGALRKLFRLKKLFLREILVVPPSGFDDSR
DHDDGIRLVEGLSGLVEACPNLEELHLVECNLHPRACGFLSGDLFEGPSPRRPPSLRVLS
LEGNPIGNEGVCLLAEAIRDNGSLVALDLDRVGCSGSGMAALADALGDNDCLESLSLVGN
EIGGERGPLRDEDERDICPGEGRDAGDGADADKAGCPFCDLLARNTRLKRVRLFQTEASQ
ISPPPPPVRESFAHVDFLLRLNRRGRRYLGDADVIPKVFPLMLESASDDADVIYSFLKSQ
SGFTKYRSLQ
>comp7224_c0_seq1; 441 192 1166 LEN=1343; translated
MGANQSHQIPPVETKSKKEKTFSTEGAGDSNEDMPIFPSPTGHEYDEIDKLQSELPSLID
EESQQQVDDYRDACNNGKGPVVACFATAEFLSMFERKHMEAFKLFENTCFRPLKDKSPNG
KEVDGTKAYPPACFNLAKMLMTGKGGVPVDRKRAYDVFDRACRGGHGGACHIQAKMLLSP
PGALGKGIPYDPYKAMDLYQGVCDMGDSVSCFTLATMLLRGDRINKMAKNASPQELQGQE
PIKQRKNEDDRSAKADEDFEYIPRDPKRAEQLLKSACRTGAHAPSCFNLTVMYQNGDDGV
PADPVKAEEYKKKTEKAVQTLGGL
>comp7226_c0_seq1; 103 1 285 LEN=285; translated
NTFTCTSALFKMVQLTKYSVLAASLAVAAVSGTTTEEMVNIDLIEADADFERLLGGTYGS
MKPRGMKGAKSSKGGKGYKGYYGDDDDDGKGGKGS
>comp7227_c0_seq1; 364 166 1725 LEN=2247; translated
MLPSSTNSFQIVESPATSCARSRIPDRGFAREGTSLMVSTERPAGFDVASSPSDCSLRDF
DPDLADMIDSENTRQRQGLELIASENFASKAVREVLGSCLTNKYSEGMVGKRYYGGNEFI
DQIESLCMARALSLYGLDSEEWGVNVQPYSGSPANFEVYTALLNPHDRIMGLDLPSGGHL
THGFQTAKKKVSATSVYFESMPYIVSPDTGLIDYDDMEYRAKMFLPKLLIAGGSAYPREW

DYKRMREIADSVGAKLMVDMAHISGLVAGRVVESPFQYADIVTTTTHKSLRGPRSGMIFA
RSEYMDSIDSAVFPMLQGGPHNHQIGALAVALKEASEPKFTEYAKAVITNAKALGKGLES
RGHKLATGGTDNHLLLWDVRPLGLTGAKVDMVLENASITANKNSLPGDLSAINPGGVRLG
TPALTTRGMDEDDFEQVAEFLHRGCKIATEAQEIADRKLKAQLESGEIKRKTKKVLLKDF
KQVLESDQDIKAKIDELRQNVEDCHPILFARMELGNFVE
>comp7227_c0_seq2; 364 166 1725 LEN=2060; translated
MLPSSTNSFQIVESPATSCARSRIPDRGFAREGTSLMVSTERPAGFDVASSPSDCSLRDF
DPDLADMIDSENTRQRQGLELIASENFASKAVREVLGSCLTNKYSEGMVGKRYYGGNEFI
DQIESLCMARALSLYGLDSEEWGVNVQPYSGSPANFEVYTALLNPHDRIMGLDLPSGGHL
THGFQTAKKKVSATSVYFESMPYIVSPDTGLIDYDDMEYRAKMFLPKLLIAGGSAYPREW
DYKRMREIADSVGAKLMVDMAHISGLVAGRVVESPFQYADIVTTTTHKSLRGPRSGMIFA
RSEYMDSIDSAVFPMLQGGPHNHQIGALAVALKEASEPKFTEYAKAVITNAKALGKGLES
RGHKLATGGTDNHLLLWDVRPLGLTGAKVDMVLENASITANKNSLPGDLSAINPGGVRLG
TPALTTRGMDEDDFEQVAEFLHRGCKIATEAQEIADRKLKAQLESGEIKRKTKKVLLKDF
KQVLESDQDIKAKIDELRQNVEDCHPILFARMELGNFVE
>comp7228_c0_seq1; 375 173 1252 LEN=1543; translated
MASRLFARRALPAAAATLAASPSFFSNPGAASCDASPAIESSKPDASEFLKASSKPKAGM
NPSATGEFHKLFPKRQLWQPRVEYPMWDEDWDGREPASTGSKDEDRRRKRKLRKEGVTRH
IILVRHGQYNEQEKEDEKRLLTPLGREQADHTGRRLREMIEGVAGTEFGGCSVKVVRVSN
MARAKETADIIASHFPEVPRAEPDPDLNEGRPAHNIPGGRASSSTVERTDESHRRIEAAF
QRYFYRAGDLPEASIEHDADAAAGNSSESANADPNSGERKEGGRHEFEVIVCHANVIRYF
LCRALQIPPEAWLRLCTFNCSLTYLTIRPTGSVNCRMLGDIGHLPYGKSTFSSHAGFNW
>comp7228_c0_seq2; 352 173 1207 LEN=1789; translated
MASRLFARRALPAAAATLAASPSFFSNPGAASCDASPAIESSKPDASEFLKASSKPKAGM
NPSATGEFHKLFPKRQLWQPRVEYPMWDEDWDGREPASTGSKDEDRRRKRKLRKEGVTRH
IILVRHGQYNEQEKEDEKRLLTPLGREQADHTGRRLREMIEGVAGTEFGGCSVKVVRVSN
MARAKETADIIASHFPEVPRAEPDPDLNEGRPAHNIPGGRASSSTVERTDESHRRIEAAF
QRYFYRAGDLPEASIEHDADAAAGNSSESANADPNSGERKEGGRHEFEVIVCHANVIRYF
LCRALQIPPEAWLRLCTFNCSLTYLTIRPTGSVNCRMLGGEFCF
>comp7228_c0_seq3; 299 538 1969 LEN=2008; minus strand; translated
MVHDKIRHQEKHETFLETKLTPEHPAVHRTGRTDRQVRQRAVEGAQPQPRLRWDLQGPAQ
KVPDDVGVADNDLELVAAPLFPFPAVGVGVCAFAGVAGSRIGVVLDAGFREVPRAVKIPL
KGRLDPSVRLVGSLDGRGRGPPTGDVVGRSSLVEVGVGFRPRDLRKVRCDDVGGFLRPGH
VRDPDDLHAAAPELGAGNALDHFPETPACVVGLFTSQRGEETLLVFLLLLVVLAVPDKDD
VAGHPLLPQLALSAAVLALLPVEAGSRPSQSSSHIGYSTCRWDAMQQNAMQHKSKEQDRI
DQHHTRILNEPQIRQLKQEGIYISSKIDLSTLIASSNISFAQYGIHCHKNAPLVAIAAVS
EIVCETPPVADGFIPAFGLEEAFRNSDASGFDDSIAGLASQLAAPGLEKKEGDAASVAAA
AGSARRANNRDAIGIWVRMQRKRKLSKERTAGCYYKKWLGLELRLGLVWSVPVEQG
>comp7228_c0_seq4; 290 343 1723 LEN=1762; minus strand; translated
MSNIPEHPAVHRTGRTDRQVRQRAVEGAQPQPRLRWDLQGPAQKVPDDVGVADNDLELVA
APLFPFPAVGVGVCAFAGVAGSRIGVVLDAGFREVPRAVKIPLKGRLDPSVRLVGSLDGR
GRGPPTGDVVGRSSLVEVGVGFRPRDLRKVRCDDVGGFLRPGHVRDPDDLHAAAPELGAG
NALDHFPETPACVVGLFTSQRGEETLLVFLLLLVVLAVPDKDDVAGHPLLPQLALSAAVL
ALLPVEAGSRPSQSSSHIGYSTCRWDAMQQNAMQHKSKEQDRIDQHHTRILNEPQIRQLK
QEGIYISSKIDLSTLIASSNISFAQYGIHCHKNAPLVAIAAVSEIVCETPPVADGFIPAF
GLEEAFRNSDASGFDDSIAGLASQLAAPGLEKKEGDAASVAAAAGSARRANNRDAIGIWV
RMQRKRKLSKERTAGCYYKKWLGLELRLGLVWSVPVEQG
>comp7229_c0_seq1; 285 176 835 LEN=932; translated
MTMVGEAFLPPNPKNSARTQHKSVLQLQSENNEETSRRDFVSTTFGFGVSAATGLVMVSG
QPALAAAASQEEMDKAKVIKGYQRLQYLLDNWEKETTVCGMGGDKLEVSCDRTPIKVMDY
LGFKSTTDPLYKGEKTLRRLYDIAPANRDSEFIEAVEIFSENAEEASSMAFVSSWGEANP
GGGKDRVELFIERAKKNVIATRNSLKTVIDILDLDASKK
>comp7229_c0_seq2; 285 179 838 LEN=935; translated
MTMVGEAFLPPNPKNSARTQHKSVLQLQSENNEETSRRDFVSTTFGFGVSAATGLVMVSG
QPALAAAASQEEMDKAKVIKGYQRLQYLLDNWEKETTVCGMGGDKLEVSCDRTPIKVMDY
LGFKSTTDPLYKGEKTLRRLYDIAPANRDSEFIEAVEIFSENAEEASSMAFVSSWGEANP
GGGKDRVELFIERAKKNVIATRNSLKTVIDILDLDASKK
>comp7234_c0_seq1; 735 325 1977 LEN=2425; translated
MKVTPNMGLQGLGIAVGTMVVLAFLTAKPSVEREVRVAMIGNSLMYYNDLPRLLEAMSNG
RLSQDSCLHGSASLKSHLYYGNGMFVKWDTGNARIWNVDNADYEGAAEAYTEYMDYYNGN

NGDDGNNYDDDAQQQQQYTYQLDSTQSHIYDFGACTVPQLLLGRDERLVEKYSDNYYTDD
YNEDDLDEDEDYDNDDNNDNGGNQRHQRQLRRERARRRMQHQSQRHETQHQLITRPSTET
QSQSNLRVLEQTYHYTYAADDAASVDDFNNNNNADDEYNTYSVVDSFTDTYALQNDGKNP
CLLSANYYFFKQSQYDEFNGGVPKWDYILINDMSRGPCCTTPRAESMELLVDVYVPWILK
TGAVPVFMVTHAYWPSTRDMSGLTDIPTFMSLTYEGYREYAEAISIYLPEHQQPKLAPVG
LAFLLVWEENPTMWANLIHMDEIHLTPTGTYLEGLIVYATLYGHLPSPRVVLNENIANLF
SNARRMTPSEHIQEAYPTLDNARYLYHIASRIMNGALPRSFKRYSNGESVDFEPNDSSSS
SSNNNNNNNN
>comp7235_c0_seq1; 448 195 1220 LEN=1395; translated
MTPPKYTRFSDYEYSRPPDSPSAAPSPFPASVDLKGLGLNANQLALLGTRLNNLQAMPAP
TEPLKLKPKNGRKNPPSTKNQKAQKKQQQLLDKKSSKDTGDDDDDDEDENNSKESSLEVA
SLHPEDQIQKSLREITSLLLKERTGGRQHYFPRPYQEKRQVVFVLANYFVLFLSLIAISA
EIQARAPGWHAAVEQQLMNVQDCSKDKESLFQCVENGDMAGLVASVILWMSRSAATKRIF
LFGFSSPNKLWTAVYESLVTSICWGFSYMFIRRGMNPDNNRDFLRRYWKDAVYGSLAGFN
AAFMKQVLKNLIPQEVIEDALQERQLKILSWLPSFKPRGEL
>comp7235_c0_seq2; 978 112 3172 LEN=3172; translated
MTTQTEIPIEHLEEKIESVEYEEPQTTEKSETTMILSDGDQPNTDPTAATTPEDAPNKSI
SFHDENTTEVVESIGFPIESDDDATGASSTDSDVDADGDAGMITDVTDVDAKVDADADPE
VDADLDGGVVAVDTSAAACLEPEGTTPIPKGKHSRRFDDSMLDVVLADSGDEKDVAAGEQ
PPIPTPLEITENRSNVTSPMLEADPTDHEQRSRSRSPRKIPREPPGSAVKRARSKHAVER
EQQMKKTTDNHHPPAEQTPTKPKDNGVQPEGAGAKKENSSDGDKLESVPSMPMETAAMDQ
QIYHEAMESTTLTSTNDRDPNAYIGGSENEQQPFESSQTPASEASGMGLETKNIKAVDNS
GGGVAVKPIASNMFAHATRTTPDTLSPDLLLSDYDPIPIPLLPPLVVPQIGSVGSYDSLS
MQGLHQPPPARLTPHNGYQHPLPQRPSFTQQPMQQQHQPTPQSMLQIPHQVATMPGGKRK
IHLHLWEQVKAGTLSNNEPEKSGFLSFRRKKGILRKRPSSDISSPIVEYGKATGAETESD
QWTNRGTLTVSWYEGTTSIELQEHVQNSVRRKLGLKDSIKLVDFRVLDESSDPPEEIVLC
PYIPDGSRLTLRFATTNDGYMTPPKYTRFSDYEYSRPPDSPSAAPSPFPASVDLKGLGLN
ANQLALLGTRLNNLQAMPAPTEPLKLKPKNGRKNPPSTKNQKAQKKQQQLLDKKSSKDTG
DDDDDDEDENNSKESSLEVASLHPEDQIQKSLREITSLLLKERTGGRQHYFPRPYQEKRQ
VVFVLANYFVLFLSLIAISAEIQARAPGWHAAVEQQLMNVQDCSKDKESLFQCVENGDMA
GLVASVILWMSRSAATKRIFLFGFSSPNKLWTAVYESLVTSICWGFSYMFIRRGMNPDNN
RDFLRRYWKDAVYGSLAGFNAAFMKQXPEEPDSAGGHRGRPPGAAAENSLVAAELQAAGR
ALTMKAKNDALLHRARTPSKQIPVLHAAATTKRGFISSCYYVTCIITKTIEILIDFFQEI
G
>comp7237_c0_seq1; 535 210 1417 LEN=1768; translated
MTSLPRAIVAFAILSSAMGFVPPKTETRIHGSTALQIKNSDRARTERLLEESMGDDWRLF
RAKLVAREKASKPSETNNIENQTPTPPSSHSTDSENEDPQASSSSTPPSSSKQHDELEKQ
GQLGELFGAAISSIFKSGNQKESTKSSNENIYRGDSIGRGIPQSELTSQDPFLSADELPI
HIKPKKYTPVDKHRWAHEIDHIEPGCVLIANEKLGGVFCQTVVLVVDHHEKTGTTGVVIN
RPLDGNLLKIASEQQTNLDLSLKLAFTSSPVTYGGPVLTEEFAVLHGFGHVEXFKKLAPG
VYIGGSEELMSEVRISRFDPRNALFIKGHAAWVSGQLEREIQKGVWYIASTSSDFLLRYA
GAPVTEDDDETDLWADILNCMGGKYAEIASKHSGRGDRRMMP
>comp7239_c0_seq3; 118 1 814 LEN=906; translated
XFNCCSMVKSFSFFRIFASSAAISSMLSITRPVVAWTVRQNCRVALSSLASATGRLTRRK
LSLETLSTLHGSSLLPGKSLRFDCLENVRRSTAKRFMVSNELGTEVSFRPGDKVQVEVVN
FGPLGASVEVIGLGHGDDVPLLPVDAPEAYGYGLIMQREISFFRSARNNVDVVRGEILPA
YVQKVRAEDGKLDISLRSYGGKAKSADAGTKILERLKEVGGRLEIGEKSSPQEINDEFPG
ISKSVFKKALGGLYKKGLVKPGPKLIKLMEK
>comp7240_c0_seq1; 959 123 2045 LEN=2377; translated
MFSSQILVFVLATSALLHPAQSFAPPSLLSASSKASSLVLNGKKSKRKGGGGGYSGGGKK
GGGQQPRQEKQSVQDQRFDAATRQFMFTLSGLSKMLPDKSKKILDNIHLSFYPGAKIGIV
GLNGSGKSTLLKIMAGVDTEYDGLARPLPGASIGYLAQEPVLEFPTVQENIDEAMKSSQA
VLDEYNEKSMKLADPDITDDEMTQIMEDIEKLNDQIEAGDMWELDRVVSRAMDSLRVPPG
DALVENLSGGEKRRTALCRLLLENHDMLLLDEPTNHLDAESIQWLETYLDKFKGTVVCIT
HDRYFLNNCAGWILELDRGKGYPHEGNYETFLDMKNKRLEEEKKAETAAAKAVANELEWI
RTNPKAKGNKSKARLNRYDELLLAAAPTELRNAGQIYIPPGPRLGNEVIEVTNLKKSFGD
RLLIDDLSFSLPPAGIVGVVGPNGAGKSTLIKMLTGLEEPDSGTIKVGDTVKMVSVGQER
MDELDSSKTVFDEISEGLDEIELGTSSIMSRAYLSWFGFKGQSQQSKVGNLSGGERNRAQ
LAKLMKAGGNLIILDEPSNDLDVEVLRSLEEGLLNFAGSAFVVSHDRYMLDRLCTHILAC
EGDSKWHYFPGNYAEYEEDRMKRLGESSIKRITYAPLVNS

>comp7241_c0_seq1; 1011 635 3853 LEN=4074; translated
MSYSQDLQQLQQQRVKVVAGYALQQRLGSGSFATVYKGIRVEQKKESQQEQPQTMSDDDE
NDSSCDSSTTQRQDKEPPRAVAIKAIARTSEKLTKKVLQNLEIEISILRTYRHPNIVCMH
DVQKTDRHFYLILEYCGGGDVQHLIRSRQKGRLSERFTRRLMRDLASGLRFLWGQELIHR
DIKPQNLLLTGRIPPEELVDNPPLENAATSCSESIHDDPNQGFSLKIADFGFARHLQTAS
LAETLCGSPLYMAPEILQHHRYDAKADLWSVGTVLFEMIAGRPPFNGENHIDLLRNIQRK
AVRLPHDVRVSKACVNLLRILLNRNPLSRAGFKEFFEACDAFVALGCGGVATGDYEGGVN
TNSSITKGGSNSKTIGGGIVGGINRIATKDLGTIHENDHSGTNPTSSDSLMTVETTAERN
QQEVAPTNTVNATSVVSTNATRTMHPHQKHQQPLPPSKISPNIGYQATSNFIPNSISMGN
NSANPQLSGRPQTFYQQPNNRNRLAPLQSSPPSSTGILYLNSRIPAPLPSPTTQNAEEQH
PQPQRHRQQTQHQSTAGYYAAGAANGNSVSQSQVSSNDDSGFVMVEHTKSPTSSQHFYAT
GSQQNLHRNNEITEHRARSSKGMLSTSPGTGKLLLGMVGRATIGFGVTVGNNNAPSSHTV
QHQQLNHTTRMTKVVDDIAAANKMLATAEDVGRRAVSVAHLGDSRAYLGMRLVFKTNEEG
SSLLSTALMEGVEEEPISCTGSTNSTDKMYDVSTDNSSSTEIMAPARRRSSTCTSTERTI
SADVSEEEEEEMPFAISPDAPSVSLPSRSNPSVYNRGTSVSSMKKSSTTKVSPQTIRNRF
SEALACYLKSLKMLKGAVGAAQRVTKDLKDIEAQLAQMNLSNPNNKIGSDVPKLKQRCEI
TSNWLCSQFSGVLERADAANAEISKLVGSGSSDVGENAGHQQTGSTTSVEELIYNHALAS
GRDGAVKHLLGQYDGARSCYRSAGLLAETLLMEVNIGADDRKILEGYVDGFAARIGELDQ
LMLQQSRMTISSNTAPTSQSQQARRGSGIVGLIGPPPAAGMTSSPFVVGSPR
>comp7241_c0_seq2; 873 109 2554 LEN=2775; translated
MKQDDAKADLWSVGTVLFEMIAGRPPFNGENHIDLLRNIQRKAVRLPHDVRVSKACVNLL
RILLNRNPLSRAGFKEFFEACDAFVALGCGGVATGDYEGGVNTNSSITKGGSNSKTIGGG
IVGGINRIATKDLGTIHENDHSGTNPTSSDSLMTVETTAERNQQEVAPTNTVNATSVVST
NATRTMHPHQKHQQPLPPSKISPNIGYQATSNFIPNSISMGNNSANPQLSGRPQTFYQQP
NNRNRLAPLQSSPPSSTGILYLNSRIPAPLPSPTTQNAEEQHPQPQRHRQQTQHQSTAGY
YAAGAANGNSVSQSQVSSNDDSGFVMVEHTKSPTSSQHFYATGSQQNLHRNNEITEHRAR
SSKGMLSTSPGTGKLLLGMVGRATIGFGVTVGNNNAPSSHTVQHQQLNHTTRMTKVVDDI
AAANKMLATAEDVGRRAVSVAHLGDSRAYLGMRLVFKTNEEGSSLLSTALMEGVEEEPIS
CTGSTNSTDKMYDVSTDNSSSTEIMAPARRRSSTCTSTERTISADVSEEEEEEMPFAISP
DAPSVSLPSRSNPSVYNRGTSVSSMKKSSTTKVSPQTIRNRFSEALACYLKSLKMLKGAV
GAAQRVTKDLKDIEAQLAQMNLSNPNNKIGSDVPKLKQRCEITSNWLCSQFSGVLERADA
ANAEISKLVGSGSSDVGENAGHQQTGSTTSVEELIYNHALASGRDGAVKHLLGQYDGARS
CYRSAGLLAETLLMEVNIGADDRKILEGYVDGFAARIGELDQLMLQQSRMTISSNTAPTS
QSQQARRGSGIVGLIGPPPAAGMTSSPFVVGSPR
>comp7242_c0_seq1; 710 230 2001 LEN=2316; translated
MAADRDGVEEEMRNPGRNGNGHPFPLLSIASSPFSSFTWRSRRMSDEATNRHRTDFVVLD
VLGSGGFGKVYKVQNKLDSKCYALKVVPMPESVDLEHPTTFSDSNPSRKDRNNEPLQLRR
VLREVEILSGIPSNDHIVRYYAAWVEKTDDENDLLSHNQEDSCYSDSCRDYQQSTTDYDE
SSVSYSSSGYRSTEKDNDEETRKYNRTRRSGPVCHLCKLSYDDWEVGFEYWGLIDSVLQP
LDLCVDCYKQSLPNNIDTSKMNIRKVRPYRRRLYILMEYCDSTLEKTMKILQQGRMGNRK
NDHLLEQTDKATSDGFQSNNNNDDFADRCWSYFRQTVKGVAHLHASGIIHRDIKPNNVFL
ANEGLAEGTVKIGDLGLATIDATRTTPVSNGSHQDFESESEEEGEEKNTETDPNNKASTG
VKQSSGVGTFLYRAPEIDTGRYNEKCDVYSLGILLVEIFSNFETAMERAKVLGDLKRTRA
TPKKTPVAAAGGDGEGLAFHVQLAHRMTALDPNDRPTCAEILDELDNLSVFRSTKGSNPS
RSSSSLSLSPSPANDEYEENPVLMEKDREIARLRNLLAAHGISYEEDDAR
>comp7243_c0_seq1; 322 95 721 LEN=787; translated
MTFKNQIALLFSFASFCCDVDAWVLKANENLPDASSRRSFFEESGRFLLLSAVTVAVPFP
ASAKYGEGTSMEIPSYIDYLIEKNTAGDYSNALYKGADPATVLKRLAESERRLGEIGDLA
ERKKWSEINGLITGPLGTLSSSMSQVVSIASTSGTSKKTKQVEQAVKKVKADVFAIGQEA
NRKNAEGCSRQAQLASTDLKGLLELAFE
>comp7246_c0_seq1; 661 473 2161 LEN=2315; translated
MDPHIGQPLMRGYEYVGRLQYISENPTLCPGNDNDGNGNDQTFDIVTPMDGLPVALVAQA
GGCSIMEKARVASTMIRPANNVQYLIIQNSSKRGAPTVLANSNQYGDEASHDSKQSQIFP
GSRYYGGSSMESLLQSLTGNEDYYDGDESFYLDFSAMDLDQPHGRVLTHLTDMSEQSSWG
HDGAINLAVMYVTFAVGDELFRAVENESARDRRRGGTRILMNSKESDVSARTVIVWMLIS
FSVCACICCCLLACIQTSFEEEEDQEAPRRPVRRRLTLEEVRARFPSFHFNPEEHNQNNN
PSHACTNDETEGSTAEDVQPKCGGYMQLSDECTICLDEFVPGVRVRELPCGHVFHSTCIA
RWLIERSAVCPLCKLDLYVEPEEDEDSDGNNNGDSDDPGGESPPGEGFASFFSSWWWGSR
TINSEGDISLENPVDGAPAANNTGEETTTAPSQDQPLGAFVEGDTDSGATSEEGESRSWW
PFSVETAPHTEEEEERLGNNRRRRPRSPGLPSAAGVLRWTMGVFGRRRQHEQQLPSQEST

DDGNMVTELTEPLVPRGSLEEV
>comp7246_c0_seq2; 608 1036 2529 LEN=2683; translated
MEKARVASTMIRPANNVQYLIIQNSSKRGAPTVLANSNQYGDEASHDSKQSQIFPGSRYY
GGSSMESLLQSLTGNEDYYDGDESFYLDFSAMDLDQPHGRVLTHLTDMSEQSSWGHDGAI
NLAVMYVTFAVGDELFRAVENESARDRRRGGTRILMNSKESDVSARTVIVWMLISFSVCA
CICCCLLACIQTSFEEEEDQEAPRRPVRRRLTLEEVRARFPSFHFNPEEHNQNNNPSHAC
TNDETEGSTAEDVQPKCGGYMQLSDECTICLDEFVPGVRVRELPCGHVFHSTCIARWLIE
RSAVCPLCKLDLYVEPEEDEDSDGNNNGDSDDPGGESPPGEGFASFFSSWWWGSRTINSE
GDISLENPVDGAPAANNTGEETTTAPSQDQPLGAFVEGDTDSGATSEEGESRSWWPFSVE
TAPHTEEEEEERLGNNRRRRPRSPGLPSAAGVLRWTMGVFGRRRQHEQQLPSQESTDDGNM
VTELTEPLVPRGSLEEV
>comp7246_c0_seq3; 544 473 2372 LEN=2526; translated
MDPHIGQPLMRGYEYVGRLQYISENPTLCPGNDNDGNGNDQTFDIVTPMDGLPVALVAQA
GGCSIMEKARVASTMIRPANNVQYLIIQNSSKRGAPTVLANSNQYGDEASHDSKQSQIFP
GSRYYGGSSMESLLQSLTGNEDYYDGDESFYLDFSAMDLDQPHGRVLTHLTDMSEQSSWG
HDGAINLAVMYVTFAVGDELFRAVENESAXGPSPGRVSEAXHKQASNAFSKVYLPKMLRT
TIEIDRLRSRHFFCVIIAESIIRNRVCCEFGTNLWFLFVAVSIGXVKNENPYELKGIRCQ
CTNRNCLDANFFQCLCVHLLLFARVHPNELRGGGGPGGTKKTRAAPIALEEVRARFPSFH
FNPEEHNQNNNPSHACTNDETEGSTAEDVQPKCGGYMQLSDECTICLDEFVPGVRVRELP
CGHVFHSTCIARWLIERSAVCPLCKLDLYVEPEEDEDSDGNNNGDSDDPGGESPPGEGFA
SFFSSWWWGSRTINSEGDISLENPVDGAPAANNTGEETTTAPSQDQPLGAFVEGDTDSGA
TSEEGESRSWWPFSVETAPHTEEEEEERLGNNRRRRPRSPGLPSAAGVLRWTMGVFGRRRQ
HEQQLPSQESTDDGNMVTELTEPLVPRGSLEEV
>comp7246_c0_seq4; 385 1712 2740 LEN=2894; translated
MNSKESDVSARTVIVWMLISFSVCACICCCLLACIQTSFEEEEDQEAPRRPVRRRLTLEE
VRARFPSFHFNPEEHNQNNNPSHACTNDETEGSTAEDVQPKCGGYMQLSDECTICLDEFV
PGVRVRELPCGHVFHSTCIARWLIERSAVCPLCKLDLYVEPEEDEDSDGNNNGDSDDPGG
ESPPGEGFASFFSSWWWGSRTINSEGDISLENPVDGAPAANNTGEETTTAPSQDQPLGAF
VEGDTDSGATSEEGESRSWWPFSVETAPHTEEEEEERLGNNRRRRPRSPGLPSAAGVLRWT
MGVFGRRRQHEQQLPSQESTDDGNMVTELTEPLVPRGSLEEV
>comp7247_c0_seq1; 324 495 1526 LEN=2478; translated
MIRQSLRSAAERTLRSPRCVFRQQRSAHRSPPKPLPTSAAGKGTAGLRKVAARASSSAAA
GTSGGGPLSRALPAFSRESLRGALPLIGNGAYMALASGFLMTDMLQLRLMLVGGYAGLVS
FHALHPKPLQIPLRWSALFVVVNAGAAALLFVDQWIGALLTDEERALYEEHFRDGLTPGQ
FYYLVRKASTREVDDGVVLTKEGKVSPYLYFIEKGQAKVYHHGTFAAYVDEGGFVNDVAF
QQMQGEEEGSDEPNRDAGAYGTVVTHGDCKVLVWDQEELREYLRERPEMDRNMKYTLSQH
LVRSLLKQREVRRRLSASDQDELEQIEMTMPGRREFRRMRTSAW
>comp7247_c0_seq2; 278 495 1737 LEN=2689; translated
MIRQSLRSAAERTLRSPRCVFRQQRSAHRSPPKPLPTSAAGKGTAGLRKVAARASSSAAA
GTSGGGPLSRALPAFSRESLRGALPLIGNGAYMVSPAPIGALNADAGRERQEGPPVGGEI
VGSLVPLPQLLRRTTNSLSYAHLFSLAKTATSFPSLFFSFLPQALASGFLMTDMLQLRLM
LVGGYAGLVSFHALHPKPLQIPLRWSALFVVVNAGAAALLFVDQWIGALLTDEERALYEE
HFRDGLTPGQFYYLVRKASTREVDDGVVLTKEGKVSPYLYFIEKGQAKVYHHGTFAAYVD
EGGFVNDVAFQQMQGEEEGSDEPNRDAGAYGTVVTHGDCKVLVWDQEELREYLRERPEMD
RNMKYTLSQHLVRSLLKQREVRRRLSASDQDELEQIEMTMPGRREFRRMRTSAW
>comp7248_c0_seq1; 112 1 341 LEN=464; translated
XLVTPCVYLISEAVEASIRNKESIMSAAVAKLMNPAFYKQQWASTCSRAYAKYHPTIREN
KATVLFHSMLAISFTMYTGTYIARVHPEKQEERAIKRQALHEYYEKHGGAPHH
>comp7251_c0_seq1; 925 140 2659 LEN=3342; translated
MSEAESDSKLAAANTIDNSGAENVSGEDRDLVERVVAKNESSVGENVSVLGSTDQKLDWV
LTNYDKKNSEPQSLEEELKRLQALRSYLLLDSEREEQFERLTALASRVMDVPIALVSLVD
LGRQWFMSNRGLGDVRETPRSSAFCSHAILSKNEILIIKDAKKDPRFVKNPLVTGPPFIR
FYAGAPLETPEGYKLGTFCIIDTKPWPDGLDLDGKQNLREFAGLAVEVMISRKKRREREE
EKNSQMIACAAHDLLTPLSGIELSLHLLKEDEDFQSKIARKHKGKMMKVETCSGIIQEIC
HSVRTTYAETKSSFEQSLMTAKQERVKISTVIDKLYTAIEPLKTDVLITIKADPDLSKEI
ISDPSKLFRCALNYLVIACKRTKKGQITLTLFSKEKPGTDKKSILFVKCEDTAPAIDVDV
YEHLFRPPSNDVALFEESKYVDSEKREQNLDLCLFSVASEMNVIGGEYGFRPRSDEDDNG
CEAYDKEIGSVTGSVFWFCVPCMELEDDPNAESEEDDNKQSSVSEDDVIAVEKSVKDDLR
SEDDSKVTRKRRALVIEDSAIIRTMLCKILAKLGFEVSEAENGMQGLEKLKSTLYDLTLI
DFLMPIMDGLDCMQQYRNWESYHRPWMTQRIIGISAHATQEDVEKGLKIGMDDYRAKPIT

AKVLSDLIKCDQQVETSRRLDELERREALIKNADKPSEDTETKKKKHEIDGRACTLLMIS
PRSEEEHTKQIQEVVKSCGWQTTTASTEREALVWLKMRTWDLVLVDETLGPAIGDFREWE
SKKRQNLQERITLMMEKVDELKGEPPKGLDALAGKPMALTAVESLLERTYLHLAGTRGK
>comp7253_c0_seq1; 371 275 1776 LEN=1776; translated
MYNSGRIDASDHGRLNDSMSSIGTTSSIRRRASTNSIPGGAMDLYYGHFSLADFQDGKRF
SADDHTTILRDTQTAMQNISSRLLLGGTSSRPTAAVQDSKKNVNKSNNESAAMDRVRLGV
GLYRNFPLARQALQNSQNRQAALKKGGHKQMNHVRGTDGWWRTLLTIEDRALDNILGPWL
VVCCNAILACLLVEVWDIQLPVEALEQWDTIYALVLKTSLAFLLVFRLNRCAMRFWEARG
YWGNMNHQTRNLVGTLLMYGTHSPNNRDAAIQWGSALCISAKNFIRSERHYEPNELAGFL
TRHQTKRLSEANHGPLYAASMCRHYIEKIFRVDADTPPGLAHAYTVRMQECESYVAGMIE
SVSGMEKIRSTPLPIAYVAHLRIFLIAYTFILPYVWVNEWSWATIPLTAVTAFALLGIEG
ASSEVEIPFSKNRTNHLALDAYCLVILESVTCLVVQDANLHVQGRKGKGSVDKFKMFRAT
EEIDEASYESSSSEEEDDDDX
>comp7254_c0_seq1; 636 78 2045 LEN=2315; translated
MTEGDSTIYPRKRFLVYFGSILVCLGFRWYGYSSRALKEIRQQLSNGFADPLIHATNASL
NDDTFCPSVLIEAGAESLSSSHVWNKLEHQILNASYGVLPRFQSLDNTMVREYTGWVGSM
FSFYTASRLRRSVMNPAPPEEIIQLLKLALEIKHHNALASDENRRSLRILVLGGSVTAGI
GCYWPDNLGVPPAKHWSIPGENCVWSYFLERLLNQVIFEGKDIVKIDNIASGGISSEFGA
LALEYQLFPDPDRVPDVVIYAFSANDAQEPNPKAVFYEYLQNFVAAAHWLNPCDNHAPLV
ILLDDFYADVPPSVALQQTGNIYMLSHWNNLMAVDYSSTVGSKVIVEDLAWAPLLSSNVQ
MHSGVGMHMGIAWTILYNLLDSMVNVCNDVGLQTIRPDRITENKANASKNILPREHDDHN
ALTIDPRISSNLEKGEPPMQHFGKVREMVKDASYVRLQLEENVNRTKEVCSELSAKGKEK
SPLRCSWSWFFNKSTNFVHSAQVRNKMNEVLLFNNGWAAEGRPVAQPRSGWYSHSPNSEF
AMKIENIAADLNFVVMLTMKSYSSKWVGSKLAVSTMVVKNSTTPDAVTNQASPVDWDNDD
SLFYIDGFHEIKTSVHFPHKVPIRGGASPGDSVIVYAKLVGGSEFKFAGMAFCAY
>comp7255_c0_seq1; 333 1 831 LEN=1285; translated
RSSRLNLSEQSTKEPIADDRLRLLTLPLPTTTTTTTTPAIRKMTRQFGSQQTGGKRRSAP
RKPPSLALAATVLVLSAPLGLPGAESFCAPNPPSAVPRRTEPVLFLADGDVSRRTALASL
SLLTGTAVLAAGPPPASAAYSSEDVGEIRAVLIEARKQLETVPALLADEKFDGVRTLLTE
APLRDCWSKTTPILKKYAEALGDTGGDELAALEGREELLQHLRFLDMAVYNNVFNPIATE
GKTGASKALIDSYYNDPTREYETSKRALDELLILSK
>comp7256_c0_seq1; 206 167 688 LEN=956; translated
MDEIVDGVKSPEGCYLIFEASSGGRLMLLYSKDRIPKNAVGFWCAGPGRSIQGFKFKQGG
GRVELIKGIAGGDQNRRKYFSGWCQFIKLAKAFDGYVIKFPNSEQGVEVDVIGFKTEEEK
AYKLDLDAGLIEVAPFDAISVVPKHNTTYHGIHNIPQQIFLESGNKAGAATTI
>comp7258_c0_seq1; 502 1291 2268 LEN=2909; translated
MLRRSTGNKVVKRLQEQANLAQSALTSLKIAEDRALSEIRGTRRDEIMEEPESKEEDYPIP
NYCPSSPEDIKARPPHLTIPEEDEHAGSMTGRSLGGNSTTKSELEIEKFYRELELEMFGE
EELPSMVGQMLEVPLKDVDTTNLMTDPADRLEYCSVNSAMSDKDLDDALLEQEQNRSMIS
EYLLEDADNDSPKLDHSTQHDNGDRDGNSNYLSQLTQPIHSDQIQPGNYSLWNKSSPLTF
PALDGPPIDTHGSVVDNQTSQHRQHETIPLHAGMTMFEDCALLGGRSNHESLYFKKKRRS
SRNKDMNAGPKVHFIPPPTHLSPSDW
>comp7258_c0_seq2; 548 639 2117 LEN=2758; translated
MASIEQHSVSSRSWDRESRRSEAGQSTQTGEISLADSRSLLEEKKKKVNFSVVTIRDYER
VVGDNPSCTIGPPVGIGWHYFETRVIDIESFEAGRSYRKVSSHLILTREEREALLLNWGA
SFHDIVEAVRSNLKIKNQRRQTVTNLGKVERIEEAFESATRKLKSALMLRRSTGNKVVKRL
QEQANLAQSALTSLKIAEDRALSEIRGTRRDEIMEEPESKEEDYPIPNYCPSSPEDIKAR
PPHLTIPEEDEHAGSMTGRSLGGNSTTKSELEIEKFYRELELEMFGEEELPSMVGQMLEV
PLKDVDTTNLMTDPADRLEYCSVNSAMSDKDLDDALLEQEQNRSMISEYLLEDADNDSPK
LDHSTQHDNGDRDGNSNYLSQLTQPIHSDQIQPGNYSLWNKSSPLTFPALDGPPIDTHGS
VVDNQTSQHRQHETIPLHAGMTMFEDCALLGGRSNHESLYFKKKRRSSRNKDMNAGPKVH
FIPPPTHLSPSDW
>comp7259_c0_seq1; 2013 225 4274 LEN=4414; translated
MAPKTIAIHRHKVDPVSPIPVRRDDFPKSQQHQQLPPSRHQRSRSQPLTLSKPMLITSAG
RSASAQGRELDDLRIRYEETKQRMEKMKRDKSENNKRLIEMSGLVKSLQDIPVGIDKAEG
STFVNVQRKIEAIGFEMKEAKKRYDELRNEESFQTDTIKEQESQIRSMEKQIQLLTERIQ
QEEQDQQEQKKMAEEKQKASTAELEQKIMLQDMEIMNLMGEIDMIKKHNRTHGQDRQNTI
GQDIKLLEANNAAKYEEIKKMEDKLAKMREEQRAQKMEQETFLKKQSEESNGFVLKPTAT
TFAEISKLEDNLATLRLEQEKSSNVADAPKKEEATEKKPASSRIQKHQPWSMDLAWKDSM
LEGNKKKEAELEVVANNDITYGEPVSPESDQRDQELLGLPSFGQKQQVEVKSATKKTENS

AFVGISSLSVASCGSSSEESESESEGSVIFLNKQRHKNPFEVSKDNNTYDVKKDNDHSDT
SATVGSSCGMSRDGDSASVESSSLSKPTMPKRSVELMPTDDGSIEAIEVGHLRKTESTES
CDGAYSDNESSVEYSSGESTTGNDTNGDSSNDSSVGEYTSGESTRTETRSVLAQIPEEGE
LEESESEWDREHTSVLSKGPSTPKTNDEPKSKDDNVSDLLLTKQLKDAEDKYENLKKDYE
EKISMSESQAKKLDEYSDKISTTESIVKKLEEENQKLRKEQSNASKKTWKDSADKKRLKR
IKEENKMLLKNMEKQNDIMMKADQNFDDLRKEHLATLEELEAKKKRYDQLVLDFTKLADR
NKDAKDYSRLQTLHNAVVMKLADMGEENENLEKQRDEAMDQIERKNAQIRGFDGIIAEYN
KVEYDLKVLRETHEETVKELHDLTEKNKEMQSSYDNDFANAQQLESLEQDKANAIAEIEK
LMKANMDLSAMEVQQNESEVRVTMLEEELKKTQEELGVAKNKSSNRQRQLKDVIAQYKAL
KEEHSGTCDKLARLETTVNGLGNGSAIQEMKKLEDAVEQAKKEAQNAVKAKEAREGDMRI
VLQHYEKLQIKFEKLKAKSESTKSETAVEEEEKKDEQDVFWPGEERDARKEKIEELEELL
KESNESLSWRDEEIAKKIEELKEARKQIEDLEIEKEQLQADVLNMQSQLAQREAKDAEDR
KKDEKDNLRTVVAEHHRLQKTYSSLQRKFEEVKSELVKVKNQAKIHEQEEKHAQKRVTNI
QTEYVNLQADYDVVVAQLERFKVEMATSVIDLKEKGTIIPDMTSKETEKNNPVIASPDIS
PKGSDVAIPLTASEENETTISDTAGKENKEVISLIDYKENEKAVPELTLEEKEKGIIDAA
SEVNDMTIVHEDSFDYESVSDDSNGIAEF
>comp7261_c0_seq1; 257 339 1151 LEN=1238; translated
MRAPSLELFILALVLGSSWNGKKSLSEALSASDSQGGRASGKSTAMEDKPRTISTVHLGY
QDKEIDIFVPGKWSRHSSANVGSERRSRNDHRGPSDFALSKLIAHSPGLVDERNVLELGS
GLGMVSAVACKYSRANHVAITDRDKSLLSLAYASCVQLQRSKASVSRCCMDWKKPSTWPQ
QDYDMLLGADILHDKRSISPLVNVIQHYLCNGAKAGARKQAVIVDPVNQVNRSDFFSAAR
RAGLIVDQTEFPGSPDQVLLSISPSDFGAR
>comp7262_c0_seq1; 218 1 1231 LEN=1288; translated
XNELVEDCHFEFTYNNEGDNLVLHLDKAKESTKMVSPISYGSCASQSTVMMIDSHLAVDQ
LKTILQIETKYRIPNASCQEASNEHRIYPYSEWRRRICQWSFKVIDHFKIDREVVSRAMS
TLDRYSYIKTSYSRNCSFNDTSKEIIDSRTFQLVAMTSLYLAIKLSDNDEGQYASCRKLT
LNSFVELSRGQFCSEEISQMECSILDELQWEVFPPTPMTSMAYLMRMMPSHESLQYTGCK
SYNLVLHVLHELARYITELSVCHSNICTGYTSSQVAYASILLSMELLTPVALPLHIRDQF
NTAVALVSHRSGGTILSSQDDKILYLQELLRNSFWPEILVEDCEYGEIGHPISMAKDFGF
IDIPHIVSPHHTIPTISSNNGTAMHCQSLSASLDKEPDLRSSPICVNRHF
>comp7263_c0_seq1; 542 468 2573 LEN=2668; translated
MGHRDGPWGWNRQEIASLLLLTHRQVIPAEASIEFLSGGNGSGTSTVSRTYDTSTDSRKA
LAELDKGNYIKVIDDSDIETVRMIFTRRDIPVDNGLIFGWEPGYLWPMDELKQSPVNDEG
DKNYDEAYHDELTAAIHDLHNMRPRHPIVSADHETKNWYYDACDDCEFEEQEPATTQKRS
GGRTSSTRPVWEGDTSKRKKLHQMLKYSDEGSEHLCVCTKDHALQPPEESRGEVARALLY
MNLRYGTLTAAHTERSFRRDGTAFLGLTLTDCPPTSATGEDDGQEATKNYSMGYFSRLVQ
WHLEDPPSQHEIERNQKICEQYQGNRNPFVDFYEESWALLDFNQIEREVCNSGDSEPDQE
GGKNANFHYDDDDDFVVTQNSTSDEDWNSDADEDEKYVCGDLMPGDISFFMVQPATDSDE
SNDFEQDEEWQRESFGLVTLVSLKPGLILYVAGVDNDNNAEVDGPMEGNHYTGDRGVLKV
EVPQKGIPAGSYFGYGKQLYLGDQWDPVLNDNEDHTFRFSVHQLYLYCIDRDHGIQNPST
MEEYKILAAMSTTGHSFGESELPYYWDKFREKHSDIIVSEVFSNGIHYGTIVLPEDASDY
ELSGGYRYVGPTYTKHDKYAKALIDEANWKPMNMLDLEKEVGGKVENEASTSIFDDSVRN
AIRDSDGMQQLIEGTRIGMNEDSSSAYGERPSIFTRELSSIW
>comp7264_c0_seq1; 476 36 1925 LEN=1967; translated
MSSKQFSSSQFICLVIAISCLATACEGFLSPSHLMATRTAHDVVQSNEKLTPSTVLFLSS
VSRNRKRSPSTFKGKKKNNSGKGNKNKKKKPGKWDRRRSKDQLERQDALAAQPRPILTRY
EDERDGEVNESRLFSSQGNILCKHFESCPGCVTDRRVGDVEIIRSAARFFSSTAIRKNRV
DVERSGEDWVIEEEDDGFYEVVVPSETTKWRTQAKLVATSKSGSQWANEGCKFGLYKRGT
HTVMEIPNCEVHHPSINRAVEVLTKATEKTGTPAYSDDNGLRYVQLQVERTTGKISLTLV
WGTSELKWTQPALSRLTKELLKSEPDLWHSMWCHCNDSPGNNIFSRNPRAWHRLSGHEFV
REPMPVGDLGWLYFSPLAFRQGNIDGFDVIANDVARAVPGGSKVCELYAGVGMLGLTSLV
YHHLNHQKYKDEVTGYGEPLTWVRCSDENPANPRCFSASVRSLPREITEYQQAETKEPKT
LGDLMKAMEAGGDDFEDQVAQRTGPKATYMVASAAEALKAGQALGADILIVDPPRKGLED
EVLEELCKPFNAKQPCVESPTMLAVPDYLVNWTNDVQTLIYVSCGFDALARDTEKLLSSG
GGWKLESATGYVLFPGSNHVETLCIFRRQ
>comp7265_c0_seq1; 1262 223 4233 LEN=4281; translated
MILARITSSCLVLAPLLLLTASRVAAADAGSTVDDPHAESVKKIFAWVHEAPDGFVTPKQ
GVSRMVEGNTDTPLIIVALEDIAKGELLVQTPWSHIIHSKTLASDDALGWYCGTTRSLAE
EMRKGADSFYAPYAMYLNEEPDNQLTHQYSPNAKKMLHQVIGTHPDEVNRSPMFDRKDTM
EQRLMPEGIDASLDVNWFGICGADPSDKIATKAASMVIQRADDYILIPAYDAYNHRNNDK

HNGKEYMNARTLTTMDKYHQTFALRDIKKGEQIFISYNMCEQCGGRIDGEFGTAEMYREY
GFVEWFPQRWWYPGFGDYHEDDNPQETAQFDLYQEEVTGNLTVVWTHLSTDAQKLARFEV
FLQREIRRLNRLKNIEWSDMYNDWTEDQWKESGITSYEWSNINLFVDANIVAMTVALEDL
RTNSGPVANDDDDDGVDKEPSREPLTISPEALKVLERNGSHYDLLEQPEKDDLAYYRYTC
DNSADFEFMGYVDTEVEIETDAGQFVNFCESFYFNTDPEAVDVCMDVDDVVQICSSYRPQ
QHEYMIHAAAKYLPKVKRVAFVGGGNSMLLHEILKYPEEDLELVVGLELDQTITRKSFKH
FHTDPHFDDPRVEWWFGDVAKSLMVLPESYWGSFDLVLVELSEKMLARNVSTSEGGEGVD
LLSALSLLLNPKTGIIAKQELYVDKLSQVFDYAIELEFESPVICSQTLALGSNGIDFFHA
PVYDHGVAVNDQSIAAMDKAHRYDLMHDFRTLPEKSCGEEERTKKTESEDEENEMPSFGV
LEVVTLEQVKKPFDSFQIVVPDGFHVVEHTTQNKEDPTMLTVTVLEEGYVTARVQRSDEG
DFSLGFDIHLWSQTHRIEEFRKALHDAYGGEVISSYKIVAGGMFGSRDETKRAGLPVPLK
ASDRCENDGGAANEESKSNPLDDVLVNSIAVQEAVSLVGKGDVVAAVFCGGTSEPCTSYD
ALSGHKSVKEAIPLYACSSTAGGNDVTELYACERKIIEELMSKLGFTSKLDMFVIDGNSS
HEMYQIVNSIFDTHNHREFLLNNHSIAVTWSPGASSKKDPWARELLDRYRKQIHNDPVKL
GEFEIRLGAKKFELGVVSTNDPQMNYGFADLEKQIQDRLVAEQSGSGIVVELRKIHGGLY
NYMEQYAPKRFSQKDYDNTLAKEHFSAQVPLGRQTIFQYELDPELKKQNFFNDHDLGLSI
VYLEKVLYGSLAATPGLFDLKVTTFASVKKHQIPVGDGCVFLLFGDNFNLVSVWDGREHL
DINFFAFGILLSAPEVKNKINSMINEVSPLKAVAIDTQPRGIGRVVNFPEDLETLSKSVL
YEYQYDDDENHSQGEL
>comp7266_c0_seq1; 286 413 1303 LEN=3245; minus strand; translated
MAISSAESNFILDGCQDDCRADGRSRRDFRLYSSVVGDFALSYGSARIFLATKENHVLVS
IKAEMAVPASSAPTEGAVEVHVDCVKGTNTQDDDLESTISNLLVPHLVDKRNLCIAPGYF
AWKLNIDLFVVNSGGGSLIDACSQGIHAALQQTLLPRISVIPATQGGENKPTLQIDSDIK
AAGKILGVGNAPIIVTVTVLKHKRTHLLIVDATTEEEICAFAKVHVVLDRGEPLSSSCRK
RKPTICALHKAGGGALPFEILQDVTSFVLEASGSSCAVESESLPHLLQDNFSIQQ
>comp7267_c0_seq1; 780 687 3599 LEN=4488; translated
MTSGTTRVSAALLHTTSNLLHQNYGEASQQRDRGGSKVQNANTTSLLSPFFVNTYNPWSS
EQKGISDANPPQDANHHHHSIDDDNDLSLSVPWGEINIVVVTDVHSWIAGNHRDDPRNWP
LDEHRNRVQDADYGDILSFYQRLGDLVKDHNSNNYGKQRDLFFVMNGDFVDGTGLSENPP
RHLMPLLKHMPWDAINVGNHELYSDKTVQFLVQRDGYDSKMGGNRSTIYPVATPPIWNGR
YLSSNTLLTETMEPMTGKRYTYLVGPNTNITLLAFGFLYDFHENGPATTTLSVEKVIQQD
WFADVLSGGRNSSTGNDYGHEAWKPFDAVLVLAHMDCLDPLVATILEGIRALLPPDKKNI
PVQFVTGHTHRRCHARLDDHSESFEAGRFLDTIGFVSFDANTRTTQATNGSNNNRQSDSN
FRHRFLDANKQSLLQTLMRSSHLSHGGNQRGLSFSEGFGTPEGRALTRAIRDTQKDLGLD
VVLGCSPRTYGIFDYETEPITRAPSSSDANPPRNDWFSESSLWGLYVANVLPRQLLRPML
ALSRQARDDSSGEEAAEAHPLFLAGTGSLRYTLFEGPVTVNDVIAISPFNNSIVEIVAKR
KGTTDSPGELLPWYGRELSLLLRVTNRLYATSAEYGMPAFVASILPQQDFVRENEGFSLP
AASAIDPDRIYRLYTTDYDSWRLISVLAILKFGEGTEDGSVDNNTSFSNDAVDKMLASEP
LPPITLGRSYETQKACLALGKNDGENENHHNIDRCYYTAELWKEFVRDAMPCDAVATSSS
GHNIRDPPLVSSNGGGSSSSEDGHRIDLLRAQLLQPSFGYSLHAQRPRQEAAPHTGDPPP
QEASVVDLAGDEKAAAALVGMASIAAIALLFFFPPRGDNEPPTRGGAPPRARFRQGRCNE
GRDHSRRRGNHRTPTAHATTNGNSDSDSDSNRNSVPDEEVQLIPLRRMDTGNLSEHSTQS
SNYGSSYGSI
>comp7268_c0_seq1; 798 256 2469 LEN=2550; translated
MLTFNRSSVDKLSAAVSNAVSNAAVASMAQVSKASETIFSNLSLSNSNNTQLTYAAGAVV
GLYMGSIVVKKSFNMIDAICNRTYIYNICWEDPAVDHQLLKITPDDVIFRICSAGDIVLD
YAIEGPSKIVVCDMNQHQLWLFELKIRMLRDPKLTYEEWWGIWGSSDVSIALSVWKRMRH
TMSAGGRKWWDGRIERVFRQGFVTTGSTGFCSKFLIPFVMFFCGFDFKEWSEKGFSHDYI
QANYHVIERSAWCMRRLFPSIIAPFAGVPPNQIGPEFYTVEFYETILKEIFMDPDFGTSN
YFYRFYLEHGYKDKSCCPRTLQEVHFEALRENASCFEWHHSTVQETMERVKPHSFTKLVL
LDHMDWMPNSMVHDEWIALQRATVPGAKILWRSAFTHMGDKPFFNNLDIYDLSPQWYPKD
RVKMYPGTFLSHMPKDPFPFIDPEPSPCQRASLARKMSTTAKMILHPLTAGLAGTSAHGD
KMSSFYAAQARGYDAVRENMLVARPEMISALGPIRNGHTWLDIGGGTGRNLHYLRAQLDR
FRTIVVLDICPELLEIGRQSALESFSPGQCERIKWVCLDVNDPNAGEELAKITGTETGRG
FDTITFSYSLSMIPEWEKALCSARSLMSNHGRVLVADFDTYTEAGTSAKDWAIRTWYKQD
GVRIDARSREVILNTVFAGDEYTNTCVRMQKKLAGVRIPHYVACCRKRTDTLHDGHRRPS
FPDFSKLENGVQEEKKE
>comp7270_c0_seq1; 412 195 935 LEN=1138; translated
MSRLAALATTVVLALFAPHVSEALSPVRPASAASRASFSSPTELRMGGRTNQQRREDRKR
YQKNNKMLDFIDKEVTPEELVGDLEEISADPGDDELASLVNCIVAAADGRKADNIVAIRV

SKVSTITSFVVICTGNSRPQNQAIASIVRDEVRDNFDMLPGATGVPEGSADSGWTILDYG
SVMVHVMTPRSRLFYNVEGKWKDQGGEYMDISAAILPNAVPQEEEASEEKGGTMEGLSEE
EDPFWS
>comp7271_c0_seq1; 358 158 1156 LEN=1255; translated
MAQTAPANQEVSNGPIFVDTQHEDLVHDAQLDYYGCKLATSSSDRTIKIYNVSEDSSELS
ATLQGHEGPVWQVSWAHPKFGVILASCSFDGSVLIHREIRPRDWSLLHAARHLHESSVNS
VAFCPHEYGLRVAAASSDGRVSILTHQPDNSWSTEYITDNPLGVNSVSWAPMGAYSDESS
TVDAAAPADSLRLVTGGCDNRIRFWLEQPETGQWVEDPSCPVSKDLGHSDWVRDVAWAPP
ILPNVNVVASCSEDRTVLIWTQNGKNKEWKASKLHKFDGPVWRLSWSTQTGHILAVSSGD
SDVSLWKKGIDGKWEQMESIEEQATSEDVVQG
>comp7271_c0_seq2; 289 158 1347 LEN=1446; translated
MAQTAPANQEVSNGPIFVDTQHEDLVHDAQLDYYGCKLATSSSDRTIKIYNVSEDSSELS
ATLQGHEGPXLASLLGASEVWRYDMTGRQKWSILNNLFVAACFVEIVVEKGLFAGQNPSF
LGMHNFLFYNTSSNSFSSGFVAMINSDSGFVQLDGSVLIHREIRPRDWSLLHAARHLHES
SVNSVAFCPHEYGLRVAAASSDGRVSILTHQPDNSWSTEYITDNPLGVNSVSWAPMGAYS
DESSTVDAAAPADSLRLVTGGCDNRIRFWLEQPETGQWVEDPSCPVSKDLGHSDWVRDVA
WAPPILPNVNVVASCSEDRTVLIWTQNGKNKEWKASKLHKFDGPVWRLSWSTQTGHILAV
SSGDSDVSLWKKGIDGKWEQMESIEEQATSEDVVQG
>comp7272_c0_seq1; 341 163 813 LEN=1065; translated
MMIKRSSIVAVLAVLSGAHAFSPEVSRRQSFEALVGGVATIVLPGVLPLLPANAVIDEET
PRVVTRMGGLLEPFQDGPRSIKMMAPSGWNKFDGEVGAYDLKWQDLVSPTENIKISSTPV
KSTTTSINALGEVQTVGDSLAAKRNAKLVSATERQTDGVLFYKFEFAISDGTHQLLQLCV
CKGKLWSIDANSTEKRWSKKADLYENVLGSFMPKLA
>comp7273_c0_seq1; 342 582 1403 LEN=1819; translated
MPSIESVPEEIRARTEFVAAIRTGIDALMGRCGFSRERAVATLLKELNRGMQQQQESLST
TIPGTTSRPTDDEIFDAMRKHKLGVDEATRAVTVSRAMRREMLSRRDITPAEAIDRLIAR
ISLDNILYESGEDDDSDDDNRGISALNMPKIDLVSSPSSSSLSPSSPSTSSTTSKTTGHF
RKNARNKKFTSQSPRKQQKTSHHHGTGNNILVGKKRGMDEMDHSGSKNDDEVETKAATGR
PLRGAKRLHRASANASSASSEASTGSSTRSVNK
>comp7273_c0_seq2; 309 244 834 LEN=1250; translated
MRKHKLGVDEATRAVTVSRAMRREMLSRRDITPAEAIDRLIARISLDNILYESGEDDDSD
DDNRGISALNMPKIDLVSSPSSSSLSPSSPSTSSTTSKTTGHFRKNARNKKFTSQSPRKQ
QKTSHHHGTGNNILVGKKRGMDEMDHSGSKNDDEVETKAATGRPLRGAKRLHRASANASS
ASSEASTGSSTRSVNK
>comp7274_c0_seq1; 464 1 933 LEN=1500; translated
FRKLNFLSRIIKPITTRALTVATRTPTSLKMAEYSTADQVARFAAAQEEKNERFLNIDSV
YNGNEIMGKRILVTGGNRGLGYEIVKQAVADGATVIVMCRSSNDELEELVGKWNVYSGID
VTDTEAVKGGMKRIKSDGGALDYVINNAGYFYEPCEKITDSSLNFEEQLKQIDICALGPL
RVNAAAVESKALADDAKLVIITSQAGSAEWRKVQNKDEGGDYGHHMSRAACNIAGVLQAE
ELRSMGYSVLMVHPGFNRTEMTAKYKHIWDIEGAVEPSVGAKRVLYEAMQSSLENTGTFV
NAEDGLQIPW
>comp7274_c0_seq2; 568 151 1140 LEN=1707; translated
MMFLRQSSIVFGFIAATSNVAAWVSQSPSVASANRAVVSSATRTPTSLKMAEYSTADQVA
RFAAAQEEKNERFLNIDSVYNGNEIMGKRILVTGGNRGLGYEIVKQAVADGATVIVMCRS
SNDELEELVGKWNVYSGIDVTDTEAVKGGMKRIKSDGGALDYVINNAGYFYEPCEKITDS
SLNFEEQLKQIDICALGPLRVNAAAVESKALADDAKLVIITSQAGSAEWRKVQNKDEGGD
YGHHMSRAACNIAGVLQAEELRSMGYSVLMVHPGFNRTEMTAKYKHIWDIEGAVEPSVGA
KRVLYEAMQSSLENTGTFVNAEDGLQIPW
>comp7275_c0_seq1; 255 59 652 LEN=692; translated
MKTVLASRCVEIPDGVDVTVKARVVGVTGKLGSLTRDFKHIPMECRLVTKMEGEEGEEVE
KKYLQVDLWFATRKQLACVRTVCSHVENMIVGVTRGFLYKMRFVYSHFPINVSTNSGNVE
IRNFLGERRVRKVLLPEGVEYVRSEDVKDQIELSGIDITAVSLTAAKIQQATNVRNKDLR
KFLDGIYVSEKGPRPEE
>comp7279_c0_seq1; 733 198 2000 LEN=2823; translated
MKCFKSKSSLLFGLLAFLSIAVNASTAERSSPTAGHFRVVLDSPRGNEKKTRLMLKNKIQ
SSVADVDLRAMVERAKKEFKTISEKTRHIGEEAKEIVETFKDSLQHYSRKELGIYAALTV
ALTVTLKTVVFSKSSSKTLDLGKISYILASTSALTATEKLSSILEFHNKYGRSAKPPELA
ITPSIVSSDGKTFIRTVKKTLKHTSYQPPSNNVPAQSRPAQVVIDTVSAFYPDSEPTIAK
SLPASHNAVIDTMSPSMRRTLKARRIRVRGGATKSALLQRLEIGGYFATWYALNIVYNIV
NKKVLNVLPAPLTVGTIQFGVGALYCALVWMLKLRPRPTLTKTGRKSVVSVGAYHMLGQL

ATMIALGAGPVSFTHIVKAVEPFFSAMVSGFYFNKWMPPQVYATLIPVVGGVGYACMKEL
NFSWLAFGAALSSNLFFALRAVVSKSALQSGSATGSNLSPPNMFGLVTMAAFLISIPVAL
FQEGSGFLSLWTAALDQVESKAQLIRAIFVSGLFHYLNNEVMYMALGSVHPVTLAVGNTM
KRVFILVASVLVFRNPVTVQAGIGSAVGVSGVLLYSLTKQYYESLESKAPASKSKGKGKR
>comp7279_c0_seq2; 733 198 2000 LEN=2641; translated
MKCFKSKSSLLFGLLAFLSIAVNASTAERSSPTAGHFRVVLDSPRGNEKKTRLMLKNKIQ
SSVADVDLRAMVERAKKEFKTISEKTRHIGEEAKEIVETFKDSLQHYSRKELGIYAALTV
ALTVTLKTVVFSKSSSKTLDLGKISYILASTSALTATEKLSSILEFHNKYGRSAKPPELA
ITPSIVSSDGKTFIRTVKKTLKHTSYQPPSNNVPAQSRPAQVVIDTVSAFYPDSEPTIAK
SLPASHNAVIDTMSPSMRRTLKARRIRVRGGATKSALLQRLEIGGYFATWYALNIVYNIV
NKKVLNVLPAPLTVGTIQFGVGALYCALVWMLKLRPRPTLTKTGRKSVVSVGAYHMLGQL
ATMIALGAGPVSFTHIVKAVEPFFSAMVSGFYFNKWMPPQVYATLIPVVGGVGYACMKEL
NFSWLAFGAALSSNLFFALRAVVSKSALQSGSATGSNLSPPNMFGLVTMAAFLISIPVAL
FQEGSGFLSLWTAALDQVESKAQLIRAIFVSGLFHYLNNEVMYMALGSVHPVTLAVGNTM
KRVFILVASVLVFRNPVTVQAGIGSAVGVSGVLLYSLTKQYYESLESKAPASKSKGKGKR
>comp7280_c0_seq1; 325 1122 2054 LEN=3440; translated
MARIRYYRSRSHLAKTGNGDSQEEVHWKHALGVVLTVVGTGIYVSSMTSADADADATGII
TGKTSPTIFSRHGESSASDIDFRSHLIGDFAAAAASAGYATYISCGRQARRVVPVYVHLP
GCVFVAMVLISVCAITMTATMNVSDVISLSASVVAPDATILTNRSDNDFCSSLFGWTCRE
YLPRSIFLGVVCGAIAVGMINWSLNHVSALQAAAANSAEPIAASIIGIVFFAEPLPNFTA
ILAASVIVIAMVLCSTDATTSGKTKNKSGDEDGDAADTLREQSAICDETTPSKGKKVISA
RARKSLPETC
>comp7281_c0_seq1; 561 1 1081 LEN=1081; translated
XVNSLNEERRKTPHGPTTAERNFGNEIYLLVRNVGMWMRRMETKAKLIEKTEWSIYILES
LQEIDEGCNAVLFWSYASWRRRHTATPHGSNRSLERENERENDSHHQNQHHRDGYVDDRN
RWGDDHHNHTDAKYDHYEGGDRCPETSRLFGDYQEAYRRNLEVTKLVRDGLGRSTNDERL
SRFLRDNYVHCKGCRATFAKRYDDLFGTRIHSPQQQKSSHNGTTATTTTTTKTTDPANQA
SNHGSRRTHHFFCTDCFDAFNPSILKKIKRDEDVKARRDLELLIEEHQQEQEGNKQQHTN
PGTAAGKSGKKRKKKKKKKAKKNNNNNNTTNEIDNGNSNGHASPEATSKDADDQQQQQQQ
Q
>comp7285_c0_seq1; 530 118 765 LEN=1287; translated
MSSIEIVTNAEREKIARPRPEENAEDAGTSEDSSPMAAAPSKKRRKLNDGSATKTEKPSS
GFLAAVSNFLSGSWFWPSSMPETKIADLSSTEEKEPATNENGEKADELSEADGEQIKNDK
EENADADRKSDESTKADDADSKDDNEKDKGDDNDEEEKTATPTAADIKDTSKKIGNEGNN
TKCSKNLANKVEKKSSSLSPKETSPKKKRSKKKKN
>comp7285_c0_seq2; 530 118 765 LEN=1151; translated
MSSIEIVTNAEREKIARPRPEENAEDAGTSEDSSPMAAAPSKKRRKLNDGSATKTEKPSS
GFLAAVSNFLSGSWFWPSSMPETKIADLSSTEEKEPATNENGEKADELSEADGEQIKNDK
EENADADRKSDESTKADDADSKDDNEKDKGDDNDEEEKTATPTAADIKDTSKKIGNEGNN
TKCSKNLANKVEKKSSSLSPKETSPKKKRSKKKKN
>comp7286_c0_seq1; 365 411 1550 LEN=2265; translated
MPFFTRIMQSPRFLACVMGGFLISVMASPSFPFVNGPLAVLLIITVTFCLLSISMGYYDD
ESIEIRNKQGTSCYKKAEYLLIFAPSFALSILLLIRLFSRNKFFTVPGLLEFGPKNGDMN
EFISNIAFLGGSGTAFGHWIQGFLASFLLRVNCKKCKKNDVDDLSFHDSDGNADDQRMAW
RCGRTPPGMNIANIAAYYGTIYPTYNFVKRCIRTPDTFATSSYMNNGMEWALGFWTGHAA
GFIADTMFYNRMRPIANASGSDSTAETVVSIISEEETRPSSAITTTTADVPVPEPTIRSE
DKRWNCPFSFSFVLLMLGWVIGTLFLTSVFGACIMTGYTWNKCEGESEECVNYNPDEFPA
IIAFIITMVPTLTVCAVLC
>comp7287_c0_seq1; 399 1 1518 LEN=2008; translated
VCSSDLQNRKGMPSAIFSKKAYQLGLHLNSEYIQSDSFRLCFLRKALFHIEDAAIQFFRY
LDVIYDMFGQVALMRPLYLTDLTERELRYLNRGQQQLFPSRDRAGRRIIAFLGCNNTEYT
LREKYRVRTYLESVMAQDETTQKLGYVSIGVIQRSGKVDLGGERNPKFFQRCNKACPVRC
SALHICFPNGHFAYYFIQIAVILFCDRKMRSLLRCHRGSTLETTCSLSGYGIPSGDIPIT
SGGKVKTKNVTSFVKARTAIEAVQKHQIETHCRHRQHQQLLRLDQRSAQHLQNAAVVGAS
PKTAGGGGYPILVRSLIDCPDSRCVVFGNKGMYEHNGNLRFRHFLIDTEMRRTTRIKNFD
ESVTSMVQFVRDIIDEAKSGESTGGVWSKGETFRFYSYKRDLFLYAEITDPLELQRRVHQ
MLRDSRKRSKGDEDLGNSAEGCPAHTIIYSNDNVASIRDSSSGDTTPTPTGLQPTPGAMG
VTSLDDSWPTEPNQNSGKRIKTSNA
>comp7287_c0_seq2; 511 1 2438 LEN=2928; translated
XFTQSMRDHGAHQTYSSCDEDSHQFGKFSEVAFGNDPRFCFRATENPEKSPTNGANPEYA

NIPLPKTMSNCPQDIQPVKKSALQSAGRIHNRTNNPDANSERSECVWEPLVHTKPNGNEN
NIVAQNNFLALGNSFDYGAESTLVDRSIFDNLRRISGSSAHKNIPRFVDGTRVSTTEYSR
TSTDMRKNKNQHHKPPDMDIEQITEKFSKVWIRLLNMGRIHEYKNDNMVKTPALLKPSTV
QVQAETIEAEKVQAKEKFEMSADEREAIANEIHGVDNCAVAENPSMIAASLLALREEIDH
HLHEDLLDNPRSNQNRKGMPSAIFSKKAYQLGLHLNSEYIQSDSFRLCFLRKALFHIEDA
AIQFFRYLDVIYDMFGQVALMRPLYLTDLTERELRYLNRGQQQLFPSRDRAGRRIIAFLG
CNNTEYTLREKYRVRTYLESVMAQDETTQKLGYVSIGVIQRSGKVDLGGERNPKFFQRCN
KACPVRCSALHICFPNGHFAYYFIQIAVILFCDRKMRSLLRCHRGSTLETTCSLSGYGIP
SGDIPITSGGKVKTKNVTSFVKARTAIEAVQKHQIETHCRHRQHQQLLRLDQRSAQHLQN
AAVVGASPKTAGGGGYPILVRSLIDCPDSRCVVFGNKGMYEHNGNLRFRHFLIDTEMRRT
TRIKNFDESVTSMVQFVRDIIDEAKSGESTGGVWSKGETFRFYSYKRDLFLYAEITDPLE
LQRRVHQMLRDSRKRSKGDEDLGNSAEGCPAHTIIYSNDNVASIRDSSSGDTTPTPTGLQ
PTPGAMGVTSLDDSWPTEPNQNSGKRIKTSNA
>comp7289_c0_seq1; 380 66 1315 LEN=1872; translated
MRNSSSSLSQLSKTAIVLVPVVVAFFLSTVPKQLGFYRWLSESISPQLVGCSPATILSED
QWGFTFGDLYGDEADNDGDAAAYXEPAGGADGPGDRSESGTGYEVSLALARLGVGVTMAC
RNPEKCASAAEKIGADEVVARRIARGRGDLPTTEIVDVSSLESVKAFCDRFLAARLAPLD
MLFLNAGIGDIGPSVDGSLRLSADGIELIFATNVVGHHLLYRLLEPLLLVGSSGESEVPA
RIVLTSSCTAYDSLYPYRVATDLETLNGVSGKSPFRYPQSKLAQFLWARELTERLSNSGG
GGRRSVYANAAHPGFVATDIWDKNPFDFPLGGCLKSLLLSAKSVMWTPEEGALTLLYLGT
AVDKLREDDIRGQYFHPQSRWMKDNYPLAGETEAYKKDLQKRLWSFLEELVAEHVA
>comp7289_c0_seq2; 123 1 522 LEN=1079; translated
RWRPCTAYDSLYPYRVATDLETLNGVSGKSPFRYPQSKLAQFLWARELTERLSNSGGGGR
RSVYANAAHPGFVATDIWDKNPFDFPLGGCLKSLLLSAKSVMWTPEEGALTLLYLGTAVD
KLREDDIRGQYFHPQSRWMKDNYPLAGETEAYKKDLQKRLWSFLEELVAEHVA
>comp7290_c0_seq1; 519 53 2080 LEN=2258; translated
MDQTKSKYPESASYQTLESNLRRREPASQLRRPKQIPLFGLLFVLSIVSTSFNILYAHYT
NVDLNQAVATRSVKAAMIYFVEQKDNIIKLSNNWGIAESNPKEQQRKFFRYDEAGPKPIA
GTARSHALPNAVPVTNIIPREYAENKEYHISQRKNSTLPHSFIKPFPTSKAGKKLKQKHP
ELYRDLGNYNWNHTYHDDYRTLYLYNPSILPLHQTVRREQQDSDKDPDWLSATDLQELTG
GDLRVRYVAVFRAYLGCNCFGRKNGDRTLMTAGEQISYMAIALLDESLDVIEGTDVLIDL
NAFYGEGIRGYFRQFAEDCRISLLKGTINLICNSQMKRIRIKRKKFSGEVKQKQESNYPP
QTPPPGYGTKEGMAFPYIFPNIHGHGLEVTLLSHNVNIHGGKNMNVFRAPVEPDFGVVNE
KPTEYDYYLQTWPVPHKYEKLIVPDGGHENDSKVPKRARRSEEHAVTPLENLPQPTFDTP
DVAHNITRCDDENNKKAMNCTDPVVNTFWENGKDHGNACCVKMLLPHKDVNERNTTIEVM
VGISHKKTSQRSNFWLLDIHKRYENVGHDQFVSRFVAYDAKPPFDIVARSGWFCLGFADE
GESVGPFGNTLAGKNKRVNLDLFDEHFNCPVIHFVSGFAEAVGNSSRAIISYGINDCHPR
MYFVEKDDIFQLLTQ
>comp7291_c1_seq1; 716 1 1852 LEN=2007; translated
XNPSITFDTLLVPSAQLTRWITHPELQHRLAKPDNWGFLKHVHPRIKMVQDYEPGEGDEP
PTDAGRHTRYKQLLLLPSSVPKGASNHTATGVPPGPQQTDGGDDVWLAGLLASPSDESGG
EEATVAWGPPRTMPIAYHQLSFQYLLKRLLPEEVHPPPSAYEQIGHVAHFNLKEKHLPFG
RLIGEALLASSTTSSGGSNHQNSNSIKTVVNKLGKVDGKFRTYECQILADAFLPDARNSA
RNDDGSTTHHHPSSSLETTVVEDGVSIRLNVAECYWCTRLSGERKELVKDIVCRSNPSSD
NPVVVADVFCGAGAVCMLLAKKIRERTRSHQQQLLPRKQKEGKGAQPPILTVLANDWNPK
AIEYMEQSIAANGMDNKEKDDGNLIQSFHRFELSCRDSYDFLFELGEELPVPMPPSPKVR
RNRKLGRKPAKNVPAAADSPRTTEDSNDRTKRIQWEQKRKPLPDHVLMNFPLEAPRFLGA
LRWWSWKRLEQGHLEKSTAHQTWFPRFHVYTFARSSEPKAGDEEEMAINVVANELLPPYV
FADETCHGNRSADGDRDENEQIEVAKTVVFRRKELNEEFGTDISTRMVRDVAPGKVVVCV
SFSVTPKLIRYMQGDYS
>comp7292_c0_seq1; 590 151 1851 LEN=2294; translated
MNYNSSEKSHFFLLALICLLTVLAAAVDFDDVDPSTVIAKASKSRSEQSPVQKDRSVAIY
DNVLSNKTADWLHLECVAWSEDAEQRKDKQRQAQHATAADPNKKSNNDDHRYLDPIIEFP
LEHPERHSPLQQFLNGLLWQMEERQHQAAPLEPGAAGQTSKRTNKKYYVEYWTRQEWQHI
LAHQDMDEGLYRQRSHQRWSERQKQPQGEASPNARGDLSSAPSGQHFDDLRHPLFGHVLY
LRVGSRVKGPTVVFGAANGGELVEQGVAGSSTMVSVPAWKGRLLRFKGDLLHAVPRPYDV
YWTFDQSEQHSIHHPRAEWERSVLLFNLWEHGQGGDDPSQTREQQQLLLVDKILDCGDGQ
WIPDRTTCAMSENQNKENVAKHTGAAHNADGEQELFSCNDAGDWEEIPVVRKTAFESPQA
IKTATATTATTAPDDDDDLFSLLLYFAKWWWNYLLLLLDDSDSKAVEHFSVPLMGDPRRT
GIDARVARMQSRKKATRDTNTNPDTHEAKPNHYSPARESMAESYWPTIVEIETDPRYQQH

SLVLVPSGEQQKRHRSRNGGDLPEEL
>comp7294_c0_seq1; 426 418 2142 LEN=2726; translated
MSKKGEVHRMVSDISSAIQSSALLSLLDEAKANGSLPEPAIAIRDDQASLSNVNNPRRRT
KRREKTTKQRDAILPKCLADIPQSTLDERIKPIKRALERVDAEKLLNLELIHPSASSRLE
GQTTLGARSKTGKNQNERNDXIEIASALDCPMSPIVNLPLSLVLAPTNRVENQLSTRAQT
CTLYKQRVYCRPISDQILPKLCVDDGEDGDDENAAMNNAHDEYRNSVVRYLHIAEIPDQY
TIGIFVFGPNQRIPLHDHPEMCVLSRVLYGDLQRLSLDLDRGEQEHNRRNTSSKSMGEVD
SVAMRDMEGDKRFDEPFVRSLDQDHHASSSWLSRSATWLRTSLNGRNFGSGGNSHNNDFD
HHQKQFPNGTKLAFKNSIDVLQAPGVVALYPFEGNLHEFVAGPHGAAVLDVLLPPYDDEC
KRDCTFYHIRDVDNATSSMSTESTSPVSSSTTSVASENSCQNQSRSLTIEEQQSNEKYRG
IETENTNKVMLGIEKHEQSFHTRRIPCLVVPTGQPETFHCISGRYRDIGDAEDDYDAMES
DDSSEESIMEGSHFDGDNKNMLHGDIIFGDDIKE
>comp7294_c0_seq2; 507 418 1977 LEN=2561; translated
MSKKGEVHRMVSDISSAIQSSALLSLLDEAKANGSLPEPAIAIRDDQASLSNVNNPRRRT
KRREKTTKQRDAILPKCLADIPQSTLDERIKPIKRALERVDAEKLLNLELIHPSASSRLE
GQTTLGARVQDGVYCRPISDQILPKLCVDDGEDGDDENAAMNNAHDEYRNSVVRYLHIAE
IPDQYTIGIFVFGPNQRIPLHDHPEMCVLSRVLYGDLQRLSLDLDRGEQEHNRRNTSSKS
MGEVDSVAMRDMEGDKRFDEPFVRSLDQDHHASSSWLSRSATWLRTSLNGRNFGSGGNSH
NNDFDHHQKQFPNGTKLAFKNSIDVLQAPGVVALYPFEGNLHEFVAGPHGAAVLDVLLPP
YDDECKRDCTFYHIRDVDNATSSMSTESTSPVSSSTTSVASENSCQNQSRSLTIEEQQSN
EKYRGIETENTNKVMLGIEKHEQSFHTRRIPCLVVPTGQPETFHCISGRYRDIGDAEDDY
DAMESDDSSEESIMEGSHFDGDNKNMLHGDIIFGDDIKE
>comp7295_c0_seq1; 123 470 975 LEN=2284; translated
MVKRKSSGGGNALSALKYCECACKDYITTGTGDEDEGEDGCPNYDFVKDVATPPLCPPEK
DLKVCDYRFKYIGCTFEVIQCVATYSCQCFGGKWHCGLLHYEGQDCEEDDPRDPRRTSGM
PGTRRMRWLNDDGVPTGDTCDPDDPLPKPPQEDSSALEDEEEDIVIPM
>comp7296_c0_seq1; 451 358 1902 LEN=1925; translated
MKYNLSKGRLEKPNASGKEKENLSKSDKHGSRTEKMPSSIASGKKSSYSSRSSSSKRRSS
SNAGGSSKGKMSSSSLRKSDPDAFKIFILLLQPKLKTFELIQLIYSSNDTTVGKIIAMIP
ENATEPALGSQKYTGLCRPKTQEEIVDHELLASEPRANTGVDSAKITMGEILVAIPDGYT
GADVAVLAKQILSNPKIVKLLKRSDPLAPKRSRRSSRRGQPPRRSRSKEHVDVMEKFDEE
DEMKQEQEMKEQEQRMKKAMEHAAQEAAAANAQIPTKSKTDIHRNIDQSSLRSSSMASTE
GSLEDEKSMESSLQESIDESYSSWSKSFDASFTSTVCSGVSKRSIRRRERQSRRMKIIYM
SVAGCFAVMLAMYFSDPHGYGRSDEEKLVSQAATTENPMGLRGILQCVFLLVFLYQVESF
IRTTSTKSDQDNNRQEKSSIIEFLQSTTAMKKLKSRFAKKLKKKSSRSRRGAVGAFDYDE
NTRTQKLRSFSLKGSNHQMRSATSLHDNSDTGSL
>comp7297_c0_seq1; 875 199 2688 LEN=2845; translated
MSTKTSIDTLESLREVQPQRRSLKRQRTRSAATTMTMTTTAFLALSAWNASPVFSFGSLA
SSTRKGMVTPTSVRFTVDEYHASHSTLTADGLLSNTPSLFRSPQHLHKLEEDYSSGGTAS
VSVSSVYGEDETRDEDHEPRRRLPAWLREDKGRRVQENIEKLRSAMLSSSSFFTESEAMK
VTYAIREAAQNDAHKMAGAAEFCLIMVETMEMGLDALVAAAFHFCECIDARERSVRWPRR
DCGHPNTNQSVDSLSHWEFRLPPIQIDSFGDHAAKIERDAARLKRLEVVASMVVHSTSHT
NNKGGGSVAKKAADNLRSLYTTEAKDWRALAIRGAACLYRLRGILSERQATERELGRKVP
LSRDANRVCREAFRIYAPLASRMGMHRLKNELEHAAFQLLYRRQHRTYESLLQQTRSSHS
ARSASRLGGSLFFFPQNPSEPNIGESMKQVLAHVQASMTKVLEGDAVFMASVEDFRVTAR
VKESYSTWKKMLRDGFDHITQVPDALALRIVLDAKKAHPSESDAVTRARERALCYYAQEL
CKTVWRPCEKDPRFKDYVANPKENGYQSLHYTAETRWNDEDWRMELQIRTGEMHKVAEFG
VASHWNYKAQAKPGQRNKEAASSNQDGSDAKSATYGTVLVEESSNDSSSQHLFGKVTIAN
NDRSSDAYLRSLQEWHWEKQNGSSPIRQTPLVLWDNEAESLERAERIRARTQRLAPYLEA
LANEQDDLQRKSVFVFVAPLDGGSGSLSSDDHEGSGDGLALPTEGTVVALPAGASILDAI
RRCEIPIEGTGNSGTNSVIRNGIPASLNAQLQNGDVLSIPSVPSACAMP
>comp7297_c0_seq2; 875 942 3431 LEN=3588; translated
MSTKTSIDTLESLREVQPQRRSLKRQRTRSAATTMTMTTTAFLALSAWNASPVFSFGSLA
SSTRKGMVTPTSVRFTVDEYHASHSTLTADGLLSNTPSLFRSPQHLHKLEEDYSSGGTAS
VSVSSVYGEDETRDEDHEPRRRLPAWLREDKGRRVQENIEKLRSAMLSSSSFFTESEAMK
VTYAIREAAQNDAHKMAGAAEFCLIMVETMEMGLDALVAAAFHFCECIDARERSVRWPRR
DCGHPNTNQSVDSLSHWEFRLPPIQIDSFGDHAAKIERDAARLKRLEVVASMVVHSTSHT
NNKGGGSVAKKAADNLRSLYTTEAKDWRALAIRGAACLYRLRGILSERQATERELGRKVP
LSRDANRVCREAFRIYAPLASRMGMHRLKNELEHAAFQLLYRRQHRTYESLLQQTRSSHS
ARSASRLGGSLFFFPQNPSEPNIGESMKQVLAHVQASMTKVLEGDAVFMASVEDFRVTAR

VKESYSTWKKMLRDGFDHITQVPDALALRIVLDAKKAHPSESDAVTRARERALCYYAQEL
CKTVWRPCEKDPRFKDYVANPKENGYQSLHYTAETRWNDEDWRMELQIRTGEMHKVAEFG
VASHWNYKAQAKPGQRNKEAASSNQDGSDAKSATYGTVLVEESSNDSSSQHLFGKVTIAN
NDRSSDAYLRSLQEWHWEKQNGSSPIRQTPLVLWDNEAESLERAERIRARTQRLAPYLEA
LANEQDDLQRKSVFVFVAPLDGGSGSLSSDDHEGSGDGLALPTEGTVVALPAGASILDAI
RRCEIPIEGTGNSGTNSVIRNGIPASLNAQLQNGDVLSIPSVPSACAMP
>comp7298_c0_seq1; 784 158 2464 LEN=2559; translated
MSLFAIRRFLVVAAIIVAAVAIGDADPDAESSPIRSEKSQEGSSSTEADVVDAREEGSVV
SSSAIEPDLPFSDINILVVSDVHSFVGGHPHEPDRNADYGDLFSFHERLKEHCEAEGKGD
LFLFNNGDWLHGTGLAMDGNATNLLPIVNAMPWDSLTMGRQEATYTDVLRDMSETMLPAF
HGKYITSNVVWNETKEPYGKRYQFLKGRNSTILVFGFLYDVASPSTTIQVVPVEEAIQQE
WFQSLLRDDYKEYEYDAIVVLAQIDVYSPLIEDIYEAIRENVDPNMPIQFIAGHSHKRER
VSSIKRDFFVHKMEPGGLFDTIGWVTIPKFEATKGWSGKSQELKDAFRQEFLNTSKTVLR
SRLGLRDEDQQGLETAGGRAISKMIHNTQERLELNQIVACPGHDYFRNISIHADNSLWKL
WREHVVRTQIFKKAEDRVIMVSKRTFRYDLRGSGKHDAMTLDDVVAIAPYMQKVIYVGDV
PDWMIRRMNNTFNTFSHHNIIPDYVLAGDIDDVKTAESYQLYTHEEDVPKIKAKLEKFNF
KDFVLKPTGQRDTLYWLNYVRNAYPCKGSENEKFKVPYFYDPNELEEESTDGKVTSDEMG
LDFDEQGSESDTDEEIVWTLPPGEEYKGYIPGKGEVKKVPVSVYENYKTQEERDKEKQEK
KAAAASRLSSKNLKSQLQERKKTHKKIVKGFAVVFAFLILFIPVVCLIMQVTGRNDDNDG
FGSGGALYDQEEMRLLKRRRRGATKKTRSRKDRSGSFHERPLKEIEII
>comp7299_c0_seq1; 139 1456 1593 LEN=2003; minus strand; translated
MWNGKLENWFSVVILQVLNEIAFEEVSHDQSQEHWCFGDVEQCGA
>comp7299_c0_seq3; 139 1334 1471 LEN=1881; minus strand; translated
MWNGKLENWFSVVILQVLNEIAFEEVSHDQSQEHWCFGDVEQCGA
>comp7300_c0_seq1; 259 189 1910 LEN=2572; translated
MAAGALGSRASRPINNGIRNGNNTFGLNKKKNMRQQNTKNNKKASYISRAIFALVLLMVI
IEYVAYTSLTLPISEPPKESEGRYFESRQKGKALTLRERMRKKRVEEKLRTRERRHKEKQ
KILETYRNTSPDPAKVQKYDAKYKNIHTSTQTDVTNNLSPEEFHDQRIKTVEHLLHMGVE
EDYDLEELPPWSLILDNFHPQTKDDEPIILGLEYCEDFRKMHSPKIIGVGPAGLFSTGTN
LIANLMTFNCDGPLKRRGNRKKFSLVQVPWGKHNPADARFHHQVPMPVVEDREAILPVVA
IRHPYTWMSAMCKHSYNTRWRHVHKKCHRGLGLENPVWKVPYGFKSYNETHNVTNSYNSL
AHMWMEWYKPYFLDEKYNQTPRLMVRHEDMVYRPEKVVSRICECVGGVNSNPIKDWEDPD
GFQYLEEGANTGGGHGIHRSGLLTAILKYGQPLRNWYDQYTATDREIMKEAFQGEVDPEL
RNIFDTFQYKLYDDVAGPTPHDIIQVERRKAMEEEKDEQVLAEAKAVAKRNGDLTASQVE
RLKAHNMKQQRKNAKKGNRGNPMHDHRKYKAAQ
>comp7301_c0_seq1; 408 83 1596 LEN=1837; translated
MDFPIRLQPLERNKRIFWNEEIEEYLAKQPTPSLNSNSIEFDEDALQLSLDAVVLELERE
LRRAFTNRKTNGCDPNLGSAVRTGKNILRWYIQIYVEHRQVQQRRHKIQDGRKMDILRSP
GILSTVVSILQWSVDNDLKLARDASLYIFYATYSHHPGDKSASNGIDHLINHESFPQIAL
EMLVANDSIPLHLSLVRNLHSMTVSFPGARATILDAKIHYDPLRATESAPWAPREPTTIN
FSKACLSLIRWLLDDESSFPTYNPEDKGAELVVEIFNCFYAMRKGQELAAPSTVISTDDG
DLSLEKLIVRILKLSPTTKSLETASAENIWKQINECKLAAISLLMDSDASFGEYLIENGC
FGTLLEVFERQVDCTVDNTRLEGSAAATLLPILIVLNRYSVANRNIQILVKDFVFPRENE
KRNNQLIXGQEKDEYESFGCTEDTLRGKLIVLLSWVDGYVKRCSAELMWTLCDSDYNEFT
TRVGLGNALPLLNAKGLSPIPIPS
>comp7302_c0_seq1; 492 157 1591 LEN=1591; translated
MVIWRTSCACLAVASLAVAAVNAFVAERASTGVPSRLLSQASDDADGPGLSRRQVGELTV
AALGLGVSFVGTRENKPTDYGLWGVLPVGTYKTKKTLIKEIVPDTIWTLDQKFGILNVQV
PIRSTVVKLKGGGLFLYNPVAATPECVNMLKELIKAHGPLKHIVVGSVALEHKAYAGVLA
QKFPTADVWLAPGQYSFPVNLPNPFLGYPLSRTKTIPQRTEDAPEEWRETFDFLTLGPFK
SRDGAFSETVFRHRESKTLIVTDTVLEVTDEVPEILTIDPAPLLYHARDTVTQVVENNEA
TRRLGWRRVALFGLFFTPSAIDIKDADVAYQERRADINPDFLGIYPWDWVRDDTLSFDAL
KGGLLCAPILQALILNRTPIQVLDFADEVSKWDIDRIIPAHFKNDLKYGGADYRKAFSFL
EASGVPKGLPKPLDADMQFLKESEAGLIDSGAIVPCPPLPGGKFTRDEILAQTVLKSLX
>comp7304_c0_seq1; 391 767 1975 LEN=3776; translated
MTNEPELVDPTTPKNEGIDACSKGTRAIGDLDLSSLGIVGGASGDESSSPLSSTTCSPVI
ARTPLSPALEEPASNDCSLPVFRDADRPAPNVIANAHSIANANTNTHSNAHSNANAPGTH
NARASPQGRSVFSRYWKKTGQQPIPLRSMRSVASTREPGSPTLAVRGSHPSPPGRPRRRS
DAECHGDDPVLRWASNADLLEMDEAGGDDDSCRGRPFGRGHPVASGAVQGQGQQQPSNCP
SPARRSILPVAPLSHPALRTTSSFSSSRRSSDTGNGLWRKSVSLPCVETHHHRLLTPSDK

NLSMPRLTPAPGASCLRPFQKYSPHRKAAGTPGTDASVSSSSAATTVASWTAASSQMGLQ
NSSSSSLASSNSVVSFLDAVDVRHFETPRETYAQKGWSEYFG
>comp7305_c0_seq1; 376 235 2067 LEN=2511; translated
MSSGGAGTSEYAAAAGCSLIEQRESLRPSRKRSTSNVLRKNHNGGSNTGIDSVLDECSLV
LFTWGRGEDGQLGLGDSSDQDEPEYVDSLRGVGIRHIACGSGHTVVLSTEGEVYTWGRGD
DGRLGHGDNGWKYVPRITRSLERQVVTKVTCGSYHTAAVTGNGDLYTWGGGMYGKLGHGN
EAGHSTPKRVDALKGLAVNQIACGSRHTAIVTSSGAIYTWGDKENGVVGHGDTTGHQYTP
KLVQRLAEKRIVQLSACAFHTGCLTADGEVFTWGEGKFGRLGHGAERNCHVPRLVETLVG
RKPRQLSCGGFHTAVVTEDGRLYTFGGGEHGQLGHNNRFNKVKPTFVEALAGVFVSQIAC
GWSHSVALTSKGKIFTWGNGDHGKLGHGSGRKVCVPNEVEKLKDYRVVRVASYNEHTAAL
VEPFESNALGPGSGAAFPVSSAYASQMRSLVNDEEFSDVTFLVEDQPIYAHRAILVQRCD
HFAAMFRSGMRESVEPTIPIVDITRNVFLLLLEYVYTDAVKVDVDYAIELYIAADIYHLD
RLRDMCCTVVRRNLNAENAGPLVQTADEKHCHTLRDVCMAYIVENFDAVSKTKGIKEVSH
GLLLEILSQR
>comp7306_c0_seq1; 392 177 1899 LEN=1899; translated
MTYGRFLEYLELGWVKQVDLYDNNRNAIVLASSPELGNRPQAIRVEIPVGASQLIQKLKE
YNIDFDAHPLPQKSLLVTIASNLILPLVFIAGLIFFFQNSENFPQNNGMSPMNLGKSTAR
FDQRPETGISFDDIAGIDEAKAEFEEIVSFLREPERYTLVGAKIPKGVLLVGPPGTGKTL
LAKAIASEANVPFYNVAGSEFVEMFIGIGAARIRDLFKKASENTPCIVFIDEIDAVGRER
GAGIGGGNDEREQTLNQLLTEMDGFKENKGVIVVGATNRVDILDSALLRPGRFDRQITVG
LPDRLGRLGILKVHARNKPLDKDVSLIQIANRTPGFSGADLANLLNESAILATRYKKDSI
TKNEVNEAVDRIIGGIAGSAMEDTKNKKLIAYHEVGHAIVGSLLQNHDAVEKVTLIPRGS
AKGLTWFAPDEDQMLISRAQLLARITTTLGGRVAERVIFGETEVTTGASSDLQQVTRVAR
QMVTRYGMSILGPIALENDNNELLFKGEMSNRIDAEVCRIVNHCEQLATRIVRDNRVIID
LIVERLLDAETLDGDEFRNLVXAIYYSSNDNEIKR
>comp7308_c0_seq1; 209 1089 1406 LEN=2082; translated
MVPLPERDMERIQLNIKEGKHPKYCGDFKMYVGNISFQSTEEDLFEIFQSIGPVGDAILV
RDDTGKPRGFGFVTMRNKADGEKAMEELDGADLNGRNLNVRPSNN
>comp7308_c0_seq2; 251 307 993 LEN=1669; translated
MILLAVAVLMTSSSVSAFNPSVPSVRTANFVLGADVAGSATEAPSGSPANSSKGSDVPKQ
SEYGKSLELPDTYASCGQCGSTYALTLEAMGPGKGGRRMECSVCGHTWFQSRDRLATLGD
GYEMVPLPERDMERIQLNIKEGKHPKYCGDFKMYVGNISFQSTEEDLFEIFQSIGPVGDA
ILVRDDTGKPRGFGFVTMRNKADGEKAMEELDGADLNGRNLNVRPSNN
>comp7310_c0_seq1; 532 35 2050 LEN=2479; translated
MPDQNATETSQRCSGTYPGLAFVTRRRSSSTGDLQKFTSEGHWYEEILNNAVAGGTNGAT
AAGAARRKSLLENFKNTYTSMNLNKSYKTGGENQRRSSIIKFPLEALTADSSVEFKNPDL
FSEVHSMAFMSASIFALAEVRKAARDGRIDTDGLDIISTPVLGGAIIKTFLRNKEFFLGE
LKDADYDFLRSVAQSTNMDSSEESGEYCLSKAEDSEHNRKENALKLINSVSIDYFGDDNI
ELECMYMVTRIPSSKQITLIFKGSTTLQDWIKDSKVIVSDIENPVGDRPEQLQMIGVHLG
FREYLYGESTTVSTENSSRSLSLTSLPDEGVEATIESFEENKVENPCTDDDNISPTANVS
RESLTSTSAILKYLGLDVSSLKGKQNNLRSSIGKNVESPFASIENNEECIGTENKIRSES
MTLSDIRENRANNRSRLCRILNEVKSLRKHYRDYKIYITGHSLGGALGLLAALEIAARFG
NVDRPVTFVGVANPRAGTEGFRDAVECLEREGKFRCVSVHGHLDIVTMLPNSFIRKSKRR
FCQSGFELVLNSESSKFYVHRSEQQNDKYFQRLGLAVFKPHKIADRHHYLTYLKELELLE
RPLHKLHLNDFYDKFVDQSLFPCSKKKMTPSVVRSSIHNWRDNVVVLEQPTKKERKRVLS
ADDFIDQELSL
>comp7310_c0_seq2; 451 35 2261 LEN=2690; translated
MPDQNATETSQRCSGTYPGLAFVTRRRSSSTGDLQKFTSEGHWYEEILNNAVAGGTNGAT
AAGAARRKSLLENFKNTYTSMNLNKSYKTGGENQRRSSIIKFPLEALTADSSVEFKNPDL
FSEVHSMAFMSACESRTRGDFAGXIDSPELVAPLWCCDLSPHRPIQIELFVYCRRISPXL
FLSTFNRNFSADMHLIVLLFLSAAIFALAEVRKAARDGRIDTDGLDIISTPVLGGAIIKT
FLRNKEFFLGELKDADYDFLRSVAQSTNMDSSEESGEYCLSKAEDSEHNRKENALKLINS
VSIDYFGDDNIELECMYMVTRIPSSKQITLIFKGSTTLQDWIKDSKVIVSDIENPVGDRP
EQLQMIGVHLGFREYLYGESTTVSTENSSRSLSLTSLPDEGVEATIESFEENKVENPCTD
DDNISPTANVSRESLTSTSAILKYLGLDVSSLKGKQNNLRSSIGKNVESPFASIENNEEC
IGTENKIRSESMTLSDIRENRANNRSRLCRILNEVKSLRKHYRDYKIYITGHSLGGALGL
LAALEIAARFGNVDRPVTFVGVANPRAGTEGFRDAVECLEREGKFRCVSVHGHLDIVTML
PNSFIRKSKRRFCQSGFELVLNSESSKFYVHRSEQQNDKYFQRLGLAVFKPHKIADRHHY
LTYLKELELLERPLHKLHLNDFYDKFVDQSLFPCSKKKMTPSVVRSSIHNWRDNVVVLEQ
PTKKERKRVLSADDFIDQELSL

>comp7310_c0_seq3; 331 35 1778 LEN=2775; translated
MPDQNATETSQRCSGTYPGLAFVTRRRSSSTGDLQKFTSEGHWYEEILNNAVAGGTNGAT
AAGAARRKSLLENFKNTYTSMNLNKSYKTGGENQRRSSIIKFPLEALTADSSVEFKNPDL
FSEVHSMAFMSACESRTRGDFAGXIDSPELVAPLWCCDLSPHRPIQIELFVYCRRISPXL
FLSTFNRNFSADMHLIVLLFLSAAIFALAEVRKAARDGRIDTDGLDIISTPVLGGAIIKT
FLRNKEFFLGELKDADYDFLRSVAQSTNMDSSEESGEYCLSKAEDSEHNRKENALKLINS
VSIDYFGDDNIELECMYMVTRIPSSKQITLIFKGSTTLQDWIKDSKVIVSDIENPVGDRP
EQLQMIGVHLGFREYLYGESTTVSTENSSRSLSLTSLPDEGVEATIESFEENKVENPCTD
DDNISPTANVSRESLTSTSAILKYLGLDVSSLKGKQNNLRSSIGKNVESPFASIENNEEC
IGTENKIRSESMTLSDIRENRANNRSRLCRILNEVKSLRKHYRDYKIYITGHSLGGALGL
LAALEIAARFGNVDRPVTFVGVANPRAGTEVSKVRLQNFAG
>comp7310_c0_seq4; 412 35 1567 LEN=2564; translated
MPDQNATETSQRCSGTYPGLAFVTRRRSSSTGDLQKFTSEGHWYEEILNNAVAGGTNGAT
AAGAARRKSLLENFKNTYTSMNLNKSYKTGGENQRRSSIIKFPLEALTADSSVEFKNPDL
FSEVHSMAFMSASIFALAEVRKAARDGRIDTDGLDIISTPVLGGAIIKTFLRNKEFFLGE
LKDADYDFLRSVAQSTNMDSSEESGEYCLSKAEDSEHNRKENALKLINSVSIDYFGDDNI
ELECMYMVTRIPSSKQITLIFKGSTTLQDWIKDSKVIVSDIENPVGDRPEQLQMIGVHLG
FREYLYGESTTVSTENSSRSLSLTSLPDEGVEATIESFEENKVENPCTDDDNISPTANVS
RESLTSTSAILKYLGLDVSSLKGKQNNLRSSIGKNVESPFASIENNEECIGTENKIRSES
MTLSDIRENRANNRSRLCRILNEVKSLRKHYRDYKIYITGHSLGGALGLLAALEIAARFG
NVDRPVTFVGVANPRAGTEVSKVRLQNFAG
>comp7311_c1_seq1; 69 1 260 LEN=1242; translated
XSSSNGNKPDGAPKRTISTARAYACTNAMEYFAELSAAFLGGLDDTNEYNKWYPFNRKQL
QKHDPRAYSLLSRLWKVDVGLTHSH
>comp7311_c1_seq2; 69 1 260 LEN=812; translated
XSSSNGNKPDGAPKRTISTARAYACTNAMEYFAELSAAFLGGLDDTNEYNKWYPFNRKQL
QKHDPRAYSLLSRLWKVDVGLTHSH
>comp7312_c0_seq1; 777 1 2995 LEN=3442; translated
RSRELLELGRCLPGGDIEQYTVALLASSTVSNRGKPRTIHKLHTLSIQLTEVTNMTETKE
TDKTAGRVLLPSYVQPQSYDLKVTPDLTAYSFDGIVSIGMVTGDSFSEEESKKITLHSKE
LMYRSAEFHAADSGKVVKADEVSCDDMNSIPVYCMIGRAIGEYNSAYFADDILSQSFPAT
ASISTLLSFRFMXNMKNTTVTFVFAESIPKSSTITLKIDFAGCLNNQMAGFYRSHYKDTD
GNDKIVASTQFEALDARRAFPCVDEPSAKATFLLTLIVPADLECFSNMPESKRVTLGGNK
VEVSFLETPKMSTYLLAYCIGEFDYVQGITKYGVLIKVYAPRGRSASCQYALDCGCKALD
SFNDFFGITYPLPKLDMVAIPEFAAGAMENWGLVTYRDVDLLIDPDTASNSQKQRVCTVV
CHELAHQWFGNLVTMAWWDDLWLNEGFASWAENYAADEIYPEYQMWDQFCSGALQTALVL
DGMLSSHPIQVPIAHAEEVEQVFDAISYCKGGSVVRMVKAVIGMDAFRQGLGNYFEKHAY
GNTETIDLWKAWEEASSMPIPELMASWTEQMGFPLLKVIGEDWQDDNVVLTLEQSWFLTD
GSEPPEDGNDKIWTIPIISCTSEGTQQDMVLMREKTATITIPLKSKSDWVKLNAGQEVPM
RVHYSDEMLARLSTAVASTDLTSPSDRVGLIQDAYALVKANQIMSPESLMKLLAAYKDED
DYVVWKGIASSLSGLDSVLSADEEMYSNFTKFAKKLVLPLVDKVGWEAKPEDGHLTSLLR
GLMINLLCKFCSDDANVAQEANNKCVAFFEDPIENAKVLSPDIKEYVFMIYLKNGGQKEY
DTVKAYYNVAKDNAERKLVLNSLGSIGDPKLKLETLDWTTSGEIKLQDFFYAIGSVSISG
KEGRTITWEYFQEHHERLKGMVGTANPSLMDACIVYSCGRFASLEKADEVEAFFKENEYP
KNERRIAQMIEAMRANGKLLVNLQASELSKEDFWDQL
>comp7312_c0_seq2; 863 1 2847 LEN=3294; translated
RSRELLELGRCLPGGDIEQYTVALLASSTVSNRGKPRTIHKLHTLSIQLTEVTNMTETKE
TDKTAGRVLLPSYVQPQSYDLKVTPDLTAYSFDGIVSIGMVTGDSFSEEESKKITLHSKE
LMYRSAEFHAADSGKVVKADEIHVNMKNTTVTFVFAESIPKSSTITLKIDFAGCLNNQMA
GFYRSHYKDTDGNDKIVASTQFEALDARRAFPCVDEPSAKATFLLTLIVPADLECFSNMP
ESKRVTLGGNKVEVSFLETPKMSTYLLAYCIGEFDYVQGITKYGVLIKVYAPRGRSASCQ
YALDCGCKALDSFNDFFGITYPLPKLDMVAIPEFAAGAMENWGLVTYRDVDLLIDPDTAS
NSQKQRVCTVVCHELAHQWFGNLVTMAWWDDLWLNEGFASWAENYAADEIYPEYQMWDQF
CSGALQTALVLDGMLSSHPIQVPIAHAEEVEQVFDAISYCKGGSVVRMVKAVIGMDAFRQ
GLGNYFEKHAYGNTETIDLWKAWEEASSMPIPELMASWTEQMGFPLLKVIGEDWQDDNVV
LTLEQSWFLTDGSEPPEDGNDKIWTIPIISCTSEGTQQDMVLMREKTATITIPLKSKSDW
VKLNAGQEVPMRVHYSDEMLARLSTAVASTDLTSPSDRVGLIQDAYALVKANQIMSPESL
MKLLAAYKDEDDYVVWKGIASSLSGLDSVLSADEEMYSNFTKFAKKLVLPLVDKVGWEAK
PEDGHLTSLLRGLMINLLCKFCSDDANVAQEANNKCVAFFEDPIENAKVLSPDIKEYVFM
IYLKNGGQKEYDTVKAYYNVAKDNAERKLVLNSLGSIGDPKLKLETLDWTTSGEIKLQDF

FYAIGSVSISGKEGRTITWEYFQEHHERLKGMVGTANPSLMDACIVYSCGRFASLEKADE
VEAFFKENEYPKNERRIAQMIEAMRANGKLLVNLQASELSKEDFWDQL
>comp7314_c0_seq1; 297 169 1055 LEN=1055; translated
MTKDDLCFMNQHVTSFAREKKAKNGTASILGEIREQLHDLWDGATMKTMETTNHHDDCNH
GCSCDSYHGHVHDDDDHKTLLMNDLCKTLSKLAQWEIDKVNQNPISCDDDDDDSSMAKRI
VRLSKTMMEFIDETEPKAETRSETKTSFLLSADQLDQIITXVPAKHLSDVLRGERFSGGH
DVLLRLVEKPHTGLDHTLLMDHGWHTSSARAAKATAARGDSNPKHPRVEALDSDDSRNDG
AANSSTLFVCGERSGIASGQREVRPVGRVPVEHADGSNQRPRPVPESEAIAIAIAI
>comp7317_c0_seq1; 483 79 1074 LEN=1191; translated
MICRSTAVLSLALVATALVGSKSFVIKPPASATRNGRAPFVAAGATEVRQGAVAVDPVVS
SETDHENKNPRLSGLALALDDGTRKSHSMAENTAFVQGFFKGISSPDSYRNLLTSLYYVY
RAMEEDVLDKADKDSVVGRLDEAPLRRLPGLEQDMEYFYGPEWRTAMDPPSGATKTYVDR
ILEITASSSPTAGDNGELSPEQMPLQEPYLFVAHQYTRYLGDLFGGQMMGNMASRSMDLP
SDGSGVAFYTFEGVDSVRDFITSWYTELYTLDLTETQKQEIVDEANLVFALNIGILEELE
GSPWAALFAMAMSRAKDFTNWSNIKETKNGQ
>comp7319_c0_seq1; 166 539 1360 LEN=1657; translated
MYPKEPLPLQIRRSNKPEDPPGKIAVRSSSSSMCAAKIVTKRDEKPGDSDIPASQCPSLE
EFIRLMQAKVSHESNNAVPAAAESSCSESGDSSCAGKTESSWSSASSFDDSEDGGSEAVE
PRHIALMKTATVEETSANSNIKYNKNQKGSRLRNKNDYGDKKISGRYTNYPSETKKNFSD
TSAMKKGSSPAISNAKATLDLSSALRGCKSSASLDLSDACKRYREAGPGVSGLGARAFRR
QDDWVGGSANELNVPEERWKVKATDRSRSNRYF
>comp7321_c0_seq1; 394 68 2259 LEN=2974; translated
MTGLAANTFPSGILSLVVYILLVASSQAEHDASNSKHDEFGSSLRGQQQRHRELKRPDYP
CILVRVETEYEQVEVEEGSIFSHFVDNAQQADVDEVRCELSEVDVAKAGGQYFVPIMGIE
NSQLNYIESGVSTLVAEGAVLMDGALQIPQGAAIEIGTVDDVEFNDGEDEGGFMRWRSLR
MQQGSRRMAQVGTKKVLVVRANAYDSQTTGSRMEISNNIFGTGGDKVTLKSQYEQCSHGK
LVFEPYDNGNGVREVNIHTDVNGKNRFDIEKAMTSAAENQMGNLANQYDHVMLCMPPGTG
SWVAYAYVNSWLSVYNDEWCQQLSTLVHEMGHNMGLAHSGKDGNPYADRSGMMGFSYKGD
DTPQQCFNPAKSYQLGWYSDCVVEWNPSRQGTWAGVVVGVADYGTNSNGKTCVIRIPRPR
NGEDLYIGYNRKKNMNSDVMDSGDKVTIIEKEDGYKVSNFIAGLHPNAGTKQVFYNFEYE
GRNLVVEFSSYGSSIDEAFVAIYFDDCEHPCWTTAPQPPTPLPTAAPVPVPSDPQEEDDD
PNKTPIIFEMLPNPRREEAEYIEIFNPHSSSINLSDYLICVQNGRNREPRCDQLMEFNLD
PGAYFMLCRNKLYVADSRTCHQVGDNLNFKDRRKQFVTLQQITNGSEVGTVTQTVVVDKL
SLRRPKKFPDYMYIXKKRTNGQILCVLLDLVAATFVVSTSIDITIVVFEEVDIDMFTEFS
TETTAPTYKL
>comp7323_c0_seq1; 482 1 1419 LEN=3106; minus strand; translated
DGTTSVVLLSAELLKRGLGDLVQKQGLHPTTILAGYRTALKAAIAYIKESILTPISLLDS
KHLLEAAKTSMSSKIIGKEGNFFAELAVDAVKSVAHTKEVSGKNGIRKKKTIYPLSAIHI
LKVHGKSSLESYLMKGGFVLNATRASQGMPTFLGDPDDETSEVKIALIDMNLQRHRMAMG
VNIQIKDPKEIERIKQRELDLTKEKIQKILDTGAKVVLTTKGIDDTCMKYFVEAGVYCAR
RCPKEDLKRLAKATGGQVVITMADMEGDESFDESALGKCRAIQETRVGDGELLKIFGCSG
SGASTIVLRGANEYLLDEIDRSLHDSLCVIKRMLESNSLVPGGGAVETALNVYLQEYALK
TLDTREQLAVEAFADALLVIPKTLAVNAAKDGAELVSKLKSIHSKSQRPDCENDDLKNYG
LDLVNGEIRDSLAAGVVEPAISKIKSLRFATEAAITILRIDDRIQINPTEQQ
>comp7324_c0_seq1; 682 1 1652 LEN=2359; translated
SSSSSNIGQATLRARVPIPVLCFPYRAPRRATNNNTREXVSASDDRRNQSLEQGHHPLWS
LNLNLDSMAQAGAGNRAQELLRRIHALYEEGYYEVSPDIVSYNSVLKAWKEEEEPEAALE
LLETMIRNEKEGAQREAFAKVRSSSDANSAKDKSDKTIRVDVISFNTVMAAFANQGNHDK
ALELLRKMQNKPDKNRGTGTEEKEDDYFYPTPDTITYNIVLYSLAQSNDPGTAAKAENLL
REMMMQKERTGLSVVDTMSFNTVLYAWSREGEKKDHNPGTTGKSGVRKRPVSPKMAAQRA
EDLLTIMEELSEAGNLSVRPDVYSYTTVIQSWSKCGEASGTDKARVVLEQMVDKGLQPNQ
LTYTALMNALNKAGQPENAESVLRQMIAANHQPDNVALSSIIDGWARVSSKDRPEAAKRA
LQILGAMKKKKDSPWMRPTALTYTSVLTALAKSGTRDACDQALDLLEEMEEAYAASGGDD
YDANNVVSSGMADAKHDKWSIRPKNIHYNCVLDAYARSSRADKAIKAQHLMTVMENHSRL
DCRPDTISYK
>comp7324_c0_seq2; 781 1 2078 LEN=2203; translated
SSSSSNIGQATLRARVPIPVLCFPYRAPRRATNNNTREXVSASDDRRNQSLEQGHHPLWS
LNLNLDSMAQAGAGNRAQELLRRIHALYEEGYYEVSPDIVSYNSVLKAWKEEEEPEAALE
LLETMIRNEKEGAQREAFAKVRSSSDANSAKDKSDKTIRVDVISFNTVMAAFANQGNHDK
ALELLRKMQNKPDKNRGTGTEEKEDDYFYPTPDTITYNIVLYSLAQSNDPGTAAKAENLL

REMMMQKERTGLSVVDTMSFNTVLYAWSREGEKKDHNPGTTGKSGVRKRPVSPKMAAQRA
EDLLTIMEELSEAGNLSVRPDVYSYTTVIQSWSKCGEASGTDKARVVLEQMVDKGLQPNQ
LTYTALMNALNKAGQPENAESVLRQMIAANHQPDNVALSSIIDGWARVSSKDRPEAAKRA
LQILGAMKKKKDSPWMRPTALTYTSVLTALAKSGTRDACDQALDLLEEMEEAYAASGGDD
YDANNVVSSGMADAKHDKWSIRPKNIHYNCVLDAYARSSRADKAIKAQHLMTVMENHSRL
DCRPDTISYNSLMMACAHATGNQQMREQSFEIALRTFKILASKGGTTGSIQQREQLLAHK
PVYELYPTSATFAHLFRACRKLLRPDNARRQTILSKSLVICQKLGLLNFLVVHQVQLACQ
SERAWRETAGDLSEYIGWKDDFKRCGKTVPREW
>comp7325_c0_seq1; 322 337 1779 LEN=2500; translated
MVLPKPVSRKDDSMPFFLGRSSLPLVRSKLFLVGTTLVLFMWSRSSLYPSNGMYSIGAES
STTNHRTIDETTRVEKPEPAQPPTKIDTGTSDRHDLISPEELGRLIDELRGDDHRLVRWD
ATKHTRKKQRRGGCDELPRALDAVASEGRGVGASDGHKALACEFATFCITDNPRQRARLG
QHEGIHAAVVGLVASPDVRASAMASHLIYIATFSNAKNHRAFVEHGAVGALGALVKSEAS
EPIQAMWAAAALQNLAASYCDTEGNGRCYWRWDKKALDRPVLVIKNSSLPMISDGSEARR
QILDDPDLVRALAGHACRGPVSGTEGPGNPFPGEHAVAGRDDRSPNIVPWAAAGALKNAA
LSLSPGFLEALDPAMPCFCRMARSDDWLEANKAGHVLDHTRRRDPCWFGPGGEDYEAPGA
ELCVDRYFVDGEGYDCSGYERAGGDECATGDATGTTRASEACCPCGGGTRGGPVRPGAGG
>comp7326_c0_seq1; 437 212 994 LEN=1080; translated
MATSTSAFVPSSNRMLSKTLPTQKIGNNKSIESVLFMADENETEWSAPTSAMIGGAGVNN
AGEPPFEIRGFSLGNLVIFSGIMITILSFAEYLSDSGADGLNVSGLGFVYGIPVFLAGAA
LKYAEIEPVPCISSPAAERIFDDKATEIIAKIKQDVTRHRYGDEAHLDTTVKKLGLVVPG
KDYPQLMELREDVEDGELTFTMVWQSIDTPYKMWADERRVKKYETFFGPGVAAEVVKIDA
EQRLVGIKLTTTSSTPAVAA
>comp7327_c0_seq2; 399 77 775 LEN=1225; translated
MGGTKRMSAEEKRKVILGIYHKEQLVYTEKEITSLAAKAGVNANSIPDIHQGMIDDALVE
KTKIGGSNYFWSFKAKKDRAAQVRYETTLKAIEELKPRVAEAEARLADAKRGREEDDDGD
GRESGSSEKDGGDEGSGAVPVRGRAKKLARLEELGKEKAALQTELDKLKENDPATLADLE
KELKLVTQAAHRWTDNIFECKSYLVKKRGMQKKEACKFLQISSDFDYPEETN
>comp7328_c0_seq1; 386 225 1739 LEN=2637; translated
MQISSNALTLYVIVASTSTYAFVHRPRLLPTHLPSIEKGGTALGYIKENYETGIGPDILR
KHKRKPDVIDAIVVEEVVLPTDVGSSQTSTRTTPTPTHARPKRSPNTRIRKELSPDFDDA
FISEDVPATAPLEPSIWDTNTAKCIQGGALRTWAIGAPEVDHTQVLLKSTHWDSPLRAQI
DVYNGPGHAPMKLAVYCAESLSQQPFSFVIPTPGFNHQSIGIKNSGPFEFPIEAVAIADV
ESAREQRKRQRERERQHTDPNTQPLQQGGYTGFYNSVDPTPARTTLGAGRHEERPTSGLG
LFAEKLRDLGDLRKIEGTSPVEYASGNEQSSSEELFEFEKNVDSVQVLIETRGLSCQARI
EVTSNGCDEILQVIELSIEDGKERPFFAVLDTSQRTSTTVRVLNLDPYSAFPITACVEPY
TINSDDDDDCNYKHDEDQAFGFDFIDASIEEDQGSDDEMIATAAASDSSAADGEGESDMY
AVPDITIPASLADYDFDEEFFFAS
>comp7329_c0_seq1; 239 1 1040 LEN=1054; minus strand; translated
XTCSITYIKYIILYLVPVARARVLAQKQVDSLSNKDVEDDDGPTNPIQLKFSDGSMIVTH
ISYLTMLPFDAVGNPLYGTDAIQGLEPWHEPMKKMTVPNESFKAIIQWHNYSLAEHLGYS
SCVNIVDGAAGMGSCDRIILDGNRPDGGIAELDSQVVRQGEADSVLIPQFISSFQFAVFL
IIIILSLPLSLSLLIKTAWMWDKYQILLYSVGGQKSPANTNIAIQTEYGMNTLIKLAIDE
LRNATKGIISKVDGKPIEIPQPSWFRGKIWPQGSLMINWNLDGMKETGWSSDAFSDTFRR
PFGEGVNIWYGNSEMSADGALHGWAEGAFAMANRSLDGIRSALDGL
>comp7330_c0_seq1; 301 257 1936 LEN=2432; translated
MKKIVTKSFFGVILLYFWVDLSNAFRQSFQKRQLACRDFLLFANEVASARVNLALPKPTN
AAPWPRNRRLRKYFCSTEYRDFEVDNRIAEDGDTDEEEEATFRSNDDVEKFFDEAERDEN
RKNFPRGKPEGFYVTNQYSIPEEGFEVLLRTVDTEKNLLKDGCTGGISREEIDRLGITGT
NITLPIALMLLDAEAYPSLSRARKSCRKGHVVVNRGSLVVNRETGKEEFDQDTCFKGRVI
DRVYPGDVIGIQCRMHGGCYPGFDTTKPKFDLPVLYEDSHCAIVNKPAGIVCYSQRDDNH
GVMTVRHALPFVLKPPKRGTAGIYLRPVGVHRLDKPTSGLLVVAKTKPALVNLSKQFEDR
RVKKTYTAVVNGIPEEPIETSITSEEALNLGIDIGDGDQASKWQLIDHALDKKSAVTVWK
PLNYTKSLKAKDTTLTLVELKPKTGRYHQLRRHMSWVRGCPLVGDVSYDGGGEATSLRGR
GLFLCSNRIRLEHPYYNTIEGRTEWEGLPEQQKWANGTLVLSEDGTTVEVHASIDLPNKF
ETFLATERRRYEKFGDDVA
>comp7330_c0_seq2; 204 1266 2039 LEN=2535; translated
MTVRHALPFVLKPPKRGTAGIYLRPVGVHRLDKPTSGLLVVAKTKPALVNLSKQFEDRRV
KKTYTAVVNGIPEEPIETSITSEEALNLGIDIGDGDQASKWQLIDHALDKKSAVTVWKPL
NYTKSLKAKDTTLTLVELKPKTGRYHQLRRHMSWVRGCPLVGDVSYDGGGEATSLRGRGL

FLCSNRIRLEHPYYNTIEGRTEWEGLPEQQKWANGTLVLSEDGTTVEVHASIDLPNKFET
FLATERRRYEKFGDDVA
>comp7331_c0_seq1; 264 50 1402 LEN=1402; minus strand; translated
MKAQRLCYFVRMEPKSTLKSPVHPFQNGTARLARTIYERAKRRAAGSRSAECGSRVALRC
IARPRASRRSGCSRLPLVRIDPDDHFALLHPVAHHGLARQPPPRAVLHHLLLRHALGVQN
VGPLHHADAAGPAEPAGPAVSLRLGVEPPGEWVVQLDAPDEHPLAQVLAREHLDGGVGLV
LLGGVLEADPDPGQAAEALDGQDKDVFVLAVERGNRSPDRLDRPVVGQALGRHGKFFFLL
RNRGLLLLWGSPPPAHQGDGRENVAGVETGSFRGSALHHPRNSRDADPVAPATRQLHQFD
PEATPVVFAPGNRDKNAVDVVGGVVLGFEPDPALVFGRFLDKPRGPPRFYHRQGQVVSIE
VHGGFSGALEDLRRDVSGRLSEEGRLRQRDYGGDTIMPARDCEAEIHRCCAAAEALHCSL
LGDGPAELCFVLVVFGRIGLWLDARIMVAAE
>comp7331_c0_seq2; 264 89 1441 LEN=1441; minus strand; translated
MKAQRLCYFVRMEPKSTLKSPVHPFQNGTARLARTIYERAKRRAAGSRSAECGSRVALRC
IARPRASRRSGCSRLPLVRIDPDDHFALLHPVAHHGLARQPPPRAVLHHLLLRHALGVQN
VGPLHHADAAGPAEPAGPAVSLRLGVEPPGEWVVQLDAPDEHPLAQVLAREHLDGGVGLV
LLGGVLEADPDPGQAAEALDGQDKDVFVLAVERGNRSPDRLDRPVVGQALGRHGKFFFLL
RNRGLLLLWGSPPPAHQGDGRENVAGVETGSFRGSALHHPRNSRDADPVAPATRQLHQFD
PEATPVVFAPGNRDKNAVDVVGGVVLGFEPDPALVFGRFLDKPRGPPRFYHRQGQVVSIE
VHGGFSGALEDLRRDVSGRLSEEGRLRQRDYGGDTIMPARDCEAEIHRCCAAAEALHCSL
LGDGPAELCFVLVVFGRIGLWLDARIMVAAE
>comp7332_c0_seq1; 345 202 1323 LEN=1424; translated
MQQDVRYRRIGGDQPYRDDPTSAPIRIGSVGTTTSNDLKKKQSYPLSPATKGLGYCCSLA
SGPREDEGPYGRYYRDDFNDFSWNCSIGTSDEDGIWLNRGDPAGSIMCVMVWILMGYSCL
TVTLLAKTGGISWYQSSVYSILVTMSLACHIKTTFTDPGAVPASAVPTEPQRMMSERLSM
CSQCQTFKPPLSHHCRICNRCISKMDHHCPWMNNCVGAGNLKHFFLFLIYTWTCNVYCLV
LLGWNYFFCASEDCEFNVVLTQLVRIMTVLCVGSFLFTSSMIMNVIYGIMTGVGTIDRLK
KKAADTMKDSIEEPVPLIDIFGIGPWWTWPLPIDPIFEDFDAVMGFATPQRLLREQILRE
QAQQGIEGHISPV
>comp7332_c0_seq2; 353 400 1464 LEN=1565; translated
MKSAPIRIGSVGTTTSNDLKKKQSYPLSPATKGLGYCCSLASGPREDEGPYGRYYRDDFN
DFSWNCSIGTSDEDGIWLNRGDPAGSIMCVMVWILMGYSCLTVTLLAKTGGISWYQSSVY
SILVTMSLACHIKTTFTDPGAVPASAVPTEPQRMMSERLSMCSQCQTFKPPLSHHCRICN
RCISKMDHHCPWMNNCVGAGNLKHFFLFLIYTWTCNVYCLVLLGWNYFFCASEDCEFNVV
LTQLVRIMTVLCVGSFLFTSSMIMNVIYGIMTGVGTIDRLKKKAADTMKDSIEEPVPLID
IFGIGPWWTWPLPIDPIFEDFDAVMGFATPQRLLREQILREQAQQGIEGHISPV
>comp7332_c0_seq3; 240 1369 1623 LEN=1724; translated
MTGVGTIDRLKKKAADTMKDSIEEPVPLIDIFGIGPWWTWPLPIDPIFEDFDAVMGFATP
QRLLREQILREQAQQGIEGHISPV
>comp7332_c0_seq4; 240 1228 1482 LEN=1583; translated
MTGVGTIDRLKKKAADTMKDSIEEPVPLIDIFGIGPWWTWPLPIDPIFEDFDAVMGFATP
QRLLREQILREQAQQGIEGHISPV
>comp7333_c0_seq1; 222 964 1485 LEN=1521; translated
MGPTSAPIVSDLVSKGYAHLTYYEHERDDVPHRASFSFLPDPVCARIAMCLMDDEFRFNA
TSYGEKLEHDIHGAKKIEMATMMGGIFSEGICLPAKGDYGEIATALYILFCGDILHQESE
VYINHSDLIQGSFSGIGISPRSNLARFTSLYRKAAKDLFTRTVNFKTEEQEER
>comp7334_c0_seq1; 746 61 2151 LEN=2578; translated
MMEMPVGQADVGVLLVVALLASAFLTNRSLTRGCSTPCTPRRMLKKNKDTGSEEIDAMET
ETSEGLDKAPVIVFHHFGSKSFHDKILLFETVSVRLETLLQFAVKDCSQRVRLIHFLPED
AGASVLHEMIHEMGHDYSATFKEAKSYKEVAKLVGSSRVCVCSGNFDKALYTIHELKANG
ADVGVLLSLSLSCMRSGSFNRADLLSHVEEILEAKILSTTKAAVKWLLPDGPSLSAAAEK
RCEETHLKIKIVPEGVVVKPELNEDHRLLISTIFKENVCKVELKKIGKGFSKAHKFFCTP
TIKTNEGAKAKGAMIFLKMGDEEEVAKELDVTRQMMMTLGNNCPQVINYAEAGETGGMLL
TLANMGDDGPKGFATLYSDLLDAAGEGKAKKQKQLSERLESAVEYVFGTLCVRLHRAKAR
SEAFSAASCLGLCYDYDGKDPTEMKENRLSSFWVLQKVWRKWGGTGKLASSVQNKMREVL
GEDEANHPTVNFGGGLELPNIVPALLQDNEKLRALAEKTAKYSQCLVHGDLHGDNIMIDS
KDNRYLIDFGRTGIGHAFEDLTWLESFCAMEYCDWNEVELADALKIVPSLAAAFTIDTCQ
AKALDDALTASLTEPRVIAMWSIVRALRVKLGDFLPDFADEKAVKVVEENADMARLLCLR
NALFLGCAPSTKPAIKKFAFALACAYGKEVVTMLSXW
>comp7335_c0_seq1; 775 110 2479 LEN=3385; translated
MVIKKETKEDVFANFRHLDDNAVPDNEETLNRVVENAMLLHGNVSAAPAANGTNRSSKIF

DDGVFAEFKLKLEQETEGKIERQRQLRDEERRRKISKEKSLRNRIWKSVRFAIDSDSYSN
SDSDSDESIIHGRPQQHDHHNHRRHHPNTNSSRRSWRTSRRSQRFDVHHGYHEHNNTRHE
HPETNGTIGNKKHSFRKRSEKRKHLSIQKEDSYNSDYDLERGGDEAFSYEKNYFSNDENE
DSDDSSDNDDDNDSAIDKEEYDSVITRSKTGMPIGGMSIRSTRRTNNSDDDDDNNTERDE
KYHWVSPHSSDLVFSEFDLDAIVKDYEKLQRNLDFGINDKQSELCRYRDHRVDEDDEGDE
NELKITAHYRNLRNLWVEYFPRTNAILFKVFVPLMFIVFFSIFLGDFLGKFEMKNEIEQN
NQIMRDKHELEKYPYEETLYFMFGLPILCFDYYLEKEGTEEIKHDSNYNWTAIVEDTSAK
EFTNETDAFSLSFNSSMLNLSELLGYELPPIYTKESYNATYDGDSNMELTLGEIKTYMAM
CEEAGADIVSRFIDFSARRMVMEAVGTMSFHWMRCWNTTELGDVNPFRPNKDQLQATTQQ
DEFYDKNWRENQKFLYEQYLSEANCSDIACRNAALRQSVENANGSEMCDINKGASAWFWF
VFMTTVGYGNQSPETTHGRLLVGCLGWIIIIIWAVVLYVAGKILGIVIDDVFRKCNCRIM
TGDIPSVIVWGAVSGLWIVAVGEIYRYWLNYTEDPHHYDWAMFVQNDAGDISTGDAYWFS
YISLLTVGK
>comp7335_c0_seq2; 945 110 3118 LEN=3182; translated
MVIKKETKEDVFANFRHLDDNAVPDNEETLNRVVENAMLLHGNVSAAPAANGTNRSSKIF
DDGVFAEFKLKLEQETEGKIERQRQLRDEERRRKISKEKSLRNRIWKSVRFAIDSDSYSN
SDSDSDESIIHGRPQQHDHHNHRRHHPNTNSSRRSWRTSRRSQRFDVHHGYHEHNNTRHE
HPETNGTIGNKKHSFRKRSEKRKHLSIQKEDSYNSDYDLERGGDEAFSYEKNYFSNDENE
DSDDSSDNDDDNDSAIDKEEYDSVITRSKTGMPIGGMSIRSTRRTNNSDDDDDNNTERDE
KYHWVSPHSSDLVFSEFDLDAIVKDYEKLQRNLDFGINDKQSELCRYRDHRVDEDDEGDE
NELKITAHYRNLRNLWVEYFPRTNAILFKVFVPLMFIVFFSIFLGDFLGKFEMKNEIEQN
NQIMRDKHELEKYPYEETLYFMFGLPILCFDYYLEKEGTEEIKHDSNYNWTAIVEDTSAK
EFTNETDAFSLSFNSSMLNLSELLGYELPPIYTKESYNATYDGDSNMELTLGEIKTYMAM
CEEAGADIVSRFIDFSARRMVMEAVGTMSFHWMRCWNTTELGDVNPFRPNKDQLQATTQQ
DEFYDKNWRENQKFLYEQYLSEANCSDIACRNAALRQSVENANGSEMCDINKGASAWFWF
VFMTTVGYGNQSPETTHGRLLVGCLGWIIIIIWAVVLYVAGKILGIVIDDVFRKCNCRIM
TGDIPSVIVWGAVSGLWIVAVGEIYRYWLNYTEDPHHYDWAMFVQNDAGDISTGDAYWFS
YISLLTVGLGDFYLDSEGFFYYDLMMWTGCFLAGFTFLSTFLGQVADVITNVFPDNGDSL
KEQLMNTNLVKRKDVQFKKENEKGIEKLEKLVEVMDDDDKELVAQRVTRIRVKKNLLIHL
LYQTKSEIEYYRKRGERYENLSYSKVCQEENMLNEVLSNTVKEREKLETYRRTEVCSAPP
PGSARARSYFEDGTRVDGVGEKLHLRSGKKSVKKRIKASTYL
>comp7337_c0_seq1; 796 231 1989 LEN=2598; translated
MAEGTTTTSASASSSRRKTRRKRNGGKKNETSAQQKGGKGGSAKSGNKKSGSGGSNNNNN
SPPPPPQIKITIRNIQNSDKFGSVKLVLEELVSKLIDSCVEKKANNQYTIELDRTAVRHL
ITEEEKIEEYRMEFLRGQENQRPETVEGGDCDPDEDDEKEKTEENKEETHNDEGANGSTV
VDGKEGVSEKRNCDKLDVILAPKMVSSLPTILARPLYVVPPRKTRRRGERGGTACVLLIG
PKIEQKKVVTAMKGNVKENRQSDSPSEPQSEDQKKQSDAATVMDDESGSIAADQQAAECG
GDATTATKALTAAPETPISYPRELAKGRLLLSNAIKALIELAAVDSKTQEFAFSGCIVEQ
SMNGKTWKMFHNNNSRPDRRDGTIEGTADYKEWLENIAKQKEELKARPKPAPGGGVAAAN
TTTATGDEVEEDGQILSSLVQHLRAKKEEAKRKKNQKKKEENSKGGKTKGEGGQPENGKK
KKKKQRNLKSHPQEGKKPALTTKAARKKEKRKKQVRAKKKRLVEAAKVAAAPMSVLKSTT
SATLKPDELVLTHSVLKTENTTKMAEAILETRGNGLDYVVPILLN
>comp7339_c0_seq1; 90 1 324 LEN=357; minus strand; translated
GSGIKRRVAAHVMDEFRQGNLRALIWNEKQSWWYVLSNETQIQRKVDRAVNYWTAVPGER
SSSWGLLREANRGKRGSQELEDNLPANKQMKPYHQAAEDHSLVNGWY
>comp7342_c0_seq1; 235 1 782 LEN=923; translated
XKYAPRHPKGGTHIVPPSVRAGFAMHDAKASRSHLFLPIPSTHVLLFLGYPVGTSIEVRY
KLRAFGIPTEHIPVSVTGTIKTGYVKQWMHAREVIEQNYFMNNSNVTIVECPRLDDVLFR
HGISFNSNPGNAAVRRLIINTYKEQEIVKVKRRKMVLAIIEEVRKNGGRFLVWNDEGWWN
ELLDFDDMIMAKVEYIIKDVRRENRISIRQQQQQLKVSSSTSIFCSASTNVQIPSSDEED
GTSDRNCNCSGLMLPAMRRR
>comp7342_c0_seq2; 272 1 1660 LEN=1801; translated
NQNQNQNQSQLSKAIQMVENDDDANVFDATISPLQQQQLLQLQSPTPSQQHGANDDYTDR
PKEDPKKVDVLLSEELLKLSCKERNDIQEEVHGVDCLAREETPELLEASLEQMARALEDN
RITPFHTKEALRRARVLFPNSYVHTRDFRLRFLRNTLFDPIWAAKKAADYLRIAGDIFGE
KALERPILFSDFTAKEKQYIRKGHCQFLPFRDRSGRRILAVVNPTLYTGDSERPLQQMSR
EERDDSMFFMQAKLLMYMVWTAGYDIETQRRGIVYLCWLDPTHQPKQNFNLSVGIPRLNM
TSVFTVRASAVHTCSPDRPFYRFFRNLLIIGSTQANRPKVRFHLGTSIEVRYKLRAFGIP
TEHIPVSVTGTIKTGYVKQWMHAREVIEQNYFMNNSNVTIVECPRLDDVLFRHGISFNSN
PGNAAVRRLIINTYKEQEIVKVKRRKMVLAIIEEVRKNGGRFLVWNDEGWWNELLDFDDM

IMAKVEYIIKDVRRENRISIRQQQQQLKVSSSTSIFCSASTNVQIPSSDEEDGTSDRNCN
CSGLMLPAMRRR
>comp7342_c0_seq3; 264 388 1149 LEN=1290; translated
MTSVFTVRASAVHTCSPDRPFYRFFRNLLIIGSTQANRPKVRFHLGTSIEVRYKLRAFGI
PTEHIPVSVTGTIKTGYVKQWMHAREVIEQNYFMNNSNVTIVECPRLDDVLFRHGISFNS
NPGNAAVRRLIINTYKEQEIVKVKRRKMVLAIIEEVRKNGGRFLVWNDEGWWNELLDFDD
MIMAKVEYIIKDVRRENRISIRQQQQQLKVSSSTSIFCSASTNVQIPSSDEEDGTSDRNC
NCSGLMLPAMRRR
>comp7346_c0_seq1; 388 1 1335 LEN=1774; translated
XEIVRDSEDQQAIVFFNRTIHDMRNPPNSSTPLLHSIDEGTPSMDSDRDPREEICQSQPK
GESDSSSSPGLGDAYETWSDSSDVIHQAFFHTIVYMSLGVVGYSFLLDTQWSIIDSLYFS
VVIFTTVGYGDLCPNSSAAGMIFTLFFALYGIVILGIFLGILGEMAVERQQKLKNEVLEH
TRKSYITSIISNSVRPSLRQSFSSIIPVGEIEEEKIEHGCSFLADVFNIAREQTPNIITL
AVLGSPIMMLENWSIIRSVYWLVVTGTTIGLGDEHPESDWSKVMCIFYIPAVVAFAGTML
GRIATFYVDQRNDKMEAEFFARALSVSDIKKMDLDGDSEVTKYEFLVFMLTTLQKVEQSD
IDEIFDLFHHLDKTNNGILTADDIAYISDRTSVLQRESDASLIKKNLAKFQYPDKGNPCV
PQLVSGNLRDPKLMWGNPCVPPAK
>comp7347_c0_seq1; 448 1 1896 LEN=2031; translated
QQNSIMMILSSALVATLSLISHTESFAPRARVRSTSTSALSRVNEKVQSALFVSTSDPIT
GDGSLPAGTTVAAVPKVAQRWRKSTKQLATLGPSSGDKEMIETLFLAGADCFRLNFSHGS
QEQKKELLMTIREIEEKYSHPIGILGDLQGPKLRVGEFSNAEGEYLVAGNTFRLDLDTAL
GDNTRVNLPHPEIIGASEVGHTLMVDDGKVKLTVIEKGADYLVCNVDVPGKISNRKGVNT
PDSILDISPLTPKDRSDLDYMLEIGVDWVALSFVQKPEDILEIRDIVDKKIPSGDLKPSI
MAKIEKPSCFESNNLARLVELCDGIMVARGDLGVECPPEDVPILQKTIIDECREQGKPVV
VATQMLESMIESPTPTRAEASDVATAIYDGADAIMLSAESAAGXGMFISSEISLNAKGTR
VVSPTVNFSLNXFFFPSRTVQYPEESVAMQQRIINRVEGDPHYRRYLDANVFGHDNTPTD
AVIVAAKQVSKTISAKSIVCFSLTGSTARRASKNRPSVPIMAMSPFKEVSRQLALSWGVY
PDLPKAGSYGYTVSEEGMFDYENPIVEEASDDLDIVLKSACRAALKKGLVSDPDDLLVVT
AGLPFGTPGAANVIRIVPAAGPSCWEGVCRA
>comp7347_c0_seq2; 153 1 372 LEN=388; translated
QQNSIMMILSSALVATLSLISHTESFAPRARVRSTSTSALSRVNEKVQSALFVSTSDPIT
GDGSLPAGTTVAAVPKVAQRWRKSTKQLATLGPSSGDKEMIETVRARQHNALKNENSCRQ
RRT
>comp7347_c0_seq3; 631 1 1785 LEN=1920; translated
QQNSIMMILSSALVATLSLISHTESFAPRARVRSTSTSALSRVNEKVQSALFVSTSDPIT
GDGSLPAGTTVAAVPKVAQRWRKSTKQLATLGPSSGDKEMIETLFLAGADCFRLNFSHGS
QEQKKELLMTIREIEEKYSHPIGILGDLQGPKLRVGEFSNAEGEYLVAGNTFRLDLDTAL
GDNTRVNLPHPEIIGASEVGHTLMVDDGKVKLTVIEKGADYLVCNVDVPGKISNRKGVNT
PDSILDISPLTPKDRSDLDYMLEIGVDWVALSFVQKPEDILEIRDIVDKKIPSGDLKPSI
MAKIEKPSCFESNNLARLVELCDGIMVARGDLGVECPPEDVPILQKTIIDECREQGKPVV
VATQMLESMIESPTPTRAEASDVATAIYDGADAIMLSAESAAGMYPEESVAMQQRIINRV
EGDPHYRRYLDANVFGHDNTPTDAVIVAAKQVSKTISAKSIVCFSLTGSTARRASKNRPS
VPIMAMSPFKEVSRQLALSWGVYPDLPKAGSYGYTVSEEGMFDYENPIVEEASDDLDIVL
KSACRAALKKGLVSDPDDLLVVTAGLPFGTPGAANVIRIVPAAGPSCWEGVCRA
>comp7348_c0_seq1; 159 1 864 LEN=1380; translated
XGRDTHFEKSQTGLQRTTHAREDETTQRRPALPPALATPQAPTHLLACFLPMGFAAGHER
LKNAVHAYWRKNPVLSPRTTAGKVVMGTFYFSIPVVLGYALVAPLADRTEAAAGERLAAA
ASGSKSEGSSGRPEKVGAGGWGGGVRLAESSARTQEINKTNLERFLKKQRRLKEDREREQ
QARADRADGTKPNQTRPNQTGGWGNPAAPSEGNAPPVHEKPGGFCRTAYPPGMPLVNREP
RKINLSGTTNRNPGPKLESATPQRIGSEFGLSSGNRWAVLNQCSTTV
>comp7349_c0_seq1; 438 408 1829 LEN=1970; translated
MTKLIVRDGQHFSSIKFFDCDTKSKEFAEVLTMVLKNNATSSLVVKGGNVNGNTTSEGHR
PFSQVCESMLGSLSNEASIAVALREGLLMNNSLKSLRLSGLNFKNEGPNTKNHGDSESCG
DEYDWCKMLIHKTTLRNLDLSGSPLSTSTATNLSNALSLNKSLQYLDLTGCYLDDQSLSK
ILKSLQEHPSLTKLNLSRNFLGKSASTLALDAVAQLLRSKCSNLACLVLSQQQHPRLNLV
TNAFDNSMYAGELEKQRHQIAFARALNALSTNTTLRMIDFSGNSHCLSDLTSLEALSSCL
VTNTGLLHVNVSSCDLTPFAIEYLAKNCIPHCSRRLKSLVLFDDETSEIDVENKGWTTAQ
SALFSALQTNKTLETFGELDGVITNFESRNRFQHLLNLNRAGRRALAANDLPLGAWSHVL
ARAGRTEIDNRDHDLCSITDTIESPTISSTKSTAASVVFALLRGPALLDNRQQ
>comp7350_c0_seq1; 454 87 1745 LEN=1759; translated

MLRRKGSWSRITNYSDRDIVVSLLRSAGHGDTLDRNVDLVLSSKVKIDVDGNVVELSLGR
NNRSTTAYQMSRQLRLEGGNYTDRSPYWDLPESVGGLRKLVKLVLYSCRSIPPSLNDRLD
LSCLELHFCDGNTMAQGQYRFLSASTTTPIAPSMSILSSIFDTPASHDGTIESKNKNDKH
ECVDRVVRGRLSLLKTLGIHGGVWNTKSIQWMKWMVSPICYNTIDAVTPSETSNHPCEAH
CSATKTTICRERCGTSHLEVVRFSWLQDDLLPALLDFLSPWKETAGCVRVGTTSRMRHLS
WIHSEMSDQGFERVLRGVVLPFHPNLSSIDVSSNRIRSLQFLLQTEPNDESSCNQEQLHQ
FNIPSYHSLRSMNLHHNPILKKKACFDPLEQQAFETLLLRFFPLLGSLTPSWEDWNPAIE
YLLRINRGGRILIDATKVSRTIASVSLASSSDQSRVVTLDSISDCTTSSRNNQDAALMQK
EEESCSAVPKSPTTTISNPRQKIPLSVWPLVLHRAYQTSANGFLPPREDATALYYLIRNG
PAISELFICNTG
>comp7351_c0_seq1; 232 53 859 LEN=1049; translated
MTIPTARTALSSVLKTTASRRRSFESIAGLRAISQSHRSNAAPRKSFSTAPMIDHPFSGP
NVAAEEIVSTRTTTRNTSADSSIKNLEDFVEKIPHVVRQRKMENIDPGAPNDPVAKLFRQ
SNLSKKAWMQYSHFDSTKPYTRNLIATDHETYTLLLLCWNSLEESPIHNHPCDGCWLQVL
EGEVQEVRYDHRLQCTMDIVYRSGQLSYITDNTGYHKVGNPSSQRAVTLHLYAPPFERCQ
CWHSPDDLPNKPVEGKSVNHSEYGRVLV
>comp7352_c0_seq1; 504 1 835 LEN=1045; translated
XNNSSSSTPASSNSNAAPFNPWGAQPQGMPMPDPDRLLAMMENPMMQQMIQQMADQNPDA
LREALEQQNPMFRQMFAGNPAAANQMIRQAMNPQAIRSMLEMQRAMGGGTGGGAGTGGSM
DFSSLLGGGPASSTPTSSSSPPQDLSSVMEQMRAMMGGAAATGGAAGFSNPWLSQQPQQT
QQQPPQHPADRYRSQLESLRAMGFDDERRCIELLQRHHGNLNRAVDALLSEPEAQAPQVE
AAATTEAQTEAQAPPAANDPSPEPPLHDPKDARDKKDD
>comp7353_c0_seq1; 481 334 1292 LEN=1292; minus strand; translated
MPPPRRGSNLAGRSRDIVSQVEDKLKLGAKLPACLLESTRSSLRVLKIRNPVGSFGRYDN
DTKRACALLEYDVRDYNKNHGDGDEIEITMEKLAKAAFMKLKDFRDFHQKIGNFRDNLRS
QTATNPTGATGKRPVAAGAKLNAANKAGKSGNKGETRIVFRDSSISSLAIRLGAFVPNSS
DVAVRAQKFFWDAVGHLKQKSKKGGVHGLRDMQRNQALYEAACFYLVATSGTESQPKIRR
RKNKPLGDSGDGPEDNQQLLLSTFLDVIKEAPSQFQTVLDYAKKLQLEIEPPSKERAGNR
GKSAGASRPRSSPRSSTSSR
>comp7356_c0_seq1; 408 91 753 LEN=1194; translated
MKFTTYSTLALATISGASAFAPSQTHPRPAVTTFMSEDAETGGKIVAIKEETVEFTAGLI
GGVAGFVVGGPIVGAVGAAAANYVSKADNEAGEIVGAVSKSALEVYNYLATLDSKYEVLA
SAQASLEGALDKLKAQETIDPAAIAKVEGALSSTKAKIEEVNDEYDLVGSGVTALGVVGD
LVEKTIAKIGELNEEYSLTDKAKTALSSAVDKAKDAASKA
>comp7356_c0_seq2; 408 91 753 LEN=1406; translated
MKFTTYSTLALATISGASAFAPSQTHPRPAVTTFMSEDAETGGKIVAIKEETVEFTAGLI
GGVAGFVVGGPIVGAVGAAAANYVSKADNEAGEIVGAVSKSALEVYNYLATLDSKYEVLA
SAQASLEGALDKLKAQETIDPAAIAKVEGALSSTKAKIEEVNDEYDLVGSGVTALGVVGD
LVEKTIAKIGELNEEYSLTDKAKTALSSAVDKAKDAASKA
>comp7357_c0_seq1; 249 692 1297 LEN=1407; translated
MILIALLPTVLSVVLLFMFSNNAAHAFTTPIMEKTRATVSSSTAQSFAPGPLDFDFLDAP
SLLLVKADTKSTMVLAKKAAASLSLPNTATAAVNPEVTAQVLNVSSHALMDFPSLYQSAT
NKKPSKLRIQYAQVAGRLMILGIGMLPHHGFTPEEILVQFFLLHANMKPIIRSIRLWNCI
NERKCAAECSLEFEELEDSLR
>comp7358_c0_seq1; 630 203 1414 LEN=1531; translated
MRIAAKSRYASGIVSAIALAVLESRGACAFVPGSRHGAPTRTRTSLNTVTTTTPVGKAEA
RAKAKAKPRLVDILEDEKGHINPQLAQLIWKWEKSQRLNLDLPDFENYSTRTGLRWVKDL
VKKTLRALHVIESTSTAASASASPHLQRRSLHDDLIQEGVIGLMQALKTFEHEARPNESF
ESFAKAQIQIALDAFTLERAKGMGNPTGTGKPLSMESTVEIADPLETESYYYNQDEWEVR
EGLVLDNGKSVQREELVEDFLDETLQYEGEDQMWIQSQSIAAPLRDSIPESLDDSQDGIT
ETSLMEDRATSPDDLALKDMILYNVDDFLGNTLEDIESQIIQMRFGLDDGVPKTQKEIAL
ELDLTIRKVRKLQKVSLAKLRARFTERYINDDTGHEDFWEDTV
>comp7359_c0_seq1; 645 1 2769 LEN=2824; translated
XNIVHSSALLLQILLSSFKARMTLIPSEQDPLLGNKEVKIAGTGEESDDDHDFVPLKDRA
WSFSHSRRQVATAGTSPVPGRRKSVAVEASVENSTNTPVVPDGSRGGKHYIFCLIYAVVN
VIIAVPSLFGYAAVIFNHPIFSSHMNALSKLLISSSLVHQLGFLMFSSLPFAIGTVQDAG
LIFLSSMANTIANTMIDDGNNEVEILSTTLVILCAGTASLGLVLVVMGKFKLADAVSFLP
MPVVGGYLAYIGYFCVLAGTSLCISQPMMDPKDWALLLDVSNLKLAIPGLITGLVLTLTC
RLATSSGTLPFVMVSIPALFLRVLLGLDISLEEARELGLVGETSPSVPVSDLIHIVDFSK
VRWDLIVEVIPTWFGMVFVVSFASCLDVAAIAIDMGQPLDTNRELATVGICNFMSGMTLG

FTGSYIFSQTIFMYRTGVHDRCIGLMIMIVYMYIVVSPVNILEIAPLFFLGSTLIFIGYD
LMSEWLWEVRHQVFLSEYFIVWFTFLSIHAVGINFGILIGILIAICDQILTTAESTGVNR
INKRSRAVYTKGDAKLIHEKAYCSFSPKIISLEVIGNLFFGSSLSVLNQIYKGIGLTGDD
QTKIDVEDLIEDSSMKYPTTPPKFVVLDLMGVTHLDASATRGCFLQLAKMAGKKEMIICA
SGLSPRIEWMFRKHEVAYETSEEEEVERAALLATMETTNNNKQLERMLIFHTAAEALEFC
ETLFLHDMNDSATKSPRSSLNESVLEQRSRAHTISTILSNFIGATEHQTEILEKLMDLRY
HNELIYNAGEVIFTRRSHSDAFYIVLNGCVANSTSSTFQVARKQRSVVSGAGRVDQLTKR
IGSDHIATLWQKGGVFGFNDFLLDRRRTFQTVATVDDTKLAVFTHSGMNTIATHDPELYS
LMQQVLLRASTLDLANCTCHDV
>comp7360_c0_seq1; 681 305 2068 LEN=2355; translated
MAQIPAHGPTNYQHLSIERKTAAPMPRFRFRFLSLLLVFLTPGTFAFVELPPLAVRHGIH
PNPCFATINKDEGEAFLPGVSCSGERIAVVGATGYIGKAVVRESIRRGYPTTAVVRPGSN
FDAASLMGNVSANVNDNDNDNASVSNQQPTNKVFVSSSHGAGEVELSPPPPLTVHRFDVS
KMEPPSPEAPLGAREDSPVLFEPGSVDVVVCCLASRNGTPRESFAVDYLASLRAAEAARA
AGARRFVLLSAYCVKSAEREEPHALQFQYAKKKLEDALAEMAEESNGAFSYVAVRPTAFF
KSVSGQLEAVRAGGPFVLFDLGAGATAACNPISESDLASALIDQVLDNTHANTHWNLGGP
DSPGLDKLGQMESMVGALGLDRPPVLVYVPIGVLHFLVGTFQWLADAFRSDKLADAAELA
RIVRYYAMEDVSRAPPNESNGTGNGTIPLPRRCACAAECPFFVLQCLLDTHRYTLVFCLS
VFVMSYPIDQCSDVEQTKRVNNTNNIPIPIFSQTHVHVHMHMHTQMLTTSPDERYGEMTT
QQHYENIAKNGQEYDPYTSAYGILKDRERILEESSKAVAASAAGMAN
>comp7360_c0_seq2; 708 305 1783 LEN=2070; translated
MAQIPAHGPTNYQHLSIERKTAAPMPRFRFRFLSLLLVFLTPGTFAFVELPPLAVRHGIH
PNPCFATINKDEGEAFLPGVSCSGERIAVVGATGYIGKAVVRESIRRGYPTTAVVRPGSN
FDAASLMGNVSANVNDNDNDNASVSNQQPTNKVFVSSSHGAGEVELSPPPPLTVHRFDVS
KMEPPSPEAPLGAREDSPVLFEPGSVDVVVCCLASRNGTPRESFAVDYLASLRAAEAARA
AGARRFVLLSAYCVKSAEREEPHALQFQYAKKKLEDALAEMAEESNGAFSYVAVRPTAFF
KSVSGQLEAVRAGGPFVLFDLGAGATAACNPISESDLASALIDQVLDNTHANTHWNLGGP
DSPGLDKLGQMESMVGALGLDRPPVLVYVPIGVLHFLVGTFQWLADAFRSDKLADAAELA
RIVRYYAMEDMLTTSPDERYGEMTTQQHYENIAKNGQEYDPYTSAYGILKDRERILEESS
KAVAASAAGMAN
>comp7362_c0_seq1; 195 245 1155 LEN=2076; translated
MAGPRGQSFYDSDGDMDGAFDNLRNTEEDKAIDNLLSASLLRLKFEDRNDLREEVHGVRS
LSPEETPELIRESLSRMDEEVEAILLASRENDFQHAYEQARYLPTTFVNDRDYRLKFLRC
DLFDPVKAADRFIVHVDYLLHLFGPXGPKGHLKADFFVNEEAAALRGGNIQLLPFRDRSG
RRVVVALSGCFSYPPETRVKLYLYFAHVAAHDCVETQRKGIILIGWCNTESLFDGKSIRF
PGSPGIKPVVLPNGRHVIDSISVRVISIHFCYPDKPIYRILRAFYVIAFGDIMRSRILFH
HGT
>comp7362_c0_seq2; 231 245 1761 LEN=1928; translated
MAGPRGQSFYDSDGDMDGAFDNLRNTEEDKAIDNLLSASLLRLKFEDRNDLREEVHGVRS
LSPEETPELIRESLSRMDEEVEAILLASRENDFQHAYEQARYLPTTFVNDRDYRLKFLRC
DLFDPVKAADRFIVHVDYLLHLFGPXGPKGHLKADFFVNEEAAALRGGNIQLLPFRDRSG
RRVVVALSGCFSYPPETRVKLYLYFAHVAAHDCVETQRKGIILIGWCNTESLFDGKSIRF
PGSPGIKPVVLPNGRHVIDSISVRVISIHFCYPDKPIYRILRAFYVIAFGDIMRSRILFH
HGTSLEMQYKLMGYGIPVGQLPSTDTGMVKVKNHIQWLKMHKLIEKDPSAYANVCECPGL
NDVLFRRAGSCLAHPGNGRFKNLIESKKDEHARSNQTGKREIAWSIVEEIENRNGRFFSW
DTKRTCWVLMANRVEIRQKVAMMIRDFNRHSKAAKNPQSNDSSTTFFKNQDGRKRKLDSM
DGFTNCTPMFRLQPFHTRSMSHGEI
>comp7362_c0_seq3; 179 245 861 LEN=2263; translated
MAGPRGQSFYDSDGDMDGAFDNLRNTEEDKAIDNLLSASLLRLKFEDRNDLREEVHGVRS
LSPEETPELIRESLSRMDEEVEAILLASRENDFQHAYEQARYLPTTFVNDRDYRLKFLRC
DLFDPVKAADRFIVHVDYLLHLFGPXGPKGHLKADFFVNEEAAALRGGNIQLLPFRDRSG
RRVVVALSGCFSYPPETRVSGFVAN
>comp7362_c0_seq4; 179 245 861 LEN=2115; translated
MAGPRGQSFYDSDGDMDGAFDNLRNTEEDKAIDNLLSASLLRLKFEDRNDLREEVHGVRS
LSPEETPELIRESLSRMDEEVEAILLASRENDFQHAYEQARYLPTTFVNDRDYRLKFLRC
DLFDPVKAADRFIVHVDYLLHLFGPXGPKGHLKADFFVNEEAAALRGGNIQLLPFRDRSG
RRVVVALSGCFSYPPETRVSGFVAN
>comp7364_c0_seq1; 711 202 3807 LEN=3896; translated
MLSLCFSFRQRQRRNRRKFRNPLGAVLGVLVYAVVNILCKMQTVAFVEVALSSGRGRKNT
PLGRTEVCNKNDSGRASEMYSSTSSTEQAIAKSNAPRRLTTDNGNIKSERNNYNDNGKKY

```
ERSPKEMNNRQRRRNQHHTTSGTRSSECQRITKELDAIRSPPGIKDWITDTIWQHQQNST
LQSPSPWAGWNVHEQVSFVKYLRQRNAYASIVPFLSGLDPNPSDREMDKDNSLRPGKSSR
TQNHLVKVYTTALFALSACTDSGQQDWNHGLDTKPMVLEILDQMDRNHIPATTLTYVAIF
KNLQGRPADVAQMIRFLEQRERNLAPKAKNPGKLWDAEVYHAAIYACRSHSNRFRRTSRS
SDNGYGSRNESMNPNFDNKDWQTAIALMQQMQSKGVSPTSQTYLAVLEVFGQHPSKMAVM
KSIIQQWKNSKAAGVCNNEEDDRIWATALNACAVAGDYCQALKFIEEMMSEGCAPNLRHC
TALLKAISGAKGYPQDQLAMTALNAMMGNETAALETQDGDNGKDYRVLLPCTDLDIVALN
TVLSIVVKAGNFEDAMVLFNRIKRGKFRNAAGGMITPDRITYHNLLAGSSDPETAKRIVK
EMRLSRRNRNGAIPPTSVTYAHAISTCQRAKTPDLSTVNQLMKWASEDGVKHTVFMYASA
IWTAQRCGDLSRCLELYEEMERTGCRANSVVMNGILTALCDRGANASALNAFHLMKDRGF
QVSPIALKRLVFLTIQSKKNQLTEKEKEATLSAILDRLDPQERTVKISSPVFEALICIHA
SRGDIDQALSVADKINGTFDAACLRAILLAYSTGPKSLWSDAVELLHTTIIEESPAPGKV
DQIALSHTLTTCSRANEFEESLSLLSLYGIPVNELPDGFPHLSIVALNALIAACGRCGRP
DLSIFVLNEISSRYGLRPDSRSYRSAMIACNKAQHAEKARNSNNSQETTNDEMDGEYALQ
WWECSLALYRRMTEEGLKPDVQTHSSIISSCEAAGEWQRALWVLQSIIDKSADEPDDATP
SLNVYCWNAALSACEKGGAWVEALDLYERMLESPFQPNVVTINSMMEALDKADQKELAQS
IYDEGLELGILHPWKETTDKDGTPIMALDLHNFSESMARAAVRKTMDNWLDEKDETGGGL
SGDFVIITGKGKHSASHPVLQRVTKQVFREEYGIEAAVDKSNSGRLVISTGDLLELYSGK
SW
>comp7366_c0_seq1; 240 103 873  LEN=1082; translated
MAPVVEETQLPGPRMTKFTCFRGFLPSIFSTAAIVLGTAANTFCENVMFRQKSGNDDLVI
FVGPWSYRAPYSPEWGGSTLVYSTCRSYSYLEEYQGFDYTFDAKTRTVQAFSIITPLVGG
LLLILTCCGTCGKSLSKSWKIIGYLLFLTSALQGITLLIENSSICYDNPVIQYLEAKNSD
LAGTFPSECKWAGGFKINVAAVLLWAGAGACCLLLPPPLVIHERPRQEQTVTYTQGPDGK
VQETHVAVVKGTPVEP
>comp7368_c0_seq1; 522 1 1528  LEN=1882; translated
XNNNNSGSDNHHGVGRSPPGMSASGDARQAAAKGPESAAAGSLSSVNPPSPIDNAASAKS
TGTPPSALEQPLQHSIPSSDSRSPPASLSTTPLTATNNNNAKESSSPVPPPAAPSVKSPI
DPSQITSTEGNKSPISPPLTSAVETAQTPLGEASEATAAPPATGGAGSIVSEYYDKAHRL
WSDRSGMSEILELKESVDAAGAAYDLASAEVISNRRNLDDSLRKWEVASGQHLQLLQRRE
SWTPEDAQRFADFVSLEITSRAELEQARHLLARSEELLTKAQLDYINKMRRRYHEEQIWQ
DQWRVLGTYGTWALICLNSCVFLGSQYFQRRRERERTETIAQLIRDSNNGNNALQAEGSQ
VPAVATDEASDAAPAPVVQTEDVAANKKEATDDSVHAREASTSEAPAKEVTGKGPYPRVT
LATERIGNTVATIGAAVNKWEHRLRTDAKEFVVSTHQKLHKALPANIAGEMPQSVSDIDV
PSAIIGASVGGITVLAASLLVSSLSGRRR
>comp7369_c0_seq1; 551 87 1958  LEN=2254; translated
MSKRTGGQSEENEIPLESQQSQSKEDGLKADGYLDEEDEDEDDDTYRLRVYGYSGDSEDS
SSSATTTKNTLQLSREFVRNINCNKRSRSVRRFSKRLSKQLSRSMDRRRSSIKESLPDTP
QGWVVFLSAVASAILAYEVRLQFSLTKPPATFCQLPSGSYVEKIYEQLTGKASSSLVGGK
GKDSRITNILSKPIQPSLFVGTRGMIASTAAYLQGGPSSSRQNFVRFREIFIMSQDGAQV
AVDWEFKGAQKQTANVEGEKNASKSEVNTSQQEILNGIIRRPVVIILHGINNDSSFGYMK
SLSSSFCNRGYVSASVNFRGCGGHRLKTPRGYNAAYTGDLRSLVRQISVRLAENVSIFIV
GNSLGANIMTKYLGEEGMAGTLPSCVSGGASLGNPLLIDSKIVRFPFNVLMALGVKKIYL
ENWKAVRALNDARSKEIHKKGFLAPTIAALDNAVAPALIRNNPMYPFEAKIGYETGEEYW
FDASSYRYIRHISVPFLNITAEDDFIVSKPSRNKLGFCLANPNVMNVETRCGGHLGWQEA
PPDGLFGGSSWADTATSDFFDAILNIKRESSSGKGIARSNKTRIESDFGRSASLPLRREK
IDNELKDLQREAIAFTKEKISRL
>comp7370_c0_seq1; 565 63 1733  LEN=1897; translated
MLISEKERTSPFSVGSGVPSVFAGNGMKNVATVNKIGAPKANNKGQSYRNINVSNNIVAH
ESDSEGFRKVFKGRNSGLPKVDILAIVDHAIEDGPTRQAKEKAANKKALSRAPIPRGSLR
KPSSFSTKPSSSVRKSLIRSLSEDLKSKSFECKADNSAYELEAENKKSDNESVISSMKKQ
EMELKNAVLRKRMDERAEKNNTIRRSASRAPIQGQEIRTKAPGSVYKNKAEILKPAVKKR
EIEPSTSITLVTERPSSSRKRGSQRNCTTESERSTIKQTDVPKKEKTKMKGGKQKHITDG
NLRQKEREENHNKMIANEKPSKRVMQLRKQLEETQQKLKRVTVVSNQELKELDQDLKKAK
EAIRFRIMKVIYTQKNKNFEVFEKYQEEVDENQRWIDEYRSANEILRDTLKELPKEIADL
KIANQTMEKANRDVAGHLEVLKKASEKLQSEKDRLLATSEKLKNEHIPRYRQQILRGELH
FEAESKAKNIYRDCLIETMKKIEQSRQKDLIEDVFALFSEKEGDVNTEFVAKFLTDHSDD
SSTDSYSDSDKCNDYE
>comp7372_c0_seq1; 1230 393 3707  LEN=3912; translated
MSSNSTGFAIPETSGFAISPTNAATPCTKPQDPKDVGVLSASSKNTAVTSTPSTSKLVAT
```

KNKPAKLTSTVEMKTKNETSSGAESKKPSTSNPSKTSSNKKDDKLQLTVKKEKTSAALVS
STTSTTTEGDATGSTKLPVHPHASWGAEDANGGIVKPHDHDVLSGRGNSVNHHPGNEYFR
SLVRHHKLAYVRCPKPQKEQFSKLIVSTVRDREPPGRFLKQNPSTKLWYNIGTQKALDKT
RQALREGAPATVAGLMLSQGLTPSAAAAAGLTPSQAVSSMSGEGEMGNKGSASTGGTNPF
LNHSHHAAAHHAAAAAAVANQQQAHQQHQQHAAMAAAAHSRLVNSQYYNAAAAAAAAGNY
TNSPKVAPPMPPSSVASSGTAILEGAGNTSDNKSSTKKTPPPPQSPTHGAINPHQQPPPS
NPYPPSANSDEALKYQAAQVIRMASAATAGNNTPTNNKTNGNGNPSNPPGSFANSAALAA
AAGTFNPVLLEHIMAQHARGTGGAGSNLPPPGGGGMDWNAGRQAPTPAGRGGDYPGQYLA
YPPPPPPPSSSGQHEDAYTKSQRQEAYIAHLQERIQQQERQLLTLCSHILLSPSGAPPMA
NANPNNGNSSVGGTNGNSHHGGISHPQFAAAAKMNAAAAVANINATTSAVKELQNYAQNN
QNTPNATASASAMIARERANEHNAVHRHAAAIAAAAAASNAGKAGVGVAPHLPVSYVVSQ
PPSSSVTVSSSPTVTPTTAGTSAATSNSSTVPAPSNSSKKNTQSSSLKMNKEKKSGGSAA
TVVTKTKPASEKSNQPTDDGDAVAALLALGIGGKSPSSSSPVGASALSSRGAGATLTGGD
KTADEAFSASASKKKRKMSGGRRSLPIKKKQIQQQQEINKNNNNNAGGSFLGPTPPQQQQ
HKQQQQNGATIPPTMNSIILAQNRGAVIPKAADMAPAPELYLYGETPIHEPHVHDVLCGR
GALTNEHPGNEWFRRLVRSNRQLYRSCPKHTKLLVAKAIVQAVQRTQRNPPGRFLEKTKT
ADGSVSNTWTPITYKSAVEKASQALREKVTPNWGAPPSNFPLSKALEAARAVAYLEKEYP
SGDGFGGTNLLALIMAVVQSAGLD
>comp7372_c0_seq2; 1230 566 3880 LEN=4085; translated
MSSNSTGFAIPETSGFAISPTNAATPCTKPQDPKDVGVLSASSKNTAVTSTPSTSKLVAT
KNKPAKLTSTVEMKTKNETSSGAESKKPSTSNPSKTSSNKKDDKLQLTVKKEKTSAALVS
STTSTTTEGDATGSTKLPVHPHASWGAEDANGGIVKPHDHDVLSGRGNSVNHHPGNEYFR
SLVRHHKLAYVRCPKPQKEQFSKLIVSTVRDREPPGRFLKQNPSTKLWYNIGTQKALDKT
RQALREGAPATVAGLMLSQGLTPSAAAAAGLTPSQAVSSMSGEGEMGNKGSASTGGTNPF
LNHSHHAAAHHAAAAAAVANQQQAHQQHQQHAAMAAAAHSRLVNSQYYNAAAAAAAAGNY
TNSPKVAPPMPPSSVASSGTAILEGAGNTSDNKSSTKKTPPPPQSPTHGAINPHQQPPPS
NPYPPSANSDEALKYQAAQVIRMASAATAGNNTPTNNKTNGNGNPSNPPGSFANSAALAA
AAGTFNPVLLEHIMAQHARGTGGAGSNLPPPGGGGMDWNAGRQAPTPAGRGGDYPGQYLA
YPPPPPPPSSSGQHEDAYTKSQRQEAYIAHLQERIQQQERQLLTLCSHILLSPSGAPPMA
NANPNNGNSSVGGTNGNSHHGGISHPQFAAAAKMNAAAAVANINATTSAVKELQNYAQNN
QNTPNATASASAMIARERANEHNAVHRHAAAIAAAAAASNAGKAGVGVAPHLPVSYVVSQ
PPSSSVTVSSSPTVTPTTAGTSAATSNSSTVPAPSNSSKKNTQSSSLKMNKEKKSGGSAA
TVVTKTKPASEKSNQPTDDGDAVAALLALGIGGKSPSSSSPVGASALSSRGAGATLTGGD
KTADEAFSASASKKKRKMSGGRRSLPIKKKQIQQQQEINKNNNNNAGGSFLGPTPPQQQQ
HKQQQQNGATIPPTMNSIILAQNRGAVIPKAADMAPAPELYLYGETPIHEPHVHDVLCGR
GALTNEHPGNEWFRRLVRSNRQLYRSCPKHTKLLVAKAIVQAVQRTQRNPPGRFLEKTKT
ADGSVSNTWTPITYKSAVEKASQALREKVTPNWGAPPSNFPLSKALEAARAVAYLEKEYP
SGDGFGGTNLLALIMAVVQSAGLD
>comp7373_c0_seq1; 472 227 1465 LEN=1566; translated
MPASTSSSVPLIKGYIPVRLNLPRQPQFVARQGEDEDDGTEDAFFFVREHQGRSAENDNA
SNNQGTTTKKGTTLFVANAPVVPGVSTKLLLKSIFGRFAEVTRVTAVQNPRAAATAVANE
DGDFGITSHATTSSSDSIFAWTDKEAAFLPTFLPPILSSTEGKYAHVVFSSAKELKKAKK
ALEDLMSASSKGKKSSRRRSKDQSNKTNKEHALIVDKLELQTLSDESMRQREEILRITVK
EASDDDDDFGDGDENTGTKKPQTKPEQPTGVLAVAQRYRESCRFLKNRSKLMEECNAVMQ
AYEEAEEAKRLAIEAARAEPDADGFVSVSYSSAVGSKIDLEQSATATTPSRRKGNKRTRK
KKEAYGAGELTDFYRFQRADNRRRTMEDLRRKFEEDVRKVKRLKQEKEYRPF
>comp7374_c0_seq1; 630 499 2931 LEN=3101; translated
MTSAETSTAMSVTEDDGYGNSSSNCNCTSSGSKRLATEEATPIAKTPPGRIDVSVLDHAV
HSSPPPSRHNLEKTNHFGVTGTPMSTCSTATNTTATTLTSSTFATERAESKAPYPNFARR
SSSLASSSSSCSRQSSSIHGIPLPSPSRSRLDRQYAKRLAASSVVFRHGSTARSPVSVSQ
DGSGITRSLDCDSSMIFRPKSILSKIDAGSNSSTGRSPRRFLFPPSPTKESPSSNIGKSH
SNTTFLRSSNSNASVSQYSTTSSTGTSHSNSSSTSLSSSRRRRNSNLSASMDLGISDRFV
PSRATSNLQNVNLWGSMNSSASQTGASSSPSSPSRRSQADADTRGDGGSNTINTGTDETS
NTAGSVNPATGRVNETSSITTQQNLHNVLLRSELLGENISPYSYAAAAAGSSYGSRPSGS
SVPLRDGMNRLRFHSPRQAYRDTIYDGTMGPTSVVNSFSLTPFGNSSSQQLMAAPQKHRR
KIAKVPFKVLDAPALQDDFYLNLVDWSSQNVLAVGLGHCVYLWSACTSKVTKLCDLTFAE
DVVTSVSWAQRGTHLAVGTNKGEVQLWDTIKEKKVRTMKGHTARVGTLAWSGPTLASGSR
DRLIFLRDVRDKSDFTRRLNAHRQEVCGLKWSYDEPAFLASGGNDNQLHVVDSRNPSAPV
HRFTDHTAAVKAIAWSPHQHGLLASGGGTADRCIRFWNTLSGSALNCVDTGSQVCNLAWS
QNCNEIVSTHGYSLNQIVVWRYPSLSKVVTLTGHTYRVLYLAMSPDGSTIVTGAGDETLR

FWQVFPGPRSDAKNNAGGLLFPNTLGSVIR
>comp7375_c0_seq1; 1130 1 2679 LEN=3162; translated
XDVDVDADADADADTNAHNDFDAEEPQEPPSLSNVRDGGADADPKPGIDFDAEDLQEPPS
LSDVRDGGADGDDNVDSNFDAKESQEPPSLSDVRDGDVNSDGDDVGDDDANDDAAAAFHV
EEPPSLSRVEEAMKRTSLMVKKHNKRRGKISTPQVATKTEAIKPLVVSEVGAGTTANSSN
TNESERLAALQRDINSLKSSLSNMNTQLRVNKPDKKTAEDEIGPDDEEASQLQQSAAPIS
PPNGEVDPRKNTSIHVNRDVPYGGRINRTLFSNSPDPVDPYASDEGDEIMKTTKSIRSKM
VEDTNITVETTELSFSHDKSNHTRSATEYRKMTPKKDLQVSDKSHGWLQEELRRRAGMKR
EINNAIEARGKHSTDGNVVIDELPSISSPPELRNSQFSLDEEVAAITANDERNLSMVDQG
KIPLDGPIPRQLKYDEQGFQPVFMYQDLQHEMMKFASETRAALREQSFTDIVQDPLSSAV
PVAFSSPMSINQSMETGKPDTNPDFQMNAVTGSTKGSKKTNGPVKINIIFSDLEAGEAPI
MLNTMDSFDGIATGDIFLSLLSDNTGRTEKSAAATWANRVHAAIWRARRMRTDLMQVSGD
AQQPIVMGSSQTVASVQRAALLHLKRDEIKESINLIEGIIFAYYSYFERSLNLQEKNMST
DTGIATIDFKPYIGTALHNLGVLNLLDGNYEEALSYFIRAVENRKGYLGEDHPHCISSLV
RLAICRYALNQFAEAHAGLEEALVCAKRKRQRSKATLEDRLQMAEILNNLGCLAYMSGQP
TTASSYYRESMDIQFGALSDSLYLGNAMSGQSISLNISIARANIGFIKLVTKDLPIAITA
LENALMEQQILLKGTNDTLIATMDHLALSNLLNGAQDKSALEAFSLDASQE
>comp7375_c0_seq2; 1269 1 2972 LEN=3057; translated
XDVDVDADADADADTNAHNDFDAEEPQEPPSLSNVRDGGADADPKPGIDFDAEDLQEPPS
LSDVRDGGADGDDNVDSNFDAKESQEPPSLSDVRDGDVNSDGDDVGDDDANDDAAAAFHV
EEPPSLSRVEEAMKRTSLMVKKHNKRRGKISTPQVATKTEAIKPLVVSEVGAGTTANSSN
TNESERLAALQRDINSLKSSLSNMNTQLRVNKPDKKTAEDEIGPDDEEASQLQQSAAPIS
PPNGEVDPRKNTSIHVNRDVPYGGRINRTLFSNSPDPVDPYASDEGDEIMKTTKSIRSKM
VEDTNITVETTELSFSHDKSNHTRSATEYRKMTPKKDLQVSDKSHGWLQEELRRRAGMKR
EINNAIEARGKHSTDGNVVIDELPSISSPPELRNSQFSLDEEVAAITANDERNLSMVDQG
KIPLDGPIPRQLKYDEQGFQPVFMYQDLQHEMMKFASETRAALREQSFTDIVQDPLSSAV
PVAFSSPMSINQSMETGKPDTNPDFQMNAVTGSTKGSKKTNGPVKINIIFSDLEAGEAPI
MLNTMDSFDGIATGDIFLSLLSDNTGRTEKSAAATWANRVHAAIWRARRMRTDLMQVSGD
AQQPIVMGSSQTVASVQRAALLHLKRDEIKESINLIEGIIFAYYSYFERSLNLQEKNMST
DTGIATIDFKPYIGTALHNLGVLNLLDGNYEEALSYFIRAVENRKGYLGEDHPHCISSLV
RLAICRYALNQFAEAHAGLEEALVCAKRKRQRSKATLEDRLQMAEILNNLGCLAYMSGQP
TTASSYYRESMDIQFGALSDSLYLGNAMSGQSISLNISIARANIGFIKLVTKDLPIAITA
LENALMEQQILLKGTNDTLIATMDHLALSNLLNGAQDKSALMYNRILTLQQREYGAHDRR
CFVTVDKINMVQGKGIQYEGAIEELRKTFSLPSPSGGPASISASSLTDTNKGQLTPPRKQ
KNIQVHKSSGKKNKKFKVLKALGSVMKKNP
>comp7376_c0_seq1; 690 448 2244 LEN=3394; translated
MSTSSLKDVTGKDSENASNSSSSNGEGGGNNSDNSALVSLGNTSFGFNFESDGGFNNSSG
SRSPSEEGDSDQGDRKPKAGNGEGNNSSGSNSSTNAQTTSTLTSCAPTGTETGRSSATNE
GKEKTTPQHLQQANGQSQLPDEKLKIAGANSIPISAGAAAAKISSDAMKQPPVAASTASV
KNESTAAISNSPSHNDAAAIAVANLNQIATDASKKTDAENDSVGKRGLKRKSDDDTGGYN
SDNEWGKGDPANLGAPDVQKSQNVKQEPTDAPKTANNTAGQNSKRSTPEGAPVKKKKQLD
DSKREERNAREKERSFRISKQINELRTLLSSGGVIVPKGTKSSVLTEAANYIRMLQQHQY
RSEIDRHQLVQQVQRIGSGALGQQAAQAVRHAAAQNGVWSLGNFGGVPPKSAMMYHNPGS
VGETHSSTSGNGNLPSKIEESDYRYVFNSCTVGMAIASMGGAFIDCNKFFCEISSYTKQE
ICSMTIFNLTARPDLQLAFDLISQMLSAPLDPSVTNTSCVLRGALKNRTDVGLSVRLIKG
HDGVAKCFCVTLINNPSSPYDTSRSIPATIEPSGPGQTHPGTKDNQPGLDVTPAFTTG
>comp7377_c0_seq1; 803 376 2355 LEN=2519; translated
MSRSGSVSTFSGLYGTNSNTGESKRWLSSIGSGTLLGVEARVHKLPSSSASKVRNYGGRA
SNGGTNENESLLRELDSFIHDATSYLIRISHTPAQDKVFPGRFTYQTDLRKPLGPTLQNS
YWNAKENYQFDYNLLRHNGAIYALSQAYARNEERHLEEARETDKLGDLGHRQRSILGAIE
RGVGYLRDNALLPVPGHKEWLAAWERTDIDDPHSEPEMAKLGGAGLAMIALGKMEAIRPD
SVSLEKELRRLGAFVESLQNPQDGSFTCKYEWKDGPNNEWVSLYYPGEAALGLVTLAELE
LEREEKAASGEKPGMVTSVHLELEHQHHNAKTAGDNDVPKLSKRWIMVATKALLYLEQLR
RDEALDDIEPDHWALLATARLLPILDRQRQALENKSEKKQADLEYWLVYNHGVRVATSIV
SDHTTEGLKKHKGCFTYDFRTCATSTRLEGLLAALTFVQESEIFLGGNSEEEGEDKKGNH
ADATELLRDRIERDVGMGIRFLLEAQQKTDQNSMRGAVPGMYSGKKPAALSKSNDDEEEG
SEDGEEDYHLAEVRVDYVQHSMSAVIAYESYLLSKTQQAKKRFHEIVHEKVRNVADPILH
HVRKKIDTATRSSTFVNYSILALLLCFVVAVIALAYCPLSILPSSRSHRTRRRRLKRRD
>comp7377_c0_seq2; 803 376 2355 LEN=2447; translated
MSRSGSVSTFSGLYGTNSNTGESKRWLSSIGSGTLLGVEARVHKLPSSSASKVRNYGGRA

SNGGTNENESLLRELDSFIHDATSYLIRISHTPAQDKVFPGRFTYQTDLRKPLGPTLQNS
YWNAKENYQFDYNLLRHNGAIYALSQAYARNEERHLEEARETDKLGDLGHRQRSILGAIE
RGVGYLRDNALLPVPGHKEWLAAWERTDIDDPHSEPEMAKLGGAGLAMIALGKMEAIRPD
SVSLEKELRRLGAFVESLQNPQDGSFTCKYEWKDGPNNEWVSLYYPGEAALGLVTLAELE
LEREEKAASGEKPGMVTSVHLELEHQHHNAKTAGDNDVPKLSKRWIMVATKALLYLEQLR
RDEALDDIEPDHWALLATARLLPILDRQRQALENKSEKKQADLEYWLVYNHGVRVATSIV
SDHTTEGLKKHKGCFTYDFRTCATSTRLEGLLAALTFVQESEIFLGGNSEEEGEDKKGNH
ADATELLRDRIERDVGMGIRFLLEAQQKTDQNSMRGAVPGMYSGKKPAALSKSNDDEEEG
SEDGEEDYHLAEVRVDYVQHSMSAVIAYESYLLSKTQQAKKRFHEIVHEKVRNVADPILH
HVRKKIDTATRSSTFVNYSILALLLCFVVAVIALAYCPLSILPSSRSHRTRRRRLKRRD
>comp7377_c0_seq3; 194 1 656 LEN=820; translated
XFANSALPLLVAALTFVQESEIFLGGNSEEEGEDKKGNHADATELLRDRIERDVGMGIRF
LLEAQQKTDQNSMRGAVPGMYSGKKPAALSKSNDDEEEGSEDGEEDYHLAEVRVDYVQHS
MSAVIAYESYLLSKTQQAKKRFHEIVHEKVRNVADPILHHVRKKIDTATRSSTFVNYSIL
ALLLCFVVAVIALAYCPLSILPSSRSHRTRRRRLKRRD
>comp7377_c0_seq4; 222 1 526 LEN=748; translated
XFANSALPLLVAALTFVQESEIFLGGNSEEEGEDKKGNHADATELLRDRIERDVGMGIRF
LLEAQQKTDQNSMRGAVPGMYSGKKPAALSKSNDDEEEGSEDGEEDYHLAEVRVDYVQHS
MSAVIAYESYLLSKTQQAKKRFHEIVHEKVRNVADPILHHVRKKIDTATPLLNLC
>comp7378_c0_seq1; 347 1 1000 LEN=1292; translated
XTTRHHTTPHHTKPKVQANPNPRQPKMNFLWQQRVLLLVSALLSATPSPAIASSFVMPSV
AGKPSGRPAFVAGRQEIGTGSCASKVLGVSPGGGGDDEETAVPQAESGAEPCATSAPLAA
SGATPLGPGAKTPPGFLRSRFPRFPWNRVPDYLTYARCLAIPVFVGLFYASLNGTSHLYT
GTIFALASATDWFDGFLARRWDISTAFGAFLDPVADKLMVSTSLVLLSGRHGKVAAIPTL
IILARELAVSALREWMAQRGERDTVKVGYQGKVKTFLTMTALTVLLYAPAEAGTASILYK
VGIPMLYMSALITITSGSVYFMAAAPALLGTKQ
>comp7378_c0_seq2; 256 384 731 LEN=1023; translated
MVSTSLVLLSGRHGKVAAIPTLIILARELAVSALREWMAQRGERDTVKVGYQGKVKTFLT
MTALTVLLYAPAEAGTASILYKVGIPMLYMSALITITSGSVYFMAAAPALLGTKQ
>comp7378_c0_seq3; 122 1 659 LEN=659; translated
XTTRHHTTPHHTKPKVQANPNPRQPKMNFLWQQRVLLLVSALLSATPSPAIASSFVMPSV
AGKPSGRPAFVAGRQEIGTGSCASKVLGVSPGGGGDDEETAVPQAESGAEPCATSAPLAA
SGATPLGPGAKTPPGFLRSRFPRFPWNRVPDYLTYARCLAIPVFVGLFYASLNGTSHLYT
GTIFALASATDWFDGFLARRWDISTACESLSLFLFVRTGSX
>comp7379_c0_seq1; 329 296 787 LEN=787; translated
MTNTAGSSSRSIITVGMLISLLPTKALLLGGNHPSGGAAATPTTRDGVCASLLGTAPRTC
STTSLCMSSYDGGWDNDNFLDSLGGGGNNAGEPSEAIEQANDEYFRQSRYGRPAVNDGNA
QQGWVPQRQQQQQQQQHLQEQLQGQLQEQLQLQQQQLQQQLQQ
>comp7380_c0_seq1; 327 1 1343 LEN=1408; translated
GCFDHHSSFIEGLQKSYTFLLVSHQSIATSLGILYRSIHSSFSYFQIELLTARTKRMKFS
SASIIAATVSVGLLDWKCADAFVVGFGSGLQSKSALMDAASSVGNTENDELLAQKKKLLS
LLGPKTFIDPVLADPITKEGVRITSSSGASLKITSPTATYGGSANTYLNLLEPVSDEPLK
KDTDPSSLLKSALRQVVPAVPPPLRGPVNSFLGDEENEYVPMRDLFTNPTVSFAYERGWR
QGFAAAGFPGPDVEAQMATDYFAPVVALANPDSVVVDMSCATGLFTRRLAKSGKYGRVMG
CDYSDSMLTEARRRIKADPELSPKSLKTSLDLVRLDVGQIPMKTESVDCLHAGAAMHCWP
DLNAAAAEIHRVLKPGGRYFATTFLSTYFSNVAAMDGQAGASPSQQAFQYFDSVETLKDL
LVGGGFDECKVNIEVLGTACVVIRAEK
>comp7382_c0_seq1; 1710 312 3689 LEN=3855; translated
MVTNSSAALSAALATVLAVTMVDAFVVPRQDASKYLVDTALDAKKKVVVIGNGMVGQRFM
ENLLEDADPEEIQLATFCEEPRAAYNRVRLTSYFETRDPSALSMTSDFDENGATSWYEEN
DVELYIGDKAVAIDVDAKTVTGKSGKTIEYDACVLATGSFPFVPPIPGKQRPGVFVYRTI
EDLEAMLAYAKDNGVTSAAVIGGGLLGLEAAKAVKDMGVTSHIIEFADILMCRQIDEGGH
NALVGKIESMGLEVHCGARTEAFVGEDGTTDMDSMSPVSALRFSNEGWDDLPVQMVVVSA
GIKPRDELARSQCTPEGGIALGERGGITVDSQMRTSADSVYAIGEIALYNNFIYGLIAPG
YEMAAVASKIVAKDLLQNAPEEEEEPGFYGADMSTKLKLLGCDVASFGVNQPKPDDEDVQ
ELVWNDPFVGVYRKLIFNKAGTALRGGIMVGDASDYDKLHKLAVSAEYDGGPAPLPEGVT
ASMLLTPPSARSDSEEEEDADLSDPNAQVCSCNDVNVGQLKSAIHELGVEEATLPAVKKC
SKAGTGCGGCEPLVKKFLVAELESMGGKLNNNICEHFDYSRQELMALIRLEEDPEKVDSF
QKILDSYGSAIAHEHGDGCETCKPAVASILSSLQNKLIMRPELIQNLDTNDRALANMQRG
GTYSVVPRVPGGELTPEQLIALGEVAKKYNLYCKITGGQRVDLFGAAKHELPDIWEELGA

AGFESGHAYGKALRTVKSCVGSTWCRYGVQDSVSFAVKVENRYKGIRAPHKIKSGVSGCA
RECAEAVGKDFGMIATENGYNLYVGGNGGATPVHAELLAADIDEETVIKYLDRYLMYYIL
TADKLERTAHWQKKLPSGVNGGGPIEHLKEVIIEDSMGICEELDKRMELLVETYHDEWAE
VVKDPELRKLYQQFANTEENIEKHDMLEFVDMRGQLRPADWPKDGQPQTNWEAPANDIFA
NSEKSWVDVGDVKEFLPNIGTSILYGDSQLAIFNNEARGEWYATQNMCPHKQAFVLSQGI
VGDKEGTPKVACPLHKKQFSLETGEGIGDEPLNILTFPIKIESGRVLTELPAIPELDAIL
GTNGLRVQKSDCIDIAGDAASLMKSIKPNLFPSKNATSVGDSINA
>comp7383_c0_seq1; 390 522 1289 LEN=1355; translated
MRIVSQLGLVLFLAGSAVEAFSPQQQSNASPPKHSVQESRRELFKKALTSVAGVTGALAV
SGLAPAPAAASGGATAGKYTTIPIAKRRYYGRVQQSVHEFLLLGPEIVQADFSGPMIQRF
FDPQGLVVVEAKRKDVNGQCTKKNGDCKGSEIRDSIYNDMKTSMYLLANAFRLNQTKAPD
NLPSVRAAKKFFKEMDLVEKAAKKKRKNDRESVVHFAAALEALDTFLDLVELPPTDSNWY
EQEFDRAVGSSARIT
>comp7383_c0_seq2; 164 522 764 LEN=827; translated
MRIVSQLGLVLFLAGSAVEAFSPQQQSNASPPKHSVQESRRELFKKALTSVAGVTGALAV
SGLAPAPAAASGGATAGKYT
>comp7383_c0_seq3; 212 522 902 LEN=1100; translated
MRIVSQLGLVLFLAGSAVEAFSPQQQSNASPPKHSVQESRRELFKKALTSVAGVTGALAV
SGLAPAPAAASGGATAGKYTTIPIAKRRYYGRVQQSVHEFLLLGPEIVQADFSGPMIQRF
FDPQGM
>comp7383_c0_seq4; 181 1 452 LEN=518; translated
XFHASWYNLLFPIDLNVLKGLVVVEAKRKDVNGQCTKKNGDCKGSEIRDSIYNDMKTSMY
LLANAFRLNQTKAPDNLPSVRAAKKFFKEMDLVEKAAKKKRKNDRESVVHFAAALEALDT
FLDLVELPPTDSNWYEQEFDRAVGSSARIT
>comp7384_c0_seq2; 116 62 298 LEN=1333; translated
MSNAGGASISTEQLKARYVGTGNADMSKHEWVTNQHRDTYASHLAHYDQLSYMAVAENQS
IGRMRLRLLDKMIQPCGP
>comp7384_c0_seq4; 124 62 337 LEN=1066; translated
MSNAGGASISTEQLKARYVGTGNADMSKHEWVTNQHRDTYASHLAHYDQLSYMAVAENQS
IGRMRLRLLDKMIQPCGPPPPKKDINRMVEN
>comp7386_c0_seq1; 484 159 1229 LEN=1921; translated
MFLVRQALATSSGSGRCCGSNGVAKRLPNPSQLLIRRQRQPVNPALFQQHRHNSCGGNAS
GTSDKKNLPATLLQRGHNSNHCSTRSFSVVPRVANHNNNHNGSRLAEADRHPEQQQQQRR
NMARSSMAKGGDADPSAKPEAKALAFLVGTLGHERDVAEGVLNALRQSGVSGDAALLSMV
RSLAGRWEVDEDAGLEALVESVTVELARSRGKTTVGFWVLPASAWPSREGEDDTAVEVDK
TRAFWVEGFEDMSLTDVAKFGDGEGADVLGEMIECACSGIMACSTCHVVVQPEWFEQAGG
EPSEAEQDMIDLAYNPKETSRLGCQVVLTPDLQNAVFKIPRGANNMMDFIPFEDKD
>comp7386_c0_seq2; 481 159 1356 LEN=2048; translated
MFLVRQALATSSGSGRCCGSNGVAKRLPNPSQLLIRRQRQPVNPALFQQHRHNSCGGNAS
GTSDKKNLPATLLQRGHNSNHCSTRSFSVVPRVANHNNNHNGSRLAEADRHPEQQQQQRR
NMARSSMAKGGDADPSAKPEAKALAFLVGTLGHERDVAEGVLNALRQSGVSGDAALLSMV
RSLAGRWEVDEDAGLEALVESVTVELARSRGKTTVGFWVLPASAWPSREGEDDTAVEVDK
TRAFWVEGFEDMVRFCVLTVVAVLAVFFGVVCVCVCDSSAGSSMFWNPFYRAAQSLTDVA
KFGDGEGADVLGEMIECACSGIMACSTCHVVVQPEWFEQAGGEPSEAEQDMIDLAYNPKE
TSRLGCQVVLTPDLQNAVFKIPRGANNMMDFIPFEDKD
>comp7387_c0_seq1; 476 549 2468 LEN=2517; translated
MAIMHTPSQTFCTAGWIDPDTPSDVLTTQPFTAIPGKIPSPTKVTKKLRKGETASPTGST
PRPSASPTQAPSSFPTSTPDSHELVFSEEFNVPGRHFGDGNDPRWTALEKNDYTNDALHY
YAASNAVTDENGELIITTEAVDTDIITQDDDHPKKKIRKTKHFKSAMLQTWNKFCFTGGI
VEAQVTLPGKSNIGGLWPAFWLLGNLARHTYVGSSQHVWPWSSNLCTEKAFSAQHISACS
EVAHFGLEKGVGRGSPEIDIFEVQPGKIKANTGVFLESSVGQPFMSASFQVAPGRPDQRP
GGGYWPGPGQWYDGLVGGKNTSLNINFYGNYNHFKDDPHPEKSDYWSDAVSFNRQLEEKH
FNSSHVYRLEWDVPTEDKDGYLHWFLDGDLVLAINGTGIREGGTGSEVSTEPMYILLNTA
VSSQWGFPTKCPANCECDEFDCNSKVYADRCGFSDGFCDMMTKGKEPPKYKIDWVRVYQD
PTKNEQKVGCSTPERPTRQYIEAHEKLYKTDLDEHPLKPIQVGRGTCNPLATDISAQACG
GEERGRCTKGNVCECREGWVGPHCLAADGYDDIDWDPPDKLSDVGFDPPKFVLNGLAVGL
SIVALGIMVSAIFRRQLLEGWTPIPEVEVKYLSRQSNKP
>comp7388_c0_seq1; 529 97 1209 LEN=1275; translated
MVRLITLFLGAACTGSATAFAPASNPSVRAEITSLSMANALIVQNKGGGHGELGFQLAKK
LAAGDKIEKITILQDDSCDREAEPFKSYSSHLPEVDVVYAKLGDESMDTNGIQDLLGGEK

FEYIFDNYSKGPEGSSKALCDVAKDWGCVKLYVYVSSAGMYQPTDETEFPMSEATTPIKE
SAGQAKMDEYIAEECKLPLVSFRPQYIYGEKSNKHDYIDWYFDRLTAGAPLLIPSPGTQK
VSLTNSEDVASLLVAPLDNEEAAVEQRFFNCGTDKLLSYDEVAYLCAEAAGIAKDDVKIQ
HYPGETFGKAKFPFRLTDFYVTPDLAKEKLGYSGASNDLQENLKWYYENYKARGKKNVDL
TKDQEILAEL
>comp7390_c0_seq1; 794 99 2994 LEN=3041; translated
MMAATSSSTAIVDALVPTTTTAGTTGFTNNLDISAFQSKRVRSRLAAIVAASKDDNDLSS
SFPSMPEDIKSVGVNNKSSQRKVRKRRSNKKKIRTIPTRKNSVPKAISSDVGRGDNDSDS
LKLHGSIPKLSDIFEGRASLNTVDQDKPEKSIKVVKTKTKKSINEESARSAATTKKSNRN
KKVRDTSPAGVAWRAGYKSSKRTQGRIQKAFNEVMPSTHNDSHSRAKLVLDTLLQTPPEF
CNSVNLVCALTYSAKALGYQRVGHVGKNIEFRNSLQETFDILHGLVVVHRAEGLFTPRQL
CNVVWAIAKHVDRDQDLLLSQGKNEDSDSPQRRLEETIDEIAKQLTTILFNEDNDDSTAI
KSTFQQPKIKLGEICMACWAYGKLRPREIPPGWPGPPQMGSVKTQDSIVADWKTEQLGTL
NKDSAASNNLGSDDSDDGSKDVSETGLLFDAMGYALCQSVNNNDLDESKLLEDCTWSELA
NVGWAFASHGRCRSPESEMLLKSVAQEASERLKEDGTIANNGNYQRFLVRDITQLLWSLG
TLQSDNFRLADDLVFLVESLTTNLQLGAKKGSSFARGRPLQRWSCADLVQTSVALAHARI
DERPLLHAIYSEGIHRLMEGASSNYNASSLTTSLSTKKTTSNRIVPMGEDRRTFHPWETS
ILLWAQARLYLTEKEGNEFAEFTEDAPLFFLDTLRNNSGSFEKSKIGPQEQANIVWSLVI
LEKYDSTEAILLIDLIFEEAAATAKNKSMQLEHAHQLWQAYFMLQKDCPEAVRRVPEWFA
SYLKEKWSLEKARFKLSSARHKSLSSTLELMGVSHINEHEEDIDVAIILQPDAMWVHETD
MDKDTSSEAAPNATDHVSVAIEFDGPNHFTRVRTEPQEPSGLPPRPNTPRALGHTVLKYR
LLKQQGWTVVRVPYYEFDKIPFWASMERQRYLQRKLKTHANIKFSEVDVSEYKALTPDRK
SRYD
>comp7391_c1_seq1; 691 145 2358 LEN=2441; translated
MPPNNRKEKIFAFCDADDDEIGESNDGQESGKNNGEEETSNTKEMGTDCSSTEDFDSDGG
SEILMDAEEDEDKPKKQSHRCAFAVAAALAVAYACEKRRQLRITKSPFLAGQIPPNSLVR
SACNKLMDTDEGVFKGPIVPTVWAGTRGGLSTSAAFLAKGPSPNVPSVLRFQEIITSSYD
GAEICLVWEVPNNNDDDKTEGQRRREEILGSSLSNRKAETSNTKIQNPTVLILHGINNSS
GYGYVRSLQRTMTDRGWNAAAVDFRGCGLAQRLSTPRSYTAAYTGDLRSLVNQISARMMG
GAECNVTAATASAPLFLVGNSLGANLLTKYLGEEGLAGTLPRSVAAGVTLGNPFVLRANR
MMFPHSVLIGAARKANYFRHRTALRMAATNDDAGCPASTRNDFRTVTRSLIYDPRIISLA
SLDRAAAPYMVRNSAHPPYETRIGYGHRGDRTTPRYSAPTKTSKTDSSRSSNETSKPVIE
PQTPEDFYWRDSSSFQQGRHVSVPLLHVQARDDPLCFANARRLMGHALQNPNVVYIETEC
GGHLGWWSEESGVPNAGWLGAPWAHKAAADFFEAVMDAGAEERFAESDKTVVASPIDDAH
NLFSRSCSSRSGVLGGEAVPQEDDKTRTGSSSSADSMRCRSGASSSDDNGDGSRRARHTL
GLRRPANSQQQQHHHHTEEQPPIAYIAVVKKKFRMLSLRQDFSPTFSEEERARRLETNRT
LERQQSIESSLRLRSRL
>comp7391_c1_seq2; 214 368 1042 LEN=1065; translated
MSTPAFSQATVLRPFGTLLQHQRGGMVGPMGGVNSFQDVGIRDYQIDESNSGNNVNGLMD
TIGFQTGTRNTNATRNLTTQNSGKKTMNNNLNGVNGFTNHQQFQQQQQQHHHHHHHSSS
QPQRANRRPTSSGSSHHHHQHHGRRSASEMSEIESYLLSSDTTQHHDDDNHTSHTDDVST
VFNPNRGGGVDAGAAPSAGSPVHQEAAMFWNRSRRSSAGGDRLY
>comp7394_c0_seq1; 253 220 777 LEN=857; translated
MSENGPERTRPYILVTGTPGTGKTTTASLIAEKTGMQHVNVGELIKQLKCYEGRDEELDT
NVLDEDKLLDAMEVILDKAAEEGKGIVVDFHVCEIFPERWFDLVLCLRTSTDVLFDRLTK
RGYNEKKRSENMEAEIMQVILEEARESYAHEIVHEVQSNTVEDMDNNVIRVEQWGKQWLQ
DKGYD
>comp7394_c0_seq2; 248 532 984 LEN=1064; translated
MQHVNVGELIKQLKCYEGRDEELDTNVLDEDKLLDAMEVILDKAAEEGKGIVVDFHVCEI
FPERWFDLVLCLRTSTDVLFDRLTKRGYNEKKRSENMEAEIMQVILEEARESYAHEIVHE
VQSNTVEDMDNNVIRVEQWGKQWLQDKGYD
>comp7395_c0_seq1; 981 312 2801 LEN=2919; translated
MKAVATMLIALGAATGAEASSAFTRISTFHICKQIDPTCNTDDQTNAETIWYYGSGSDLH
LVYTDSESENLGFVDISNPSSPAADGIVPLGGEPTTVRVIGDYAVVGVNTSPDFVNPTGK
LAVVDLESKTIVHEIELPGQPDAVSVSSNWKDNDGSPLYIAVAIENERDEDLGDGVPPQL
PAGLVSIITVTDLASPESWTTVDVPMANDPSVVGACLYPDDPEPEYVAIHPDNTKVVVSL
QENNCNVVIDLASASITAAFDAGSVTLDSIDTTEDGLILQGKPSLSIERAGEDVQEHELL
REPDGIAWIGSSDYFATANEGDLDGGSRGWSIVDSTSGEVVYDSGLEMEWETARVGHYPD
ERSGNKGNEPENVFYSAFGEKEYVFVLSERSSVVFVYLMNGPTEPELVQILPVGTGPEGI
TTIPSQNLVAIASEVDERGDKIRSSITIFELTDGATPEYPTLVSASKGNSPSIPFAALSG

LAADLTNENILYTIEDSFYNKNRIMTIDTSSFPAVVTTEQRIMDSDGVLAECLAGNFSSM
ALQVDVASMINSEDNTVNIDPEGISVSVDGGFWIASEGKGTVGDEDKPFAFPNLLLKTTE
EAVITDCFLLPDDGSFADQLRFGFEGVAEDGKMVVVAVQRAWGDEPNPRLAIYDVSNDSW
KSVFYPLDEAESQNGGWVGLSDISSLGDGKFLVLERDNQGGPDAAIKKIYEIDLGDYSMA
DGVTIEKKLVKDLVSDLLASQGQILEKVEGLAIAKNGMMWINTDNDGVDDSSGESLLMSV
GIFDETGMTSSSDSDVDDITTSGAHAGFSIALLTVAACLAPVLNTLVGL
>comp7396_c0_seq1; 233 334 723 LEN=723; minus strand; translated
MSVNPRLLDKTKTSLVLQQQGLHRPSIPKVSENSTLGASVGATSYASKNTITDNPSAMDH
HSQCTTPYQTTLGEVTGLAGVLDPTLLSTAQKLRLQQLLQHNIQQQQLLQLQKLQAQQLQ
VQQLQAQKLQ
>comp7396_c0_seq2; 834 1 2856 LEN=2856; minus strand; translated
AQQLQVQQLQAQKLQQHLLNLHQPQQQRHRPLLETAIATTTTAPSDDDVCLPPLKLTDLK
PSILVIFKRLIKEHATEYSSHATTHSKKTAAEKLTKSSEETKNNISSKDEVVDQIYCSLS
GFCQPYPNLMVDFPSRERMSELIRSSLEFTLELRASQSSSSLAAAKSKLTKKKKSDTHEN
HTQDGATTLASSPLPPSMSVRQKDAIVLAAASATRTKELAGANTAKHSGGPNTPLMDPGS
VDRENIGGGAVLLSPSGIQVAPEAMAPLKNDALSVAHQVAIVAAHQQQLAAQLWKQHRQQ
QQQEQQNREVCNKALGRALGVPKPNTNGLTCFHEDVLAATYKSSAGGLSESNYQAQALCN
AMGLPPISTSSASLEHLHIAKLKSSVVNDSNKNLSLSGSGITATNPTPMTEEDFCRIIHG
LKASLPSFSQNVAARPTATNHAGVVPSELNRTITHGTLLDRSGACSPGLMATARELELAR
ISLALATAAGIATNSTAASSAEQQTTLARVAQSSVGPITSGPLEAATEGIINSNALVADD
RSTDLAGMMATARQLRLATISLAEATAAGIATNPTLTSSVEPQSTLAQPVRNRFGPIRSS
PSESGVVGIPNPNVLESGTTVLTPVCRFSFPIVVFQEVDRRFLREFECALRQQLVIFEAE
MADVEAYRRNTIKYTDNDNGVSGERRPKSIRISQIGIRCRHCATKGGKSNTQRAFWYPTS
LNEVDTYSEKIYEEHFRSNCSAIHSVNLLKDLKMLEDKKNDVSSRKRIKNPFGDKYWSVM
LSNMGIYEDKVNVMLRVNRGKKNGVTDSASAVVTSPVPSNNITARDIKREQKKKRAVSEE
SNAPRRNISGKAPAVIESSDGEQEEINKGRNSIAKKRNLSRHNEGHRSSLNSSADESSSC
AKEENTTKKTRSDGNSKSSSSRKKMAEQRVKLPNRRTKLVPHSETPNRKERA
>comp7396_c0_seq3; 169 1 697 LEN=697; minus strand; translated
XGVTLSNISEKIYEEHFRSNCSAIHSVNLLKDLKMLEDKKNDVSSRKRIKNPFGDKYWSV
MLSNMGIYEDKVNVMLRVNRGKKNGVTDSASAVVTSPVPSNNITARDIKREQKKKRAVSE
ESNAPRRNISGKAPAVIESSDGEQEEINKGRNSIAKKRNLSRHNEGHRSSLNSSADESSS
CAKEENTTKKTRSDGNSKSSSSRKKMAEQRVKLPNRRTKLVPHSETPNRKERA
>comp7397_c0_seq1; 664 149 1351 LEN=1444; translated
MKFSAVVMSVALAASQTSAFVAPSARSSAMSRLYSSEMKEETAAPTTTTEDAAPDMKEFE
DKVVEETASELKAAATAAAVAAPAAPVTAADYVLKTIDKSKIVPGRNAETDRSVAVPFLK
RPPKLDGTQAGDFGFDPLGLTEEFDLFTMQEAELRHARLAMLAVVGWPLSELFAPSWMLQ
NGCAPSVLNGFNPVSAVAVLAAFGAFGFFEYKTALRRNDNTAFGKMHRADLADCKDGEYG
VAGDYNFDPLGLYSSIGNDAYARKGLREVEISHGRSAMLGITAFAAWEALTGHPIVENSM
FFHPNFELPALVAAYYAFGFFYERDEESADTFFRYKLSSEGNARLENMKIGGGESAAPSL
PFDVDAIKEIPDKISATVENLQQQYSKLENAYMDNVVKKD
>comp7397_c0_seq2; 679 149 1372 LEN=1465; translated
MKFSAVVMSVALAASQTSAFVAPSARSSAMSRLYSSEMKEETAAPTTTTEDAAPASPIPA
ADMKEFEDKVVEETASELKAAATAAAVAAPAAPVTAADYVLKTIDKSKIVPGRNAETDRS
VAVPFLKRPPKLDGTQAGDFGFDPLGLTEEFDLFTMQEAELRHARLAMLAVVGWPLSELF
APSWMLQNGCAPSVLNGFNPVSAVAVLAAFGAFGFFEYKTALRRNDNTAFGKMHRADLAD
CKDGEYGVAGDYNFDPLGLYSSIGNDAYARKGLREVEISHGRSAMLGITAFAAWEALTGH
PIVENSMFFHPNFELPALVAAYYAFGFFYERDEESADTFFRYKLSSEGNARLENMKIGGG
ESAAPSLPFDVDAIKEIPDKISATVENLQQQYSKLENAYMDNVVKKD
>comp7398_c0_seq1; 891 251 2371 LEN=2527; translated
MGTGAAKKTSHSWHRWTMLATALLLQGSATAKGVVQLKLNPRHVELERRRQRERNLHPVA
TAIEEEDEGQRHNEEQYRRRGKAQQVGALYEGYGTHYIDLWVGTPPQRQTVIVDTGSGIT
AFPCGACSDCGAPKYHVDALFVEKDSSSFAKSTCASKSDCIMSRSSCSGDKCLVSMAYAE
GSRWNAYEAVDRCYVAGPHETPLVAPEGASLEGDDMNPRHAADLAFDMTFGCQTLVTGLF
KTQLADGIMGMSNQASTYWSQMFEAQKMGPDRQFALCFSRPVHIAREGTEAGAMTLGGVD
TRLHRTPMVYTPNFNTGRATFFSTRIRRMMLRDGKYGTSVQSTADNPNLGLTVLDIDPSI
LNKGGTIVDSGTTDTYFNSAIGGEFKRVFRKITGREHTNSGLDLTEEEVLALPTILIQLI
SNDETNGHVDDMFHTPGLAGAMDKPNKSDVILAIPPTHYMEYDEKSKKYTSRFYTTERSG
NVLGANAMMGHDIFFDVDTKRIGWAESDCDYTAKVRENGYDFDITGELKKAEAAGTAAKE
SKECSSISSGAKCQKVEGCTWGWGKCSKVGDAPPKETPEEPKETPAETKETPAEPKPTPA
PLTPDYMVQEIPPLRTWVEDHLVAIGSFVVLSLLLCCIYCLFCRSESSRGRYEKASLEPV

SIEMRNGNGMRKIQSFQDEPDDDDSHVENGNGSKFRDEPEFEGDFM
>comp7398_c0_seq2; 822 129 2027 LEN=2183; translated
MRQCITQRRDLPMDRAQGYGTHYIDLWVGTPPQRQTVIVDTGSGITAFPCGACSDCGAPK
YHVDALFVEKDSSSFAKSTCASKSDCIMSRSSCSGDKCLVSMAYAEGSRWNAYEAVDRCY
VAGPHETPLVAPEGASLEGDDMNPRHAADLAFDMTFGCQTLVTGLFKTQLADGIMGMSNQ
ASTYWSQMFEAQKMGPDRQFALCFSRPVHIAREGTEAGAMTLGGVDTRLHRTPMVYTPNF
NTGRATFFSTRIRRMMLRDGKYGTSVQSTADNPNLGLTVLDIDPSILNKGGTIVDSGTTD
TYFNSAIGGEFKRVFRKITGREHTNSGLDLTEEEVLALPTILIQLISNDETNGHVDDMFH
TPGLAGAMDKPNKSDVILAIPPTHYMEYDEKSKKYTSRFYTTERSGNVLGANAMMGHDIF
FDVDTKRIGWAESDCDYTAKVRENGYDFDITGELKKAEAAGTAAKESKECSSISSGAKCQ
KVEGCTWGWGKCSKVGDAPPKETPEEPKETPAETKETPAEPKPTPAPLTPDYMVQEIPPL
RTWVEDHLVAIGSFVVLSLLLCCIYCLFCRSESSRGRYEKASLEPVSIEMRNGNGMRKIQ
SFQDEPDDDDSHVENGNGSKFRDEPEFEGDFM
>comp7398_c0_seq3; 125 251 563 LEN=563; translated
MGTGAAKKTSHSWHRWTMLATALLLQGSATAKGVVQLKLNPRHVELERRRQRERNLHPVA
TAIEEEDEGQRHNEEQYRRRGKAQQVGALYEVSSLVVWHAVARIX
>comp7401_c0_seq1; 464 179 1609 LEN=1663; translated
MTVPHNNDNEDSSGALANHGIPLSSSGVESNDFFLGDYRHNREILDNLEADPTDGEVDED
RLIYAATKKFATINMDIQNRESFRARRPLVVSSSALRCDDSDSSGELICPDTAPQRTTRM
AEMRMNFFRIFQQDNSYLEVSREEIRRVRAMHKRFAEGSEFSFNYNVLLIIASILAALGL
GSDSVATIIASMLVSPLMGPVMGMAYSATIQDFTLFRTAFMTELVSLLACIAVGGLVTCC
MIPFVDVPDEWPTPEMESRALMTSFWIGIPVAFFSGLGVAVSVLDEQTSSLVGVAISASL
LPPAINAGMLWTTYFFNEAGTNEEIGDDAQHRFFREGVISLGLTLANIALIILSSMIMFR
VKERVNIKRKKIFWTDLGLARCIYRNLAVYDNPADAPNPVRRMARRVSLYFHKGSTNRAS
NNEQSSEISFRSNGSNYSSGFCTDVDVPQGSEDRRHKKNLSVVLEEKRSTSSSTSV
>comp7404_c0_seq1; 368 113 1075 LEN=1392; translated
MARIFTGKPTTFLRTQTYSTQTTHRRCTVLDWVTISEEHGIYVADLGMSPAQCDALVTTT
EQACKGHYAAYTYAKQTLGCREYPALARAVVSPVHSMVYAITQRFRGEGGPPGEENDREE
AAEAKDGGDGRPPNGGTTGGDAPAGALDSAEPSLSRPGDGEPRPPARVLQLDEREPHIVK
YDVTKKERQKLDMHTDKSEFTFLIALSNGCGMDYDGGGTYFECLDATVHIQRGHALVFPG
KLRHRGTKITRGLRFLLVGFLVDKPLPLQASDAPAEEKTKTEHKPERRDAGGDAFPAEGG
SPARTVSQPAKQVLPAVDWC
>comp7405_c0_seq1; 512 1 882 LEN=1115; translated
DDEDDDLYTEKELDELIETIKVQDAFLFLKSRGWDDKLLPLVDMFNHRNGPQRNVEVTSV
HDGGGDIAAYTTRDVAAGEQLQYTYSECFDSTCAYSAVMYNYGSQPILADYGFVELYPQR
WYLGGADDDVVAPEADDDDYSTVVDDGKVQDLTVEVTTDENDDSKIVLRWIFRTPNTASL
QWIKEQLDRLKSIEAEVTRRVEGLGTIYEKGHPITDLYNLEHEGETLLDYHRAYVQVLEL
ALKHADDPVAVTAENHRDHMVELRRTVTQWTVDELRKHELLSSSSSRNGADEL
>comp7407_c0_seq1; 781 161 2318 LEN=2496; translated
MAPSPMKTPGKASGPKKVYLFNCPYKEKASAKKAGAKWDASAGCWYVLSSQNLEAFNKWH
PNENKYLVCSYDEKDDAKRAGAKWDATCKQWYIAANQDESRFARWLPSSAGNRATKATKL
IKGKKSEIKTTKTPTKLSTKSQLATIPRINGDMTVAQLQTECKARDPSIKGITTKRKDWF
LEHLKVGTPWISAEVPYLDYEPESPPXKAKQKLTTSALKASKSNSDTKKNNSAVARISAD
MTVAQLQIECRARDSGIIGISNKNKNWLLEHLDVGSKWVSQGWKEKDLQNLISANKKKAS
SQTKQQSSSKHKDSVDCRDIEGMARQTSSKNNSKKRLGDFPVNNITIPQQVGKKAKKDST
SAKKPSVKLVAESVPDASLQRKRPAKKEQTSSANKKHKIKDGNLKSADVVLLSTRKKEGP
KSATTVEAETIASSTYTRITSKMTLSTLKKEAIARNVEIAKQKSLHVHKFPRVHRCAMAK
SNRLMMNGNARDGECDLCYQKRERHFFVVGRSSTAIYTCEKCDFDICQKCFDEENRSESE
KEKIRKKRQREREEETRRIQEREEQRAQRWDSNLRFKNTITKVKGKHLDAESGLKFTVWC
SDGYDYDGRHSYEGEPTKEFDSVWKTKEEANSRAEYYSSAKILGGXPPEDIEDNEVVKSK
LVDGLKNYTVAPDDSSRWTVGVVPTEALVHLPNSSMNRHNLDRGSSMHDDFLYNEQTCF
>comp7409_c0_seq1; 152 110 397 LEN=397; translated
MTPNFLRLVRLLLCLDAAARALSFGNHDEVRRSRNPLGNTGSSATTNLYAEGSGKCHREE
HRVSRRDAIAATVKAASLTTGMATAEVTWRSTAYRG
>comp7409_c0_seq2; 586 110 1530 LEN=1687; translated
MTPNFLRLVRLLLCLDAAARALSFGNHDEVRRSRNPLGNTGSSATTNLYAEGSGKCHREE
HRVSRRDAIAATVKAASLTTGMATAEVTWRSLVEDACLAHAVGTPNSADASFLPPGTLRT
IEAGKAAIINDWLSPEETKALCQDARDCFRGGHFTNFILSRNPKKADRFANDRWIMPSFA
SSQGIRDGPFVDSAVGNLQVRESLRRKMAAVKAALANGLTDRPTLSNDVAQTHEMEYLRY
GEGAXSESAHLELKRAHGSRLPLKKNASRRSVTWLVYLNDDWTPEDGGQLRLHERASDSI

VPVGARNANDLQVGWLREIPETGKGEQPVFLNPFCRKRDGSMDENESCMLYTFDDNSGEN
KRRELSKQPFPNAALYLGGGDKLARNFMVDDPEDRKRFHLIDAPKSLVSELAKTETPPEG
EDGGERIHDILPRAGTLVLFDSVSLPHEVLRTNRERFGVQGWFHETIQQQTQQ
>comp7409_c0_seq3; 634 110 1543 LEN=1700; translated
MTPNFLRLVRLLLCLDAAARALSFGNHDEVRRSRNPLGNTGSSATTNLYAEGSGKCHREE
HRVSRRDAIAATVKAASLTTGMATAEVTWRSLVEDACLAHAVGTPNSADASFLPPGTLRT
IEAGKAAIINDWLSPEETKALCQDARDCFRGGHFTNFILSRNPKKADRFANDRWIMPSFA
SSQGIRDGPFVDSAVGNLQVRESLRRKMAAVKAALANGLTDRPTLSNDVAQTHEMEYLRY
GEGALLNRHTDEHHLELKRAHGSRLPLKKNASRRSVTWLVYLNDDWTPEDGGQLRLHERA
SDSIVPVGARNANDLQVGWLREIPETGKGEQPVFLNPFCRKRDGSMDENESCMLYTFDDN
SGENKRRELSKQPFPNAALYLGGGDKLARNFMVDDPEDRKRFHLIDAPKSLVSELAKTET
PPEGEDGGERIHDILPRAGTLVLFDSVSLPHEVLRTNRERFGVQGWFHETIQQQTQQ
>comp7410_c0_seq2; 236 249 476 LEN=476; translated
MEAEDLAPEDIETVVEEFDKDGDGELDFCEFKTMVKYLTRDSKQRSSIVMDDIMLQLGSV
EEGLDEEGDDGDDDDD
>comp7410_c0_seq3; 263 439 795 LEN=795; translated
MTSPEERALEDERKLVFESFDKHDLDDSGKLSRAELISALQEVMEAEDLAPEDIETVVEE
FDKDGDGELDFCEFKTMVKYLTRDSKQRSSIVMDDIMLQLGSVEEGLDEEGDDGDDDDD
>comp7411_c0_seq2; 832 1 1968 LEN=2016; translated
XPALALFLISLCVFSTTSSKDDDAKFMGLAESVHPVRRAEIAFKLLGKAEEFVAYYELNR
FGDVKIGSGKDDEKDGKNESRSSLSSLTGDDVSIGTDRIFFSKSLPHLCASVVGFSAVEA
ALELGHFVDEDDIDKGGADPNAAGLVAVQTLFRDSSEKYERSLVTELGNLLRRRAVGANL
AELVRASCLMAAFRSALKVVHPSSTTRRFDKELLAMDVDILMIALKVAQDEQLKATTAIV
QADRNQPLVVPYKYPTKKEGNFPDPEEVGLPFGLAELKQQRKRTSMDLTGSTTSFGNDKE
SEECLTFSQSVPLVVRSIHARAIACASFALSQEELGQSFLQKKGSGSACYVLDCVEECVN
VAAVGMKDADNSSDEGDIETAVQVMANVAALQHSLPRLFGTLMRGMCHVGMIRSDQLAET
FQYAEATLKGADKACDNQVGTMYSKVYECARGKIDAHLTYALEGFNWVQKTCRDMPNAYC
EGLIGYIRNVFDSLGPMDEGSRAGLHFSCCGHVAERLVKILSDRALEDGSRSDGGLPPIA
KIDPFGLYNMSSDVDEFKKFATSTGVPQLADCFDELKSITSAMLDPDLPNLLRPENSDAR
KRKYPYLSLEKVGNILEKYSSPGMTLMGGGKVPAGMLTLDKKEVVALLNLVRSYK
>comp7412_c0_seq1; 991 228 2339 LEN=2419; translated
MGKRKTAKTGDKALYGSREQLGNQLGSKRSGRHGHGHGHGQDHEGERKRRTKTGENPIYG
EVDRFHNEKDYDVLRLDRETRGQTNSEDEDLAGTTRSVLDLAAGETTSEEEDGDNDGDDS
LSSSDGSGGWHNDQGGTDPYWSSEEDDSVSSSNRSIDEGDSEPASKDDPRNWGKRKSLYY
HGDTADLEIGQEEEDAYLEEEAAQEIQASRFEEMDDYDFVLSENDNEDGNDGAESKNEMT
TTKKQHEEPTTIRYVSRLSKKETRKLLEQQFPELLPMVTYFSNIVEDVKVRTKVGVTALL
DGEGTAESVGATIKGQQYLLTKAMVQKSLALNSVVYILLRLALVGDEDATTNIRAHPIMD
QLKKFTSLLGRLEEDVQRKTSDIALQLDNLIEAAALVEYLGGNDGVGSAVRGTCDGSETN
AKRNDDRNLEQPKRGAIDDGDELSNGRSDGGDGDDFEEEKRAAVARQKQKDFERTLNEAR
FGLRPNEANTSGRASRKTICMPMVDDFGDLERDHATSNSHLASAVNTIEQRSRKRRPAPV
MAEDLDDIDEYDDRVVHGIKMMEEELGKLDQNGDYERMDDAETSDGQDPDAGEPGFYDRI
AAQSQERKFLRTSLYGVVPKFPRVDGEVVGERSIGRVILKNRGLVAHKAKINRNPRVKKR
EQYRRALIRRKGAVREVREAGEGNRYGGEQTGIKSRISRSRKL
>comp7412_c0_seq2; 461 306 1448 LEN=1528; translated
MVQKSLALNSVVYILLRLALVGDEDATTNIRAHPIMDQLKKFTSLLGRLEEDVQRKTSDI
ALQLDNLIEAAALVEYLGGNDGVGSAVRGTCDGSETNAKRNDDRNLEQPKRGAIDDGDEL
SNGRSDGGDGDDFEEEKRAAVARQKQKDFERTLNEARFGLRPNEANTSGRASRKTICMPM
VDDFGDLERDHATSNSHLASAVNTIEQRSRKRRPAPVMAEDLDDIDEYDDRVVHGIKMME
EELGKLDQNGDYERMDDAETSDGQDPDAGEPGFYDRIAAQSQERKFLRTSLYGVVPKFPR
VDGEVVGERSIGRVILKNRGLVAHKAKINRNPRVKKREQYRRALIRRKGAVREVREAGEG
NRYGGEQTGIKSRISRSRKL
>comp7413_c0_seq1; 86 1 283 LEN=585; translated
XRERNPTRDLGKTADLEHVEGDGPAPGNRPGLSQLASLWKGKRGGNEIQHALENQPSPQG
AVVGDQGGHRRLAWQERRPGGAPEVHSHEEGRIQW
>comp7413_c0_seq2; 724 144 1904 LEN=2175; translated
MTGTQILQRLKERFEEVSTRYLPCVDFLVQCQQELRKGLQVATTKRYVHSVFRDTLTSRE
FHTQYISKLPDRFYRKNNGKMTPDHLREAYKELQKLVANSKDVESQGCEAVKNTFLGGMK
DGESWGLRKWLSKHGGALHICNDTECMSTSCQKLDRELETTRKLAERLRPMAAKSLKKLK
SDIPASYQEQSSAHPYLPFFHRLECALRAMSNFDPEDDDVICIIDDDEVEELKAKAAAAS
SSSSSSNKAKRRSSAAGKSKTSESMNGNRGGTKRKHQQVELDIIDPGDDSDVEVLEQKPP

PRRTSYKNNKRSELSTETNASRLEVDGISHNEKTRTVDEIFKETDNELEELFKTLEDDPH
EDMFYDHMPKTDNISKYDAYQLADGLDSLATLFDTNQHDKVRPDDFKQESFWDRSYPFAS
ALRLFSEILRSPDCSGNFLESVDDLERKQEGIRPYKSIVKHPLCFRDICCALLEDFNQID
NSITCSDGKLTSEKLQIWNMWKGMDLLQAIDLVFLNSLAYGKANEAGTKSNTRSKTNRLR
KVLWSGIKEVIDASLGKNGGQEERRKCTPTRRGESSGFVVYKGVST
>comp7414_c0_seq1; 573 162 1112 LEN=1487; translated
METVSLGKFALNPYPIHHPEGKDVALIHLKQEEEALKILTEDLGVKILRLRDLDEIYDKG
DEVTFDGYVVAEANQADSQTFSGGNDSDDASKSSNDEEEDDRVFFPYSEIGQLSFHTRDR
FFATTPEPLPEGLCGGPVLDSNDMVCGIVEGIVSKDHKNKDIAGSAALMPNYVMAPFIDF
AERLMLQKILPKNLFQKAVTAKTTGQLGGGIFQKDAEGNVQPSGNSLSYEEAFDRAVDQL
KQNHSEEEVNAILNTIQRERKEVLEIMDKEGGELDDVVERVRQRTLQVREMIHEEYRKEH
ANDADPSVVEAEIVER
>comp7414_c0_seq2; 625 126 1565 LEN=1940; translated
MRFTSVLFQAAKKGTRSSTSGRNIKSLALSVYFKQPDLGMKDYEILSRASAFLLAVDPDP
SSGKTSGPYDHLEKNERDRILKMHKQGSKYTPAGHPGDQRSLQELGWLEYAPREVRPTVH
CVCSSHVLSPYLWKDYYPQDWLNKVRQEHCAYSLEVFDNDPETMETVSLGKFALNPYPIH
HPEGKDVALIHLKQEEEALKILTEDLGVKILRLRDLDEIYDKGDEVTFDGYVVAEANQAD
SQTFSGGNDSDDASKSSNDEEEDDRVFFPYSEIGQLSFHTRDRFFATTPEPLPEGLCGGP
VLDSNDMVCGIVEGIVSKDHKNKDIAGSAALMPNYVMAPFIDFAERLMLQKILPKNLFQK
AVTAKTTGQLGGGIFQKDAEGNVQPSGNSLSYEEAFDRAVDQLKQNHSEEEVNAILNTIQ
RERKEVLEIMDKEGGELDDVVERVRQRTLQVREMIHEEYRKEHANDADPSVVEAEIVER
>comp7415_c0_seq1; 379 1184 2555 LEN=2555; minus strand; translated
MAASLCVTLLFLLSVKAKGANRERQQLPKNDFNDTATGDGSTGENERGPVLFPAPGPSAR
IEYPIRATGNSPYARLPDDERCRWLESGPDIWAANDTGTEKKHTDNPRFNATTLDAMFRR
IVRIYREDEDLKGRLTVASSPDALPKKVKSDYAKSEDTDADADDDDGFVDGYEDEGYYYY
DDDEIVDEDPFDLLEGDGGPWVLWIDNFLTDKESNQLAKLGAMEGYQKSTIFDDDDSYMG
GDGAYDYSAEDKEDGTGTKKEAIQESQTAAATNPVPKMGRTSETAWCQSPFCVGDEIYRS
VTARIHDLVQIDSSHSESLQLLHYNVGQYYADHHDYYFIEDPTEWSQDGGRILTVFLYLN
DVQGGGETSFPRLNLTIAPQKGRAVLWPSVLDSDPRSIDWRTEHAALPVARGEKHGANLW
FHLKPIRRKWFRYECDAPRTRGKTYGDVIWFDDDDAMX
>comp7417_c0_seq1; 148 410 717 LEN=717; translated
MNPGASGTETMEATGRGNRHGACARTAREGRSTGIPLRSAFRTERPRKQTSVSARWWLAV
AAALAVLATGPSRRAFAFRPPGPLPAARKGPAALRNNNNNDNX
>comp7418_c0_seq1; 104 1 211 LEN=538; translated
XKGGKGSKGGYWEVVCDNGPSYGPSYGYGGKGGKGGKGSKGGYYGGSKYGYDNSWYGYDY
GYGNKYGRGPW
>comp7421_c0_seq1; 37 127 441 LEN=1119; translated
MSAPRTFDEATEIAADPAILKVGHSEKSKLYGYFKIASTASVAPEGPRPGFLSVTDRTKW
DAWKSSGETIPGGASEEDIVKAAKLRYTNVVRVAMGAGELESLS
>comp7422_c0_seq1; 145 63 557 LEN=962; translated
MEDLSTMRGTNQMIRELVEASWETSDLPDMLLMDFGAWVDQLTELNARLAGMDTQSNANY
TLERLGCAKFSPSIALSCAEAVKPDSCKCTRNVVSRDGMHWCMETIGGRVTAGFACLIQC
SLSIEGDKKSRFRSCQQSCNEQFMSLRKASSLLTNHSVSGGALG
>comp7422_c0_seq2; 496 1 1840 LEN=2245; translated
XIAASAGQPMDIPITPCDIMKKLLEKKRIVLAETGLVNSSANYGNNDTNNGGQDEVADEV
TVGIAGKISENVADELANQITDNITDQTTSRQQKDDPSFVFSAFLVLYGVVFIFSSLRID
LGQPQLEVDREKQQSRAPSQTDIHRNHALWKNGTASTRATTPPQETGIEKHQSGNGGPEE
NRVRDIIGKKDDAGNTGTKPFLTYRLELDKPYSVTYGPHTRCWIGVETNKWGRKPIHRTG
LSKPLSDILNVTAYVKTNLRIISVGDSVGIQFHELLEEALEPPGVTANRTGVDPVLGGGY
RTVYQNAWGDHESVSVSAPVHGGGALAAFRMTGLFLKEGEGGKPPNAGPNQTSAAGGWRR
EHLRQILDHNYTATAVGGSHRRGESTGGTTTQRTDSFDVMIYRIPHGWLPASSITRARLE
ASMRLARELLGVRTVVVPSLYLNNNVQTMEDLSTMRGTNQMIRELVEASWETSDLPDMLL
MDFGAWVDQLTELNARLAGMDTQSNANYTLERLGCAKFSPSIALSCAEAVKPDSCKCTRN
VVSRDGMHWCMETIGGRVTAGFACLIQCSLSIEGDKKSRFRSCQQSCNEQFMSLRKASSL
LTNHSVSGGALG
>comp7423_c0_seq1; 483 377 2050 LEN=3228; translated
MSTPLNFKGELSSNGGGSYGTHSGSTGNNTTRQRRNPADYGPFSSPHASFDDPASFPPYA
ELPFTSLKRRRQLQENNSFARWVMKGVLLSPCAVLVLWGVAAVLFAGTHHKAGHDSNGSS
GHRNGSARQHSRSGRRMLPNLFGNGQPSQQLYMAGNPPLQDQQGNLIYNVNGNAMMMAPP
QLQQQVVQGISPVSGGYPQQQQFQQPQIVSMQQQGNALPVVPPLGNGMNANGVPANSLAQ

GQTSMVQQQQQFLGMQQLSGAAQPQQVLMATDQGLAAPVQTSSLVQQQQQGLGPPGAVAL
QQAMASTGDIPQGMDQNMNIVQAAAPAMQQQQTSNQSGNQNVIQTPSAMQLASGSSNAAS
DQLQASPSKQAVYFYDPKDTAMSQTGEMIQMPTIVYDTNGRAIPVSELQHQAPIYVQPPA
RGASLTDATSSDAAGSGGIGGQTELVSPSTRGASLVSPSSREASISMPKSWGASTSQDQT
IIVATVAVMALLVGALSARRLRSKSFLSACIENEALEDDVAYDDAYTTTAAASGAMGNDS
SYNTFGGWKGDLEKFDV
>comp7424_c0_seq1; 398 32 1681 LEN=1681; translated
MEETTRVDLSRSTSSSSSDSRLPPLLIFNHNPKAGGGSIKDVLNELKPIKIICKCKPFNA
RGDARRKNLCCREADTVDARKSDALFVNVDEFSRTPLYLMQNDESIGRSSFVIGSIREPC
SQYLSLWSYGSQGEGYFMQRFITKEPANLYYGQNPPYFNTSEDVERFRNWMSHDLVTGLV
GDRIWASYATSLNKWKNPSEGVLQSVDCWVVVENFRETLLNCLRAYETKGGLVDWSLPTV
SNLVRQEQEFQKAENERHNSRRLYTNSKDKNDALHDPRSKHHSPCAMYFDNSTEHLVQDG
KEAFVYGLFGYKGCCEPGNSFLGARTASAVDEETTTNTGNLPAPPNVWSNPLEIALSLGL
VAVALYFGLARRTTGRNTTSTTQNSMVVAAGSKSASTDSEEREQELQQFLPKTSSASAAR
KETGSAIVPTAVTRRSQQDGRTHVGGPSGEIVWAEAWRRATRSSKEQVFPWLSGPGRAGR
YKPVSVEATDHTDRTALLLGWSAGPRLELARHGWCTSTDWCCTISSNKEKHLSFVEGRCD
TPPPTVSPSX
>comp7426_c0_seq1; 430 237 1442 LEN=1629; translated
MANVPGTTRDEKNDNGLSPVVAPDSSSLLEATGADITPNPPNPPNVIQAPEEEIFFDDQA
DLPPMEKKVVGAAGQLTTAEMLELTLDRNFQMSRLATQYYYTRHFWMMFLPSVSITMISG
IMAFMIKSEITMSETVSNWIAFLVGSFSIMNVFLQNLSNELNYDHCCKSQRAVSLGLKRI
LAEMEFHKSTTGGIPIEKIHEIEKTMNTIMGQNDATVPAAITSAYELAKTRLSLRLFPPV
RFDDNDPYASNKLSWSQTMHTVHNELLCAIANSPGWPLRIPNAEEVVQIAIQRVVEILKE
EQHNELYKALLDGQELIRVKNIENKRKRKKERLRSAIGAFSGIASAMRKNEKIKGWSSNR
TTDESTALLEKAPENSGGQKYWSMLANGTSNHDNNYDSSIP
>comp7427_c0_seq1; 634 266 2523 LEN=3506; translated
MAIDKSVDKSIQKTKAVNVIGNNYAAVFYRPVARFLEGGXSSLSKSSSNDNMDEDEDIFY
EYRENHQTKTIGKRELQQREQKGAWALLAALEKAKEAQYVEVASLITSLETLLPREGQQG
TDENGEEGARQPVQCSSRSNKLPRFSVEDTKSLTGDDVVALLEATRSGRLIEIHTIRALI
EAATVVFRSRHPDRLVELPPLSKLQQVQVVGDLHGSLNDLATVLALMQSRSGEPCASNRI
LFNGDLADRGEHGVEVICIVCALKLAYPEFVFVNRGNHEDLALAIAYGLAAEITRKYGAG
CRKVLRPTLDAFFRALPLATVIERDALIVHAGPPPPSSDGSGLADLLLRDHPILTGCGLS
RTVVDTGCKNDNSSSNNKLQKEEKVSKQIIESMLWSDPLVDECEGTLHDFGDVENDISGT
GSNRWVPNISRGAGYKYDASVVRNLLNREGLSRMVRSHEPVRDGCVRYVIDNSSYNNSSC
PKHKNKPMELFTVFSASRYPYKEGFNQGAVLELKANGEHSVLRYATEDDEPLEVAMTIAS
SSVTEENSRESRNATREYACAIDSDDIRHTLLEALRYNRTAIRNSLRLASVKHQTIPFET
VVDIVIETLNLGNEGNHLKQIGAKTTLAKAMGIQCPDTELPPEHVDVDRCFEVLLANPTD
RATRPQRSLRSYDSYPWLFSVFELLDTNQDGFLSRSEWDECVRTINSKLPEGLGIAADRT
WEVLDTEGYGKITMAEWERLGHAIESDIARLD
>comp7429_c0_seq1; 329 202 1329 LEN=1961; translated
MRLISFFTYLFCNARNPLVGKAAAFAPLSLQAHVASRRKHPFIVGSMTTLTTSSTSAMTI
GITGESNPTSTFATATQYTIDDSICSPTKAETLEKAVYKACRSLEVYMEKKPTAAHTRRA
FEVLVEMIAEEGSNGYEGIILDSGCGTGKSTKLLASSIYPQHLVIGVDRSLARLTKTKDN
RQGGGQSGNNDERKGGNCENSPHTDKREAYCHRLSNNAYLVRAELVDFWRCCIEHGWTTE
DEKGGGDNSLKISHHYMLYPNPYPTKARLSSRWYAHPSFPLILKLGIQTMVIRSNWEGYL
KEFSQAIVMANAFYVGHSDEDSLHGGSNLALPYISSAKKGPTERTDKAMALTNFESKYDN
VGESTYELILERAHT
>comp7431_c0_seq1; 1268 262 4855 LEN=4910; translated
MNTNNGNSNYNGFPSIADAGNGVVYSGIEGSYGESYSYGEQQCEDGSGEYYYNEYPMPQQ
QQALYNPYQQQHSEEYGDEYGMTTPYQQQHNEEYGMATSGAWNQQCAEKRGDYVYASEFS
PEVYDHAISALAYDSTYDCMYLTSTTQSMSSSRHTKVKNHRASLLLNYSSNTQTRDYFLY
SSVAGHPEAPLAHLRAIYESIYGFSKVAVSTTSGVQQRGGQSSFYPPPHAYIPAYGGGEV
AEIRSYHNHHVQHNEHNQNRGHIGITELLPLEGGYVASVSPSAVRIHSHGGLQVHDSELS
GMISGTIHPHCSSAIPDDGNVAATHITVGGLSMKMTGALGTDNTKTAVNRNNSRVVQPSL
HCLDLWQGLRVVVSRSFKATNHKSNGNSQVGVTCMATSHDKGSVVAGCTDGNIRLFDGSL
RELATVRSHLGGVSSIAVSPDGNLVATTGYSSRACPKNSIDGMPFCSSLYTFPDPTIYVY
DIRYLGRGGIPHPFSGMKGTPRHVCFVPDMEGCASNRLLVASGKNGGGMQIMTPFEKQNE
NTISFSLPPLQQRESITAMSKPKKKRELALGTSMGRVMEYRLTGYIENKTIRRKADLAMP
PFYPDAPAVSLEPSLLQGSPSLRNGTTDEMRSLFSTYILQSTPNVTSIGESFEDAMEVFG
SLAGMPISIDQRREVNDTLLKEATTGEKDYMLTVPTSKLEIDILANQNTVSNNYKGKKAA

KGLANPNKLLYNDKLYSLCYKEGWNGKGKQPQHHHHRGKRSNGASSKQKGIPPRYQLQVR
PGFKASGAFDASQHNDTGLFPGWDYGSSMPNAWVSPVLLLFYFIPEIRKAALAAQFNGKT
IGTKAYEKSLSPELGFLFHQIESLSRYGLLYPHRGNKFKPKIGAWIPSNFVTFLSTMDAA
EQMAVLDDSPAAVDLPRRPESFYRFLAYQLDKEIFSTSSSSSDLKKSAAKPKLMDSLNGL
DFLSSNQFIESKSTPPTQSMTRALTLDLNYDIFRPGTKRKSIRFGELLQHSLCRSTRLRA
WNDKSRAYETIVQRKIATSLPQILTLSCACAGRKEQDGLWAWRMNNGGEPWLAETIEVEL
LADGNVAVTEWHKTESGDGITSSTFRGKSSLPPEVGKLVLKASSKQRFRYRLQSVLSYVR
SYEEDSGDDEASGHHVLHSRVSADYKRRVLKSQAKEARKLAEEVSALNSPEEIELQELLL
SSGITAEDYVCRAESVEEQLTSLDEEVNGSLSNDWIIYNGYKVSKTVVEDARAFHVPFKE
PVLVVFHAVDENGMPLRNTNEENSESQQVAKSVSASVMNPSSLSKNSGDILTDRCLQRLQ
KGRPLAFDAEFVSVQEEESAVTDTGQKEVLCDTRHALGRISIFDCPREETIVDDHVLPRE
PVVDFLTRFSGIVEEDLDVARSKHRIISTRSAYLQLRYLLDQSCIFVGHGLKQDFSTVNL
VVPPNQILDTVKIFHIPGRRYVSLRFLANFVFGRDMQQDTHDSVEDARTAYELYCKALKW
KKEGIWEKSLQELYSYGEKTSWKLGIKDRF
>comp7434_c0_seq1; 189 1 869 LEN=869; minus strand; translated
XQEREHAFVEDEEISTQLELVKALIDANPDALKTCISSCGCTMDRTRKENRNVTDHTEYG
YLPLHMACASLCEIQNYRTQSVTGGAGDRIFQLQRQQQHRILELQLGVIRILLDRHLSG
AFHNENATKELPLHIIVRWILRNCIYDNSTTNNNPNTCSNNICERNETETTPTPTEIKTK
STDHGHPRSDHGCDYPNGFYVNEKEEQYDSGFHRSDYCCQAECIATFLVMANPNGVNVQD
YTGETPLGIVQNHLRAIDTNTGTQYTTSDLKEPHDSLSSVGTRFRYIHDE
>comp7436_c0_seq1; 551 323 1663 LEN=1892; translated
MTWHGTALVMMRYKRAAASGILCLERTSLASNNALLNQAAQRKFGFGVLSGPGRSAALND
DKEKGEILCIGRKLTNFSRSFWSCRGGEMDPRDKSLAKKDSVLSDDAKRESSSSSDTTNI
DTNMSTTRTTTPVGSHTYQVSVGVEQGFRSYMEDEHYVSNDGDFAAVFDGHGGGKVSRYL
RKNLYANVQAFLPLVLVEKQEDLSVDGNTGGTEEKLKSTRPRNPTVNDYANALESALEKV
DSEILKISHWSFQGSTALVVWLHEKQEERNTGDDNMSPDTQRTILAANIGDSRAVLCRDN
KAWDLSRDHKPNDPTEKSRIESLGGSVIWCGETDQFGAPIEHQGIYRVNGNLALSRAIGD
RSERPHVTAEPEIISVPIEEGDEFIVVASDGLWDVMNSGDAVNYVKLSLKAGISKKKIAS
MMVEEAMRLGTWDNVTVVIIWIDRKL
>comp7437_c0_seq1; 437 11 1362 LEN=1910; translated
MVRALLPVQRFLFGLQIFVGHVCDFQGFDFVLYSASIMIAFGFLNPLANPTFSSLFENSS
XIKTTHNYYIDPSCSFQLIEAHESTLRALVLTADGSKLATASHKGTILRVWDVATSQNIH
EFRRGVERANITCLAFSWDDQWIACSSDKGTTHIFYLDNESSGLKKDKDKNSSKSKGSIF
ASTGSSLLSSGSKLLMGSFSSSTAKSPSKSVCQIRGVPHPLACAFIGDAPHLIAVAGWDA
DGNGVLLVSEFAAHQEARRVAYHVLVKNASTADETEEERRRRRARGWVPGDETSKNGSGH
DFSENNPRMHFGHLQISDEMAEESHRFQTQTTEDDFCEVIVQPRQEPIIEDKPPINPSSP
LSSSNTQQDLEEQQTSPSSSEKGTEQKTSSDGDASNGIDGENNDIIASNTKAGASGDKET
SECKGTDLKTEADPEPIATNSCSDHDDDTE
>comp7437_c0_seq2; 473 399 2171 LEN=2719; translated
MDDREVFMDANSNAVADNSNINKSNTGTSSSSPDFIDQTLNLPAHHHPSQRSQLLTLSFN
QDGGCLAVGTANGFSVHNLHPQYNLSVERSLRGGIGQIEMLFRCNLMALVGGGSDPHAAP
HRVLIWDDHVPKEIGELSFRQIVLRVKLRKDAIAVALRDRVYVYHLADLSLRDKIYTADN
PYGLLSLSTHVQDMVLACPSVTTGHVRVELYGMRKTMLIEAHESTLRALVLTADGSKLAT
ASHKGTILRVWDVATSQNIHEFRRGVERANITCLAFSWDDQWIACSSDKGTTHIFYLDNE
SSGLKKDKDKNSSKSKGSIFASTGSSLLSSGSKLLMGSFSSSTAKSPSKSVCQIRGVPHP
LACAFIGDAPHLIAVAGWDADGNGVLLVSEFAAHQEARRVAYHVLVKNASTADETEEERR
RRRARGWVPGDETSKNGSGHDFSENNPRMHFGHLQISDEMAEESHRFQTQTTEDDFCEVI
VQPRQEPIIEDKPPINPSSPLSSSNTQQDLEEQQTSPSSSEKGTEQKTSSDGDASNGIDG
ENNDIIASNTKAGASGDKETSECKGTDLKTEADPEPIATNSCSDHDDDTE
>comp7438_c0_seq1; 254 250 1431 LEN=2591; translated
MGVGGWSAAFGSTYEGLALFRILLGTLLAAELILRFRFLHVFYTDEGTKPLRLMLPNIDD
LYRLVCYHCHFGTLFQQQMLLVIQVLVAVCLAIGYKTKMAAILSFYMYTSLILRNTWLYF
ILDRYFYYLLFYSMFLPLDERWSLAAVLKPRHANATATATKSIREKNVFINPATVALKML
VVWIYFDAGSGKYMDPKKGWTYHADPLPALDTYARHTVPAQHLYGMIGPEGLRLMTPLVV
WVELLCAPVALAGSYLGSAKTVWFAIGVISMMHLGISLCIRNSNLLSYVACCAWLVFLPL
GWKNADRTNGDITHDPRKKRDSGATTTPIRAQLFARGPTSVVTWVMVTAMVGGNIWFETI
APGCTTESSRLIWSTVFQNRWNVFIGAEEVSLH
>comp7438_c0_seq2; 249 250 1857 LEN=2432; translated
MGVGGWSAAFGSTYEGLALFRILLGTLLAAELILRFRFLHVFYTDEGTKPLRLMLPNIDD
LYRLVCYHCHFGTLFQQQMLLVIQVLVAVCLAIGYKTKMAAILSFYMYTSLILRNTWLYF

ILDRYFYYLLFYSMFLPLDERWSLAAVLKPRHANATATATKSIREKNVFINPATVALKML
VVWIYFDAGSGKYMDPKKGWTYHADPLPALDTYARHTVPAQHLYGMIGPEGLRLMTPLVV
WVELLCAPVALAGSYLGSAKTVWFAIGVISMMHLGISLCIRNSNLLSYVACCAWLVFLPL
GWKNADRTNGDITHDPRKKRDSGATTTPIRAQLFARGPTSVVTWVMVTAMVGGNIWFETI
APGCTTESSRLIWSTVFQNRWNVFIGAEEYVTWEIAPGRLQDGSVVDVWGQTDIVNWNMP
GSGAPCTSTSRPGRWRSFPYLAELDGEEAEALWGYLCQEWDRNNGVSENKNPGRKLLRYN
FFMLQADVLPNMGFSSTRKRLIYSHECSSAHKNDRDGLQNQTYGGKEISSDGQEL
>comp7439_c0_seq1; 524 29 2121 LEN=2793; translated
MASPTTLSARDKAQAFVDNFNRSYEEKHVAFENQFWGTKMALSSTEESPYSTELLTKTKA
EMEDLLSDPAVIKEAKEQQEALANAKGEDVQEVLKVLDVIVRTCRCNEFPTPESKAVREE
TNKIEGSLEKSRNEMTLGYTNEEGEFIKASSVGLRTVLRTAPSESTRKSAYDGLRSIGAF
VCSNGFLEIVRLRNKLAHLLGFEDYYDYTVTNAEGFSKSKLFEILDGLEEGTRPLMAKAR
KDLEELHGVKALDPWNTSYMMAGSVVKKMVSLPCVSCLLGHRISLLLLKHVNPESGKKKN
IENTCEVGSHACYHWLSPFVDPYFPFSKSVENYLRSYAAMNIGYSNATMTLDLLDRKNKY
SNGFCHWPVPAWIKPDGTHVPSQTNFTSLADPAAVGSGLTALNTLMHEAGHAAHFANIAQ
PSPLFSQERAPTSVAYAENQSMFLDSLVGDSAWRAKYAKDLQGTPIPFAIIEEDIRAKHP
FAVFQLRAMLSVSYFEKALYELPEAELTAEKVQSLADEVEIRIQGGLGARPLLSVPHLVS
DEASCYYQGYTLAEMSVHQTRKYFKDKYGYIVDNPNVGPTLTESYWKCGNSKPFLQIVKD
LTGKELSGDDWVDALKEDLEEHIAQERKEYDGMISKCEASSAEEKDGEEPLDLAMTVKFV
DGDHVISDSSQSGLLSACKEFEAFVAARVAAAKASS
>comp7439_c0_seq2; 615 29 1966 LEN=2638; translated
MASPTTLSARDKAQAFVDNFNRSYEEKHVAFENQFWGTKMALSSTEESPYSTELLTKTKA
EMEDLLSDPAVIKEAKEQQEALANAKGEDVQEVLKVLDVIVRTCRCNEFPTPESKAVREE
TNKIEGSLEKSRNEMTLGYTNEEGEFIKASSVGLRTVLRTAPSESTRKSAYDGLRSIGAF
VCSNGFLEIVRLRNKLAHLLGFEDYYDYTVTNAEGFSKSKLFEILDGLEEGTRPLMAKAR
KDLEELHGVKALDPWNTSYMMAGSVVKKMDPYFPFSKSVENYLRSYAAMNIGYSNATMTL
DLLDRKNKYSNGFCHWPVPAWIKPDGTHVPSQTNFTSLADPAAVGSGLTALNTLMHEAGH
AAHFANIAQPSPLFSQERAPTSVAYAENQSMFLDSLVGDSAWRAKYAKDLQGTPIPFAII
EEDIRAKHPFAVFQLRAMLSVSYFEKALYELPEAELTAEKVQSLADEVEIRIQGGLGARP
LLSVPHLVSDEASCYYQGYTLAEMSVHQTRKYFKDKYGYIVDNPNVGPTLTESYWKCGNS
KPFLQIVKDLTGKELSGDDWVDALKEDLEEHIAQERKEYDGMISKCEASSAEEKDGEEPL
DLAMTVKFVDGDHVISDSSQSGLLSACKEFEAFVAARVAAAKASS
>comp7439_c0_seq3; 264 191 643 LEN=1315; translated
MFNLQLAEMSVHQTRKYFKDKYGYIVDNPNVGPTLTESYWKCGNSKPFLQIVKDLTGKEL
SGDDWVDALKEDLEEHIAQERKEYDGMISKCEASSAEEKDGEEPLDLAMTVKFVDGDHVI
SDSSQSGLLSACKEFEAFVAARVAAAKASS
>comp7440_c0_seq1; 533 1 1418 LEN=1602; translated
NNNNNNNNGEEANNEEENNAEGEEENNGEEEKEQGDDQDEGDEEAGPEIDFLKCAAFTVE
PKLVDTQSMLQSGAISESAAAALEASSITKMTPKLNTQESVVFFTYGYGADDQDRKLYMA
GIDDWVAASYGYGQICHAIDQDDVDYVFSRIPSVFSVHRYSSHTWYAGFNCRSDGTGFQP
RLFLDATCTTFSTTMNEYYPFGSDTGEDQANQDYTTQVASDQTKYMVQNIQDTLEYAQYC
EESDFCDTVLGSSVEVTTCGGNGDDDAANGRNLAESGNAYQIEYGASSSIQNACPSIKAS
MGLGEDYDYTSYQLEWLAVLWSNTENGRQAPDRSRYPSRWVYPAACLLAGAVSVAALFLL
VSRARAKKNWNKAGCSGSESDDSSGKKEPLVGNHXPPPPPQRQLGGLHGPQRDRVQVLGA
QAQDPPGRPENLPEPLGEPATPLPAGGDGVRVGRTAHRLSGEQDTAQHIIKL
>comp7441_c0_seq1; 609 55 1320 LEN=1530; translated
MGKKAKSTKGALRAKERKLDKSTLPPPPISVSEEVGSAKLLDEPSLPGSGYKALGQDGYD
DGGVADLTLVIDLDDNVLRRKRGETDYFTSLKADLLPRVTKGPMAHLPRREQKIKLAKLL
FSDEETEEDKTPSEVWADNYDPEDDDDYETKKNRYDNLASARMALSLSSVLREPACASLS
LSEISKRRLACYGKAGGEDALRCAIKAIEIASTGFWDRDEIELECEESPKVDPNHEKNAT
APGLSHPDSLKLRPIRVSELCLRSAYLHKGNALAALGREAEARETYLKVMPMLEPEPRCG
RLDWERVSIYVNVGNTFSREGDFEKADEQYAKAEALGREHITASEGNQIDGMGIVVIAMR
ARAFALKRAGREDEGKKVLKDVIAMQINLNAENEKKKAEQKAEEEKKAAELKAAAEANAS
A
>comp7442_c0_seq1; 368 119 784 LEN=1008; translated
MFAVAVIAGCRQSVDAFSTVRRSVDHVGNTLQPRTAPVTLFGILDDLMEEENSCEEEEGK
NDDDERLERLYHSLIFSGDVKSEISRRLEACTDPGFSEYLVASSKSTRDRDEQQGLEELI
GLIEEVKTTAAMEAAAEKEAALAKAEKDKELRTEAEAQTAINASESKKLTNADLLKKANQ
IDAAIALSDDEKPSDFISDCREVVNLSRGFNDSGQMRVGGG
>comp7443_c0_seq1; 267 103 1130 LEN=1130; translated

MPAIYLISGGARSGKSGYAQELCEYLSPNPIYLATSTVFENDNDFAQRVEKHQRDRGDQW
ITIEEPLEPSKHLEKMKGRVVLVDCCTLWLTNYMMQEGLFSLDENENDKLKKNGSAQDAA
ERALRKIEKEFDKMTEPWDCTYVIVTNEVGSGTHAHDHVTRKFVDAQGWFNQYVAKRAEA
VVHMVCGVPNIVRGPLSKPSLSTAVRASGGLAAPTLHEAQRADMLDHYLSKRSIAMDPKG
YFKFRIDRDKKVIFASYHSCIINEKGEFFDLHGNRLTCHGKSPEPVRTWECRTAKELTTE
LLEKWDMADKVLSVGHGGYIGREAQKAEDALYTGETTDKTREX
>comp7443_c0_seq2; 46 715 1343 LEN=1343; translated
MTEPWDCTYVIVTNEVGSGTHAHDHVTRKFVDAQGWFNQYVAKRAEAVVHMVCGVPNIVR
GPLSKPSLSTAVRASGGLAAPTLHEAQRADMLDHYLSKRSIAMDPKGYFKFRIDRDKKVI
FASYHSCIINEKGEFFDLHGNRLTCHGKSPEPVRTWECRTAKELTTELLEKWDMADKVLS
VGHGGYIGREAQKAEDALYTGETTDKTREX
>comp7446_c0_seq1; 481 64 1072 LEN=1072; translated
MTNPPAGAATPSRNPARHNYANTHTPIASAKRPRSSMDKSALKNRTAGLSVPSTPLGKFG
SADANGNGNDRADAPLTPVPSMVTTQSVSSPVASTPRAMDRANLSPMHPARPTIRSEEED
SDRVEGATNMVDAIFSPMLKLLGAGEAGEEEEGQDREGGQELEVVAEHDEAEAEEHQHRP
ANNEDPAAEVEVPPEDAAVVETDDGSSQGTEYEDEEEFNPYAFIKSLPHYDDVKSLCPAD
RLPAKRDDAPPITLVLDLDETLVHCTVEQVDDADLTFPVDFHGMTYTVHVRLRPYLNQFL
EAIADKFEVVVFTASQQVYADALLDLIDPSKWLGLAX
>comp7446_c0_seq2; 623 64 1356 LEN=1615; translated
MTNPPAGAATPSRNPARHNYANTHTPIASAKRPRSSMDKSALKNRTAGLSVPSTPLGKFG
SADANGNGNDRADAPLTPVPSMVTTQSVSSPVASTPRAMDRANLSPMHPARPTIRSEEED
SDRVEGATNMVDAIFSPMLKLLGAGEAGEEEEGQDREGGQELEVVAEHDEAEAEEHQHRP
ANNEDPAAEVEVPPEDAAVVETDDGSSQGTEYEDEEEFNPYAFIKSLPHYDDVKSLCPAD
RLPAKRDDAPPITLVLDLDETLVHCTVEQVDDADLTFPVDFHGMTYTVHVRLRPYLNQFL
EAIADKFEVVVFTASQQVYADALLDLIDPNHEYIKYRMFRESCLPVEGNYLKDLNVLGRD
LKKAVLVDNSPHAFGYQVNNGIPIESWFDDKNDTELLKLERFLRTLQNVEDVRPVVRRTF
RTYELIANAP
>comp7447_c0_seq1; 521 1 1746 LEN=1876; translated
NNNNNNNQNYGDRYKFVTYESFNPLLFASTLAYCAIVTLLVVLRMKLHRKSNKKRKRLGL
PPASRENRKRSKNGDDEEDNIITDEEIHRYVLPSKQEQSVKEAAKVVRCDRETERIMGLC
KPFWMEAFVSGICDVFNTALIGKVLGVDSLAIFYIVTVPTSFTETIIGAVLETVSSLGGQ
SIGVGSYKLTGQYCQISIILYTILYIPHAILWITCLKPTLRWFAPDLGEEVIEEGYKYFL
VSLVGTYIESYSTVLHSLLEVSDHYGYGTKSEMVHNLAGVLGFFLYVKANPAGQKSIVMV
EVIWTCLTFAFLIIDLMVAKRMKWFDDFWKGILRSVAMKNWEAVWLVLKTAAPLSMGNII
MTVEWELLAIFAASMGGIQVAAWGIVGTIWGILEYATACVASAGEIRVAKLLGNGNPRLA
KLSAYKCLFLGNAFASIMSILFLVGLPYIPSWFTDDGELHGLVANVLPYCAIGNLTLTIG
SLAWTLVGAQGRYSVATFHGCIGSLLITIPCAALSTYRLGWELPSLAASVVIGYMTSGAF
NSITLFLSDWEYISYRVMIKNGATEPVEEDEDYEDQDEEPRW
>comp7447_c0_seq2; 644 1 2427 LEN=4518; translated
NNNNNNNQNYGDRYKFVTYESFNPLLFASTLAYCAIVTLLVVLRMKLHRKSNKKRKRLGL
PPASRENRKRSKNGDDEEDNIITDEEIHRYVLPSKQEQSVKEAAKVVRCDRETERIMGLC
KPFWMEAFVSGICDVFNTALIGKVLGVDSLAIFYIVTVPTSFTETIIGAVLETVSSLGGQ
SIGVGSYKLTGQYCQISIILYTILYIPHAILWITCLKPTLRWFAPDLGEEVIEEGYKYFL
VSLVGTYIESYSTVLHSLLEVSDHYGYGTKSEMVHNLAGVLGFFLYVKANPAGQKSIVMV
EVIWTCLTFAFLIIDLMVAKRMKWFDDFWKGILRSVAMKNWEAVWLVLKTAAPLSMGNII
MTVEWELLAIFAASMGGIQVAAWGIVGTIWGILEYATACVASAGEIRVAKLLGNGNPRLA
KLSAYKCLFLGNAFASIMSILFLVGLPYIPSWFTDDGELHGLVANVLPYCAIGNLTLTIG
SLAWTLVGAQGRYSVATFHGCIGSLLITIPCAALSTYRLGWELPSLAASVVIGYMTSGAF
NSITLFLSDWEYISYRVMIKNGATEPVEEDEDYEDQDEEPRWNVFNFMKTSSLDGNDSHR
NTNRTLLVDTLTRNTSSLSVEDDGSNSASSKRSLERNYENGPVTNTSSWWASLGVDQSEV
REVLIGPGKLGYTIQSTKRGPMIVRCPKESNQSFIGPMRGTSHRGRRAIIISGDLFLGDI
IVQIDEIPTDKMCGRELGKILSKRSNRESRKLIVSHREGTGLATVYQPGPYGSAAITGVL
KNAKEIQNKEFEDEGVVGAGYFNMGWFS
>comp7447_c0_seq3; 644 1 2427 LEN=4655; translated
NNNNNNNQNYGDRYKFVTYESFNPLLFASTLAYCAIVTLLVVLRMKLHRKSNKKRKRLGL
PPASRENRKRSKNGDDEEDNIITDEEIHRYVLPSKQEQSVKEAAKVVRCDRETERIMGLC
KPFWMEAFVSGICDVFNTALIGKVLGVDSLAIFYIVTVPTSFTETIIGAVLETVSSLGGQ
SIGVGSYKLTGQYCQISIILYTILYIPHAILWITCLKPTLRWFAPDLGEEVIEEGYKYFL
VSLVGTYIESYSTVLHSLLEVSDHYGYGTKSEMVHNLAGVLGFFLYVKANPAGQKSIVMV
EVIWTCLTFAFLIIDLMVAKRMKWFDDFWKGILRSVAMKNWEAVWLVLKTAAPLSMGNII

MTVEWELLAIFAASMGGIQVAAWGIVGTIWGILEYATACVASAGEIRVAKLLGNGNPRLA
KLSAYKCLFLGNAFASIMSILFLVGLPYIPSWFTDDGELHGLVANVLPYCAIGNLTLTIG
SLAWTLVGAQGRYSVATFHGCIGSLLITIPCAALSTYRLGWELPSLAASVVIGYMTSGAF
NSITLFLSDWEYISYRVMIKNGATEPVEEDEDYEDQDEEPRWNVFNFMKTSSLDGNDSHR
NTNRTLLVDTLTRNTSSLSVEDDGSNSASSKRSLERNYENGPVTNTSSWWASLGVDQSEV
REVLIGPGKLGYTIQSTKRGPMIVRCPKESNQSFIGPMRGTSHRGRRAIIISGDLFLGDI
IVQIDEIPTDKMCGRELGKILSKRSNRESRKLIVSHREGTGLATVYQPGPYGSAAITGVL
KNAKEIQNKEFEDEGVVGAGYFNMGWFS
>comp7449_c0_seq1; 361 167 1399 LEN=1770; translated
MSPKKSSGGGKVLGLFPKKKKNQQQEQSKKSKQKRSASNDGRSTTSKSVASQEQWDRSRA
NTEAFSSANRASKRSLVALAYETTTSPSFRRERRKTGKQQPFPNGSASDSNSAKRSKENV
PPPSTRSRMRSAARKVSGNKTTVETSITDKKRVSRGGGWFSFLNPARSSFFQKLCDDTFD
SIDVDGSGKIDESELYRGLLLIHLKLGIYFGAPACKPLSAENAVTVFQQLDTNRDGSLDK
DEFRSVLTLLMGNVLFRIIFQFVCTLVMVPMLAQTFLEQVSFGWTWLVAVAVPLWKEYLP
LLECALALDLVKKYSSLALSRGLELFETTVPLETIESIQKTIEPIQNTVLESWETMVMAN
VRDIPQETLDSLPLTIVSTMLTLIIVPYSILKTDEFFQFLARKISGGKKG
>comp7450_c0_seq1; 204 192 512 LEN=1046; translated
MTKSNQNEMESCRSSLGIRCLPVINDQSEFLTILVSSLRSLFGELESHSAEVKVSRVPDT
QEAEYHFILECNKQSTDALRASLTMVTTPAYLERNIYVFDIFDISS
>comp7450_c0_seq2; 184 240 449 LEN=1030; minus strand; translated
MRKDGDESPVSMETIFLDWSKDPAMSSKGGFDIVEALSPDGIYGLIEQGKLYGKIMEDQG
KFDSLEKLT
>comp7451_c0_seq1; 251 89 547 LEN=1077; translated
MNRLISILVLLMNSGVFVFARPSNDRCIDSITLVESEKVNGDNSNANYDYNNQGVCGPRS
DRRAVWYEINGVGKEVTVNVCTNNDKLTDFGIFRSCNSQNCKGFPPQQMVVTDCAADESN
QYSFLAKNGESYYIHVRSDVVFEGEGSDVSSV
>comp7451_c0_seq2; 236 275 795 LEN=1000; translated
MTITTKEFAVLGVTVEQSGTKSMELEKKXTVNVCTNNDKLTDFGIFRSCNSQNCKGFPPQ
QMVVTDCAADESNQYSFLAKNGESYYIHVRSDVVFEGEGSDFTIWYTEVADDVTSTPETL
PYTEEQTESTNATSTSEEQTMSTTSGSESPKTFLNVGISSMIAGFGYGLLSLL
>comp7451_c0_seq3; 307 89 751 LEN=956; translated
MNRLISILVLLMNSGVFVFARPSNDRCIDSITLVESEKVNGDNSNANYDYNNQGVCGPRS
DRRAVWYEINGVGKEVTVNVCTNNDKLTDFGIFRSCNSQNCKGFPPQQMVVTDCAADESN
QYSFLAKNGESYYIHVRSDVVFEGEGSDFTIWYTEVADDVTSTPETLPYTEEQTESTNAT
STSEEQTMSTTSGSESPKTFLNVGISSMIAGFGYGLLSLL
>comp7452_c0_seq1; 244 268 1227 LEN=1562; translated
MMTTNTKIVLYTFPPIPKSYSMTPFGLKTESFLRANDIPYEKVYTTSFGNNGTIPYLRIF
SSDDESDESSFEEIPDSNAILSRLSKDPRFEIRSDANLTTEQKAIGHACLRMLEEHTSQA
GFYFRYALKMPEFCEATQLRERVFMGDESKLGNFIYNQFKSKMPGGWMKKAKVRGFVRYS
SPAVVWAMTSEDLKVLEDLLSQHVYFFGQSHPGTLDCTVFGHLSQFLYIDIDFPQKKYLK
ESCPNLLRFMEHFKTTHFPDWETLCLKQPNDALDPDNPRMKALAKMKQKAILVGATALLA
IVGVGGRYFSSVFSSSQEL
>comp7453_c0_seq1; 638 1 1851 LEN=1971; translated
QQQQQQPYNQRRGVFLEGSHSTPKKNNVFLSPVPSQTSFNPKTICSVPLSNNSPVQLQIL
QMQWTQQQQQLQQRYSNVSMLSNNQSDAPGSICRNDDPIAQTNEQSCPISSANKDSDPFR
ISSNDHPSQQNSAPITEAPKLNAKNSSLQPETAIRPMFPALESKTSQSRGFQSIEQQKKK
VEKQNMSQIEMLREAYISSVSPKASCTSKNVNIADRTMITATPGLKSNSRGNSGMKHQLV
SPLPMQVPTSEPKVVTTPIDVSVESSALQFCKPREKGVVNNIQSSSEAVTLDSVTSNQMM
MDCKMSHTHIPNFLAGFDKVANPKKMNHTNPIVTNNQLPELSPAYYTSKSFDDFHRFLGK
GLPPGVSTSIKFPDLEPLNVTKQGPLVSSNHSADEVLKSSLQFKPRTPLNLPSAANNTDG
TSSGTSERRCYGMPLAADFKEVTGMFPDKKPPKLPQTEGVRNDVNSSSRHSMRADHSPNN
VVLDHQEHLSRSSFPQELQPQPQGKAEDNPSDFRIEDIMLEEIGAIGGSDPDATPYPHTM
FGDGSGIVLDSGAPASDGRPCSSDAYSVSGGGIVSDESIGALSEGSSRIQKRKRDVLPEN
DLVYVTRKERNCIYGL
>comp7454_c0_seq1; 375 146 1447 LEN=2033; translated
MEDSGISVRMGSKIAEGGFSVIFHASDTASDASPSQKYVLKRICCHDGESRSACIKESKV
HYAIMREQKNNQQHSSKIIAAGGGGMGGMAGSTTETIRSSLSSHIMPLLGMTFSENNTIC
YMLFPFLPDSLRREVDRRIFEPMEEAIGNGKNKNGSAMTQSQKNQKTKDTLRMPPWSETA
VLRWFEQLCDAVILMHDAGYTHRDIKLDNVLLQSAATQQTNKRGNNDSRSGVVLMDYGSA
GPLTRSLASRRDVLEISDEASQHTTISYRPPELFAGELRVADTNTNAAISKDIDGDDVFD

PESVLDYRKVDSWMLGCTLFAIFFGASPGESEFSRSTGQLLIVDPSHNKMLGQMPPWPDE
ETPSSRWYSSDTKELLDWILTKDRGDRPTVKQIRRRVREILSQKPALFAGDGSGILDVES
QMIDMTFSTKNML
>comp7456_c0_seq1; 404 84 2021 LEN=2378; translated
MQAMMPSQHQRDLSVRDQEIARLQNQVNALSNQLESAMQQQQQLRRYPQQSSDRNSRKKS
HKRTNKMSPHAVHIQHVDGGKLGSNVIRTGRQRMGRNASFYTQRVMASLTMPEGEKYASN
PIIMRIMNMFERPNENVEYLRGKLFATDLFKLCQKVRQILEREPRVVYLQSPCYIFGDIH
GNLEDLHFFSDNIWRLGMNLTAGNFLFLGDYVDRGMNCLAVVAYLLAMKLQNPQKVFLLR
GNHETRDVNGWEEHYGERSFIWQCQDRFDRFGGDIGYRIWEACNQVFDRLPLAAVVDQDI
FCVHGGIPRPVCMGNPRLRGASTTRIQDILNVPKVAGINPPYEHEDDVYQQVASDCIWSD
PASEEQERISVDPHTGYGESLRGGGAICFGNKAVTDFLQEQGFSYIMRAHEAHAEGVAVS
KGARVFTVFSTSKDHNQGSQALAGCILVDDDRLQVINRSPAYKNQYVHRRDSLSLQTLSH
LEIAKRIKLGLVTPDDTADYEQSDDESWDDQAAEEQNPGVASTGGSDDDSIGLPVDDPYG
YRELLGQQGKTQQEQQYTFAQGRRSSIDLSQMSTNLHDSQSEINMELSASSIRMGSSSRS
LQESLRSNNDIIHQAVAEGEDNNEDEDSTVNGDDLDADADVSMEN
>comp7457_c0_seq1; 1202 93 2627 LEN=3087; translated
MSWLWGTKKDDSMEEDLADEGIQSEEEIHTDSSDESEPEDHSVDGKVTTEETVDDYDGES
SDESAYDNSSDEDDEGDYDEDDDEDDDSLVVNLIESMTSDDDTVDENLEEADLLTSDDEE
DDETVARVEGGTQENRKTDKVDENETSHPPVDDTDDDDDDGDDDDDDKNGDPVTSFWEKQ
SLLVLAAEHDRVDILKKLLKEENEEKDSLLNTGIPPLHLAITFGSVNTAQSLLRMGSDPS
VRPNVDVVLEERRSQPKDSKVDIANIRRFDNVTAWELAFGNALYDEKSMSNKSSGSWSMF
GSTMSGESEGMNEAAPTAKSTNGAQTIVKPVDMAPSKREGIRHAFTAEALRSVGGDEAHR
LKQLLDSGMPSDIDIGGKDLYGWAVDMGSLKCEELLRPDESPDQDGGSTNASPGGDDAPS
DLSSSFVVHRPDVQETIPQLKNRLDELESLSSALSTCLDNLAEEVSVCHGLLLMGGGASA
LASHVRSLRDLKEQKLYEMENARLECQDAERELADLVHSSGDIGKEIDHIANFNFLNYGV
EKNAGVHTSHIKLQQNENDSDGDGAIDDPSINKEAEAEAEKLNIKAQIAAIENKIRKLRA
SITDLSEENTRDLAEVQRRGLEGGINLVRGLREELRDFDFYLSEARSMKAVCKSKISMII
AHVSKKNINNTEGSLDKVPVARRGTTNHSVGYDVRNDATIANDATIGSKSMDYSTDEDDD
EIEEAVLVESPSKIDALQKEESSKETAFDSPKMQTPIQVSEEKRSQESTVNNVDSSTVDG
KSASQRISSGESREITVIHPGNKGLFTVDLWEVILRIIGFESAANRRSLENKAHHGSSTT
VMII
>comp7457_c0_seq2; 1069 93 2740 LEN=3200; translated
MSWLWGTKKDDSMEEDLADEGIQSEEEIHTDSSDESEPEDHSVDGKVTTEETVDDYDGES
SDESAYDNSSDEDDEGDYDEDDDEDDDSLVVNLIESMTSDDDTVDENLEEADLLTSDDEE
DDETVARVEGGTQENRKTDKVDENETSHPPVDDTDDDDDDGDDDDDDKNGDPVTSFWEKQ
SLLVLAAEHDRVDILKKLLKEENEEKDSLLNTGIPPLHLAITFGSVNTAQSLLRMGSDPS
VRPNVDVVLEERRSQPKDSKVDIANIRRFDNVTAWELAFGNALYDEKSMSNKSSGSWSMF
[truncated: 441,569 more chars]
